# Evolutionary Patterns and Genotype-Specific Amino Acid Mutations of Tick-Borne Encephalitis Virus

**DOI:** 10.3390/ijms26030954

**Published:** 2025-01-23

**Authors:** Ruichen Wang, Anqi Gu, Fan Li, Qian Ma, Qikai Yin, Kai Nie, Shihong Fu, Qianqian Cui, Songtao Xu, Hao Li, Huanyu Wang

**Affiliations:** 1 National Key Laboratory of Intelligent Tracking and Forecasting for Infectious Diseases, NHC Key Laboratory of Biosafety, National Institute for Viral Disease Control and Prevention, Chinese Center for Disease Control and Prevention, Beijing 102206, China; wangrc96@163.com (R.W.); 17852850389@163.com (A.G.); lifan@ivdc.chinacdc.cn (F.L.); yinqk@ivdc.chinacdc.cn (Q.Y.); niekai@ivdc.chinacdc.cn (K.N.); fush@ivdc.chinacdc.cn (S.F.); cuiqq@ivdc.chinacdc.cn (Q.C.); xust@ivdc.chinacdc.cn (S.X.); 2 Chinese Center for Disease Control and Prevention, Beijing 102206, China; maqian@chinacdc.cn

**Keywords:** TBEV, genetic evolution, amino acid mutations, protein structure

## Abstract

Tick-borne encephalitis virus (TBEV) is a significant tick-borne flavivirus responsible for severe human diseases. Here, we analyzed the genetic diversity and evolutionary dynamics of TBEV using 263 genome sequences from the NCBI database and identified key amino acid mutations. TBEV sequences were classified into five genotypes—Baikalian, European, Far-Eastern, Himalaya, and Siberian—showing ORF nucleotide similarity of 81.5% to 88.0% and amino acid similarity of 93.0% to 96.4%. Extensive recombination between genotypes was not observed. Entropy analyses revealed highly variable sites distributed across the Baikalian (n = 2), European (n = 3), Far-Eastern (n = 5), and Siberian (n = 13) genotypes. Each genotype exhibited specific amino acid mutations. Positive selection analysis identified sites under selection in the full dataset (n = 2), as well as in the European (n = 6), Far-Eastern (n = 7), and Siberian (n = 4) genotypes. By integrating highly variable sites, shared genotype-specific mutations, and positively selected sites, we identified 37 key amino acid positions, primarily located on the surfaces of viral proteins. These positions may have a potential impact on protein function and pathogenicity, though further studies are required to validate and evaluate these effects comprehensively. This study provides the first comprehensive analysis of mutational landscapes across TBEV genotypes, uncovering potential critical mutations that may shape viral biology and pathogenicity, and offers valuable insights for further exploration of TBEV characteristics.

## 1. Introduction

Tick-borne encephalitis (TBE) is a natural focal infectious disease caused by bites from ticks carrying the tick-borne encephalitis virus (TBEV), characterized primarily by central nervous system symptoms and potentially leading to fatal outcomes [1]. TBE is primarily endemic in the vast regions of Europe and Asia between latitudes 39° N and 65° N, with approximately 10,000–12,000 cases reported globally each year [2,3]. The annual incidence rate of TBE varies significantly across different regions. In Europe, the reported incidence is approximately 2.19 cases per 100,000 population [4]. In Russia, the annual incidence is lower, estimated at 0.67 cases per 100,000 population [5]. In China, the incidence ranges from 0.09 to 0.44 cases per 100,000 population [6]. TBEV is primarily transmitted by ticks, with at least 24 tick species capable of spreading the virus. Among them, *Ixodes persulcatus* and *Ixodes ricinus* are the main vectors of TBEV [7]. TBEV has also been reported to spread through the consumption of unpasteurized milk and dairy products [8,9]. The host range of TBEV is broad, encompassing ticks and various vertebrate hosts such as cattle, sheep, dogs, horses, deer, and rodents [7]. Humans serve as terminal hosts for TBEV [10].

TBEV has been classified by the International Committee on Taxonomy of Viruses (ICTV) under the family *Flaviviridae* and the genus *Flavivirus* (recently renamed as *Orthoflavivirus*) [11]. TBEV is an enveloped, single-stranded positive-sense RNA virus with a genome approximately 11 kb in length. It contains a single open reading frame (ORF) that encodes three structural proteins (anchC (the precursor of the C protein), PreM, and E) and eight non-structural proteins (NS1, NS2a, NS2b, NS3, NS4a, 2K, NS4b, and NS5) [12]. Based on the genetic diversity of viral genome sequences, TBEV is classified into five main genotypes [13], including the traditionally recognized European, Siberian, and Far-Eastern genotypes, as well as the recently defined Baikalian [14] and Himalaya [15] genotypes. The virulence of different TBEV genotypes varies significantly, with case fatality rates among the three traditional genotypes ranked from highest to lowest as Far-Eastern (5.0–20.0%), Siberian (6.0–8.0%), and European (0.5–2.0%) [16]. Currently, there is no conclusive evidence that the Baikalian and Himalaya genotypes infect or cause disease in humans.

The evolutionary characteristics of TBEV have long been a focus of research. TBEV is considered the slowest-evolving tick-borne flavivirus, with divergence times among its genotypes dating back to ancient periods (95% HPD, 6373–13,208 years) [17]. However, even a few mutations in TBEV can lead to significant changes in its virological properties, including plaque size, virulence, neuroinvasiveness in mice, drug resistance, and host adaptability [18,19,20,21]. Therefore, this study conducted an in-depth investigation into the evolutionary patterns of TBEV and the mutational differences among its various genotypes. The findings provide valuable insights into the evolutionary characteristics of different TBEV genotypes. Additionally, as live TBEV research typically requires BSL-3 laboratory conditions, this study identifies key amino acid sites that can serve as references for future investigations into the virological properties of TBEV.

## 2. Results

### 2.1. The Phylogenetic Tree of TBEV

**Figure 1:**
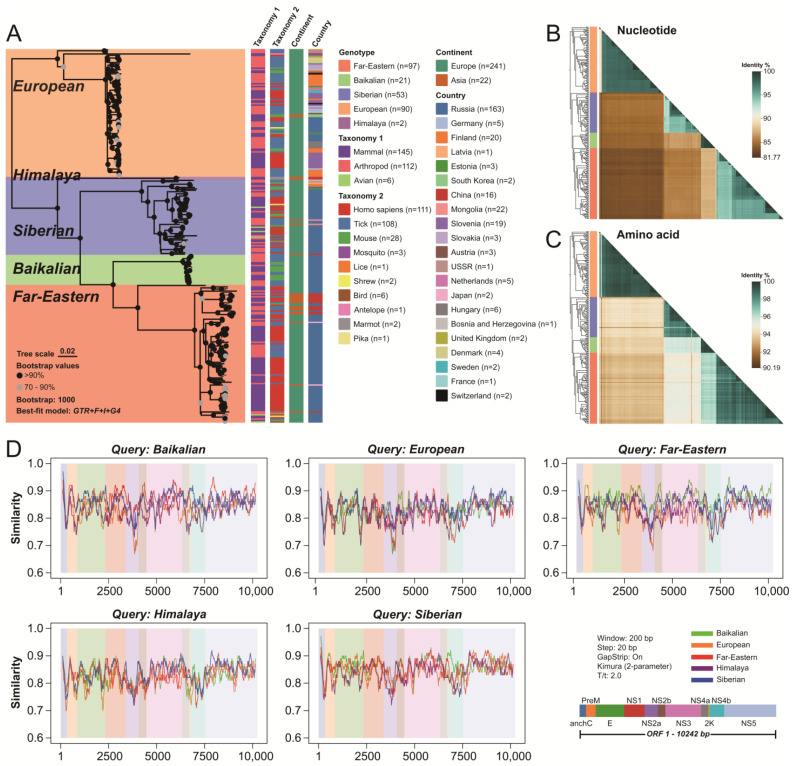
Phylogenetic and similarity analysis of five TBEV genotypes. (**A**) Maximum likelihood (ML) tree illustrating the genetic relationships among the five TBEV genotypes, with detailed information on genotypes, isolates, and locations of isolation. The number of sequences for each category is shown in the legend. Heatmaps of pairwise nucleotide similarity (**B**) and amino acid similarity (**C**). The color gradient reflects the degree of similarity. (**D**) Nucleotide similarity within different genome regions across groups, detected using Simplot software (Version: 3.5.1). The background shading indicates distinct genomic regions.

A phylogenetic tree was reconstructed based on the sequences included in this study. The analyzed TBEV sequences clearly clustered into five evolutionary branches (bootstrap support > 99%), corresponding to the European, Himalaya, Siberian, Baikalian, and Far-Eastern genotypes (Figure 1A). In terms of host origin, TBEV is predominantly found in mammals (humans and rodents) and arthropods (ticks), but has also been detected in birds. Occasionally, TBEV has been reported in mosquitoes, lice, shrews, antelopes, marmots, and pikas. Human infections have been reported for the European, Far-Eastern, and Siberian genotypes, while the Baikalian genotype (found in ticks and rodents) and the Himalaya genotype (found in marmots) have not yet been associated with human infection. TBEV strains from different hosts did not form distinct host-specific clusters, but were interspersed across the phylogenetic tree. This finding highlights the broad host range of TBEV and suggests that the virus lacks strong host specificity.

From an administrative regional perspective, TBEV is primarily distributed across Europe and Asia, including continental countries and island nations such as the UK and Japan (Figure 1A). Notably, for simplicity in statistical analysis, TBEV from Russia’s Far East was grouped with Europe. When examining the administrative distribution of genotypes, it was observed that the European, Siberian, and Far-Eastern genotypes are found in more than one country, indicating cross-regional transmission for these three genotypes. The Far-Eastern genotype is primarily distributed in Russia and China, with sporadic detections in Germany. The European genotype exhibits the widest spread, having been detected in at least 15 countries across Europe and Asia. Furthermore, European genotype sequences from different countries did not form distinct country-specific clusters, suggesting that this genotype may be associated with more frequent long-distance transmission events compared to other genotypes. The Siberian genotype is also found in multiple countries across Europe and Asia, but it is primarily concentrated in Russia. Among the sequences analyzed in this study, the Baikalian genotype was exclusively detected in Russia, while the Himalaya genotype was only found in China.

Analysis based on species and administrative regions revealed that the main differences among TBEV sequences lie between genotypes. Therefore, subsequent analyses were conducted using genotypes as the grouping criterion.

### 2.2. Genotypic Similarity Analysis of TBEV

The ORF regions of TBEV strains from different genotypes were compared for nucleotide and amino acid similarity. Nucleotide similarity among TBEV strains ranged from 81.7% to 100.0% (Figure 1B and Table 1), while amino acid similarity ranged from 90.2% to 100.0% (Figure 1C). Between different genotypes, nucleotide similarity was 81.5–88.0%, and amino acid similarity was 93.0–96.4%. Specifically, at the nucleotide level, the European and Himalaya genotypes exhibited the greatest divergence (81.5%), while the European and Far-Eastern genotypes showed the highest similarity (88.0%) (Figure 1B). At the amino acid level, the European and Himalaya genotypes again displayed the greatest divergence (93.0%), while the Far-Eastern and Baikalian genotypes exhibited the highest similarity (96.4%) (Figure 1C). These findings underscore the significant differences among TBEV genotypes.

A sliding window analysis was performed to examine the distribution of nucleotide similarity across the genomes of different TBEV genotypes. Nucleotide similarity in each genome window between genotypes was generally below 95%, suggesting the absence of widespread recombination events between genotypes (Figure 1D). However, recombination analyses of individual sequences revealed potential intra-genotype recombination events in the European (n = 3), Far-Eastern (n = 10), and Siberian (n = 12) genotypes (Supplementary Table S2). Overall, the NS2a region was identified as the most divergent region at the nucleotide level, while the NS5 region exhibited the highest nucleotide similarity (Figure 1D). Further analysis revealed that similarity levels between a given genotype and others were not consistent across all genome regions. For instance, the Siberian genotype displayed the greatest divergence from the Himalaya genotype in the NS2a region, while showing higher similarity with the Himalaya genotype in the early part of the NS1 region. This variation in similarity levels across genome regions was observed to varying degrees for all genotypes and was also evident in amino acid analyses (Supplementary Figure S1). These findings highlight the necessity of conducting in-depth analyses of different regions across TBEV genotypes.

### 2.3. Variability of Amino Acid Sites Across Different Genotypes of TBEV

**Figure 2:**
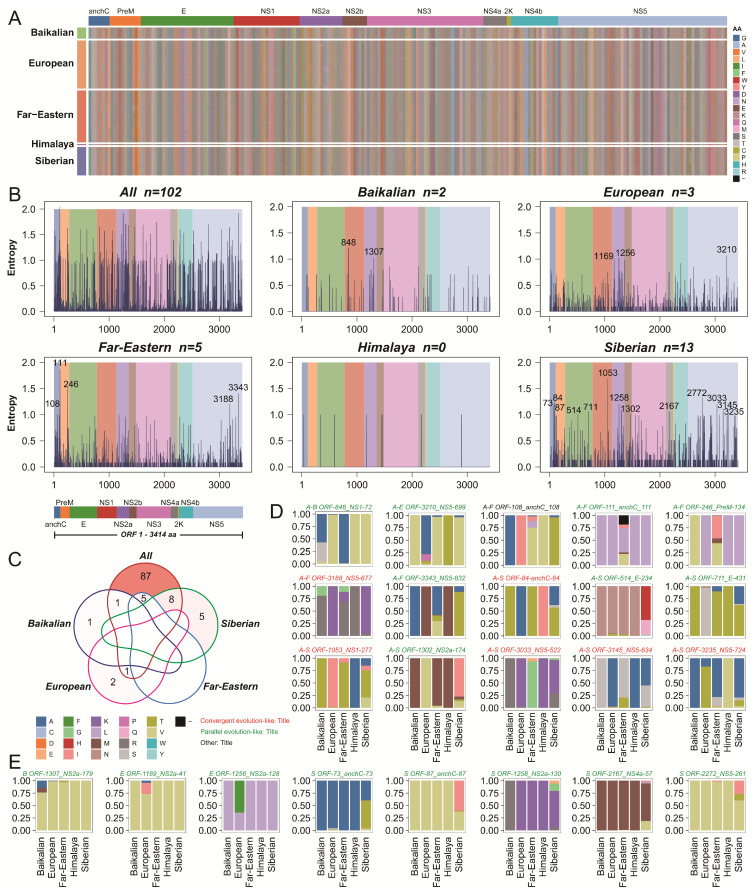
Mutation landscape and analysis of hypervariable sites across the five TBEV genotypes. (**A**) The mutation landscape of the analyzed sequences is displayed as a heatmap, where different colors represent distinct nucleotides. (**B**) Shannon entropy was calculated for each amino acid position across different datasets, with entropy values greater than 1 identified as hypervariable sites, which are annotated accordingly. (**C**) The sharing of hypervariable sites across different datasets is visualized. Different lines represent different genotypes, and the intensity of the background color indicates the quantity. (**D**) Amino acid composition of shared hypervariable sites is illustrated using stacked bar charts, with colors representing different amino acids. (**E**) Amino acid composition of non-shared hypervariable sites is similarly shown as stacked bar charts. The evolutionary patterns of these sites are indicated: red denotes convergence-like evolution, green indicates parallel-like evolution, and black represents other types.

Amino acid mutations can lead to differences in viral characteristics, so we further analyzed the mutational patterns among different TBEV genotypes from multiple perspectives. The mutation landscape of TBEV revealed that, while most positions showed consistent amino acid usage across genotypes, some loci exhibited variation (Figure 2A and Supplementary Data S2). Information entropy analysis identified numerous high-variability amino acid sites (Entropy > 1) (Figure 2B). These highly variable sites are often closely associated with changes in virological properties. Across the entire dataset, 102 high-variability sites were identified. These included 2, 3, 5, and 13 sites in the Baikalian, European, Far-Eastern, and Siberian genotypes, respectively. Due to the limited sequence data available for the Himalaya genotype, entropy analysis could not provide meaningful results for this group.

In the full dataset, highly variable sites were distributed across all protein regions except the 2K protein. However, the distribution of high-variability sites differed among individual genotypes. For the Baikalian genotype, high-variability sites were located in NS1 (n = 1) and NS2a (n = 1). For the European genotype, they were found in NS1 (n = 1), NS2a (n = 1), and NS5 (n = 1). The Far-Eastern genotype exhibited high-variability sites in anchC (n = 2), PreM (n = 1), and NS5 (n = 2). In the Siberian genotype, these sites were distributed across anchC (n = 3), E (n = 2), NS1 (n = 1), NS2a (n = 2), NS4a (n = 1), and NS5 (n = 4). Notably, high-variability sites were not shared across genotypes, although some overlap was observed between genotype-specific sites and those identified in the full dataset (Figure 2C–E).

The mutation patterns at shared sites revealed two main modes. The first is convergent evolution-like, exemplified by anchC-84 in the Siberian genotype. The primary mutations, 84T and 84A, were conserved in the Baikalian (T), Far-Eastern (T), and European (A) genotypes, while the minor mutation 84S was not observed in other genotypes. The second mode is parallel evolution-like, as observed in anchC-111 in the Far-Eastern genotype. While other genotypes exhibited strong conservation of L (>90%) at this site, the Far-Eastern genotype showed a more diverse pattern, including gap, V, I, and M mutations. A unique case was identified in the Far-Eastern genotype at anchC-108, a high-variability site where the primary amino acid V and the mutations A and I were found in other genotypes, but a unique mutation L was also present, indicating a complex mutation pattern.

Based on these observations, we identified six convergent evolution-like sites (one in the Far-Eastern genotype and five in the Siberian genotype) and sixteen parallel evolution-like sites (two in the Baikalian genotype, three in the European genotype, three in the Far-Eastern genotype, and eight in the Siberian genotype). These findings highlight diverse mutational patterns across TBEV genotypes, which may have implications for viral adaptation and evolution.

### 2.4. Genotype-Specific Amino Acid Mutations

**Figure 3:**
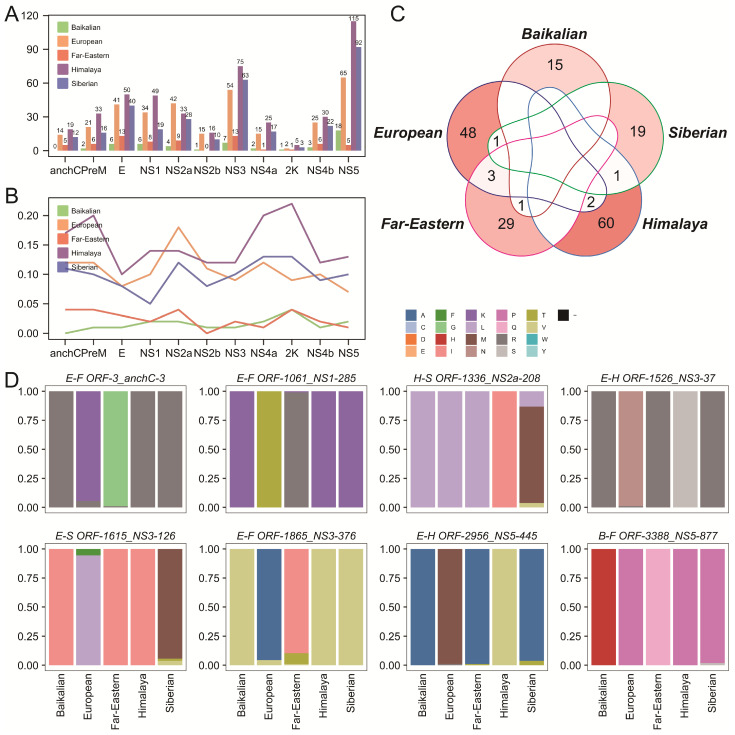
Analysis of genotype-specific amino acid mutations across the five TBEV genotypes. (**A**) The number of genotype-specific amino acid mutations in different protein-coding regions is displayed for each genotype. (**B**) The proportion of genotype-specific amino acid mutations relative to the length of the corresponding protein-coding region. (**C**) The sharing patterns of positions with genotype-specific amino acid mutations among the five genotypes. Different lines represent different genotypes, and the intensity of the background color indicates the quantity. (**D**) The amino acid composition of shared mutation sites is illustrated.

This study also focused on genotype-specific amino acid mutations, which may play a critical role in the virological differences among TBEV genotypes. A total of 50, 314, 67, 450, and 322 genotype-specific mutation sites were identified in the Baikalian, European, Far-Eastern, Himalaya, and Siberian genotypes, respectively. These genotype-specific mutation sites were distributed across all 11 gene regions (Figure 3A), but the proportion of mutations within each region varied among genotypes (Figure 3B). In general, all genotypes exhibited relatively fewer mutation sites in the 2K protein region, though the proportion of mutations in this region was higher. Conversely, the NS5 protein region contained a larger number of mutation sites, but these did not represent the highest proportions. The high number of mutations complicated further analysis, as some genotype-specific mutations may occur randomly. Therefore, we focused on genotype-specific mutations with a prevalence greater than 60%, which can be considered as parallel evolution-like sites. After filtering, the number of genotype-specific mutation sites was reduced to 16, 54, 33, 63, and 22 for the Baikalian, European, Far-Eastern, Himalaya, and Siberian genotypes, respectively. Some mutation sites were shared among genotypes, such as anchC-3, NS1-285, and NS3-376 shared between the European and Far-Eastern genotypes, NS3-37 and NS5-445 shared between the European and Himalaya genotypes, NS3-126 shared between the European and Siberian genotypes, NS2a-208 shared between the European and Siberian genotypes, and NS5-877 shared between the Baikalian and Far-Eastern genotypes (Figure 3C,D). Notably, shared mutation sites were also observed between genotypes with distant evolutionary relationships (Figure 1A and Figure 3D), suggesting that these shared sites likely result from convergent or parallel evolution under similar selective pressures rather than direct genetic inheritance.

### 2.5. Selective Pressure Analysis of Different TBEV Genotypes

**Figure 4:**
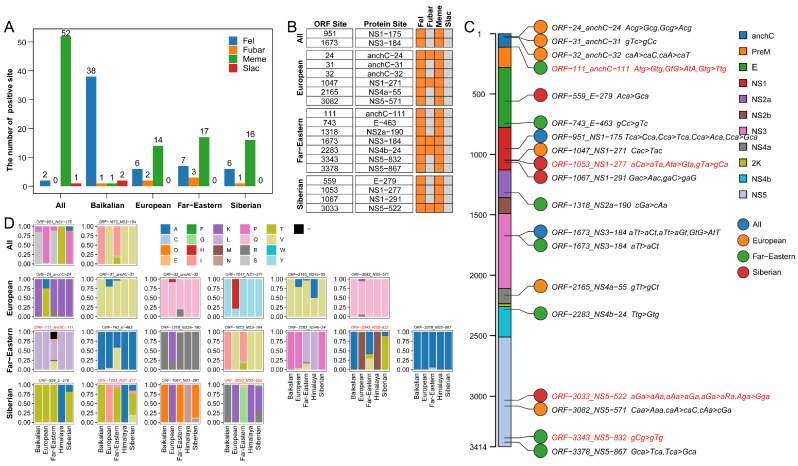
Selection pressure analysis of the five TBEV genotypes. (**A**) The number of positively selected sites identified across different datasets using various algorithms. (**B**) Positively selected sites confirmed by two or more algorithms. Orange indicates positive, while gray indicates negative. (**C**) Schematic representation of the positions of positively selected sites within genotypes, along with the major codon mutation patterns at these sites. (**D**) Amino acid composition of positively selected sites. The red annotations in (**C**,**D**) indicating sites that were also identified as highly variable sites.

Both information entropy analysis and genotype-specific amino acid mutations have identified numerous key amino acid sites, prompting further investigation into the evolutionary forces driving these mutations. Selection pressure analysis revealed the presence of positively selected sites at both the overall TBEV and genotype-specific levels, with consistent results across multiple algorithms (Figure 4A). To minimize overestimation, only sites identified as under positive selection by at least two algorithms were included in further analysis (Figure 4B). At the overall TBEV level, two positively selected sites were identified: NS1-175 and NS3-184. At the genotype level, six positively selected sites were detected in the European genotype (anchC-24, anchC-31, anchC-32, NS1-271, NS4a-55, and NS5-571), seven in the Far-Eastern genotype (anchC-111, E-463, NS2a-190, NS3-184, NS4b-24, NS5-832, and NS5-867), and four in the Siberian genotype (E-279, NS1-277, NS1-291, and NS5-522). No positively selected sites were identified in the Baikalian genotype, and due to limited sequence data, selection pressure analysis was not feasible for the Himalaya genotype.

Across the genome, positively selected sites were distributed across eight proteins: anchC (n = 4), E (n = 2), NS1 (n = 4), NS2a (n = 1), NS3 (n = 1), NS4a (n = 1), NS4b (n = 1), and NS5 (n = 1), with no positive selection detected in the PreM and 2K proteins (Figure 4C). Notably, NS3-184 was identified as a positively selected site at both the overall TBEV level and in the Far-Eastern genotype. Four sites were identified as both high-variability sites (Figure 2B) and positively selected sites (Figure 4D): anchC-111 (ORF-111) in the Far-Eastern genotype, NS1-277 (ORF-1053) and NS5-522 (ORF-3033) in the Siberian genotype, and NS5-832 (ORF-3343) in the Far-Eastern genotype. However, none of the genotype-specific amino acid mutation sites were found to be under positive selection.

### 2.6. Mapping of Mutation Sites on Protein Structure

**Figure 5:**
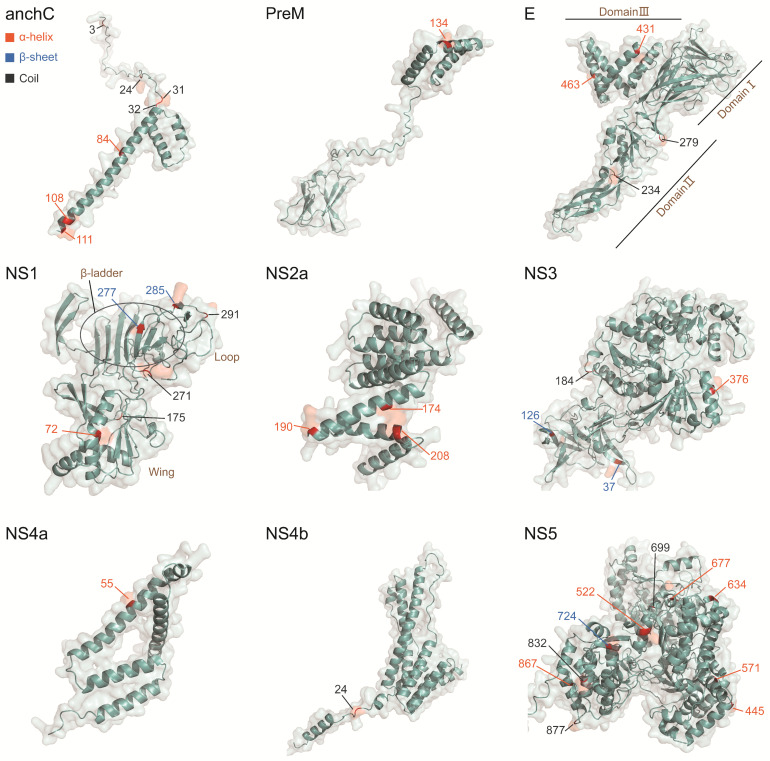
Structural mapping of selected key amino acid sites on the protein structure. The text labeling the key sites is annotated with secondary structure elements: red represents alpha helices, blue indicates beta sheets, and black denotes random coils. Key amino acid sites are highlighted on the structure.

By incorporating all high-variability sites across genotypes (n = 23), shared genotype-specific amino acid mutation sites (n = 8), and all positively selected sites (n = 19), a comprehensive dataset of 37 key amino acid mutation sites associated with TBEV genotypes was compiled (with overlaps between methods). These sites are distributed across nine TBEV proteins, including anchC (n = 7), PreM (n = 1), E (n = 4), NS1 (n = 6), NS2a (n = 3), NS3 (n = 4), NS4a (n = 1), NS4b (n = 1), and NS5 (n = 10). Mapping these sites onto protein structures enabled further analysis of the potential impacts of mutations (Figure 5).

In terms of location, the key sites were predominantly found on the protein surfaces. Structurally, these sites were distributed across all three major secondary structures, with a higher number of sites located on α-helices (n = 18) and coils (n = 14), while fewer were associated with β-sheets (n = 5). In the structural proteins anchC, PreM, and E, all key sites were located on α-helices and coils, with no sites associated with β-sheets. On the E protein, two key sites (E-234 and E-279) were identified in the coil structure of Domain II, while two others (E-463 and E-431) were located on the α-helix structure of Domain III. No key sites were found in Domain I of the E protein.

Among the non-structural proteins, NS5 had the highest number of key mutation sites, which were distributed across all three major domains of this protein. In the extracellular protein NS1, the β-ladder region contained the key site NS1-277, the Wing region contained NS1-72, and the Loop region contained NS1-291. In NS3, the β-ladder region also harbored two key sites: NS3-37 and NS3-126.

Overall, the majority of the identified key sites are located within structural domains closely associated with protein function, highlighting their potential biological significance and the value of further investigation into their roles in viral evolution and pathogenesis.

## 3. Discussion

This study analyzed 263 TBEV genome sequences to investigate their genetic diversity and amino acid characteristics. The results revealed significant genetic and evolutionary differences among the five genotypes: Baikalian, European, Far-Eastern, Himalaya, and Siberian. High-variability amino acid sites were identified in four genotypes (excluding Himalaya), and genotype-specific mutations were observed in all five genotypes, with some sites shared between genotypes. Selection pressure analysis revealed positively selected sites at the overall TBEV level and in specific genotypes (European, Far-Eastern, and Siberian). By integrating high-variability sites, shared genotype-specific mutation sites, and positively selected sites, a total of 37 key amino acid sites were identified.

TBEV has evolved relatively independently. The divergence of TBEV genotypes occurred hundreds or even thousands of years ago, leading to the general consensus that the genotypes have evolved independently. However, a recent study suggests that the Baikalian genotype may have originated from a recombination event between the Siberian and Far-Eastern genotypes [22]. The data and methods used in this study did not identify sufficient evidence for inter-genotype recombination events (Supplementary Table S2), even under the condition that a single algorithm hit was sufficient for positive recombination detection. Additionally, the nucleotide similarity between the Baikalian genotype and the other four genotypes was below 95% across nearly all genomic regions. Therefore, while it is possible that the origin of the Baikalian genotype may have some potential historical relationship with the Siberian and Far-Eastern genotypes, Baikalian has maintained relatively independent evolution over the course of hundreds or even thousands of years, and this is even more evident for the other genotypes. However, potential recombination events occasionally occur within genotypes, particularly in the European, Far-Eastern, and Siberian genotypes, consistent with previous studies [23].

TBEV genotypes exhibit both unique and shared evolutionary characteristics. In this study, high-variability site analysis, genotype-specific amino acid mutation analysis, and selection pressure analysis were used to identify amino acid sites critical for each genotype. Some key sites showed variations specific to a single genotype, such as anchC-111 in the Far-Eastern genotype. Others were shared between certain genotypes, for instance, shared evolutionary sites were observed between the European and Far-Eastern, European and Himalaya, European and Siberian, Himalaya and Siberian, and Baikalian and Far-Eastern genotypes during the genotype-specific amino acid mutation analysis. This indicates that while each genotype has unique mutational features, similar evolutionary events may occur at the same sites across genotypes, displaying convergent or parallel evolutionary characteristics. Convergent and parallel evolution events play a crucial role in understanding how different subtypes or evolutionary branches acquire distinct or similar virological features, which is essential for decoding a virus’s adaptive strategies [24,25,26]. Strict convergent evolution refers to the acquisition of identical amino acid mutations at the same site across different genotypes or evolutionary branches, fitting a convergent evolution model, as previously reported in studies on SARS-CoV-2 [27]. In contrast, strict parallel evolution refers to the acquisition of different amino acid mutations at the same site across genotypes or branches, also conforming to a convergent model, as demonstrated in studies on highly pathogenic avian influenza [28]. However, the limited data availability for TBEV makes it challenging to conduct comprehensive analyses of strict convergent or parallel evolution. Therefore, this study adopted approaches resembling convergent and parallel evolution to define potential evolutionary events.

For example, anchC-111 in the Far-Eastern genotype, NS1-277 and NS5-522 in the Siberian genotype, and NS5-832 in the Far-Eastern genotype were identified as both high-variability sites and positively selected sites. Among these, NS1-277 and NS5-522 were classified as convergent evolution-like sites, while anchC-111 and NS5-832 were classified as parallel evolution-like sites. All genotype-specific amino acid mutation sites identified in this study were categorized as parallel evolution-like sites. These findings highlight that TBEV genotypes exhibit both unique and shared evolutionary characteristics, with evidence of potential convergent and parallel evolutionary processes shaping their diversity.

The key amino acid mutation sites identified in TBEV are closely associated with virological characteristics. The final dataset included 37 amino acid sites distributed across nine proteins, with most located on the protein surface and some positioned within α-helices or β-sheets, structures closely linked to protein tertiary structure. This underscores the importance of the selected sites. A literature review revealed no experimental studies directly targeting the 37 sites identified in this study. However, mutations at sites proximal to those identified here, or mutations in similar flaviviruses, have demonstrated biological significance.

For example, the D277A mutation in the E protein of TBEV has been linked to enhanced host receptor binding or cell fusion capabilities [29]. In this study, E-279, a nearby site, was identified as a key mutation, suggesting that similar experimental studies on E-279 may yield important insights. Likewise, the E-426 site in the E protein of TBEV has been associated with plaque size formation [30], potentially impacting viral virulence or growth characteristics. This study identified E-431, a nearby site, as a key mutation, further suggesting its potential biological relevance. In addition, mutations in the E protein, including D67G, T68A, E84K, F119V, E122G, A123K, N154L/Q, S158R, G159R, K171E, D181Y, E201K, D203G, D277A, D308K, T310K, K311E, G368R, Y384H, H390Y, T426I, D483E, and H496R, are also considered to influence the neuroinvasiveness and neurovirulence of TBEV [21]. Although none of our final selected sites overlap with these mutations, we have provided the amino acid composition at these sites for different TBEV genotypes in Supplementary Data S2, which may help to understand the occurrence of these mutations. Langat virus (LGTV), a tick-borne flavivirus closely related to TBEV, requires the NS5 region spanning amino acids 355–735 for its IFN inhibition function [31]. TBEV’s NS5 protein has also been shown to inhibit IFN signaling [32]. Within this region, this study identified seven key amino acid sites (NS5-455, NS5-522, NS5-571, NS5-634, NS5-677, NS5-699, and NS5-724). Notably, NS5-522 was identified as a positively selected site in the Siberian genotype, while NS5-571 was a positively selected site in the European genotype. These findings suggest that these sites may be under selective pressure related to IFN inhibition, highlighting their potential importance in further experimental studies.

This study included all currently available TBEV sequences with detailed background information. However, the uneven distribution of sequences across genotypes and time points limited certain analyses, such as the identification of convergent and parallel evolution sites. Additionally, the potential key sites identified through bioinformatics approaches in this study still require experimental validation to confirm their functional relevance. Given that live TBEV research must be conducted in BSL-3 or higher biosafety laboratories, further experimental studies face certain practical challenges. Moreover, TBEV sequences derived from terminal or incidental hosts may harbor mutations associated with host-specific adaptive evolution, warranting further investigation in future studies. As Supplementary Data for this research, mutation information categorized by host, country, and temporal factors is available in Supplementary Data S2 for reference by the scientific community.

## 4. Materials and Methods

### 4.1. Dataset Collection and Preprocessing

The full name of the virus, tick-borne encephalitis virus, was used to retrieve its complete or near-complete genome sequences (greater than 90% of full length) from the NCBI database, with data up to 31 December 2023. A total of 263 sequences, which included information on collection date, geographic location, and host, were obtained and are provided in Supplementary Table S1. The ORF regions were extracted for analysis.

### 4.2. Phylogenetic Analysis

Multiple sequence alignment was performed using Mafft software (Version: 7.450, 23 August 2019) [33]. The most appropriate substitution model was determined using ModelFinder software (Version: 2.1.4) according to Bayesian information criterion (BIC) [34]. A maximum likelihood (ML) tree was generated using IQ-TREE software (Version: 2.1.4, 30 April 2021) with 1000 bootstrap replicates [35,36]. The resulting phylogenetic tree was visualized using Chiplot (www.chiplot.online, accessed on 1 December 2024) [37].

### 4.3. Recombination Analysis

Recombination detection was performed using RDP5 (Version: Beta 5.64), employing seven different detection models: RDP, GENECONV, Bootscan, Maxchi, Chimaera, SiSscan, and 3Seq [38]. A potential recombination event was considered if one model provided supporting evidence with a *p*-value threshold of *p* < 0.01. This loose criterion is aimed at identifying potential recombination events between genotypes as thoroughly as possible.

### 4.4. Shannon Entropy Calculation

The algorithm calculates the Shannon entropy for each position in a multiple sequence alignment (MSA), which quantifies the degree of variability at each nucleotide or amino acid position across all sequences. Entropy is computed by measuring the frequency distribution of characters at each position. In Equation (1), *H* represents the information entropy at a specific position in an amino acid sequence, quantifying the uncertainty or variability at that position. The term *i* denotes the index of each possible amino acid at the given position in the sequence, where *i* ranges from 1 to *n*, the total number of possible amino acids and gap. The term *p_i_* is the probability of the *i*-th amino acid or gap occurring at that position, calculated based on the frequency of that amino acid or gap across multiple sequences. Higher entropy values indicate greater variability and diversity at a given position, suggesting that the site is less conserved, while lower entropy values indicate that the position is more conserved across sequences. The calculation of Shannon entropy was carried out using a custom Python (Version: 3.10) script developed for this purpose.

(1) $$H=-\sum_{i=1}^{n}p_{i}log2(i)$$

### 4.5. Genotype-Specific Amino Acid Analysis

A specific genotype is first extracted from the dataset, and its amino acid sequence is aligned with those of other genotypes. The analysis focuses on identifying amino acid differences between the selected subtype and the other genotypes. A unique amino acid substitution at a given position in the selected genotype, absent in all other sequences at the same position, is considered a genotype-specific amino acid for that particular genotype.

### 4.6. Selective Pressure Analysis

Selection pressure on internal branches was assessed using the FEL, FUBAR, MEME, and SLAC algorithms implemented in HyPhy (Version: 2.5.48, 2 March 2023) [39]. The required tree file for the analyses was generated using IQ-TREE software (Version: 2.1.4, 30 April 2021) [35]. For the FEL, MEME, and SLAC algorithms, results were considered significant when *p* < 0.1; for the FUBAR algorithm, results were considered significant when the posterior probability was greater than 0.9. Only sites identified as positively selected by two or more algorithms were included in the final results.

### 4.7. Evolutionary Patterns Analysis

Due to the limited availability of TBEV sequences and their uneven distribution across the timeline, it is difficult to apply strict definitions for analyzing convergent or parallel evolution in TBEV. Therefore, a relaxed definition was adopted to identify convergent evolution-like or parallel evolution-like patterns. For mutations resembling parallel evolution, in simple terms, the mutation type at a specified highly variable site in a given genotype does not occur in other genotypes. For mutations resembling convergent evolution, in simple terms, the mutation type at a specified highly variable site in a given genotype corresponds to the predominant amino acid (>90%) at the same site in other genotypes.

### 4.8. Protein Structure Prediction

The consensus sequences of the TBEV dataset used in this study were generated using Geneious Prime software (Version: 2022.2.2, Biomatters), with the most frequent amino acids included in the consensus sequence. From the consensus sequences, the amino acid sequences for each protein were extracted. Subsequently, protein structures were predicted using the AlphaFold Server (https://alphafoldserver.com/, accessed on 8 December 2024). The predicted protein structures were then visualized and annotated using PyMOL software (Version: 3.0.3, 12 March 2024, Schrödinger, Inc., New York, NY, USA). The sequences used for structure prediction and the prediction results are provided in Supplementary Data S1.

### 4.9. Software

In the methods described above, several key software tools not previously mentioned were used for specific tasks. Sequence preprocessing, including information extraction, filtering, grouping, and mutation statistics, was performed using the SeqProcessor web application developed by our team (https://sp-app.streamlit.app/, accessed on 11 November 2024) [40]. Pairwise sequence identity was calculated using BioEdit (Version: 7.7), and the average distance between groups was computed using MEGA7 (Version: 7.0) [41]. The sliding window similarity analysis between different genotype groups was performed using SimPlot (Version: 3.5.1) [42]. For data visualization, ggplot2 v3.4.4 package in R v4.3.0 was used to create basic plots, while ComplexHeatmap v2.16.0 package [43] was utilized to generate heatmaps. ggVennDiagram v1.5.2 package was employed to create Venn diagrams, and trackViewer v1.36.2 package [44] was used to annotate important positions on the genome. The images were stitched together using Adobe Illustrator 2024 software, without any modification of the original data that could potentially affect the analysis or interpretation of the results.

## 5. Conclusions

This study applied three different methods to analyze mutations in 263 full-genome sequences of TBEV, constructing a mutation characteristic database for different TBEV genotypes and selecting 37 key amino acid sites. These results provide critical insights into the genetic diversity, evolutionary characteristics, and virological properties of different TBEV genotypes. They also offer important directions for future research on functional sites across TBEV genotypes.

## Figures and Tables

**Table 1:** The mean nucleotide and amino acid similarity between genotypes.

Number	TBEV Genotypes	TBEV Genotypes				
		1	2	3	4	5
1	European	***	81.5%	82.6%	82.1%	88.0%
2	Himalaya	93.0%	***	82.9%	82.6%	81.7%
3	Siberian	94.2%	93.9%	***	85.0%	83.6%
4	Baikalian	94.0%	93.9%	95.8%	***	86.1%
5	Far-Eastern	93.5%	93.3%	95.1%	96.4%	***

Note: ‘***’ indicates 100%; the upper right quadrant indicates the nucleotide similarity; the lower left quadrant indicates the amino acid similarity.

## Data Availability

All available data are provided in the manuscript and Supplementary Materials.

## Supplementary Materials

The supporting information can be downloaded at: https://www.mdpi.com/article/10.3390/ijms26030954/s1.
