# Supplementary figures and images for: Evolutionary Patterns and Genotype-Specific Amino Acid Mutations of Tick-Borne Encephalitis Virus
Source: Int J Mol Sci. 2025 Jan 23;26(3):954. doi: 10.3390/ijms26030954 (PMC11817229; doi:10.3390/ijms26030954)

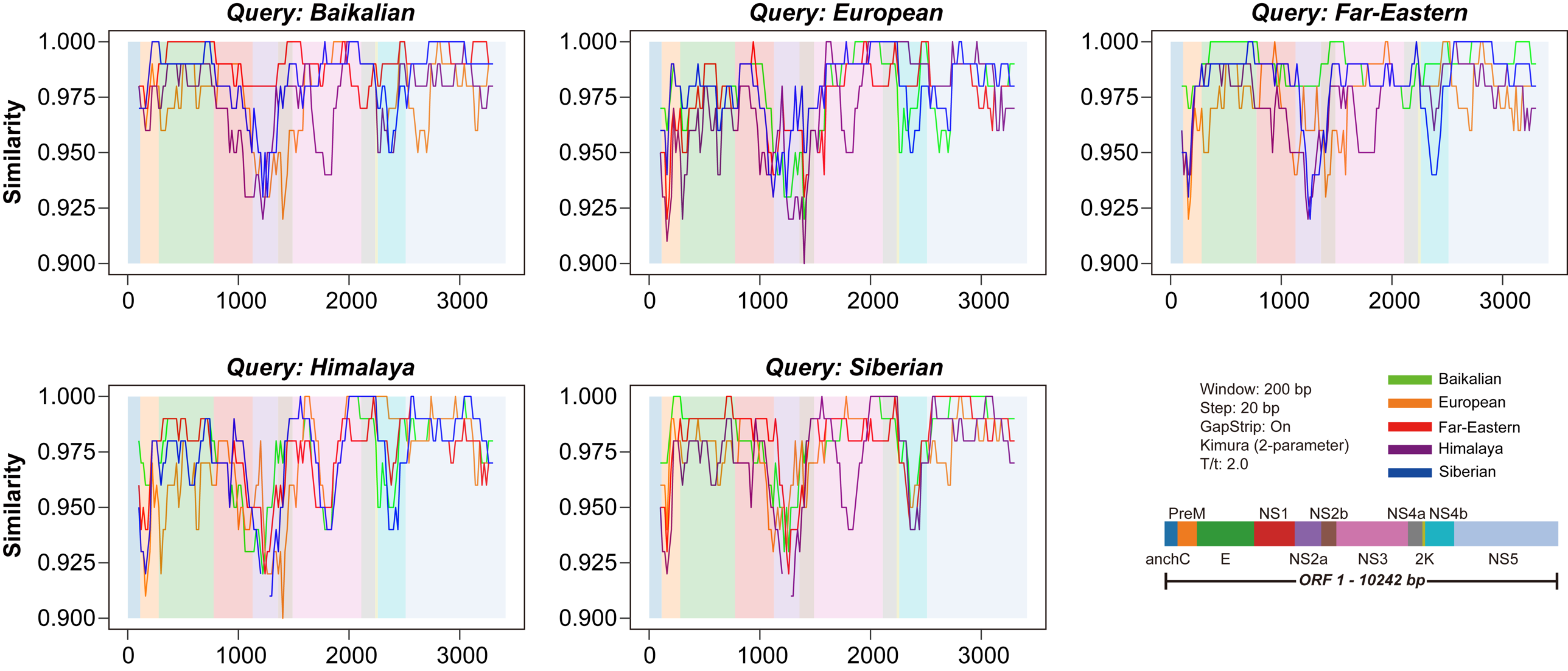

Supplement: Supplementary file 1 [file ijms-26-00954-s001.zip › ijms-3423653-supplementary/Supplementary Figure S1.tif]
