# Supplementary material for: Evolutionary Patterns and Genotype-Specific Amino Acid Mutations of Tick-Borne Encephalitis Virus
Source: Int J Mol Sci. 2025 Jan 23;26(3):954. doi: 10.3390/ijms26030954 (PMC11817229; doi:10.3390/ijms26030954)
Supplement: Supplementary file 1 [file ijms-26-00954-s001.zip › ijms-3423653-supplementary/Supplementary Data S2.zip!/country/country_aa_mutation.pdf]

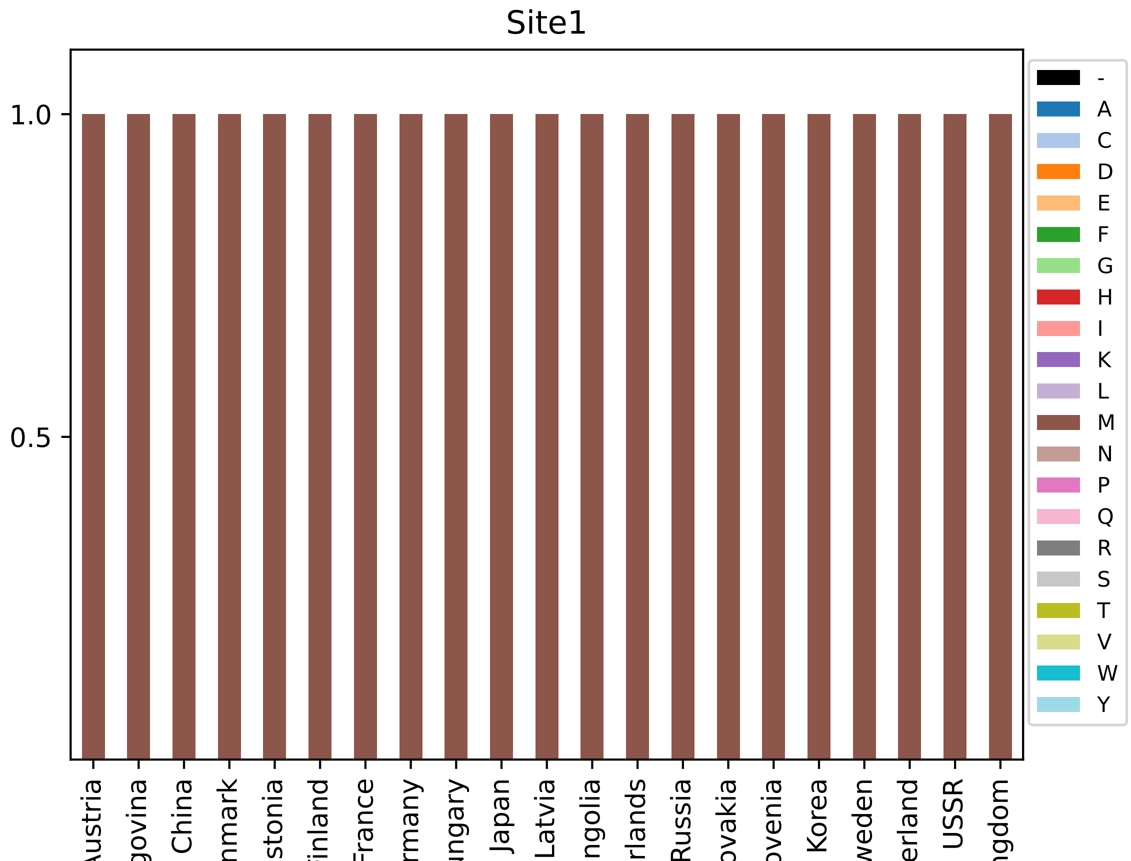
Site1
1.0
0.5
China
Japan
Latvia
Russia
Korea
USSR
-
A
C
D
E
F
G
H
I
K
L
M
N
P
Q
R
S
T
V
W
Y

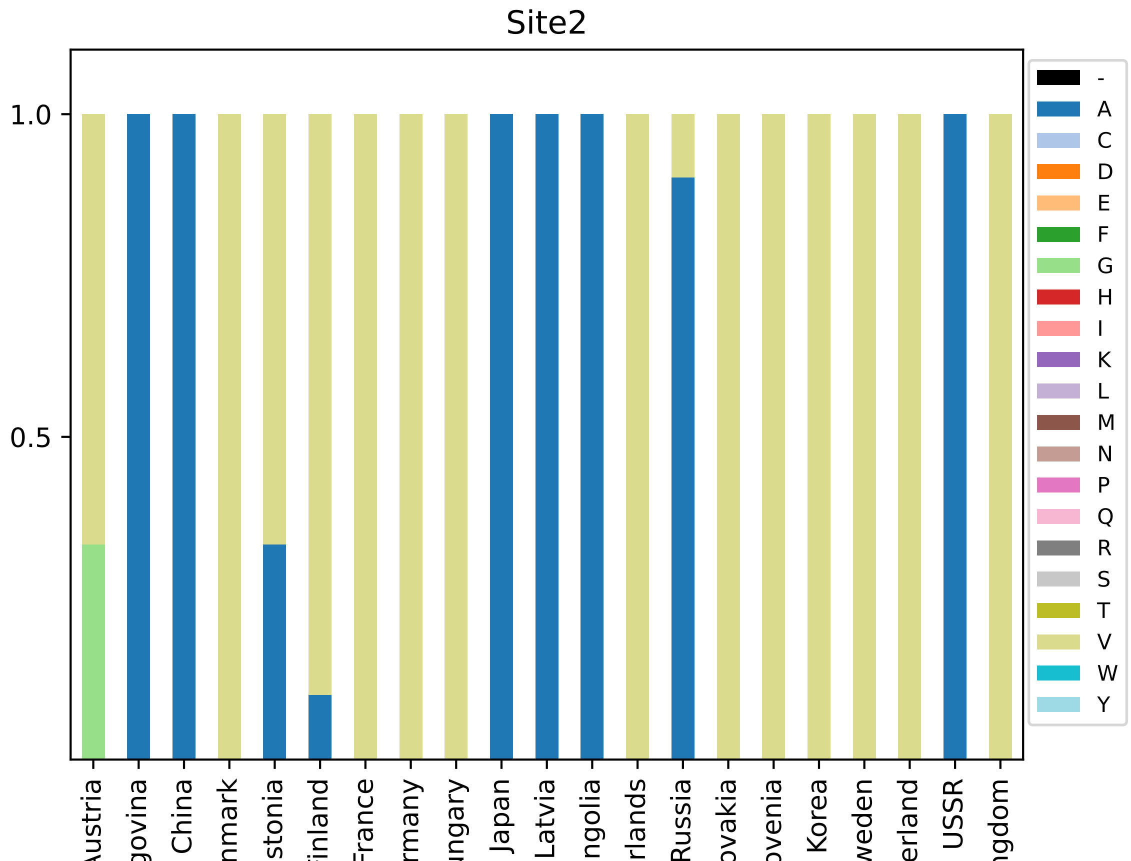
Site2
1.0
0.5
-
A
C
D
E
F
G
H
I
K
L
M
N
P
Q
R
S
T
V
W
Y
China
Japan
Latvia
Russia
Korea
USSR

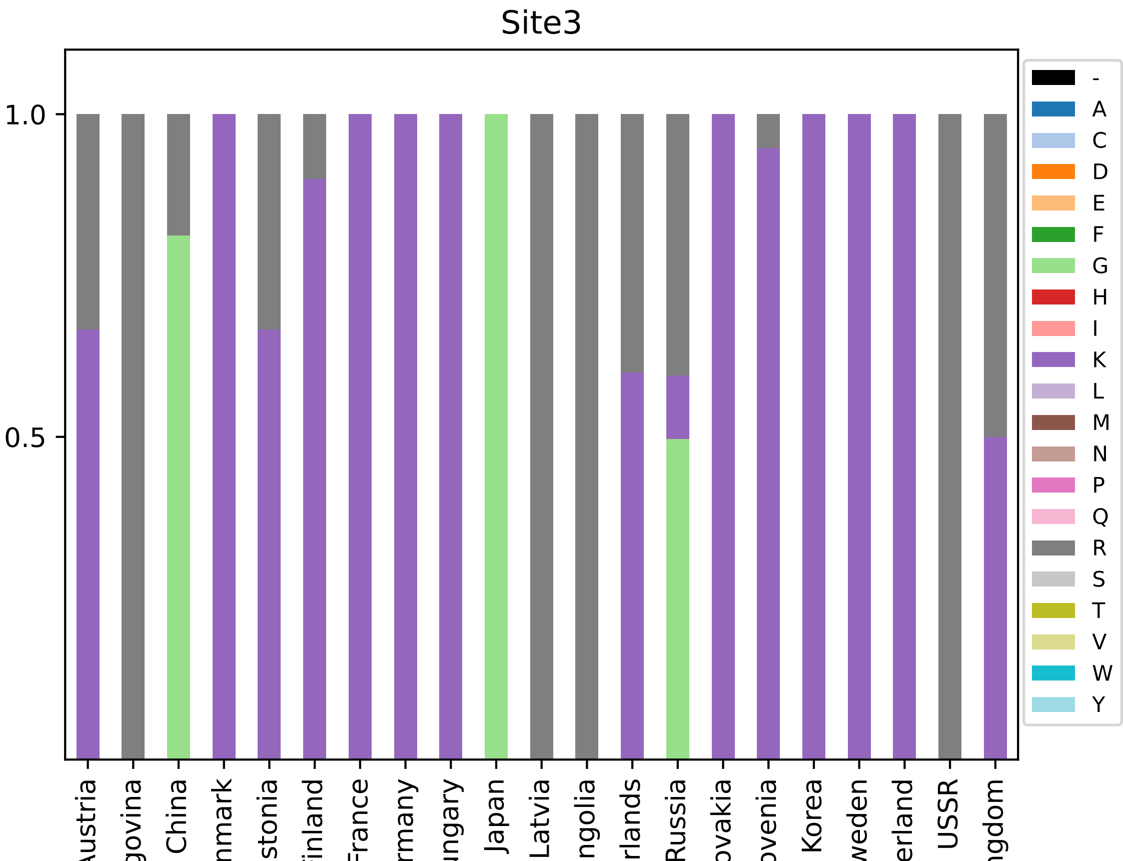
Site3
1.0
0.5
-
A
C
D
E
F
G
H
I
K
L
M
N
P
Q
R
S
T
V
W
Y
China
Japan
Latvia
Russia
Korea
USSR

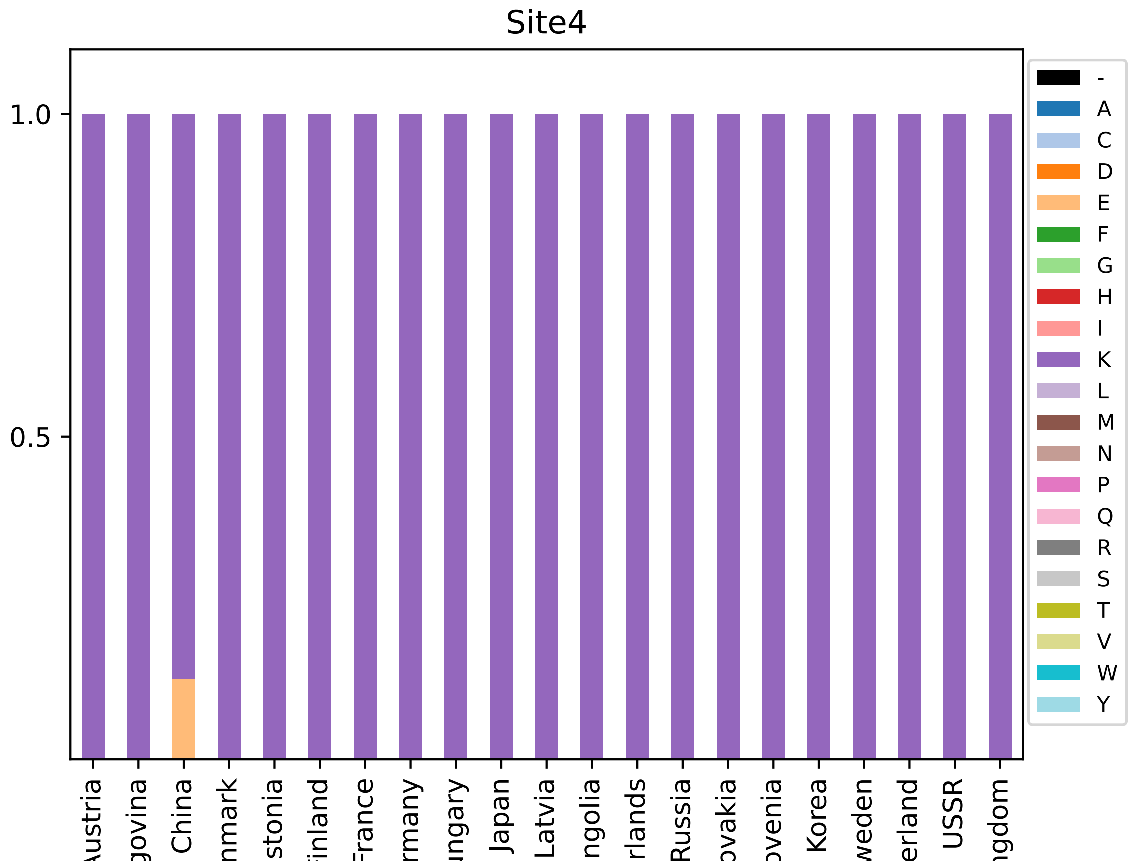
Site4
1.0
0.5
-
A
C
D
E
F
G
H
I
K
L
M
N
P
Q
R
S
T
V
W
Y
Austria
govina
China
nmark
stonia
inland
France
rmany
ungary
Japan
Latvia
ngolia
rlands
Russia
ovakia
ovenia
Korea
weden
erland
USSR
ngdom

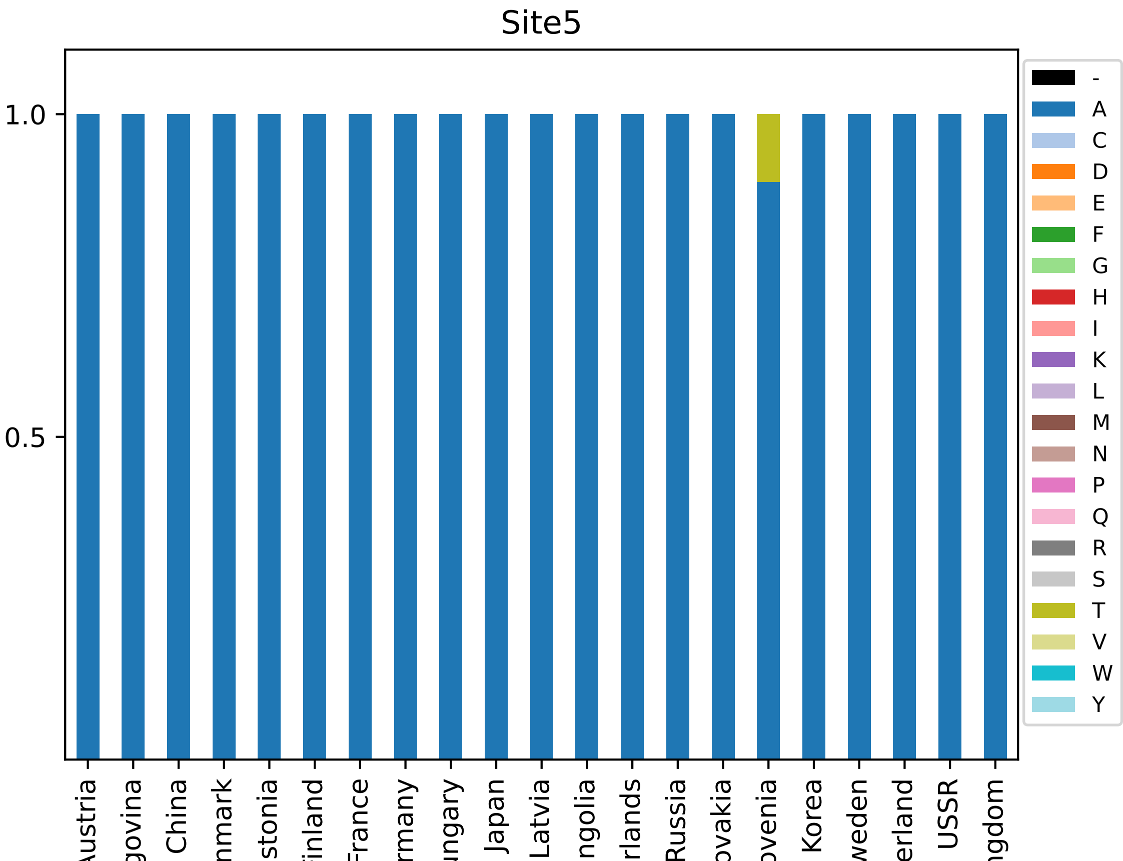
Site5
1.0
0.5
-
A
C
D
E
F
G
H
I
K
L
M
N
P
Q
R
S
T
V
W
Y
China
Japan
Latvia
Russia
Korea
USSR

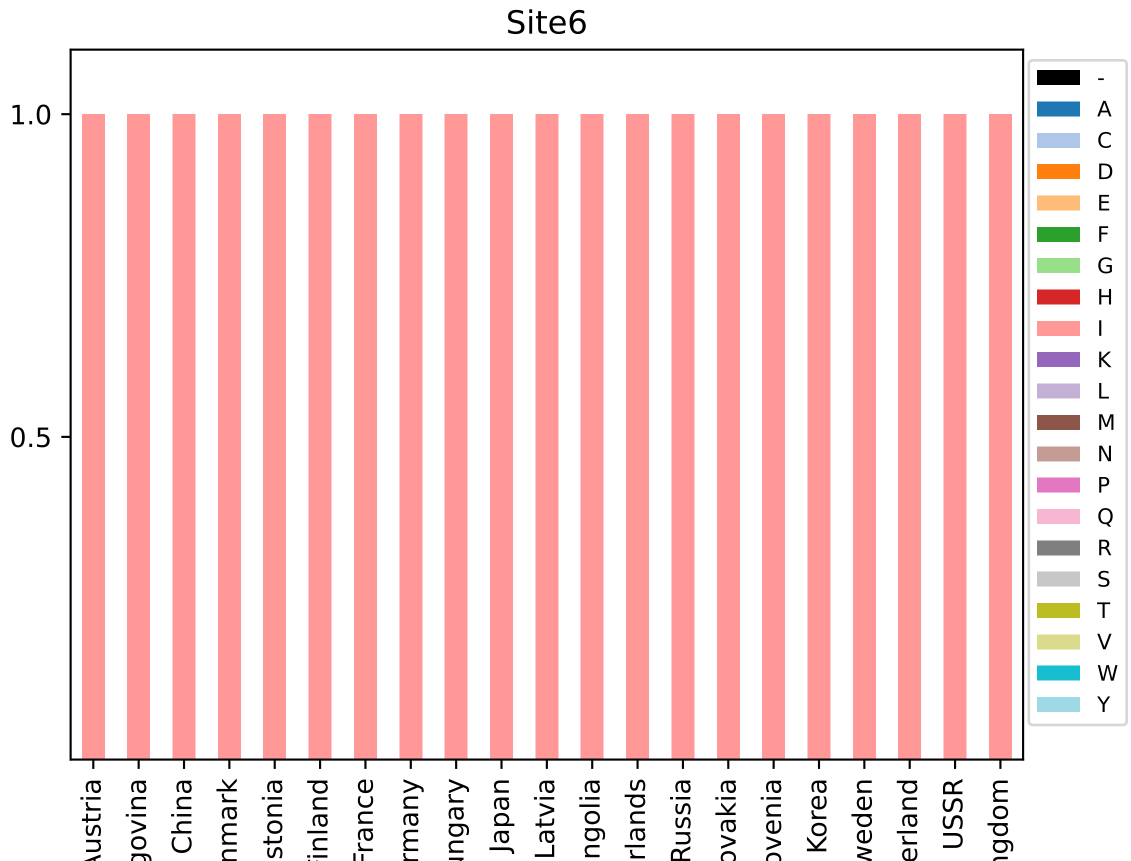

Site6
1.0
0.5
-
A
C
D
E
F
G
H
I
K
L
M
N
P
Q
R
S
T
V
W
Y
China
Japan
Latvia
Russia
Korea
USSR

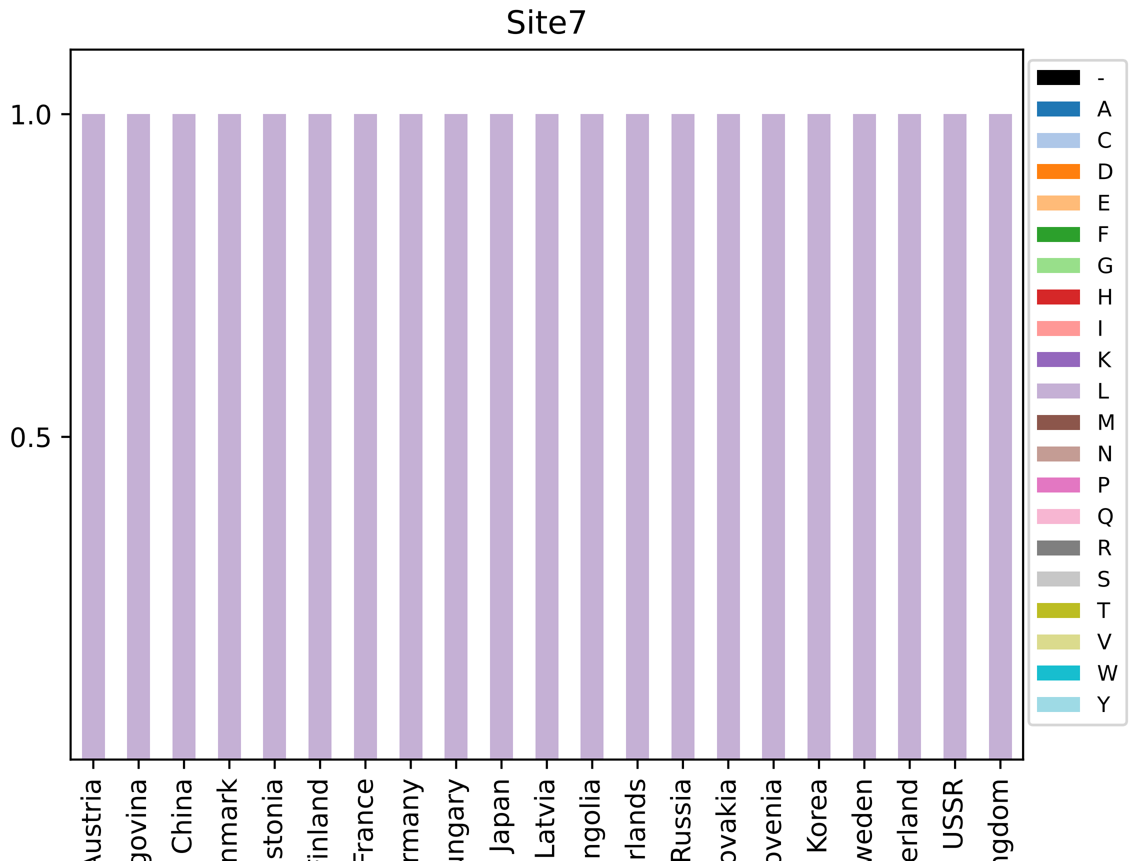
Site7
1.0
0.5
-
A
C
D
E
F
G
H
I
K
L
M
N
P
Q
R
S
T
V
W
Y
China
Japan
Latvia
Russia
Korea
USSR

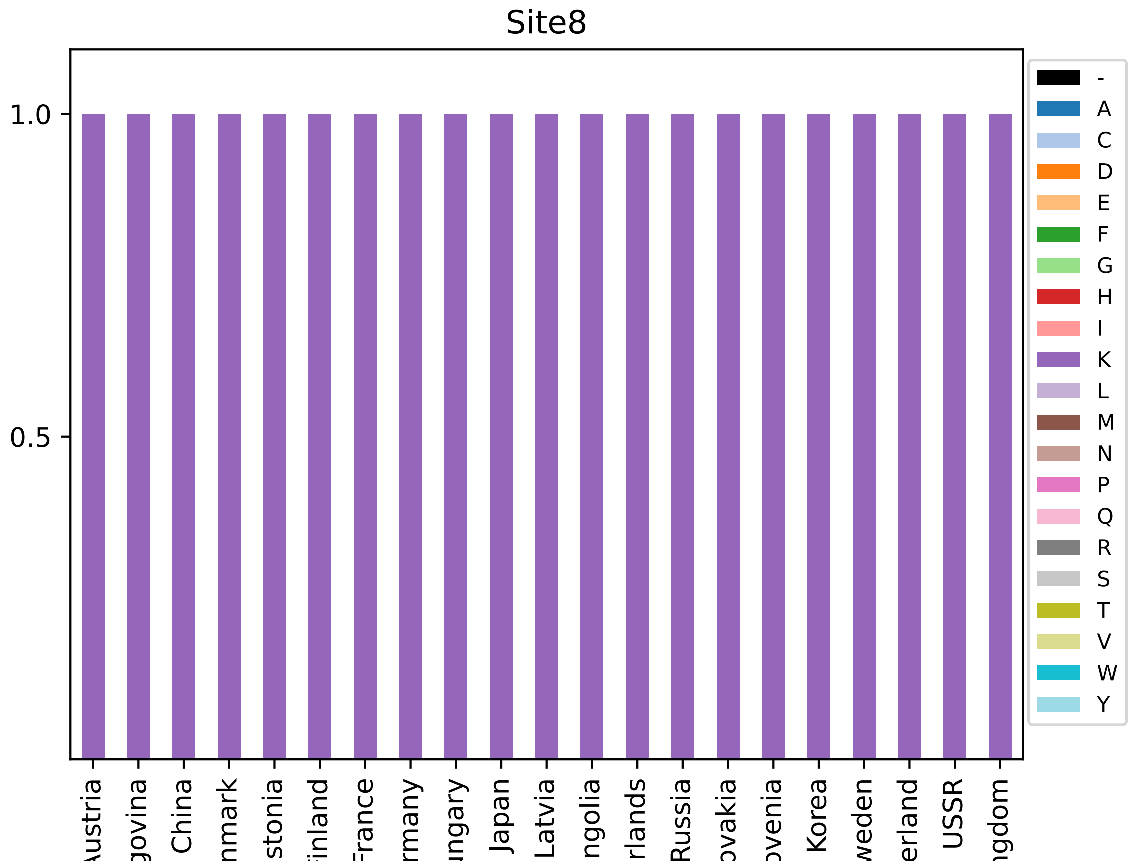

Site8
1.0
0.5
-
A
C
D
E
F
G
H
I
K
L
M
N
P
Q
R
S
T
V
W
Y
China
Japan
Latvia
Russia
Korea
USSR

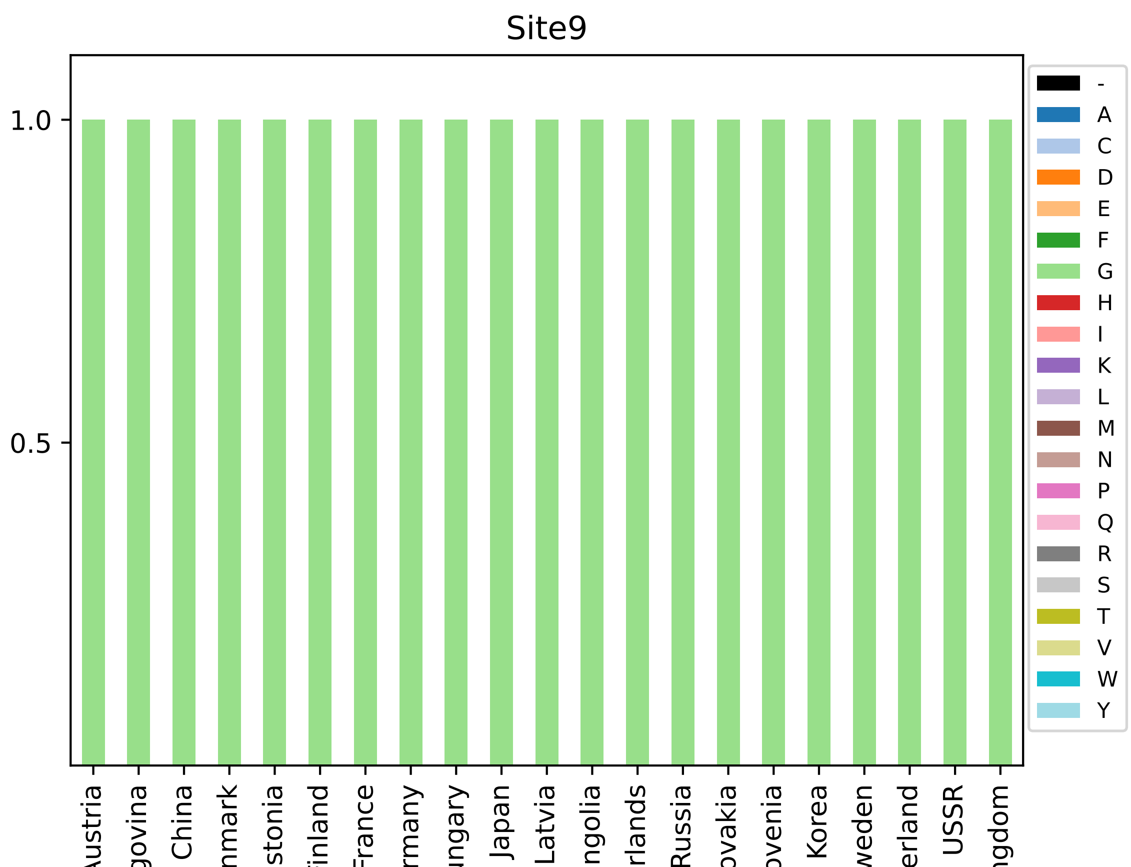

Site9
1.0
0.5
-
A
C
D
E
F
G
H
I
K
L
M
N
P
Q
R
S
T
V
W
Y
Austria
govina
China
nmark
stonia
inland
France
rmany
ungary
Japan
Latvia
ngolia
rlands
Russia
ovakia
ovenia
Korea
weden
erland
USSR
ngdom

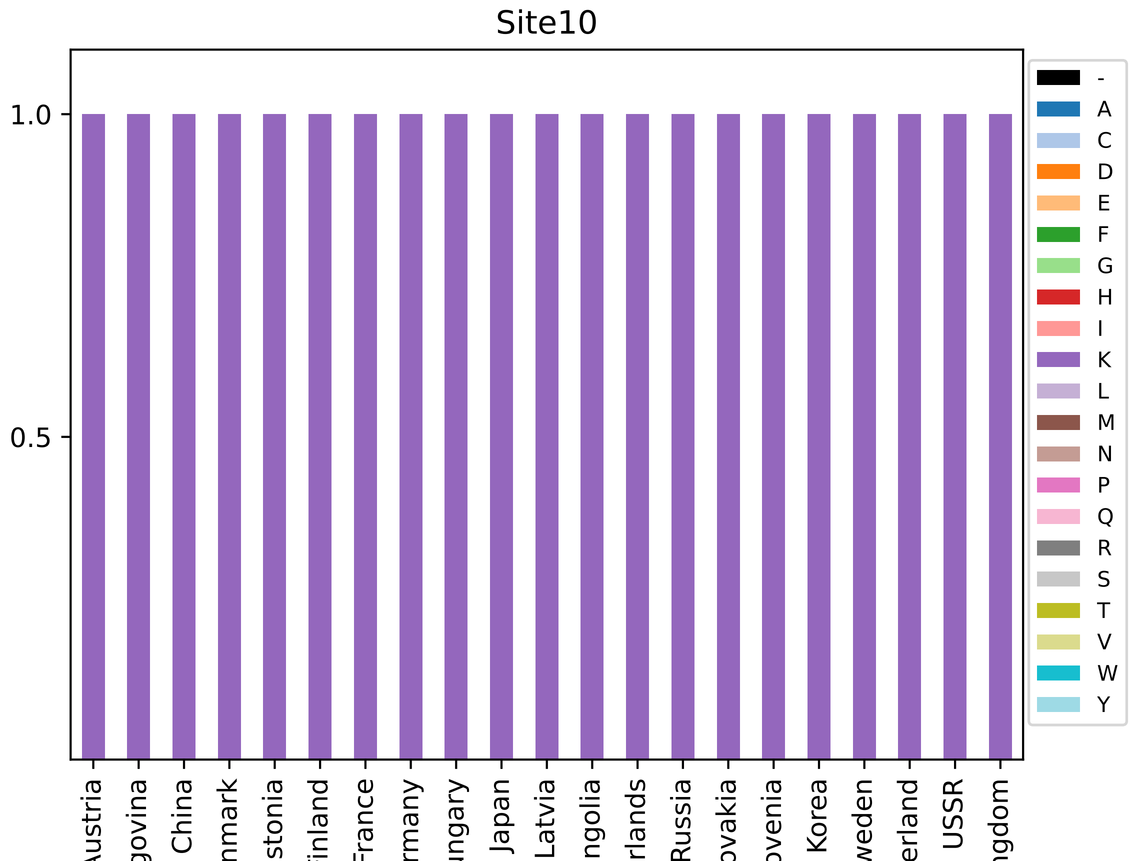
Site10
1.0
0.5
China
Japan
Latvia
Russia
Korea
USSR
-
A
C
D
E
F
G
H
I
K
L
M
N
P
Q
R
S
T
V
W
Y

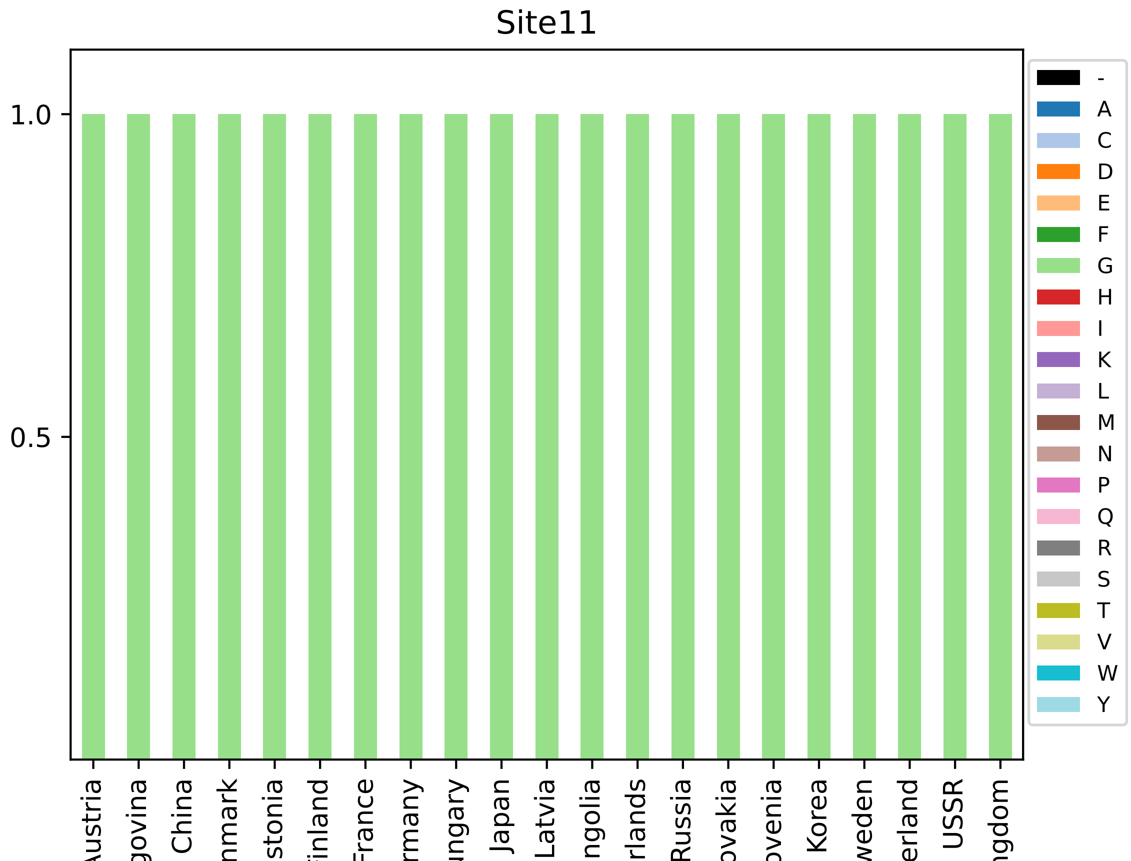

Site11
1.0
0.5
-
A
C
D
E
F
G
H
I
K
L
M
N
P
Q
R
S
T
V
W
Y
China
Japan
Latvia
Russia
Korea
USSR

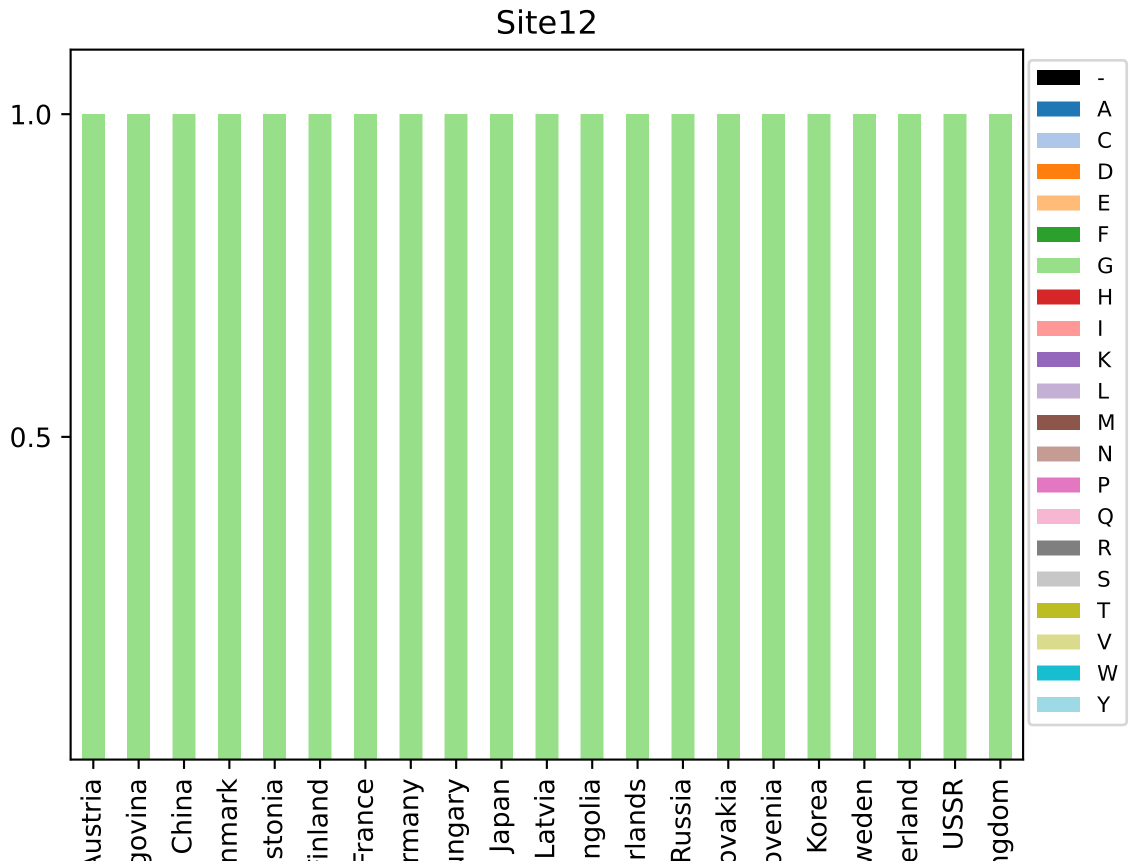
Site12
1.0
0.5
-
A
C
D
E
F
G
H
I
K
L
M
N
P
Q
R
S
T
V
W
Y
China
Japan
Latvia
Russia
Korea
USSR

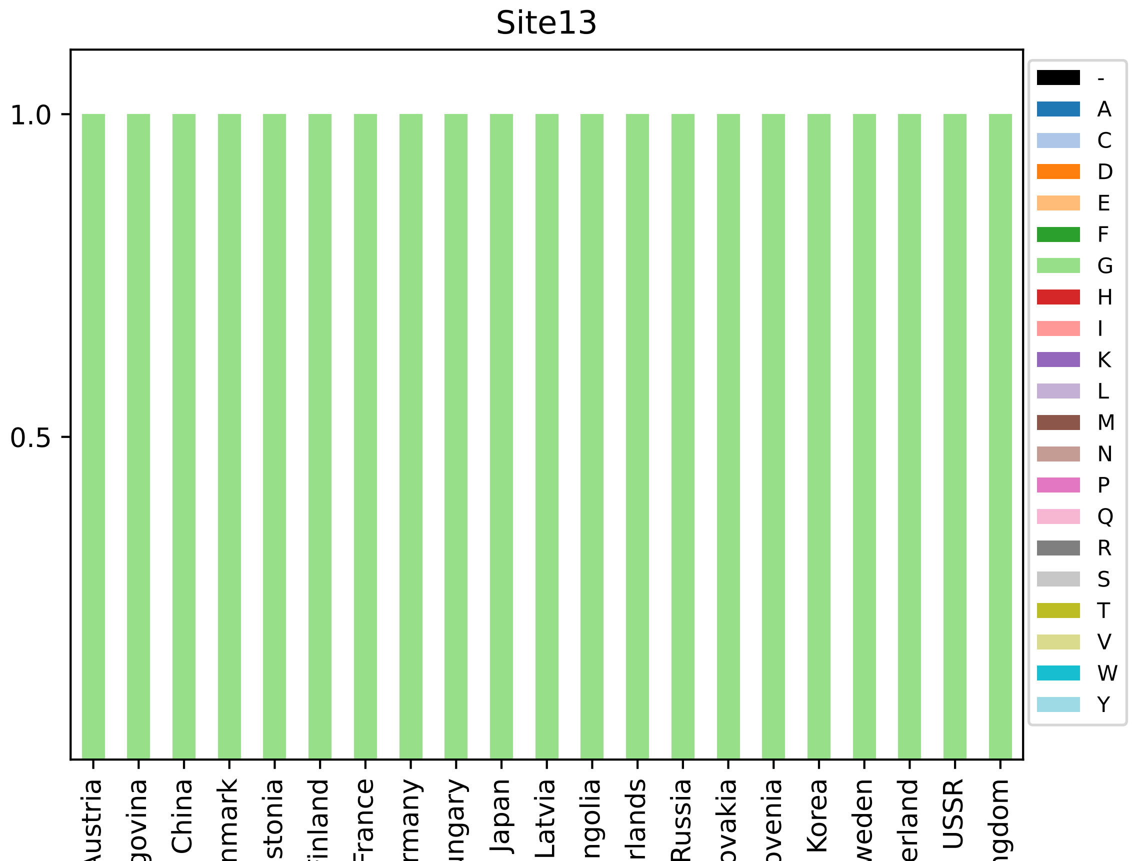
Site13
1.0
0.5
-
A
C
D
E
F
G
H
I
K
L
M
N
P
Q
R
S
T
V
W
Y
China
Japan
Latvia
Russia
Korea
USSR

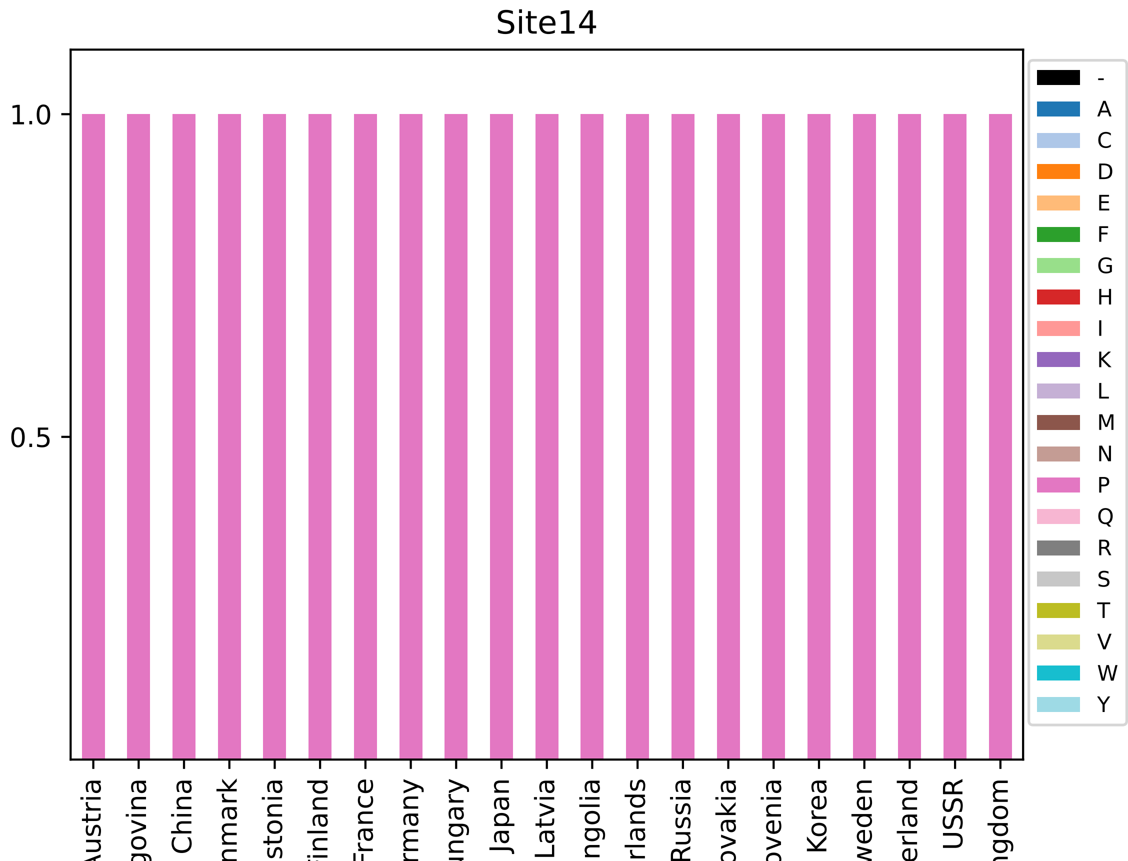
Site14
1.0
0.5
-
A
C
D
E
F
G
H
I
K
L
M
N
P
Q
R
S
T
V
W
Y
China
Japan
Latvia
Russia
Korea
USSR

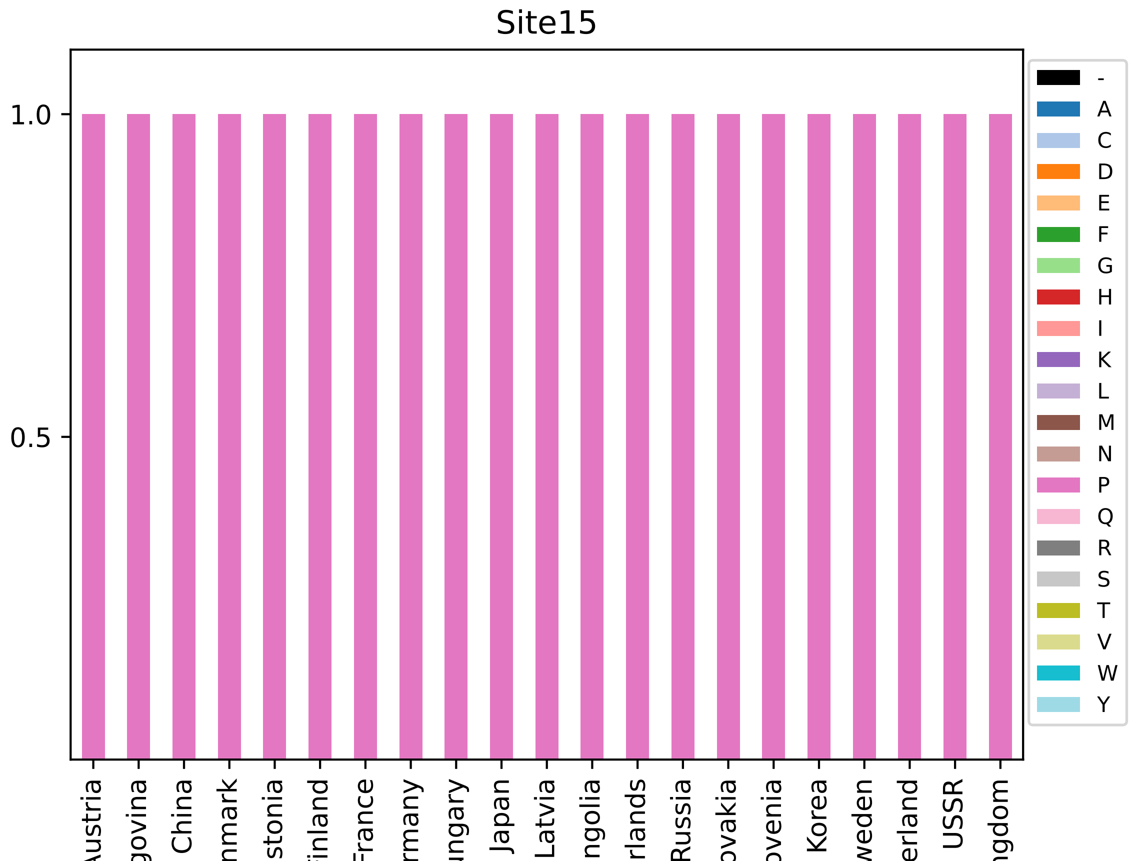

Site15
1.0
0.5
-
A
C
D
E
F
G
H
I
K
L
M
N
P
Q
R
S
T
V
W
Y
Austria
China
Japan
Latvia
Russia
Korea
USSR

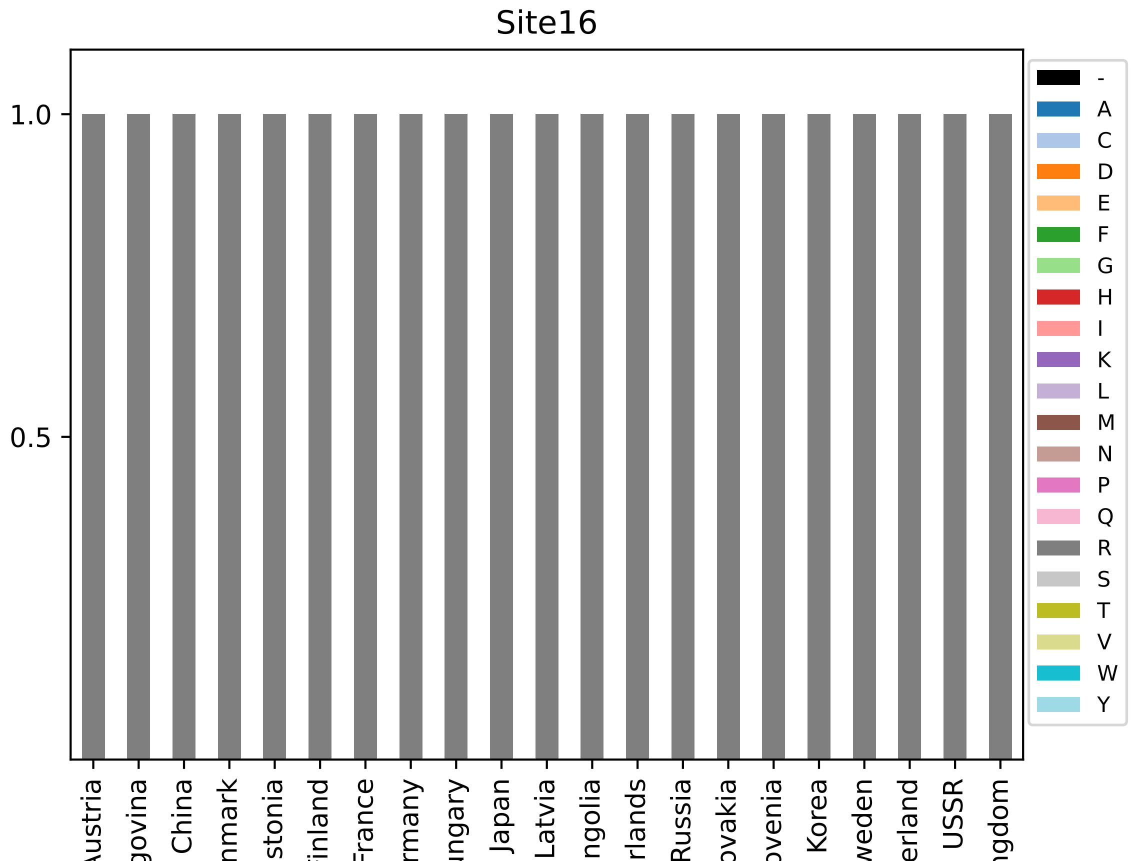
Site16
1.0
0.5
-
A
C
D
E
F
G
H
I
K
L
M
N
P
Q
R
S
T
V
W
Y
Austria
China
Finland
France
Japan
Latvia
Russia
Korea
USSR

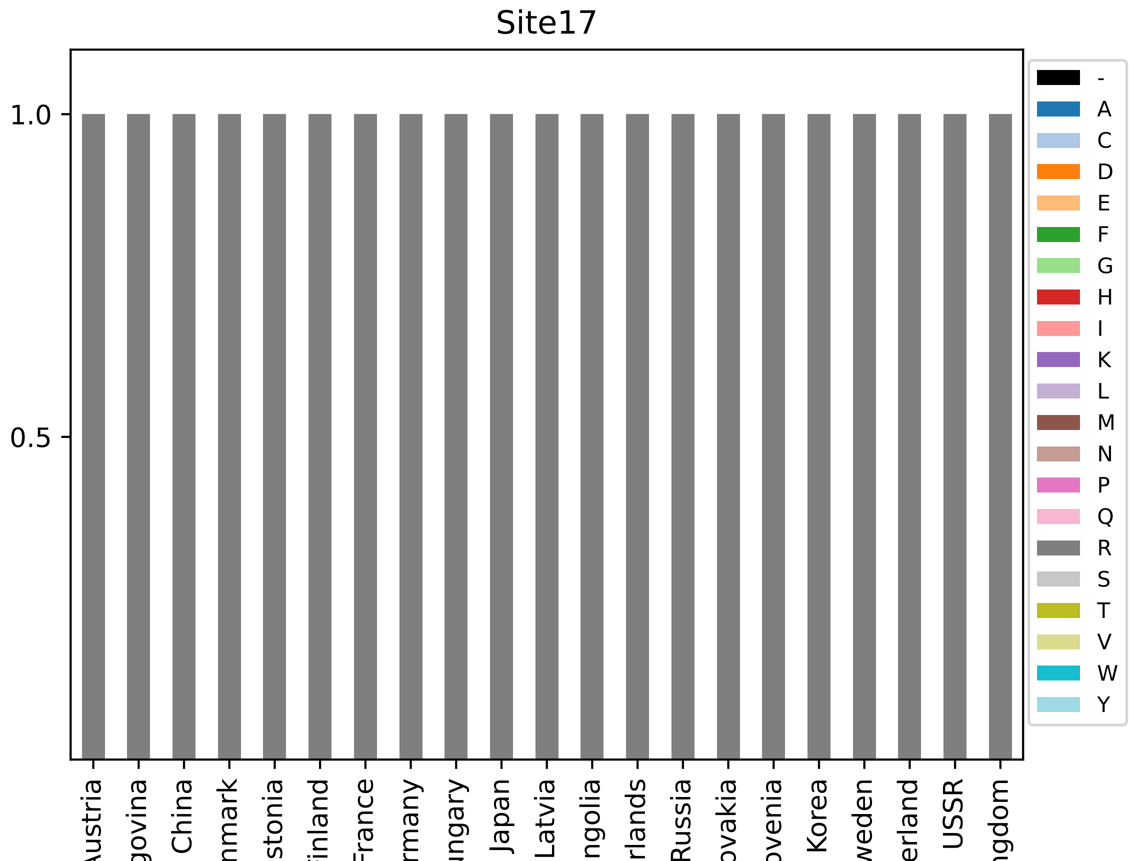
Site17
1.0
0.5
-
A
C
D
E
F
G
H
I
K
L
M
N
P
Q
R
S
T
V
W
Y
Austria
govina
China
nmark
stonia
inland
France
rmany
ungary
Japan
Latvia
ngolia
rlands
Russia
ovakia
ovenia
Korea
weden
erland
USSR
ngdom

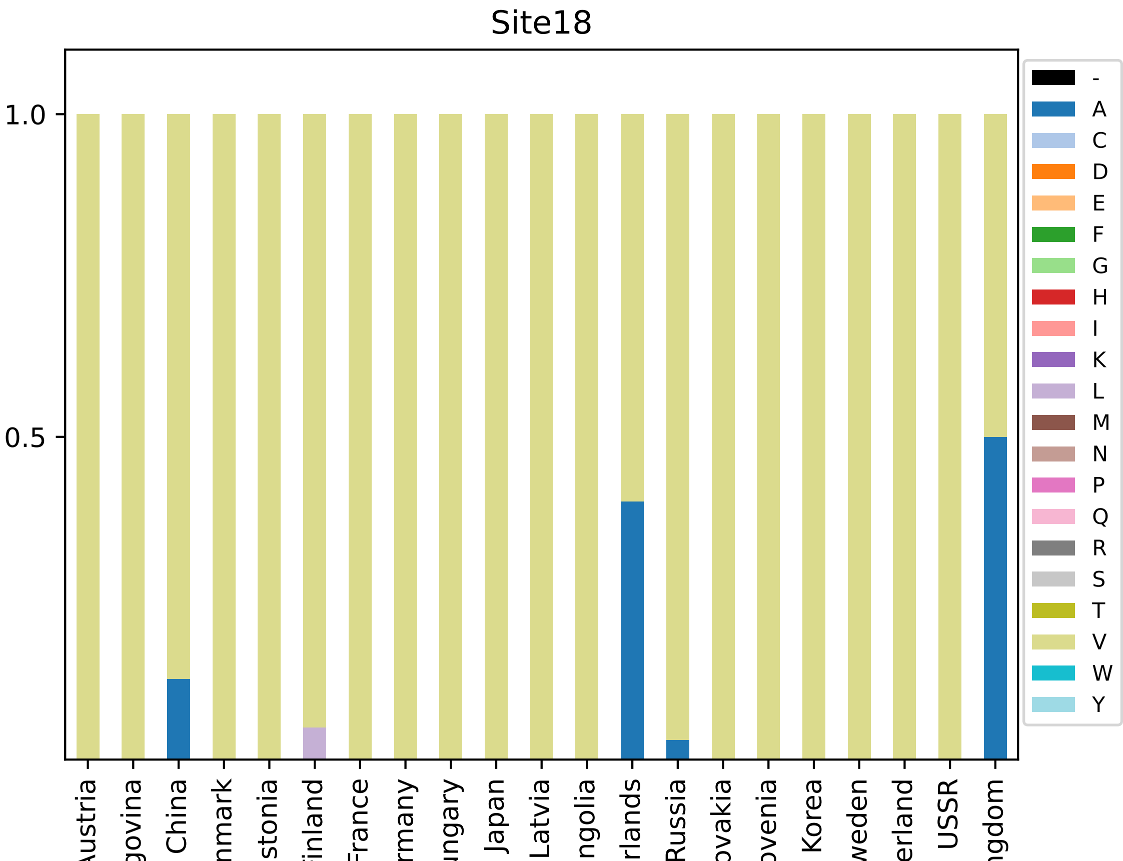

Site18
1.0
0.5
-
A
C
D
E
F
G
H
I
K
L
M
N
P
Q
R
S
T
V
W
Y
China
Japan
Latvia
Russia
Korea
USSR

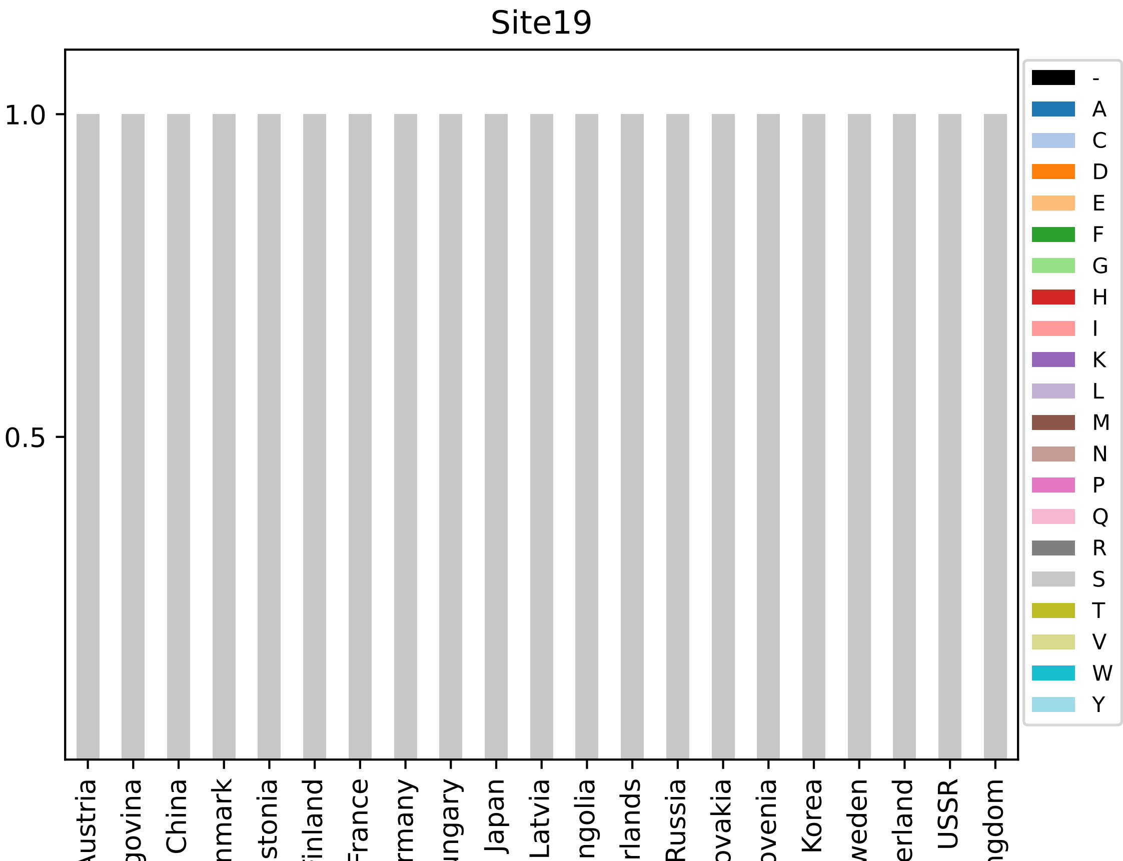
Site19
1.0
0.5
-
A
C
D
E
F
G
H
I
K
L
M
N
P
Q
R
S
T
V
W
Y
China
Japan
Latvia
Korea
USSR

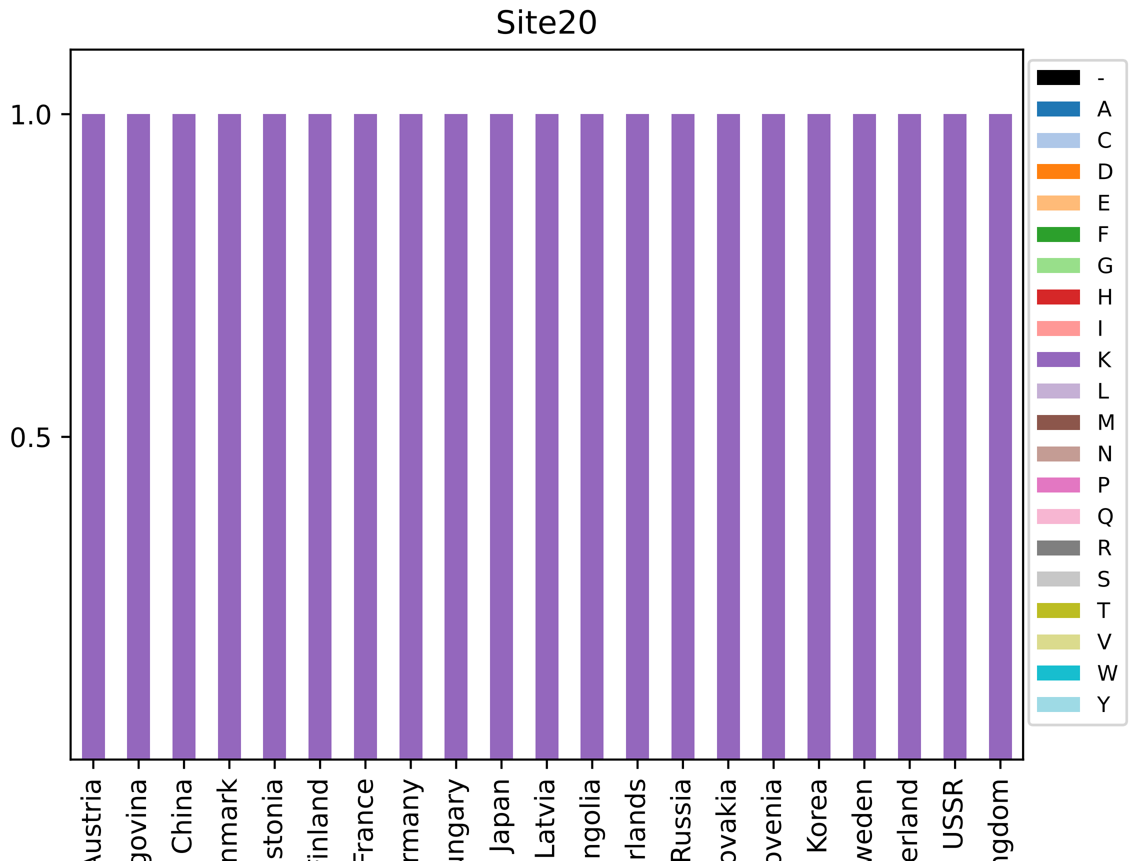
Site20
1.0
0.5
-
A
C
D
E
F
G
H
I
K
L
M
N
P
Q
R
S
T
V
W
Y
China
Japan
Latvia
Russia
Korea
USSR

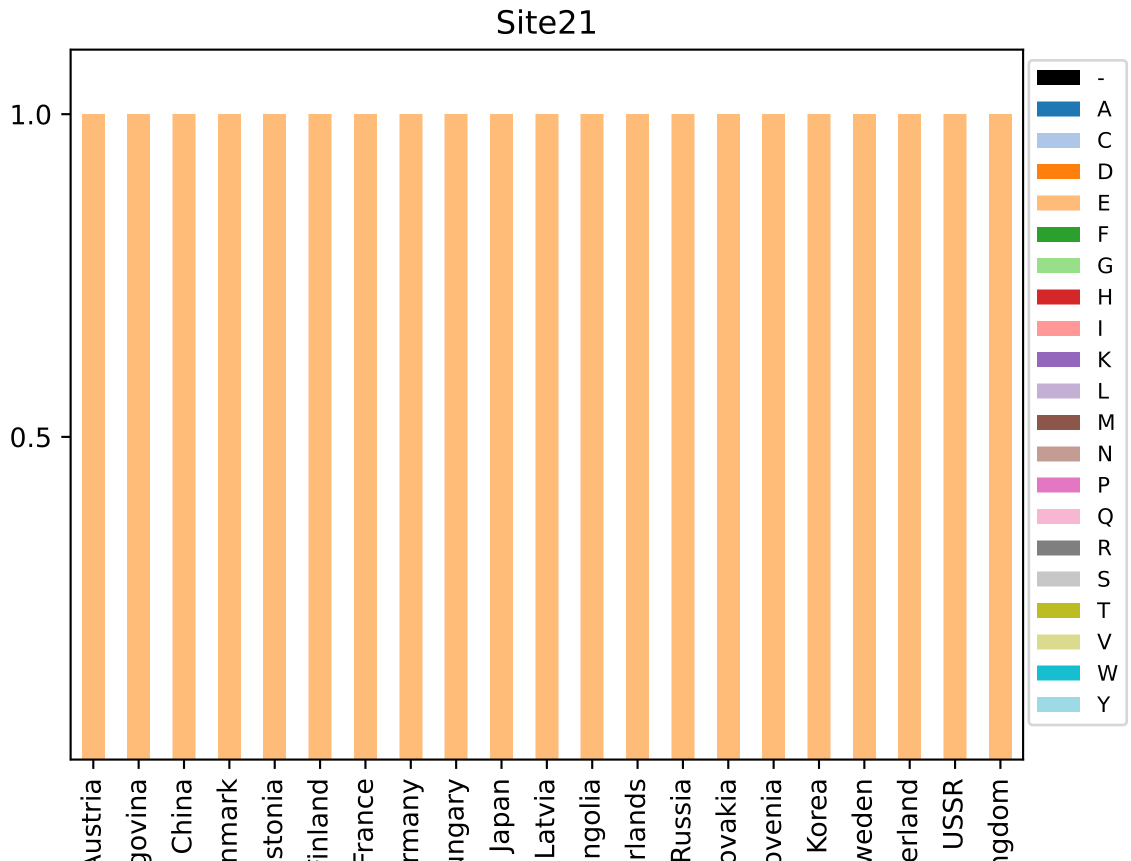
Site21
1.0
0.5
-
A
C
D
E
F
G
H
I
K
L
M
N
P
Q
R
S
T
V
W
Y
Austria
govina
China
nmark
stonia
Finland
France
rmany
ungary
Japan
Latvia
ngolia
rlands
Russia
ovakia
ovenia
Korea
weden
erland
USSR
ngdom

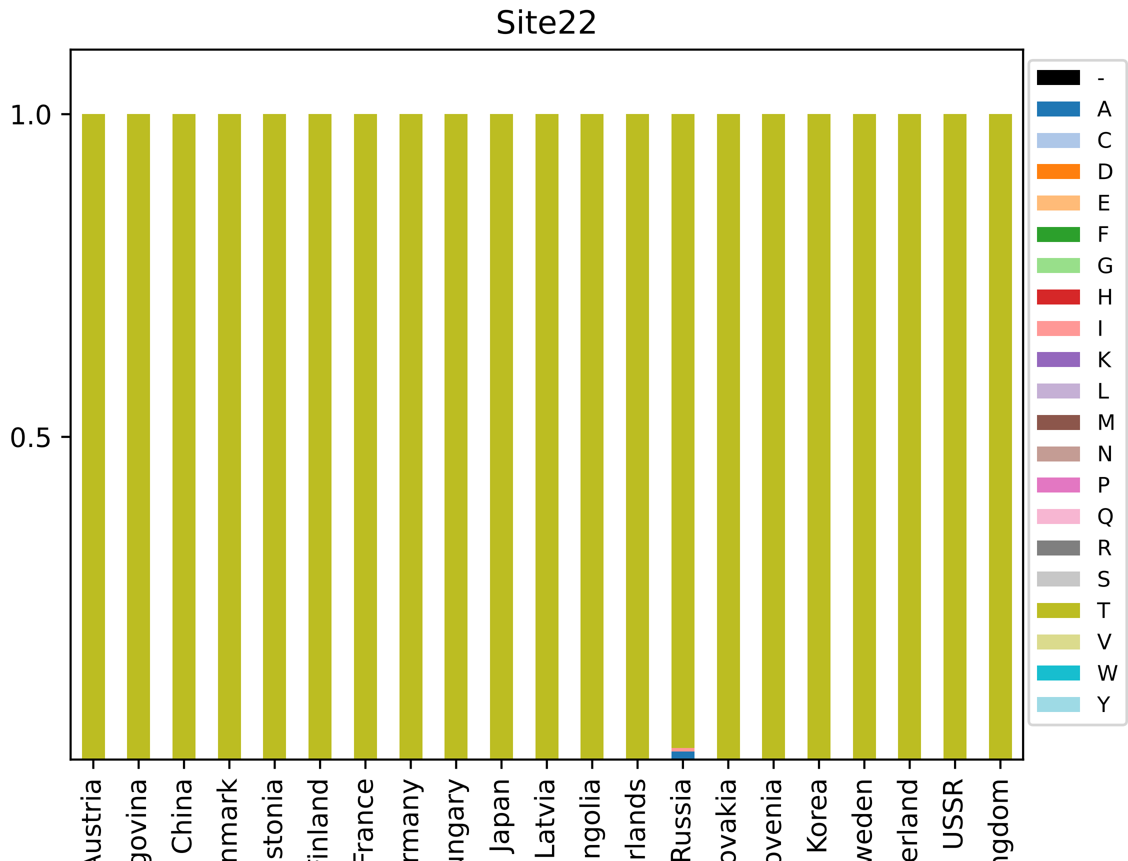

Site22
1.0
0.5
-
A
C
D
E
F
G
H
I
K
L
M
N
P
Q
R
S
T
V
W
Y
China
Japan
Latvia
Russia
Korea
USSR

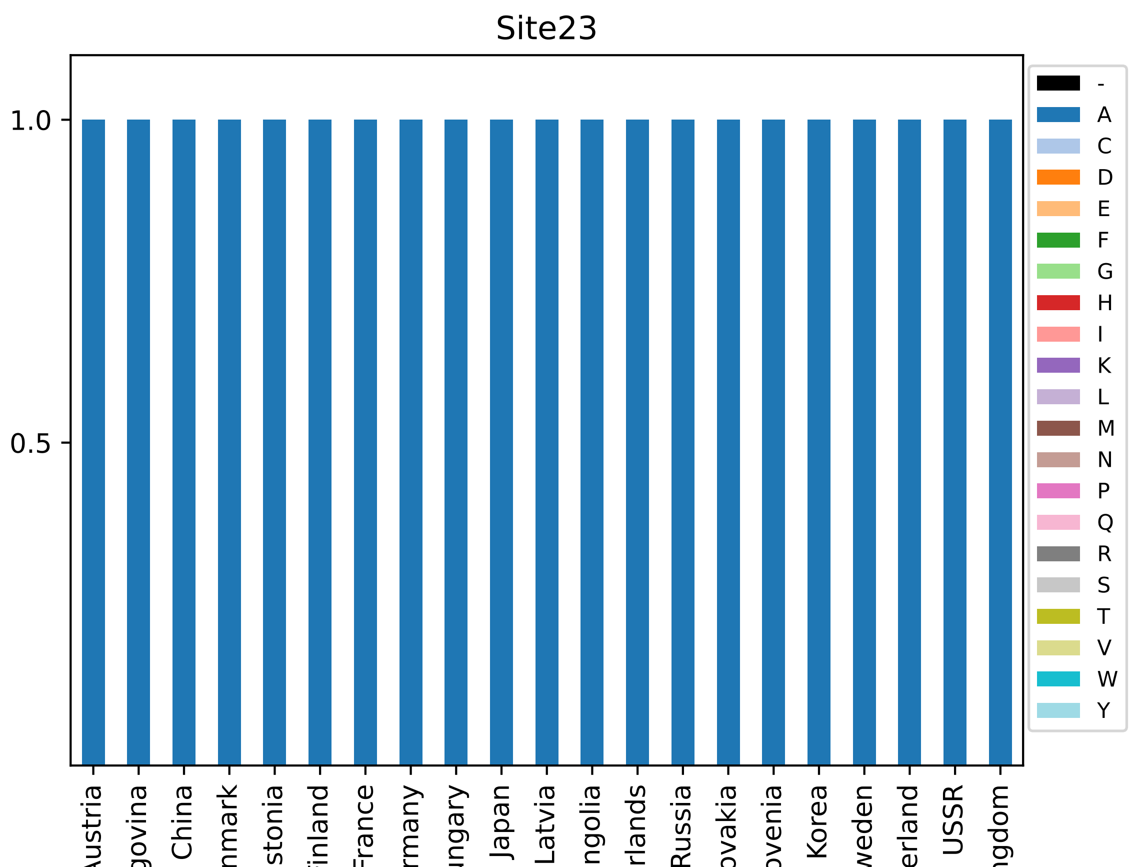

Site23
1.0
0.5
Austria
govina
China
nmark
stonia
inland
France
rmany
ungary
Japan
Latvia
ngolia
rlands
Russia
ovakia
ovenia
Korea
weden
erland
USSR
ngdom
-
A
C
D
E
F
G
H
I
K
L
M
N
P
Q
R
S
T
V
W
Y

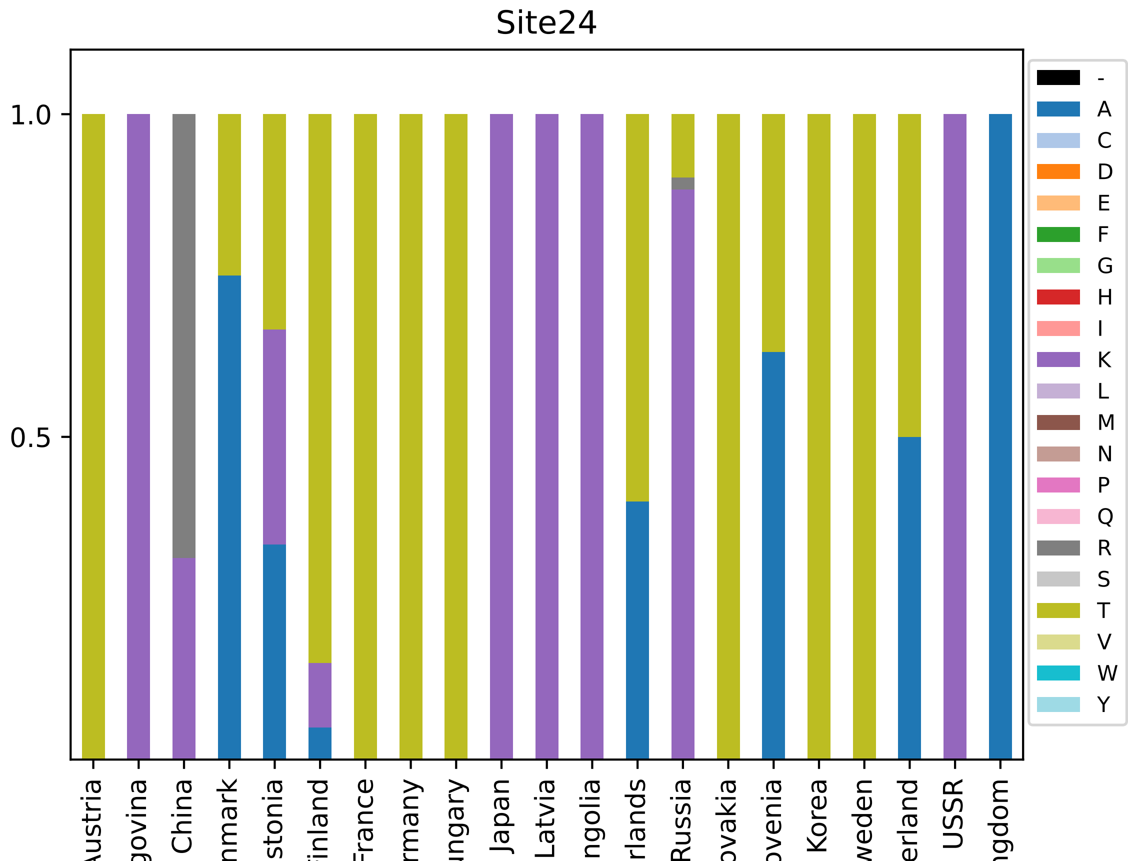
Site24
1.0
0.5
-
A
C
D
E
F
G
H
I
K
L
M
N
P
Q
R
S
T
V
W
Y
China
Japan
Latvia
Russia
Korea
USSR

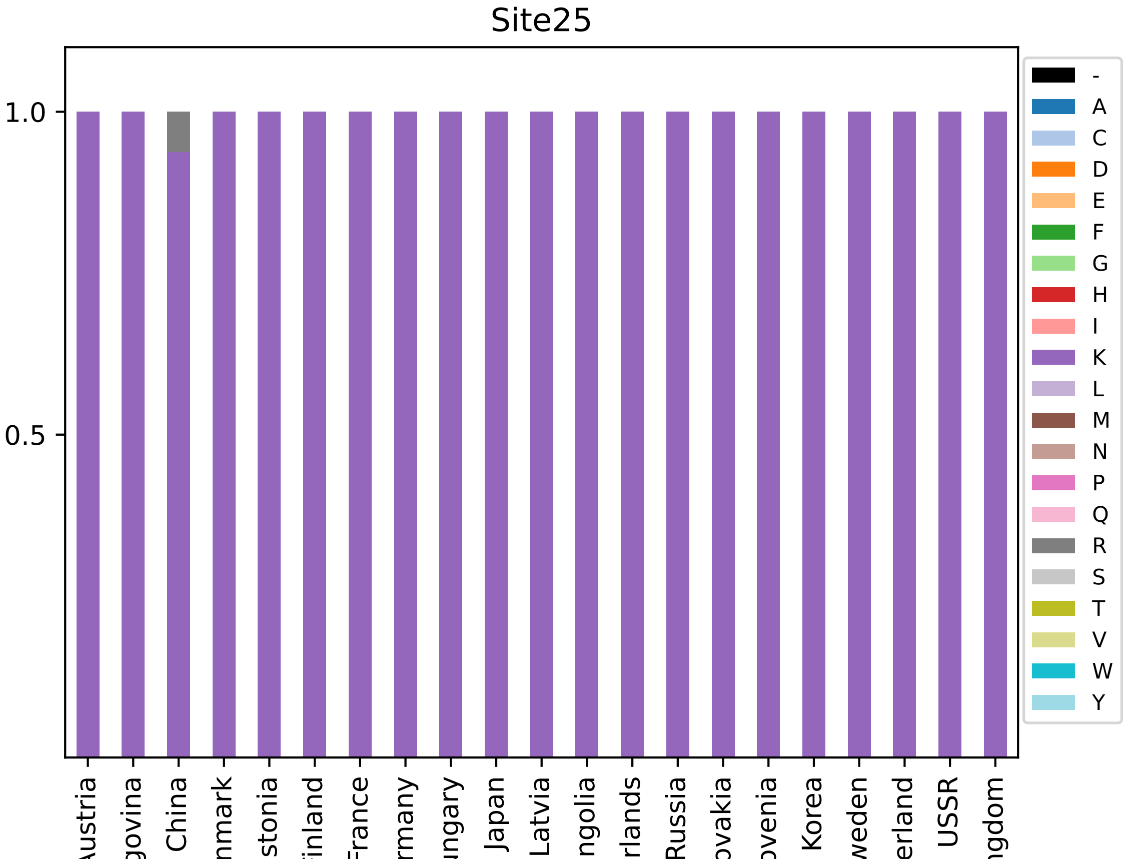
Site25
1.0
0.5
-
A
C
D
E
F
G
H
I
K
L
M
N
P
Q
R
S
T
V
W
Y
China
Japan
Latvia
Russia
Korea
USSR

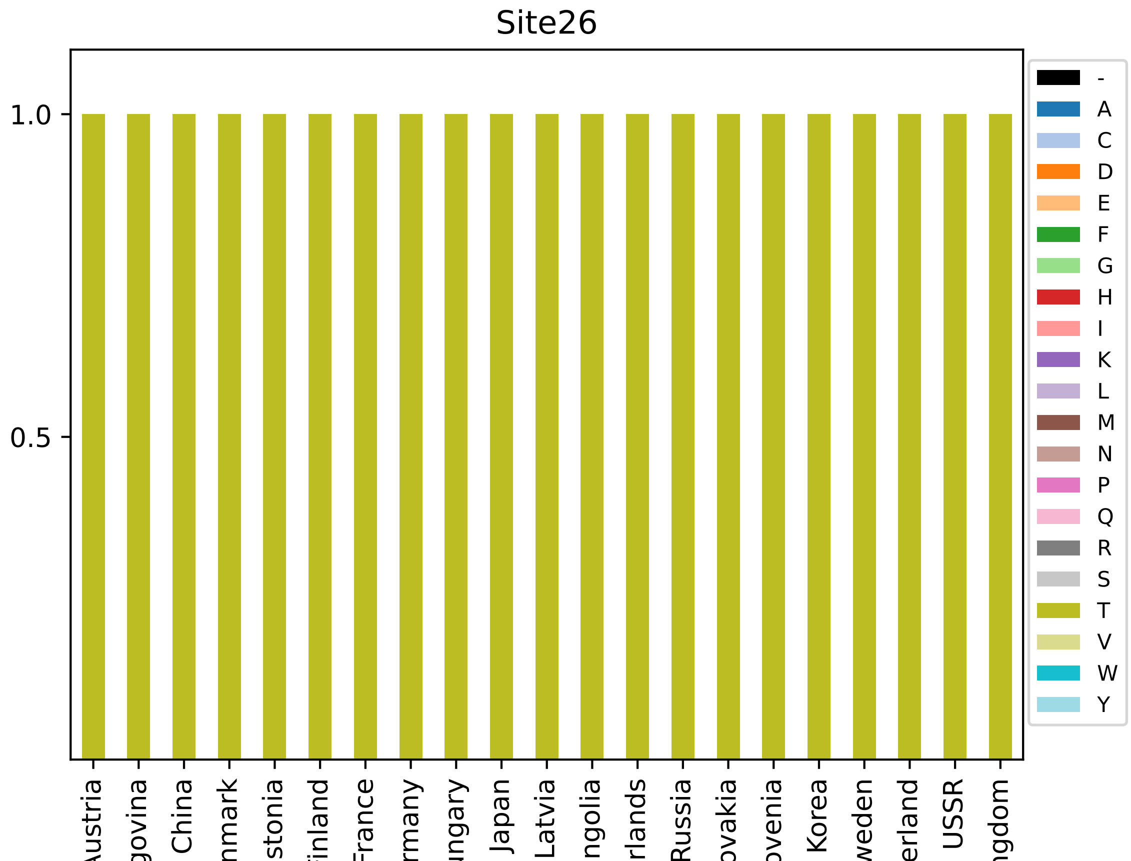
Site26
1.0
0.5
-
A
C
D
E
F
G
H
I
K
L
M
N
P
Q
R
S
T
V
W
Y
Austria
govina
China
nmark
stonia
Finland
France
rmany
ungary
Japan
Latvia
ngolia
rlands
Russia
ovakia
ovenia
Korea
weden
erland
USSR
ngdom

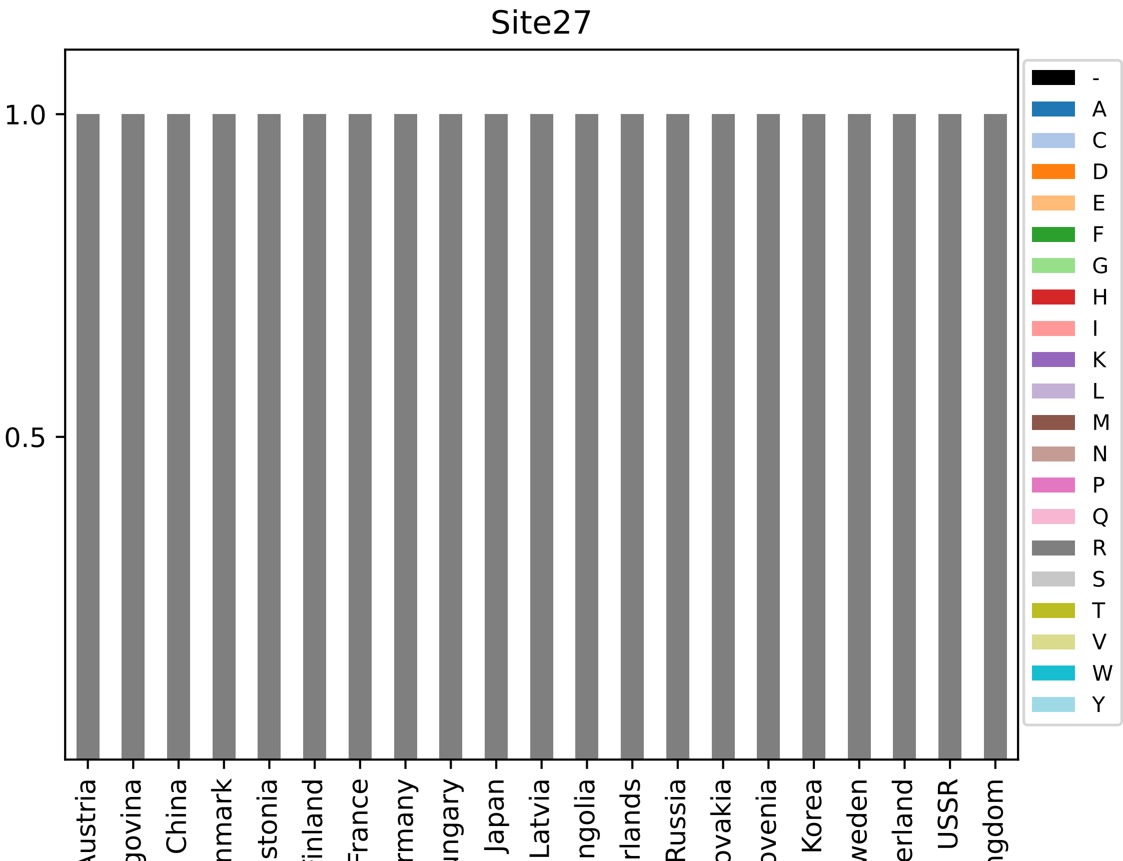

Site27
1.0
0.5
-
A
C
D
E
F
G
H
I
K
L
M
N
P
Q
R
S
T
V
W
Y
China
Japan
Latvia
Russia
Korea
USSR

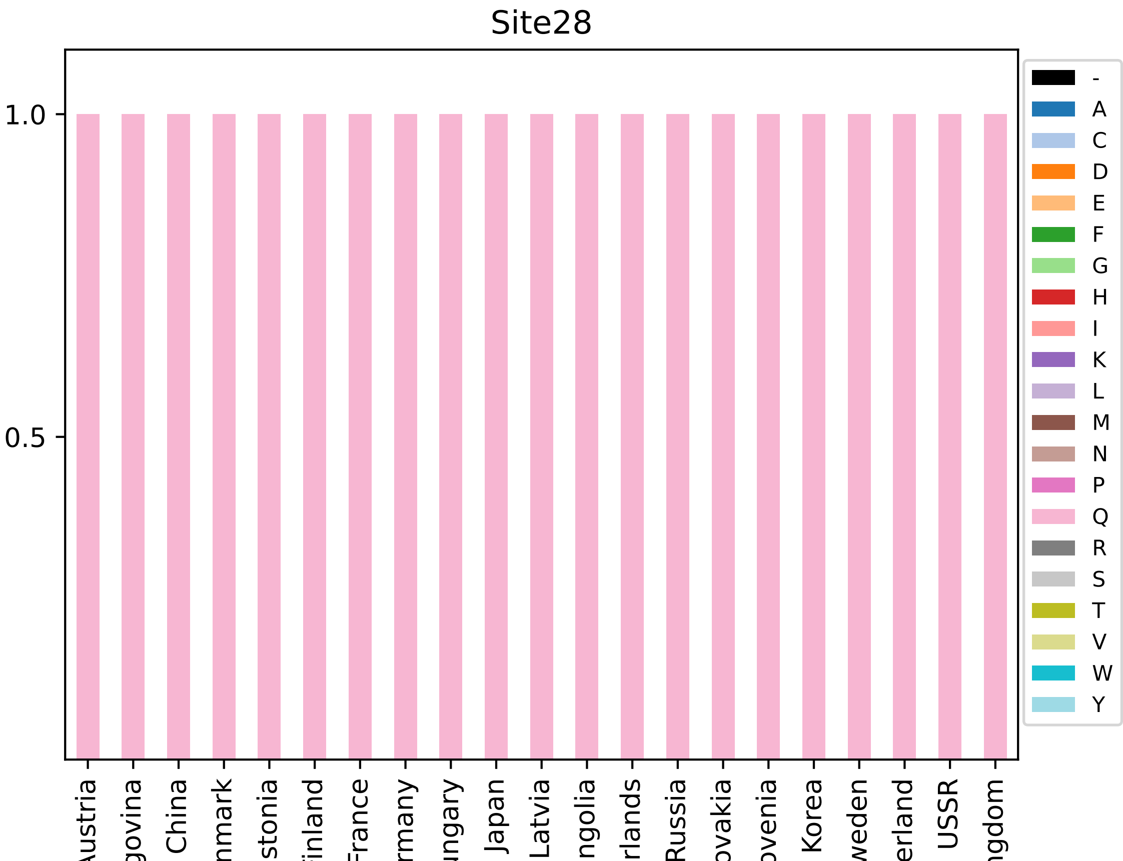
Site28
1.0
0.5
-
A
C
D
E
F
G
H
I
K
L
M
N
P
Q
R
S
T
V
W
Y
China
Japan
Latvia
Russia
Korea
USSR

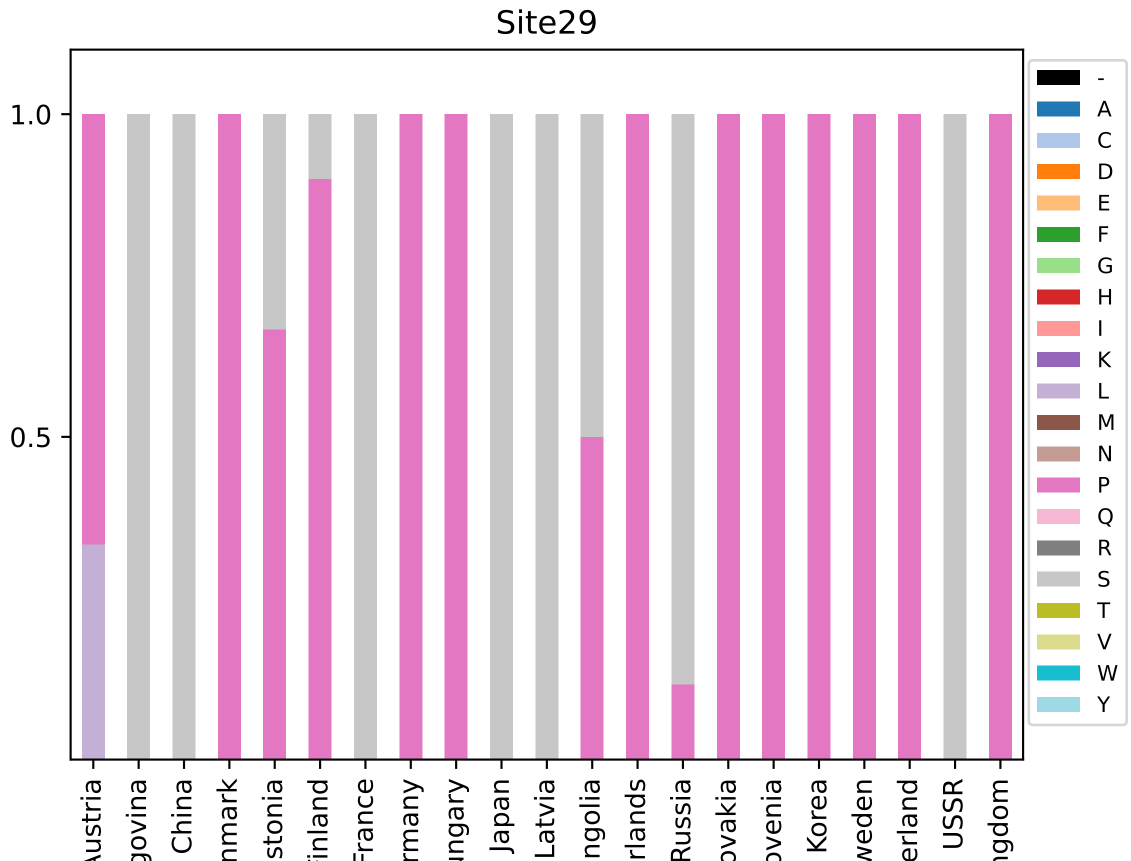

Site29
1.0
0.5
-
A
C
D
E
F
G
H
I
K
L
M
N
P
Q
R
S
T
V
W
Y
Austria
China
Estonia
Finland
France
Hungary
Japan
Latvia
Russia
Korea
Sweden
USSR

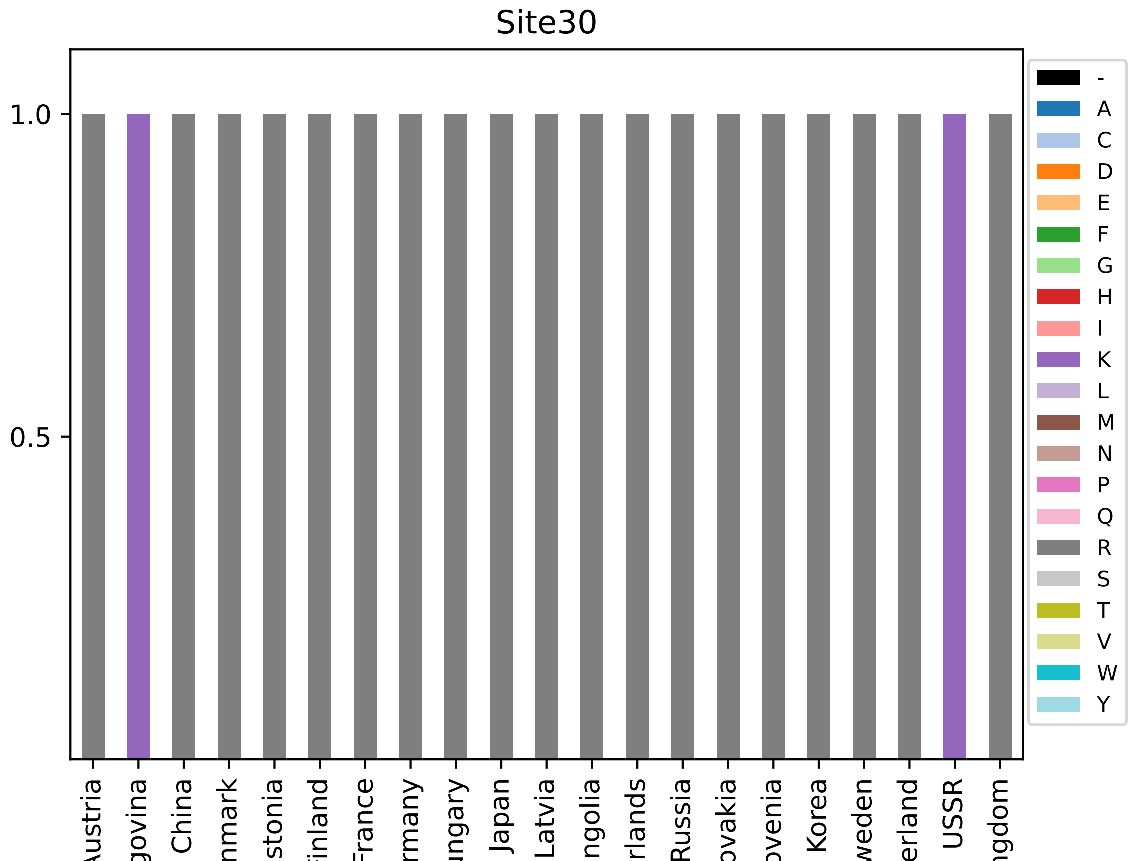
Site30
1.0
0.5
-
A
C
D
E
F
G
H
I
K
L
M
N
P
Q
R
S
T
V
W
Y
China
Japan
Latvia
Russia
Korea
USSR

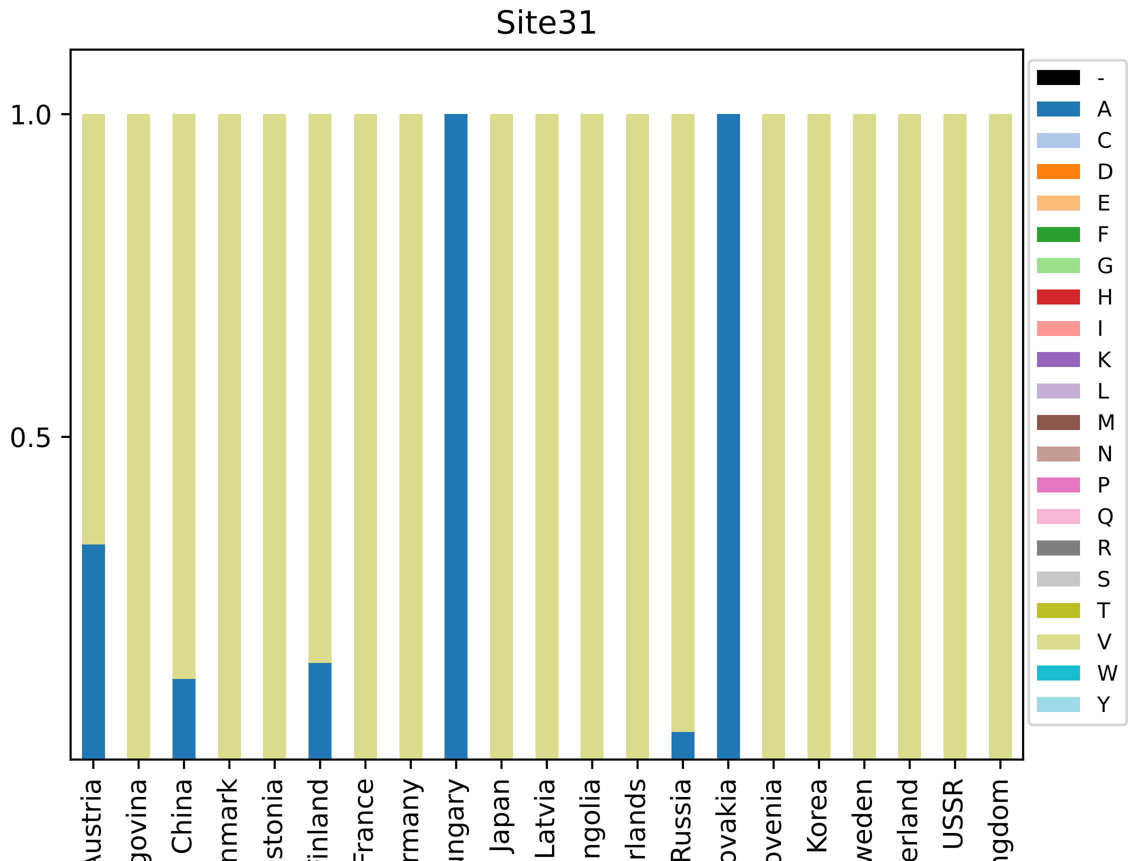

Site31
1.0
0.5
-
A
C
D
E
F
G
H
I
K
L
M
N
P
Q
R
S
T
V
W
Y
China
Japan
Latvia
Russia
Korea
USSR

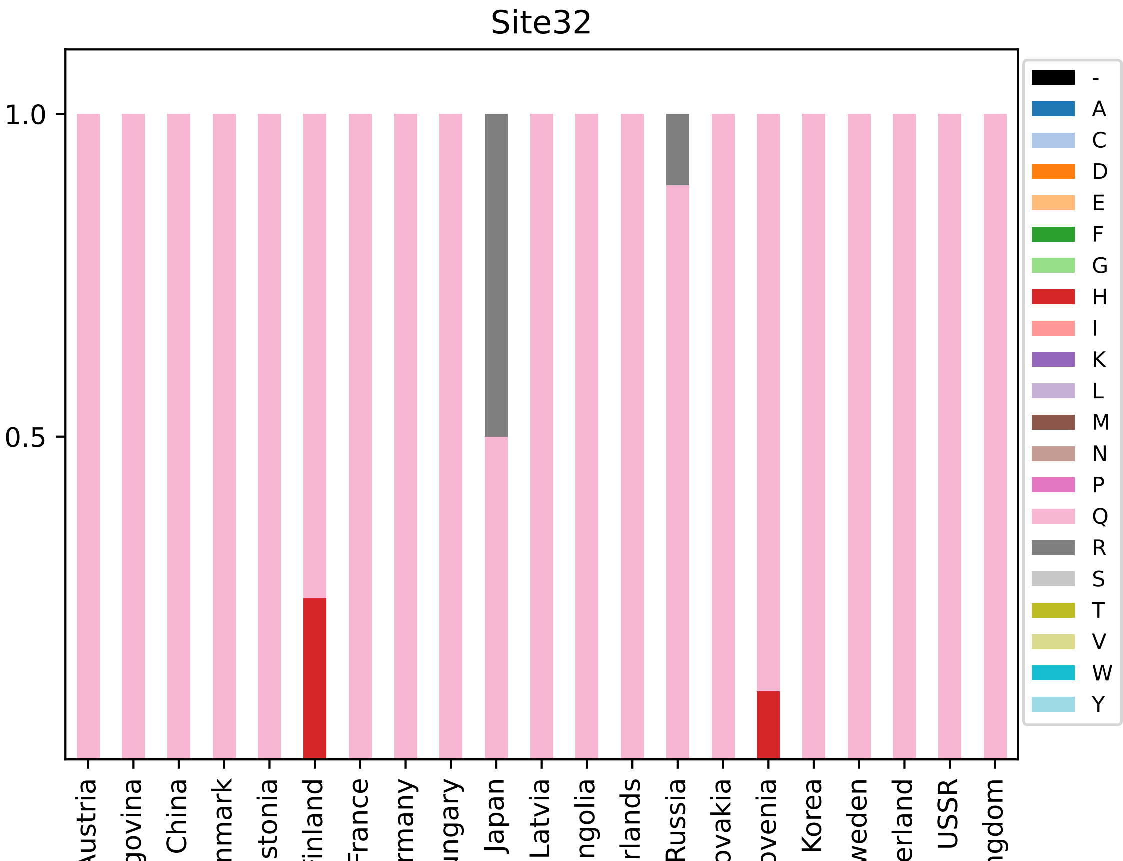

Site32
1.0
0.5
Austria
govina
China
nmark
stonia
Finland
France
rmany
ungary
Japan
Latvia
ngolia
rlands
Russia
ovakia
ovenia
Korea
weden
erland
USSR
ngdom
-
A
C
D
E
F
G
H
I
K
L
M
N
P
Q
R
S
T
V
W
Y

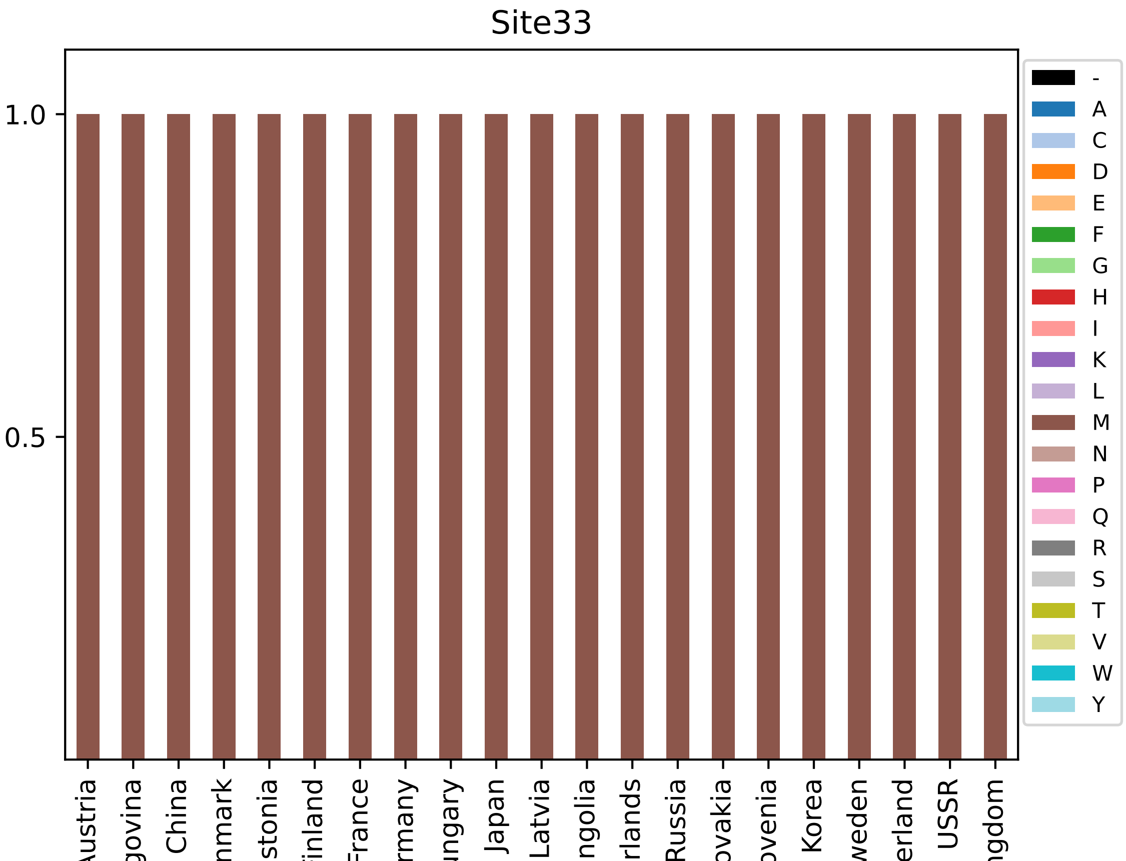

Site33
1.0
0.5
-
A
C
D
E
F
G
H
I
K
L
M
N
P
Q
R
S
T
V
W
Y
China
Japan
Latvia
Russia
Korea
USSR

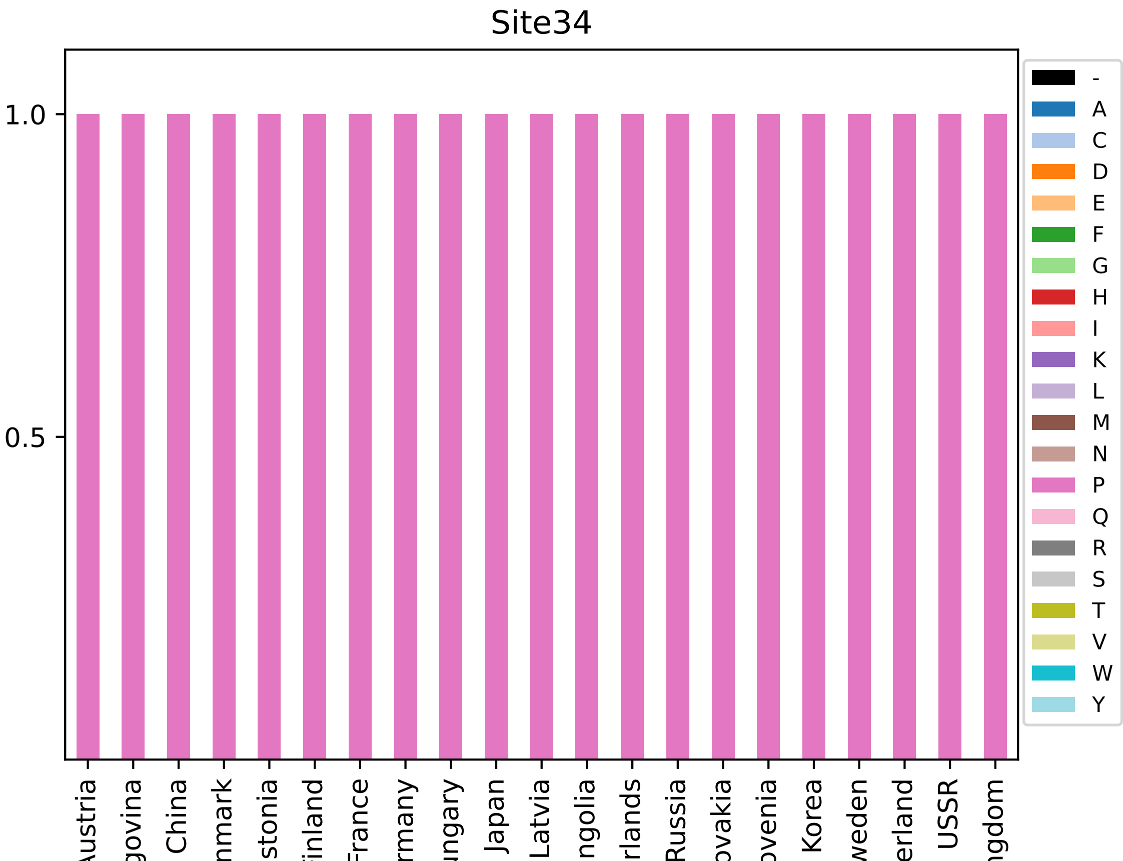
Site34
1.0
0.5
China
Japan
Latvia
Russia
Korea
USSR
-
A
C
D
E
F
G
H
I
K
L
M
N
P
Q
R
S
T
V
W
Y

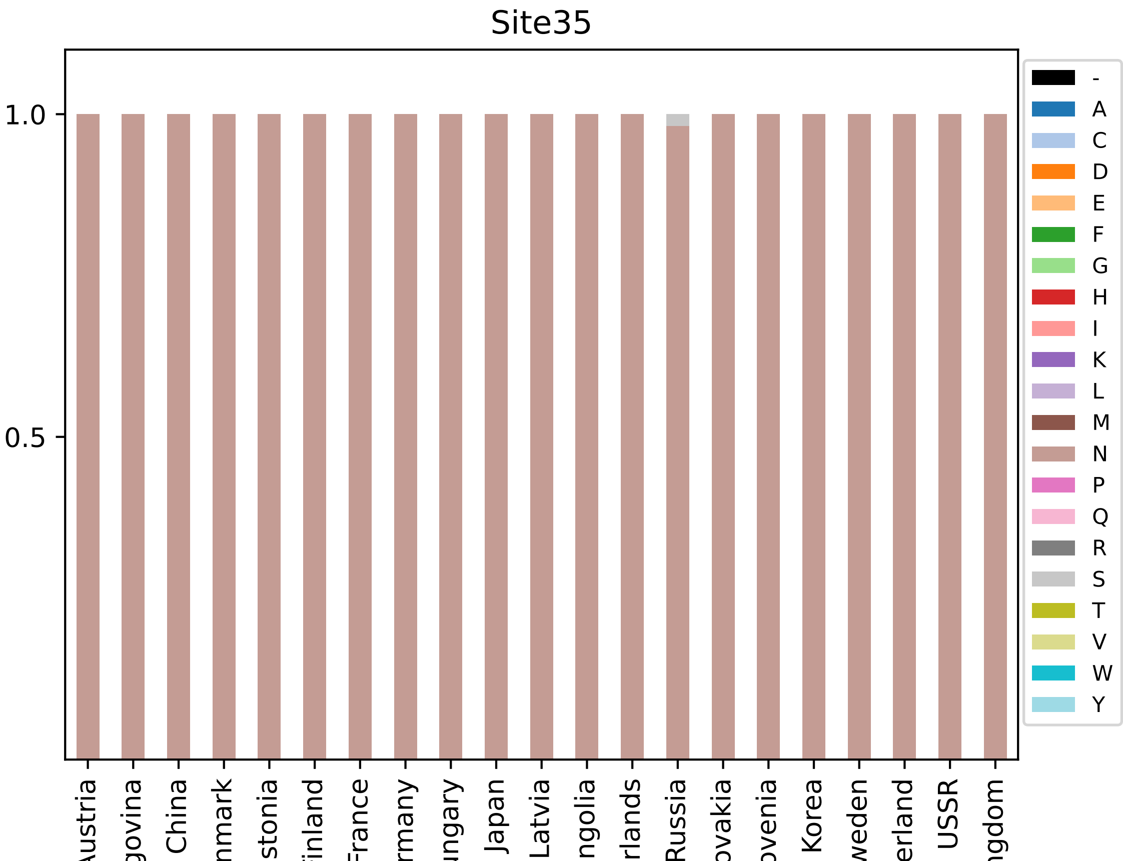
Site35
1.0
0.5
Austria
govina
China
nmark
stonia
Finland
France
rmany
ungary
Japan
Latvia
ngolia
rlands
Russia
ovakia
ovenia
Korea
weden
erland
USSR
ngdom
-
A
C
D
E
F
G
H
I
K
L
M
N
P
Q
R
S
T
V
W
Y

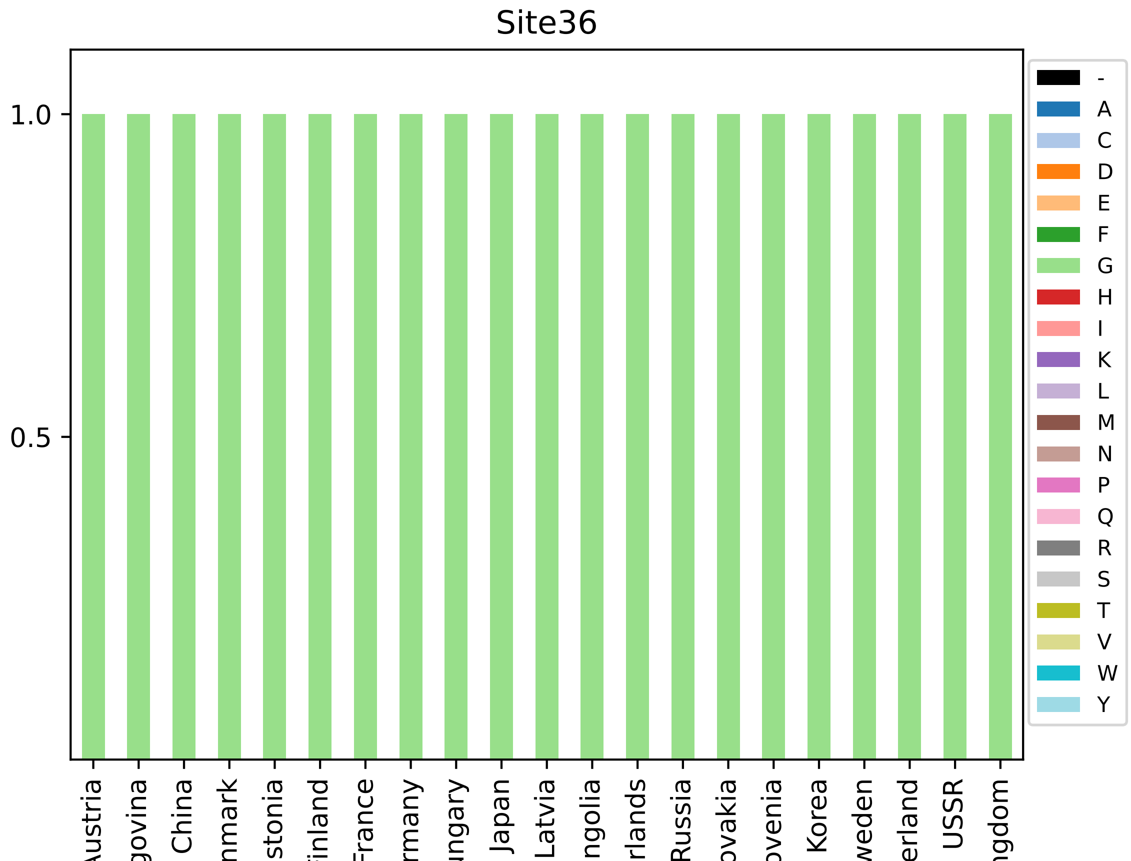

Site36
1.0
0.5
-
A
C
D
E
F
G
H
I
K
L
M
N
P
Q
R
S
T
V
W
Y
China
Japan
Latvia
Russia
Korea
USSR

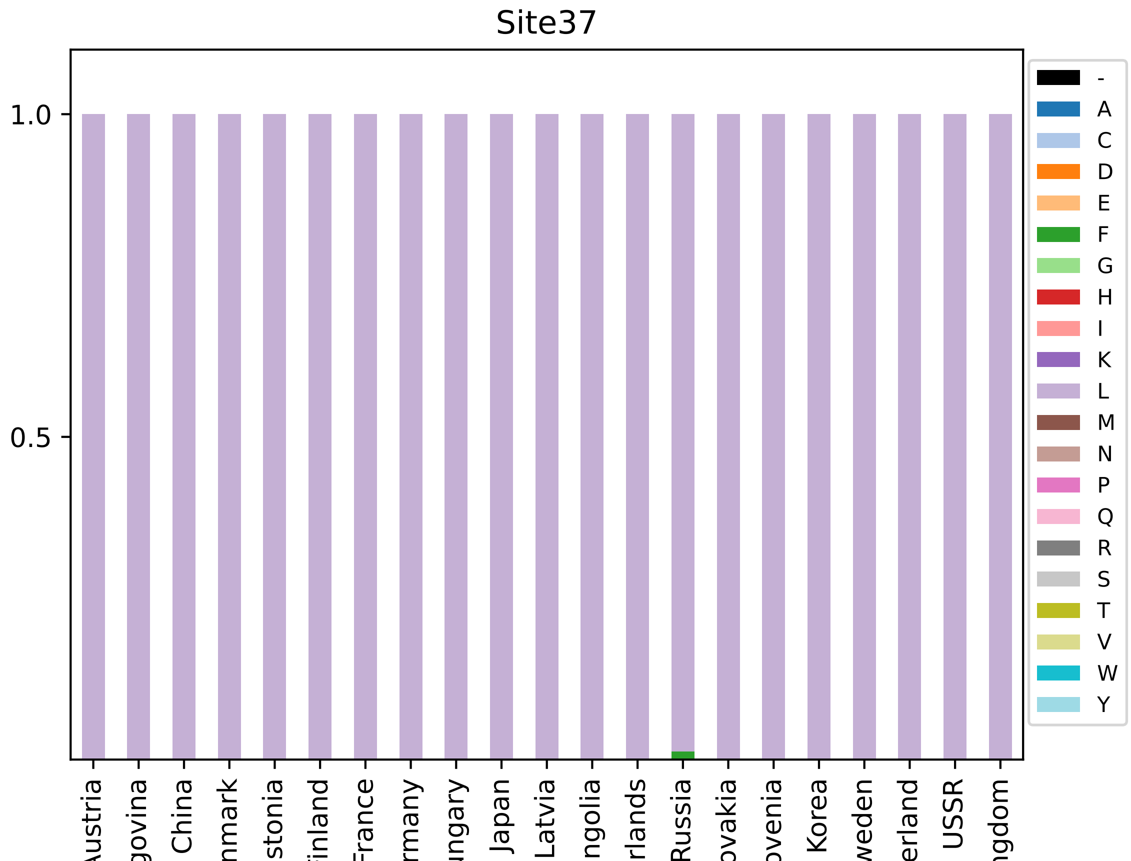

Site37
1.0
0.5
-
A
C
D
E
F
G
H
I
K
L
M
N
P
Q
R
S
T
V
W
Y
China
Finland
France
Japan
Latvia
Russia
Korea
USSR

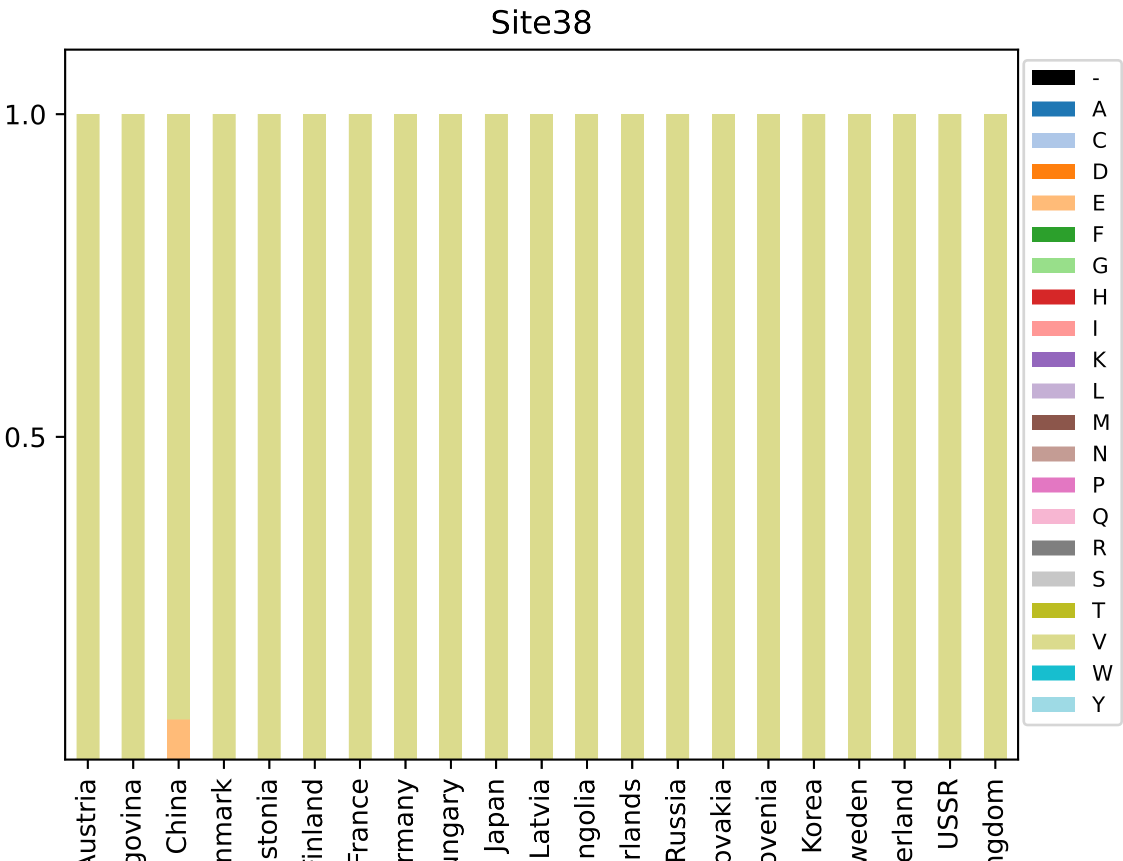
Site38
1.0
0.5
-
A
C
D
E
F
G
H
I
K
L
M
N
P
Q
R
S
T
V
W
Y
China
Japan
Latvia
Russia
Korea
USSR

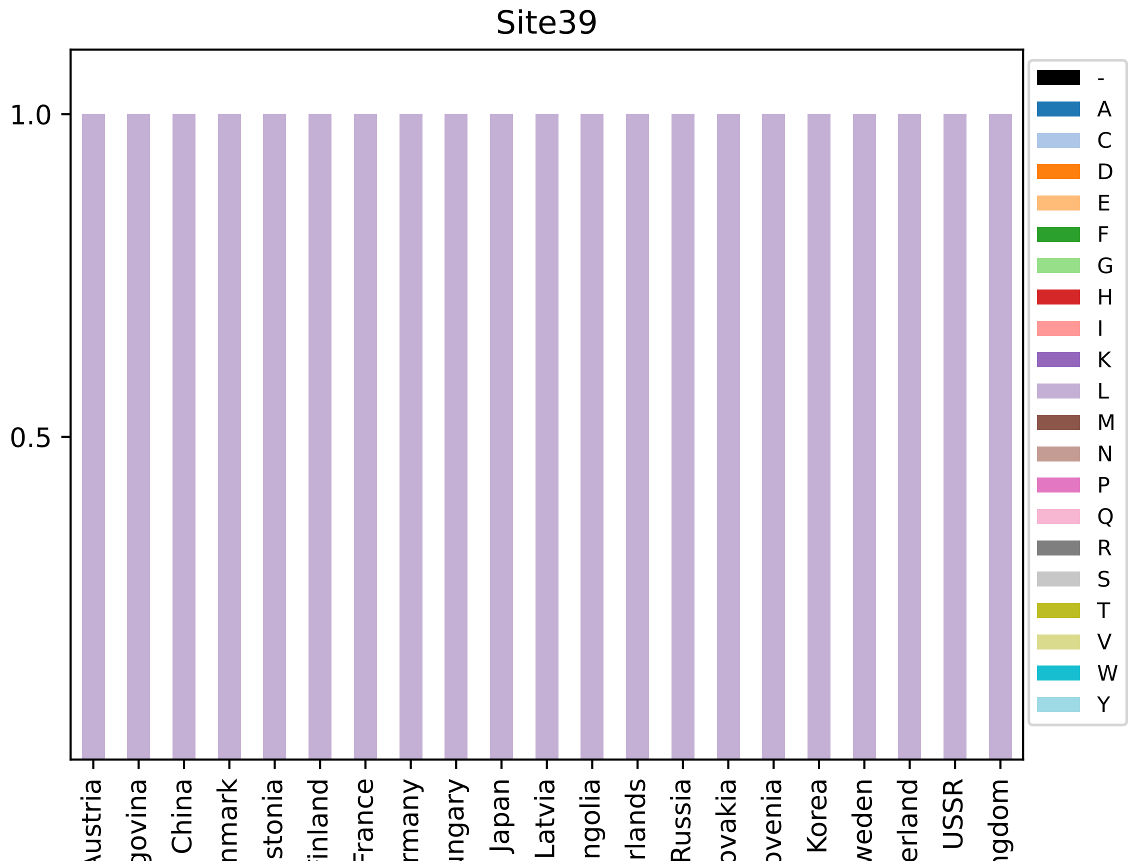

Site39
1.0
0.5
-
A
C
D
E
F
G
H
I
K
L
M
N
P
Q
R
S
T
V
W
Y
China
Japan
Latvia
Russia
Korea
USSR

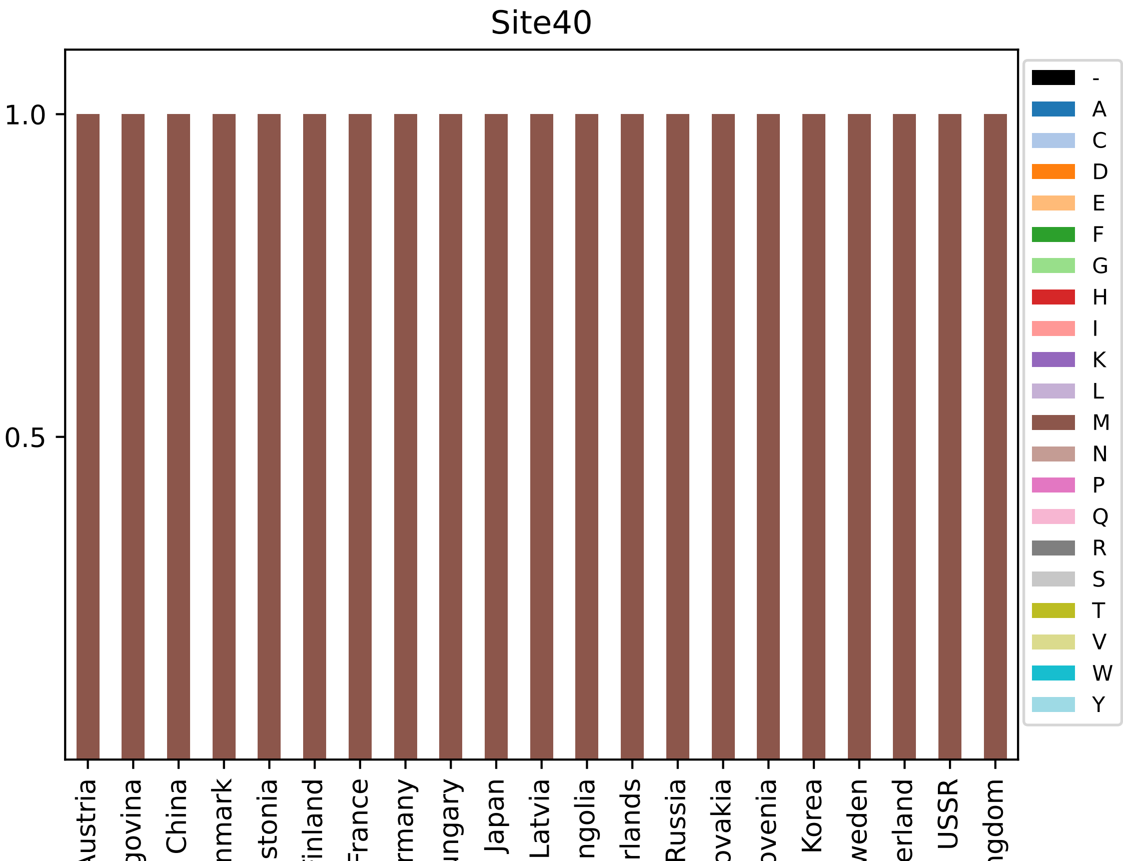

Site40
1.0
0.5
-
A
C
D
E
F
G
H
I
K
L
M
N
P
Q
R
S
T
V
W
Y
China
Japan
Latvia
Russia
Korea
USSR

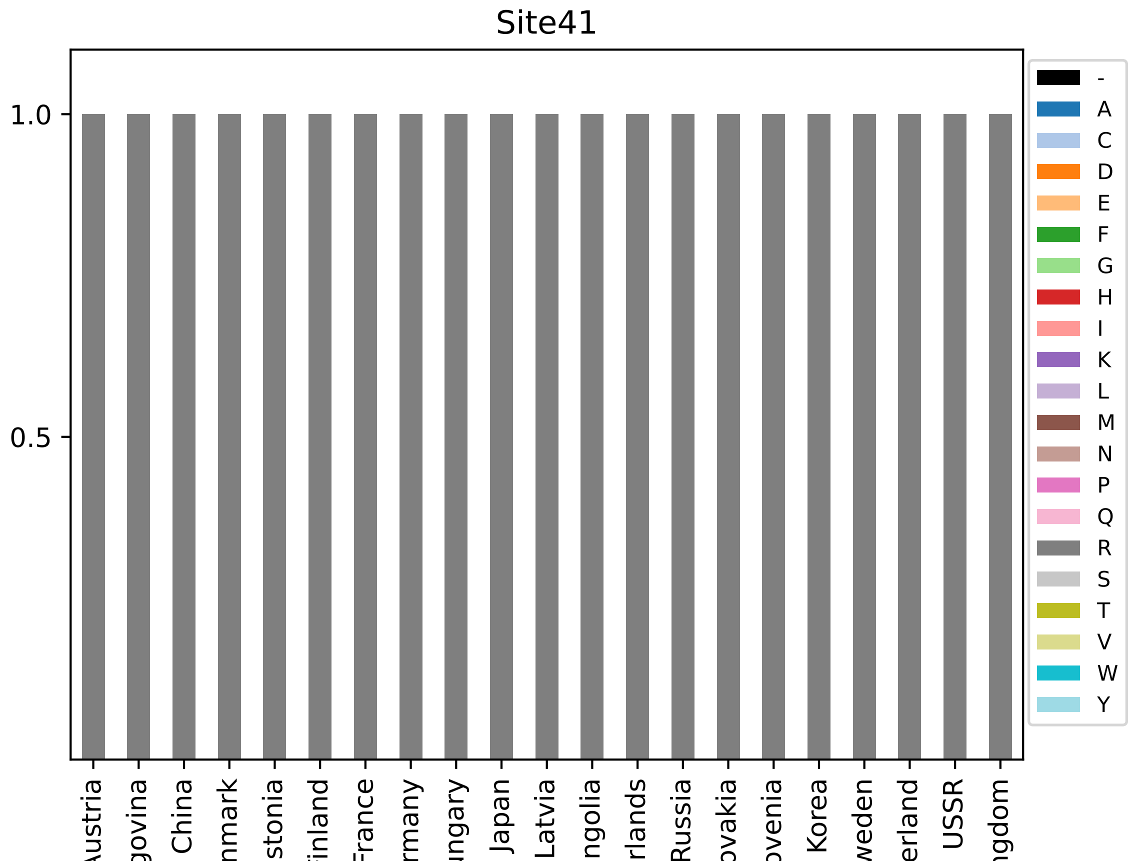
Site41
1.0
0.5
-
A
C
D
E
F
G
H
I
K
L
M
N
P
Q
R
S
T
V
W
Y
Austria
govina
China
nmark
stonia
inland
France
rmany
ungary
Japan
Latvia
ngolia
rlands
Russia
ovakia
ovenia
Korea
weden
erland
USSR
ngdom

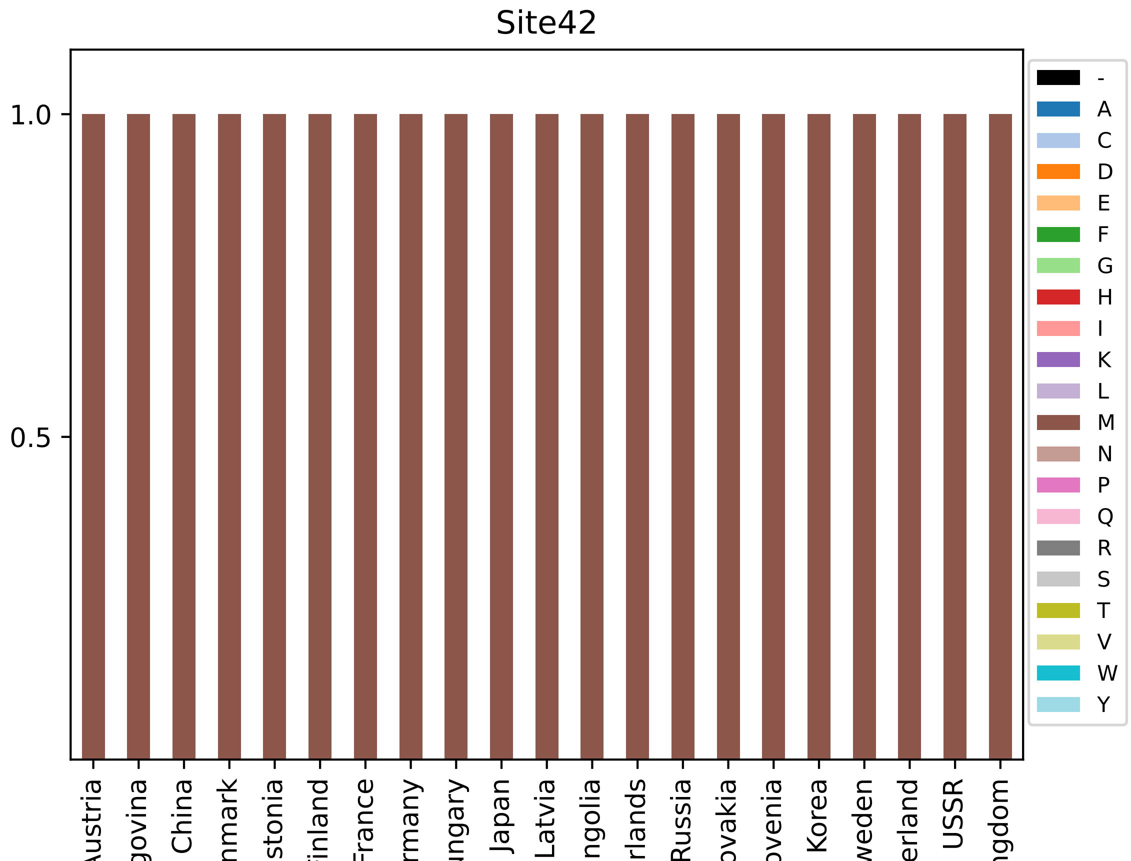
Site42
1.0
0.5
China
Japan
Latvia
Russia
Korea
USSR
-
A
C
D
E
F
G
H
I
K
L
M
N
P
Q
R
S
T
V
W
Y

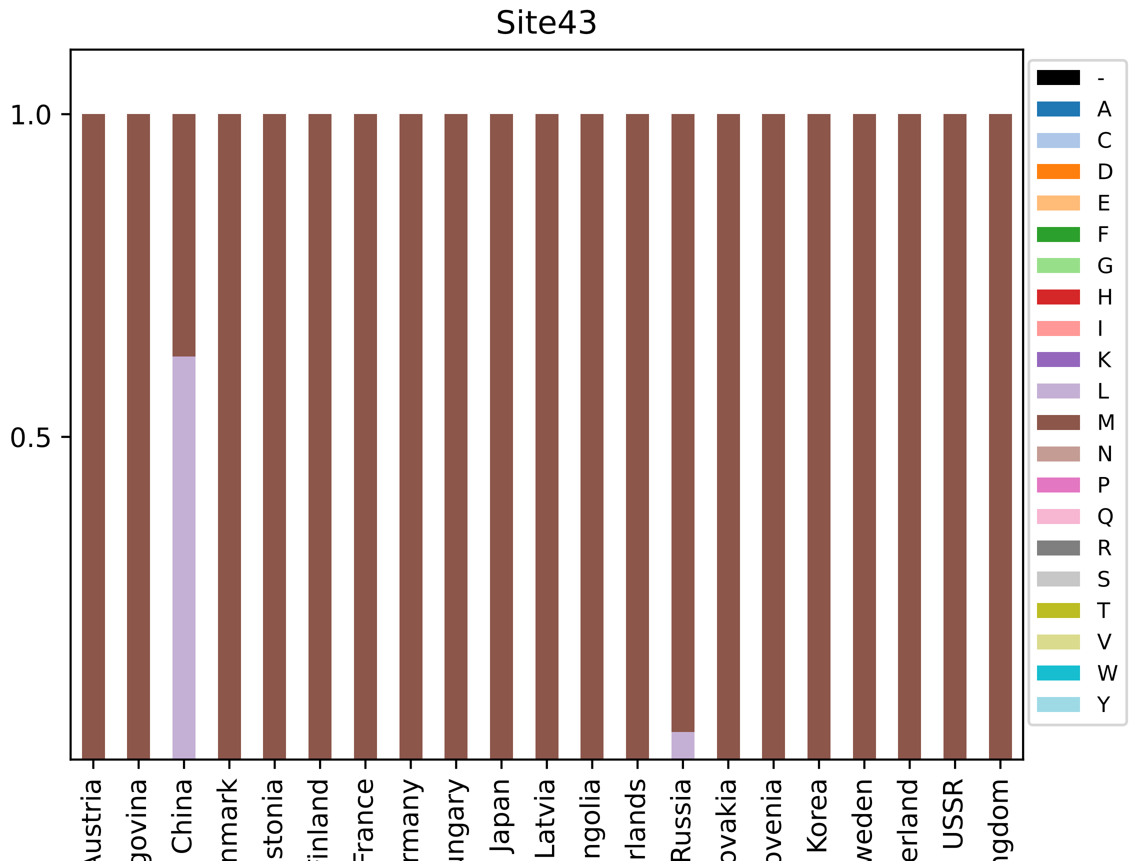
Site43
1.0
0.5
China
Japan
Latvia
Russia
Korea
USSR
-
A
C
D
E
F
G
H
I
K
L
M
N
P
Q
R
S
T
V
W
Y

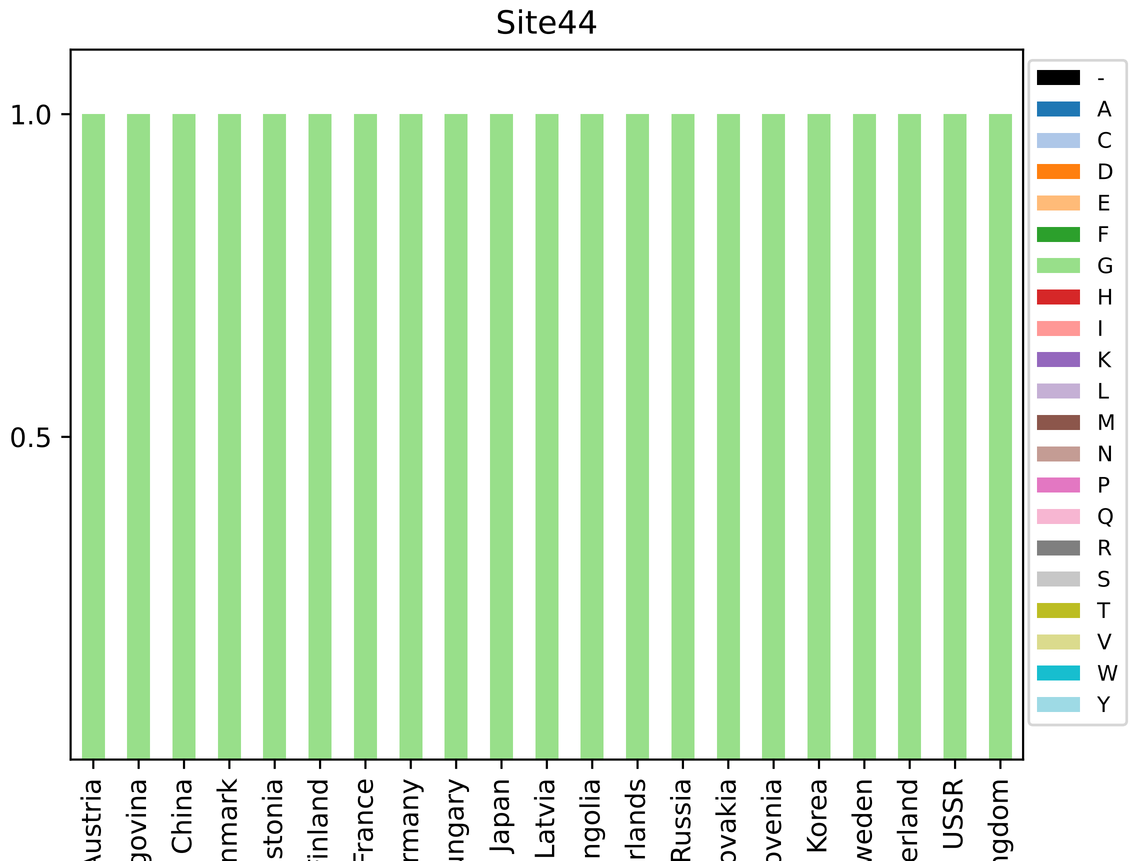
Site44
1.0
0.5
Austria
govina
China
nmark
stonia
Finland
France
rmany
ungary
Japan
Latvia
ngolia
rlands
Russia
ovakia
ovenia
Korea
weden
erland
USSR
ngdom
-
A
C
D
E
F
G
H
I
K
L
M
N
P
Q
R
S
T
V
W
Y

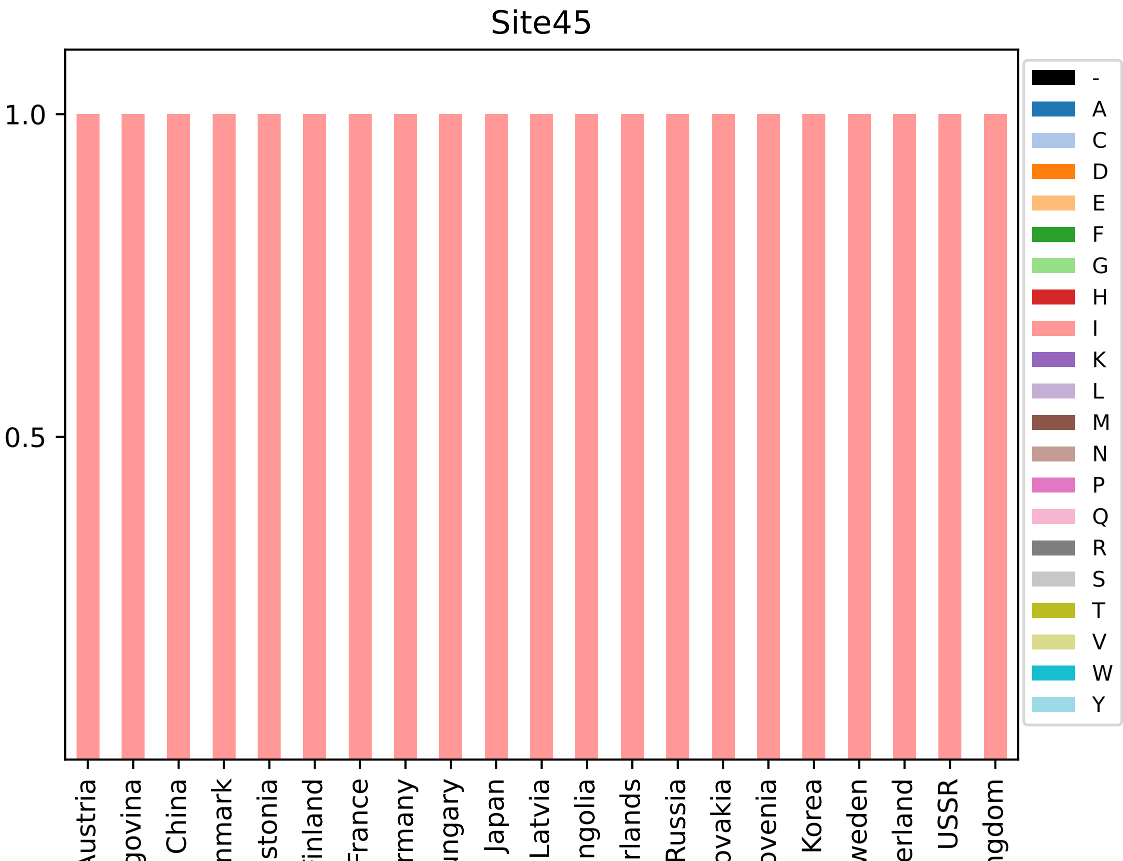

Site45
1.0
0.5
-
A
C
D
E
F
G
H
I
K
L
M
N
P
Q
R
S
T
V
W
Y
Austria
China
Japan
Latvia
Russia
Korea
USSR

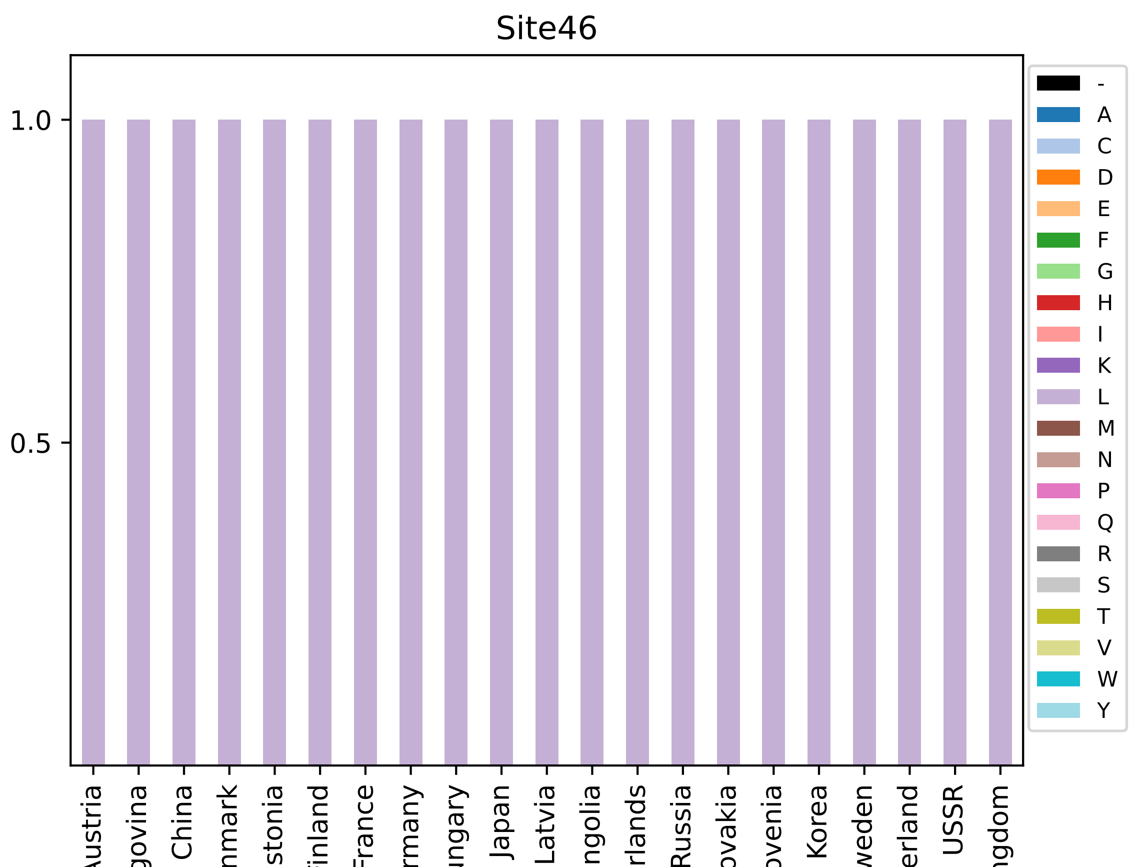

Site46
1.0
0.5
China
Japan
Latvia
Russia
Korea
USSR
-
A
C
D
E
F
G
H
I
K
L
M
N
P
Q
R
S
T
V
W
Y

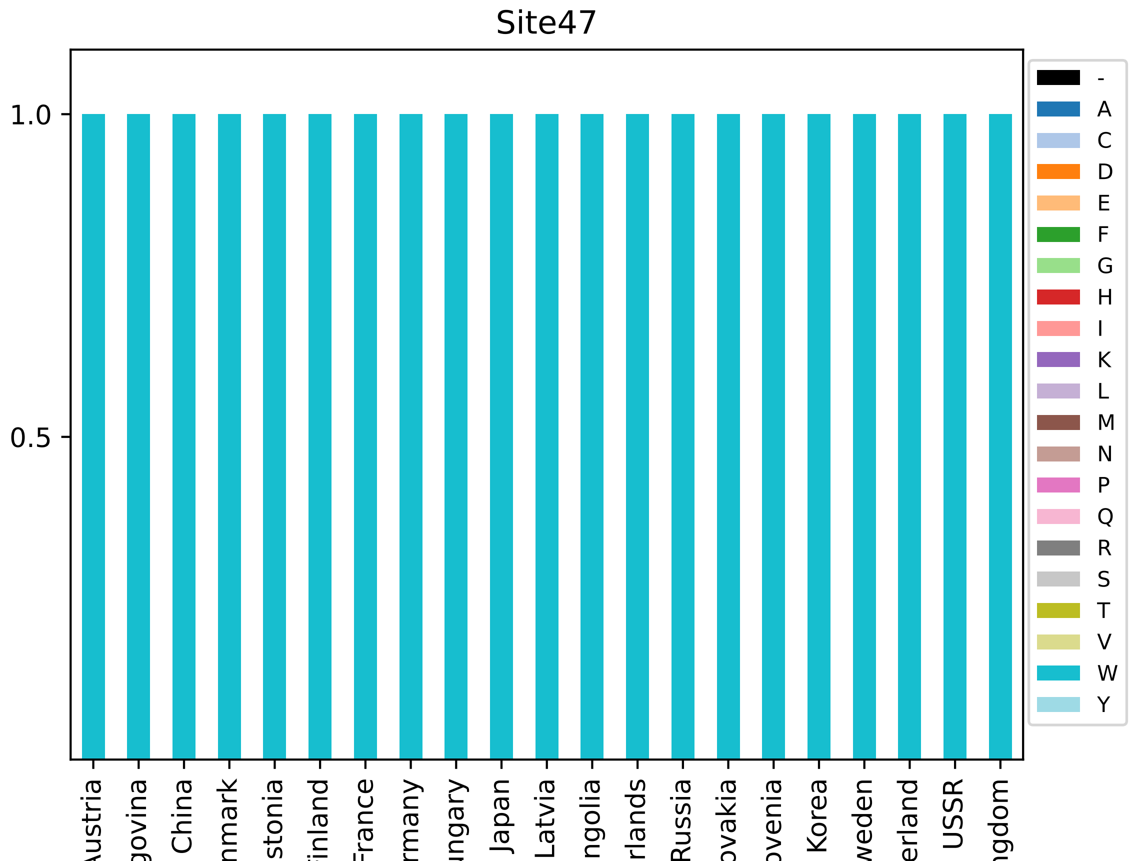
Site47
1.0
0.5
-
A
C
D
E
F
G
H
I
K
L
M
N
P
Q
R
S
T
V
W
Y
China
Japan
Latvia
Russia
Korea
USSR

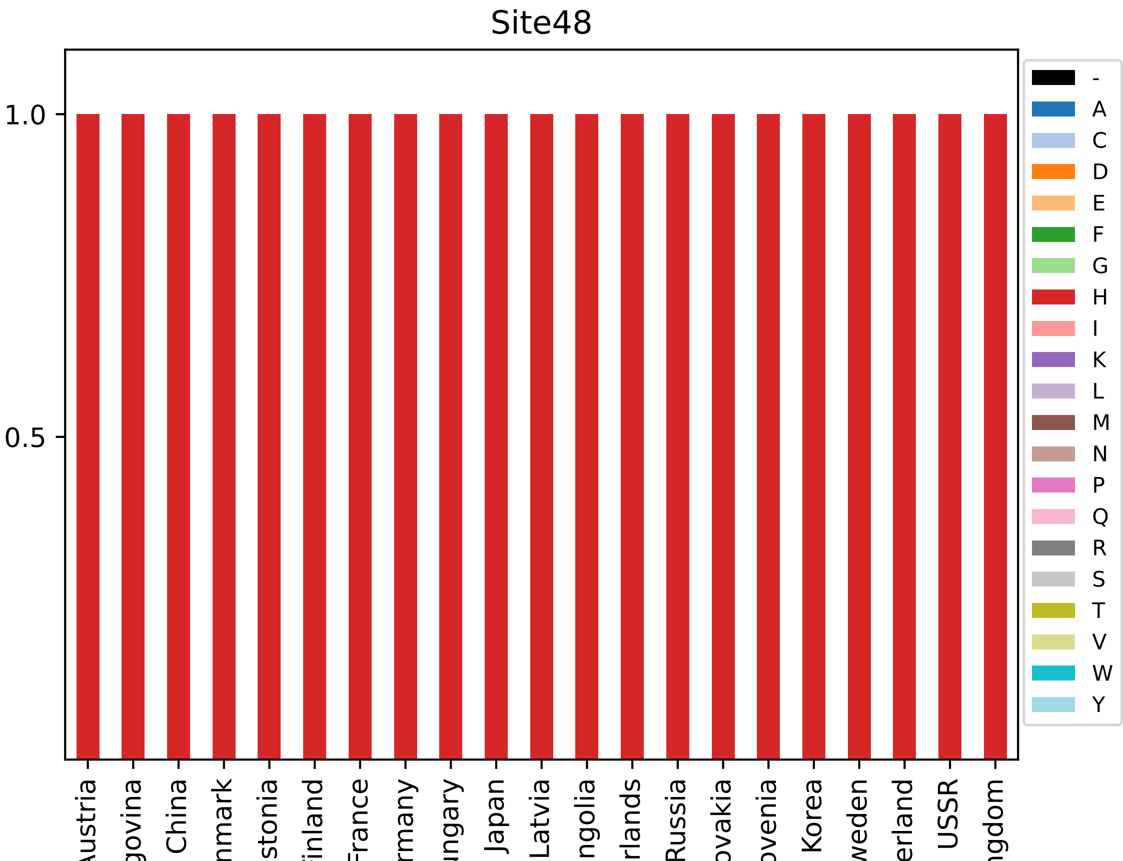

Site48
1.0
0.5
-
A
C
D
E
F
G
H
I
K
L
M
N
P
Q
R
S
T
V
W
Y
Austria
govina
China
nmark
stonia
inland
France
rmany
ungary
Japan
Latvia
ngolia
rlands
Russia
ovakia
ovenia
Korea
weden
erland
USSR
ngdom

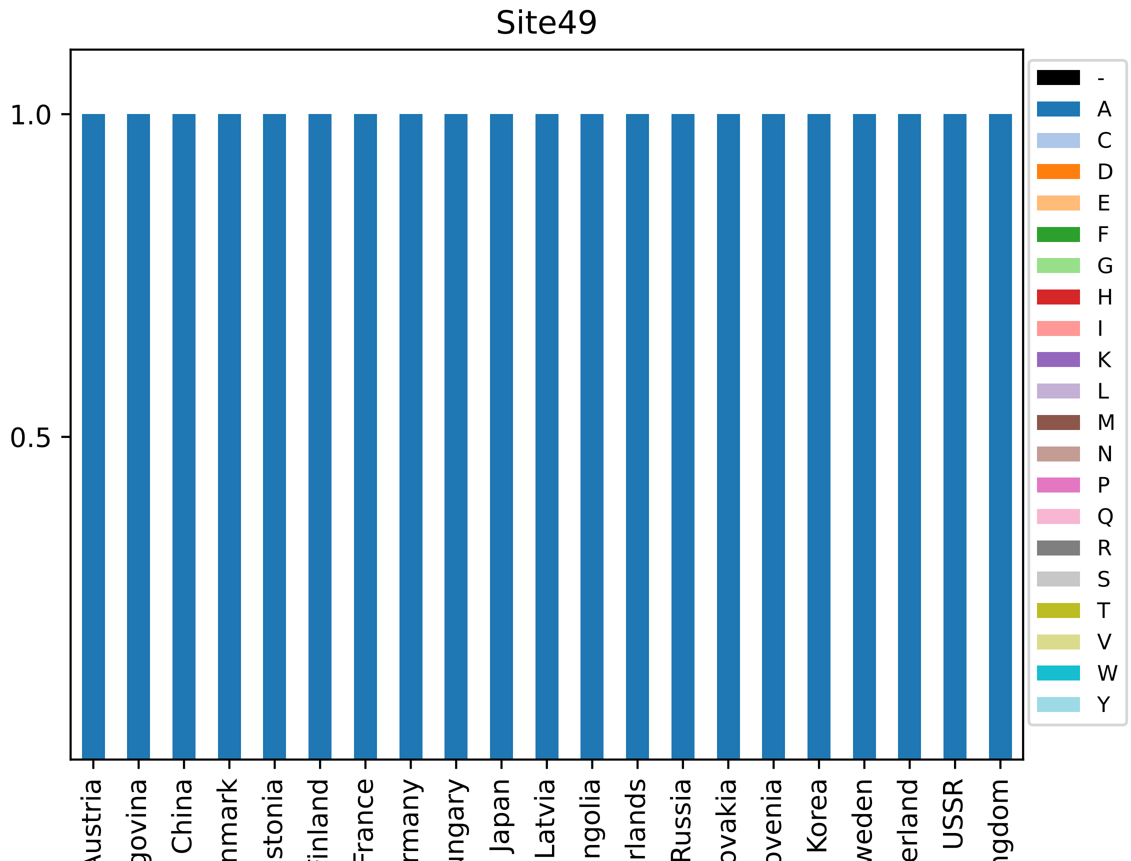
Site49
1.0
0.5
Austria
govina
China
nmark
stonia
Finland
France
rmany
ungary
Japan
Latvia
ngolia
rlands
Russia
ovakia
ovenia
Korea
weden
erland
USSR
ngdom
-
A
C
D
E
F
G
H
I
K
L
M
N
P
Q
R
S
T
V
W
Y

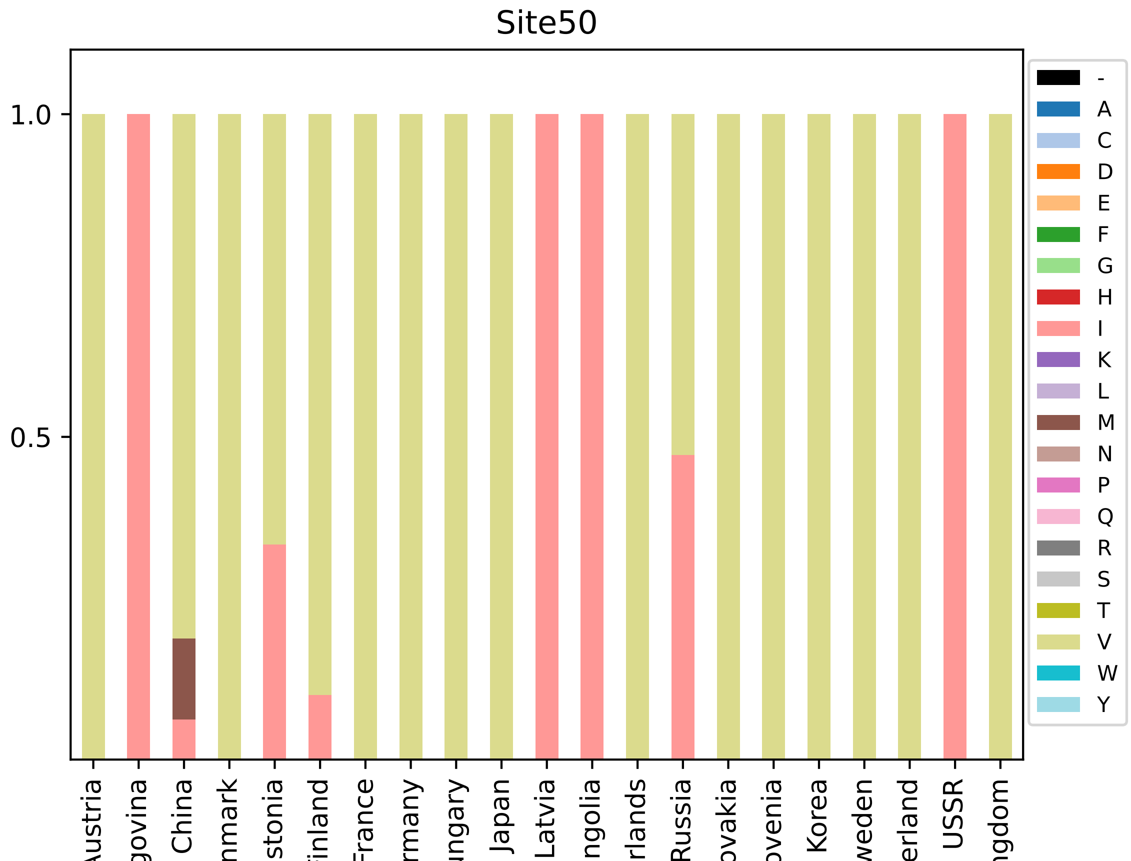
Site50
1.0
0.5
-
A
C
D
E
F
G
H
I
K
L
M
N
P
Q
R
S
T
V
W
Y
China
Japan
Latvia
Russia
Korea
USSR

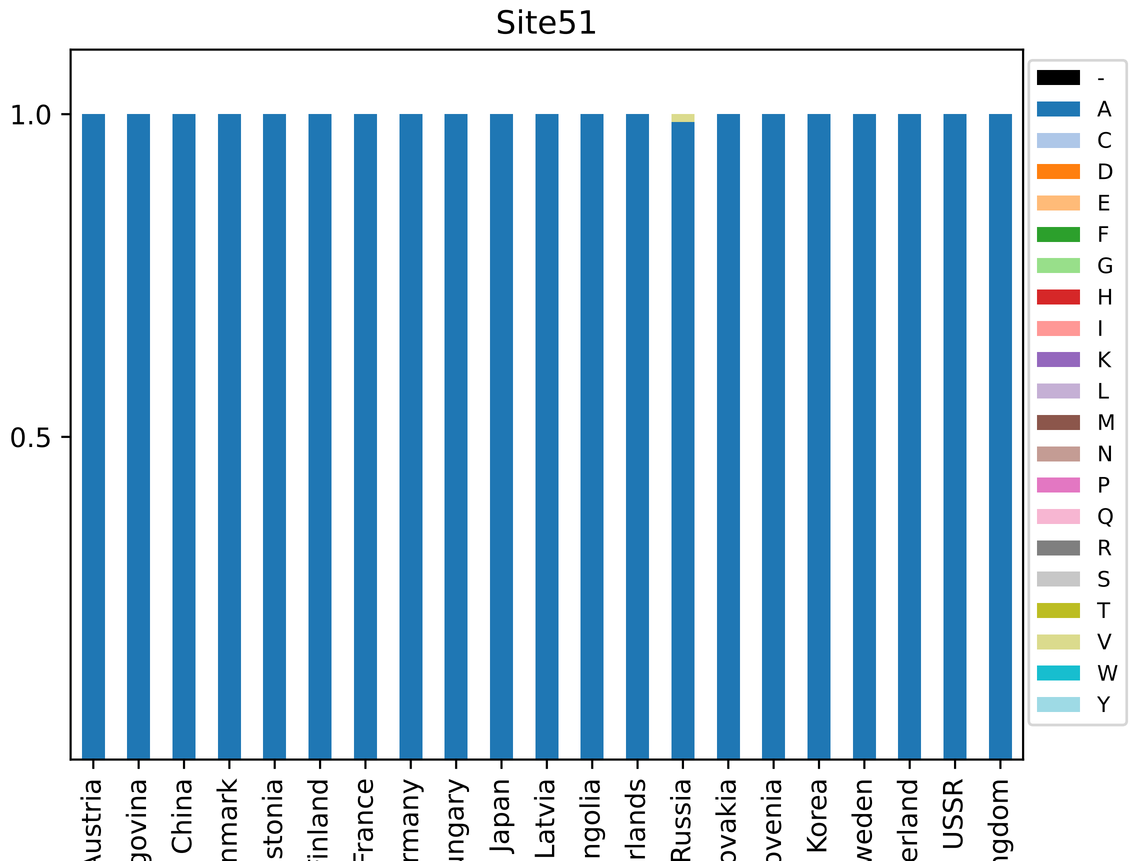
Site51
1.0
0.5
-
A
C
D
E
F
G
H
I
K
L
M
N
P
Q
R
S
T
V
W
Y
China
Japan
Latvia
Russia
Korea
USSR

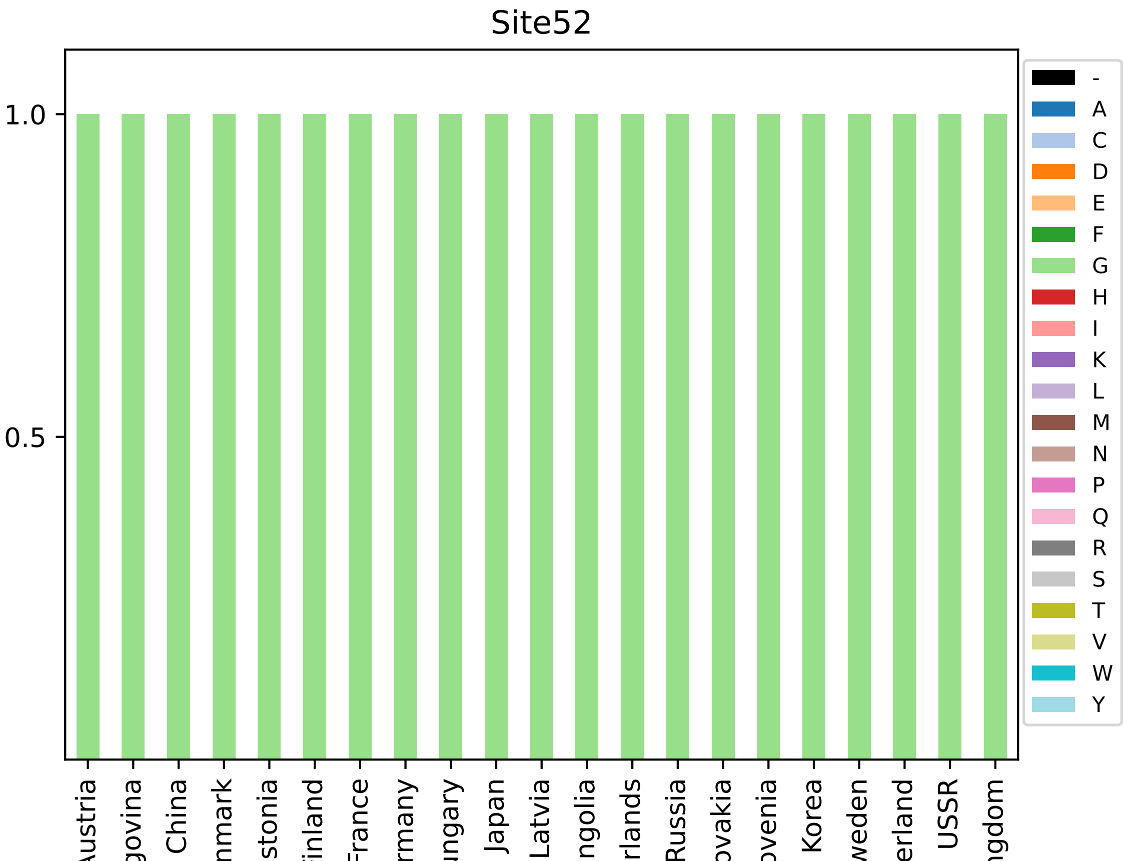
Site52
1.0
0.5
-
A
C
D
E
F
G
H
I
K
L
M
N
P
Q
R
S
T
V
W
Y
Austria
govina
China
nmark
stonia
Finland
France
rmany
ungary
Japan
Latvia
ngolia
rlands
Russia
ovakia
ovenia
Korea
weden
erland
USSR
ngdom

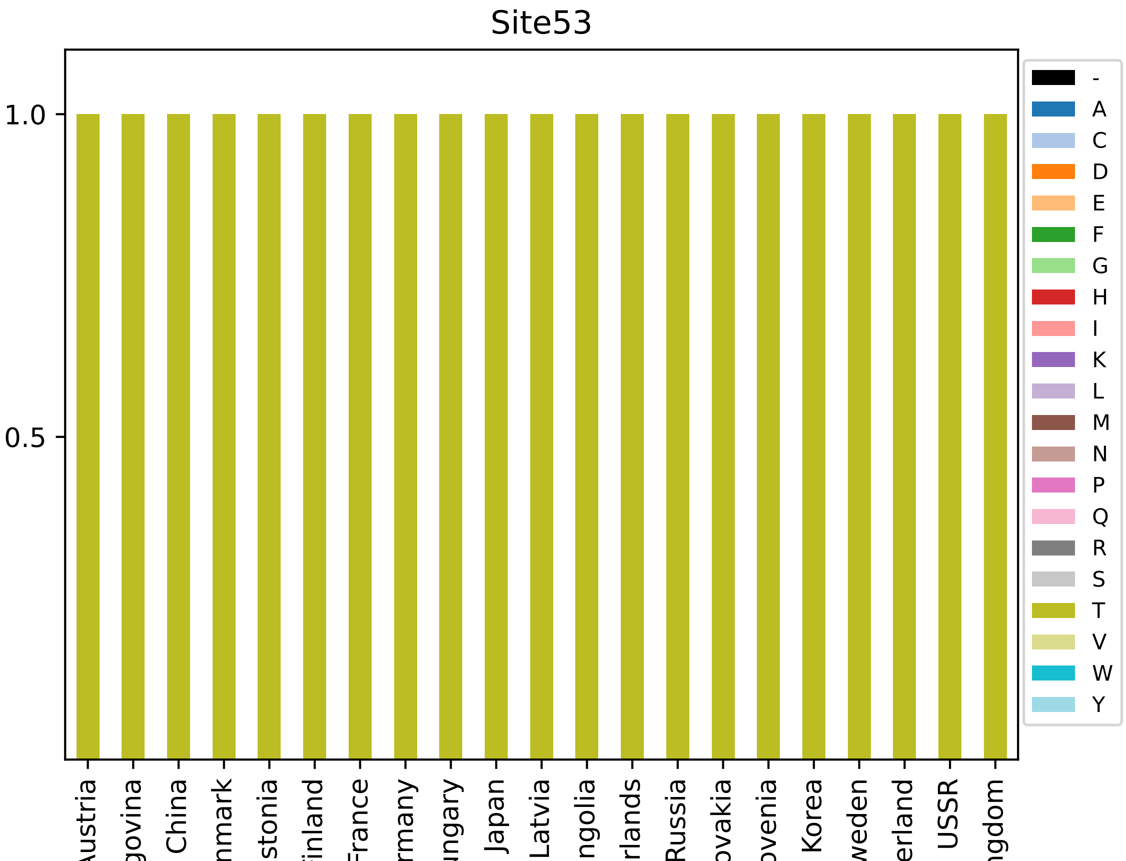

Site53
1.0
0.5
-
A
C
D
E
F
G
H
I
K
L
M
N
P
Q
R
S
T
V
W
Y
China
Japan
Latvia
Russia
Korea
USSR

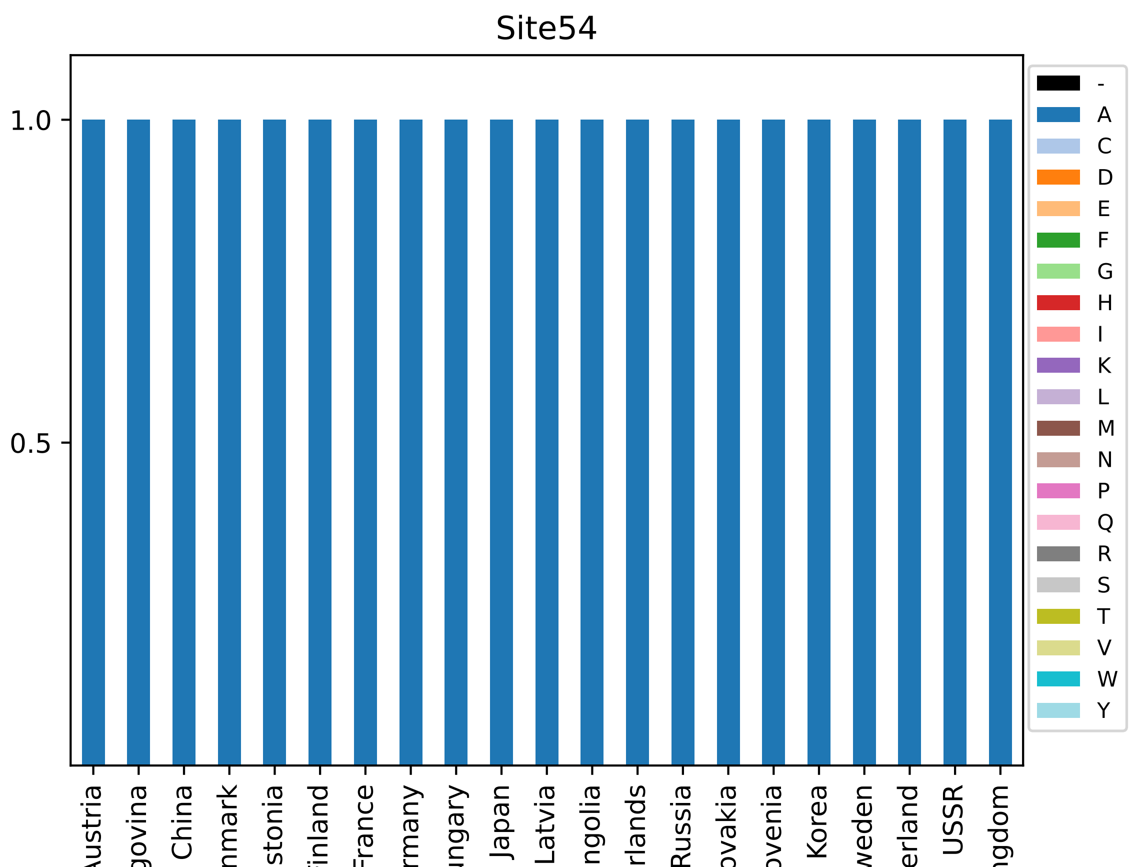

Site54
1.0
0.5
-
A
C
D
E
F
G
H
I
K
L
M
N
P
Q
R
S
T
V
W
Y
Austria
govina
China
nmark
stonia
inland
France
rmany
ungary
Japan
Latvia
ngolia
rlands
Russia
ovakia
ovenia
Korea
weden
erland
USSR
ngdom

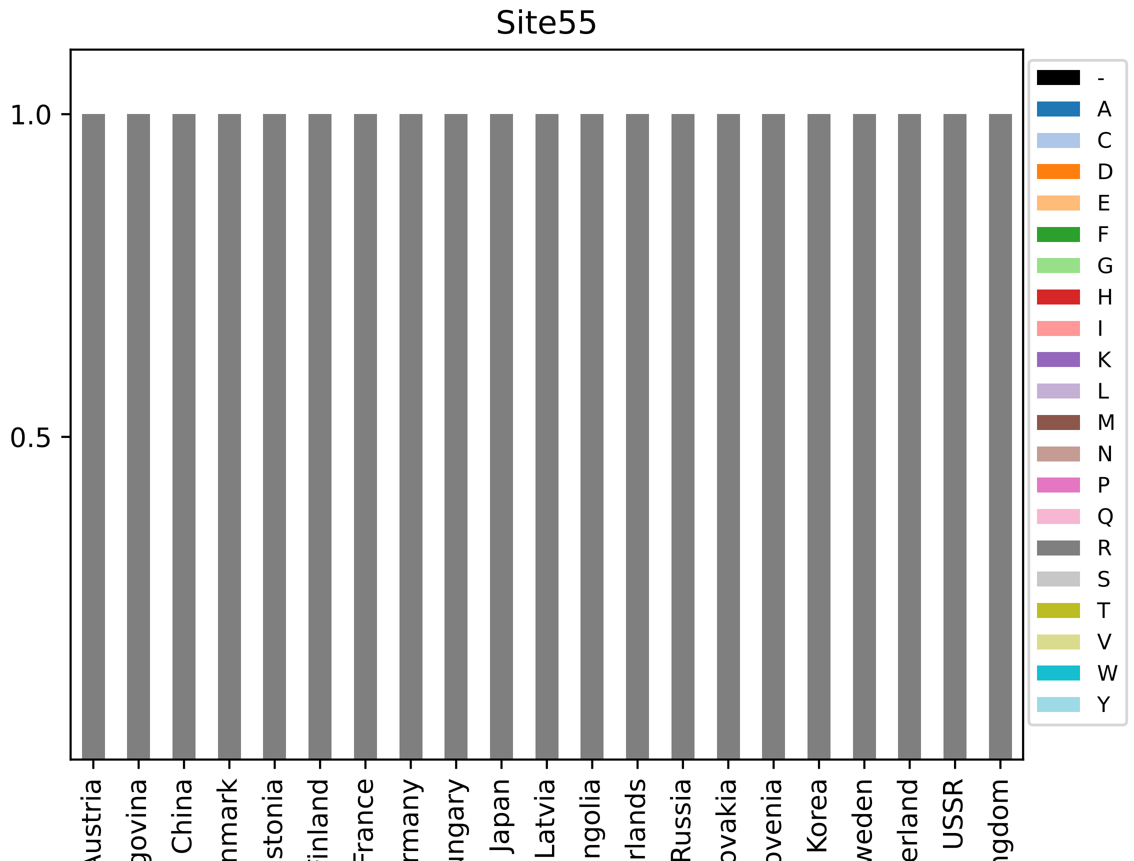
Site55
1.0
0.5
-
A
C
D
E
F
G
H
I
K
L
M
N
P
Q
R
S
T
V
W
Y
Austria
govina
China
nmark
stonia
Finland
France
rmany
ungary
Japan
Latvia
ngolia
rlands
Russia
ovakia
ovenia
Korea
weden
erland
USSR
ngdom

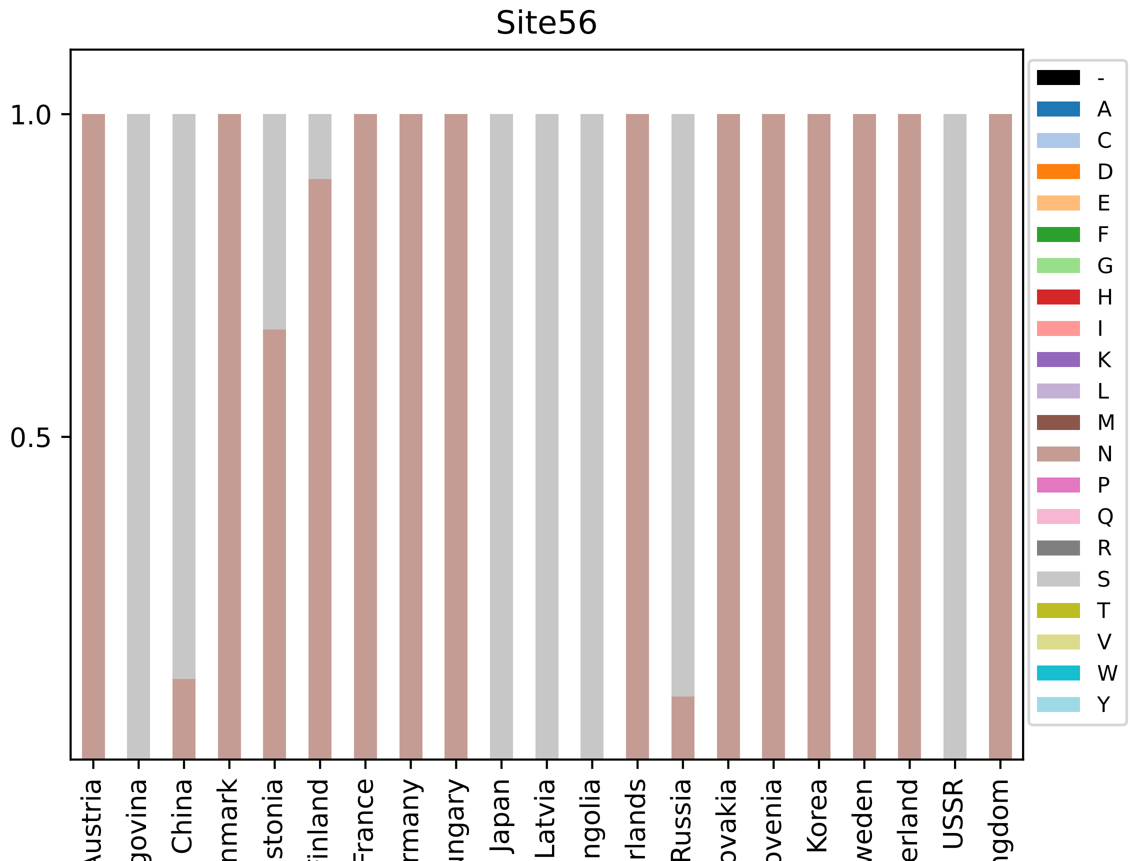

Site56
1.0
0.5
-
A
C
D
E
F
G
H
I
K
L
M
N
P
Q
R
S
T
V
W
Y
China
Japan
Latvia
Russia
Korea
USSR

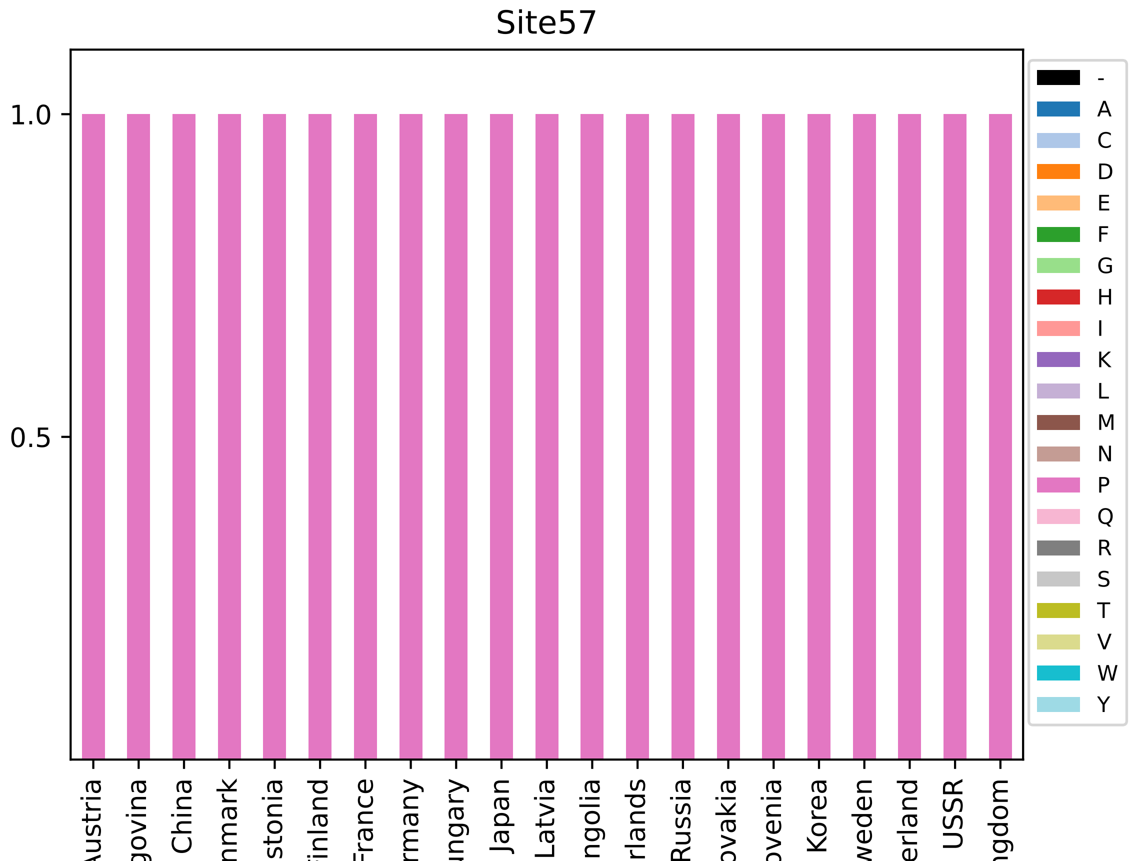

Site57
1.0
0.5
-
A
C
D
E
F
G
H
I
K
L
M
N
P
Q
R
S
T
V
W
Y
China
Japan
Latvia
Russia
Korea
USSR

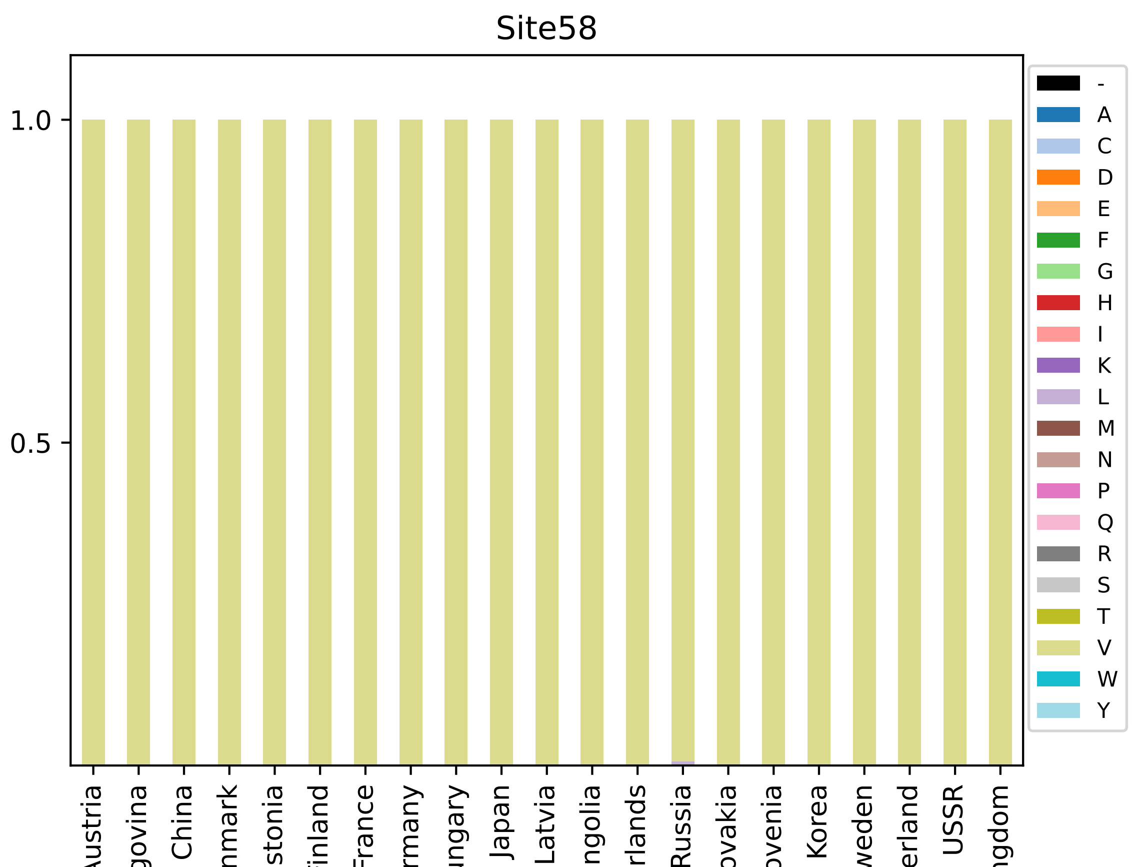

Site58
1.0
0.5
-
A
C
D
E
F
G
H
I
K
L
M
N
P
Q
R
S
T
V
W
Y
Austria
China
Japan
Latvia
Russia
Korea
USSR

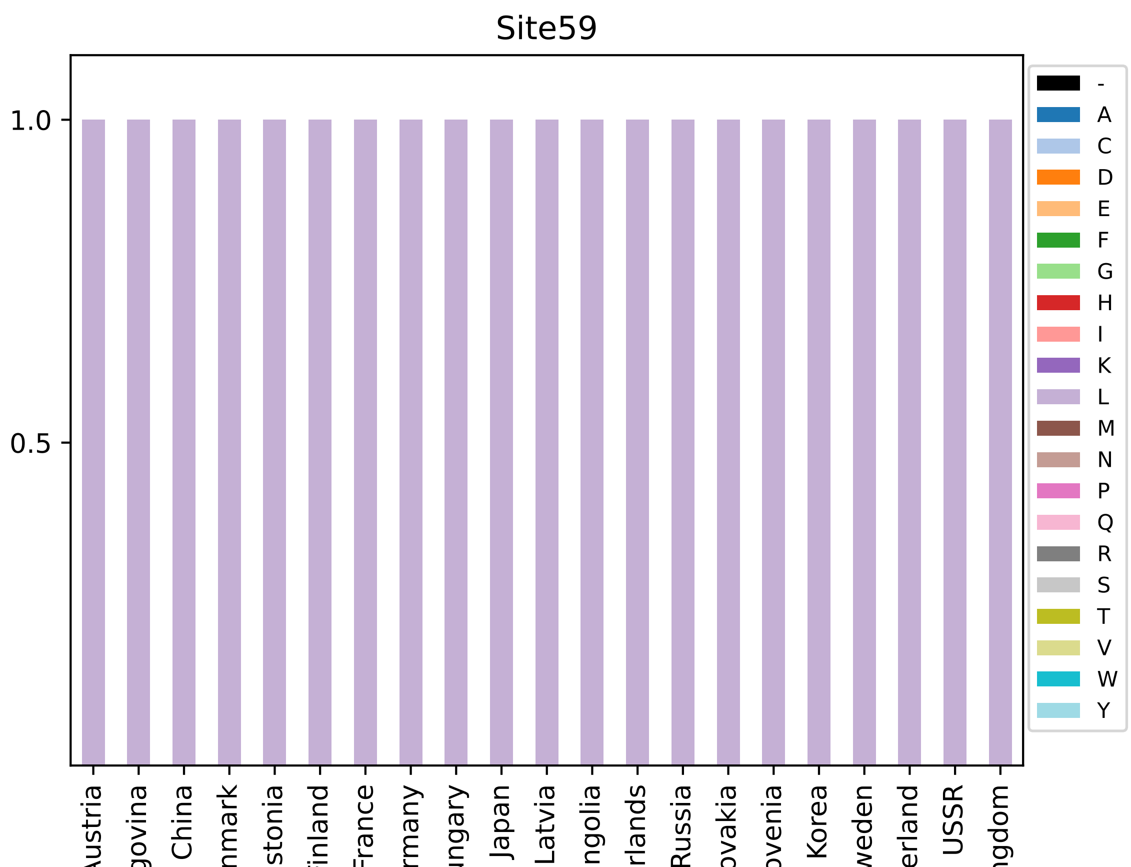
Site59
1.0
0.5
Austria
govina
China
nmark
stonia
Finland
France
rmany
ungary
Japan
Latvia
ngolia
rlands
Russia
ovakia
ovenia
Korea
weden
erland
USSR
ngdom
-
A
C
D
E
F
G
H
I
K
L
M
N
P
Q
R
S
T
V
W
Y

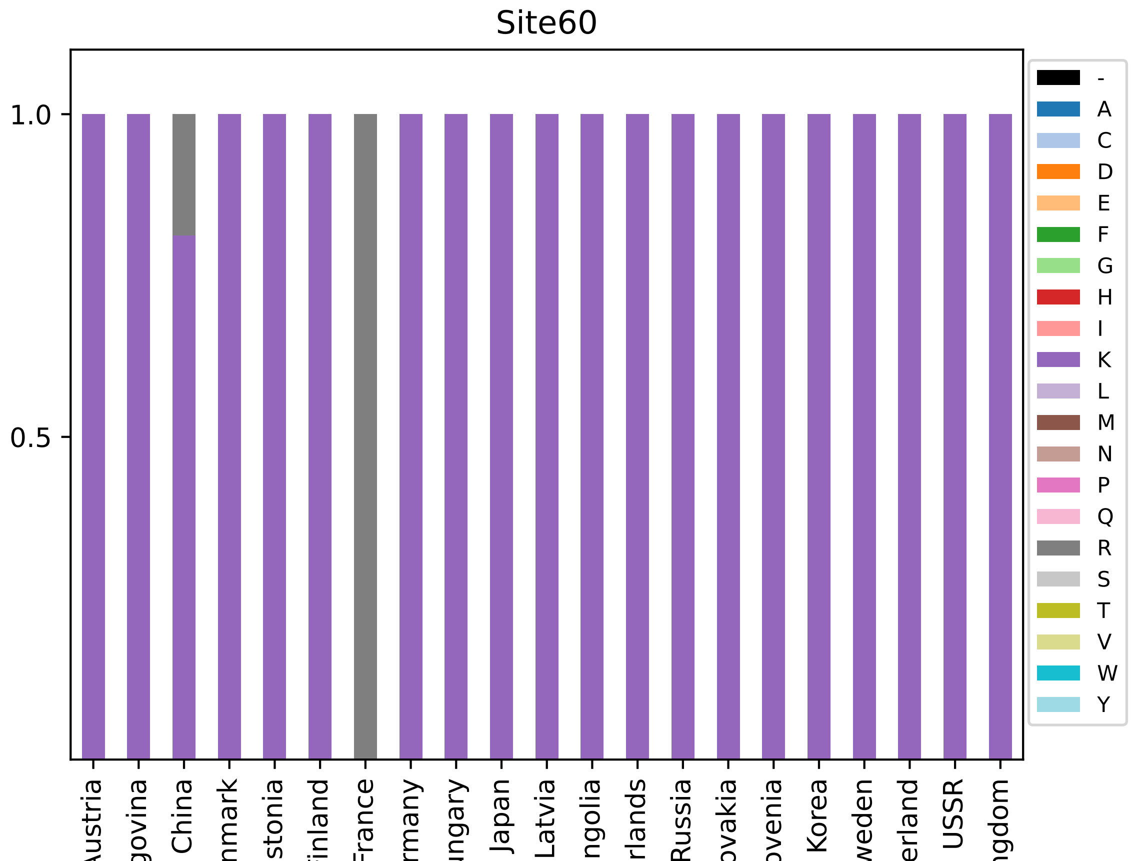

Site60
1.0
0.5
-
A
C
D
E
F
G
H
I
K
L
M
N
P
Q
R
S
T
V
W
Y
Austria
govina
China
nmark
stonia
Finland
France
rmany
ungary
Japan
Latvia
ngolia
rlands
Russia
ovakia
ovenia
Korea
weden
erland
USSR
ngdom

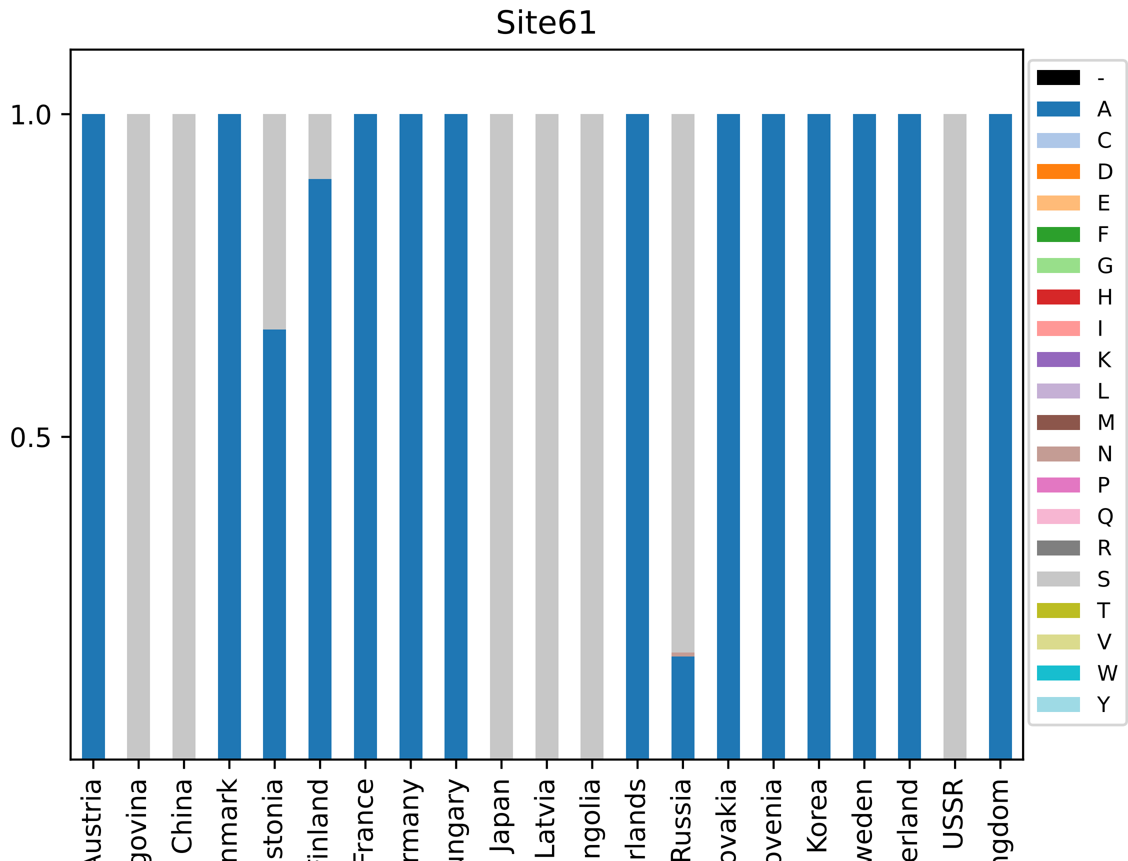
Site61
1.0
0.5
-
A
C
D
E
F
G
H
I
K
L
M
N
P
Q
R
S
T
V
W
Y
China
Japan
Latvia
Russia
Korea
USSR

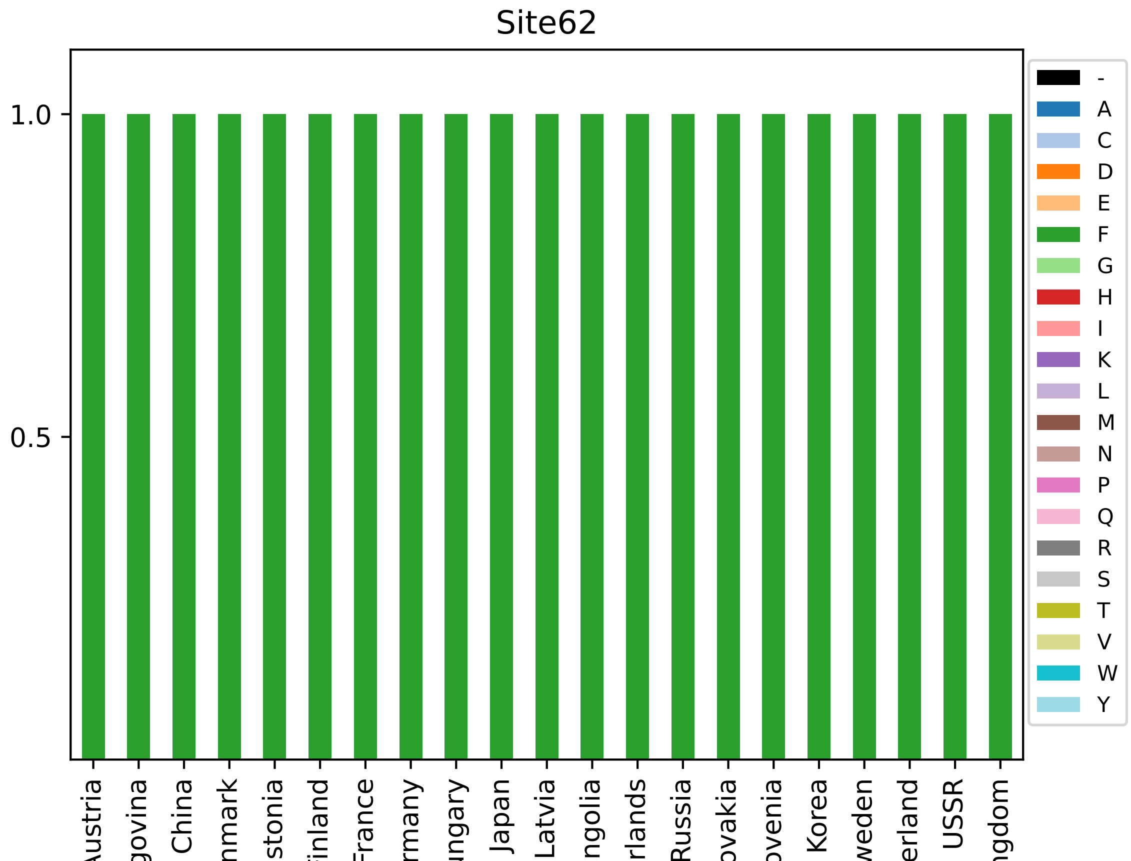
Site62
1.0
0.5
-
A
C
D
E
F
G
H
I
K
L
M
N
P
Q
R
S
T
V
W
Y
China
Japan
Latvia
Russia
Korea
USSR

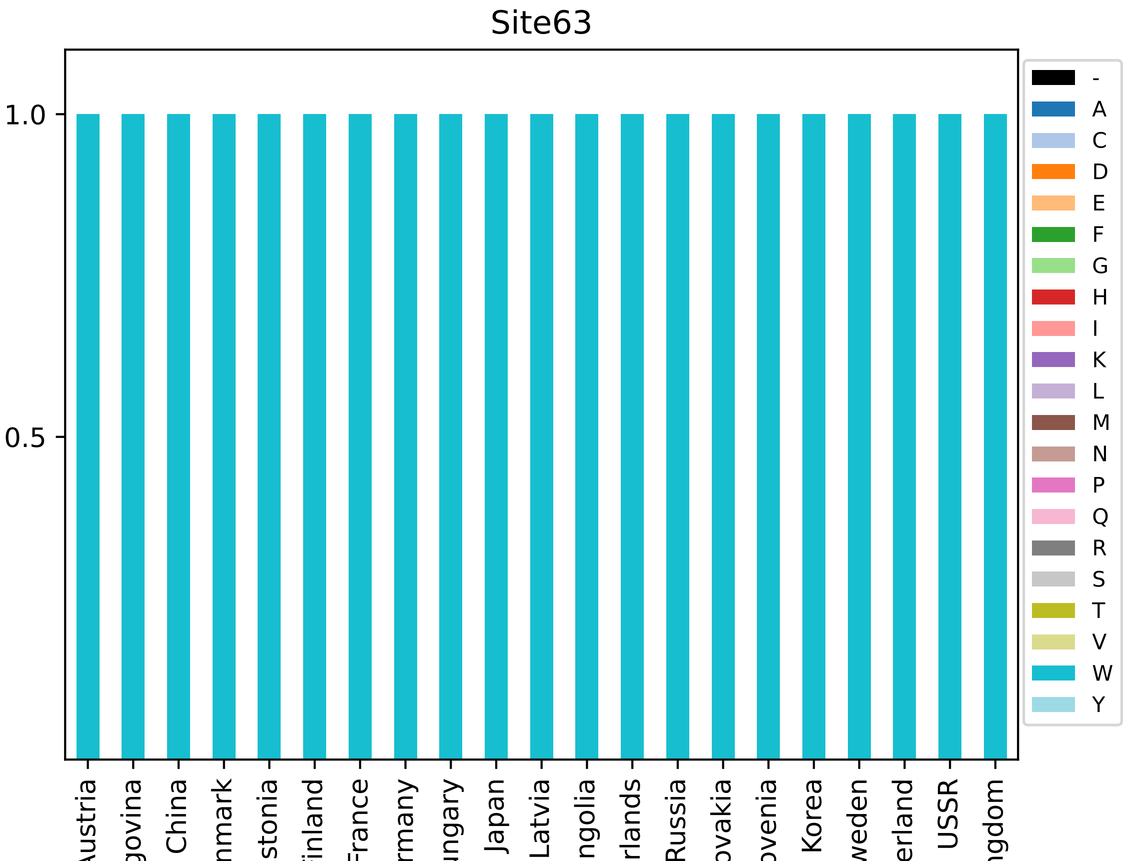
Site63
1.0
0.5
-
A
C
D
E
F
G
H
I
K
L
M
N
P
Q
R
S
T
V
W
Y
China
Japan
Latvia
Russia
Korea
USSR

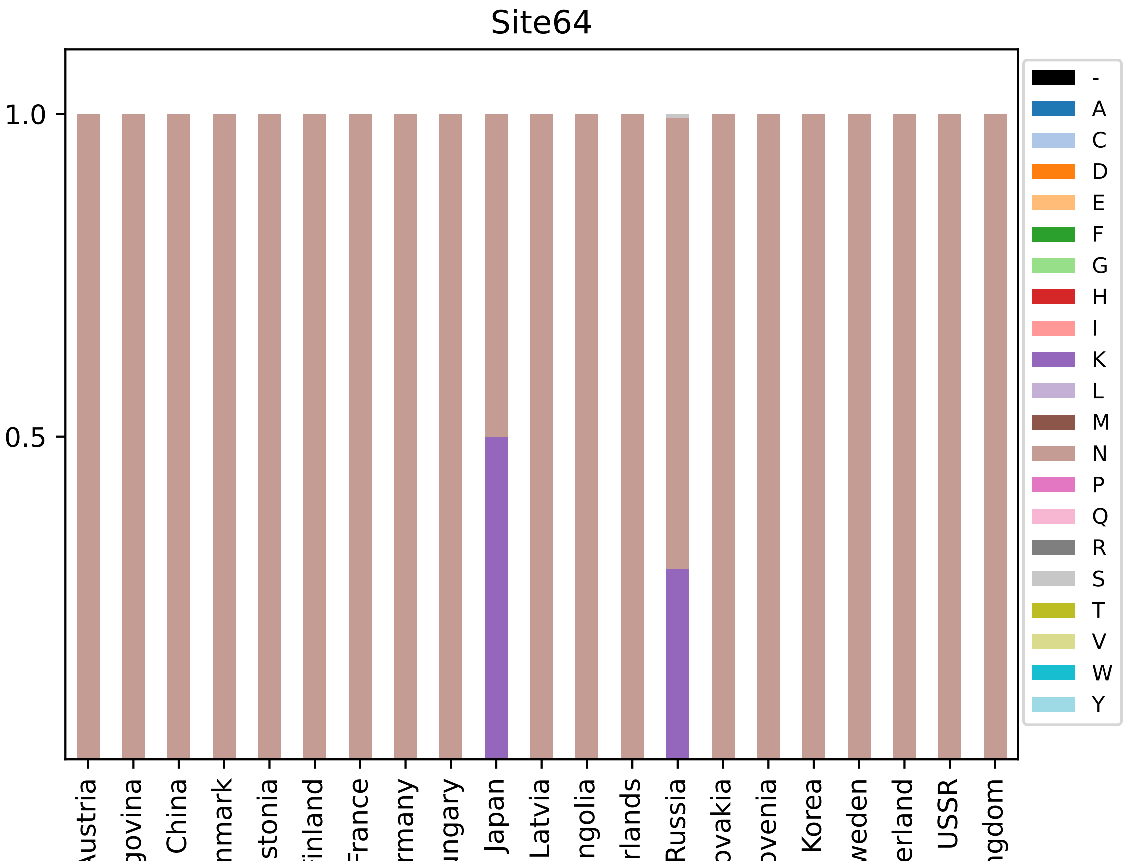
Site64
1.0
0.5
-
A
C
D
E
F
G
H
I
K
L
M
N
P
Q
R
S
T
V
W
Y
ustria
govina
China
nmark
stonia
inland
rance
rmany
ungary
Japan
Latvia
ngolia
rlands
Russia
ovakia
ovenia
Korea
weden
erland
USSR
ngdom

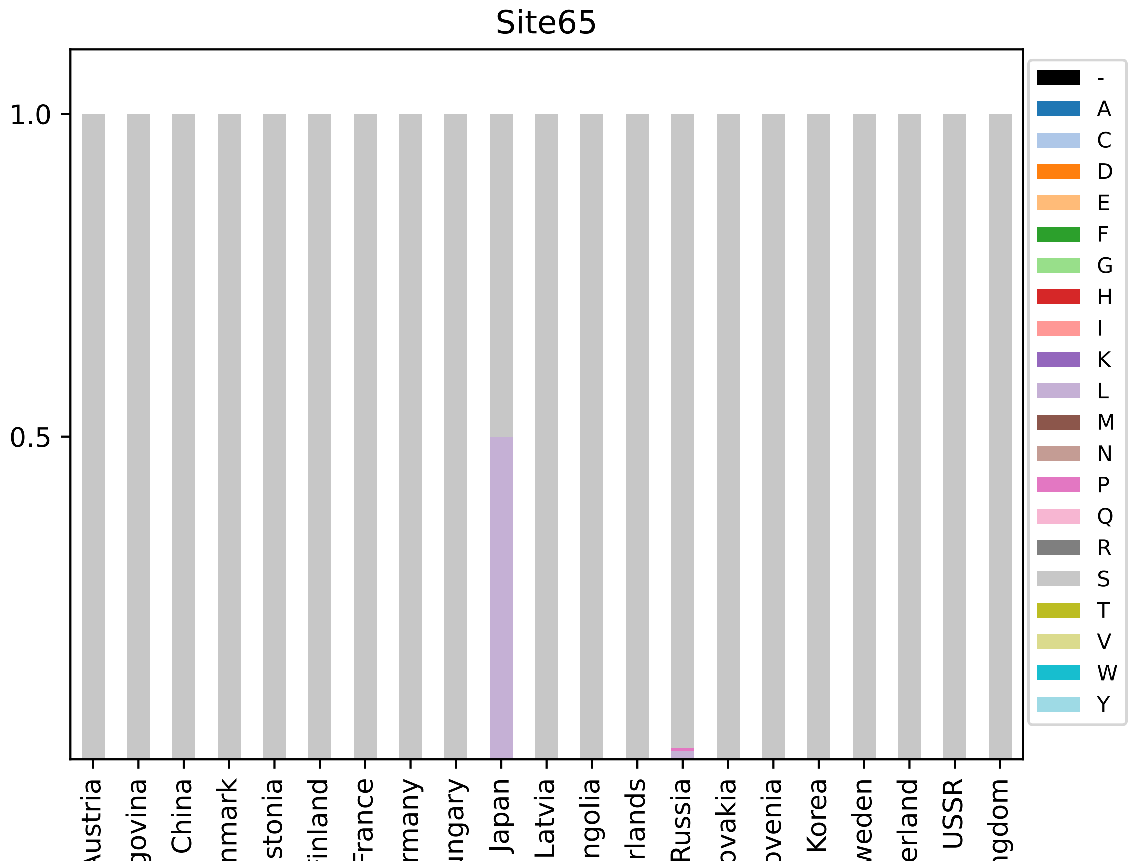
Site65
1.0
0.5
-
A
C
D
E
F
G
H
I
K
L
M
N
P
Q
R
S
T
V
W
Y
Austria
China
Finland
France
Japan
Latvia
Russia
Korea
USSR

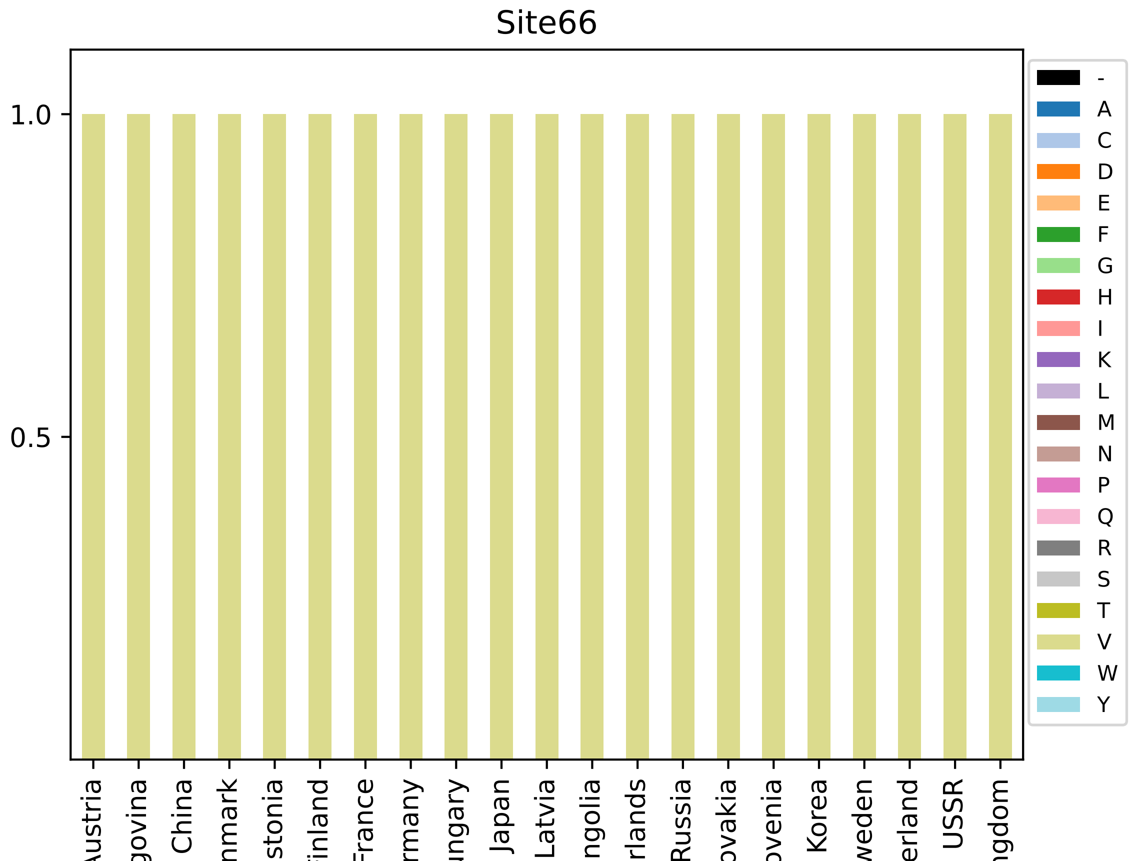
Site66
1.0
0.5
-
A
C
D
E
F
G
H
I
K
L
M
N
P
Q
R
S
T
V
W
Y
Austria
China
Japan
Latvia
Russia
Korea
USSR

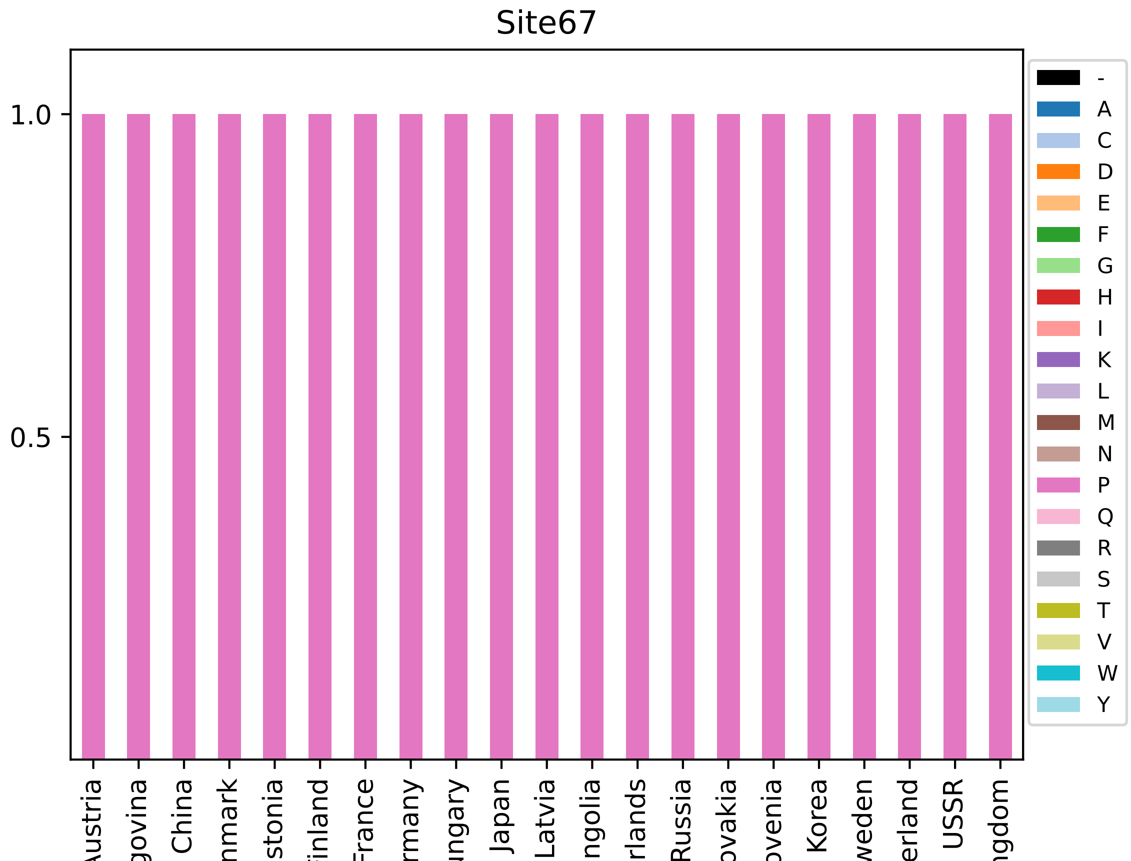
Site67
1.0
0.5
-
A
C
D
E
F
G
H
I
K
L
M
N
P
Q
R
S
T
V
W
Y
Austria
China
Japan
Latvia
Russia
Korea
USSR

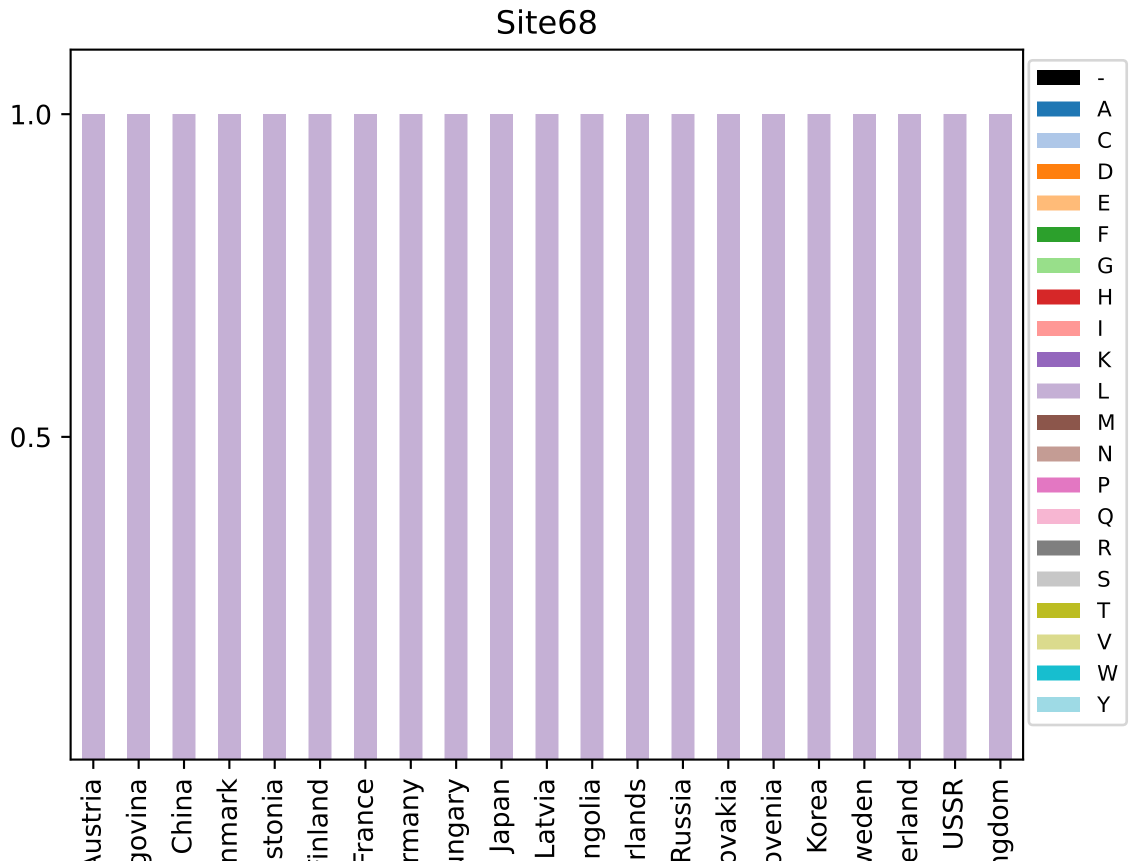
Site68
1.0
0.5
-
A
C
D
E
F
G
H
I
K
L
M
N
P
Q
R
S
T
V
W
Y
China
Japan
Latvia
Russia
Korea
USSR

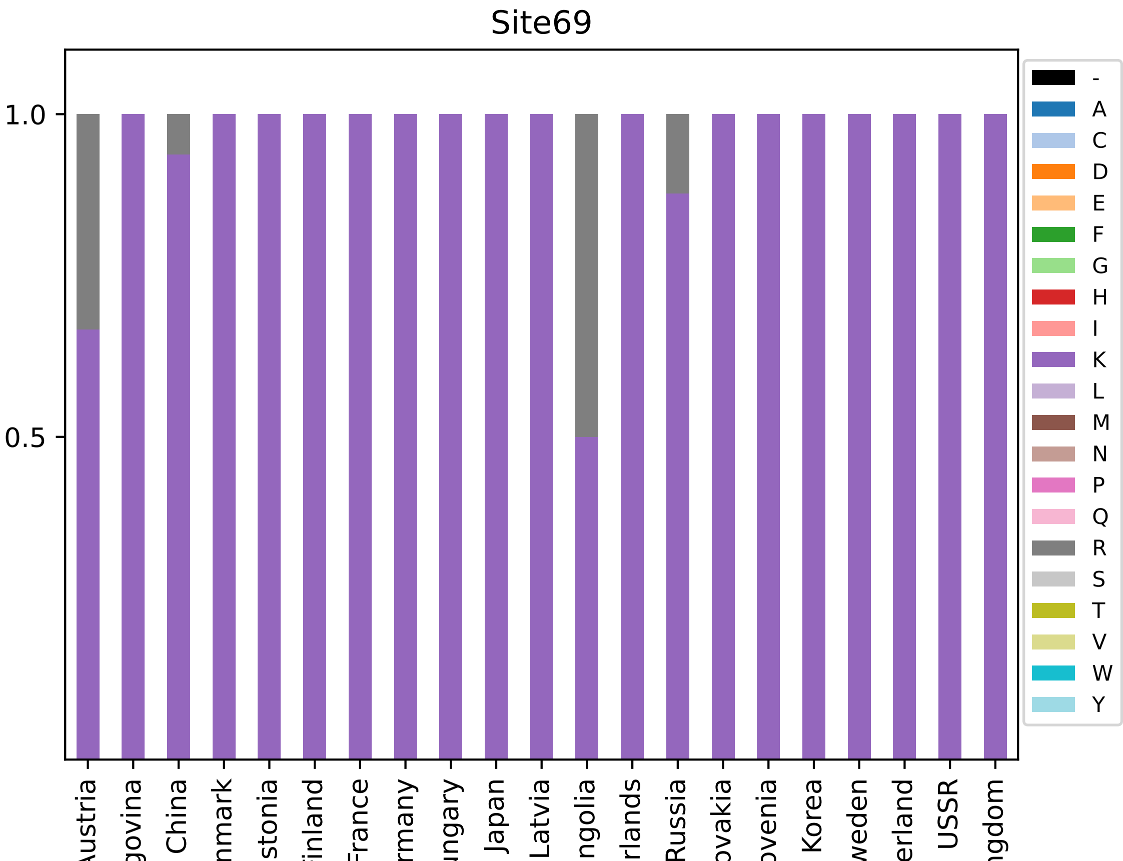
Site69
1.0
0.5
-
A
C
D
E
F
G
H
I
K
L
M
N
P
Q
R
S
T
V
W
Y
China
Japan
Latvia
Russia
Korea
USSR

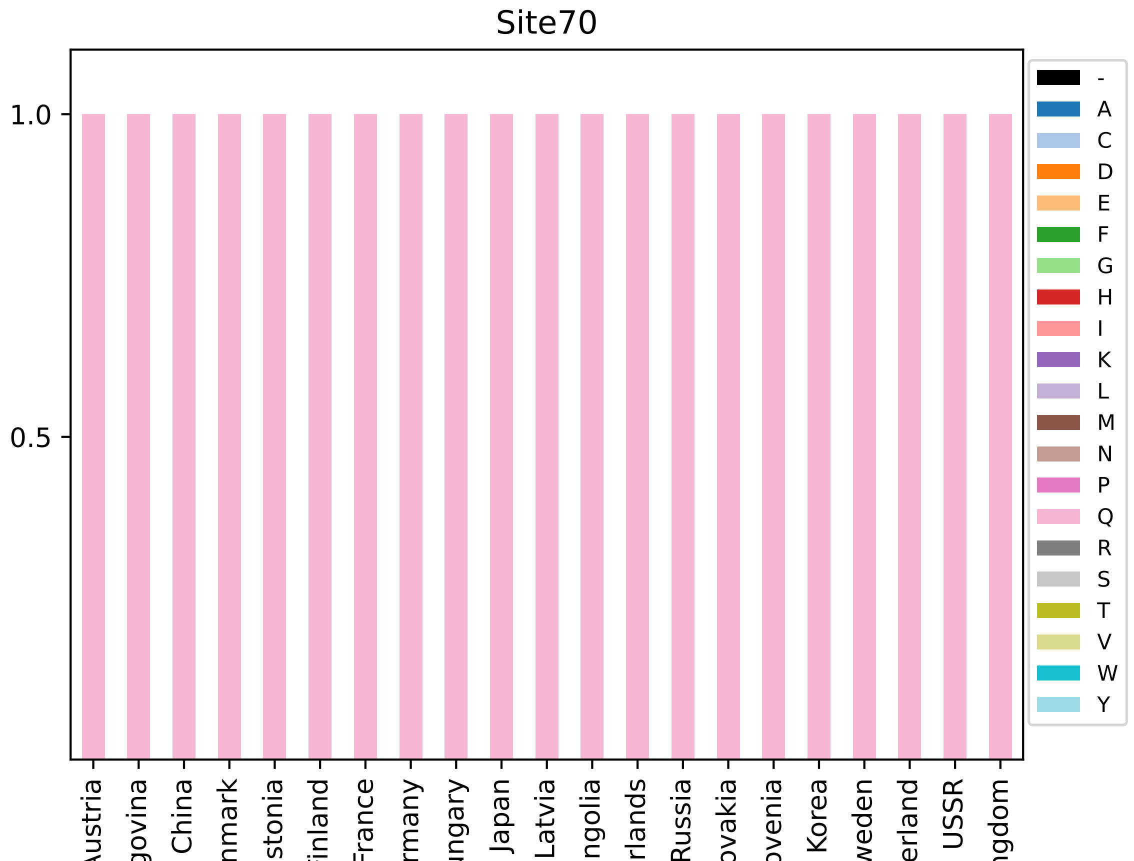

Site70
1.0
0.5
-
A
C
D
E
F
G
H
I
K
L
M
N
P
Q
R
S
T
V
W
Y
Austria
govina
China
nmark
stonia
inland
France
rmany
ungary
Japan
Latvia
ngolia
rlands
Russia
ovakia
ovenia
Korea
weden
erland
USSR
ngdom

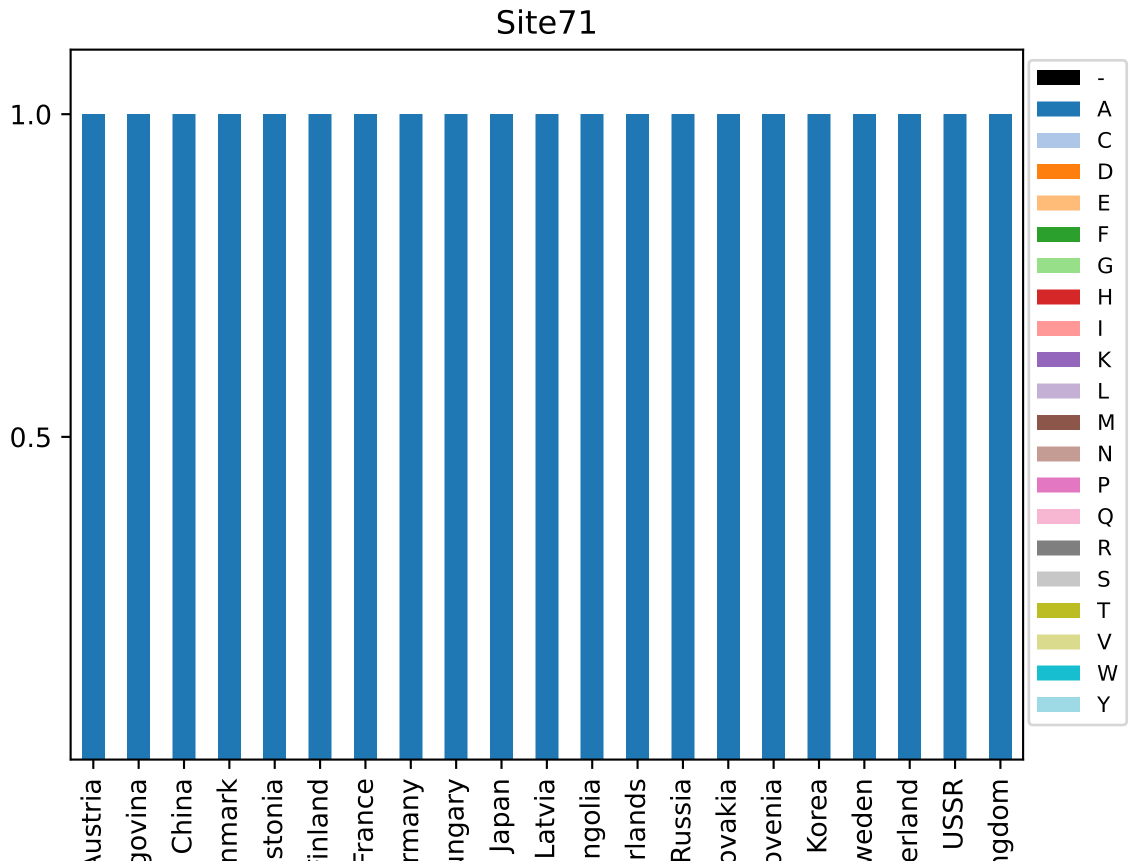
Site71
1.0
0.5
-
A
C
D
E
F
G
H
I
K
L
M
N
P
Q
R
S
T
V
W
Y
China
Japan
Latvia
Russia
Korea
USSR

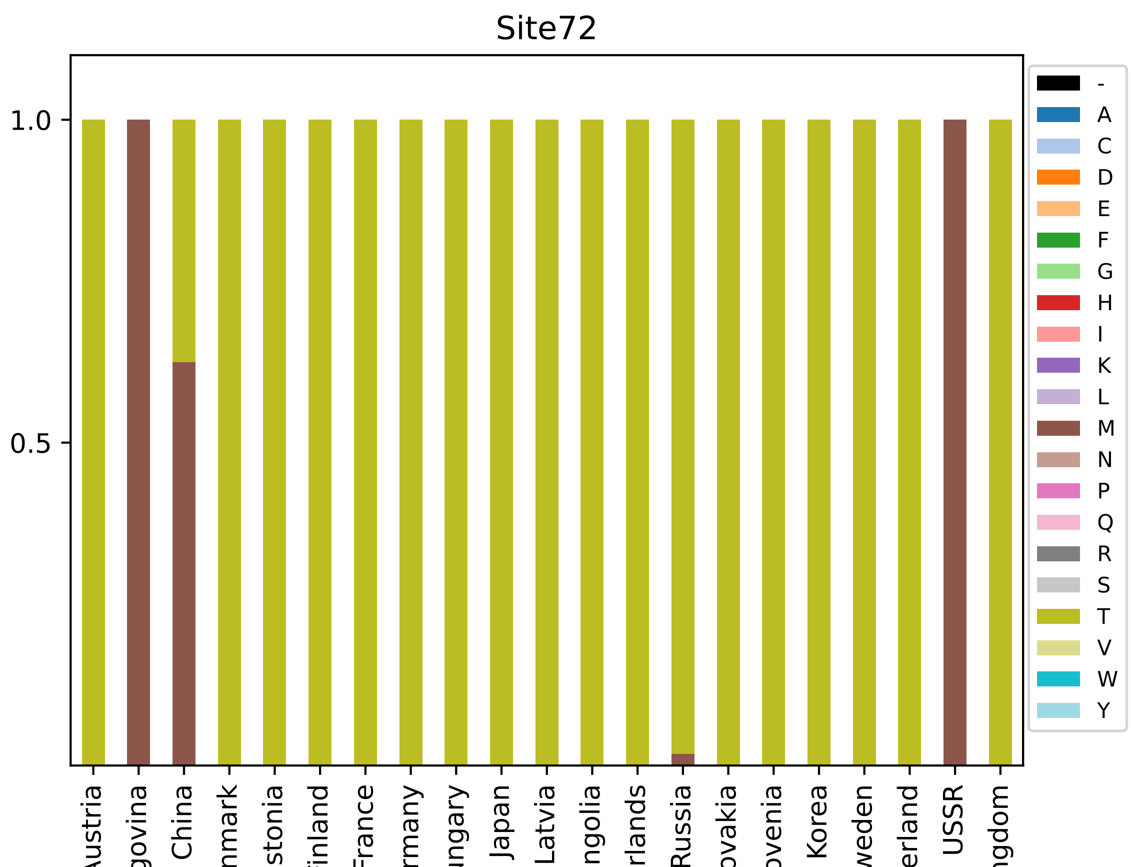
Site72
1.0
0.5
-
A
C
D
E
F
G
H
I
K
L
M
N
P
Q
R
S
T
V
W
Y
Austria
govina
China
nmark
stonia
inland
France
rmany
ungary
Japan
Latvia
ngolia
rlands
Russia
ovakia
ovenia
Korea
weden
erland
USSR
ngdom

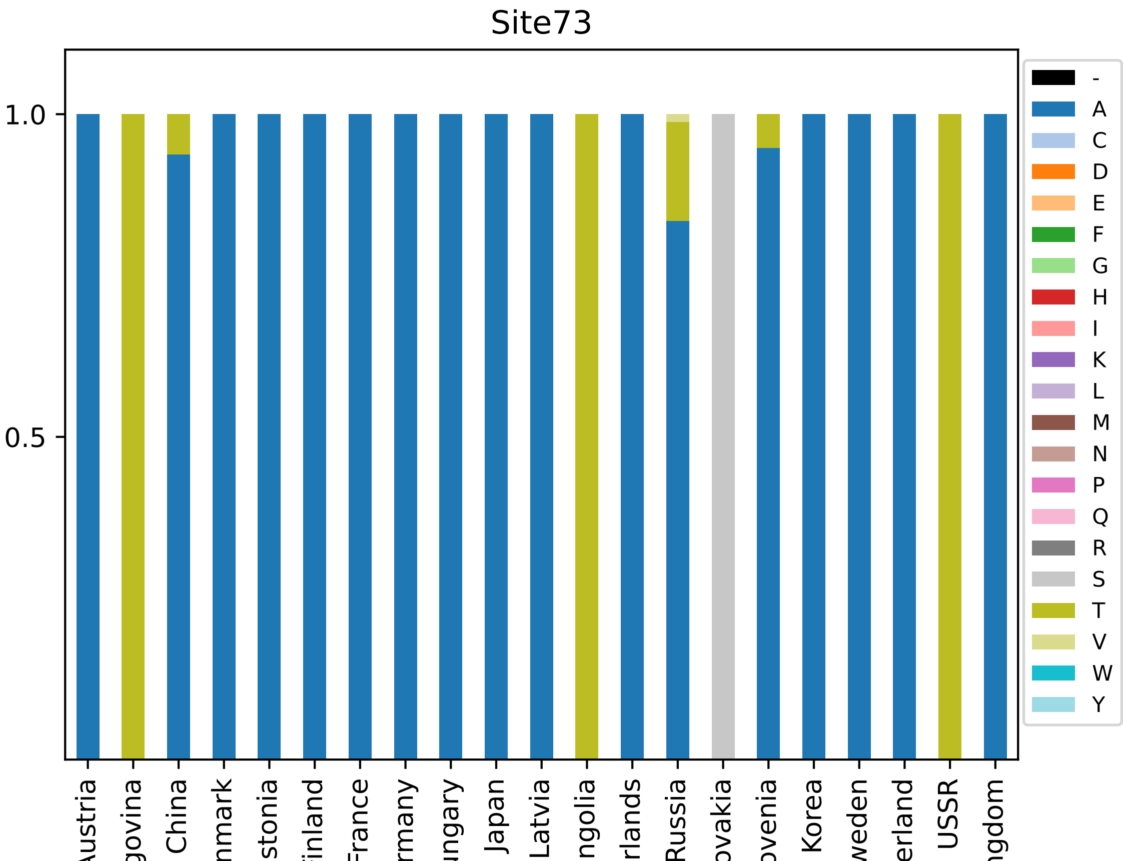

Site73
1.0
0.5
-
A
C
D
E
F
G
H
I
K
L
M
N
P
Q
R
S
T
V
W
Y
Austria
China
Japan
Latvia
Russia
Korea
USSR

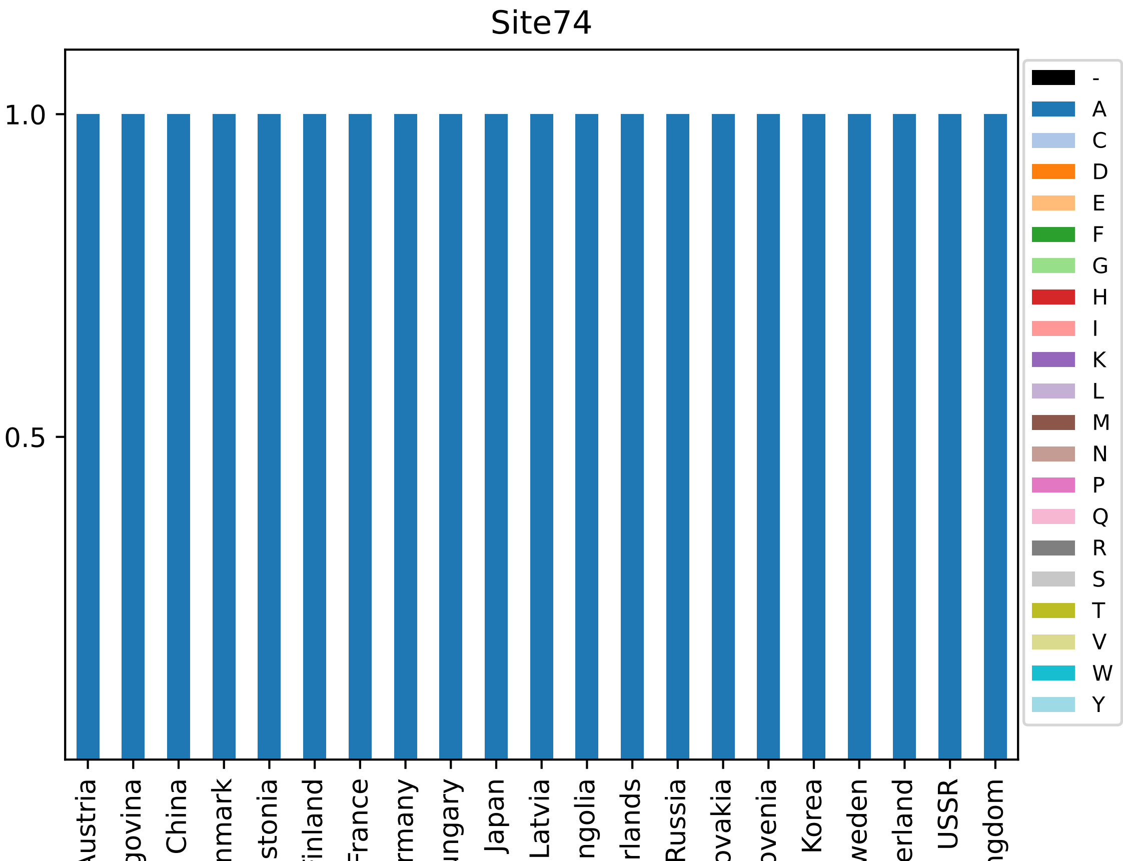
Site74
1.0
0.5
-
A
C
D
E
F
G
H
I
K
L
M
N
P
Q
R
S
T
V
W
Y
China
Japan
Latvia
Russia
Korea
USSR

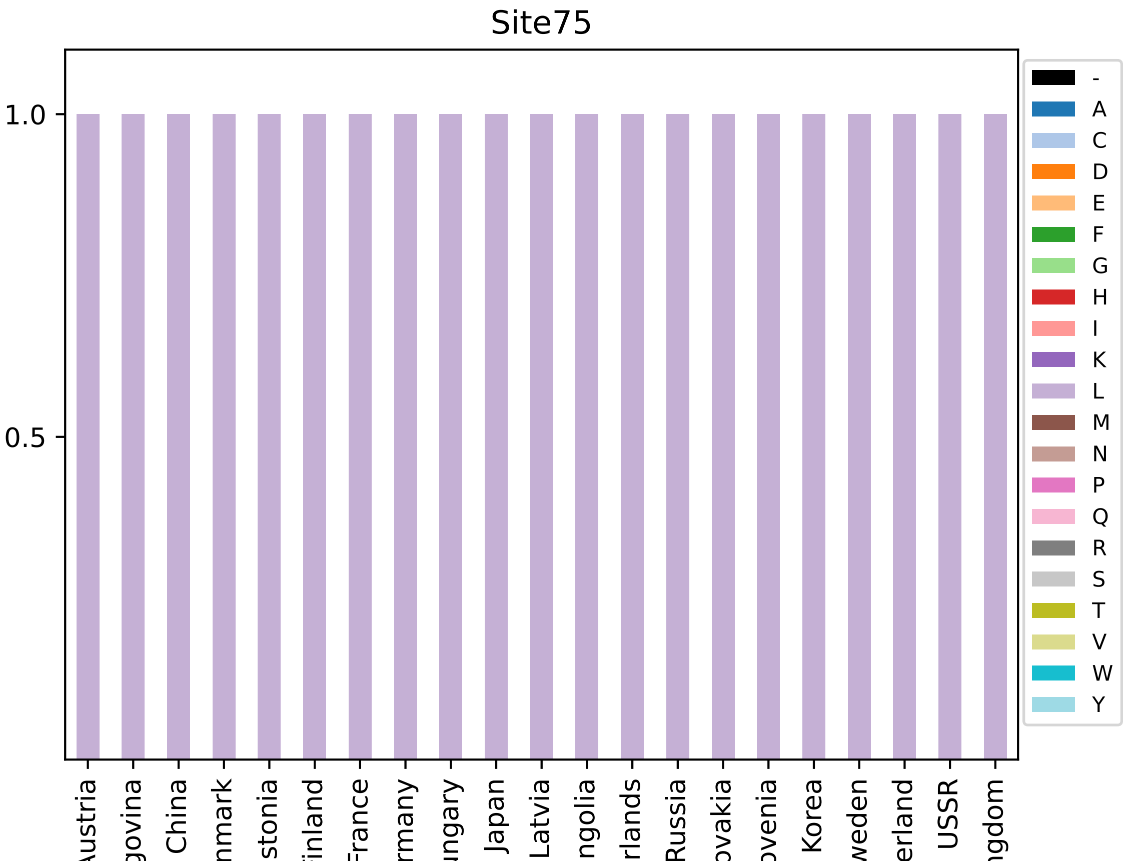

Site75
1.0
0.5
-
A
C
D
E
F
G
H
I
K
L
M
N
P
Q
R
S
T
V
W
Y
China
Japan
Latvia
Russia
Korea
USSR

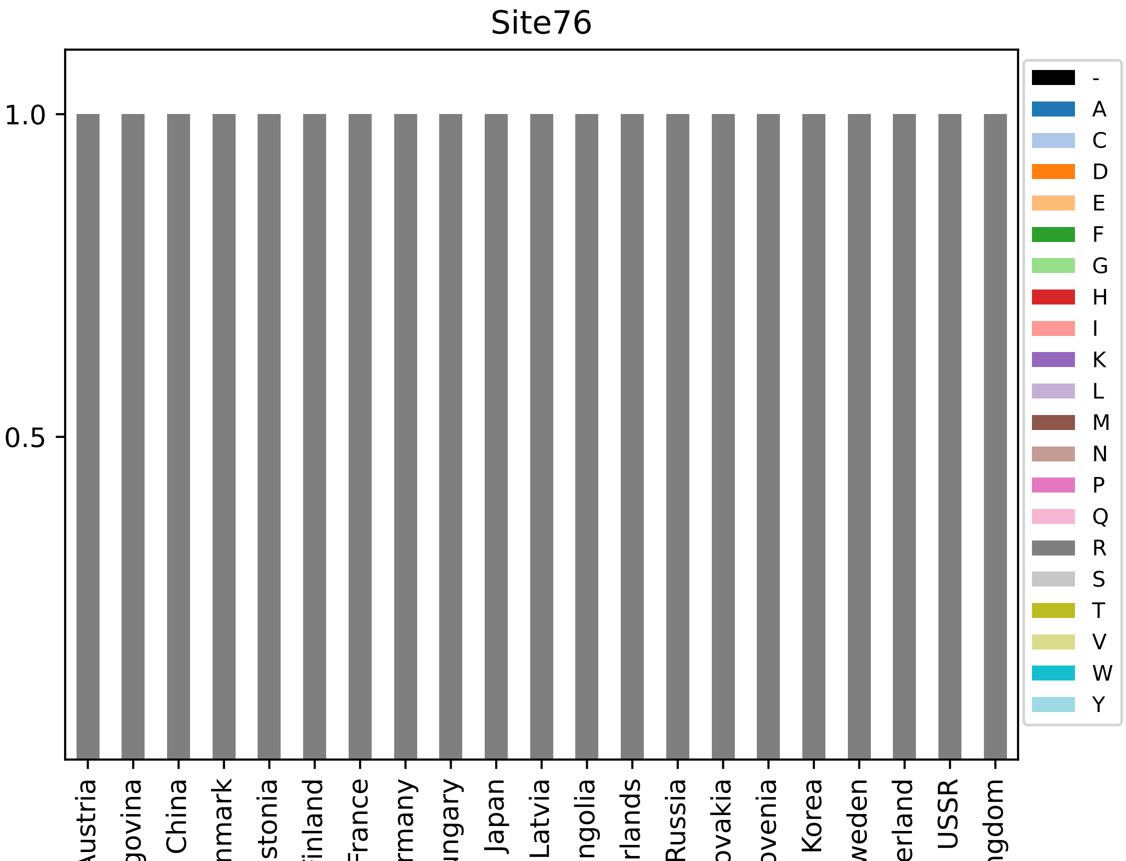

Site76
1.0
0.5
-
A
C
D
E
F
G
H
I
K
L
M
N
P
Q
R
S
T
V
W
Y
Austria
China
Japan
Latvia
Korea
USSR

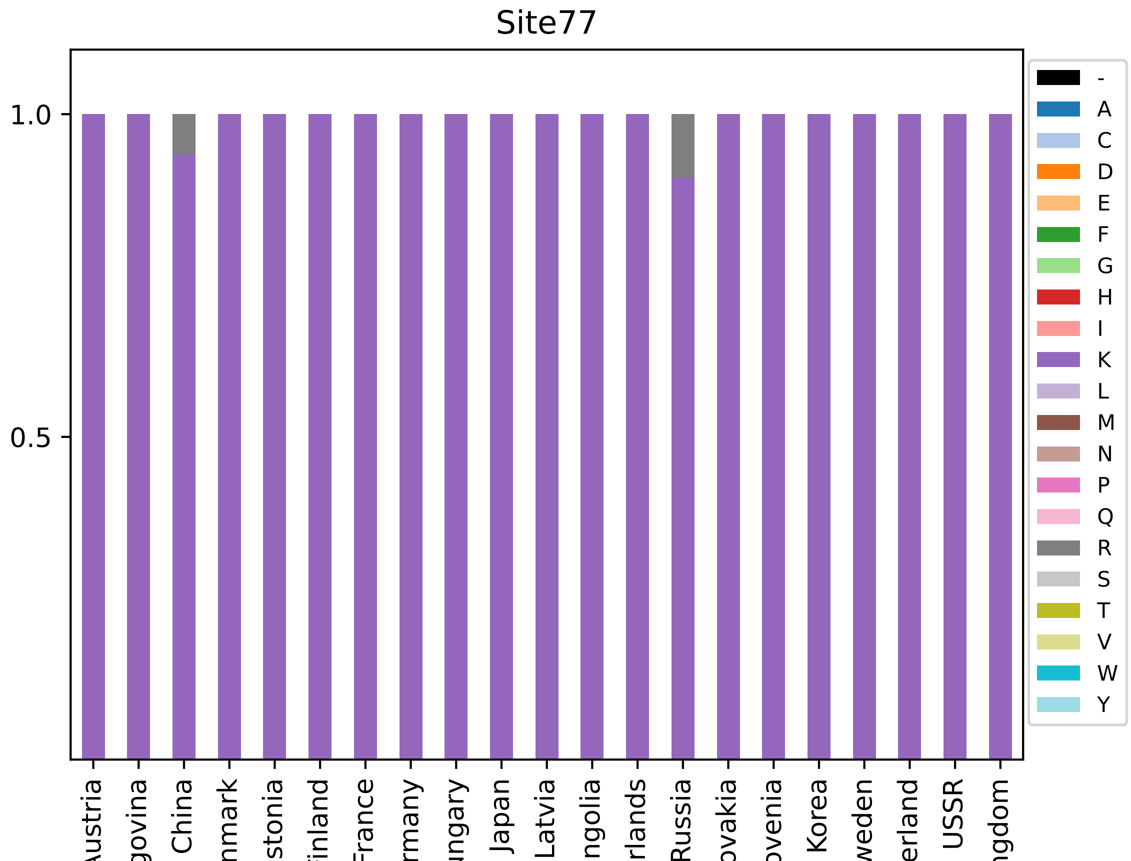
Site77
1.0
0.5
-
A
C
D
E
F
G
H
I
K
L
M
N
P
Q
R
S
T
V
W
Y
Austria
govina
China
nmark
stonia
inland
France
rmany
ungary
Japan
Latvia
ngolia
rlands
Russia
ovakia
ovenia
Korea
weden
erland
USSR
ngdom

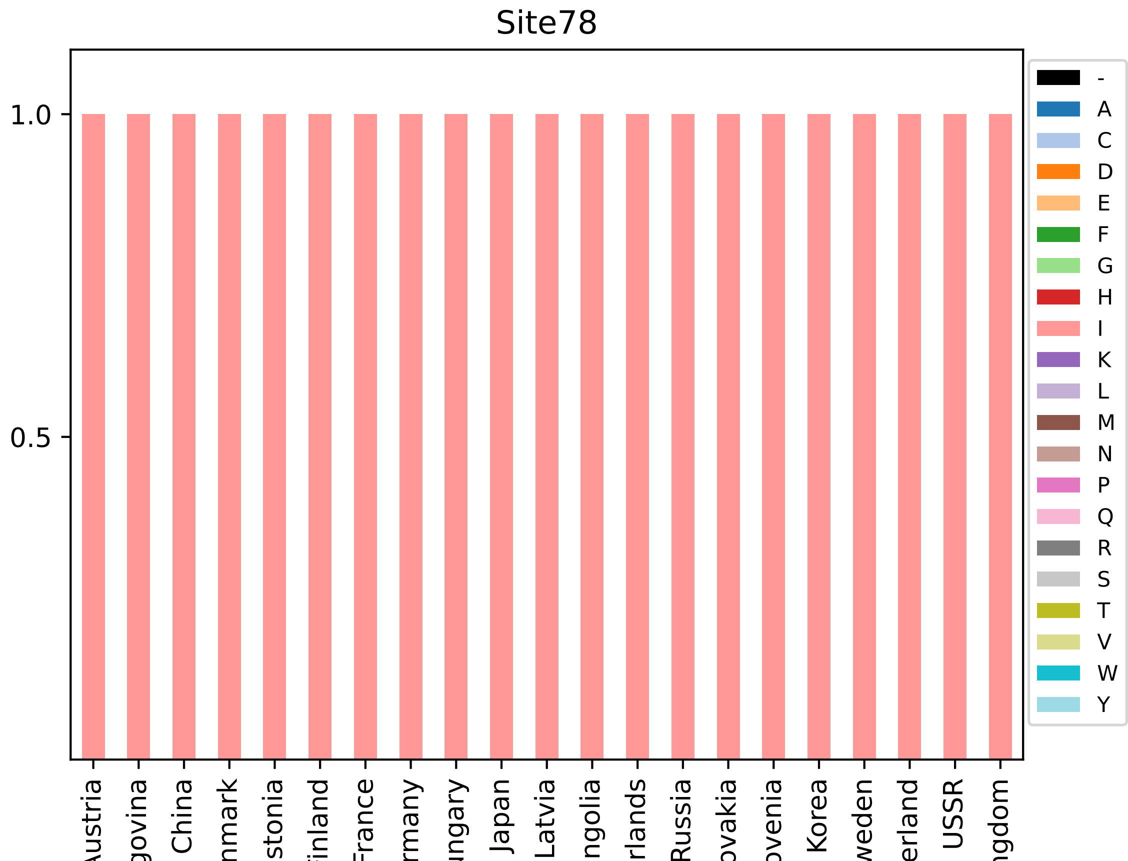
Site78
1.0
0.5
-
A
C
D
E
F
G
H
I
K
L
M
N
P
Q
R
S
T
V
W
Y
China
Japan
Latvia
Russia
Korea
USSR

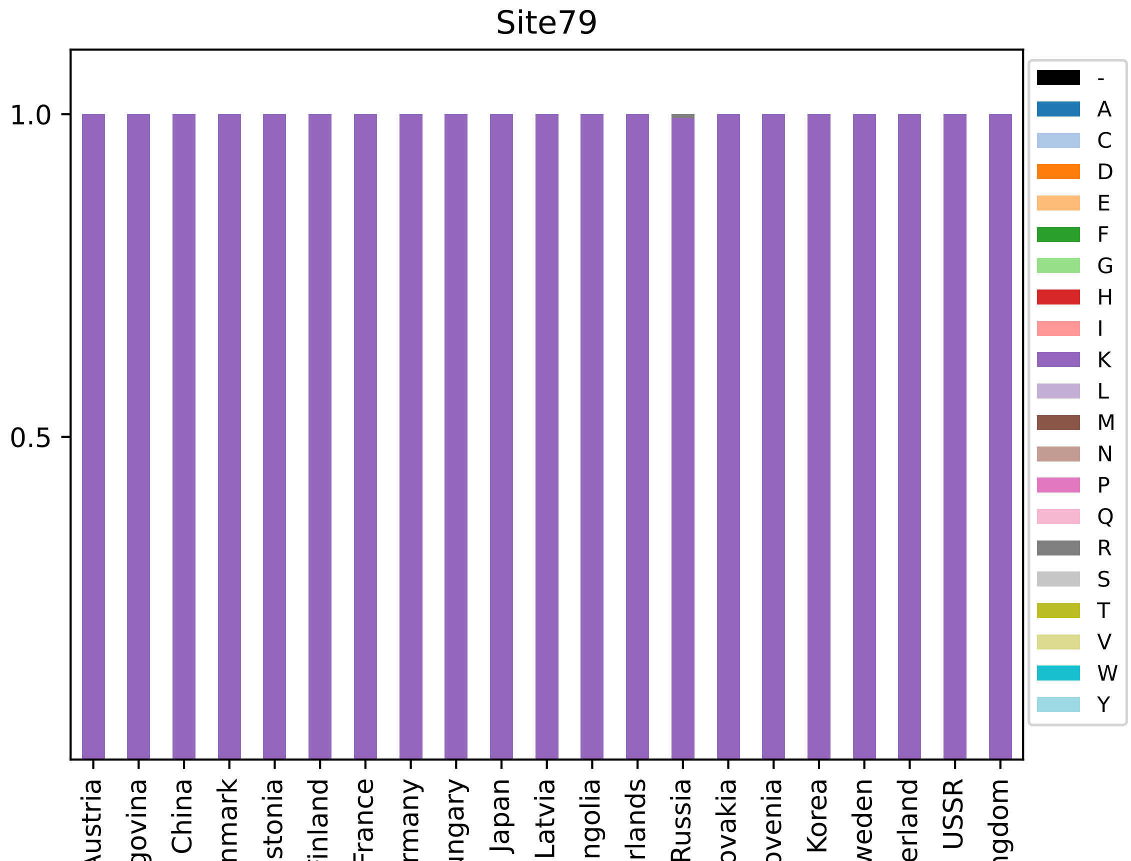

Site79
1.0
0.5
-
A
C
D
E
F
G
H
I
K
L
M
N
P
Q
R
S
T
V
W
Y
China
Japan
Latvia
Russia
Korea
USSR

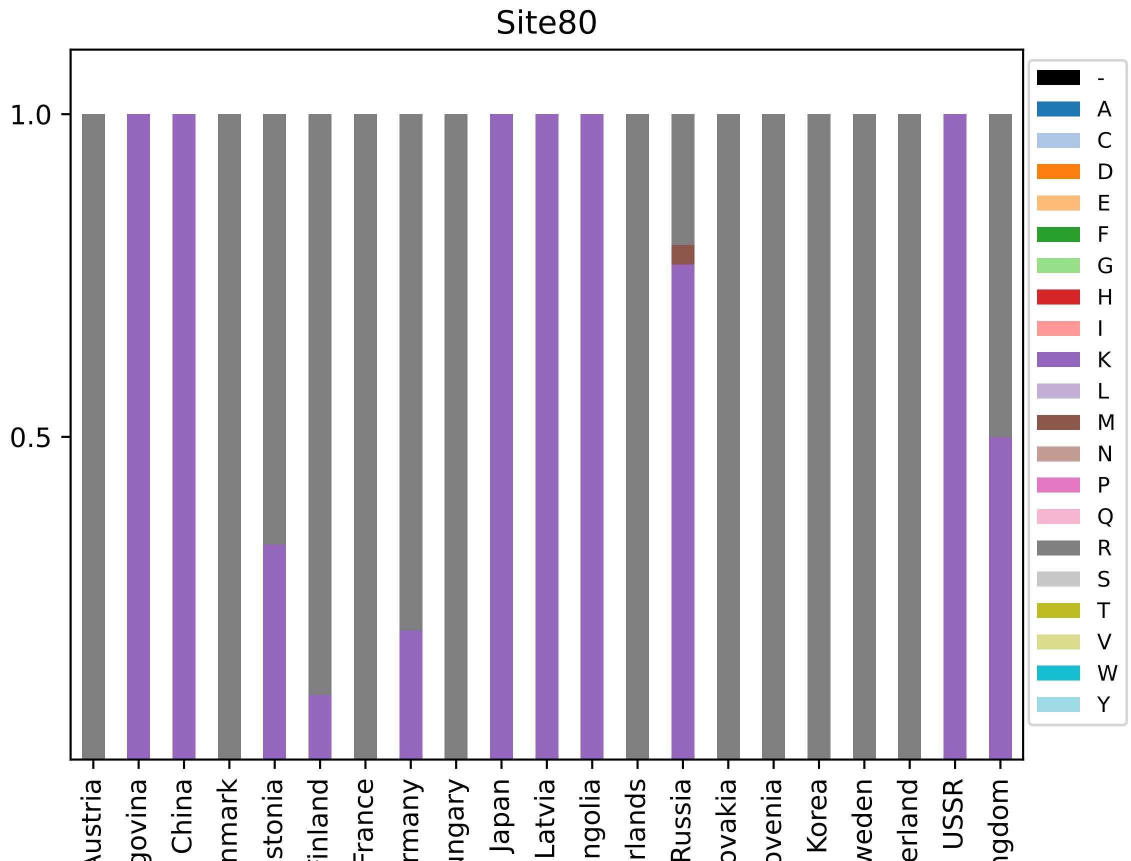
Site80
1.0
0.5
-
A
C
D
E
F
G
H
I
K
L
M
N
P
Q
R
S
T
V
W
Y
Austria
govina
China
nmark
stonia
Finland
France
rmany
ungary
Japan
Latvia
ngolia
rlands
Russia
ovakia
ovenia
Korea
weden
erland
USSR
ngdom

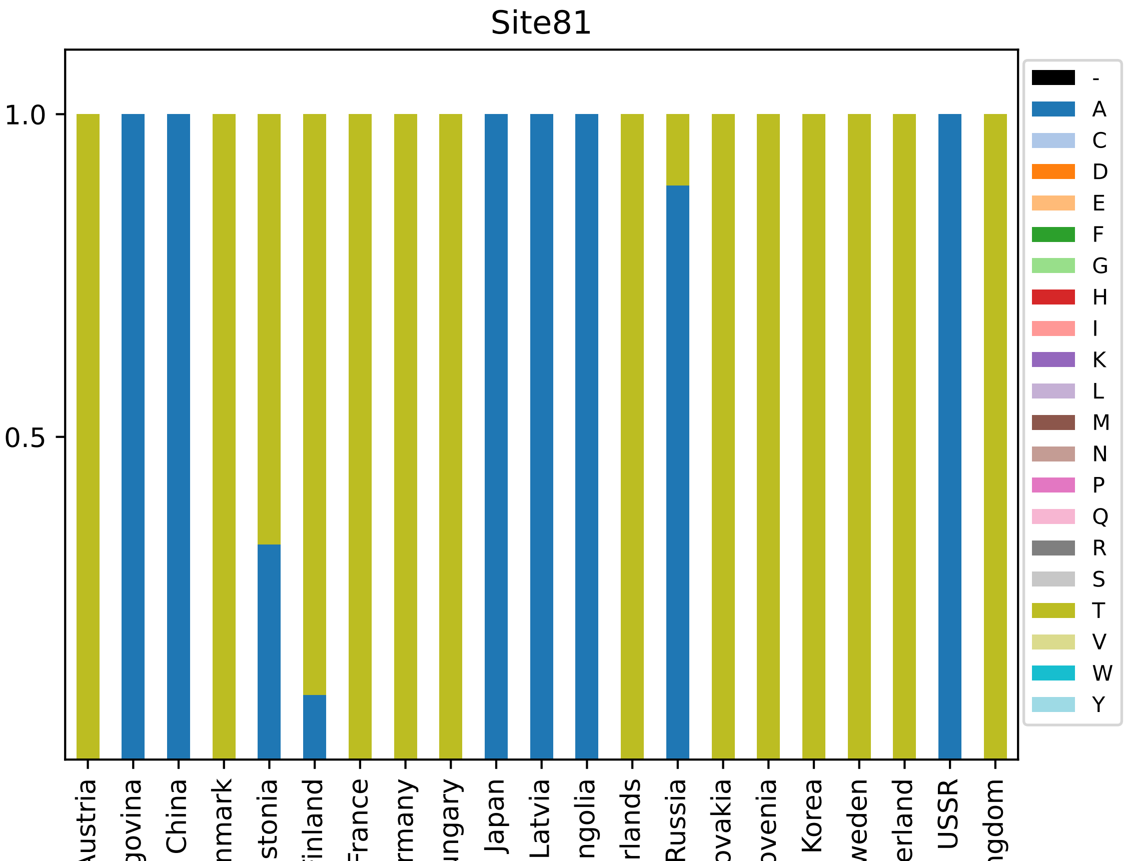
Site81
1.0
0.5
-
A
C
D
E
F
G
H
I
K
L
M
N
P
Q
R
S
T
V
W
Y
China
Japan
Latvia
Russia
Korea
USSR

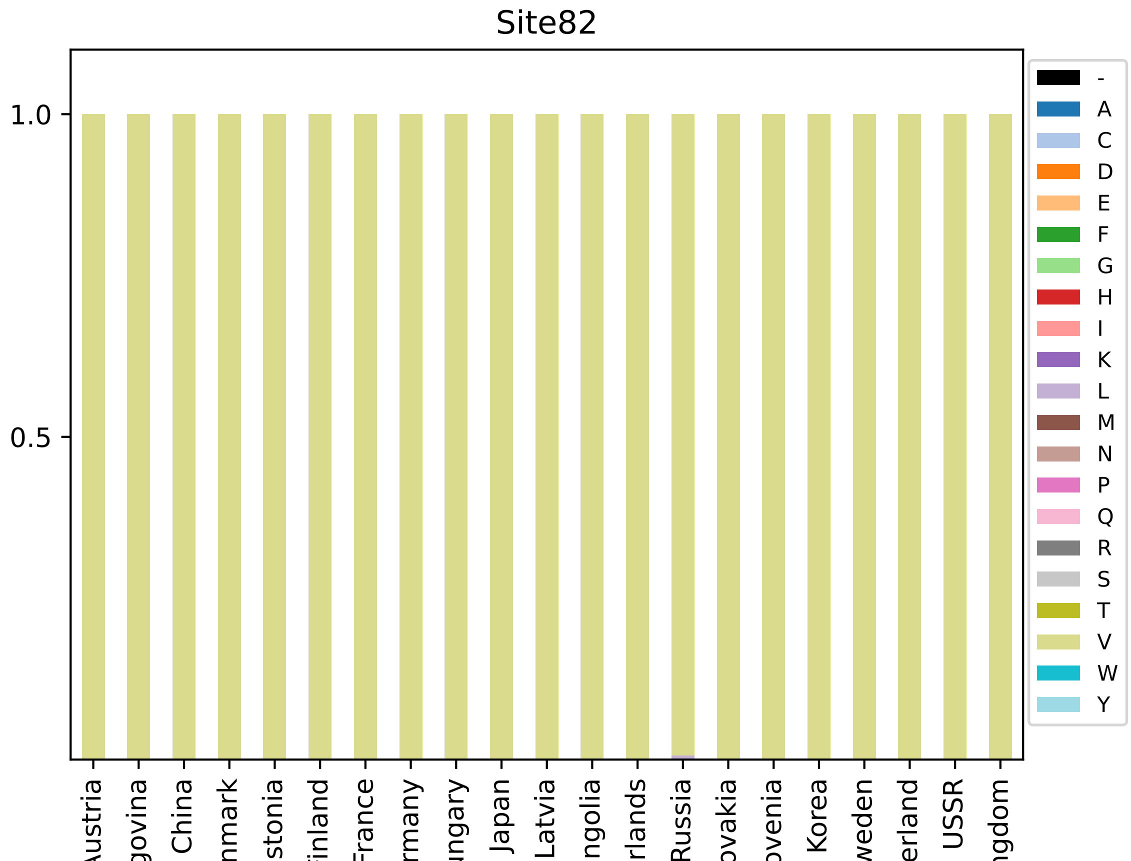
Site82
1.0
0.5
-
A
C
D
E
F
G
H
I
K
L
M
N
P
Q
R
S
T
V
W
Y
Austria
China
Finland
France
Japan
Latvia
Russia
Korea
USSR

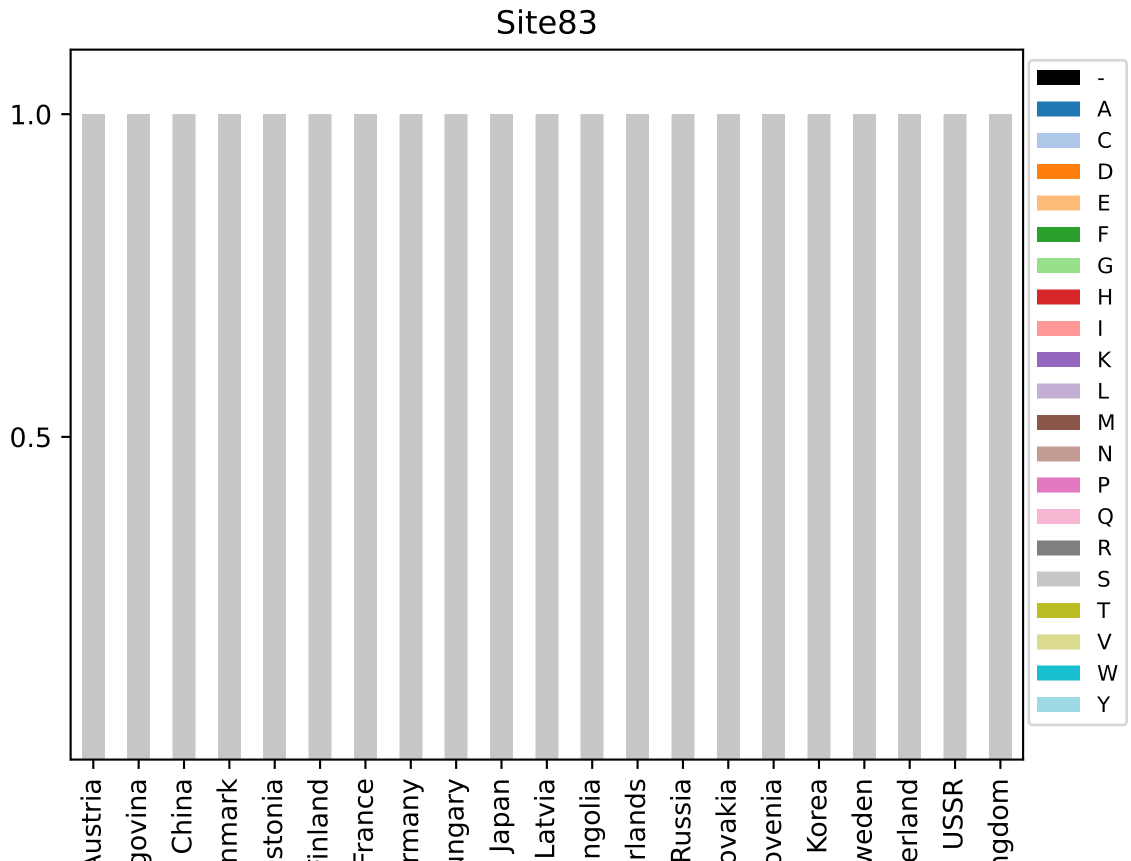
Site83
1.0
0.5
-
A
C
D
E
F
G
H
I
K
L
M
N
P
Q
R
S
T
V
W
Y
Austria
China
Finland
France
Japan
Latvia
Russia
Korea
USSR

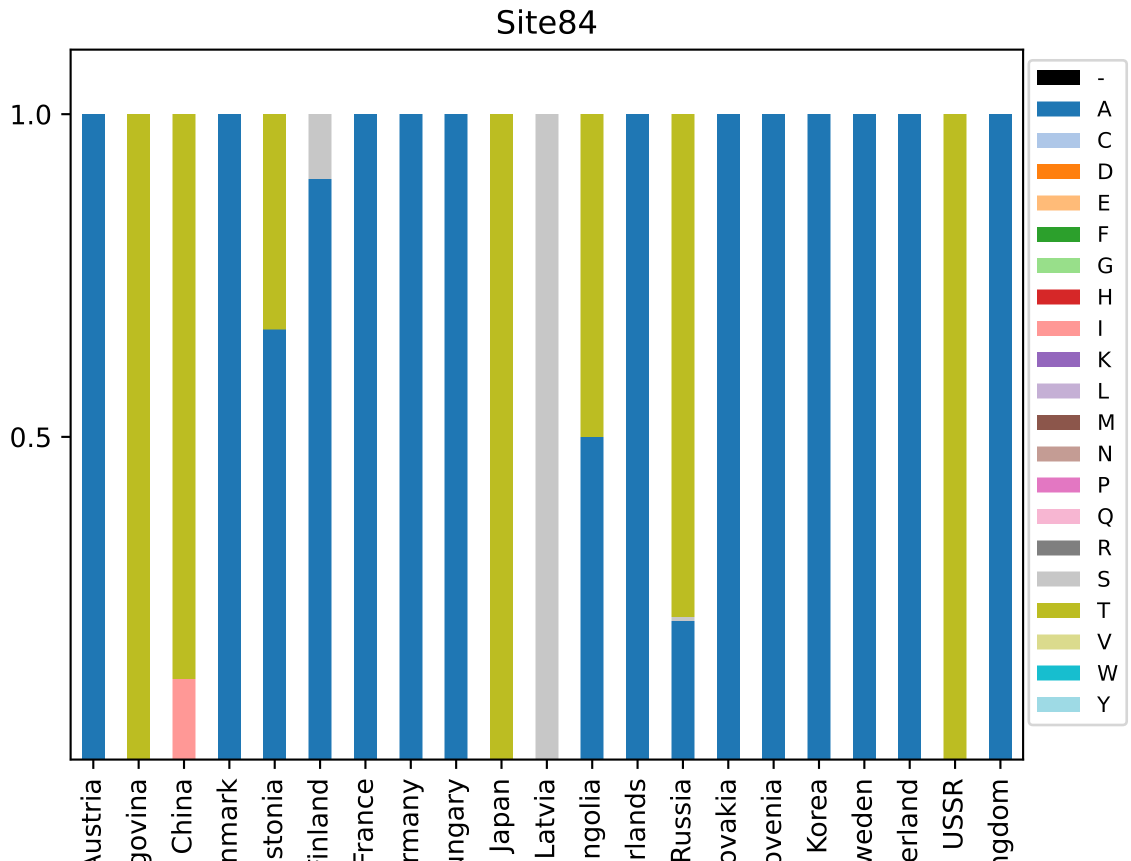

Site84
1.0
0.5
-
A
C
D
E
F
G
H
I
K
L
M
N
P
Q
R
S
T
V
W
Y
Austria
govina
China
nmark
stonia
Finland
France
rmany
ungary
Japan
Latvia
ngolia
rlands
Russia
ovakia
ovenia
Korea
weden
erland
USSR
ngdom

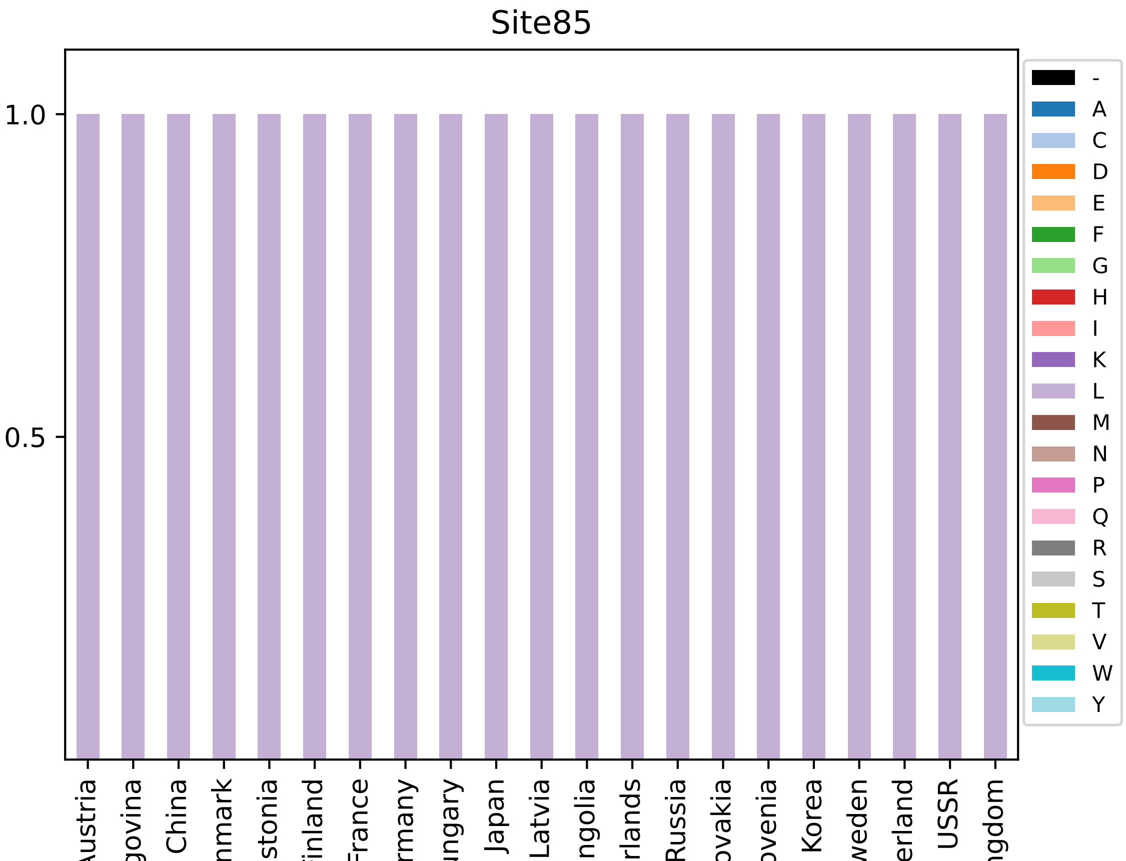

Site85
1.0
0.5
-
A
C
D
E
F
G
H
I
K
L
M
N
P
Q
R
S
T
V
W
Y
Austria
govina
China
nmark
stonia
Finland
France
rmany
ungary
Japan
Latvia
ngolia
rlands
Russia
ovakia
ovenia
Korea
weden
erland
USSR
ngdom

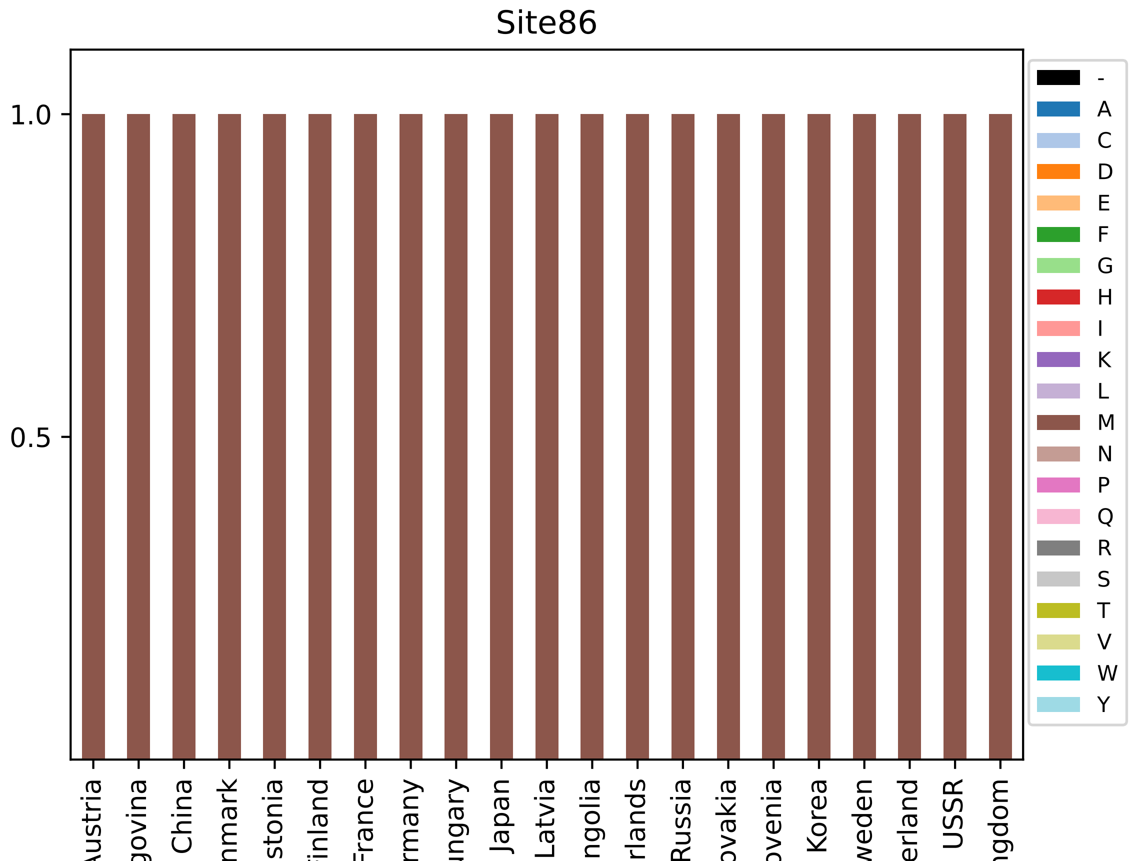
Site86
1.0
0.5
-
A
C
D
E
F
G
H
I
K
L
M
N
P
Q
R
S
T
V
W
Y
China
Japan
Latvia
Russia
Korea
USSR

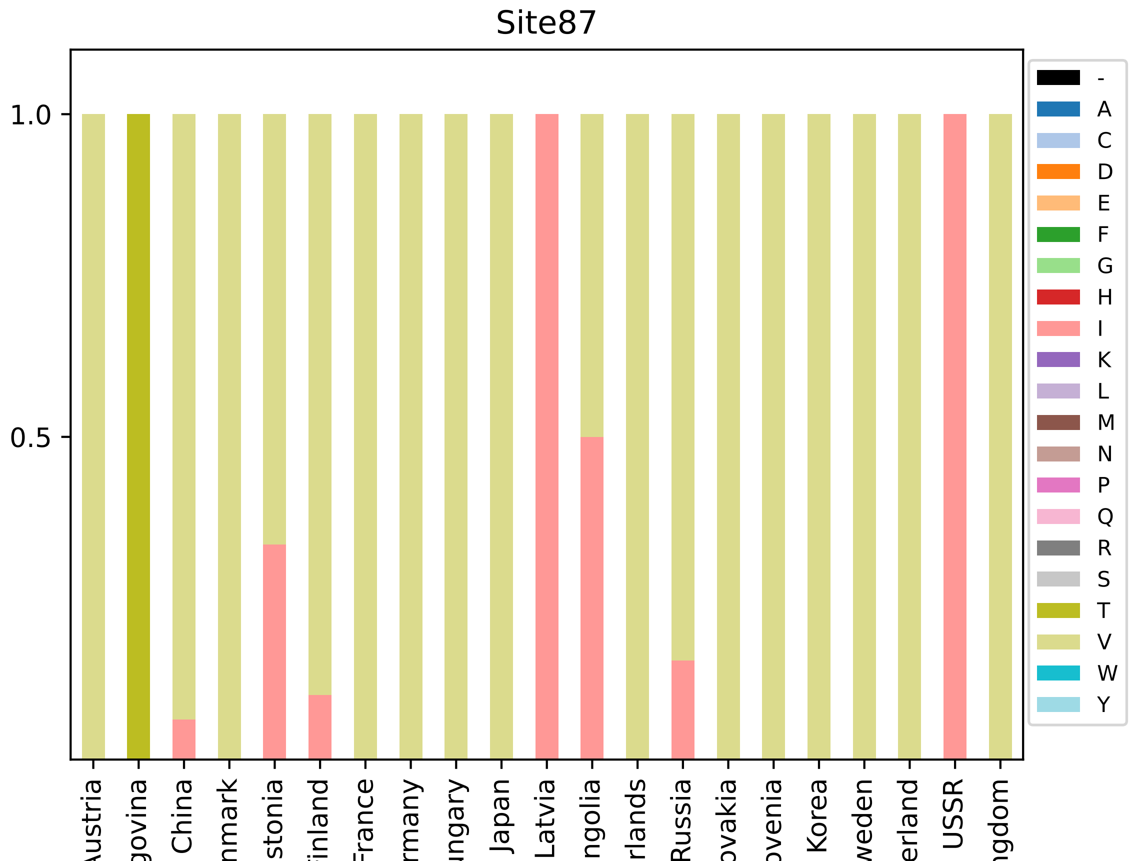

Site87
1.0
0.5
-
A
C
D
E
F
G
H
I
K
L
M
N
P
Q
R
S
T
V
W
Y
ustria
govina
China
nmark
stonia
inland
rance
rmany
ungary
Japan
Latvia
ngolia
rlands
Russia
ovakia
ovenia
Korea
weden
erland
USSR
ngdom

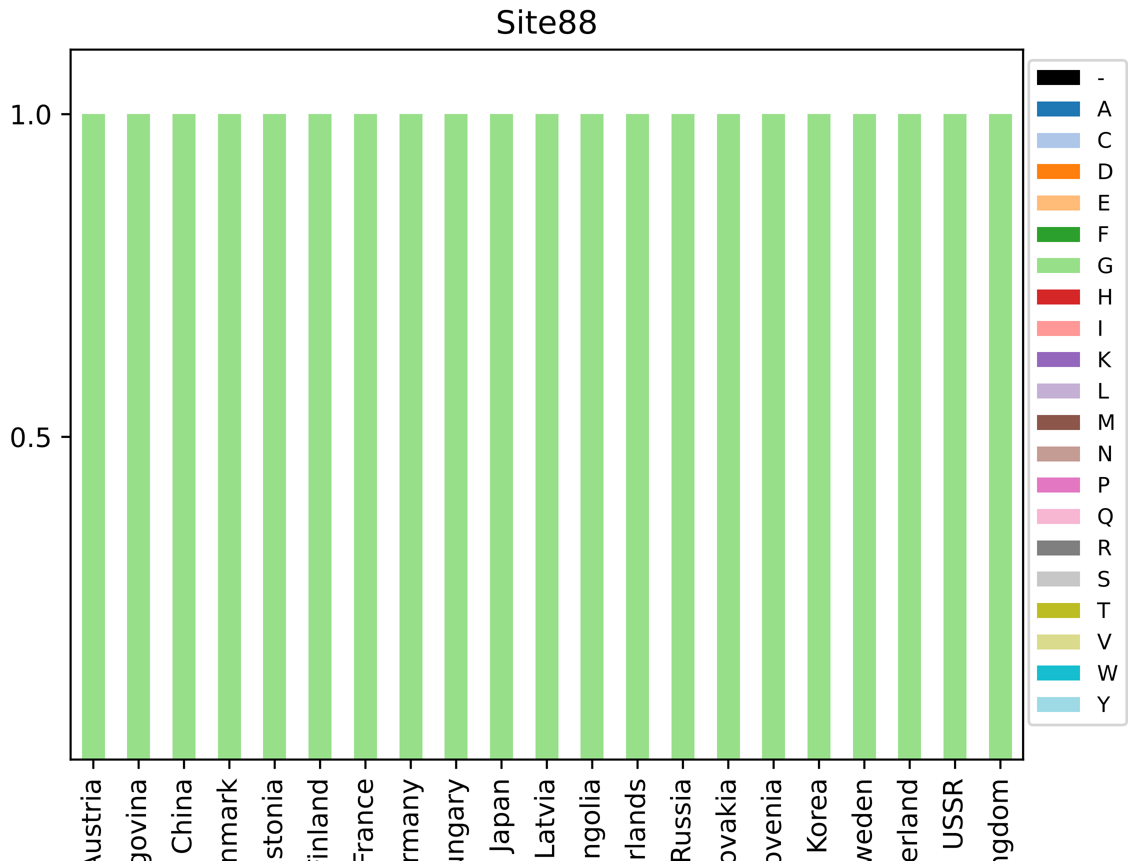

Site88
1.0
0.5
Austria
govina
China
nmark
stonia
Finland
France
rmany
ungary
Japan
Latvia
ngolia
rlands
Russia
ovakia
ovenia
Korea
weden
erland
USSR
ngdom
-
A
C
D
E
F
G
H
I
K
L
M
N
P
Q
R
S
T
V
W
Y

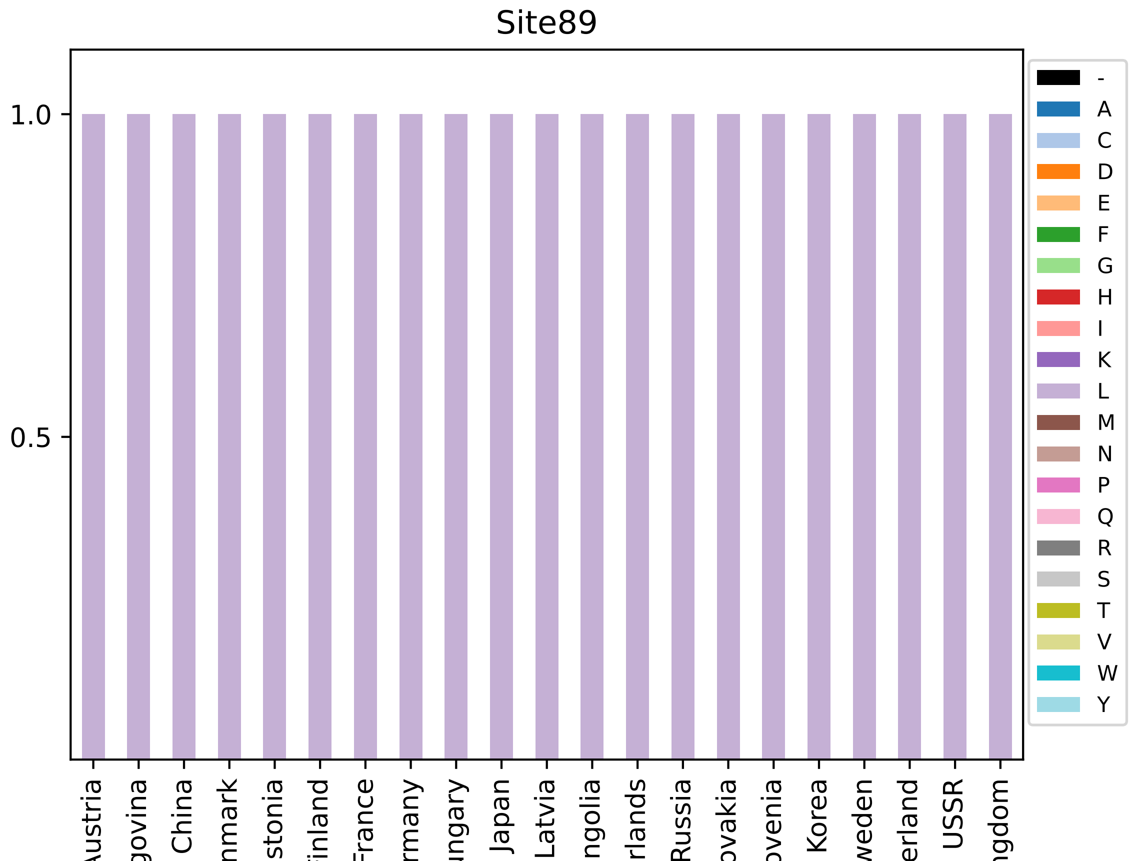

Site89
1.0
0.5
China
Japan
Latvia
Russia
Korea
USSR
-
A
C
D
E
F
G
H
I
K
L
M
N
P
Q
R
S
T
V
W
Y

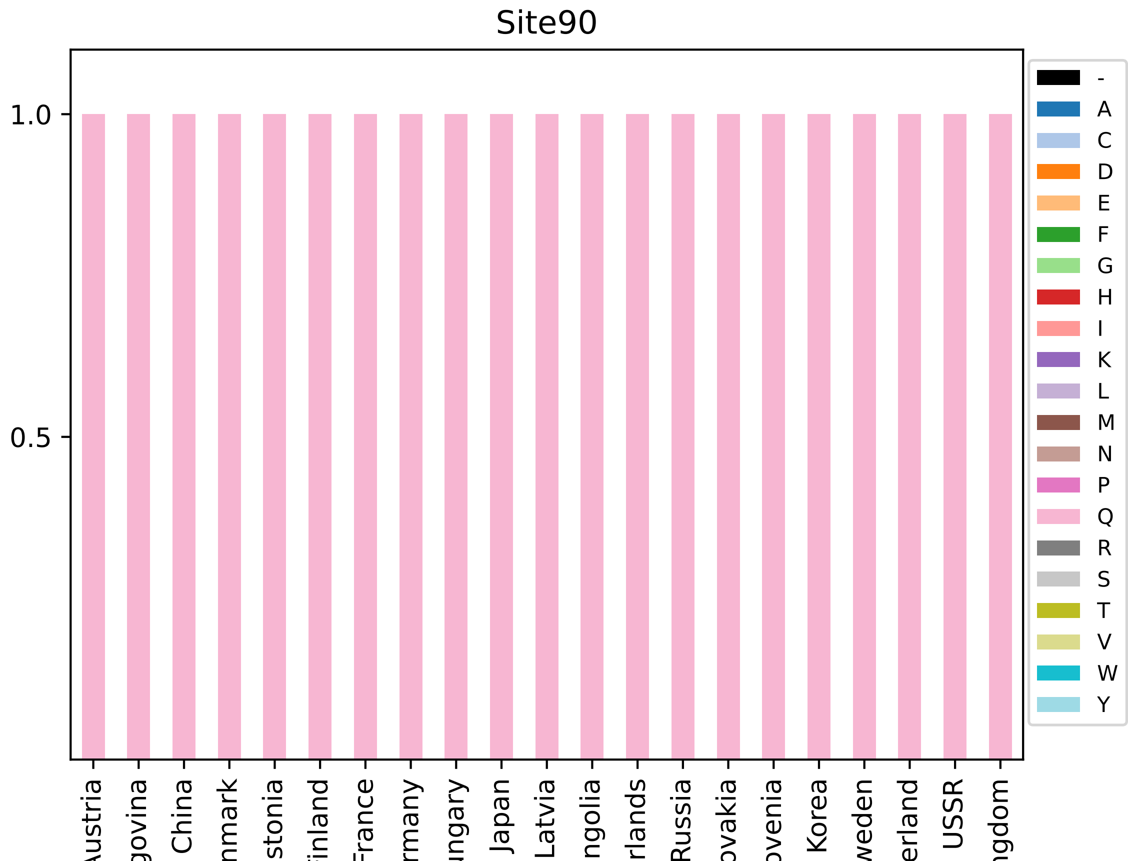

Site90
1.0
0.5
-
A
C
D
E
F
G
H
I
K
L
M
N
P
Q
R
S
T
V
W
Y
Austria
govina
China
nmark
stonia
Finland
France
rmany
ungary
Japan
Latvia
ngolia
rlands
Russia
ovakia
ovenia
Korea
weden
erland
USSR
ngdom

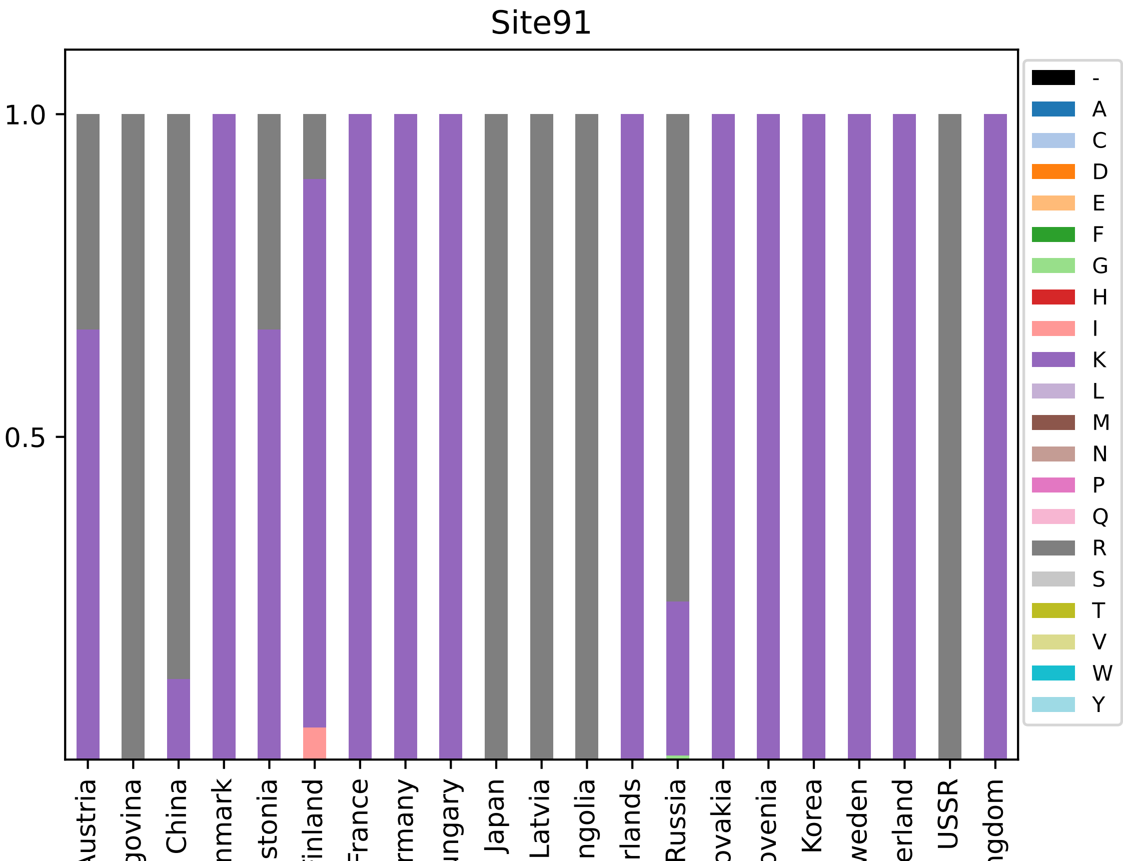
Site91
1.0
0.5
-
A
C
D
E
F
G
H
I
K
L
M
N
P
Q
R
S
T
V
W
Y
China
Japan
Latvia
Russia
Korea
USSR

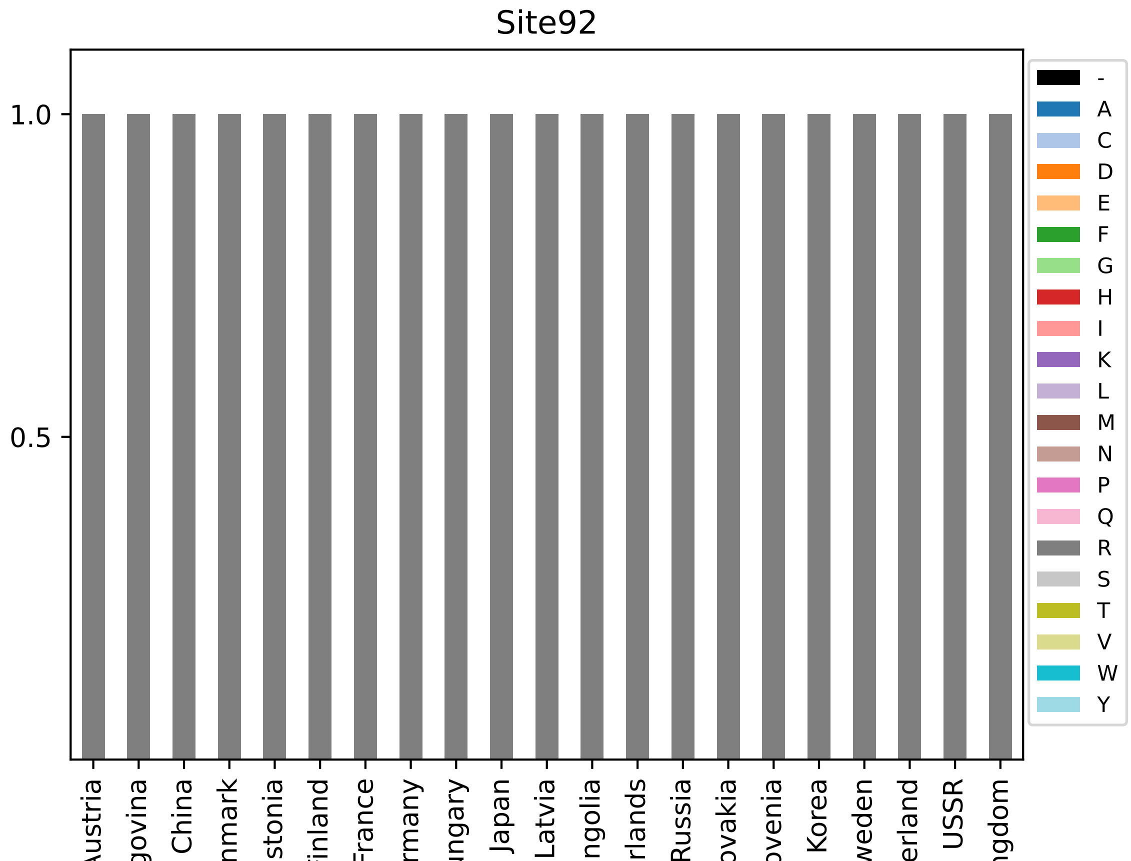

Site92
1.0
0.5
-
A
C
D
E
F
G
H
I
K
L
M
N
P
Q
R
S
T
V
W
Y
Austria
China
Finland
France
Japan
Latvia
Russia
Korea
USSR

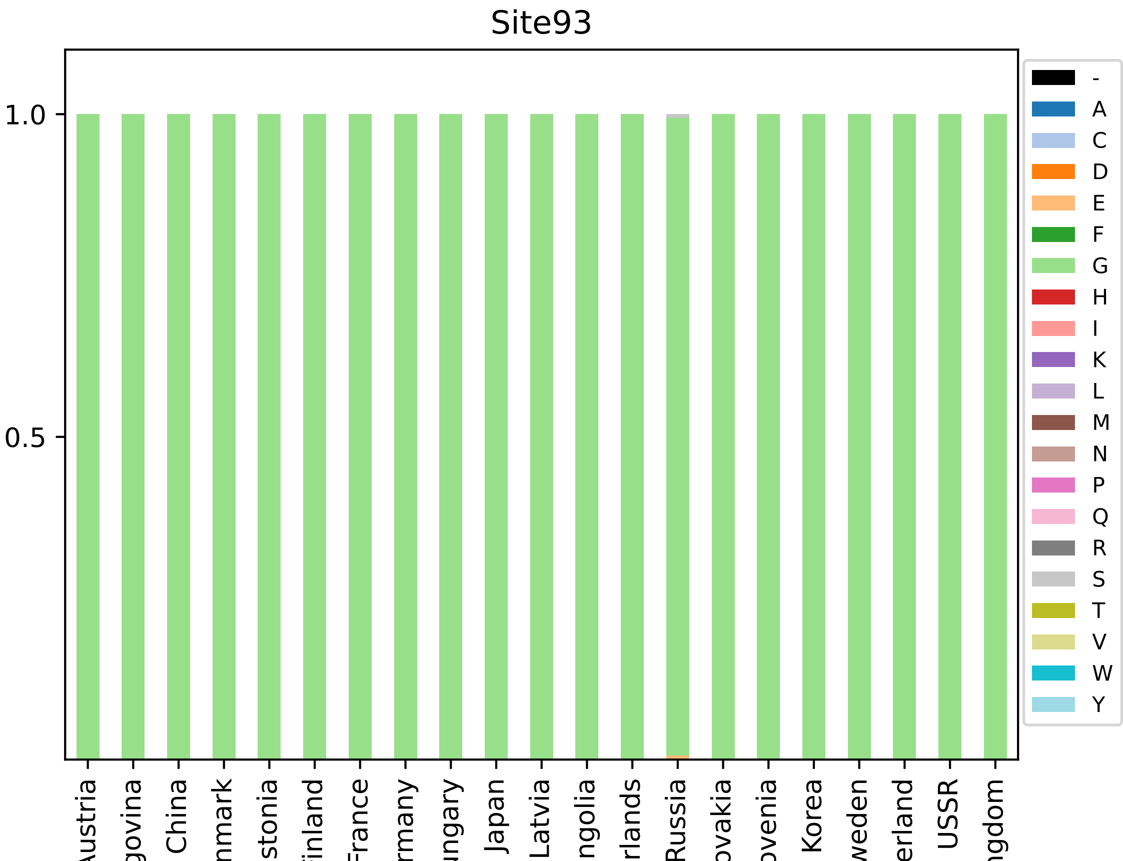
Site93
1.0
0.5
-
A
C
D
E
F
G
H
I
K
L
M
N
P
Q
R
S
T
V
W
Y
China
Japan
Latvia
Russia
Korea
USSR

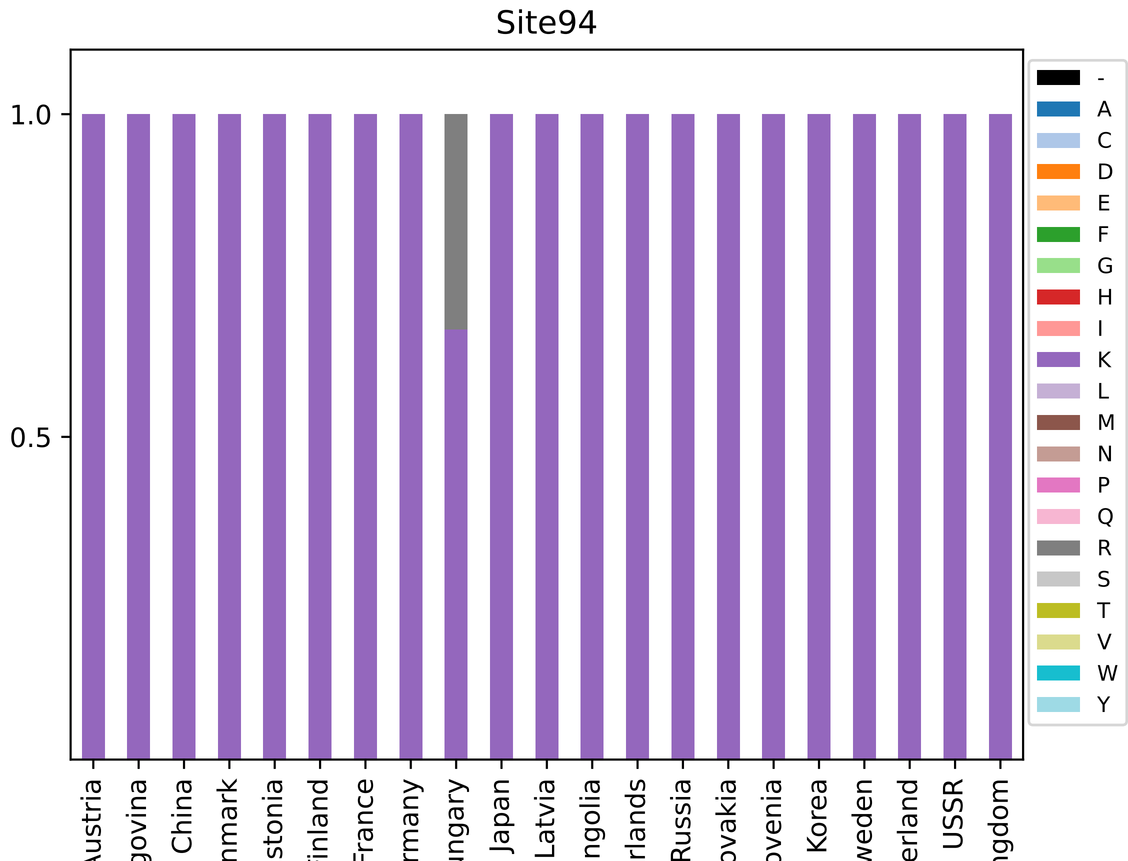

Site94
1.0
0.5
-
A
C
D
E
F
G
H
I
K
L
M
N
P
Q
R
S
T
V
W
Y
China
Finland
France
Japan
Latvia
Russia
Korea
USSR

Site95

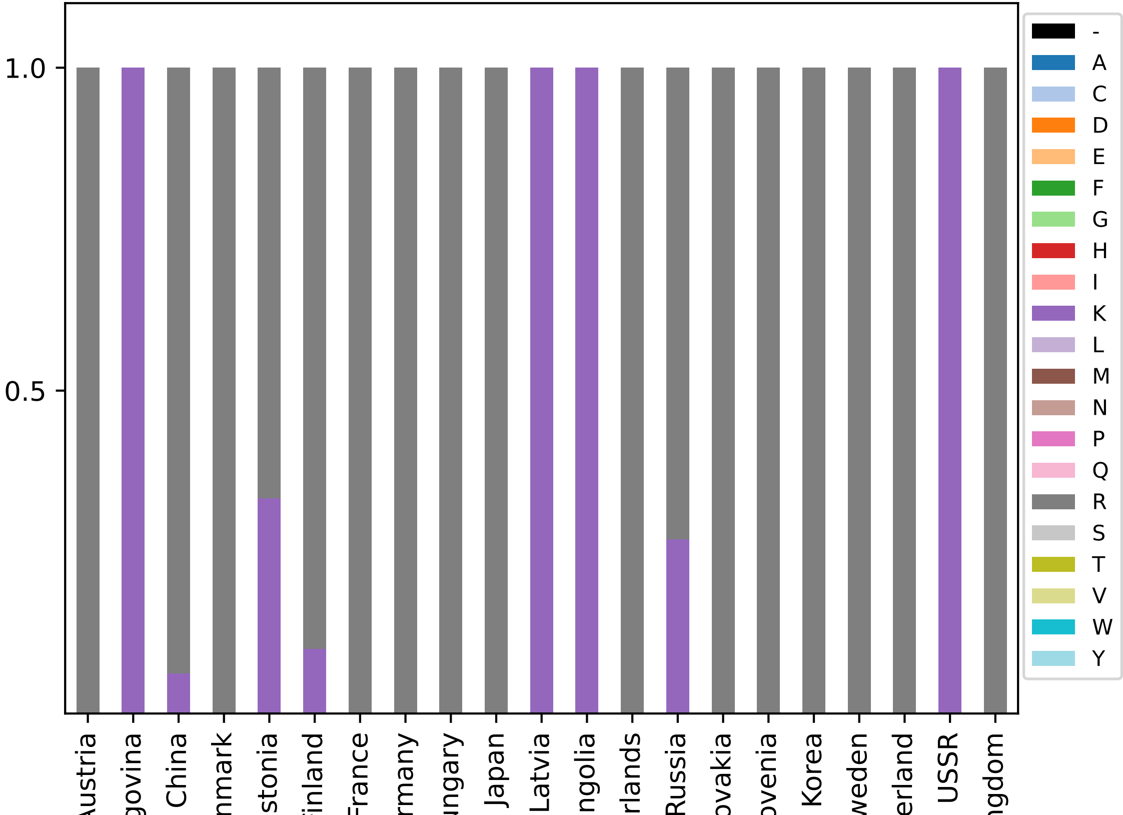
1.0
0.5
-
A
C
D
E
F
G
H
I
K
L
M
N
P
Q
R
S
T
V
W
Y
Austria
govina
China
nmark
stonia
Finland
France
rmany
ungary
Japan
Latvia
ngolia
rlands
Russia
ovakia
ovenia
Korea
weden
erland
USSR
ngdom

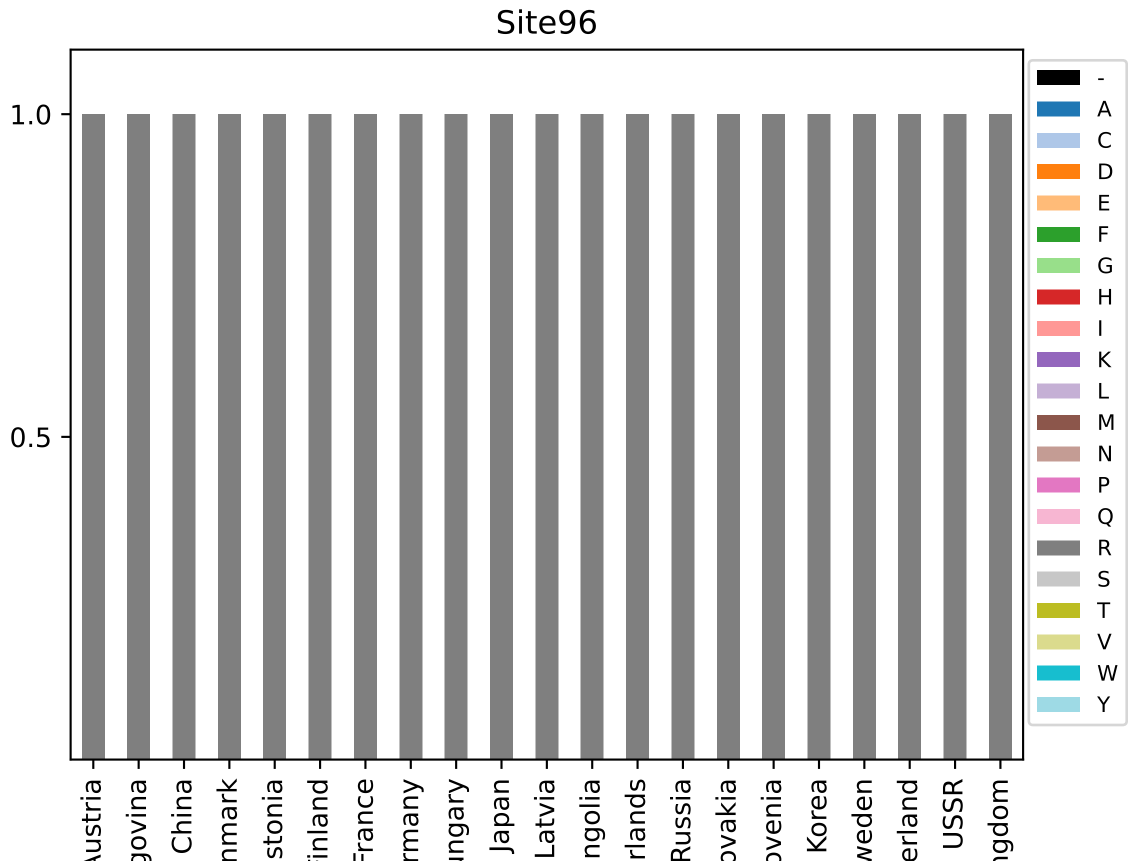

Site96
1.0
0.5
Austria
govina
China
nmark
stonia
Finland
France
rmany
ungary
Japan
Latvia
ngolia
rlands
Russia
ovakia
ovenia
Korea
weden
erland
USSR
ngdom
-
A
C
D
E
F
G
H
I
K
L
M
N
P
Q
R
S
T
V
W
Y

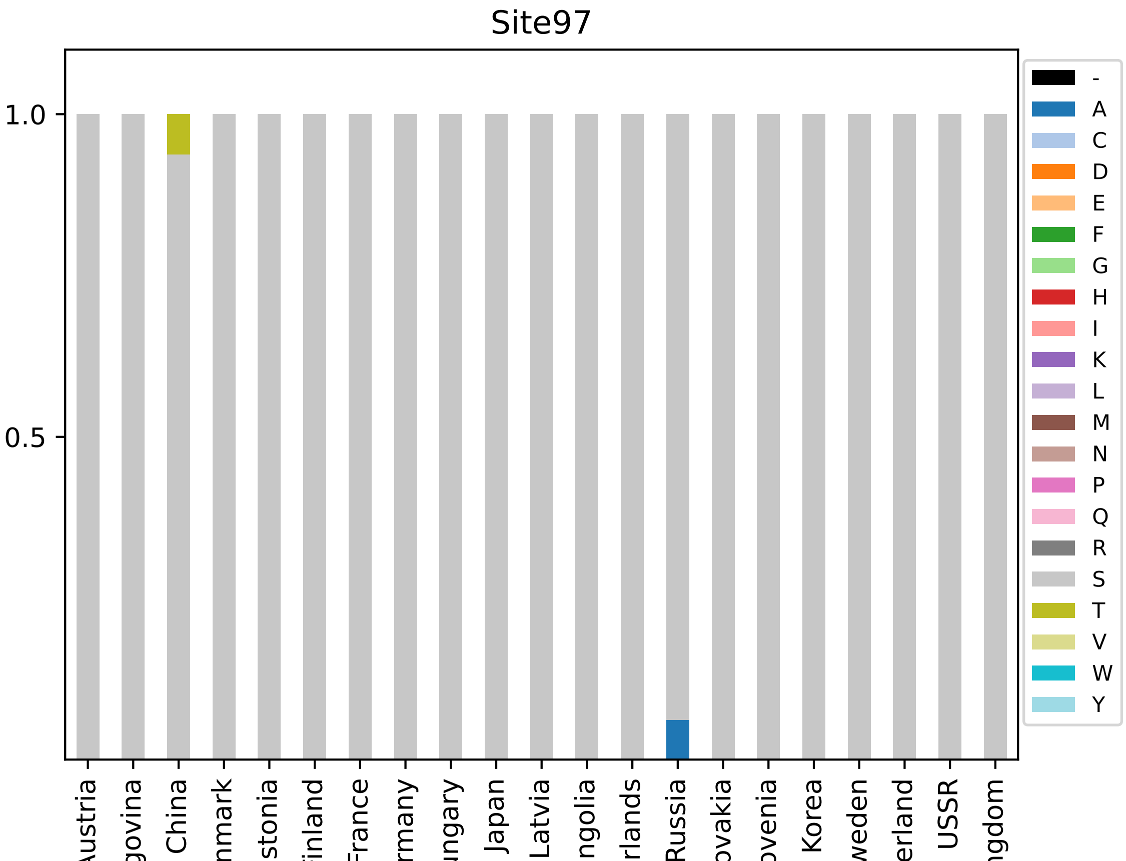
Site97
1.0
0.5
-
A
C
D
E
F
G
H
I
K
L
M
N
P
Q
R
S
T
V
W
Y
Austria
China
Finland
France
Japan
Latvia
Russia
Korea
USSR

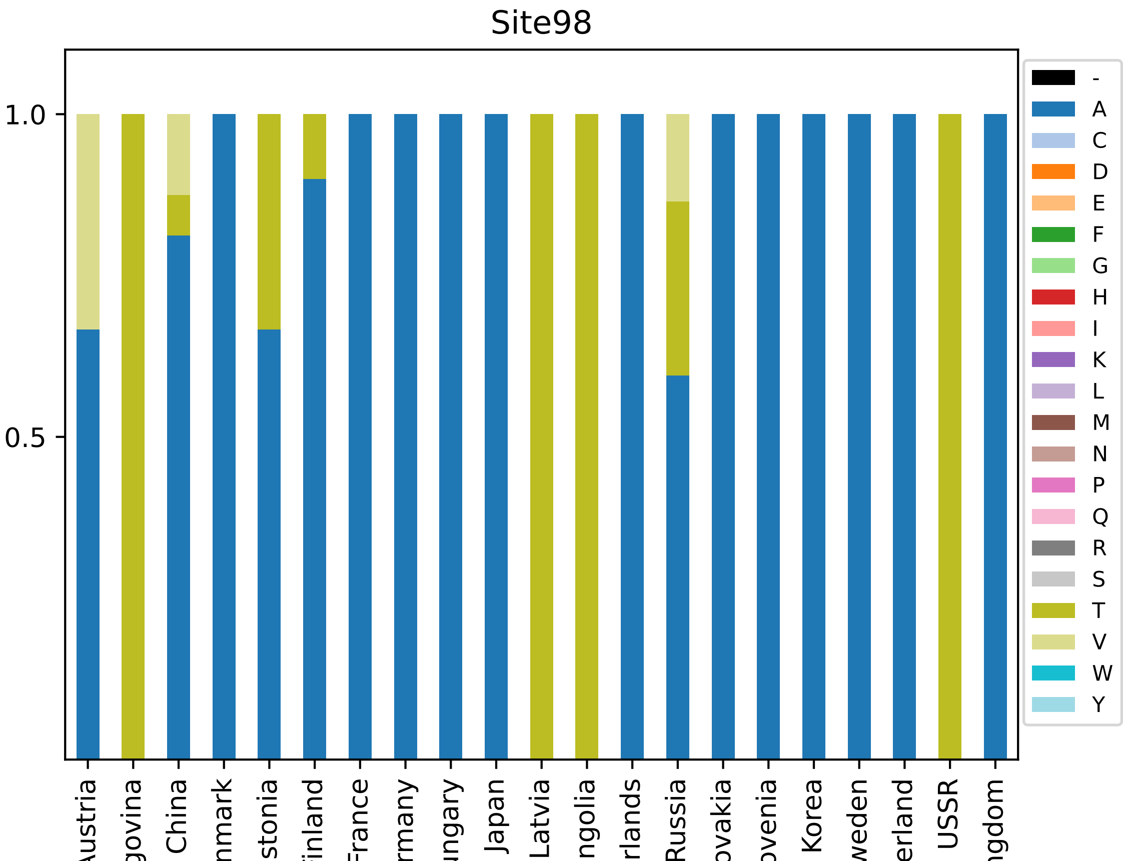
Site98
1.0
0.5
-
A
C
D
E
F
G
H
I
K
L
M
N
P
Q
R
S
T
V
W
Y
China
France
Japan
Latvia
Russia
Korea
USSR

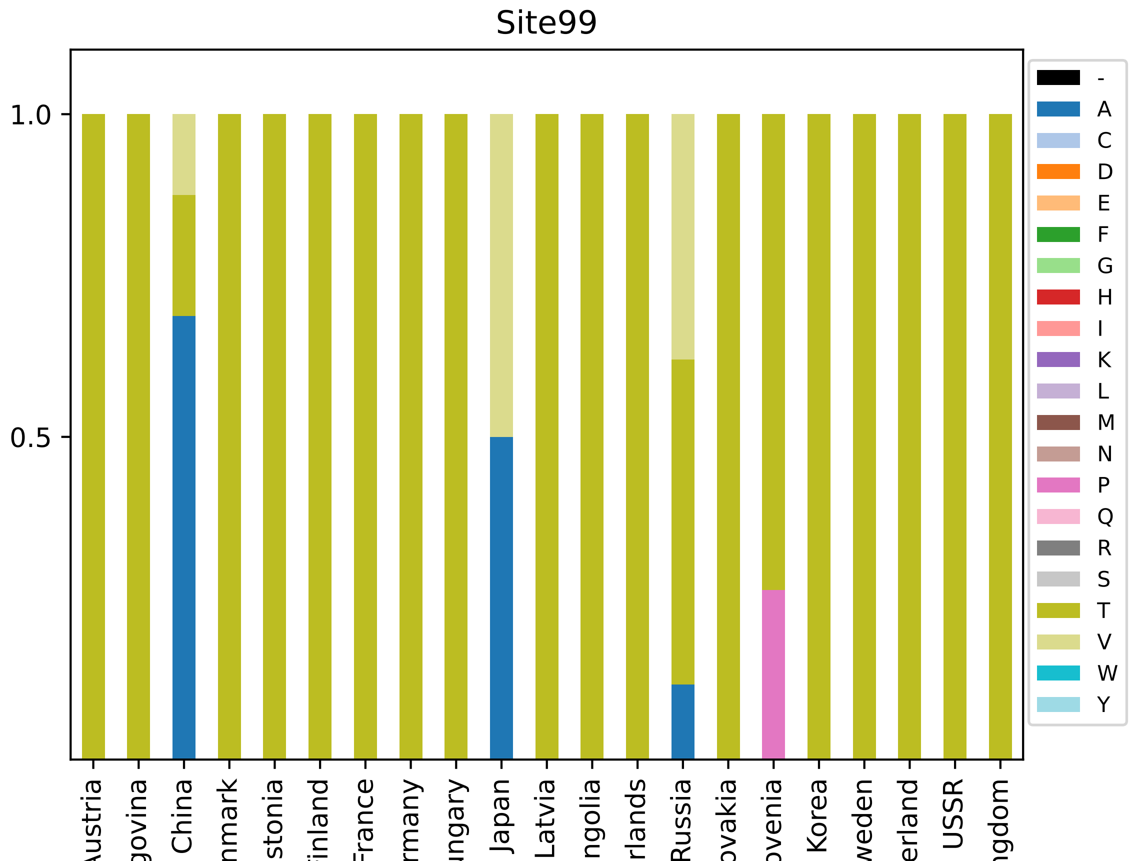
Site99
1.0
0.5
-
A
C
D
E
F
G
H
I
K
L
M
N
P
Q
R
S
T
V
W
Y
China
Japan
Latvia
Russia
Korea
USSR

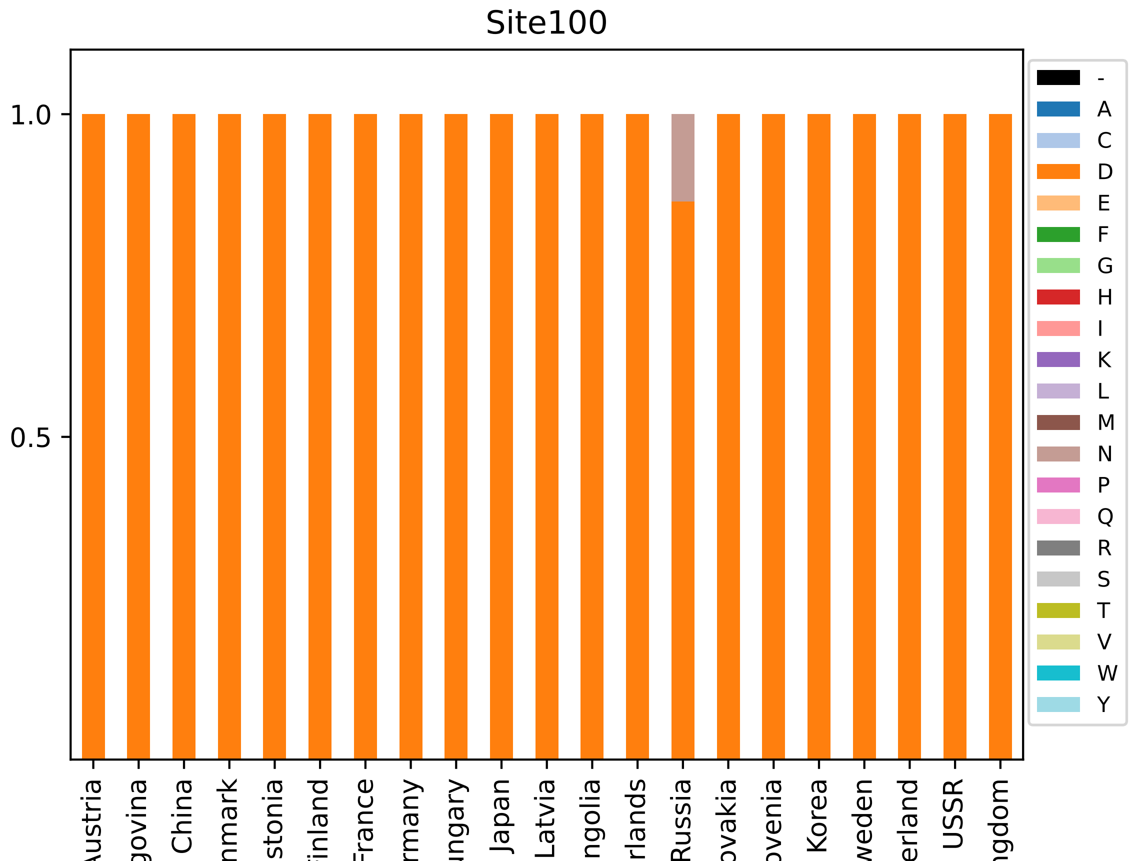

Site100
1.0
0.5
-
A
C
D
E
F
G
H
I
K
L
M
N
P
Q
R
S
T
V
W
Y
Austria
govina
China
nmark
stonia
inland
France
rmany
ungary
Japan
Latvia
ngolia
rlands
Russia
ovakia
ovenia
Korea
weden
erland
USSR
ngdom

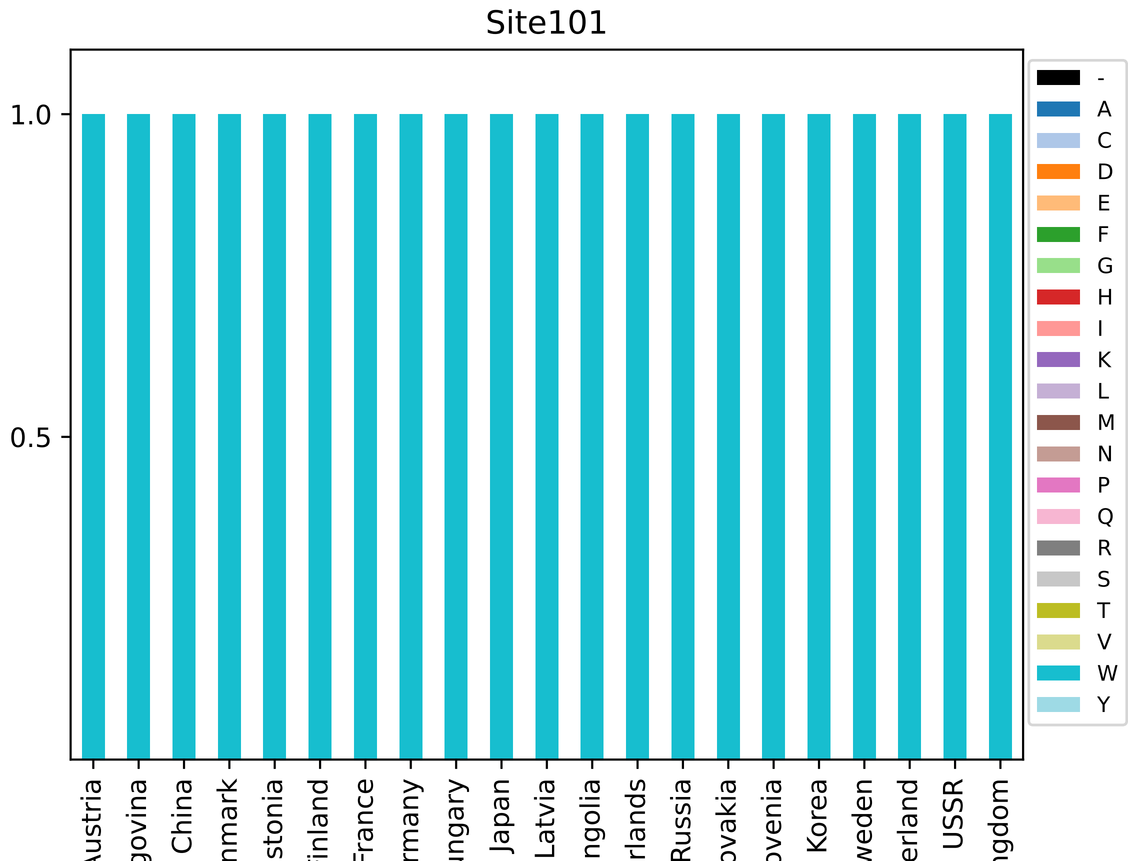
Site101
1.0
0.5
-
A
C
D
E
F
G
H
I
K
L
M
N
P
Q
R
S
T
V
W
Y
China
Japan
Latvia
Russia
Korea
USSR

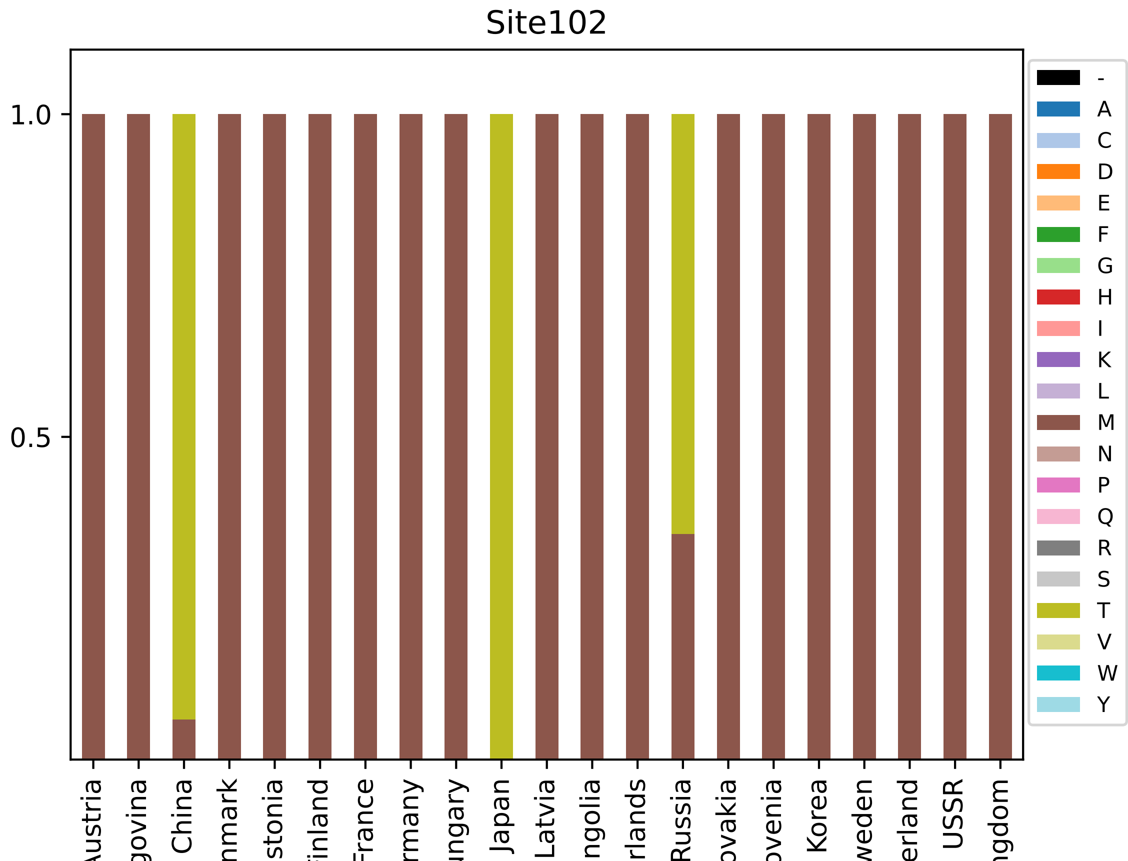

Site102
1.0
0.5
-
A
C
D
E
F
G
H
I
K
L
M
N
P
Q
R
S
T
V
W
Y
Austria
govina
China
nmark
stonia
inland
France
rmany
ungary
Japan
Latvia
ngolia
rlands
Russia
ovakia
ovenia
Korea
weden
erland
USSR
ngdom

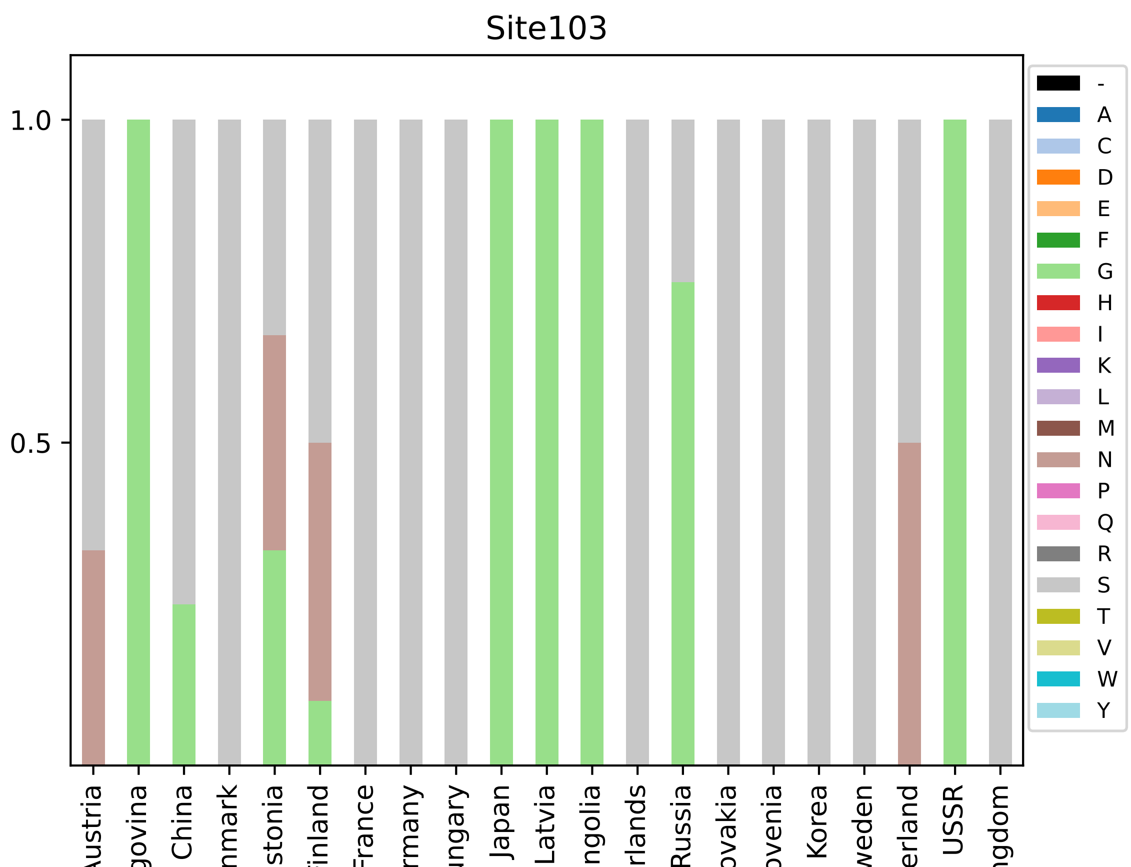

Site103
1.0
0.5
-
A
C
D
E
F
G
H
I
K
L
M
N
P
Q
R
S
T
V
W
Y
Austria
govina
China
nmark
stonia
Finland
France
rmany
ungary
Japan
Latvia
ngolia
rlands
Russia
ovakia
ovenia
Korea
weden
erland
USSR
ngdom

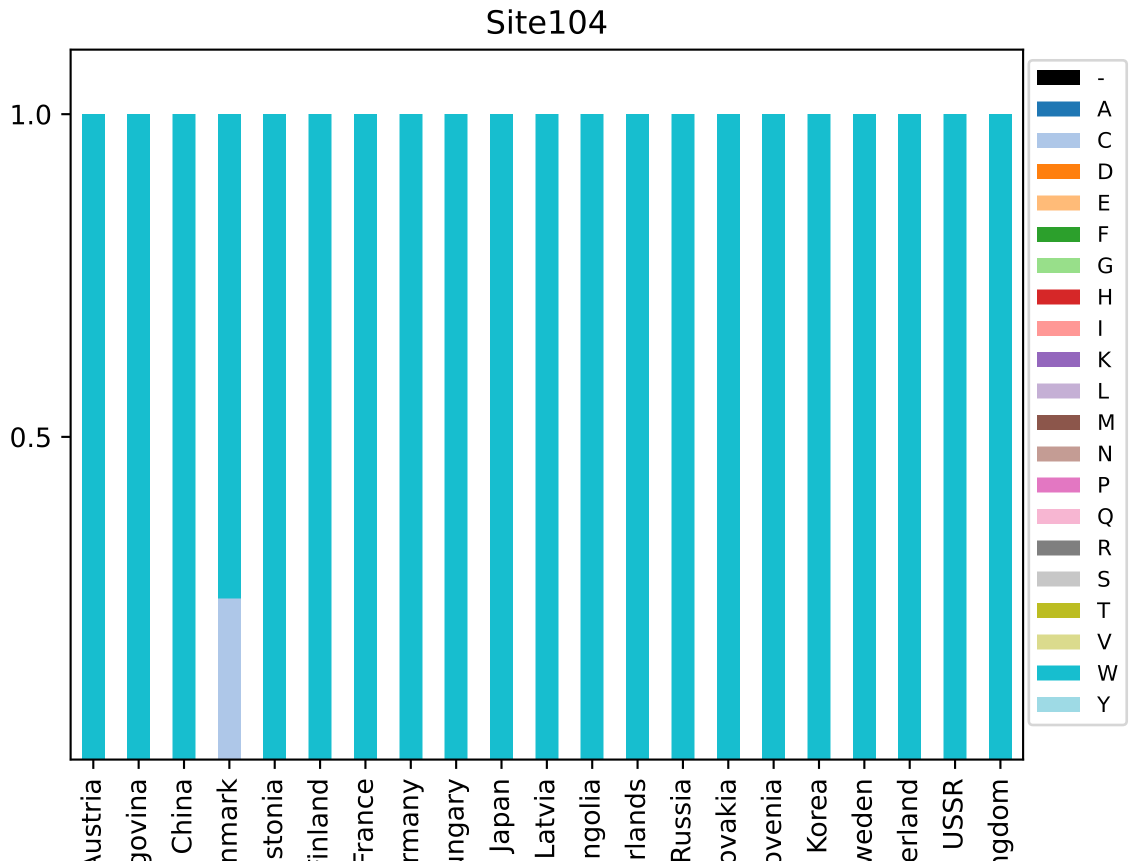
Site104
1.0
0.5
-
A
C
D
E
F
G
H
I
K
L
M
N
P
Q
R
S
T
V
W
Y
Austria
China
Japan
Latvia
Russia
Korea
USSR

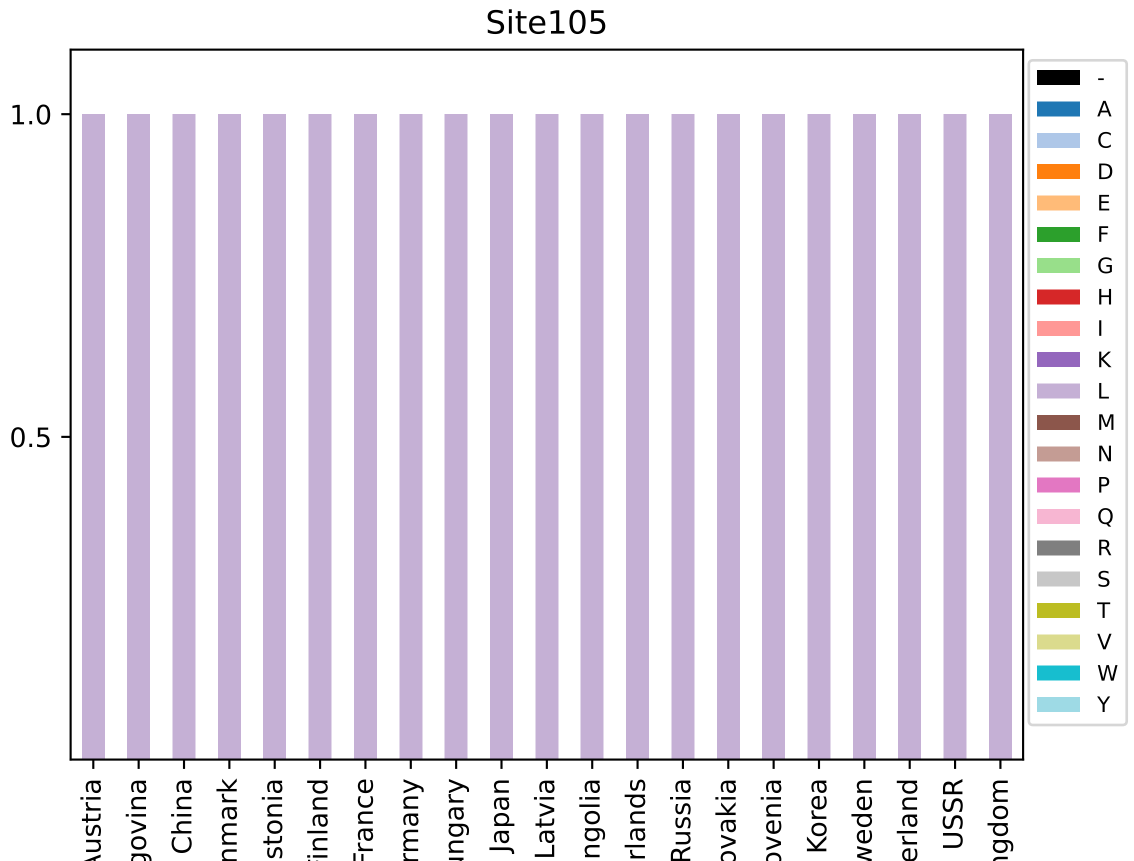
Site105
1.0
0.5
-
A
C
D
E
F
G
H
I
K
L
M
N
P
Q
R
S
T
V
W
Y
China
Japan
Latvia
Russia
Korea
USSR

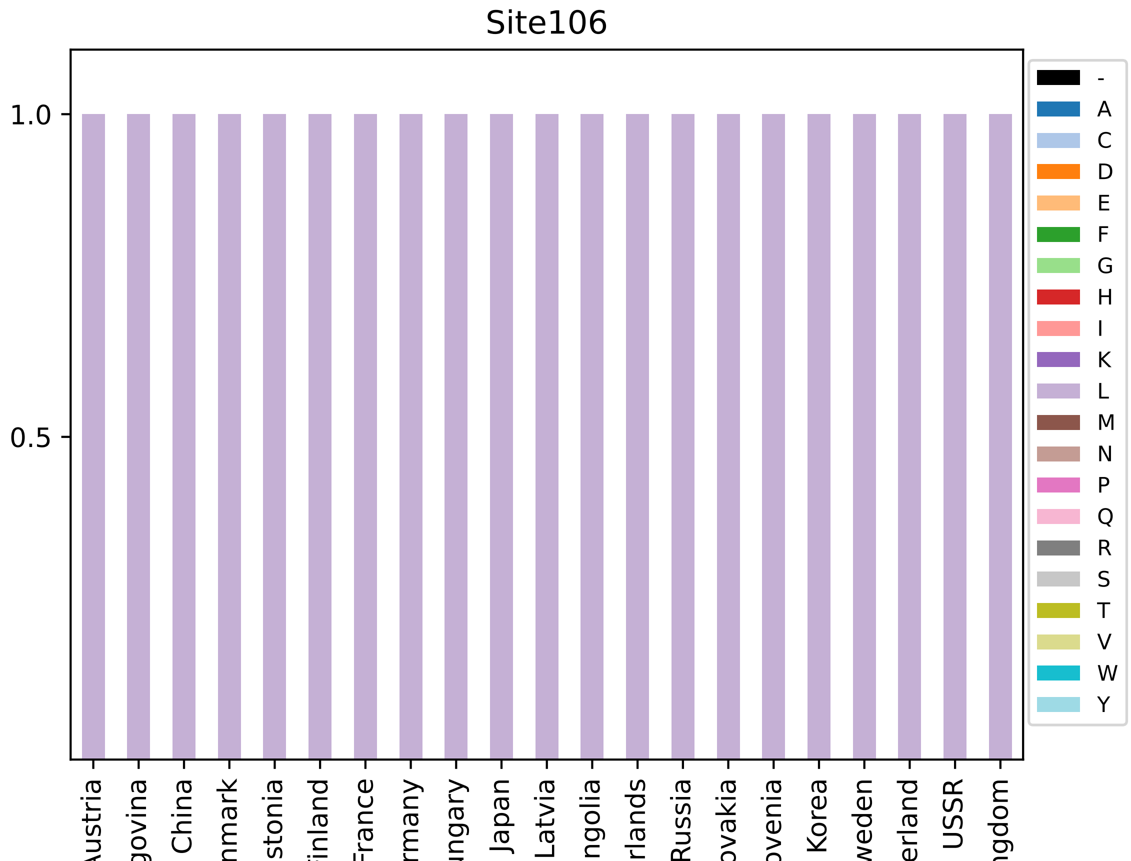

Site106
1.0
0.5
-
A
C
D
E
F
G
H
I
K
L
M
N
P
Q
R
S
T
V
W
Y
China
Japan
Latvia
Russia
Korea
USSR

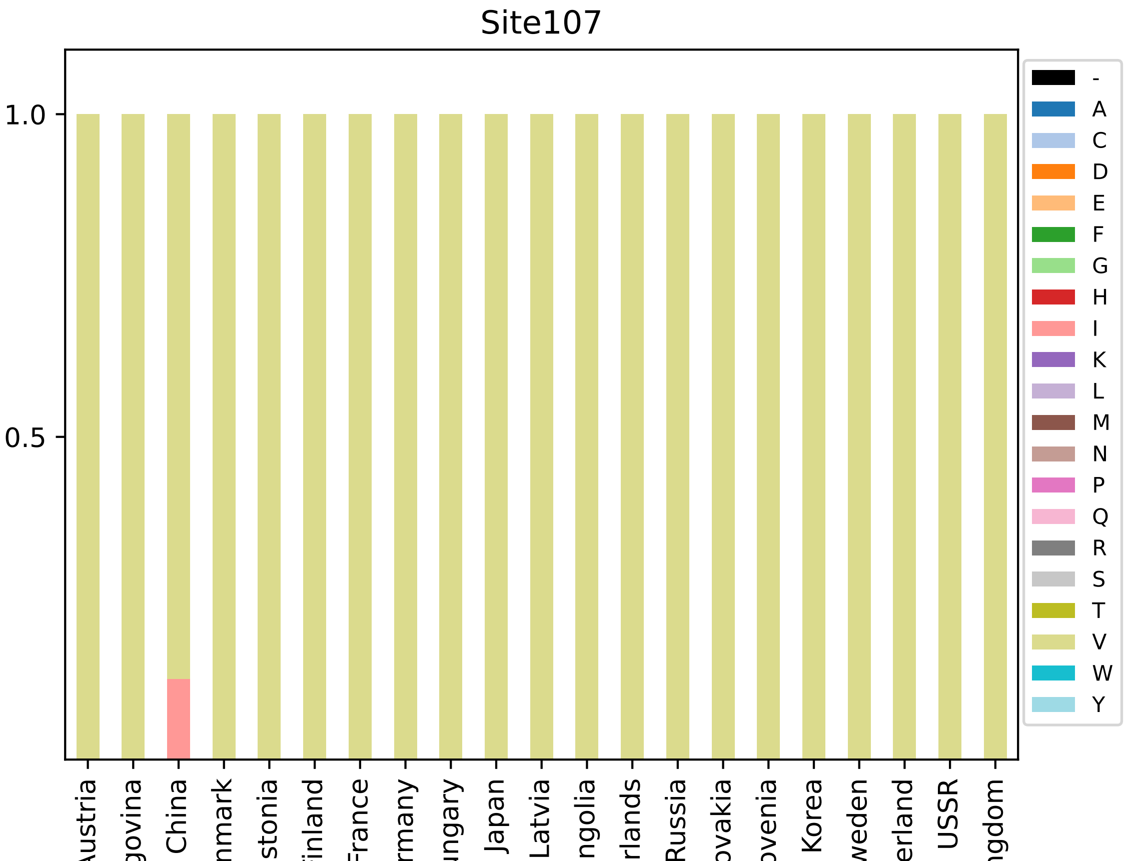
Site107
1.0
0.5
-
A
C
D
E
F
G
H
I
K
L
M
N
P
Q
R
S
T
V
W
Y
Austria
govina
China
nmark
stonia
Finland
France
rmany
ungary
Japan
Latvia
ngolia
rlands
Russia
ovakia
ovenia
Korea
weden
erland
USSR
ngdom

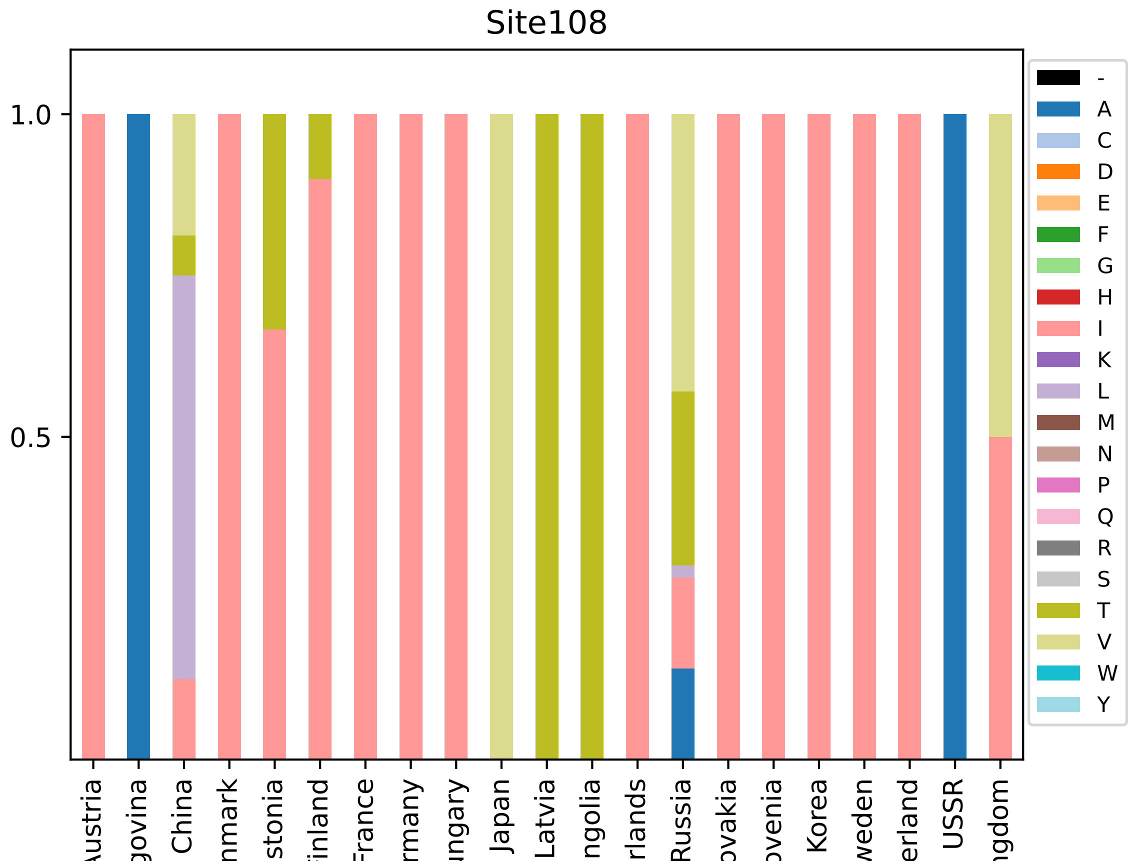

Site108
1.0
0.5
-
A
C
D
E
F
G
H
I
K
L
M
N
P
Q
R
S
T
V
W
Y
China
Finland
France
Japan
Latvia
Russia
Korea
USSR

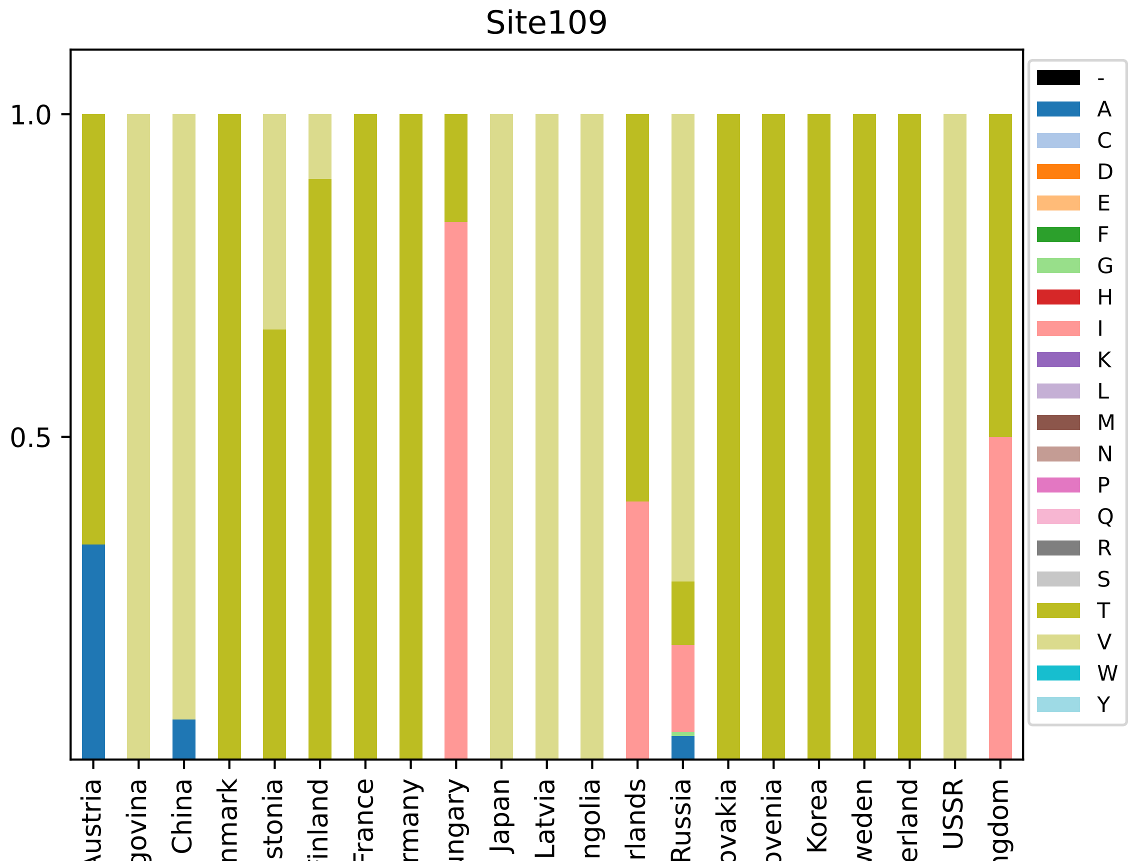
Site109
1.0
0.5
-
A
C
D
E
F
G
H
I
K
L
M
N
P
Q
R
S
T
V
W
Y
China
Japan
Latvia
Russia
Korea
USSR

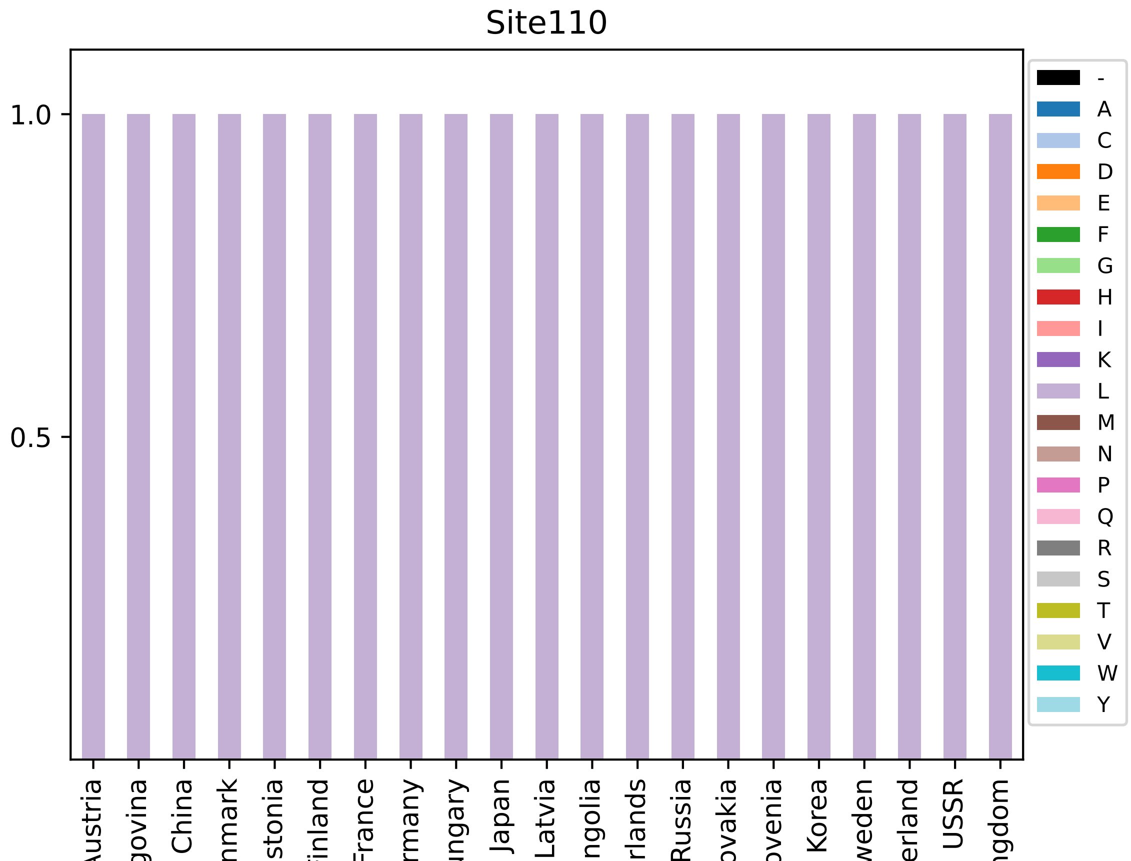
Site110
1.0
0.5
-
A
C
D
E
F
G
H
I
K
L
M
N
P
Q
R
S
T
V
W
Y
Austria
China
Estonia
Finland
France
Japan
Latvia
Russia
Korea
USSR

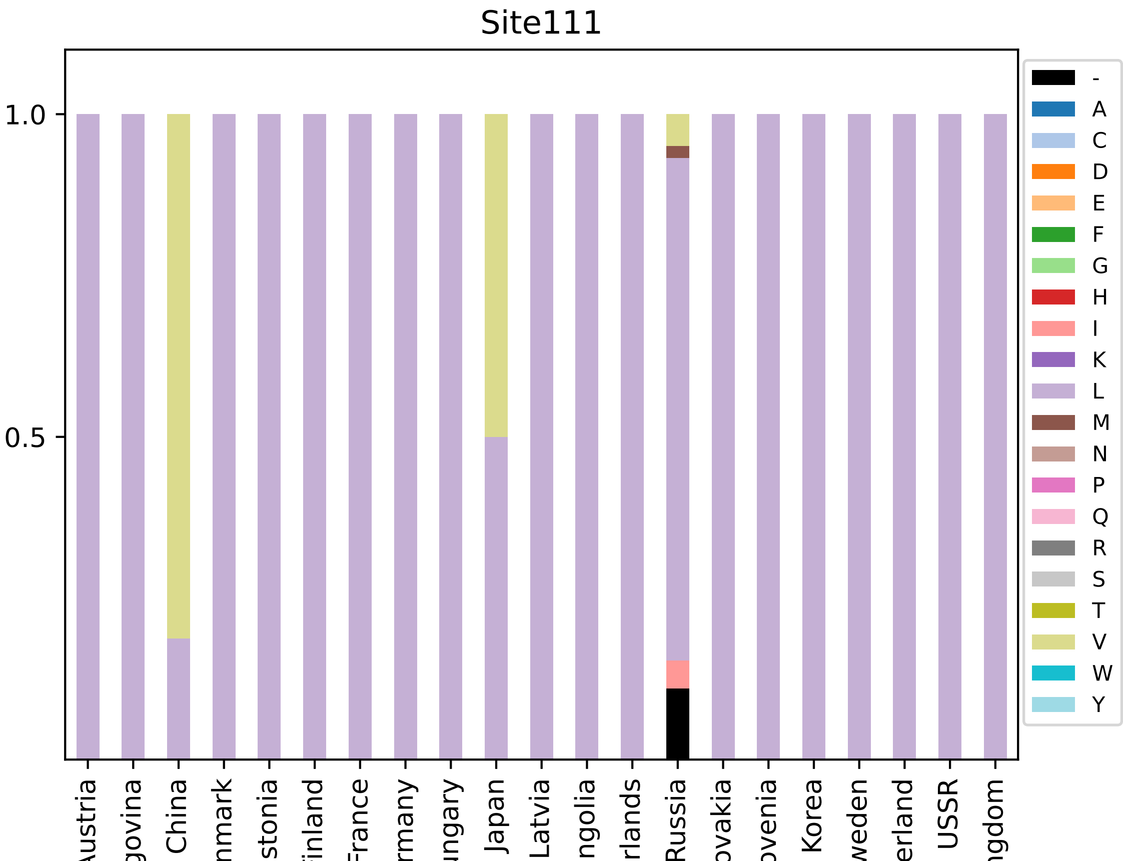

Site111
1.0
0.5
-
A
C
D
E
F
G
H
I
K
L
M
N
P
Q
R
S
T
V
W
Y
Austria
govina
China
nmark
stonia
inland
France
rmany
ungary
Japan
Latvia
ngolia
rlands
Russia
ovakia
ovenia
Korea
weden
erland
USSR
ngdom

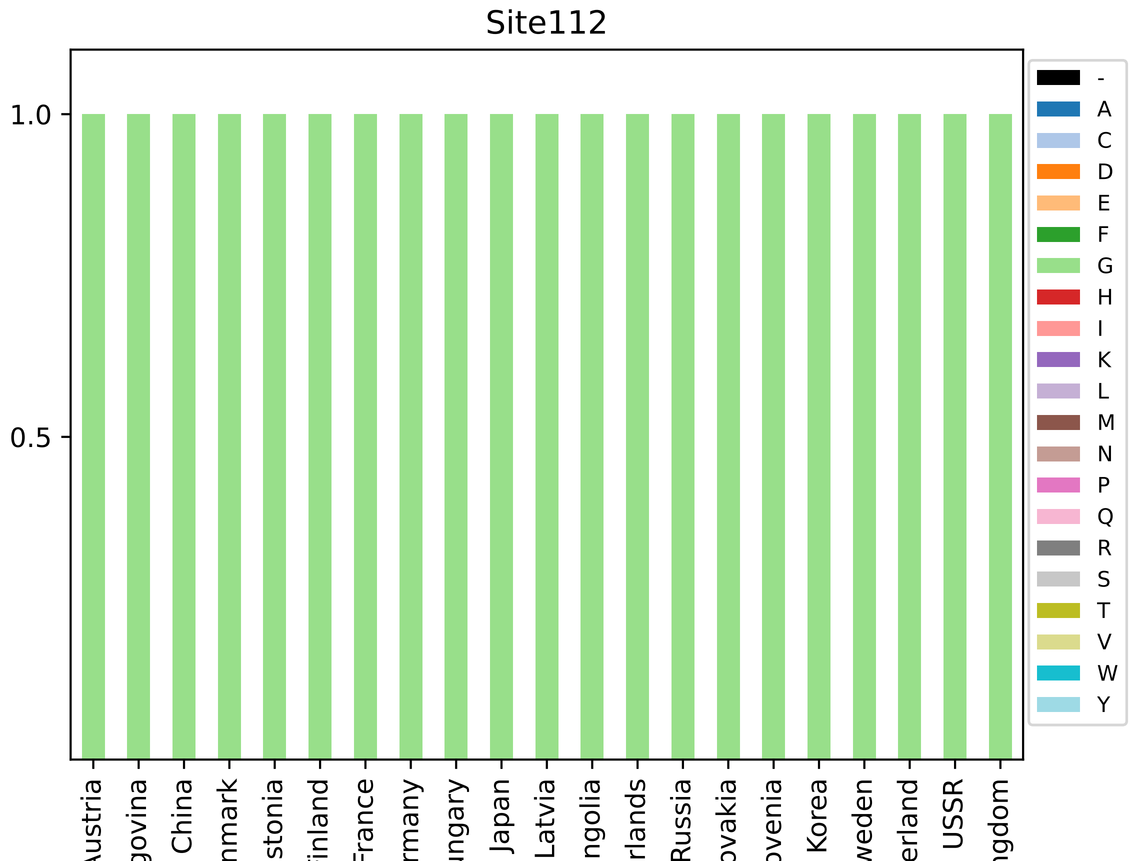
Site112
1.0
0.5
-
A
C
D
E
F
G
H
I
K
L
M
N
P
Q
R
S
T
V
W
Y
China
Japan
Latvia
Russia
Korea
USSR

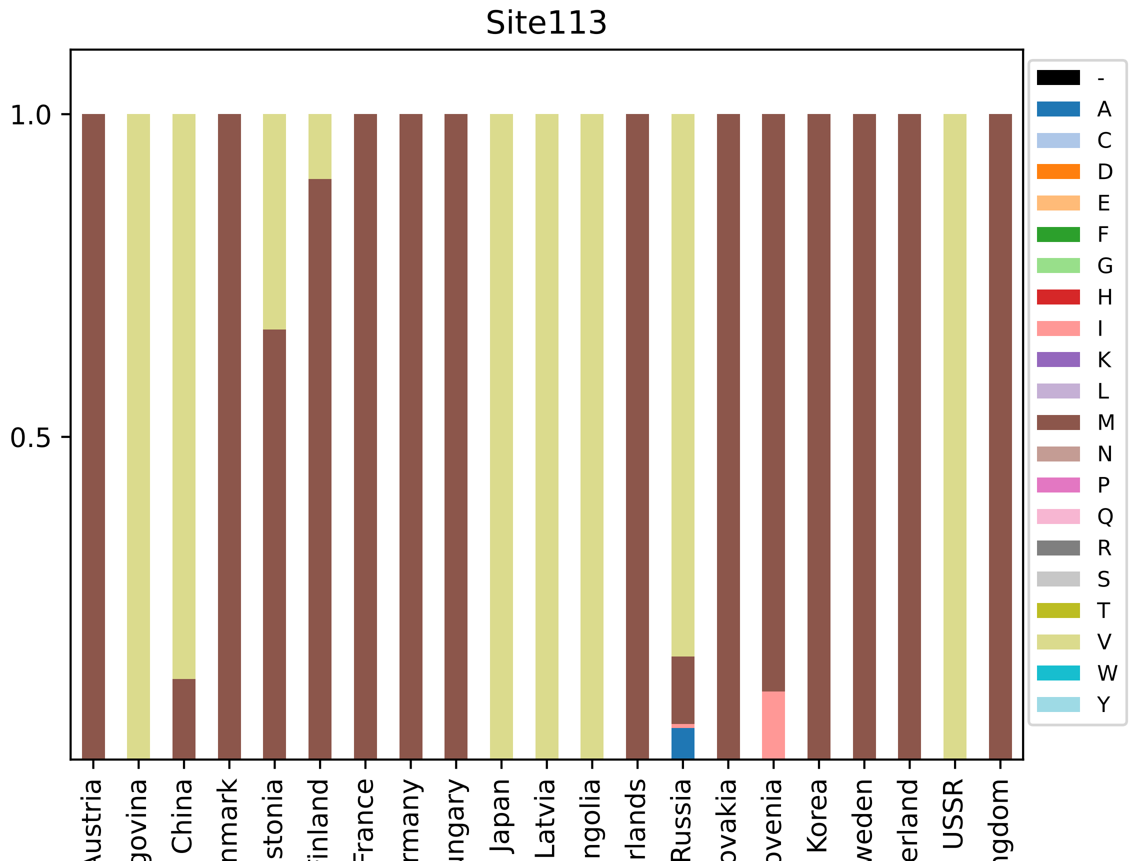
Site113
1.0
0.5
Austria
govina
China
nmark
stonia
Finland
France
rmany
ungary
Japan
Latvia
ngolia
rlands
Russia
ovakia
ovenia
Korea
weden
erland
USSR
ngdom
-
A
C
D
E
F
G
H
I
K
L
M
N
P
Q
R
S
T
V
W
Y

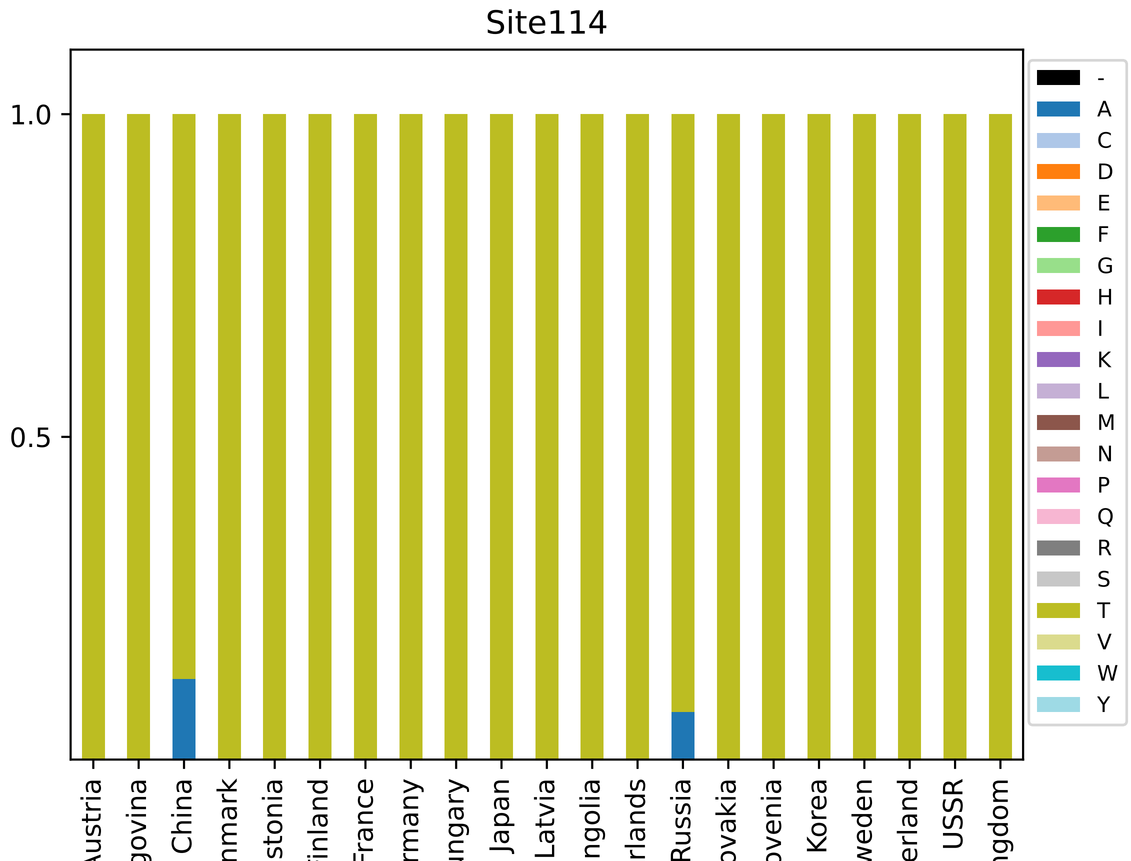

Site114
1.0
0.5
-
A
C
D
E
F
G
H
I
K
L
M
N
P
Q
R
S
T
V
W
Y
Austria
govina
China
nmark
stonia
Finland
France
rmany
ungary
Japan
Latvia
ngolia
rlands
Russia
ovakia
ovenia
Korea
weden
erland
USSR
ngdom

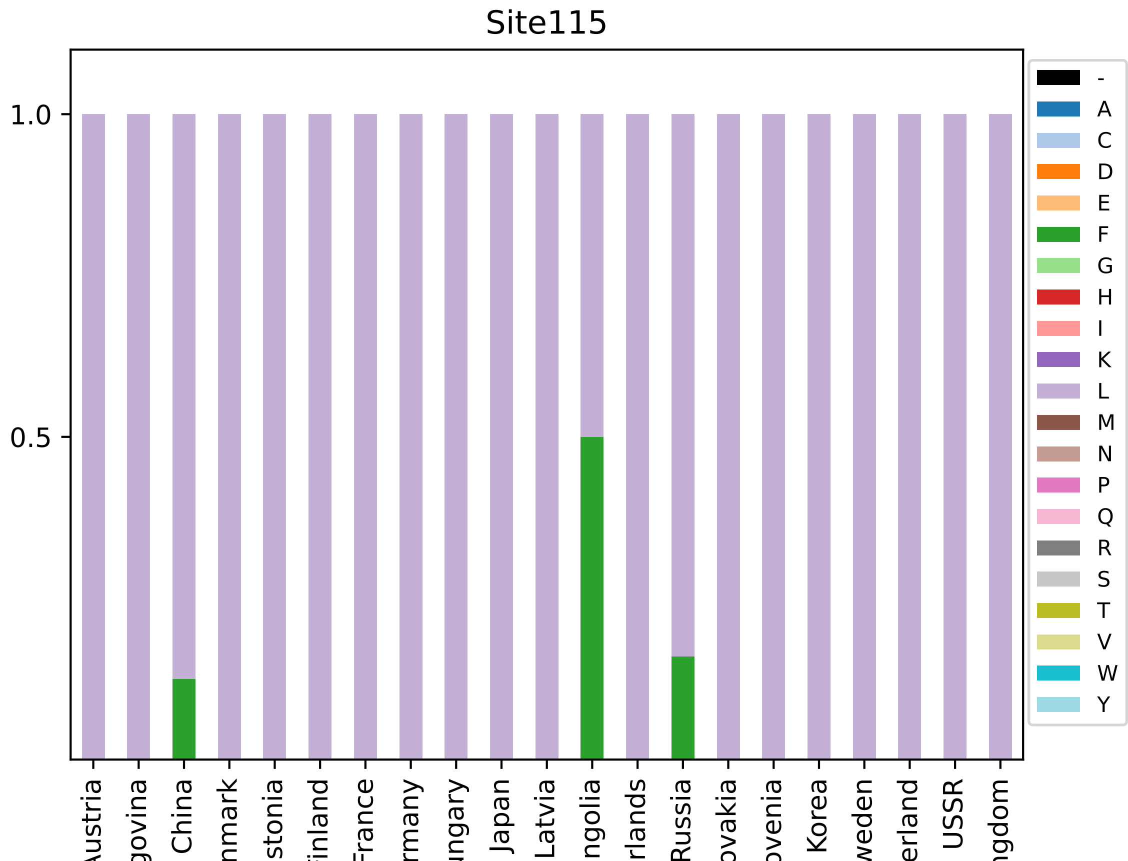
Site115
1.0
0.5
-
A
C
D
E
F
G
H
I
K
L
M
N
P
Q
R
S
T
V
W
Y
China
Japan
Latvia
Russia
Korea
USSR

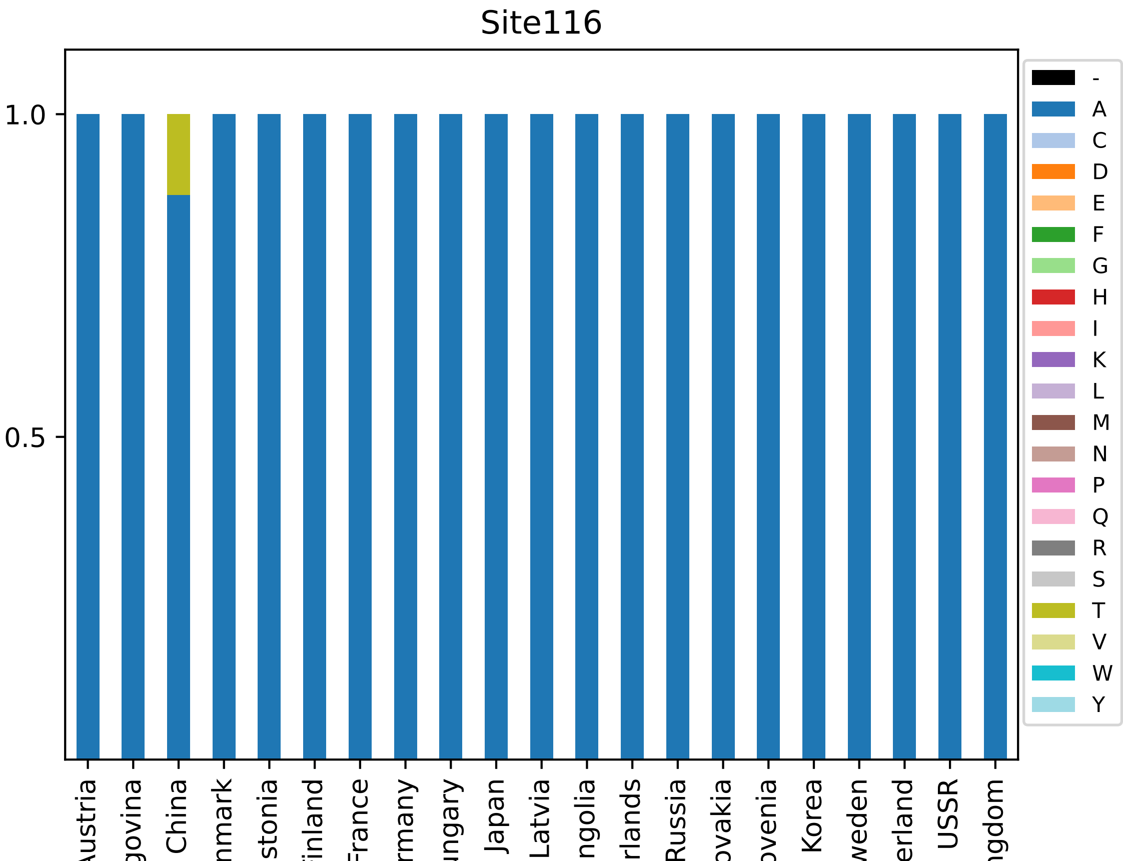
Site116
1.0
0.5
-
A
C
D
E
F
G
H
I
K
L
M
N
P
Q
R
S
T
V
W
Y
Austria
govina
China
nmark
stonia
inland
France
rmany
ungary
Japan
Latvia
ngolia
rlands
Russia
ovakia
ovenia
Korea
weden
erland
USSR
ngdom

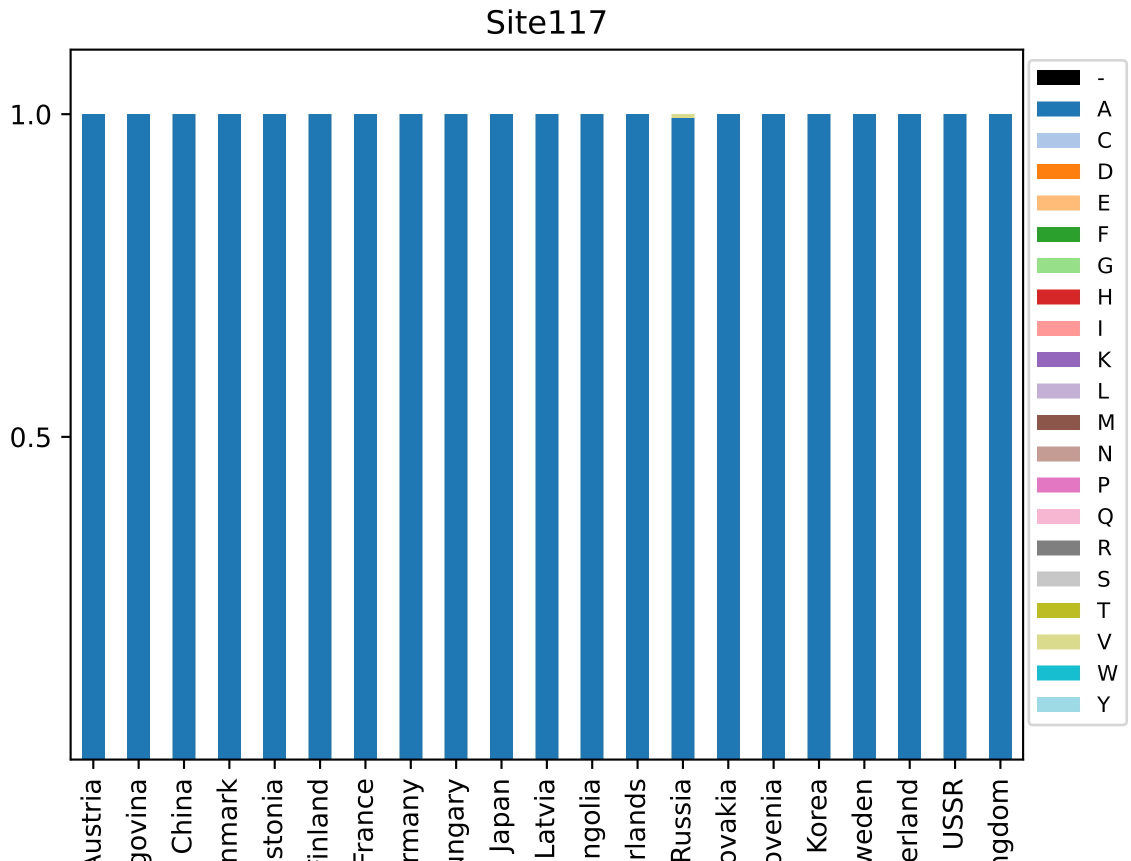
Site117
1.0
0.5
-
A
C
D
E
F
G
H
I
K
L
M
N
P
Q
R
S
T
V
W
Y
China
Japan
Latvia
Russia
Korea
USSR

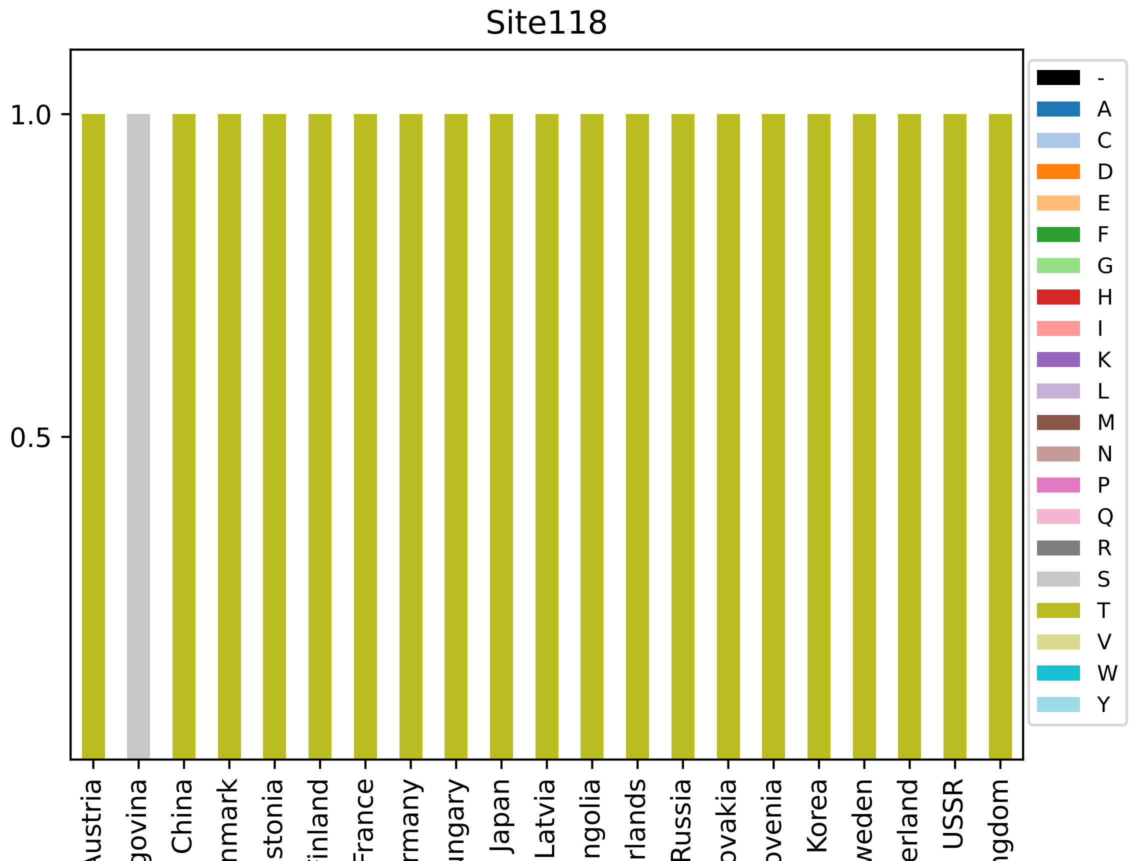
Site118
1.0
0.5
-
A
C
D
E
F
G
H
I
K
L
M
N
P
Q
R
S
T
V
W
Y
China
Japan
Latvia
Russia
Korea
USSR

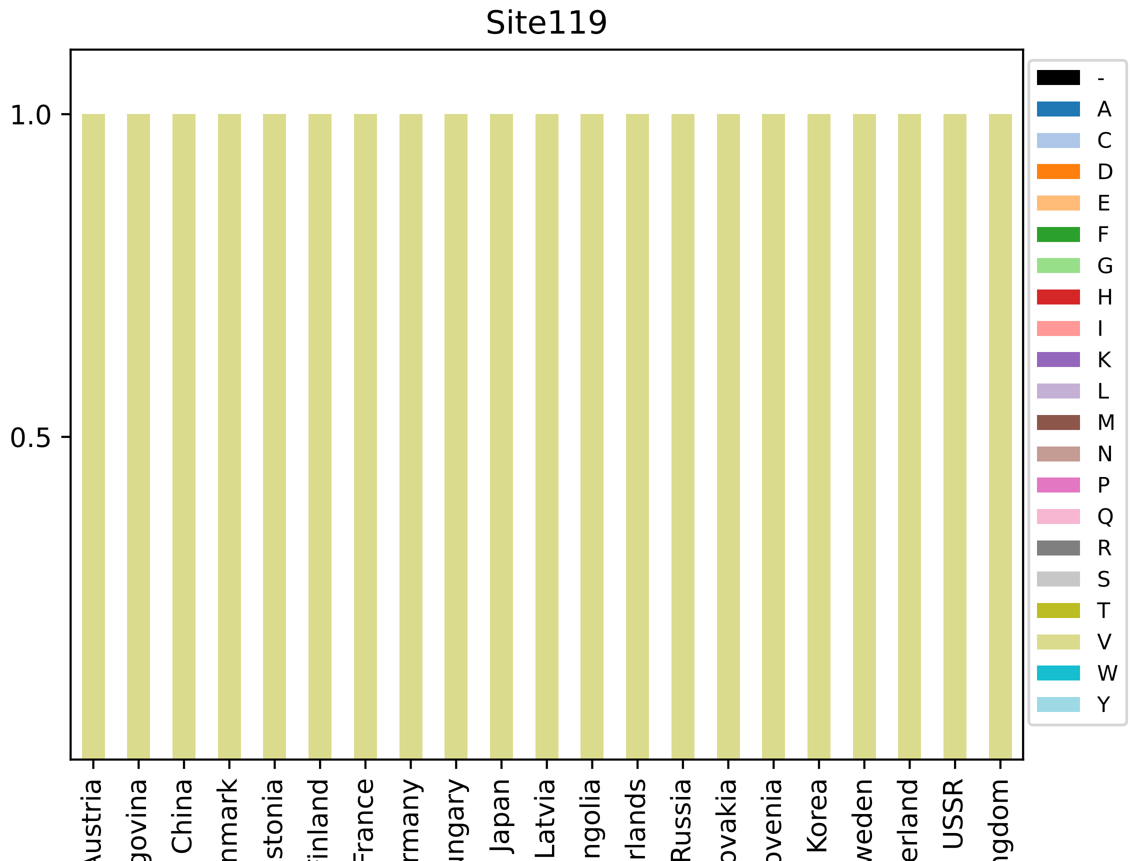
Site119
1.0
0.5
-
A
C
D
E
F
G
H
I
K
L
M
N
P
Q
R
S
T
V
W
Y
ustria
govina
China
nmark
stonia
inland
rance
rmany
ungary
Japan
Latvia
ngolia
rlands
Russia
ovakia
ovenia
Korea
weden
erland
USSR
ngdom

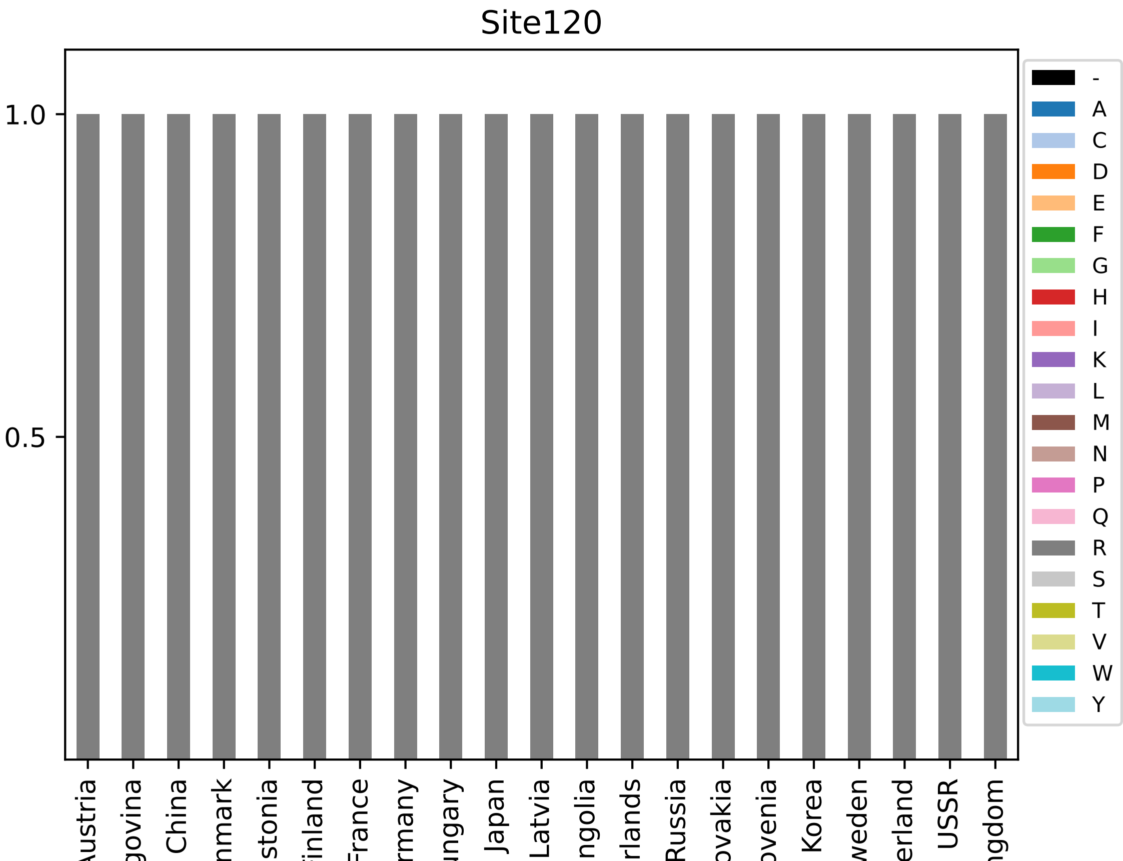
Site120
1.0
0.5
Austria
govina
China
nmark
stonia
inland
France
rmany
ungary
Japan
Latvia
ngolia
rlands
Russia
ovakia
ovenia
Korea
weden
erland
USSR
ngdom
-
A
C
D
E
F
G
H
I
K
L
M
N
P
Q
R
S
T
V
W
Y

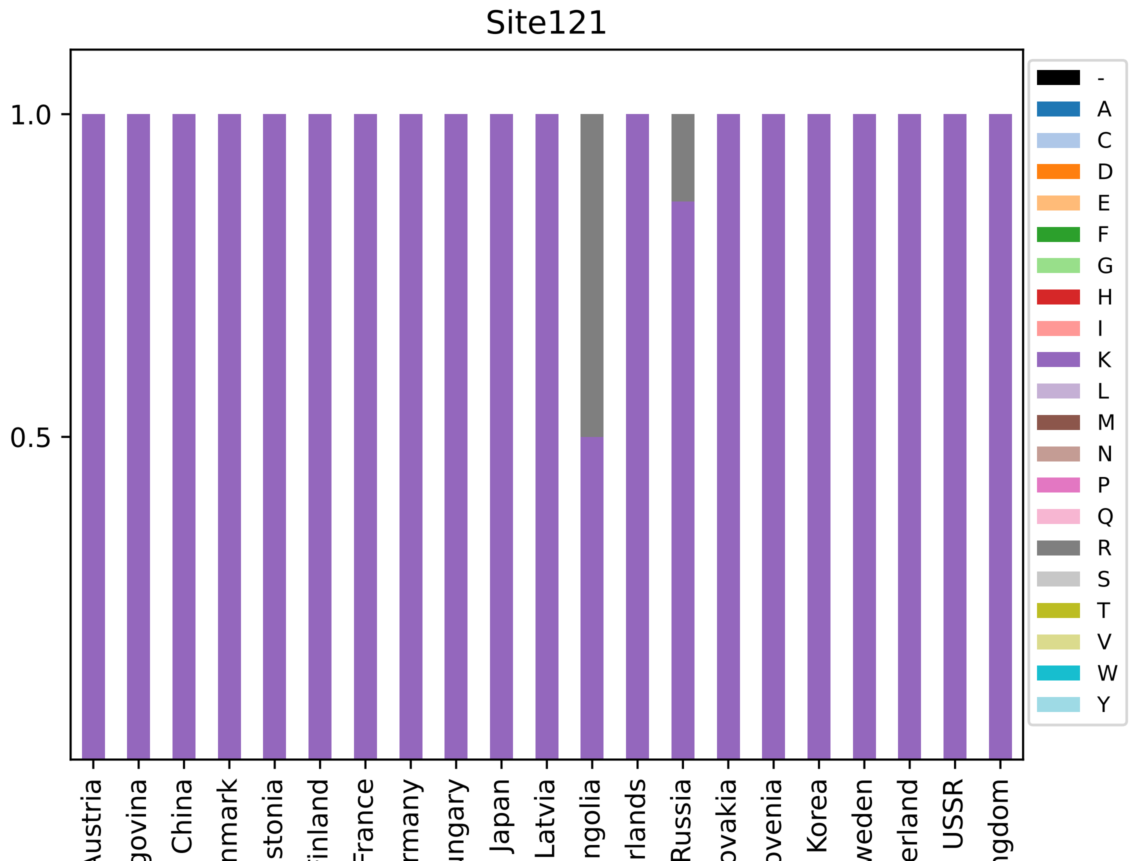
Site121
1.0
0.5
-
A
C
D
E
F
G
H
I
K
L
M
N
P
Q
R
S
T
V
W
Y
Austria
China
Estonia
Finland
France
Japan
Latvia
Russia
Korea
USSR

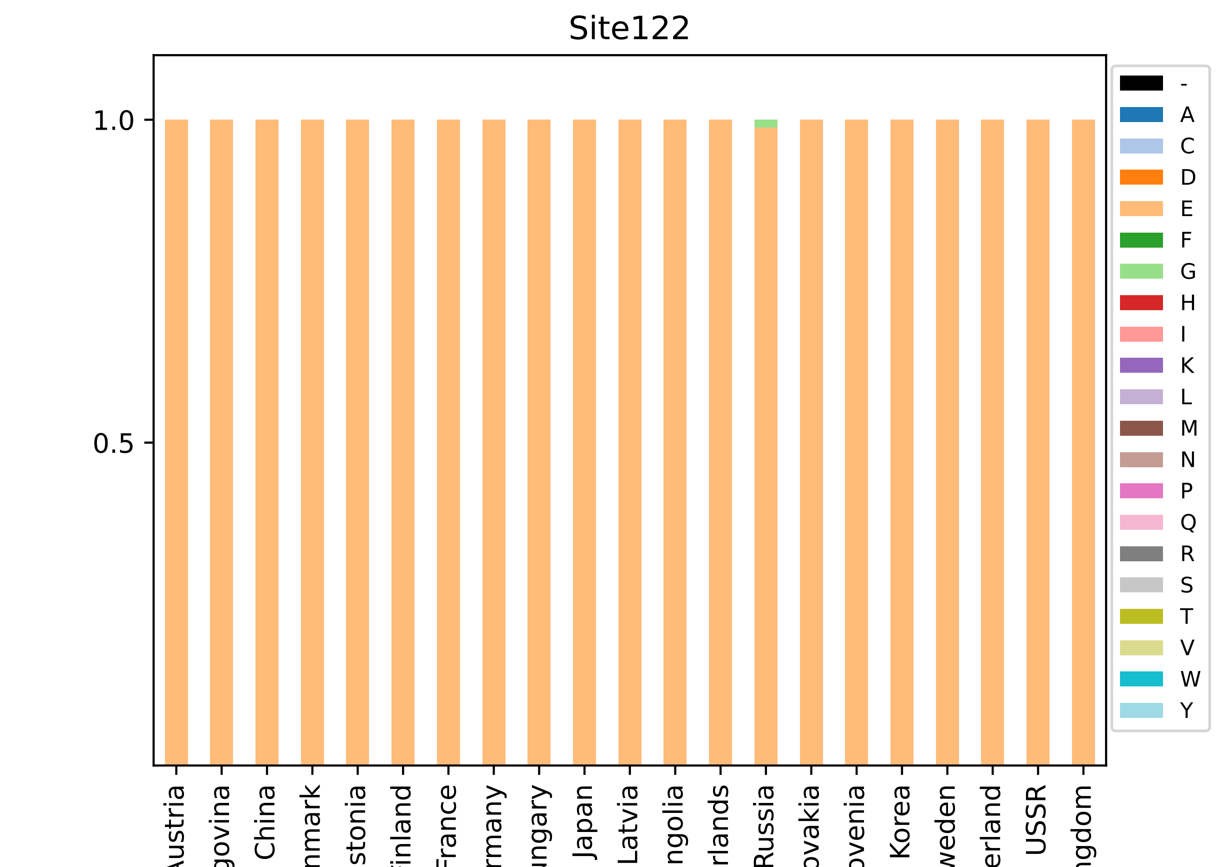

Site122
1.0
0.5
-
A
C
D
E
F
G
H
I
K
L
M
N
P
Q
R
S
T
V
W
Y
Austria
govina
China
nmark
stonia
Finland
France
rmany
ungary
Japan
Latvia
ngolia
rlands
Russia
ovakia
ovenia
Korea
weden
erland
USSR
ngdom

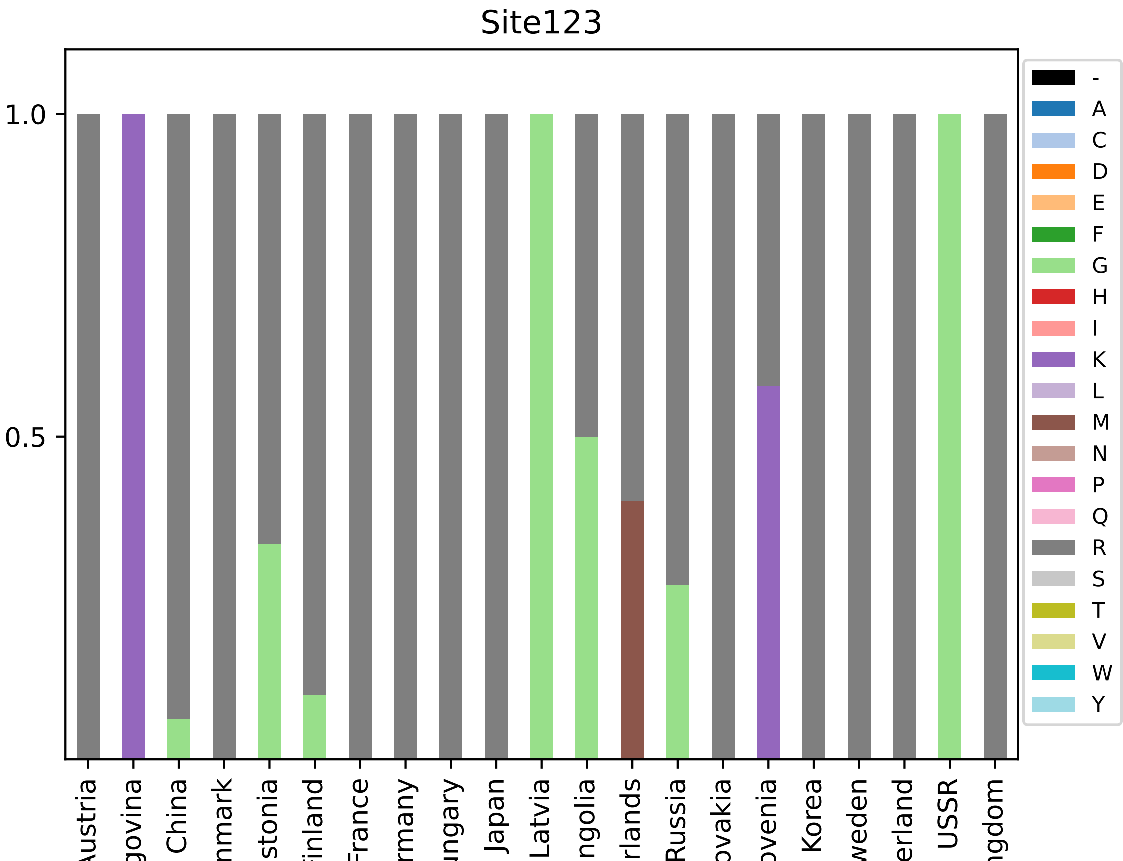
Site123
1.0
0.5
Austria
govina
China
nmark
stonia
inland
France
rmany
ungary
Japan
Latvia
ngolia
rlands
Russia
ovakia
ovenia
Korea
weden
erland
USSR
ngdom
-
A
C
D
E
F
G
H
I
K
L
M
N
P
Q
R
S
T
V
W
Y

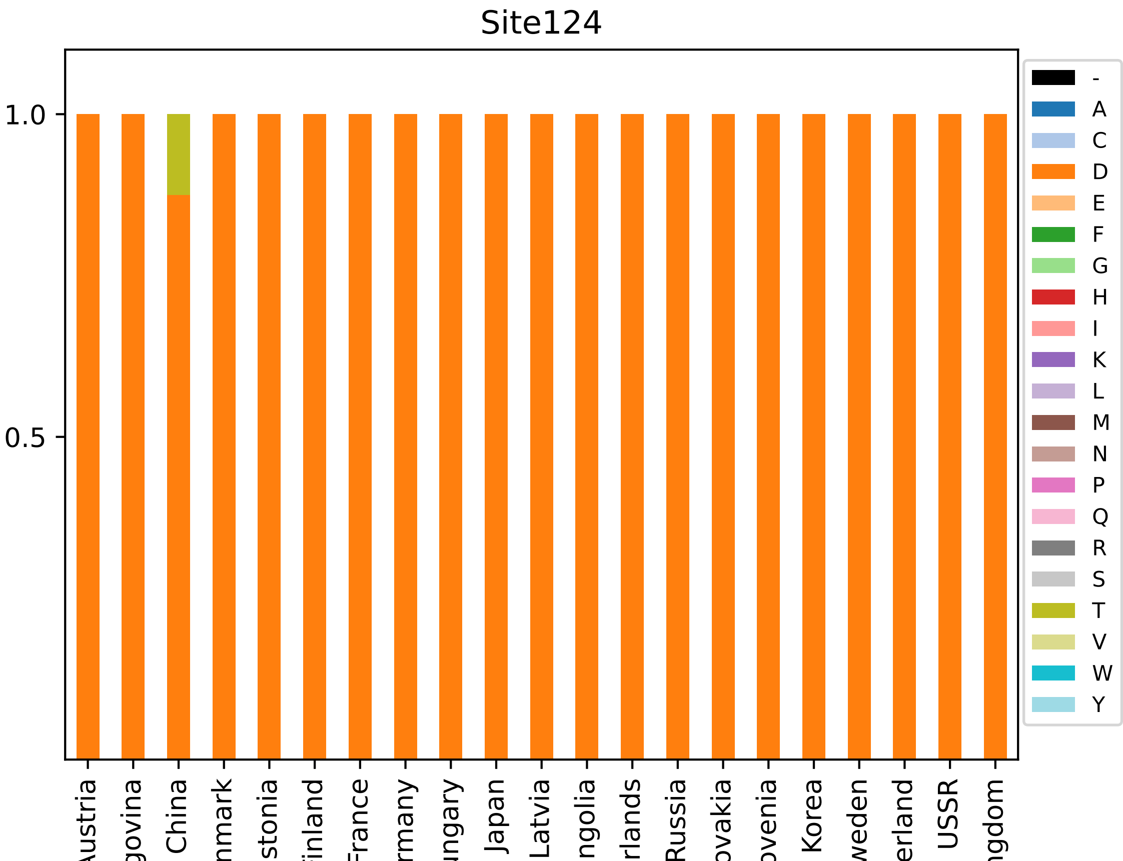

Site124
1.0
0.5
-
A
C
D
E
F
G
H
I
K
L
M
N
P
Q
R
S
T
V
W
Y
China
Japan
Latvia
Russia
Korea
USSR

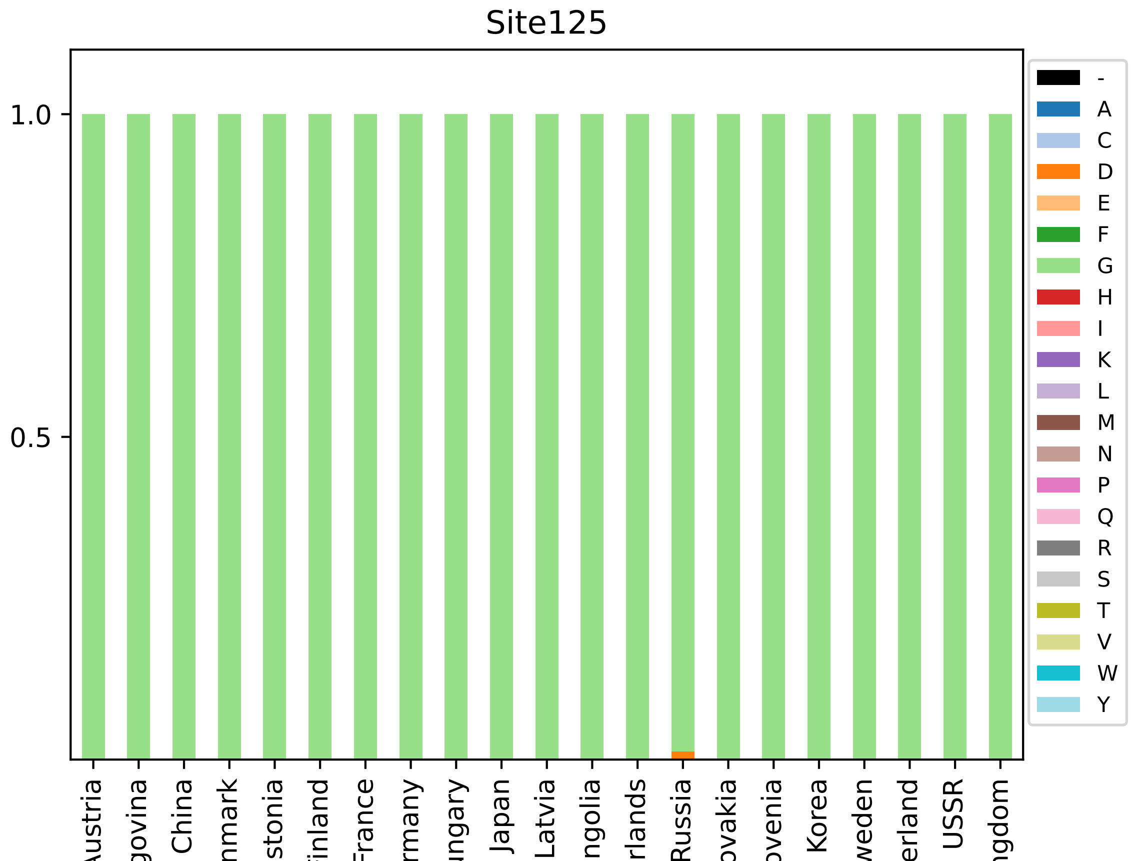
Site125
1.0
0.5
-
A
C
D
E
F
G
H
I
K
L
M
N
P
Q
R
S
T
V
W
Y
Austria
China
Japan
Latvia
Russia
Korea
USSR

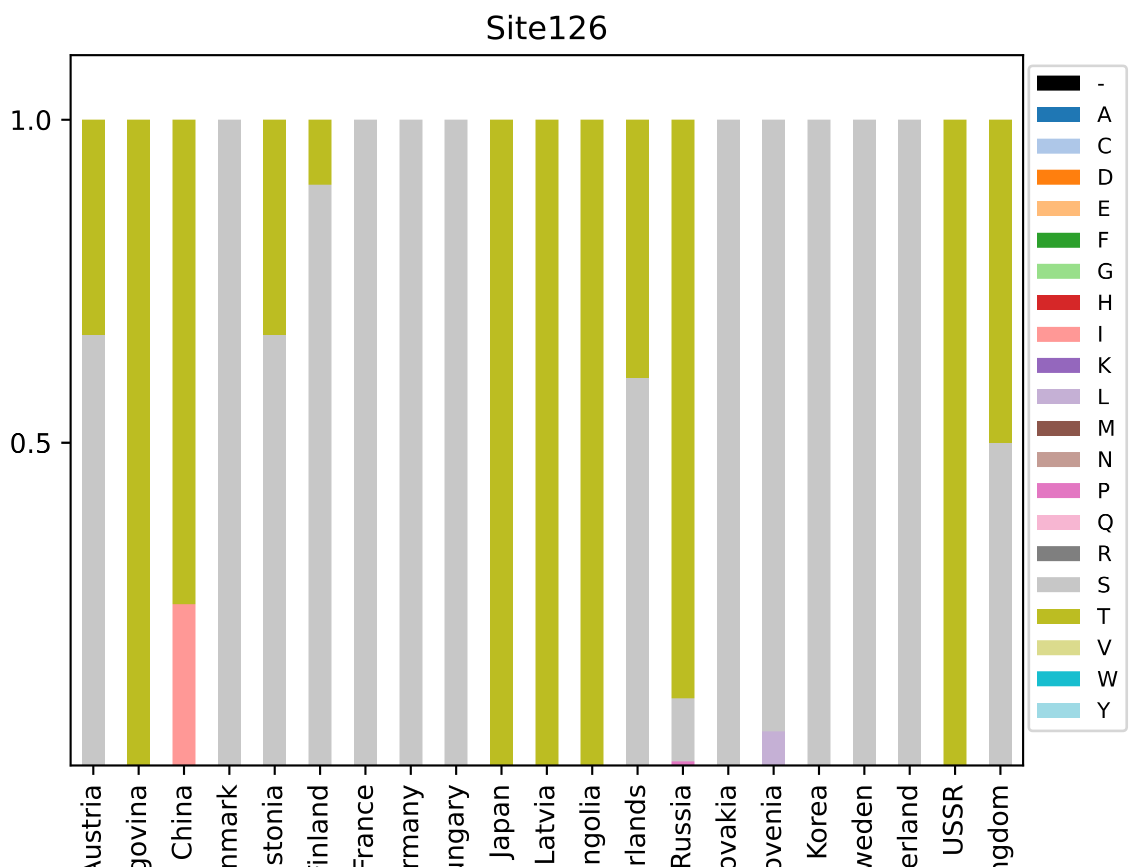

Site126
1.0
0.5
-
A
C
D
E
F
G
H
I
K
L
M
N
P
Q
R
S
T
V
W
Y
Austria
China
Estonia
Finland
France
Japan
Latvia
Russia
Korea
USSR

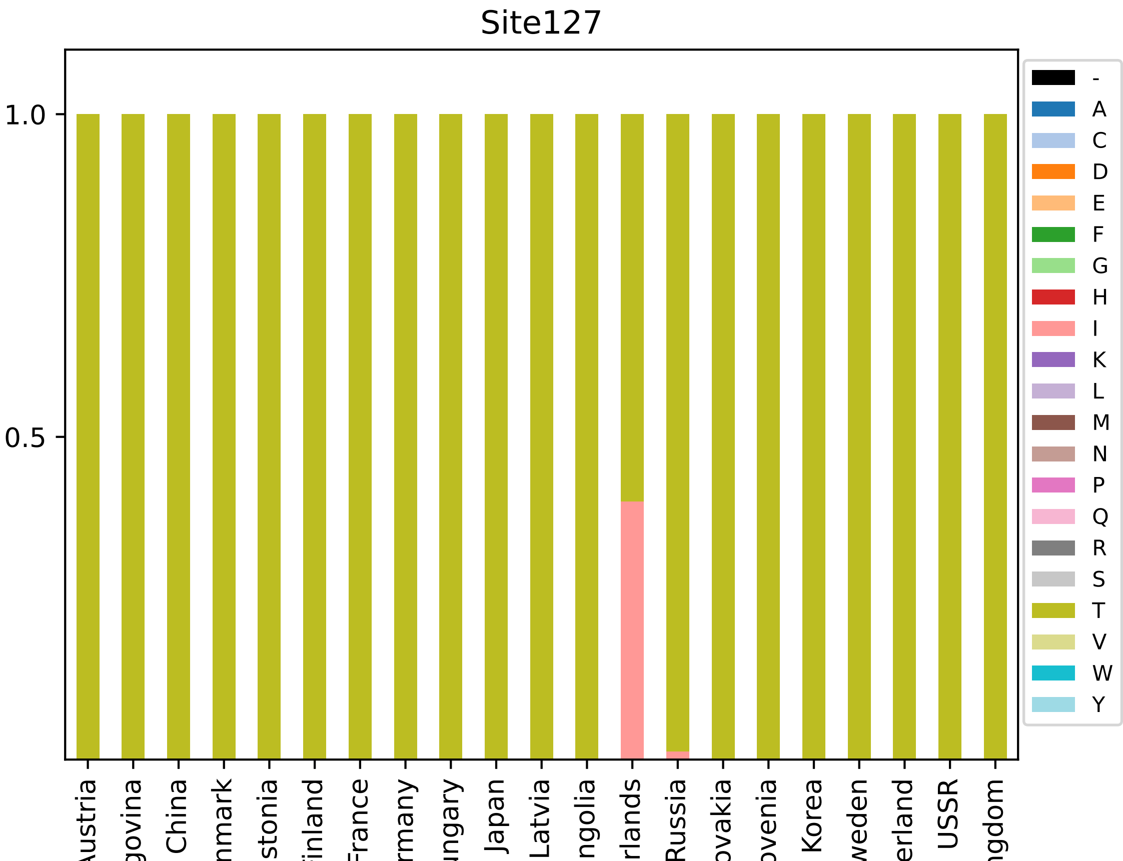

Site127
1.0
0.5
-
A
C
D
E
F
G
H
I
K
L
M
N
P
Q
R
S
T
V
W
Y
China
Japan
Latvia
Russia
Korea
USSR

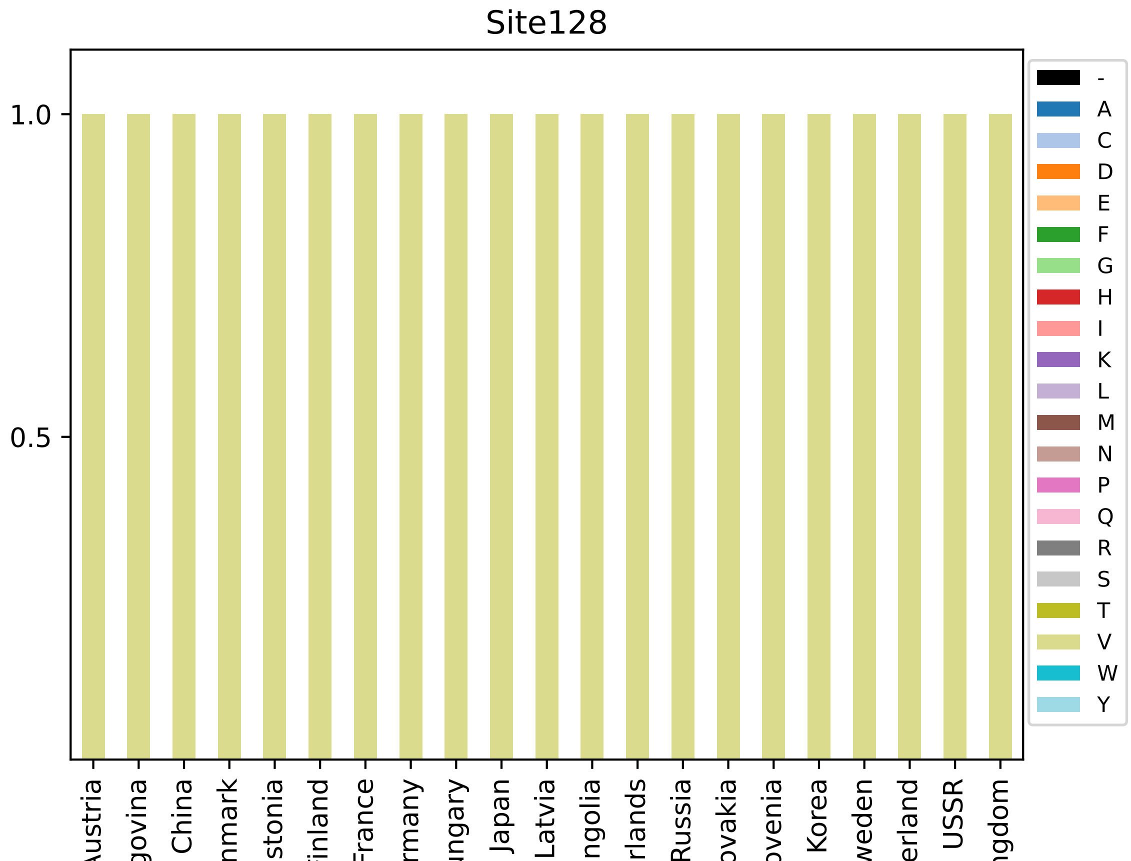
Site128
1.0
0.5
-
A
C
D
E
F
G
H
I
K
L
M
N
P
Q
R
S
T
V
W
Y
Austria
govina
China
nmark
stonia
Finland
France
rmany
ungary
Japan
Latvia
ngolia
rlands
Russia
ovakia
ovenia
Korea
weden
erland
USSR
ngdom

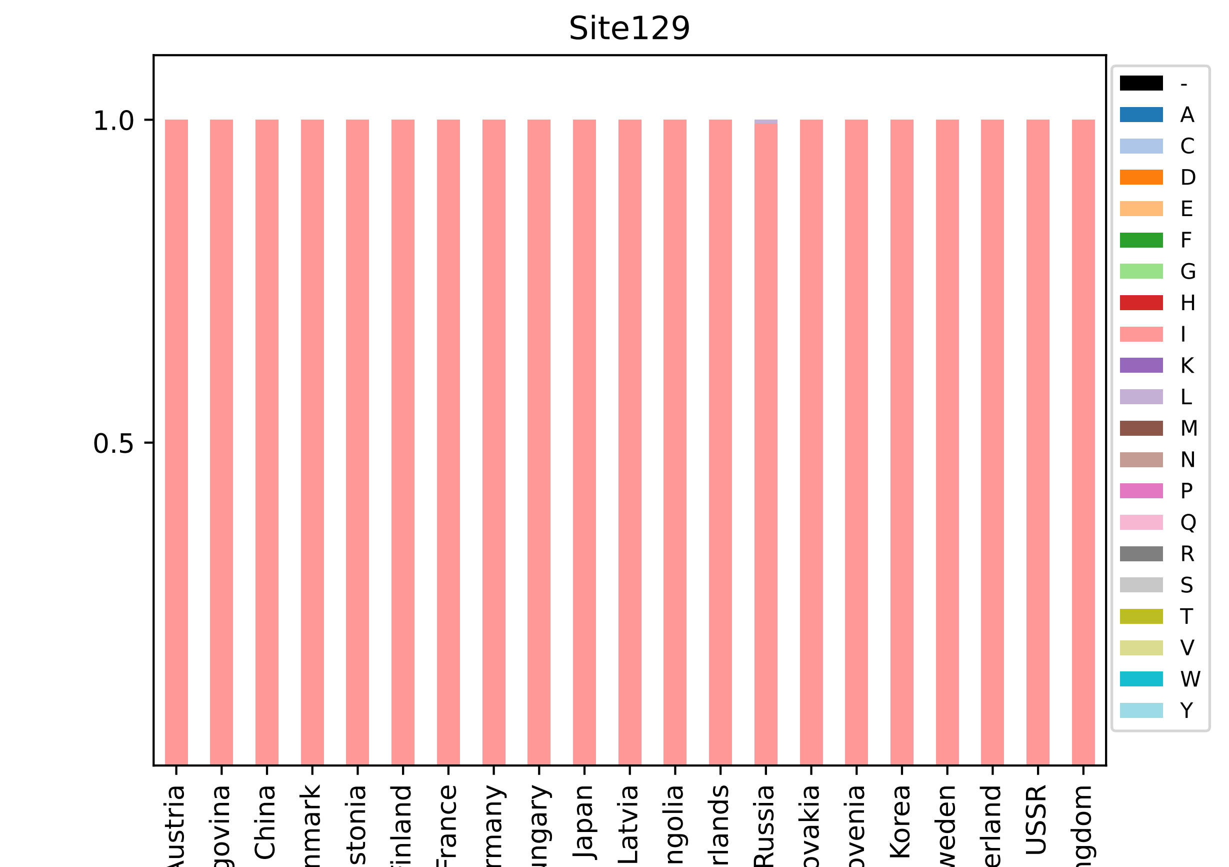

Site129
1.0
0.5
-
A
C
D
E
F
G
H
I
K
L
M
N
P
Q
R
S
T
V
W
Y
Austria
govina
China
nmark
stonia
Finland
France
rmany
ungary
Japan
Latvia
ngolia
rlands
Russia
ovakia
ovenia
Korea
weden
erland
USSR
ngdom

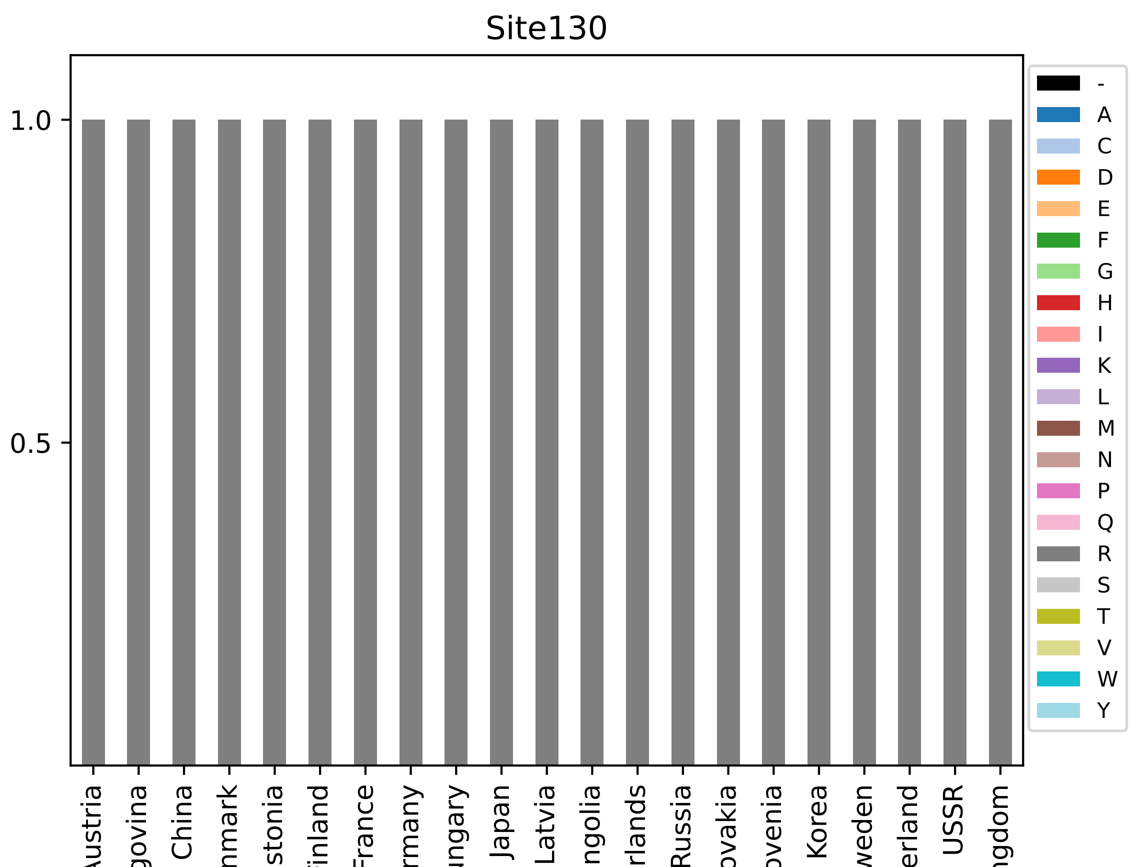
Site130
1.0
0.5
-
A
C
D
E
F
G
H
I
K
L
M
N
P
Q
R
S
T
V
W
Y
Austria
govina
China
nmark
stonia
Finland
France
rmany
ungary
Japan
Latvia
ngolia
rlands
Russia
ovakia
ovenia
Korea
weden
erland
USSR
ngdom

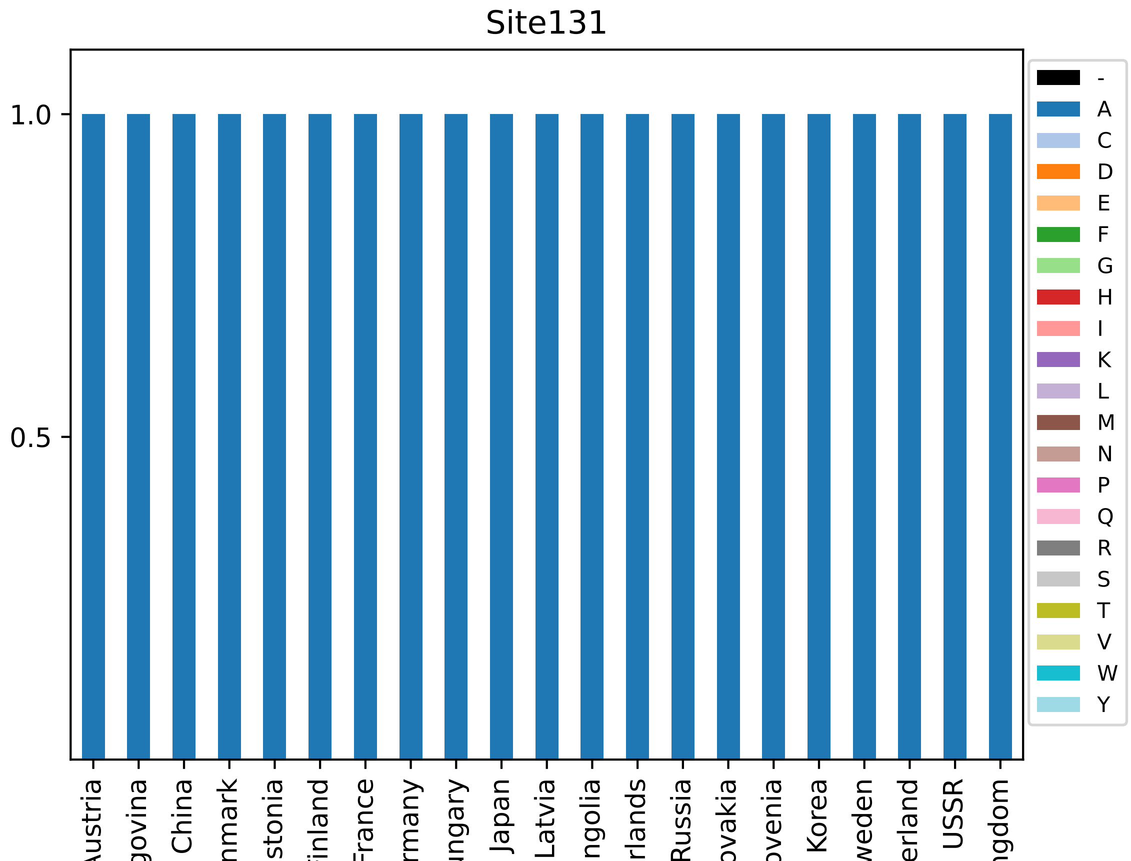

Site131
1.0
0.5
-
A
C
D
E
F
G
H
I
K
L
M
N
P
Q
R
S
T
V
W
Y
Austria
govina
China
nmark
stonia
Finland
France
rmany
ungary
Japan
Latvia
ngolia
rlands
Russia
ovakia
ovenia
Korea
weden
erland
USSR
ngdom

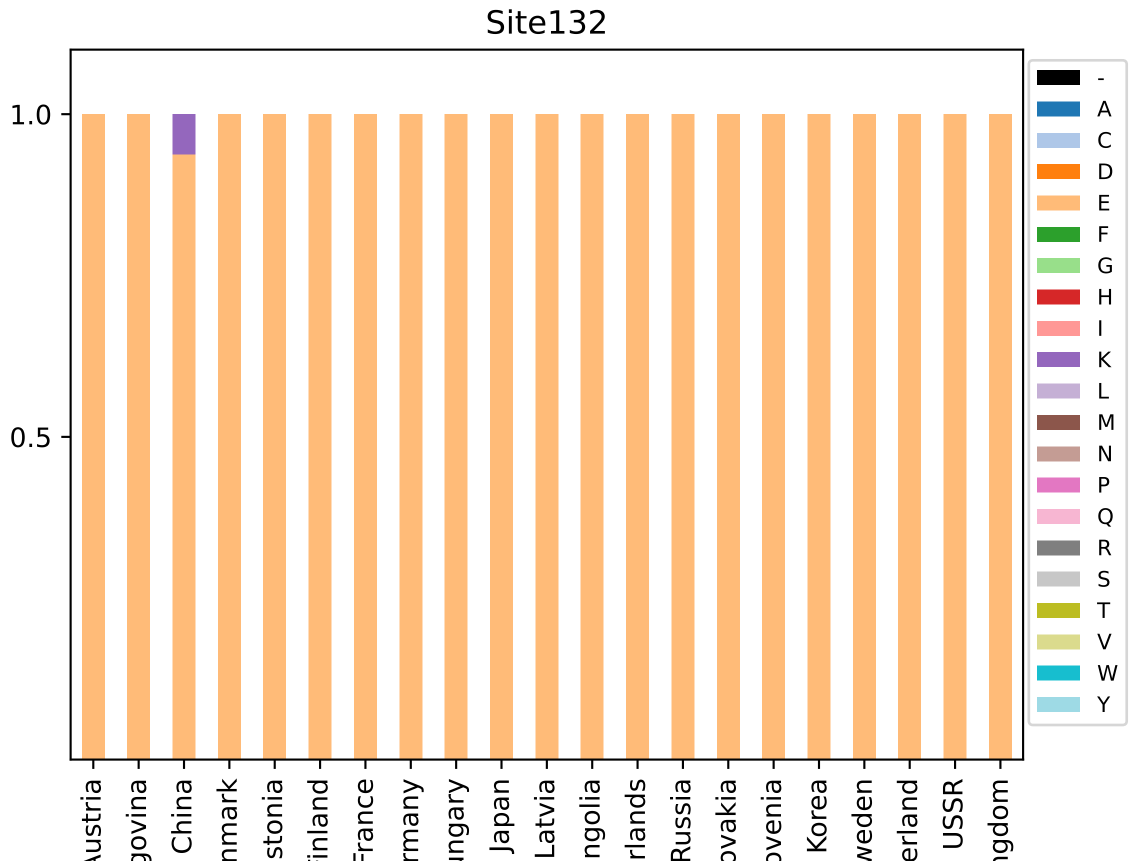

Site132
1.0
0.5
-
A
C
D
E
F
G
H
I
K
L
M
N
P
Q
R
S
T
V
W
Y
China
Japan
Latvia
Russia
Korea
USSR

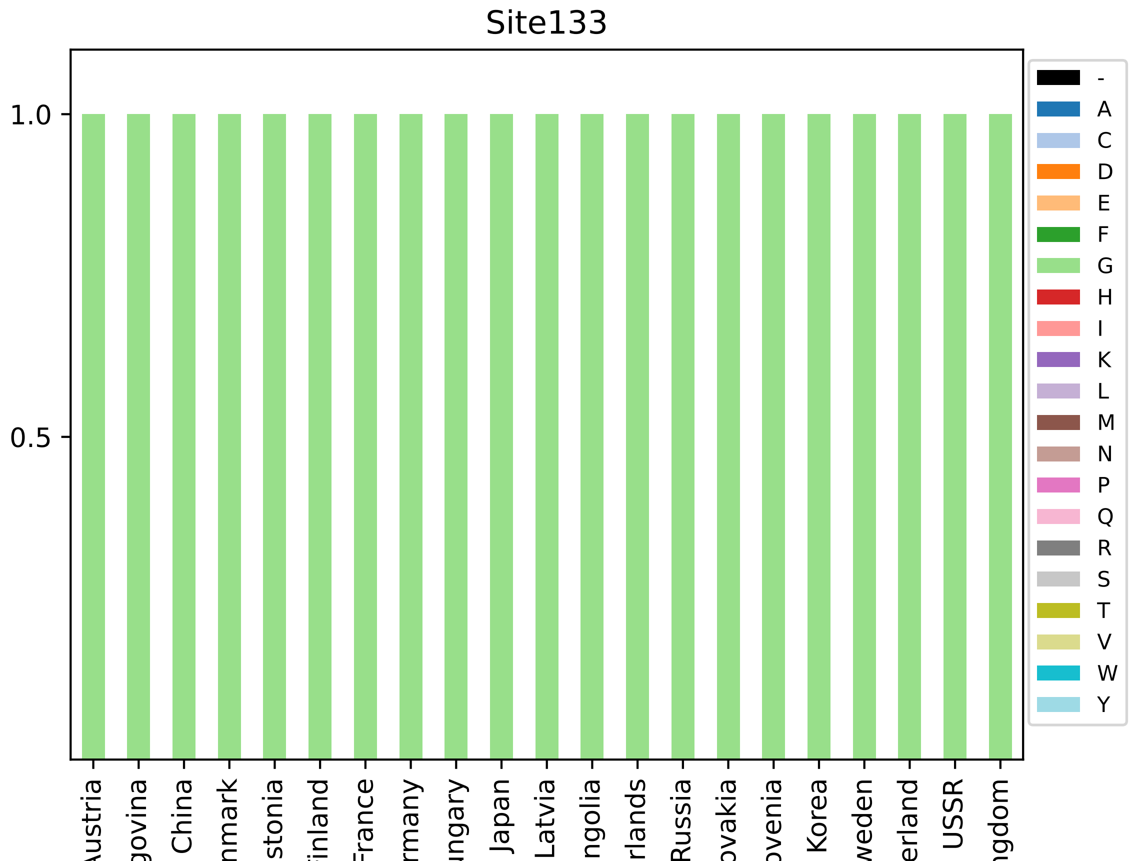
Site133
1.0
0.5
-
A
C
D
E
F
G
H
I
K
L
M
N
P
Q
R
S
T
V
W
Y
China
Japan
Latvia
Russia
Korea
USSR

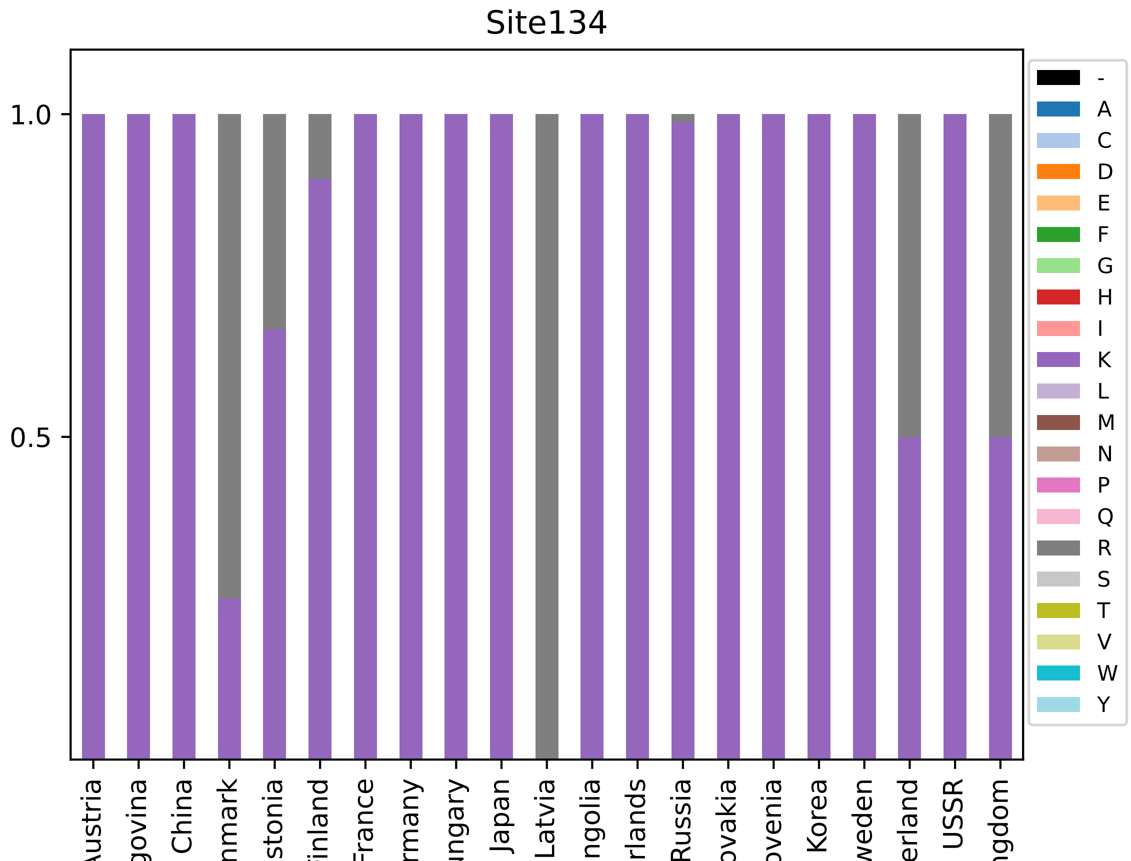

Site134
1.0
0.5
-
A
C
D
E
F
G
H
I
K
L
M
N
P
Q
R
S
T
V
W
Y
Austria
govina
China
nmark
stonia
Finland
France
rmany
ungary
Japan
Latvia
ngolia
rlands
Russia
ovakia
ovenia
Korea
weden
erland
USSR
ngdom

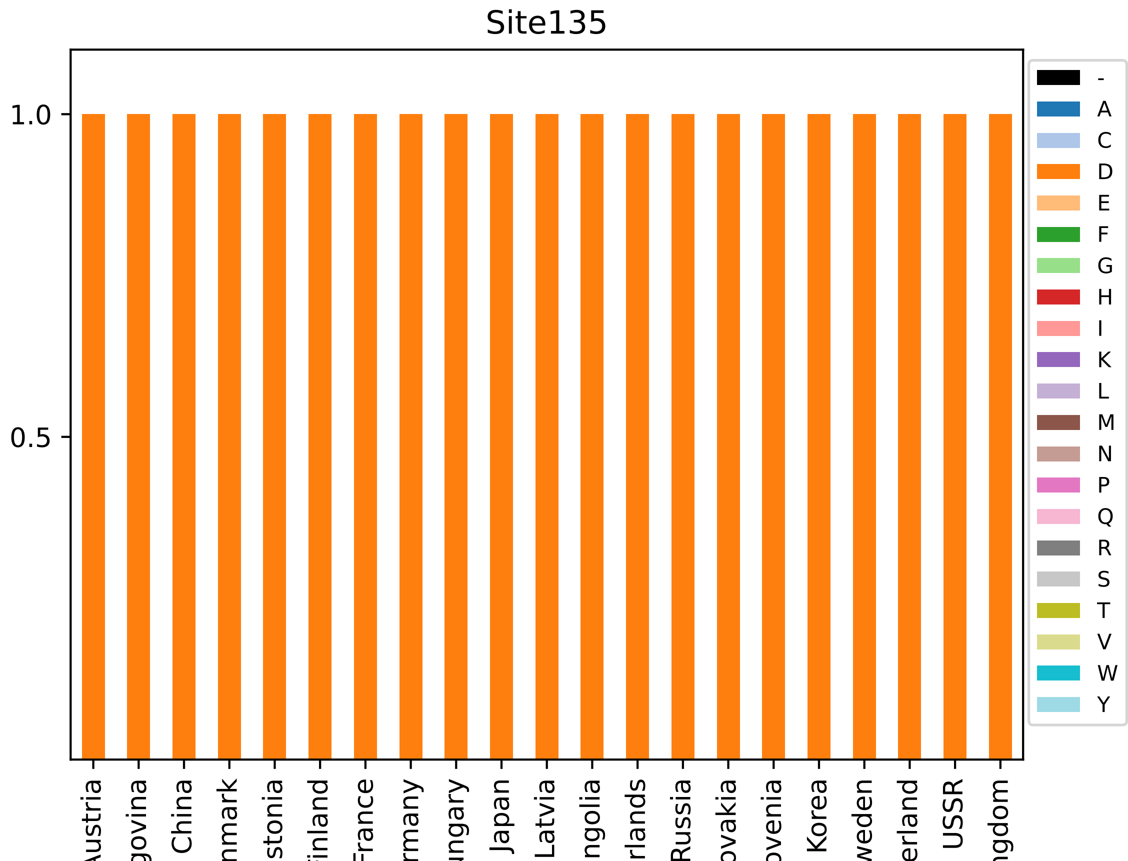
Site135
1.0
0.5
-
A
C
D
E
F
G
H
I
K
L
M
N
P
Q
R
S
T
V
W
Y
China
Japan
Latvia
Russia
Korea
USSR

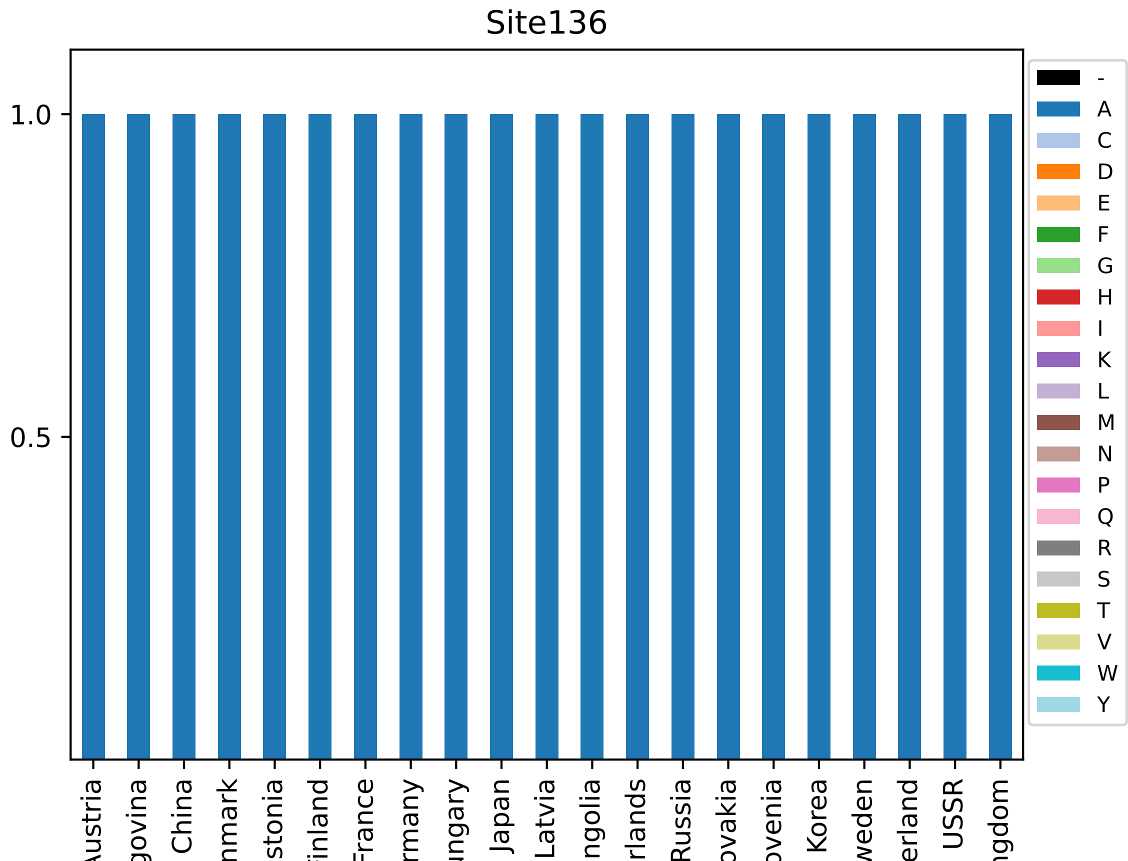
Site136
1.0
0.5
-
A
C
D
E
F
G
H
I
K
L
M
N
P
Q
R
S
T
V
W
Y
China
Japan
Latvia
Russia
Korea
USSR

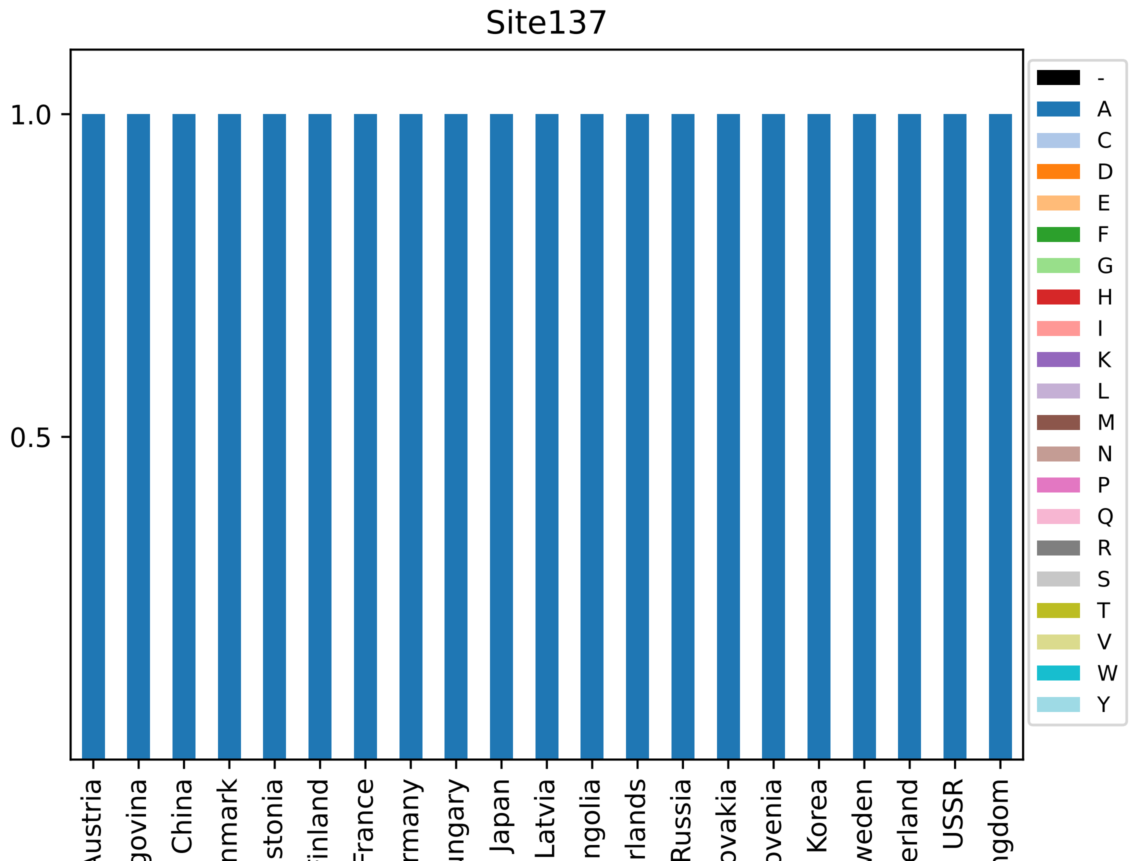

Site137
1.0
0.5
China
Japan
Latvia
Russia
Korea
USSR
-
A
C
D
E
F
G
H
I
K
L
M
N
P
Q
R
S
T
V
W
Y

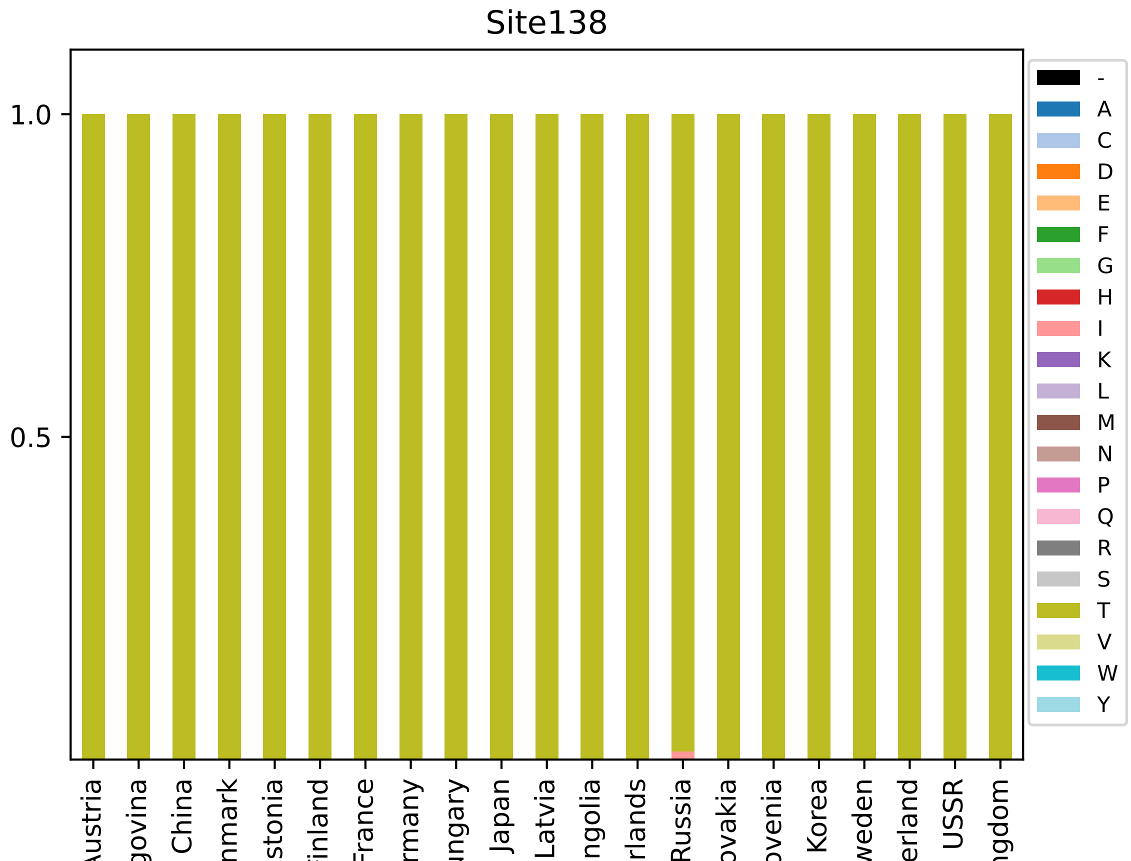

Site138
1.0
0.5
-
A
C
D
E
F
G
H
I
K
L
M
N
P
Q
R
S
T
V
W
Y
ustria
govina
China
nmark
stonia
inland
rance
rmany
ungary
Japan
Latvia
ngolia
rlands
Russia
ovakia
ovenia
Korea
weden
erland
USSR
ngdom

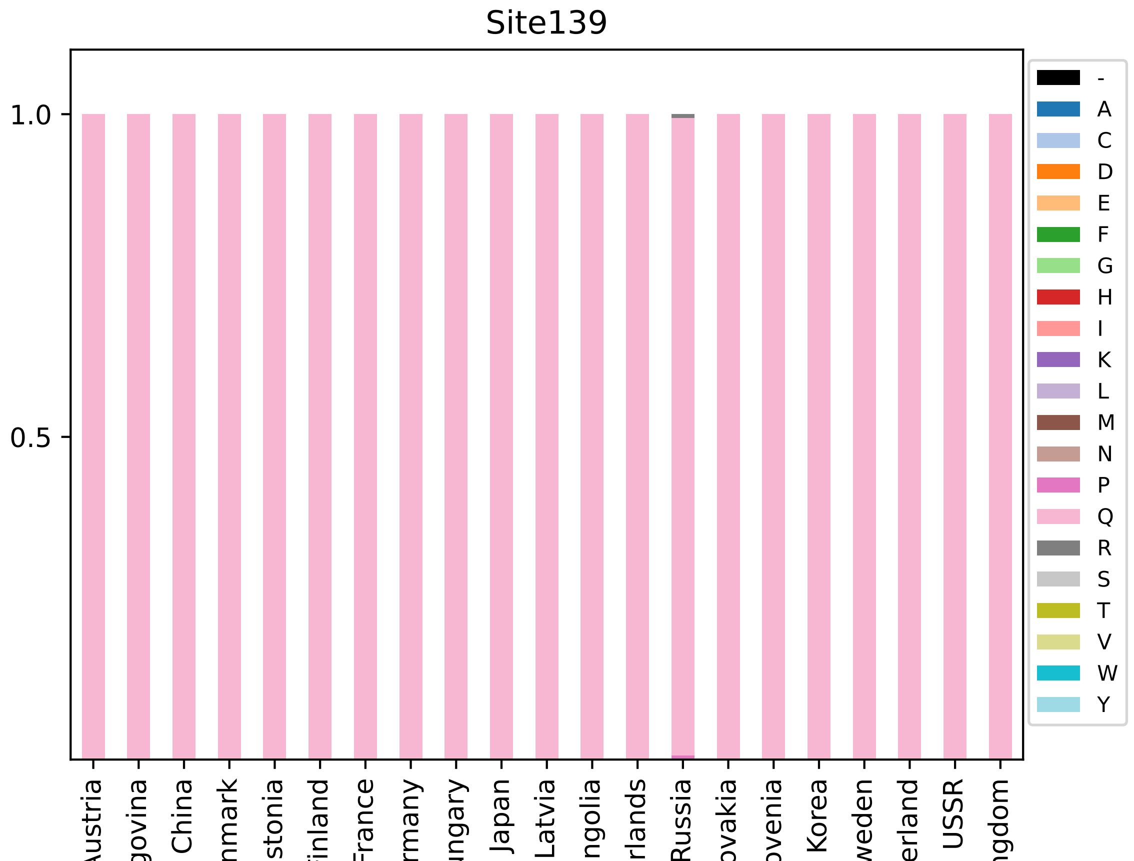
Site139
1.0
0.5
-
A
C
D
E
F
G
H
I
K
L
M
N
P
Q
R
S
T
V
W
Y
Austria
China
Finland
France
Japan
Latvia
Russia
Korea
USSR

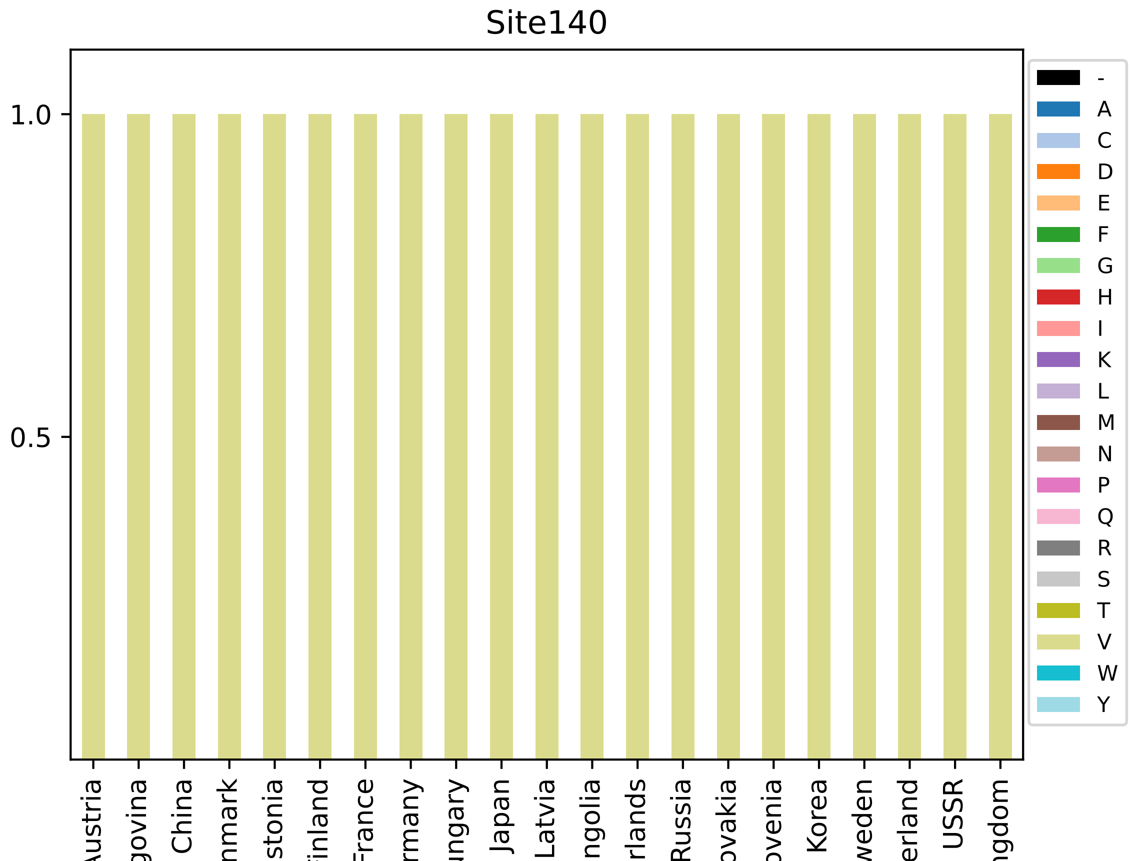
Site140
1.0
0.5
-
A
C
D
E
F
G
H
I
K
L
M
N
P
Q
R
S
T
V
W
Y
China
Japan
Latvia
Russia
Korea
USSR

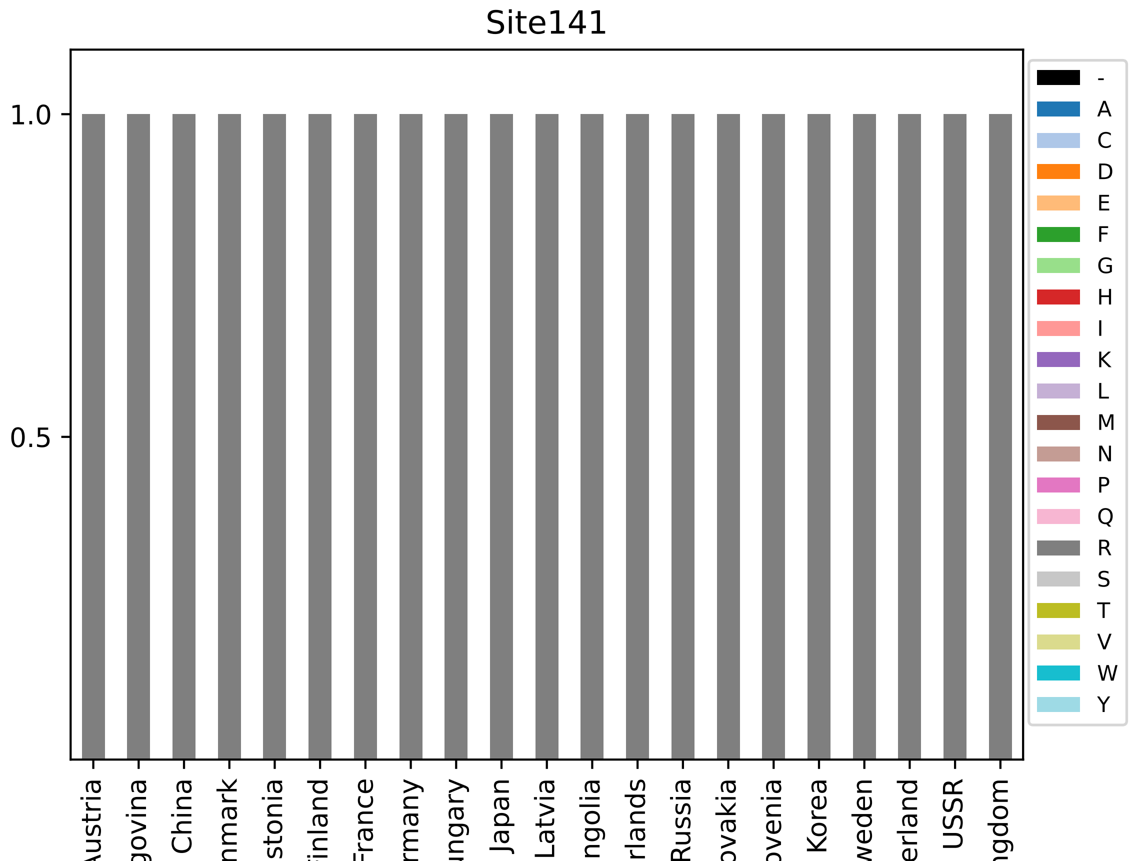

Site141
1.0
0.5
-
A
C
D
E
F
G
H
I
K
L
M
N
P
Q
R
S
T
V
W
Y
Austria
govina
China
nmark
stonia
inland
France
rmany
ungary
Japan
Latvia
ngolia
rlands
Russia
ovakia
ovenia
Korea
weden
erland
USSR
ngdom

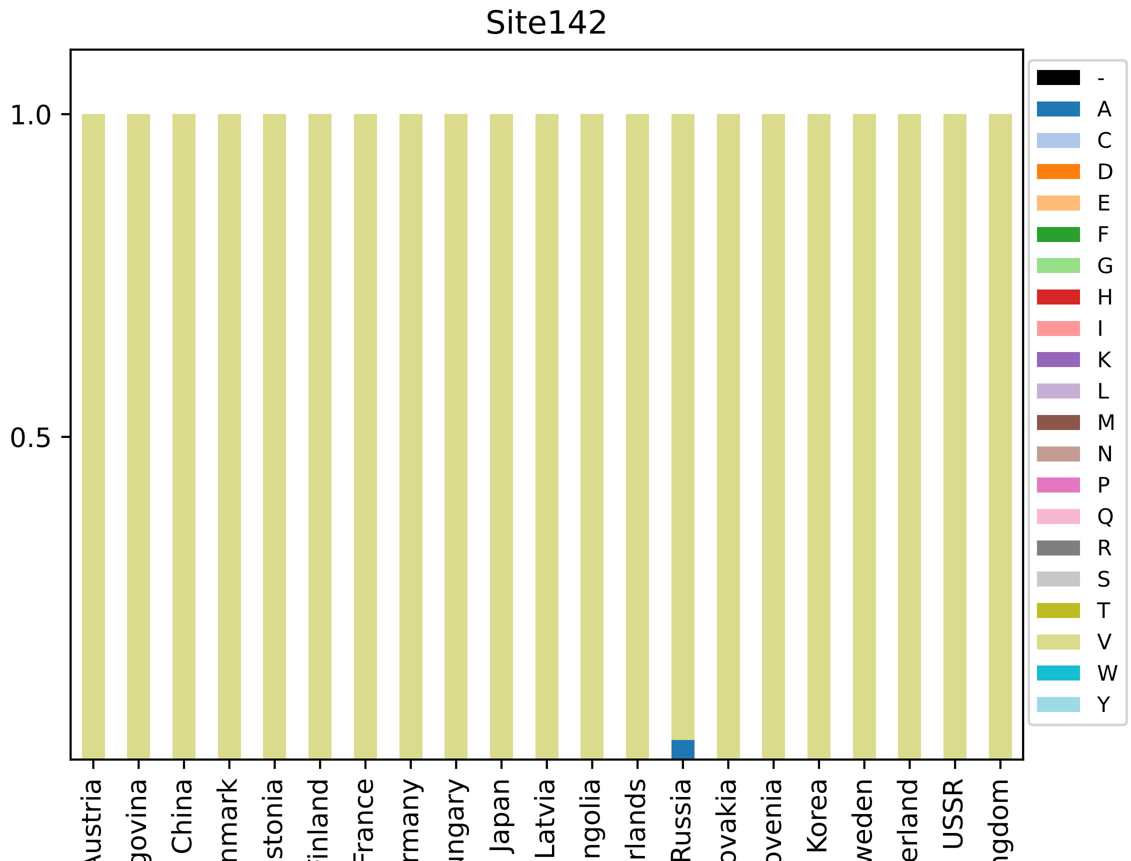

Site142
1.0
0.5
-
A
C
D
E
F
G
H
I
K
L
M
N
P
Q
R
S
T
V
W
Y
Austria
govina
China
nmark
stonia
inland
France
rmany
ungary
Japan
Latvia
ngolia
rlands
Russia
ovakia
ovenia
Korea
weden
erland
USSR
ngdom

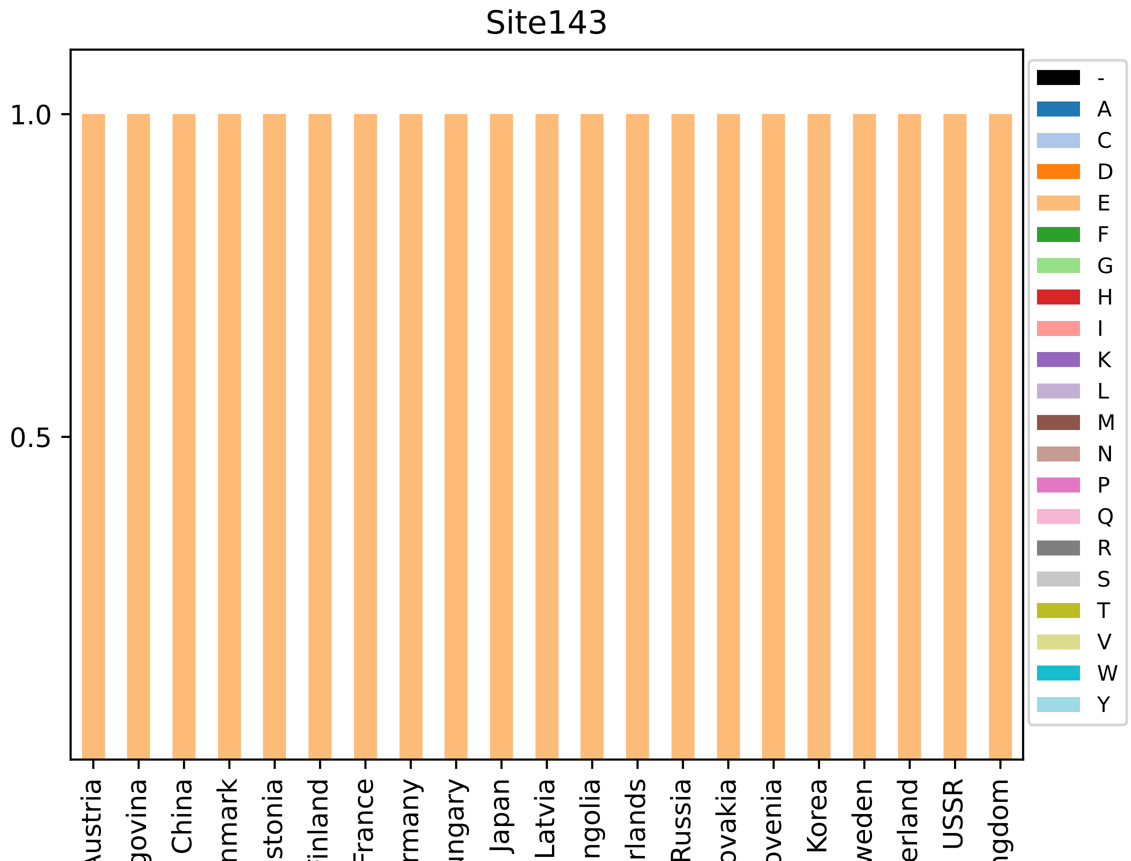

Site143
1.0
0.5
-
A
C
D
E
F
G
H
I
K
L
M
N
P
Q
R
S
T
V
W
Y
China
Japan
Latvia
Russia
Korea
USSR

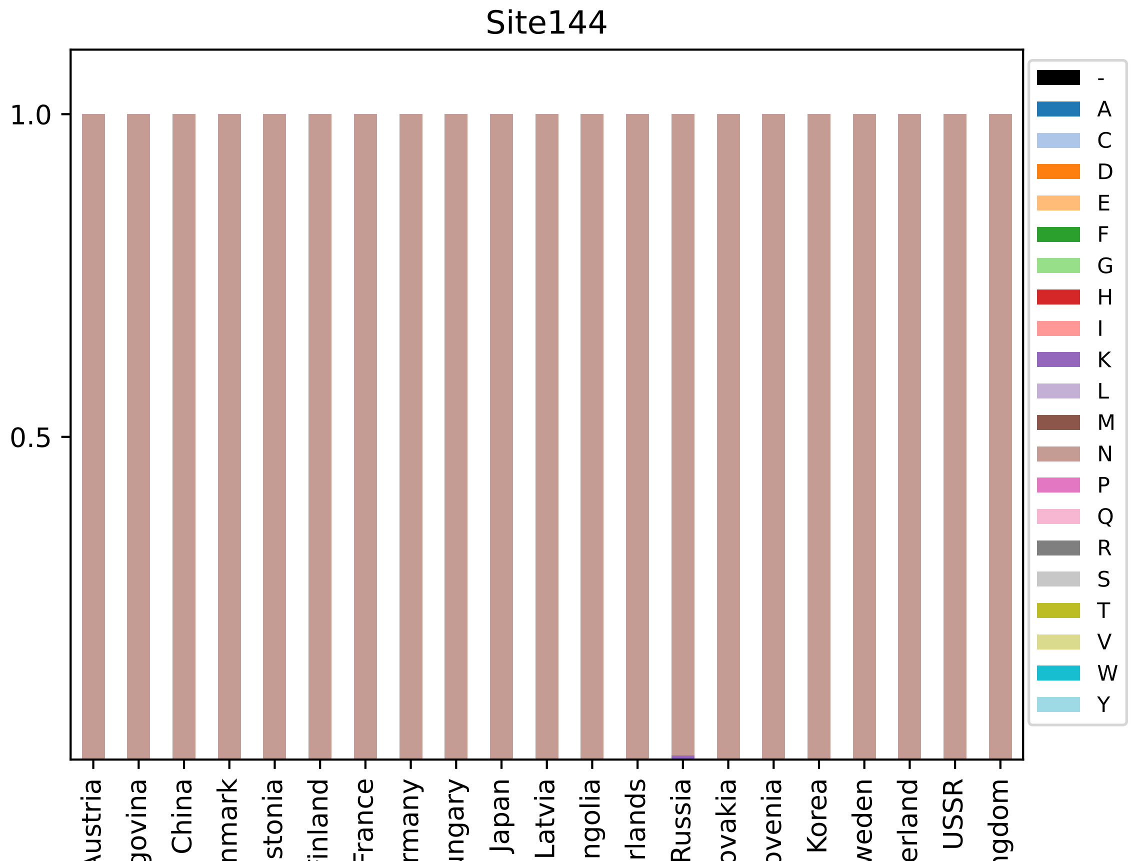

Site144
1.0
0.5
-
A
C
D
E
F
G
H
I
K
L
M
N
P
Q
R
S
T
V
W
Y
Austria
govina
China
nmark
stonia
inland
France
rmany
ungary
Japan
Latvia
ngolia
rlands
Russia
ovakia
ovenia
Korea
weden
erland
USSR
ngdom

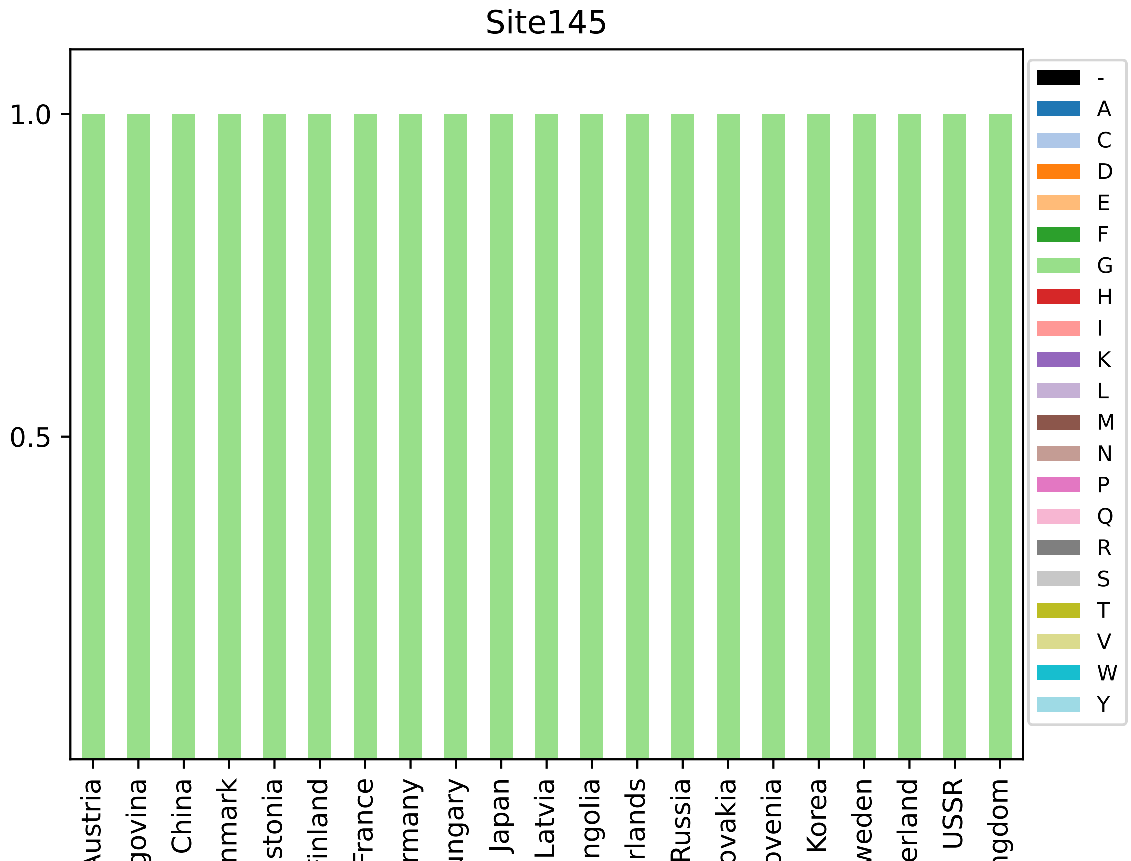
Site145
1.0
0.5
-
A
C
D
E
F
G
H
I
K
L
M
N
P
Q
R
S
T
V
W
Y
China
Japan
Latvia
Russia
Korea
USSR

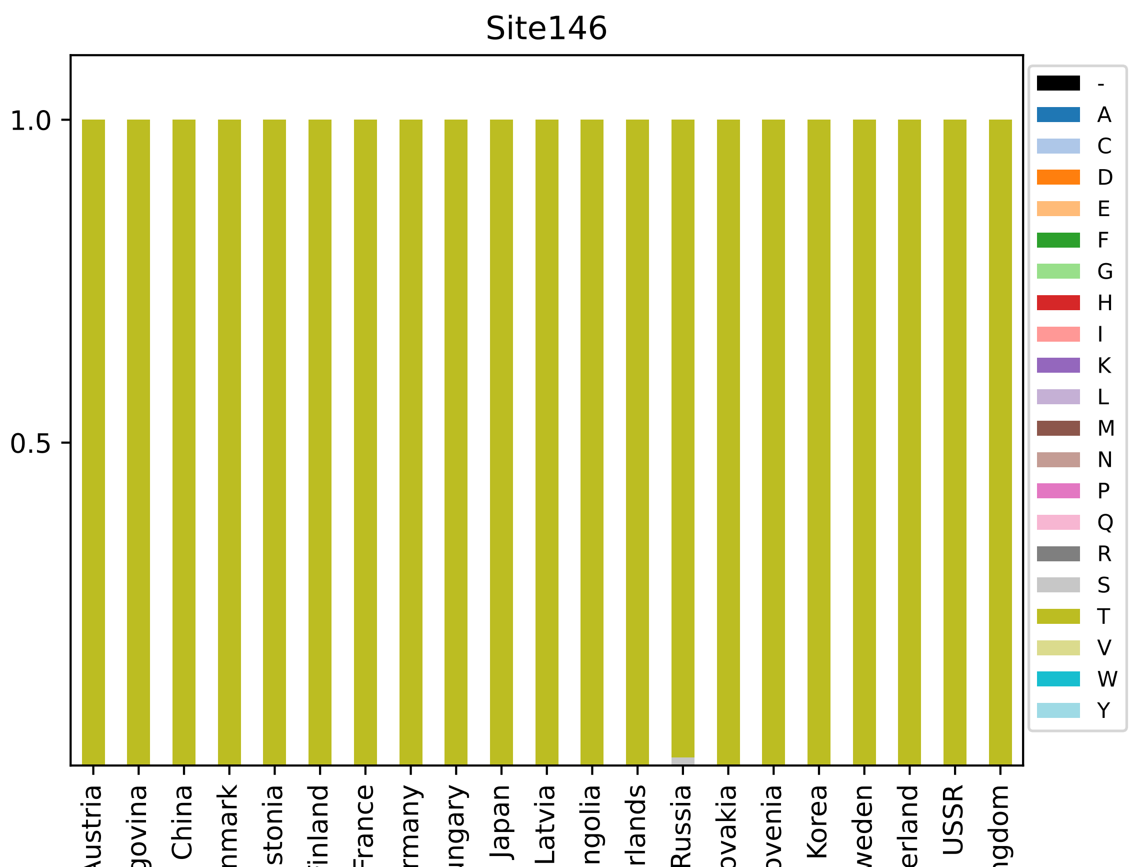

Site146
1.0
0.5
-
A
C
D
E
F
G
H
I
K
L
M
N
P
Q
R
S
T
V
W
Y
Austria
govina
China
nmark
stonia
inland
France
rmany
ungary
Japan
Latvia
ngolia
rlands
Russia
ovakia
ovenia
Korea
weden
erland
USSR
ngdom

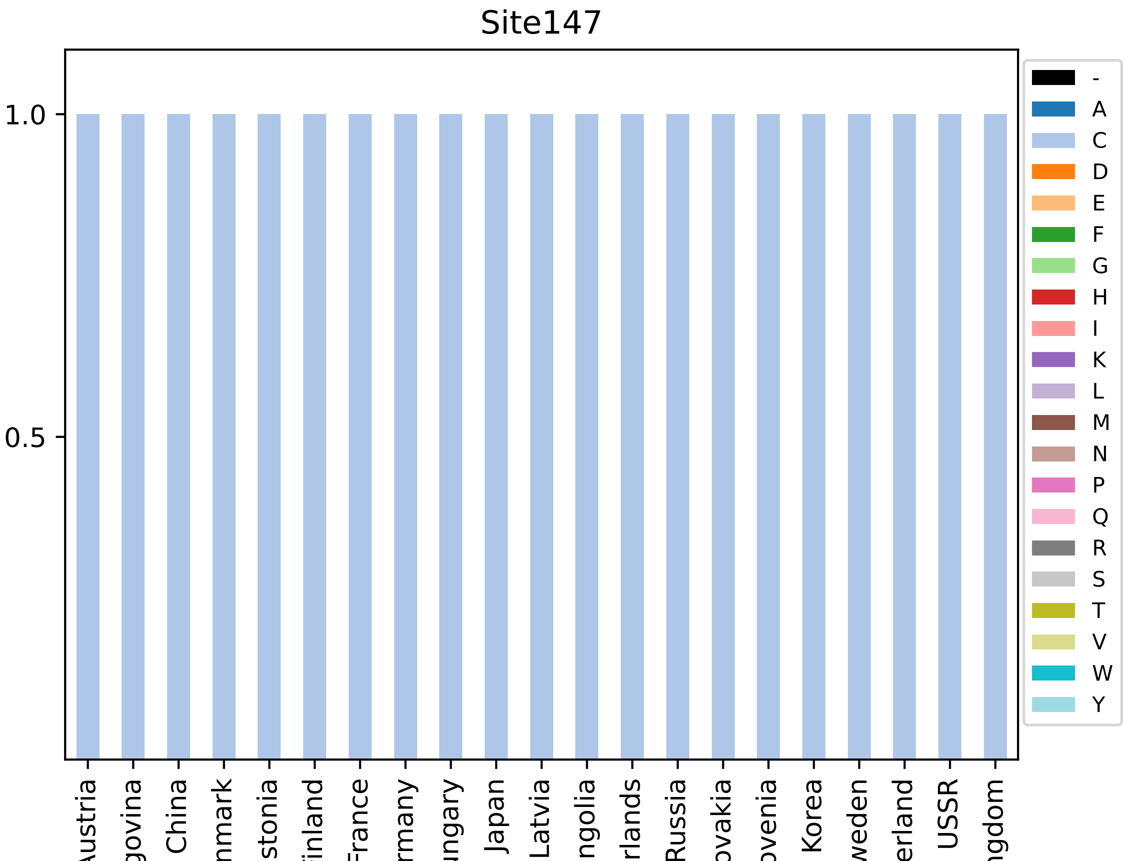

Site147
1.0
0.5
Austria
govina
China
nmark
stonia
Finland
France
rmany
ungary
Japan
Latvia
ngolia
rlands
Russia
ovakia
ovenia
Korea
weden
erland
USSR
ngdom
-
A
C
D
E
F
G
H
I
K
L
M
N
P
Q
R
S
T
V
W
Y

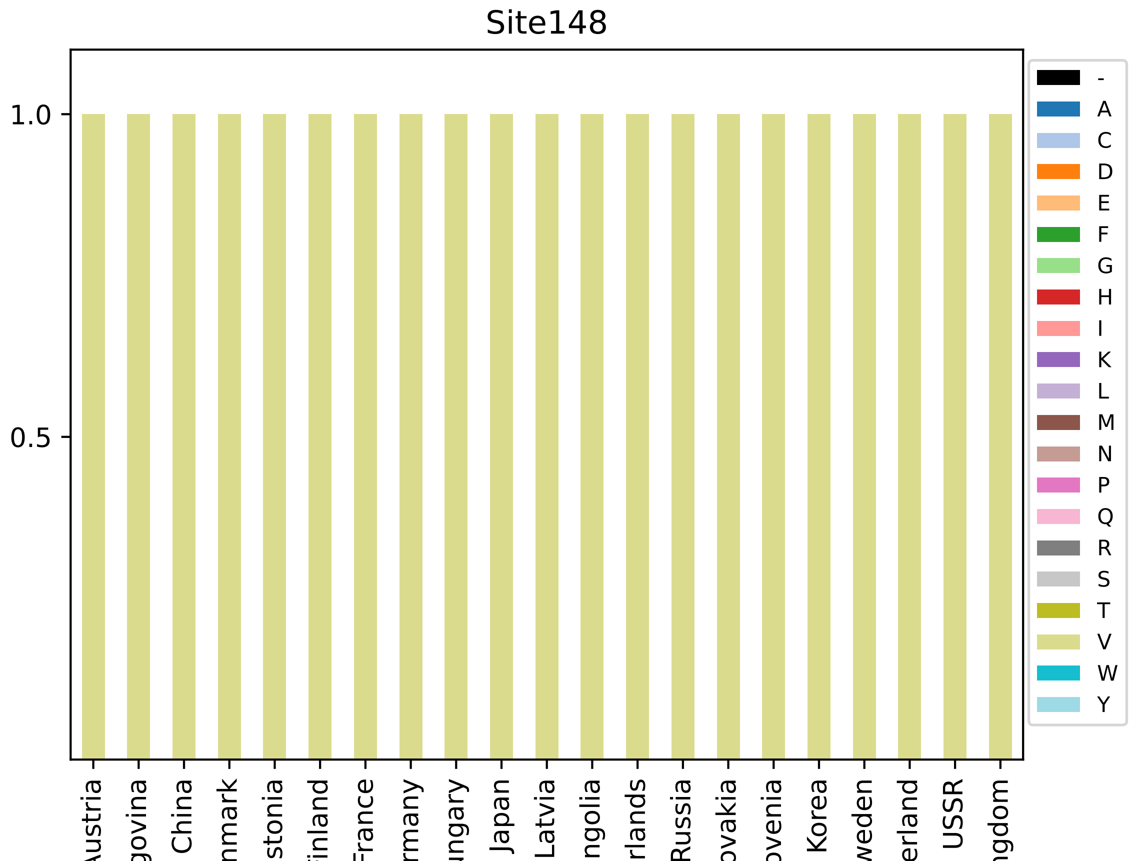

Site148
1.0
0.5
-
A
C
D
E
F
G
H
I
K
L
M
N
P
Q
R
S
T
V
W
Y
Austria
govina
China
nmark
stonia
Finland
France
rmany
ungary
Japan
Latvia
ngolia
rlands
Russia
ovakia
ovenia
Korea
weden
erland
USSR
ngdom

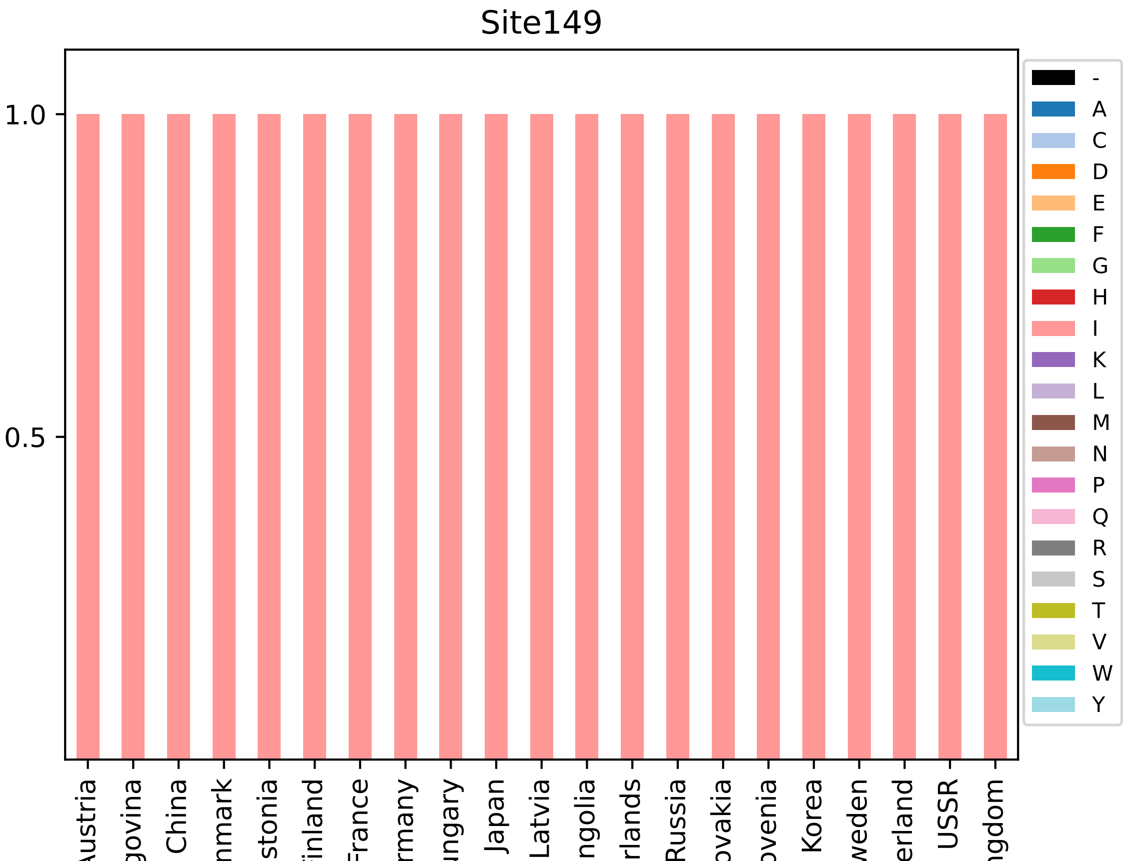
Site149
1.0
0.5
-
A
C
D
E
F
G
H
I
K
L
M
N
P
Q
R
S
T
V
W
Y
ustria
govina
China
nmark
stonia
inland
rance
rmany
ungary
Japan
Latvia
ngolia
rlands
Russia
ovakia
ovenia
Korea
weden
erland
USSR
ngdom

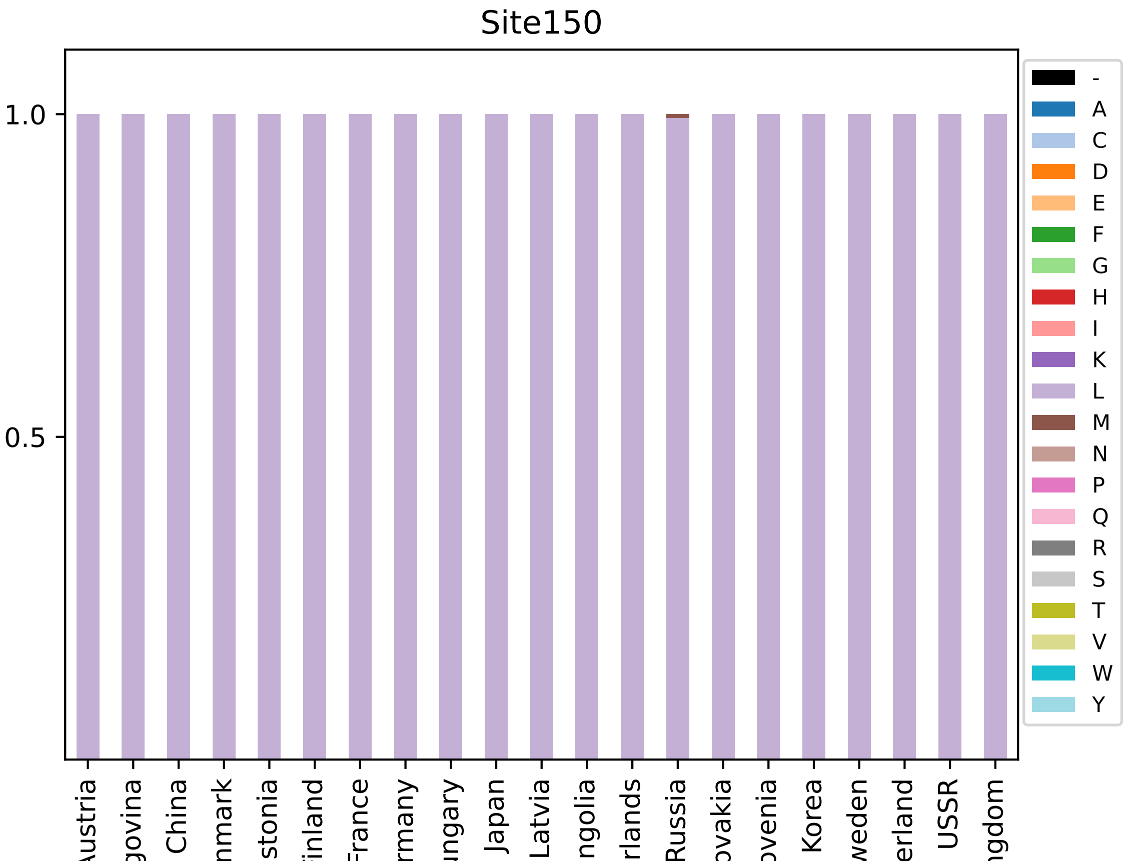
Site150
1.0
0.5
-
A
C
D
E
F
G
H
I
K
L
M
N
P
Q
R
S
T
V
W
Y
China
Japan
Latvia
Russia
Korea
USSR

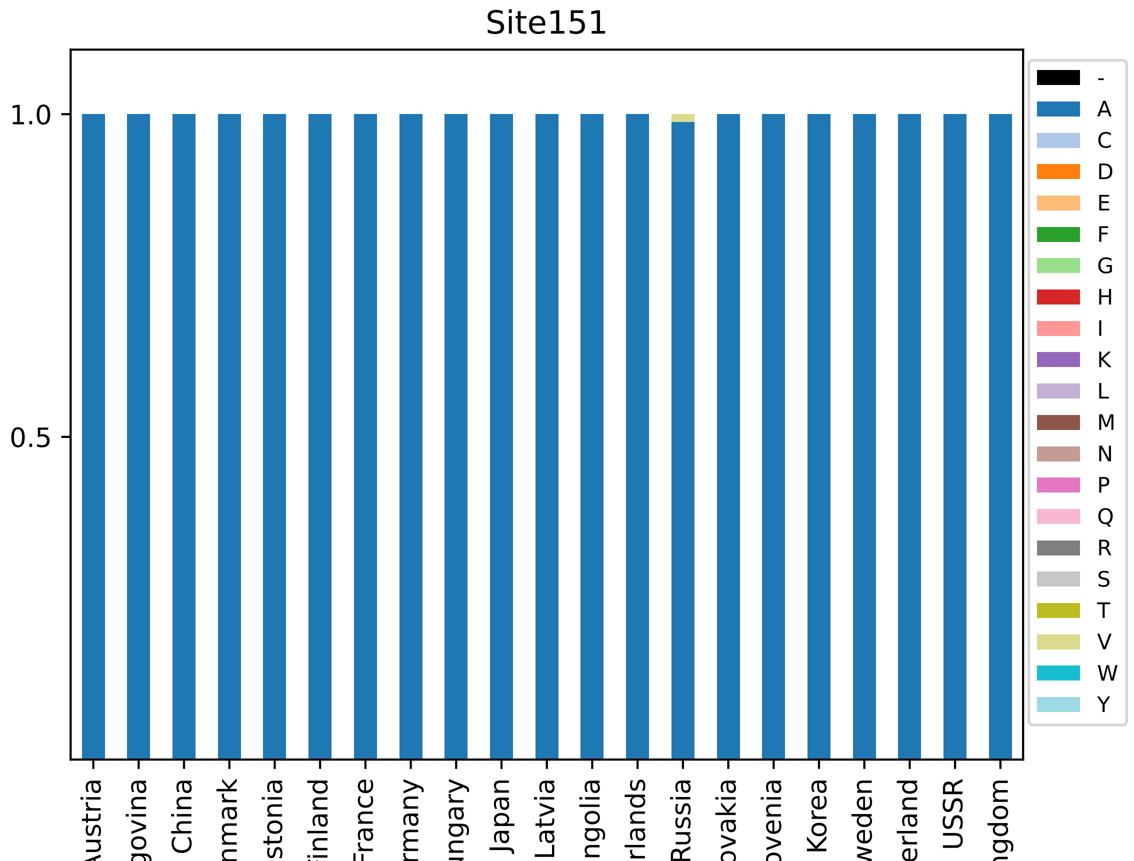
Site151
1.0
0.5
-
A
C
D
E
F
G
H
I
K
L
M
N
P
Q
R
S
T
V
W
Y
Austria
govina
China
nmark
stonia
Finland
France
rmany
ungary
Japan
Latvia
ngolia
rlands
Russia
ovakia
ovenia
Korea
weden
erland
USSR
ngdom

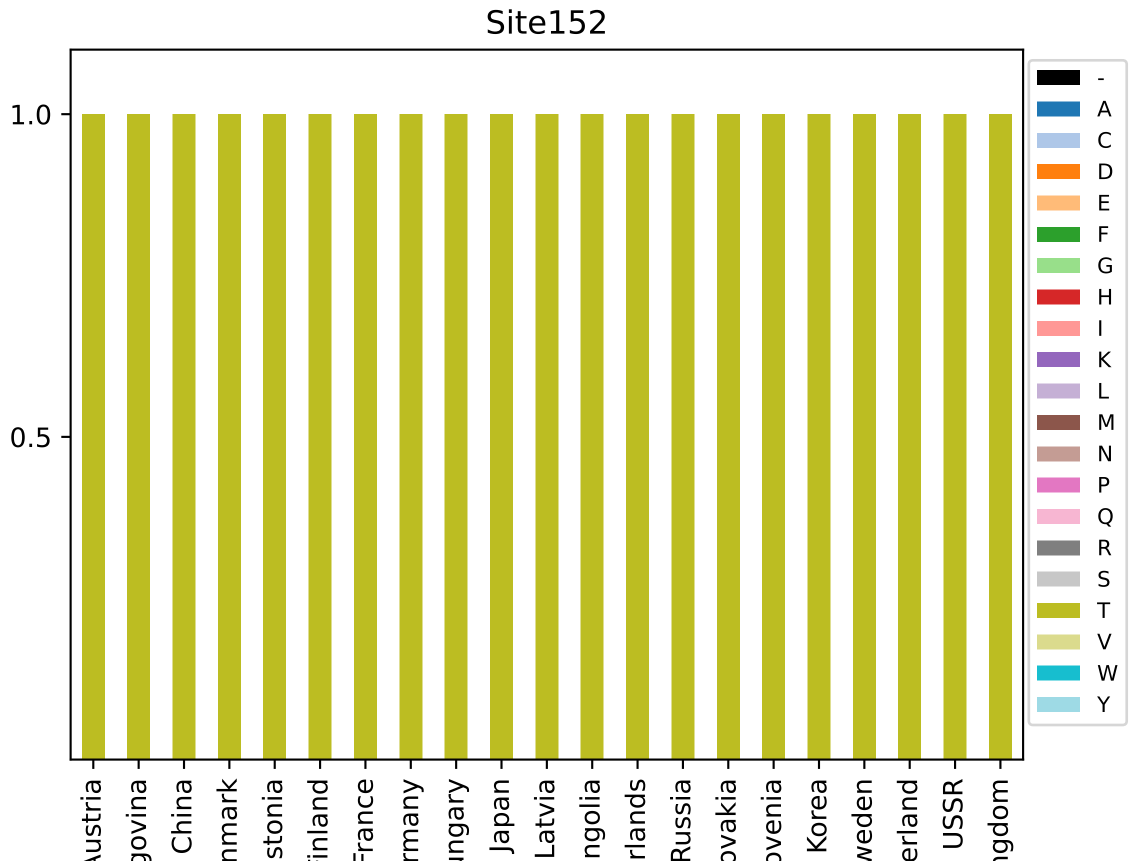

Site152
1.0
0.5
-
A
C
D
E
F
G
H
I
K
L
M
N
P
Q
R
S
T
V
W
Y
China
Japan
Latvia
Russia
Korea
USSR

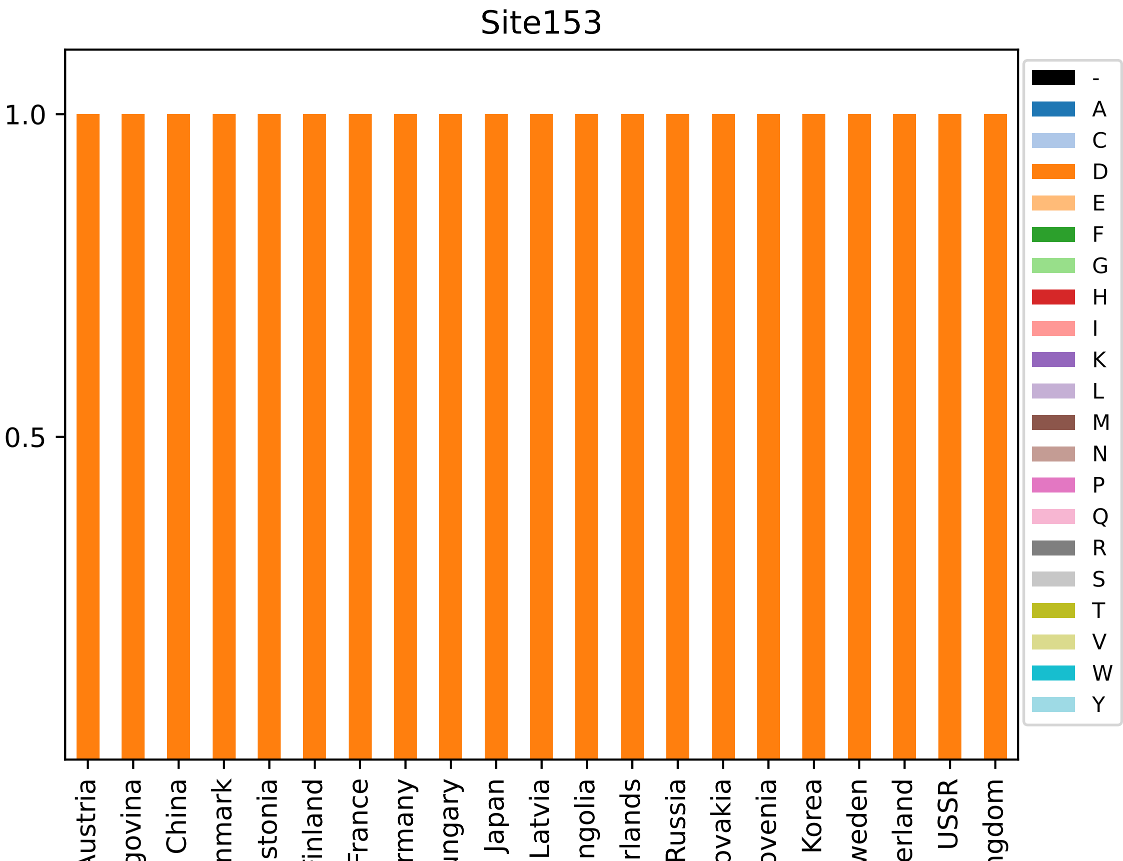

Site153
1.0
0.5
-
A
C
D
E
F
G
H
I
K
L
M
N
P
Q
R
S
T
V
W
Y
China
Japan
Latvia
Russia
Korea
USSR

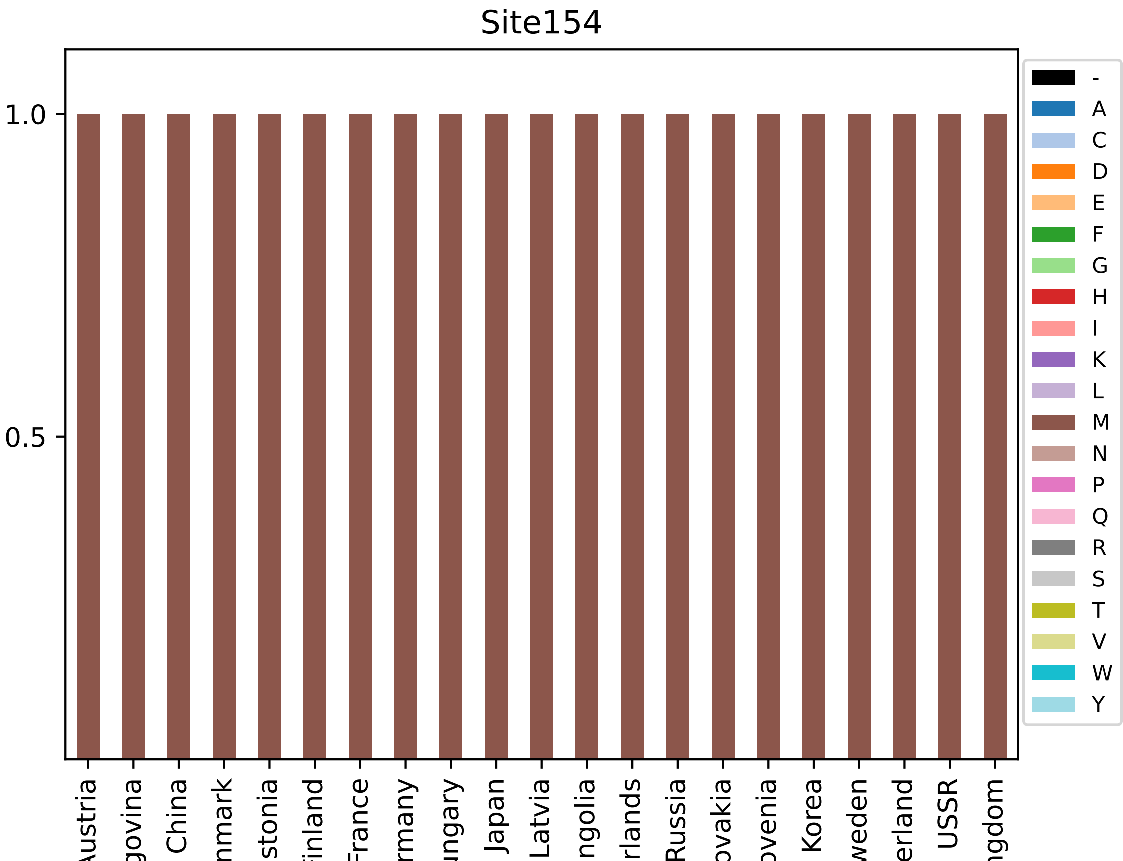
Site154
1.0
0.5
-
A
C
D
E
F
G
H
I
K
L
M
N
P
Q
R
S
T
V
W
Y
China
Japan
Latvia
Russia
Korea
USSR

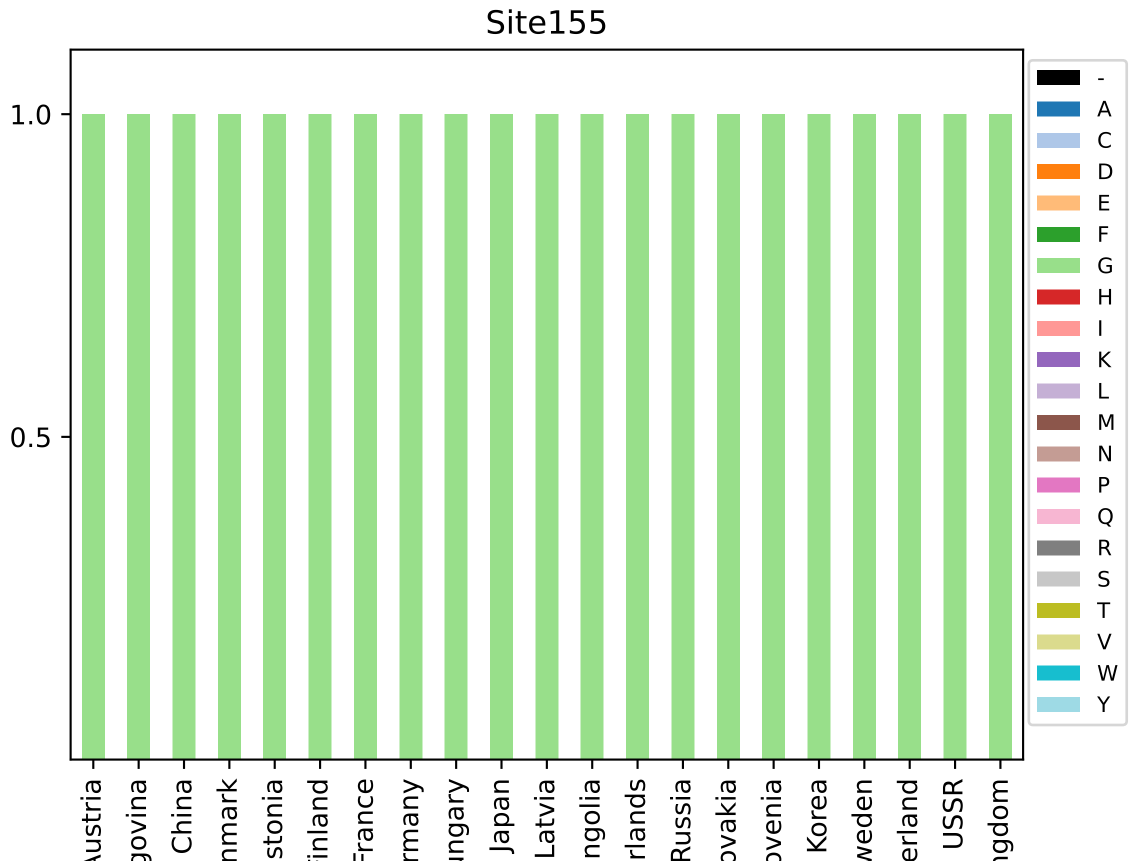

Site155
1.0
0.5
-
A
C
D
E
F
G
H
I
K
L
M
N
P
Q
R
S
T
V
W
Y
China
Japan
Latvia
Russia
Korea
USSR

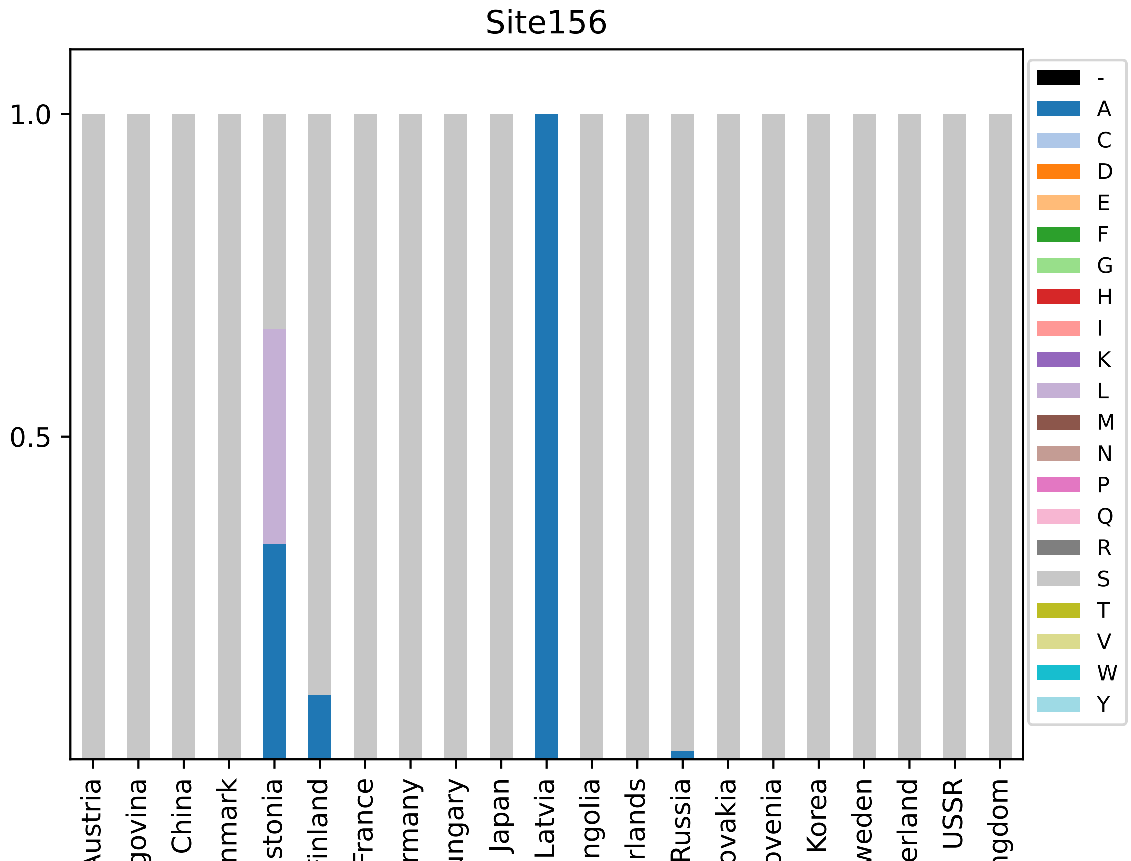
Site156
1.0
0.5
-
A
C
D
E
F
G
H
I
K
L
M
N
P
Q
R
S
T
V
W
Y
Austria
govina
China
nmark
stonia
inland
France
rmany
ungary
Japan
Latvia
ngolia
rlands
Russia
ovakia
ovenia
Korea
weden
erland
USSR
ngdom

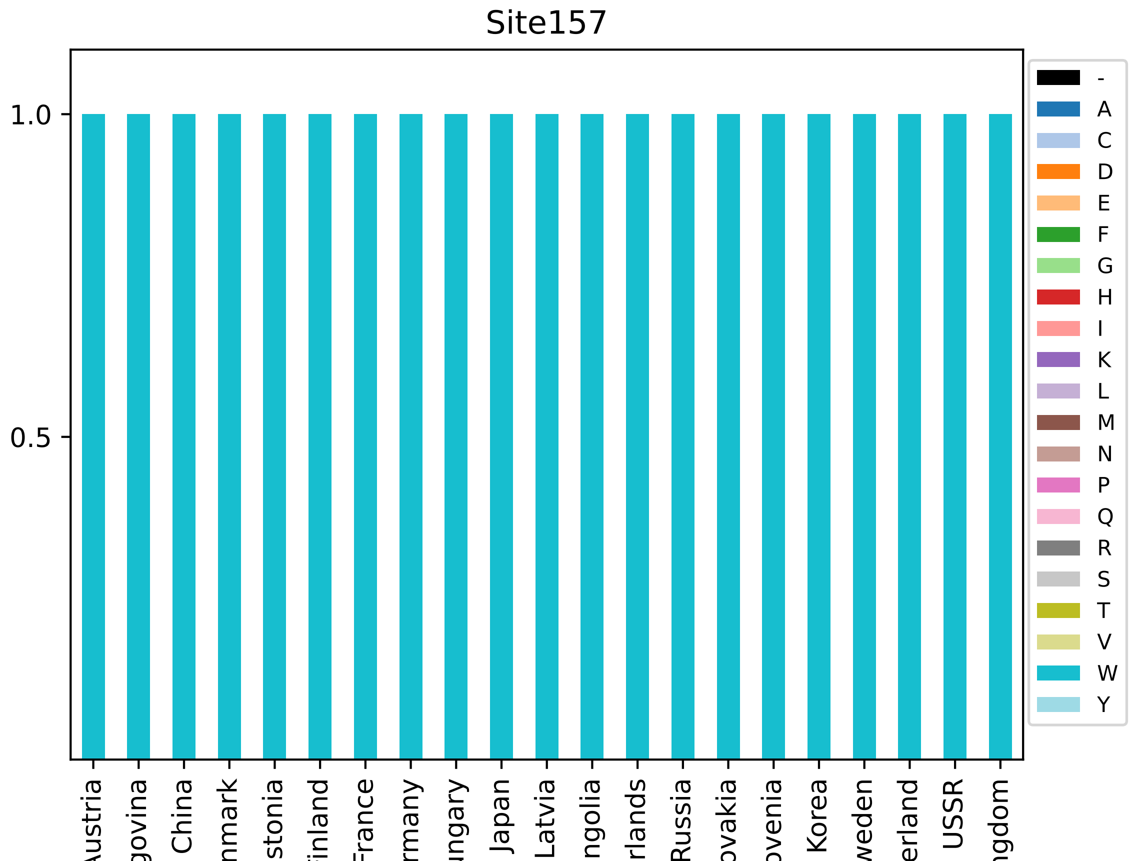
Site157
1.0
0.5
-
A
C
D
E
F
G
H
I
K
L
M
N
P
Q
R
S
T
V
W
Y
China
Japan
Latvia
Russia
Korea
USSR

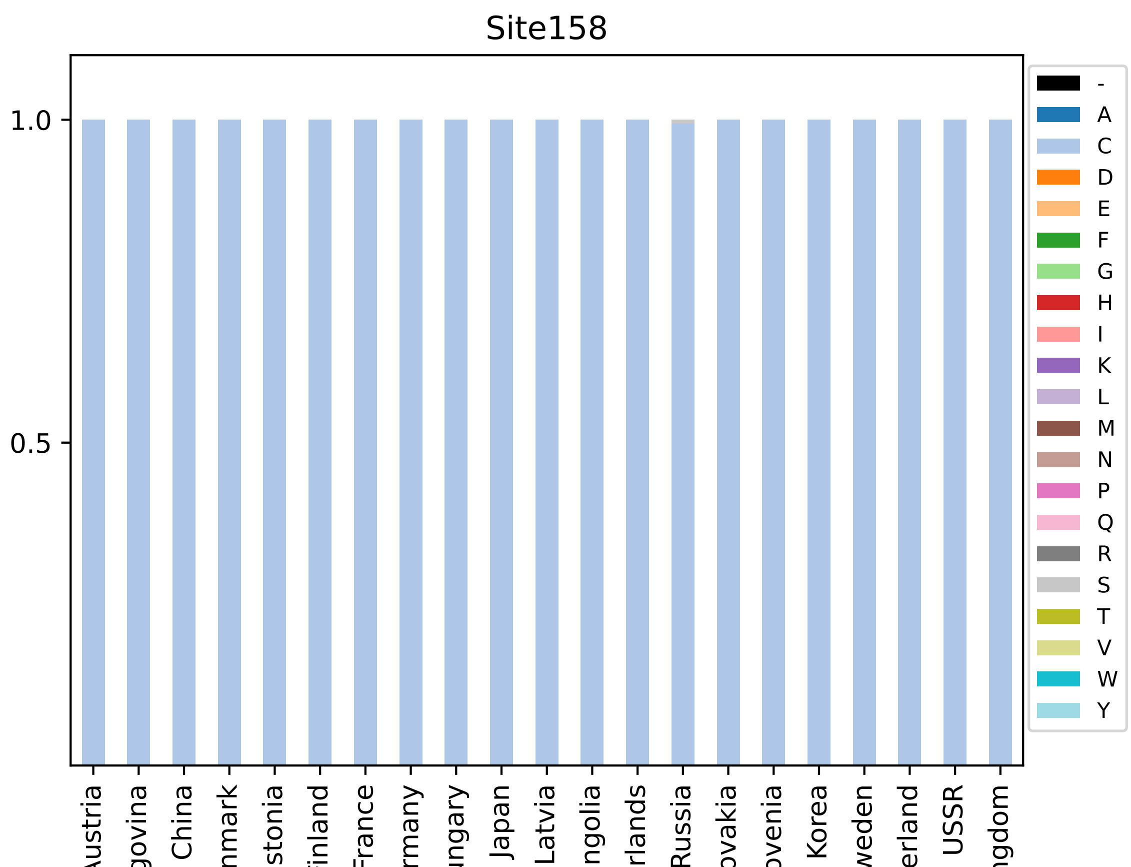

Site158
1.0
0.5
-
A
C
D
E
F
G
H
I
K
L
M
N
P
Q
R
S
T
V
W
Y
China
Japan
Latvia
Russia
Korea
USSR

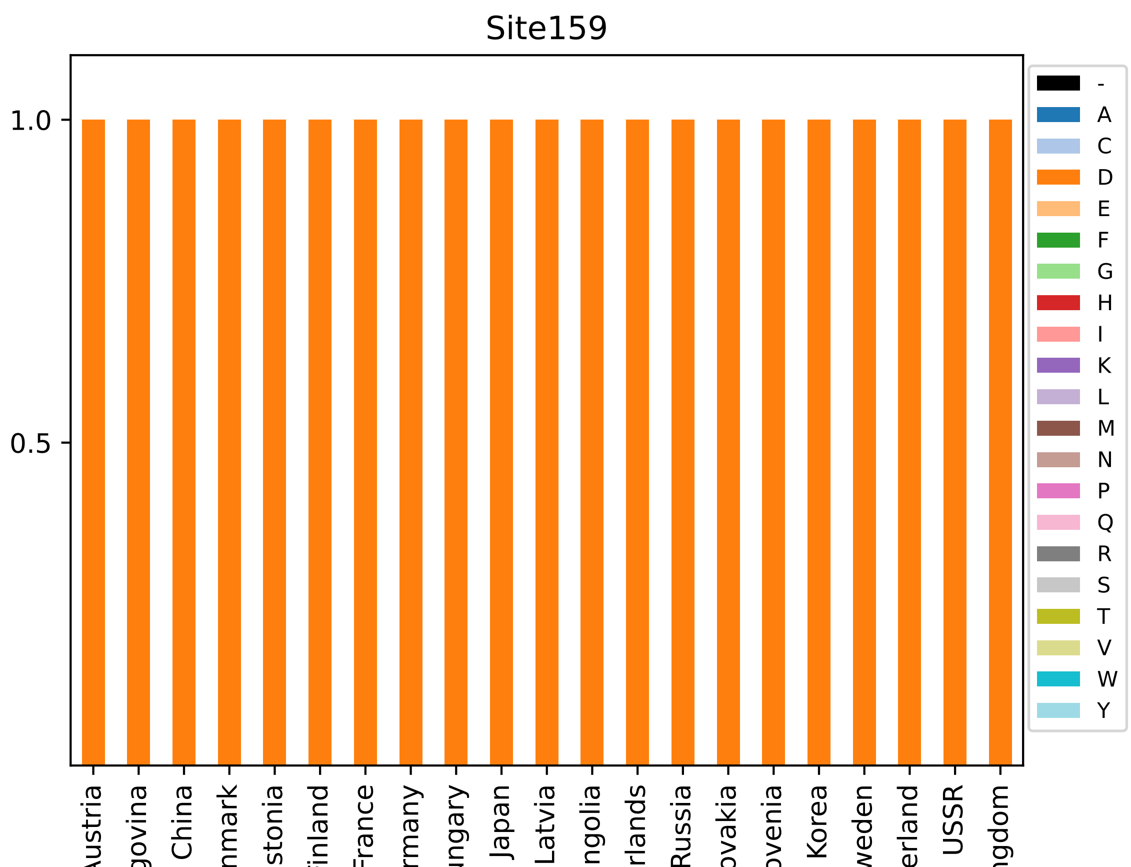

Site159
1.0
0.5
-
A
C
D
E
F
G
H
I
K
L
M
N
P
Q
R
S
T
V
W
Y
Austria
China
Japan
Latvia
Russia
Korea
USSR

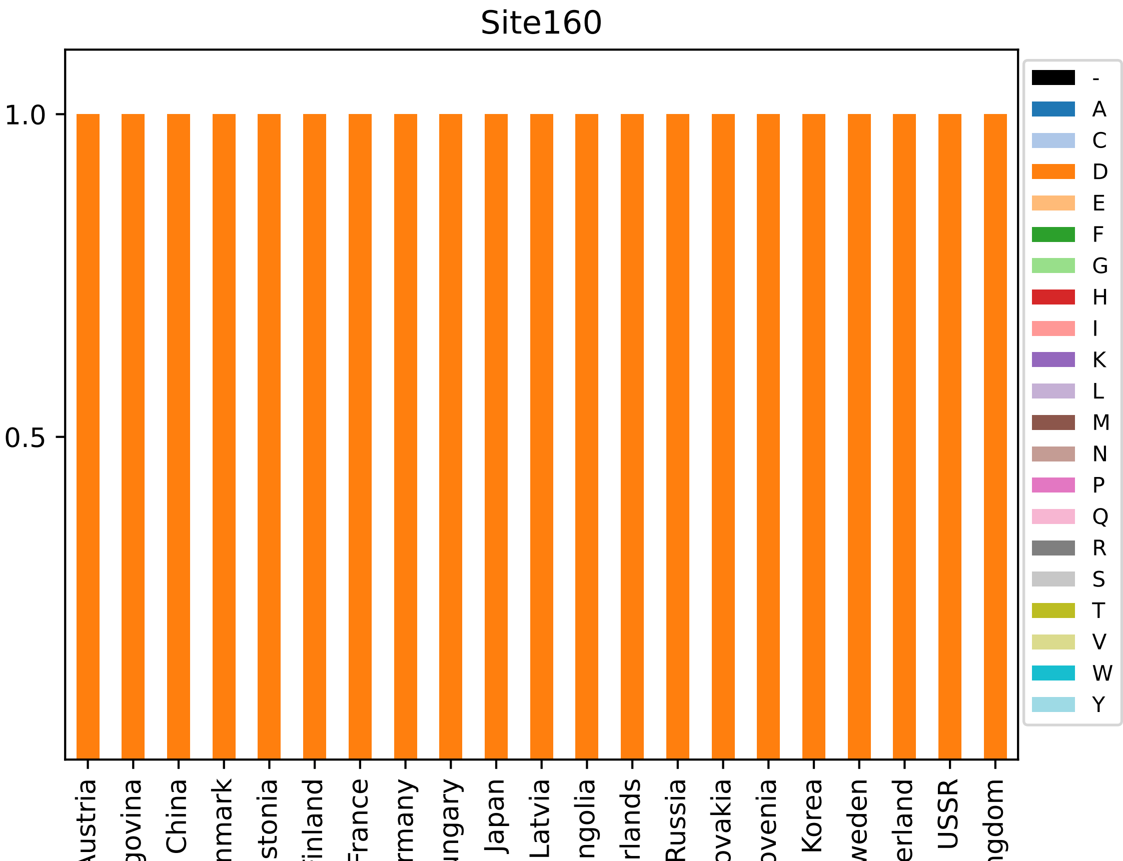

Site160
1.0
0.5
-
A
C
D
E
F
G
H
I
K
L
M
N
P
Q
R
S
T
V
W
Y
China
Japan
Latvia
Russia
Korea
USSR

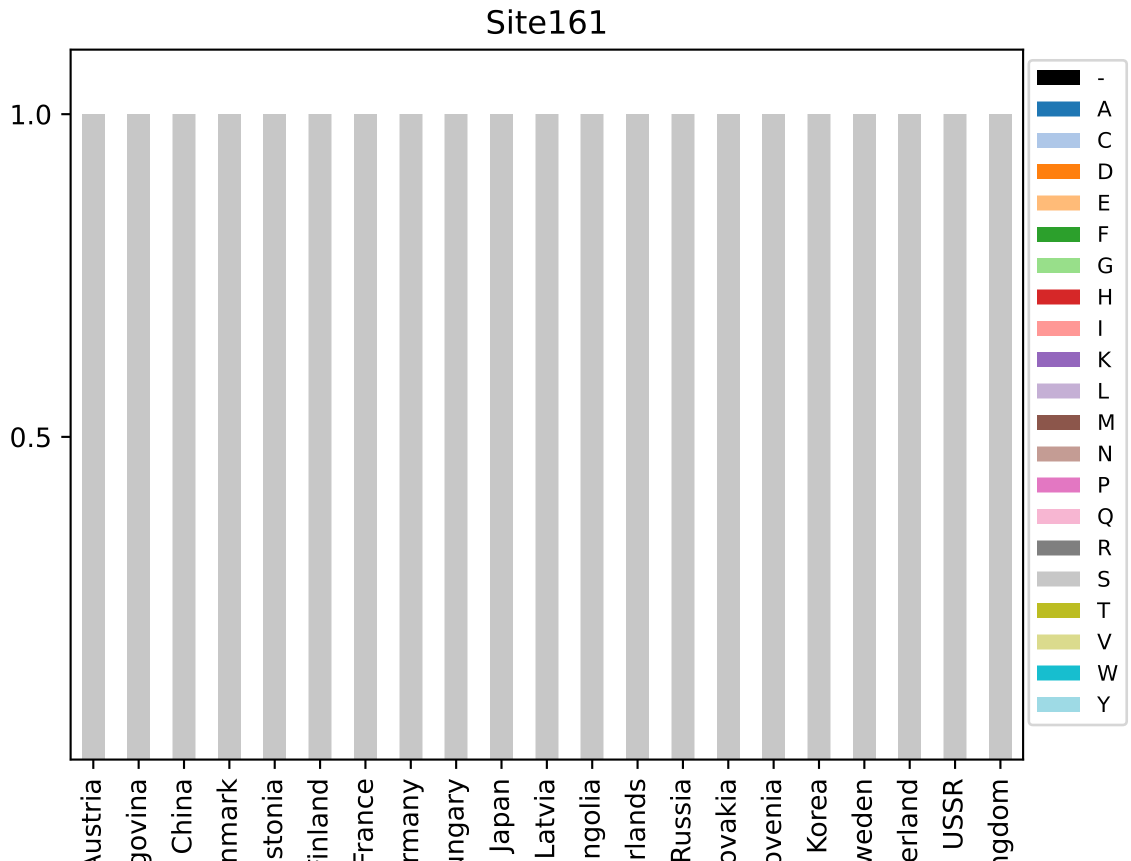

Site161
1.0
0.5
Austria
govina
China
nmark
stonia
inland
France
rmany
ungary
Japan
Latvia
ngolia
rlands
Russia
ovakia
ovenia
Korea
weden
erland
USSR
ngdom
-
A
C
D
E
F
G
H
I
K
L
M
N
P
Q
R
S
T
V
W
Y

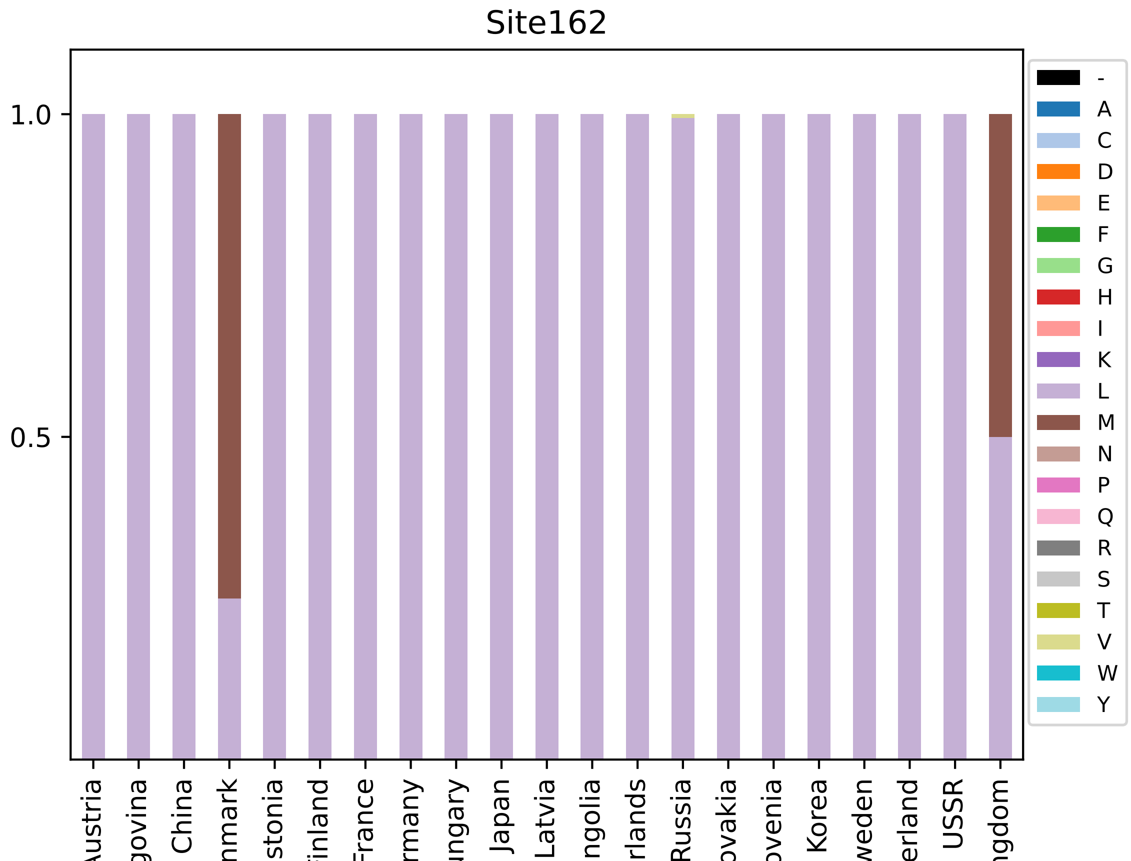
Site162
1.0
0.5
-
A
C
D
E
F
G
H
I
K
L
M
N
P
Q
R
S
T
V
W
Y
Austria
China
Japan
Latvia
Russia
Korea
USSR

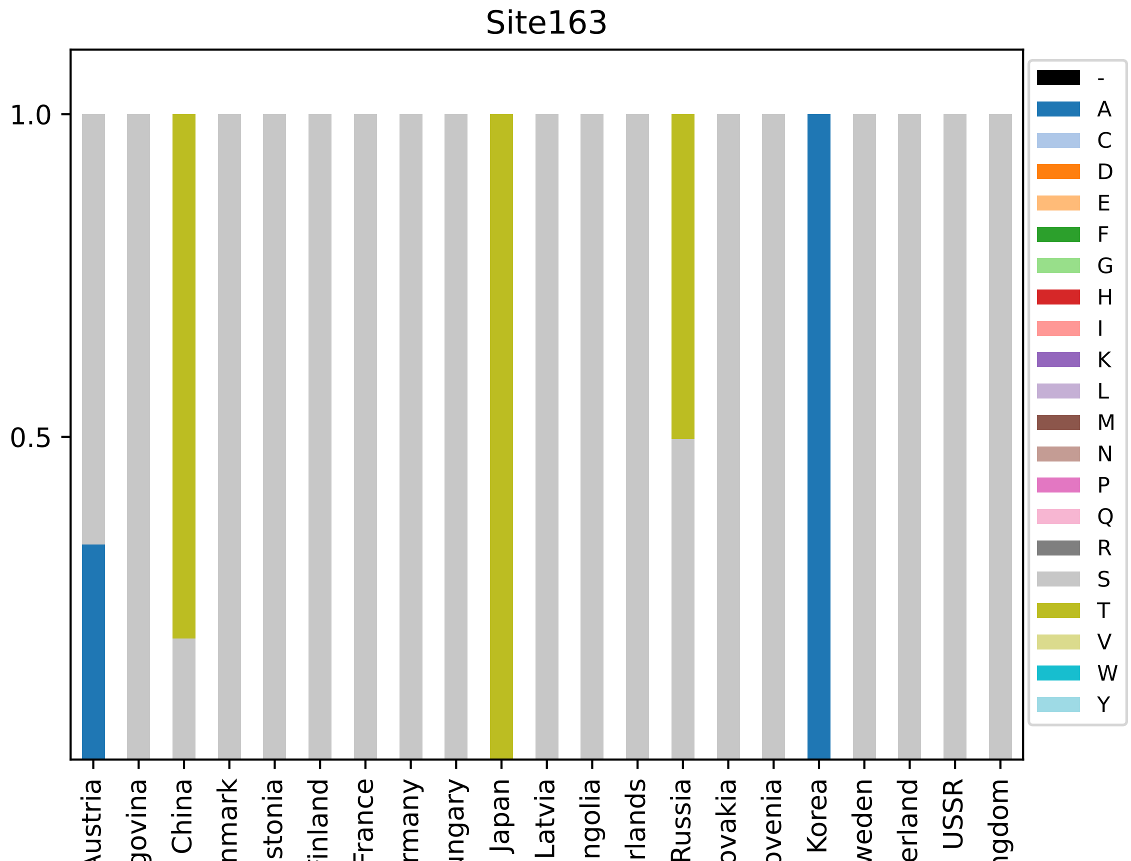
Site163
1.0
0.5
-
A
C
D
E
F
G
H
I
K
L
M
N
P
Q
R
S
T
V
W
Y
ustria
govina
China
nmark
stonia
inland
rance
rmany
ungary
Japan
Latvia
ngolia
rlands
Russia
ovakia
ovenia
Korea
weden
erland
USSR
ngdom

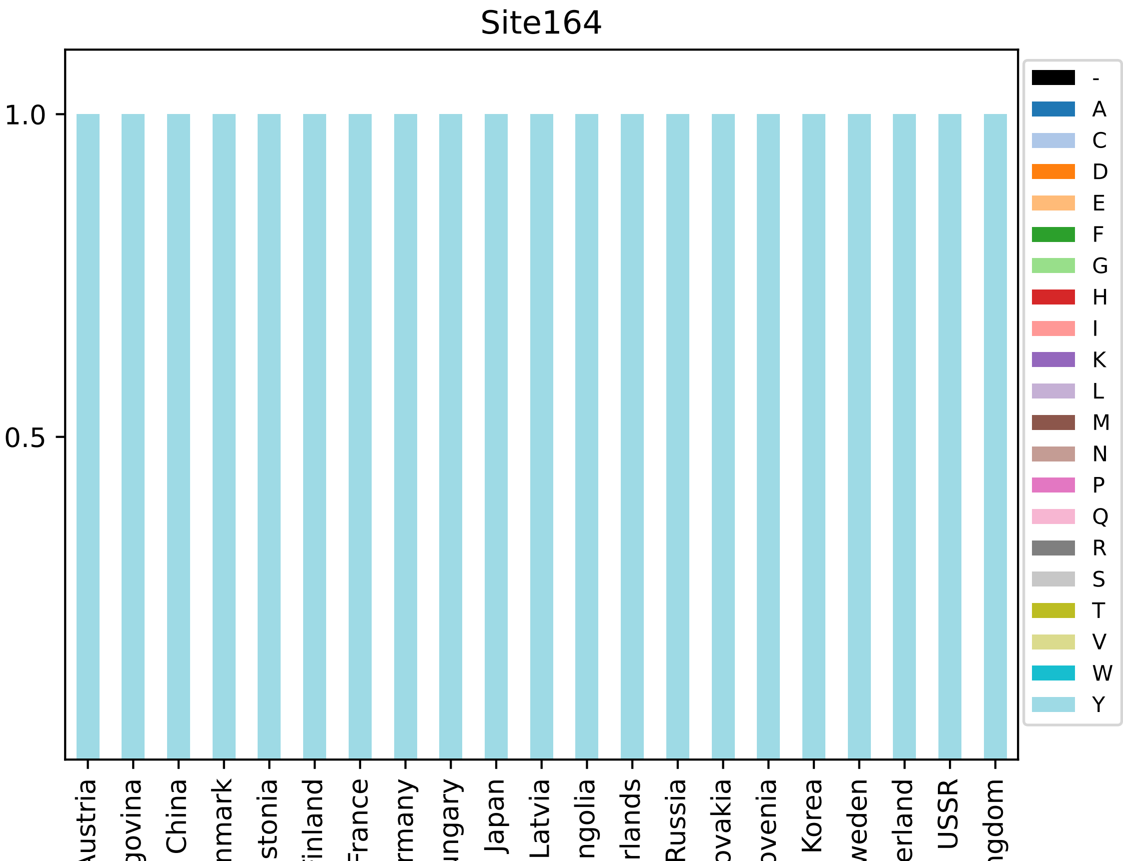
Site164
1.0
0.5
-
A
C
D
E
F
G
H
I
K
L
M
N
P
Q
R
S
T
V
W
Y
Austria
govina
China
nmark
stonia
Finland
France
rmany
ungary
Japan
Latvia
ngolia
rlands
Russia
ovakia
ovenia
Korea
weden
erland
USSR
ngdom

Site165

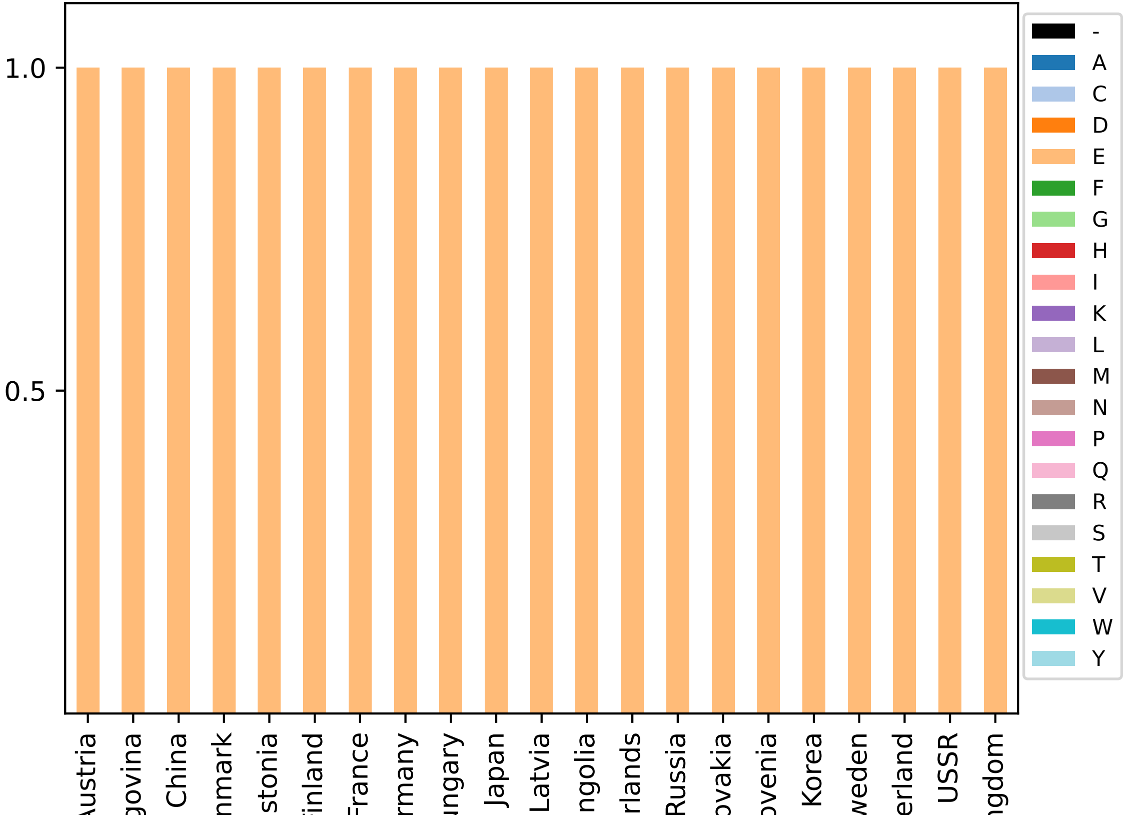
1.0
0.5
China
Japan
Latvia
Russia
Korea
USSR
-
A
C
D
E
F
G
H
I
K
L
M
N
P
Q
R
S
T
V
W
Y

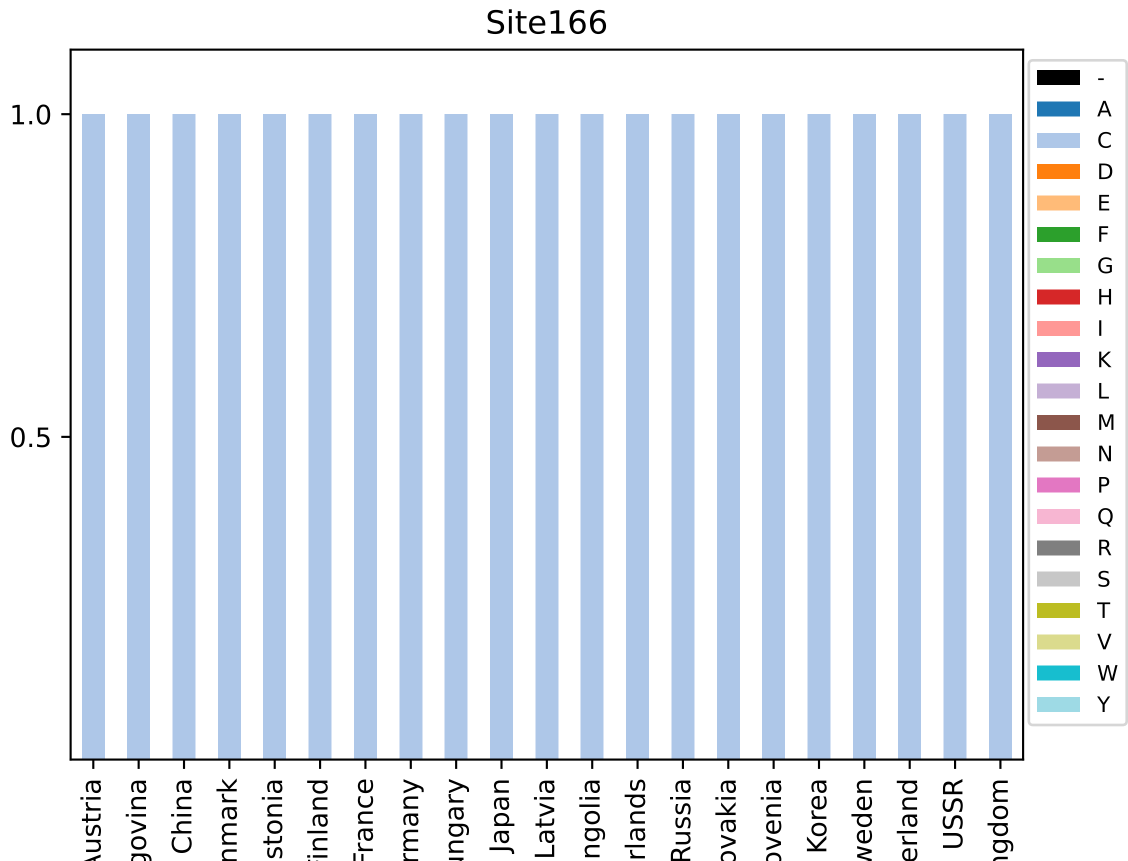
Site166
1.0
0.5
-
A
C
D
E
F
G
H
I
K
L
M
N
P
Q
R
S
T
V
W
Y
China
Japan
Latvia
Russia
Korea
USSR

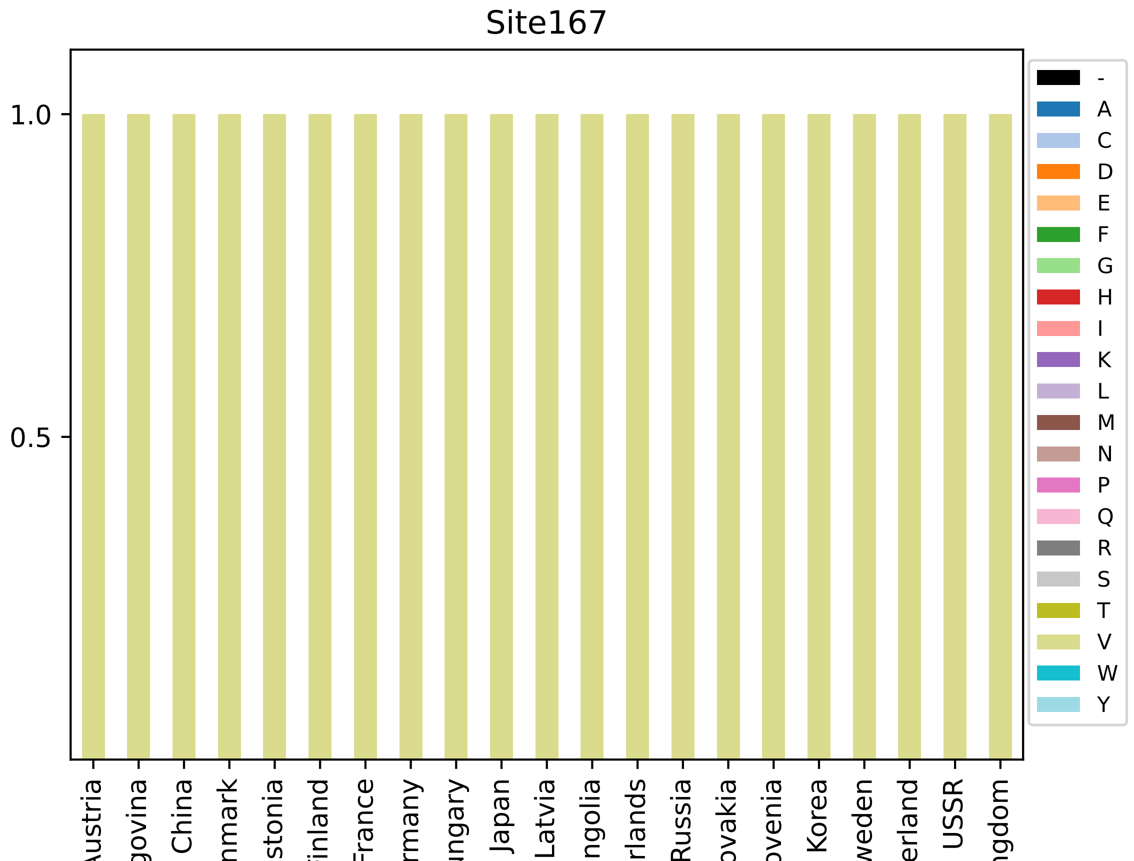
Site167
1.0
0.5
-
A
C
D
E
F
G
H
I
K
L
M
N
P
Q
R
S
T
V
W
Y
Austria
China
Japan
Latvia
Russia
Korea
USSR

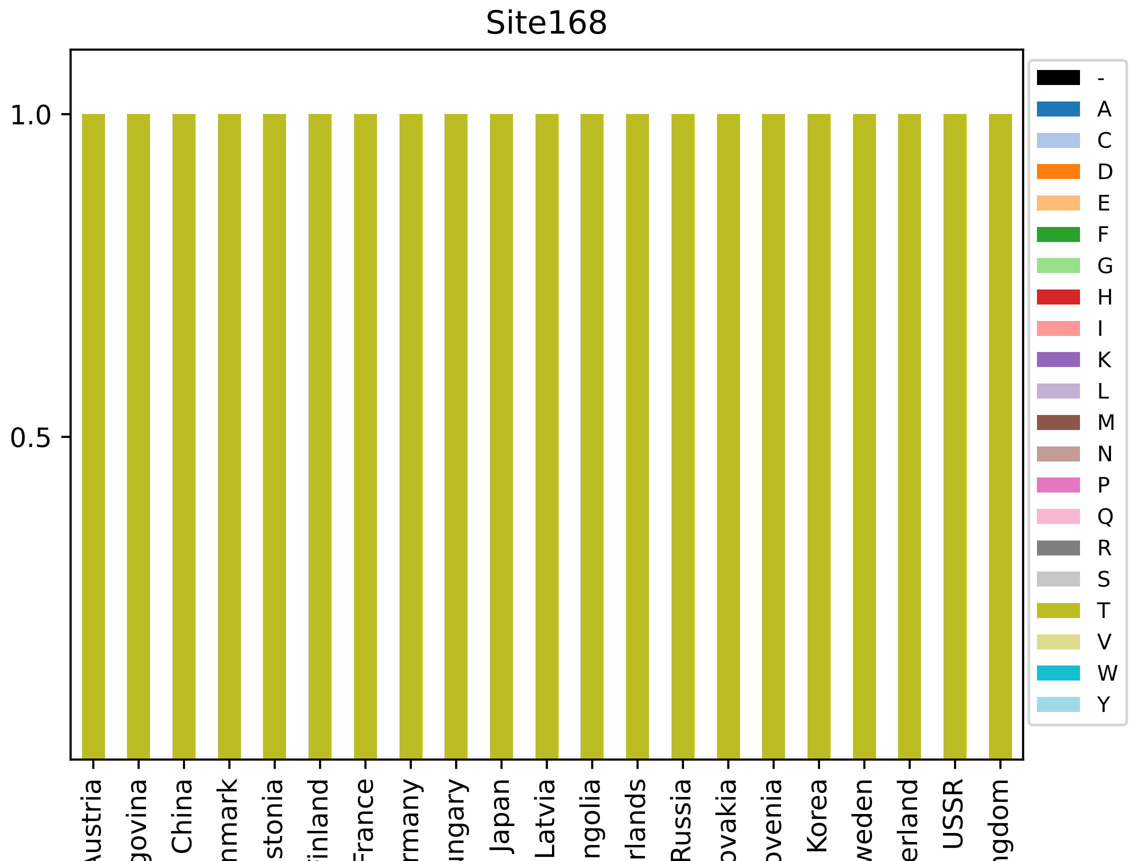
Site168
1.0
0.5
-
A
C
D
E
F
G
H
I
K
L
M
N
P
Q
R
S
T
V
W
Y
China
Japan
Latvia
Russia
Korea
USSR

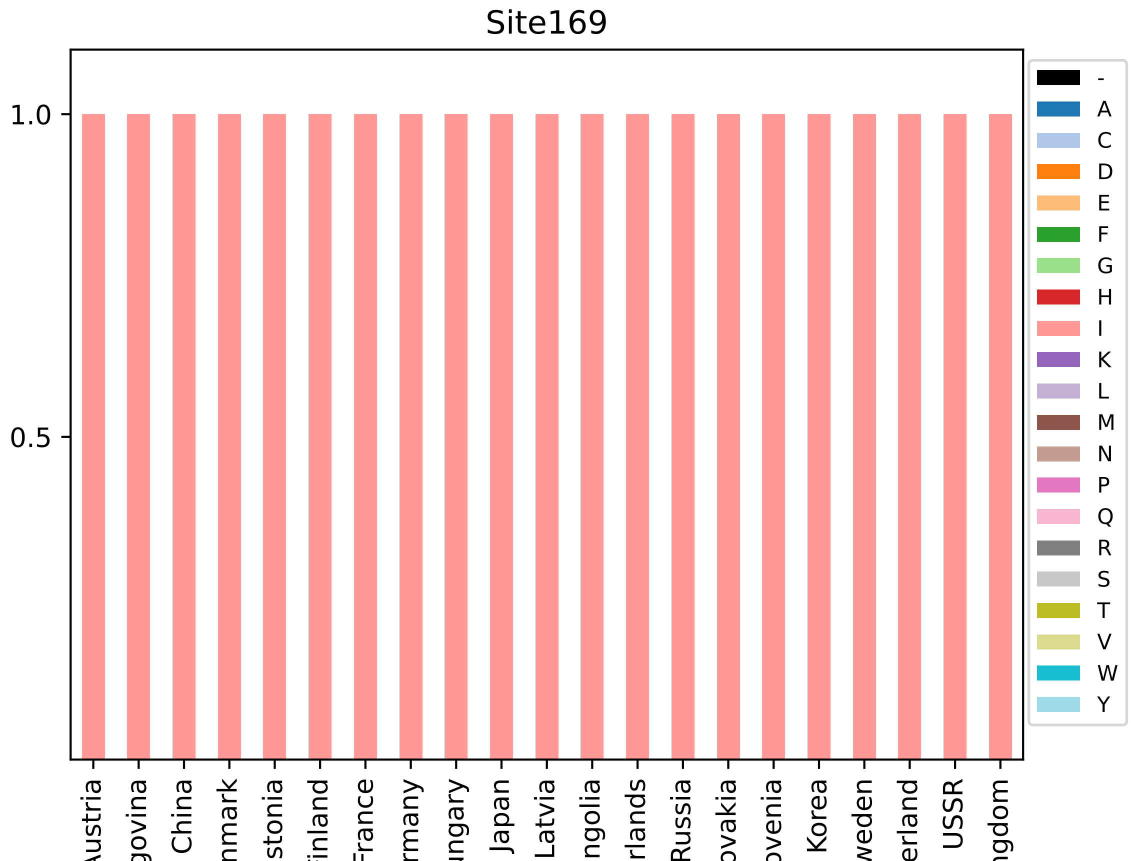
Site169
1.0
0.5
-
A
C
D
E
F
G
H
I
K
L
M
N
P
Q
R
S
T
V
W
Y
China
Japan
Latvia
Russia
Korea
USSR

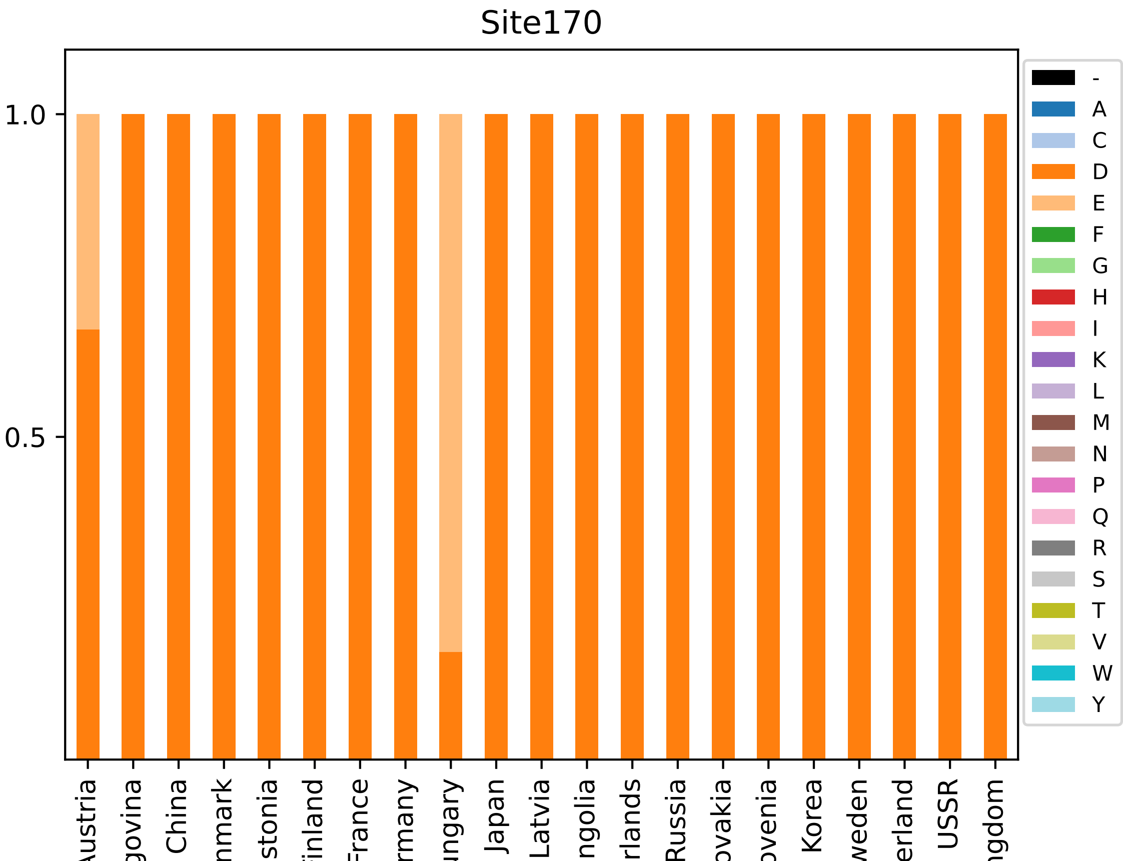

Site170
1.0
0.5
-
A
C
D
E
F
G
H
I
K
L
M
N
P
Q
R
S
T
V
W
Y
China
Japan
Latvia
Russia
Korea
USSR

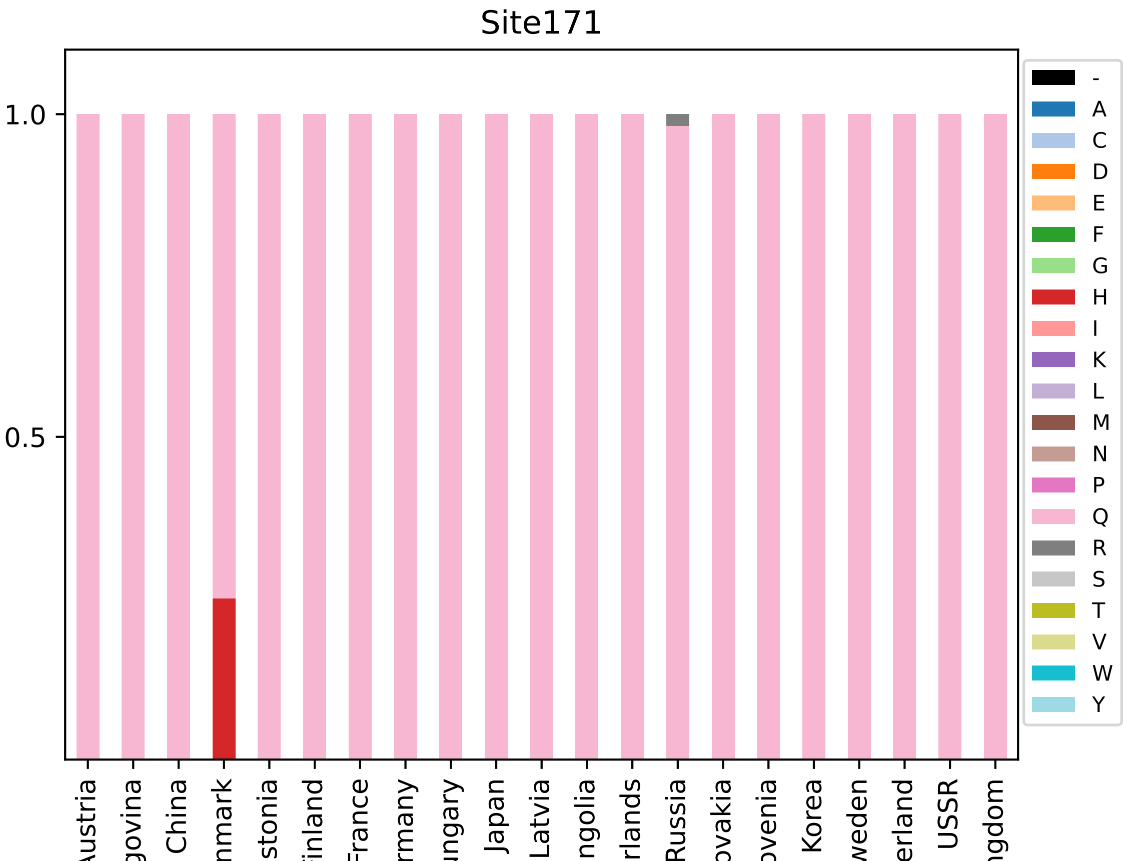
Site171
1.0
0.5
-
A
C
D
E
F
G
H
I
K
L
M
N
P
Q
R
S
T
V
W
Y
Austria
China
Japan
Latvia
Russia
Korea
USSR

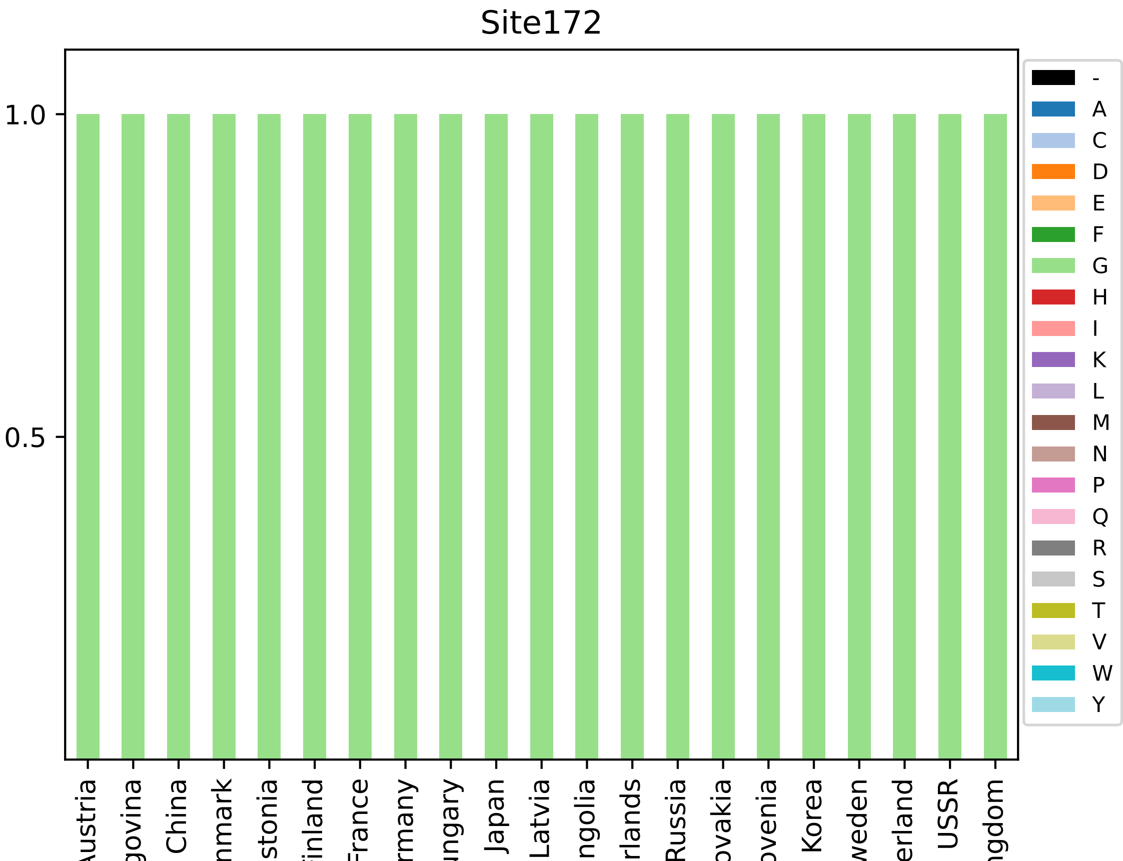

Site172
1.0
0.5
-
A
C
D
E
F
G
H
I
K
L
M
N
P
Q
R
S
T
V
W
Y
China
Japan
Latvia
Russia
Korea
USSR

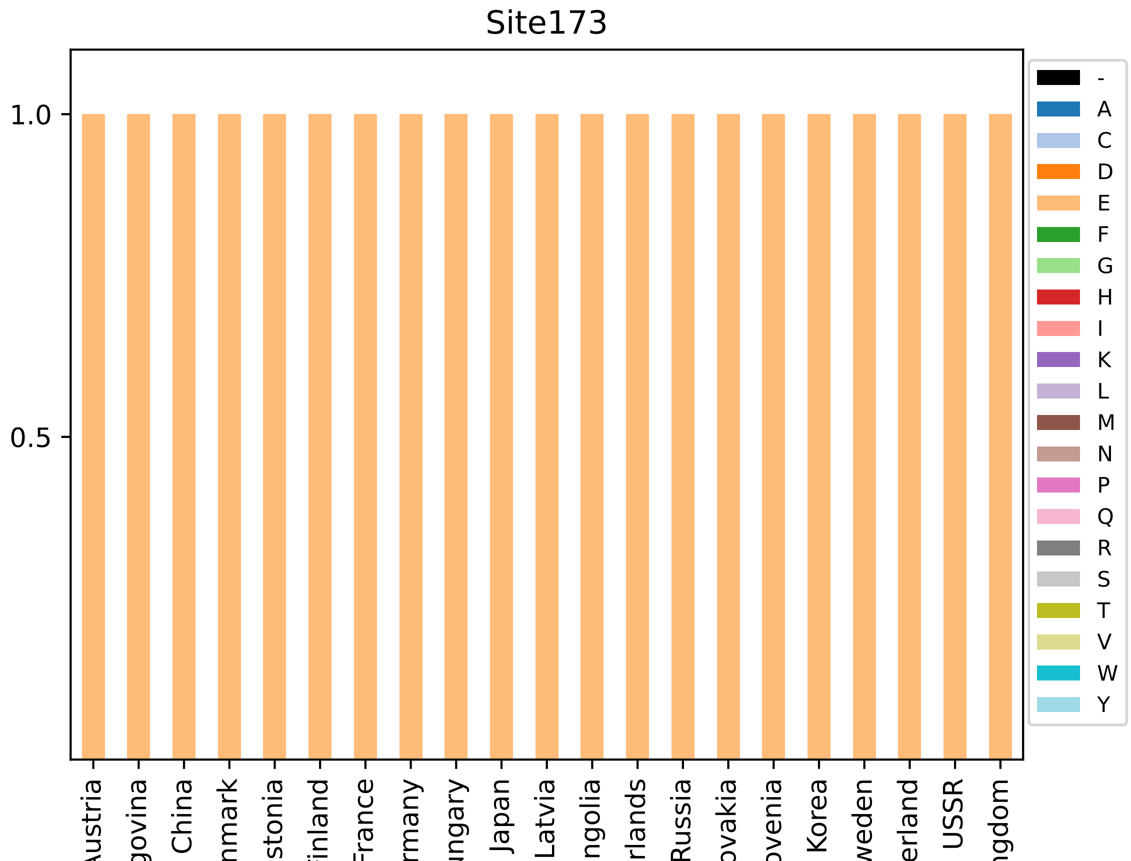

Site173
1.0
0.5
-
A
C
D
E
F
G
H
I
K
L
M
N
P
Q
R
S
T
V
W
Y
China
Japan
Latvia
Russia
Korea
USSR

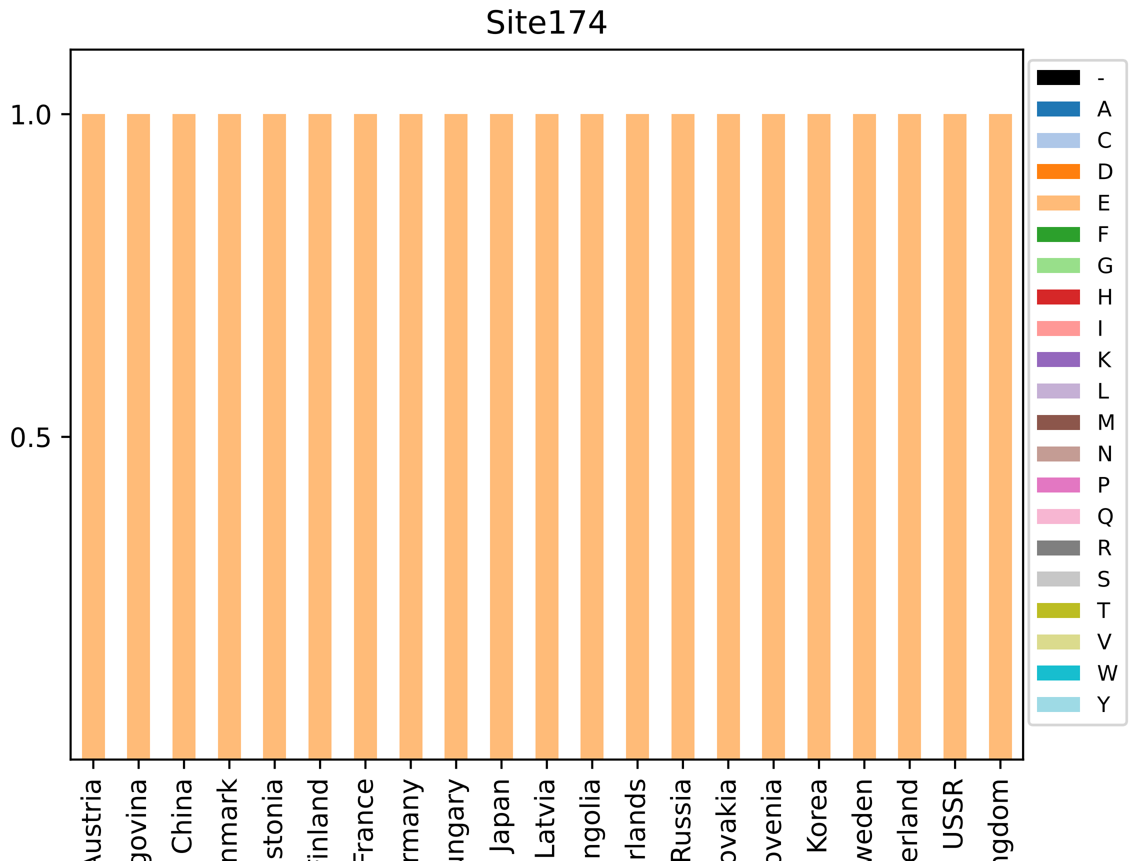
Site174
1.0
0.5
-
A
C
D
E
F
G
H
I
K
L
M
N
P
Q
R
S
T
V
W
Y
Austria
govina
China
nmark
stonia
inland
France
rmany
ungary
Japan
Latvia
ngolia
rlands
Russia
ovakia
ovenia
Korea
weden
erland
USSR
ngdom

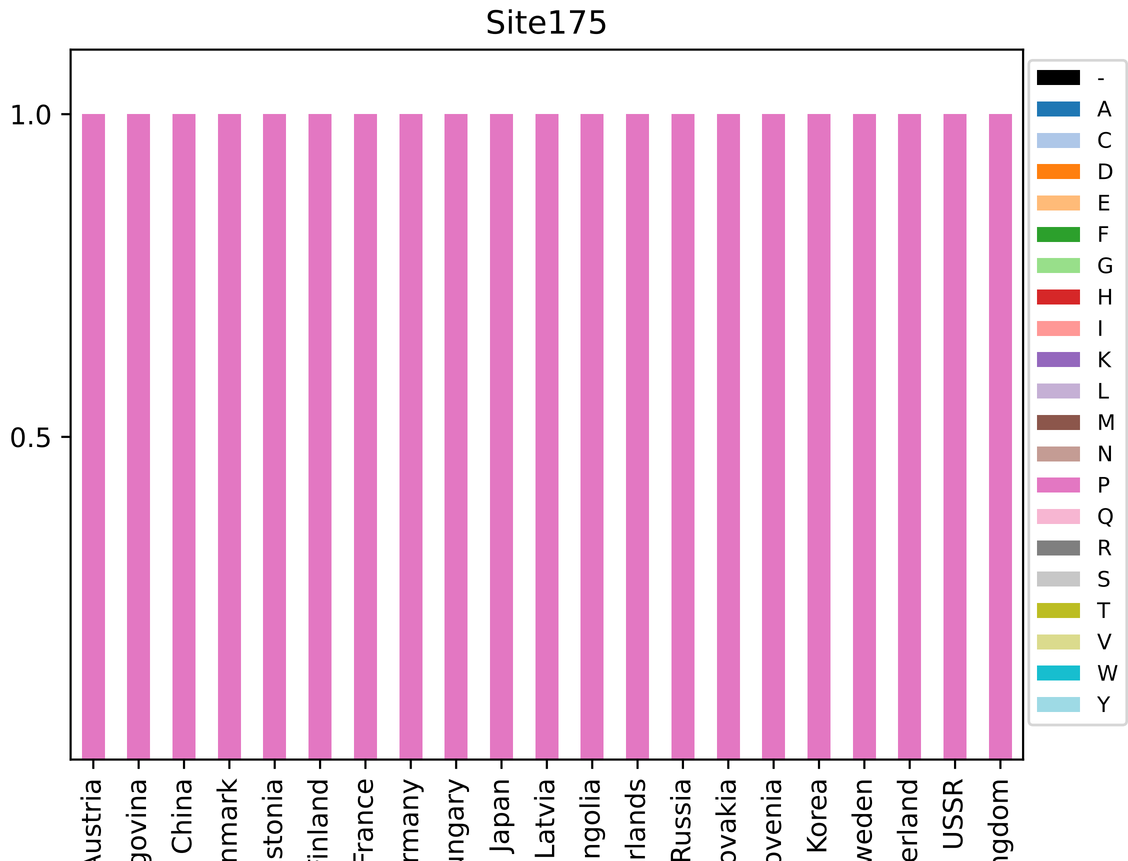

Site175
1.0
0.5
-
A
C
D
E
F
G
H
I
K
L
M
N
P
Q
R
S
T
V
W
Y
China
Japan
Latvia
Russia
Korea
USSR

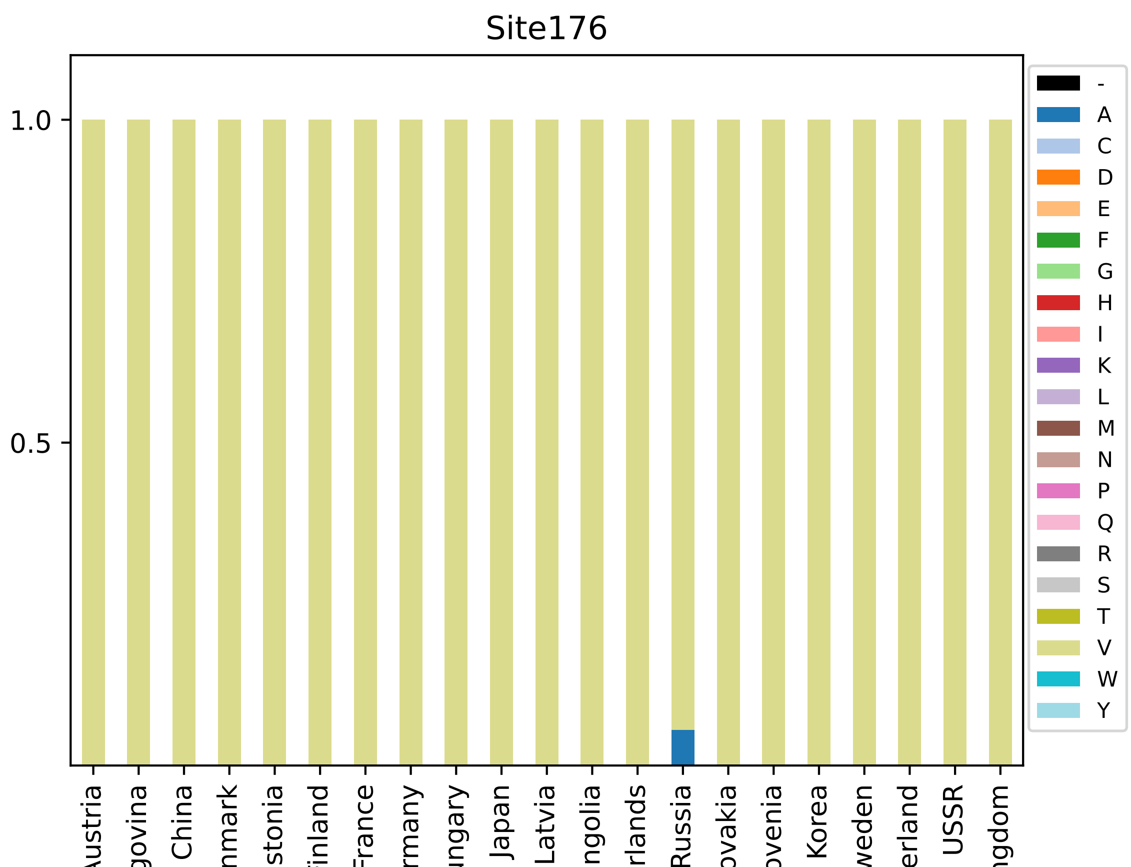

Site176
1.0
0.5
-
A
C
D
E
F
G
H
I
K
L
M
N
P
Q
R
S
T
V
W
Y
China
Japan
Latvia
Russia
Korea
USSR

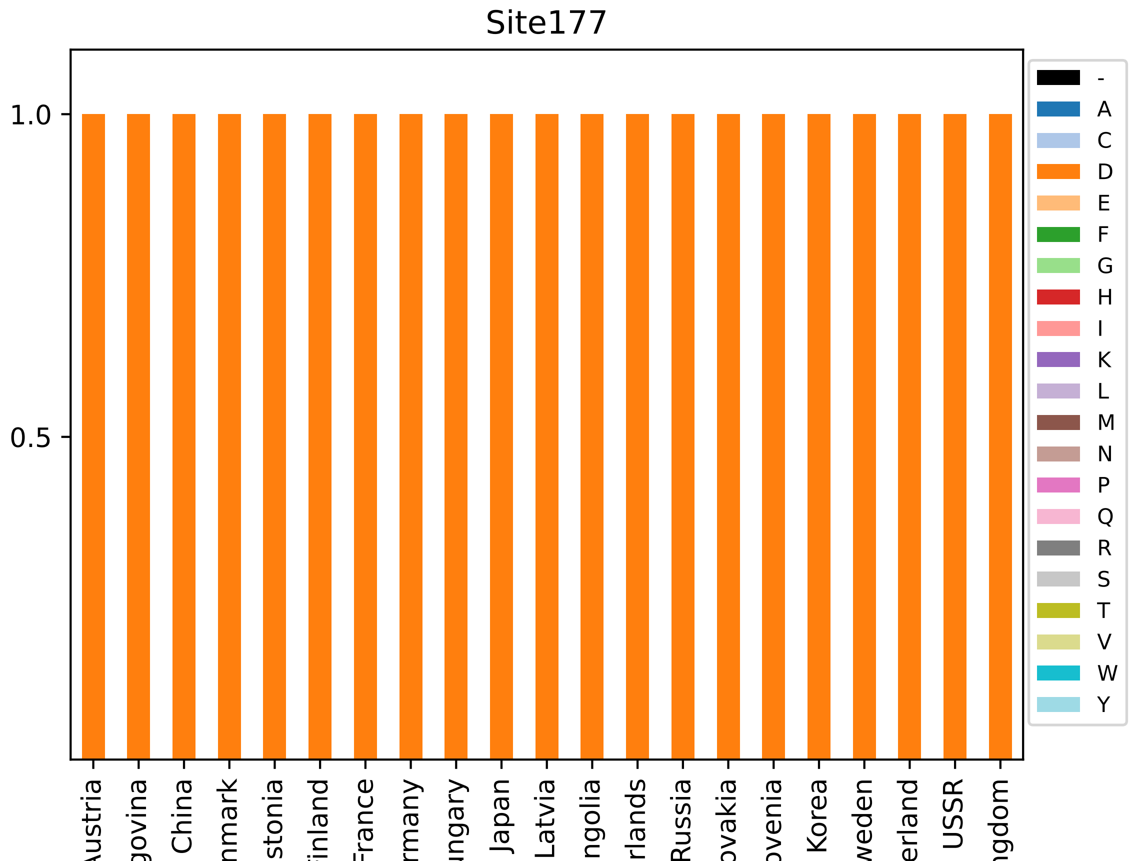
Site177
1.0
0.5
China
Japan
Latvia
Russia
Korea
USSR
-
A
C
D
E
F
G
H
I
K
L
M
N
P
Q
R
S
T
V
W
Y

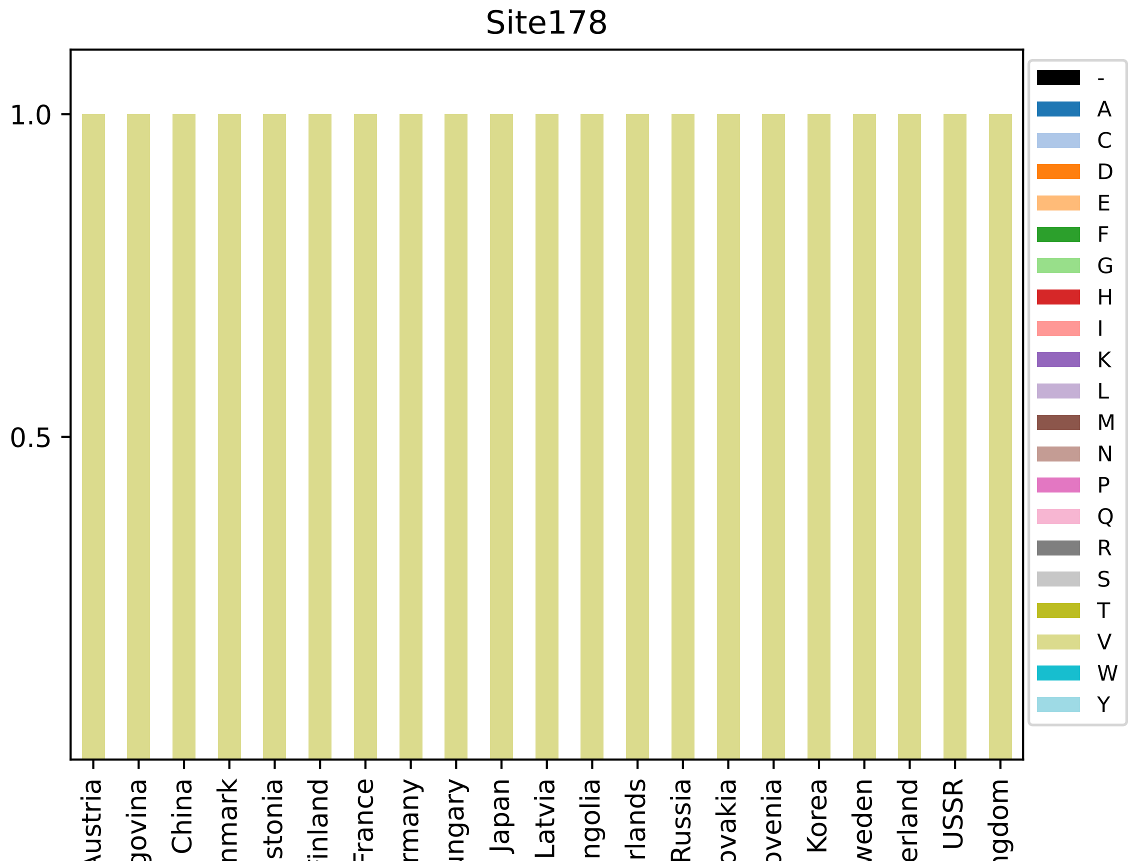

Site178
1.0
0.5
-
A
C
D
E
F
G
H
I
K
L
M
N
P
Q
R
S
T
V
W
Y
Austria
China
Japan
Latvia
Russia
Korea
USSR

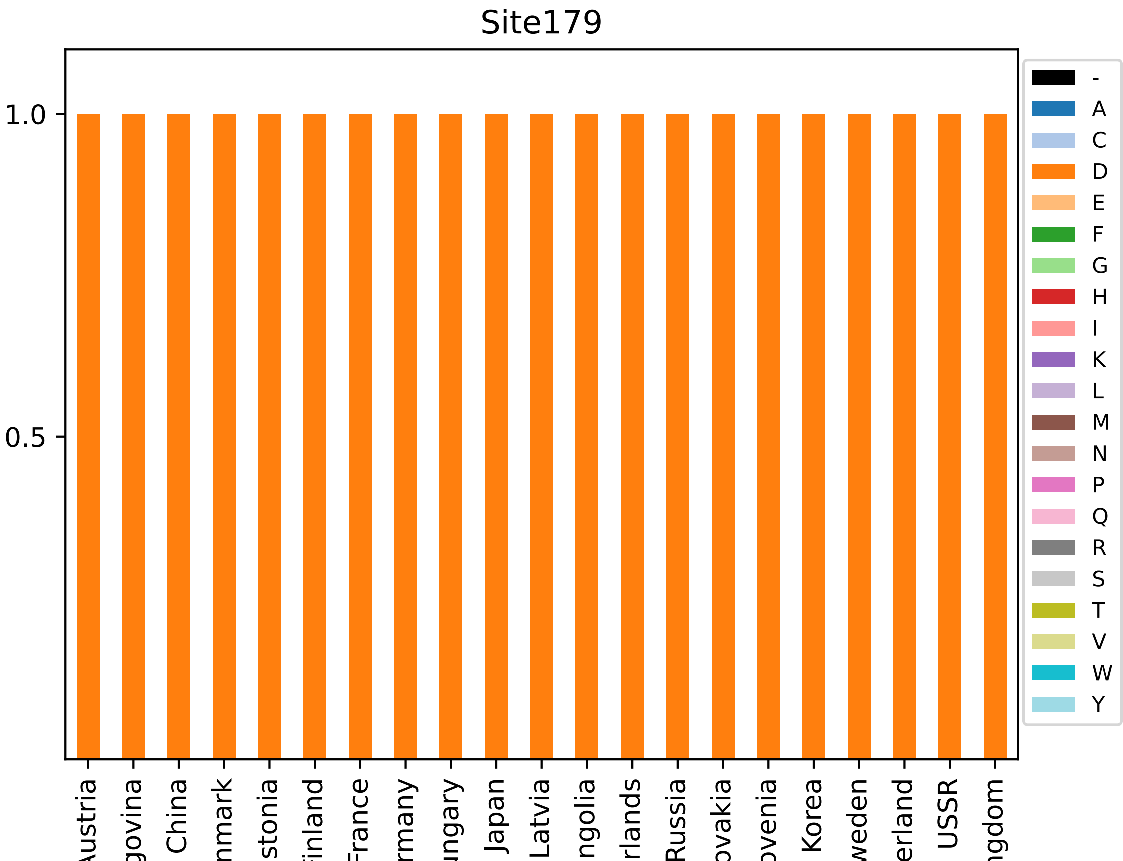
Site179
1.0
0.5
-
A
C
D
E
F
G
H
I
K
L
M
N
P
Q
R
S
T
V
W
Y
China
Japan
Latvia
Russia
Korea
USSR

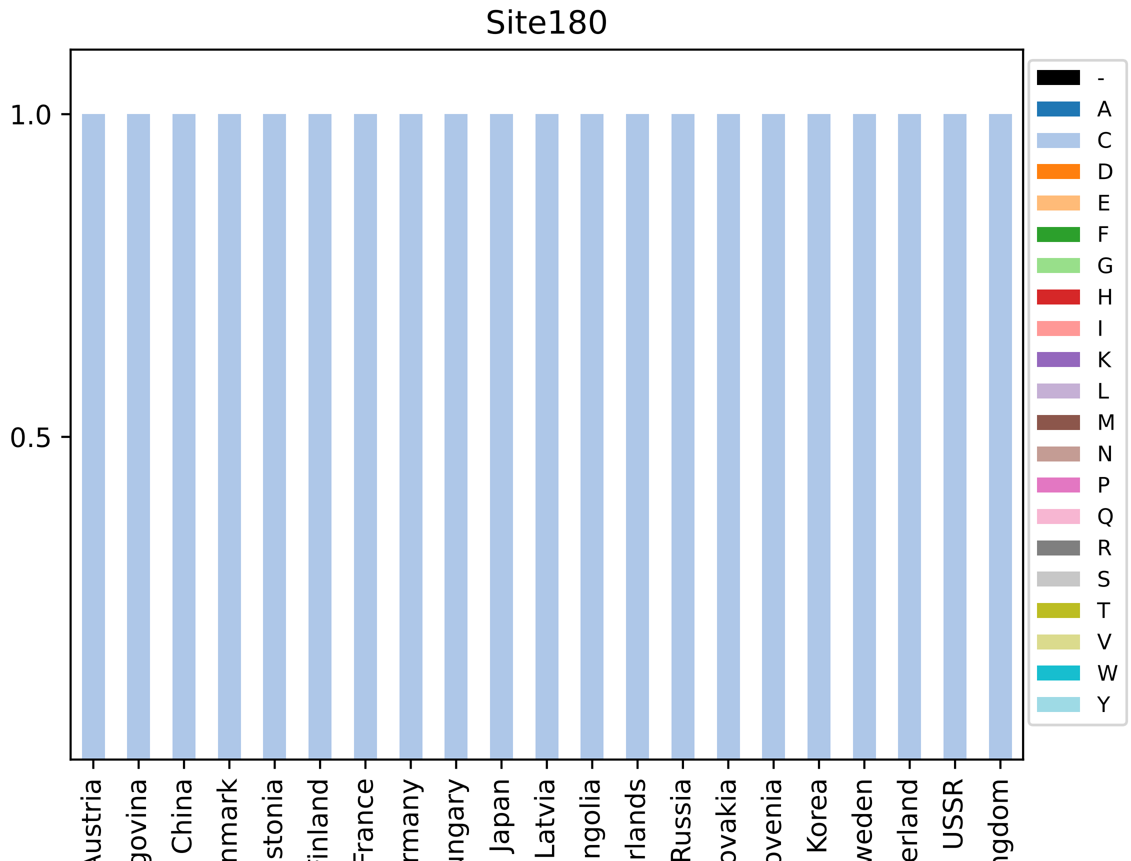
Site180
1.0
0.5
-
A
C
D
E
F
G
H
I
K
L
M
N
P
Q
R
S
T
V
W
Y
Austria
govina
China
nmark
stonia
inland
France
rmany
ungary
Japan
Latvia
ngolia
rlands
Russia
ovakia
ovenia
Korea
weden
erland
USSR
ngdom

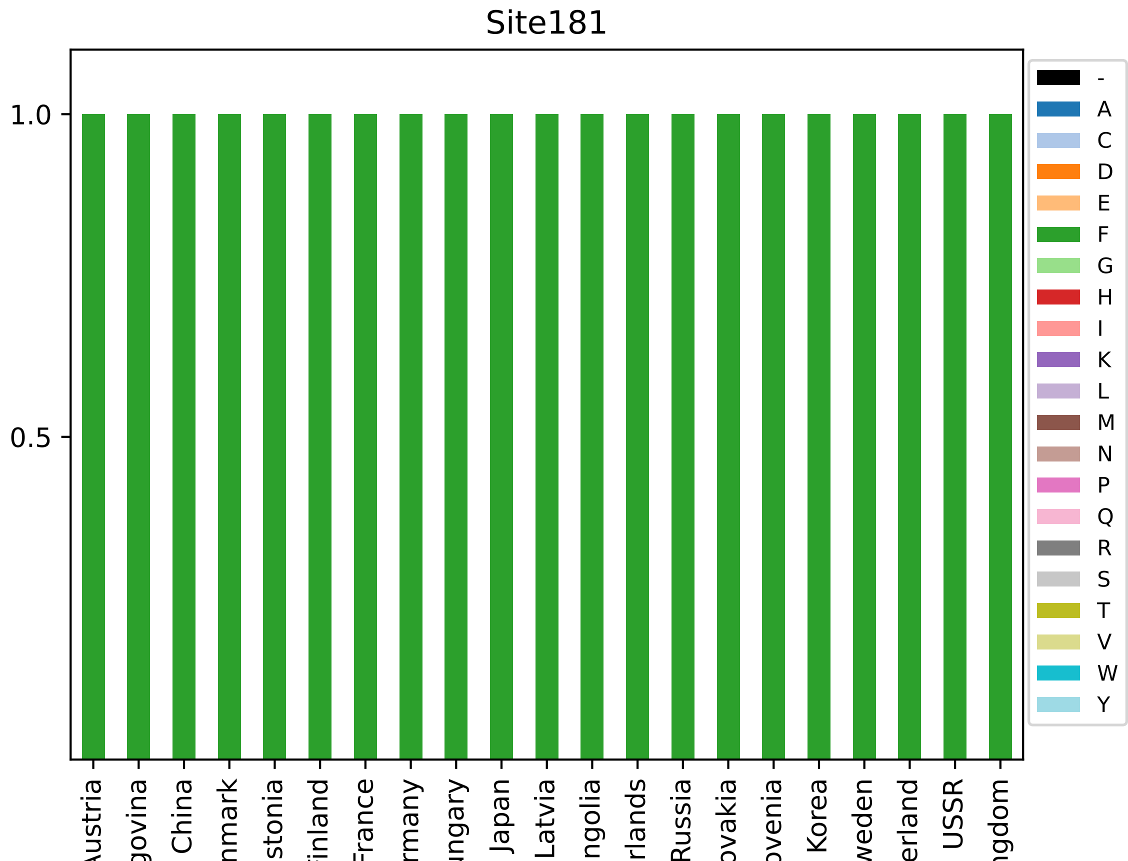

Site181
1.0
0.5
-
A
C
D
E
F
G
H
I
K
L
M
N
P
Q
R
S
T
V
W
Y
Austria
govina
China
nmark
stonia
Finland
France
rmany
ungary
Japan
Latvia
ngolia
rlands
Russia
ovakia
ovenia
Korea
weden
erland
USSR
ngdom

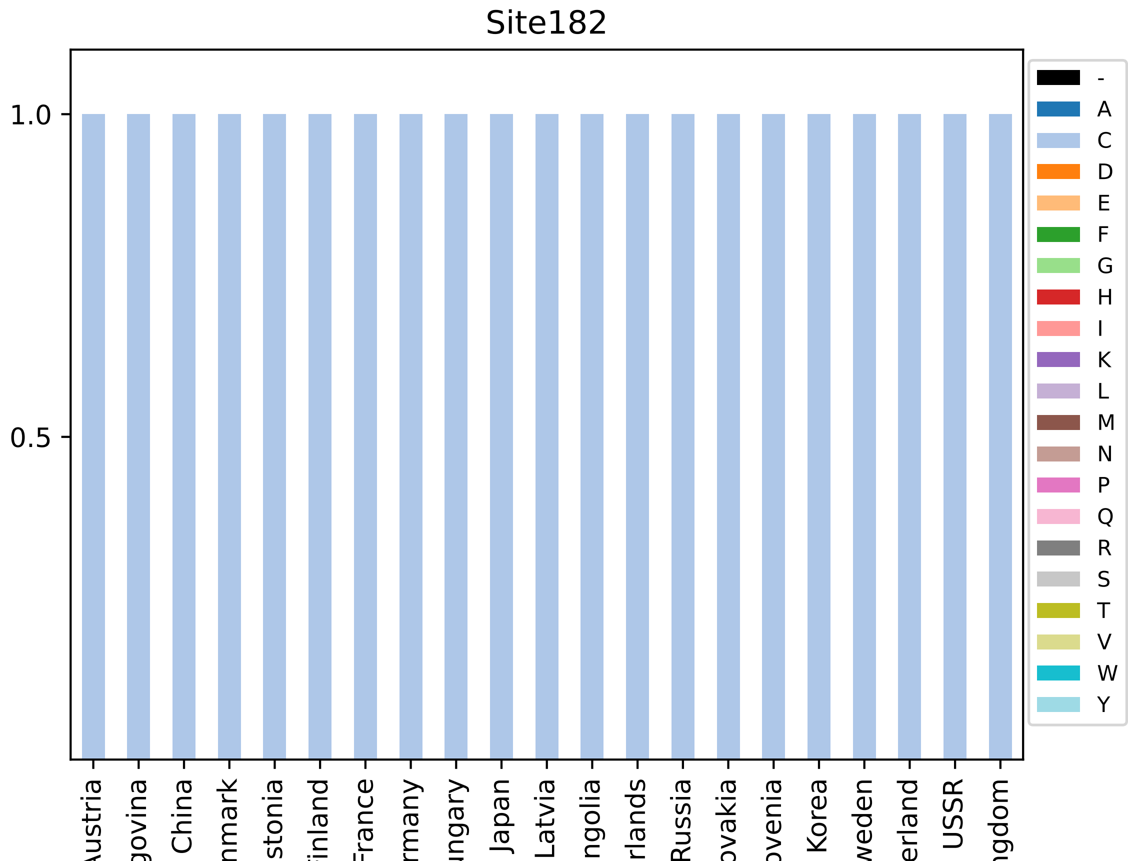
Site182
1.0
0.5
-
A
C
D
E
F
G
H
I
K
L
M
N
P
Q
R
S
T
V
W
Y
China
Japan
Latvia
Russia
Korea
USSR

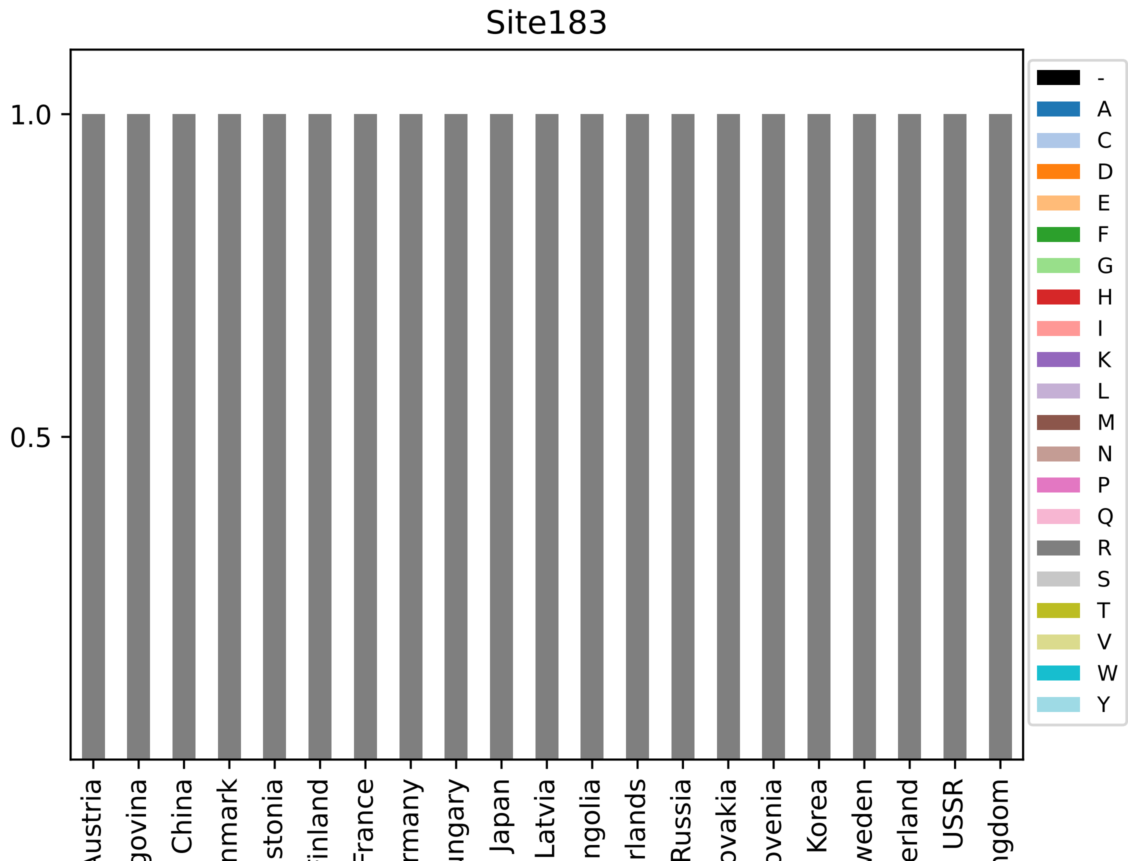

Site183
1.0
0.5
-
A
C
D
E
F
G
H
I
K
L
M
N
P
Q
R
S
T
V
W
Y
Austria
China
Japan
Latvia
Russia
Korea
USSR

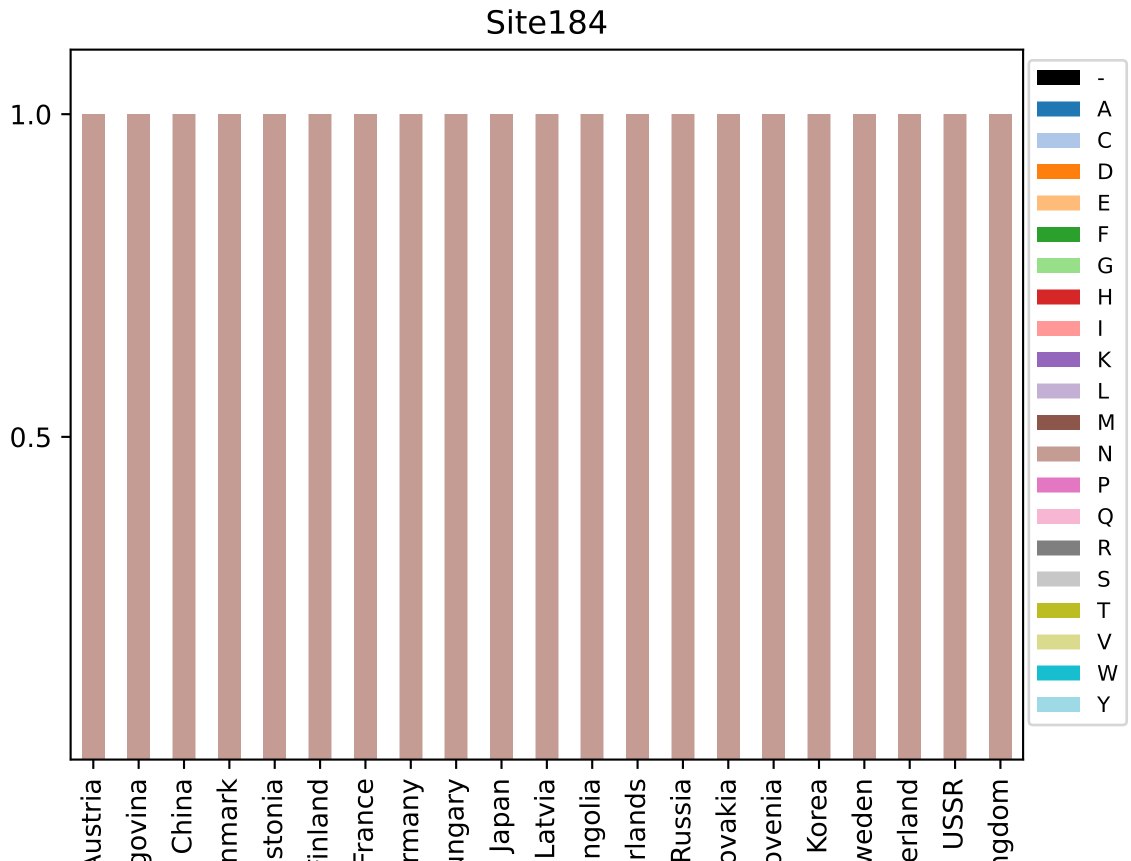
Site184
1.0
0.5
-
A
C
D
E
F
G
H
I
K
L
M
N
P
Q
R
S
T
V
W
Y
China
Japan
Latvia
Russia
Korea
USSR

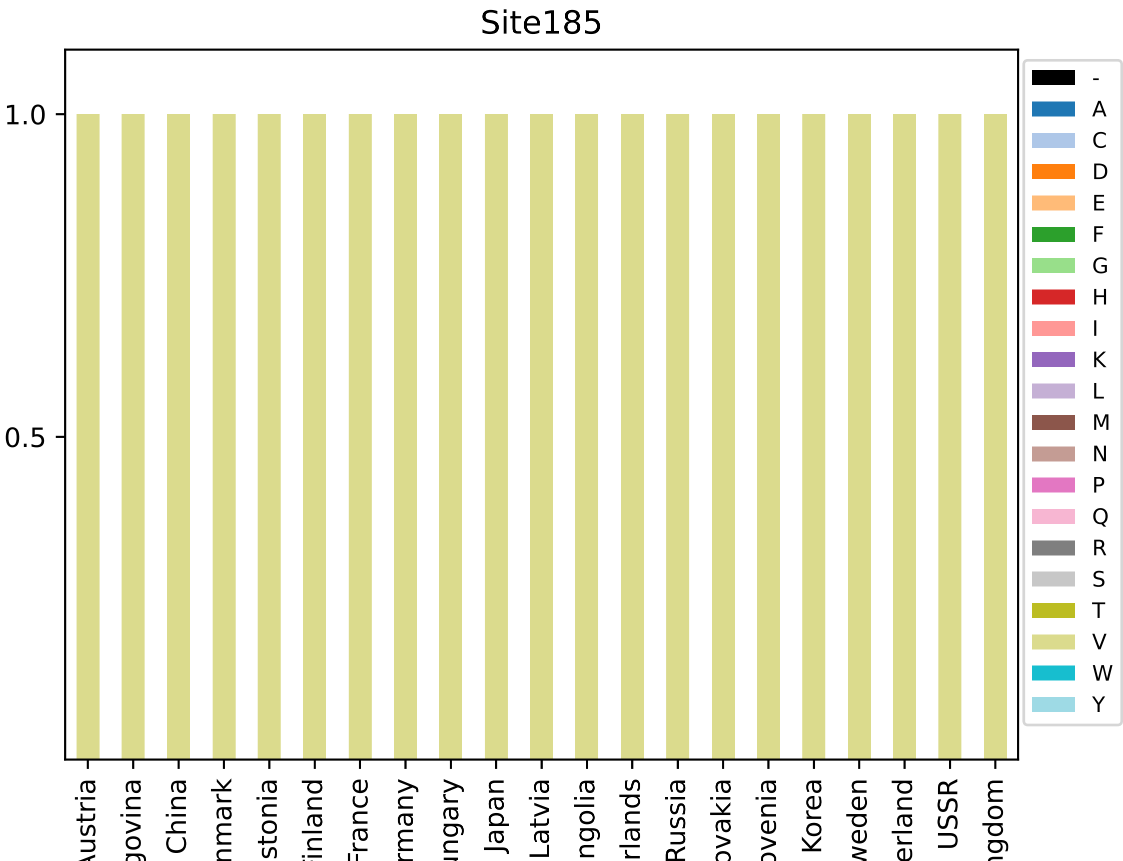
Site185
1.0
0.5
-
A
C
D
E
F
G
H
I
K
L
M
N
P
Q
R
S
T
V
W
Y
China
Japan
Latvia
Russia
Korea
USSR

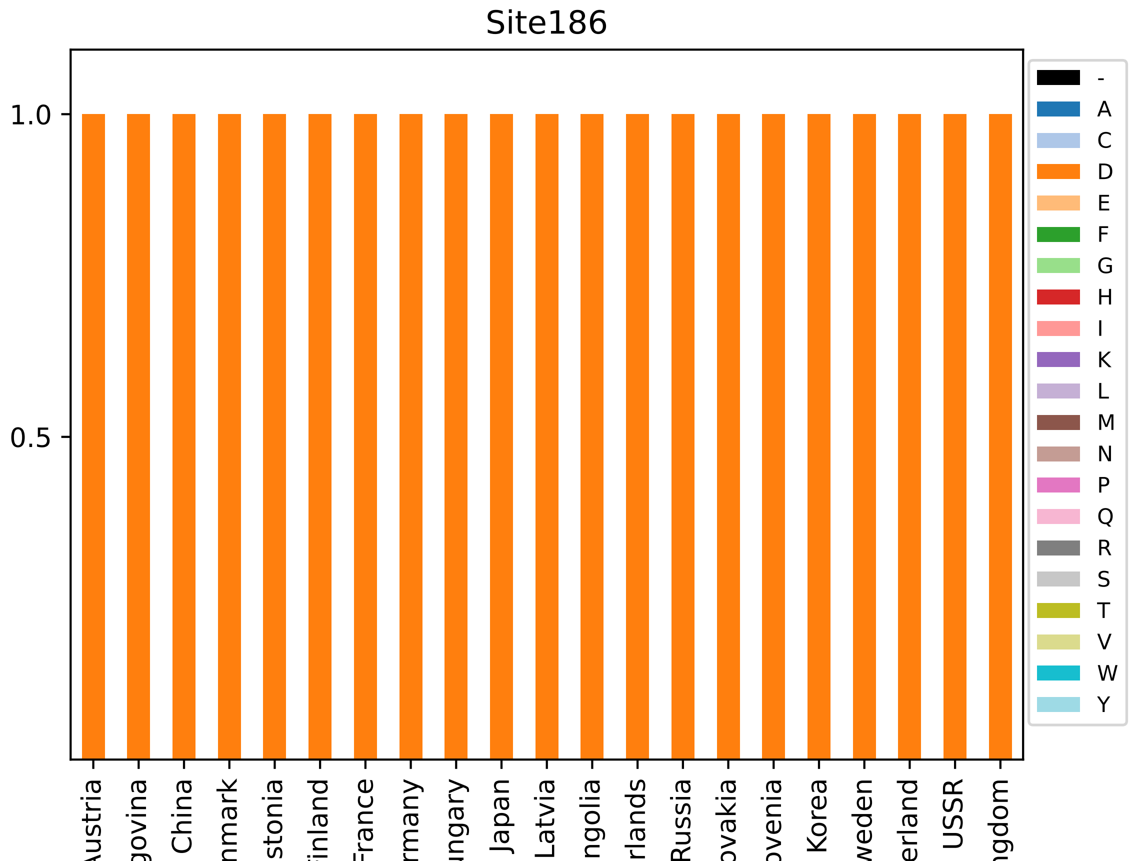
Site186
1.0
0.5
-
A
C
D
E
F
G
H
I
K
L
M
N
P
Q
R
S
T
V
W
Y
China
Japan
Latvia
Russia
Korea
USSR

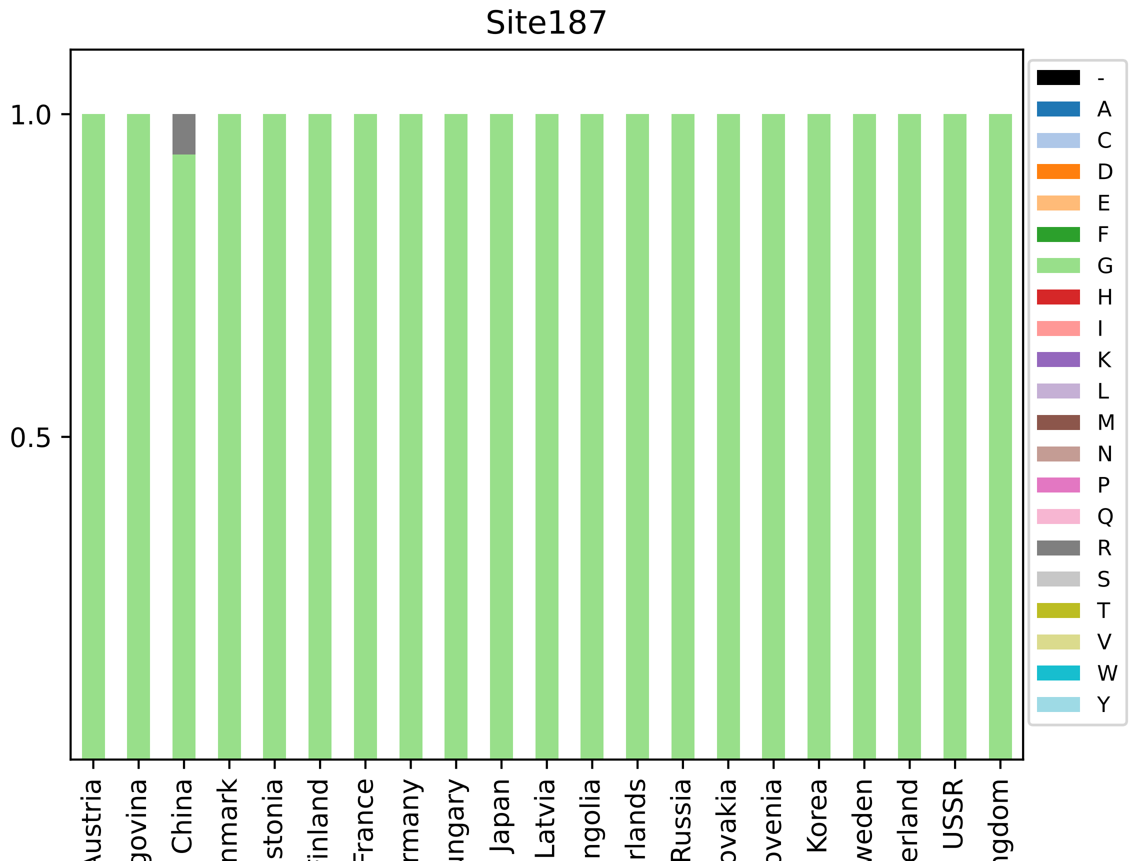
Site187
1.0
0.5
-
A
C
D
E
F
G
H
I
K
L
M
N
P
Q
R
S
T
V
W
Y
Austria
govina
China
nmark
stonia
Finland
France
rmany
ungary
Japan
Latvia
ngolia
rlands
Russia
ovakia
ovenia
Korea
weden
erland
USSR
ngdom

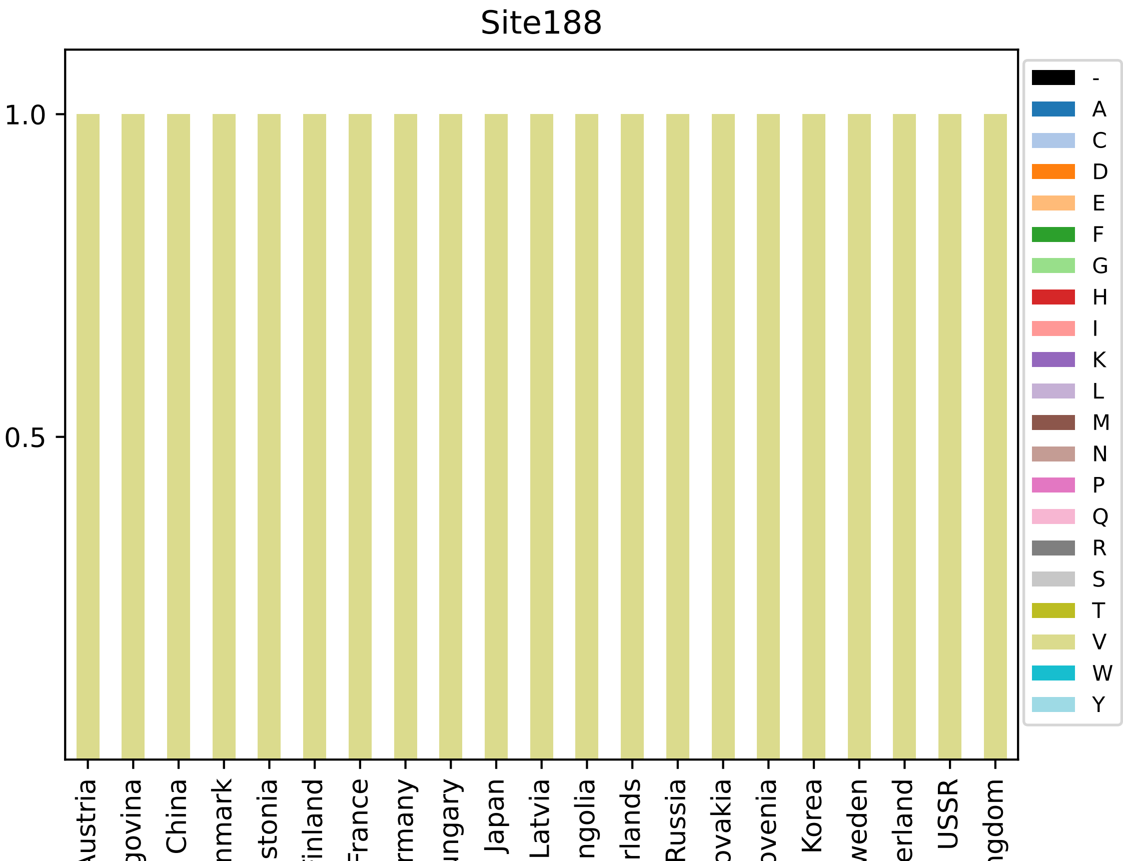

Site188
1.0
0.5
-
A
C
D
E
F
G
H
I
K
L
M
N
P
Q
R
S
T
V
W
Y
Austria
govina
China
nmark
stonia
Finland
France
rmany
ungary
Japan
Latvia
ngolia
rlands
Russia
ovakia
ovenia
Korea
weden
erland
USSR
ngdom

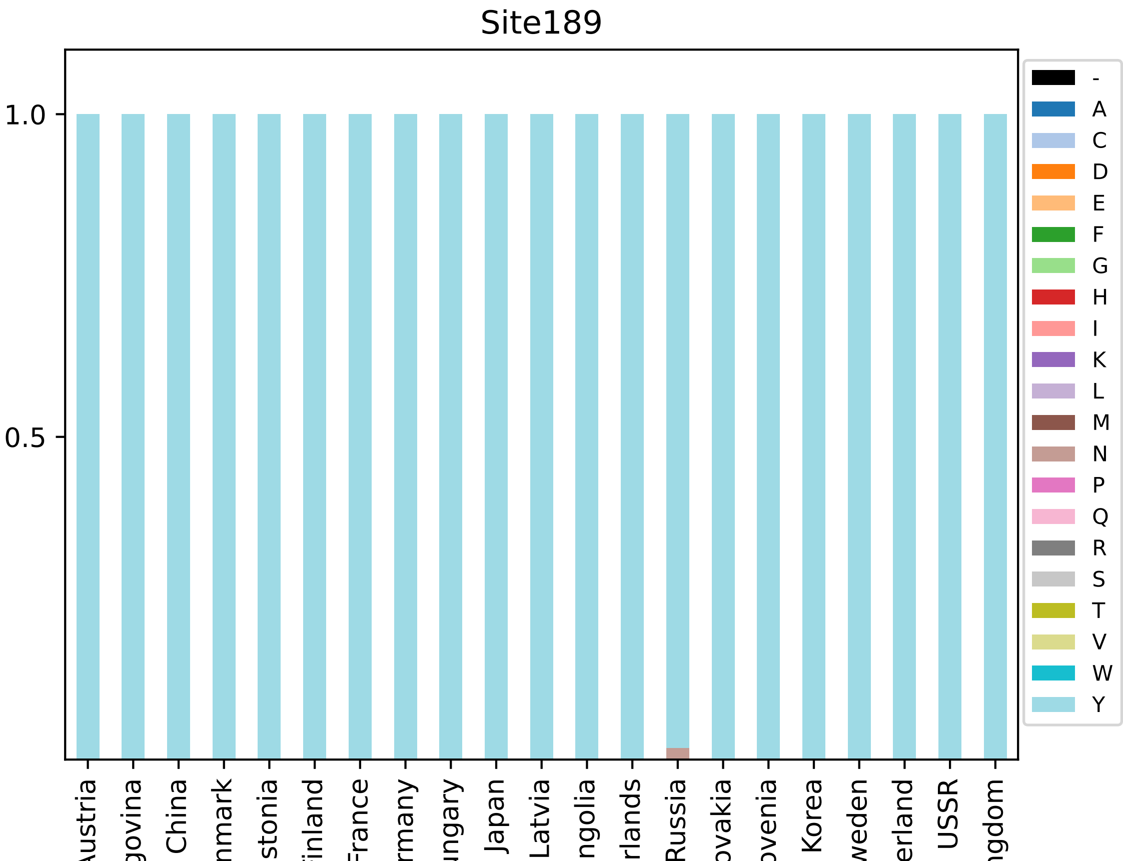

Site189
1.0
0.5
-
A
C
D
E
F
G
H
I
K
L
M
N
P
Q
R
S
T
V
W
Y
Austria
govina
China
nmark
stonia
Finland
France
rmany
ungary
Japan
Latvia
ngolia
rlands
Russia
ovakia
ovenia
Korea
weden
erland
USSR
ngdom

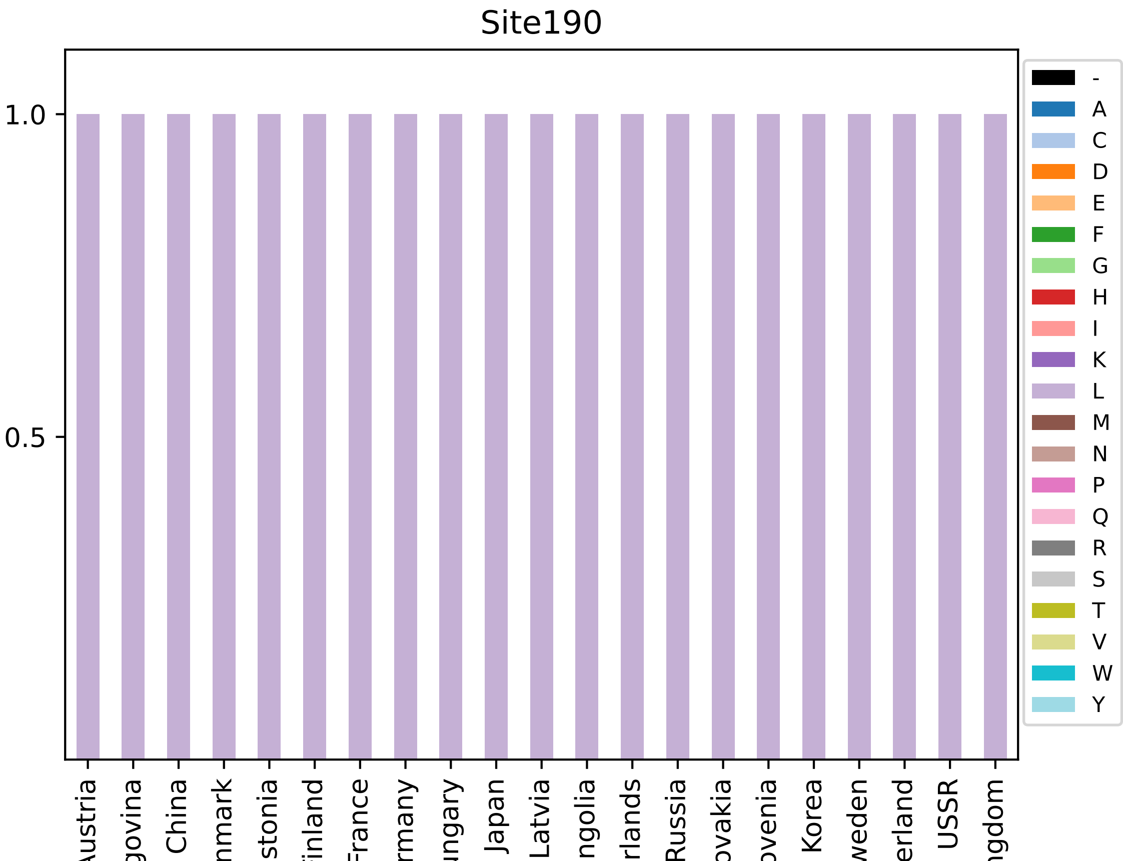
Site190
1.0
0.5
-
A
C
D
E
F
G
H
I
K
L
M
N
P
Q
R
S
T
V
W
Y
China
Japan
Latvia
Russia
Korea
USSR

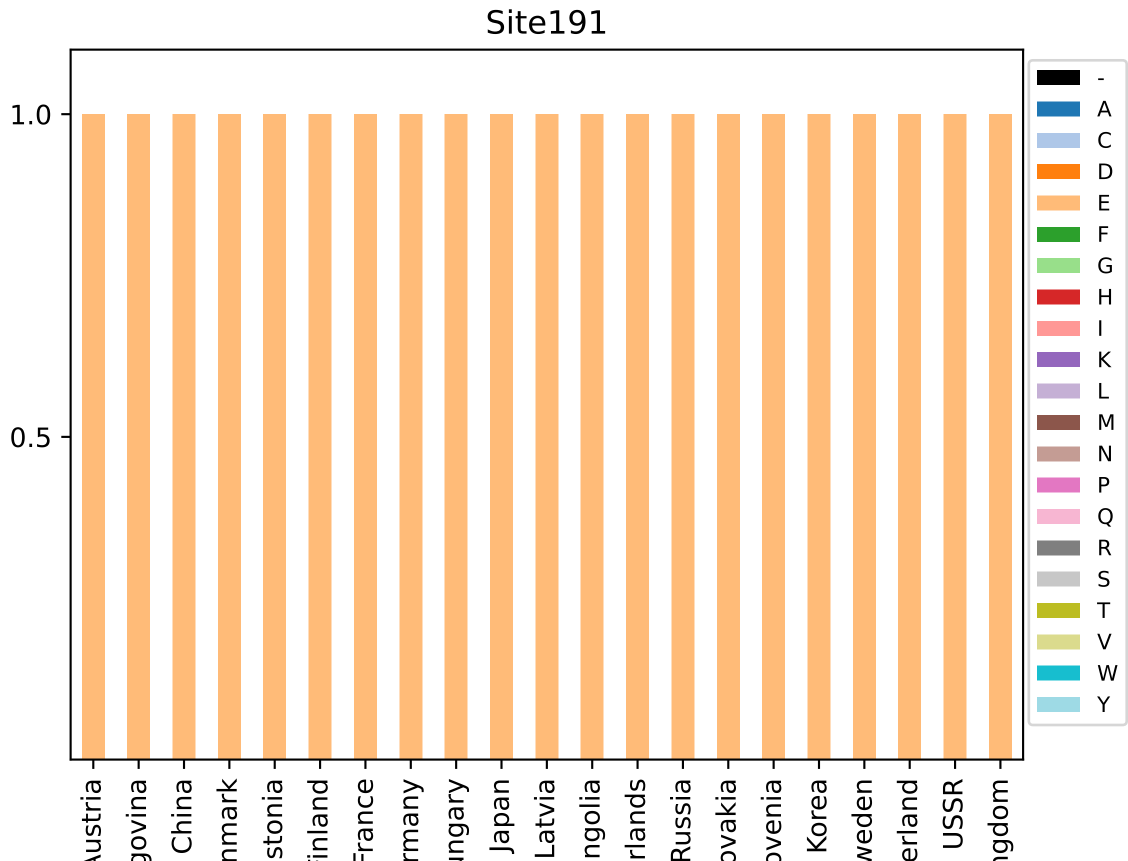
Site191
1.0
0.5
-
A
C
D
E
F
G
H
I
K
L
M
N
P
Q
R
S
T
V
W
Y
China
Japan
Latvia
Korea
USSR

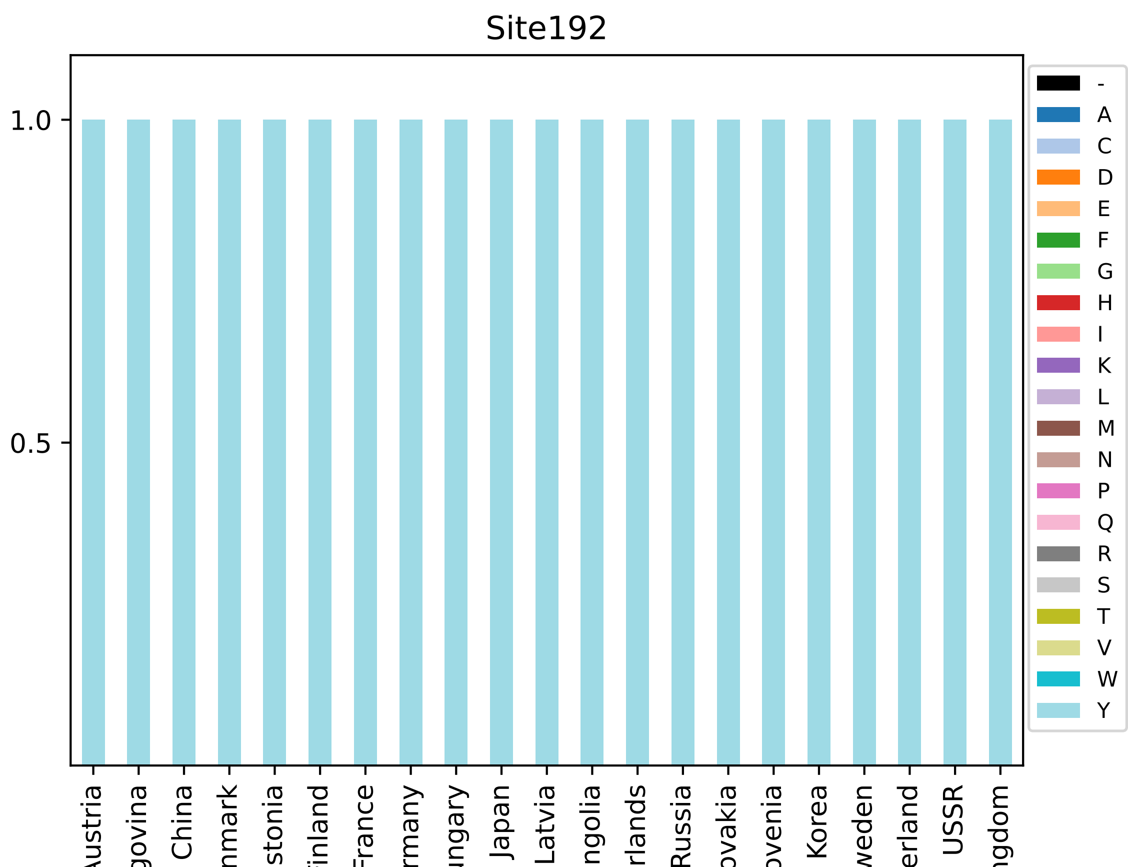

Site192
1.0
0.5
-
A
C
D
E
F
G
H
I
K
L
M
N
P
Q
R
S
T
V
W
Y
Austria
govina
China
nmark
stonia
Finland
France
rmany
ungary
Japan
Latvia
ngolia
rlands
Russia
ovakia
ovenia
Korea
weden
erland
USSR
ngdom

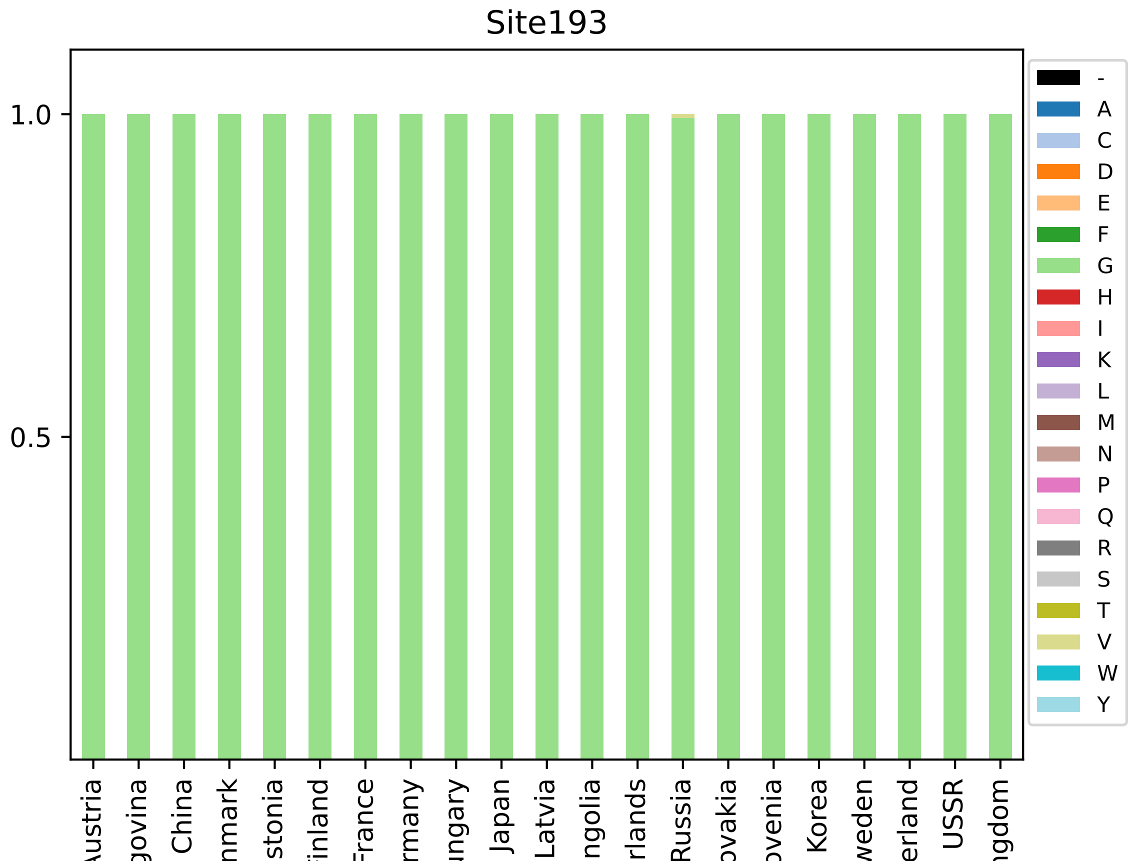
Site193
1.0
0.5
-
A
C
D
E
F
G
H
I
K
L
M
N
P
Q
R
S
T
V
W
Y
ustria
govina
China
nmark
stonia
inland
rance
rmany
ungary
Japan
Latvia
ngolia
rlands
Russia
ovakia
ovenia
Korea
weden
erland
USSR
ngdom

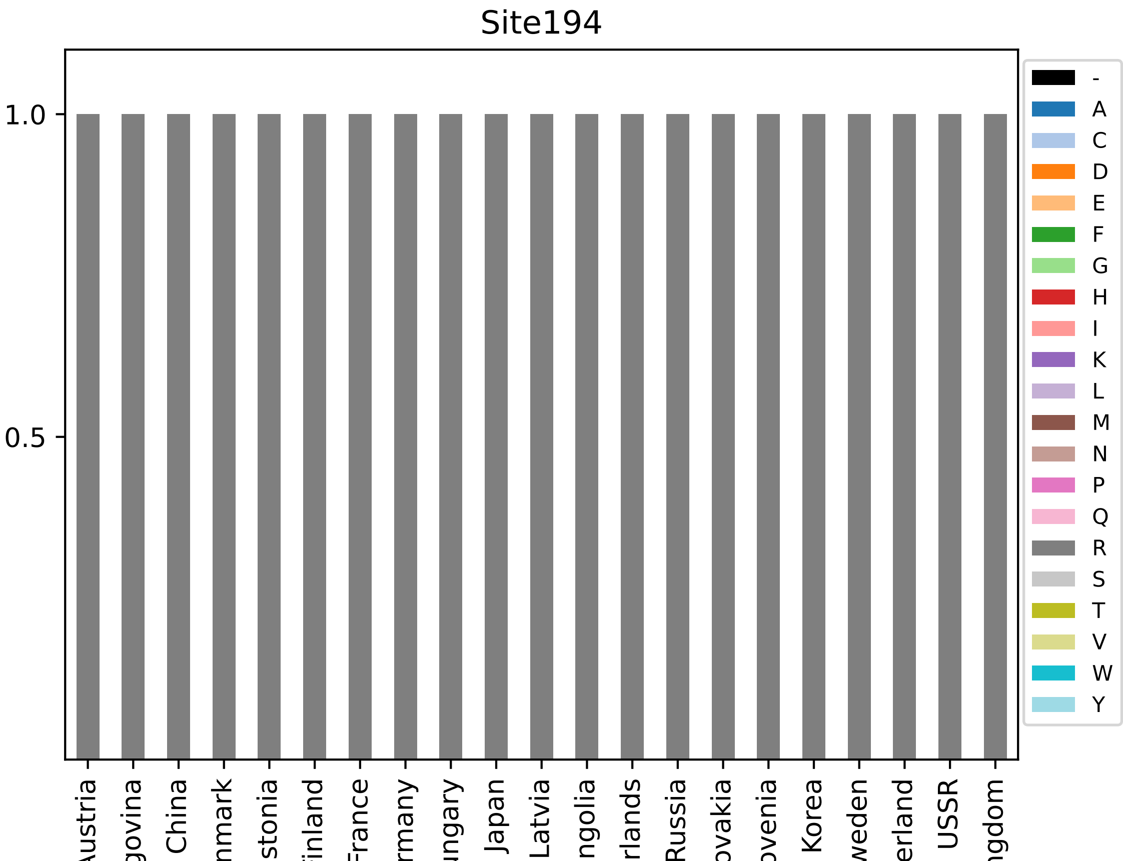
Site194
1.0
0.5
-
A
C
D
E
F
G
H
I
K
L
M
N
P
Q
R
S
T
V
W
Y
Austria
govina
China
nmark
stonia
Finland
France
rmany
ungary
Japan
Latvia
ngolia
rlands
Russia
ovakia
ovenia
Korea
weden
erland
USSR
ngdom

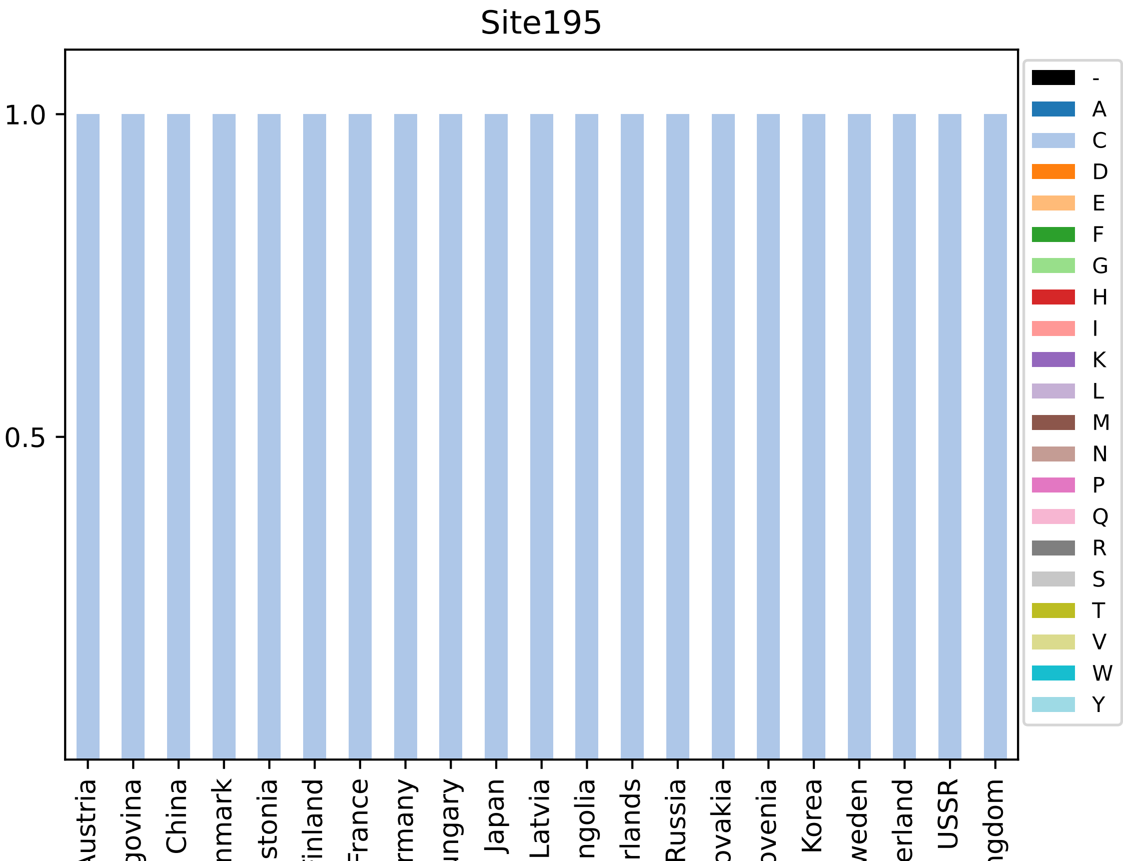
Site195
1.0
0.5
-
A
C
D
E
F
G
H
I
K
L
M
N
P
Q
R
S
T
V
W
Y
China
Japan
Latvia
Russia
Korea
USSR

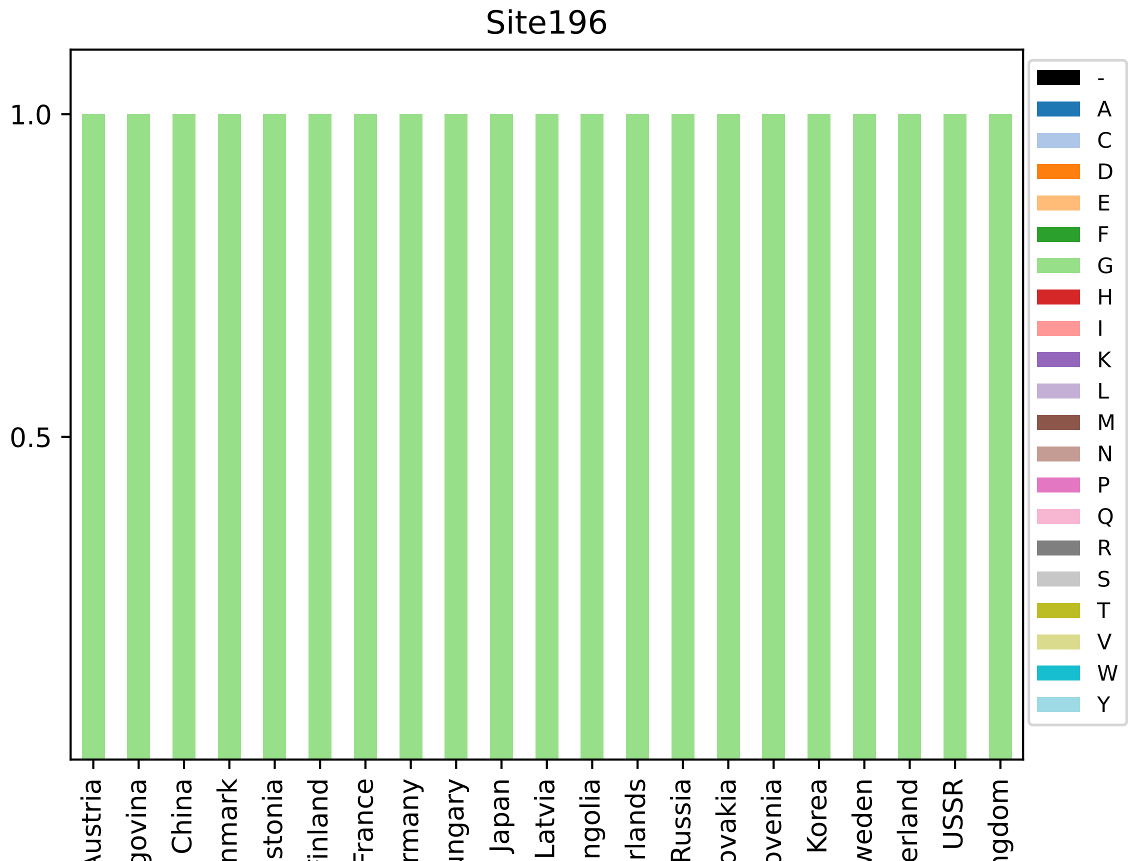
Site196
1.0
0.5
-
A
C
D
E
F
G
H
I
K
L
M
N
P
Q
R
S
T
V
W
Y
China
Japan
Latvia
Russia
Korea
USSR

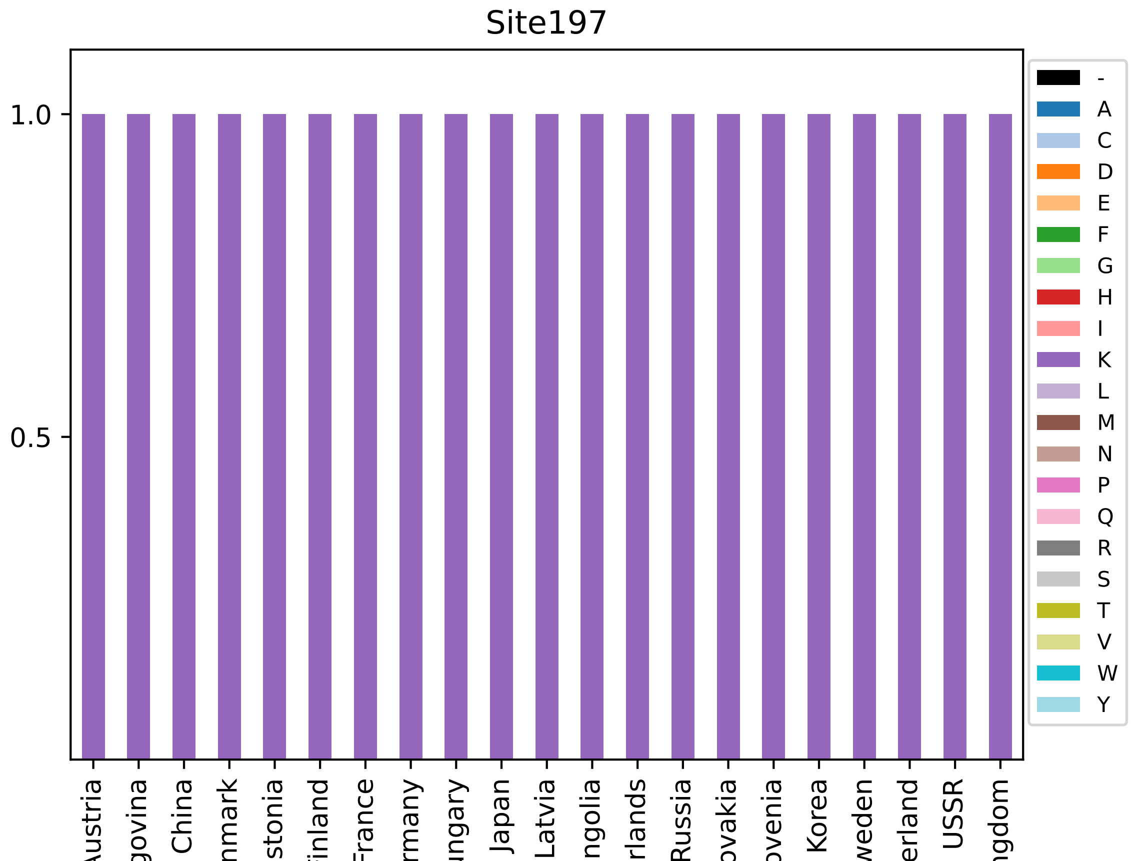

Site197
1.0
0.5
-
A
C
D
E
F
G
H
I
K
L
M
N
P
Q
R
S
T
V
W
Y
Austria
govina
China
nmark
stonia
inland
France
rmany
ungary
Japan
Latvia
ngolia
rlands
Russia
ovakia
ovenia
Korea
weden
erland
USSR
ngdom

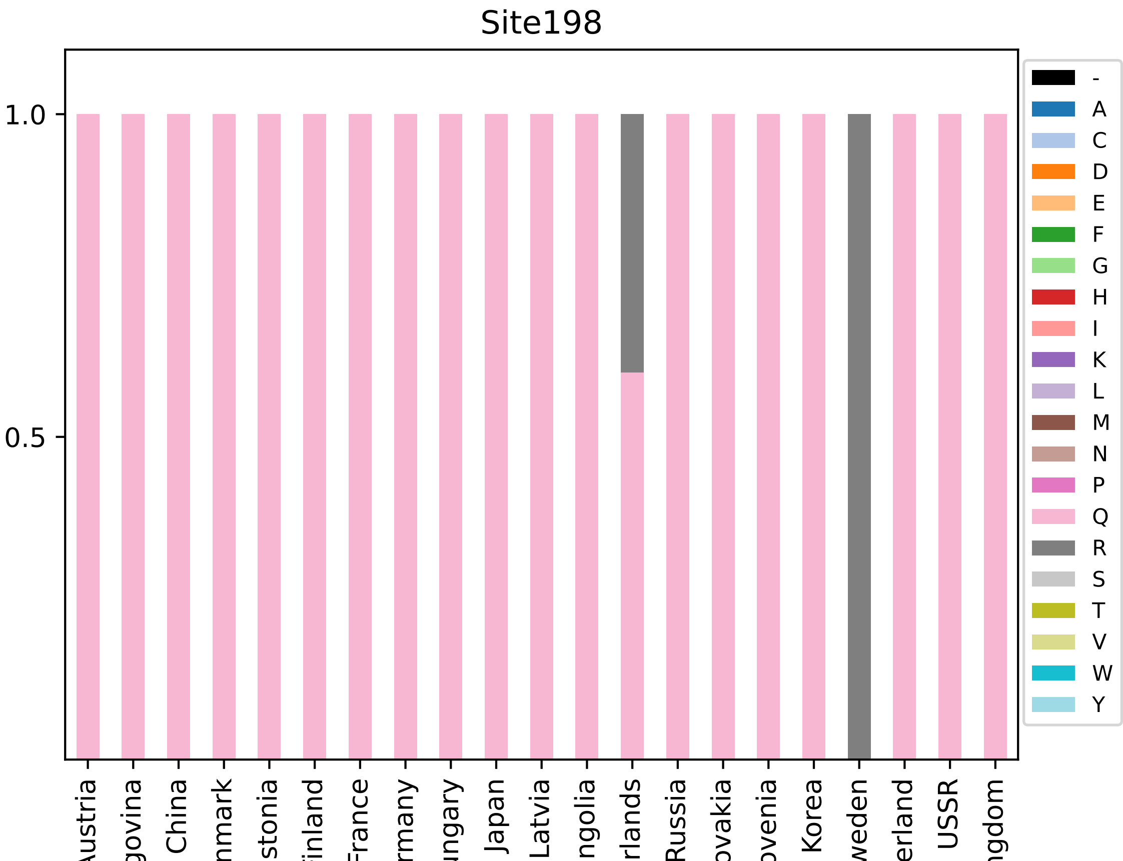
Site198
1.0
0.5
-
A
C
D
E
F
G
H
I
K
L
M
N
P
Q
R
S
T
V
W
Y
Austria
govina
China
nmark
stonia
inland
France
rmany
ungary
Japan
Latvia
ngolia
rlands
Russia
ovakia
ovenia
Korea
weden
erland
USSR
ngdom

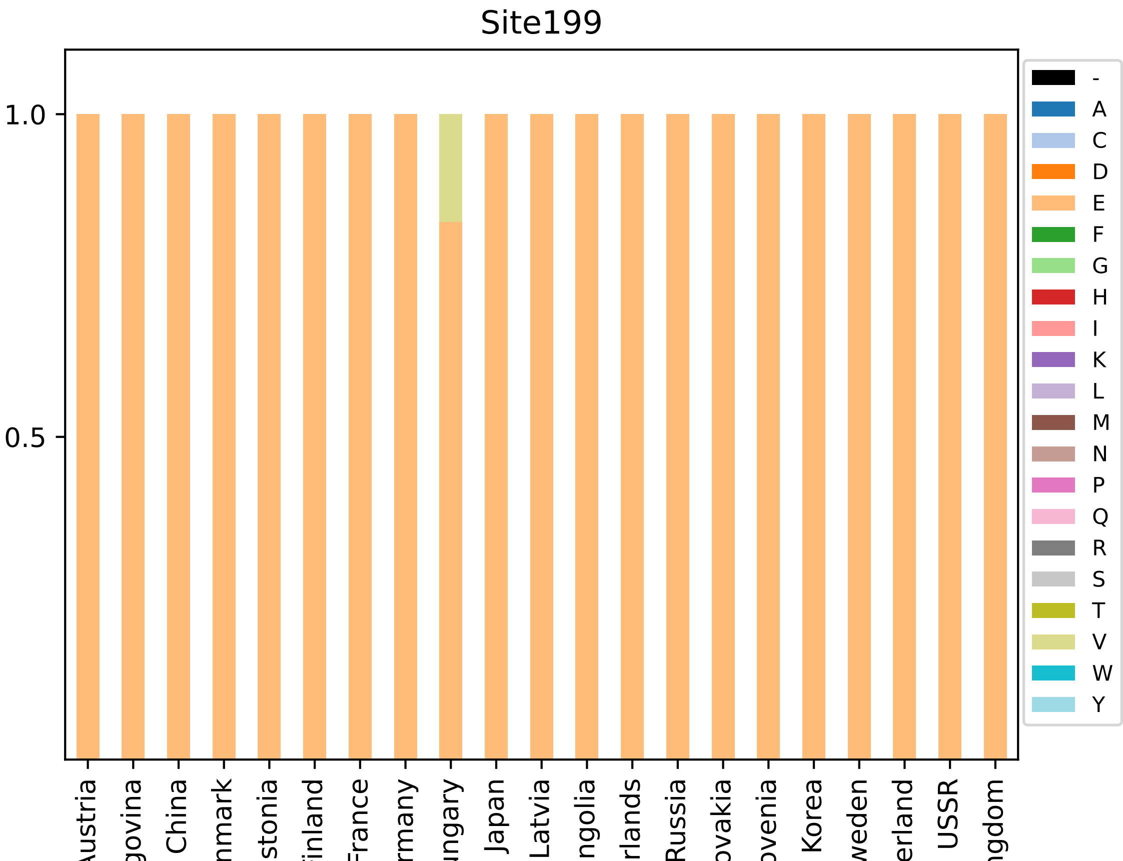

Site199
1.0
0.5
-
A
C
D
E
F
G
H
I
K
L
M
N
P
Q
R
S
T
V
W
Y
China
Japan
Latvia
Russia
Korea
USSR

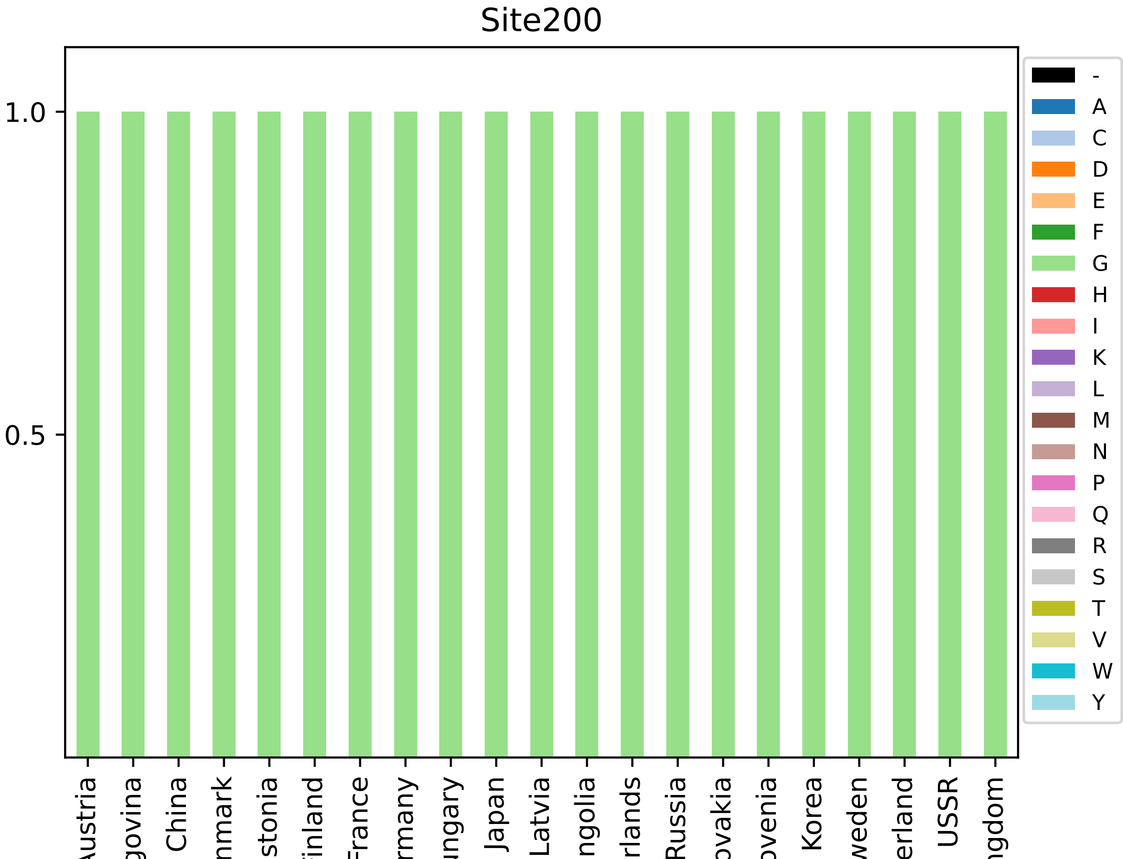

Site200
1.0
0.5
-
A
C
D
E
F
G
H
I
K
L
M
N
P
Q
R
S
T
V
W
Y
China
Japan
Latvia
Russia
Korea
USSR

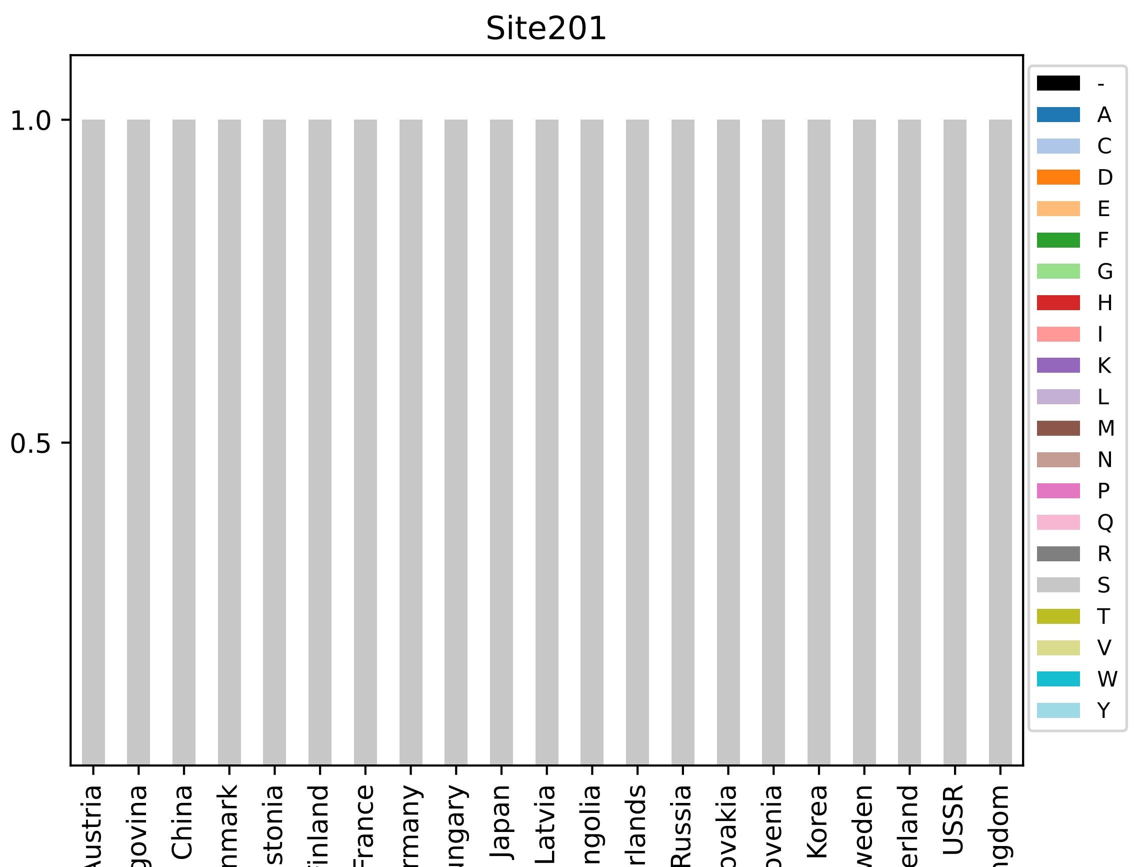

Site201
1.0
0.5
-
A
C
D
E
F
G
H
I
K
L
M
N
P
Q
R
S
T
V
W
Y
Austria
govina
China
nmark
stonia
inland
France
rmany
ungary
Japan
Latvia
ngolia
rlands
Russia
ovakia
ovenia
Korea
weden
erland
USSR
ngdom

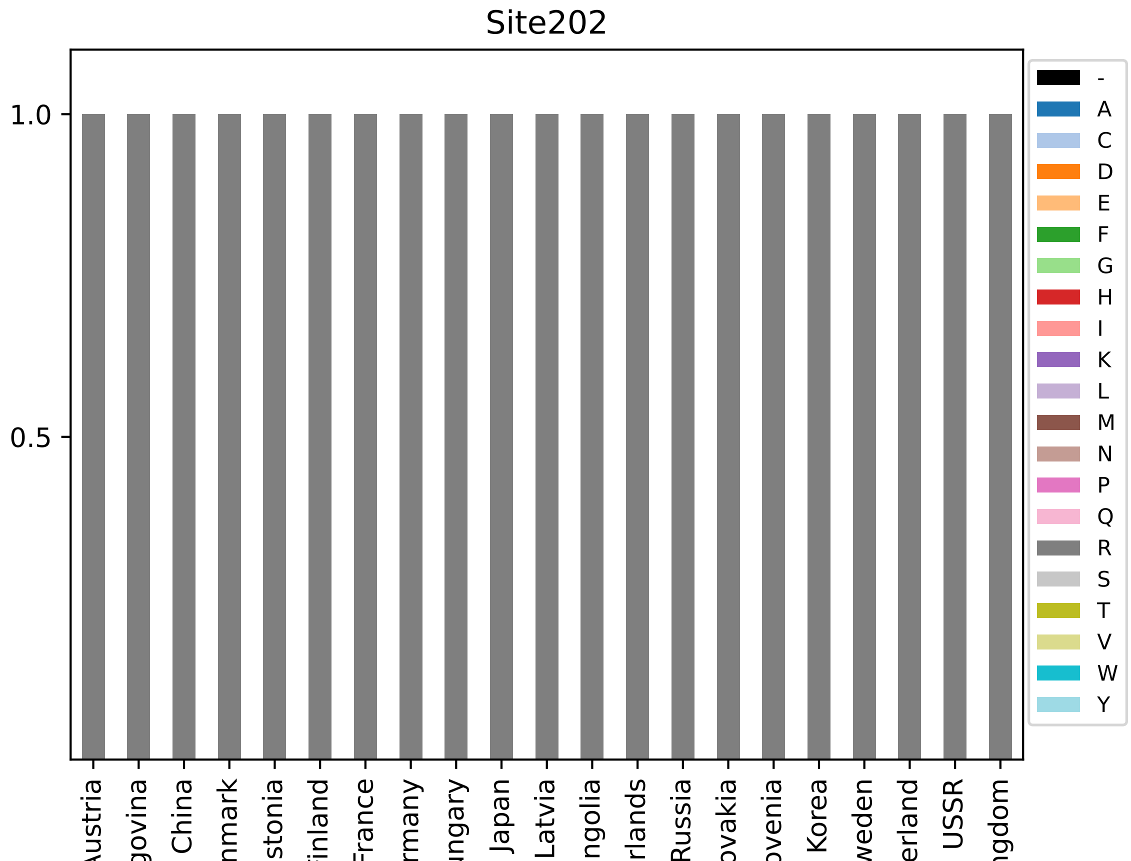
Site202
1.0
0.5
-
A
C
D
E
F
G
H
I
K
L
M
N
P
Q
R
S
T
V
W
Y
Austria
China
Japan
Latvia
Russia
Korea
USSR

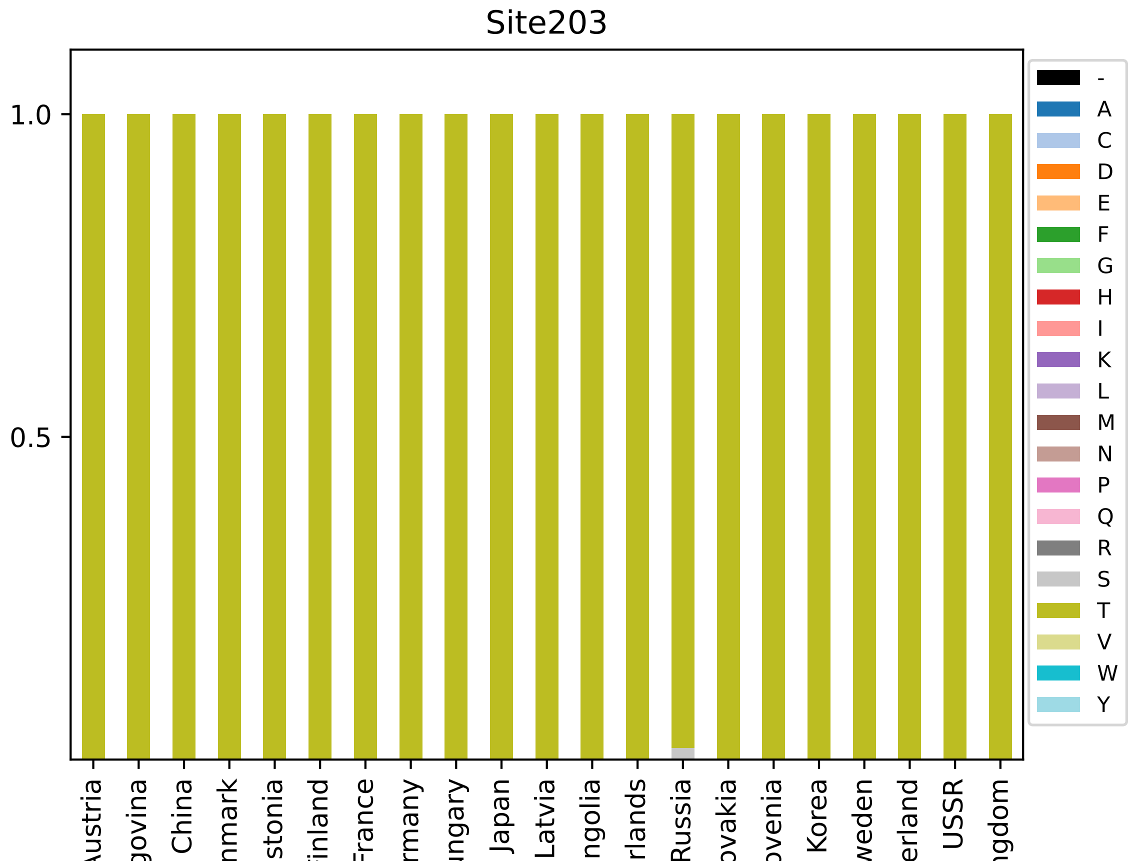
Site203
1.0
0.5
-
A
C
D
E
F
G
H
I
K
L
M
N
P
Q
R
S
T
V
W
Y
Austria
govina
China
nmark
stonia
Finland
France
rmany
ungary
Japan
Latvia
ngolia
rlands
Russia
ovakia
ovenia
Korea
weden
erland
USSR
ngdom

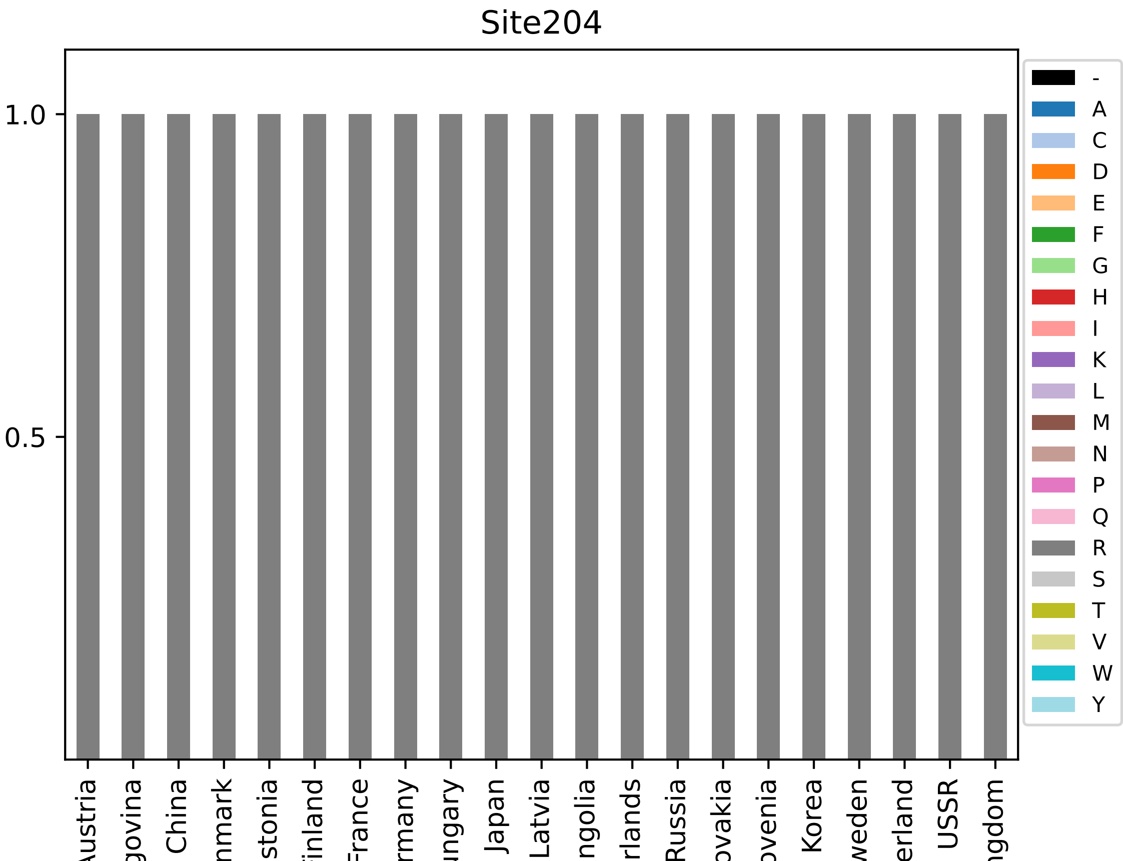

Site204
1.0
0.5
China
Japan
Latvia
Russia
Korea
USSR
-
A
C
D
E
F
G
H
I
K
L
M
N
P
Q
R
S
T
V
W
Y

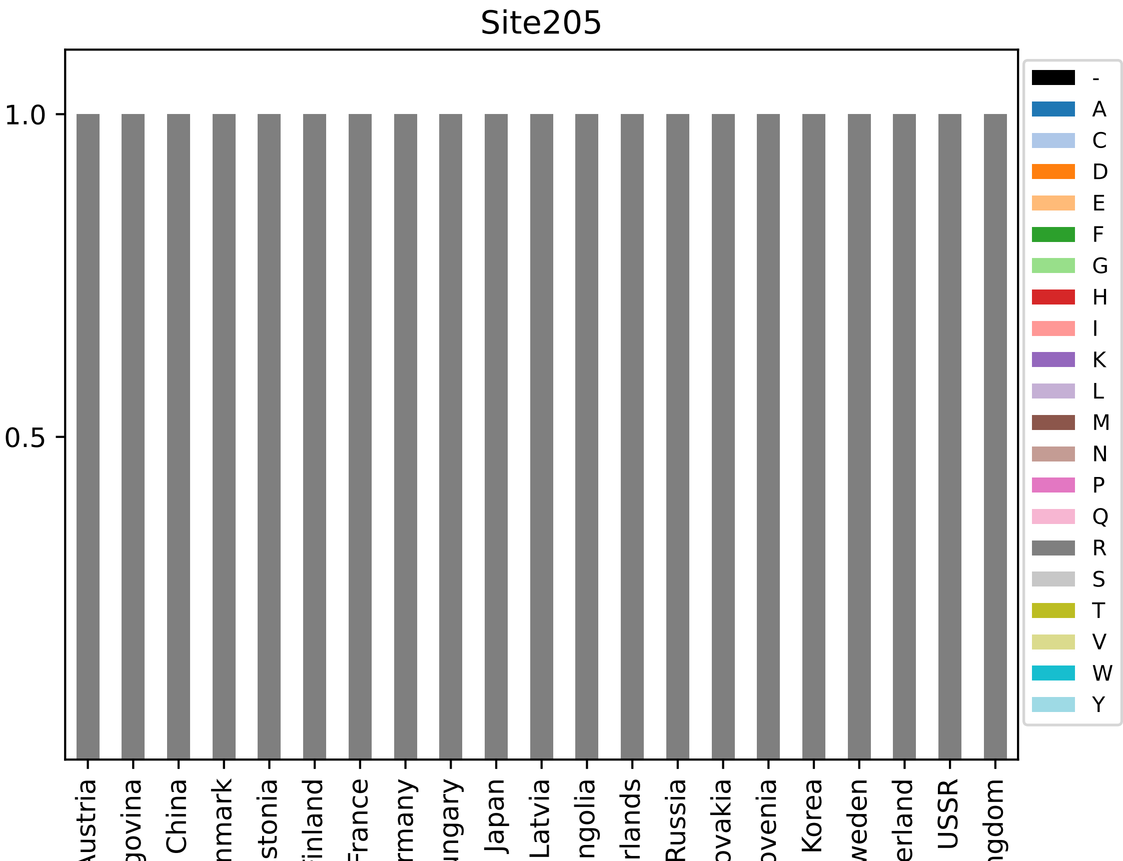

Site205
1.0
0.5
Austria
govina
China
nmark
stonia
Finland
France
rmany
ungary
Japan
Latvia
ngolia
rlands
Russia
ovakia
ovenia
Korea
weden
erland
USSR
ngdom
-
A
C
D
E
F
G
H
I
K
L
M
N
P
Q
R
S
T
V
W
Y

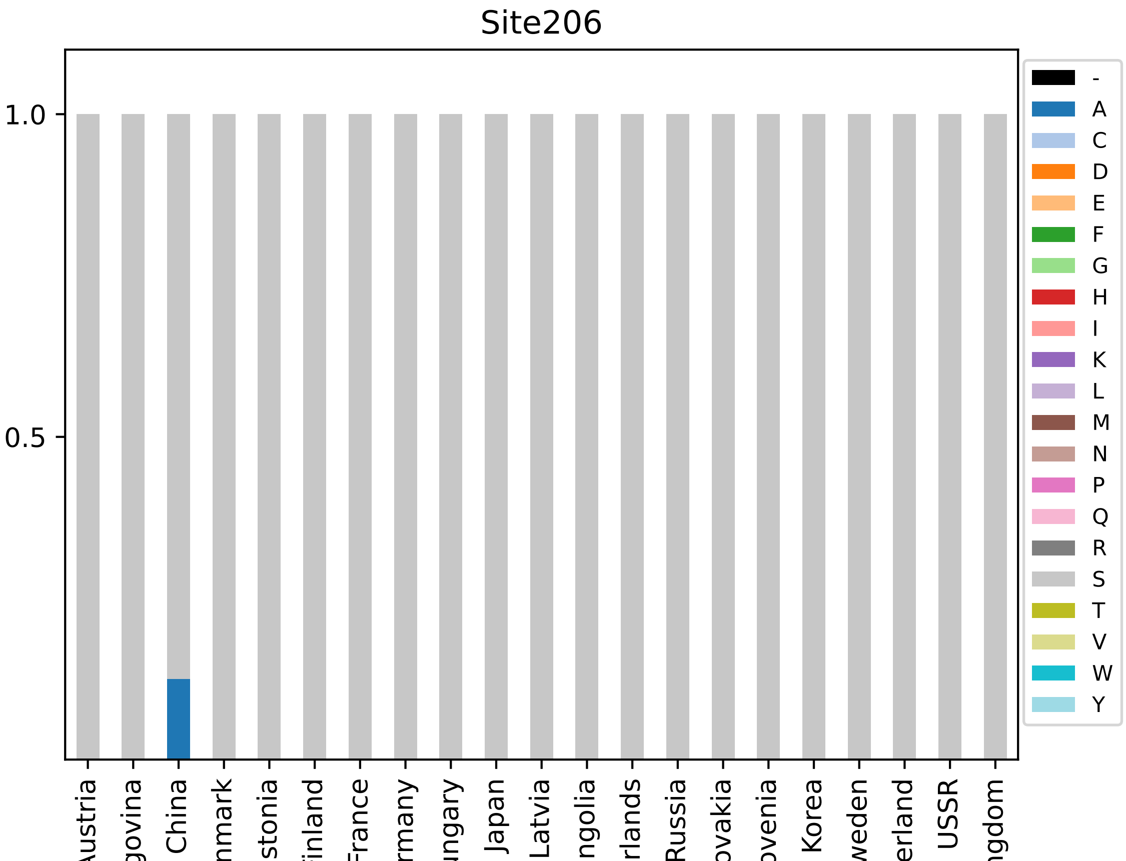
Site206
1.0
0.5
China
Japan
Latvia
Russia
Korea
USSR
-
A
C
D
E
F
G
H
I
K
L
M
N
P
Q
R
S
T
V
W
Y

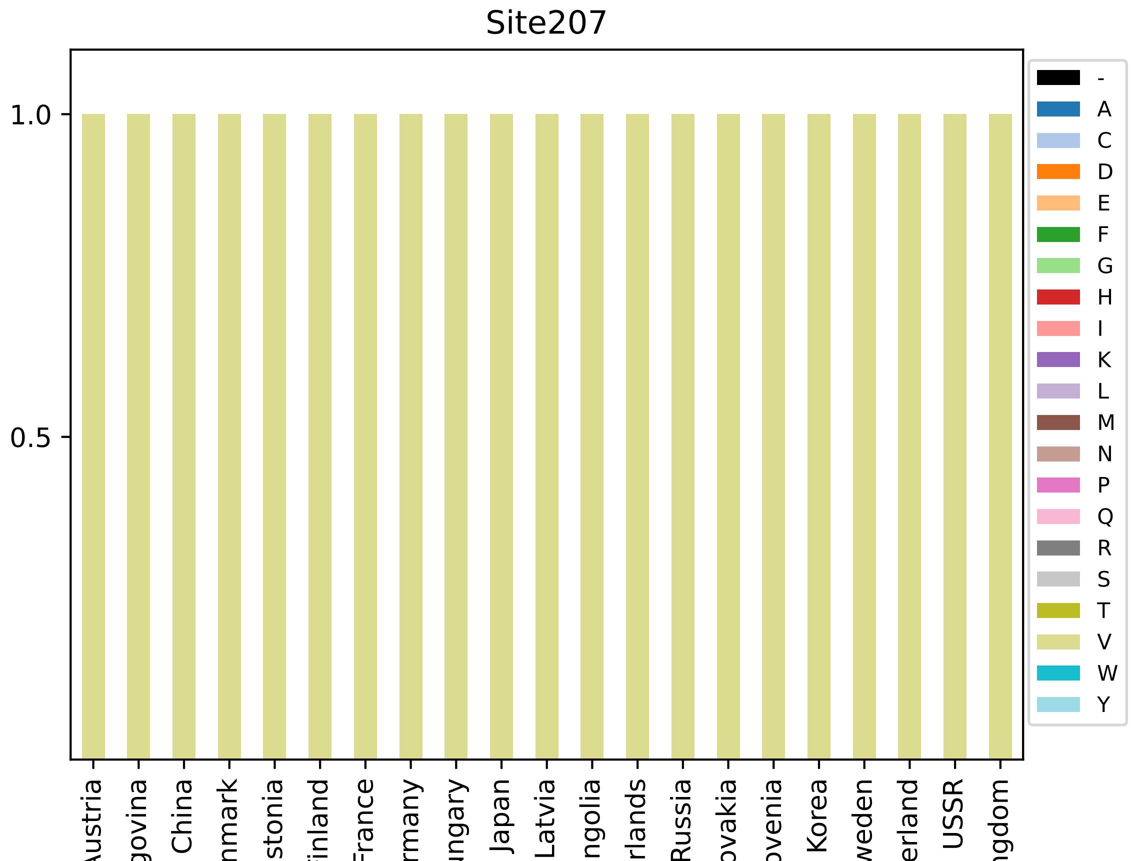

Site207
1.0
0.5
-
A
C
D
E
F
G
H
I
K
L
M
N
P
Q
R
S
T
V
W
Y
Austria
China
Japan
Latvia
Russia
Korea
USSR

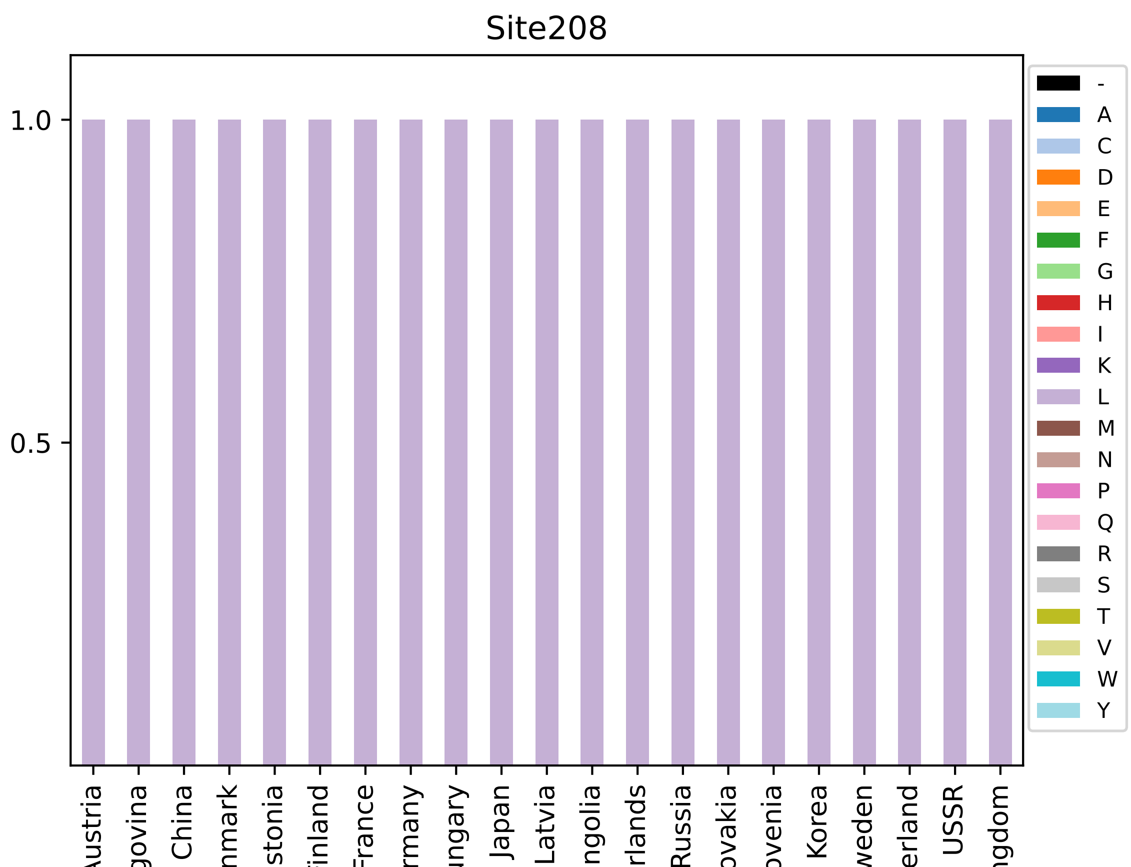
Site208
1.0
0.5
-
A
C
D
E
F
G
H
I
K
L
M
N
P
Q
R
S
T
V
W
Y
Austria
govina
China
nmark
stonia
inland
France
rmany
ungary
Japan
Latvia
ngolia
rlands
Russia
ovakia
ovenia
Korea
weden
erland
USSR
ngdom

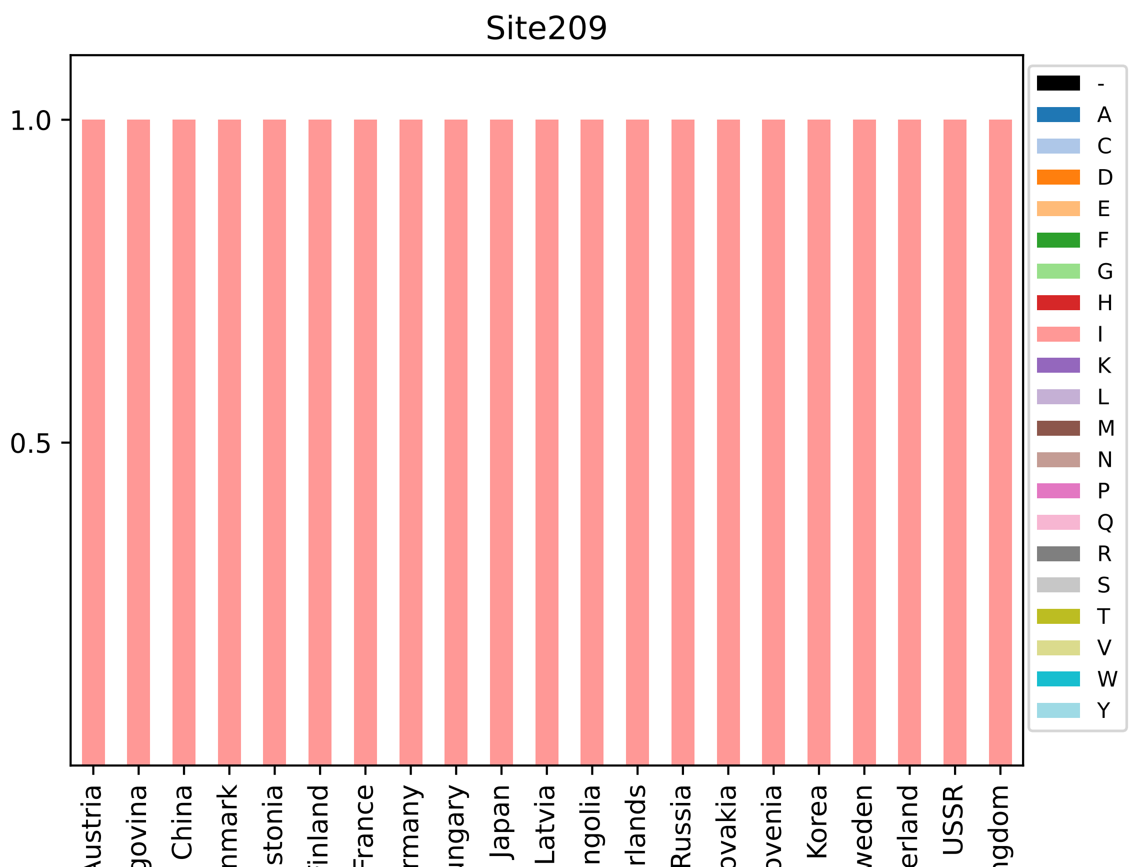

Site209
1.0
0.5
-
A
C
D
E
F
G
H
I
K
L
M
N
P
Q
R
S
T
V
W
Y
China
Japan
Latvia
Russia
Korea
USSR

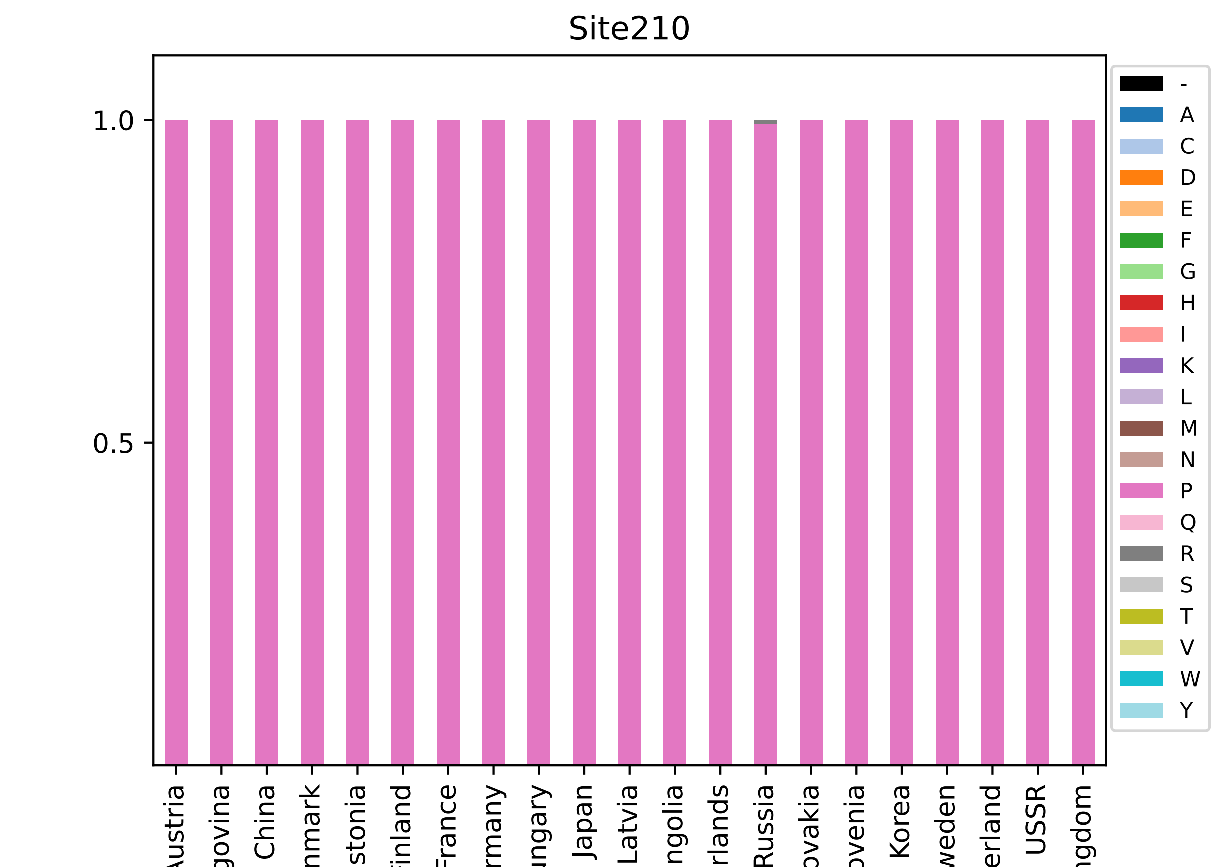

Site210
1.0
0.5
Austria
govina
China
nmark
stonia
Finland
France
rmany
ungary
Japan
Latvia
ngolia
rlands
Russia
ovakia
ovenia
Korea
weden
erland
USSR
ngdom
-
A
C
D
E
F
G
H
I
K
L
M
N
P
Q
R
S
T
V
W
Y

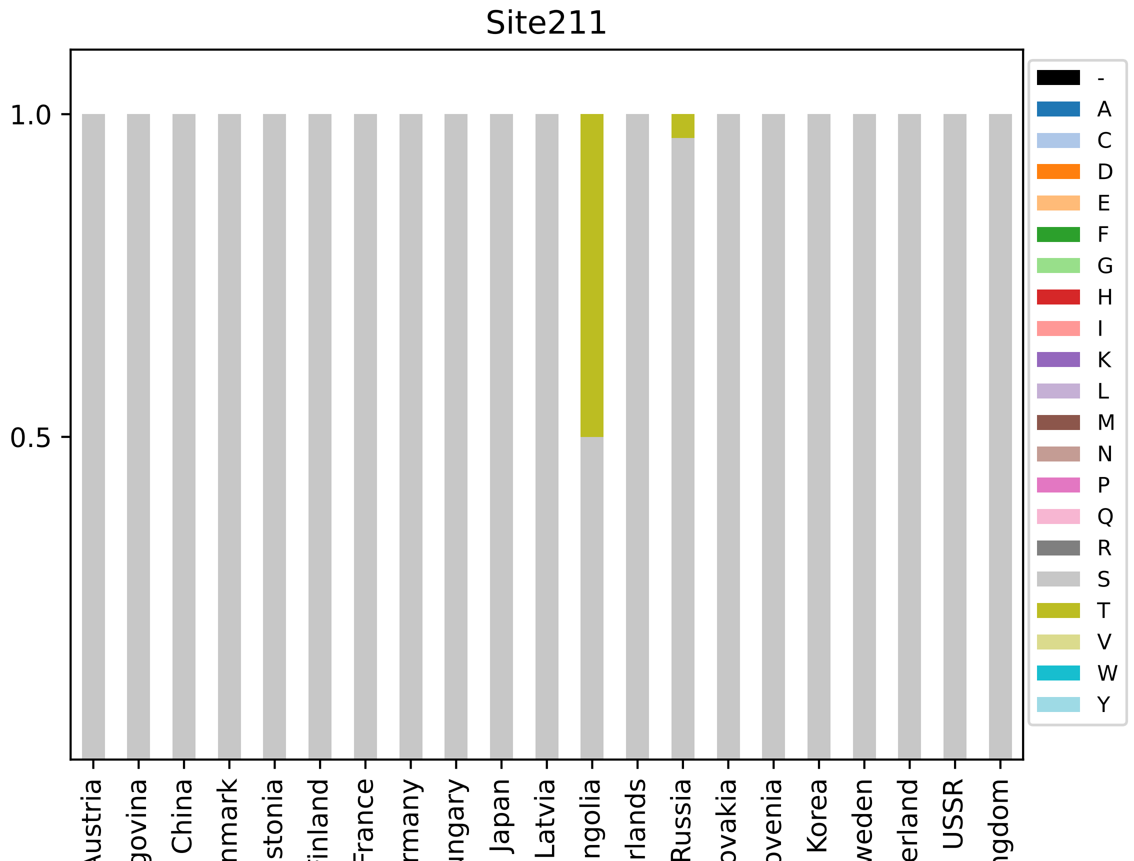
Site211
1.0
0.5
-
A
C
D
E
F
G
H
I
K
L
M
N
P
Q
R
S
T
V
W
Y
Austria
China
Japan
Latvia
Russia
Korea
USSR

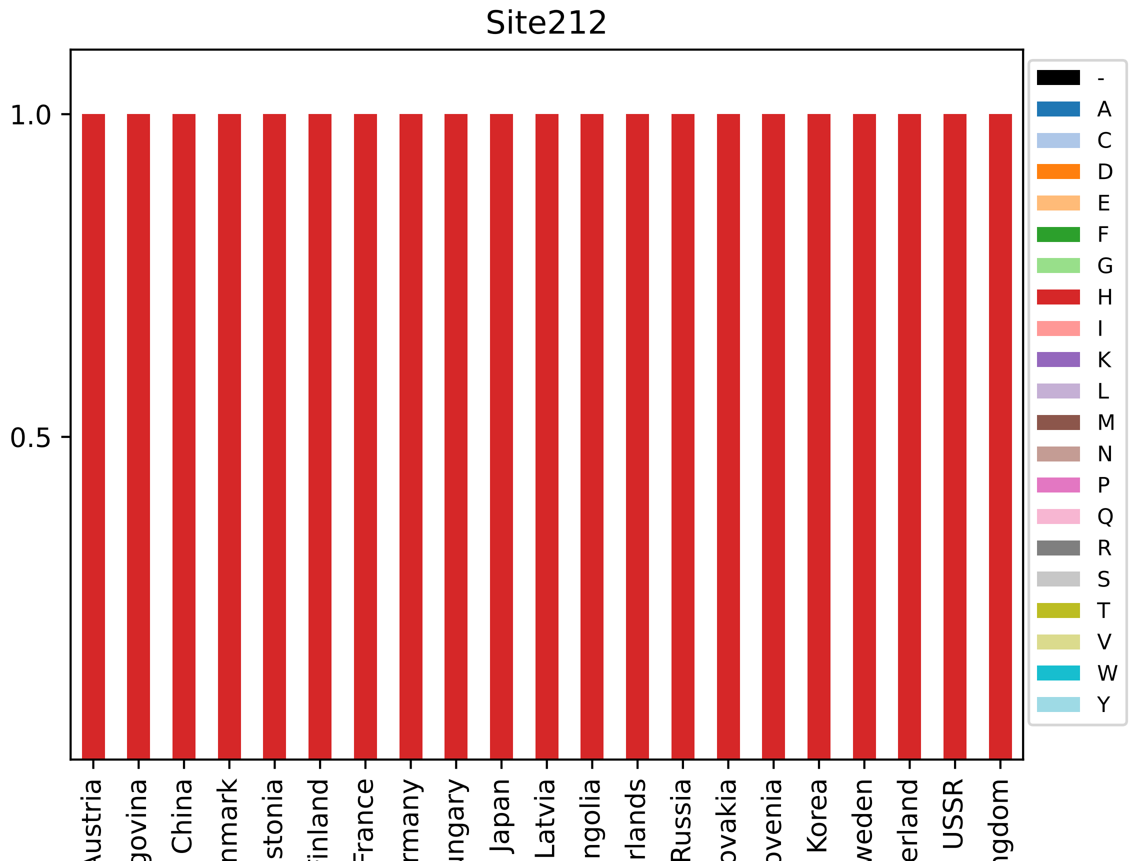
Site212
1.0
0.5
China
Japan
Latvia
Russia
Korea
USSR
-
A
C
D
E
F
G
H
I
K
L
M
N
P
Q
R
S
T
V
W
Y

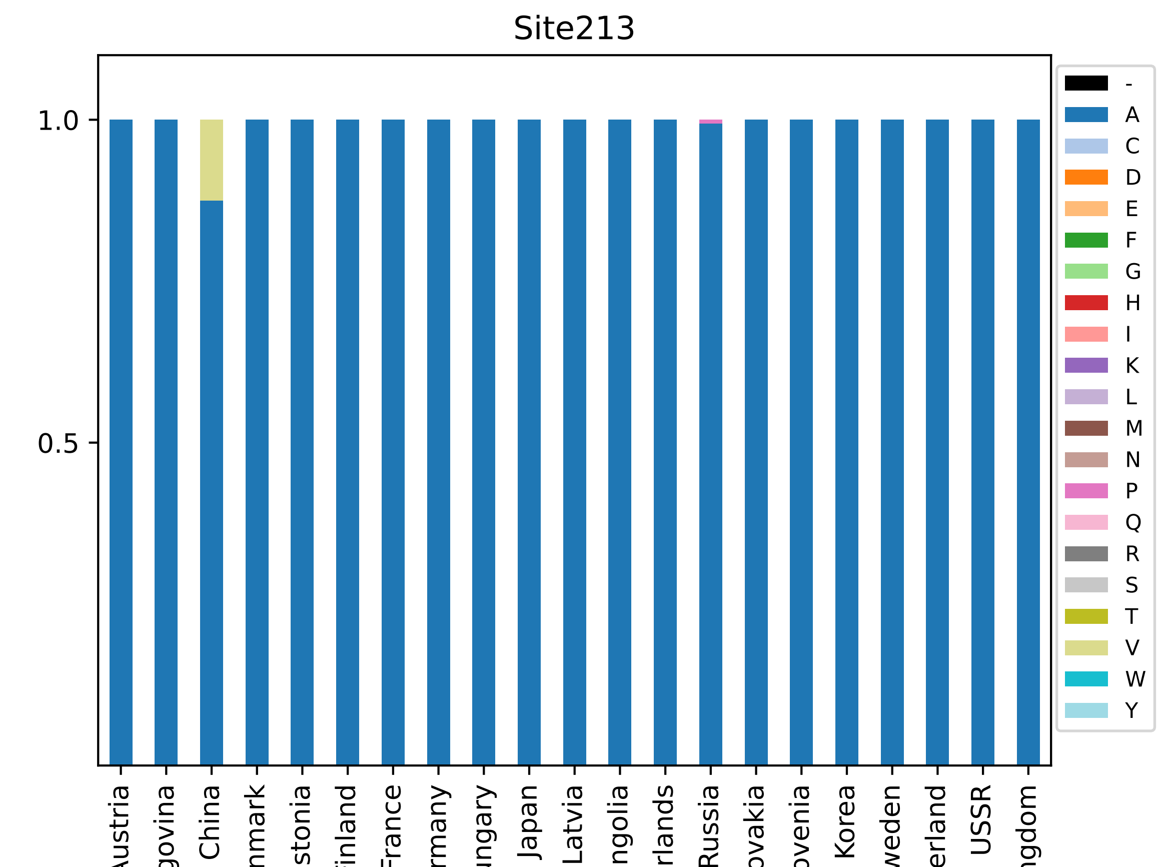
Site213
1.0
0.5
-
A
C
D
E
F
G
H
I
K
L
M
N
P
Q
R
S
T
V
W
Y
China
Japan
Latvia
Russia
Korea
USSR

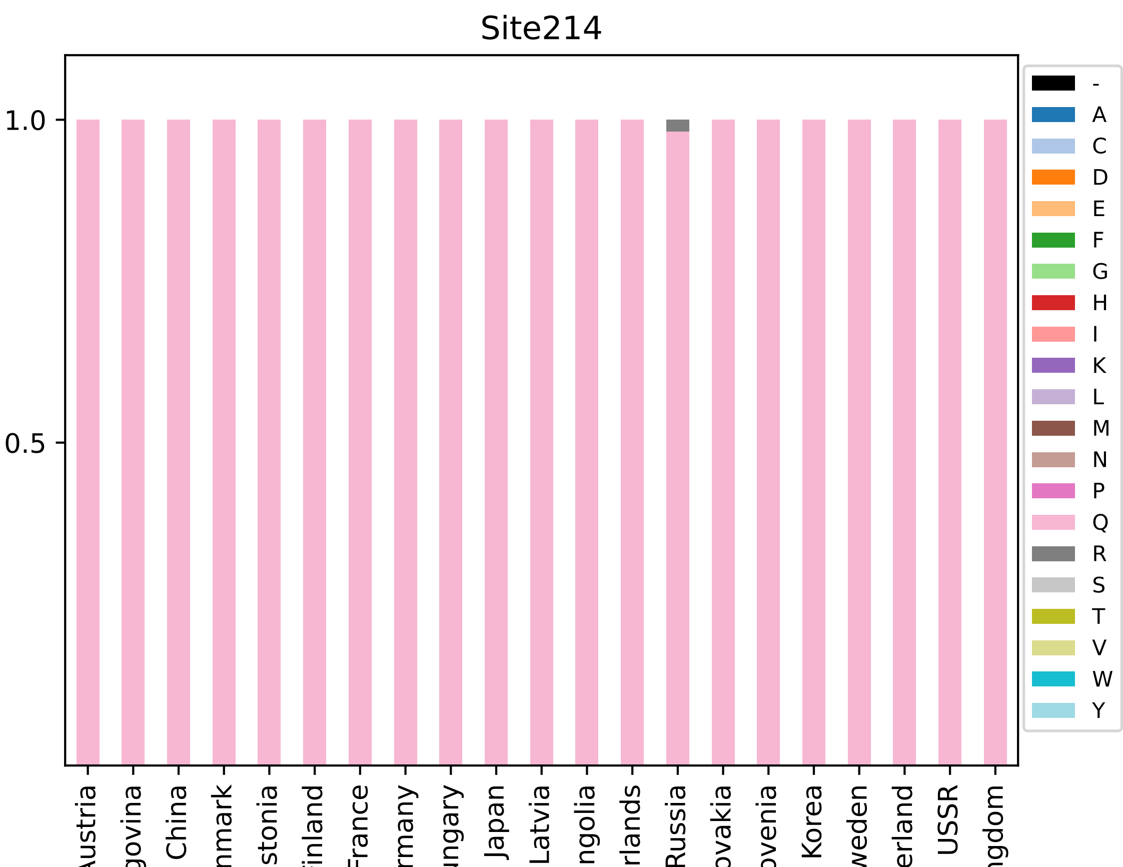
Site214
1.0
0.5
-
A
C
D
E
F
G
H
I
K
L
M
N
P
Q
R
S
T
V
W
Y
China
Japan
Latvia
Russia
Korea
USSR

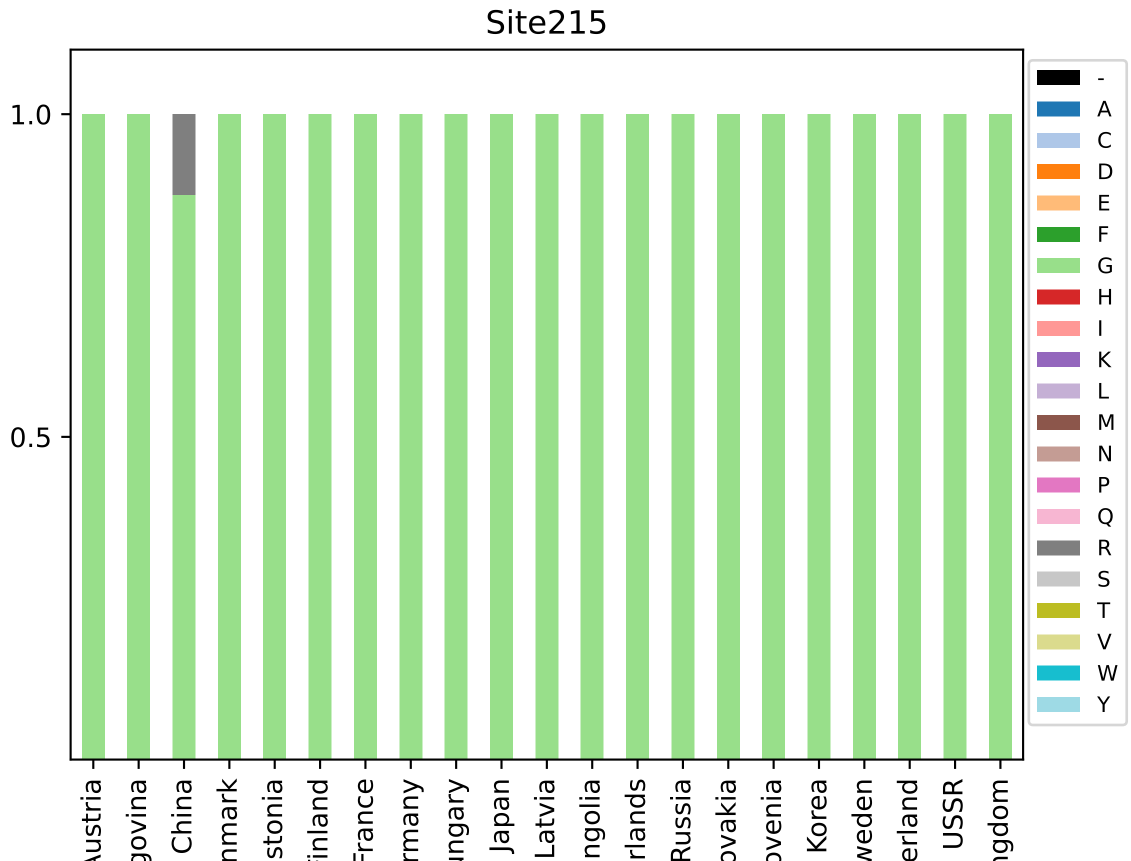
Site215
1.0
0.5
-
A
C
D
E
F
G
H
I
K
L
M
N
P
Q
R
S
T
V
W
Y
China
Japan
Latvia
Russia
Korea
USSR

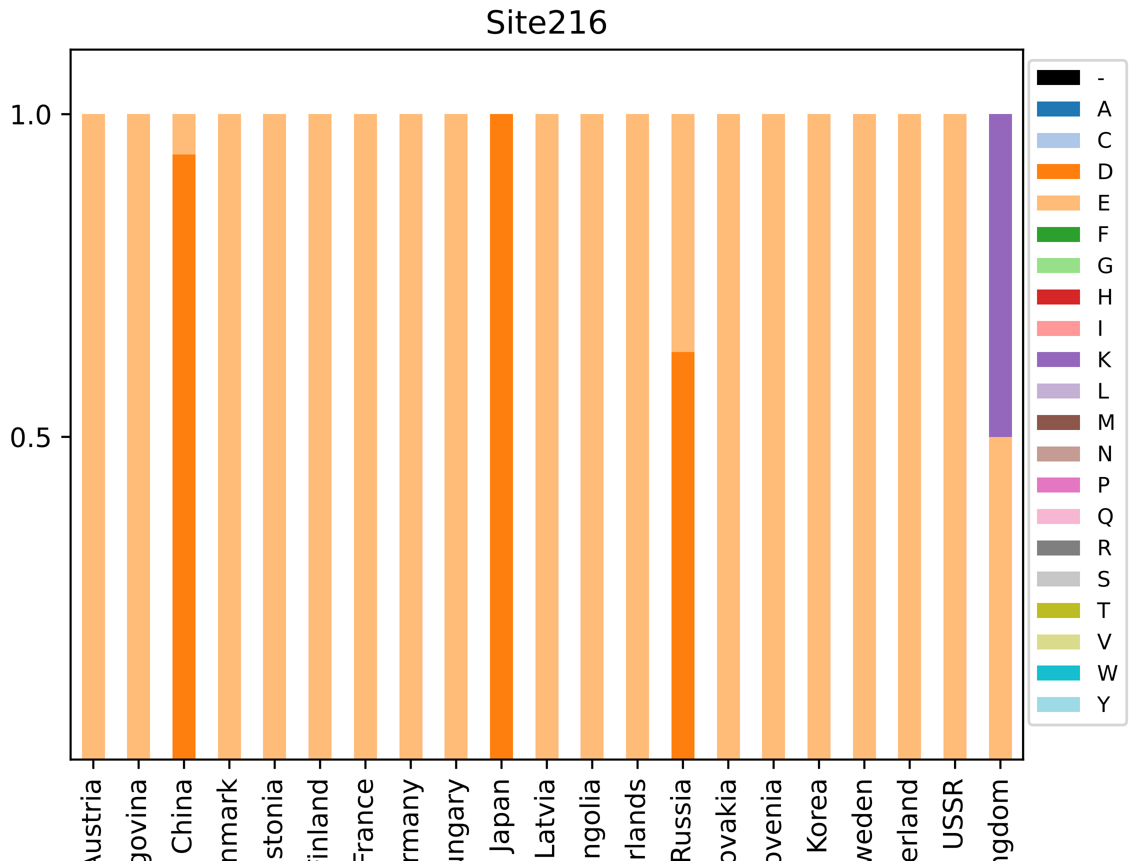

Site216
1.0
0.5
-
A
C
D
E
F
G
H
I
K
L
M
N
P
Q
R
S
T
V
W
Y
China
Japan
Latvia
Russia
Korea
USSR

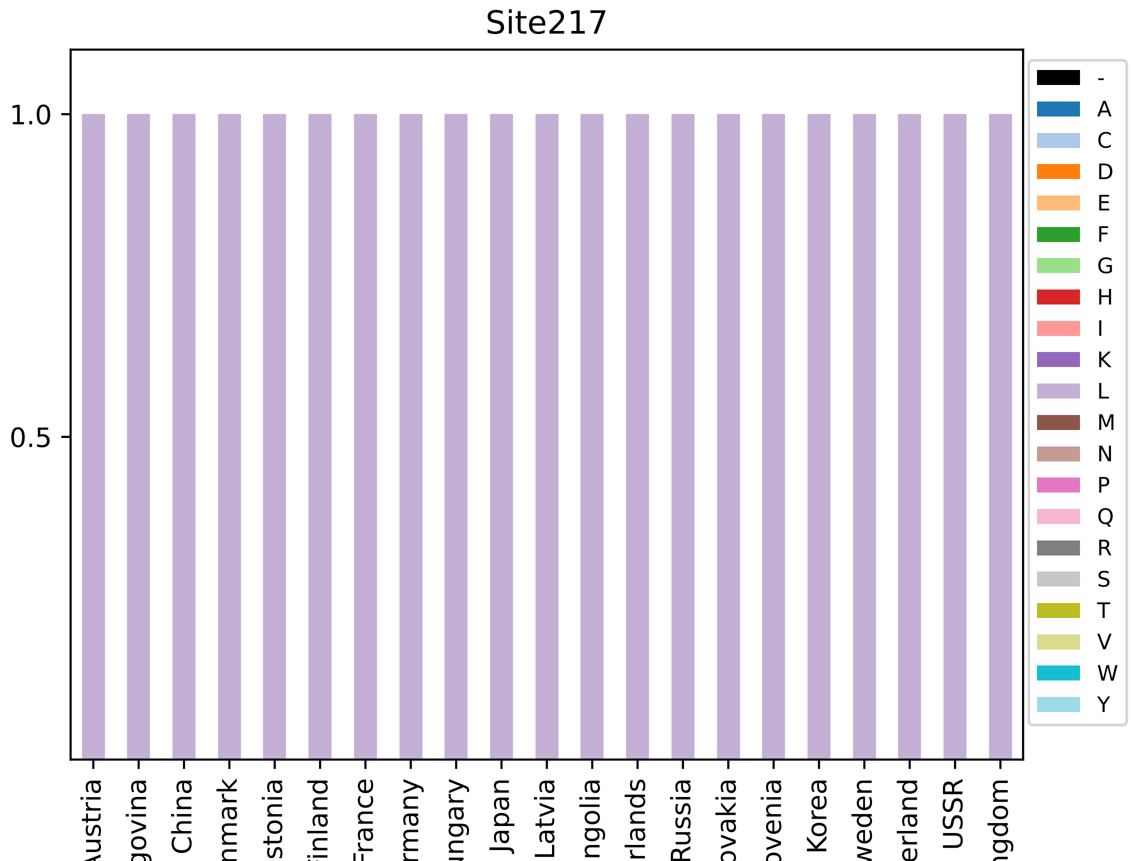

Site217
1.0
0.5
-
A
C
D
E
F
G
H
I
K
L
M
N
P
Q
R
S
T
V
W
Y
Austria
govina
China
nmark
stonia
inland
France
rmany
ungary
Japan
Latvia
ngolia
rlands
Russia
ovakia
ovenia
Korea
weden
erland
USSR
ngdom

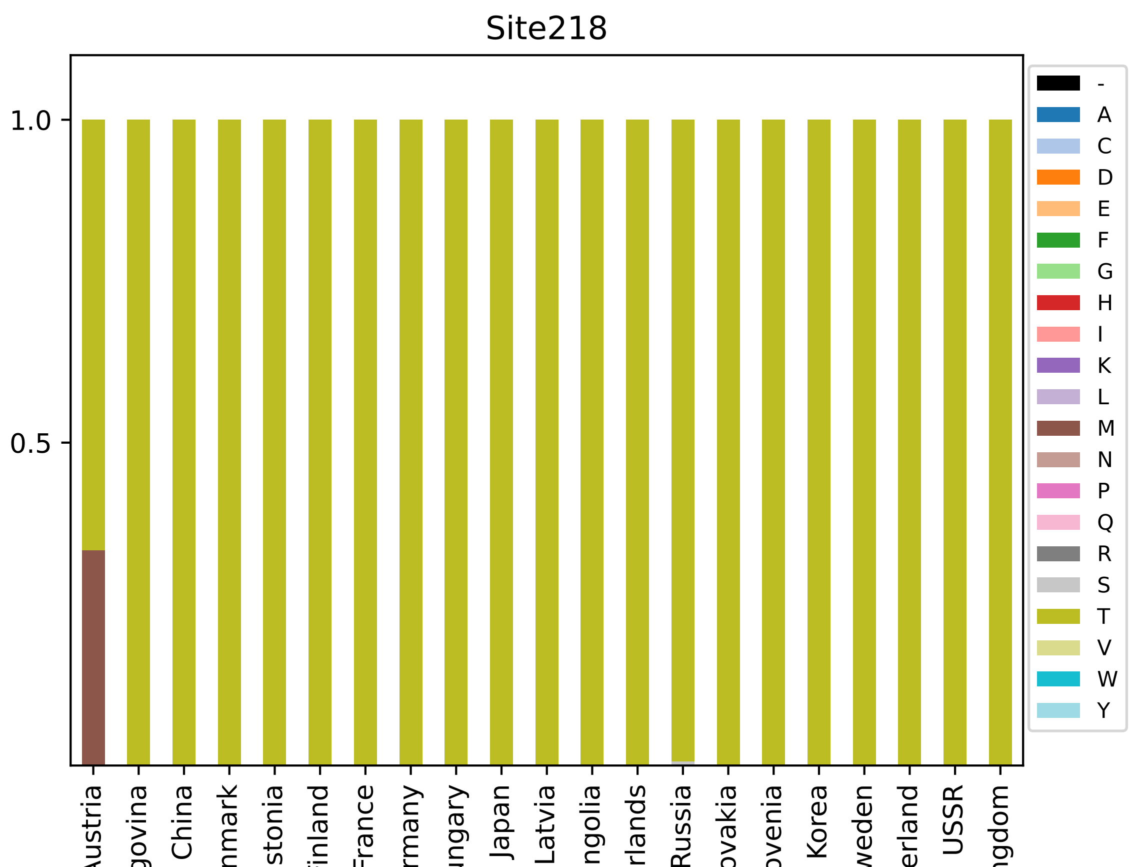

Site218
1.0
0.5
-
A
C
D
E
F
G
H
I
K
L
M
N
P
Q
R
S
T
V
W
Y
Austria
govina
China
nmark
stonia
inland
France
rmany
ungary
Japan
Latvia
ngolia
rlands
Russia
ovakia
ovenia
Korea
weden
erland
USSR
ngdom

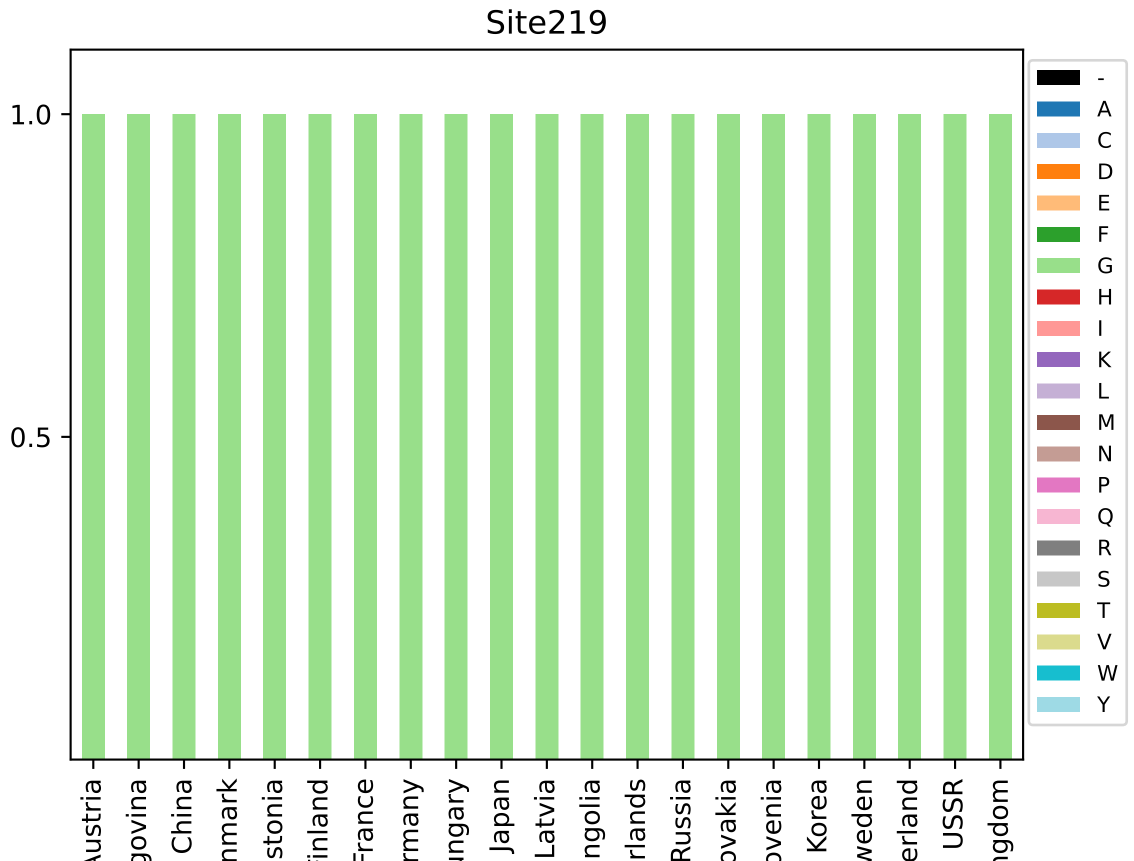
Site219
1.0
0.5
China
Japan
Latvia
Korea
USSR
-
A
C
D
E
F
G
H
I
K
L
M
N
P
Q
R
S
T
V
W
Y

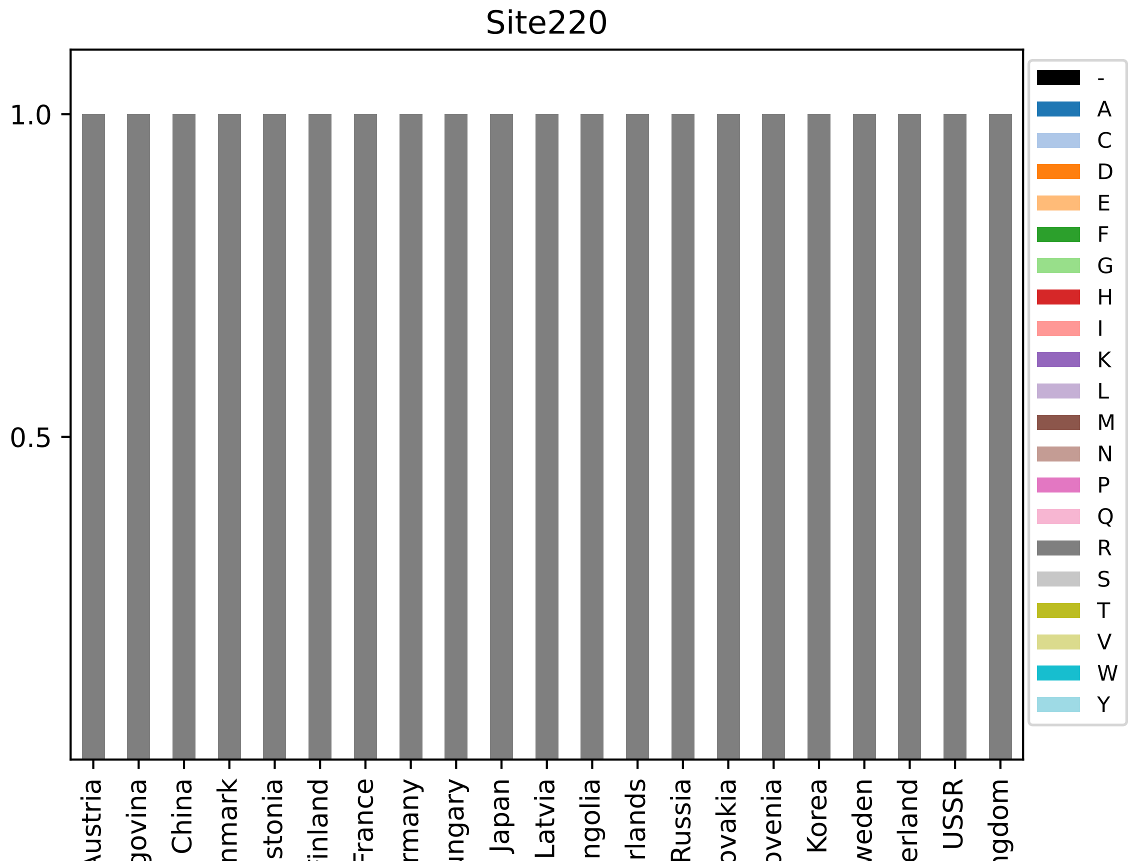

Site220
1.0
0.5
-
A
C
D
E
F
G
H
I
K
L
M
N
P
Q
R
S
T
V
W
Y
Austria
govina
China
nmark
stonia
inland
France
rmany
ungary
Japan
Latvia
ngolia
rlands
Russia
ovakia
ovenia
Korea
weden
erland
USSR
ngdom

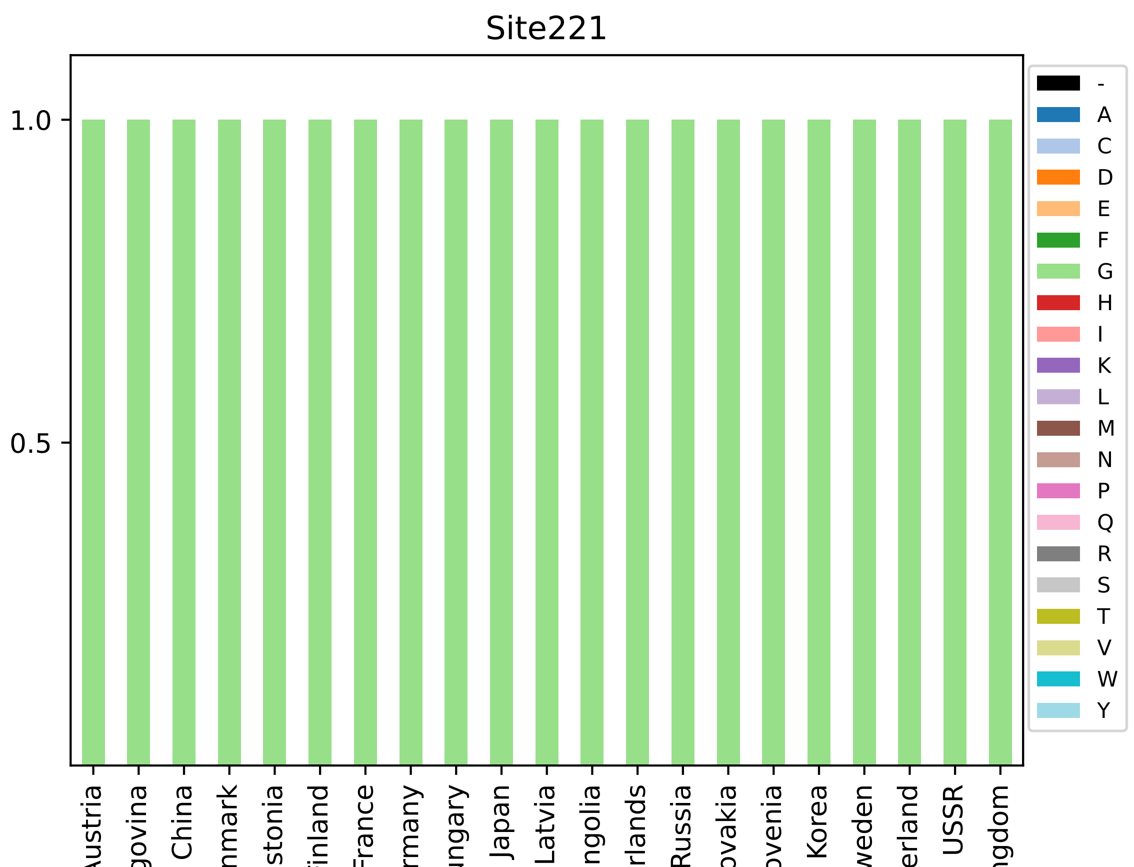

Site221
1.0
0.5
-
A
C
D
E
F
G
H
I
K
L
M
N
P
Q
R
S
T
V
W
Y
China
Japan
Latvia
Russia
Korea
USSR

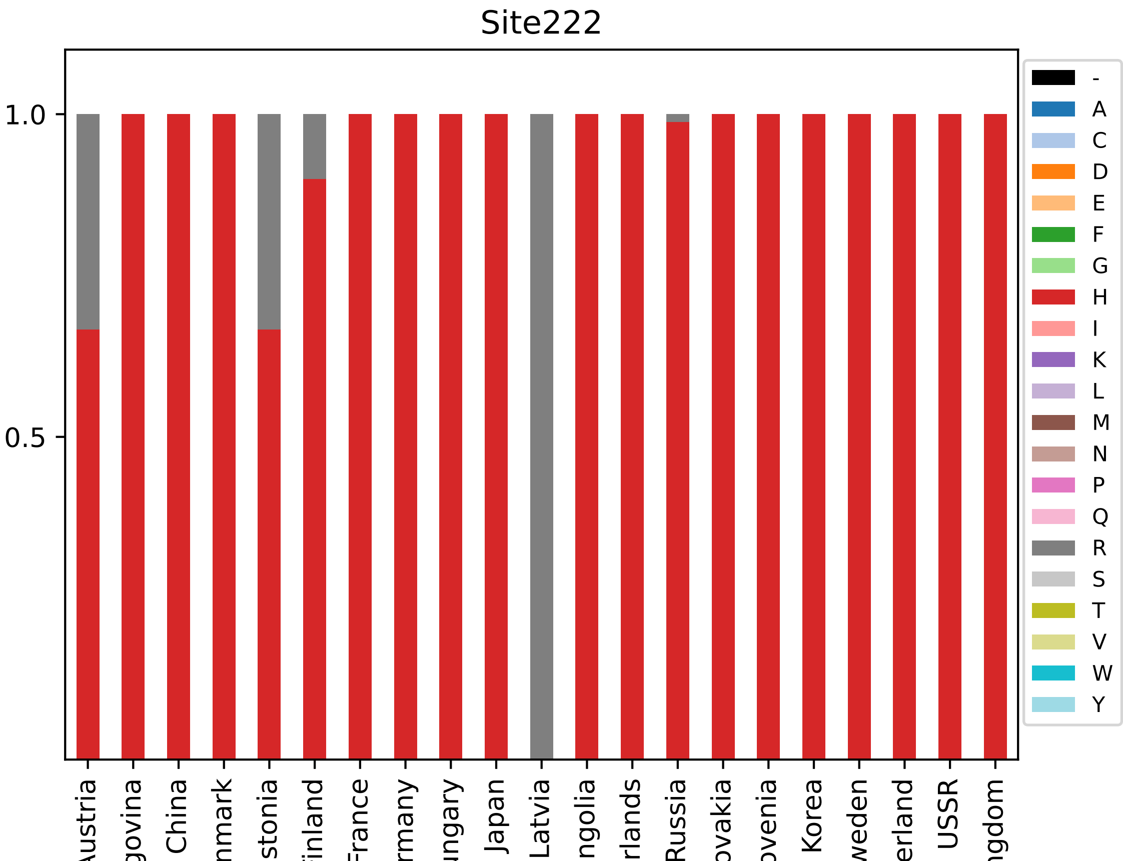

Site222
1.0
0.5
-
A
C
D
E
F
G
H
I
K
L
M
N
P
Q
R
S
T
V
W
Y
China
Japan
Latvia
Russia
Korea
USSR

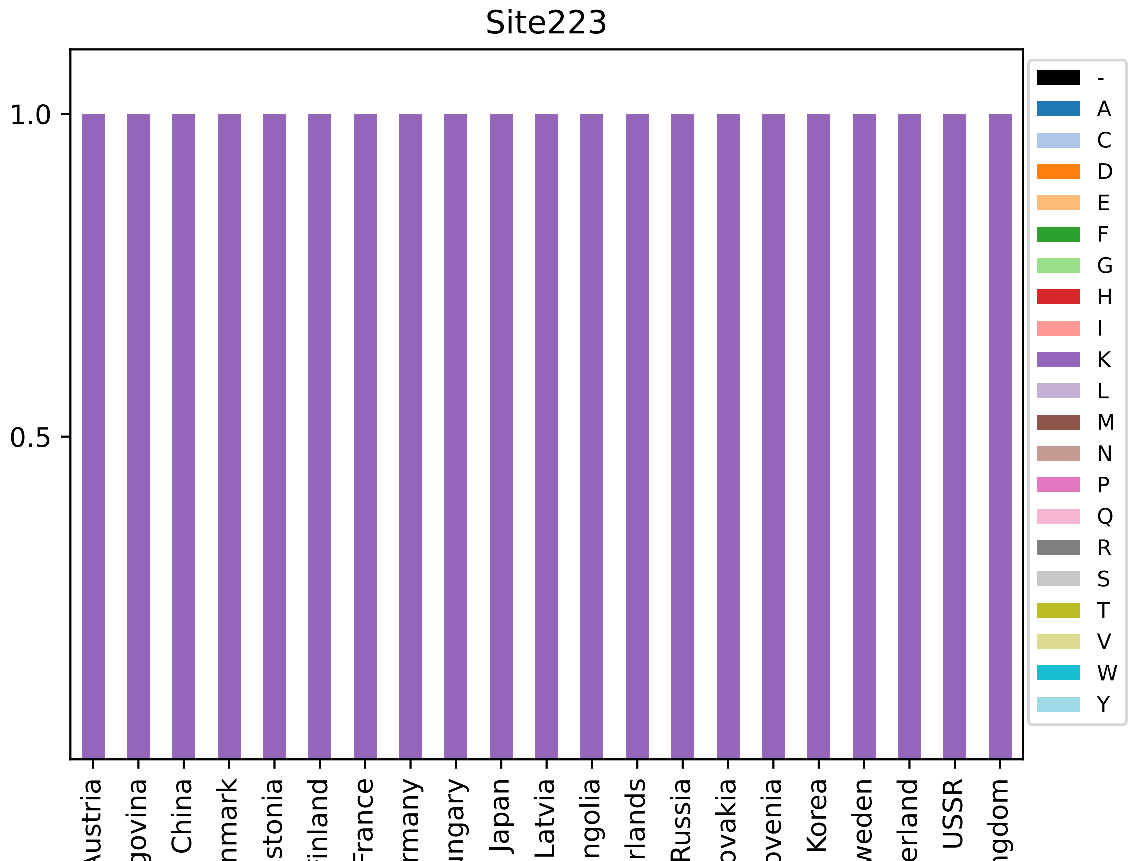
Site223
1.0
0.5
Austria
govina
China
nmark
stonia
inland
France
rmany
ungary
Japan
Latvia
ngolia
rlands
Russia
ovakia
ovenia
Korea
weden
erland
USSR
ngdom
-
A
C
D
E
F
G
H
I
K
L
M
N
P
Q
R
S
T
V
W
Y

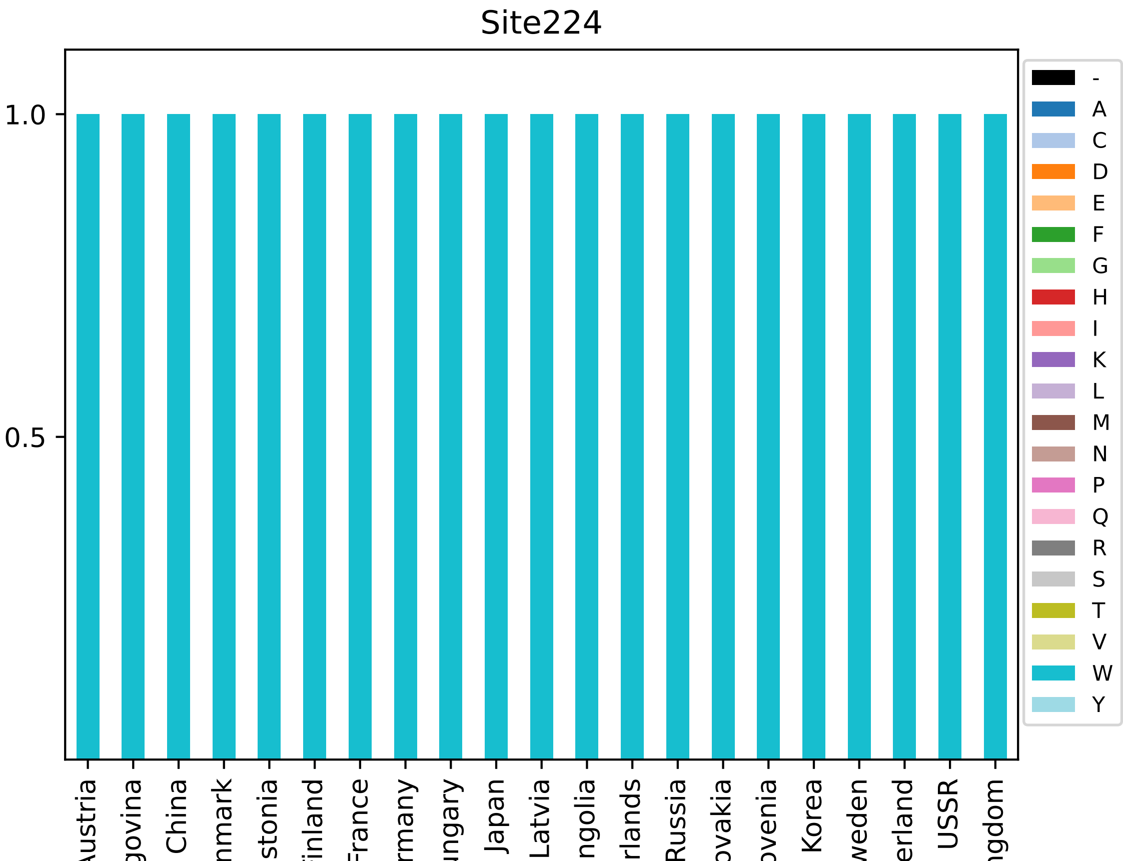
Site224
1.0
0.5
-
A
C
D
E
F
G
H
I
K
L
M
N
P
Q
R
S
T
V
W
Y
China
Japan
Latvia
Russia
Korea
USSR

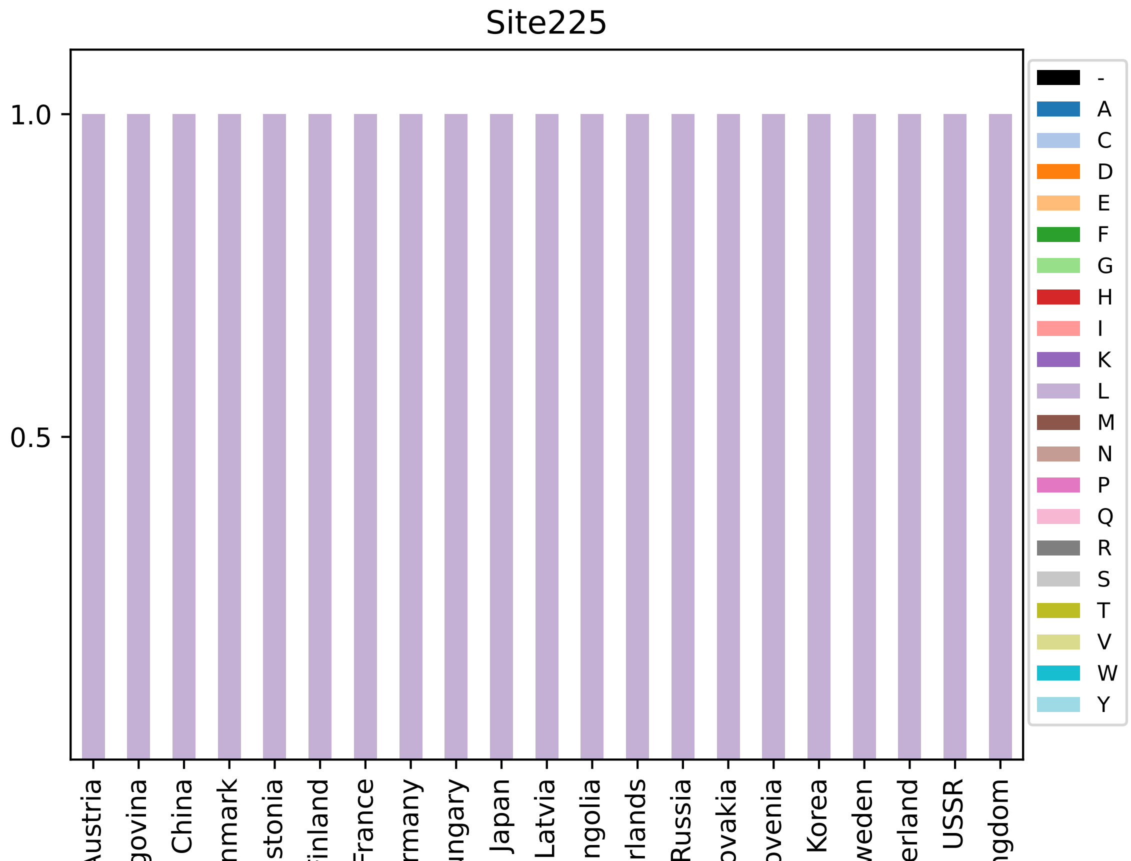

Site225
1.0
0.5
-
A
C
D
E
F
G
H
I
K
L
M
N
P
Q
R
S
T
V
W
Y
Austria
govina
China
nmark
stonia
Finland
France
rmany
ungary
Japan
Latvia
ngolia
rlands
Russia
ovakia
ovenia
Korea
weden
erland
USSR
ngdom

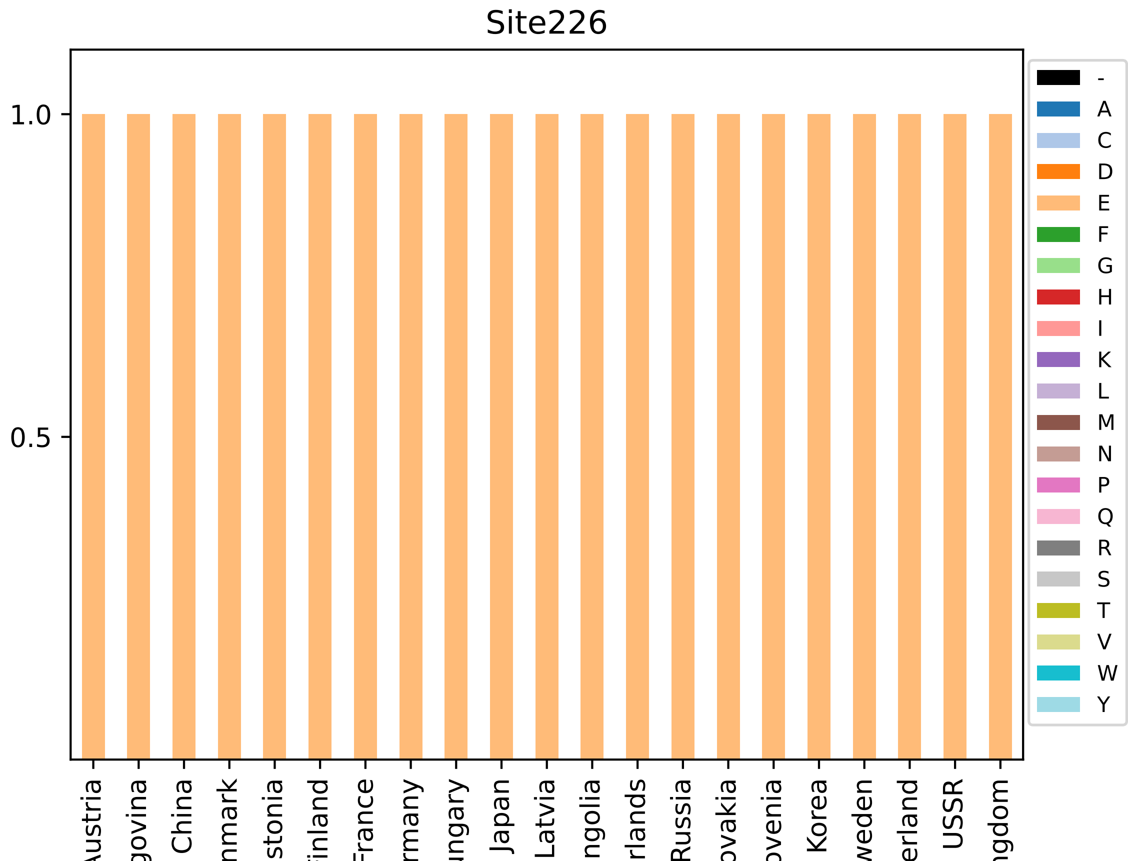
Site226
1.0
0.5
-
A
C
D
E
F
G
H
I
K
L
M
N
P
Q
R
S
T
V
W
Y
China
Japan
Latvia
Russia
Korea
USSR

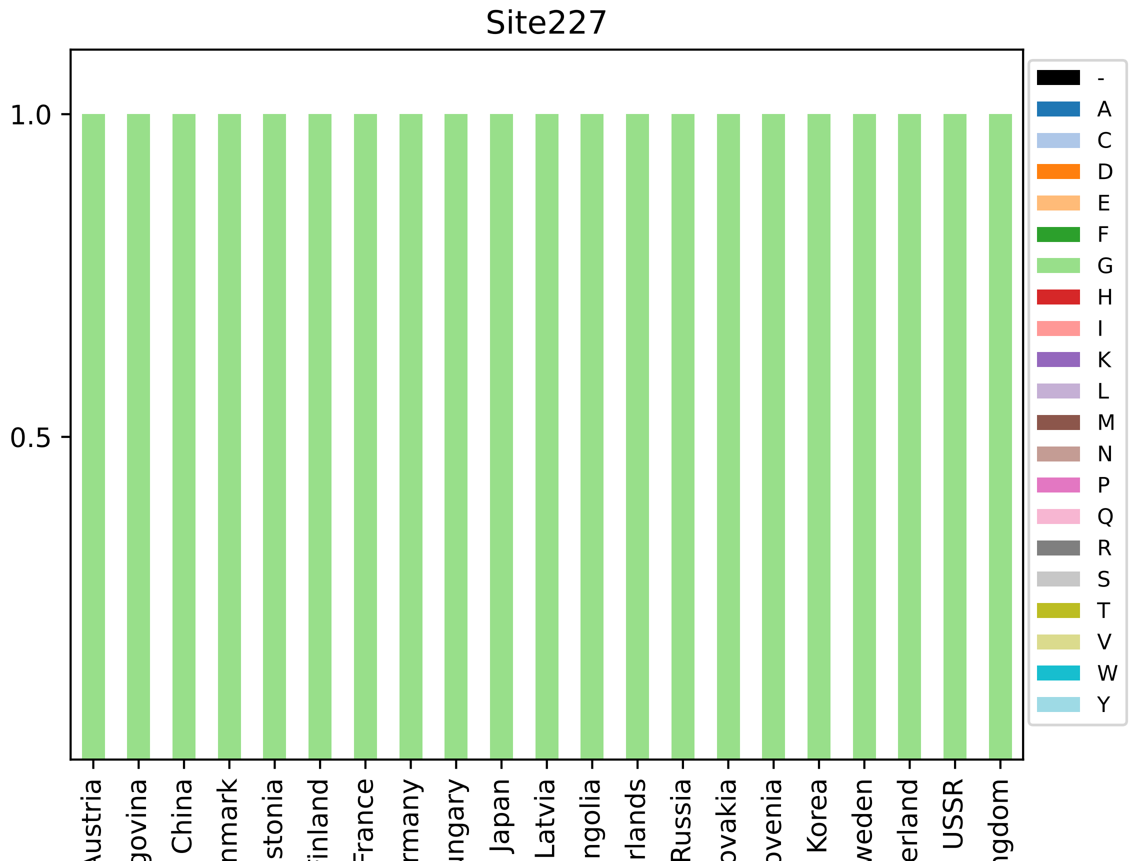

Site227
1.0
0.5
-
A
C
D
E
F
G
H
I
K
L
M
N
P
Q
R
S
T
V
W
Y
China
Japan
Latvia
Russia
Korea
USSR

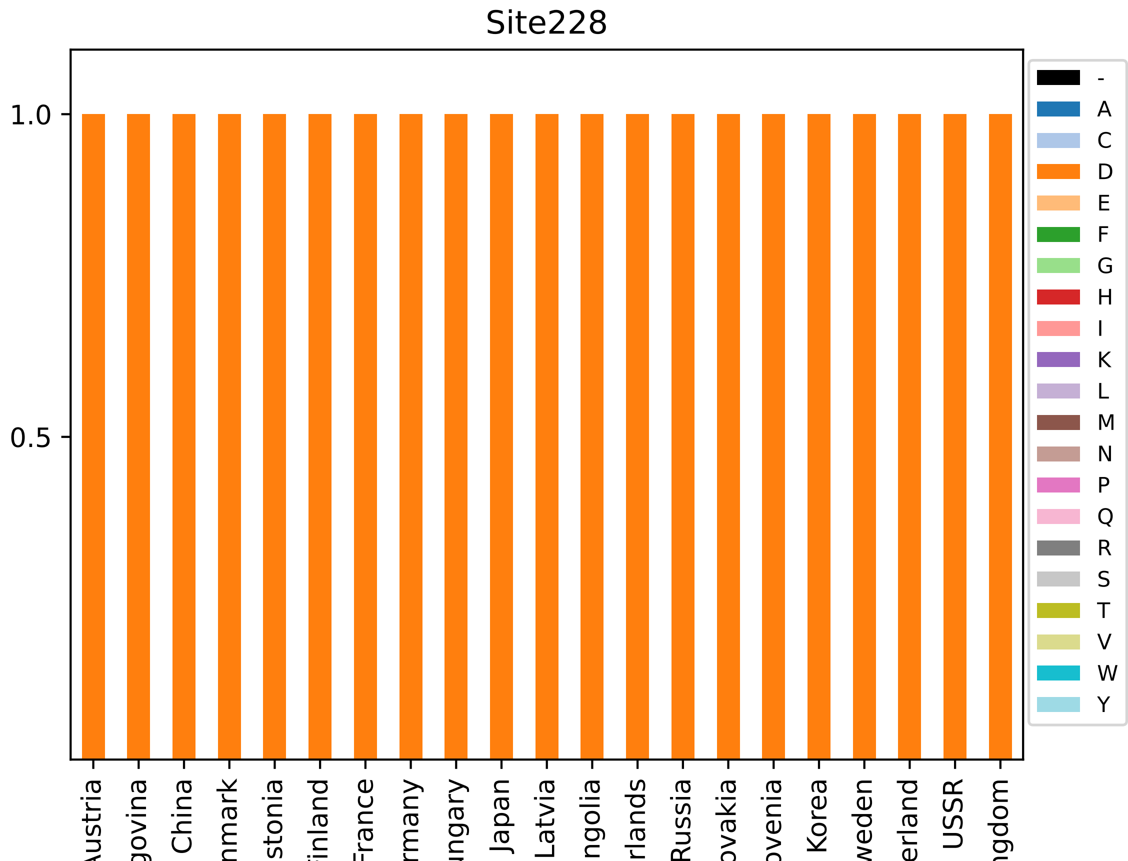
Site228
1.0
0.5
-
A
C
D
E
F
G
H
I
K
L
M
N
P
Q
R
S
T
V
W
Y
China
Japan
Latvia
Russia
Korea
USSR

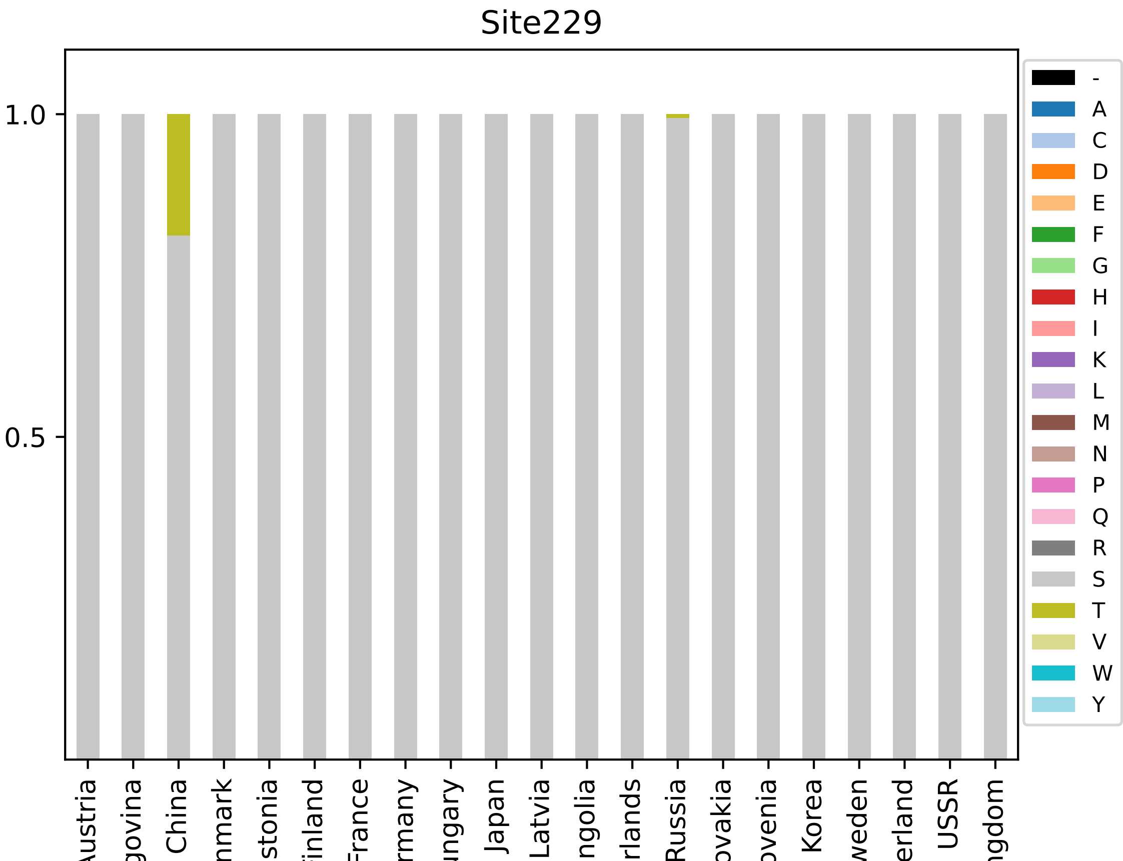

Site229
1.0
0.5
Austria
govina
China
nmark
stonia
Finland
France
rmany
ungary
Japan
Latvia
ngolia
rlands
Russia
ovakia
ovenia
Korea
weden
erland
USSR
ngdom
-
A
C
D
E
F
G
H
I
K
L
M
N
P
Q
R
S
T
V
W
Y

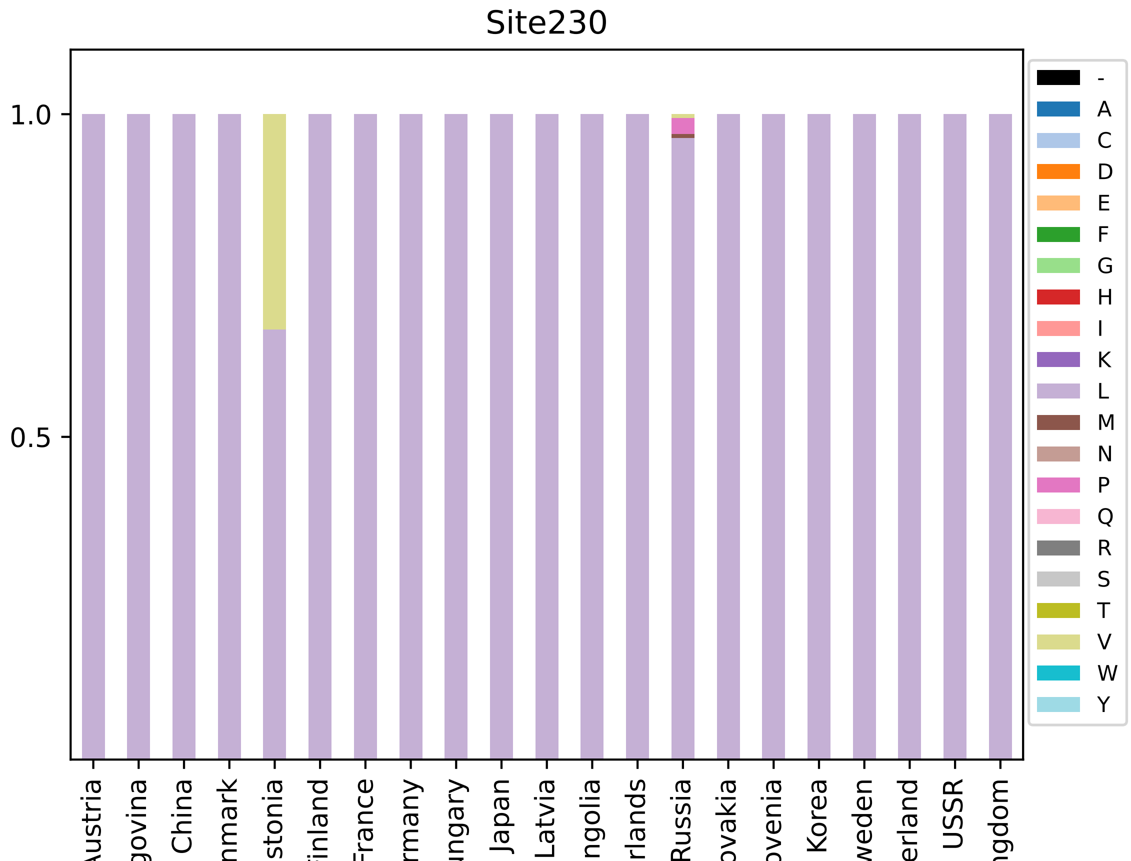
Site230
1.0
0.5
-
A
C
D
E
F
G
H
I
K
L
M
N
P
Q
R
S
T
V
W
Y
Austria
govina
China
nmark
stonia
Finland
France
rmany
ungary
Japan
Latvia
ngolia
rlands
Russia
ovakia
ovenia
Korea
weden
erland
USSR
ngdom

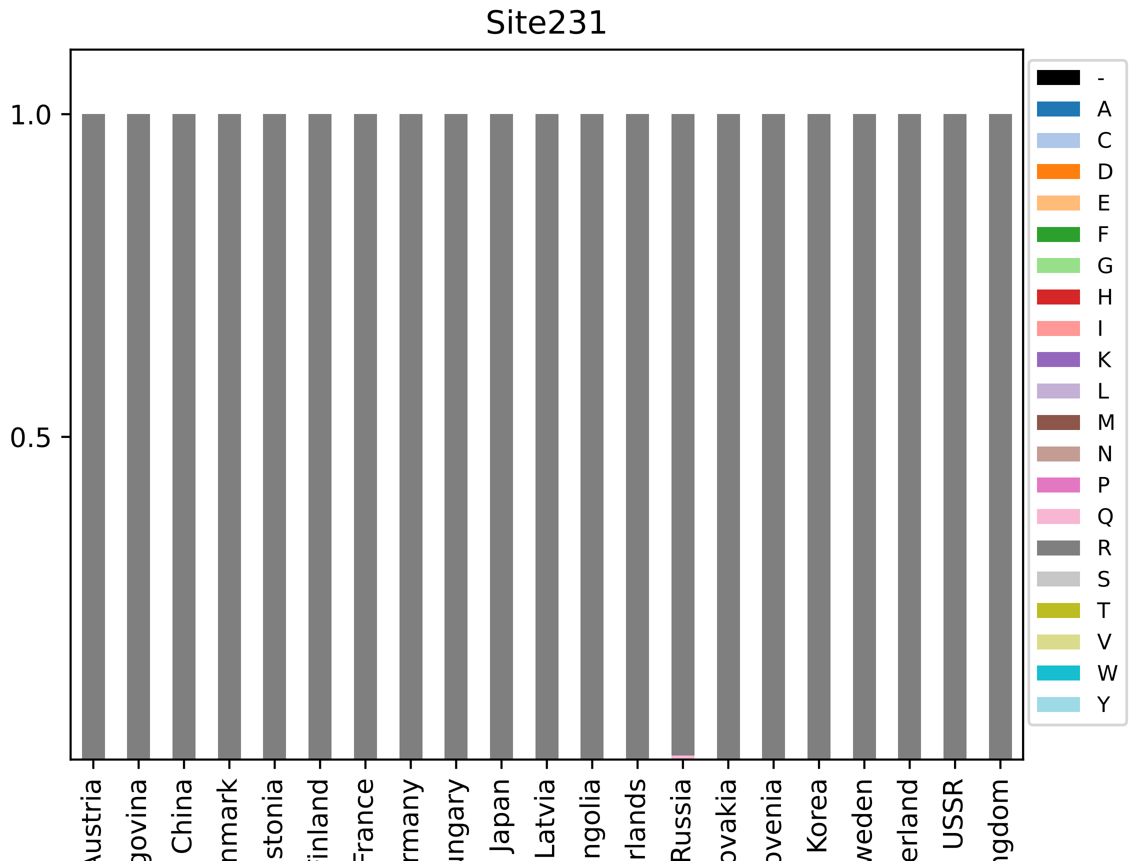

Site231
1.0
0.5
-
A
C
D
E
F
G
H
I
K
L
M
N
P
Q
R
S
T
V
W
Y
China
Japan
Latvia
Russia
Korea
USSR

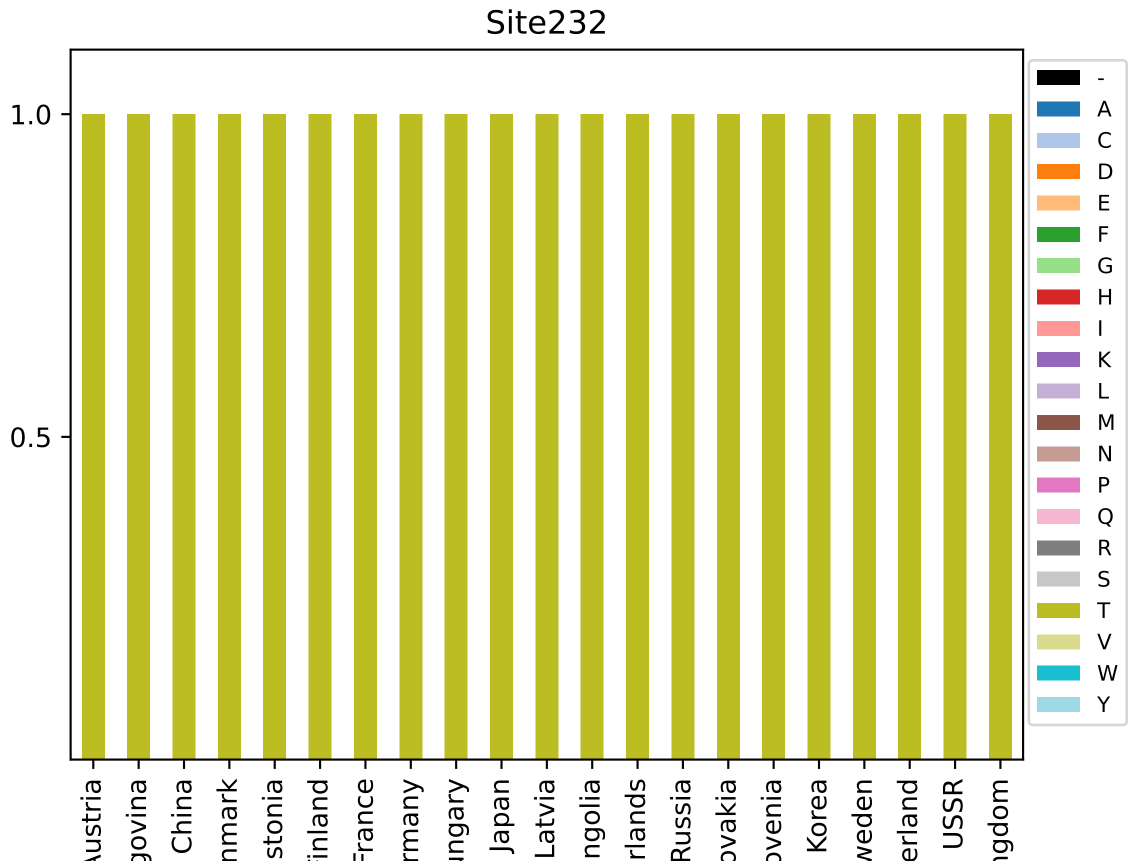
Site232
1.0
0.5
-
A
C
D
E
F
G
H
I
K
L
M
N
P
Q
R
S
T
V
W
Y
Austria
govina
China
nmark
stonia
Finland
France
rmany
ungary
Japan
Latvia
ngolia
rlands
Russia
ovakia
ovenia
Korea
weden
erland
USSR
ngdom

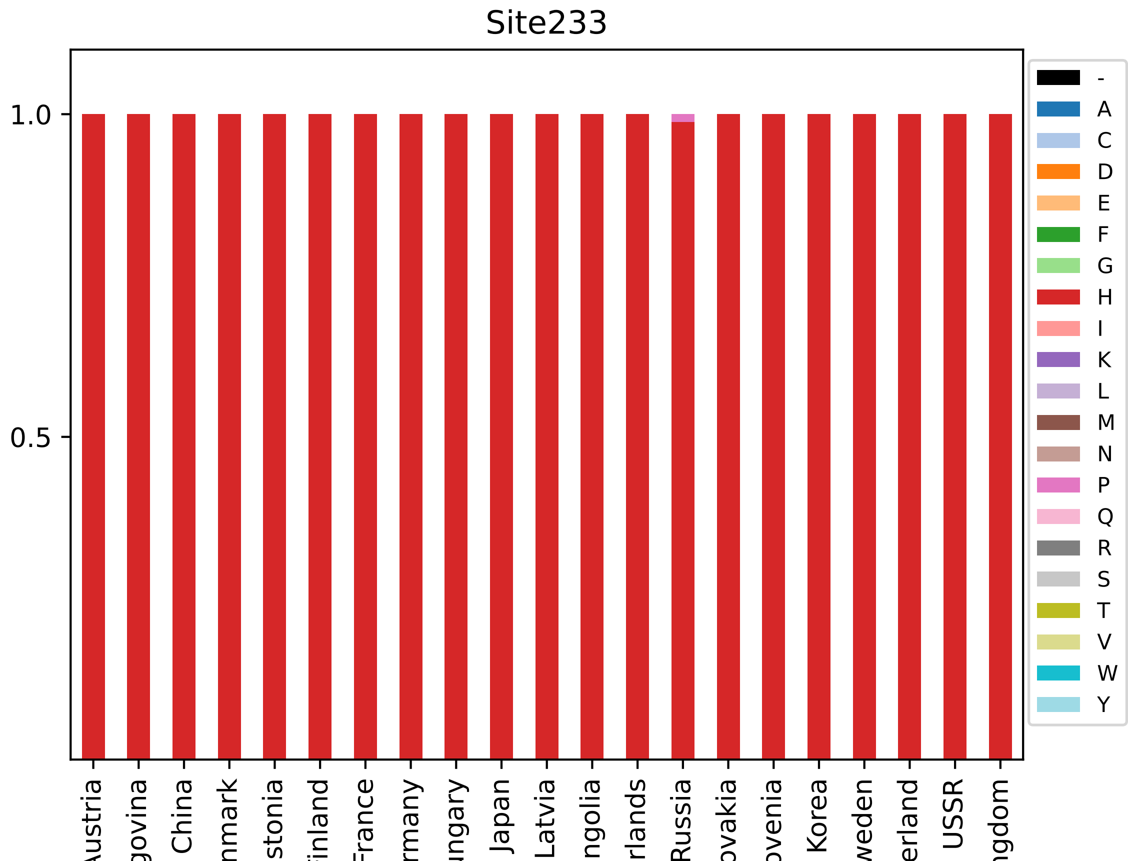

Site233
1.0
0.5
-
A
C
D
E
F
G
H
I
K
L
M
N
P
Q
R
S
T
V
W
Y
Austria
govina
China
nmark
stonia
inland
France
rmany
ungary
Japan
Latvia
ngolia
rlands
Russia
ovakia
ovenia
Korea
weden
erland
USSR
ngdom

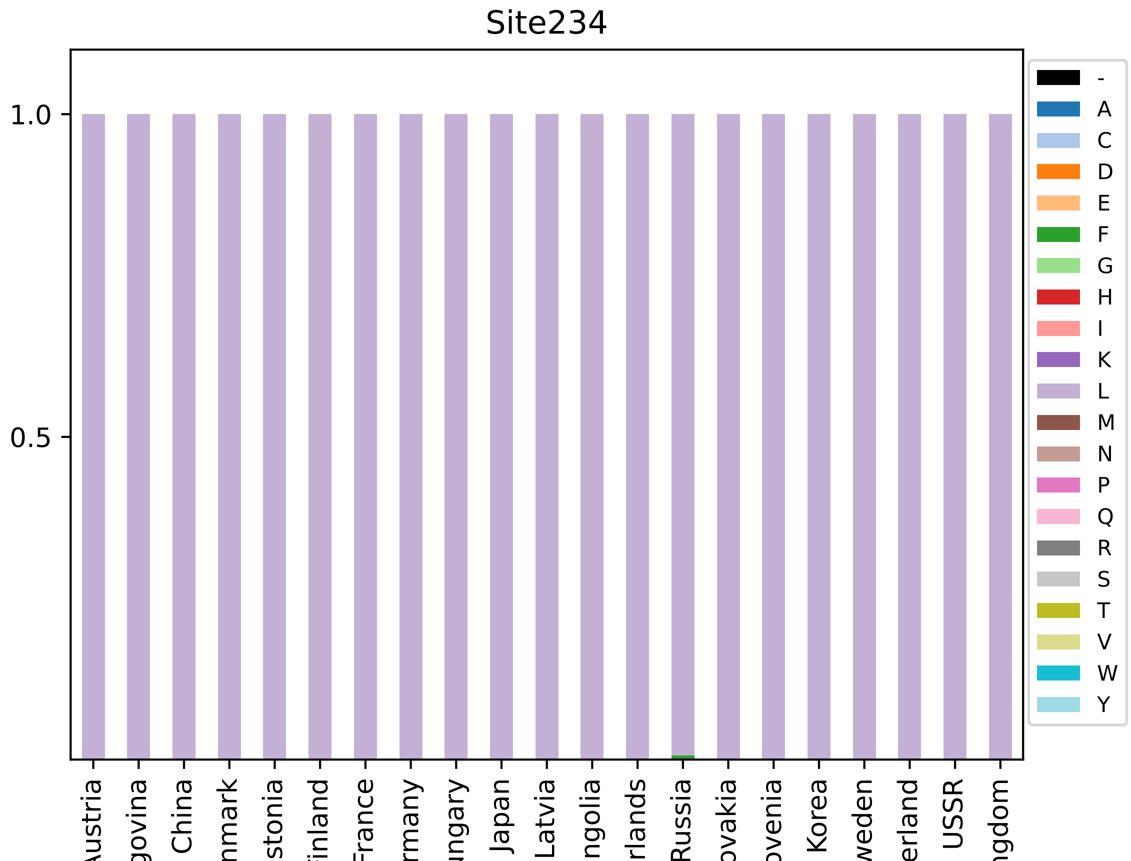

Site234
1.0
0.5
-
A
C
D
E
F
G
H
I
K
L
M
N
P
Q
R
S
T
V
W
Y
Austria
govina
China
nmark
stonia
inland
France
rmany
ungary
Japan
Latvia
ngolia
rlands
Russia
ovakia
ovenia
Korea
weden
erland
USSR
ngdom

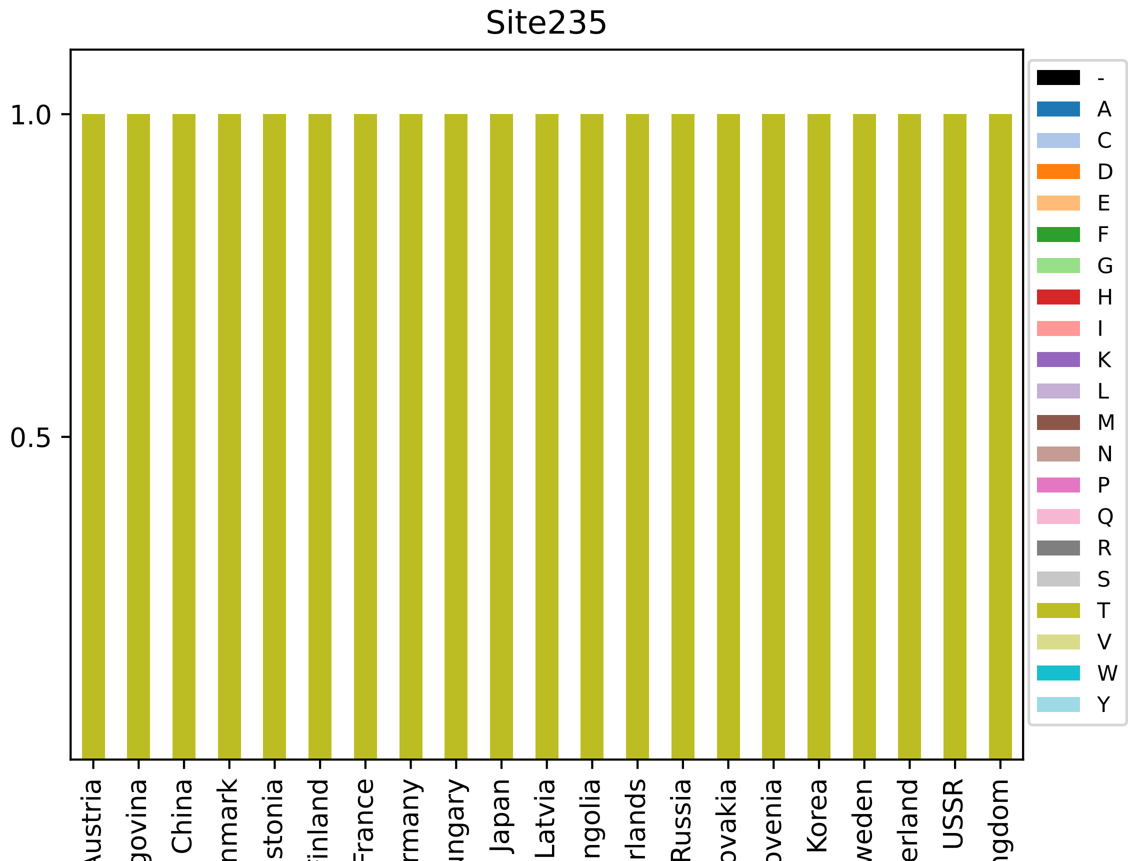
Site235
1.0
0.5
-
A
C
D
E
F
G
H
I
K
L
M
N
P
Q
R
S
T
V
W
Y
China
Japan
Latvia
Russia
Korea
USSR

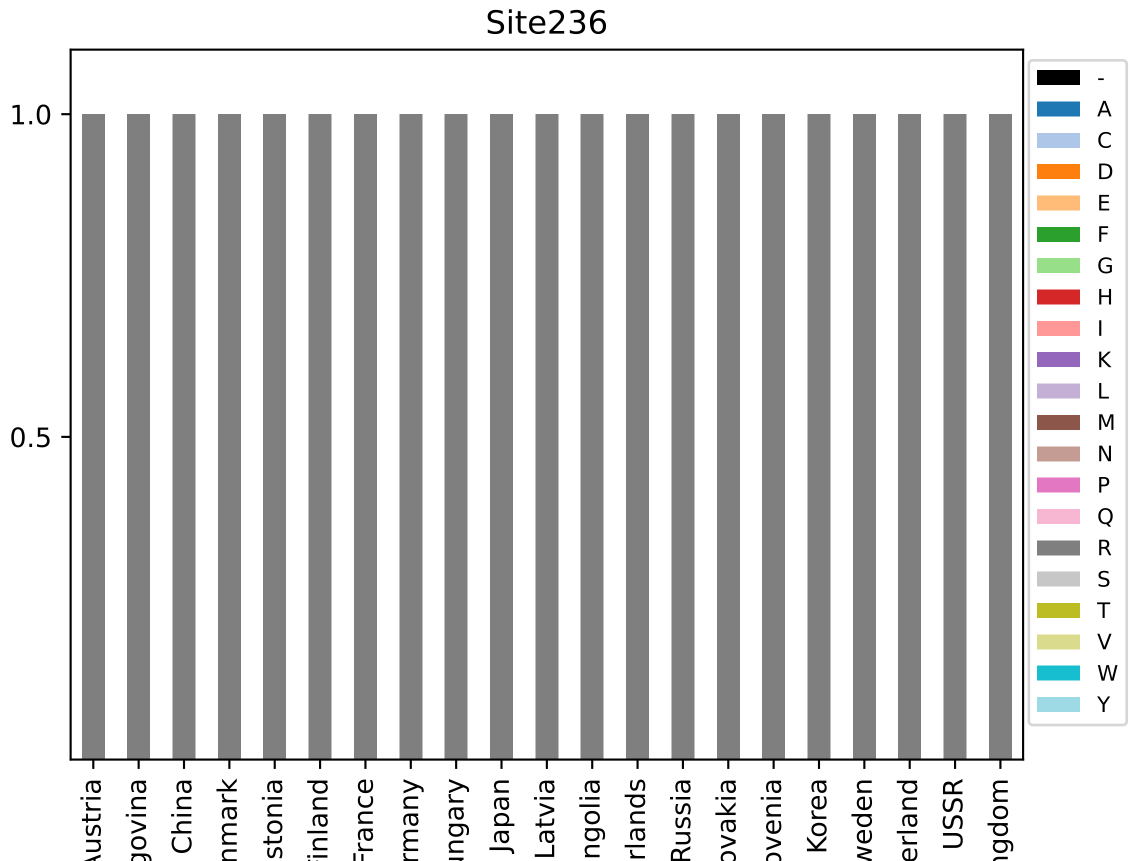

Site236
1.0
0.5
-
A
C
D
E
F
G
H
I
K
L
M
N
P
Q
R
S
T
V
W
Y
ustria
govina
China
nmark
stonia
inland
rance
rmany
ungary
Japan
Latvia
ngolia
rlands
Russia
ovakia
ovenia
Korea
weden
erland
USSR
ngdom

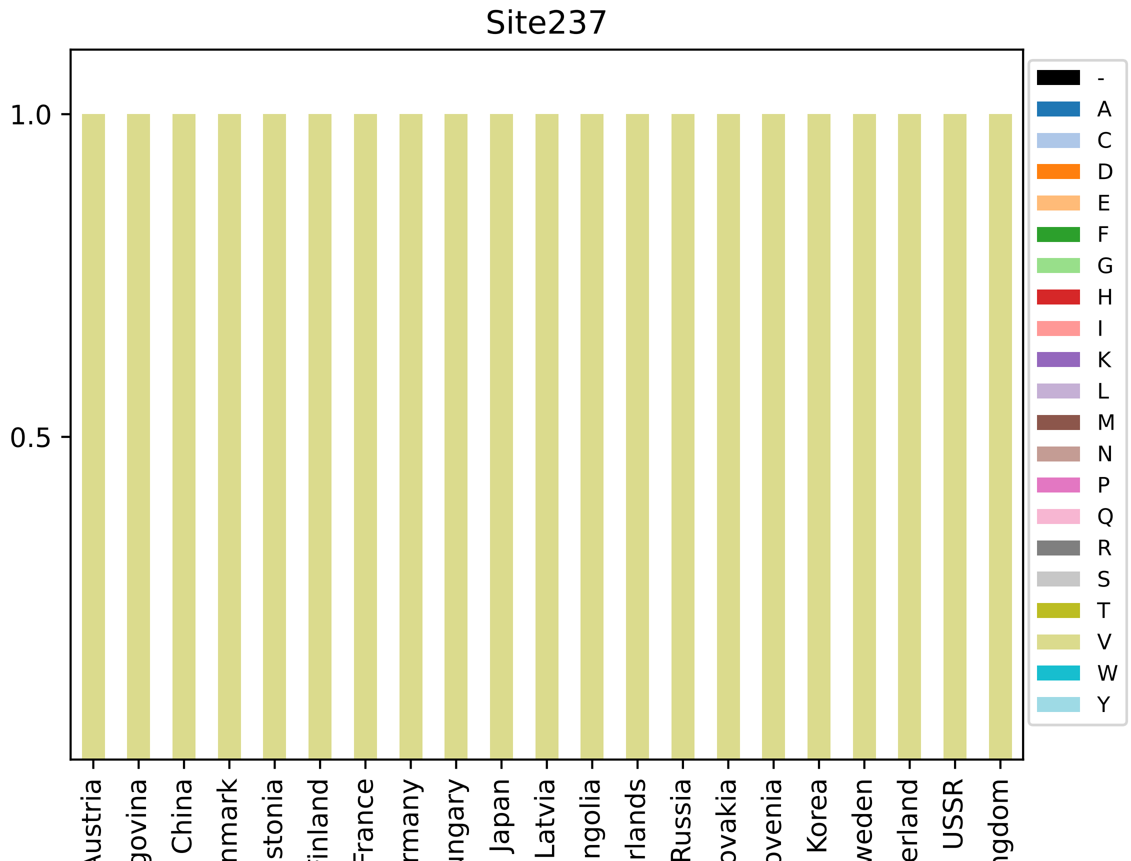

Site237
1.0
0.5
ustria
govina
China
nmark
stonia
inland
rance
rmany
ungary
Japan
Latvia
ngolia
rlands
Russia
ovakia
ovenia
Korea
weden
erland
USSR
ngdom
-
A
C
D
E
F
G
H
I
K
L
M
N
P
Q
R
S
T
V
W
Y

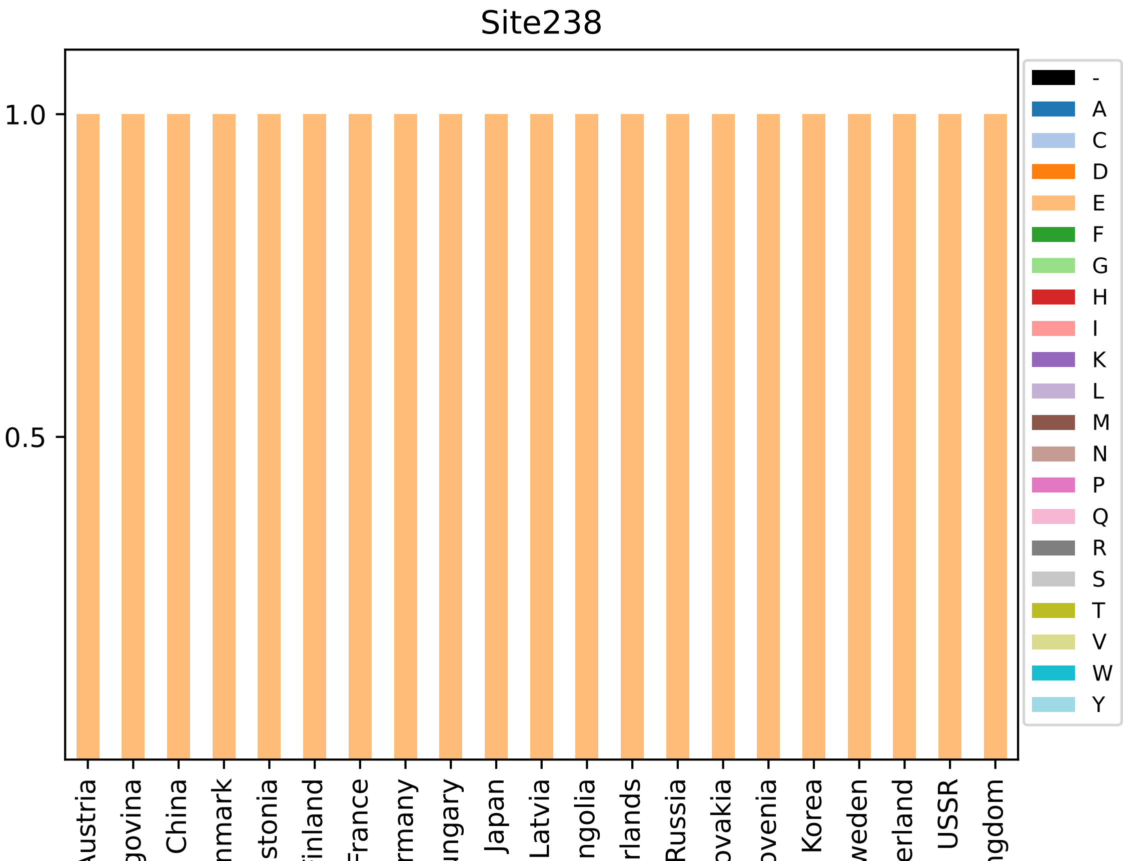
Site238
1.0
0.5
-
A
C
D
E
F
G
H
I
K
L
M
N
P
Q
R
S
T
V
W
Y
China
Japan
Latvia
Russia
Korea
USSR

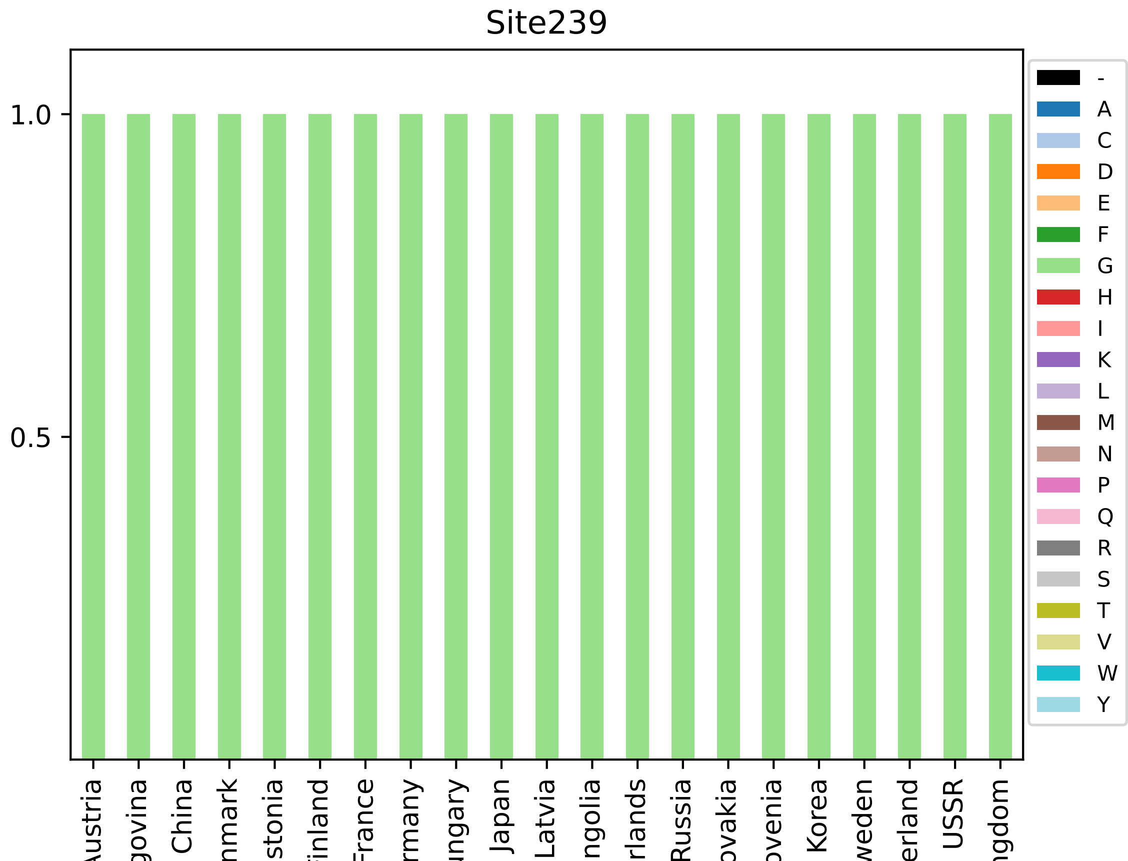
Site239
1.0
0.5
-
A
C
D
E
F
G
H
I
K
L
M
N
P
Q
R
S
T
V
W
Y
China
Japan
Latvia
Russia
Korea
USSR

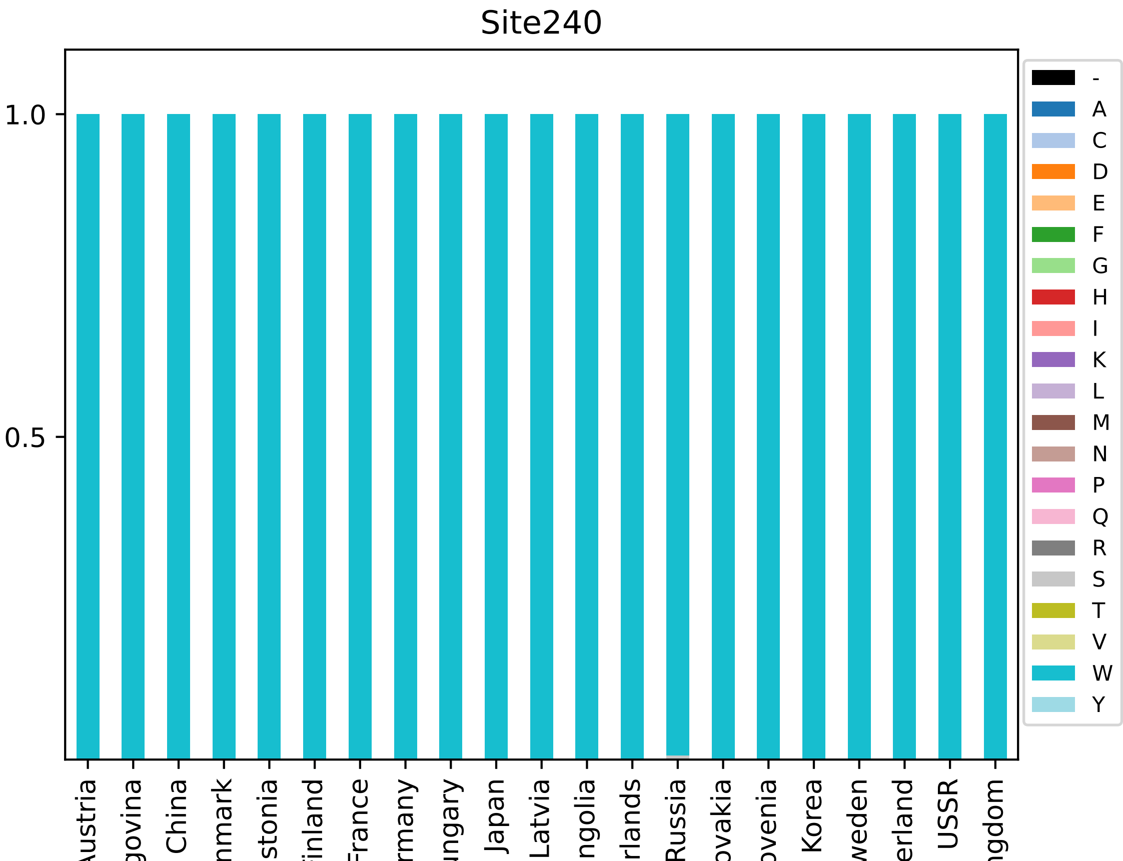
Site240
1.0
0.5
-
A
C
D
E
F
G
H
I
K
L
M
N
P
Q
R
S
T
V
W
Y
Austria
China
Japan
Latvia
Russia
Korea
USSR

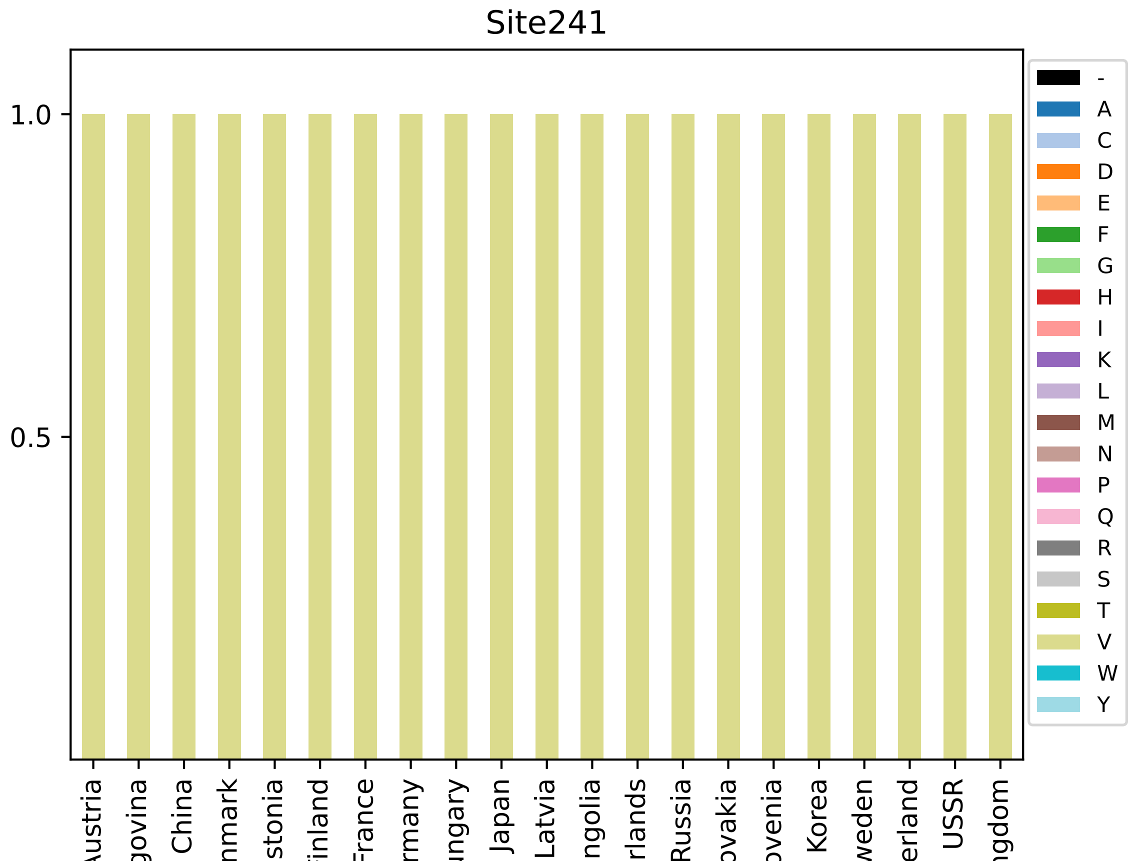
Site241
1.0
0.5
Austria
govina
China
nmark
stonia
Finland
France
rmany
ungary
Japan
Latvia
ngolia
rlands
Russia
ovakia
ovenia
Korea
weden
erland
USSR
ngdom
-
A
C
D
E
F
G
H
I
K
L
M
N
P
Q
R
S
T
V
W
Y

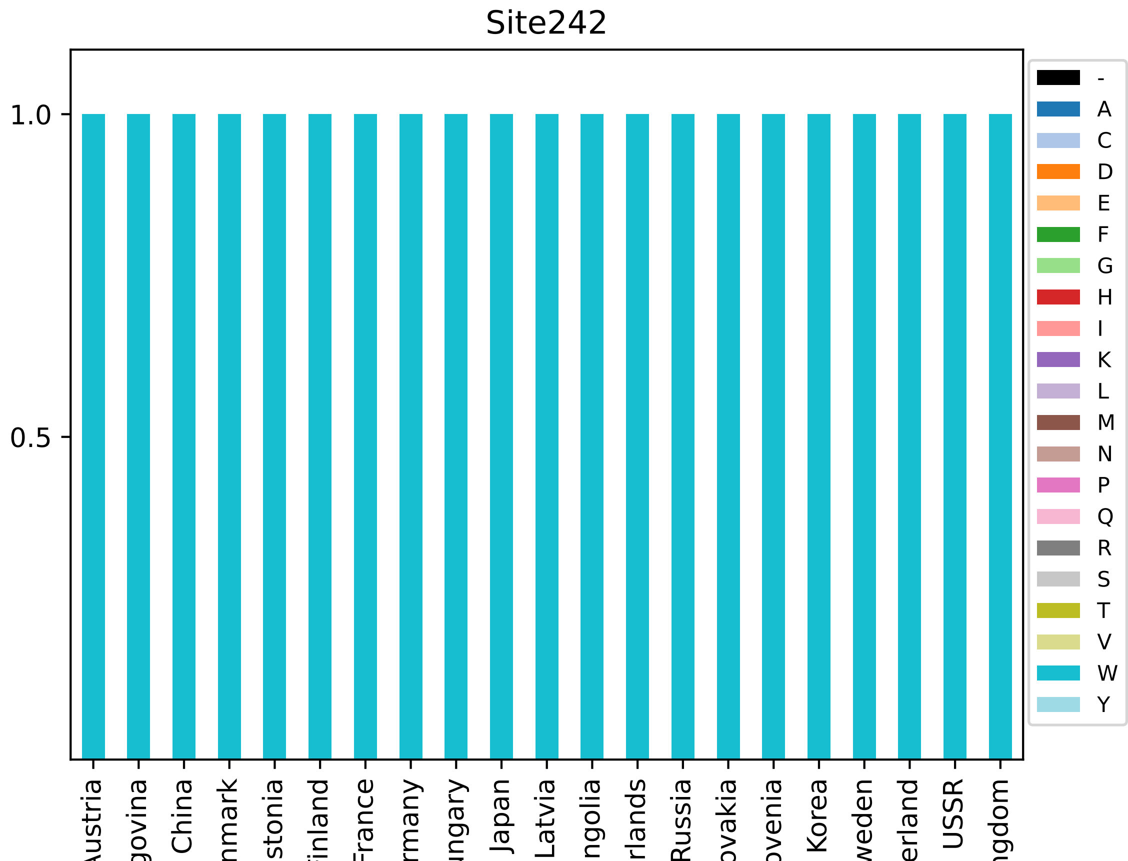
Site242
1.0
0.5
China
Japan
Latvia
Russia
Korea
USSR
-
A
C
D
E
F
G
H
I
K
L
M
N
P
Q
R
S
T
V
W
Y

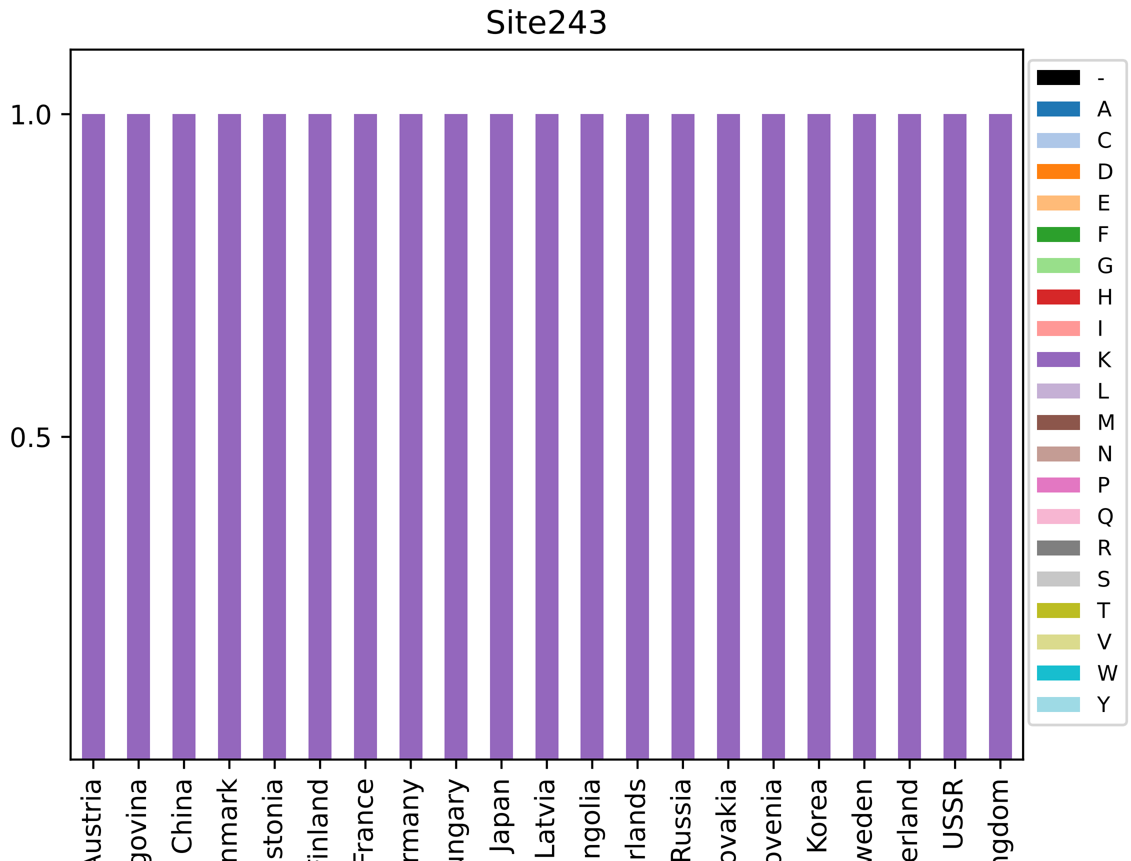
Site243
1.0
0.5
-
A
C
D
E
F
G
H
I
K
L
M
N
P
Q
R
S
T
V
W
Y
China
Japan
Latvia
Russia
Korea
USSR

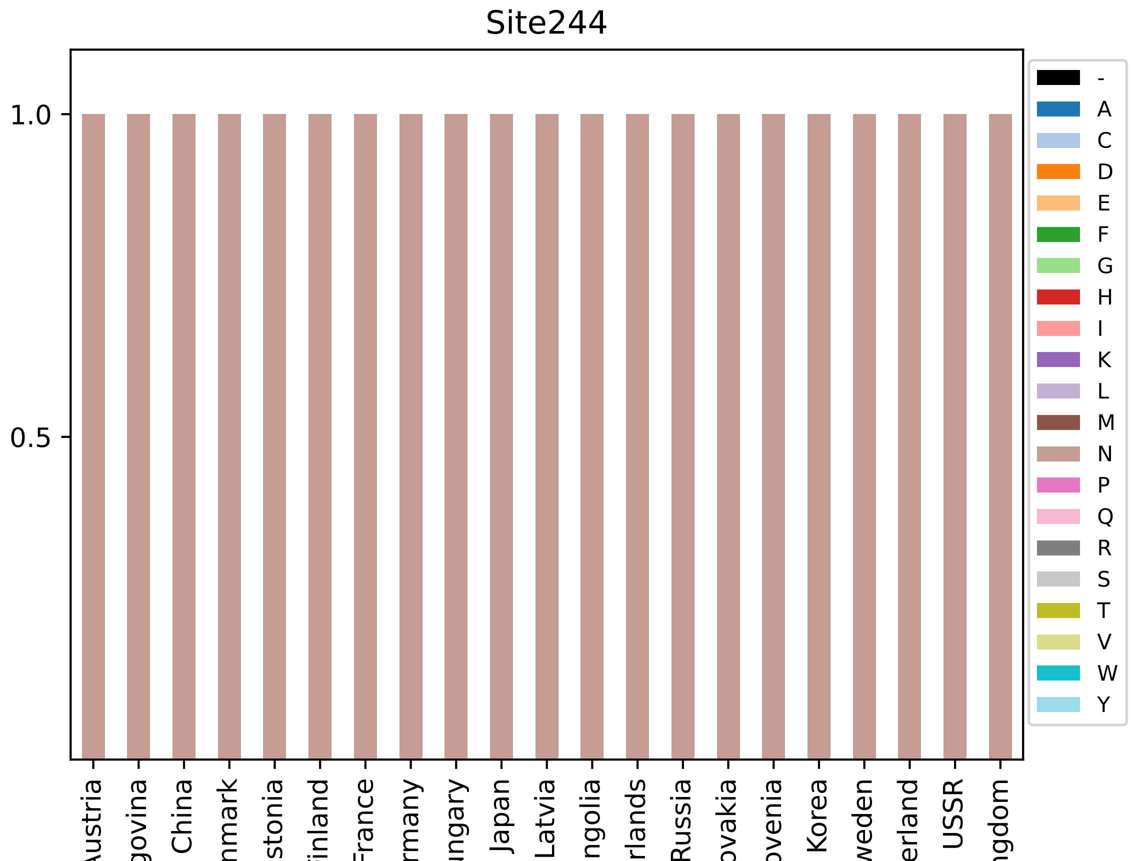
Site244
1.0
0.5
-
A
C
D
E
F
G
H
I
K
L
M
N
P
Q
R
S
T
V
W
Y
China
Japan
Latvia
Russia
Korea
USSR

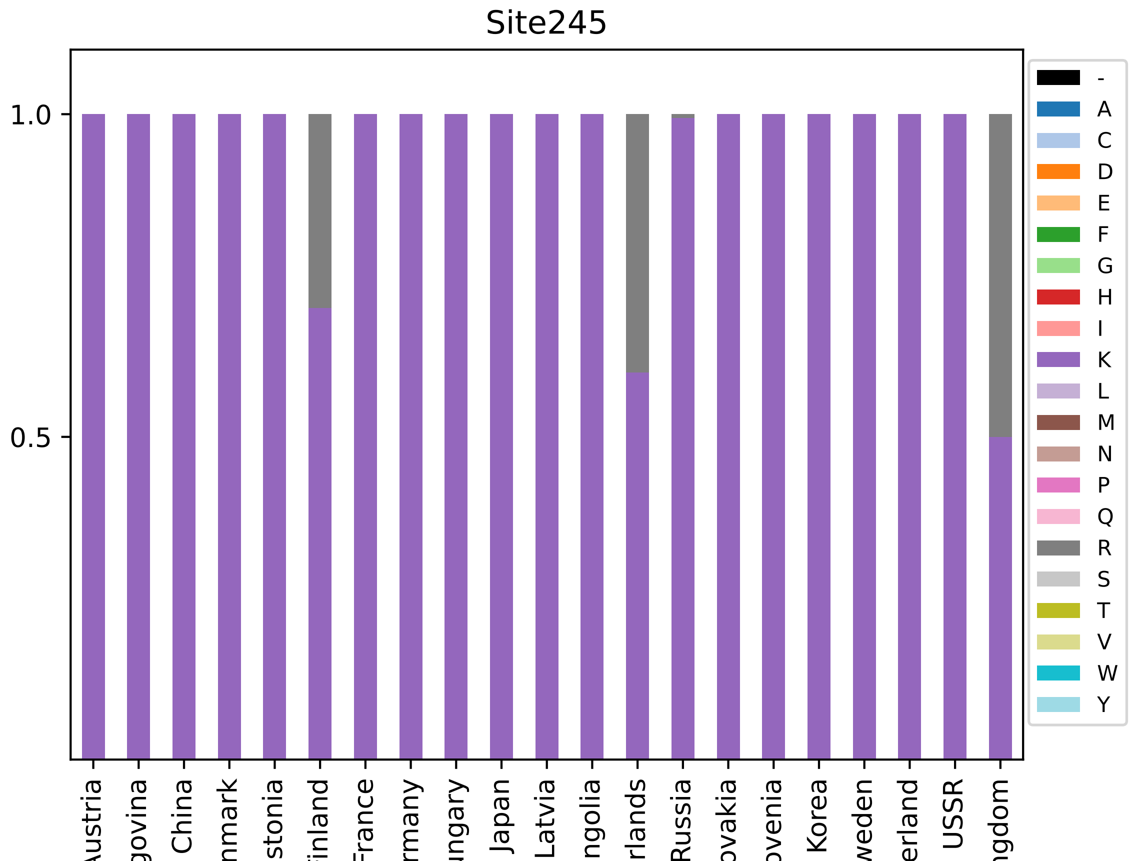

Site245
1.0
0.5
-
A
C
D
E
F
G
H
I
K
L
M
N
P
Q
R
S
T
V
W
Y
China
Finland
Japan
Latvia
Russia
Korea
USSR

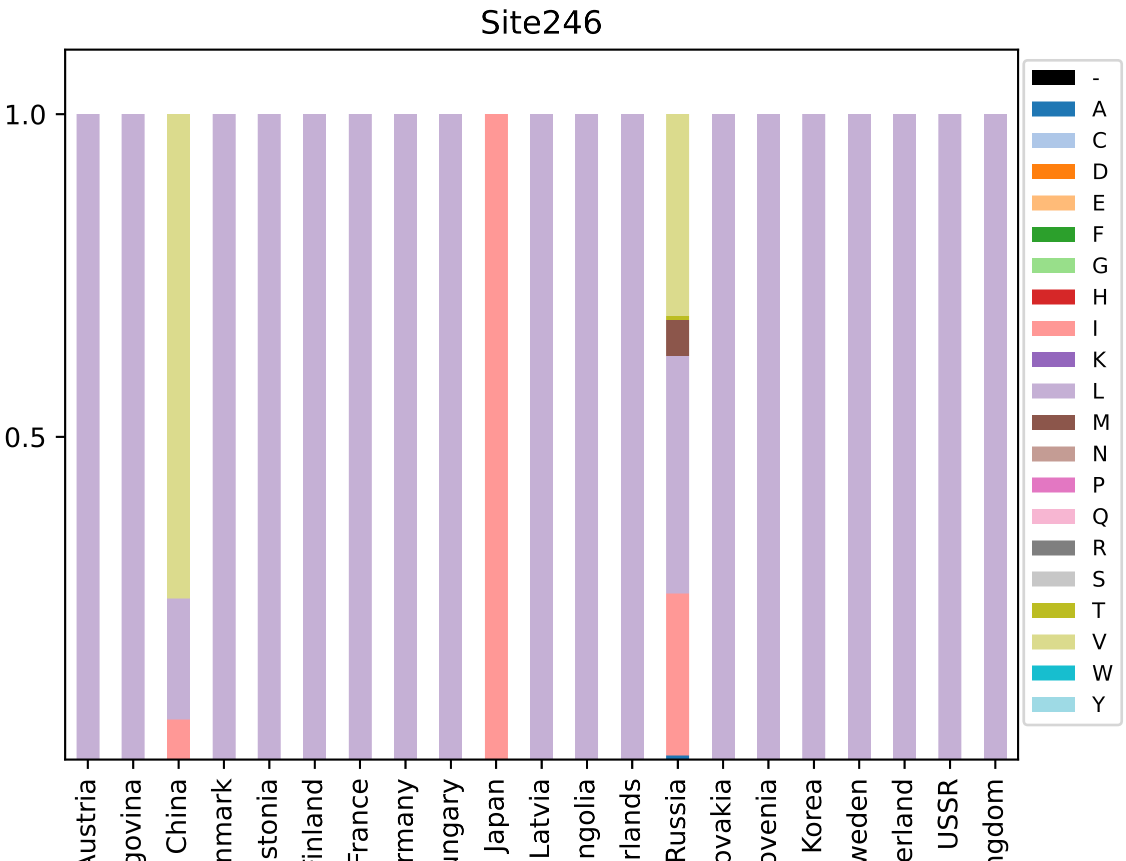

Site246
1.0
0.5
-
A
C
D
E
F
G
H
I
K
L
M
N
P
Q
R
S
T
V
W
Y
Austria
govina
China
nmark
stonia
inland
France
rmany
ungary
Japan
Latvia
ngolia
rlands
Russia
ovakia
ovenia
Korea
weden
erland
USSR
ngdom

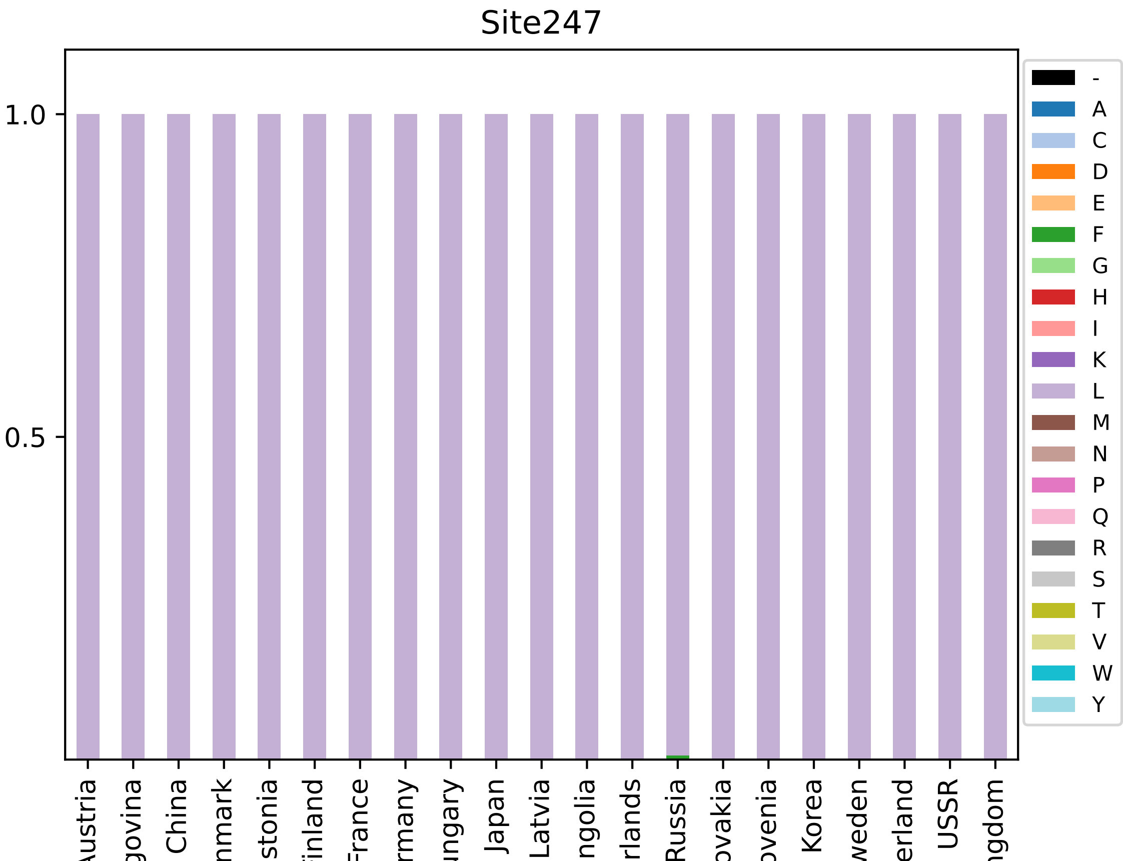

Site247
1.0
0.5
-
A
C
D
E
F
G
H
I
K
L
M
N
P
Q
R
S
T
V
W
Y
Austria
govina
China
nmark
stonia
inland
France
rmany
ungary
Japan
Latvia
ngolia
rlands
Russia
ovakia
ovenia
Korea
weden
erland
USSR
ngdom

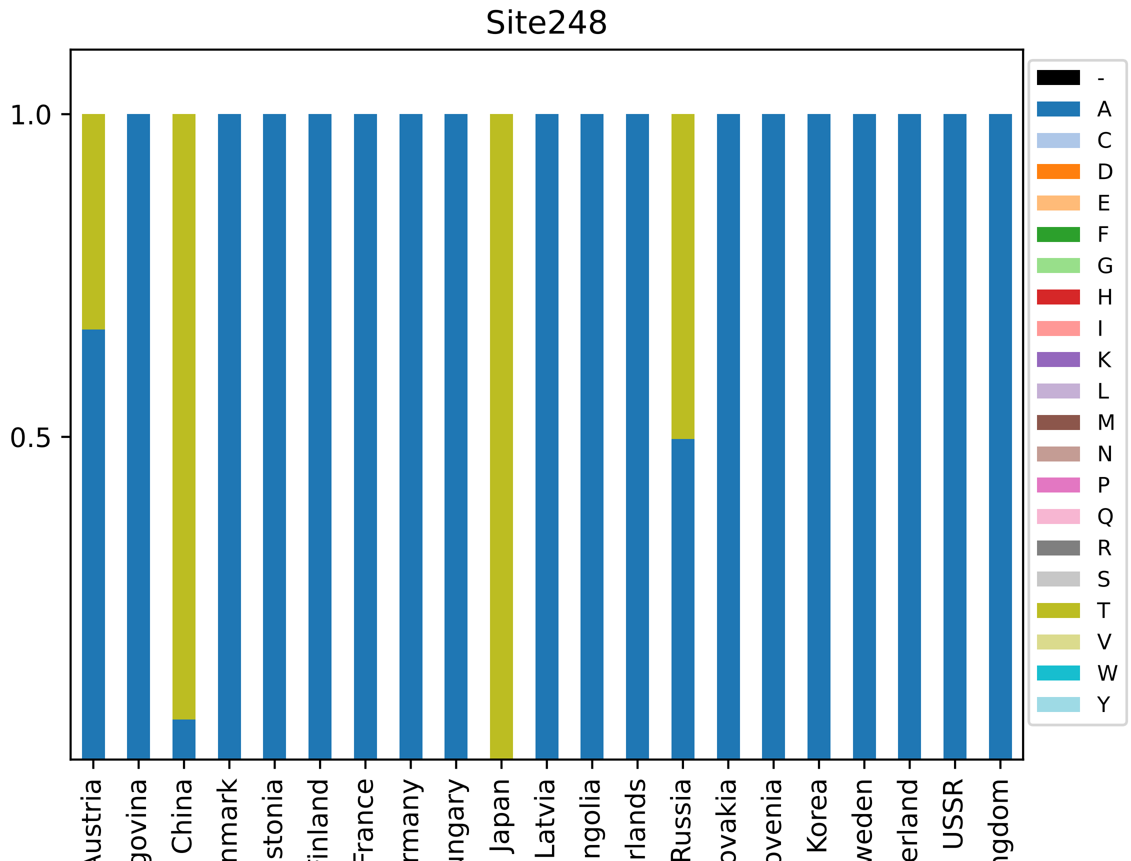
Site248
1.0
0.5
-
A
C
D
E
F
G
H
I
K
L
M
N
P
Q
R
S
T
V
W
Y
China
Japan
Latvia
Russia
Korea
USSR

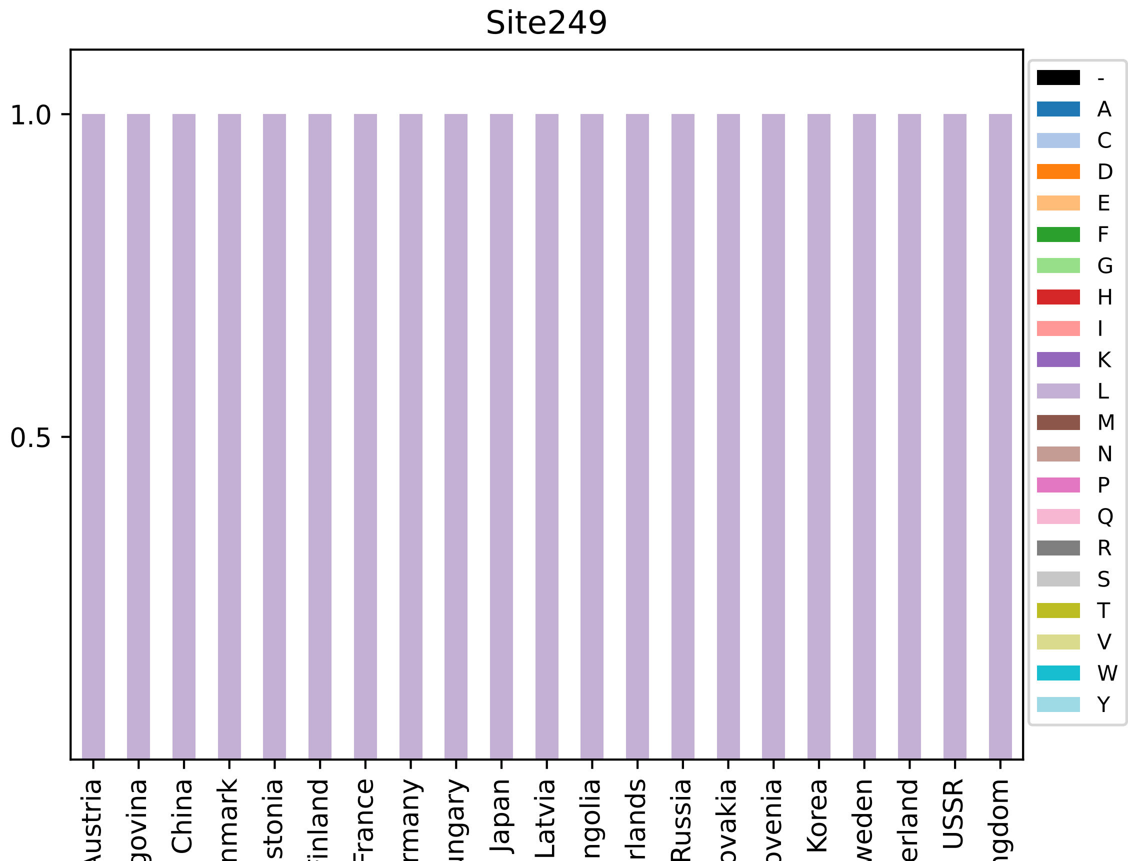
Site249
1.0
0.5
China
Japan
Latvia
Russia
Korea
USSR
-
A
C
D
E
F
G
H
I
K
L
M
N
P
Q
R
S
T
V
W
Y

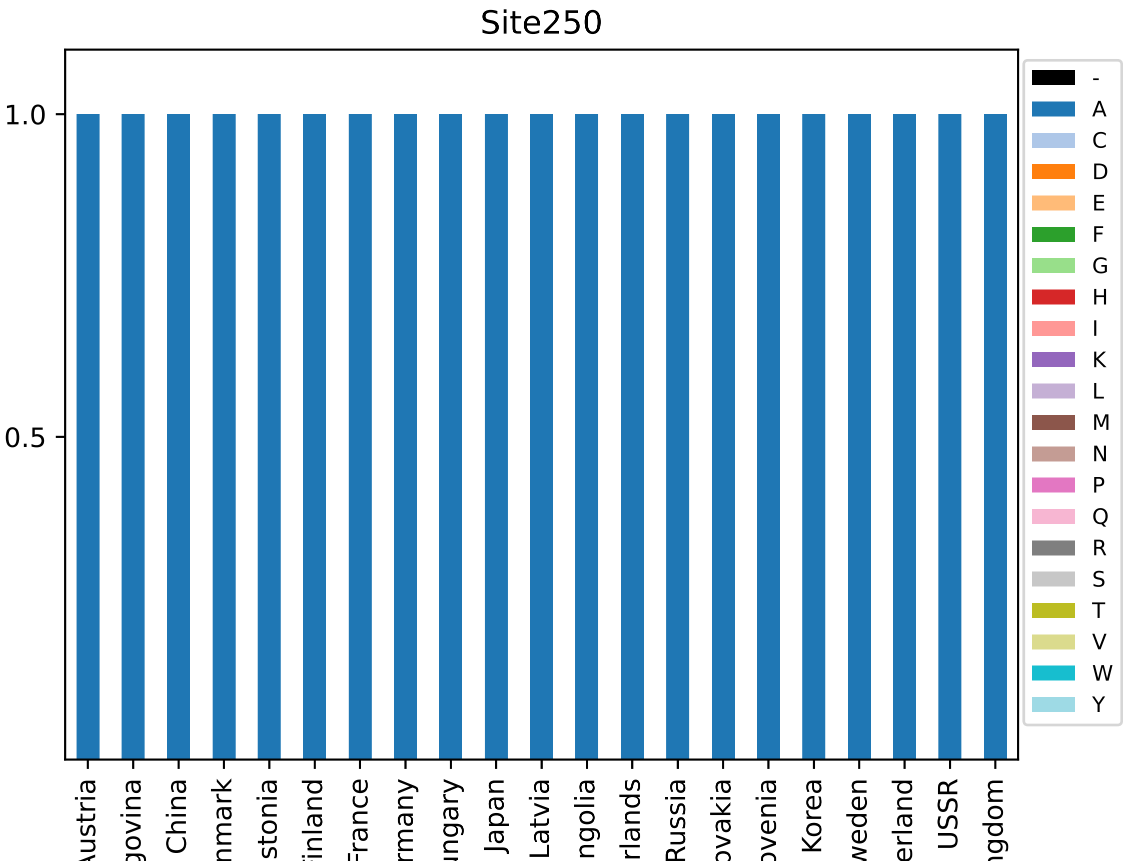
Site250
1.0
0.5
-
A
C
D
E
F
G
H
I
K
L
M
N
P
Q
R
S
T
V
W
Y
China
Japan
Latvia
Russia
Korea
USSR

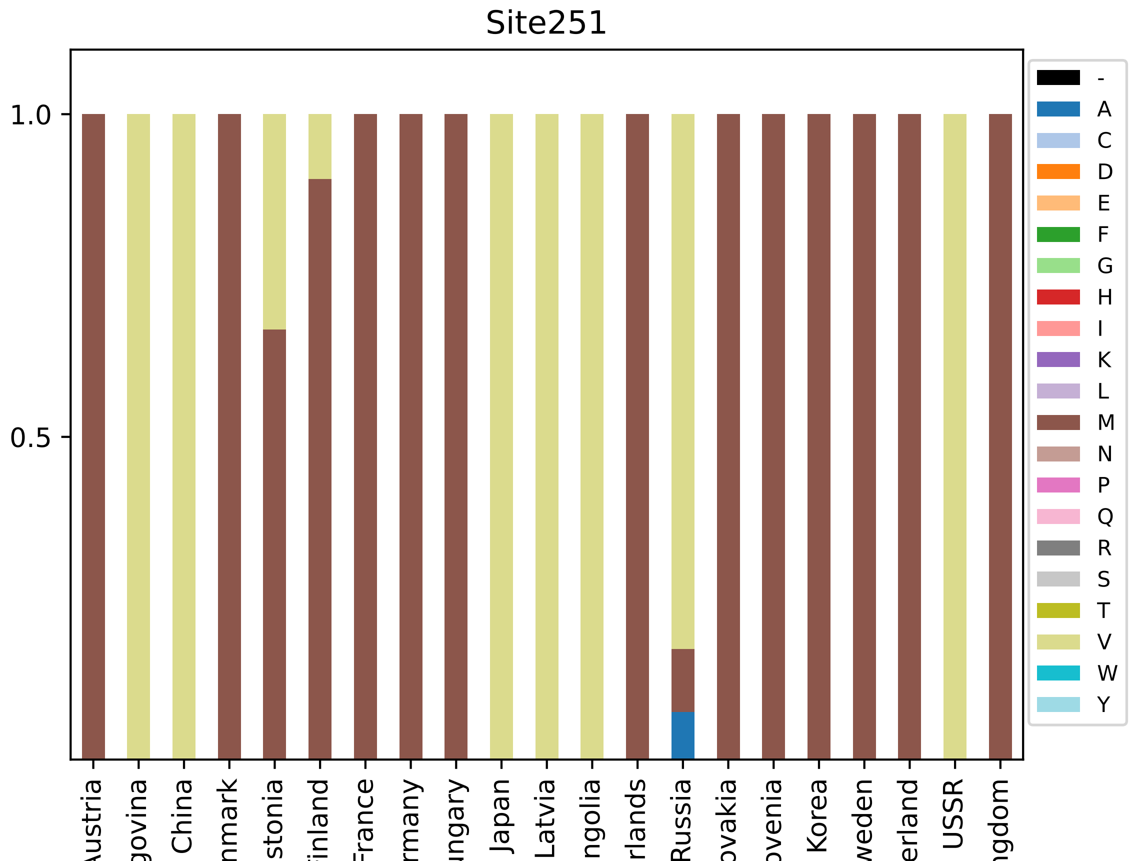
Site251
1.0
0.5
-
A
C
D
E
F
G
H
I
K
L
M
N
P
Q
R
S
T
V
W
Y
China
Japan
Latvia
Russia
Korea
USSR

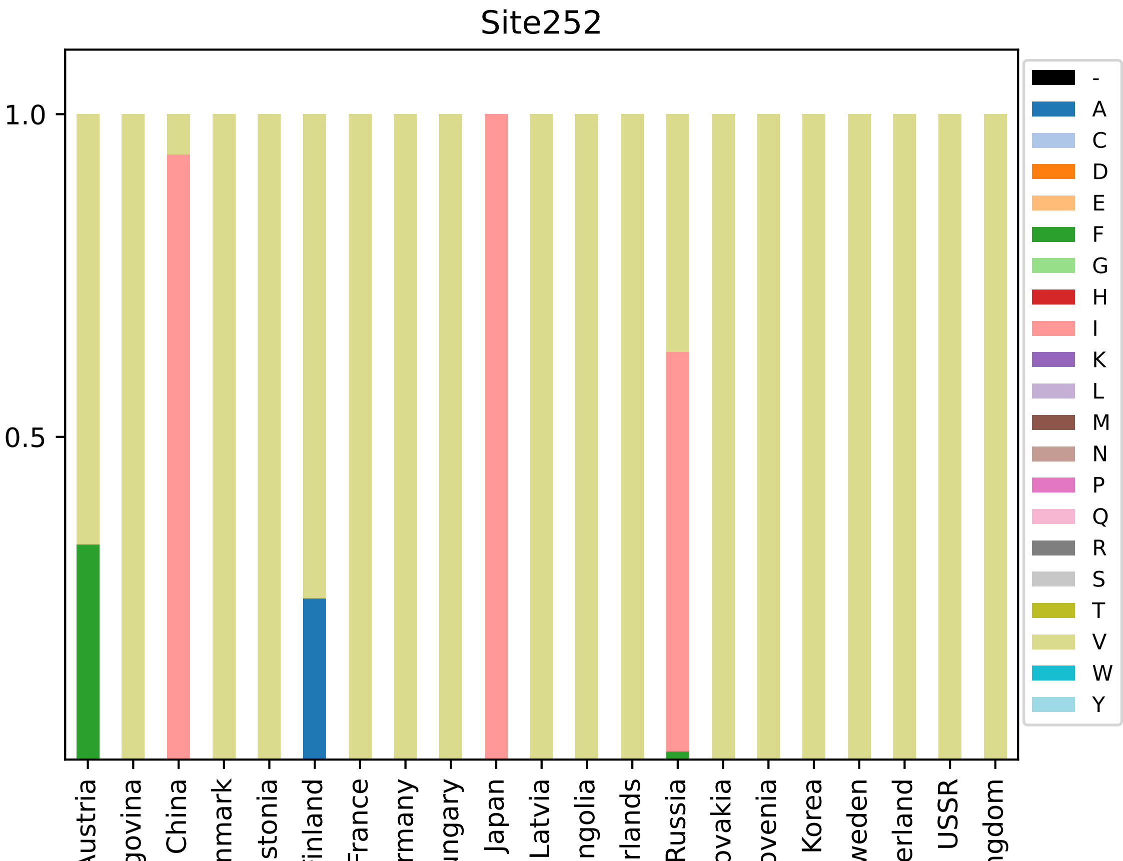

Site252
1.0
0.5
-
A
C
D
E
F
G
H
I
K
L
M
N
P
Q
R
S
T
V
W
Y
China
Japan
Latvia
Russia
Korea
USSR

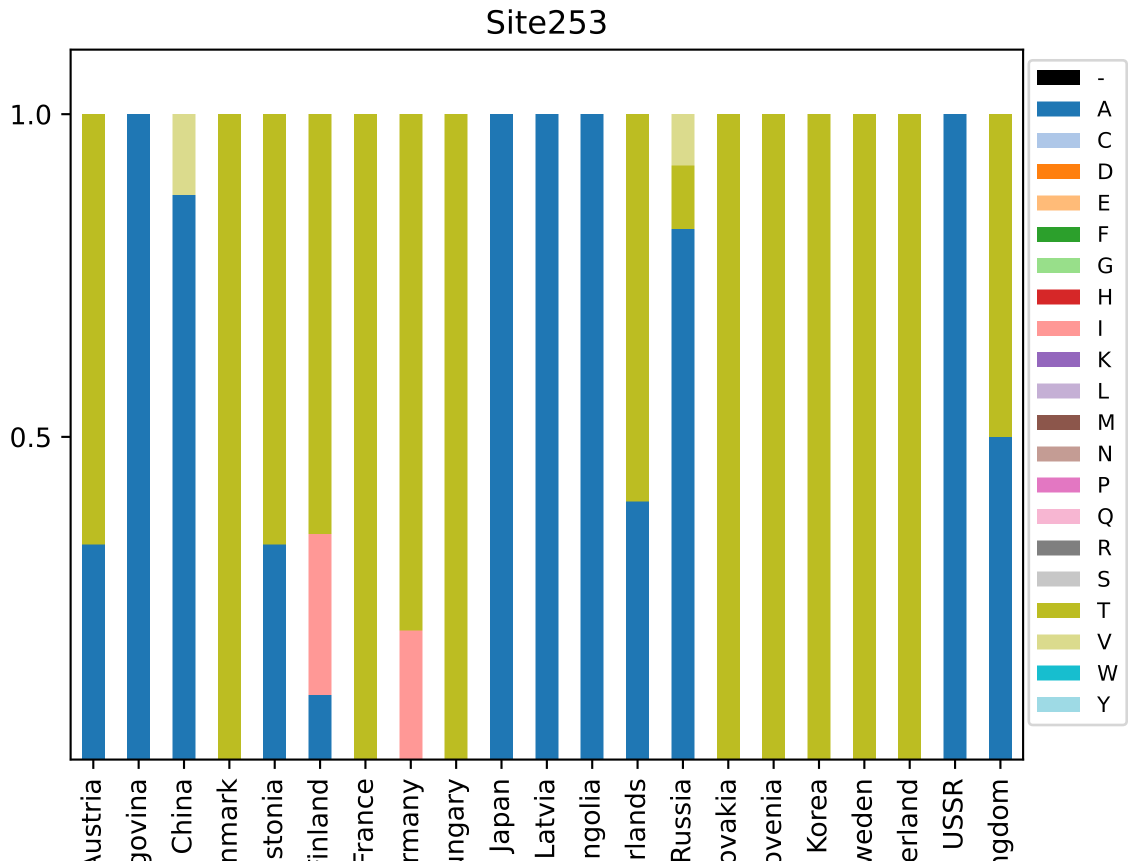
Site253
1.0
0.5
-
A
C
D
E
F
G
H
I
K
L
M
N
P
Q
R
S
T
V
W
Y
China
Japan
Latvia
Russia
Korea
USSR

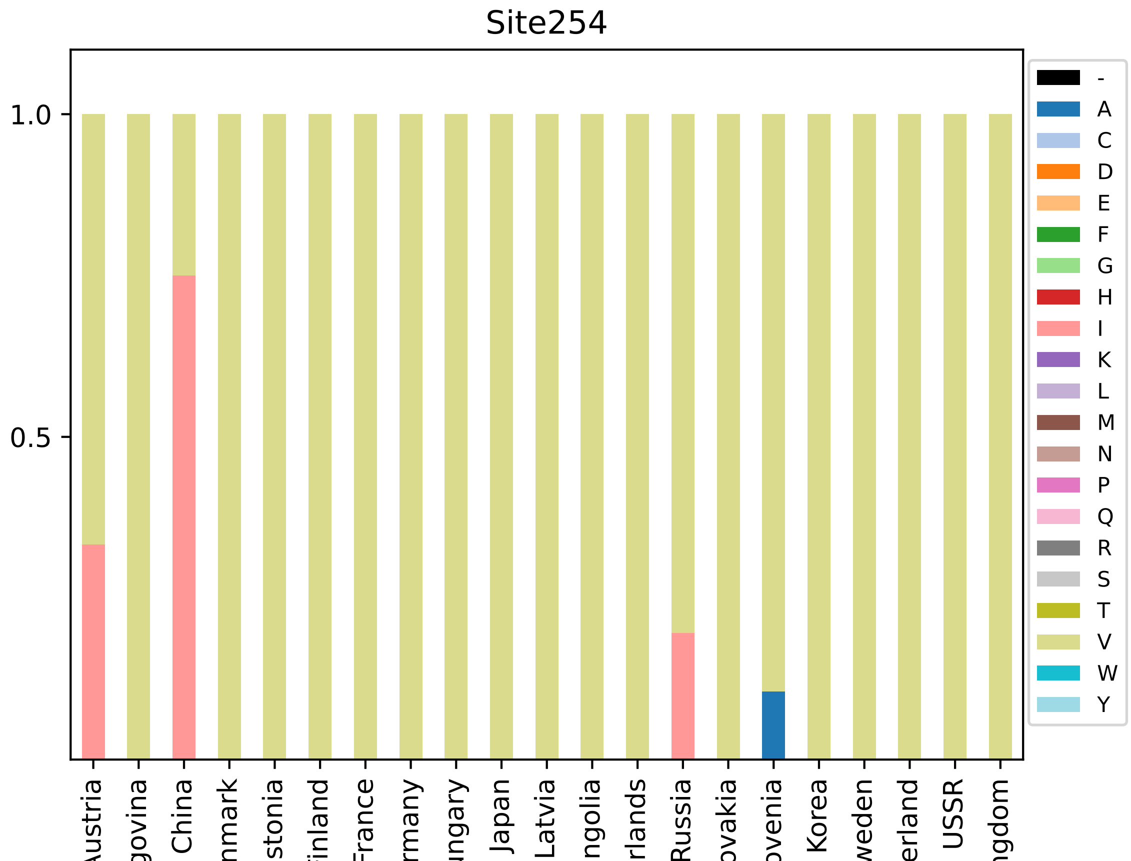

Site254
1.0
0.5
China
Japan
Latvia
Russia
Korea
USSR
-
A
C
D
E
F
G
H
I
K
L
M
N
P
Q
R
S
T
V
W
Y

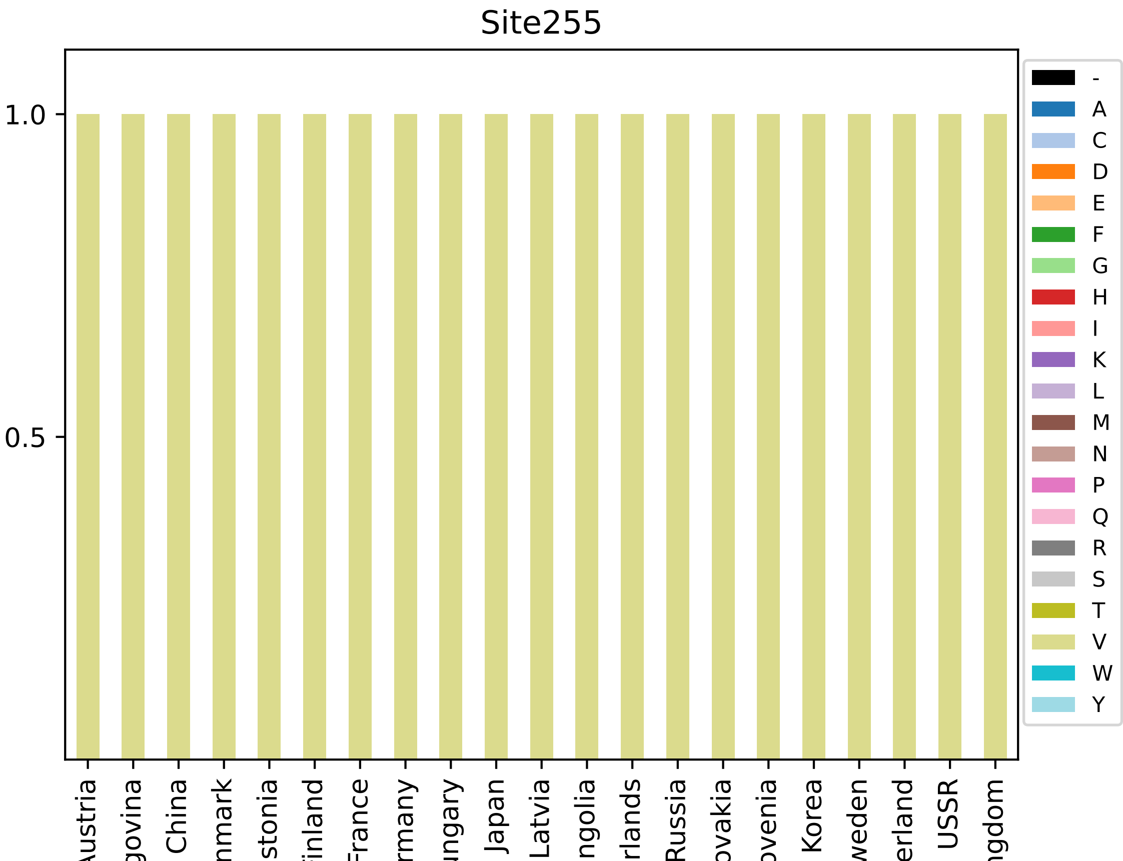
Site255
1.0
0.5
-
A
C
D
E
F
G
H
I
K
L
M
N
P
Q
R
S
T
V
W
Y
China
Japan
Latvia
Russia
Korea
USSR

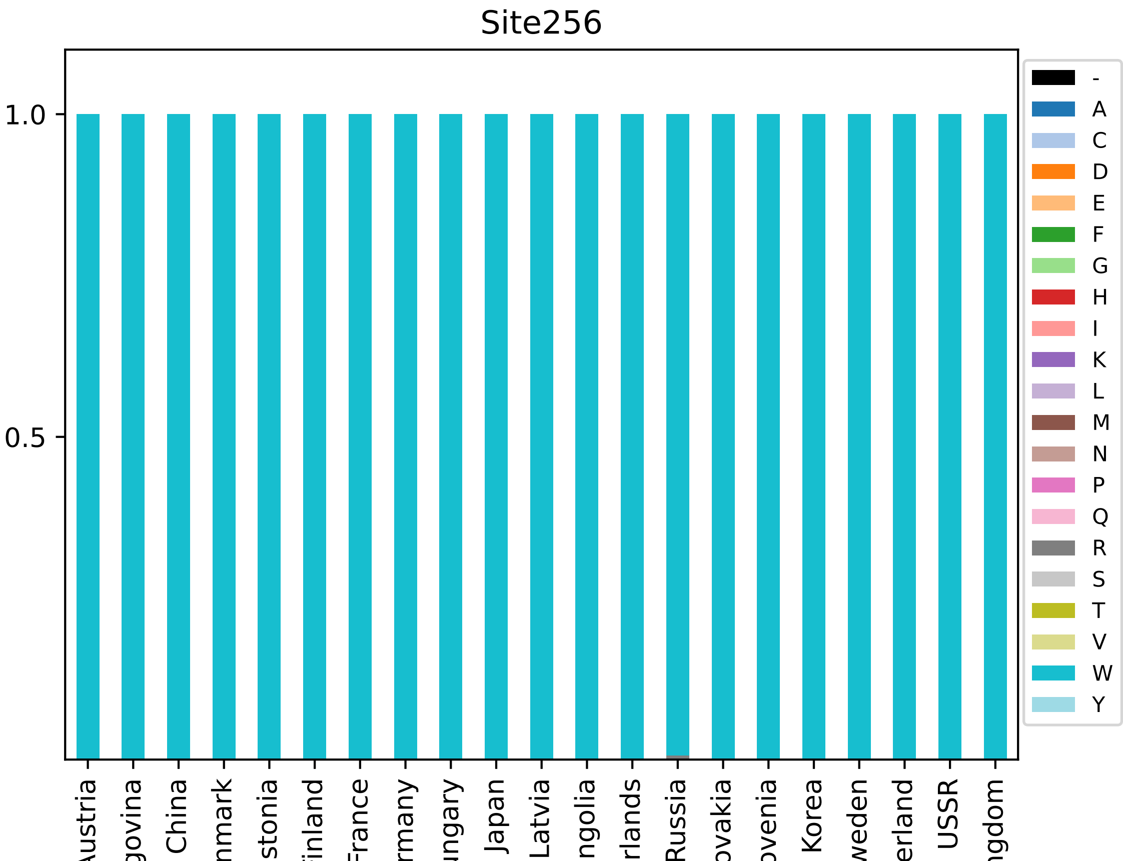
Site256
1.0
0.5
-
A
C
D
E
F
G
H
I
K
L
M
N
P
Q
R
S
T
V
W
Y
China
France
Japan
Latvia
Russia
Korea
USSR

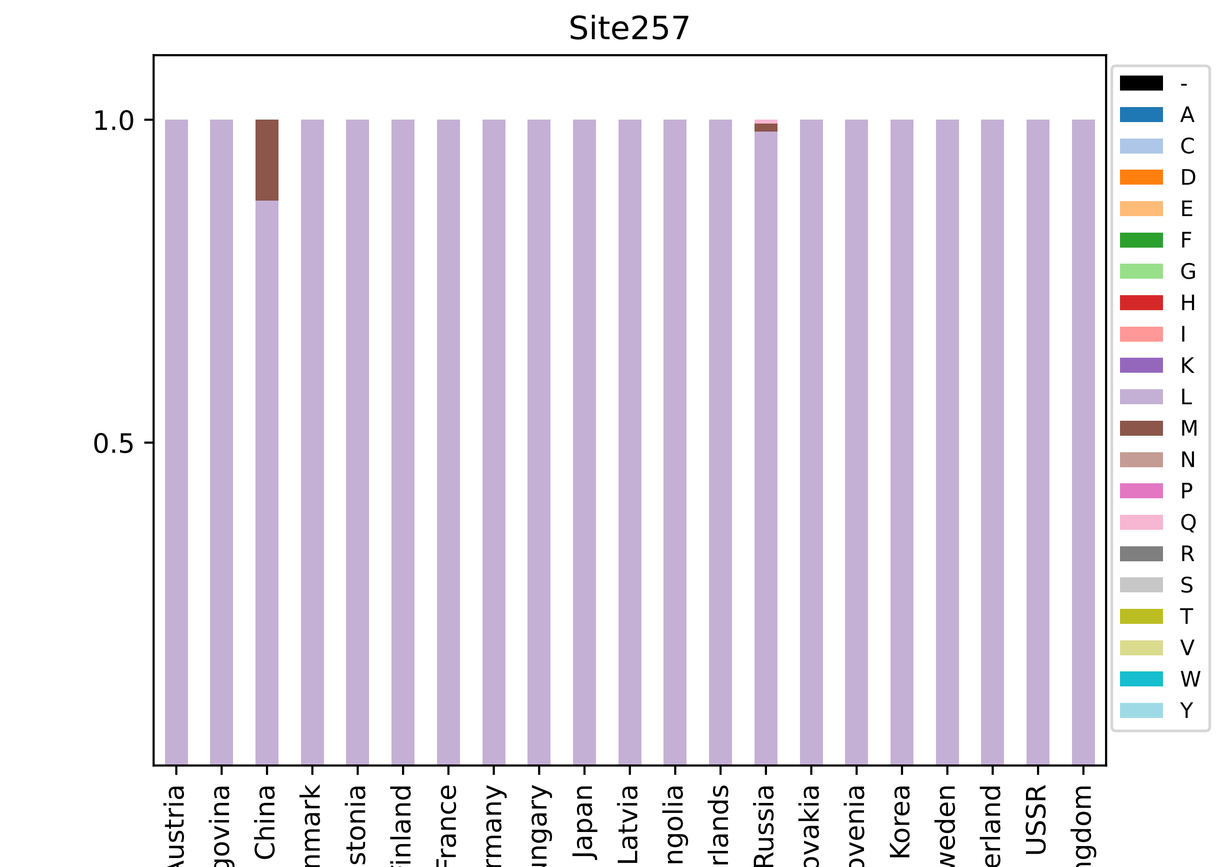

Site257
1.0
0.5
-
A
C
D
E
F
G
H
I
K
L
M
N
P
Q
R
S
T
V
W
Y
Austria
govina
China
nmark
stonia
inland
France
rmany
ungary
Japan
Latvia
ngolia
rlands
Russia
ovakia
ovenia
Korea
weden
erland
USSR
ngdom

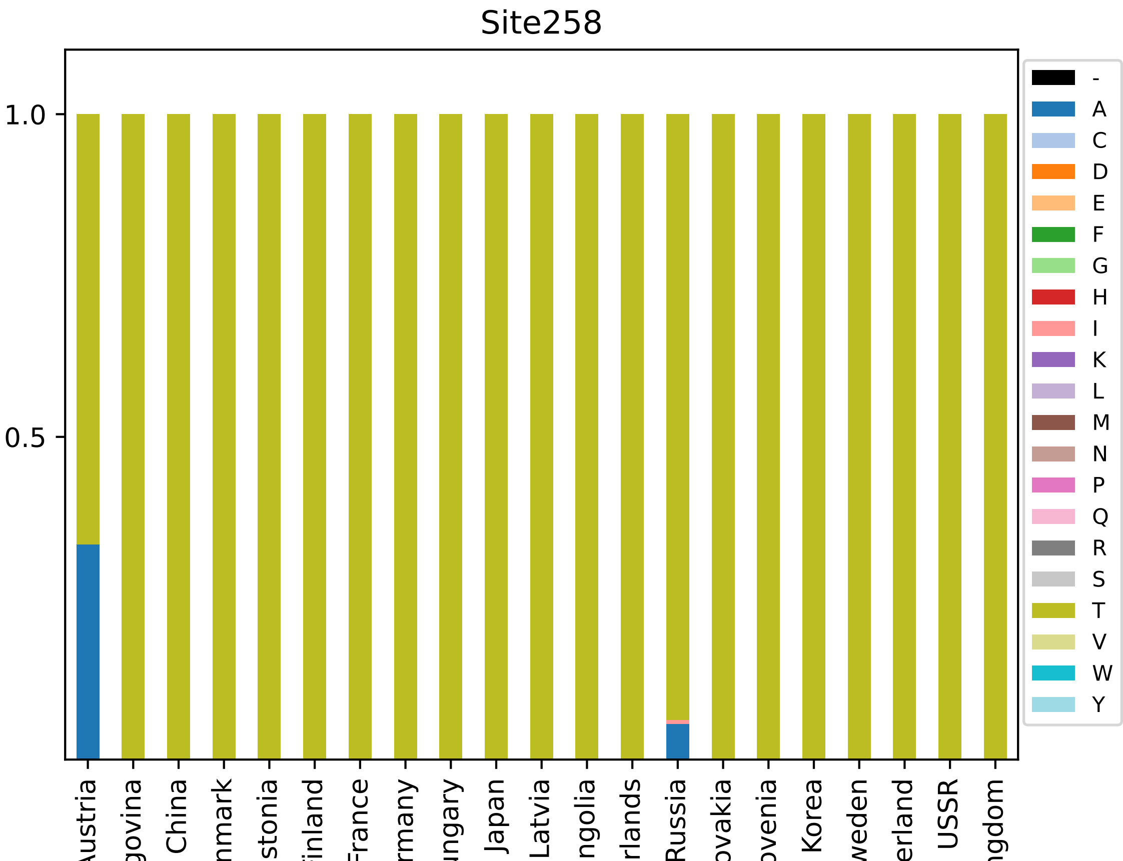

Site258
1.0
0.5
-
A
C
D
E
F
G
H
I
K
L
M
N
P
Q
R
S
T
V
W
Y
China
Japan
Latvia
Russia
Korea
USSR

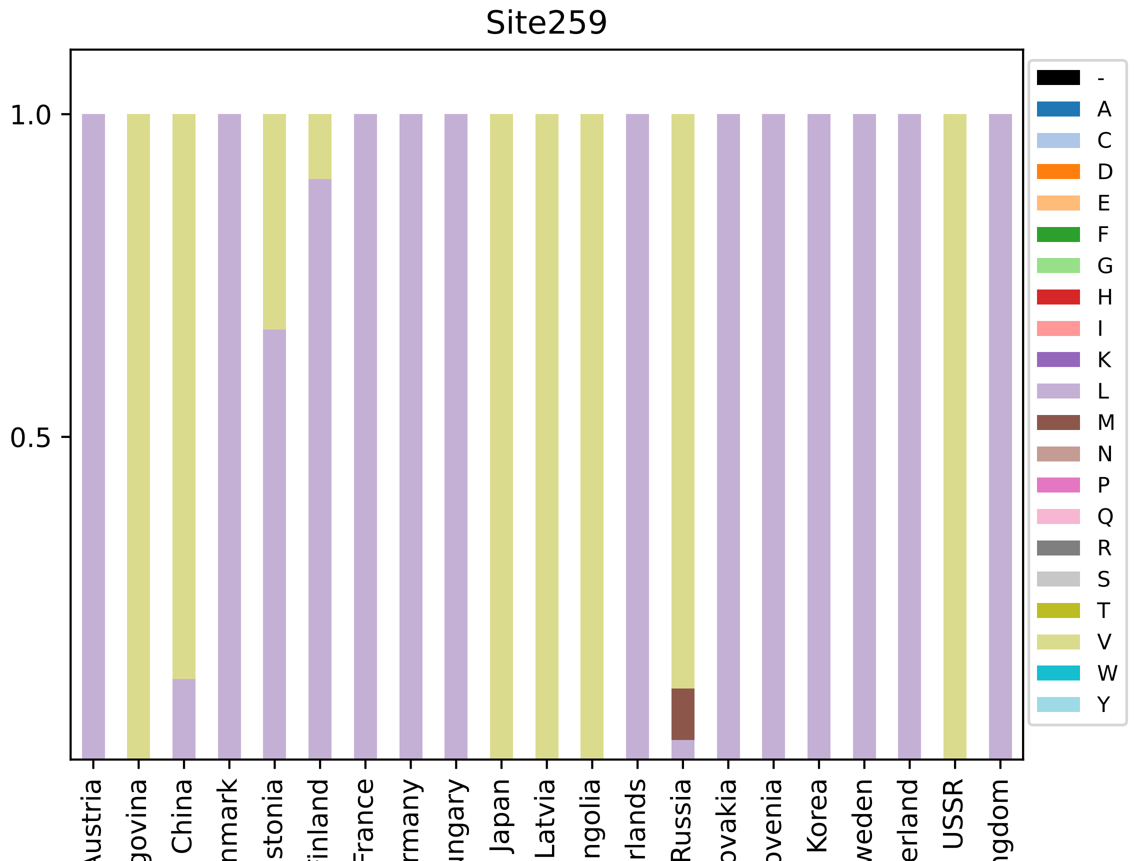
Site259
1.0
0.5
-
A
C
D
E
F
G
H
I
K
L
M
N
P
Q
R
S
T
V
W
Y
Austria
govina
China
nmark
stonia
inland
rance
rmany
ungary
Japan
Latvia
ngolia
rlands
Russia
ovakia
ovenia
Korea
weden
erland
USSR
ngdom

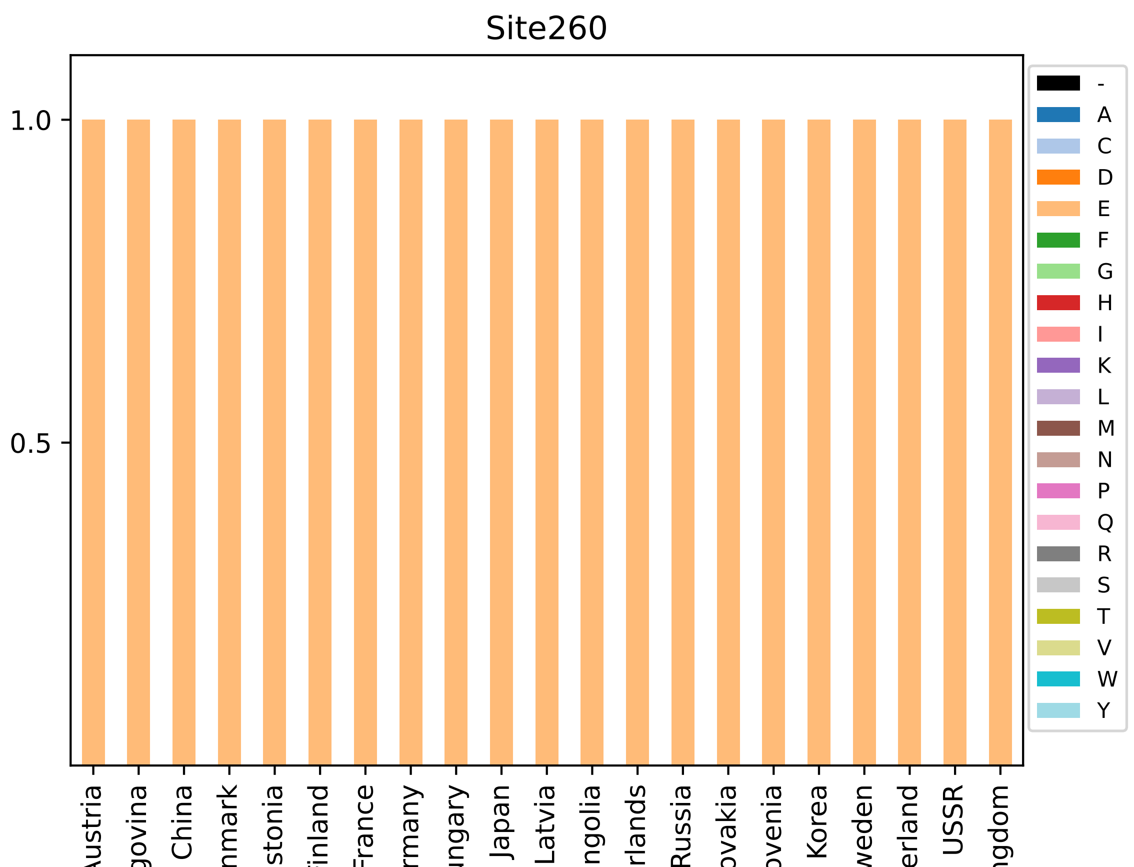
Site260
1.0
0.5
-
A
C
D
E
F
G
H
I
K
L
M
N
P
Q
R
S
T
V
W
Y
China
Japan
Latvia
Russia
Korea
USSR

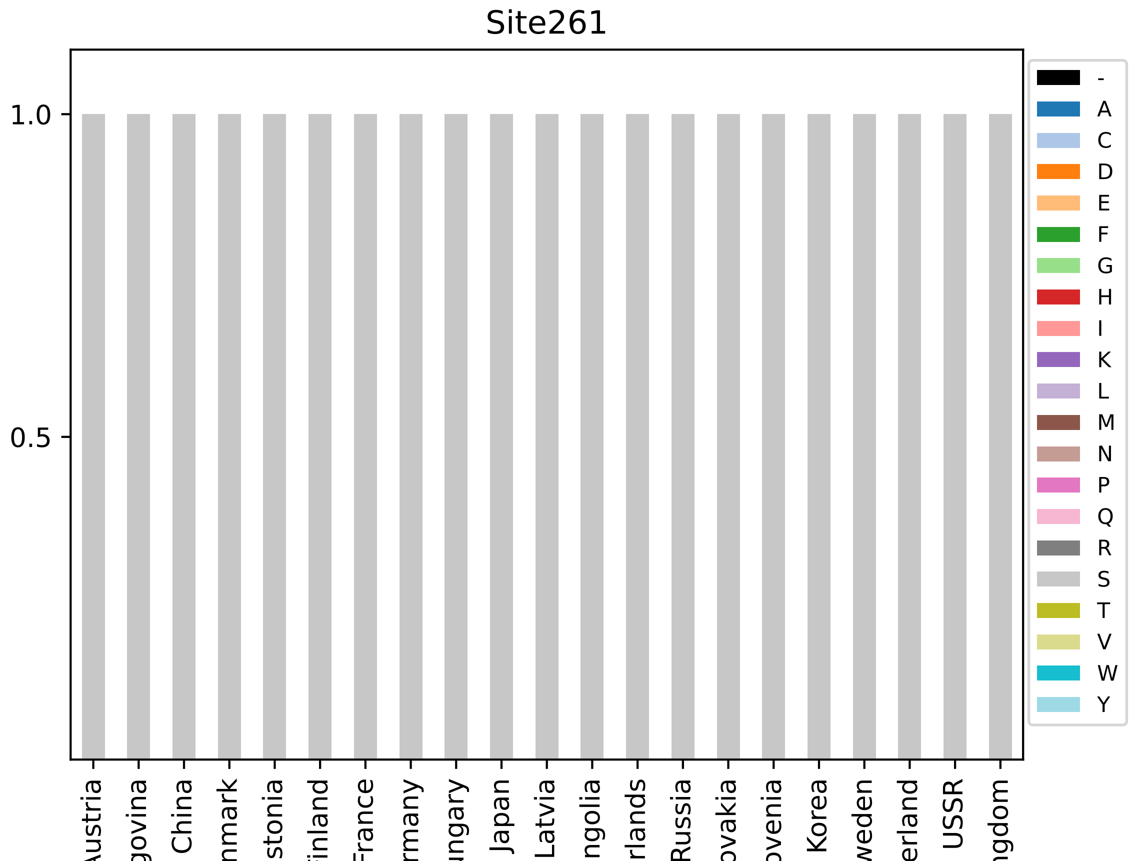

Site261
1.0
0.5
-
A
C
D
E
F
G
H
I
K
L
M
N
P
Q
R
S
T
V
W
Y
China
Japan
Latvia
Russia
Korea
USSR

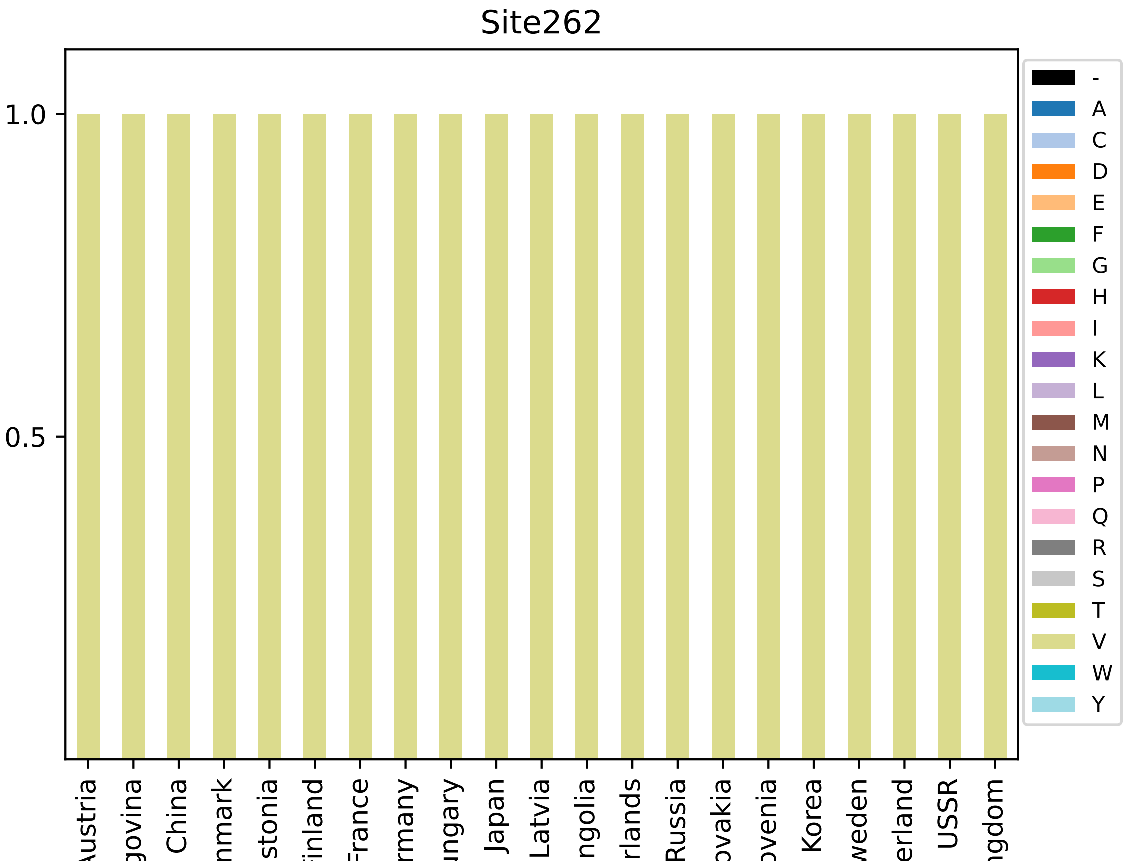
Site262
1.0
0.5
-
A
C
D
E
F
G
H
I
K
L
M
N
P
Q
R
S
T
V
W
Y
China
Japan
Latvia
Russia
Korea
USSR

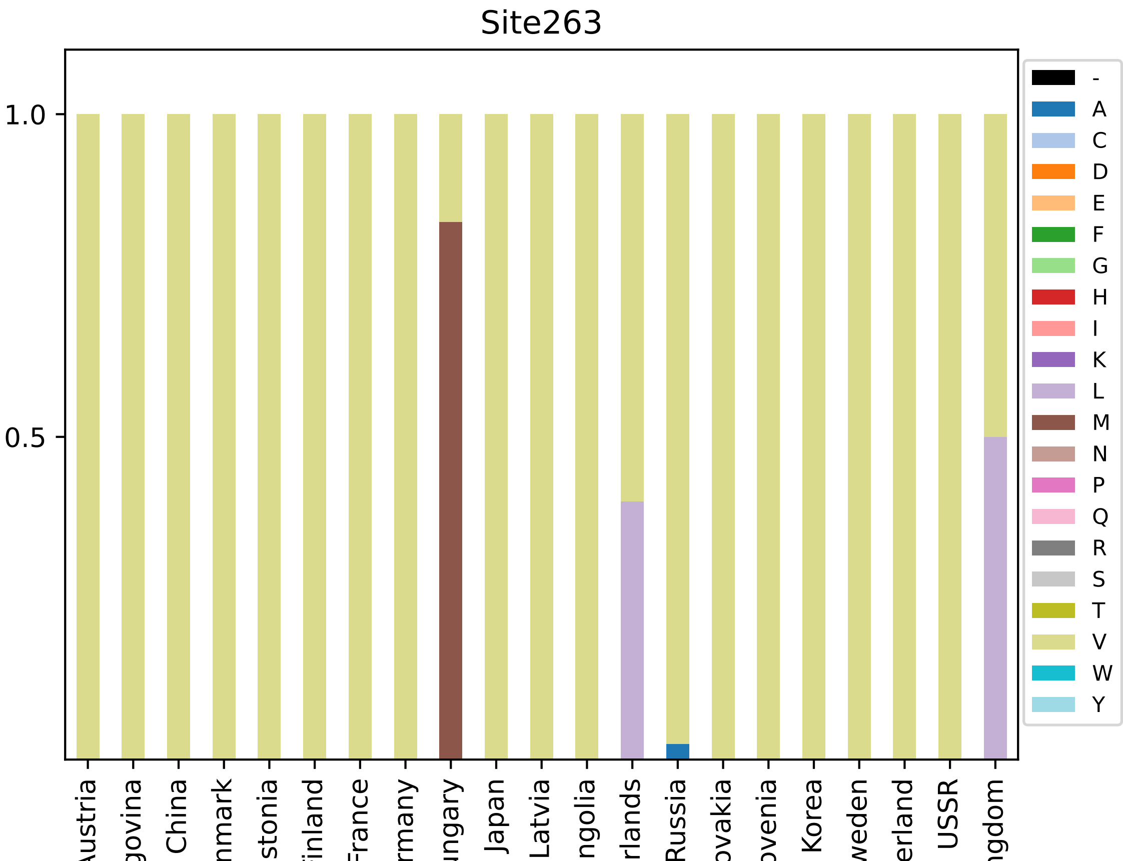

Site263
1.0
0.5
-
A
C
D
E
F
G
H
I
K
L
M
N
P
Q
R
S
T
V
W
Y
China
Japan
Latvia
Russia
Korea
USSR

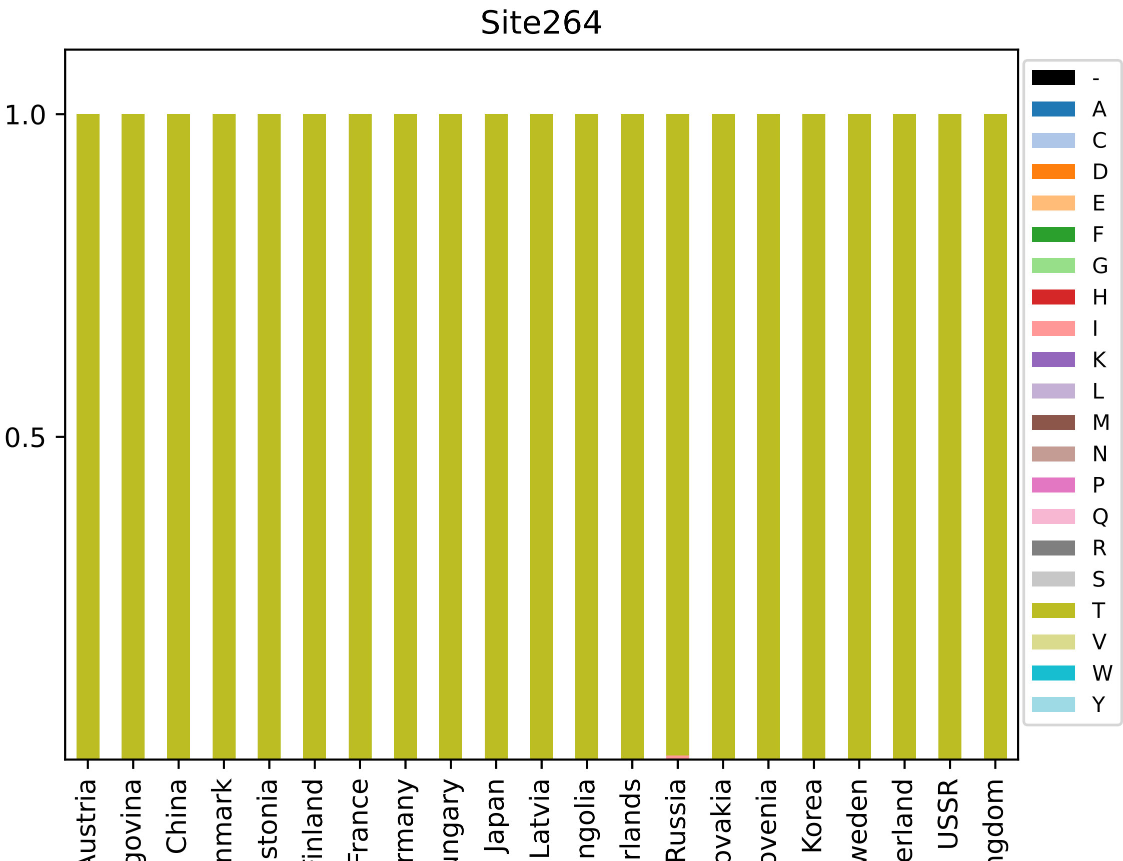
Site264
1.0
0.5
-
A
C
D
E
F
G
H
I
K
L
M
N
P
Q
R
S
T
V
W
Y
China
Japan
Latvia
Russia
Korea
USSR

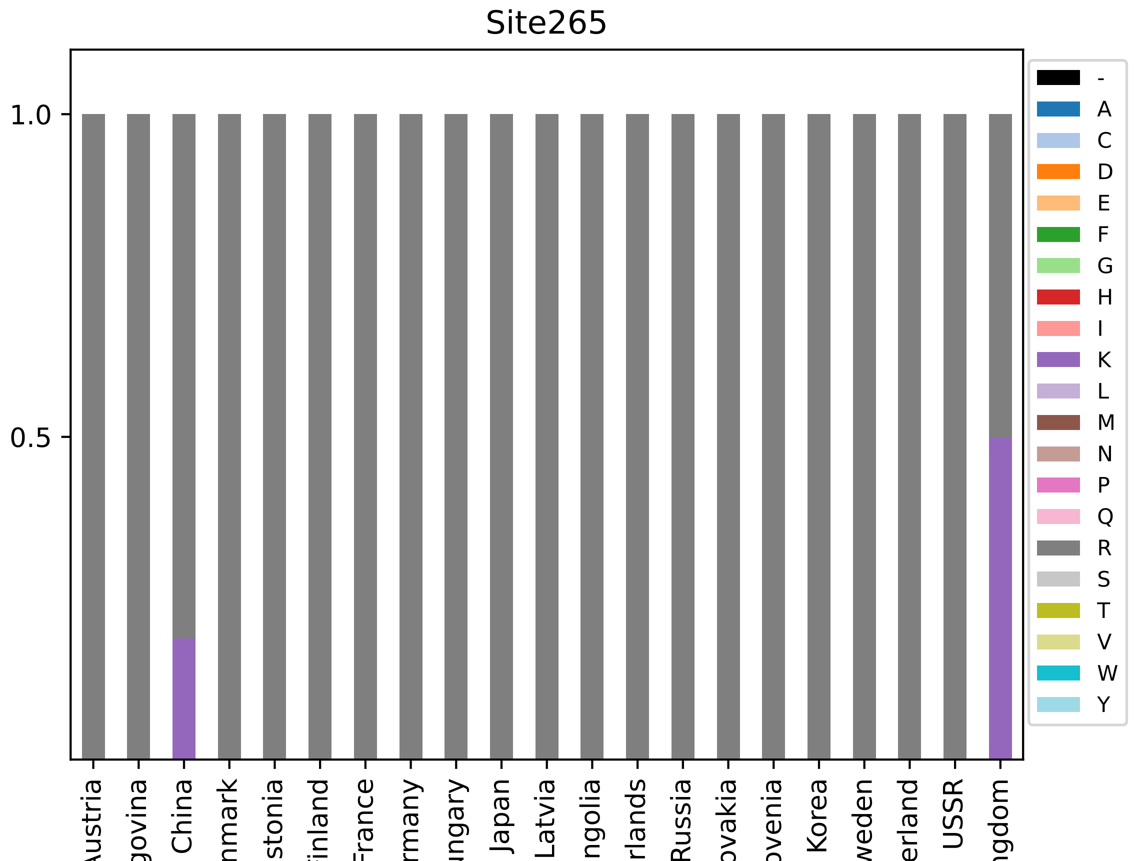

Site265
1.0
0.5
-
A
C
D
E
F
G
H
I
K
L
M
N
P
Q
R
S
T
V
W
Y
China
Japan
Latvia
Russia
Korea
USSR

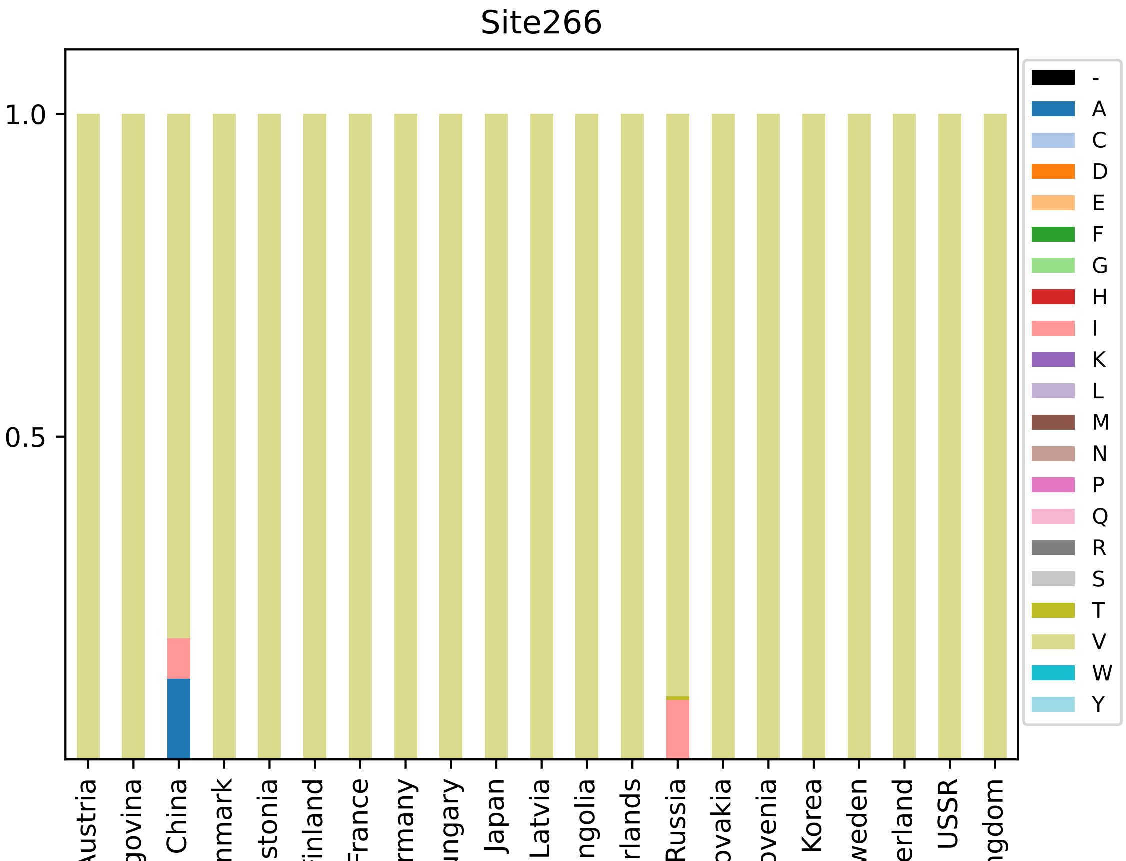
Site266
1.0
0.5
-
A
C
D
E
F
G
H
I
K
L
M
N
P
Q
R
S
T
V
W
Y
China
Japan
Latvia
Russia
Korea
USSR

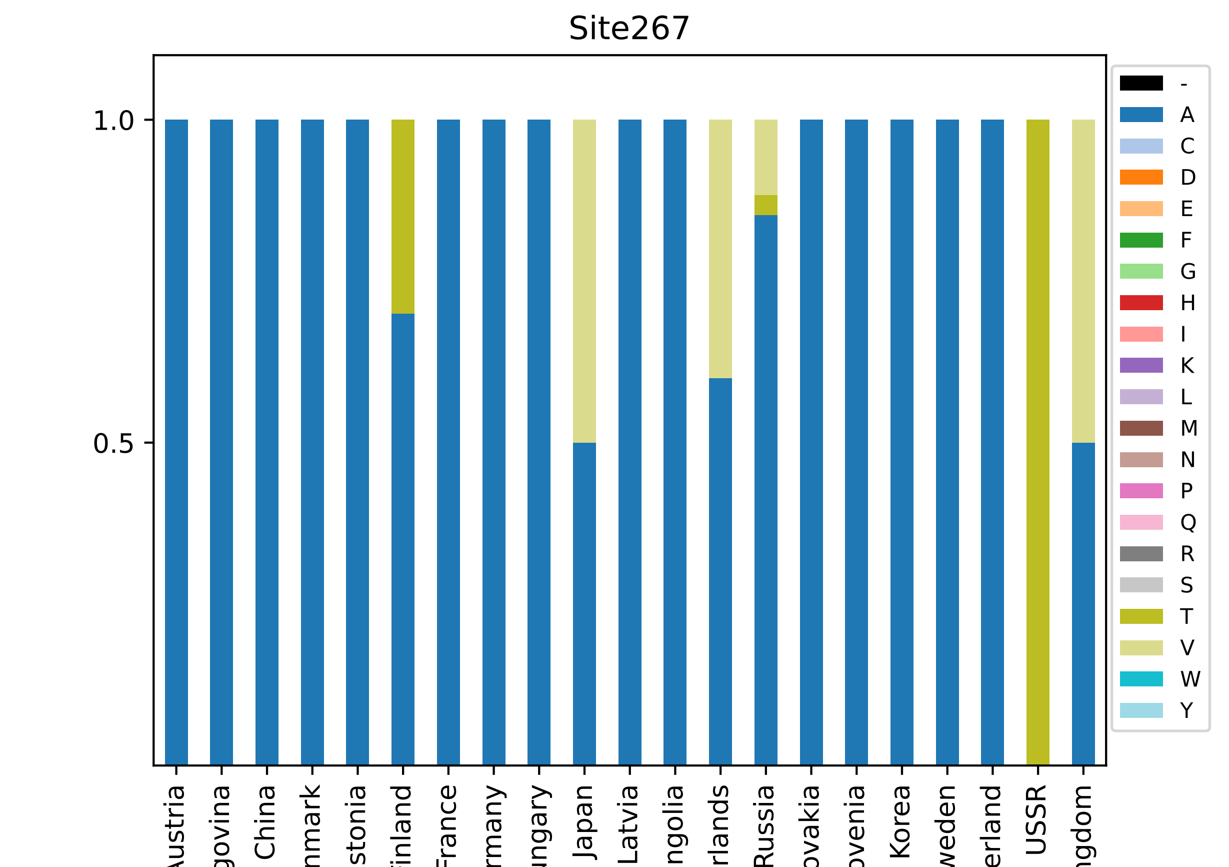

Site267
1.0
0.5
-
A
C
D
E
F
G
H
I
K
L
M
N
P
Q
R
S
T
V
W
Y
ustria
govina
China
nmark
stonia
inland
rance
rmany
ungary
Japan
Latvia
ngolia
rlands
Russia
ovakia
ovenia
Korea
weden
erland
USSR
ngdom

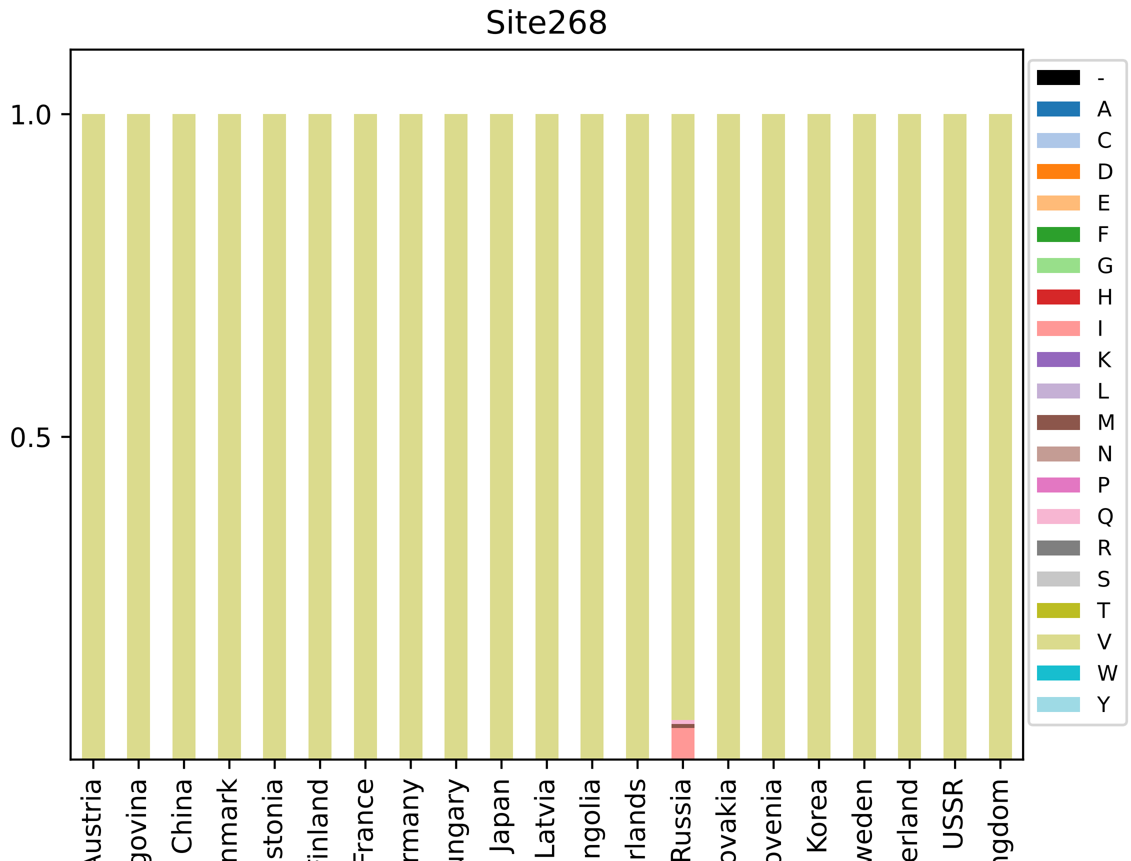

Site268
1.0
0.5
-
A
C
D
E
F
G
H
I
K
L
M
N
P
Q
R
S
T
V
W
Y
Austria
govina
China
nmark
stonia
inland
France
rmany
ungary
Japan
Latvia
ngolia
rlands
Russia
ovakia
ovenia
Korea
weden
erland
USSR
ngdom

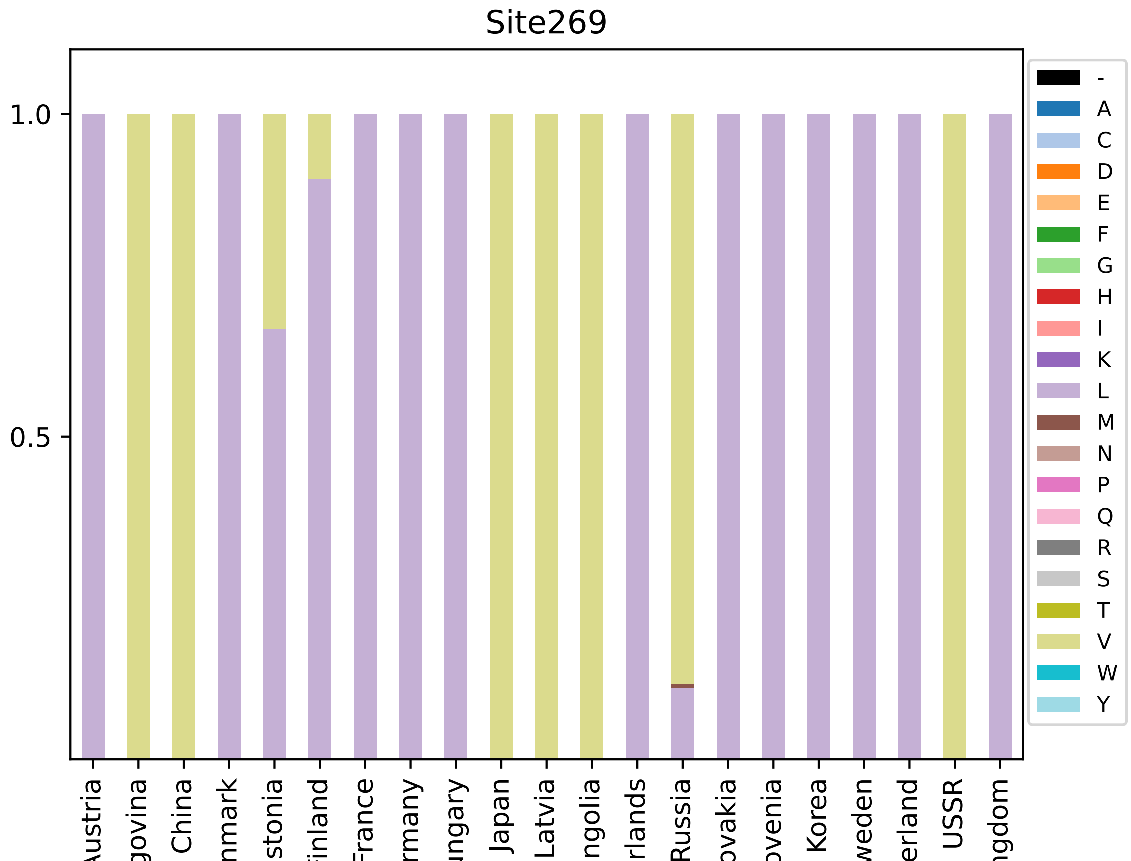

Site269
1.0
0.5
-
A
C
D
E
F
G
H
I
K
L
M
N
P
Q
R
S
T
V
W
Y
China
Japan
Latvia
Russia
Korea
USSR

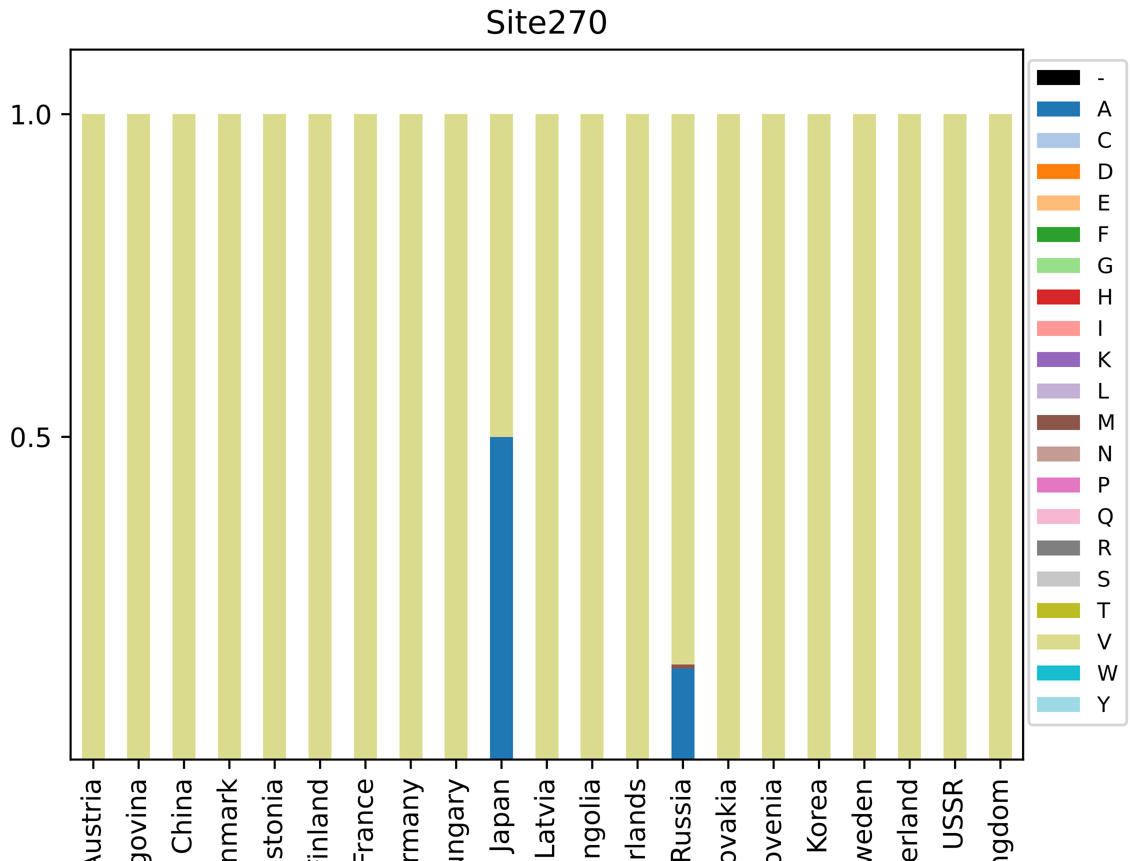

Site270
1.0
0.5
-
A
C
D
E
F
G
H
I
K
L
M
N
P
Q
R
S
T
V
W
Y
Austria
govina
China
nmark
stonia
Finland
France
rmany
ungary
Japan
Latvia
ngolia
rlands
Russia
ovakia
ovenia
Korea
weden
erland
USSR
ngdom

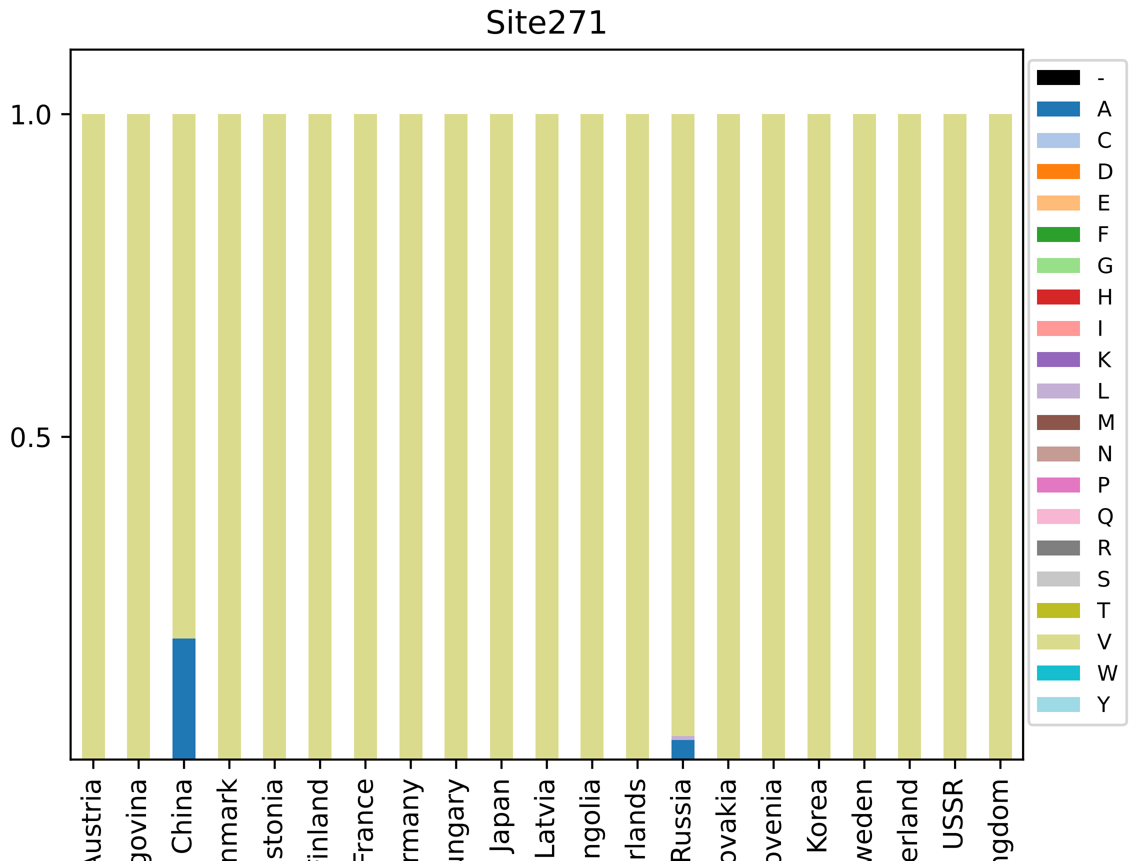

Site271
1.0
0.5
-
A
C
D
E
F
G
H
I
K
L
M
N
P
Q
R
S
T
V
W
Y
China
Japan
Latvia
Russia
Korea
USSR

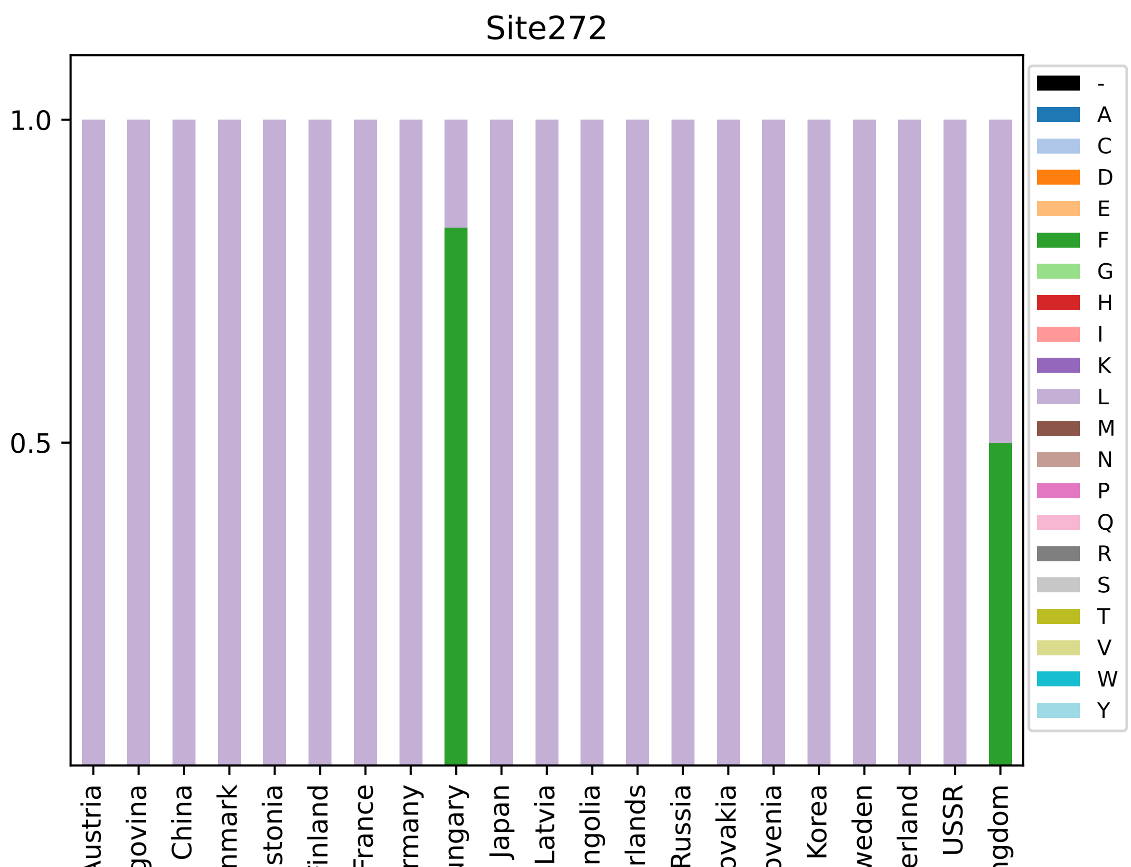
Site272
1.0
0.5
-
A
C
D
E
F
G
H
I
K
L
M
N
P
Q
R
S
T
V
W
Y
China
Japan
Latvia
Russia
Korea
USSR

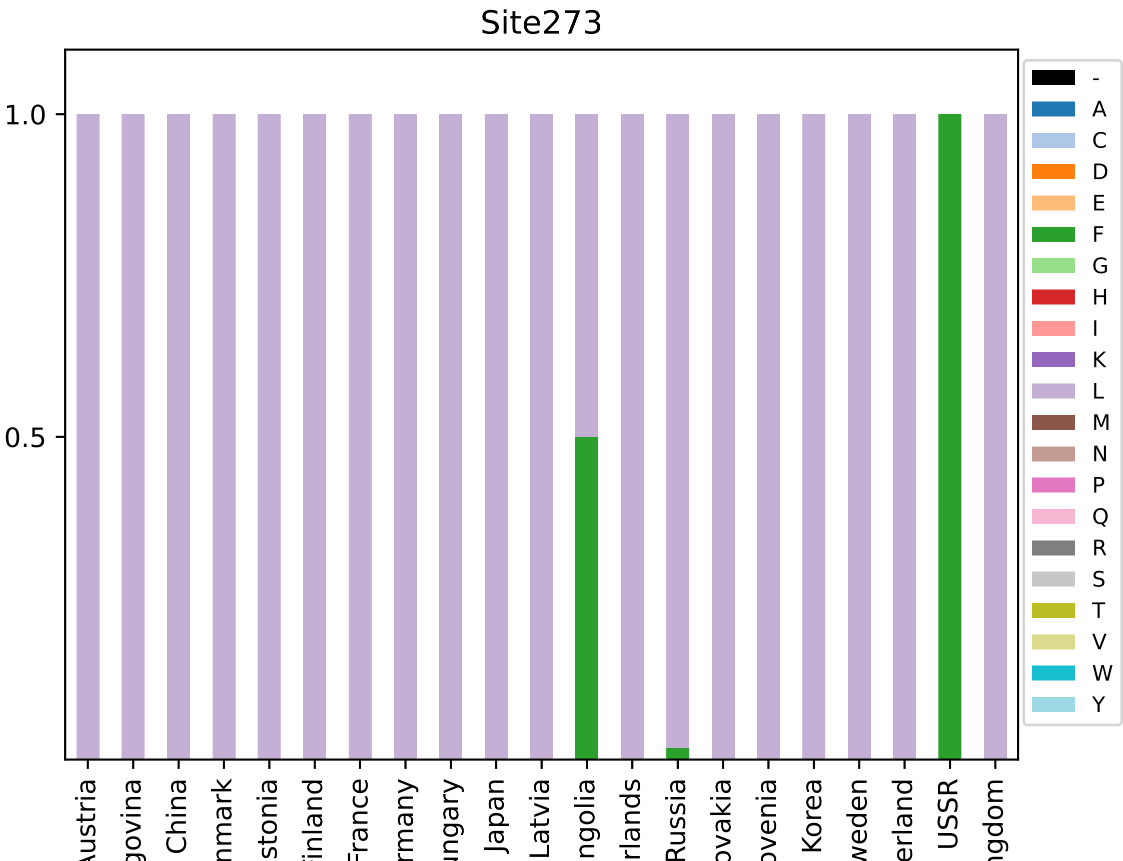

Site273
1.0
0.5
-
A
C
D
E
F
G
H
I
K
L
M
N
P
Q
R
S
T
V
W
Y
Austria
China
Japan
Latvia
Russia
Korea
USSR

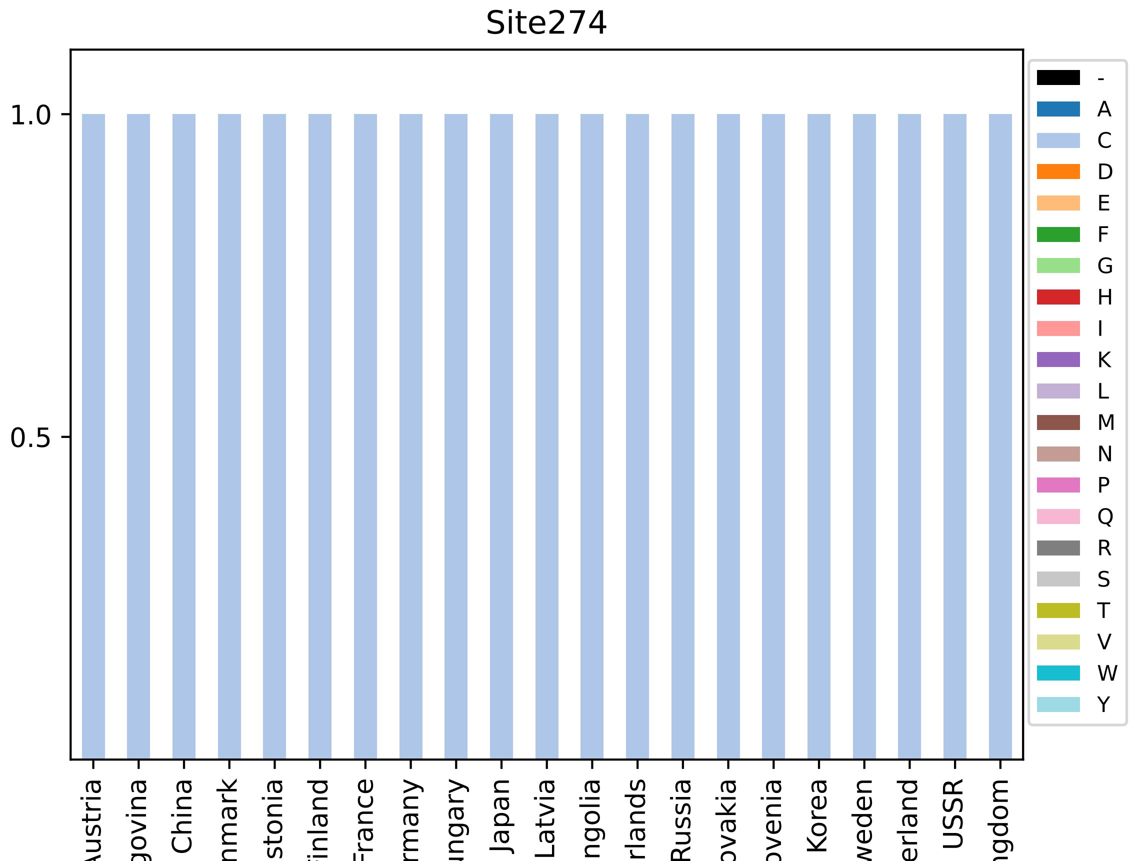

Site274
1.0
0.5
-
A
C
D
E
F
G
H
I
K
L
M
N
P
Q
R
S
T
V
W
Y
China
Japan
Latvia
Russia
Korea
USSR

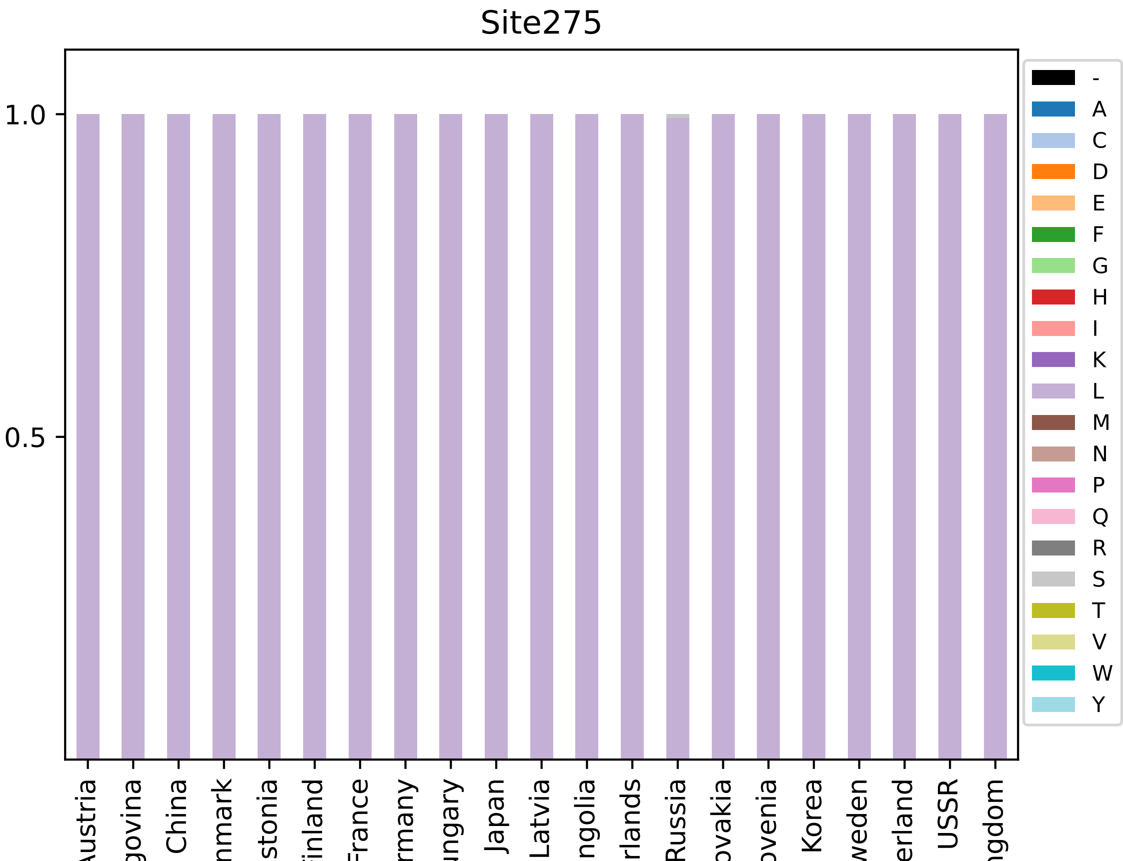
Site275
1.0
0.5
-
A
C
D
E
F
G
H
I
K
L
M
N
P
Q
R
S
T
V
W
Y
China
Japan
Latvia
Russia
Korea
USSR

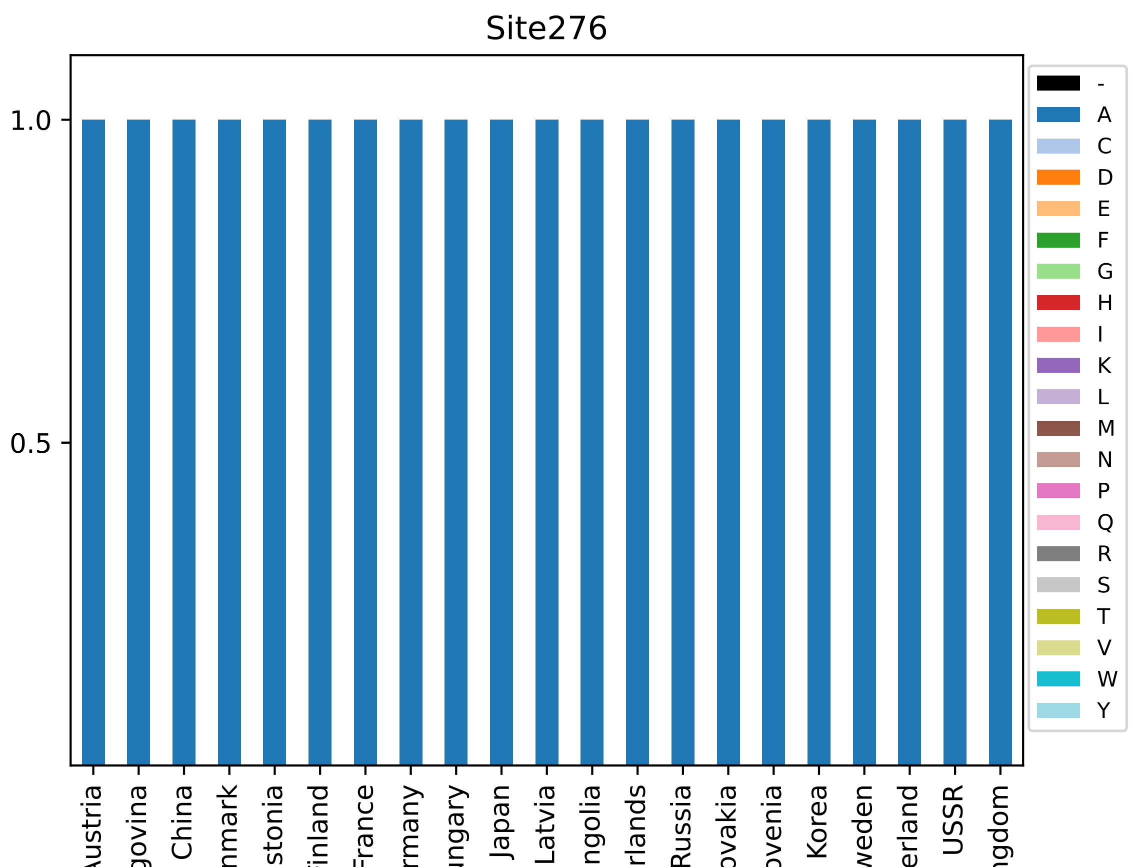

Site276
1.0
0.5
-
A
C
D
E
F
G
H
I
K
L
M
N
P
Q
R
S
T
V
W
Y
Austria
China
Japan
Latvia
Russia
Korea
USSR

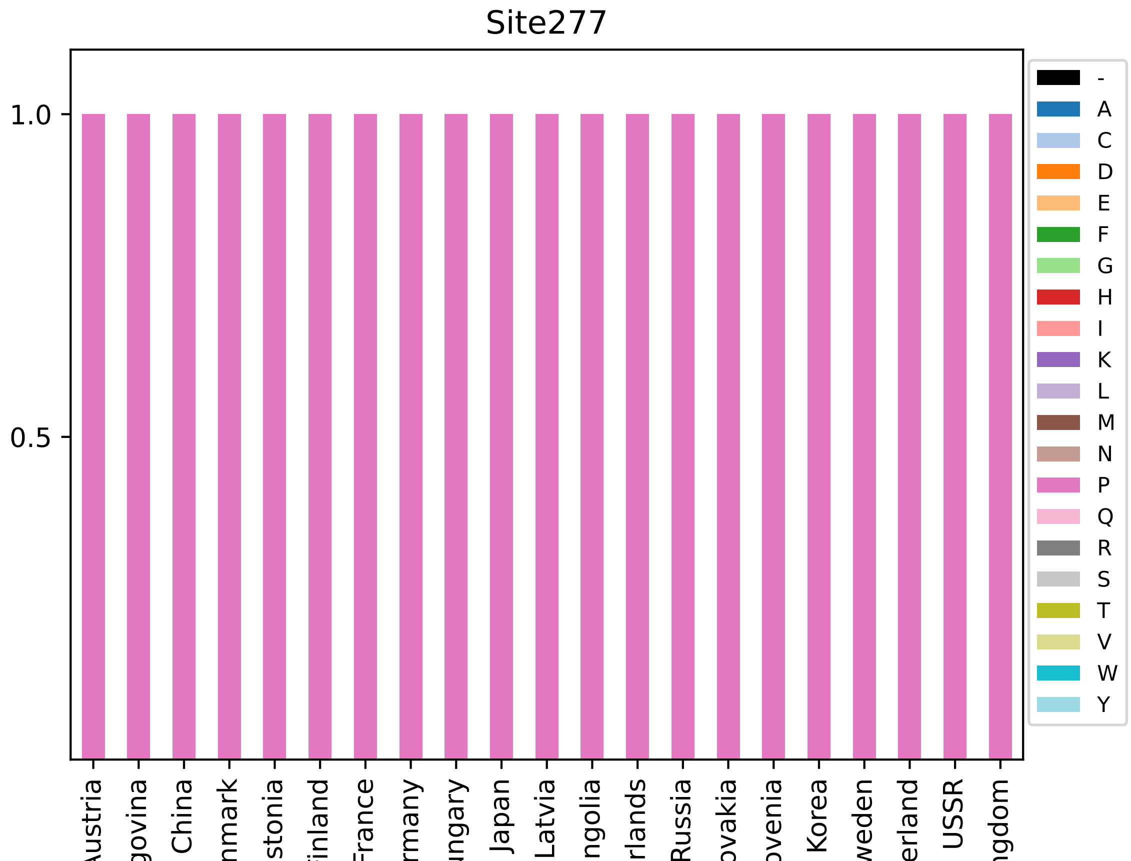
Site277
1.0
0.5
-
A
C
D
E
F
G
H
I
K
L
M
N
P
Q
R
S
T
V
W
Y
China
Japan
Latvia
Russia
Korea
USSR

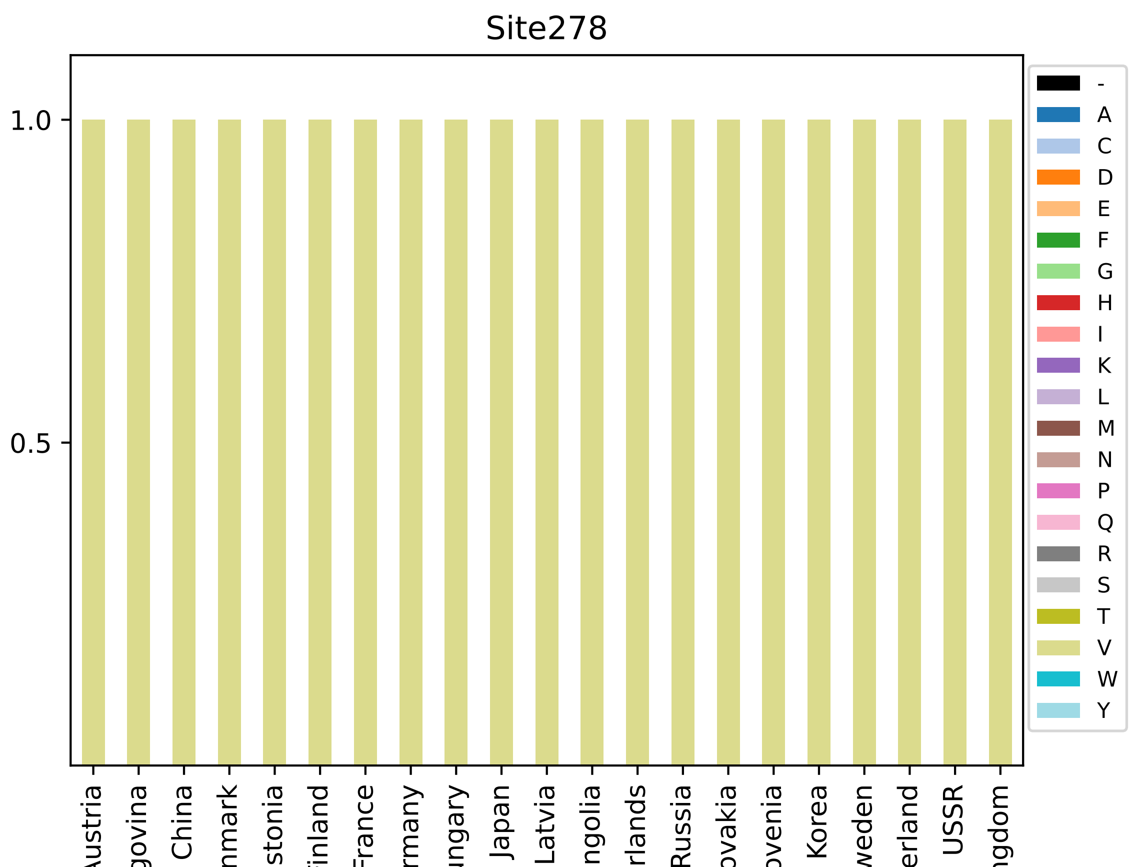
Site278
1.0
0.5
-
A
C
D
E
F
G
H
I
K
L
M
N
P
Q
R
S
T
V
W
Y
China
Japan
Latvia
Russia
Korea
USSR

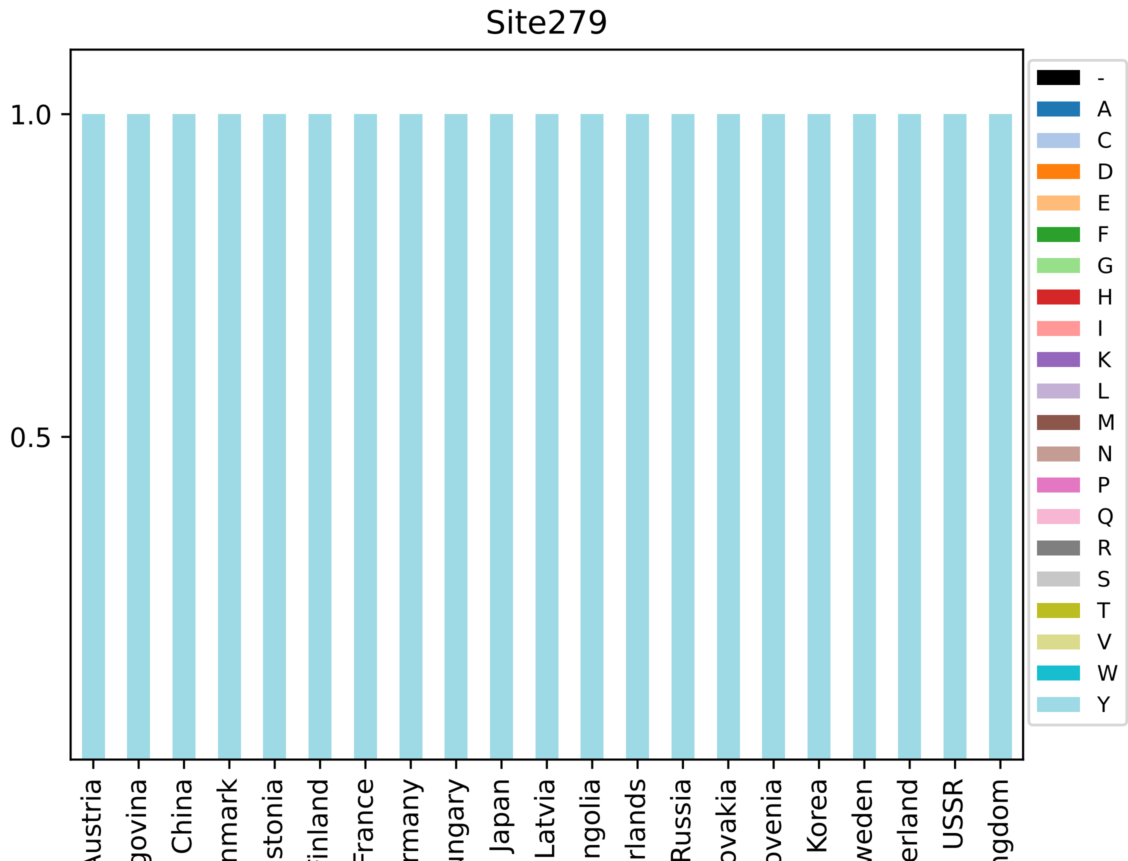

Site279
1.0
0.5
-
A
C
D
E
F
G
H
I
K
L
M
N
P
Q
R
S
T
V
W
Y
Austria
China
Japan
Latvia
Russia
Korea
USSR

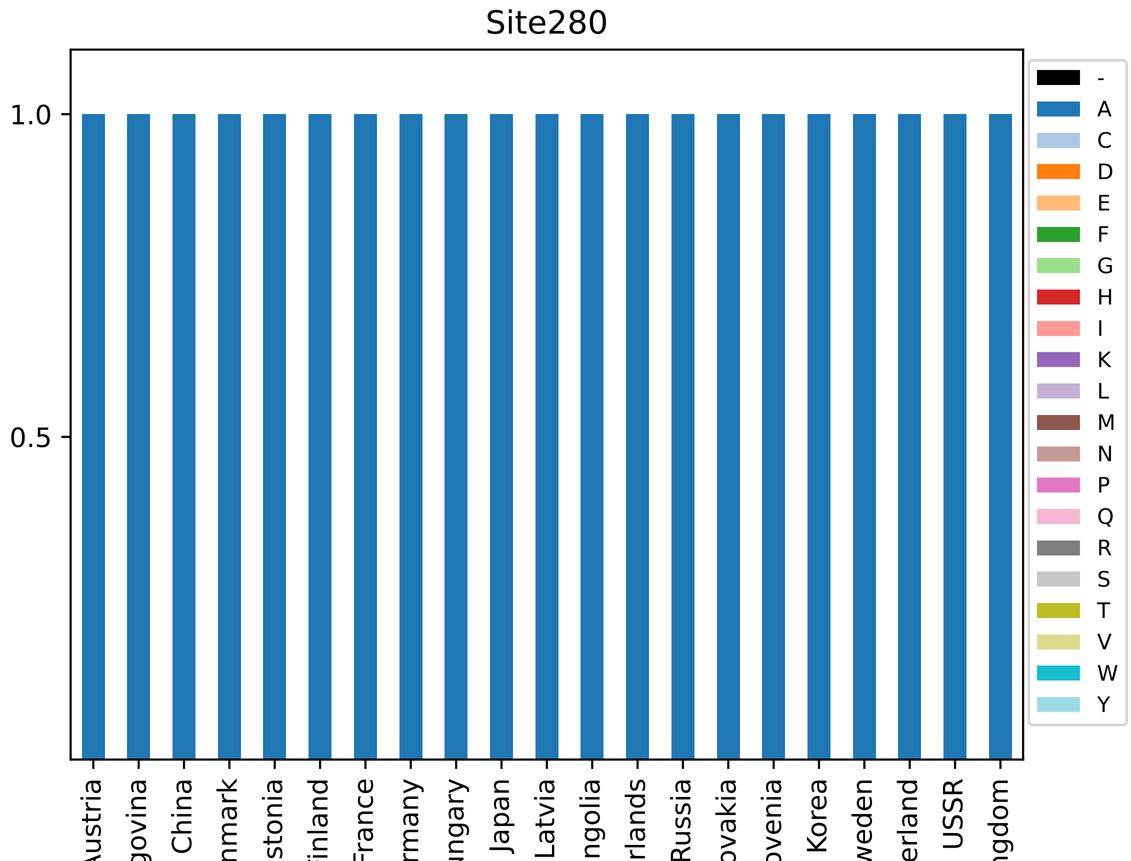

Site280
1.0
0.5
-
A
C
D
E
F
G
H
I
K
L
M
N
P
Q
R
S
T
V
W
Y
China
Japan
Latvia
Russia
Korea
USSR

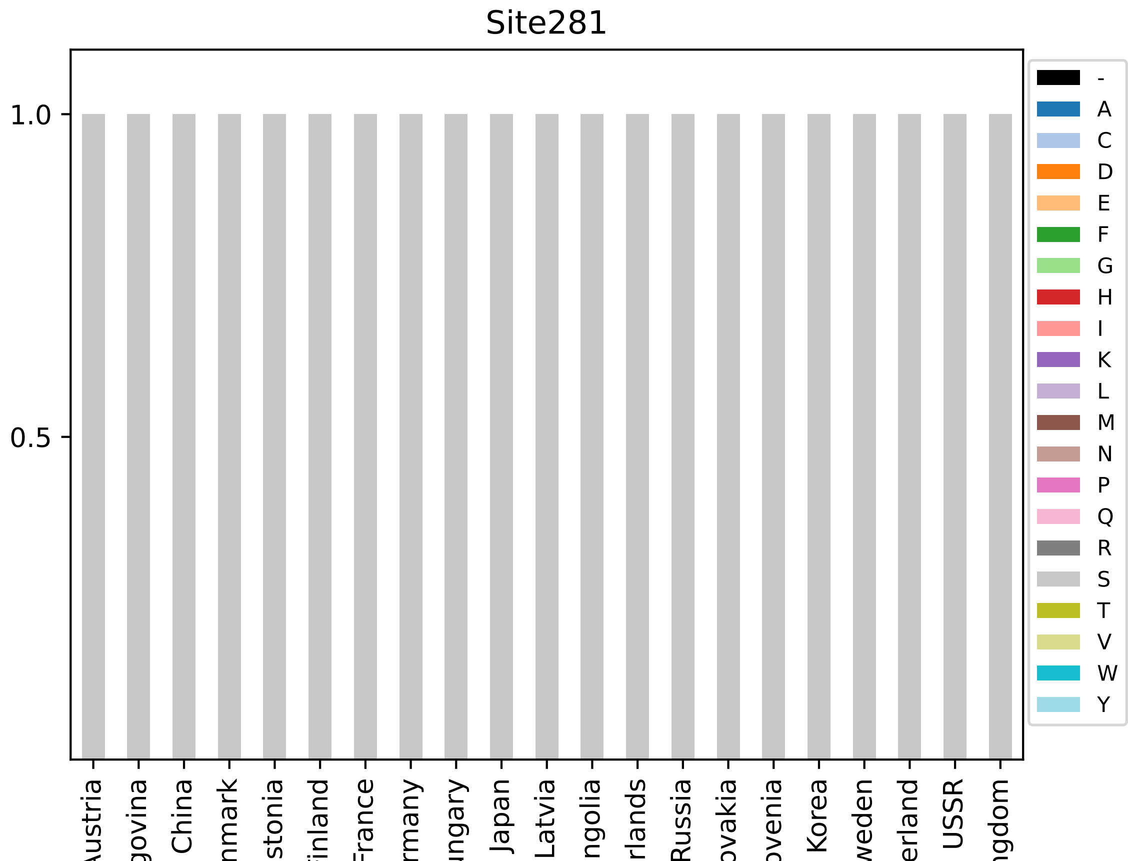

Site281
1.0
0.5
-
A
C
D
E
F
G
H
I
K
L
M
N
P
Q
R
S
T
V
W
Y
China
Japan
Latvia
Russia
Korea
USSR

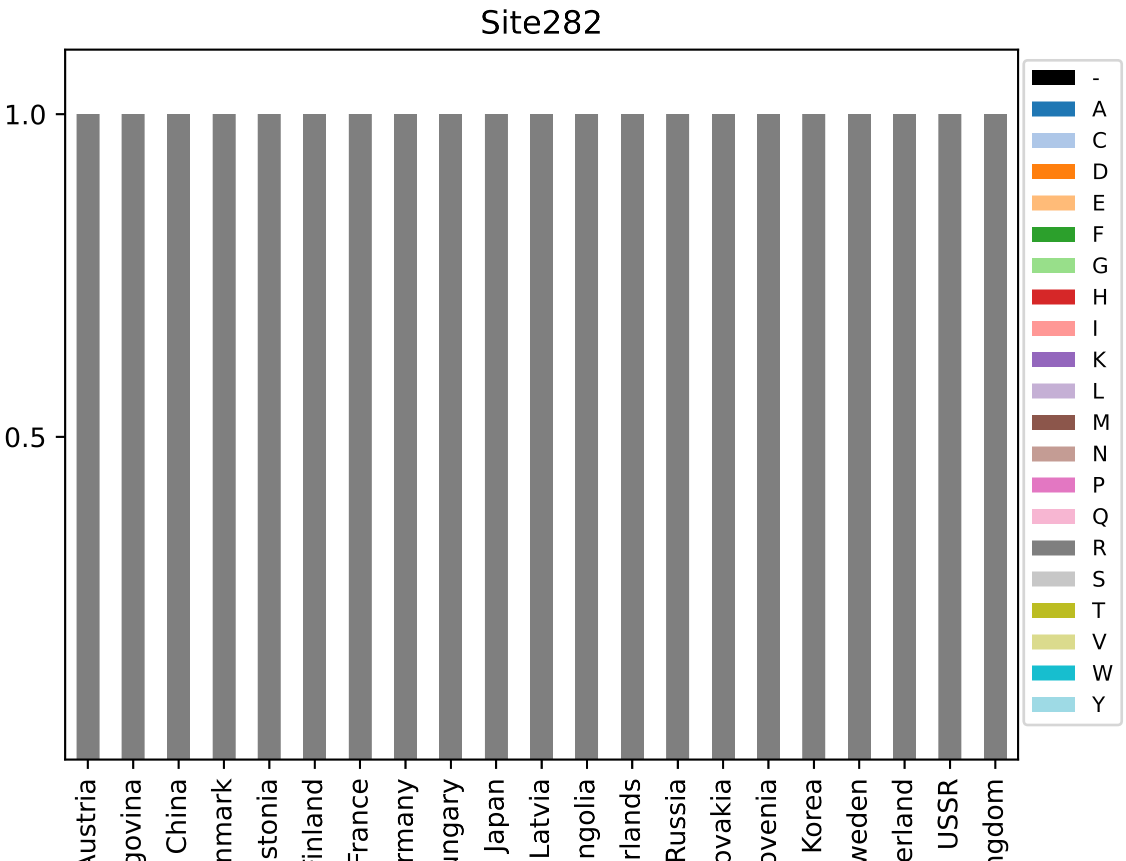
Site282
1.0
0.5
-
A
C
D
E
F
G
H
I
K
L
M
N
P
Q
R
S
T
V
W
Y
China
Japan
Latvia
Russia
Korea
USSR

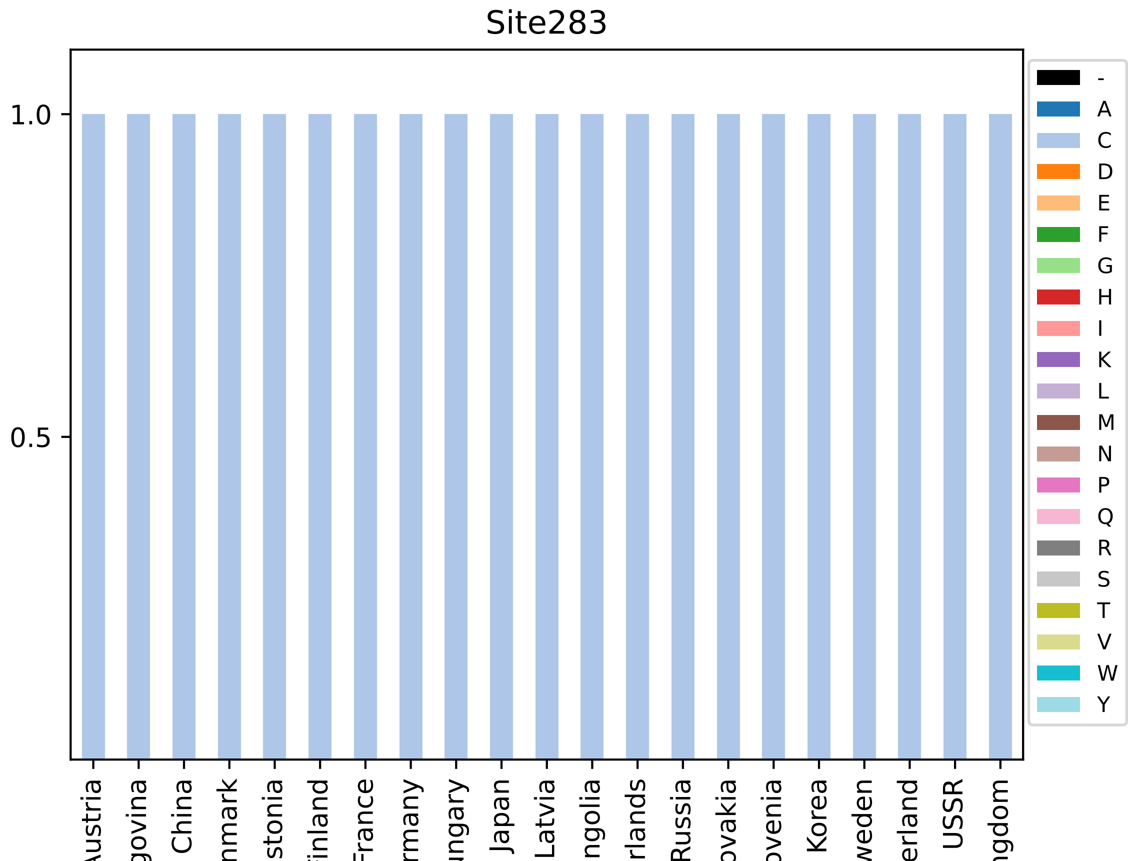

Site283
1.0
0.5
-
A
C
D
E
F
G
H
I
K
L
M
N
P
Q
R
S
T
V
W
Y
Austria
govina
China
nmark
stonia
Finland
France
rmany
ungary
Japan
Latvia
ngolia
rlands
Russia
ovakia
ovenia
Korea
weden
erland
USSR
ngdom

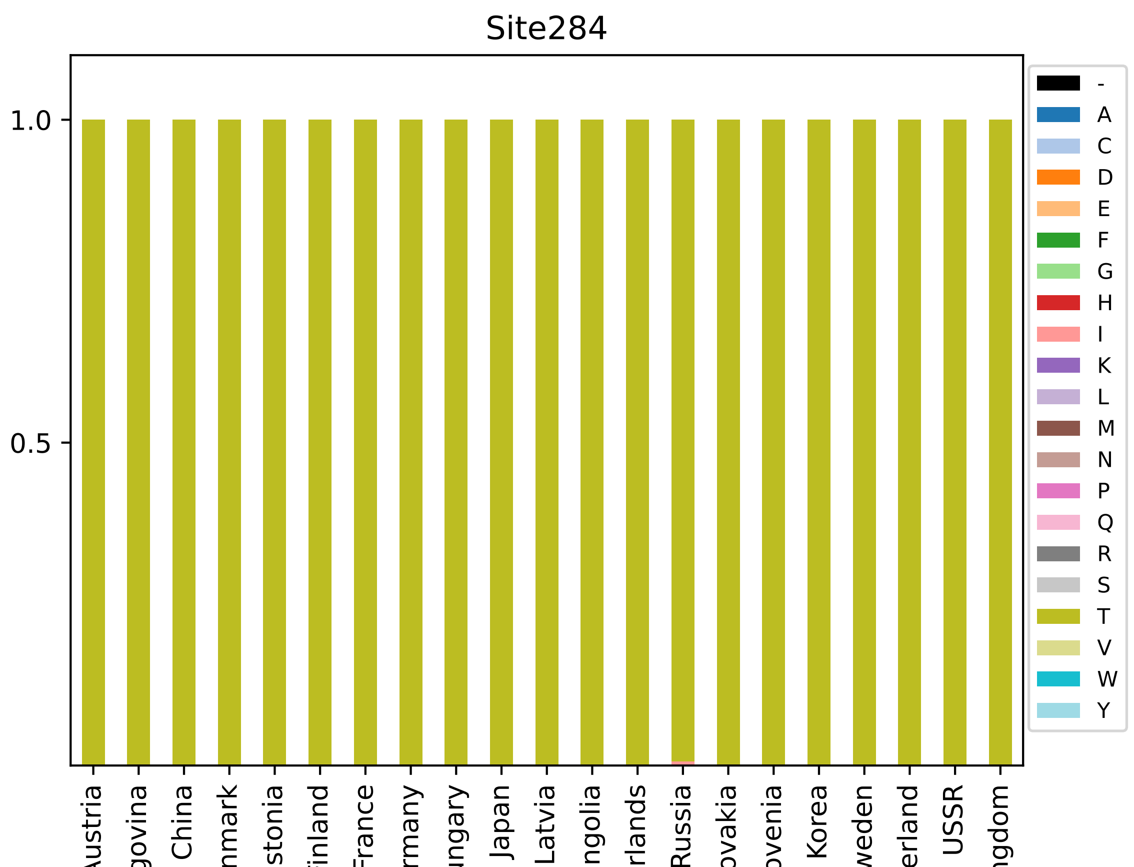

Site284
1.0
0.5
-
A
C
D
E
F
G
H
I
K
L
M
N
P
Q
R
S
T
V
W
Y
China
Japan
Latvia
Russia
Korea
USSR

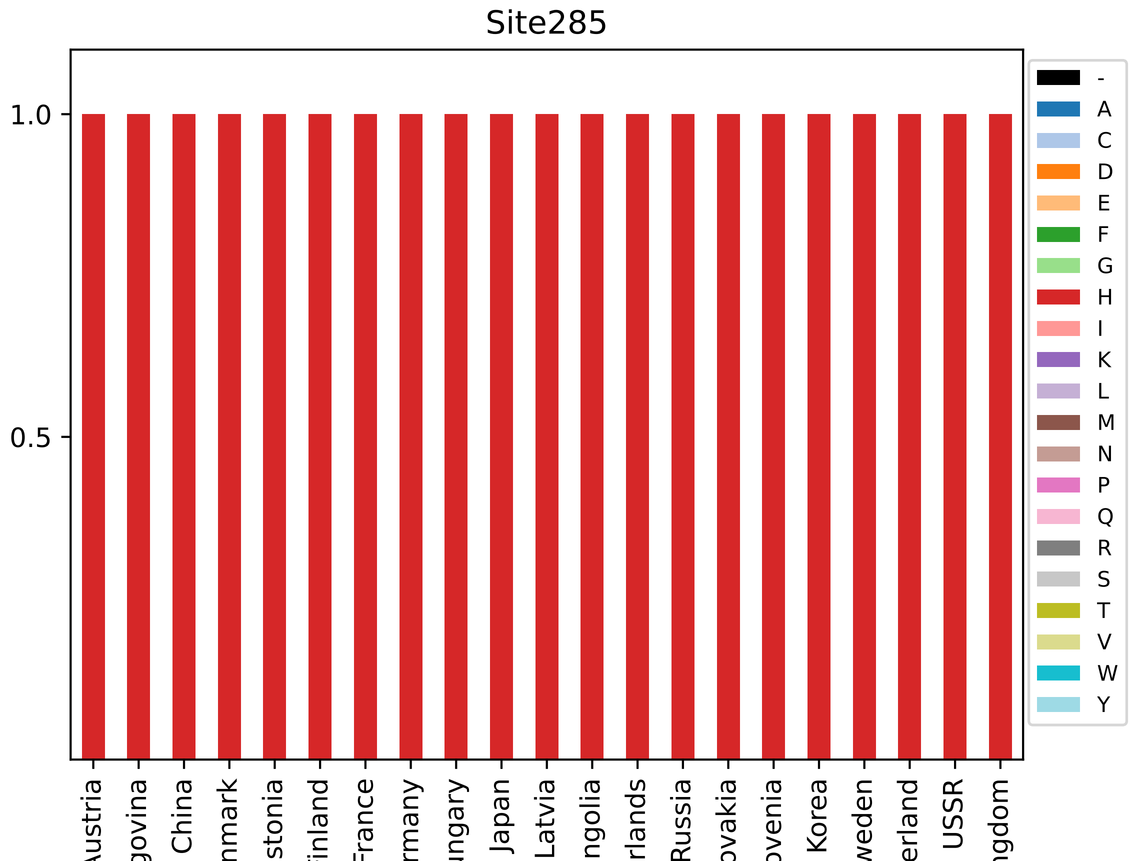
Site285
1.0
0.5
-
A
C
D
E
F
G
H
I
K
L
M
N
P
Q
R
S
T
V
W
Y
Austria
govina
China
nmark
stonia
Finland
France
rmany
ungary
Japan
Latvia
ngolia
rlands
Russia
ovakia
ovenia
Korea
weden
erland
USSR
ngdom

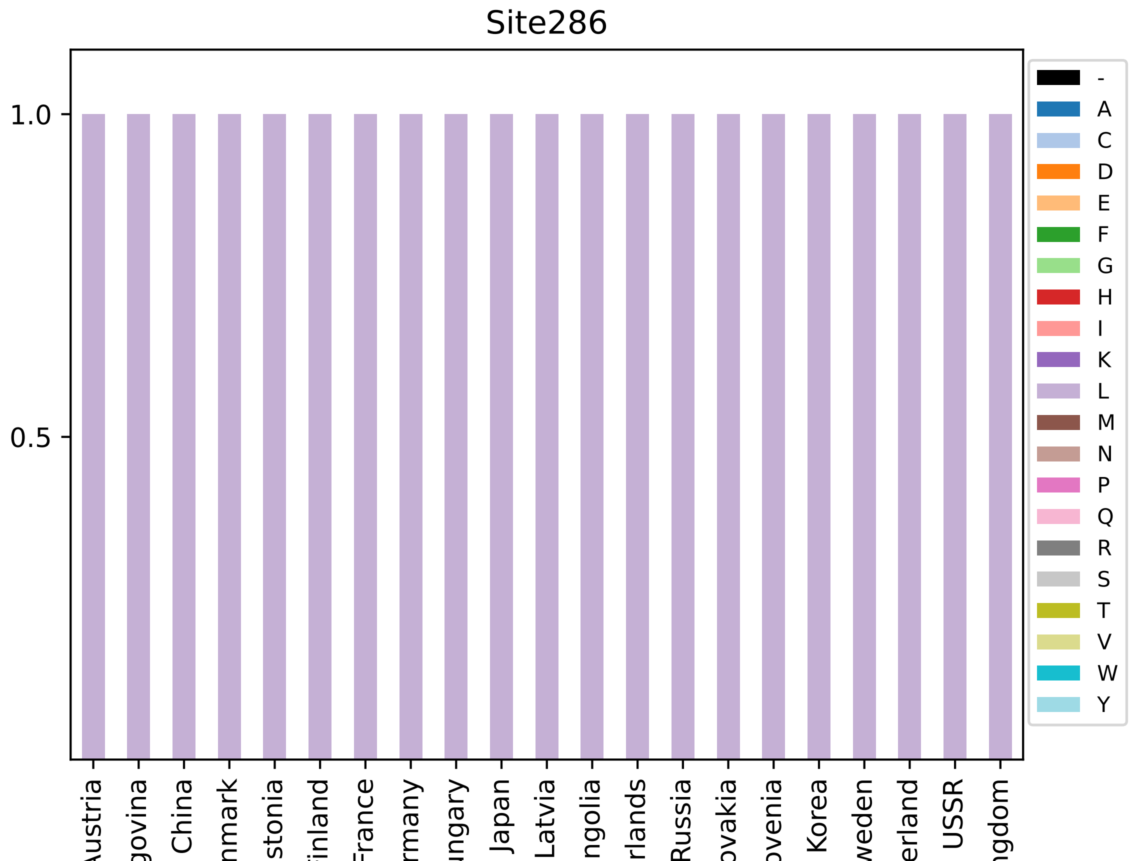

Site286
1.0
0.5
-
A
C
D
E
F
G
H
I
K
L
M
N
P
Q
R
S
T
V
W
Y
Austria
govina
China
nmark
stonia
inland
France
rmany
ungary
Japan
Latvia
ngolia
rlands
Russia
ovakia
ovenia
Korea
weden
erland
USSR
ngdom

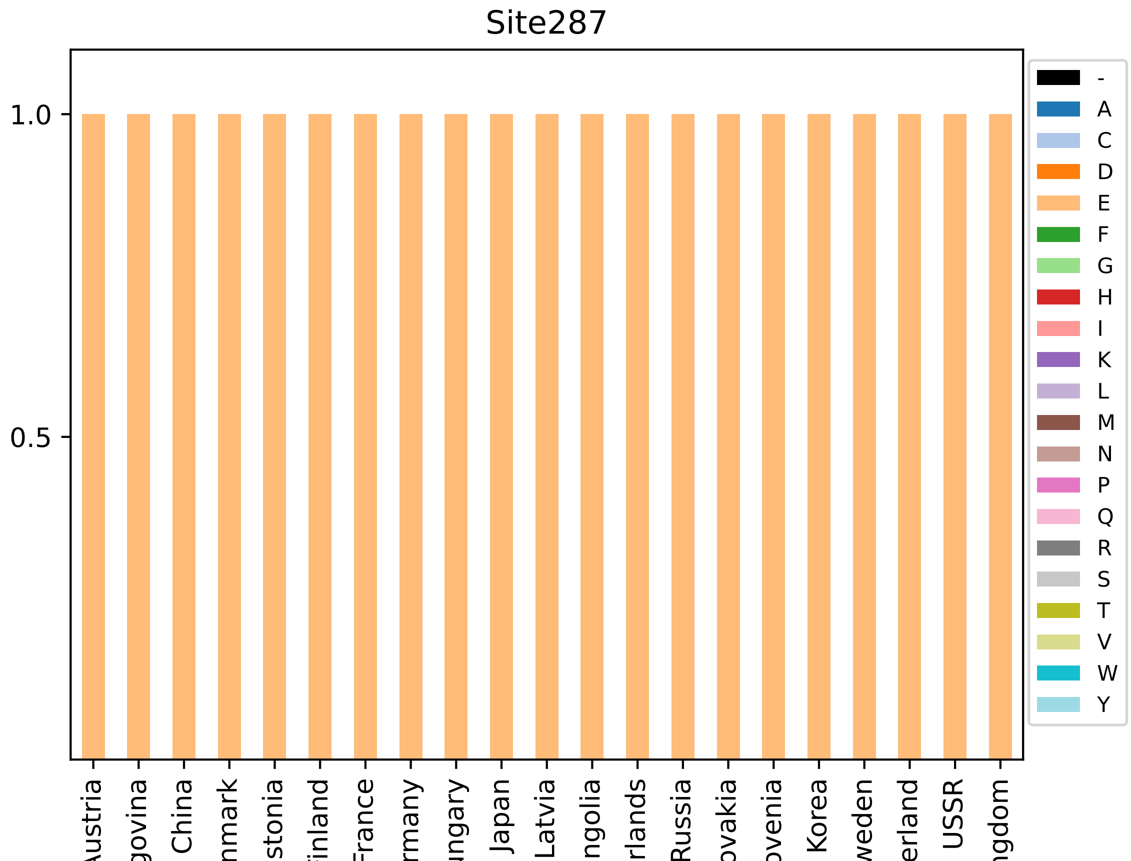

Site287
1.0
0.5
-
A
C
D
E
F
G
H
I
K
L
M
N
P
Q
R
S
T
V
W
Y
Austria
China
Japan
Latvia
Russia
Korea
USSR

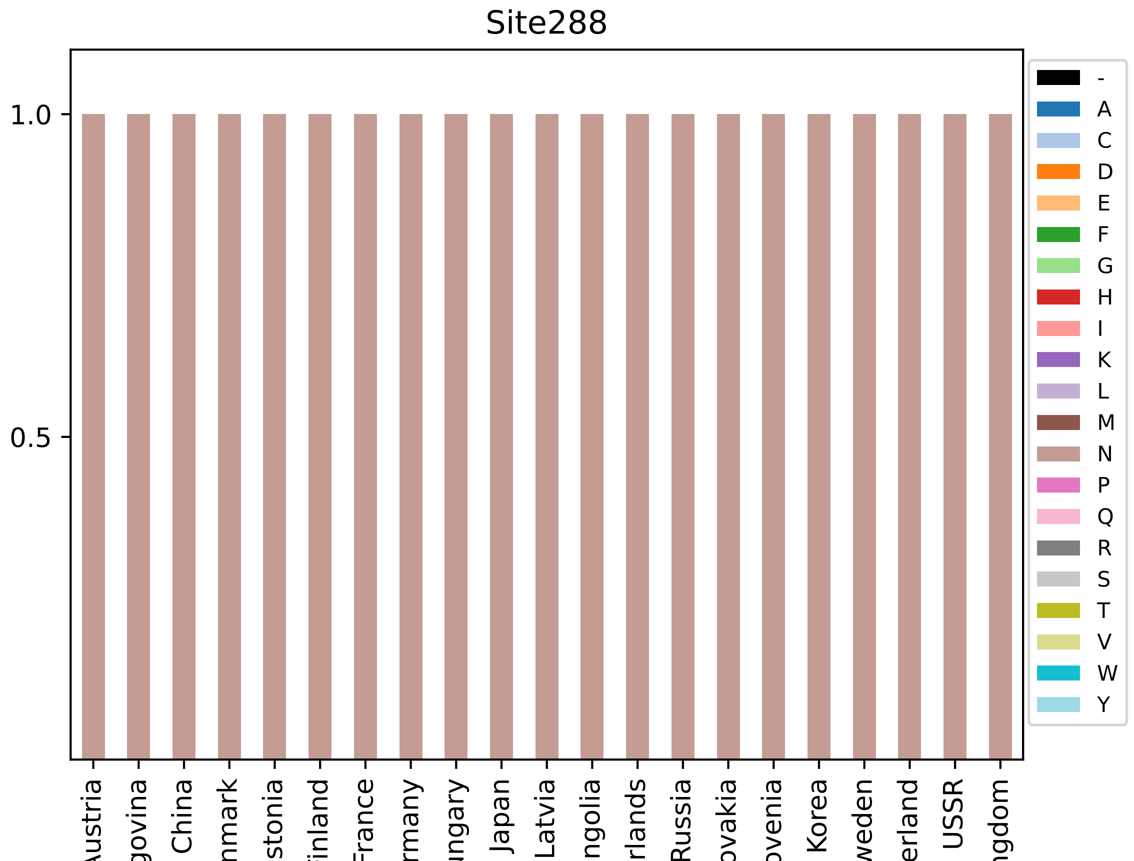
Site288
1.0
0.5
China
Japan
Latvia
Russia
Korea
USSR
-
A
C
D
E
F
G
H
I
K
L
M
N
P
Q
R
S
T
V
W
Y

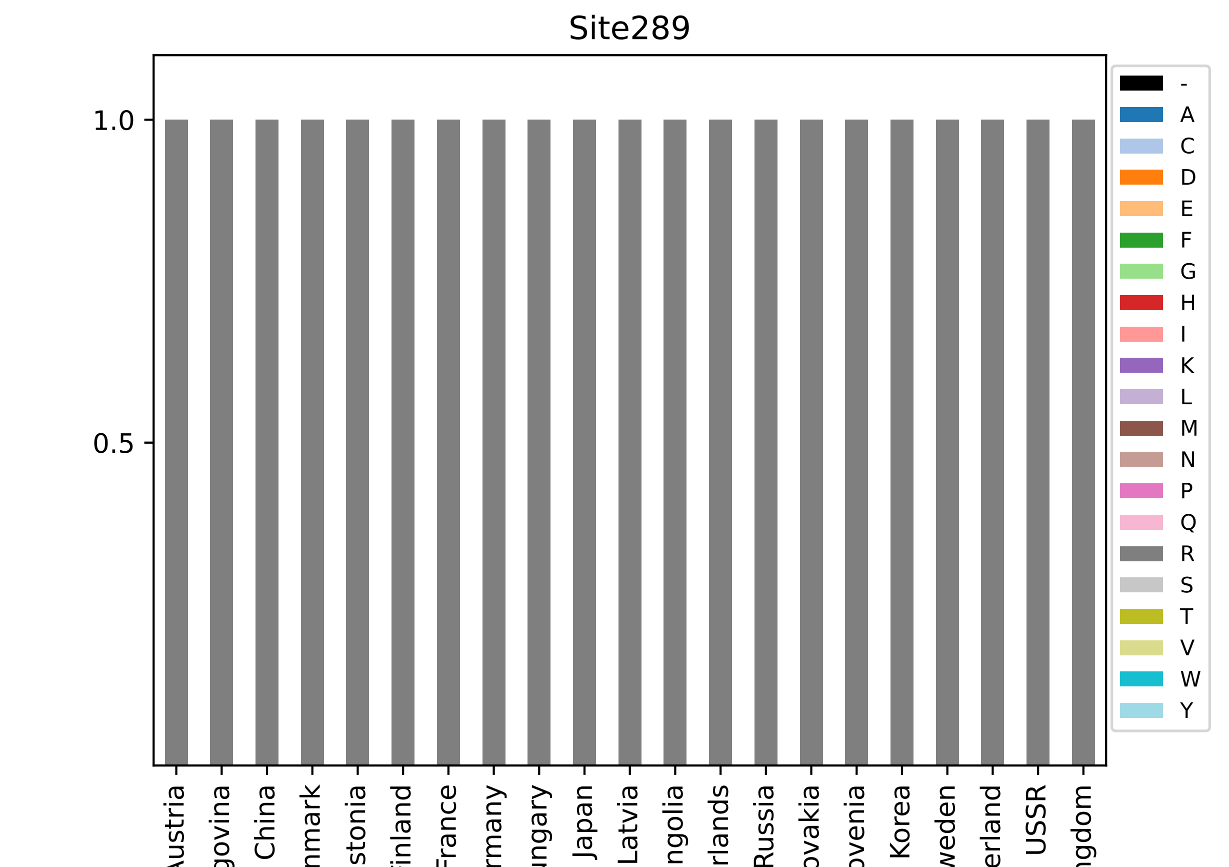

Site289
1.0
0.5
-
A
C
D
E
F
G
H
I
K
L
M
N
P
Q
R
S
T
V
W
Y
Austria
China
Finland
France
Japan
Latvia
Russia
Korea
USSR

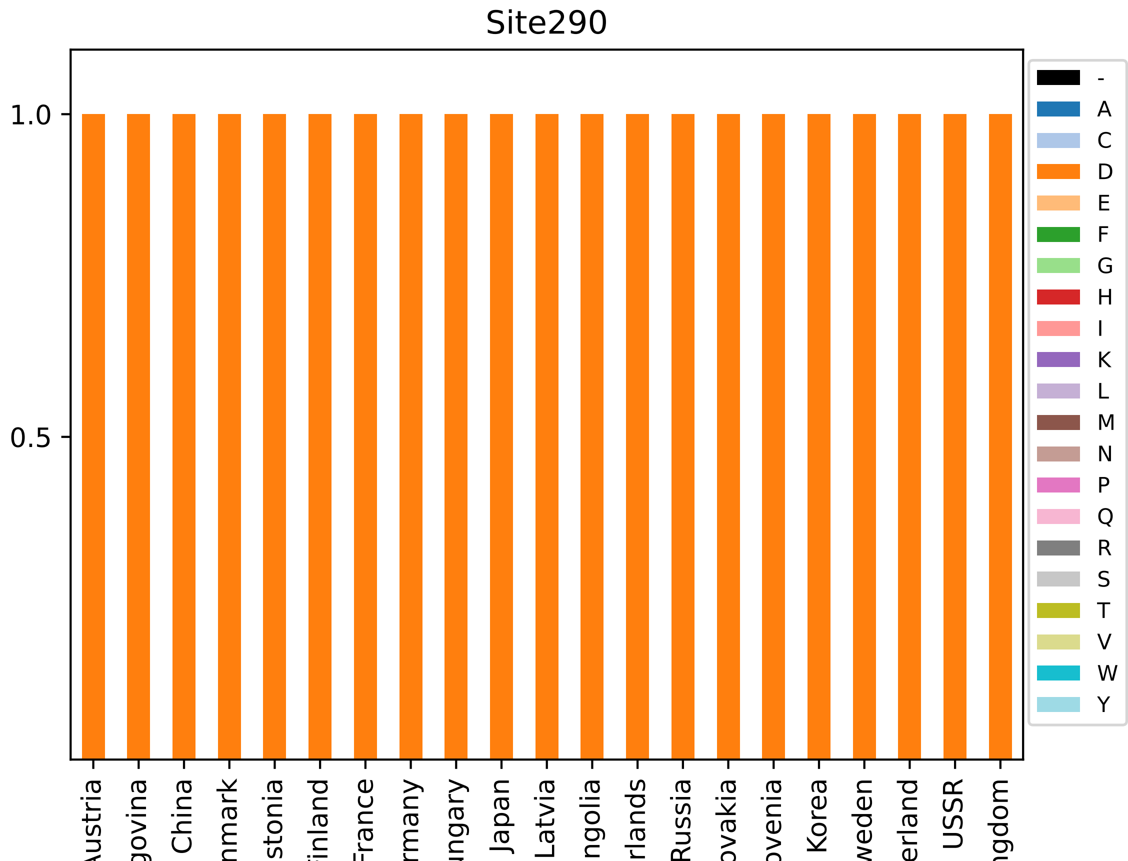

Site290
1.0
0.5
-
A
C
D
E
F
G
H
I
K
L
M
N
P
Q
R
S
T
V
W
Y
Austria
govina
China
nmark
stonia
inland
France
rmany
ungary
Japan
Latvia
ngolia
rlands
Russia
ovakia
ovenia
Korea
weden
erland
USSR
ngdom

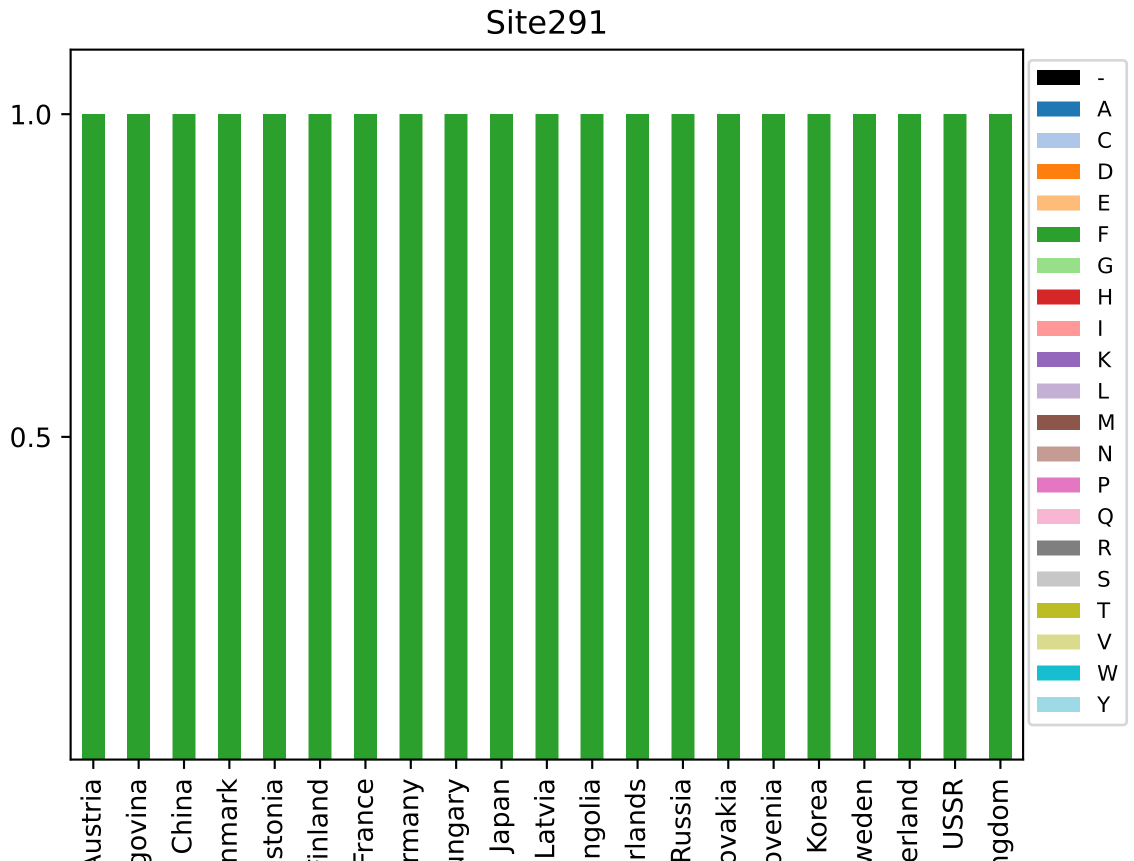

Site291
1.0
0.5
-
A
C
D
E
F
G
H
I
K
L
M
N
P
Q
R
S
T
V
W
Y
Austria
China
Japan
Latvia
Russia
Korea
USSR

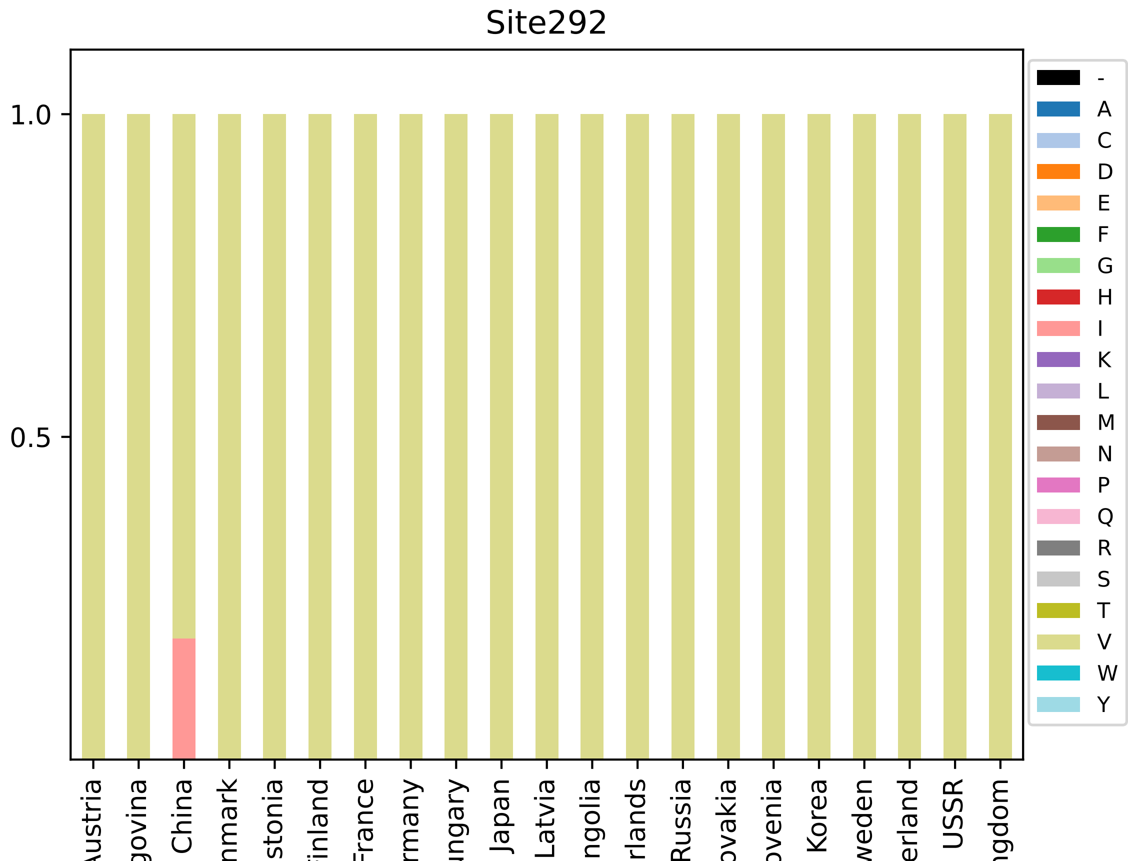
Site292
1.0
0.5
-
A
C
D
E
F
G
H
I
K
L
M
N
P
Q
R
S
T
V
W
Y
Austria
govina
China
nmark
stonia
Finland
France
rmany
ungary
Japan
Latvia
ngolia
rlands
Russia
ovakia
ovenia
Korea
weden
erland
USSR
ngdom

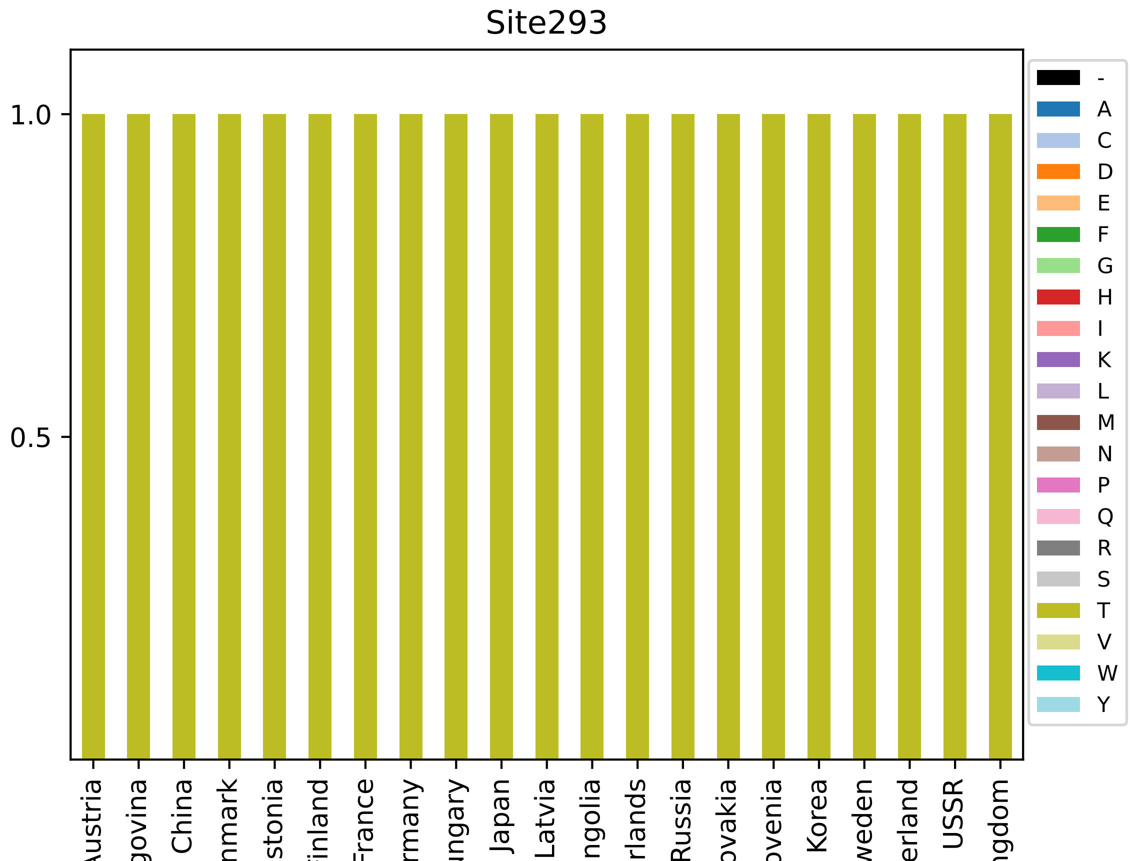
Site293
1.0
0.5
-
A
C
D
E
F
G
H
I
K
L
M
N
P
Q
R
S
T
V
W
Y
China
Japan
Latvia
Russia
Korea
USSR

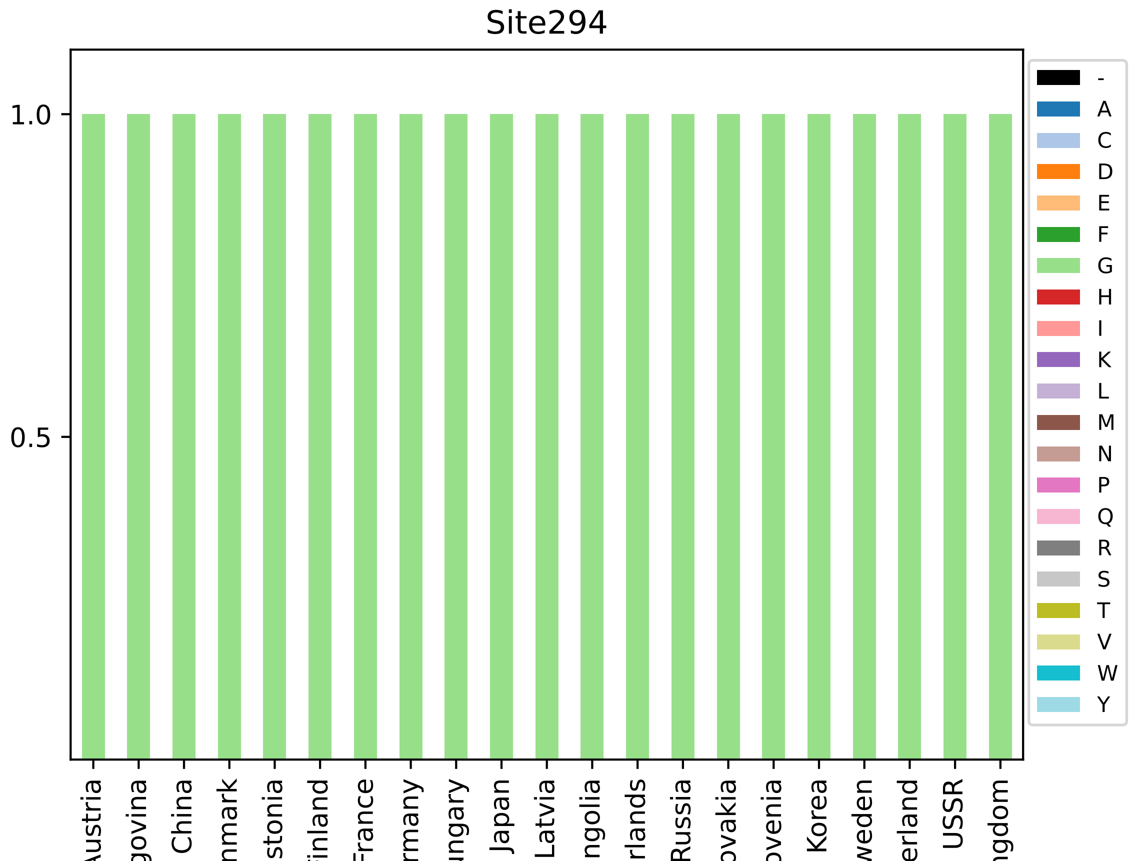
Site294
1.0
0.5
-
A
C
D
E
F
G
H
I
K
L
M
N
P
Q
R
S
T
V
W
Y
China
Japan
Latvia
Russia
Korea
USSR

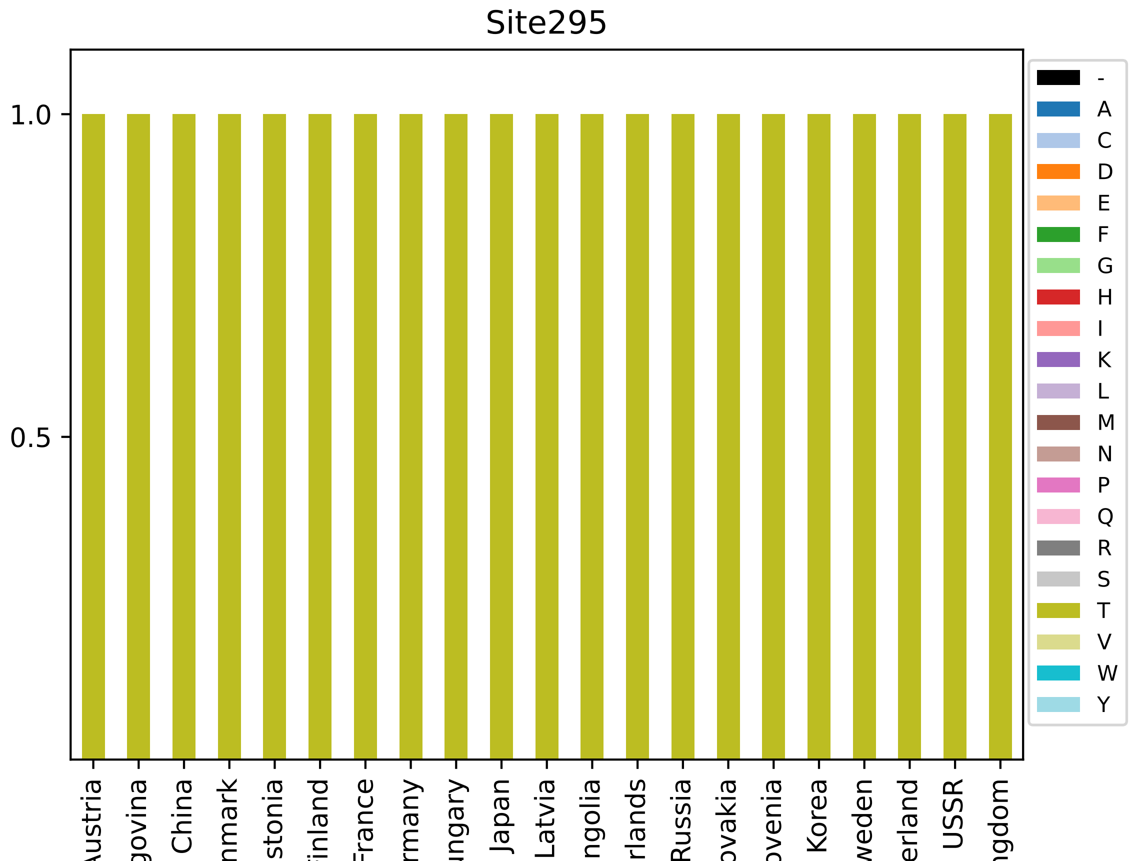
Site295
1.0
0.5
-
A
C
D
E
F
G
H
I
K
L
M
N
P
Q
R
S
T
V
W
Y
China
Japan
Latvia
Russia
Korea
USSR

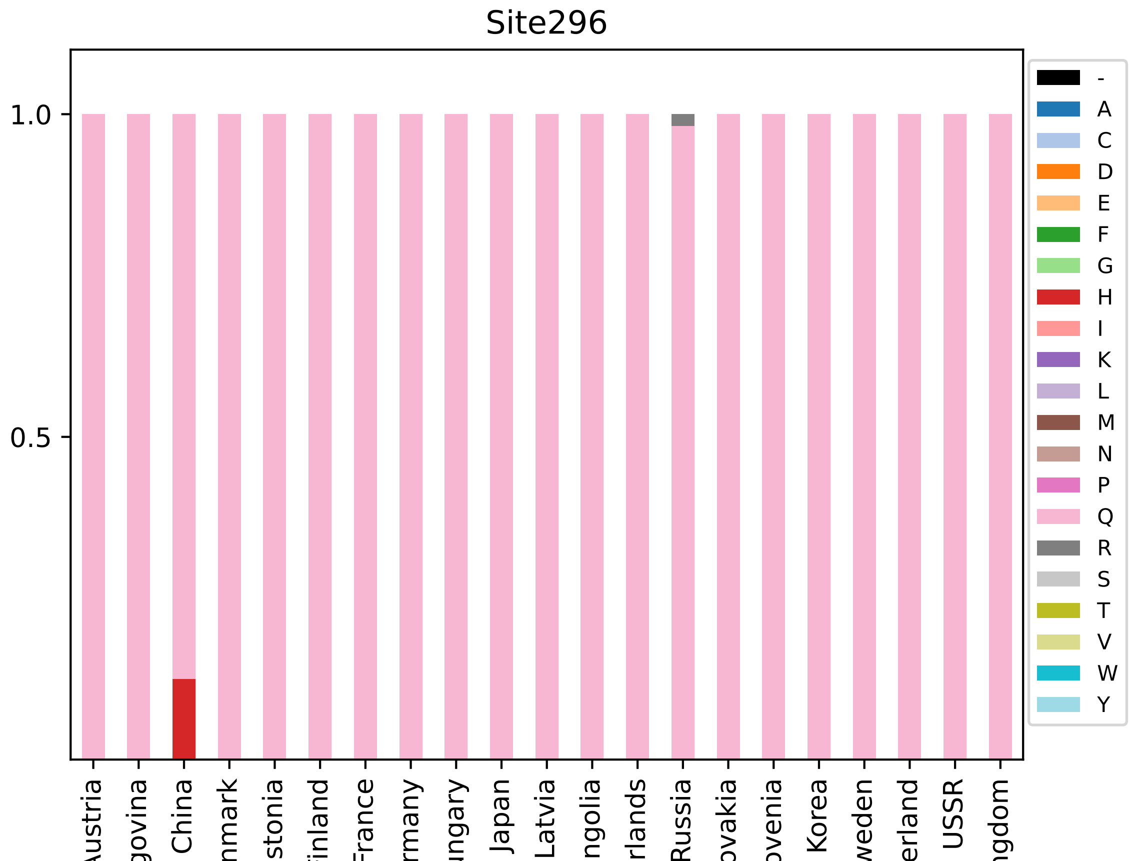

Site296
1.0
0.5
-
A
C
D
E
F
G
H
I
K
L
M
N
P
Q
R
S
T
V
W
Y
China
Japan
Latvia
Russia
Korea
USSR

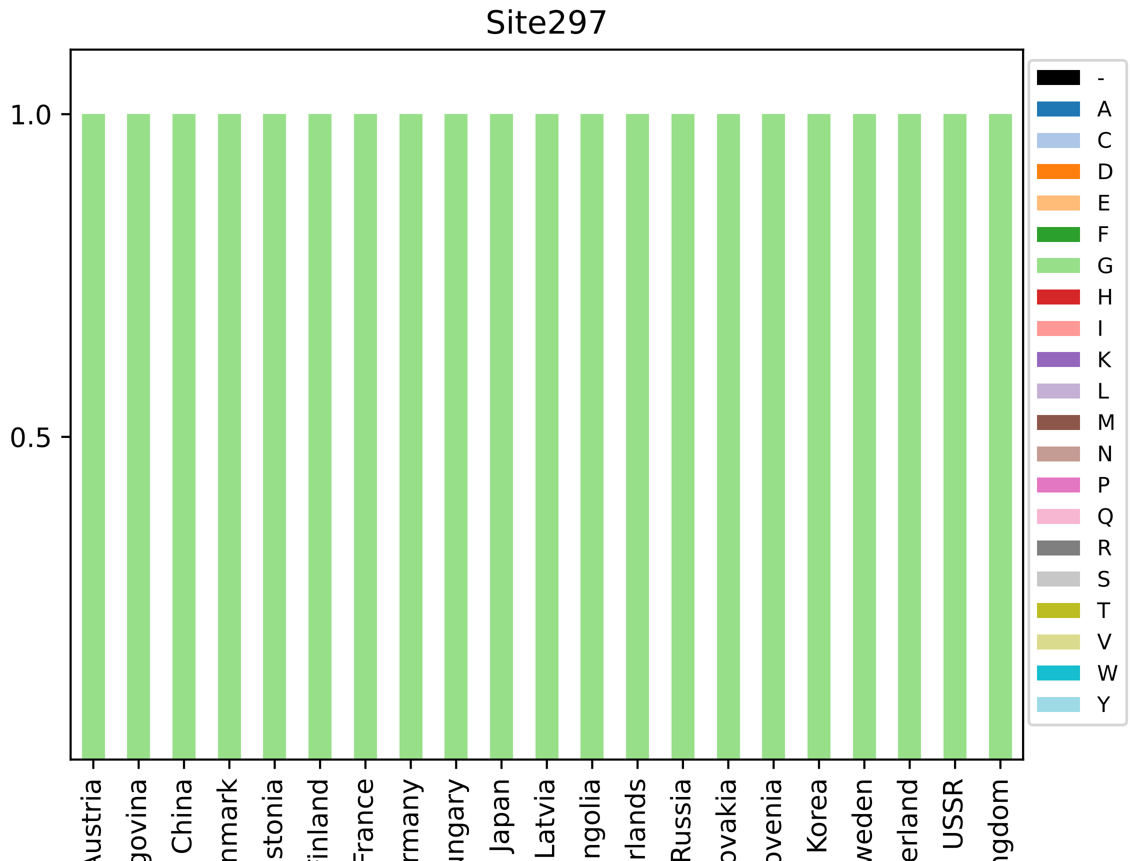

Site297
1.0
0.5
-
A
C
D
E
F
G
H
I
K
L
M
N
P
Q
R
S
T
V
W
Y
Austria
govina
China
nmark
stonia
inland
France
rmany
ungary
Japan
Latvia
ngolia
rlands
Russia
ovakia
ovenia
Korea
weden
erland
USSR
ngdom

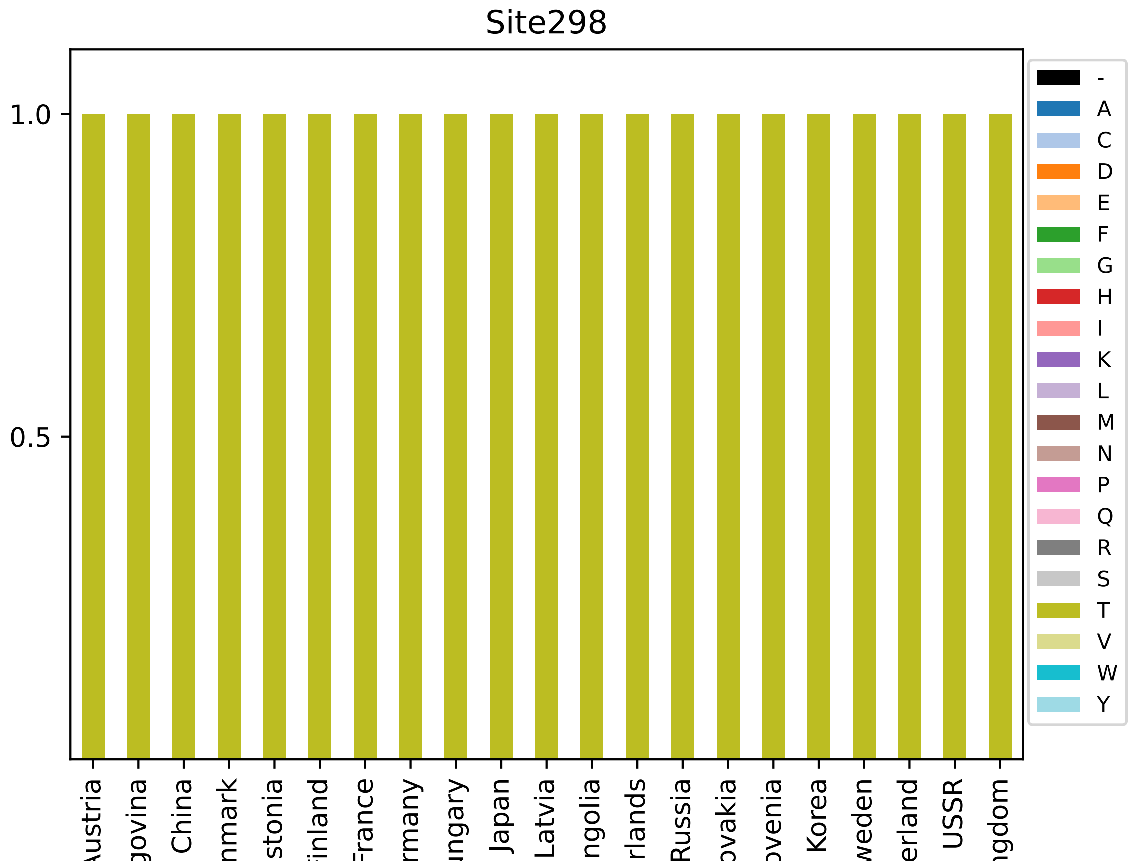

Site298
1.0
0.5
-
A
C
D
E
F
G
H
I
K
L
M
N
P
Q
R
S
T
V
W
Y
Austria
govina
China
nmark
stonia
Finland
France
rmany
ungary
Japan
Latvia
ngolia
rlands
Russia
ovakia
ovenia
Korea
weden
erland
USSR
ngdom

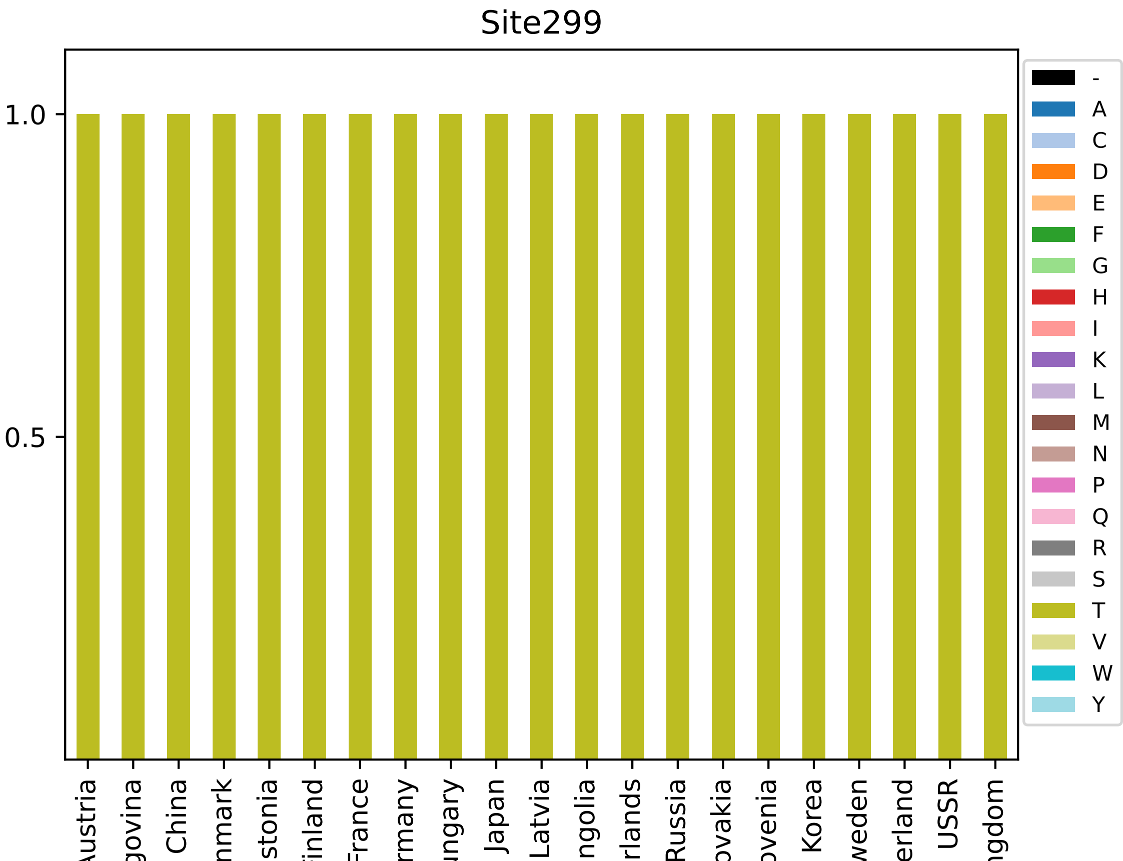
Site299
1.0
0.5
-
A
C
D
E
F
G
H
I
K
L
M
N
P
Q
R
S
T
V
W
Y
China
Japan
Latvia
Russia
Korea
USSR

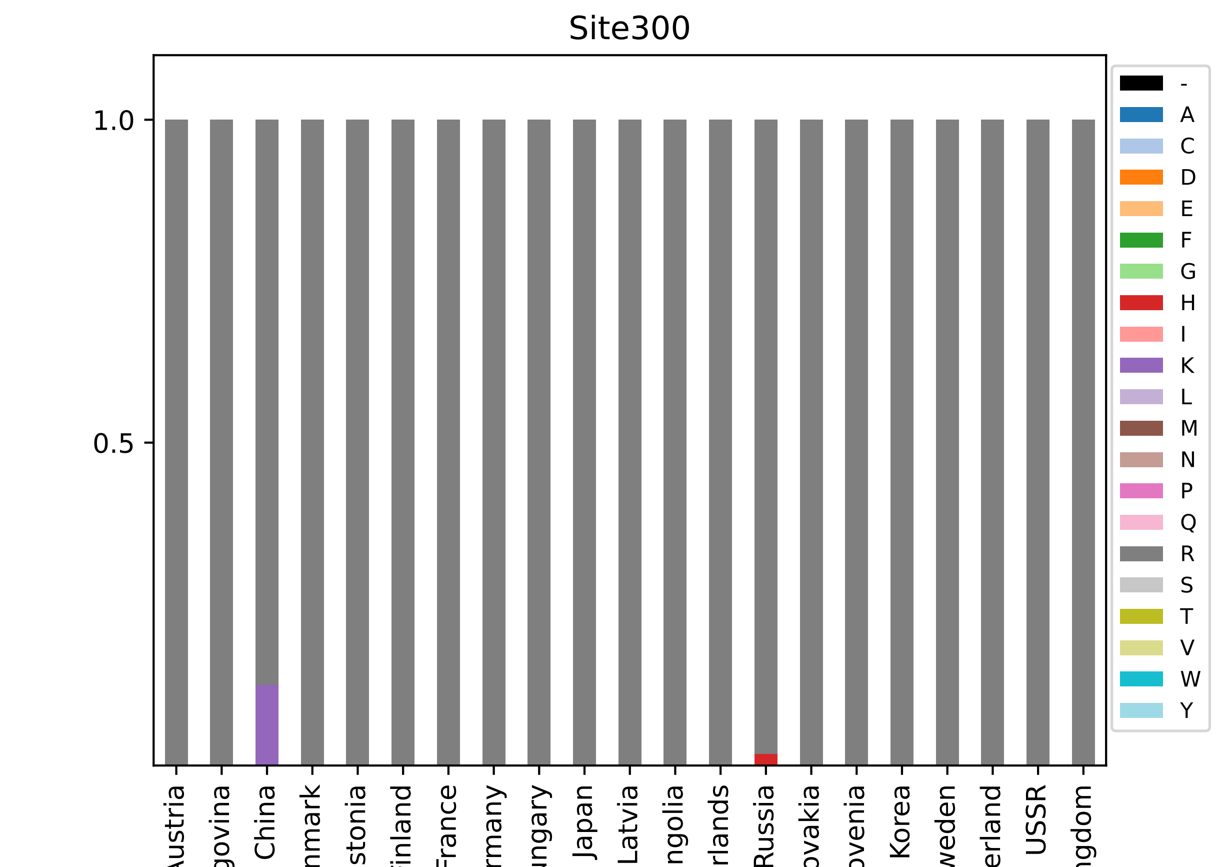
Site300
1.0
0.5
-
A
C
D
E
F
G
H
I
K
L
M
N
P
Q
R
S
T
V
W
Y
Austria
govina
China
nmark
stonia
Finland
France
rmany
ungary
Japan
Latvia
ngolia
rlands
Russia
ovakia
ovenia
Korea
weden
erland
USSR
ngdom

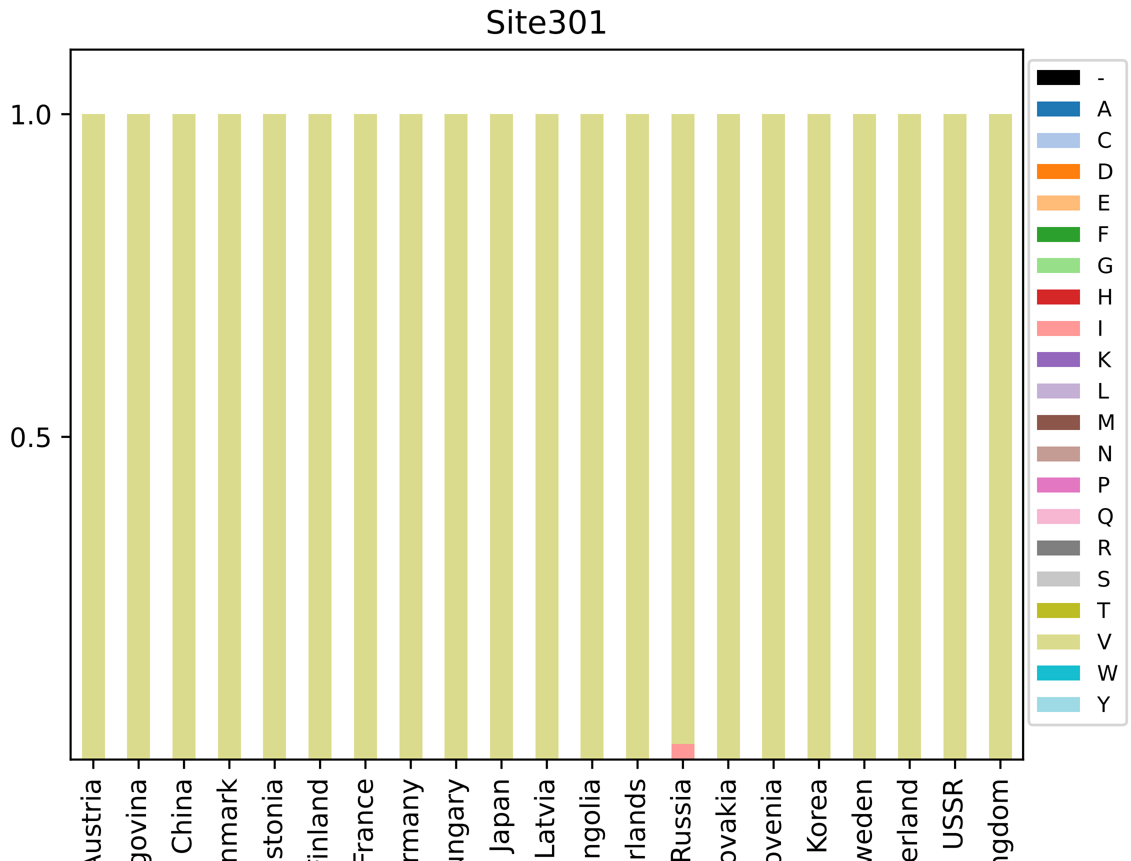

Site301
1.0
0.5
-
A
C
D
E
F
G
H
I
K
L
M
N
P
Q
R
S
T
V
W
Y
China
Japan
Latvia
Russia
Korea
USSR

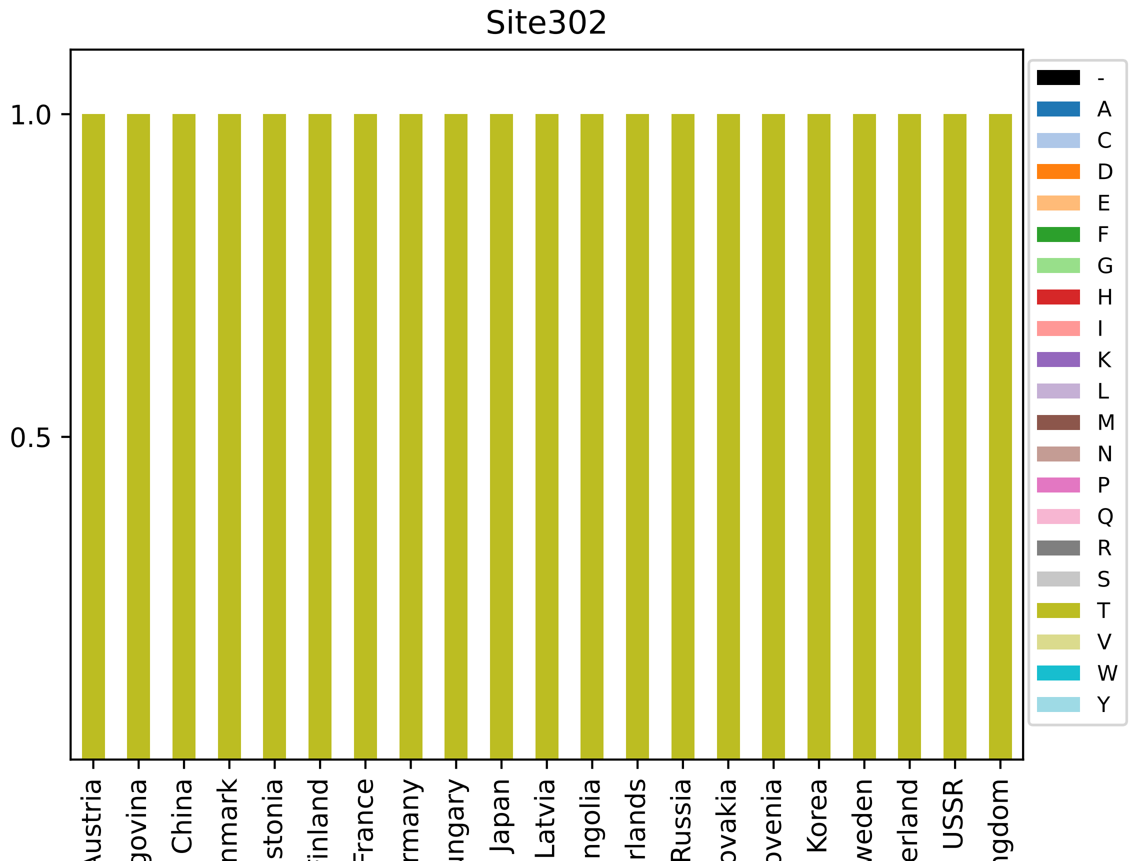

Site302
1.0
0.5
-
A
C
D
E
F
G
H
I
K
L
M
N
P
Q
R
S
T
V
W
Y
Austria
govina
China
nmark
stonia
inland
France
rmany
ungary
Japan
Latvia
ngolia
rlands
Russia
ovakia
ovenia
Korea
weden
erland
USSR
ngdom

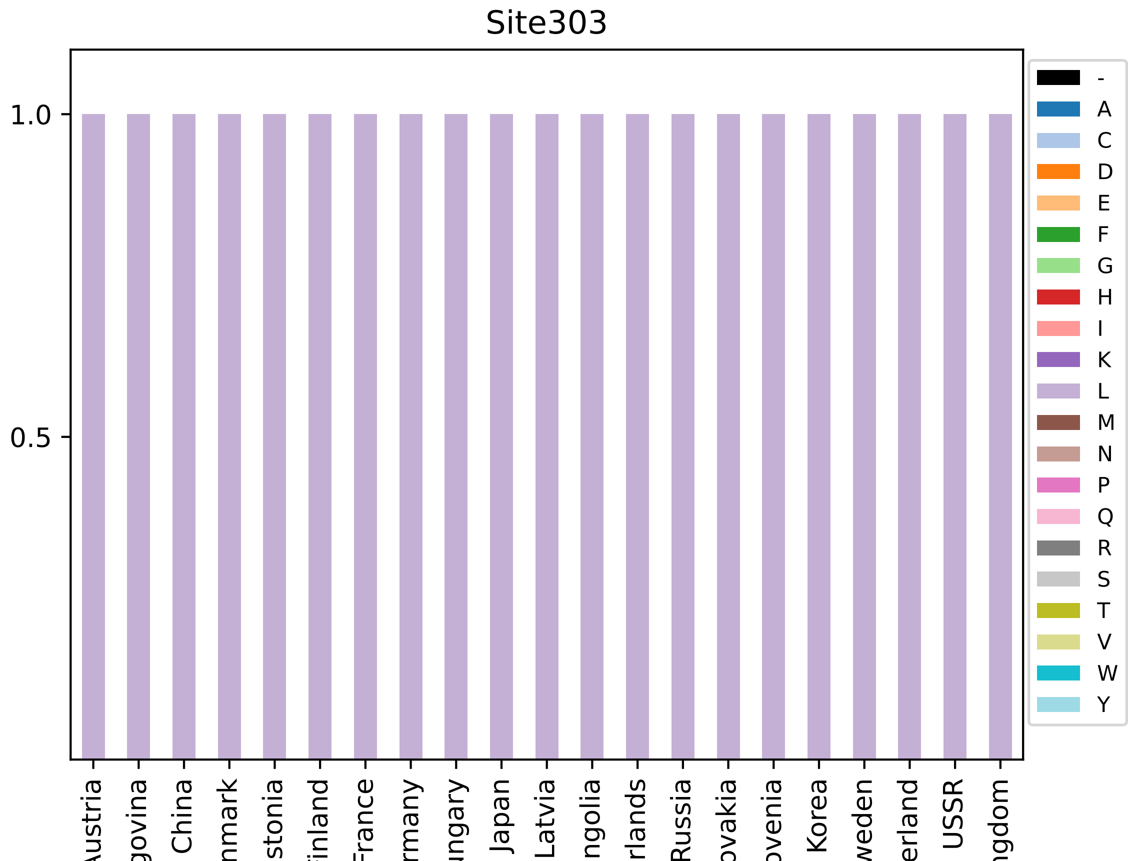
Site303
1.0
0.5
-
A
C
D
E
F
G
H
I
K
L
M
N
P
Q
R
S
T
V
W
Y
China
Japan
Latvia
Russia
Korea
USSR

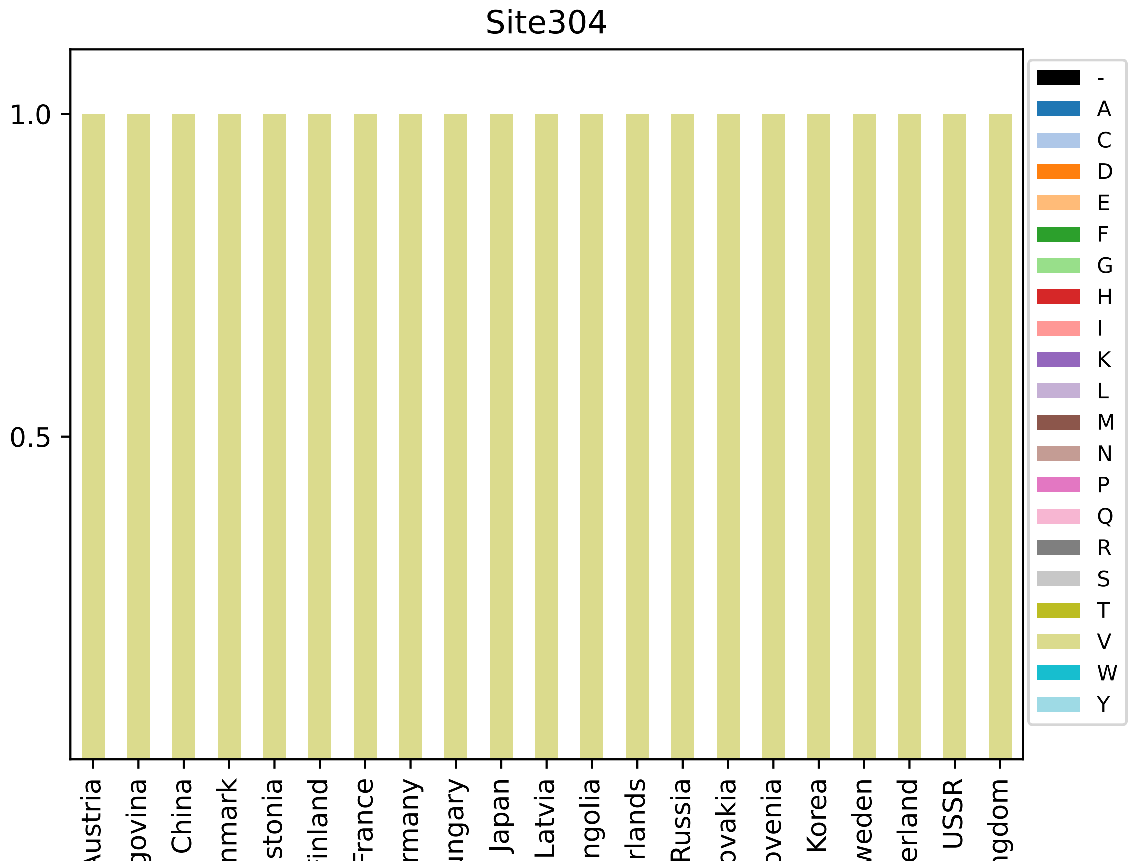
Site304
1.0
0.5
-
A
C
D
E
F
G
H
I
K
L
M
N
P
Q
R
S
T
V
W
Y
China
Japan
Latvia
Russia
Korea
USSR

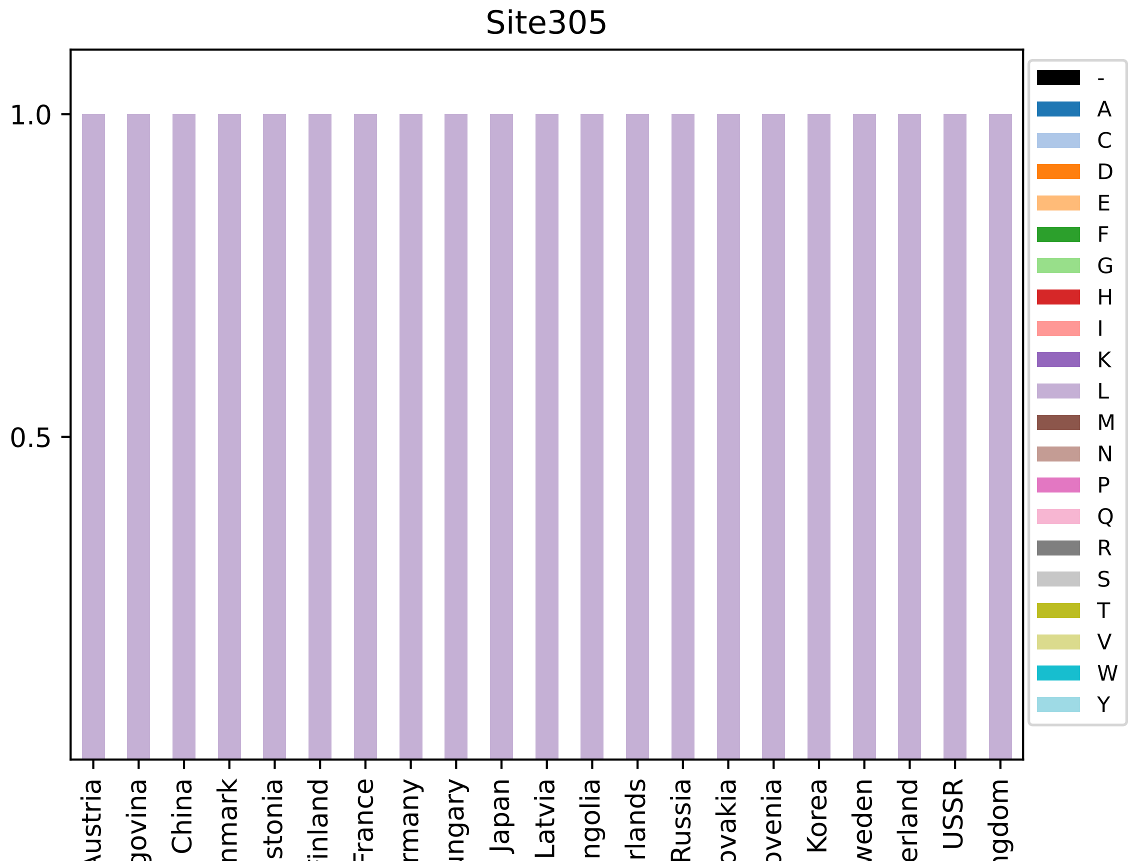
Site305
1.0
0.5
-
A
C
D
E
F
G
H
I
K
L
M
N
P
Q
R
S
T
V
W
Y
China
Japan
Latvia
Russia
Korea
USSR

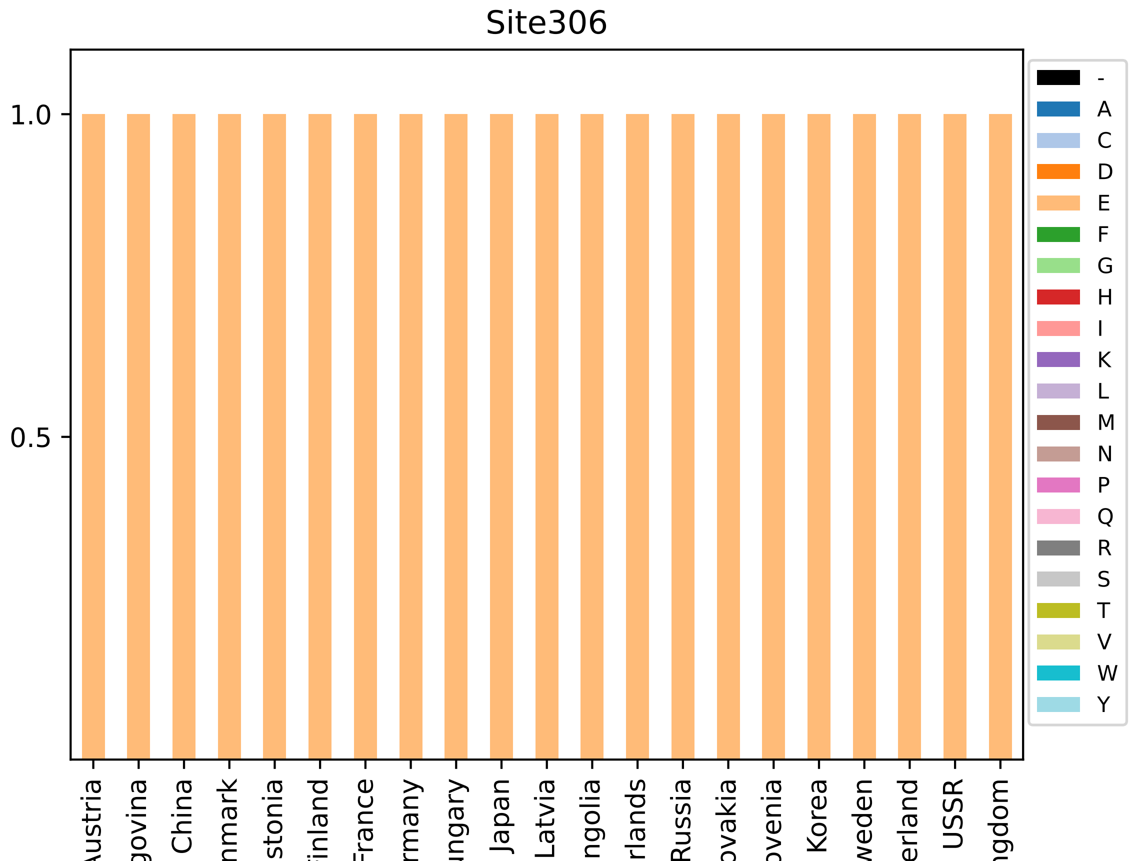
Site306
1.0
0.5
-
A
C
D
E
F
G
H
I
K
L
M
N
P
Q
R
S
T
V
W
Y
China
Japan
Latvia
Russia
Korea
USSR

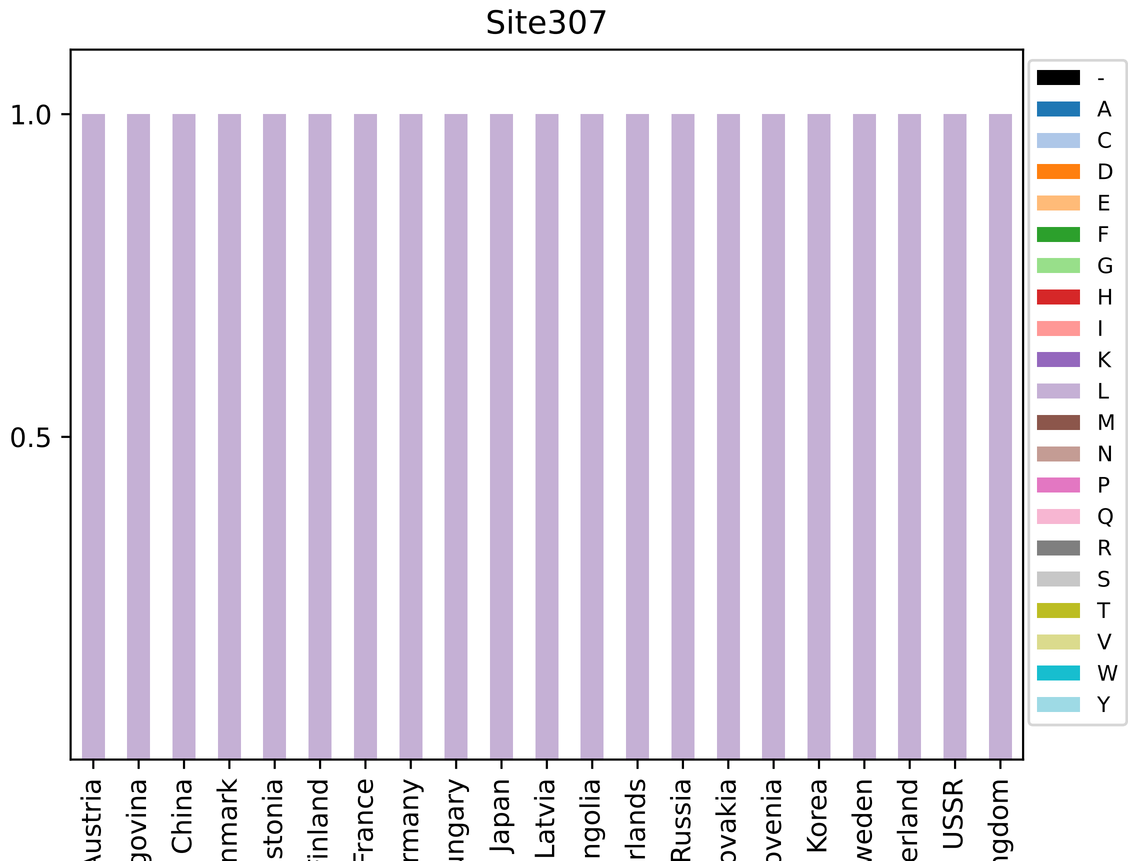

Site307
1.0
0.5
-
A
C
D
E
F
G
H
I
K
L
M
N
P
Q
R
S
T
V
W
Y
Austria
govina
China
nmark
stonia
inland
France
rmany
ungary
Japan
Latvia
ngolia
rlands
Russia
ovakia
ovenia
Korea
weden
erland
USSR
ngdom

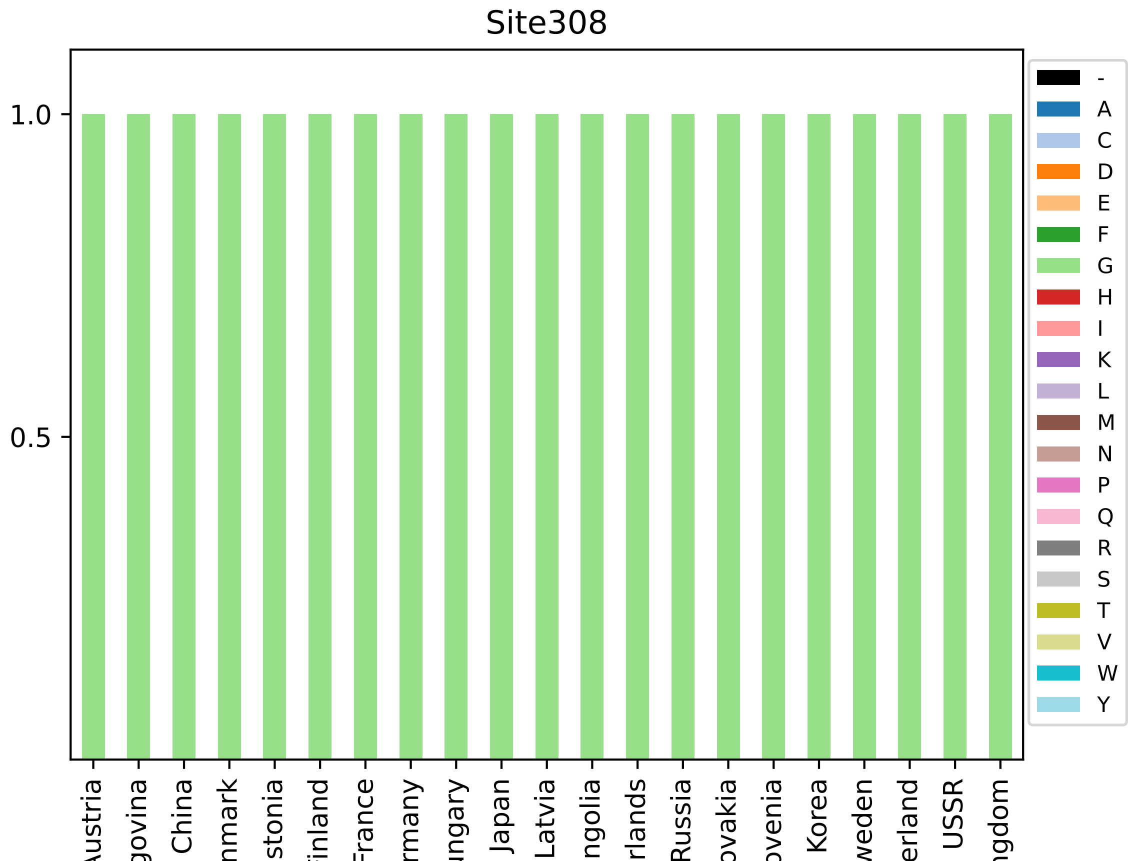
Site308
1.0
0.5
China
Japan
Latvia
Russia
Korea
USSR
-
A
C
D
E
F
G
H
I
K
L
M
N
P
Q
R
S
T
V
W
Y

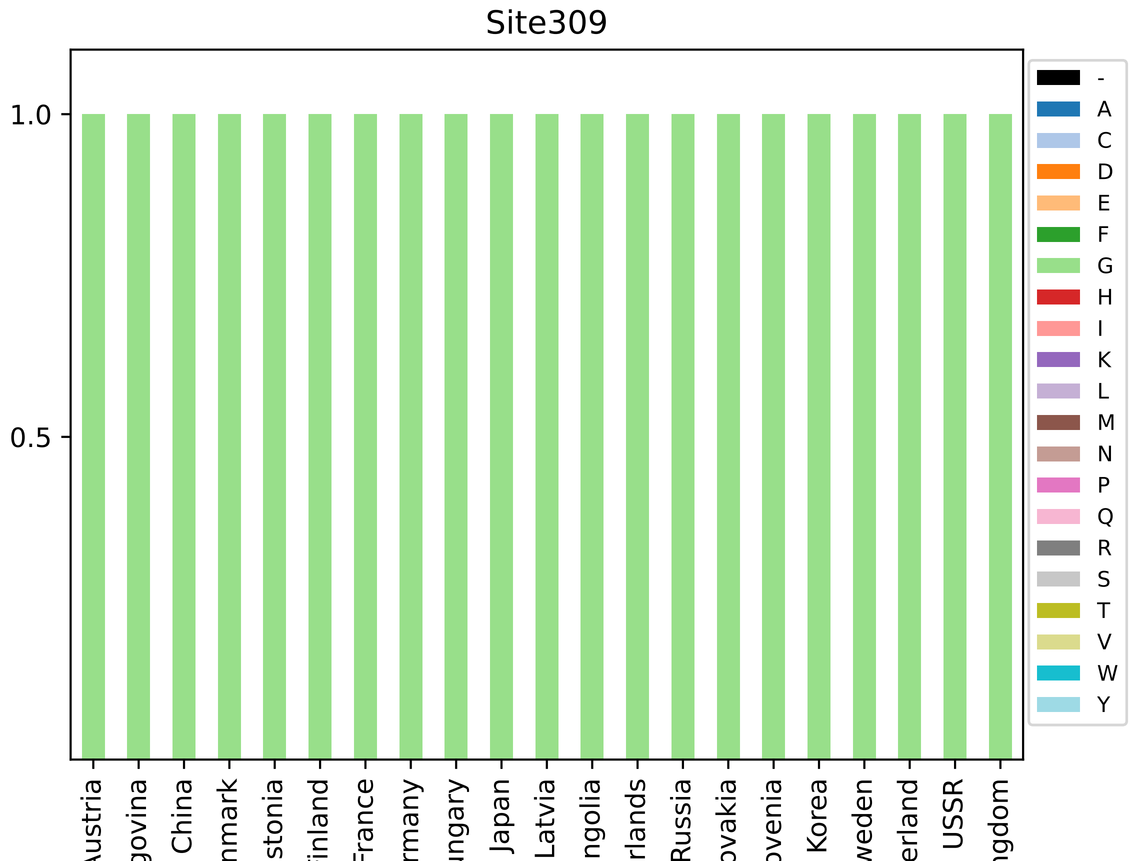
Site309
1.0
0.5
-
A
C
D
E
F
G
H
I
K
L
M
N
P
Q
R
S
T
V
W
Y
China
Japan
Latvia
Russia
Korea
USSR

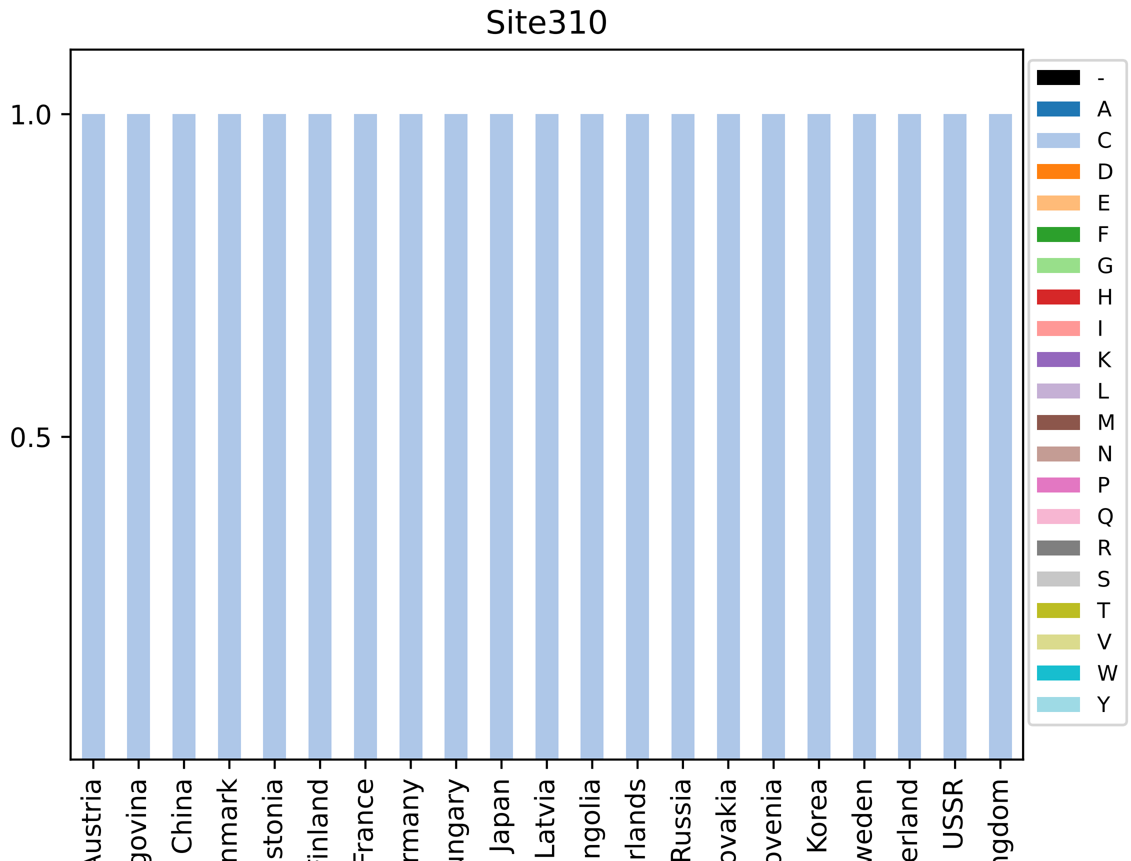

Site310
1.0
0.5
-
A
C
D
E
F
G
H
I
K
L
M
N
P
Q
R
S
T
V
W
Y
China
Japan
Latvia
Russia
Korea
USSR

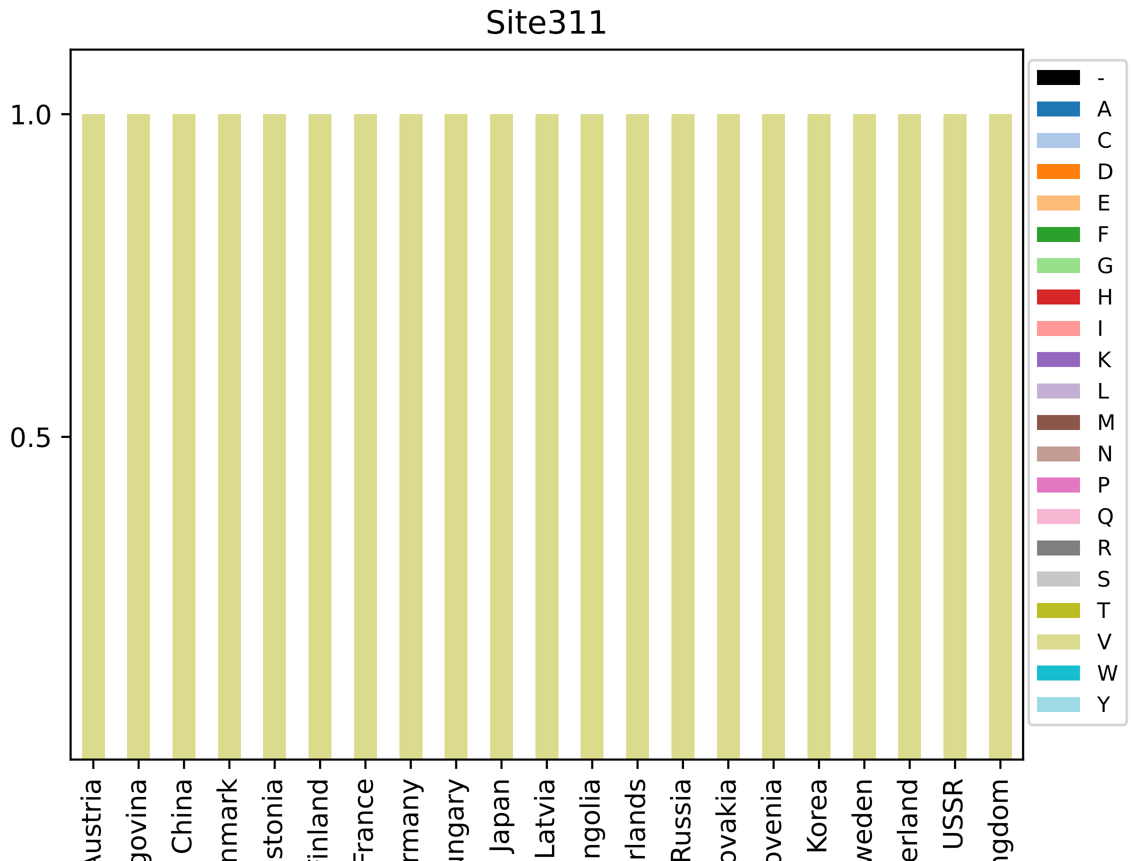
Site311
1.0
0.5
-
A
C
D
E
F
G
H
I
K
L
M
N
P
Q
R
S
T
V
W
Y
China
Japan
Latvia
Russia
Korea
USSR

Site312

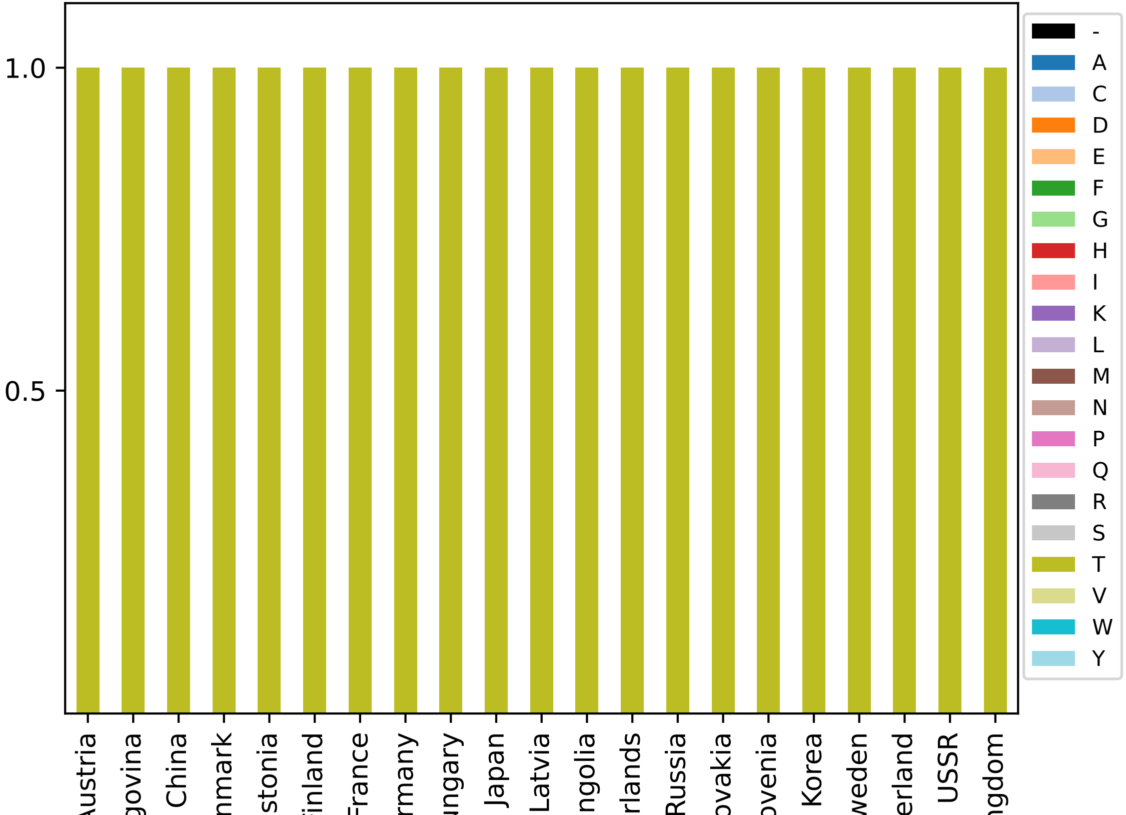

1.0
0.5
-
A
C
D
E
F
G
H
I
K
L
M
N
P
Q
R
S
T
V
W
Y
Austria
China
Japan
Latvia
Russia
Korea
USSR

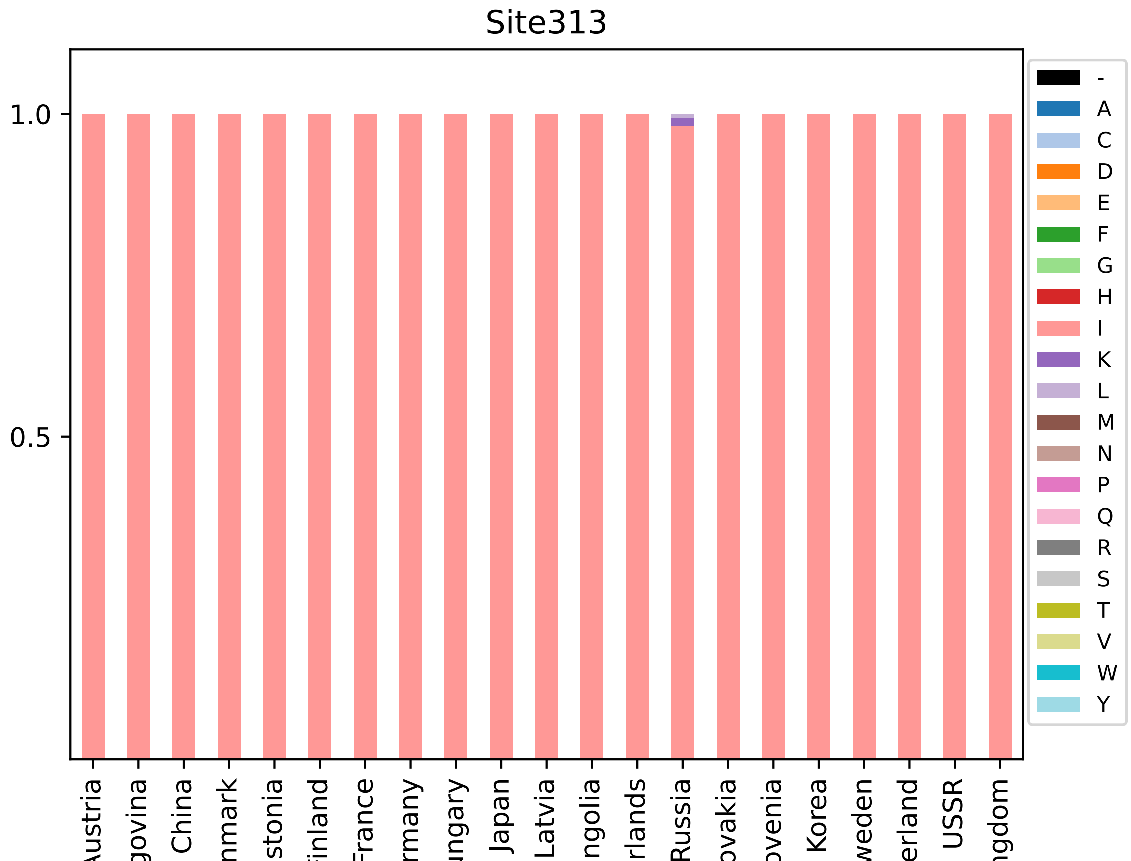
Site313
1.0
0.5
-
A
C
D
E
F
G
H
I
K
L
M
N
P
Q
R
S
T
V
W
Y
China
Japan
Latvia
Russia
Korea
USSR

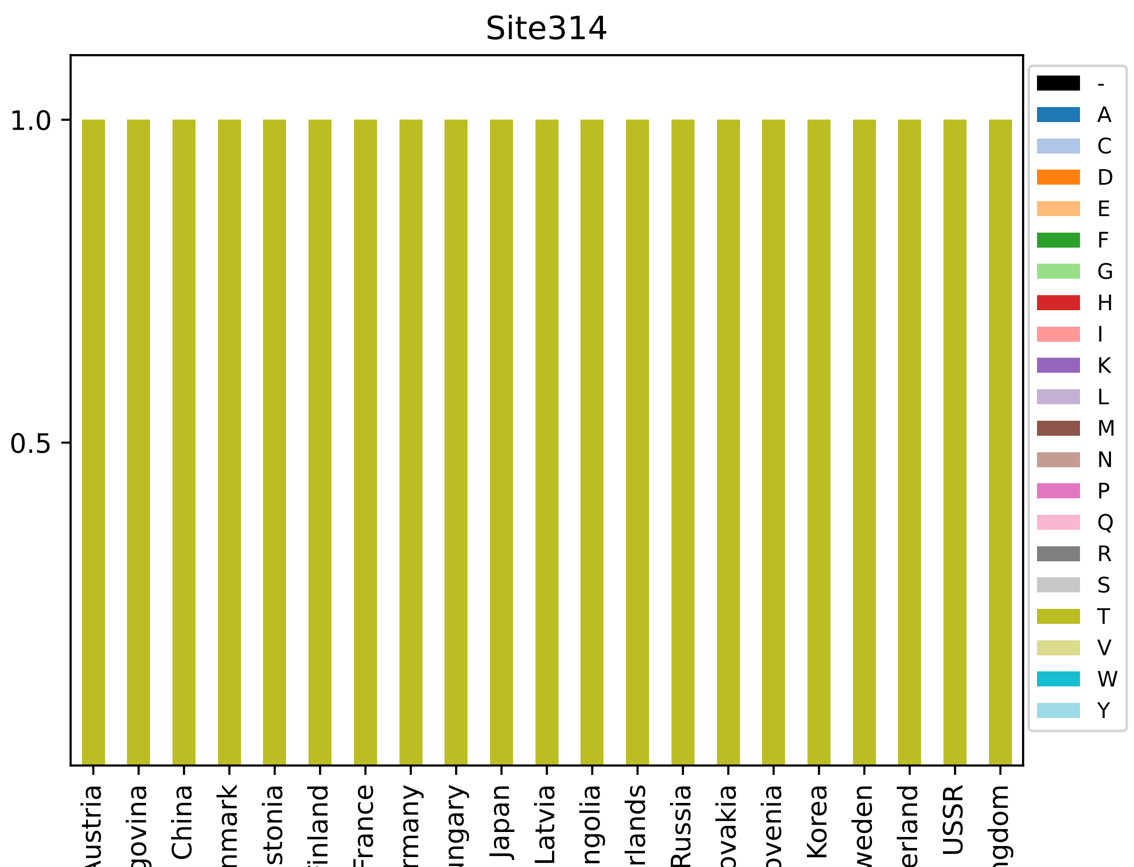
Site314
1.0
0.5
-
A
C
D
E
F
G
H
I
K
L
M
N
P
Q
R
S
T
V
W
Y
China
Japan
Latvia
Russia
Korea
USSR

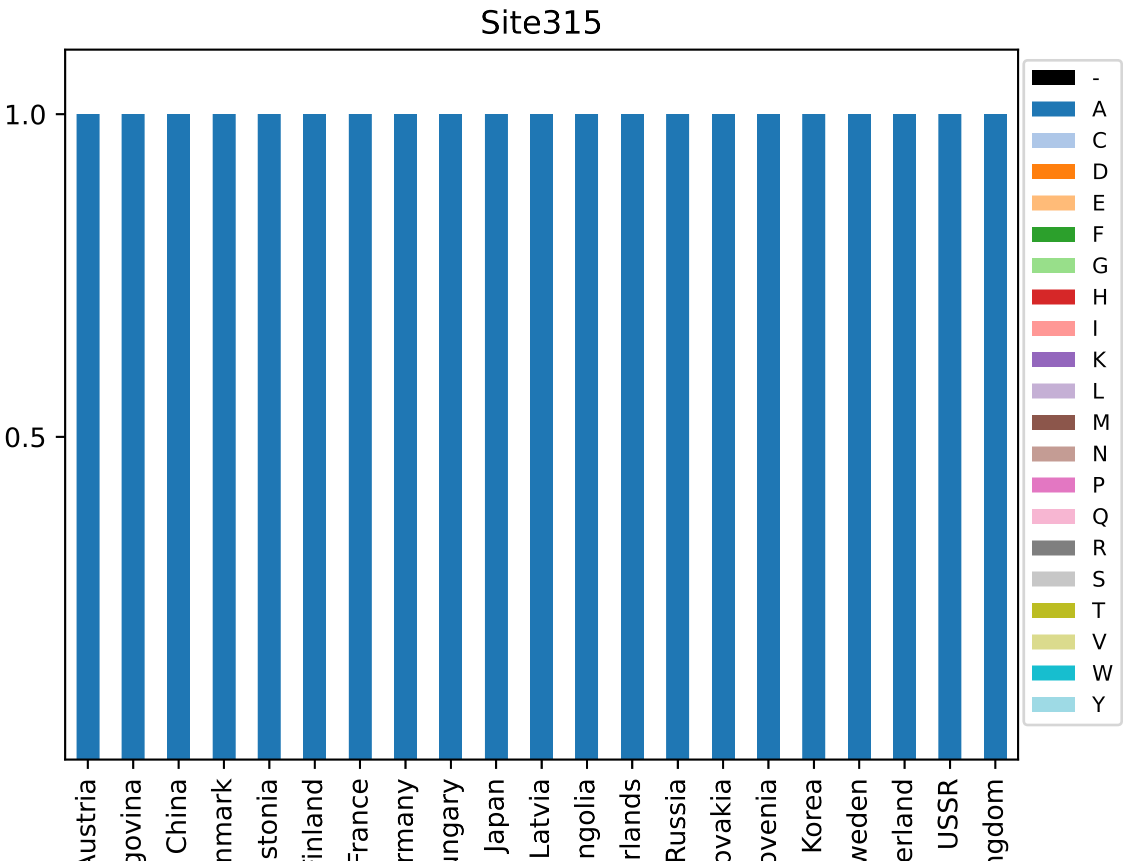
Site315
1.0
0.5
-
A
C
D
E
F
G
H
I
K
L
M
N
P
Q
R
S
T
V
W
Y
China
Japan
Latvia
Russia
Korea
USSR

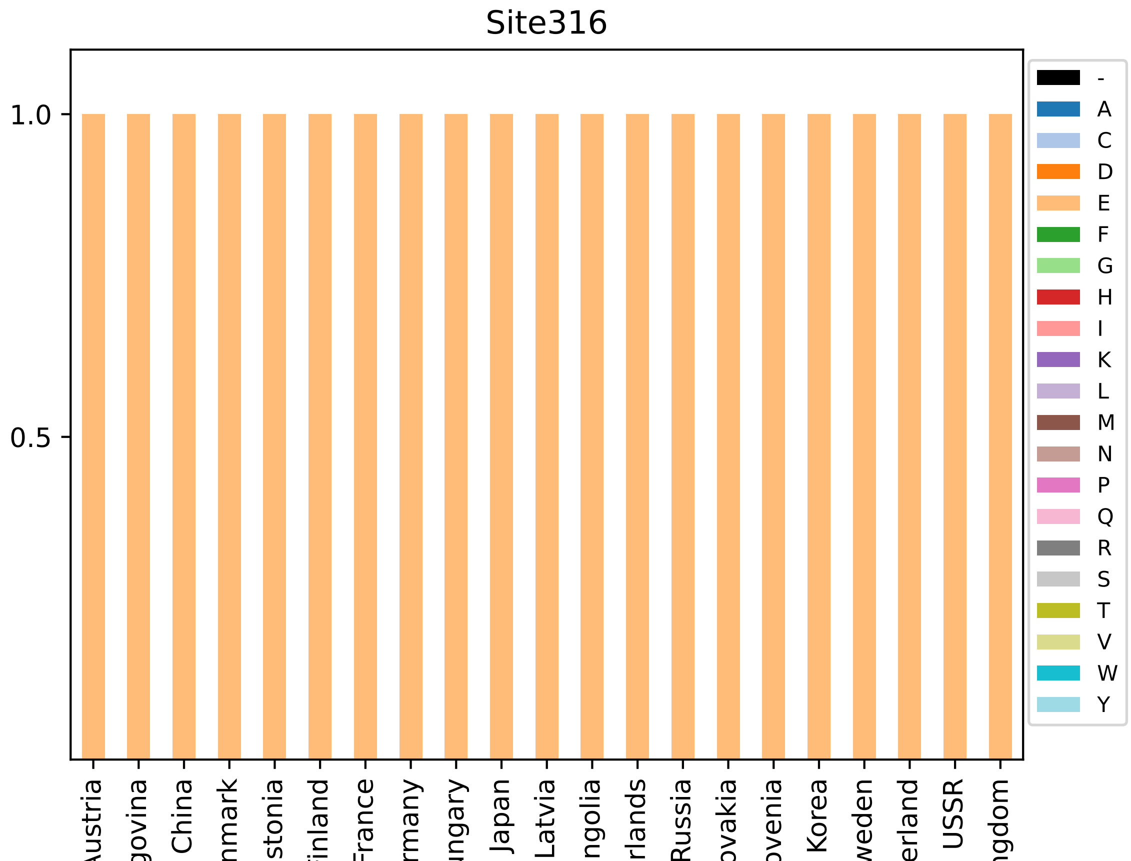
Site316
1.0
0.5
-
A
C
D
E
F
G
H
I
K
L
M
N
P
Q
R
S
T
V
W
Y
China
Japan
Latvia
Russia
Korea
USSR

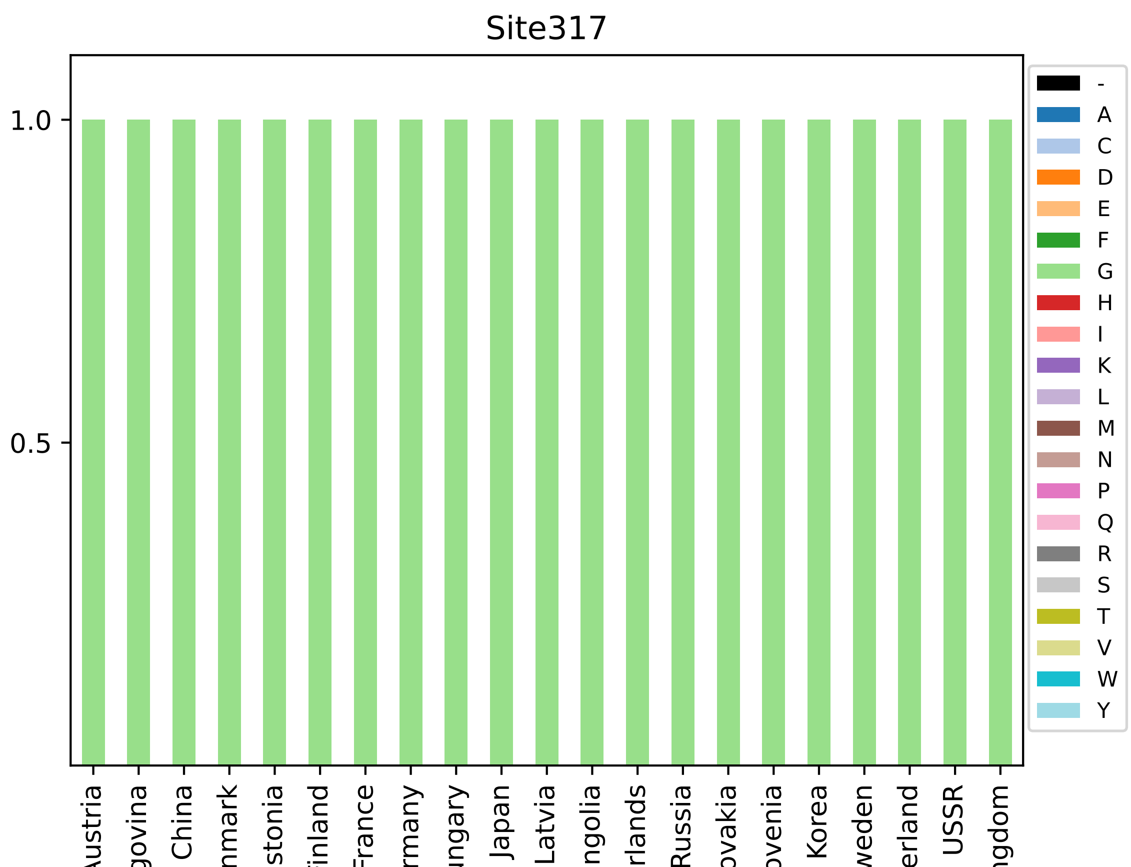

Site317
1.0
0.5
-
A
C
D
E
F
G
H
I
K
L
M
N
P
Q
R
S
T
V
W
Y
Austria
govina
China
nmark
stonia
Finland
France
rmany
ungary
Japan
Latvia
ngolia
rlands
Russia
ovakia
ovenia
Korea
weden
erland
USSR
ngdom

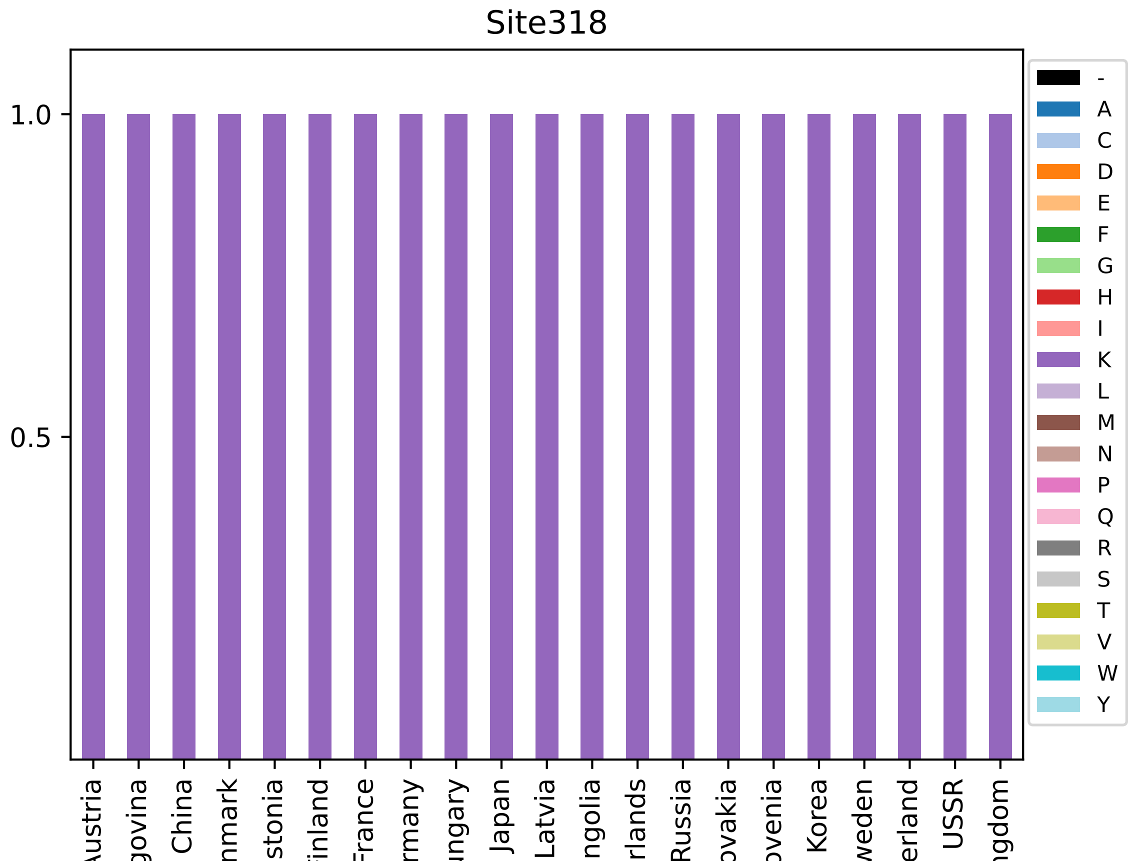

Site318
1.0
0.5
-
A
C
D
E
F
G
H
I
K
L
M
N
P
Q
R
S
T
V
W
Y
China
Japan
Latvia
Russia
Korea
USSR

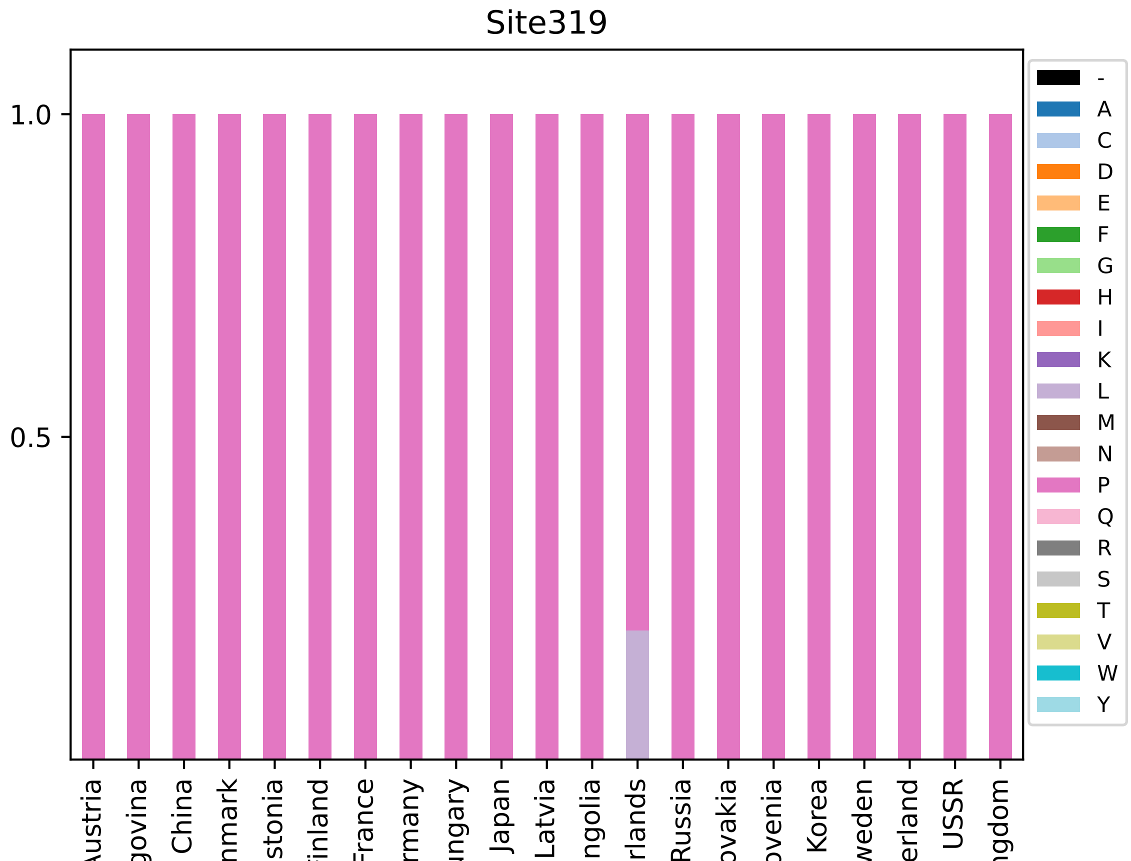
Site319
1.0
0.5
-
A
C
D
E
F
G
H
I
K
L
M
N
P
Q
R
S
T
V
W
Y
China
Japan
Latvia
Russia
Korea
USSR

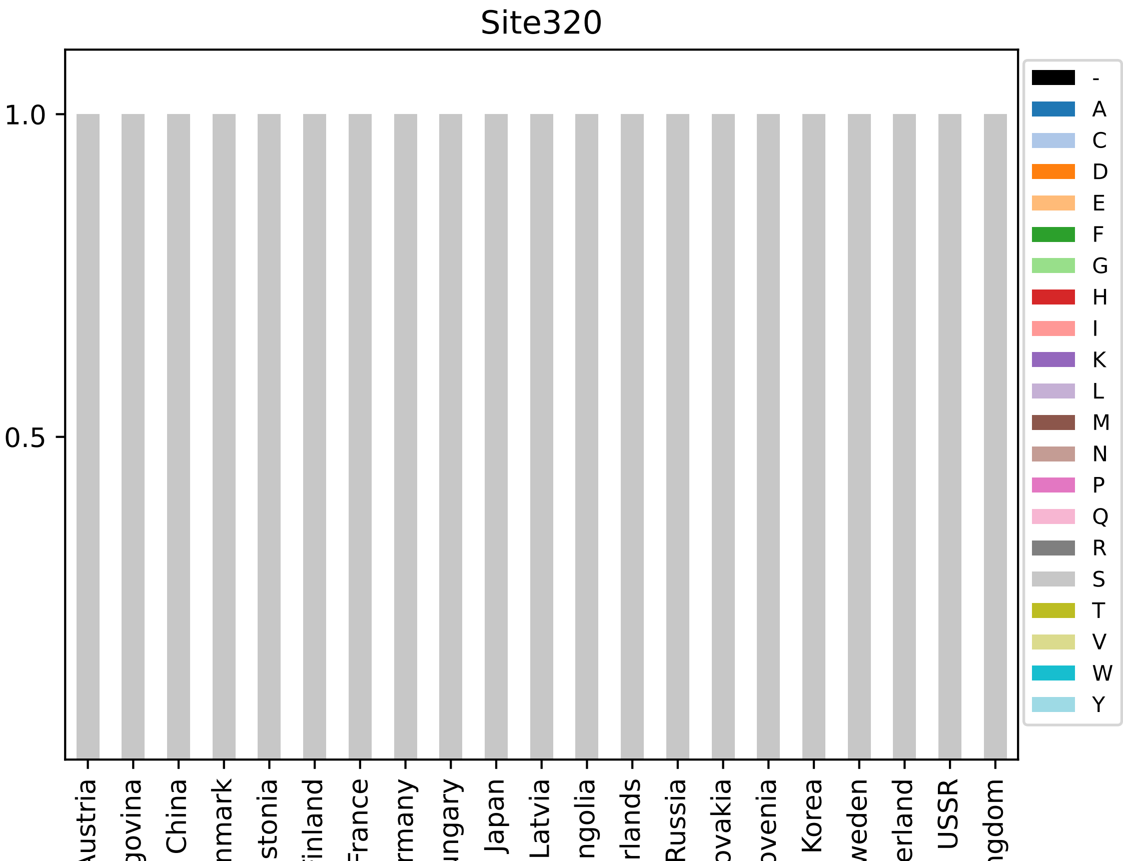

Site320
1.0
0.5
-
A
C
D
E
F
G
H
I
K
L
M
N
P
Q
R
S
T
V
W
Y
Austria
China
Estonia
Finland
France
Japan
Latvia
Russia
Korea
USSR

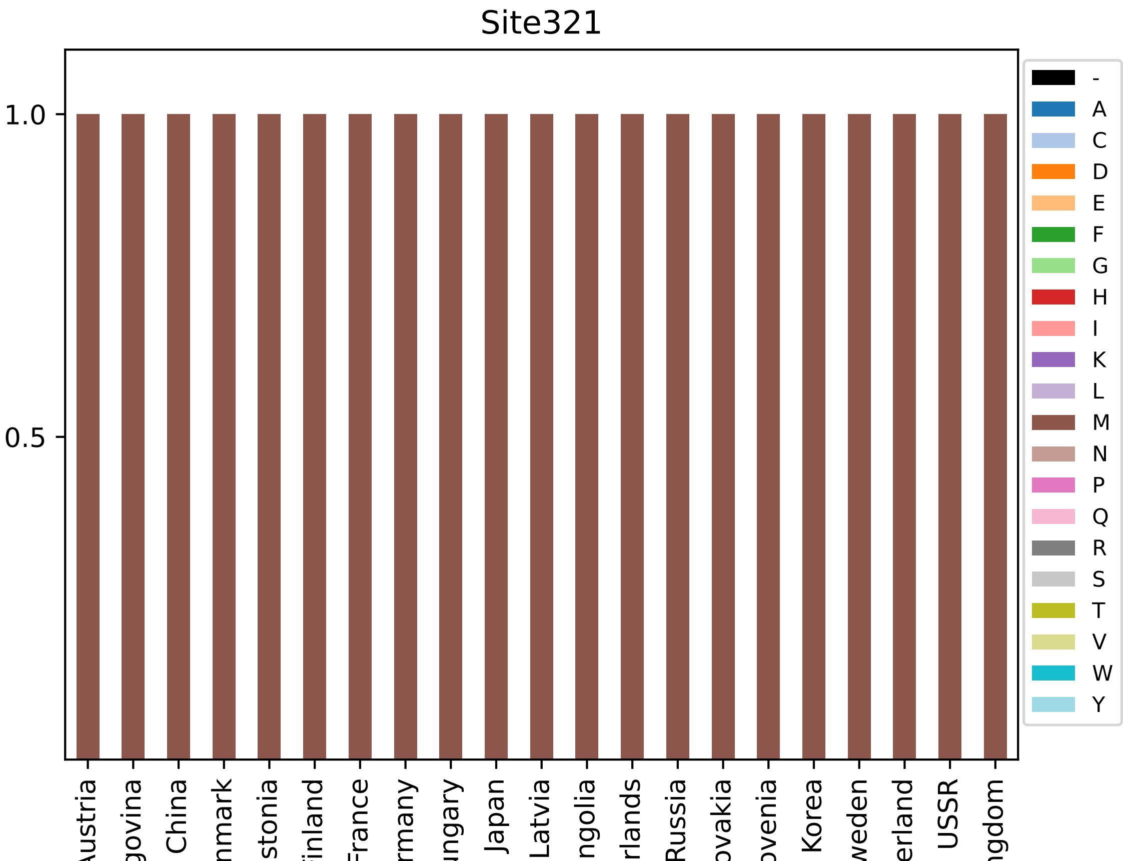
Site321
1.0
0.5
-
A
C
D
E
F
G
H
I
K
L
M
N
P
Q
R
S
T
V
W
Y
China
Japan
Latvia
Russia
Korea
USSR

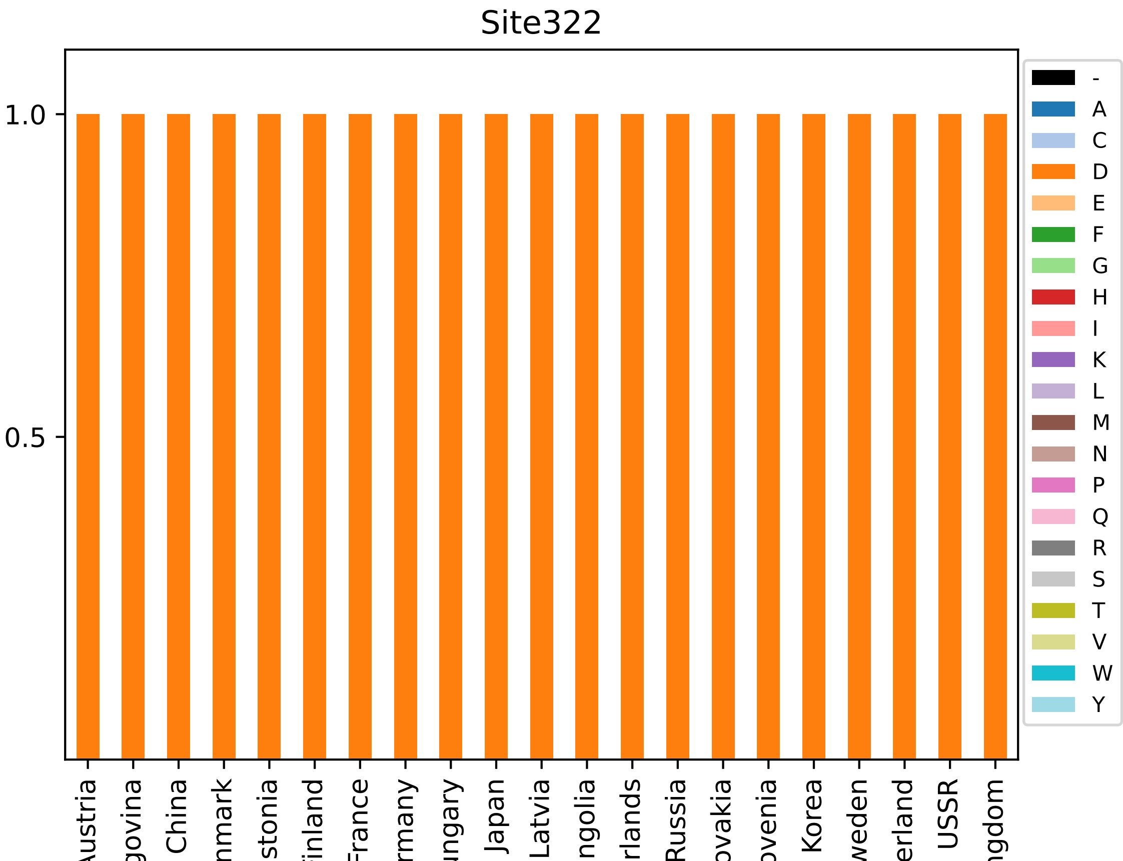

Site322
1.0
0.5
-
A
C
D
E
F
G
H
I
K
L
M
N
P
Q
R
S
T
V
W
Y
Austria
govina
China
nmark
stonia
Finland
France
rmany
ungary
Japan
Latvia
ngolia
rlands
Russia
ovakia
ovenia
Korea
weden
erland
USSR
ngdom

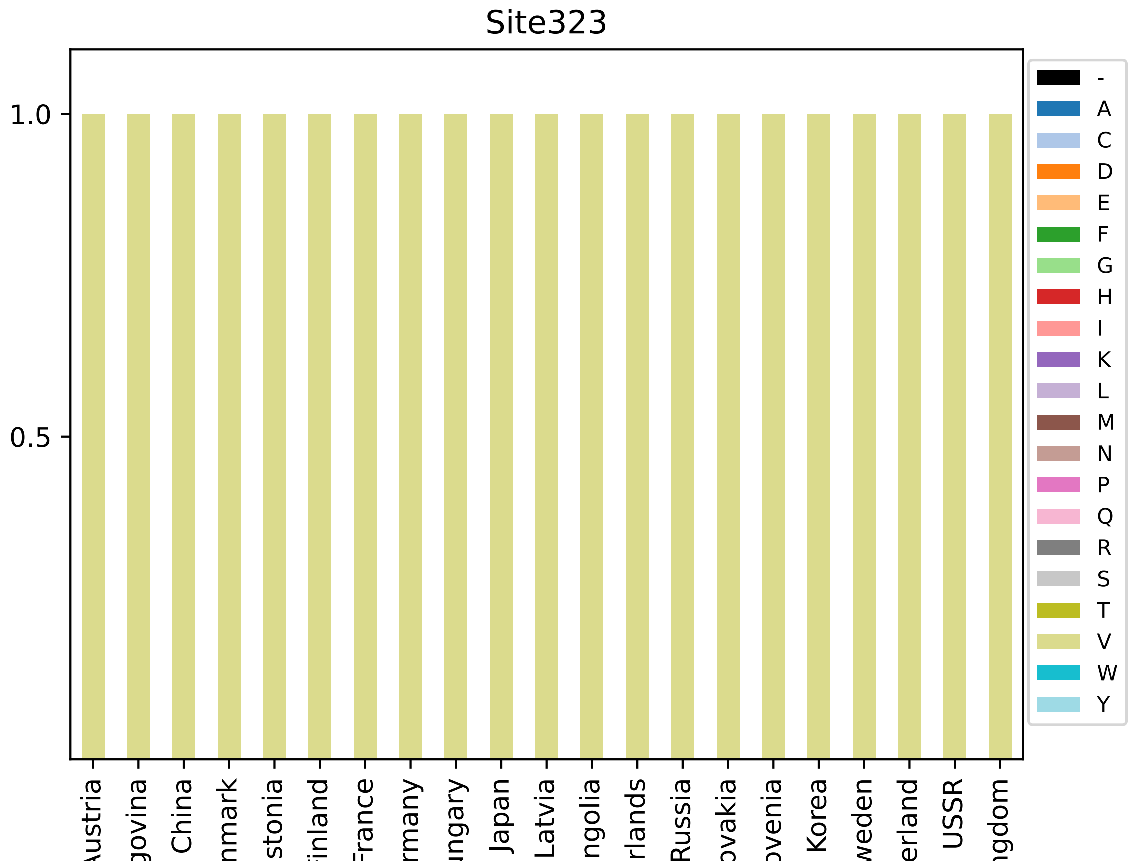

Site323
1.0
0.5
-
A
C
D
E
F
G
H
I
K
L
M
N
P
Q
R
S
T
V
W
Y
Austria
govina
China
nmark
stonia
Finland
France
rmany
ungary
Japan
Latvia
ngolia
rlands
Russia
ovakia
ovenia
Korea
weden
erland
USSR
ngdom

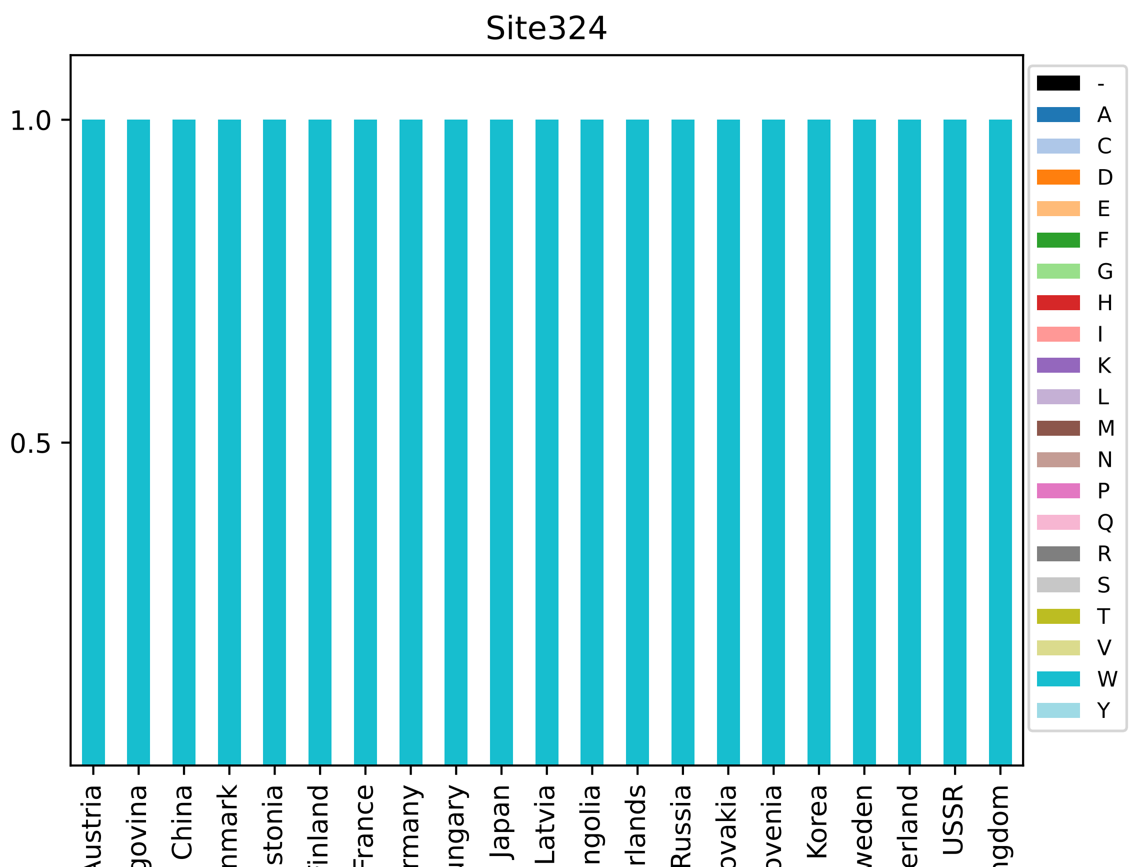
Site324
1.0
0.5
-
A
C
D
E
F
G
H
I
K
L
M
N
P
Q
R
S
T
V
W
Y
China
Japan
Latvia
Russia
Korea
USSR

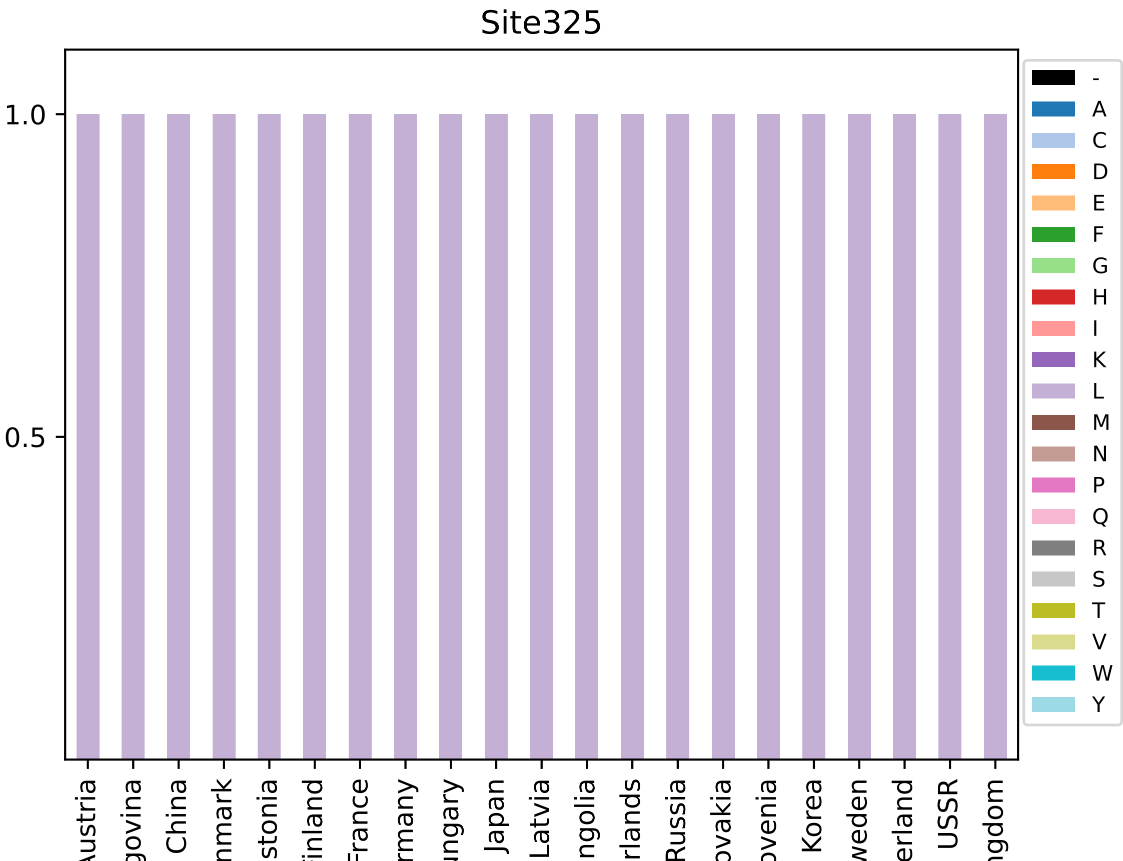

Site325
1.0
0.5
-
A
C
D
E
F
G
H
I
K
L
M
N
P
Q
R
S
T
V
W
Y
China
Japan
Latvia
Russia
Korea
USSR

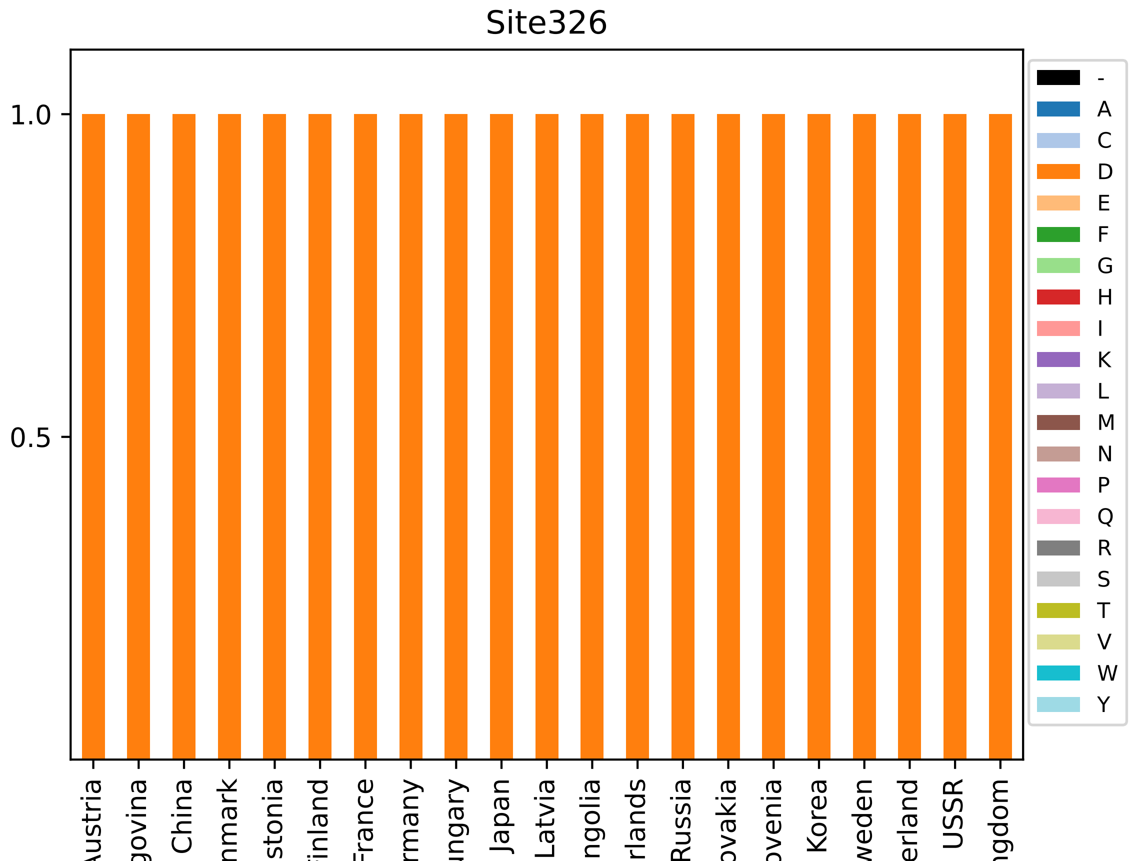

Site326
1.0
0.5
-
A
C
D
E
F
G
H
I
K
L
M
N
P
Q
R
S
T
V
W
Y
China
Japan
Latvia
Russia
Korea
USSR

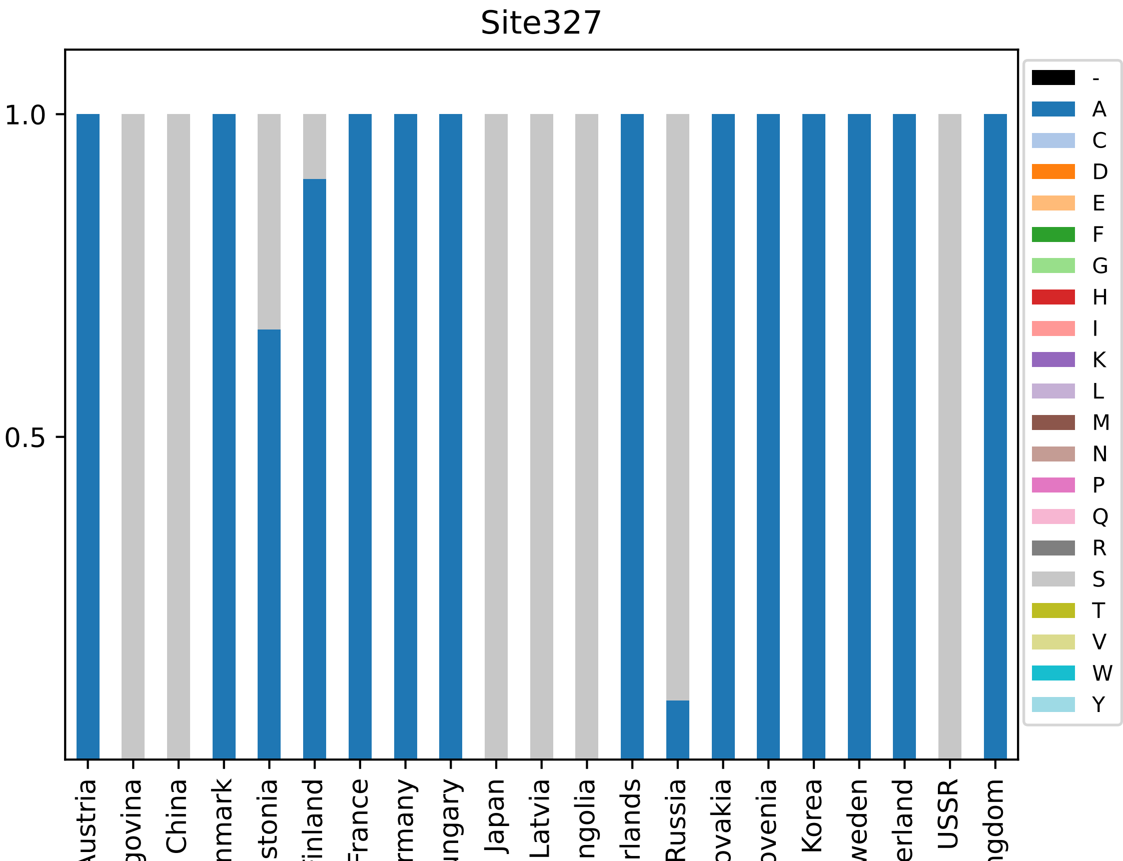

Site327
1.0
0.5
-
A
C
D
E
F
G
H
I
K
L
M
N
P
Q
R
S
T
V
W
Y
Austria
govina
China
nmark
stonia
inland
France
rmany
ungary
Japan
Latvia
ngolia
rlands
Russia
ovakia
ovenia
Korea
weden
erland
USSR
ngdom

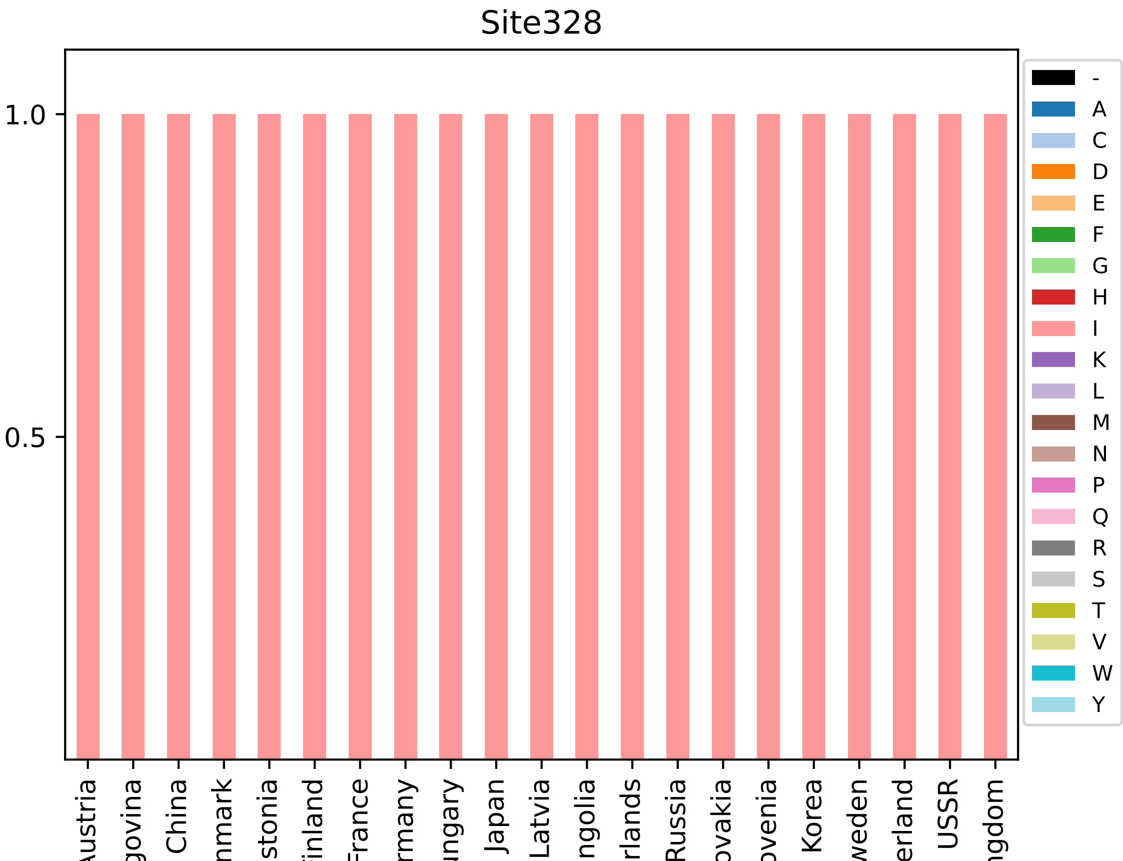

Site328
1.0
0.5
-
A
C
D
E
F
G
H
I
K
L
M
N
P
Q
R
S
T
V
W
Y
China
Japan
Latvia
Russia
Korea
USSR

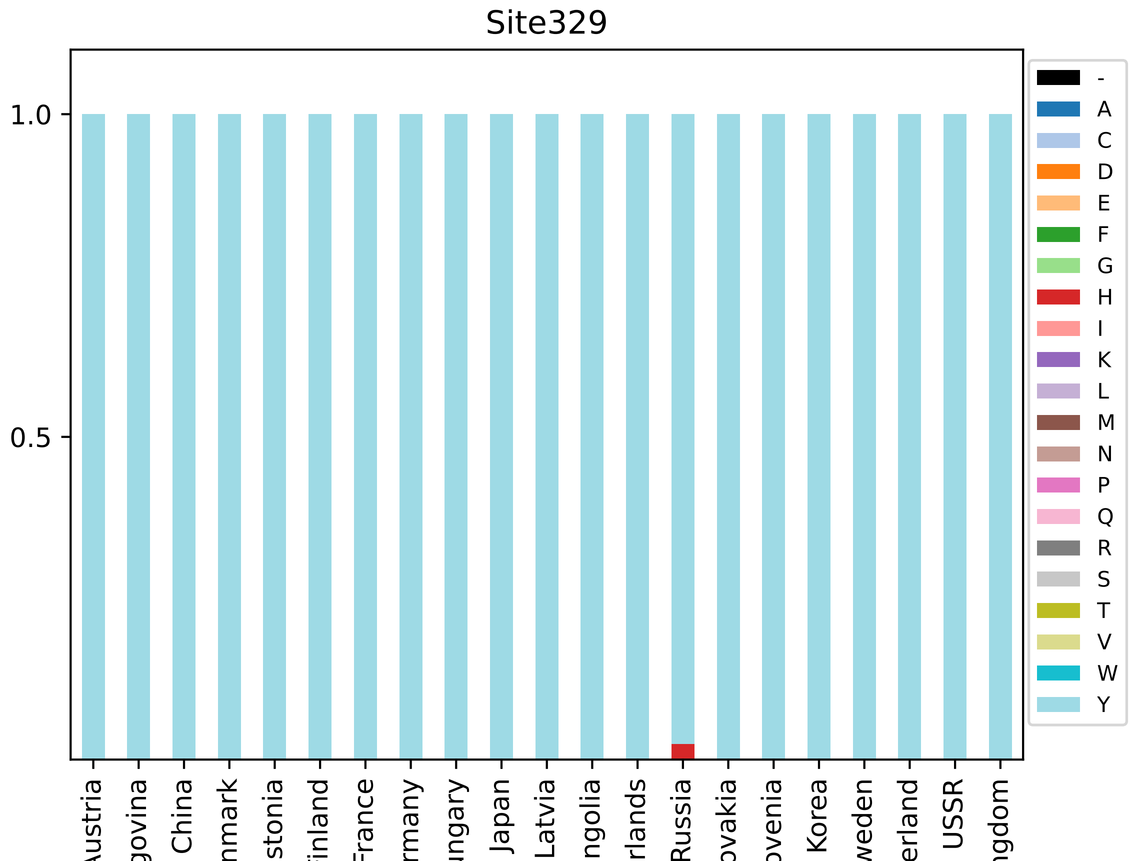

Site329
1.0
0.5
-
A
C
D
E
F
G
H
I
K
L
M
N
P
Q
R
S
T
V
W
Y
Austria
govina
China
nmark
stonia
Finland
France
rmany
ungary
Japan
Latvia
ngolia
rlands
Russia
ovakia
ovenia
Korea
weden
erland
USSR
ngdom

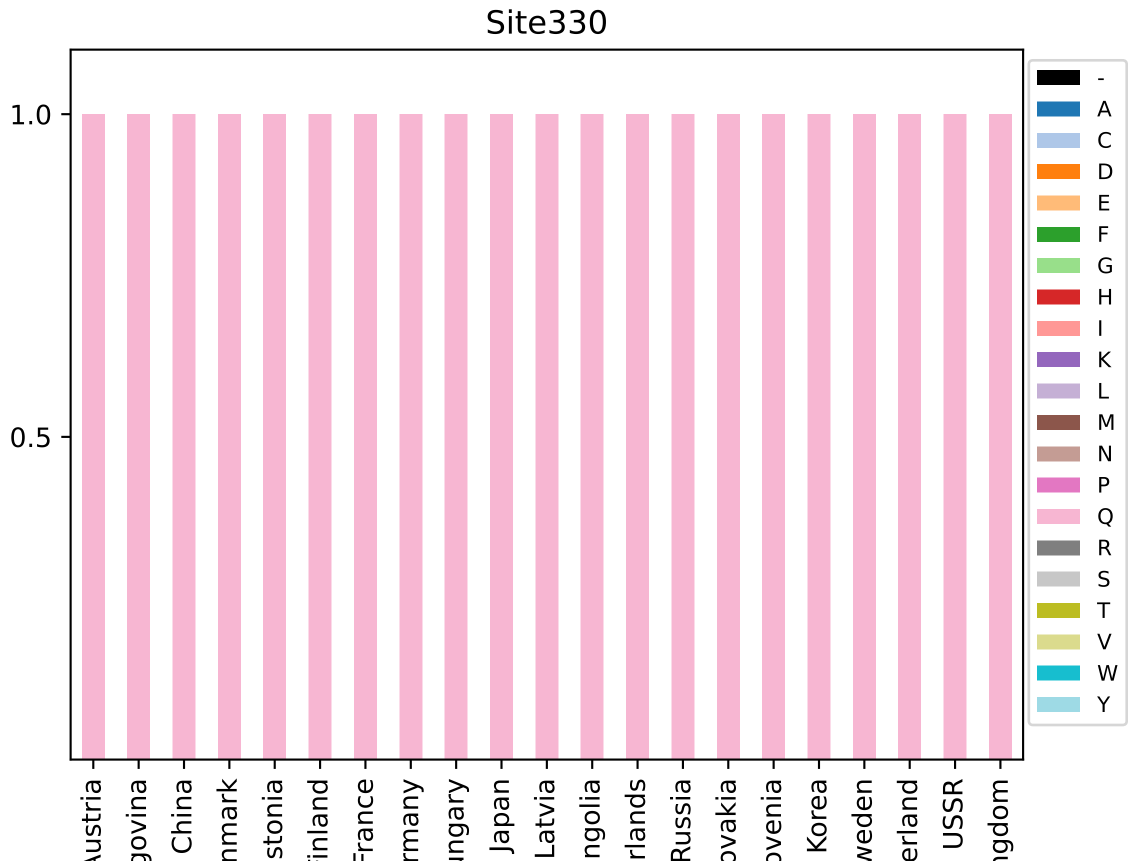
Site330
1.0
0.5
-
A
C
D
E
F
G
H
I
K
L
M
N
P
Q
R
S
T
V
W
Y
China
Japan
Latvia
Russia
Korea
USSR

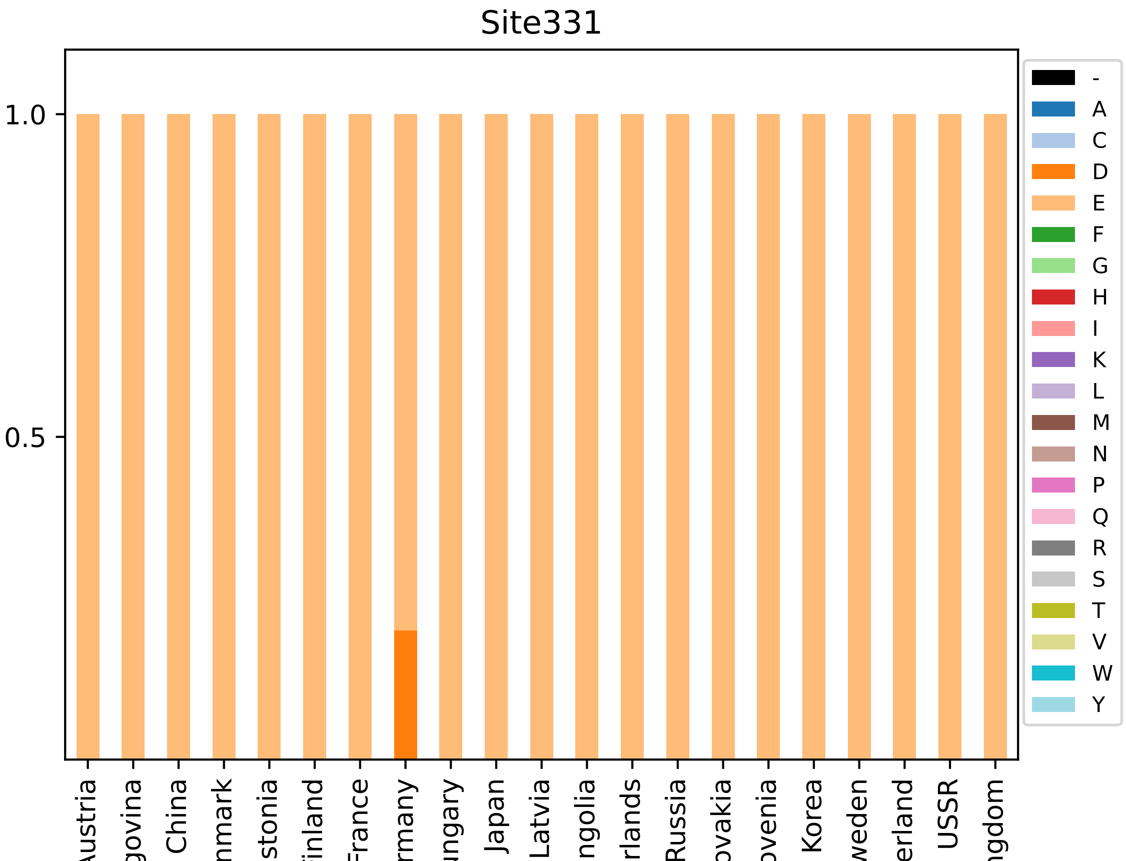

Site331
1.0
0.5
-
A
C
D
E
F
G
H
I
K
L
M
N
P
Q
R
S
T
V
W
Y
Austria
govina
China
nmark
stonia
Finland
France
rmany
ungary
Japan
Latvia
ngolia
rlands
Russia
ovakia
ovenia
Korea
weden
erland
USSR
ngdom

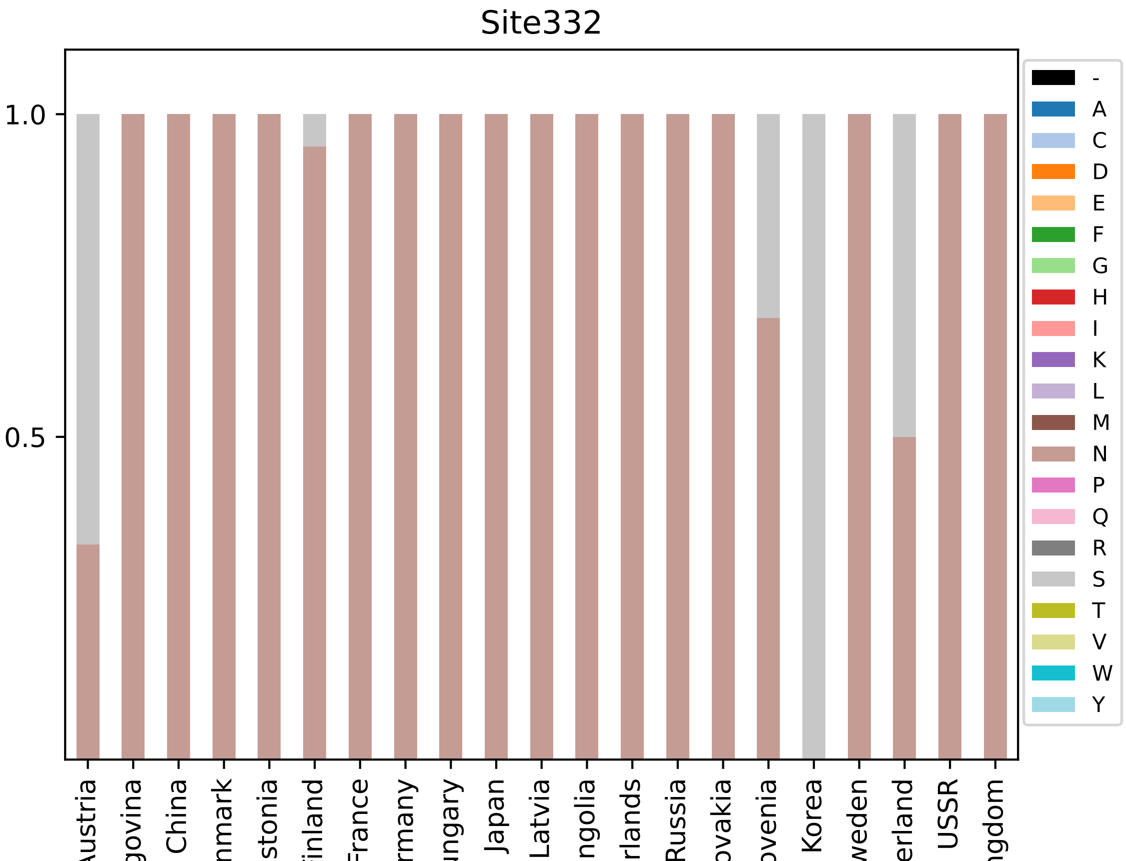

Site332
1.0
0.5
-
A
C
D
E
F
G
H
I
K
L
M
N
P
Q
R
S
T
V
W
Y
China
Japan
Latvia
Russia
Korea
USSR

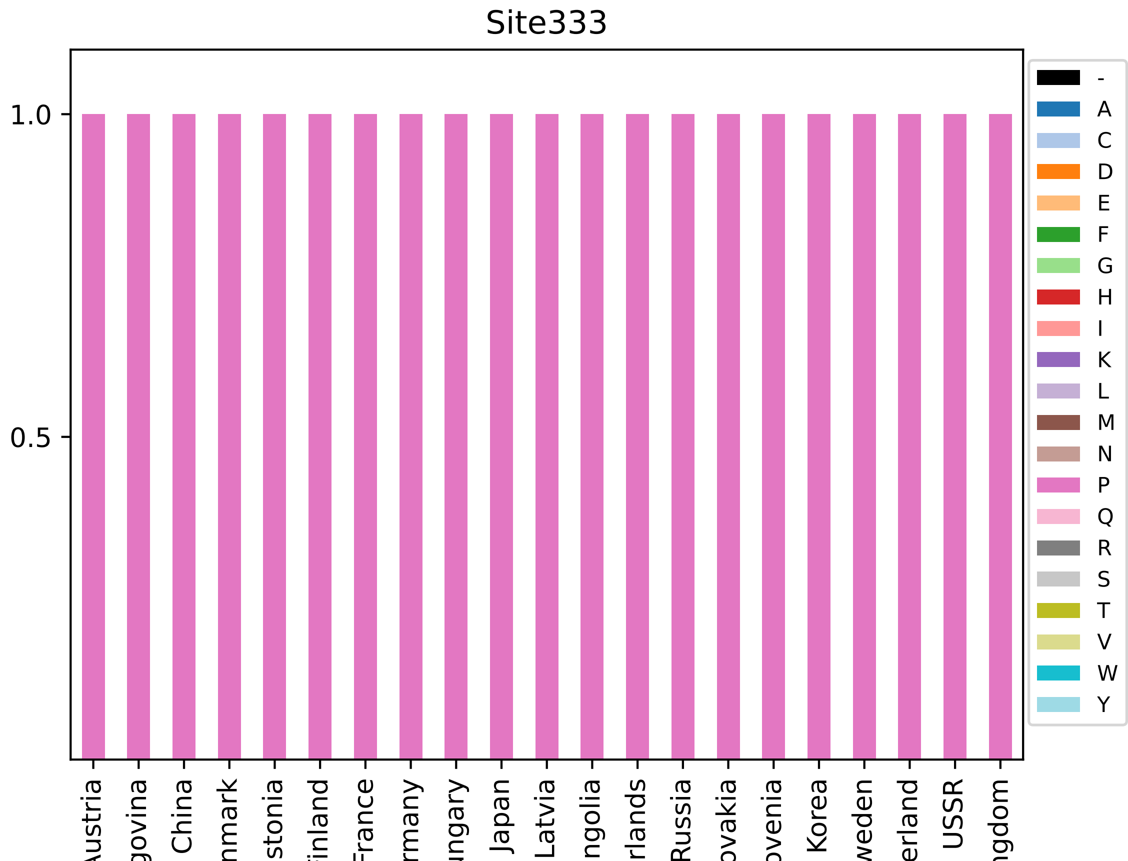

Site333
1.0
0.5
-
A
C
D
E
F
G
H
I
K
L
M
N
P
Q
R
S
T
V
W
Y
China
Japan
Latvia
Russia
Korea
USSR

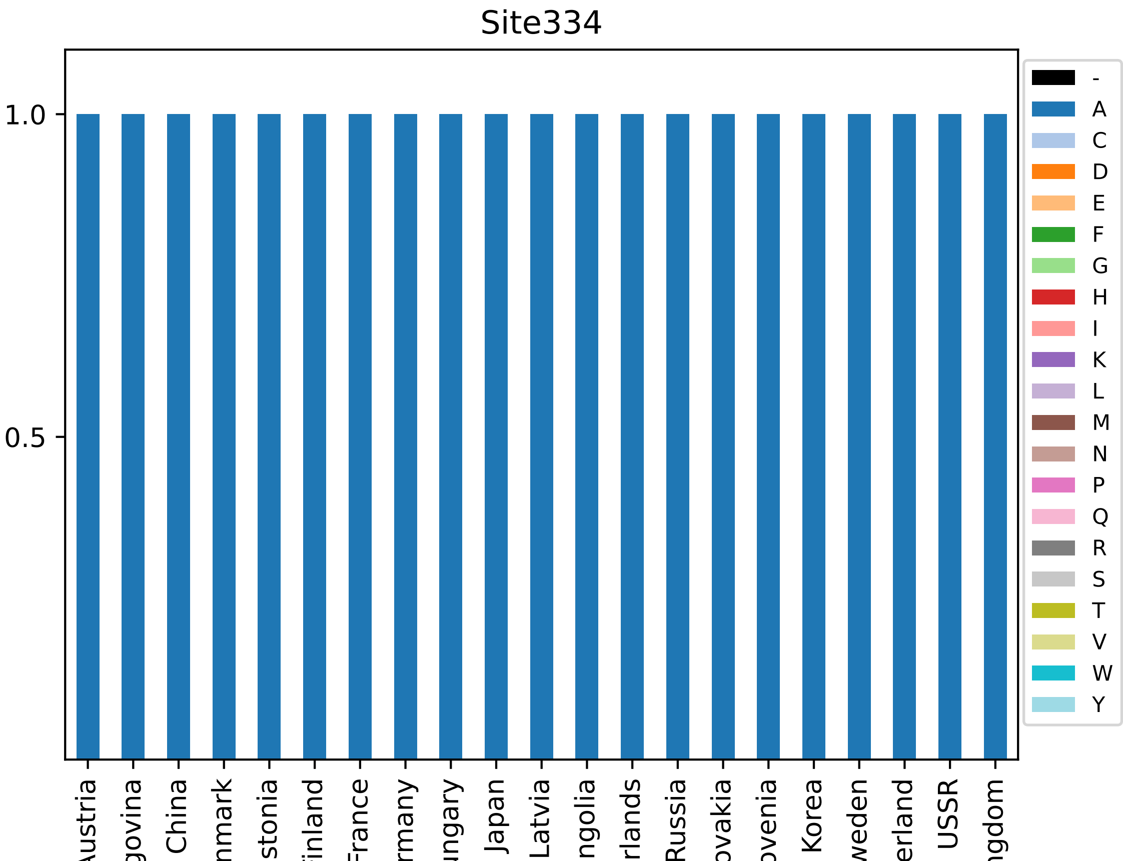

Site334
1.0
0.5
-
A
C
D
E
F
G
H
I
K
L
M
N
P
Q
R
S
T
V
W
Y
China
Japan
Latvia
Russia
Korea
USSR

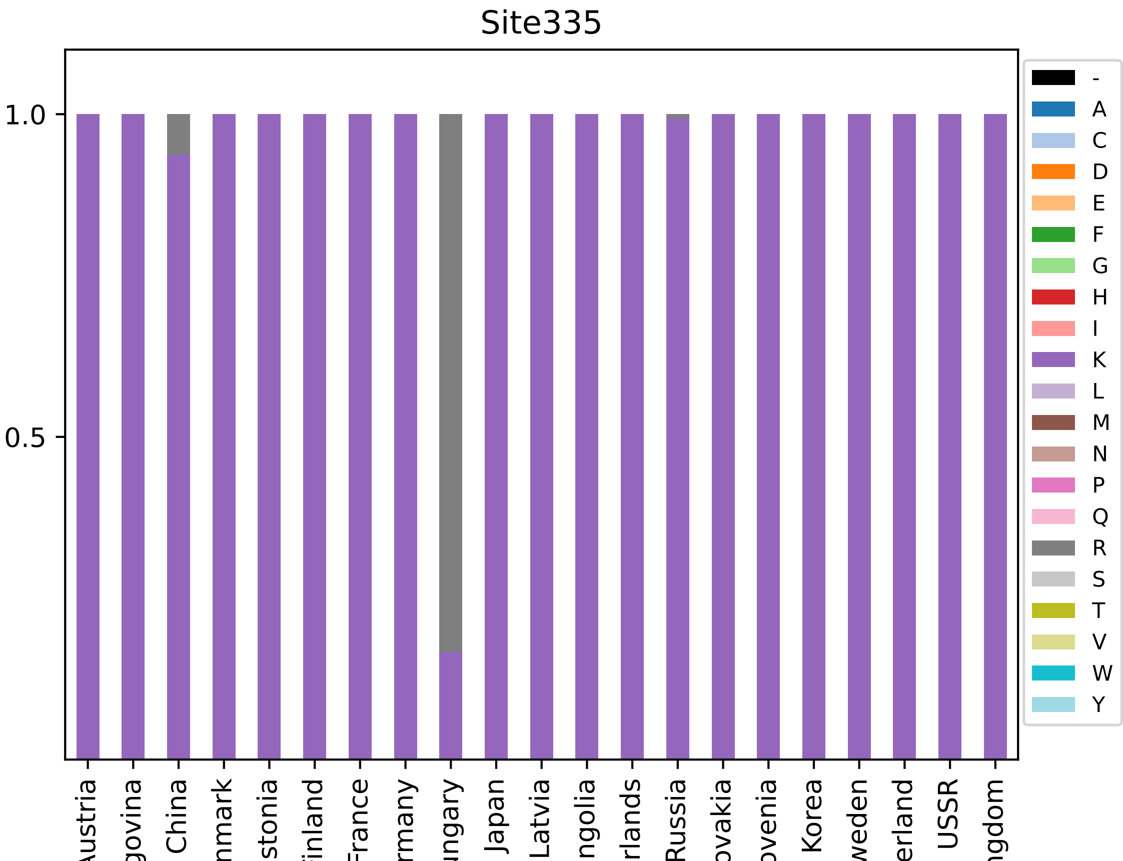

Site335
1.0
0.5
-
A
C
D
E
F
G
H
I
K
L
M
N
P
Q
R
S
T
V
W
Y
China
Japan
Latvia
Russia
Korea
USSR

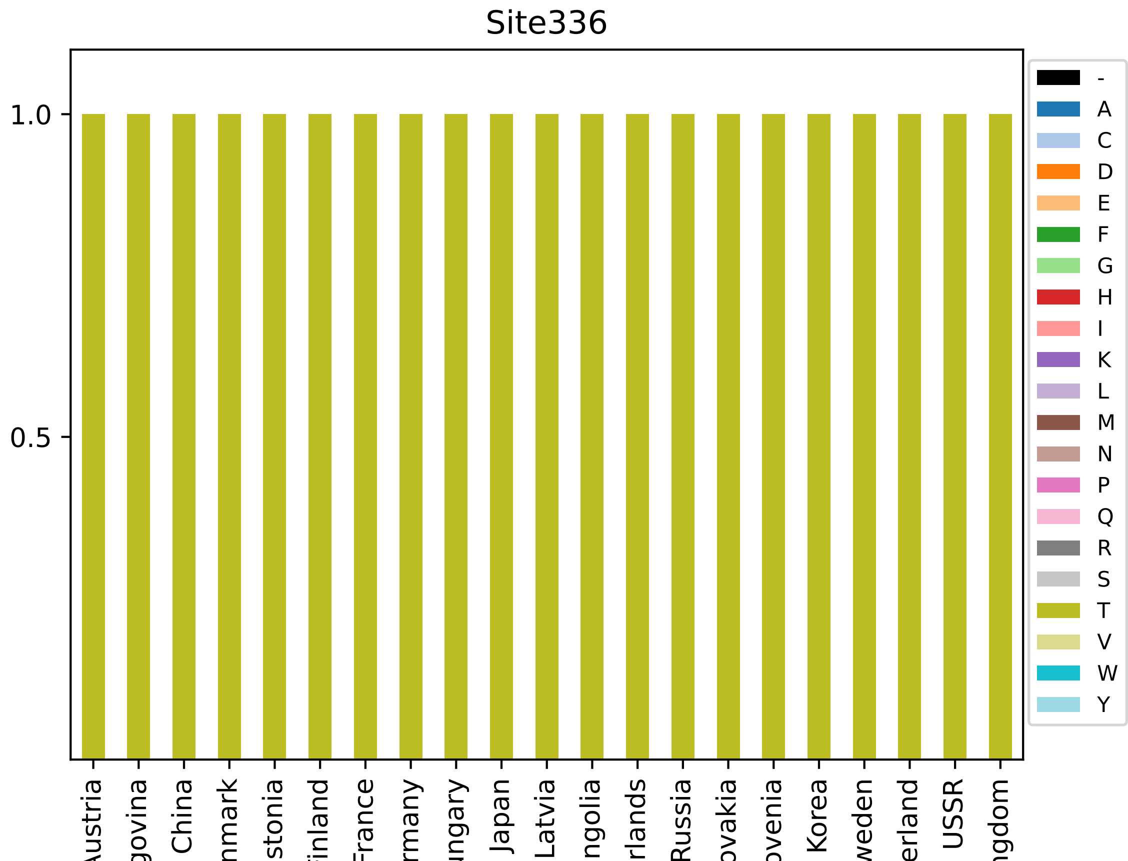

Site336
1.0
0.5
-
A
C
D
E
F
G
H
I
K
L
M
N
P
Q
R
S
T
V
W
Y
Austria
govina
China
nmark
stonia
Finland
France
rmany
ungary
Japan
Latvia
ngolia
rlands
Russia
ovakia
ovenia
Korea
weden
erland
USSR
ngdom

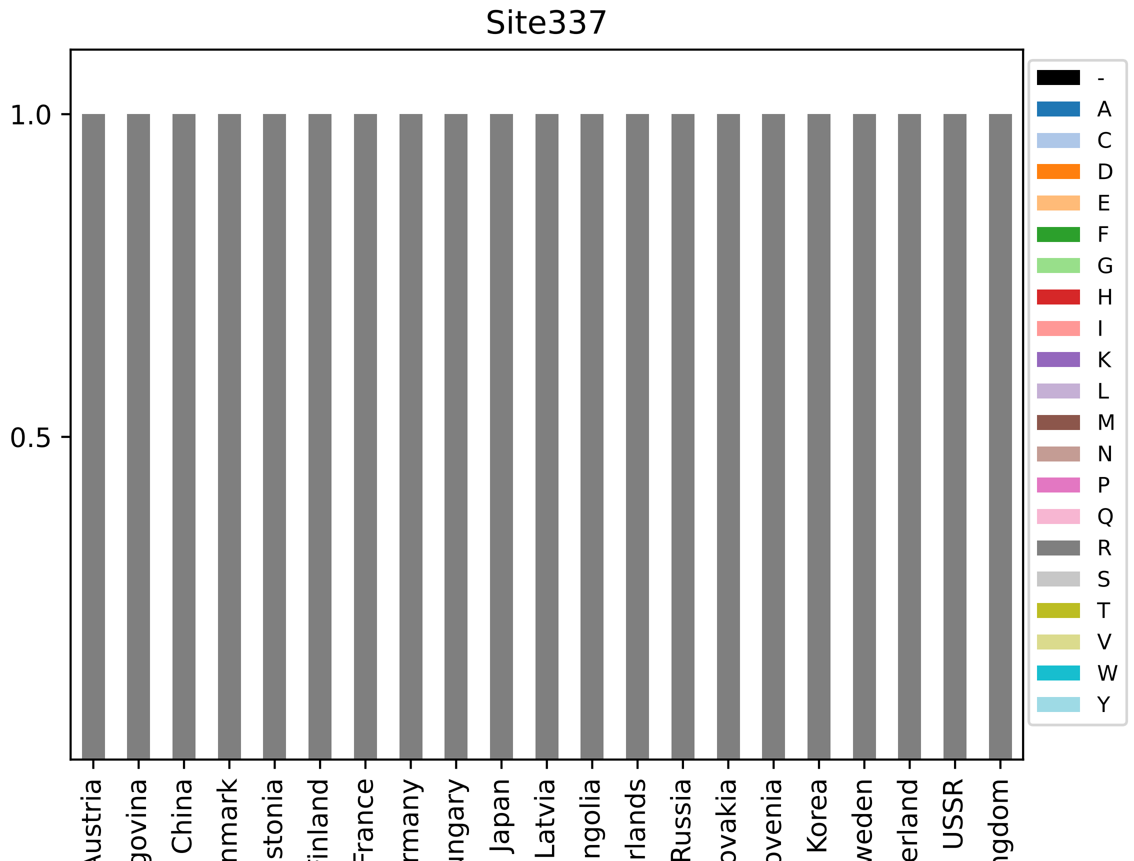
Site337
1.0
0.5
-
A
C
D
E
F
G
H
I
K
L
M
N
P
Q
R
S
T
V
W
Y
China
Japan
Latvia
Russia
Korea
USSR

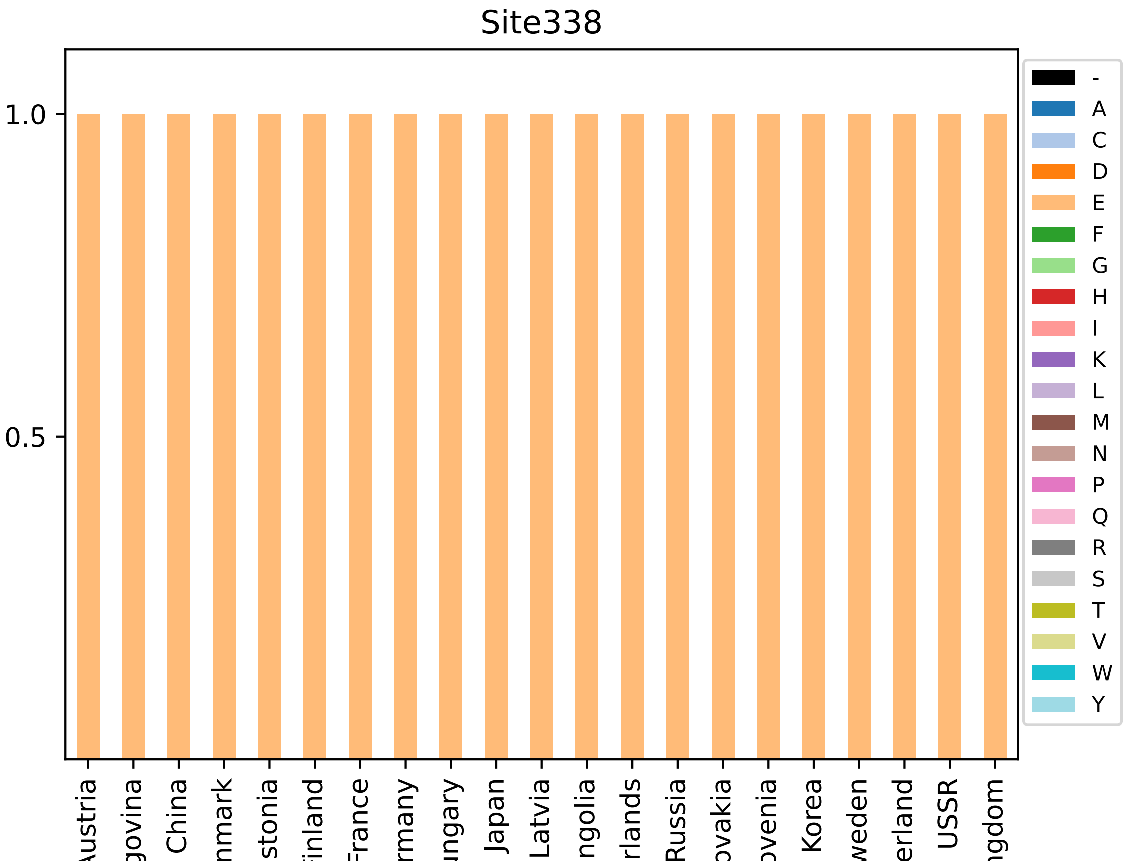

Site338
1.0
0.5
-
A
C
D
E
F
G
H
I
K
L
M
N
P
Q
R
S
T
V
W
Y
China
Japan
Latvia
Russia
Korea
USSR

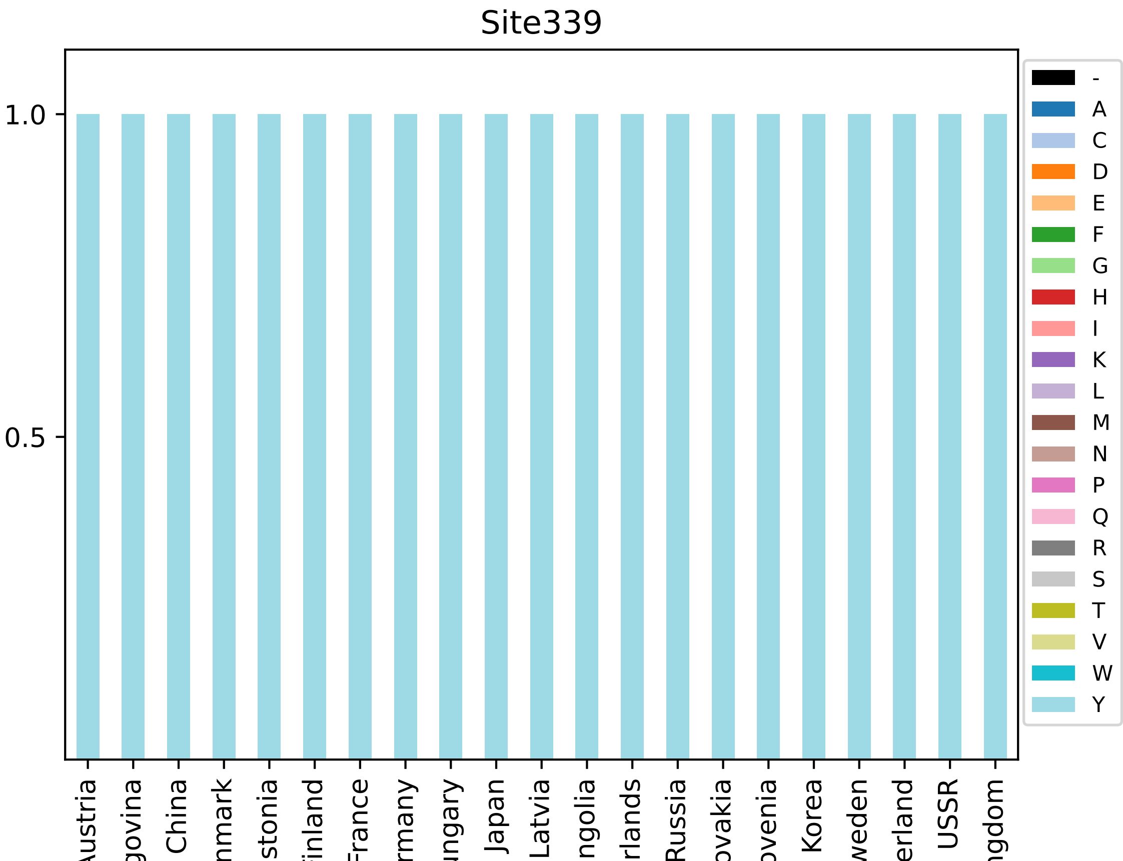
Site339
1.0
0.5
China
Japan
Latvia
Russia
Korea
USSR
-
A
C
D
E
F
G
H
I
K
L
M
N
P
Q
R
S
T
V
W
Y

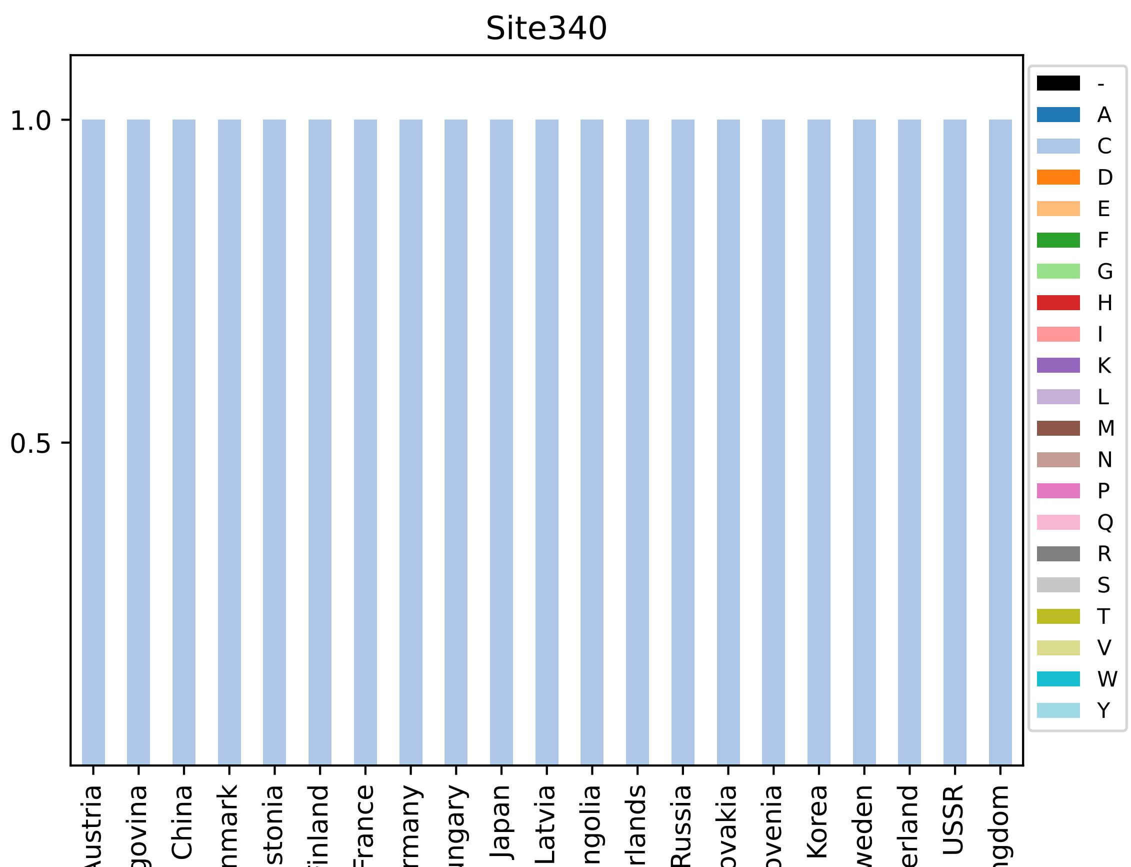

Site340
1.0
0.5
China
Japan
Latvia
Russia
Korea
USSR
-
A
C
D
E
F
G
H
I
K
L
M
N
P
Q
R
S
T
V
W
Y

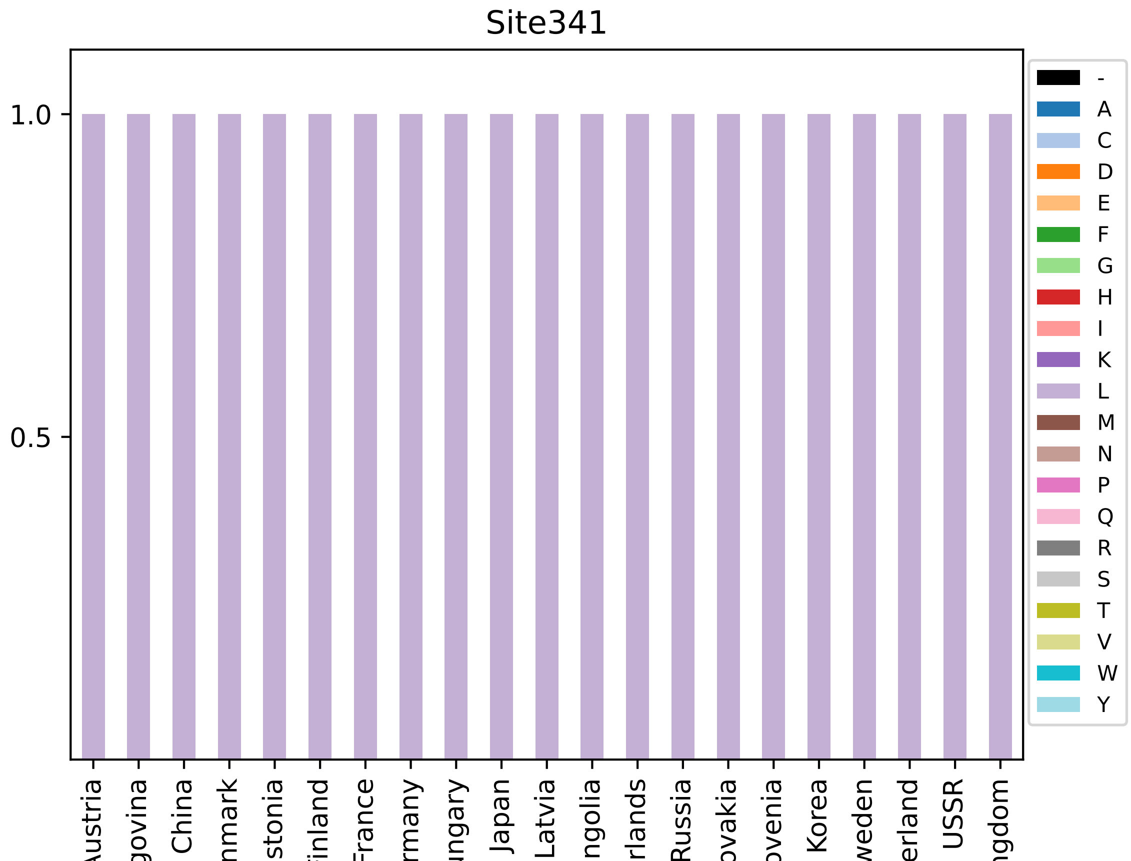

Site341
1.0
0.5
Austria
govina
China
nmark
stonia
Finland
France
rmany
ungary
Japan
Latvia
ngolia
rlands
Russia
ovakia
ovenia
Korea
weden
erland
USSR
ngdom
-
A
C
D
E
F
G
H
I
K
L
M
N
P
Q
R
S
T
V
W
Y

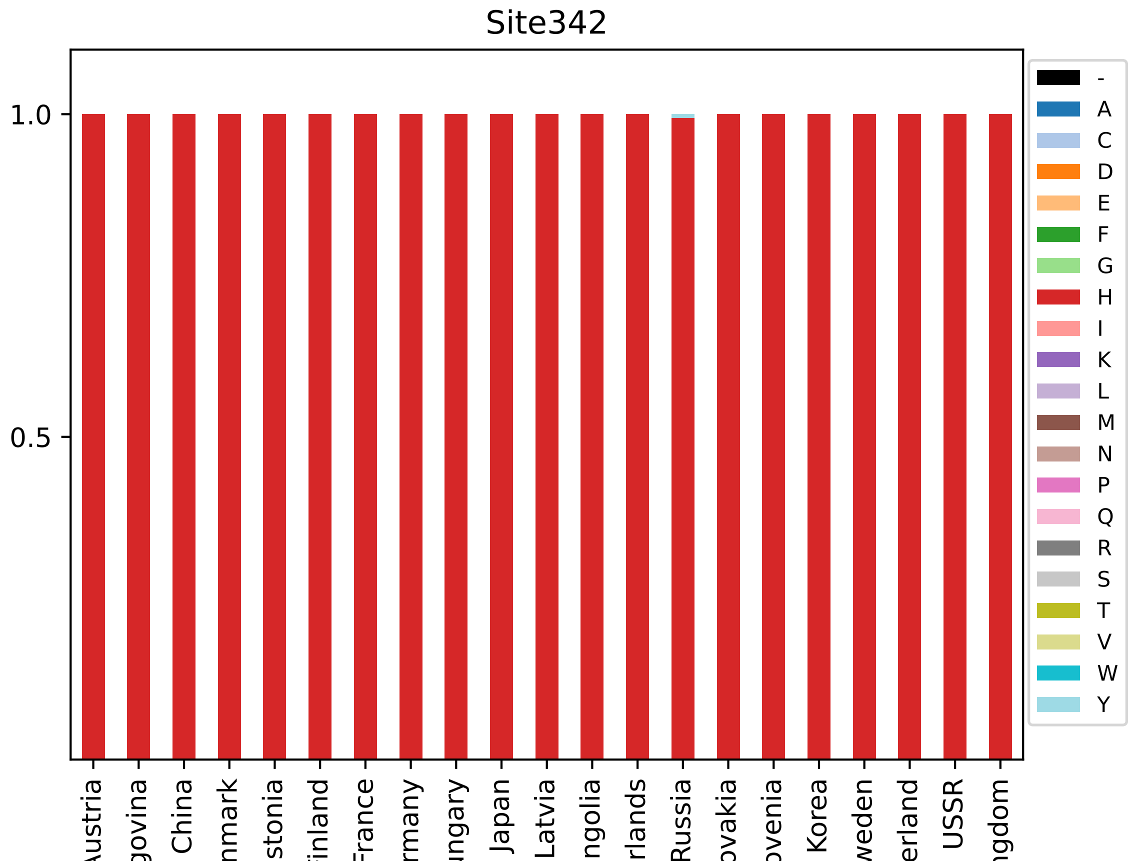

Site342
1.0
0.5
-
A
C
D
E
F
G
H
I
K
L
M
N
P
Q
R
S
T
V
W
Y
Austria
China
Japan
Latvia
Russia
Korea
USSR

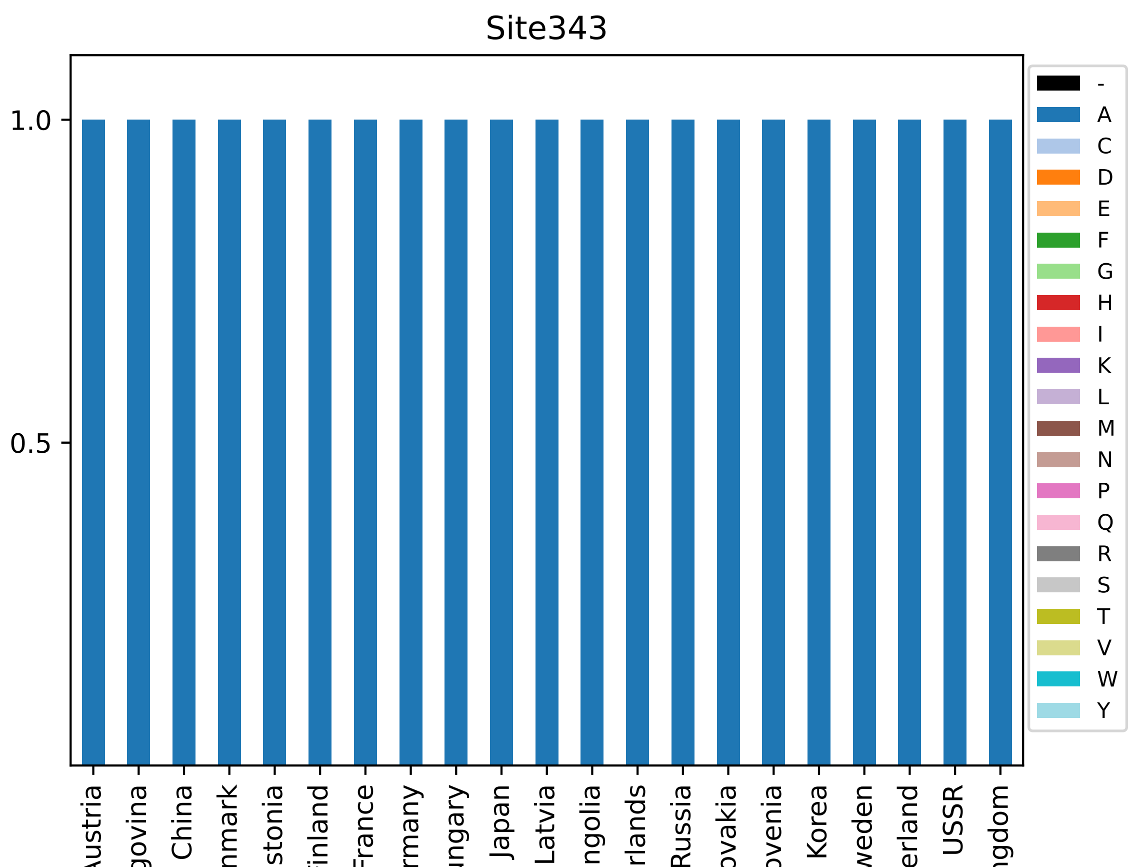
Site343
1.0
0.5
Austria
govina
China
nmark
stonia
Finland
France
rmany
ungary
Japan
Latvia
ngolia
rlands
Russia
ovakia
ovenia
Korea
weden
erland
USSR
ngdom
-
A
C
D
E
F
G
H
I
K
L
M
N
P
Q
R
S
T
V
W
Y

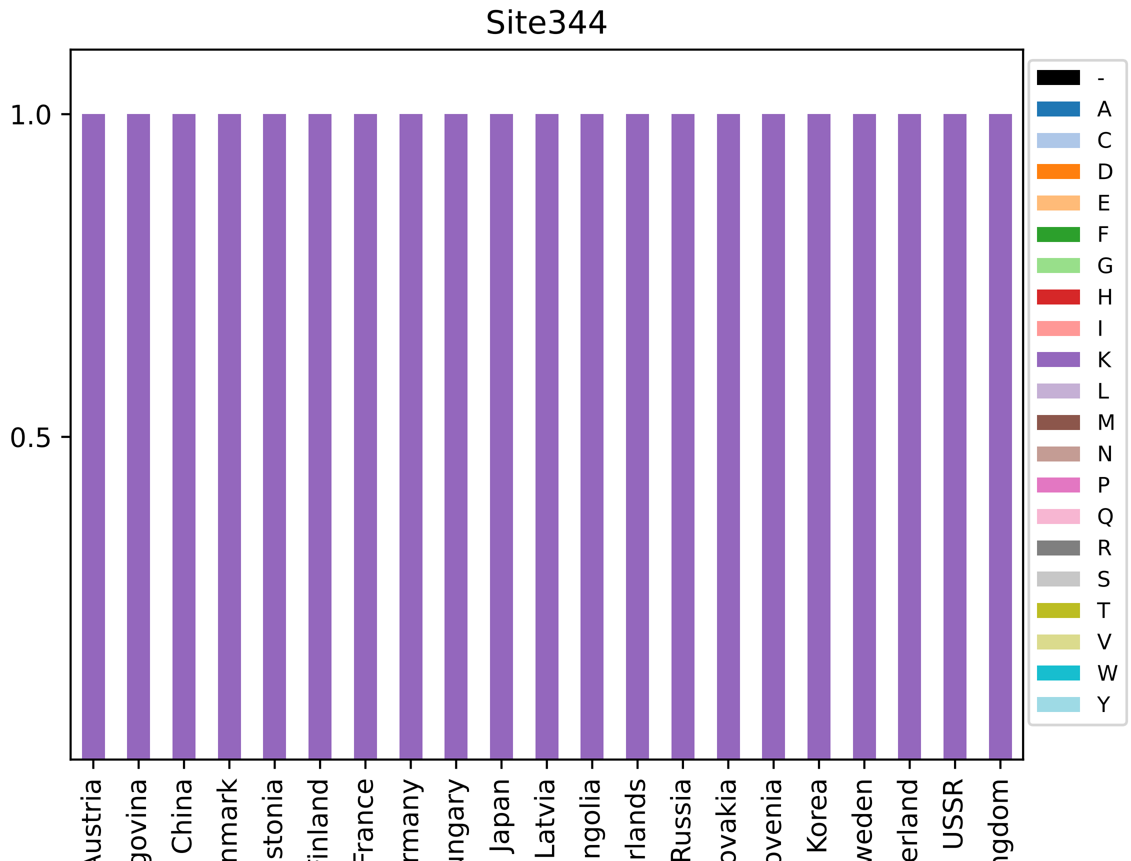
Site344
1.0
0.5
Austria
govina
China
nmark
stonia
inland
France
rmany
ungary
Japan
Latvia
ngolia
rlands
Russia
ovakia
ovenia
Korea
weden
erland
USSR
ngdom
-
A
C
D
E
F
G
H
I
K
L
M
N
P
Q
R
S
T
V
W
Y

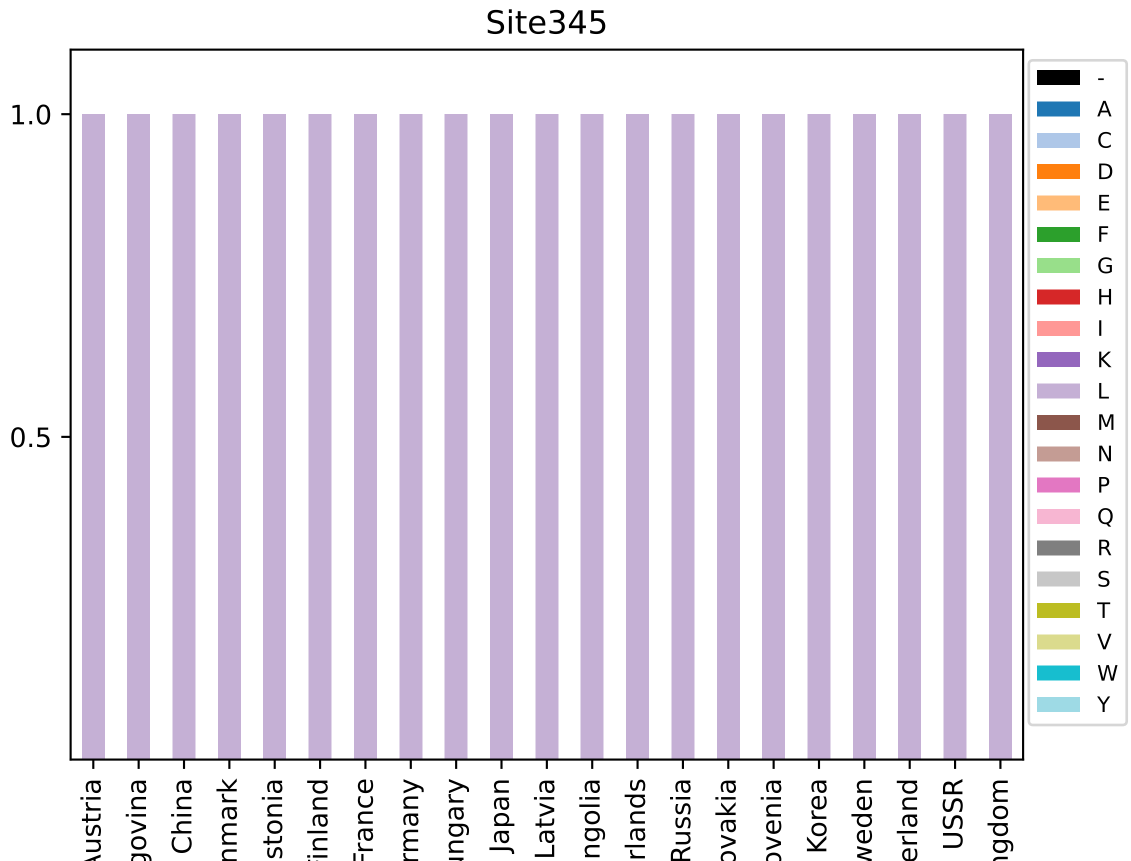

Site345
1.0
0.5
-
A
C
D
E
F
G
H
I
K
L
M
N
P
Q
R
S
T
V
W
Y
Austria
China
Finland
France
Japan
Latvia
Russia
Korea
USSR

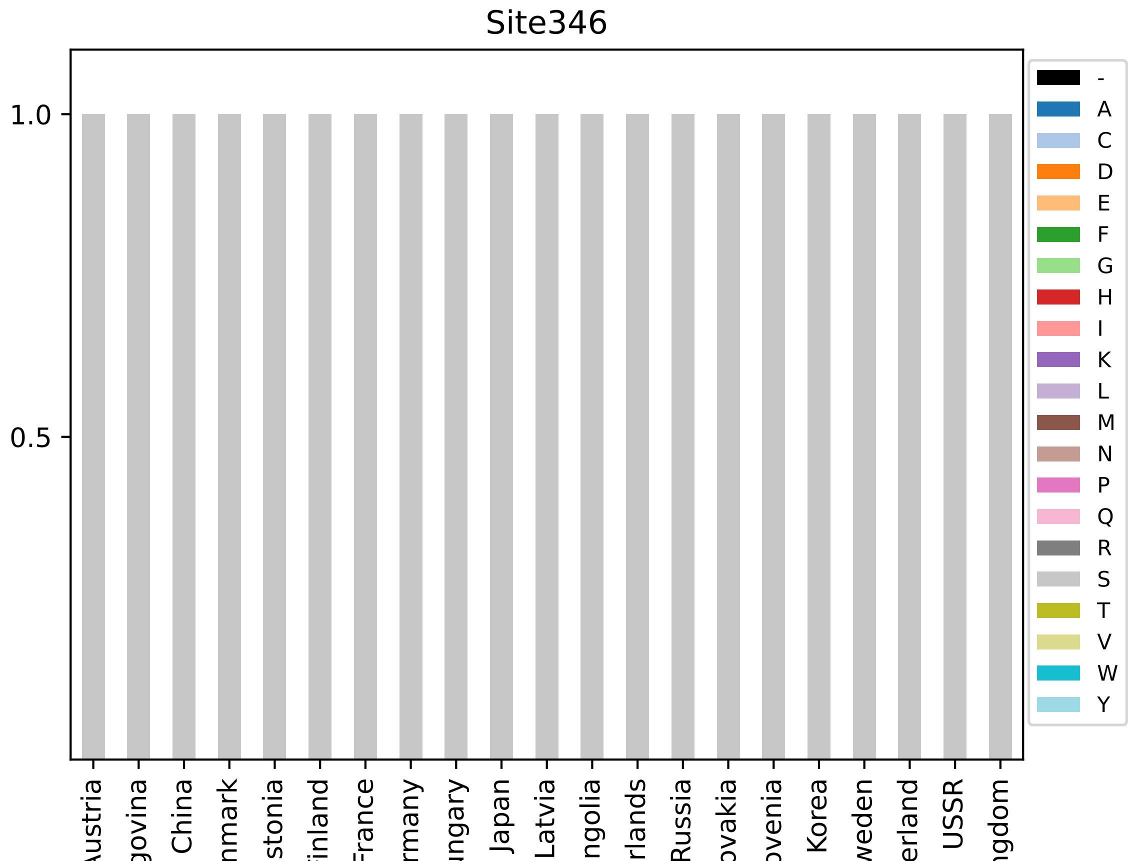

Site346
1.0
0.5
-
A
C
D
E
F
G
H
I
K
L
M
N
P
Q
R
S
T
V
W
Y
Austria
govina
China
nmark
stonia
Finland
France
rmany
ungary
Japan
Latvia
ngolia
rlands
Russia
ovakia
ovenia
Korea
weden
erland
USSR
ngdom

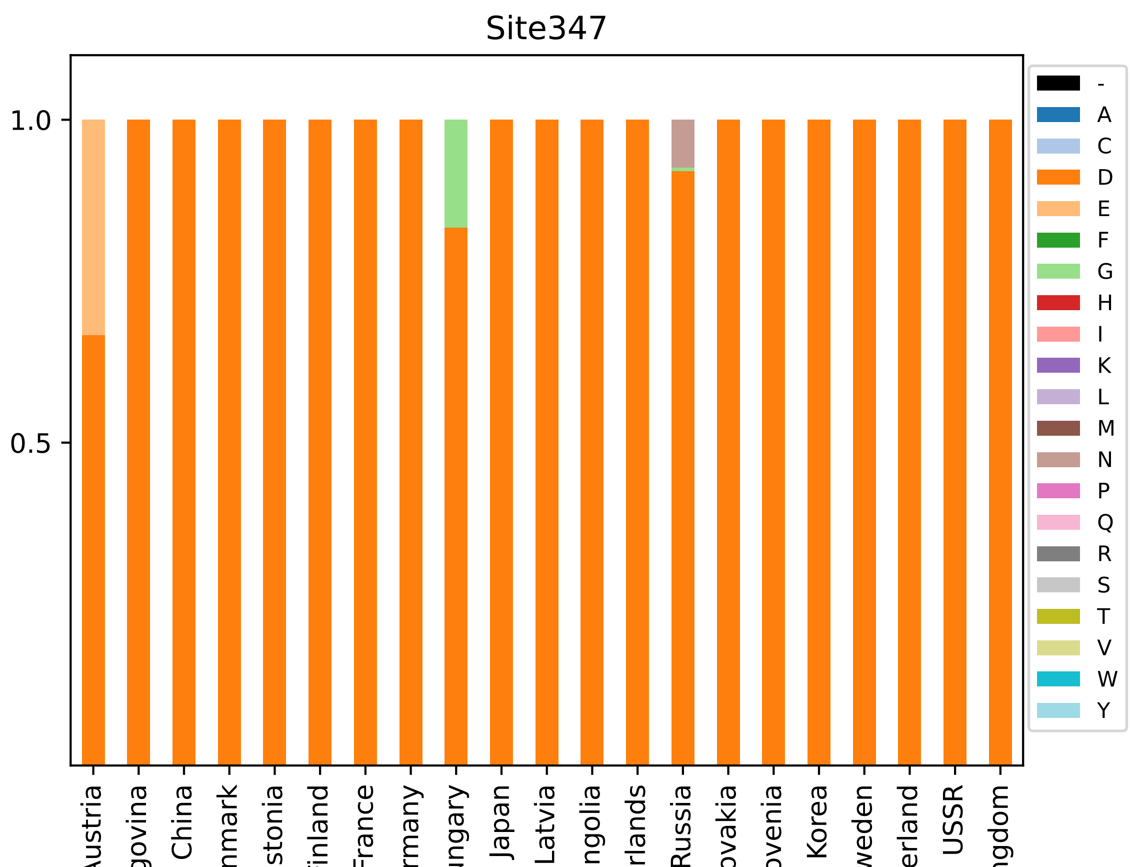

Site347
1.0
0.5
-
A
C
D
E
F
G
H
I
K
L
M
N
P
Q
R
S
T
V
W
Y
Austria
govina
China
nmark
stonia
Finland
France
rmany
ungary
Japan
Latvia
ngolia
rlands
Russia
ovakia
ovenia
Korea
weden
erland
USSR
ngdom

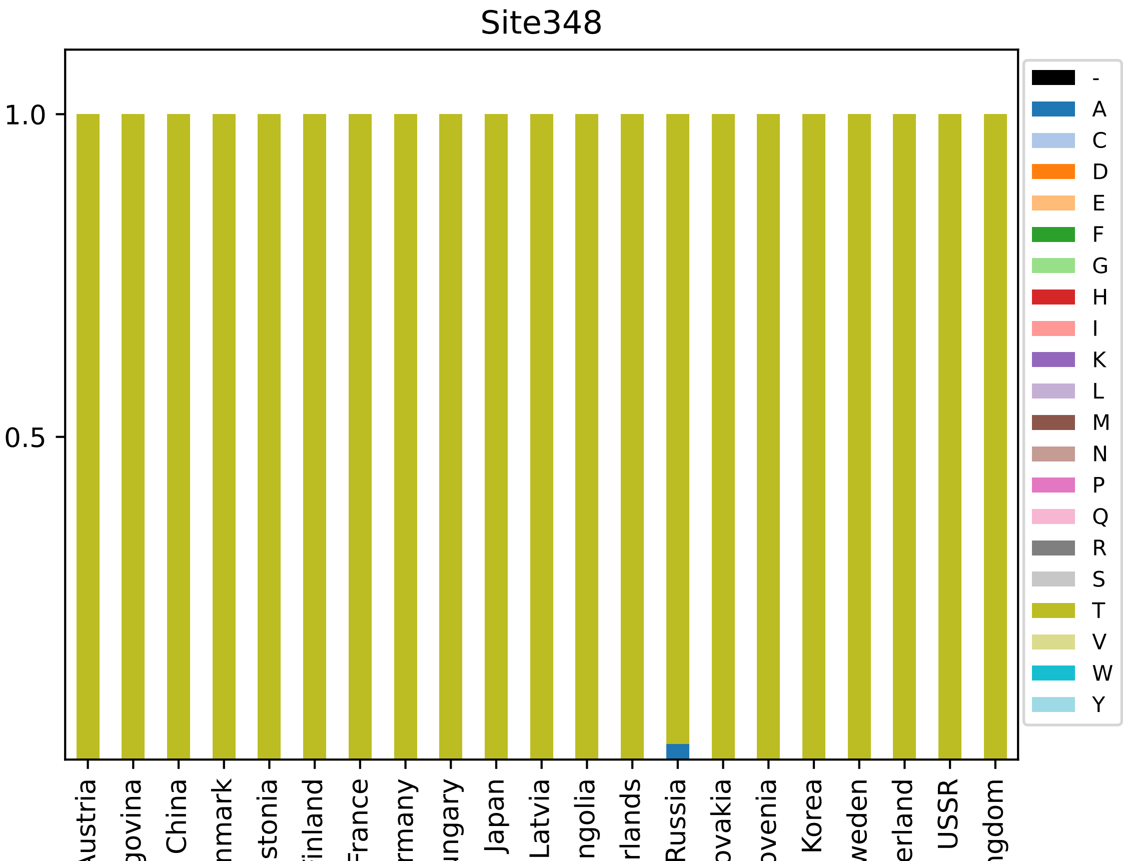

Site348
1.0
0.5
-
A
C
D
E
F
G
H
I
K
L
M
N
P
Q
R
S
T
V
W
Y
China
Japan
Latvia
Russia
Korea
USSR

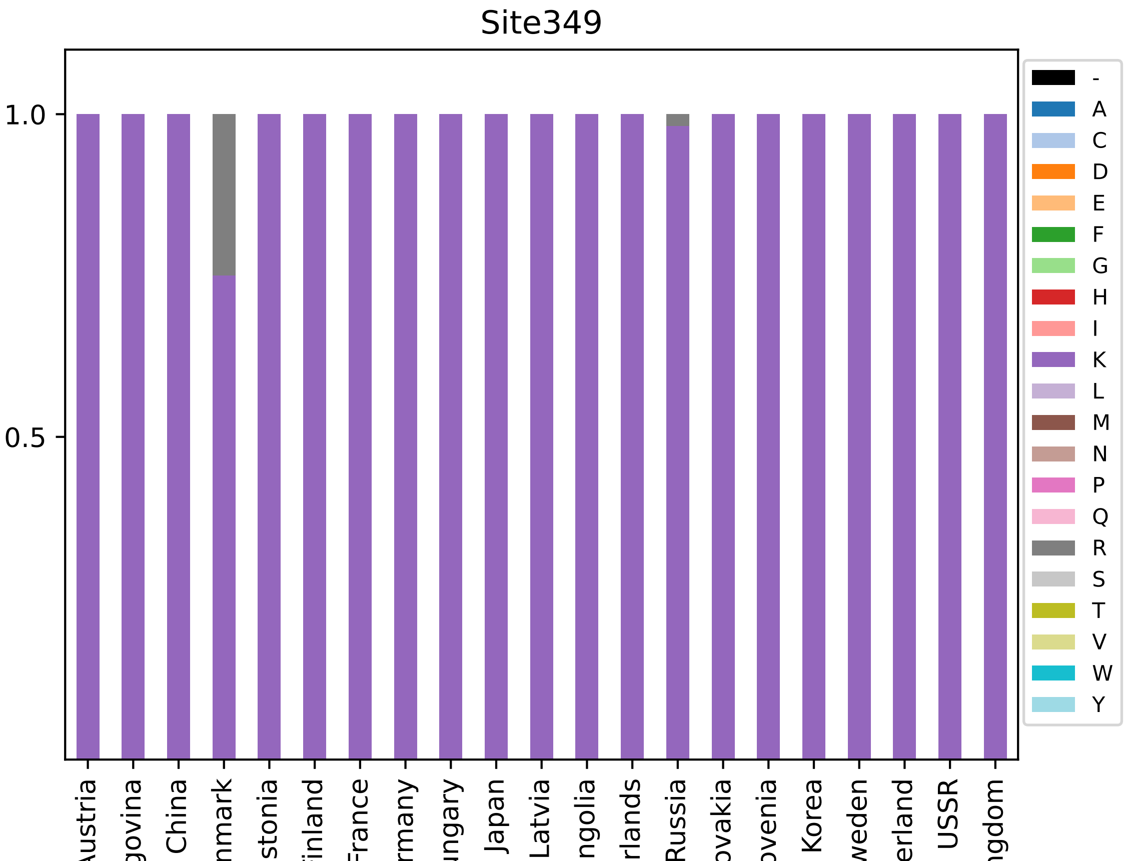
Site349
1.0
0.5
-
A
C
D
E
F
G
H
I
K
L
M
N
P
Q
R
S
T
V
W
Y
China
Finland
Japan
Latvia
Russia
Korea
USSR

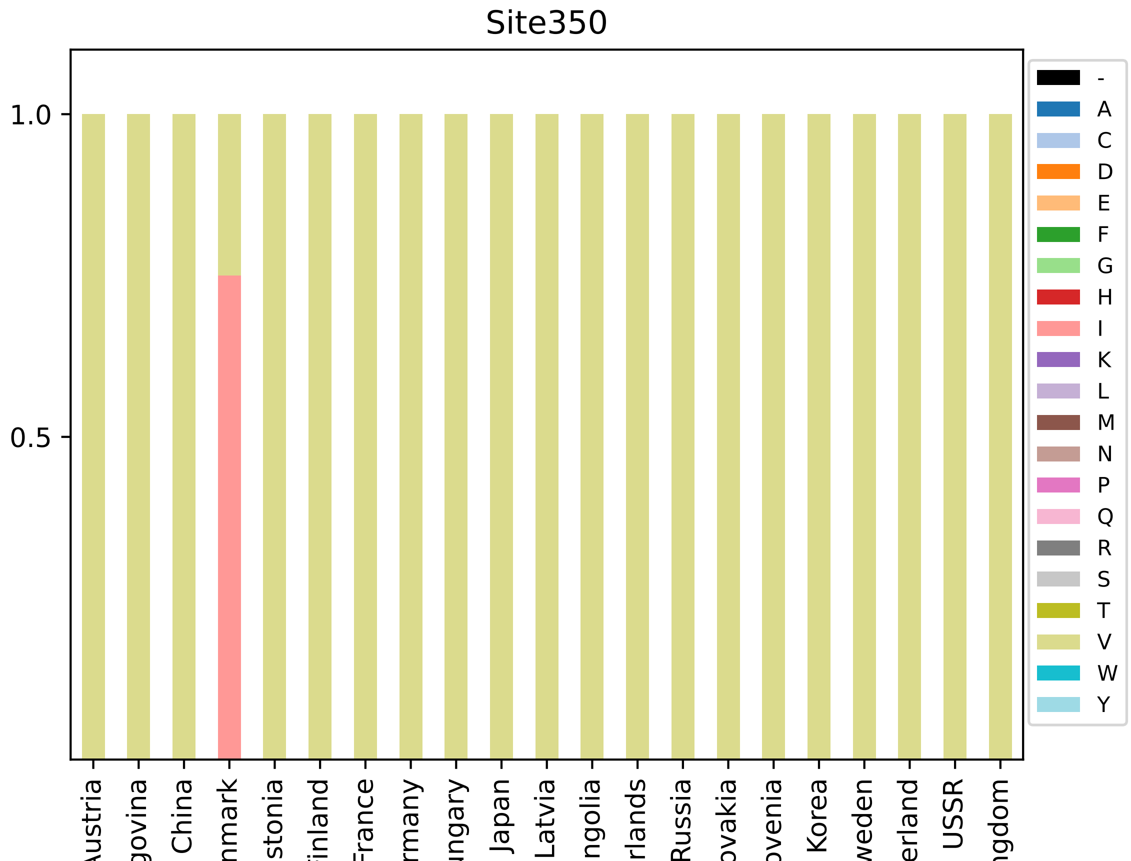
Site350
1.0
0.5
-
A
C
D
E
F
G
H
I
K
L
M
N
P
Q
R
S
T
V
W
Y
Austria
govina
China
nmark
stonia
inland
France
rmany
ungary
Japan
Latvia
ngolia
rlands
Russia
ovakia
ovenia
Korea
weden
erland
USSR
ngdom

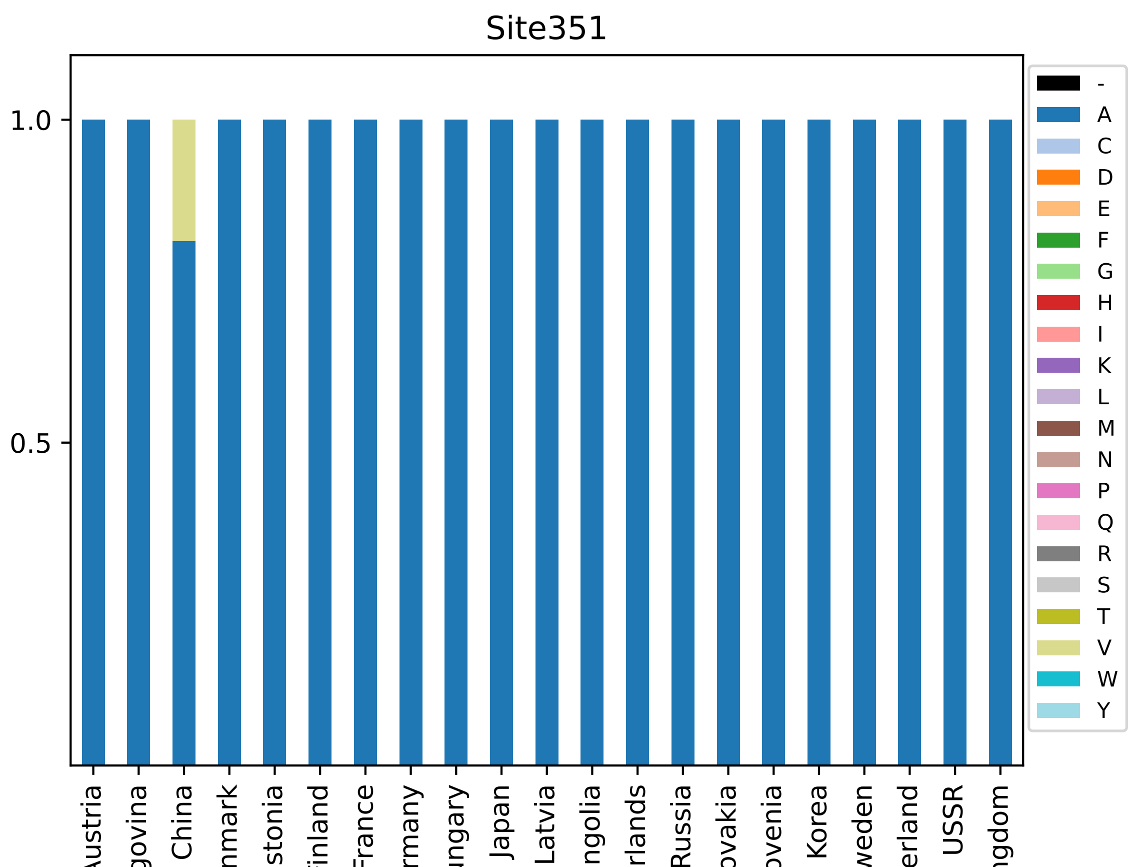

Site351
1.0
0.5
-
A
C
D
E
F
G
H
I
K
L
M
N
P
Q
R
S
T
V
W
Y
Austria
govina
China
nmark
stonia
Finland
France
rmany
ungary
Japan
Latvia
ngolia
rlands
Russia
ovakia
ovenia
Korea
weden
erland
USSR
ngdom

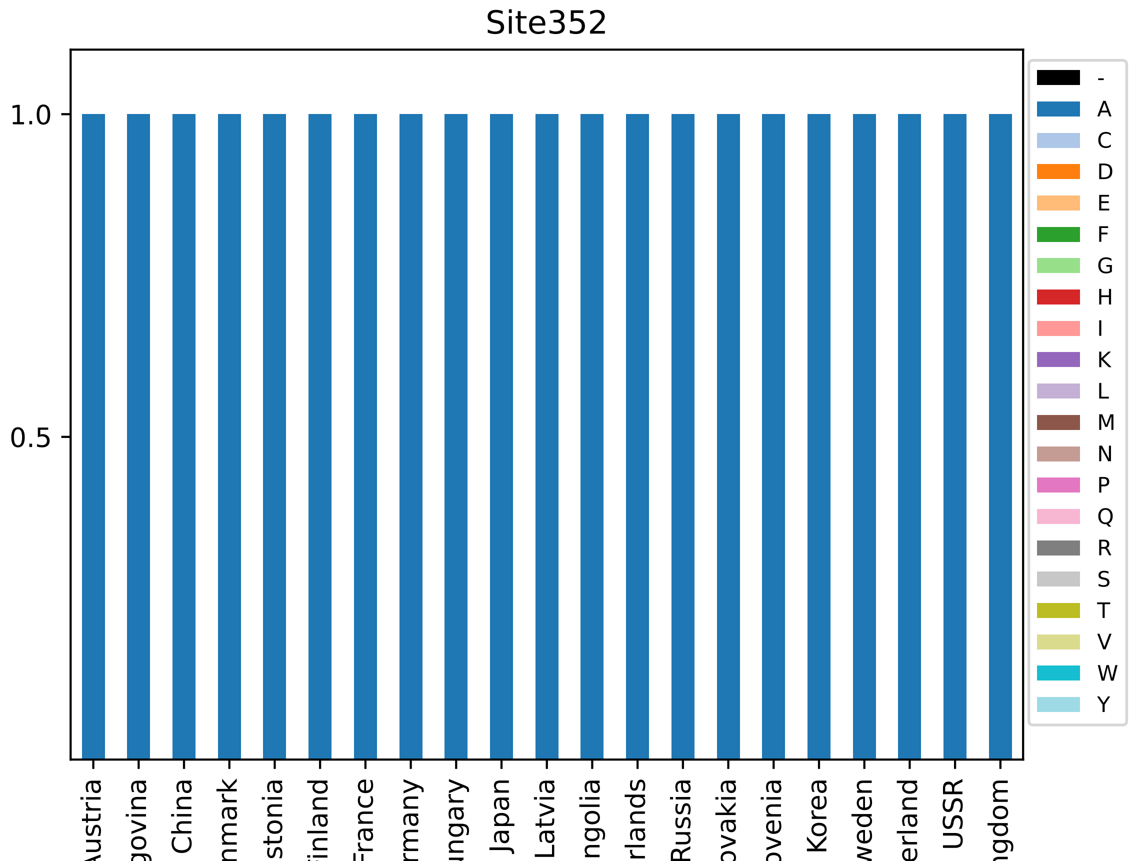

Site352
1.0
0.5
Austria
govina
China
nmark
stonia
Finland
France
rmany
ungary
Japan
Latvia
ngolia
rlands
Russia
ovakia
ovenia
Korea
weden
erland
USSR
ngdom
-
A
C
D
E
F
G
H
I
K
L
M
N
P
Q
R
S
T
V
W
Y

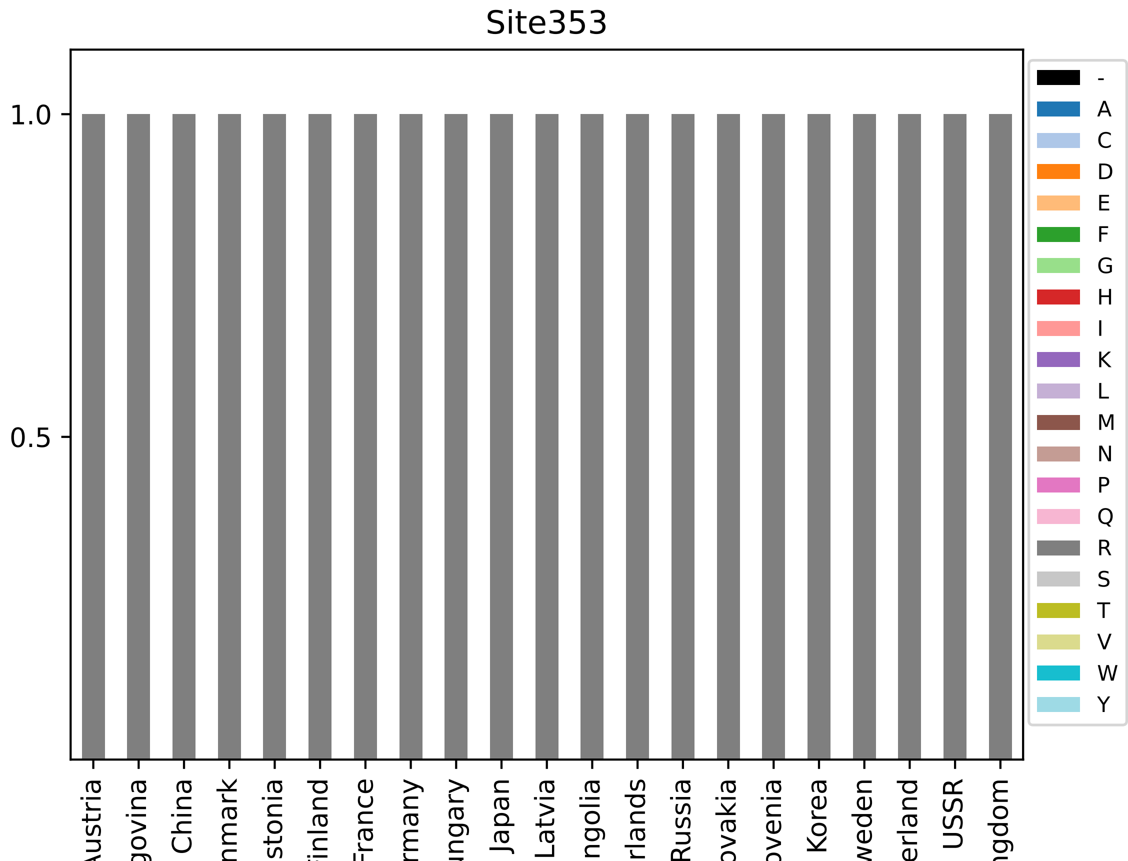
Site353
1.0
0.5
-
A
C
D
E
F
G
H
I
K
L
M
N
P
Q
R
S
T
V
W
Y
Austria
govina
China
nmark
stonia
inland
France
rmany
ungary
Japan
Latvia
ngolia
rlands
Russia
ovakia
ovenia
Korea
weden
erland
USSR
ngdom

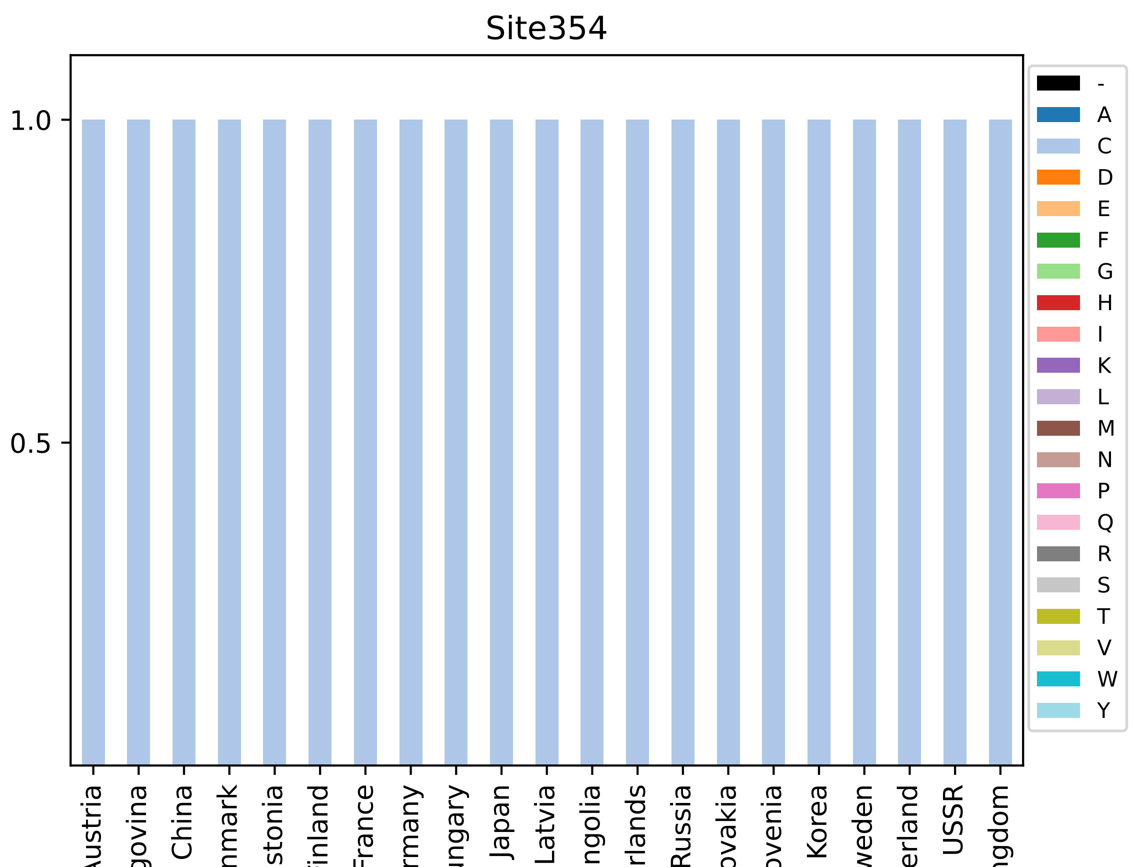
Site354
1.0
0.5
-
A
C
D
E
F
G
H
I
K
L
M
N
P
Q
R
S
T
V
W
Y
Austria
China
Finland
France
Japan
Latvia
Russia
Korea
USSR

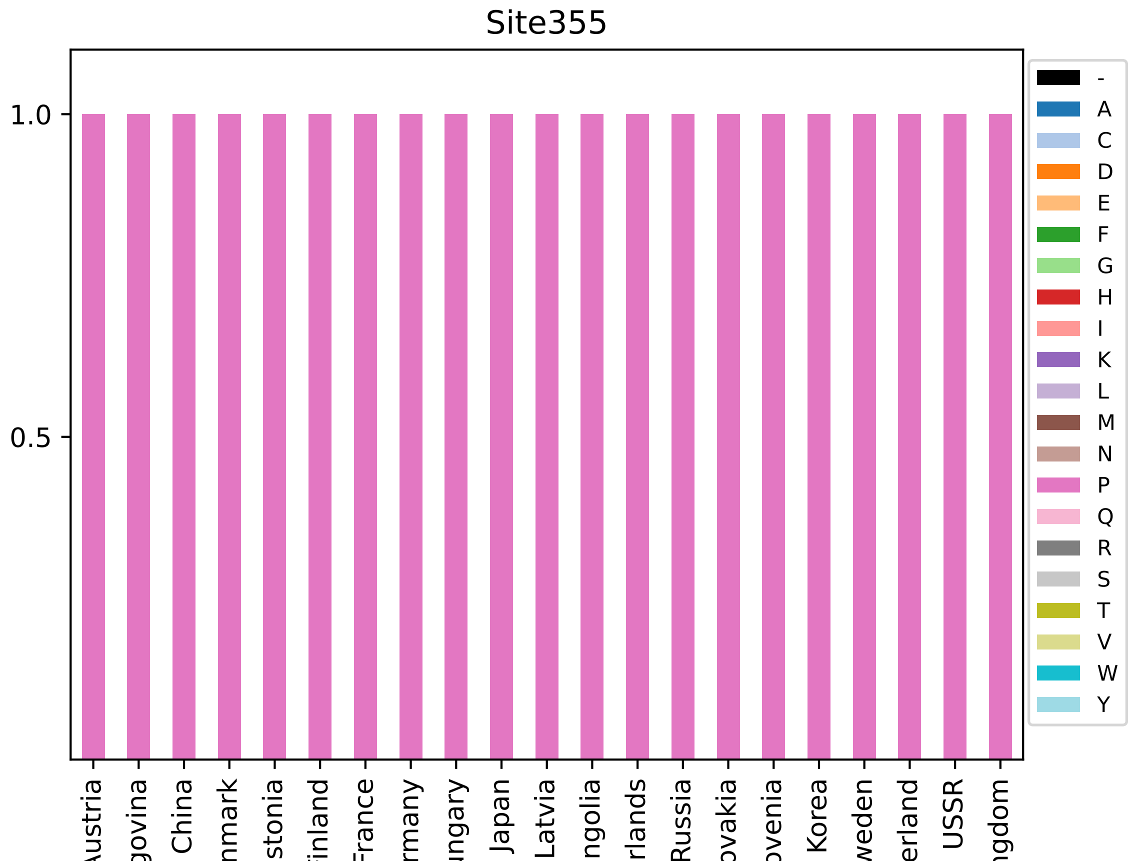
Site355
1.0
0.5
-
A
C
D
E
F
G
H
I
K
L
M
N
P
Q
R
S
T
V
W
Y
China
Japan
Latvia
Russia
Korea
USSR

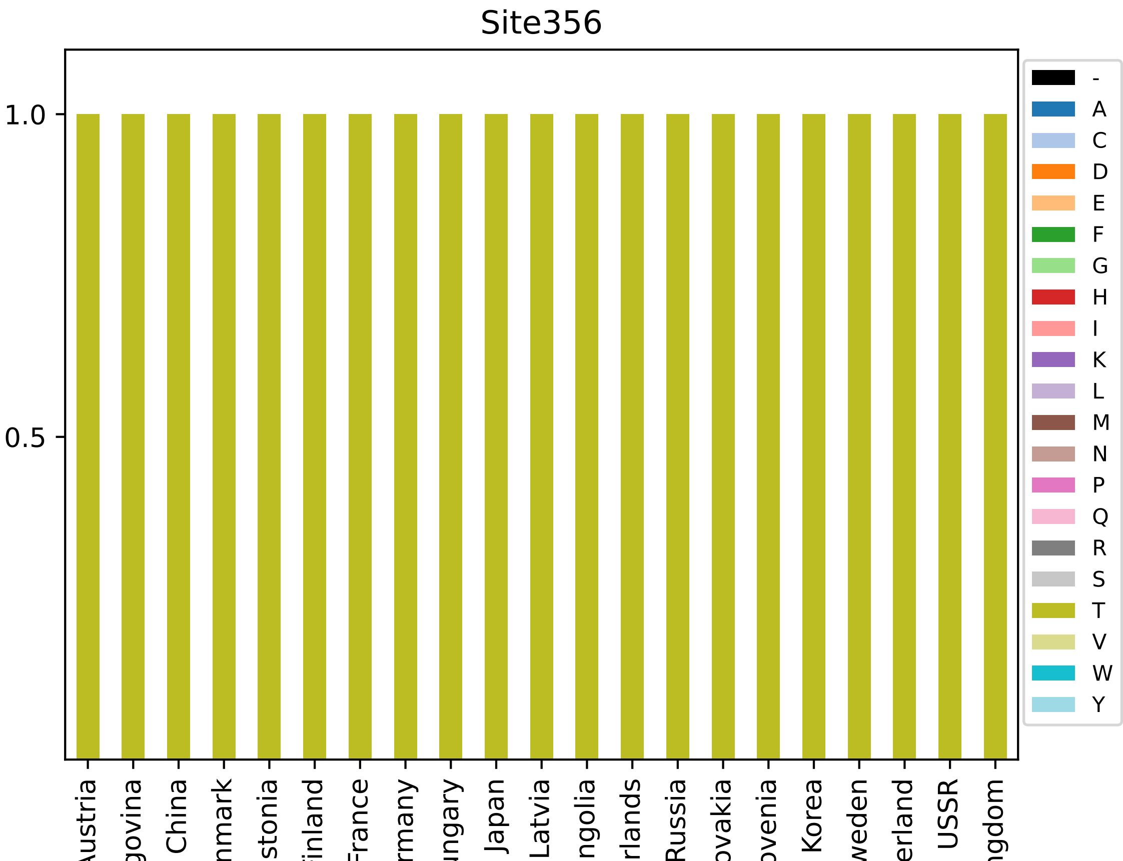
Site356
1.0
0.5
-
A
C
D
E
F
G
H
I
K
L
M
N
P
Q
R
S
T
V
W
Y
China
Japan
Latvia
Russia
Korea
USSR

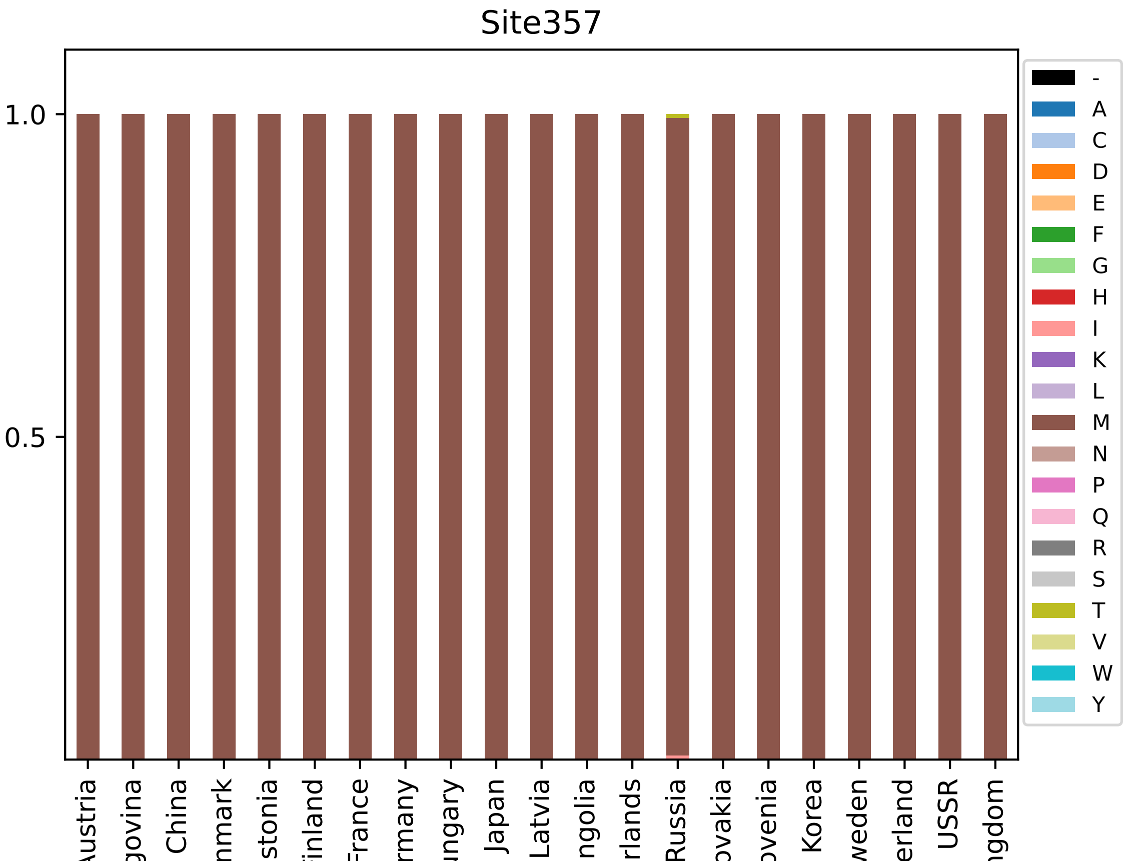
Site357
1.0
0.5
-
A
C
D
E
F
G
H
I
K
L
M
N
P
Q
R
S
T
V
W
Y
China
Japan
Latvia
Russia
Korea
USSR

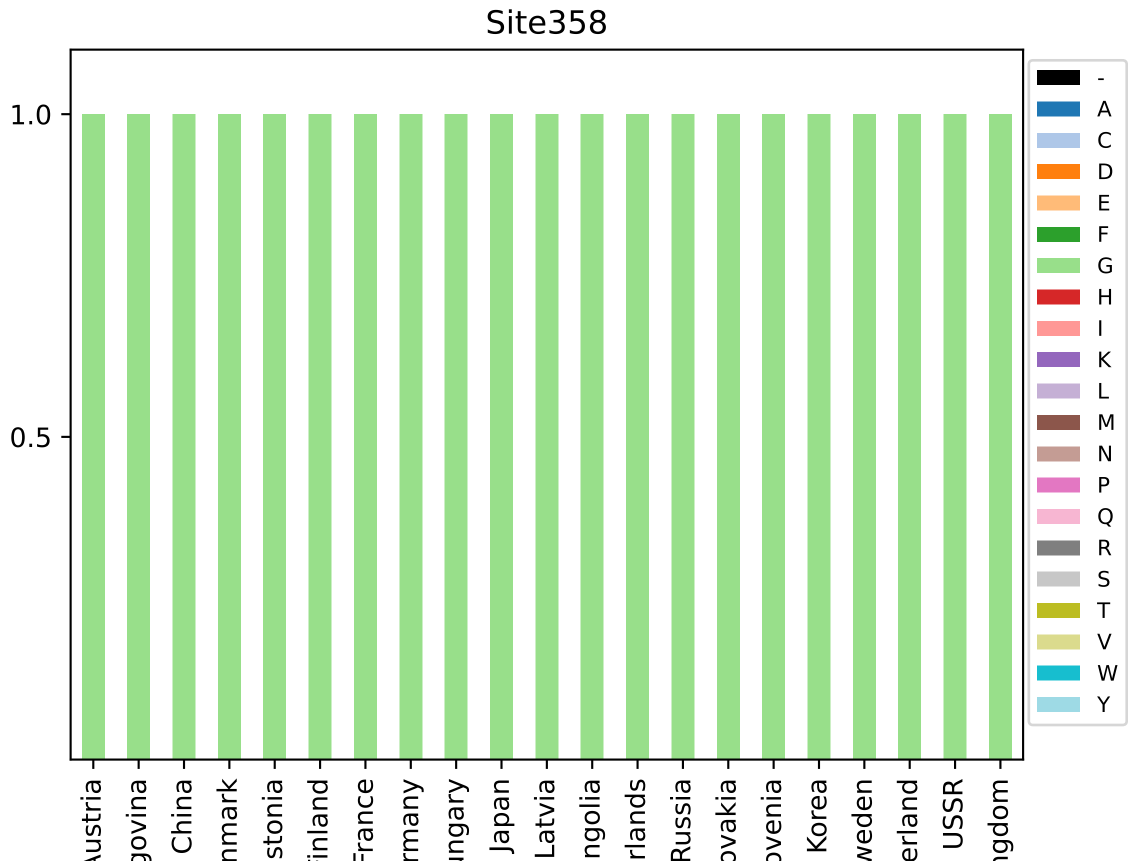

Site358
1.0
0.5
-
A
C
D
E
F
G
H
I
K
L
M
N
P
Q
R
S
T
V
W
Y
China
Japan
Latvia
Russia
Korea
USSR

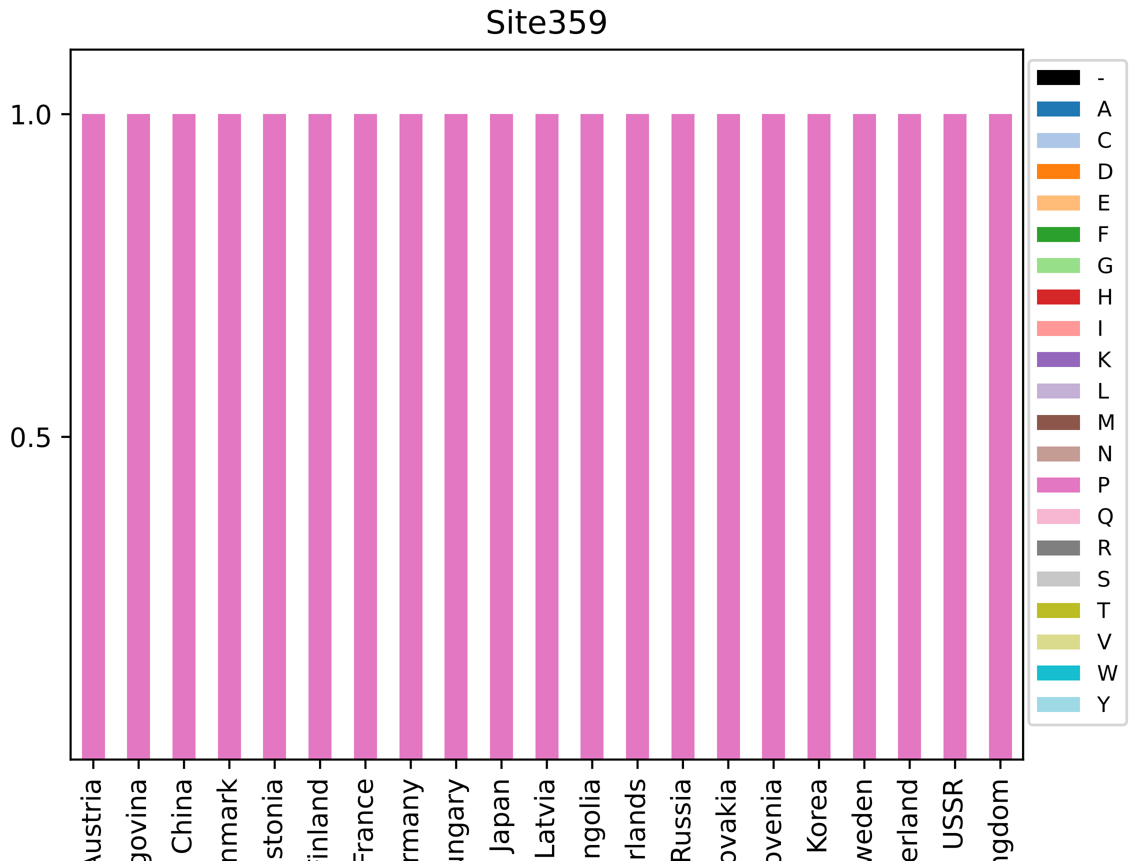
Site359
1.0
0.5
Austria
govina
China
nmark
stonia
inland
France
rmany
ungary
Japan
Latvia
ngolia
rlands
Russia
ovakia
ovenia
Korea
weden
erland
USSR
ngdom
-
A
C
D
E
F
G
H
I
K
L
M
N
P
Q
R
S
T
V
W
Y

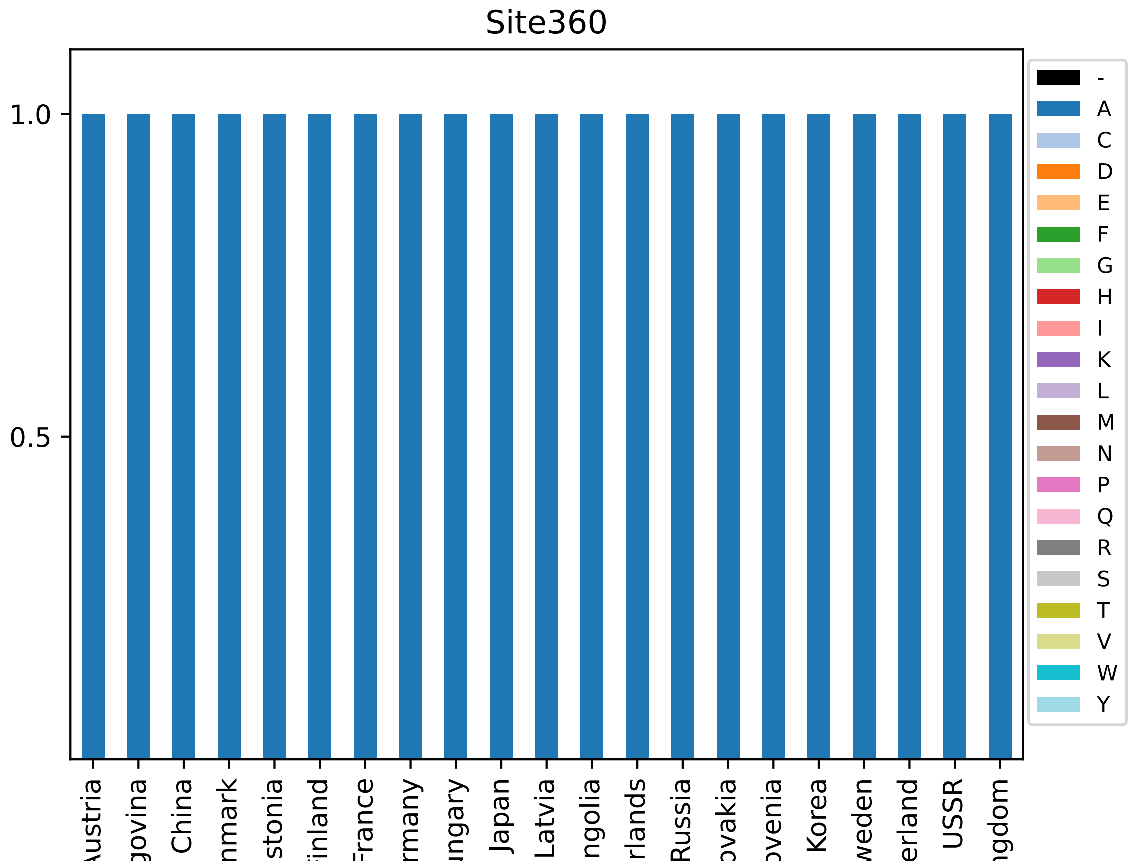

Site360
1.0
0.5
-
A
C
D
E
F
G
H
I
K
L
M
N
P
Q
R
S
T
V
W
Y
China
Japan
Latvia
Russia
Korea
USSR

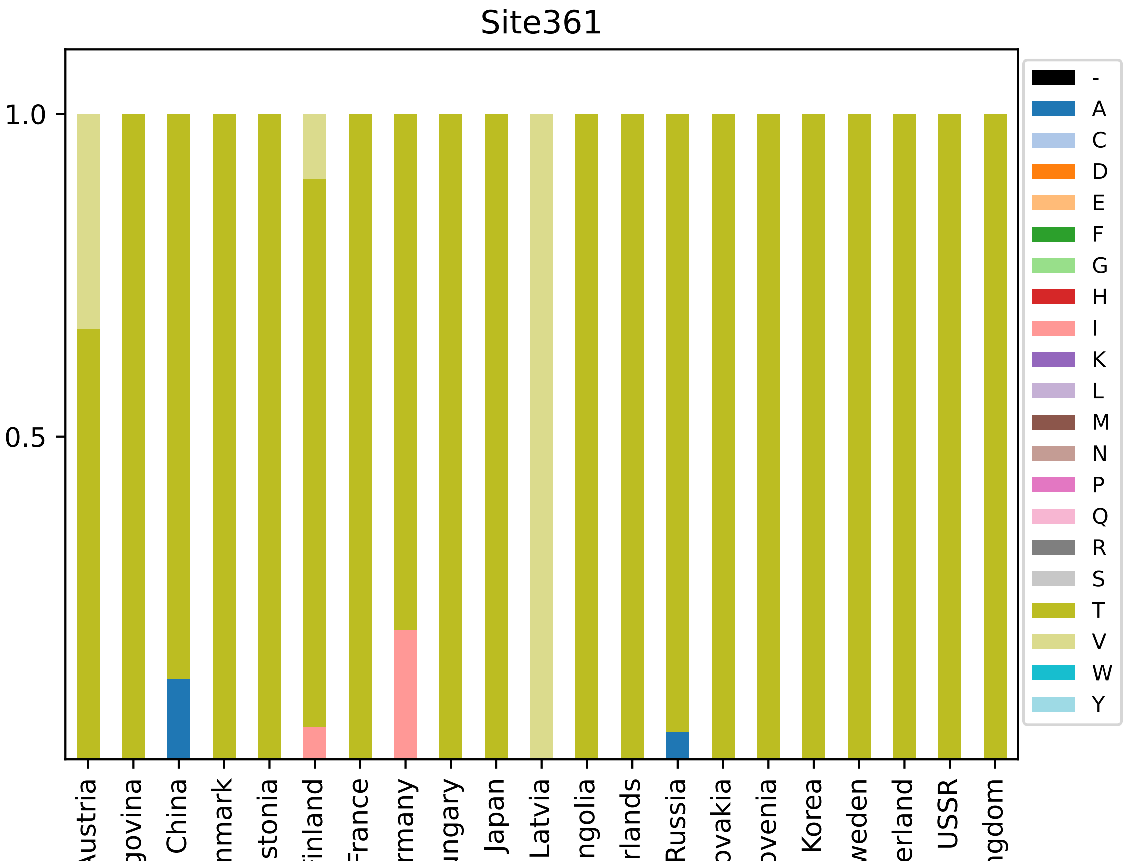
Site361
1.0
0.5
-
A
C
D
E
F
G
H
I
K
L
M
N
P
Q
R
S
T
V
W
Y
China
Japan
Latvia
Russia
Korea
USSR

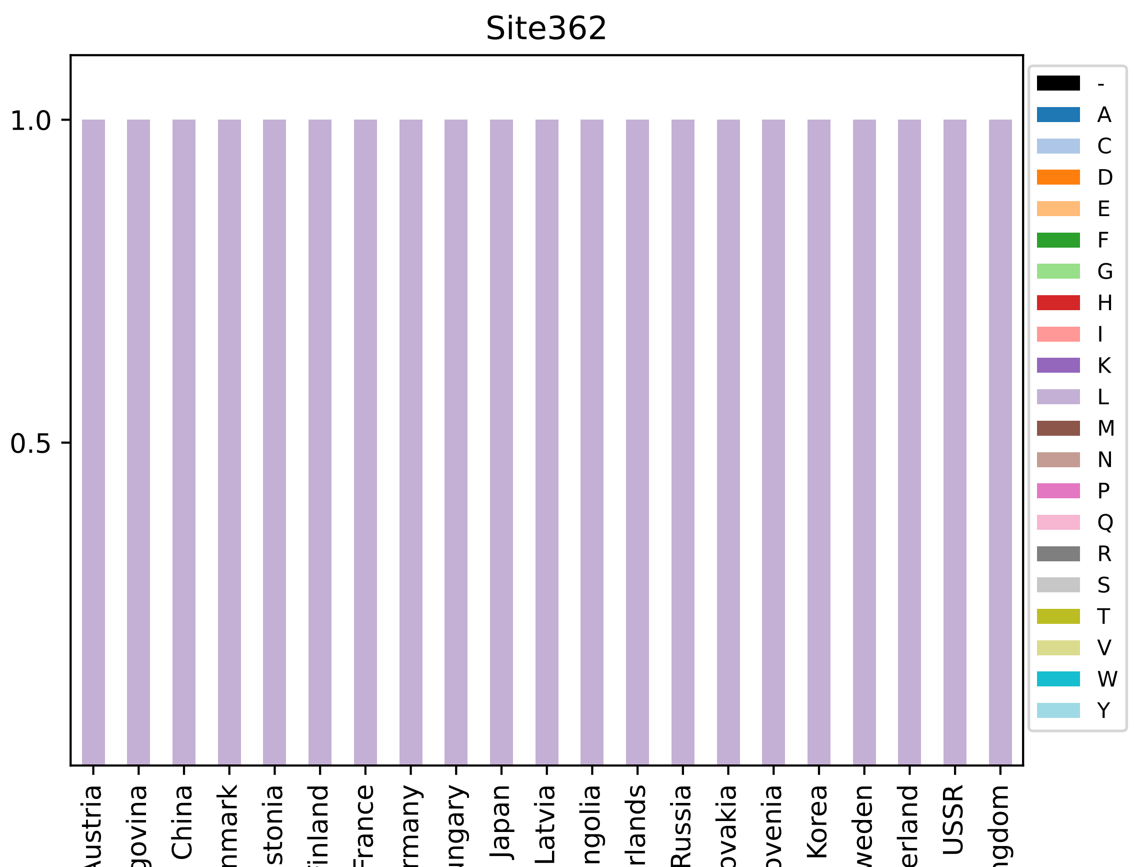
Site362
1.0
0.5
-
A
C
D
E
F
G
H
I
K
L
M
N
P
Q
R
S
T
V
W
Y
Austria
China
Japan
Latvia
Russia
Korea
USSR

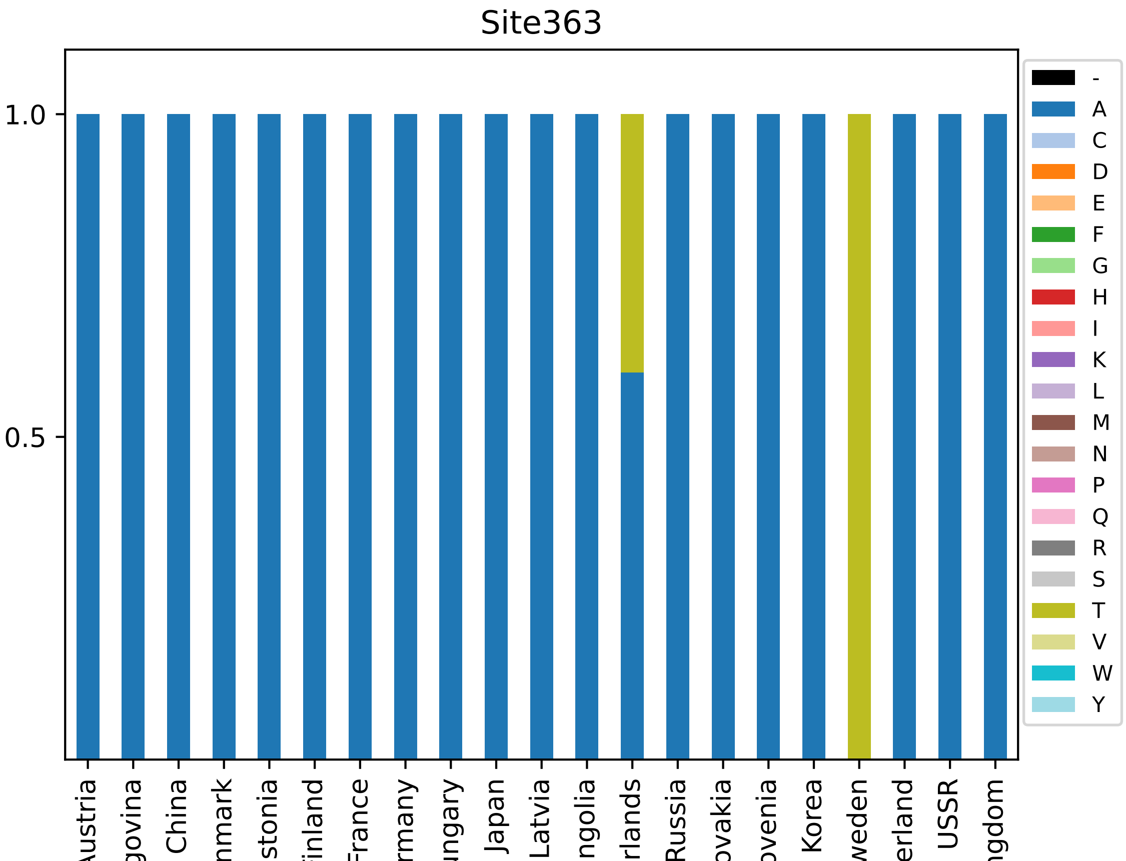
Site363
1.0
0.5
-
A
C
D
E
F
G
H
I
K
L
M
N
P
Q
R
S
T
V
W
Y
China
Japan
Latvia
Russia
Korea
USSR

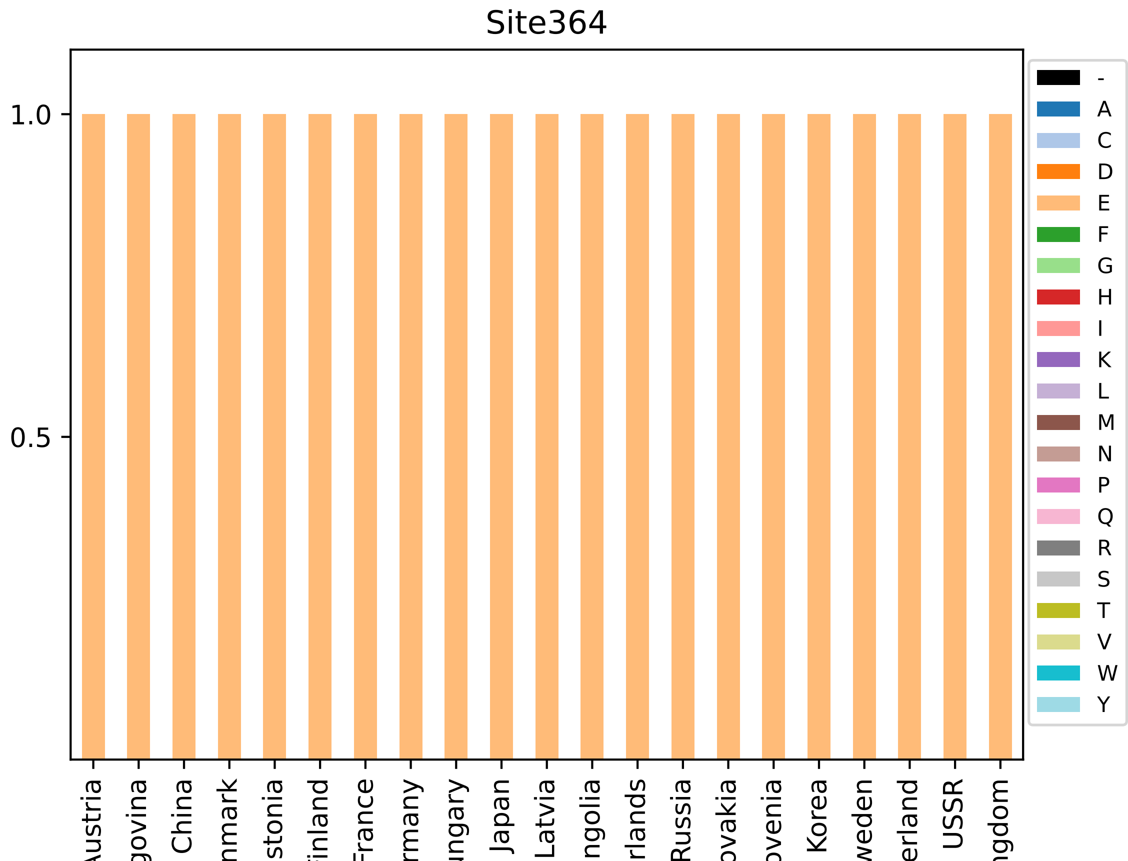

Site364
1.0
0.5
-
A
C
D
E
F
G
H
I
K
L
M
N
P
Q
R
S
T
V
W
Y
Austria
govina
China
nmark
stonia
inland
France
rmany
ungary
Japan
Latvia
ngolia
rlands
Russia
ovakia
ovenia
Korea
weden
erland
USSR
ngdom

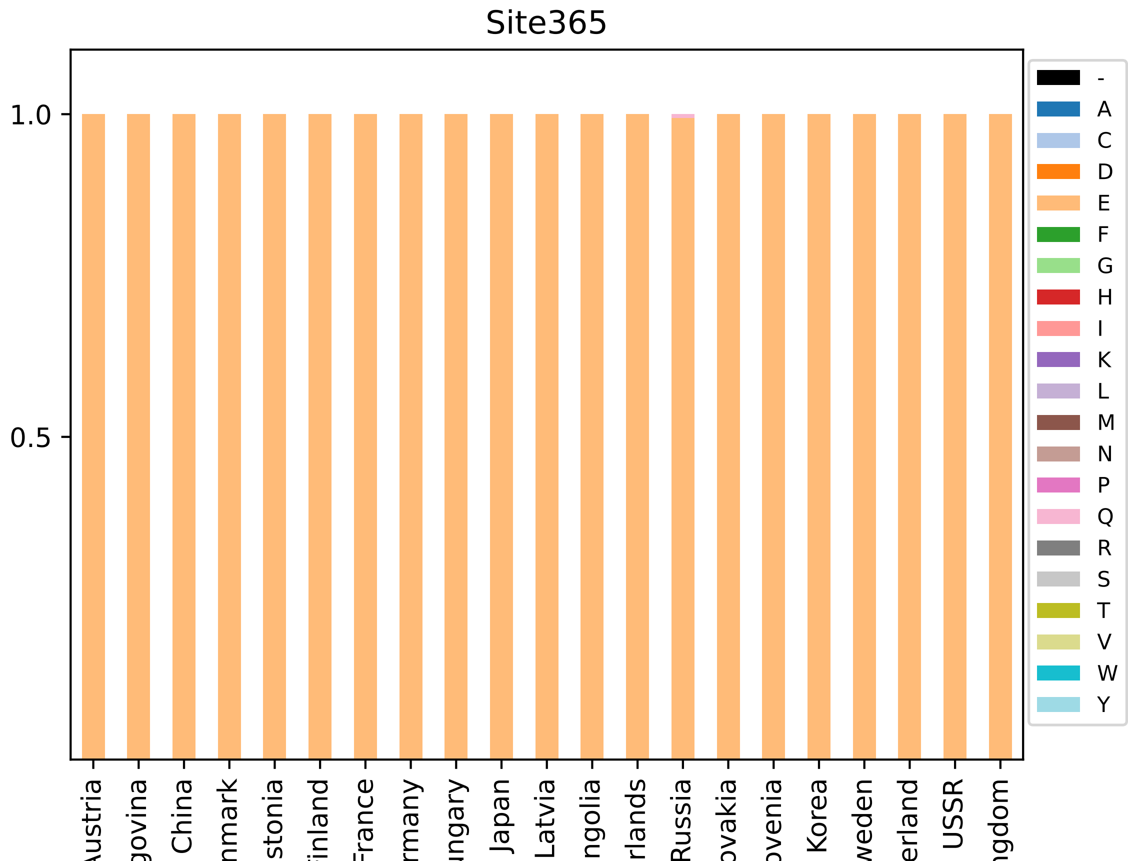

Site365
1.0
0.5
-
A
C
D
E
F
G
H
I
K
L
M
N
P
Q
R
S
T
V
W
Y
Austria
govina
China
nmark
stonia
inland
France
rmany
ungary
Japan
Latvia
ngolia
rlands
Russia
ovakia
ovenia
Korea
weden
erland
USSR
ngdom

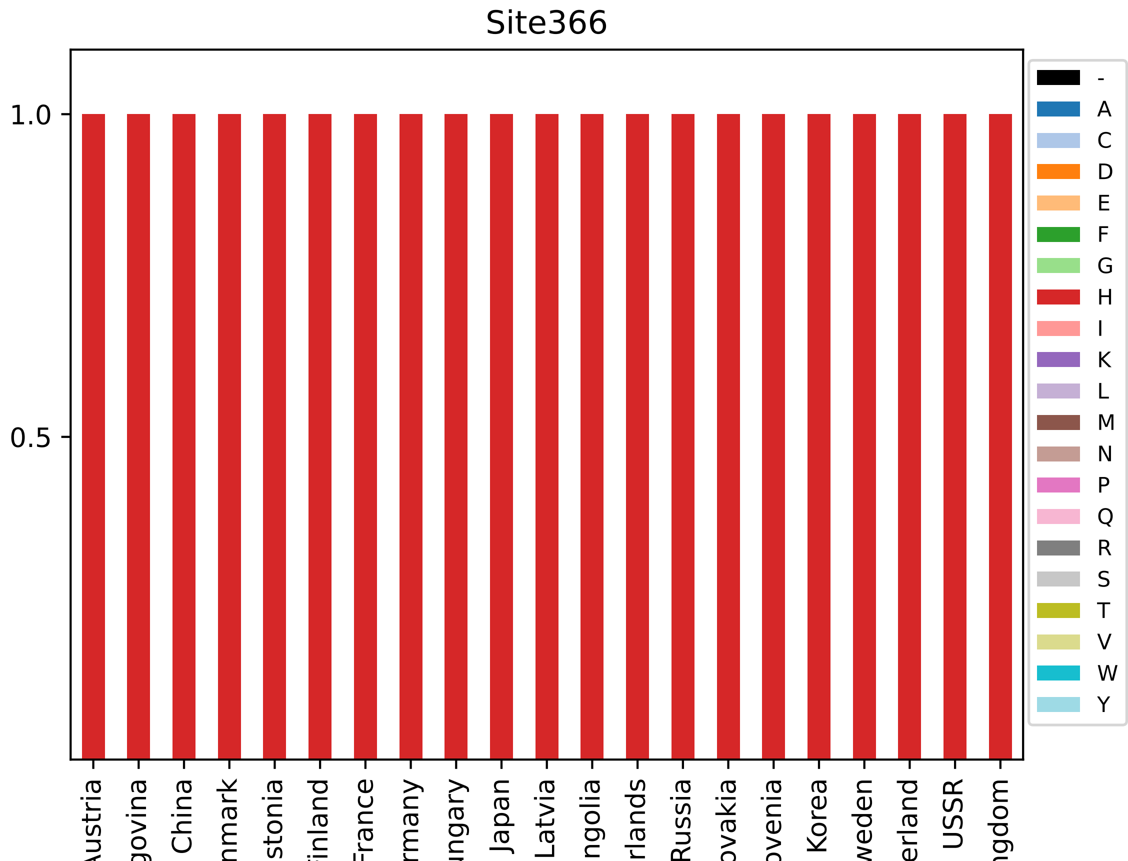
Site366
1.0
0.5
China
Japan
Latvia
Russia
Korea
USSR
-
A
C
D
E
F
G
H
I
K
L
M
N
P
Q
R
S
T
V
W
Y

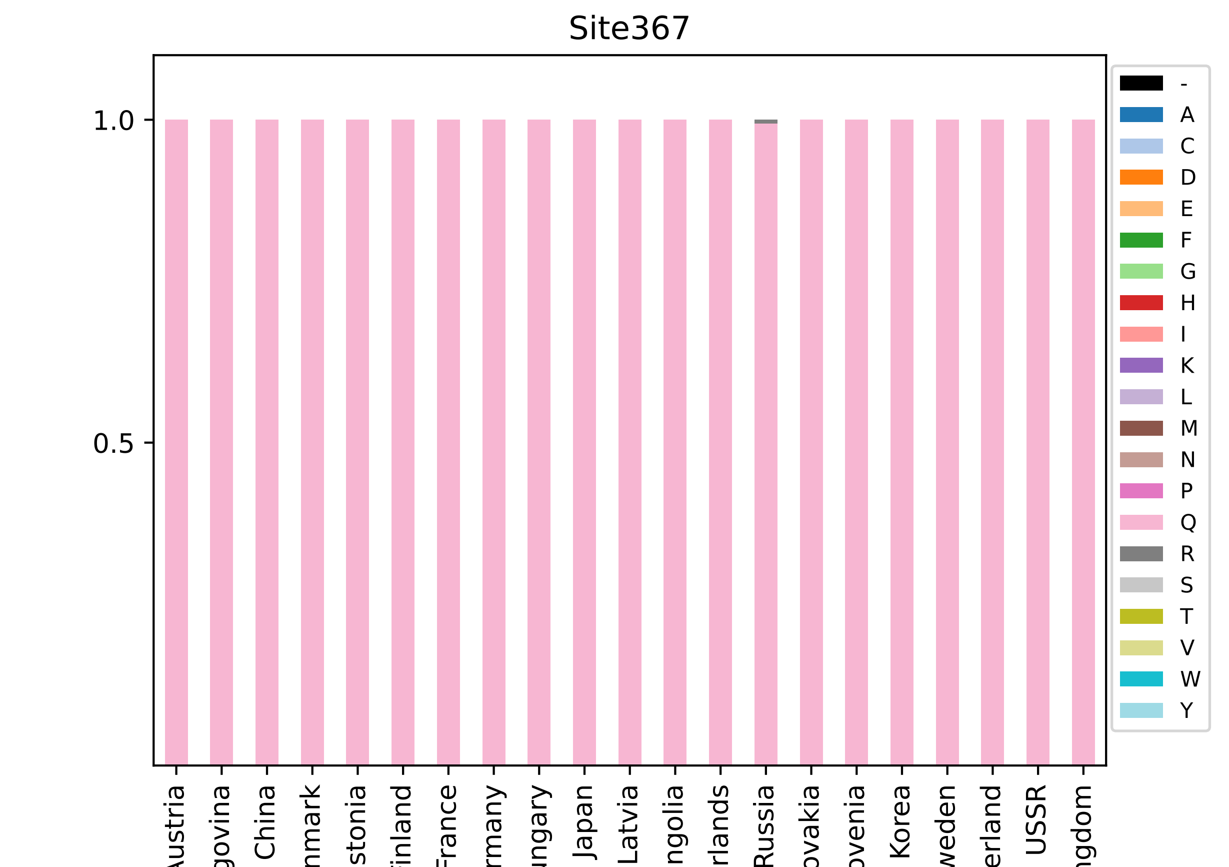

Site367
1.0
0.5
-
A
C
D
E
F
G
H
I
K
L
M
N
P
Q
R
S
T
V
W
Y
Austria
govina
China
nmark
stonia
Finland
France
rmany
ungary
Japan
Latvia
ngolia
rlands
Russia
ovakia
ovenia
Korea
weden
erland
USSR
ngdom

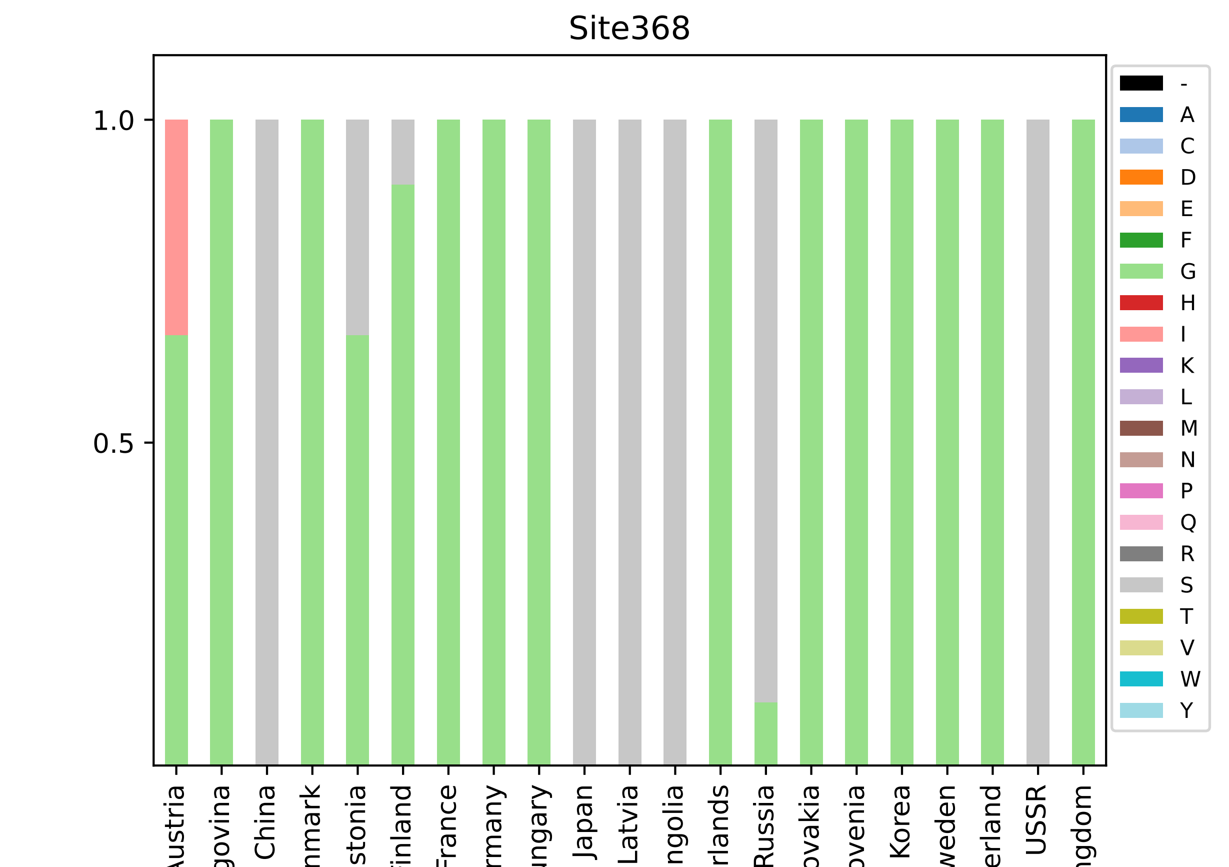

Site368
1.0
0.5
-
A
C
D
E
F
G
H
I
K
L
M
N
P
Q
R
S
T
V
W
Y
Austria
govina
China
nmark
stonia
Finland
France
rmany
ungary
Japan
Latvia
ngolia
rlands
Russia
ovakia
ovenia
Korea
weden
erland
USSR
ngdom

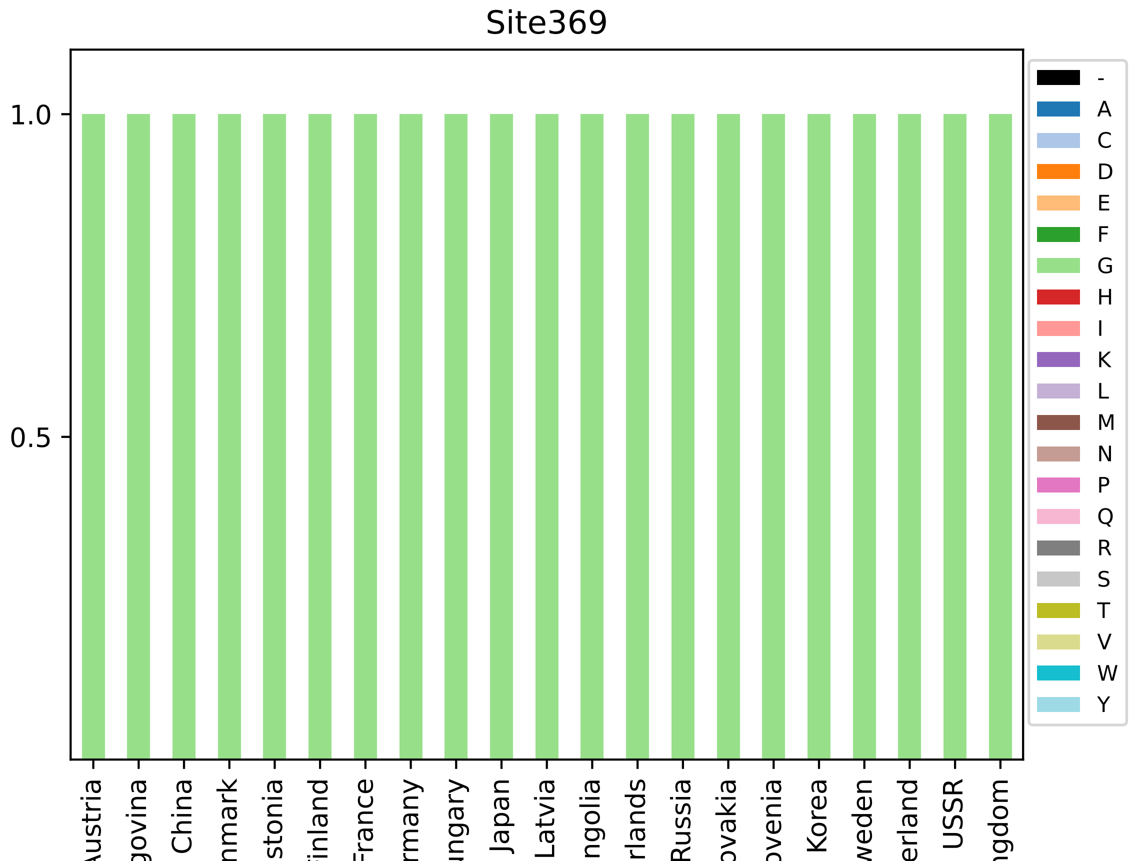

Site369
1.0
0.5
-
A
C
D
E
F
G
H
I
K
L
M
N
P
Q
R
S
T
V
W
Y
China
Japan
Latvia
Russia
Korea
USSR

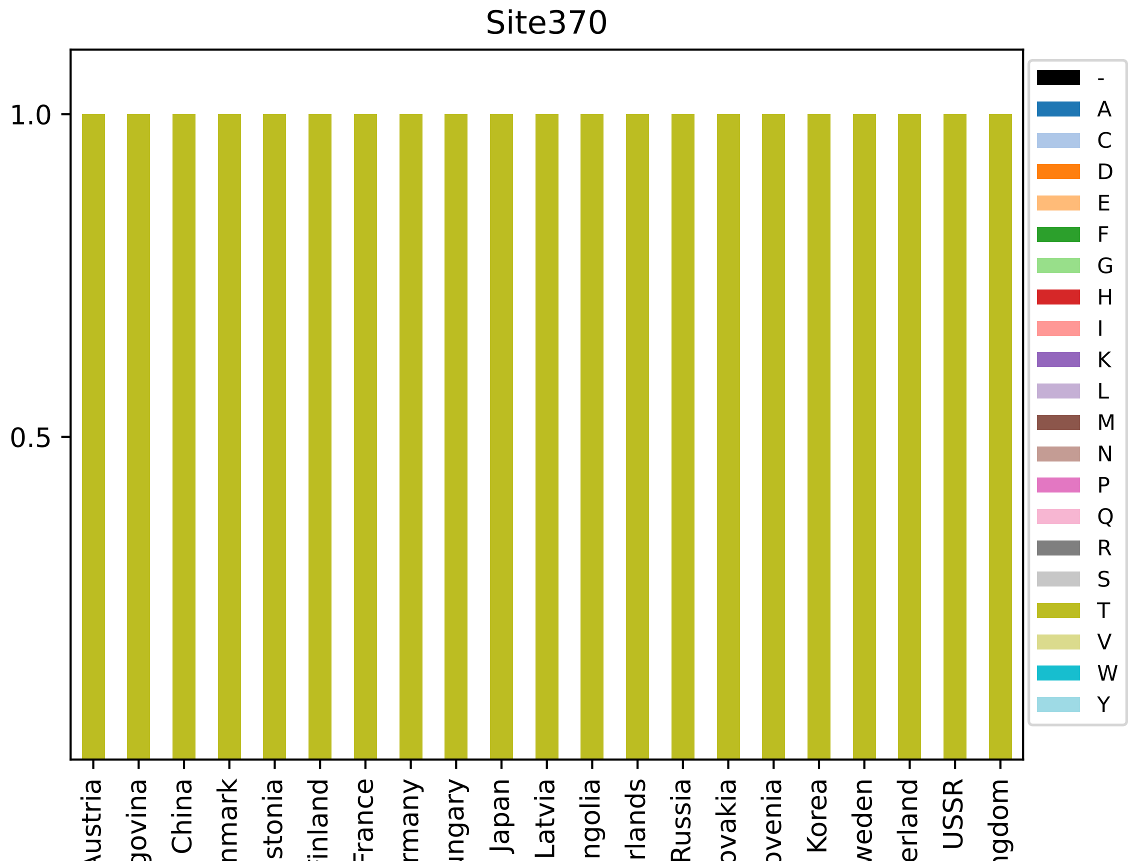
Site370
1.0
0.5
-
A
C
D
E
F
G
H
I
K
L
M
N
P
Q
R
S
T
V
W
Y
China
Japan
Latvia
Russia
Korea
USSR

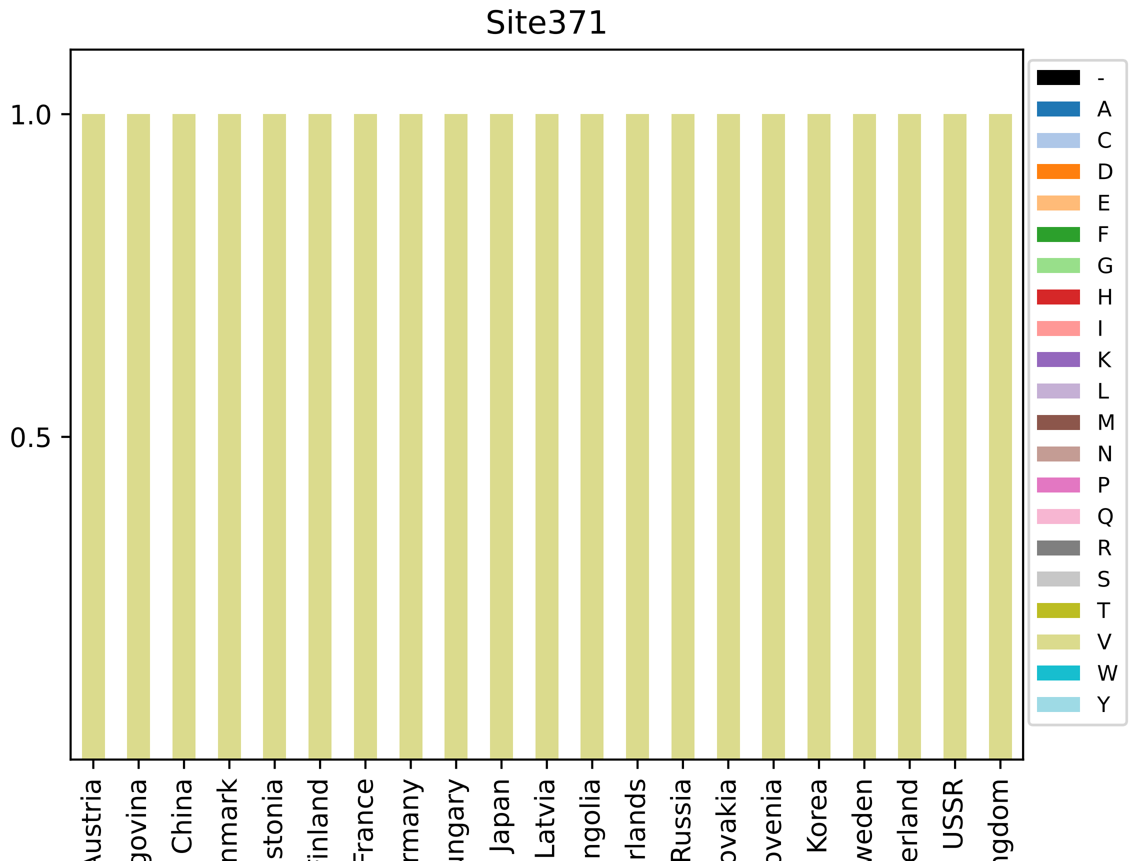
Site371
1.0
0.5
-
A
C
D
E
F
G
H
I
K
L
M
N
P
Q
R
S
T
V
W
Y
Austria
govina
China
nmark
stonia
inland
France
rmany
ungary
Japan
Latvia
ngolia
rlands
Russia
ovakia
ovenia
Korea
weden
erland
USSR
ngdom

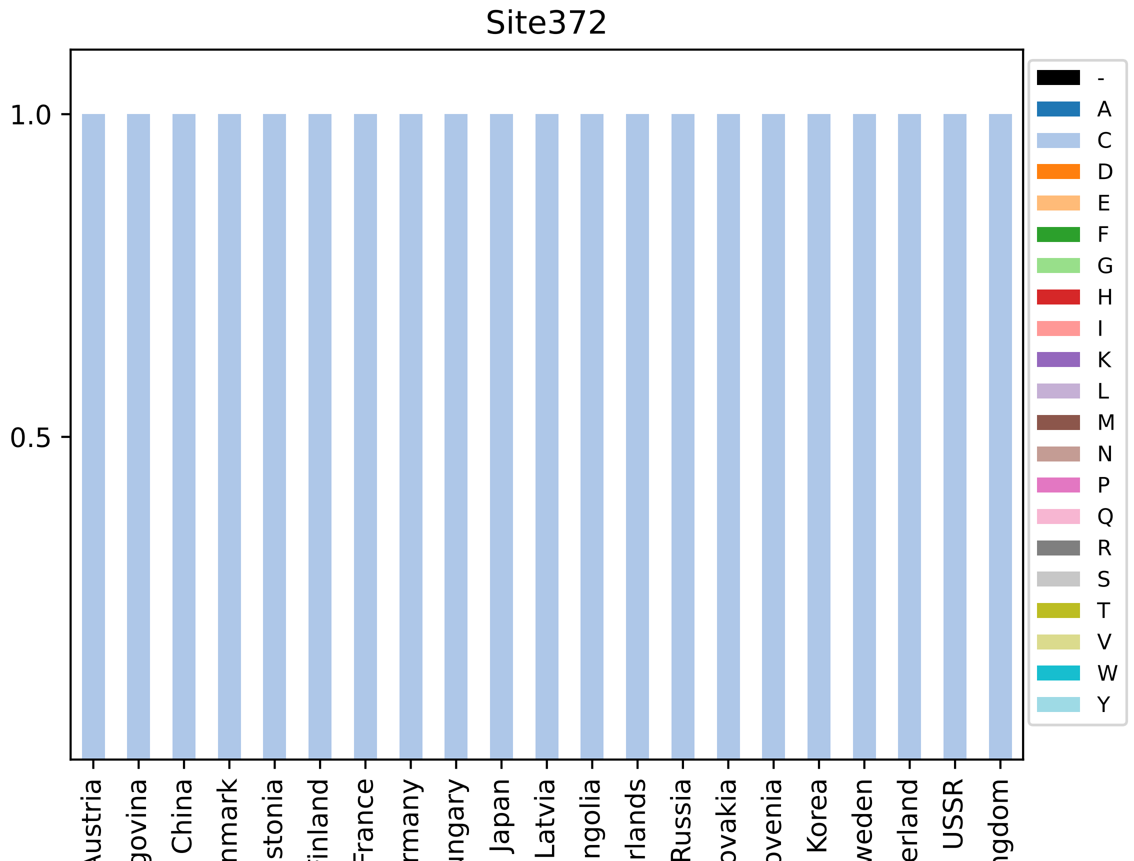
Site372
1.0
0.5
-
A
C
D
E
F
G
H
I
K
L
M
N
P
Q
R
S
T
V
W
Y
China
Japan
Latvia
Russia
Korea
USSR

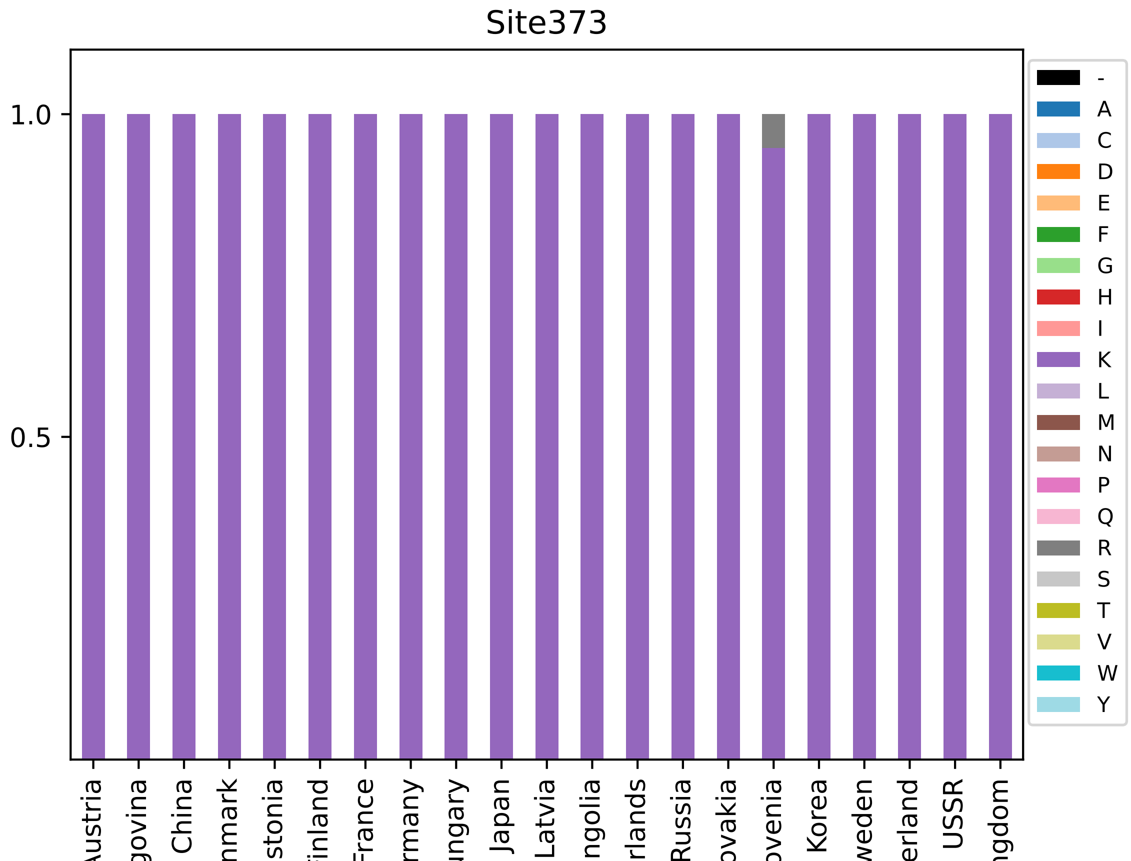
Site373
1.0
0.5
-
A
C
D
E
F
G
H
I
K
L
M
N
P
Q
R
S
T
V
W
Y
China
Japan
Latvia
Russia
Korea
USSR

Site374

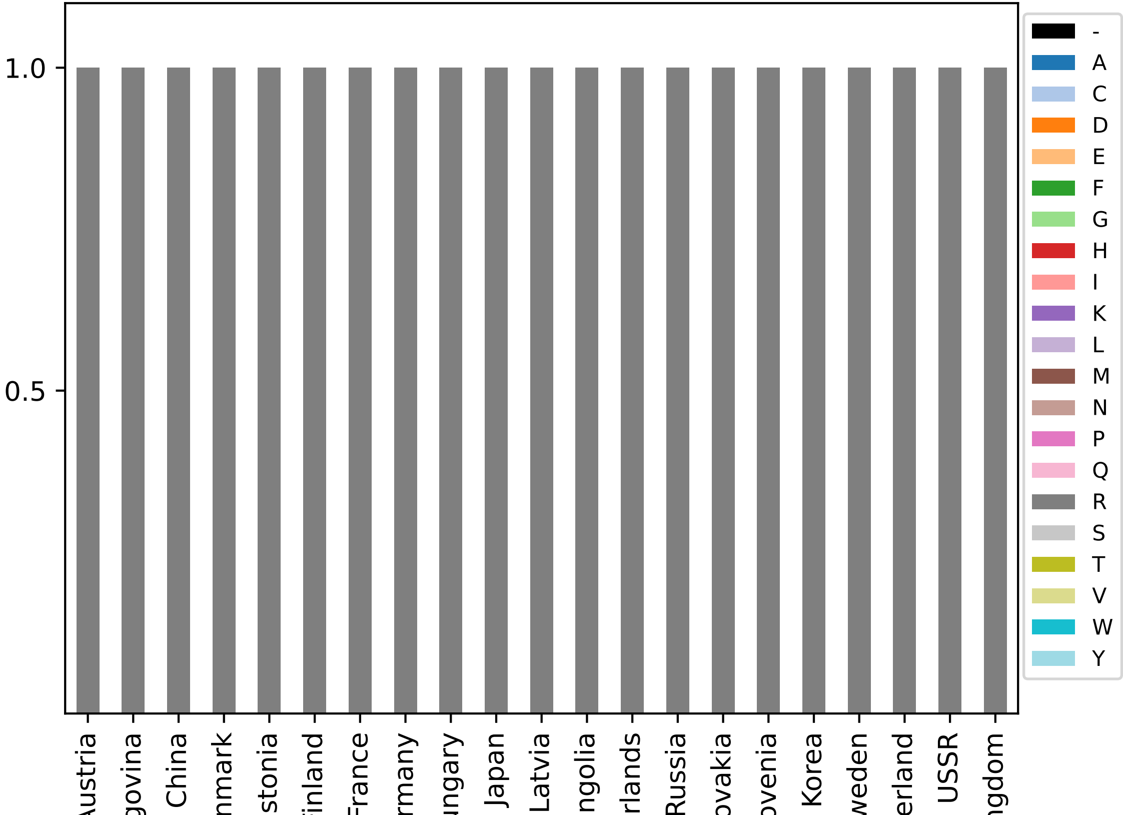
1.0
0.5
-
A
C
D
E
F
G
H
I
K
L
M
N
P
Q
R
S
T
V
W
Y
Austria
govina
China
nmark
stonia
Finland
France
rmany
ungary
Japan
Latvia
ngolia
rlands
Russia
ovakia
ovenia
Korea
weden
erland
USSR
ngdom

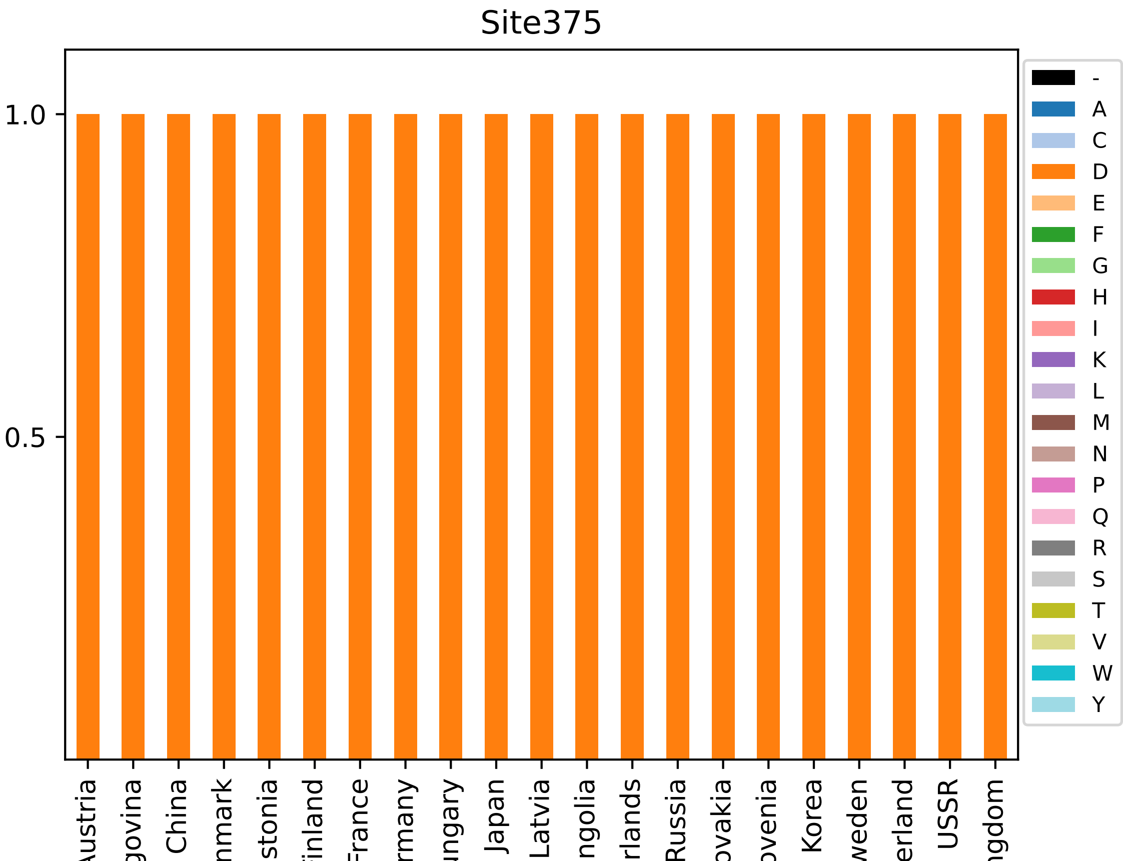

Site375
1.0
0.5
-
A
C
D
E
F
G
H
I
K
L
M
N
P
Q
R
S
T
V
W
Y
Austria
govina
China
nmark
stonia
Finland
France
rmany
ungary
Japan
Latvia
ngolia
rlands
Russia
ovakia
ovenia
Korea
weden
erland
USSR
ngdom

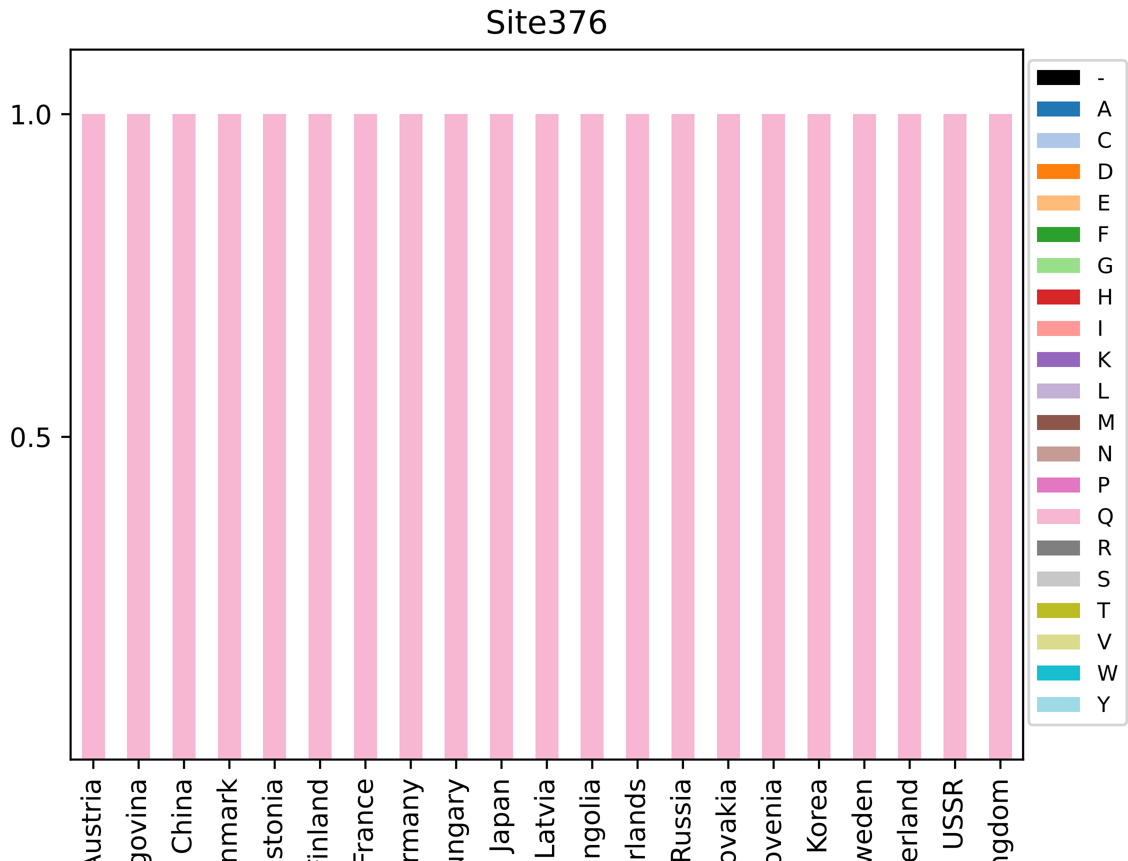

Site376
1.0
0.5
-
A
C
D
E
F
G
H
I
K
L
M
N
P
Q
R
S
T
V
W
Y
China
Japan
Latvia
Russia
Korea
USSR

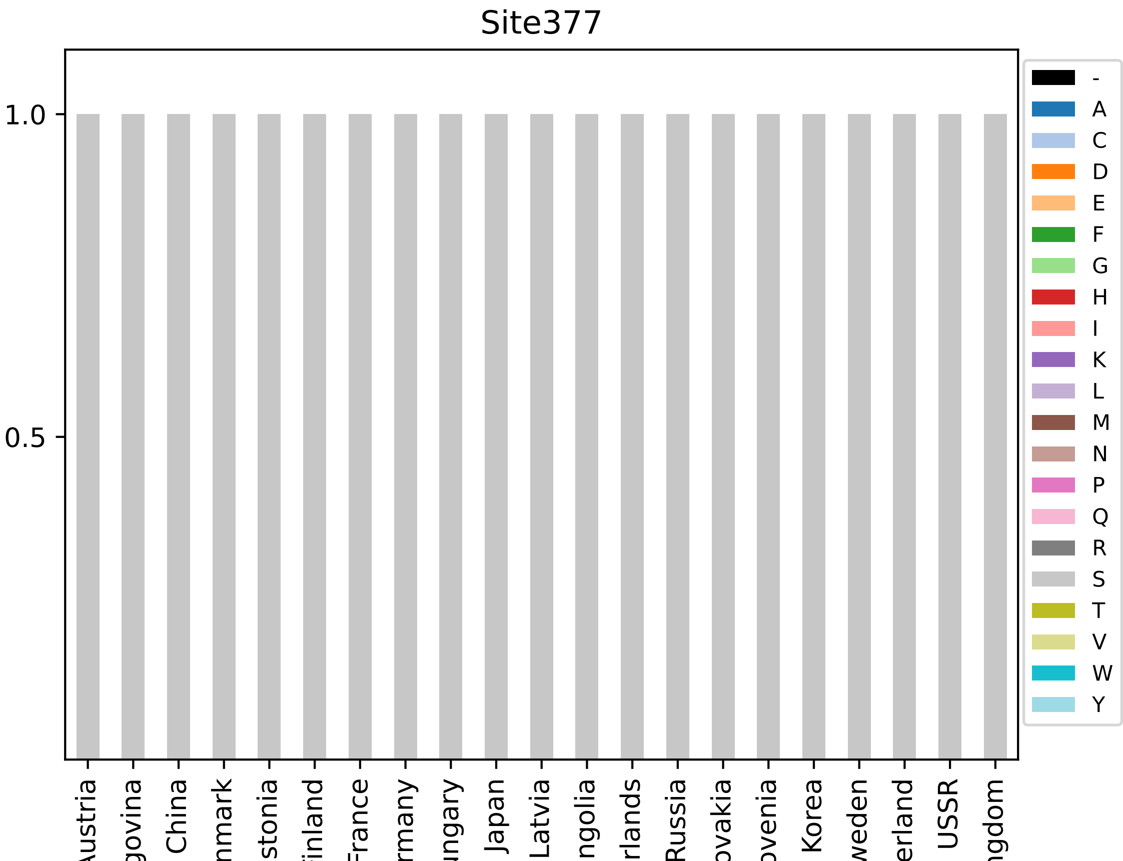

Site377
1.0
0.5
-
A
C
D
E
F
G
H
I
K
L
M
N
P
Q
R
S
T
V
W
Y
China
Japan
Latvia
Russia
Korea
USSR

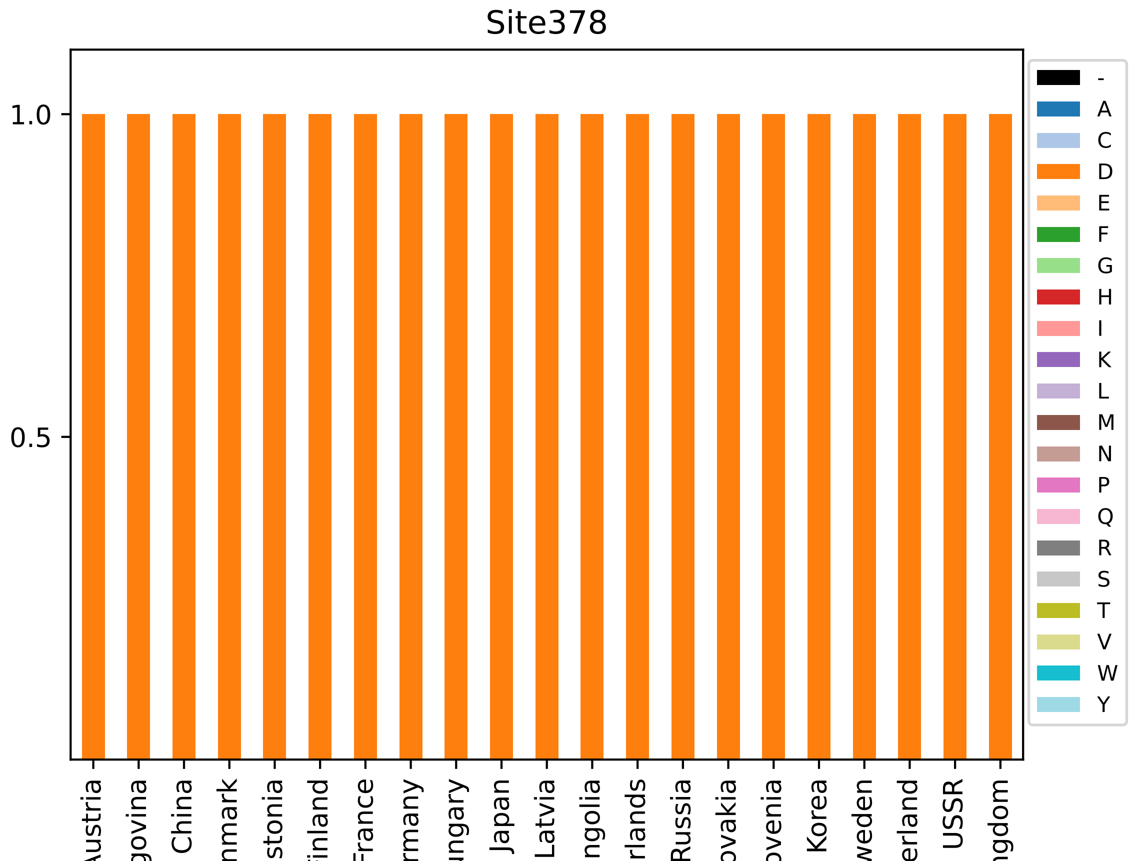
Site378
1.0
0.5
-
A
C
D
E
F
G
H
I
K
L
M
N
P
Q
R
S
T
V
W
Y
Austria
govina
China
nmark
stonia
inland
France
rmany
ungary
Japan
Latvia
ngolia
rlands
Russia
ovakia
ovenia
Korea
weden
erland
USSR
ngdom

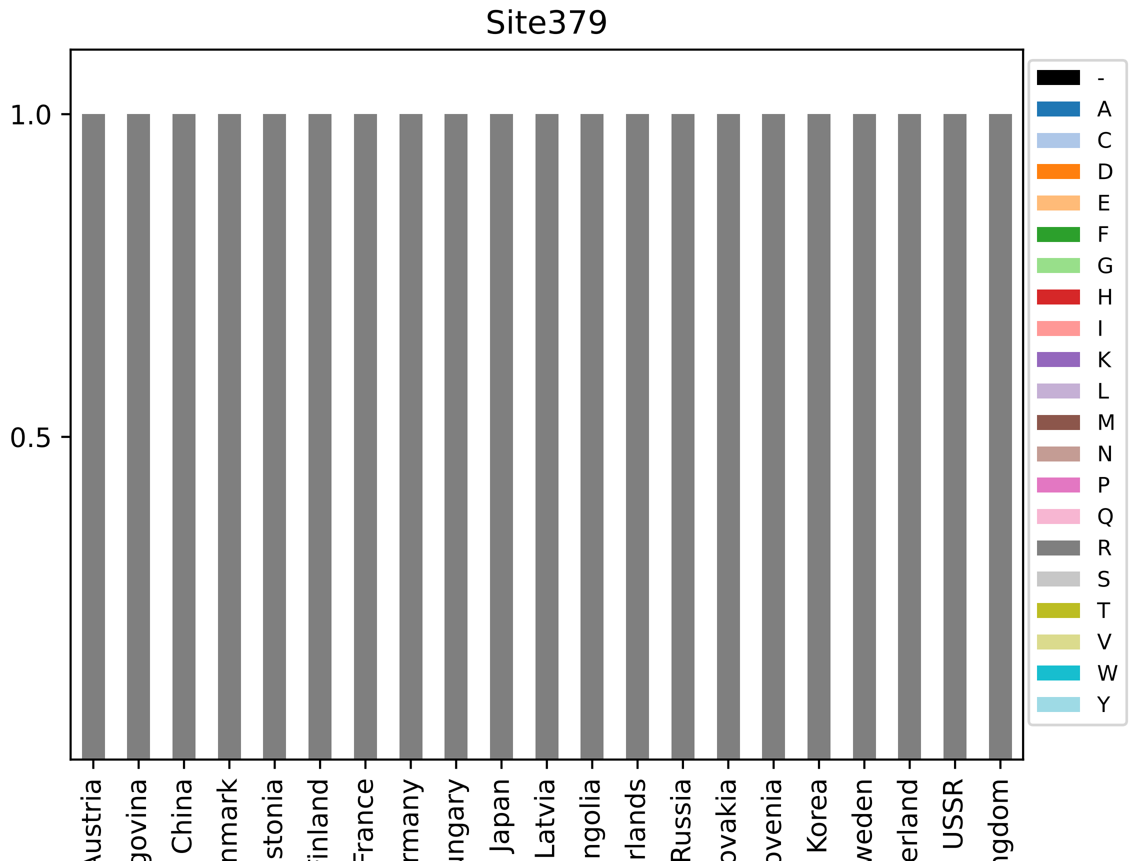
Site379
1.0
0.5
-
A
C
D
E
F
G
H
I
K
L
M
N
P
Q
R
S
T
V
W
Y
China
Japan
Latvia
Russia
Korea
USSR

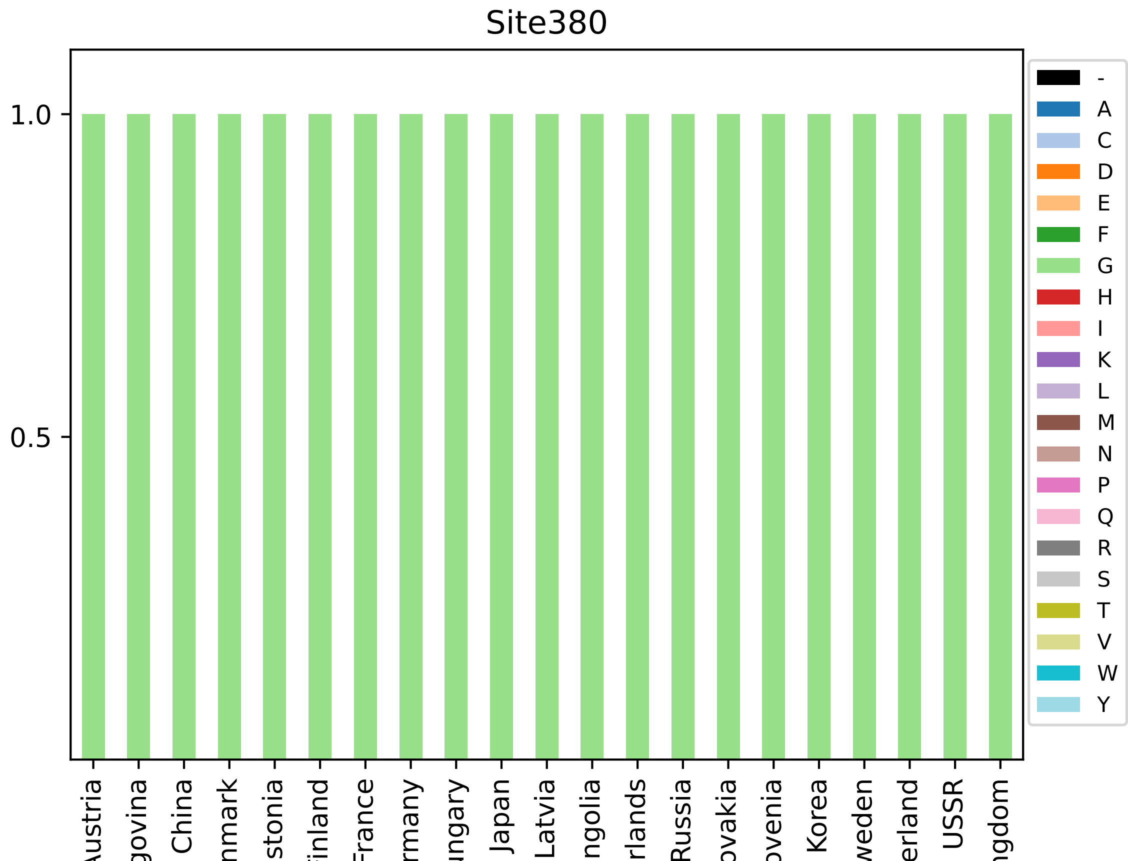
Site380
1.0
0.5
-
A
C
D
E
F
G
H
I
K
L
M
N
P
Q
R
S
T
V
W
Y
China
Japan
Latvia
Russia
Korea
USSR

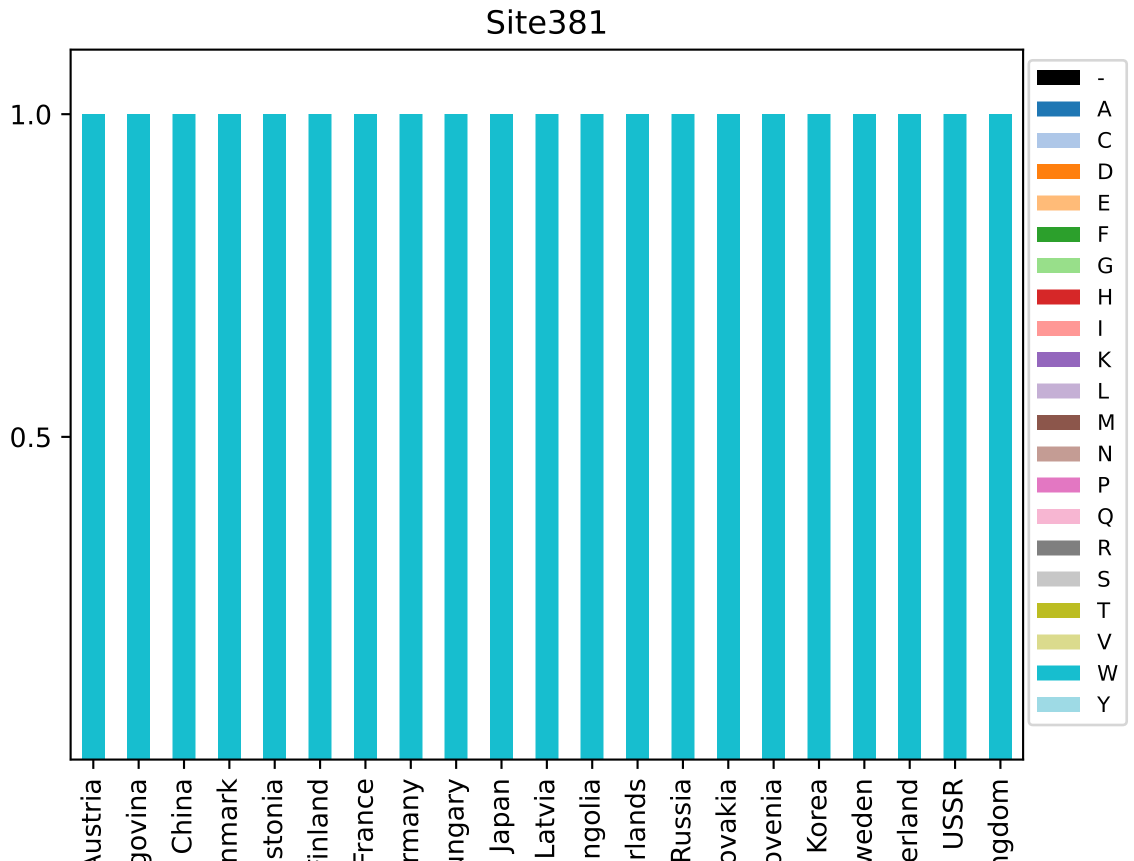
Site381
1.0
0.5
-
A
C
D
E
F
G
H
I
K
L
M
N
P
Q
R
S
T
V
W
Y
China
Japan
Latvia
Russia
Korea
USSR

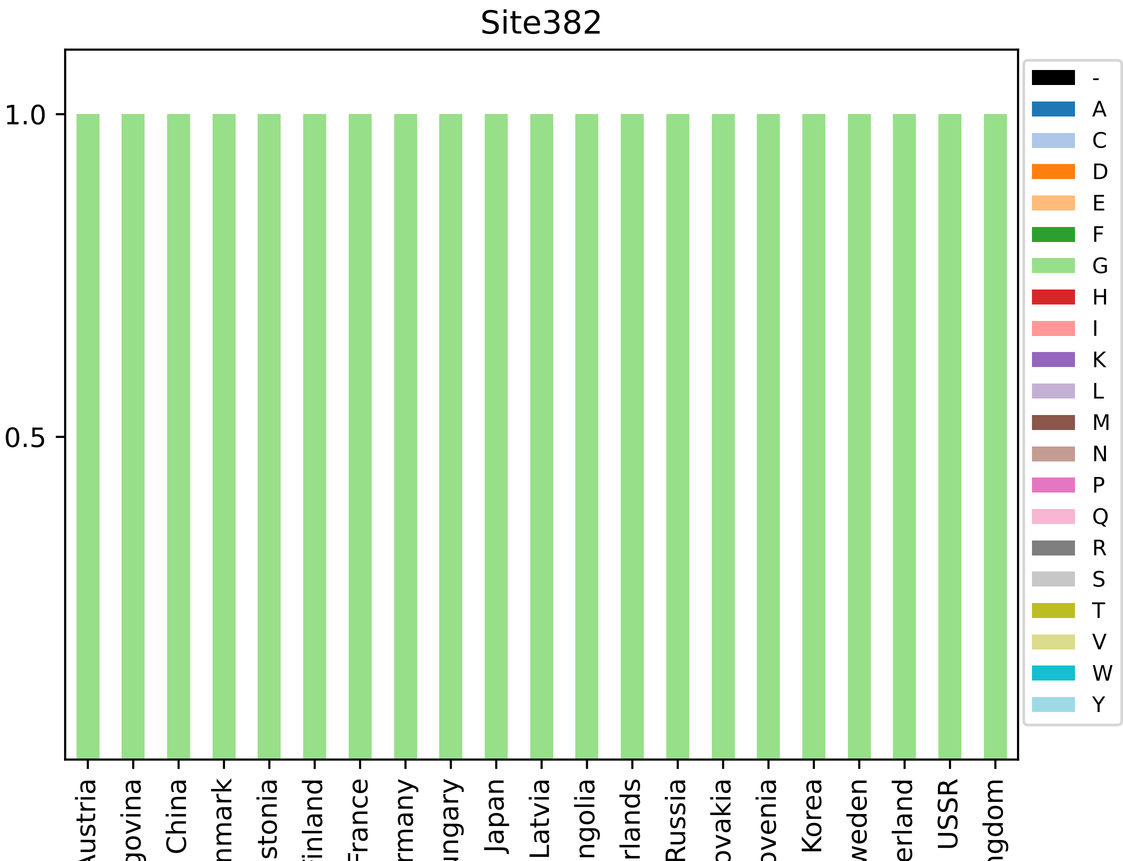

Site382
1.0
0.5
-
A
C
D
E
F
G
H
I
K
L
M
N
P
Q
R
S
T
V
W
Y
China
Japan
Latvia
Russia
Korea
USSR

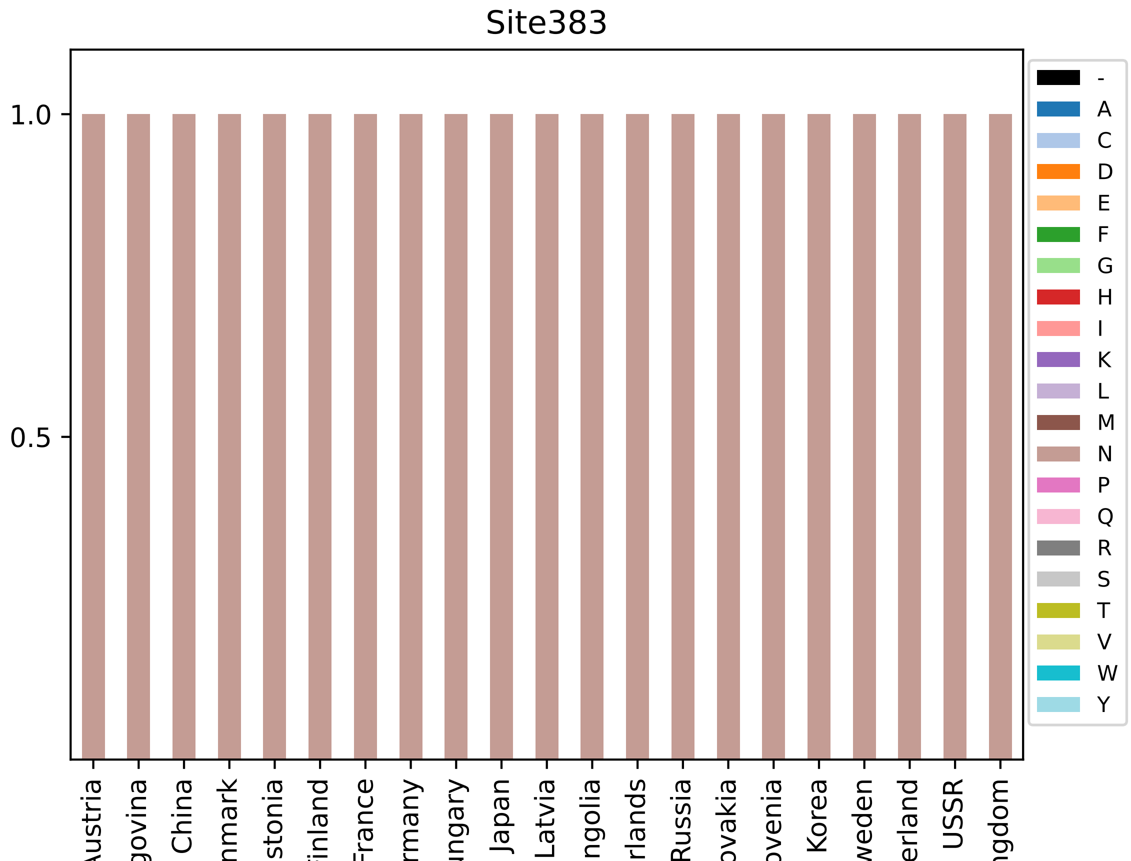
Site383
1.0
0.5
-
A
C
D
E
F
G
H
I
K
L
M
N
P
Q
R
S
T
V
W
Y
China
Japan
Latvia
Russia
Korea
USSR

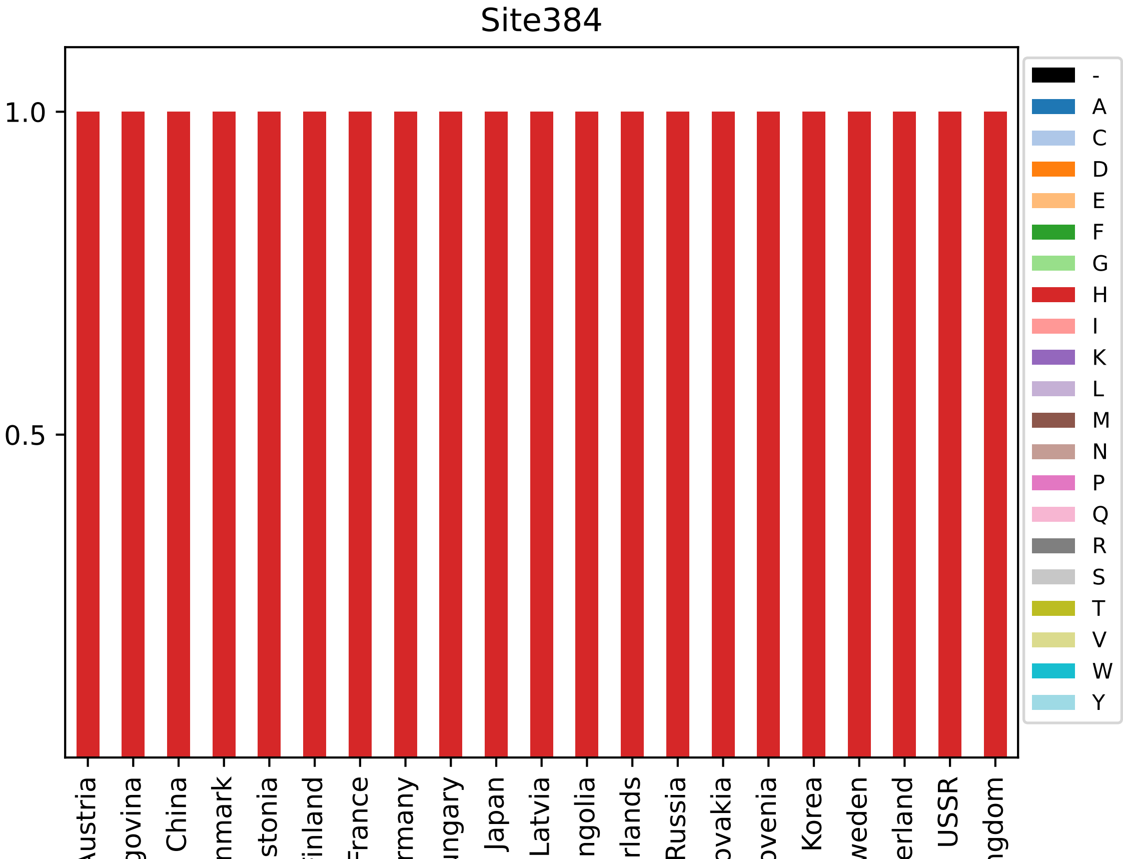

Site384
1.0
0.5
-
A
C
D
E
F
G
H
I
K
L
M
N
P
Q
R
S
T
V
W
Y
Austria
China
Japan
Latvia
Russia
Korea
USSR

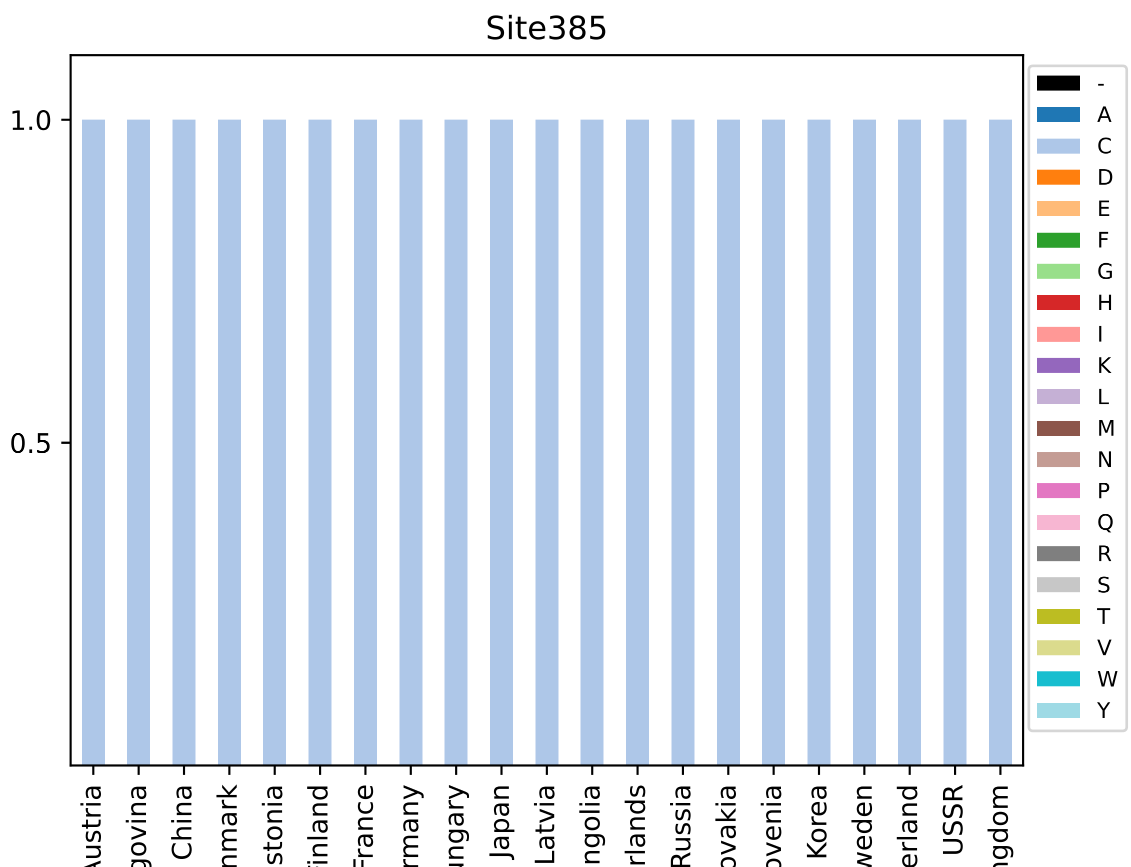
Site385
1.0
0.5
-
A
C
D
E
F
G
H
I
K
L
M
N
P
Q
R
S
T
V
W
Y
ustria
govina
China
nmark
stonia
inland
rance
rmany
ungary
Japan
Latvia
ngolia
rlands
Russia
ovakia
ovenia
Korea
weden
erland
USSR
ngdom

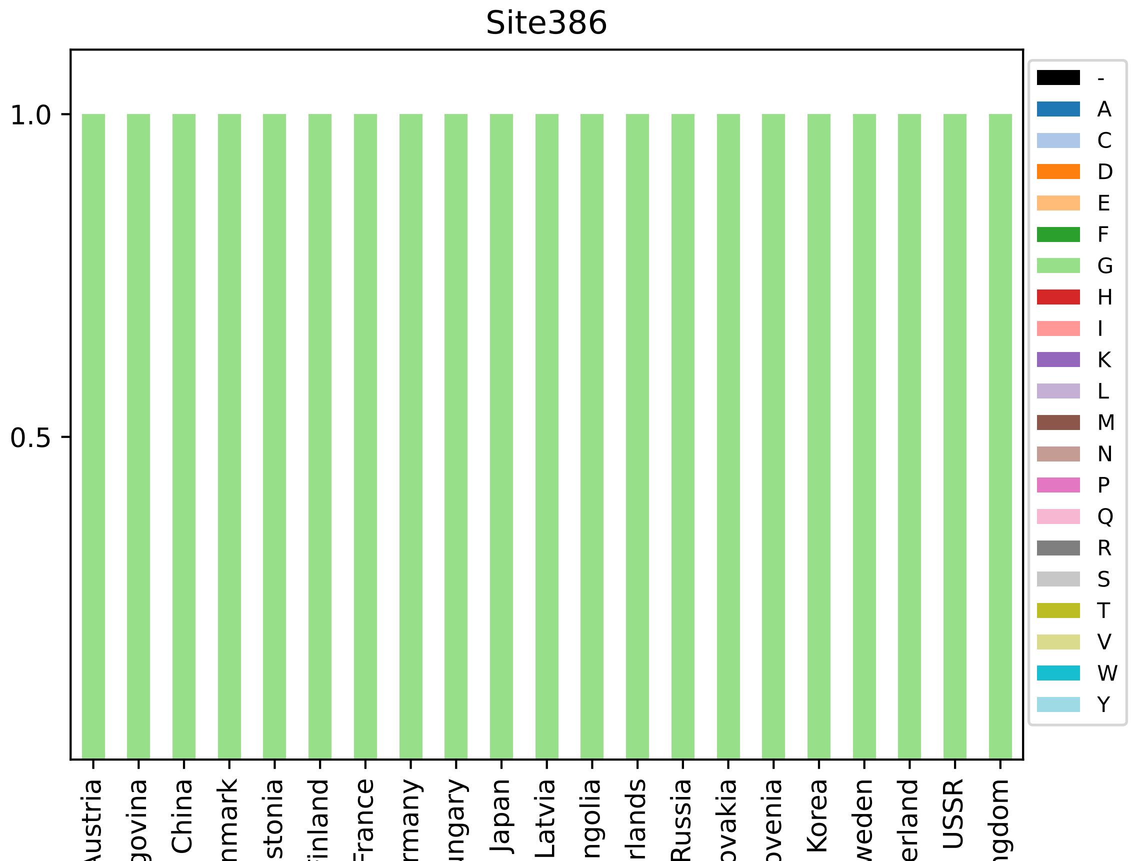
Site386
1.0
0.5
-
A
C
D
E
F
G
H
I
K
L
M
N
P
Q
R
S
T
V
W
Y
China
Japan
Latvia
Russia
Korea
USSR

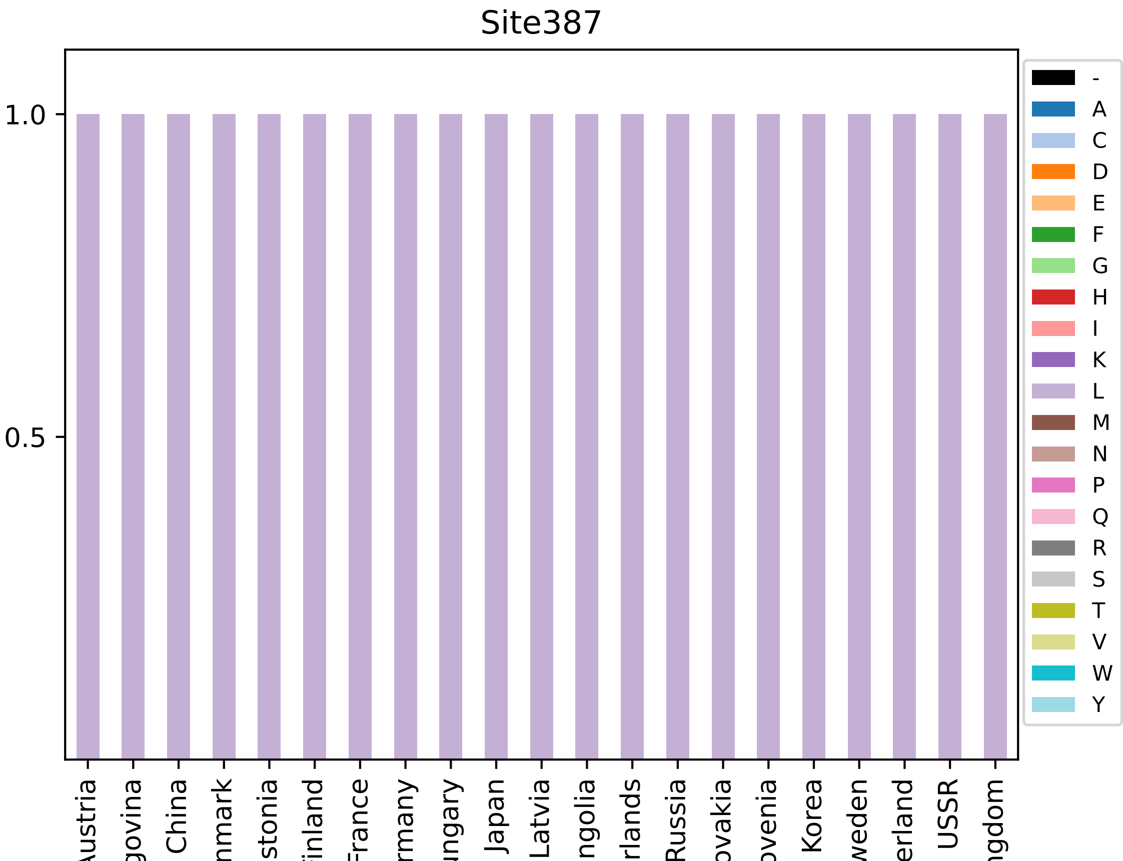
Site387
1.0
0.5
-
A
C
D
E
F
G
H
I
K
L
M
N
P
Q
R
S
T
V
W
Y
China
Japan
Latvia
Russia
Korea
USSR

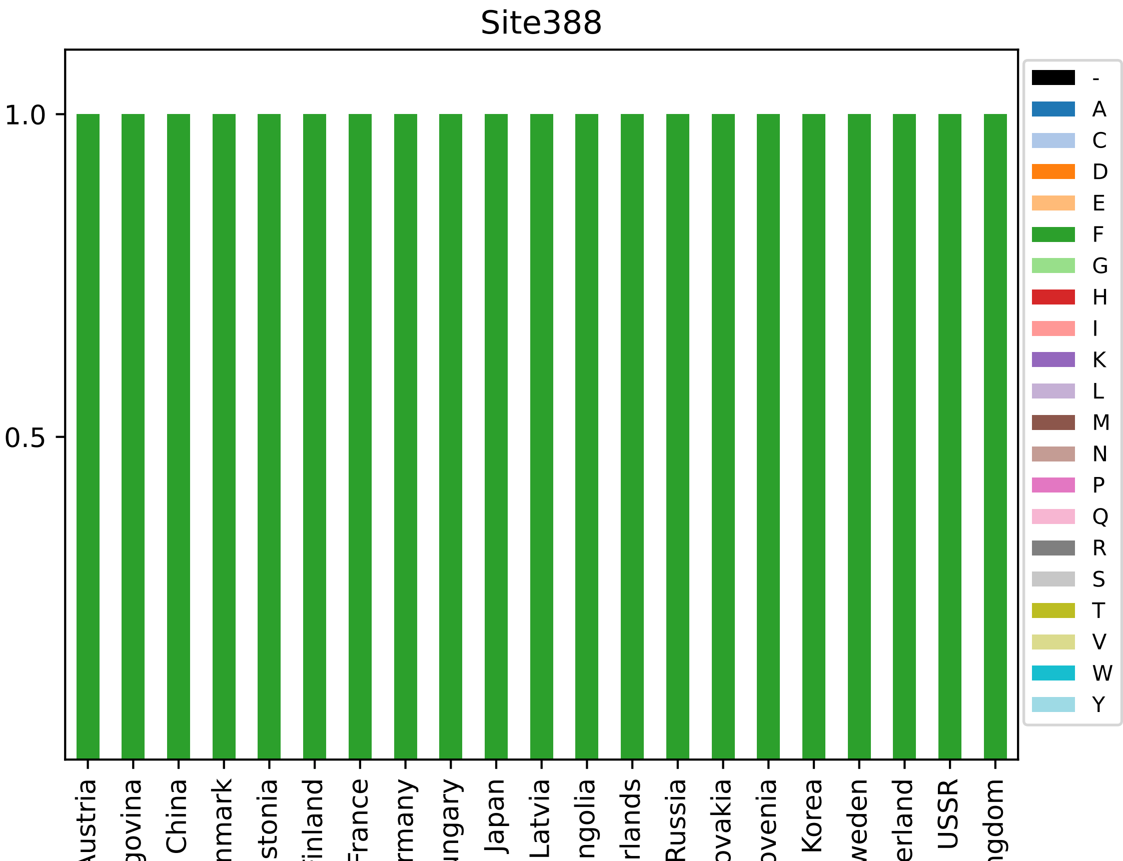
Site388
1.0
0.5
-
A
C
D
E
F
G
H
I
K
L
M
N
P
Q
R
S
T
V
W
Y
Austria
govina
China
nmark
stonia
inland
France
rmany
ungary
Japan
Latvia
ngolia
rlands
Russia
ovakia
ovenia
Korea
weden
erland
USSR
ngdom

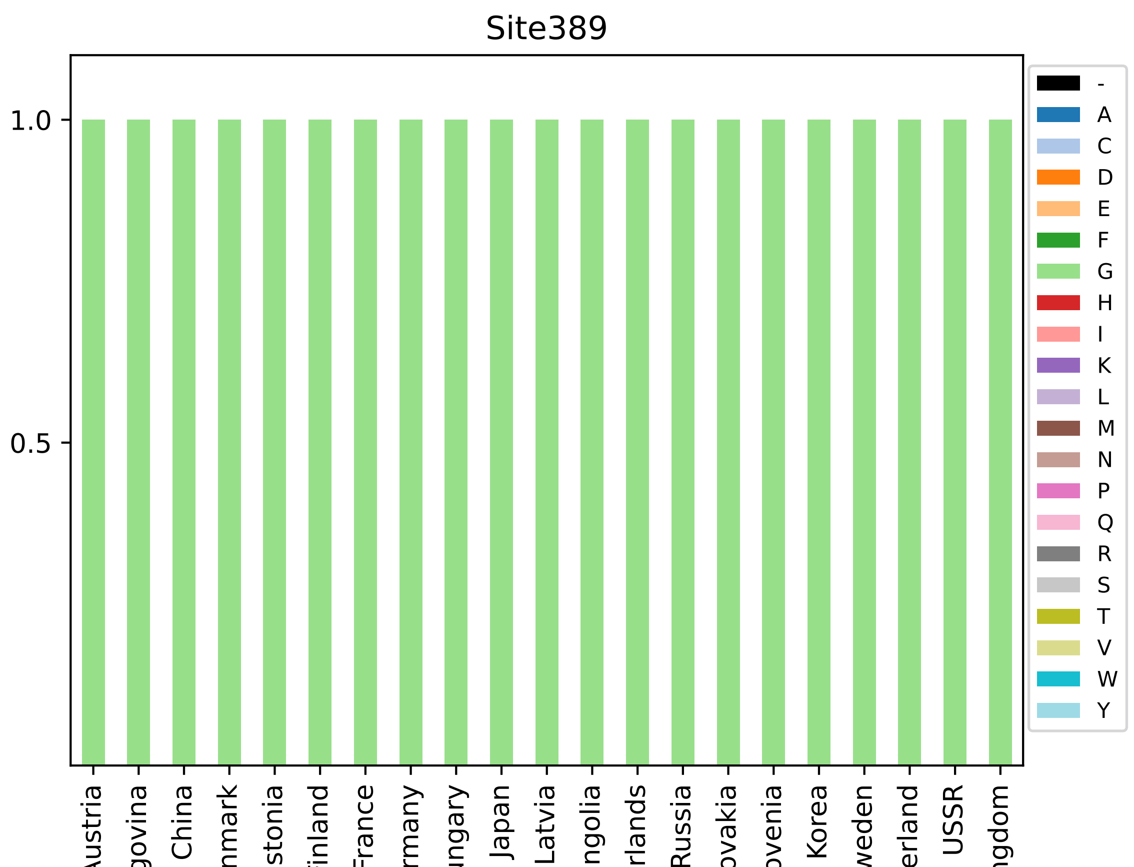

Site389
1.0
0.5
-
A
C
D
E
F
G
H
I
K
L
M
N
P
Q
R
S
T
V
W
Y
China
Japan
Latvia
Russia
Korea
USSR

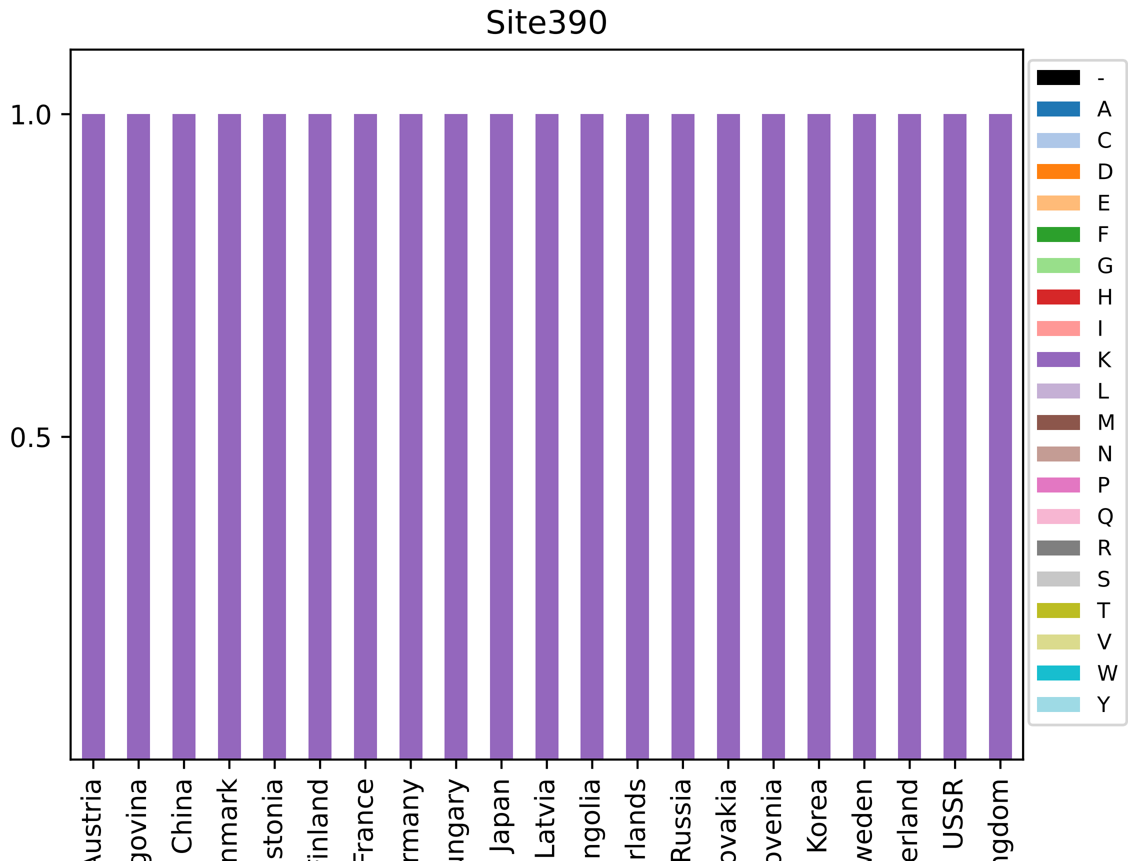

Site390
1.0
0.5
-
A
C
D
E
F
G
H
I
K
L
M
N
P
Q
R
S
T
V
W
Y
China
Japan
Latvia
Russia
Korea
USSR

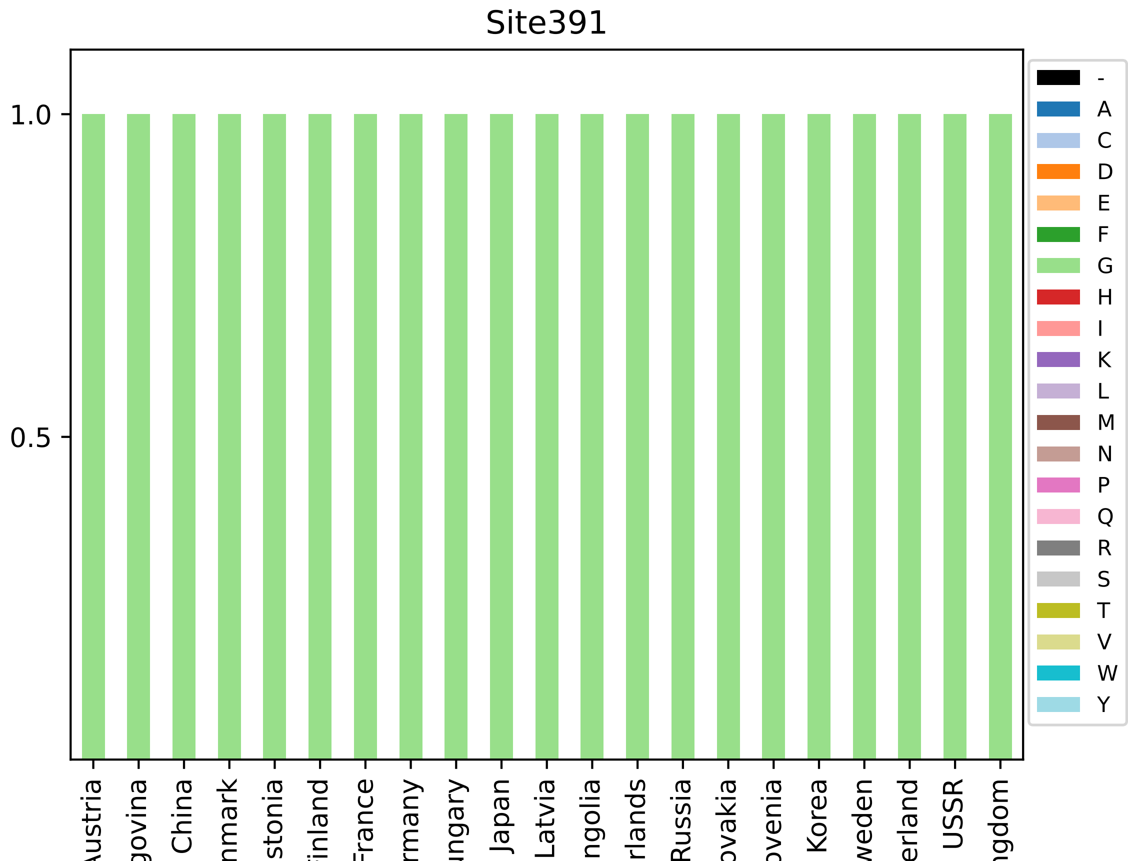

Site391
1.0
0.5
-
A
C
D
E
F
G
H
I
K
L
M
N
P
Q
R
S
T
V
W
Y
China
Japan
Latvia
Russia
Korea
USSR

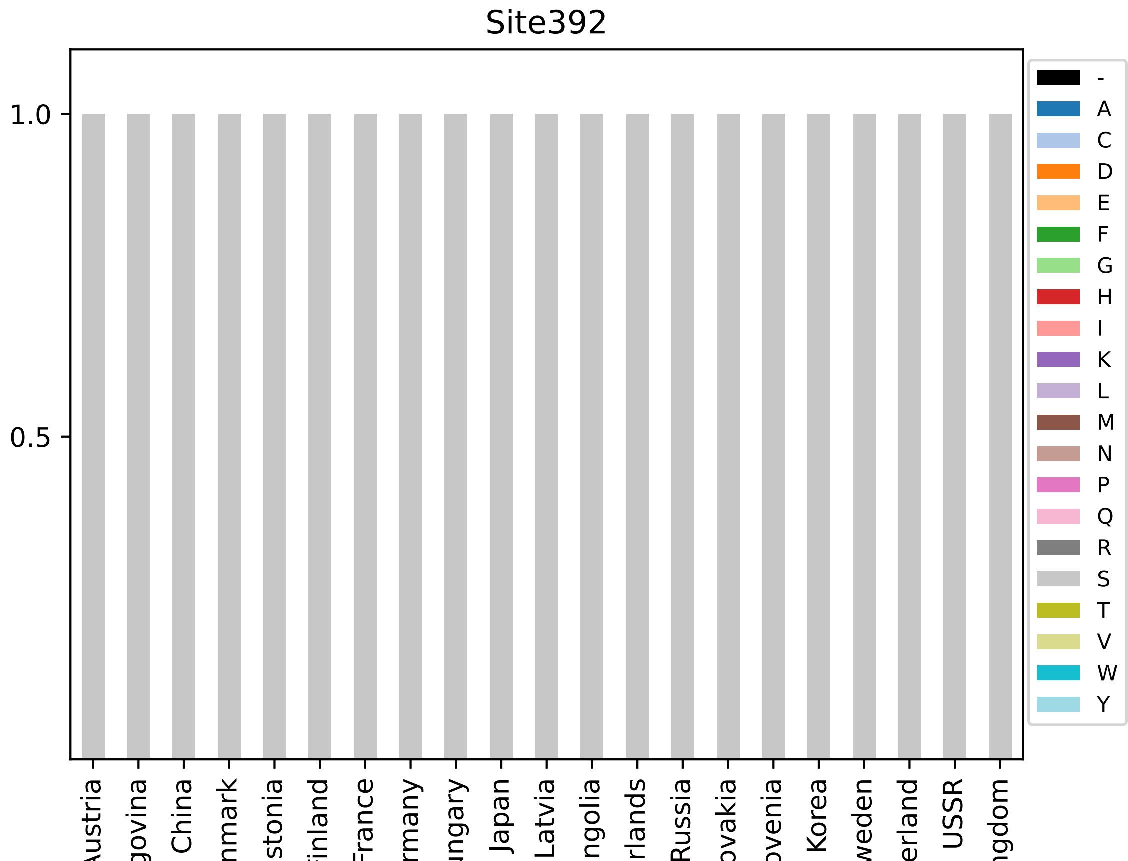

Site392
1.0
0.5
-
A
C
D
E
F
G
H
I
K
L
M
N
P
Q
R
S
T
V
W
Y
Austria
govina
China
nmark
stonia
Finland
France
rmany
ungary
Japan
Latvia
ngolia
rlands
Russia
ovakia
ovenia
Korea
weden
erland
USSR
ngdom

Site393

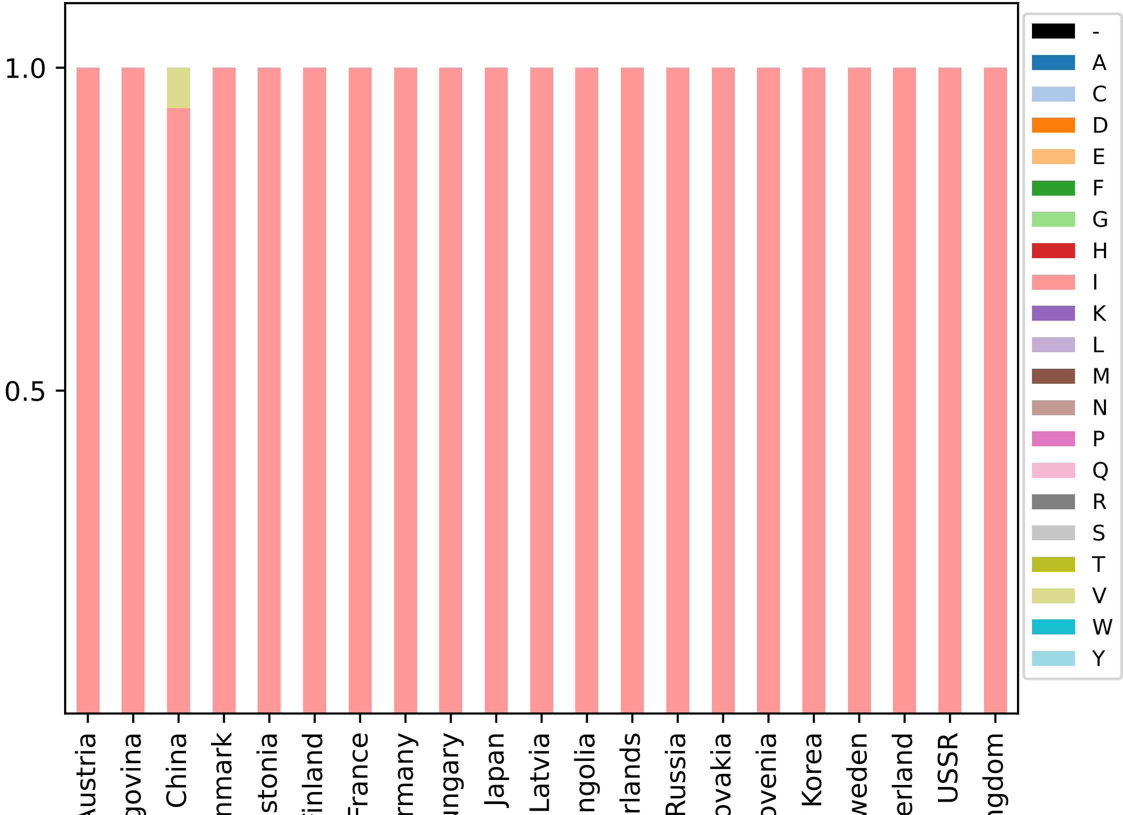
1.0
0.5
China
Japan
Latvia
Russia
Korea
USSR
-
A
C
D
E
F
G
H
I
K
L
M
N
P
Q
R
S
T
V
W
Y

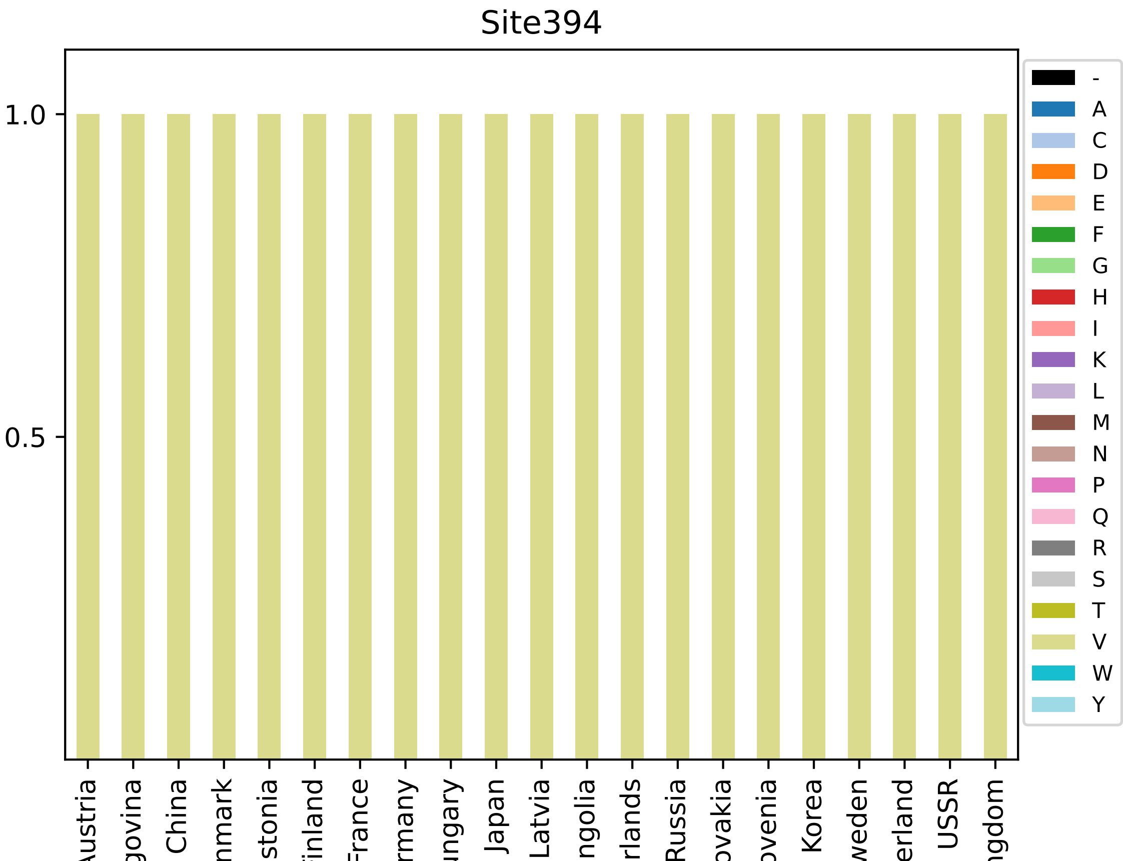
Site394
1.0
0.5
-
A
C
D
E
F
G
H
I
K
L
M
N
P
Q
R
S
T
V
W
Y
China
Japan
Latvia
Russia
Korea
USSR

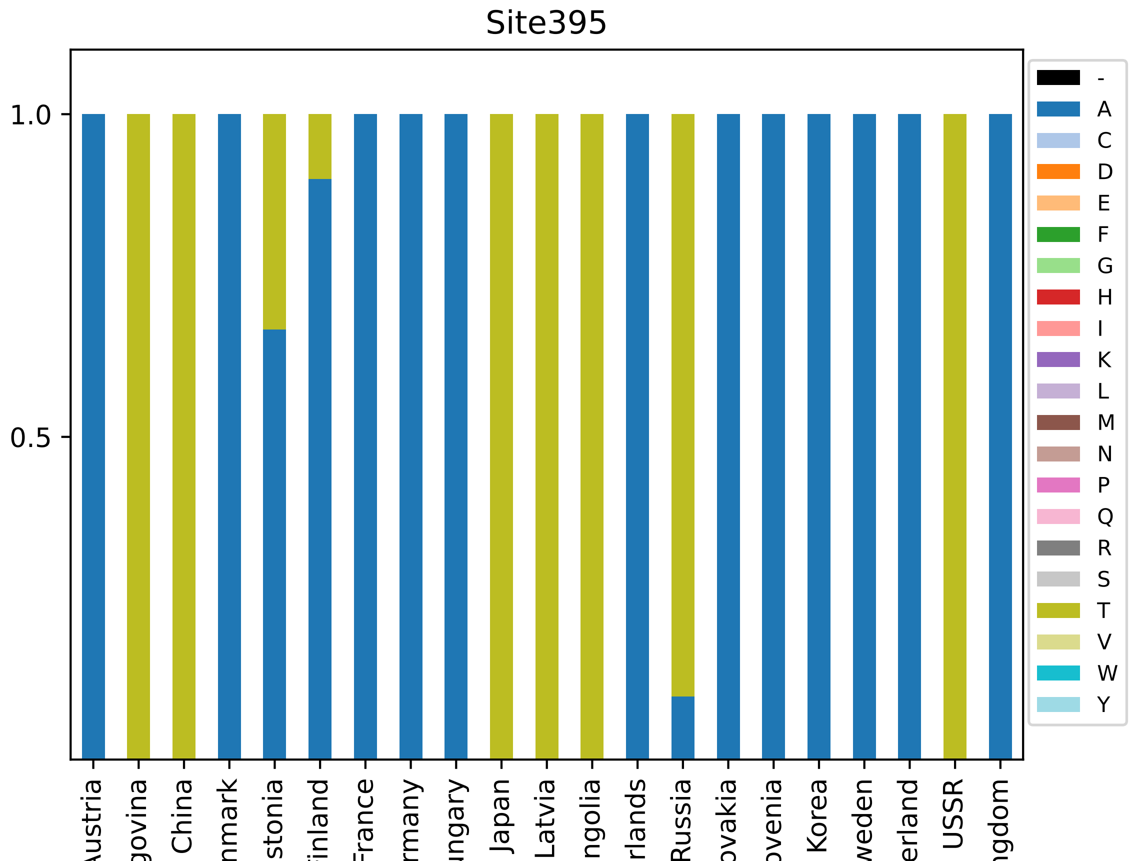
Site395
1.0
0.5
-
A
C
D
E
F
G
H
I
K
L
M
N
P
Q
R
S
T
V
W
Y
China
Japan
Latvia
Russia
Korea
USSR

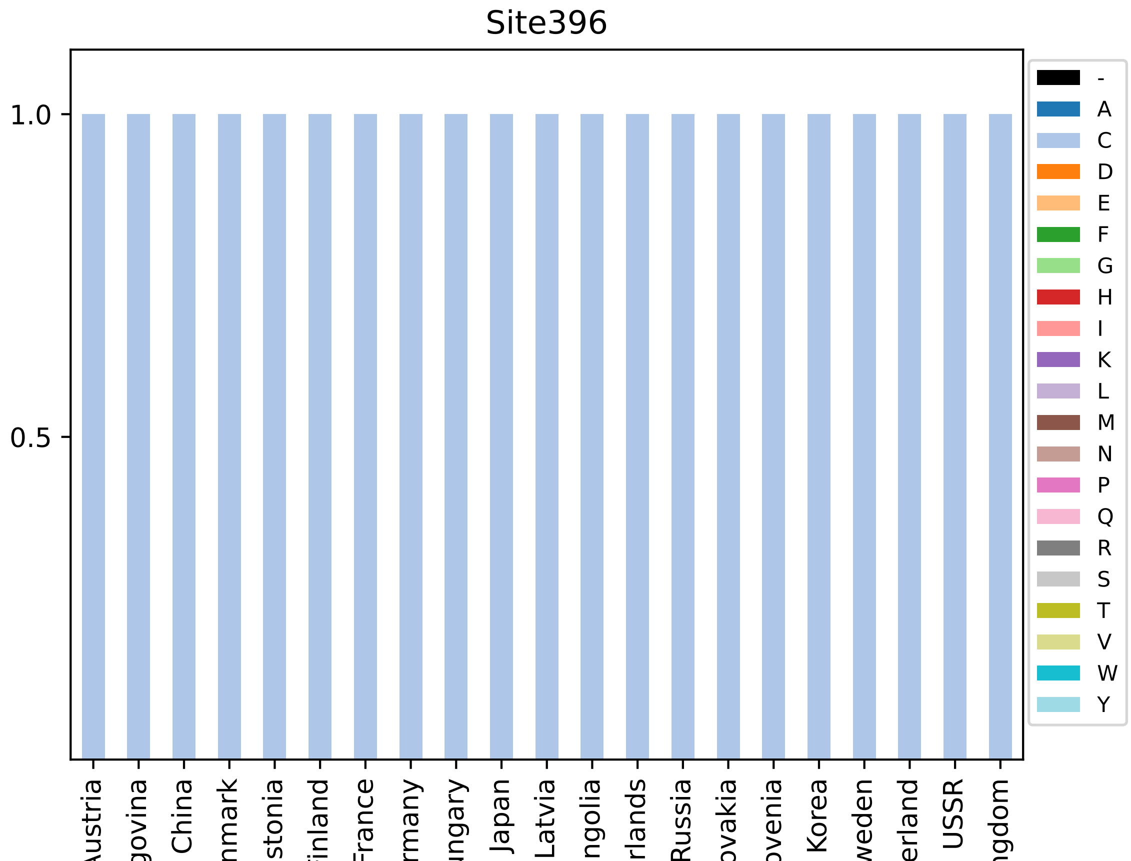
Site396
1.0
0.5
China
Japan
Latvia
Russia
Korea
USSR
-
A
C
D
E
F
G
H
I
K
L
M
N
P
Q
R
S
T
V
W
Y

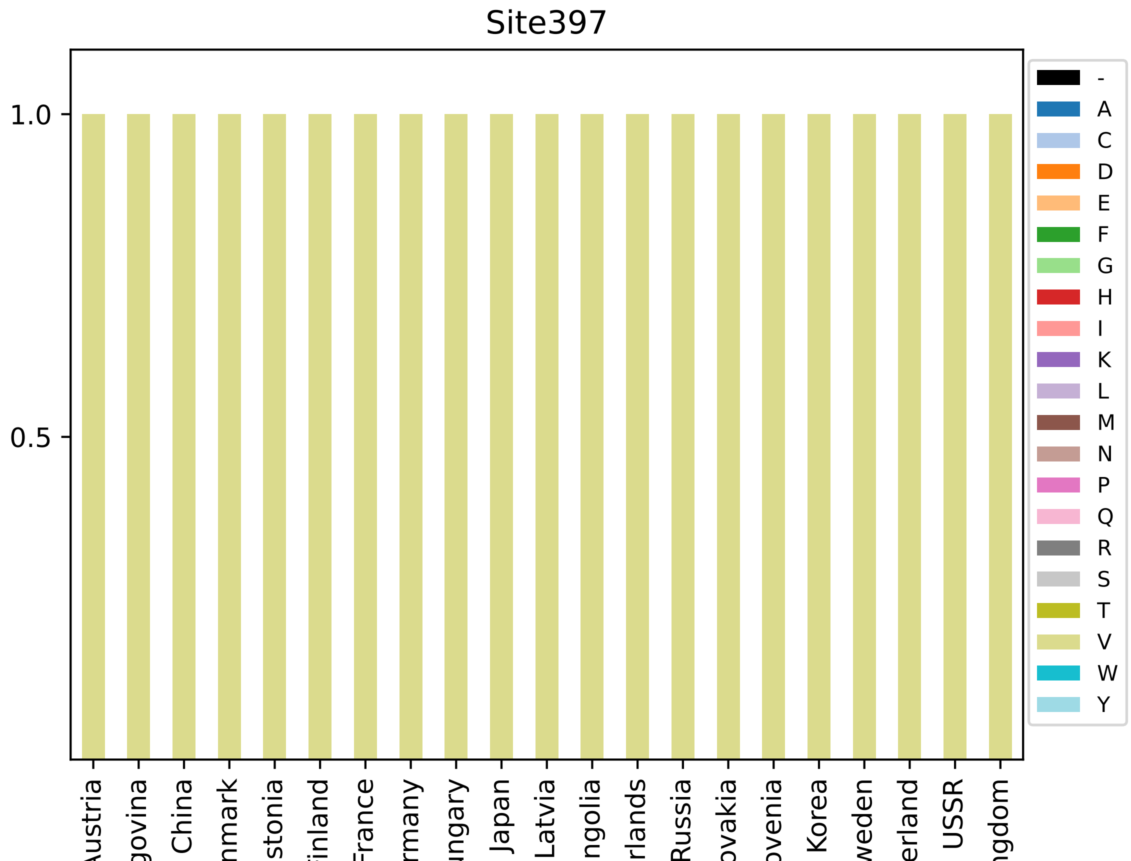
Site397
1.0
0.5
-
A
C
D
E
F
G
H
I
K
L
M
N
P
Q
R
S
T
V
W
Y
China
Japan
Latvia
Russia
Korea
USSR

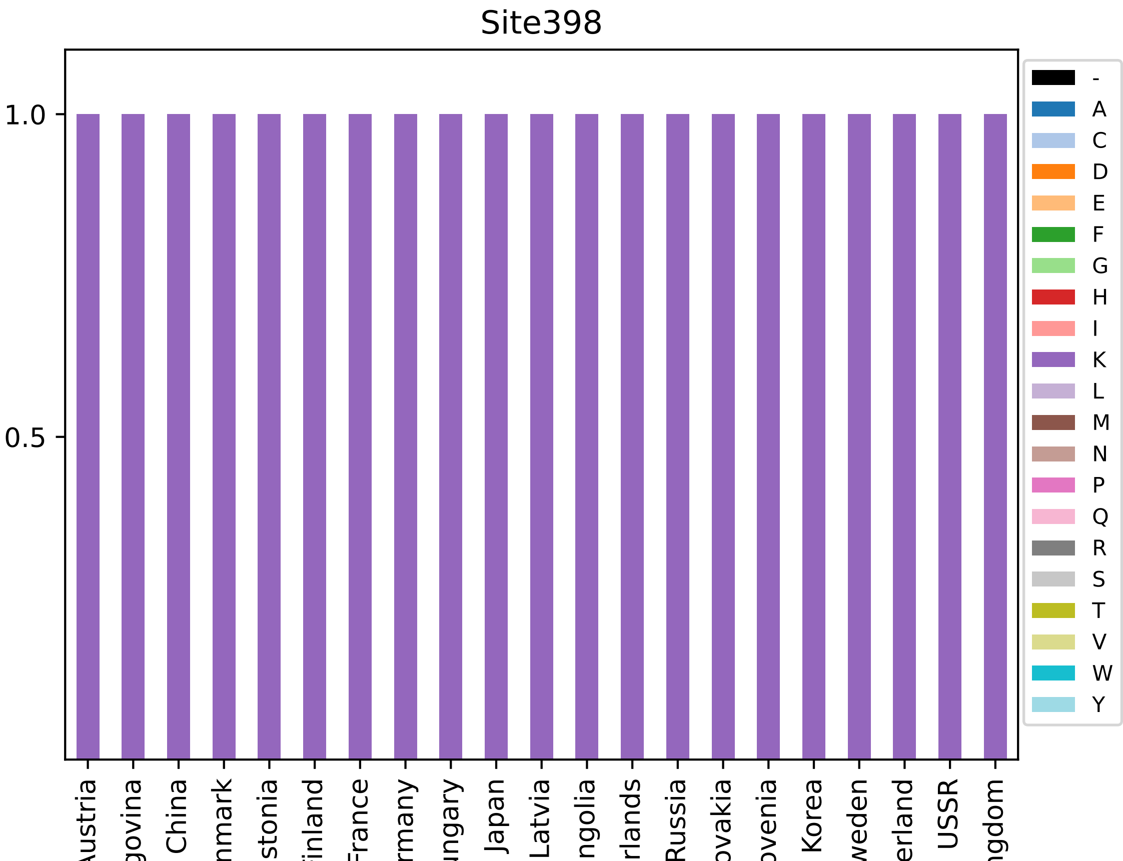

Site398
1.0
0.5
-
A
C
D
E
F
G
H
I
K
L
M
N
P
Q
R
S
T
V
W
Y
Austria
China
Japan
Latvia
Russia
Korea
USSR

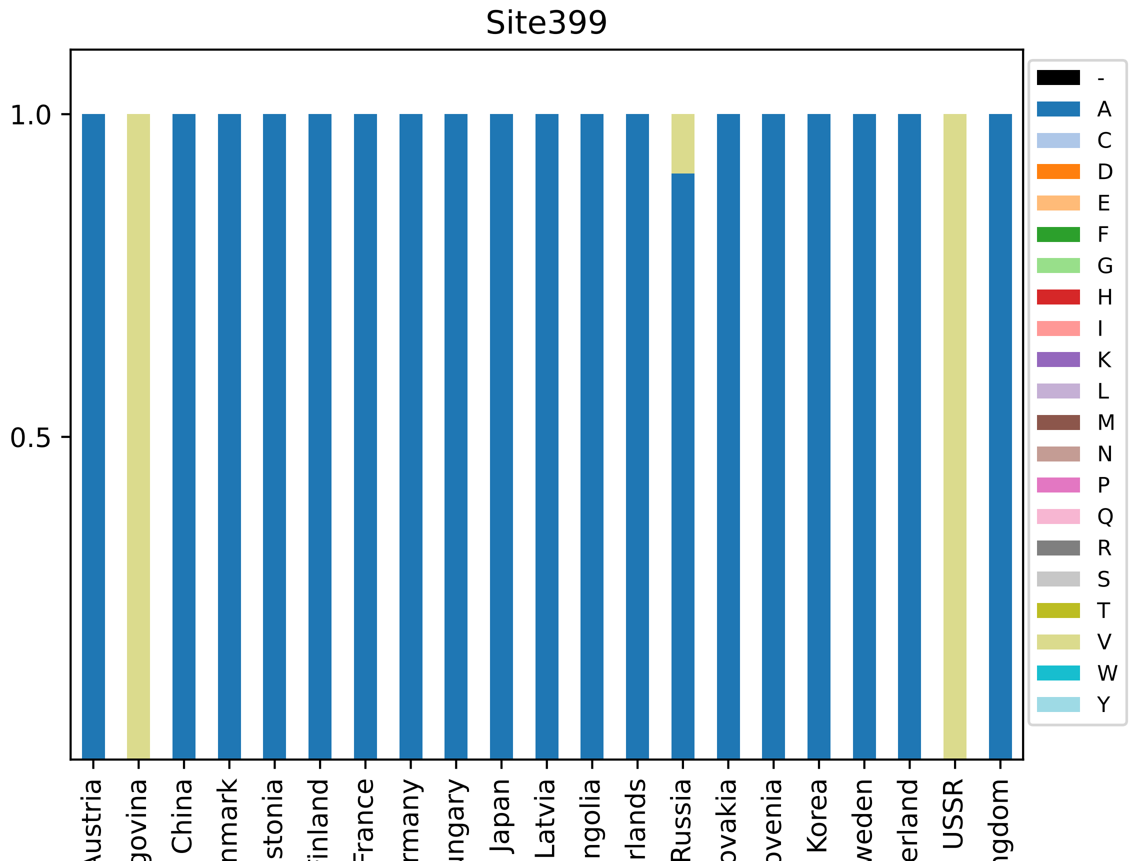

Site399
1.0
0.5
-
A
C
D
E
F
G
H
I
K
L
M
N
P
Q
R
S
T
V
W
Y
Austria
China
Japan
Latvia
Russia
Korea
USSR

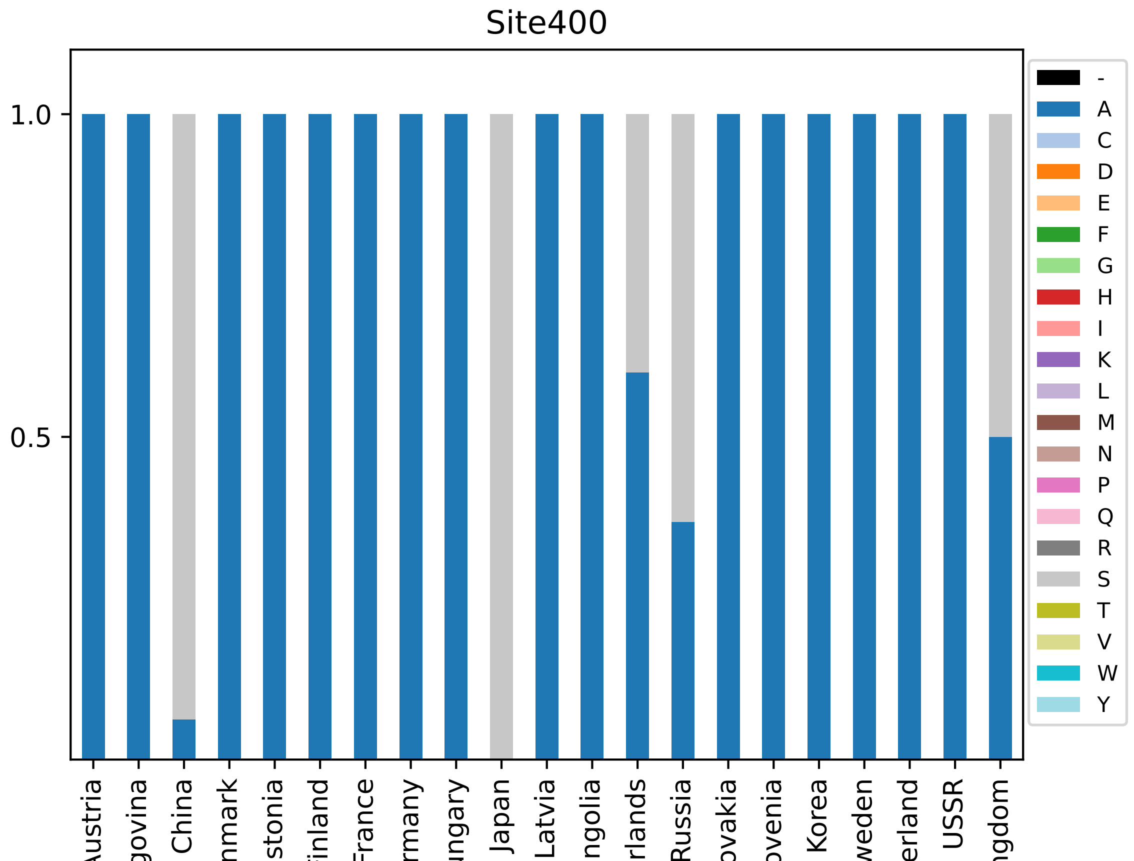
Site400
1.0
0.5
-
A
C
D
E
F
G
H
I
K
L
M
N
P
Q
R
S
T
V
W
Y
China
Japan
Latvia
Russia
Korea
USSR

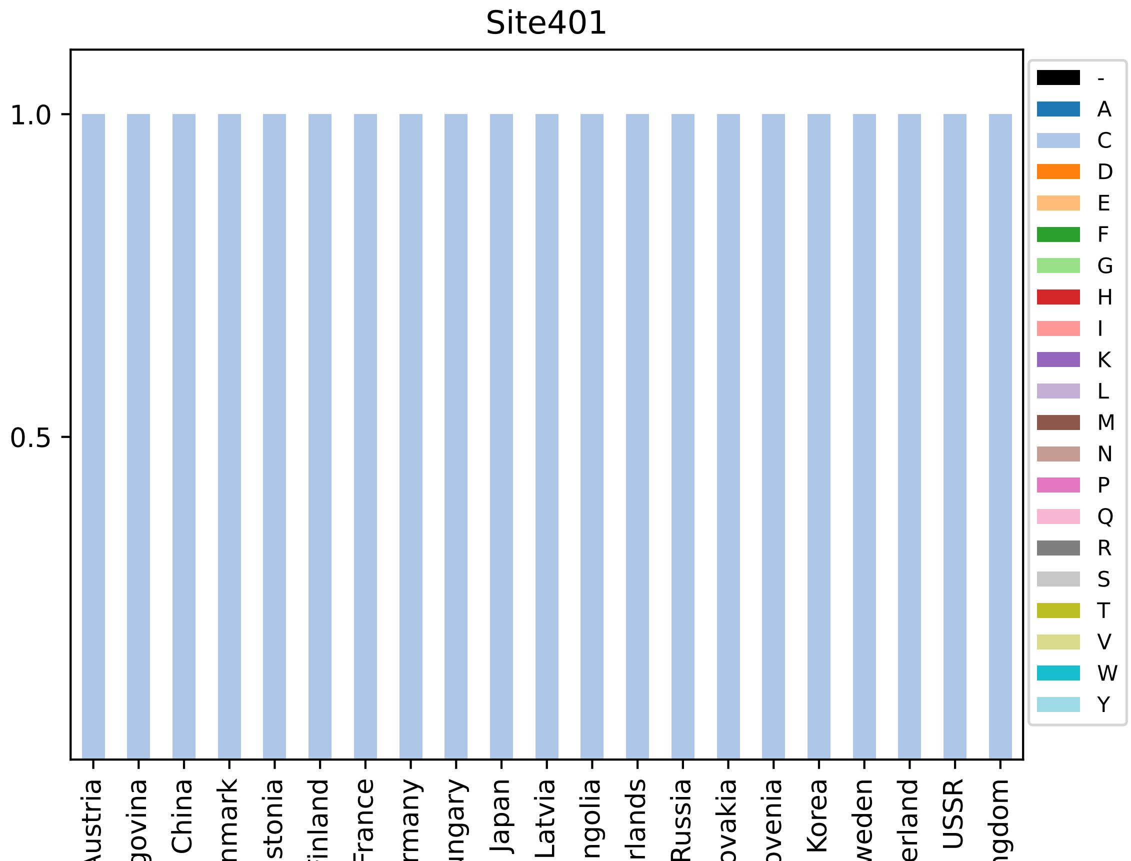

Site401
1.0
0.5
-
A
C
D
E
F
G
H
I
K
L
M
N
P
Q
R
S
T
V
W
Y
China
Japan
Latvia
Russia
Korea
USSR

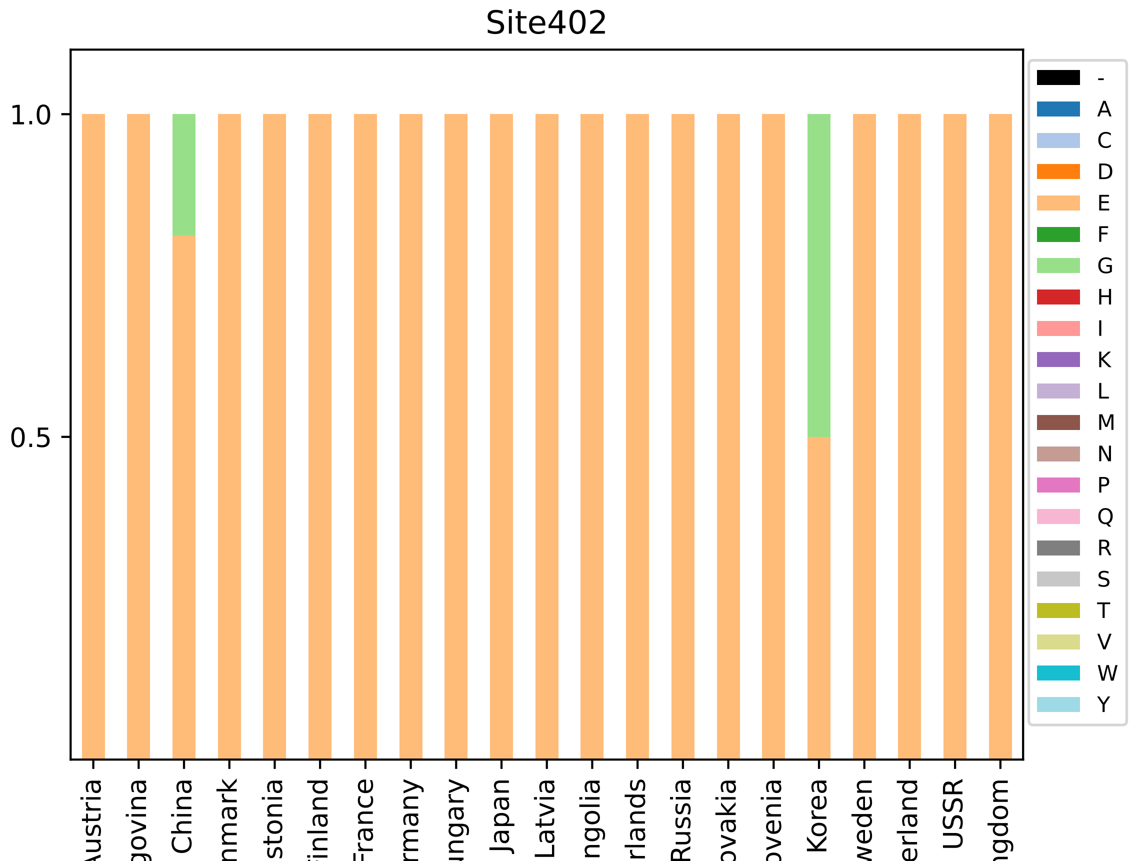
Site402
1.0
0.5
-
A
C
D
E
F
G
H
I
K
L
M
N
P
Q
R
S
T
V
W
Y
China
Japan
Latvia
Russia
Korea
USSR

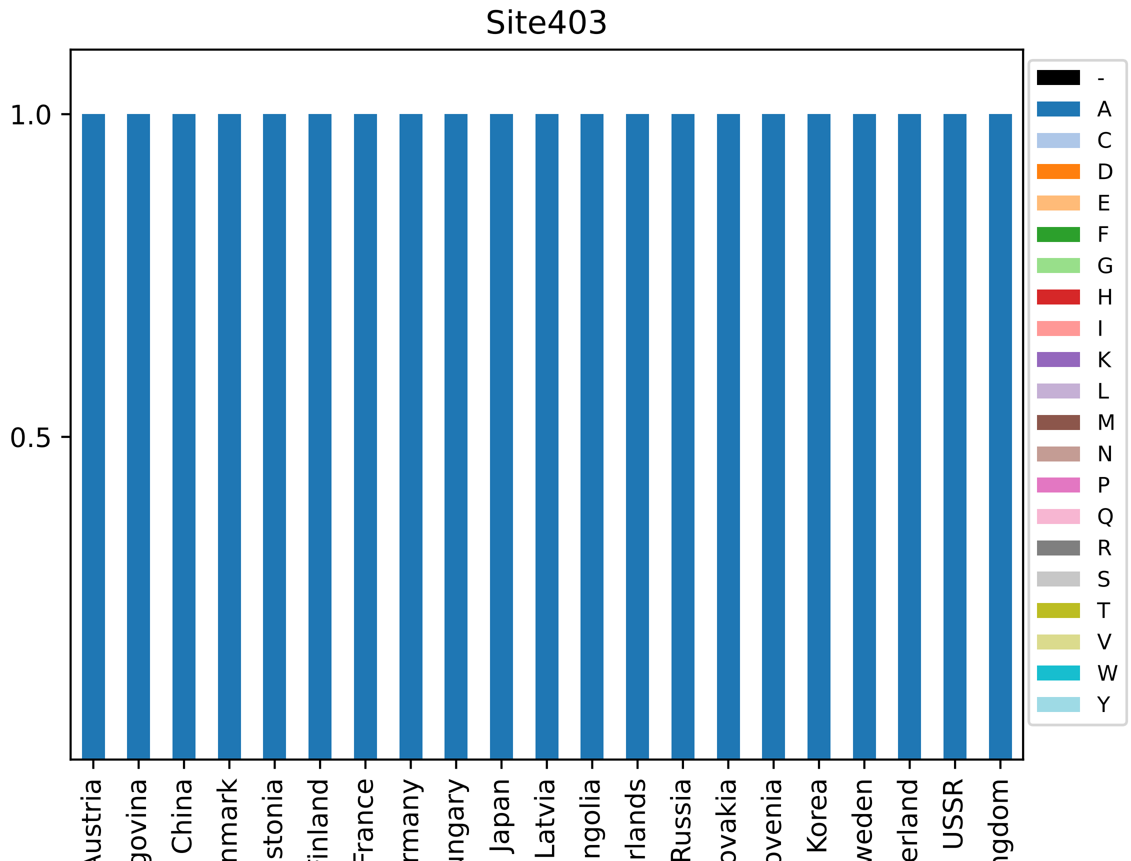
Site403
1.0
0.5
-
A
C
D
E
F
G
H
I
K
L
M
N
P
Q
R
S
T
V
W
Y
Austria
govina
China
nmark
stonia
inland
France
rmany
ungary
Japan
Latvia
ngolia
rlands
Russia
ovakia
ovenia
Korea
weden
erland
USSR
ngdom

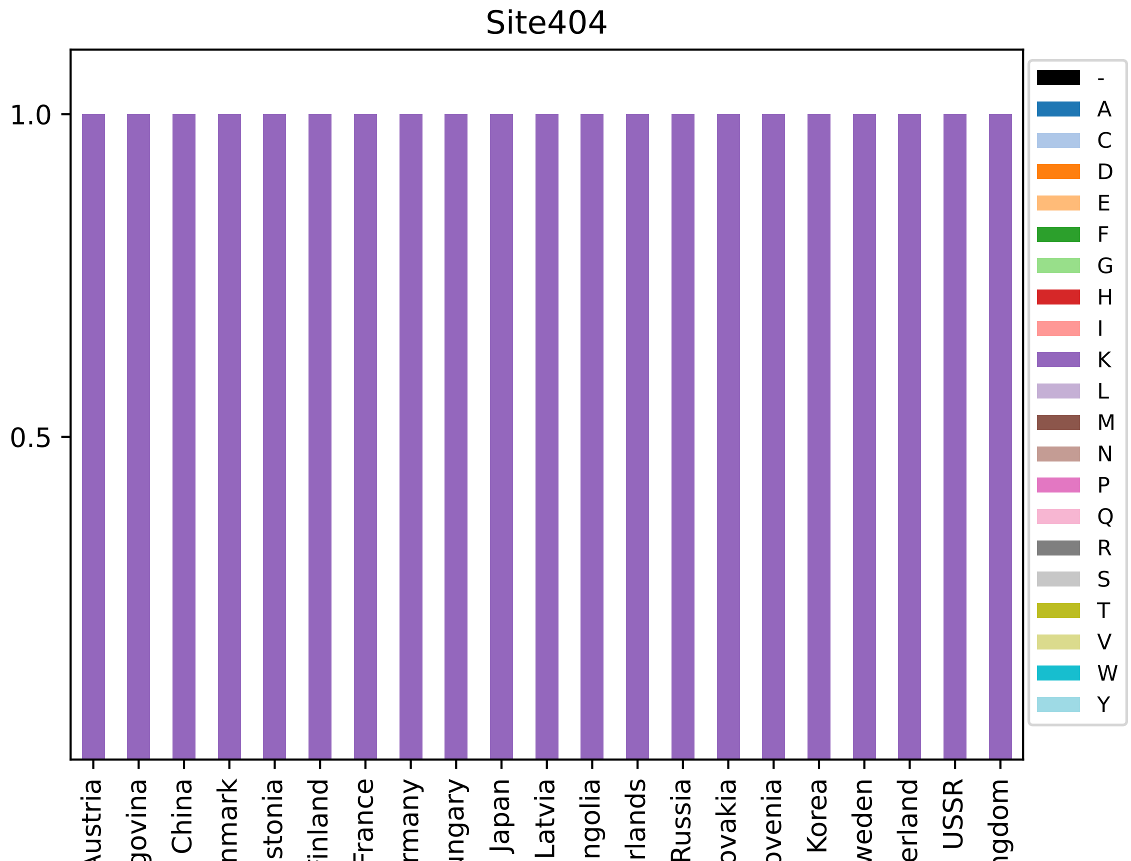
Site404
1.0
0.5
-
A
C
D
E
F
G
H
I
K
L
M
N
P
Q
R
S
T
V
W
Y
China
Japan
Latvia
Russia
Korea
USSR

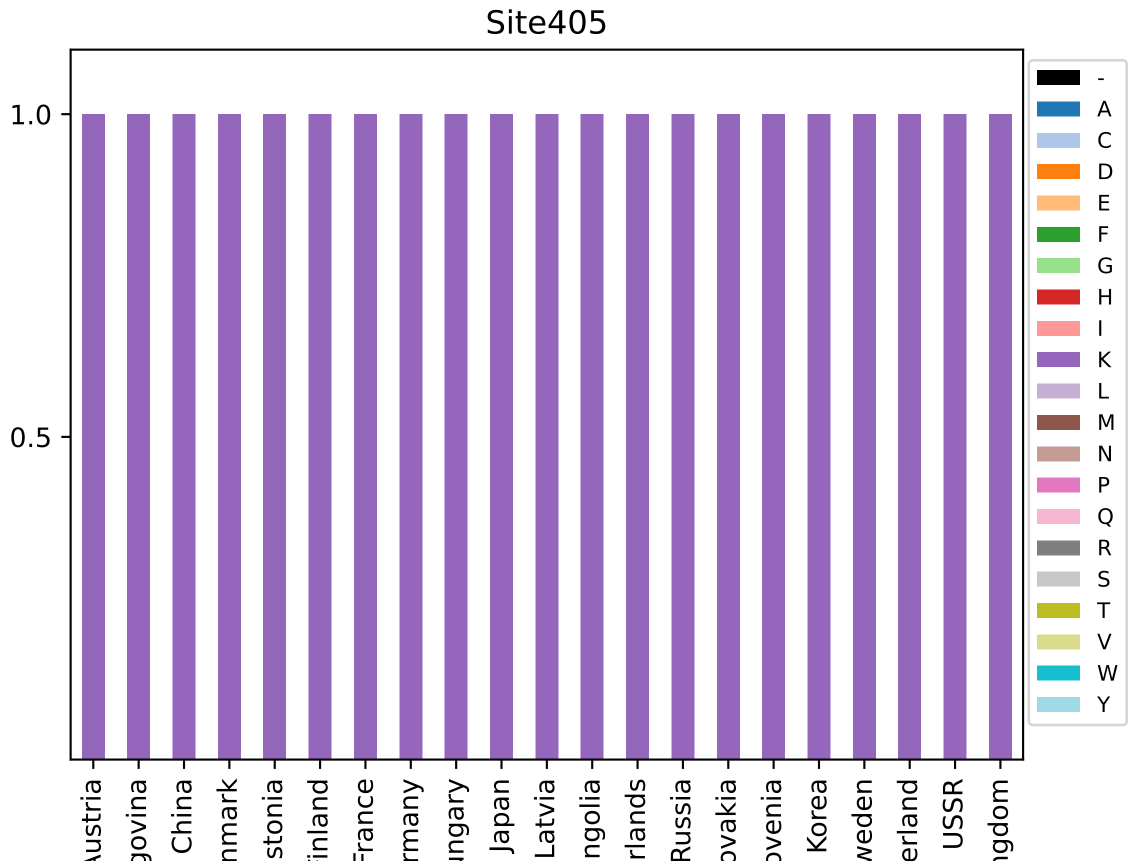

Site405
1.0
0.5
China
Japan
Latvia
Russia
Korea
USSR
-
A
C
D
E
F
G
H
I
K
L
M
N
P
Q
R
S
T
V
W
Y

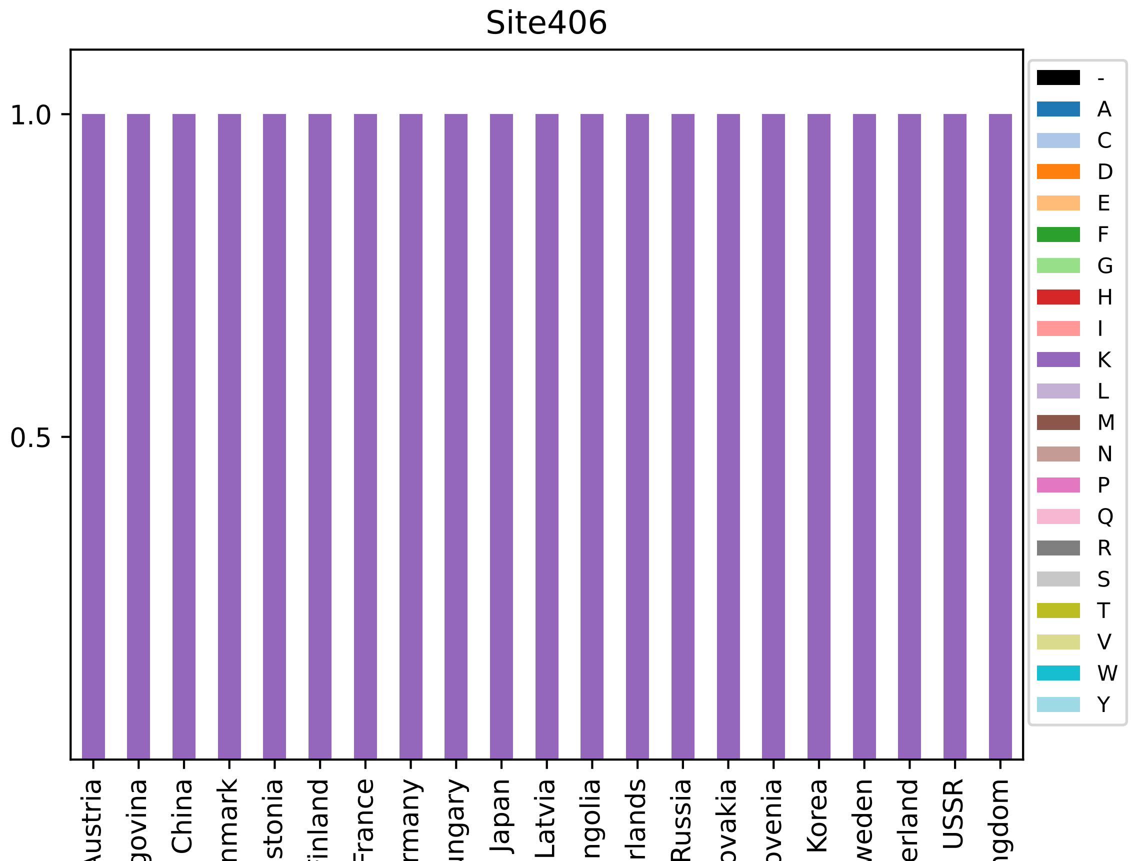
Site406
1.0
0.5
-
A
C
D
E
F
G
H
I
K
L
M
N
P
Q
R
S
T
V
W
Y
China
Japan
Latvia
Russia
Korea
USSR

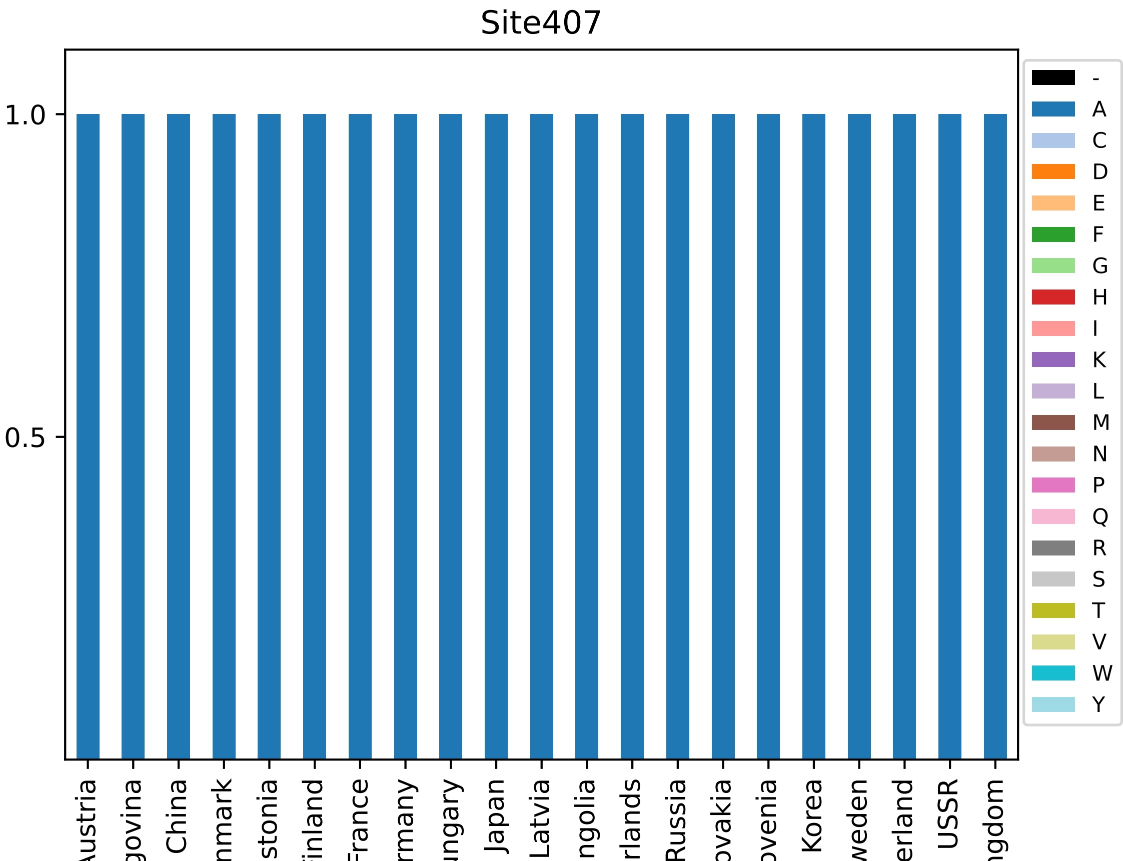
Site407
1.0
0.5
-
A
C
D
E
F
G
H
I
K
L
M
N
P
Q
R
S
T
V
W
Y
Austria
govina
China
nmark
stonia
inland
France
rmany
ungary
Japan
Latvia
ngolia
rlands
Russia
ovakia
ovenia
Korea
weden
erland
USSR
ngdom

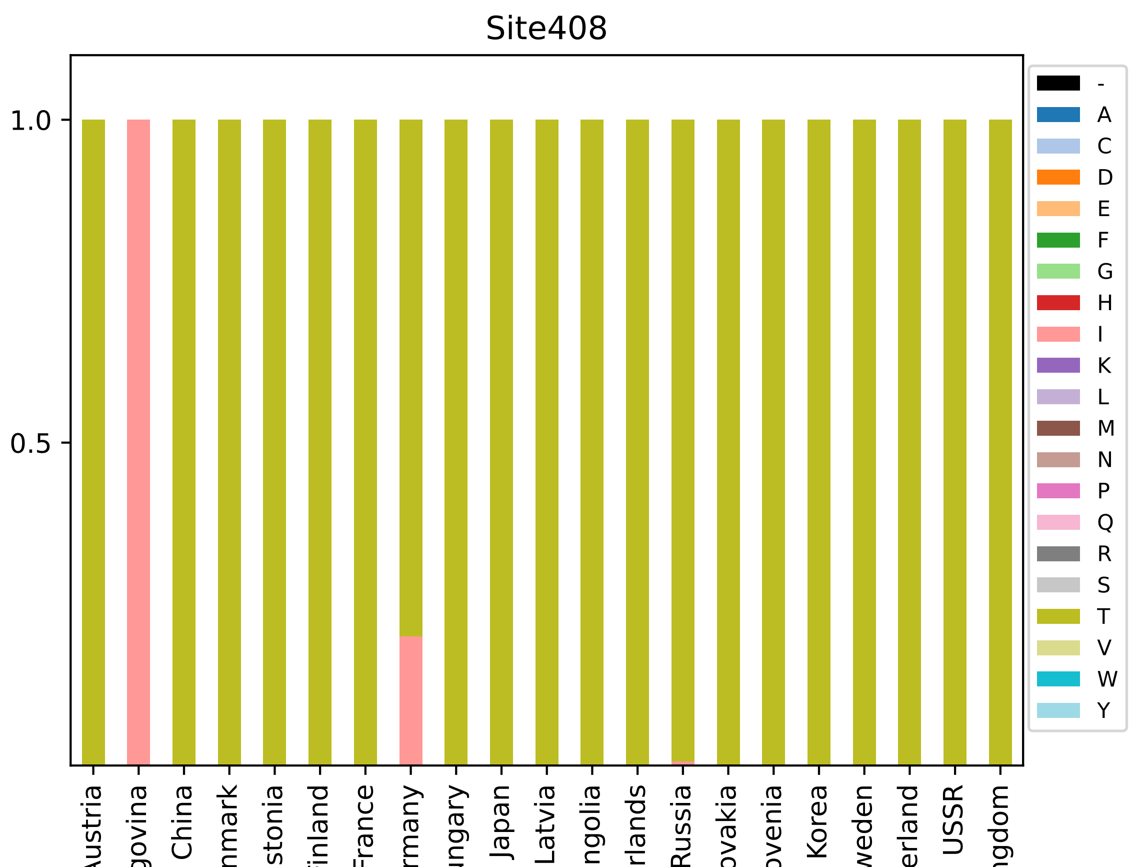
Site408
1.0
0.5
-
A
C
D
E
F
G
H
I
K
L
M
N
P
Q
R
S
T
V
W
Y
China
Japan
Latvia
Russia
Korea
USSR

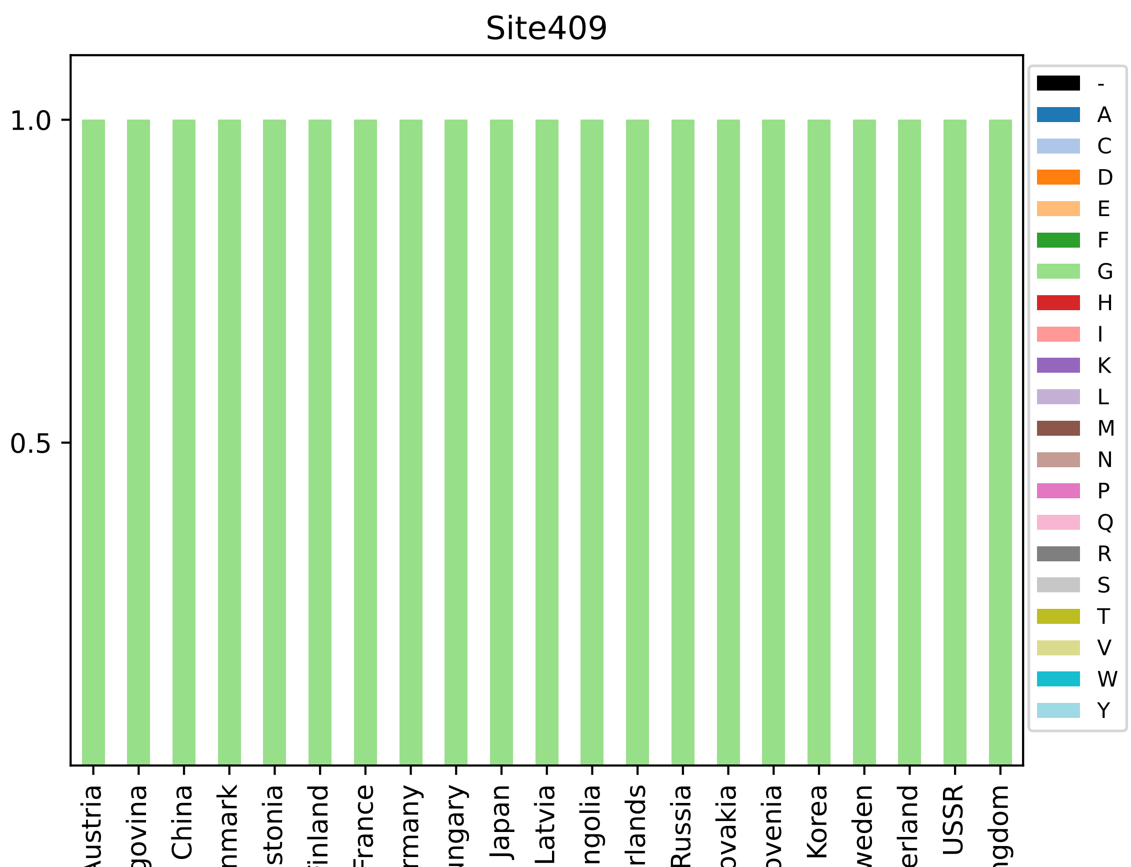

Site409
1.0
0.5
-
A
C
D
E
F
G
H
I
K
L
M
N
P
Q
R
S
T
V
W
Y
China
Japan
Latvia
Russia
Korea
USSR

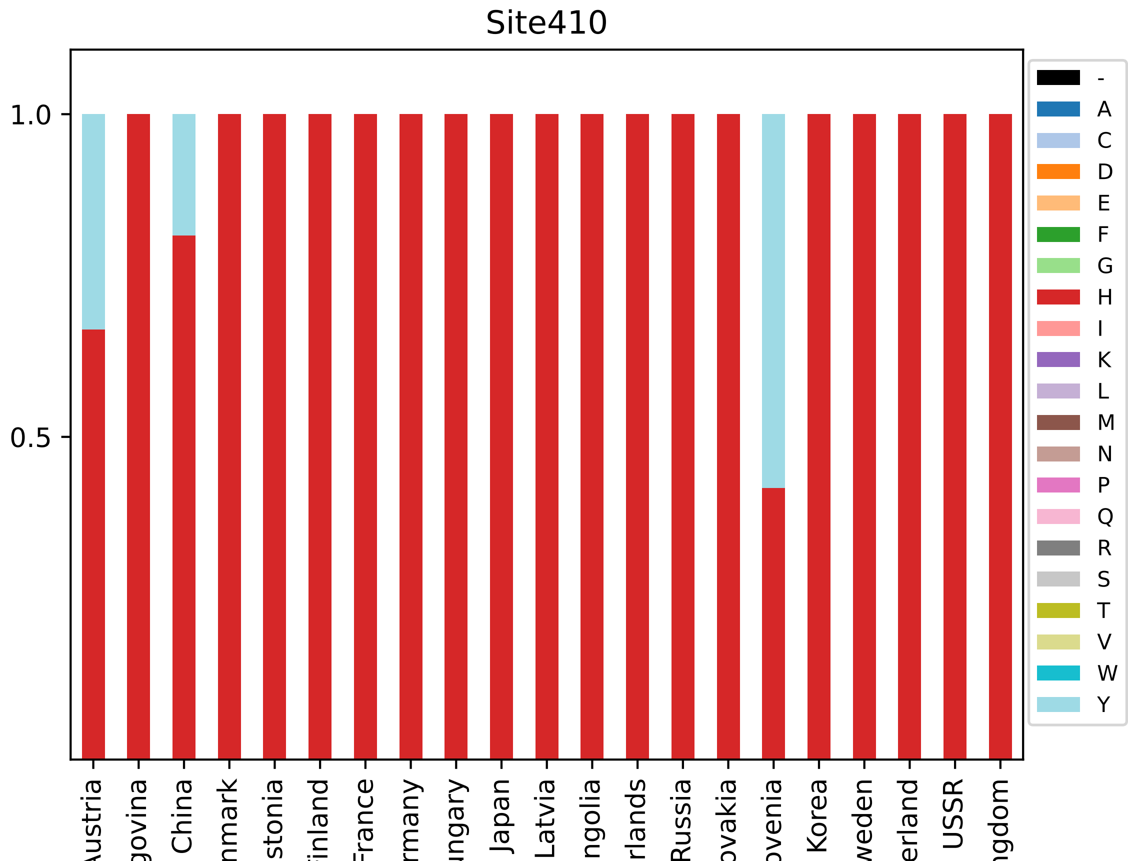
Site410
1.0
0.5
-
A
C
D
E
F
G
H
I
K
L
M
N
P
Q
R
S
T
V
W
Y
China
Japan
Latvia
Russia
Korea
USSR

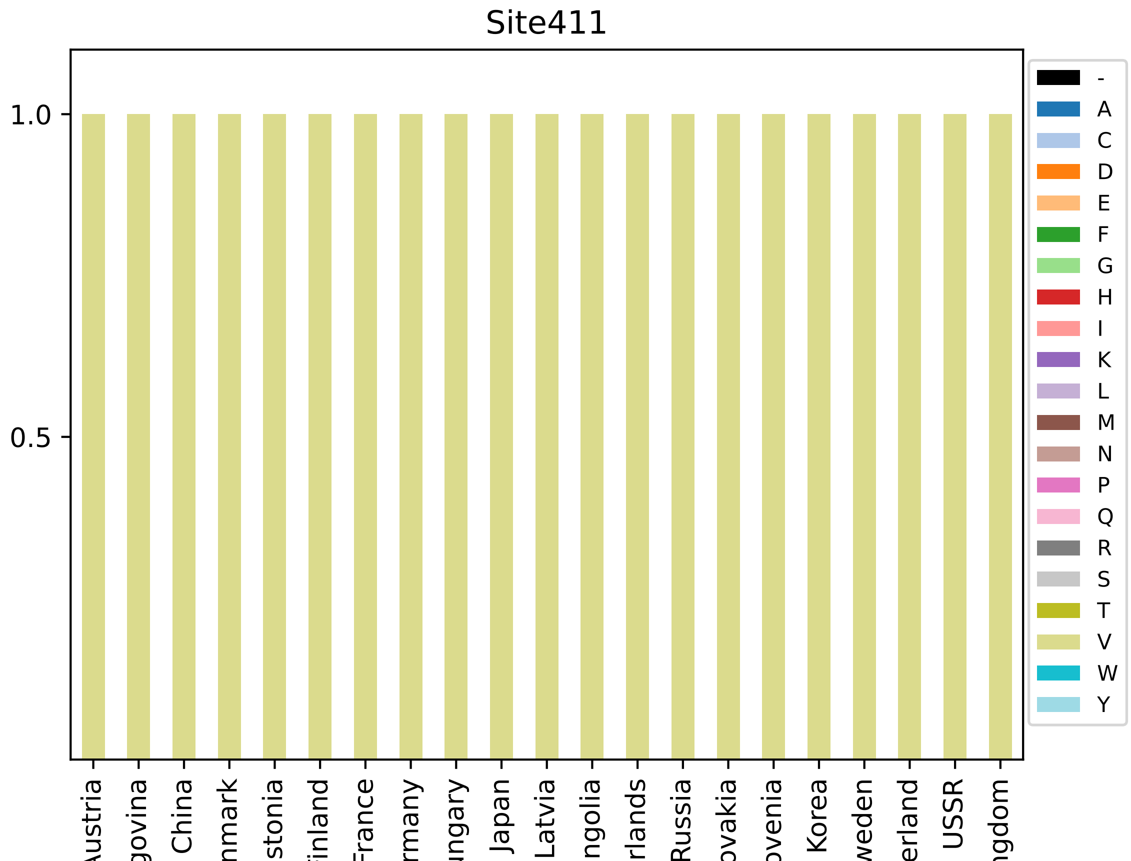
Site411
1.0
0.5
-
A
C
D
E
F
G
H
I
K
L
M
N
P
Q
R
S
T
V
W
Y
China
Japan
Latvia
Russia
Korea
USSR

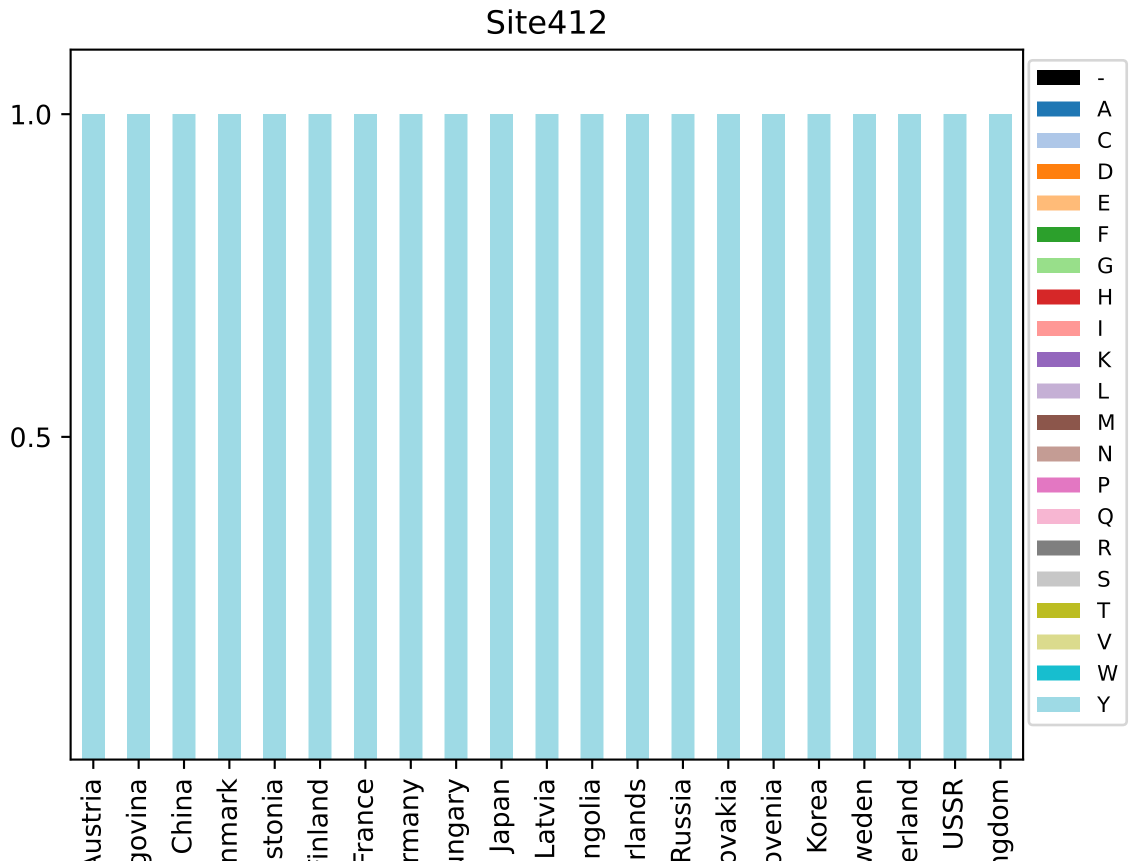

Site412
1.0
0.5
-
A
C
D
E
F
G
H
I
K
L
M
N
P
Q
R
S
T
V
W
Y
China
Japan
Latvia
Russia
Korea
USSR

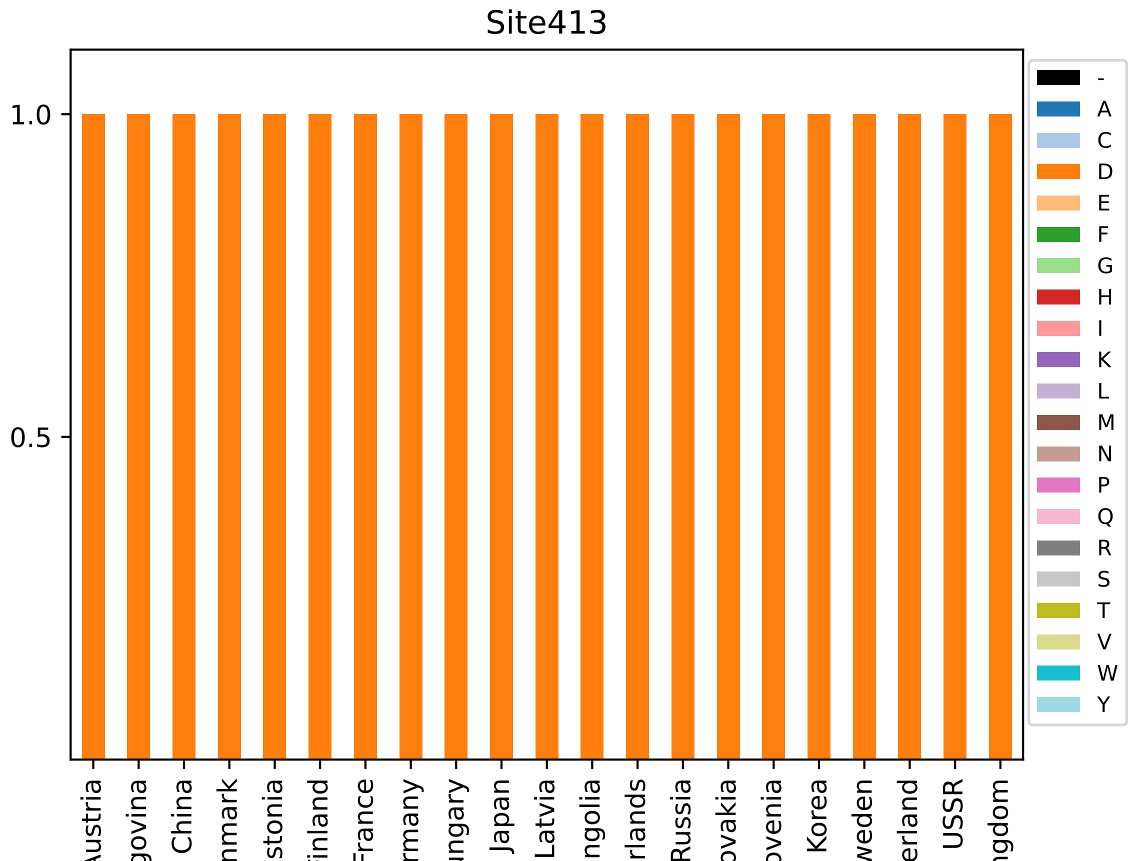

Site413
1.0
0.5
-
A
C
D
E
F
G
H
I
K
L
M
N
P
Q
R
S
T
V
W
Y
Austria
govina
China
nmark
stonia
inland
France
rmany
ungary
Japan
Latvia
ngolia
rlands
Russia
ovakia
ovenia
Korea
weden
erland
USSR
ngdom

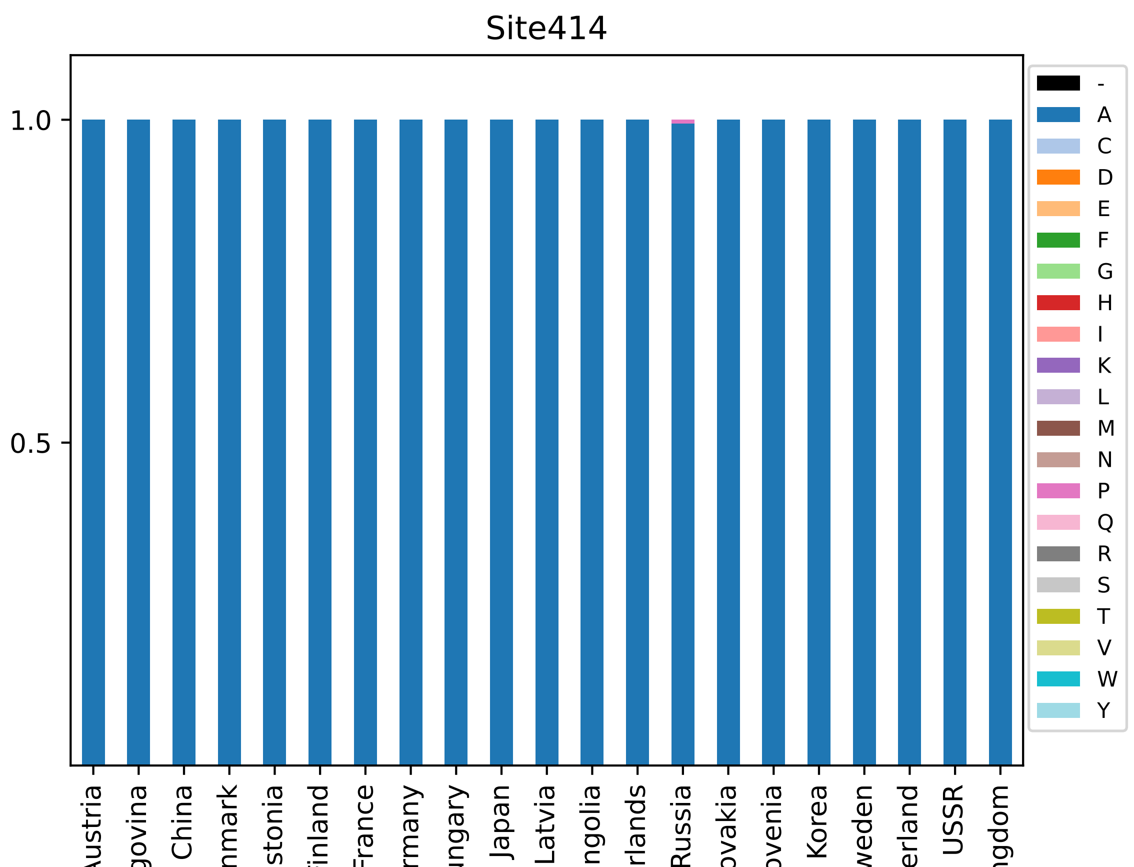
Site414
1.0
0.5
-
A
C
D
E
F
G
H
I
K
L
M
N
P
Q
R
S
T
V
W
Y
Austria
govina
China
nmark
stonia
Finland
France
rmany
ungary
Japan
Latvia
ngolia
rlands
Russia
ovakia
ovenia
Korea
weden
erland
USSR
ngdom

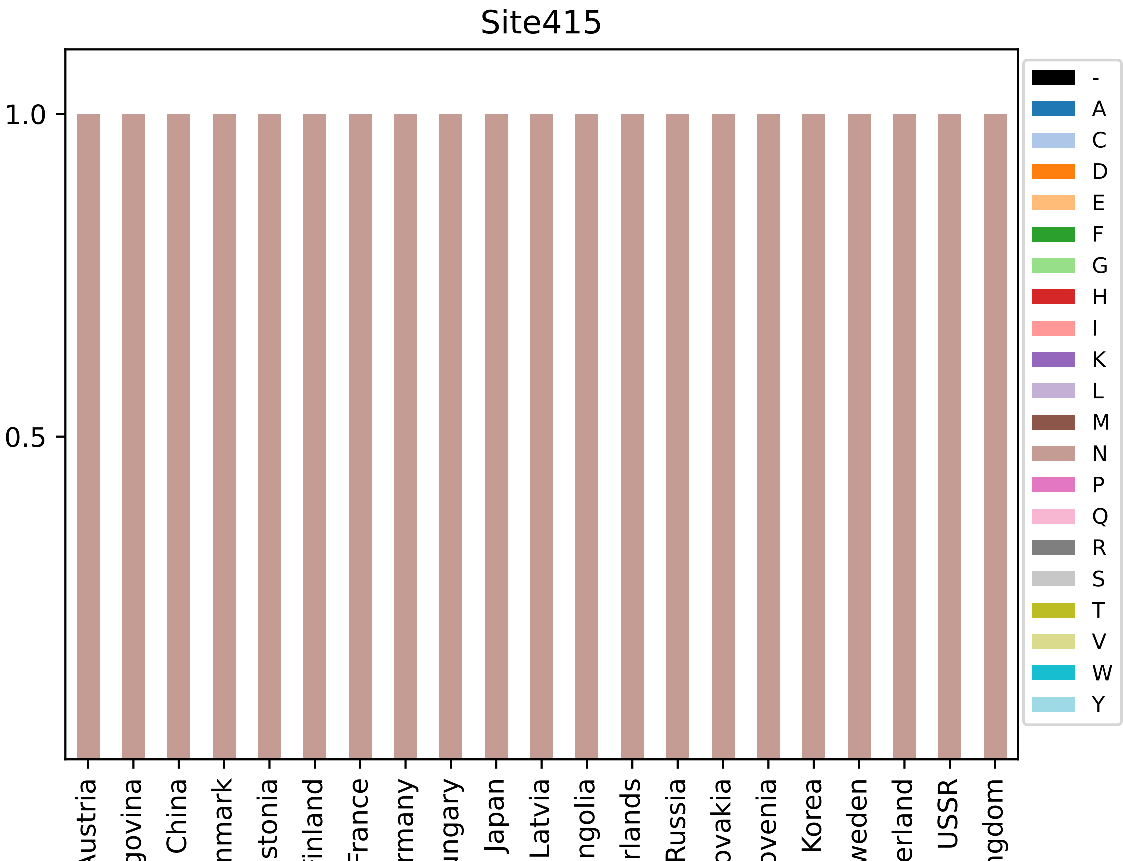
Site415
1.0
0.5
-
A
C
D
E
F
G
H
I
K
L
M
N
P
Q
R
S
T
V
W
Y
China
Japan
Latvia
Russia
Korea
USSR

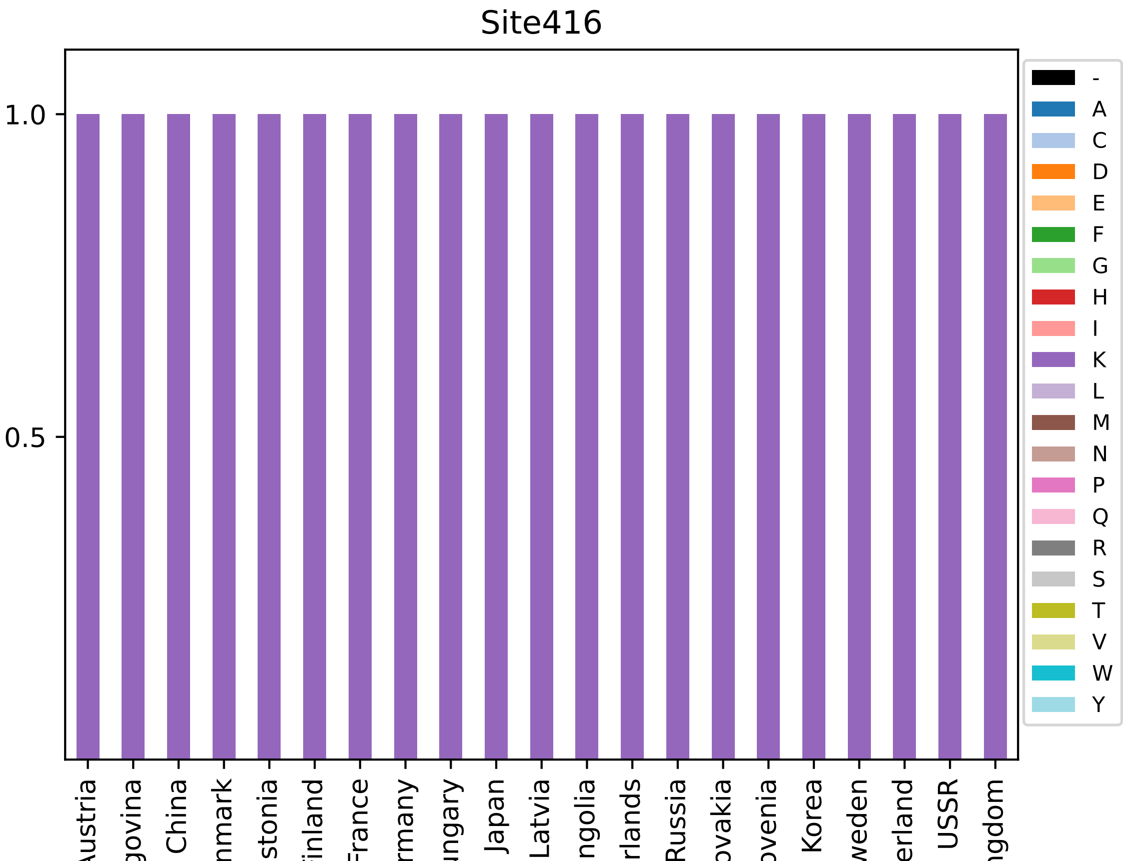
Site416
1.0
0.5
-
A
C
D
E
F
G
H
I
K
L
M
N
P
Q
R
S
T
V
W
Y
China
Japan
Latvia
Russia
Korea
USSR

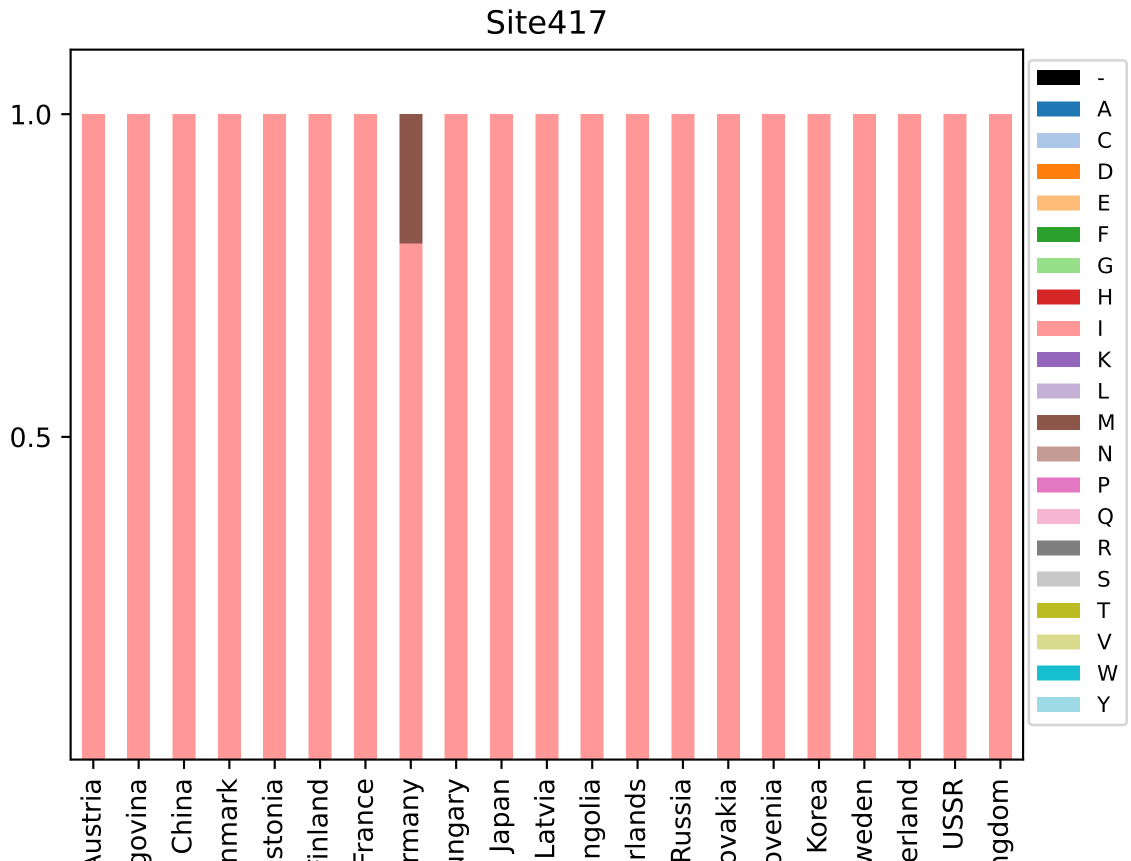

Site417
1.0
0.5
-
A
C
D
E
F
G
H
I
K
L
M
N
P
Q
R
S
T
V
W
Y
China
France
Japan
Latvia
Russia
Korea
USSR

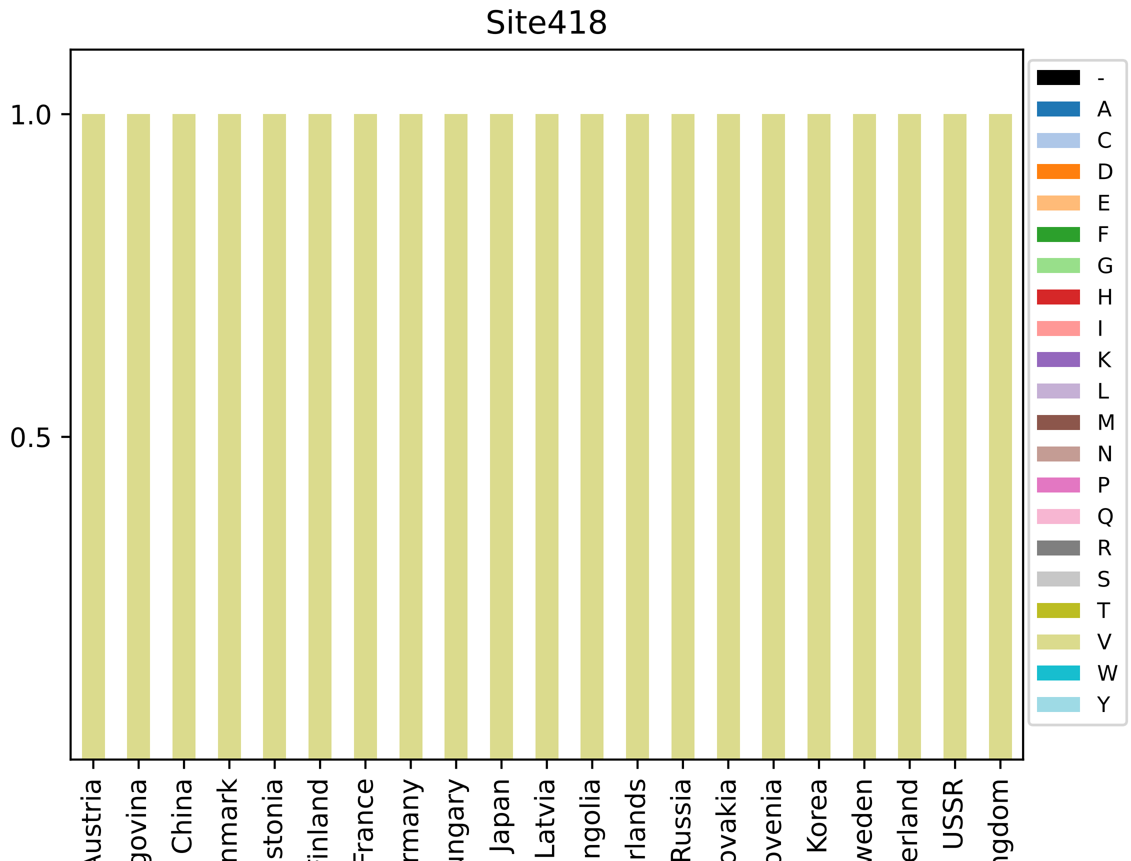

Site418
1.0
0.5
-
A
C
D
E
F
G
H
I
K
L
M
N
P
Q
R
S
T
V
W
Y
Austria
China
Japan
Latvia
Russia
Korea
USSR

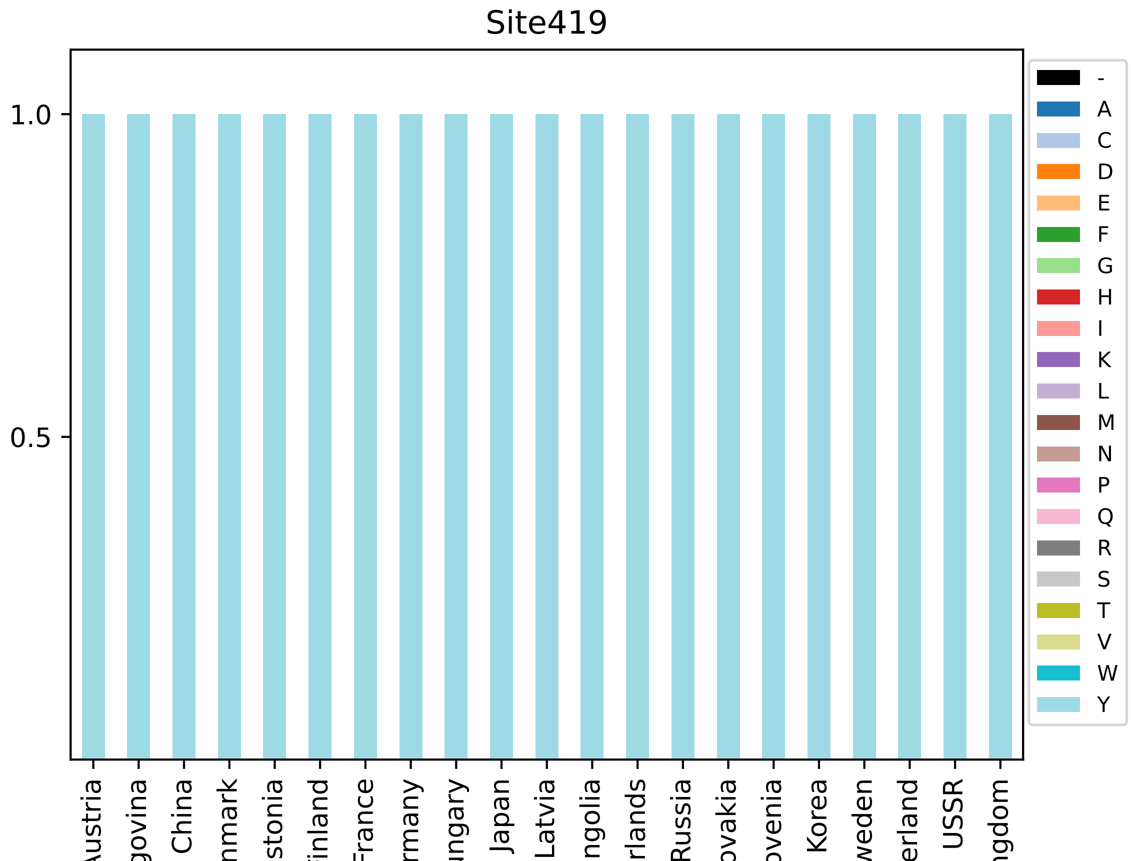
Site419
1.0
0.5
-
A
C
D
E
F
G
H
I
K
L
M
N
P
Q
R
S
T
V
W
Y
Austria
China
Japan
Latvia
Russia
Korea
USSR

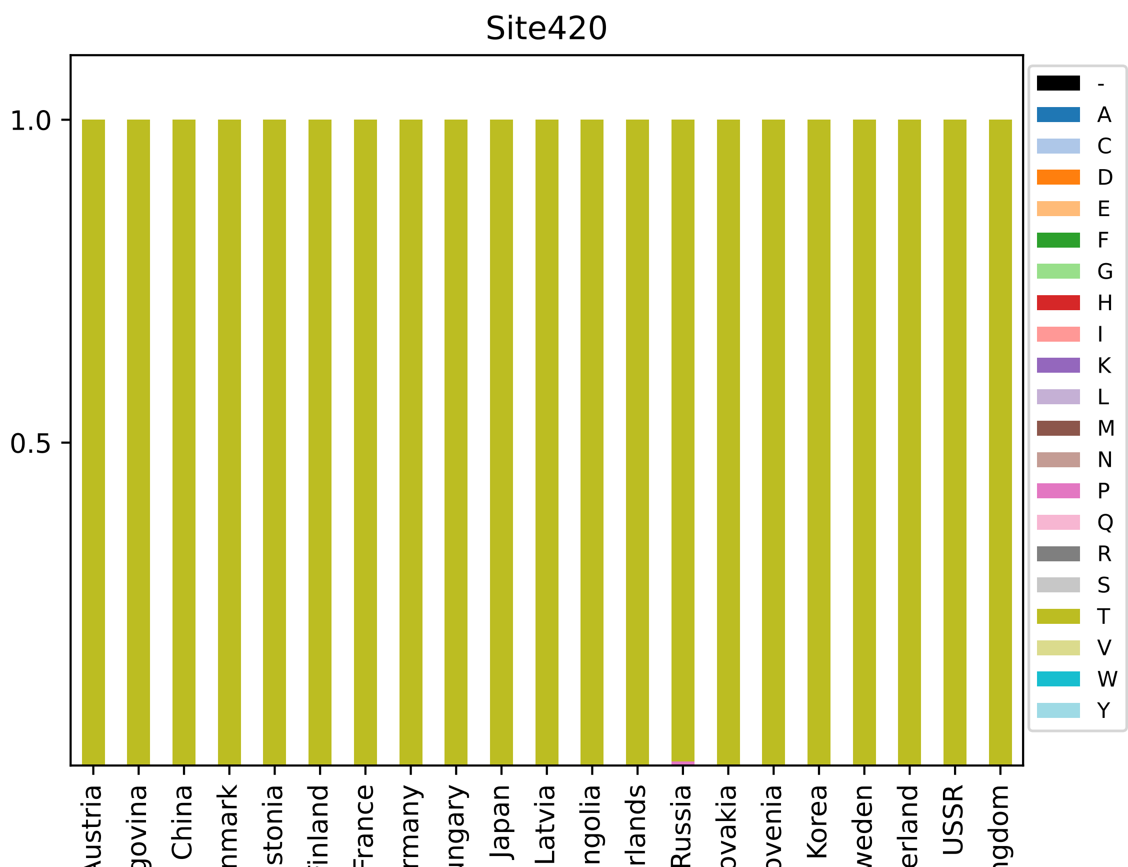

Site420
1.0
0.5
-
A
C
D
E
F
G
H
I
K
L
M
N
P
Q
R
S
T
V
W
Y
Austria
govina
China
nmark
stonia
inland
France
rmany
ungary
Japan
Latvia
ngolia
rlands
Russia
ovakia
ovenia
Korea
weden
erland
USSR
ngdom

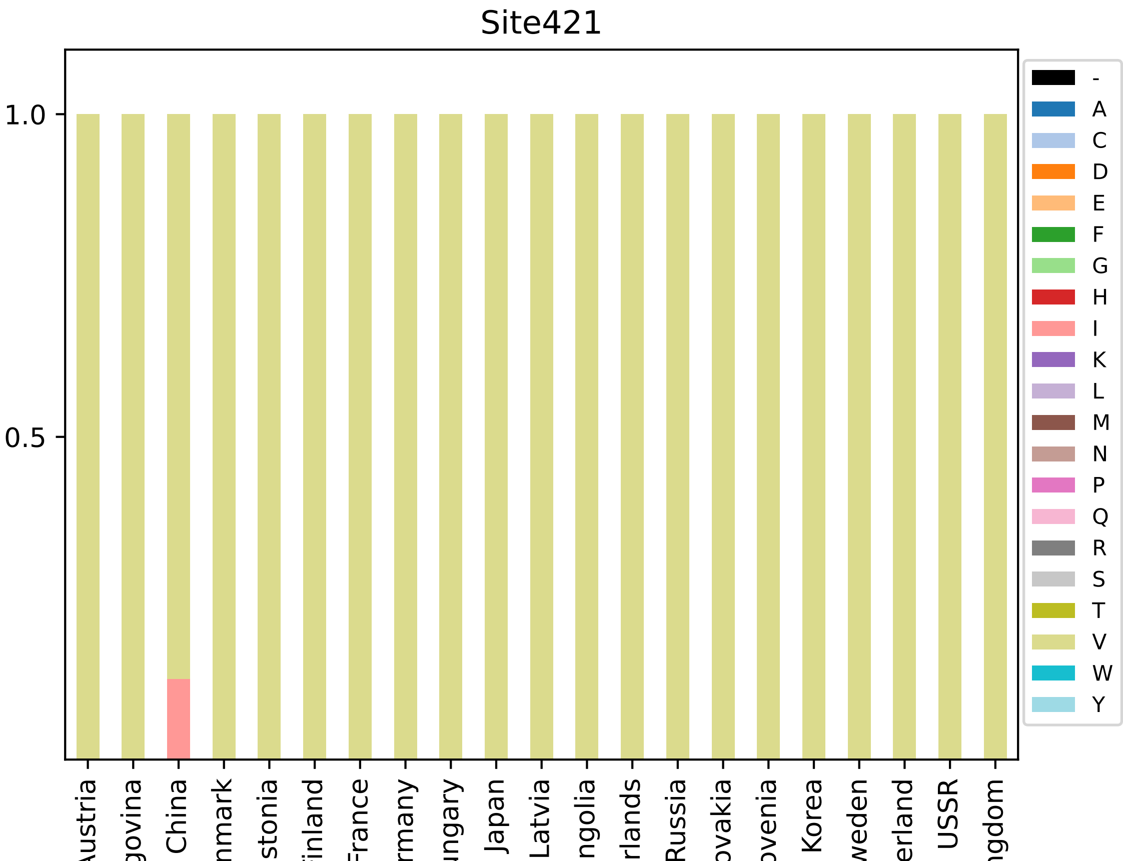
Site421
1.0
0.5
Austria
govina
China
nmark
stonia
inland
France
rmany
ungary
Japan
Latvia
ngolia
rlands
Russia
ovakia
ovenia
Korea
weden
erland
USSR
ngdom
-
A
C
D
E
F
G
H
I
K
L
M
N
P
Q
R
S
T
V
W
Y

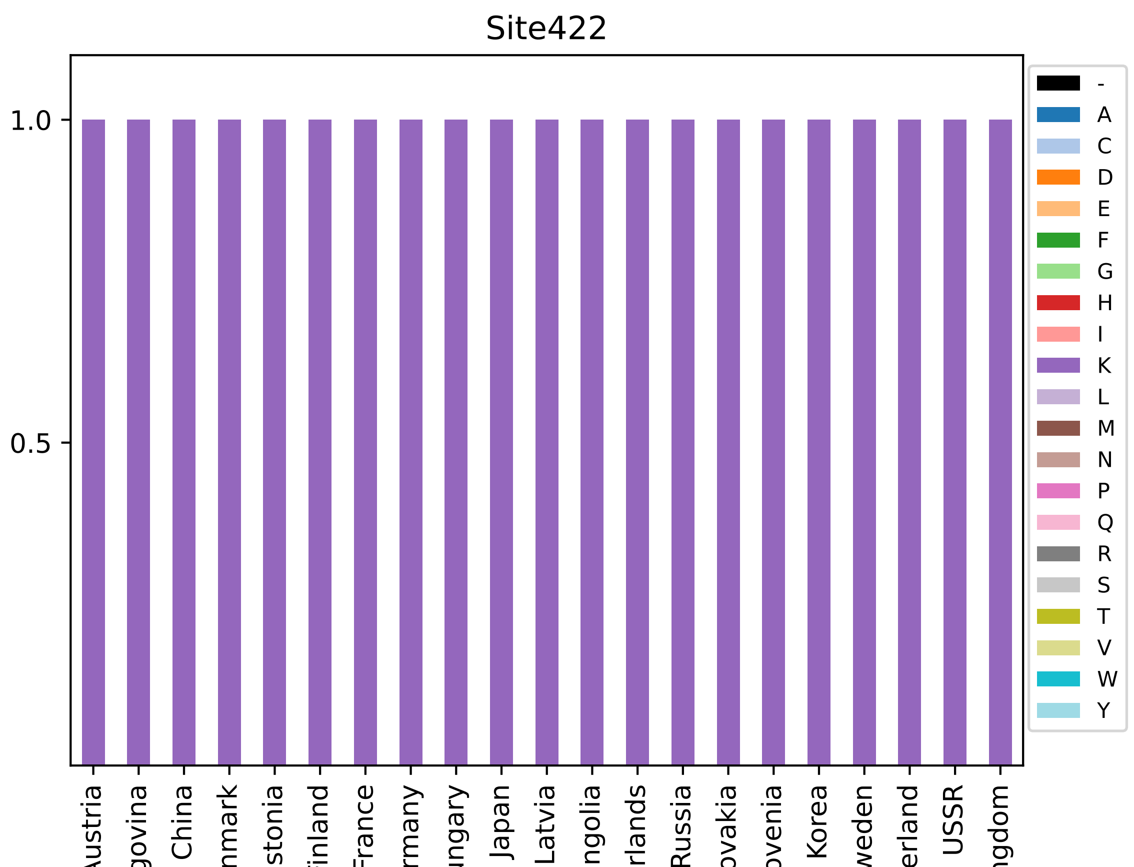
Site422
1.0
0.5
-
A
C
D
E
F
G
H
I
K
L
M
N
P
Q
R
S
T
V
W
Y
China
Japan
Latvia
Russia
Korea
USSR

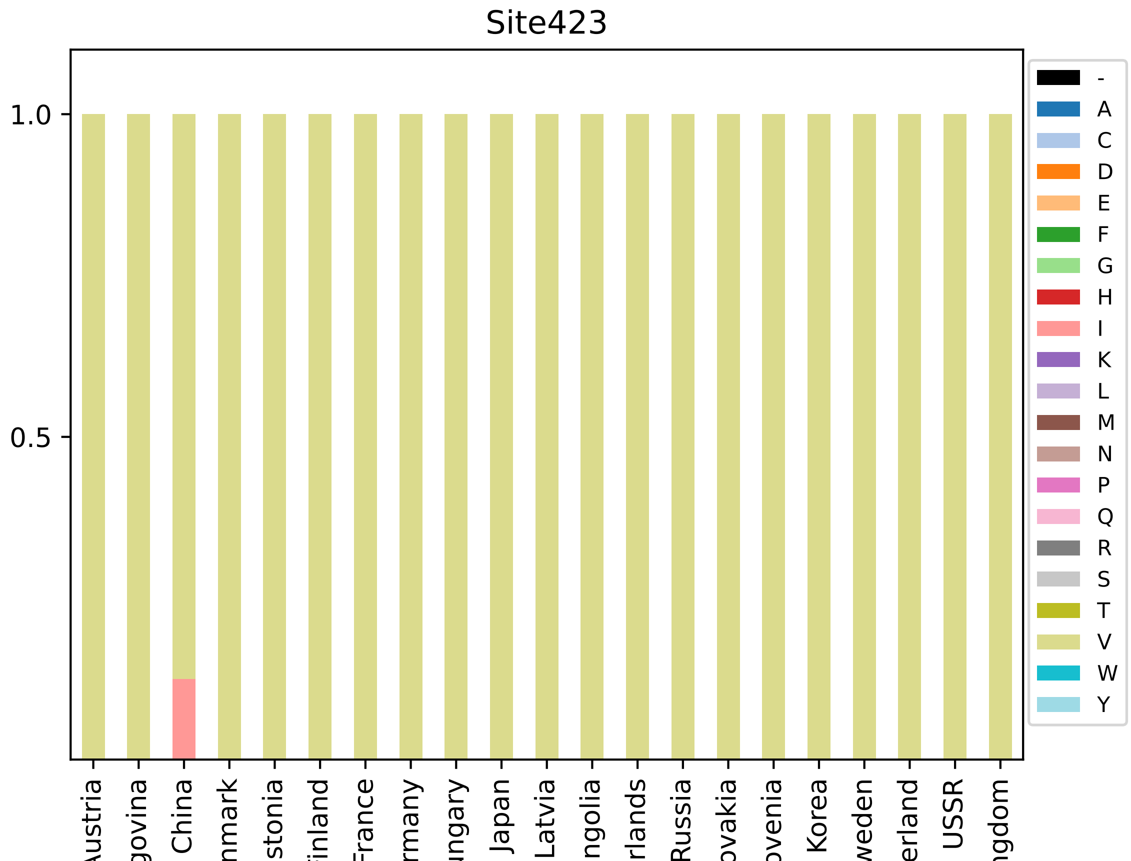

Site423
1.0
0.5
-
A
C
D
E
F
G
H
I
K
L
M
N
P
Q
R
S
T
V
W
Y
China
Japan
Latvia
Russia
Korea
USSR

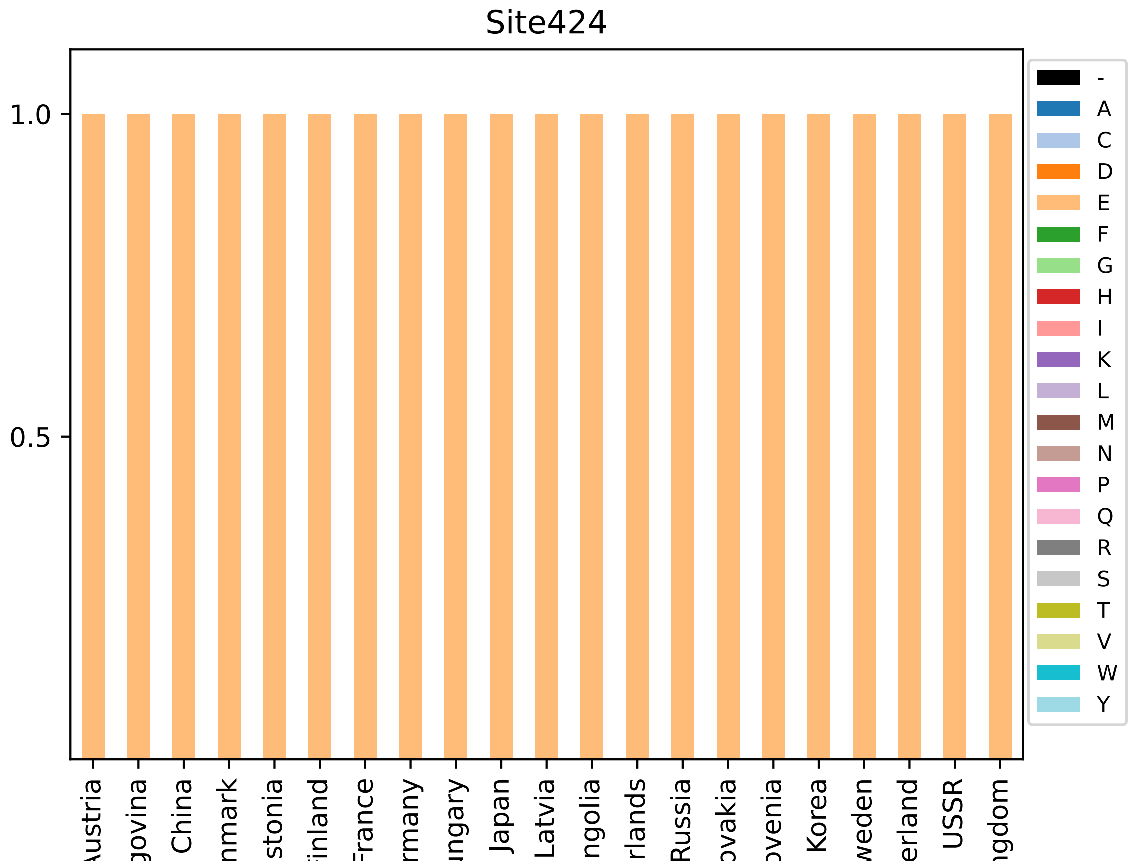
Site424
1.0
0.5
-
A
C
D
E
F
G
H
I
K
L
M
N
P
Q
R
S
T
V
W
Y
Austria
govina
China
nmark
stonia
Finland
France
rmany
ungary
Japan
Latvia
ngolia
rlands
Russia
ovakia
ovenia
Korea
weden
erland
USSR
ngdom

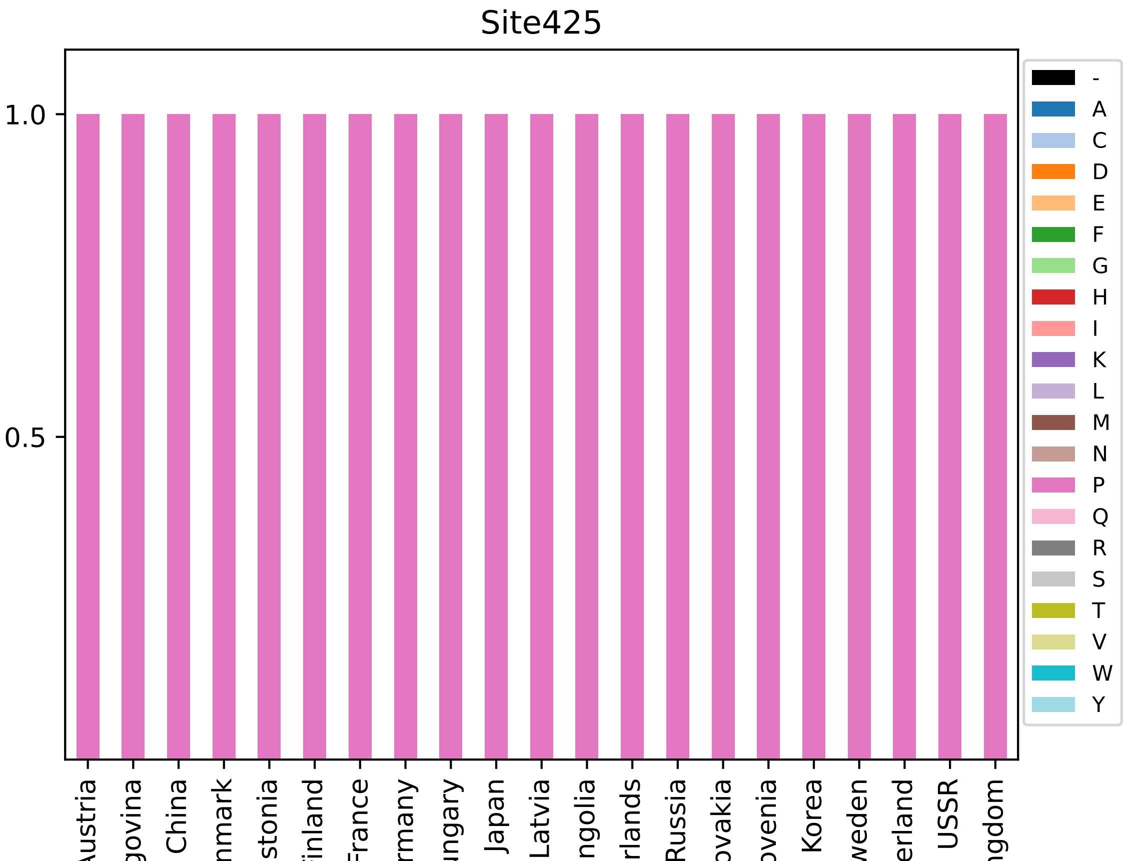

Site425
1.0
0.5
-
A
C
D
E
F
G
H
I
K
L
M
N
P
Q
R
S
T
V
W
Y
China
Japan
Latvia
Russia
Korea
USSR

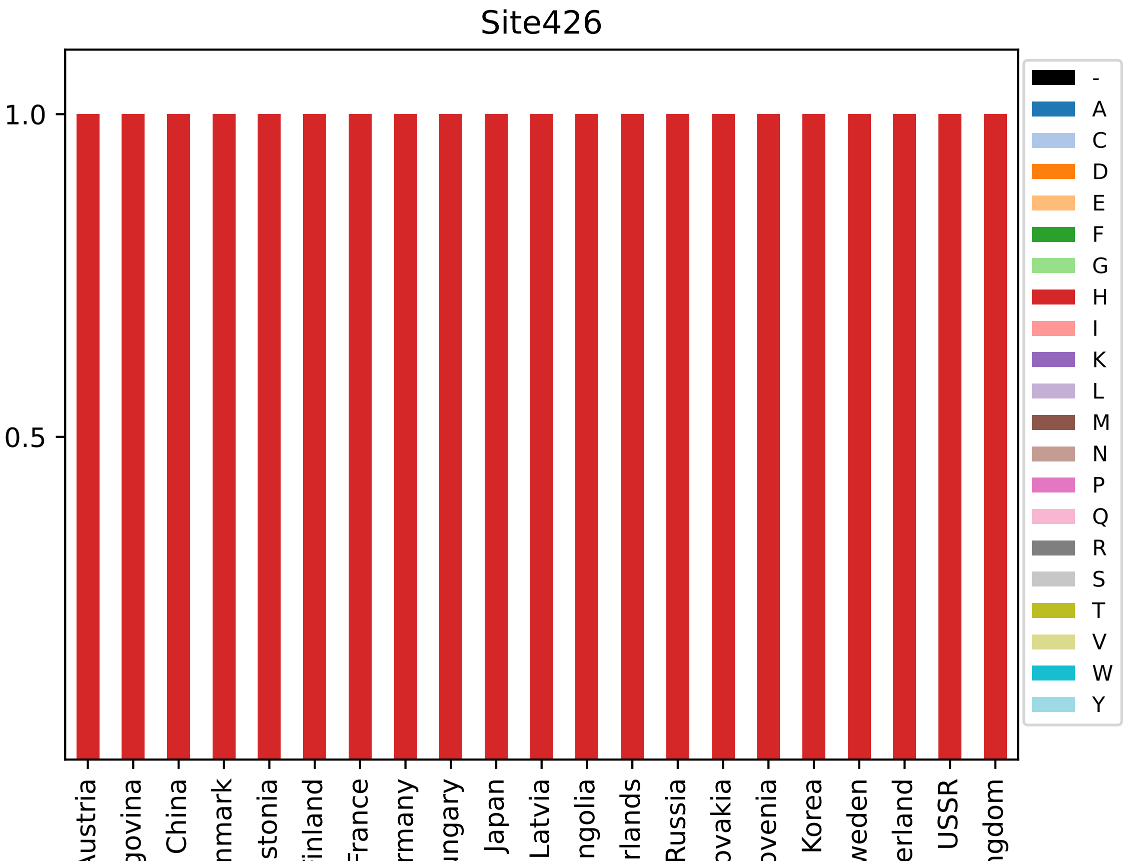
Site426
1.0
0.5
-
A
C
D
E
F
G
H
I
K
L
M
N
P
Q
R
S
T
V
W
Y
Austria
govina
China
nmark
stonia
inland
France
rmany
ungary
Japan
Latvia
ngolia
rlands
Russia
ovakia
ovenia
Korea
weden
erland
USSR
ngdom

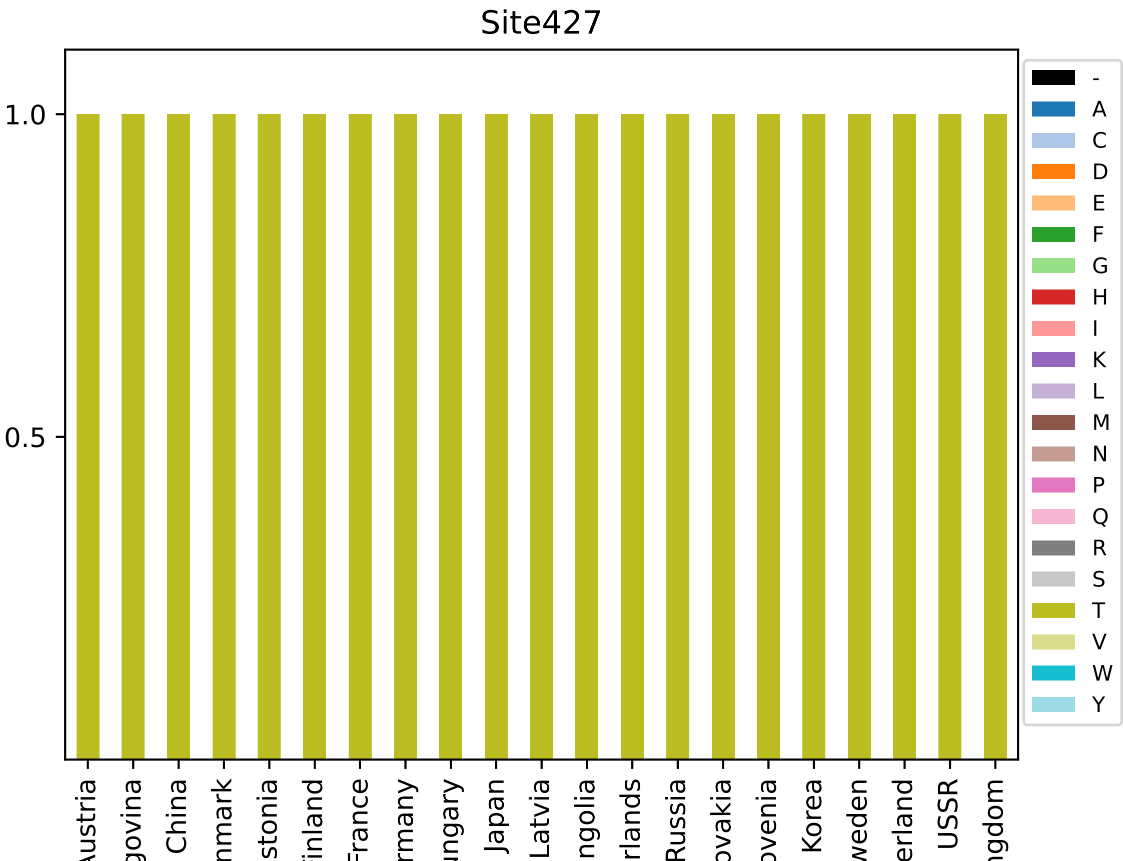

Site427
1.0
0.5
-
A
C
D
E
F
G
H
I
K
L
M
N
P
Q
R
S
T
V
W
Y
China
Japan
Latvia
Russia
Korea
USSR

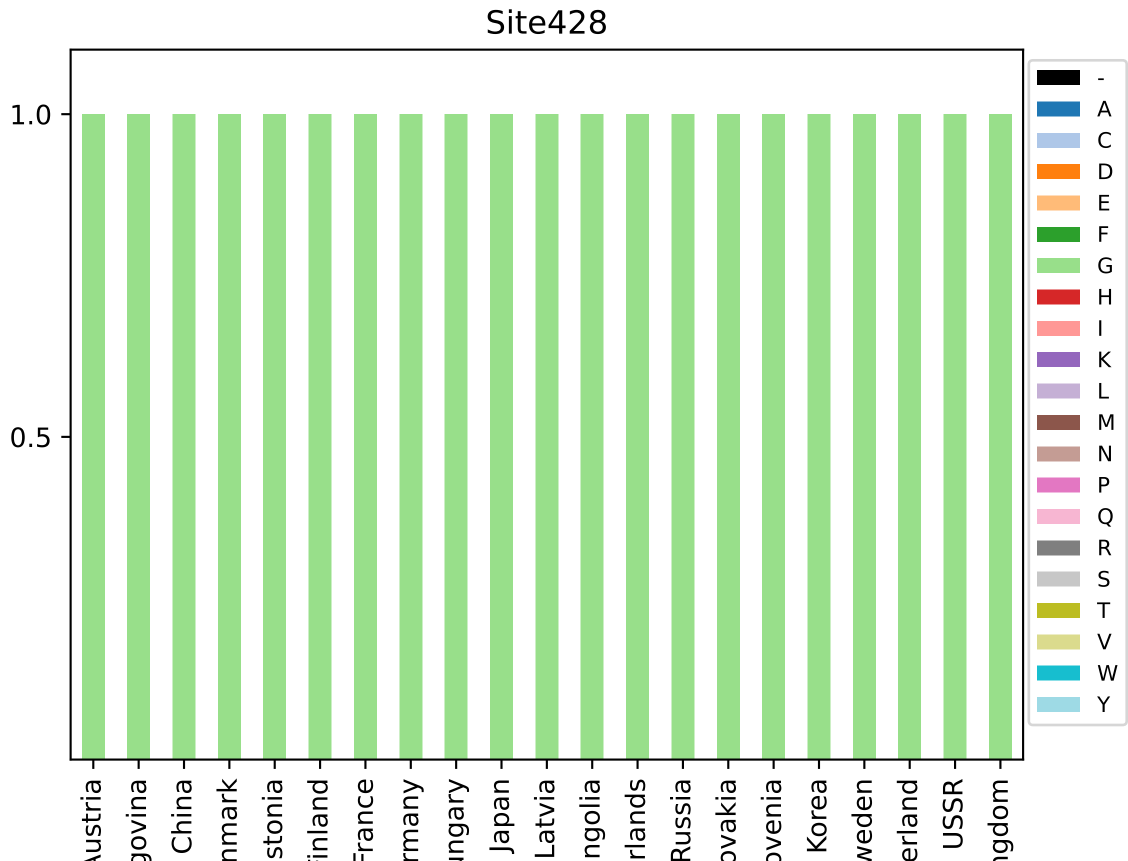

Site428
1.0
0.5
-
A
C
D
E
F
G
H
I
K
L
M
N
P
Q
R
S
T
V
W
Y
China
Japan
Latvia
Russia
Korea
USSR

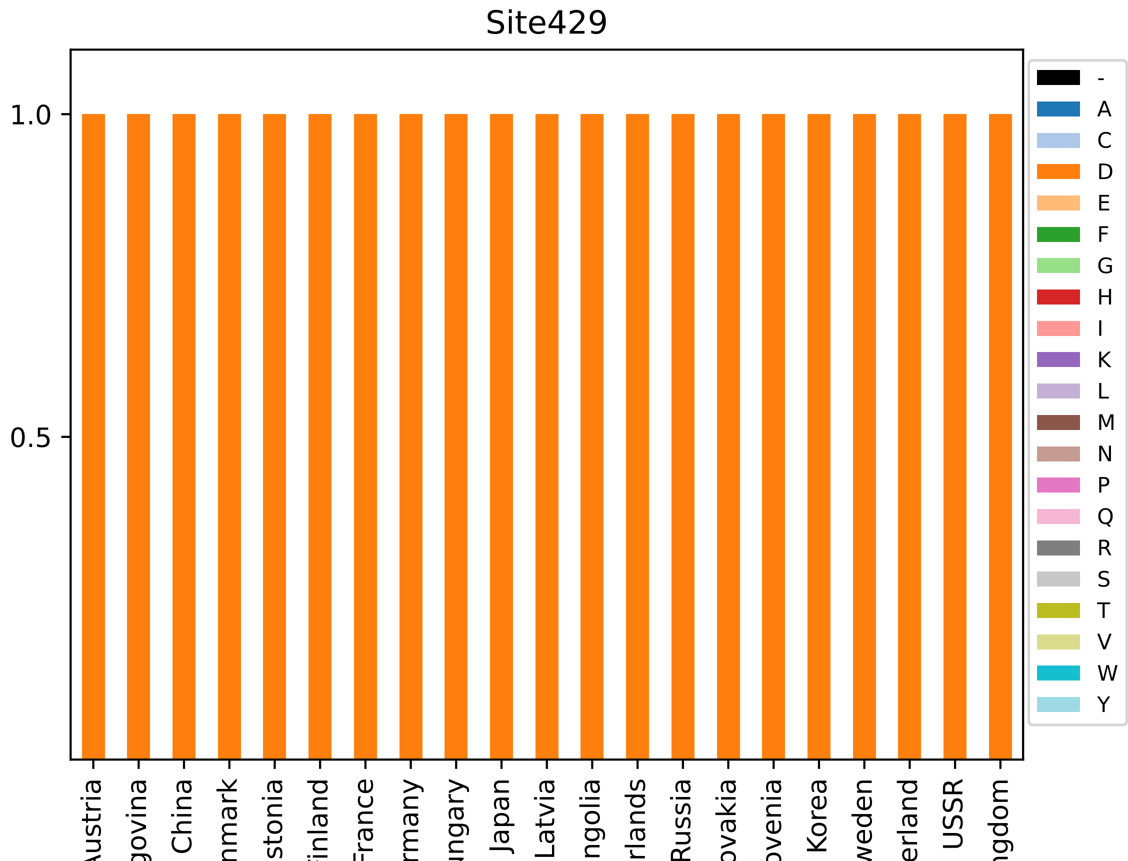

Site429
1.0
0.5
-
A
C
D
E
F
G
H
I
K
L
M
N
P
Q
R
S
T
V
W
Y
Austria
govina
China
nmark
stonia
Finland
France
rmany
ungary
Japan
Latvia
ngolia
rlands
Russia
ovakia
ovenia
Korea
weden
erland
USSR
ngdom

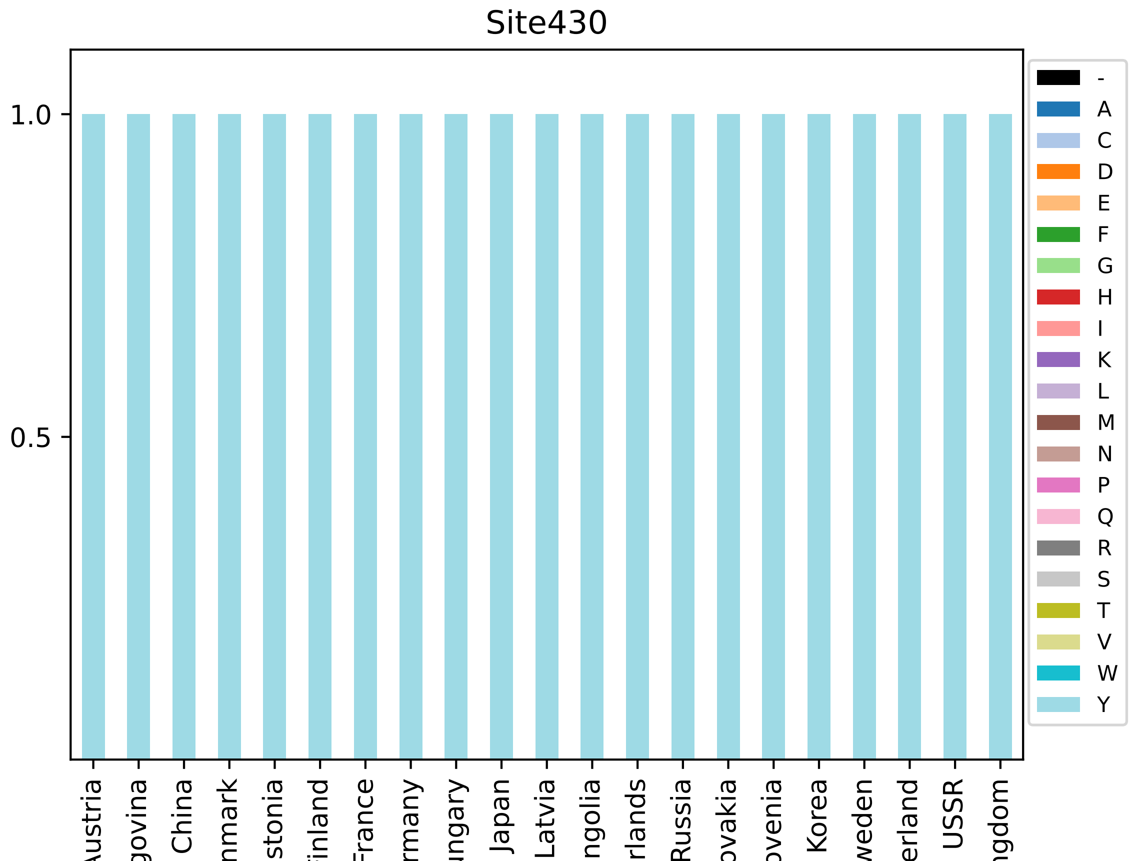
Site430
1.0
0.5
-
A
C
D
E
F
G
H
I
K
L
M
N
P
Q
R
S
T
V
W
Y
Austria
govina
China
nmark
stonia
inland
France
rmany
ungary
Japan
Latvia
ngolia
rlands
Russia
ovakia
ovenia
Korea
weden
erland
USSR
ngdom

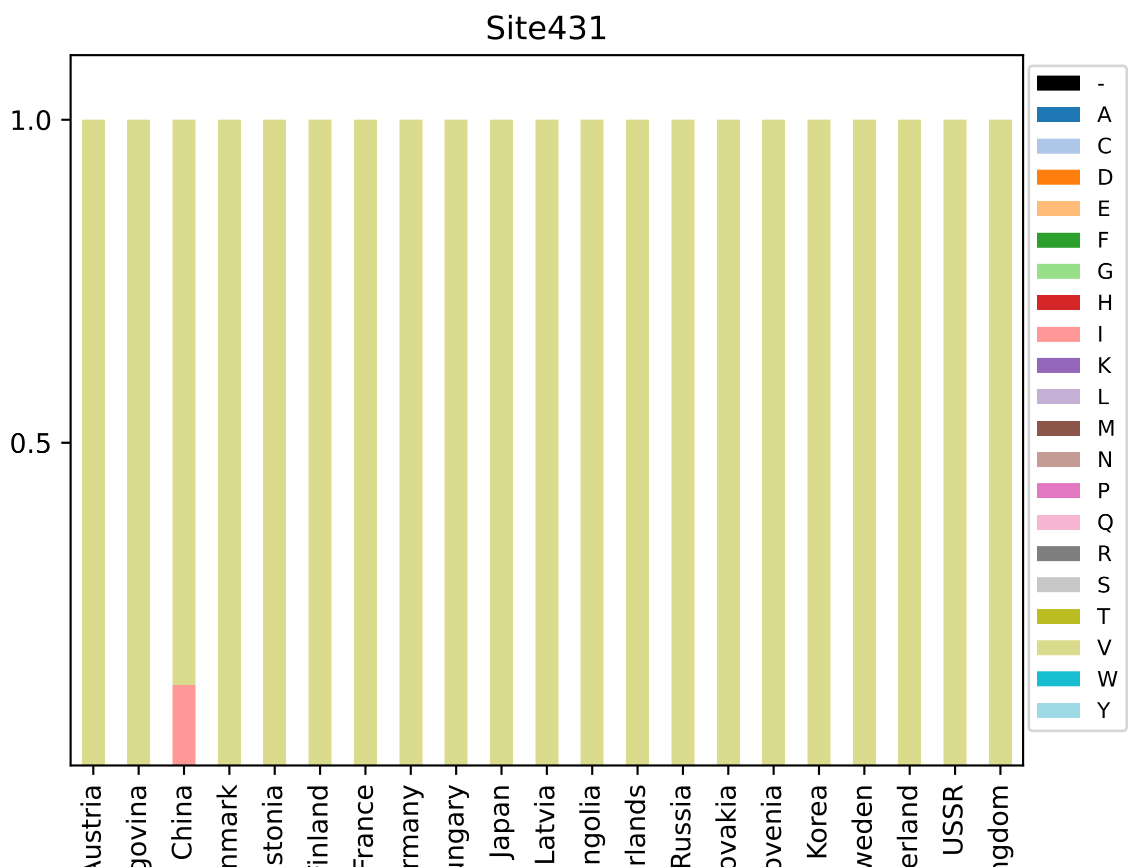

Site431
1.0
0.5
-
A
C
D
E
F
G
H
I
K
L
M
N
P
Q
R
S
T
V
W
Y
China
Japan
Latvia
Russia
Korea
USSR

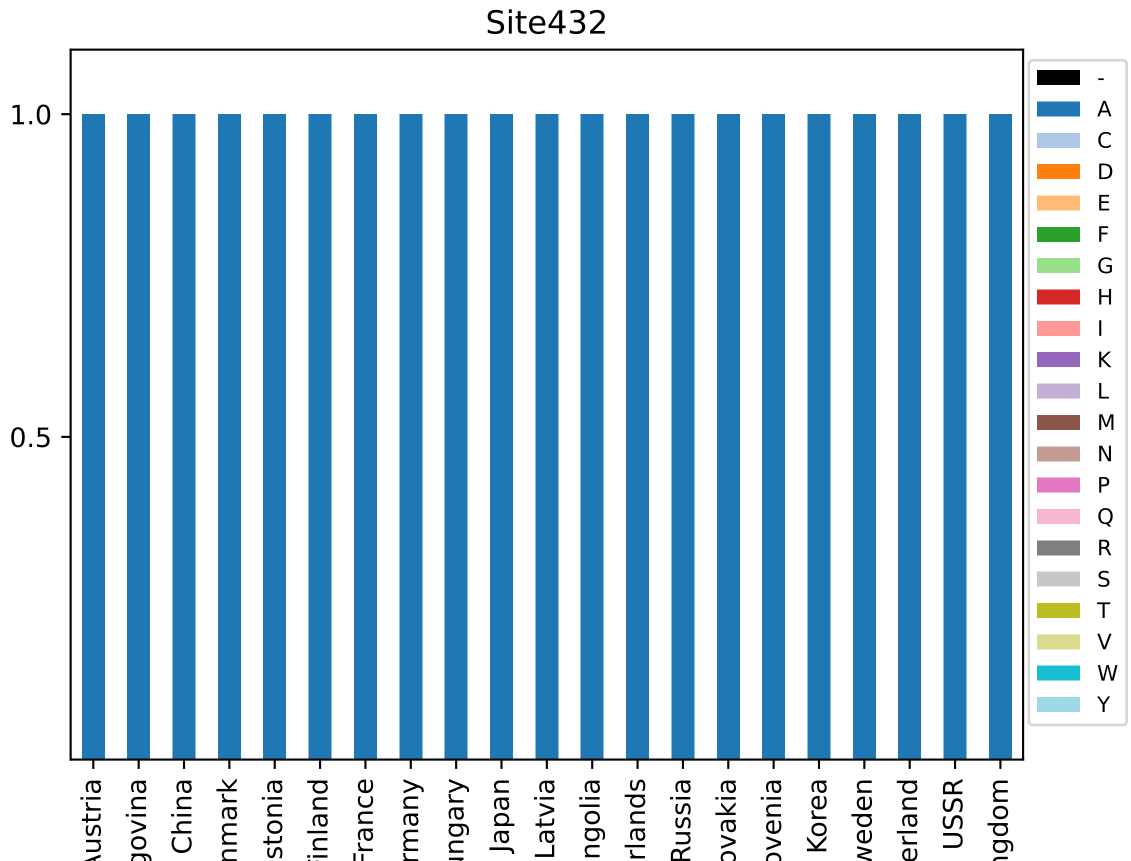
Site432
1.0
0.5
Austria
govina
China
nmark
stonia
inland
France
rmany
ungary
Japan
Latvia
ngolia
rlands
Russia
ovakia
ovenia
Korea
weden
erland
USSR
ngdom
-
A
C
D
E
F
G
H
I
K
L
M
N
P
Q
R
S
T
V
W
Y

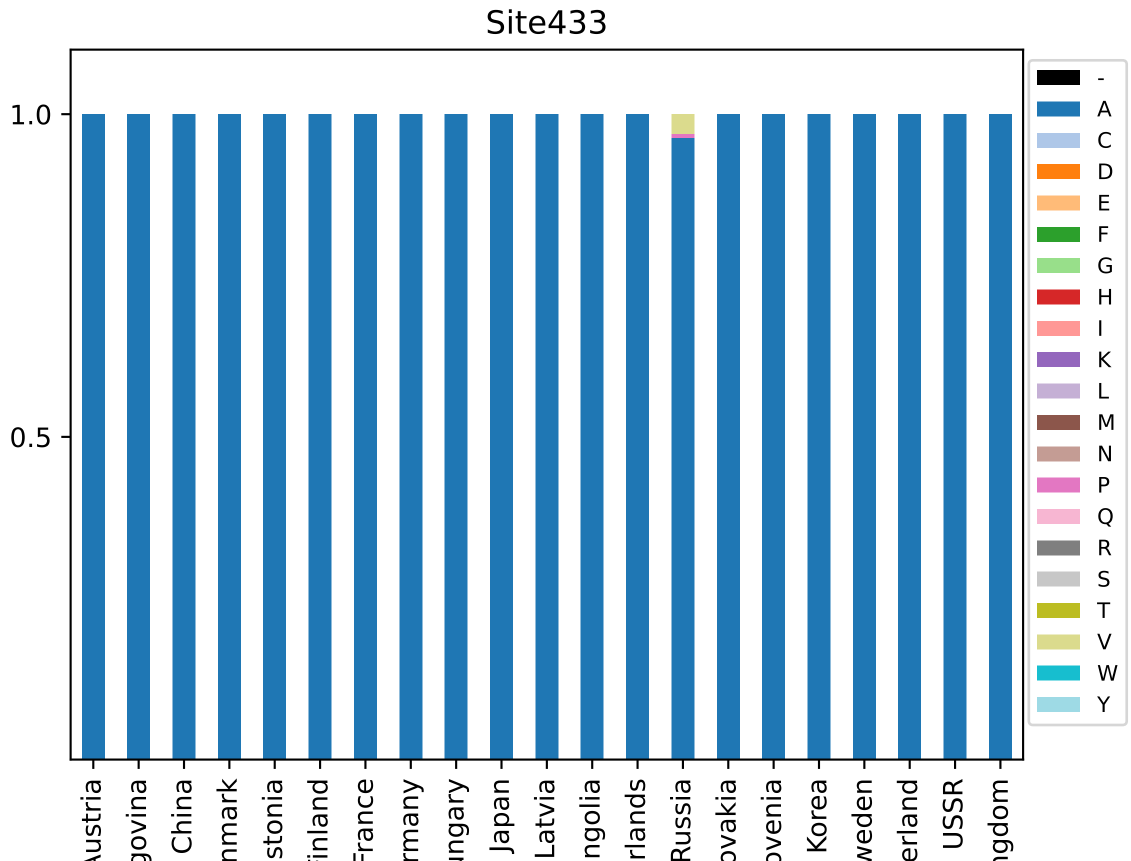

Site433
1.0
0.5
-
A
C
D
E
F
G
H
I
K
L
M
N
P
Q
R
S
T
V
W
Y
ustria
govina
China
nmark
stonia
inland
rance
rmany
ungary
Japan
Latvia
ngolia
rlands
Russia
ovakia
ovenia
Korea
weden
erland
USSR
ngdom

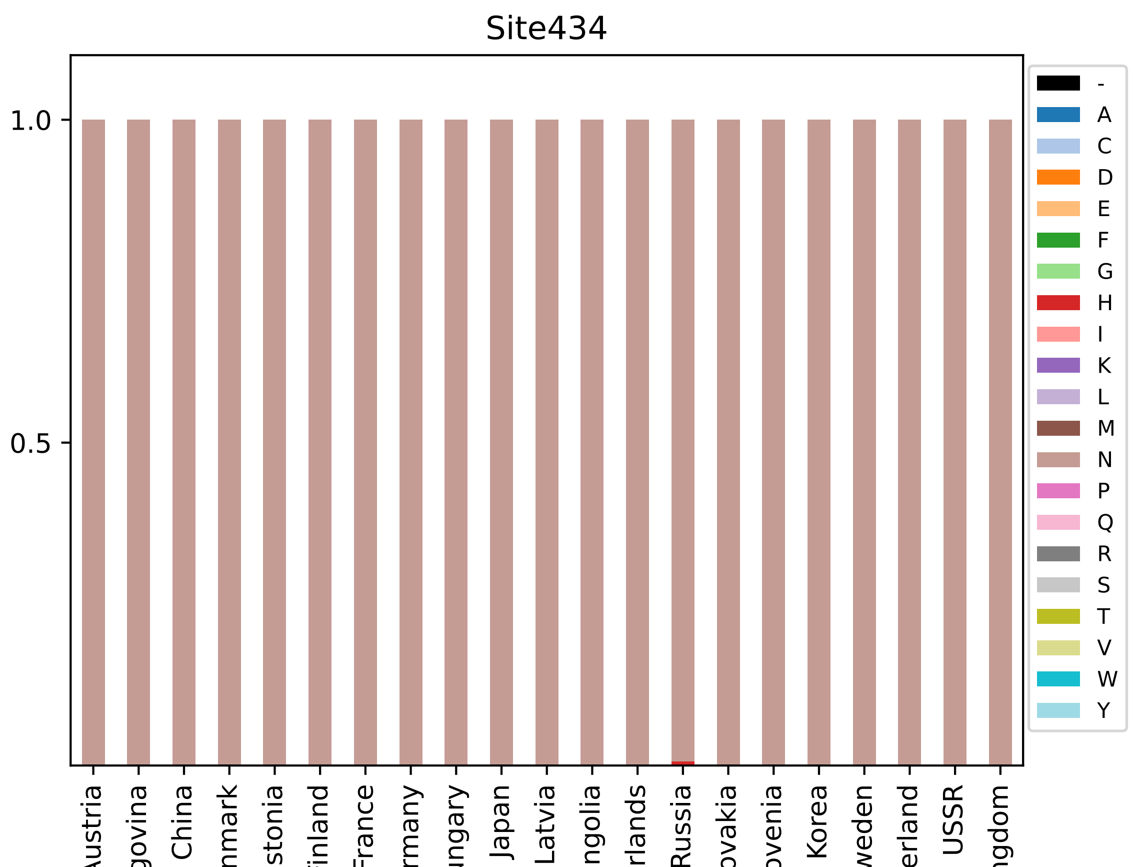

Site434
1.0
0.5
-
A
C
D
E
F
G
H
I
K
L
M
N
P
Q
R
S
T
V
W
Y
Austria
China
Japan
Latvia
Russia
Korea
USSR

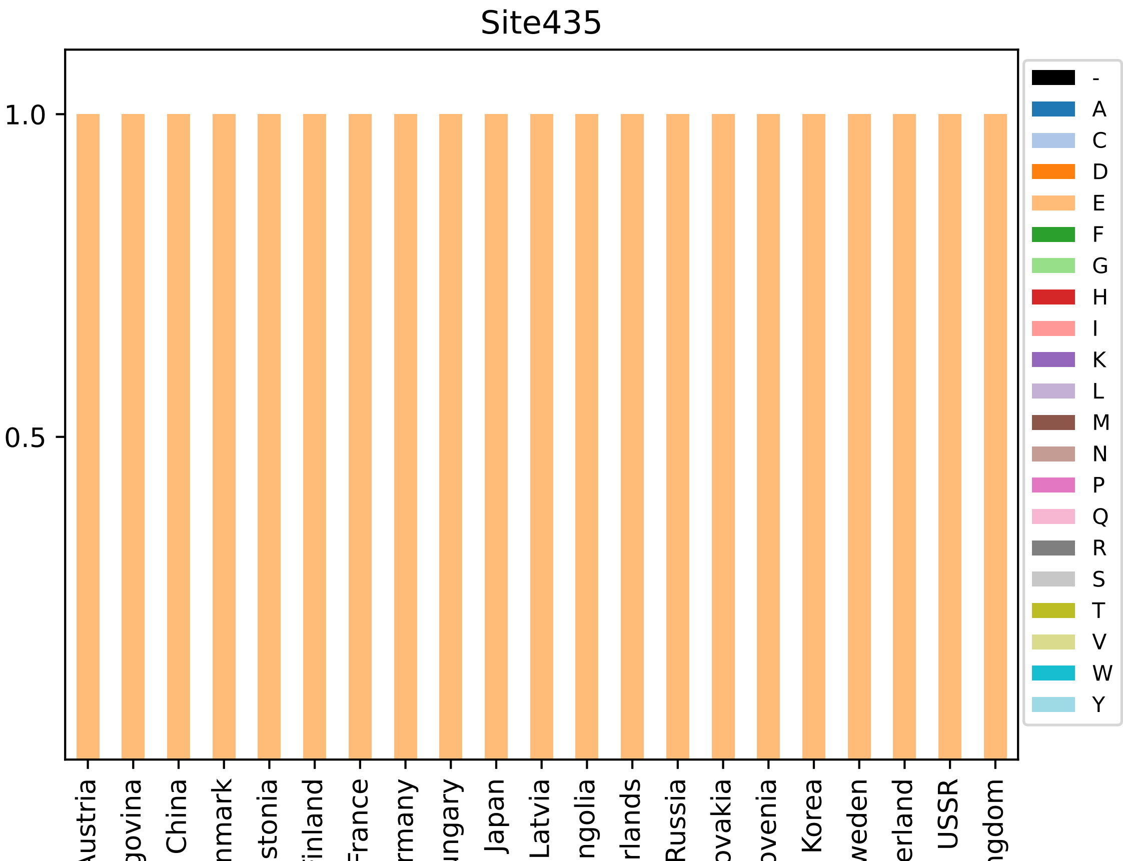

Site435
1.0
0.5
-
A
C
D
E
F
G
H
I
K
L
M
N
P
Q
R
S
T
V
W
Y
Austria
China
Japan
Latvia
Russia
Korea
USSR

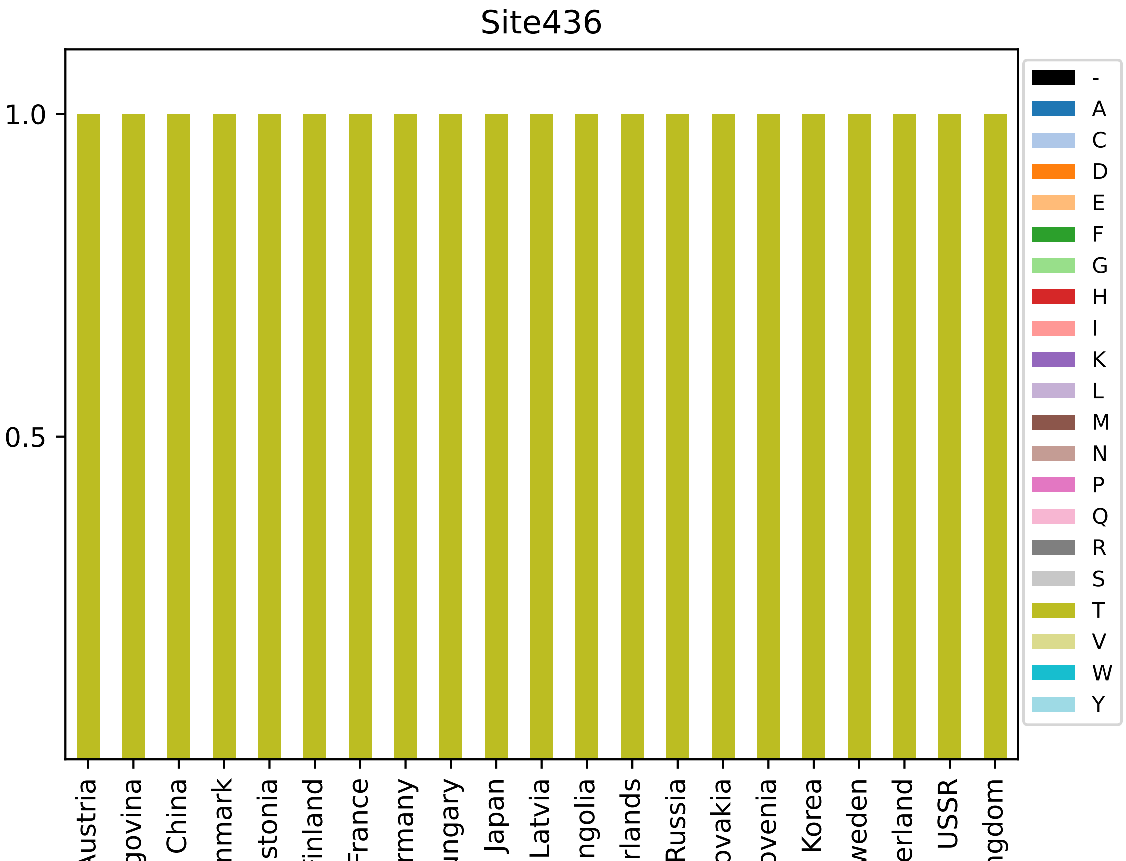

Site436
1.0
0.5
-
A
C
D
E
F
G
H
I
K
L
M
N
P
Q
R
S
T
V
W
Y
Austria
China
Japan
Latvia
Russia
Korea
USSR

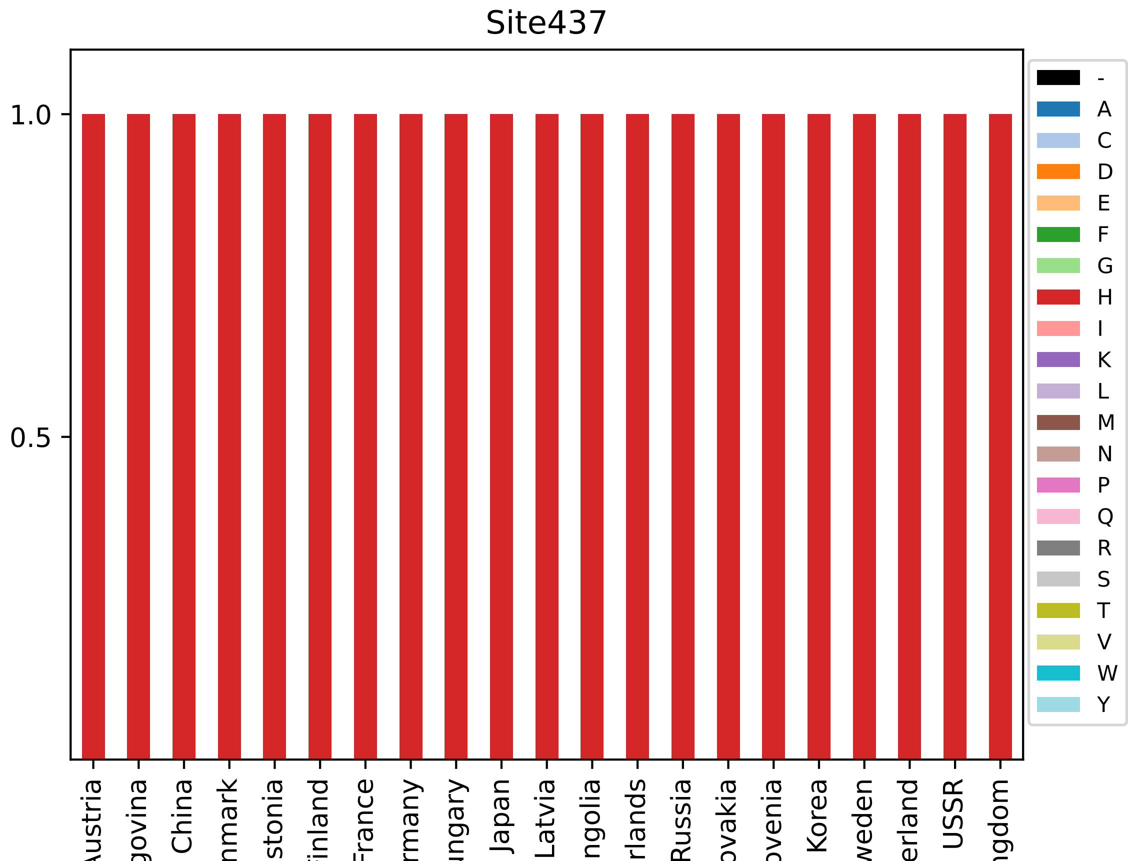

Site437
1.0
0.5
-
A
C
D
E
F
G
H
I
K
L
M
N
P
Q
R
S
T
V
W
Y
China
Japan
Latvia
Russia
Korea
USSR

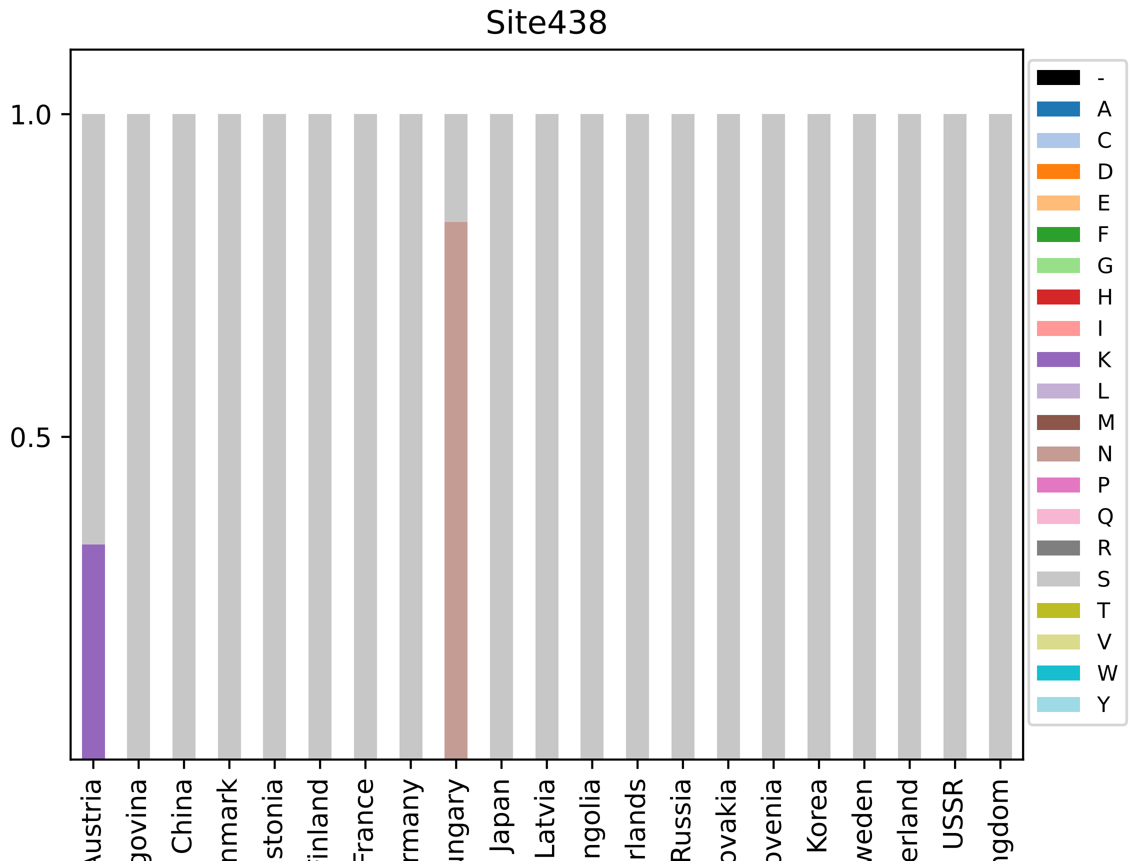
Site438
1.0
0.5
-
A
C
D
E
F
G
H
I
K
L
M
N
P
Q
R
S
T
V
W
Y
Austria
govina
China
nmark
stonia
Finland
France
rmany
ungary
Japan
Latvia
ngolia
rlands
Russia
ovakia
ovenia
Korea
weden
erland
USSR
ngdom

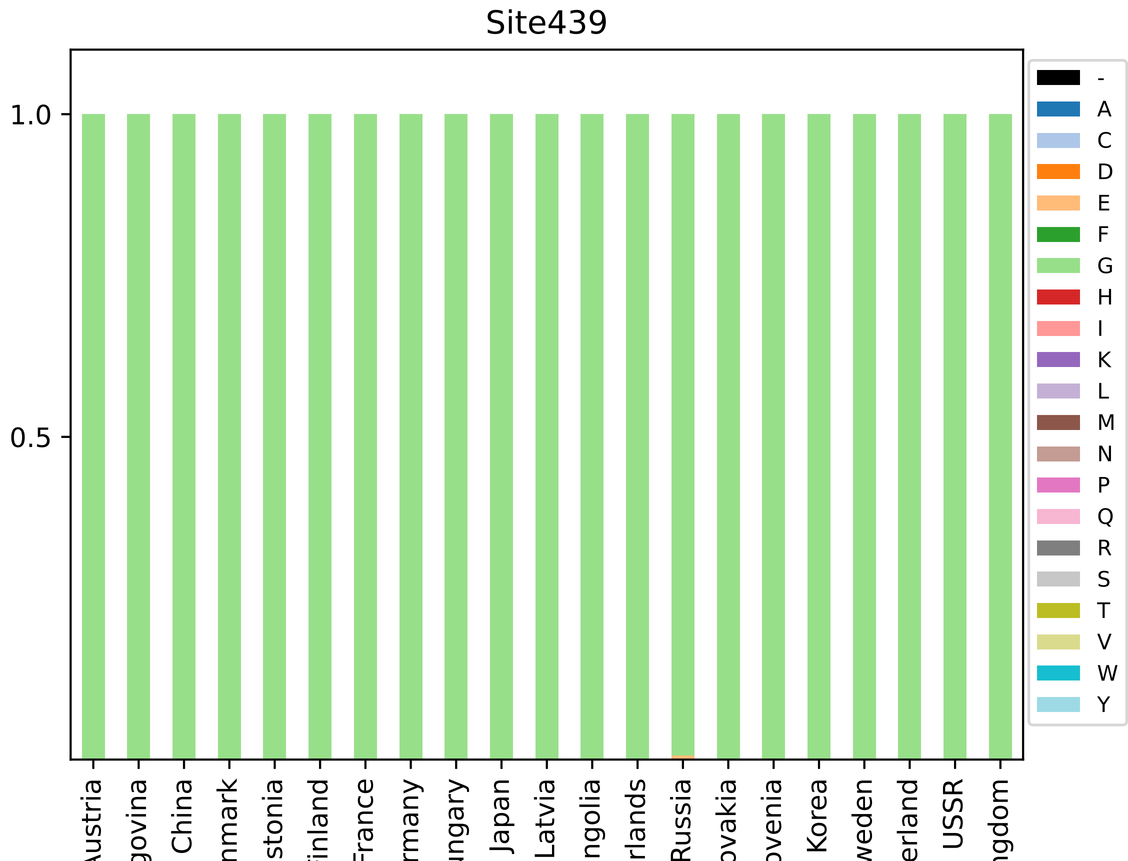
Site439
1.0
0.5
-
A
C
D
E
F
G
H
I
K
L
M
N
P
Q
R
S
T
V
W
Y
China
Japan
Latvia
Russia
Korea
USSR

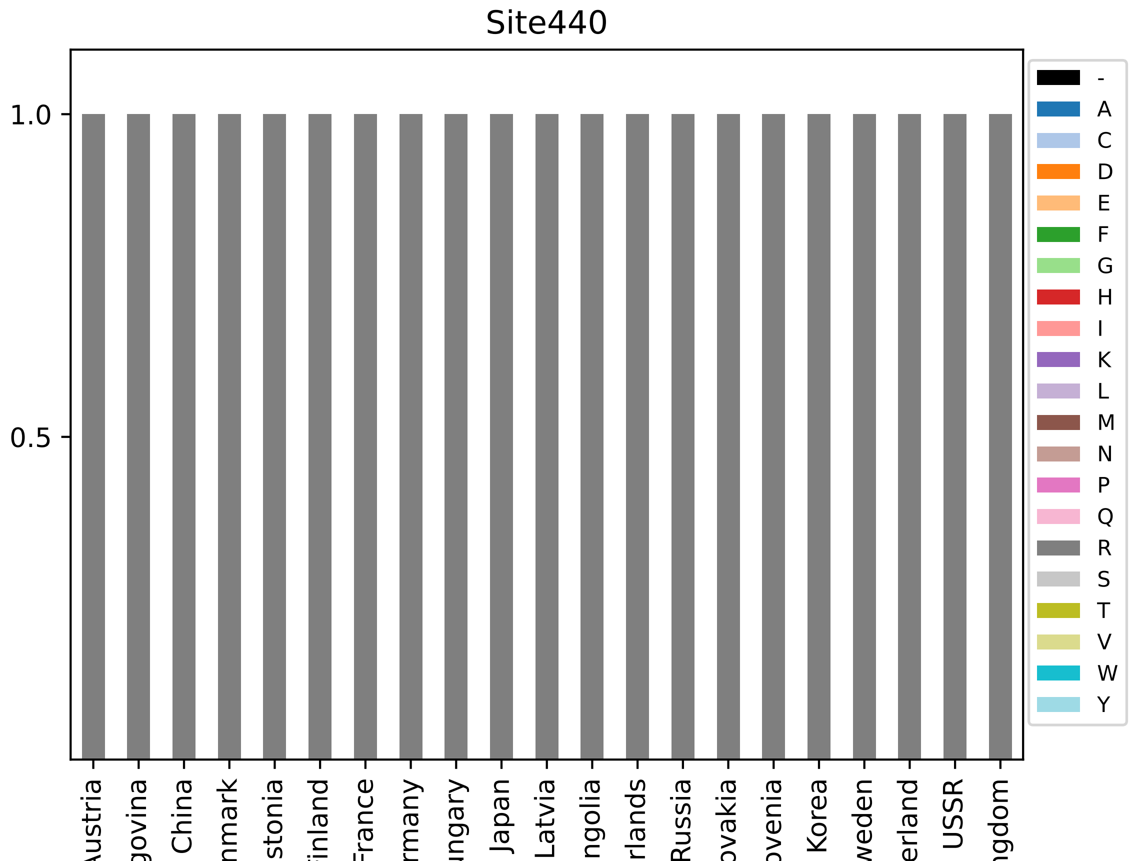

Site440
1.0
0.5
-
A
C
D
E
F
G
H
I
K
L
M
N
P
Q
R
S
T
V
W
Y
Austria
govina
China
nmark
stonia
Finland
France
rmany
ungary
Japan
Latvia
ngolia
rlands
Russia
ovakia
ovenia
Korea
weden
erland
USSR
ngdom

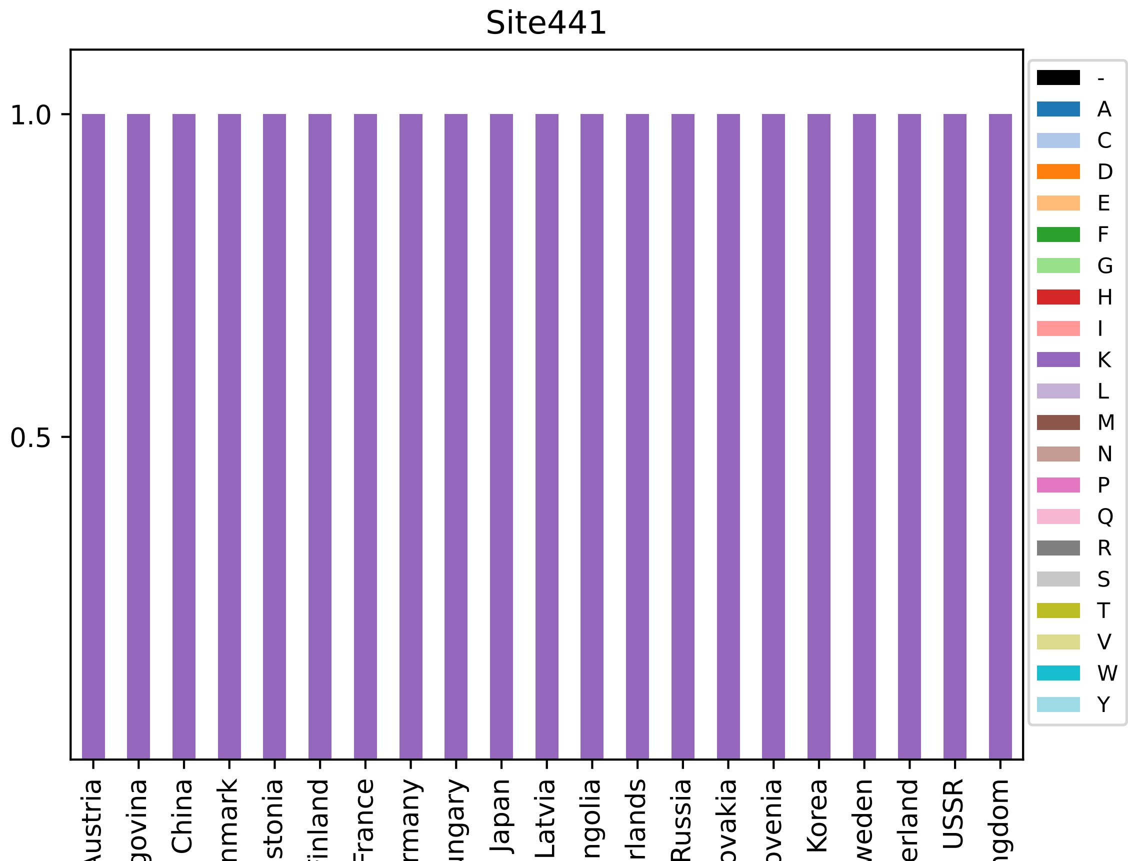

Site441
1.0
0.5
-
A
C
D
E
F
G
H
I
K
L
M
N
P
Q
R
S
T
V
W
Y
China
Japan
Latvia
Russia
Korea
USSR

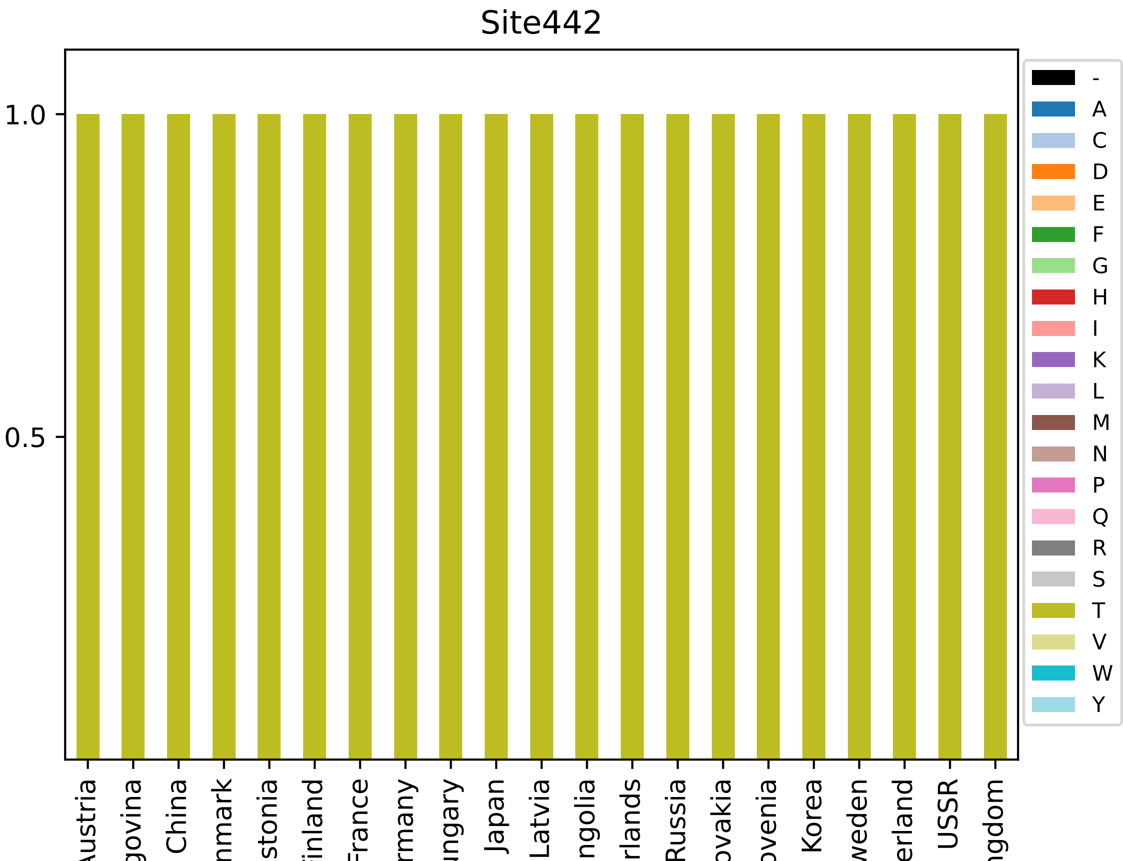

Site442
1.0
0.5
-
A
C
D
E
F
G
H
I
K
L
M
N
P
Q
R
S
T
V
W
Y
Austria
govina
China
nmark
stonia
Finland
France
rmany
ungary
Japan
Latvia
ngolia
rlands
Russia
ovakia
ovenia
Korea
weden
erland
USSR
ngdom

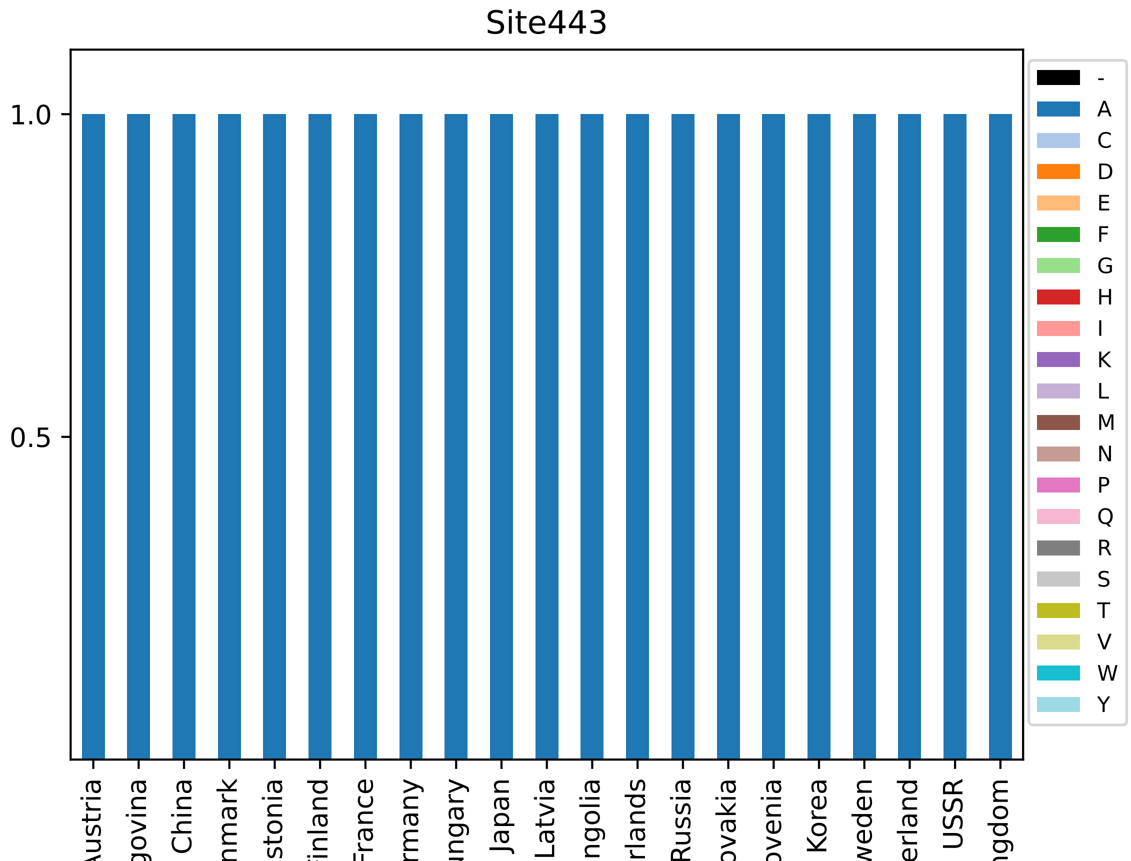

Site443
1.0
0.5
-
A
C
D
E
F
G
H
I
K
L
M
N
P
Q
R
S
T
V
W
Y
China
Japan
Latvia
Russia
Korea
USSR

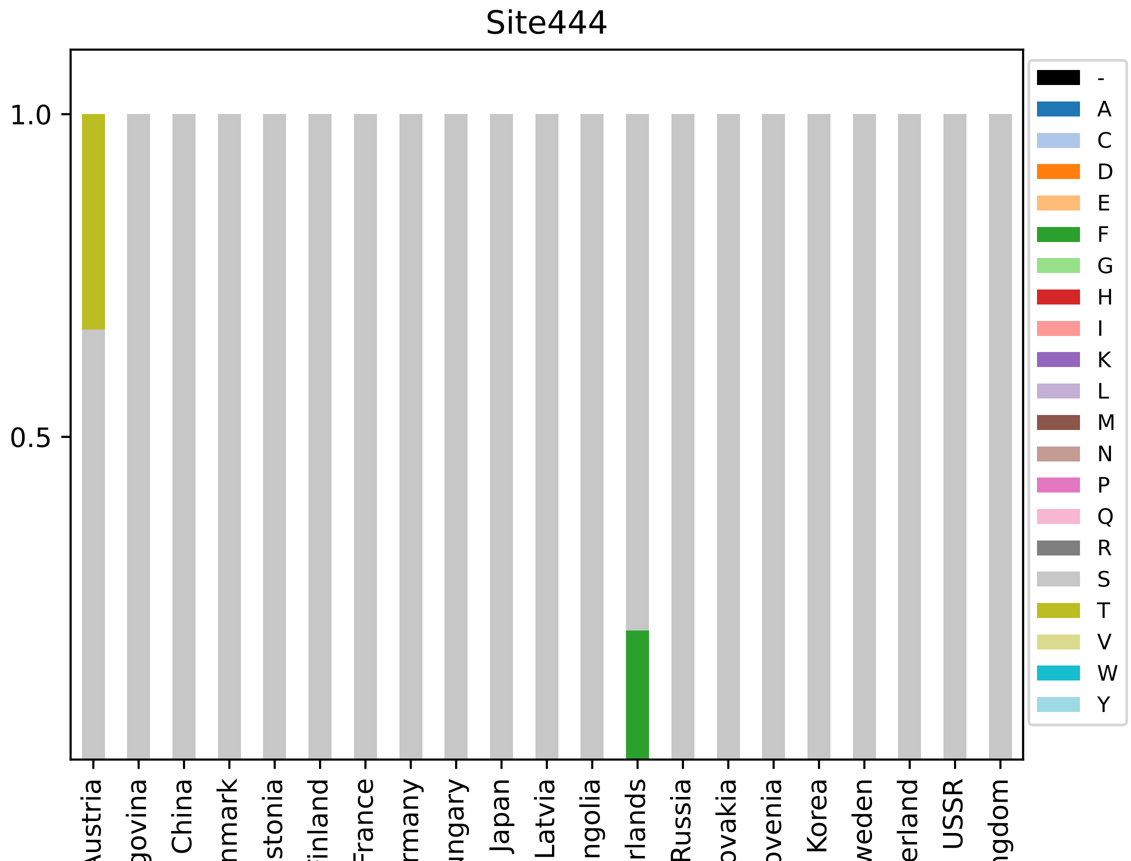

Site444
1.0
0.5
-
A
C
D
E
F
G
H
I
K
L
M
N
P
Q
R
S
T
V
W
Y
Austria
China
Japan
Latvia
Russia
Korea
USSR

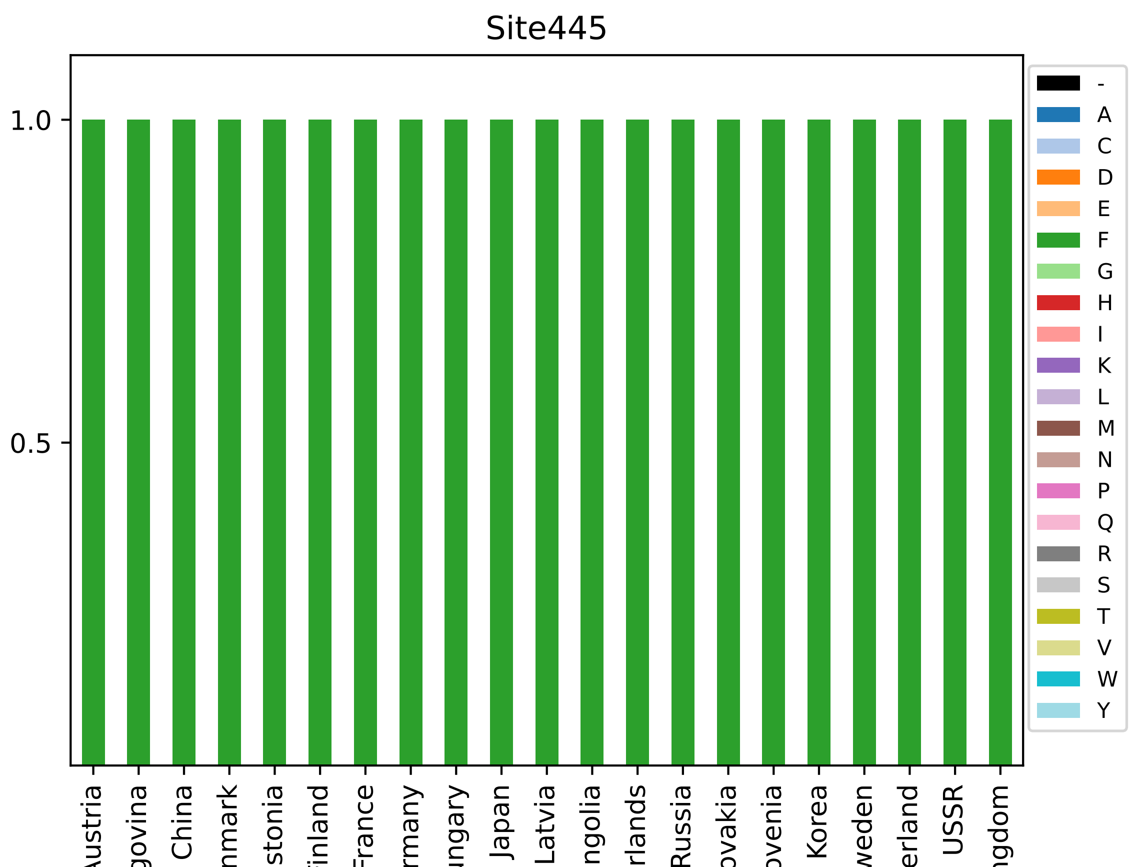

Site445
1.0
0.5
-
A
C
D
E
F
G
H
I
K
L
M
N
P
Q
R
S
T
V
W
Y
Japan
Latvia
Russia
Korea
USSR
China

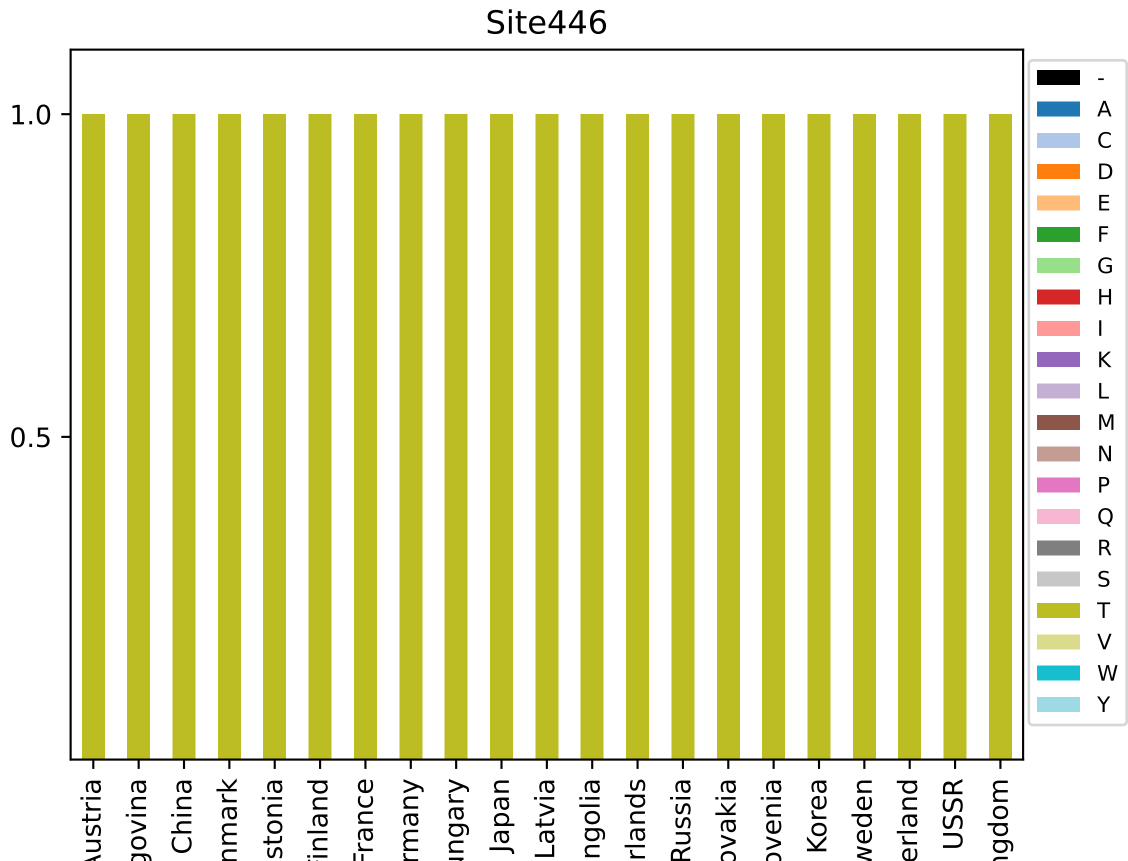
Site446
1.0
0.5
-
A
C
D
E
F
G
H
I
K
L
M
N
P
Q
R
S
T
V
W
Y
China
Japan
Latvia
Russia
Korea
USSR

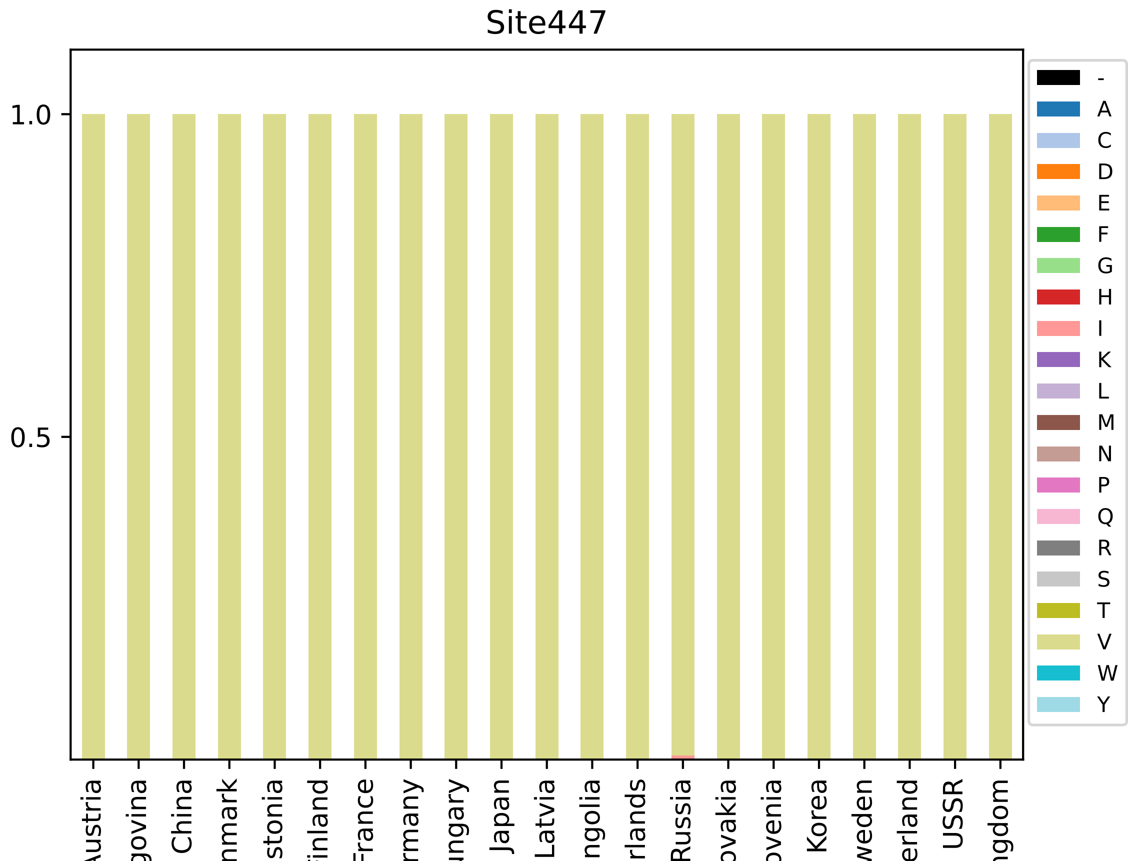

Site447
1.0
0.5
-
A
C
D
E
F
G
H
I
K
L
M
N
P
Q
R
S
T
V
W
Y
China
Japan
Latvia
Russia
Korea
USSR

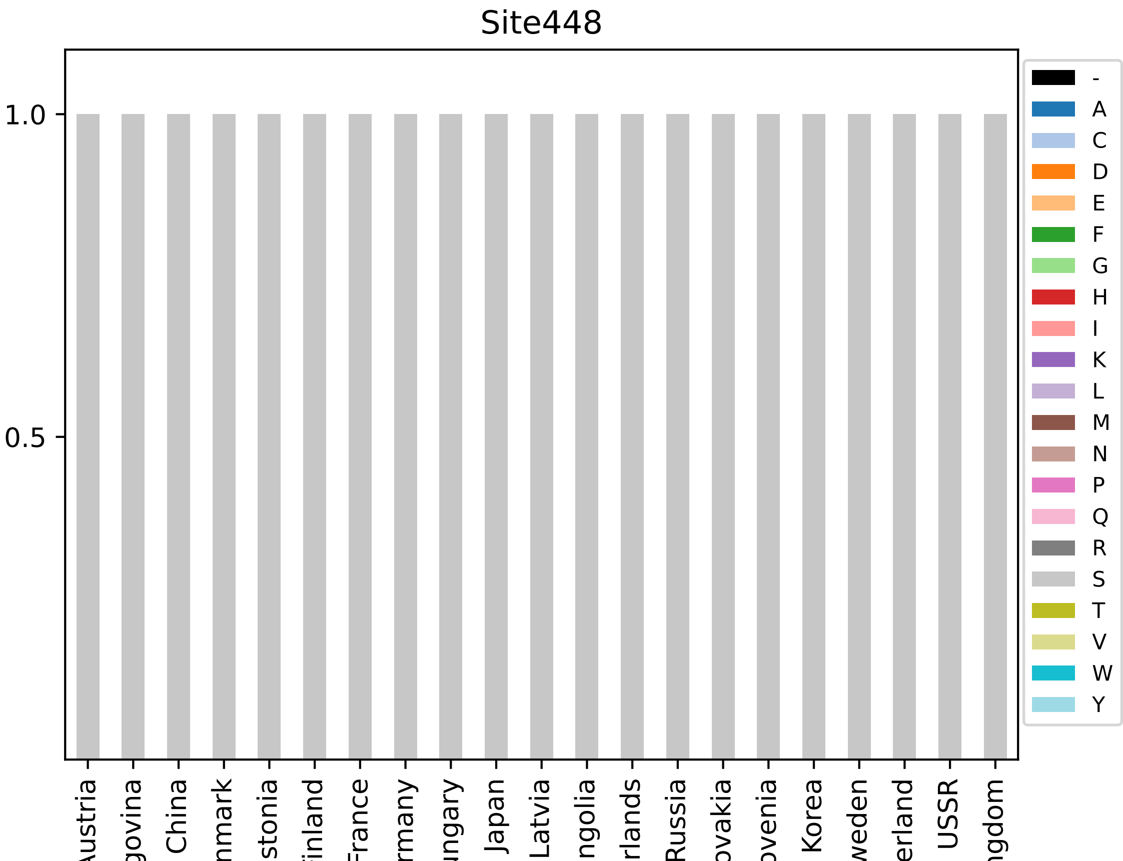

Site448
1.0
0.5
-
A
C
D
E
F
G
H
I
K
L
M
N
P
Q
R
S
T
V
W
Y
China
Japan
Latvia
Russia
Korea
USSR

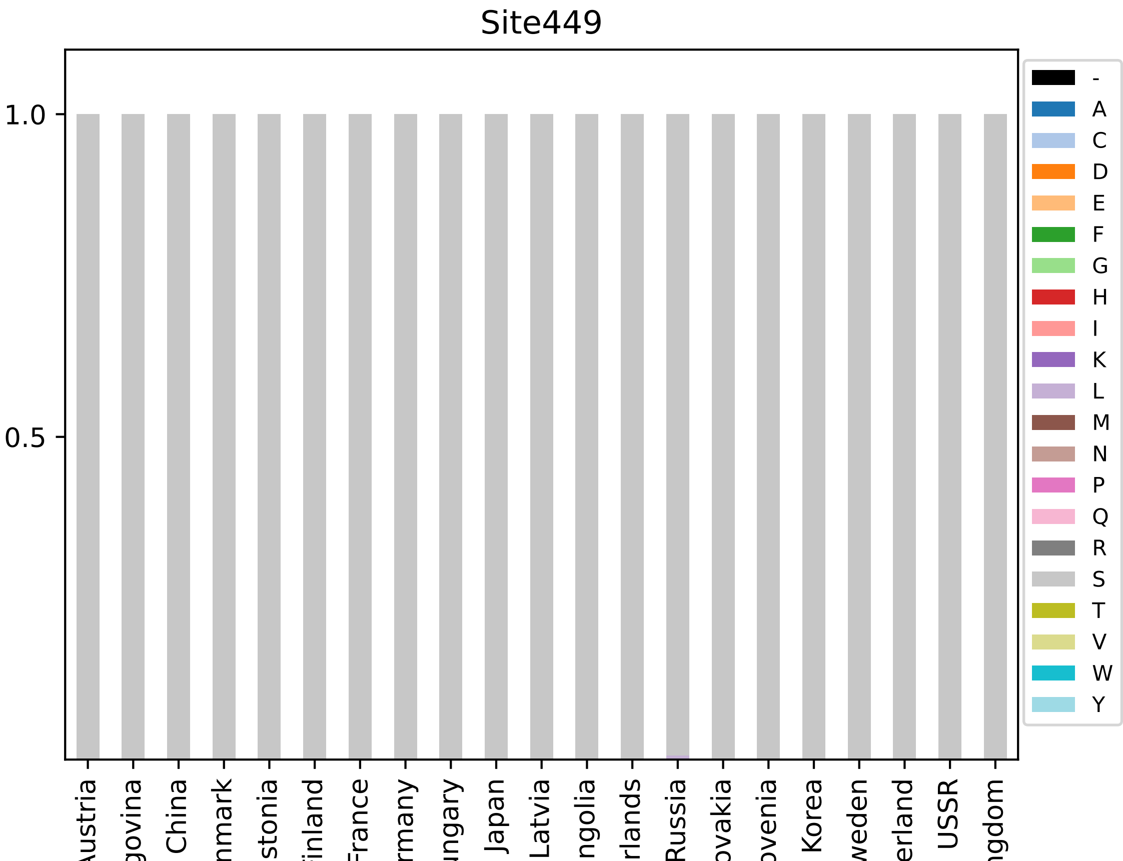

Site449
1.0
0.5
-
A
C
D
E
F
G
H
I
K
L
M
N
P
Q
R
S
T
V
W
Y
Austria
govina
China
nmark
stonia
inland
France
rmany
ungary
Japan
Latvia
ngolia
rlands
Russia
ovakia
ovenia
Korea
weden
erland
USSR
ngdom

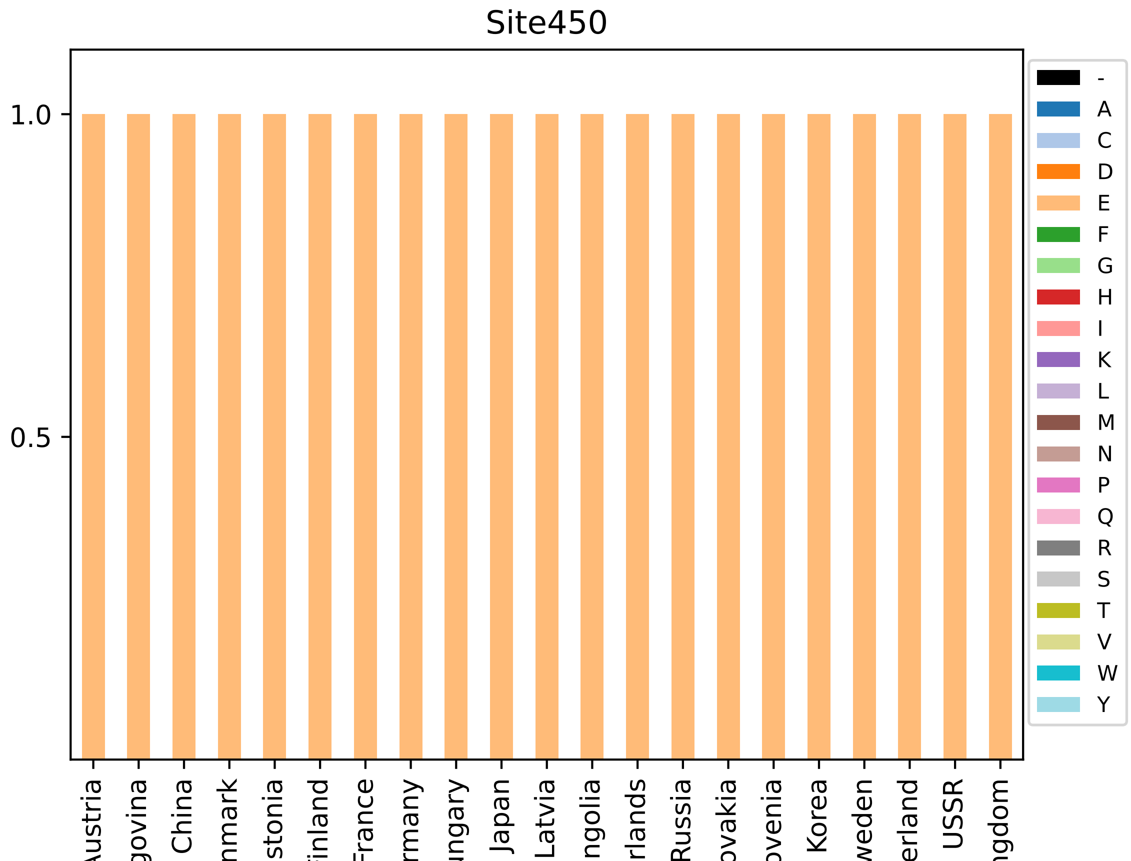
Site450
1.0
0.5
-
A
C
D
E
F
G
H
I
K
L
M
N
P
Q
R
S
T
V
W
Y
China
Japan
Latvia
Korea
USSR

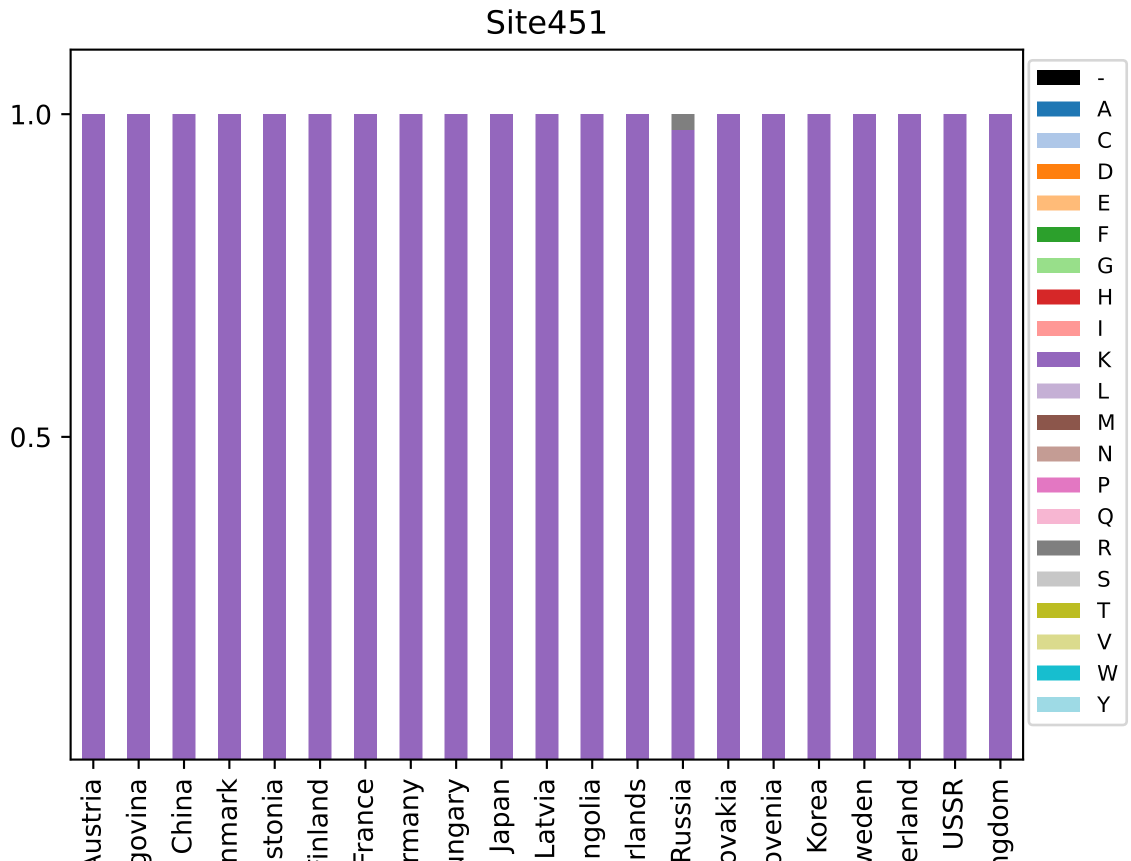

Site451
1.0
0.5
-
A
C
D
E
F
G
H
I
K
L
M
N
P
Q
R
S
T
V
W
Y
Austria
govina
China
nmark
stonia
inland
France
rmany
ungary
Japan
Latvia
ngolia
rlands
Russia
ovakia
ovenia
Korea
weden
erland
USSR
ngdom

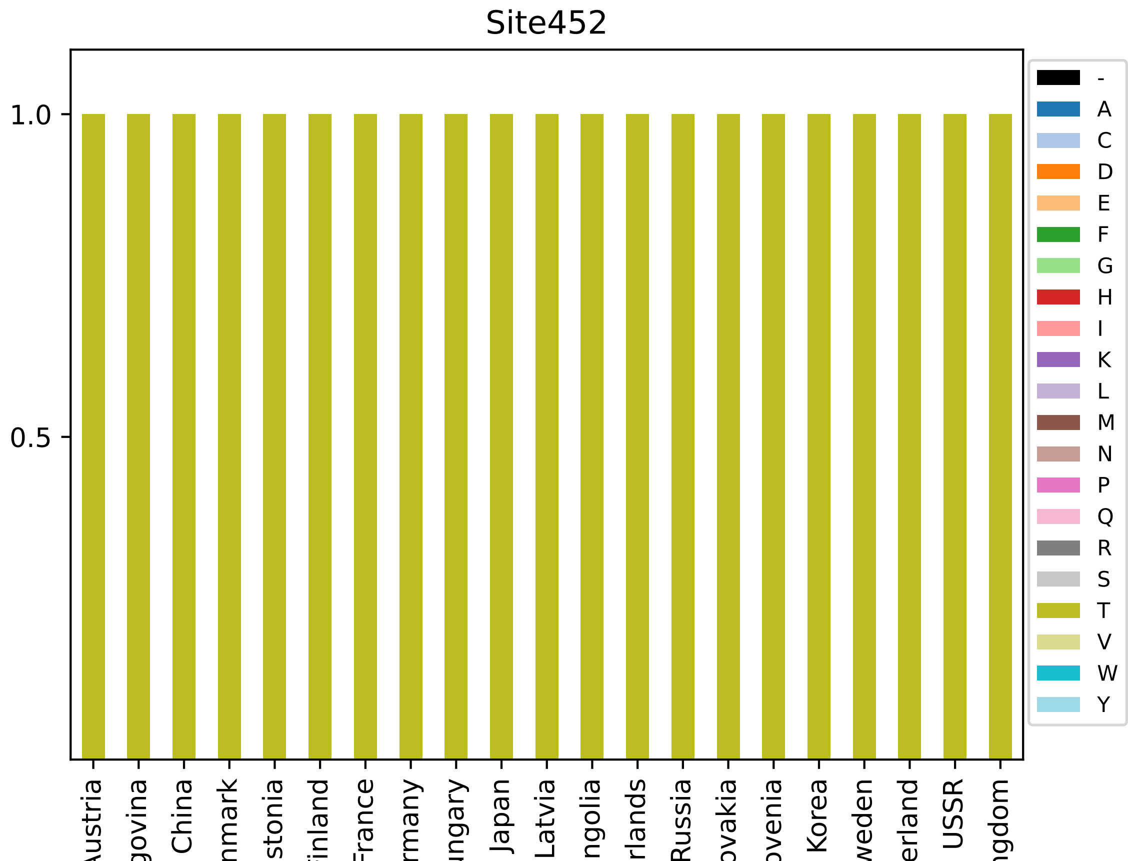

Site452
1.0
0.5
Austria
govina
China
nmark
stonia
Finland
France
rmany
ungary
Japan
Latvia
ngolia
rlands
Russia
ovakia
ovenia
Korea
weden
erland
USSR
ngdom
-
A
C
D
E
F
G
H
I
K
L
M
N
P
Q
R
S
T
V
W
Y

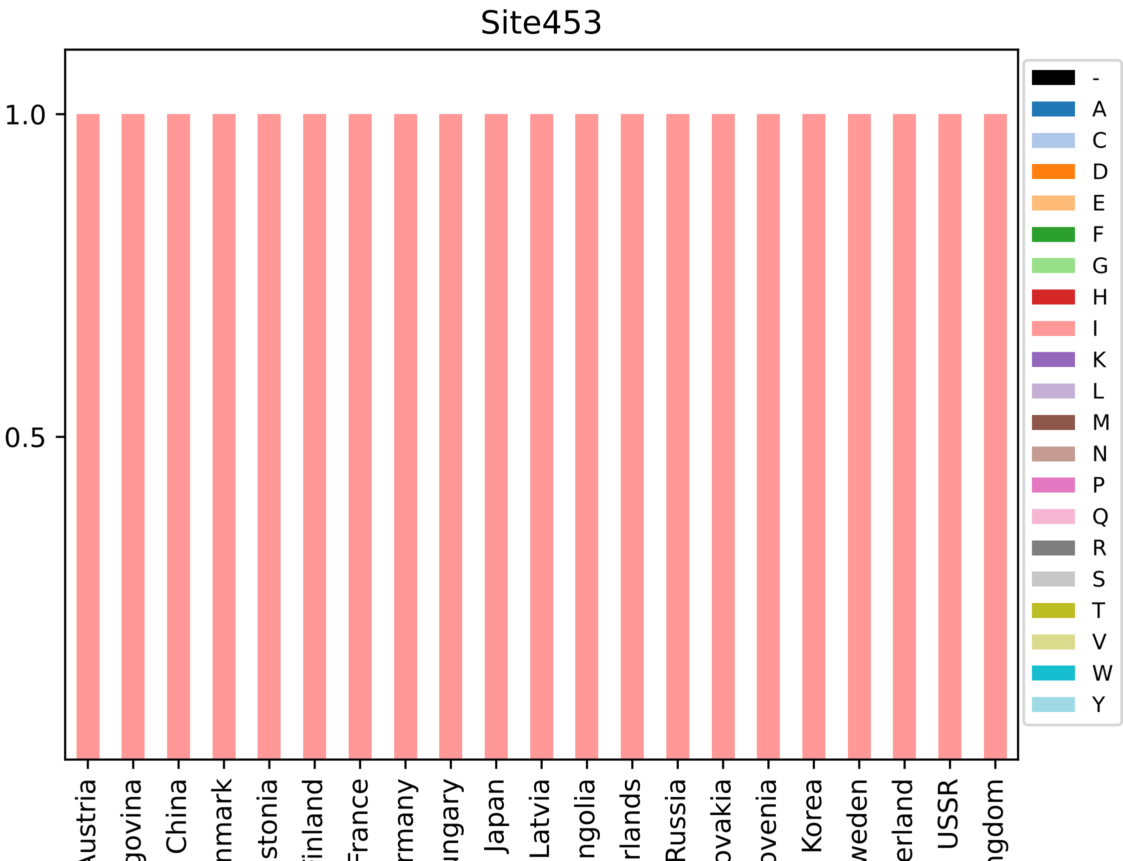
Site453
1.0
0.5
-
A
C
D
E
F
G
H
I
K
L
M
N
P
Q
R
S
T
V
W
Y
China
Japan
Latvia
Russia
Korea
USSR

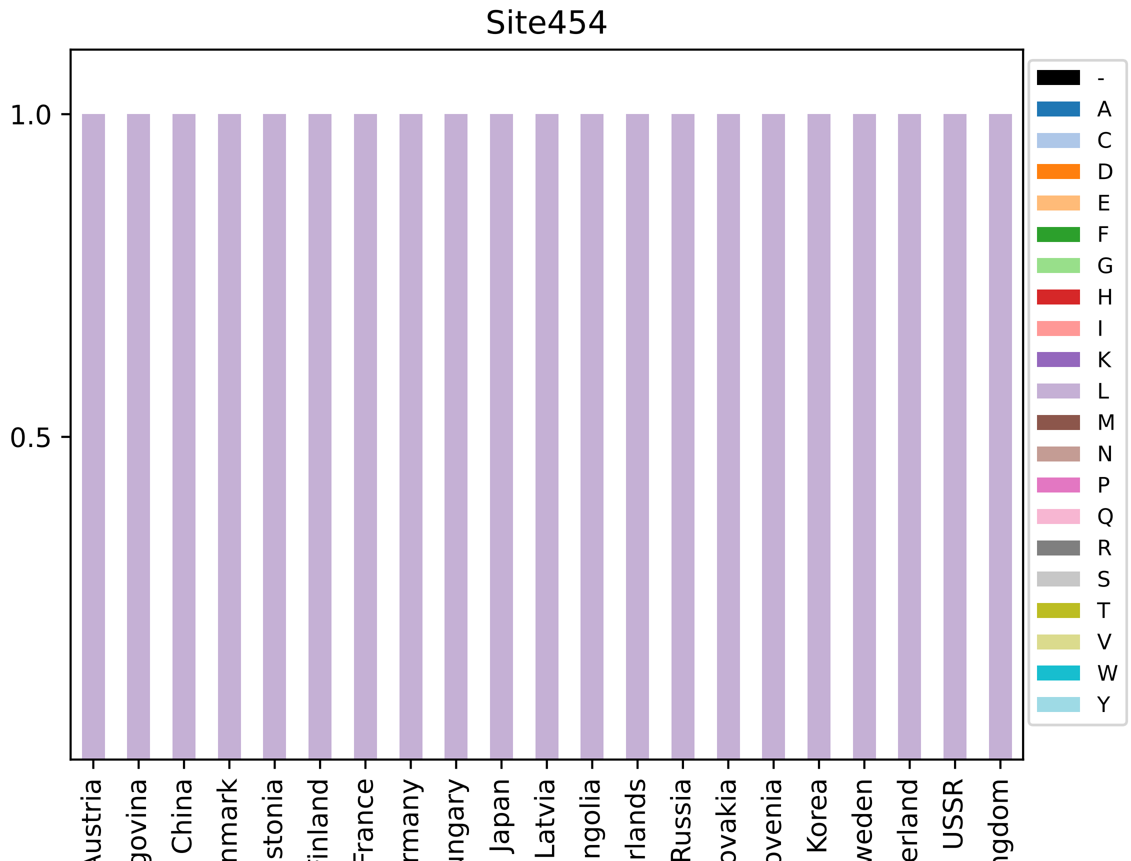
Site454
1.0
0.5
-
A
C
D
E
F
G
H
I
K
L
M
N
P
Q
R
S
T
V
W
Y
China
Japan
Latvia
Russia
Korea
USSR

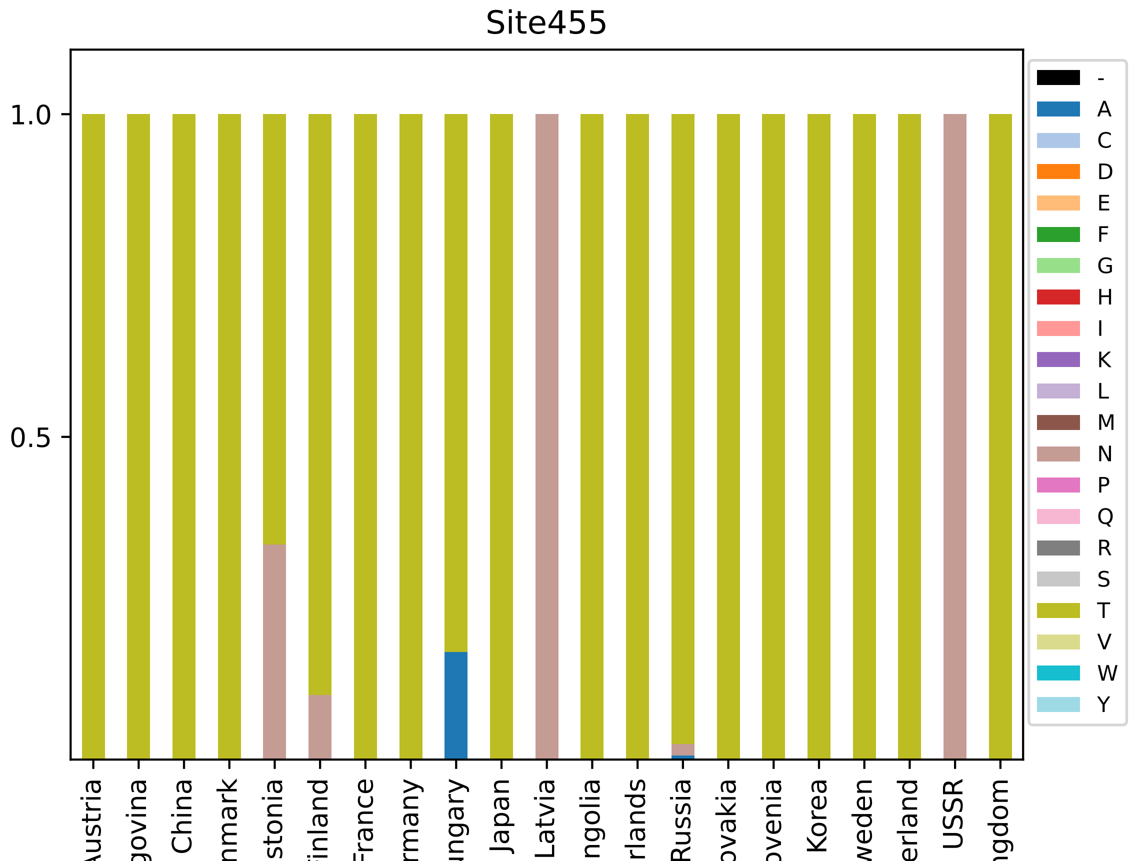

Site455
1.0
0.5
-
A
C
D
E
F
G
H
I
K
L
M
N
P
Q
R
S
T
V
W
Y
Austria
China
Finland
France
Japan
Latvia
Russia
Korea
USSR

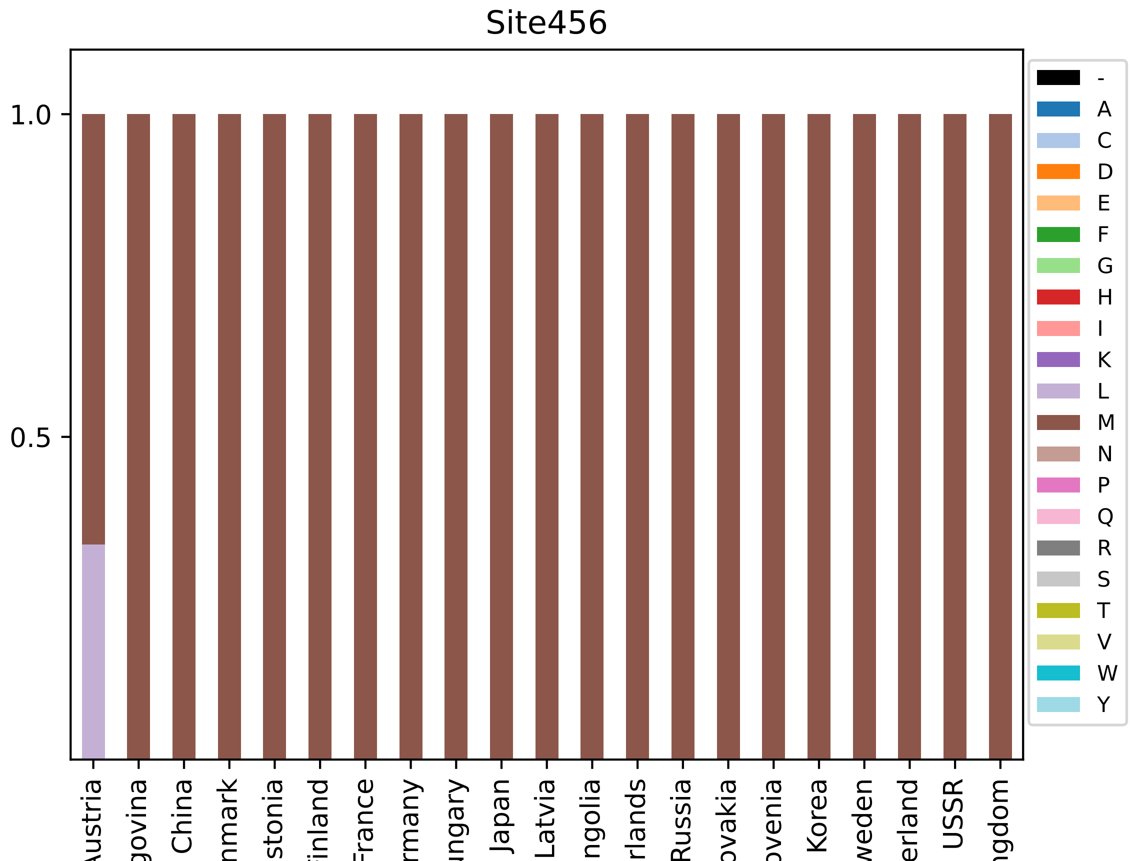

Site456
1.0
0.5
-
A
C
D
E
F
G
H
I
K
L
M
N
P
Q
R
S
T
V
W
Y
Austria
govina
China
nmark
stonia
inland
France
rmany
ungary
Japan
Latvia
ngolia
rlands
Russia
ovakia
ovenia
Korea
weden
erland
USSR
ngdom

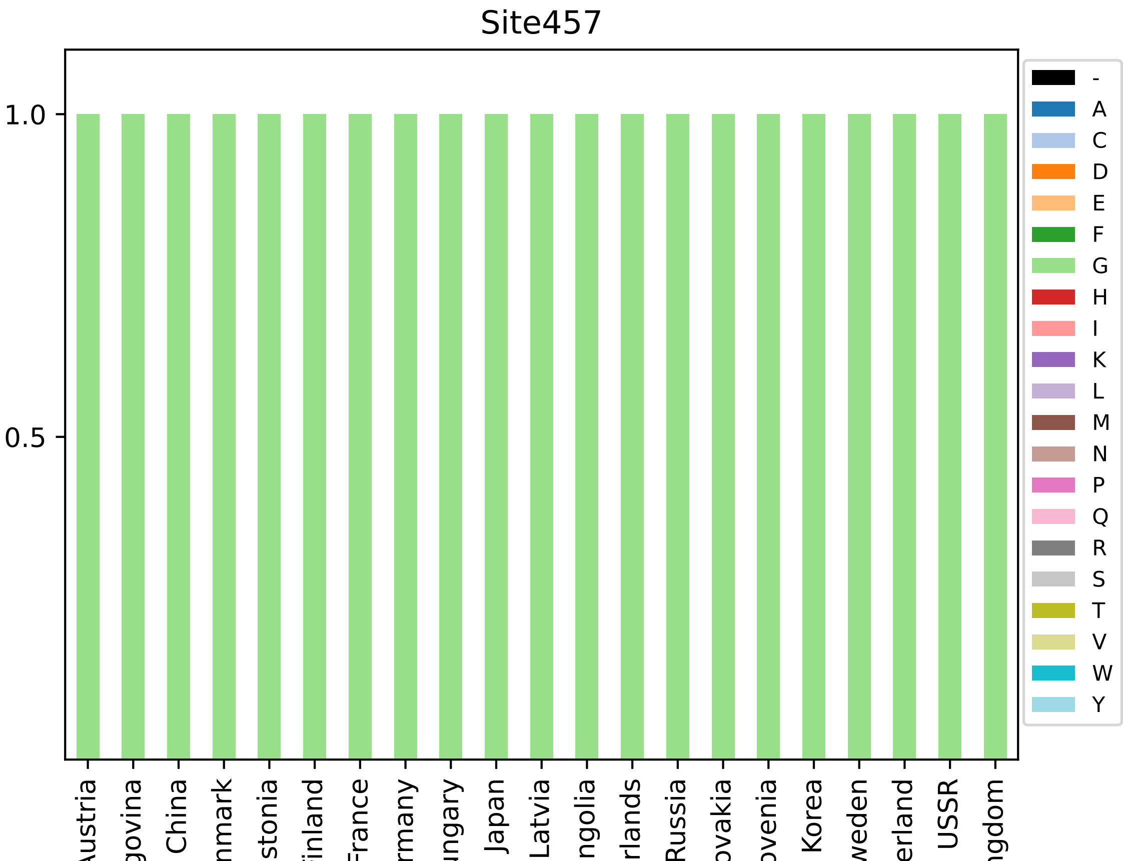
Site457
1.0
0.5
-
A
C
D
E
F
G
H
I
K
L
M
N
P
Q
R
S
T
V
W
Y
China
Japan
Latvia
Russia
Korea
USSR

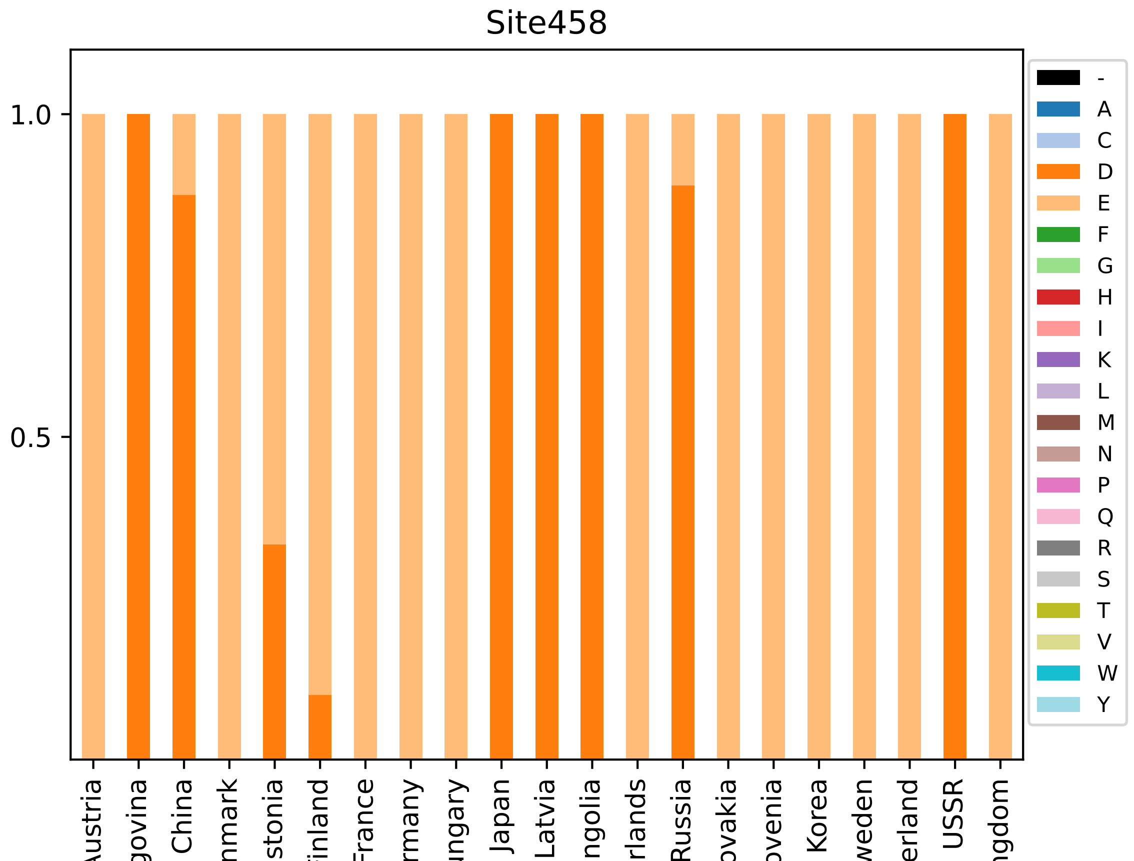

Site458
1.0
0.5
-
A
C
D
E
F
G
H
I
K
L
M
N
P
Q
R
S
T
V
W
Y
Austria
govina
China
nmark
stonia
inland
France
rmany
ungary
Japan
Latvia
ngolia
rlands
Russia
ovakia
ovenia
Korea
weden
erland
USSR
ngdom

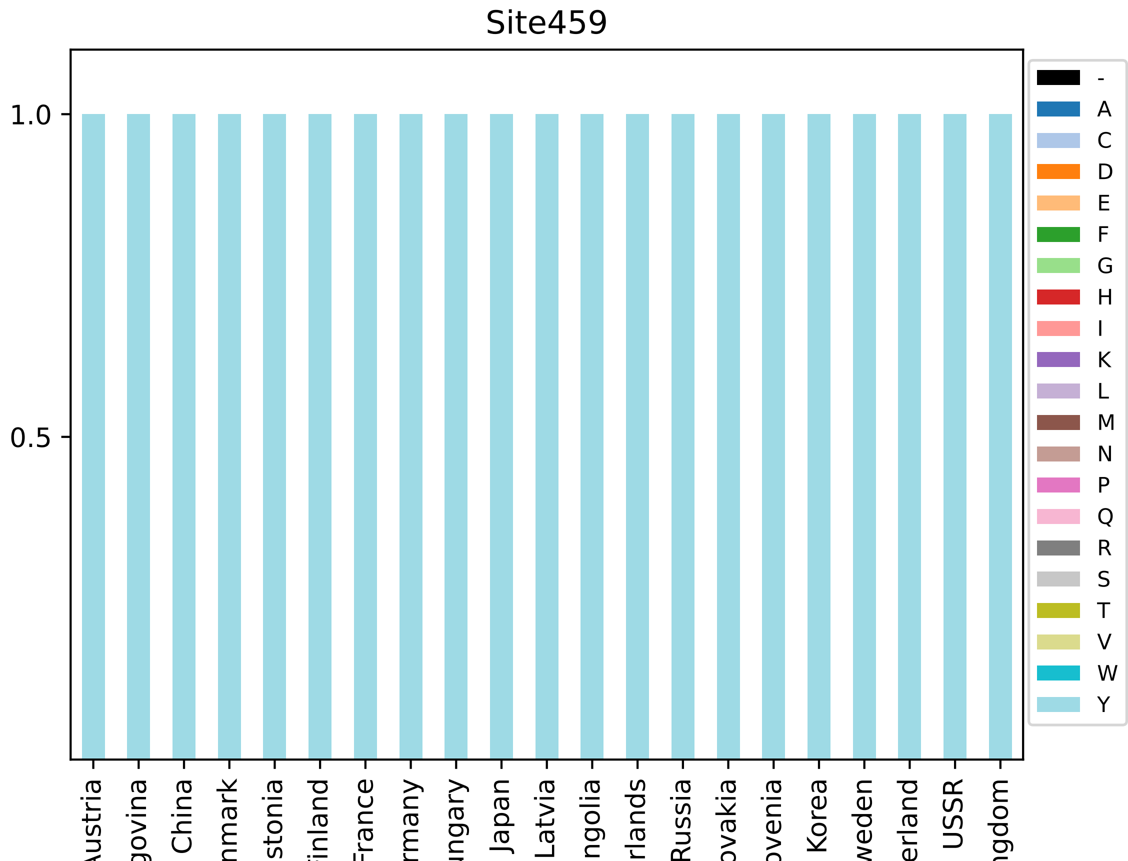

Site459
1.0
0.5
-
A
C
D
E
F
G
H
I
K
L
M
N
P
Q
R
S
T
V
W
Y
China
Japan
Latvia
Russia
Korea
USSR

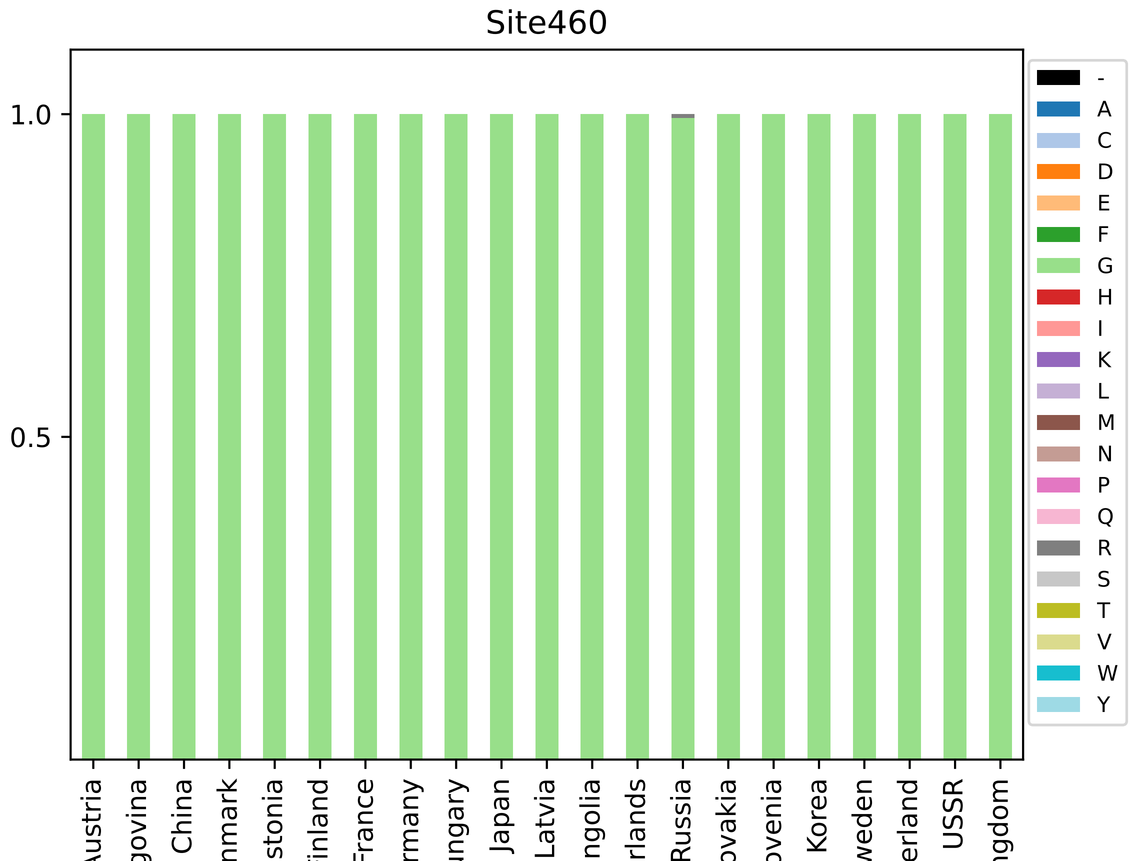

Site460
1.0
0.5
-
A
C
D
E
F
G
H
I
K
L
M
N
P
Q
R
S
T
V
W
Y
Austria
govina
China
nmark
stonia
inland
France
rmany
ungary
Japan
Latvia
ngolia
rlands
Russia
ovakia
ovenia
Korea
weden
erland
USSR
ngdom

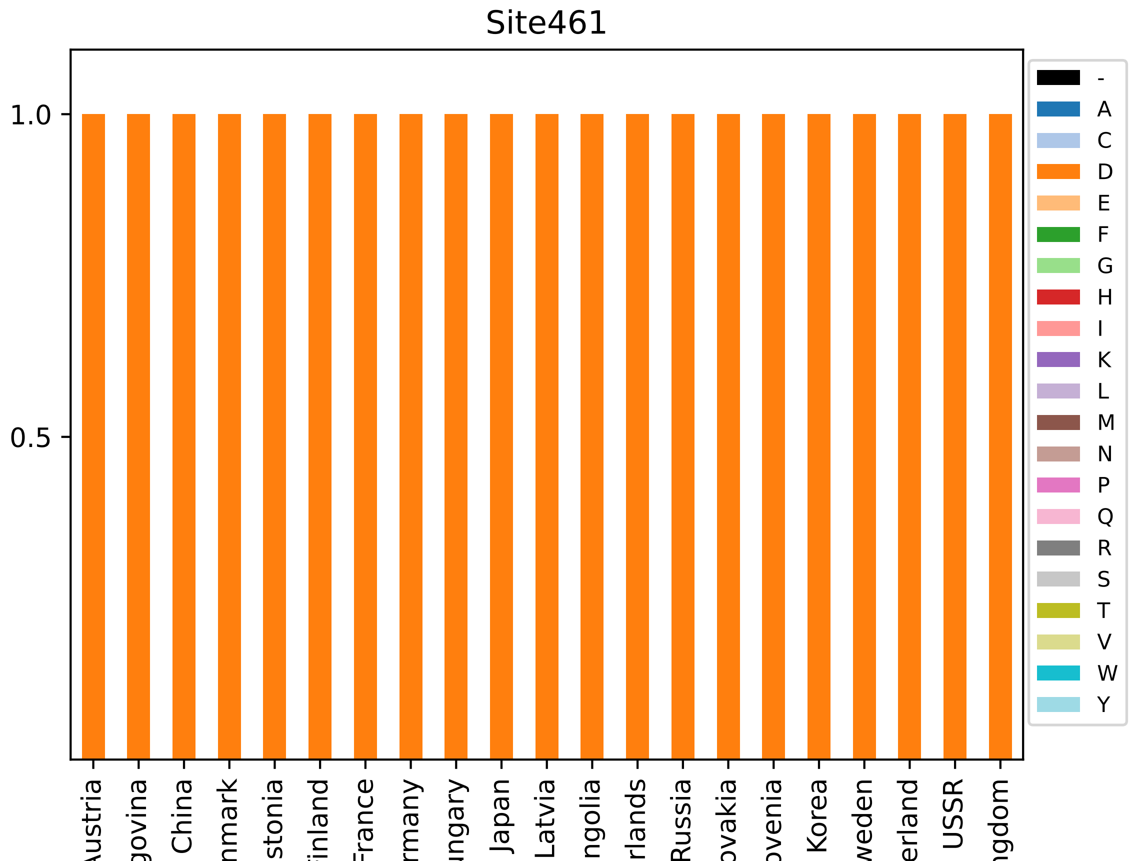
Site461
1.0
0.5
-
A
C
D
E
F
G
H
I
K
L
M
N
P
Q
R
S
T
V
W
Y
China
Japan
Latvia
Russia
Korea
USSR

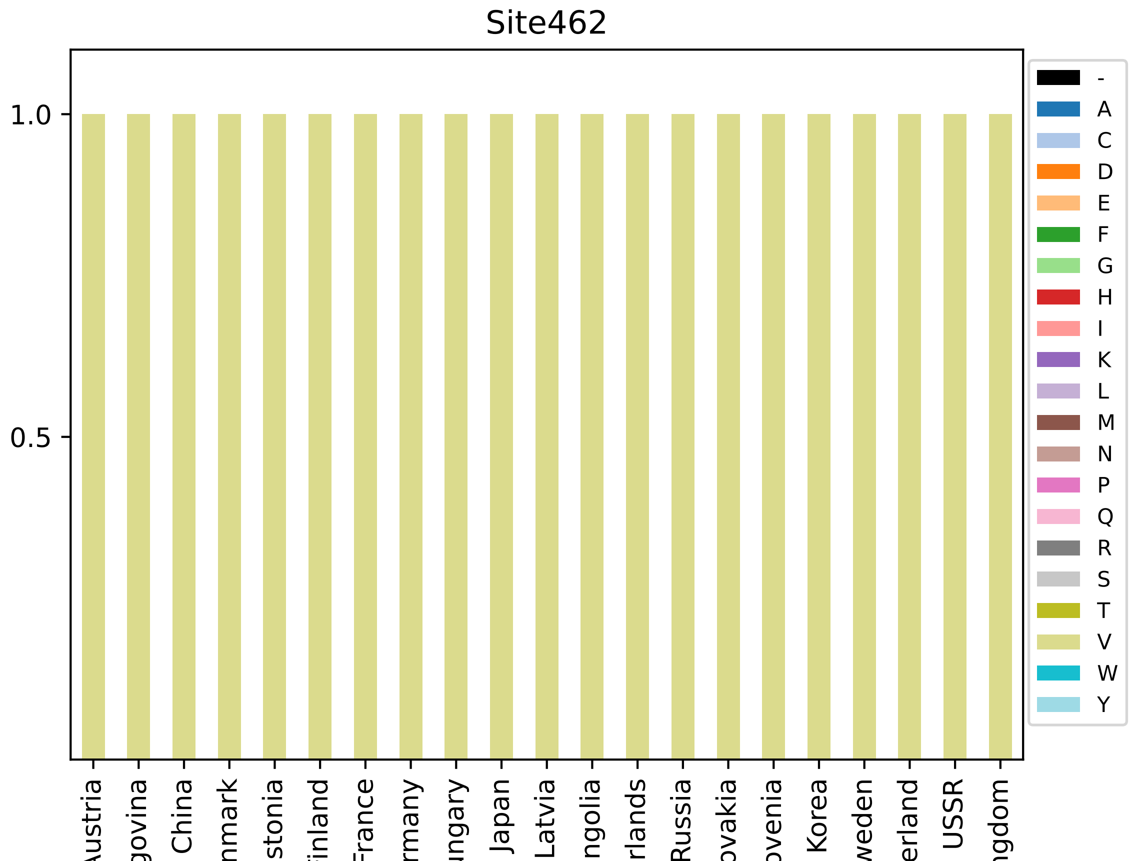

Site462
1.0
0.5
-
A
C
D
E
F
G
H
I
K
L
M
N
P
Q
R
S
T
V
W
Y
Austria
China
Japan
Latvia
Russia
Korea
USSR

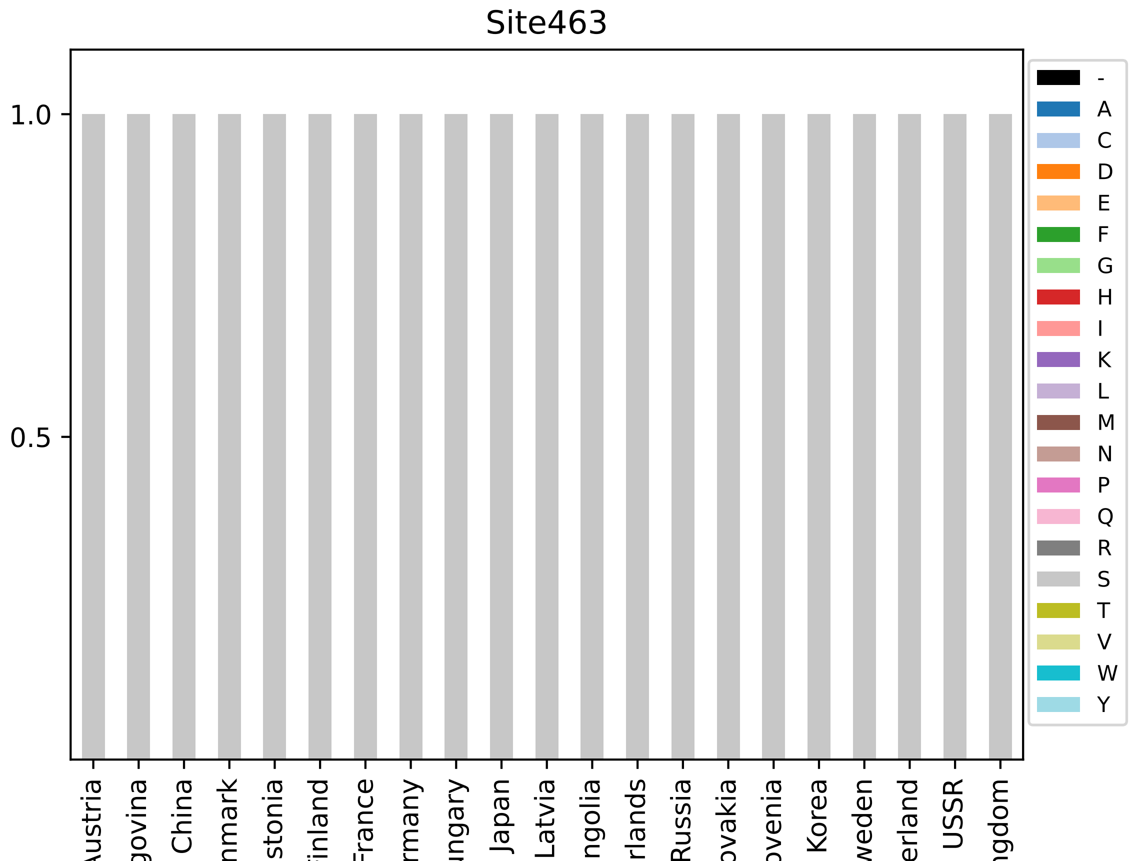

Site463
1.0
0.5
-
A
C
D
E
F
G
H
I
K
L
M
N
P
Q
R
S
T
V
W
Y
Austria
China
Finland
France
Japan
Latvia
Russia
Korea
USSR

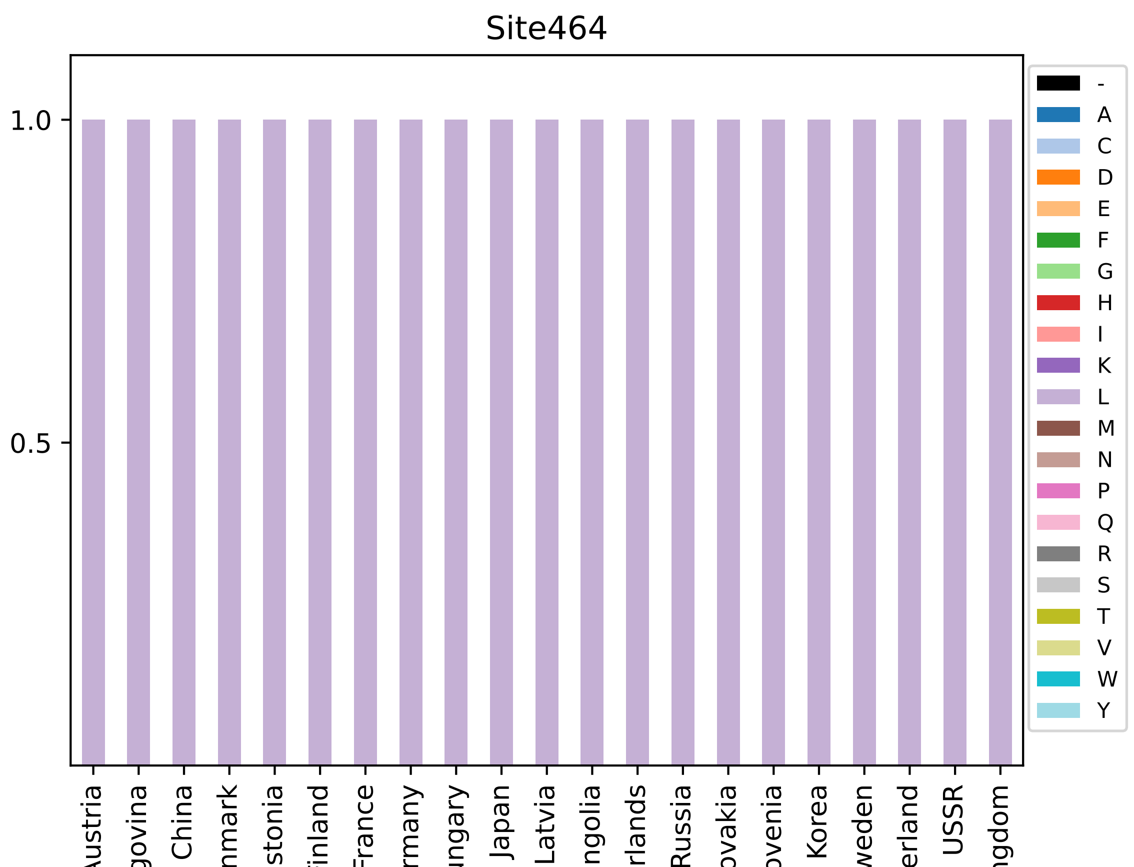
Site464
1.0
0.5
-
A
C
D
E
F
G
H
I
K
L
M
N
P
Q
R
S
T
V
W
Y
China
Japan
Latvia
Russia
Korea
USSR

Site465

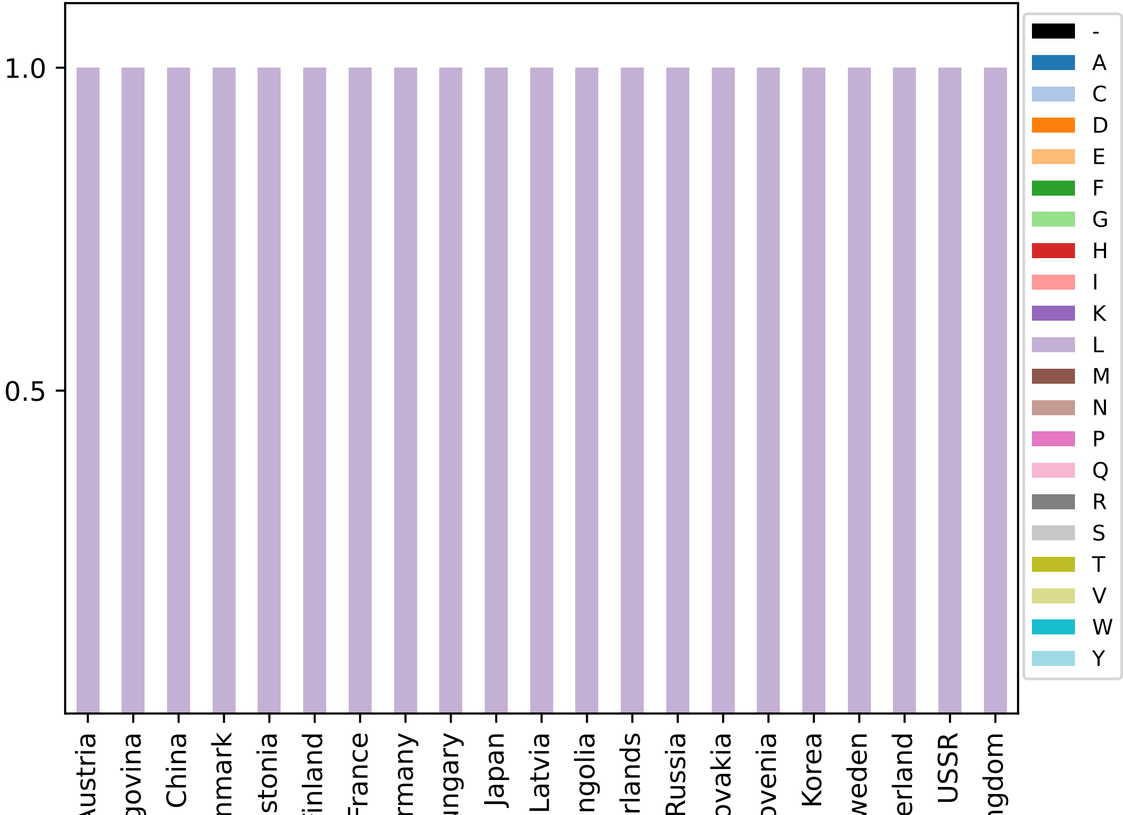

1.0
0.5
Austria
govina
China
nmark
stonia
Finland
France
rmany
ungary
Japan
Latvia
ngolia
rlands
Russia
ovakia
ovenia
Korea
weden
erland
USSR
ngdom
-
A
C
D
E
F
G
H
I
K
L
M
N
P
Q
R
S
T
V
W
Y

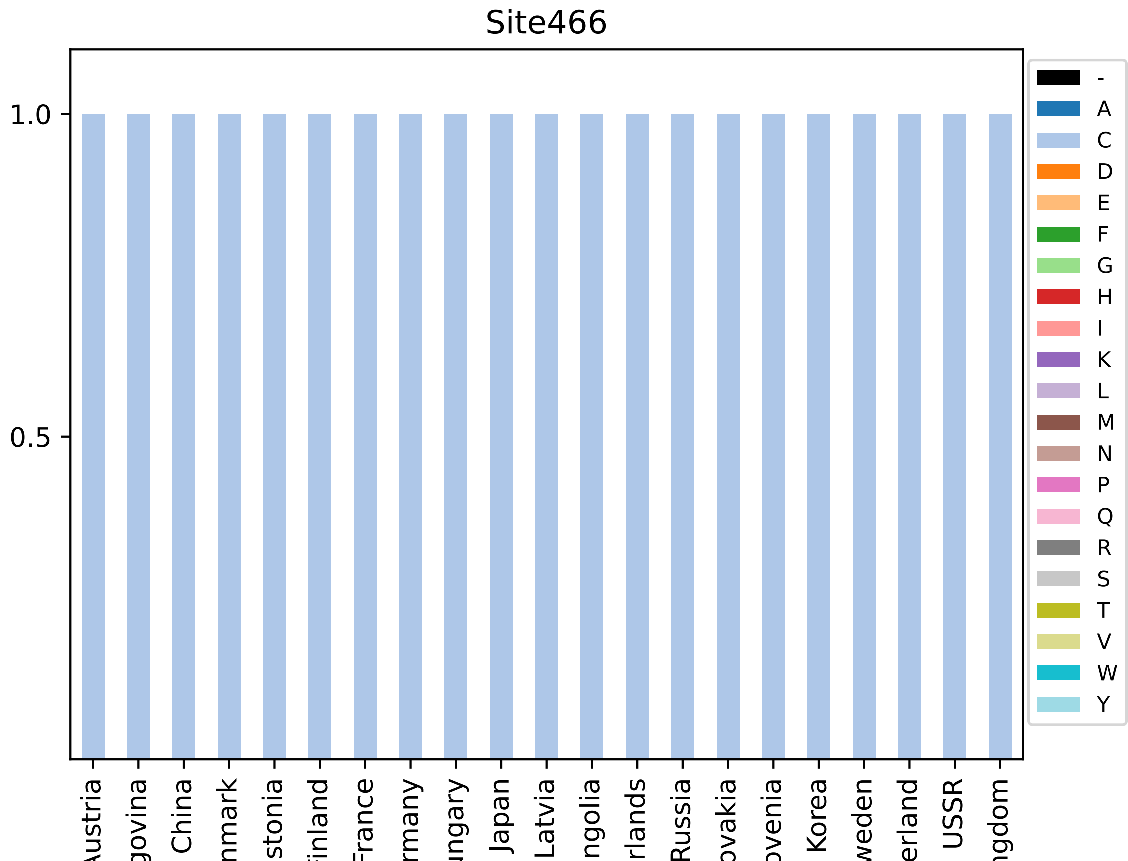
Site466
1.0
0.5
-
A
C
D
E
F
G
H
I
K
L
M
N
P
Q
R
S
T
V
W
Y
China
Japan
Latvia
Russia
Korea
USSR

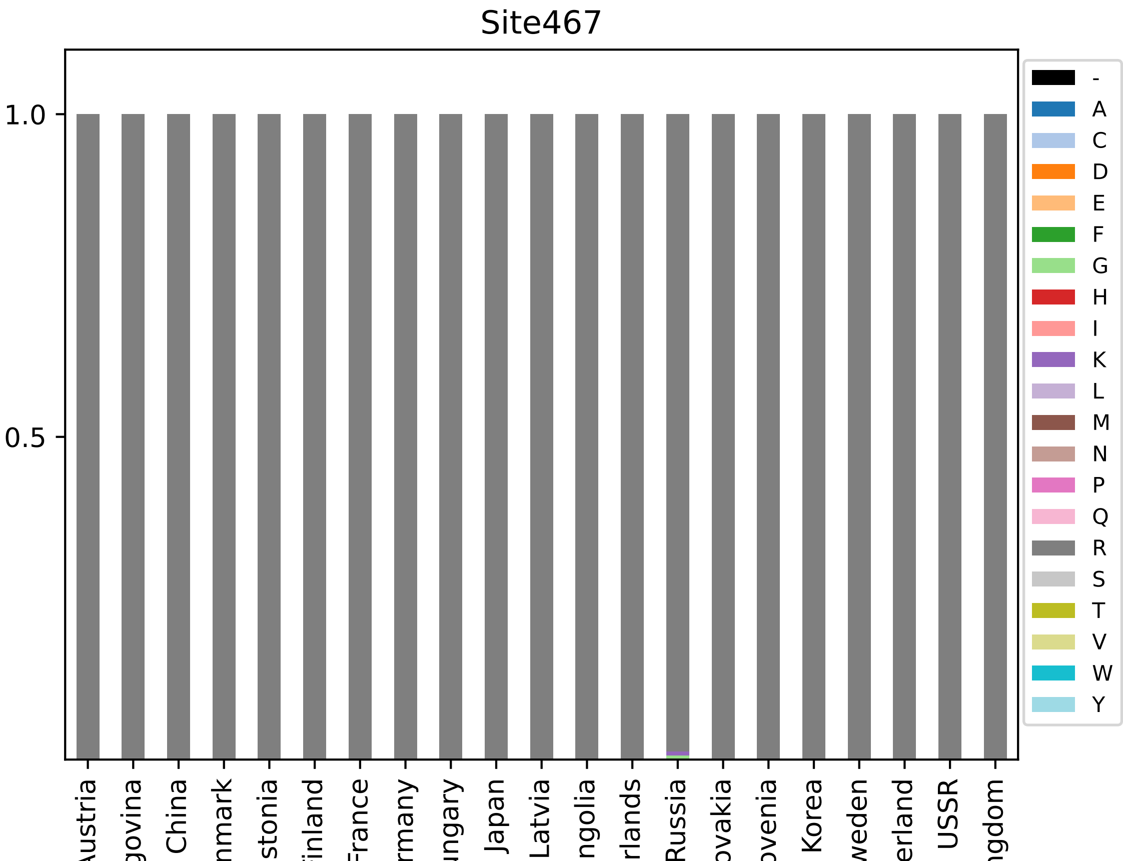
Site467
1.0
0.5
-
A
C
D
E
F
G
H
I
K
L
M
N
P
Q
R
S
T
V
W
Y
China
Japan
Latvia
Russia
Korea
USSR

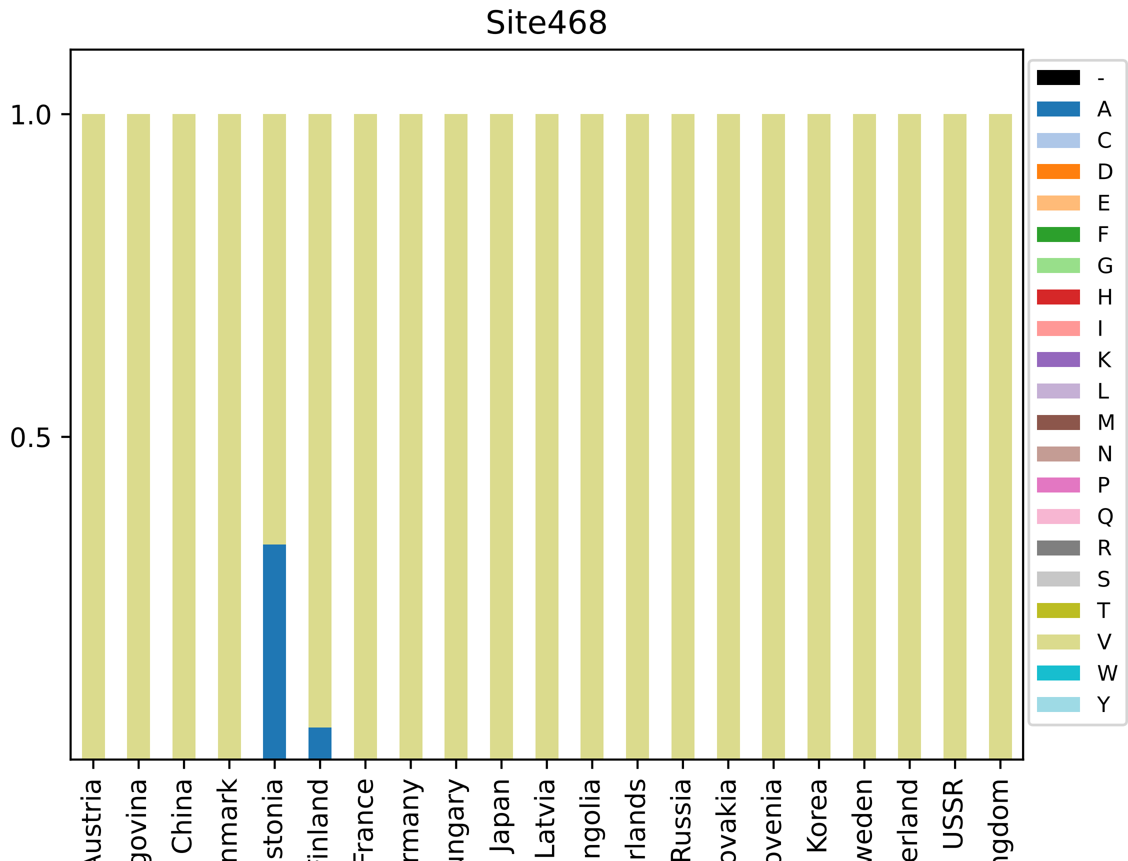
Site468
1.0
0.5
-
A
C
D
E
F
G
H
I
K
L
M
N
P
Q
R
S
T
V
W
Y
Austria
China
Japan
Latvia
Russia
Korea
USSR

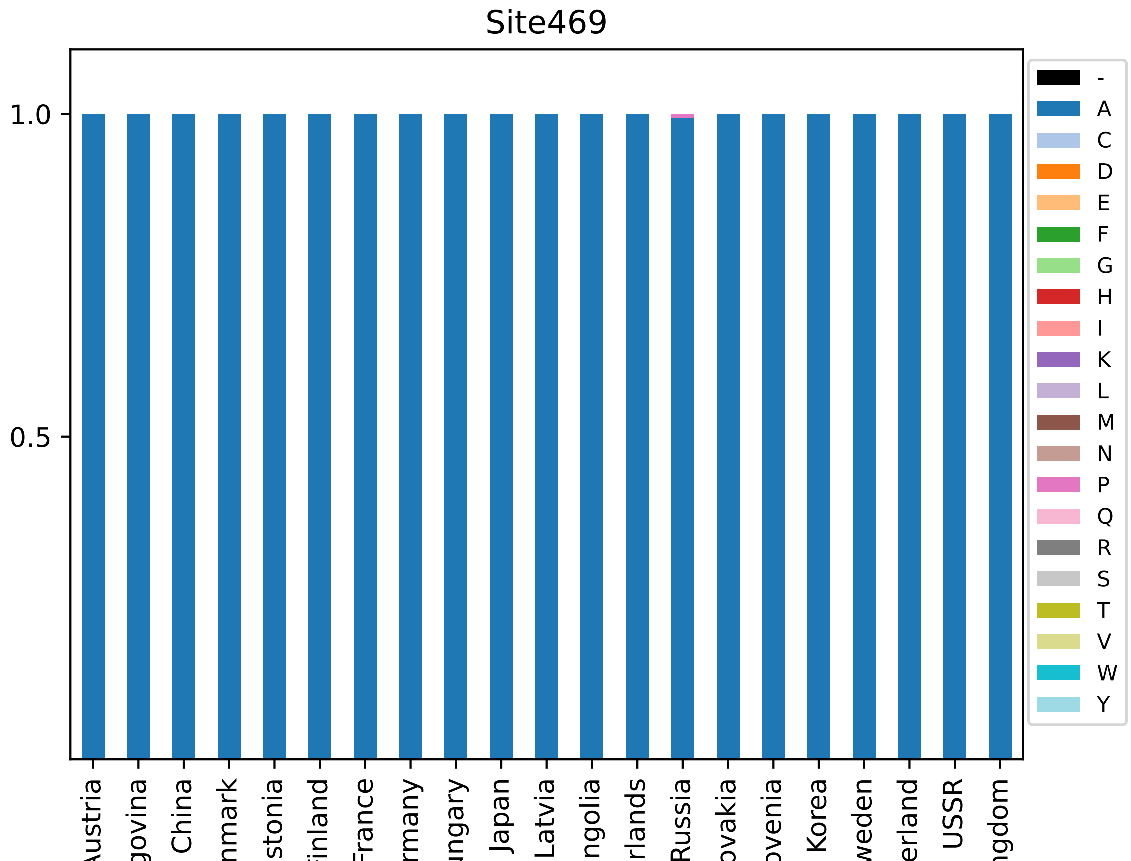
Site469
1.0
0.5
-
A
C
D
E
F
G
H
I
K
L
M
N
P
Q
R
S
T
V
W
Y
Austria
govina
China
nmark
stonia
inland
France
rmany
ungary
Japan
Latvia
ngolia
rlands
Russia
ovakia
ovenia
Korea
weden
erland
USSR
ngdom

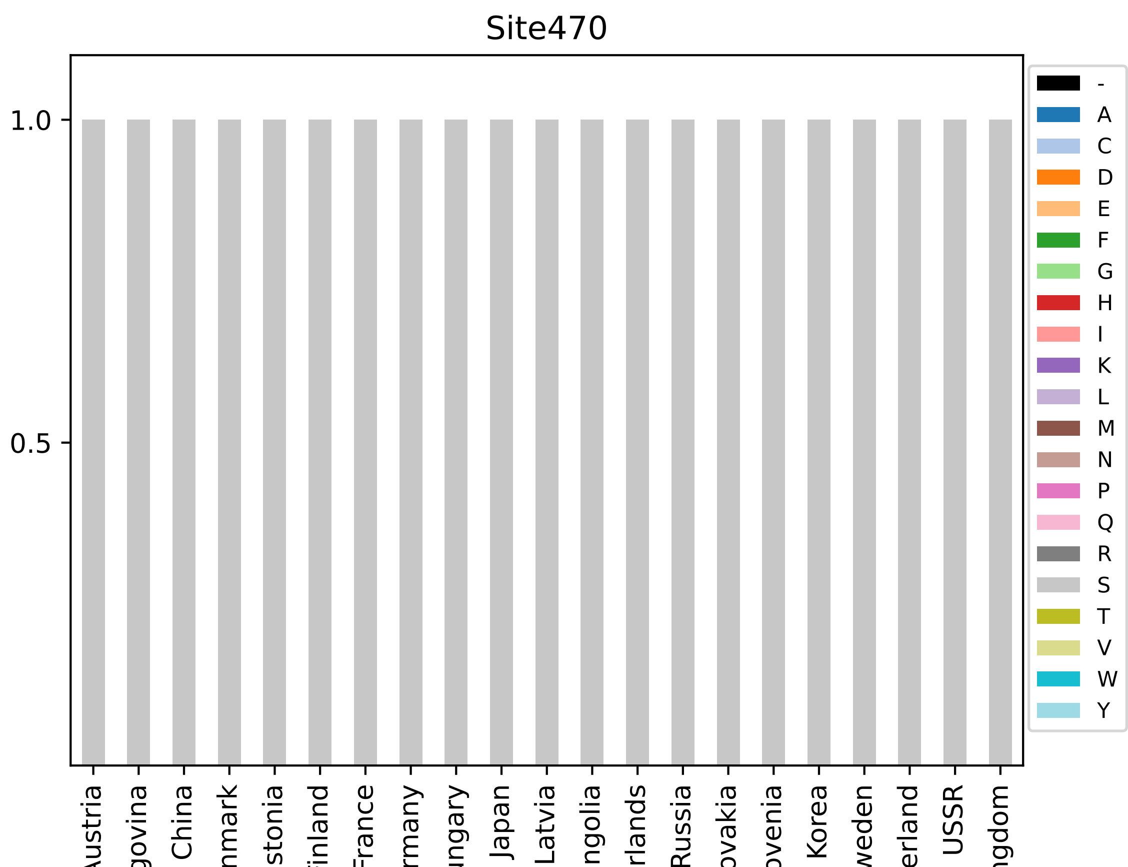
Site470
1.0
0.5
-
A
C
D
E
F
G
H
I
K
L
M
N
P
Q
R
S
T
V
W
Y
China
Japan
Latvia
Russia
Korea
USSR

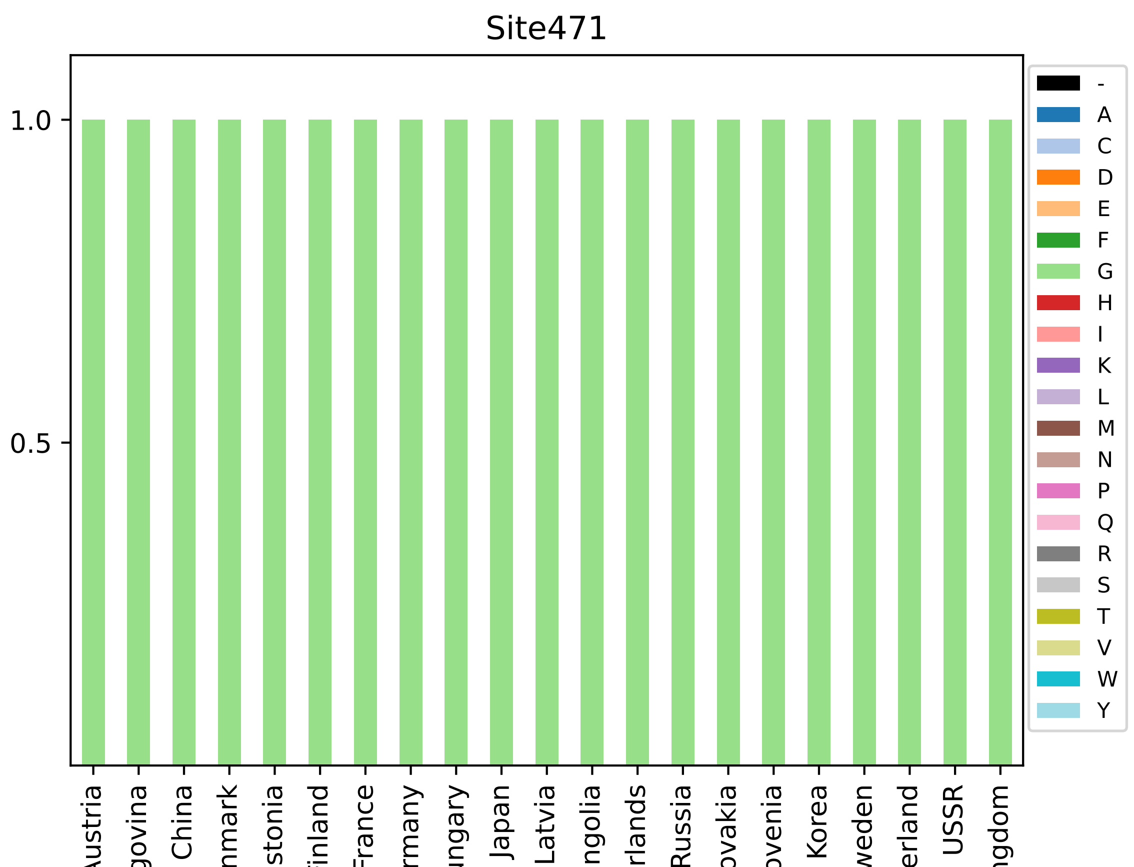

Site471
1.0
0.5
Austria
govina
China
nmark
stonia
Finland
France
rmany
ungary
Japan
Latvia
ngolia
rlands
Russia
ovakia
ovenia
Korea
weden
erland
USSR
ngdom
-
A
C
D
E
F
G
H
I
K
L
M
N
P
Q
R
S
T
V
W
Y

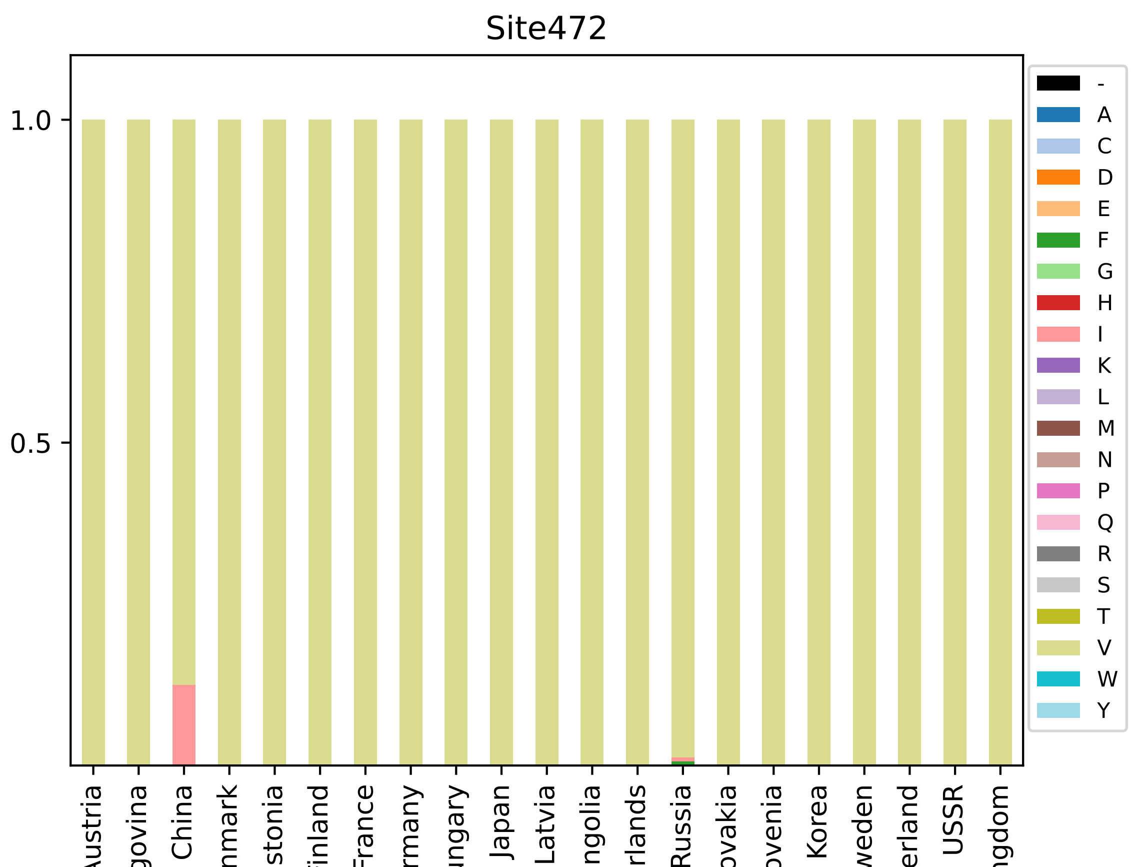
Site472
1.0
0.5
-
A
C
D
E
F
G
H
I
K
L
M
N
P
Q
R
S
T
V
W
Y
China
Japan
Latvia
Russia
Korea
USSR

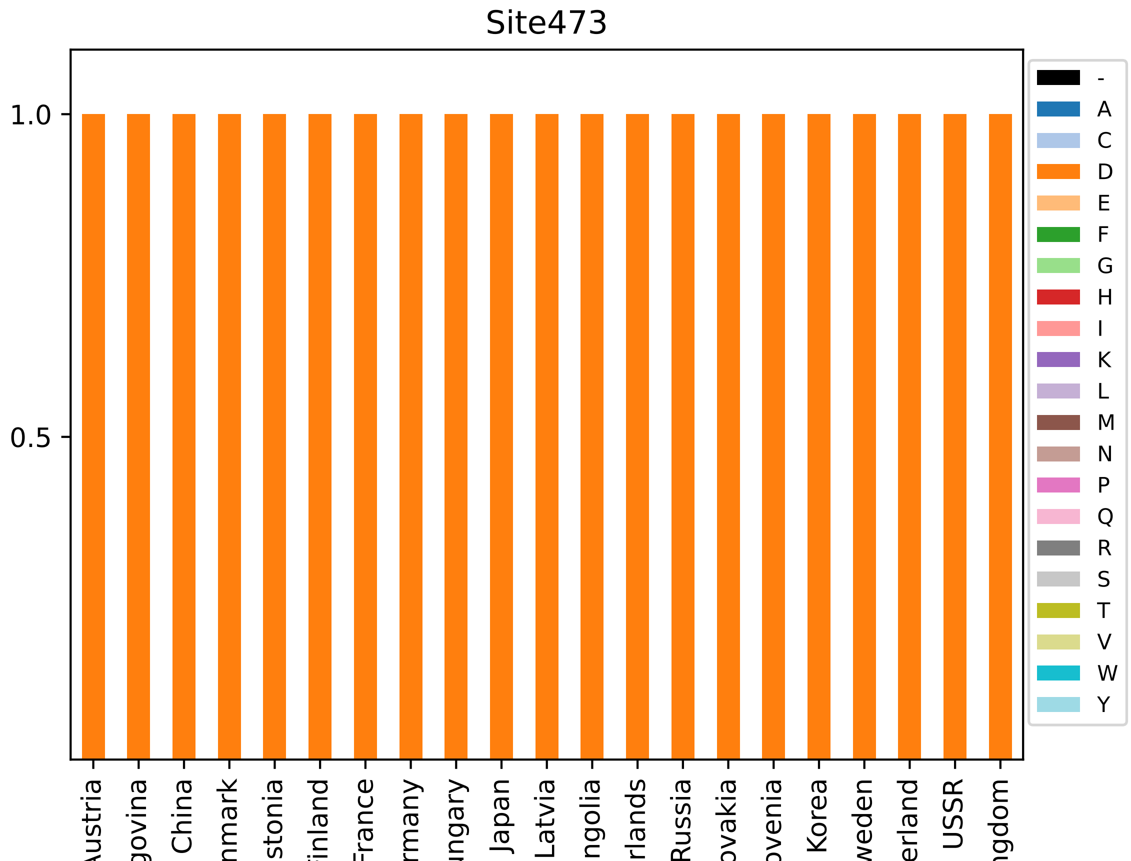

Site473
1.0
0.5
-
A
C
D
E
F
G
H
I
K
L
M
N
P
Q
R
S
T
V
W
Y
China
Japan
Latvia
Russia
Korea
USSR

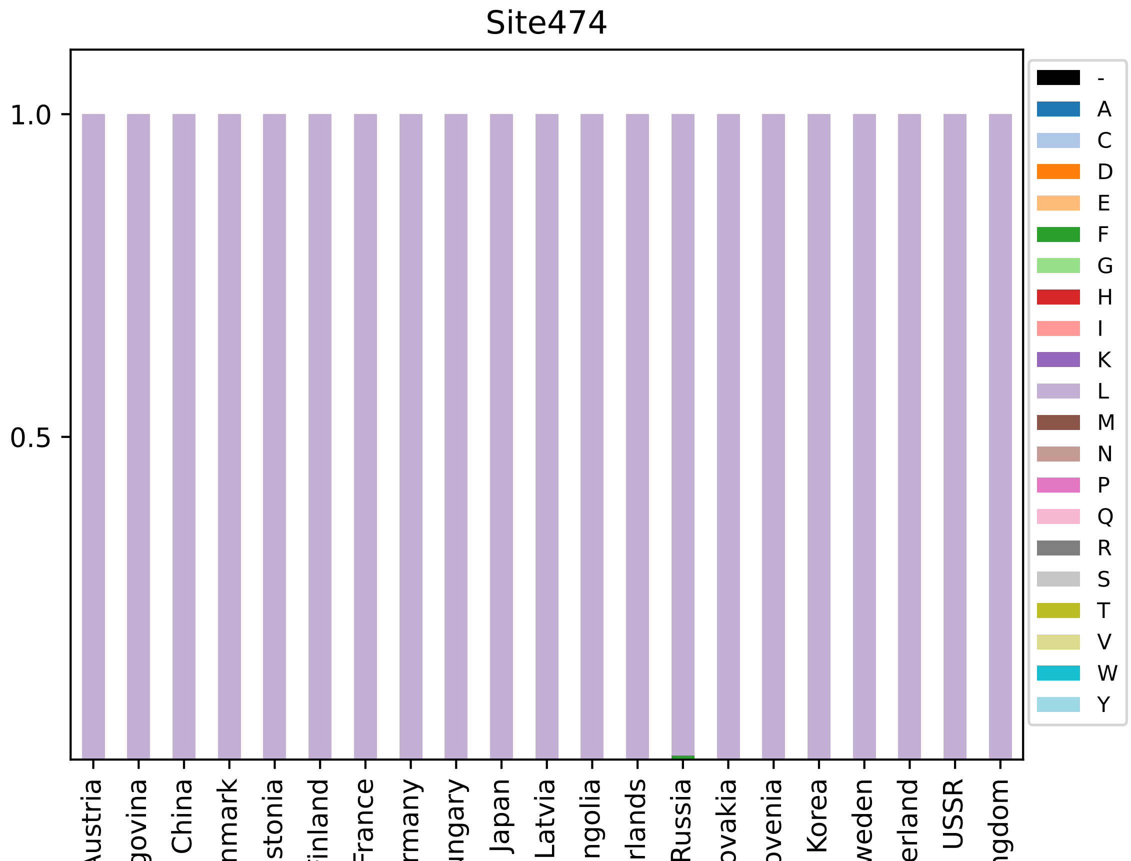

Site474
1.0
0.5
-
A
C
D
E
F
G
H
I
K
L
M
N
P
Q
R
S
T
V
W
Y
Austria
China
Finland
France
Japan
Latvia
Russia
Korea
USSR

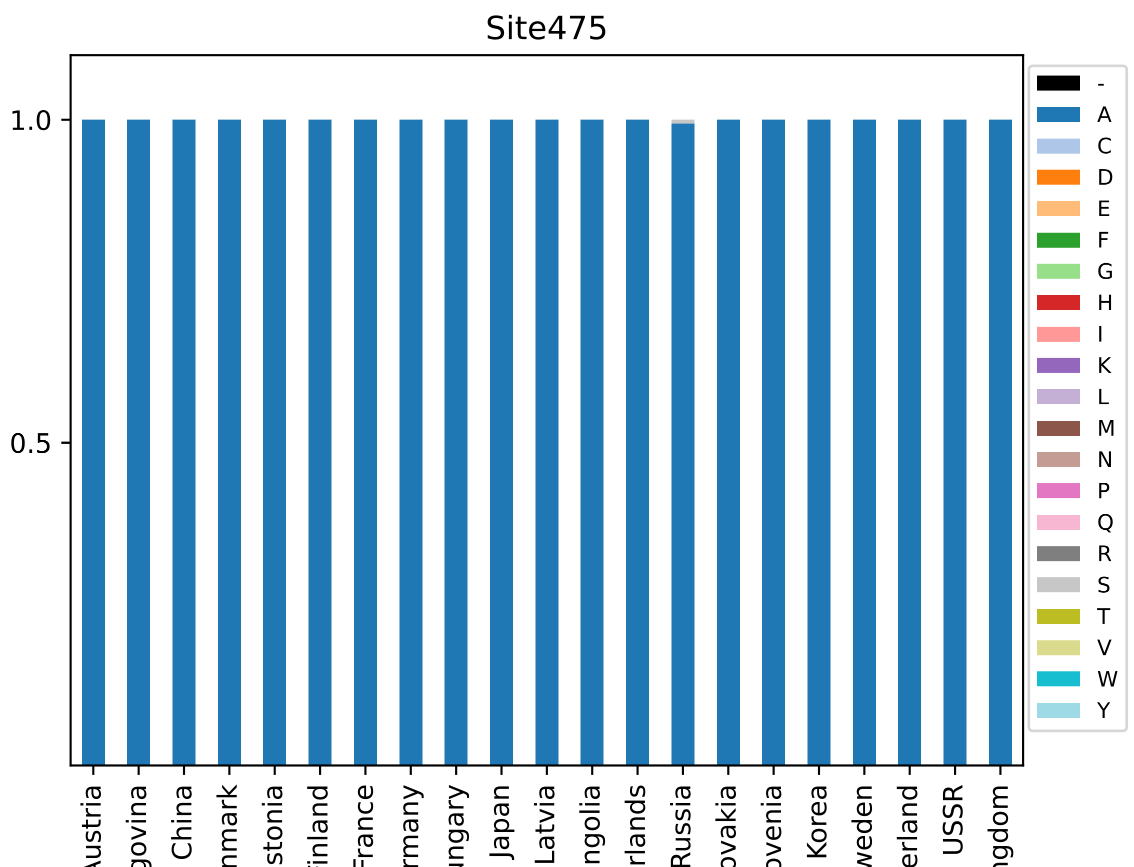
Site475
1.0
0.5
-
A
C
D
E
F
G
H
I
K
L
M
N
P
Q
R
S
T
V
W
Y
Austria
govina
China
nmark
stonia
inland
France
rmany
ungary
Japan
Latvia
ngolia
rlands
Russia
ovakia
ovenia
Korea
weden
erland
USSR
ngdom

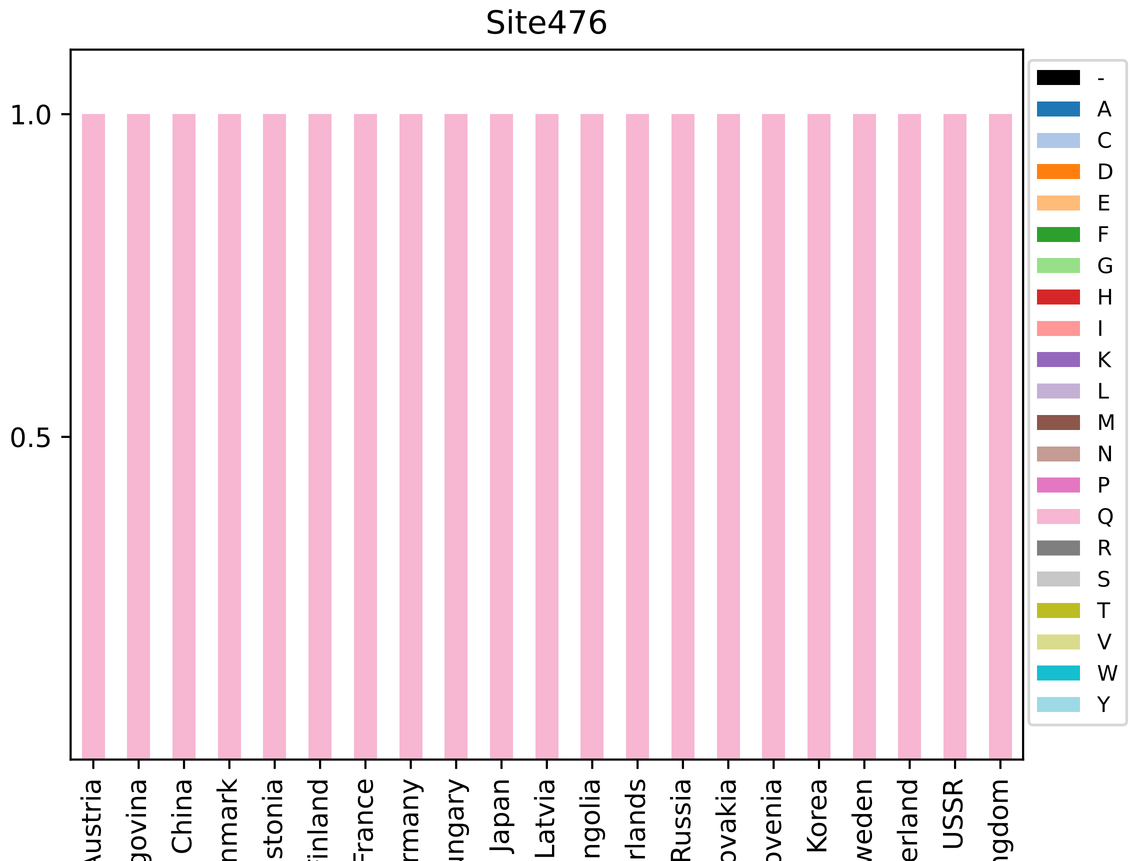

Site476
1.0
0.5
Austria
govina
China
nmark
stonia
inland
France
rmany
ungary
Japan
Latvia
ngolia
rlands
Russia
ovakia
ovenia
Korea
weden
erland
USSR
ngdom
-
A
C
D
E
F
G
H
I
K
L
M
N
P
Q
R
S
T
V
W
Y

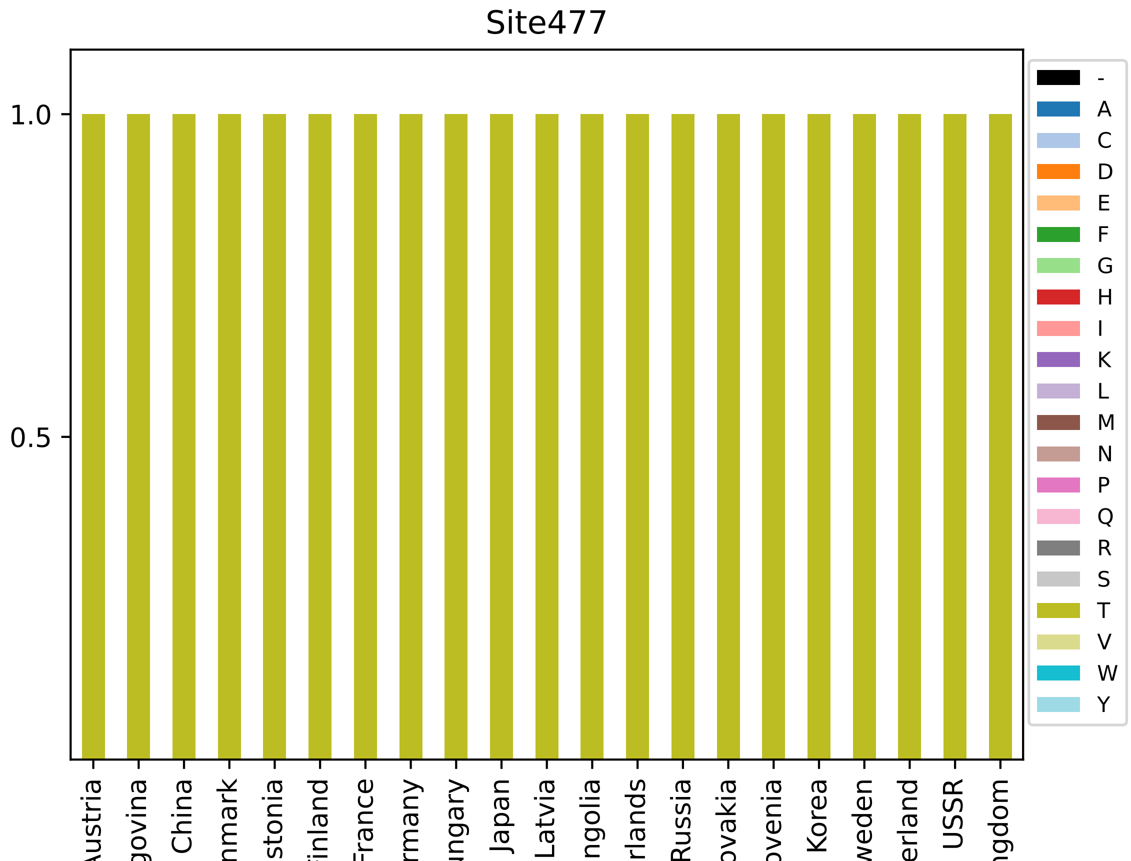
Site477
1.0
0.5
-
A
C
D
E
F
G
H
I
K
L
M
N
P
Q
R
S
T
V
W
Y
China
Japan
Latvia
Russia
Korea
USSR

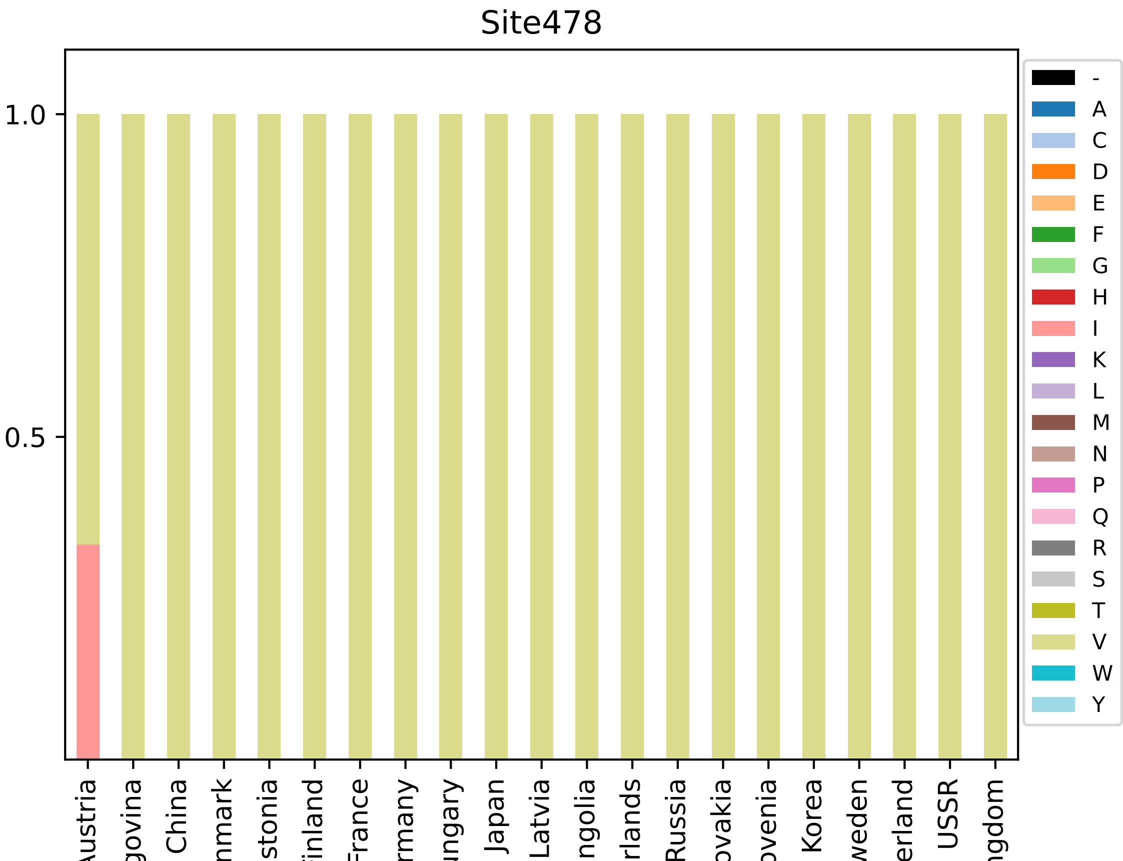

Site478
1.0
0.5
-
A
C
D
E
F
G
H
I
K
L
M
N
P
Q
R
S
T
V
W
Y
China
Latvia
Japan
Korea
USSR

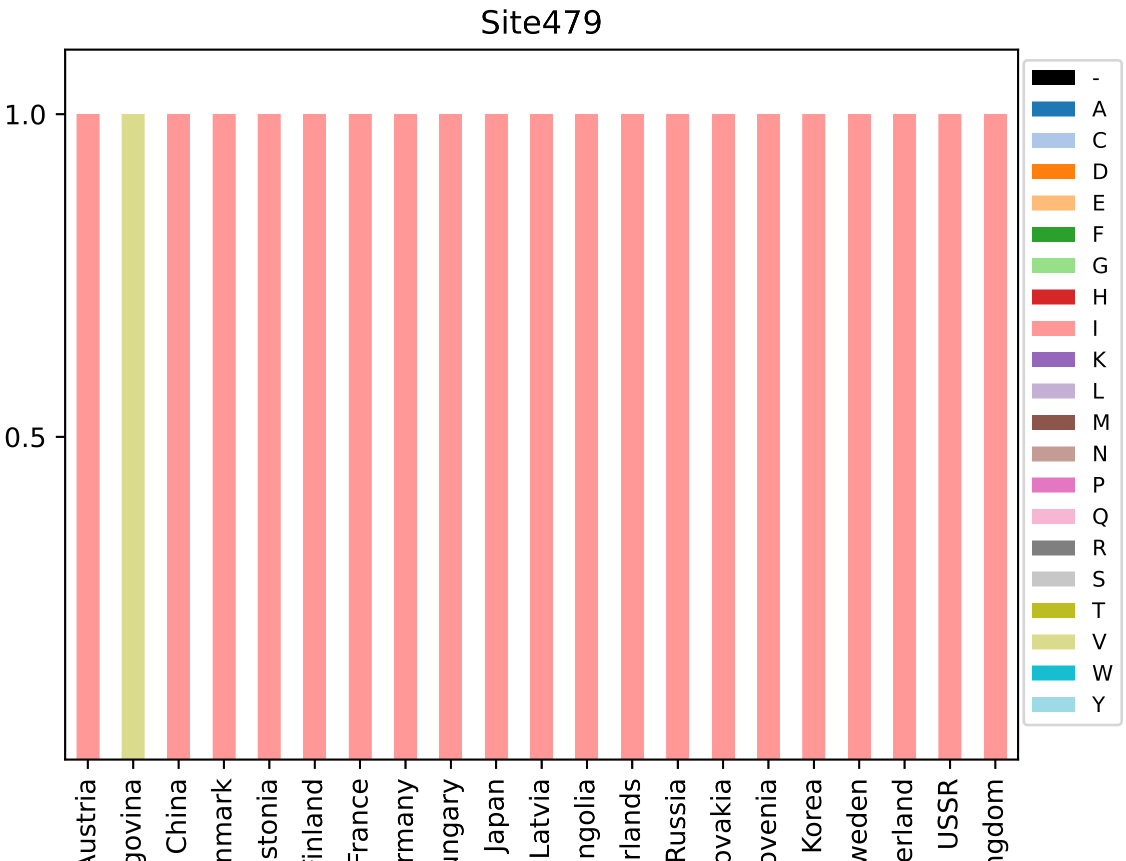

Site479
1.0
0.5
-
A
C
D
E
F
G
H
I
K
L
M
N
P
Q
R
S
T
V
W
Y
China
Japan
Latvia
Russia
Korea
USSR

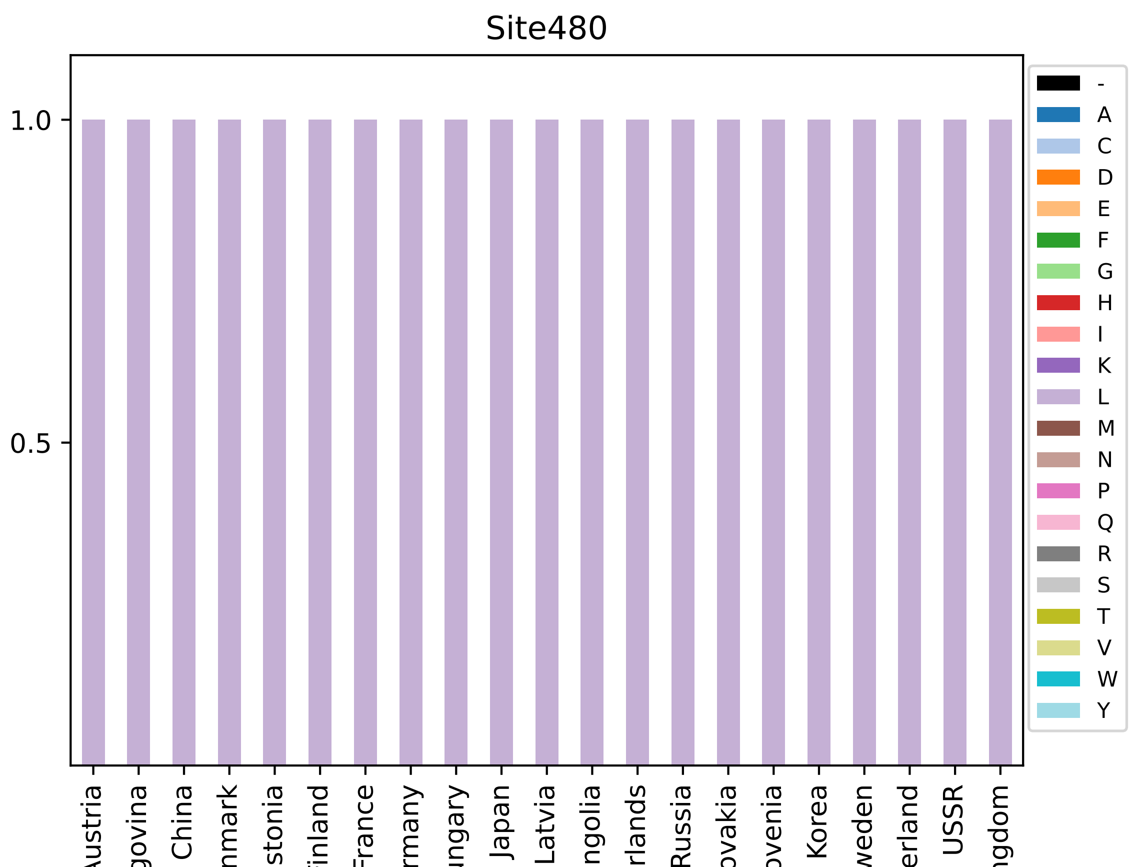
Site480
1.0
0.5
-
A
C
D
E
F
G
H
I
K
L
M
N
P
Q
R
S
T
V
W
Y
China
Japan
Latvia
Russia
Korea
USSR

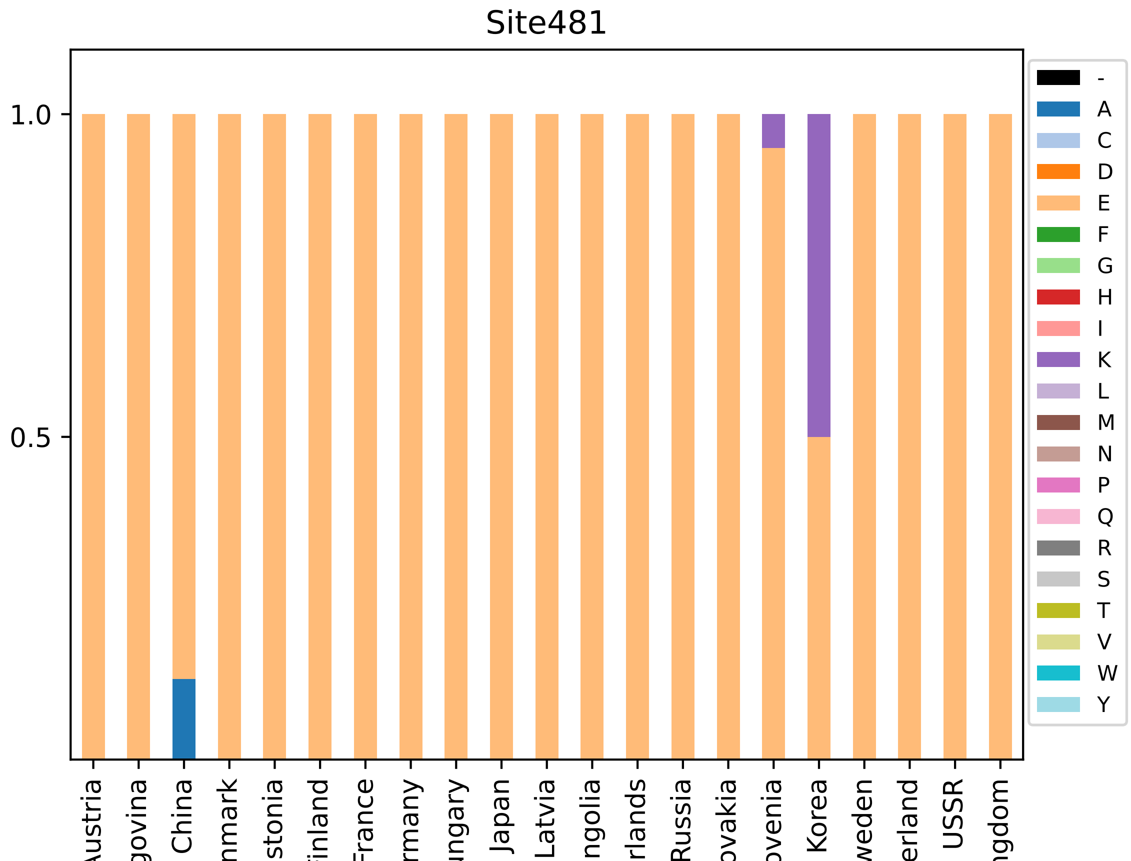

Site481
1.0
0.5
-
A
C
D
E
F
G
H
I
K
L
M
N
P
Q
R
S
T
V
W
Y
China
Japan
Latvia
Korea
USSR

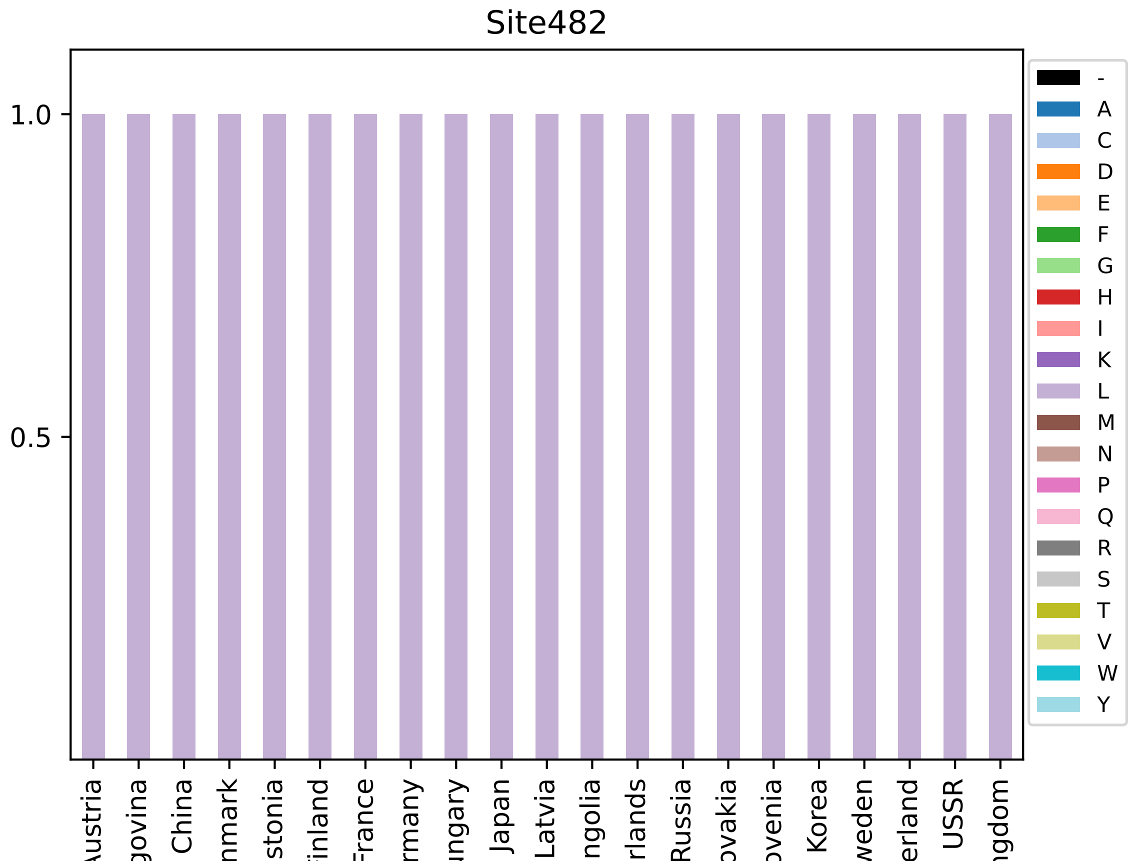

Site482
1.0
0.5
-
A
C
D
E
F
G
H
I
K
L
M
N
P
Q
R
S
T
V
W
Y
China
Japan
Latvia
Russia
Korea
USSR

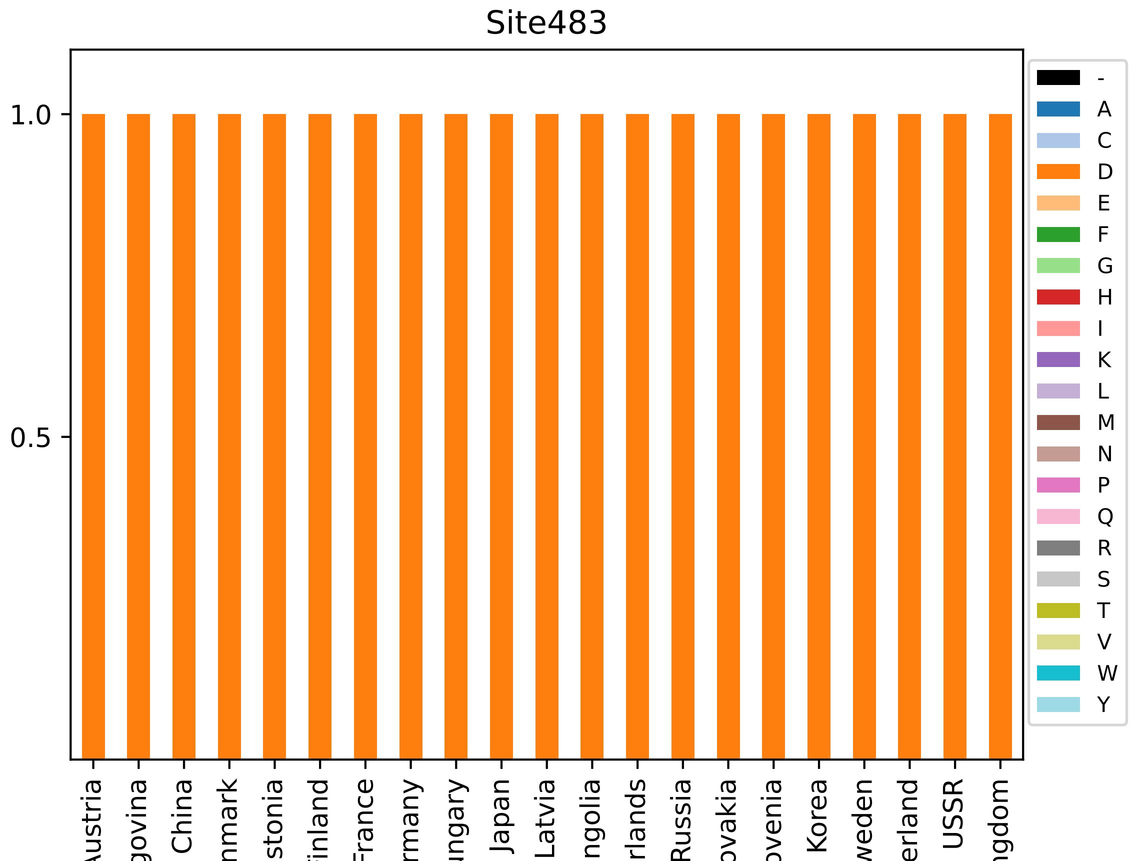

Site483
1.0
0.5
-
A
C
D
E
F
G
H
I
K
L
M
N
P
Q
R
S
T
V
W
Y
Austria
China
Japan
Latvia
Russia
Korea
USSR

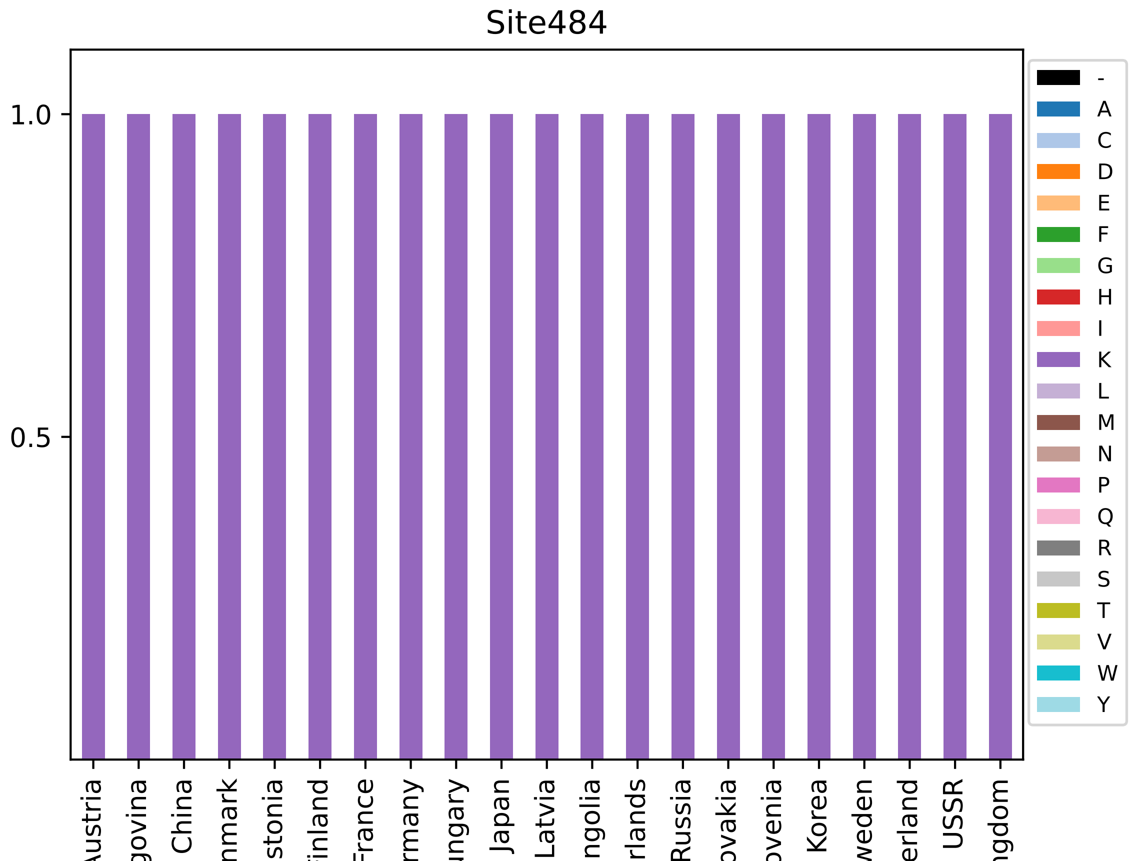
Site484
1.0
0.5
-
A
C
D
E
F
G
H
I
K
L
M
N
P
Q
R
S
T
V
W
Y
Austria
govina
China
nmark
stonia
inland
France
rmany
ungary
Japan
Latvia
ngolia
rlands
Russia
ovakia
ovenia
Korea
weden
erland
USSR
ngdom

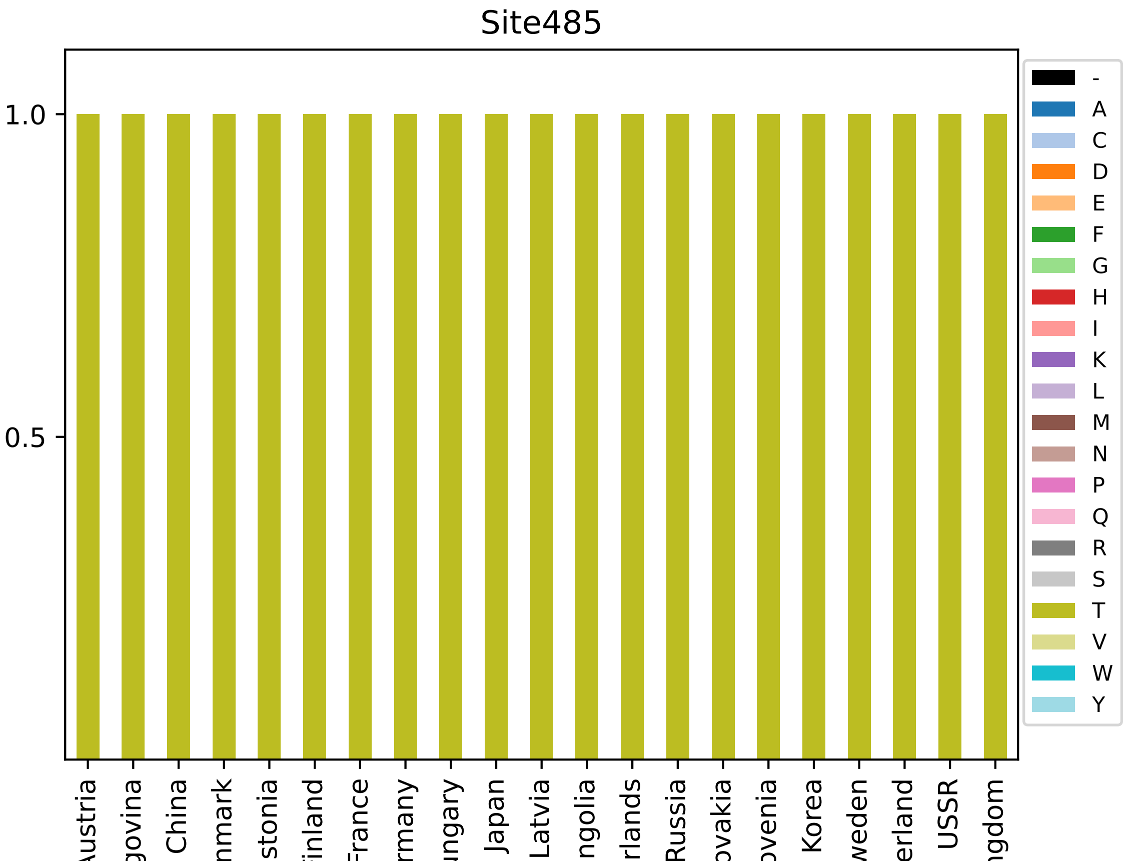
Site485
1.0
0.5
-
A
C
D
E
F
G
H
I
K
L
M
N
P
Q
R
S
T
V
W
Y
China
Japan
Latvia
Russia
Korea
USSR

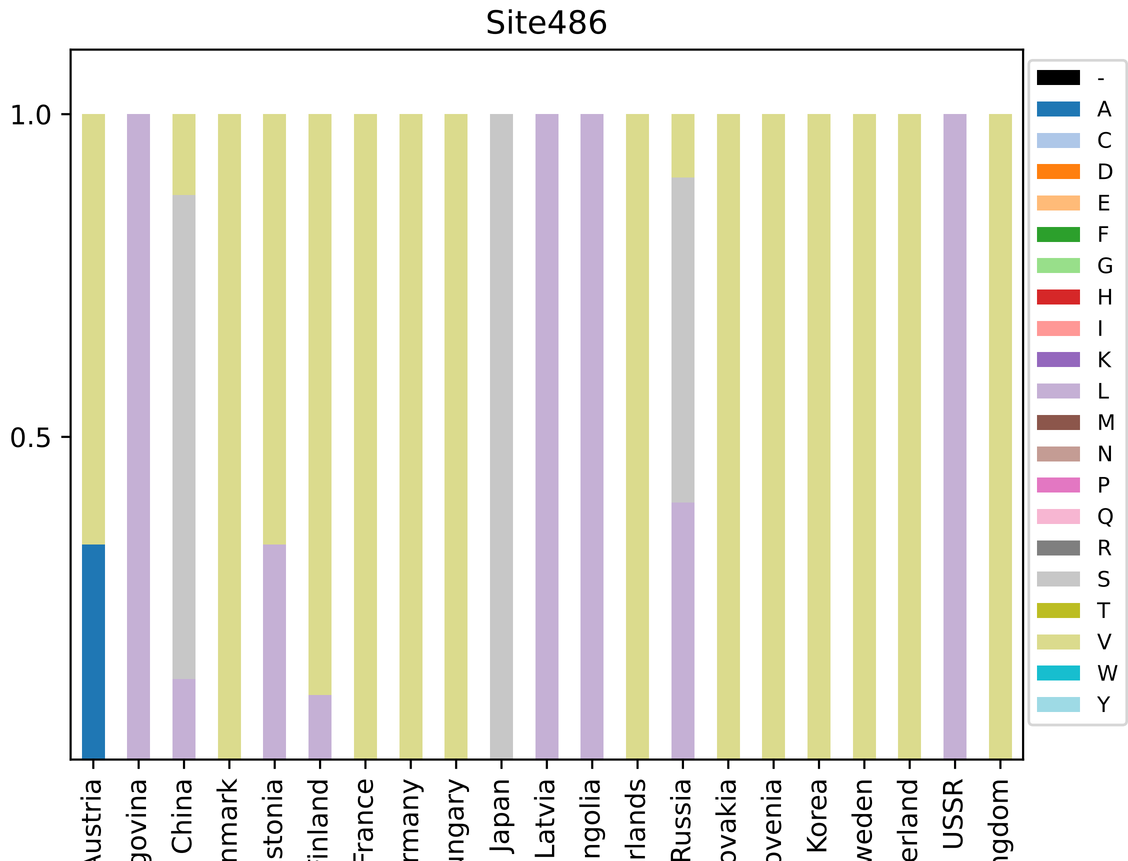

Site486
1.0
0.5
-
A
C
D
E
F
G
H
I
K
L
M
N
P
Q
R
S
T
V
W
Y
Austria
govina
China
nmark
stonia
Finland
France
rmany
ungary
Japan
Latvia
ngolia
rlands
Russia
ovakia
ovenia
Korea
weden
erland
USSR
ngdom

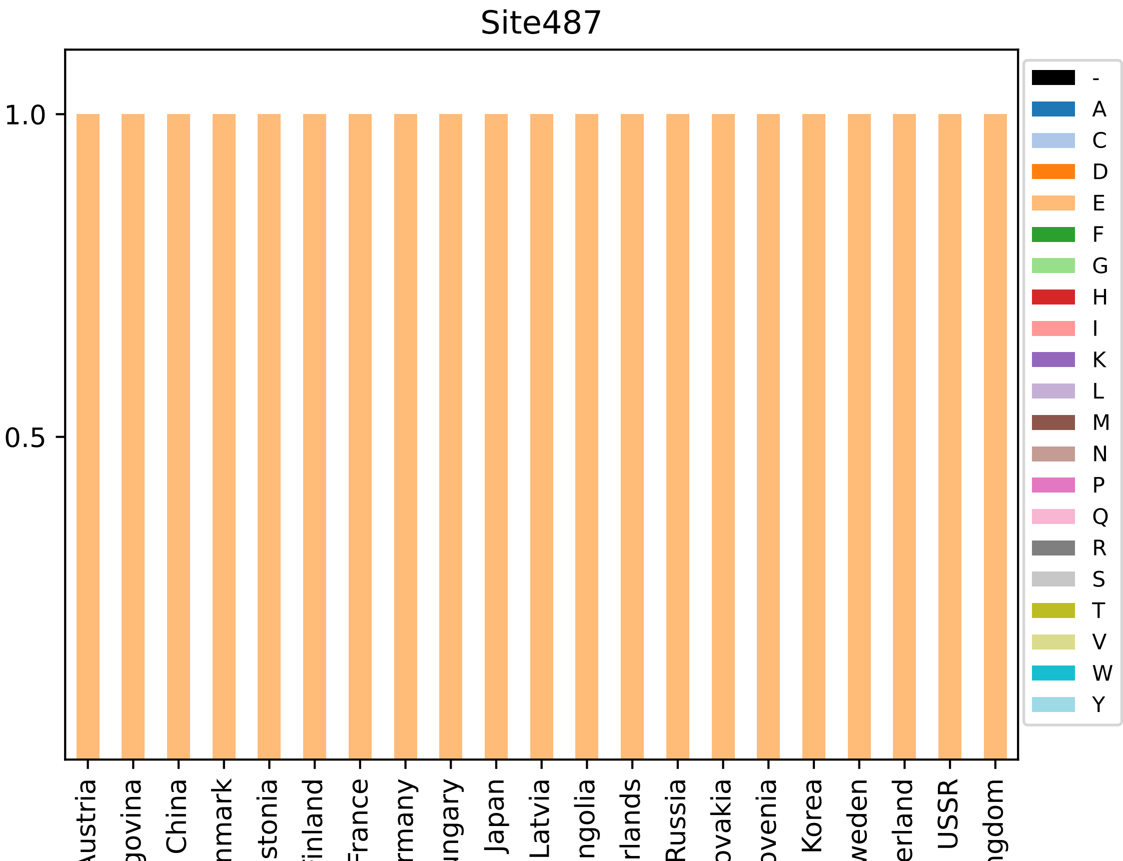

Site487
1.0
0.5
Austria
govina
China
nmark
stonia
inland
France
rmany
ungary
Japan
Latvia
ngolia
rlands
Russia
ovakia
ovenia
Korea
weden
erland
USSR
ngdom
-
A
C
D
E
F
G
H
I
K
L
M
N
P
Q
R
S
T
V
W
Y

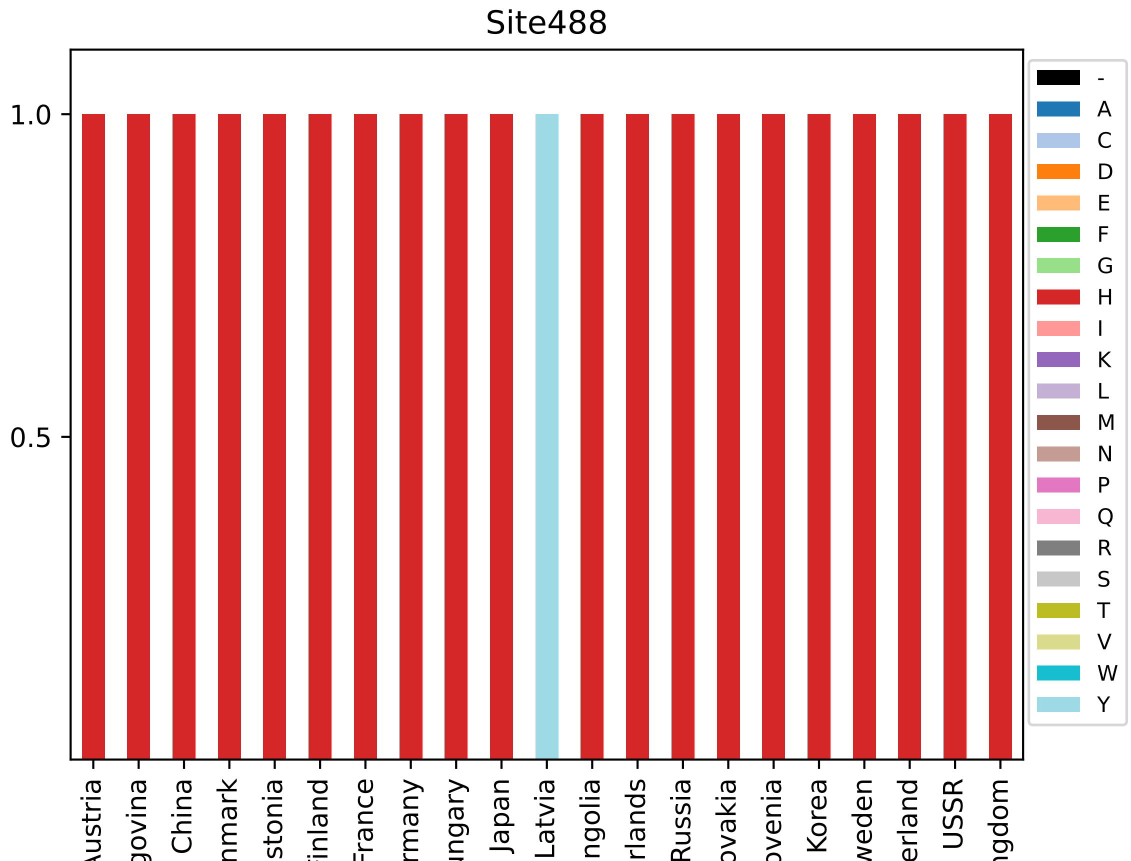

Site488
1.0
0.5
-
A
C
D
E
F
G
H
I
K
L
M
N
P
Q
R
S
T
V
W
Y
China
Japan
Latvia
Russia
Korea
USSR

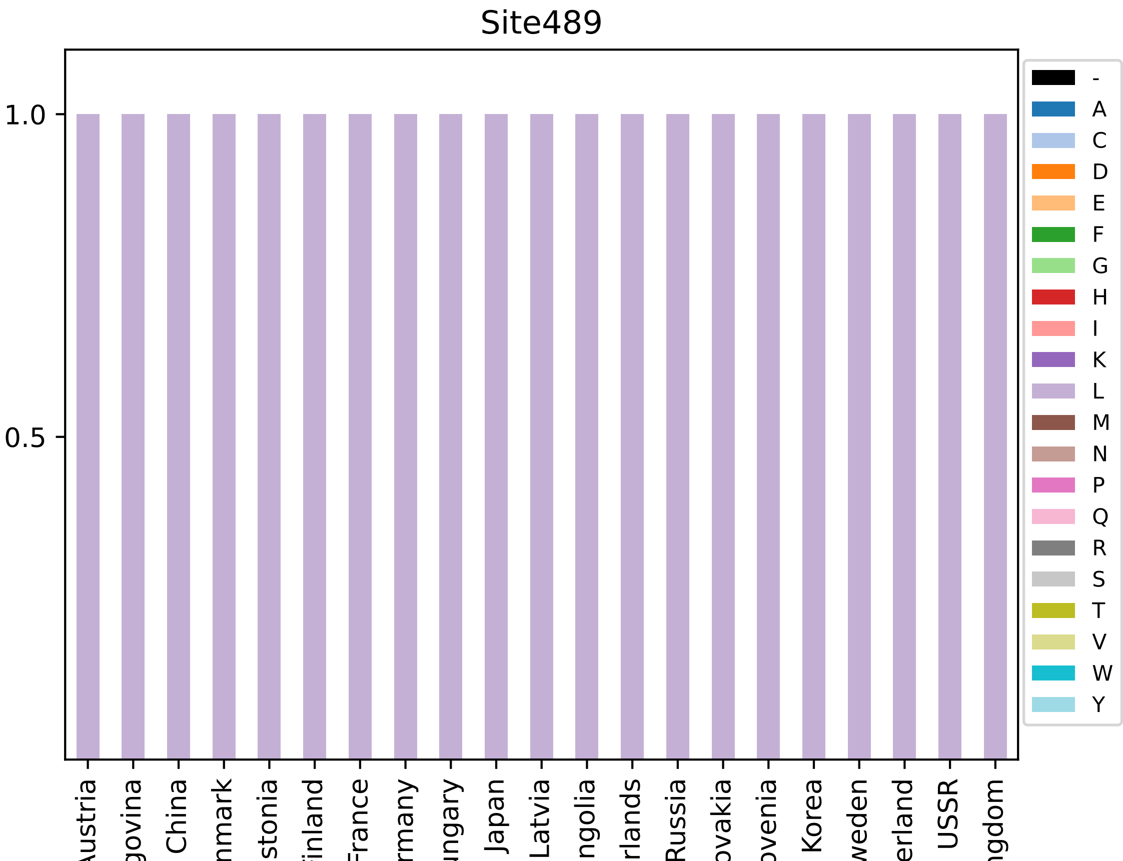

Site489
1.0
0.5
-
A
C
D
E
F
G
H
I
K
L
M
N
P
Q
R
S
T
V
W
Y
China
Japan
Latvia
Russia
Korea
USSR

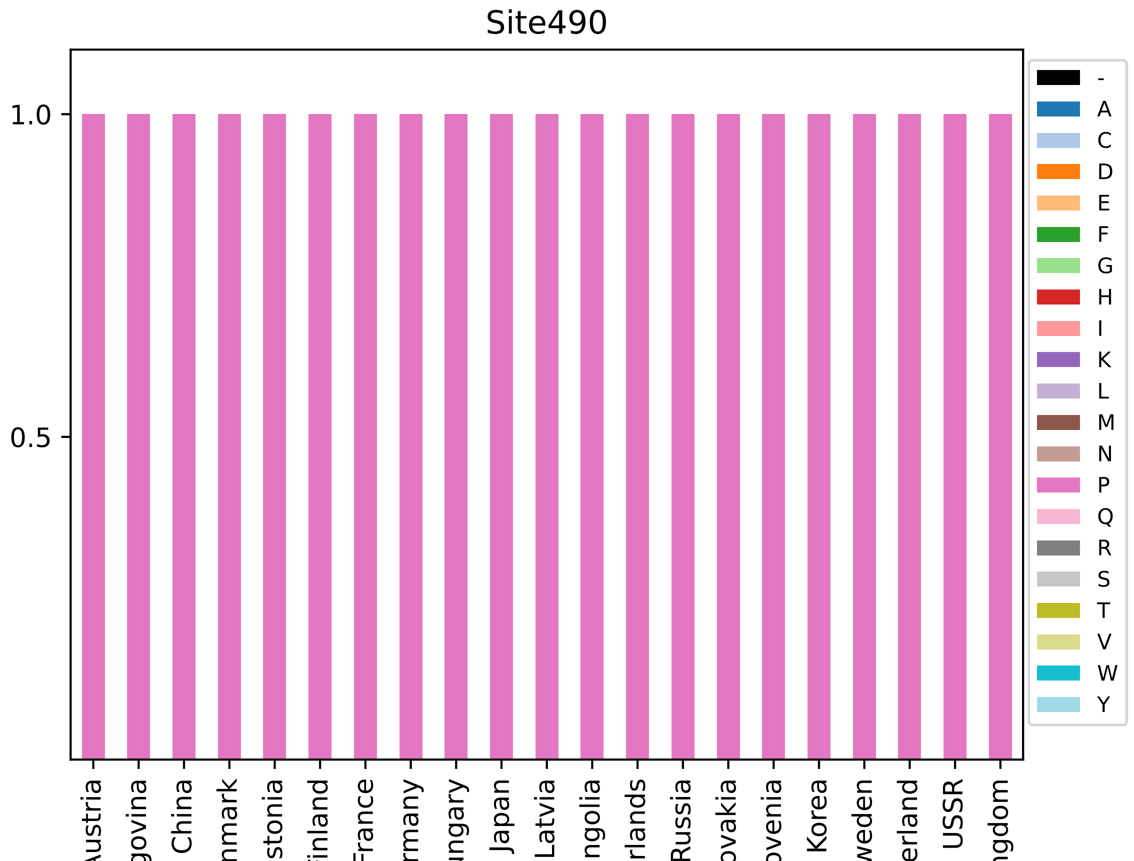
Site490
1.0
0.5
-
A
C
D
E
F
G
H
I
K
L
M
N
P
Q
R
S
T
V
W
Y
Austria
China
Japan
Latvia
Russia
Korea
USSR

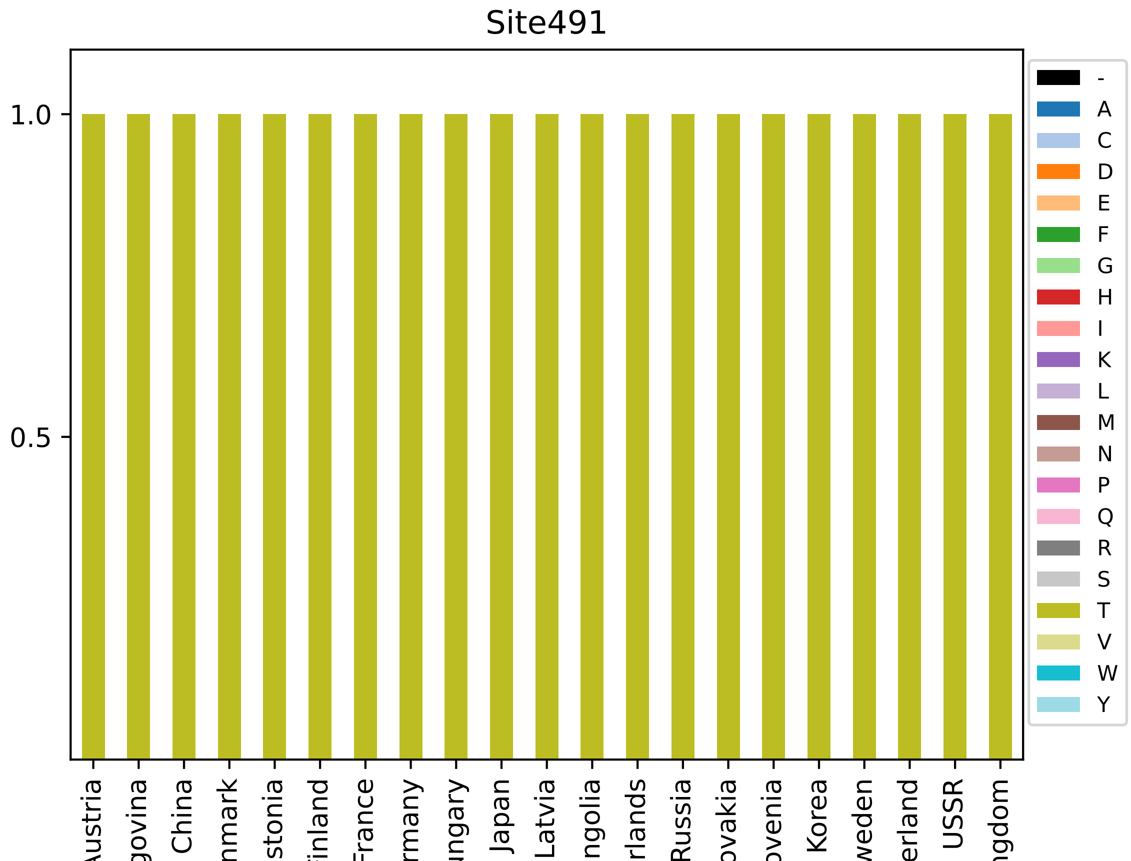

Site491
1.0
0.5
-
A
C
D
E
F
G
H
I
K
L
M
N
P
Q
R
S
T
V
W
Y
China
Japan
Latvia
Russia
Korea
USSR

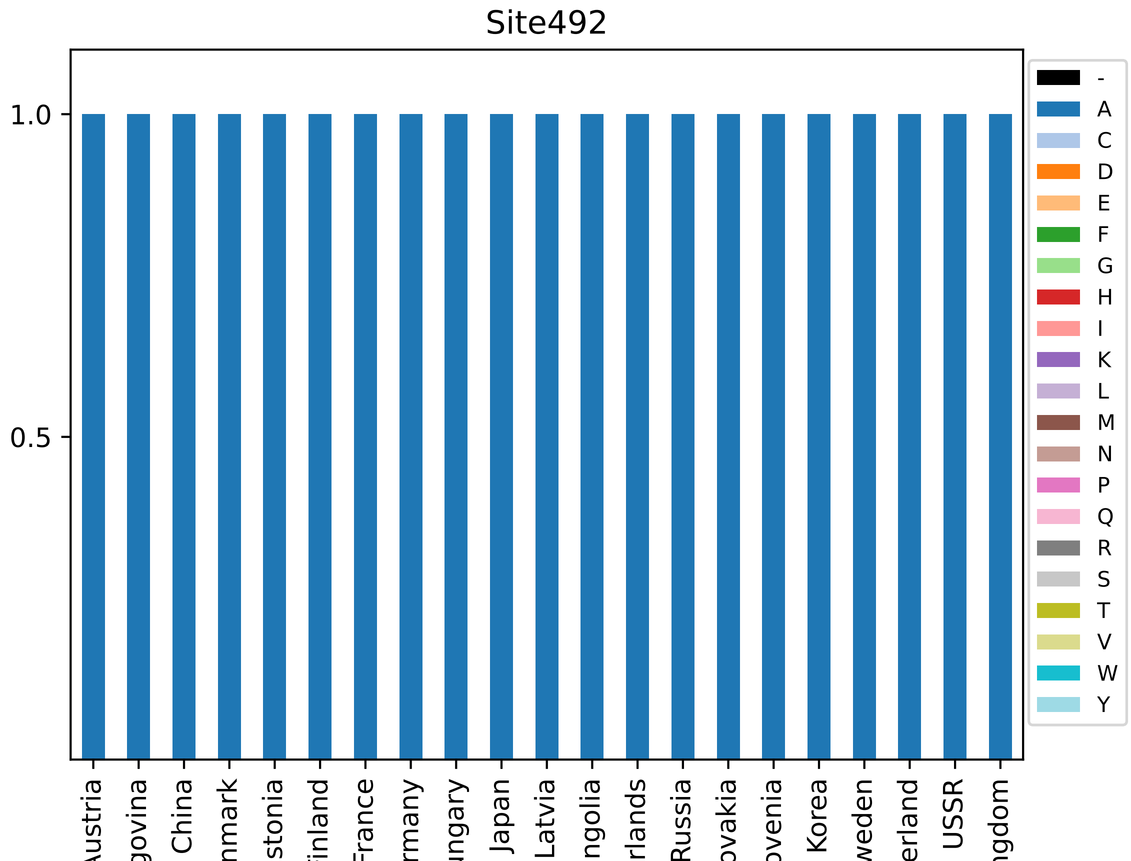
Site492
1.0
0.5
-
A
C
D
E
F
G
H
I
K
L
M
N
P
Q
R
S
T
V
W
Y
China
Japan
Latvia
Russia
Korea
USSR

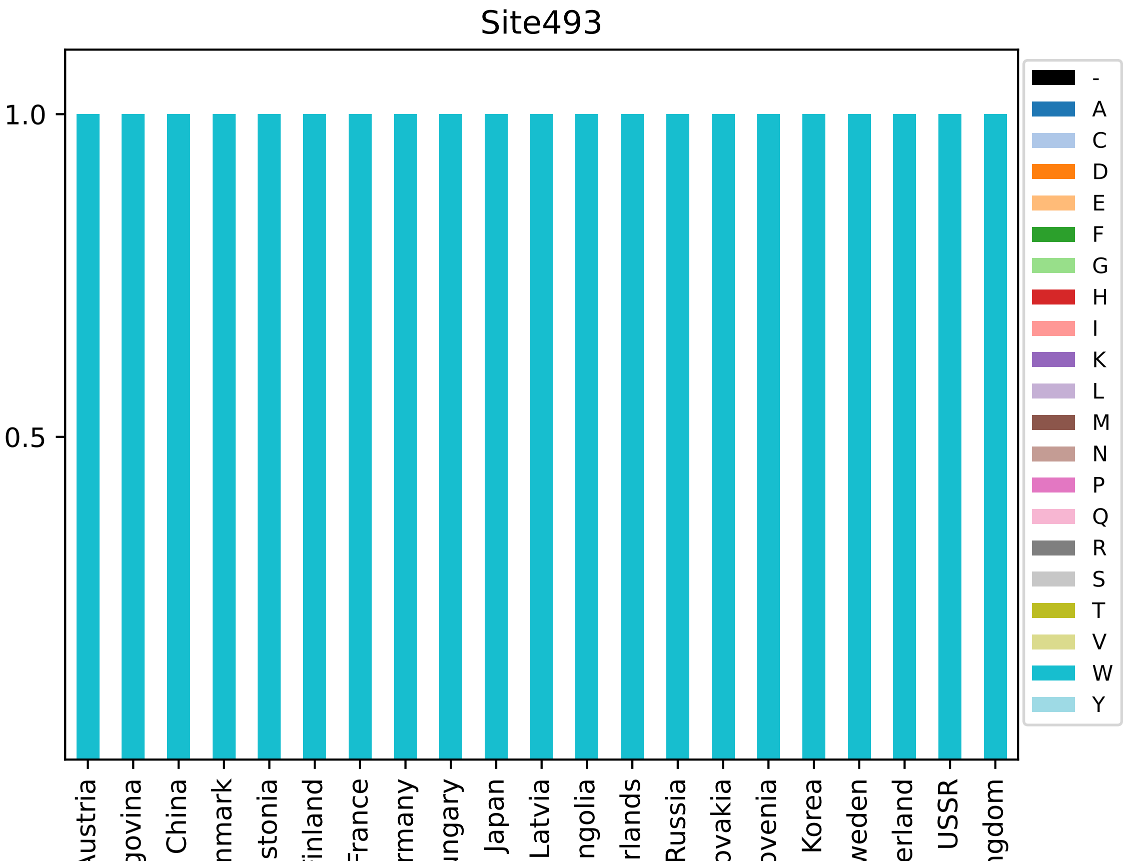

Site493
1.0
0.5
-
A
C
D
E
F
G
H
I
K
L
M
N
P
Q
R
S
T
V
W
Y
China
Japan
Latvia
Russia
Korea
USSR

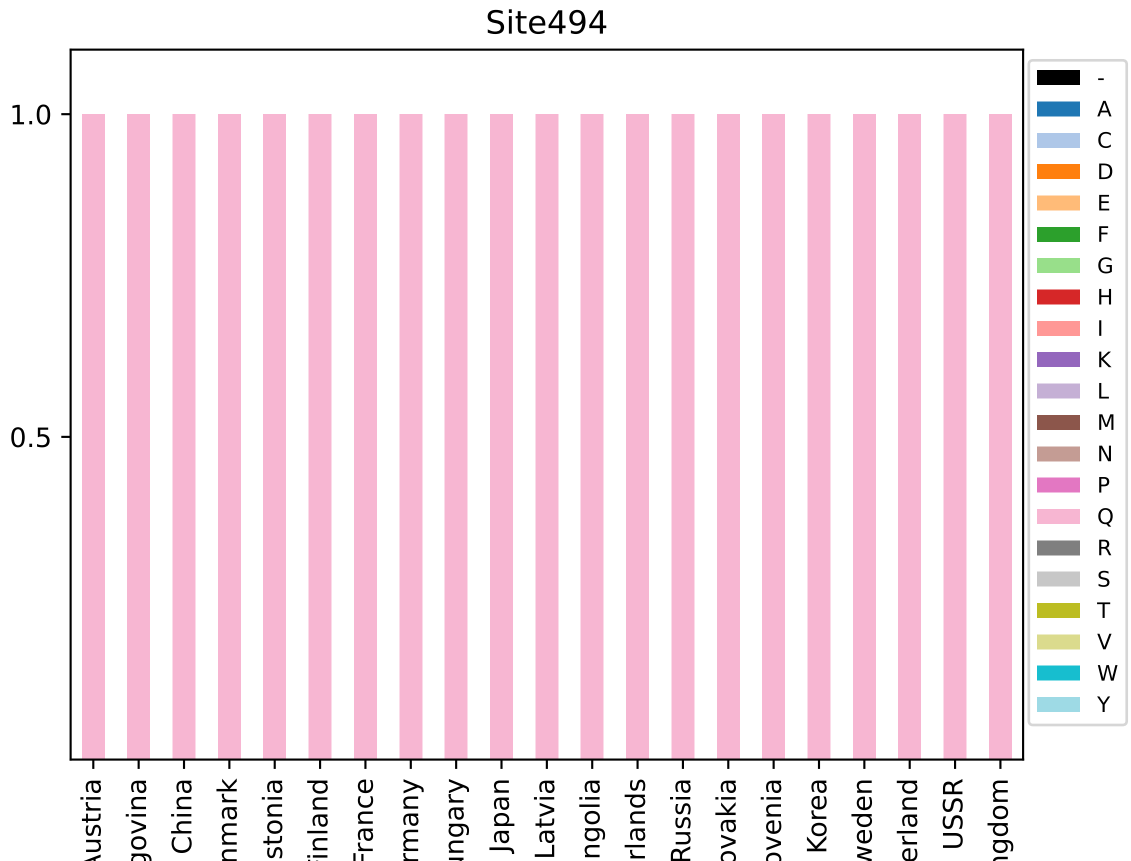

Site494
1.0
0.5
-
A
C
D
E
F
G
H
I
K
L
M
N
P
Q
R
S
T
V
W
Y
Austria
China
Estonia
Finland
France
Germany
Hungary
Japan
Latvia
Mongolia
Russia
Slovenia
Korea
Sweden
USSR

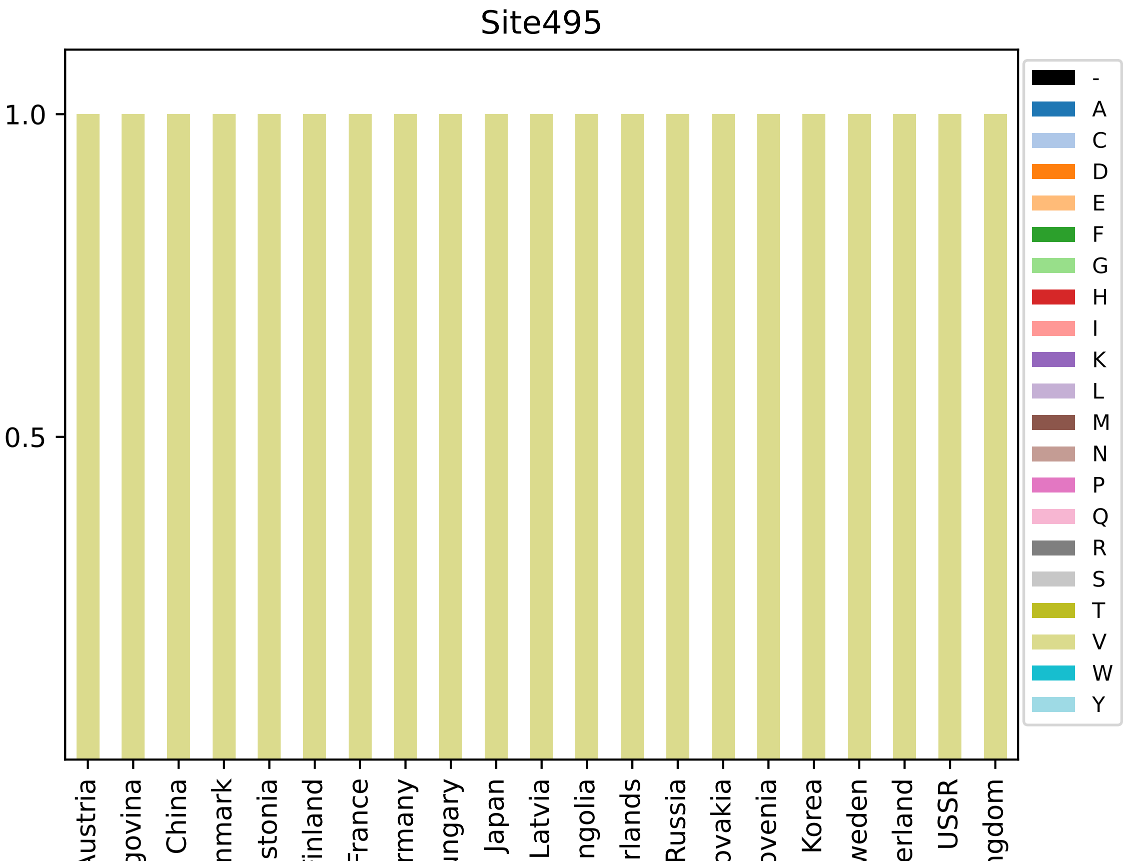
Site495
1.0
0.5
-
A
C
D
E
F
G
H
I
K
L
M
N
P
Q
R
S
T
V
W
Y
China
Japan
Latvia
Russia
Korea
USSR

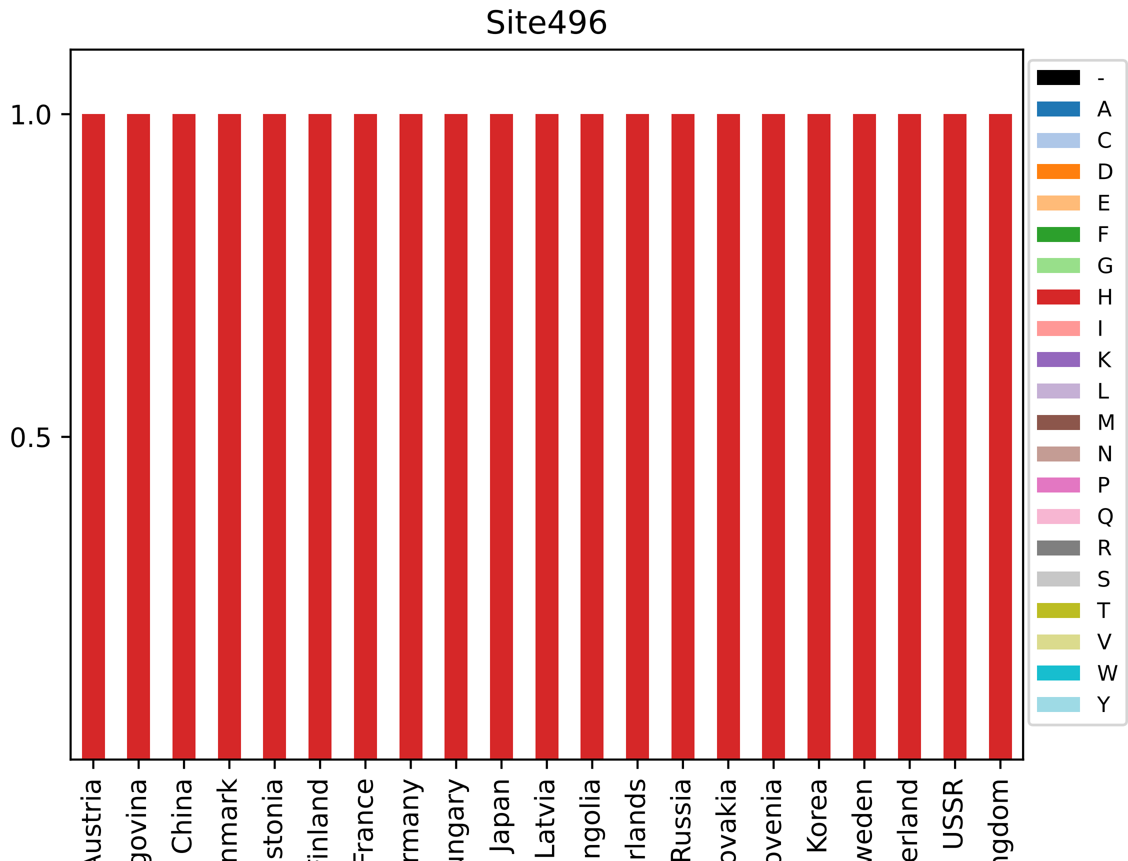

Site496
1.0
0.5
-
A
C
D
E
F
G
H
I
K
L
M
N
P
Q
R
S
T
V
W
Y
Austria
govina
China
nmark
stonia
Finland
France
rmany
ungary
Japan
Latvia
ngolia
rlands
Russia
ovakia
ovenia
Korea
weden
erland
USSR
ngdom

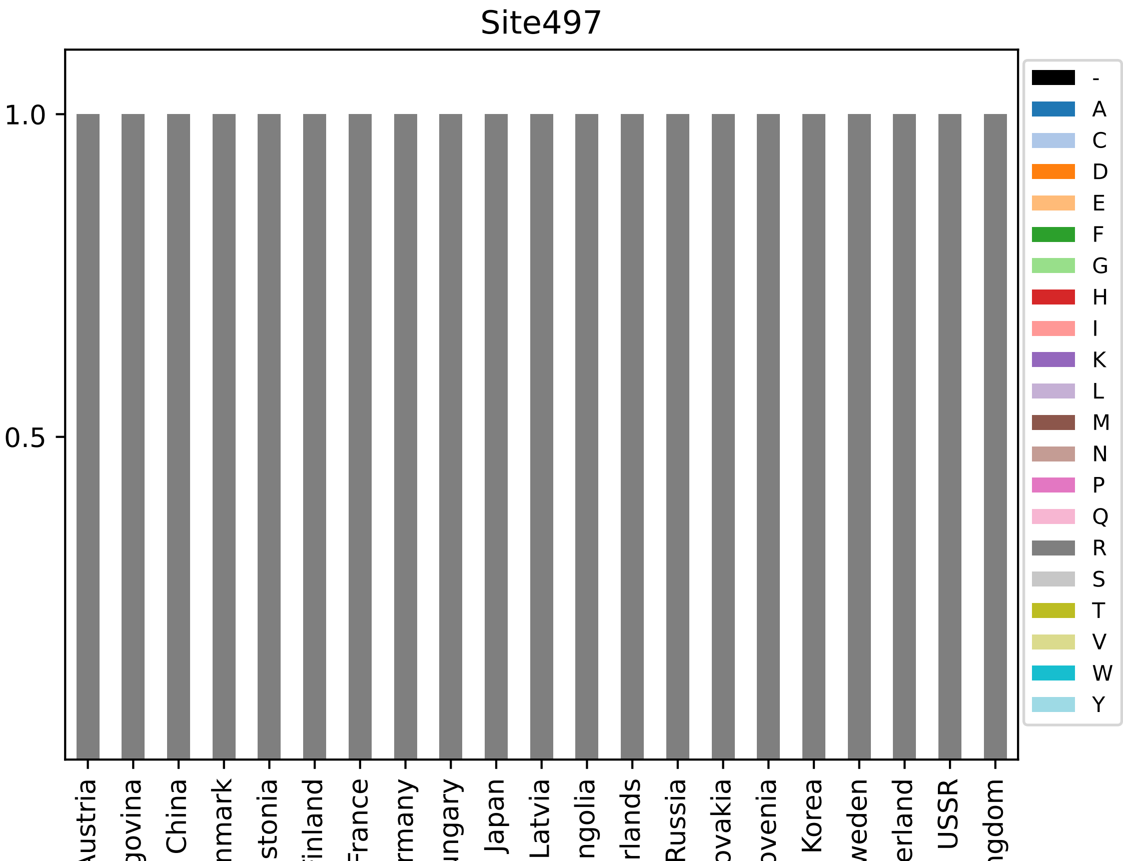
Site497
1.0
0.5
-
A
C
D
E
F
G
H
I
K
L
M
N
P
Q
R
S
T
V
W
Y
China
Japan
Latvia
Russia
Korea
USSR

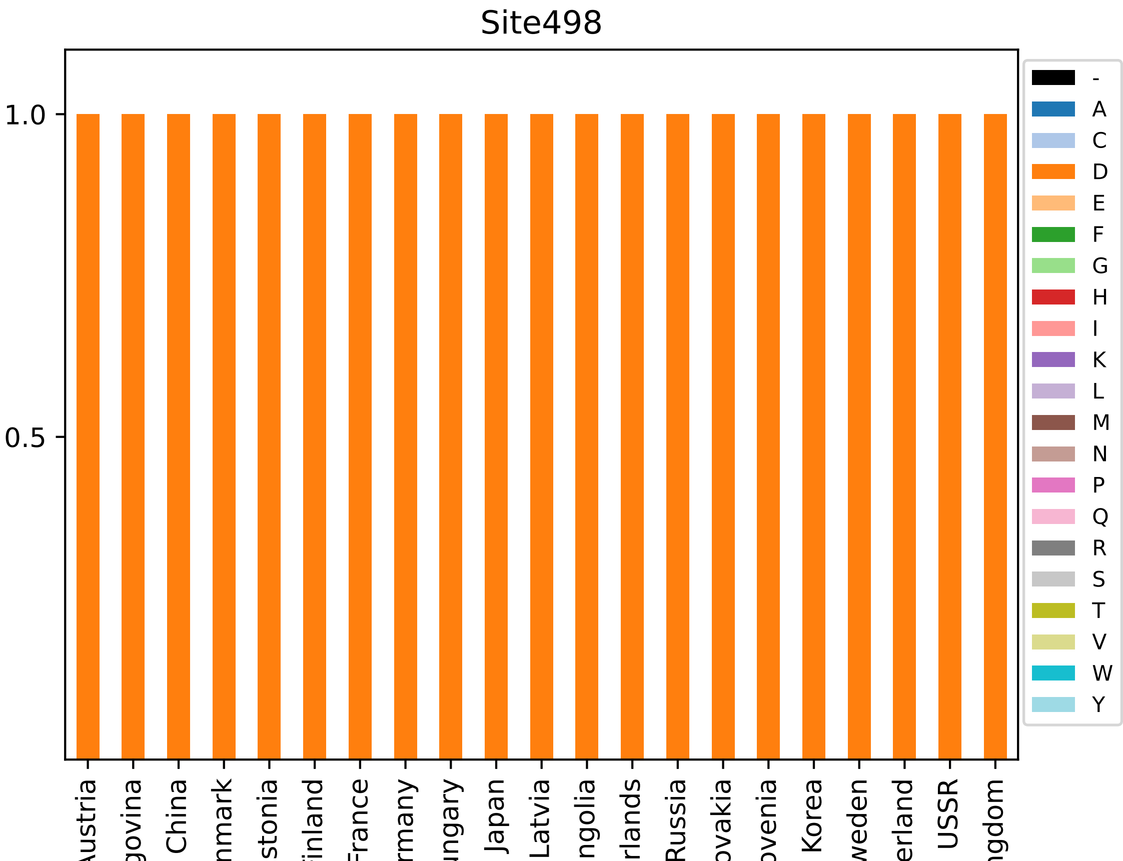

Site498
1.0
0.5
-
A
C
D
E
F
G
H
I
K
L
M
N
P
Q
R
S
T
V
W
Y
Austria
govina
China
nmark
stonia
inland
France
rmany
ungary
Japan
Latvia
ngolia
rlands
Russia
ovakia
ovenia
Korea
weden
erland
USSR
ngdom

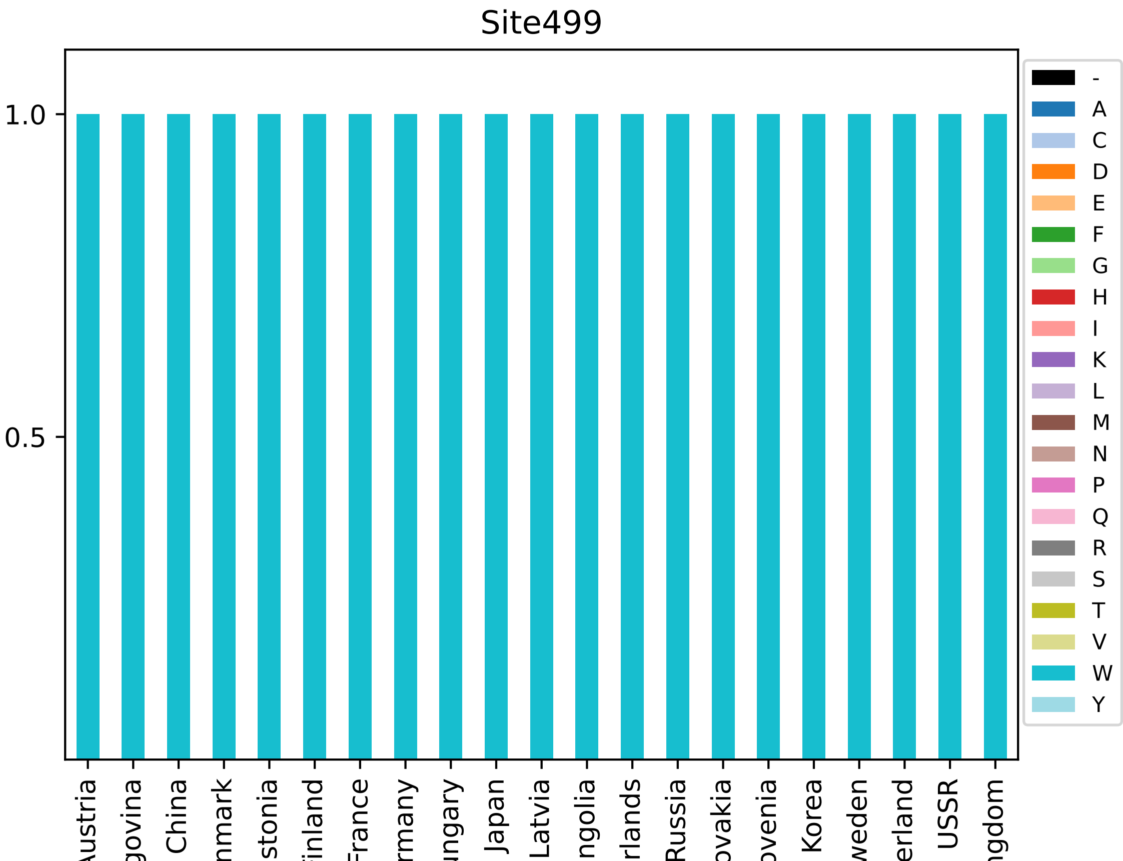

Site499
1.0
0.5
-
A
C
D
E
F
G
H
I
K
L
M
N
P
Q
R
S
T
V
W
Y
China
Japan
Latvia
Russia
Korea
USSR

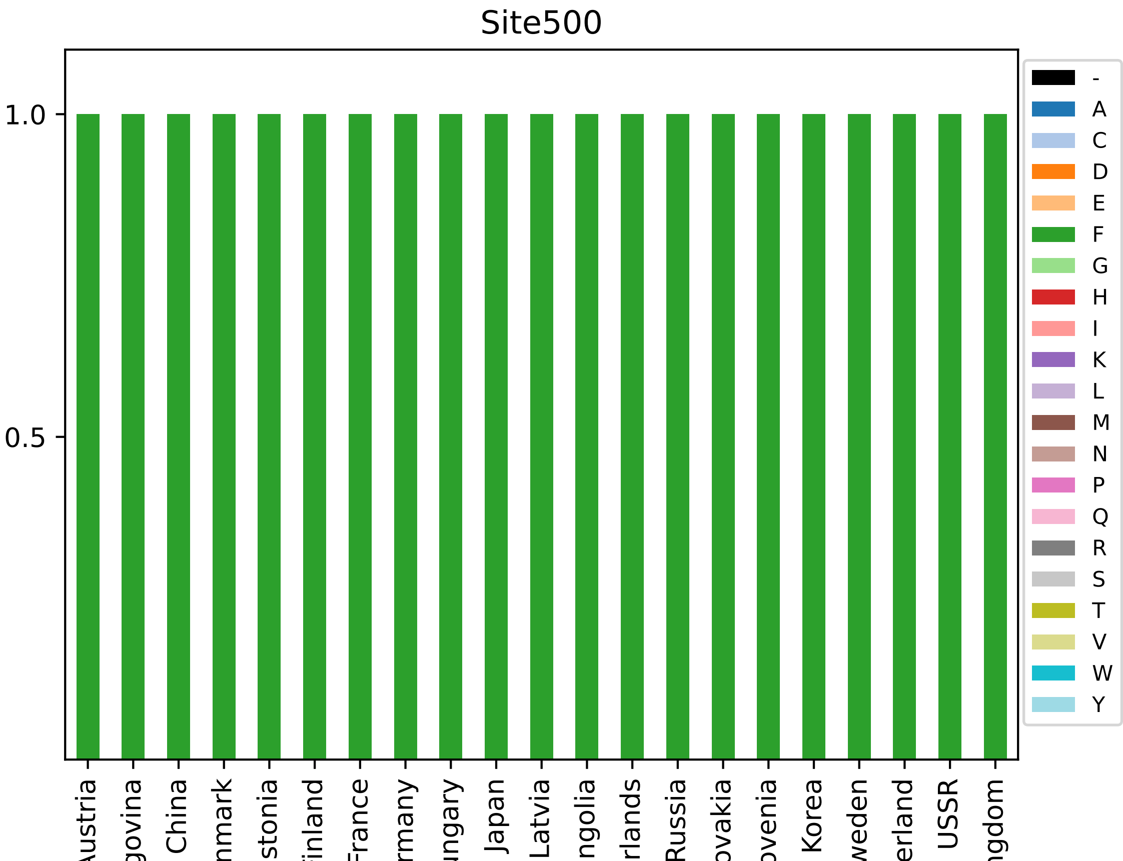
Site500
1.0
0.5
-
A
C
D
E
F
G
H
I
K
L
M
N
P
Q
R
S
T
V
W
Y
China
Japan
Latvia
Russia
Korea
USSR

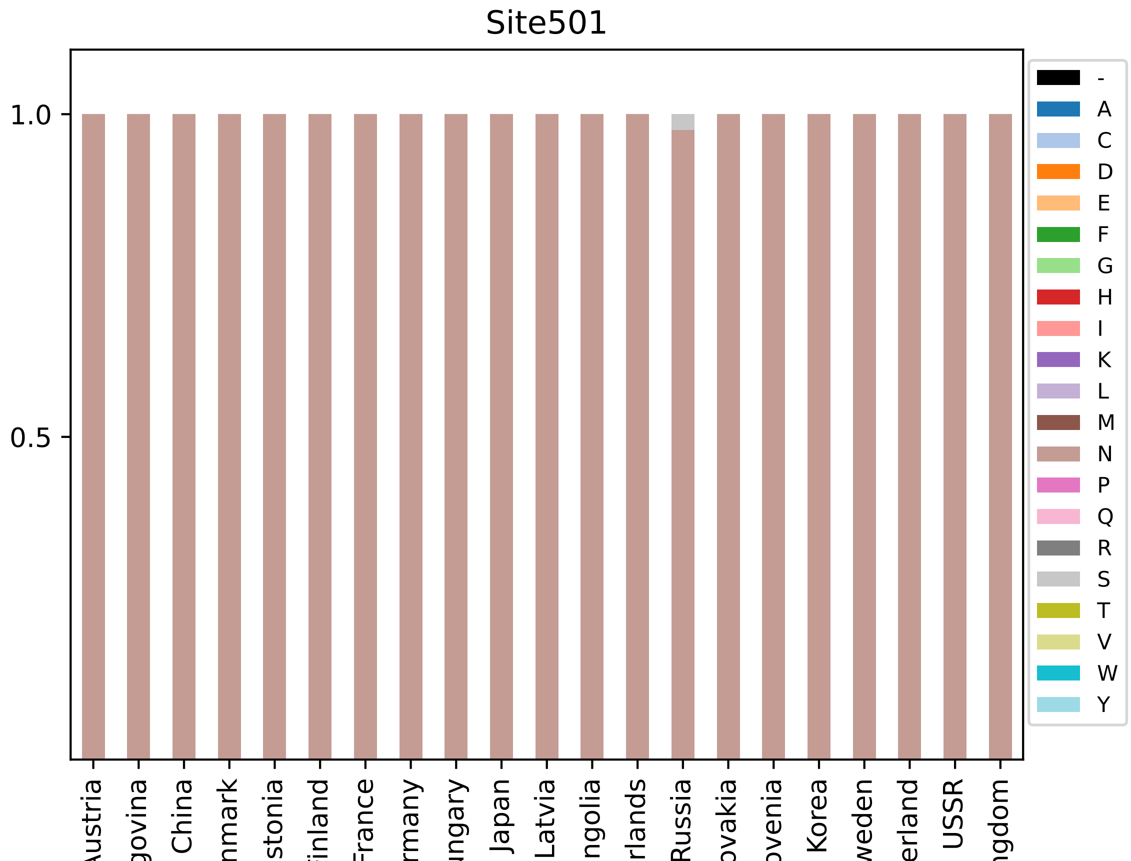
Site501
1.0
0.5
-
A
C
D
E
F
G
H
I
K
L
M
N
P
Q
R
S
T
V
W
Y
Austria
China
Japan
Latvia
Russia
Korea
USSR

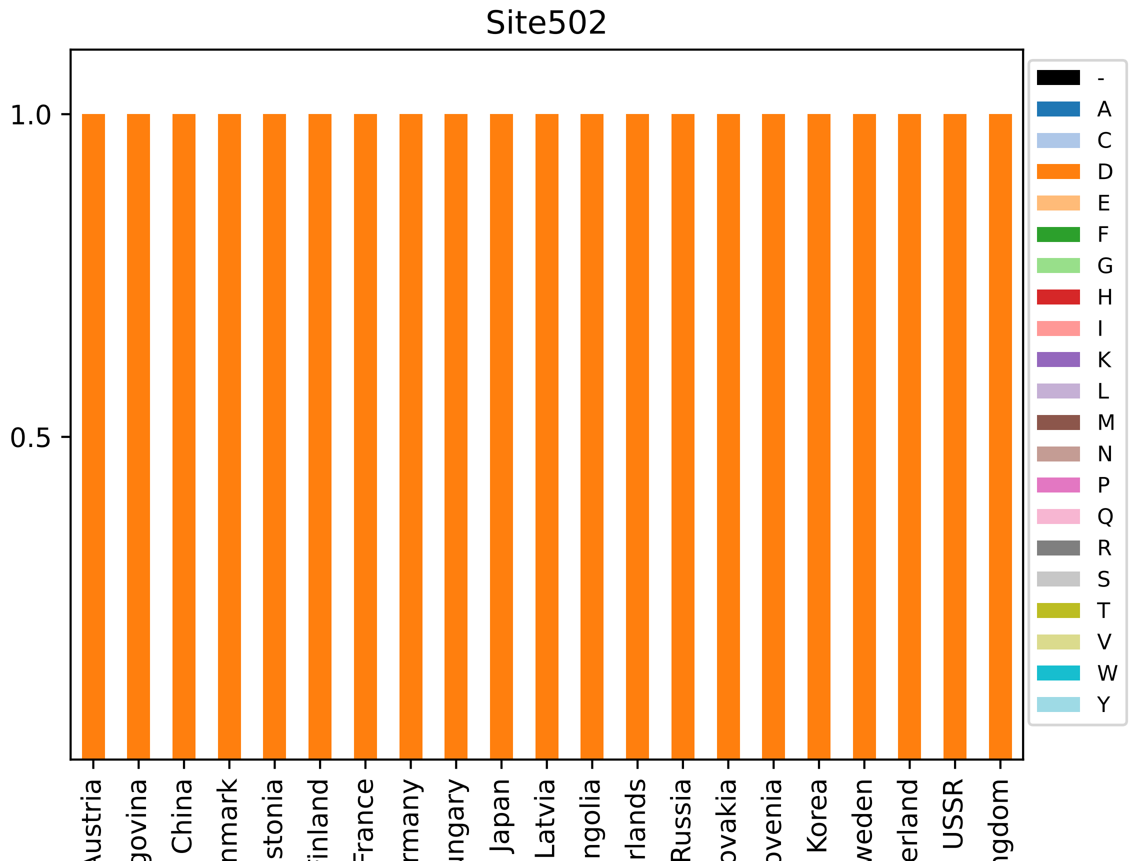

Site502
1.0
0.5
-
A
C
D
E
F
G
H
I
K
L
M
N
P
Q
R
S
T
V
W
Y
China
Japan
Latvia
Russia
Korea
USSR

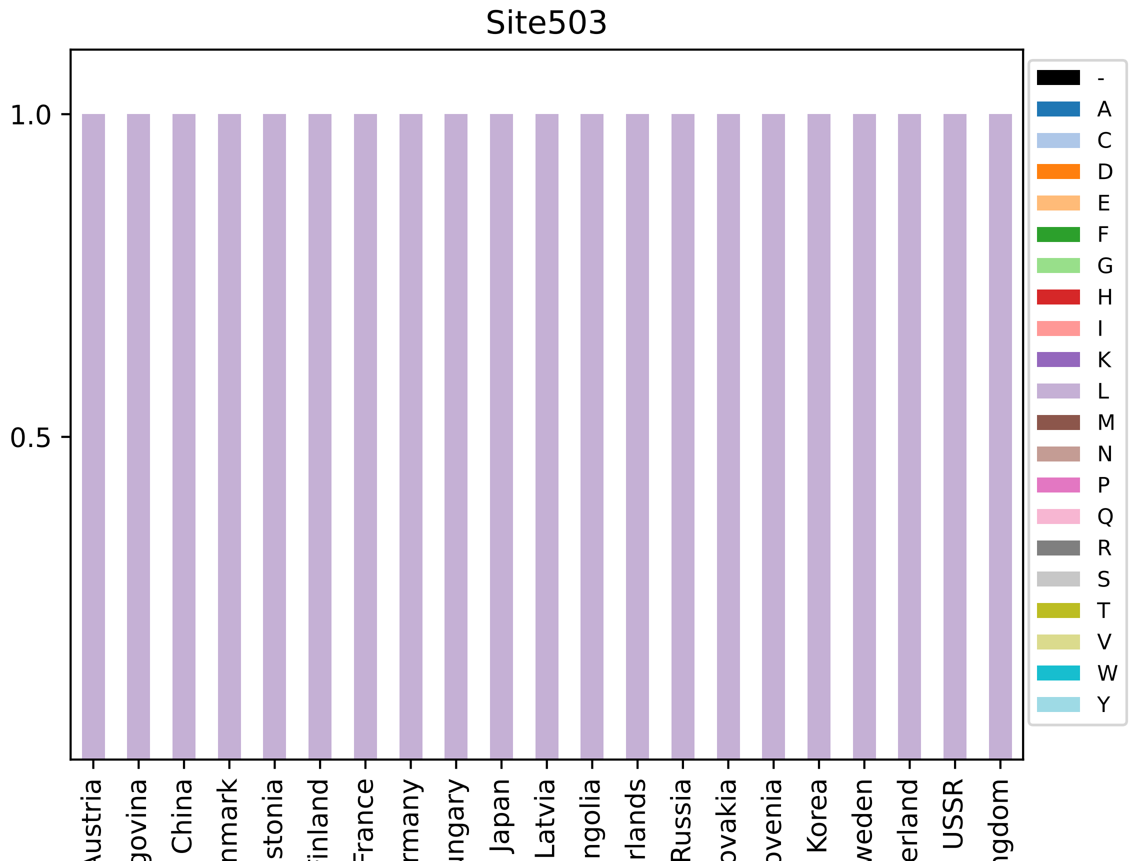

Site503
1.0
0.5
-
A
C
D
E
F
G
H
I
K
L
M
N
P
Q
R
S
T
V
W
Y
China
Japan
Latvia
Russia
Korea
USSR

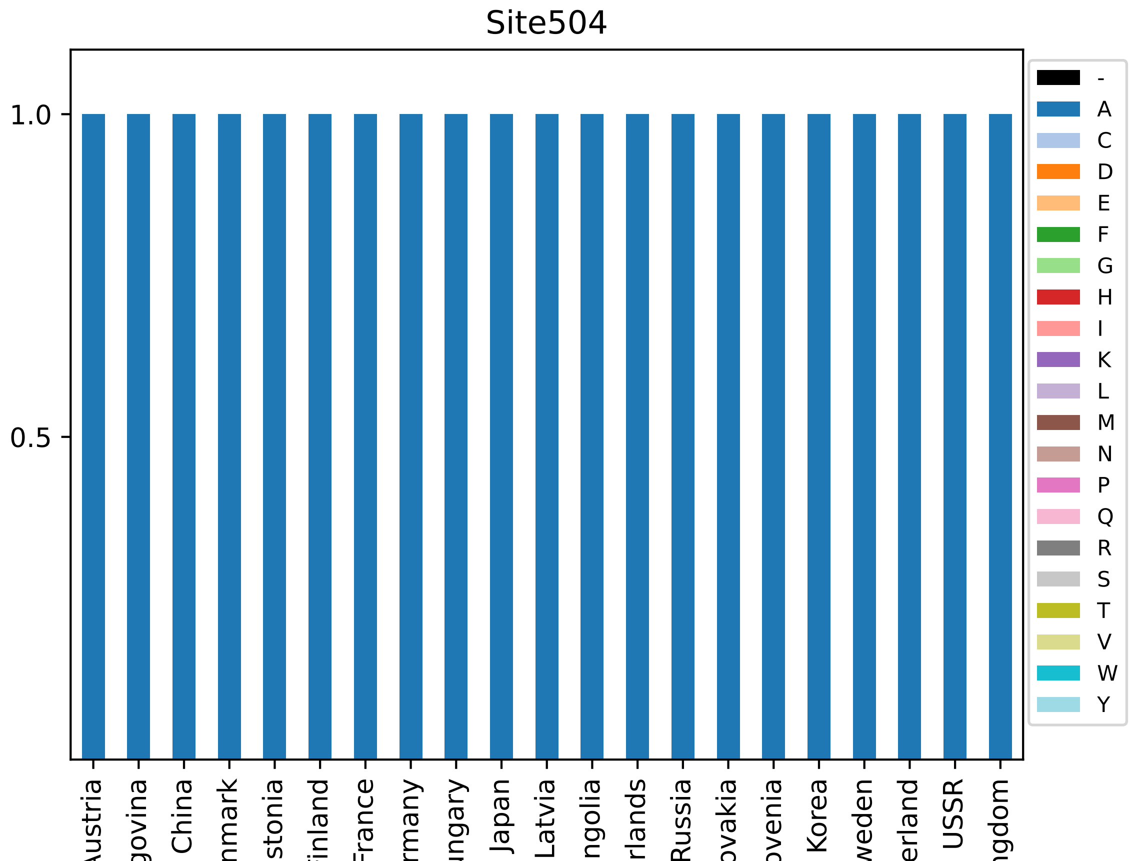
Site504
1.0
0.5
Austria
govina
China
nmark
stonia
Finland
France
rmany
ungary
Japan
Latvia
ngolia
rlands
Russia
ovakia
ovenia
Korea
weden
erland
USSR
ngdom
-
A
C
D
E
F
G
H
I
K
L
M
N
P
Q
R
S
T
V
W
Y

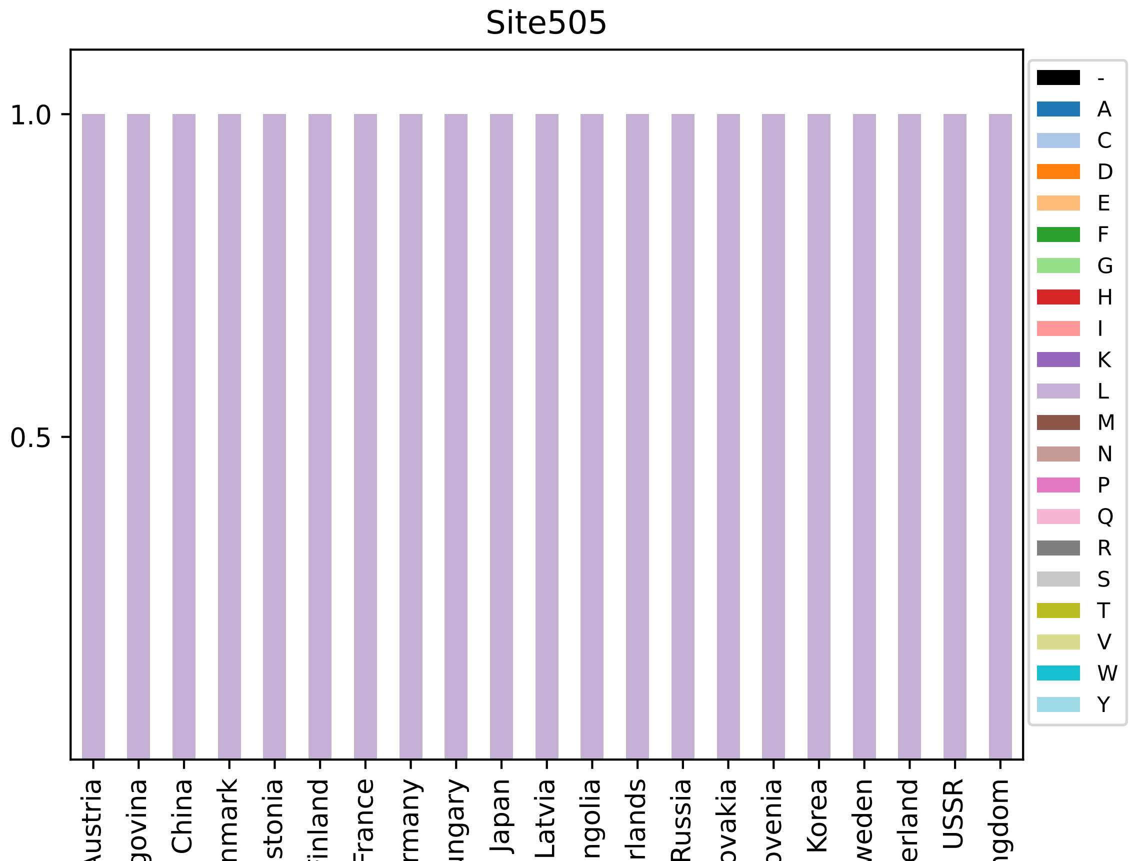

Site505
1.0
0.5
-
A
C
D
E
F
G
H
I
K
L
M
N
P
Q
R
S
T
V
W
Y
Austria
China
Finland
France
Japan
Latvia
Russia
Korea
USSR

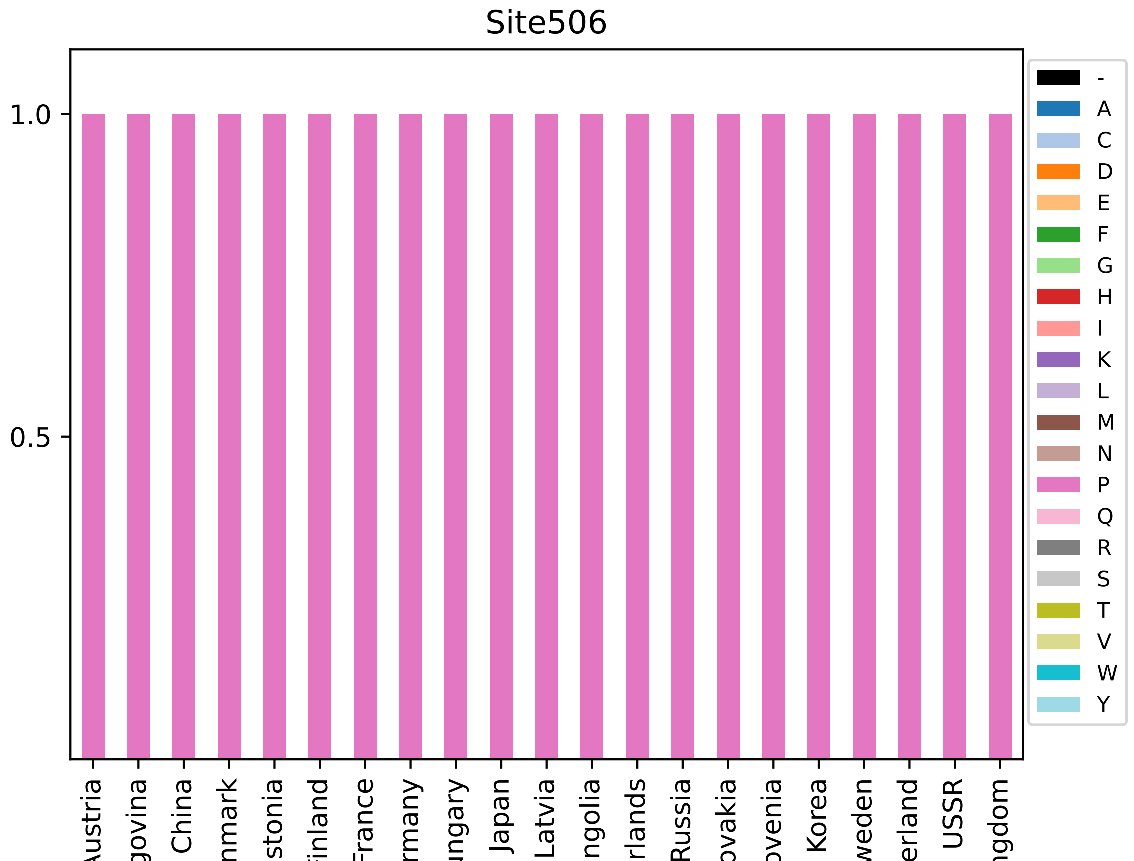

Site506
1.0
0.5
-
A
C
D
E
F
G
H
I
K
L
M
N
P
Q
R
S
T
V
W
Y
China
Japan
Latvia
Russia
Korea
USSR

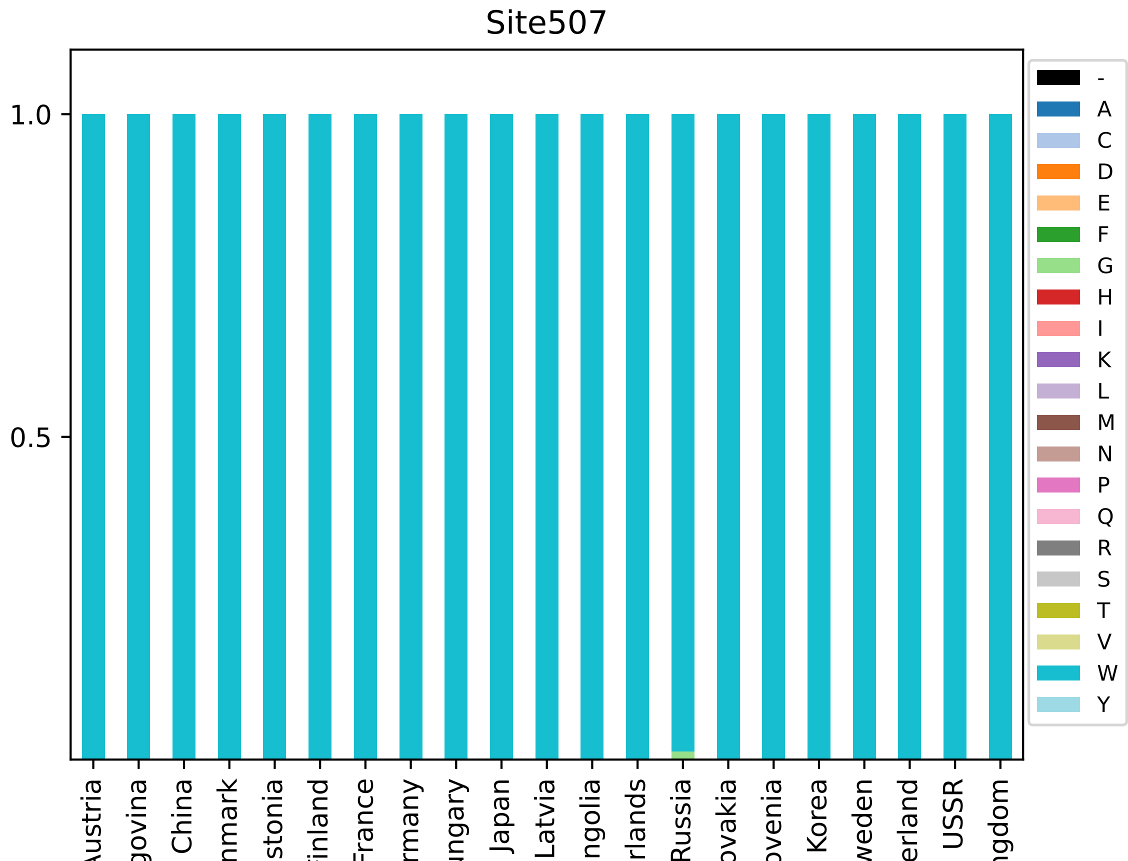
Site507
1.0
0.5
-
A
C
D
E
F
G
H
I
K
L
M
N
P
Q
R
S
T
V
W
Y
China
Japan
Latvia
Russia
Korea
USSR

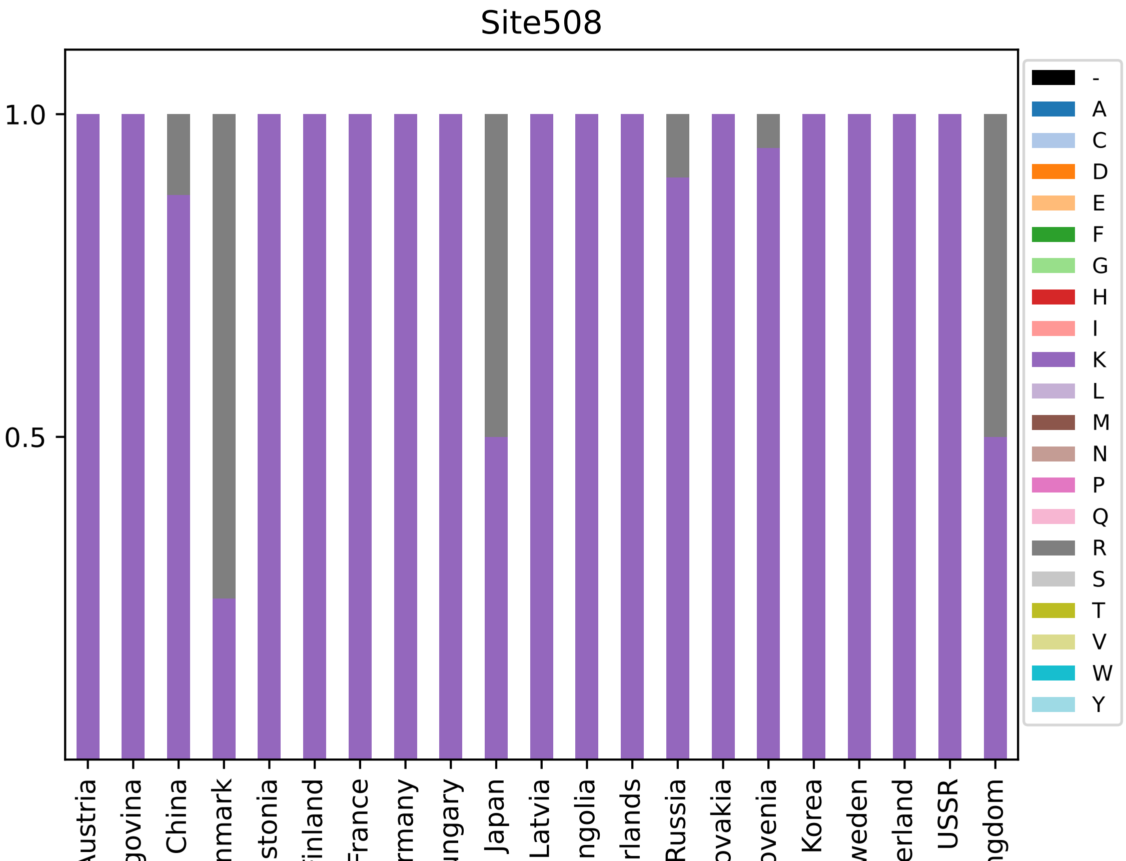

Site508
1.0
0.5
-
A
C
D
E
F
G
H
I
K
L
M
N
P
Q
R
S
T
V
W
Y
Austria
China
Japan
Latvia
Russia
Korea
USSR

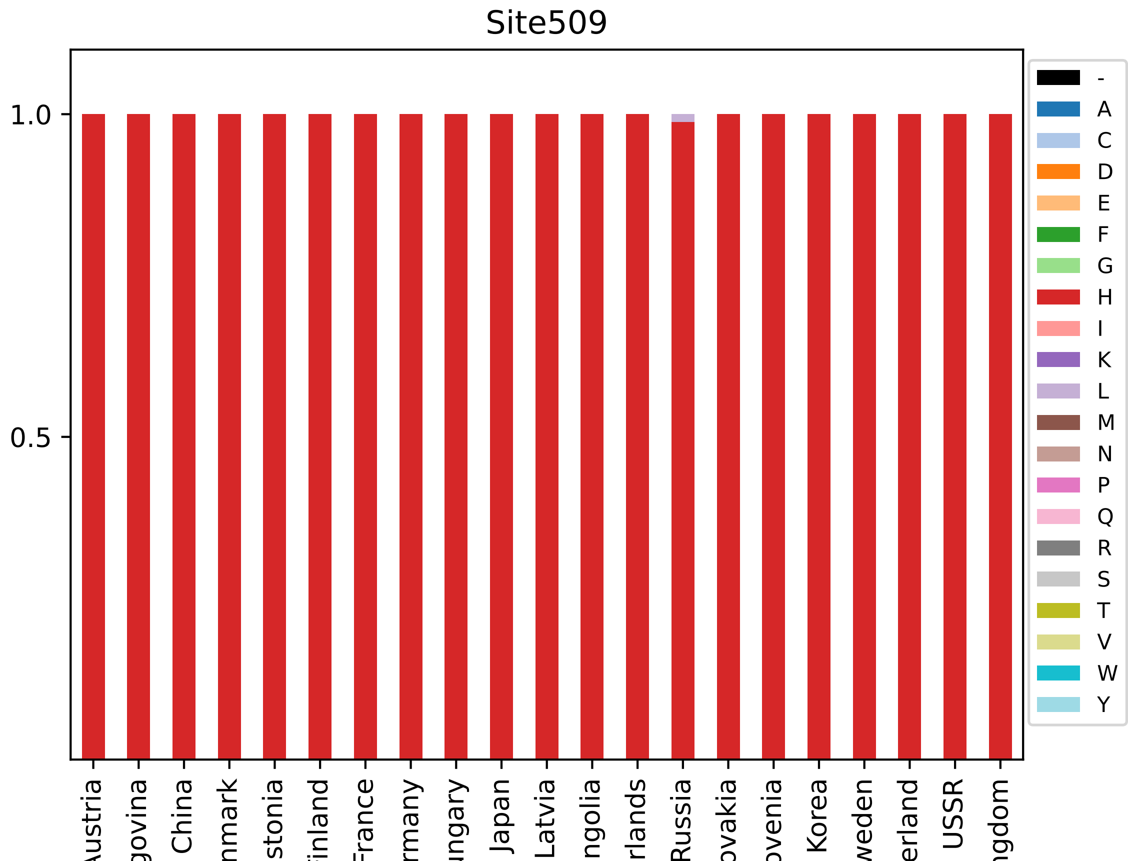
Site509
1.0
0.5
-
A
C
D
E
F
G
H
I
K
L
M
N
P
Q
R
S
T
V
W
Y
China
Japan
Latvia
Russia
Korea
USSR

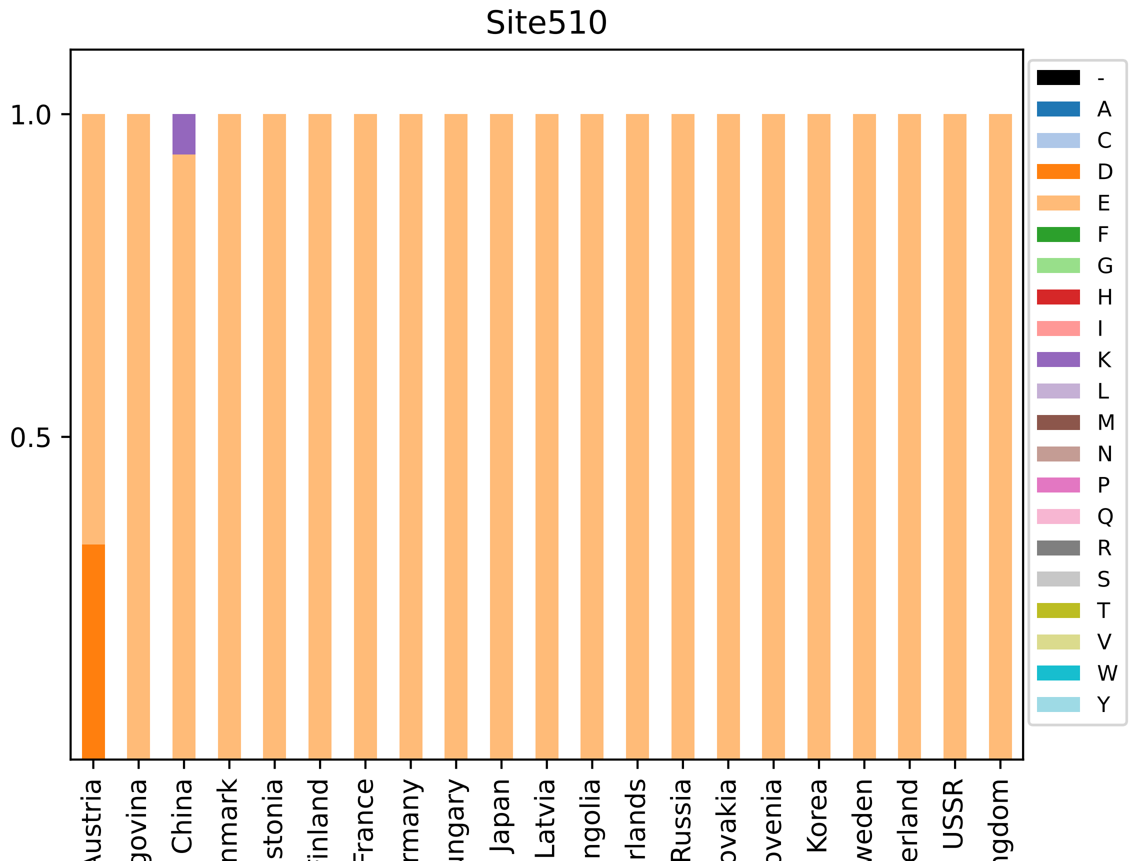
Site510
1.0
0.5
-
A
C
D
E
F
G
H
I
K
L
M
N
P
Q
R
S
T
V
W
Y
China
Japan
Latvia
Russia
Korea
USSR

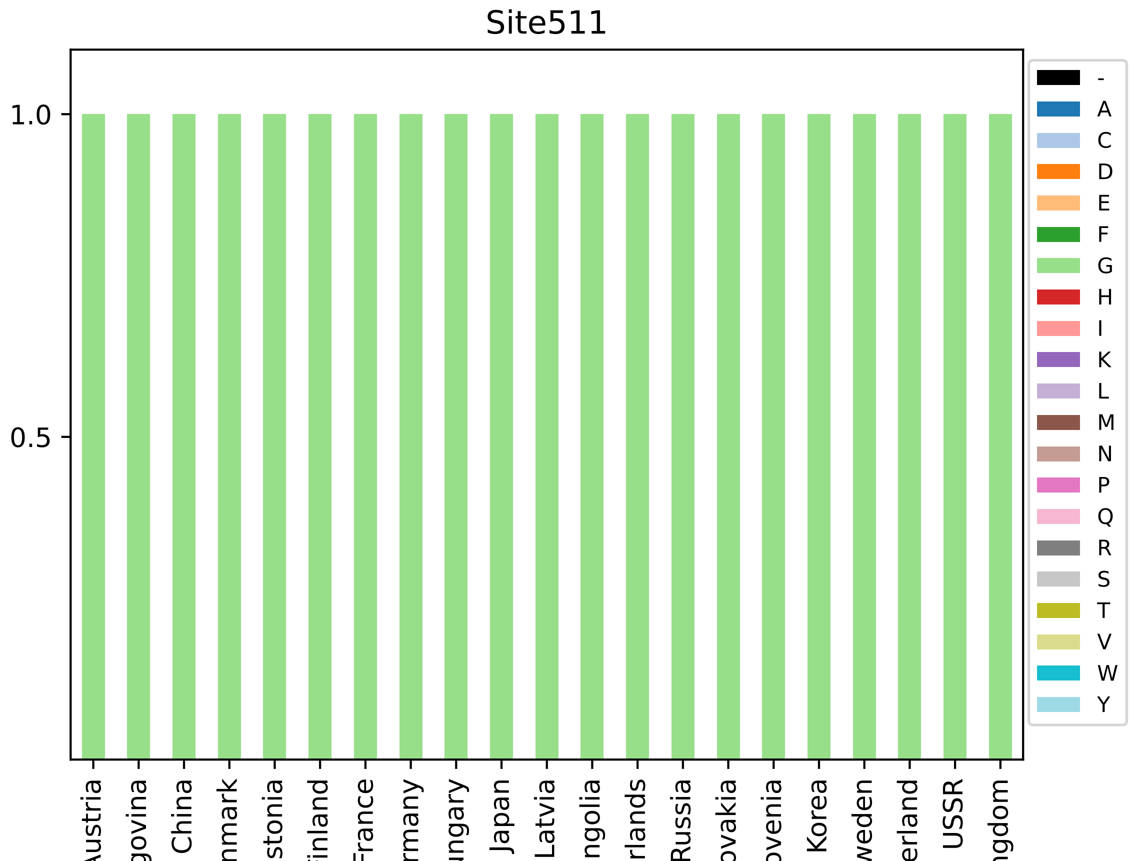
Site511
1.0
0.5
-
A
C
D
E
F
G
H
I
K
L
M
N
P
Q
R
S
T
V
W
Y
Austria
China
Japan
Latvia
Russia
Korea
USSR

Site512

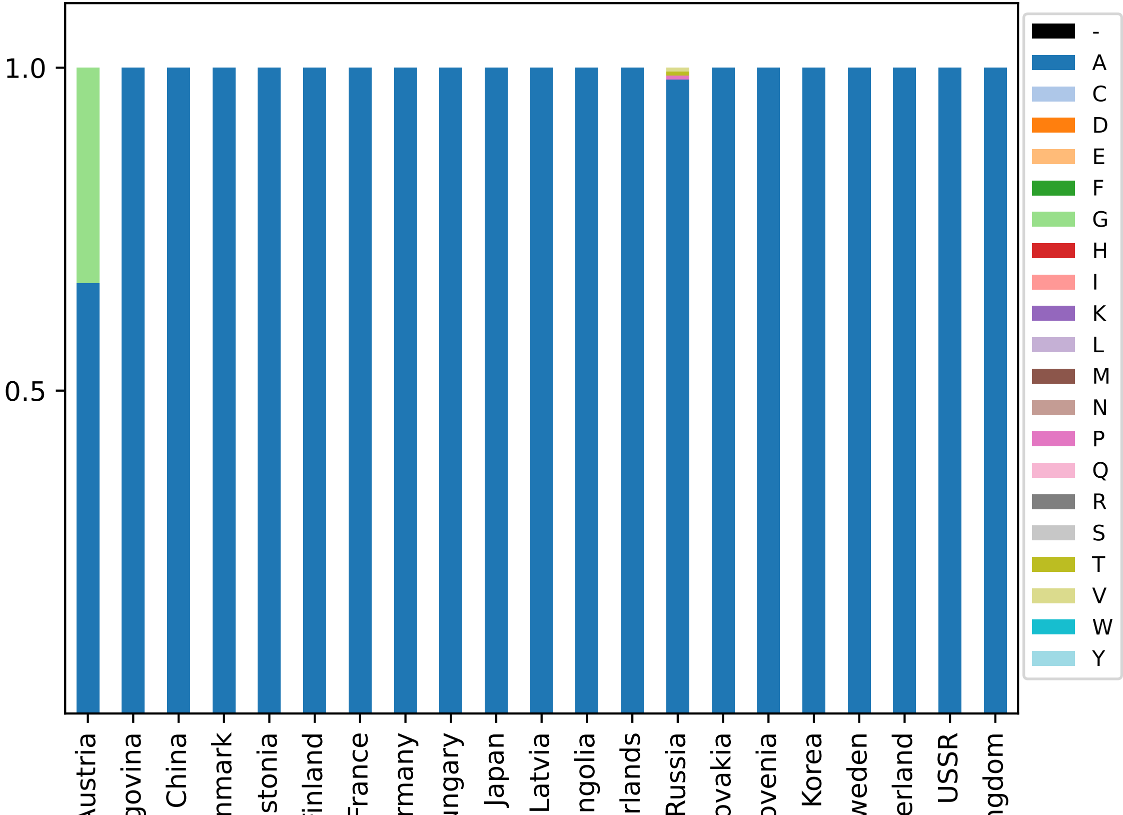

1.0
0.5
Austria
govina
China
nmark
stonia
Finland
France
rmany
ungary
Japan
Latvia
ngolia
rlands
Russia
ovakia
ovenia
Korea
weden
erland
USSR
ngdom
-
A
C
D
E
F
G
H
I
K
L
M
N
P
Q
R
S
T
V
W
Y

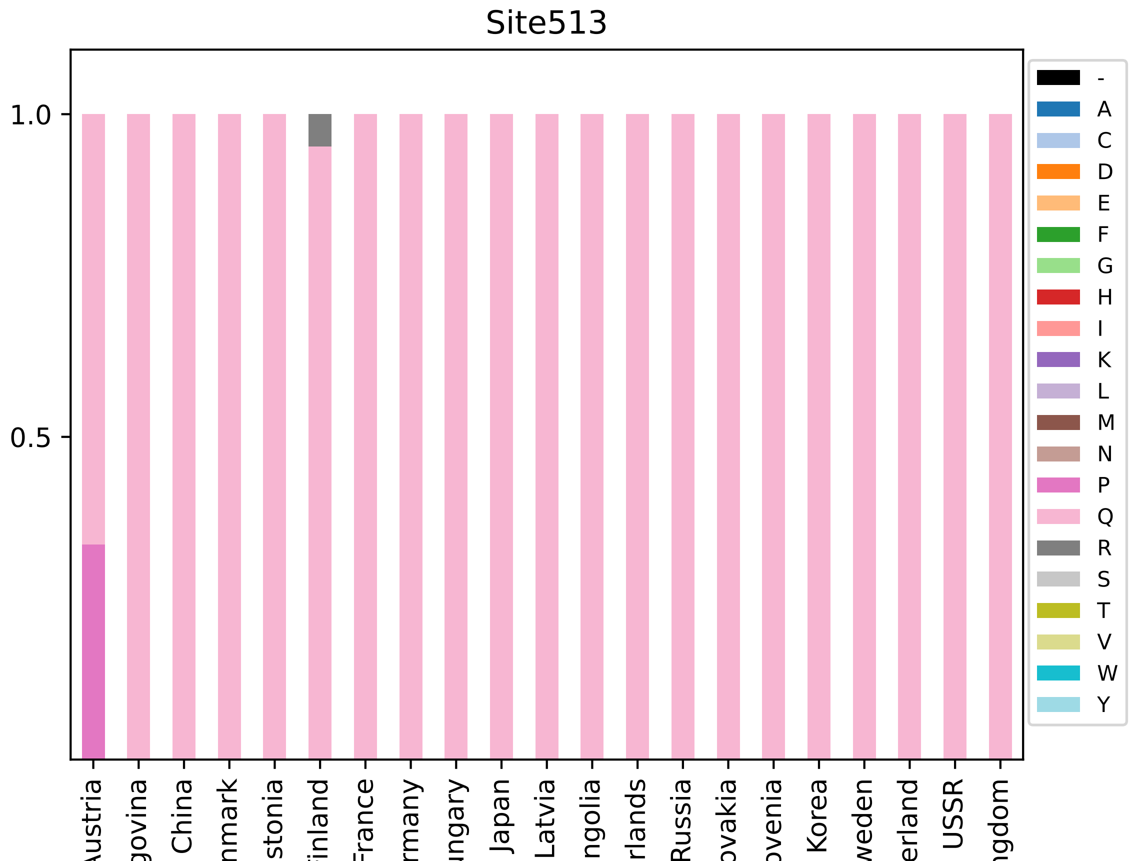
Site513
1.0
0.5
-
A
C
D
E
F
G
H
I
K
L
M
N
P
Q
R
S
T
V
W
Y
China
Japan
Latvia
Russia
Korea
USSR

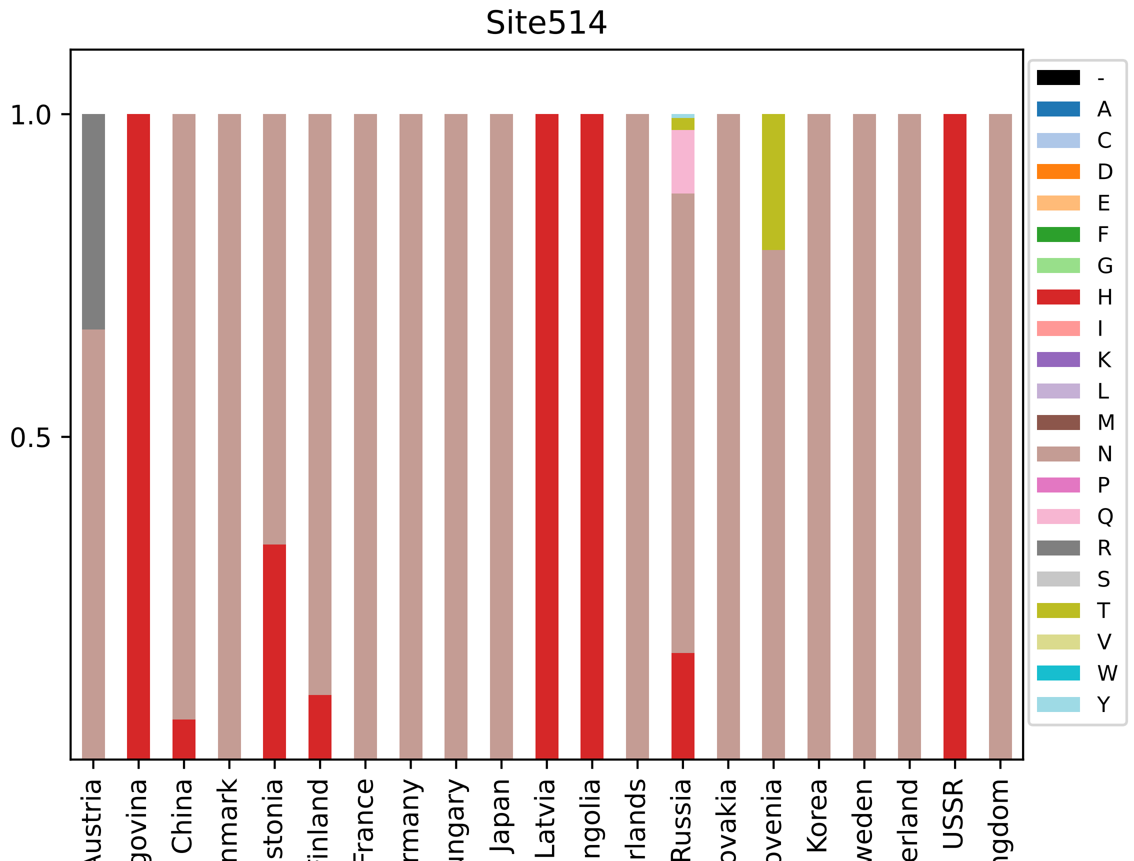
Site514
1.0
0.5
-
A
C
D
E
F
G
H
I
K
L
M
N
P
Q
R
S
T
V
W
Y
Austria
govina
China
nmark
stonia
Finland
France
rmany
ungary
Japan
Latvia
ngolia
rlands
Russia
ovakia
ovenia
Korea
weden
erland
USSR
ngdom

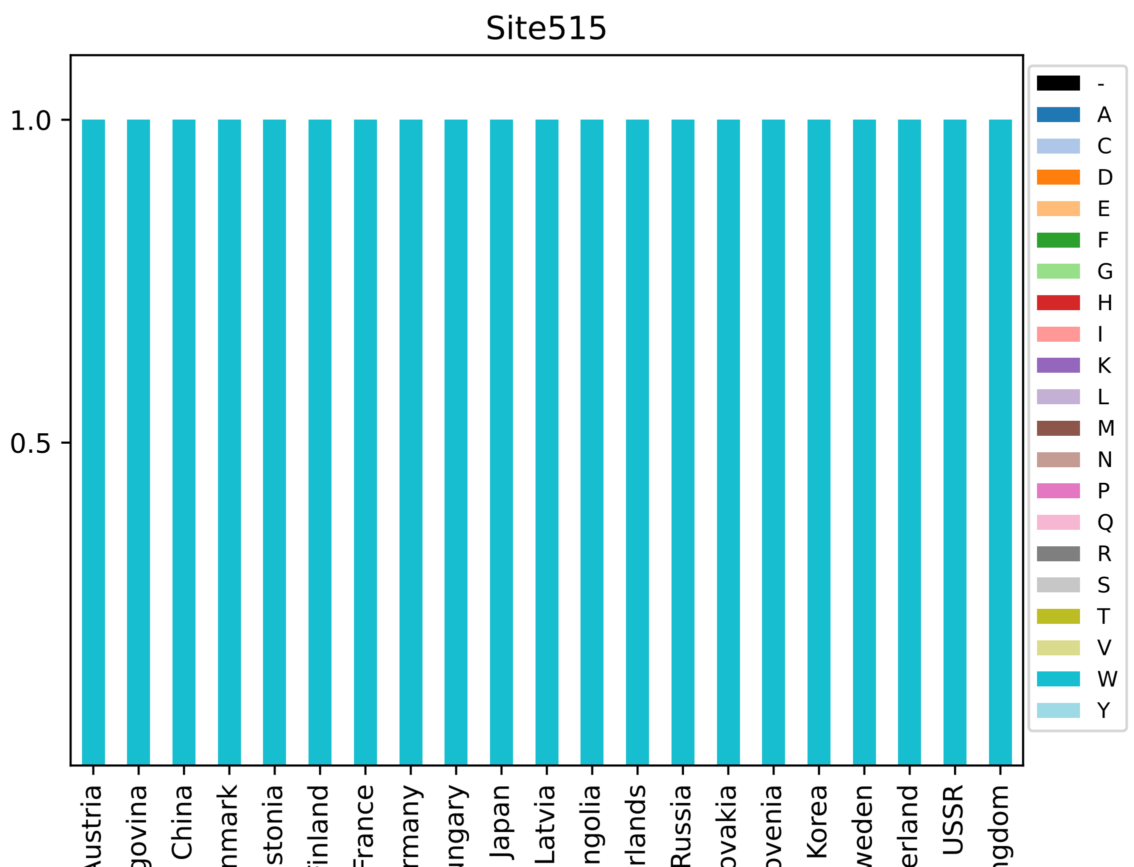

Site515
1.0
0.5
-
A
C
D
E
F
G
H
I
K
L
M
N
P
Q
R
S
T
V
W
Y
China
Japan
Latvia
Russia
Korea
USSR

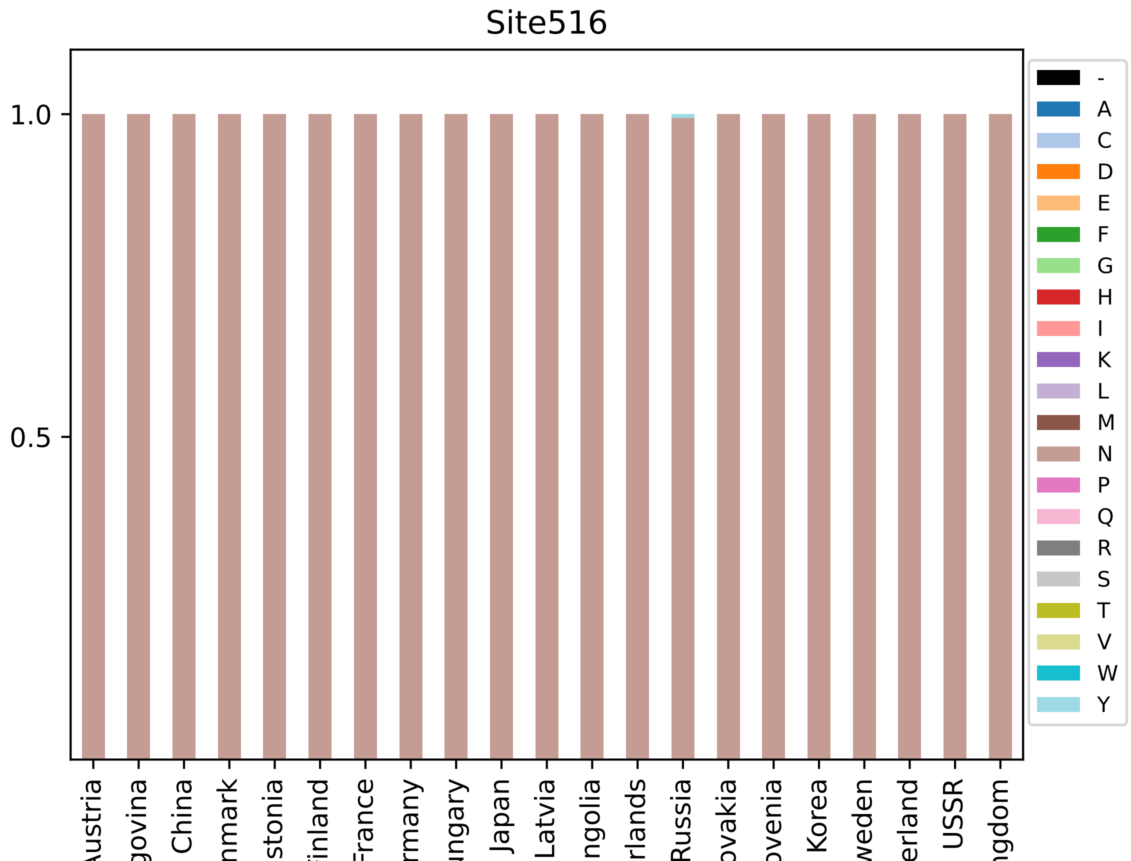
Site516
1.0
0.5
-
A
C
D
E
F
G
H
I
K
L
M
N
P
Q
R
S
T
V
W
Y
China
Japan
Latvia
Russia
Korea
USSR

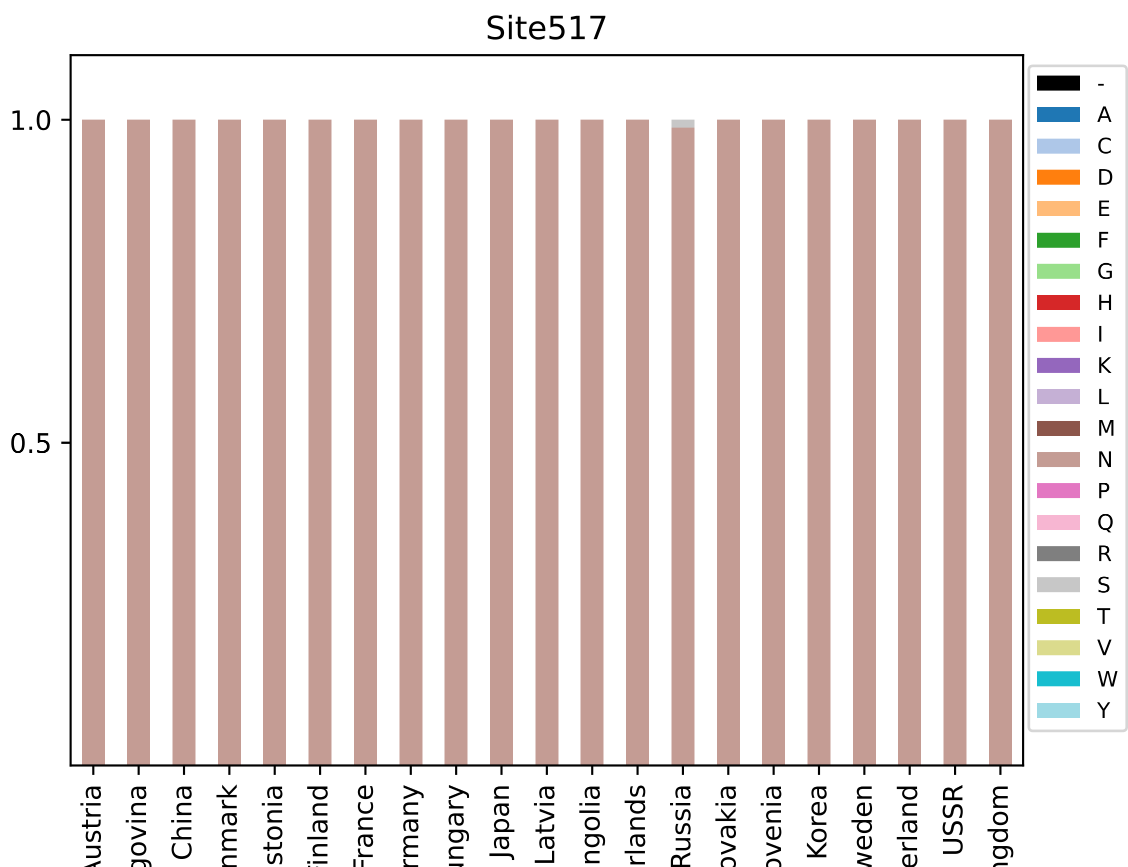

Site517
1.0
0.5
-
A
C
D
E
F
G
H
I
K
L
M
N
P
Q
R
S
T
V
W
Y
Japan
Latvia
Russia
Korea
USSR

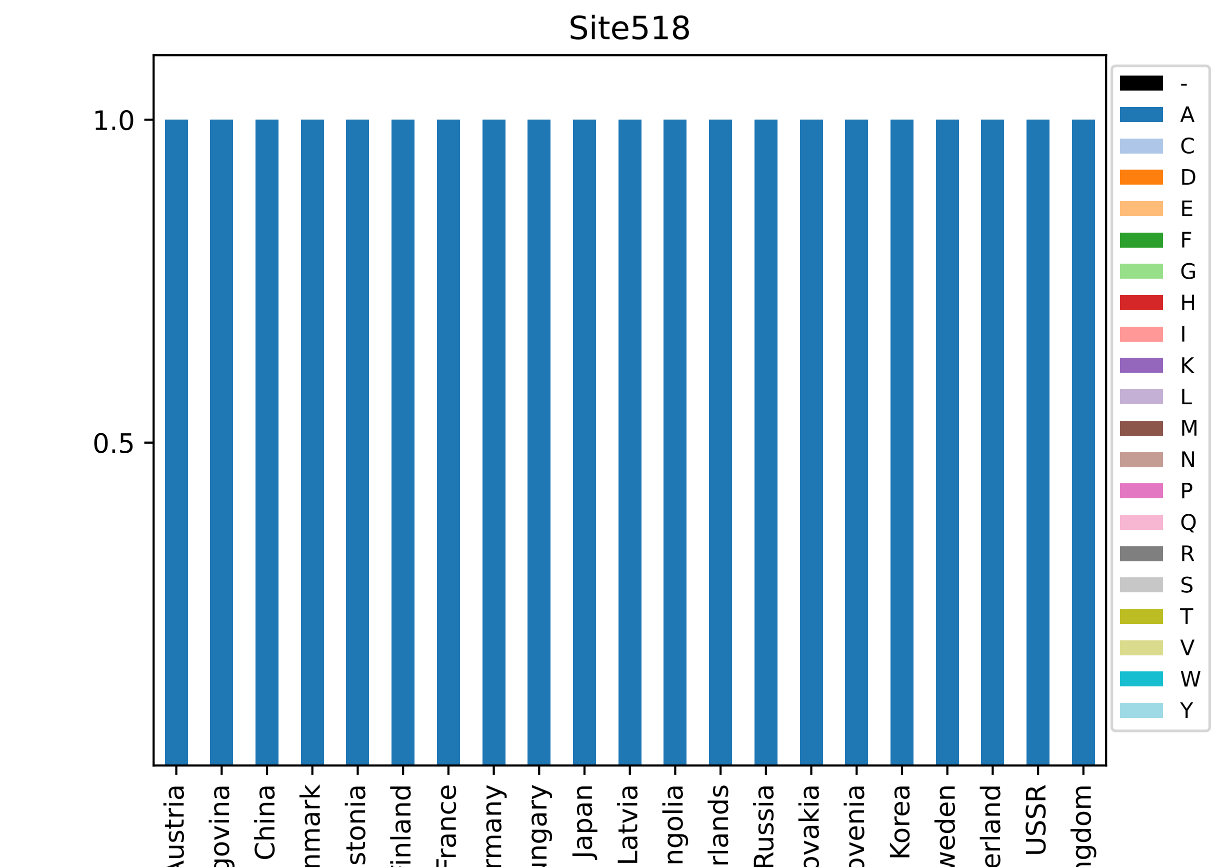

Site518
1.0
0.5
-
A
C
D
E
F
G
H
I
K
L
M
N
P
Q
R
S
T
V
W
Y
Austria
govina
China
nmark
stonia
inland
France
rmany
ungary
Japan
Latvia
ngolia
rlands
Russia
ovakia
ovenia
Korea
weden
erland
USSR
ngdom

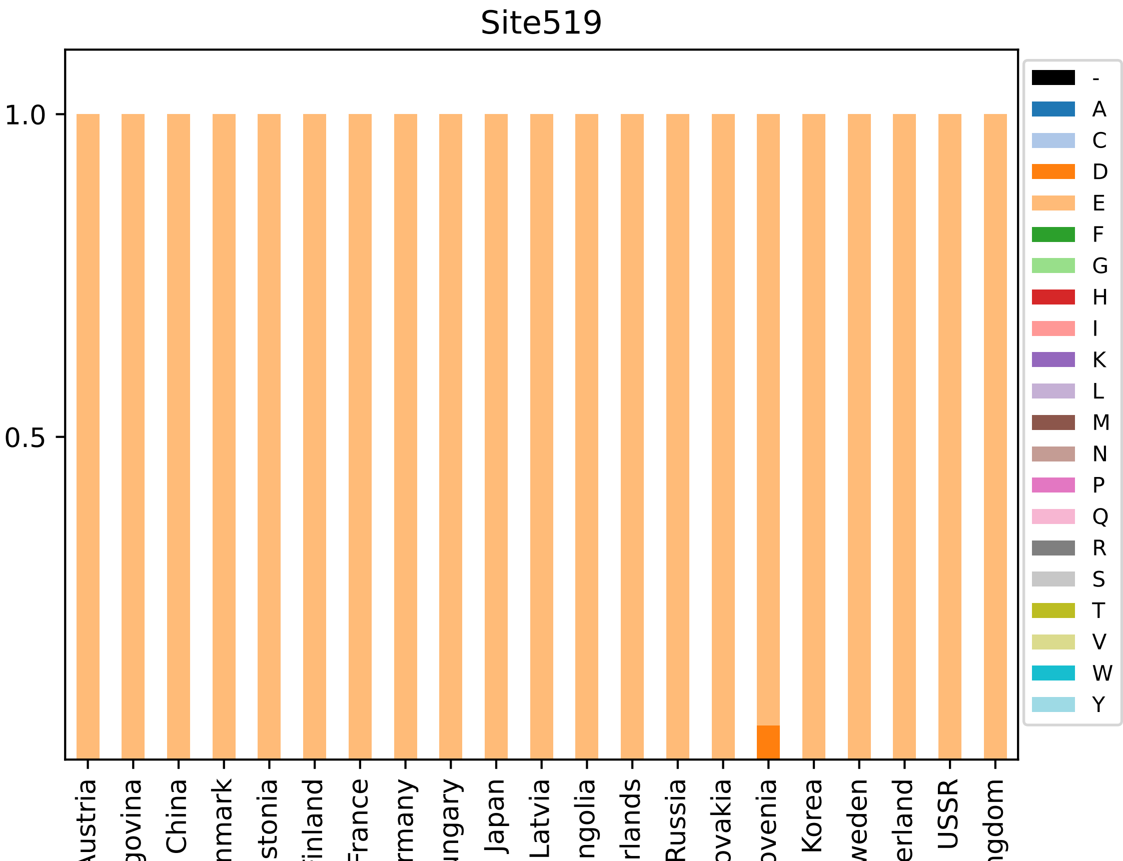

Site519
1.0
0.5
-
A
C
D
E
F
G
H
I
K
L
M
N
P
Q
R
S
T
V
W
Y
China
Japan
Latvia
Russia
Korea
USSR

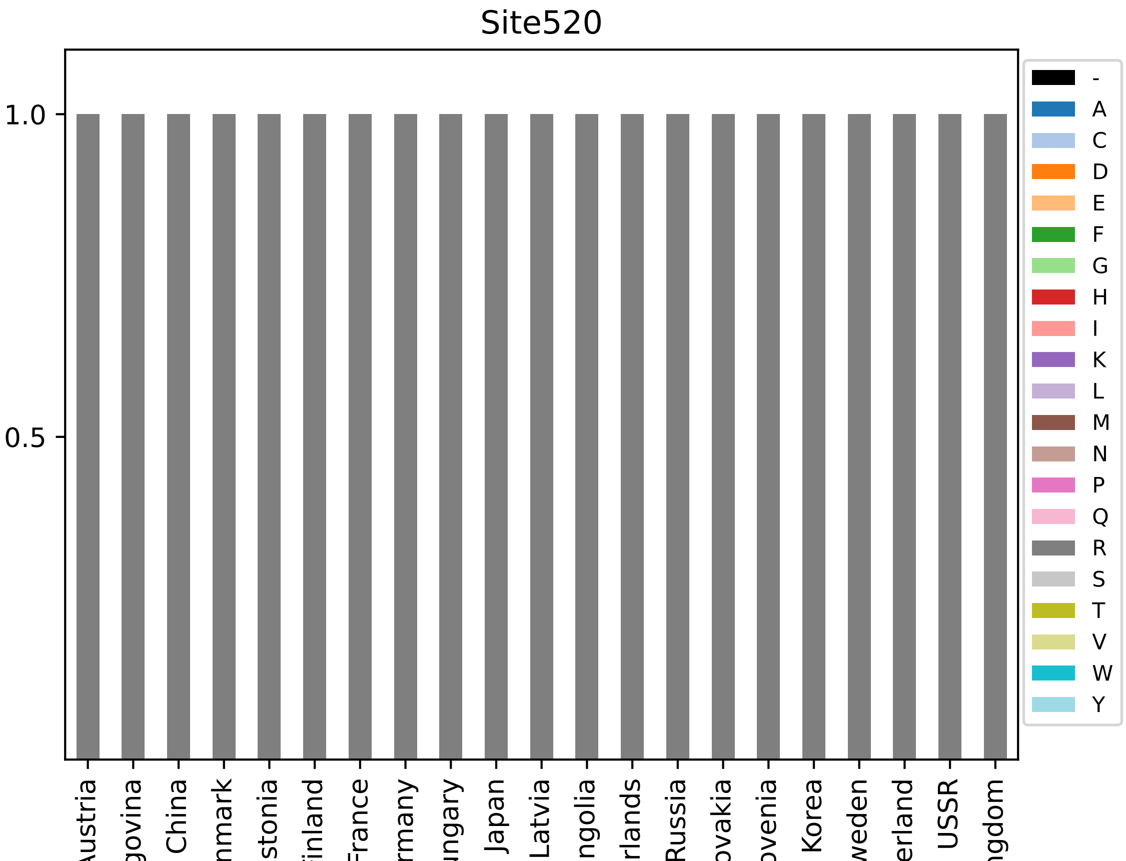
Site520
1.0
0.5
-
A
C
D
E
F
G
H
I
K
L
M
N
P
Q
R
S
T
V
W
Y
China
Japan
Latvia
Russia
Korea
USSR

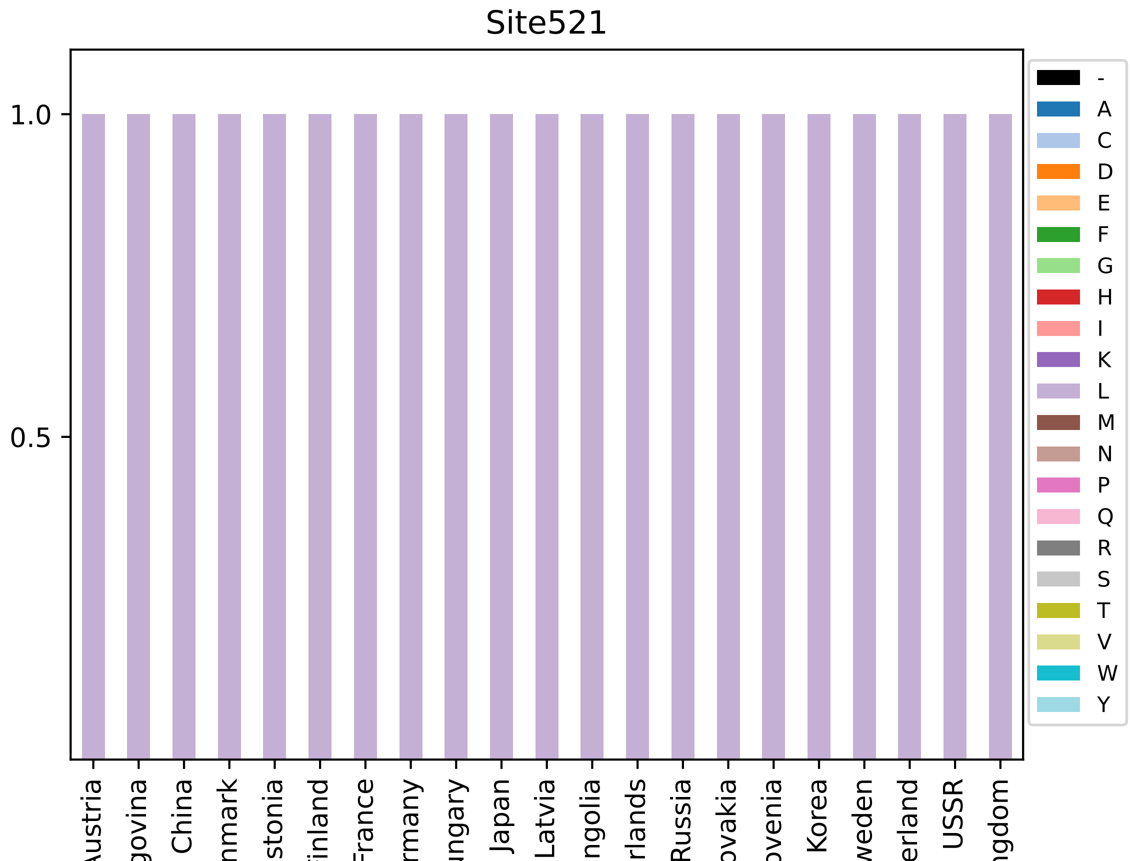

Site521
1.0
0.5
-
A
C
D
E
F
G
H
I
K
L
M
N
P
Q
R
S
T
V
W
Y
China
Japan
Latvia
Russia
Korea
USSR

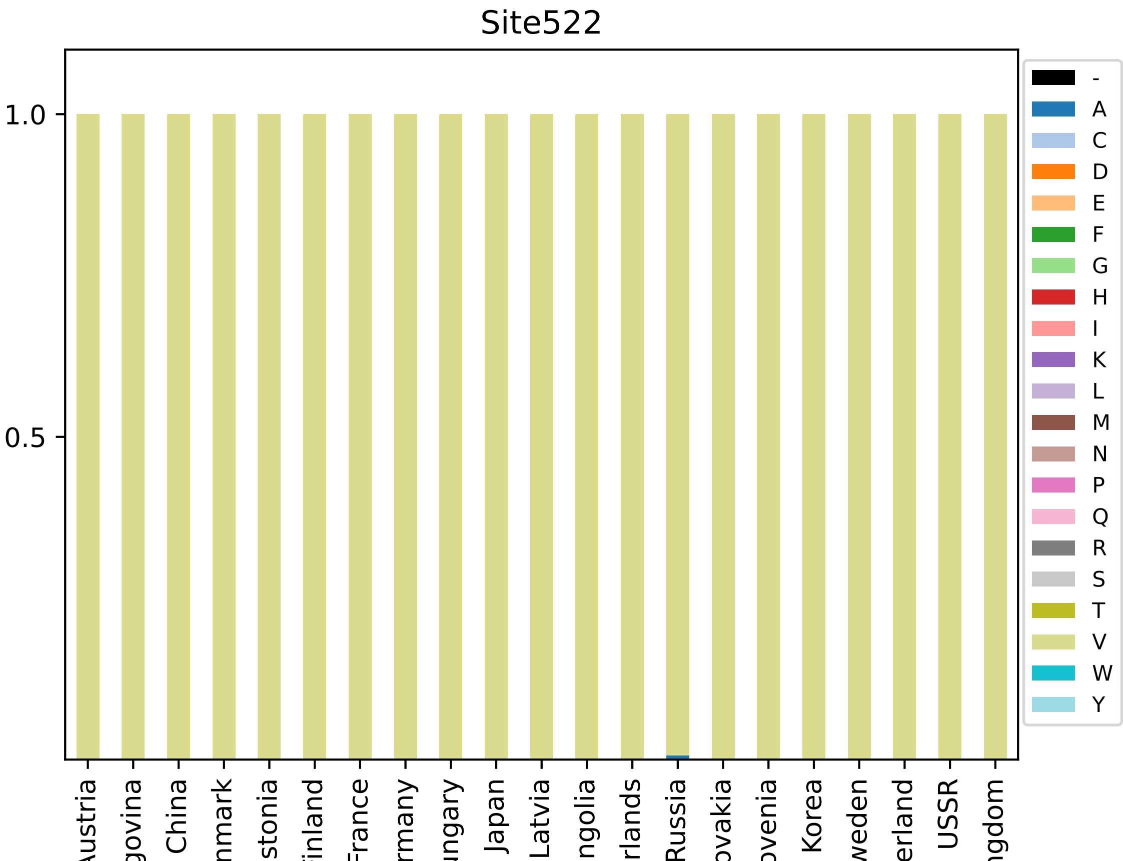

Site522
1.0
0.5
-
A
C
D
E
F
G
H
I
K
L
M
N
P
Q
R
S
T
V
W
Y
Austria
China
Japan
Latvia
Russia
Korea
USSR

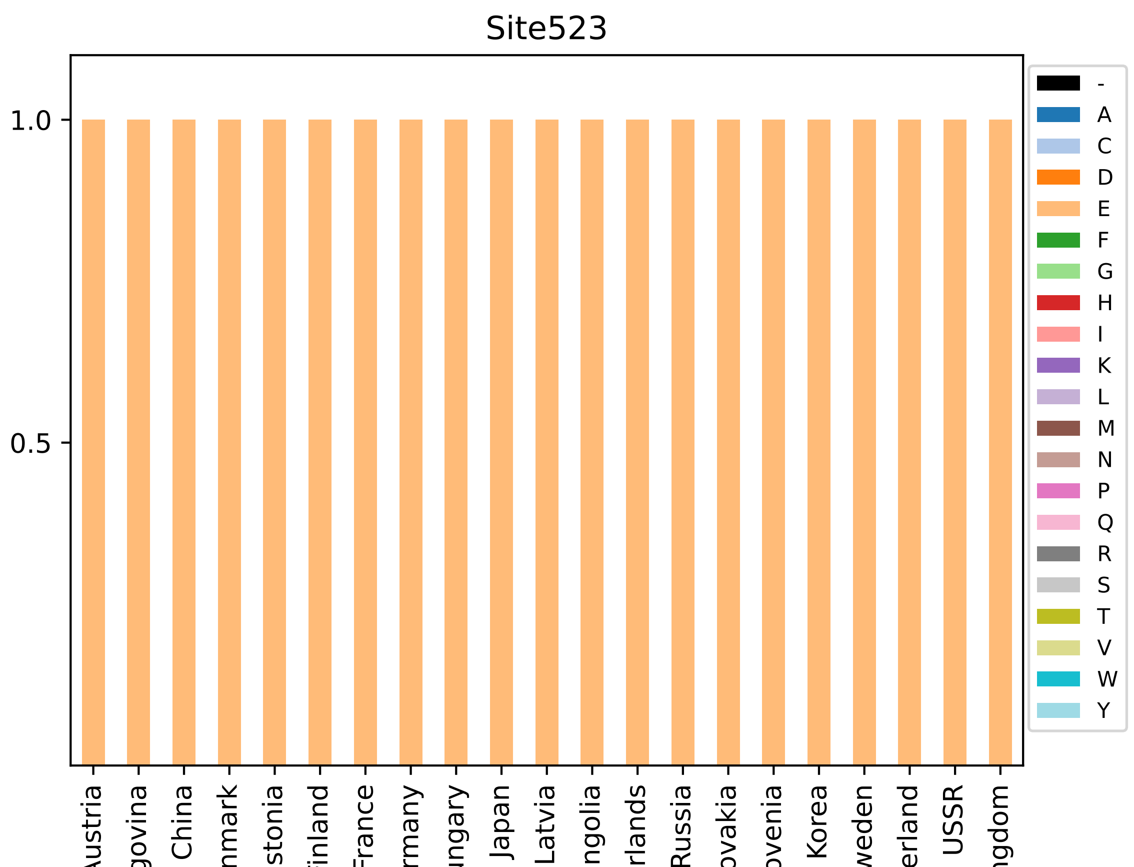

Site523
1.0
0.5
-
A
C
D
E
F
G
H
I
K
L
M
N
P
Q
R
S
T
V
W
Y
Austria
China
Japan
Latvia
Russia
Korea
USSR

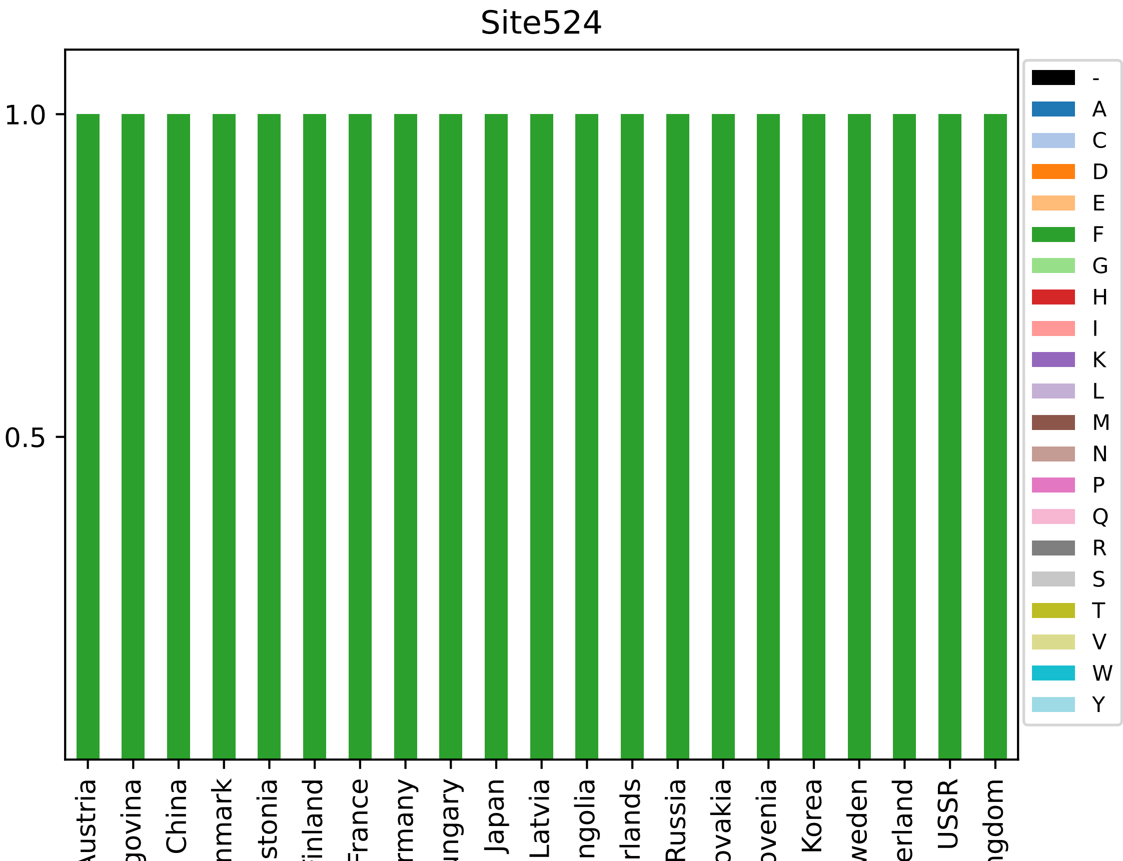
Site524
1.0
0.5
-
A
C
D
E
F
G
H
I
K
L
M
N
P
Q
R
S
T
V
W
Y
Austria
China
Japan
Latvia
Russia
Korea
USSR

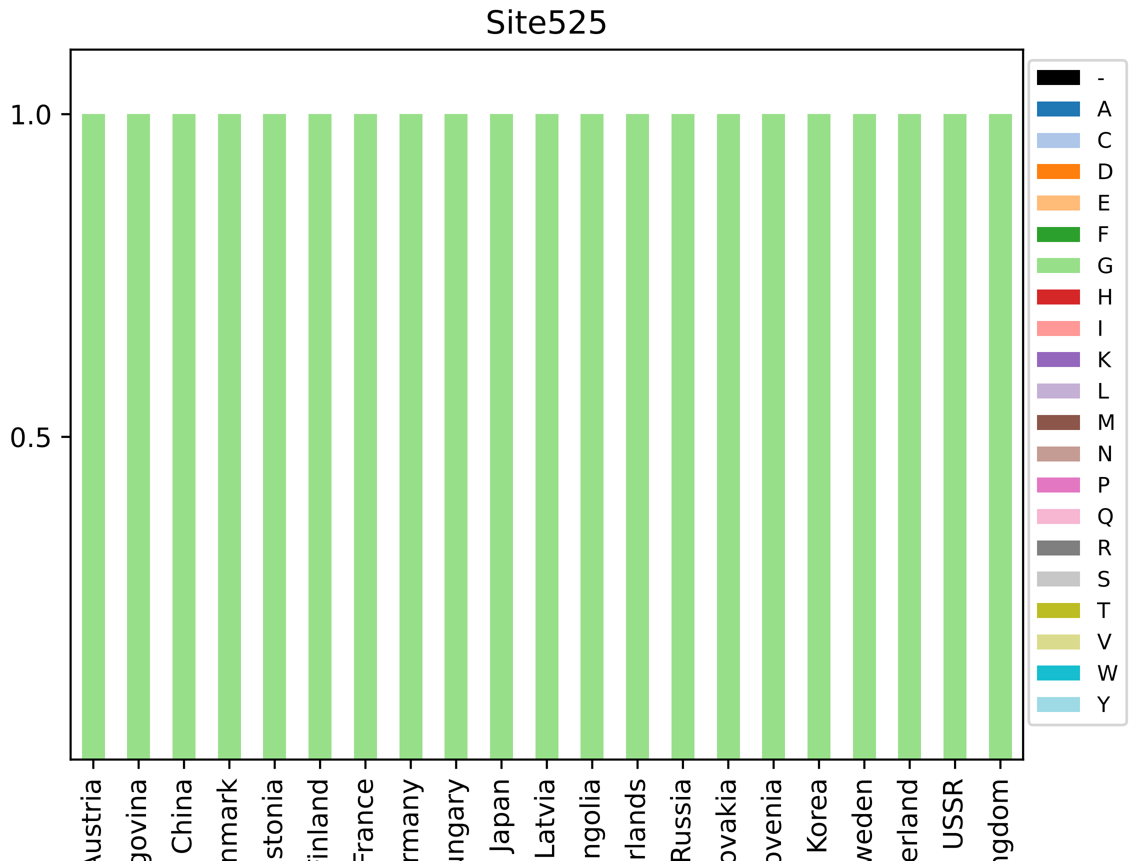

Site525
1.0
0.5
-
A
C
D
E
F
G
H
I
K
L
M
N
P
Q
R
S
T
V
W
Y
China
Japan
Latvia
Russia
Korea
USSR

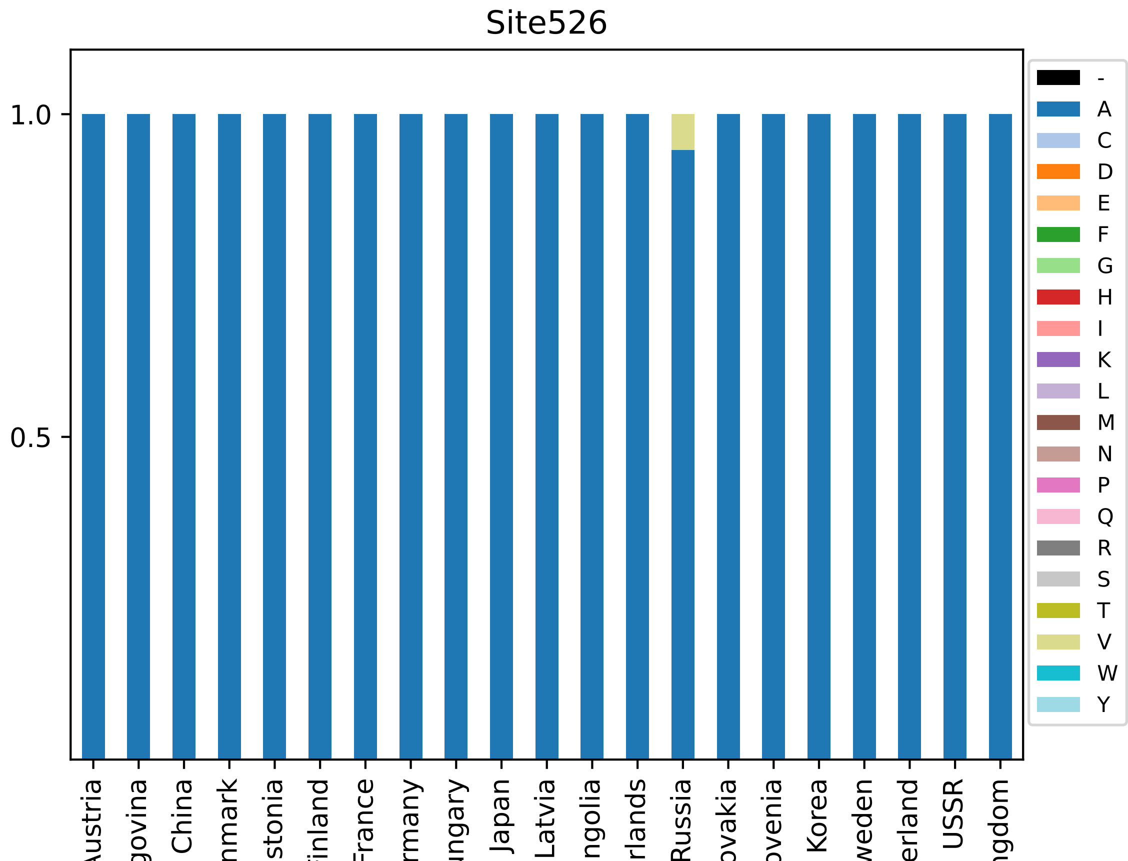
Site526
1.0
0.5
-
A
C
D
E
F
G
H
I
K
L
M
N
P
Q
R
S
T
V
W
Y
Austria
govina
China
nmark
stonia
Finland
France
rmany
ungary
Japan
Latvia
ngolia
rlands
Russia
ovakia
ovenia
Korea
weden
erland
USSR
ngdom

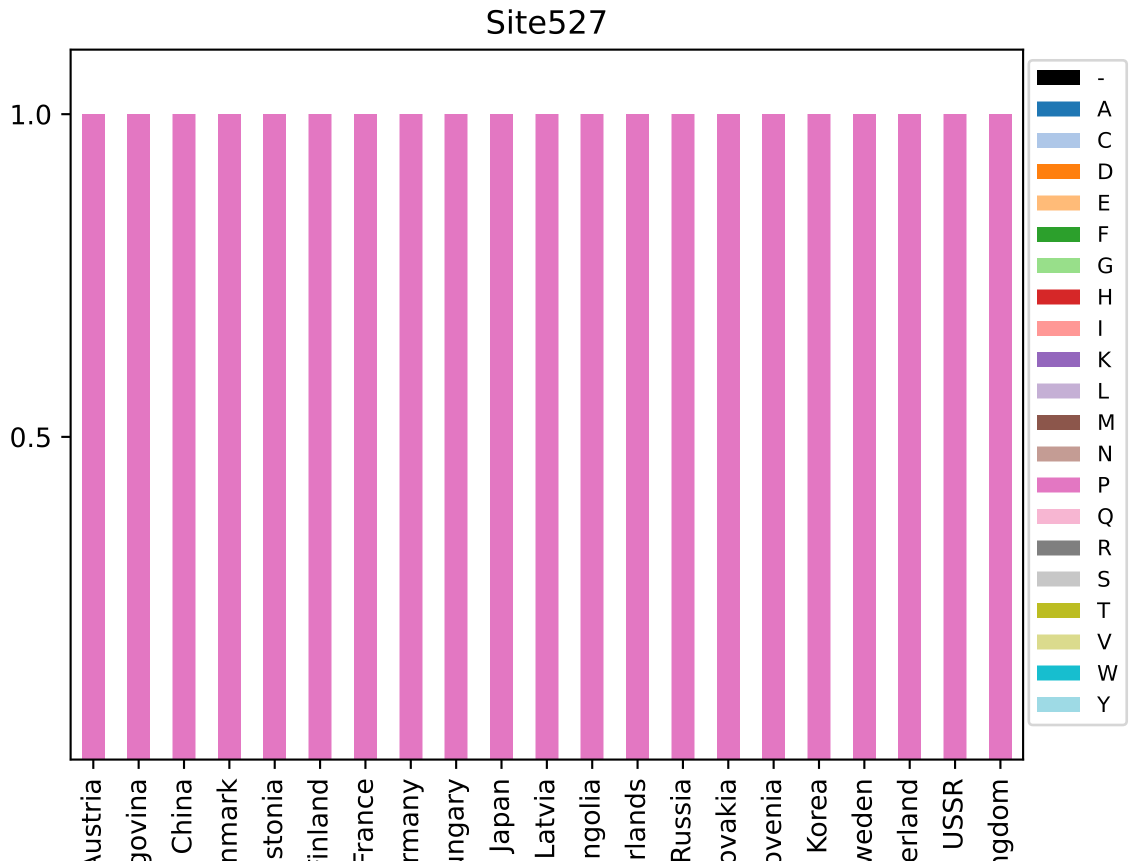

Site527
1.0
0.5
Austria
govina
China
nmark
stonia
Finland
France
rmany
ungary
Japan
Latvia
ngolia
rlands
Russia
ovakia
ovenia
Korea
weden
erland
USSR
ngdom
-
A
C
D
E
F
G
H
I
K
L
M
N
P
Q
R
S
T
V
W
Y

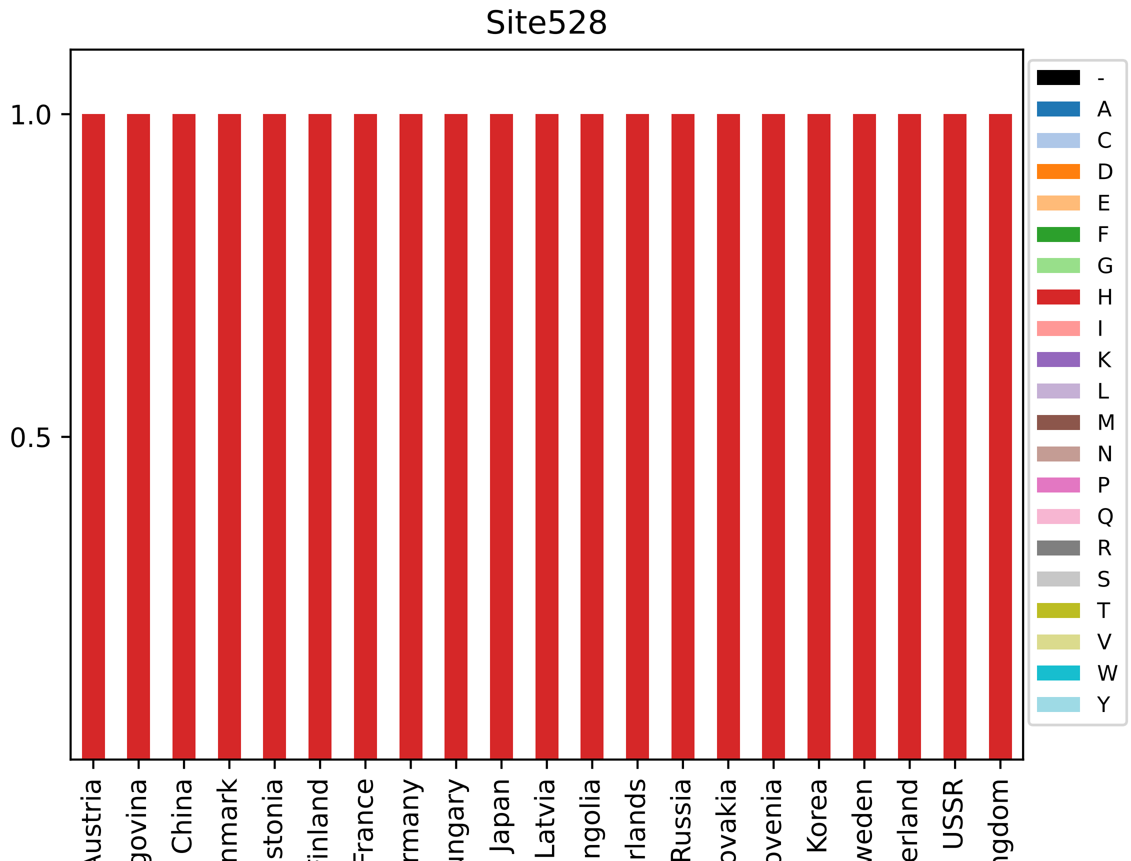

Site528
1.0
0.5
-
A
C
D
E
F
G
H
I
K
L
M
N
P
Q
R
S
T
V
W
Y
China
Japan
Latvia
Russia
Korea
USSR

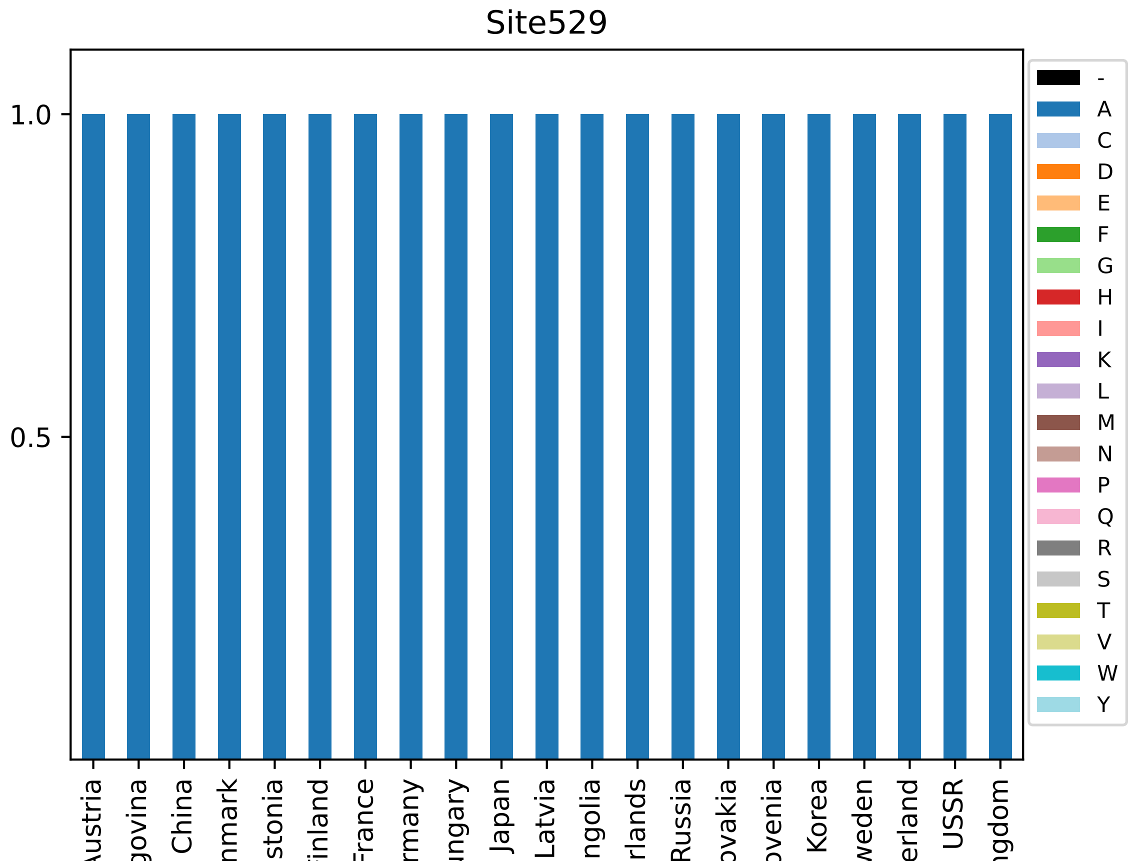
Site529
1.0
0.5
-
A
C
D
E
F
G
H
I
K
L
M
N
P
Q
R
S
T
V
W
Y
China
Japan
Latvia
Russia
Korea
USSR

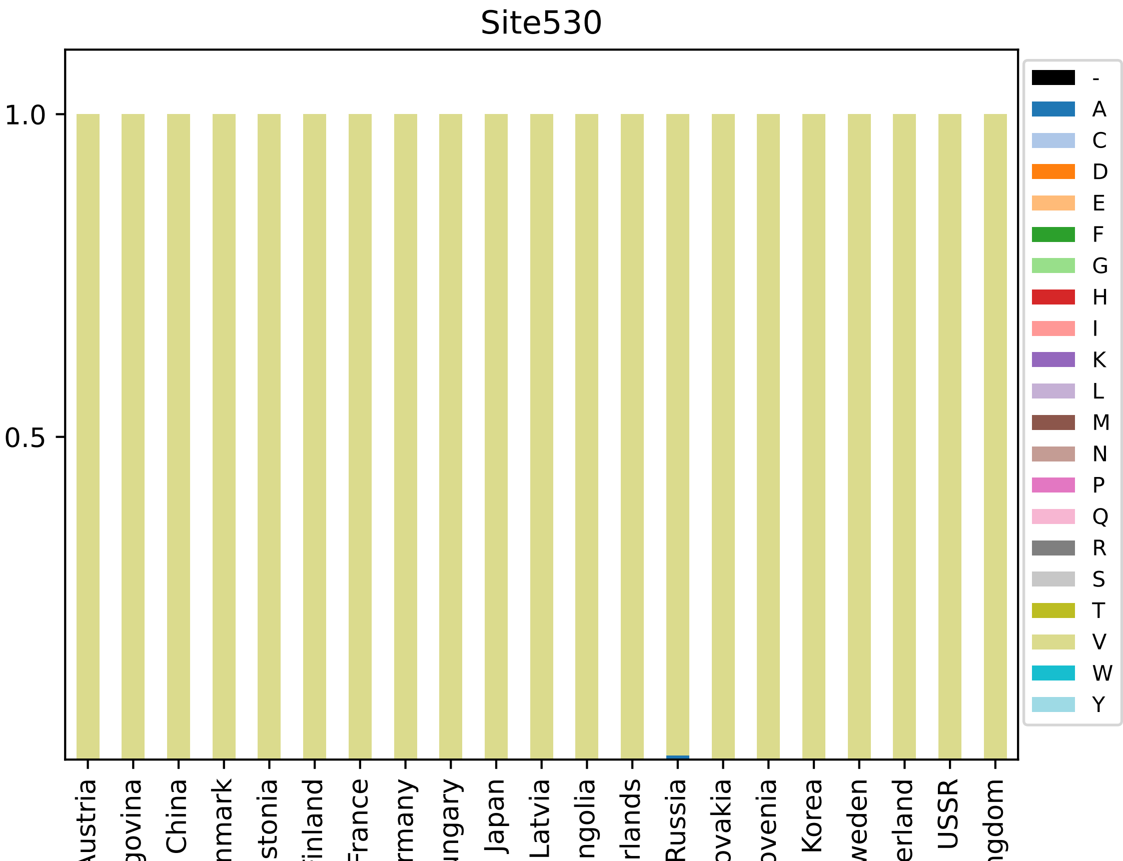

Site530
1.0
0.5
-
A
C
D
E
F
G
H
I
K
L
M
N
P
Q
R
S
T
V
W
Y
Austria
govina
China
nmark
stonia
inland
France
rmany
ungary
Japan
Latvia
ngolia
rlands
Russia
ovakia
ovenia
Korea
weden
erland
USSR
ngdom

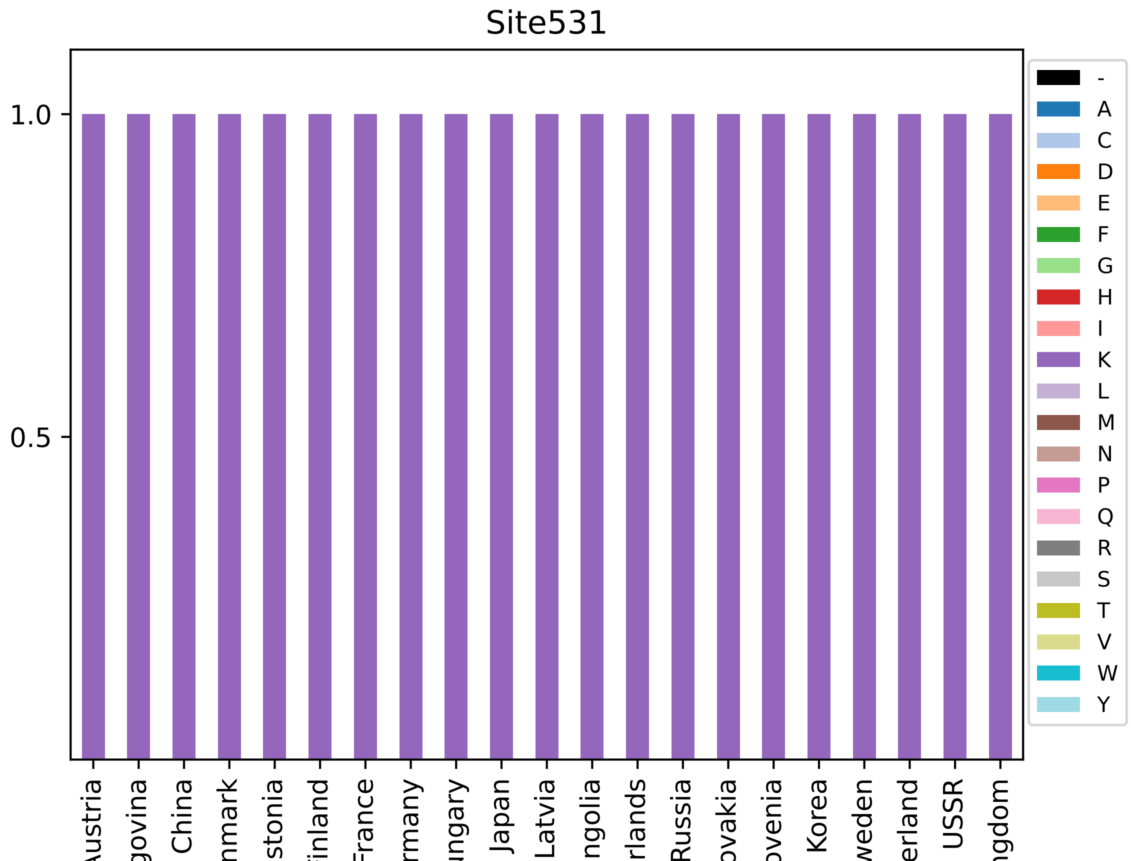

Site531
1.0
0.5
-
A
C
D
E
F
G
H
I
K
L
M
N
P
Q
R
S
T
V
W
Y
China
Japan
Latvia
Russia
Korea
USSR

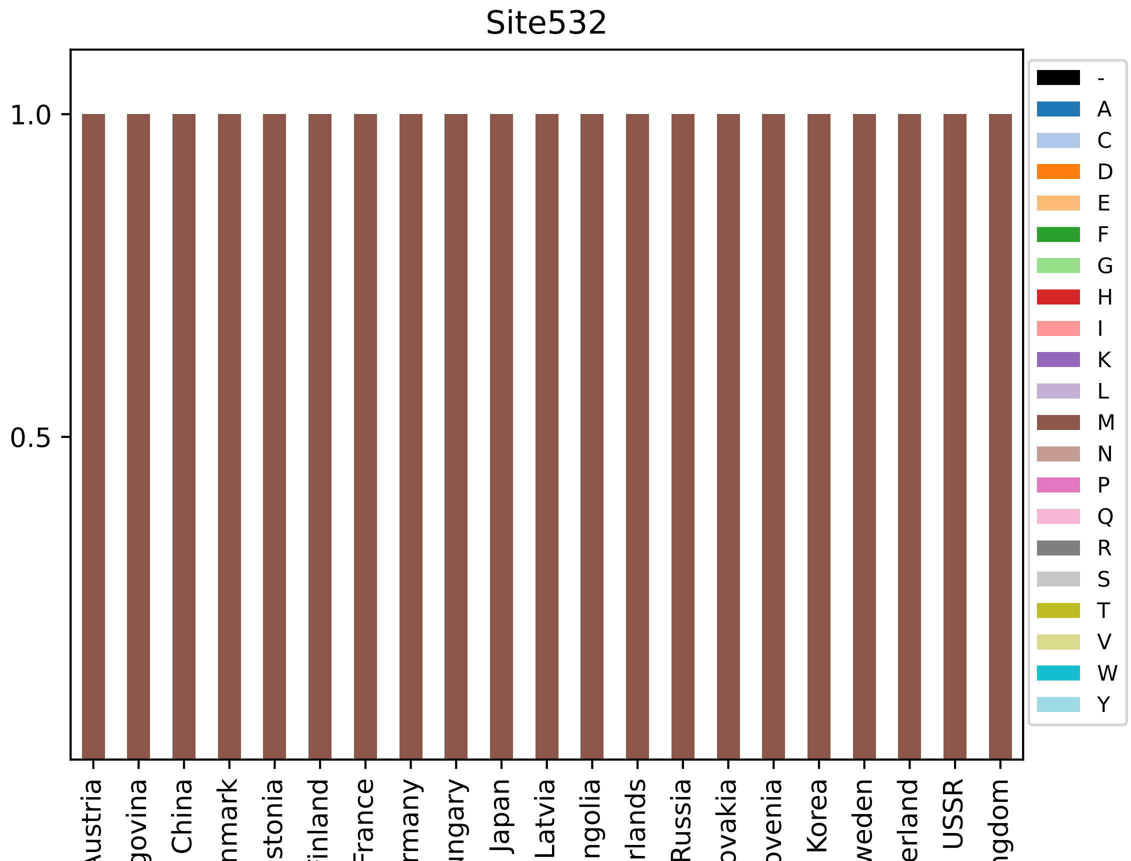
Site532
1.0
0.5
-
A
C
D
E
F
G
H
I
K
L
M
N
P
Q
R
S
T
V
W
Y
China
Japan
Latvia
Russia
Korea
USSR

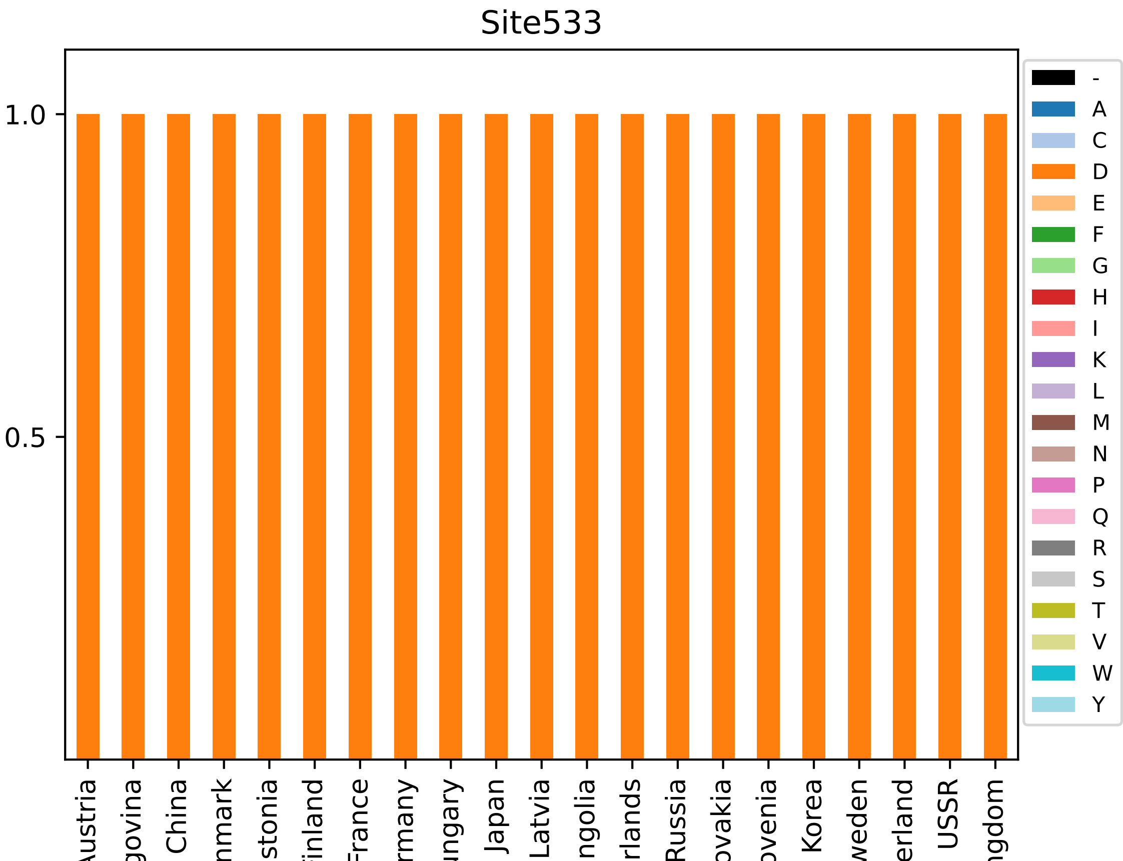

Site533
1.0
0.5
-
A
C
D
E
F
G
H
I
K
L
M
N
P
Q
R
S
T
V
W
Y
China
Japan
Latvia
Russia
Korea
USSR

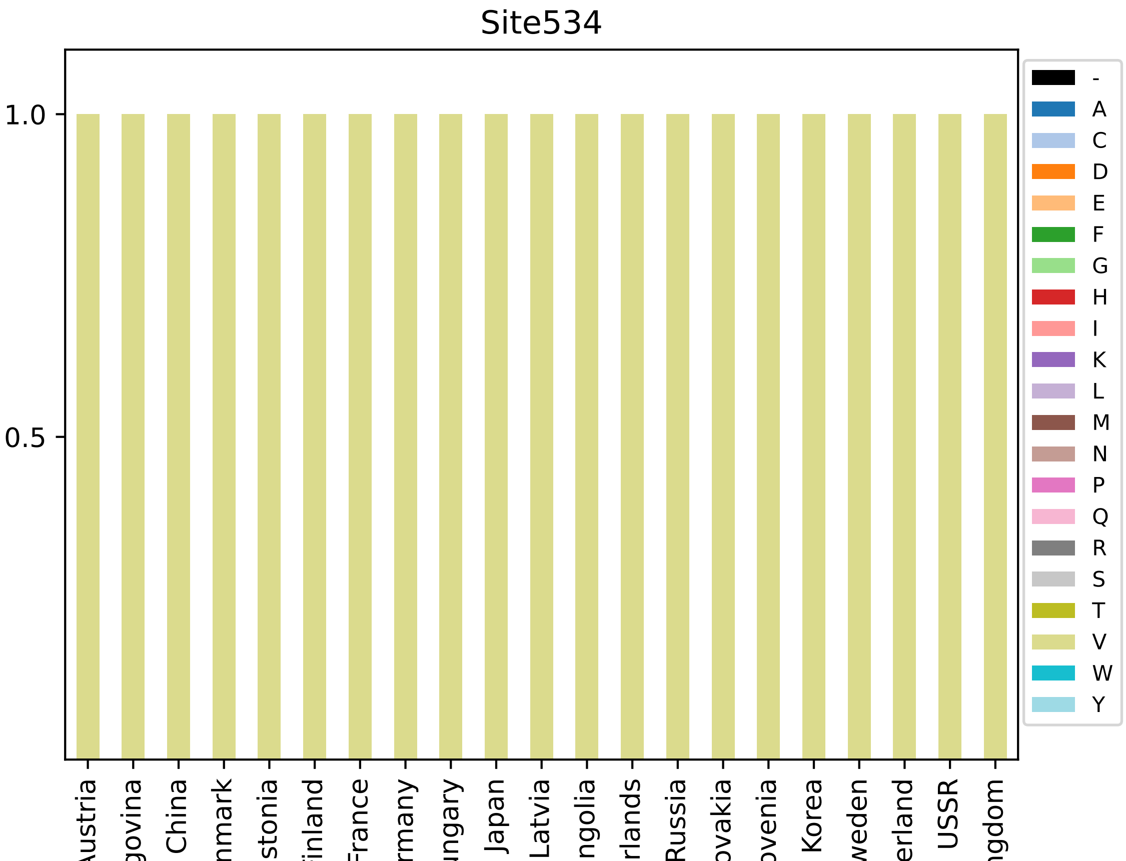

Site534
1.0
0.5
Austria
China
Japan
Latvia
Russia
Korea
USSR
-
A
C
D
E
F
G
H
I
K
L
M
N
P
Q
R
S
T
V
W
Y

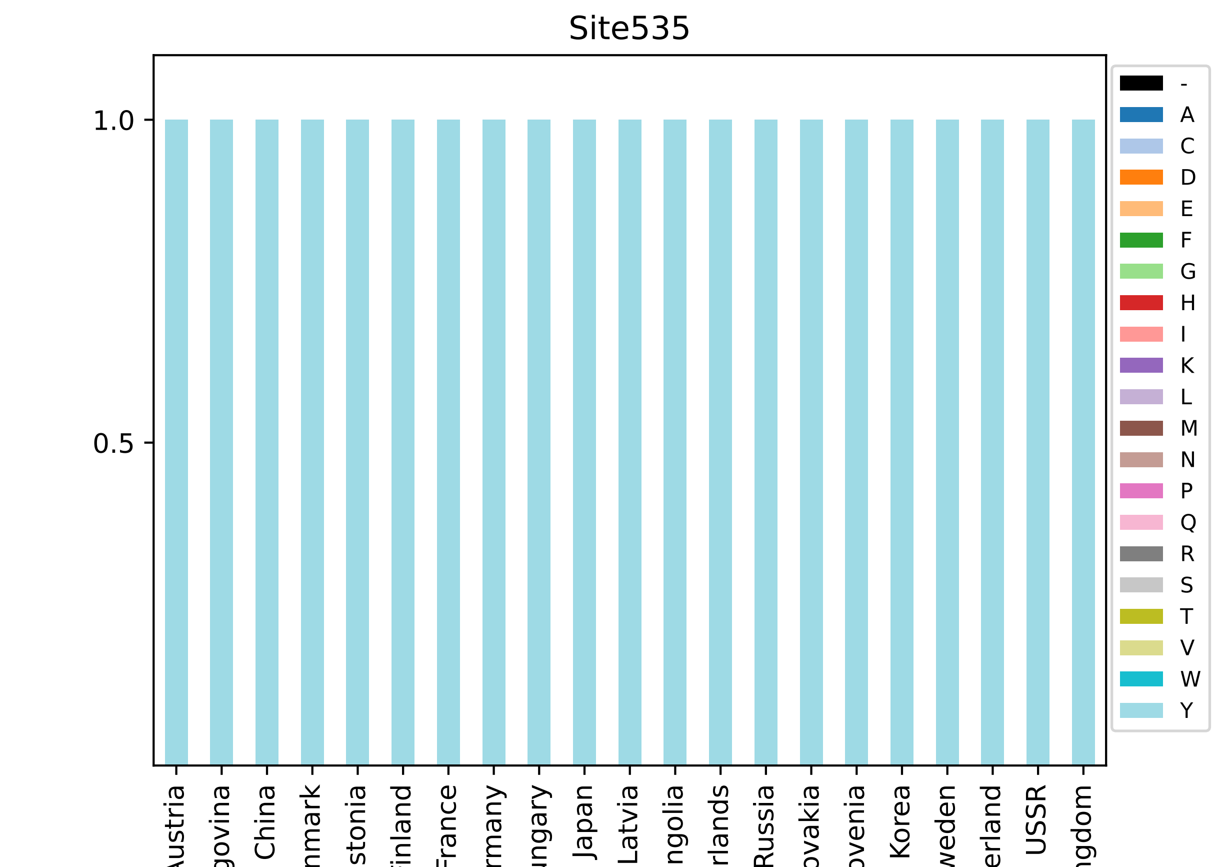

Site535
1.0
0.5
-
A
C
D
E
F
G
H
I
K
L
M
N
P
Q
R
S
T
V
W
Y
Austria
govina
China
nmark
stonia
inland
France
rmany
ungary
Japan
Latvia
ngolia
rlands
Russia
ovakia
ovenia
Korea
weden
erland
USSR
ngdom

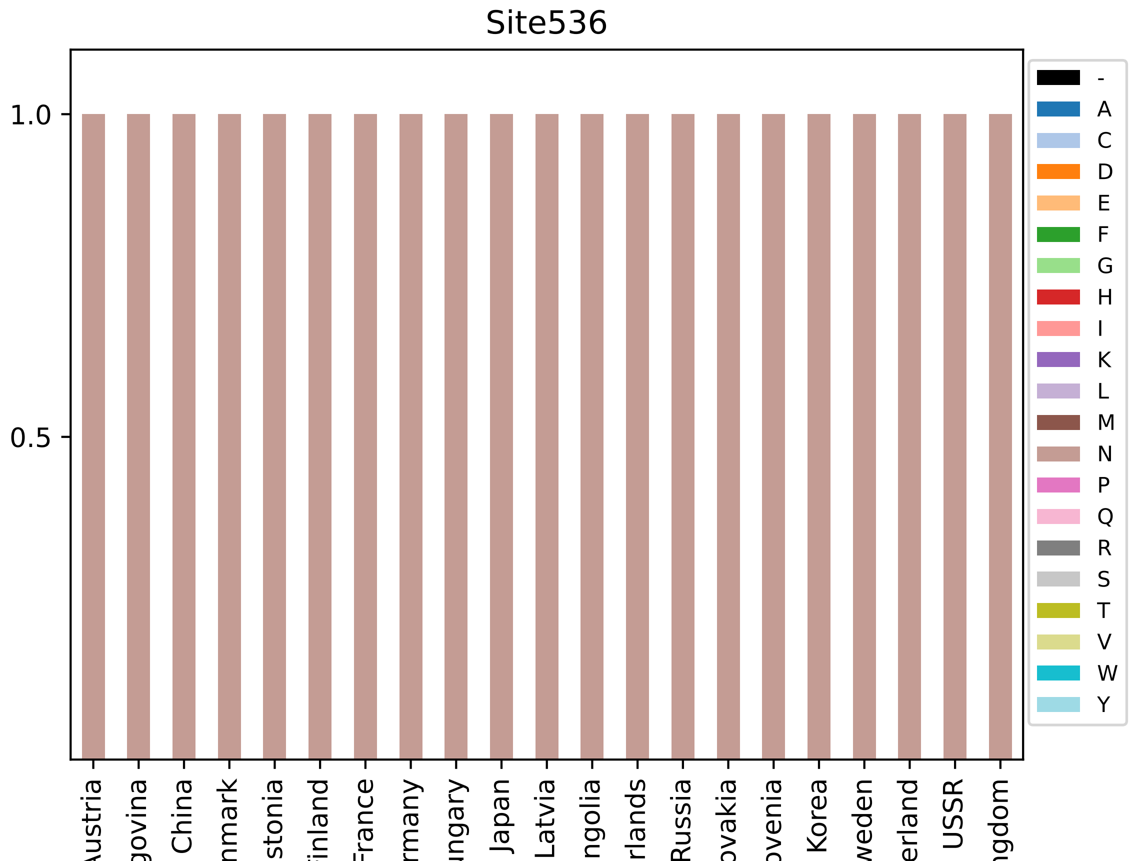
Site536
1.0
0.5
China
Japan
Latvia
Russia
Korea
USSR
-
A
C
D
E
F
G
H
I
K
L
M
N
P
Q
R
S
T
V
W
Y

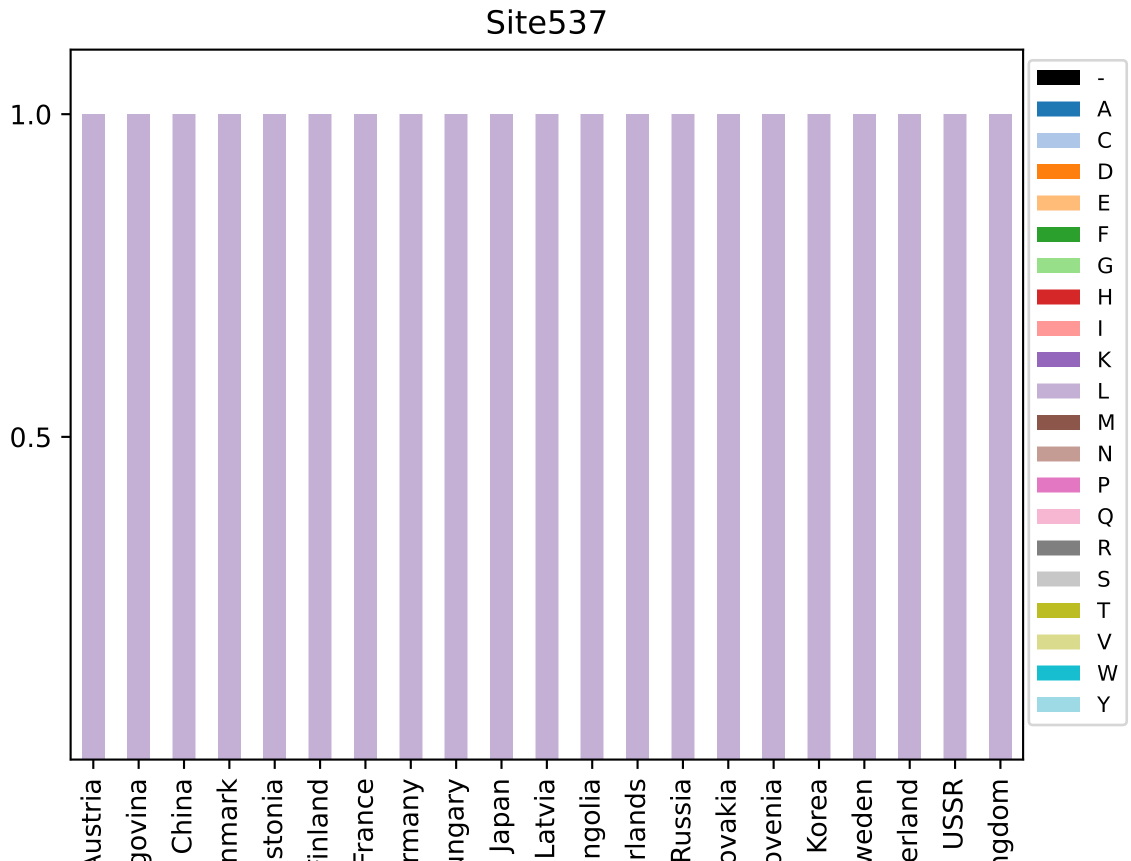
Site537
1.0
0.5
-
A
C
D
E
F
G
H
I
K
L
M
N
P
Q
R
S
T
V
W
Y
China
Japan
Latvia
Russia
Korea
USSR

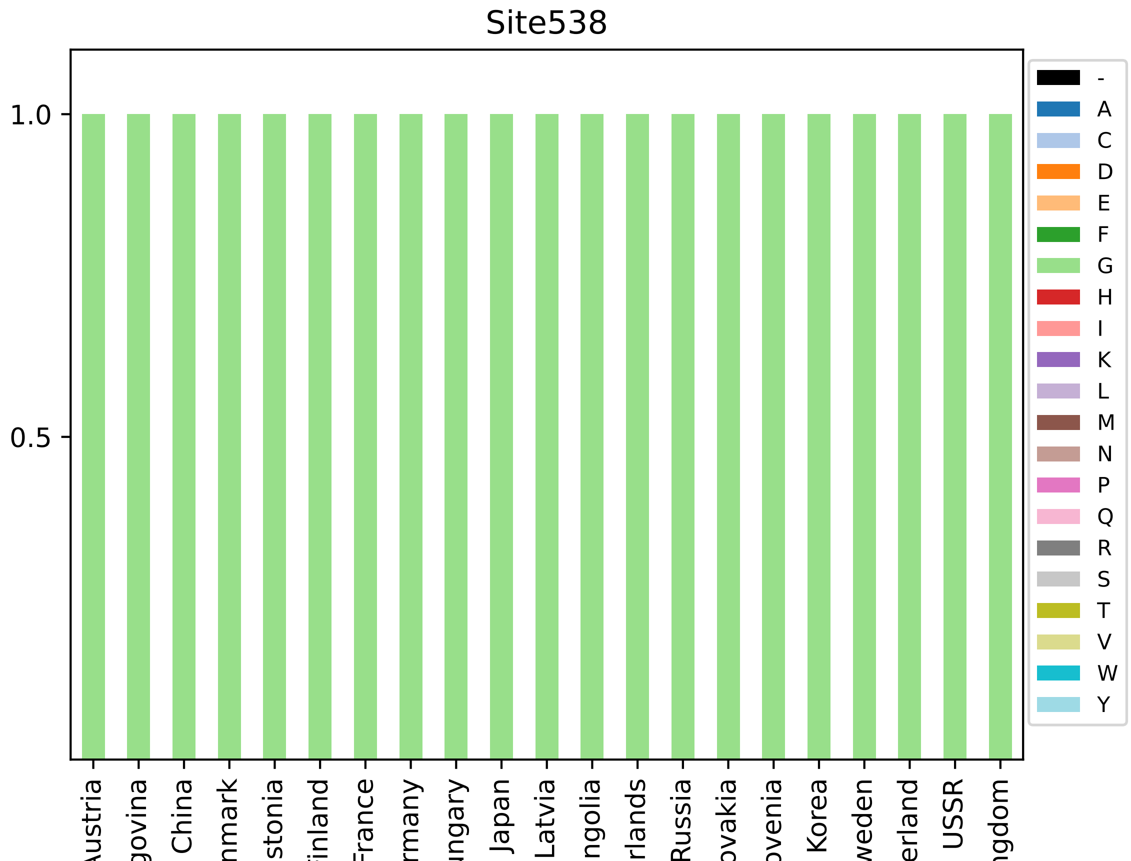

Site538
1.0
0.5
-
A
C
D
E
F
G
H
I
K
L
M
N
P
Q
R
S
T
V
W
Y
Austria
govina
China
nmark
stonia
inland
France
rmany
ungary
Japan
Latvia
ngolia
rlands
Russia
ovakia
ovenia
Korea
weden
erland
USSR
ngdom

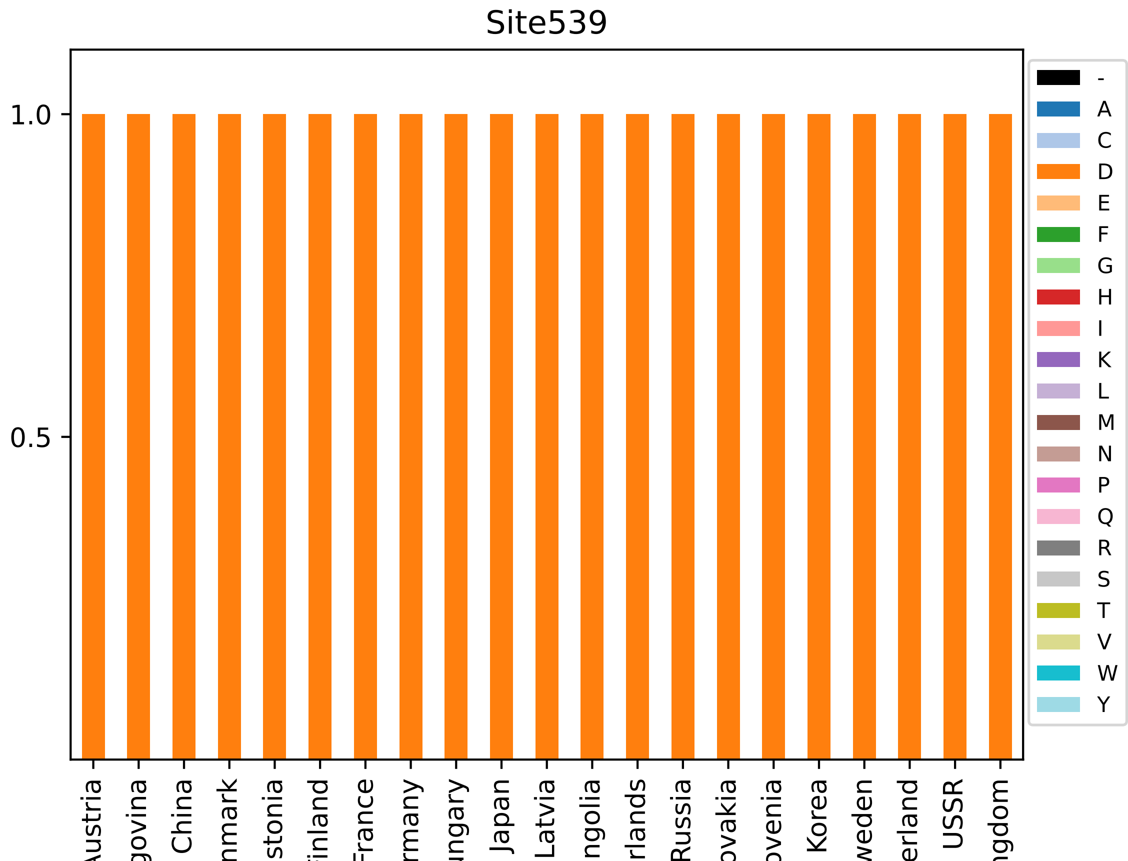

Site539
1.0
0.5
-
A
C
D
E
F
G
H
I
K
L
M
N
P
Q
R
S
T
V
W
Y
Austria
govina
China
nmark
stonia
Finland
France
rmany
ungary
Japan
Latvia
ngolia
rlands
Russia
ovakia
ovenia
Korea
weden
erland
USSR
ngdom

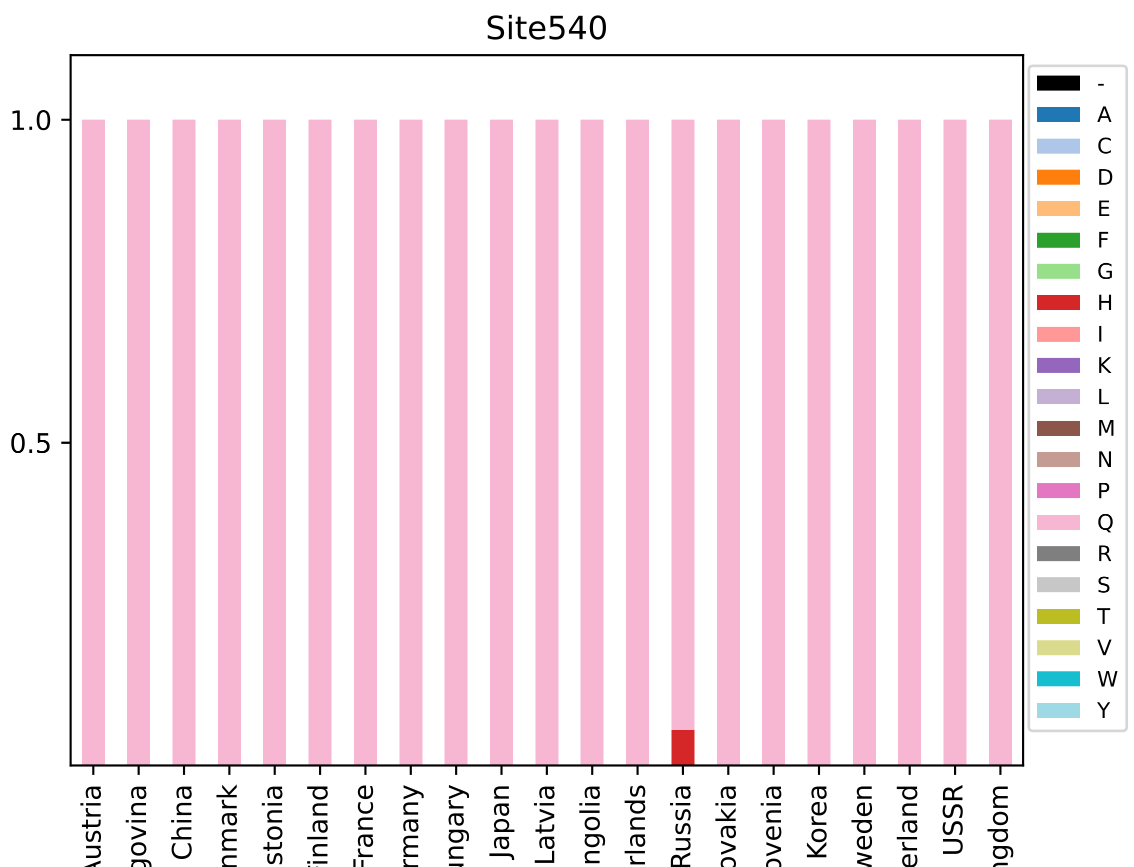

Site540
1.0
0.5
-
A
C
D
E
F
G
H
I
K
L
M
N
P
Q
R
S
T
V
W
Y
Austria
govina
China
nmark
stonia
inland
France
rmany
ungary
Japan
Latvia
ngolia
rlands
Russia
ovakia
ovenia
Korea
weden
erland
USSR
ngdom

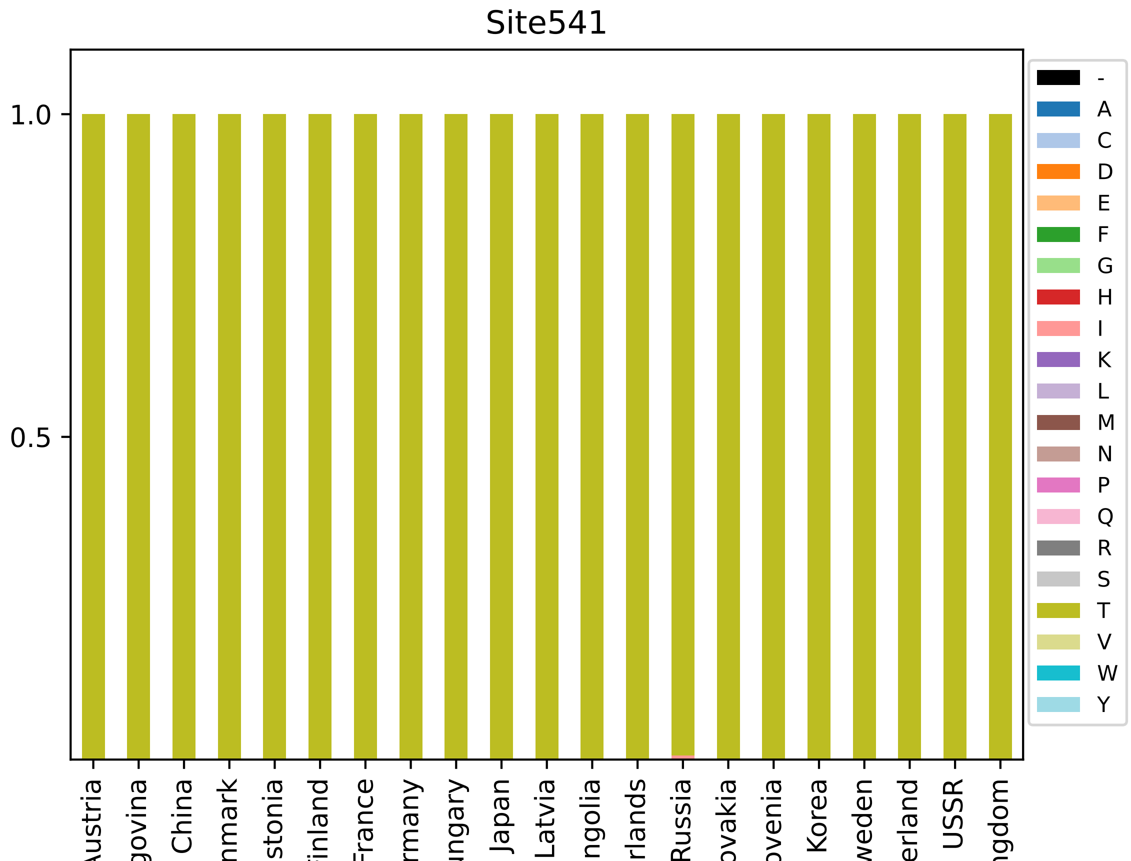
Site541
1.0
0.5
-
A
C
D
E
F
G
H
I
K
L
M
N
P
Q
R
S
T
V
W
Y
China
Japan
Latvia
Russia
Korea
USSR

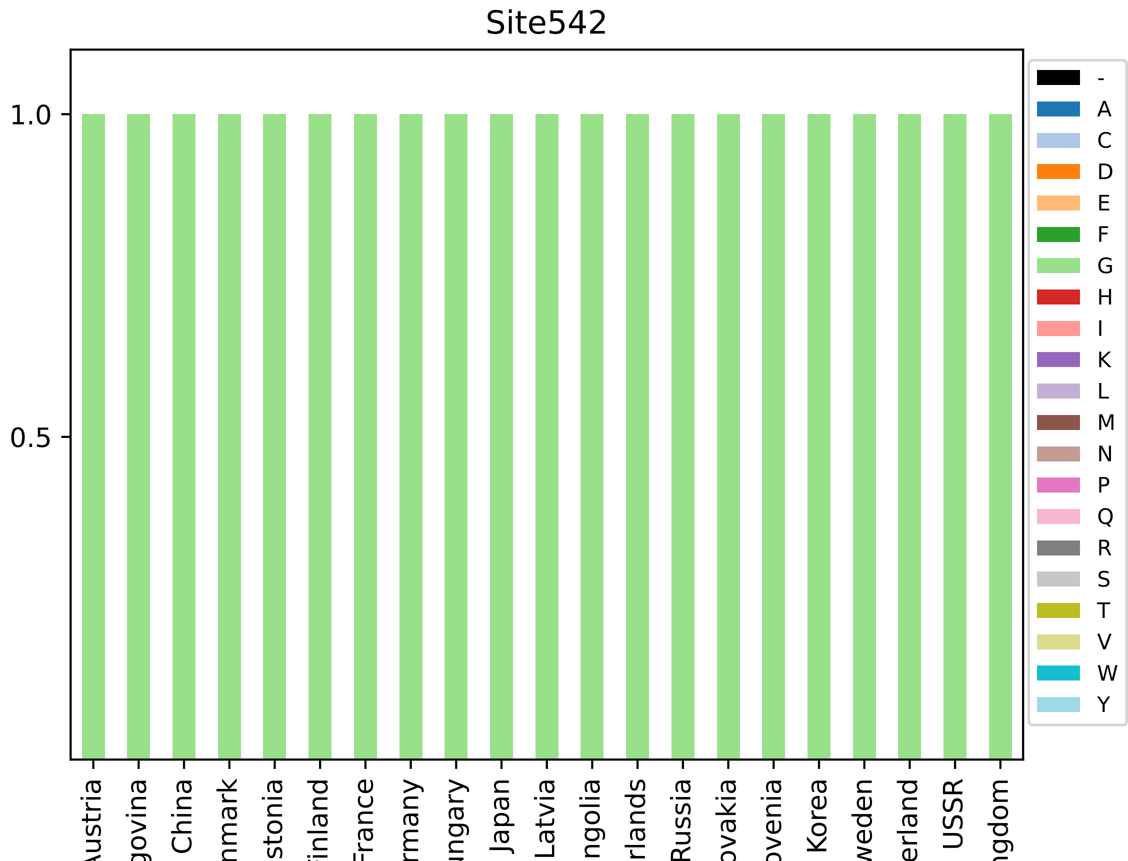
Site542
1.0
0.5
-
A
C
D
E
F
G
H
I
K
L
M
N
P
Q
R
S
T
V
W
Y
China
Japan
Latvia
Russia
Korea
USSR

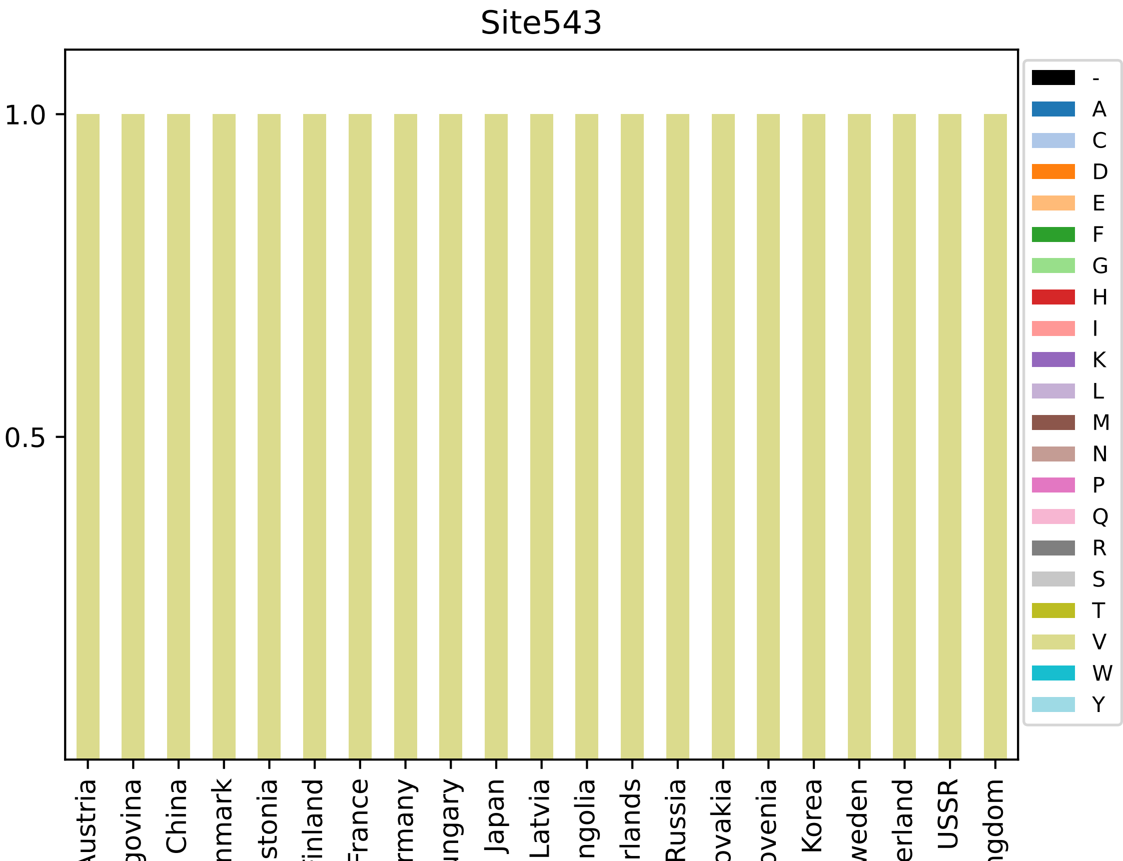

Site543
1.0
0.5
Austria
govina
China
nmark
stonia
inland
France
rmany
ungary
Japan
Latvia
ngolia
rlands
Russia
ovakia
ovenia
Korea
weden
erland
USSR
ngdom
-
A
C
D
E
F
G
H
I
K
L
M
N
P
Q
R
S
T
V
W
Y

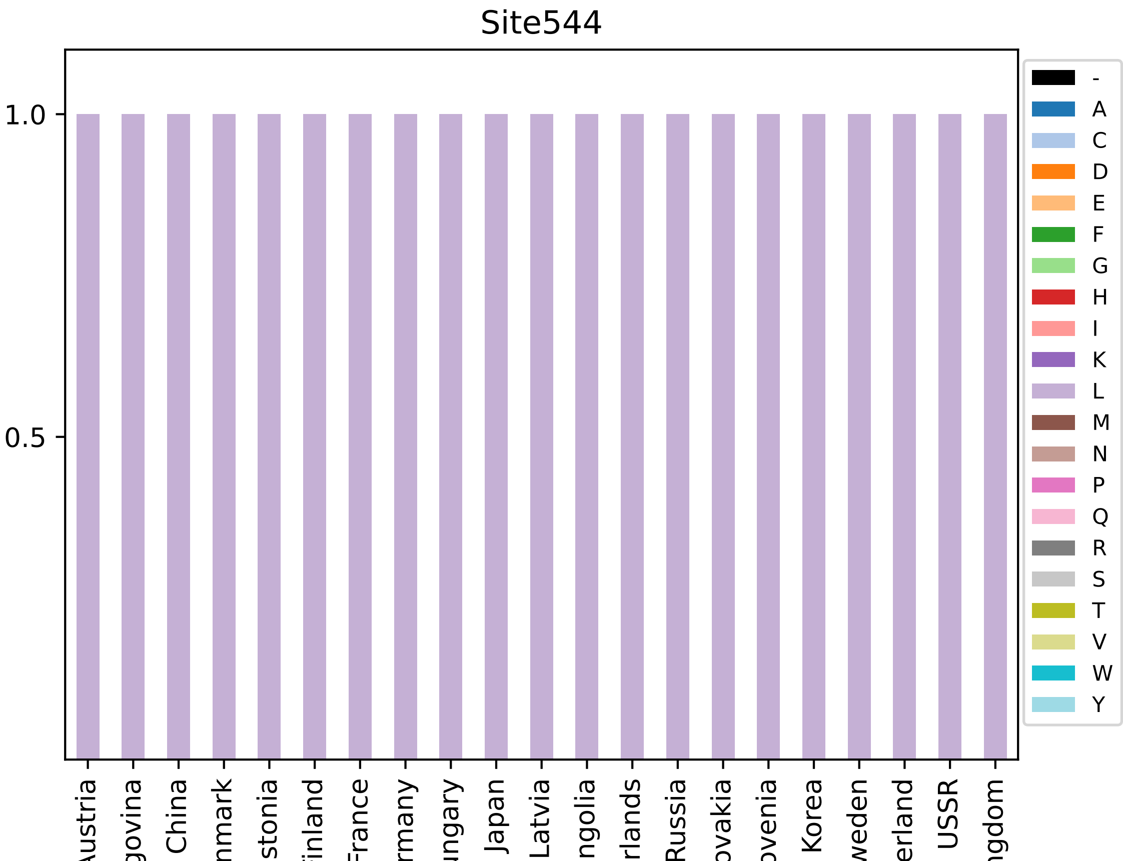
Site544
1.0
0.5
-
A
C
D
E
F
G
H
I
K
L
M
N
P
Q
R
S
T
V
W
Y
China
Japan
Latvia
Russia
Korea
USSR

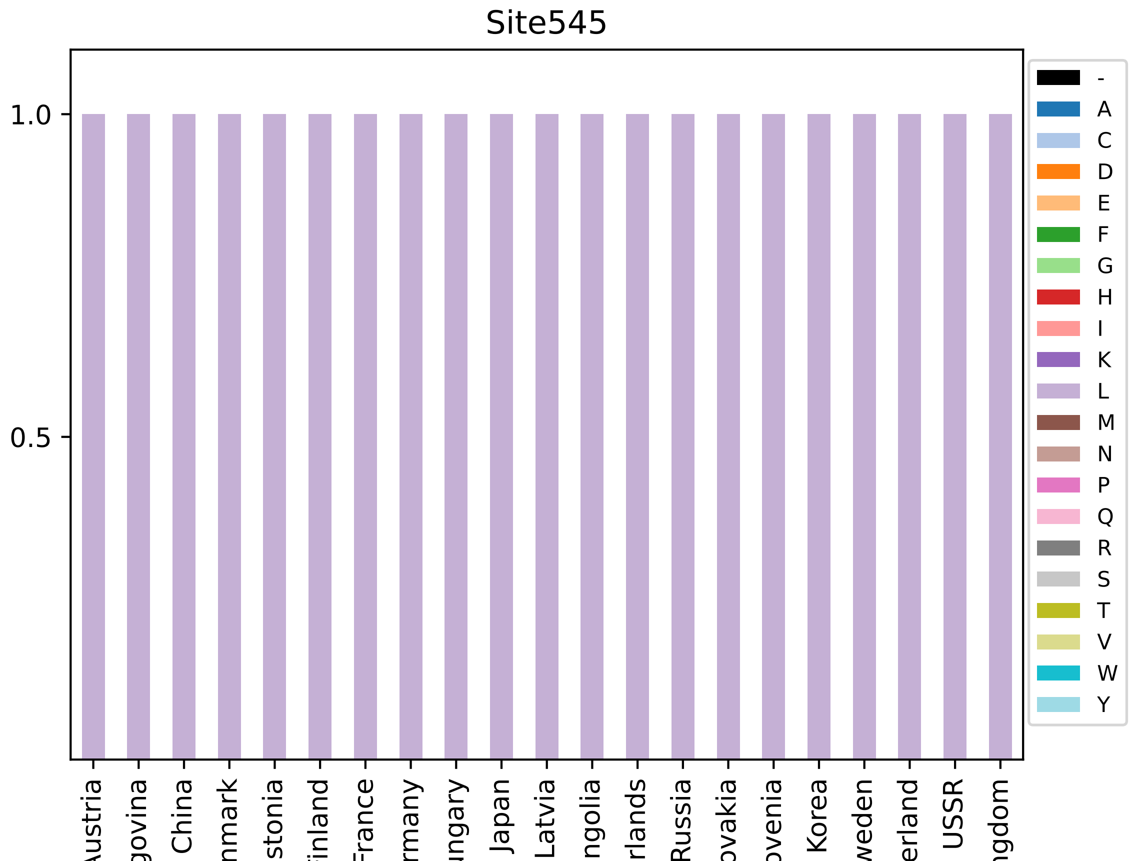

Site545
1.0
0.5
-
A
C
D
E
F
G
H
I
K
L
M
N
P
Q
R
S
T
V
W
Y
China
Japan
Latvia
Russia
Korea
USSR

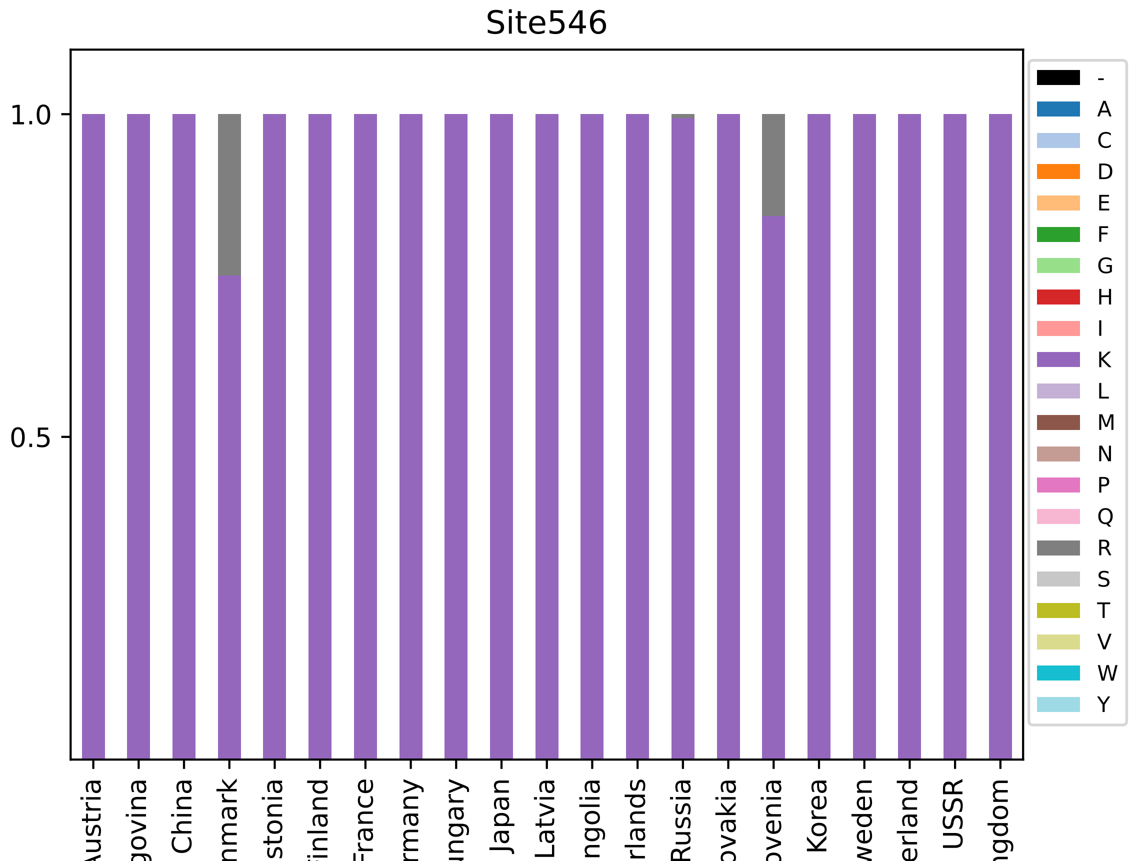

Site546
1.0
0.5
-
A
C
D
E
F
G
H
I
K
L
M
N
P
Q
R
S
T
V
W
Y
China
Japan
Latvia
Russia
Korea
USSR

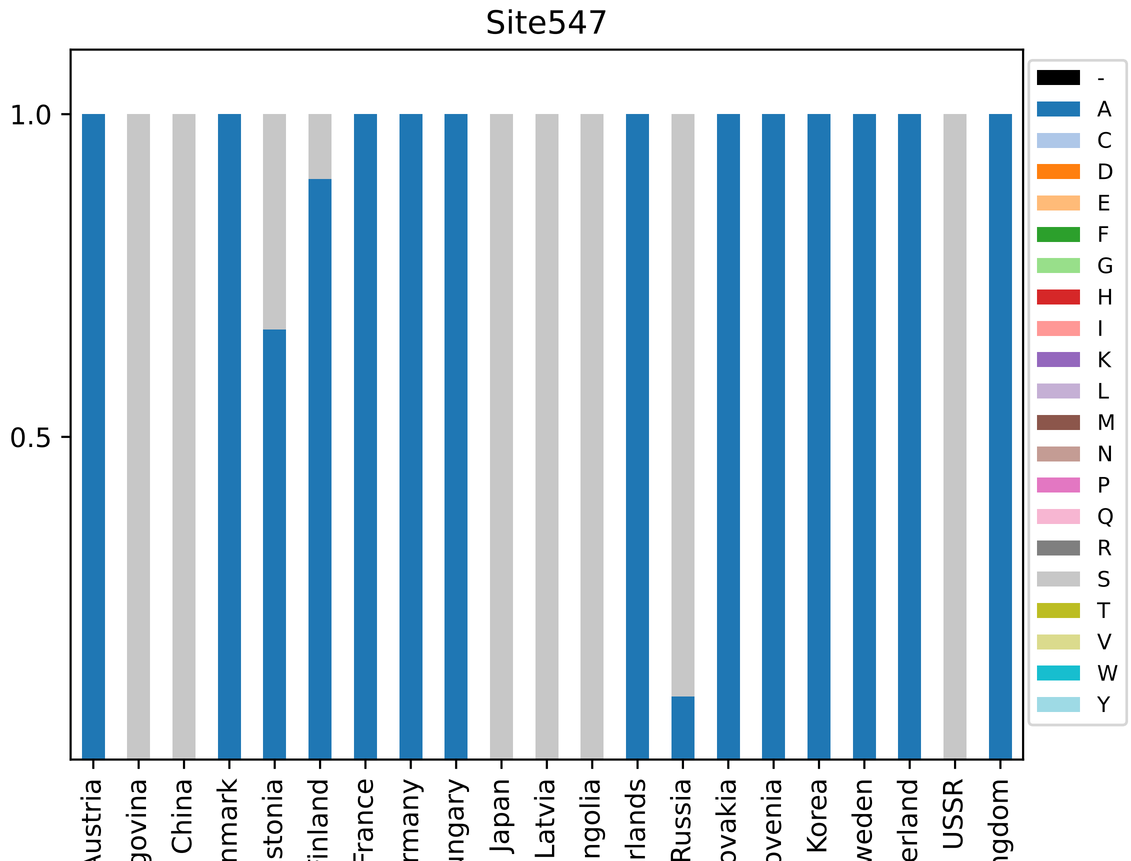

Site547
1.0
0.5
-
A
C
D
E
F
G
H
I
K
L
M
N
P
Q
R
S
T
V
W
Y
Austria
govina
China
nmark
stonia
inland
France
rmany
ungary
Japan
Latvia
ngolia
rlands
Russia
ovakia
ovenia
Korea
weden
erland
USSR
ngdom

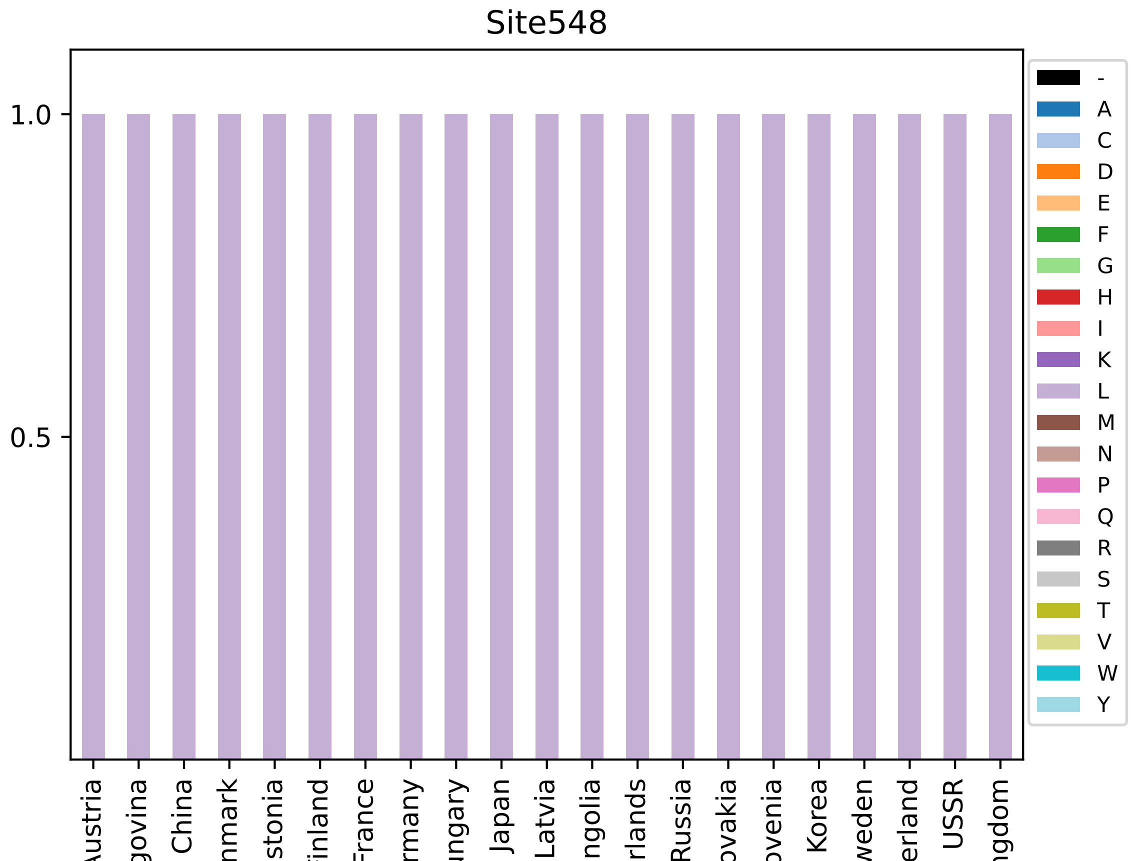
Site548
1.0
0.5
-
A
C
D
E
F
G
H
I
K
L
M
N
P
Q
R
S
T
V
W
Y
China
Japan
Latvia
Russia
Korea
USSR

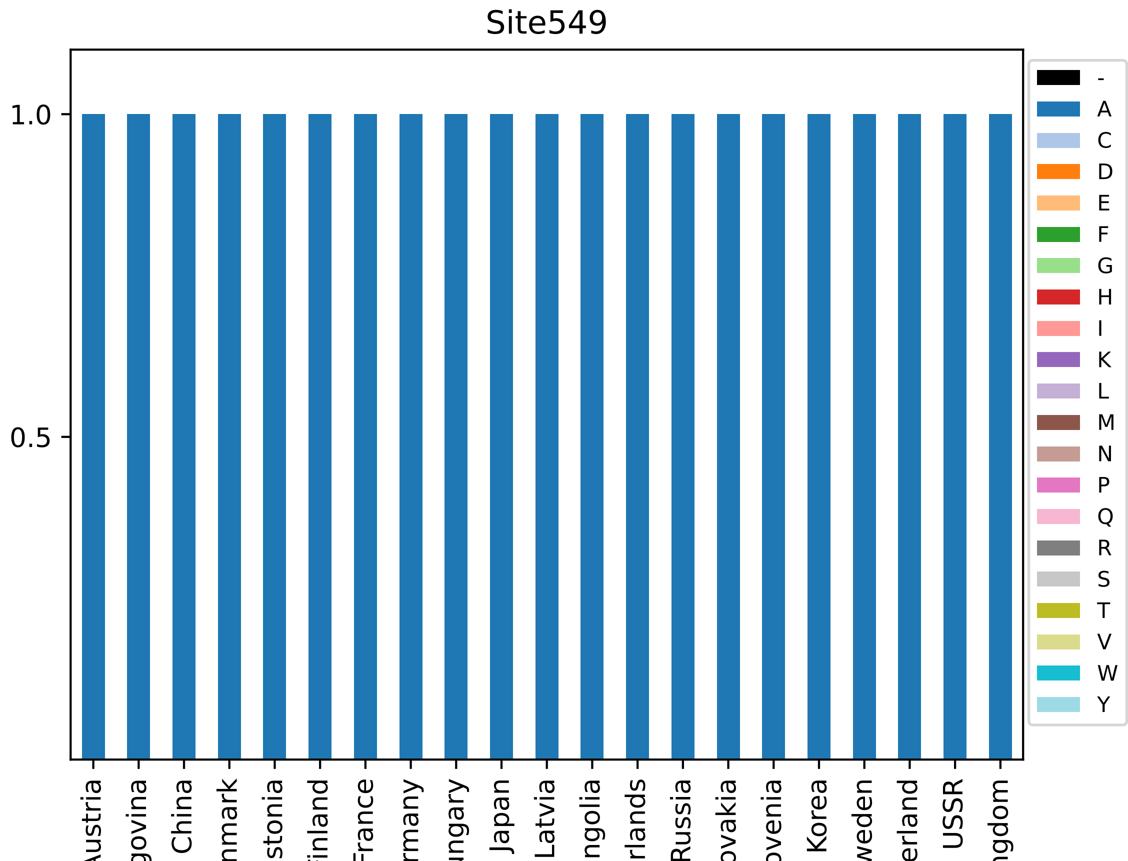
Site549
1.0
0.5
-
A
C
D
E
F
G
H
I
K
L
M
N
P
Q
R
S
T
V
W
Y
Austria
govina
China
nmark
stonia
Finland
France
rmany
ungary
Japan
Latvia
ngolia
rlands
Russia
ovakia
ovenia
Korea
weden
erland
USSR
ngdom

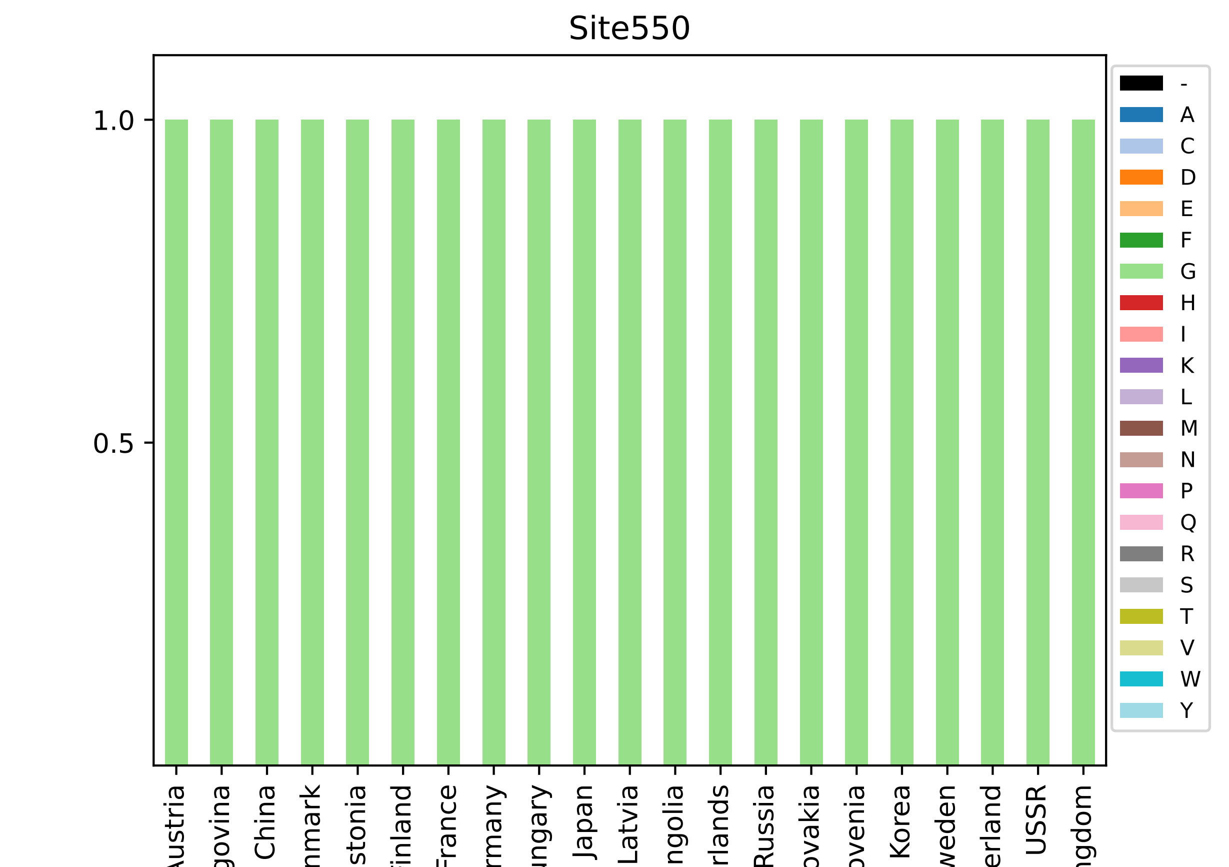

Site550
1.0
0.5
-
A
C
D
E
F
G
H
I
K
L
M
N
P
Q
R
S
T
V
W
Y
Austria
govina
China
nmark
stonia
Finland
France
rmany
ungary
Japan
Latvia
ngolia
rlands
Russia
ovakia
ovenia
Korea
weden
erland
USSR
ngdom

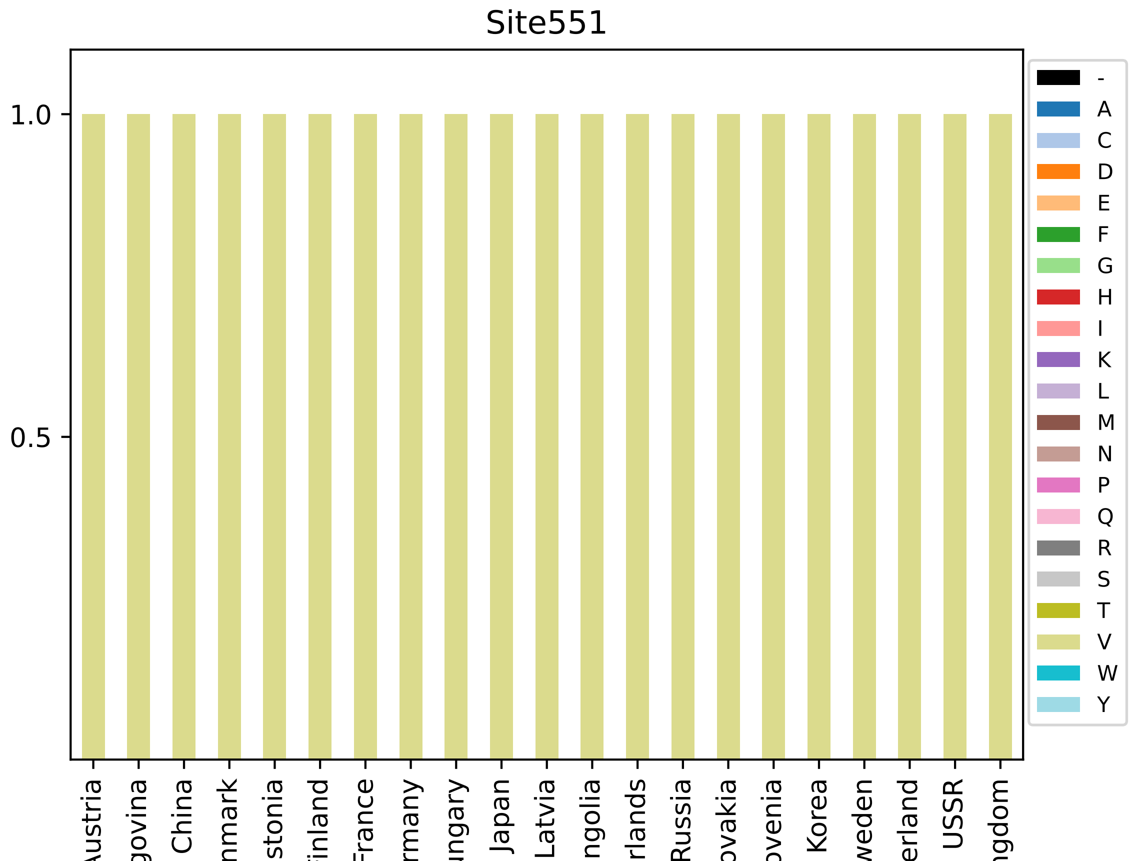
Site551
1.0
0.5
-
A
C
D
E
F
G
H
I
K
L
M
N
P
Q
R
S
T
V
W
Y
Austria
govina
China
nmark
stonia
inland
France
rmany
ungary
Japan
Latvia
ngolia
rlands
Russia
ovakia
ovenia
Korea
weden
erland
USSR
ngdom

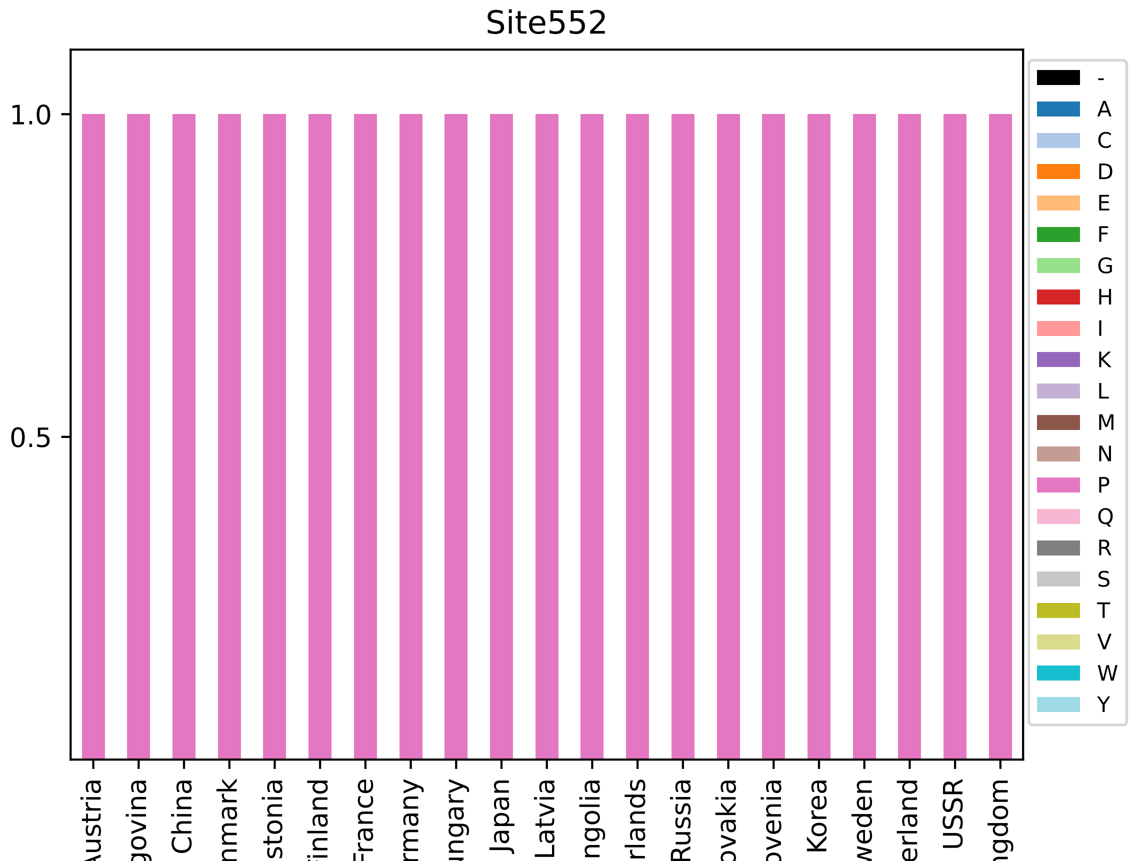
Site552
1.0
0.5
China
Japan
Latvia
Russia
Korea
USSR
-
A
C
D
E
F
G
H
I
K
L
M
N
P
Q
R
S
T
V
W
Y

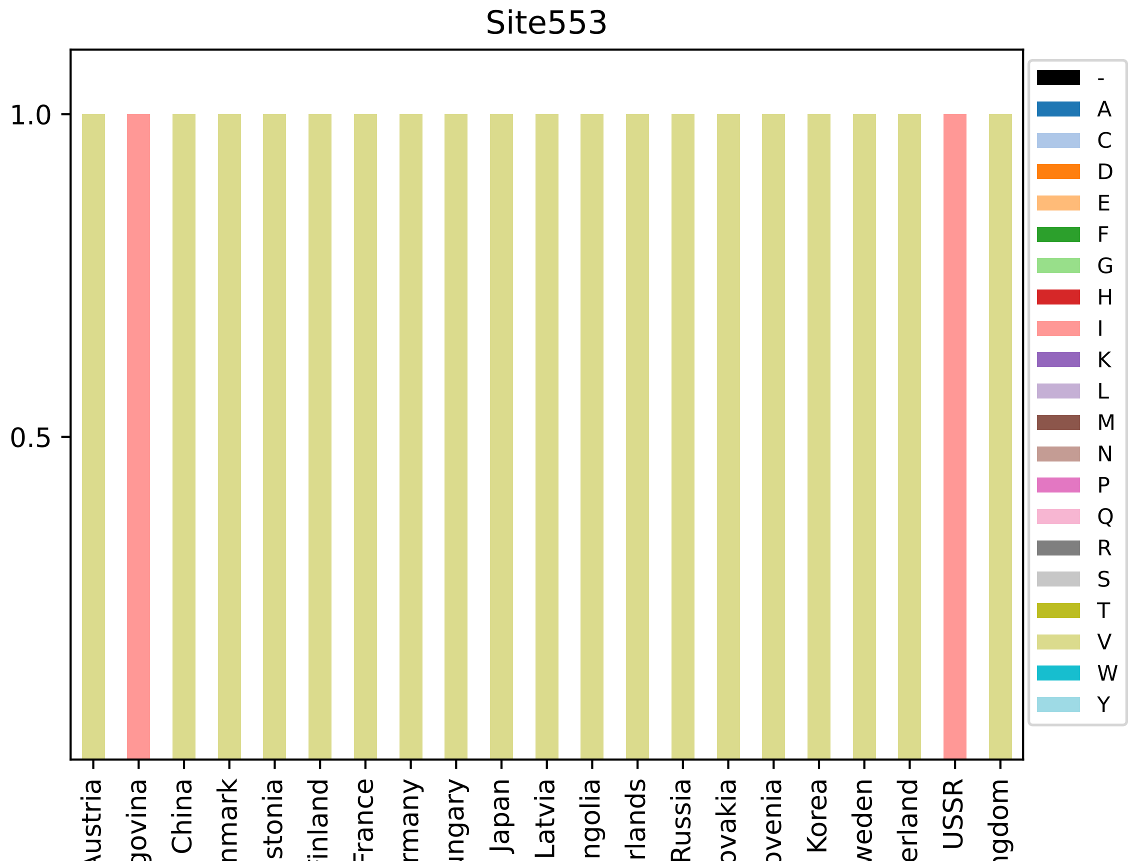

Site553
1.0
0.5
China
Japan
Latvia
Russia
Korea
USSR
-
A
C
D
E
F
G
H
I
K
L
M
N
P
Q
R
S
T
V
W
Y

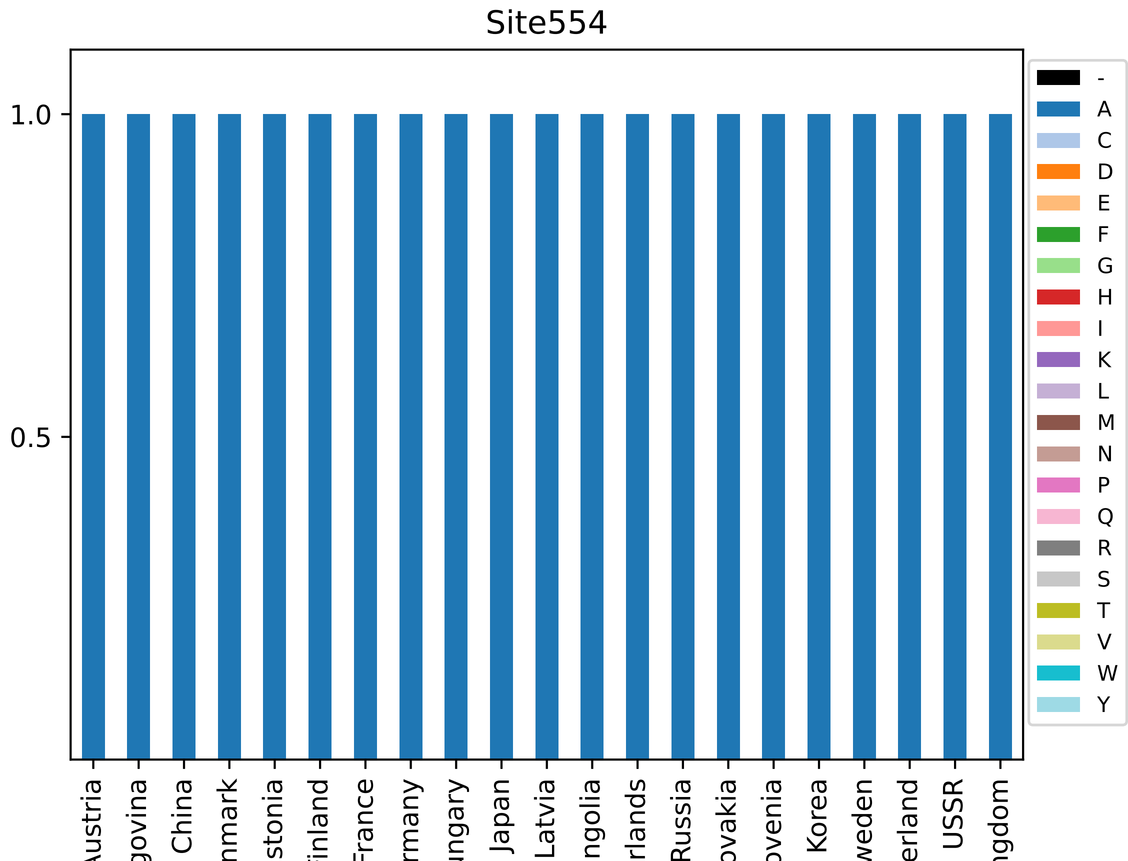

Site554
1.0
0.5
-
A
C
D
E
F
G
H
I
K
L
M
N
P
Q
R
S
T
V
W
Y
Austria
govina
China
nmark
stonia
Finland
France
rmany
ungary
Japan
Latvia
ngolia
rlands
Russia
ovakia
ovenia
Korea
weden
erland
USSR
ngdom

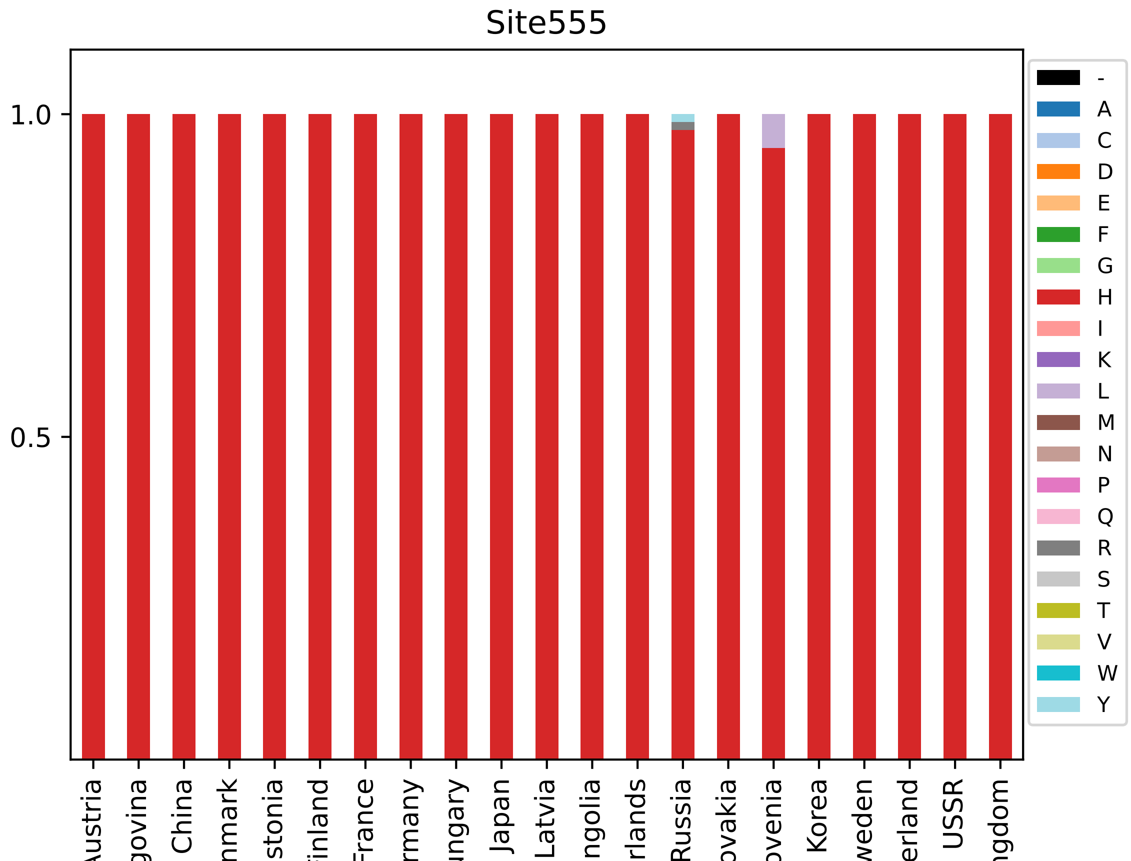

Site555
1.0
0.5
-
A
C
D
E
F
G
H
I
K
L
M
N
P
Q
R
S
T
V
W
Y
Austria
govina
China
nmark
stonia
Finland
France
rmany
ungary
Japan
Latvia
ngolia
rlands
Russia
ovakia
ovenia
Korea
weden
erland
USSR
ngdom

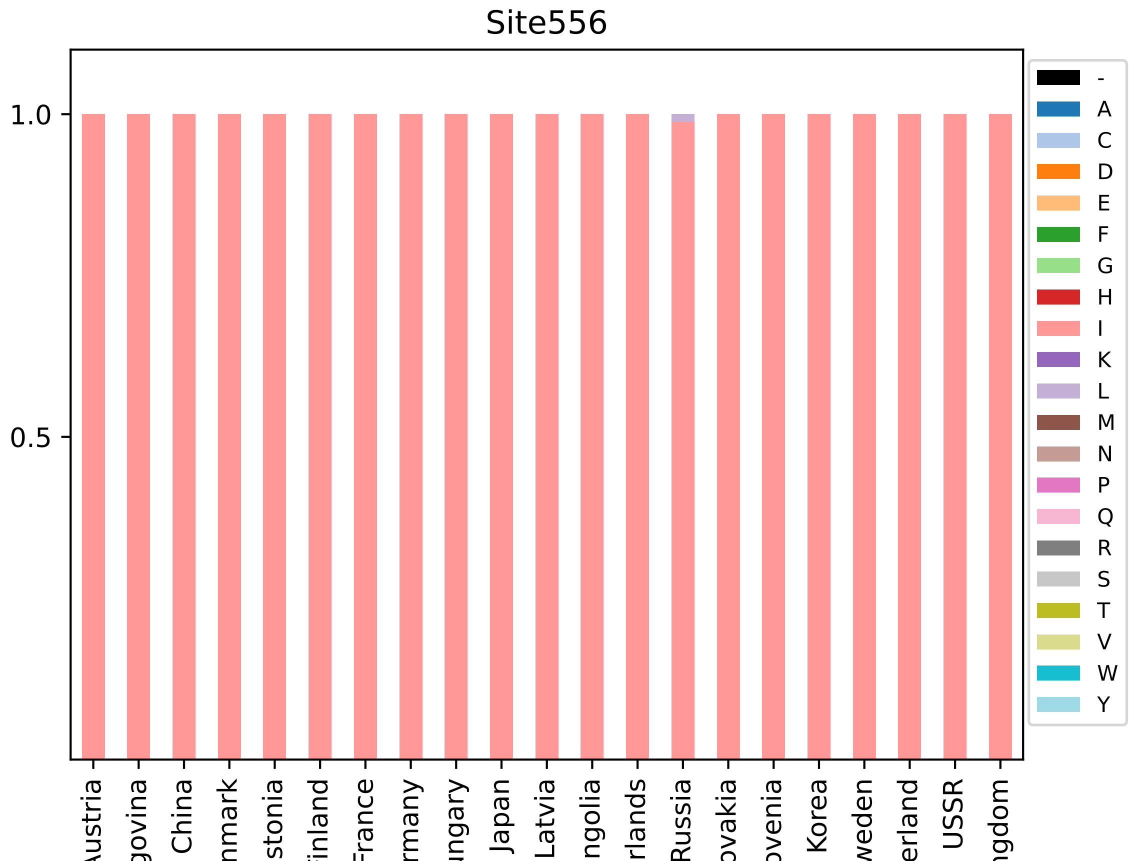
Site556
1.0
0.5
-
A
C
D
E
F
G
H
I
K
L
M
N
P
Q
R
S
T
V
W
Y
China
Japan
Latvia
Russia
Korea
USSR

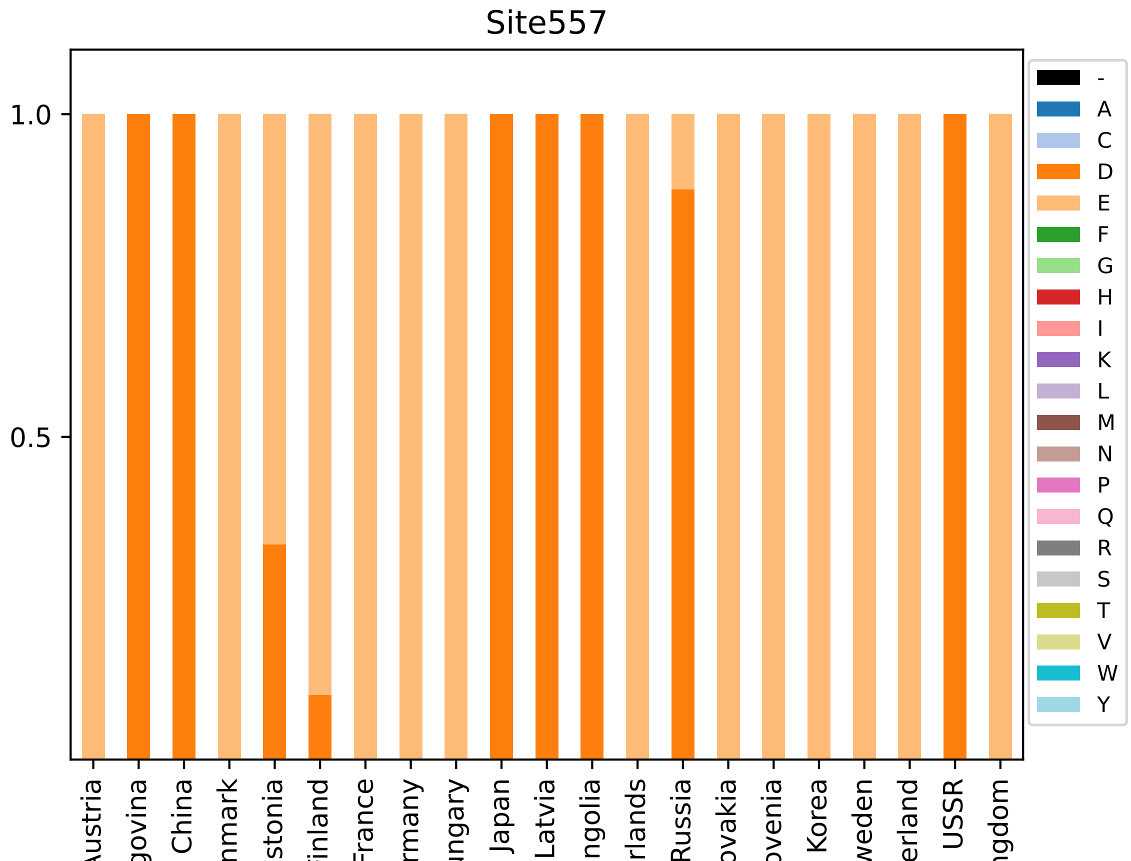
Site557
1.0
0.5
-
A
C
D
E
F
G
H
I
K
L
M
N
P
Q
R
S
T
V
W
Y
China
Japan
Latvia
Russia
Korea
USSR

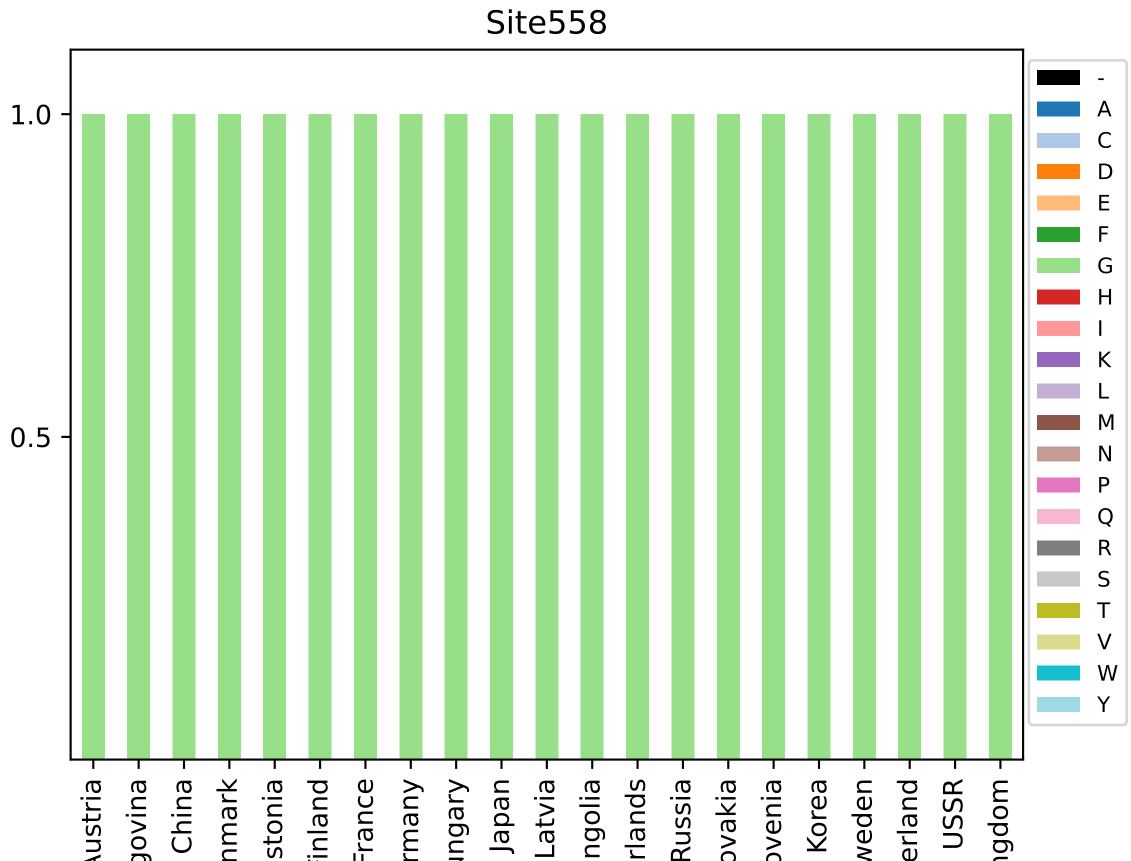
Site558
1.0
0.5
-
A
C
D
E
F
G
H
I
K
L
M
N
P
Q
R
S
T
V
W
Y
Austria
govina
China
nmark
stonia
inland
France
rmany
ungary
Japan
Latvia
ngolia
rlands
Russia
ovakia
ovenia
Korea
weden
erland
USSR
ngdom

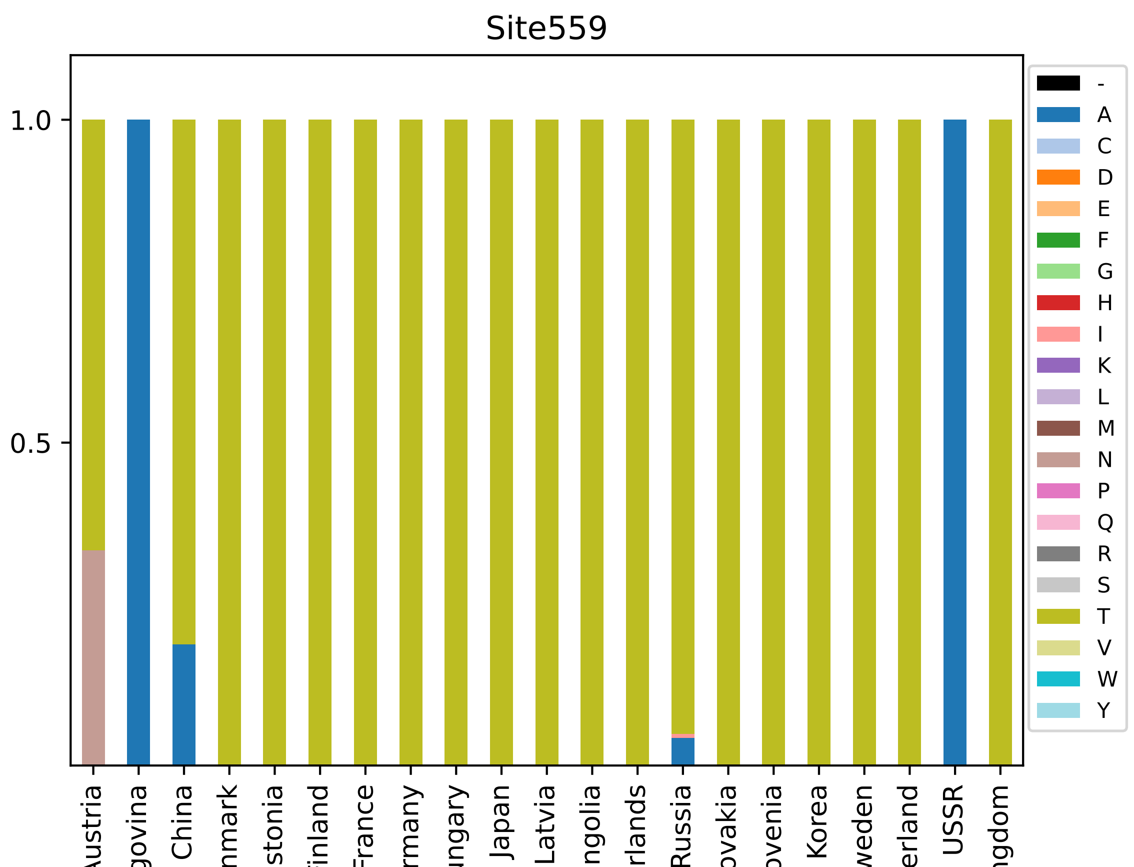

Site559
1.0
0.5
-
A
C
D
E
F
G
H
I
K
L
M
N
P
Q
R
S
T
V
W
Y
Austria
govina
China
nmark
stonia
Finland
France
rmany
ungary
Japan
Latvia
ngolia
rlands
Russia
ovakia
ovenia
Korea
weden
erland
USSR
ngdom

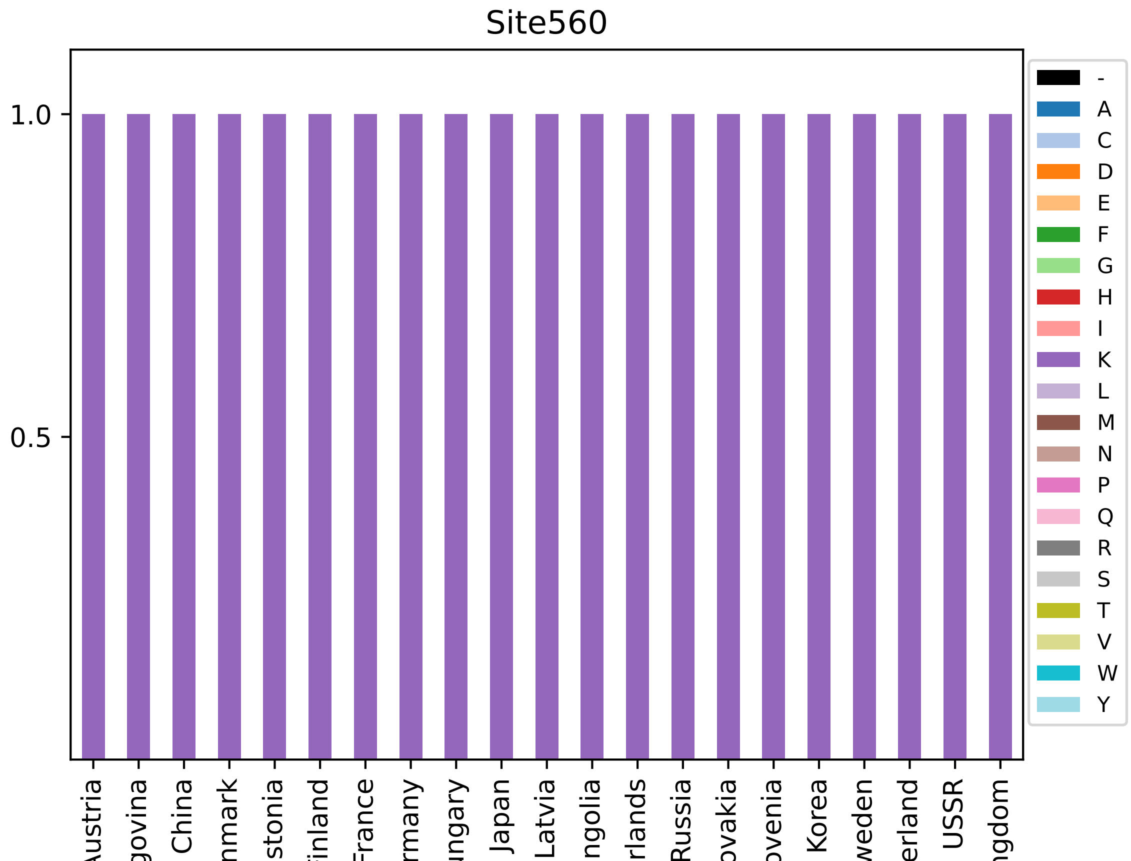

Site560
1.0
0.5
-
A
C
D
E
F
G
H
I
K
L
M
N
P
Q
R
S
T
V
W
Y
China
Japan
Latvia
Korea
USSR

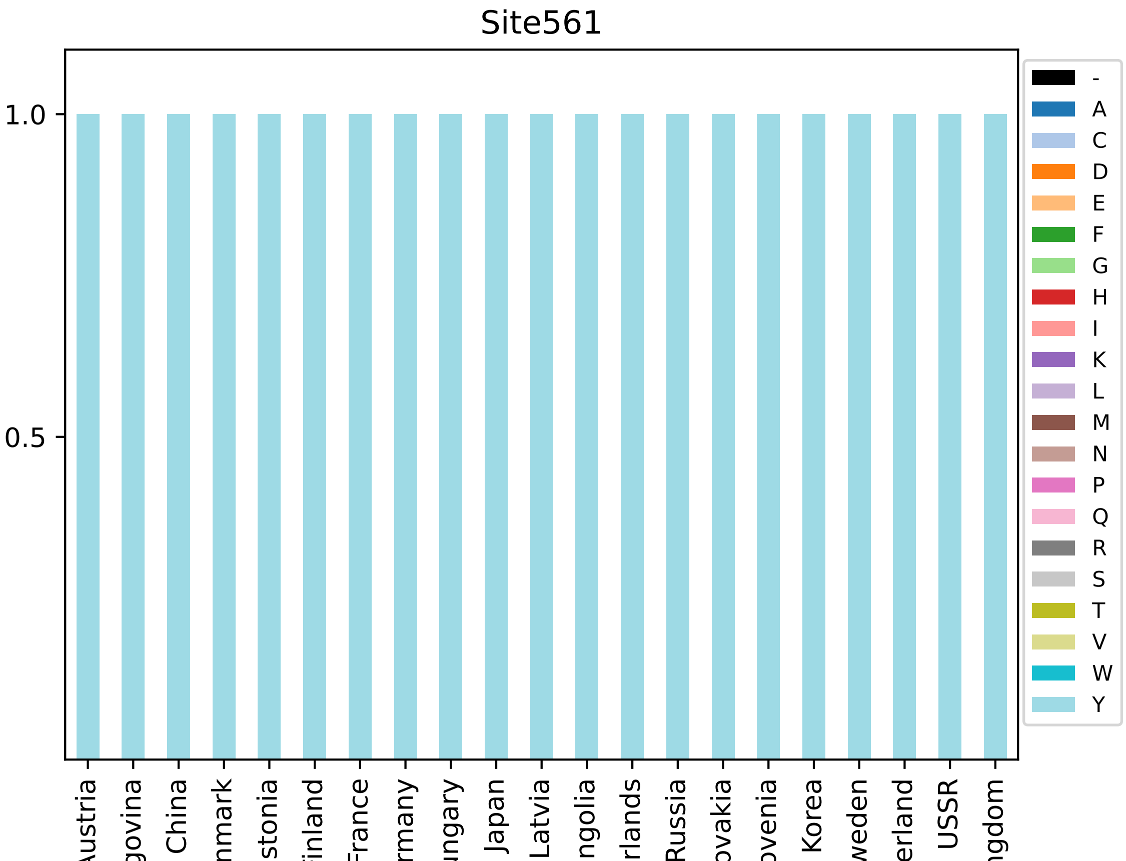
Site561
1.0
0.5
-
A
C
D
E
F
G
H
I
K
L
M
N
P
Q
R
S
T
V
W
Y
China
Japan
Latvia
Russia
Korea
USSR

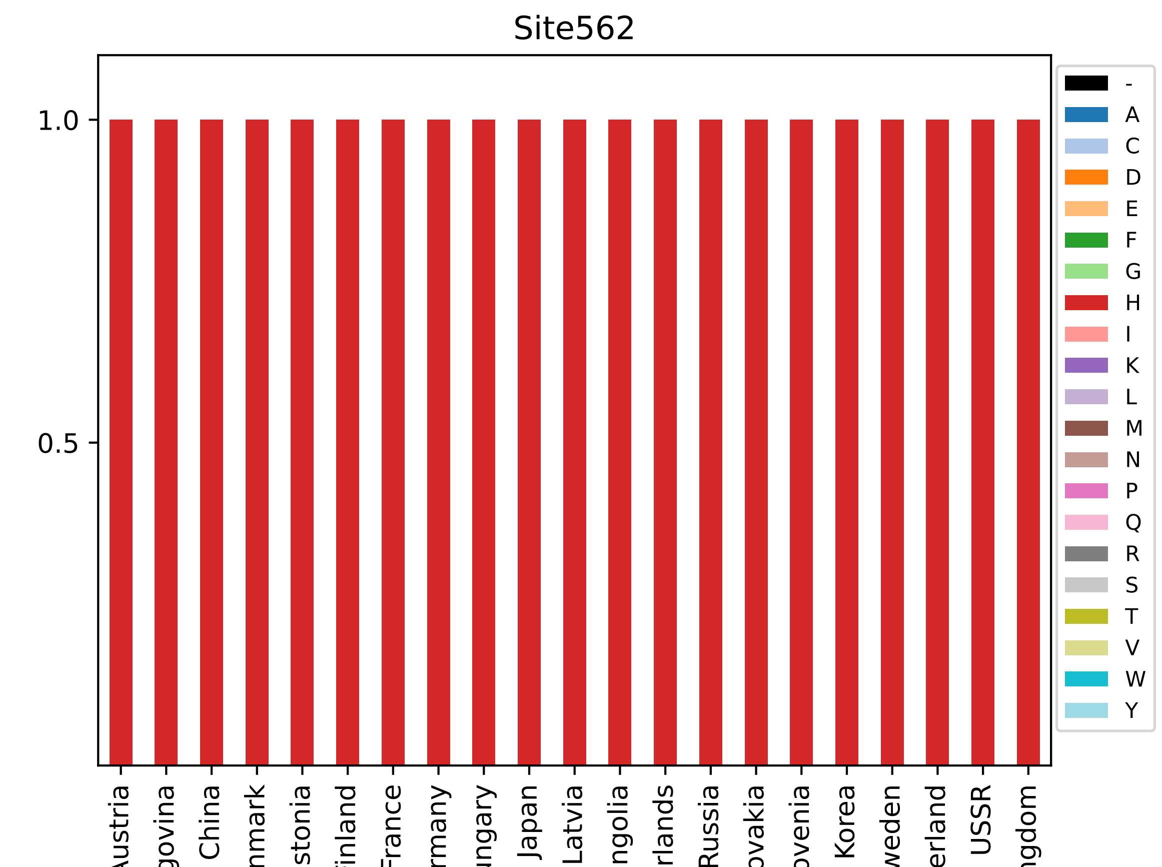
Site562
1.0
0.5
-
A
C
D
E
F
G
H
I
K
L
M
N
P
Q
R
S
T
V
W
Y
Austria
China
Japan
Latvia
Russia
Korea
USSR

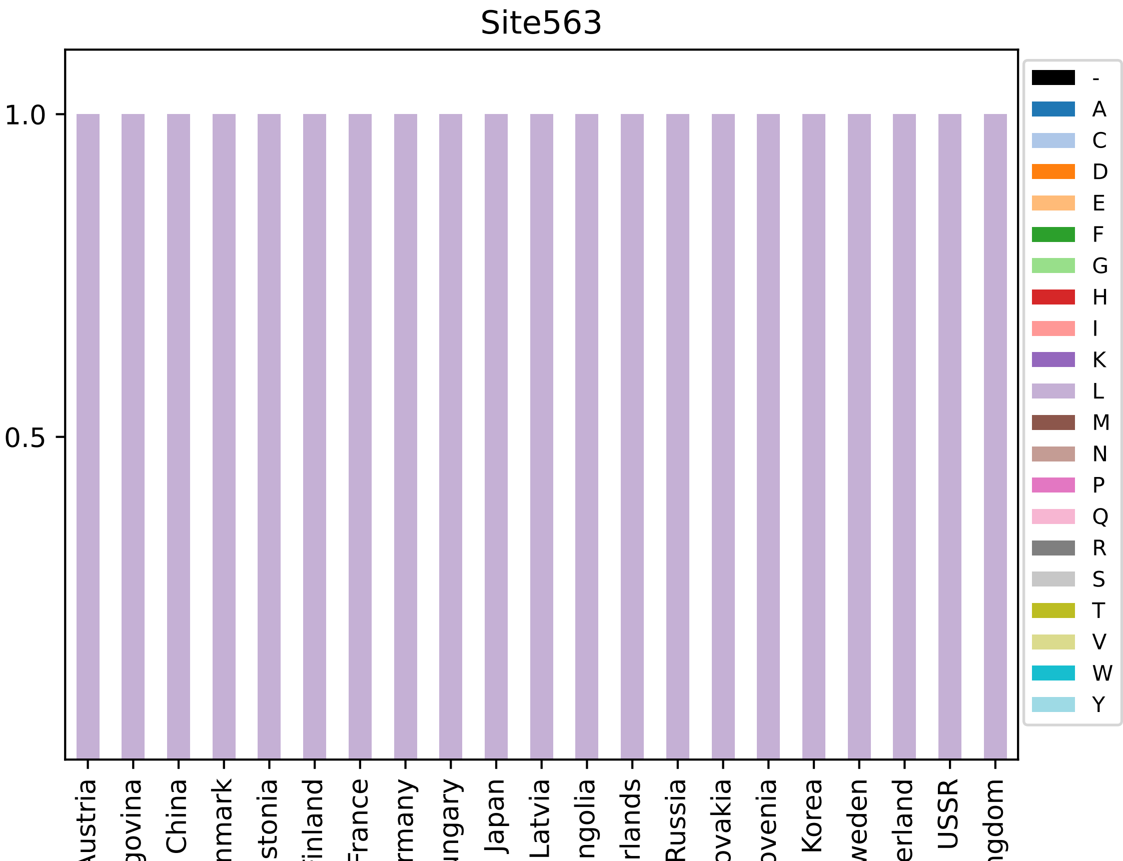

Site563
1.0
0.5
-
A
C
D
E
F
G
H
I
K
L
M
N
P
Q
R
S
T
V
W
Y
China
Japan
Latvia
Russia
Korea
USSR

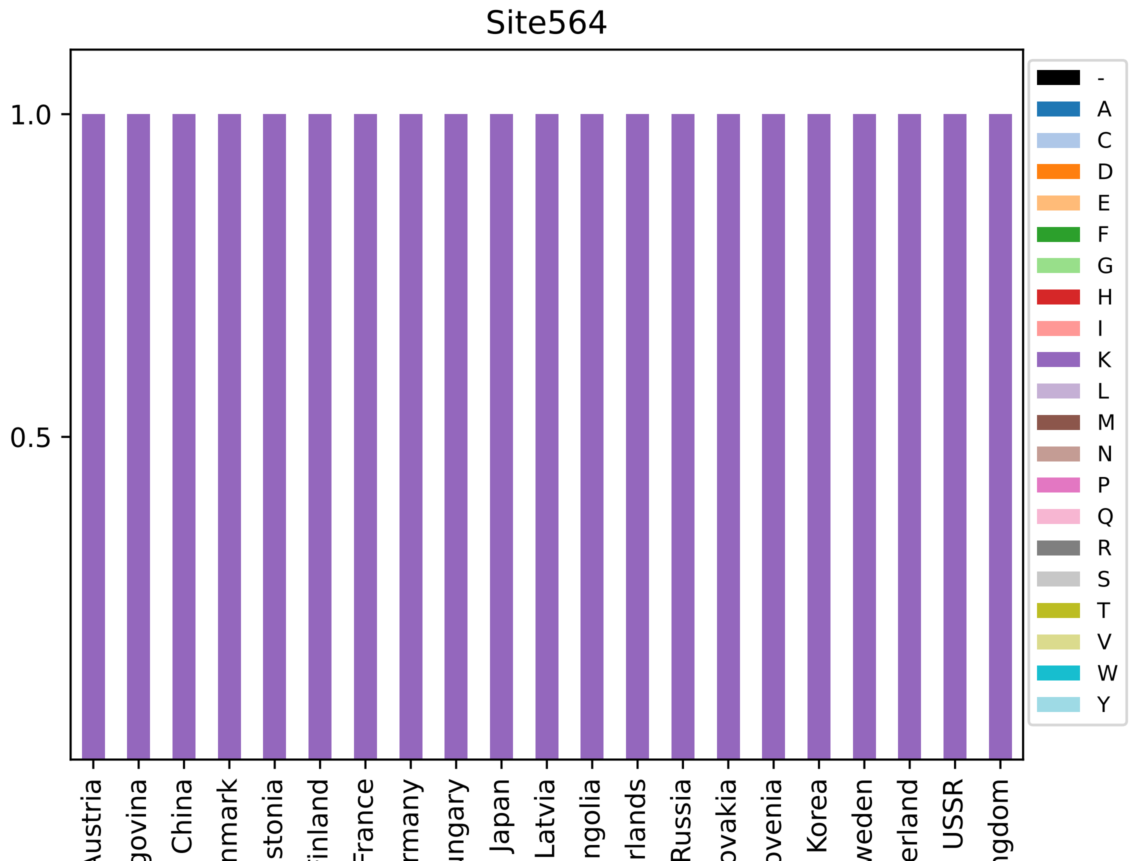
Site564
1.0
0.5
-
A
C
D
E
F
G
H
I
K
L
M
N
P
Q
R
S
T
V
W
Y
China
Japan
Latvia
Russia
Korea
USSR

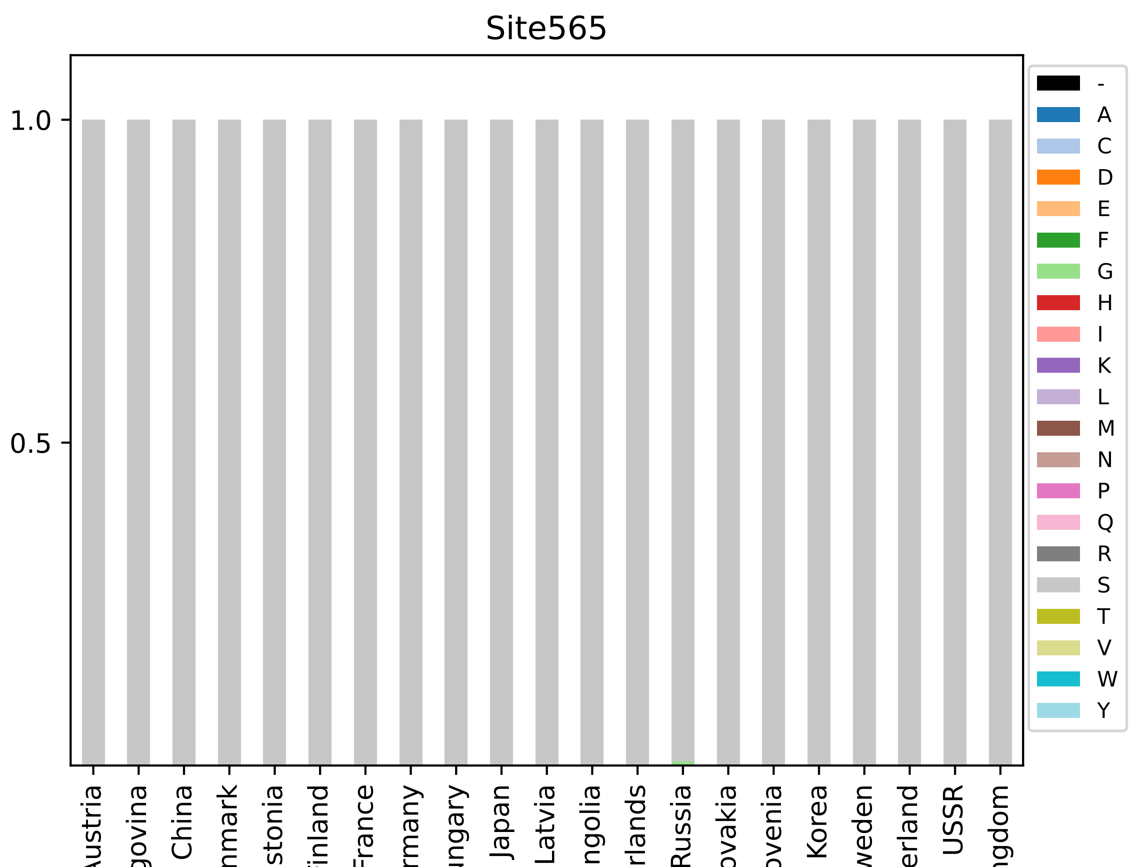
Site565
1.0
0.5
-
A
C
D
E
F
G
H
I
K
L
M
N
P
Q
R
S
T
V
W
Y
Austria
China
Japan
Latvia
Russia
Korea
USSR

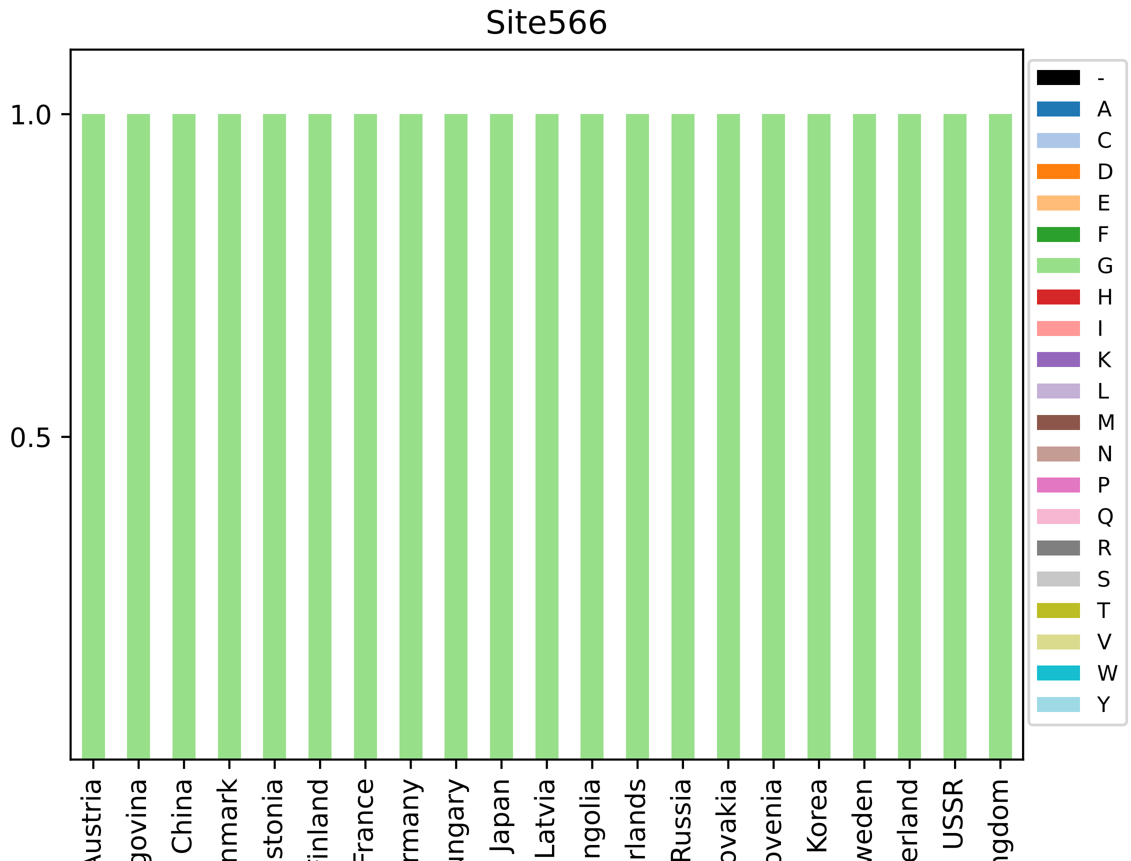
Site566
1.0
0.5
-
A
C
D
E
F
G
H
I
K
L
M
N
P
Q
R
S
T
V
W
Y
Austria
govina
China
nmark
stonia
Finland
France
rmany
ungary
Japan
Latvia
ngolia
rlands
Russia
ovakia
ovenia
Korea
weden
erland
USSR
ngdom

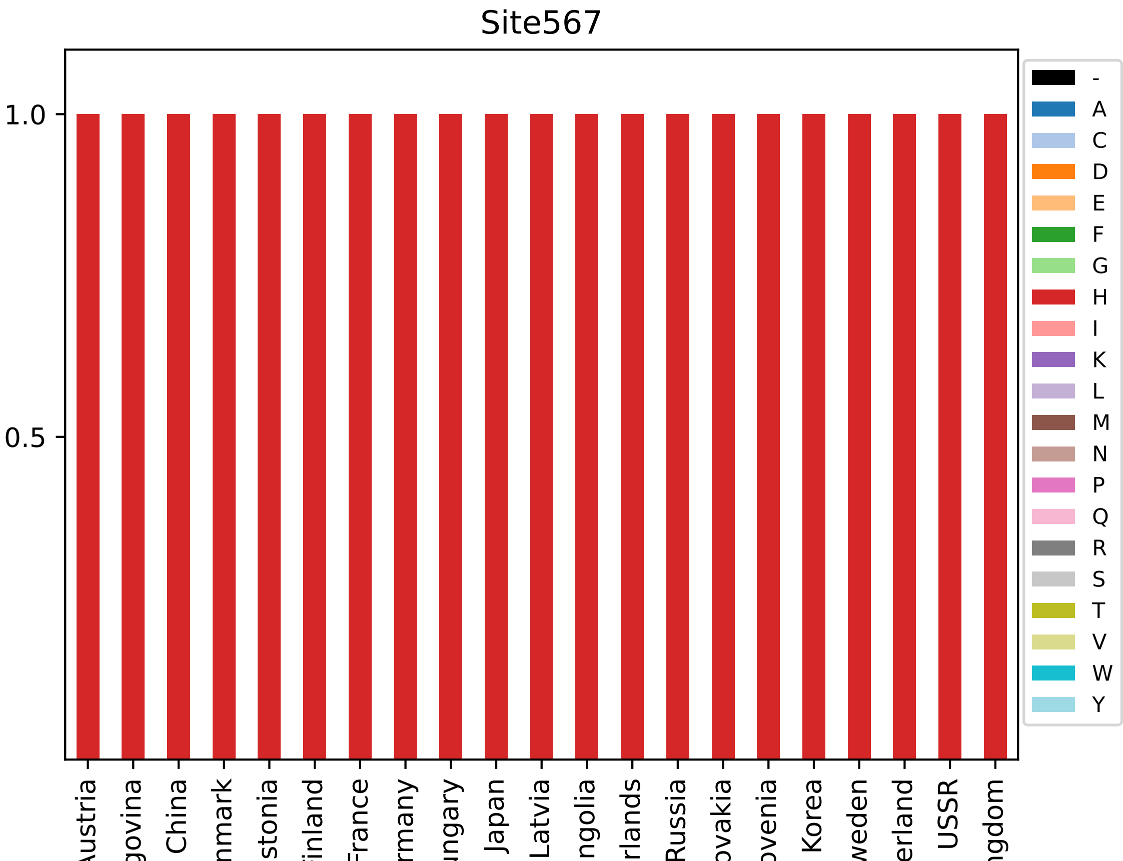

Site567
1.0
0.5
-
A
C
D
E
F
G
H
I
K
L
M
N
P
Q
R
S
T
V
W
Y
China
Japan
Latvia
Russia
Korea
USSR

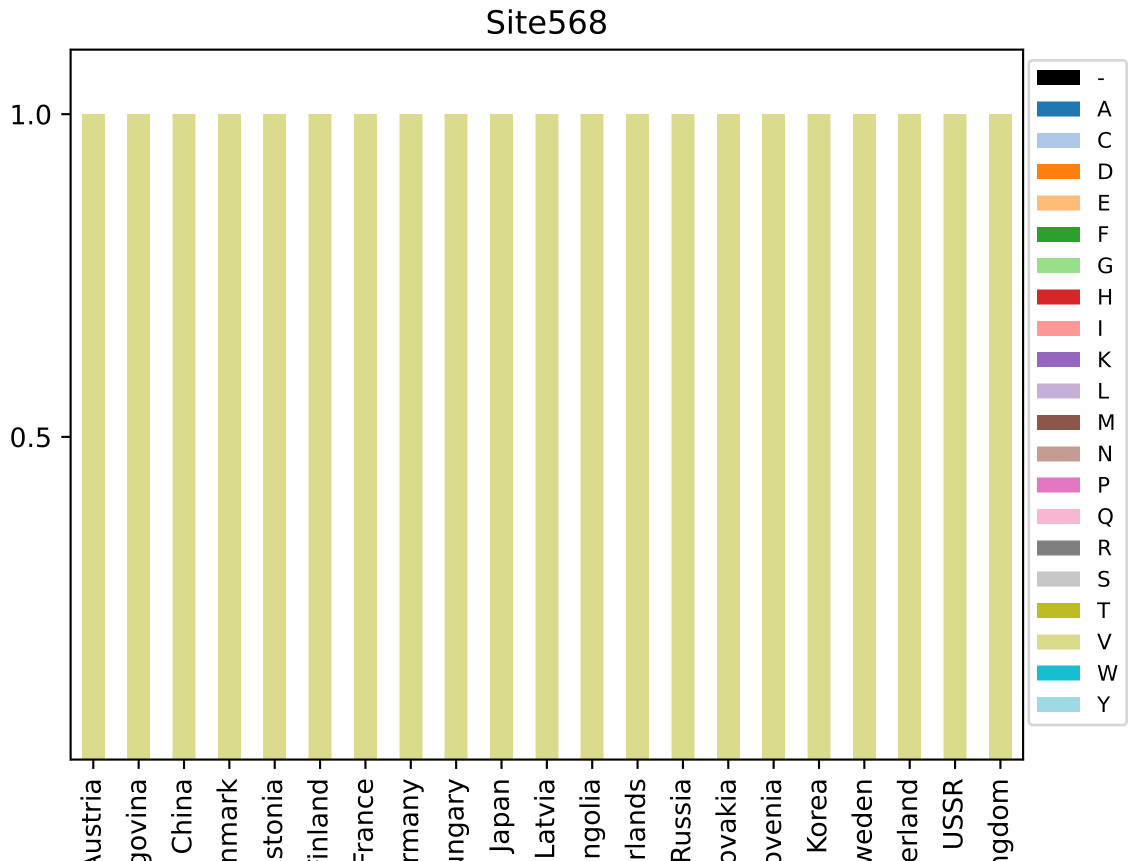
Site568
1.0
0.5
-
A
C
D
E
F
G
H
I
K
L
M
N
P
Q
R
S
T
V
W
Y
Austria
govina
China
nmark
stonia
Finland
France
rmany
ungary
Japan
Latvia
ngolia
rlands
Russia
ovakia
ovenia
Korea
weden
erland
USSR
ngdom

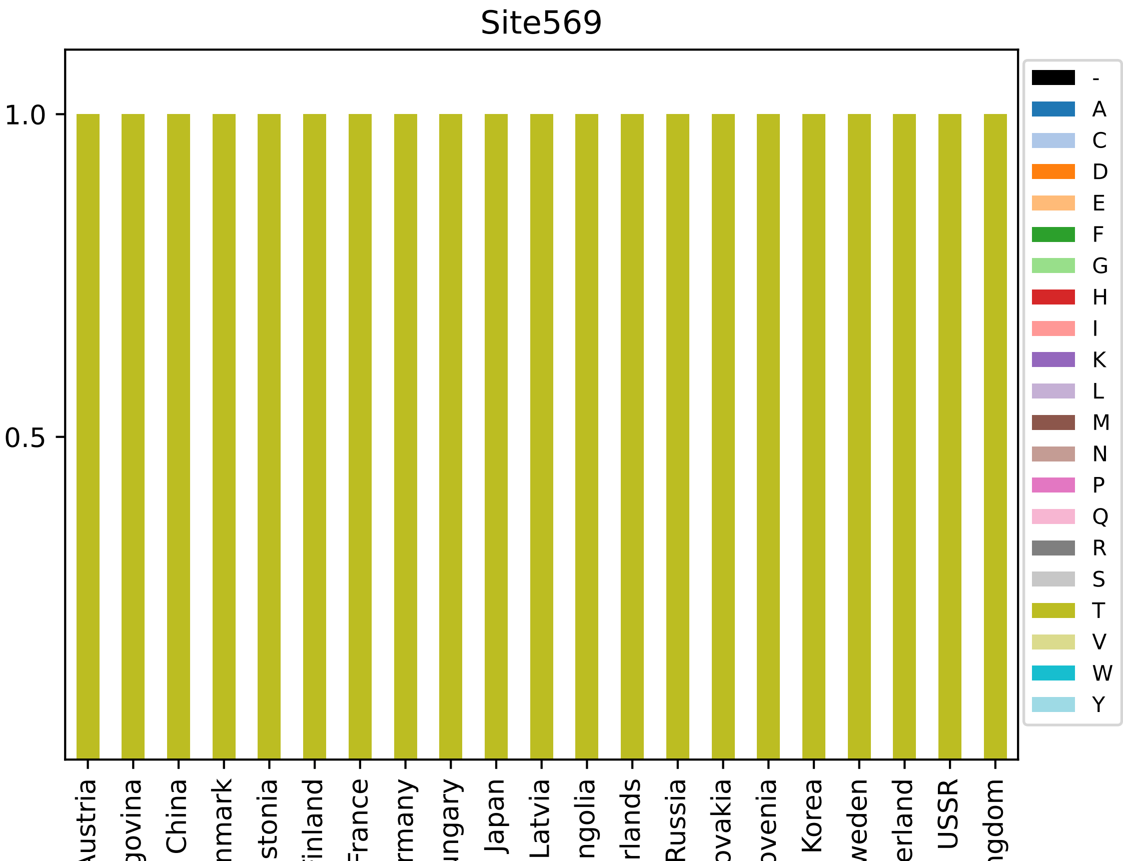

Site569
1.0
0.5
-
A
C
D
E
F
G
H
I
K
L
M
N
P
Q
R
S
T
V
W
Y
China
Japan
Latvia
Russia
Korea
USSR

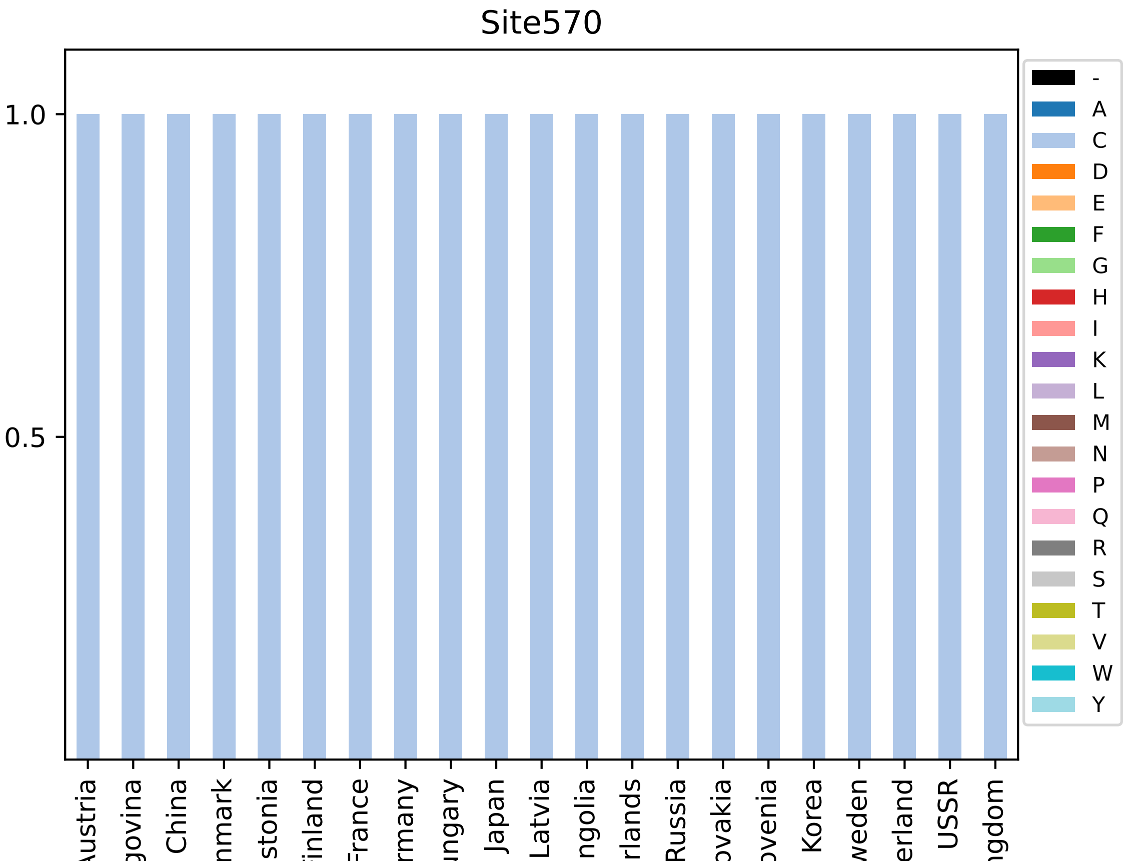
Site570
1.0
0.5
-
A
C
D
E
F
G
H
I
K
L
M
N
P
Q
R
S
T
V
W
Y
China
Japan
Latvia
Russia
Korea
USSR

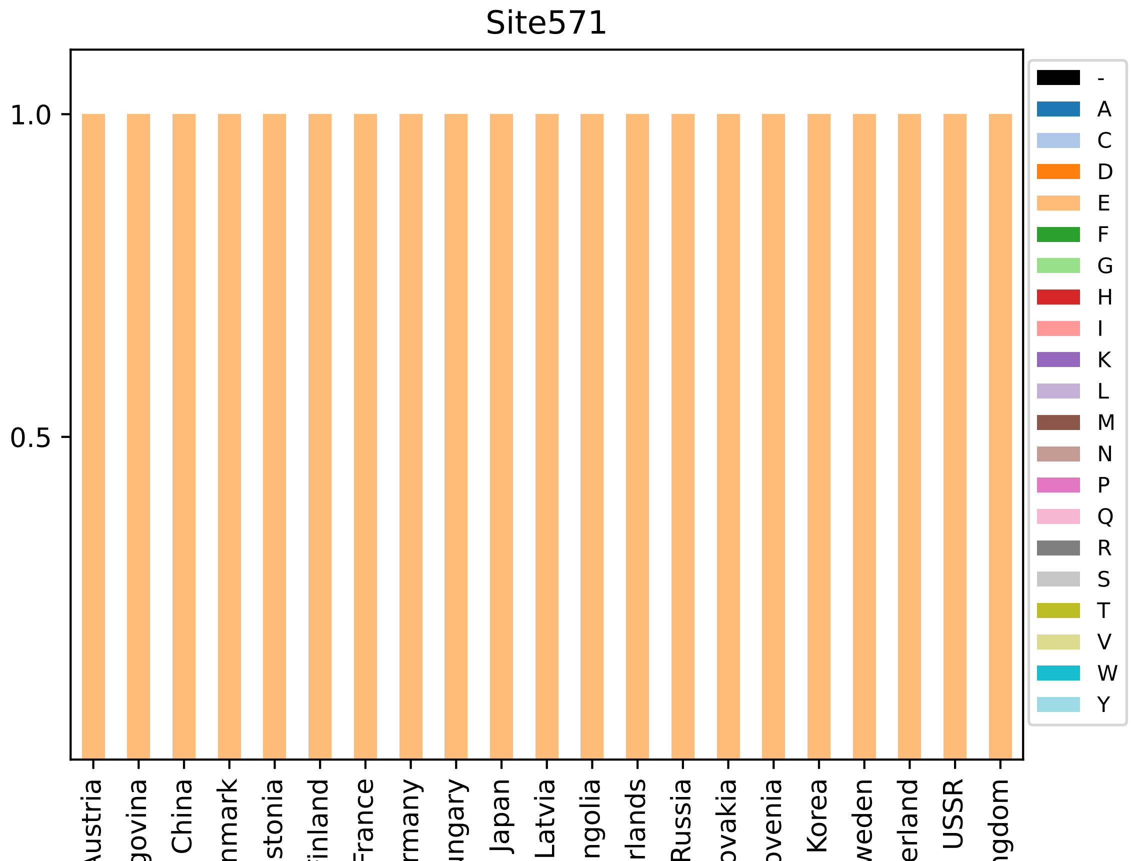
Site571
1.0
0.5
China
Japan
Latvia
Russia
Korea
USSR
-
A
C
D
E
F
G
H
I
K
L
M
N
P
Q
R
S
T
V
W
Y

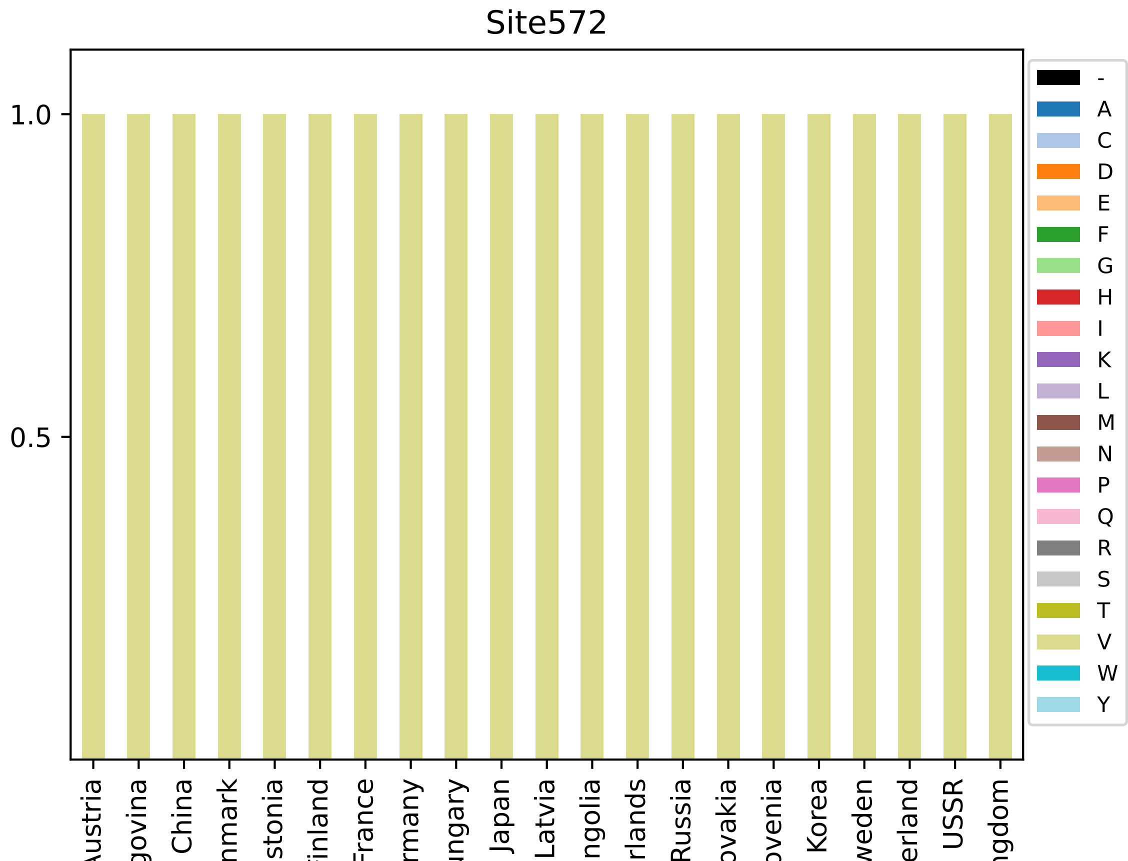

Site572
1.0
0.5
-
A
C
D
E
F
G
H
I
K
L
M
N
P
Q
R
S
T
V
W
Y
China
Japan
Latvia
Russia
Korea
USSR

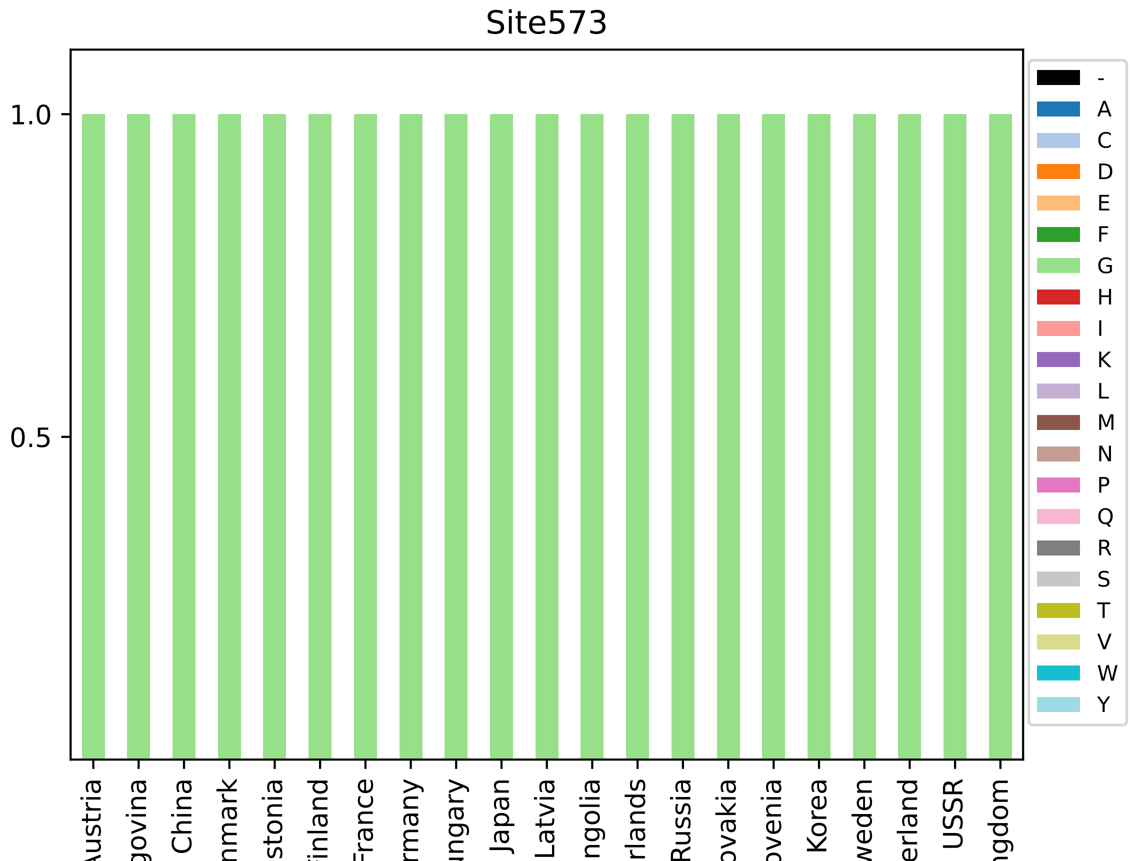
Site573
1.0
0.5
China
Japan
Latvia
Russia
Korea
USSR
-
A
C
D
E
F
G
H
I
K
L
M
N
P
Q
R
S
T
V
W
Y

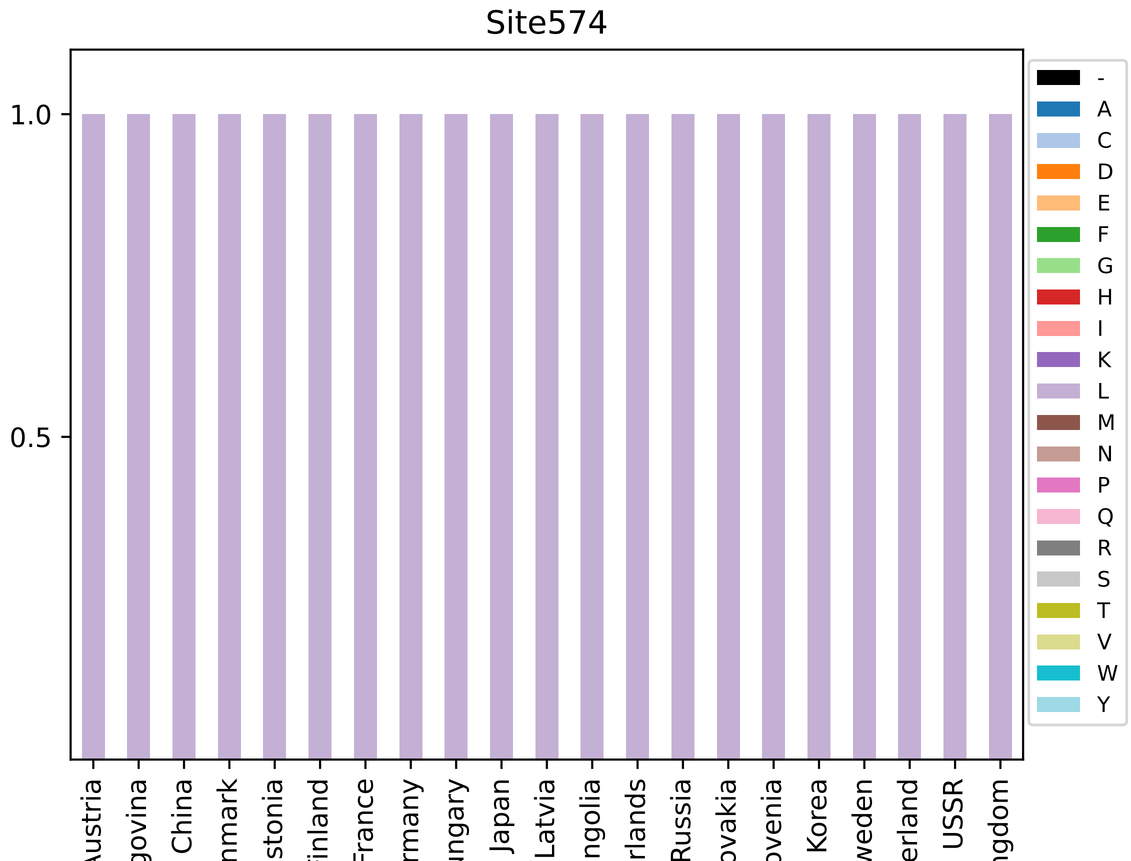
Site574
1.0
0.5
-
A
C
D
E
F
G
H
I
K
L
M
N
P
Q
R
S
T
V
W
Y
China
Japan
Latvia
Russia
Korea
USSR

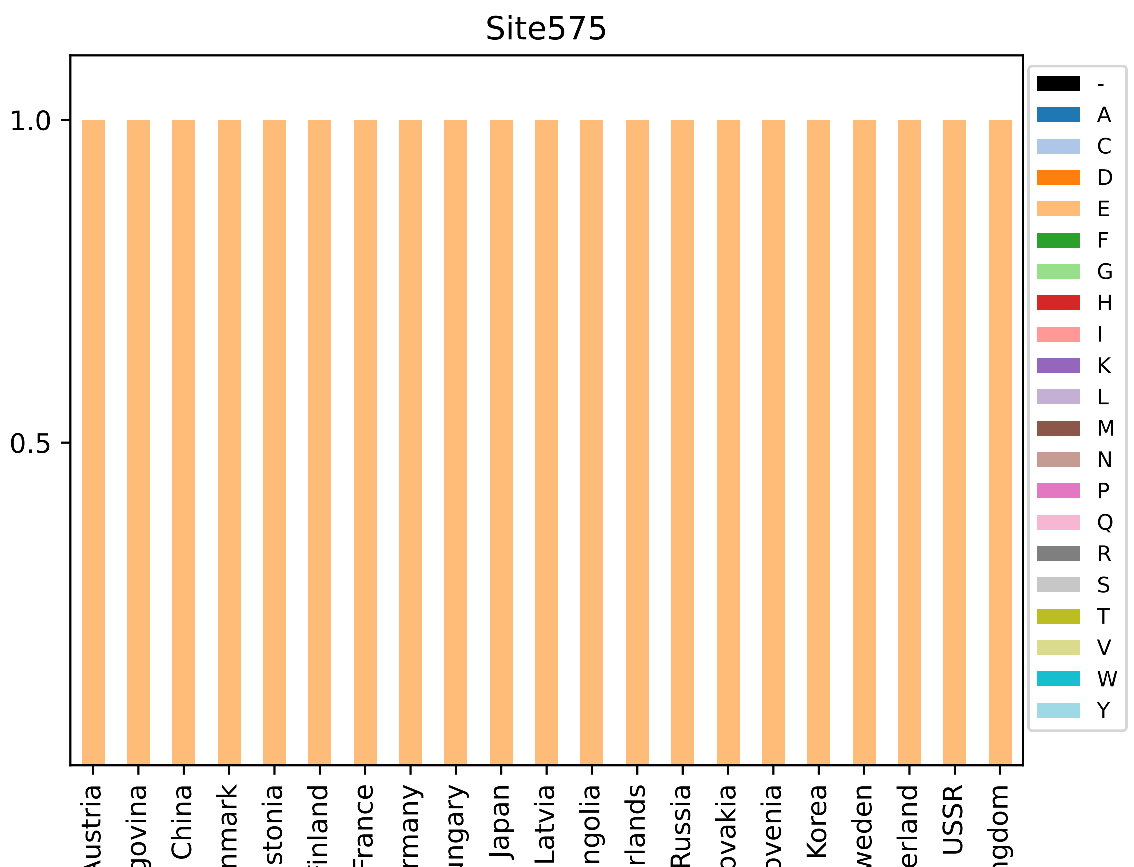

Site575
1.0
0.5
-
A
C
D
E
F
G
H
I
K
L
M
N
P
Q
R
S
T
V
W
Y
China
Japan
Latvia
Russia
Korea
USSR

Site576

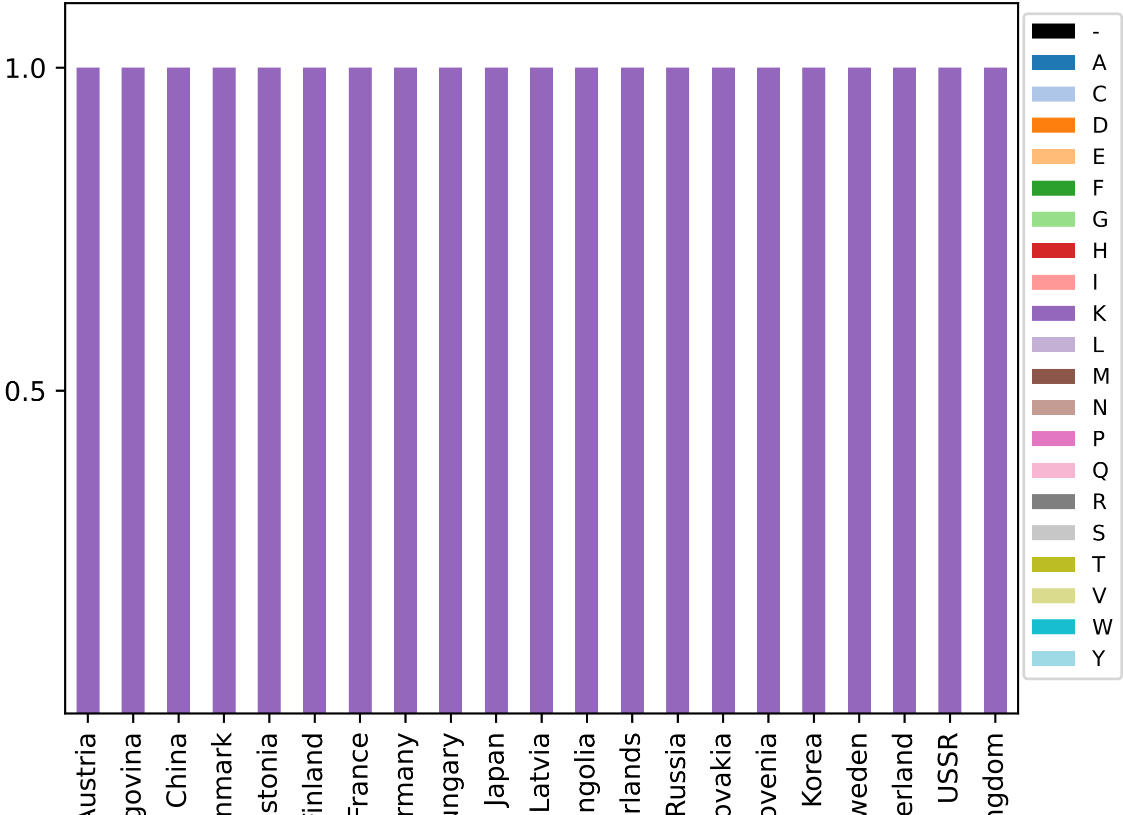

1.0
0.5
-
A
C
D
E
F
G
H
I
K
L
M
N
P
Q
R
S
T
V
W
Y
China
Japan
Latvia
Russia
Korea
USSR

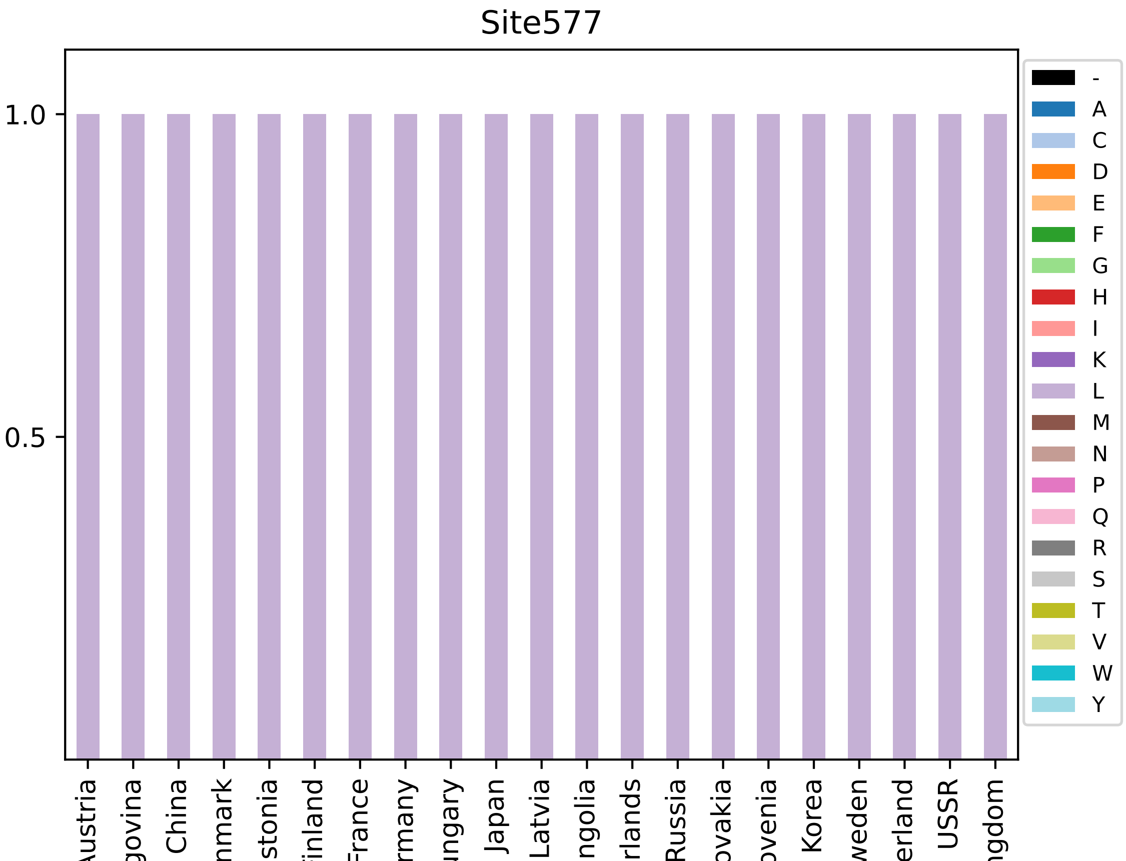
Site577
1.0
0.5
-
A
C
D
E
F
G
H
I
K
L
M
N
P
Q
R
S
T
V
W
Y
China
Japan
Latvia
Russia
Korea
USSR

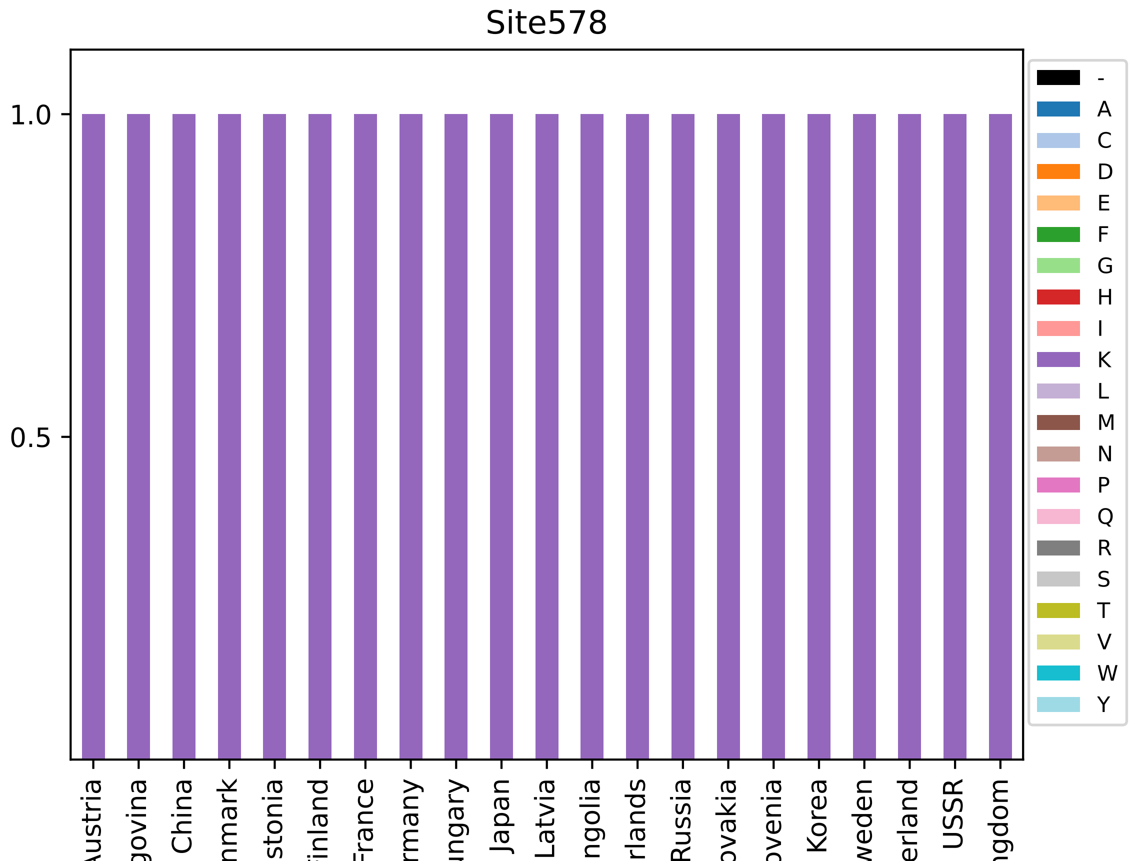
Site578
1.0
0.5
-
A
C
D
E
F
G
H
I
K
L
M
N
P
Q
R
S
T
V
W
Y
Austria
China
Japan
Latvia
Russia
Korea
USSR

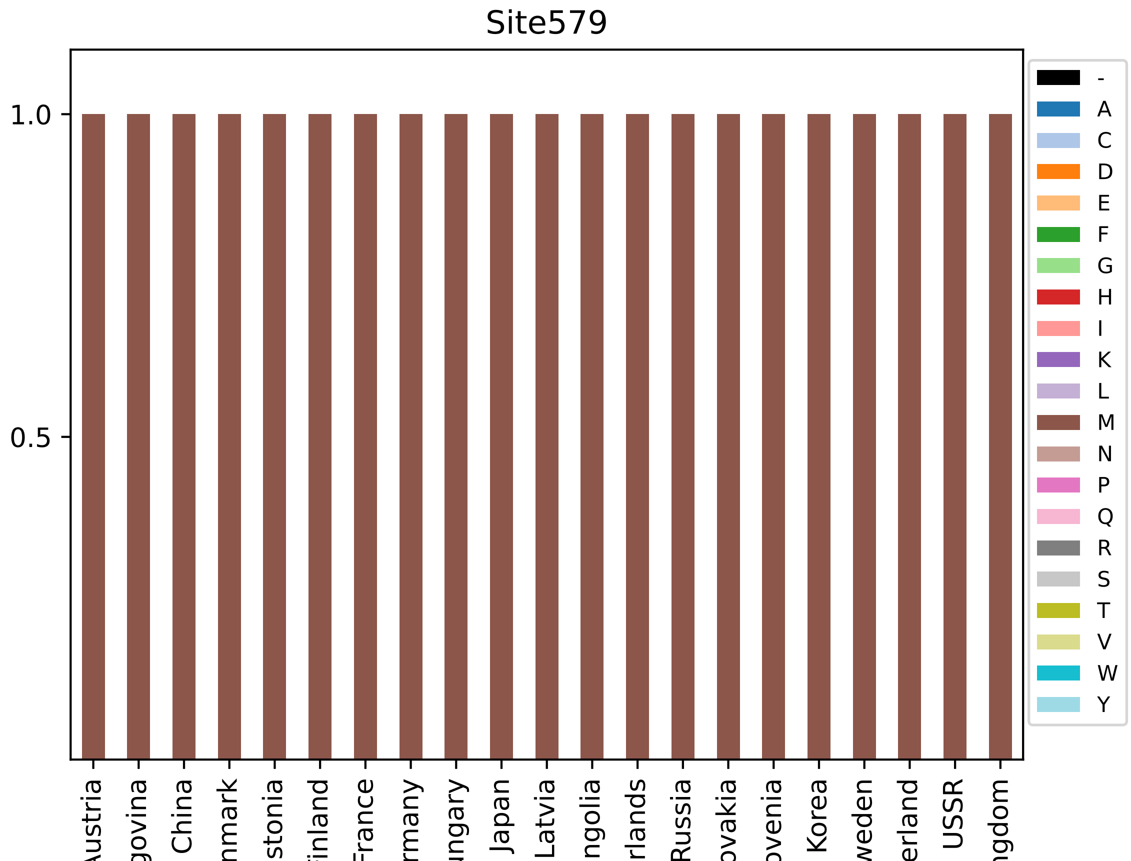

Site579
1.0
0.5
-
A
C
D
E
F
G
H
I
K
L
M
N
P
Q
R
S
T
V
W
Y
Austria
govina
China
nmark
stonia
inland
France
rmany
ungary
Japan
Latvia
ngolia
rlands
Russia
ovakia
ovenia
Korea
weden
erland
USSR
ngdom

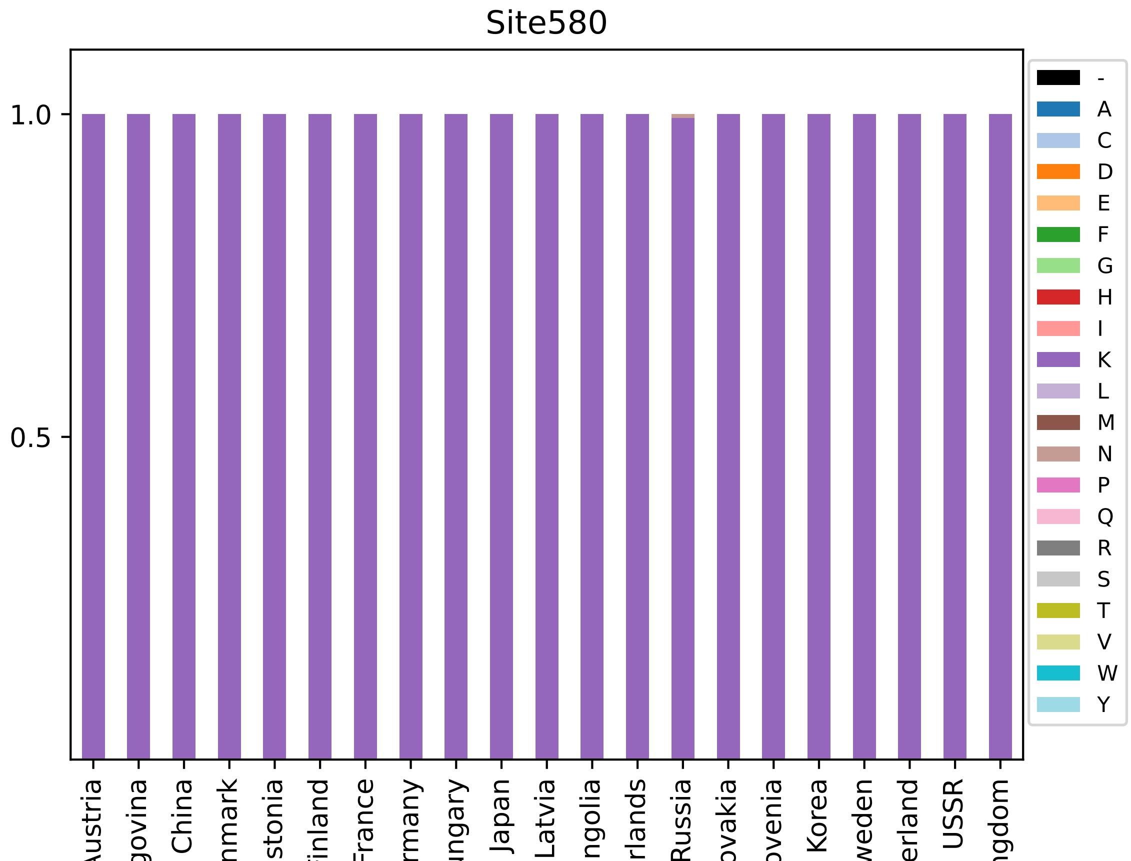
Site580
1.0
0.5
-
A
C
D
E
F
G
H
I
K
L
M
N
P
Q
R
S
T
V
W
Y
Austria
govina
China
nmark
stonia
inland
France
rmany
ungary
Japan
Latvia
ngolia
rlands
Russia
ovakia
ovenia
Korea
weden
erland
USSR
ngdom

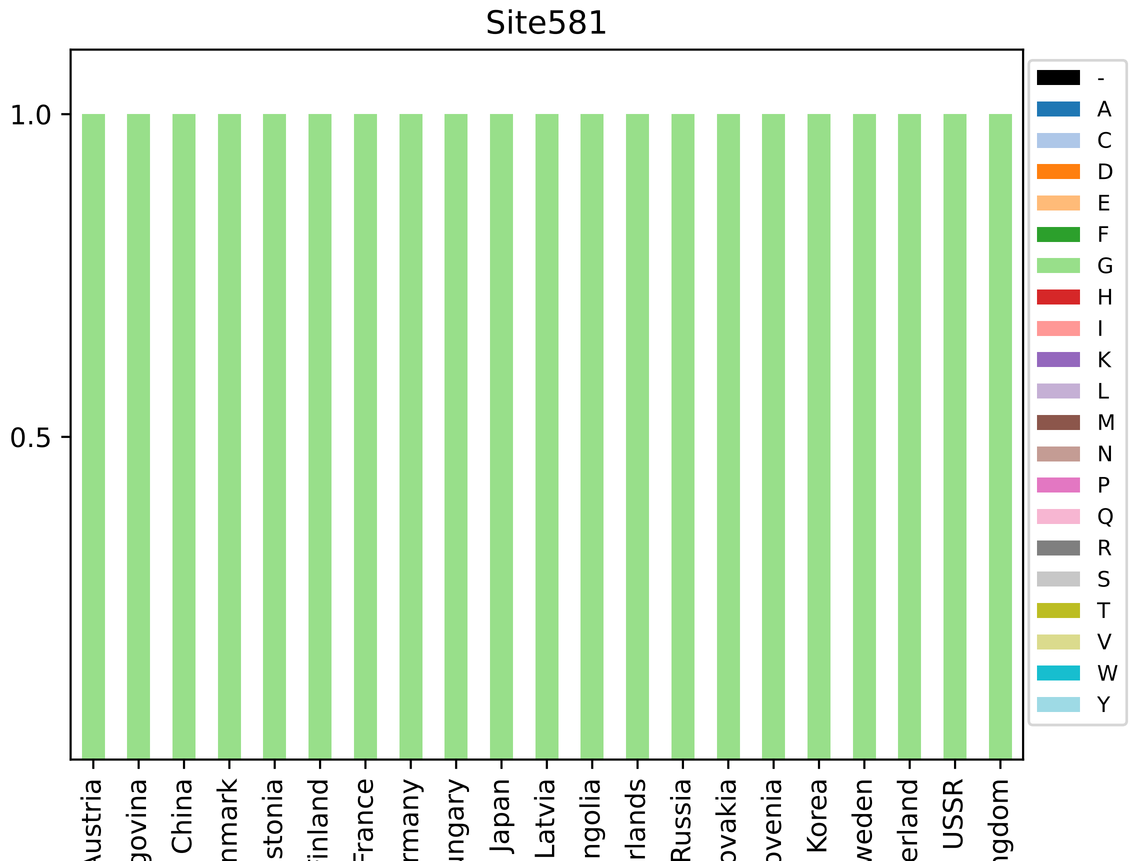
Site581
1.0
0.5
China
Japan
Latvia
Russia
Korea
USSR
-
A
C
D
E
F
G
H
I
K
L
M
N
P
Q
R
S
T
V
W
Y

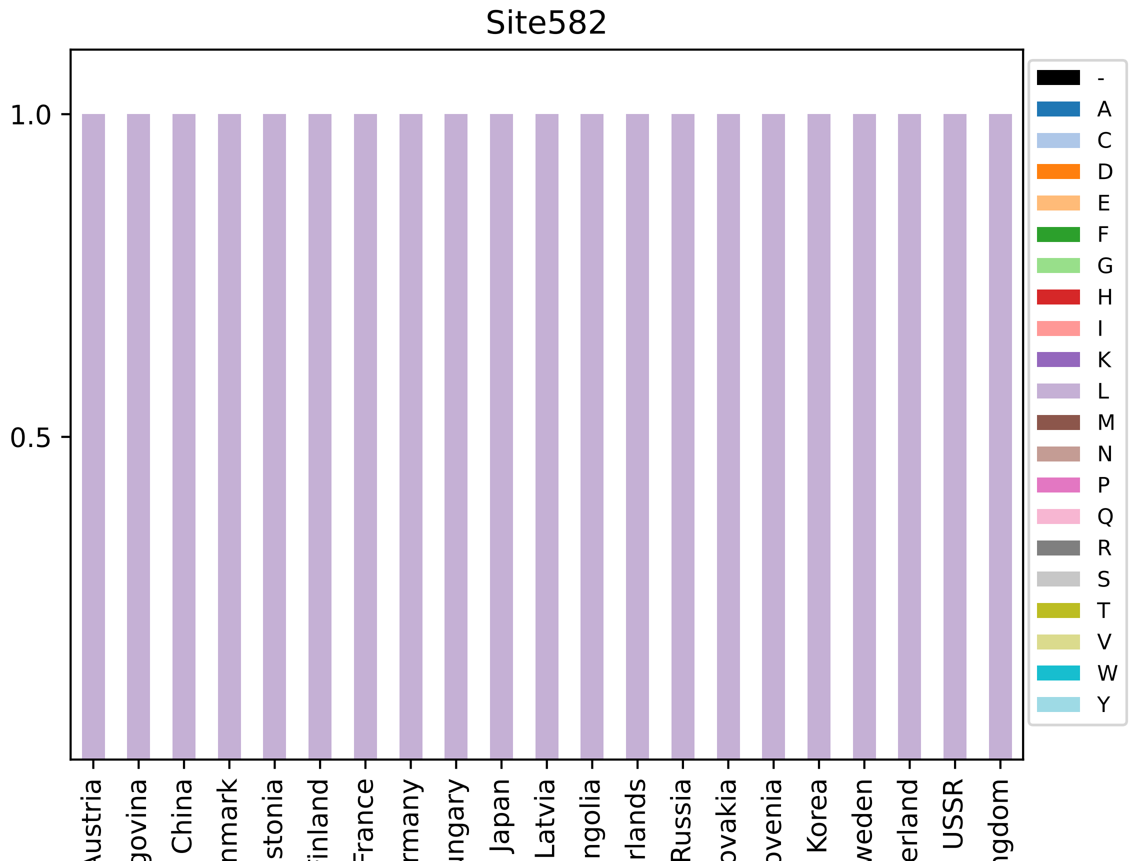
Site582
1.0
0.5
-
A
C
D
E
F
G
H
I
K
L
M
N
P
Q
R
S
T
V
W
Y
Austria
China
Japan
Latvia
Russia
Korea
USSR

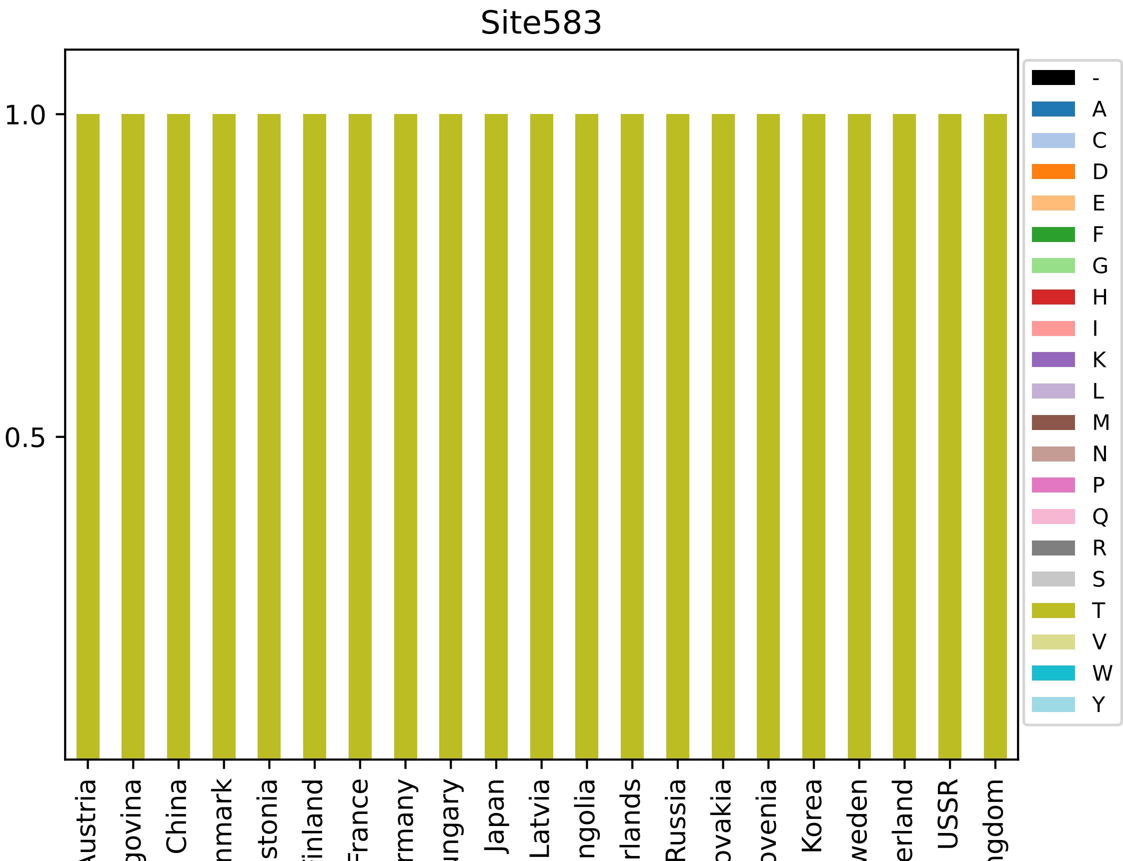
Site583
1.0
0.5
-
A
C
D
E
F
G
H
I
K
L
M
N
P
Q
R
S
T
V
W
Y
Austria
govina
China
nmark
stonia
inland
France
rmany
ungary
Japan
Latvia
ngolia
rlands
Russia
ovakia
ovenia
Korea
weden
erland
USSR
ngdom

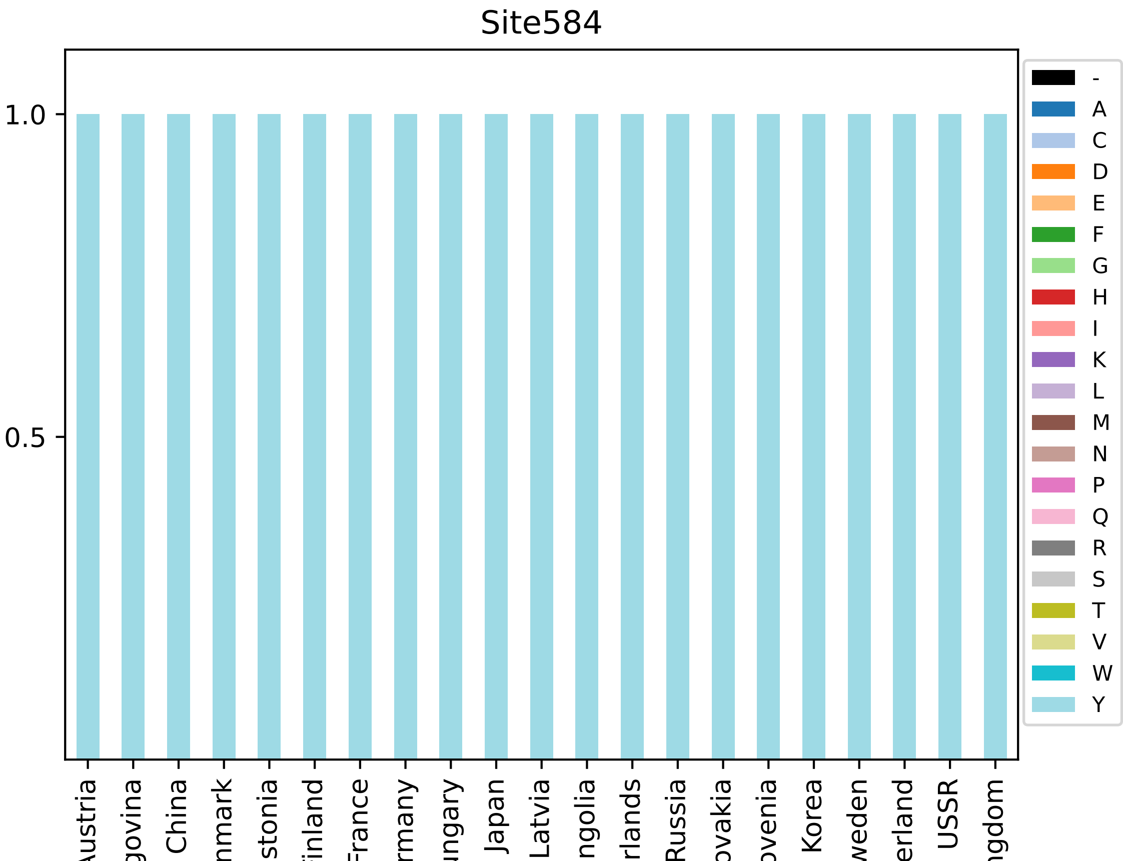

Site584
1.0
0.5
-
A
C
D
E
F
G
H
I
K
L
M
N
P
Q
R
S
T
V
W
Y
China
Japan
Latvia
Russia
Korea
USSR

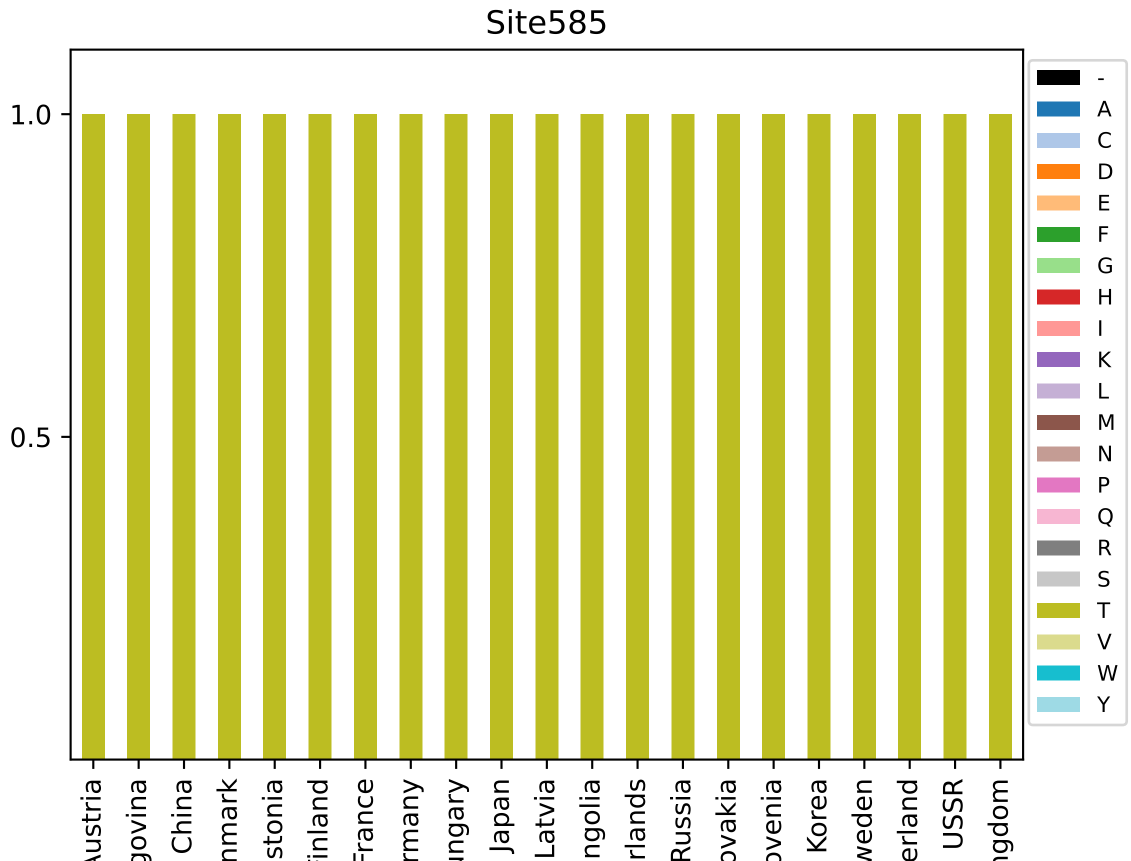
Site585
1.0
0.5
-
A
C
D
E
F
G
H
I
K
L
M
N
P
Q
R
S
T
V
W
Y
Austria
govina
China
nmark
stonia
Finland
France
rmany
ungary
Japan
Latvia
ngolia
rlands
Russia
ovakia
ovenia
Korea
weden
erland
USSR
ngdom

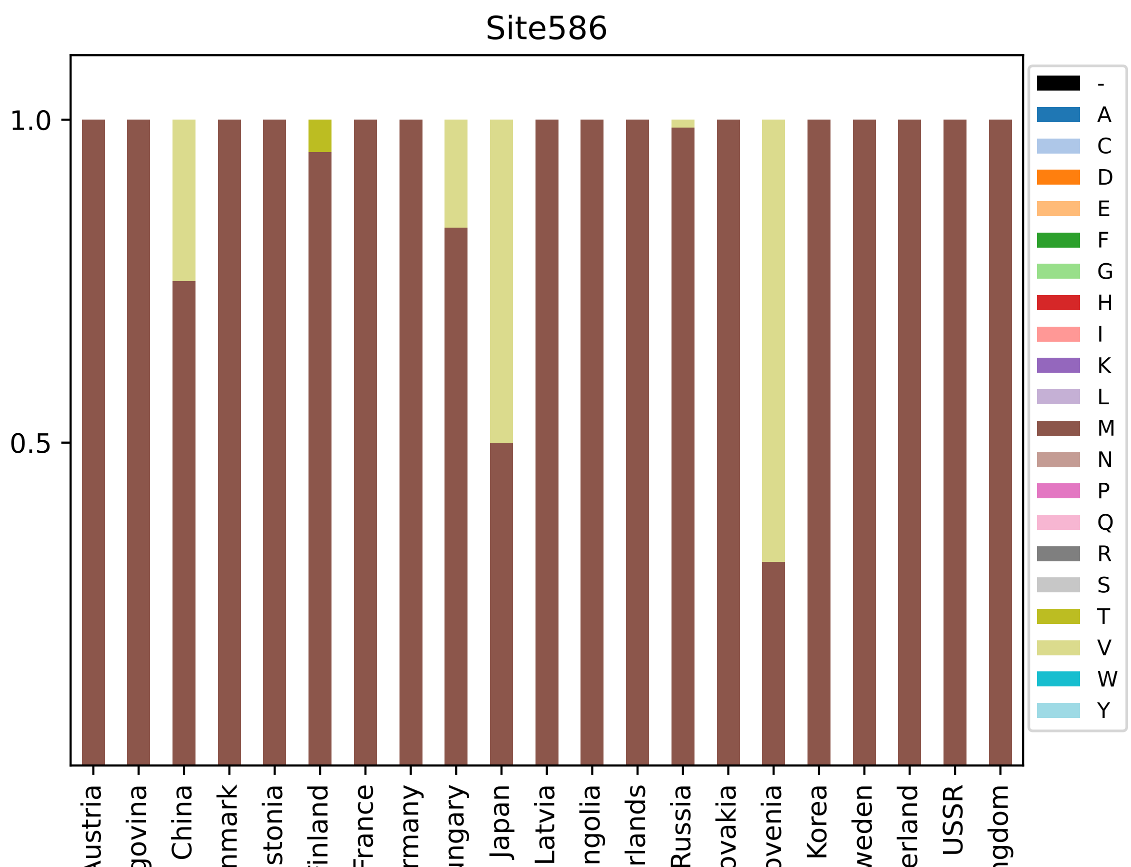

Site586
1.0
0.5
-
A
C
D
E
F
G
H
I
K
L
M
N
P
Q
R
S
T
V
W
Y
ustria
govina
China
nmark
stonia
inland
rance
rmany
ungary
Japan
Latvia
ngolia
rlands
Russia
ovakia
ovenia
Korea
weden
erland
USSR
ngdom

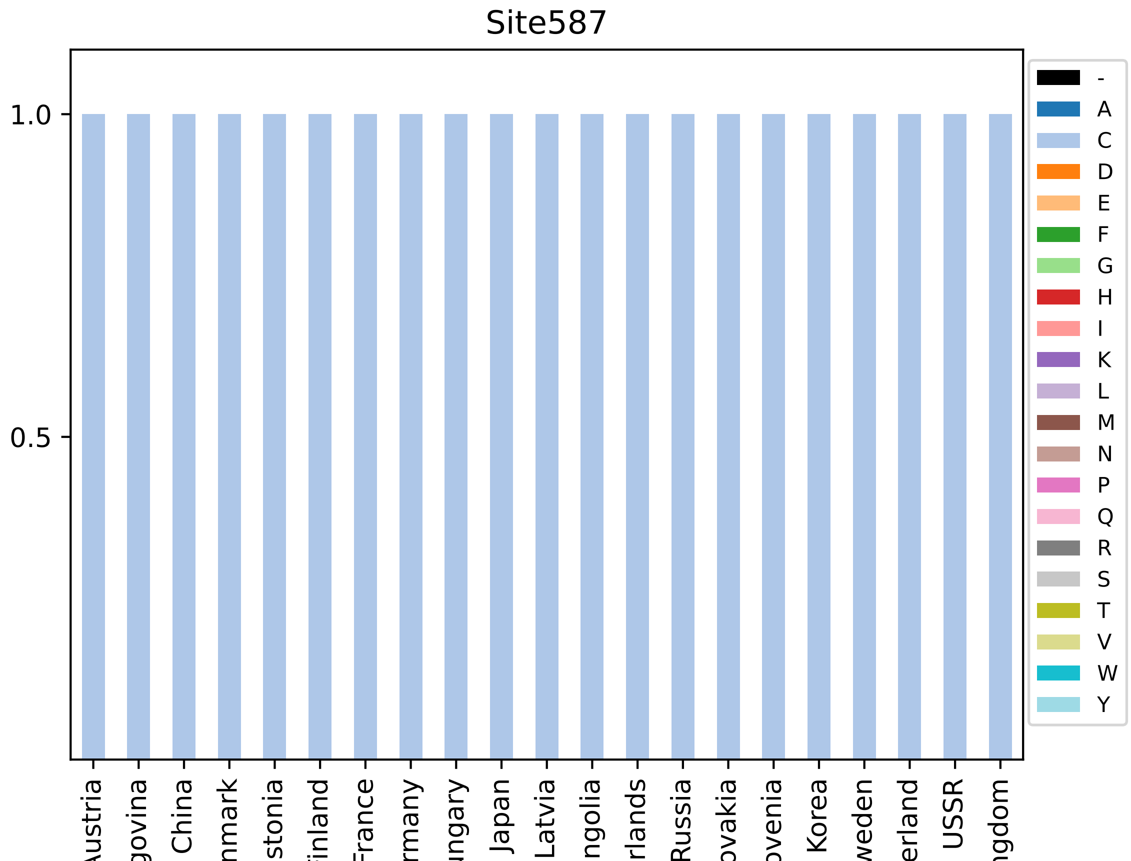
Site587
1.0
0.5
-
A
C
D
E
F
G
H
I
K
L
M
N
P
Q
R
S
T
V
W
Y
China
Japan
Latvia
Russia
Korea
USSR

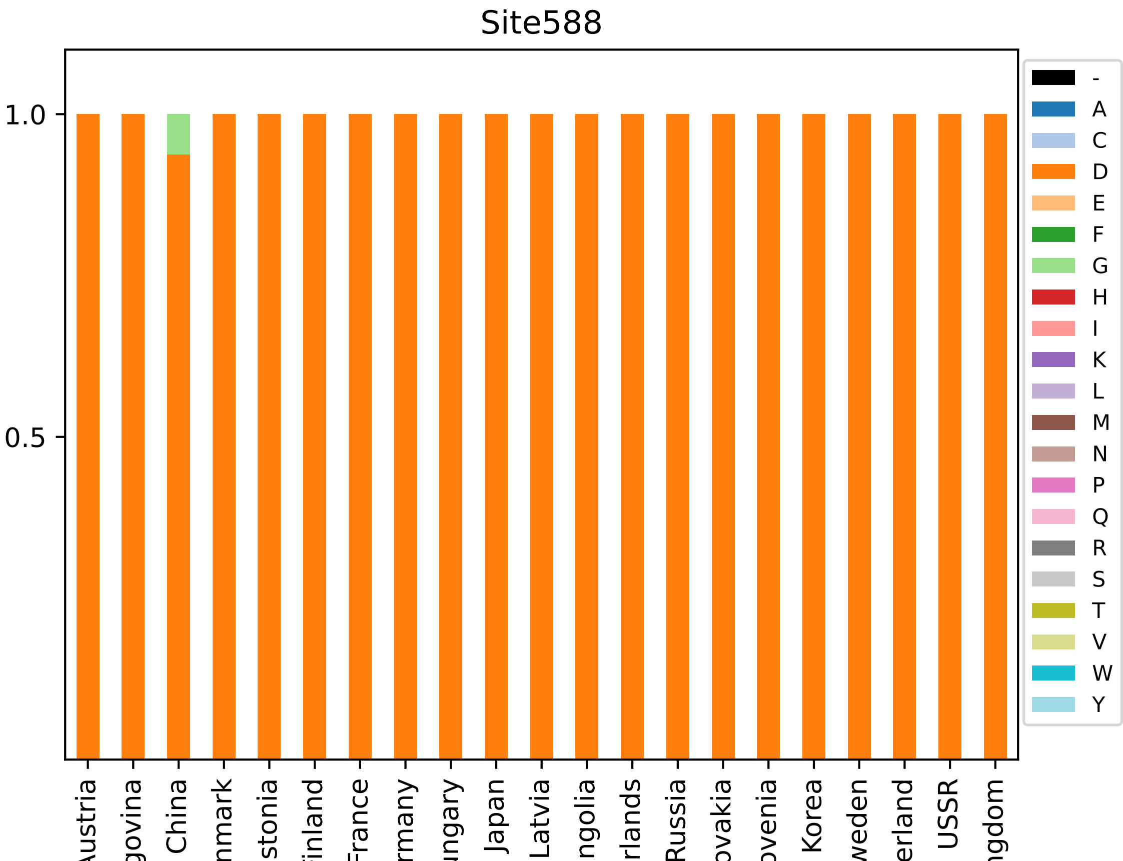

Site588
1.0
0.5
-
A
C
D
E
F
G
H
I
K
L
M
N
P
Q
R
S
T
V
W
Y
China
Japan
Latvia
Russia
Korea
USSR

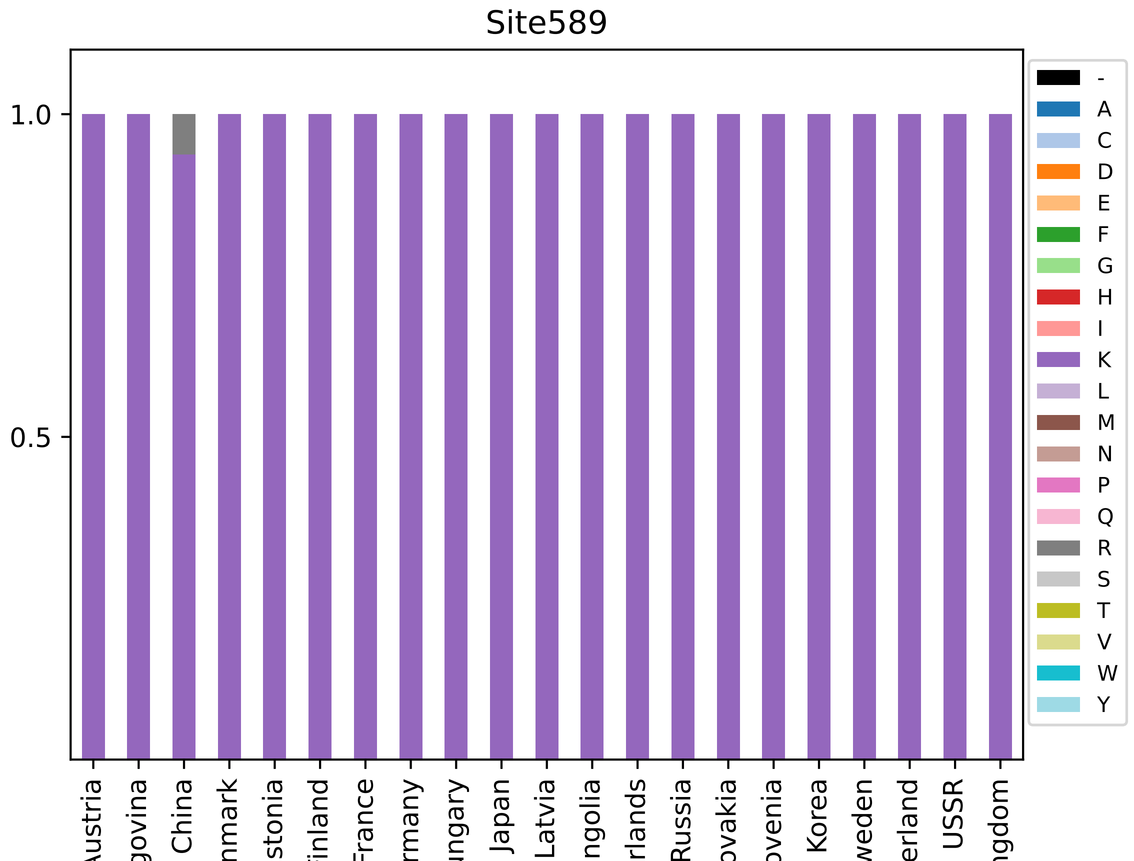
Site589
1.0
0.5
-
A
C
D
E
F
G
H
I
K
L
M
N
P
Q
R
S
T
V
W
Y
China
Japan
Latvia
Russia
Korea
USSR

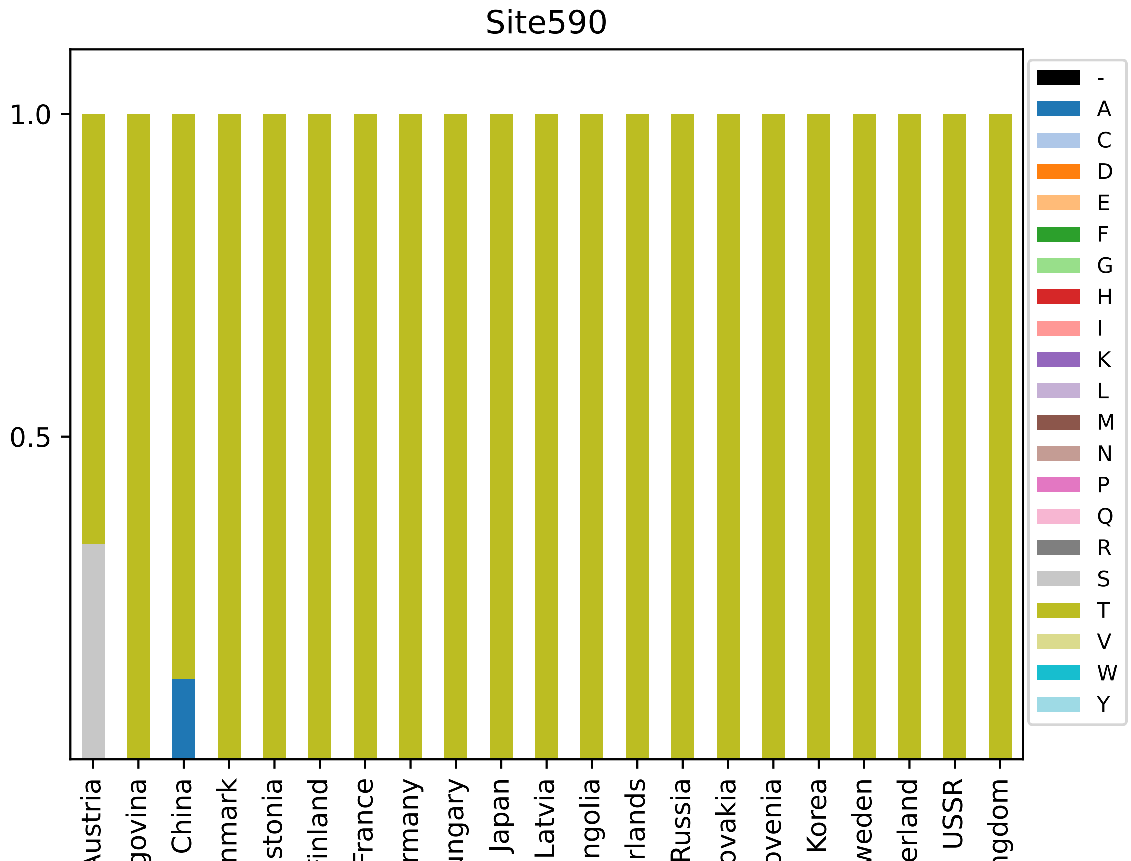

Site590
1.0
0.5
-
A
C
D
E
F
G
H
I
K
L
M
N
P
Q
R
S
T
V
W
Y
Austria
govina
China
nmark
stonia
inland
France
rmany
ungary
Japan
Latvia
ngolia
rlands
Russia
ovakia
ovenia
Korea
weden
erland
USSR
ngdom

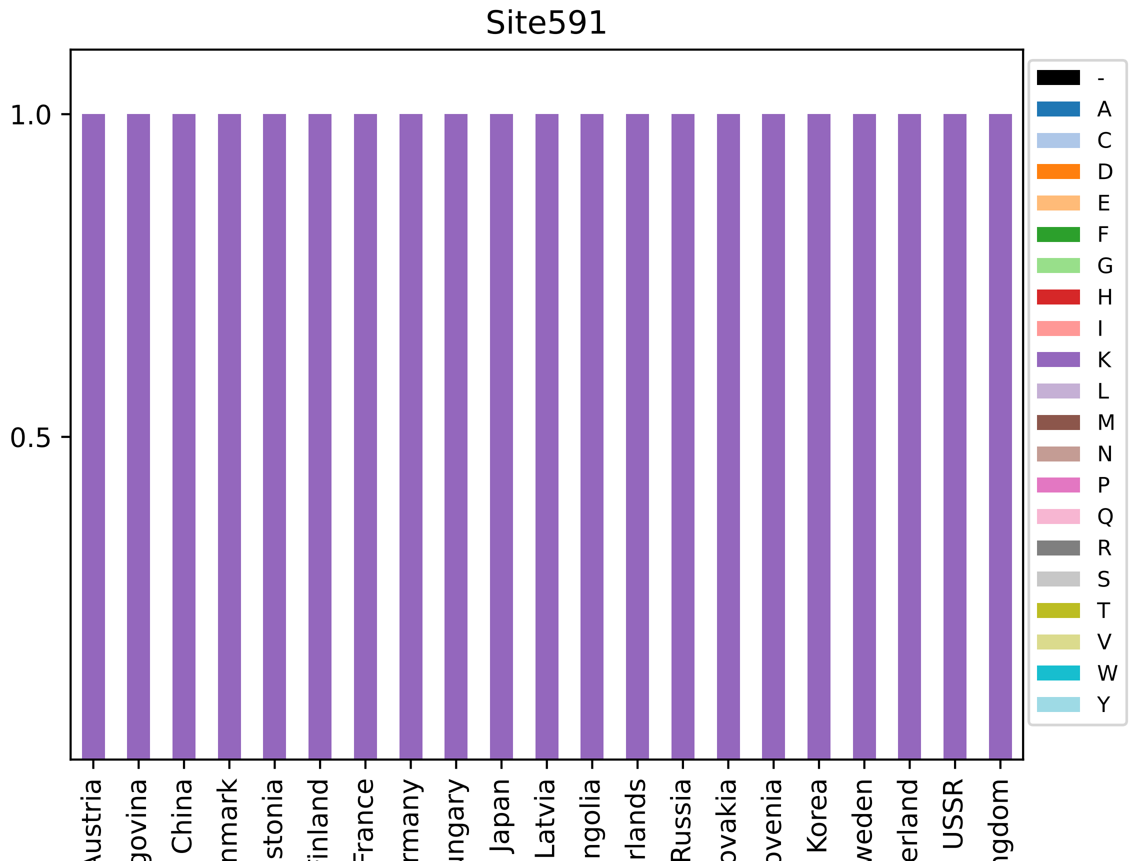

Site591
1.0
0.5
-
A
C
D
E
F
G
H
I
K
L
M
N
P
Q
R
S
T
V
W
Y
China
Japan
Latvia
Russia
Korea
USSR

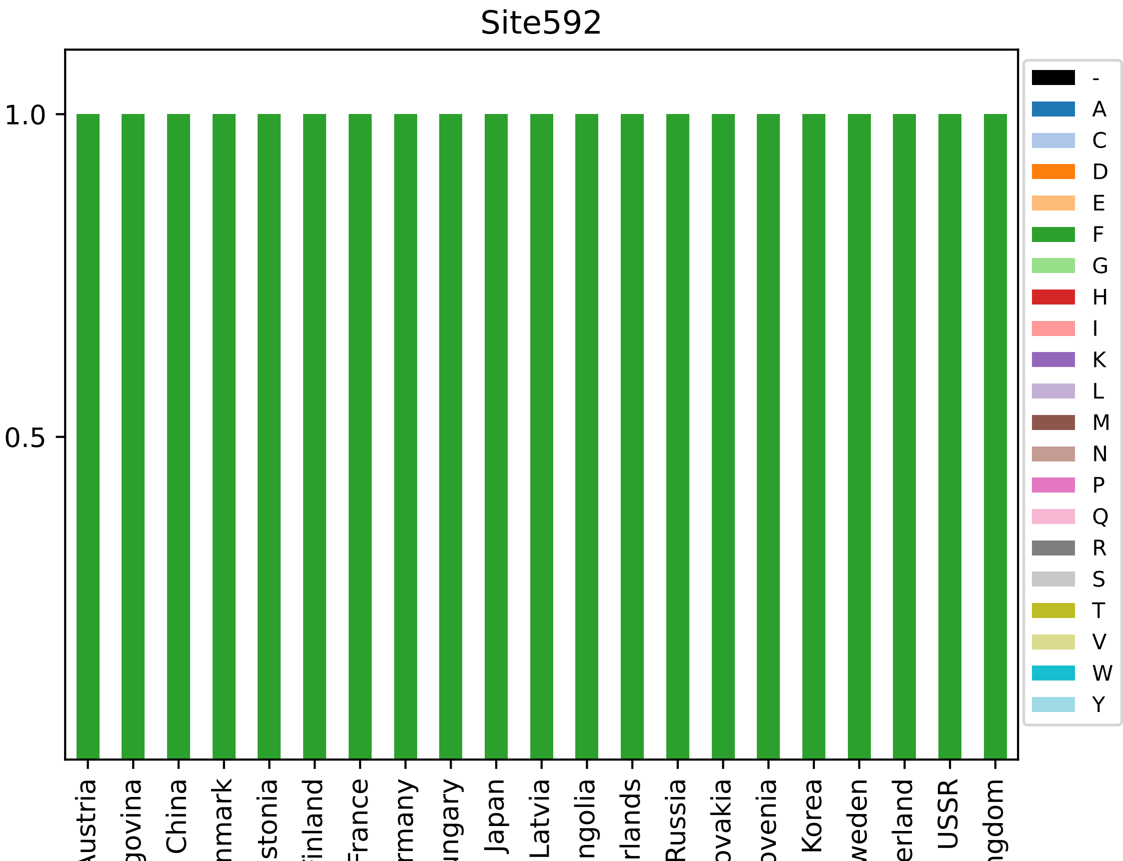
Site592
1.0
0.5
Austria
govina
China
nmark
stonia
Finland
France
rmany
ungary
Japan
Latvia
ngolia
rlands
Russia
ovakia
ovenia
Korea
weden
erland
USSR
ngdom
-
A
C
D
E
F
G
H
I
K
L
M
N
P
Q
R
S
T
V
W
Y

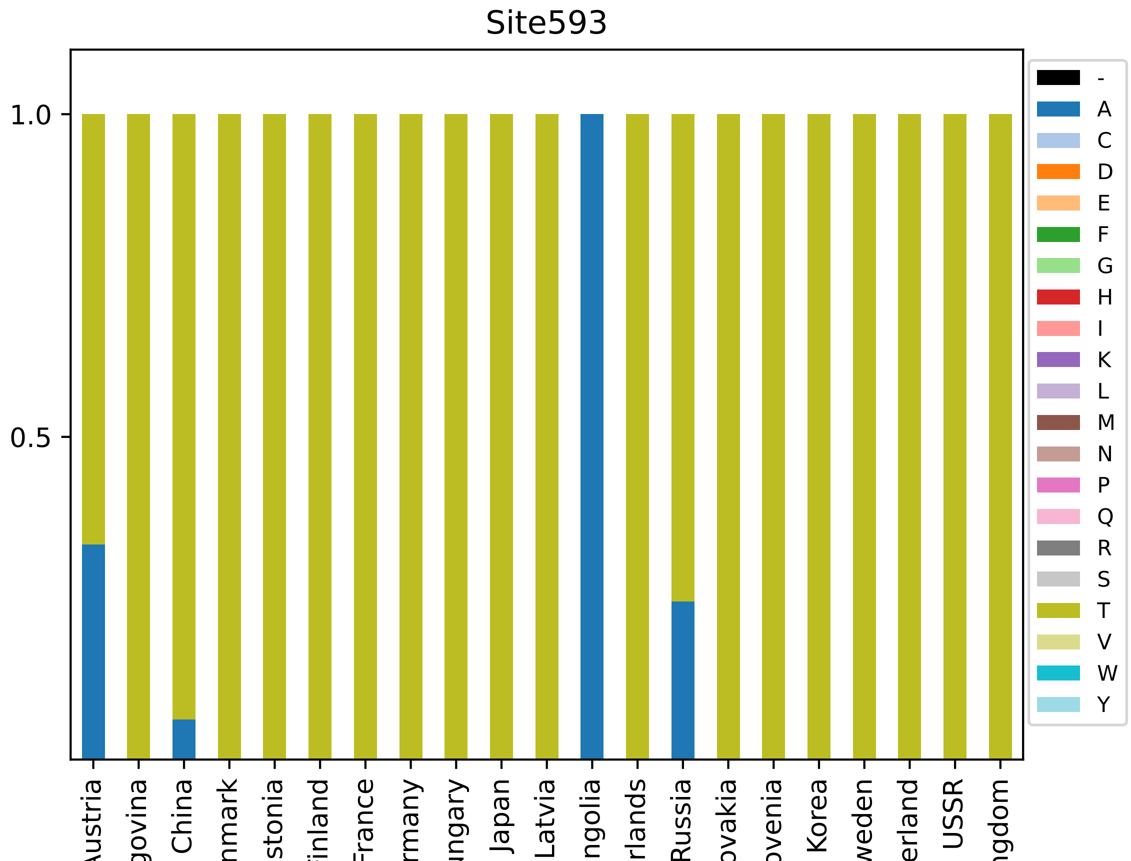

Site593
1.0
0.5
-
A
C
D
E
F
G
H
I
K
L
M
N
P
Q
R
S
T
V
W
Y
Austria
govina
China
nmark
stonia
inland
France
rmany
ungary
Japan
Latvia
ngolia
rlands
Russia
ovakia
ovenia
Korea
weden
erland
USSR
ngdom

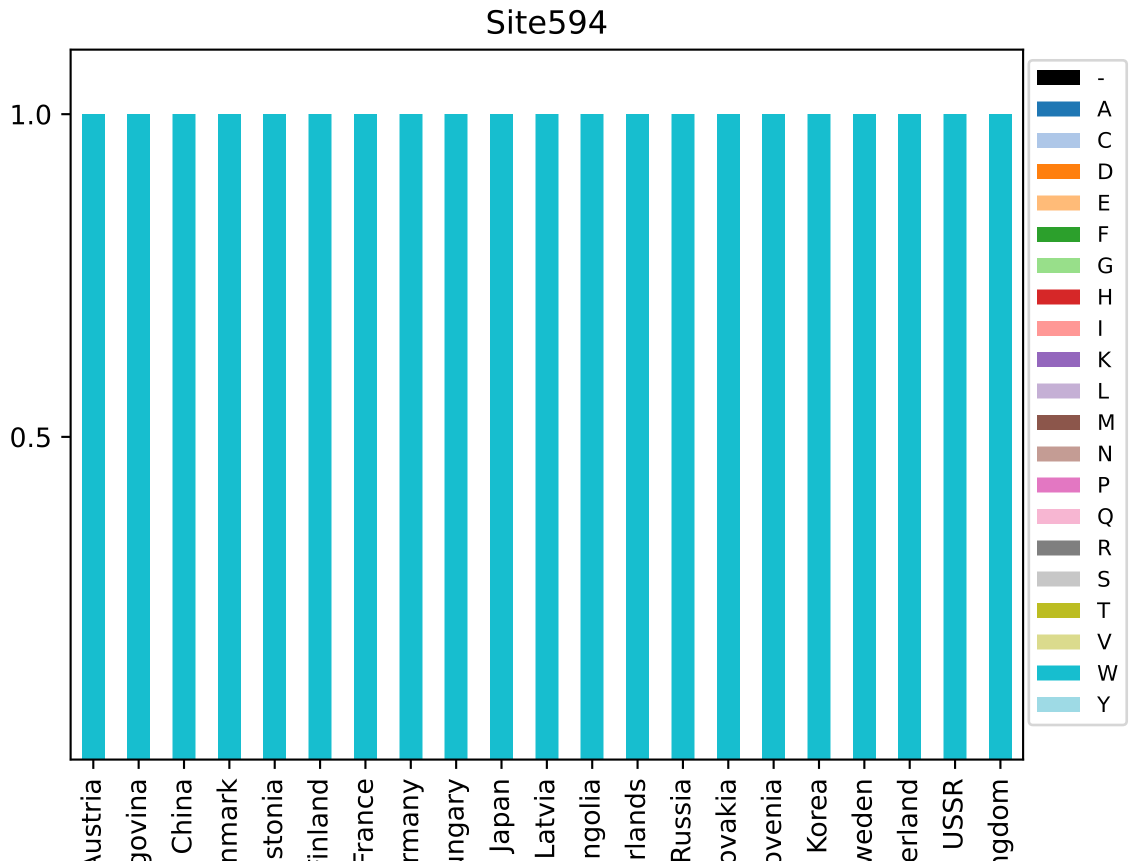
Site594
1.0
0.5
-
A
C
D
E
F
G
H
I
K
L
M
N
P
Q
R
S
T
V
W
Y
China
Japan
Latvia
Russia
Korea
USSR

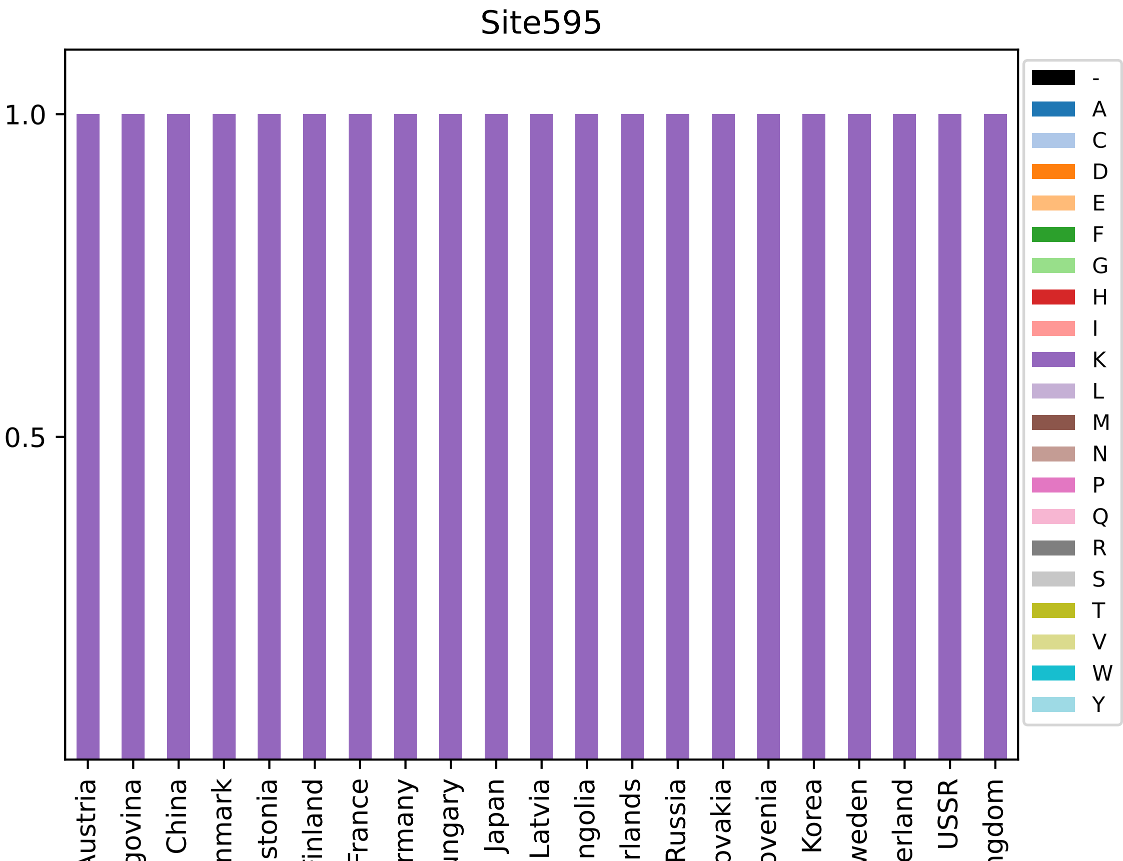

Site595
1.0
0.5
-
A
C
D
E
F
G
H
I
K
L
M
N
P
Q
R
S
T
V
W
Y
China
Japan
Latvia
Russia
Korea
USSR

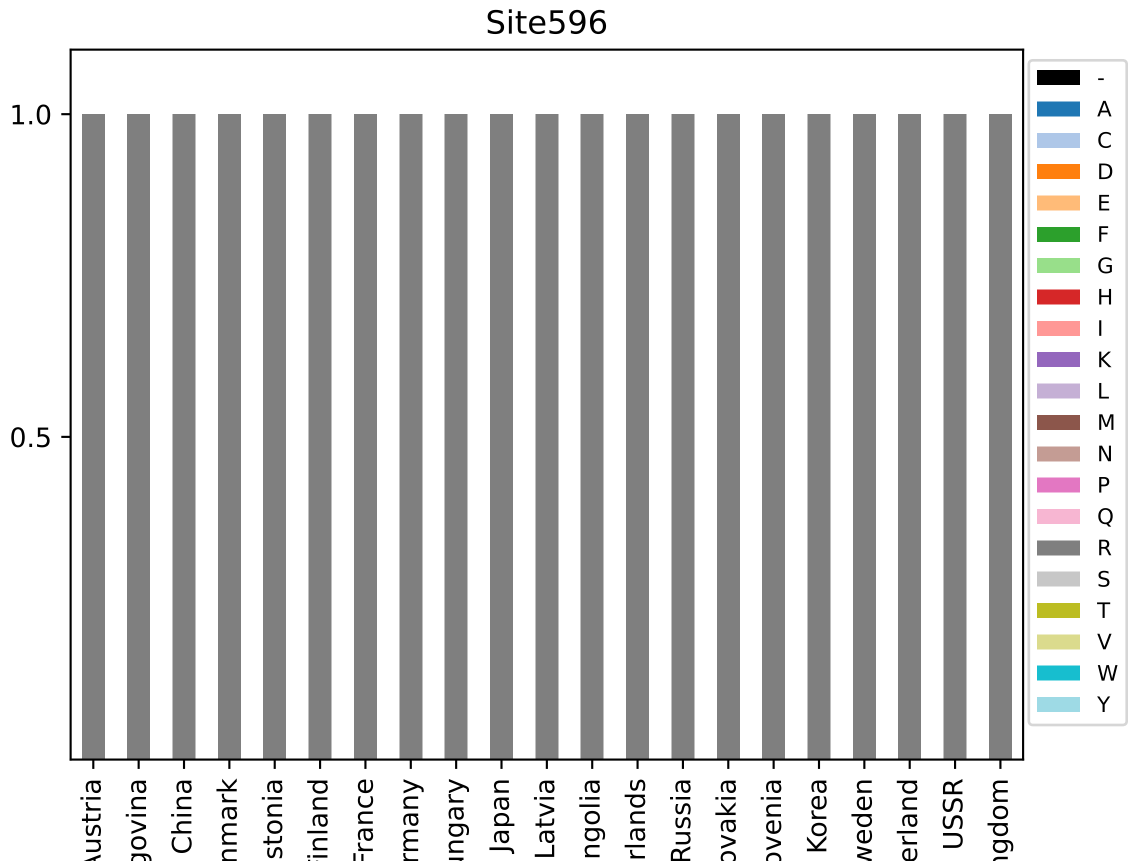

Site596
1.0
0.5
-
A
C
D
E
F
G
H
I
K
L
M
N
P
Q
R
S
T
V
W
Y
China
Japan
Latvia
Russia
Korea
USSR

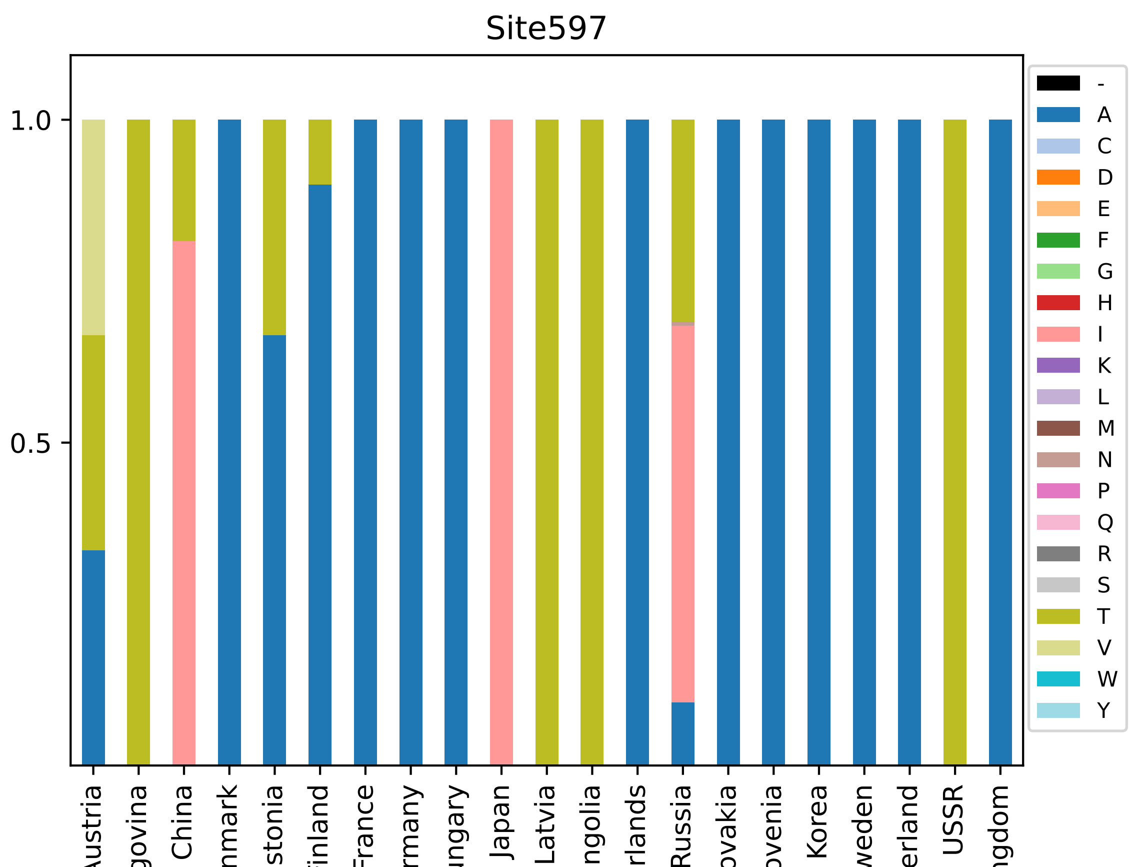

Site597
1.0
0.5
-
A
C
D
E
F
G
H
I
K
L
M
N
P
Q
R
S
T
V
W
Y
Austria
China
Japan
Latvia
Russia
Korea
USSR

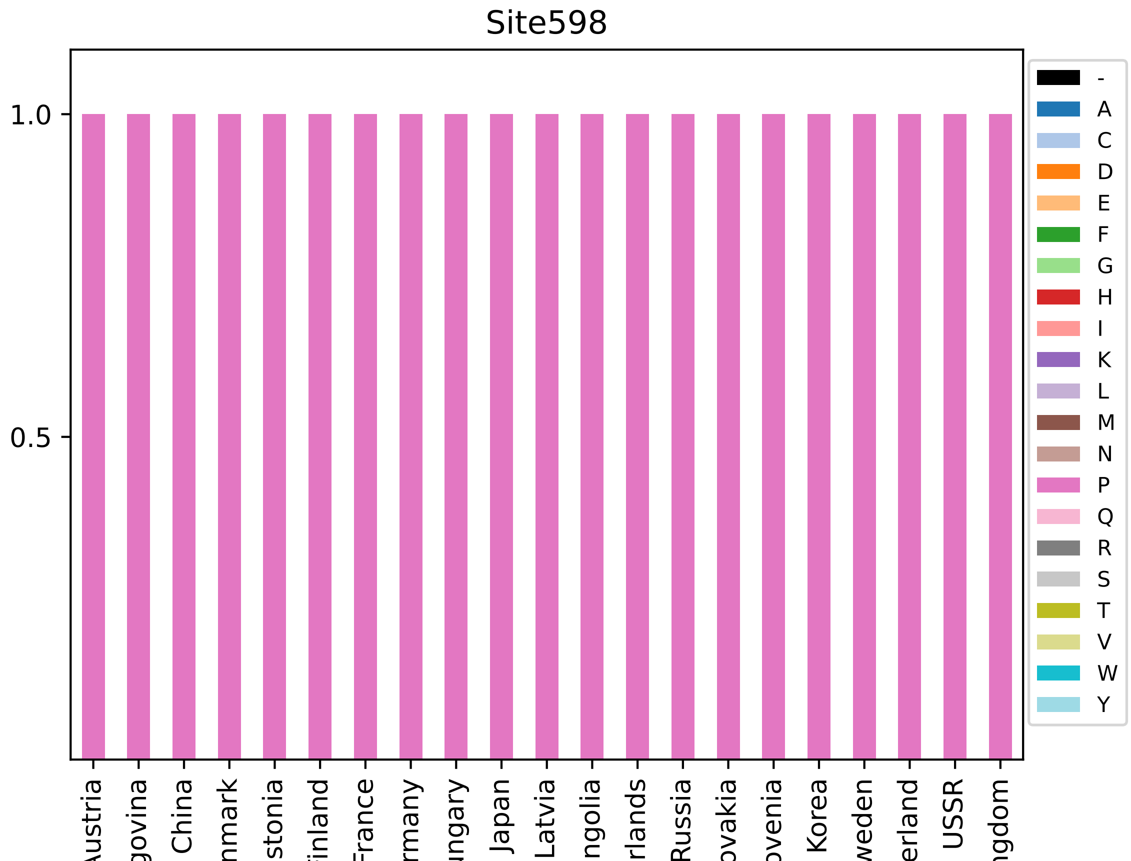
Site598
1.0
0.5
-
A
C
D
E
F
G
H
I
K
L
M
N
P
Q
R
S
T
V
W
Y
China
Japan
Latvia
Russia
Korea
USSR

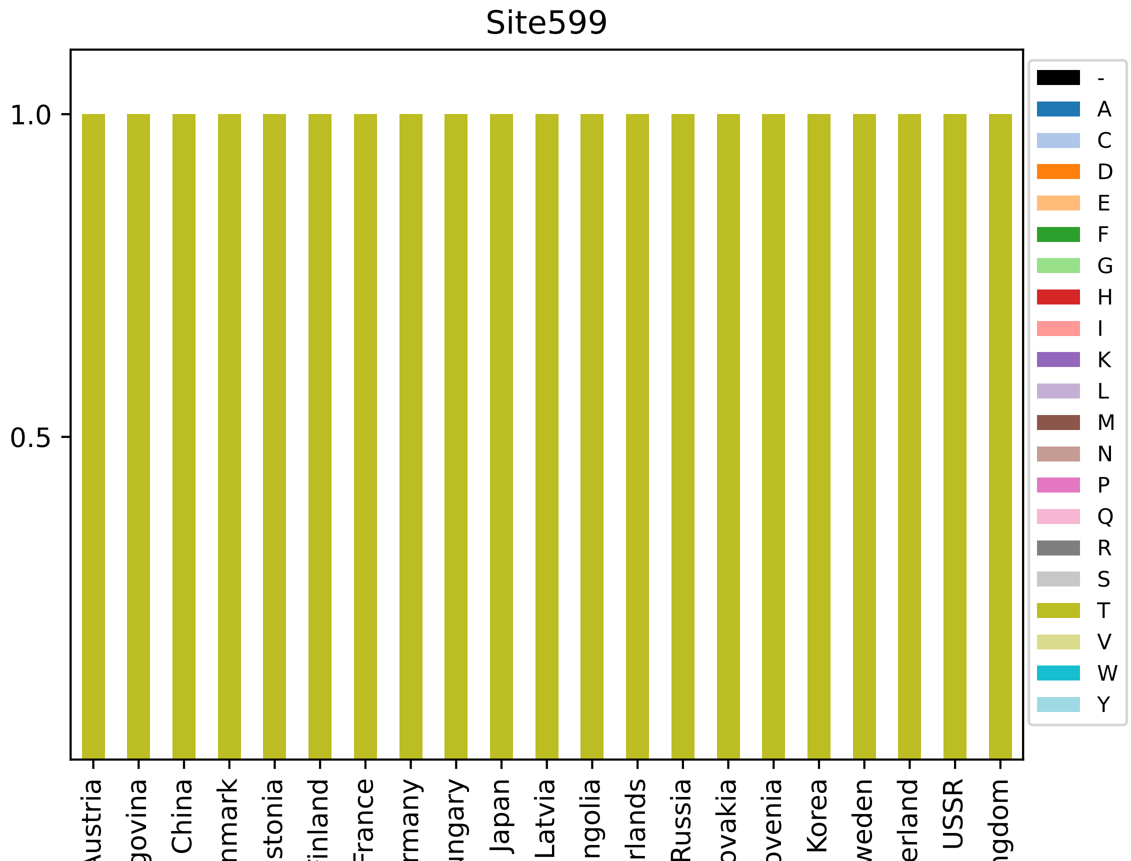
Site599
1.0
0.5
-
A
C
D
E
F
G
H
I
K
L
M
N
P
Q
R
S
T
V
W
Y
China
Japan
Latvia
Russia
Korea
USSR

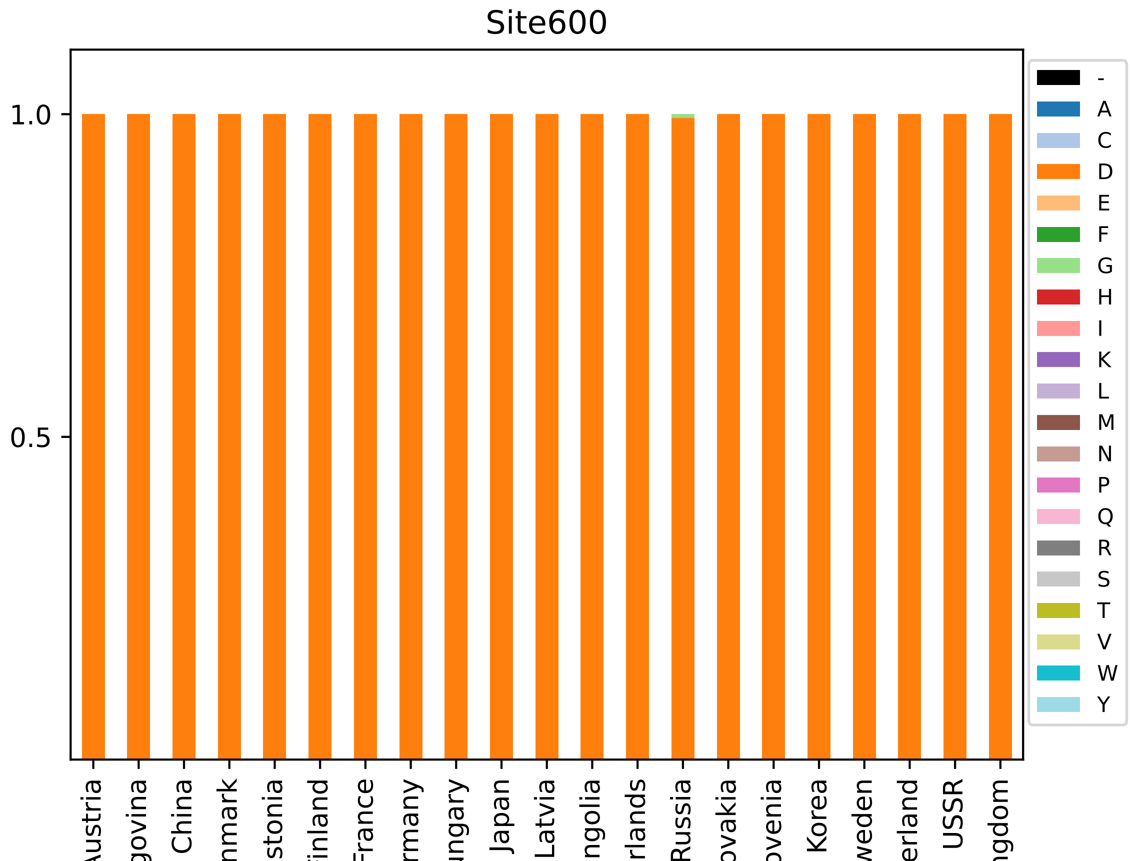
Site600
1.0
0.5
-
A
C
D
E
F
G
H
I
K
L
M
N
P
Q
R
S
T
V
W
Y
China
Japan
Latvia
Russia
Korea
USSR

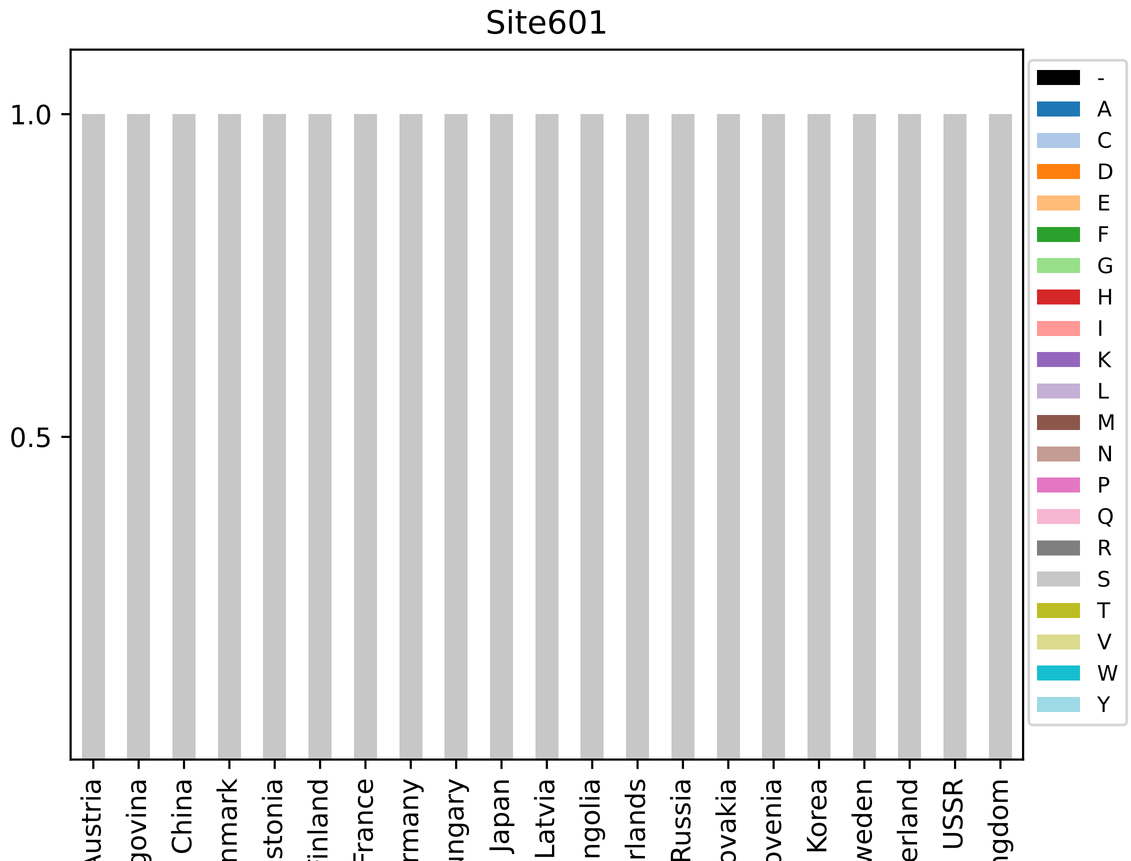

Site601
1.0
0.5
-
A
C
D
E
F
G
H
I
K
L
M
N
P
Q
R
S
T
V
W
Y
China
Japan
Latvia
Russia
Korea
USSR

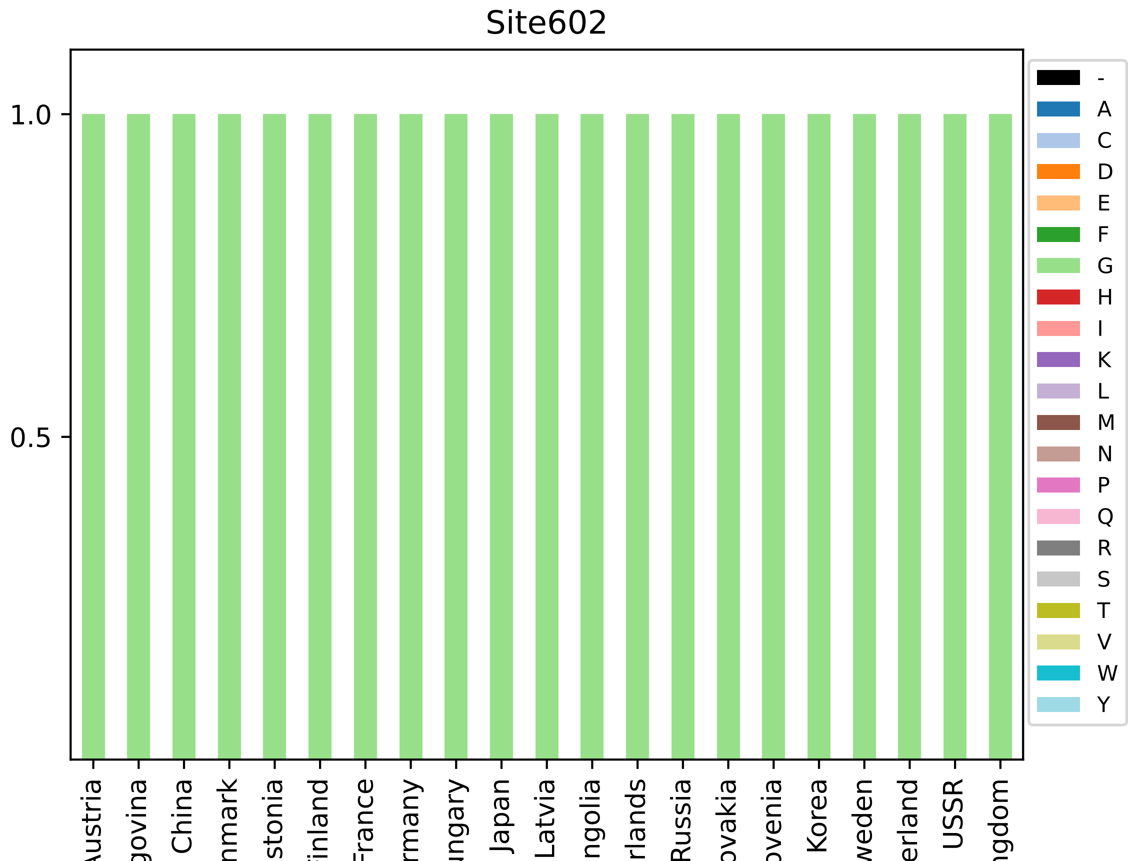

Site602
1.0
0.5
-
A
C
D
E
F
G
H
I
K
L
M
N
P
Q
R
S
T
V
W
Y
China
Japan
Latvia
Russia
Korea
USSR

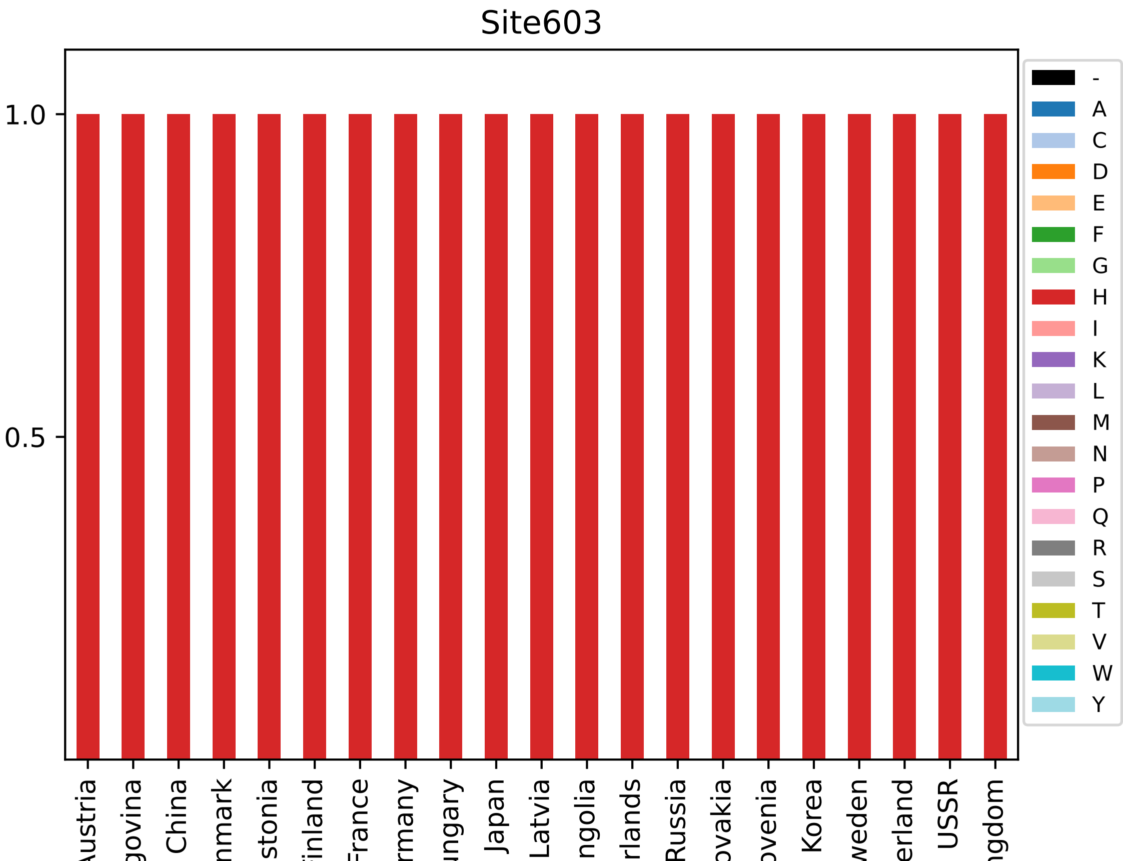

Site603
1.0
0.5
-
A
C
D
E
F
G
H
I
K
L
M
N
P
Q
R
S
T
V
W
Y
Austria
China
Japan
Latvia
Russia
Korea
USSR

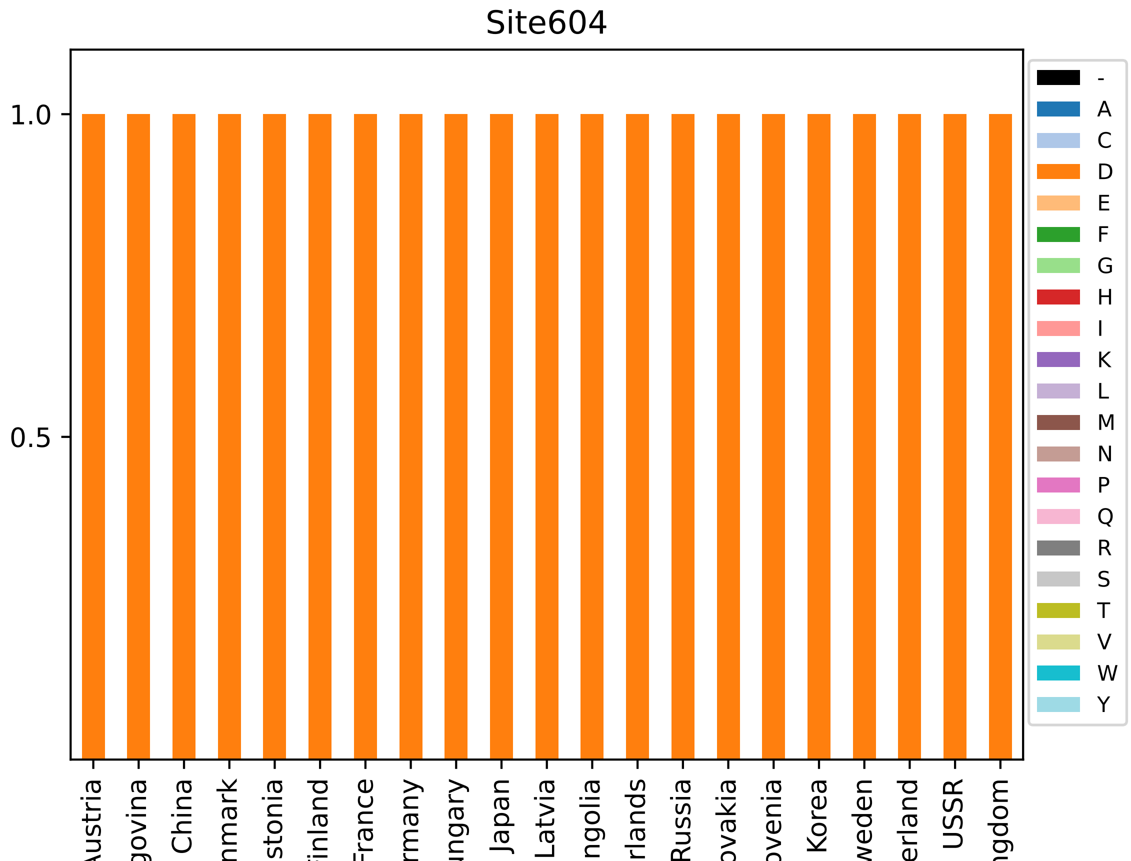
Site604
1.0
0.5
-
A
C
D
E
F
G
H
I
K
L
M
N
P
Q
R
S
T
V
W
Y
China
Japan
Latvia
Russia
Korea
USSR

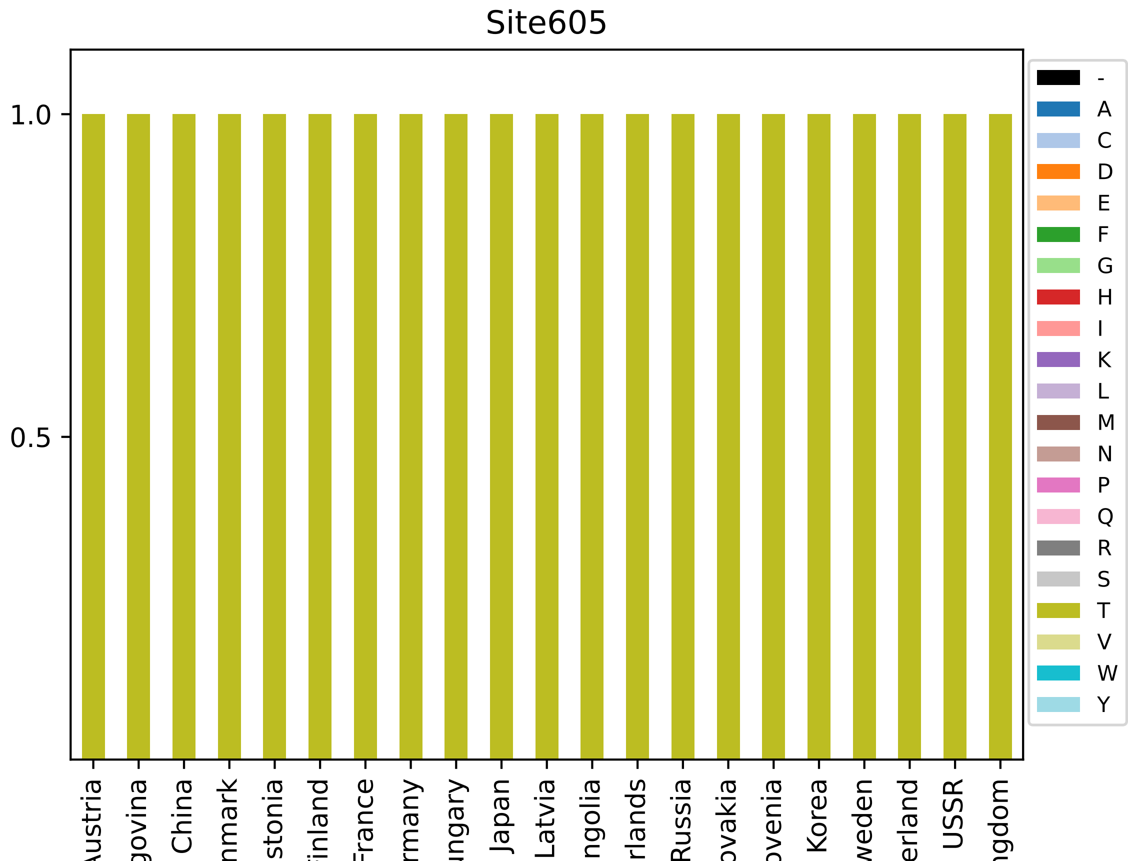

Site605
1.0
0.5
-
A
C
D
E
F
G
H
I
K
L
M
N
P
Q
R
S
T
V
W
Y
China
Japan
Latvia
Russia
Korea
USSR

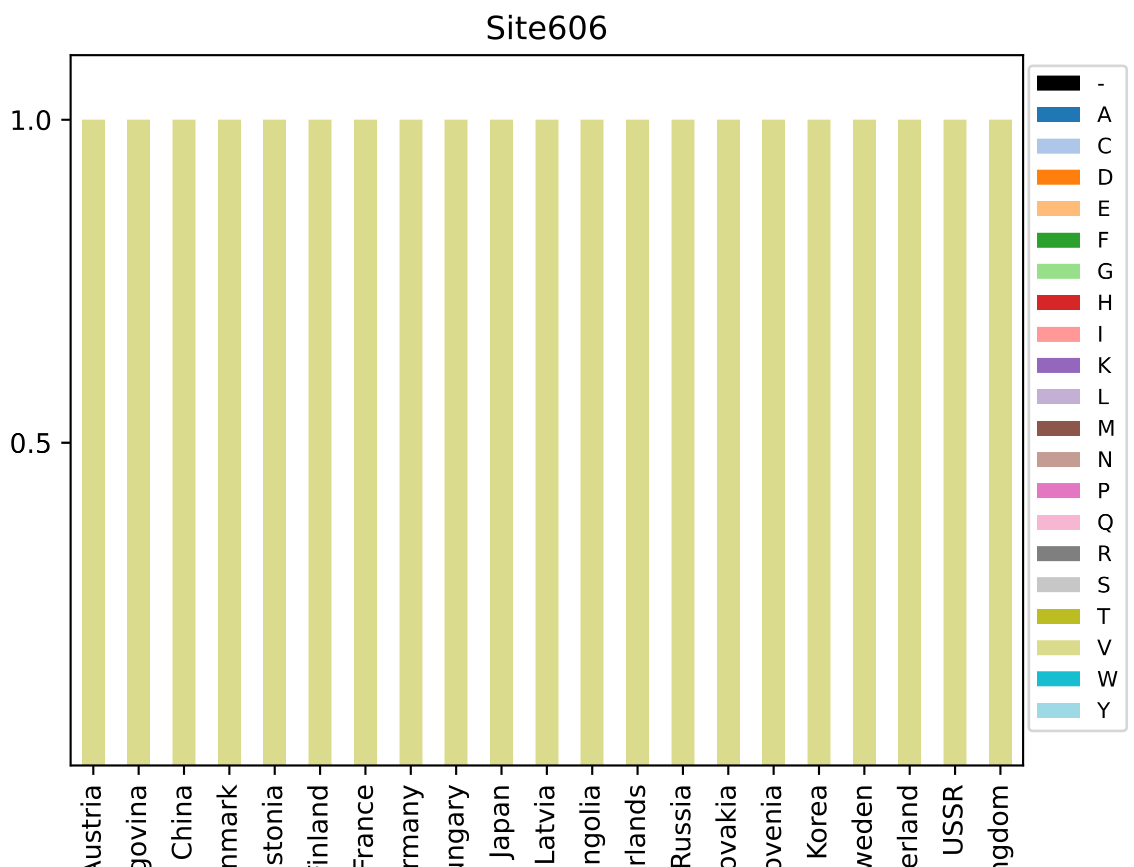

Site606
1.0
0.5
-
A
C
D
E
F
G
H
I
K
L
M
N
P
Q
R
S
T
V
W
Y
Austria
govina
China
nmark
stonia
Finland
France
rmany
ungary
Japan
Latvia
ngolia
rlands
Russia
ovakia
ovenia
Korea
weden
erland
USSR
ngdom

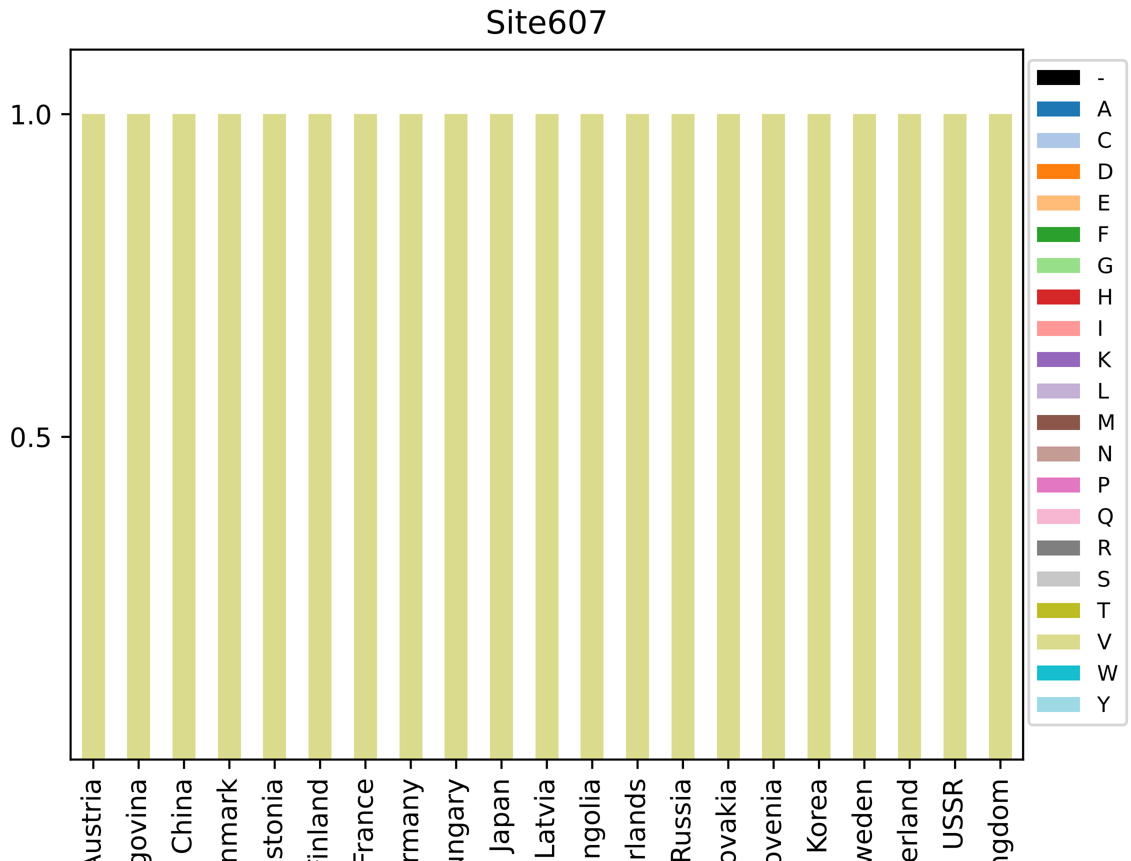

Site607
1.0
0.5
-
A
C
D
E
F
G
H
I
K
L
M
N
P
Q
R
S
T
V
W
Y
Austria
China
Japan
Latvia
Russia
Korea
USSR

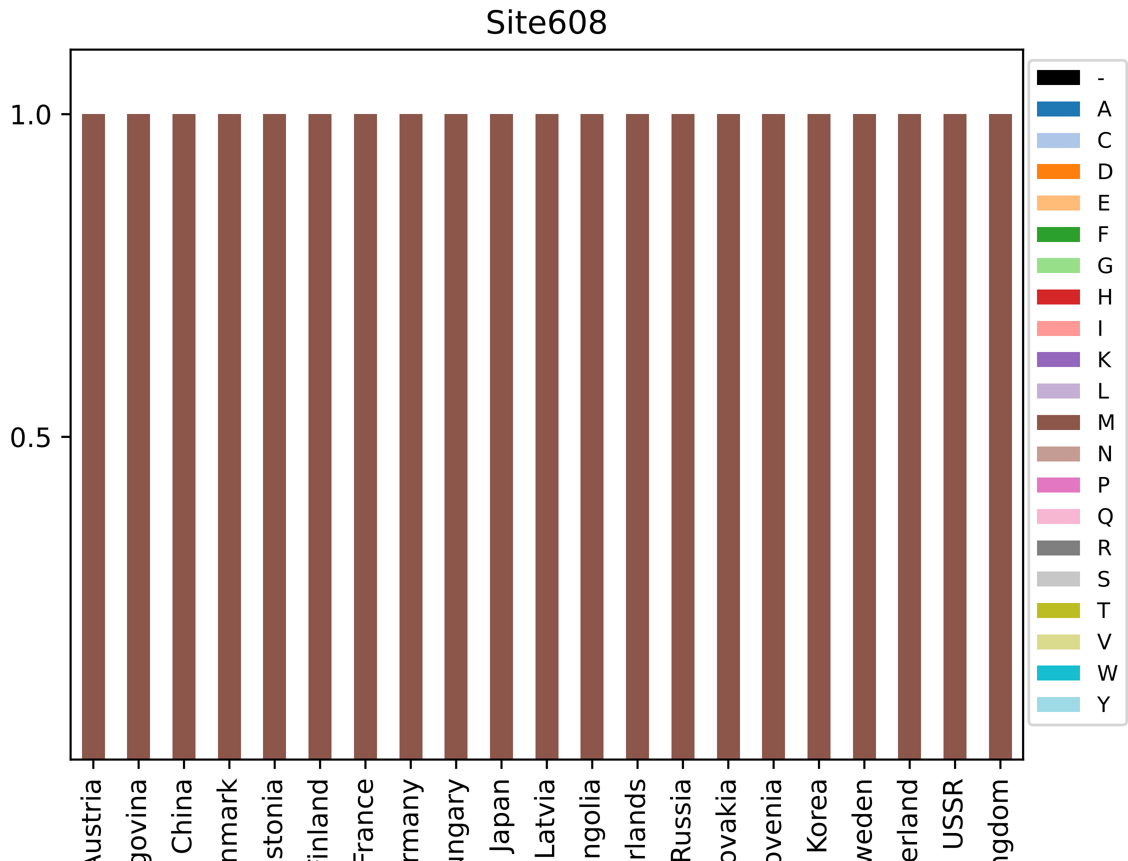
Site608
1.0
0.5
-
A
C
D
E
F
G
H
I
K
L
M
N
P
Q
R
S
T
V
W
Y
China
Japan
Latvia
Russia
Korea
USSR

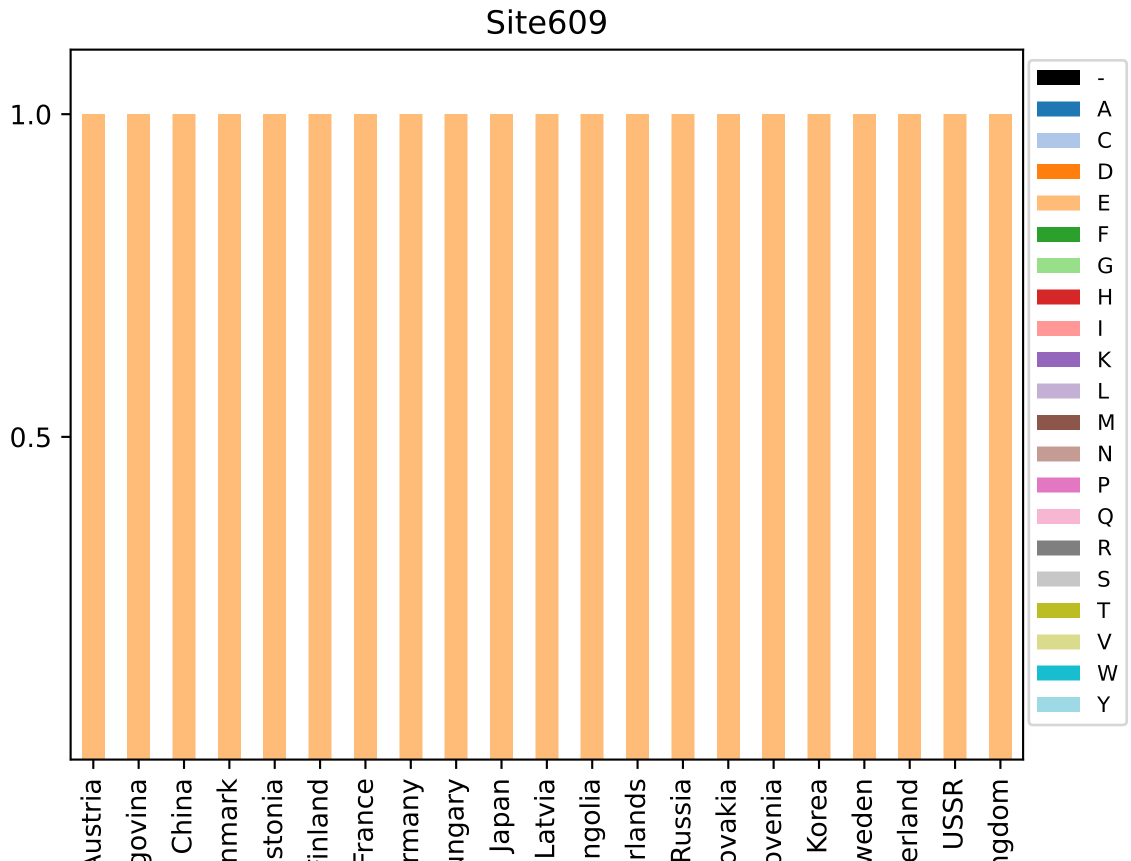
Site609
1.0
0.5
-
A
C
D
E
F
G
H
I
K
L
M
N
P
Q
R
S
T
V
W
Y
China
Japan
Latvia
Russia
Korea
USSR

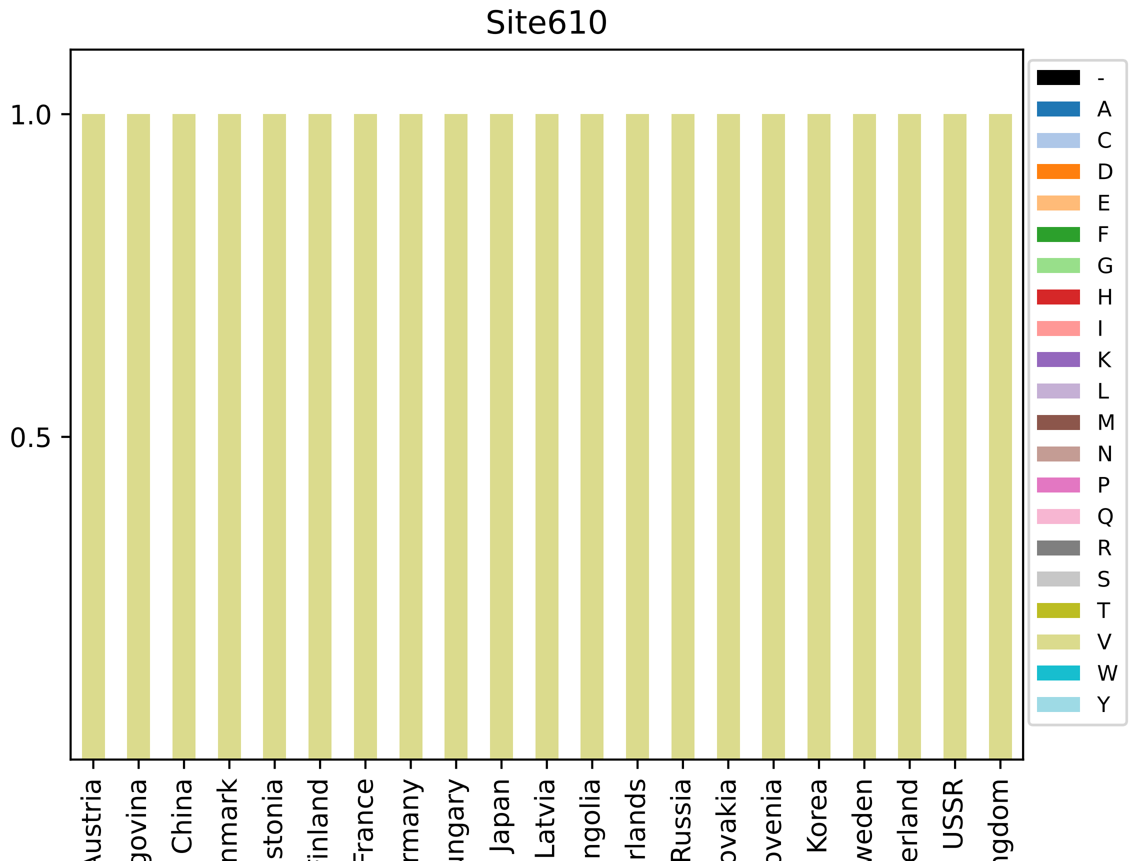

Site610
1.0
0.5
-
A
C
D
E
F
G
H
I
K
L
M
N
P
Q
R
S
T
V
W
Y
Austria
China
Japan
Latvia
Russia
Korea
USSR

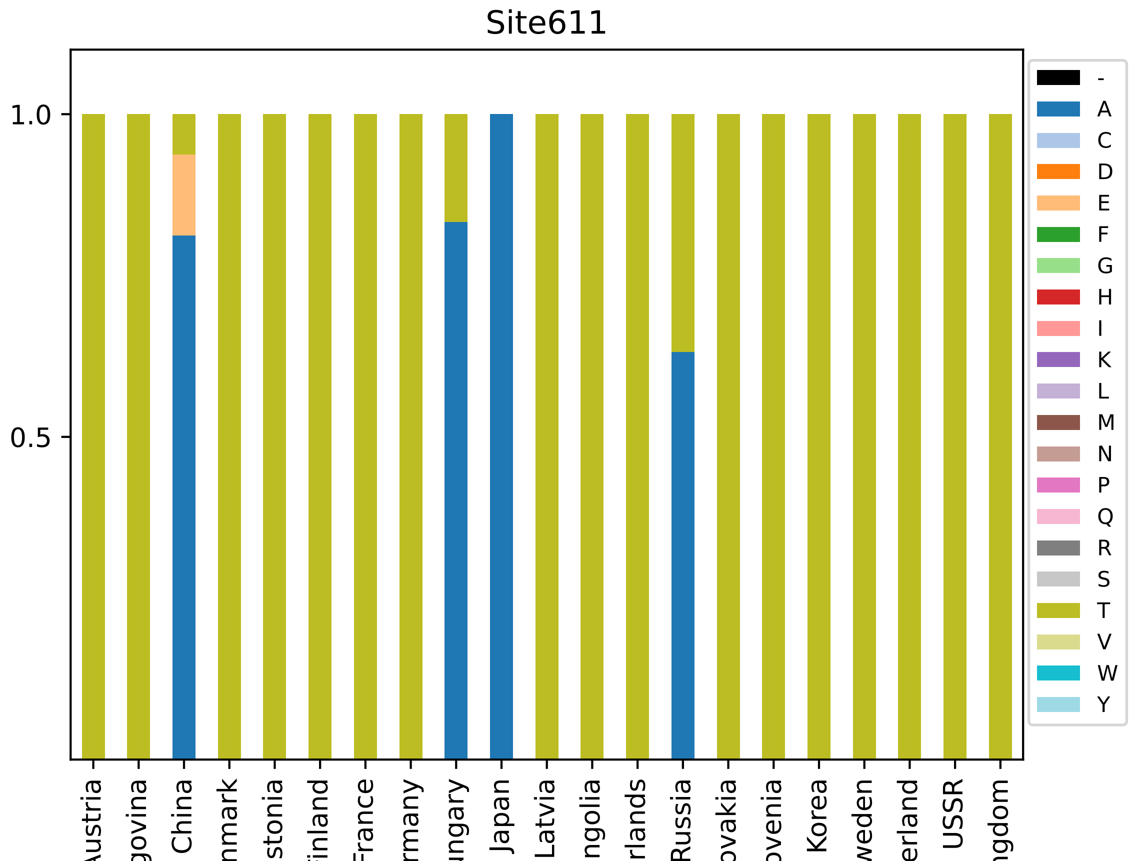
Site611
1.0
0.5
-
A
C
D
E
F
G
H
I
K
L
M
N
P
Q
R
S
T
V
W
Y
China
Japan
Latvia
Russia
Korea
USSR

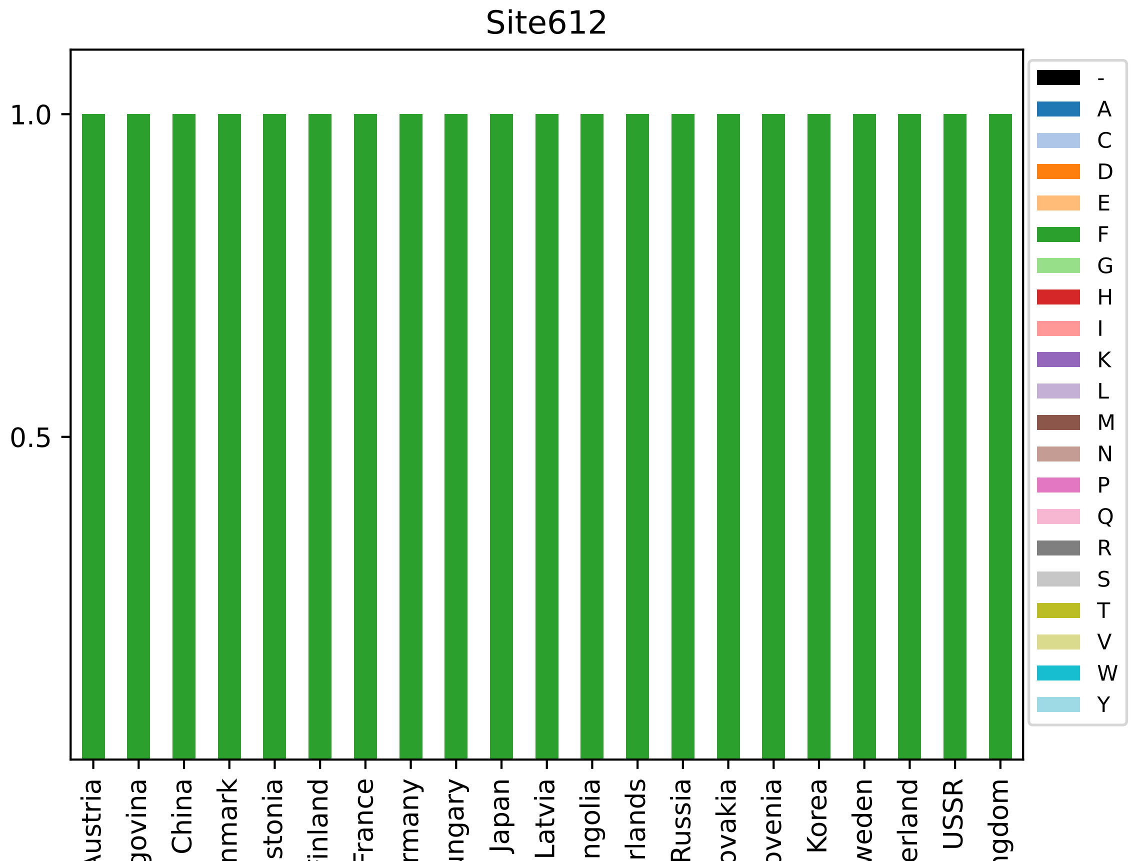
Site612
1.0
0.5
-
A
C
D
E
F
G
H
I
K
L
M
N
P
Q
R
S
T
V
W
Y
Austria
govina
China
nmark
stonia
Finland
France
rmany
ungary
Japan
Latvia
ngolia
rlands
Russia
ovakia
ovenia
Korea
weden
erland
USSR
ngdom

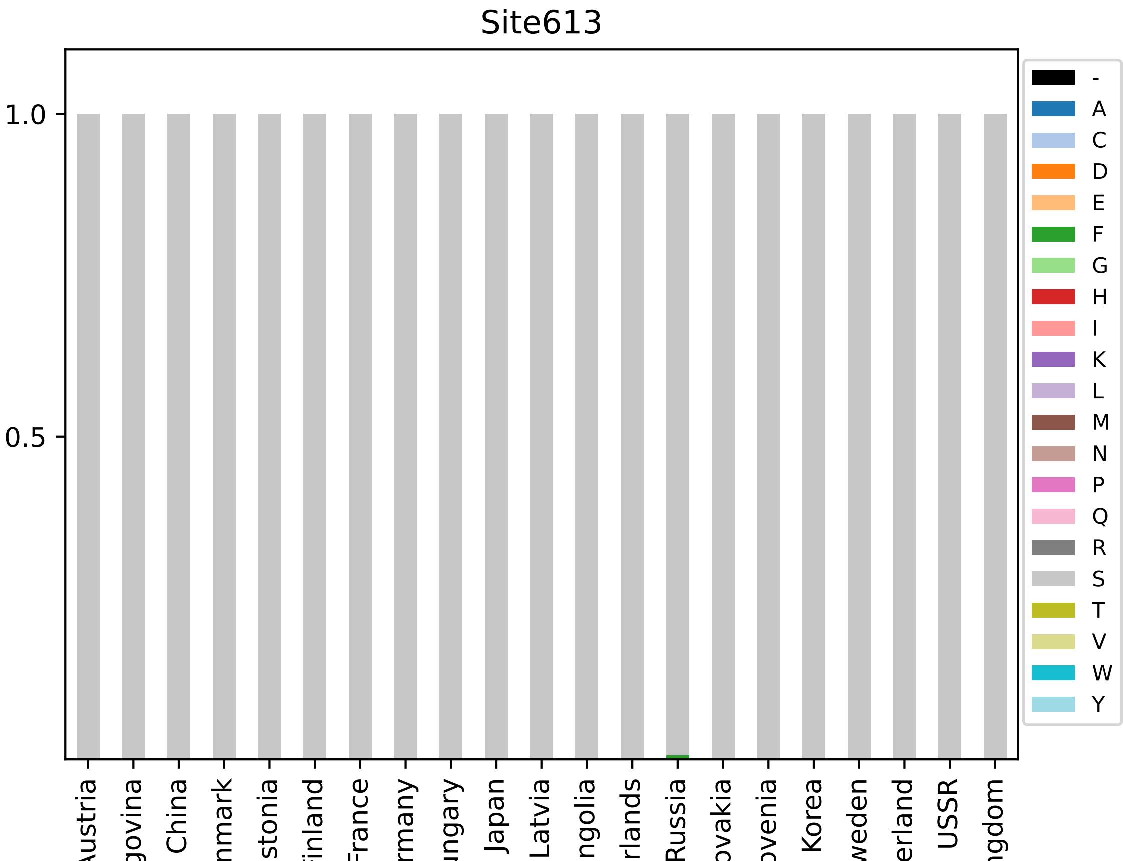
Site613
1.0
0.5
-
A
C
D
E
F
G
H
I
K
L
M
N
P
Q
R
S
T
V
W
Y
Austria
China
Japan
Latvia
Russia
Korea
USSR

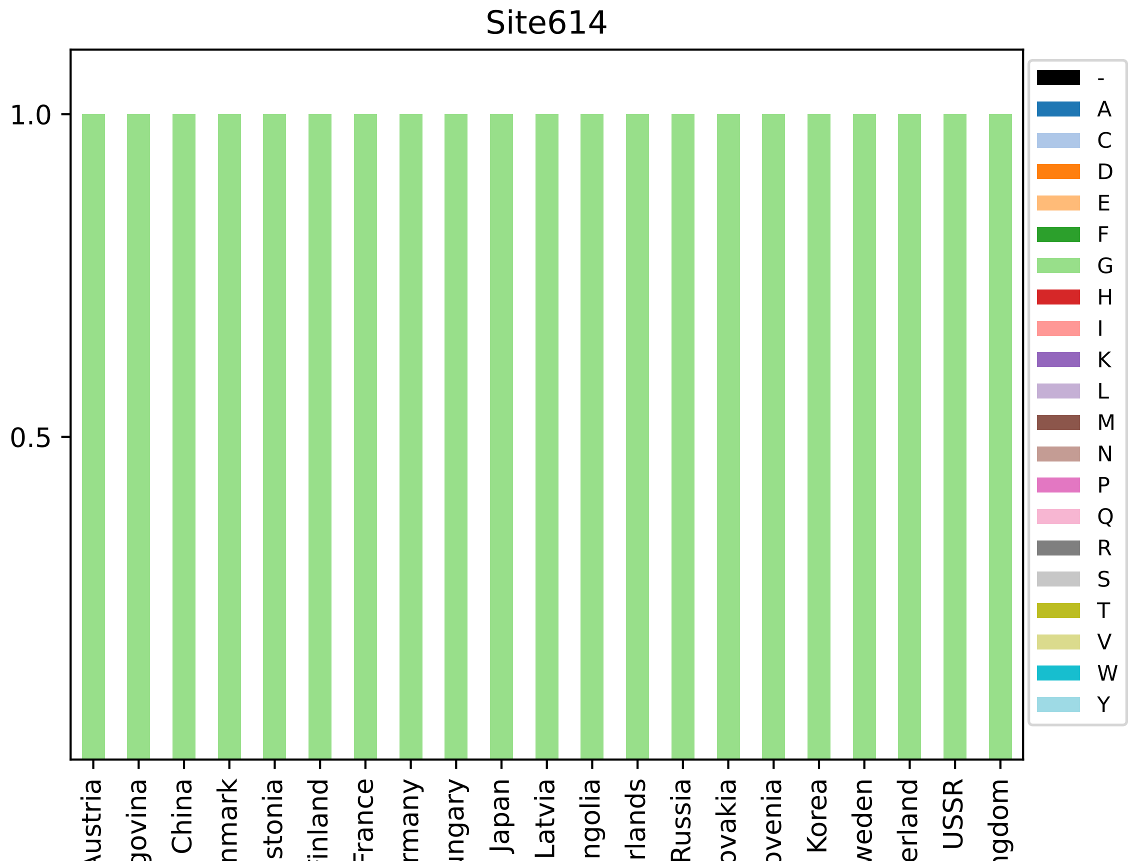

Site614
1.0
0.5
-
A
C
D
E
F
G
H
I
K
L
M
N
P
Q
R
S
T
V
W
Y
China
Japan
Latvia
Russia
Korea
USSR

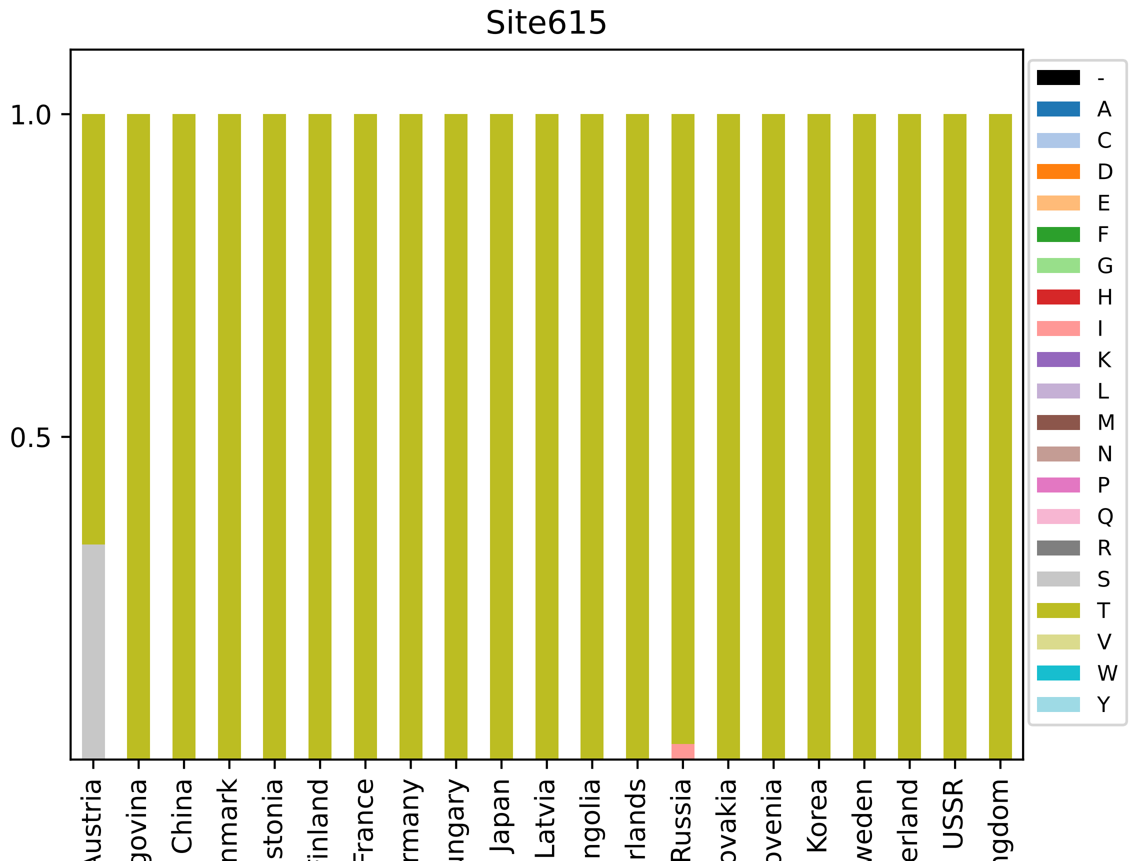
Site615
1.0
0.5
-
A
C
D
E
F
G
H
I
K
L
M
N
P
Q
R
S
T
V
W
Y
Austria
govina
China
nmark
stonia
inland
France
rmany
ungary
Japan
Latvia
ngolia
rlands
Russia
ovakia
ovenia
Korea
weden
erland
USSR
ngdom

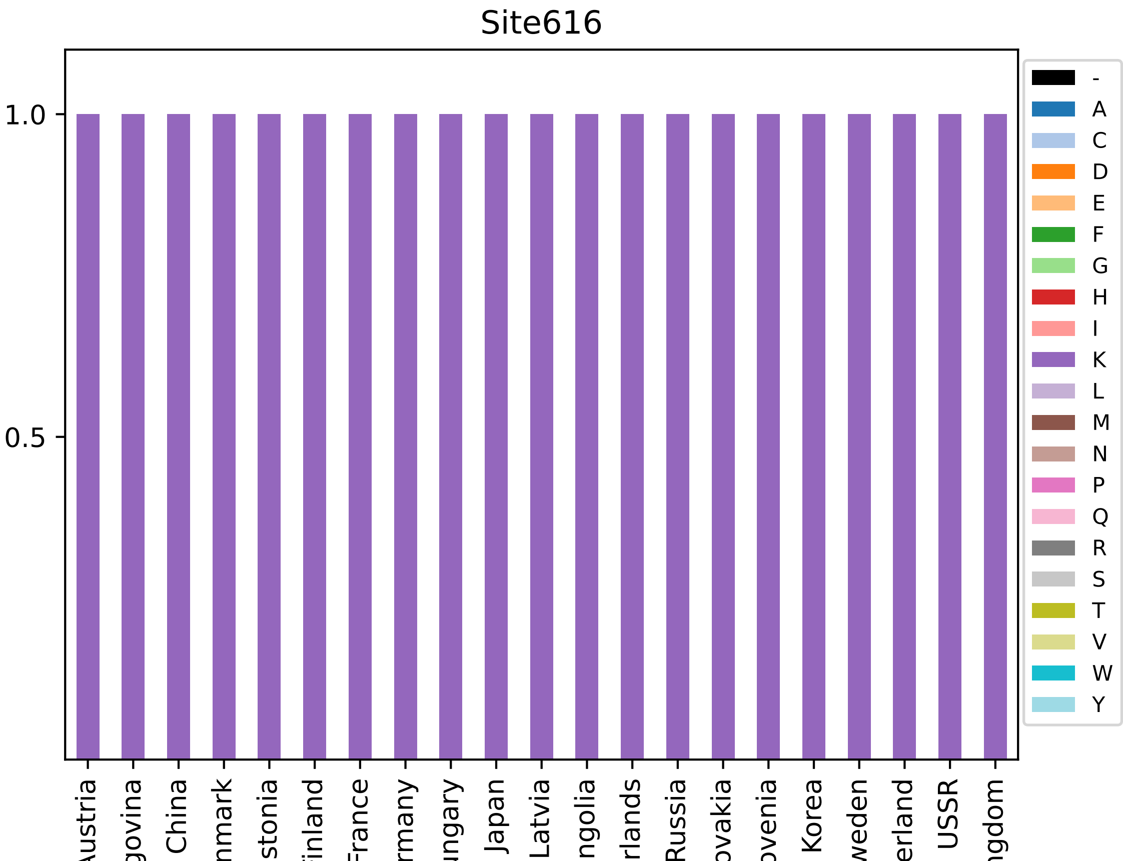

Site616
1.0
0.5
-
A
C
D
E
F
G
H
I
K
L
M
N
P
Q
R
S
T
V
W
Y
China
Finland
France
Japan
Latvia
Russia
Korea
USSR

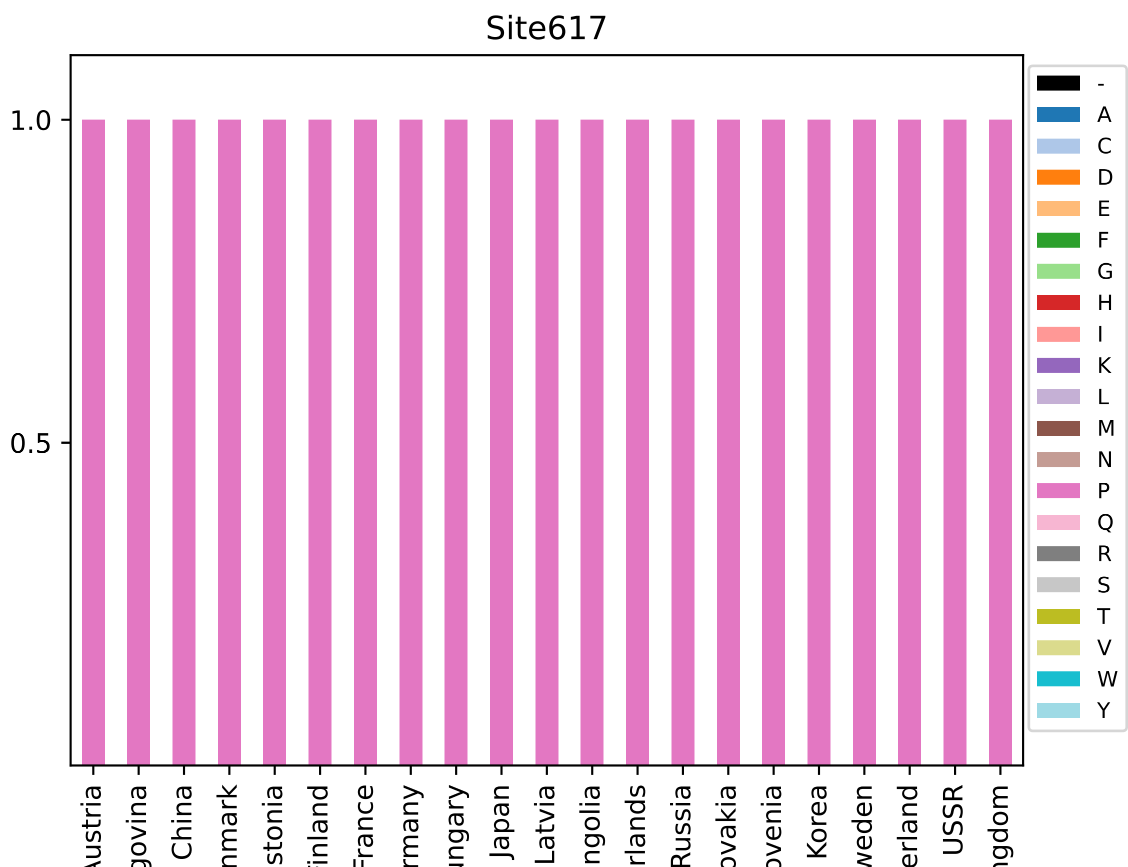
Site617
1.0
0.5
-
A
C
D
E
F
G
H
I
K
L
M
N
P
Q
R
S
T
V
W
Y
China
Japan
Latvia
Russia
Korea
USSR

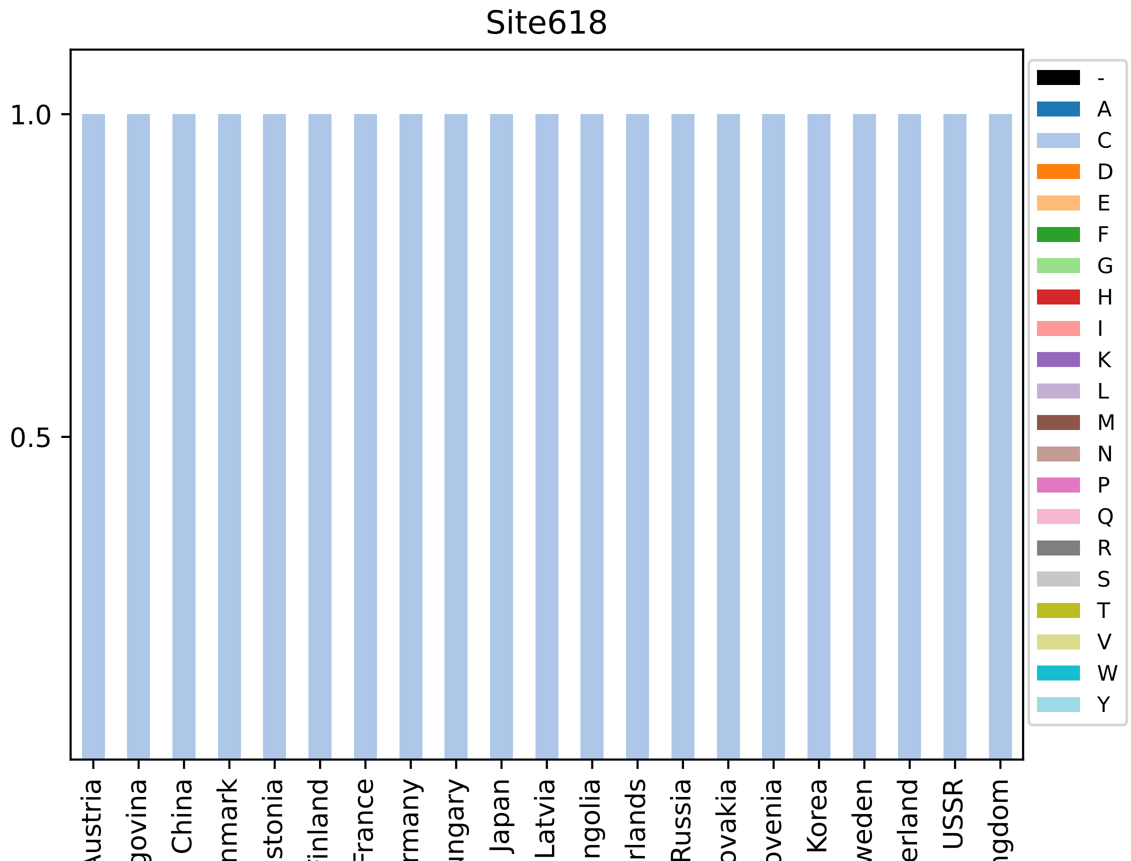
Site618
1.0
0.5
China
Japan
Latvia
Russia
Korea
USSR
-
A
C
D
E
F
G
H
I
K
L
M
N
P
Q
R
S
T
V
W
Y

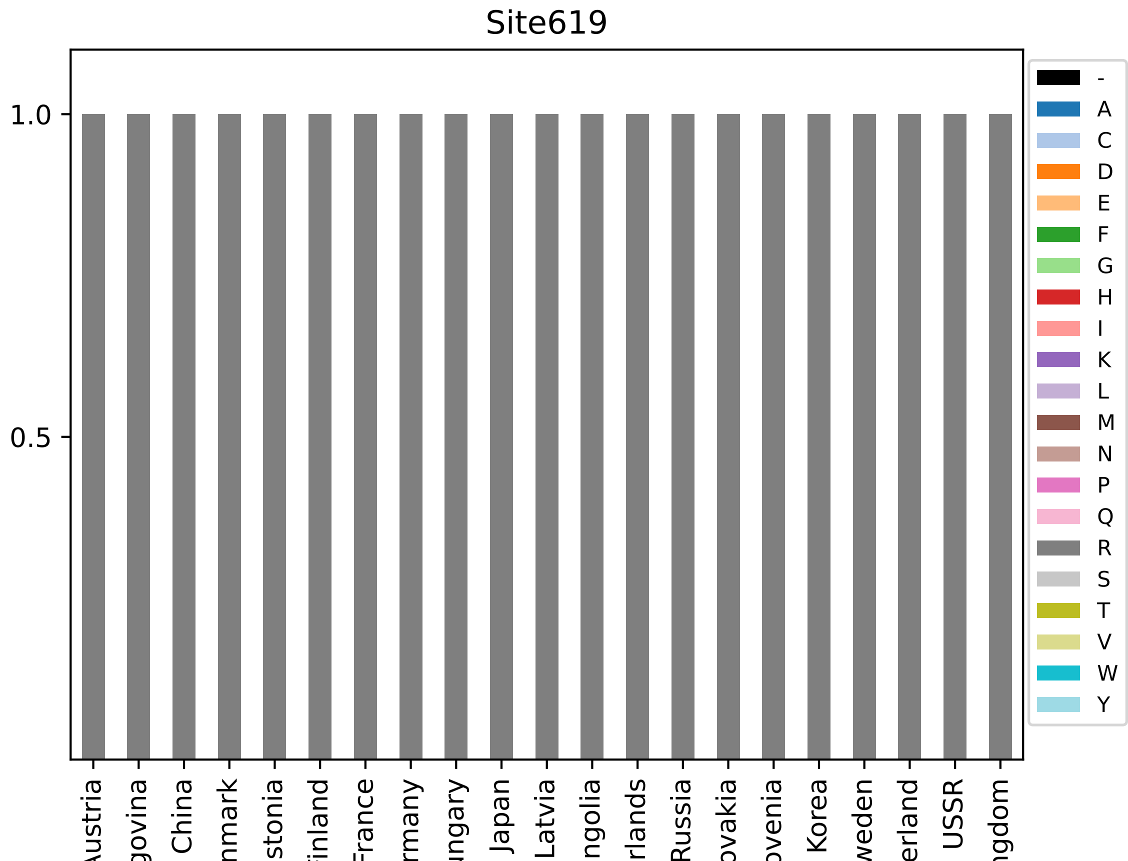
Site619
1.0
0.5
-
A
C
D
E
F
G
H
I
K
L
M
N
P
Q
R
S
T
V
W
Y
China
Japan
Latvia
Russia
Korea
USSR

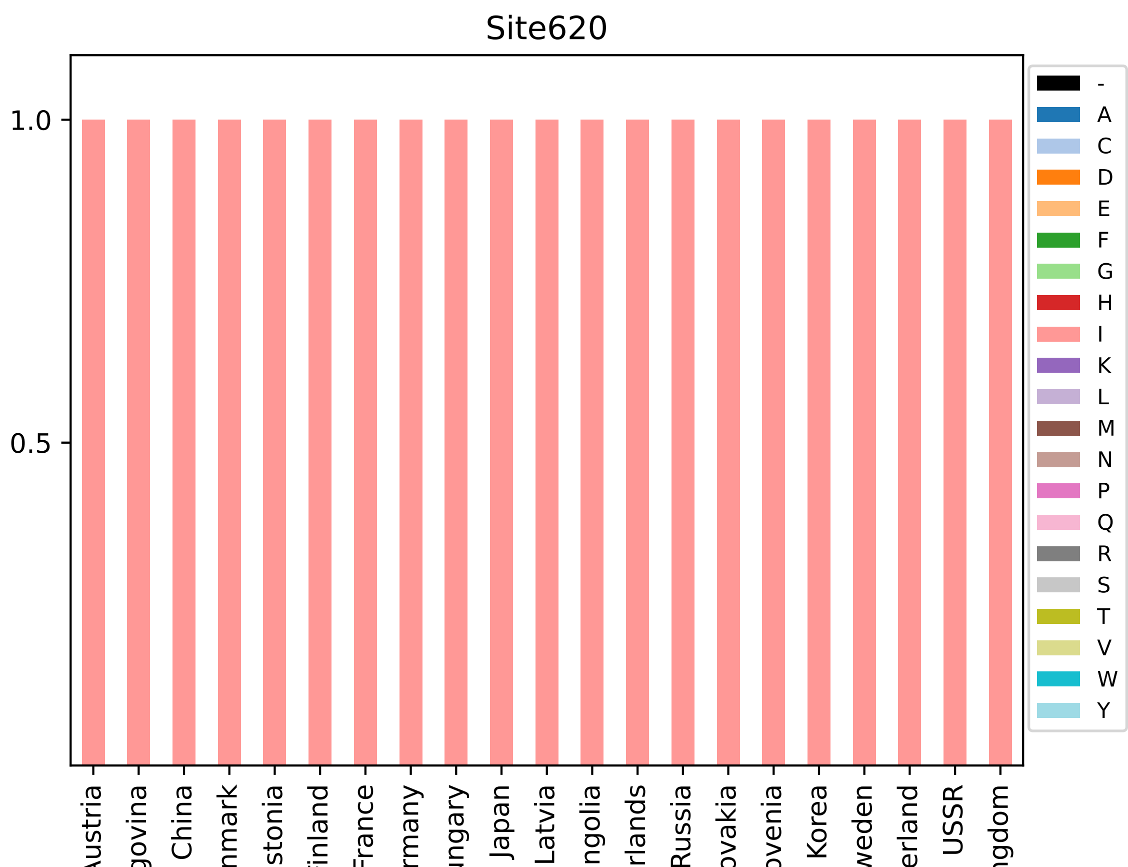
Site620
1.0
0.5
-
A
C
D
E
F
G
H
I
K
L
M
N
P
Q
R
S
T
V
W
Y
China
Japan
Latvia
Russia
Korea
USSR

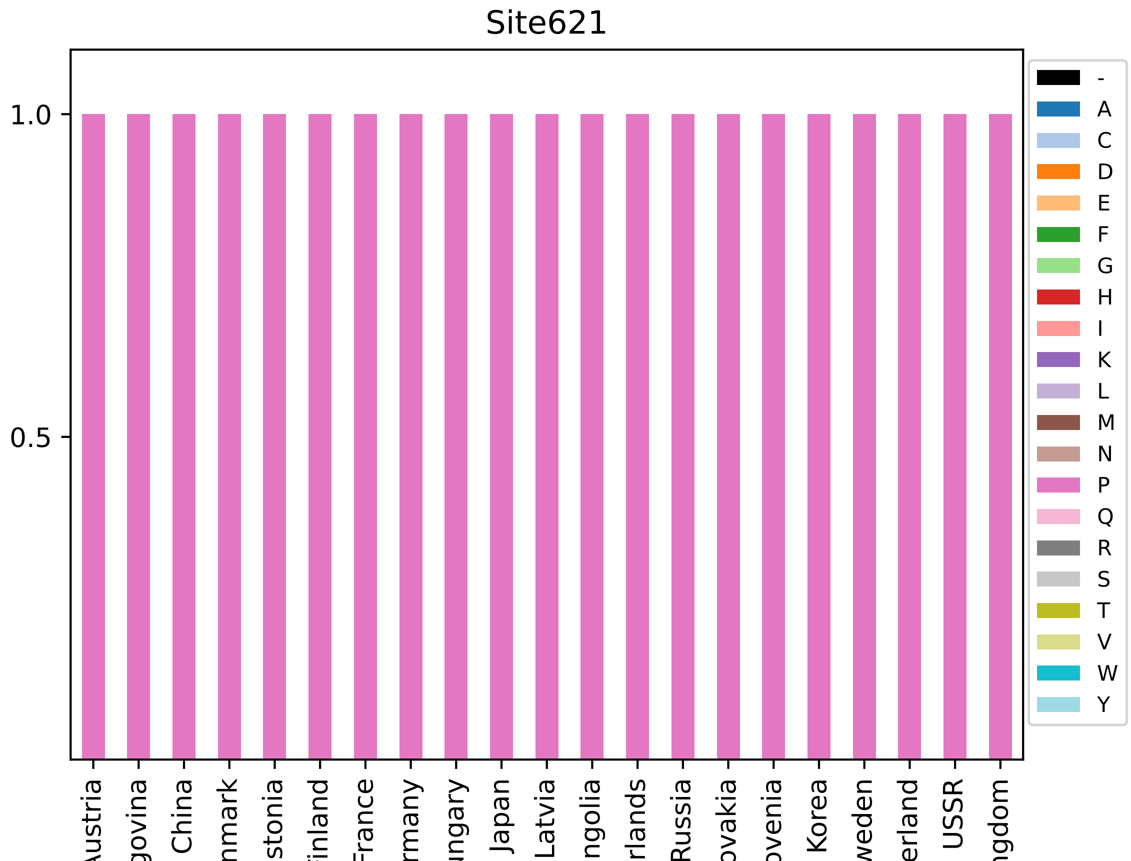
Site621
1.0
0.5
-
A
C
D
E
F
G
H
I
K
L
M
N
P
Q
R
S
T
V
W
Y
China
Japan
Latvia
Russia
Korea
USSR

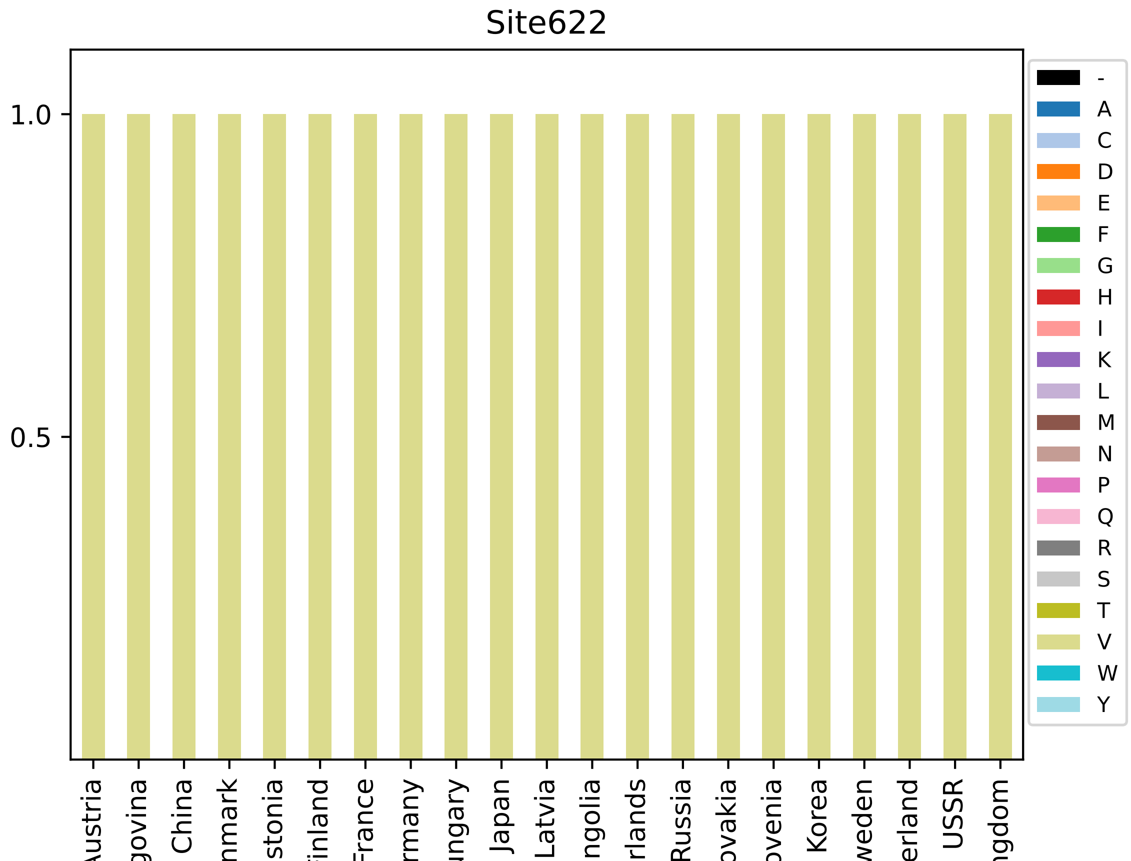
Site622
1.0
0.5
-
A
C
D
E
F
G
H
I
K
L
M
N
P
Q
R
S
T
V
W
Y
China
Japan
Latvia
Russia
Korea
USSR

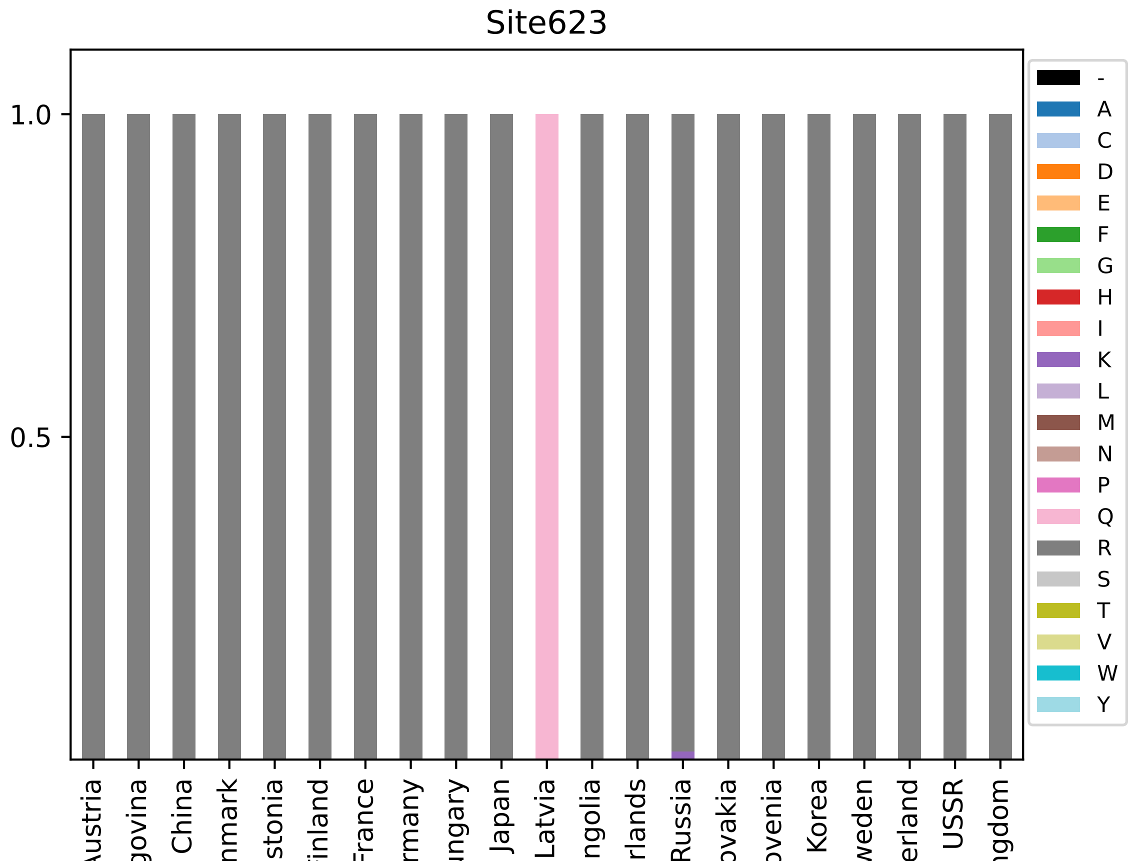
Site623
1.0
0.5
-
A
C
D
E
F
G
H
I
K
L
M
N
P
Q
R
S
T
V
W
Y
Japan
Latvia
Russia
Korea
USSR
China

Site624

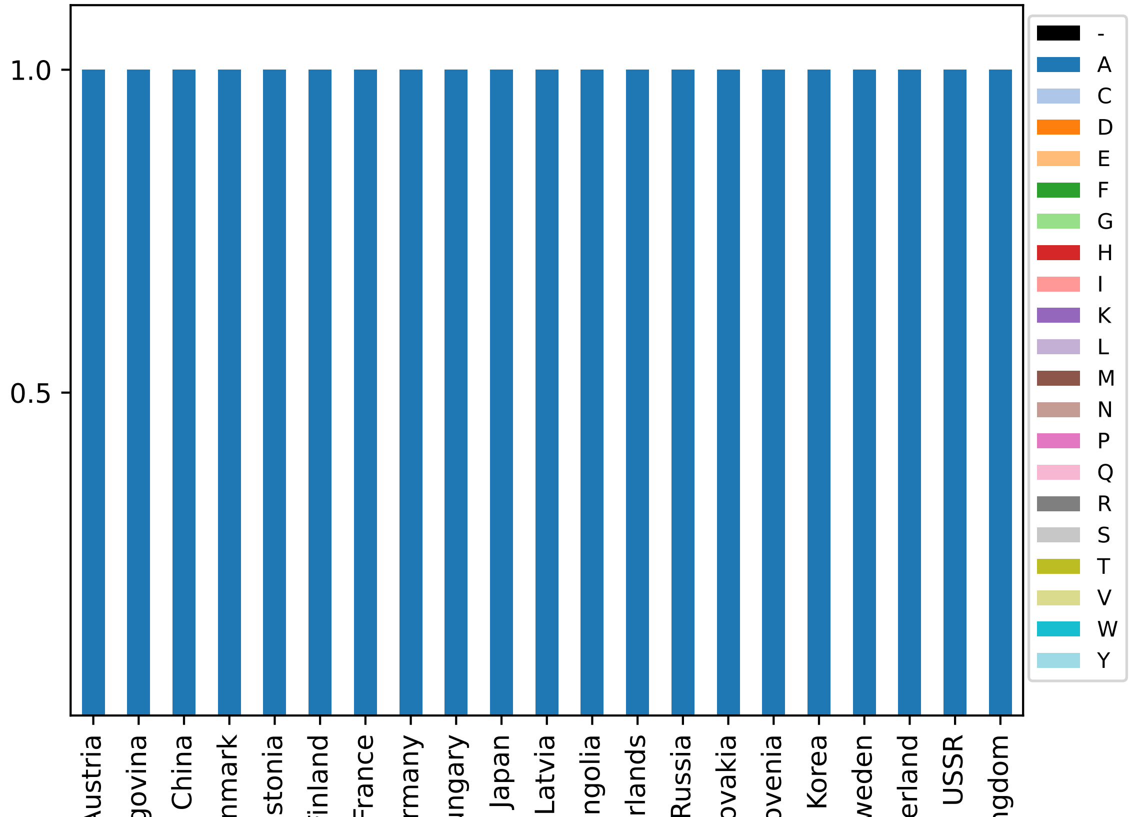
1.0
0.5
-
A
C
D
E
F
G
H
I
K
L
M
N
P
Q
R
S
T
V
W
Y
China
Japan
Latvia
Russia
Korea
USSR

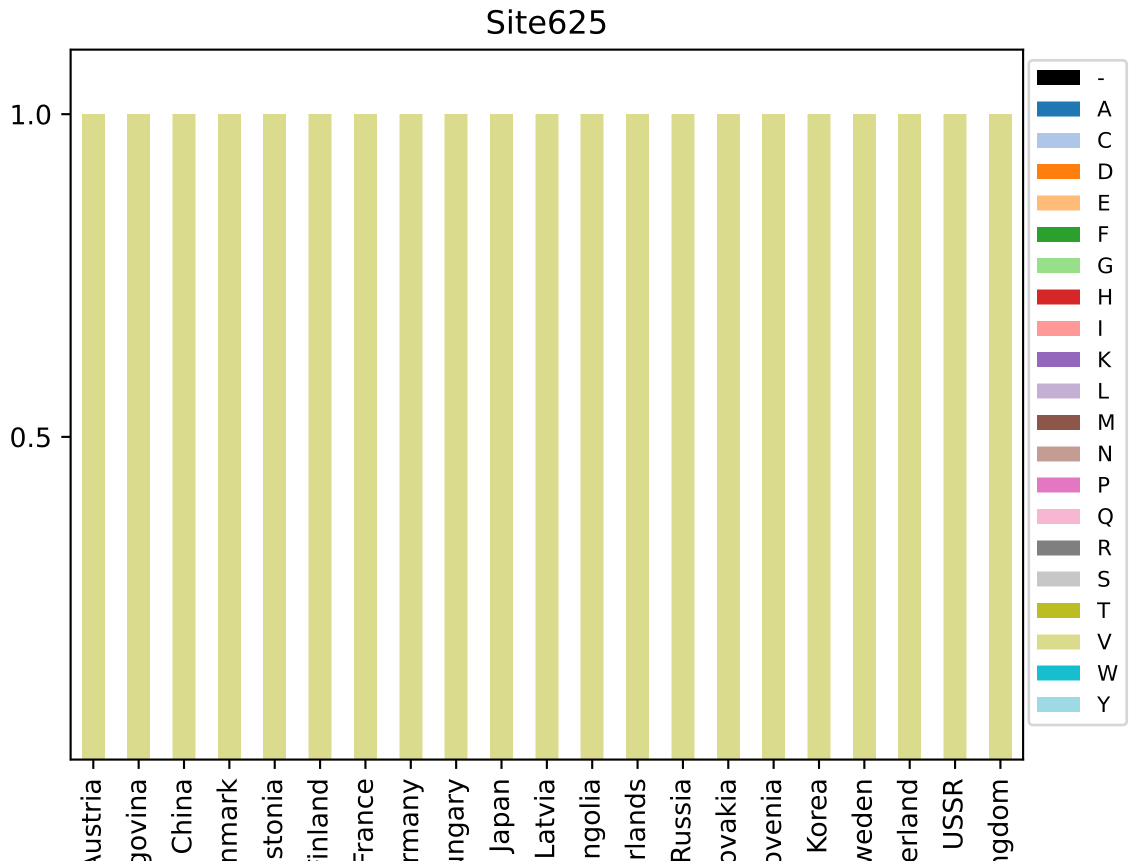
Site625
1.0
0.5
-
A
C
D
E
F
G
H
I
K
L
M
N
P
Q
R
S
T
V
W
Y
China
Japan
Latvia
Russia
Korea
USSR

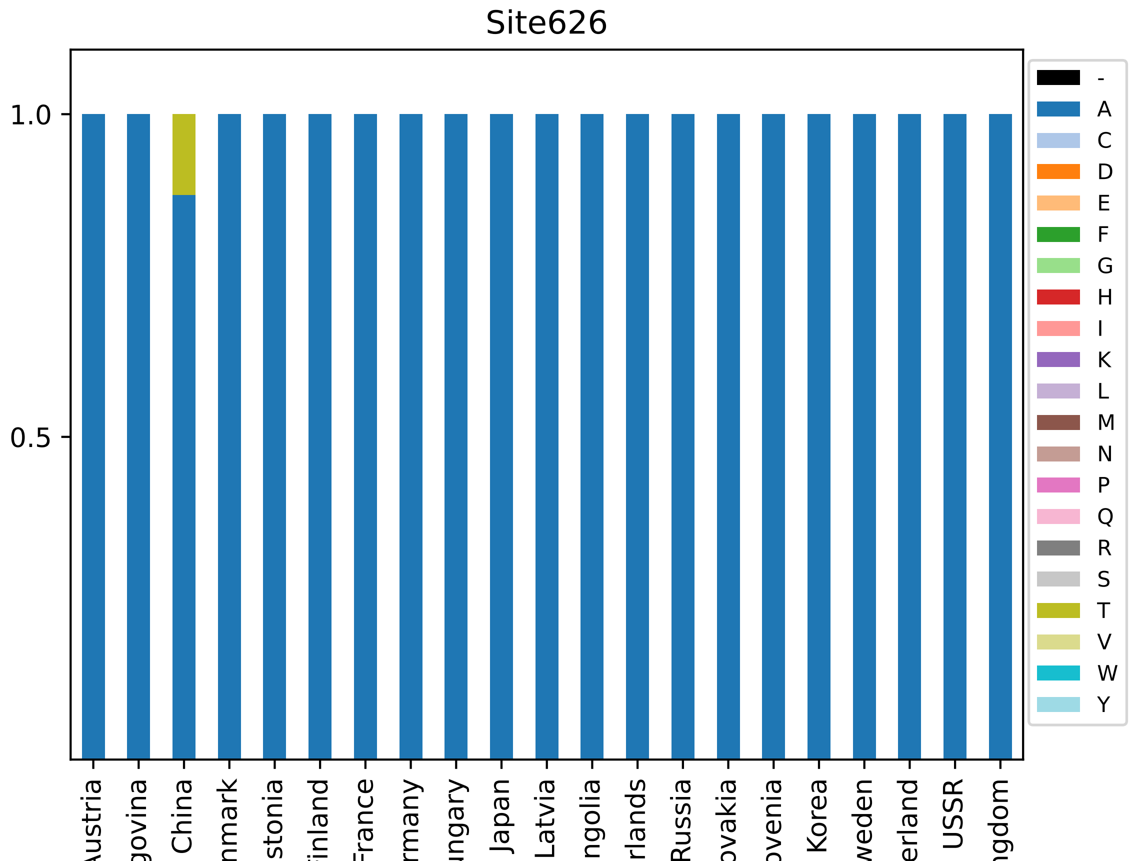

Site626
1.0
0.5
-
A
C
D
E
F
G
H
I
K
L
M
N
P
Q
R
S
T
V
W
Y
China
Japan
Latvia
Russia
Korea
USSR

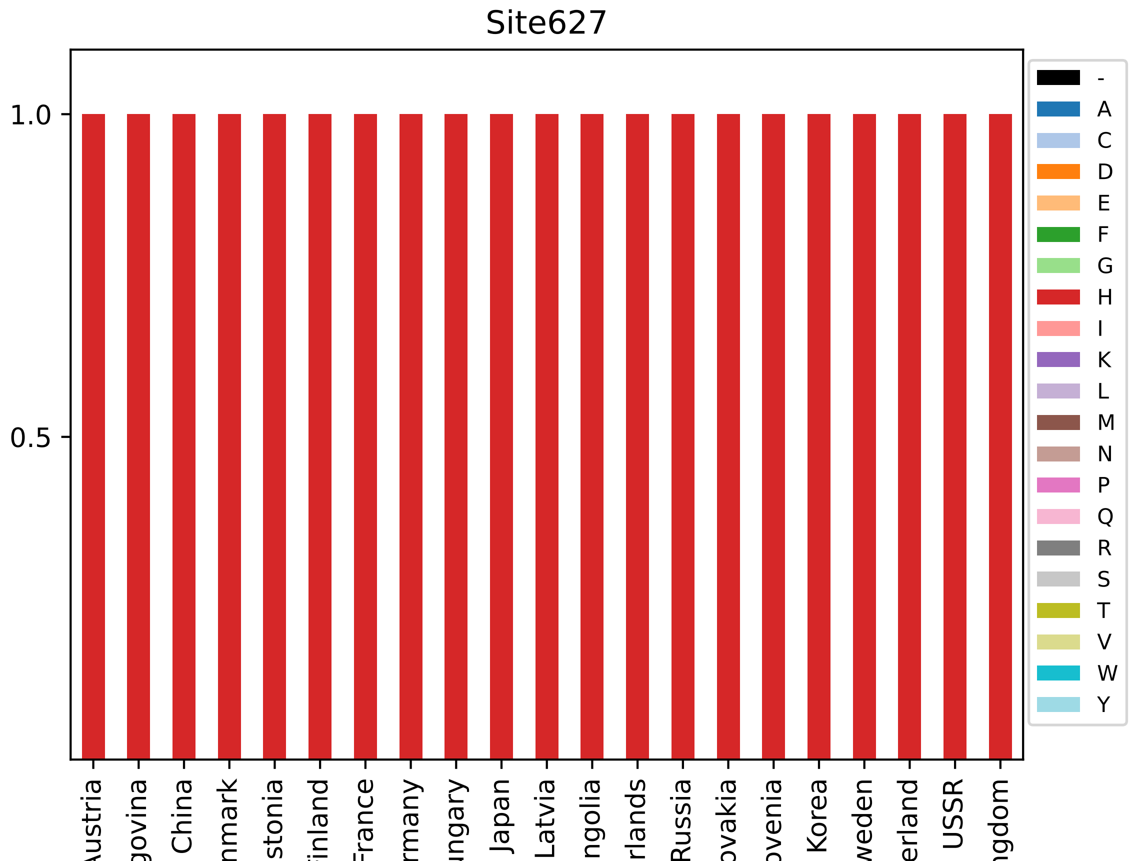

Site627
1.0
0.5
-
A
C
D
E
F
G
H
I
K
L
M
N
P
Q
R
S
T
V
W
Y
China
Japan
Latvia
Russia
Korea
USSR

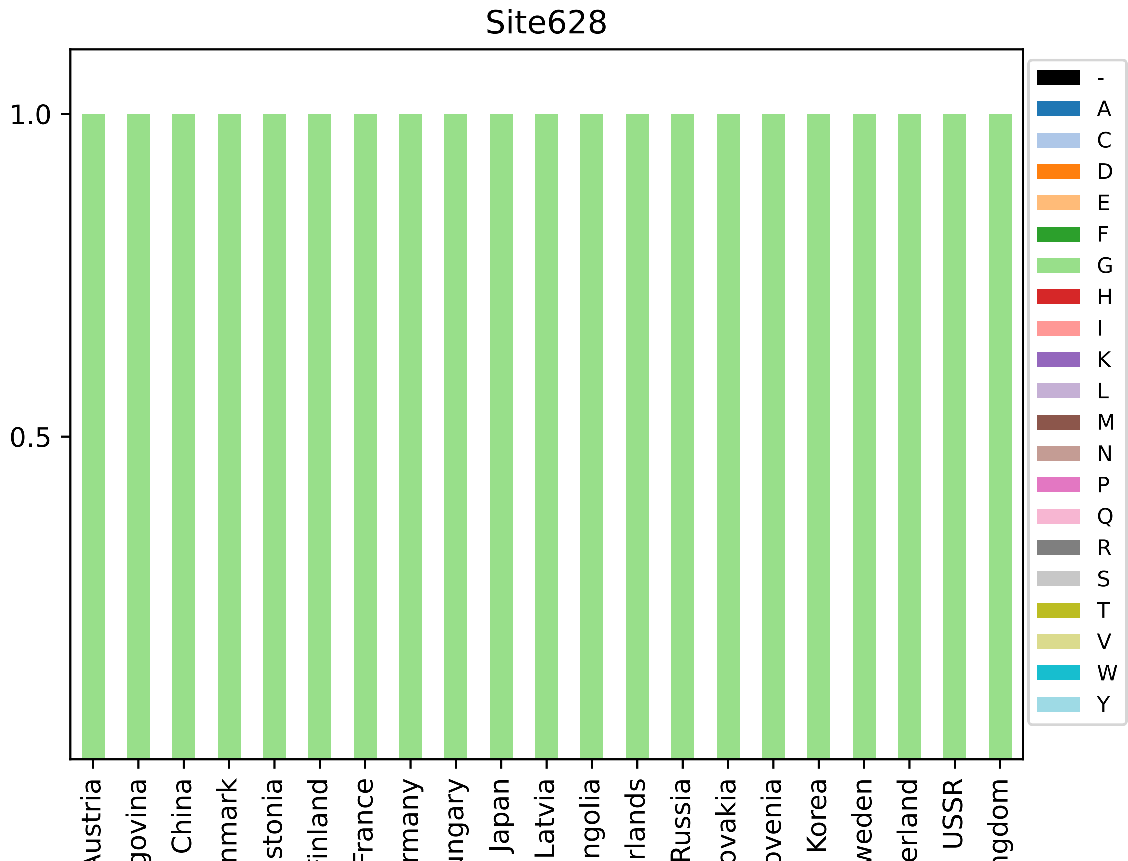
Site628
1.0
0.5
China
Japan
Latvia
Russia
Korea
USSR
-
A
C
D
E
F
G
H
I
K
L
M
N
P
Q
R
S
T
V
W
Y

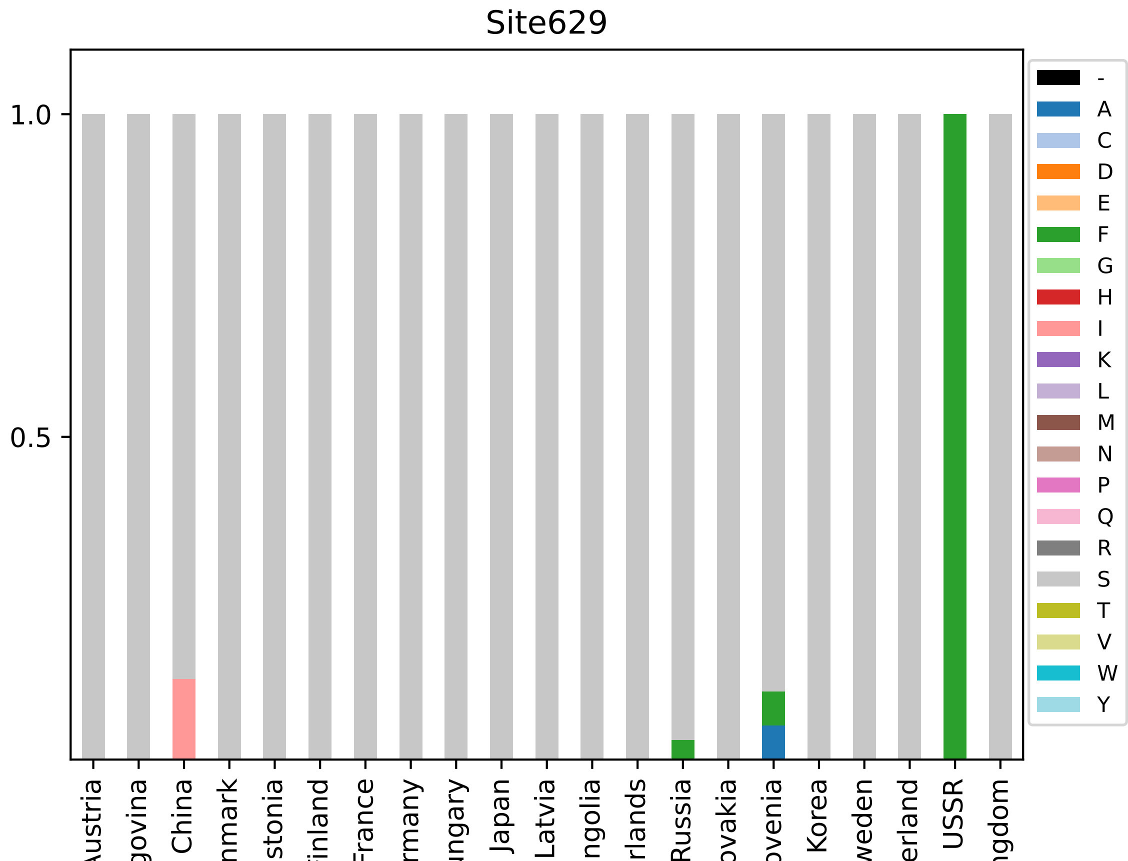

Site629
1.0
0.5
-
A
C
D
E
F
G
H
I
K
L
M
N
P
Q
R
S
T
V
W
Y
Austria
govina
China
nmark
stonia
Finland
France
rmany
ungary
Japan
Latvia
ngolia
rlands
Russia
ovakia
ovenia
Korea
weden
erland
USSR
ngdom

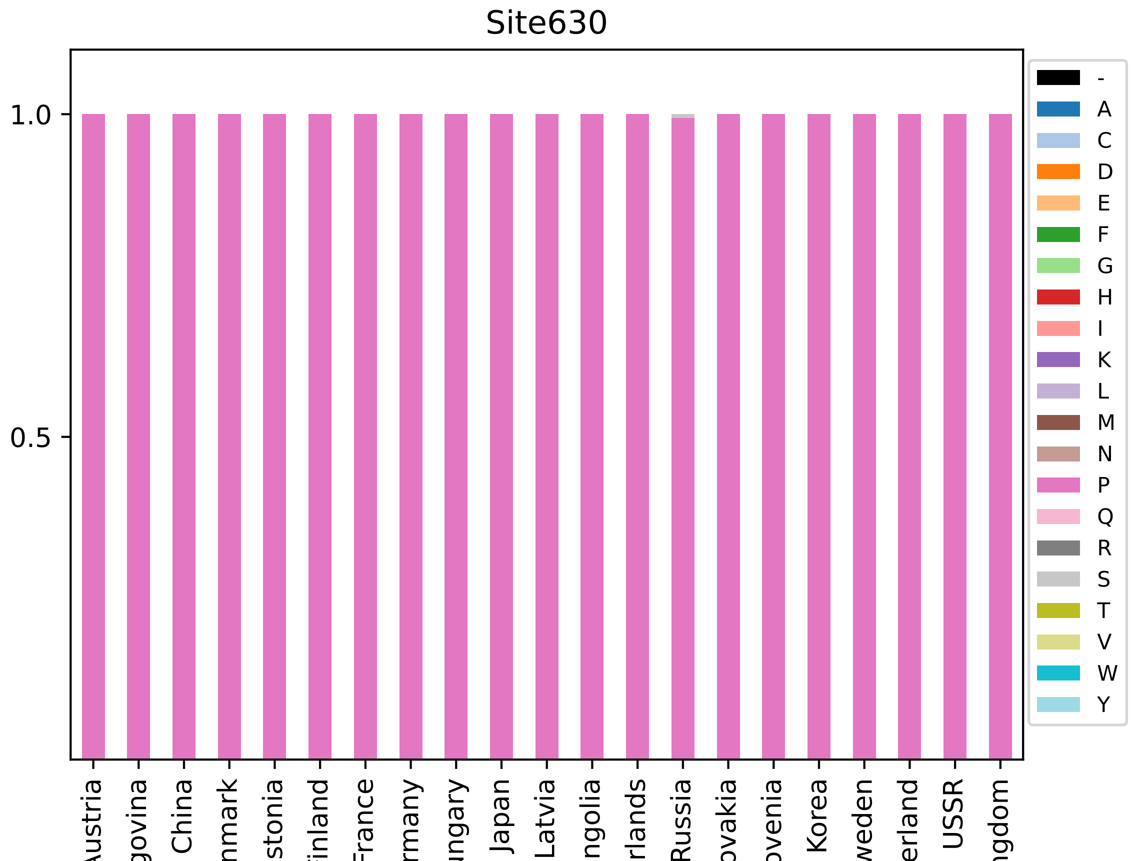

Site630
1.0
0.5
-
A
C
D
E
F
G
H
I
K
L
M
N
P
Q
R
S
T
V
W
Y
Austria
govina
China
nmark
stonia
inland
France
rmany
ungary
Japan
Latvia
ngolia
rlands
Russia
ovakia
ovenia
Korea
weden
erland
USSR
ngdom

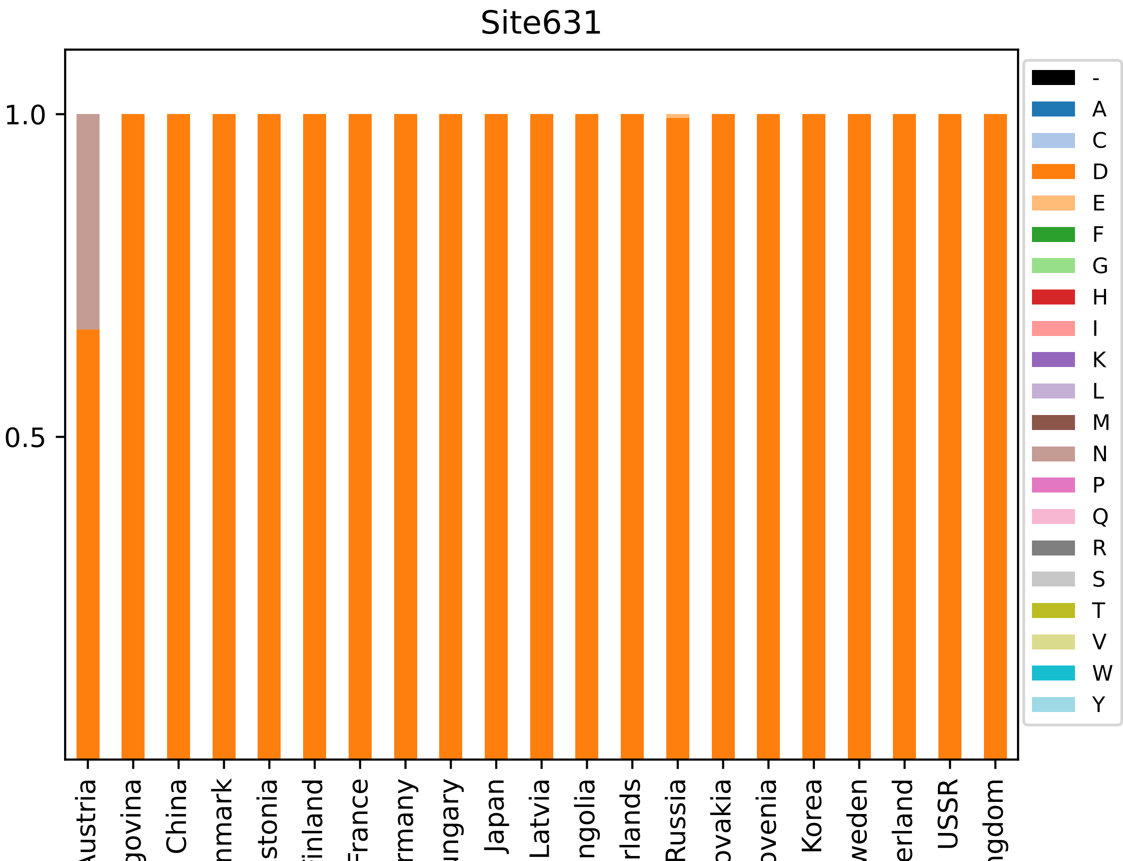
Site631
1.0
0.5
-
A
C
D
E
F
G
H
I
K
L
M
N
P
Q
R
S
T
V
W
Y
Austria
govina
China
nmark
stonia
inland
France
rmany
ungary
Japan
Latvia
ngolia
rlands
Russia
ovakia
ovenia
Korea
weden
erland
USSR
ngdom

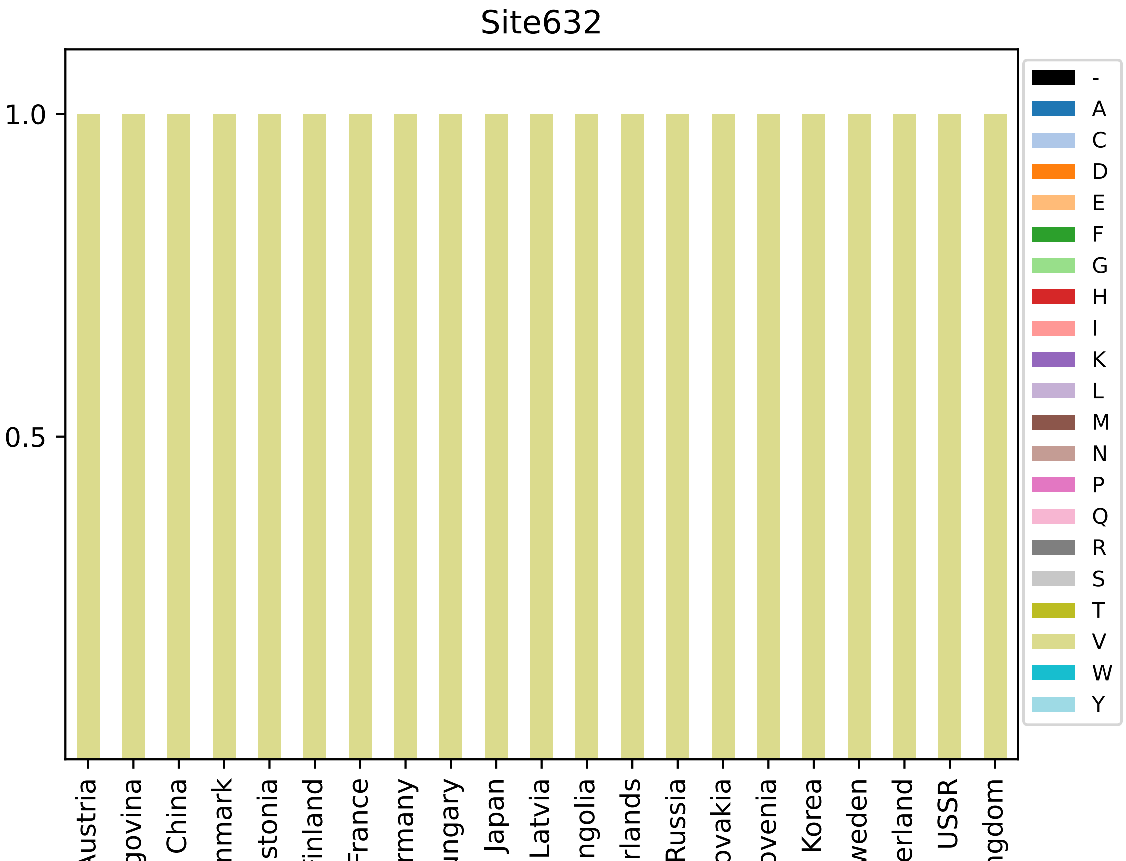
Site632
1.0
0.5
-
A
C
D
E
F
G
H
I
K
L
M
N
P
Q
R
S
T
V
W
Y
Austria
China
Japan
Latvia
Russia
Korea
USSR

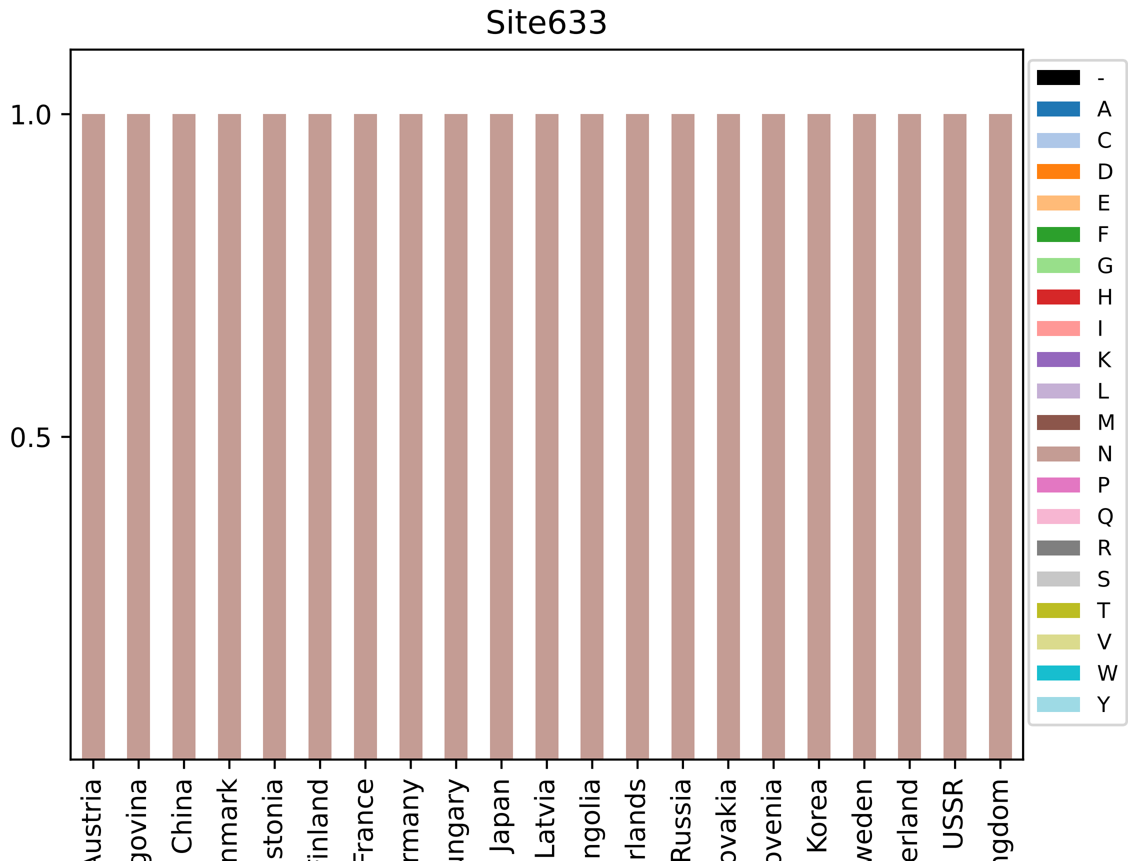
Site633
1.0
0.5
-
A
C
D
E
F
G
H
I
K
L
M
N
P
Q
R
S
T
V
W
Y
China
Japan
Latvia
Russia
Korea
USSR

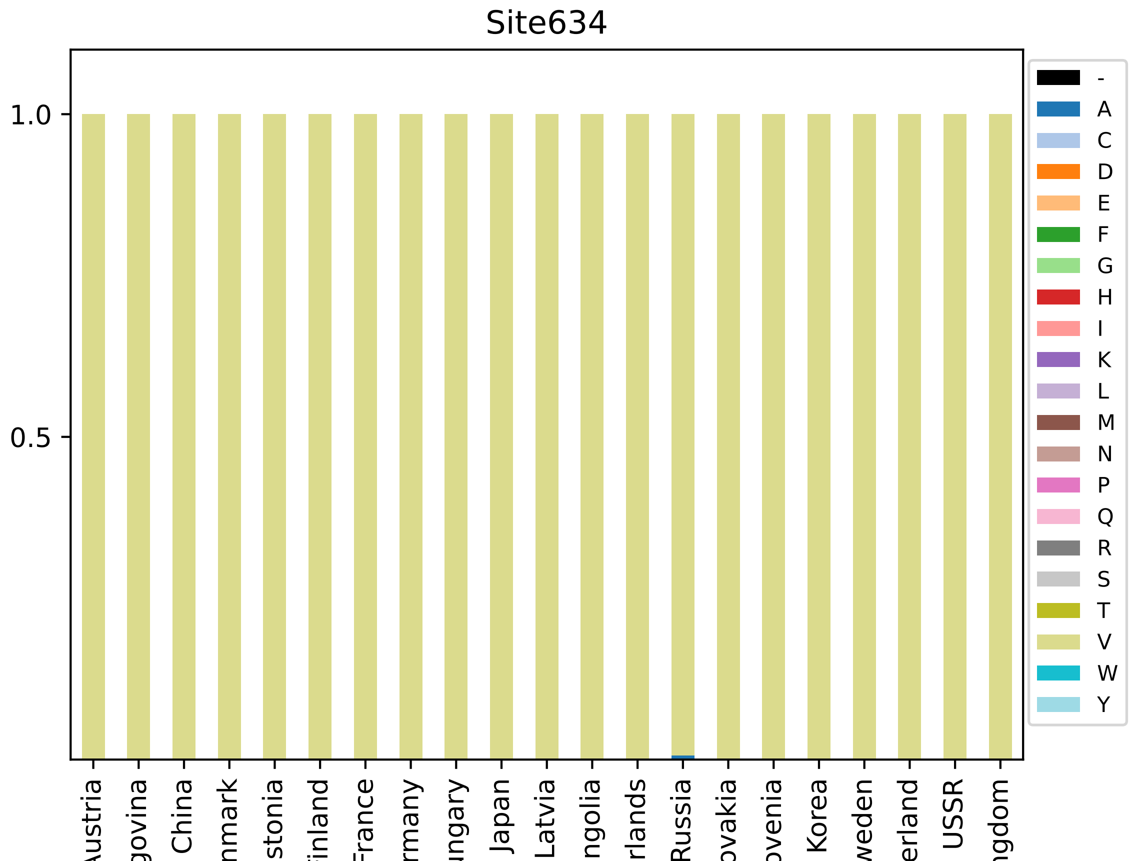
Site634
1.0
0.5
-
A
C
D
E
F
G
H
I
K
L
M
N
P
Q
R
S
T
V
W
Y
Austria
China
Japan
Latvia
Russia
Korea
USSR

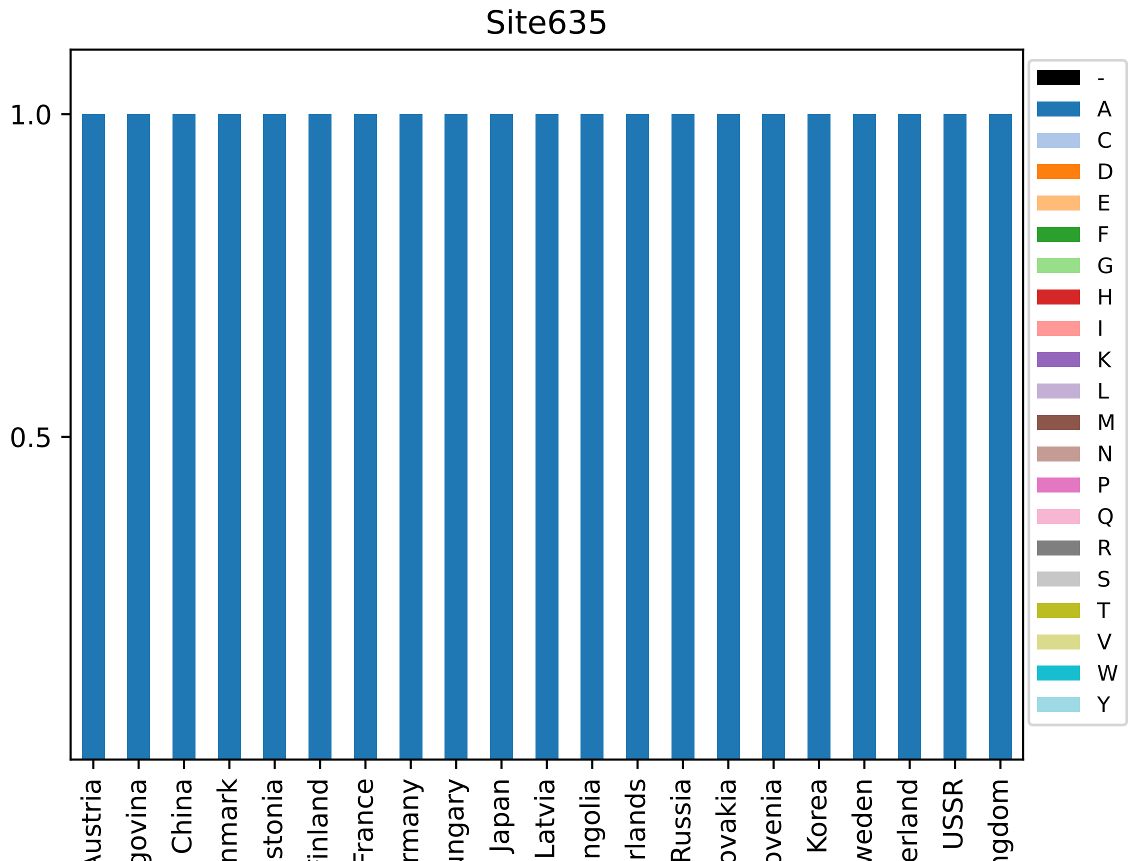
Site635
1.0
0.5
Austria
govina
China
nmark
stonia
Finland
France
rmany
ungary
Japan
Latvia
ngolia
rlands
Russia
ovakia
ovenia
Korea
weden
erland
USSR
ngdom
-
A
C
D
E
F
G
H
I
K
L
M
N
P
Q
R
S
T
V
W
Y

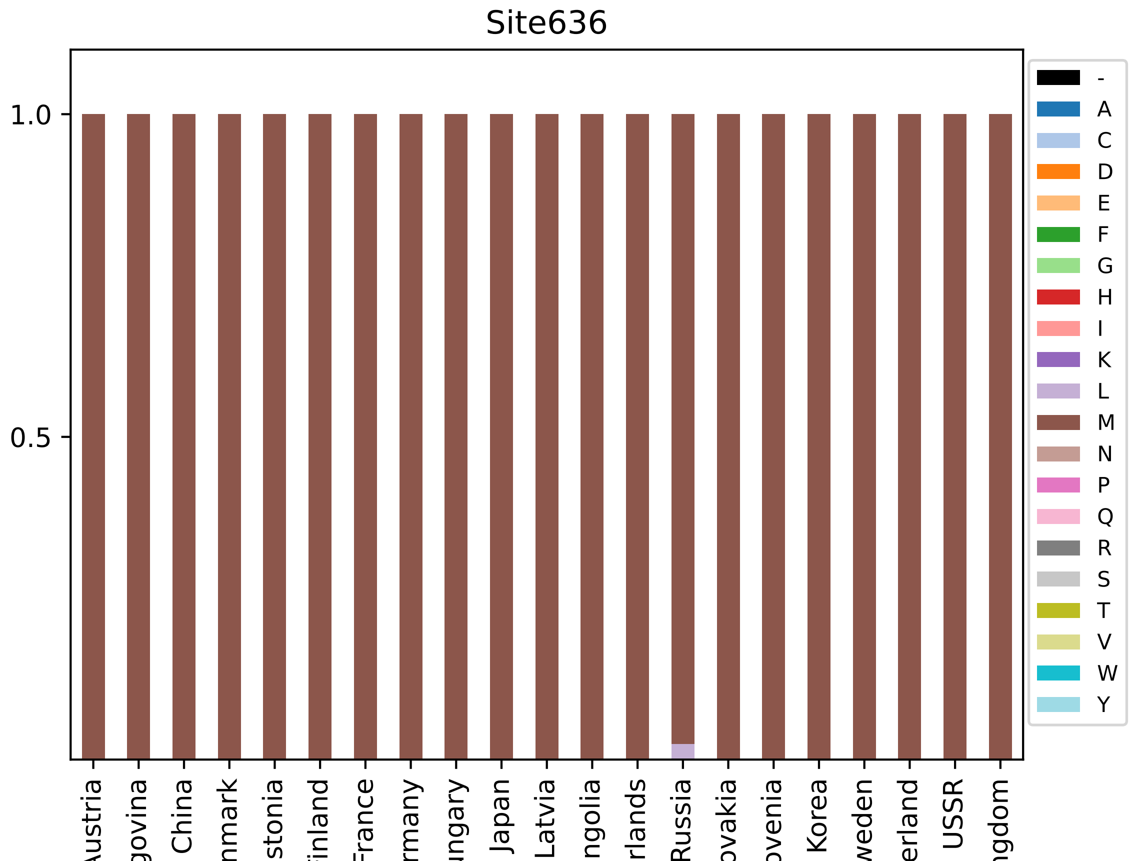
Site636
1.0
0.5
-
A
C
D
E
F
G
H
I
K
L
M
N
P
Q
R
S
T
V
W
Y
Austria
China
Japan
Latvia
Russia
Korea
USSR

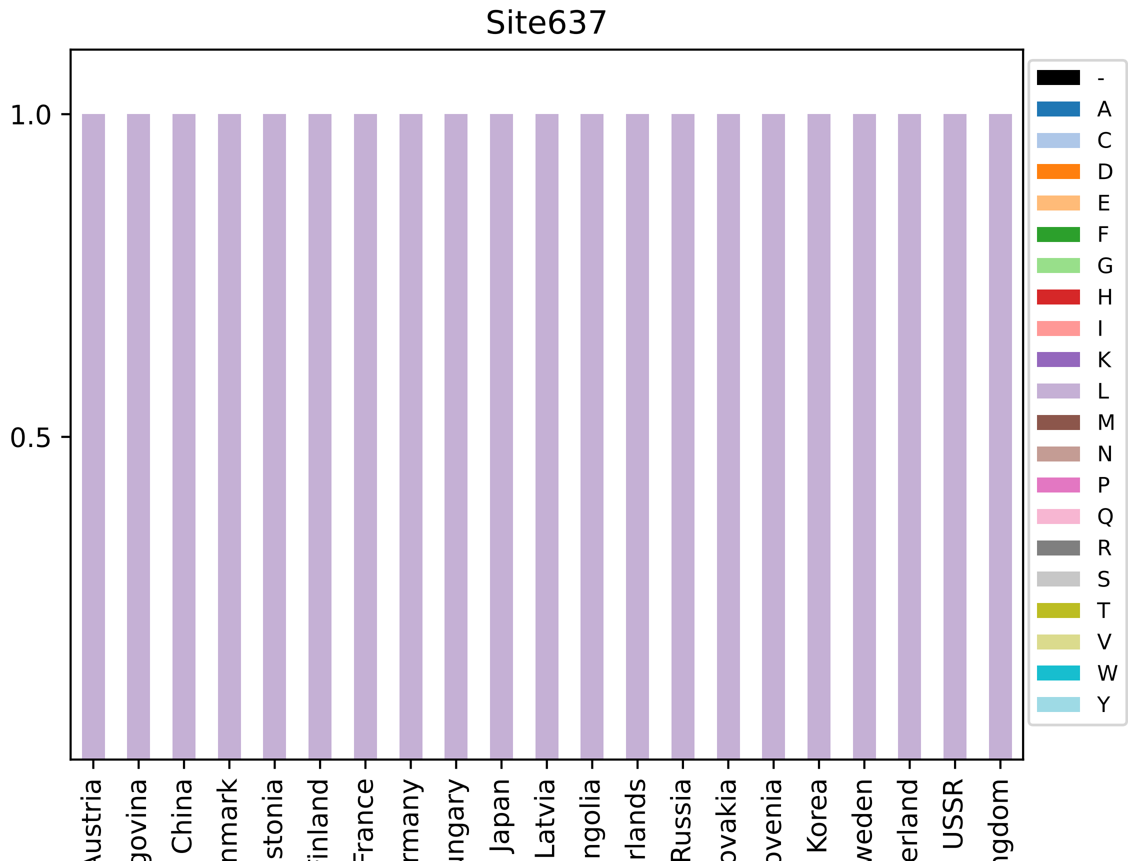
Site637
1.0
0.5
Austria
govina
China
nmark
stonia
Finland
France
rmany
ungary
Japan
Latvia
ngolia
rlands
Russia
ovakia
ovenia
Korea
weden
erland
USSR
ngdom
-
A
C
D
E
F
G
H
I
K
L
M
N
P
Q
R
S
T
V
W
Y

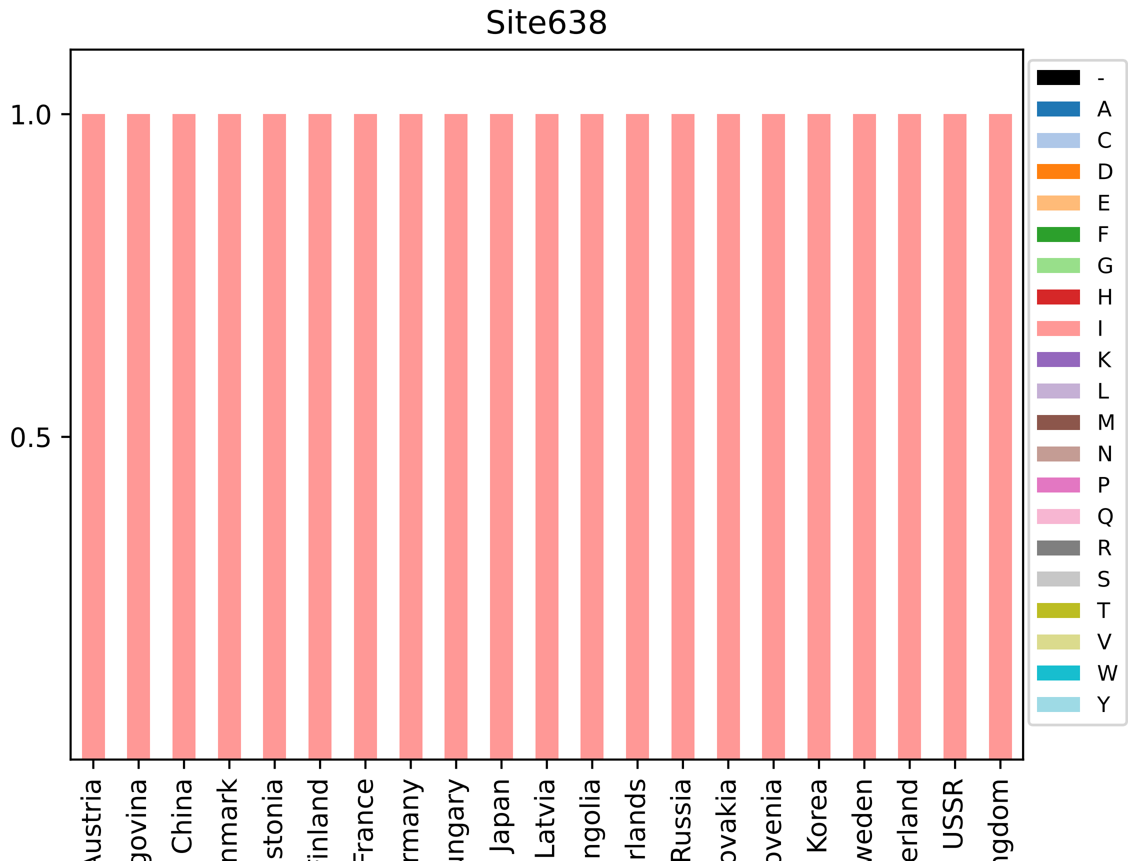

Site638
1.0
0.5
Austria
govina
China
nmark
stonia
inland
France
rmany
ungary
Japan
Latvia
ngolia
rlands
Russia
ovakia
ovenia
Korea
weden
erland
USSR
ngdom
-
A
C
D
E
F
G
H
I
K
L
M
N
P
Q
R
S
T
V
W
Y

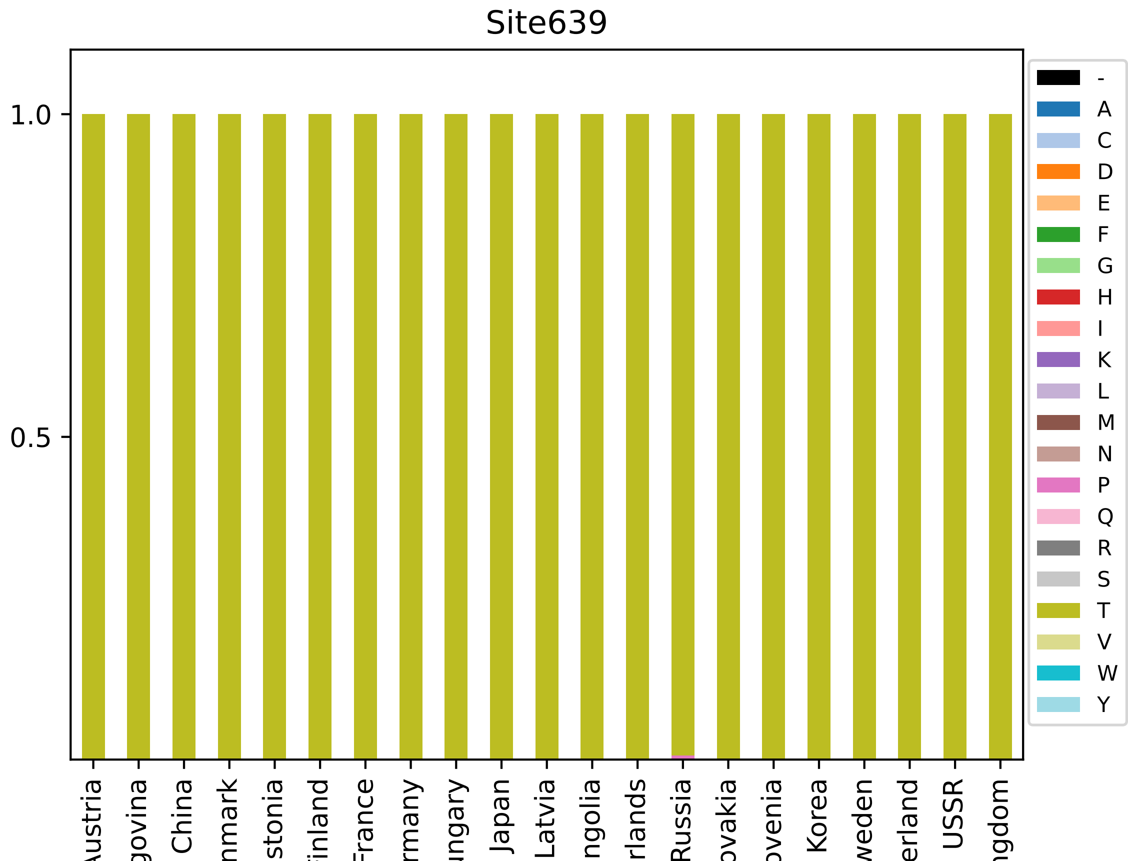

Site639
1.0
0.5
-
A
C
D
E
F
G
H
I
K
L
M
N
P
Q
R
S
T
V
W
Y
Austria
govina
China
nmark
stonia
inland
France
rmany
ungary
Japan
Latvia
ngolia
rlands
Russia
ovakia
ovenia
Korea
weden
erland
USSR
ngdom

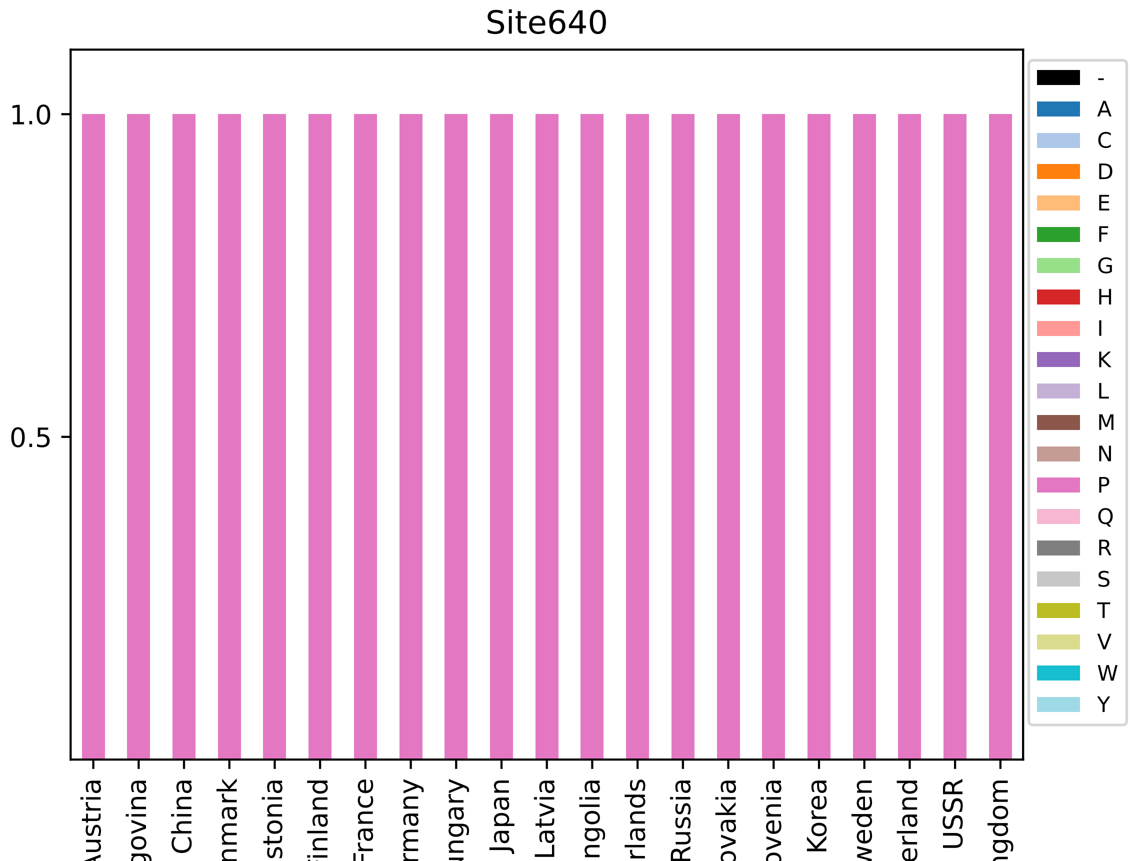

Site640
1.0
0.5
-
A
C
D
E
F
G
H
I
K
L
M
N
P
Q
R
S
T
V
W
Y
China
Japan
Latvia
Russia
Korea
USSR

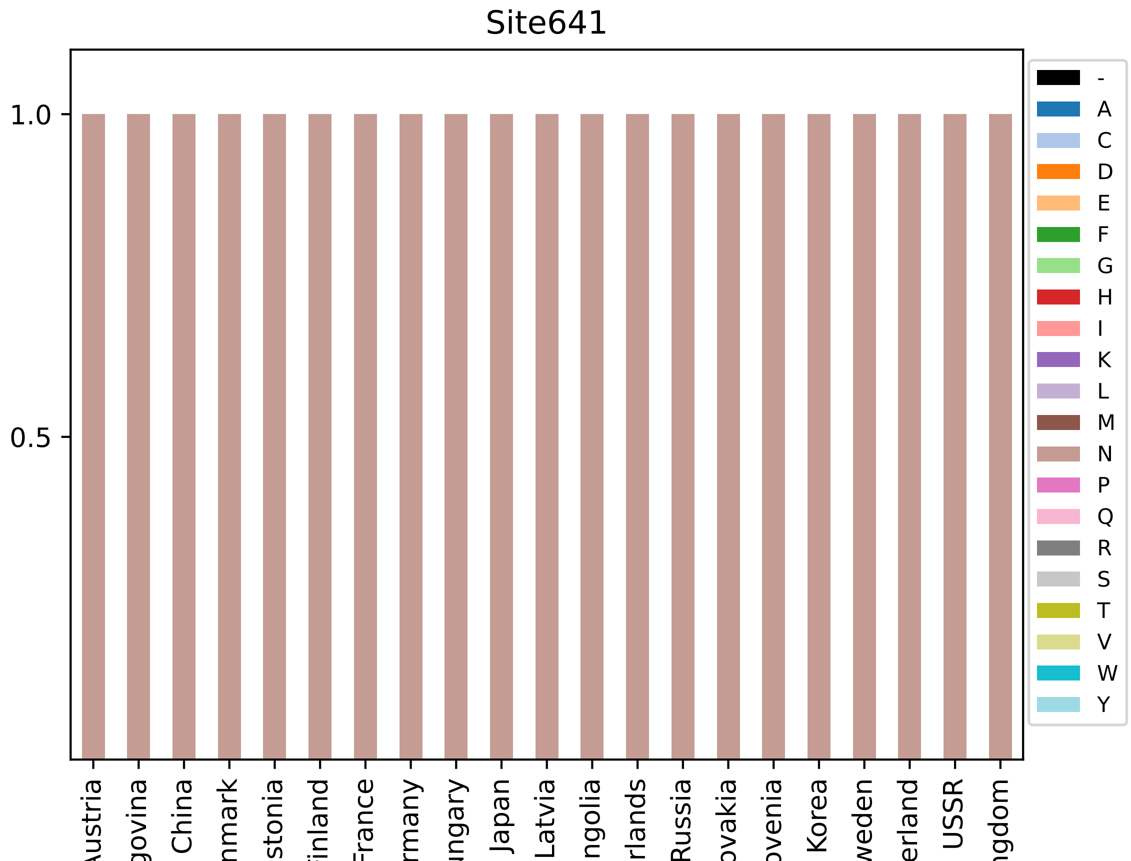
Site641
1.0
0.5
-
A
C
D
E
F
G
H
I
K
L
M
N
P
Q
R
S
T
V
W
Y
Austria
govina
China
nmark
stonia
inland
France
rmany
ungary
Japan
Latvia
ngolia
rlands
Russia
ovakia
ovenia
Korea
weden
erland
USSR
ngdom

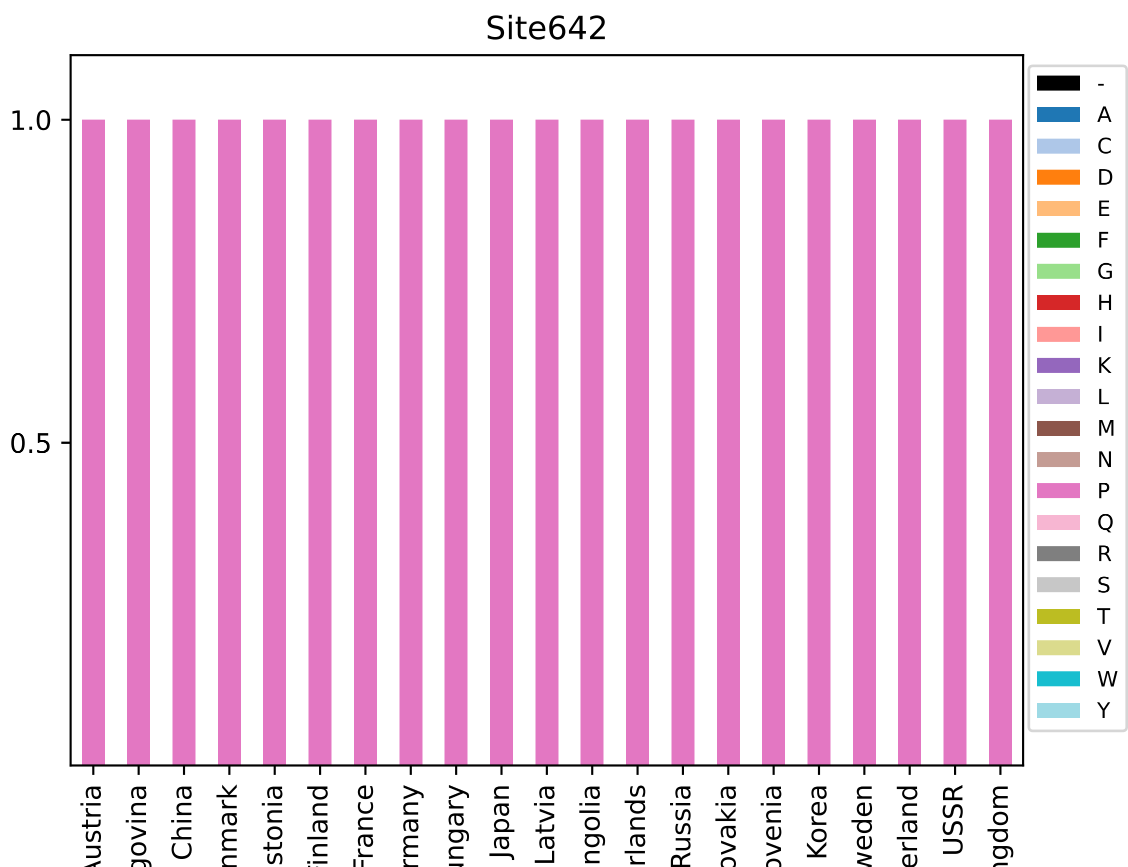

Site642
1.0
0.5
-
A
C
D
E
F
G
H
I
K
L
M
N
P
Q
R
S
T
V
W
Y
China
Japan
Latvia
Russia
Korea
USSR

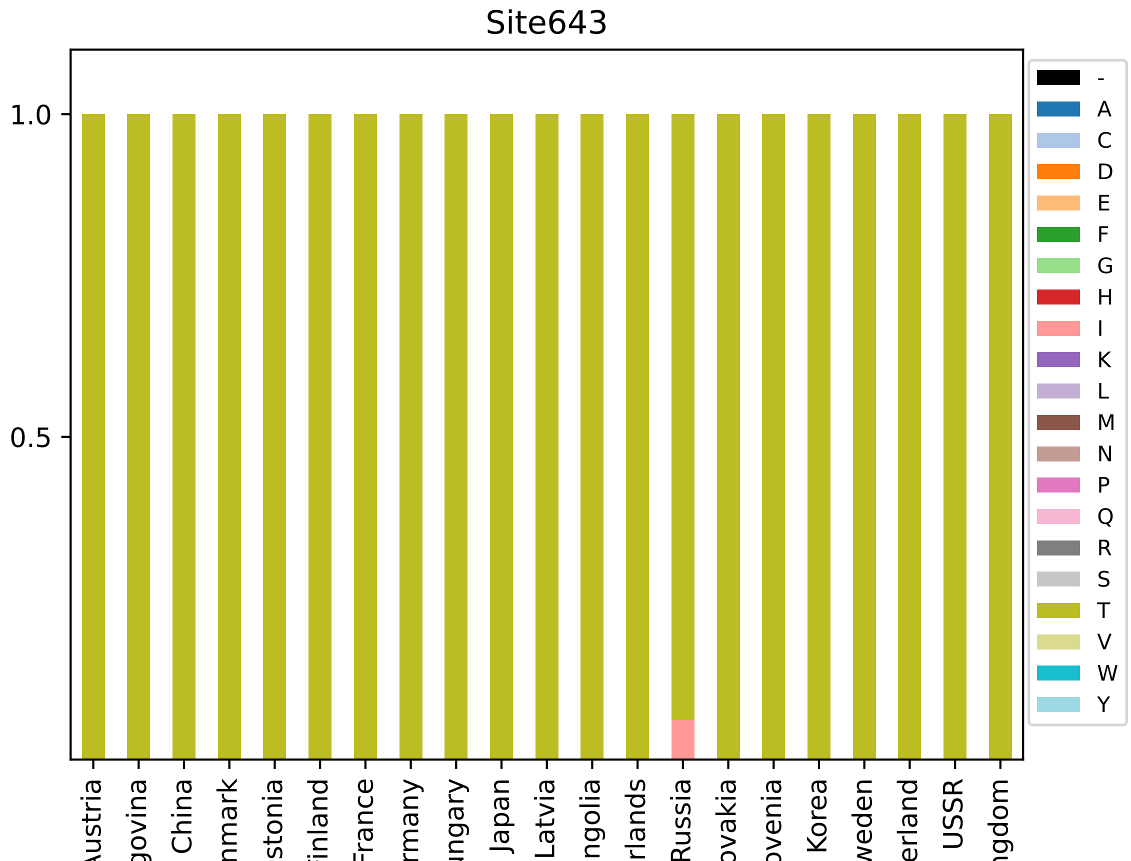

Site643
1.0
0.5
-
A
C
D
E
F
G
H
I
K
L
M
N
P
Q
R
S
T
V
W
Y
Austria
govina
China
nmark
stonia
inland
France
rmany
ungary
Japan
Latvia
ngolia
rlands
Russia
ovakia
ovenia
Korea
weden
erland
USSR
ngdom

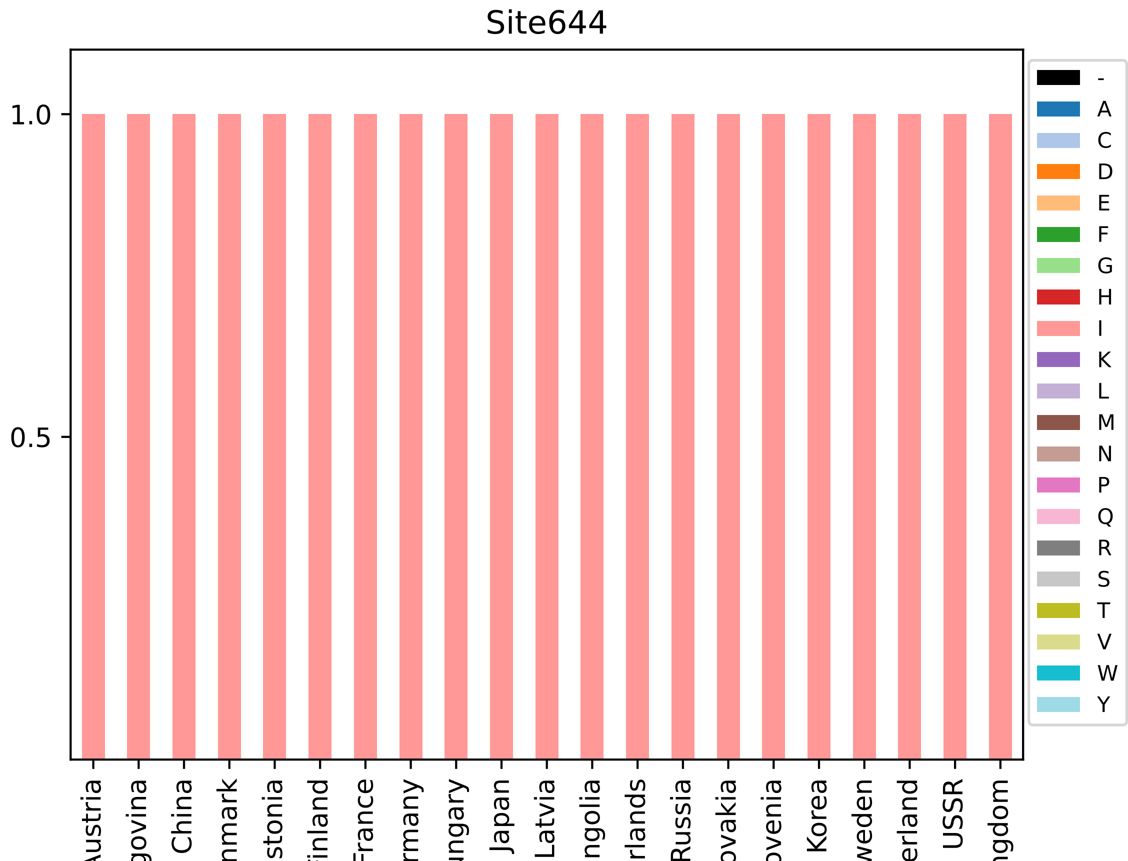

Site644
1.0
0.5
-
A
C
D
E
F
G
H
I
K
L
M
N
P
Q
R
S
T
V
W
Y
Austria
govina
China
nmark
stonia
Finland
France
rmany
ungary
Japan
Latvia
ngolia
rlands
Russia
ovakia
ovenia
Korea
weden
erland
USSR
ngdom

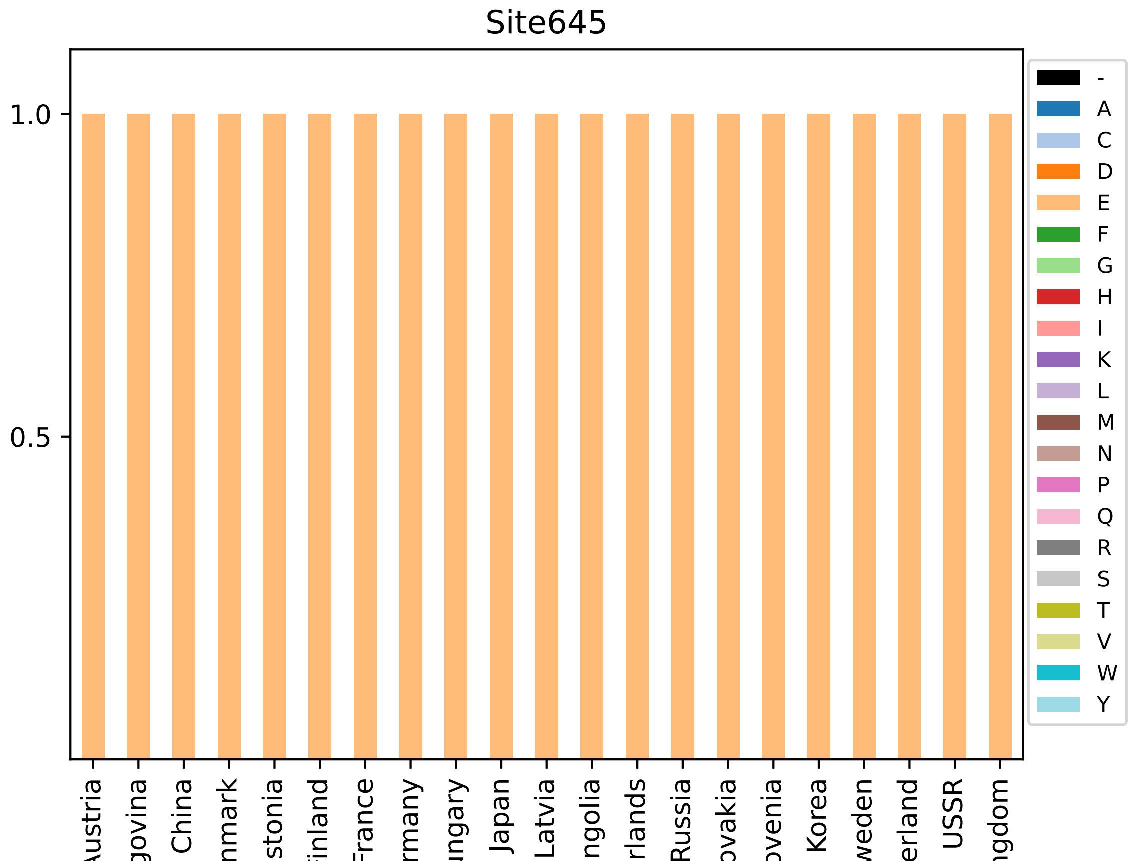

Site645
1.0
0.5
-
A
C
D
E
F
G
H
I
K
L
M
N
P
Q
R
S
T
V
W
Y
Austria
China
Japan
Latvia
Russia
Korea
USSR

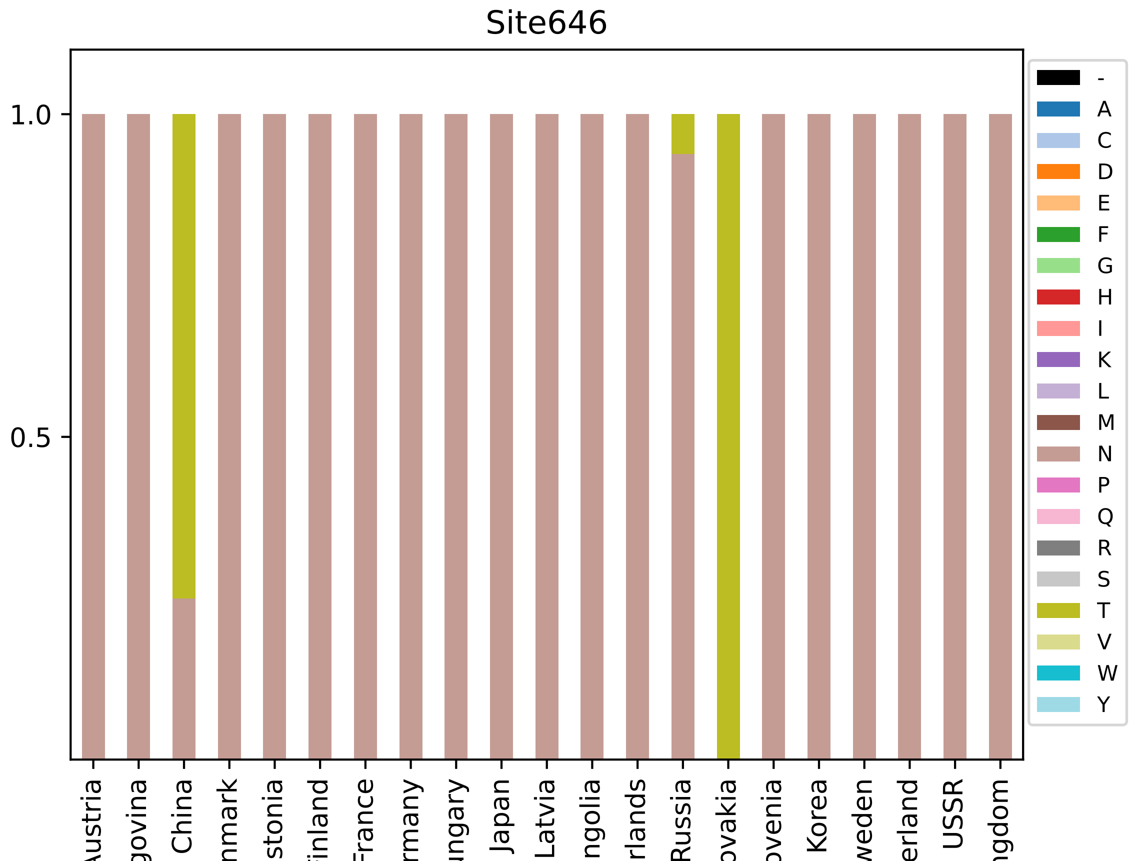

Site646
1.0
0.5
-
A
C
D
E
F
G
H
I
K
L
M
N
P
Q
R
S
T
V
W
Y
Austria
China
Japan
Latvia
Russia
Korea
USSR

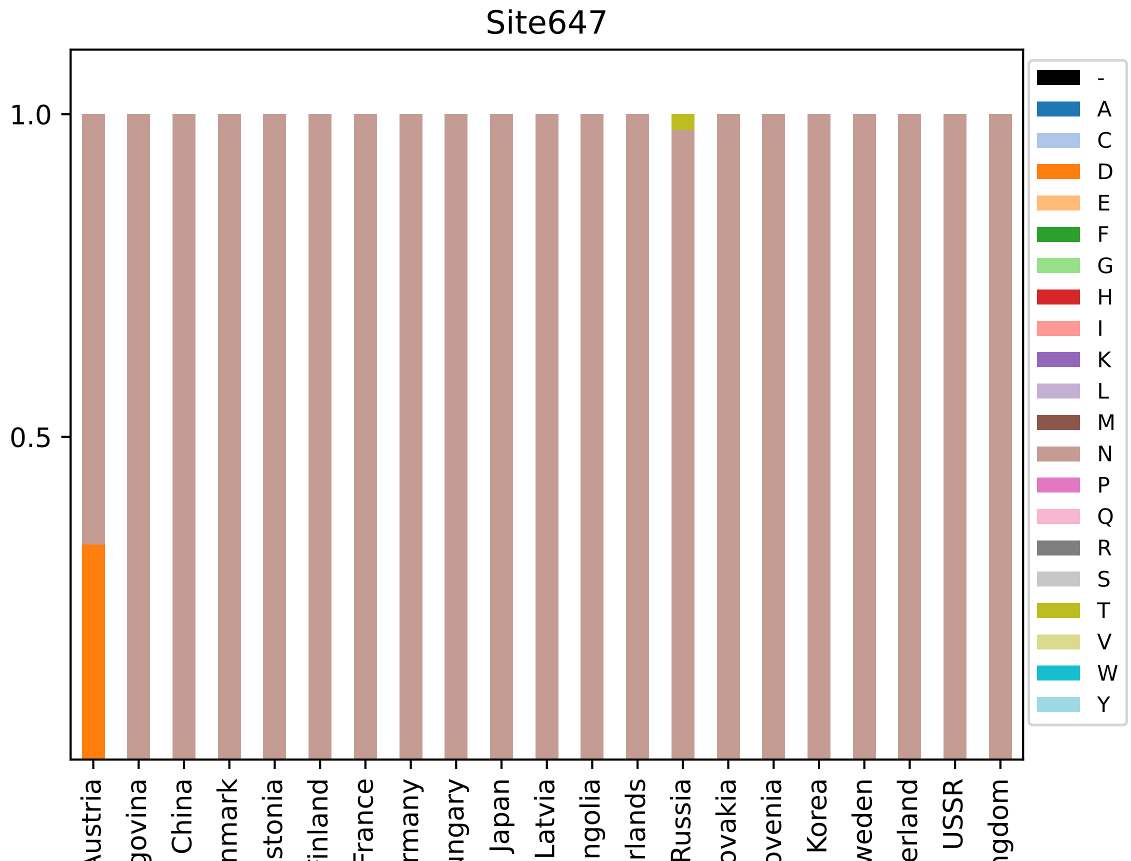

Site647
1.0
0.5
-
A
C
D
E
F
G
H
I
K
L
M
N
P
Q
R
S
T
V
W
Y
China
Japan
Latvia
Russia
Korea
USSR

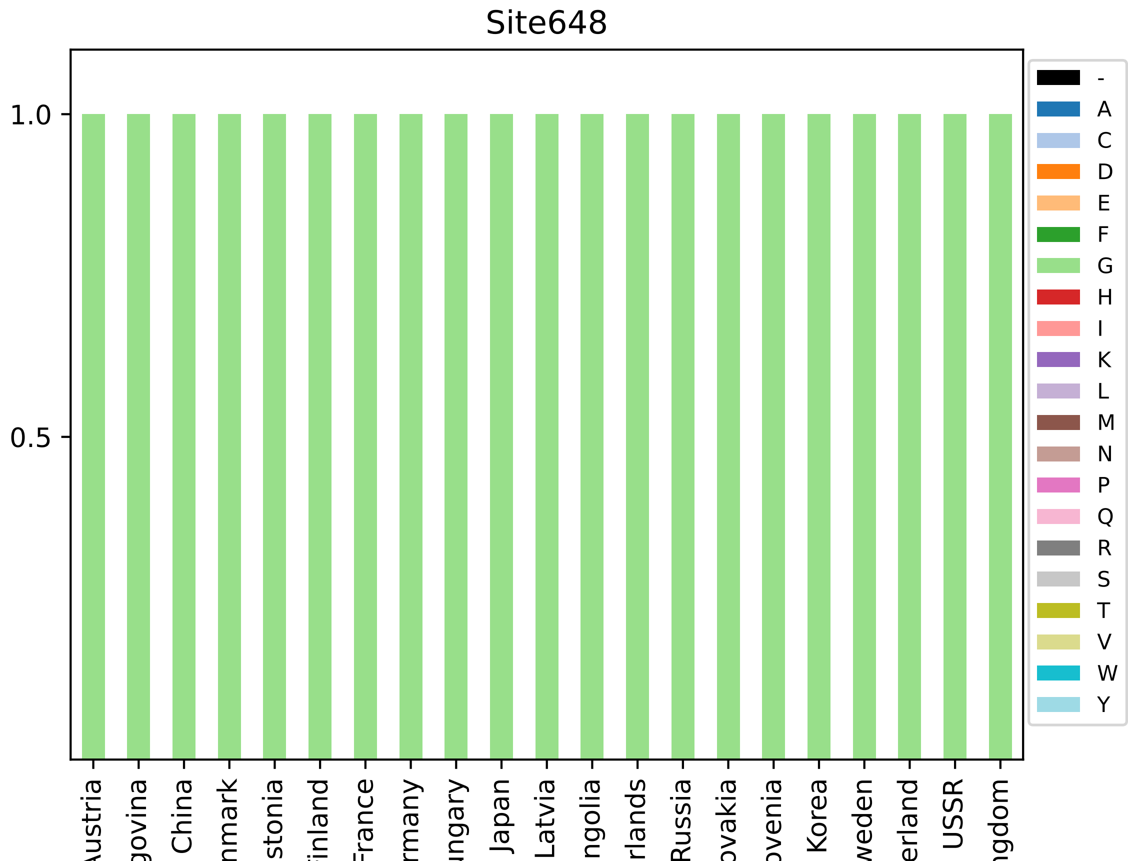

Site648
1.0
0.5
-
A
C
D
E
F
G
H
I
K
L
M
N
P
Q
R
S
T
V
W
Y
China
Japan
Latvia
Russia
Korea
USSR

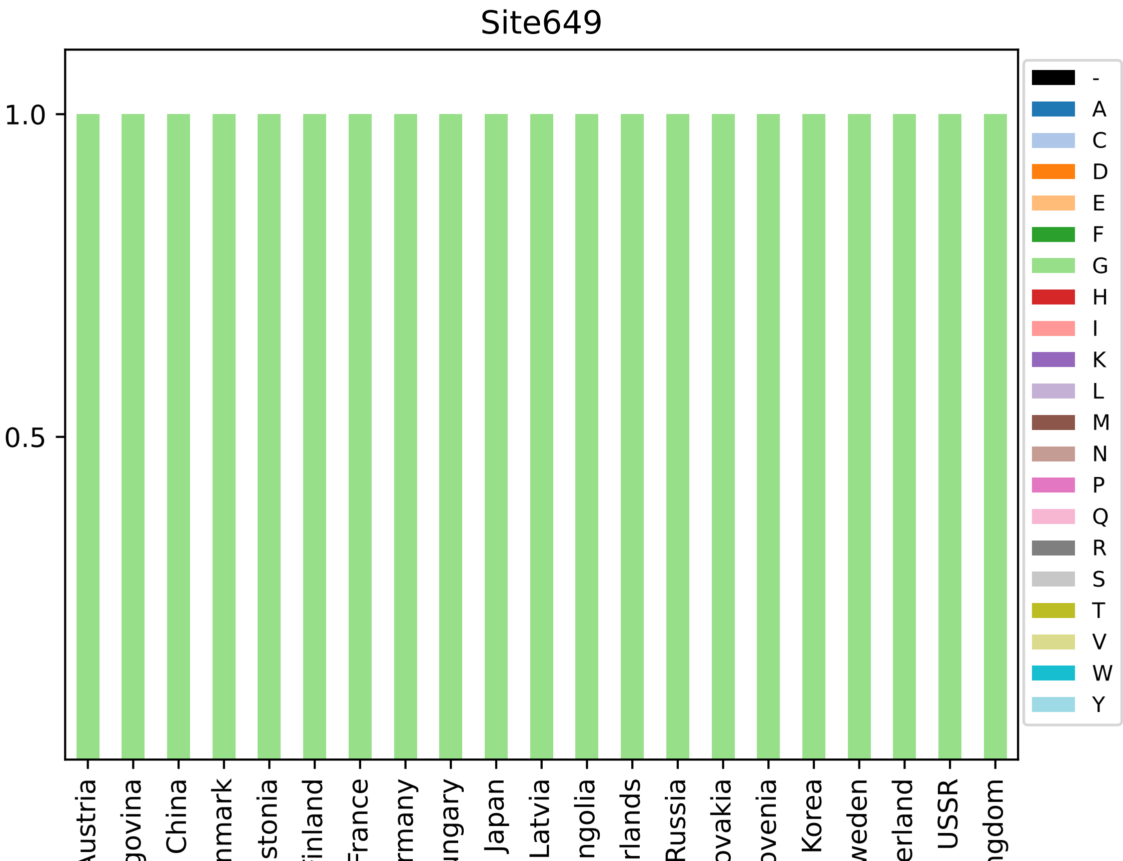
Site649
1.0
0.5
-
A
C
D
E
F
G
H
I
K
L
M
N
P
Q
R
S
T
V
W
Y
China
Japan
Latvia
Russia
Korea
USSR

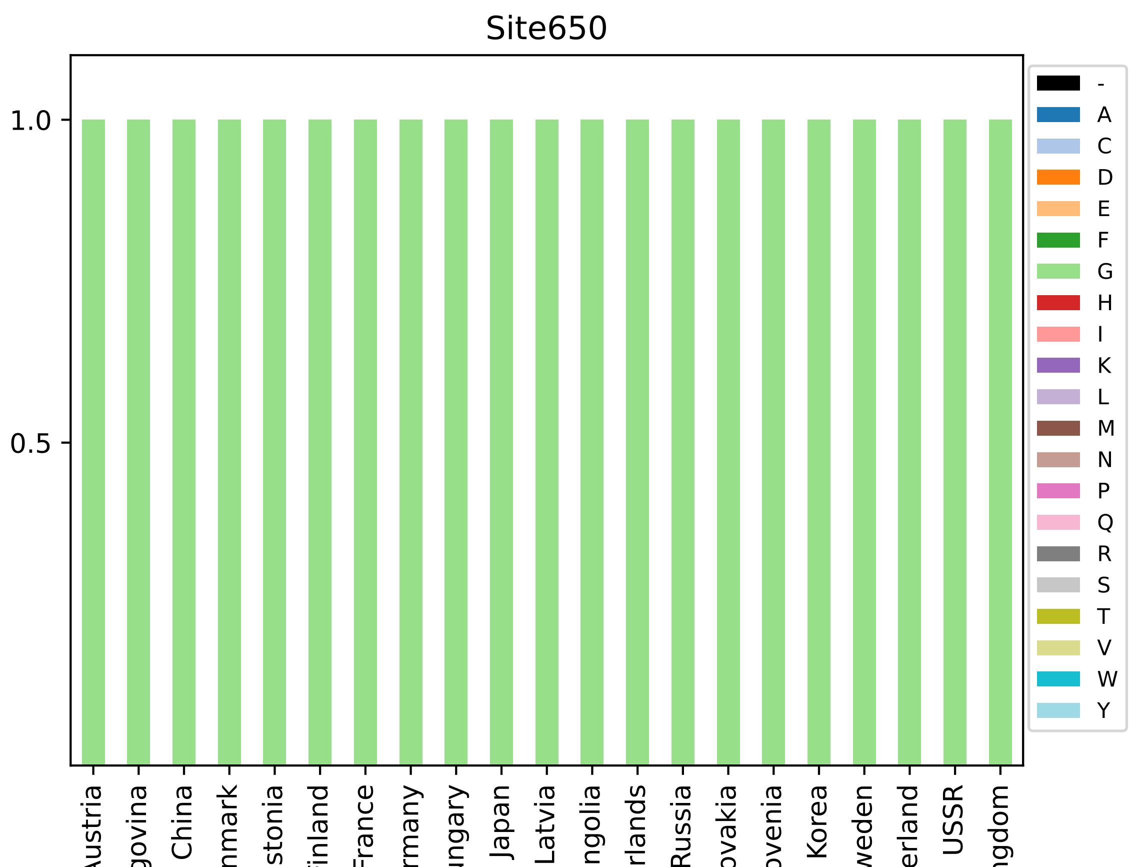
Site650
1.0
0.5
-
A
C
D
E
F
G
H
I
K
L
M
N
P
Q
R
S
T
V
W
Y
Austria
China
Finland
France
Japan
Latvia
Russia
Korea
USSR

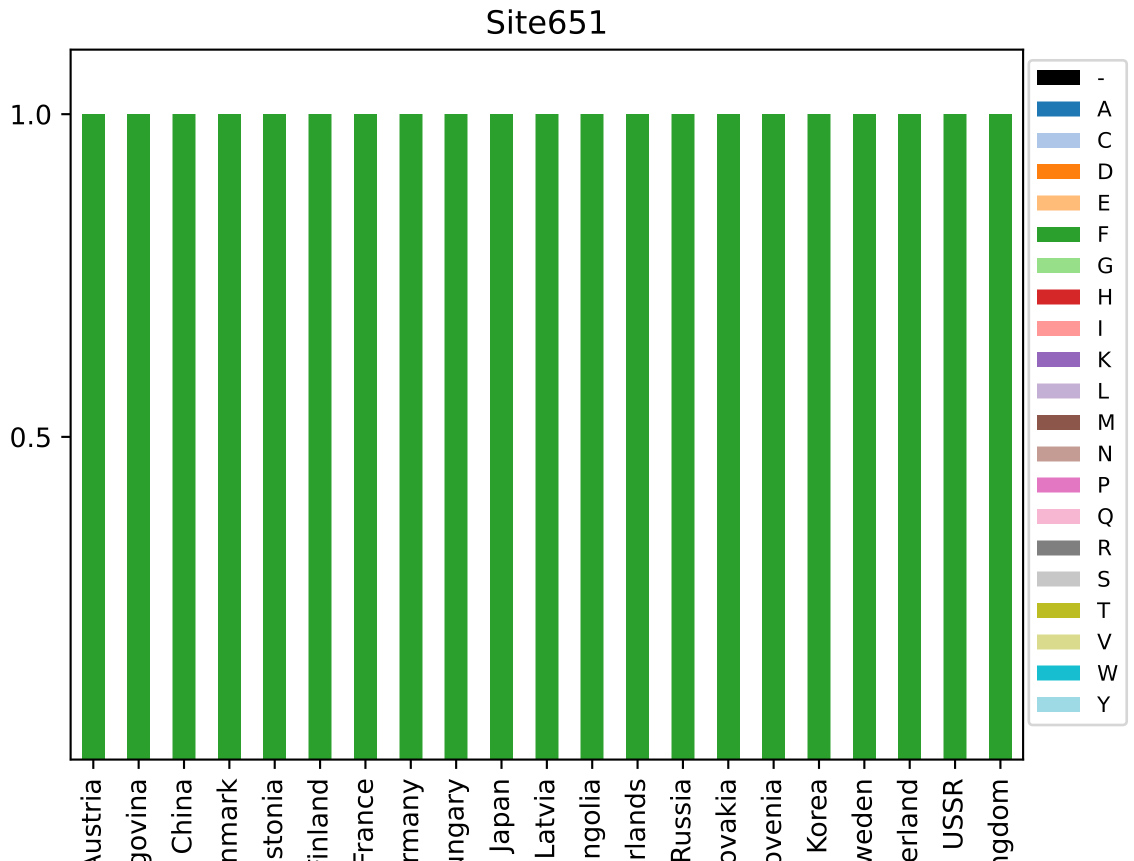
Site651
1.0
0.5
-
A
C
D
E
F
G
H
I
K
L
M
N
P
Q
R
S
T
V
W
Y
China
Japan
Latvia
Russia
Korea
USSR

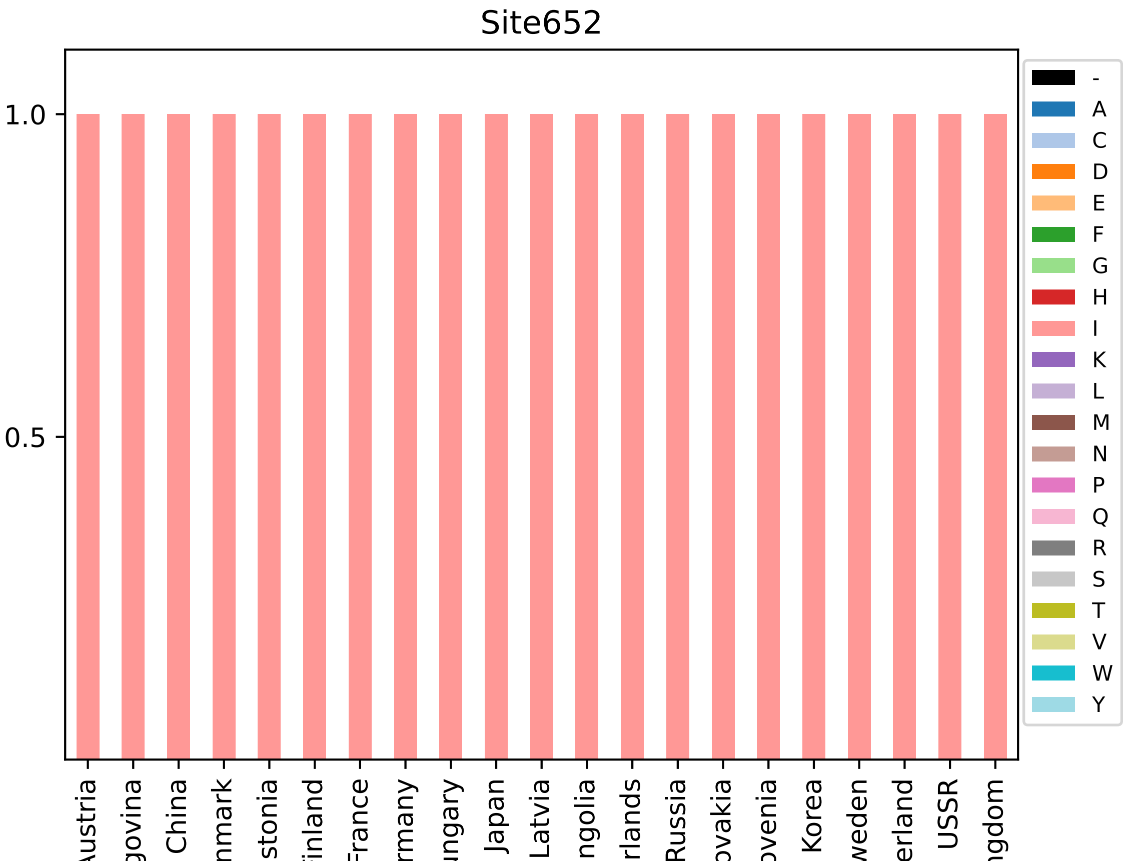

Site652
1.0
0.5
-
A
C
D
E
F
G
H
I
K
L
M
N
P
Q
R
S
T
V
W
Y
China
Japan
Latvia
Russia
Korea
USSR

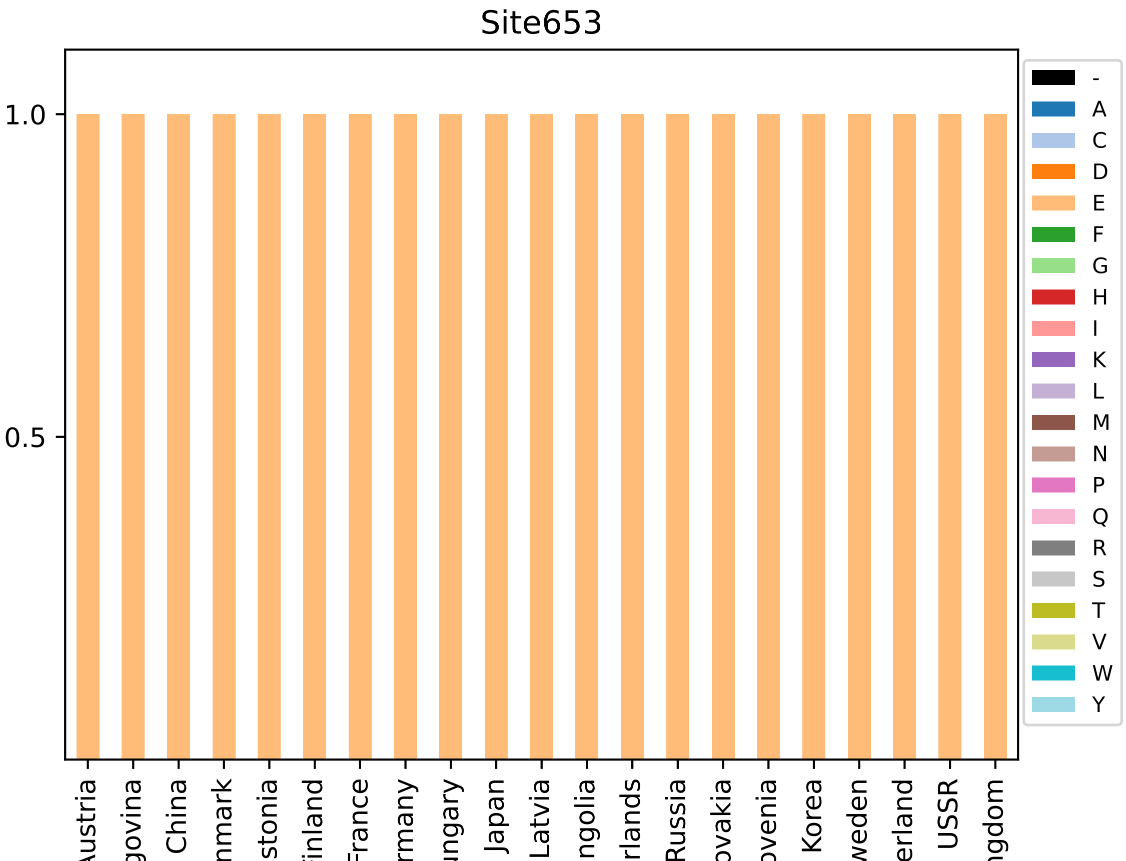

Site653
1.0
0.5
-
A
C
D
E
F
G
H
I
K
L
M
N
P
Q
R
S
T
V
W
Y
ustria
govina
China
nmark
stonia
inland
rance
rmany
ungary
Japan
Latvia
ngolia
rlands
Russia
ovakia
ovenia
Korea
weden
erland
USSR
ngdom

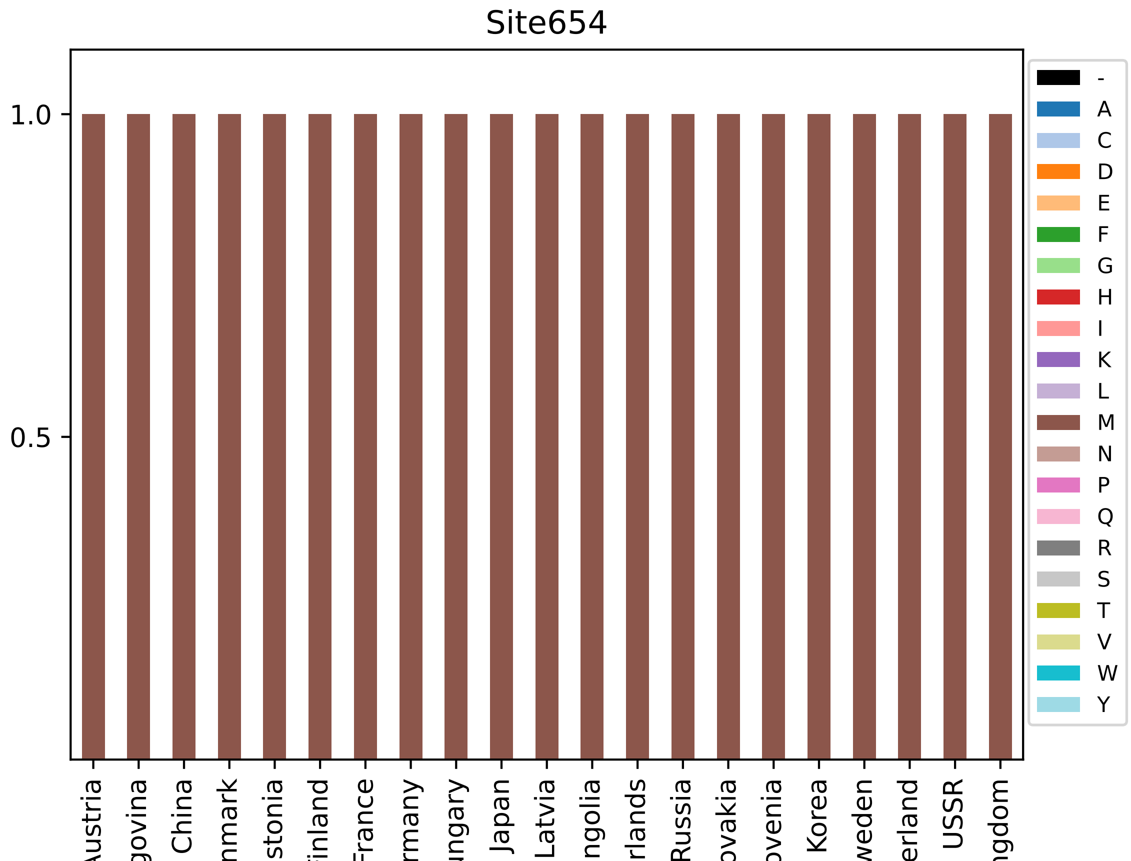

Site654
1.0
0.5
-
A
C
D
E
F
G
H
I
K
L
M
N
P
Q
R
S
T
V
W
Y
China
Japan
Latvia
Russia
Korea
USSR

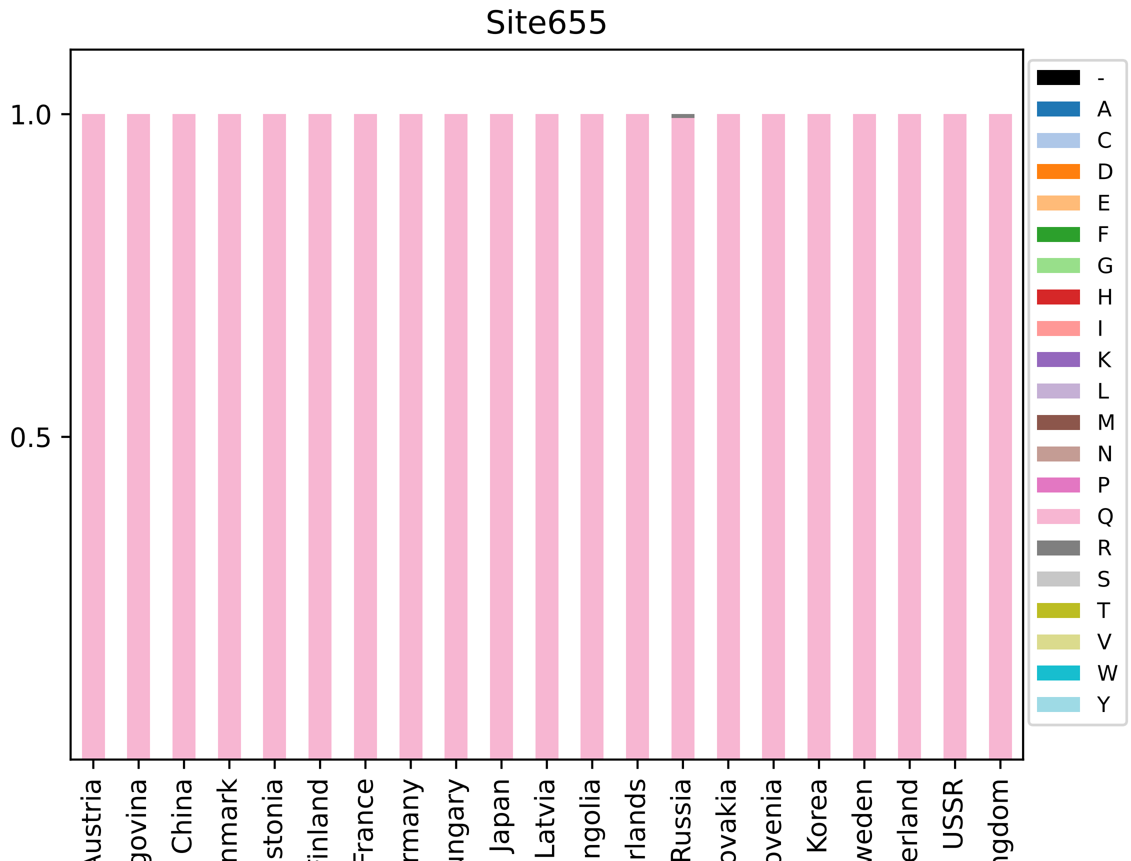
Site655
1.0
0.5
-
A
C
D
E
F
G
H
I
K
L
M
N
P
Q
R
S
T
V
W
Y
China
Japan
Latvia
Russia
Korea
USSR

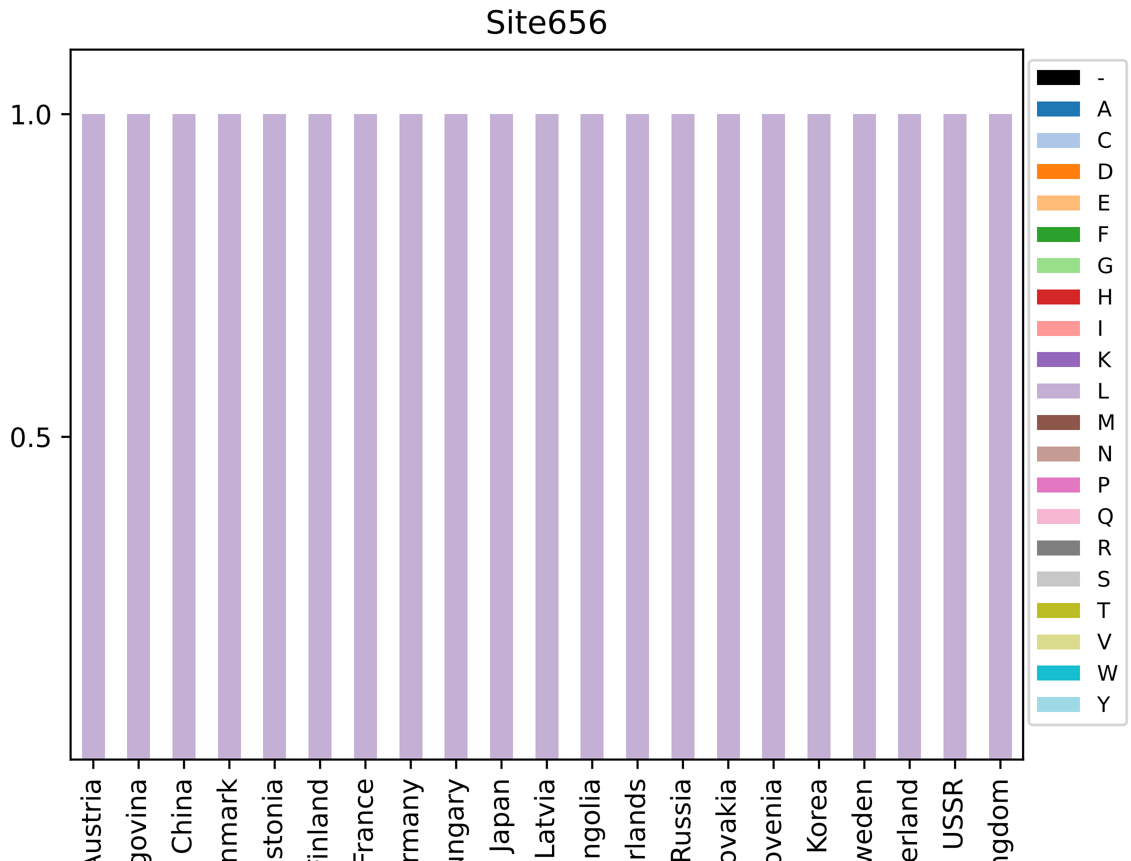
Site656
1.0
0.5
-
A
C
D
E
F
G
H
I
K
L
M
N
P
Q
R
S
T
V
W
Y
Austria
govina
China
nmark
stonia
inland
France
rmany
ungary
Japan
Latvia
ngolia
rlands
Russia
ovakia
ovenia
Korea
weden
erland
USSR
ngdom

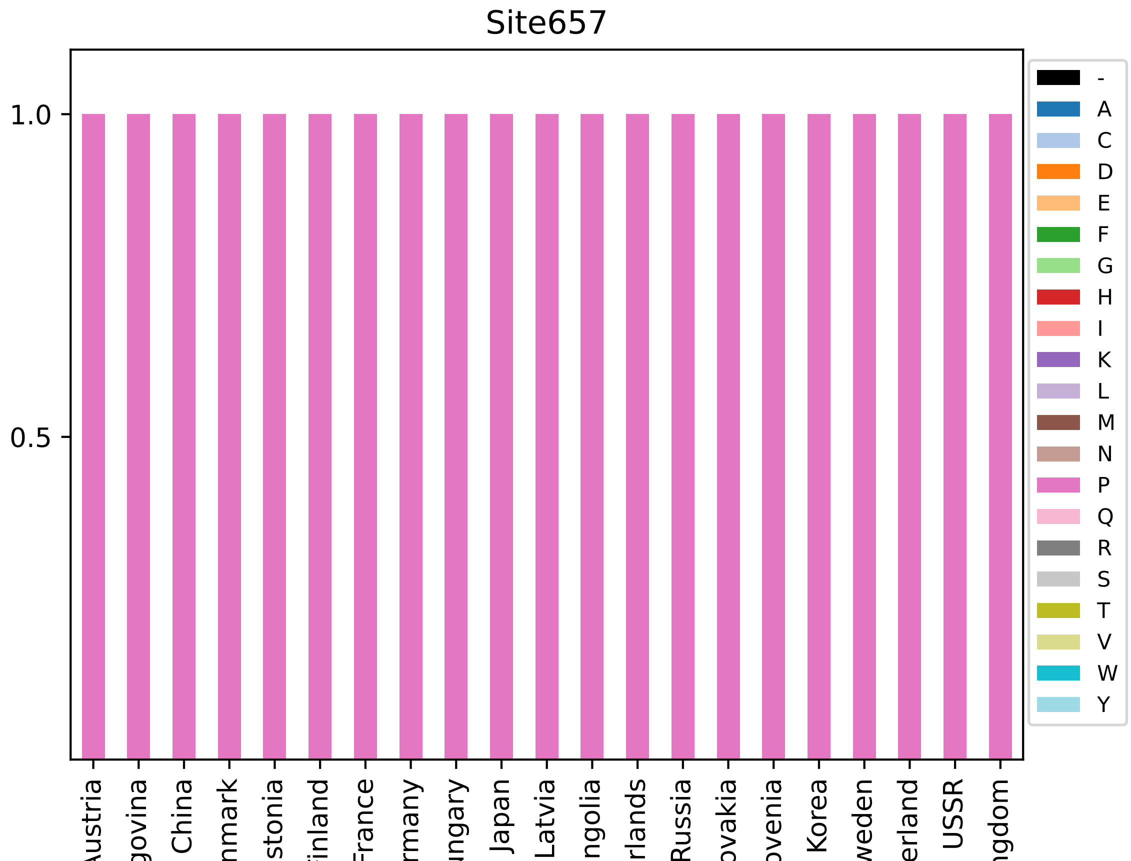
Site657
1.0
0.5
-
A
C
D
E
F
G
H
I
K
L
M
N
P
Q
R
S
T
V
W
Y
Austria
China
Finland
France
Japan
Latvia
Russia
Korea
USSR

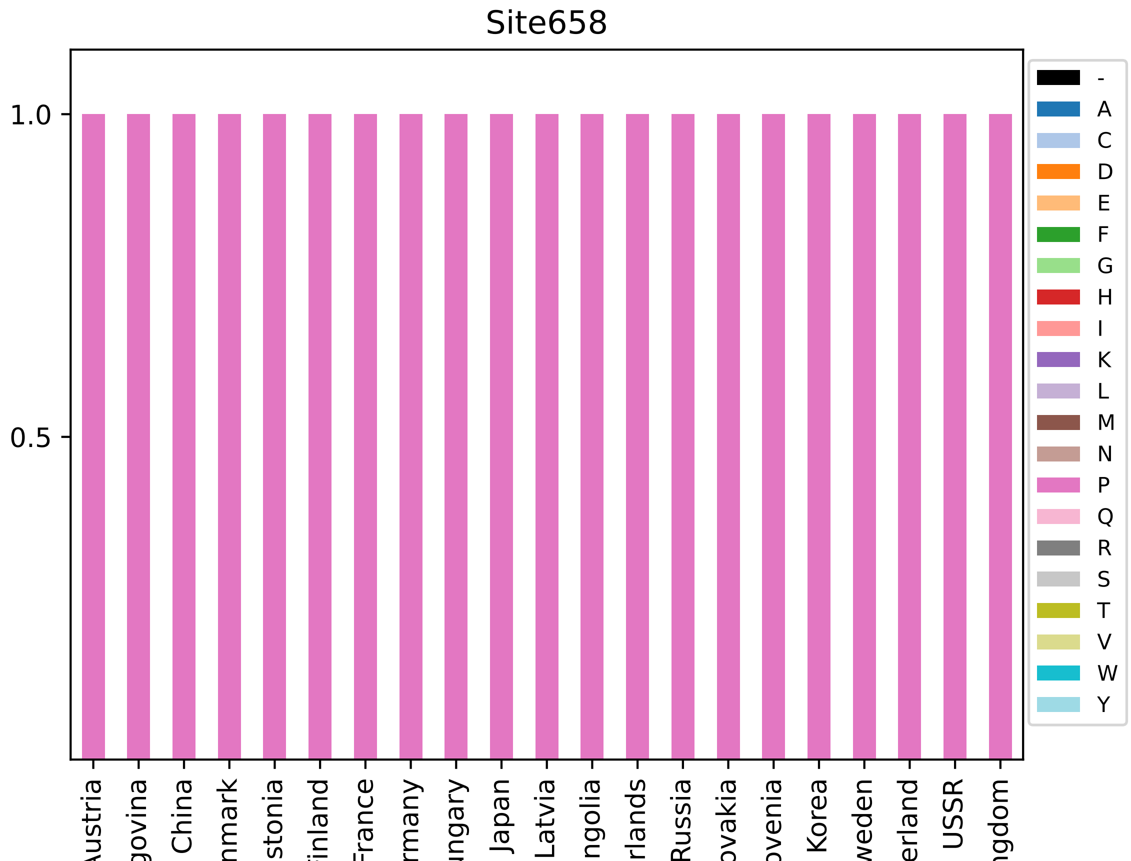
Site658
1.0
0.5
-
A
C
D
E
F
G
H
I
K
L
M
N
P
Q
R
S
T
V
W
Y
Austria
govina
China
nmark
stonia
inland
France
rmany
ungary
Japan
Latvia
ngolia
rlands
Russia
ovakia
ovenia
Korea
weden
erland
USSR
ngdom

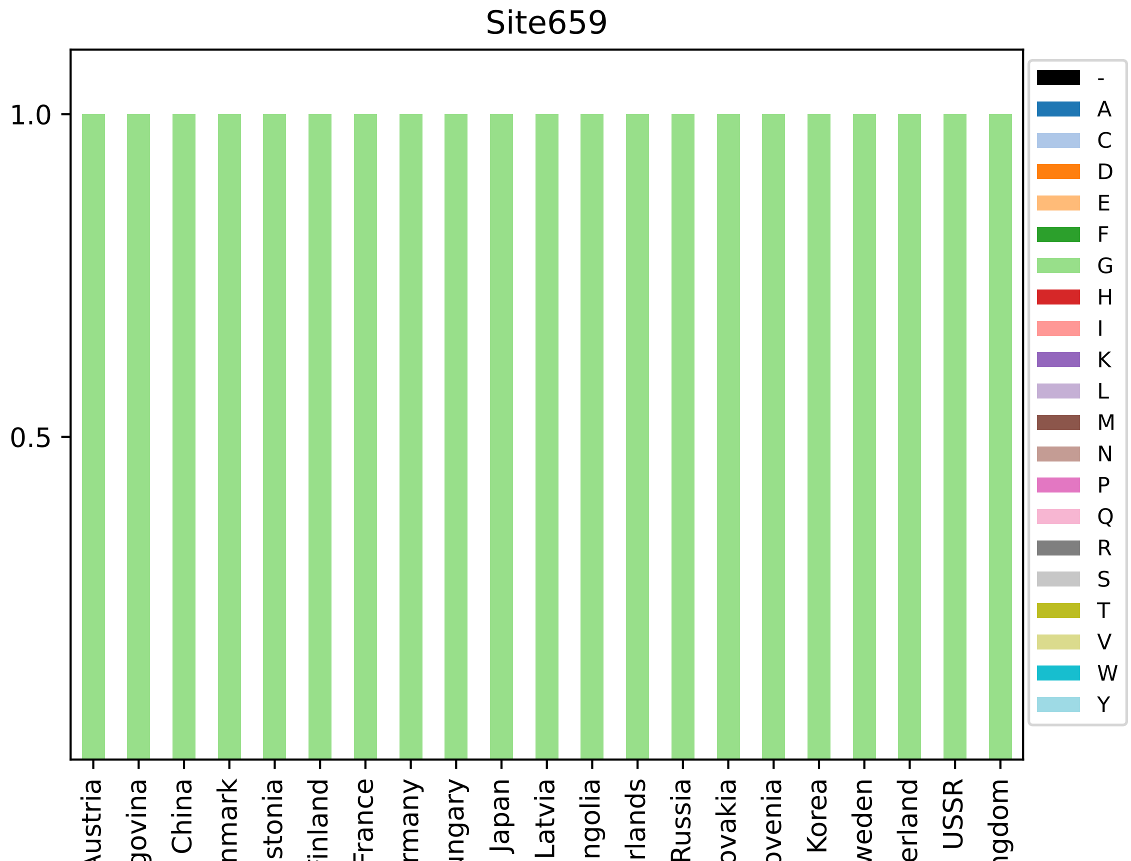

Site659
1.0
0.5
-
A
C
D
E
F
G
H
I
K
L
M
N
P
Q
R
S
T
V
W
Y
Austria
China
Japan
Latvia
Russia
Korea
USSR

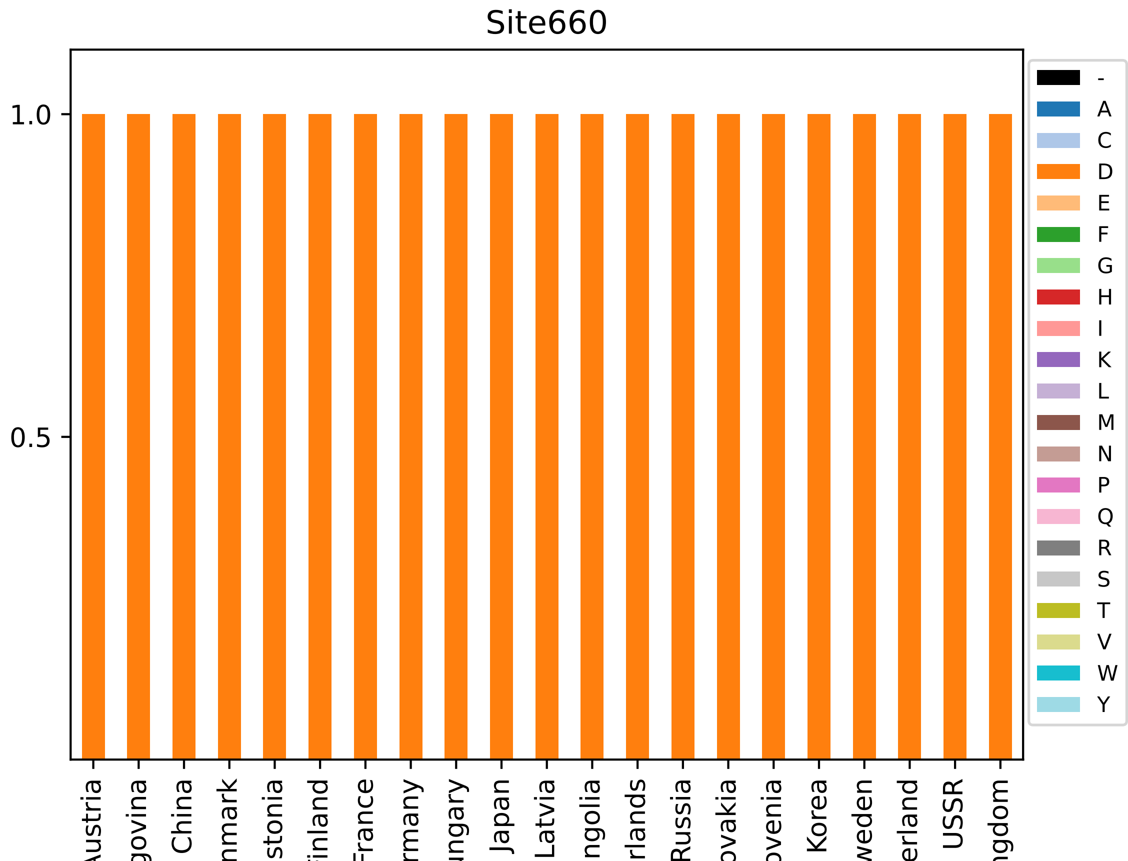
Site660
1.0
0.5
-
A
C
D
E
F
G
H
I
K
L
M
N
P
Q
R
S
T
V
W
Y
China
Japan
Latvia
Russia
Korea
USSR

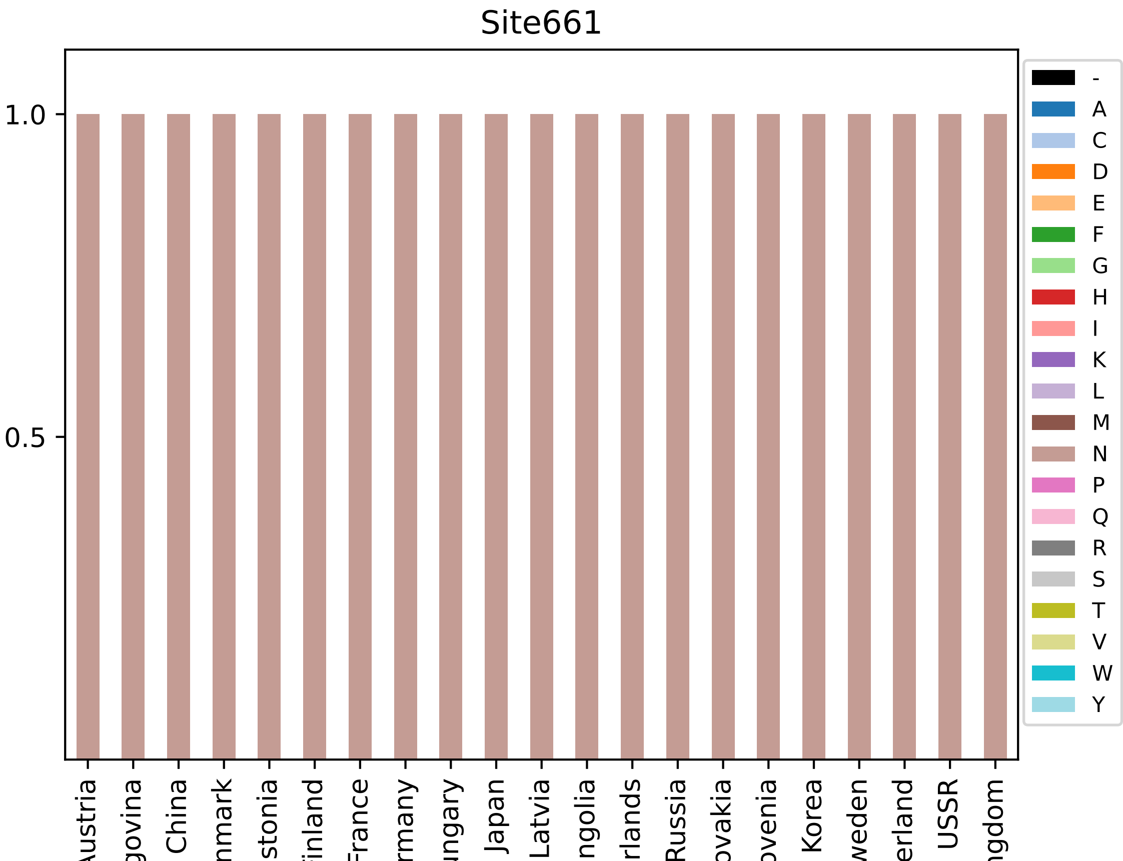
Site661
1.0
0.5
-
A
C
D
E
F
G
H
I
K
L
M
N
P
Q
R
S
T
V
W
Y
Austria
govina
China
nmark
stonia
inland
France
rmany
ungary
Japan
Latvia
ngolia
rlands
Russia
ovakia
ovenia
Korea
weden
erland
USSR
ngdom

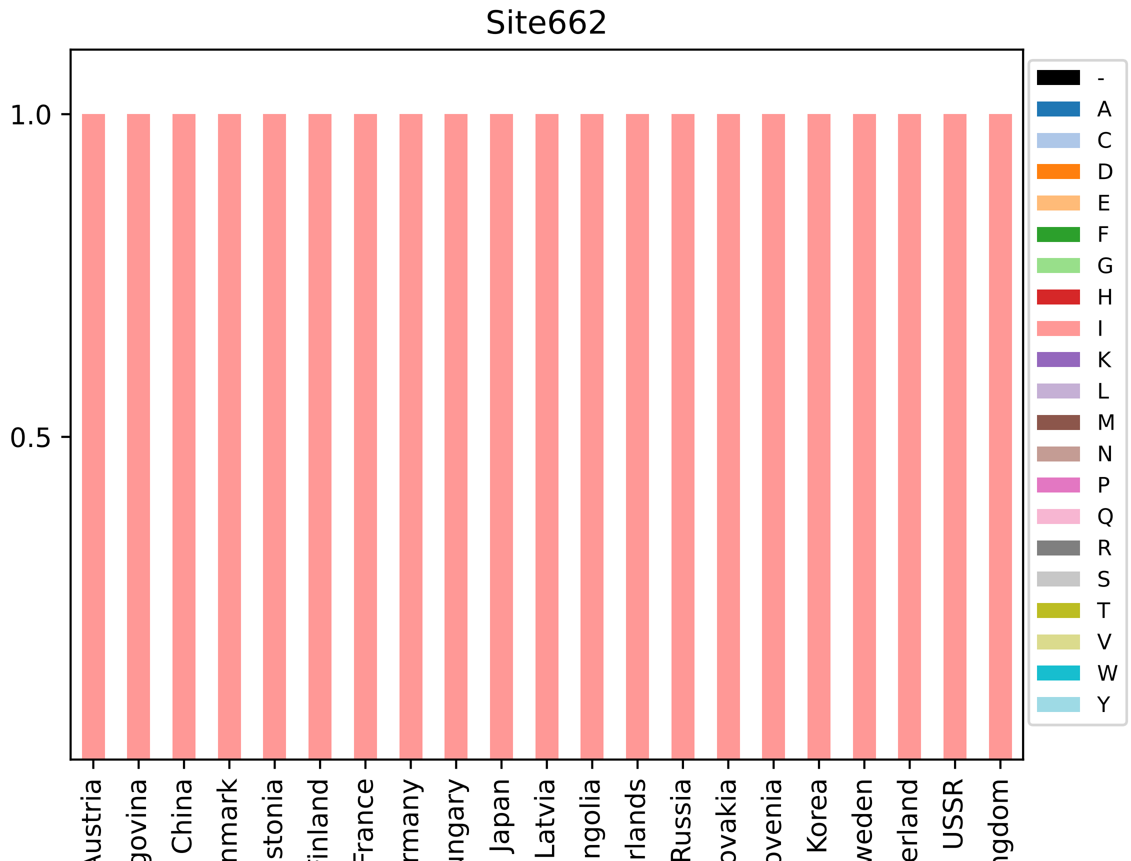
Site662
1.0
0.5
-
A
C
D
E
F
G
H
I
K
L
M
N
P
Q
R
S
T
V
W
Y
China
Japan
Latvia
Russia
Korea
USSR

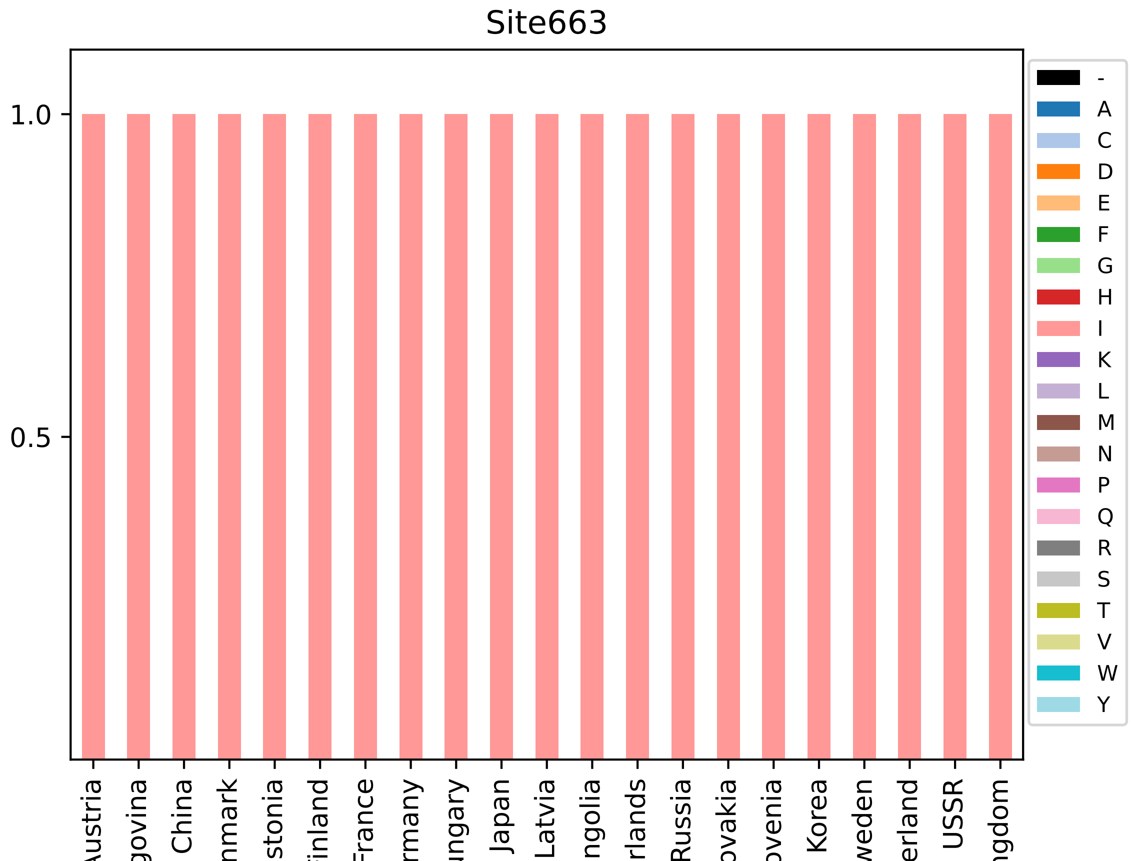

Site663
1.0
0.5
-
A
C
D
E
F
G
H
I
K
L
M
N
P
Q
R
S
T
V
W
Y
China
Japan
Latvia
Russia
Korea
USSR

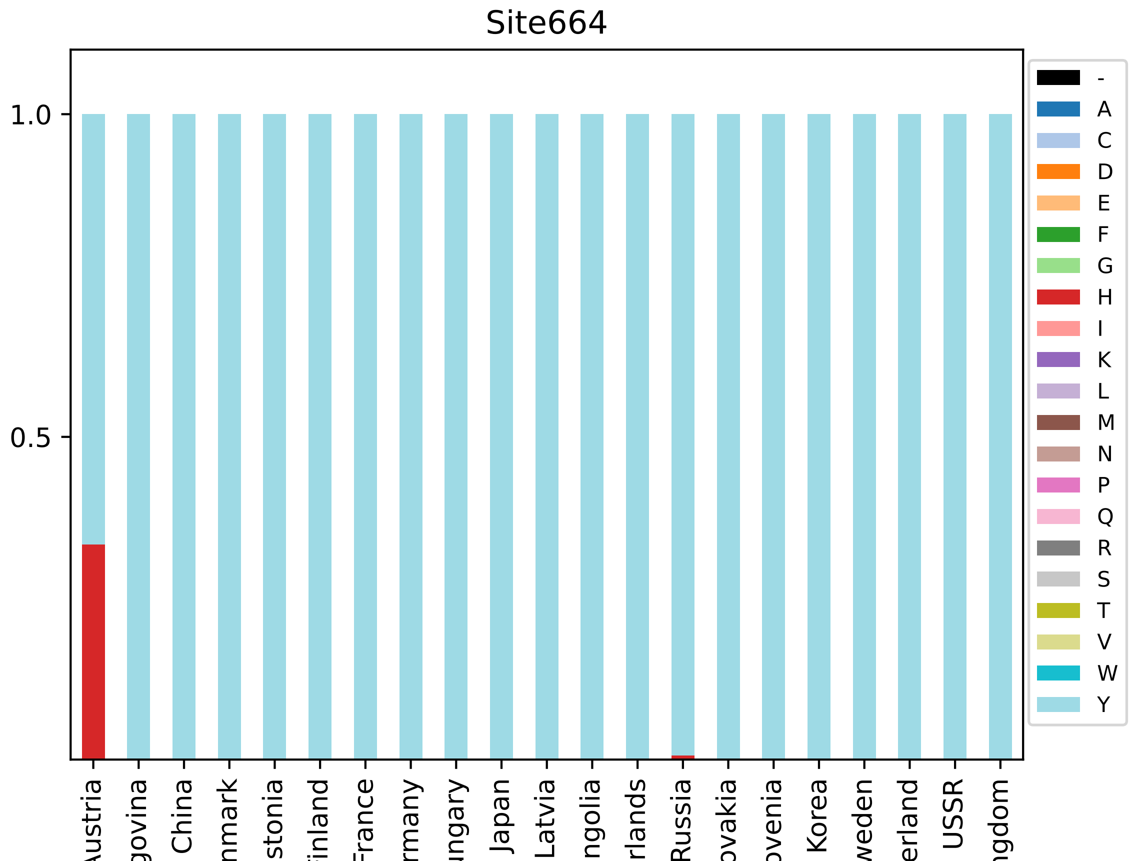

Site664
1.0
0.5
Austria
govina
China
nmark
stonia
inland
France
rmany
ungary
Japan
Latvia
ngolia
rlands
Russia
ovakia
ovenia
Korea
weden
erland
USSR
ngdom
-
A
C
D
E
F
G
H
I
K
L
M
N
P
Q
R
S
T
V
W
Y

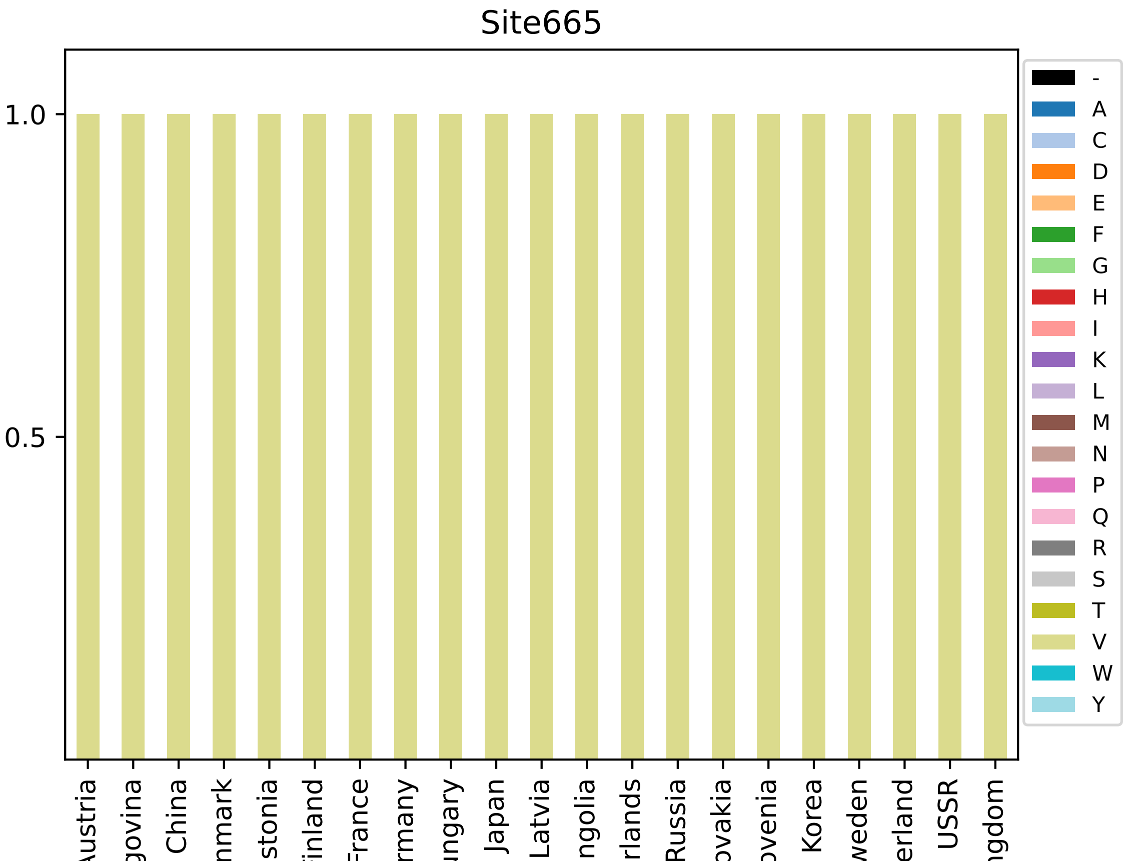
Site665
1.0
0.5
-
A
C
D
E
F
G
H
I
K
L
M
N
P
Q
R
S
T
V
W
Y
Austria
China
Japan
Latvia
Russia
Korea
USSR

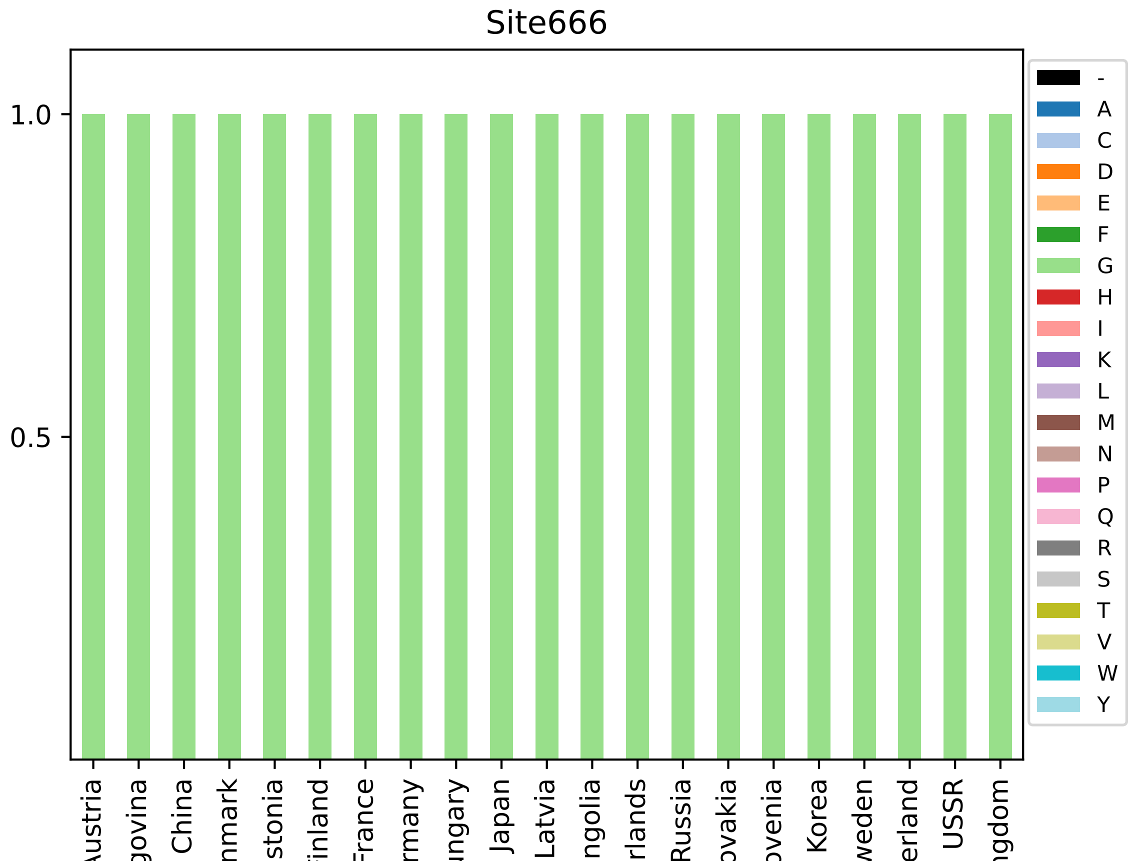
Site666
1.0
0.5
-
A
C
D
E
F
G
H
I
K
L
M
N
P
Q
R
S
T
V
W
Y
China
Japan
Latvia
Russia
Korea
USSR

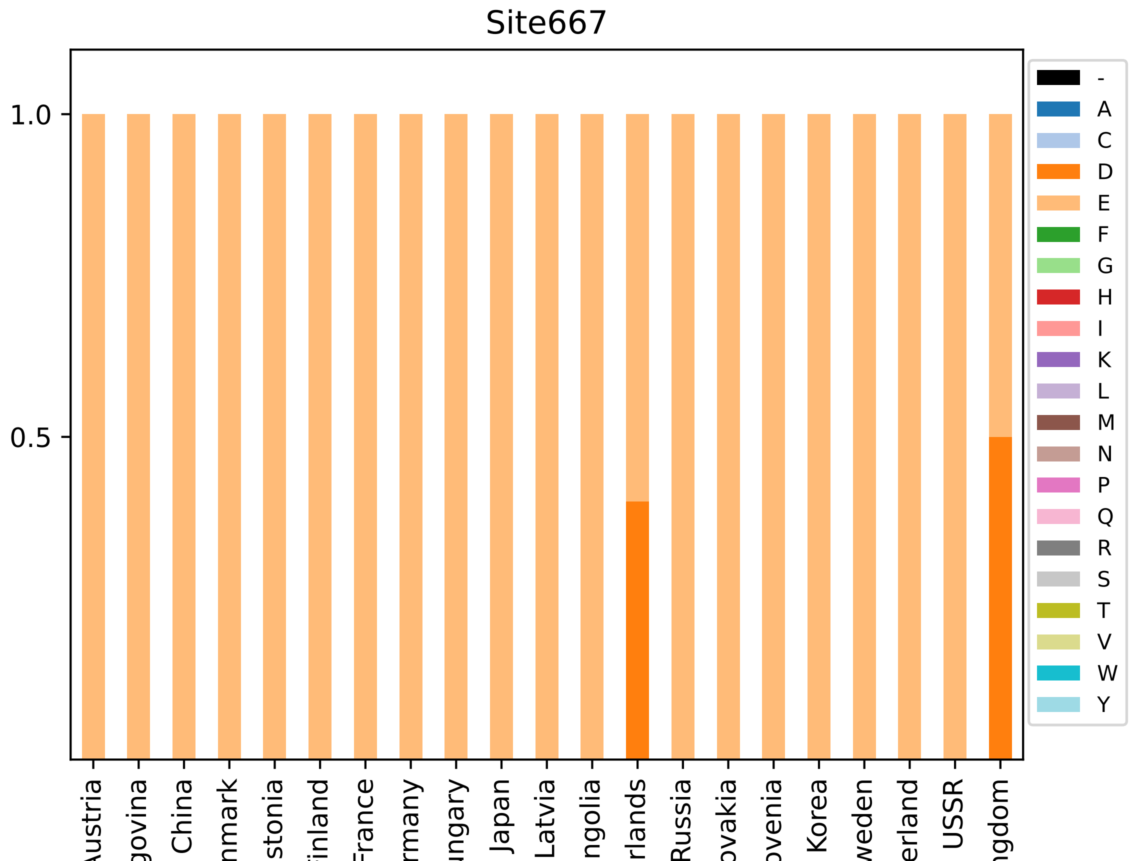

Site667
1.0
0.5
Austria
govina
China
nmark
stonia
Finland
France
rmany
ungary
Japan
Latvia
ngolia
rlands
Russia
ovakia
ovenia
Korea
weden
erland
USSR
ngdom
-
A
C
D
E
F
G
H
I
K
L
M
N
P
Q
R
S
T
V
W
Y

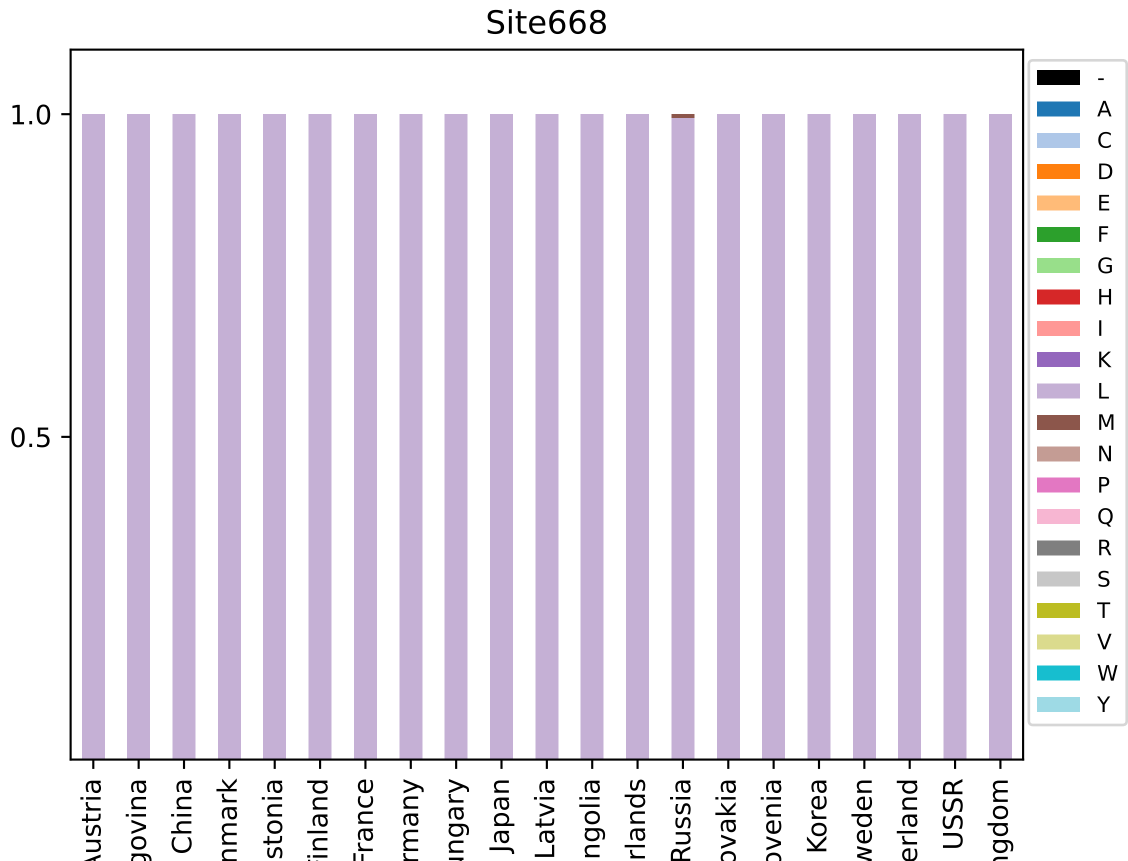

Site668
1.0
0.5
-
A
C
D
E
F
G
H
I
K
L
M
N
P
Q
R
S
T
V
W
Y
Austria
China
Japan
Latvia
Russia
Korea
USSR

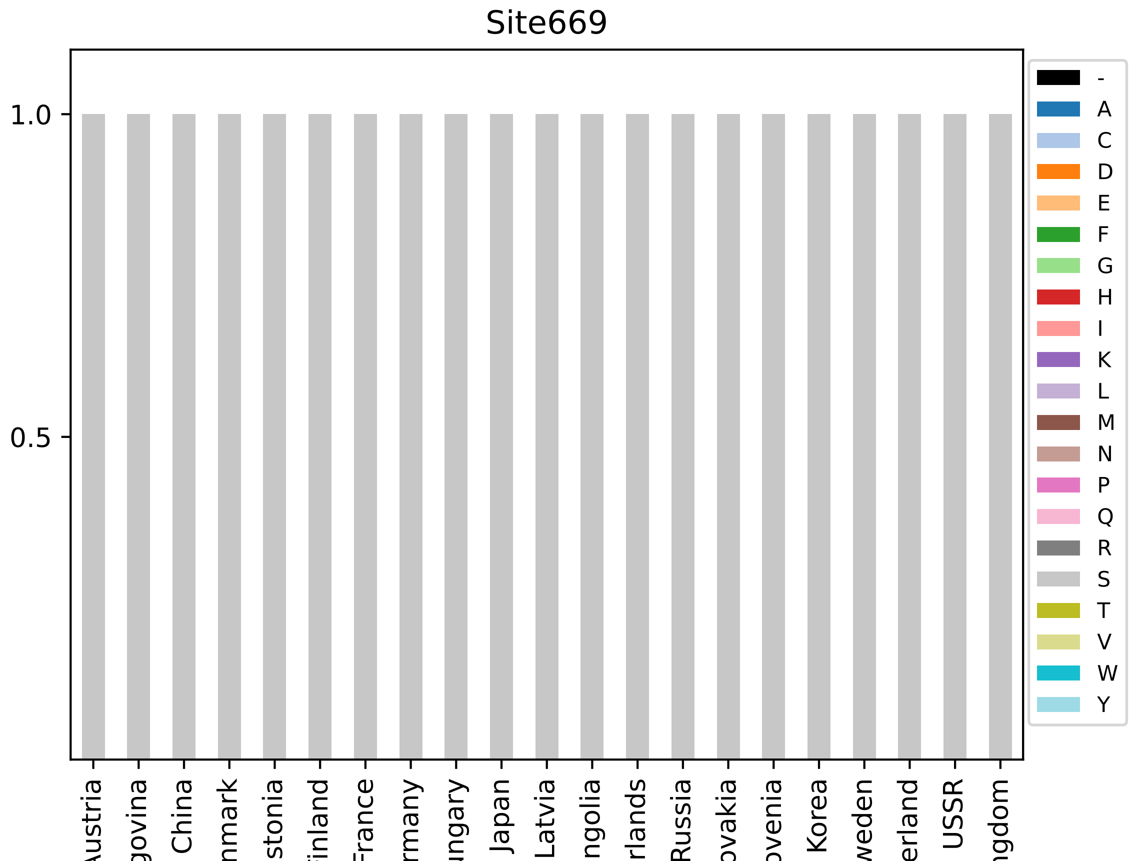
Site669
1.0
0.5
-
A
C
D
E
F
G
H
I
K
L
M
N
P
Q
R
S
T
V
W
Y
Austria
govina
China
nmark
stonia
Finland
France
rmany
ungary
Japan
Latvia
ngolia
rlands
Russia
ovakia
ovenia
Korea
weden
erland
USSR
ngdom

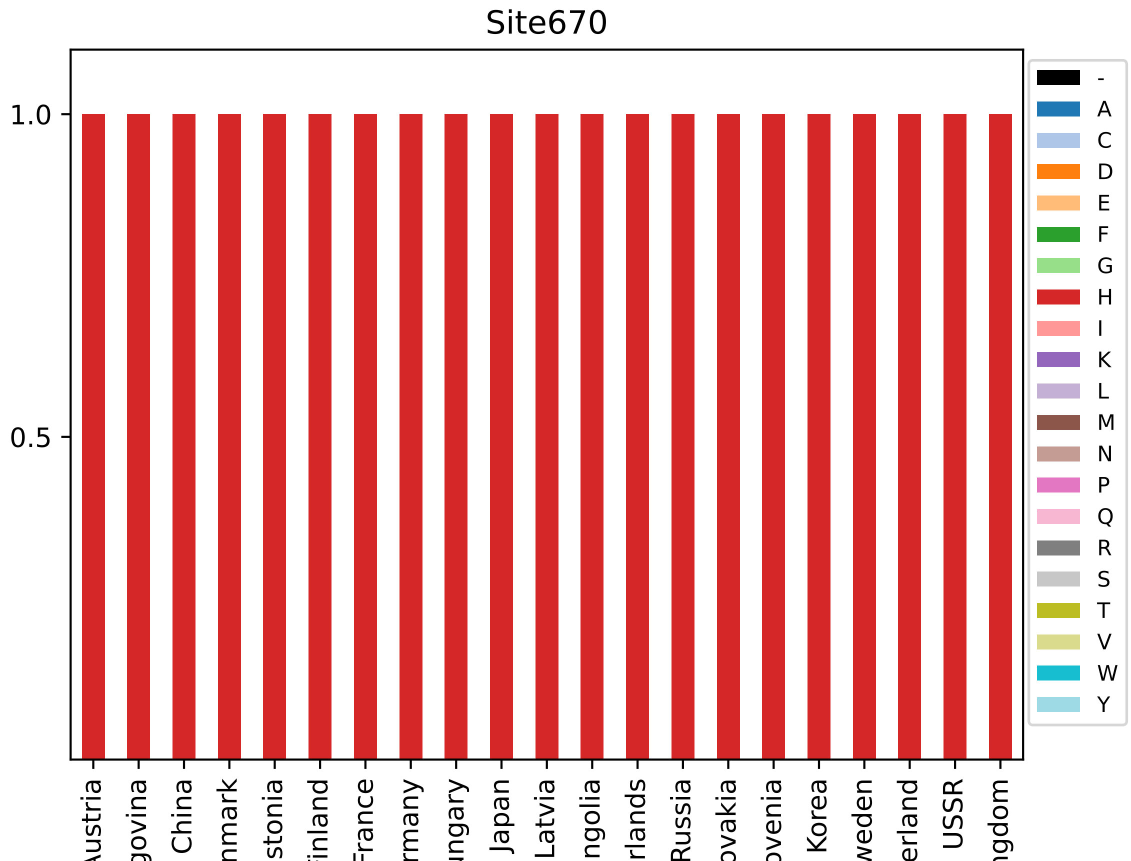

Site670
1.0
0.5
-
A
C
D
E
F
G
H
I
K
L
M
N
P
Q
R
S
T
V
W
Y
China
Japan
Latvia
Russia
Korea
USSR

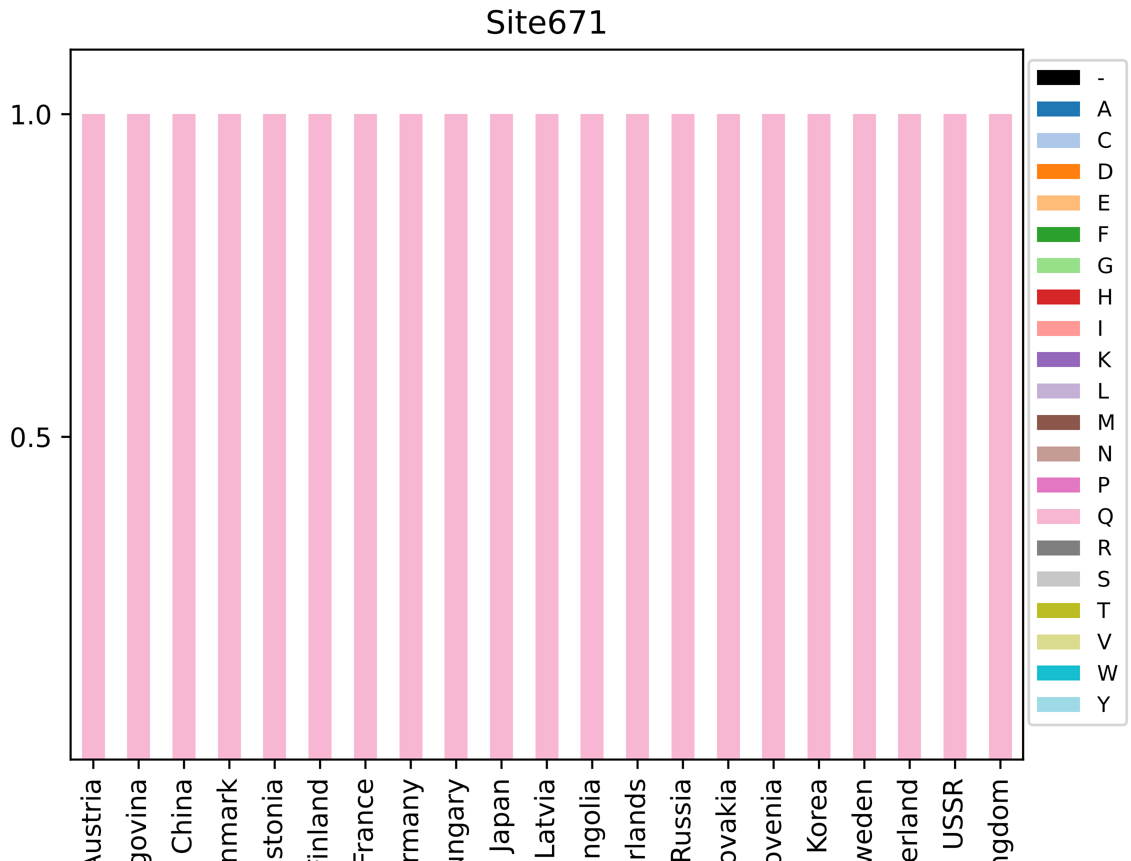
Site671
1.0
0.5
-
A
C
D
E
F
G
H
I
K
L
M
N
P
Q
R
S
T
V
W
Y
China
Japan
Latvia
Russia
Korea
USSR

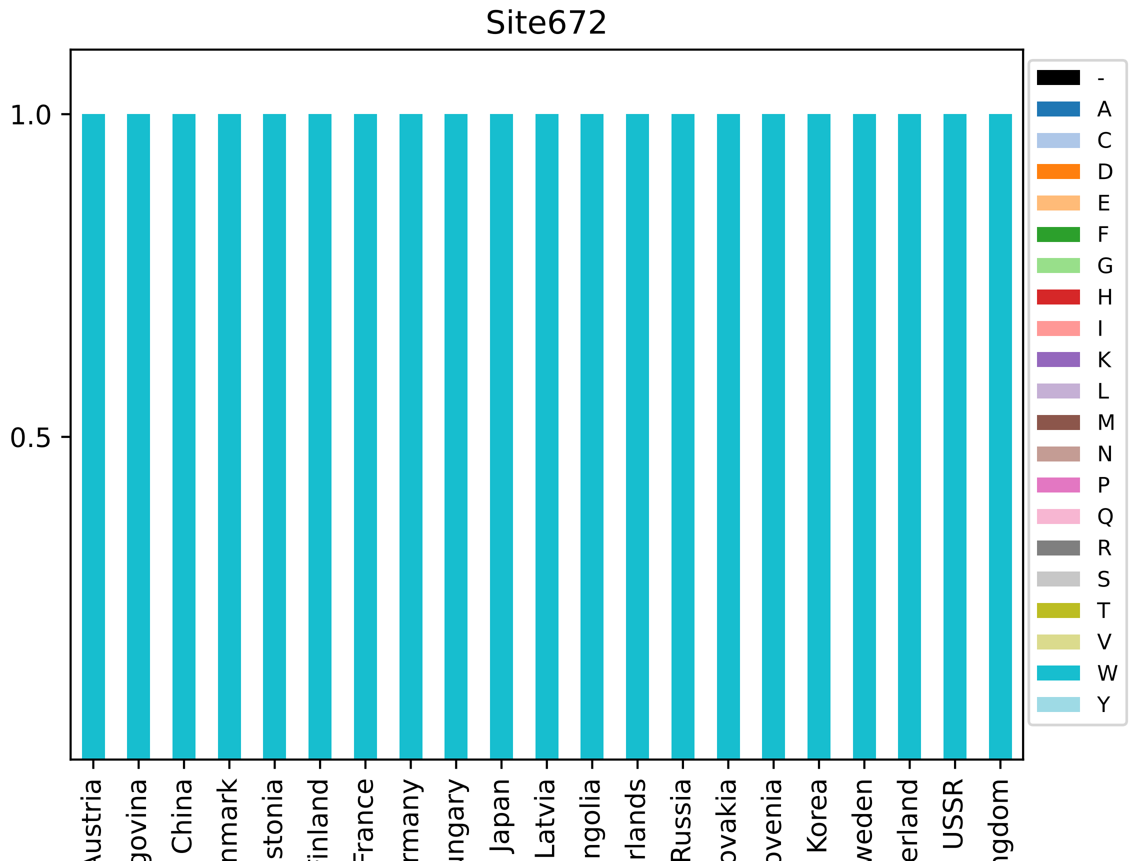

Site672
1.0
0.5
China
Japan
Latvia
Russia
Korea
USSR
-
A
C
D
E
F
G
H
I
K
L
M
N
P
Q
R
S
T
V
W
Y

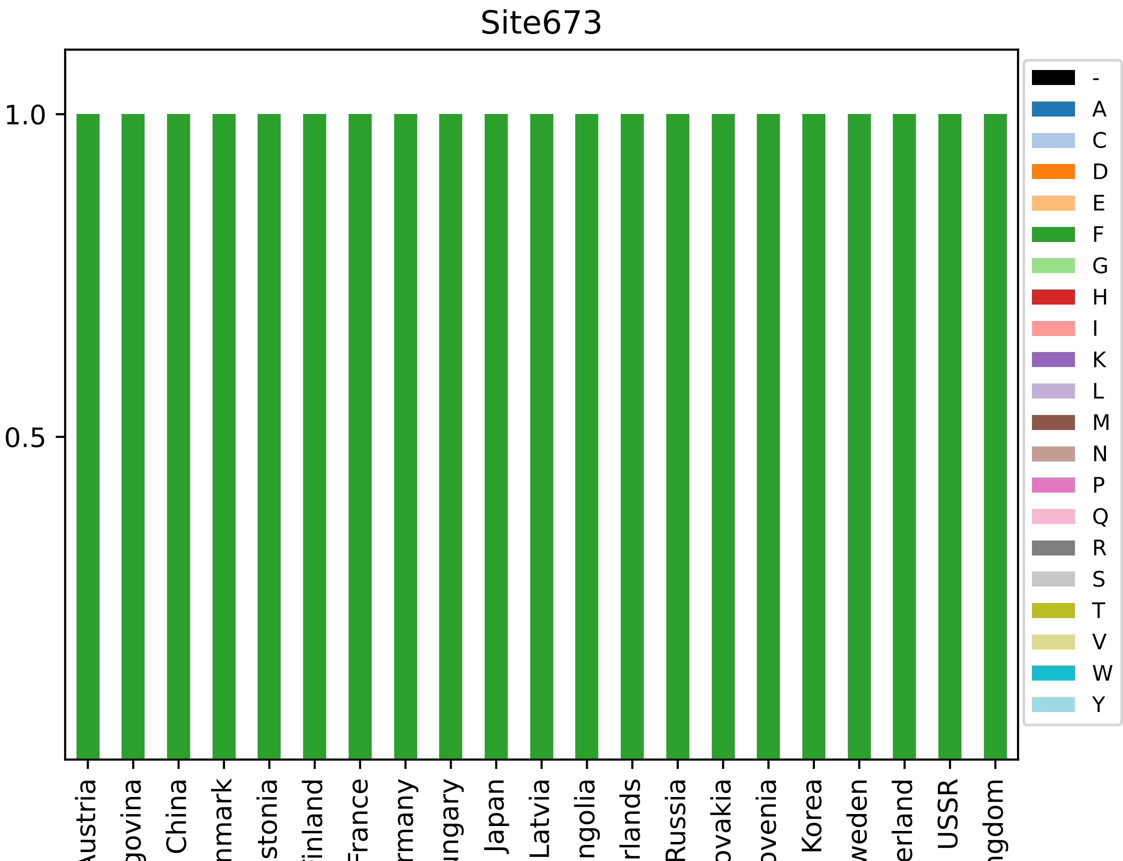
Site673
1.0
0.5
-
A
C
D
E
F
G
H
I
K
L
M
N
P
Q
R
S
T
V
W
Y
Austria
govina
China
nmark
stonia
inland
France
rmany
ungary
Japan
Latvia
ngolia
rlands
Russia
ovakia
ovenia
Korea
weden
erland
USSR
ngdom

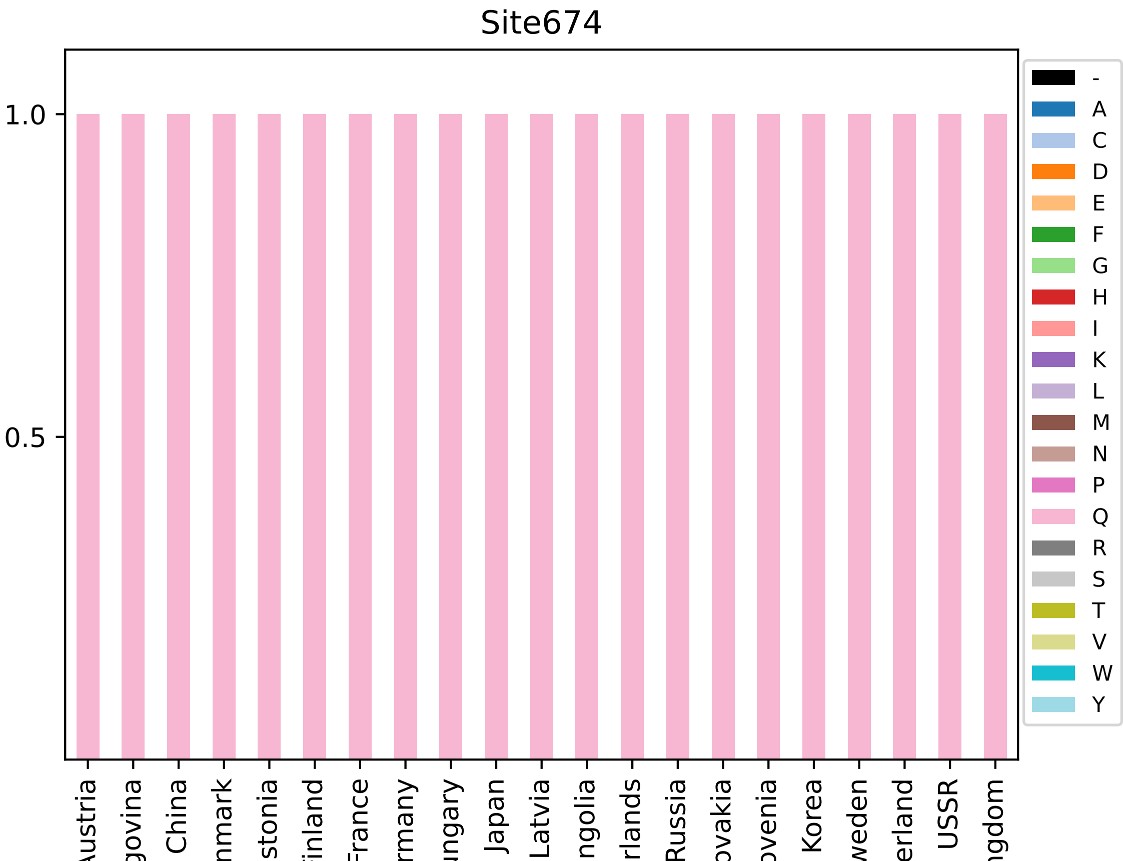

Site674
1.0
0.5
-
A
C
D
E
F
G
H
I
K
L
M
N
P
Q
R
S
T
V
W
Y
Austria
govina
China
nmark
stonia
Finland
France
rmany
ungary
Japan
Latvia
ngolia
rlands
Russia
ovakia
ovenia
Korea
weden
erland
USSR
ngdom

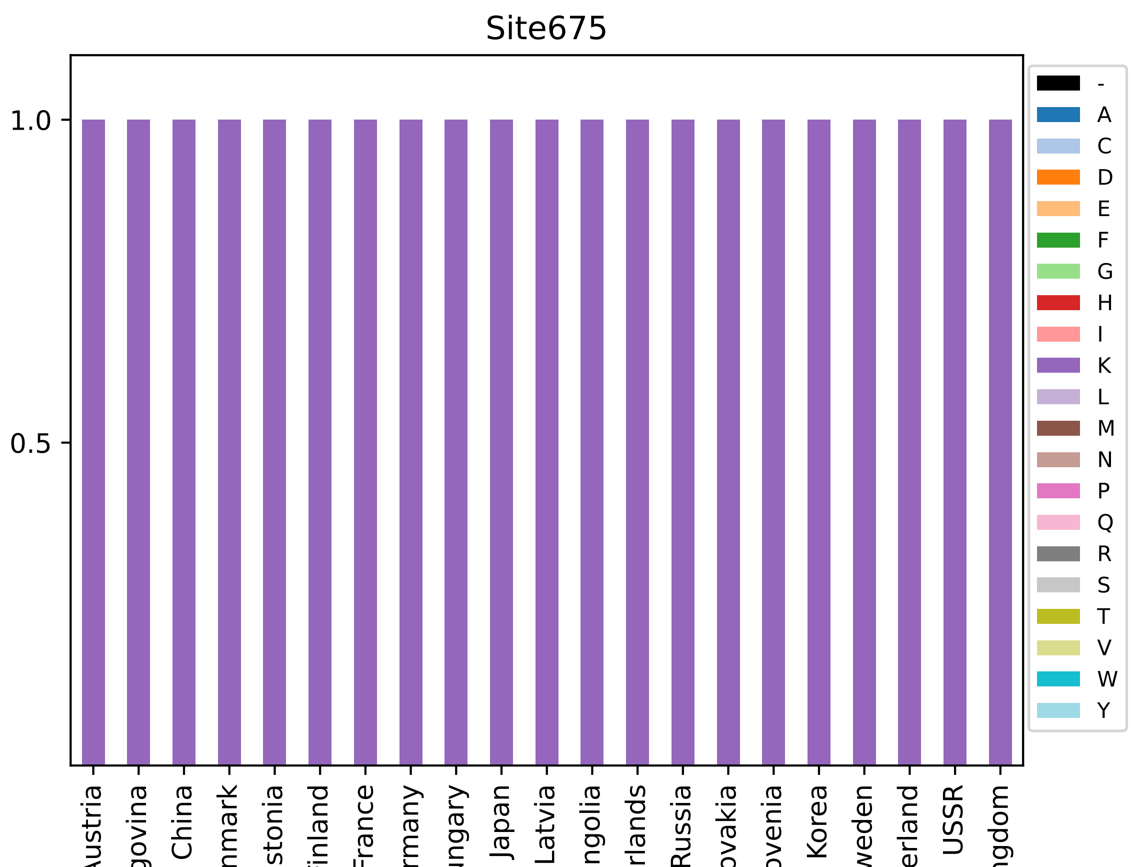

Site675
1.0
0.5
-
A
C
D
E
F
G
H
I
K
L
M
N
P
Q
R
S
T
V
W
Y
China
Japan
Latvia
Russia
Korea
USSR

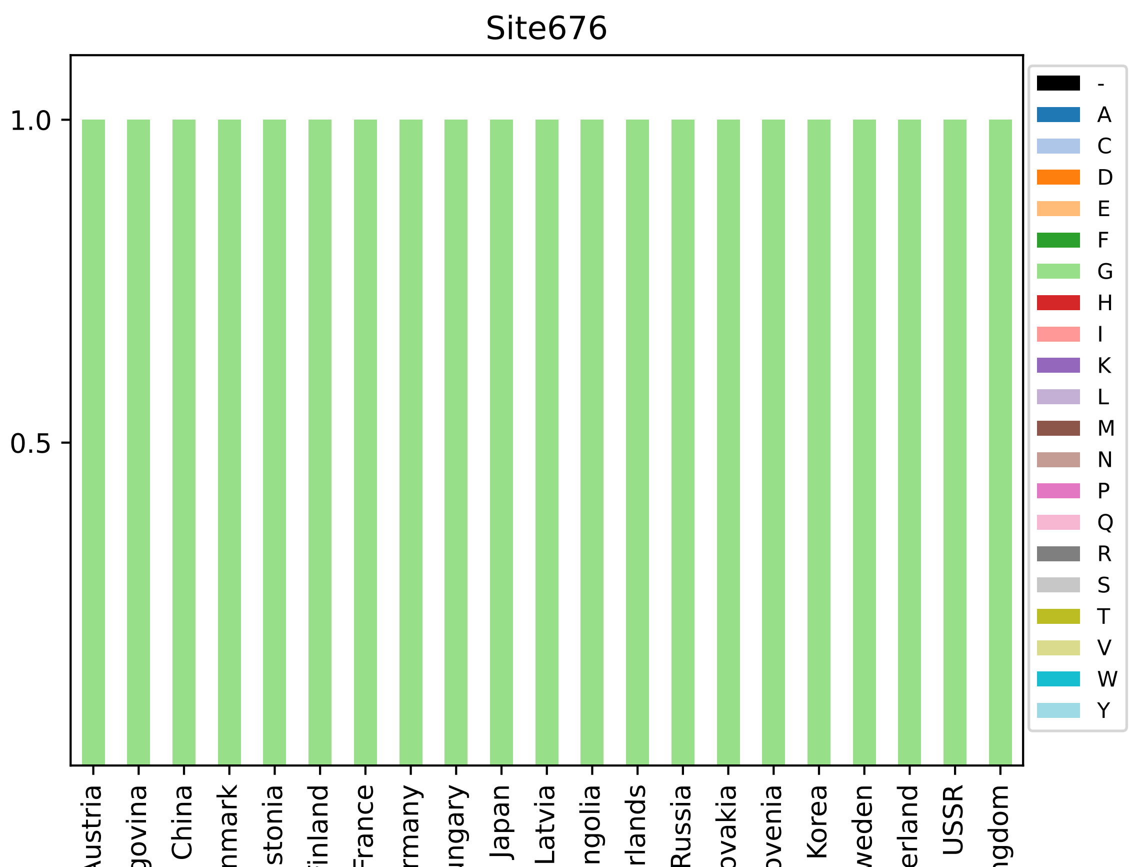
Site676
1.0
0.5
-
A
C
D
E
F
G
H
I
K
L
M
N
P
Q
R
S
T
V
W
Y
China
Japan
Latvia
Russia
Korea
USSR

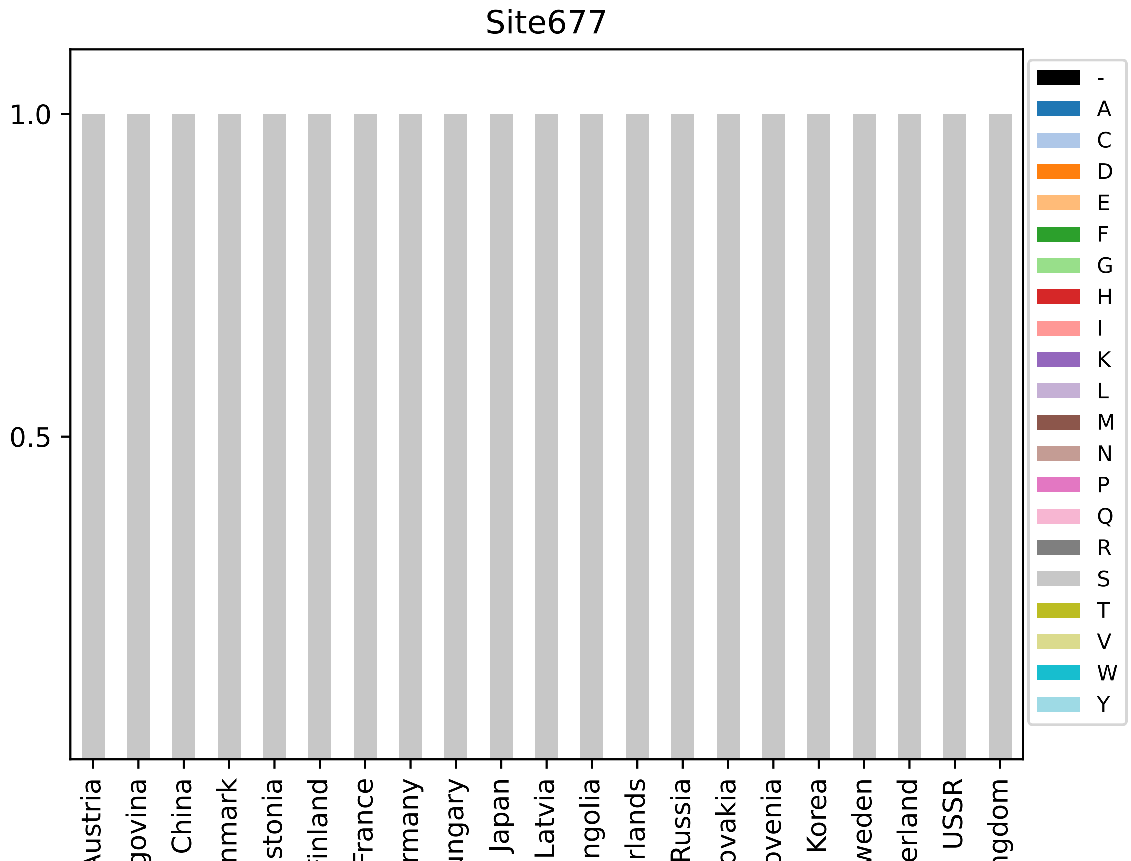

Site677
1.0
0.5
Austria
govina
China
nmark
stonia
inland
France
rmany
ungary
Japan
Latvia
ngolia
rlands
Russia
ovakia
ovenia
Korea
weden
erland
USSR
ngdom
-
A
C
D
E
F
G
H
I
K
L
M
N
P
Q
R
S
T
V
W
Y

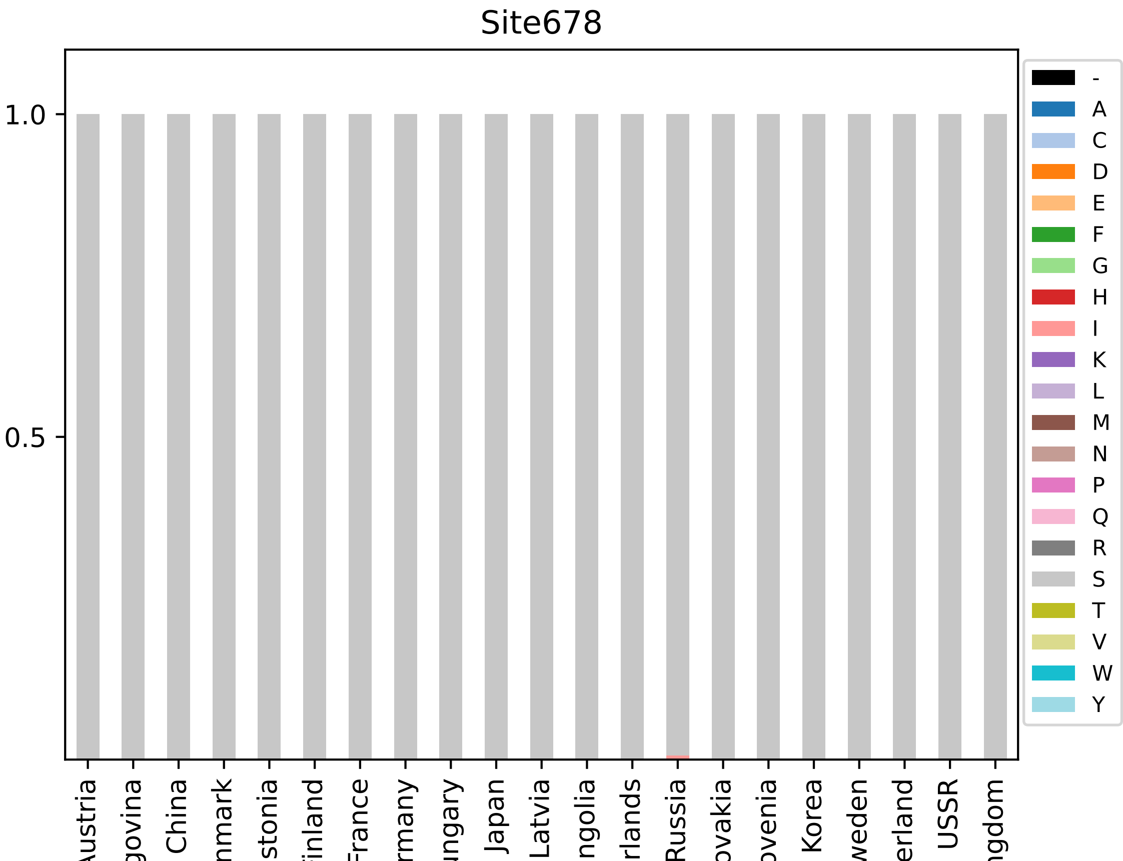
Site678
1.0
0.5
-
A
C
D
E
F
G
H
I
K
L
M
N
P
Q
R
S
T
V
W
Y
Austria
govina
China
nmark
stonia
Finland
France
rmany
ungary
Japan
Latvia
ngolia
rlands
Russia
ovakia
ovenia
Korea
weden
erland
USSR
ngdom

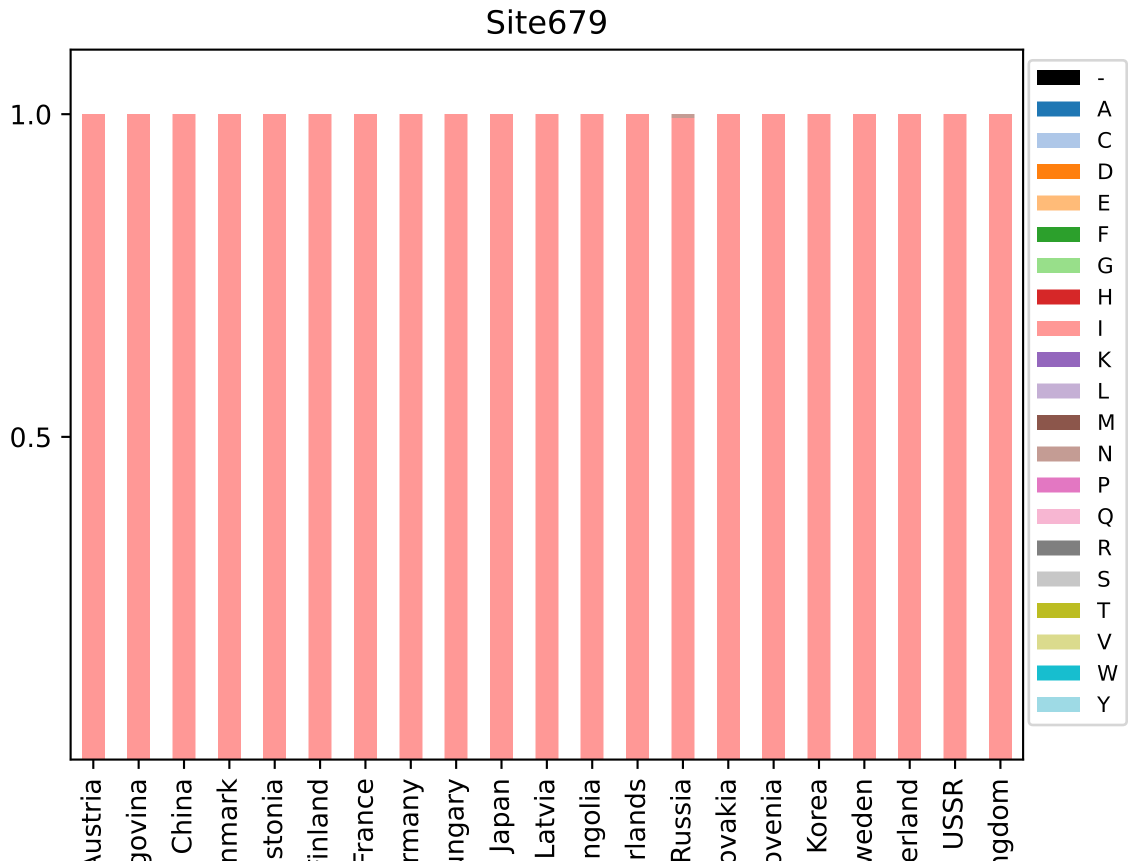

Site679
1.0
0.5
-
A
C
D
E
F
G
H
I
K
L
M
N
P
Q
R
S
T
V
W
Y
China
Japan
Latvia
Russia
Korea
USSR

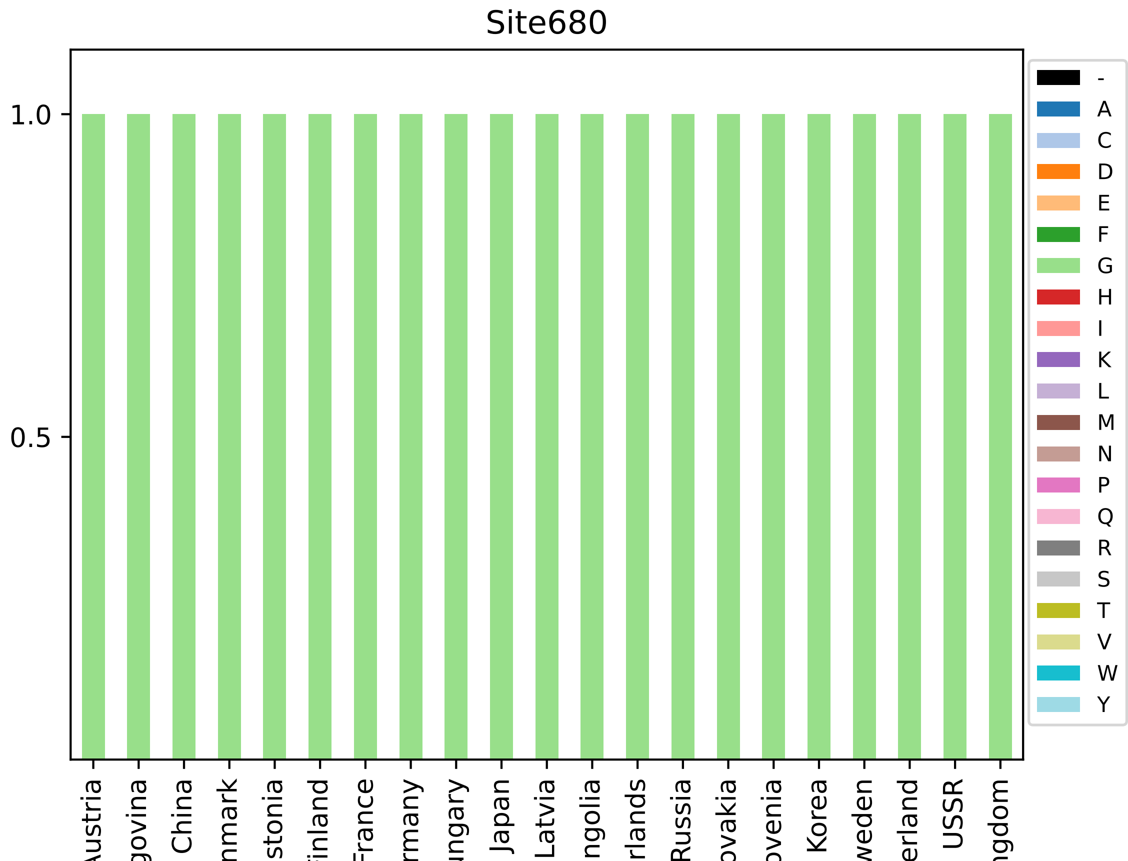

Site680
1.0
0.5
-
A
C
D
E
F
G
H
I
K
L
M
N
P
Q
R
S
T
V
W
Y
China
France
Japan
Latvia
Russia
Korea
USSR

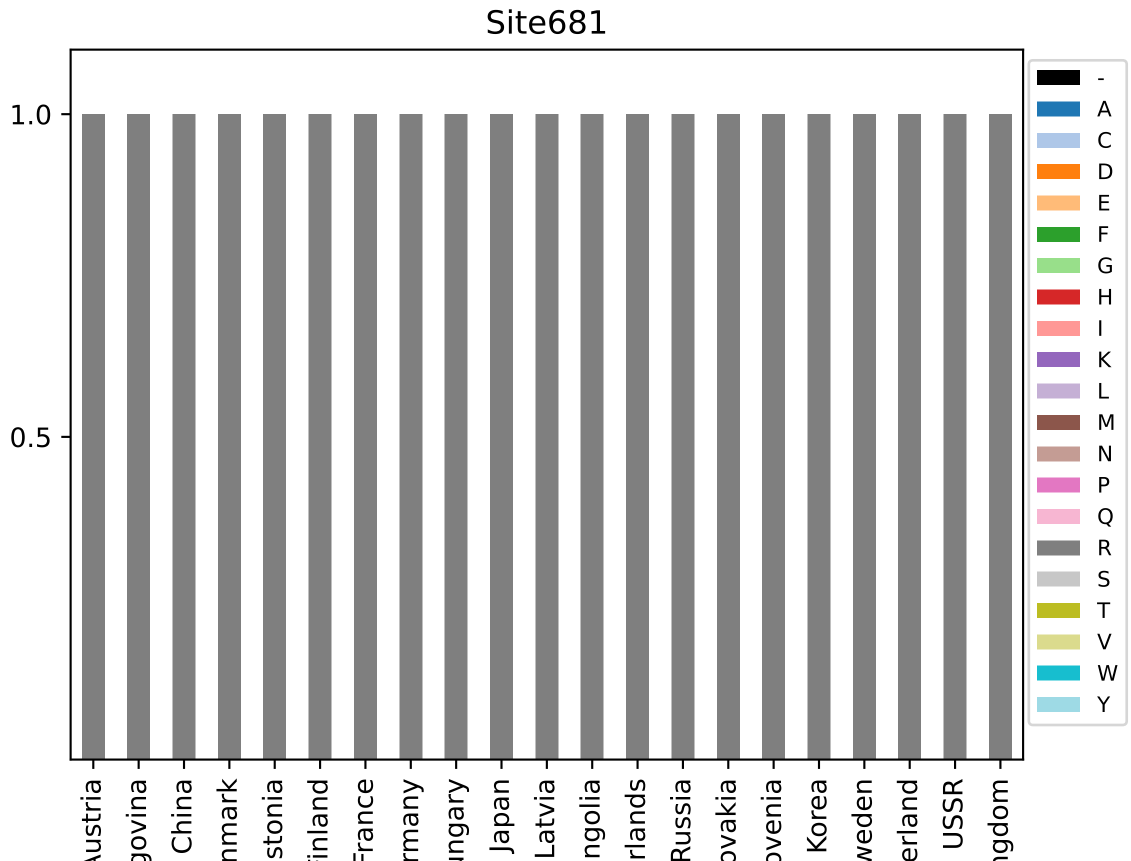
Site681
1.0
0.5
-
A
C
D
E
F
G
H
I
K
L
M
N
P
Q
R
S
T
V
W
Y
China
Japan
Latvia
Russia
Korea
USSR

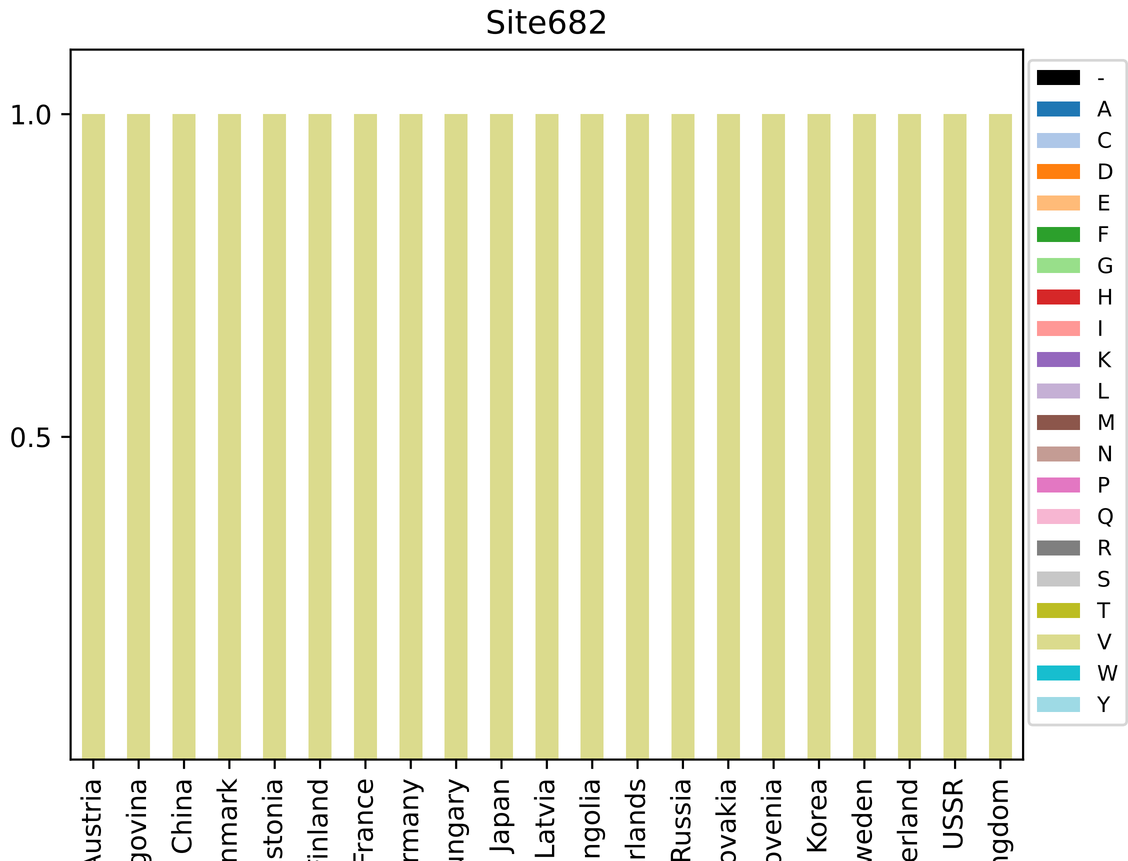
Site682
1.0
0.5
-
A
C
D
E
F
G
H
I
K
L
M
N
P
Q
R
S
T
V
W
Y
Austria
govina
China
nmark
stonia
inland
France
rmany
ungary
Japan
Latvia
ngolia
rlands
Russia
ovakia
ovenia
Korea
weden
erland
USSR
ngdom

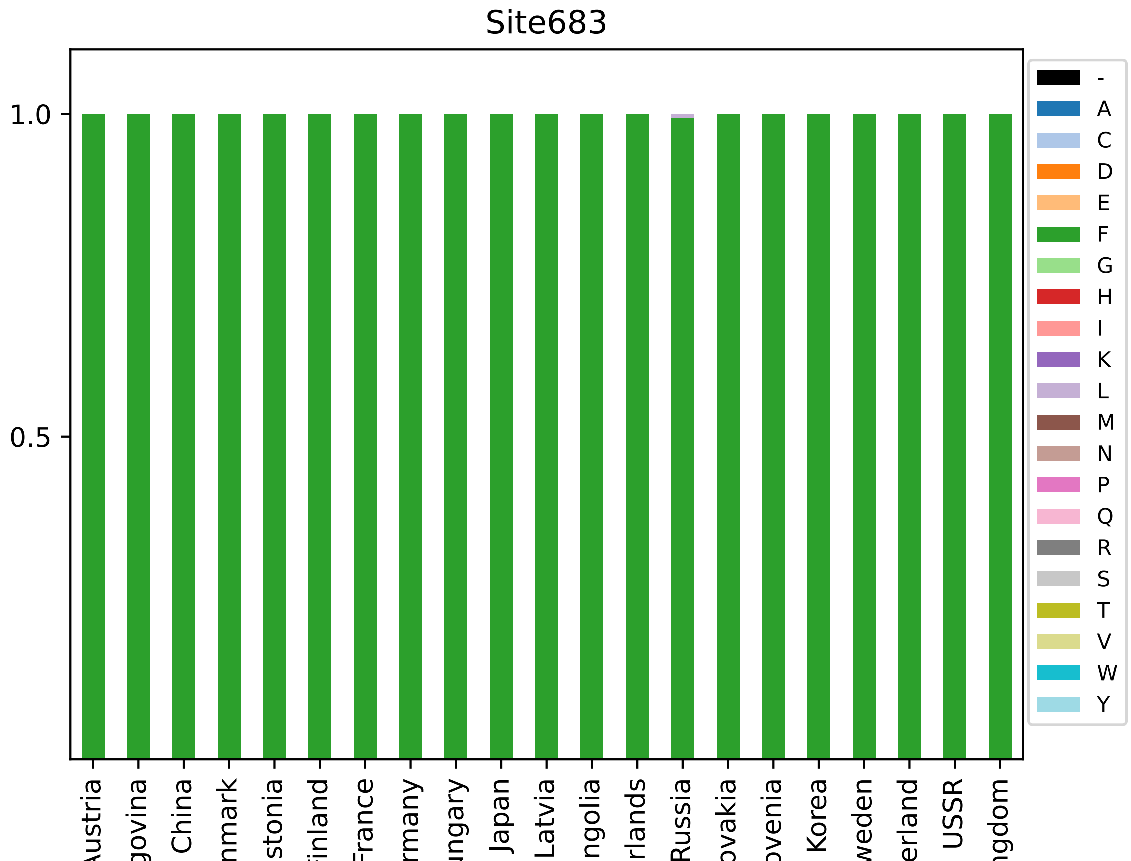

Site683
1.0
0.5
-
A
C
D
E
F
G
H
I
K
L
M
N
P
Q
R
S
T
V
W
Y
China
Japan
Latvia
Russia
Korea
USSR

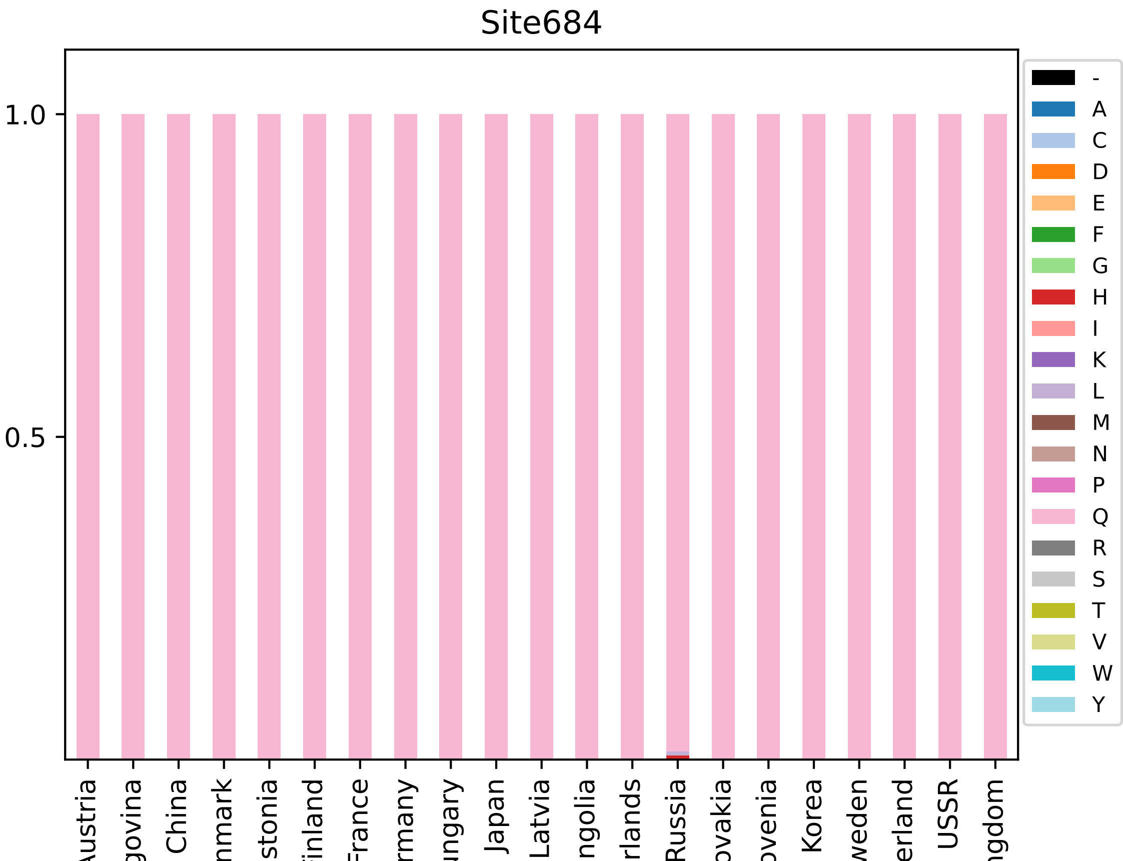

Site684
1.0
0.5
-
A
C
D
E
F
G
H
I
K
L
M
N
P
Q
R
S
T
V
W
Y
China
Japan
Latvia
Russia
Korea
USSR

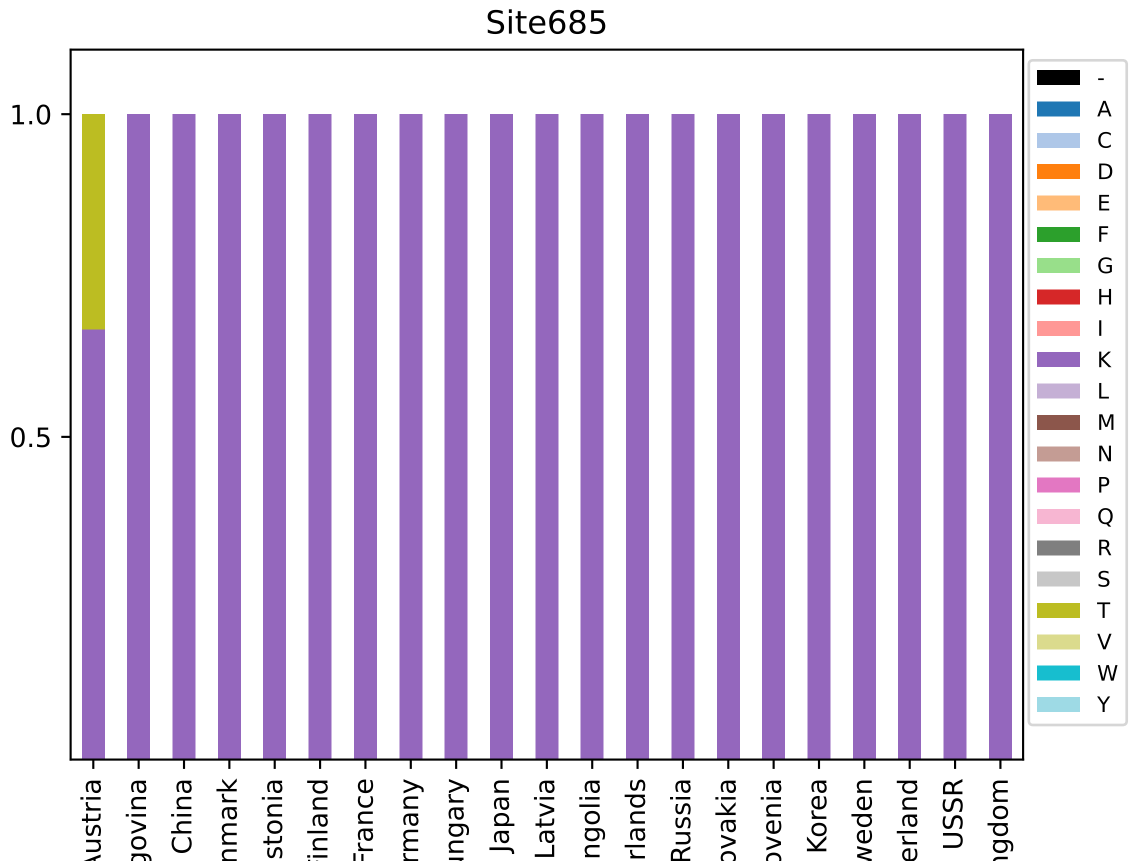

Site685
1.0
0.5
Austria
govina
China
nmark
stonia
Finland
France
rmany
ungary
Japan
Latvia
ngolia
rlands
Russia
ovakia
ovenia
Korea
weden
erland
USSR
ngdom
-
A
C
D
E
F
G
H
I
K
L
M
N
P
Q
R
S
T
V
W
Y

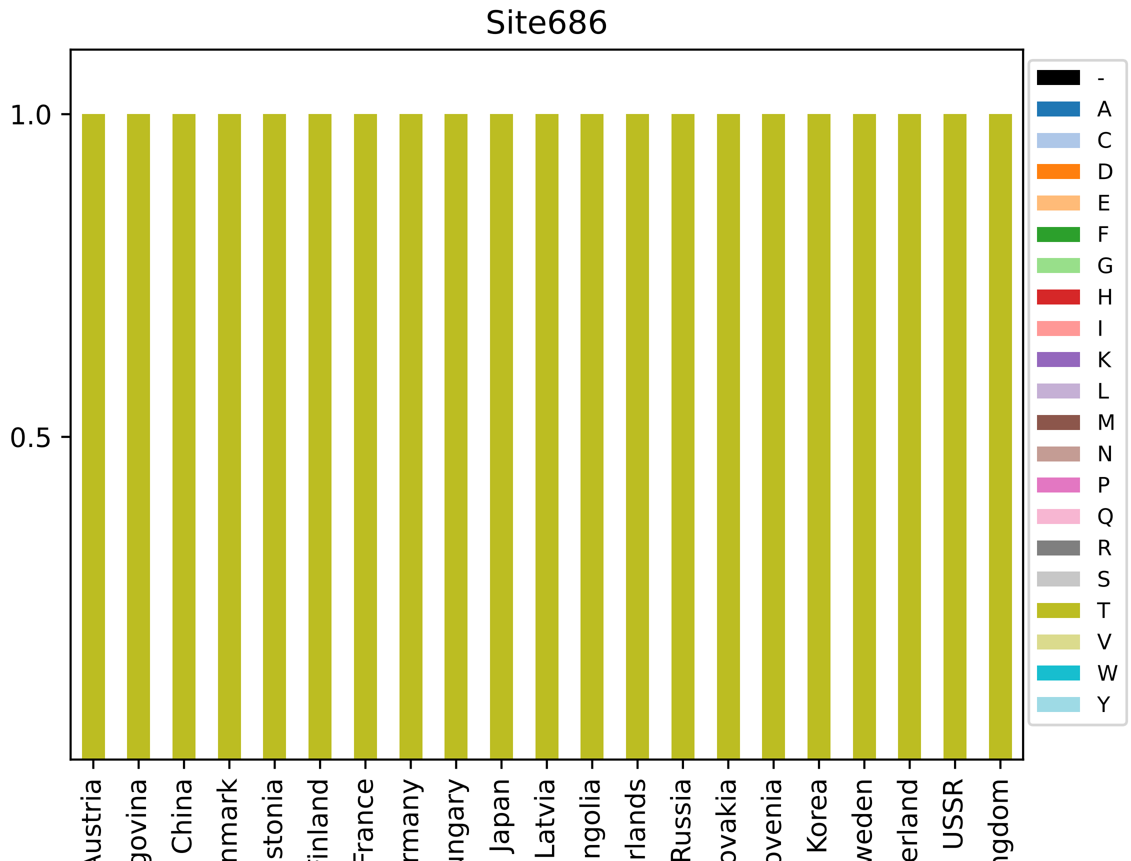
Site686
1.0
0.5
-
A
C
D
E
F
G
H
I
K
L
M
N
P
Q
R
S
T
V
W
Y
Japan
Latvia
Russia
Korea
USSR
China

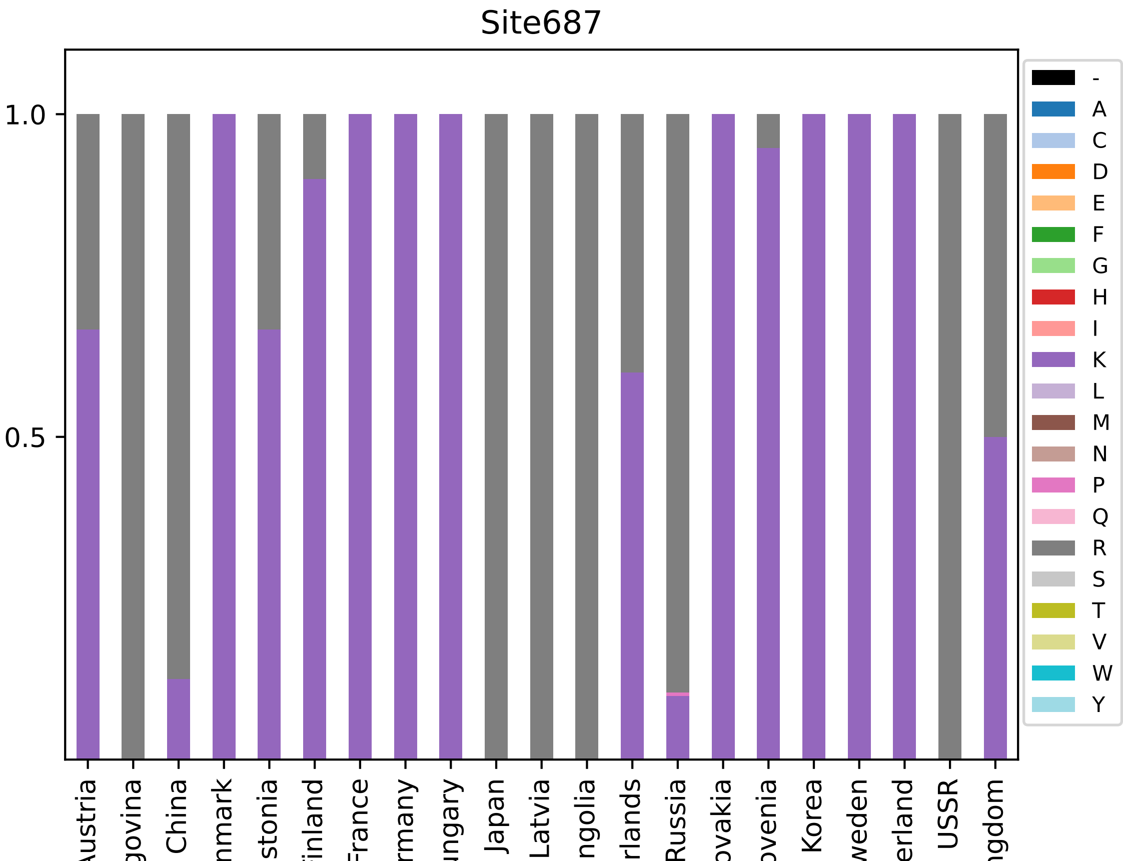
Site687
1.0
0.5
-
A
C
D
E
F
G
H
I
K
L
M
N
P
Q
R
S
T
V
W
Y
Austria
govina
China
nmark
stonia
Finland
France
rmany
ungary
Japan
Latvia
ngolia
rlands
Russia
ovakia
ovenia
Korea
weden
erland
USSR
ngdom

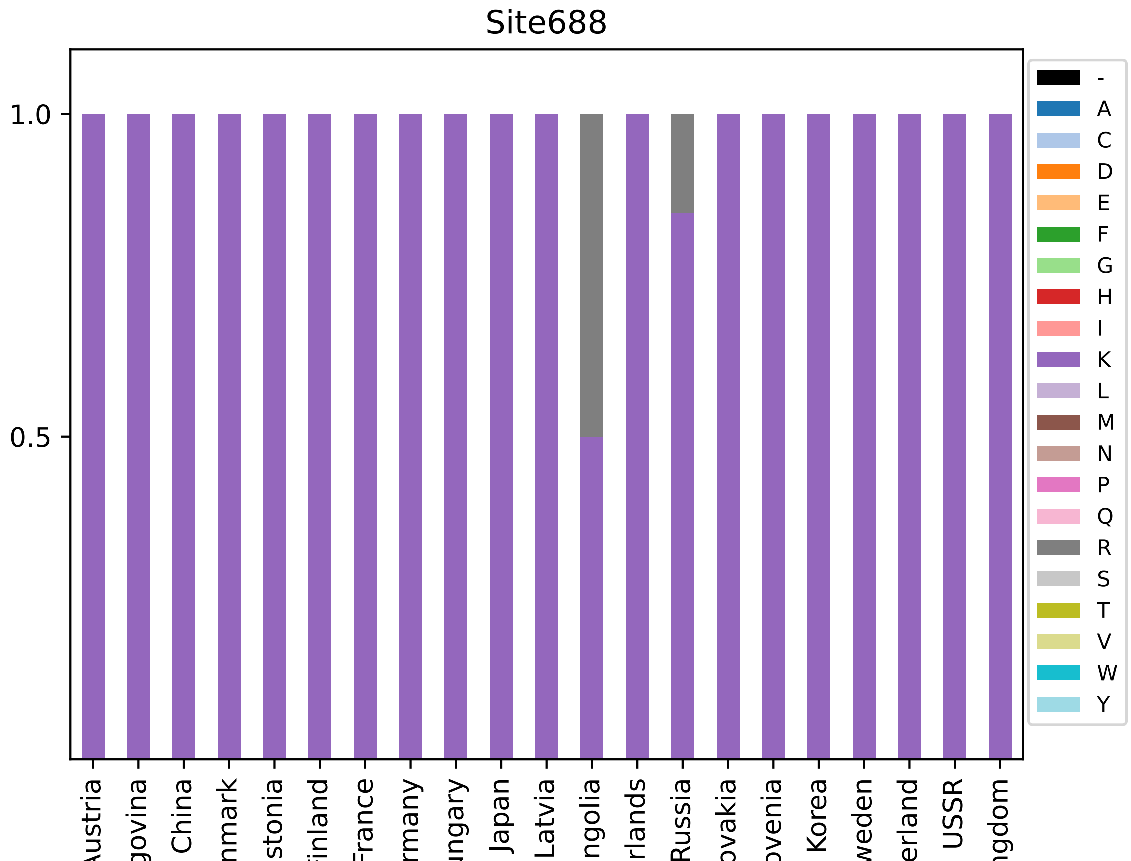

Site688
1.0
0.5
-
A
C
D
E
F
G
H
I
K
L
M
N
P
Q
R
S
T
V
W
Y
China
Japan
Latvia
Russia
Korea
USSR

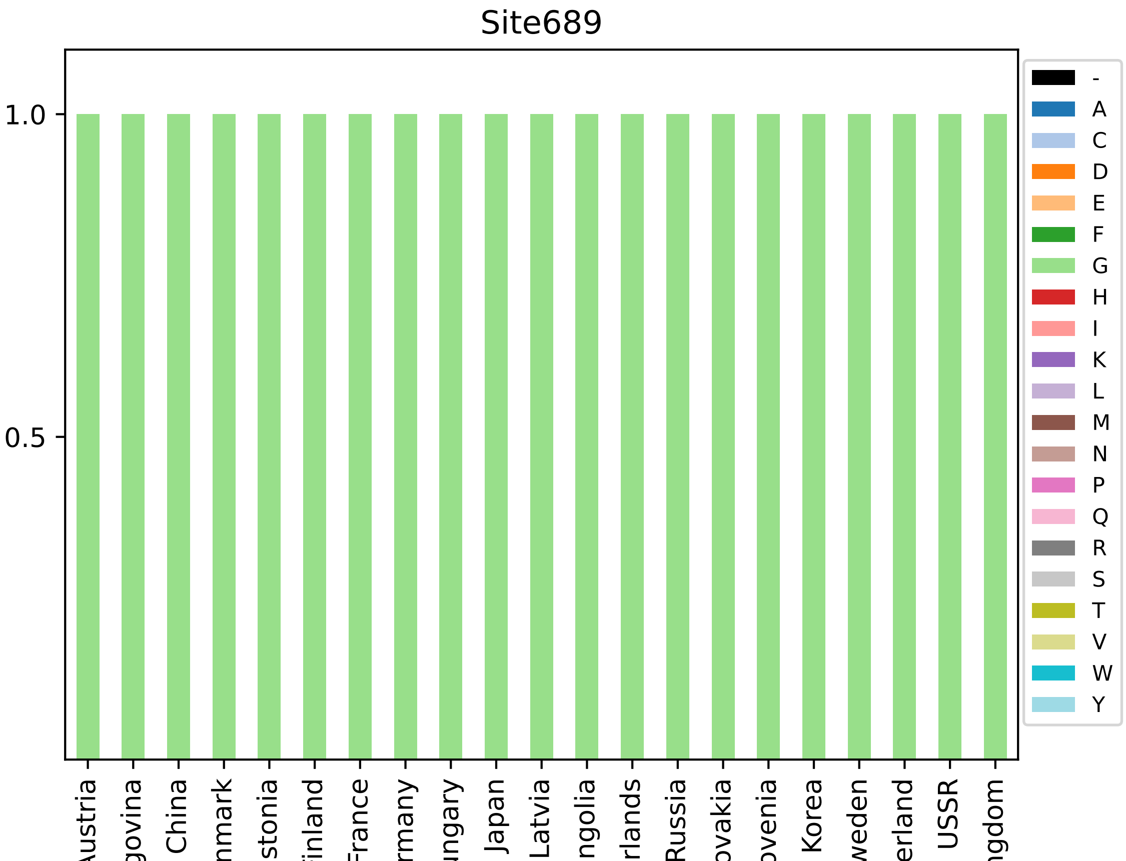

Site689
1.0
0.5
-
A
C
D
E
F
G
H
I
K
L
M
N
P
Q
R
S
T
V
W
Y
China
Japan
Latvia
Russia
Korea
USSR

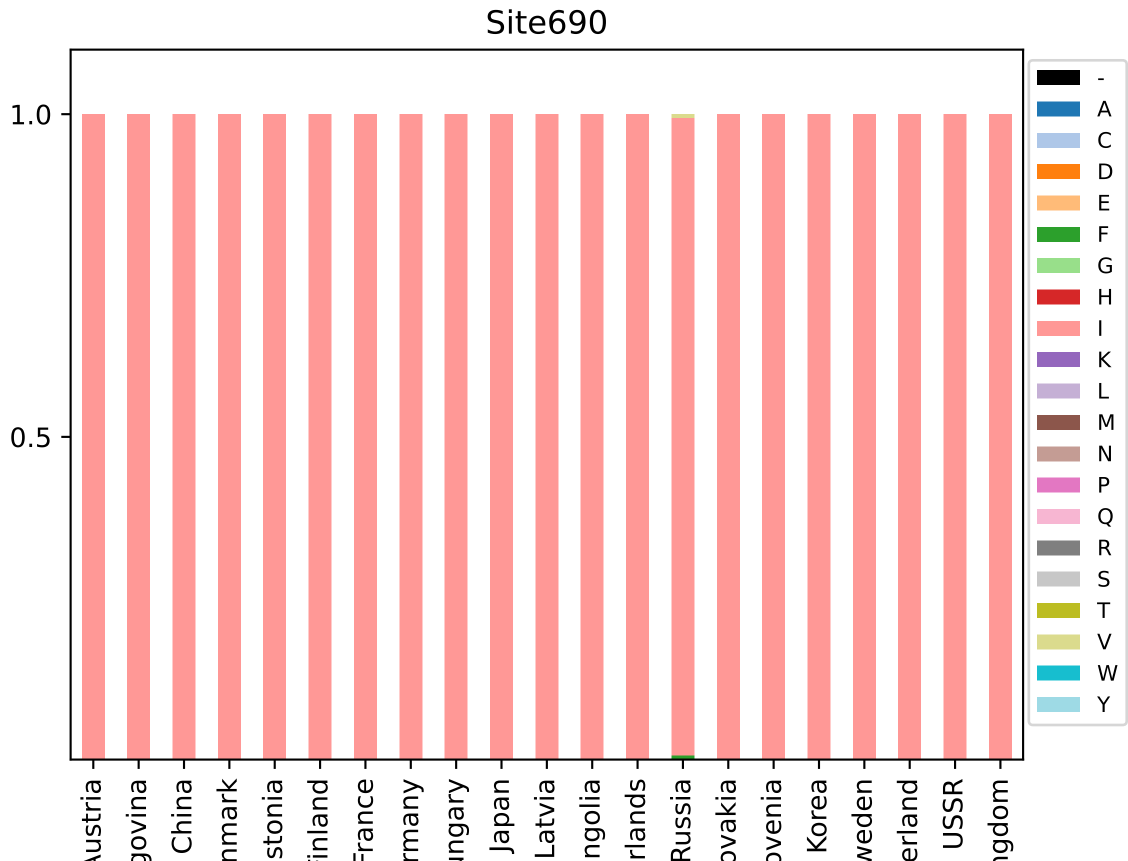

Site690
1.0
0.5
-
A
C
D
E
F
G
H
I
K
L
M
N
P
Q
R
S
T
V
W
Y
Austria
govina
China
nmark
stonia
inland
France
rmany
ungary
Japan
Latvia
ngolia
rlands
Russia
ovakia
ovenia
Korea
weden
erland
USSR
ngdom

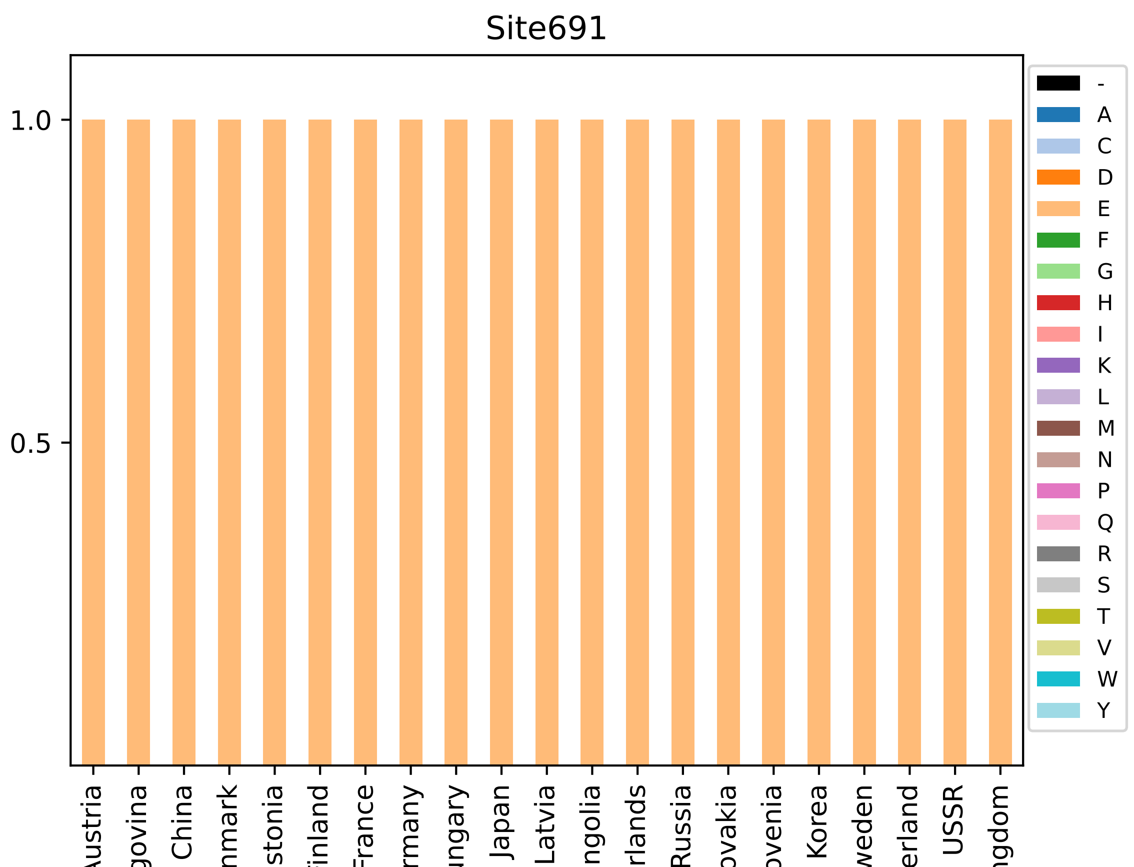
Site691
1.0
0.5
-
A
C
D
E
F
G
H
I
K
L
M
N
P
Q
R
S
T
V
W
Y
China
Japan
Latvia
Russia
Korea
USSR

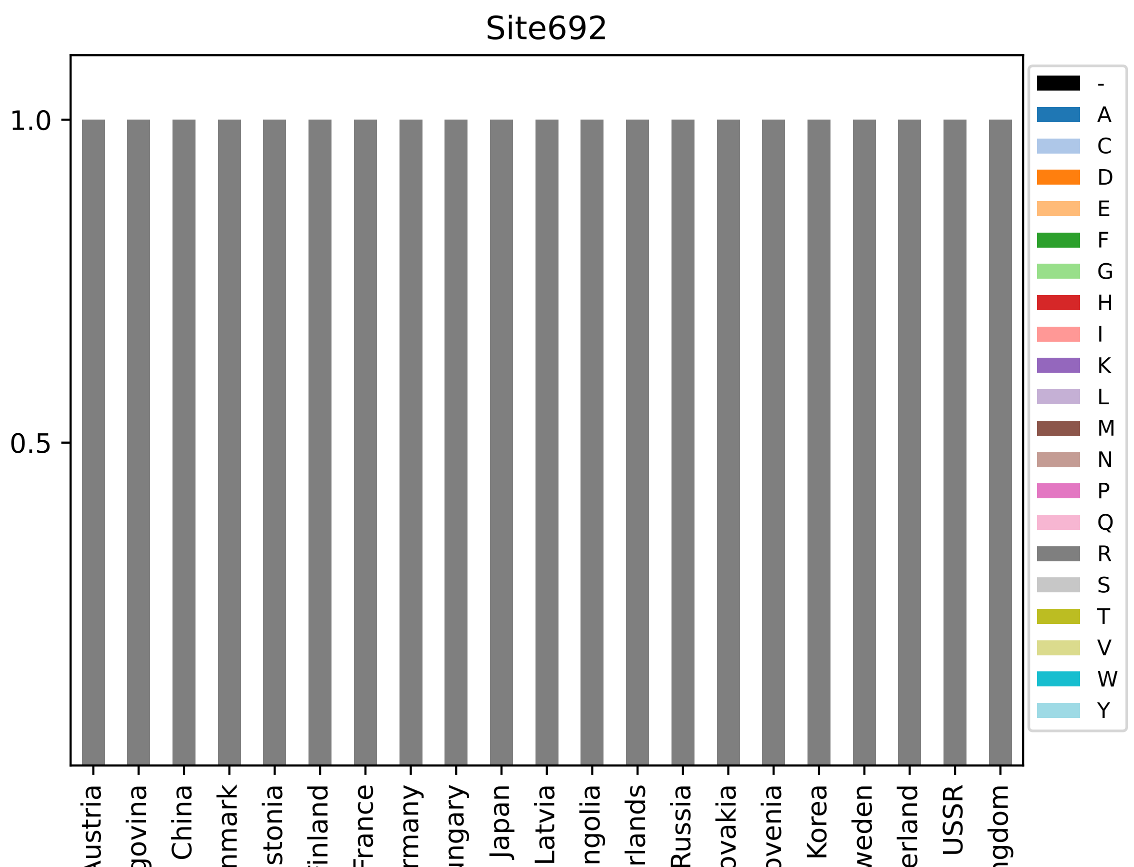
Site692
1.0
0.5
-
A
C
D
E
F
G
H
I
K
L
M
N
P
Q
R
S
T
V
W
Y
Austria
China
Japan
Latvia
Russia
Korea
USSR

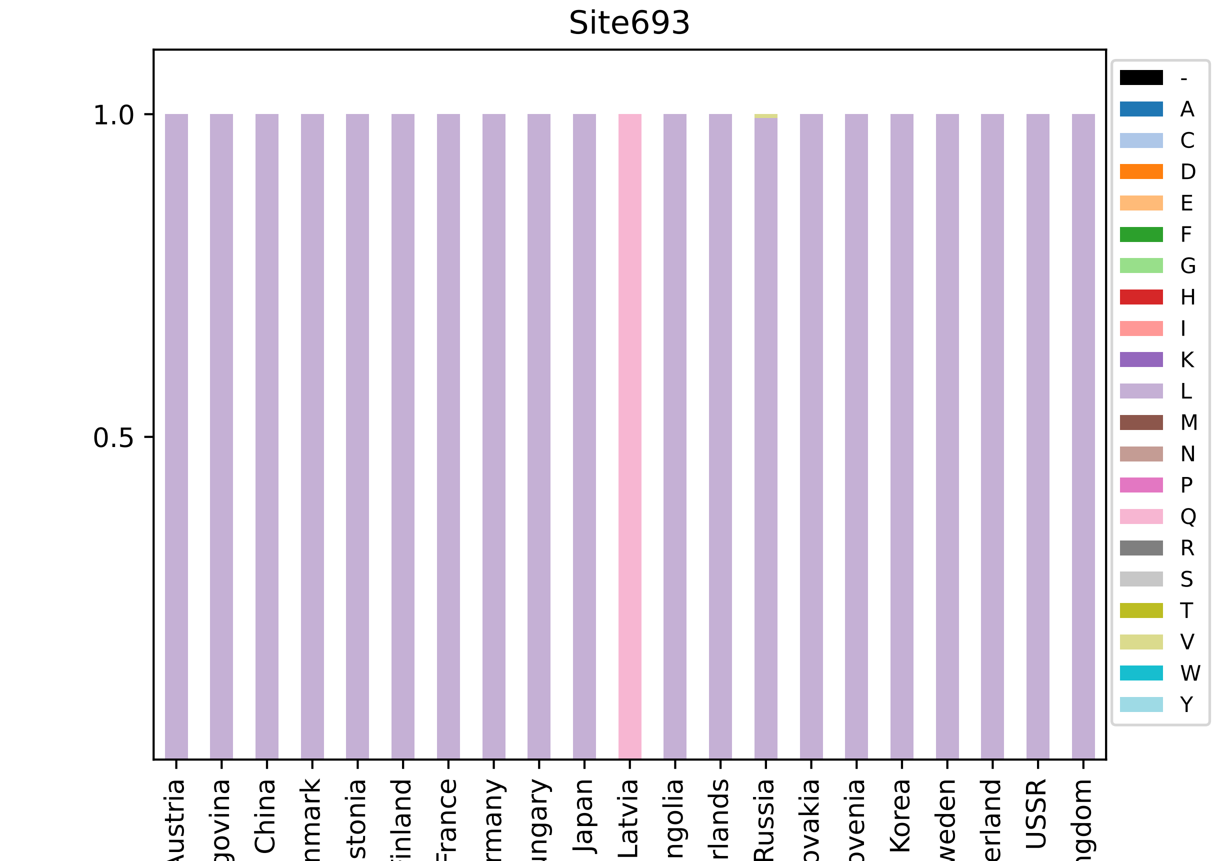
Site693
1.0
0.5
-
A
C
D
E
F
G
H
I
K
L
M
N
P
Q
R
S
T
V
W
Y
Austria
China
Finland
France
Japan
Latvia
Russia
Korea
USSR

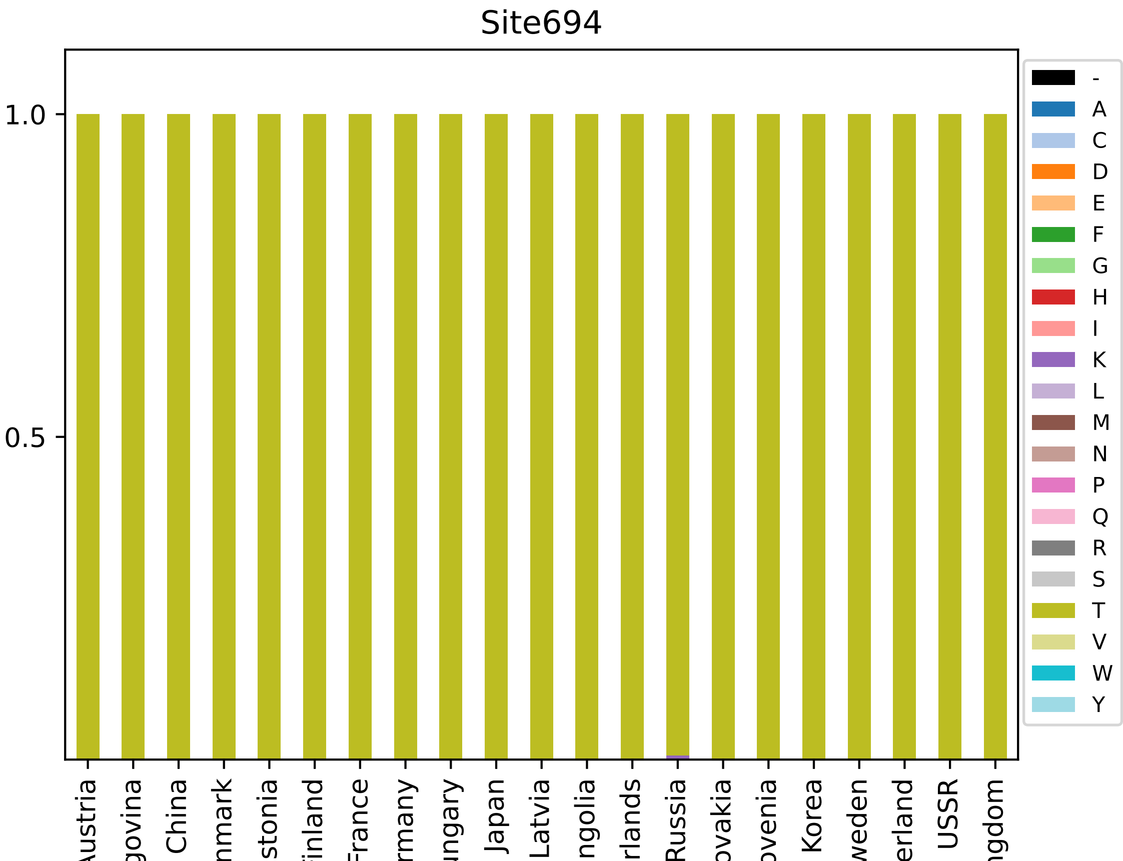

Site694
1.0
0.5
-
A
C
D
E
F
G
H
I
K
L
M
N
P
Q
R
S
T
V
W
Y
Austria
govina
China
nmark
stonia
Finland
France
rmany
ungary
Japan
Latvia
ngolia
rlands
Russia
ovakia
ovenia
Korea
weden
erland
USSR
ngdom

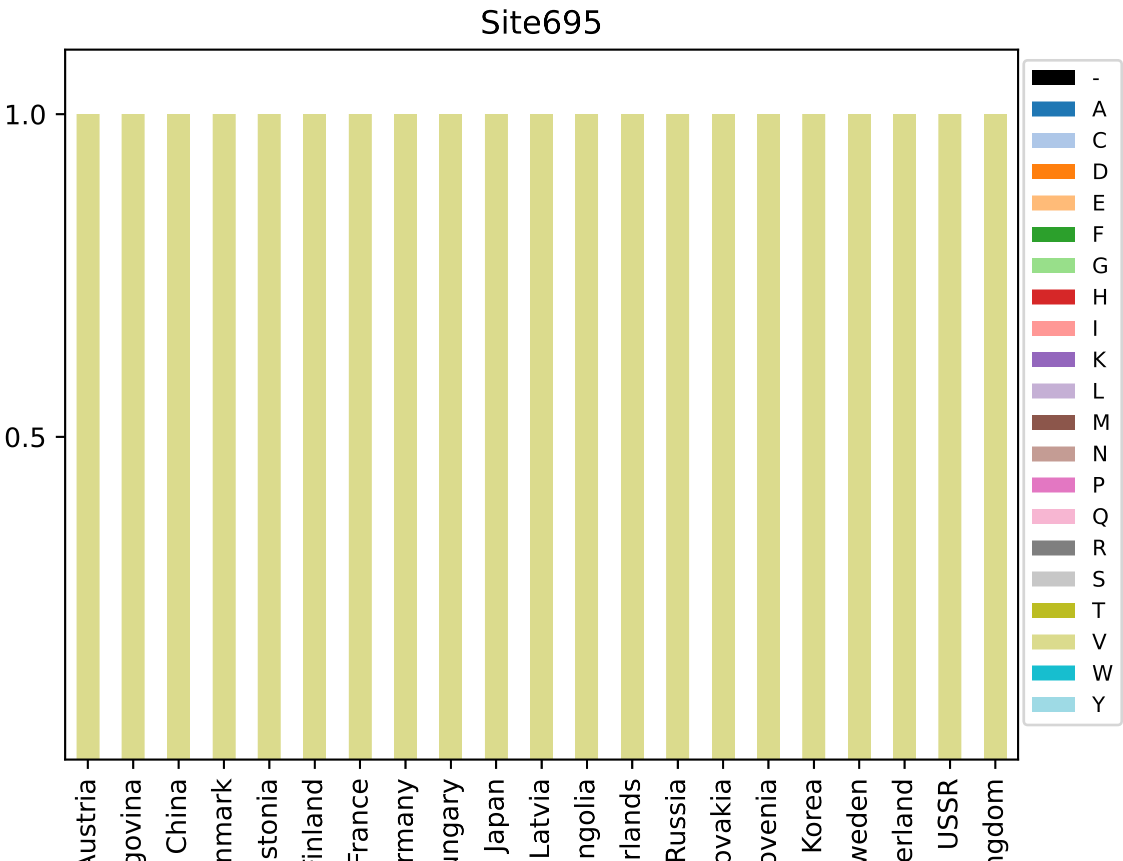

Site695
1.0
0.5
-
A
C
D
E
F
G
H
I
K
L
M
N
P
Q
R
S
T
V
W
Y
China
Japan
Latvia
Russia
Korea
USSR

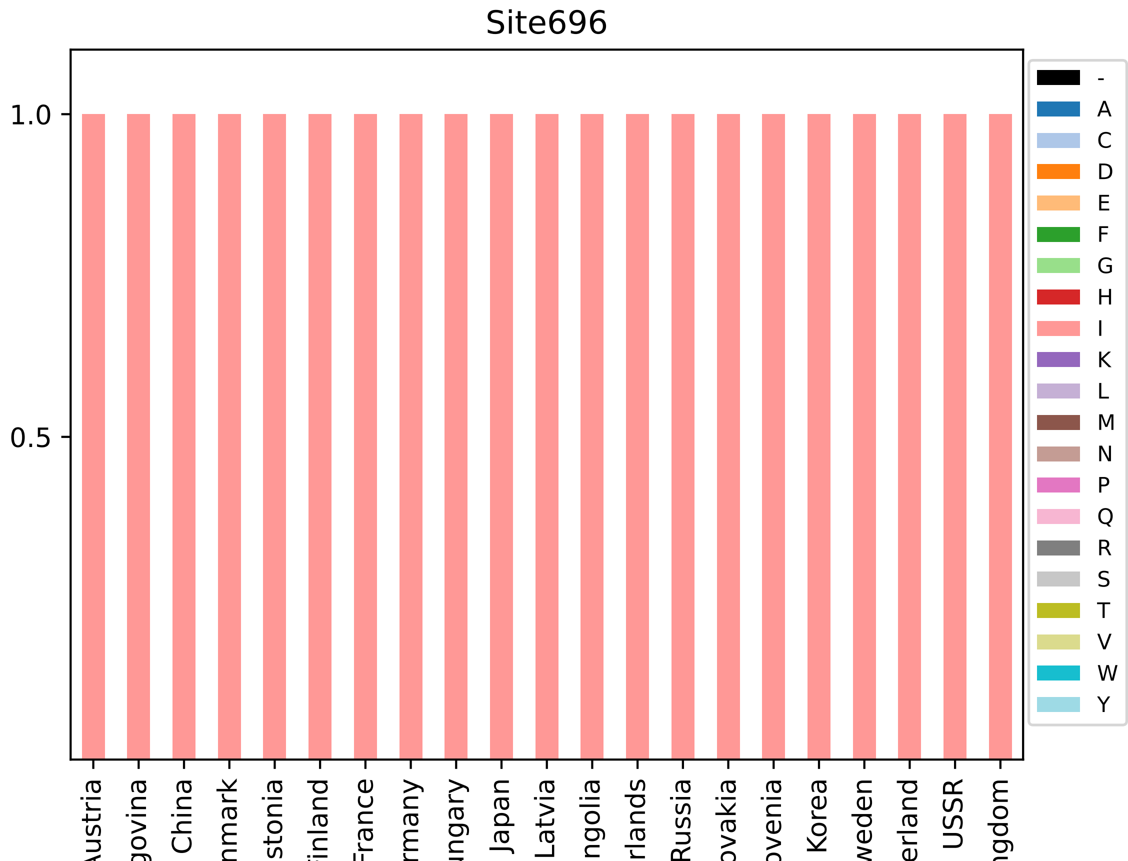
Site696
1.0
0.5
-
A
C
D
E
F
G
H
I
K
L
M
N
P
Q
R
S
T
V
W
Y
China
Japan
Latvia
Russia
Korea
USSR

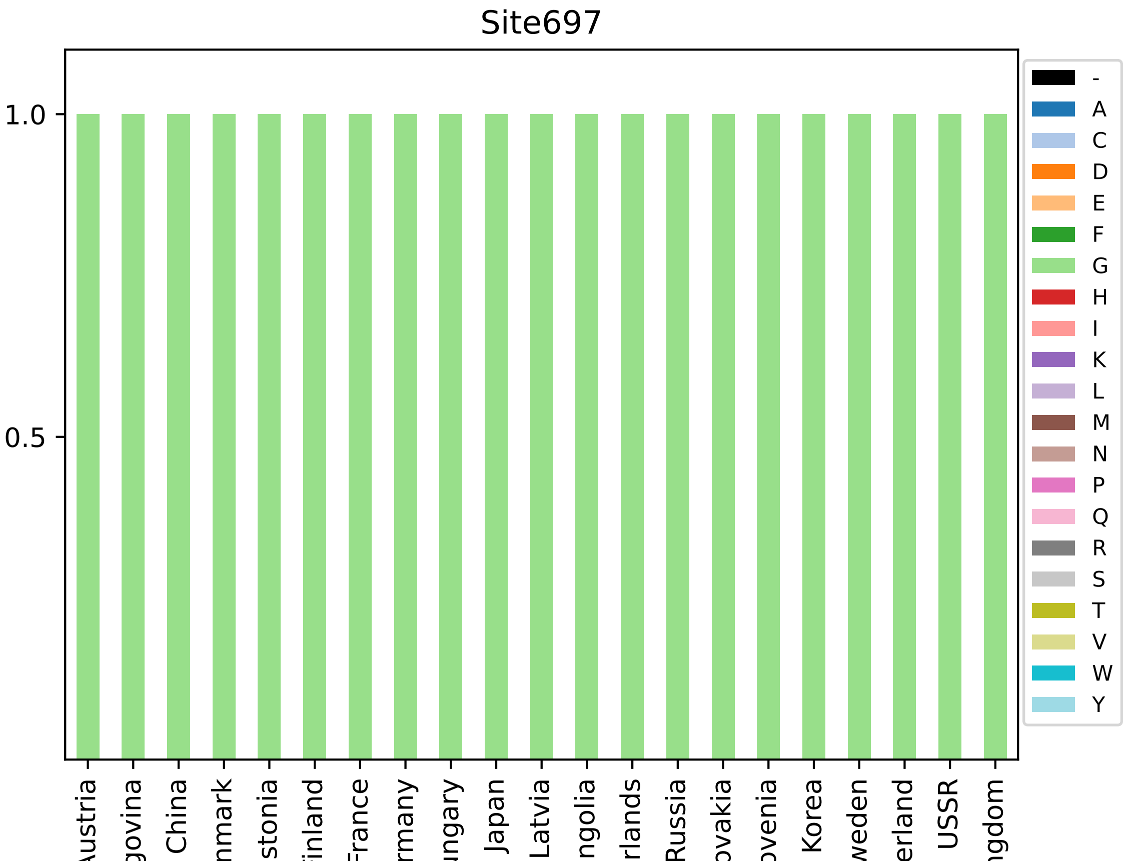
Site697
1.0
0.5
-
A
C
D
E
F
G
H
I
K
L
M
N
P
Q
R
S
T
V
W
Y
China
Japan
Latvia
Russia
Korea
USSR

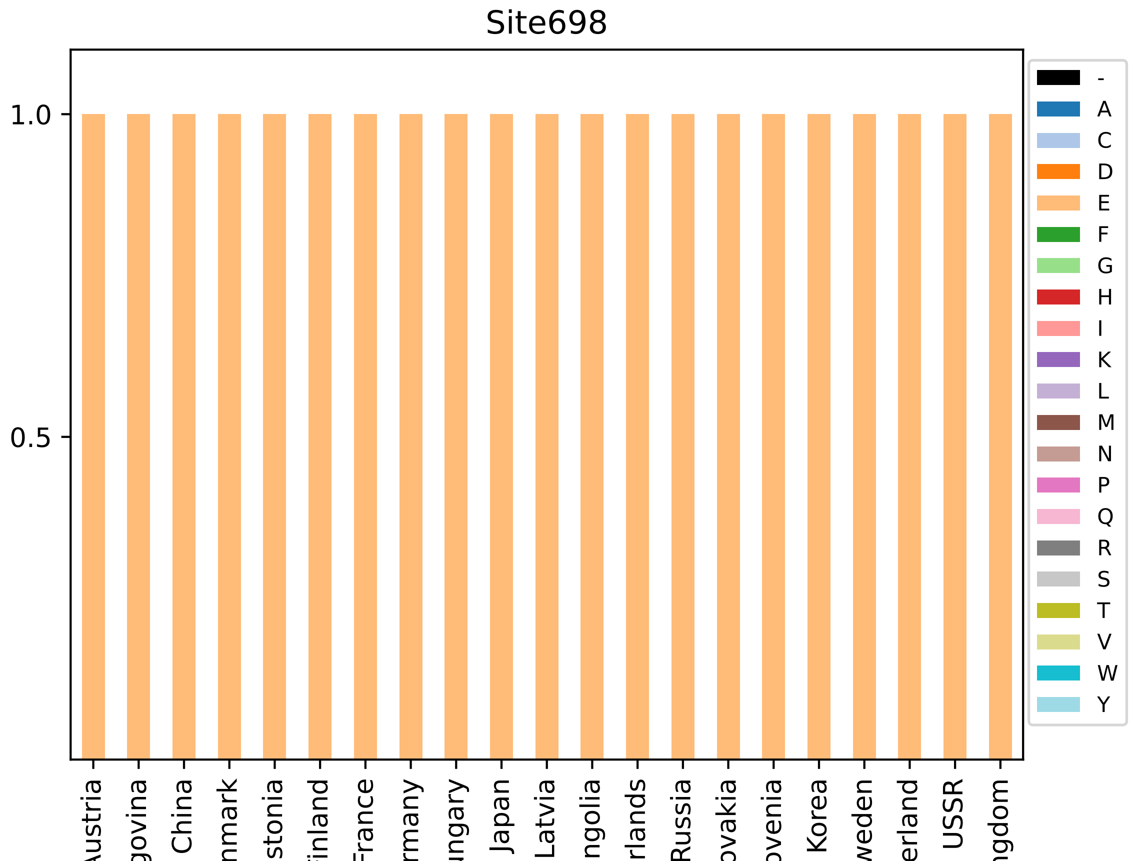

Site698
1.0
0.5
-
A
C
D
E
F
G
H
I
K
L
M
N
P
Q
R
S
T
V
W
Y
China
Japan
Latvia
Russia
Korea
USSR

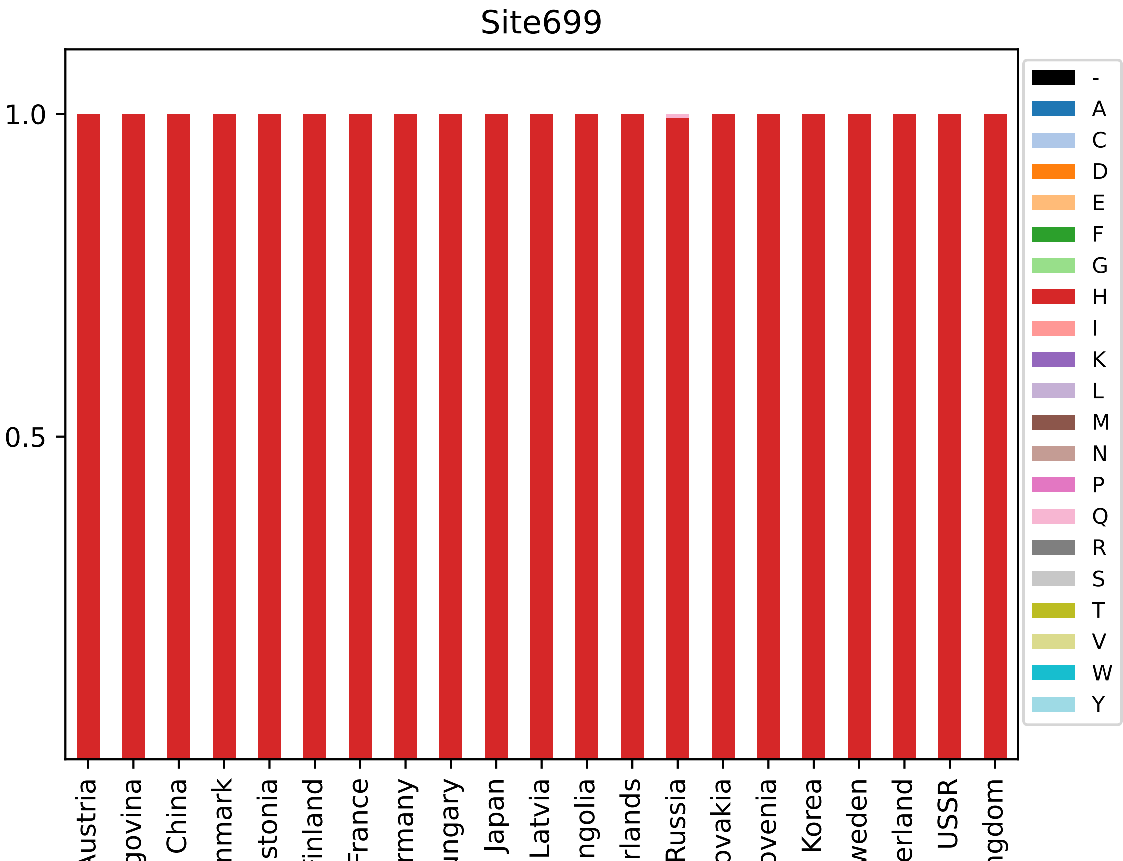

Site699
1.0
0.5
-
A
C
D
E
F
G
H
I
K
L
M
N
P
Q
R
S
T
V
W
Y
Austria
China
Japan
Latvia
Russia
Korea
USSR

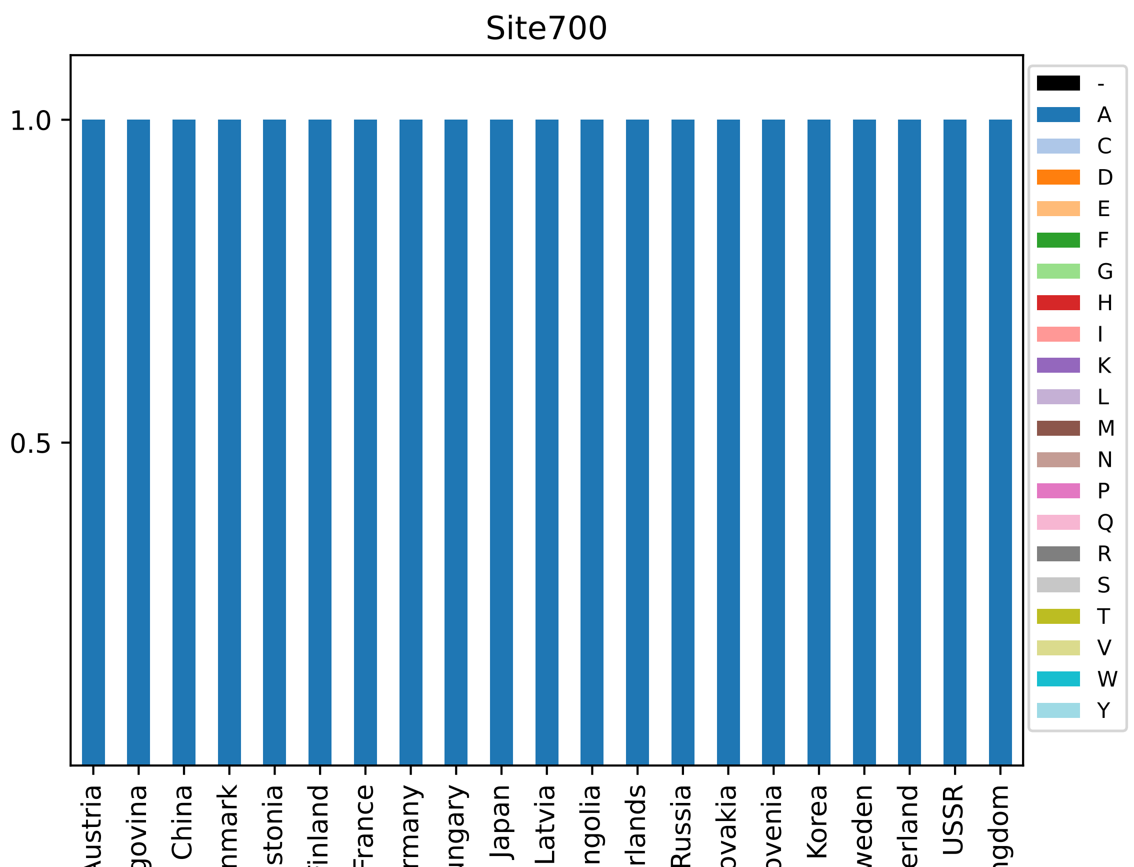
Site700
1.0
0.5
Austria
govina
China
nmark
stonia
Finland
France
rmany
ungary
Japan
Latvia
ngolia
rlands
Russia
ovakia
ovenia
Korea
weden
erland
USSR
ngdom
-
A
C
D
E
F
G
H
I
K
L
M
N
P
Q
R
S
T
V
W
Y

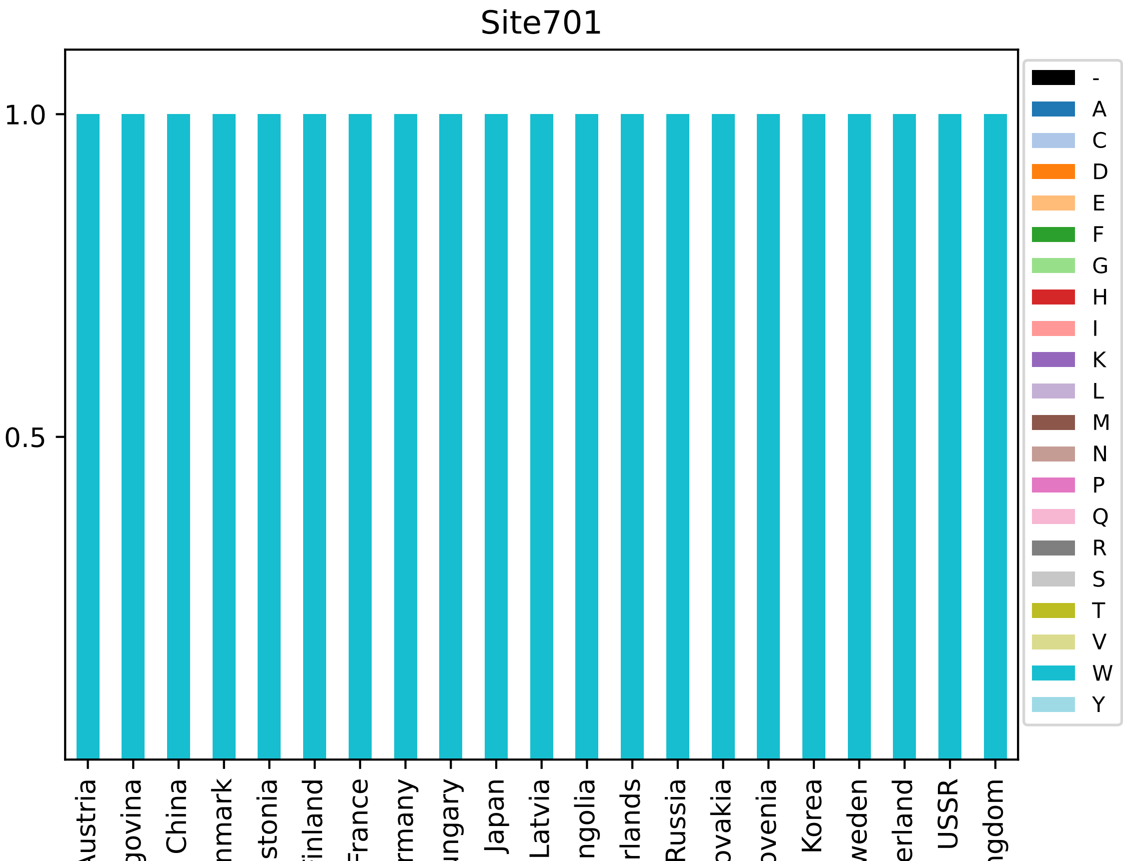
Site701
1.0
0.5
-
A
C
D
E
F
G
H
I
K
L
M
N
P
Q
R
S
T
V
W
Y
China
Japan
Latvia
Russia
Korea
USSR

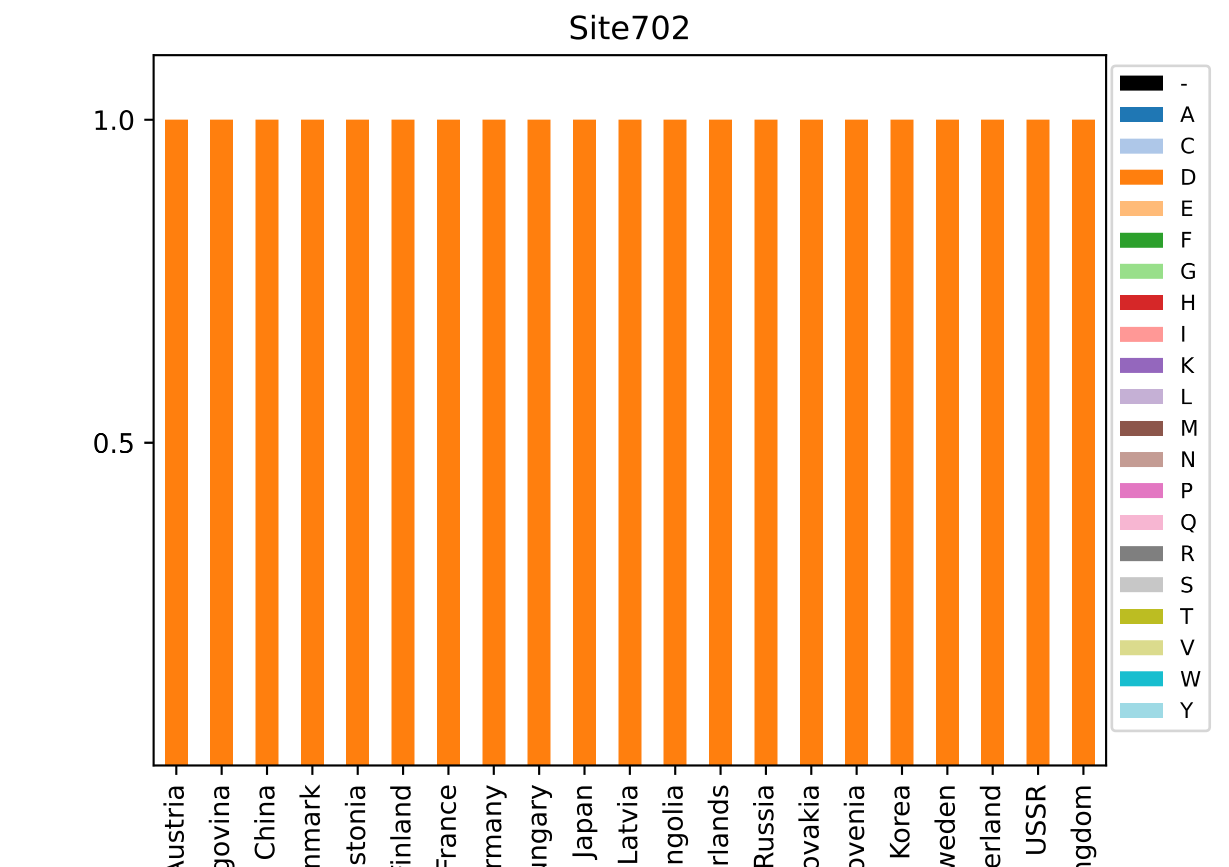
Site702
1.0
0.5
-
A
C
D
E
F
G
H
I
K
L
M
N
P
Q
R
S
T
V
W
Y
Austria
govina
China
nmark
stonia
inland
France
rmany
ungary
Japan
Latvia
ngolia
rlands
Russia
ovakia
ovenia
Korea
weden
erland
USSR
ngdom

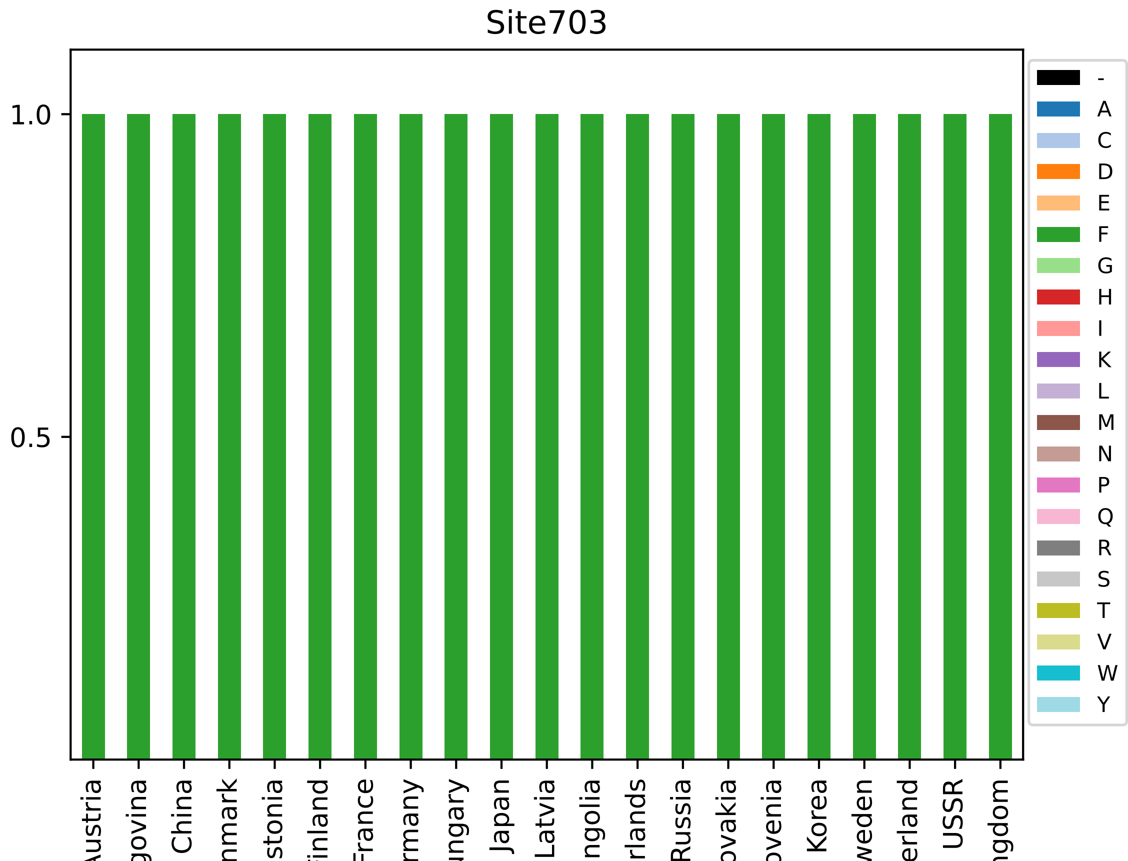

Site703
1.0
0.5
-
A
C
D
E
F
G
H
I
K
L
M
N
P
Q
R
S
T
V
W
Y
China
Japan
Latvia
Russia
Korea
USSR

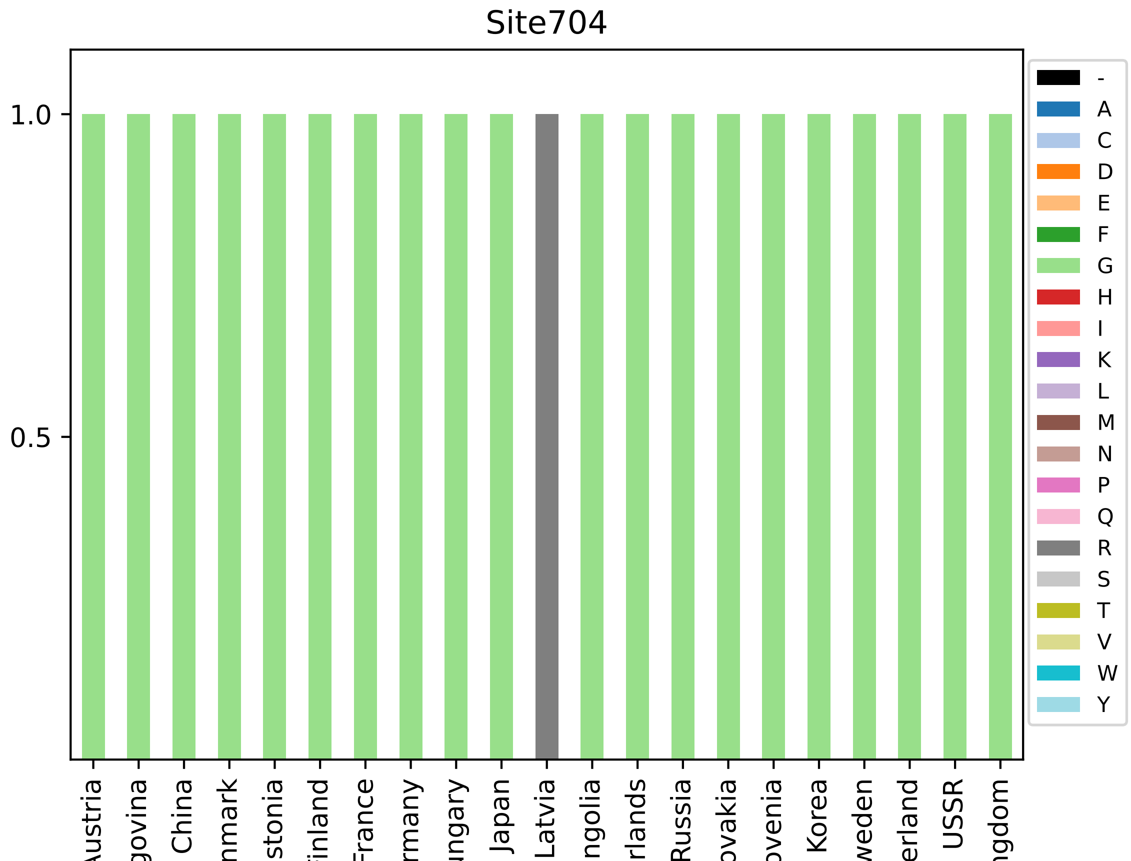

Site704
1.0
0.5
-
A
C
D
E
F
G
H
I
K
L
M
N
P
Q
R
S
T
V
W
Y
Austria
govina
China
nmark
stonia
inland
France
rmany
ungary
Japan
Latvia
ngolia
rlands
Russia
ovakia
ovenia
Korea
weden
erland
USSR
ngdom

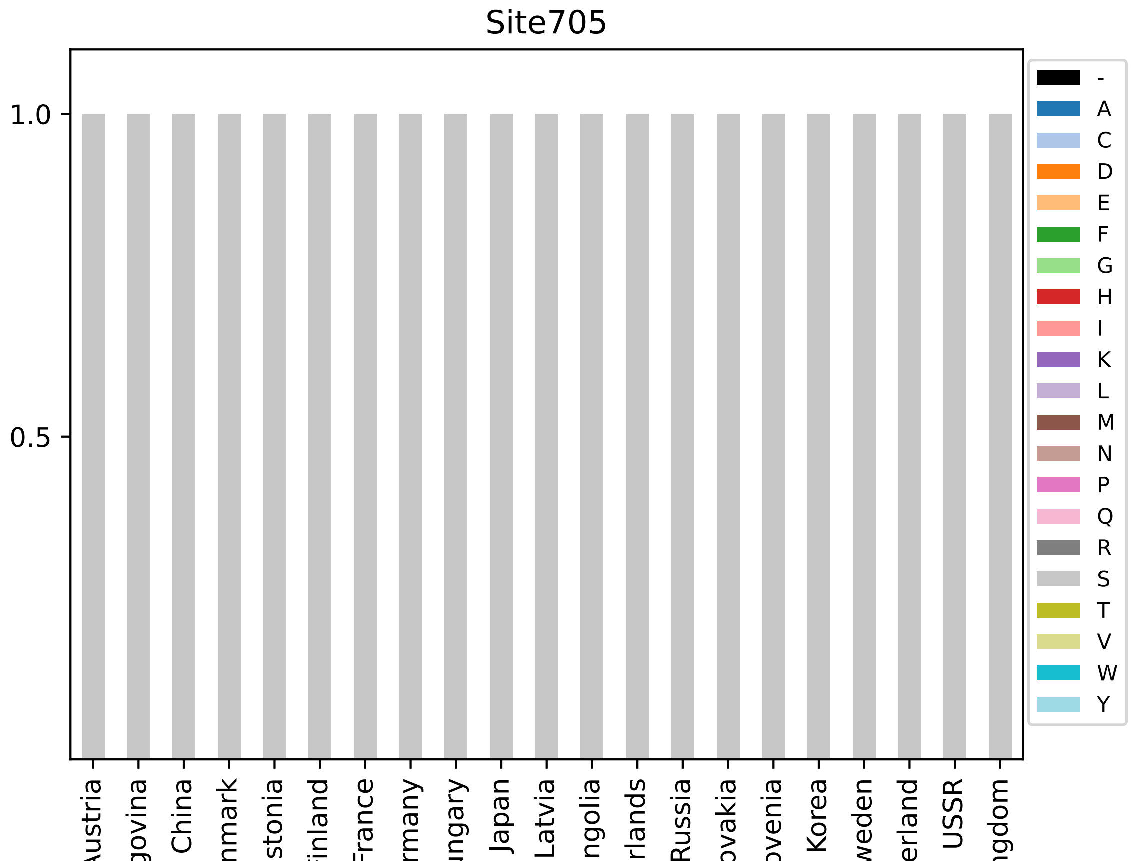
Site705
1.0
0.5
Austria
govina
China
nmark
stonia
inland
rance
rmany
ungary
Japan
Latvia
ngolia
rlands
Russia
ovakia
ovenia
Korea
weden
erland
USSR
ngdom
-
A
C
D
E
F
G
H
I
K
L
M
N
P
Q
R
S
T
V
W
Y

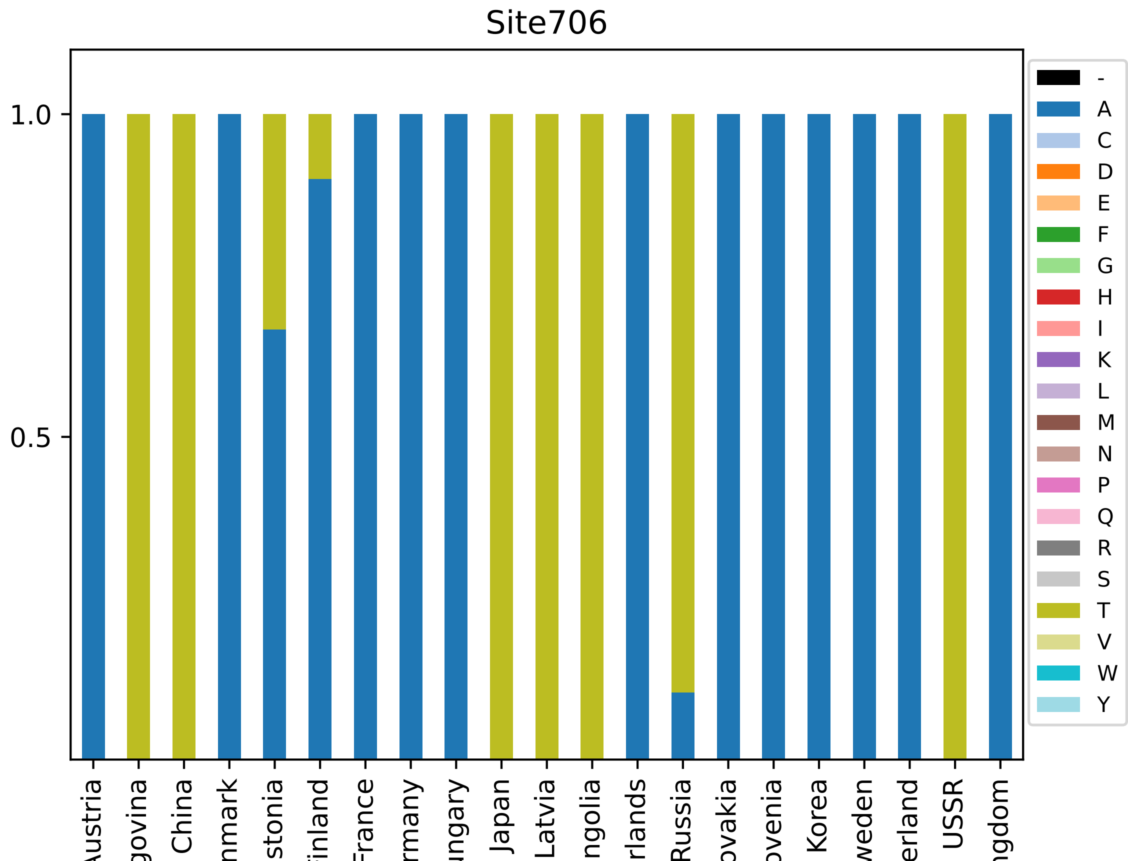
Site706
1.0
0.5
China
Japan
Latvia
Russia
Korea
USSR
-
A
C
D
E
F
G
H
I
K
L
M
N
P
Q
R
S
T
V
W
Y

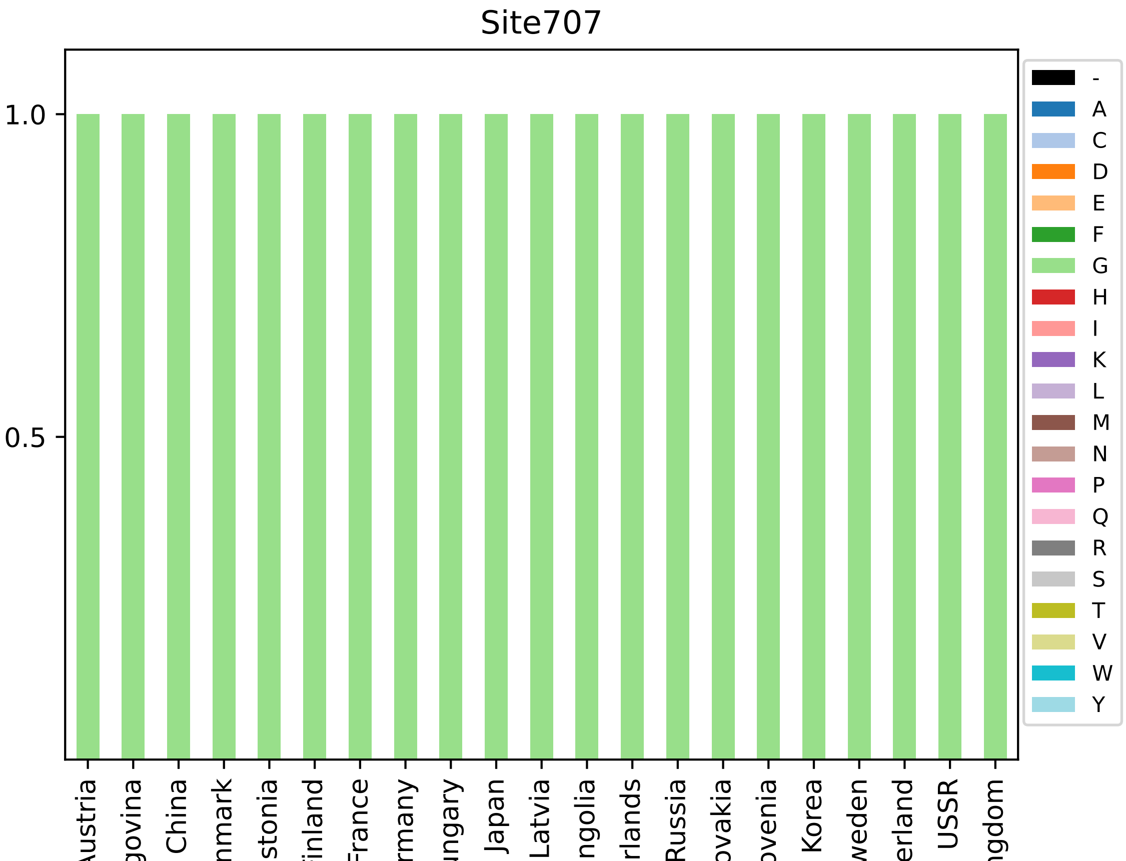

Site707
1.0
0.5
-
A
C
D
E
F
G
H
I
K
L
M
N
P
Q
R
S
T
V
W
Y
China
Japan
Latvia
Russia
Korea
USSR

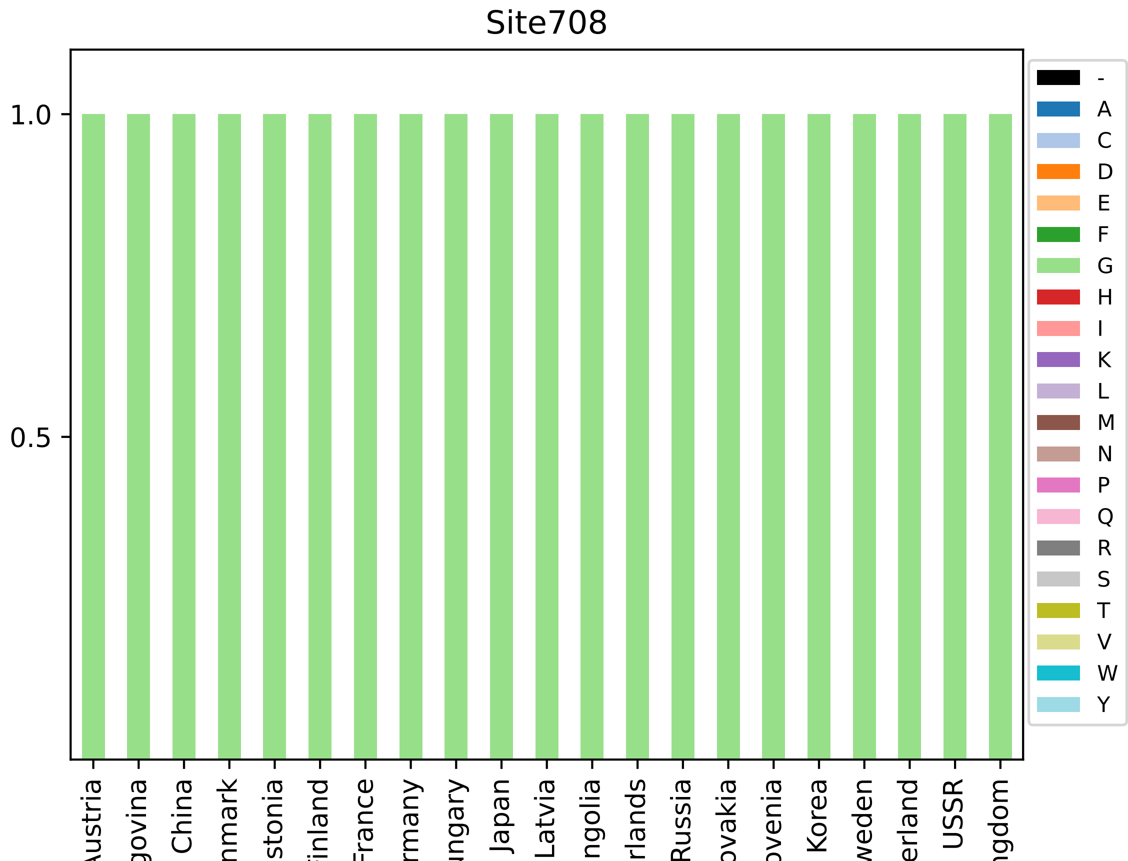
Site708
1.0
0.5
-
A
C
D
E
F
G
H
I
K
L
M
N
P
Q
R
S
T
V
W
Y
China
Japan
Latvia
Russia
Korea
USSR

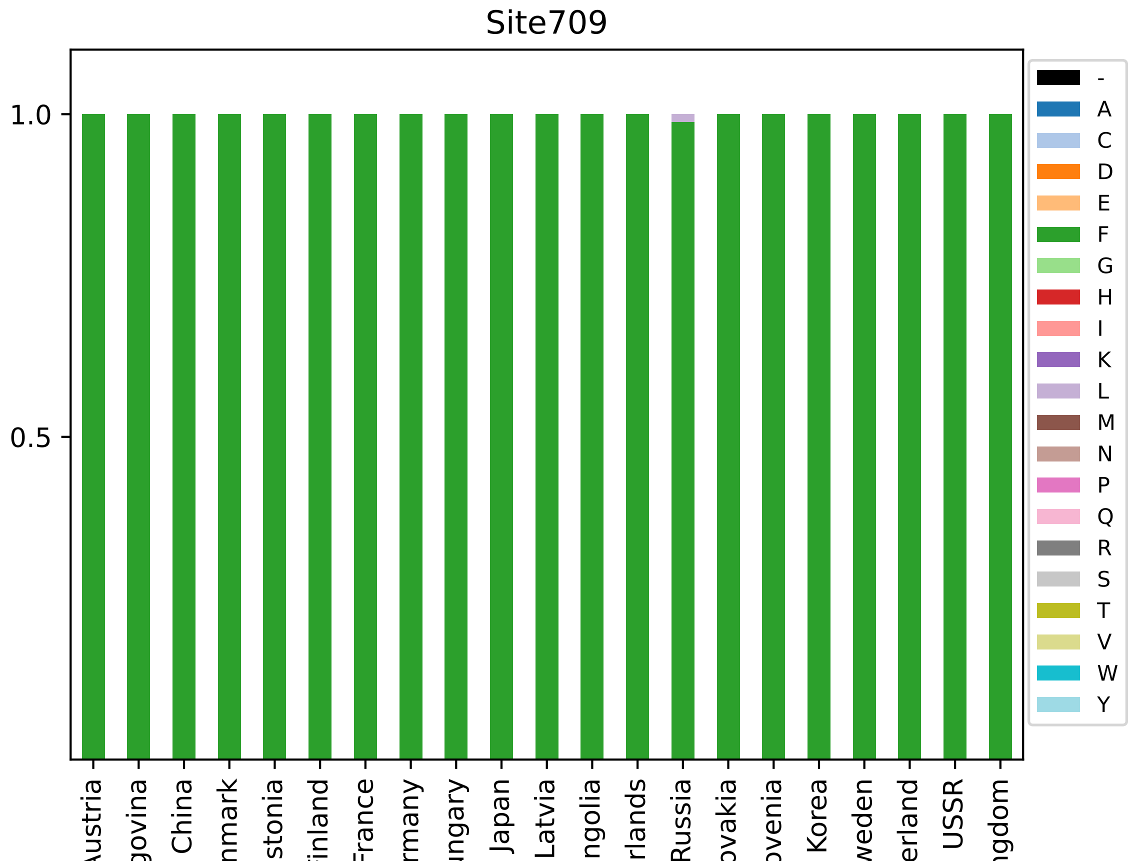
Site709
1.0
0.5
-
A
C
D
E
F
G
H
I
K
L
M
N
P
Q
R
S
T
V
W
Y
China
Japan
Latvia
Russia
Korea
USSR

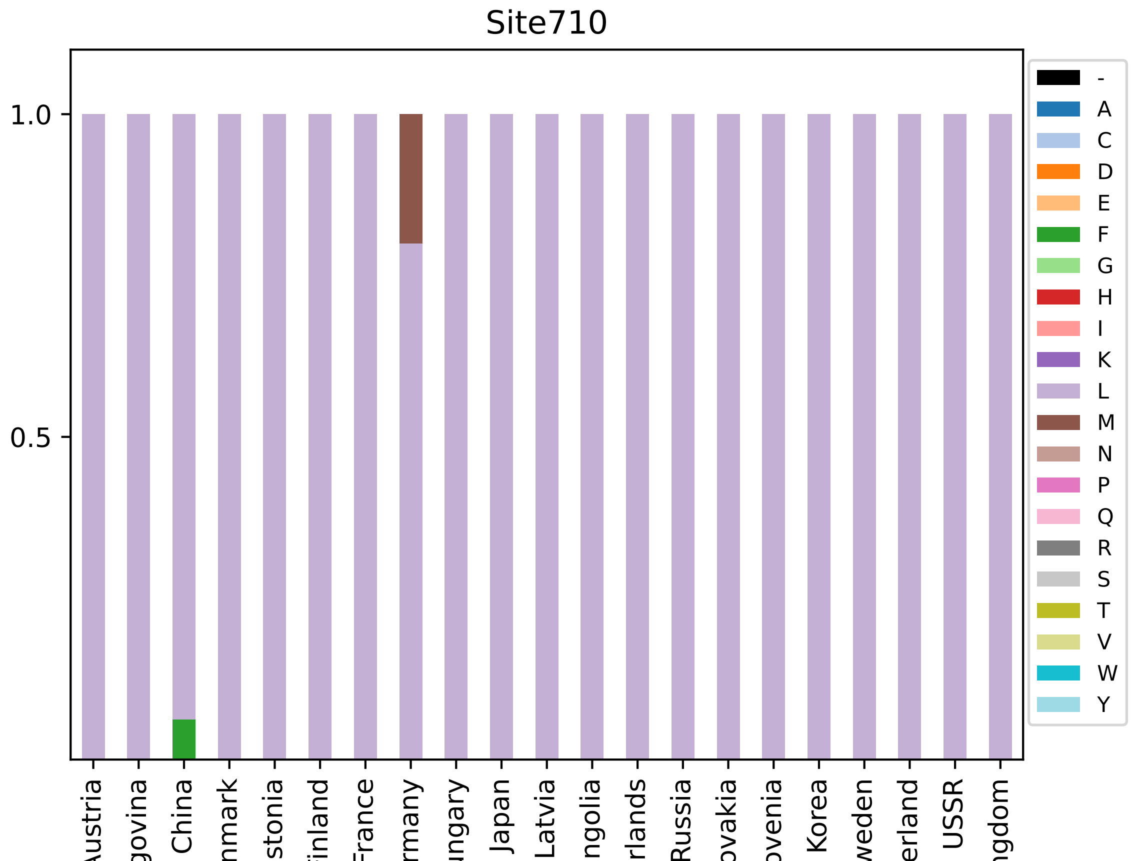
Site710
1.0
0.5
-
A
C
D
E
F
G
H
I
K
L
M
N
P
Q
R
S
T
V
W
Y
China
Japan
Latvia
Russia
Korea
USSR

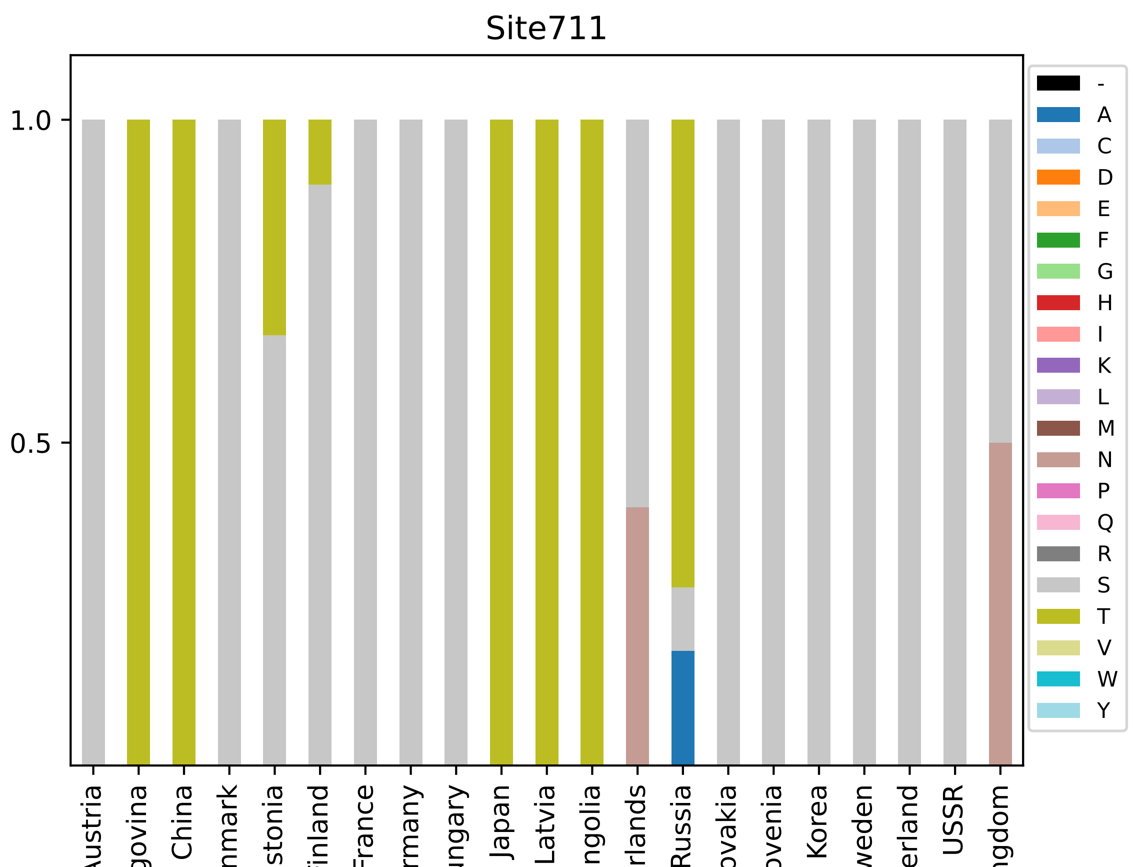

Site711
1.0
0.5
-
A
C
D
E
F
G
H
I
K
L
M
N
P
Q
R
S
T
V
W
Y
Japan
Latvia
Russia
Korea
USSR
China

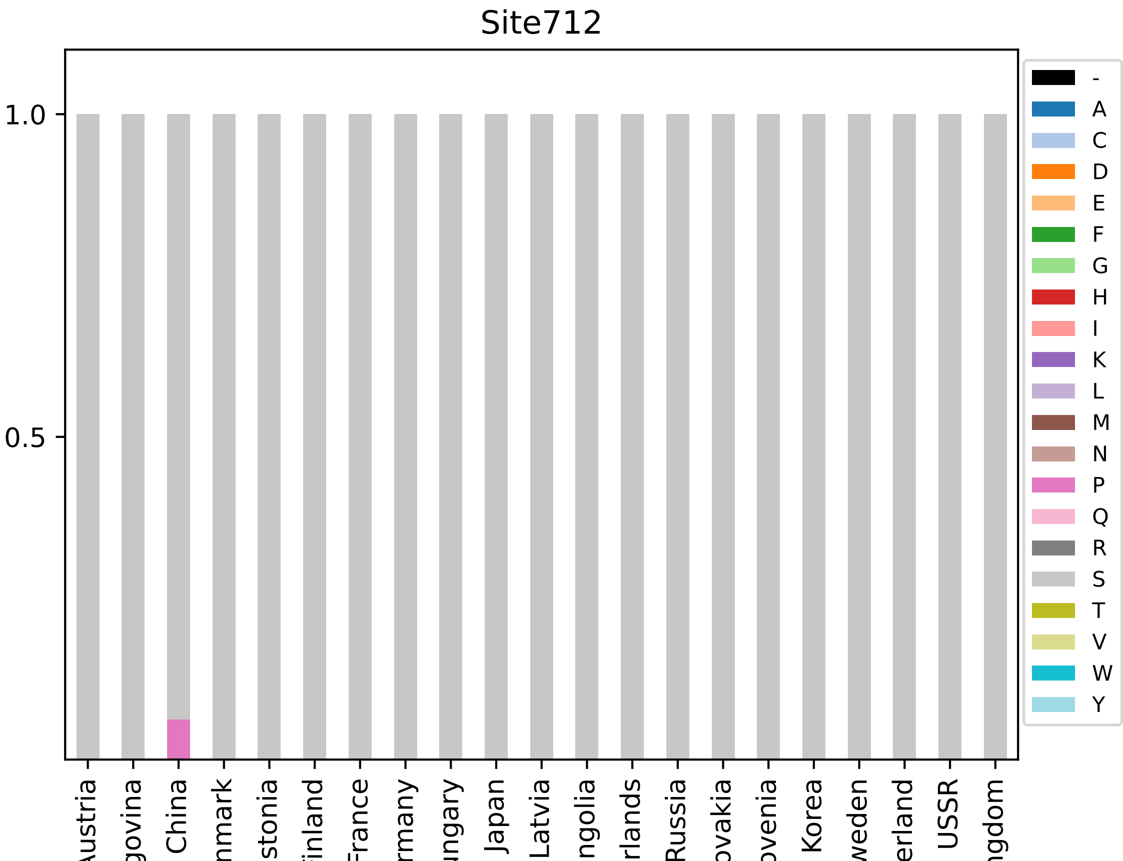
Site712
1.0
0.5
-
A
C
D
E
F
G
H
I
K
L
M
N
P
Q
R
S
T
V
W
Y
Austria
govina
China
nmark
stonia
inland
France
rmany
ungary
Japan
Latvia
ngolia
rlands
Russia
ovakia
ovenia
Korea
weden
erland
USSR
ngdom

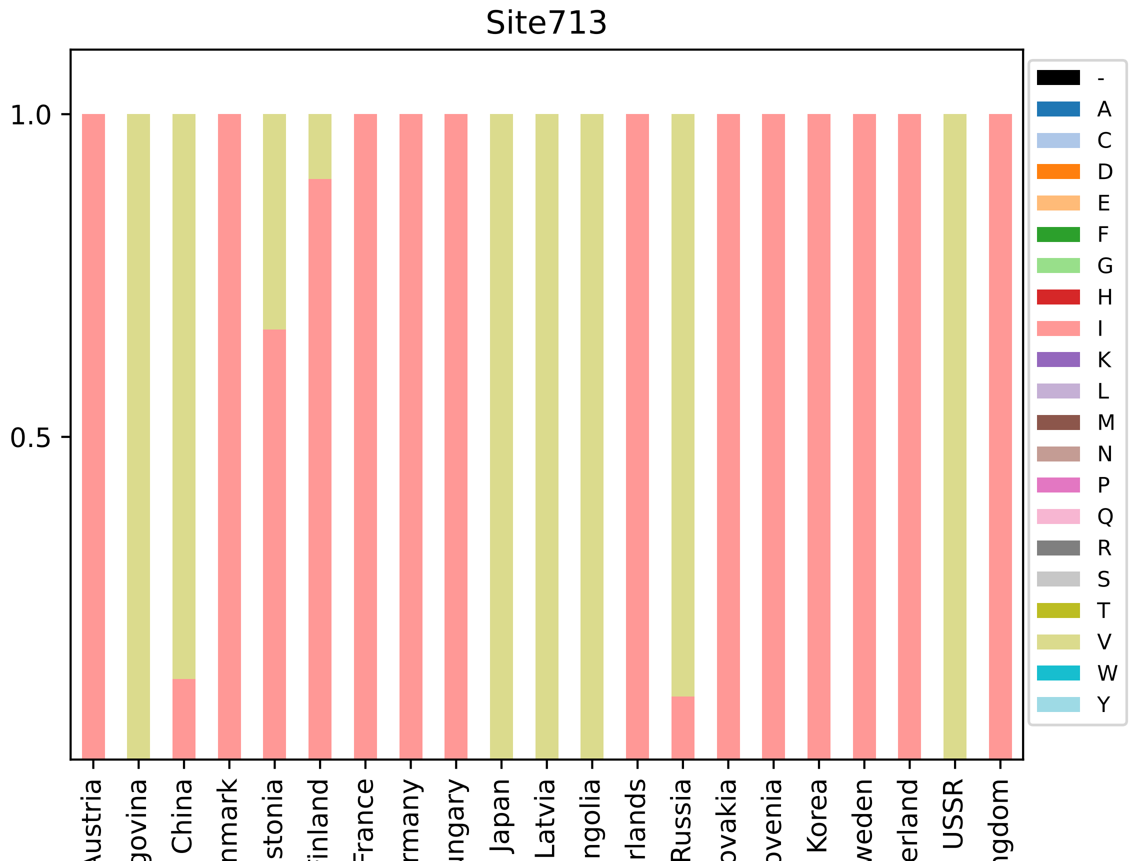

Site713
1.0
0.5
-
A
C
D
E
F
G
H
I
K
L
M
N
P
Q
R
S
T
V
W
Y
Austria
govina
China
nmark
stonia
inland
France
rmany
ungary
Japan
Latvia
ngolia
rlands
Russia
ovakia
ovenia
Korea
weden
erland
USSR
ngdom

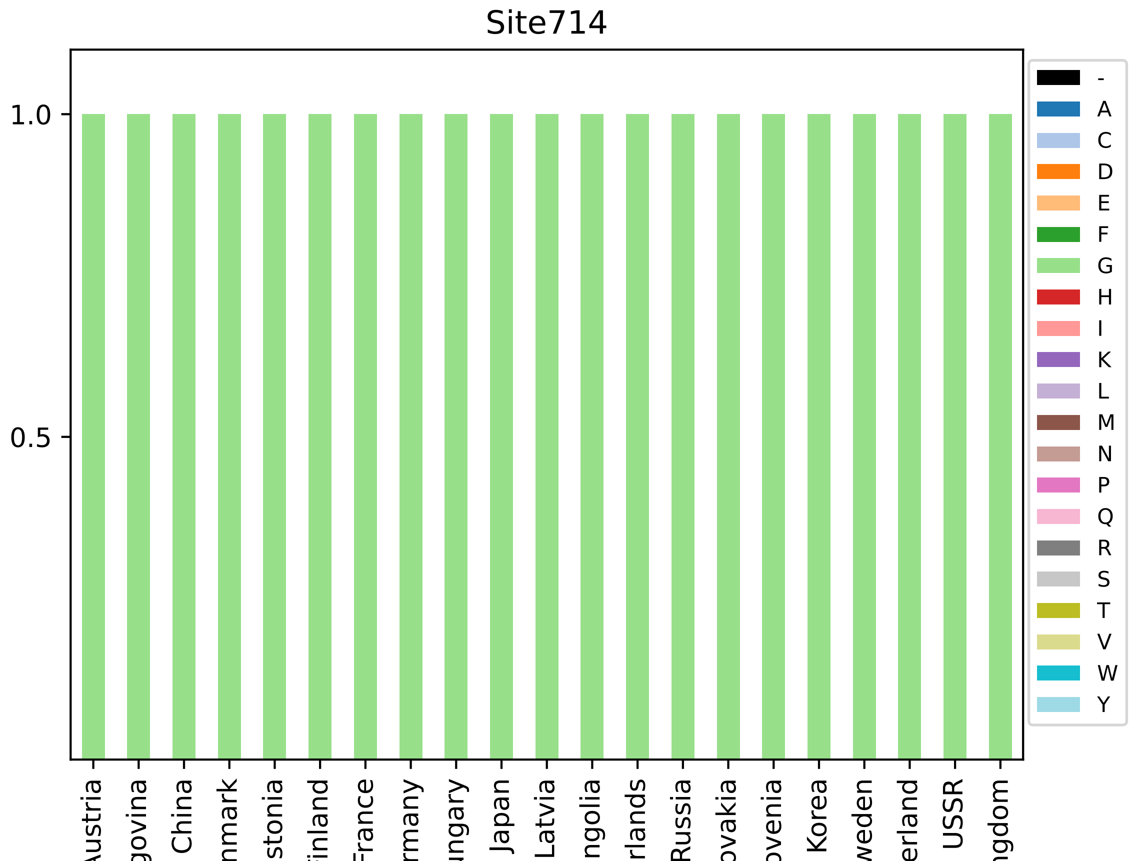

Site714
1.0
0.5
-
A
C
D
E
F
G
H
I
K
L
M
N
P
Q
R
S
T
V
W
Y
China
Japan
Latvia
Russia
Korea
USSR

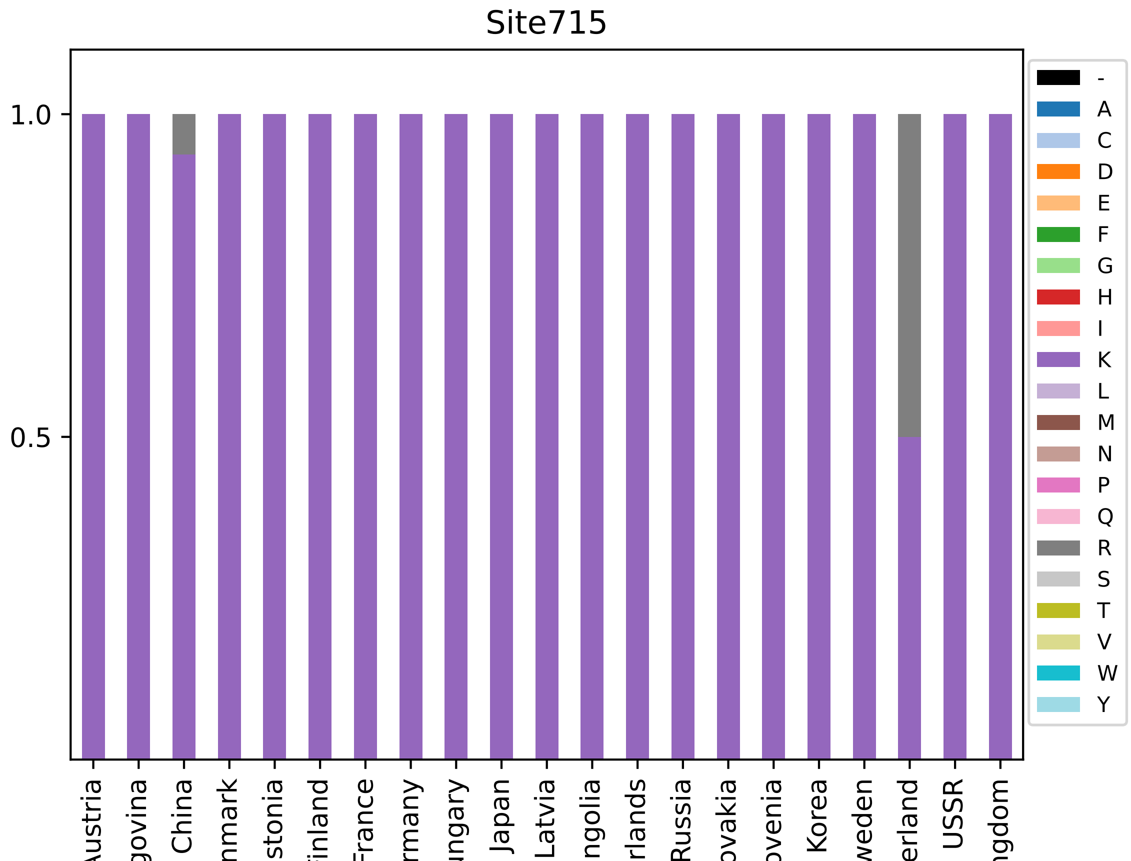

Site715
1.0
0.5
-
A
C
D
E
F
G
H
I
K
L
M
N
P
Q
R
S
T
V
W
Y
China
Japan
Latvia
Russia
Korea
USSR

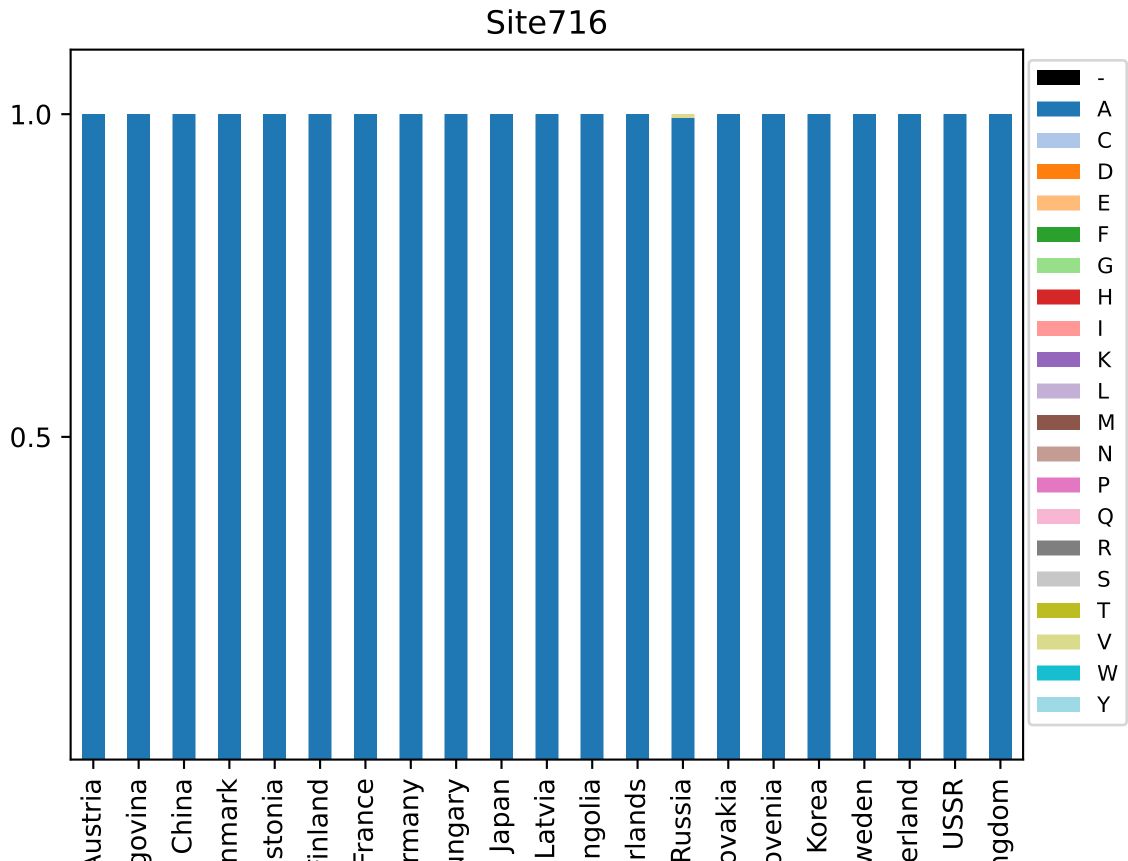

Site716
1.0
0.5
-
A
C
D
E
F
G
H
I
K
L
M
N
P
Q
R
S
T
V
W
Y
Austria
govina
China
nmark
stonia
inland
France
rmany
ungary
Japan
Latvia
ngolia
rlands
Russia
ovakia
ovenia
Korea
weden
erland
USSR
ngdom

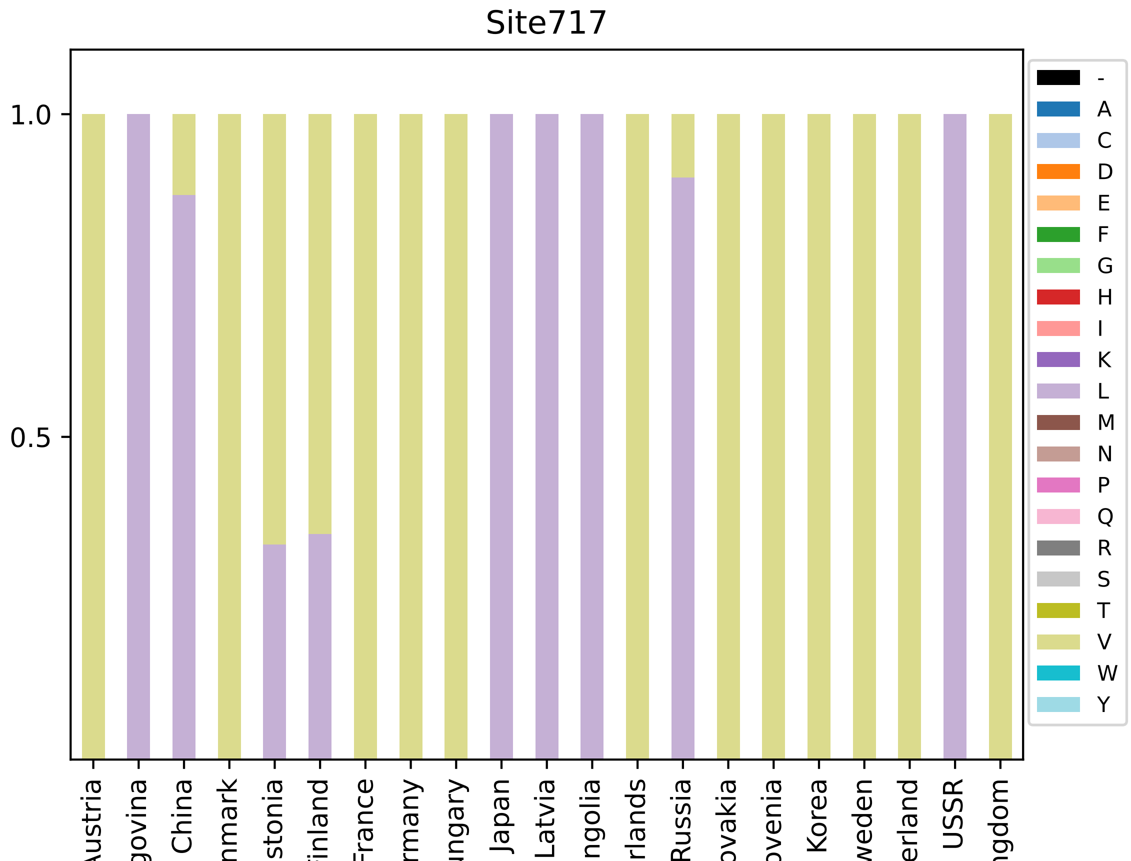
Site717
1.0
0.5
-
A
C
D
E
F
G
H
I
K
L
M
N
P
Q
R
S
T
V
W
Y
Austria
govina
China
nmark
stonia
Finland
France
rmany
ungary
Japan
Latvia
ngolia
rlands
Russia
ovakia
ovenia
Korea
weden
erland
USSR
ngdom

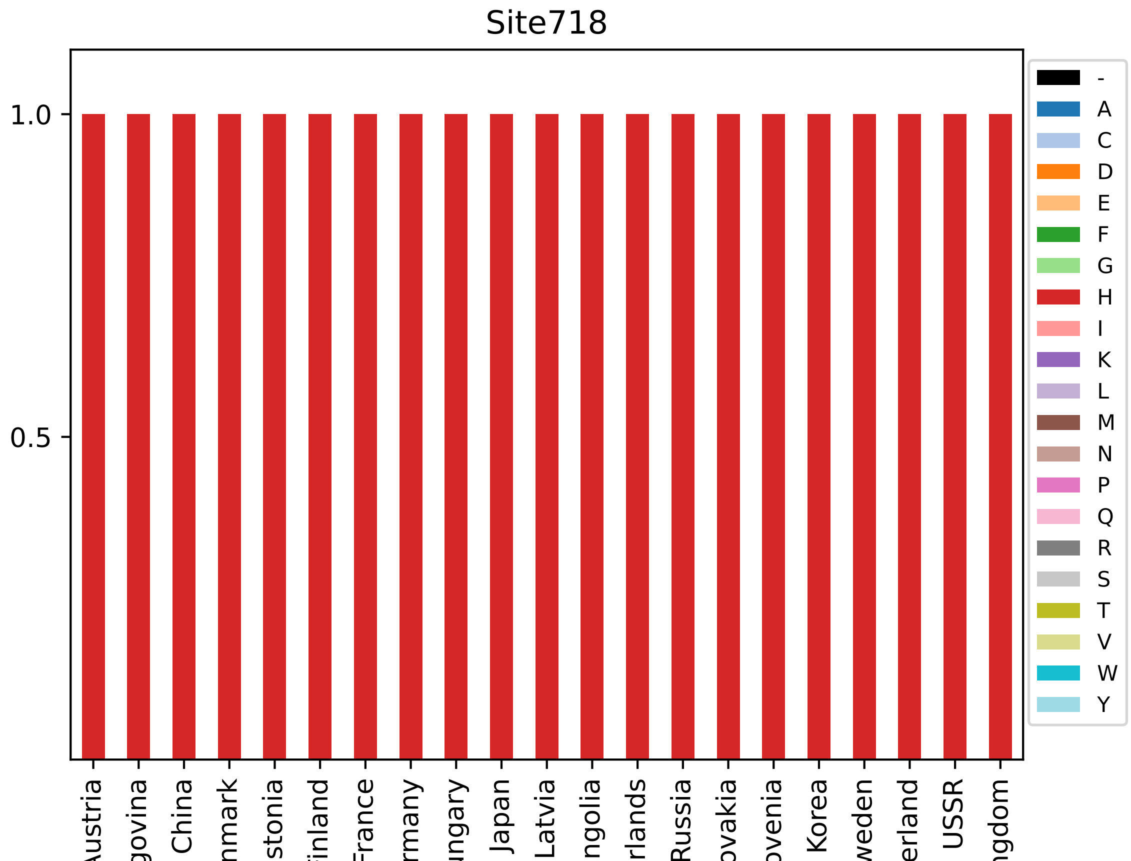
Site718
1.0
0.5
-
A
C
D
E
F
G
H
I
K
L
M
N
P
Q
R
S
T
V
W
Y
Austria
govina
China
nmark
stonia
inland
France
rmany
ungary
Japan
Latvia
ngolia
rlands
Russia
ovakia
ovenia
Korea
weden
erland
USSR
ngdom

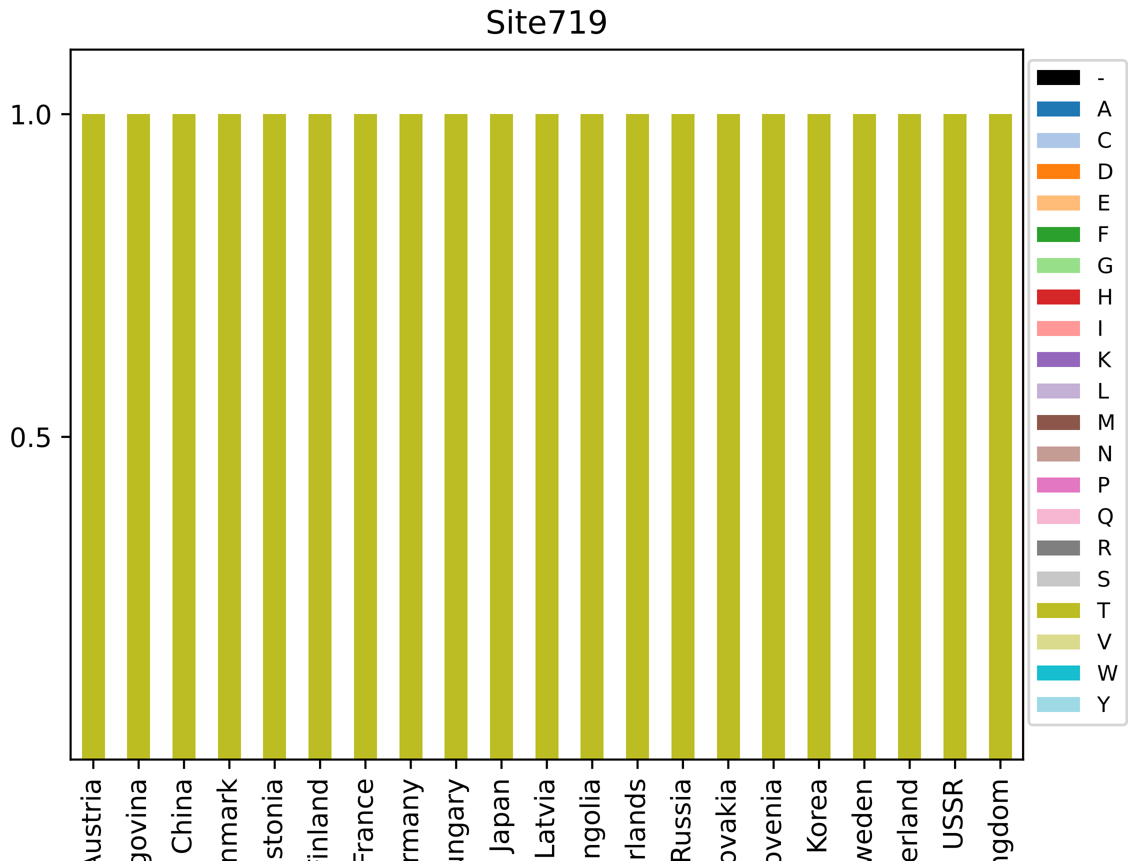
Site719
1.0
0.5
-
A
C
D
E
F
G
H
I
K
L
M
N
P
Q
R
S
T
V
W
Y
China
Japan
Latvia
Russia
Korea
USSR

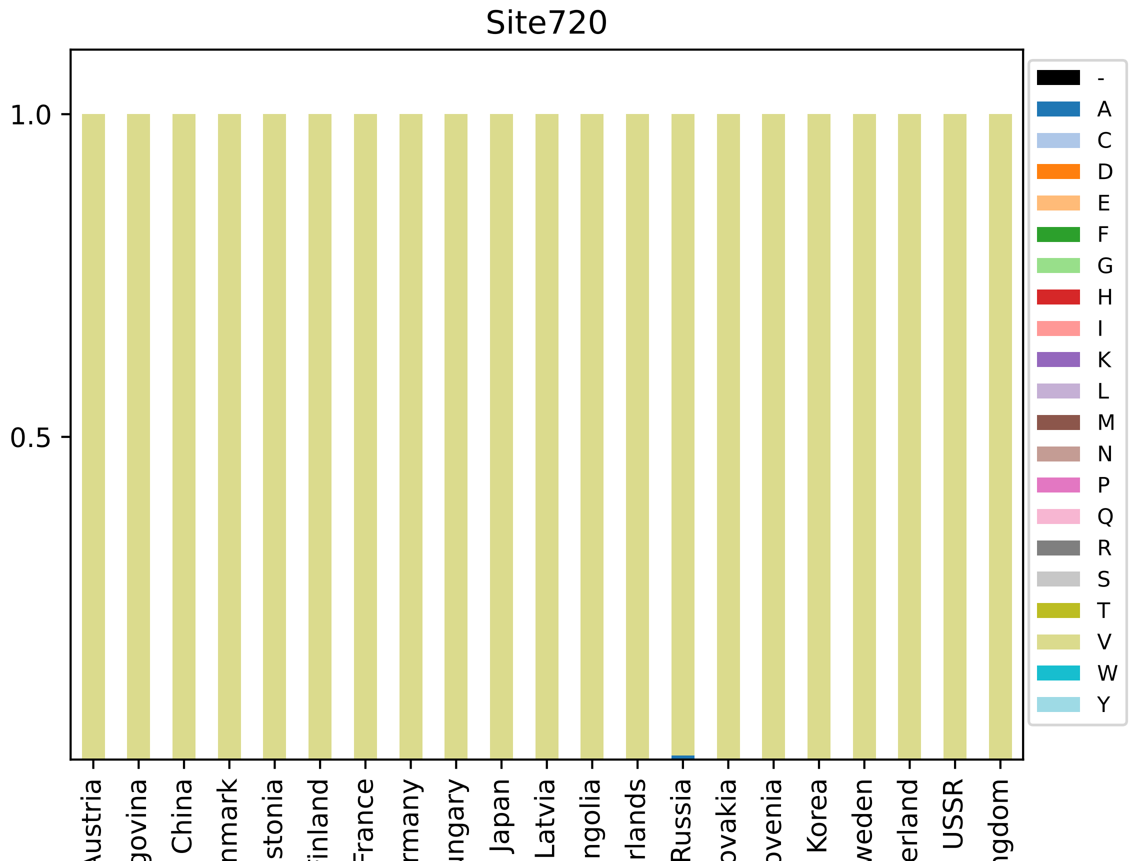
Site720
1.0
0.5
-
A
C
D
E
F
G
H
I
K
L
M
N
P
Q
R
S
T
V
W
Y
Austria
govina
China
nmark
stonia
Finland
France
rmany
ungary
Japan
Latvia
ngolia
rlands
Russia
ovakia
ovenia
Korea
weden
erland
USSR
ngdom

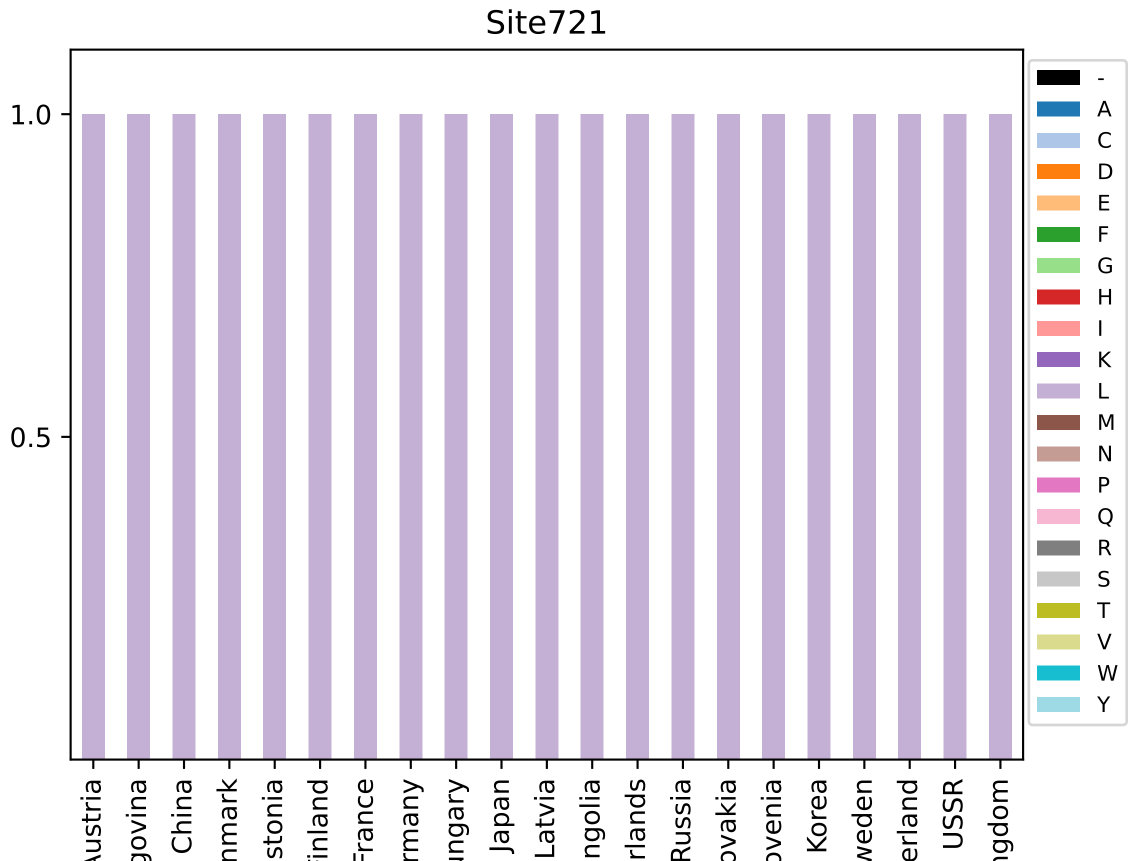
Site721
1.0
0.5
-
A
C
D
E
F
G
H
I
K
L
M
N
P
Q
R
S
T
V
W
Y
China
Japan
Latvia
Russia
Korea
USSR

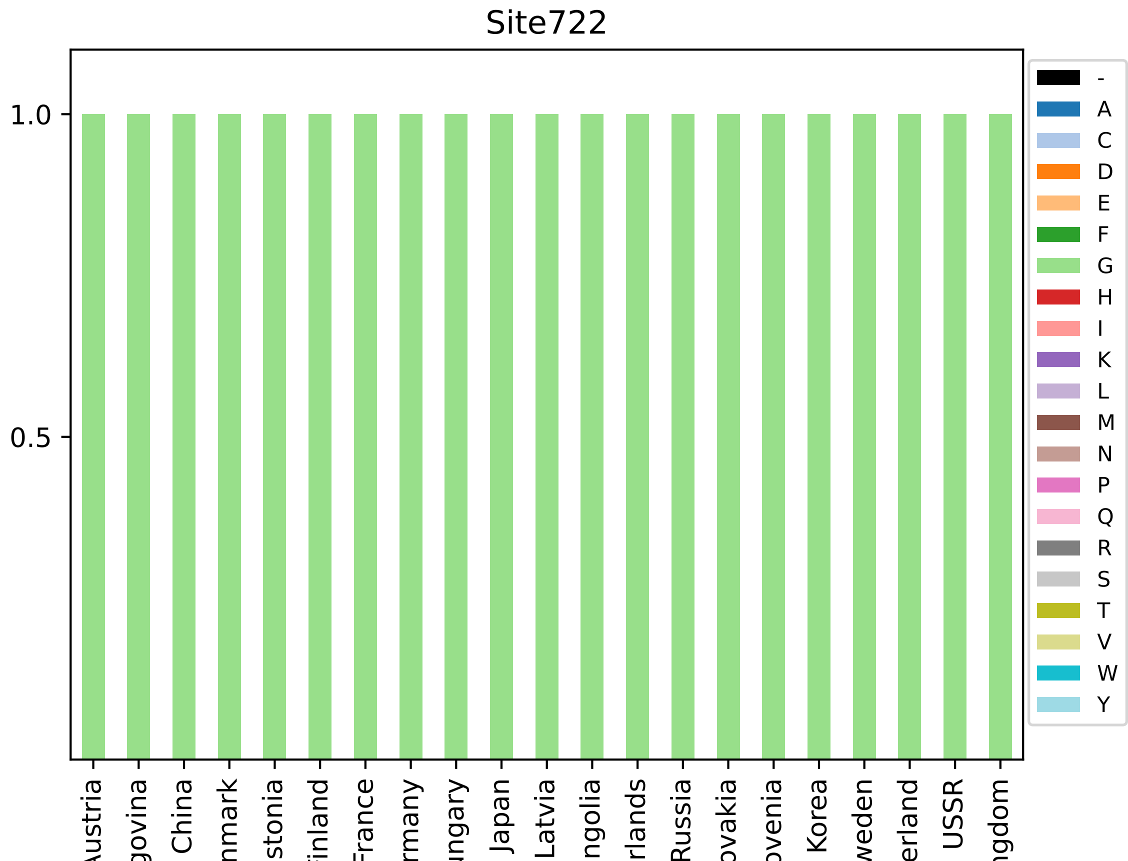
Site722
1.0
0.5
-
A
C
D
E
F
G
H
I
K
L
M
N
P
Q
R
S
T
V
W
Y
Austria
govina
China
nmark
stonia
Finland
France
rmany
ungary
Japan
Latvia
ngolia
rlands
Russia
ovakia
ovenia
Korea
weden
erland
USSR
ngdom

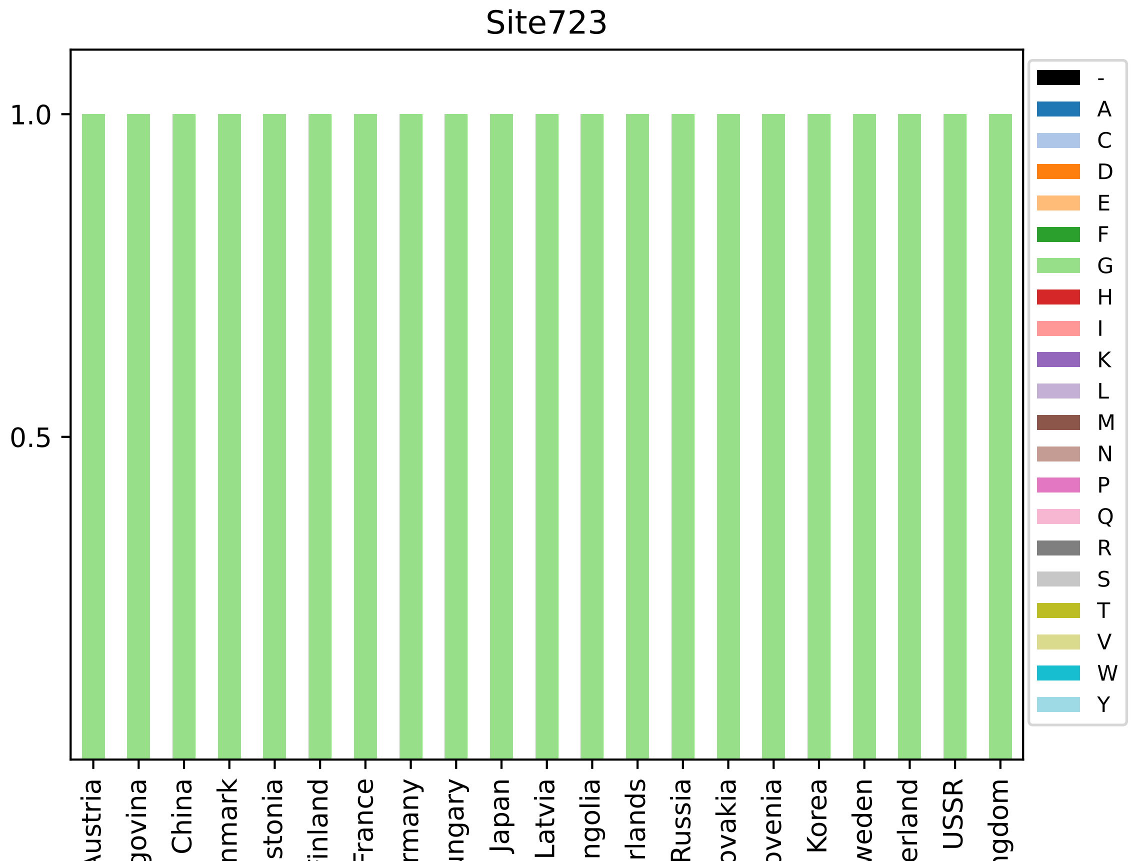
Site723
1.0
0.5
-
A
C
D
E
F
G
H
I
K
L
M
N
P
Q
R
S
T
V
W
Y
Austria
China
Japan
Latvia
Russia
Korea
USSR

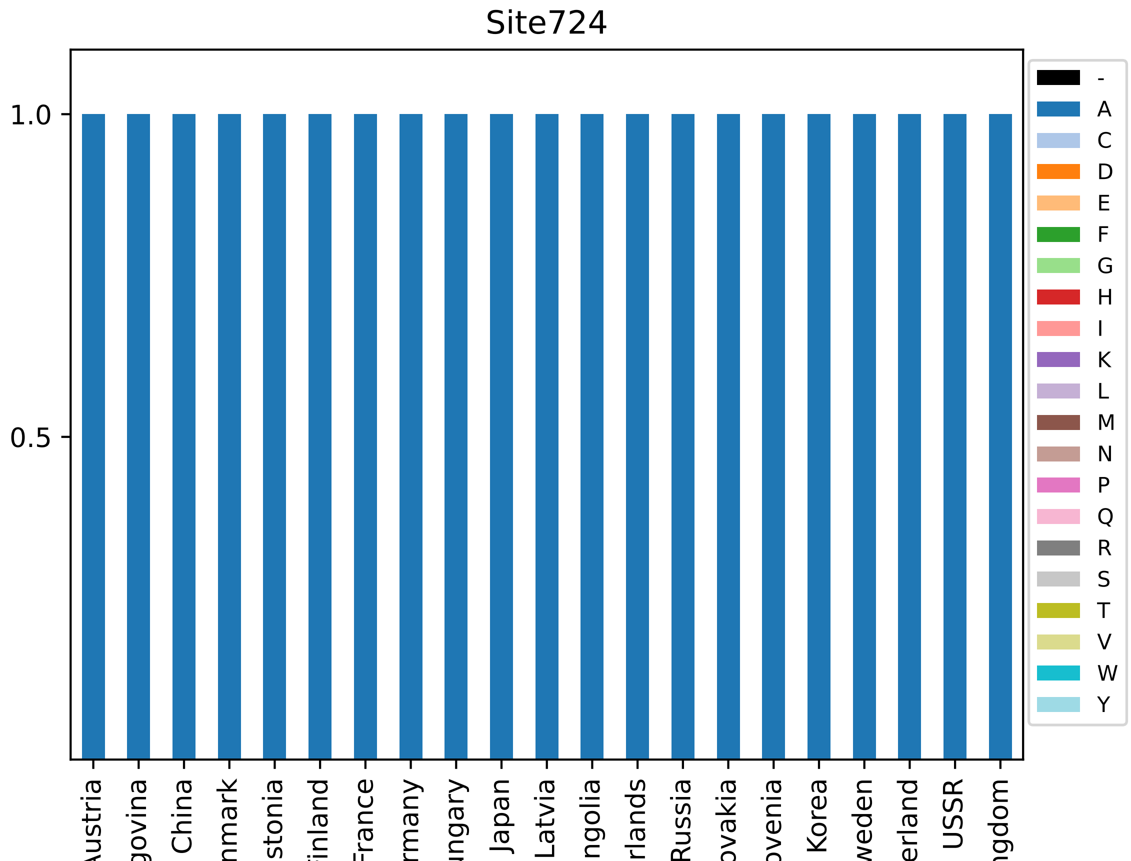
Site724
1.0
0.5
-
A
C
D
E
F
G
H
I
K
L
M
N
P
Q
R
S
T
V
W
Y
China
Japan
Latvia
Russia
Korea
USSR

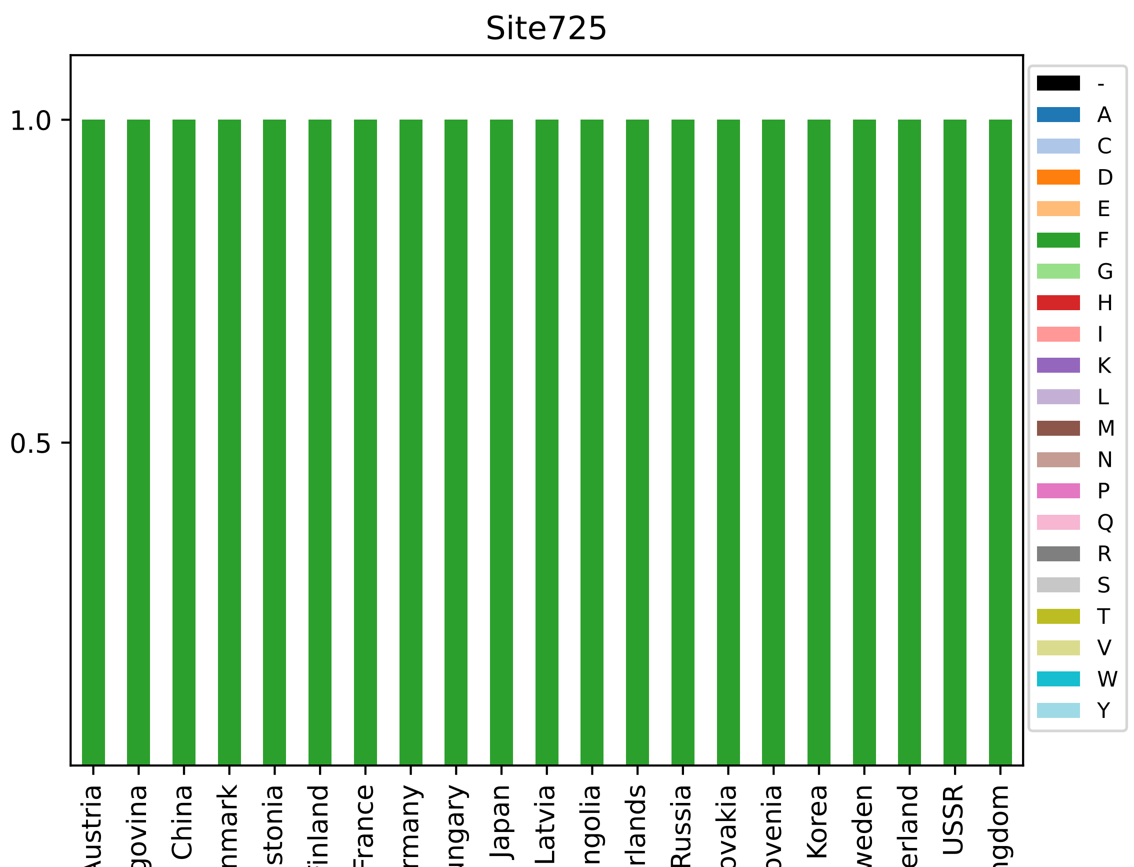
Site725
1.0
0.5
-
A
C
D
E
F
G
H
I
K
L
M
N
P
Q
R
S
T
V
W
Y
Austria
govina
China
nmark
stonia
Finland
France
rmany
ungary
Japan
Latvia
ngolia
rlands
Russia
ovakia
ovenia
Korea
weden
erland
USSR
ngdom

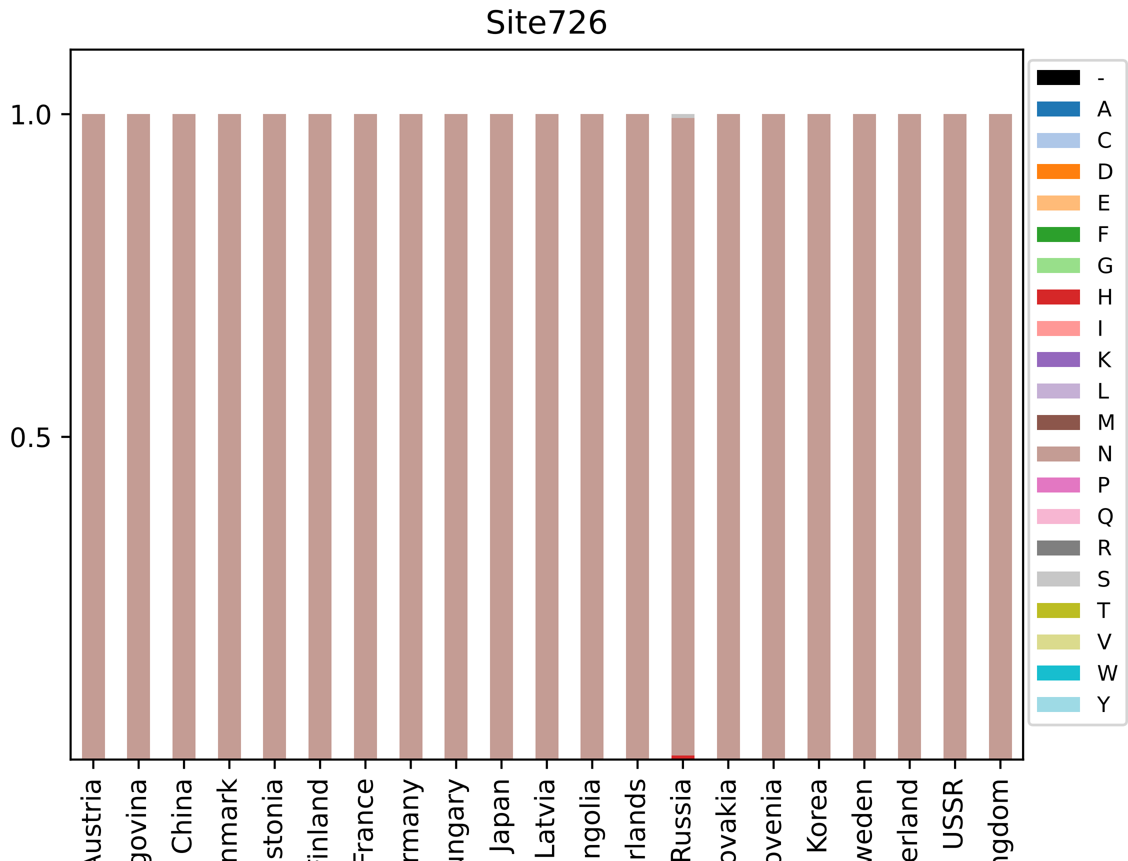
Site726
1.0
0.5
Austria
govina
China
nmark
stonia
Finland
France
rmany
ungary
Japan
Latvia
ngolia
rlands
Russia
ovakia
ovenia
Korea
weden
erland
USSR
ngdom
-
A
C
D
E
F
G
H
I
K
L
M
N
P
Q
R
S
T
V
W
Y

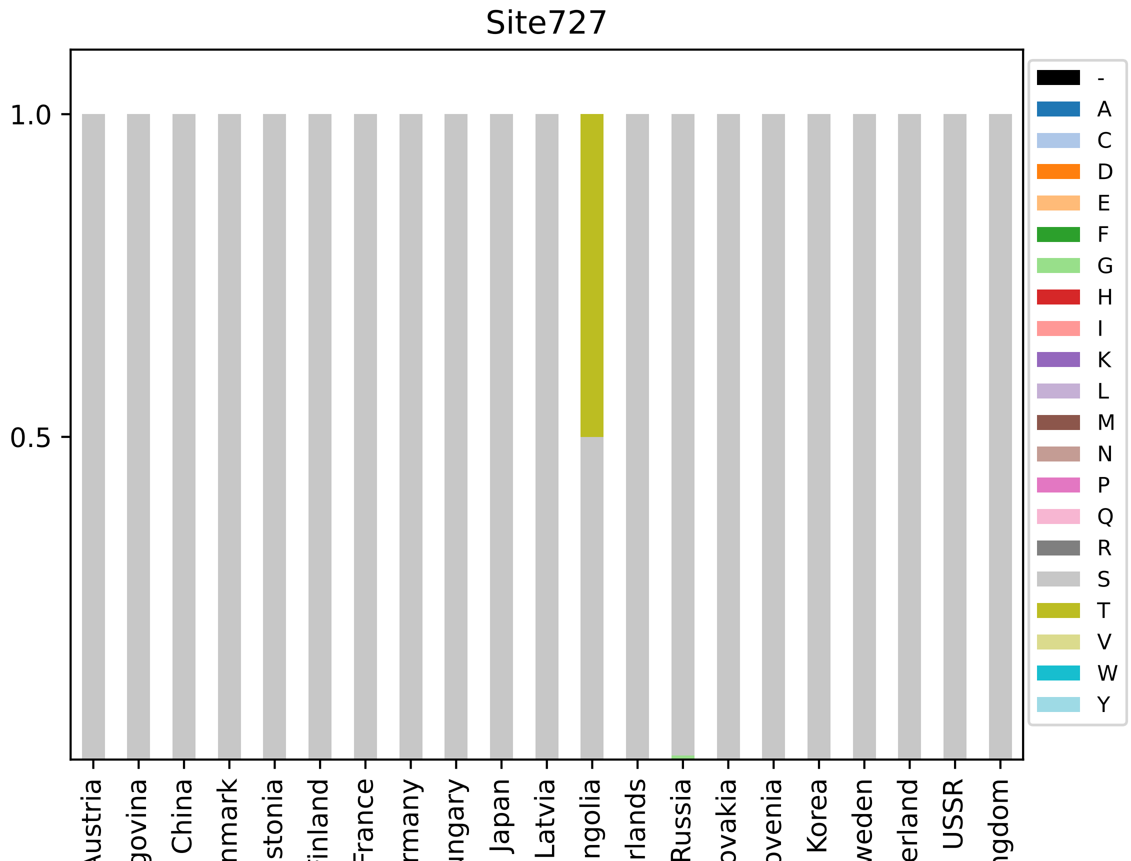

Site727
1.0
0.5
-
A
C
D
E
F
G
H
I
K
L
M
N
P
Q
R
S
T
V
W
Y
Austria
govina
China
nmark
stonia
Finland
France
rmany
ungary
Japan
Latvia
ngolia
rlands
Russia
ovakia
ovenia
Korea
weden
erland
USSR
ngdom

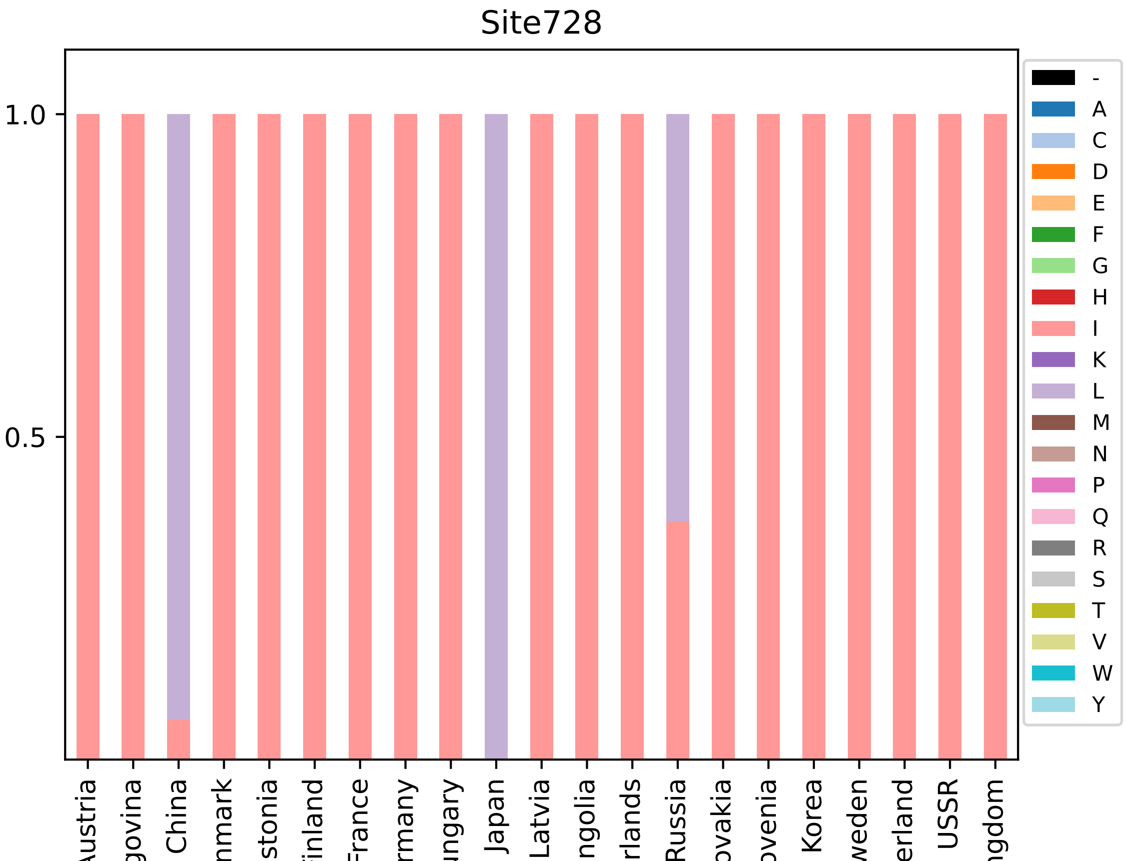

Site728
1.0
0.5
-
A
C
D
E
F
G
H
I
K
L
M
N
P
Q
R
S
T
V
W
Y
China
Japan
Latvia
Russia
Korea
USSR

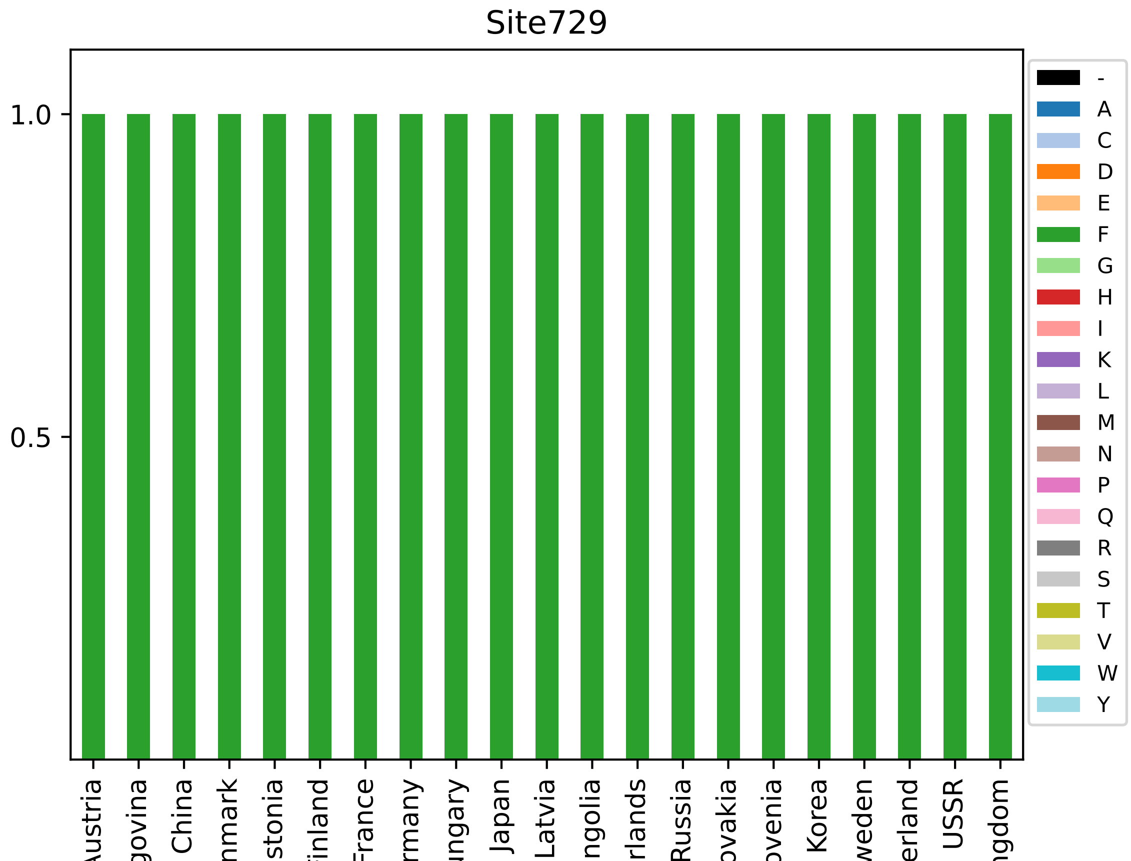
Site729
1.0
0.5
-
A
C
D
E
F
G
H
I
K
L
M
N
P
Q
R
S
T
V
W
Y
Austria
China
Japan
Latvia
Russia
Korea
USSR

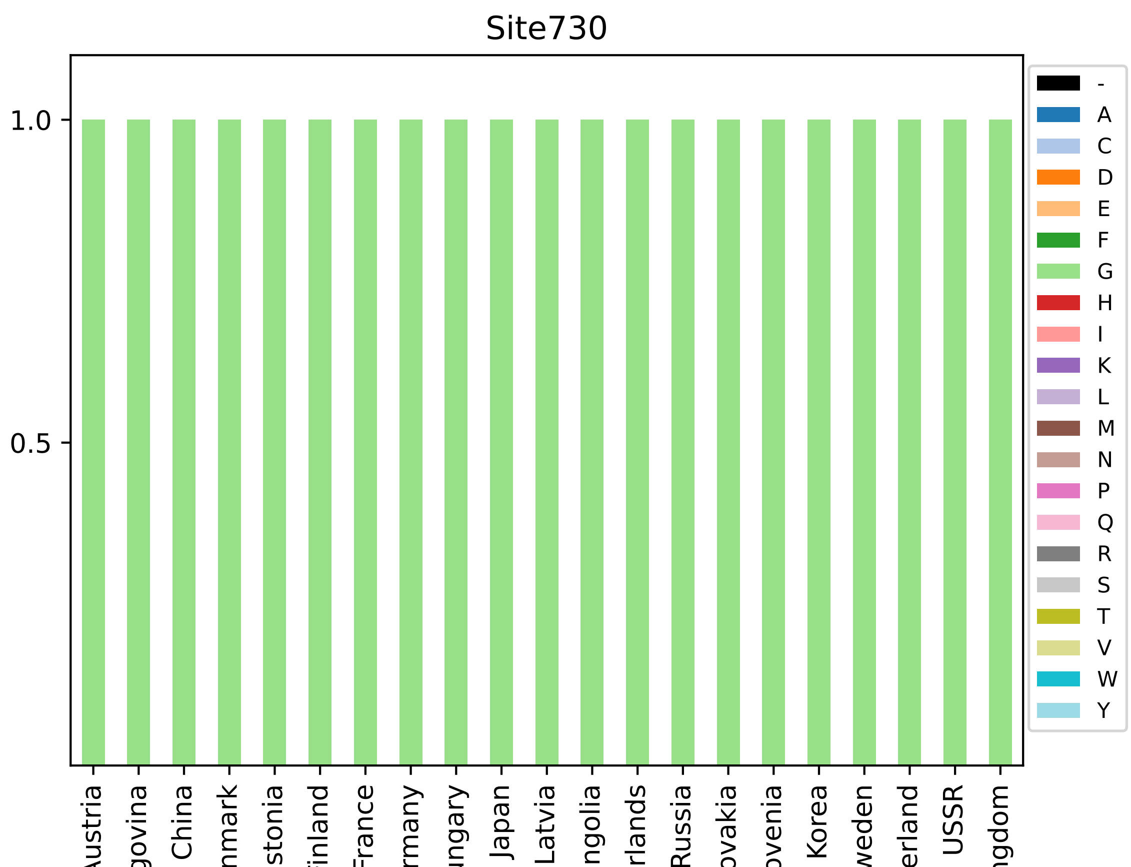

Site730
1.0
0.5
-
A
C
D
E
F
G
H
I
K
L
M
N
P
Q
R
S
T
V
W
Y
Austria
govina
China
nmark
stonia
Finland
France
rmany
ungary
Japan
Latvia
ngolia
rlands
Russia
ovakia
ovenia
Korea
weden
erland
USSR
ngdom

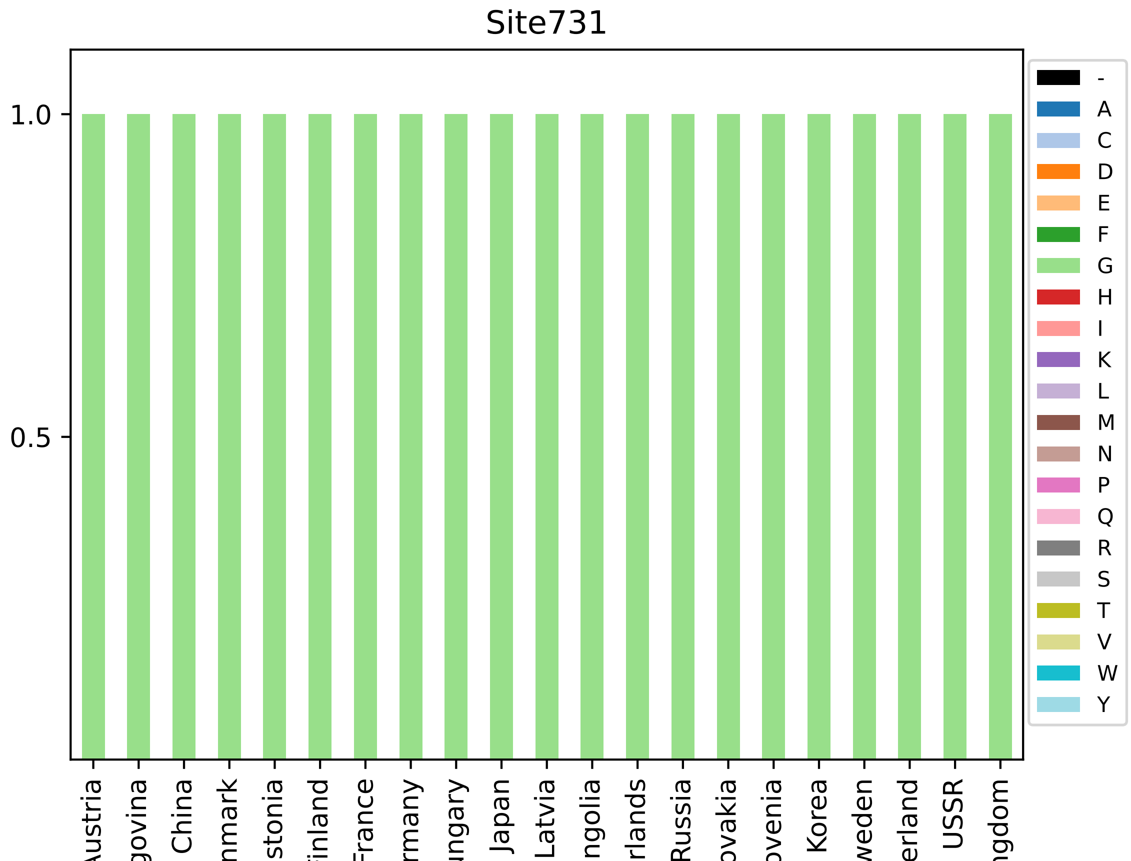
Site731
1.0
0.5
-
A
C
D
E
F
G
H
I
K
L
M
N
P
Q
R
S
T
V
W
Y
Austria
China
Japan
Latvia
Russia
Korea
USSR

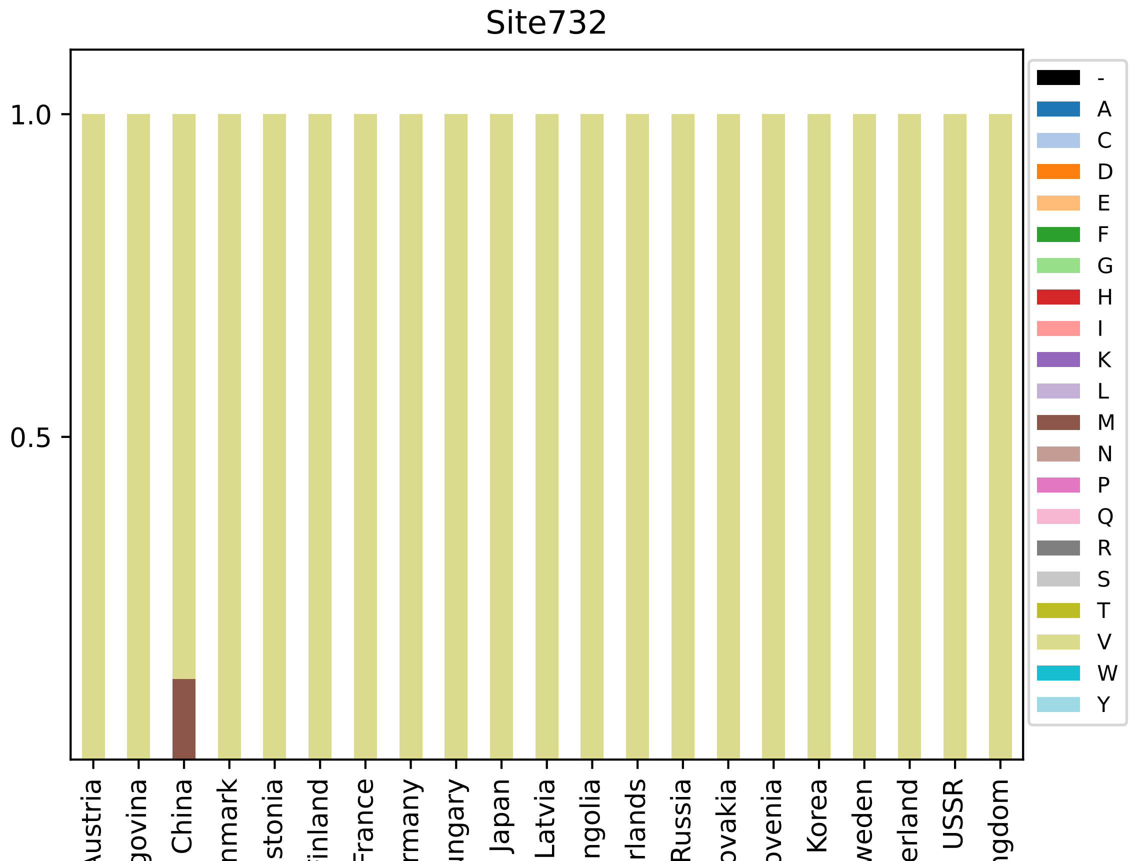
Site732
1.0
0.5
China
Japan
Latvia
Russia
Korea
USSR
-
A
C
D
E
F
G
H
I
K
L
M
N
P
Q
R
S
T
V
W
Y

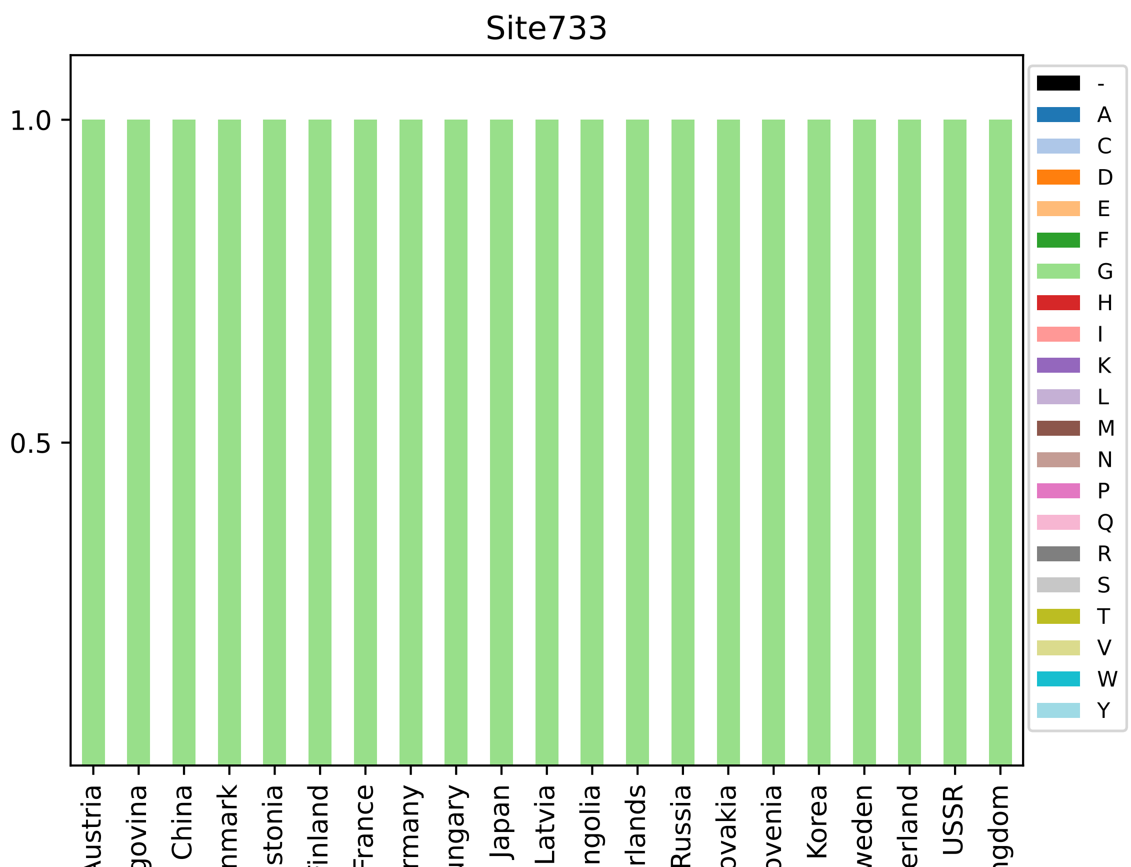

Site733
1.0
0.5
-
A
C
D
E
F
G
H
I
K
L
M
N
P
Q
R
S
T
V
W
Y
China
Japan
Latvia
Russia
Korea
USSR

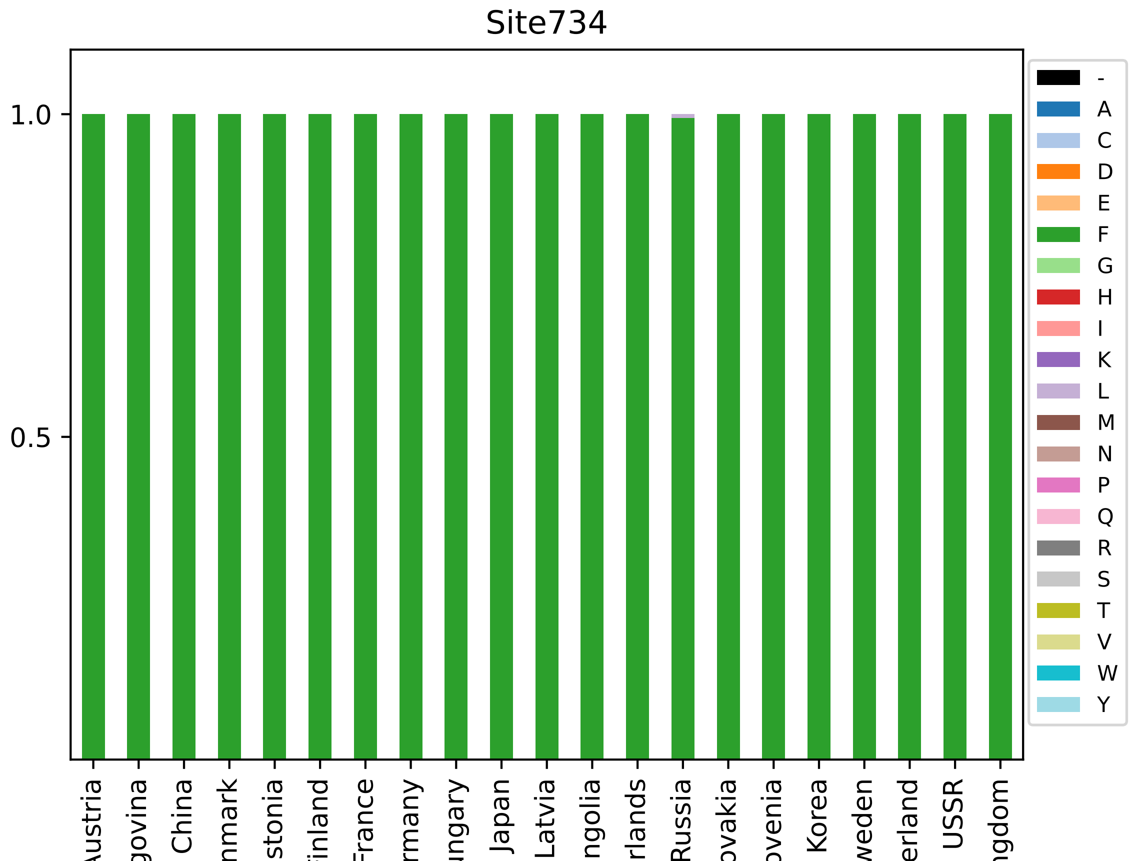
Site734
1.0
0.5
-
A
C
D
E
F
G
H
I
K
L
M
N
P
Q
R
S
T
V
W
Y
Austria
govina
China
nmark
stonia
inland
France
rmany
ungary
Japan
Latvia
ngolia
rlands
Russia
ovakia
ovenia
Korea
weden
erland
USSR
ngdom

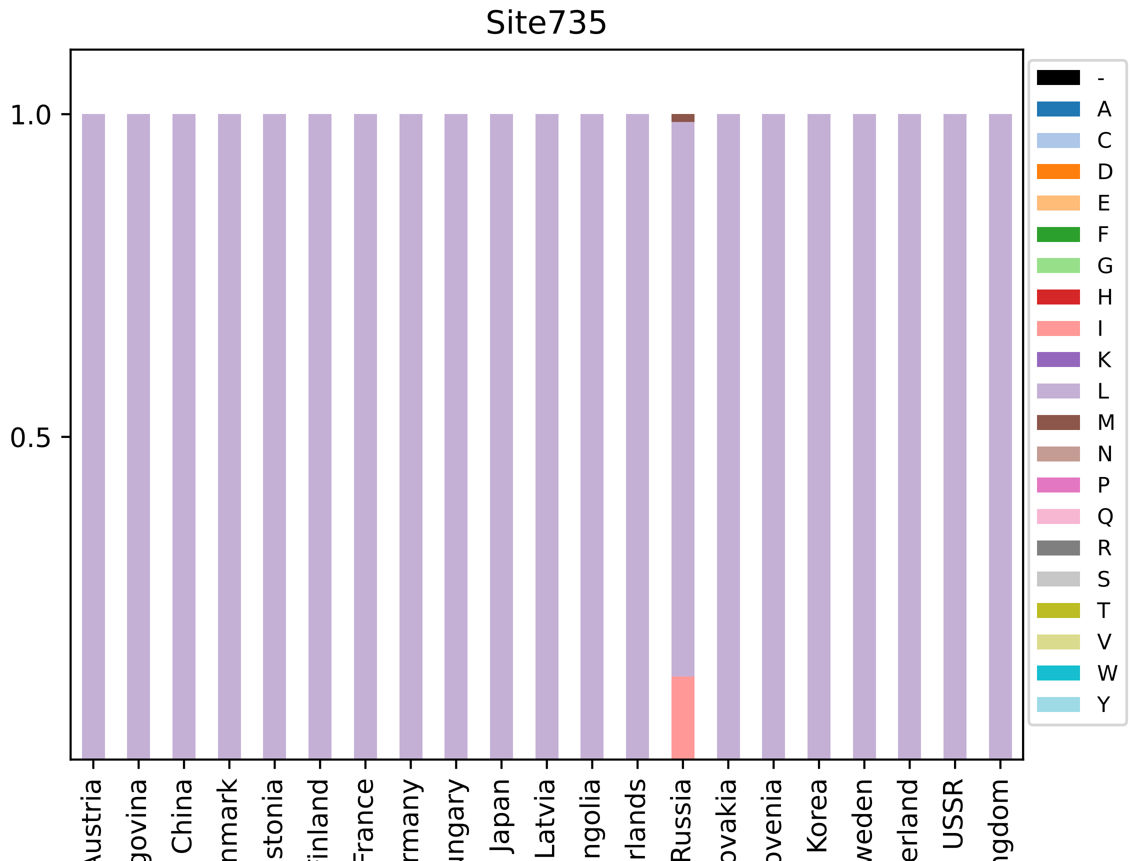
Site735
1.0
0.5
-
A
C
D
E
F
G
H
I
K
L
M
N
P
Q
R
S
T
V
W
Y
Austria
China
Japan
Latvia
Russia
Korea
USSR

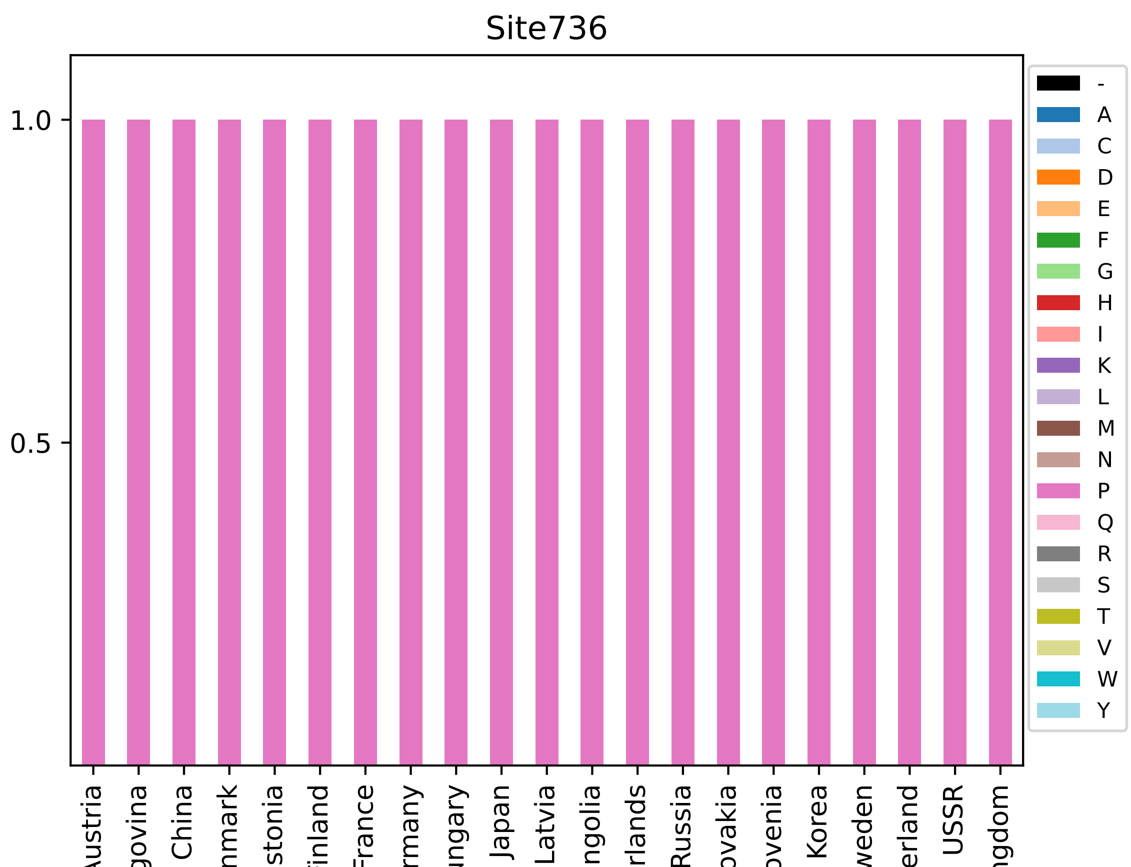

Site736
1.0
0.5
-
A
C
D
E
F
G
H
I
K
L
M
N
P
Q
R
S
T
V
W
Y
Austria
govina
China
nmark
stonia
Finland
France
rmany
ungary
Japan
Latvia
ngolia
rlands
Russia
ovakia
ovenia
Korea
weden
erland
USSR
ngdom

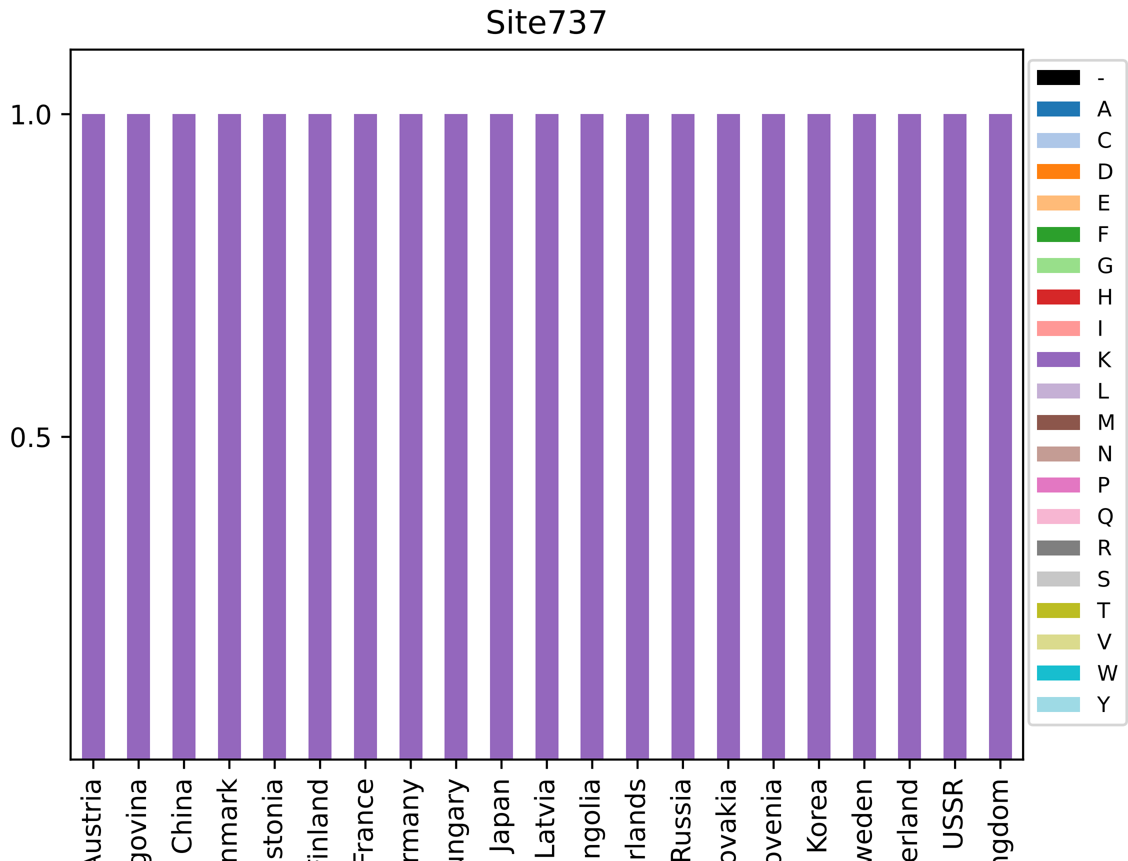

Site737
1.0
0.5
-
A
C
D
E
F
G
H
I
K
L
M
N
P
Q
R
S
T
V
W
Y
Austria
govina
China
nmark
stonia
inland
France
rmany
ungary
Japan
Latvia
ngolia
rlands
Russia
ovakia
ovenia
Korea
weden
erland
USSR
ngdom

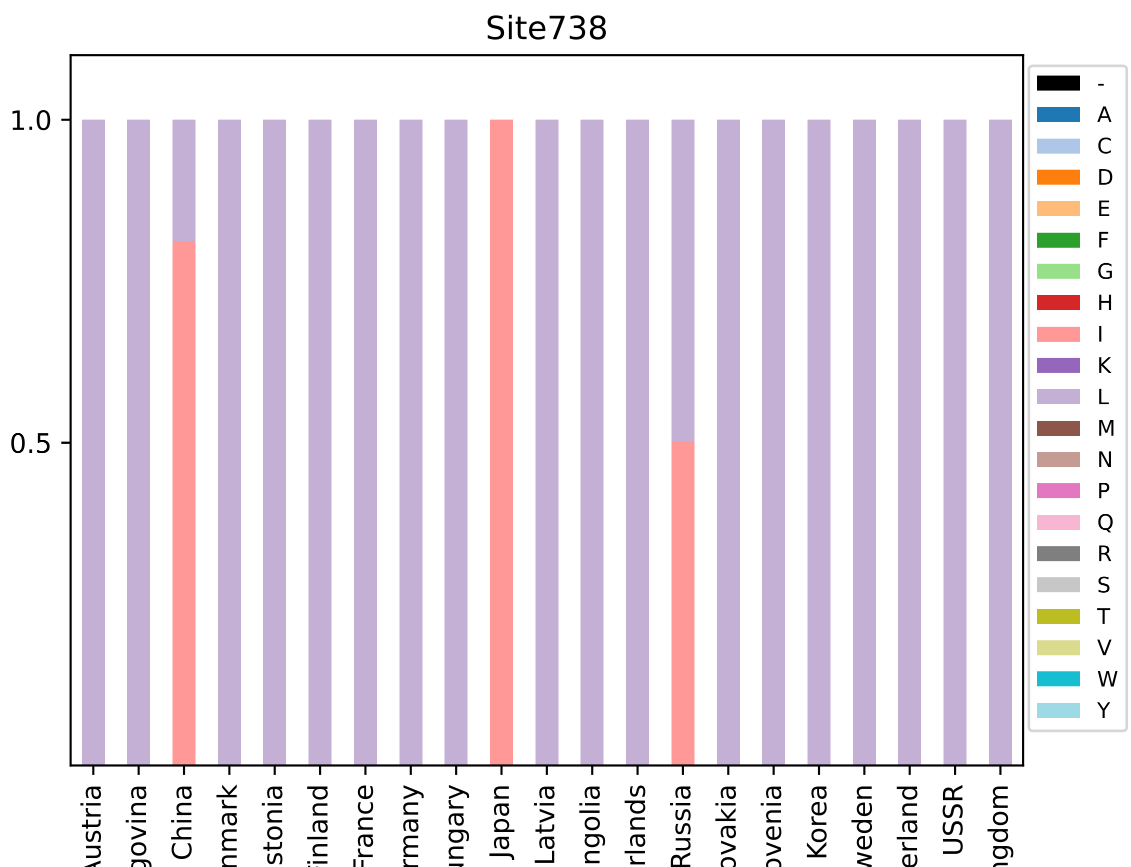
Site738
1.0
0.5
-
A
C
D
E
F
G
H
I
K
L
M
N
P
Q
R
S
T
V
W
Y
Austria
govina
China
nmark
stonia
inland
France
rmany
ungary
Japan
Latvia
ngolia
rlands
Russia
ovakia
ovenia
Korea
weden
erland
USSR
ngdom

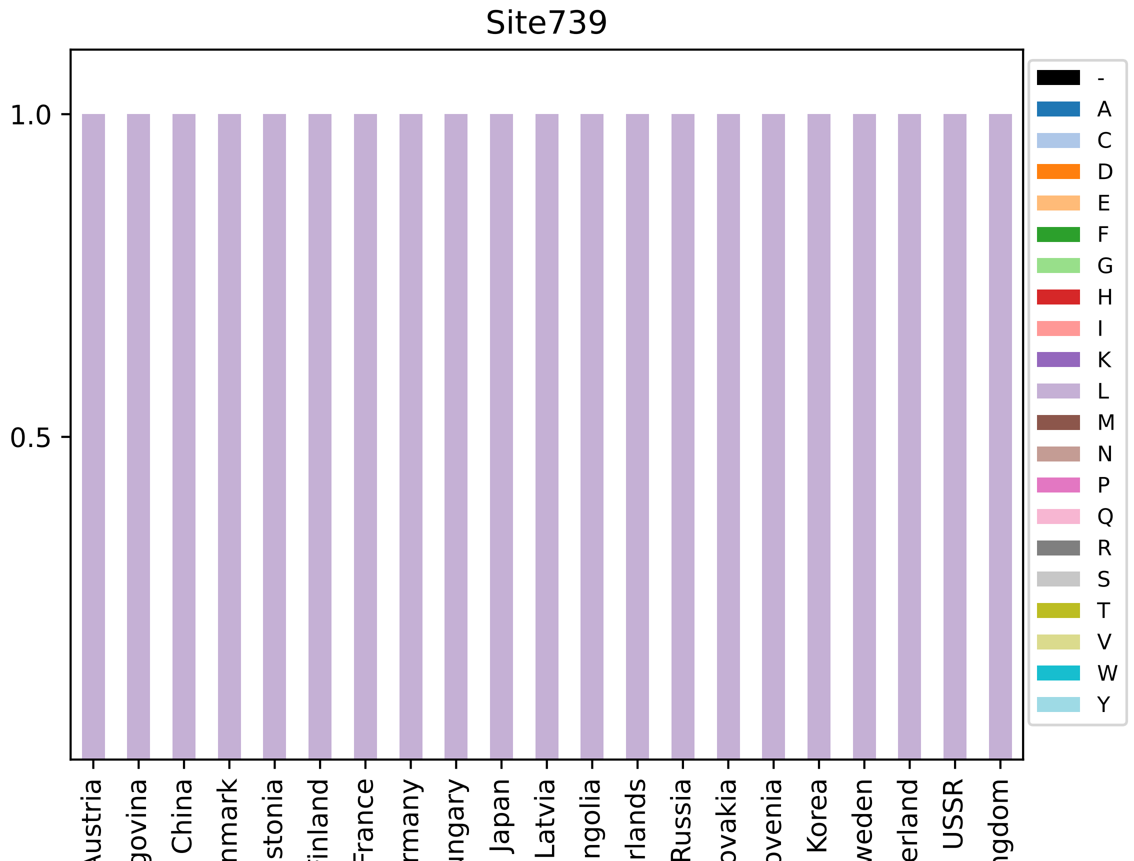

Site739
1.0
0.5
-
A
C
D
E
F
G
H
I
K
L
M
N
P
Q
R
S
T
V
W
Y
China
Japan
Latvia
Russia
Korea
USSR

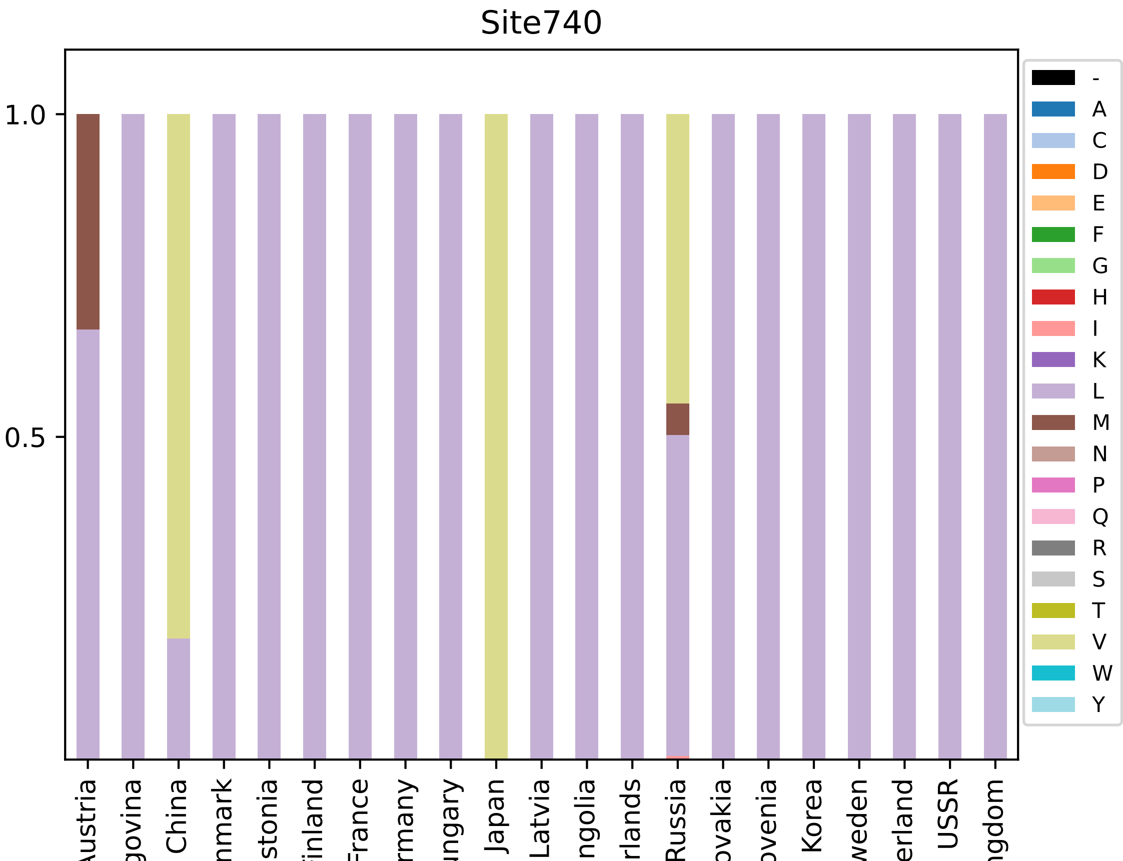

Site740
1.0
0.5
-
A
C
D
E
F
G
H
I
K
L
M
N
P
Q
R
S
T
V
W
Y
China
Japan
Latvia
Russia
Korea
USSR

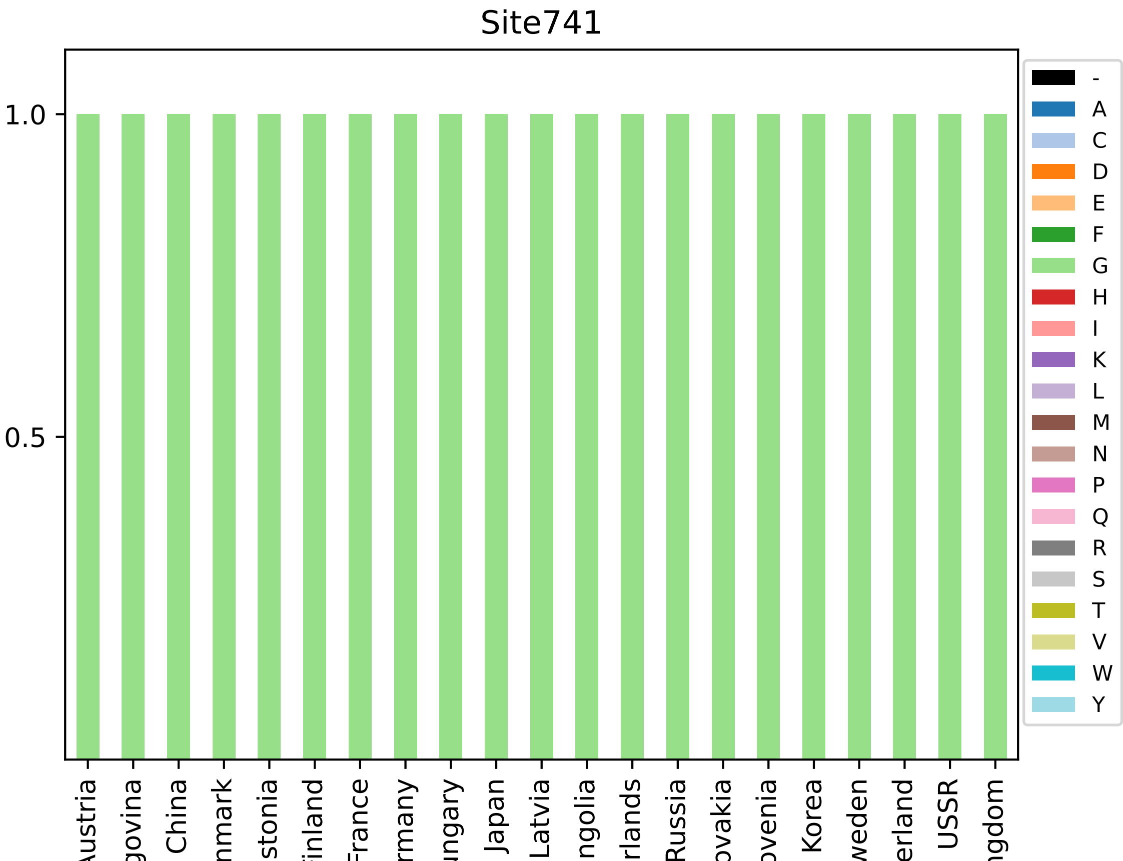

Site741
1.0
0.5
-
A
C
D
E
F
G
H
I
K
L
M
N
P
Q
R
S
T
V
W
Y
Austria
govina
China
nmark
stonia
Finland
France
rmany
ungary
Japan
Latvia
ngolia
rlands
Russia
ovakia
ovenia
Korea
weden
erland
USSR
ngdom

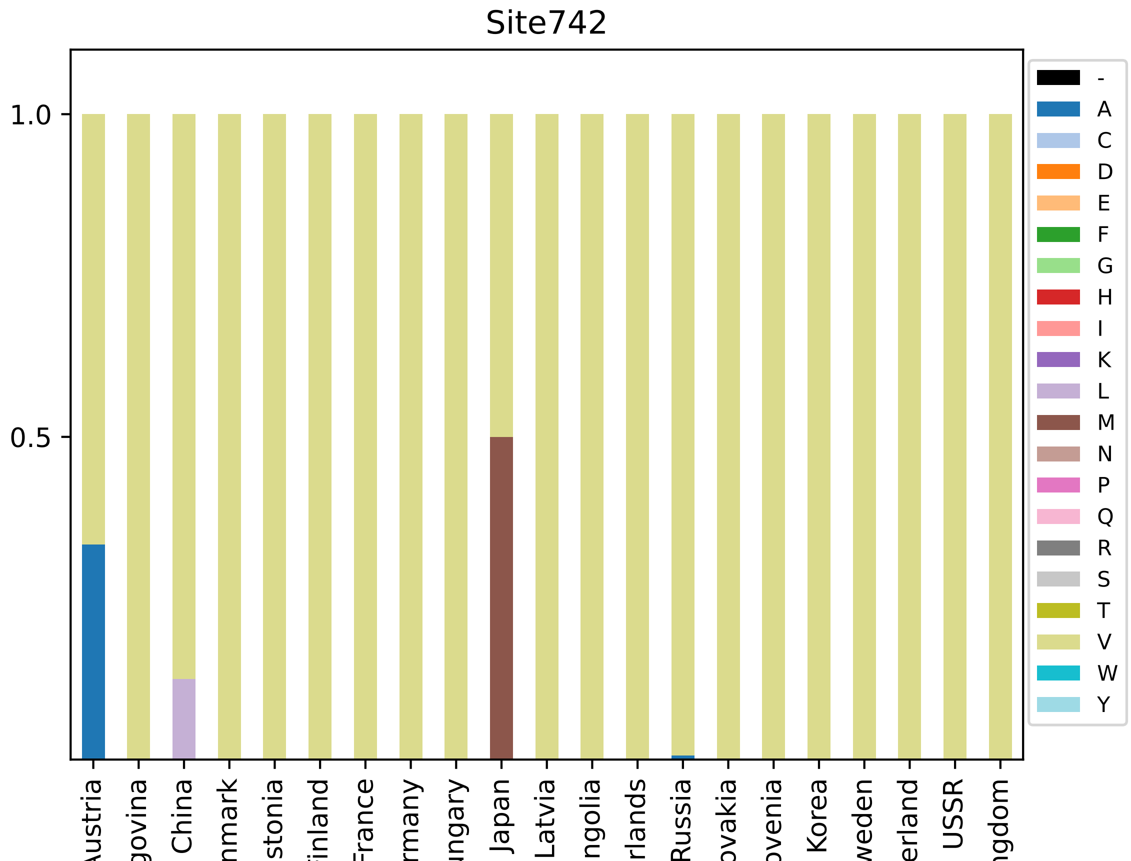

Site742
1.0
0.5
-
A
C
D
E
F
G
H
I
K
L
M
N
P
Q
R
S
T
V
W
Y
Austria
govina
China
nmark
stonia
inland
France
rmany
ungary
Japan
Latvia
ngolia
rlands
Russia
ovakia
ovenia
Korea
weden
erland
USSR
ngdom

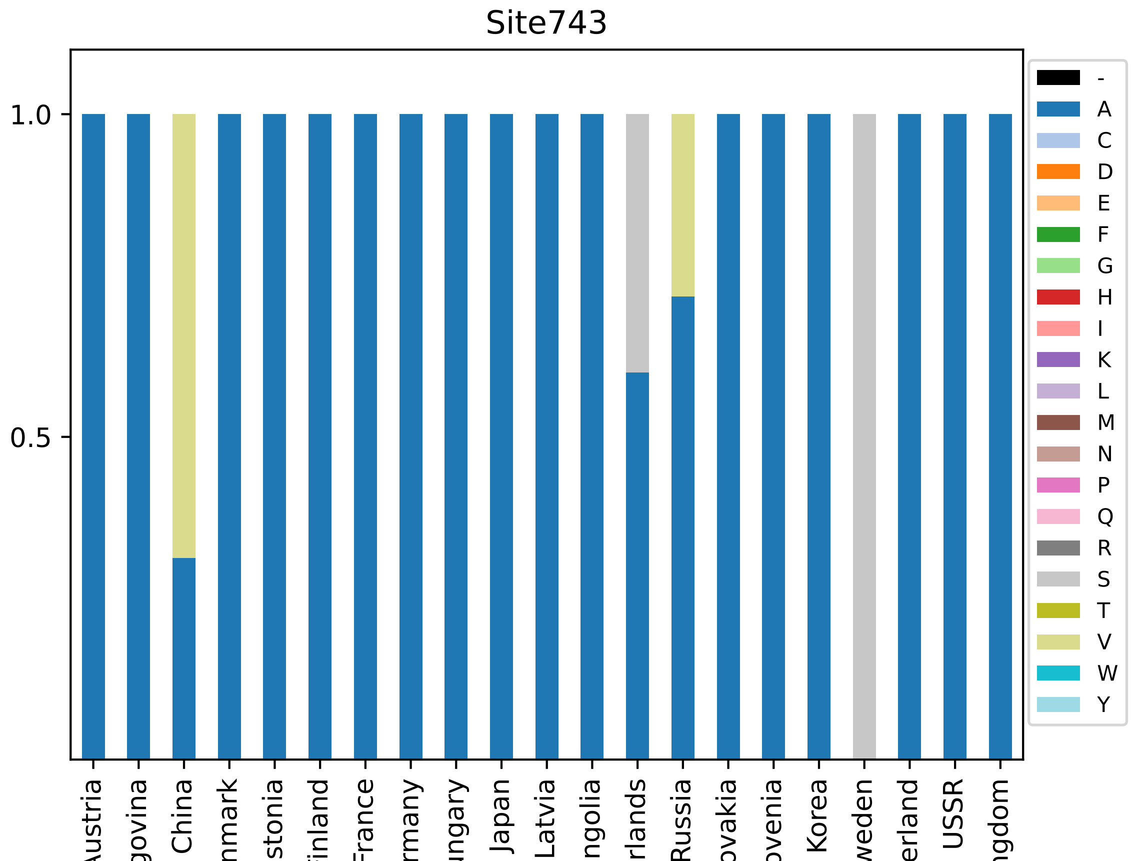
Site743
1.0
0.5
-
A
C
D
E
F
G
H
I
K
L
M
N
P
Q
R
S
T
V
W
Y
China
Japan
Latvia
Russia
Korea
USSR

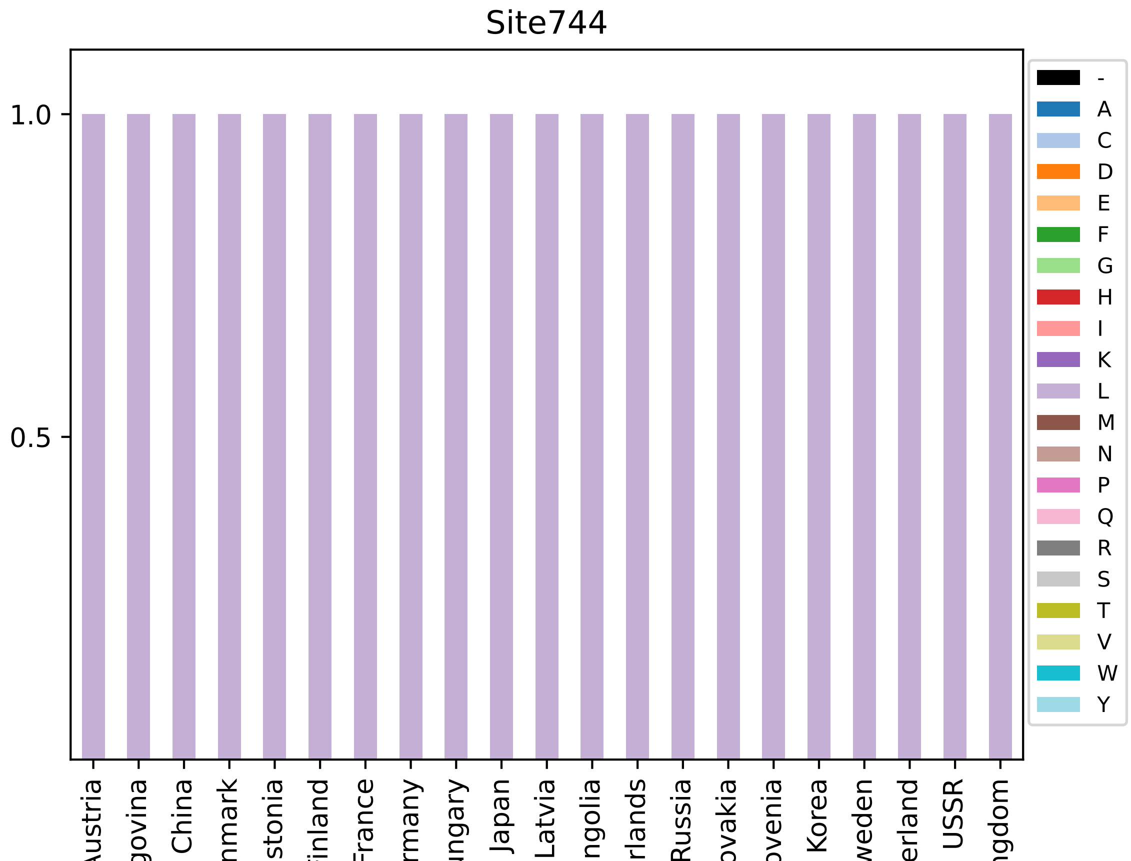

Site744
1.0
0.5
-
A
C
D
E
F
G
H
I
K
L
M
N
P
Q
R
S
T
V
W
Y
Austria
govina
China
nmark
stonia
Finland
France
rmany
ungary
Japan
Latvia
ngolia
rlands
Russia
ovakia
ovenia
Korea
weden
erland
USSR
ngdom

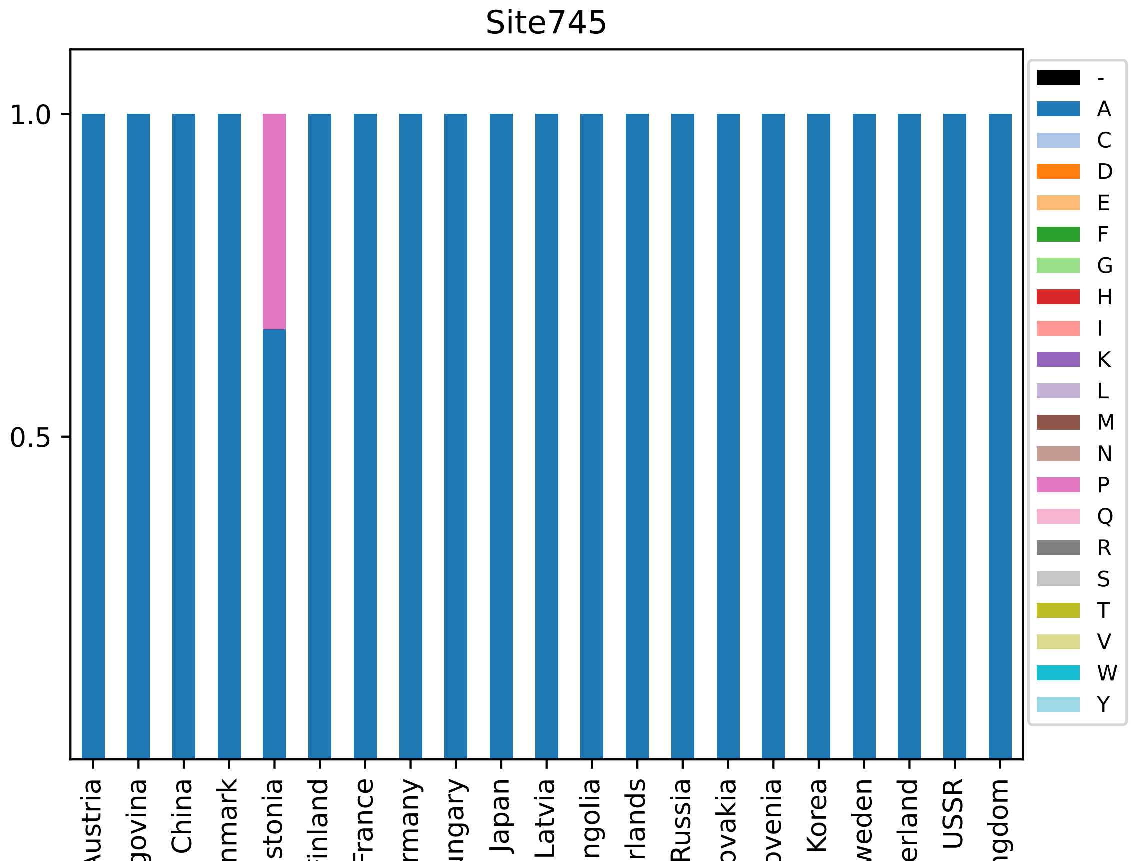

Site745
1.0
0.5
-
A
C
D
E
F
G
H
I
K
L
M
N
P
Q
R
S
T
V
W
Y
China
Japan
Latvia
Russia
Korea
USSR

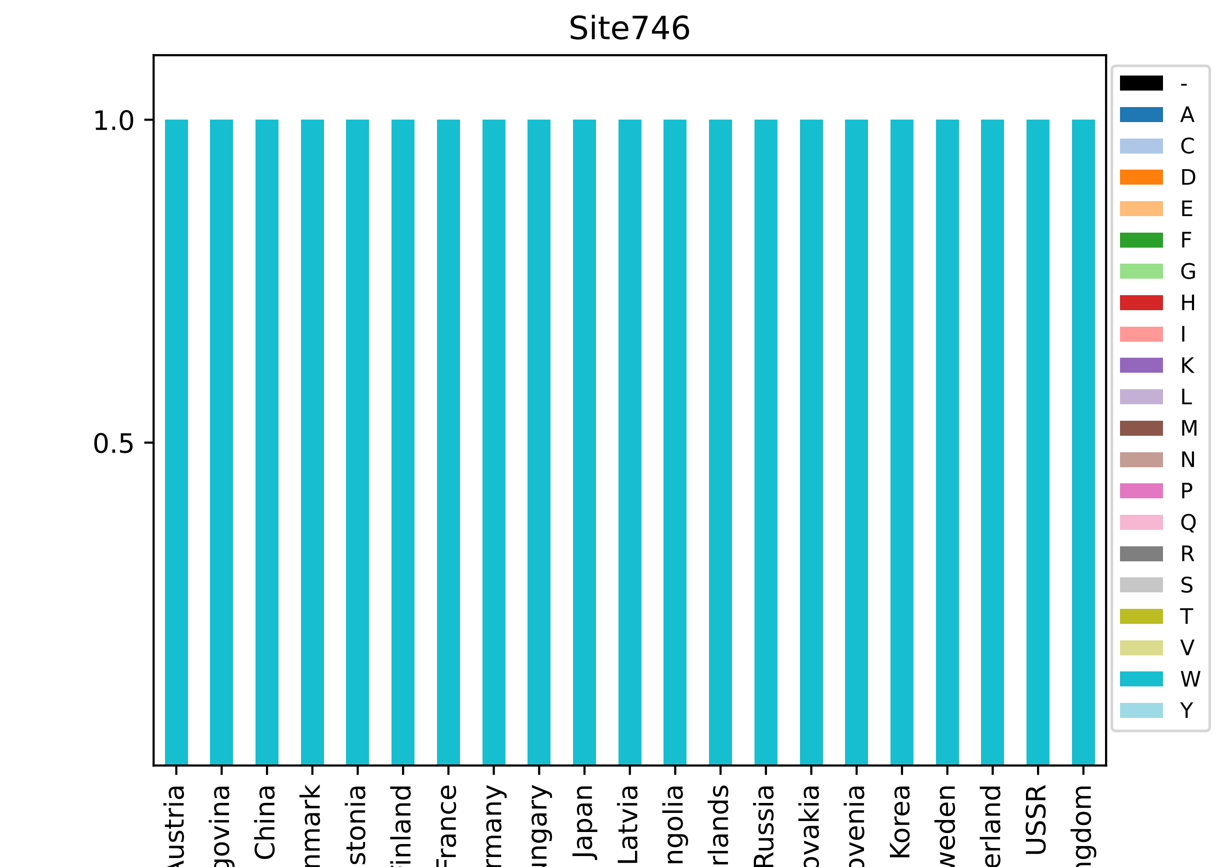

Site746
1.0
0.5
-
A
C
D
E
F
G
H
I
K
L
M
N
P
Q
R
S
T
V
W
Y
Austria
govina
China
nmark
stonia
Finland
France
rmany
ungary
Japan
Latvia
ngolia
rlands
Russia
ovakia
ovenia
Korea
weden
erland
USSR
ngdom

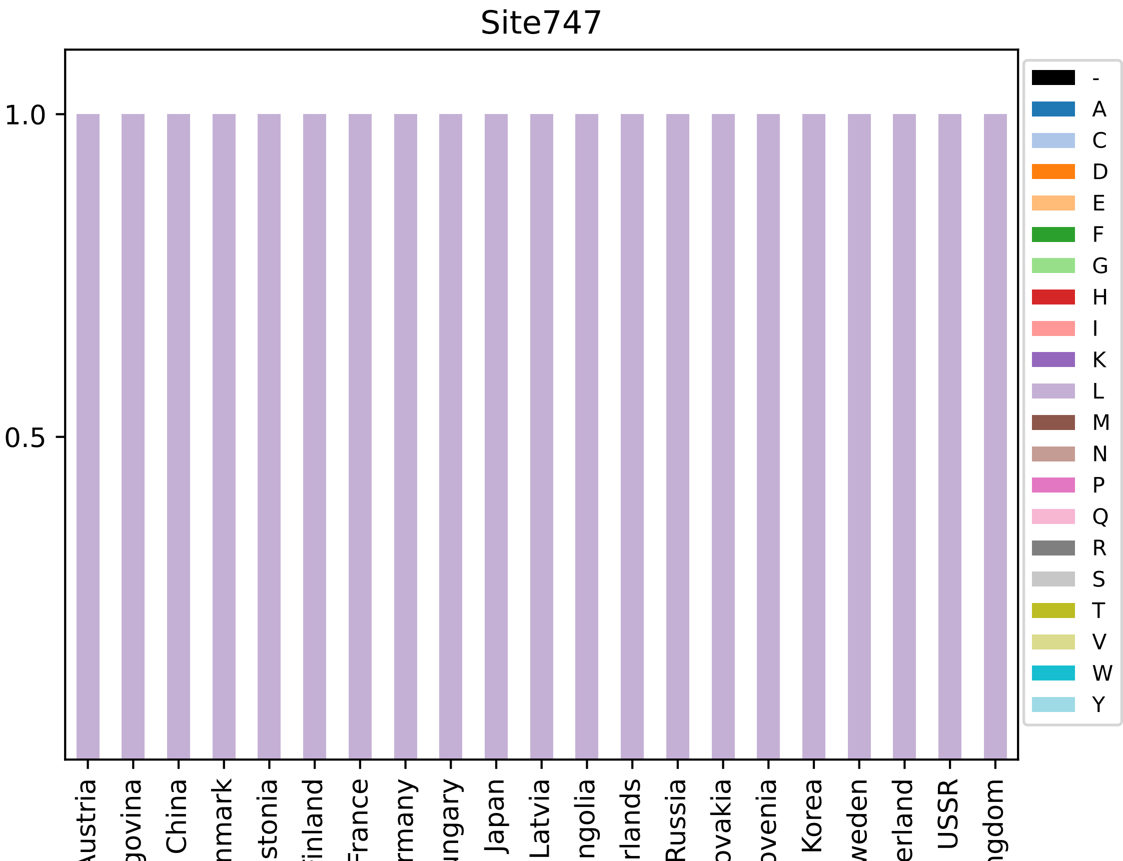

Site747
1.0
0.5
-
A
C
D
E
F
G
H
I
K
L
M
N
P
Q
R
S
T
V
W
Y
Austria
China
Japan
Latvia
Russia
Korea
USSR

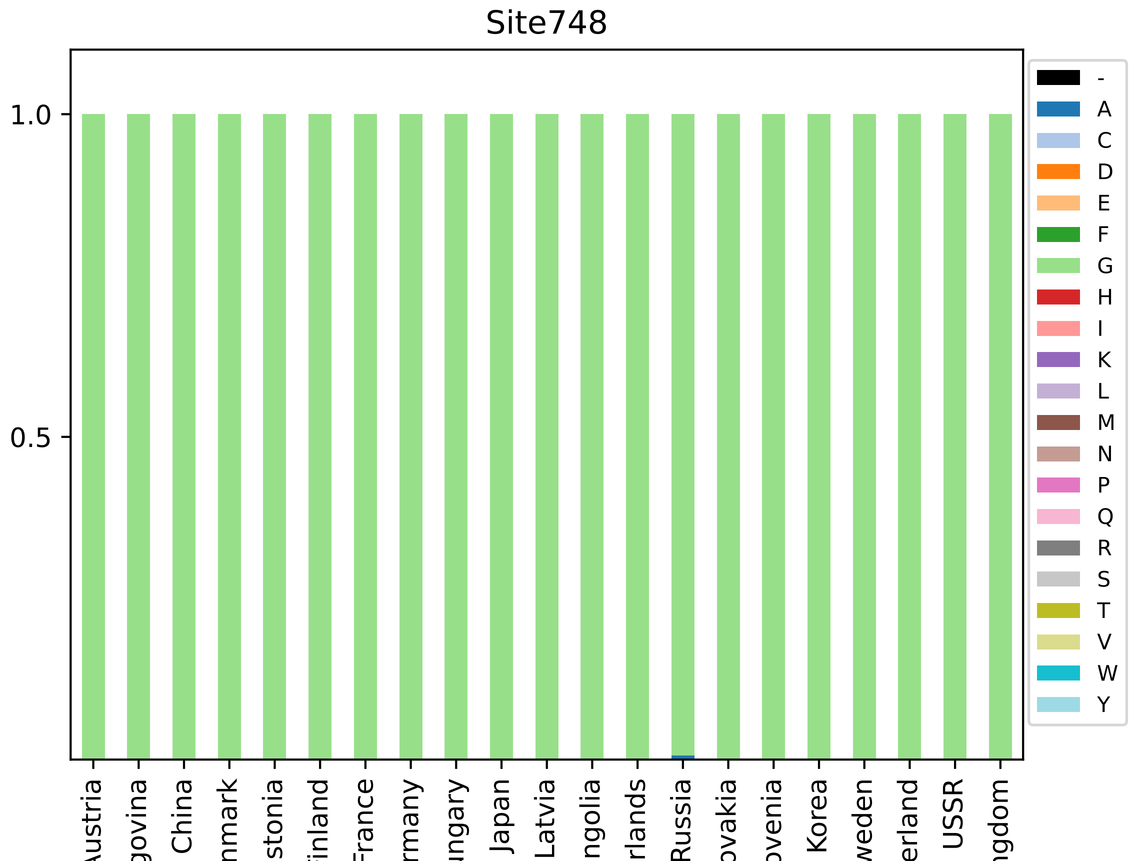

Site748
1.0
0.5
-
A
C
D
E
F
G
H
I
K
L
M
N
P
Q
R
S
T
V
W
Y
China
Japan
Latvia
Russia
Korea
USSR

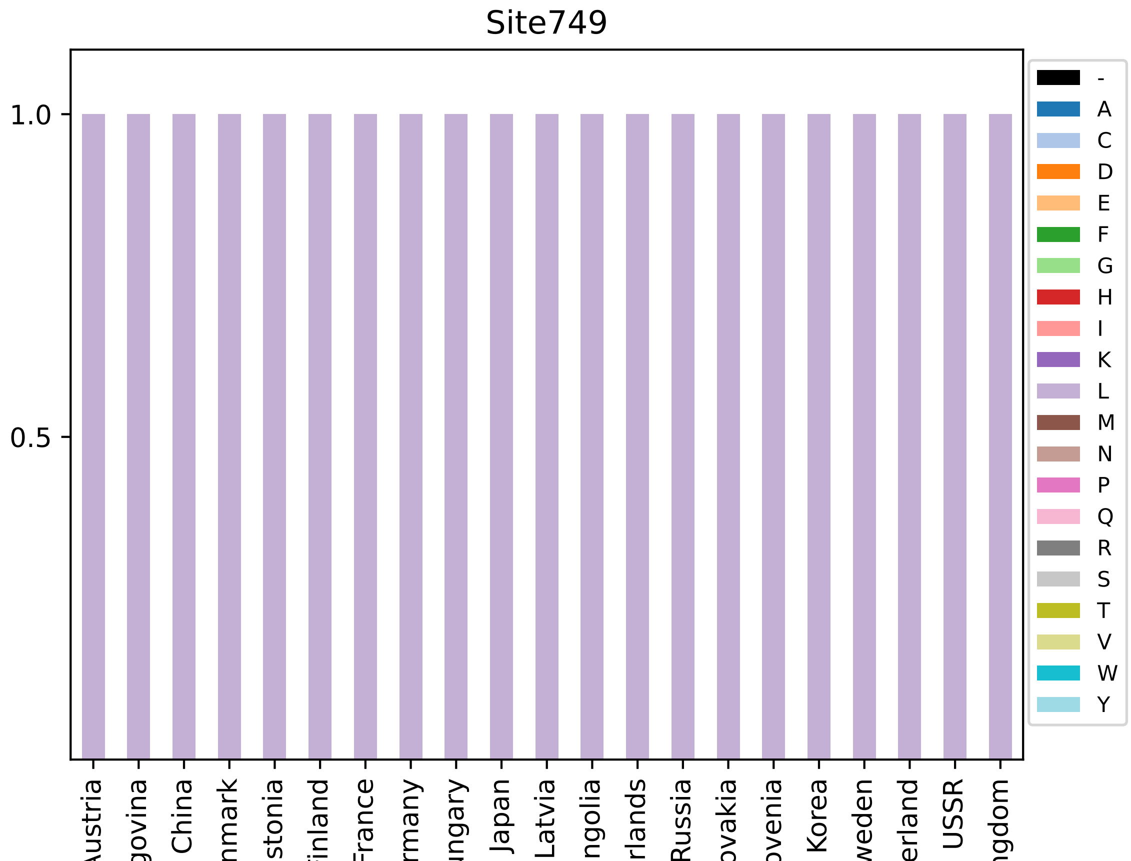
Site749
1.0
0.5
-
A
C
D
E
F
G
H
I
K
L
M
N
P
Q
R
S
T
V
W
Y
Austria
govina
China
nmark
stonia
Finland
France
rmany
ungary
Japan
Latvia
ngolia
rlands
Russia
ovakia
ovenia
Korea
weden
erland
USSR
ngdom

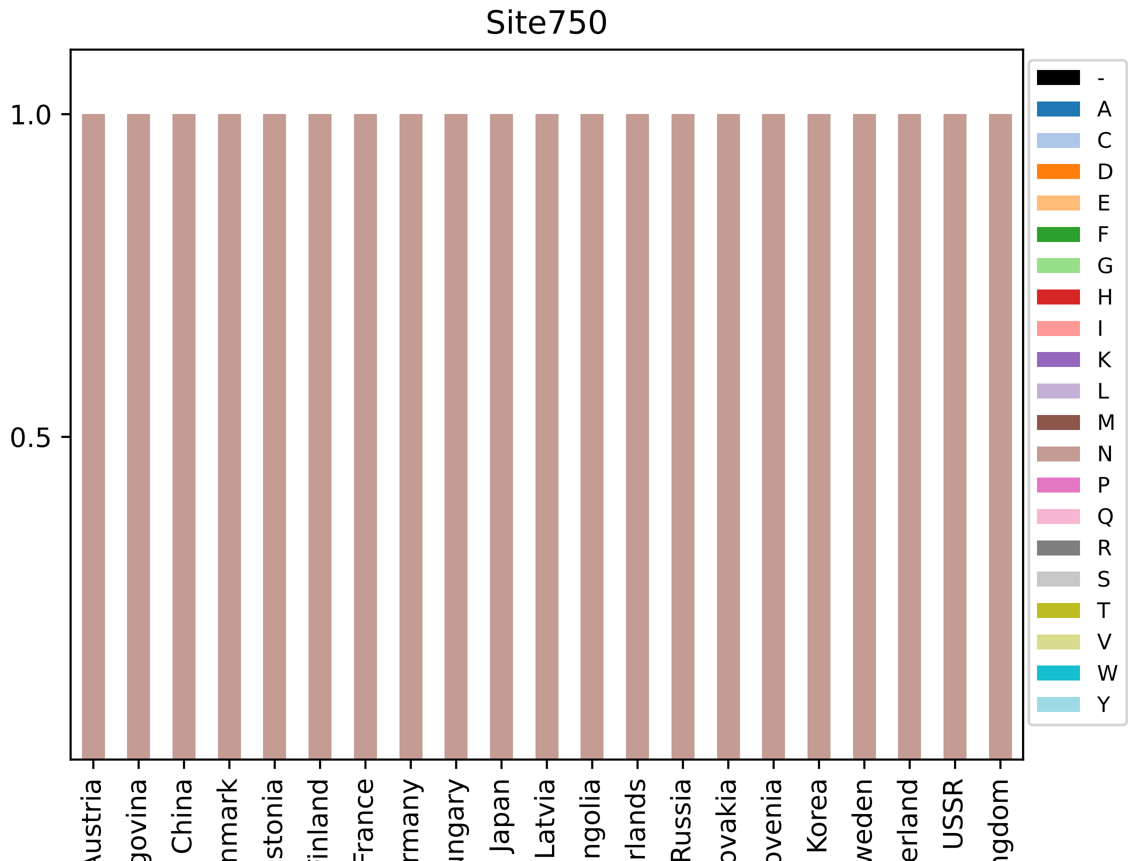
Site750
1.0
0.5
-
A
C
D
E
F
G
H
I
K
L
M
N
P
Q
R
S
T
V
W
Y
China
Japan
Latvia
Russia
Korea
USSR

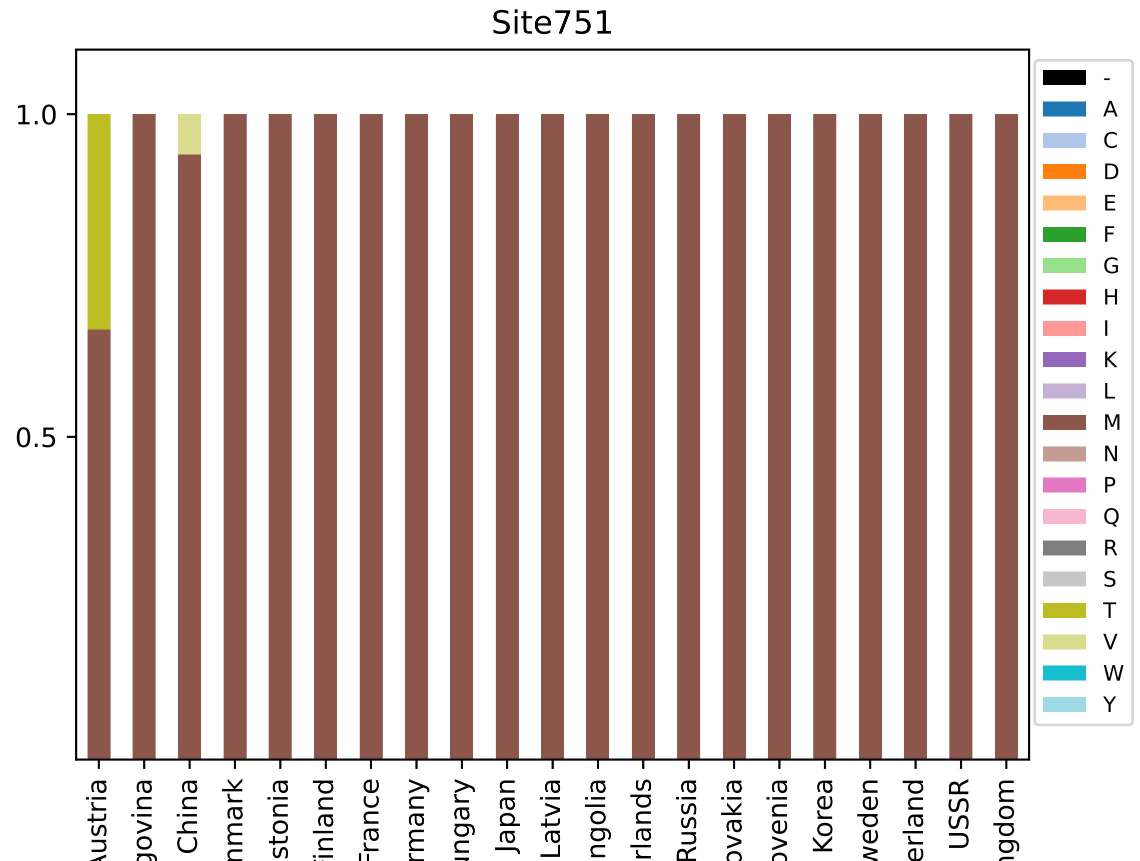
Site751
1.0
0.5
Austria
govina
China
nmark
stonia
inland
France
rmany
ungary
Japan
Latvia
ngolia
rlands
Russia
ovakia
ovenia
Korea
weden
erland
USSR
ngdom
-
A
C
D
E
F
G
H
I
K
L
M
N
P
Q
R
S
T
V
W
Y

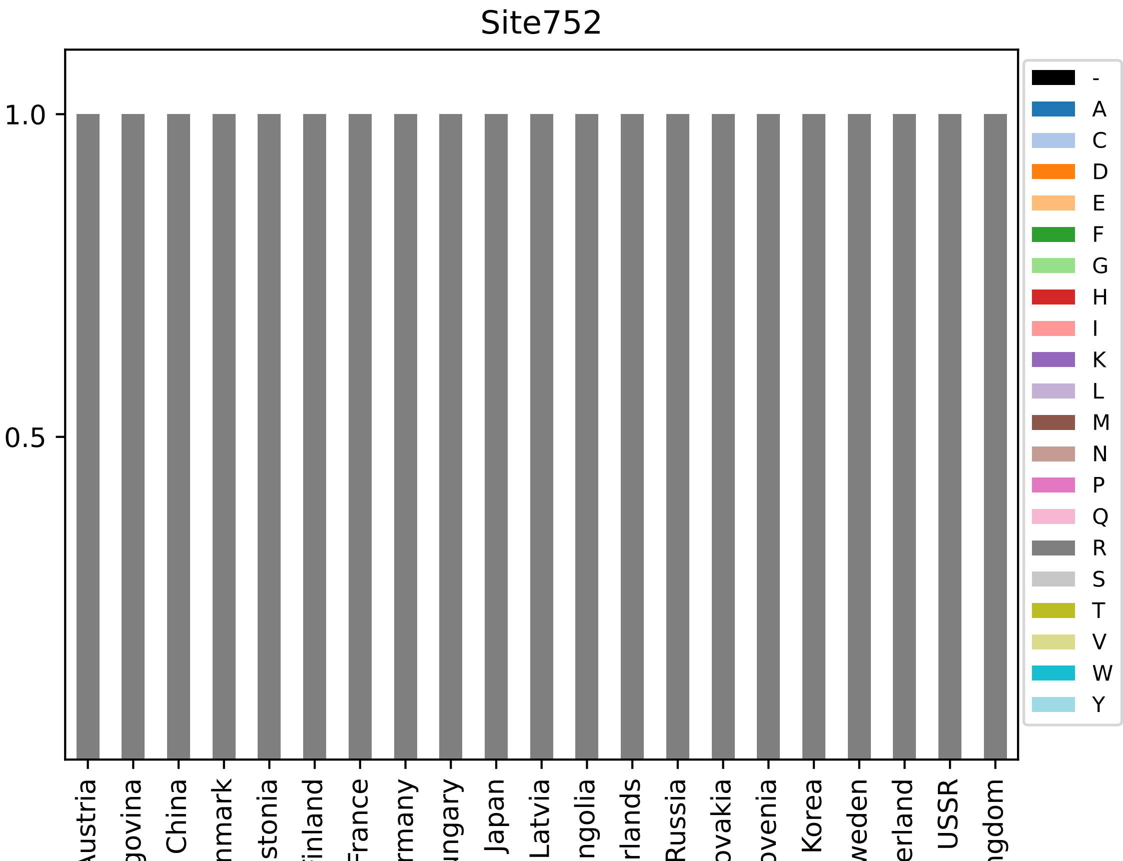

Site752
1.0
0.5
-
A
C
D
E
F
G
H
I
K
L
M
N
P
Q
R
S
T
V
W
Y
China
Japan
Latvia
Russia
Korea
USSR

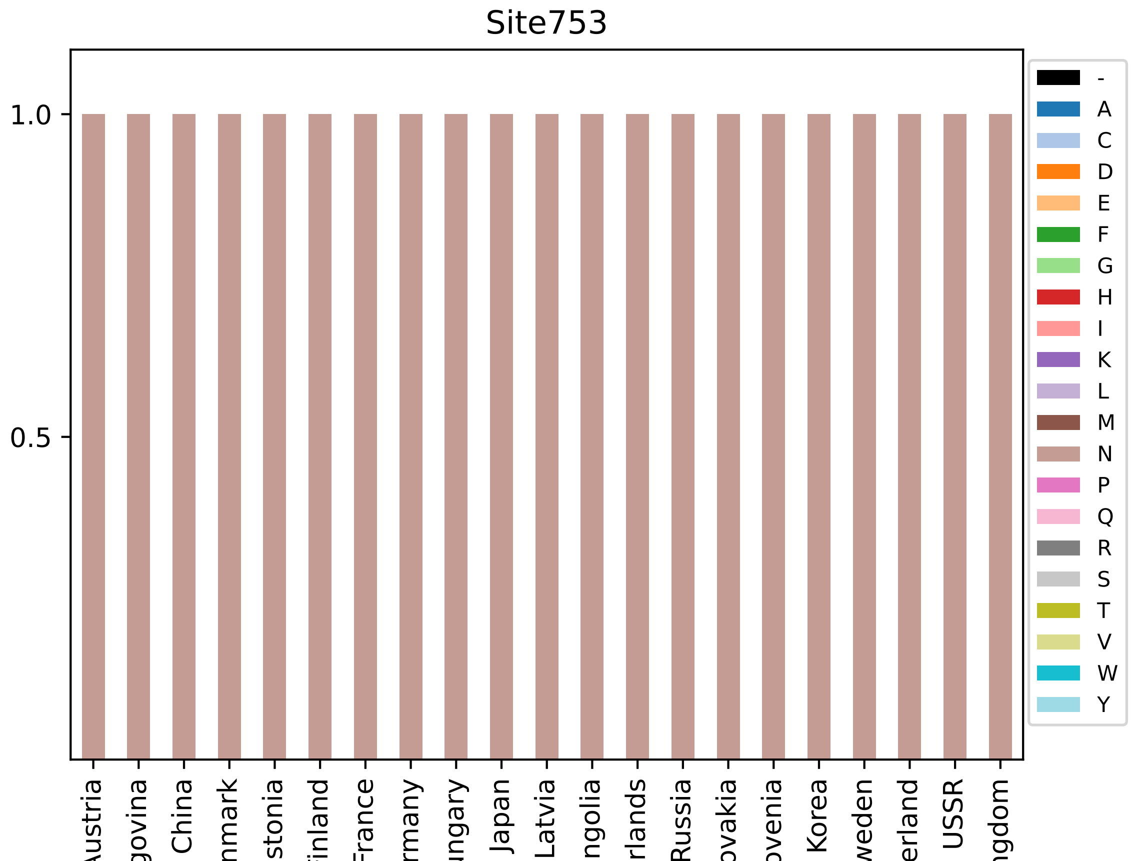

Site753
1.0
0.5
-
A
C
D
E
F
G
H
I
K
L
M
N
P
Q
R
S
T
V
W
Y
Austria
China
Finland
France
Japan
Latvia
Russia
Korea
USSR

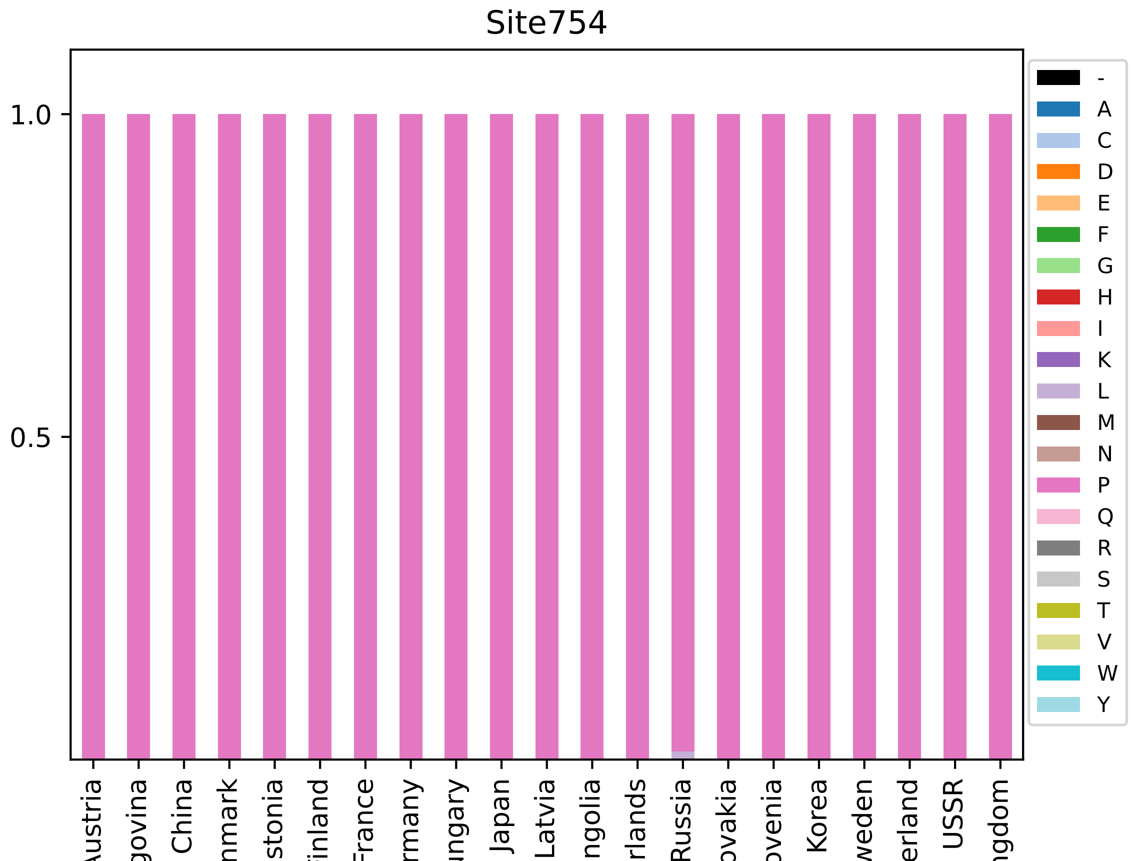

Site754
1.0
0.5
-
A
C
D
E
F
G
H
I
K
L
M
N
P
Q
R
S
T
V
W
Y
Austria
govina
China
nmark
stonia
inland
France
rmany
ungary
Japan
Latvia
ngolia
rlands
Russia
ovakia
ovenia
Korea
weden
erland
USSR
ngdom

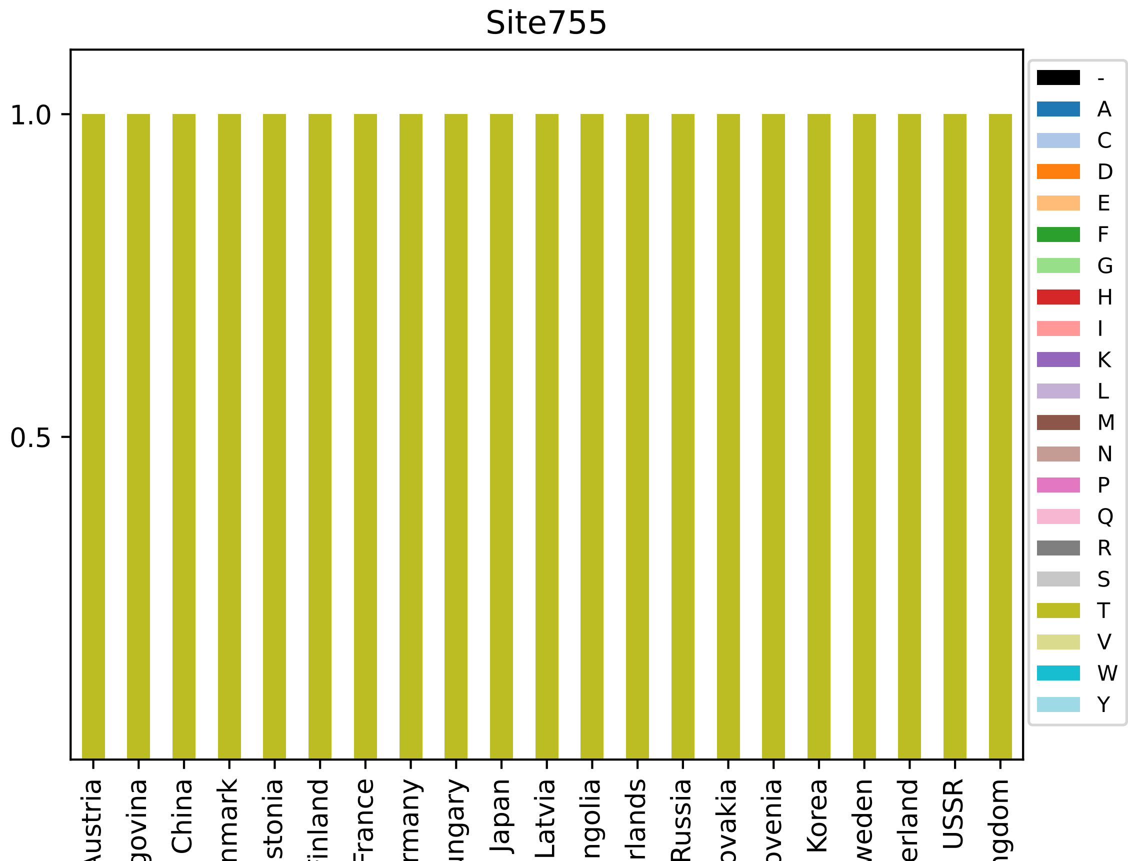

Site755
1.0
0.5
-
A
C
D
E
F
G
H
I
K
L
M
N
P
Q
R
S
T
V
W
Y
China
Japan
Latvia
Russia
Korea
USSR

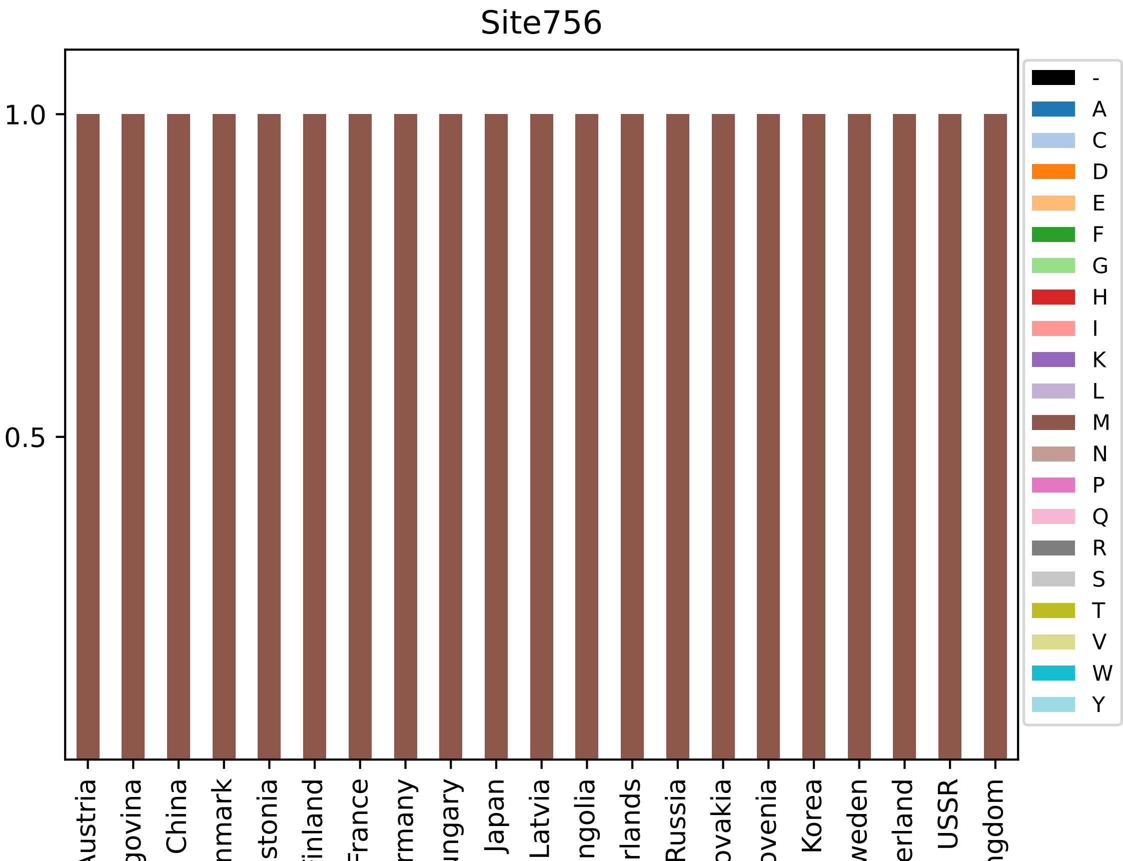

Site756
1.0
0.5
-
A
C
D
E
F
G
H
I
K
L
M
N
P
Q
R
S
T
V
W
Y
China
Japan
Latvia
Russia
Korea
USSR

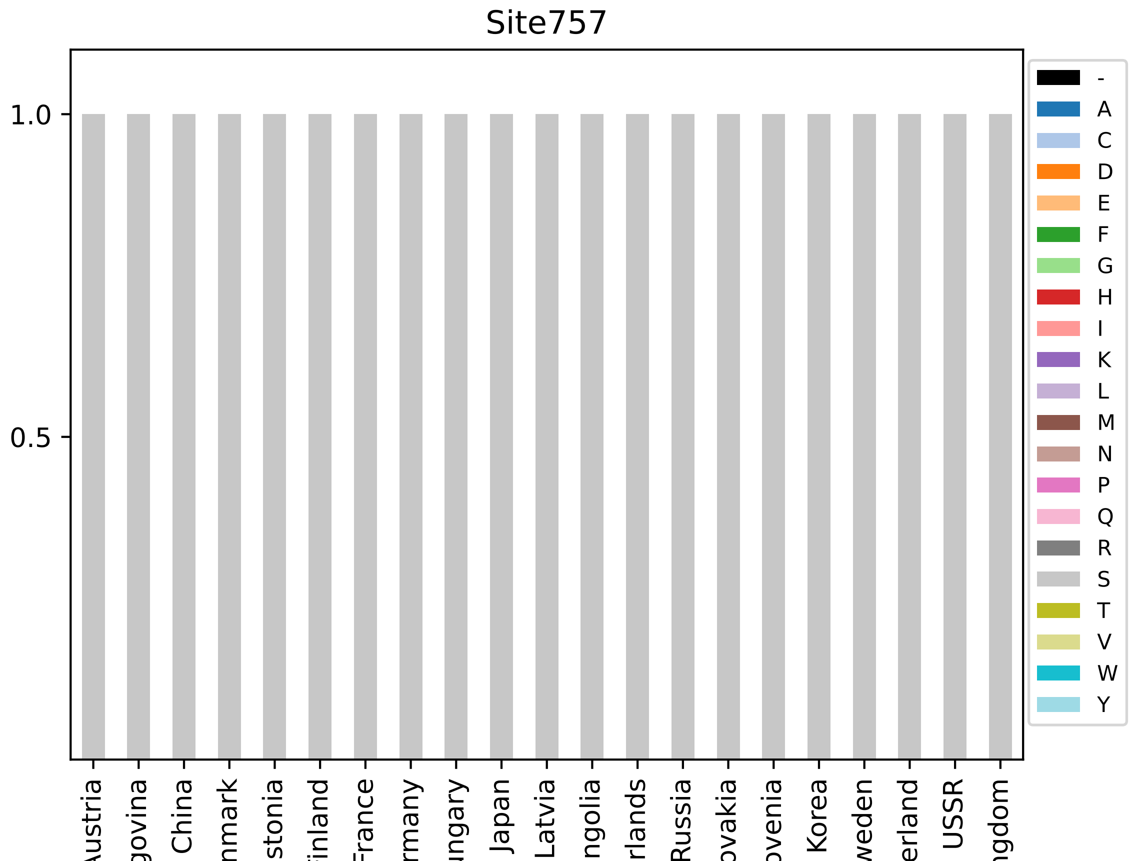

Site757
1.0
0.5
-
A
C
D
E
F
G
H
I
K
L
M
N
P
Q
R
S
T
V
W
Y
China
Japan
Latvia
Russia
Korea
USSR

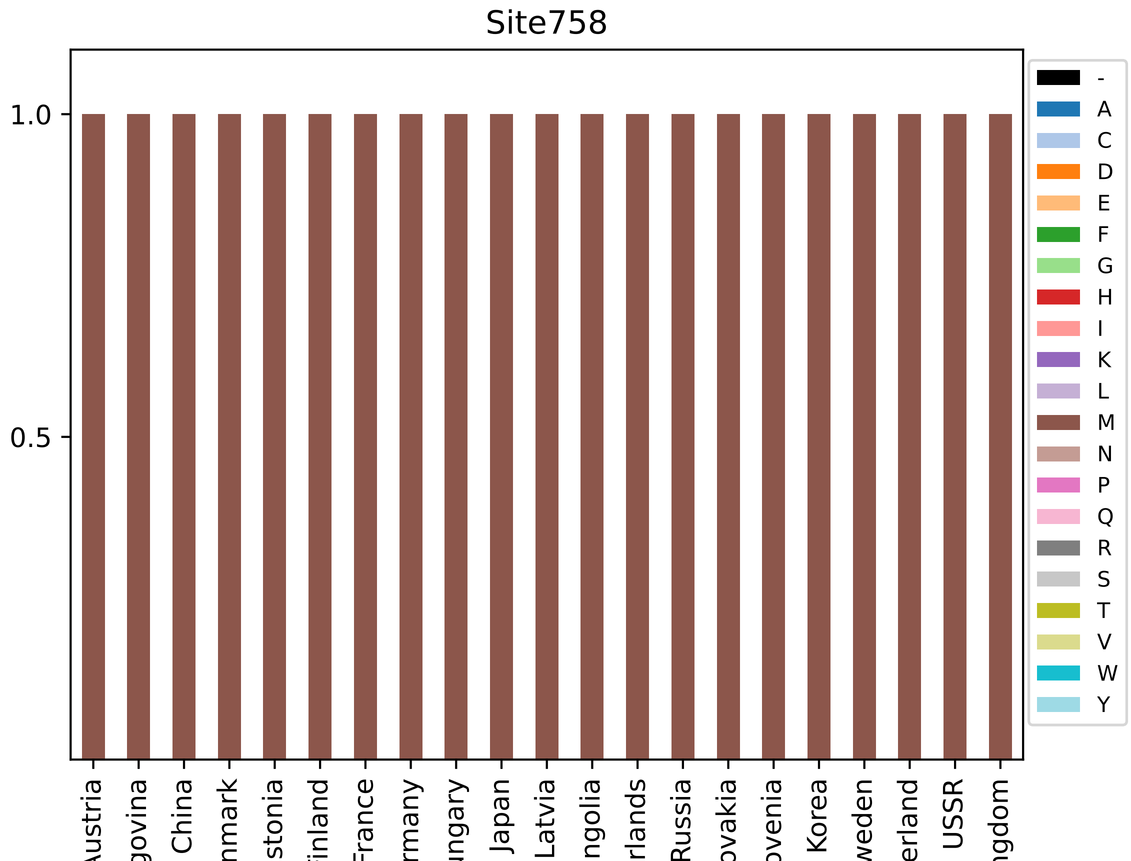

Site758
1.0
0.5
-
A
C
D
E
F
G
H
I
K
L
M
N
P
Q
R
S
T
V
W
Y
China
Japan
Latvia
Russia
Korea
USSR

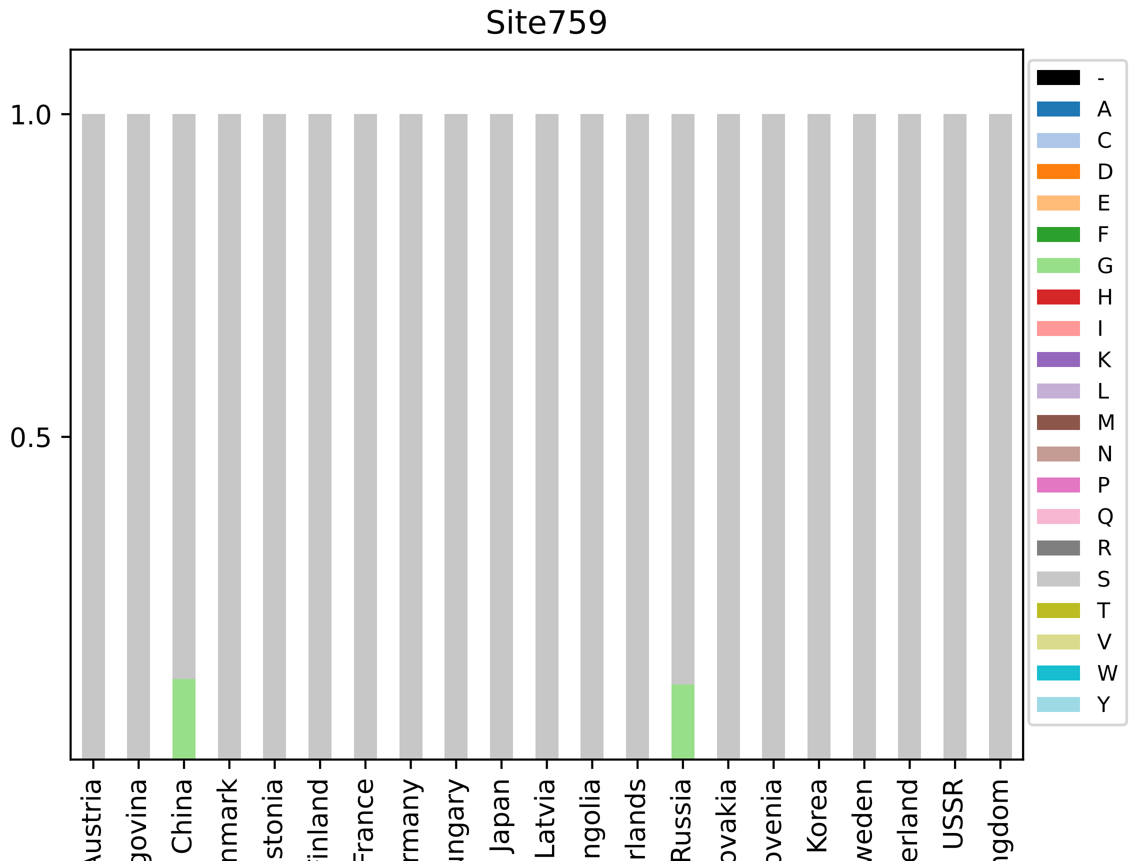

Site759
1.0
0.5
-
A
C
D
E
F
G
H
I
K
L
M
N
P
Q
R
S
T
V
W
Y
Austria
govina
China
nmark
stonia
inland
France
rmany
ungary
Japan
Latvia
ngolia
rlands
Russia
ovakia
ovenia
Korea
weden
erland
USSR
ngdom

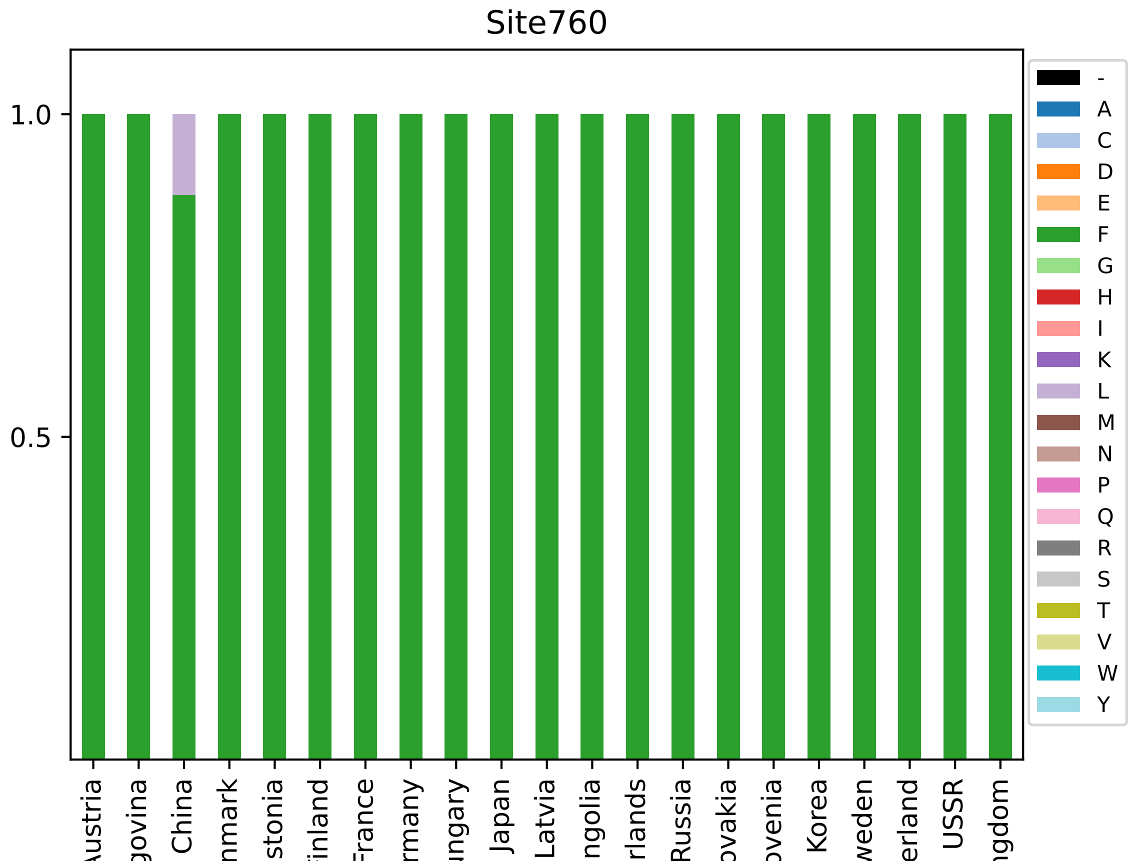

Site760
1.0
0.5
-
A
C
D
E
F
G
H
I
K
L
M
N
P
Q
R
S
T
V
W
Y
Austria
govina
China
nmark
stonia
inland
France
rmany
ungary
Japan
Latvia
ngolia
rlands
Russia
ovakia
ovenia
Korea
weden
erland
USSR
ngdom

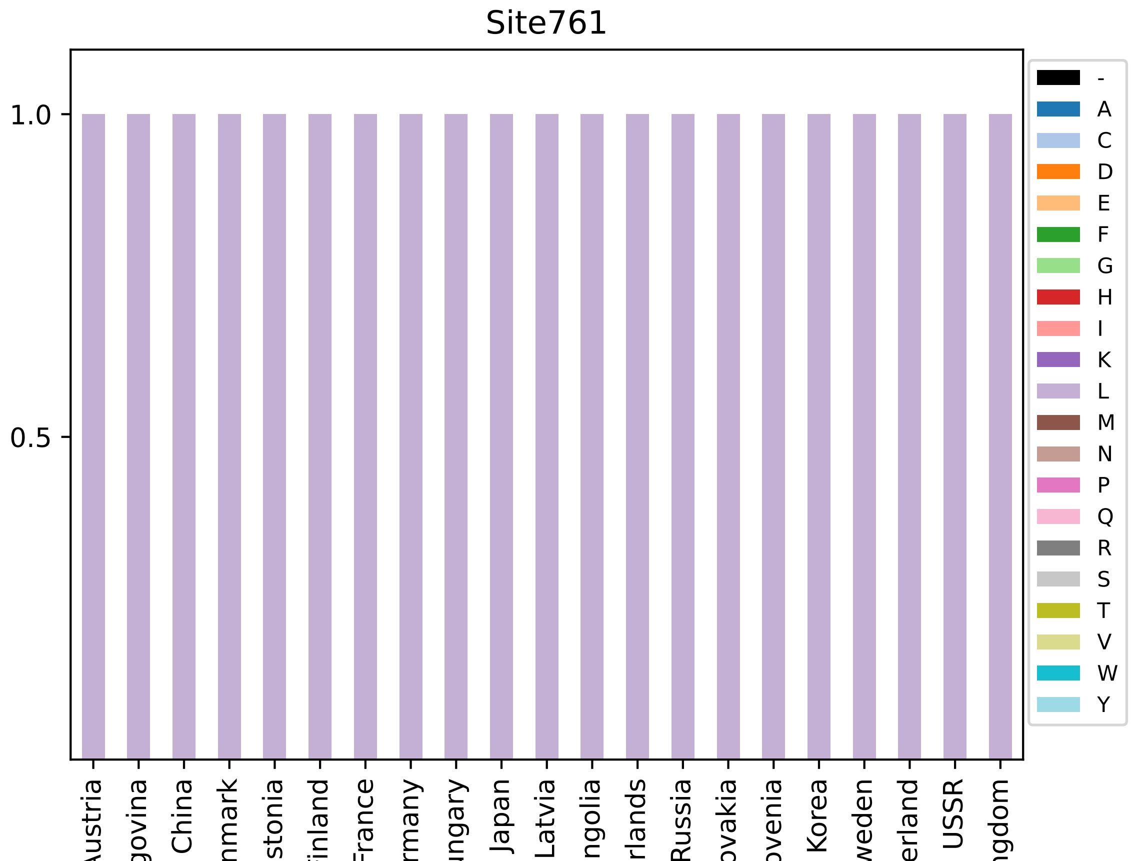

Site761
1.0
0.5
-
A
C
D
E
F
G
H
I
K
L
M
N
P
Q
R
S
T
V
W
Y
China
Japan
Latvia
Russia
Korea
USSR

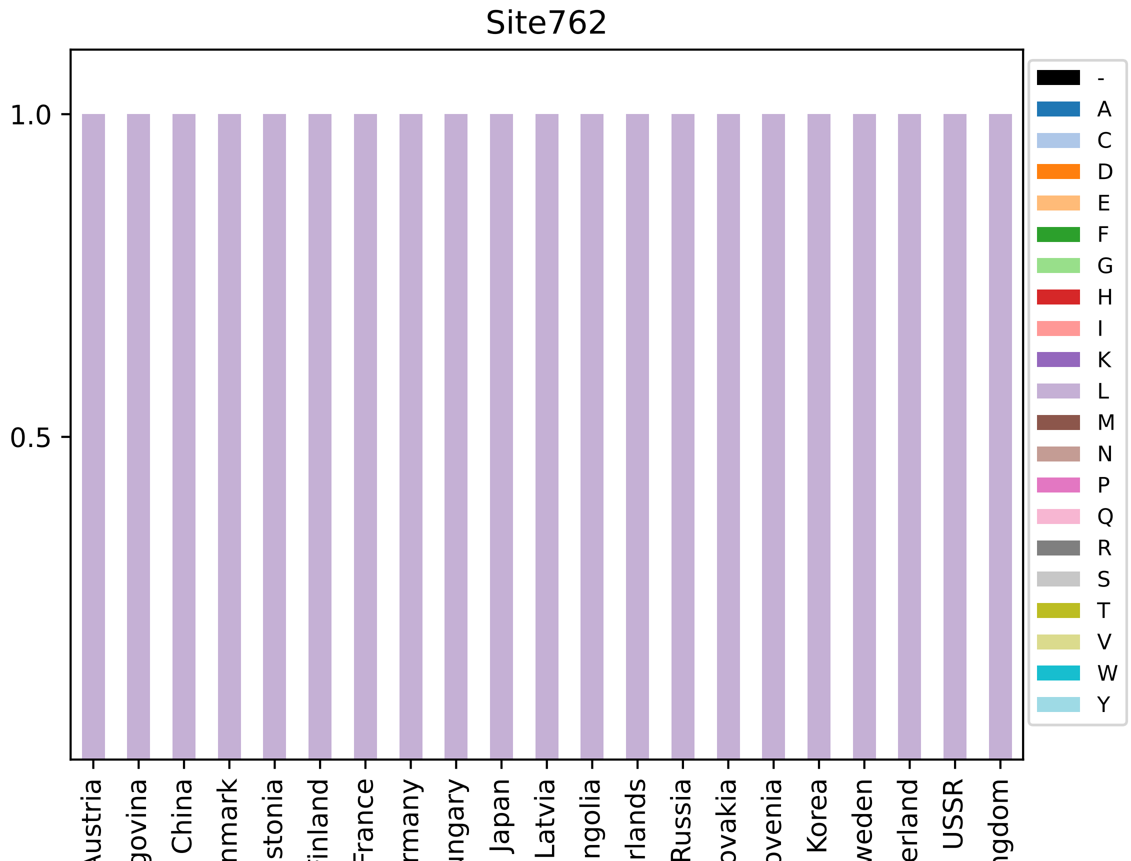
Site762
1.0
0.5
-
A
C
D
E
F
G
H
I
K
L
M
N
P
Q
R
S
T
V
W
Y
Austria
govina
China
nmark
stonia
Finland
France
rmany
ungary
Japan
Latvia
ngolia
rlands
Russia
ovakia
ovenia
Korea
weden
erland
USSR
ngdom

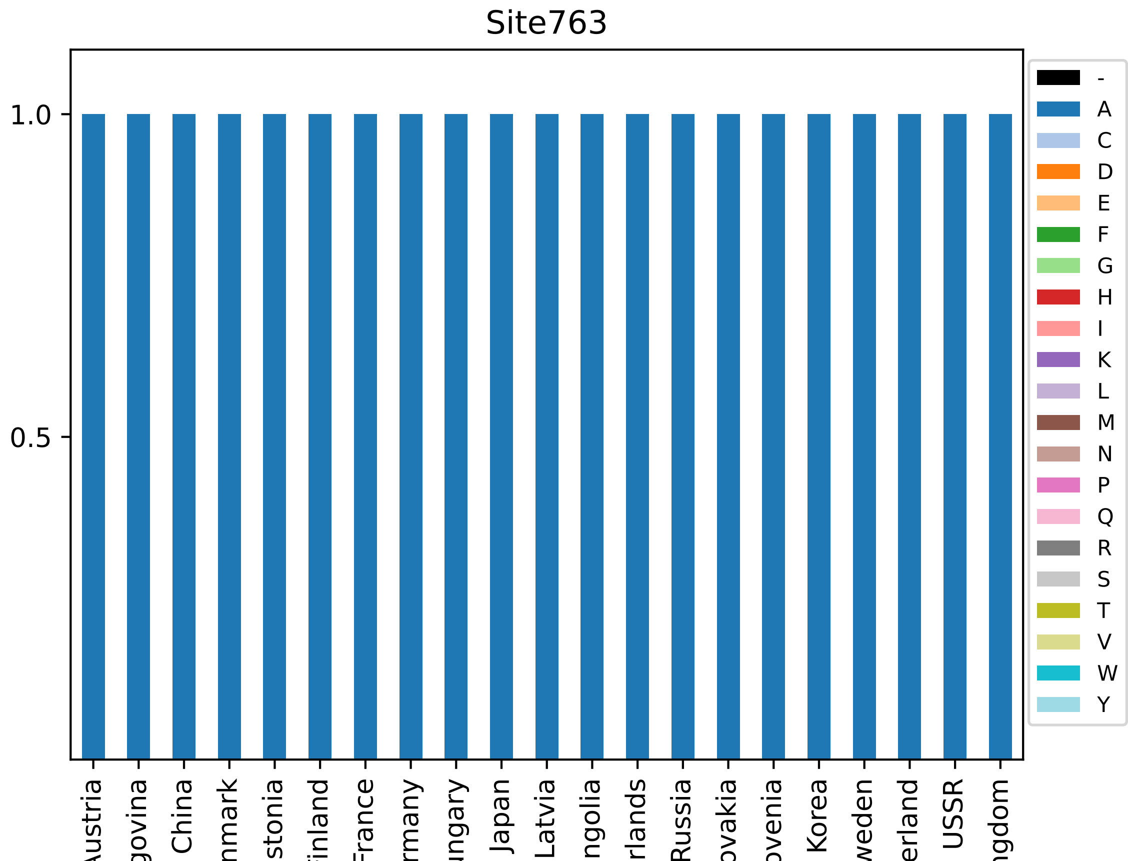

Site763
1.0
0.5
-
A
C
D
E
F
G
H
I
K
L
M
N
P
Q
R
S
T
V
W
Y
Austria
China
Finland
France
Japan
Latvia
Russia
Korea
USSR

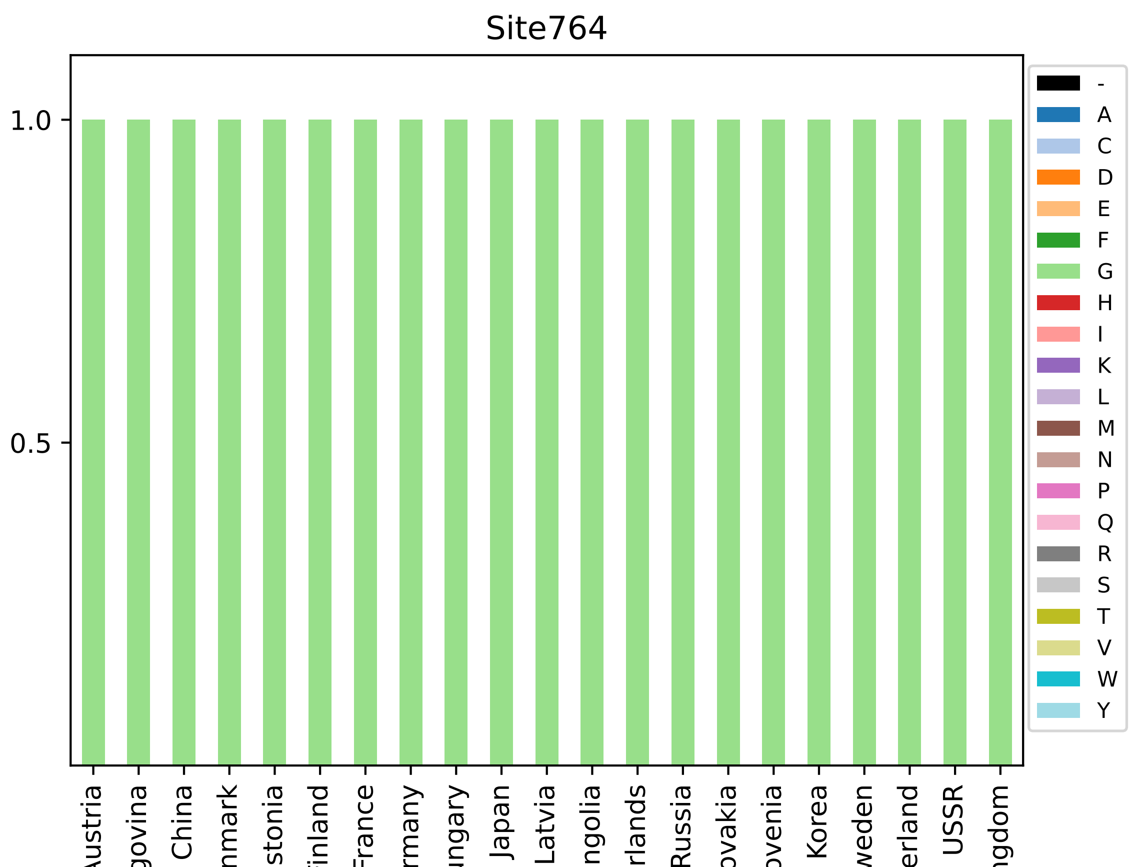

Site764
1.0
0.5
-
A
C
D
E
F
G
H
I
K
L
M
N
P
Q
R
S
T
V
W
Y
China
Japan
Latvia
Russia
Korea
USSR

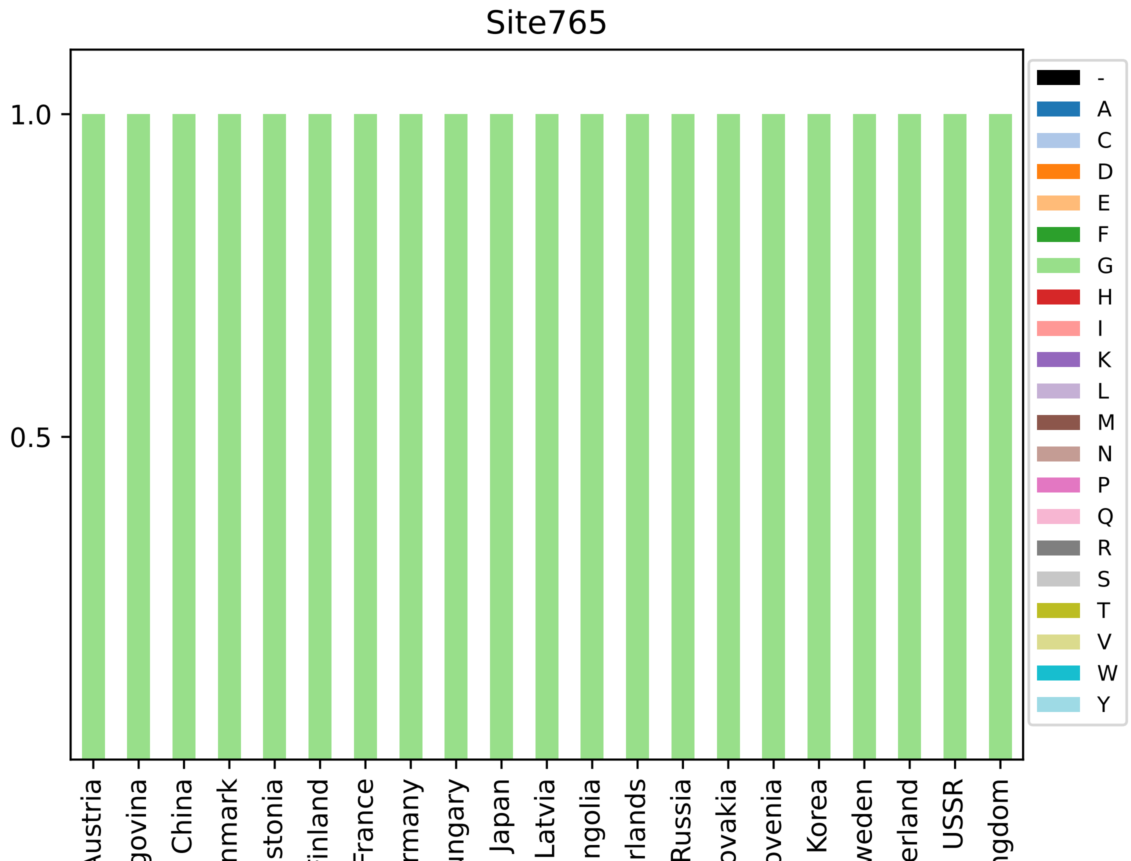
Site765
1.0
0.5
-
A
C
D
E
F
G
H
I
K
L
M
N
P
Q
R
S
T
V
W
Y
China
Japan
Latvia
Russia
Korea
USSR

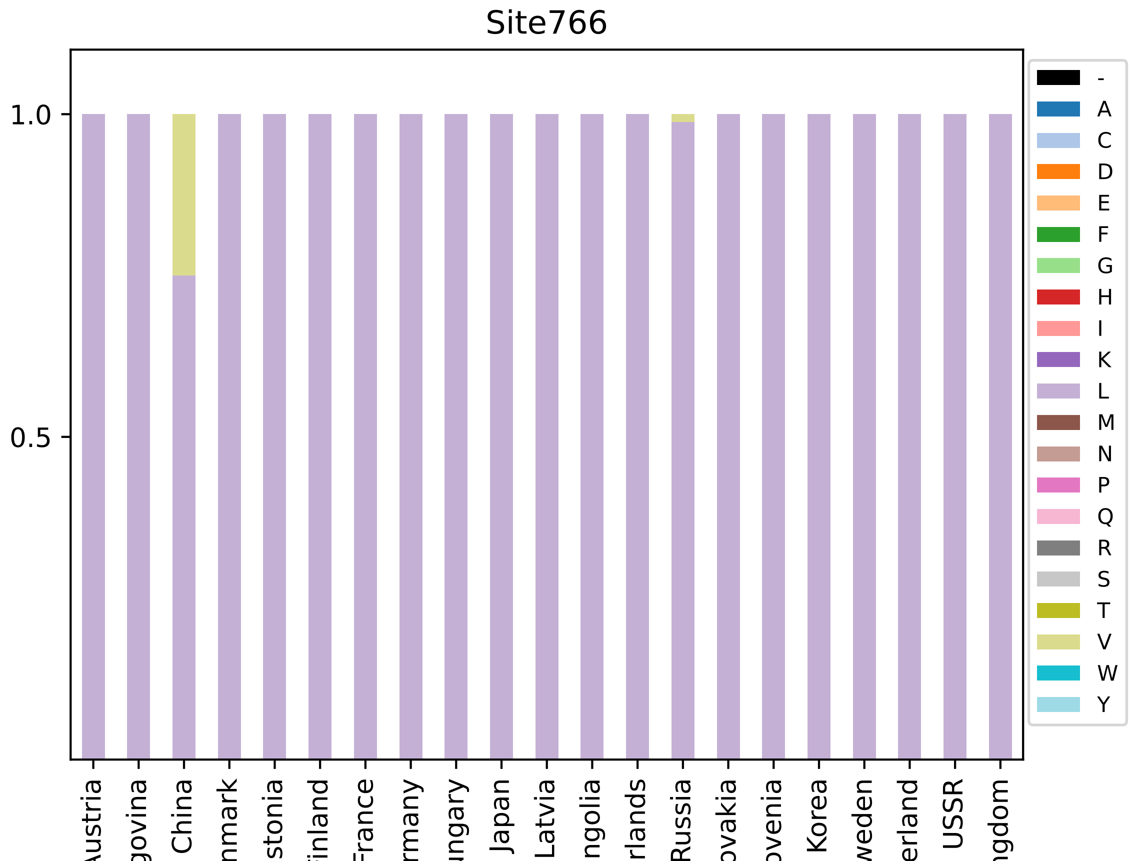

Site766
1.0
0.5
-
A
C
D
E
F
G
H
I
K
L
M
N
P
Q
R
S
T
V
W
Y
China
Japan
Latvia
Russia
Korea
USSR

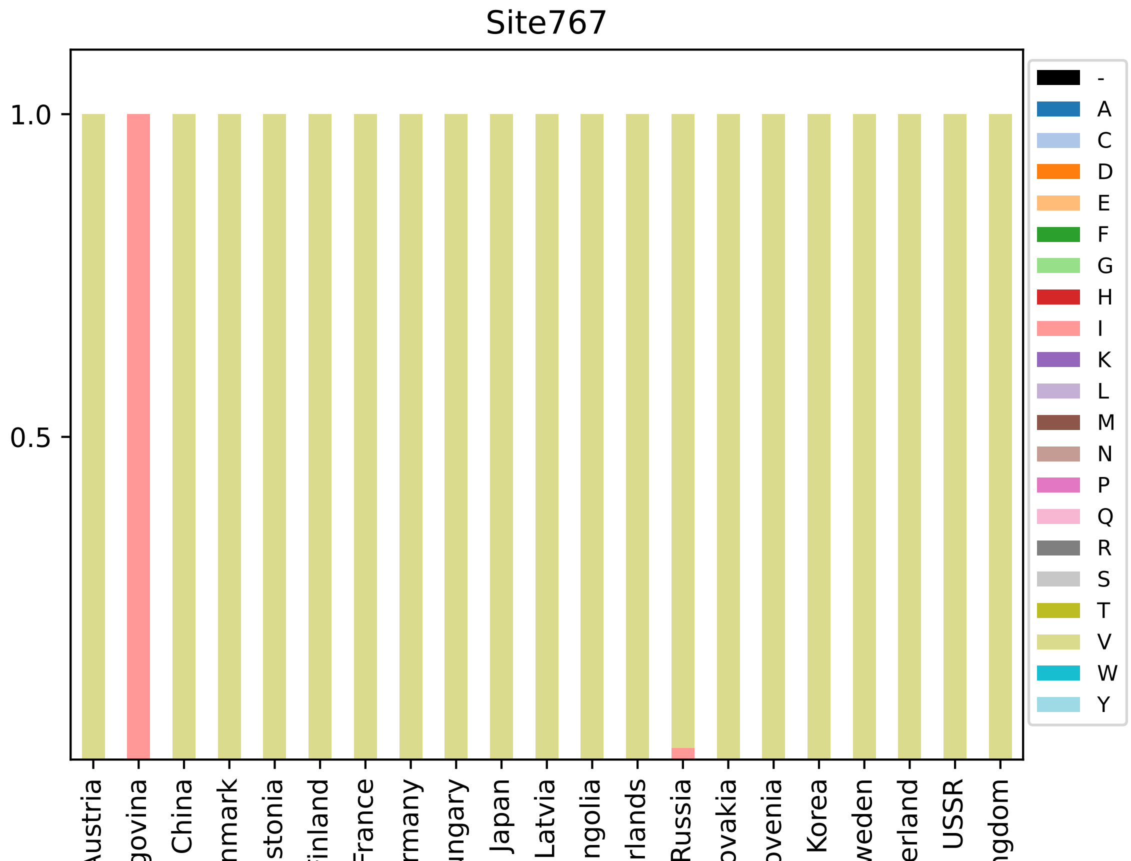
Site767
1.0
0.5
Austria
govina
China
nmark
stonia
Finland
France
rmany
ungary
Japan
Latvia
ngolia
rlands
Russia
ovakia
ovenia
Korea
weden
erland
USSR
ngdom
-
A
C
D
E
F
G
H
I
K
L
M
N
P
Q
R
S
T
V
W
Y

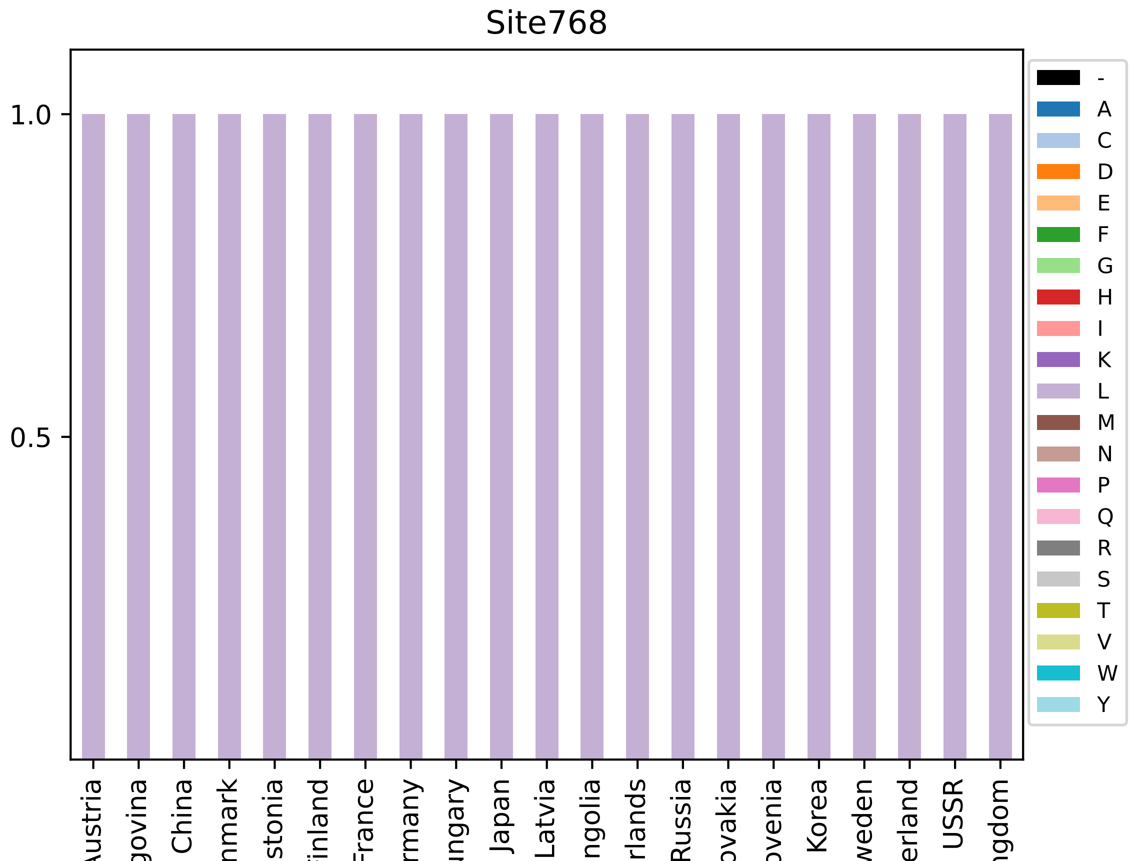

Site768
1.0
0.5
-
A
C
D
E
F
G
H
I
K
L
M
N
P
Q
R
S
T
V
W
Y
Austria
govina
China
nmark
stonia
inland
France
rmany
ungary
Japan
Latvia
ngolia
rlands
Russia
ovakia
ovenia
Korea
weden
erland
USSR
ngdom

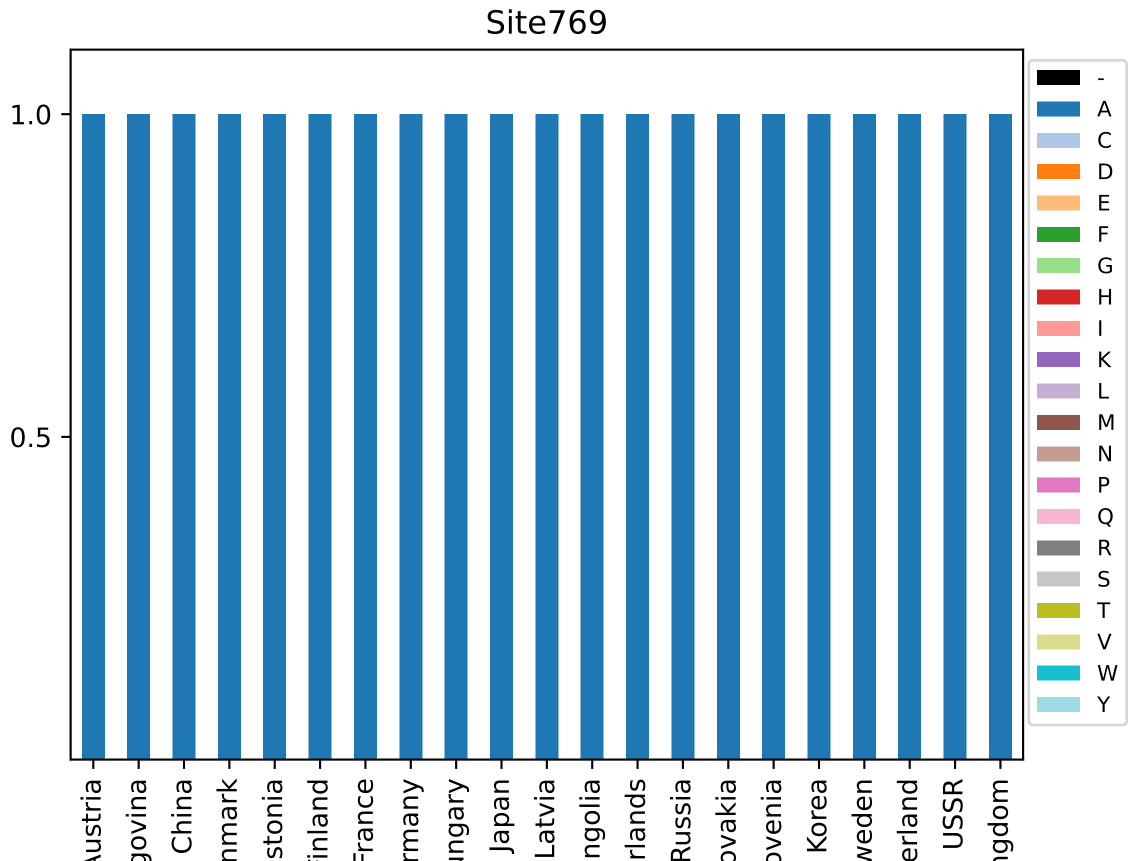

Site769
1.0
0.5
China
Japan
Latvia
Russia
Korea
USSR
-
A
C
D
E
F
G
H
I
K
L
M
N
P
Q
R
S
T
V
W
Y

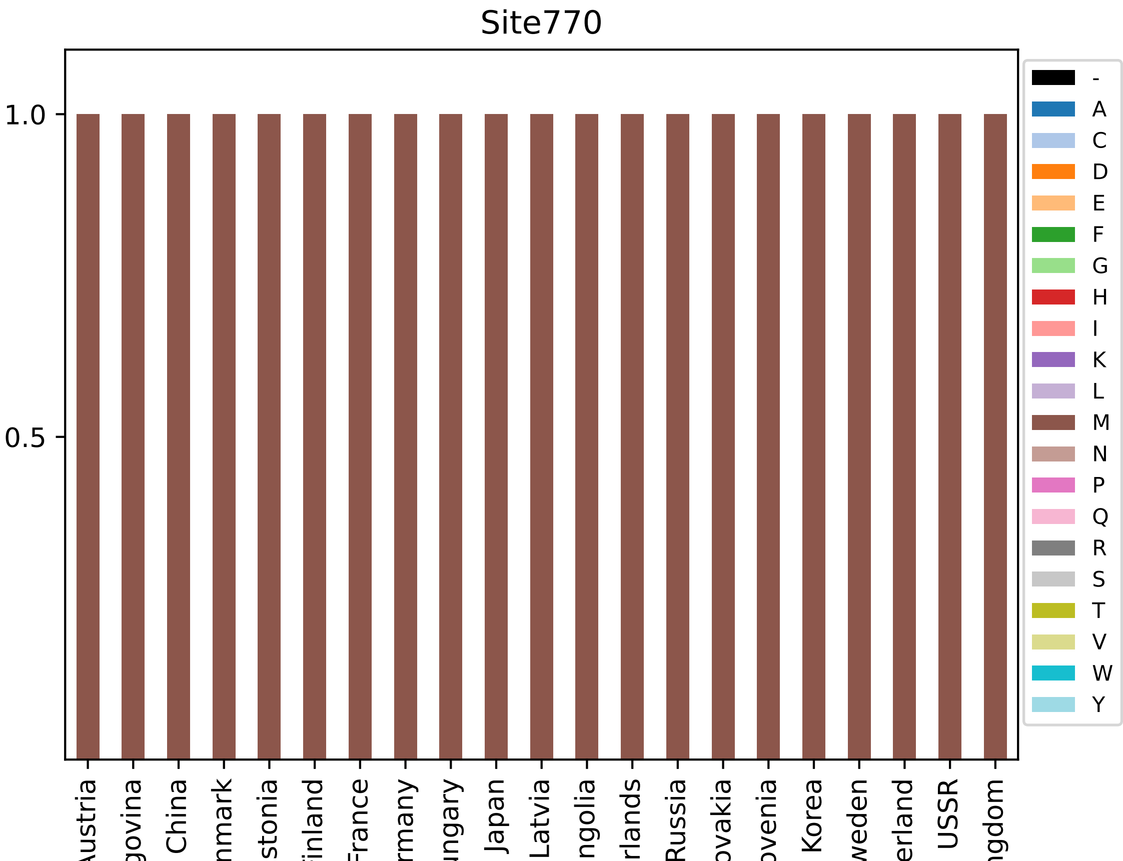
Site770
1.0
0.5
-
A
C
D
E
F
G
H
I
K
L
M
N
P
Q
R
S
T
V
W
Y
China
Japan
Latvia
Russia
Korea
USSR

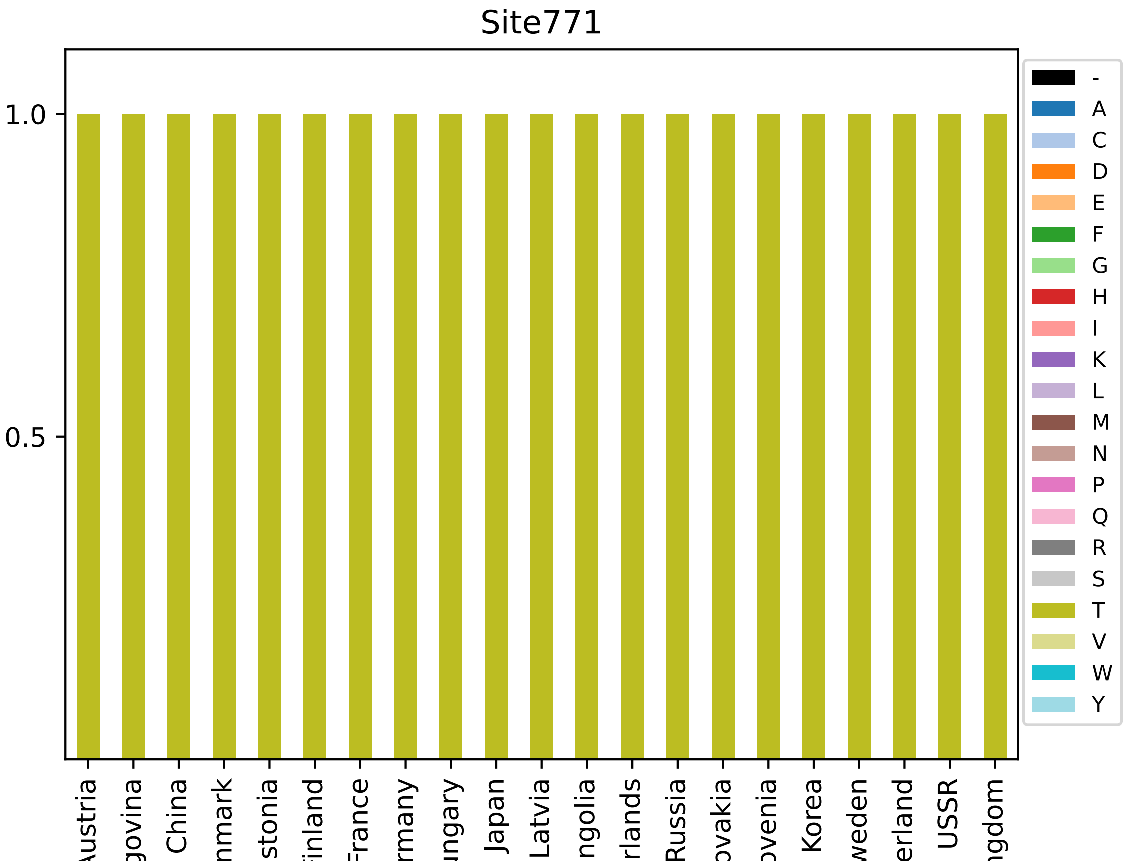

Site771
1.0
0.5
-
A
C
D
E
F
G
H
I
K
L
M
N
P
Q
R
S
T
V
W
Y
China
Japan
Latvia
Russia
Korea
USSR

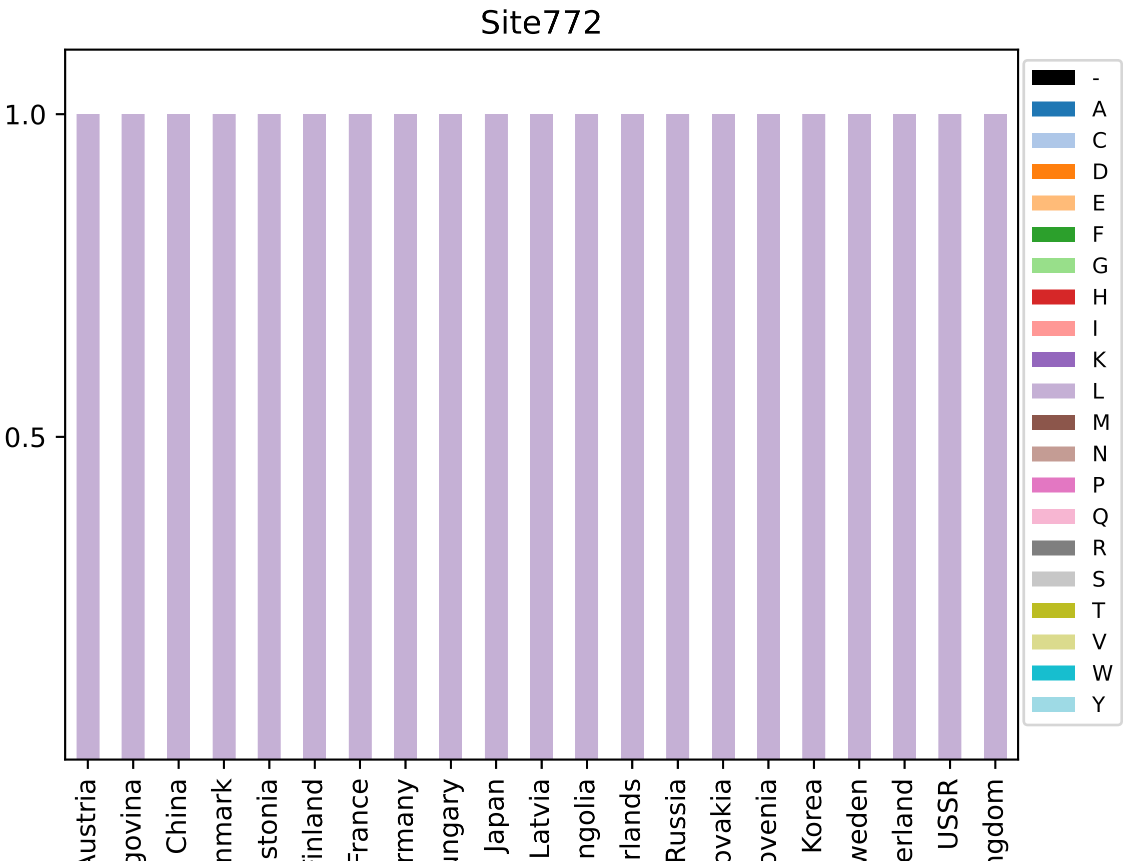

Site772
1.0
0.5
Austria
govina
China
nmark
stonia
Finland
France
rmany
ungary
Japan
Latvia
ngolia
rlands
Russia
ovakia
ovenia
Korea
weden
erland
USSR
ngdom
-
A
C
D
E
F
G
H
I
K
L
M
N
P
Q
R
S
T
V
W
Y

Site773

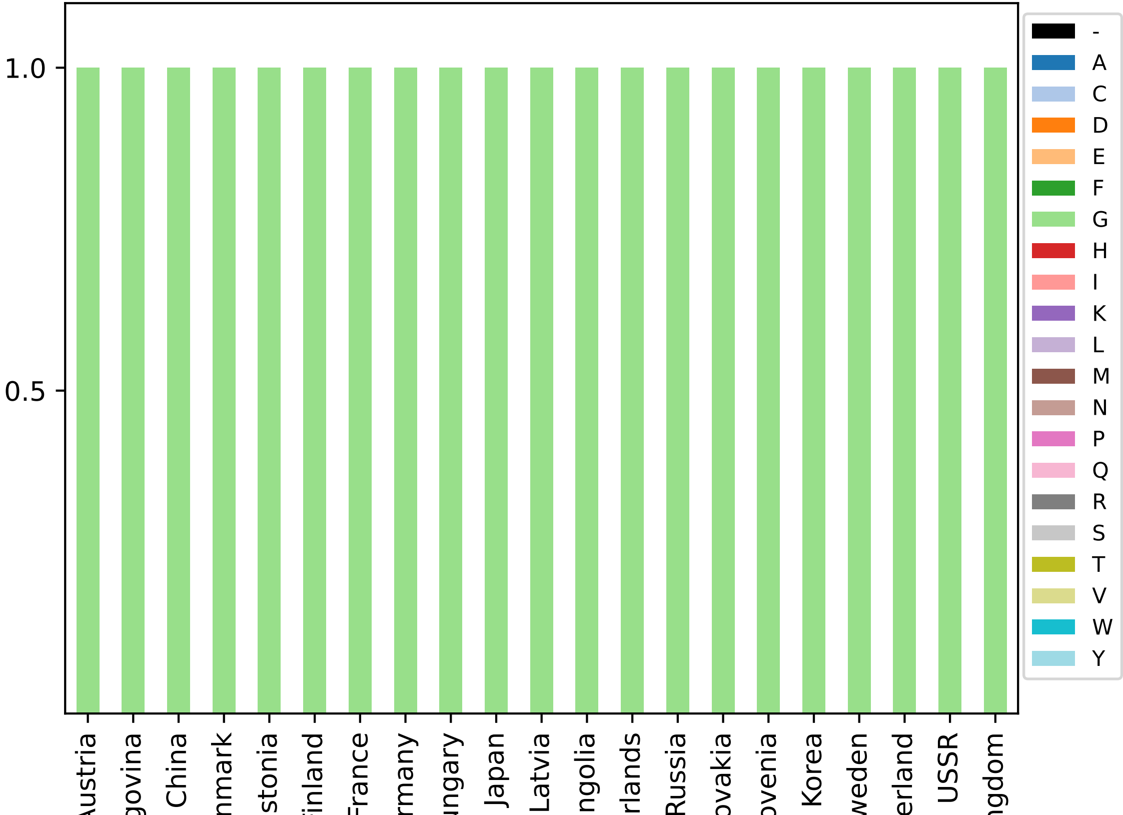

Site774

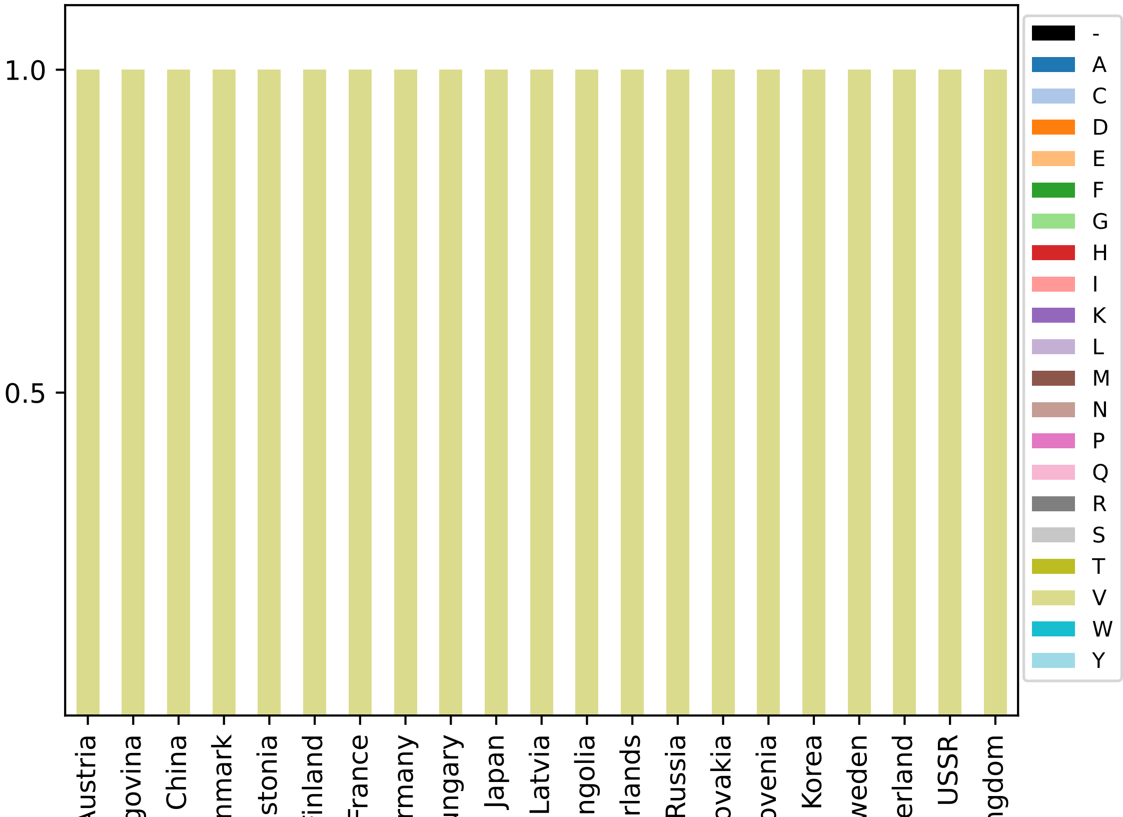
1.0
0.5
-
A
C
D
E
F
G
H
I
K
L
M
N
P
Q
R
S
T
V
W
Y
China
Japan
Latvia
Russia
Korea
USSR

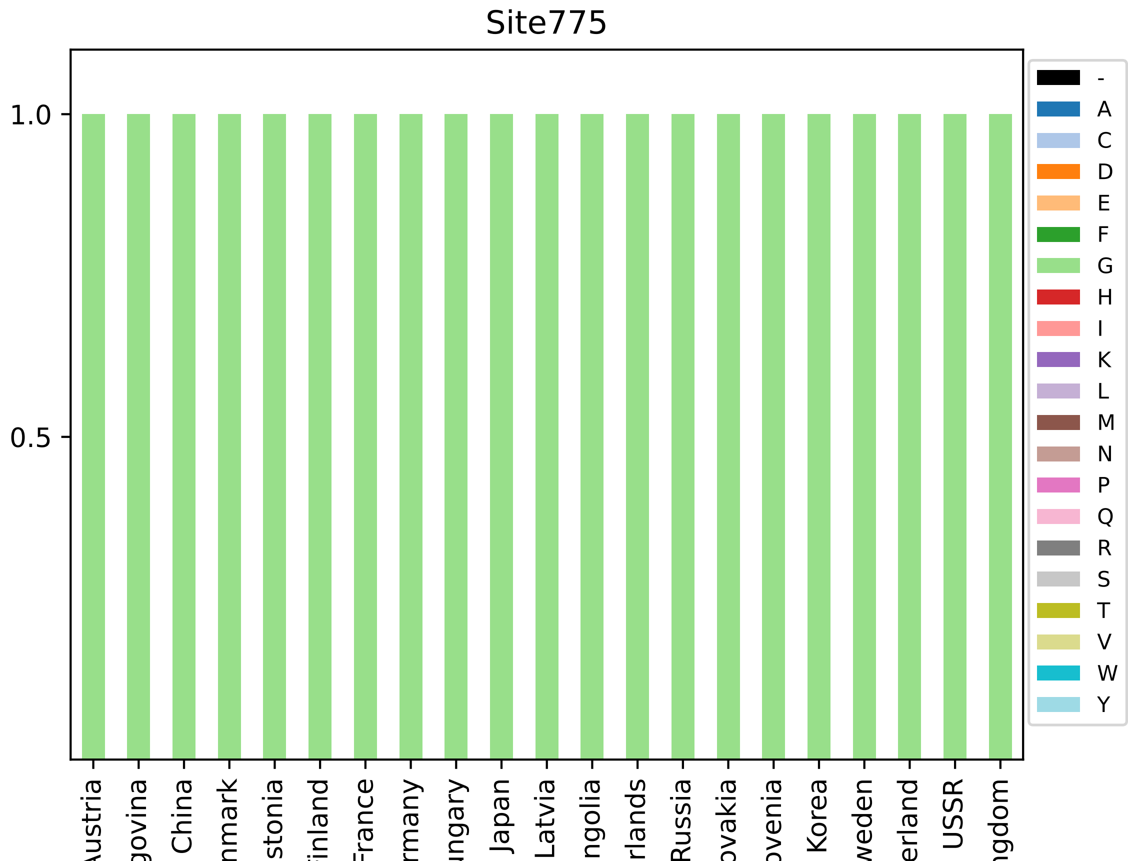
Site775
1.0
0.5
-
A
C
D
E
F
G
H
I
K
L
M
N
P
Q
R
S
T
V
W
Y
China
Japan
Latvia
Russia
Korea
USSR

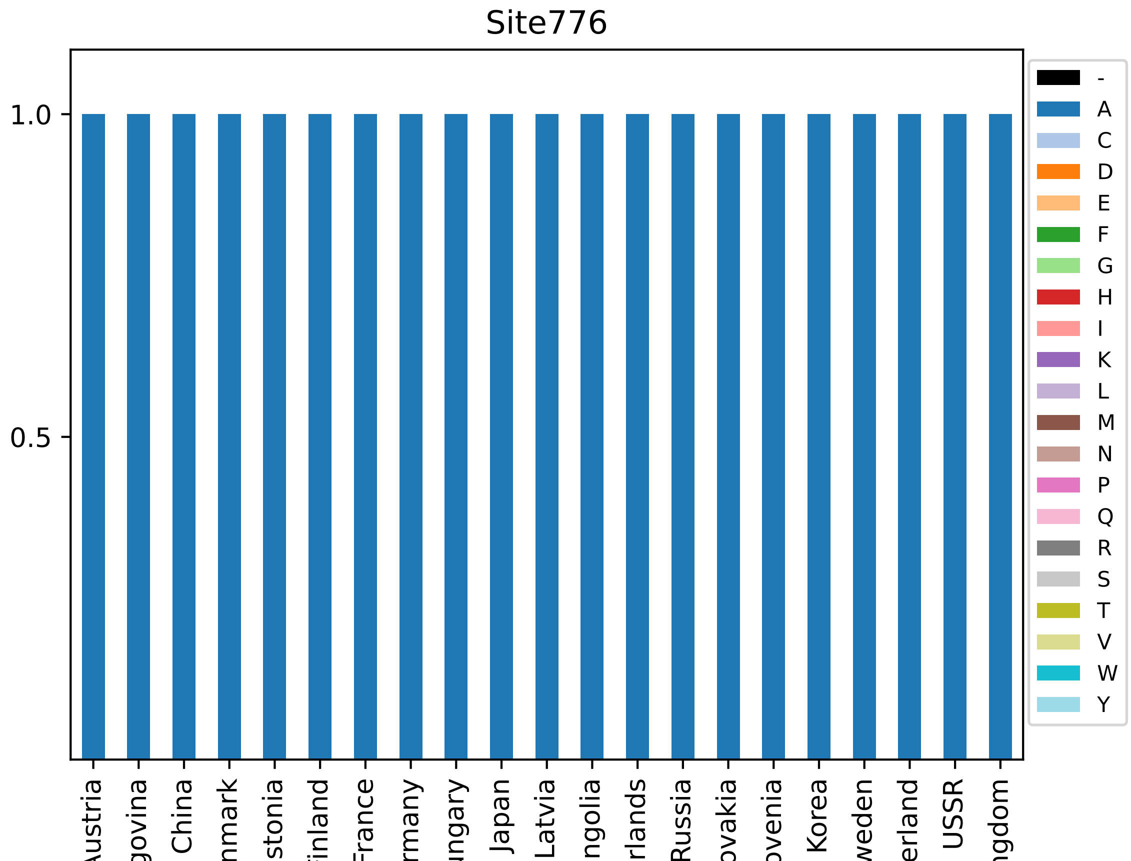

Site776
1.0
0.5
China
Japan
Latvia
Russia
Korea
USSR
-
A
C
D
E
F
G
H
I
K
L
M
N
P
Q
R
S
T
V
W
Y

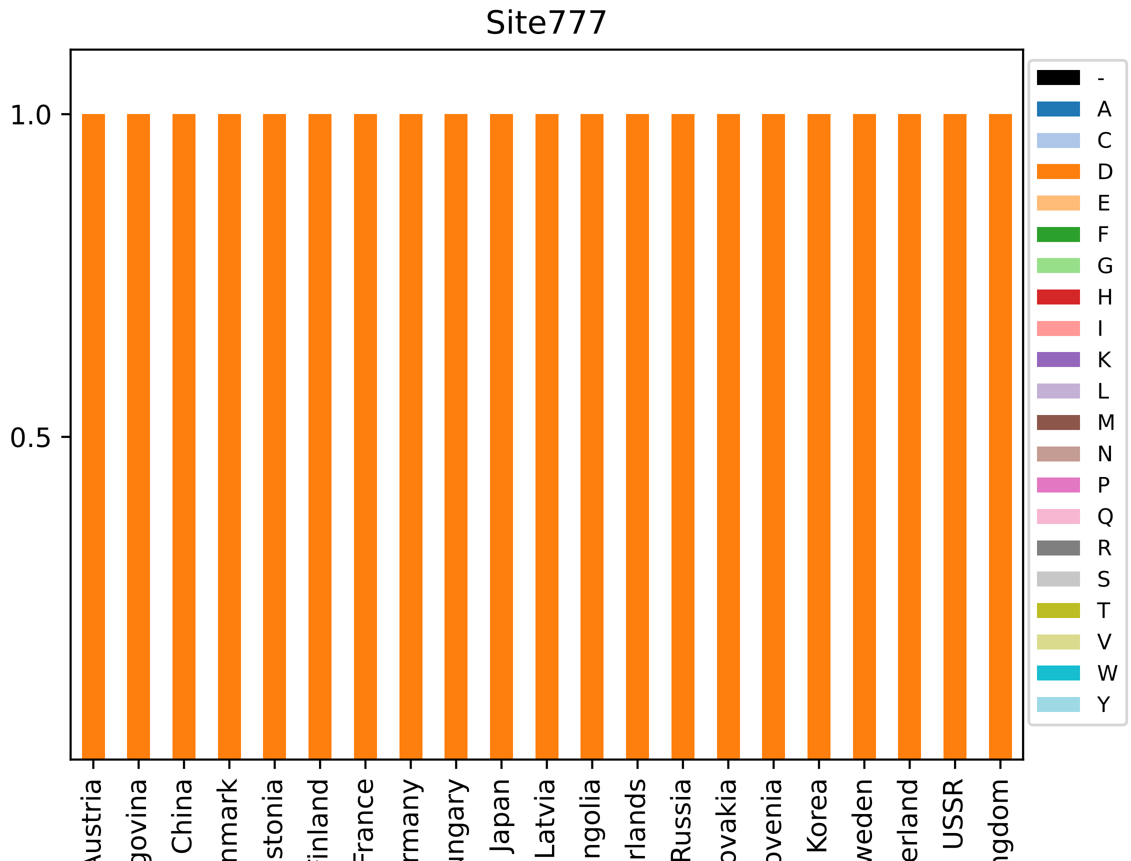

Site777
1.0
0.5
-
A
C
D
E
F
G
H
I
K
L
M
N
P
Q
R
S
T
V
W
Y
Austria
China
Japan
Latvia
Russia
Korea
USSR

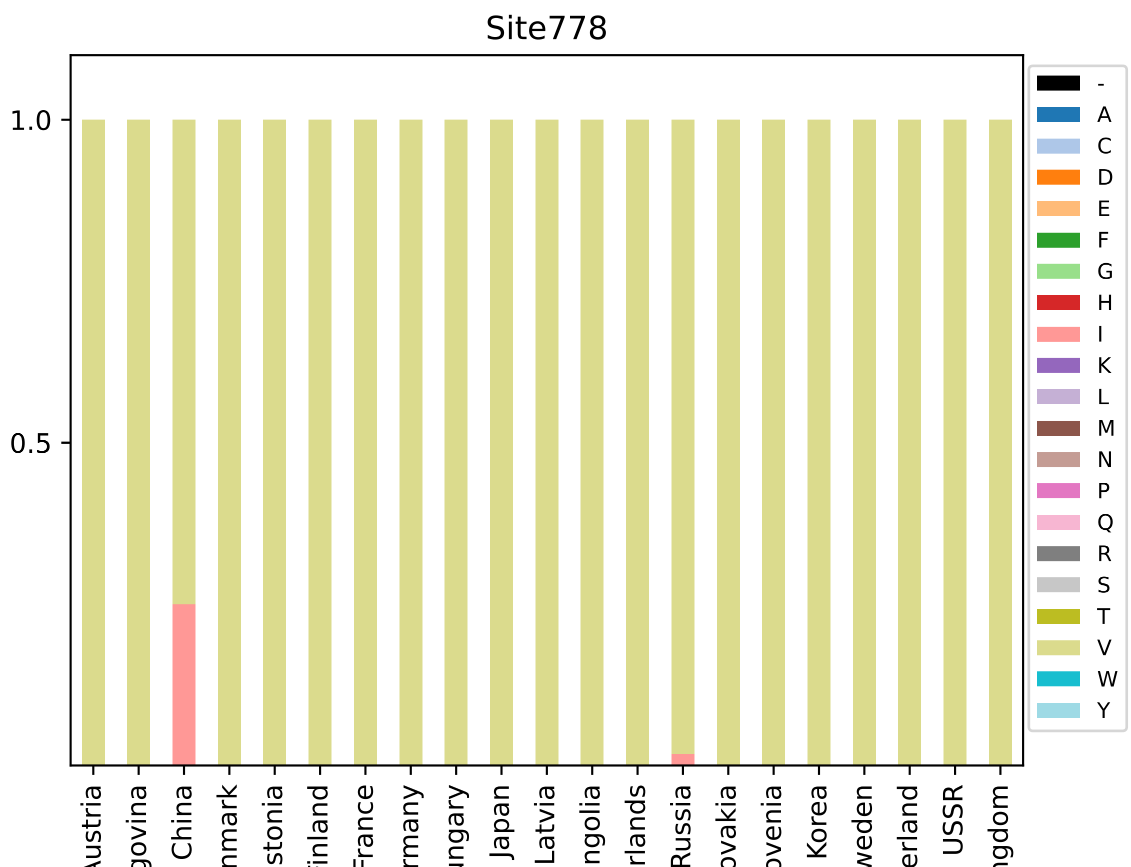

Site778
1.0
0.5
-
A
C
D
E
F
G
H
I
K
L
M
N
P
Q
R
S
T
V
W
Y
Austria
govina
China
nmark
stonia
Finland
France
rmany
ungary
Japan
Latvia
ngolia
rlands
Russia
ovakia
ovenia
Korea
weden
erland
USSR
ngdom

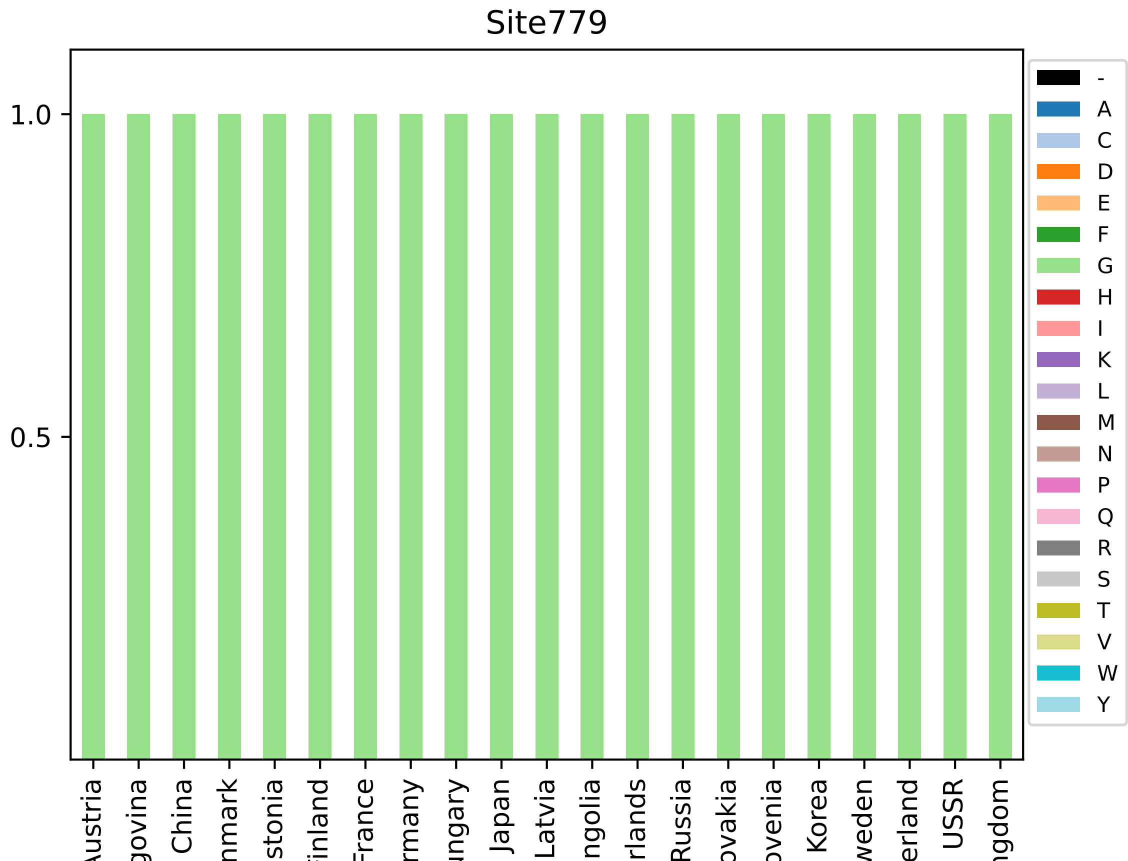

Site779
1.0
0.5
-
A
C
D
E
F
G
H
I
K
L
M
N
P
Q
R
S
T
V
W
Y
China
Japan
Latvia
Russia
Korea
USSR

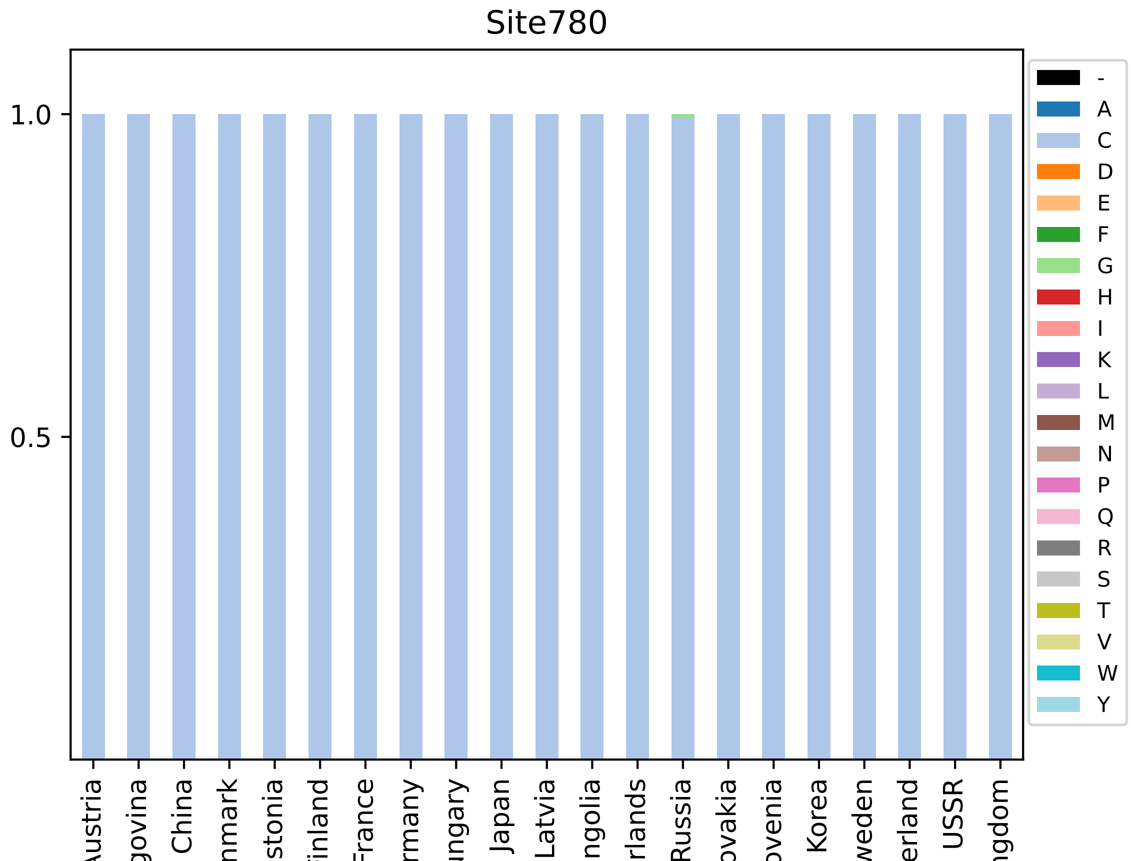

Site780
1.0
0.5
-
A
C
D
E
F
G
H
I
K
L
M
N
P
Q
R
S
T
V
W
Y
Austria
govina
China
nmark
stonia
Finland
France
rmany
ungary
Japan
Latvia
ngolia
rlands
Russia
ovakia
ovenia
Korea
weden
erland
USSR
ngdom

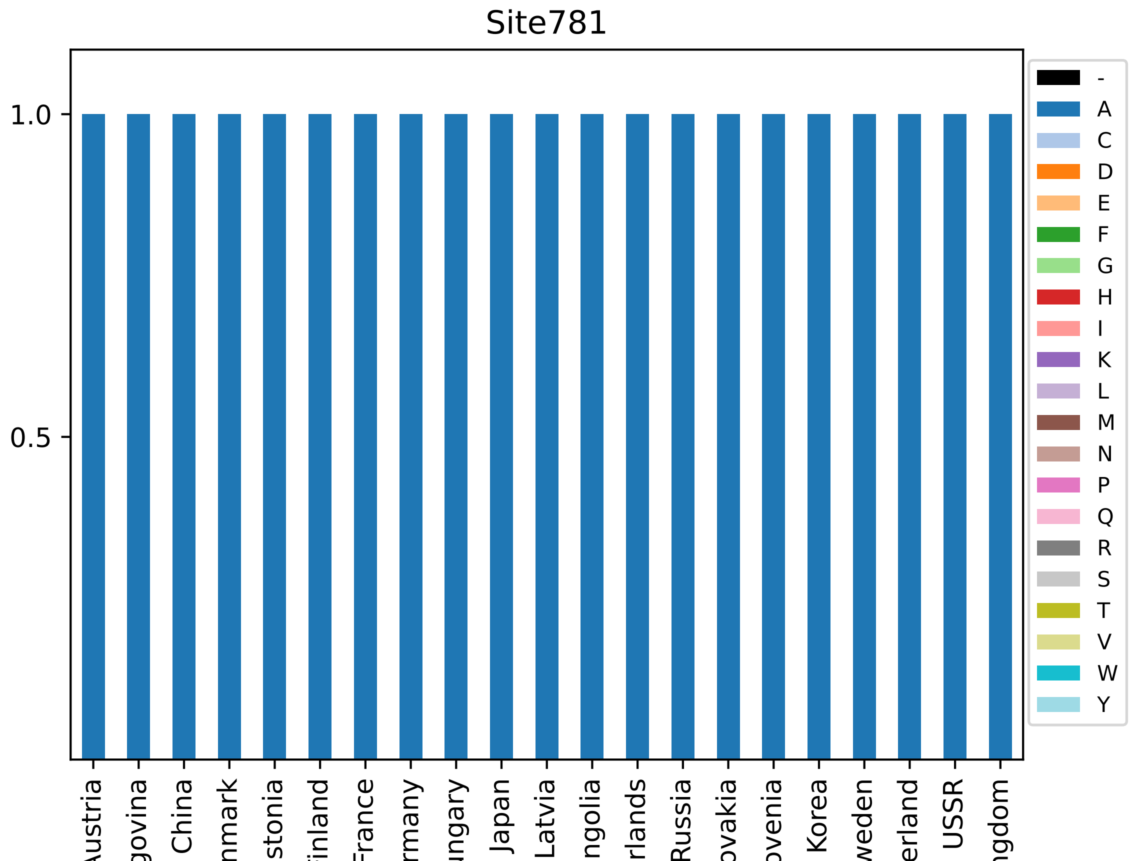

Site781
1.0
0.5
-
A
C
D
E
F
G
H
I
K
L
M
N
P
Q
R
S
T
V
W
Y
China
Japan
Latvia
Russia
Korea
USSR

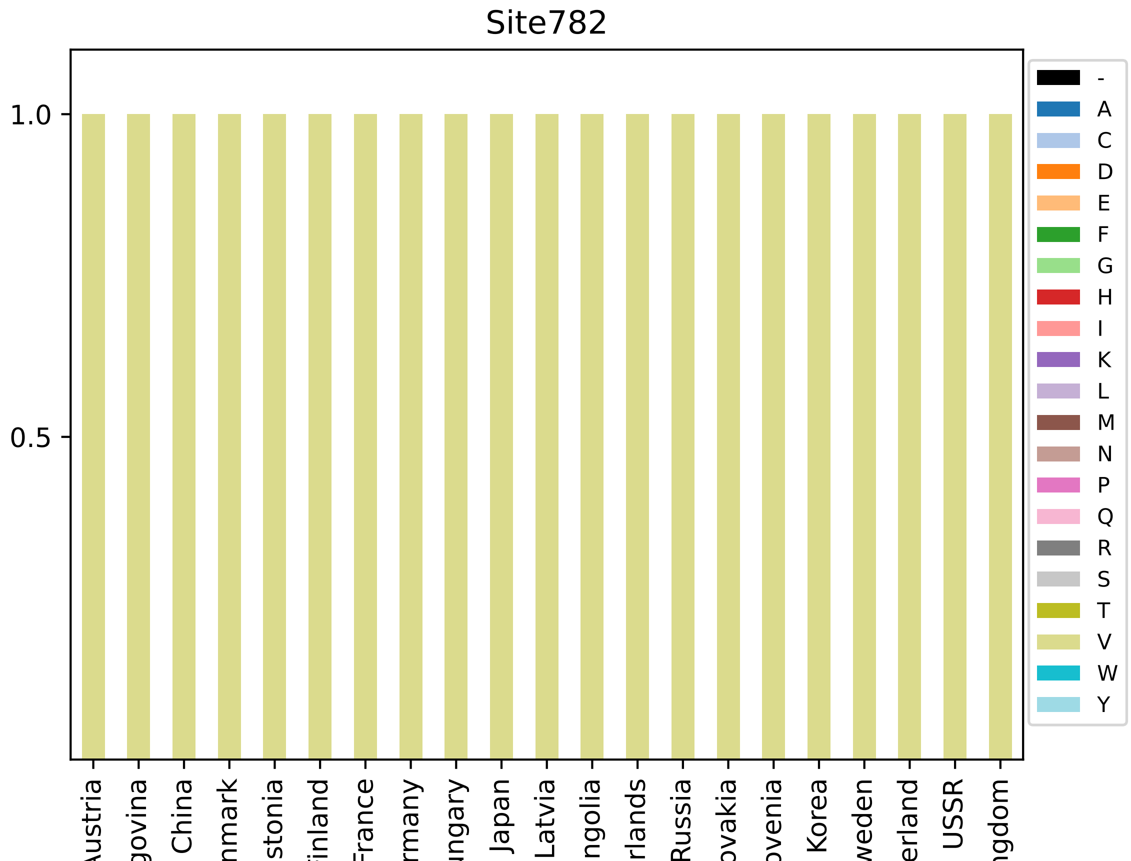

Site782
1.0
0.5
-
A
C
D
E
F
G
H
I
K
L
M
N
P
Q
R
S
T
V
W
Y
China
Japan
Latvia
Russia
Korea
USSR

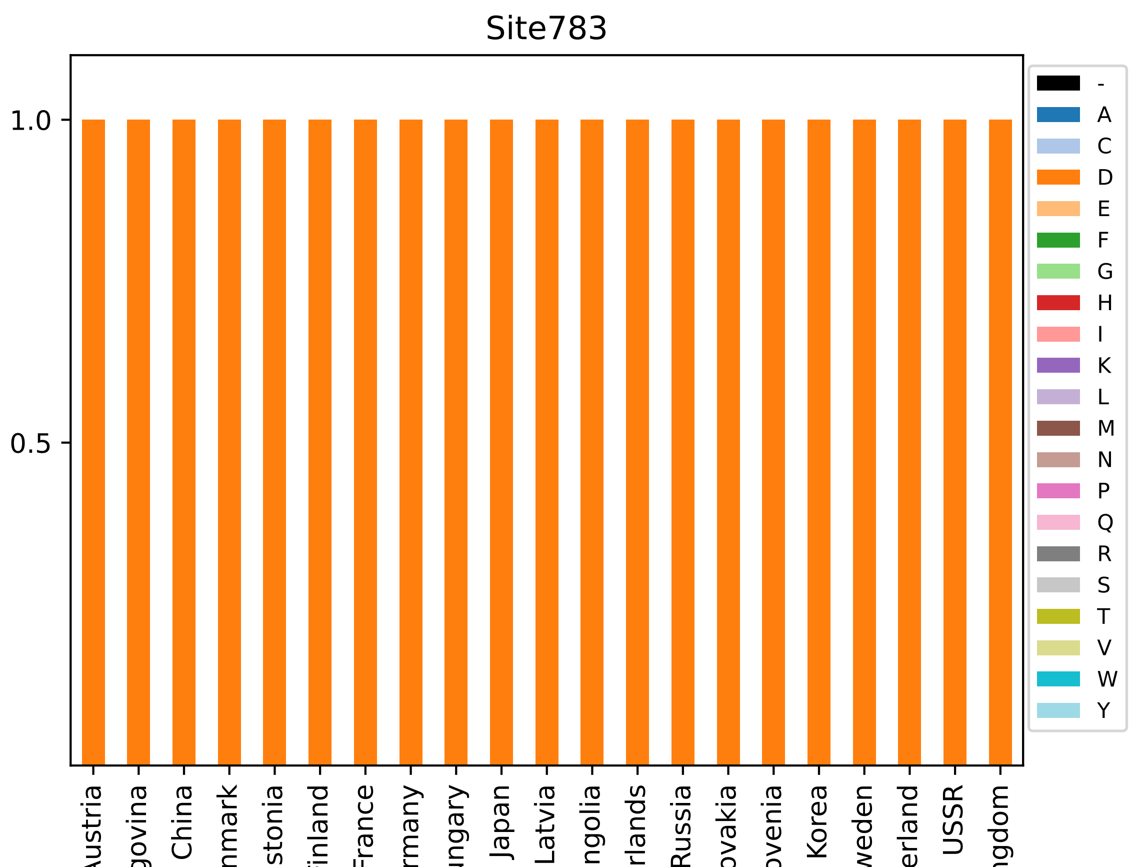
Site783
1.0
0.5
-
A
C
D
E
F
G
H
I
K
L
M
N
P
Q
R
S
T
V
W
Y
China
Japan
Latvia
Russia
Korea
USSR

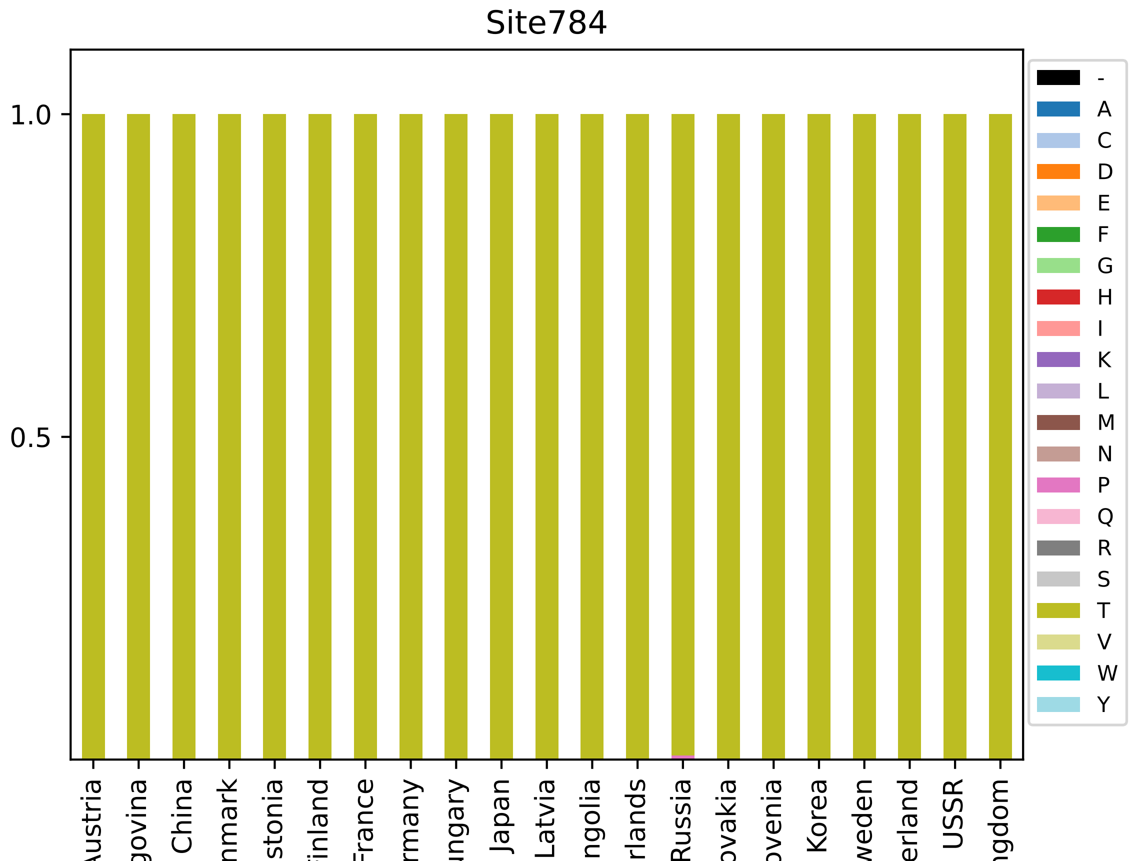

Site784
1.0
0.5
-
A
C
D
E
F
G
H
I
K
L
M
N
P
Q
R
S
T
V
W
Y
Austria
govina
China
nmark
stonia
inland
France
rmany
ungary
Japan
Latvia
ngolia
rlands
Russia
ovakia
ovenia
Korea
weden
erland
USSR
ngdom

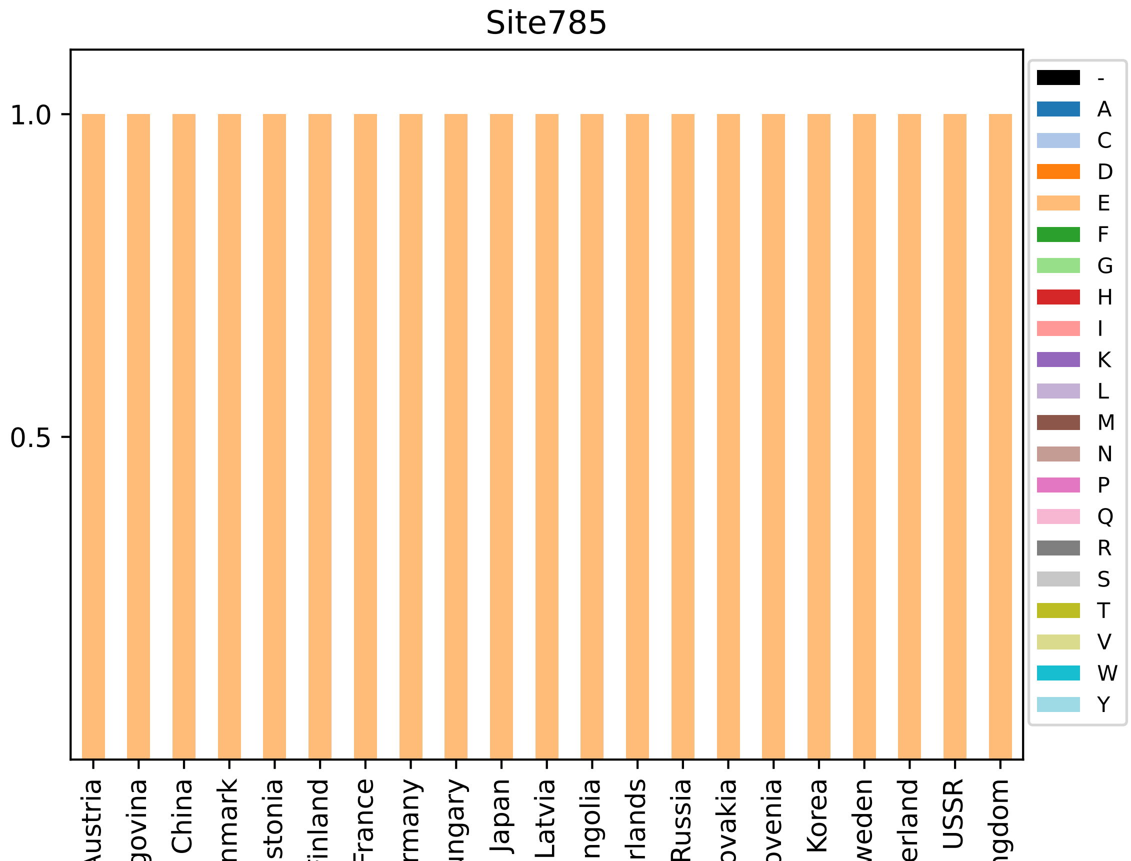

Site785
1.0
0.5
-
A
C
D
E
F
G
H
I
K
L
M
N
P
Q
R
S
T
V
W
Y
China
Japan
Latvia
Russia
Korea
USSR

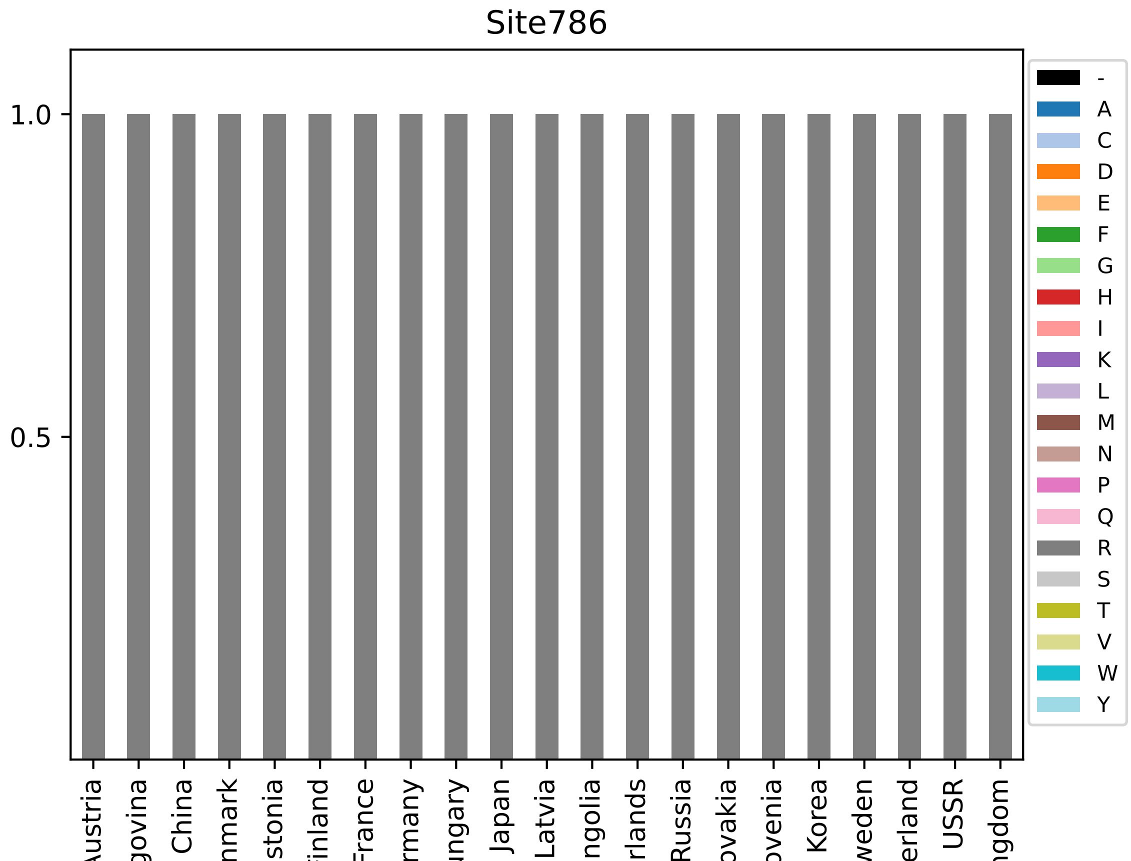

Site786
1.0
0.5
-
A
C
D
E
F
G
H
I
K
L
M
N
P
Q
R
S
T
V
W
Y
Austria
govina
China
nmark
stonia
Finland
France
rmany
ungary
Japan
Latvia
ngolia
rlands
Russia
ovakia
ovenia
Korea
weden
erland
USSR
ngdom

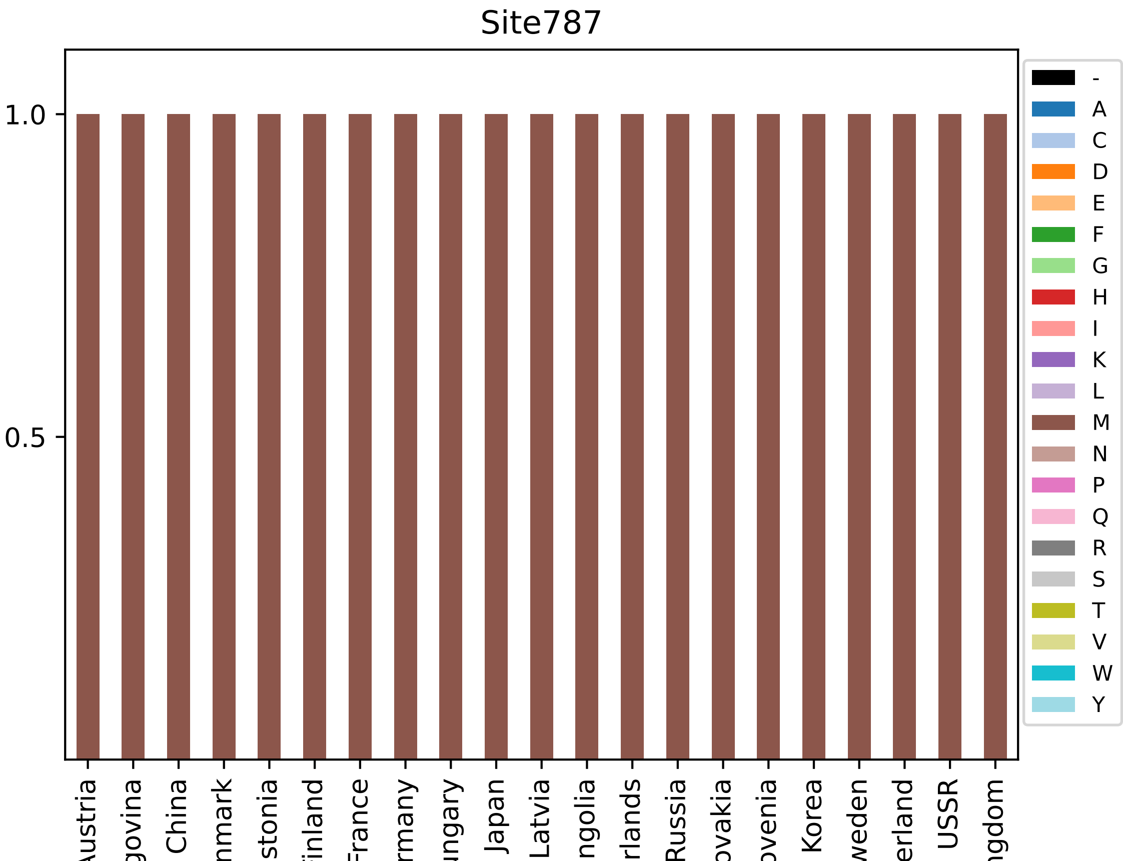
Site787
1.0
0.5
-
A
C
D
E
F
G
H
I
K
L
M
N
P
Q
R
S
T
V
W
Y
Austria
China
Japan
Latvia
Russia
Korea
USSR

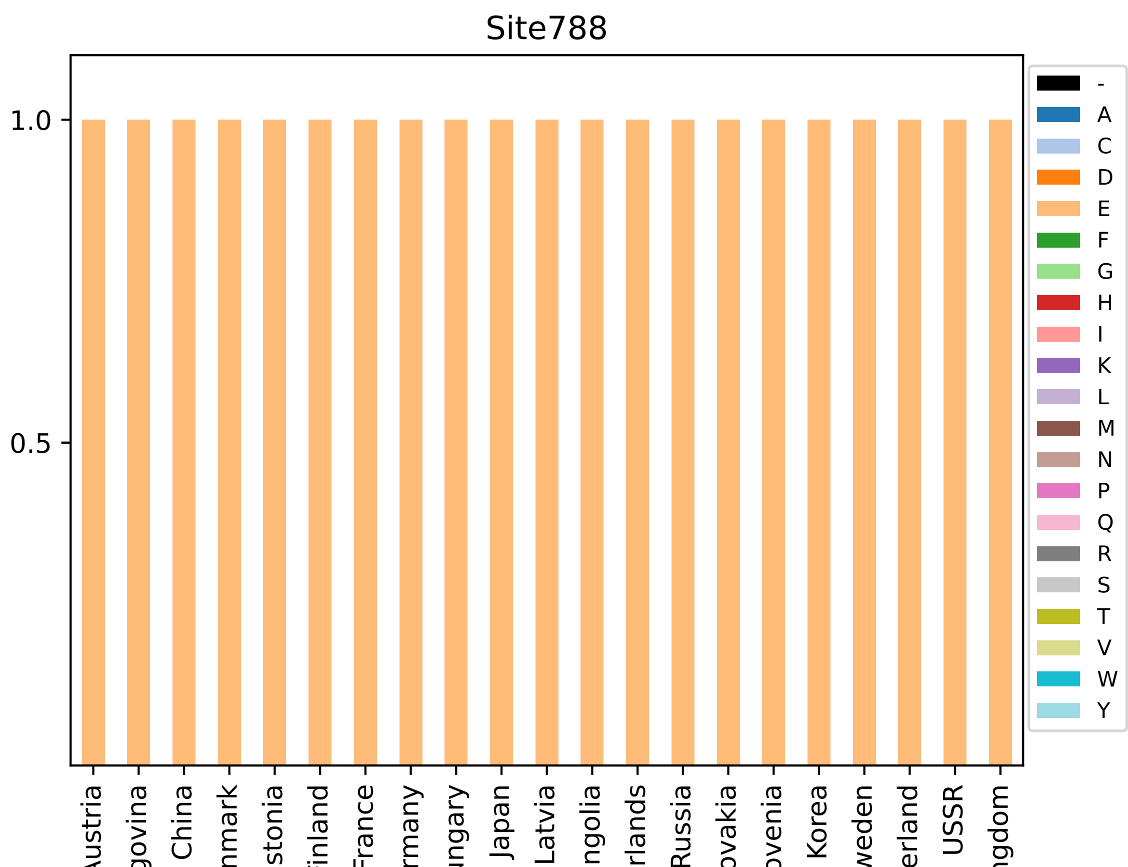
Site788
1.0
0.5
-
A
C
D
E
F
G
H
I
K
L
M
N
P
Q
R
S
T
V
W
Y
China
Japan
Latvia
Russia
Korea
USSR

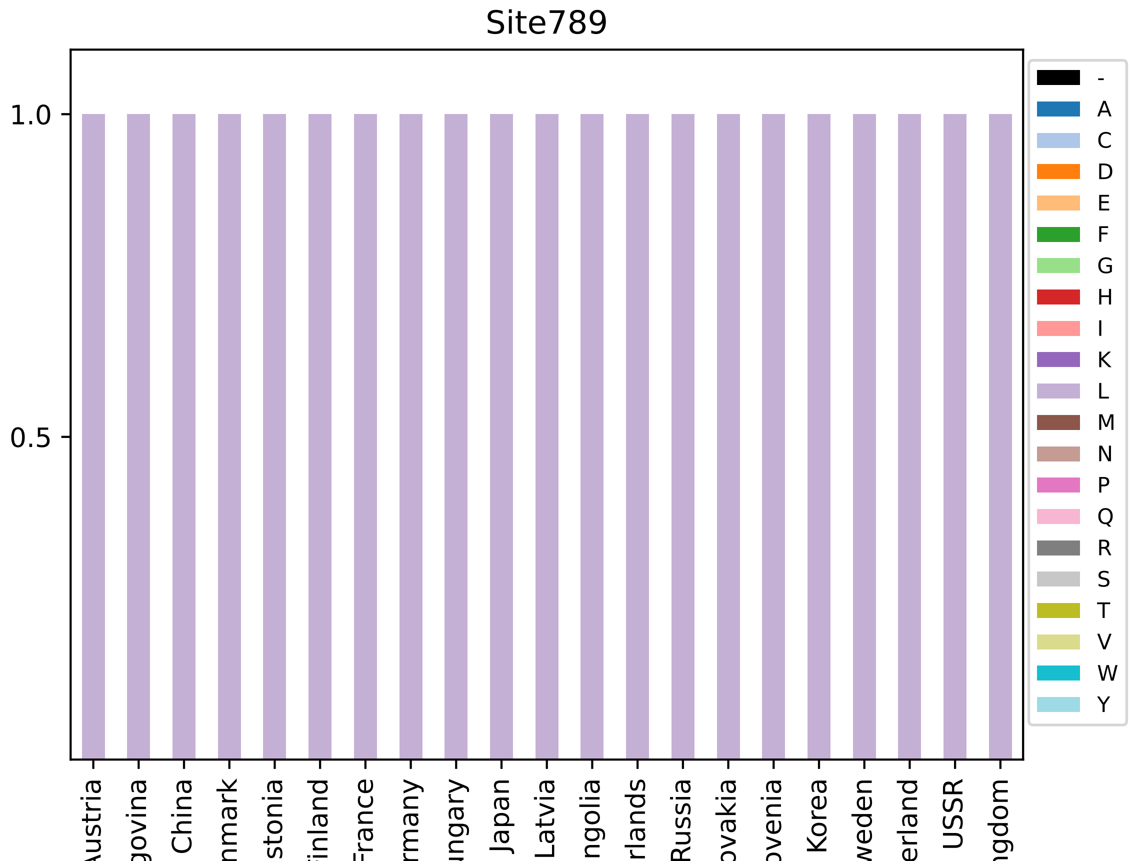
Site789
1.0
0.5
Austria
govina
China
nmark
stonia
inland
France
rmany
ungary
Japan
Latvia
ngolia
rlands
Russia
ovakia
ovenia
Korea
weden
erland
USSR
ngdom
-
A
C
D
E
F
G
H
I
K
L
M
N
P
Q
R
S
T
V
W
Y

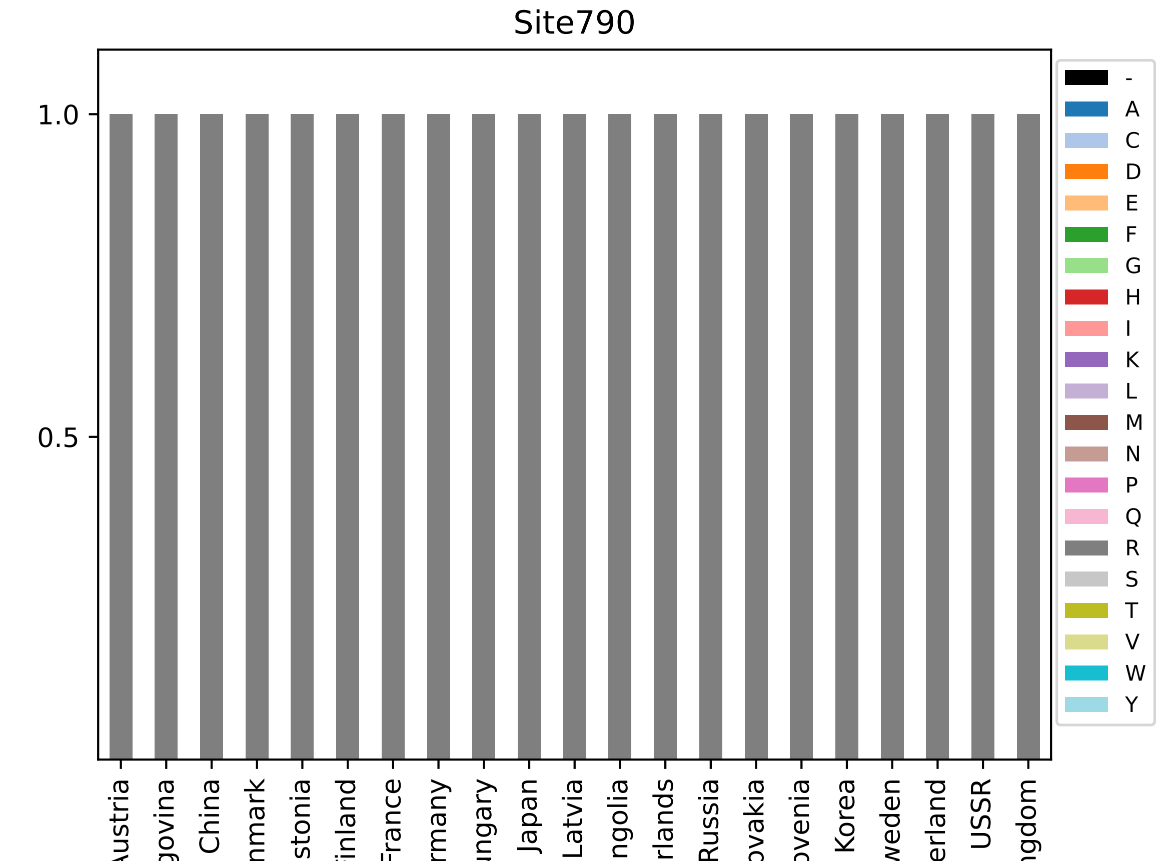
Site790
1.0
0.5
Austria
govina
China
nmark
stonia
Finland
France
rmany
ungary
Japan
Latvia
ngolia
rlands
Russia
ovakia
ovenia
Korea
weden
erland
USSR
ngdom
-
A
C
D
E
F
G
H
I
K
L
M
N
P
Q
R
S
T
V
W
Y

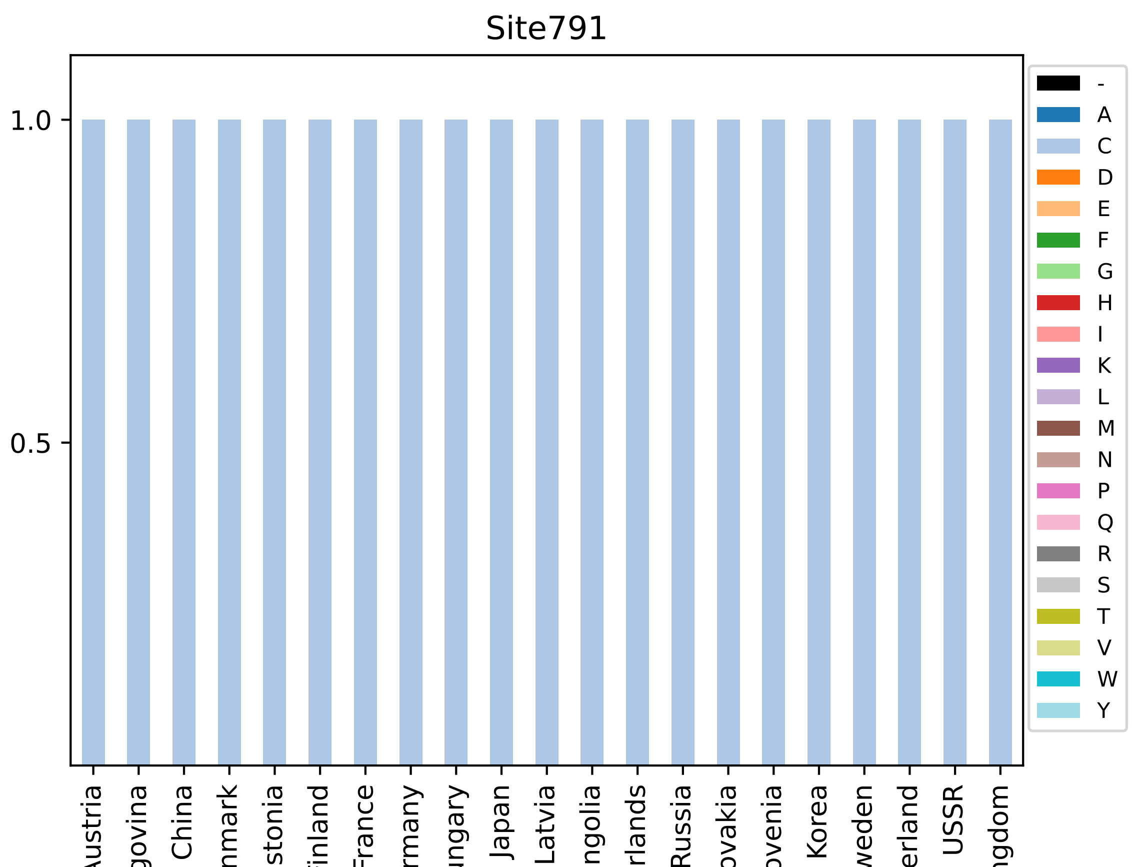

Site791
1.0
0.5
-
A
C
D
E
F
G
H
I
K
L
M
N
P
Q
R
S
T
V
W
Y
China
Japan
Latvia
Russia
Korea
USSR

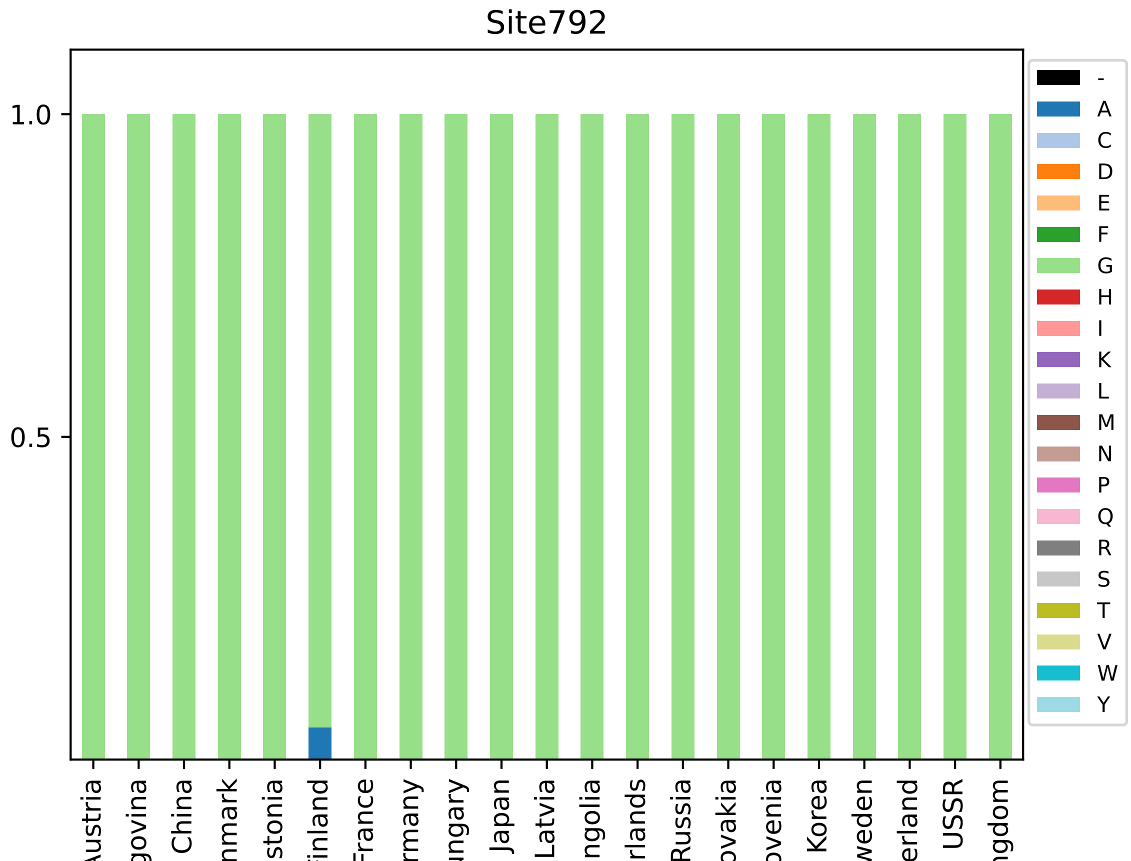

Site792
1.0
0.5
-
A
C
D
E
F
G
H
I
K
L
M
N
P
Q
R
S
T
V
W
Y
Austria
govina
China
nmark
stonia
Finland
France
rmany
ungary
Japan
Latvia
ngolia
rlands
Russia
ovakia
ovenia
Korea
weden
erland
USSR
ngdom

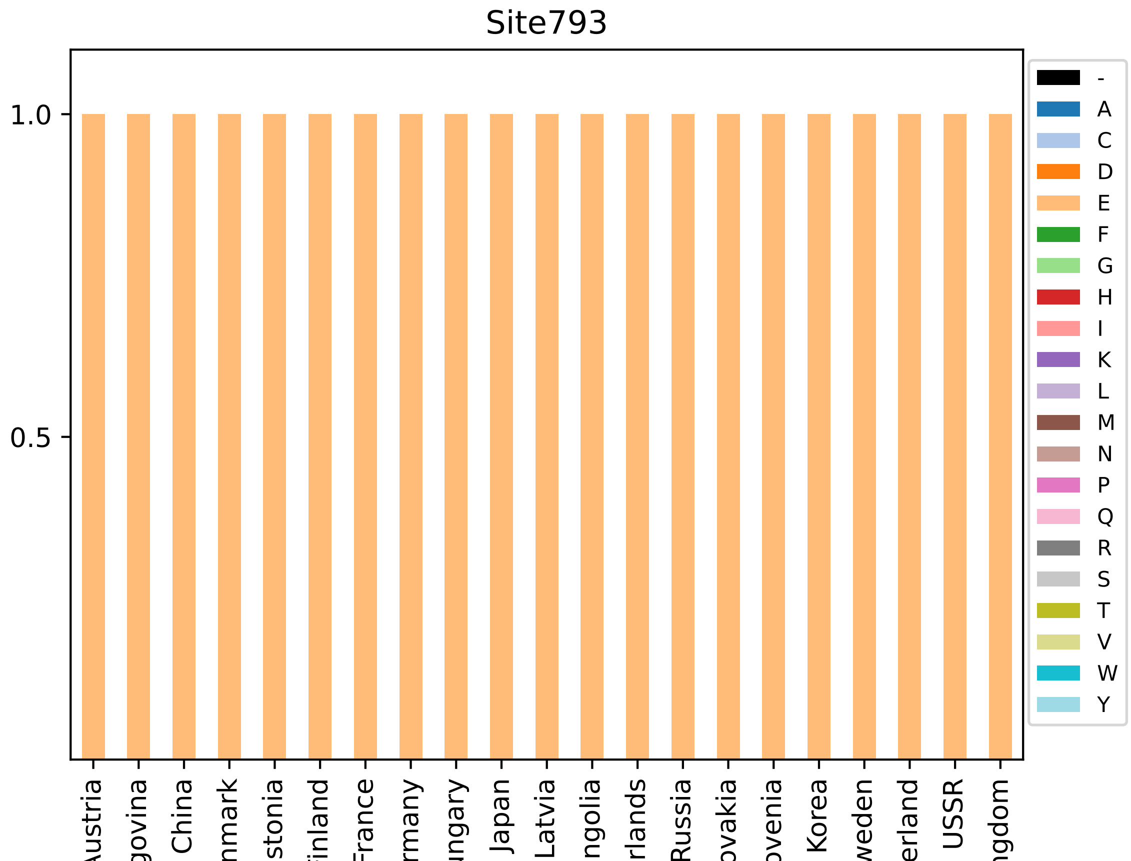

Site793
1.0
0.5
-
A
C
D
E
F
G
H
I
K
L
M
N
P
Q
R
S
T
V
W
Y
China
Japan
Latvia
Russia
Korea
USSR

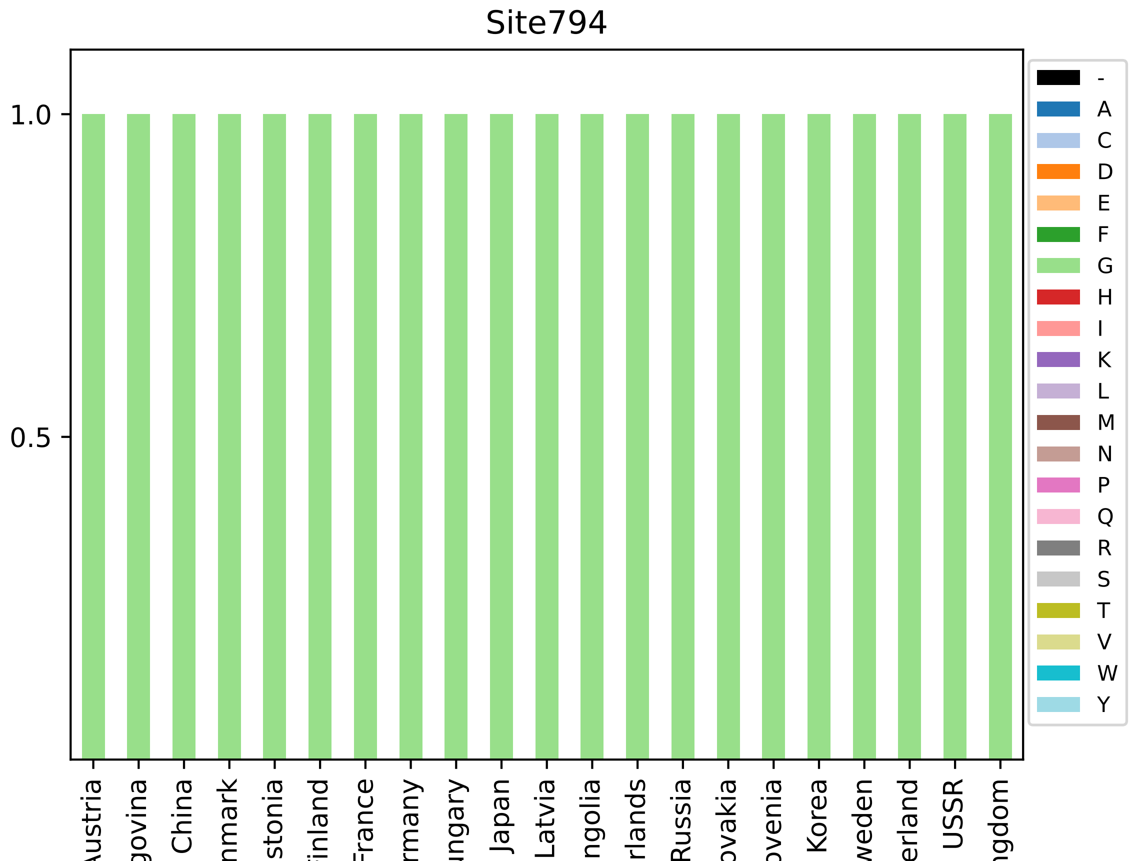

Site794
1.0
0.5
-
A
C
D
E
F
G
H
I
K
L
M
N
P
Q
R
S
T
V
W
Y
China
Japan
Latvia
Russia
Korea
USSR

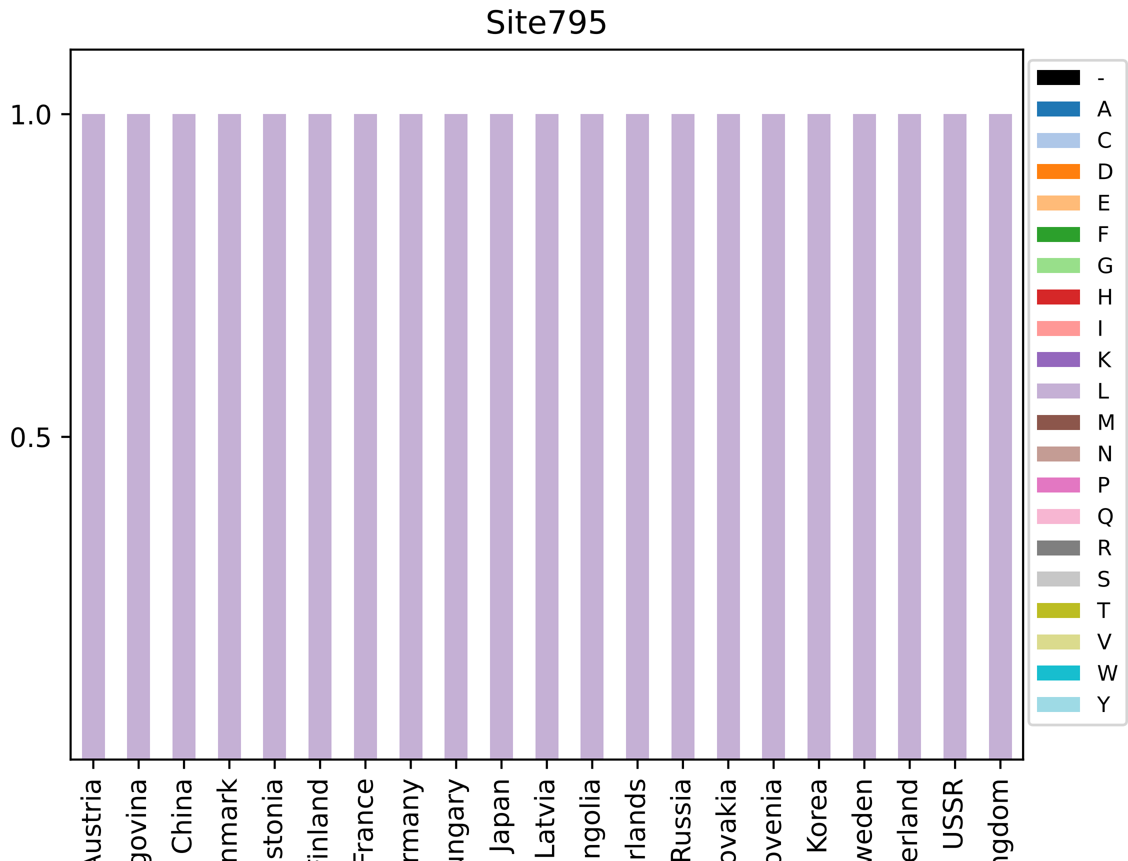

Site795
1.0
0.5
-
A
C
D
E
F
G
H
I
K
L
M
N
P
Q
R
S
T
V
W
Y
Austria
China
Finland
France
Japan
Latvia
Russia
Korea
USSR

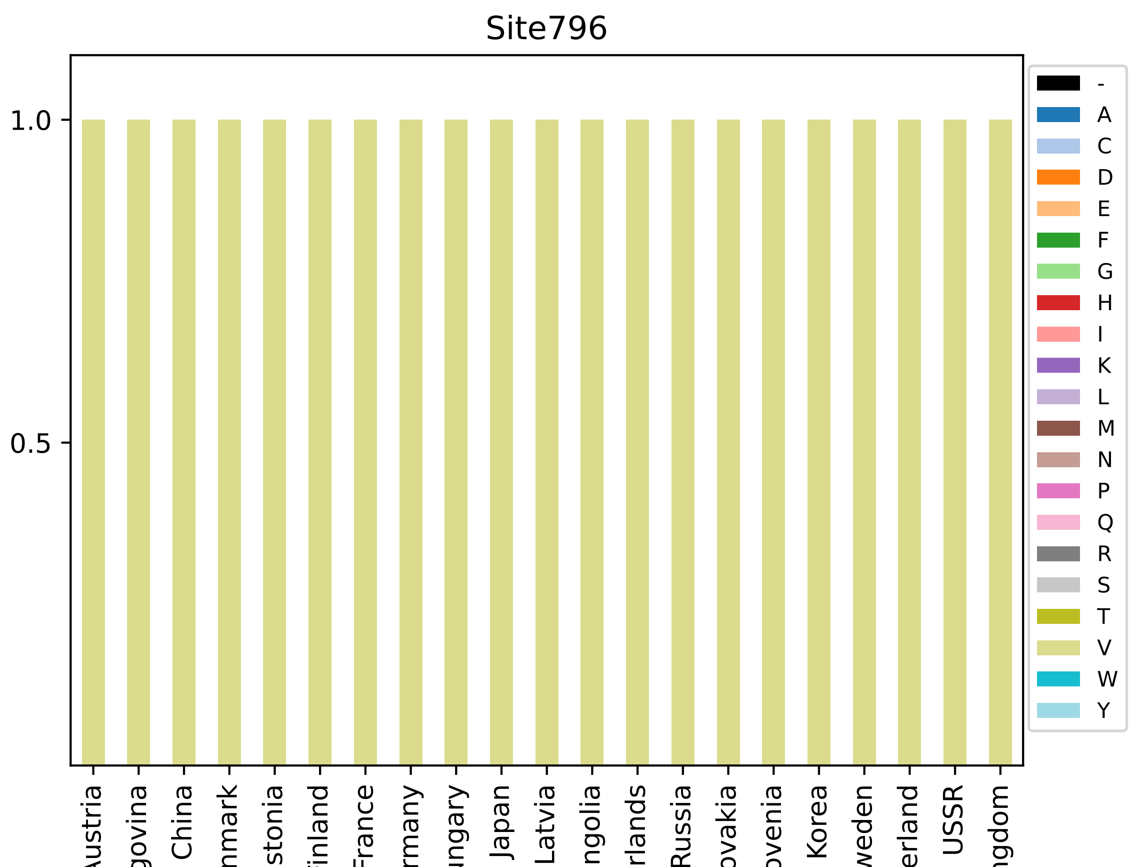
Site796
1.0
0.5
-
A
C
D
E
F
G
H
I
K
L
M
N
P
Q
R
S
T
V
W
Y
China
Japan
Latvia
Russia
Korea
USSR

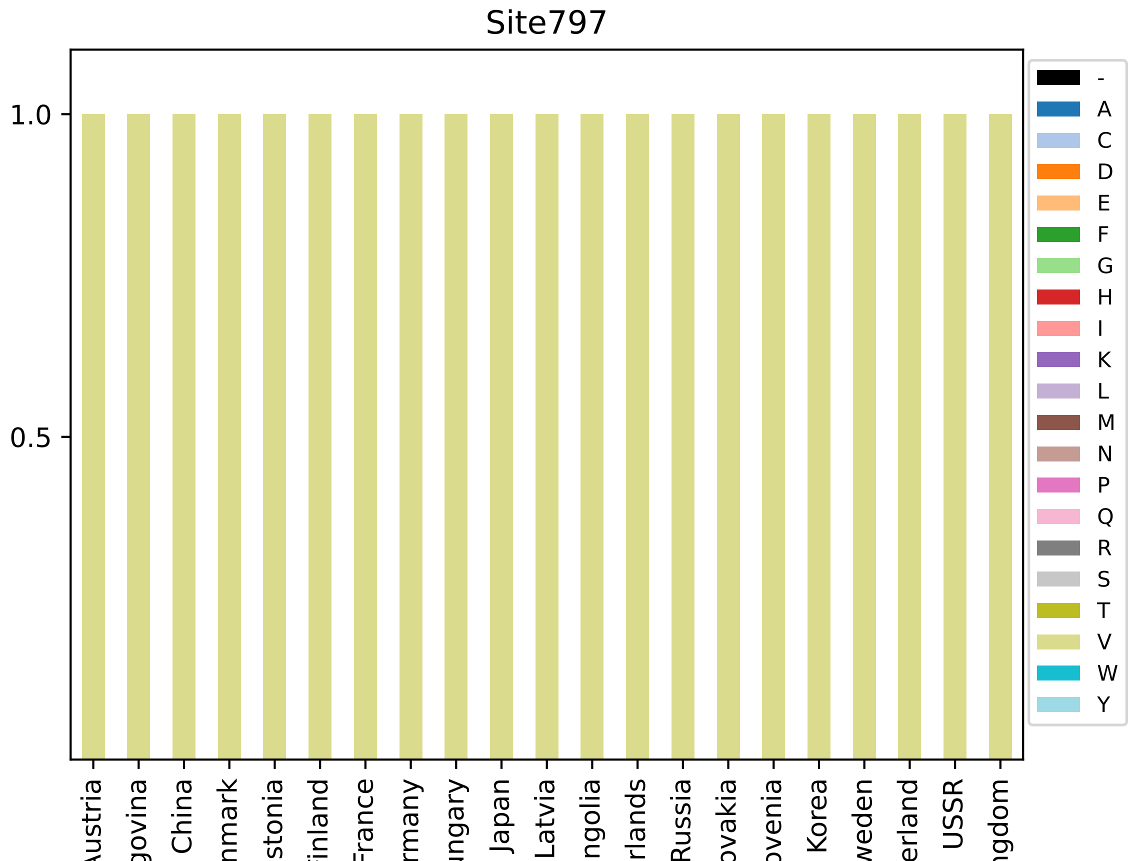
Site797
1.0
0.5
-
A
C
D
E
F
G
H
I
K
L
M
N
P
Q
R
S
T
V
W
Y
Austria
China
Japan
Latvia
Russia
Korea
USSR

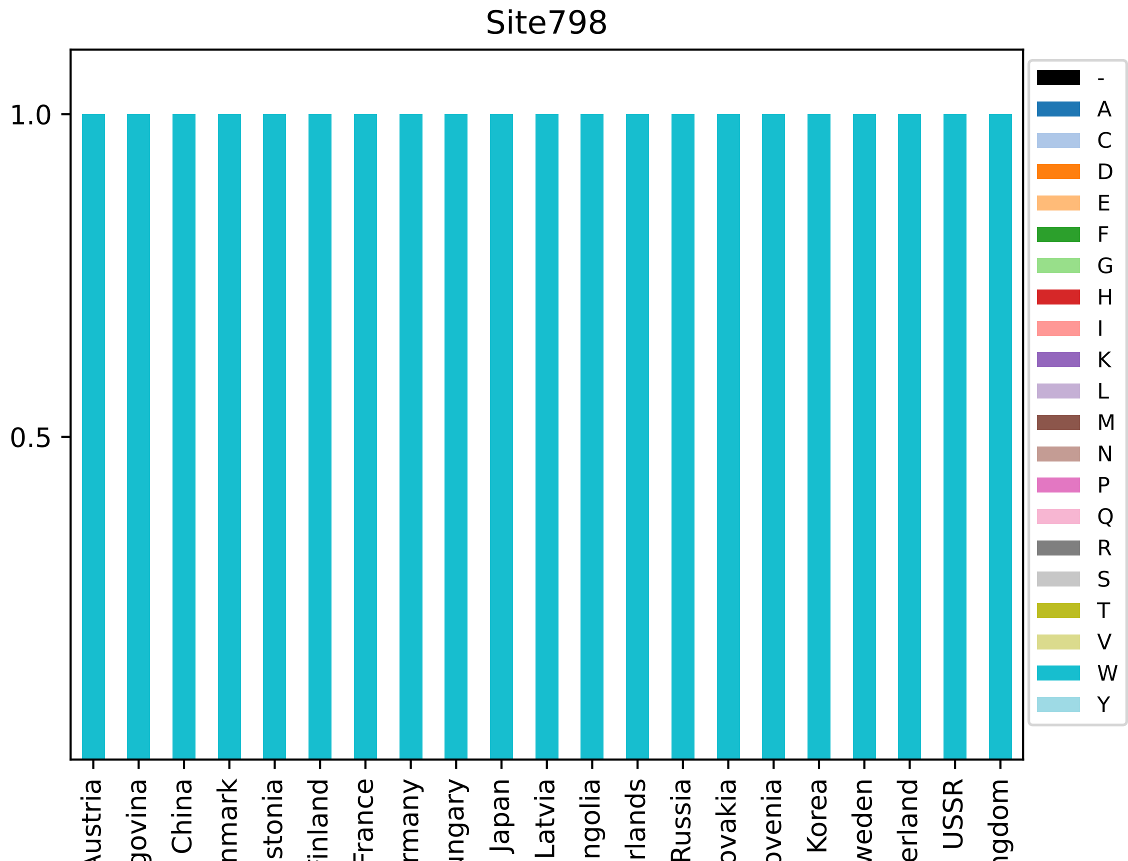

Site798
1.0
0.5
-
A
C
D
E
F
G
H
I
K
L
M
N
P
Q
R
S
T
V
W
Y
China
Japan
Latvia
Russia
Korea
USSR

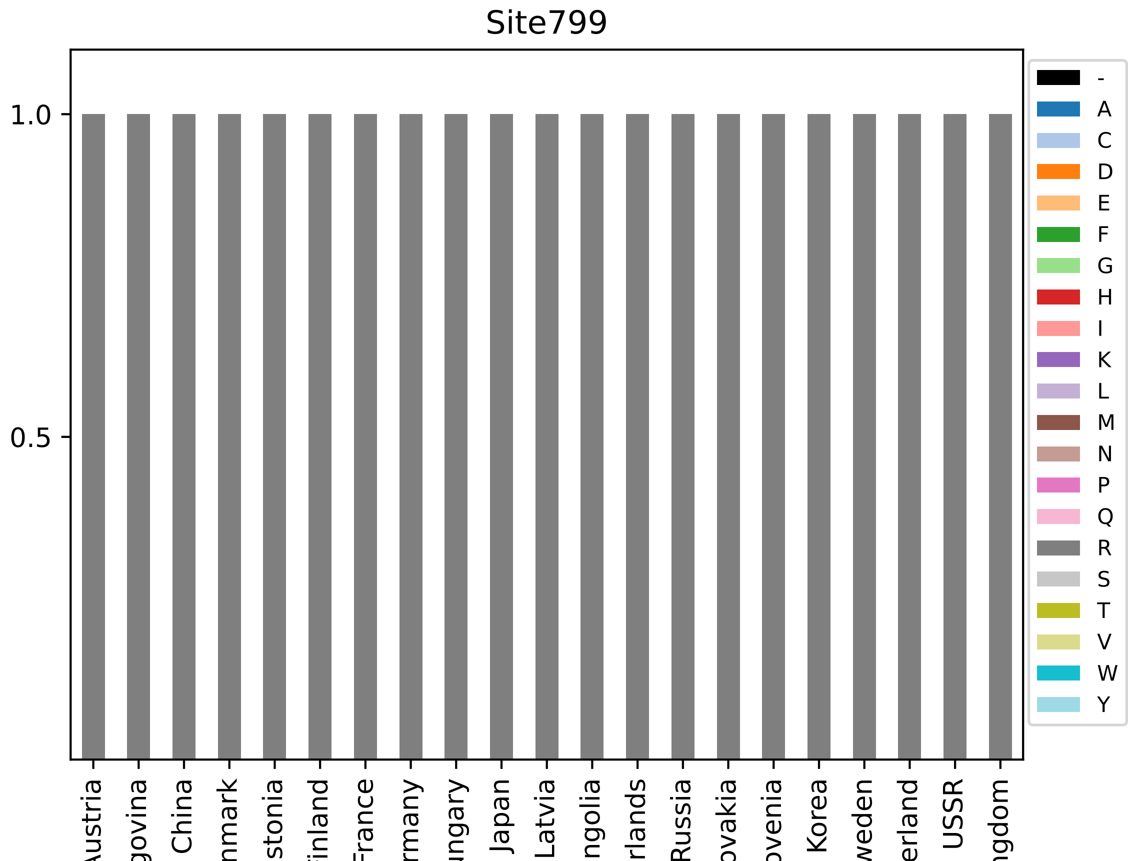
Site799
1.0
0.5
-
A
C
D
E
F
G
H
I
K
L
M
N
P
Q
R
S
T
V
W
Y
China
Japan
Latvia
Russia
Korea
USSR

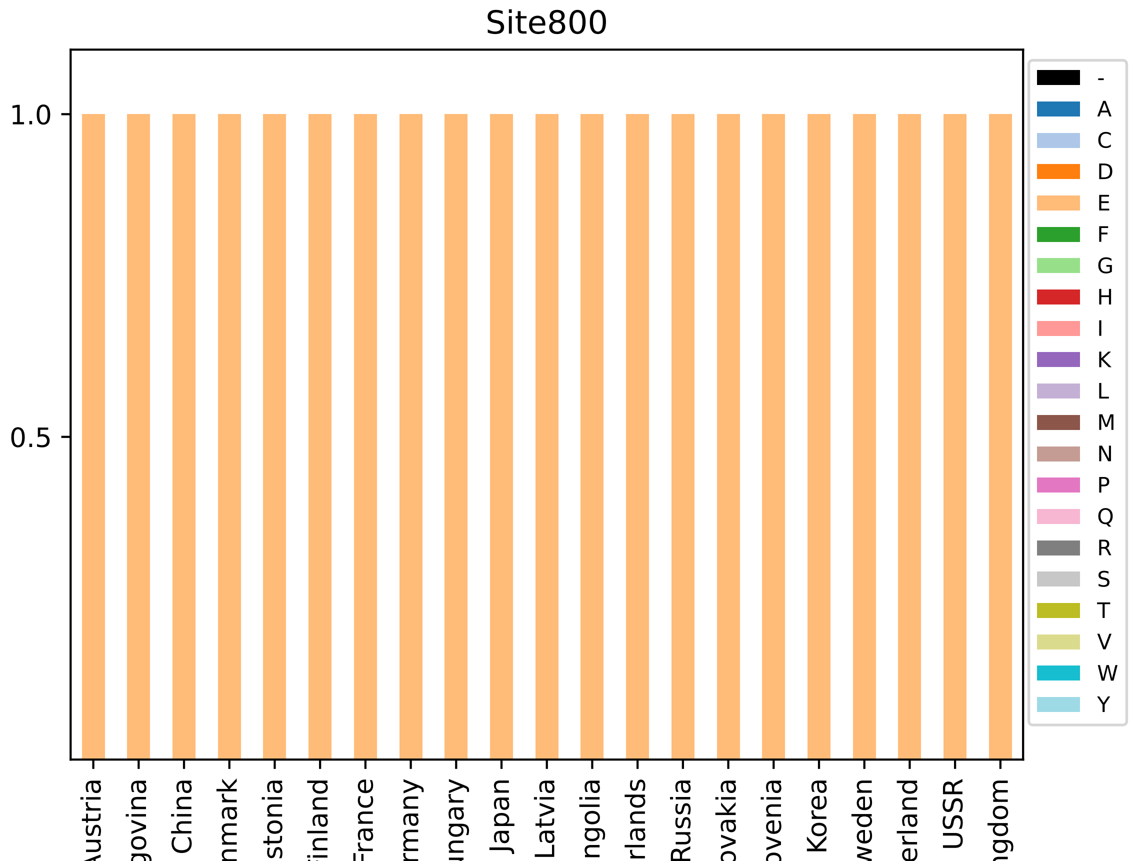

Site800
1.0
0.5
-
A
C
D
E
F
G
H
I
K
L
M
N
P
Q
R
S
T
V
W
Y
Austria
govina
China
nmark
stonia
inland
France
rmany
ungary
Japan
Latvia
ngolia
rlands
Russia
ovakia
ovenia
Korea
weden
erland
USSR
ngdom

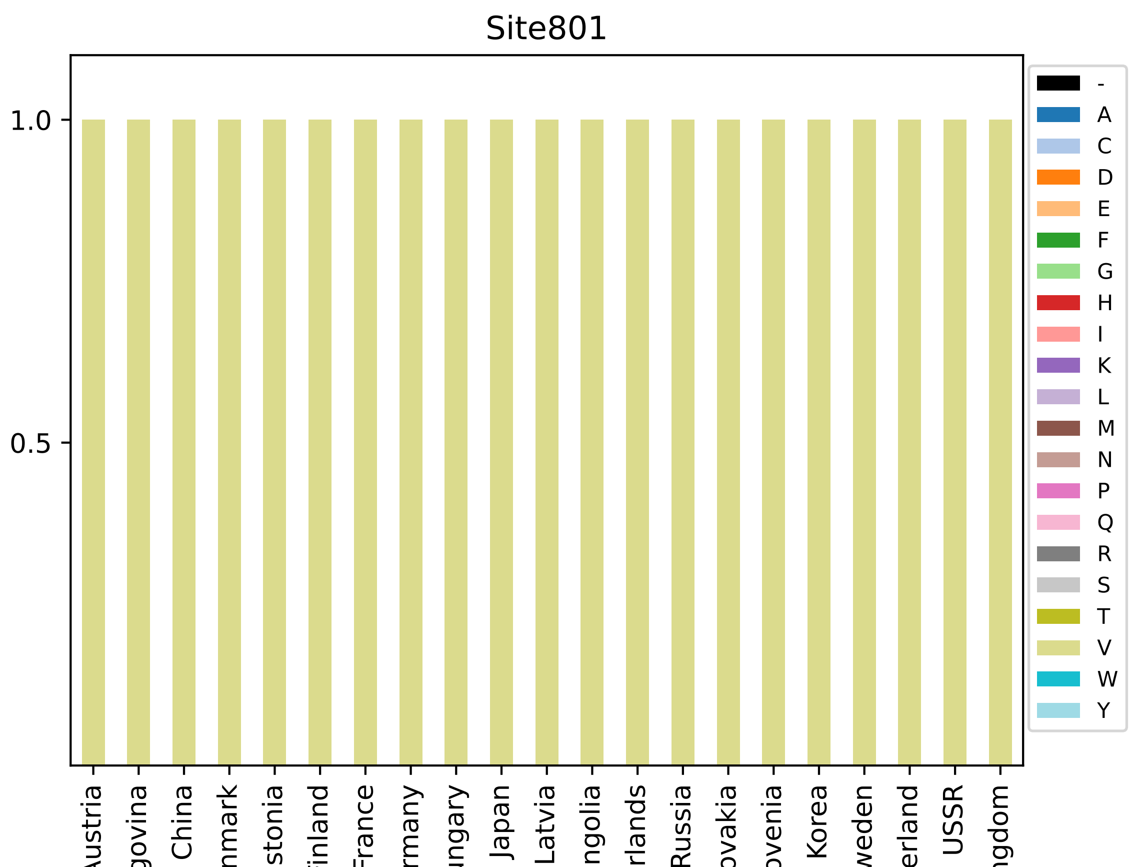
Site801
1.0
0.5
-
A
C
D
E
F
G
H
I
K
L
M
N
P
Q
R
S
T
V
W
Y
China
Japan
Latvia
Russia
Korea
USSR

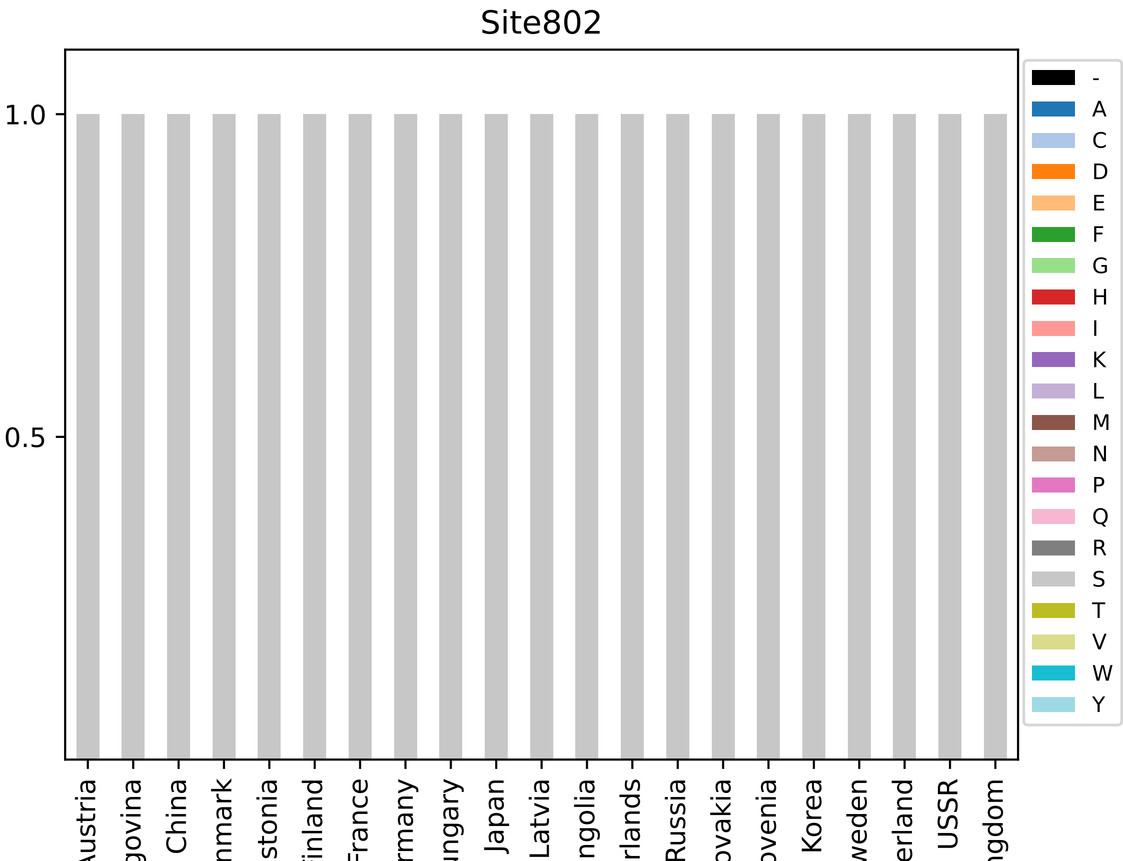
Site802
1.0
0.5
-
A
C
D
E
F
G
H
I
K
L
M
N
P
Q
R
S
T
V
W
Y
China
Japan
Latvia
Russia
Korea
USSR

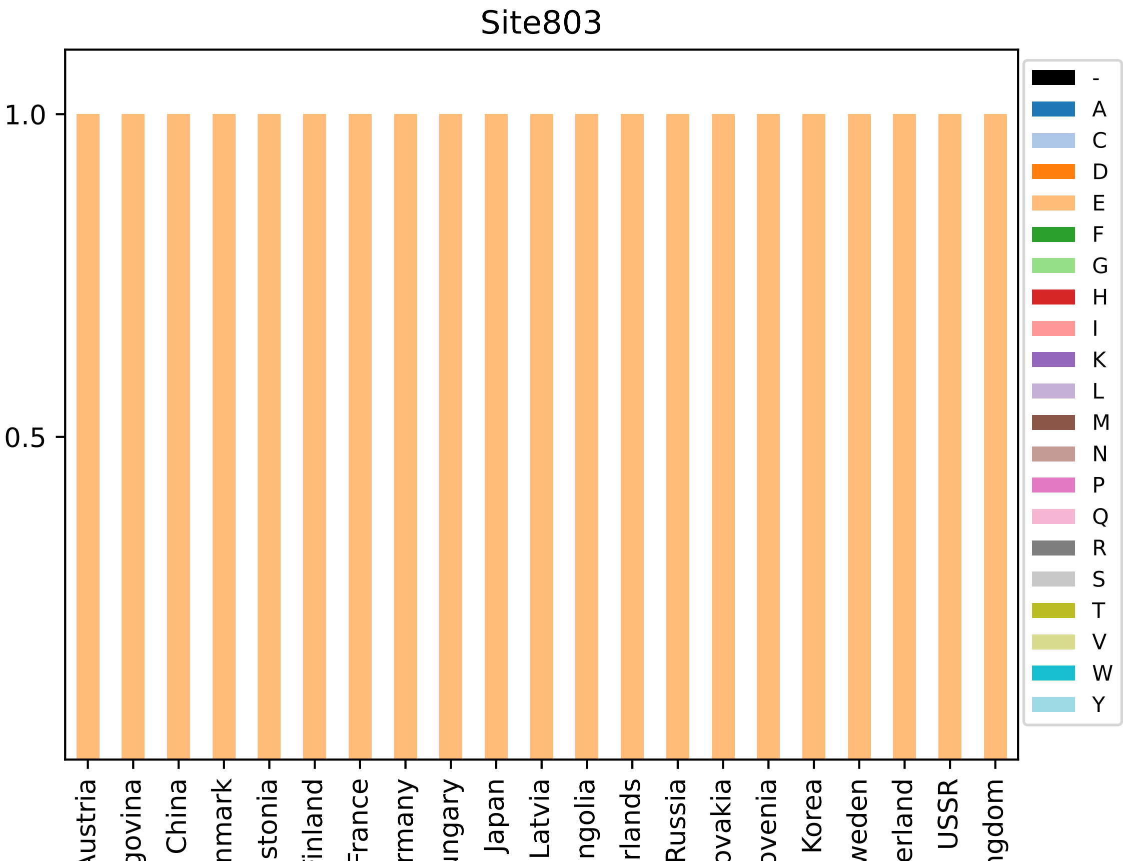
Site803
1.0
0.5
Austria
govina
China
nmark
stonia
Finland
France
rmany
ungary
Japan
Latvia
ngolia
rlands
Russia
ovakia
ovenia
Korea
weden
erland
USSR
ngdom
-
A
C
D
E
F
G
H
I
K
L
M
N
P
Q
R
S
T
V
W
Y

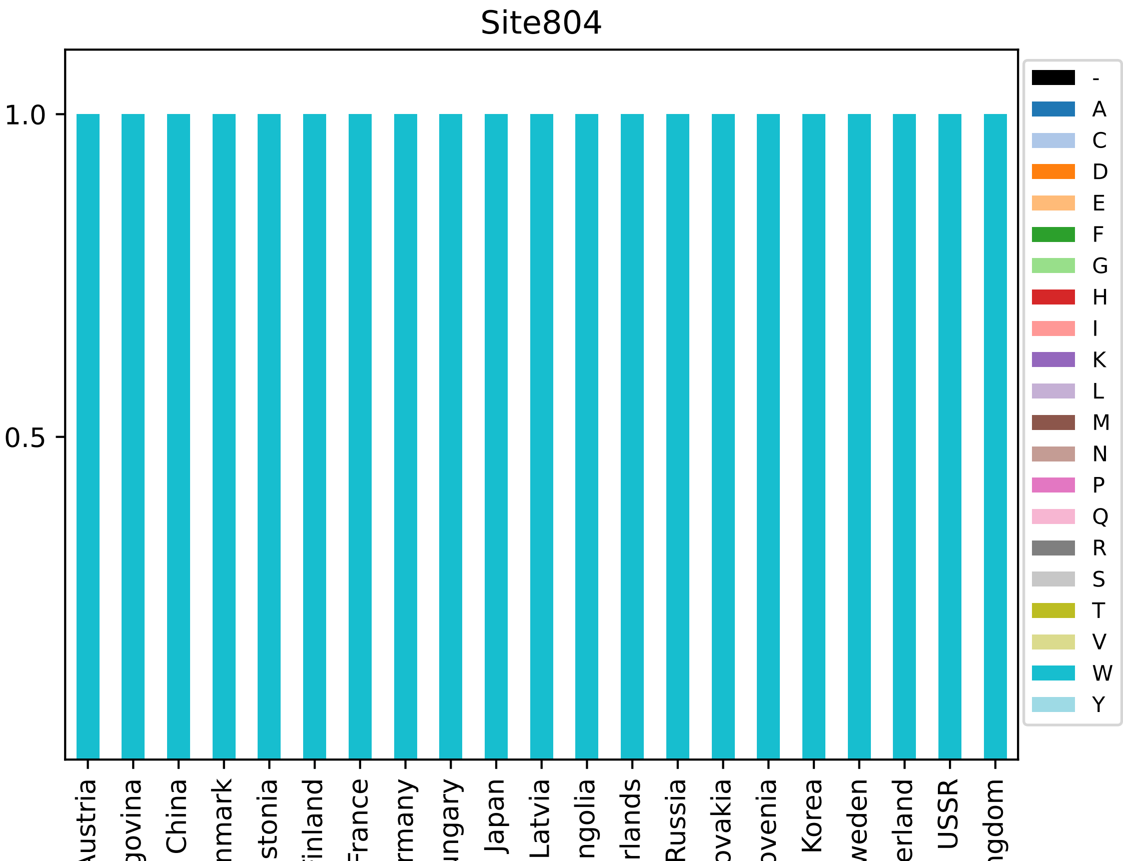
Site804
1.0
0.5
-
A
C
D
E
F
G
H
I
K
L
M
N
P
Q
R
S
T
V
W
Y
Austria
govina
China
nmark
stonia
inland
France
rmany
ungary
Japan
Latvia
ngolia
rlands
Russia
ovakia
ovenia
Korea
weden
erland
USSR
ngdom

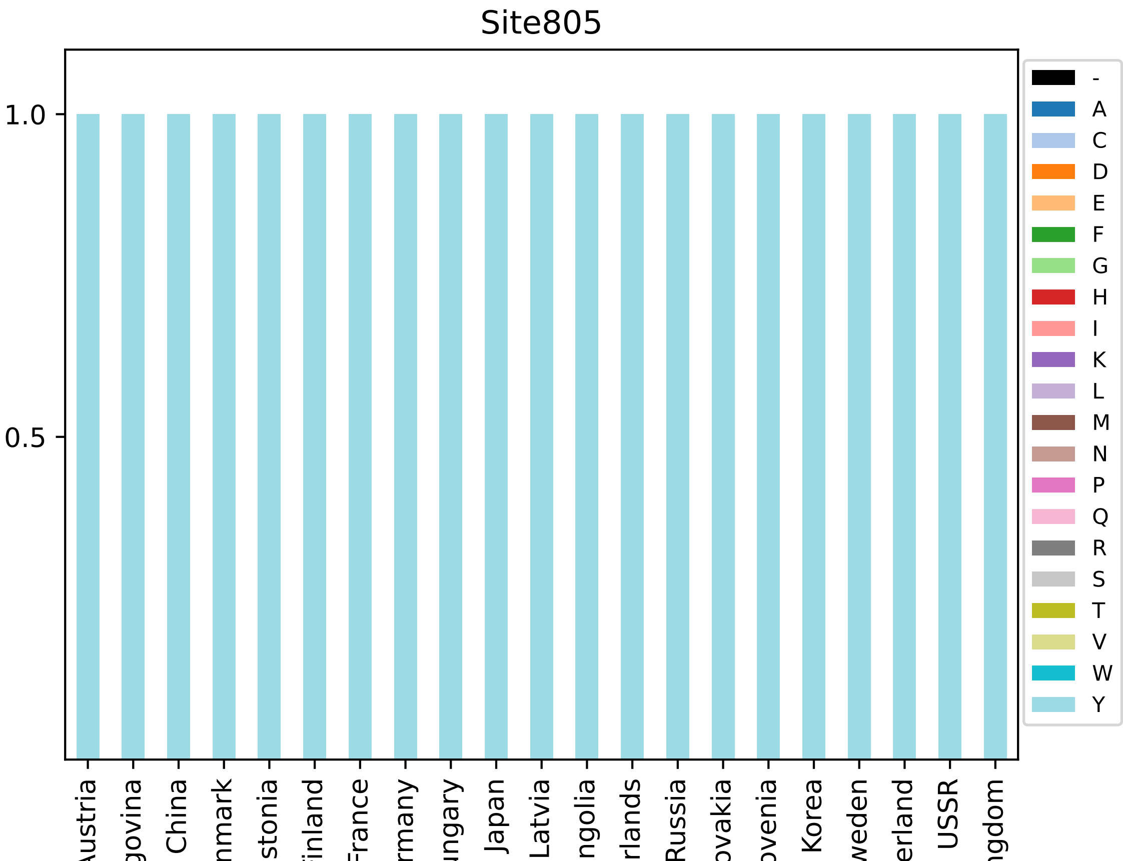

Site805
1.0
0.5
-
A
C
D
E
F
G
H
I
K
L
M
N
P
Q
R
S
T
V
W
Y
China
Japan
Latvia
Russia
Korea
USSR

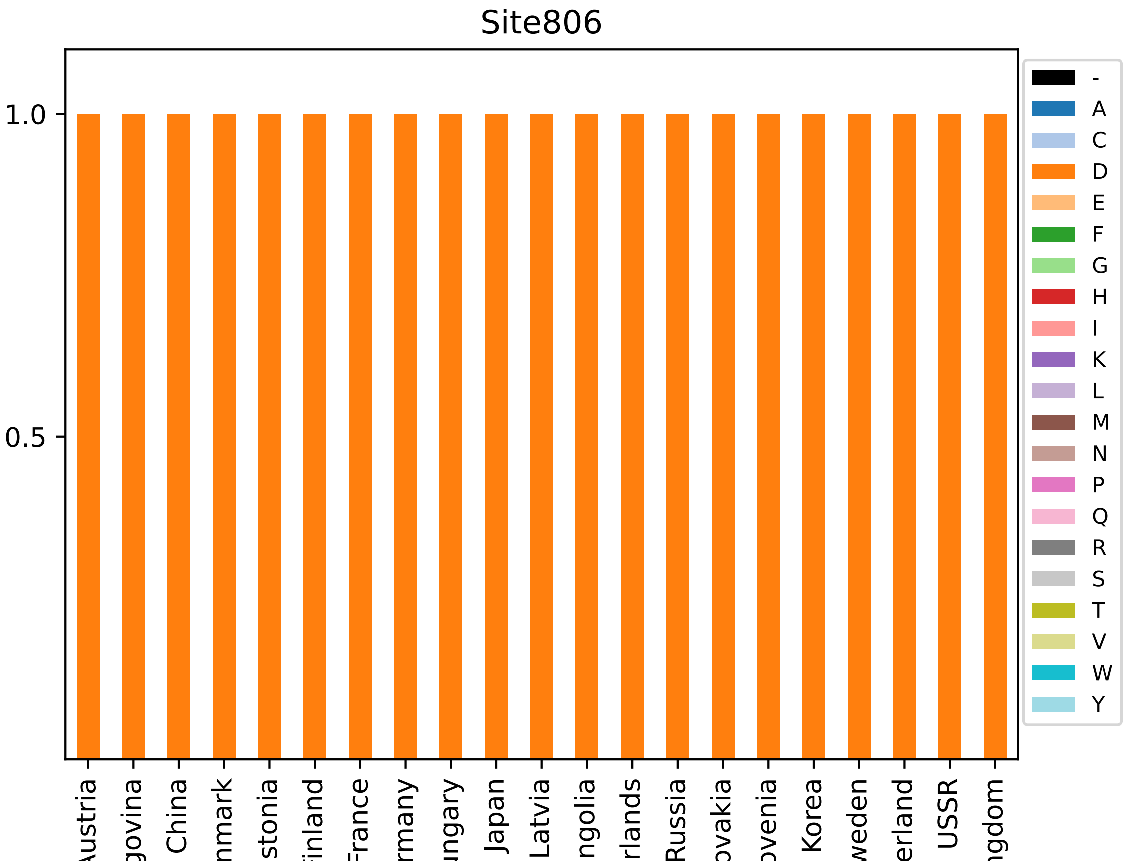
Site806
1.0
0.5
-
A
C
D
E
F
G
H
I
K
L
M
N
P
Q
R
S
T
V
W
Y
Austria
govina
China
nmark
stonia
Finland
France
rmany
ungary
Japan
Latvia
ngolia
rlands
Russia
ovakia
ovenia
Korea
weden
erland
USSR
ngdom

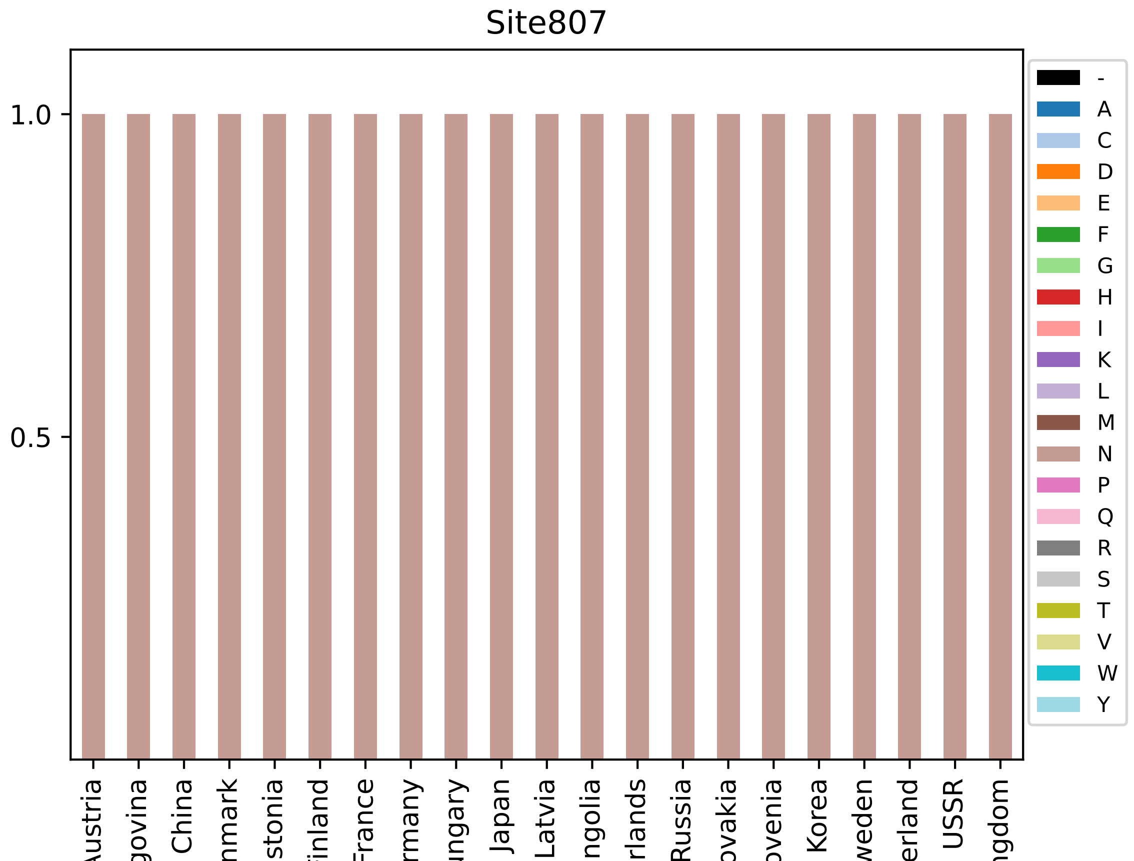

Site807
1.0
0.5
-
A
C
D
E
F
G
H
I
K
L
M
N
P
Q
R
S
T
V
W
Y
Austria
China
Japan
Latvia
Russia
Korea
USSR

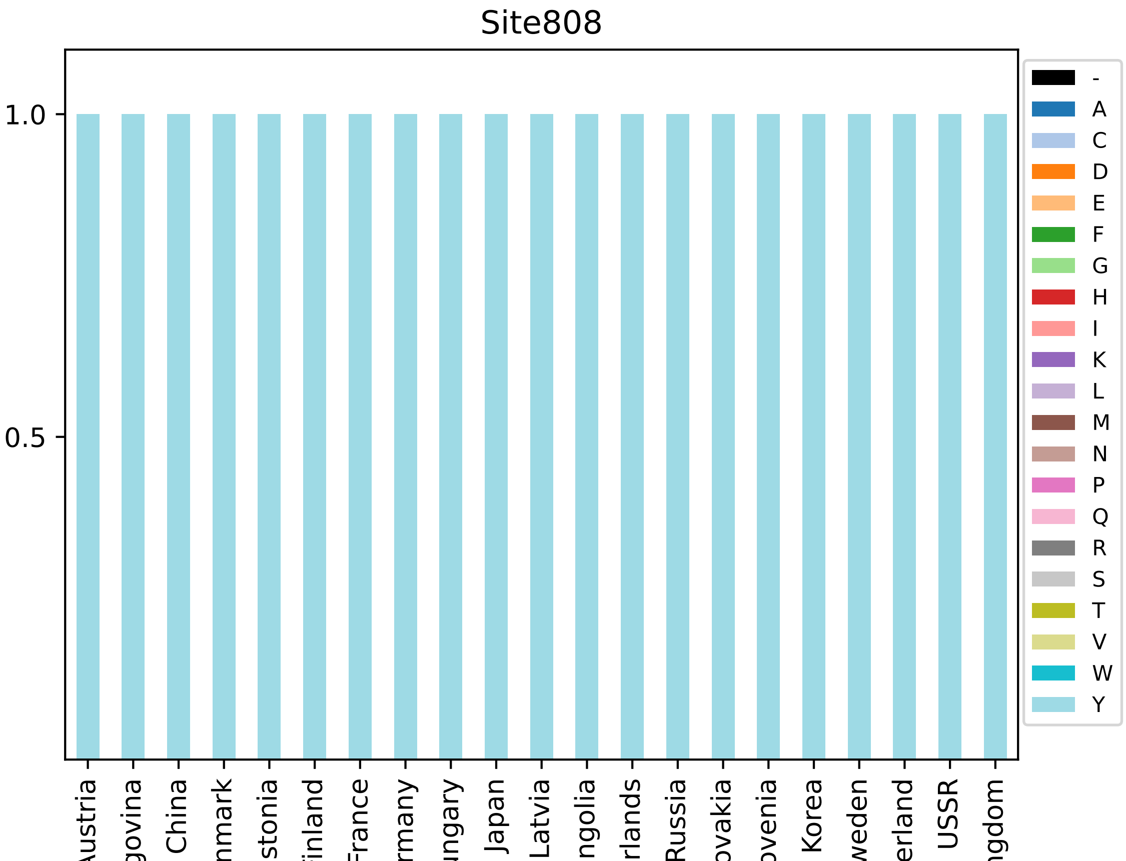

Site808
1.0
0.5
-
A
C
D
E
F
G
H
I
K
L
M
N
P
Q
R
S
T
V
W
Y
Austria
China
Japan
Latvia
Russia
Korea
USSR

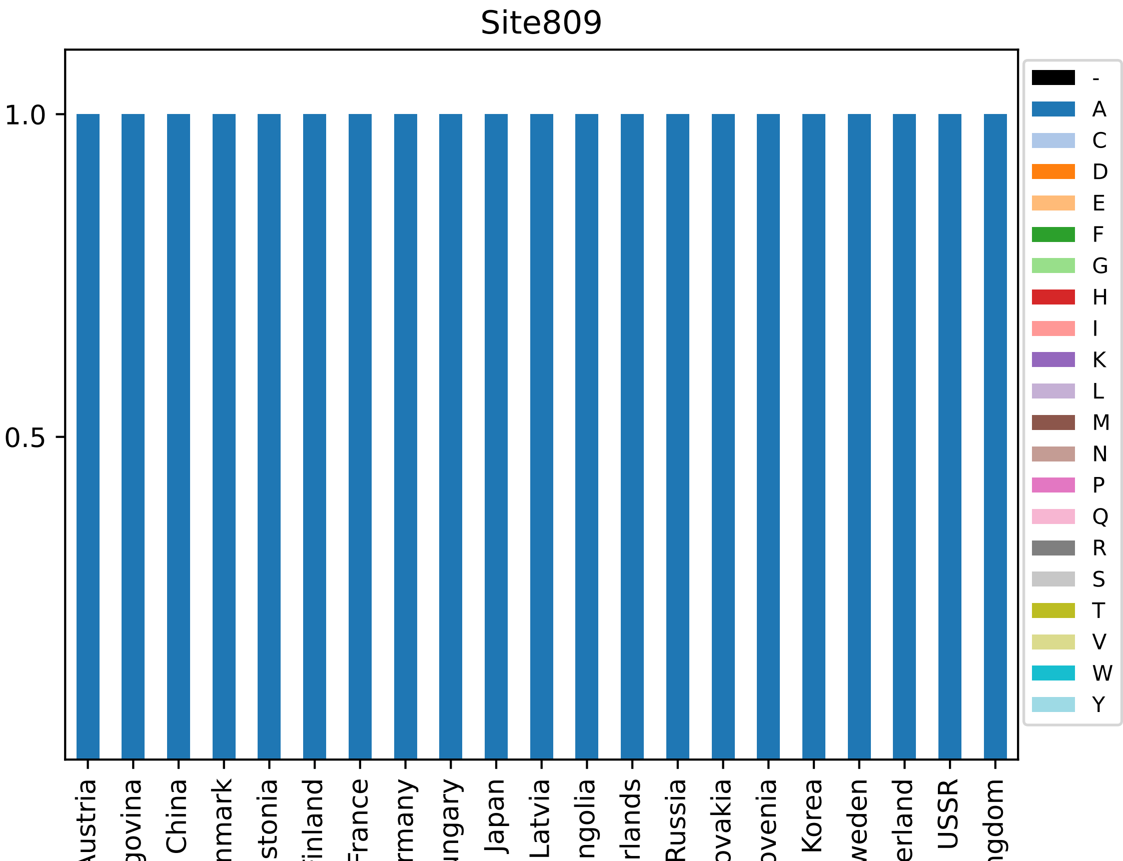
Site809
1.0
0.5
-
A
C
D
E
F
G
H
I
K
L
M
N
P
Q
R
S
T
V
W
Y
China
Japan
Latvia
Russia
Korea
USSR

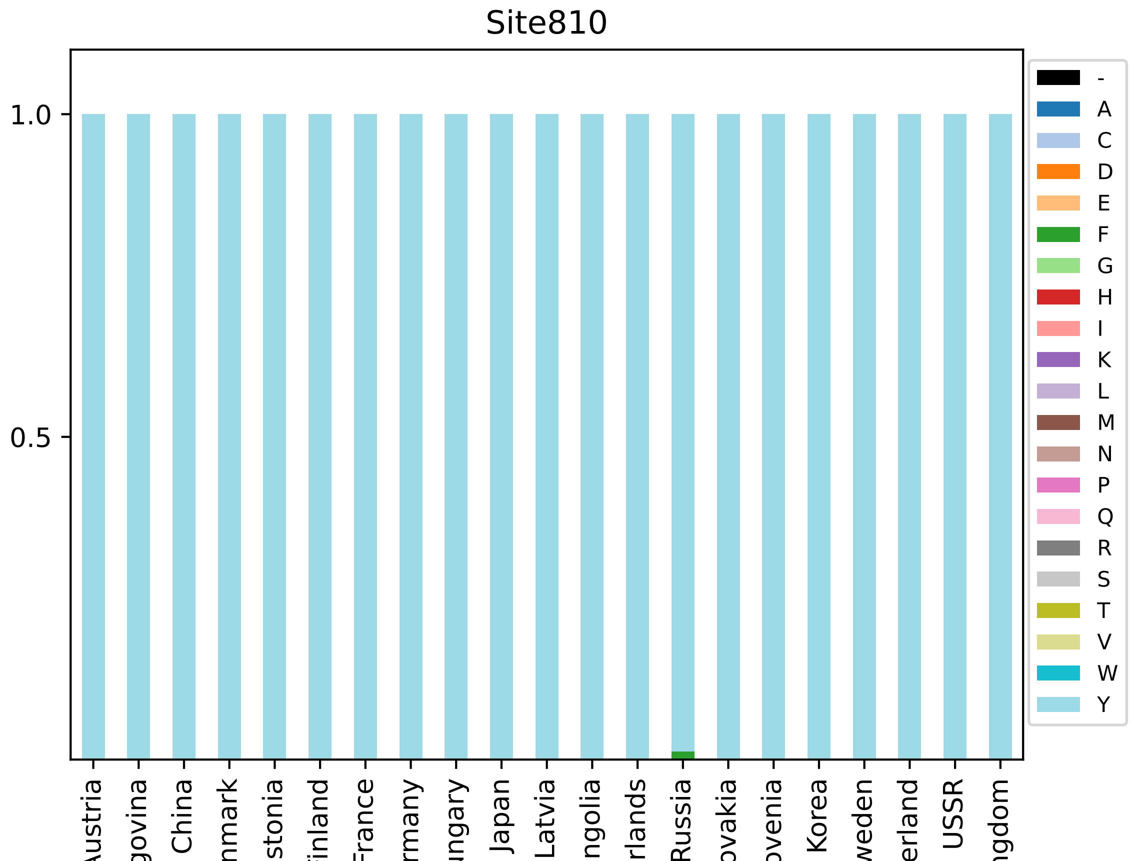
Site810
1.0
0.5
Austria
govina
China
nmark
stonia
inland
France
rmany
ungary
Japan
Latvia
ngolia
rlands
Russia
ovakia
ovenia
Korea
weden
erland
USSR
ngdom
-
A
C
D
E
F
G
H
I
K
L
M
N
P
Q
R
S
T
V
W
Y

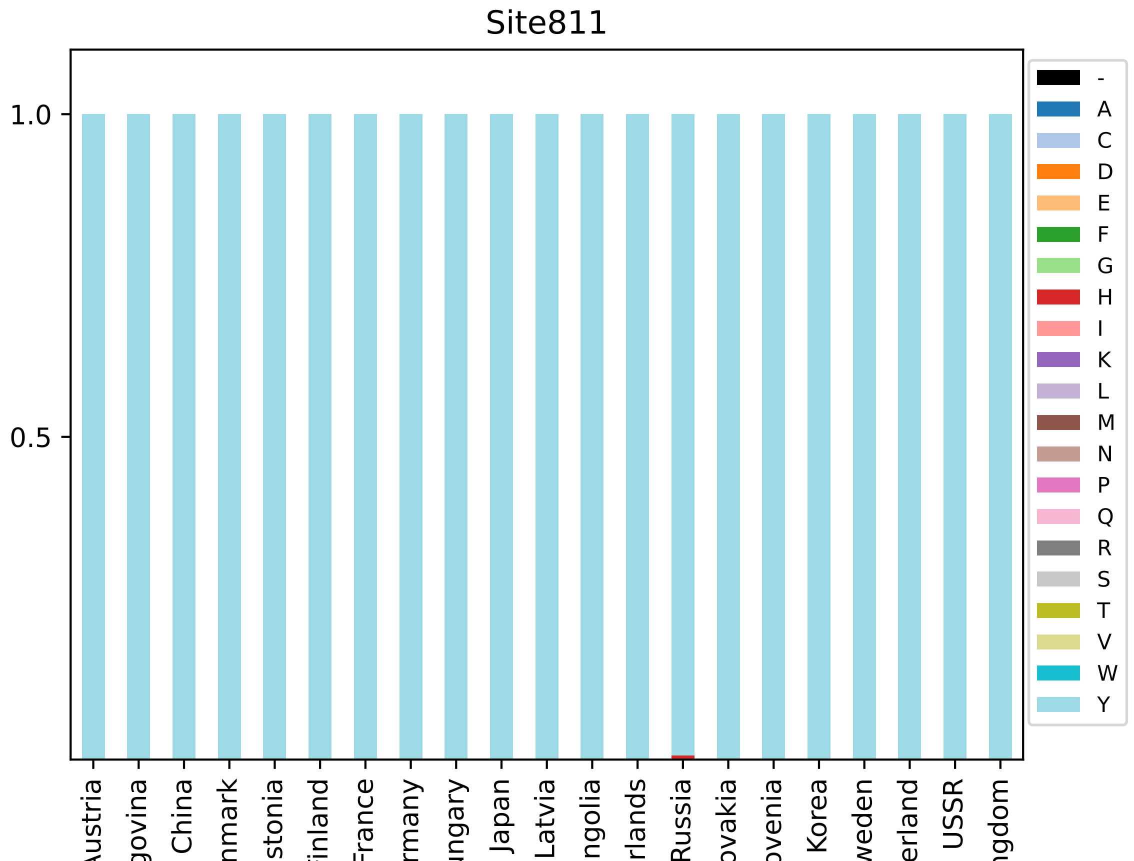
Site811
1.0
0.5
-
A
C
D
E
F
G
H
I
K
L
M
N
P
Q
R
S
T
V
W
Y
China
Japan
Latvia
Russia
Korea
USSR

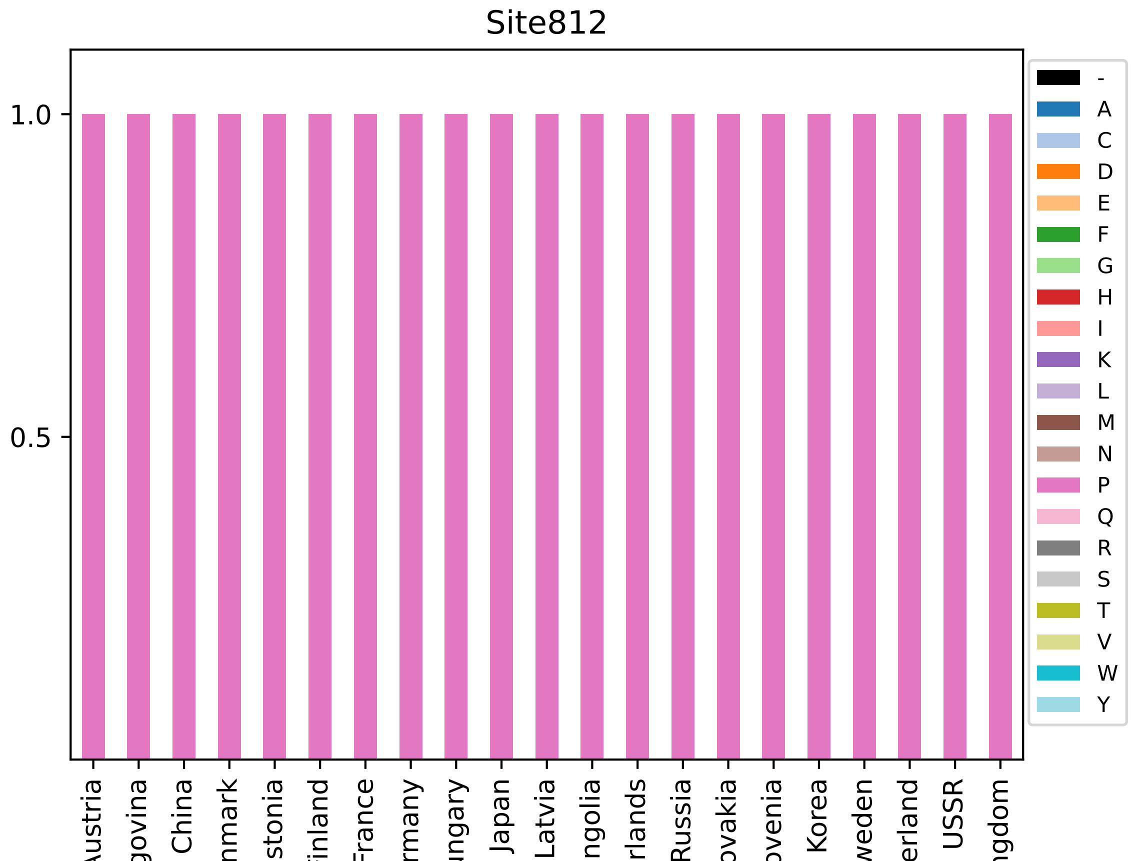
Site812
1.0
0.5
Austria
govina
China
nmark
stonia
inland
France
rmany
ungary
Japan
Latvia
ngolia
rlands
Russia
ovakia
ovenia
Korea
weden
erland
USSR
ngdom
-
A
C
D
E
F
G
H
I
K
L
M
N
P
Q
R
S
T
V
W
Y

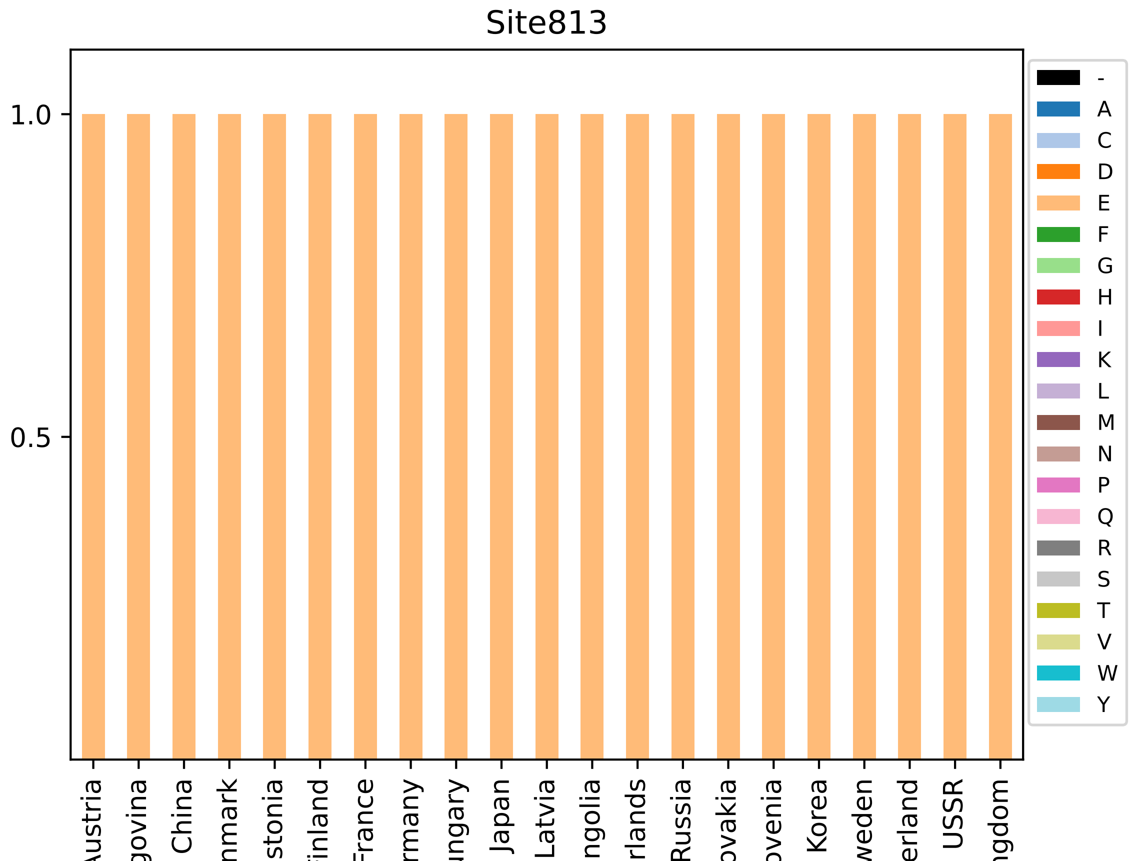
Site813
1.0
0.5
-
A
C
D
E
F
G
H
I
K
L
M
N
P
Q
R
S
T
V
W
Y
China
Japan
Latvia
Russia
Korea
USSR

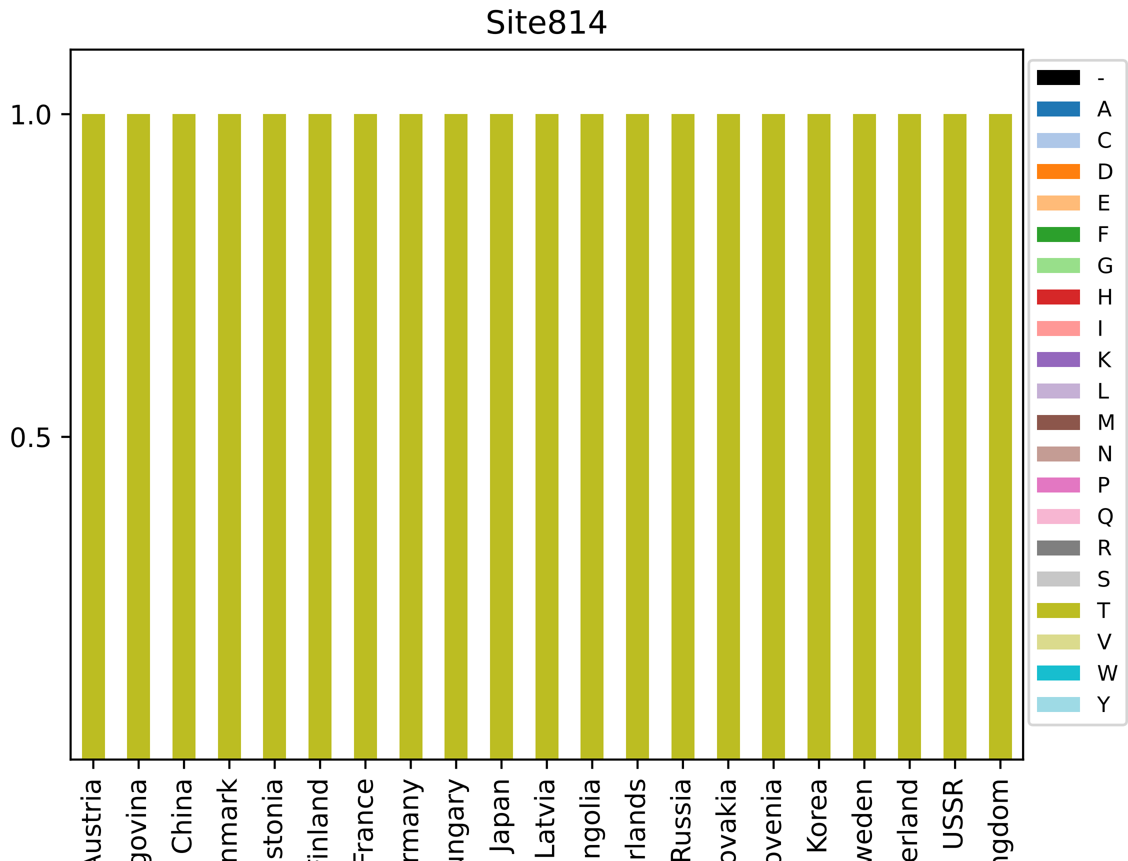
Site814
1.0
0.5
-
A
C
D
E
F
G
H
I
K
L
M
N
P
Q
R
S
T
V
W
Y
Austria
govina
China
nmark
stonia
inland
France
rmany
ungary
Japan
Latvia
ngolia
rlands
Russia
ovakia
ovenia
Korea
weden
erland
USSR
ngdom

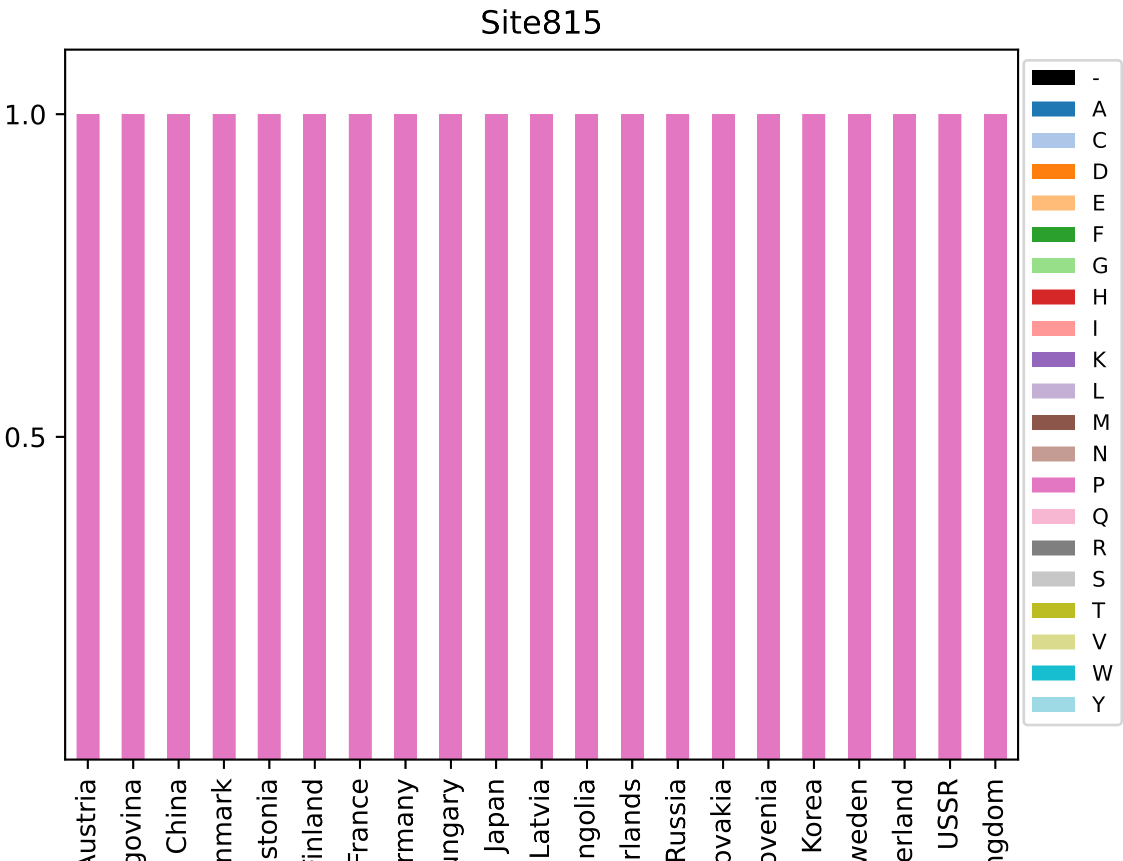

Site815
1.0
0.5
-
A
C
D
E
F
G
H
I
K
L
M
N
P
Q
R
S
T
V
W
Y
Austria
China
Japan
Latvia
Russia
Korea
USSR

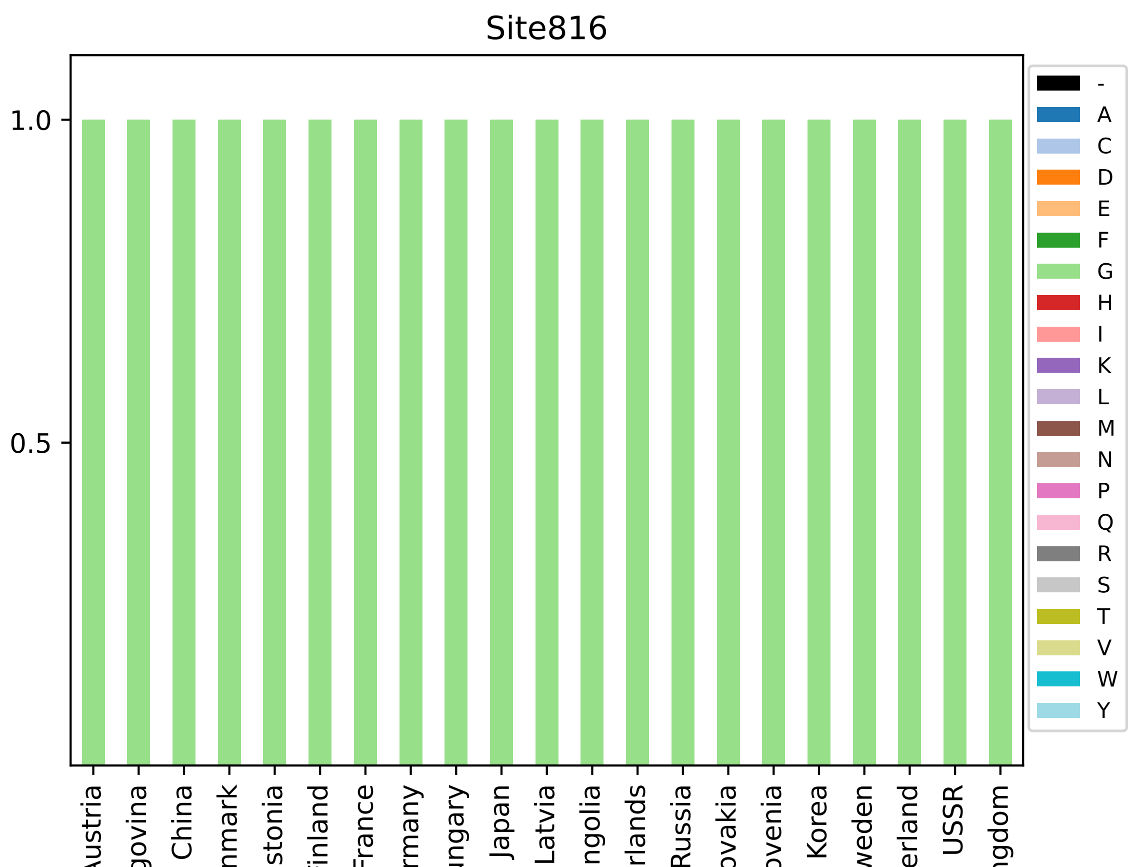
Site816
1.0
0.5
-
A
C
D
E
F
G
H
I
K
L
M
N
P
Q
R
S
T
V
W
Y
China
Japan
Latvia
Russia
Korea
USSR

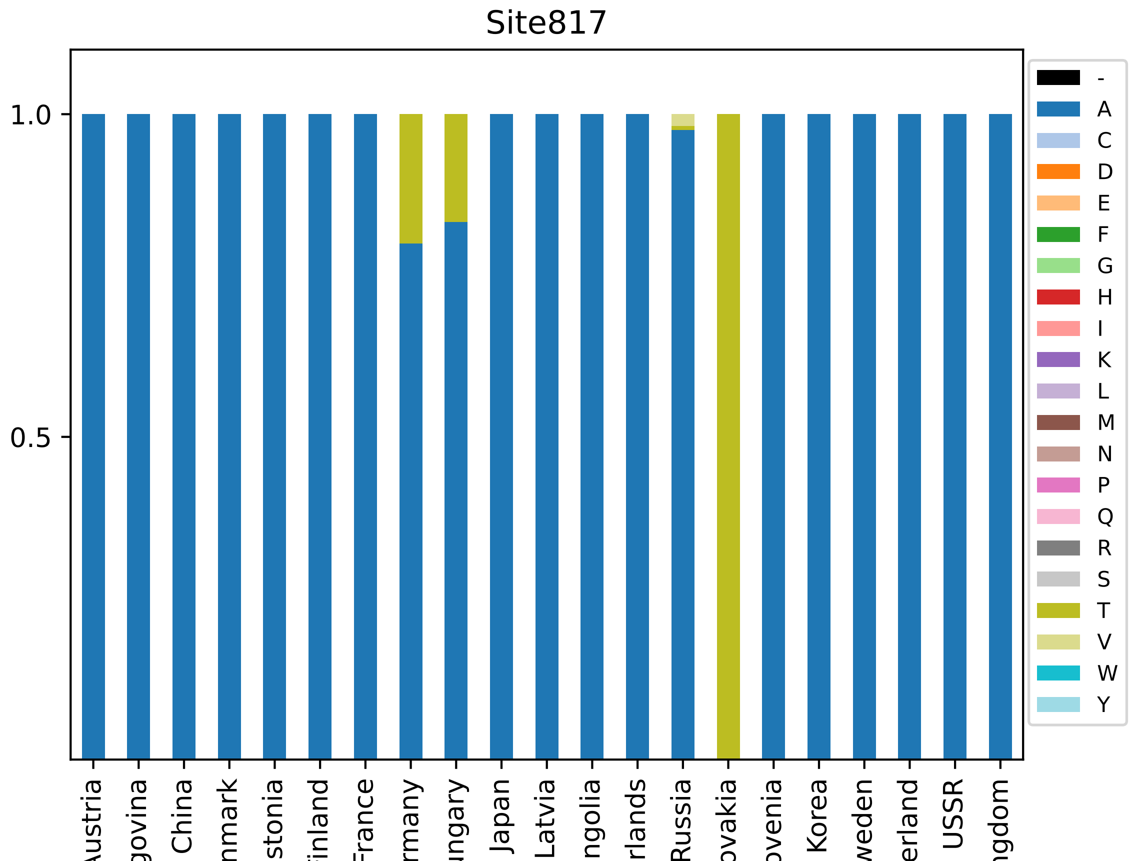
Site817
1.0
0.5
-
A
C
D
E
F
G
H
I
K
L
M
N
P
Q
R
S
T
V
W
Y
China
Japan
Latvia
Russia
Korea
USSR

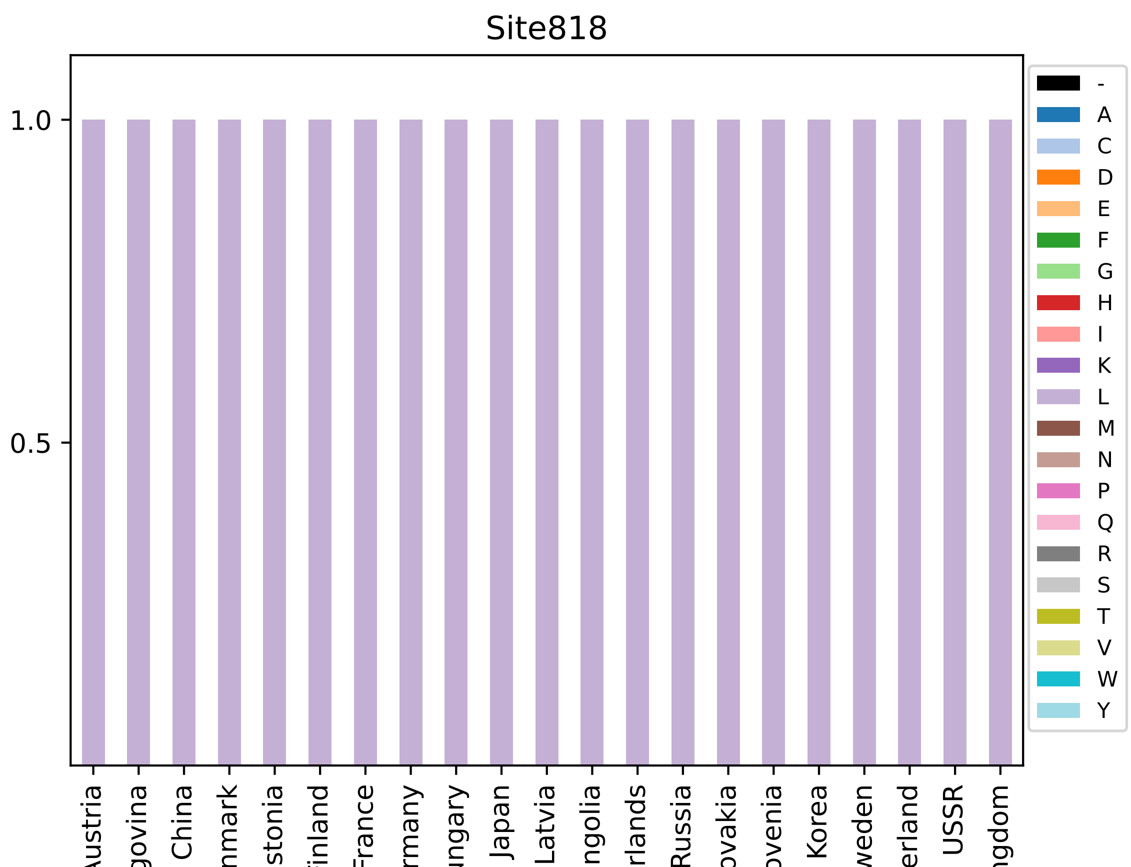
Site818
1.0
0.5
-
A
C
D
E
F
G
H
I
K
L
M
N
P
Q
R
S
T
V
W
Y
China
Japan
Latvia
Russia
Korea
USSR

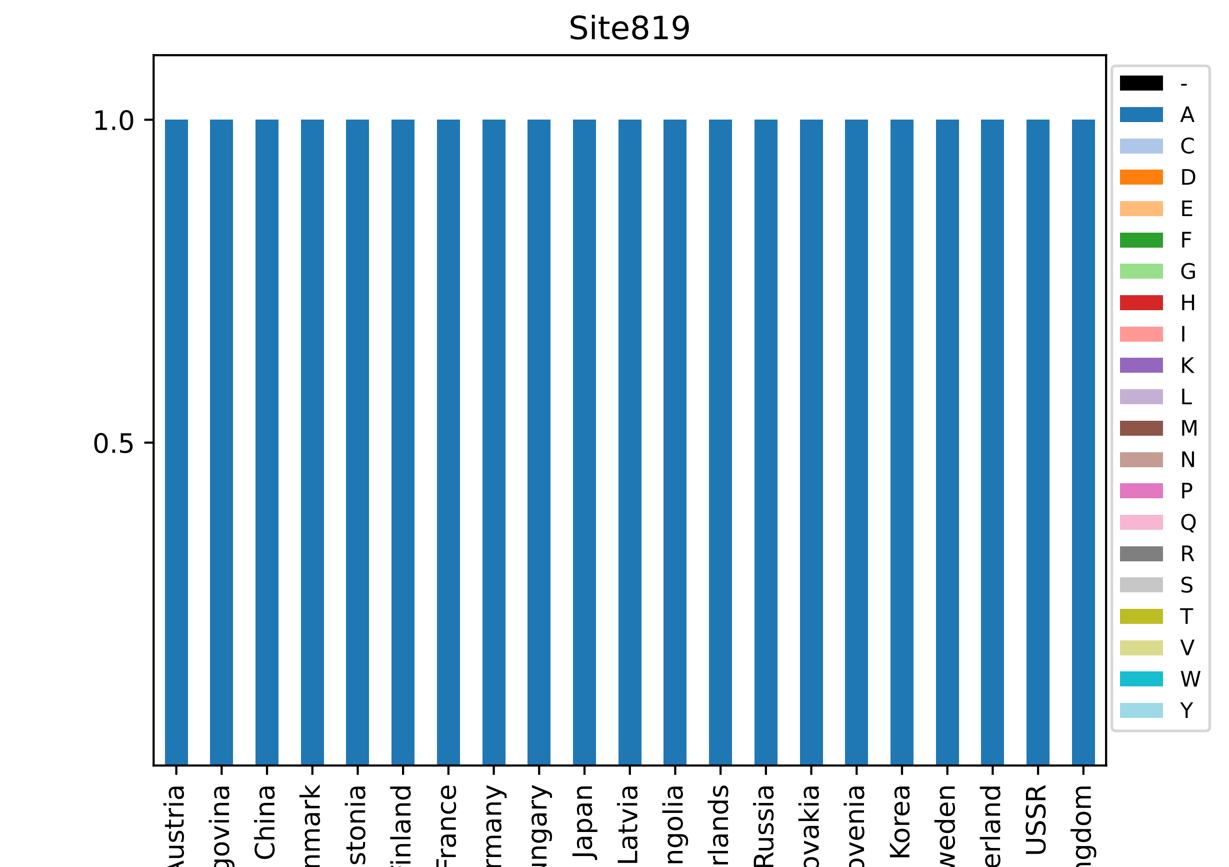

Site819
1.0
0.5
-
A
C
D
E
F
G
H
I
K
L
M
N
P
Q
R
S
T
V
W
Y
Austria
govina
China
nmark
stonia
inland
France
rmany
ungary
Japan
Latvia
ngolia
rlands
Russia
ovakia
ovenia
Korea
weden
erland
USSR
ngdom

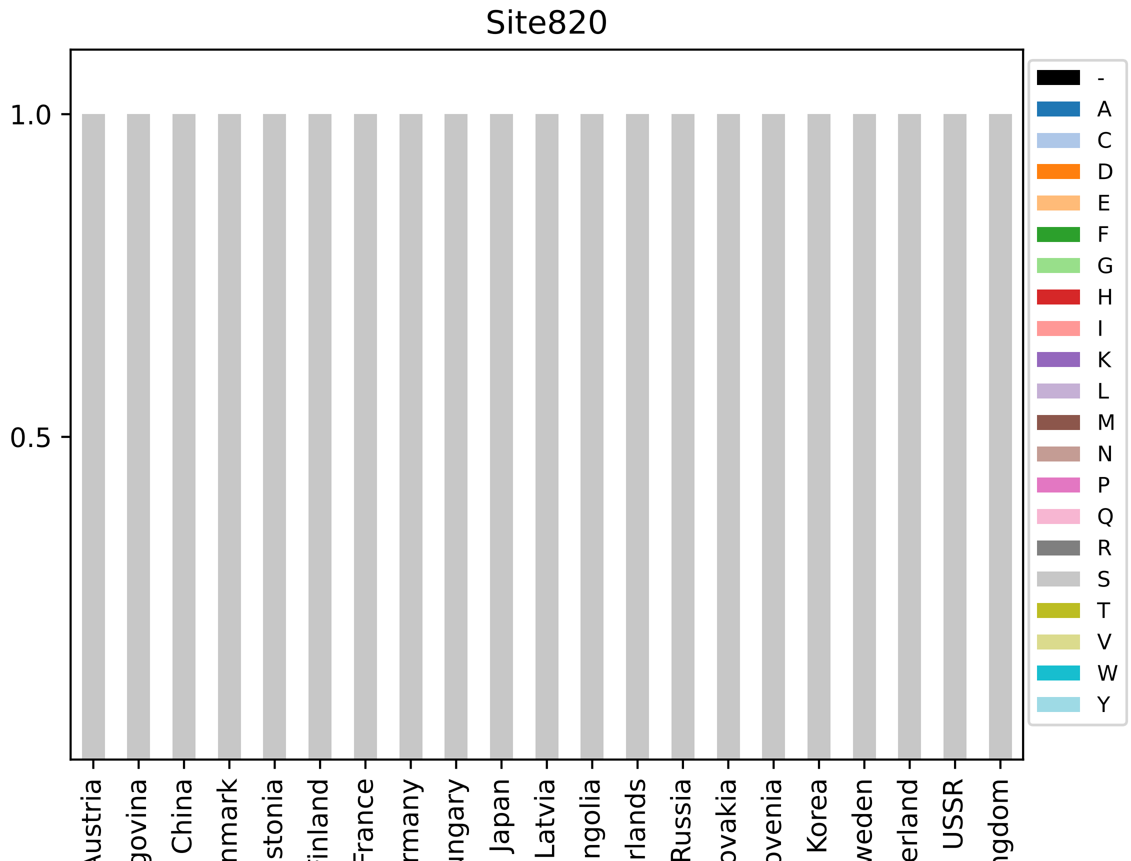
Site820
1.0
0.5
-
A
C
D
E
F
G
H
I
K
L
M
N
P
Q
R
S
T
V
W
Y
Austria
govina
China
nmark
stonia
inland
France
rmany
ungary
Japan
Latvia
ngolia
rlands
Russia
ovakia
ovenia
Korea
weden
erland
USSR
ngdom

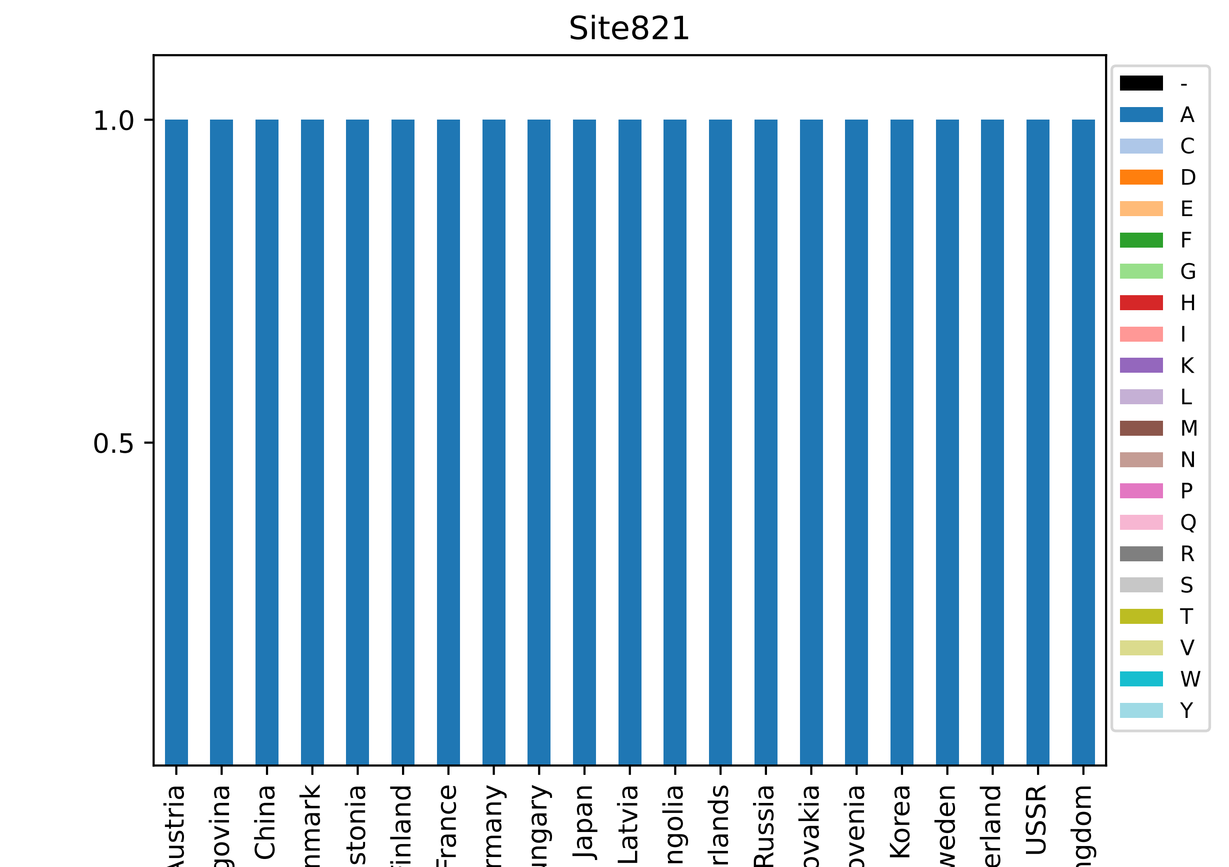
Site821
1.0
0.5
-
A
C
D
E
F
G
H
I
K
L
M
N
P
Q
R
S
T
V
W
Y
Austria
govina
China
nmark
stonia
Finland
France
rmany
ungary
Japan
Latvia
ngolia
rlands
Russia
ovakia
ovenia
Korea
weden
erland
USSR
ngdom

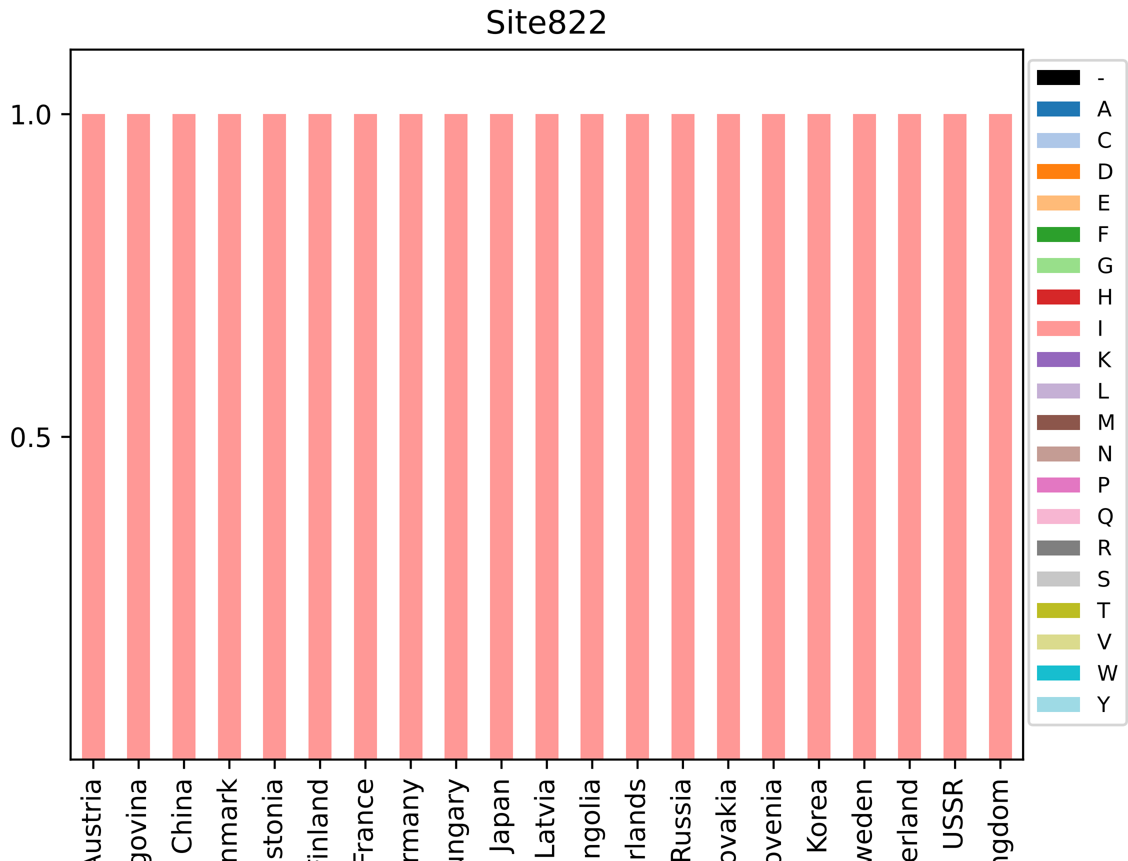

Site822
1.0
0.5
Austria
govina
China
nmark
stonia
Finland
France
rmany
ungary
Japan
Latvia
ngolia
rlands
Russia
ovakia
ovenia
Korea
weden
erland
USSR
ngdom
-
A
C
D
E
F
G
H
I
K
L
M
N
P
Q
R
S
T
V
W
Y

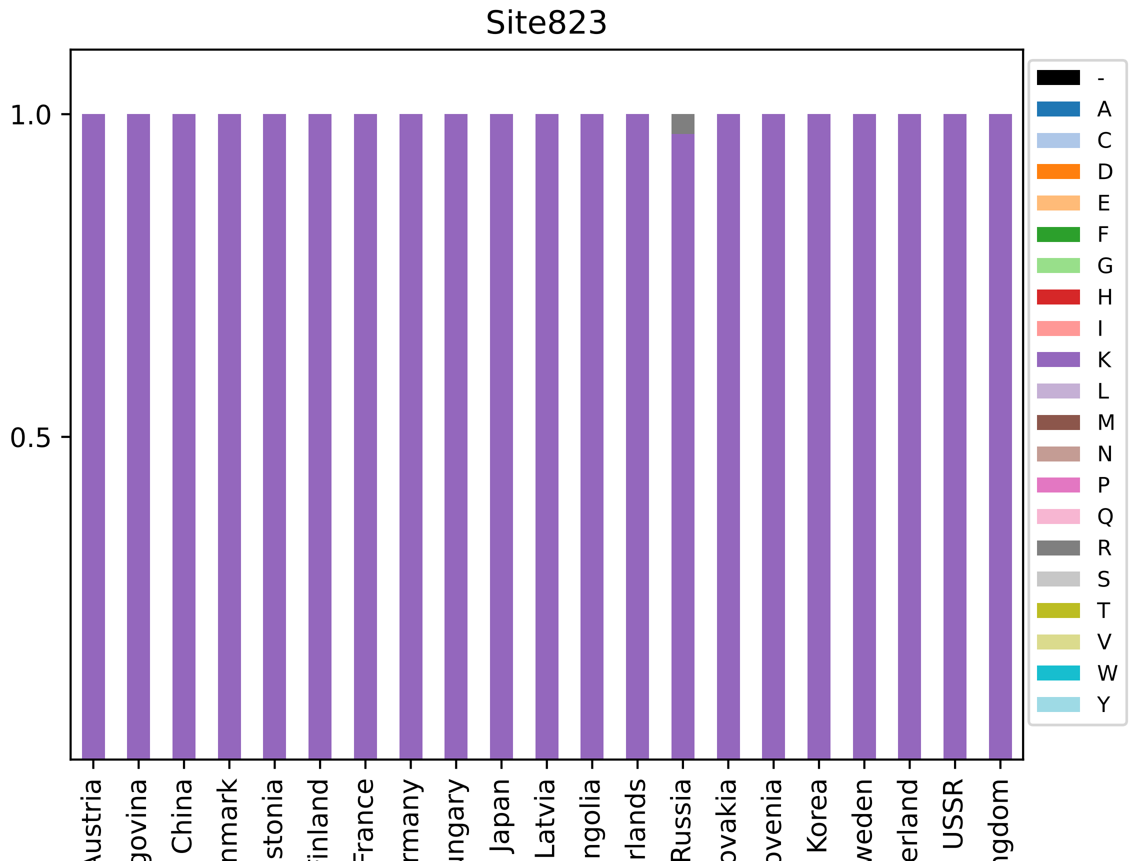
Site823
1.0
0.5
-
A
C
D
E
F
G
H
I
K
L
M
N
P
Q
R
S
T
V
W
Y
China
Japan
Latvia
Russia
Korea
USSR

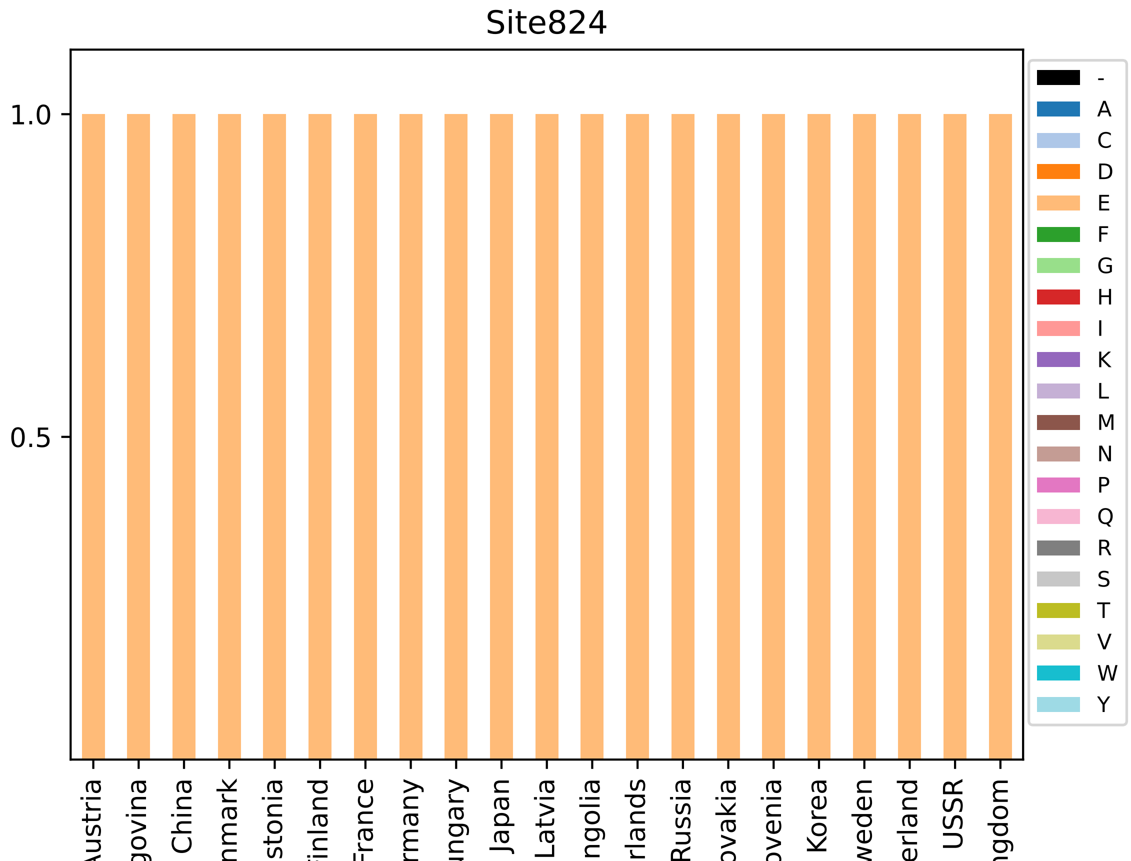
Site824
1.0
0.5
-
A
C
D
E
F
G
H
I
K
L
M
N
P
Q
R
S
T
V
W
Y
China
Japan
Latvia
Russia
Korea
USSR

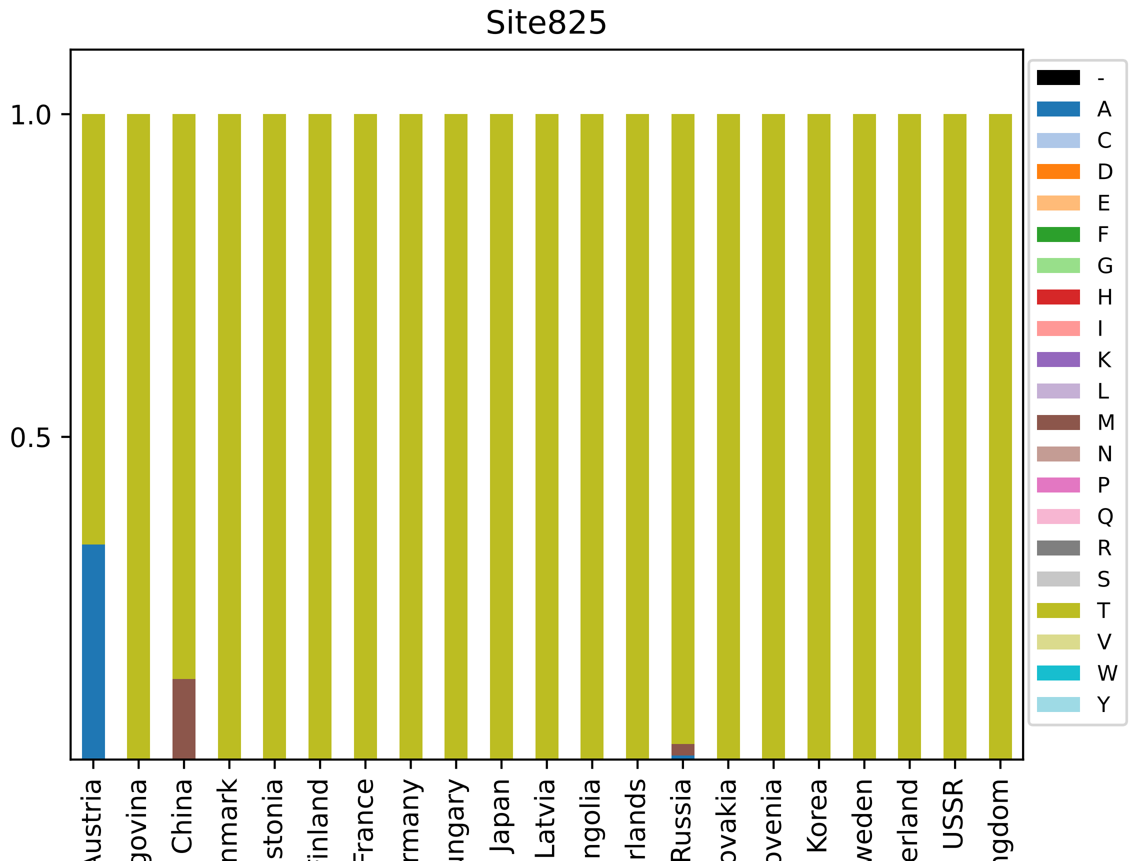
Site825
1.0
0.5
-
A
C
D
E
F
G
H
I
K
L
M
N
P
Q
R
S
T
V
W
Y
China
Japan
Latvia
Russia
Korea
USSR

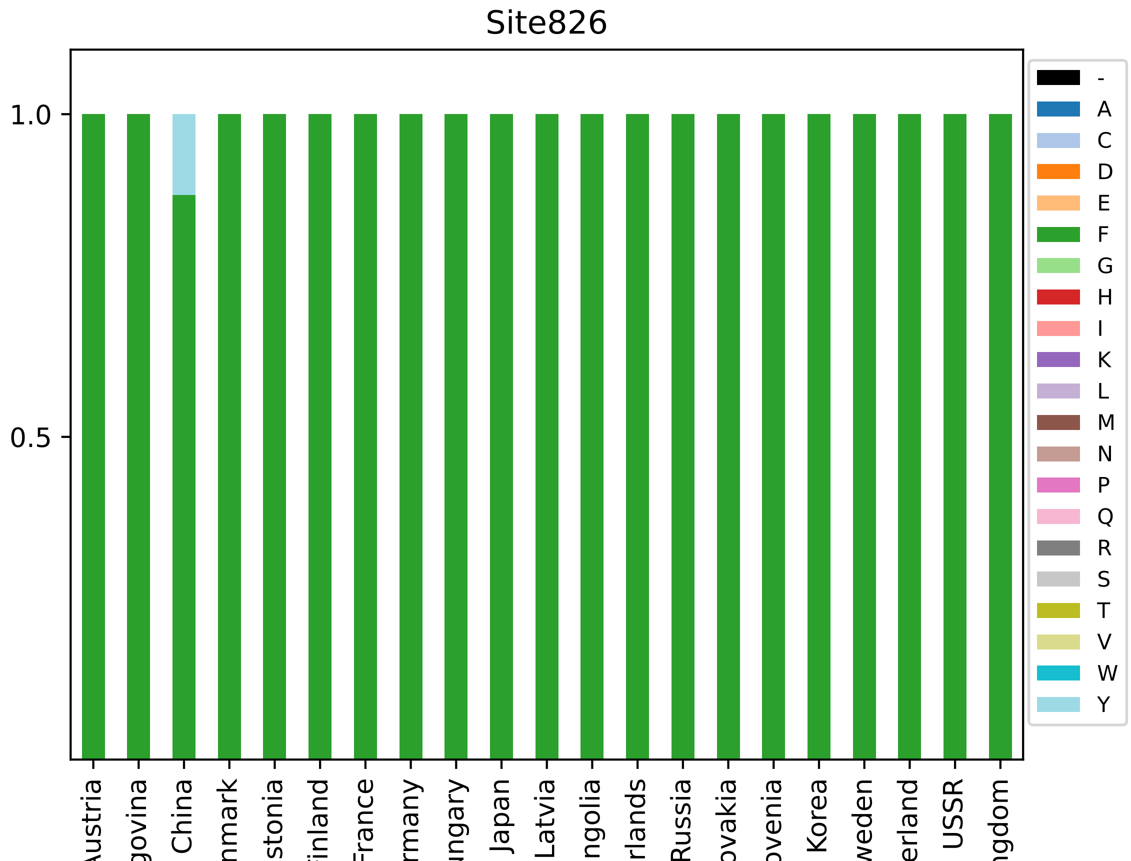

Site826
1.0
0.5
-
A
C
D
E
F
G
H
I
K
L
M
N
P
Q
R
S
T
V
W
Y
Austria
govina
China
nmark
stonia
inland
France
rmany
ungary
Japan
Latvia
ngolia
rlands
Russia
ovakia
ovenia
Korea
weden
erland
USSR
ngdom

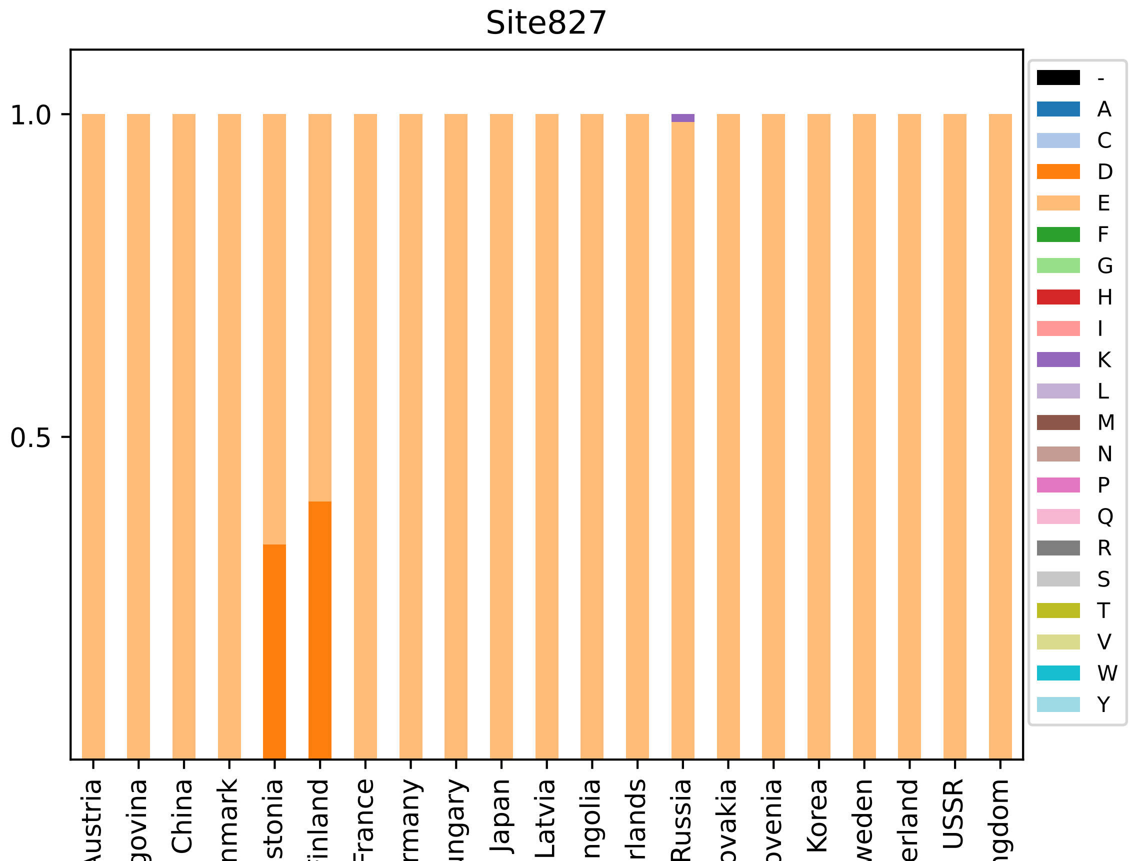

Site827
1.0
0.5
-
A
C
D
E
F
G
H
I
K
L
M
N
P
Q
R
S
T
V
W
Y
China
Finland
France
Japan
Latvia
Russia
Korea
USSR

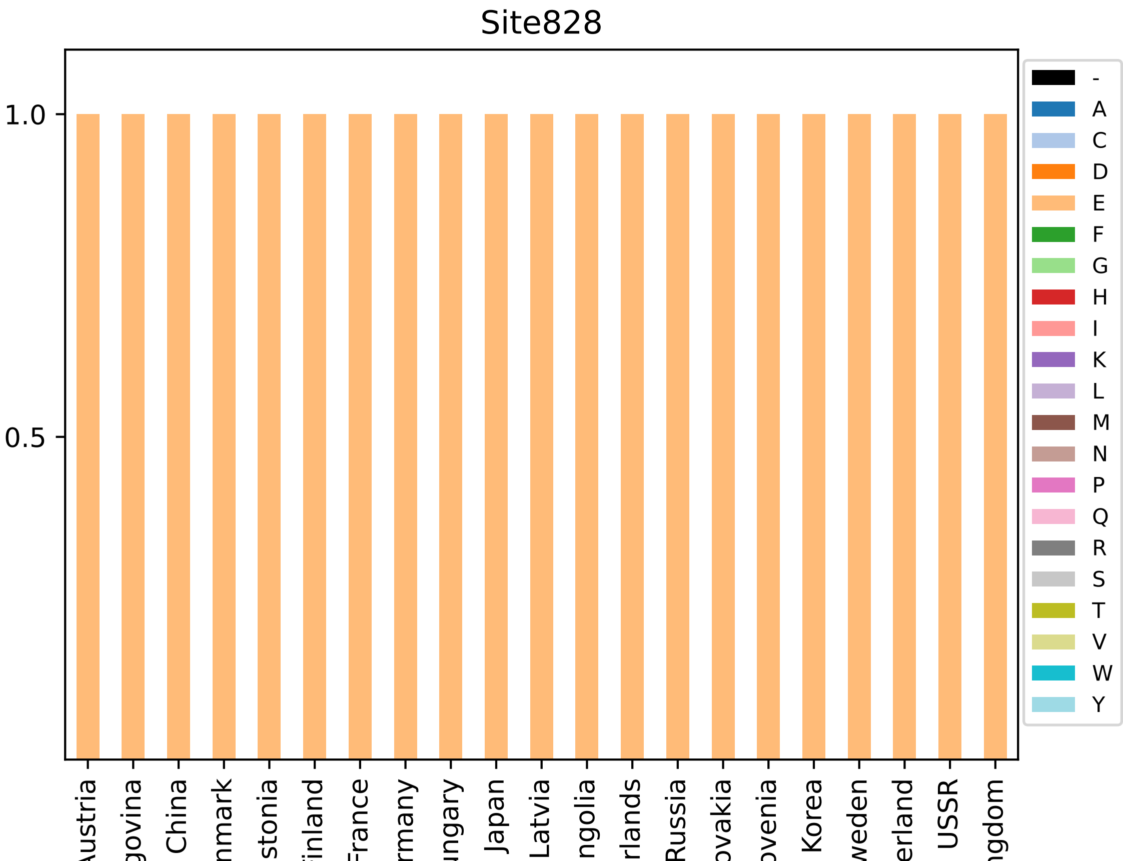
Site828
1.0
0.5
-
A
C
D
E
F
G
H
I
K
L
M
N
P
Q
R
S
T
V
W
Y
China
Japan
Latvia
Russia
Korea
USSR

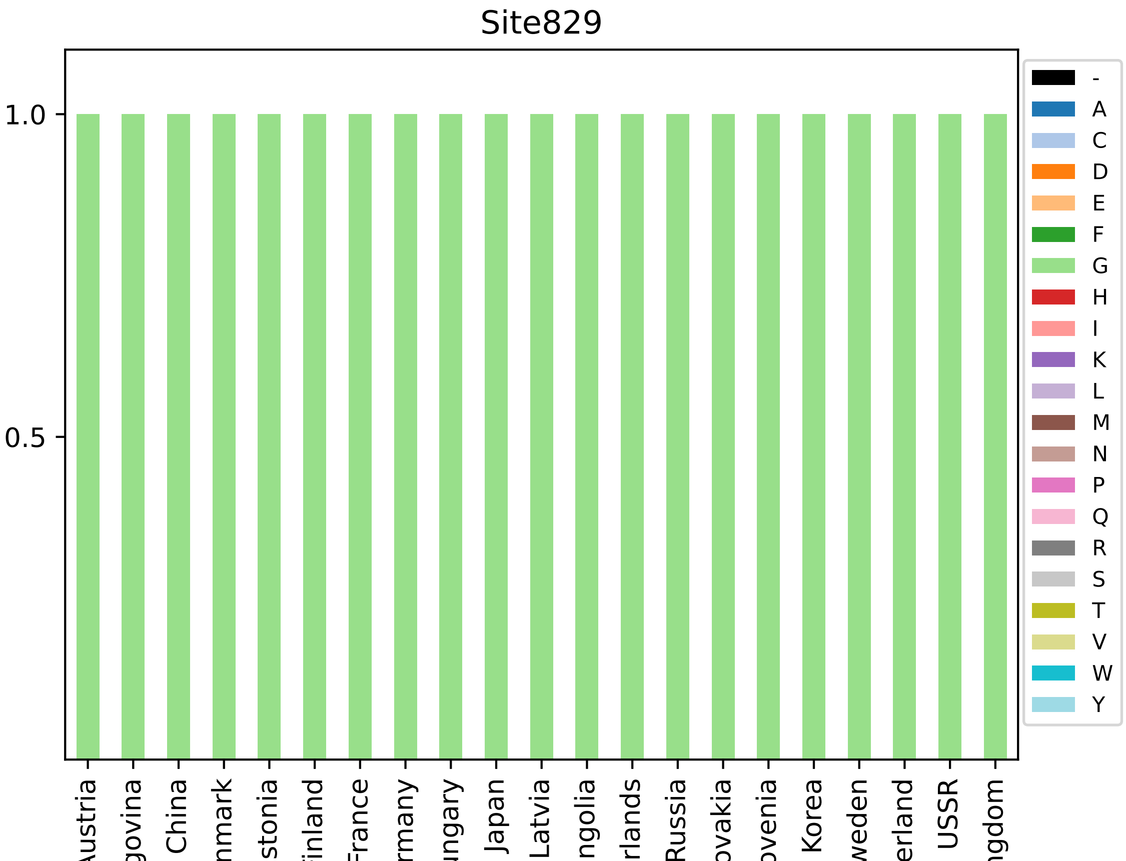

Site829
1.0
0.5
-
A
C
D
E
F
G
H
I
K
L
M
N
P
Q
R
S
T
V
W
Y
China
Japan
Latvia
Russia
Korea
USSR

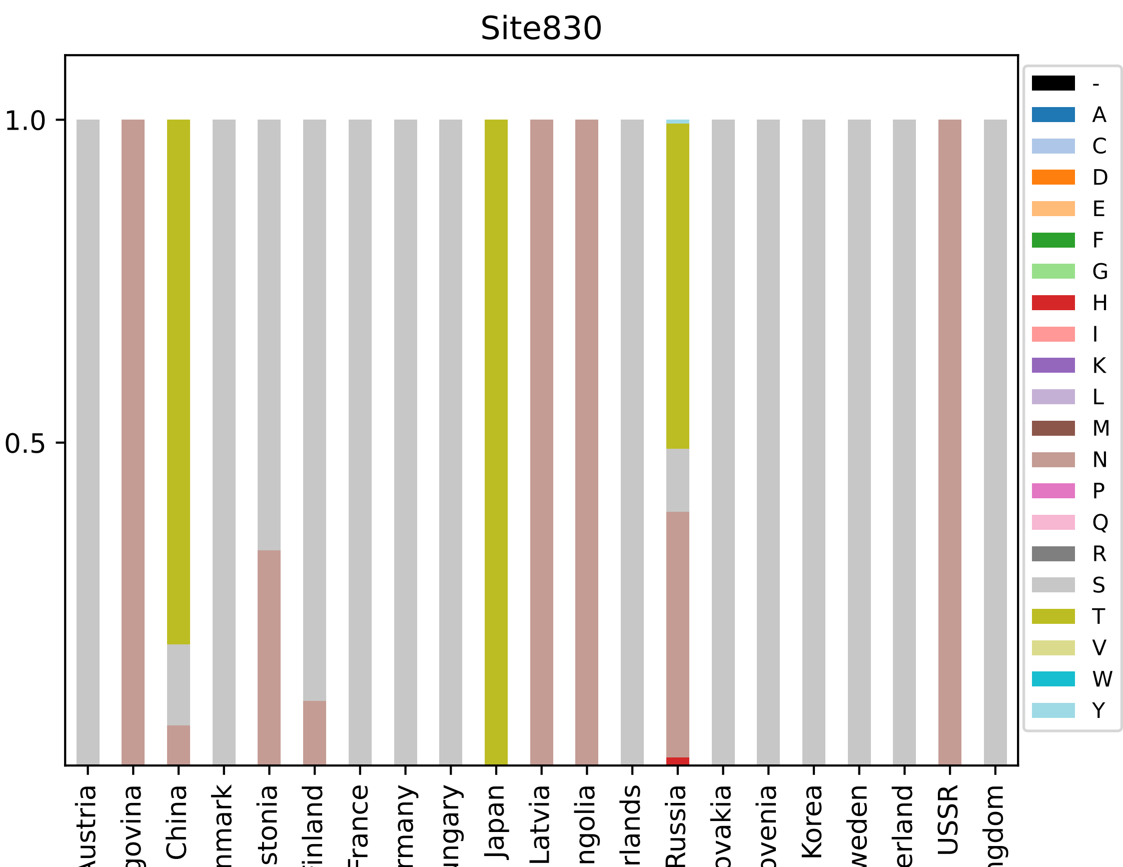

Site830
1.0
0.5
Austria
govina
China
nmark
stonia
Finland
France
rmany
ungary
Japan
Latvia
ngolia
rlands
Russia
ovakia
ovenia
Korea
weden
erland
USSR
ngdom
-
A
C
D
E
F
G
H
I
K
L
M
N
P
Q
R
S
T
V
W
Y

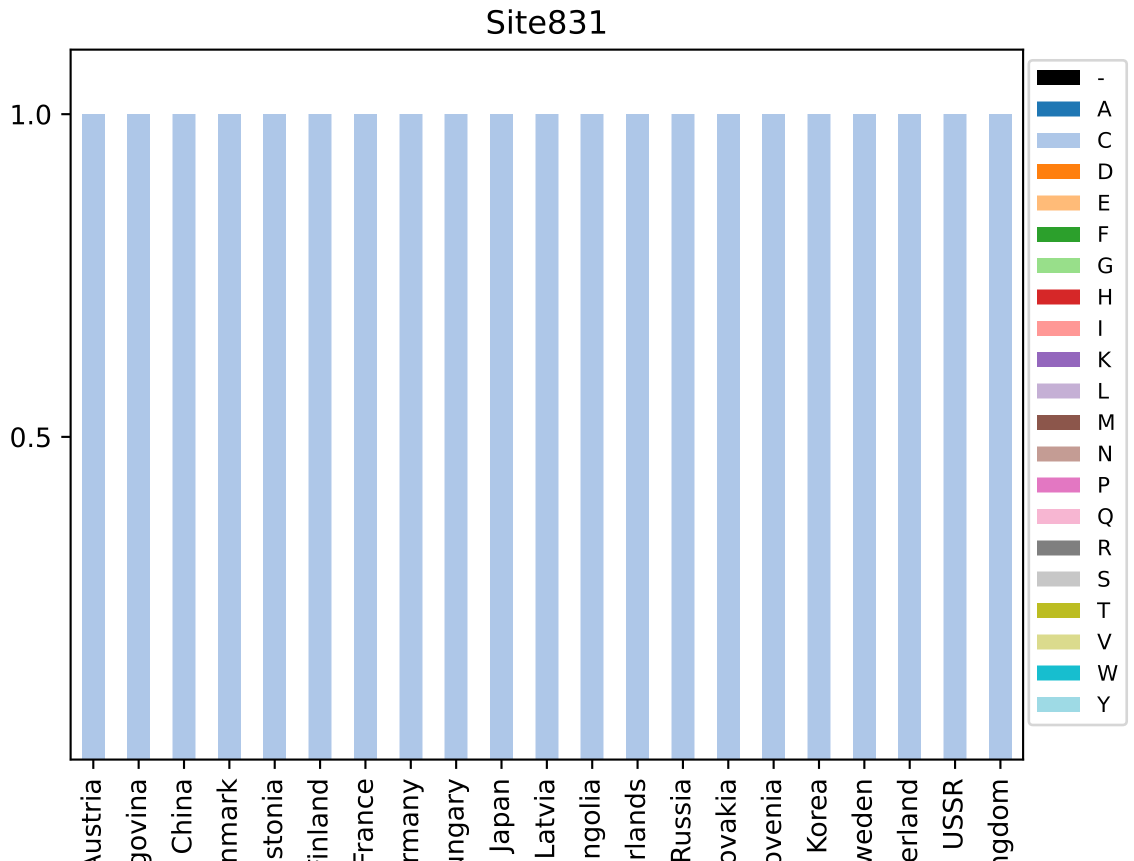
Site831
1.0
0.5
Austria
govina
China
nmark
stonia
inland
France
rmany
ungary
Japan
Latvia
ngolia
rlands
Russia
ovakia
ovenia
Korea
weden
erland
USSR
ngdom
-
A
C
D
E
F
G
H
I
K
L
M
N
P
Q
R
S
T
V
W
Y

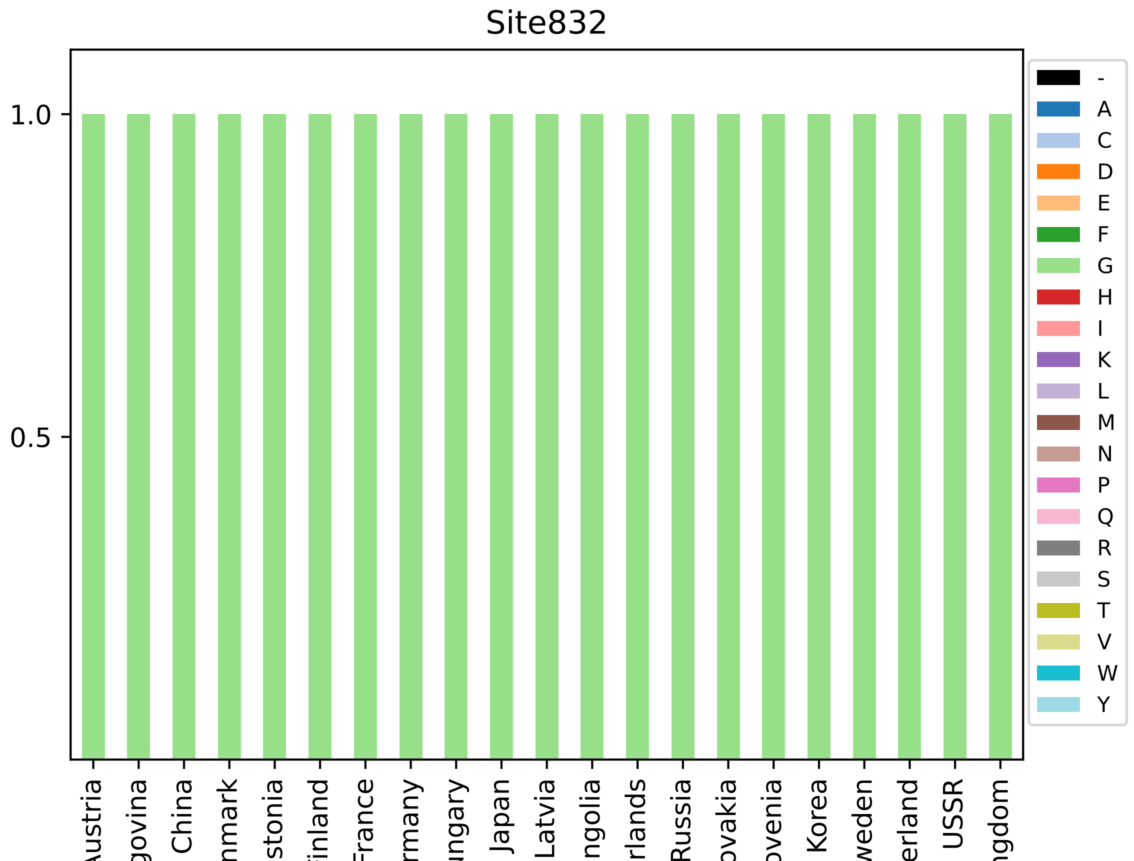
Site832
1.0
0.5
-
A
C
D
E
F
G
H
I
K
L
M
N
P
Q
R
S
T
V
W
Y
Austria
China
Japan
Latvia
Russia
Korea
USSR

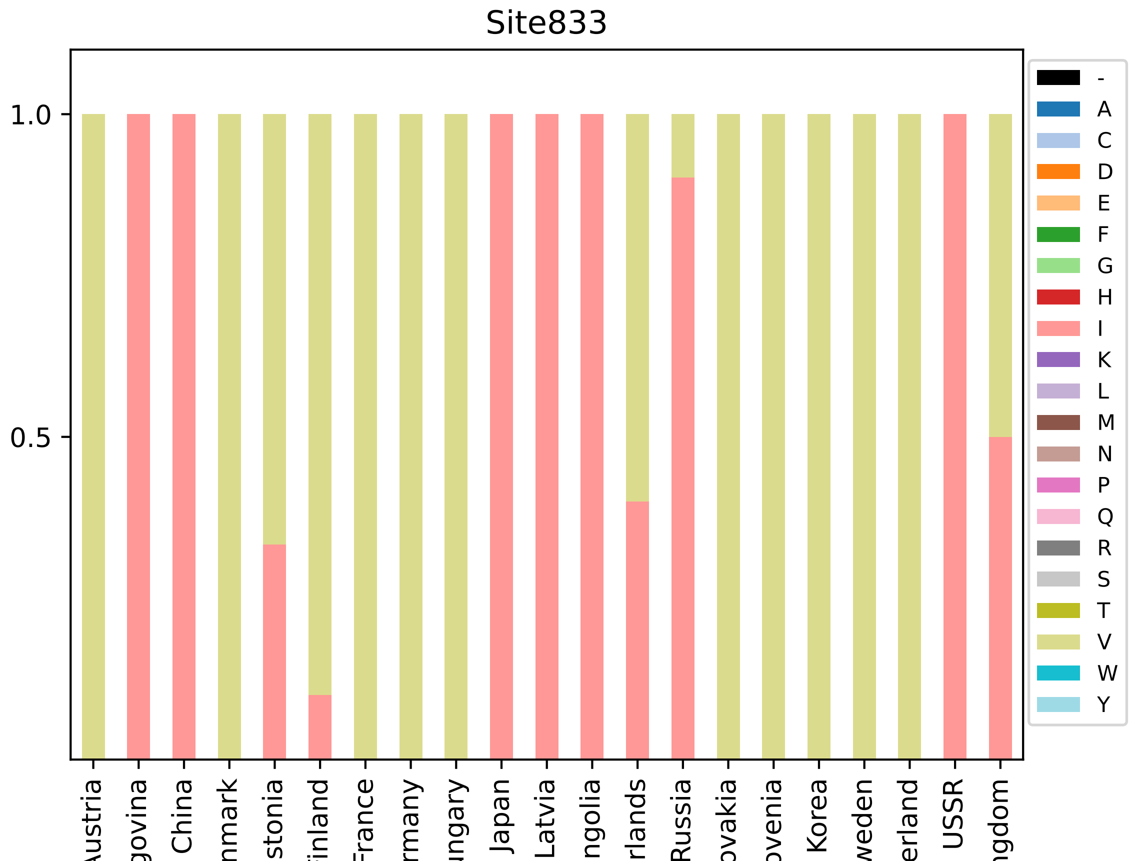
Site833
1.0
0.5
-
A
C
D
E
F
G
H
I
K
L
M
N
P
Q
R
S
T
V
W
Y
China
Japan
Latvia
Russia
Korea
USSR

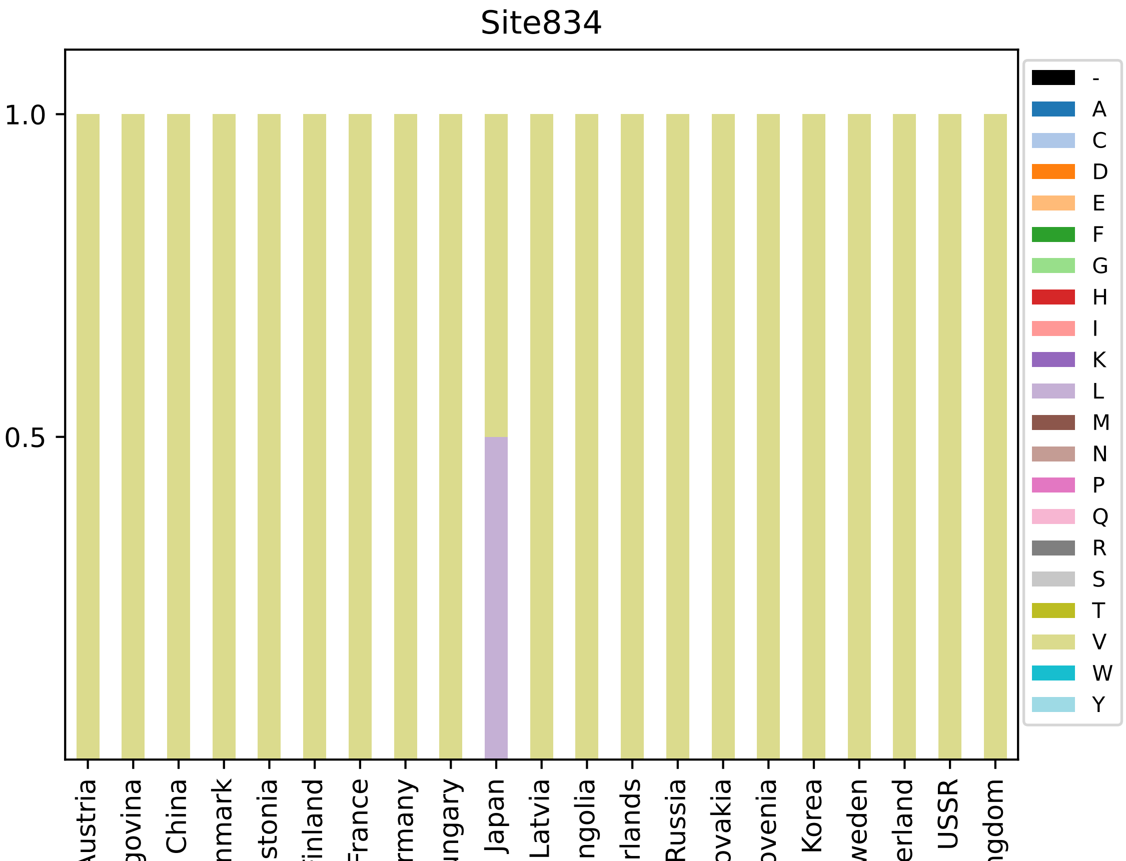

Site834
1.0
0.5
-
A
C
D
E
F
G
H
I
K
L
M
N
P
Q
R
S
T
V
W
Y
Austria
China
Japan
Latvia
Russia
Korea
USSR

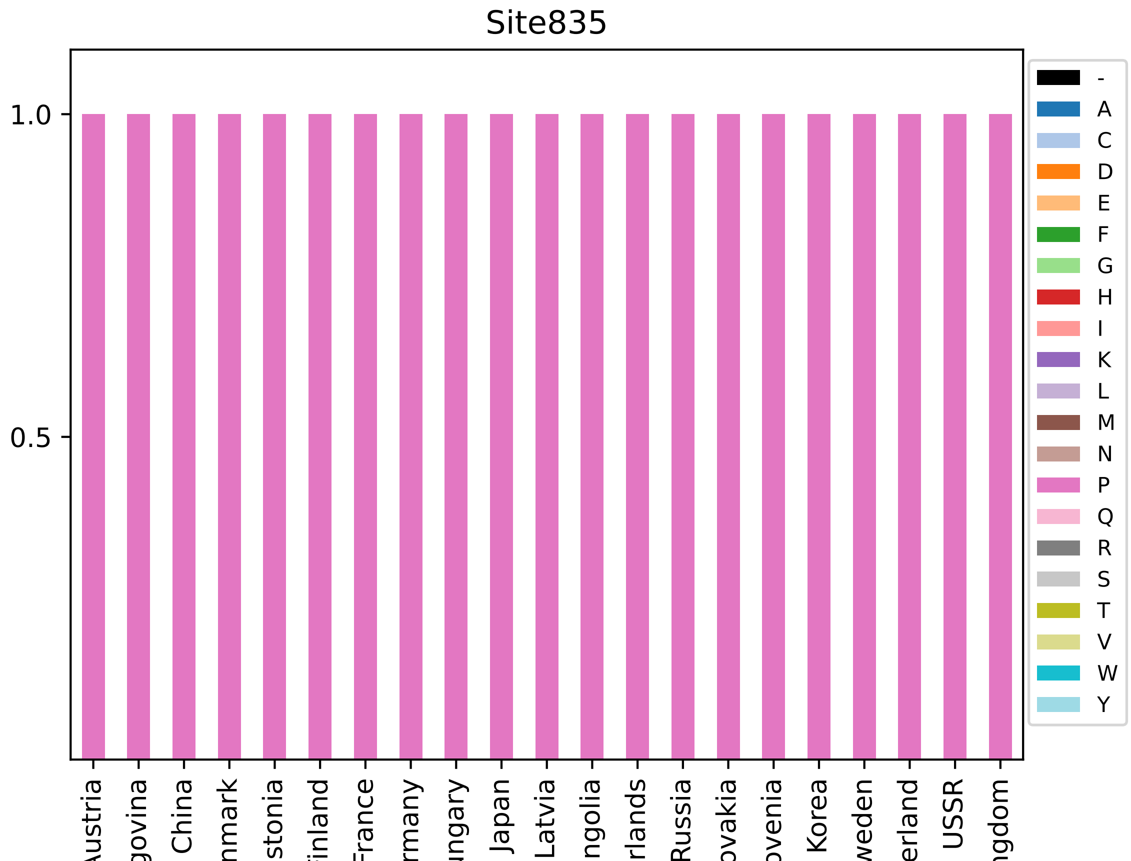
Site835
1.0
0.5
-
A
C
D
E
F
G
H
I
K
L
M
N
P
Q
R
S
T
V
W
Y
China
Japan
Latvia
Russia
Korea
USSR

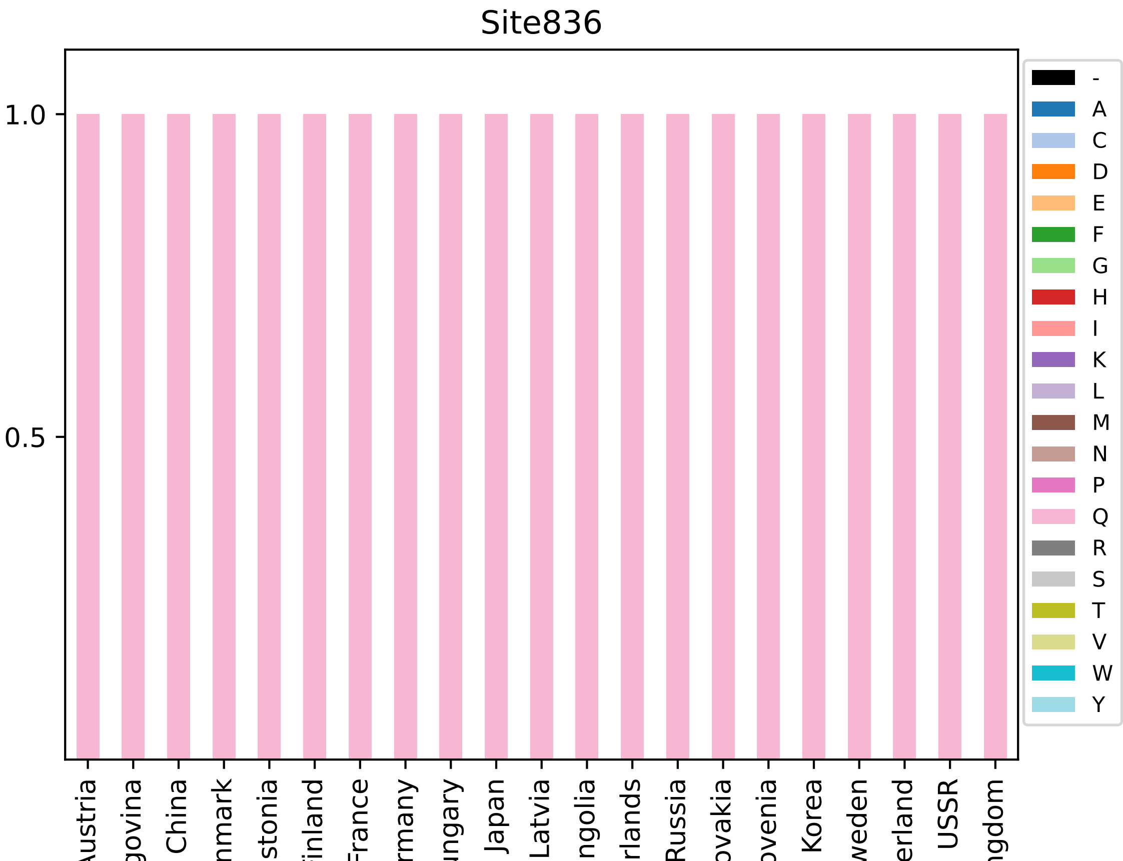

Site836
1.0
0.5
-
A
C
D
E
F
G
H
I
K
L
M
N
P
Q
R
S
T
V
W
Y
China
Japan
Latvia
Russia
Korea
USSR

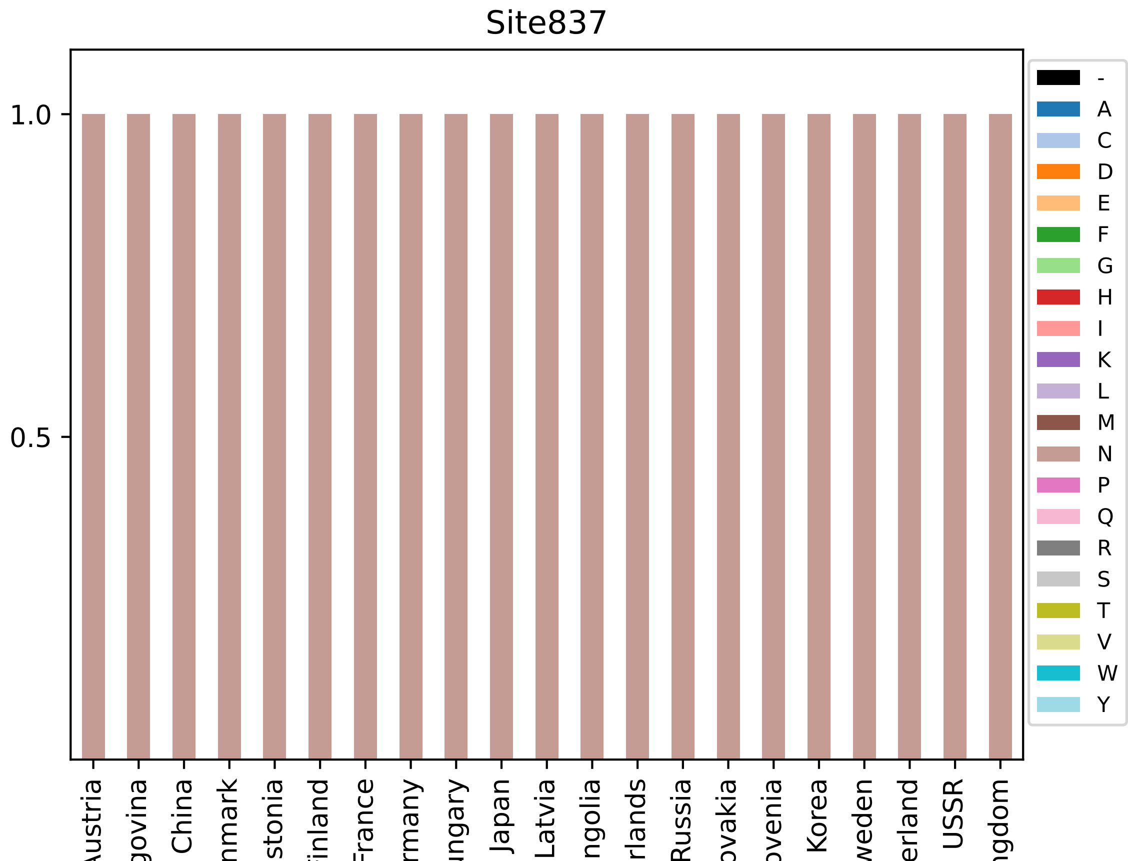

Site837
1.0
0.5
-
A
C
D
E
F
G
H
I
K
L
M
N
P
Q
R
S
T
V
W
Y
Austria
govina
China
nmark
stonia
Finland
France
rmany
ungary
Japan
Latvia
ngolia
rlands
Russia
ovakia
ovenia
Korea
weden
erland
USSR
ngdom

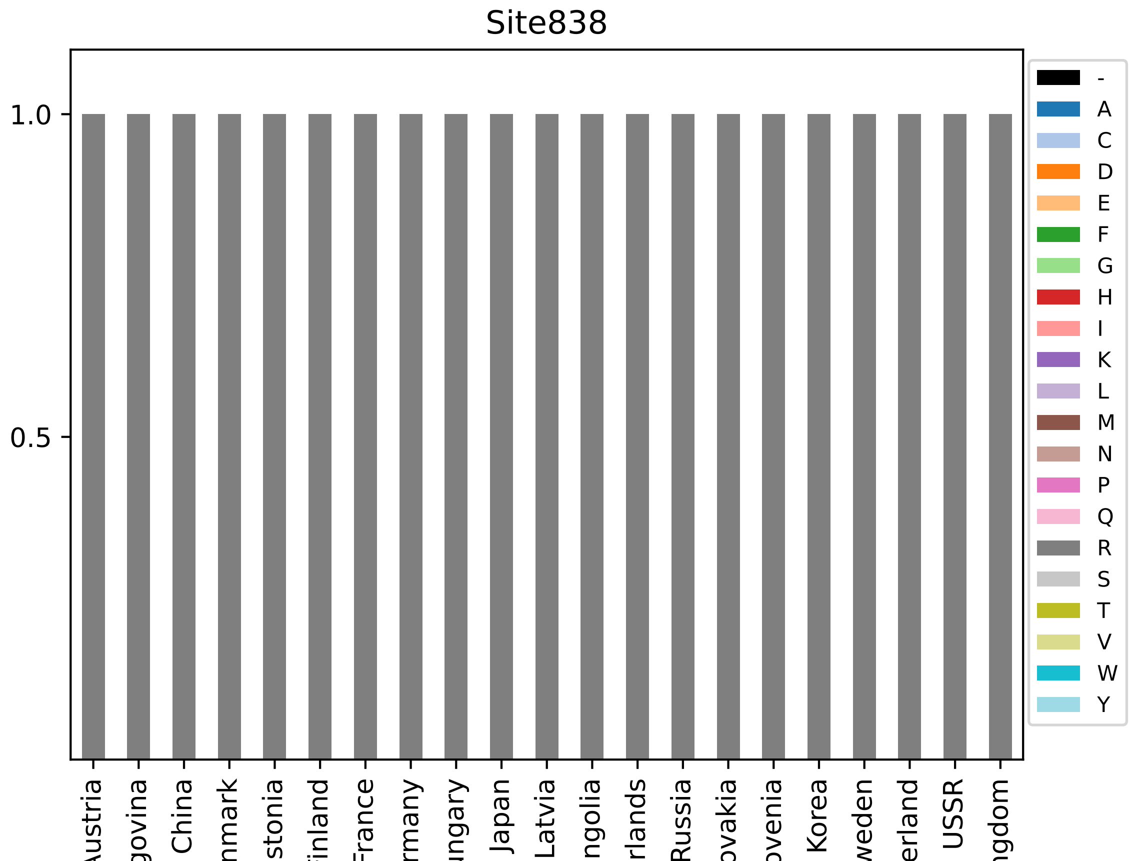

Site838
1.0
0.5
-
A
C
D
E
F
G
H
I
K
L
M
N
P
Q
R
S
T
V
W
Y
Austria
govina
China
nmark
stonia
inland
France
rmany
ungary
Japan
Latvia
ngolia
rlands
Russia
ovakia
ovenia
Korea
weden
erland
USSR
ngdom

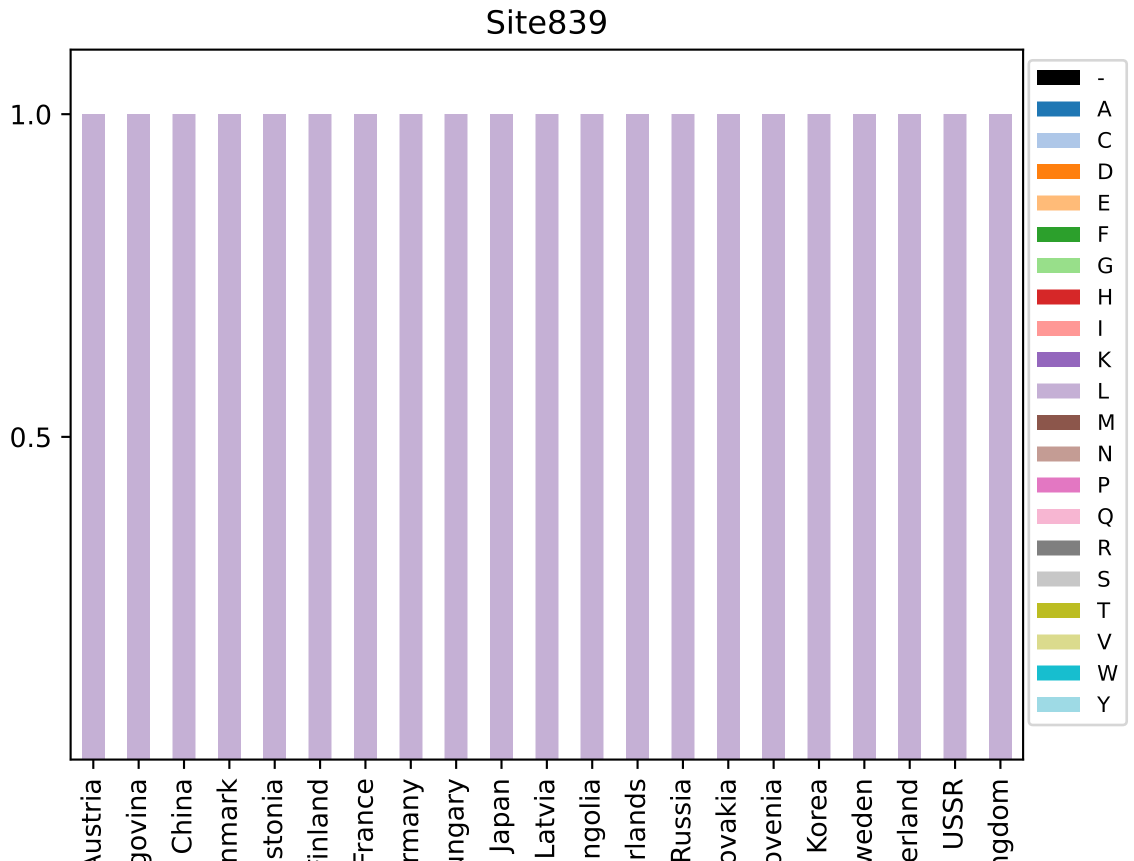
Site839
1.0
0.5
-
A
C
D
E
F
G
H
I
K
L
M
N
P
Q
R
S
T
V
W
Y
China
Japan
Latvia
Russia
Korea
USSR

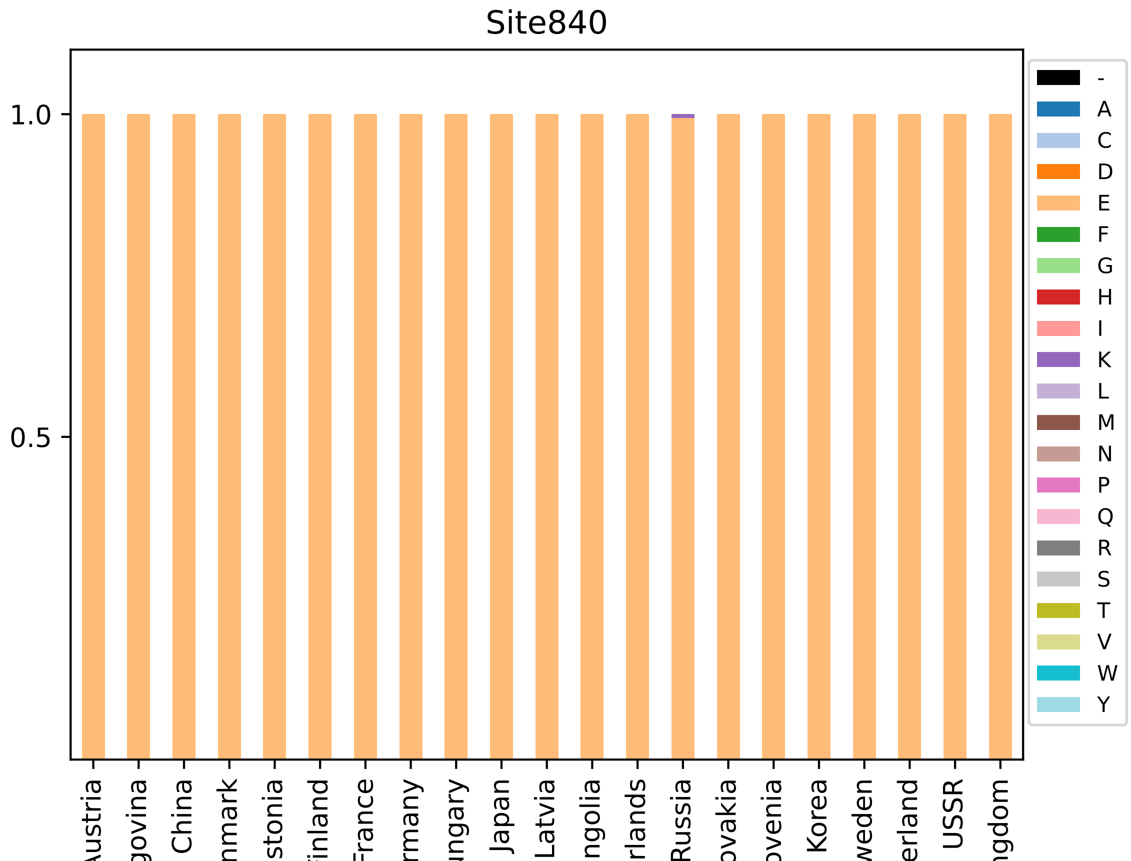

Site840
1.0
0.5
-
A
C
D
E
F
G
H
I
K
L
M
N
P
Q
R
S
T
V
W
Y
Austria
govina
China
nmark
stonia
inland
France
rmany
ungary
Japan
Latvia
ngolia
rlands
Russia
ovakia
ovenia
Korea
weden
erland
USSR
ngdom

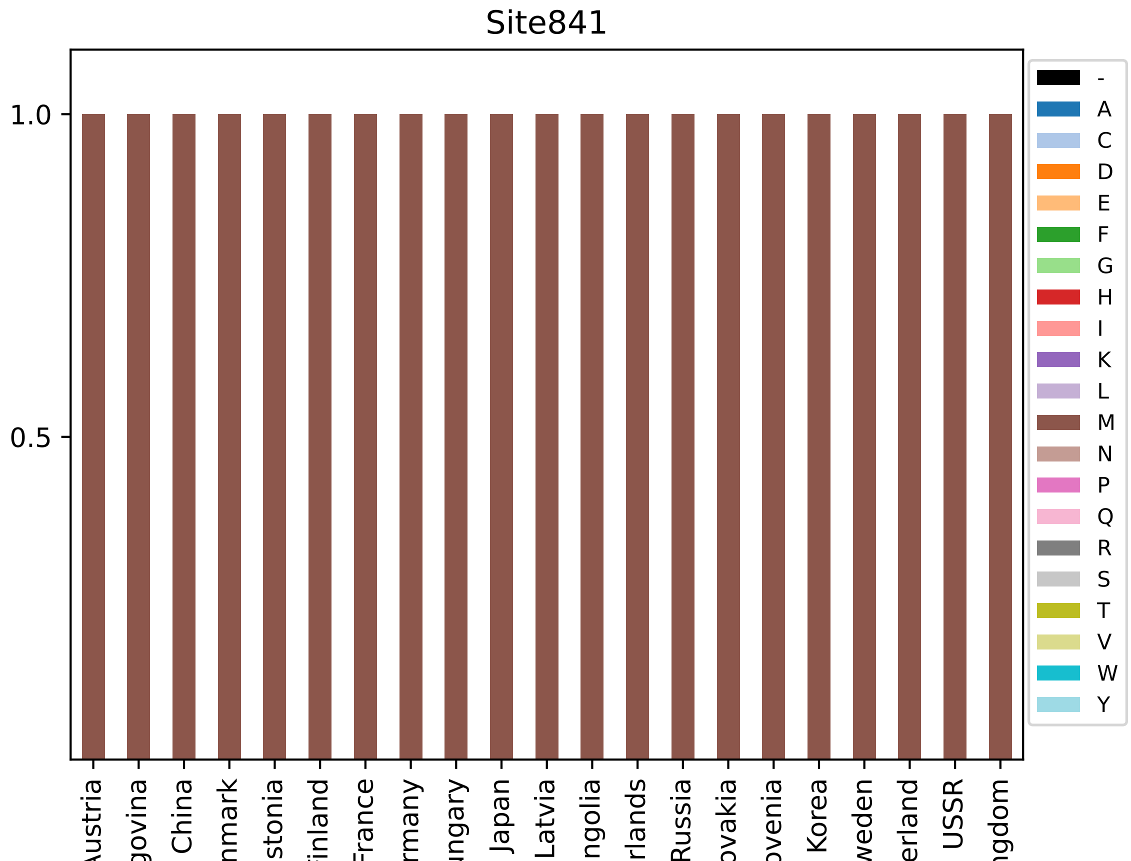

Site841
1.0
0.5
-
A
C
D
E
F
G
H
I
K
L
M
N
P
Q
R
S
T
V
W
Y
Austria
govina
China
nmark
stonia
inland
France
rmany
ungary
Japan
Latvia
ngolia
rlands
Russia
ovakia
ovenia
Korea
weden
erland
USSR
ngdom

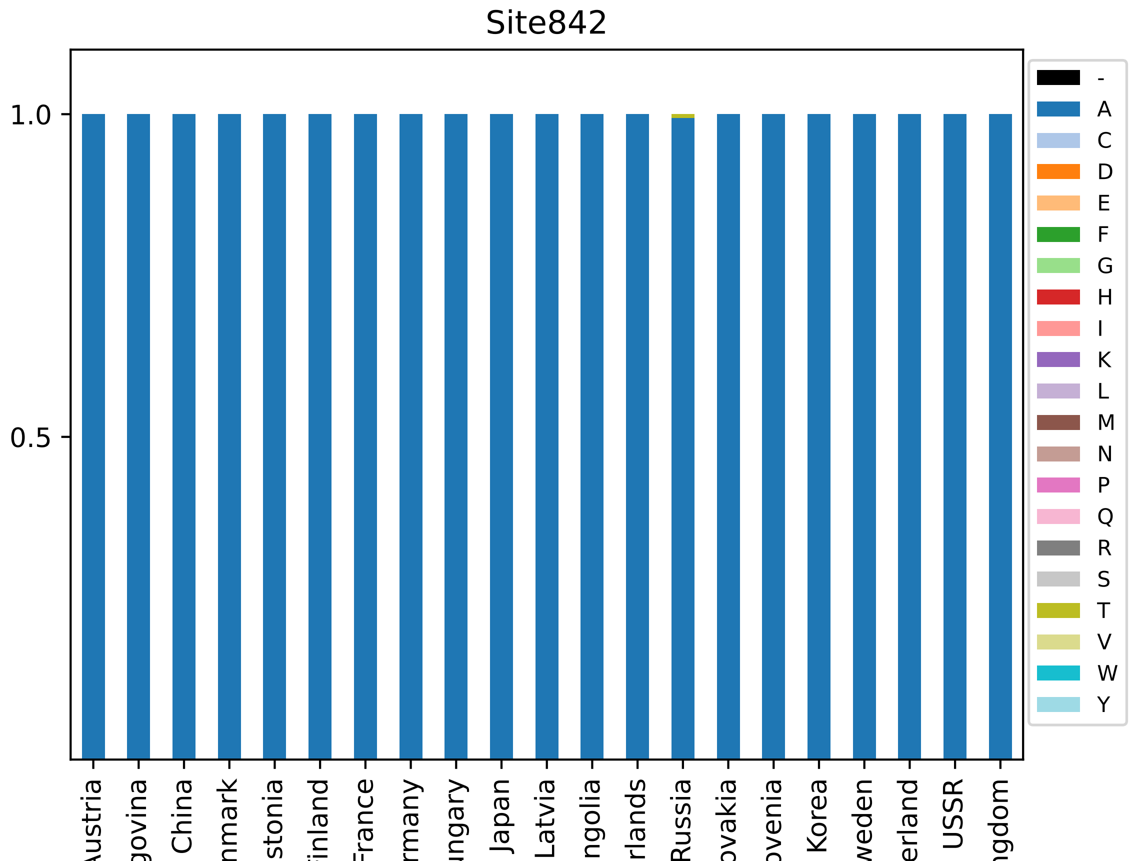
Site842
1.0
0.5
-
A
C
D
E
F
G
H
I
K
L
M
N
P
Q
R
S
T
V
W
Y
China
Japan
Latvia
Russia
Korea
USSR

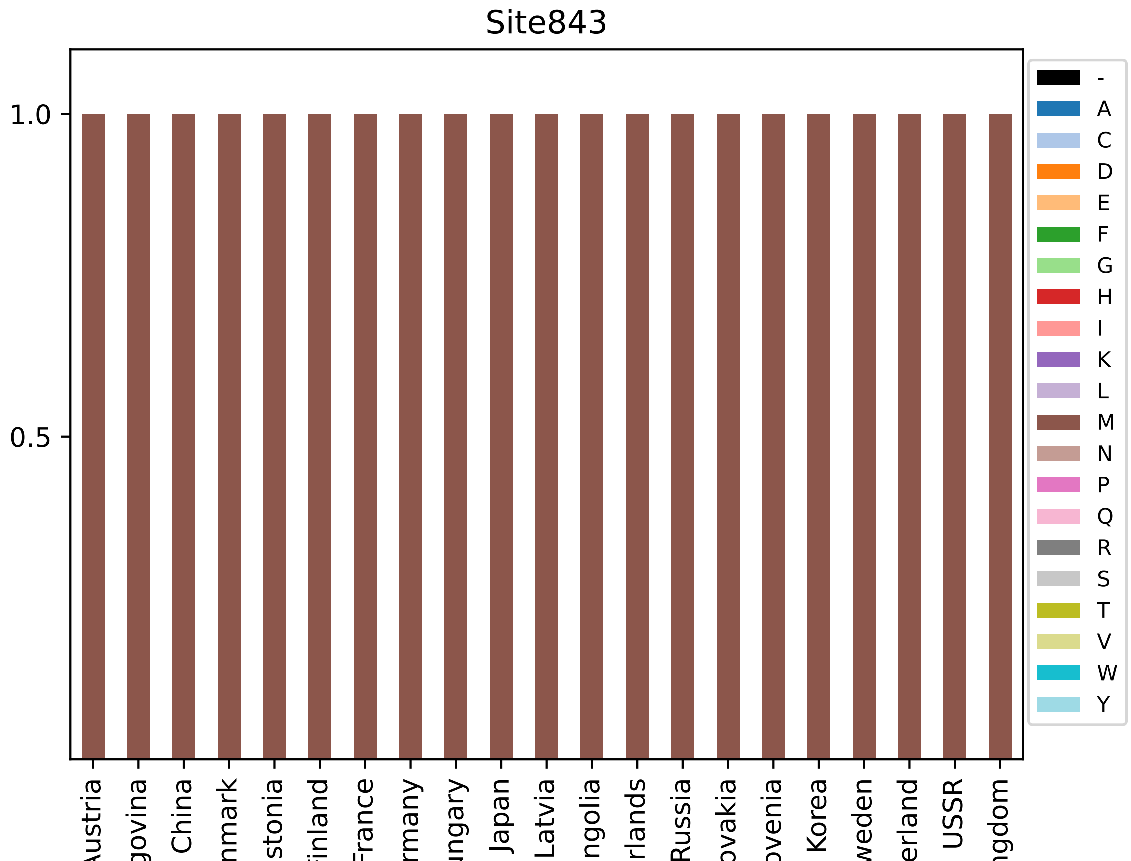

Site843
1.0
0.5
-
A
C
D
E
F
G
H
I
K
L
M
N
P
Q
R
S
T
V
W
Y
Austria
govina
China
nmark
stonia
Finland
France
rmany
ungary
Japan
Latvia
ngolia
rlands
Russia
ovakia
ovenia
Korea
weden
erland
USSR
ngdom

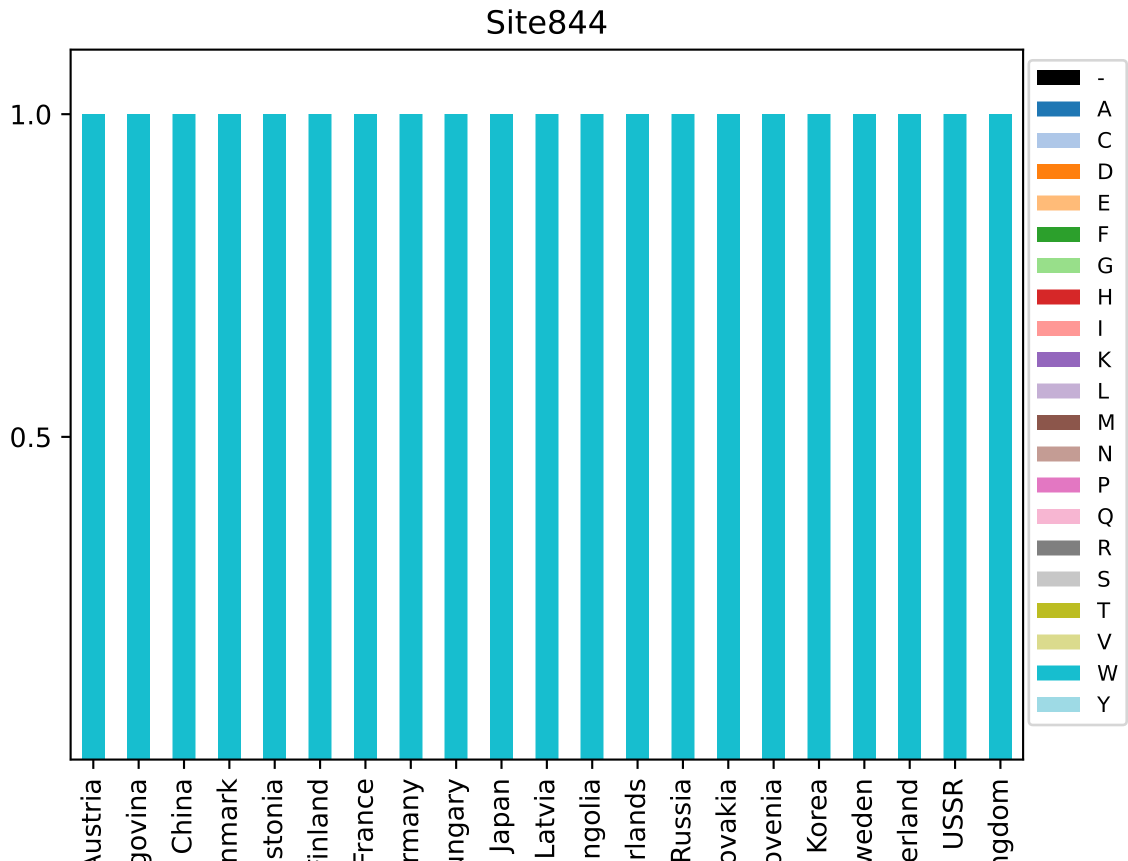

Site844
1.0
0.5
-
A
C
D
E
F
G
H
I
K
L
M
N
P
Q
R
S
T
V
W
Y
China
Japan
Latvia
Russia
Korea
USSR

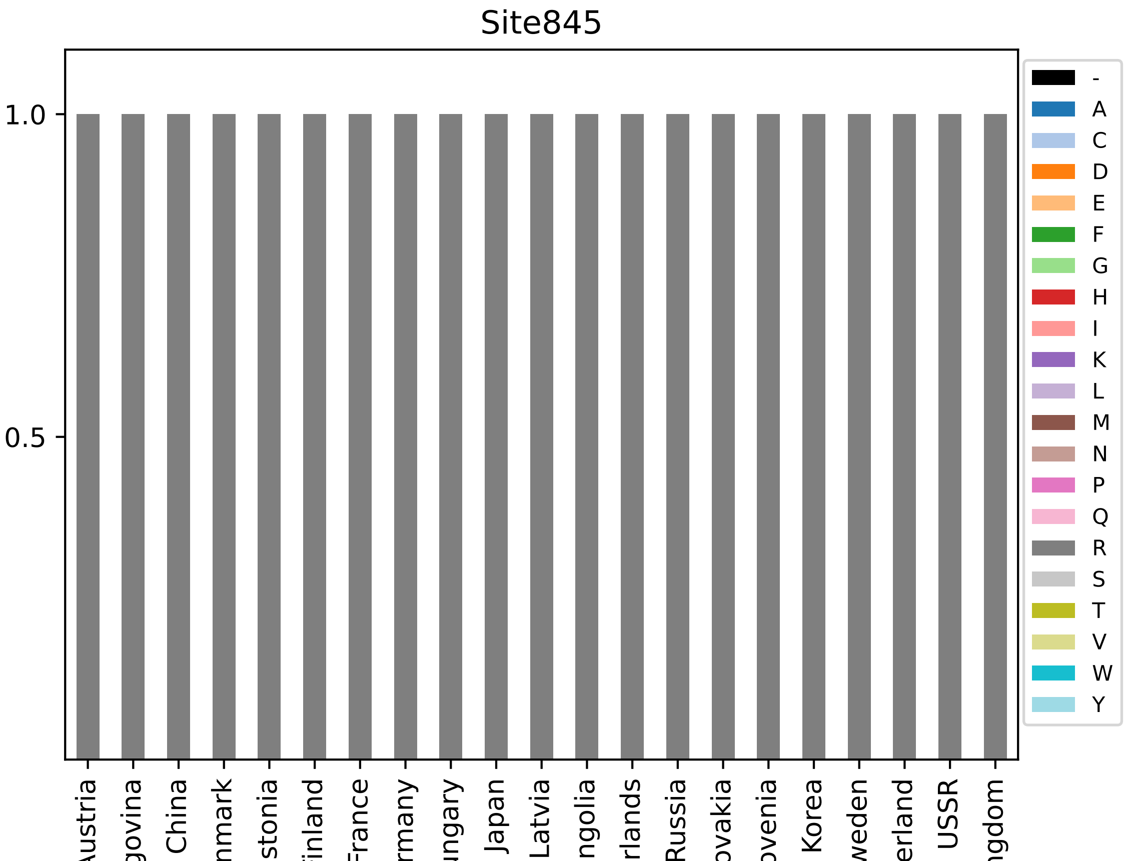
Site845
1.0
0.5
-
A
C
D
E
F
G
H
I
K
L
M
N
P
Q
R
S
T
V
W
Y
Austria
China
Japan
Latvia
Russia
Korea
USSR

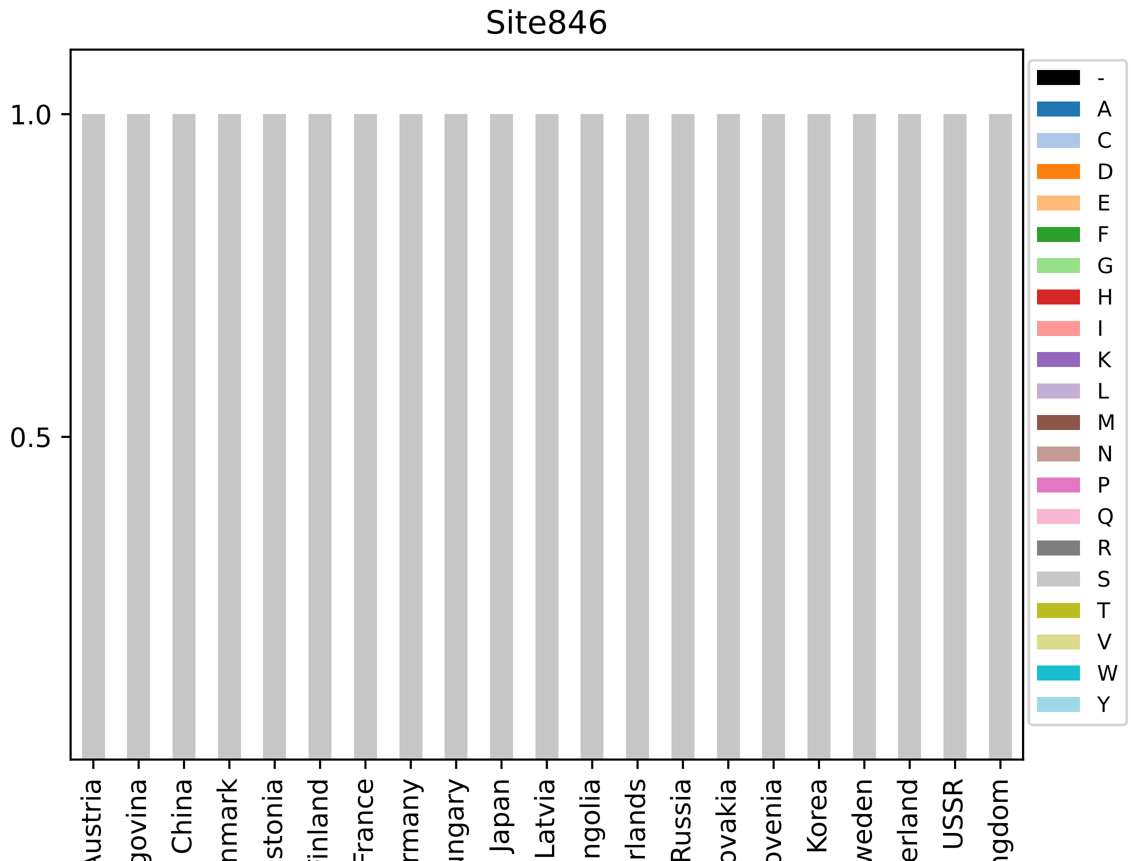
Site846
1.0
0.5
-
A
C
D
E
F
G
H
I
K
L
M
N
P
Q
R
S
T
V
W
Y
Austria
China
Finland
France
Japan
Latvia
Russia
Korea
USSR

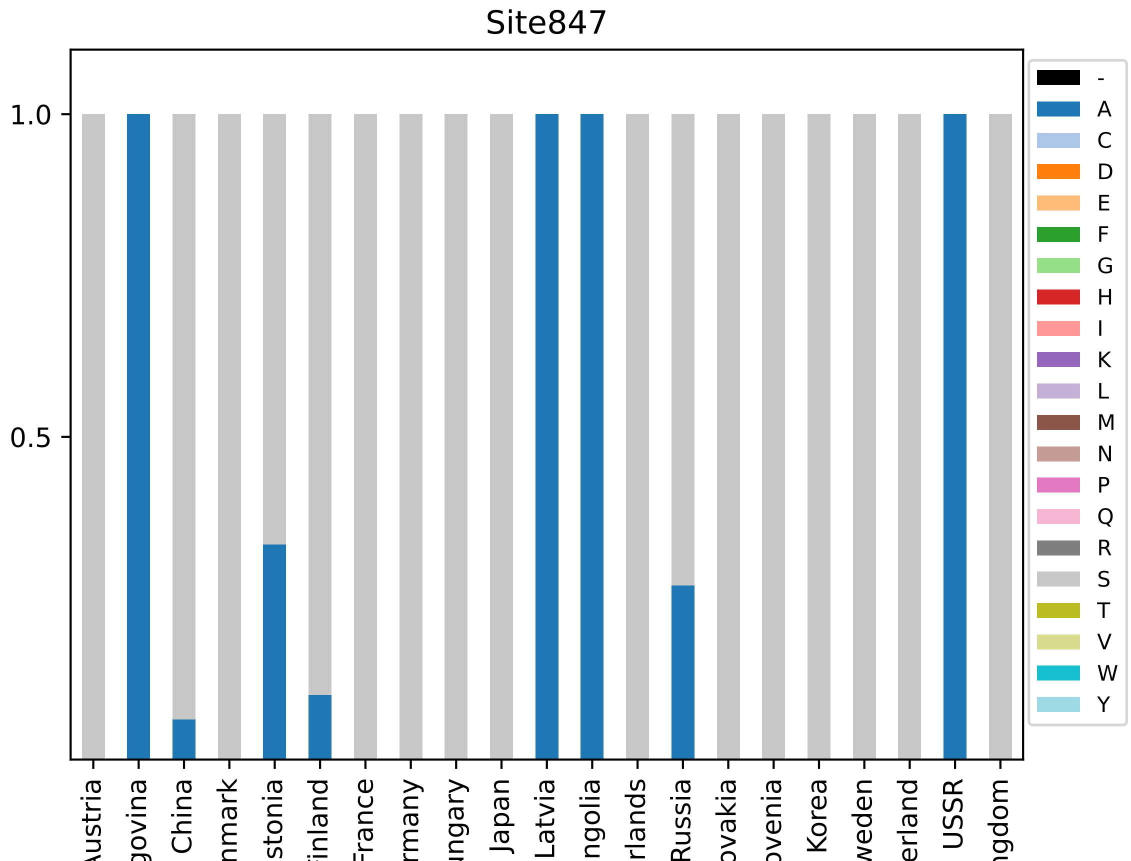

Site847
1.0
0.5
-
A
C
D
E
F
G
H
I
K
L
M
N
P
Q
R
S
T
V
W
Y
Austria
govina
China
nmark
stonia
inland
France
rmany
ungary
Japan
Latvia
ngolia
rlands
Russia
ovakia
ovenia
Korea
weden
erland
USSR
ngdom

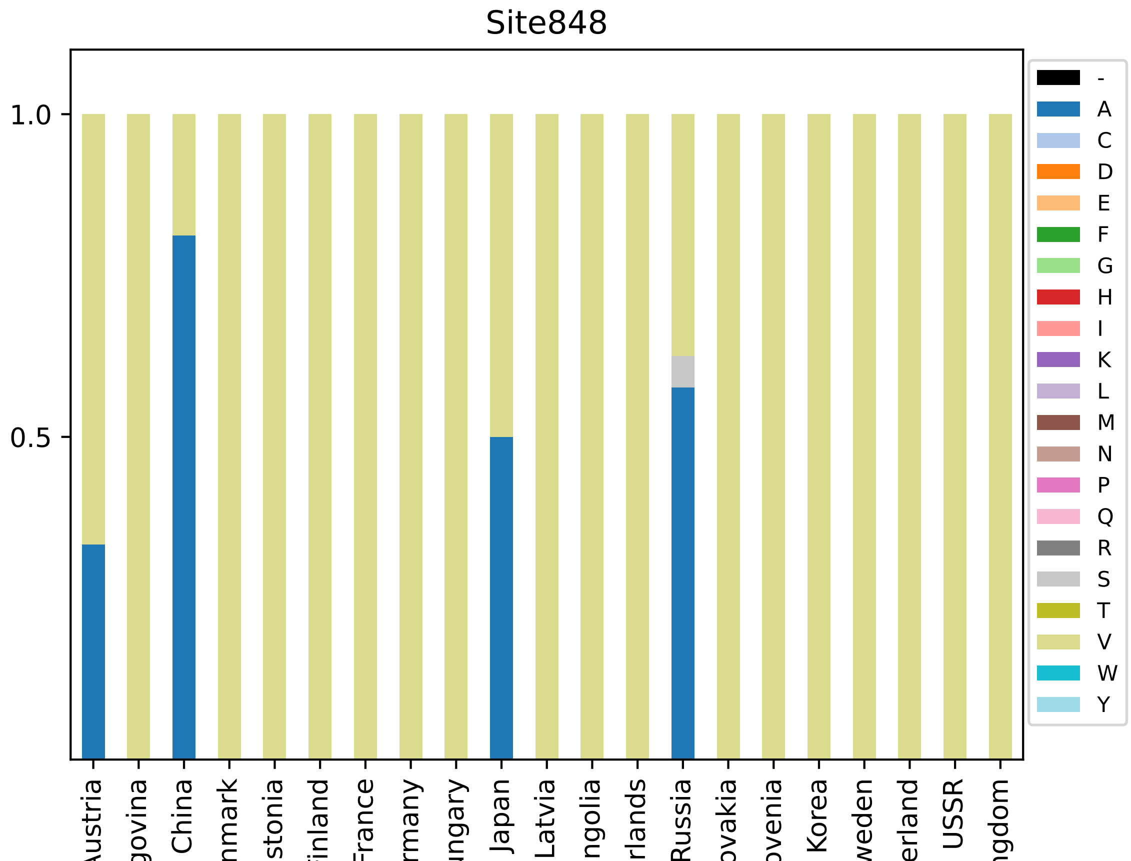
Site848
1.0
0.5
-
A
C
D
E
F
G
H
I
K
L
M
N
P
Q
R
S
T
V
W
Y
Austria
govina
China
nmark
stonia
inland
rance
rmany
ungary
Japan
Latvia
ngolia
rlands
Russia
ovakia
ovenia
Korea
weden
erland
USSR
ngdom

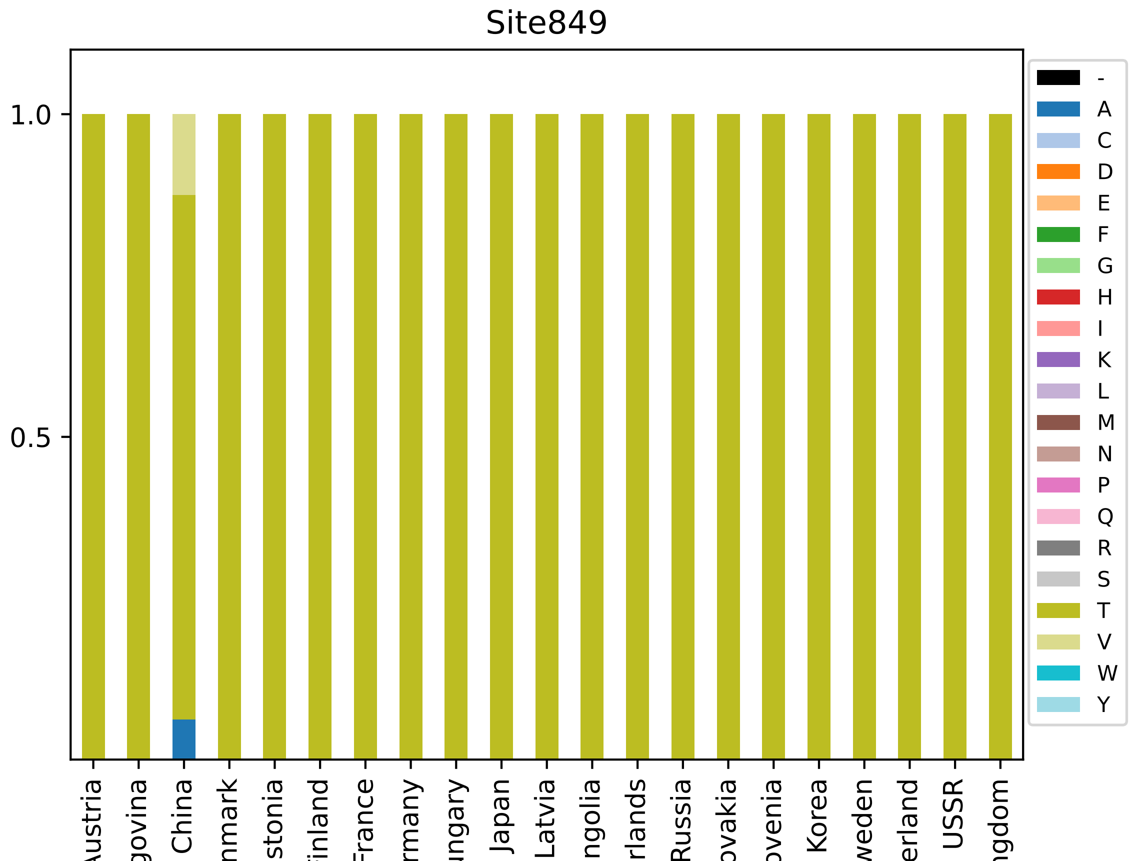
Site849
1.0
0.5
Austria
govina
China
nmark
stonia
inland
France
rmany
ungary
Japan
Latvia
ngolia
rlands
Russia
ovakia
ovenia
Korea
weden
erland
USSR
ngdom
-
A
C
D
E
F
G
H
I
K
L
M
N
P
Q
R
S
T
V
W
Y

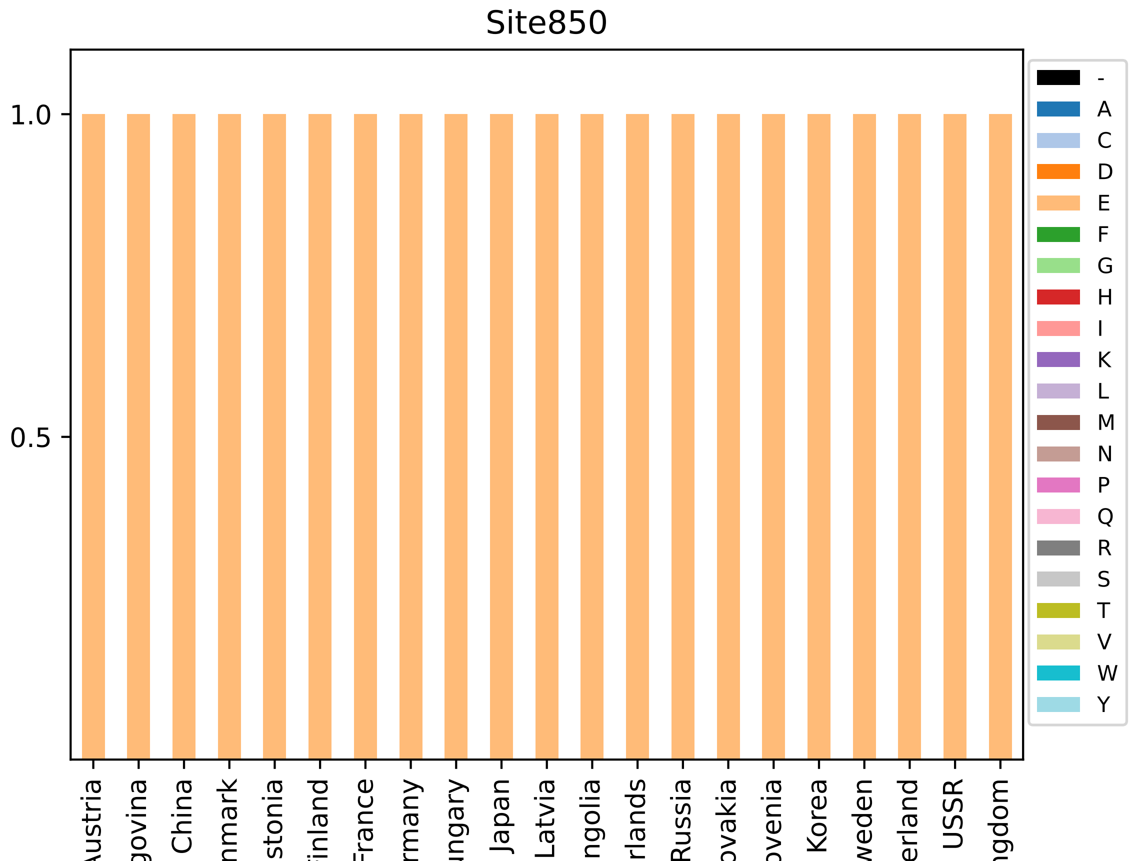

Site850
1.0
0.5
-
A
C
D
E
F
G
H
I
K
L
M
N
P
Q
R
S
T
V
W
Y
Austria
China
Japan
Latvia
Russia
Korea
USSR

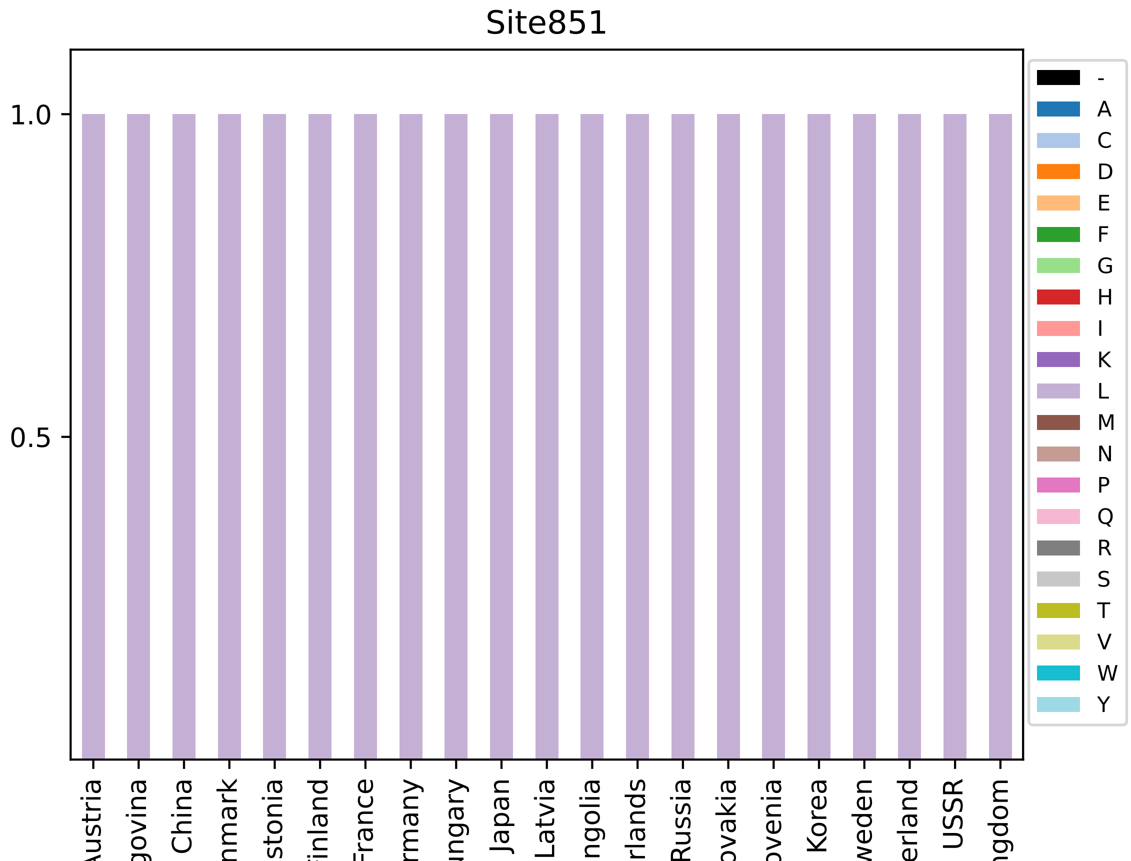
Site851
1.0
0.5
-
A
C
D
E
F
G
H
I
K
L
M
N
P
Q
R
S
T
V
W
Y
China
Japan
Latvia
Russia
Korea
USSR

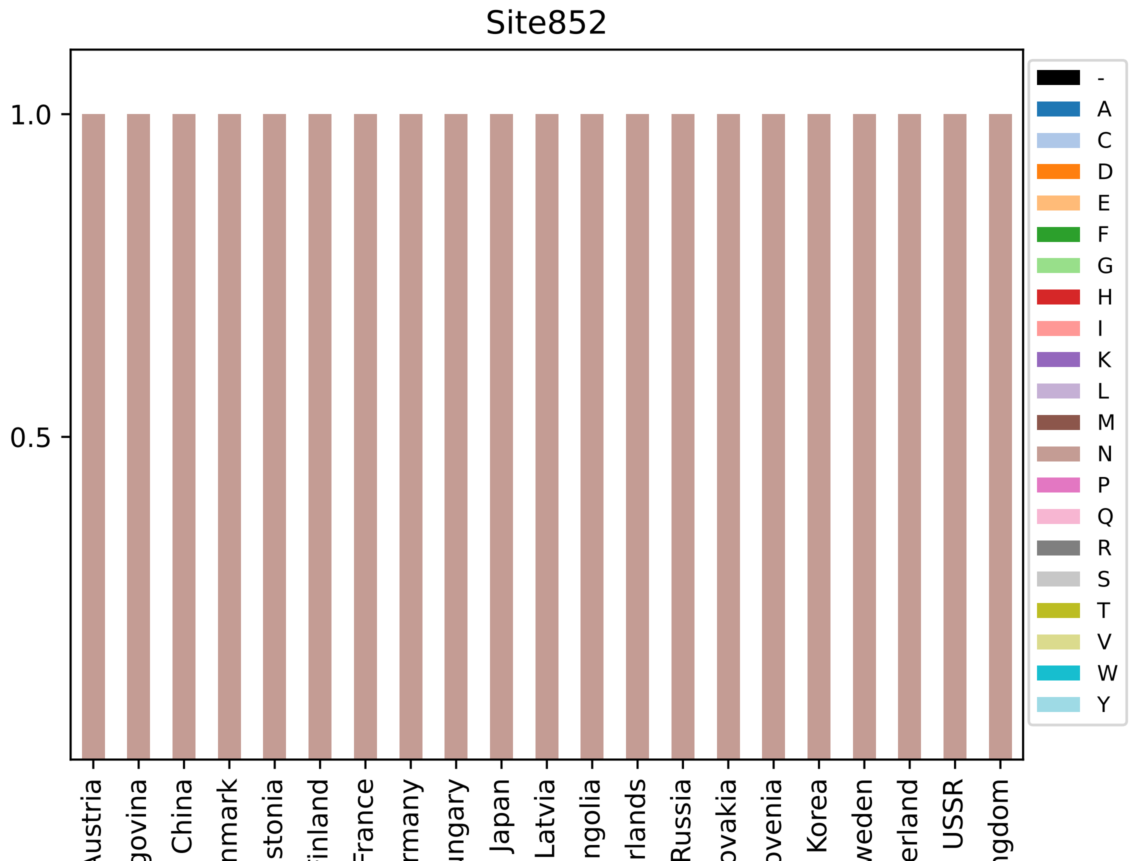
Site852
1.0
0.5
China
Japan
Latvia
Russia
Korea
USSR
-
A
C
D
E
F
G
H
I
K
L
M
N
P
Q
R
S
T
V
W
Y

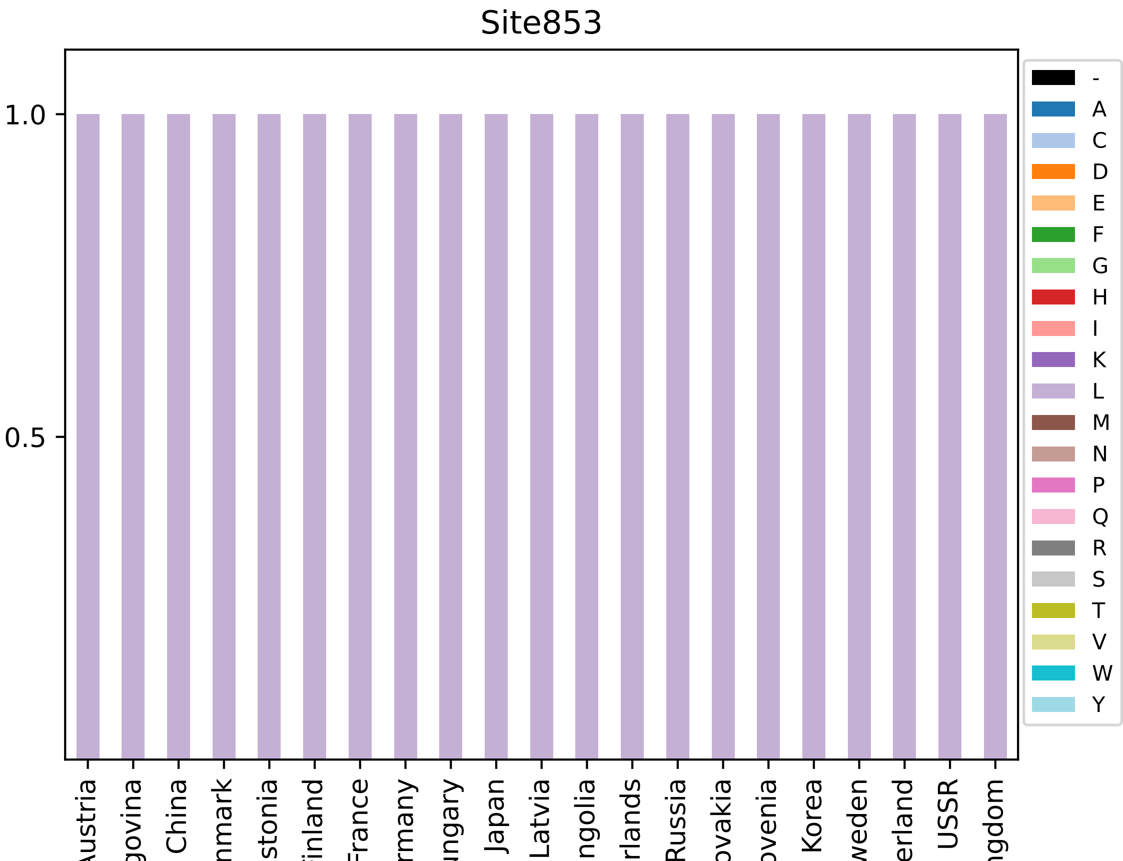

Site853
1.0
0.5
-
A
C
D
E
F
G
H
I
K
L
M
N
P
Q
R
S
T
V
W
Y
China
Japan
Latvia
Russia
Korea
USSR

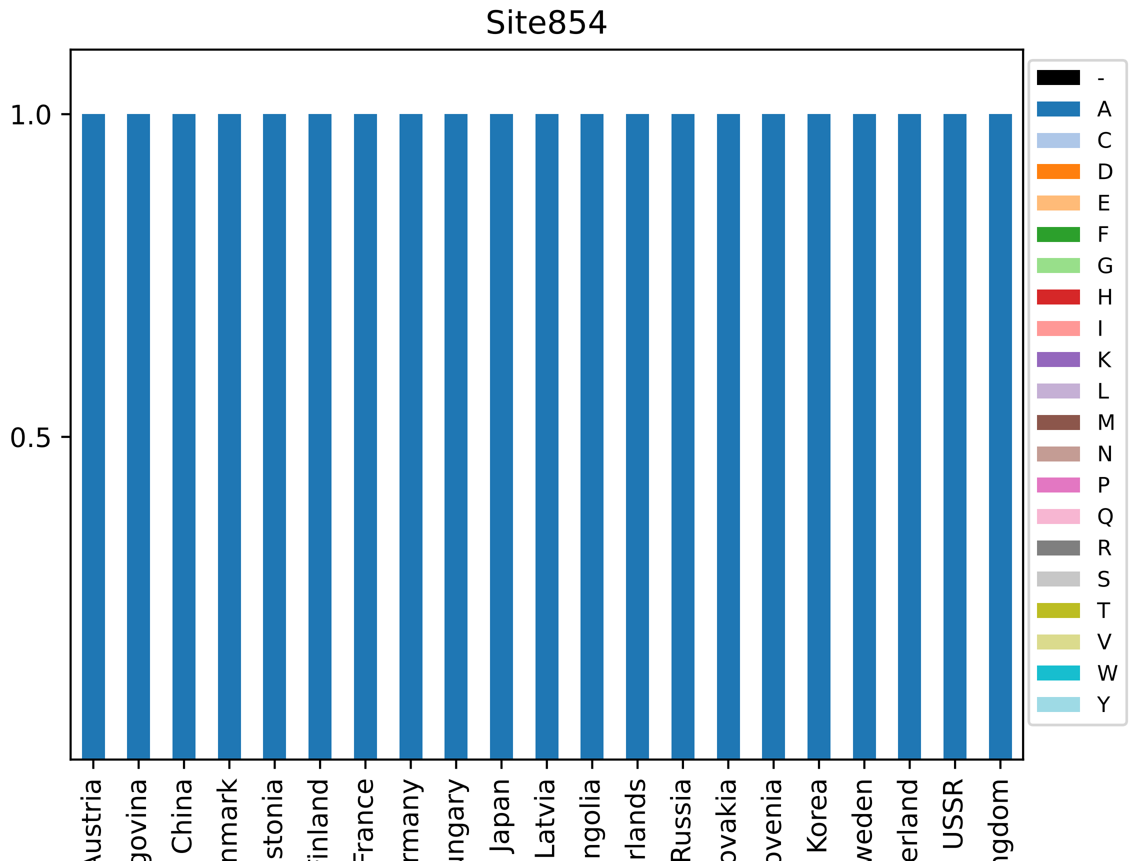
Site854
1.0
0.5
-
A
C
D
E
F
G
H
I
K
L
M
N
P
Q
R
S
T
V
W
Y
China
Japan
Latvia
Russia
Korea
USSR

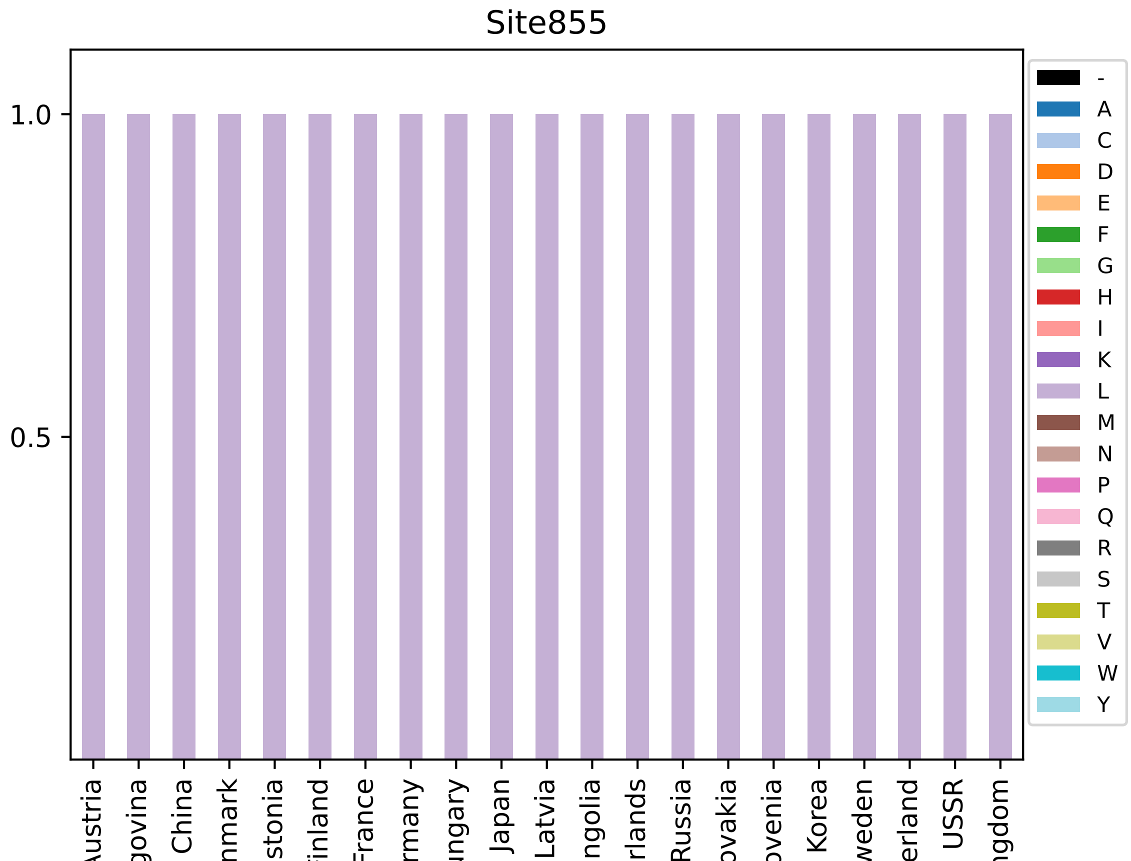

Site855
1.0
0.5
-
A
C
D
E
F
G
H
I
K
L
M
N
P
Q
R
S
T
V
W
Y
Austria
govina
China
nmark
stonia
inland
France
rmany
ungary
Japan
Latvia
ngolia
rlands
Russia
ovakia
ovenia
Korea
weden
erland
USSR
ngdom

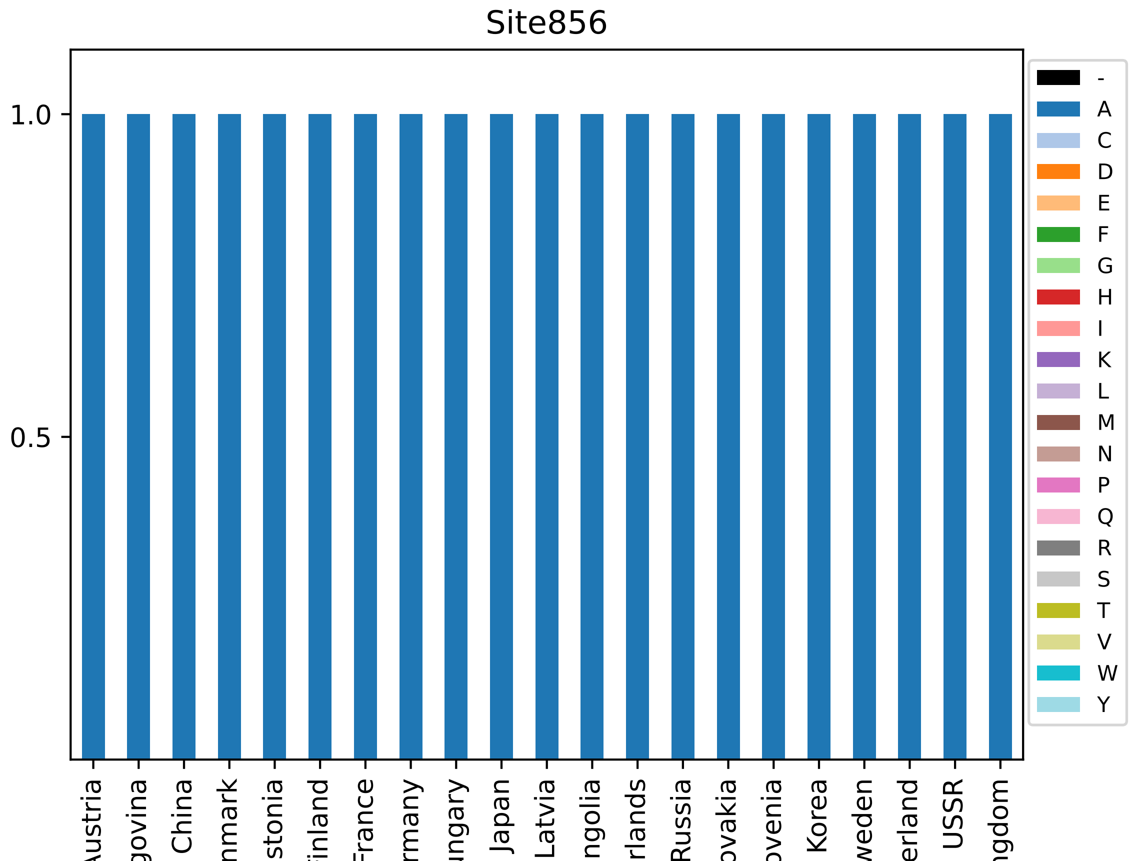

Site856
1.0
0.5
-
A
C
D
E
F
G
H
I
K
L
M
N
P
Q
R
S
T
V
W
Y
Austria
govina
China
nmark
stonia
inland
France
rmany
ungary
Japan
Latvia
ngolia
rlands
Russia
ovakia
ovenia
Korea
weden
erland
USSR
ngdom

Site857

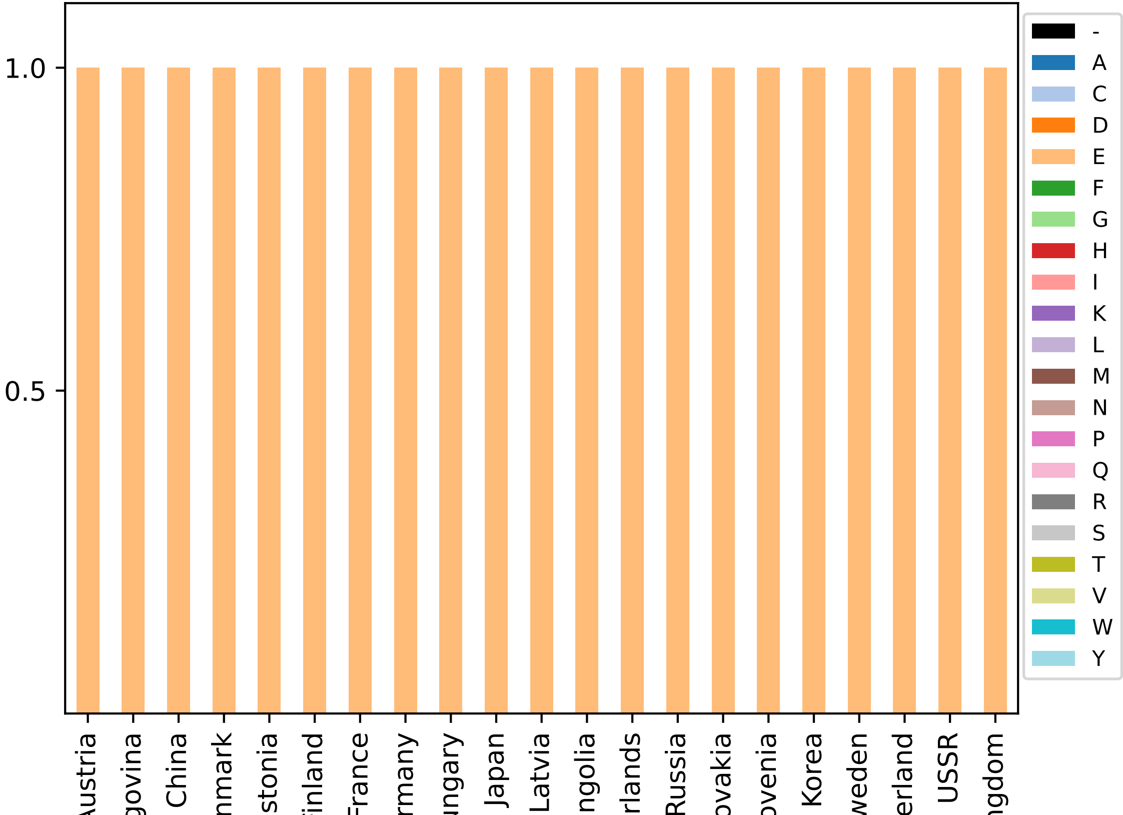

1.0
0.5
-
A
C
D
E
F
G
H
I
K
L
M
N
P
Q
R
S
T
V
W
Y
Austria
govina
China
nmark
stonia
Finland
France
rmany
ungary
Japan
Latvia
ngolia
rlands
Russia
ovakia
ovenia
Korea
weden
erland
USSR
ngdom

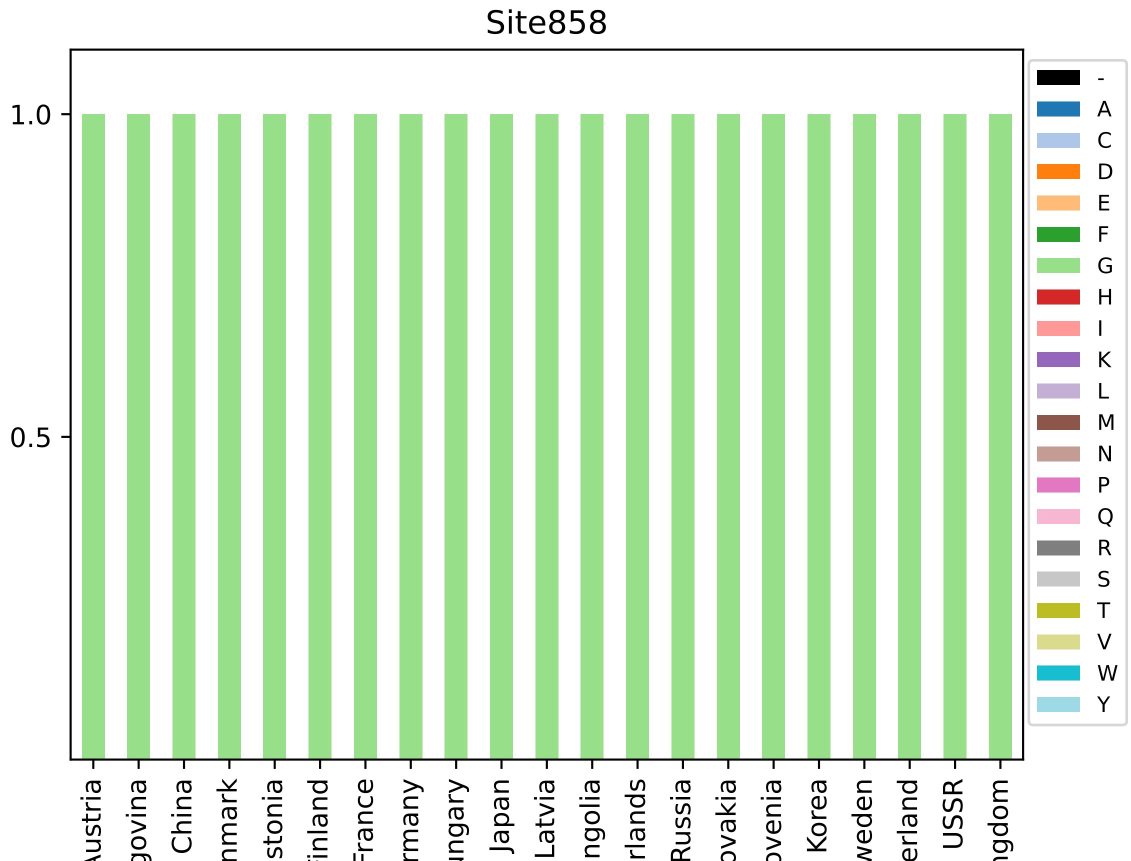
Site858
1.0
0.5
-
A
C
D
E
F
G
H
I
K
L
M
N
P
Q
R
S
T
V
W
Y
China
Japan
Latvia
Russia
Korea
USSR

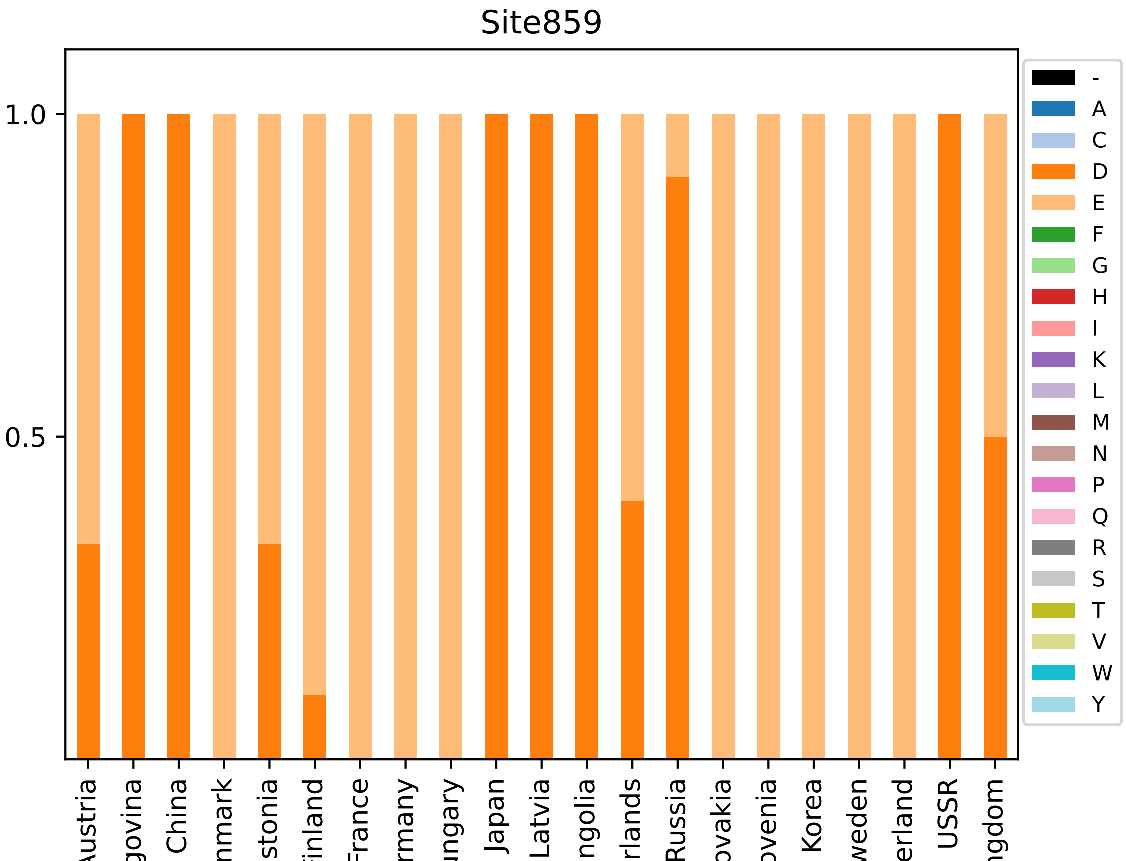
Site859
1.0
0.5
-
A
C
D
E
F
G
H
I
K
L
M
N
P
Q
R
S
T
V
W
Y
China
Japan
Latvia
Russia
Korea
USSR

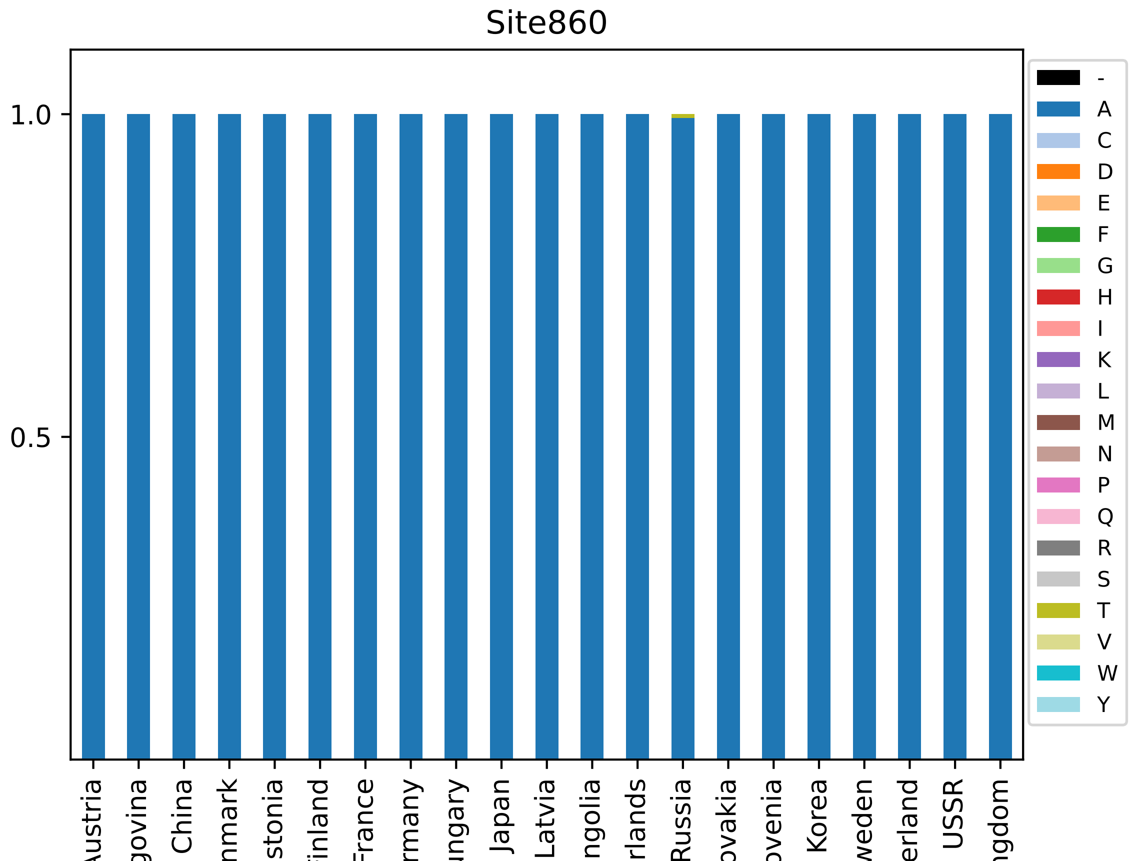
Site860
1.0
0.5
-
A
C
D
E
F
G
H
I
K
L
M
N
P
Q
R
S
T
V
W
Y
Austria
govina
China
nmark
stonia
Finland
France
rmany
ungary
Japan
Latvia
ngolia
rlands
Russia
ovakia
ovenia
Korea
weden
erland
USSR
ngdom

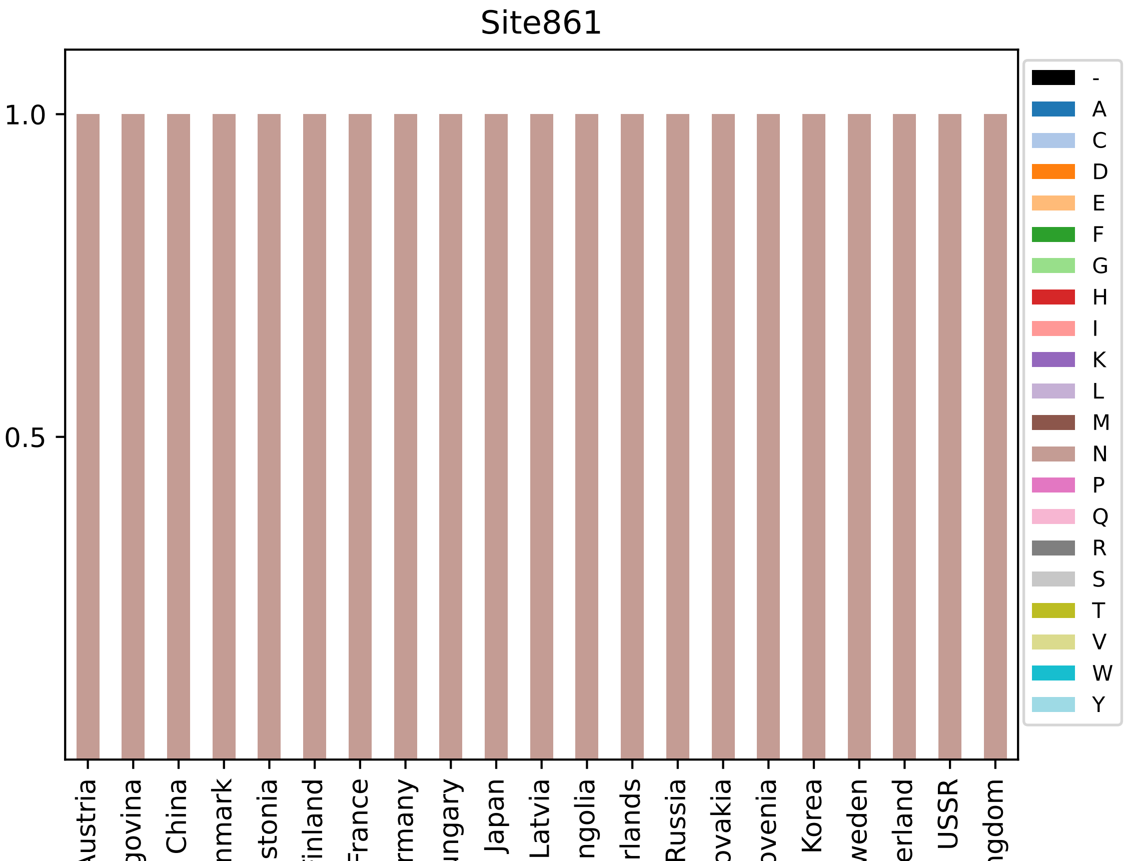
Site861
1.0
0.5
-
A
C
D
E
F
G
H
I
K
L
M
N
P
Q
R
S
T
V
W
Y
Austria
China
Finland
France
Japan
Latvia
Russia
Korea
USSR

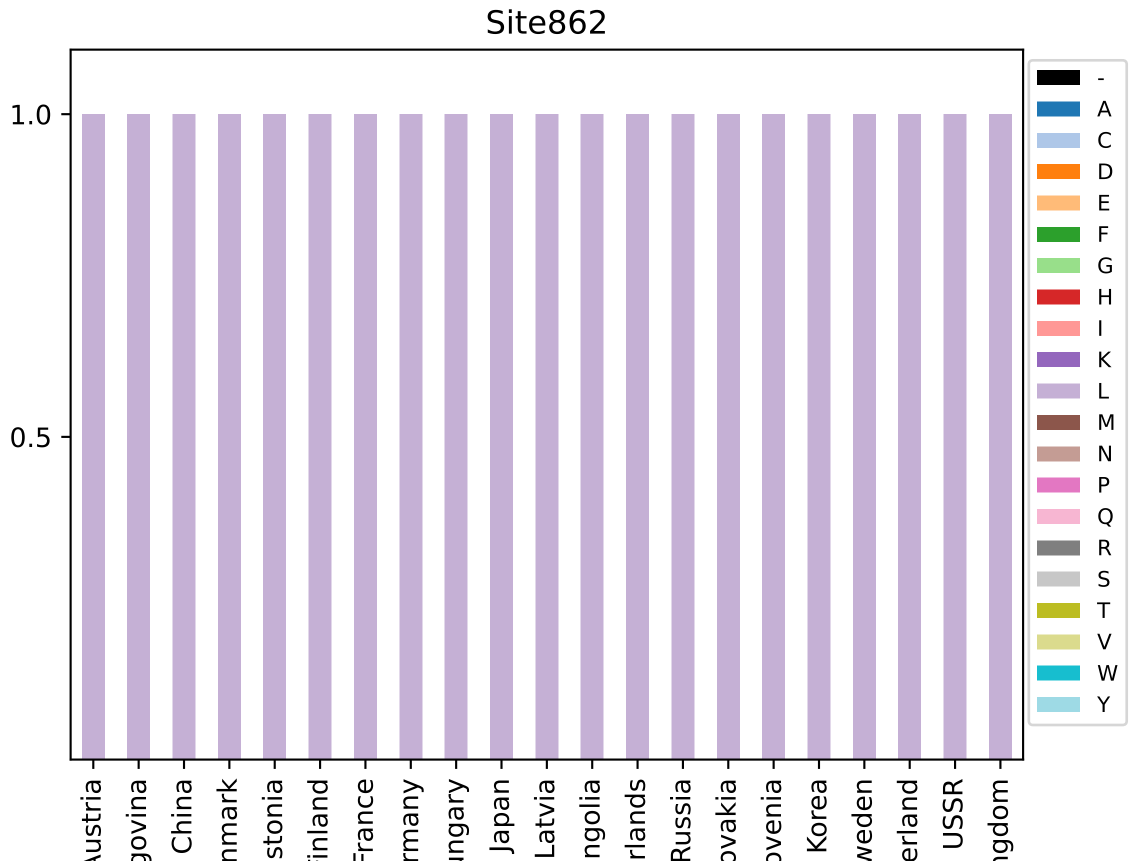

Site862
1.0
0.5
-
A
C
D
E
F
G
H
I
K
L
M
N
P
Q
R
S
T
V
W
Y
Austria
China
Japan
Latvia
Russia
Korea
USSR

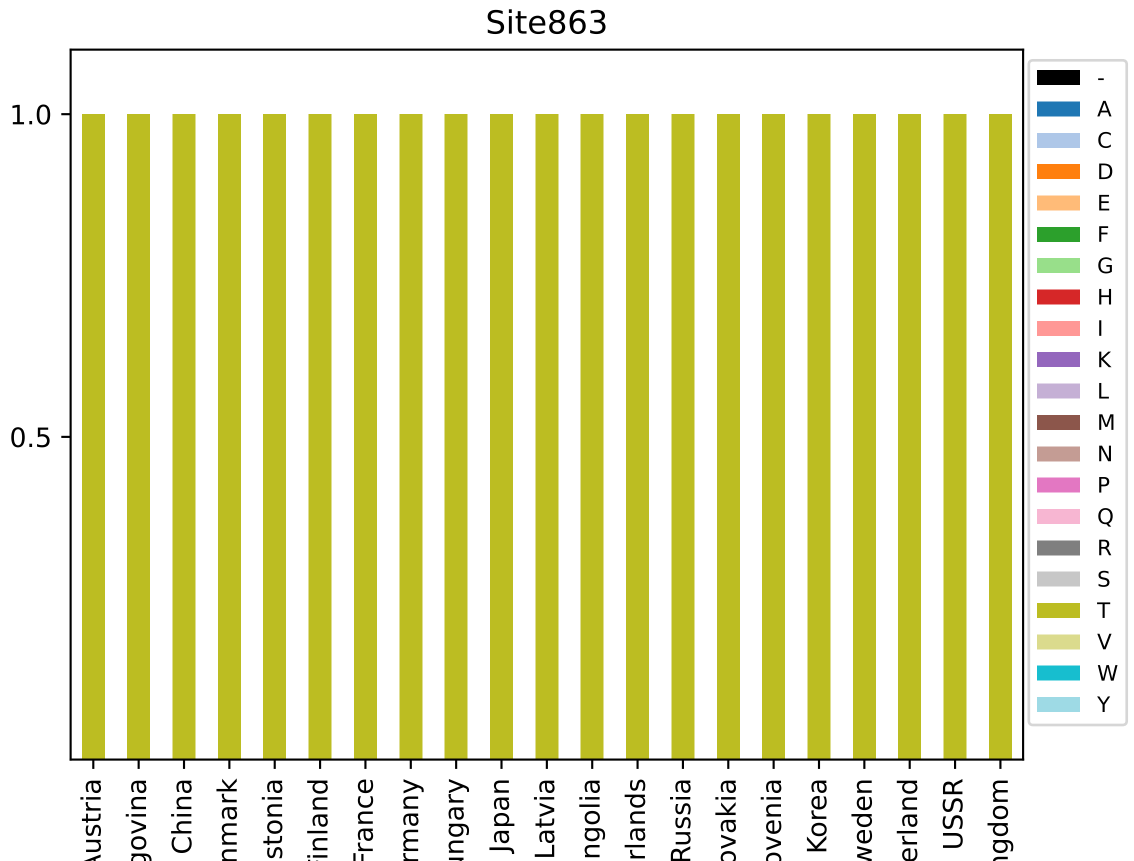

Site863
1.0
0.5
-
A
C
D
E
F
G
H
I
K
L
M
N
P
Q
R
S
T
V
W
Y
Austria
govina
China
nmark
stonia
inland
France
rmany
ungary
Japan
Latvia
ngolia
rlands
Russia
ovakia
ovenia
Korea
weden
erland
USSR
ngdom

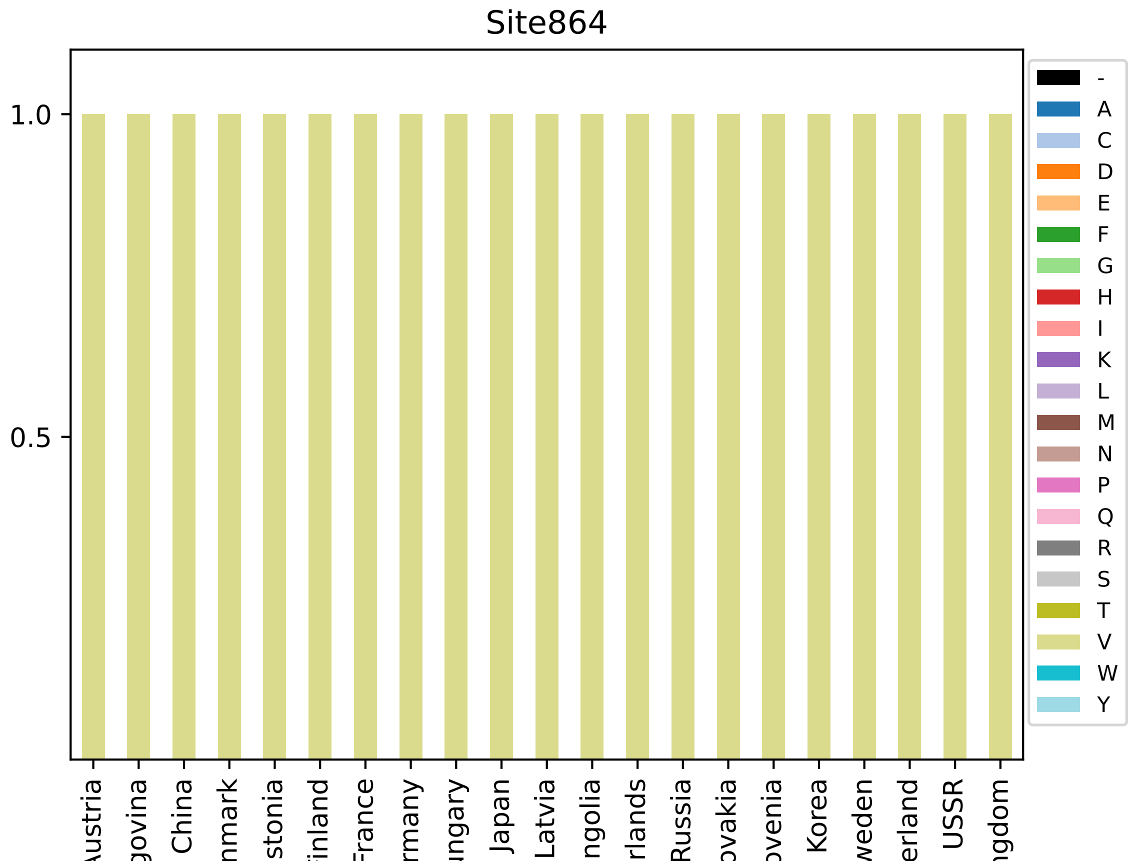

Site864
1.0
0.5
-
A
C
D
E
F
G
H
I
K
L
M
N
P
Q
R
S
T
V
W
Y
China
Japan
Latvia
Russia
Korea
USSR

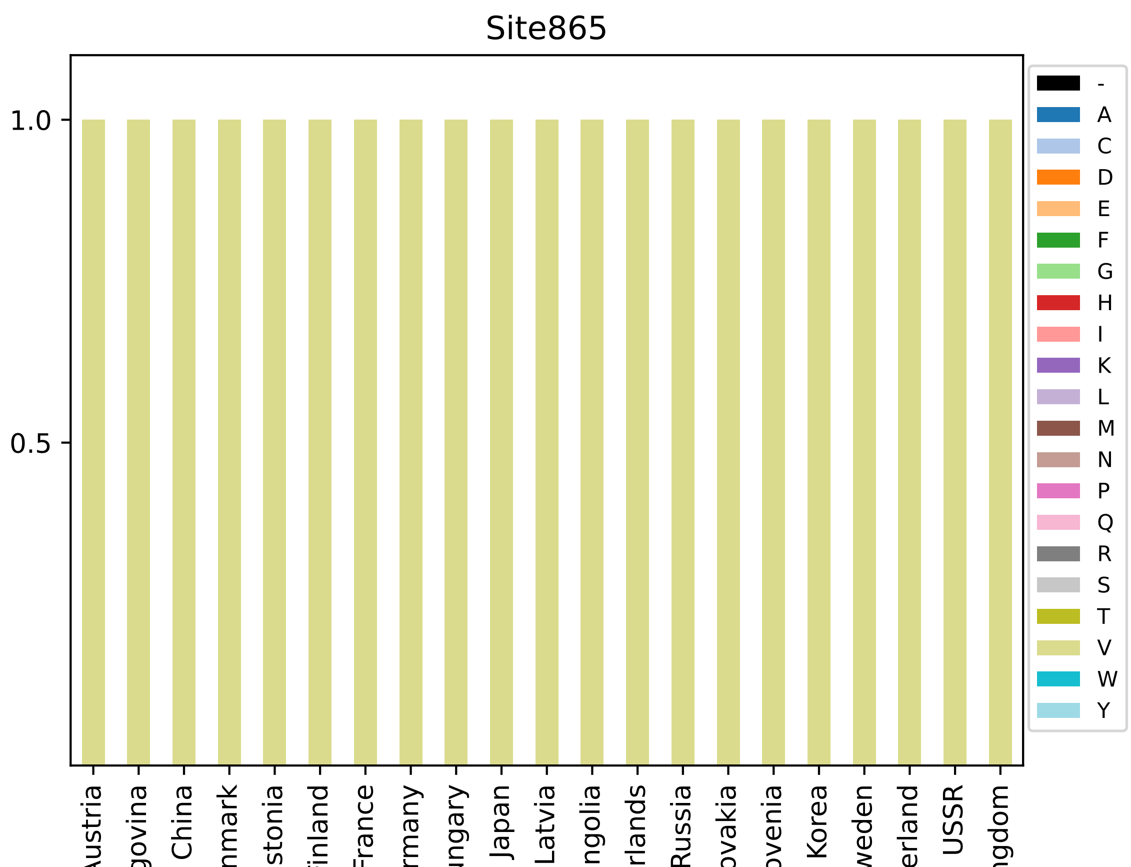
Site865
1.0
0.5
-
A
C
D
E
F
G
H
I
K
L
M
N
P
Q
R
S
T
V
W
Y
China
Japan
Latvia
Russia
Korea
USSR

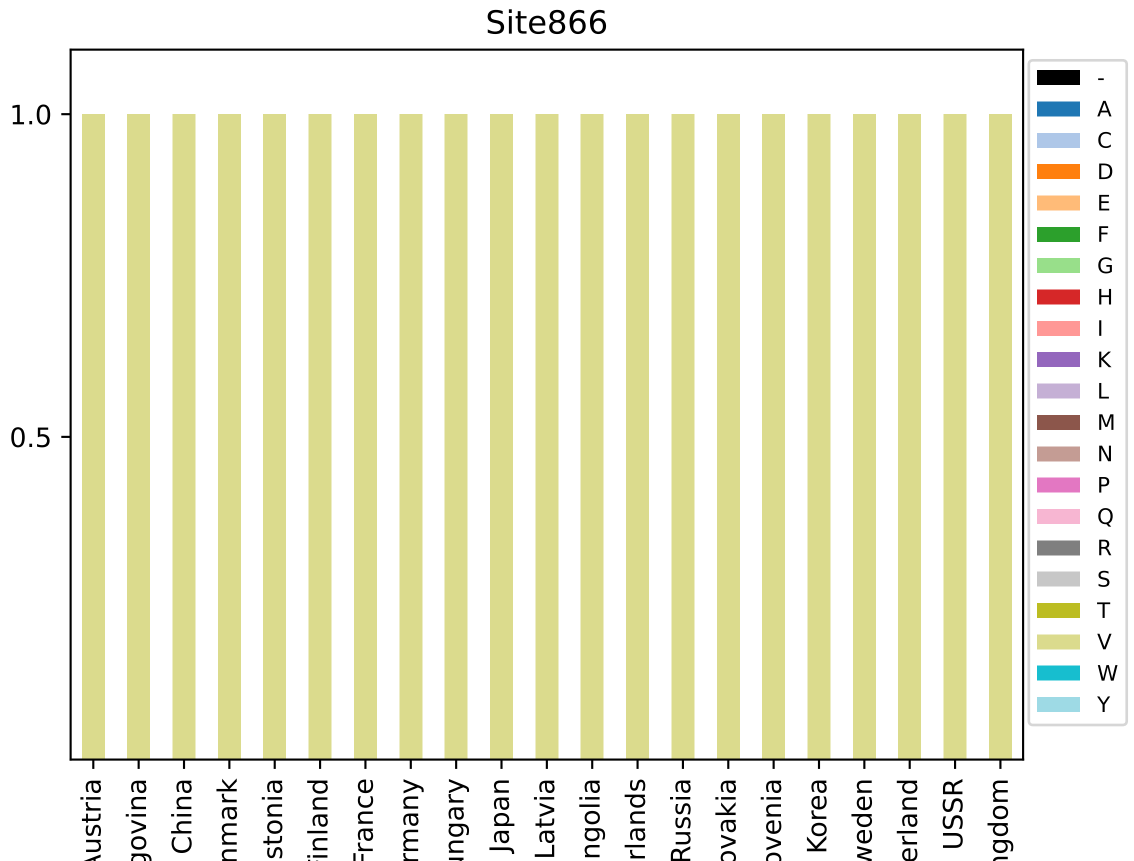
Site866
1.0
0.5
-
A
C
D
E
F
G
H
I
K
L
M
N
P
Q
R
S
T
V
W
Y
China
Japan
Latvia
Russia
Korea
USSR

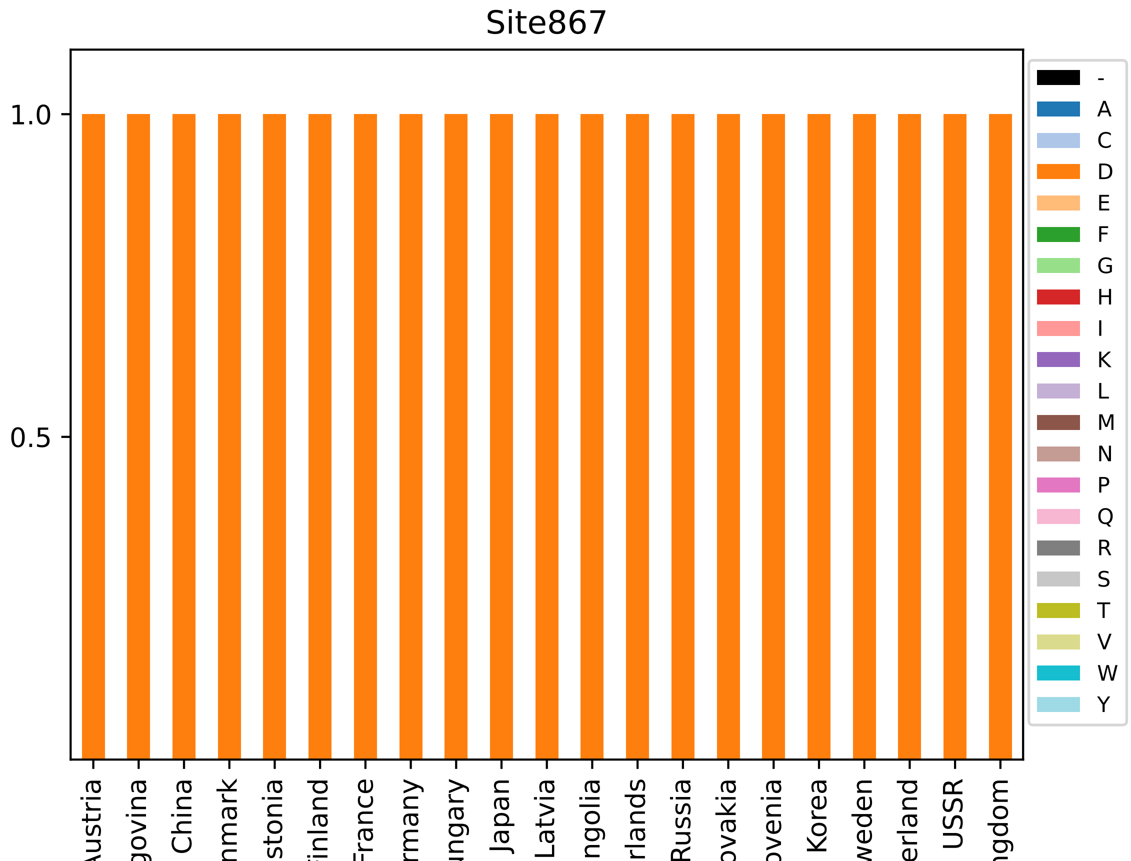

Site867
1.0
0.5
-
A
C
D
E
F
G
H
I
K
L
M
N
P
Q
R
S
T
V
W
Y
Austria
govina
China
nmark
stonia
inland
France
rmany
ungary
Japan
Latvia
ngolia
rlands
Russia
ovakia
ovenia
Korea
weden
erland
USSR
ngdom

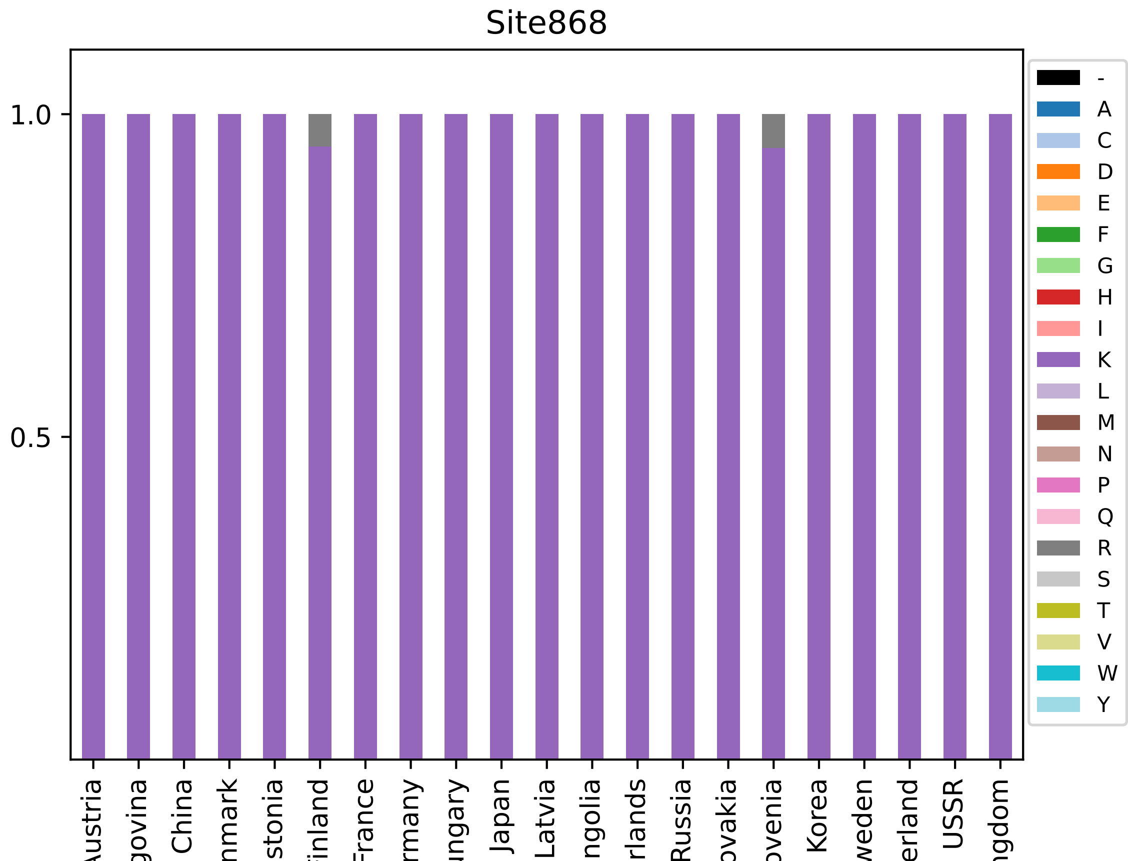

Site868
1.0
0.5
-
A
C
D
E
F
G
H
I
K
L
M
N
P
Q
R
S
T
V
W
Y
Austria
govina
China
nmark
stonia
inland
France
rmany
ungary
Japan
Latvia
ngolia
rlands
Russia
ovakia
ovenia
Korea
weden
erland
USSR
ngdom

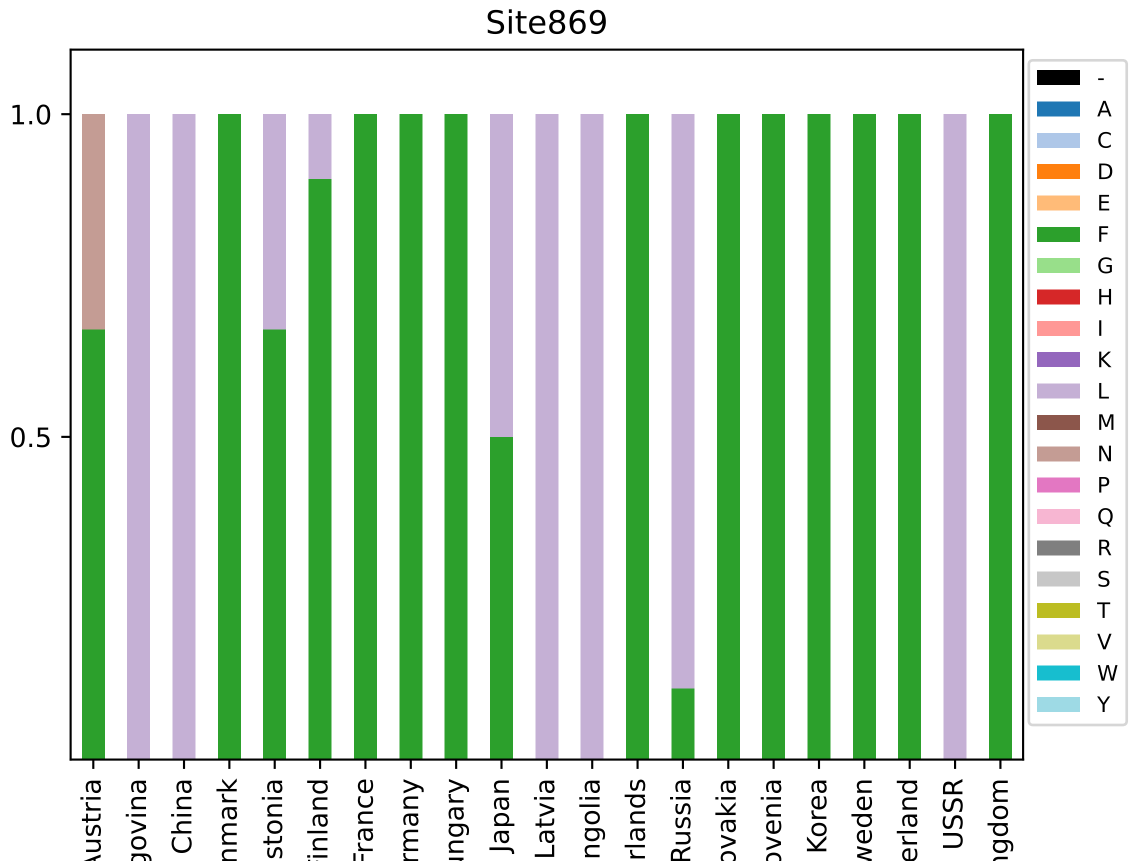
Site869
1.0
0.5
-
A
C
D
E
F
G
H
I
K
L
M
N
P
Q
R
S
T
V
W
Y
Austria
govina
China
nmark
stonia
inland
France
rmany
ungary
Japan
Latvia
ngolia
rlands
Russia
ovakia
ovenia
Korea
weden
erland
USSR
ngdom

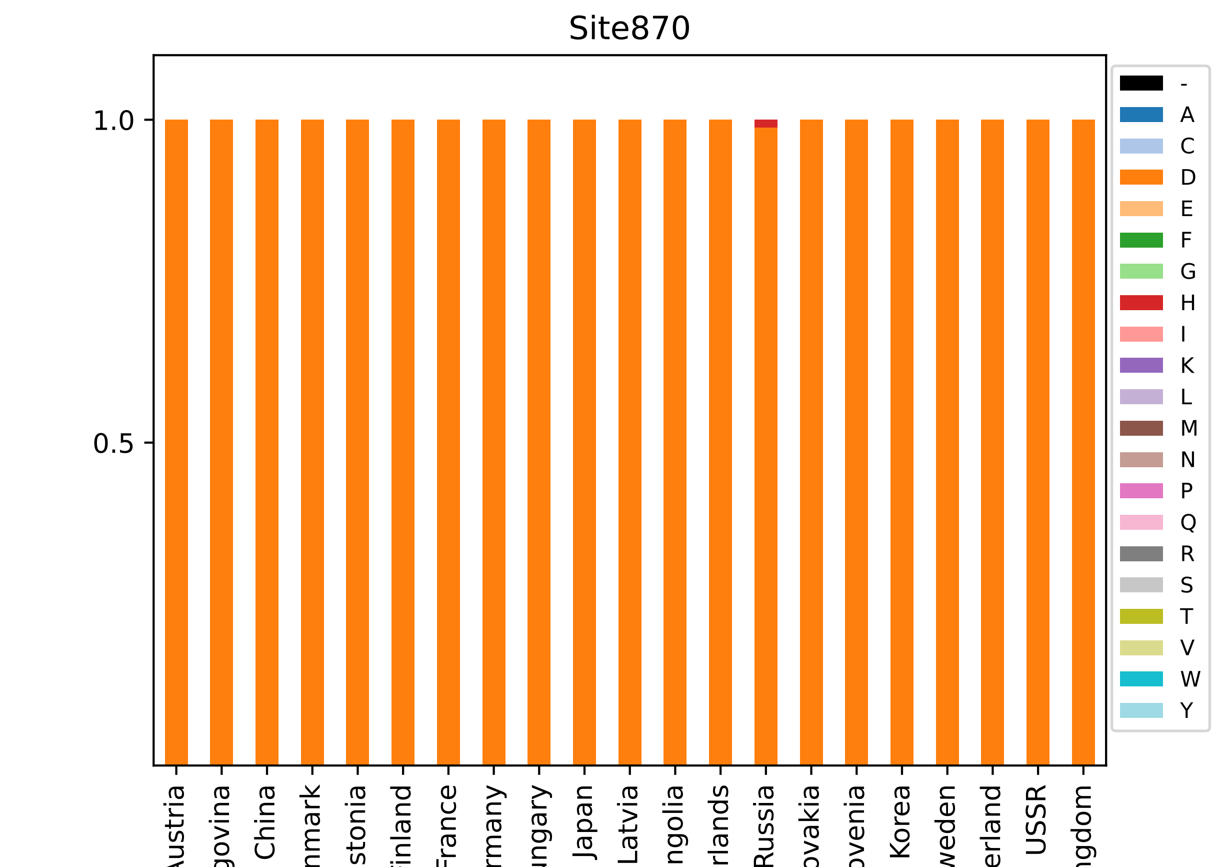

Site870
1.0
0.5
-
A
C
D
E
F
G
H
I
K
L
M
N
P
Q
R
S
T
V
W
Y
Austria
govina
China
nmark
stonia
Finland
France
rmany
ungary
Japan
Latvia
ngolia
rlands
Russia
ovakia
ovenia
Korea
weden
erland
USSR
ngdom

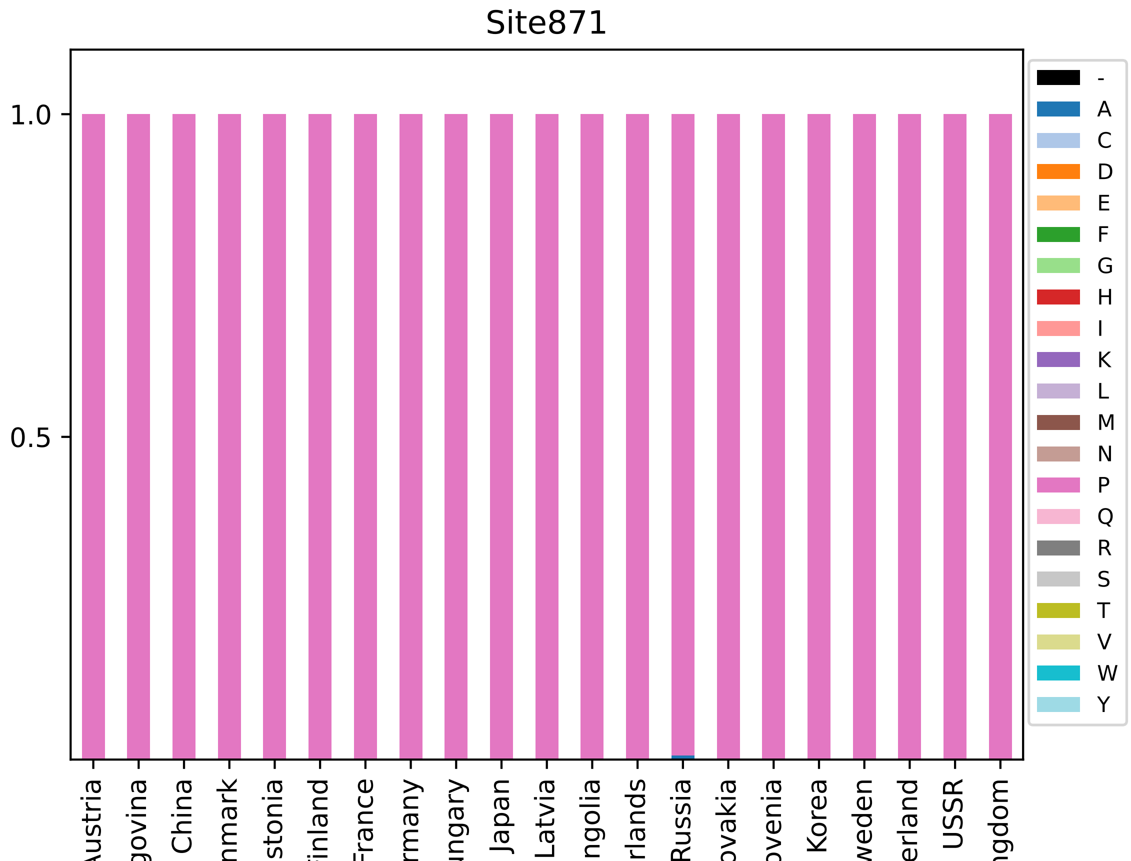
Site871
1.0
0.5
-
A
C
D
E
F
G
H
I
K
L
M
N
P
Q
R
S
T
V
W
Y
Austria
China
Finland
France
Japan
Latvia
Russia
Korea
USSR

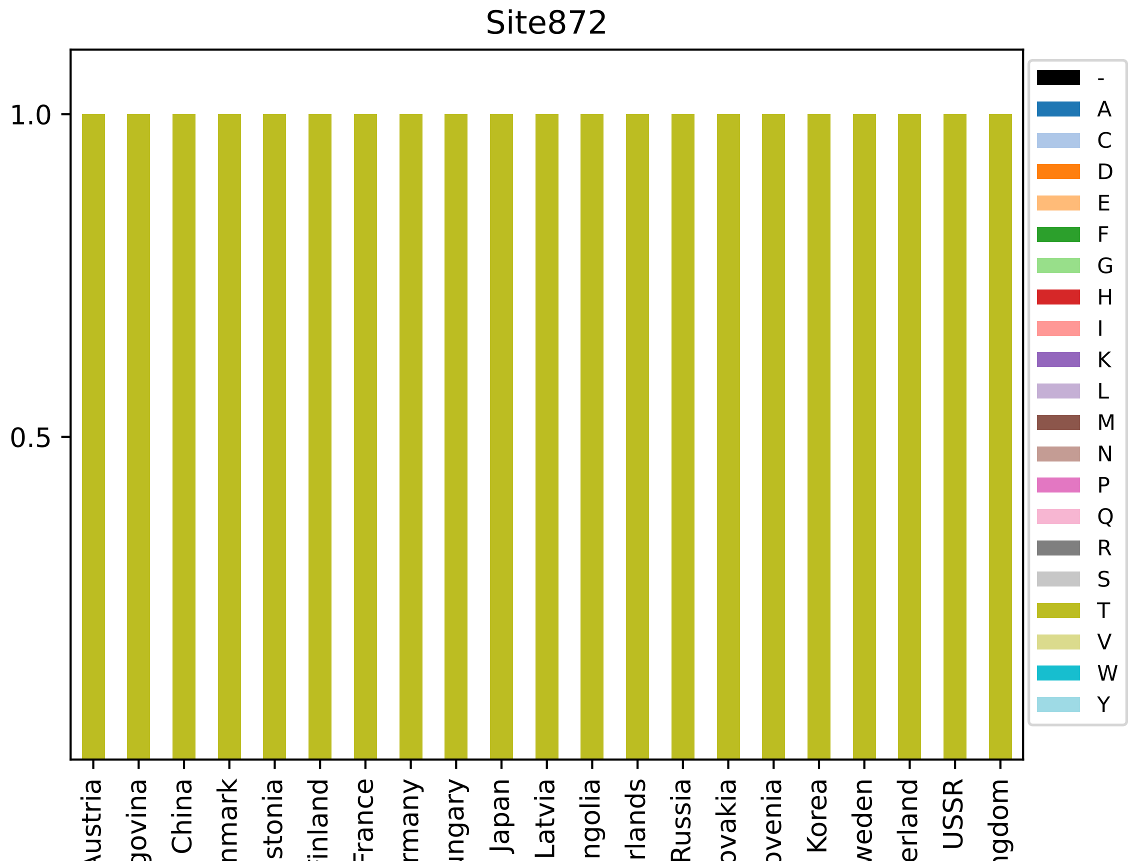
Site872
1.0
0.5
-
A
C
D
E
F
G
H
I
K
L
M
N
P
Q
R
S
T
V
W
Y
ustria
govina
China
nmark
stonia
inland
rance
rmany
ungary
Japan
Latvia
ngolia
rlands
Russia
ovakia
ovenia
Korea
weden
erland
USSR
ngdom

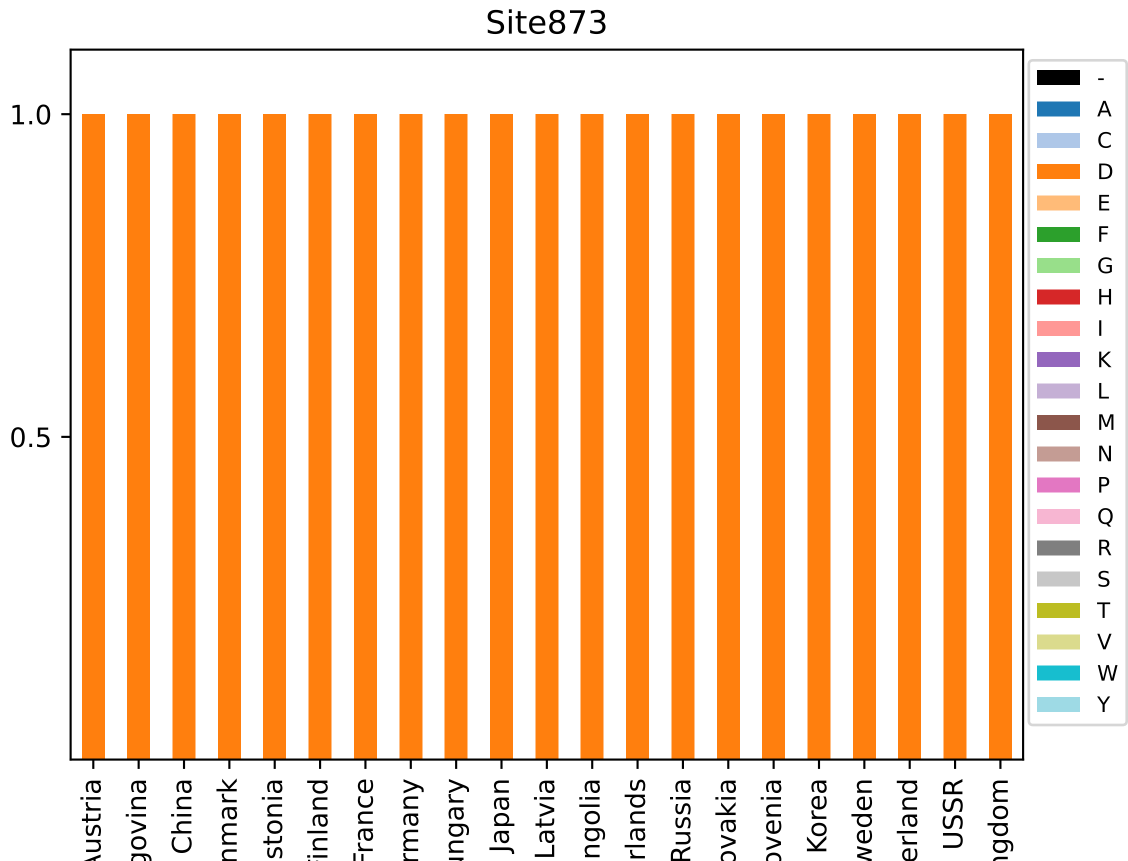

Site873
1.0
0.5
-
A
C
D
E
F
G
H
I
K
L
M
N
P
Q
R
S
T
V
W
Y
China
Japan
Latvia
Russia
Korea
USSR

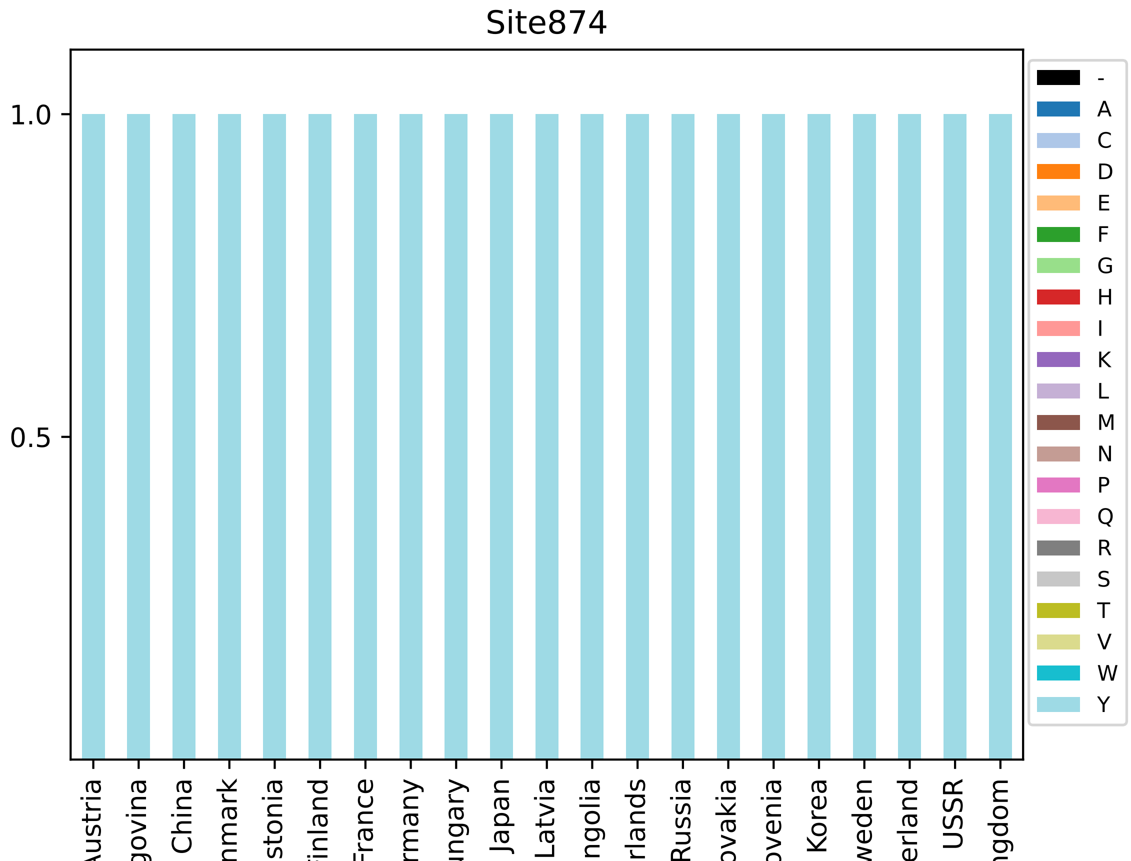

Site874
1.0
0.5
-
A
C
D
E
F
G
H
I
K
L
M
N
P
Q
R
S
T
V
W
Y
China
Japan
Latvia
Russia
Korea
USSR

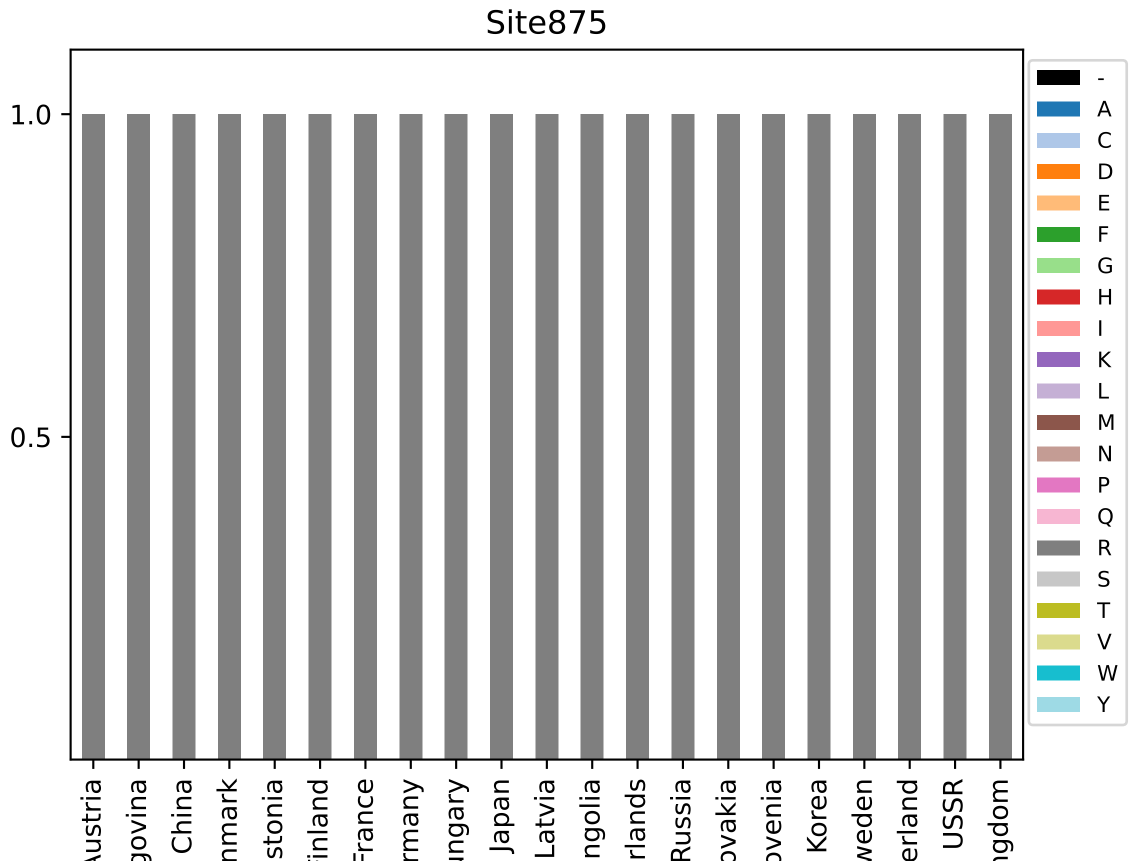

Site875
1.0
0.5
-
A
C
D
E
F
G
H
I
K
L
M
N
P
Q
R
S
T
V
W
Y
China
Japan
Latvia
Russia
Korea
USSR

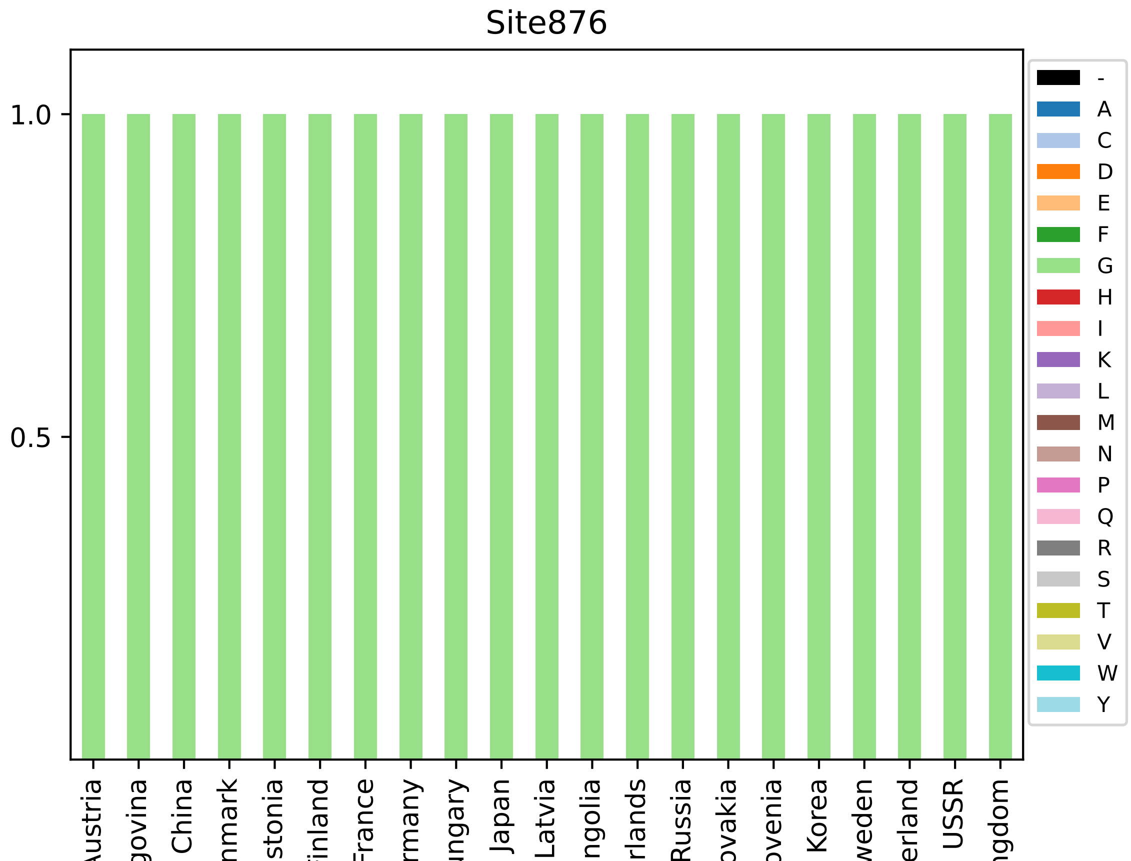
Site876
1.0
0.5
-
A
C
D
E
F
G
H
I
K
L
M
N
P
Q
R
S
T
V
W
Y
China
Japan
Latvia
Russia
Korea
USSR

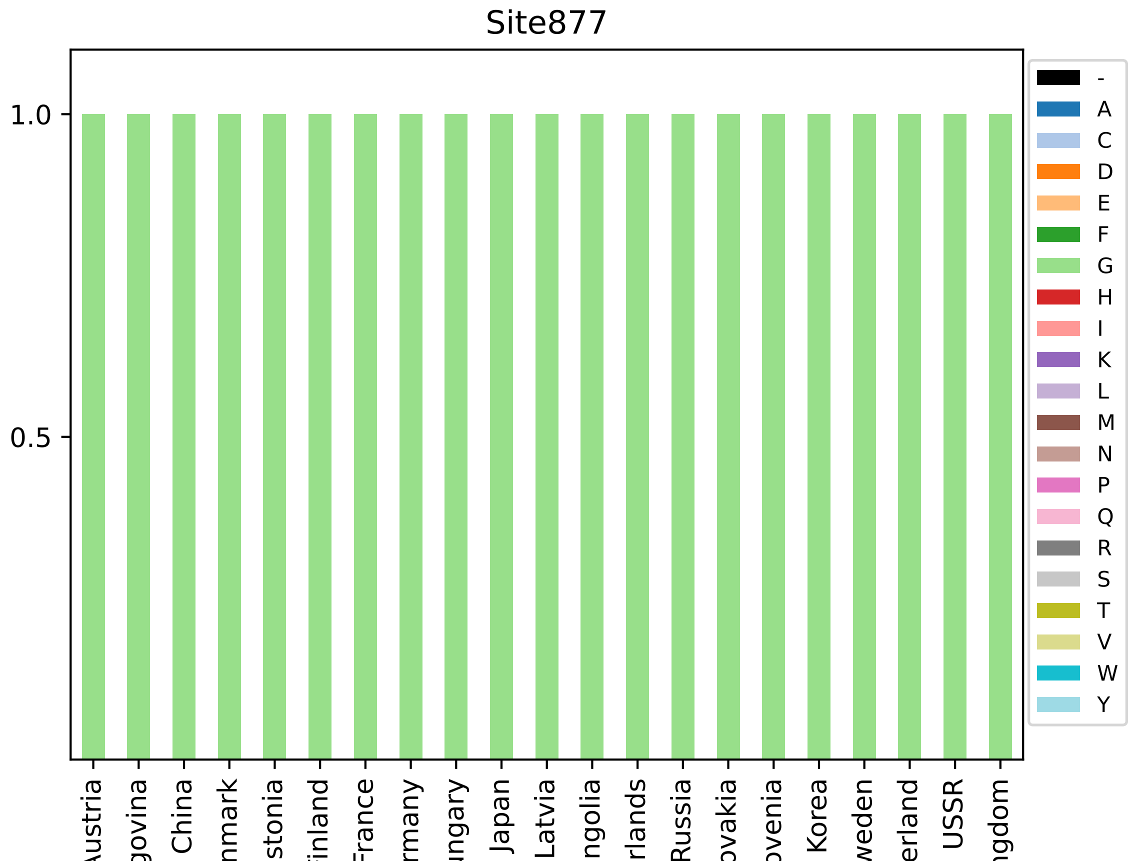

Site877
1.0
0.5
-
A
C
D
E
F
G
H
I
K
L
M
N
P
Q
R
S
T
V
W
Y
ustria
govina
China
nmark
stonia
inland
rance
rmany
ungary
Japan
Latvia
ngolia
rlands
Russia
ovakia
ovenia
Korea
weden
erland
USSR
ngdom

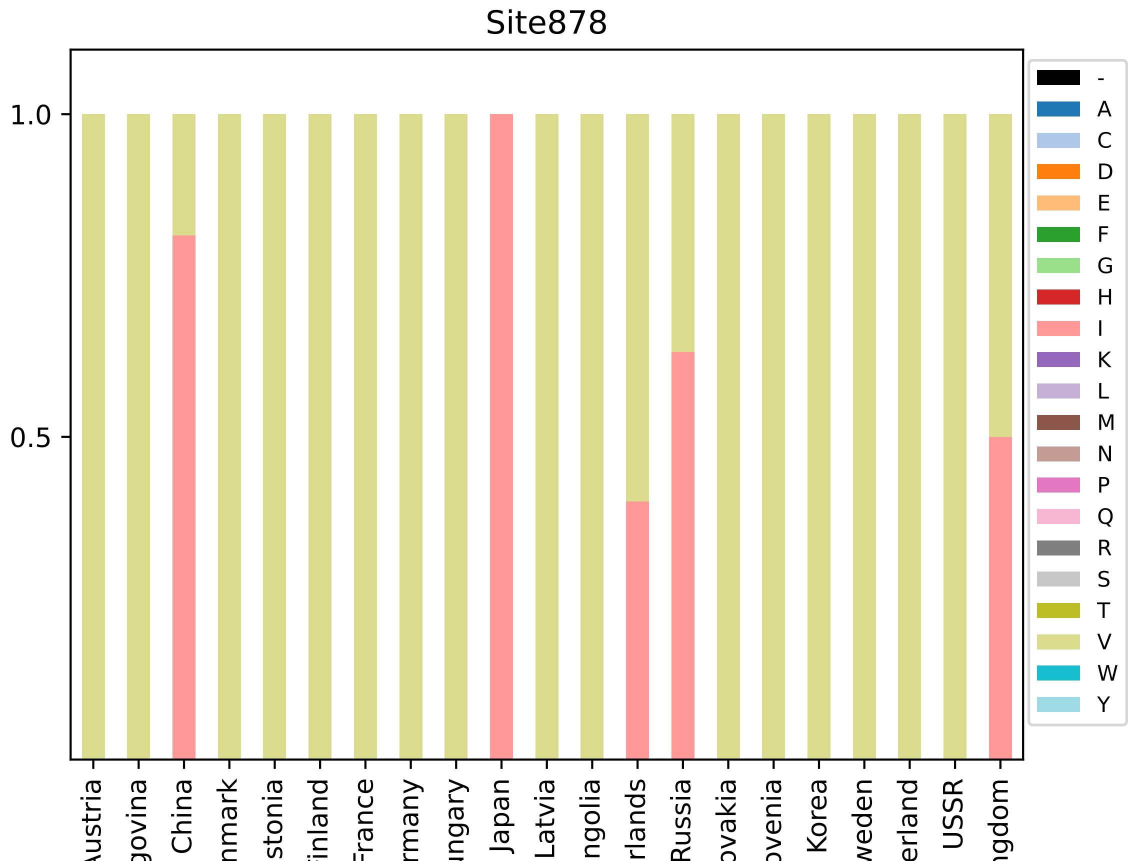

Site878
1.0
0.5
-
A
C
D
E
F
G
H
I
K
L
M
N
P
Q
R
S
T
V
W
Y
China
Japan
Latvia
Russia
Korea
USSR

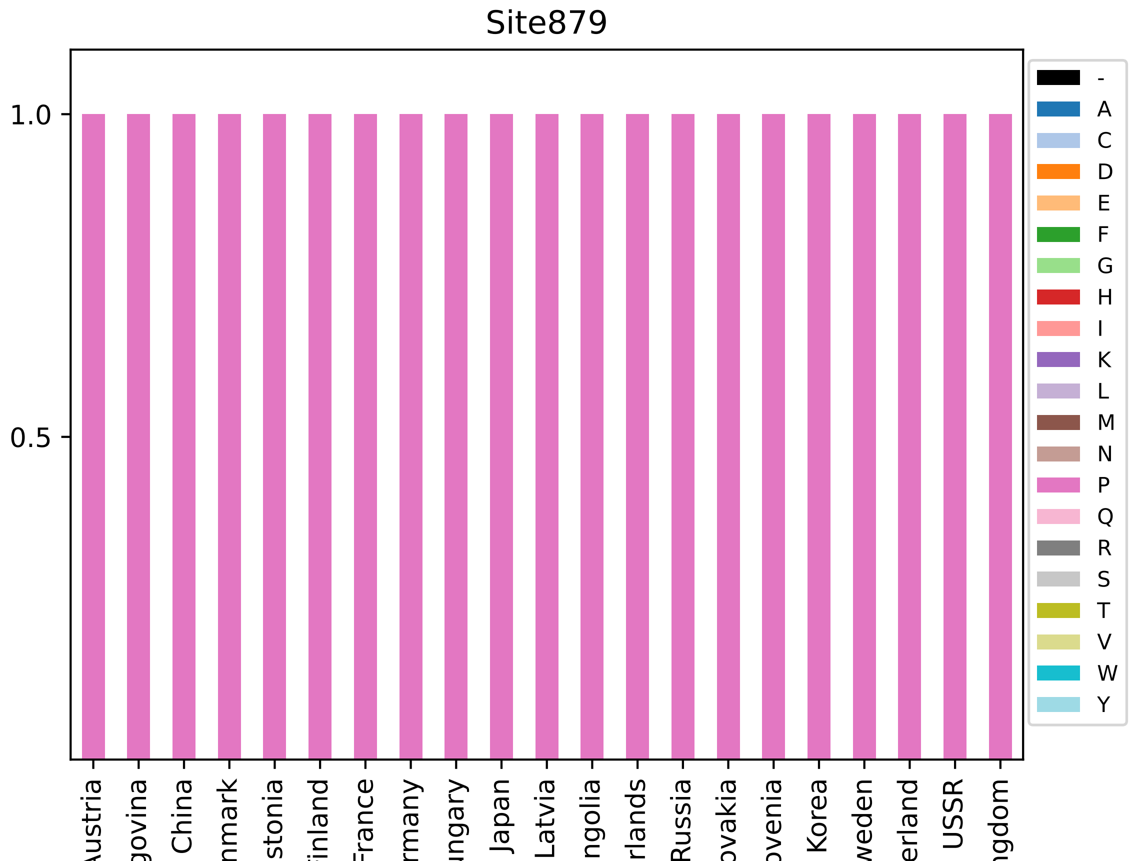

Site879
1.0
0.5
-
A
C
D
E
F
G
H
I
K
L
M
N
P
Q
R
S
T
V
W
Y
Austria
China
Japan
Latvia
Russia
Korea
USSR

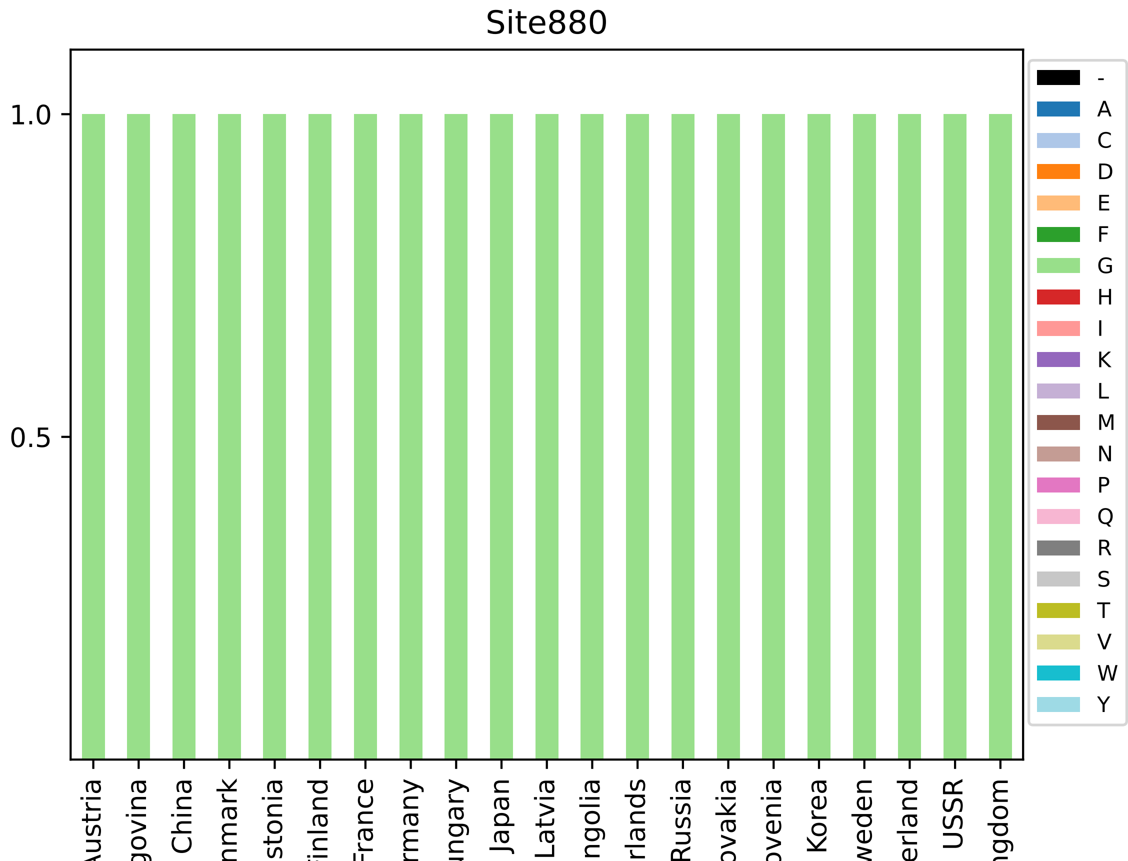

Site880
1.0
0.5
-
A
C
D
E
F
G
H
I
K
L
M
N
P
Q
R
S
T
V
W
Y
Austria
China
Japan
Latvia
Russia
Korea
USSR

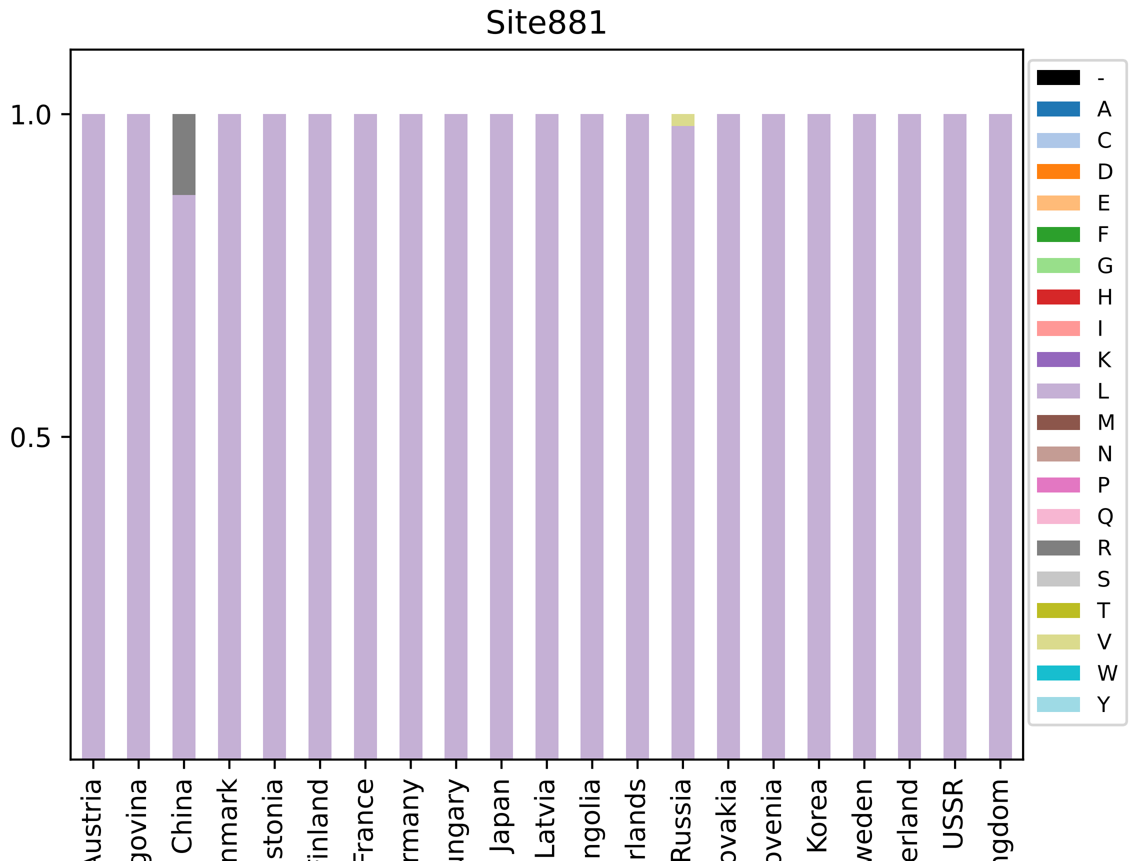
Site881
1.0
0.5
-
A
C
D
E
F
G
H
I
K
L
M
N
P
Q
R
S
T
V
W
Y
China
Japan
Latvia
Russia
Korea
USSR

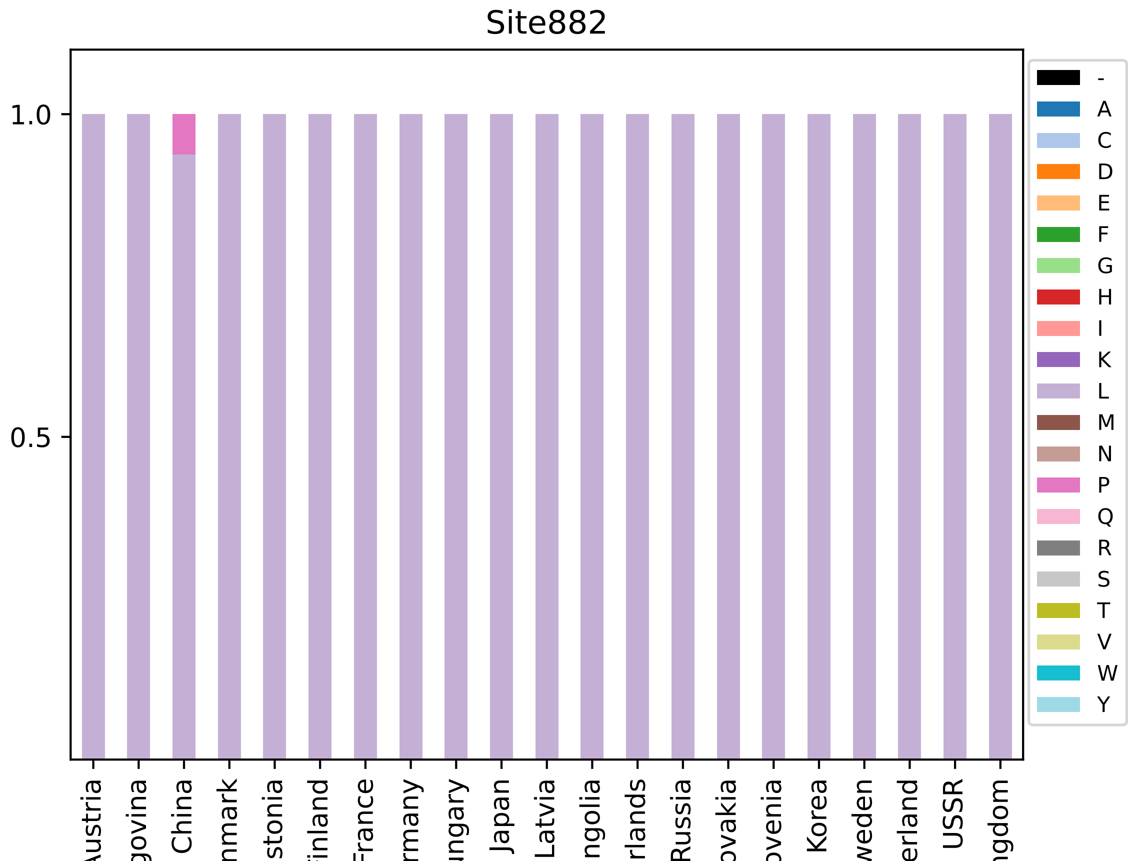
Site882
1.0
0.5
-
A
C
D
E
F
G
H
I
K
L
M
N
P
Q
R
S
T
V
W
Y
China
Japan
Latvia
Russia
Korea
USSR

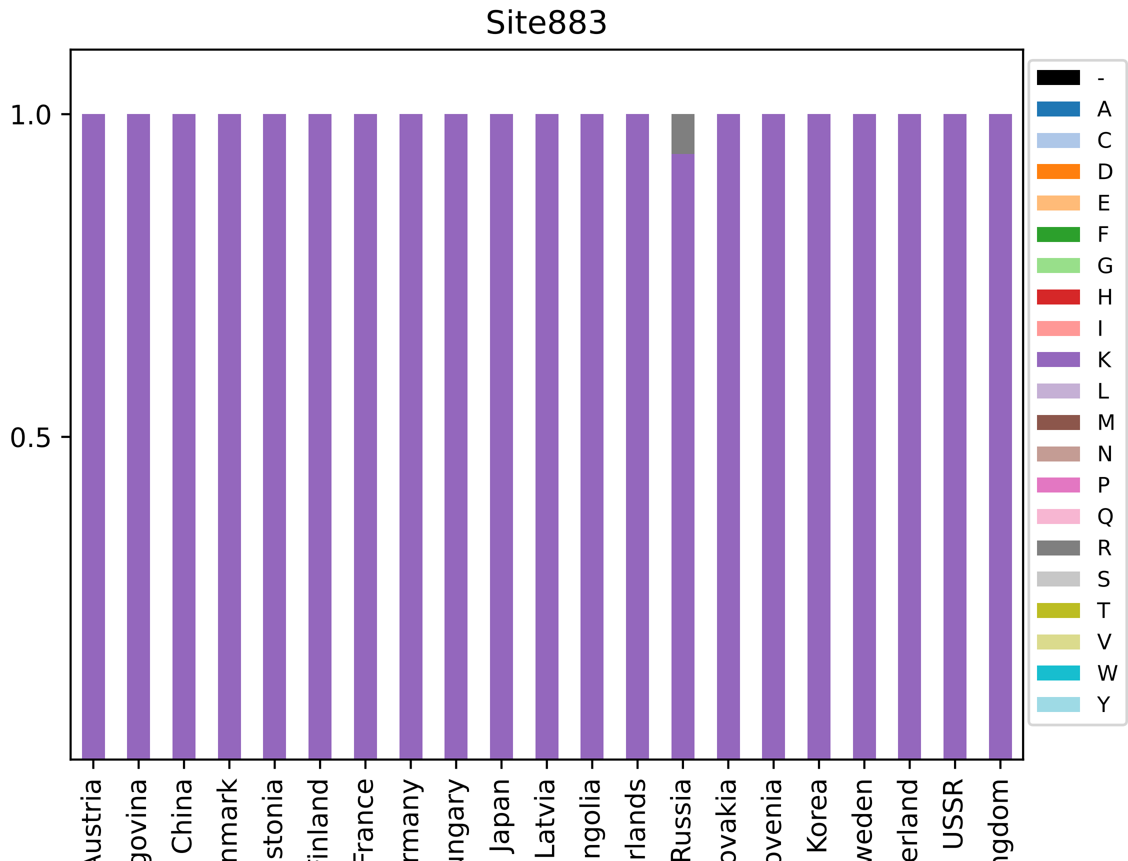
Site883
1.0
0.5
-
A
C
D
E
F
G
H
I
K
L
M
N
P
Q
R
S
T
V
W
Y
China
Japan
Latvia
Russia
Korea
USSR

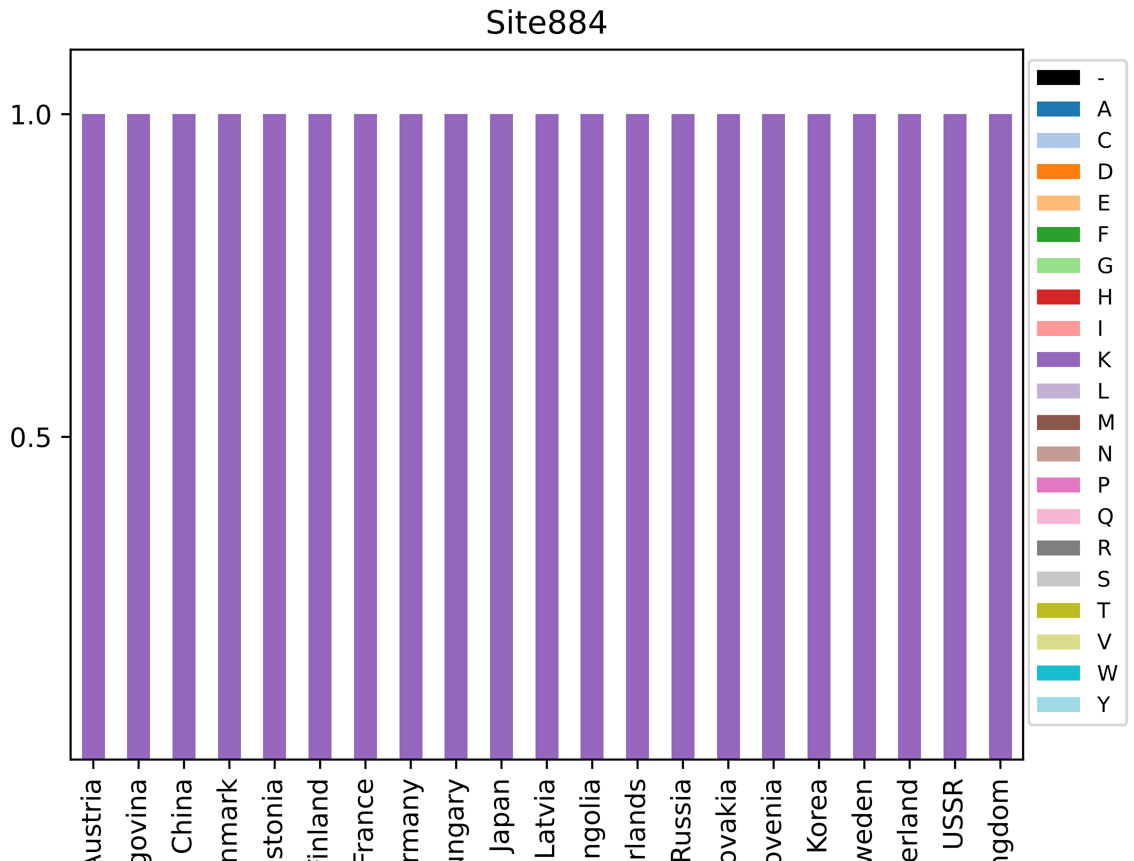

Site884
1.0
0.5
-
A
C
D
E
F
G
H
I
K
L
M
N
P
Q
R
S
T
V
W
Y
Austria
govina
China
nmark
stonia
inland
France
rmany
ungary
Japan
Latvia
ngolia
rlands
Russia
ovakia
ovenia
Korea
weden
erland
USSR
ngdom

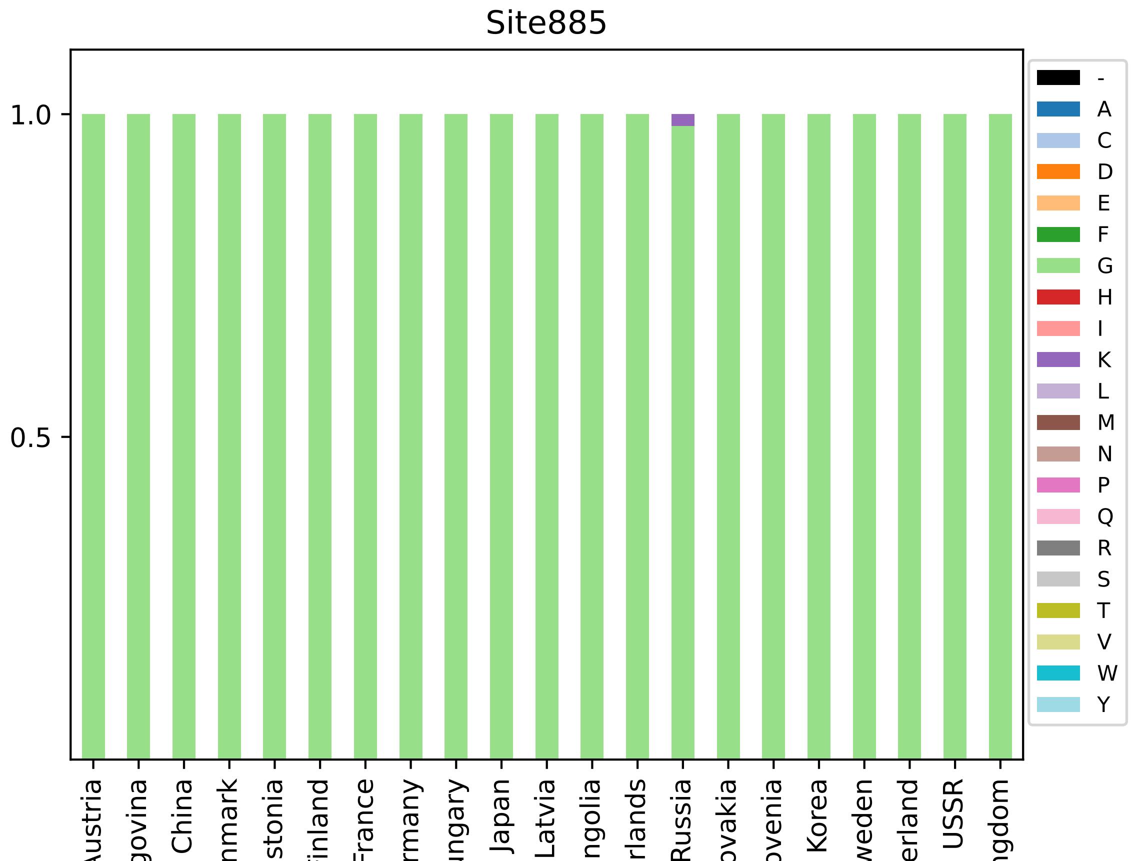
Site885
1.0
0.5
-
A
C
D
E
F
G
H
I
K
L
M
N
P
Q
R
S
T
V
W
Y
Austria
govina
China
nmark
stonia
Finland
France
rmany
ungary
Japan
Latvia
ngolia
rlands
Russia
ovakia
ovenia
Korea
weden
erland
USSR
ngdom

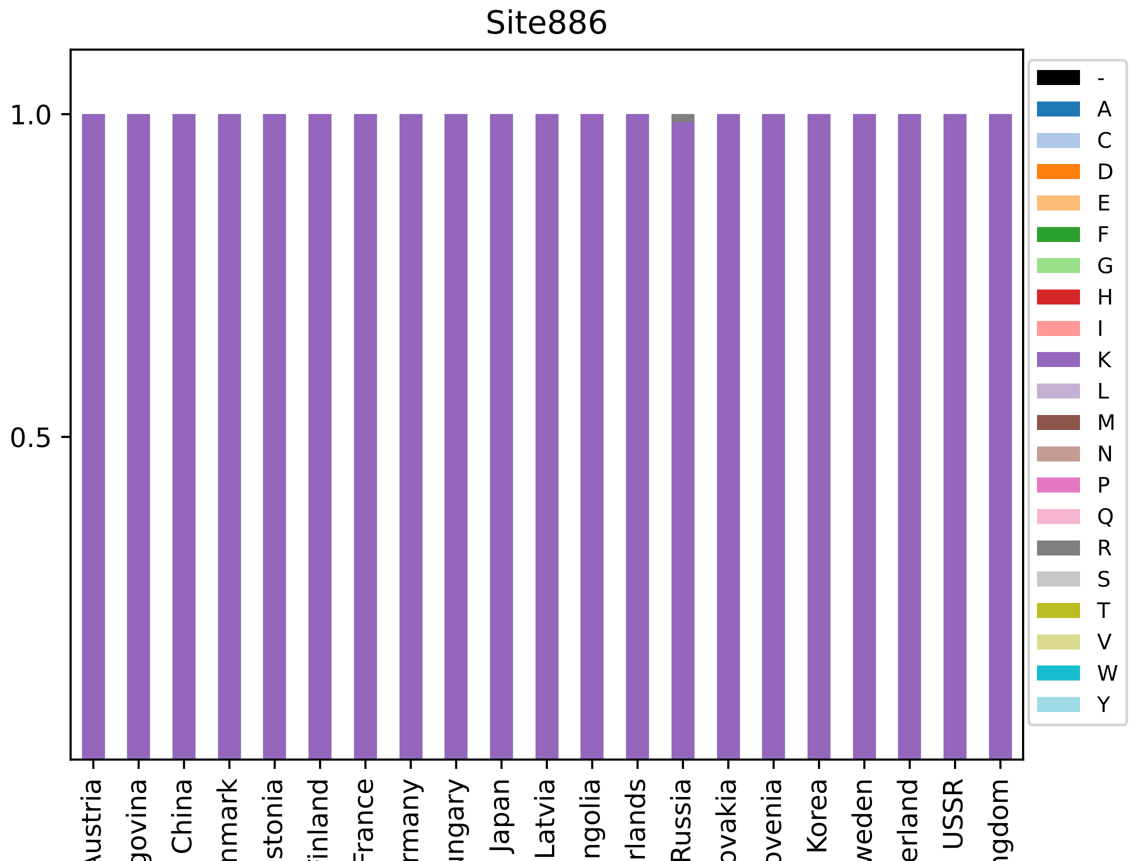

Site886
1.0
0.5
-
A
C
D
E
F
G
H
I
K
L
M
N
P
Q
R
S
T
V
W
Y
China
Japan
Latvia
Russia
Korea
USSR

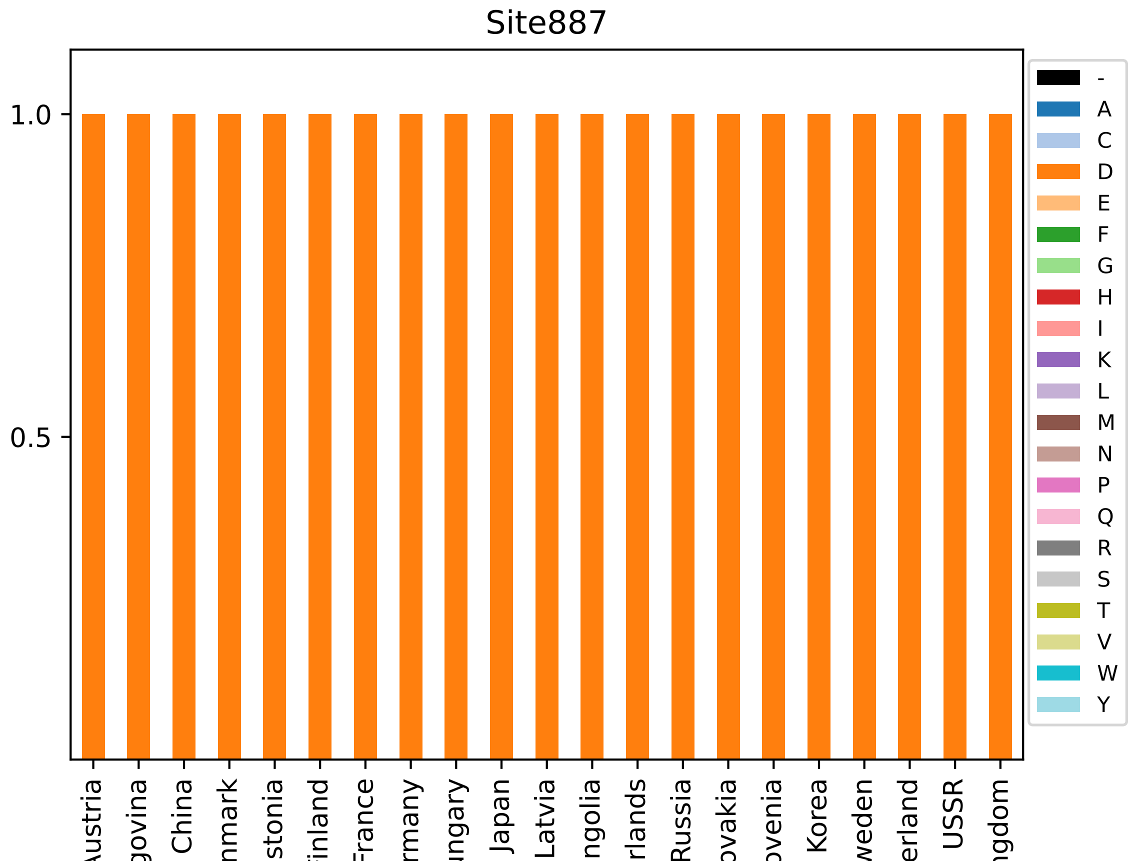
Site887
1.0
0.5
-
A
C
D
E
F
G
H
I
K
L
M
N
P
Q
R
S
T
V
W
Y
Austria
China
Japan
Latvia
Russia
Korea
USSR

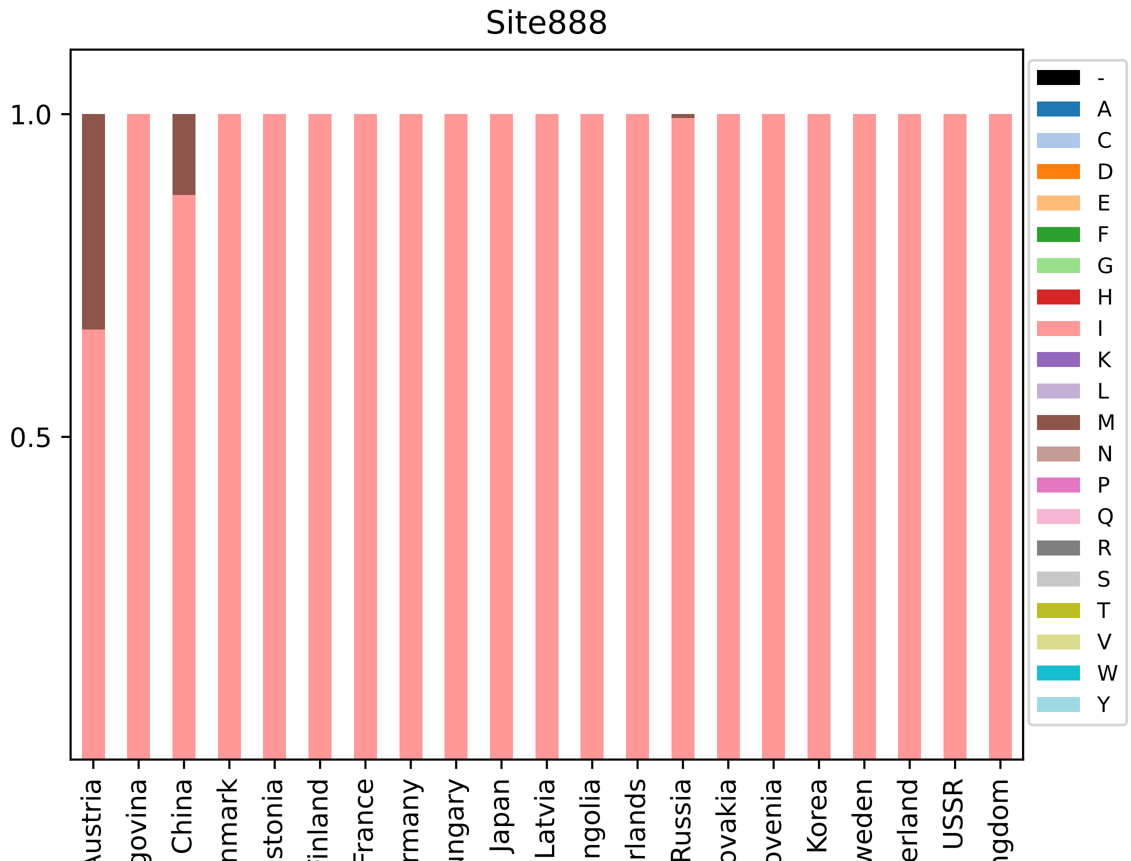

Site888
1.0
0.5
-
A
C
D
E
F
G
H
I
K
L
M
N
P
Q
R
S
T
V
W
Y
China
Japan
Latvia
Russia
Korea
USSR

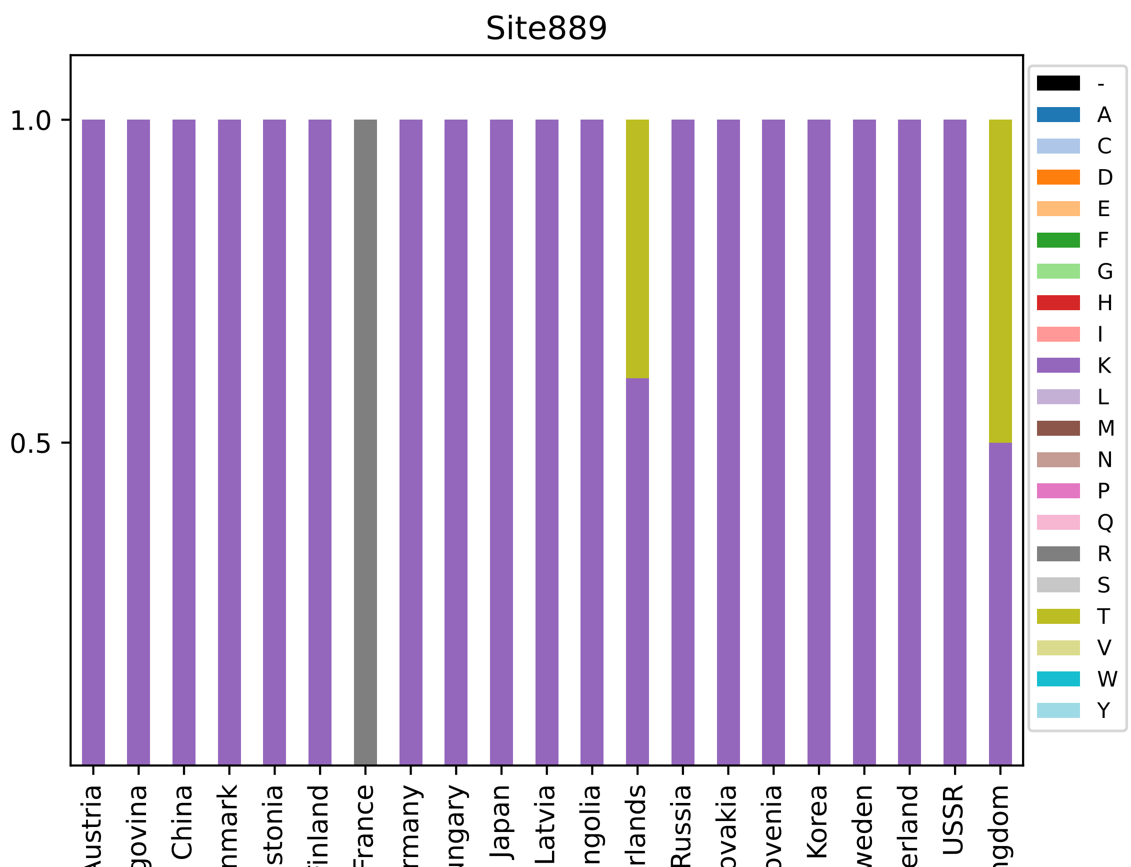

Site889
1.0
0.5
-
A
C
D
E
F
G
H
I
K
L
M
N
P
Q
R
S
T
V
W
Y
China
Japan
Latvia
Russia
Korea
USSR

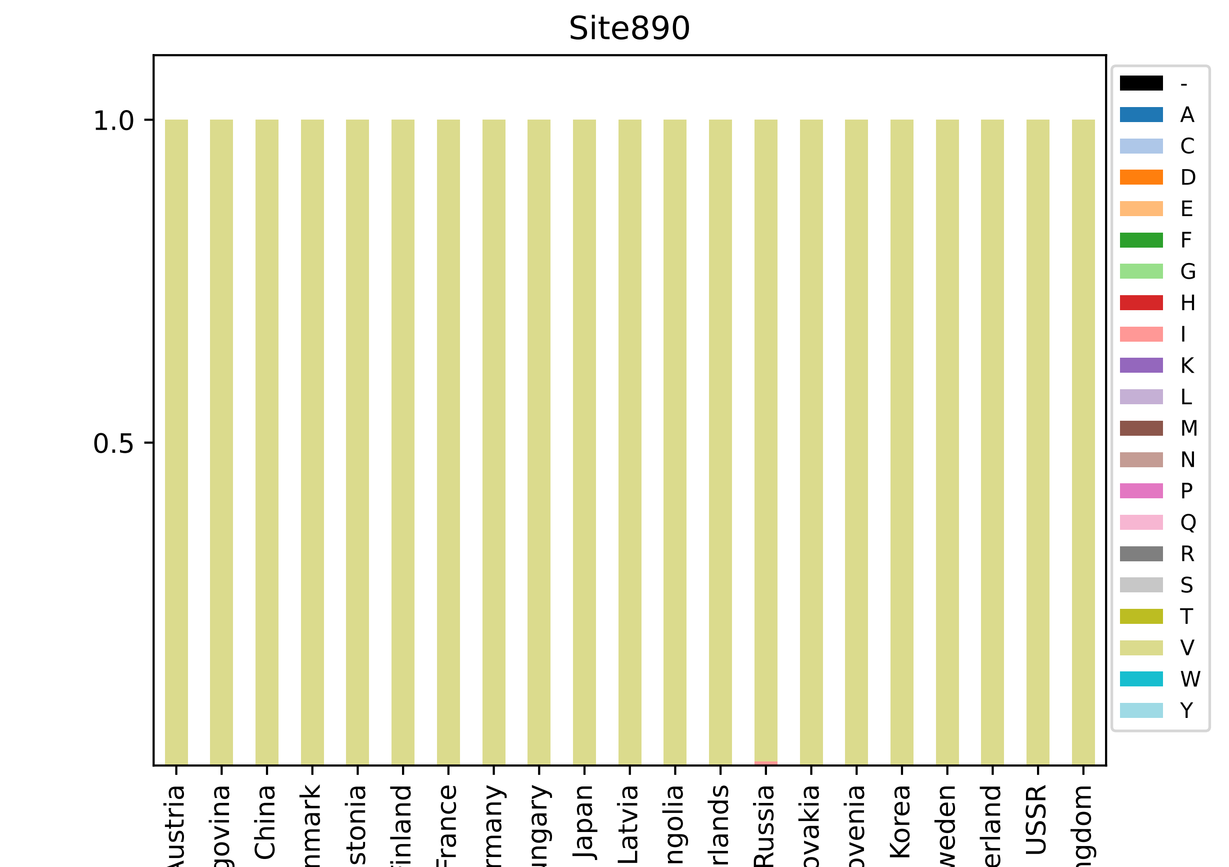

Site890
1.0
0.5
-
A
C
D
E
F
G
H
I
K
L
M
N
P
Q
R
S
T
V
W
Y
Austria
China
Japan
Latvia
Russia
Korea
USSR

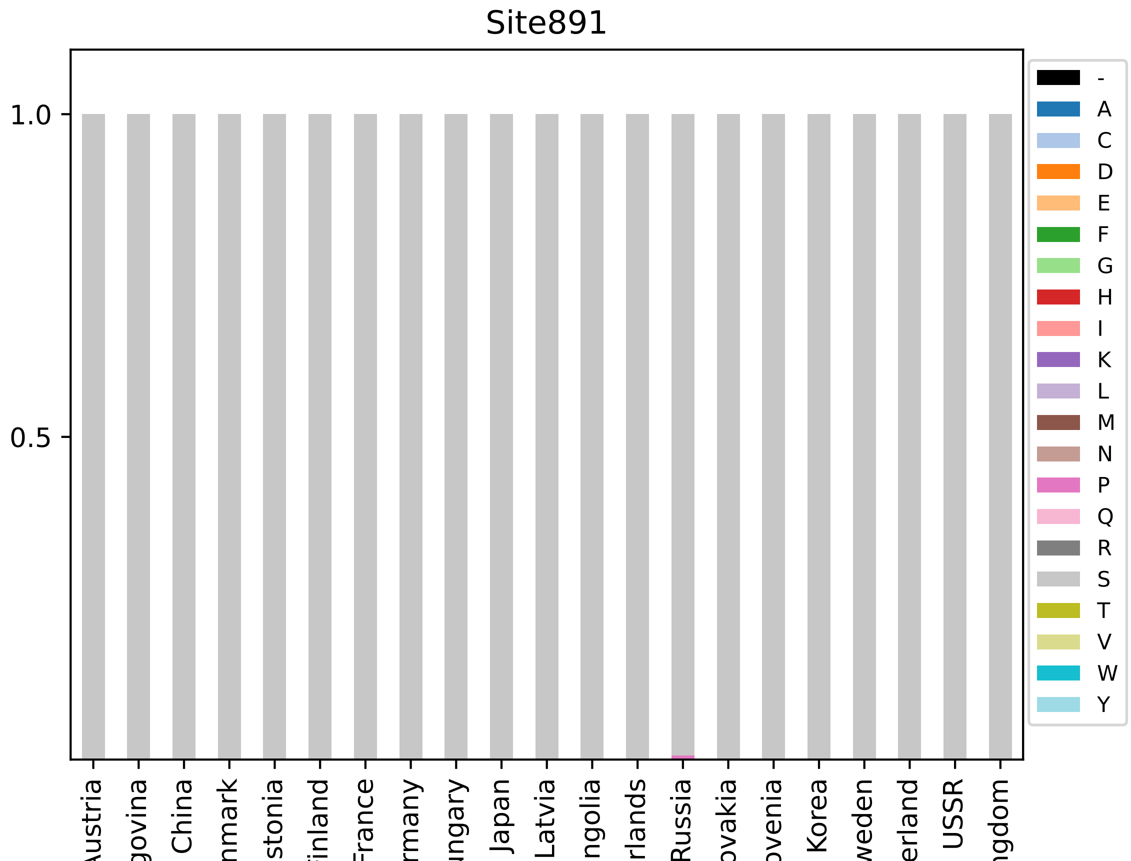

Site891
1.0
0.5
-
A
C
D
E
F
G
H
I
K
L
M
N
P
Q
R
S
T
V
W
Y
Austria
govina
China
nmark
stonia
Finland
France
rmany
ungary
Japan
Latvia
ngolia
rlands
Russia
ovakia
ovenia
Korea
weden
erland
USSR
ngdom

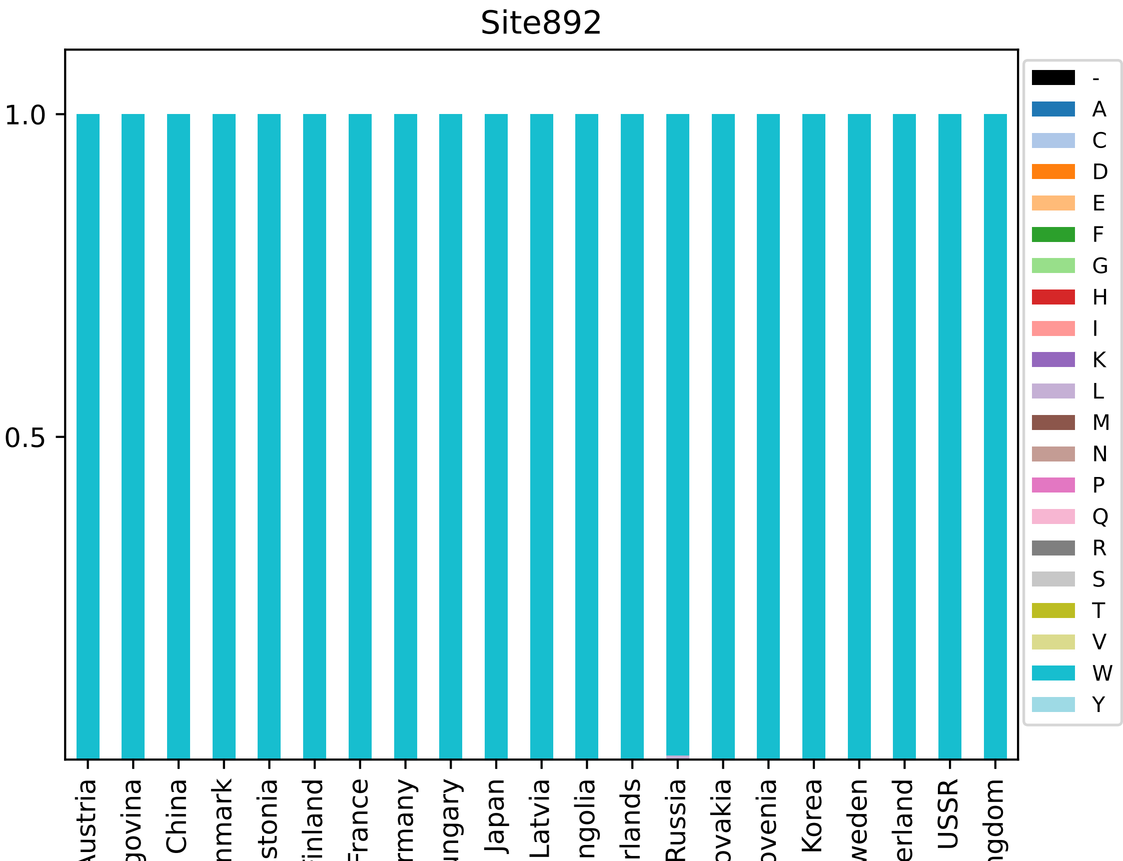
Site892
1.0
0.5
-
A
C
D
E
F
G
H
I
K
L
M
N
P
Q
R
S
T
V
W
Y
China
Japan
Latvia
Russia
Korea
USSR

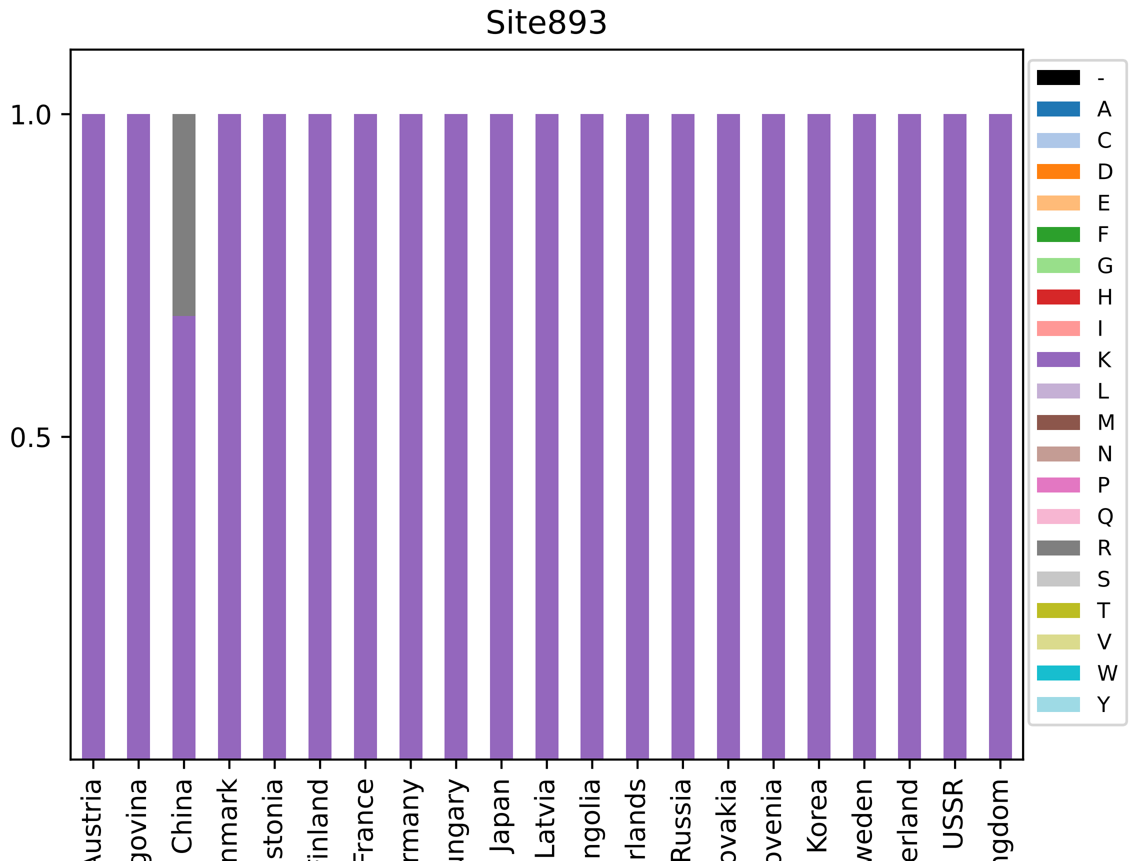

Site893
1.0
0.5
-
A
C
D
E
F
G
H
I
K
L
M
N
P
Q
R
S
T
V
W
Y
China
Japan
Latvia
Russia
Korea
USSR

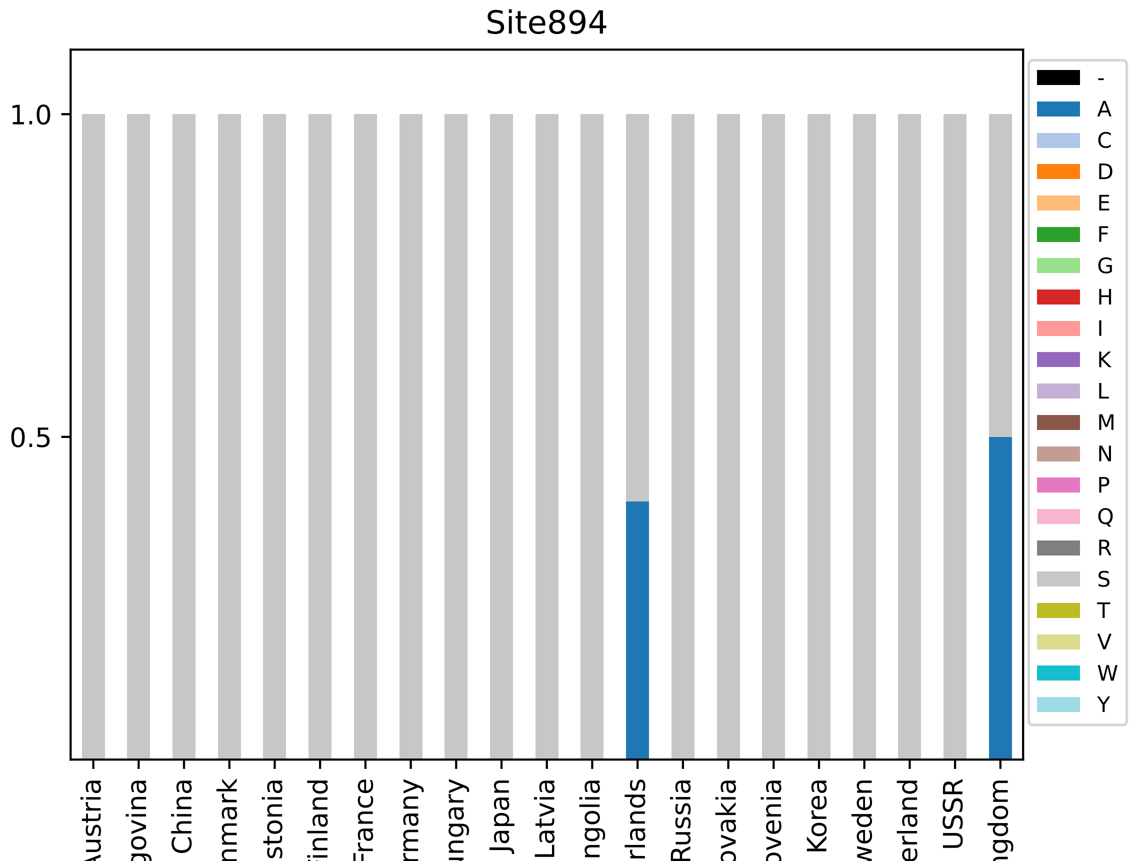

Site894
1.0
0.5
-
A
C
D
E
F
G
H
I
K
L
M
N
P
Q
R
S
T
V
W
Y
Austria
govina
China
nmark
stonia
Finland
France
rmany
ungary
Japan
Latvia
ngolia
rlands
Russia
ovakia
ovenia
Korea
weden
erland
USSR
ngdom

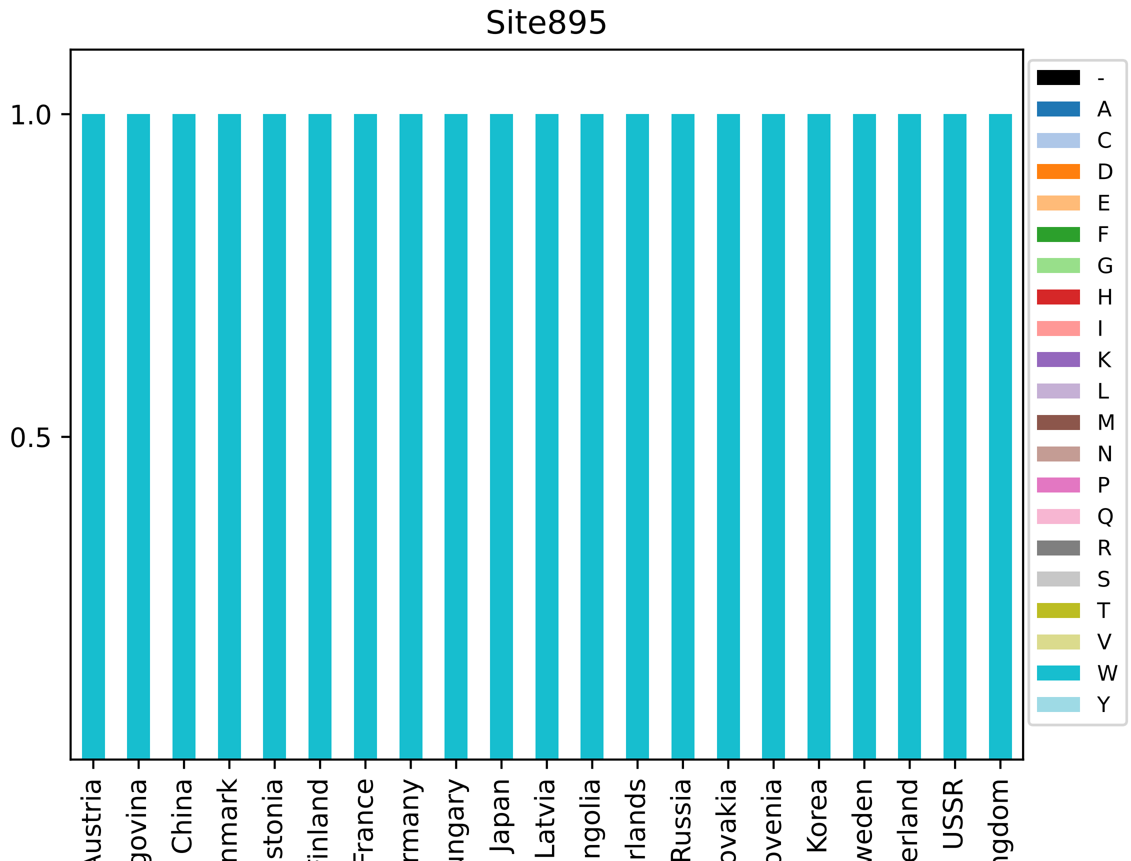
Site895
1.0
0.5
-
A
C
D
E
F
G
H
I
K
L
M
N
P
Q
R
S
T
V
W
Y
Austria
China
Japan
Latvia
Russia
Korea
USSR

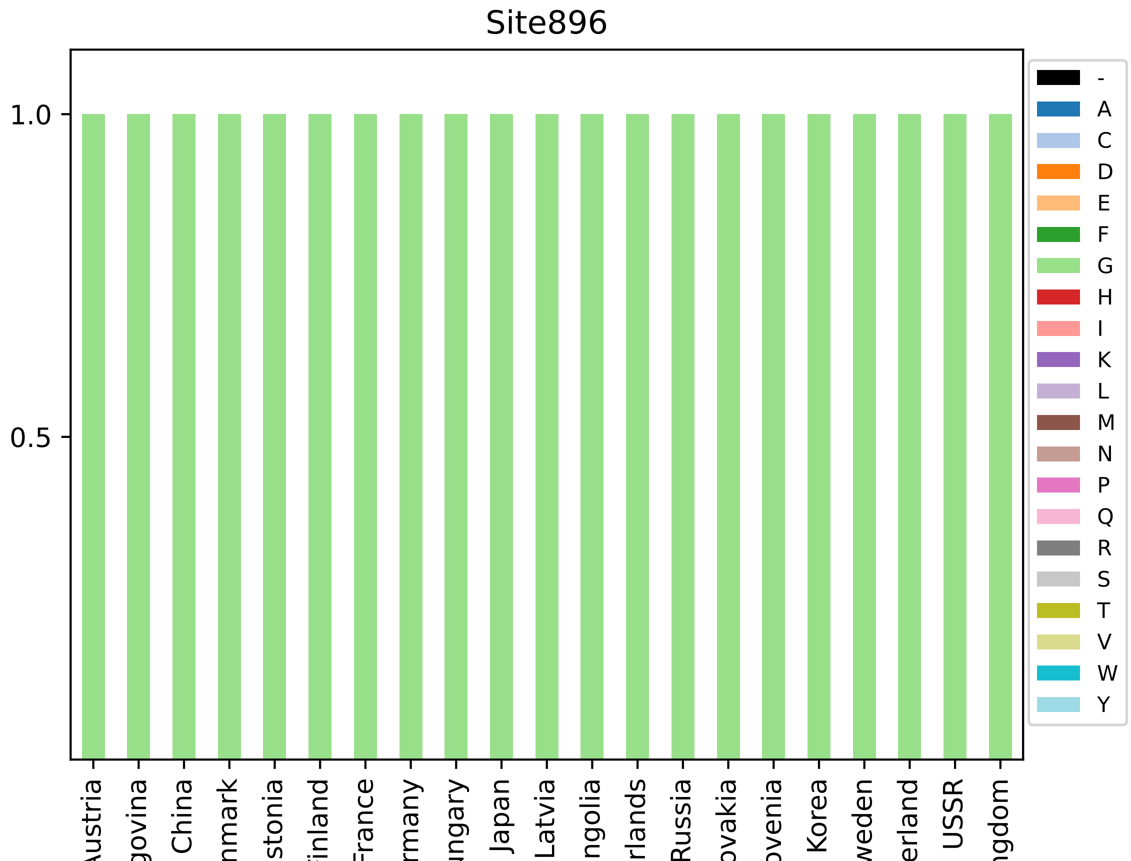

Site896
1.0
0.5
-
A
C
D
E
F
G
H
I
K
L
M
N
P
Q
R
S
T
V
W
Y
ustria
govina
China
nmark
stonia
inland
rance
rmany
ngary
Japan
Latvia
ngolia
rlands
Russia
ovakia
ovenia
Korea
weden
erland
USSR
ngdom

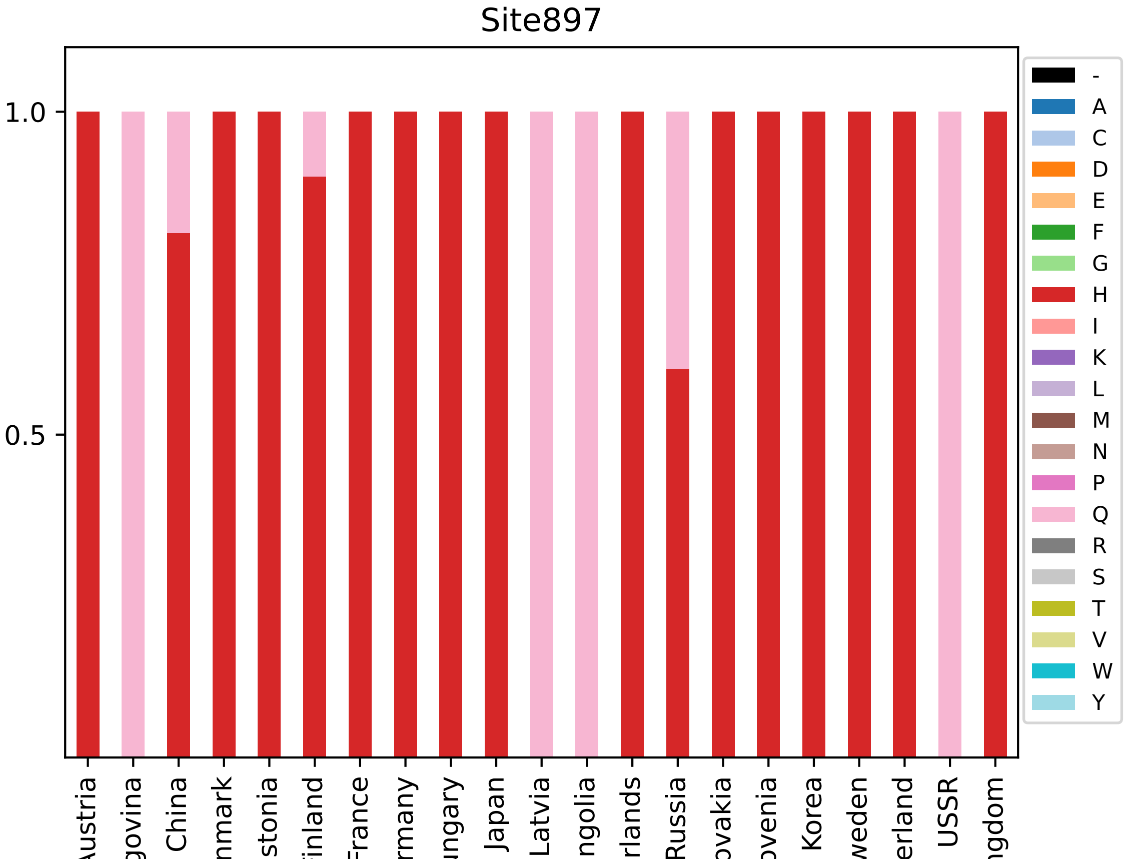
Site897
1.0
0.5
-
A
C
D
E
F
G
H
I
K
L
M
N
P
Q
R
S
T
V
W
Y
China
Japan
Latvia
Russia
Korea
USSR

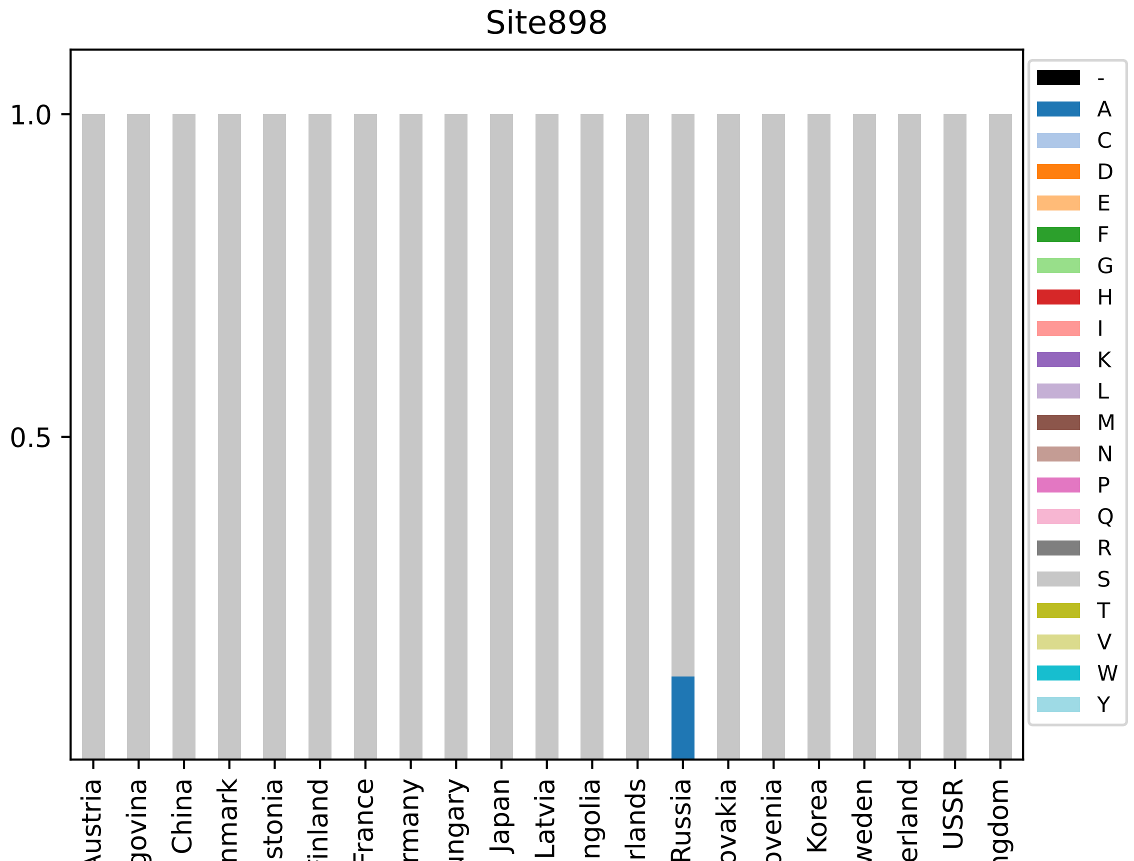

Site898
1.0
0.5
-
A
C
D
E
F
G
H
I
K
L
M
N
P
Q
R
S
T
V
W
Y
China
Japan
Latvia
Russia
Korea
USSR

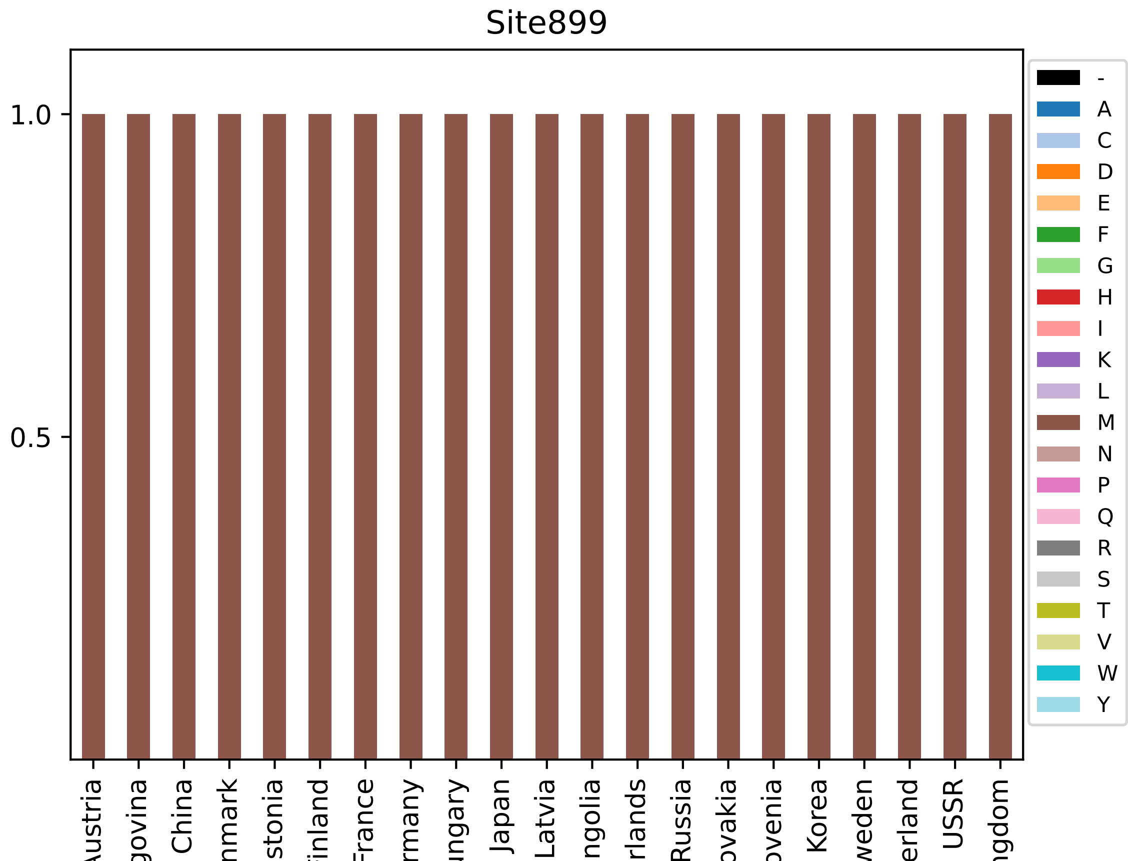

Site899
1.0
0.5
-
A
C
D
E
F
G
H
I
K
L
M
N
P
Q
R
S
T
V
W
Y
Austria
China
Finland
France
Japan
Latvia
Russia
Korea
USSR

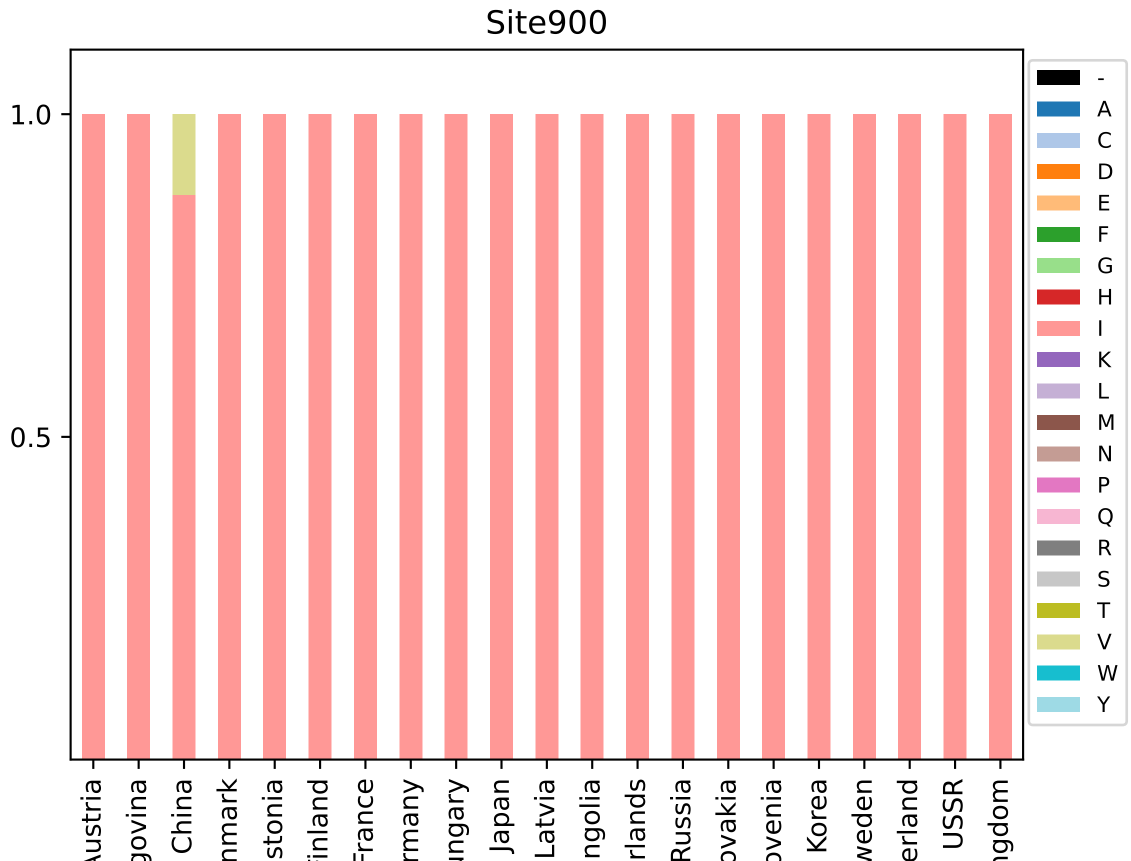

Site900
1.0
0.5
-
A
C
D
E
F
G
H
I
K
L
M
N
P
Q
R
S
T
V
W
Y
Austria
China
Finland
France
Japan
Latvia
Russia
Korea
USSR

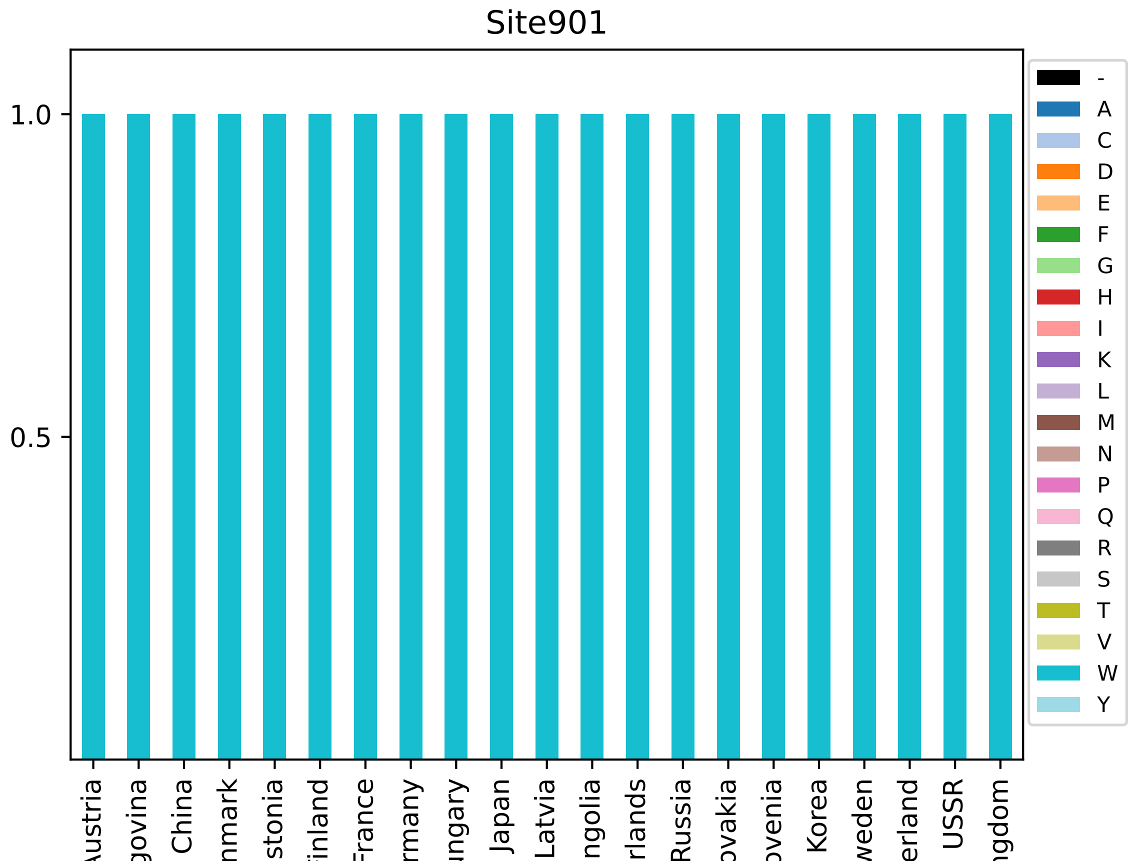
Site901
1.0
0.5
-
A
C
D
E
F
G
H
I
K
L
M
N
P
Q
R
S
T
V
W
Y
Austria
govina
China
nmark
stonia
inland
France
rmany
ungary
Japan
Latvia
ngolia
rlands
Russia
ovakia
ovenia
Korea
weden
erland
USSR
ngdom

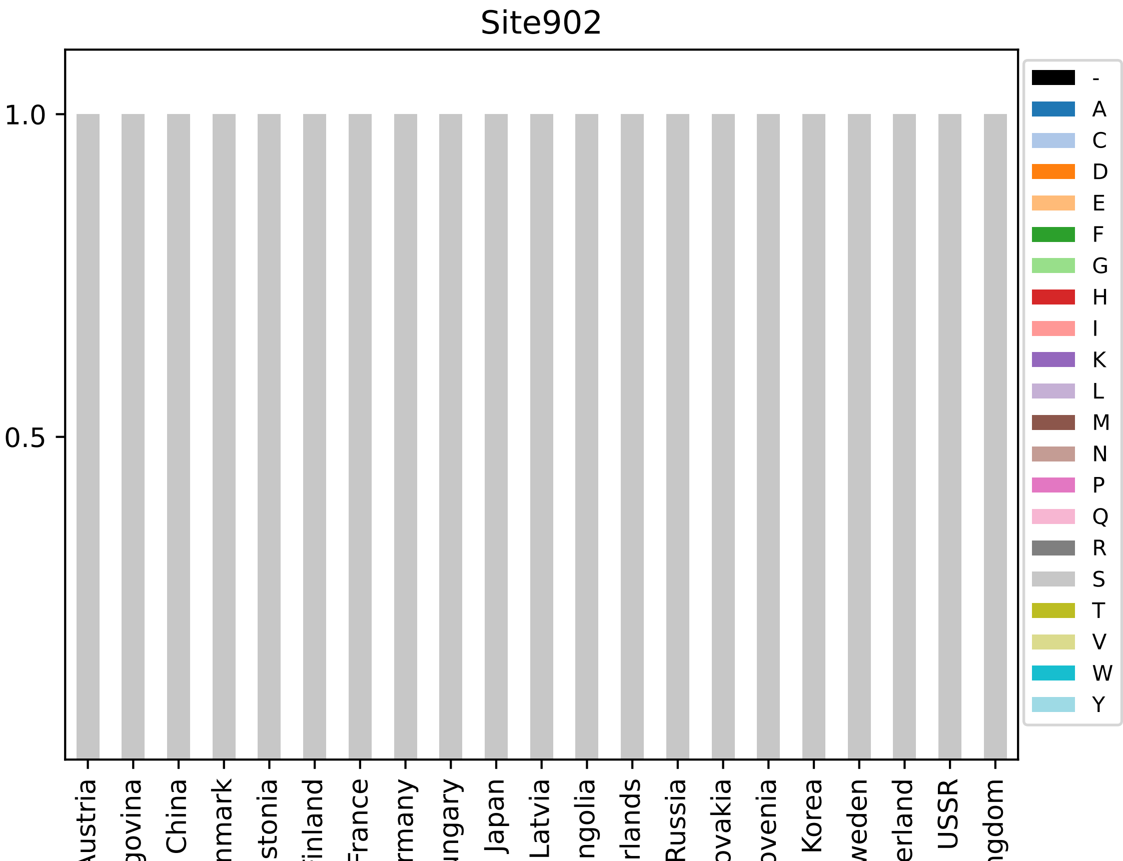

Site902
1.0
0.5
-
A
C
D
E
F
G
H
I
K
L
M
N
P
Q
R
S
T
V
W
Y
Austria
govina
China
nmark
stonia
Finland
France
rmany
ungary
Japan
Latvia
ngolia
rlands
Russia
ovakia
ovenia
Korea
weden
erland
USSR
ngdom

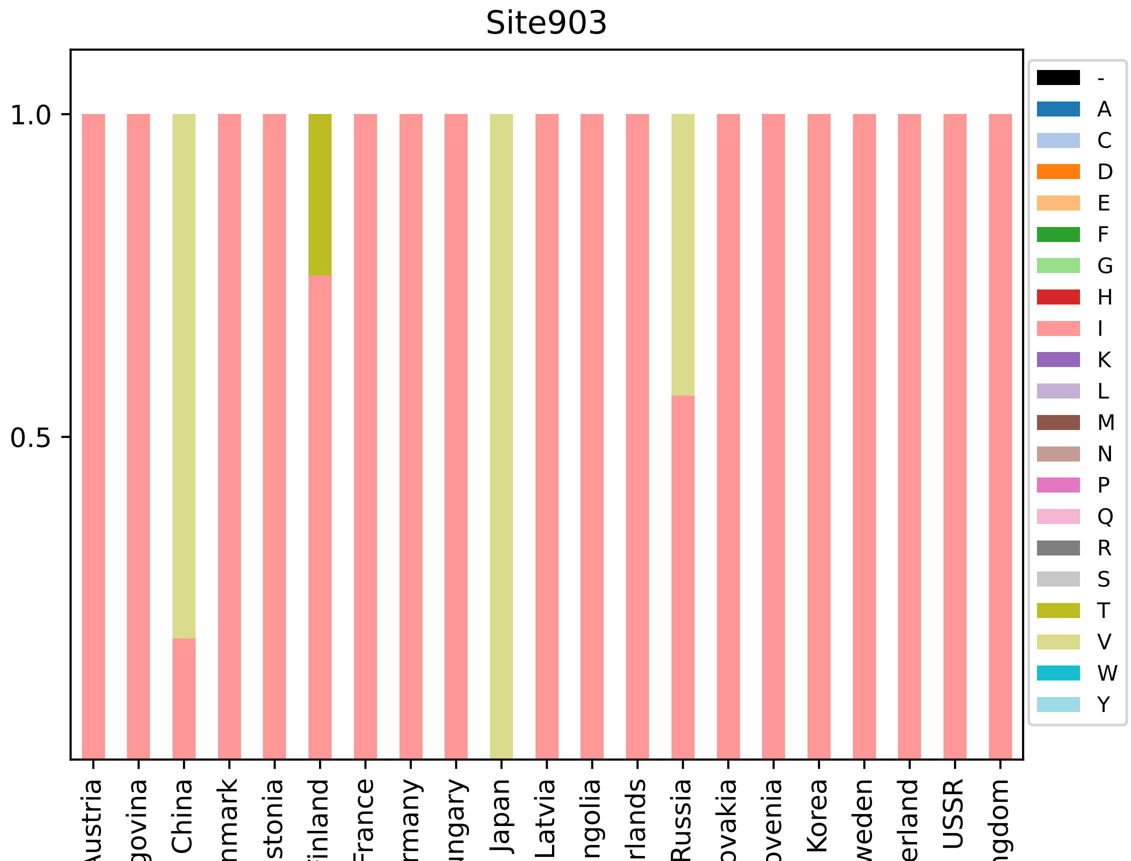

Site903
1.0
0.5
-
A
C
D
E
F
G
H
I
K
L
M
N
P
Q
R
S
T
V
W
Y
China
Finland
France
Japan
Latvia
Russia
Korea
USSR

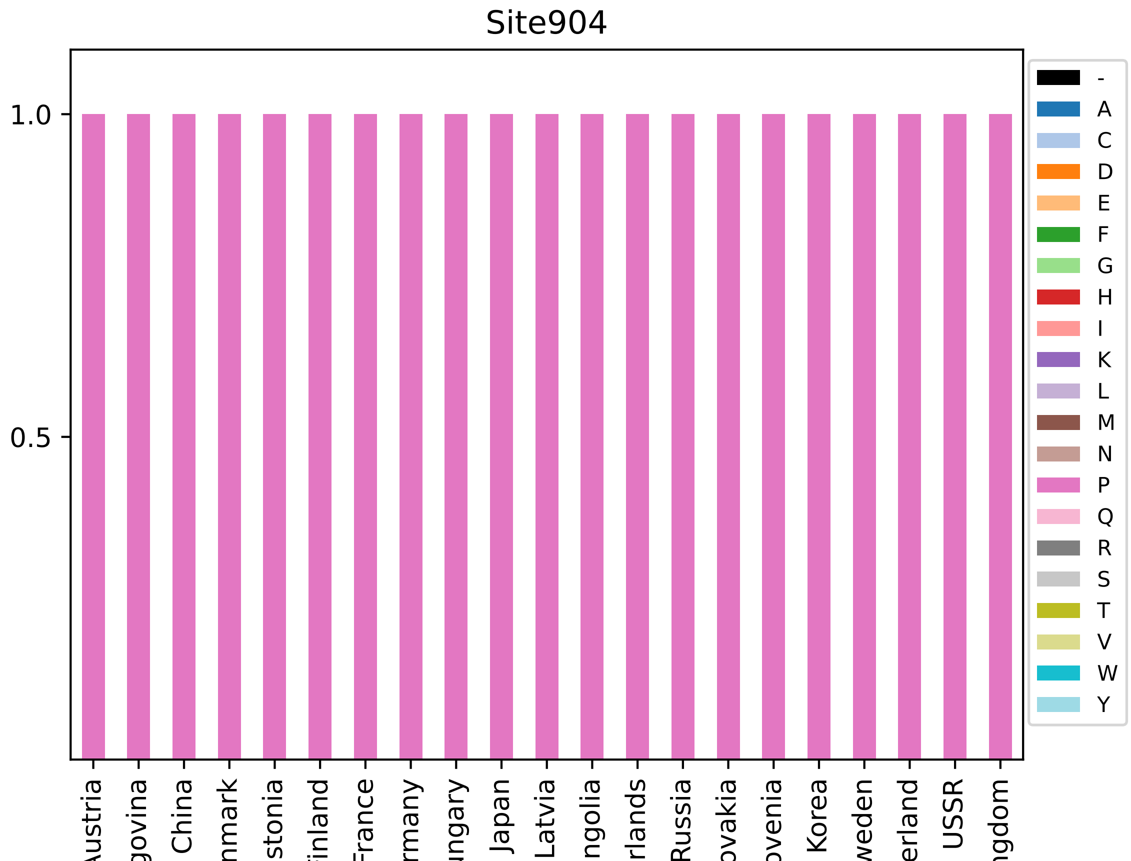

Site904
1.0
0.5
-
A
C
D
E
F
G
H
I
K
L
M
N
P
Q
R
S
T
V
W
Y
China
Japan
Latvia
Russia
Korea
USSR

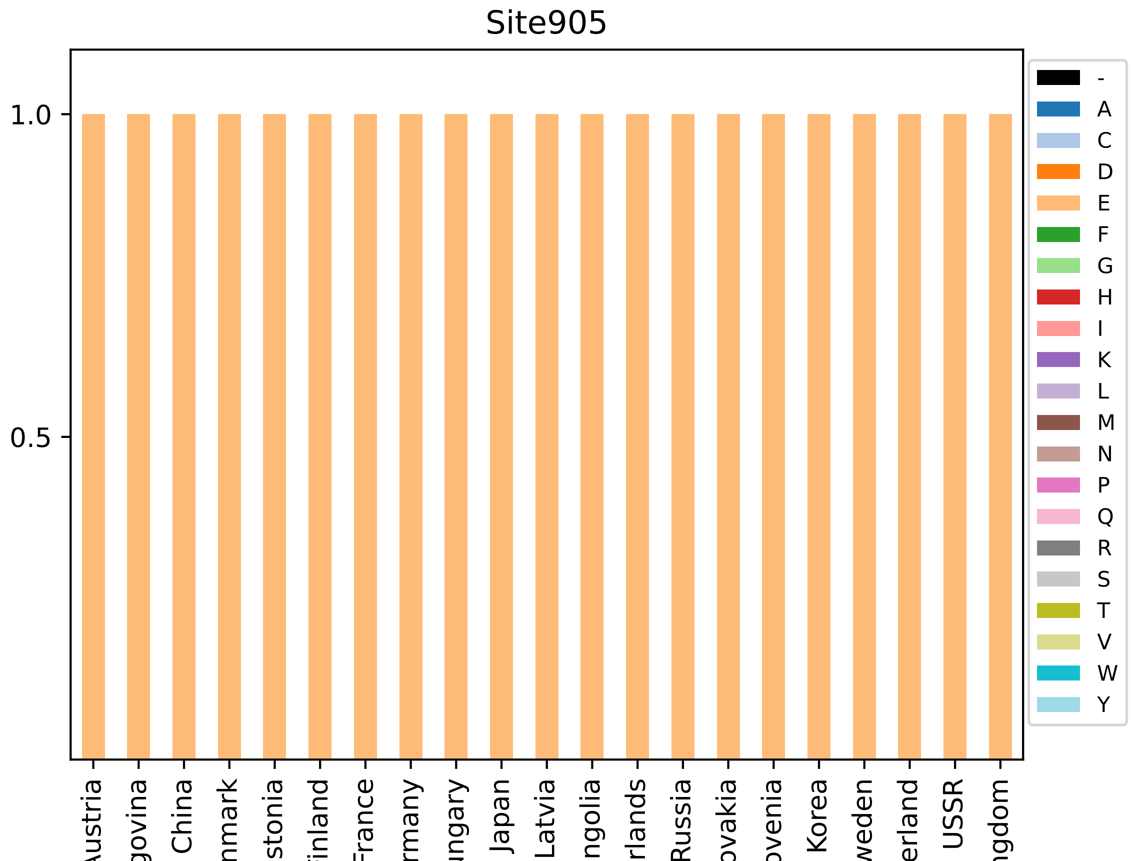

Site905
1.0
0.5
-
A
C
D
E
F
G
H
I
K
L
M
N
P
Q
R
S
T
V
W
Y
China
Japan
Latvia
Russia
Korea
USSR

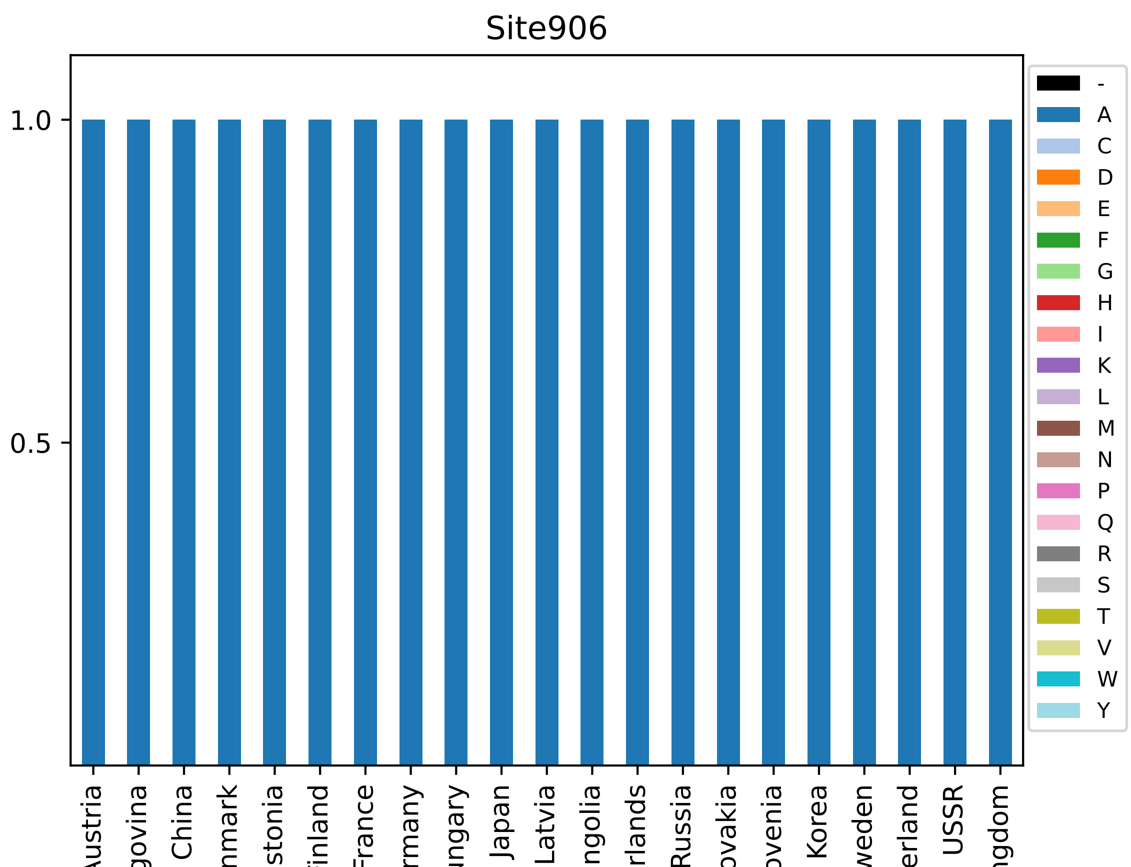
Site906
1.0
0.5
-
A
C
D
E
F
G
H
I
K
L
M
N
P
Q
R
S
T
V
W
Y
China
Japan
Latvia
Russia
Korea
USSR

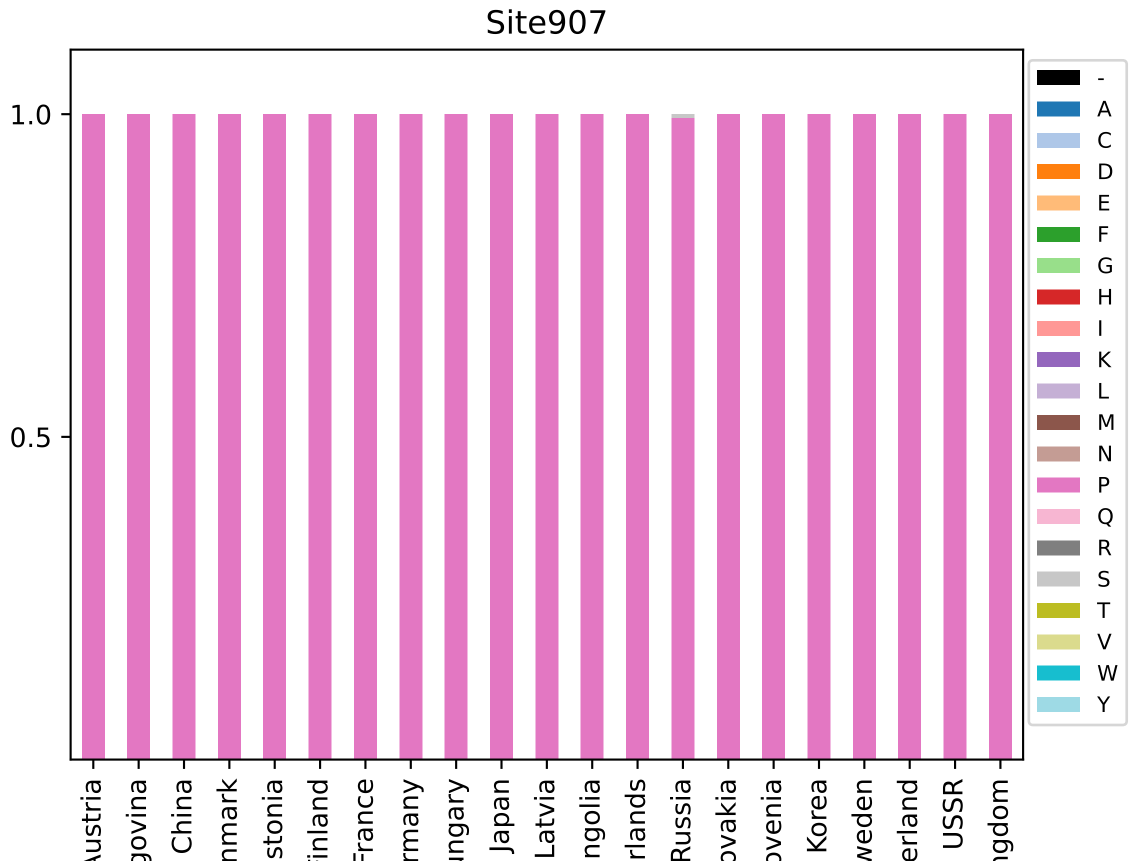
Site907
1.0
0.5
-
A
C
D
E
F
G
H
I
K
L
M
N
P
Q
R
S
T
V
W
Y
China
Japan
Latvia
Russia
Korea
USSR

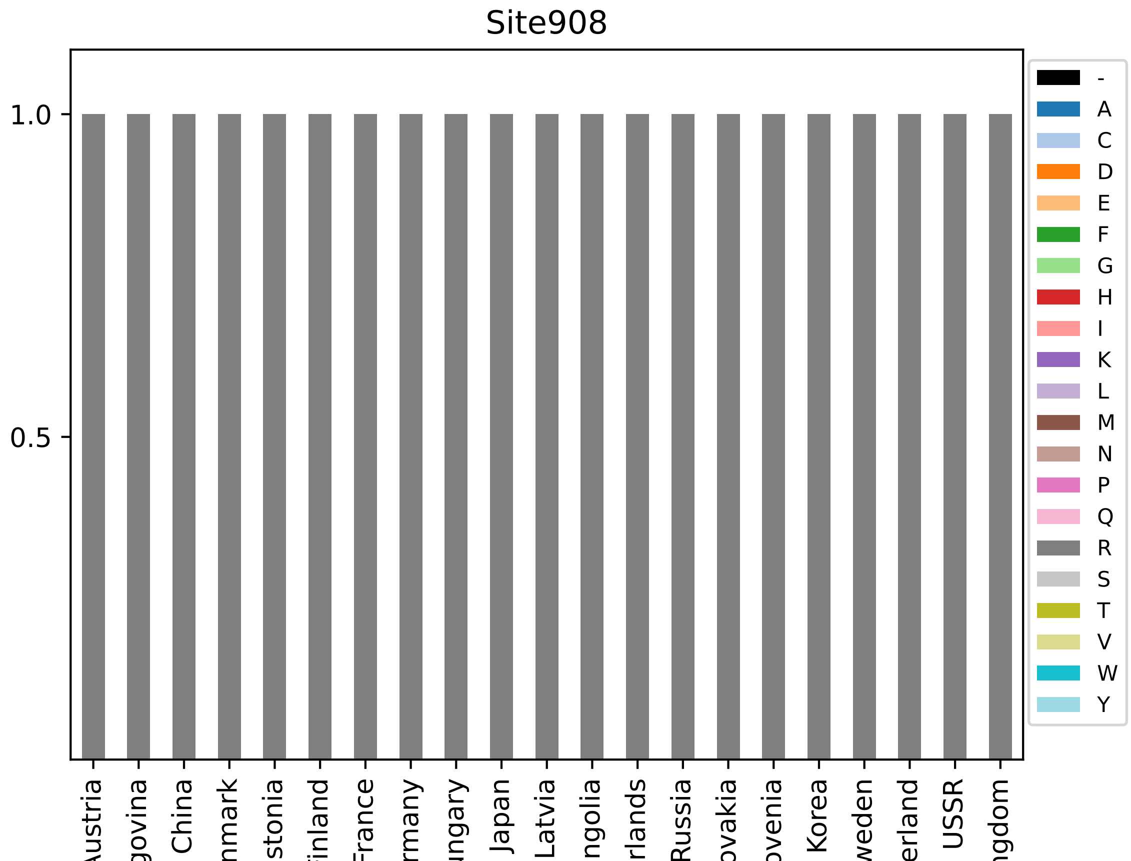
Site908
1.0
0.5
-
A
C
D
E
F
G
H
I
K
L
M
N
P
Q
R
S
T
V
W
Y
China
Japan
Latvia
Russia
Korea
USSR

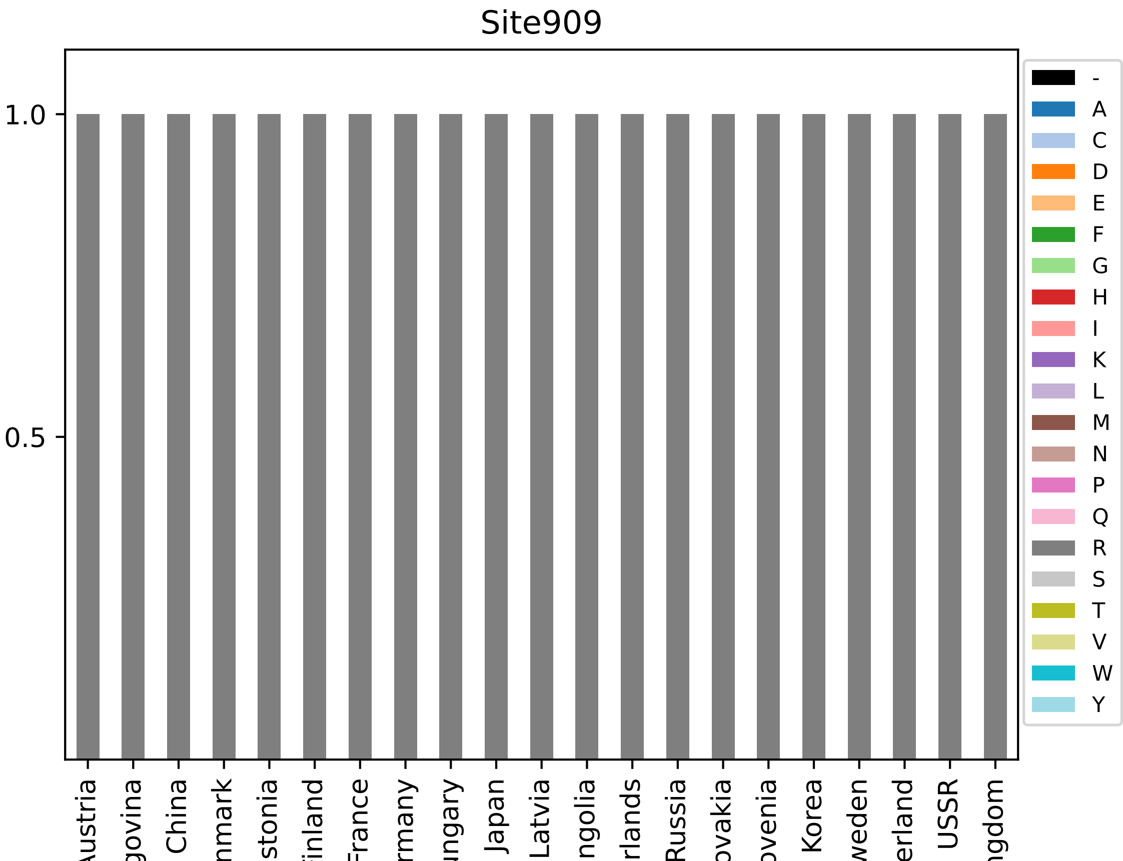

Site909
1.0
0.5
-
A
C
D
E
F
G
H
I
K
L
M
N
P
Q
R
S
T
V
W
Y
China
Japan
Latvia
Russia
Korea
USSR

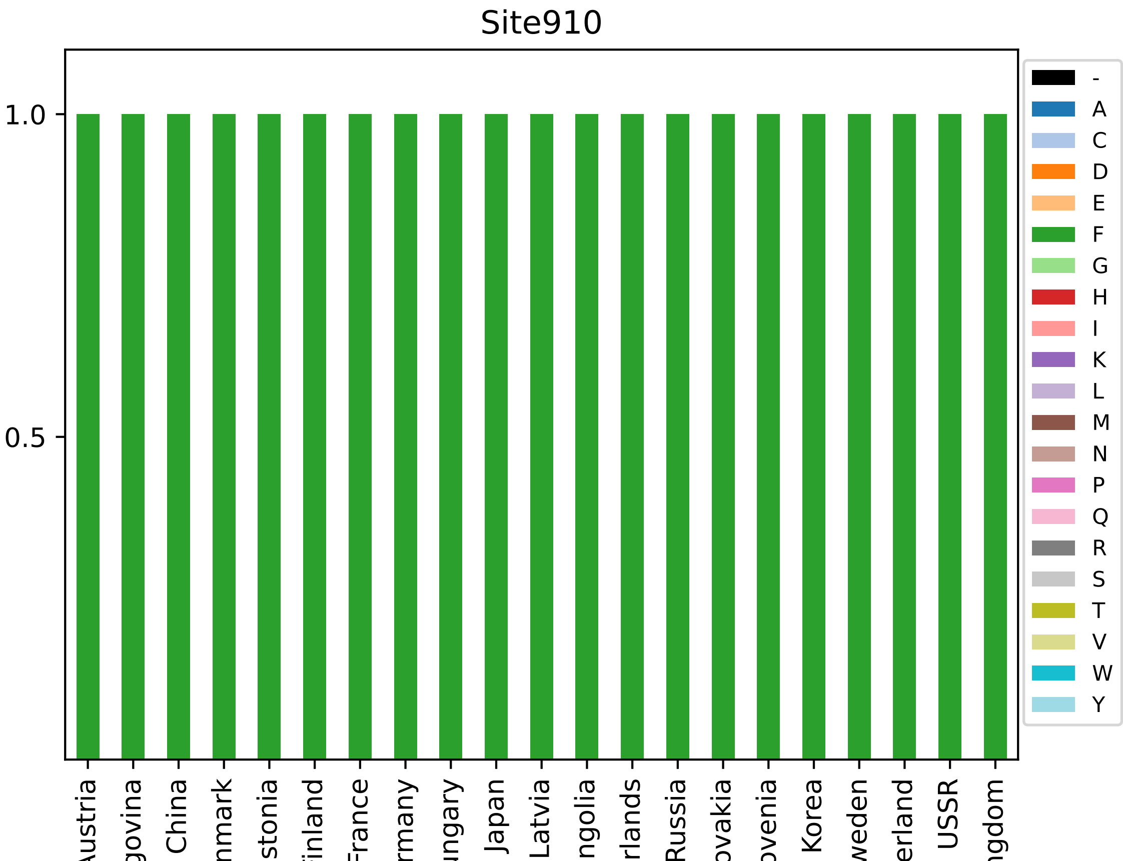
Site910
1.0
0.5
-
A
C
D
E
F
G
H
I
K
L
M
N
P
Q
R
S
T
V
W
Y
Austria
govina
China
nmark
stonia
inland
France
rmany
ungary
Japan
Latvia
ngolia
rlands
Russia
ovakia
ovenia
Korea
weden
erland
USSR
ngdom

Site911

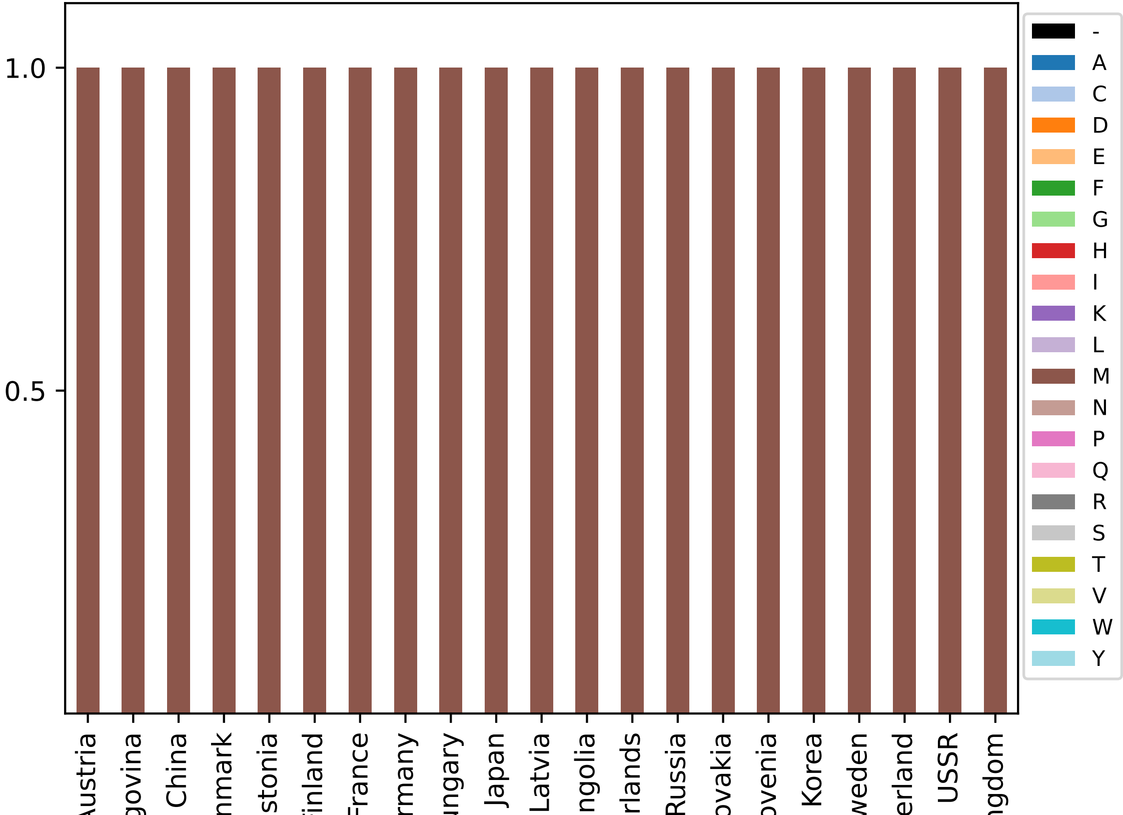

1.0
0.5
-
A
C
D
E
F
G
H
I
K
L
M
N
P
Q
R
S
T
V
W
Y
China
Japan
Latvia
Russia
Korea
USSR

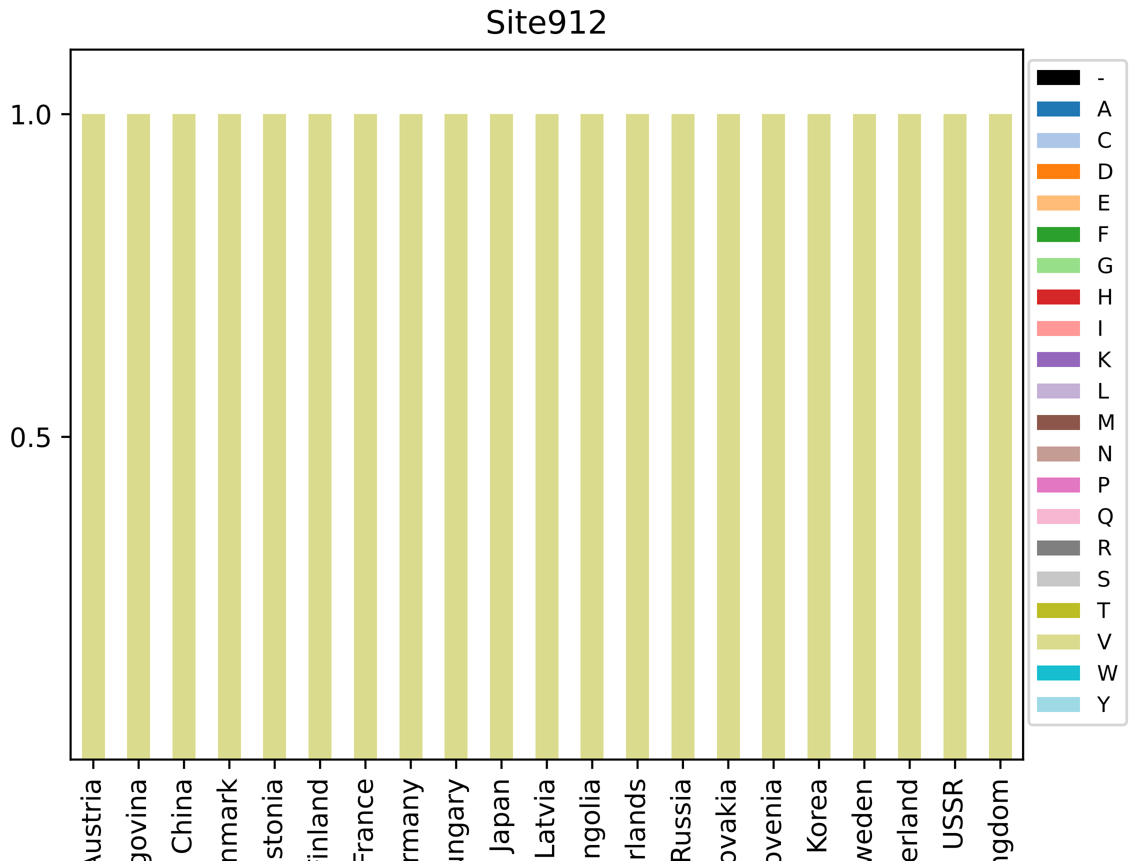
Site912
1.0
0.5
-
A
C
D
E
F
G
H
I
K
L
M
N
P
Q
R
S
T
V
W
Y
China
Japan
Latvia
Russia
Korea
USSR

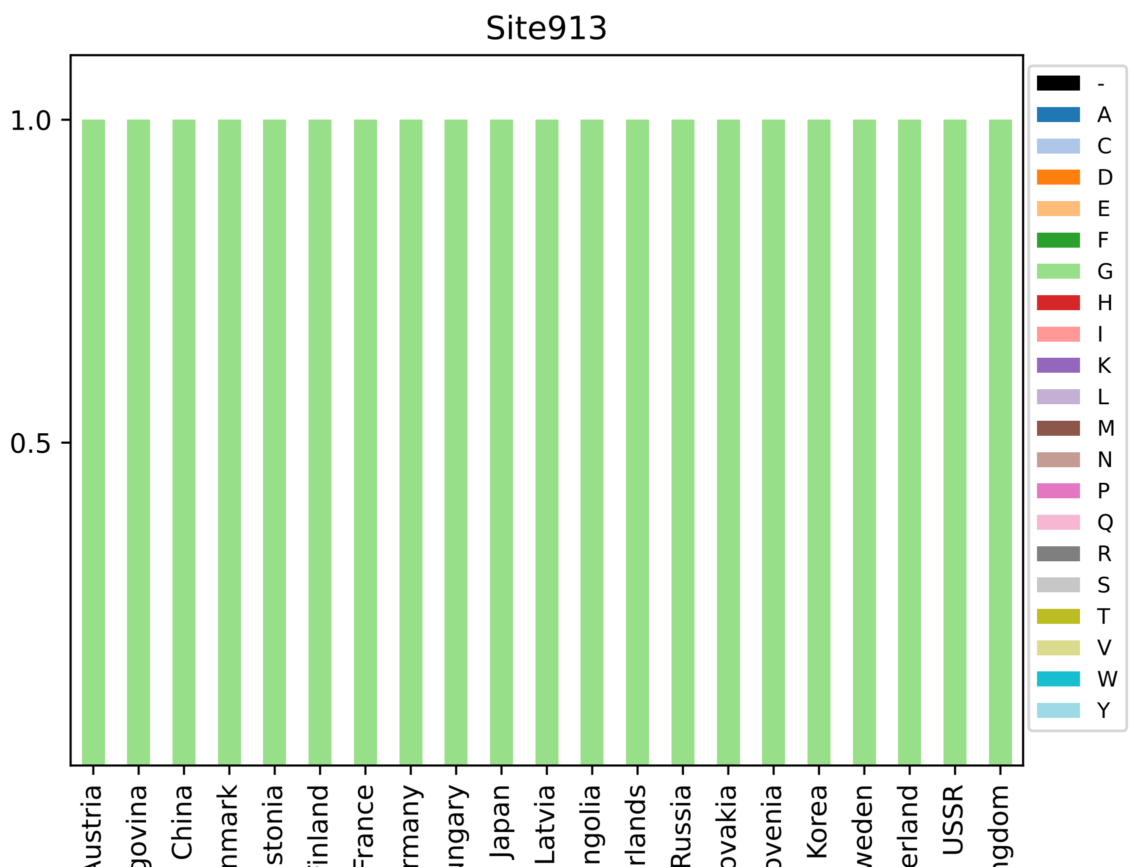

Site913
1.0
0.5
-
A
C
D
E
F
G
H
I
K
L
M
N
P
Q
R
S
T
V
W
Y
China
Japan
Latvia
Russia
Korea
USSR

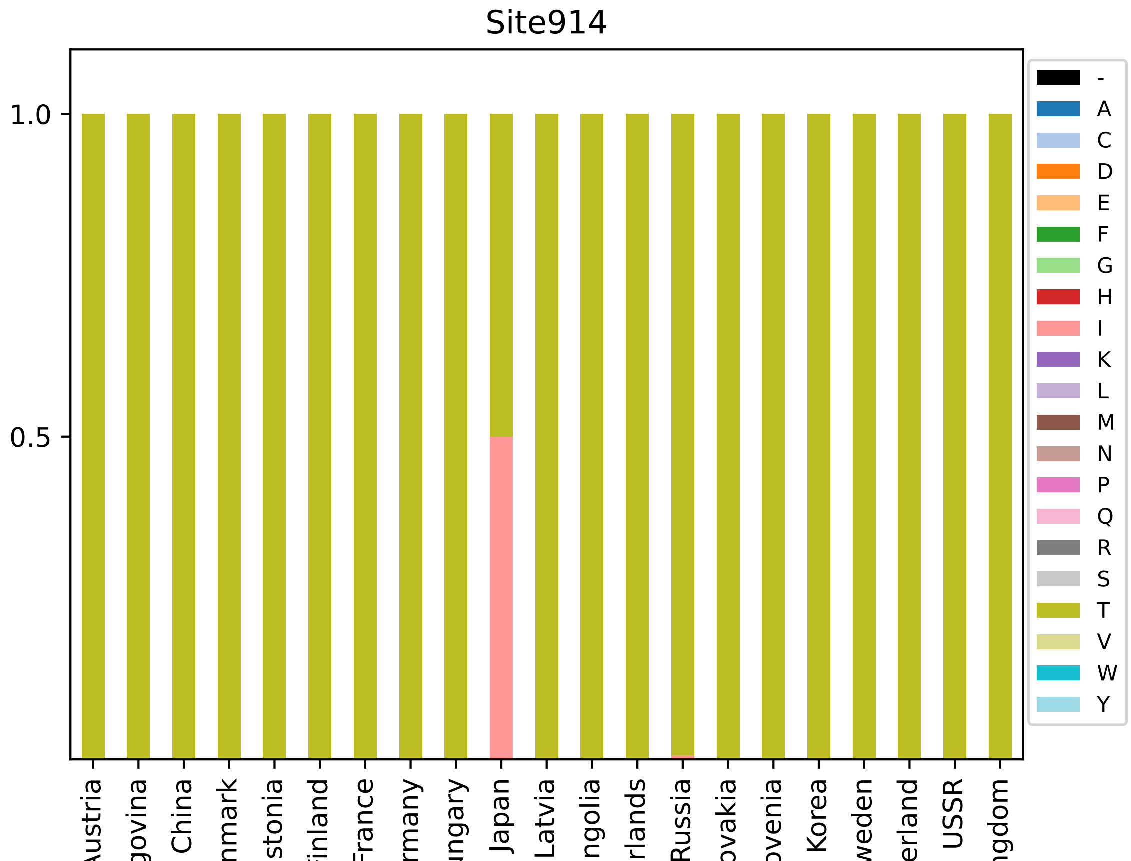

Site914
1.0
0.5
China
Japan
Latvia
Russia
Korea
USSR
-
A
C
D
E
F
G
H
I
K
L
M
N
P
Q
R
S
T
V
W
Y

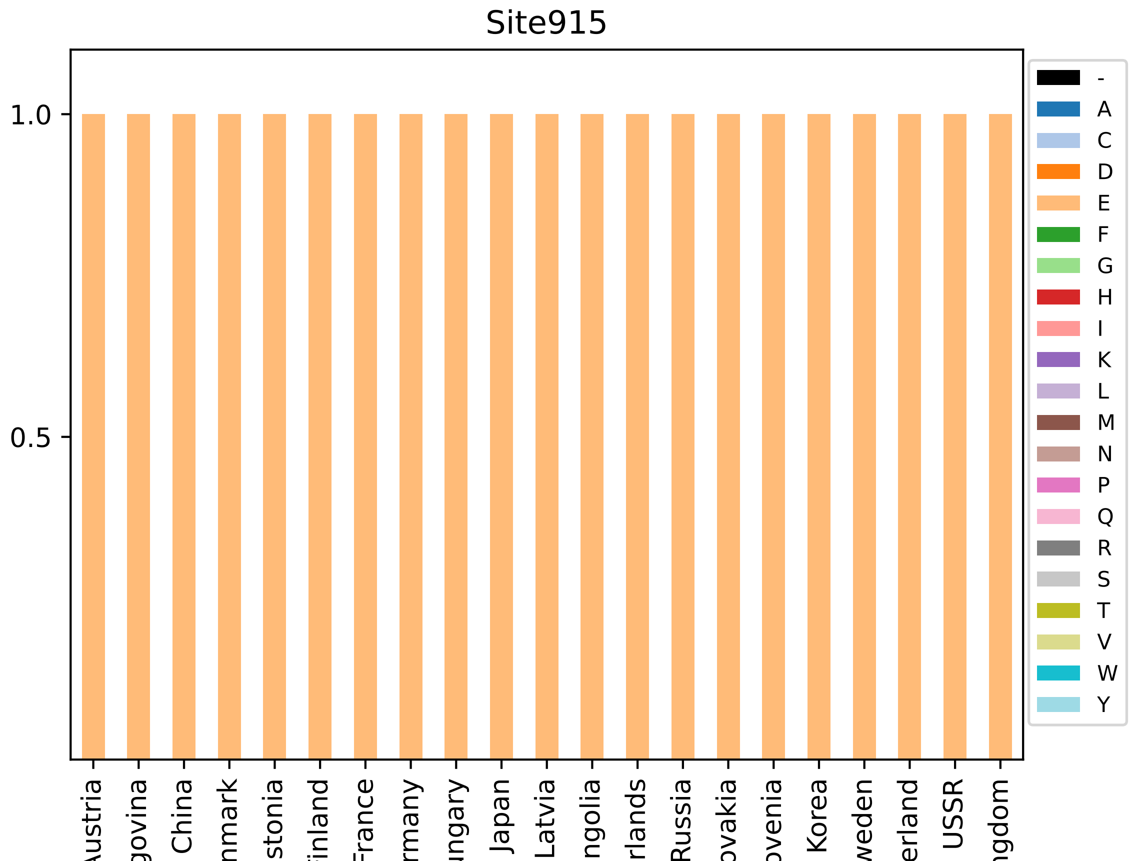
Site915
1.0
0.5
-
A
C
D
E
F
G
H
I
K
L
M
N
P
Q
R
S
T
V
W
Y
China
Japan
Latvia
Russia
Korea
USSR

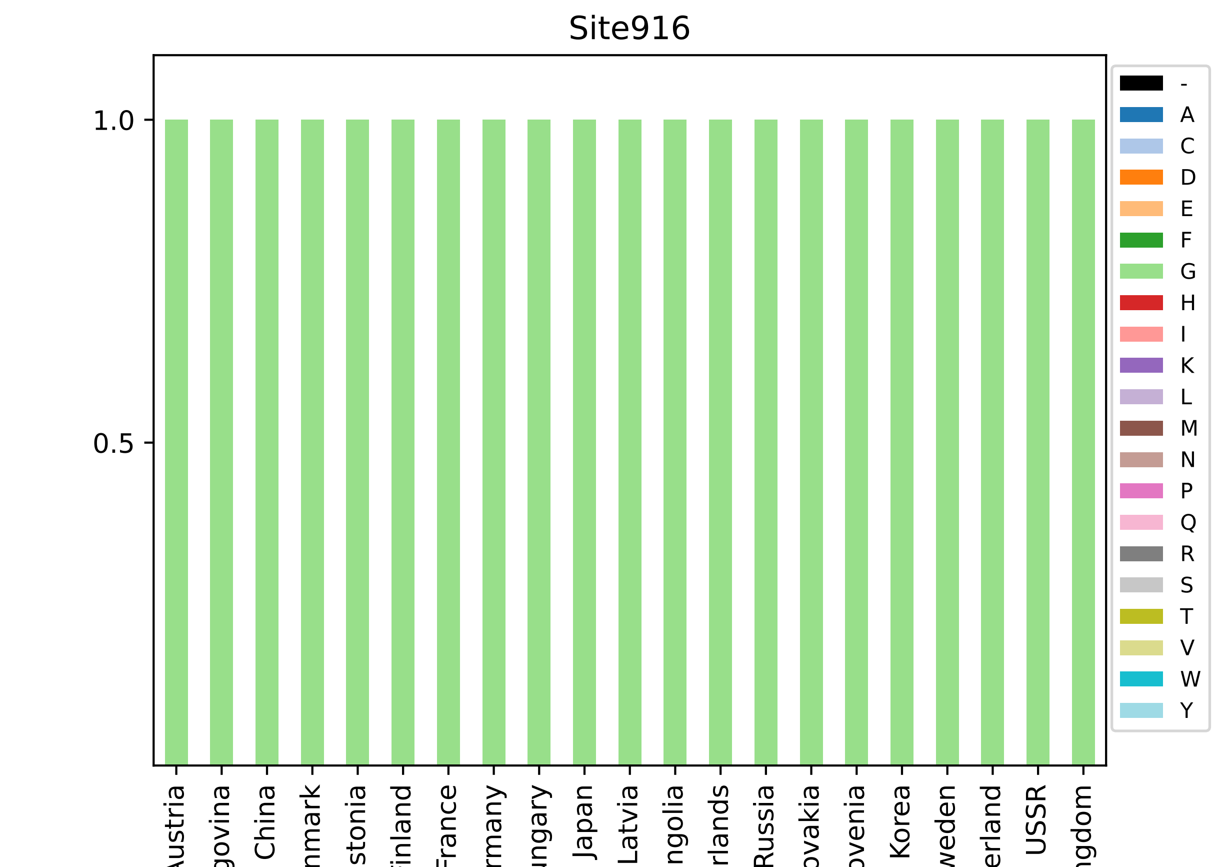
Site916
1.0
0.5
-
A
C
D
E
F
G
H
I
K
L
M
N
P
Q
R
S
T
V
W
Y
China
Japan
Latvia
Russia
Korea
USSR

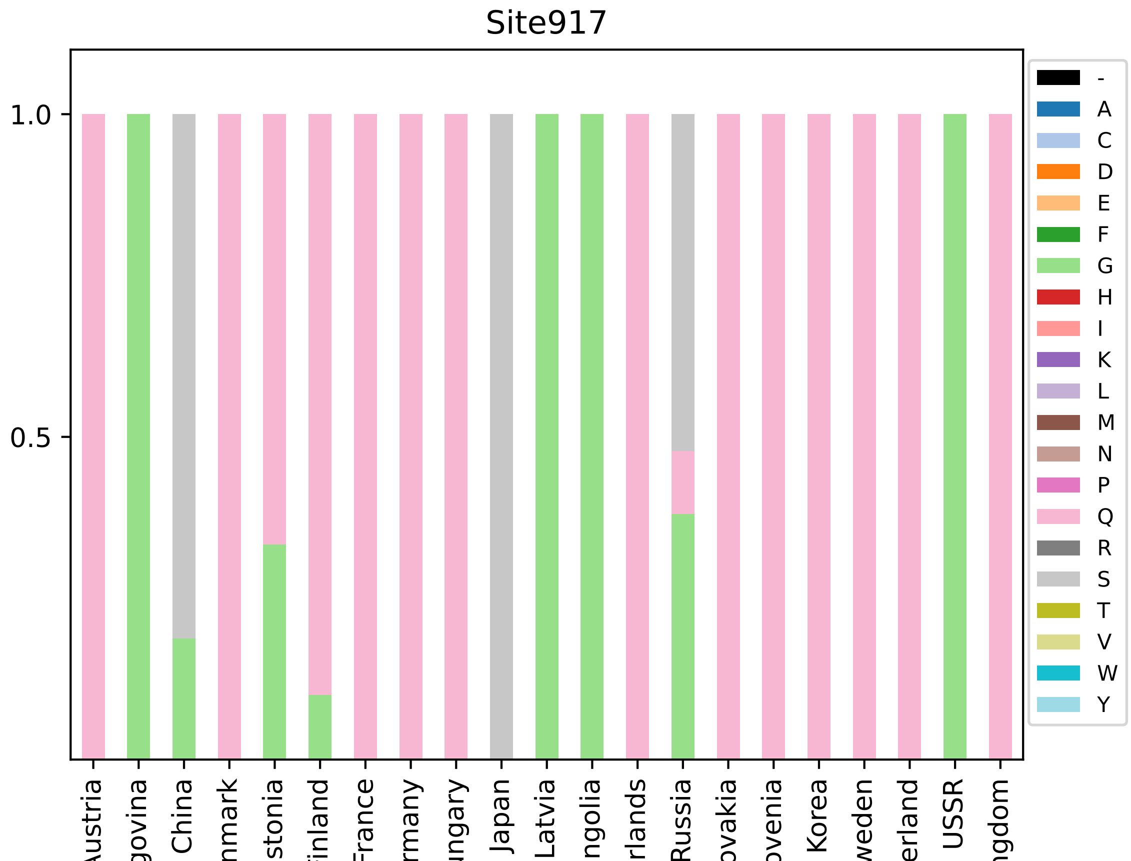

Site917
1.0
0.5
-
A
C
D
E
F
G
H
I
K
L
M
N
P
Q
R
S
T
V
W
Y
China
Japan
Latvia
Russia
Korea
USSR

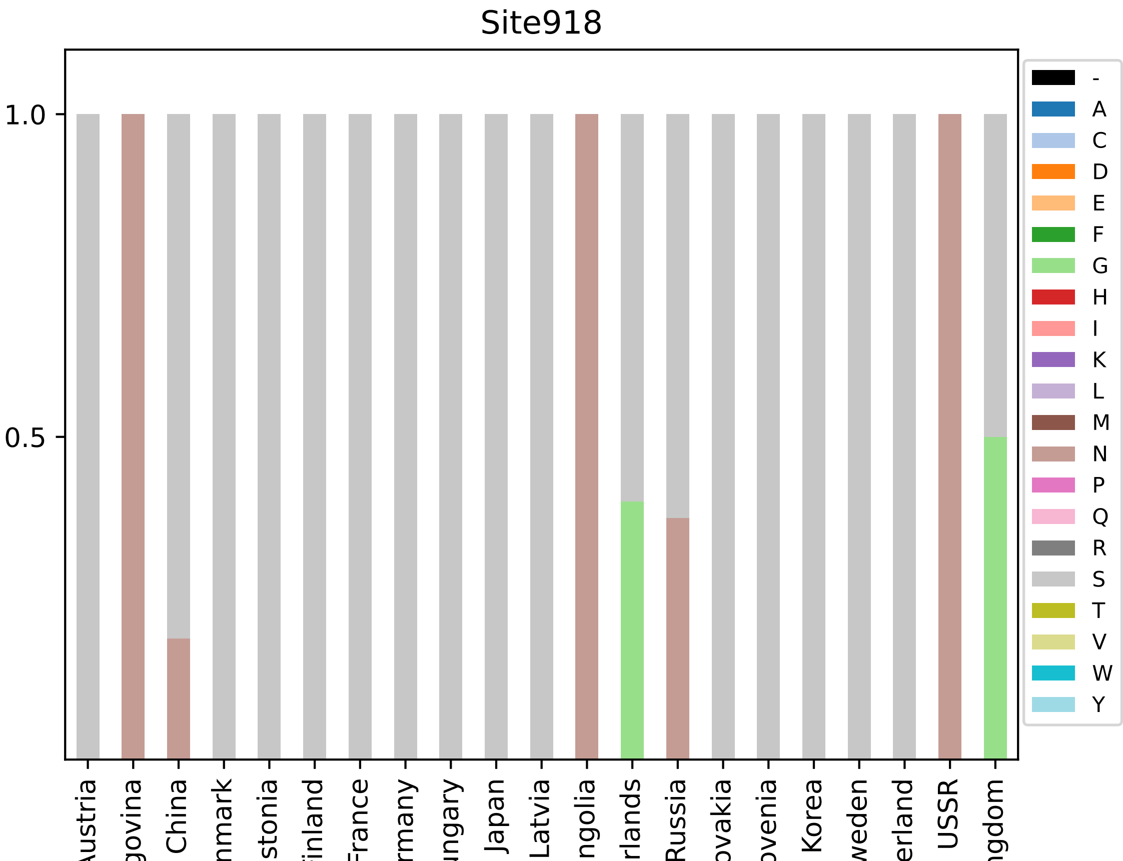

Site918
1.0
0.5
-
A
C
D
E
F
G
H
I
K
L
M
N
P
Q
R
S
T
V
W
Y
Austria
China
Russia
Japan
Latvia
Korea
USSR
France

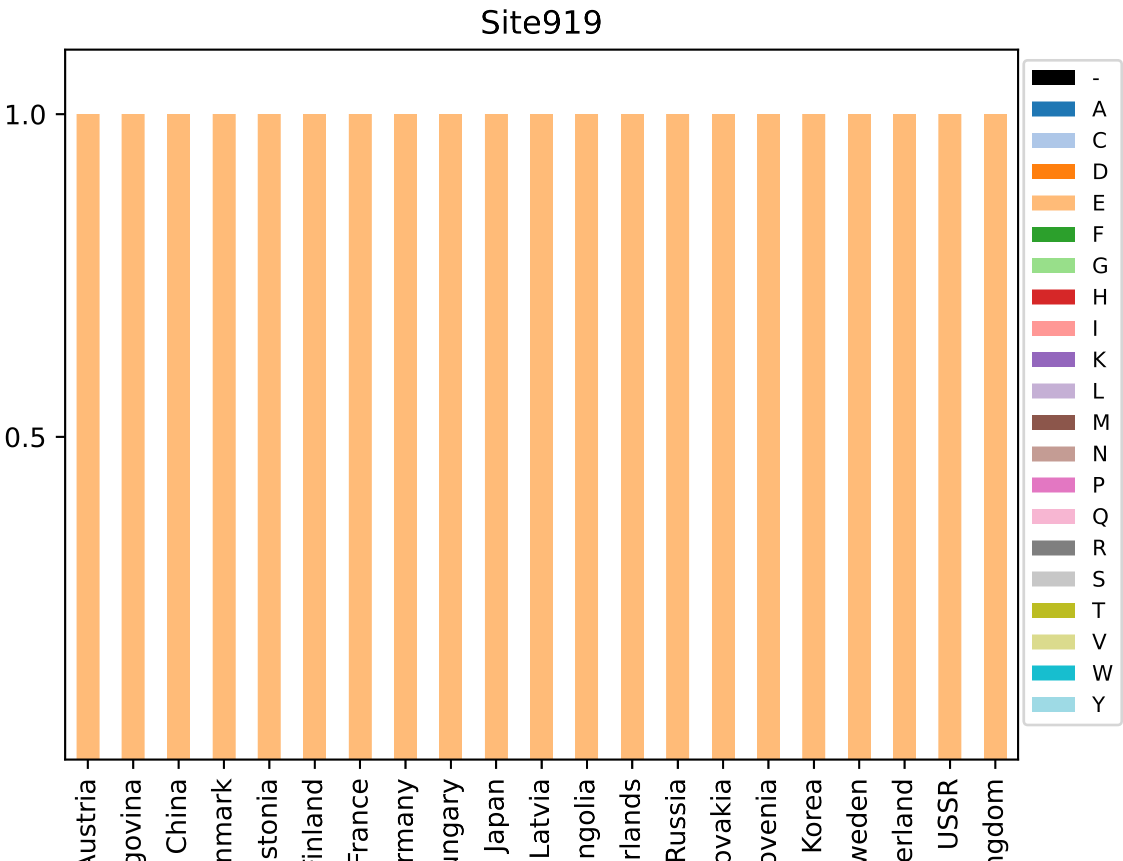
Site919
1.0
0.5
-
A
C
D
E
F
G
H
I
K
L
M
N
P
Q
R
S
T
V
W
Y
China
Japan
Latvia
Russia
Korea
USSR

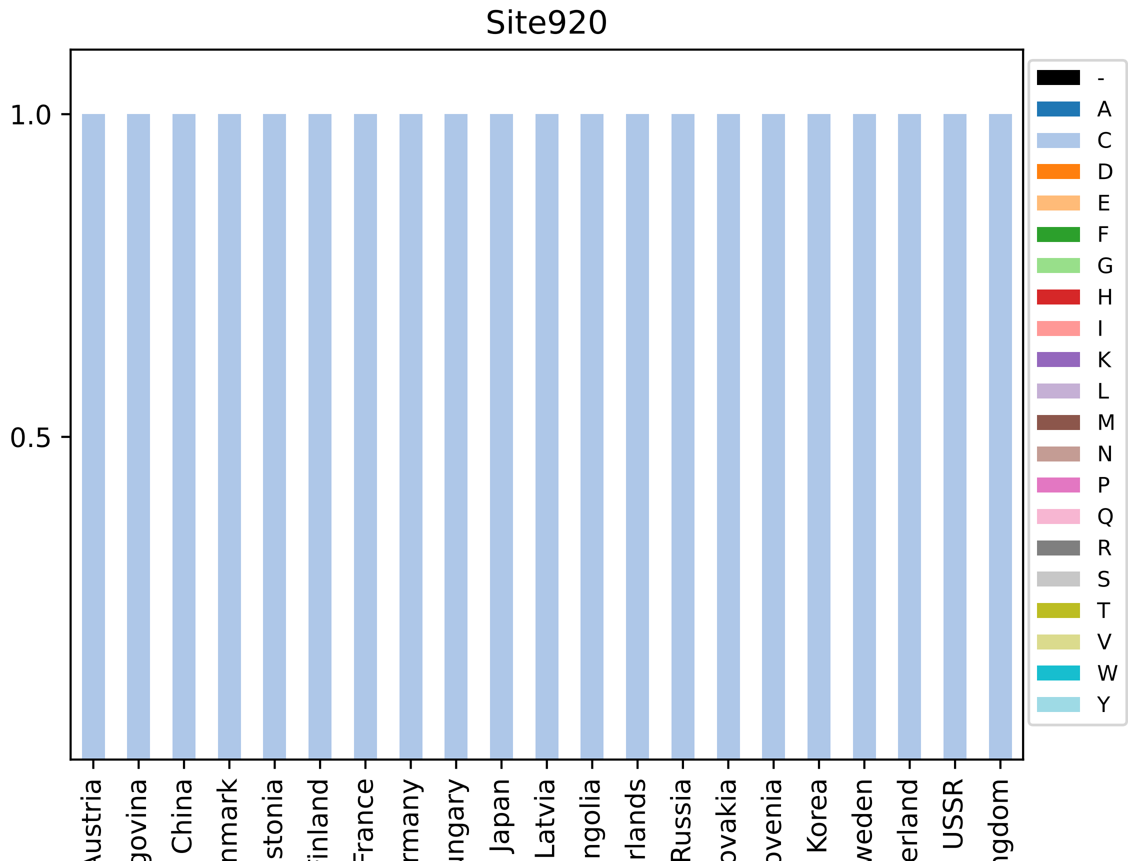
Site920
1.0
0.5
-
A
C
D
E
F
G
H
I
K
L
M
N
P
Q
R
S
T
V
W
Y
China
Japan
Latvia
Russia
Korea
USSR

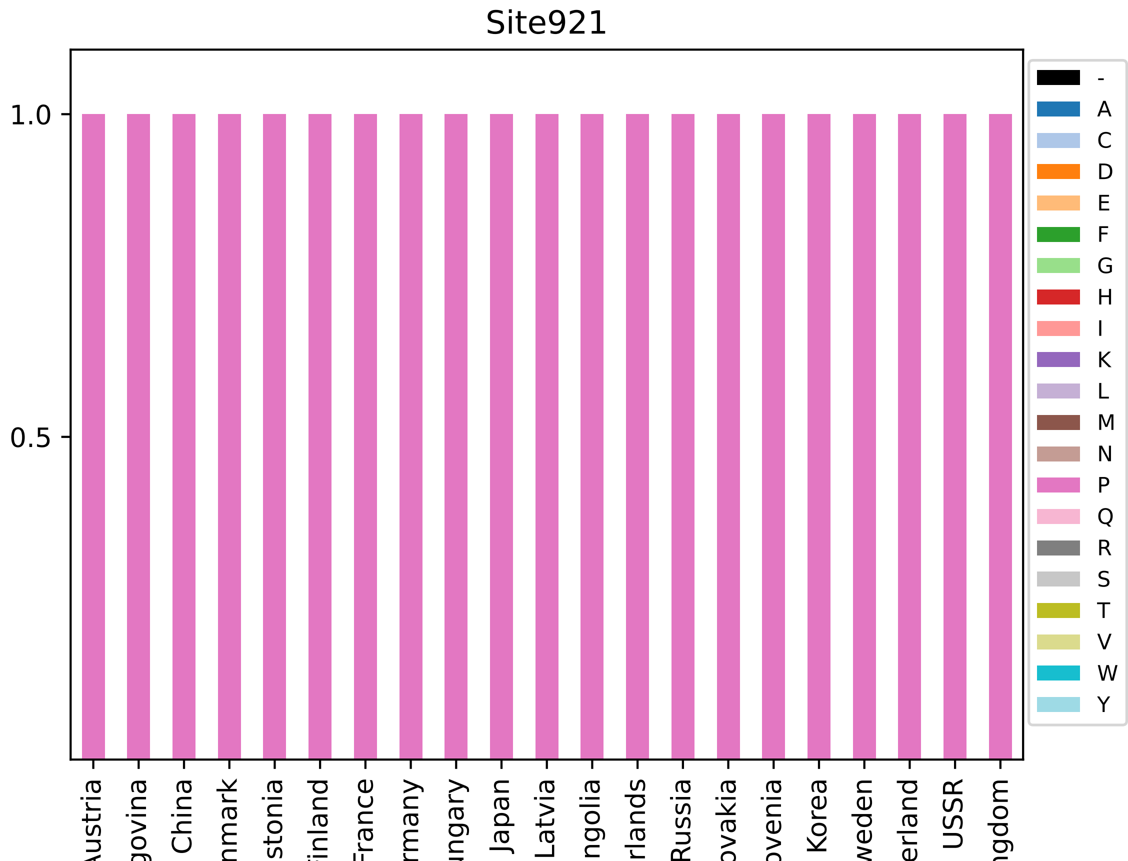
Site921
1.0
0.5
-
A
C
D
E
F
G
H
I
K
L
M
N
P
Q
R
S
T
V
W
Y
Austria
China
Japan
Latvia
Russia
Korea
USSR

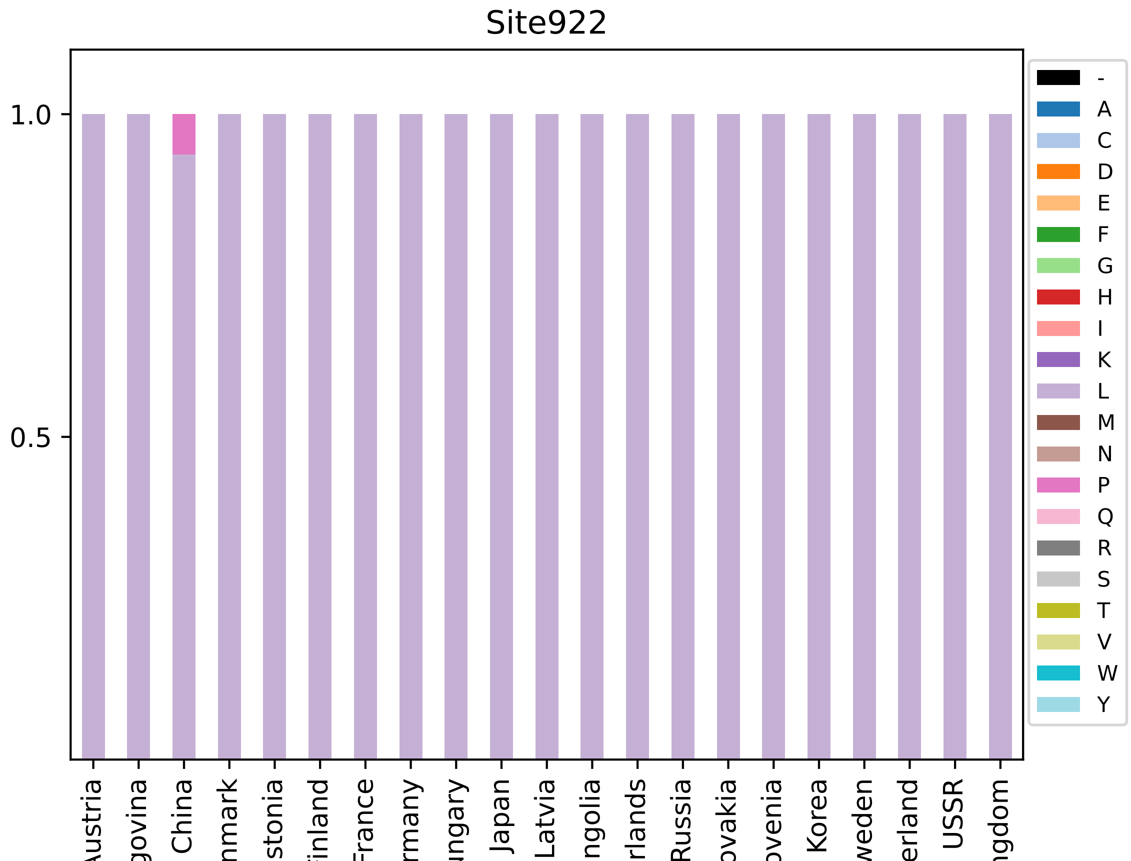

Site922
1.0
0.5
-
A
C
D
E
F
G
H
I
K
L
M
N
P
Q
R
S
T
V
W
Y
China
Japan
Latvia
Russia
Korea
USSR

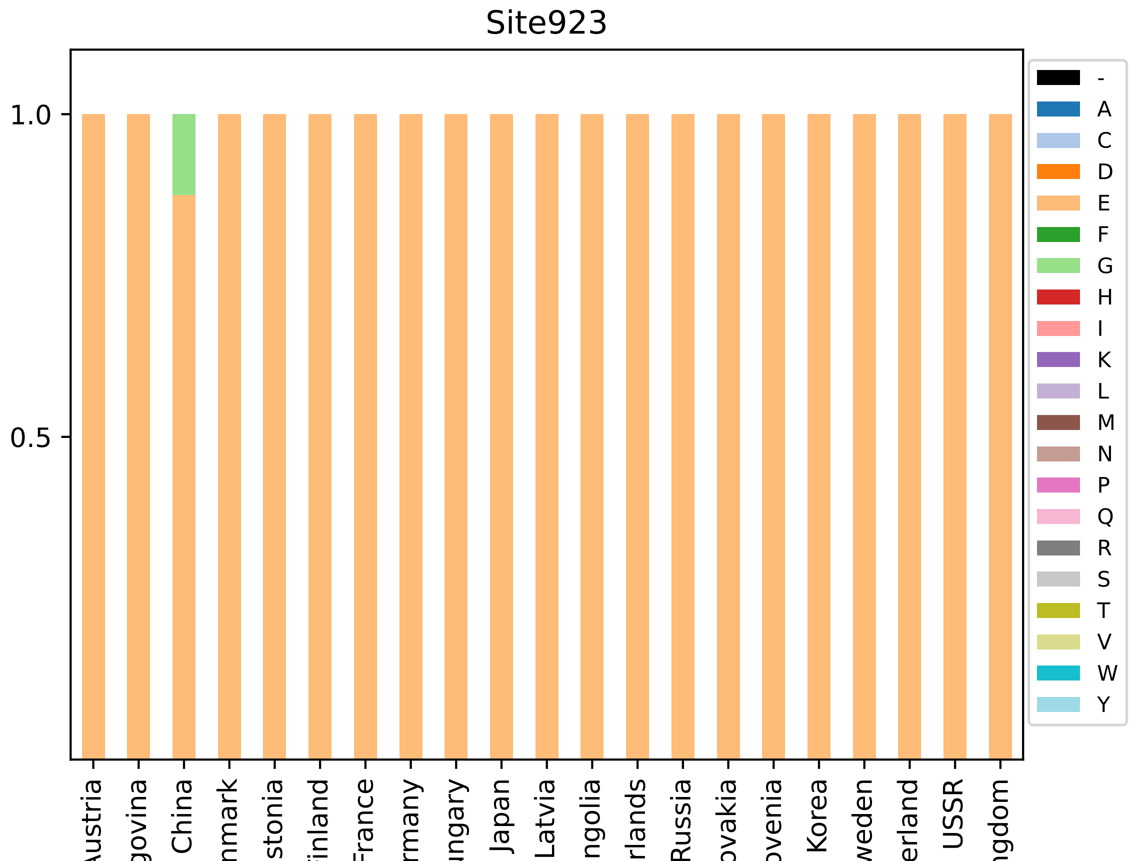

Site923
1.0
0.5
-
A
C
D
E
F
G
H
I
K
L
M
N
P
Q
R
S
T
V
W
Y
Austria
govina
China
nmark
stonia
inland
France
rmany
ungary
Japan
Latvia
ngolia
rlands
Russia
ovakia
ovenia
Korea
weden
erland
USSR
ngdom

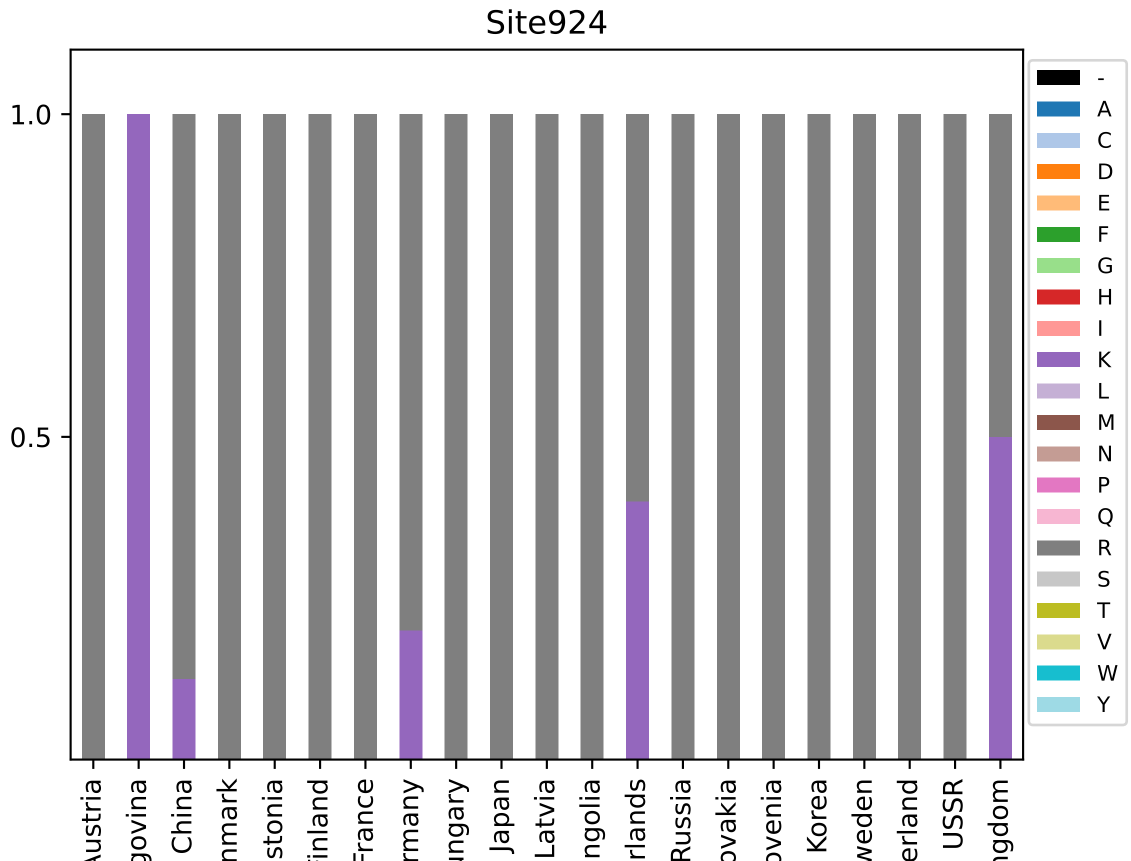
Site924
1.0
0.5
Austria
govina
China
nmark
stonia
Finland
France
rmany
ungary
Japan
Latvia
ngolia
rlands
Russia
ovakia
ovenia
Korea
weden
erland
USSR
ngdom
-
A
C
D
E
F
G
H
I
K
L
M
N
P
Q
R
S
T
V
W
Y

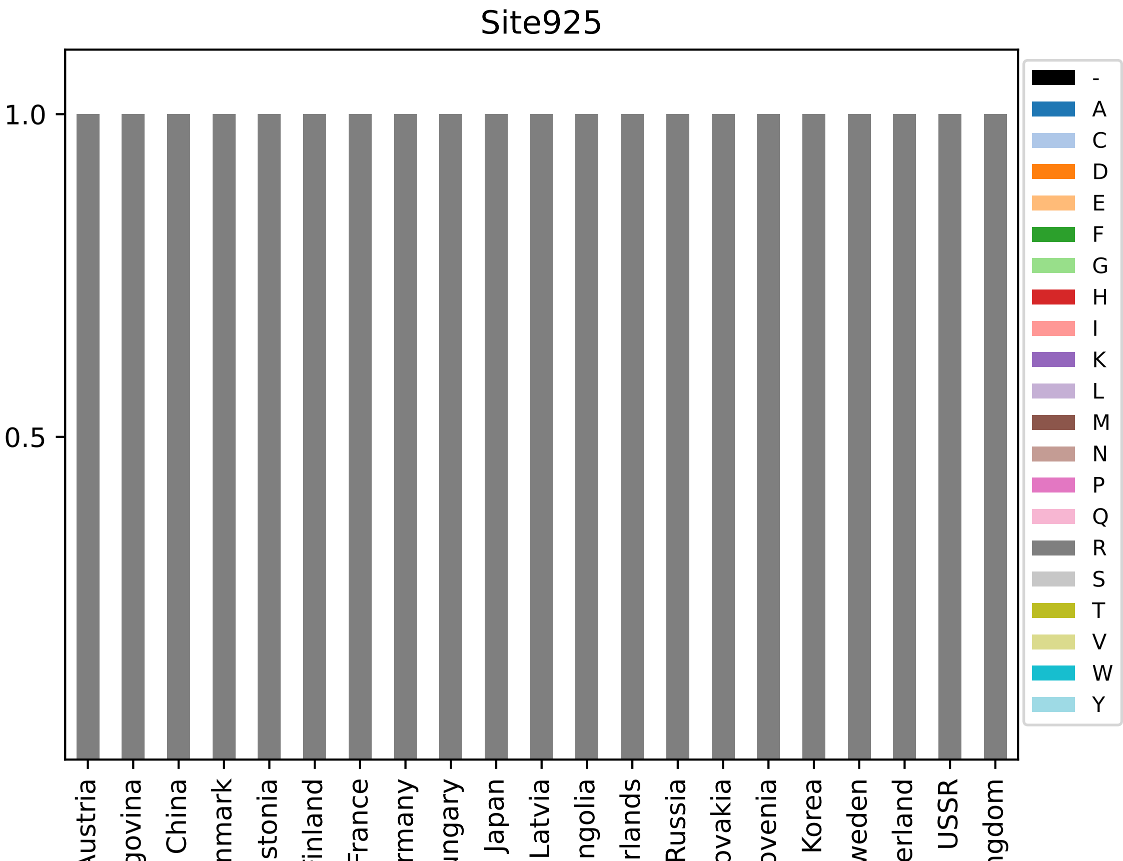
Site925
1.0
0.5
-
A
C
D
E
F
G
H
I
K
L
M
N
P
Q
R
S
T
V
W
Y
China
Japan
Latvia
Russia
Korea
USSR

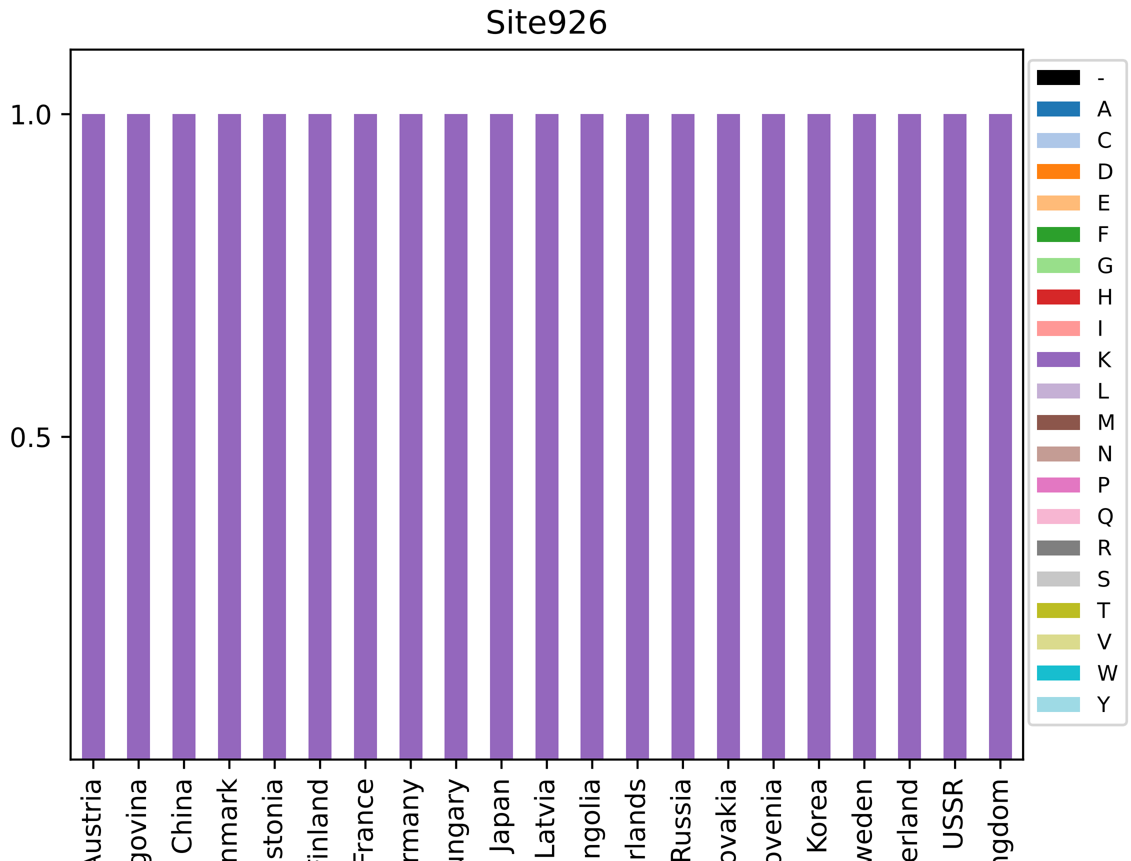

Site926
1.0
0.5
-
A
C
D
E
F
G
H
I
K
L
M
N
P
Q
R
S
T
V
W
Y
China
Japan
Latvia
Russia
Korea
USSR

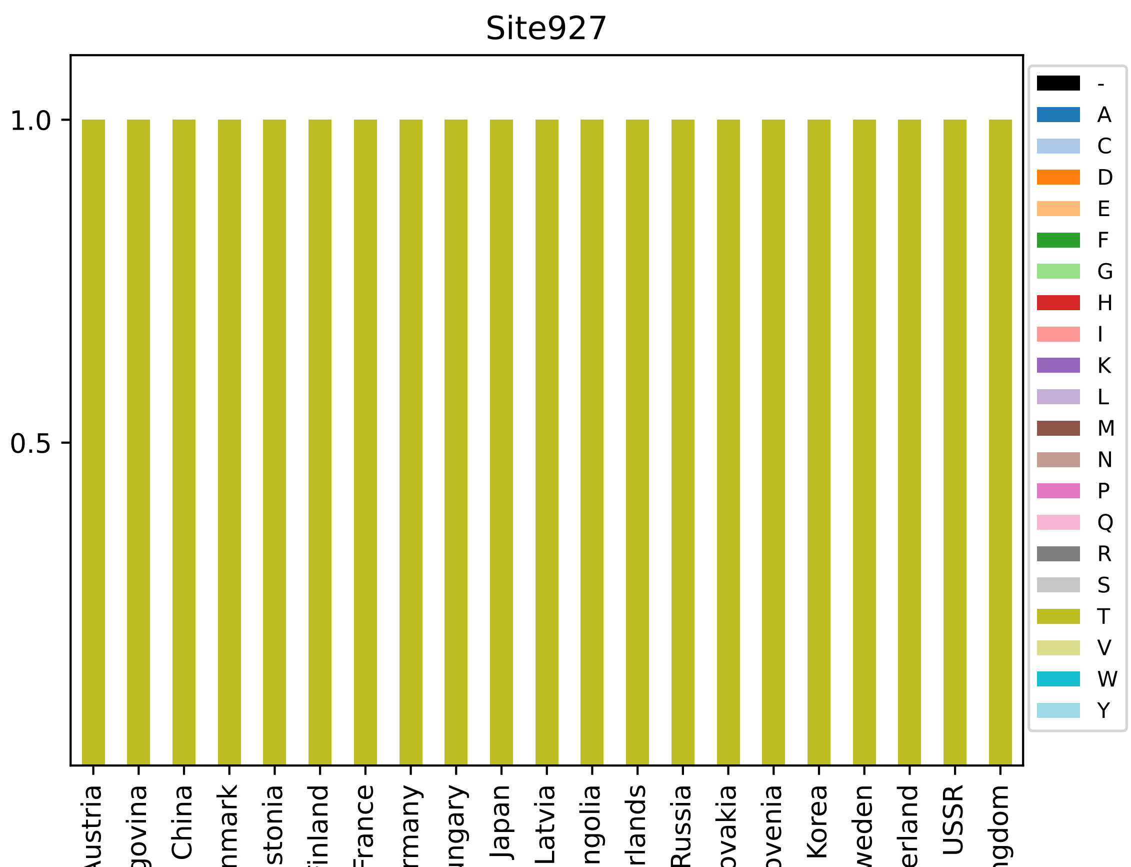

Site927
1.0
0.5
-
A
C
D
E
F
G
H
I
K
L
M
N
P
Q
R
S
T
V
W
Y
China
Japan
Latvia
Russia
Korea
USSR

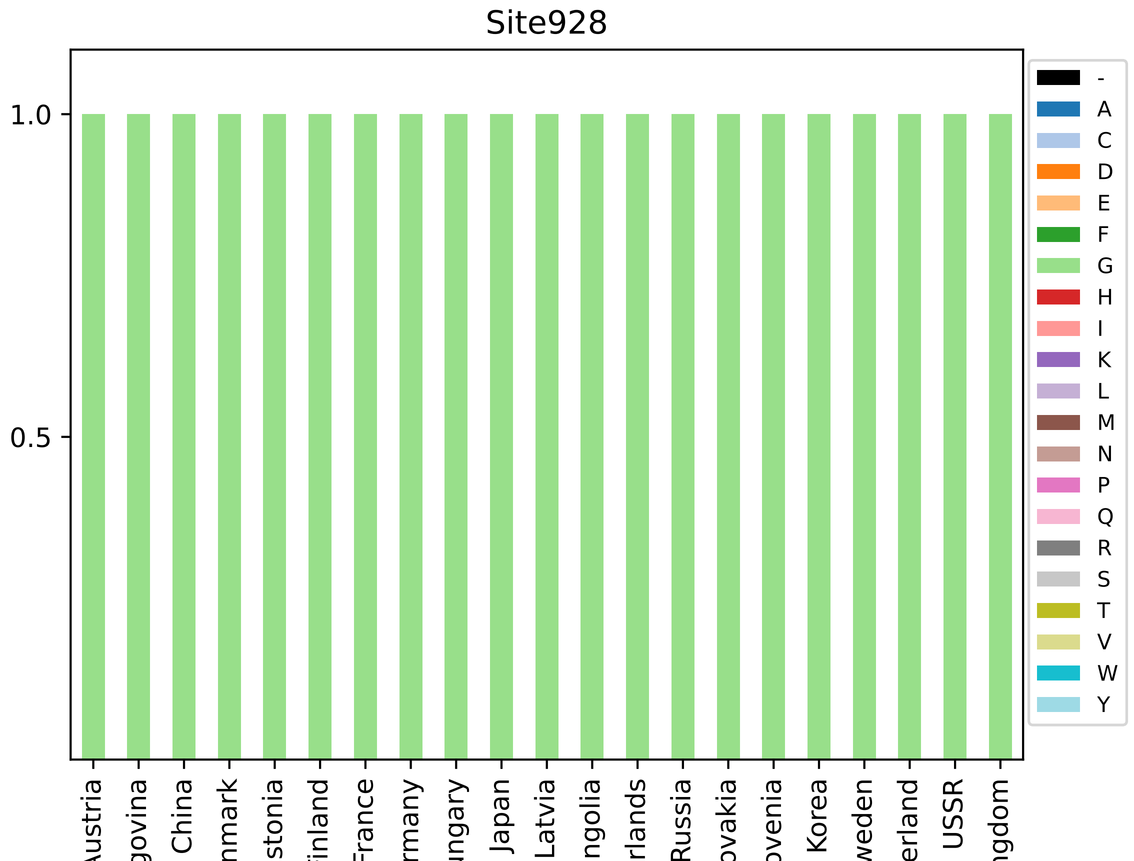
Site928
1.0
0.5
-
A
C
D
E
F
G
H
I
K
L
M
N
P
Q
R
S
T
V
W
Y
China
Japan
Latvia
Russia
Korea
USSR

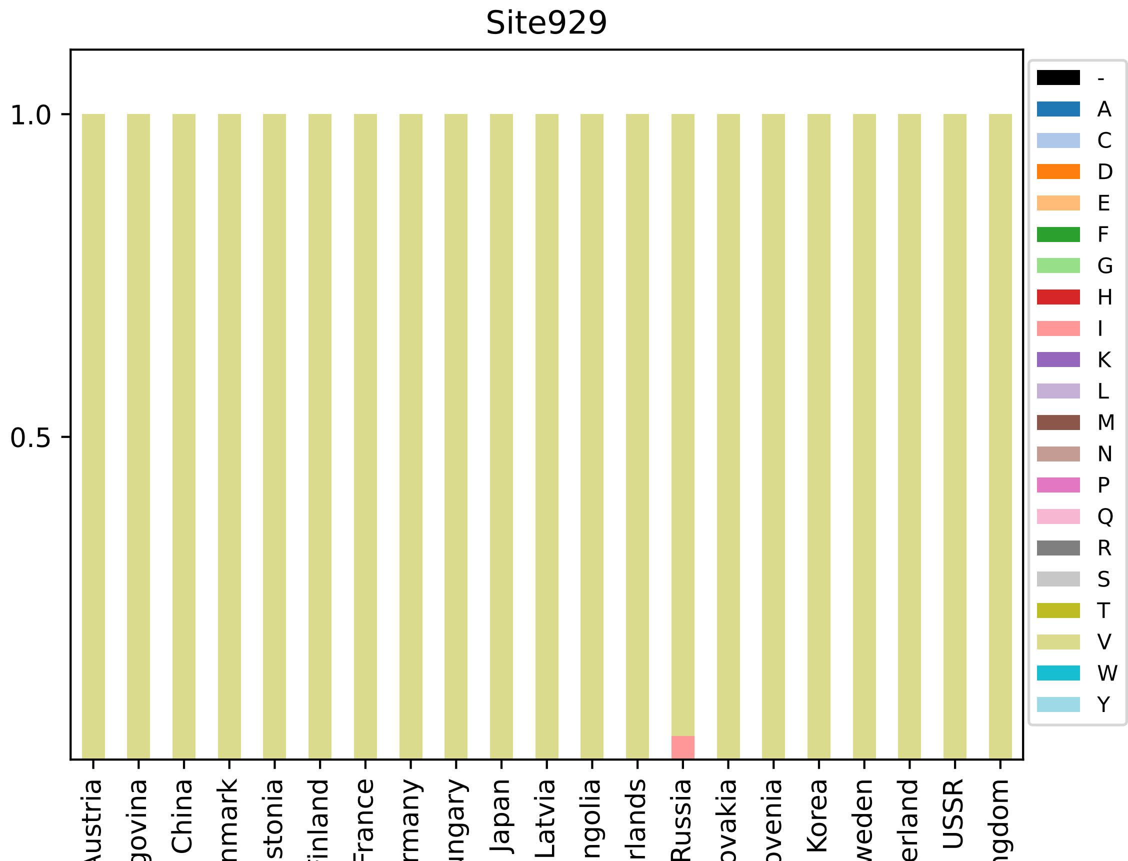

Site929
1.0
0.5
China
Japan
Latvia
Russia
Korea
USSR
-
A
C
D
E
F
G
H
I
K
L
M
N
P
Q
R
S
T
V
W
Y

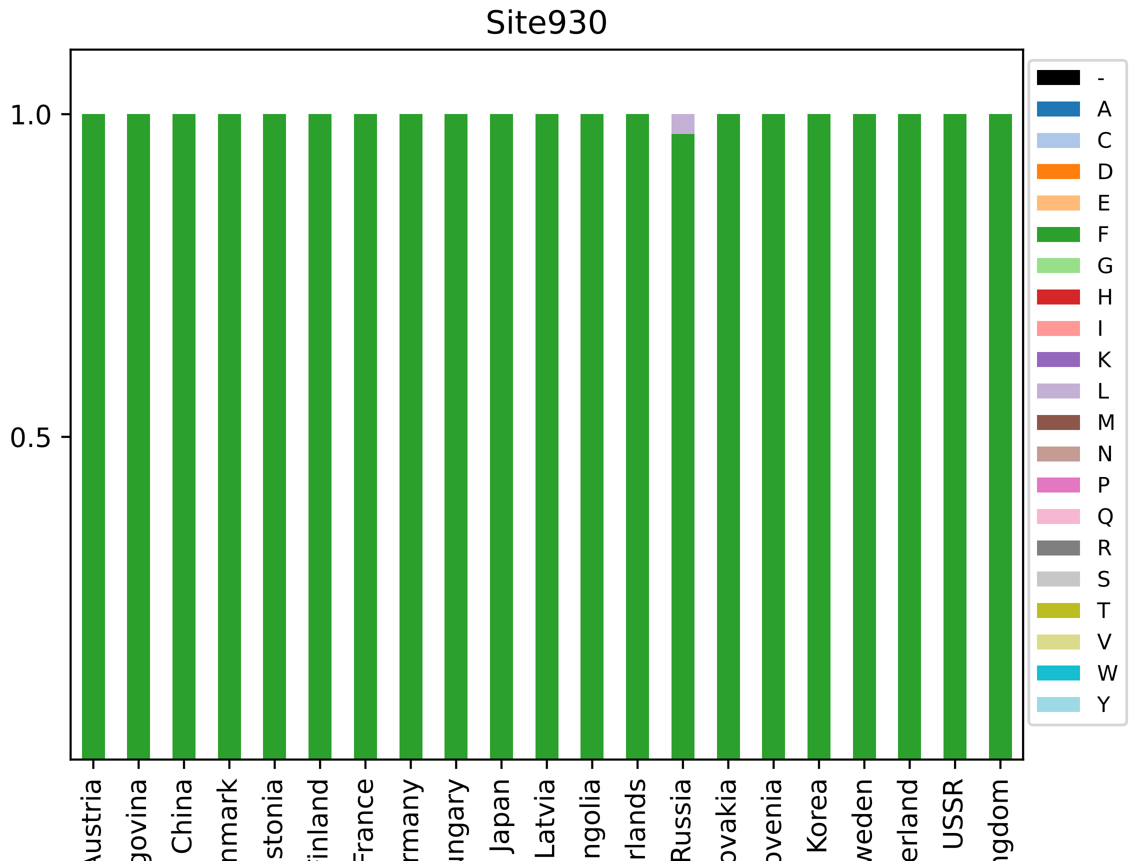
Site930
1.0
0.5
-
A
C
D
E
F
G
H
I
K
L
M
N
P
Q
R
S
T
V
W
Y
China
Japan
Latvia
Russia
Korea
USSR

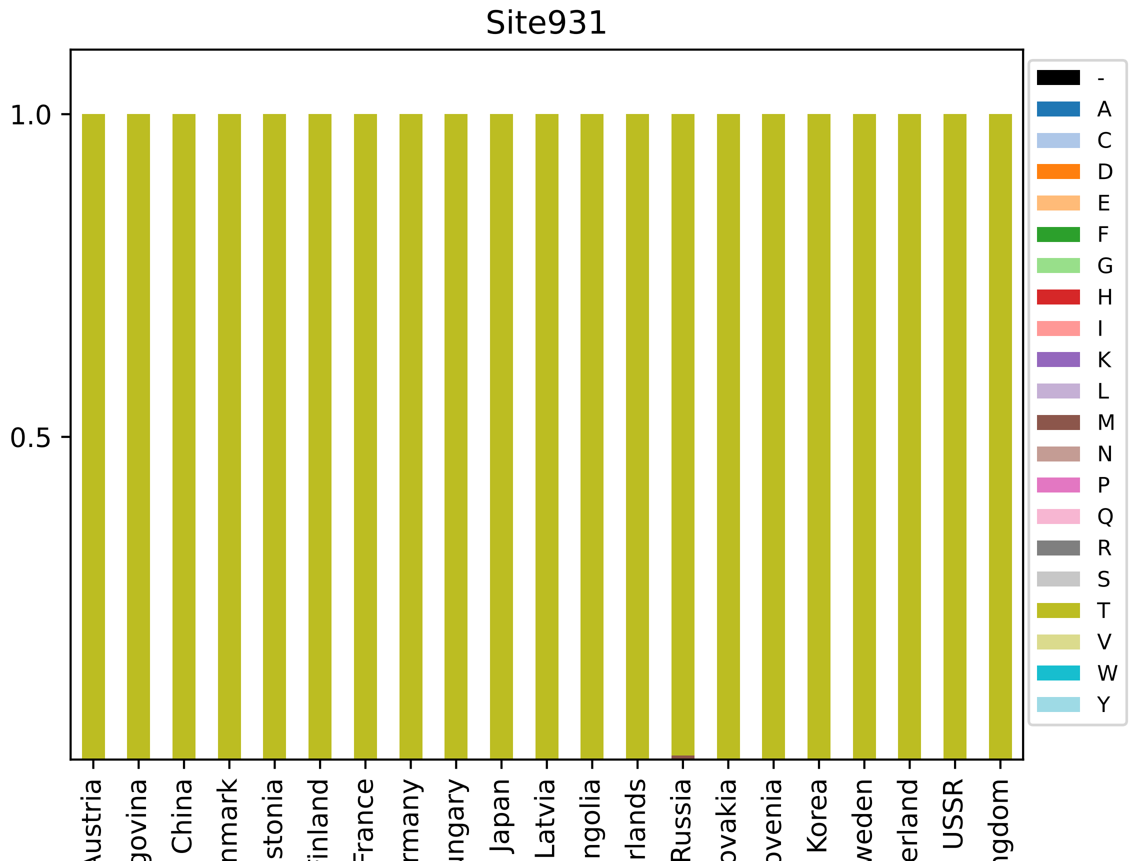
Site931
1.0
0.5
-
A
C
D
E
F
G
H
I
K
L
M
N
P
Q
R
S
T
V
W
Y
China
Japan
Latvia
Russia
Korea
USSR

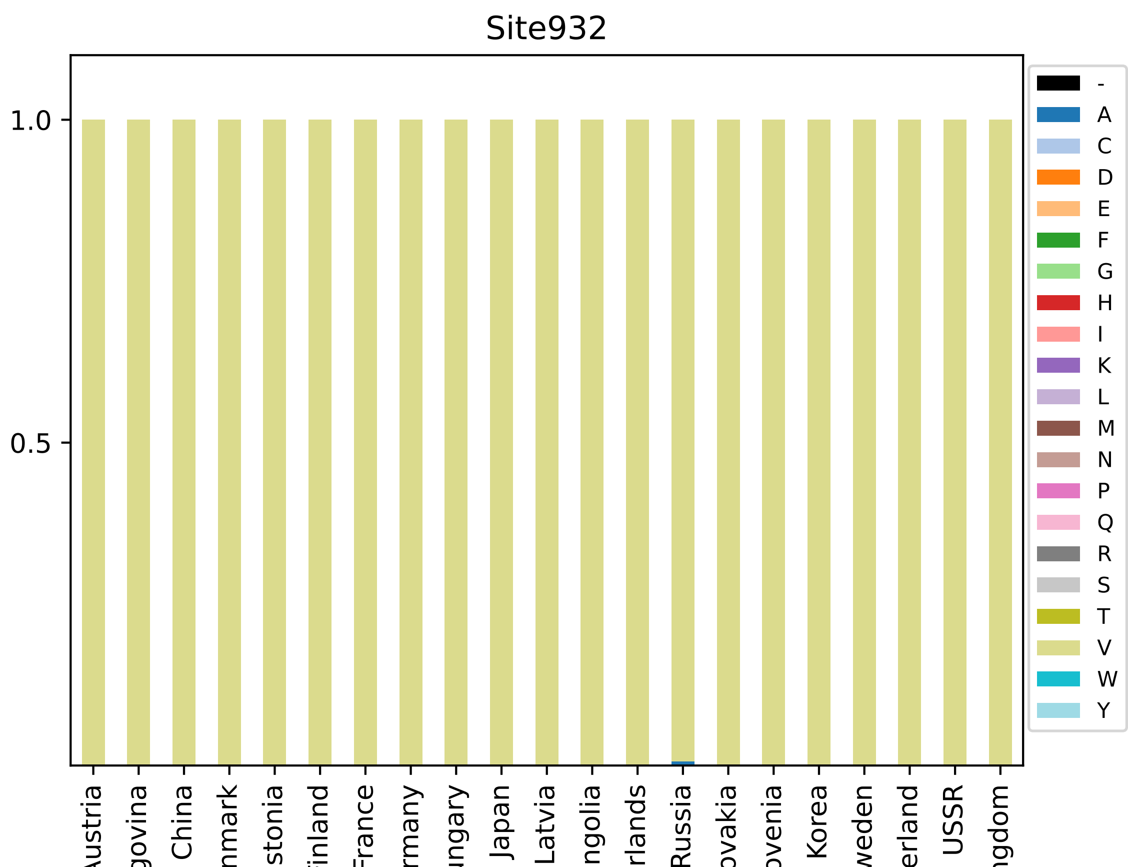
Site932
1.0
0.5
-
A
C
D
E
F
G
H
I
K
L
M
N
P
Q
R
S
T
V
W
Y
Austria
govina
China
nmark
stonia
Finland
France
rmany
ungary
Japan
Latvia
ngolia
rlands
Russia
ovakia
ovenia
Korea
weden
erland
USSR
ngdom

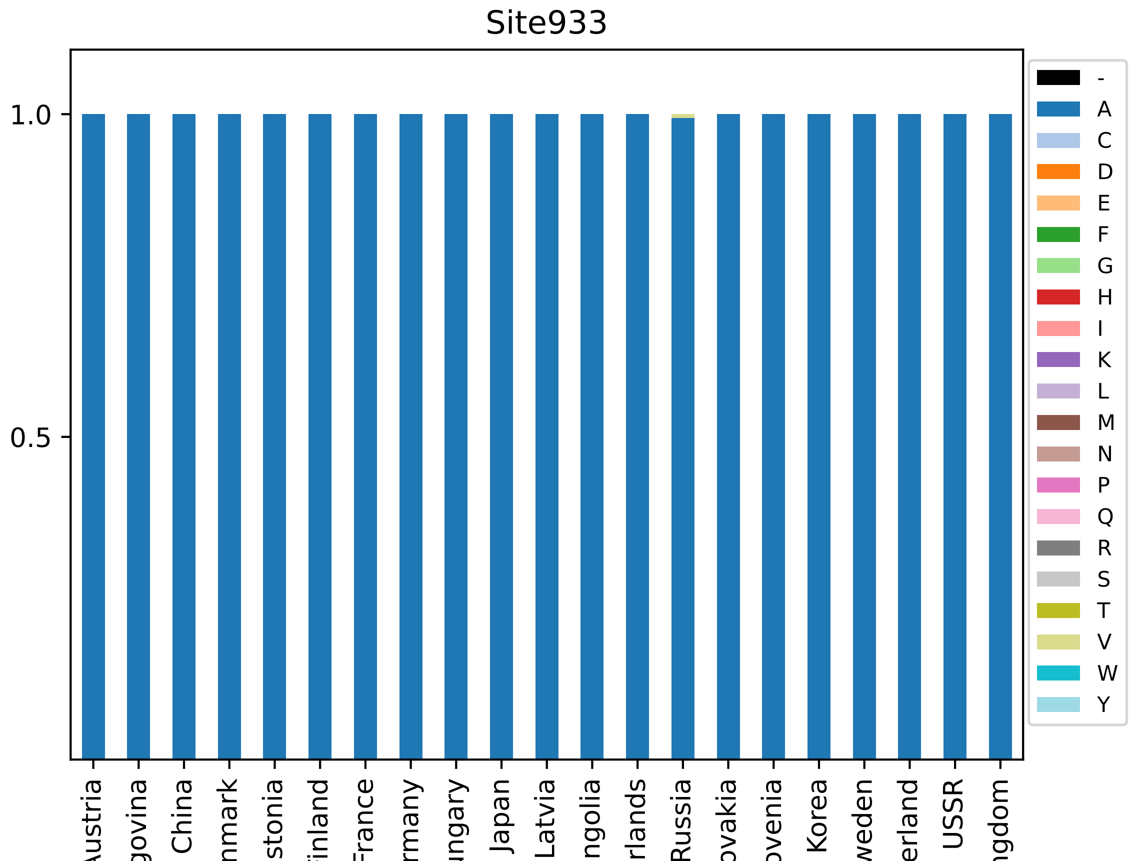

Site933
1.0
0.5
-
A
C
D
E
F
G
H
I
K
L
M
N
P
Q
R
S
T
V
W
Y
China
Japan
Latvia
Russia
Korea
USSR

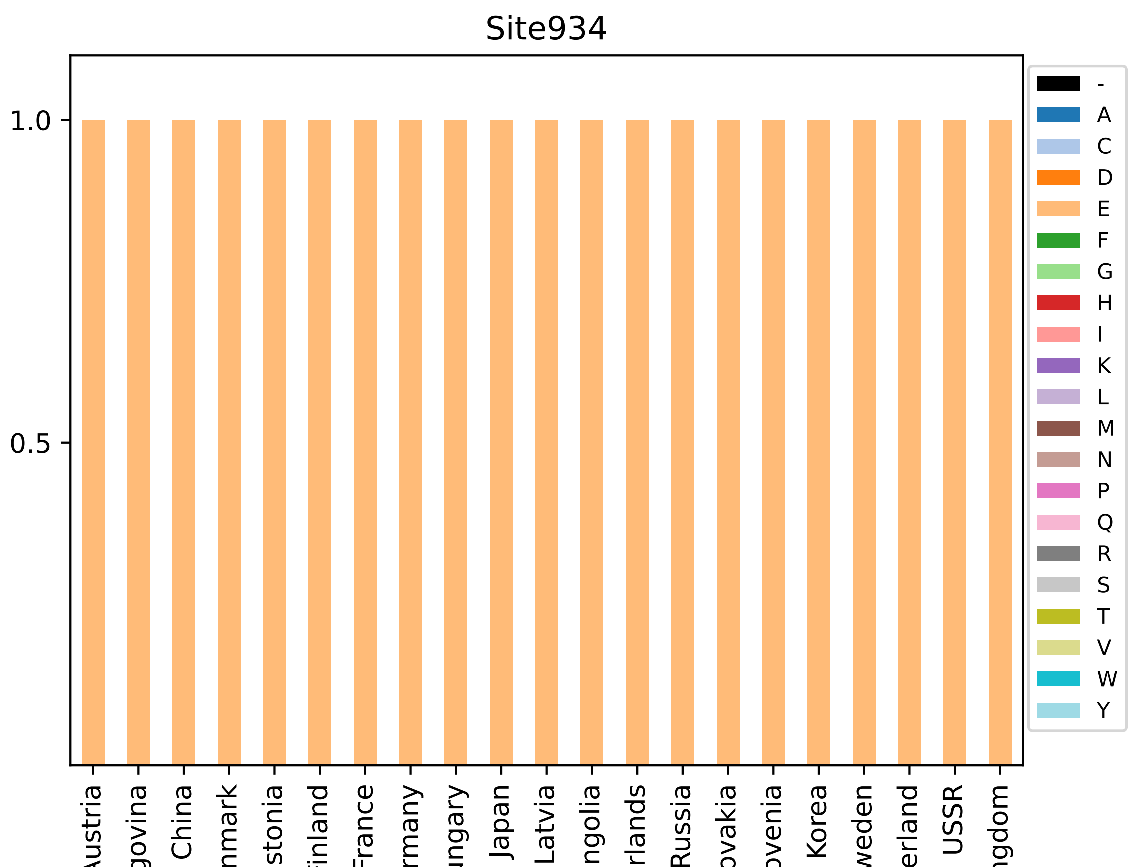

Site934
1.0
0.5
Austria
govina
China
nmark
stonia
Finland
France
rmany
ungary
Japan
Latvia
ngolia
rlands
Russia
ovakia
ovenia
Korea
weden
erland
USSR
ngdom
-
A
C
D
E
F
G
H
I
K
L
M
N
P
Q
R
S
T
V
W
Y

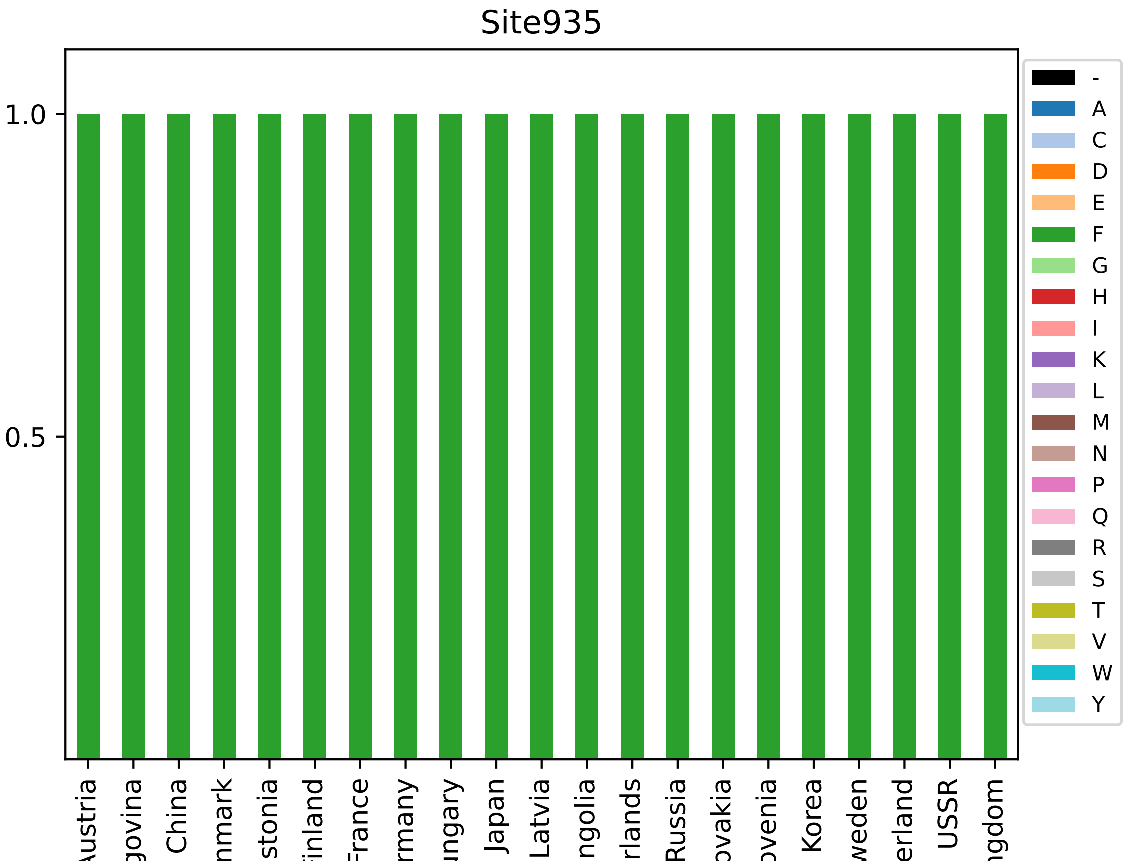

Site935
1.0
0.5
China
Japan
Latvia
Russia
Korea
USSR
-
A
C
D
E
F
G
H
I
K
L
M
N
P
Q
R
S
T
V
W
Y

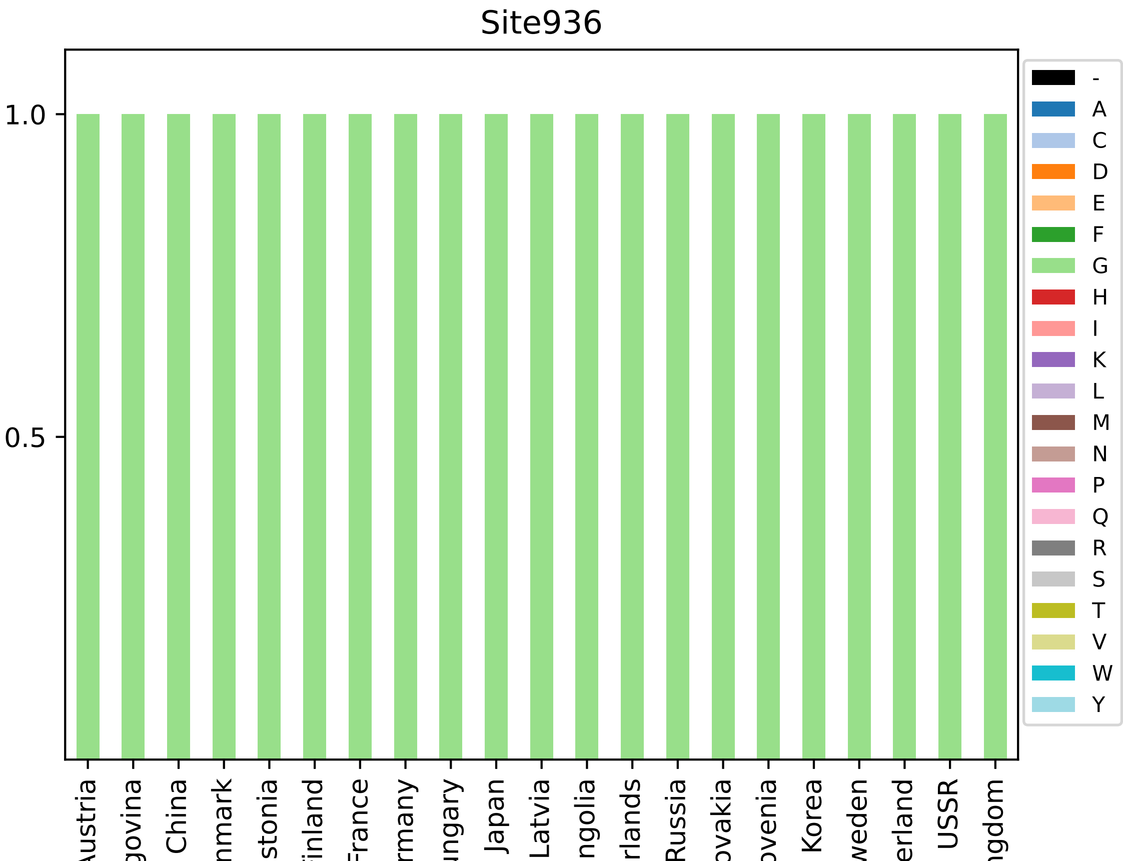
Site936
1.0
0.5
-
A
C
D
E
F
G
H
I
K
L
M
N
P
Q
R
S
T
V
W
Y
China
Japan
Latvia
Russia
Korea
USSR

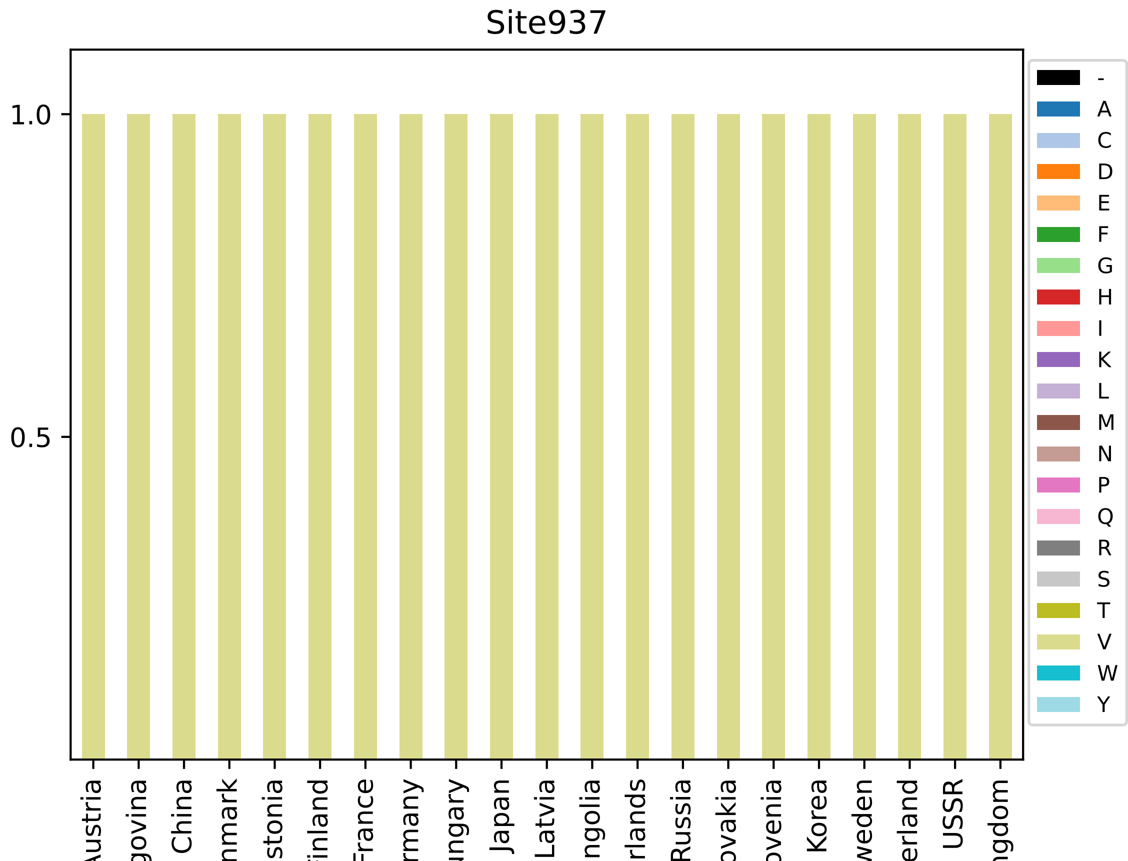

Site937
1.0
0.5
-
A
C
D
E
F
G
H
I
K
L
M
N
P
Q
R
S
T
V
W
Y
China
Japan
Latvia
Russia
Korea
USSR

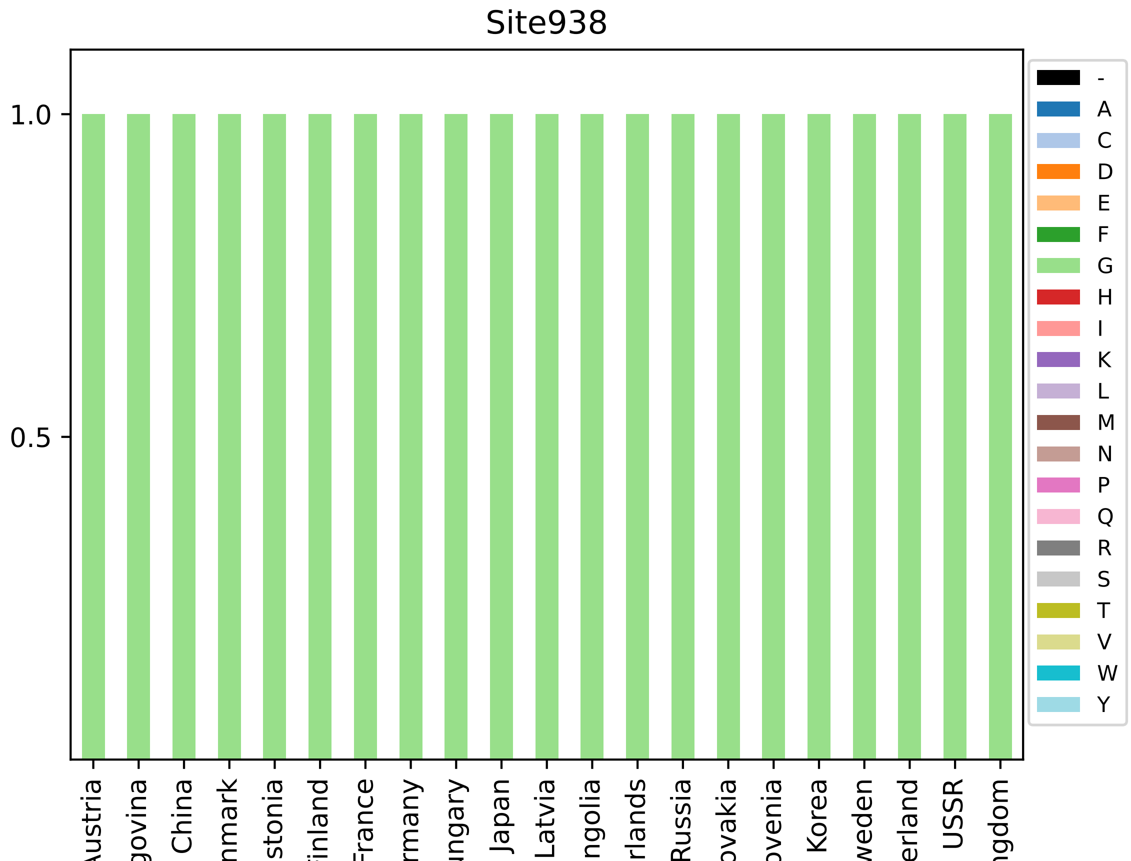

Site938
1.0
0.5
-
A
C
D
E
F
G
H
I
K
L
M
N
P
Q
R
S
T
V
W
Y
China
Japan
Latvia
Russia
Korea
USSR

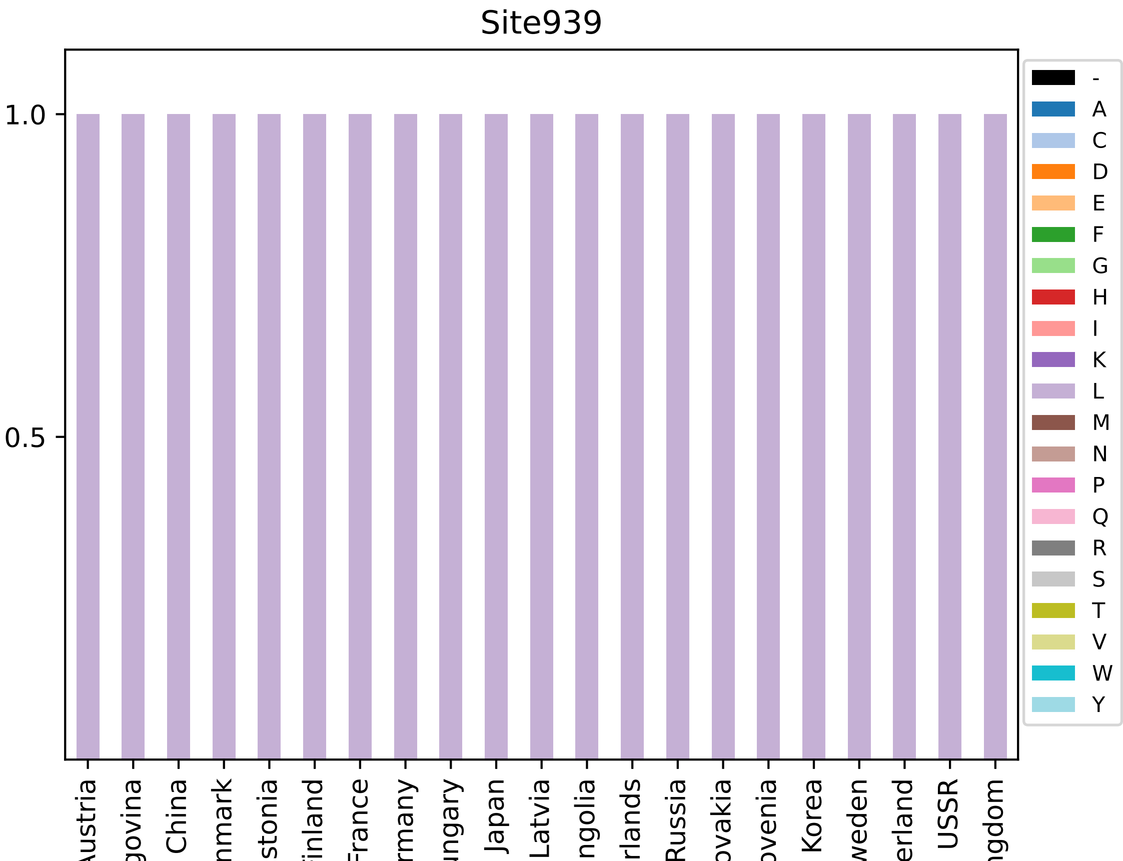
Site939
1.0
0.5
-
A
C
D
E
F
G
H
I
K
L
M
N
P
Q
R
S
T
V
W
Y
China
Japan
Latvia
Russia
Korea
USSR

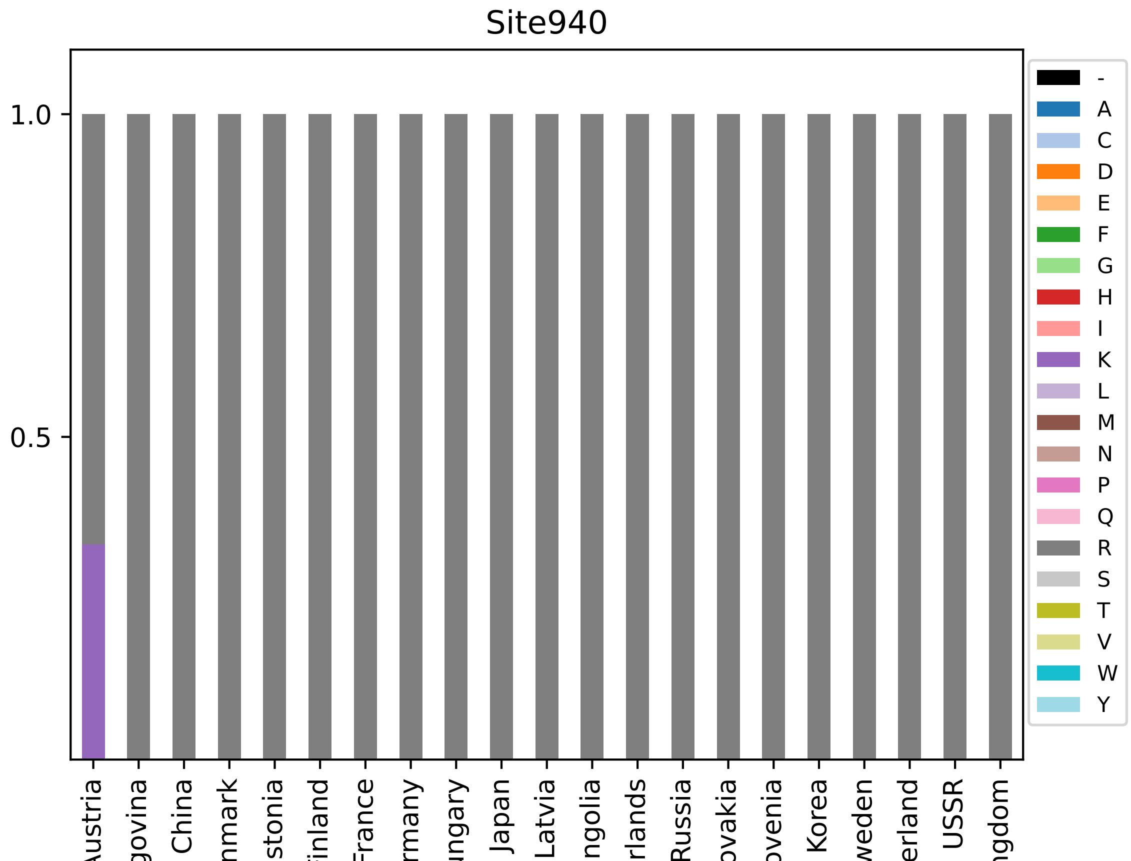

Site940
1.0
0.5
-
A
C
D
E
F
G
H
I
K
L
M
N
P
Q
R
S
T
V
W
Y
China
Japan
Latvia
Russia
Korea
USSR

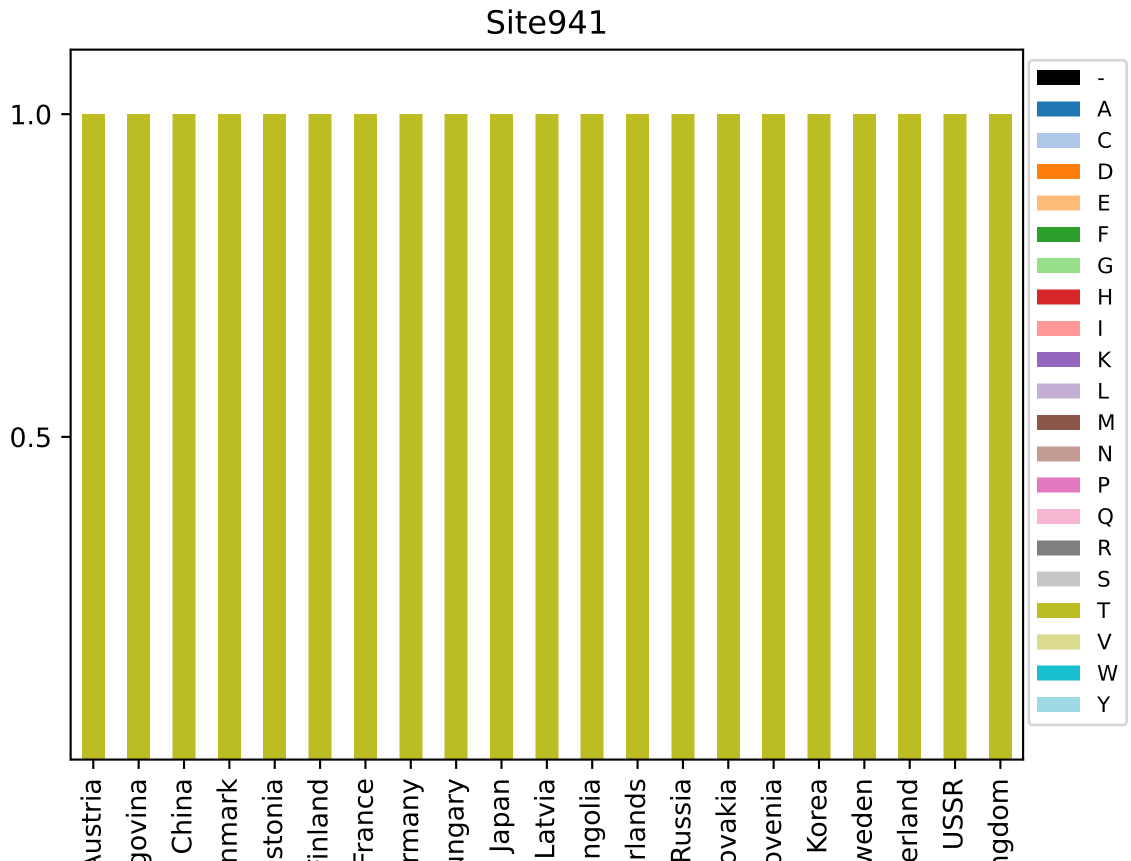

Site941
1.0
0.5
-
A
C
D
E
F
G
H
I
K
L
M
N
P
Q
R
S
T
V
W
Y
Austria
govina
China
nmark
stonia
inland
France
rmany
ungary
Japan
Latvia
ngolia
rlands
Russia
ovakia
ovenia
Korea
weden
erland
USSR
ngdom

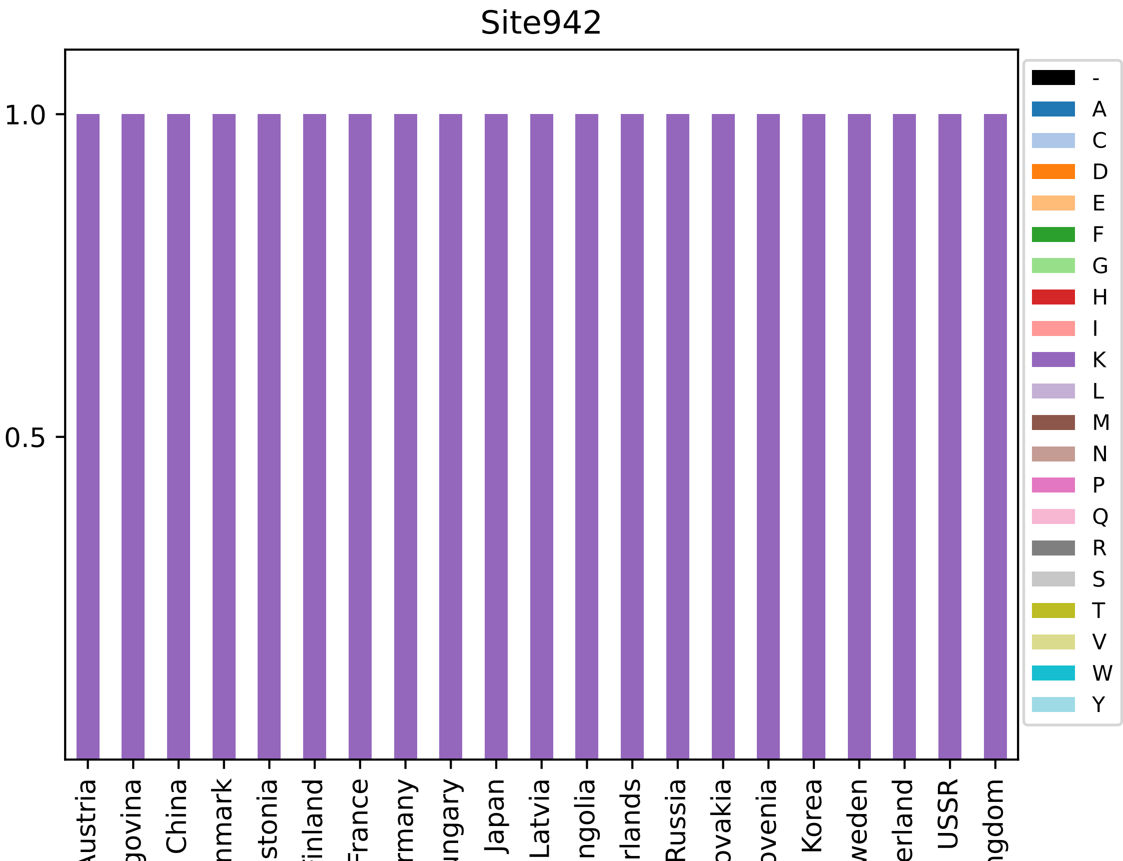

Site942
1.0
0.5
-
A
C
D
E
F
G
H
I
K
L
M
N
P
Q
R
S
T
V
W
Y
China
Japan
Latvia
Russia
Korea
USSR

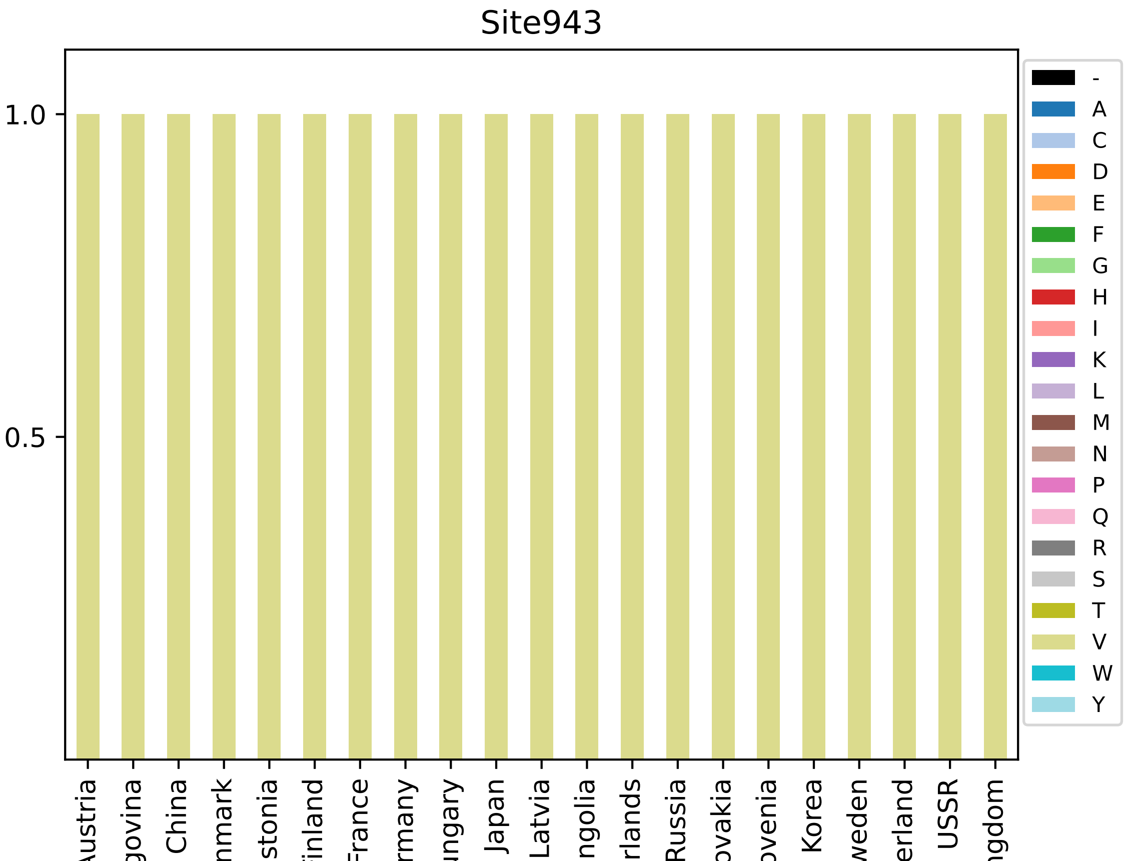
Site943
1.0
0.5
-
A
C
D
E
F
G
H
I
K
L
M
N
P
Q
R
S
T
V
W
Y
Austria
China
Japan
Latvia
Russia
Korea
USSR

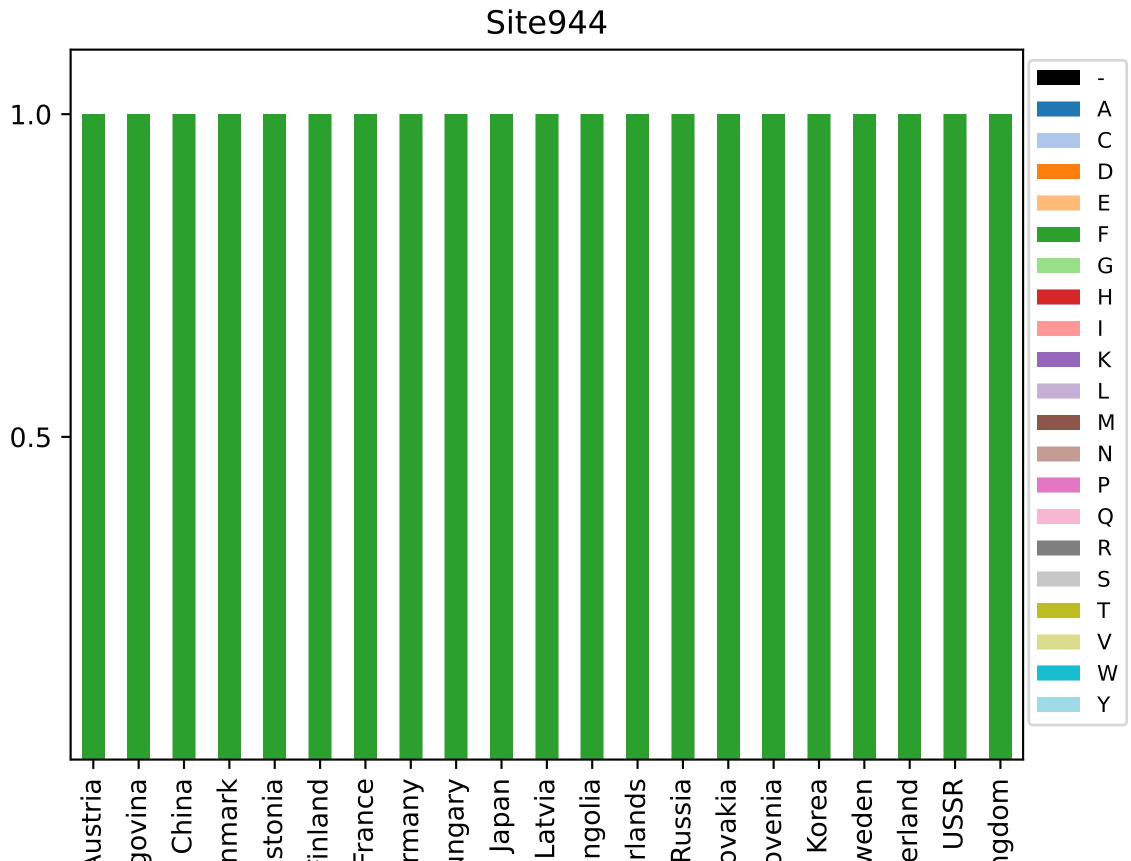

Site944
1.0
0.5
-
A
C
D
E
F
G
H
I
K
L
M
N
P
Q
R
S
T
V
W
Y
China
Japan
Latvia
Russia
Korea
USSR

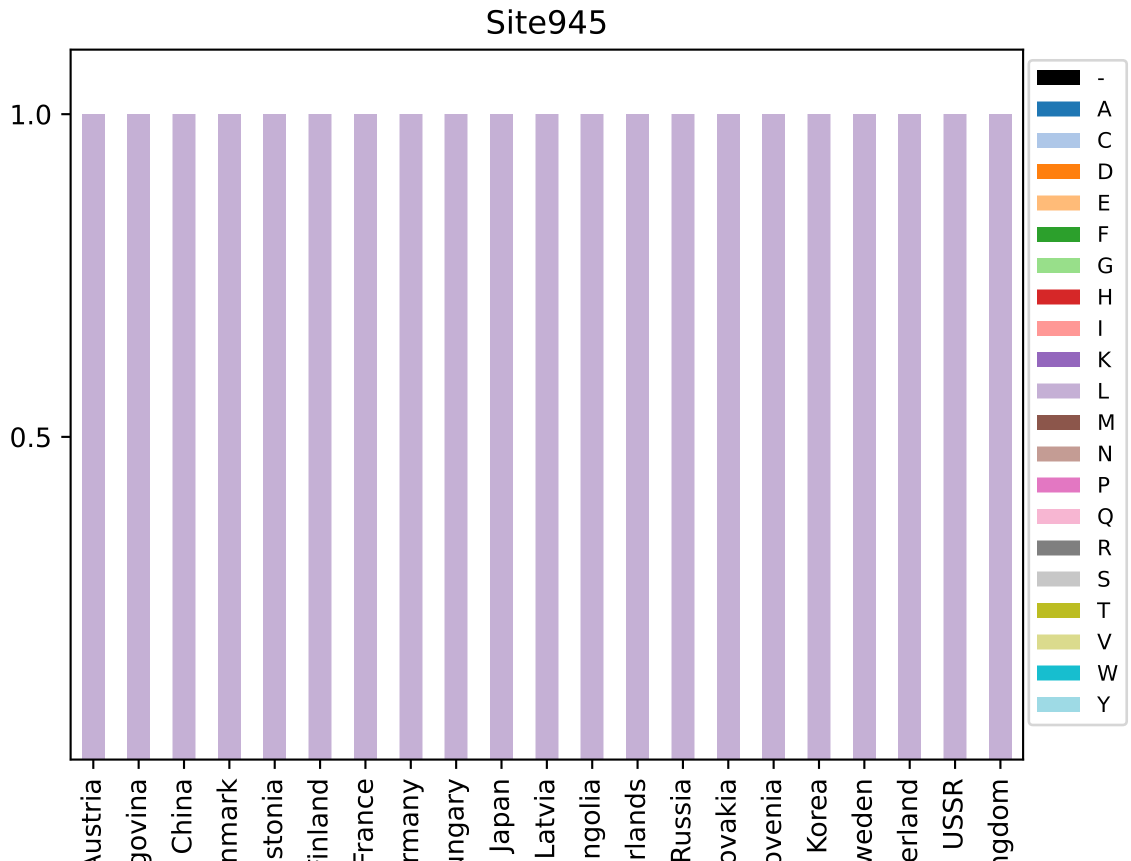

Site945
1.0
0.5
-
A
C
D
E
F
G
H
I
K
L
M
N
P
Q
R
S
T
V
W
Y
Austria
govina
China
nmark
stonia
inland
France
rmany
ungary
Japan
Latvia
ngolia
rlands
Russia
ovakia
ovenia
Korea
weden
erland
USSR
ngdom

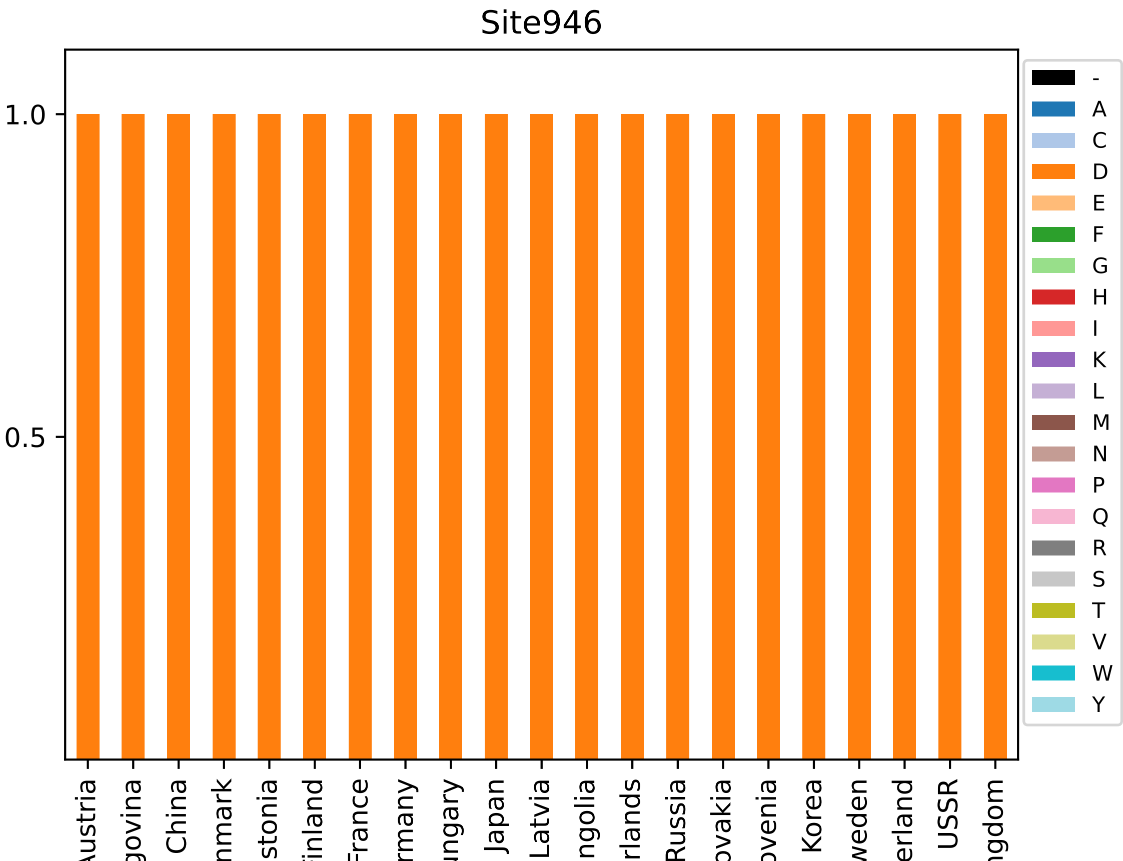
Site946
1.0
0.5
-
A
C
D
E
F
G
H
I
K
L
M
N
P
Q
R
S
T
V
W
Y
Austria
govina
China
nmark
stonia
inland
France
rmany
ungary
Japan
Latvia
ngolia
rlands
Russia
ovakia
ovenia
Korea
weden
erland
USSR
ngdom

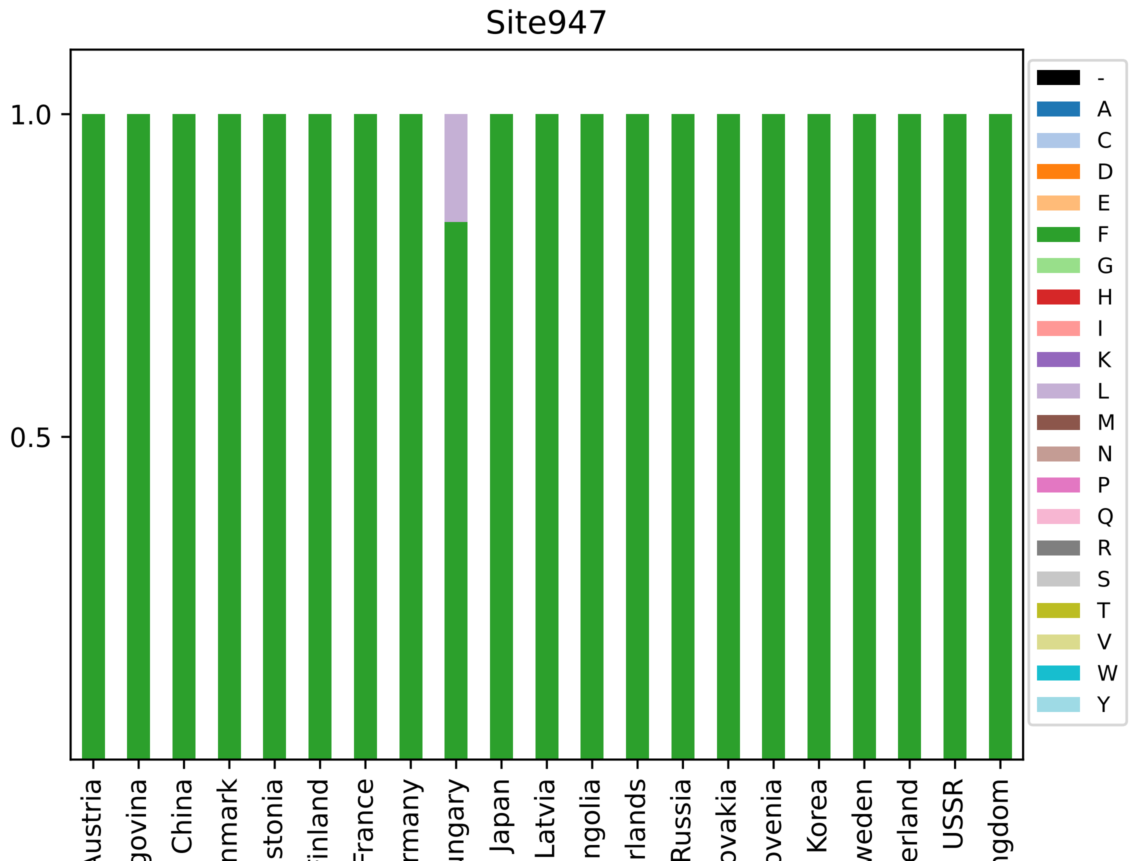
Site947
1.0
0.5
-
A
C
D
E
F
G
H
I
K
L
M
N
P
Q
R
S
T
V
W
Y
Austria
govina
China
nmark
stonia
Finland
France
rmany
ungary
Japan
Latvia
ngolia
rlands
Russia
ovakia
ovenia
Korea
weden
erland
USSR
ngdom

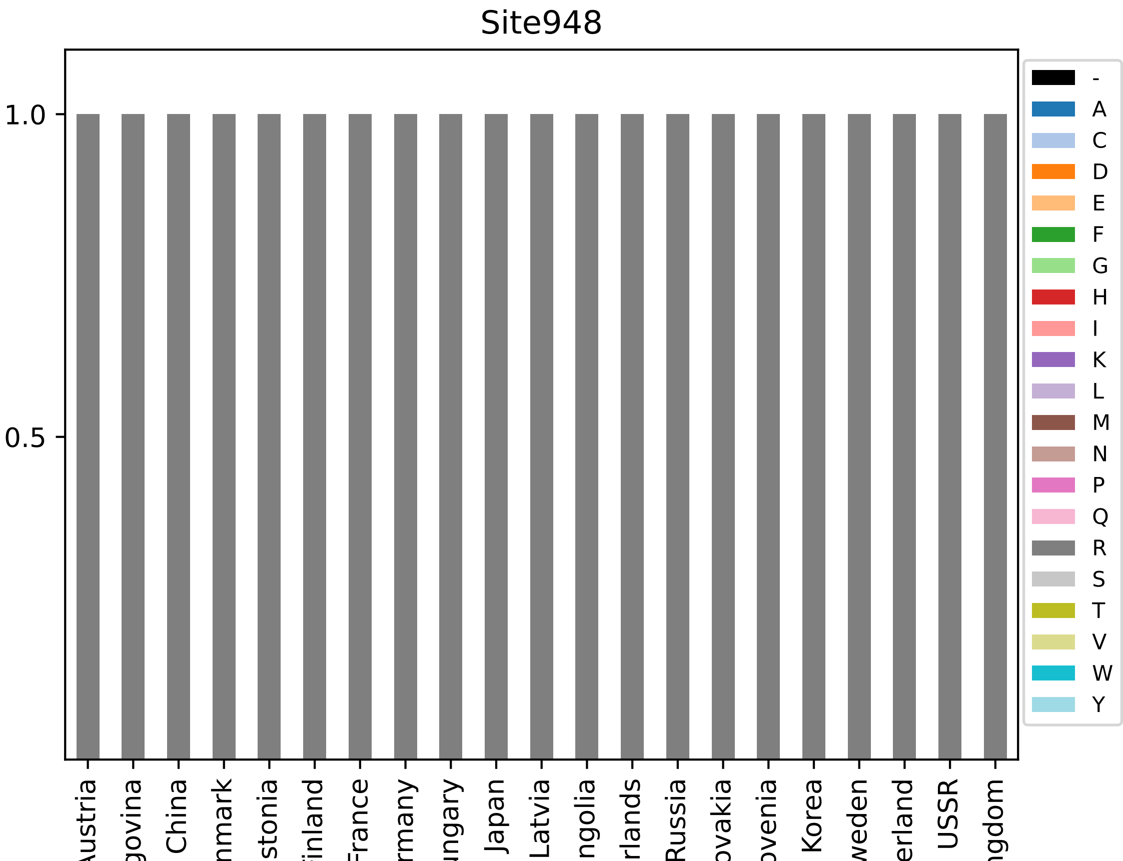

Site948
1.0
0.5
-
A
C
D
E
F
G
H
I
K
L
M
N
P
Q
R
S
T
V
W
Y
Austria
China
Japan
Latvia
Russia
Korea
USSR

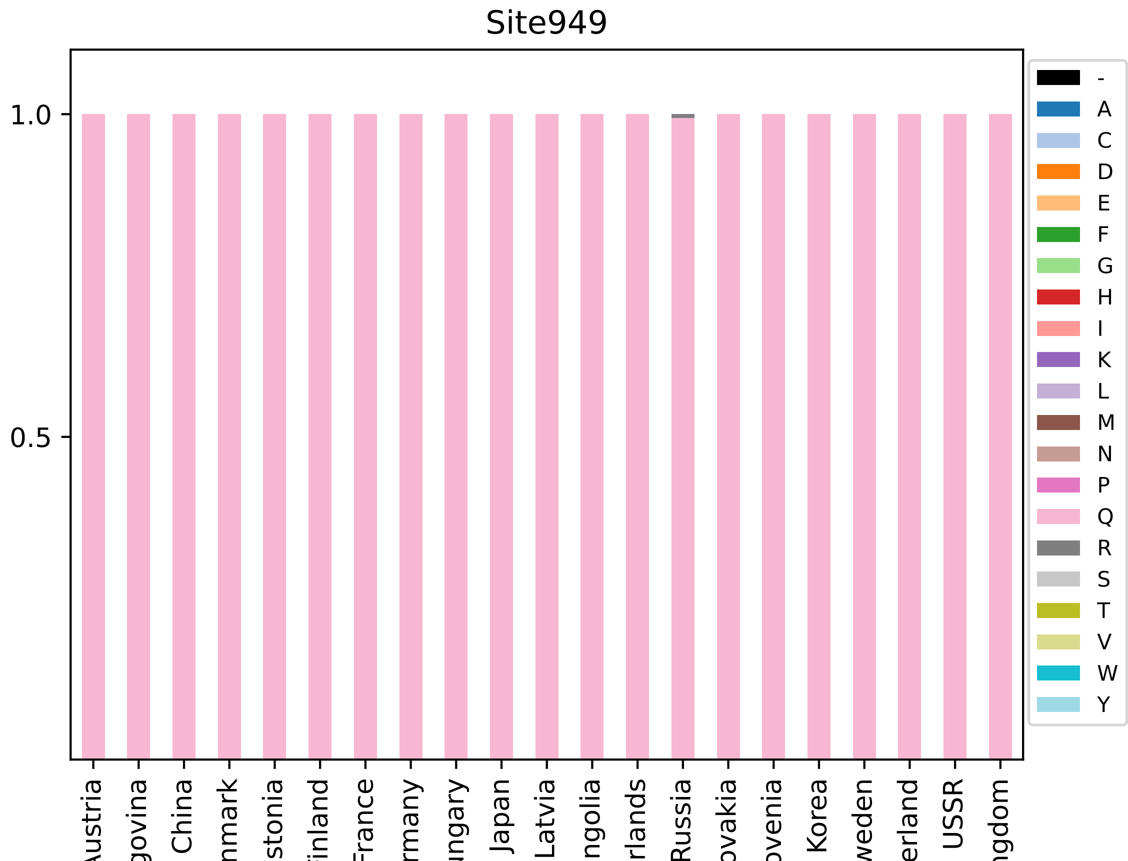

Site949
1.0
0.5
-
A
C
D
E
F
G
H
I
K
L
M
N
P
Q
R
S
T
V
W
Y
Austria
govina
China
nmark
stonia
Finland
France
rmany
ungary
Japan
Latvia
ngolia
rlands
Russia
ovakia
ovenia
Korea
weden
erland
USSR
ngdom

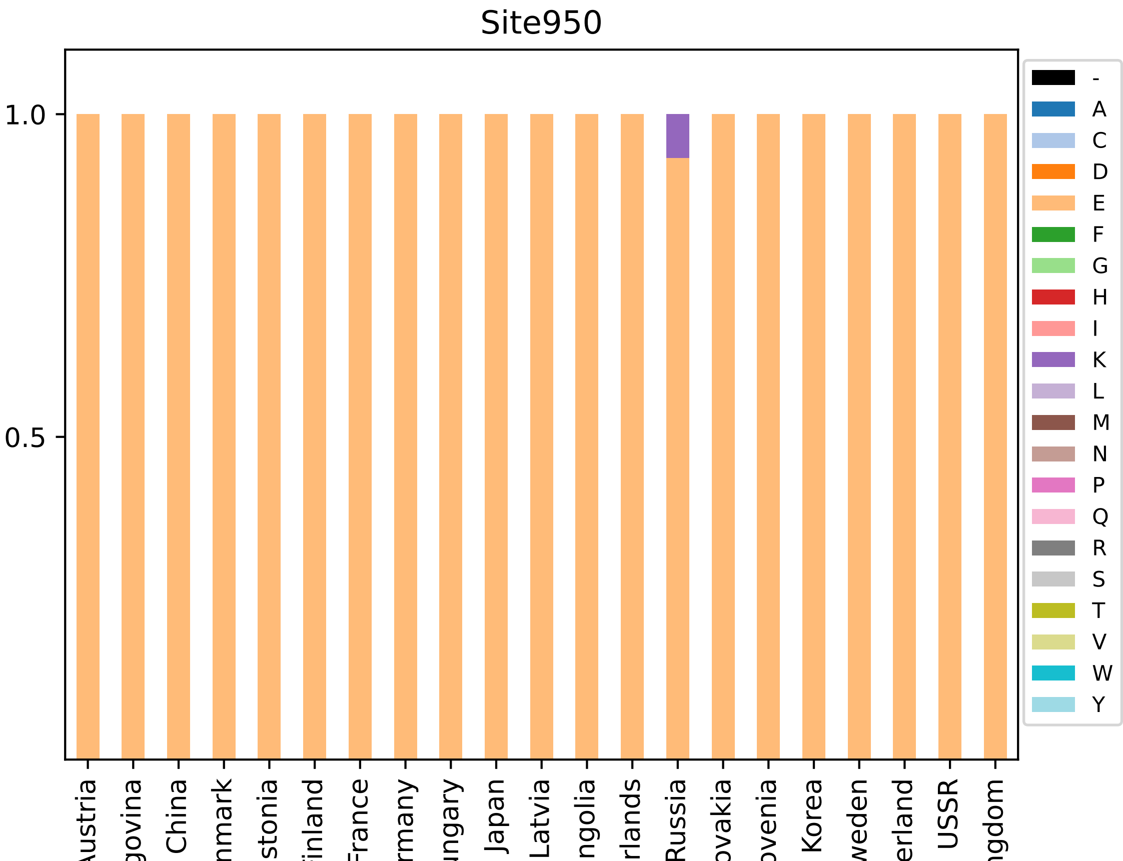
Site950
1.0
0.5
-
A
C
D
E
F
G
H
I
K
L
M
N
P
Q
R
S
T
V
W
Y
Austria
govina
China
nmark
stonia
Finland
France
rmany
ungary
Japan
Latvia
ngolia
rlands
Russia
ovakia
ovenia
Korea
weden
erland
USSR
ngdom

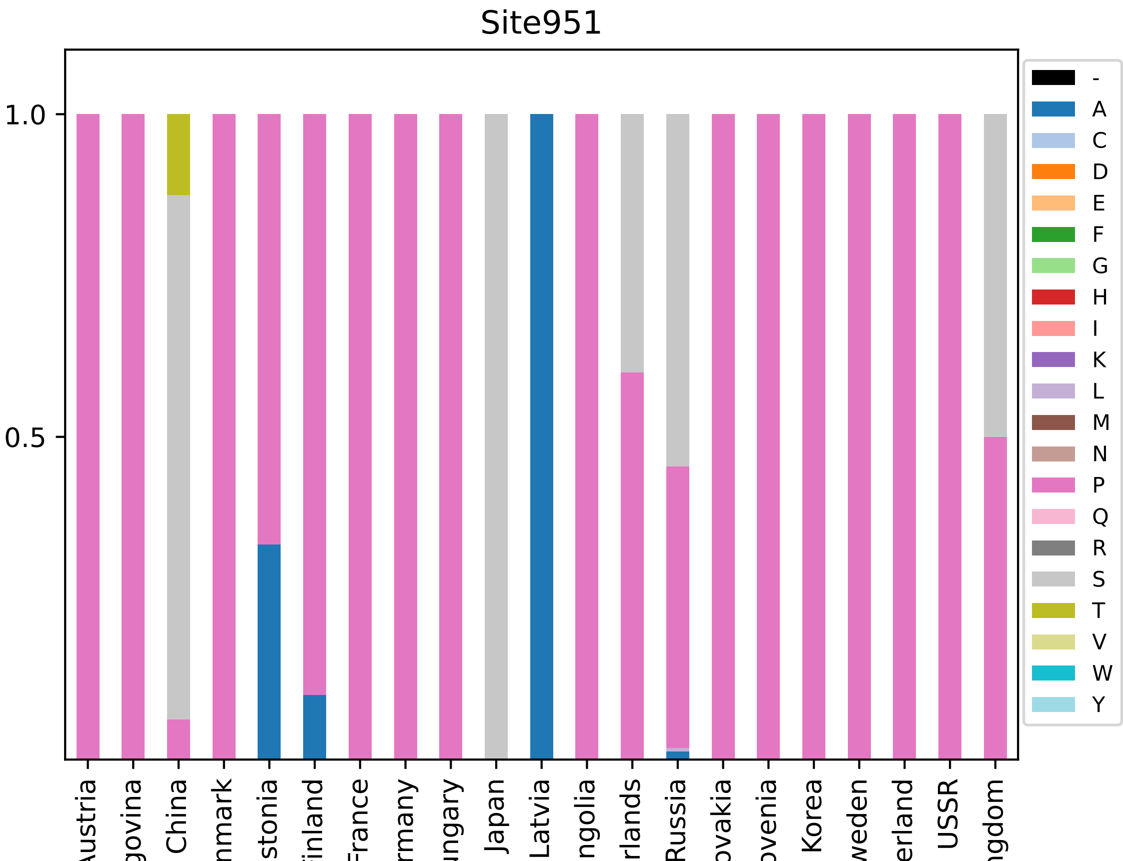
Site951
1.0
0.5
-
A
C
D
E
F
G
H
I
K
L
M
N
P
Q
R
S
T
V
W
Y
Austria
govina
China
nmark
stonia
inland
France
rmany
ungary
Japan
Latvia
ngolia
rlands
Russia
ovakia
ovenia
Korea
weden
erland
USSR
ngdom

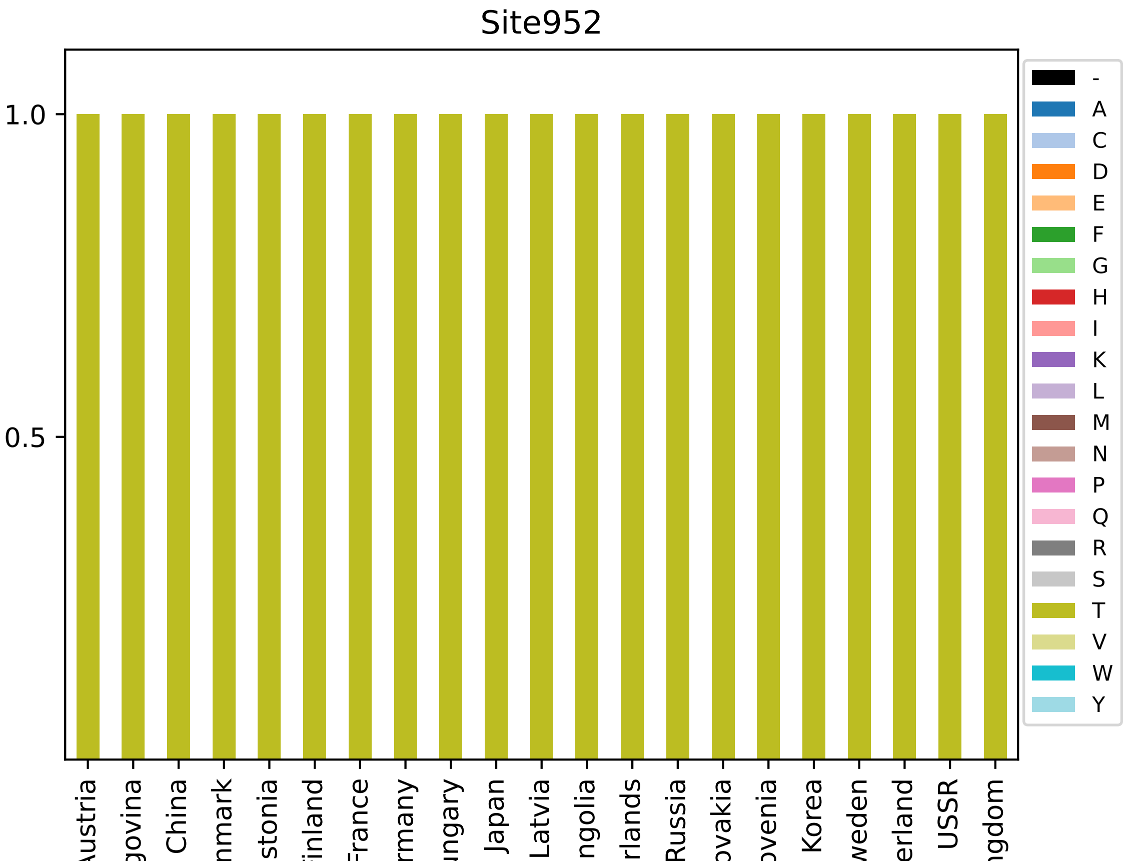

Site952
1.0
0.5
-
A
C
D
E
F
G
H
I
K
L
M
N
P
Q
R
S
T
V
W
Y
Austria
govina
China
nmark
stonia
inland
France
rmany
ungary
Japan
Latvia
ngolia
rlands
Russia
ovakia
ovenia
Korea
weden
erland
USSR
ngdom

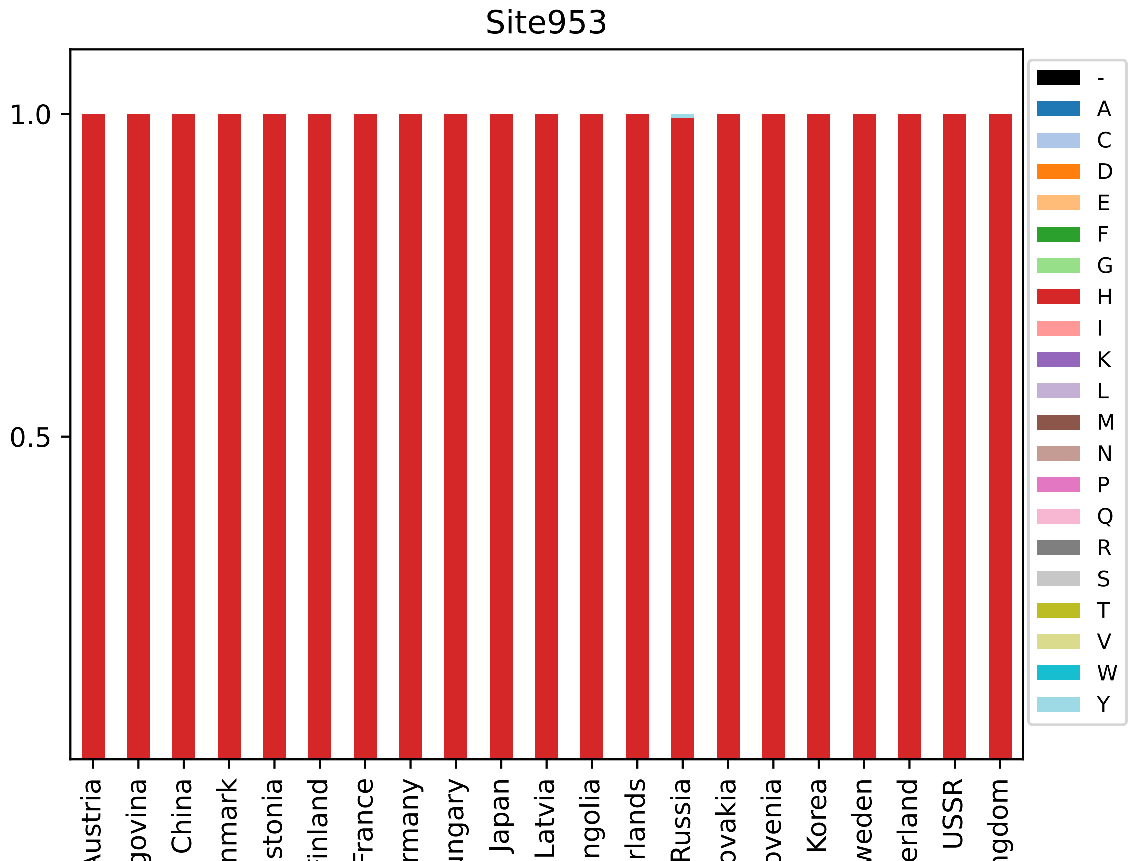
Site953
1.0
0.5
-
A
C
D
E
F
G
H
I
K
L
M
N
P
Q
R
S
T
V
W
Y
China
Japan
Latvia
Russia
Korea
USSR

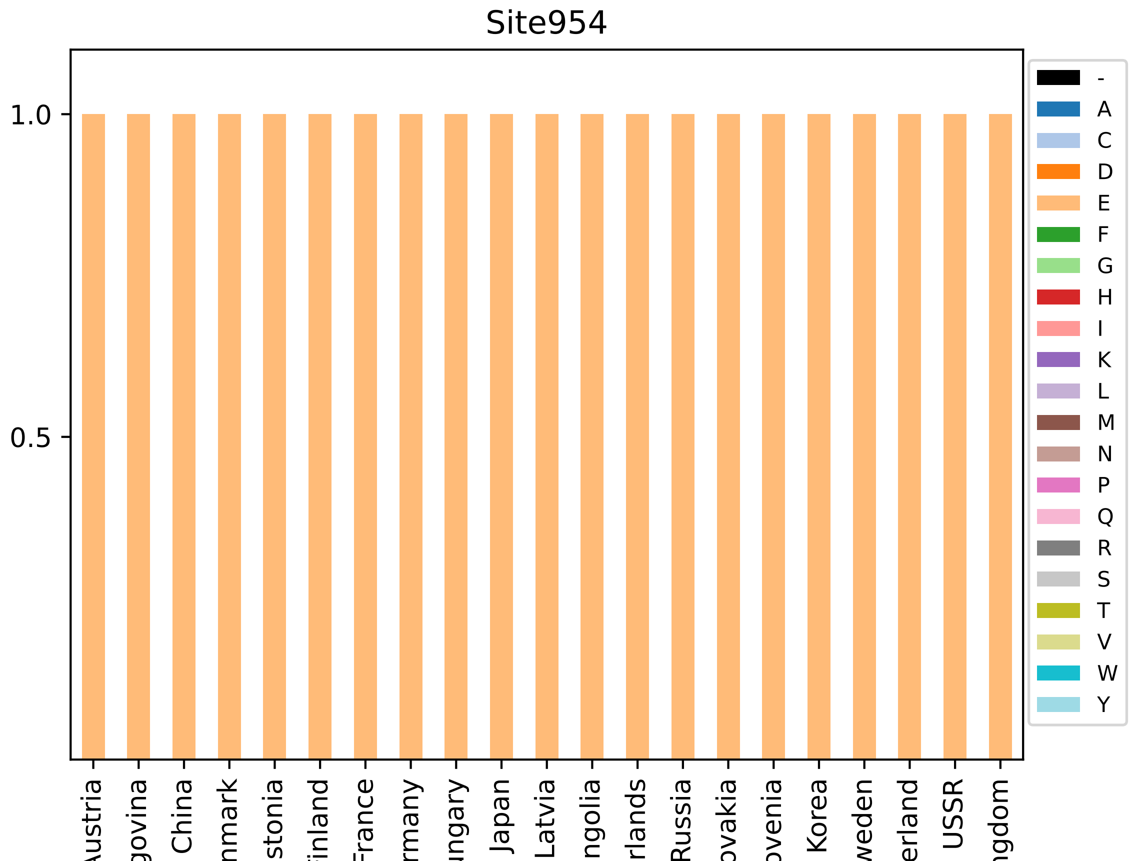

Site954
1.0
0.5
-
A
C
D
E
F
G
H
I
K
L
M
N
P
Q
R
S
T
V
W
Y
Austria
govina
China
nmark
stonia
inland
France
rmany
ungary
Japan
Latvia
ngolia
rlands
Russia
ovakia
ovenia
Korea
weden
erland
USSR
ngdom

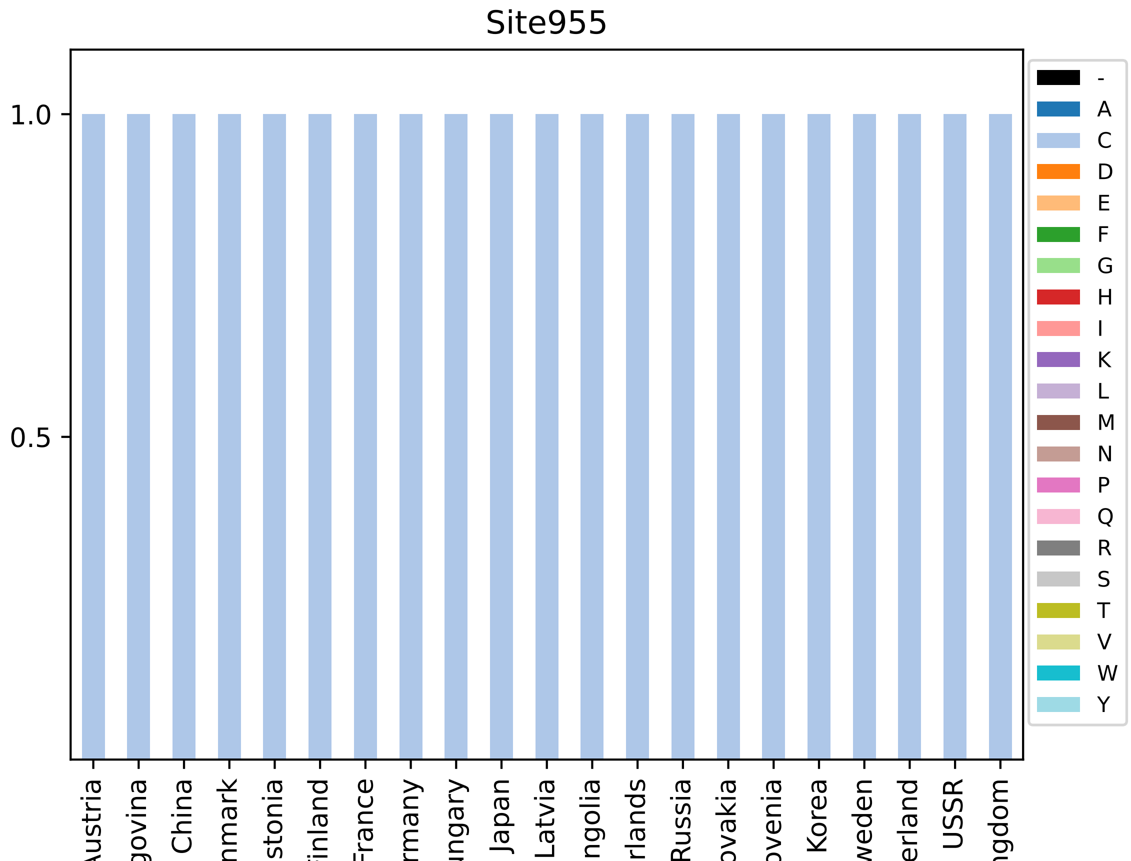
Site955
1.0
0.5
-
A
C
D
E
F
G
H
I
K
L
M
N
P
Q
R
S
T
V
W
Y
Austria
China
Japan
Latvia
Russia
Korea
USSR

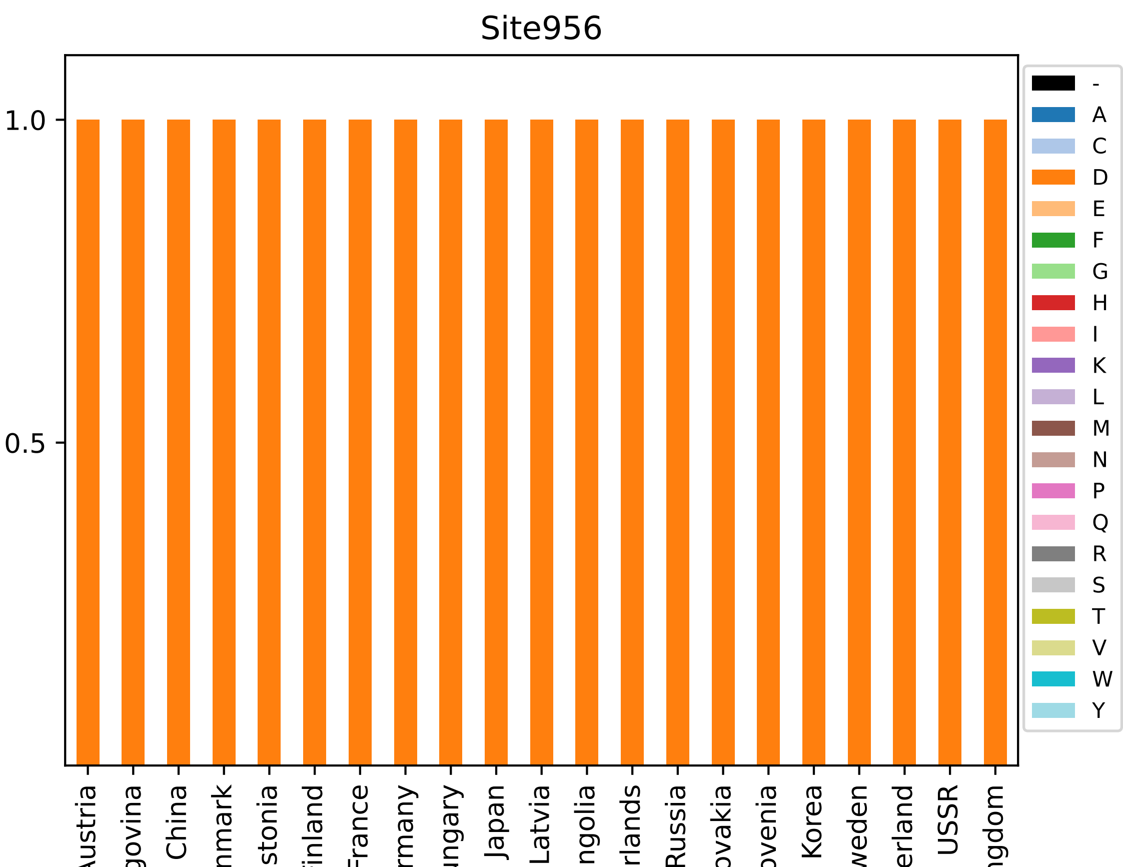
Site956
1.0
0.5
-
A
C
D
E
F
G
H
I
K
L
M
N
P
Q
R
S
T
V
W
Y
China
Japan
Latvia
Russia
Korea
USSR

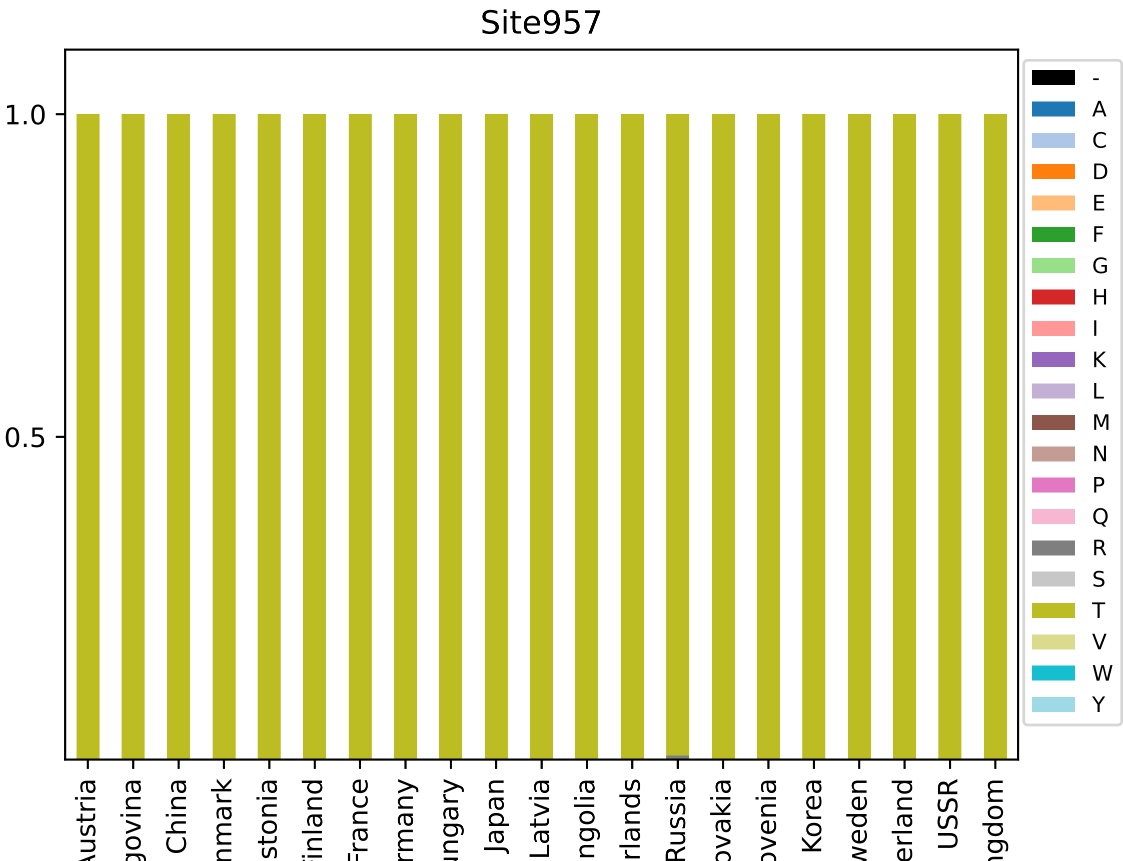
Site957
1.0
0.5
-
A
C
D
E
F
G
H
I
K
L
M
N
P
Q
R
S
T
V
W
Y
Austria
govina
China
nmark
stonia
inland
France
rmany
ungary
Japan
Latvia
ngolia
rlands
Russia
ovakia
ovenia
Korea
weden
erland
USSR
ngdom

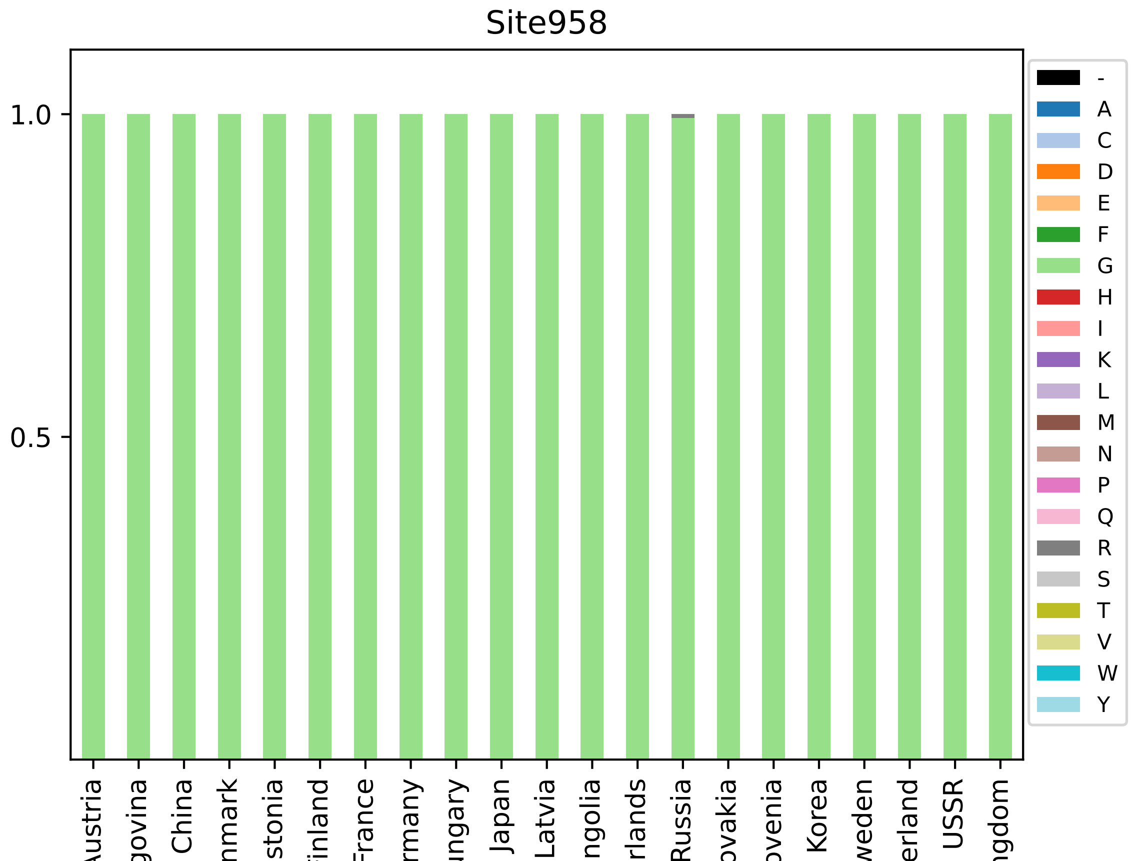

Site958
1.0
0.5
China
Japan
Latvia
Russia
Korea
USSR
-
A
C
D
E
F
G
H
I
K
L
M
N
P
Q
R
S
T
V
W
Y

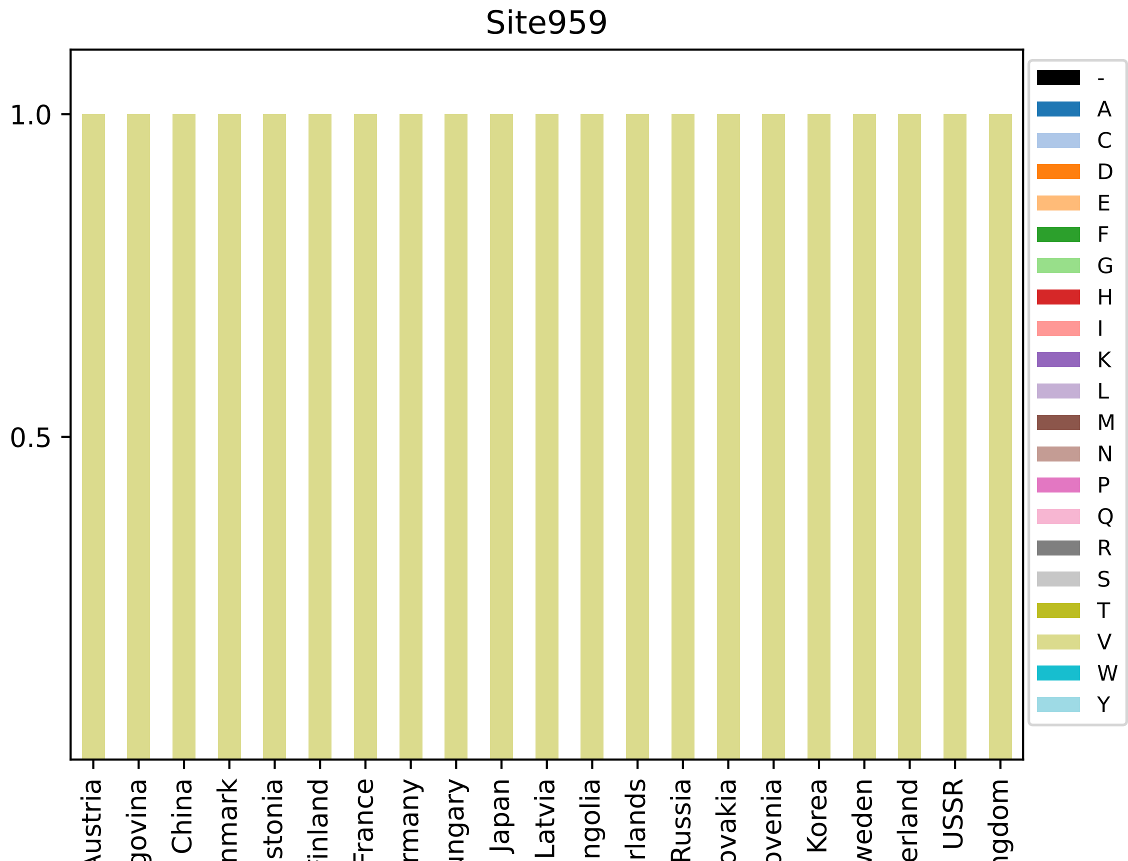

Site959
1.0
0.5
-
A
C
D
E
F
G
H
I
K
L
M
N
P
Q
R
S
T
V
W
Y
China
Japan
Latvia
Russia
Korea
USSR

Site960

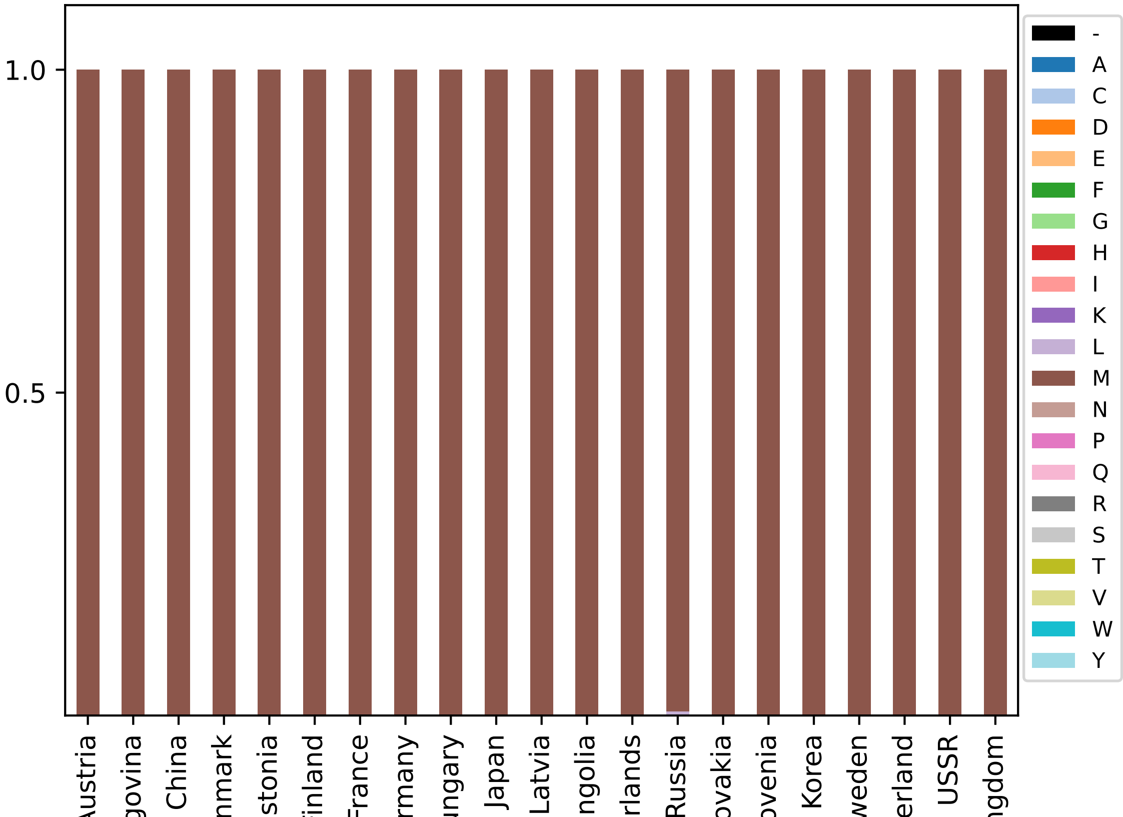
1.0
0.5
-
A
C
D
E
F
G
H
I
K
L
M
N
P
Q
R
S
T
V
W
Y
China
Japan
Latvia
Russia
Korea
USSR

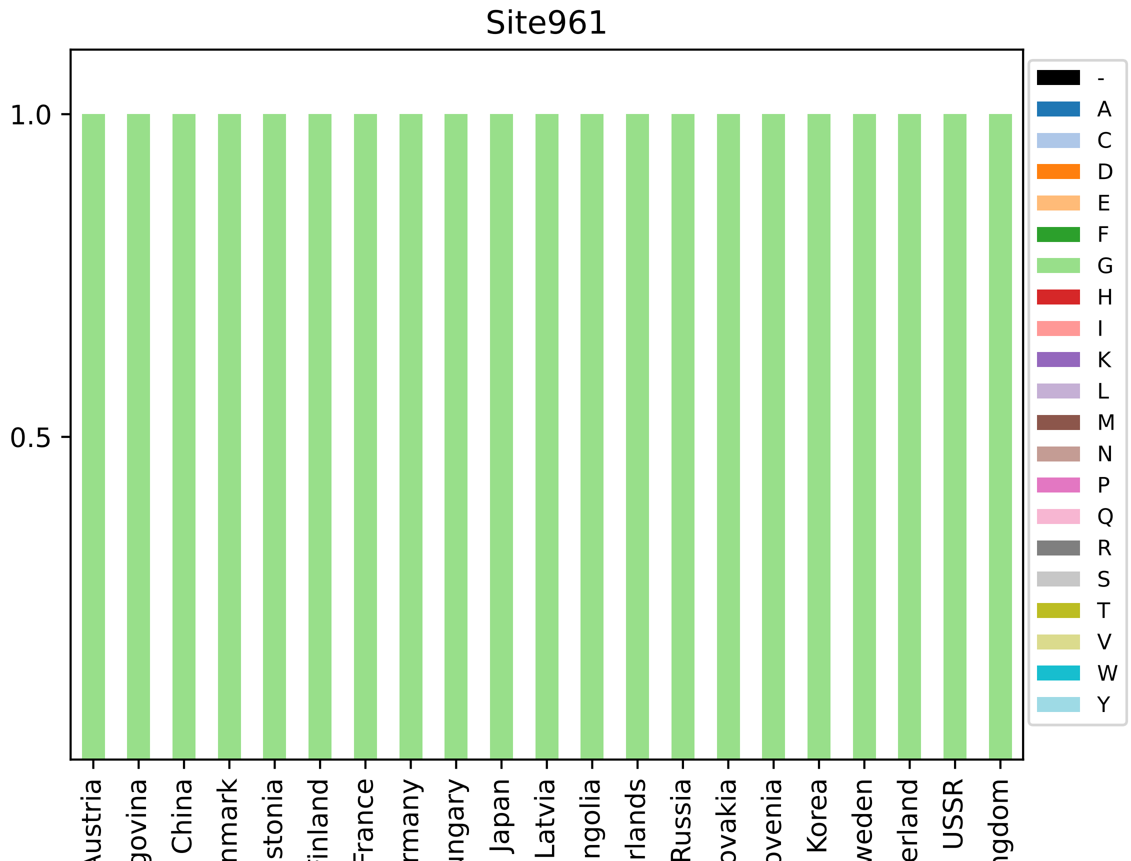
Site961
1.0
0.5
-
A
C
D
E
F
G
H
I
K
L
M
N
P
Q
R
S
T
V
W
Y
China
Japan
Latvia
Russia
Korea
USSR

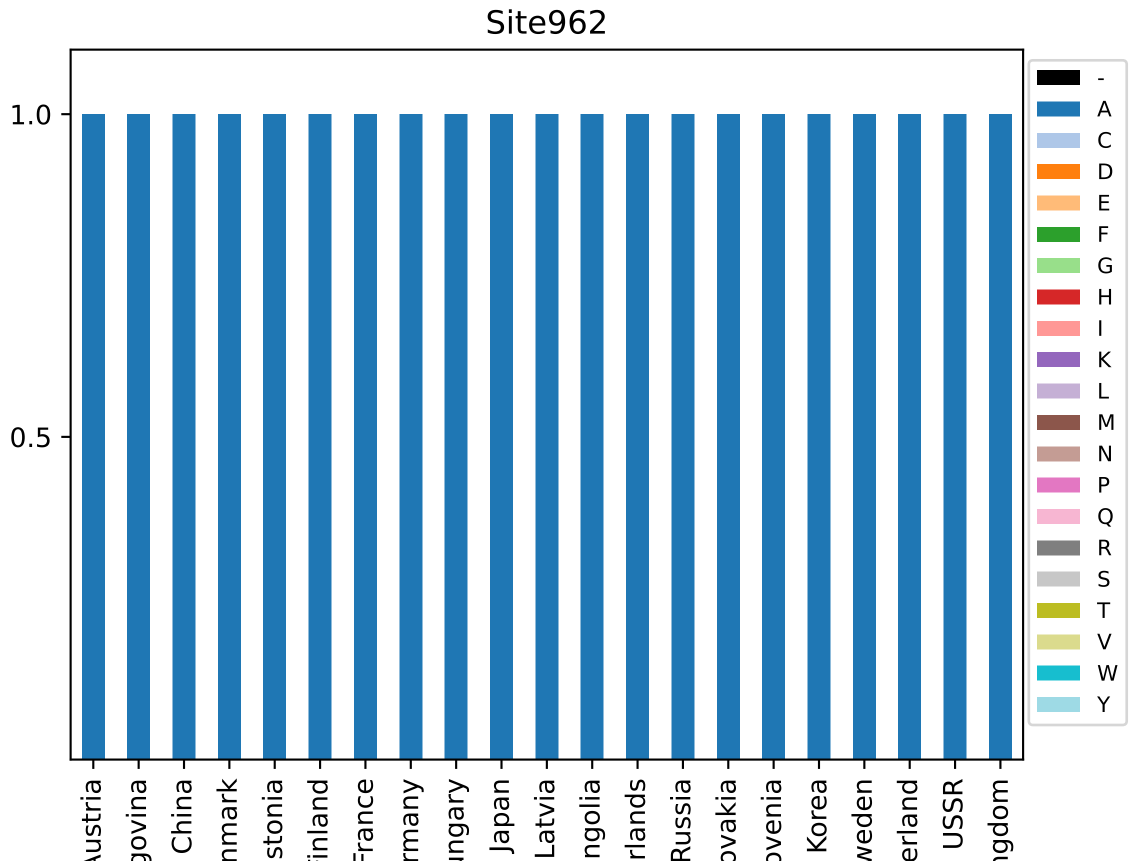

Site962
1.0
0.5
-
A
C
D
E
F
G
H
I
K
L
M
N
P
Q
R
S
T
V
W
Y
Austria
govina
China
nmark
stonia
Finland
France
rmany
ungary
Japan
Latvia
ngolia
rlands
Russia
ovakia
ovenia
Korea
weden
erland
USSR
ngdom

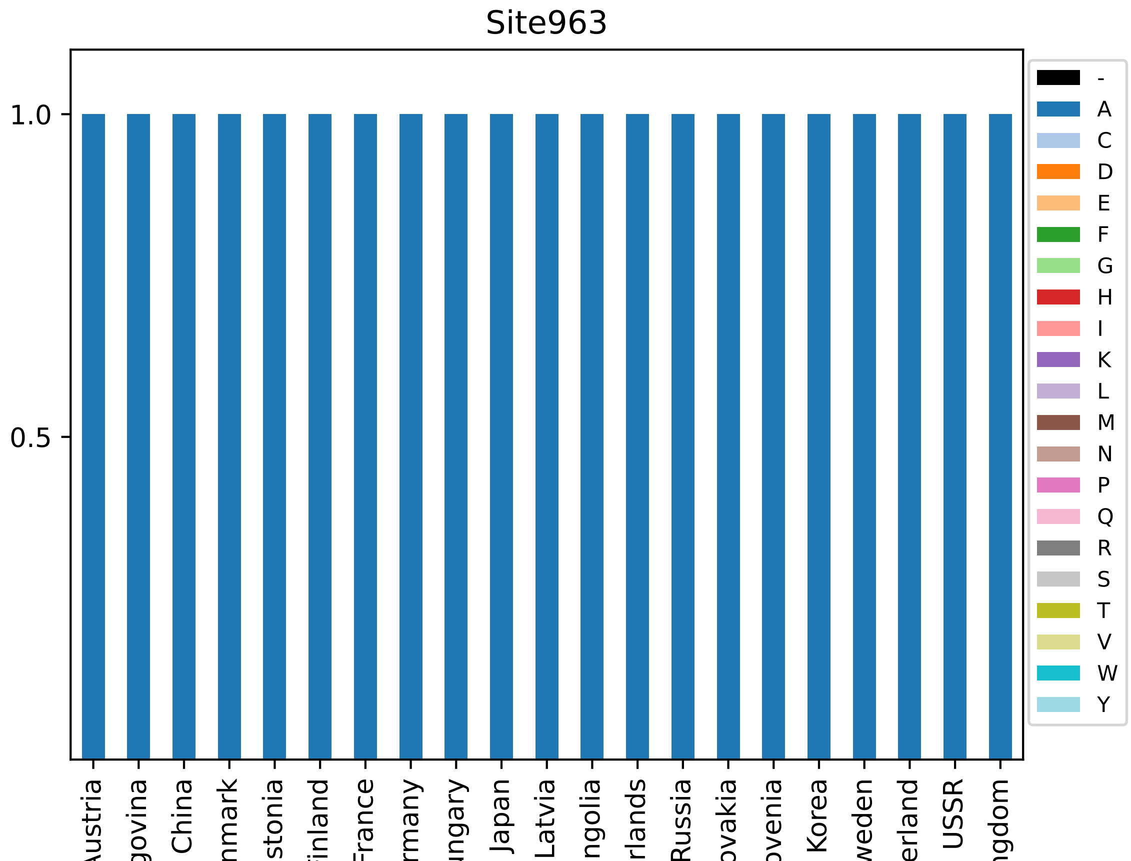

Site963
1.0
0.5
Austria
govina
China
nmark
stonia
inland
France
rmany
ungary
Japan
Latvia
ngolia
rlands
Russia
ovakia
ovenia
Korea
weden
erland
USSR
ngdom
-
A
C
D
E
F
G
H
I
K
L
M
N
P
Q
R
S
T
V
W
Y

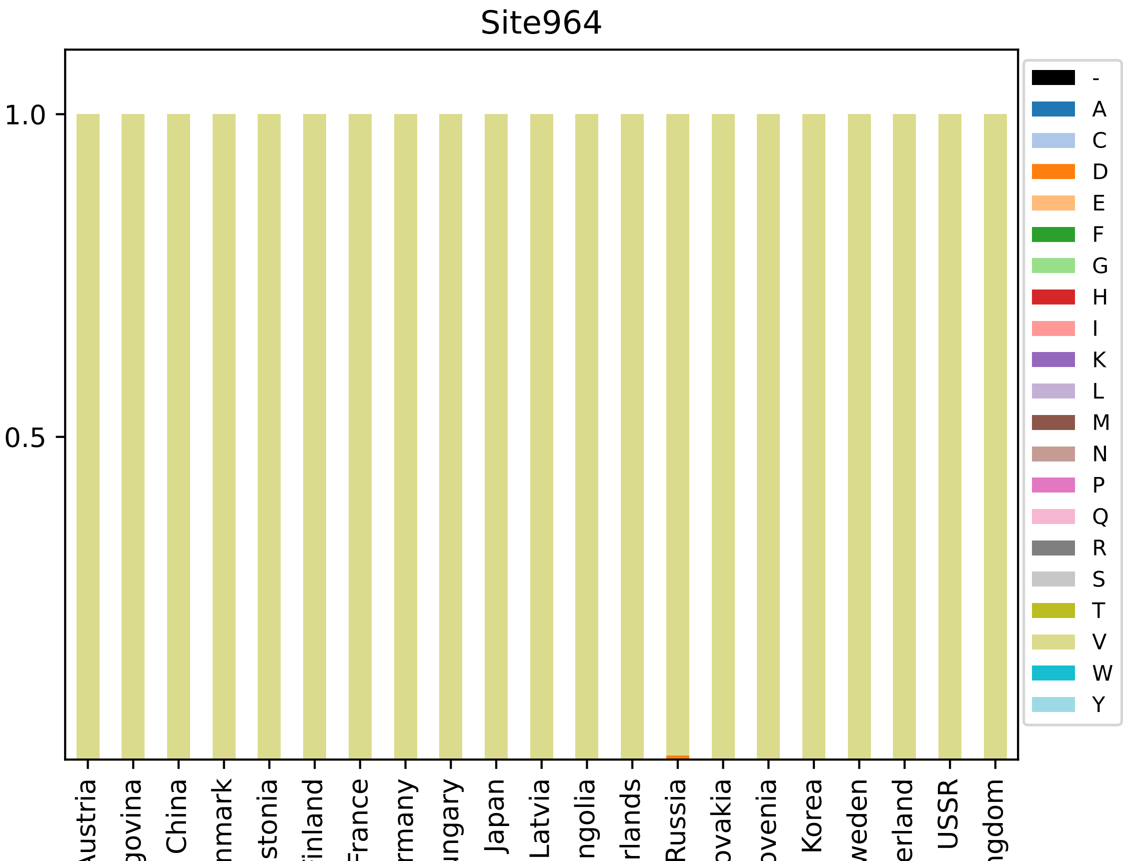

Site964
1.0
0.5
-
A
C
D
E
F
G
H
I
K
L
M
N
P
Q
R
S
T
V
W
Y
Austria
China
Japan
Latvia
Russia
Korea
USSR

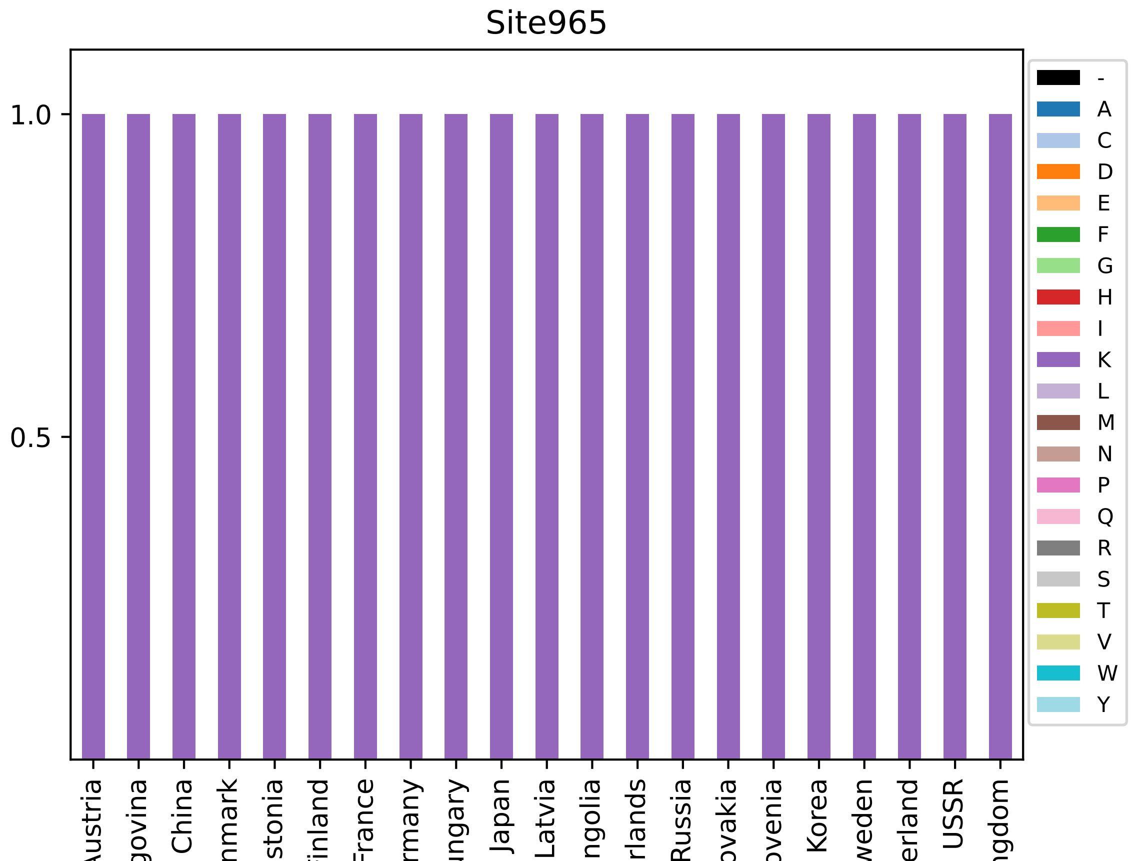
Site965
1.0
0.5
-
A
C
D
E
F
G
H
I
K
L
M
N
P
Q
R
S
T
V
W
Y
Austria
China
Finland
France
Japan
Latvia
Russia
Korea
USSR

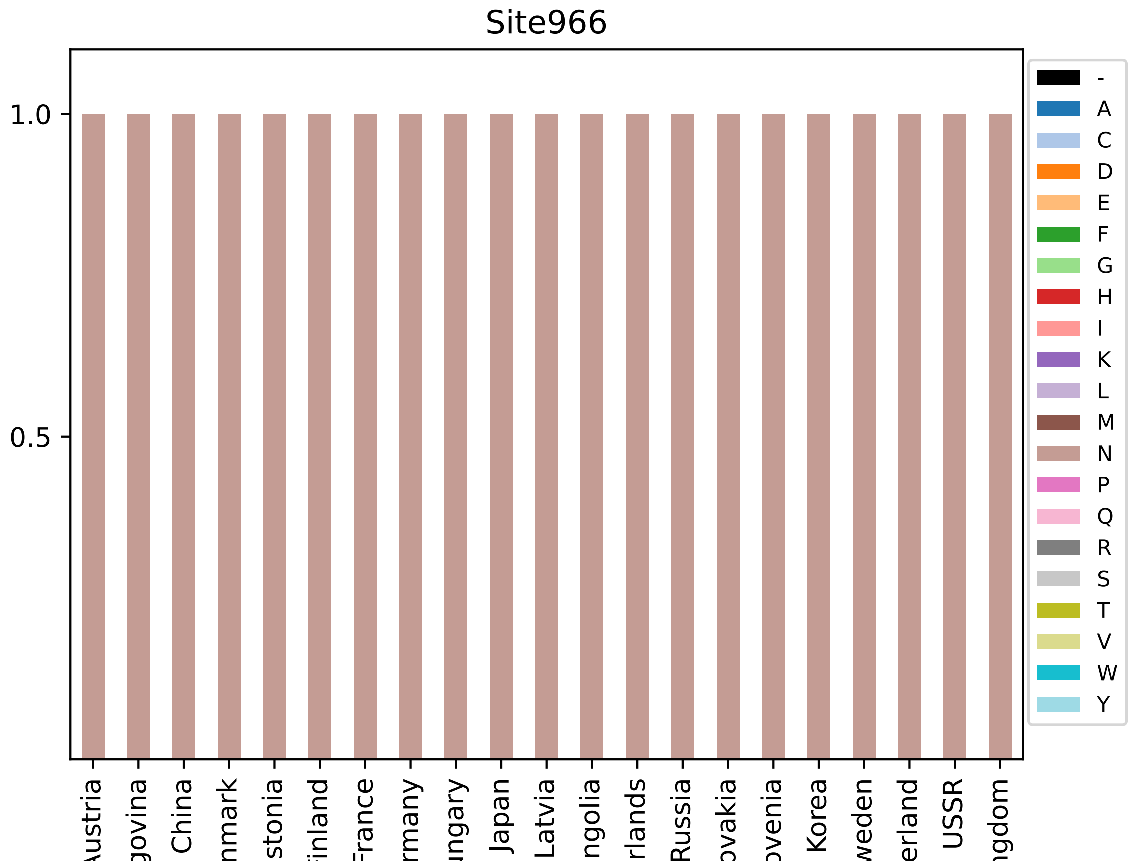

Site966
1.0
0.5
-
A
C
D
E
F
G
H
I
K
L
M
N
P
Q
R
S
T
V
W
Y
Japan
Latvia
Russia
Korea
USSR
China

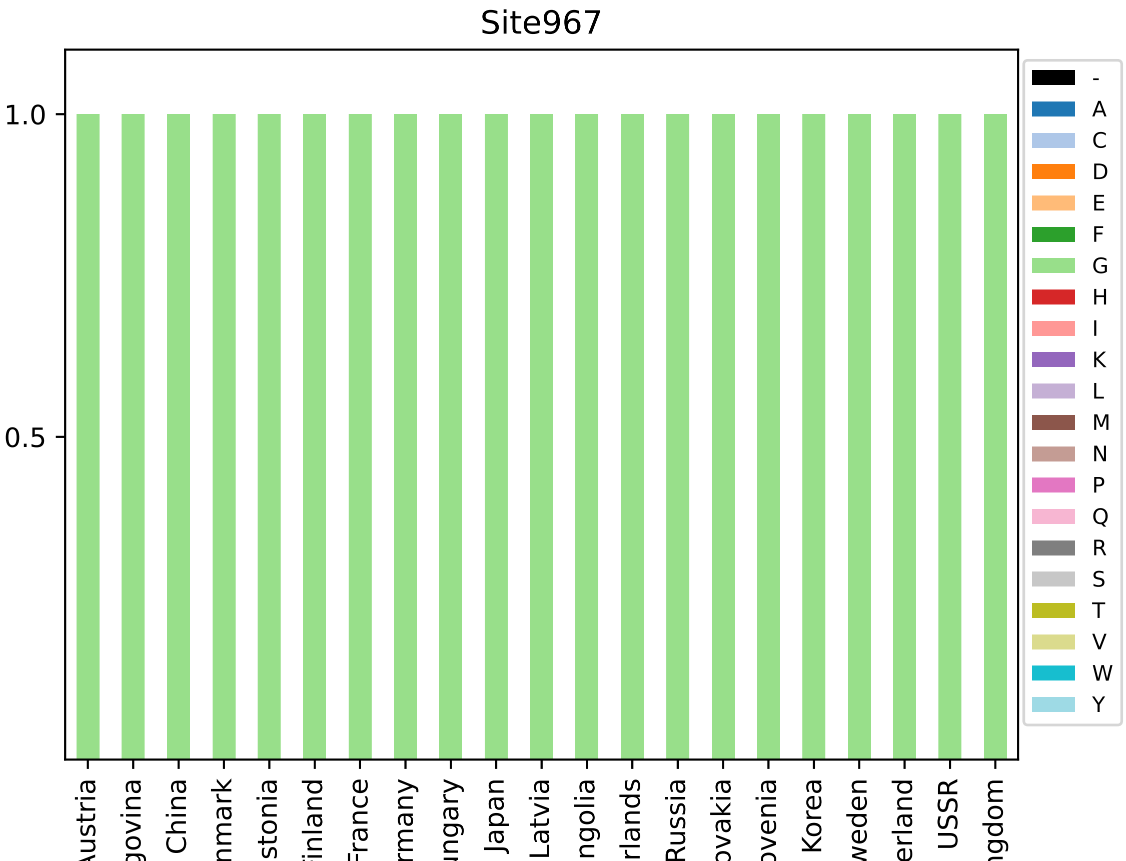

Site967
1.0
0.5
-
A
C
D
E
F
G
H
I
K
L
M
N
P
Q
R
S
T
V
W
Y
ustria
govina
China
nmark
stonia
inland
rance
rmany
ungary
Japan
Latvia
ngolia
rlands
Russia
ovakia
ovenia
Korea
weden
erland
USSR
ngdom

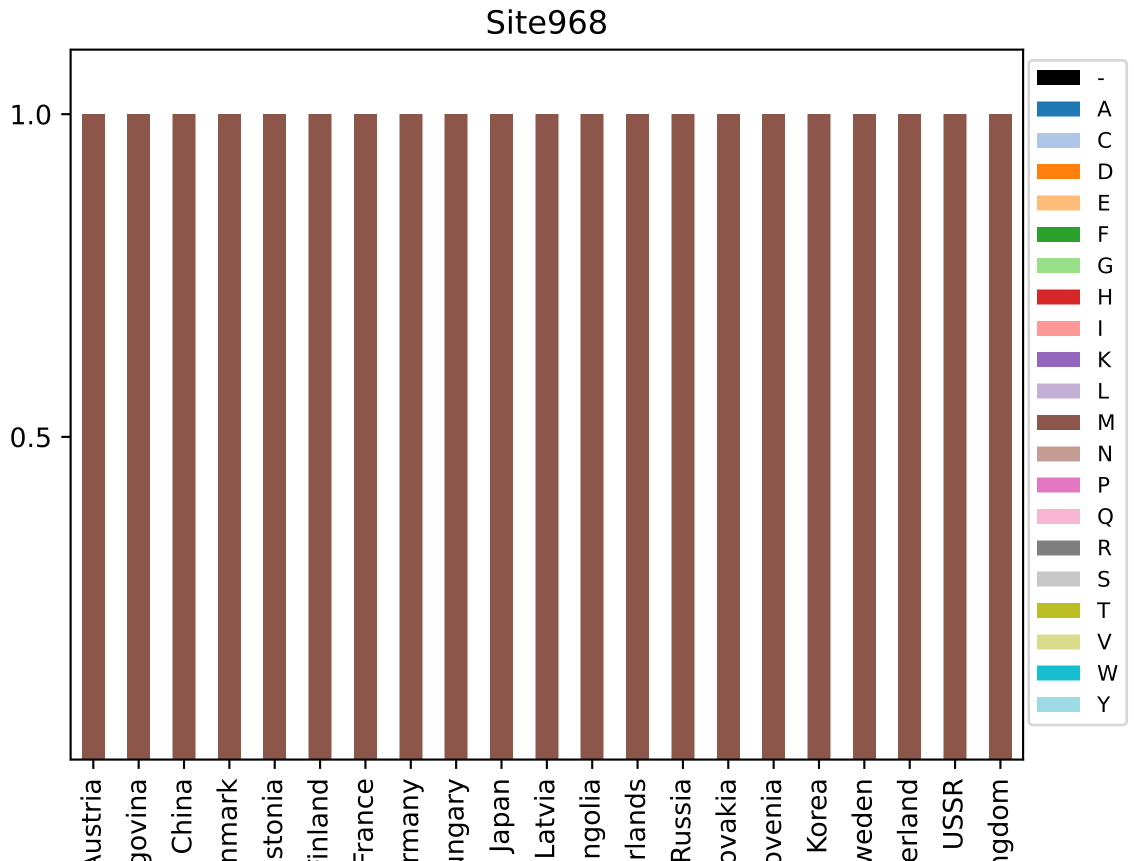
Site968
1.0
0.5
-
A
C
D
E
F
G
H
I
K
L
M
N
P
Q
R
S
T
V
W
Y
China
Japan
Latvia
Russia
Korea
USSR

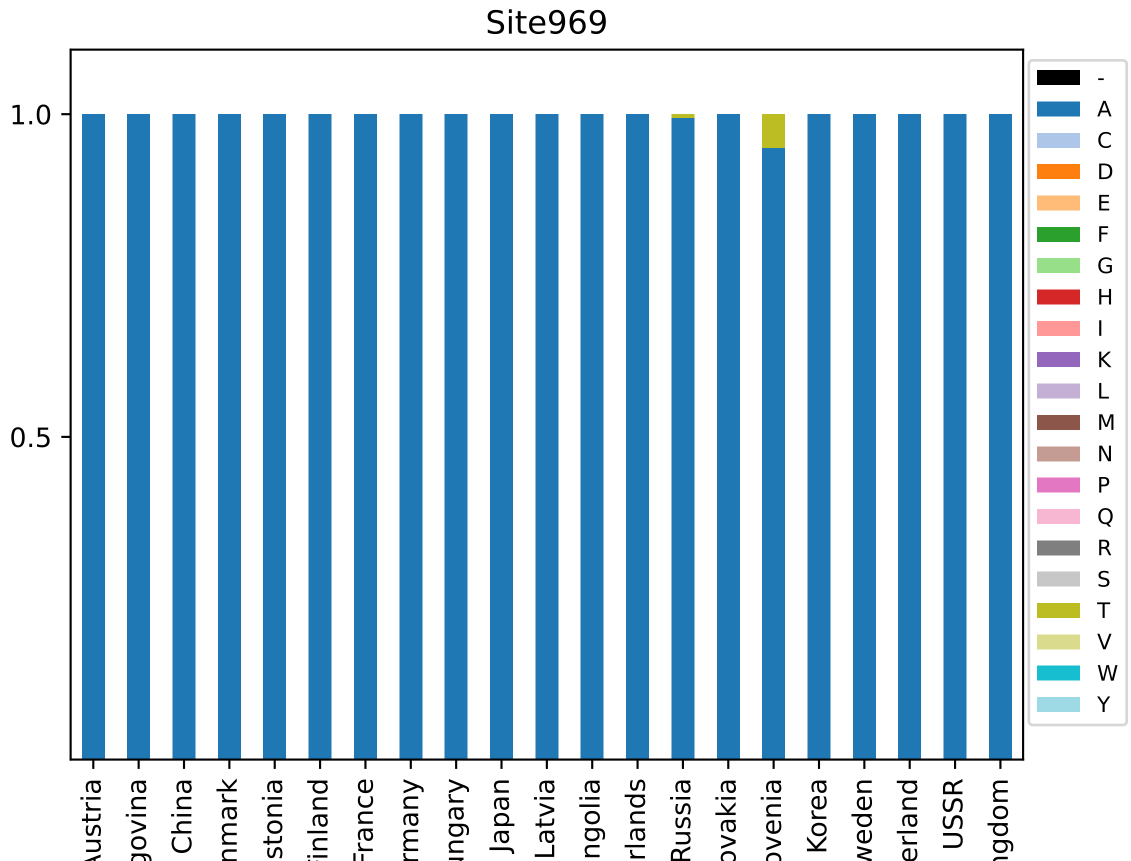
Site969
1.0
0.5
-
A
C
D
E
F
G
H
I
K
L
M
N
P
Q
R
S
T
V
W
Y
Austria
govina
China
nmark
stonia
Finland
France
rmany
ungary
Japan
Latvia
ngolia
rlands
Russia
ovakia
ovenia
Korea
weden
erland
USSR
ngdom

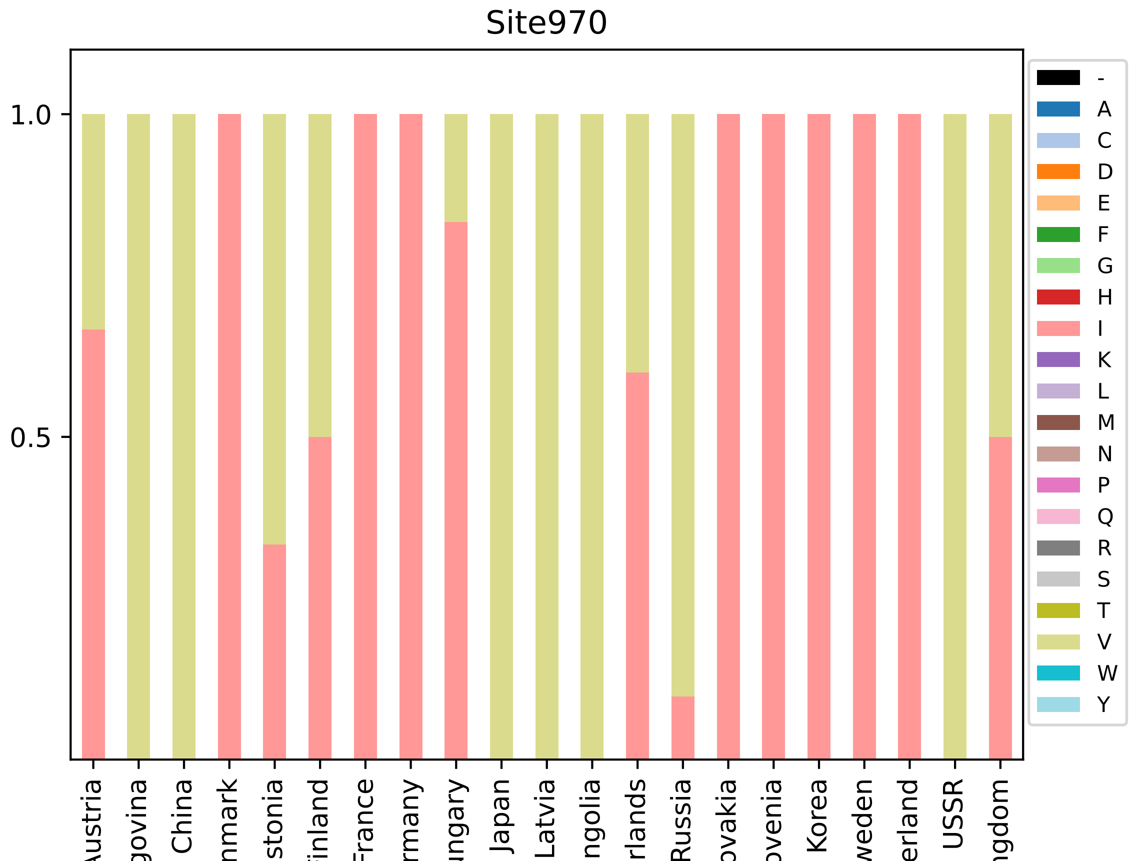
Site970
1.0
0.5
-
A
C
D
E
F
G
H
I
K
L
M
N
P
Q
R
S
T
V
W
Y
China
Japan
Latvia
Russia
Korea
USSR

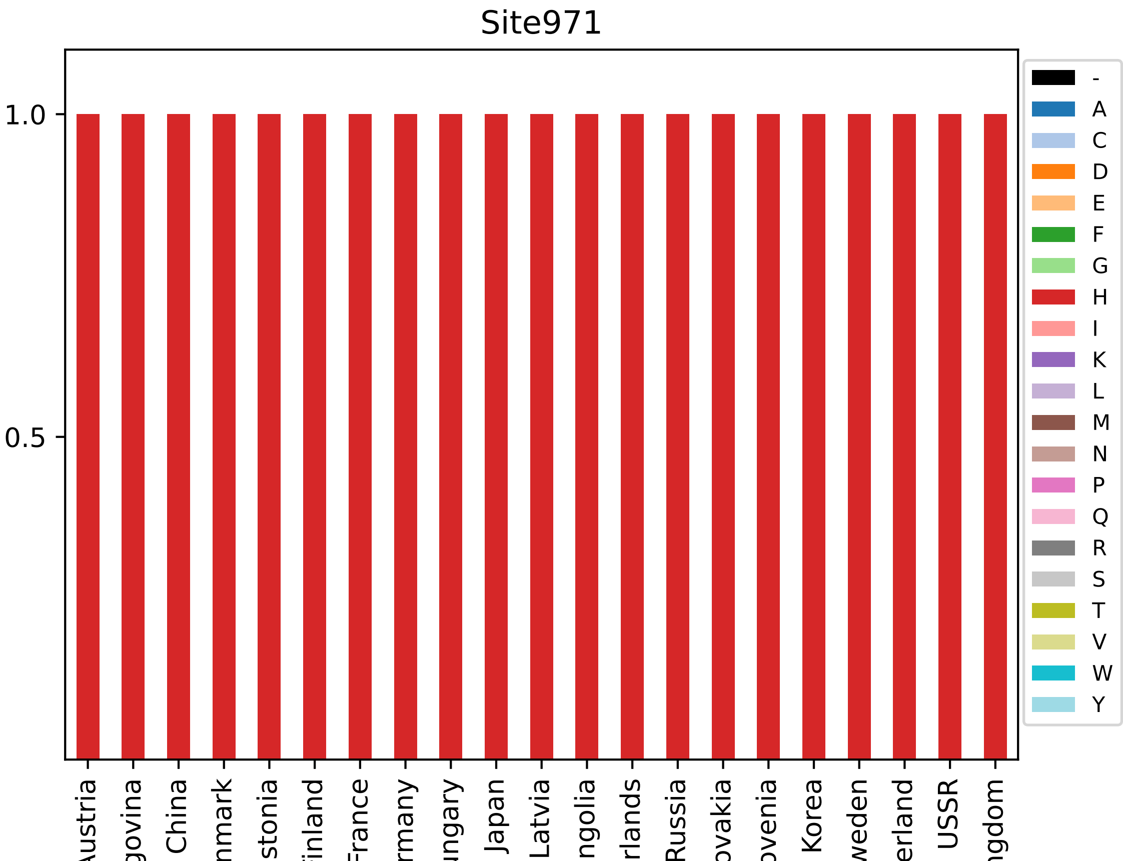
Site971
1.0
0.5
-
A
C
D
E
F
G
H
I
K
L
M
N
P
Q
R
S
T
V
W
Y
Austria
govina
China
nmark
stonia
Finland
France
rmany
ungary
Japan
Latvia
ngolia
rlands
Russia
ovakia
ovenia
Korea
weden
erland
USSR
ngdom

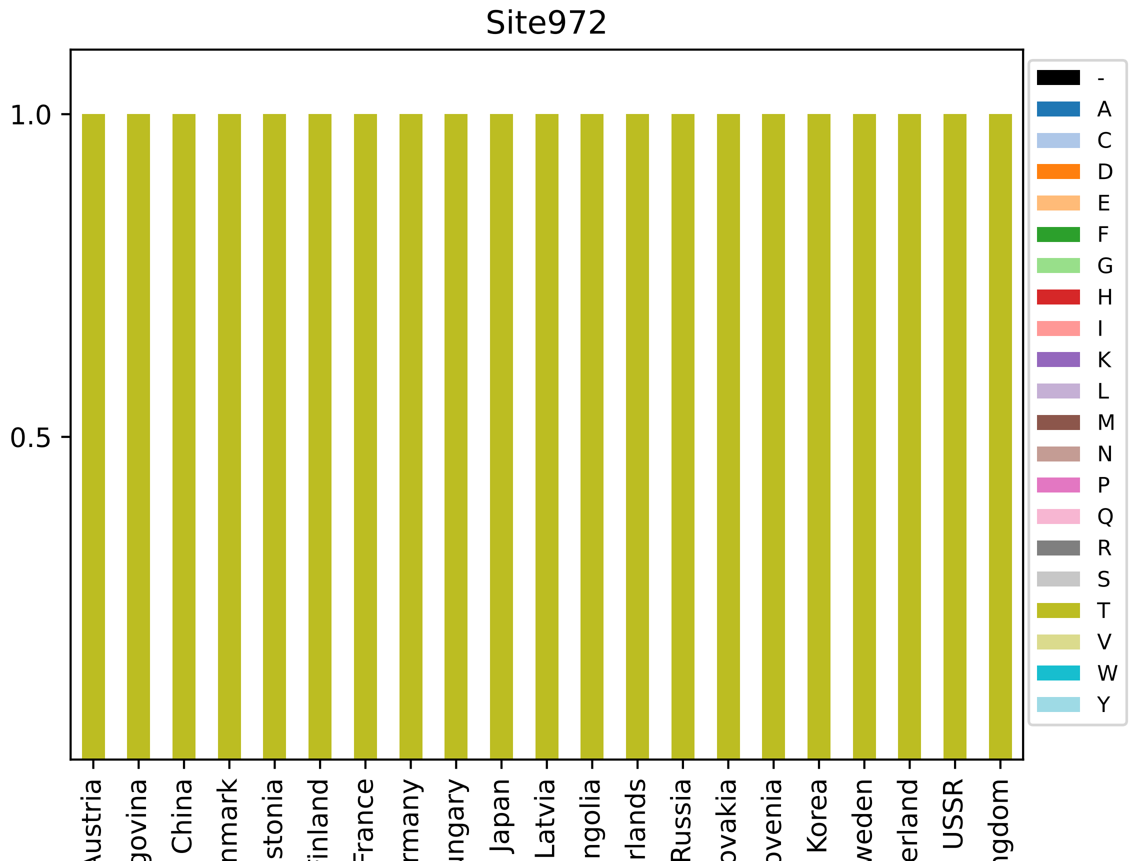

Site972
1.0
0.5
-
A
C
D
E
F
G
H
I
K
L
M
N
P
Q
R
S
T
V
W
Y
Austria
govina
China
nmark
stonia
Finland
France
rmany
ungary
Japan
Latvia
ngolia
rlands
Russia
ovakia
ovenia
Korea
weden
erland
USSR
ngdom

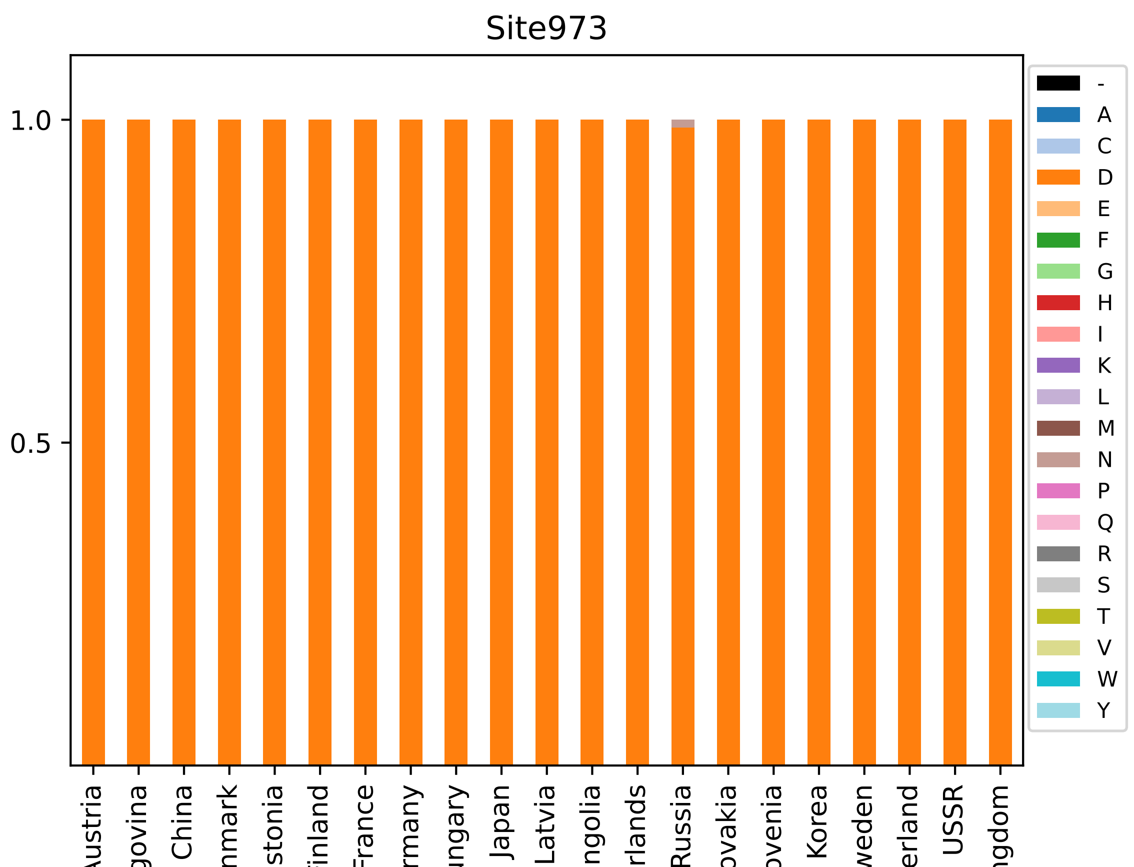

Site973
1.0
0.5
-
A
C
D
E
F
G
H
I
K
L
M
N
P
Q
R
S
T
V
W
Y
Austria
China
Japan
Latvia
Russia
Korea
USSR

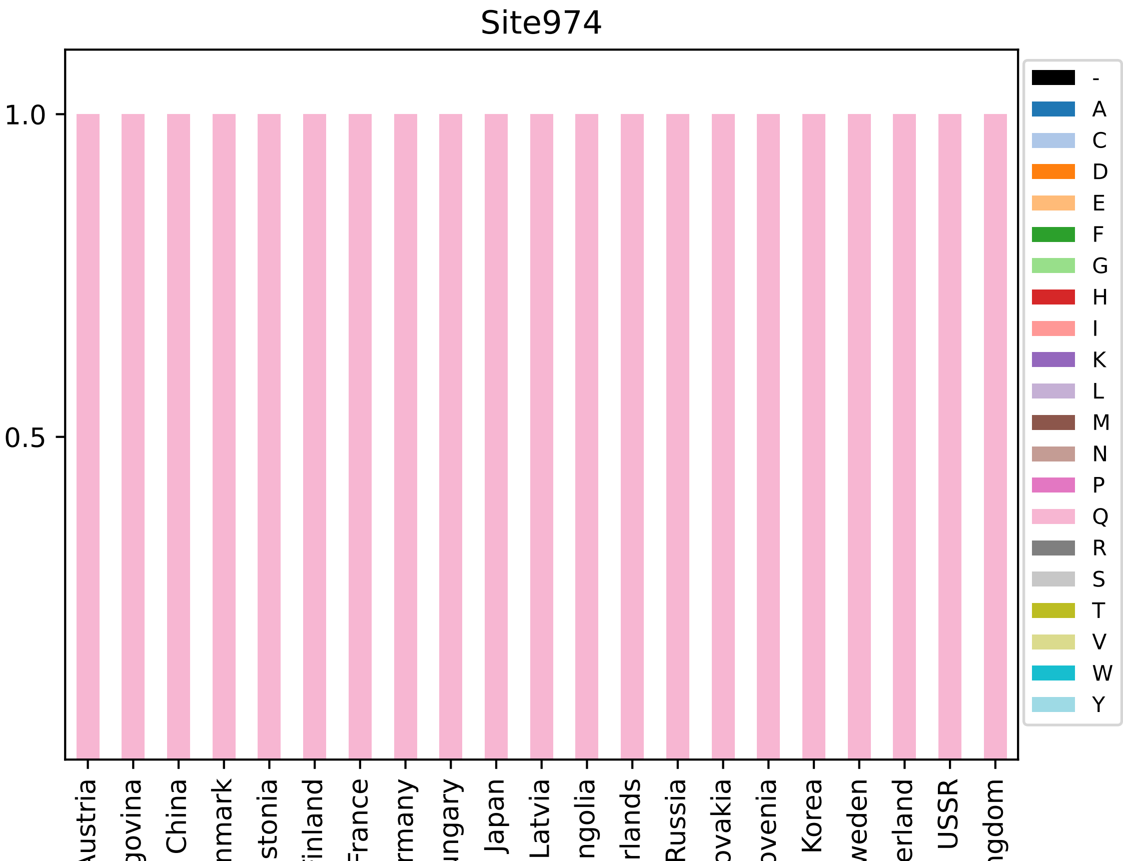

Site974
1.0
0.5
-
A
C
D
E
F
G
H
I
K
L
M
N
P
Q
R
S
T
V
W
Y
China
Japan
Latvia
Russia
Korea
USSR

Site975

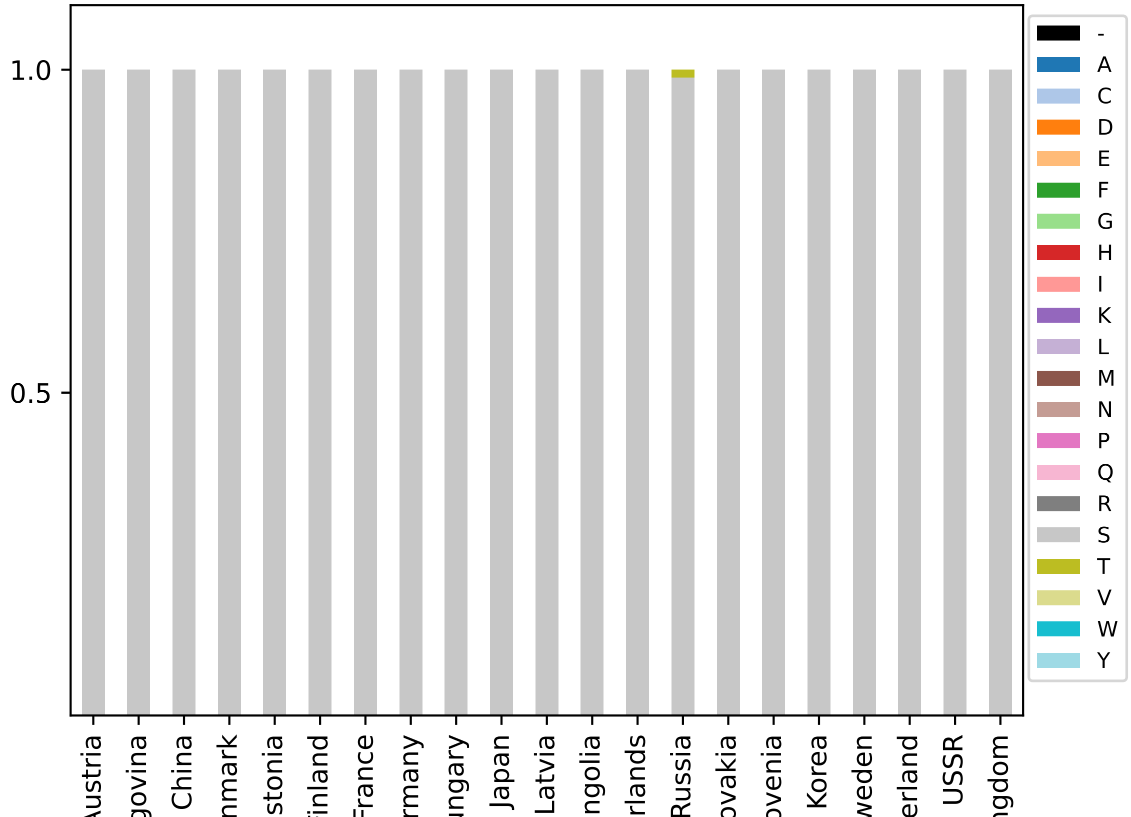

1.0
0.5
Austria
govina
China
nmark
stonia
Finland
France
rmany
ungary
Japan
Latvia
ngolia
rlands
Russia
ovakia
ovenia
Korea
weden
erland
USSR
ngdom
-
A
C
D
E
F
G
H
I
K
L
M
N
P
Q
R
S
T
V
W
Y

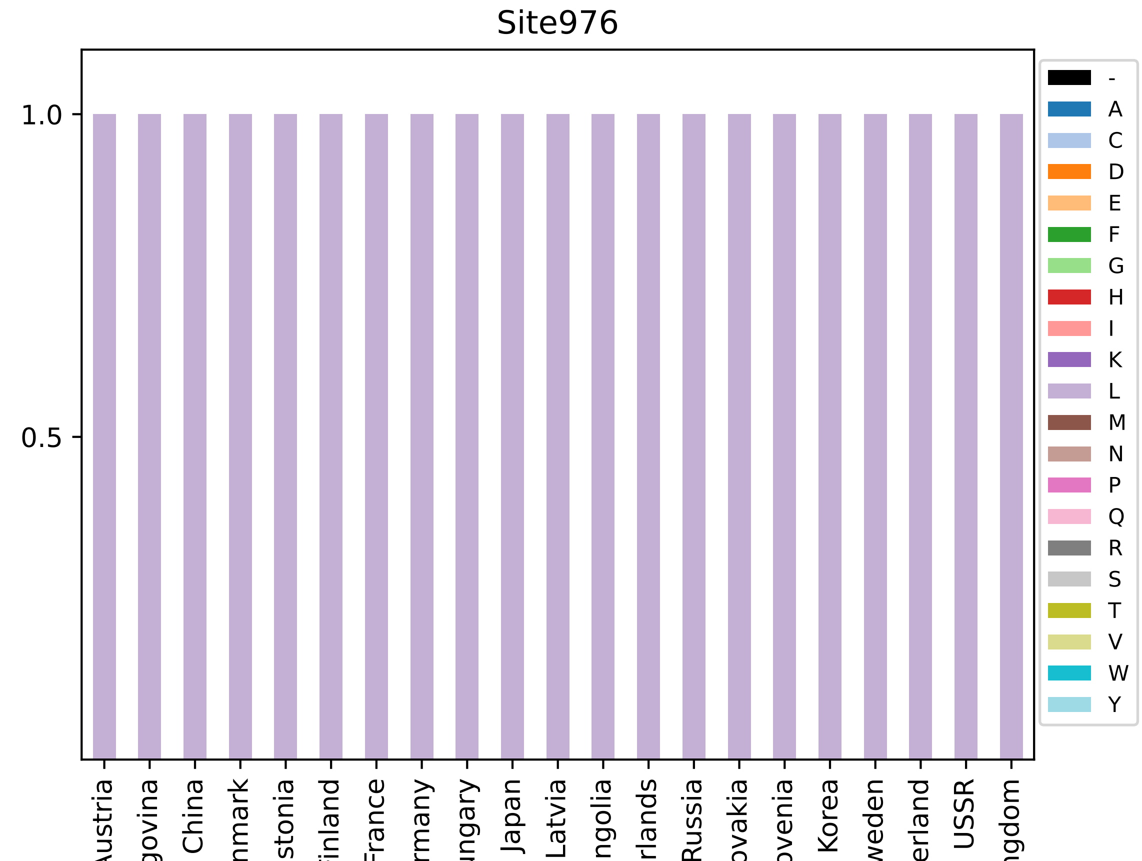

Site976
1.0
0.5
-
A
C
D
E
F
G
H
I
K
L
M
N
P
Q
R
S
T
V
W
Y
China
Japan
Latvia
Russia
Korea
USSR

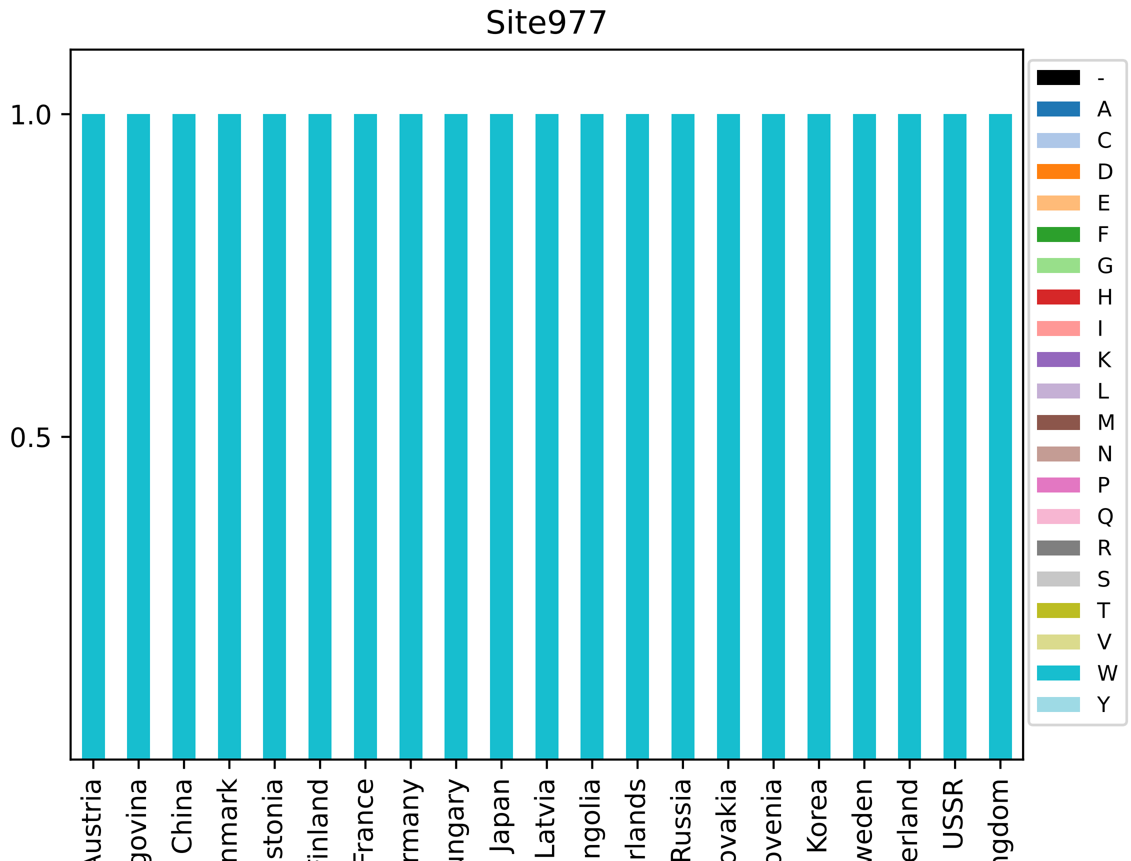

Site977
1.0
0.5
-
A
C
D
E
F
G
H
I
K
L
M
N
P
Q
R
S
T
V
W
Y
China
Japan
Latvia
Russia
Korea
USSR

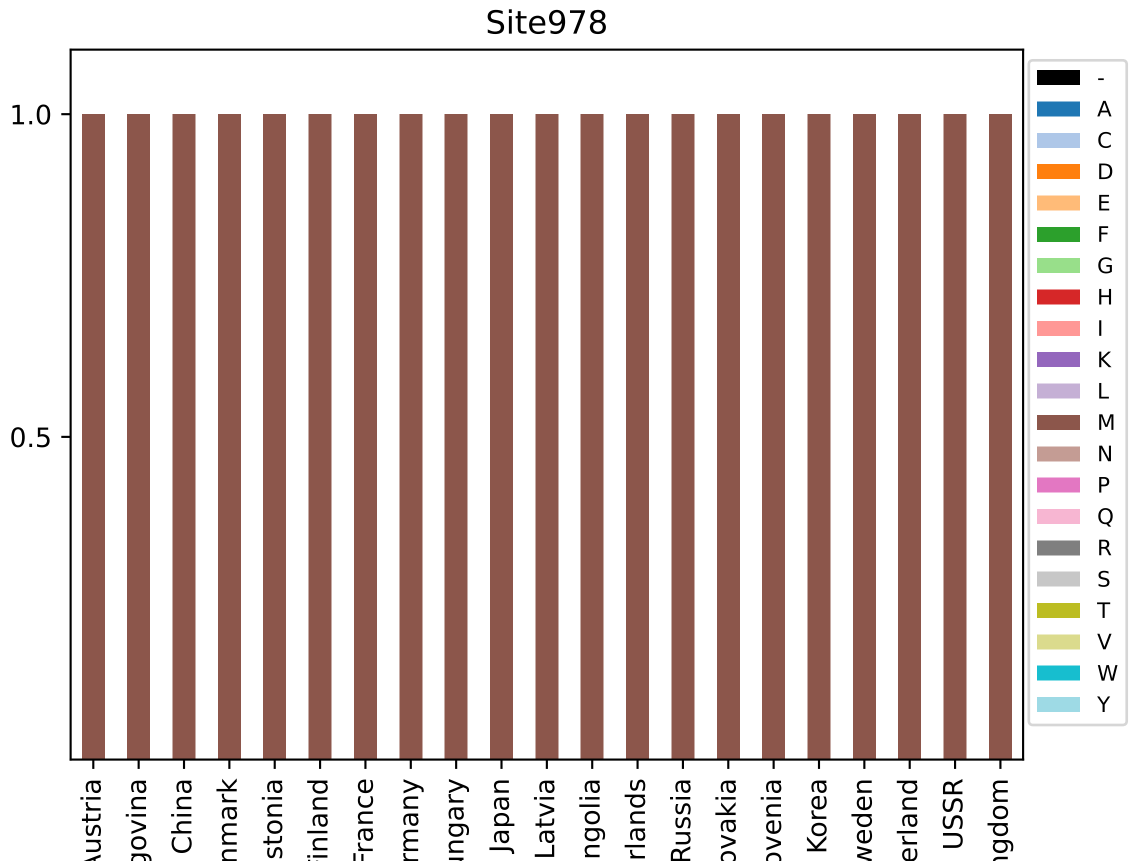

Site978
1.0
0.5
-
A
C
D
E
F
G
H
I
K
L
M
N
P
Q
R
S
T
V
W
Y
Japan
Latvia
Russia
Korea
USSR
China

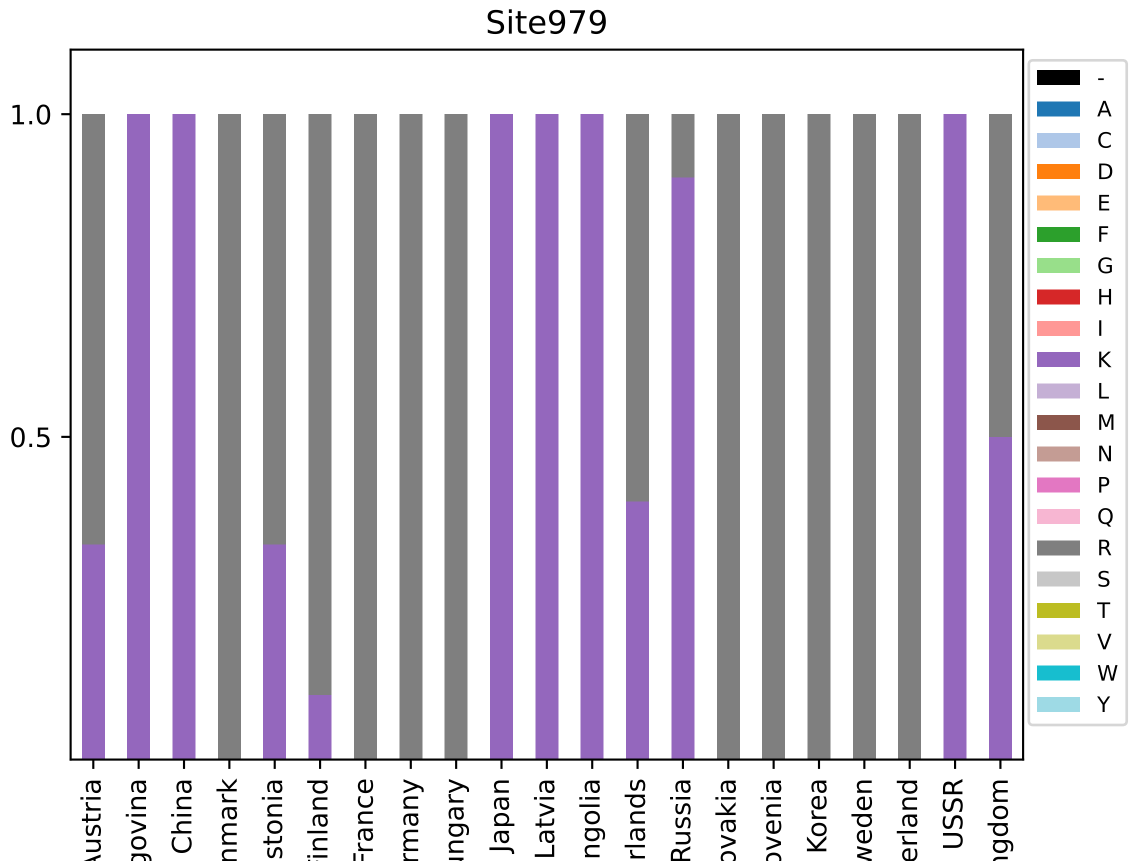
Site979
1.0
0.5
Austria
govina
China
nmark
stonia
inland
France
rmany
ungary
Japan
Latvia
ngolia
rlands
Russia
ovakia
ovenia
Korea
weden
erland
USSR
ngdom
-
A
C
D
E
F
G
H
I
K
L
M
N
P
Q
R
S
T
V
W
Y

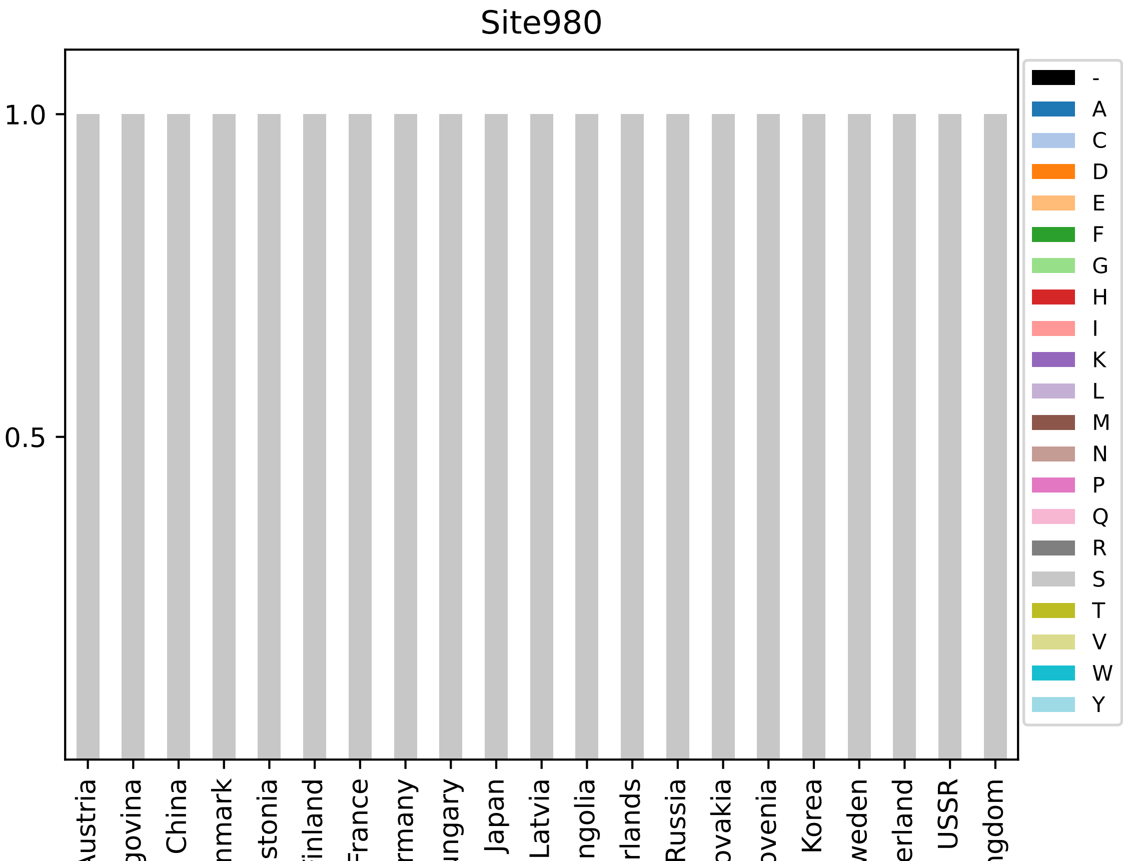
Site980
1.0
0.5
-
A
C
D
E
F
G
H
I
K
L
M
N
P
Q
R
S
T
V
W
Y
Japan
Latvia
Russia
Korea
USSR

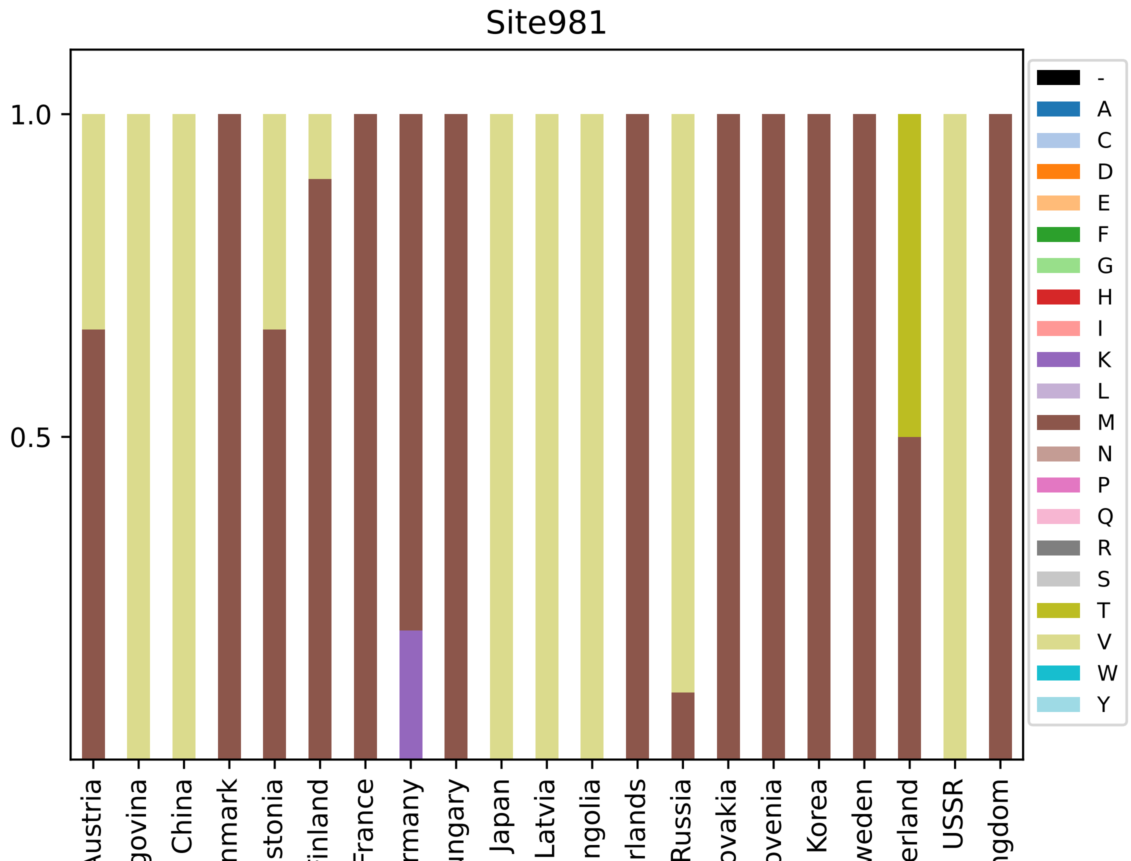

Site981
1.0
0.5
China
Japan
Latvia
Russia
Korea
USSR
-
A
C
D
E
F
G
H
I
K
L
M
N
P
Q
R
S
T
V
W
Y

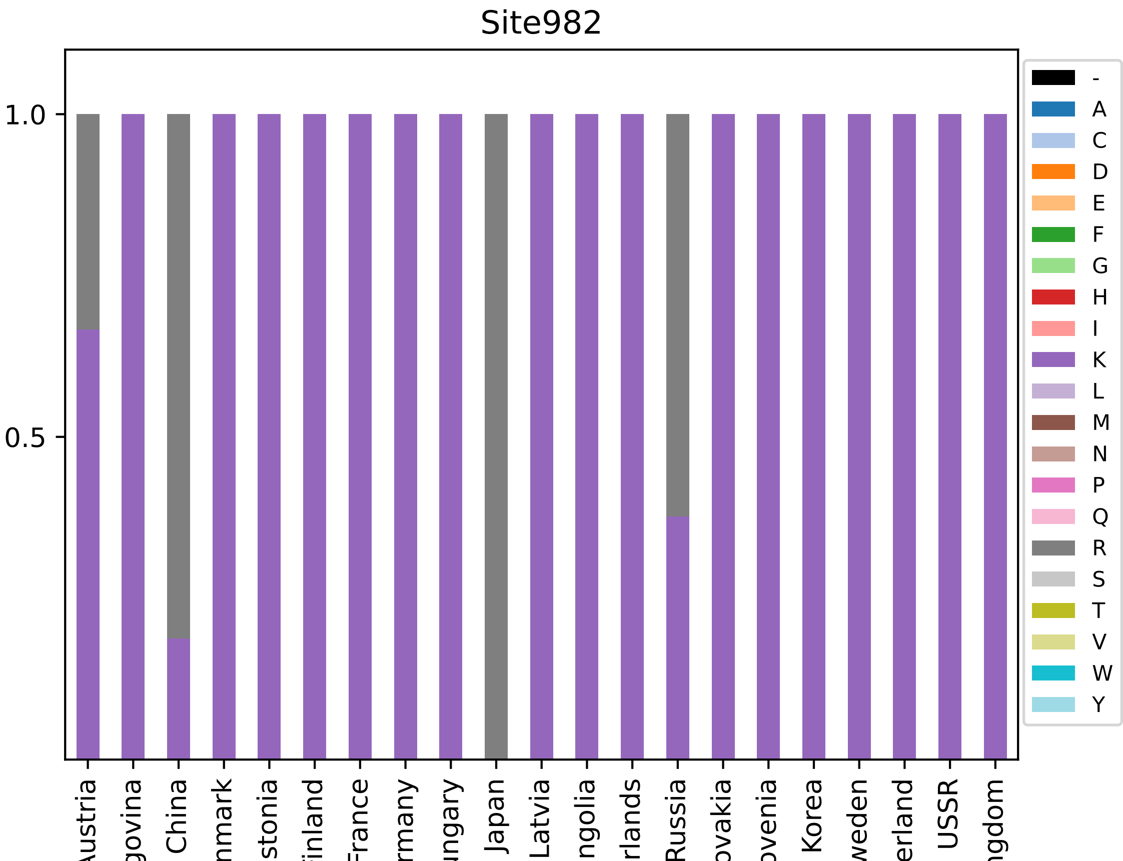

Site982
1.0
0.5
-
A
C
D
E
F
G
H
I
K
L
M
N
P
Q
R
S
T
V
W
Y
China
Japan
Latvia
Russia
Korea
USSR

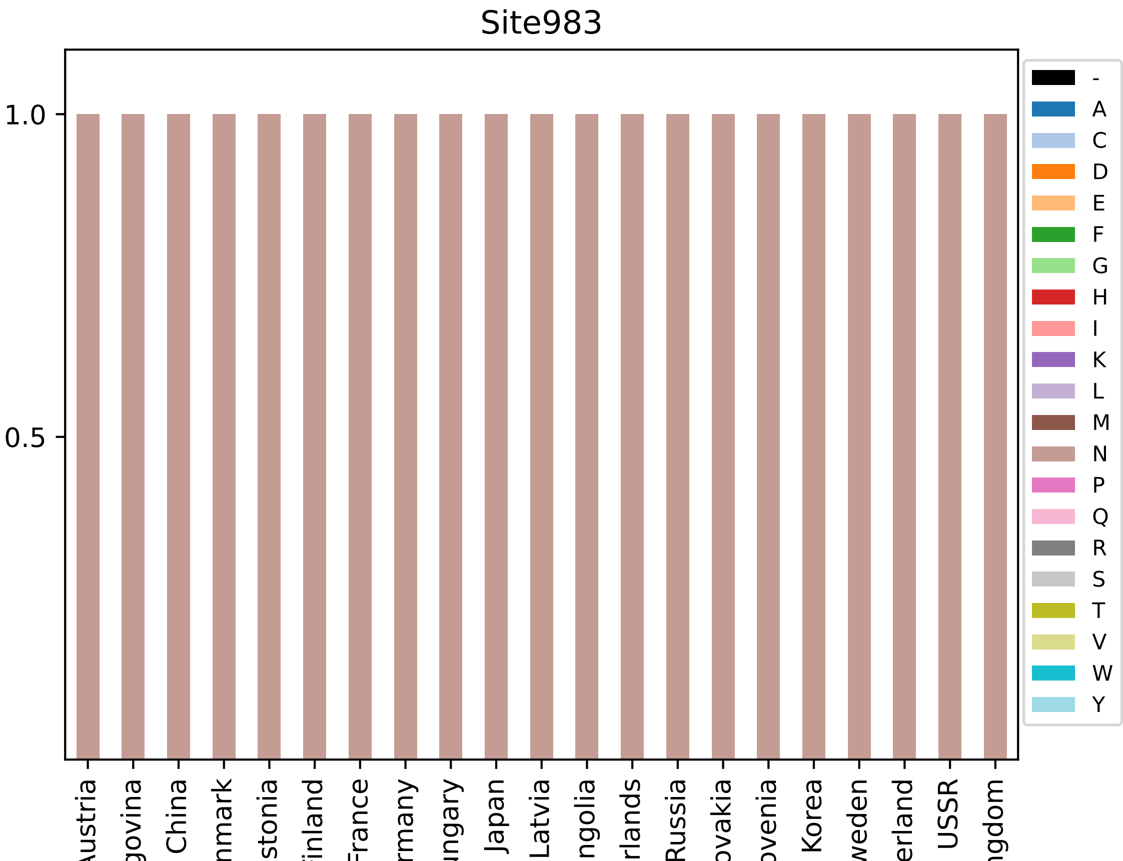
Site983
1.0
0.5
-
A
C
D
E
F
G
H
I
K
L
M
N
P
Q
R
S
T
V
W
Y
Austria
China
Japan
Latvia
Russia
Korea
USSR

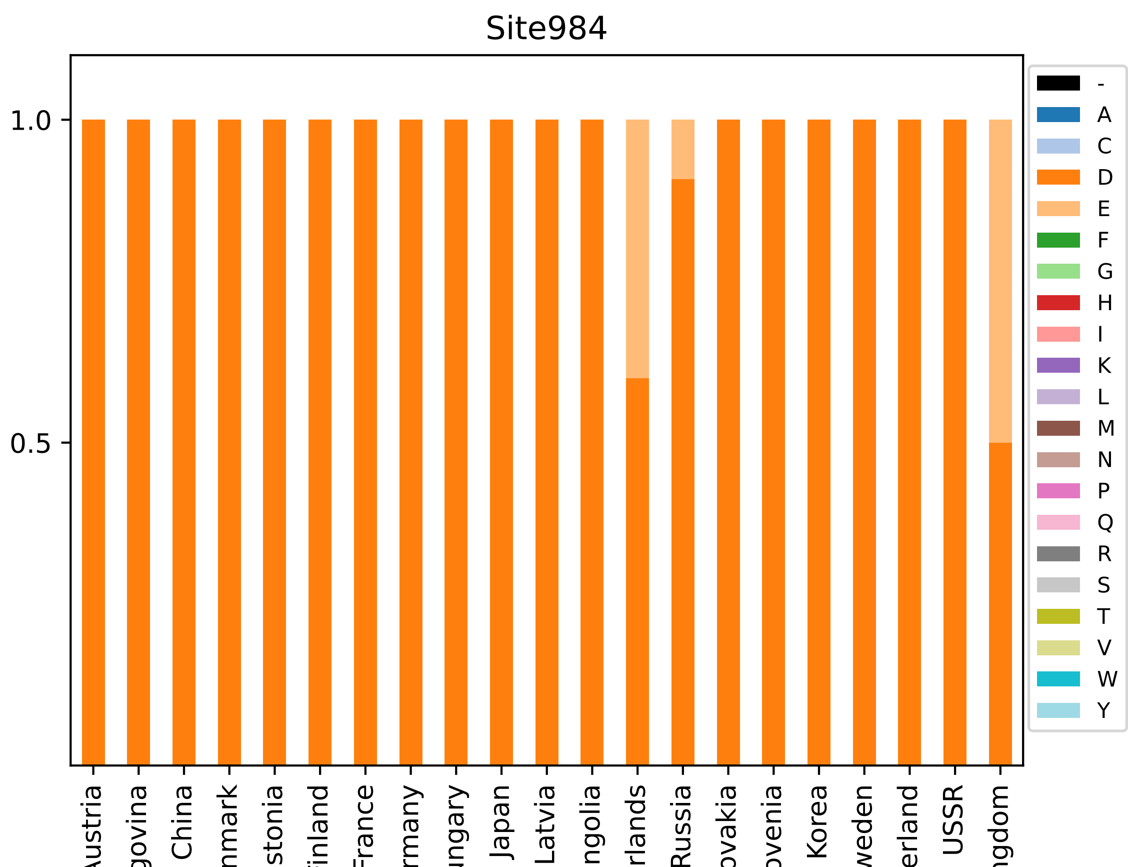

Site984
1.0
0.5
-
A
C
D
E
F
G
H
I
K
L
M
N
P
Q
R
S
T
V
W
Y
China
Japan
Latvia
Russia
Korea
USSR

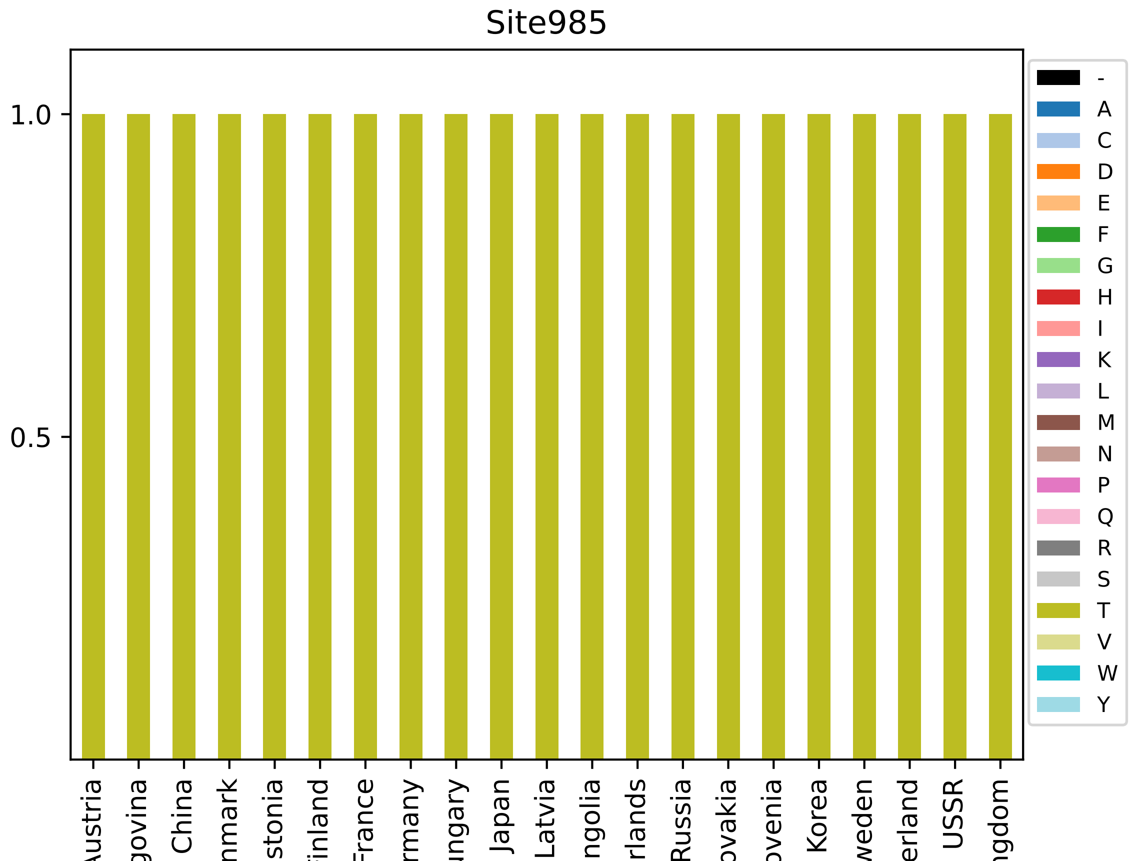

Site985
1.0
0.5
Austria
govina
China
nmark
stonia
inland
France
rmany
ungary
Japan
Latvia
ngolia
rlands
Russia
ovakia
ovenia
Korea
weden
erland
USSR
ngdom
-
A
C
D
E
F
G
H
I
K
L
M
N
P
Q
R
S
T
V
W
Y

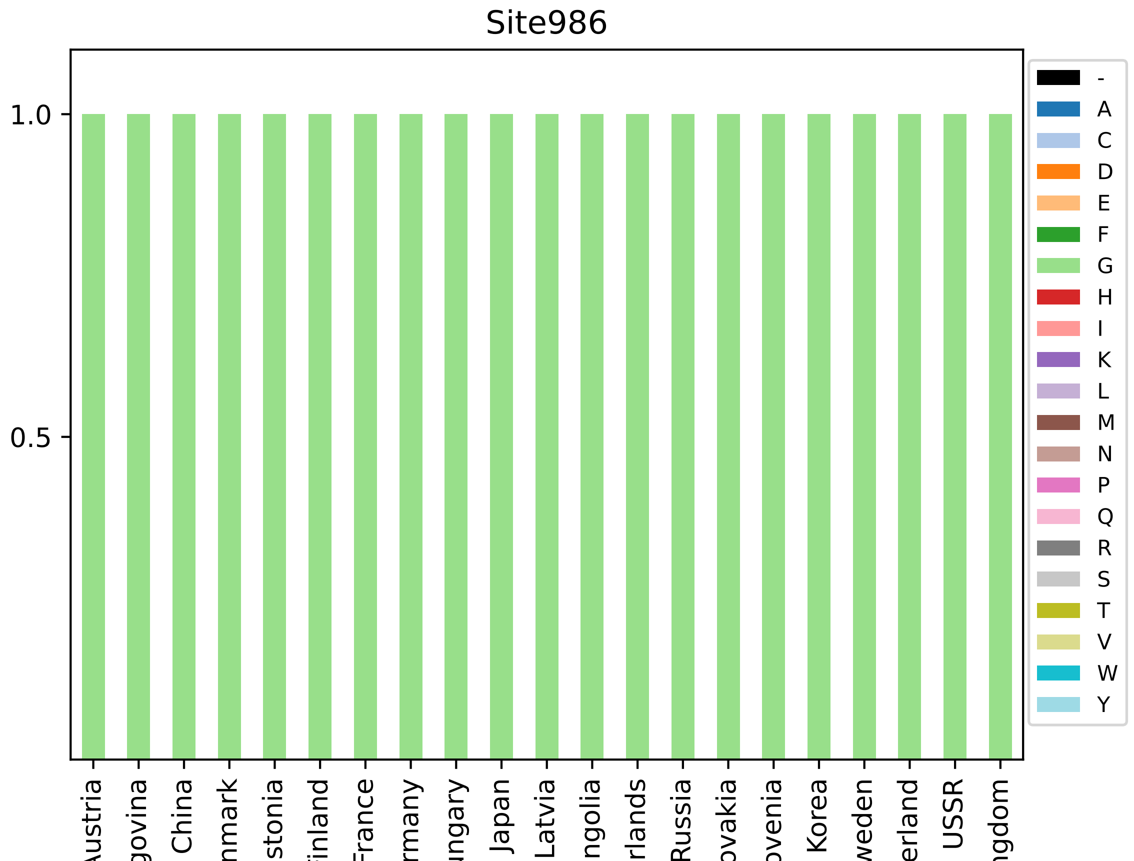
Site986
1.0
0.5
-
A
C
D
E
F
G
H
I
K
L
M
N
P
Q
R
S
T
V
W
Y
Austria
China
Japan
Latvia
Russia
Korea
USSR

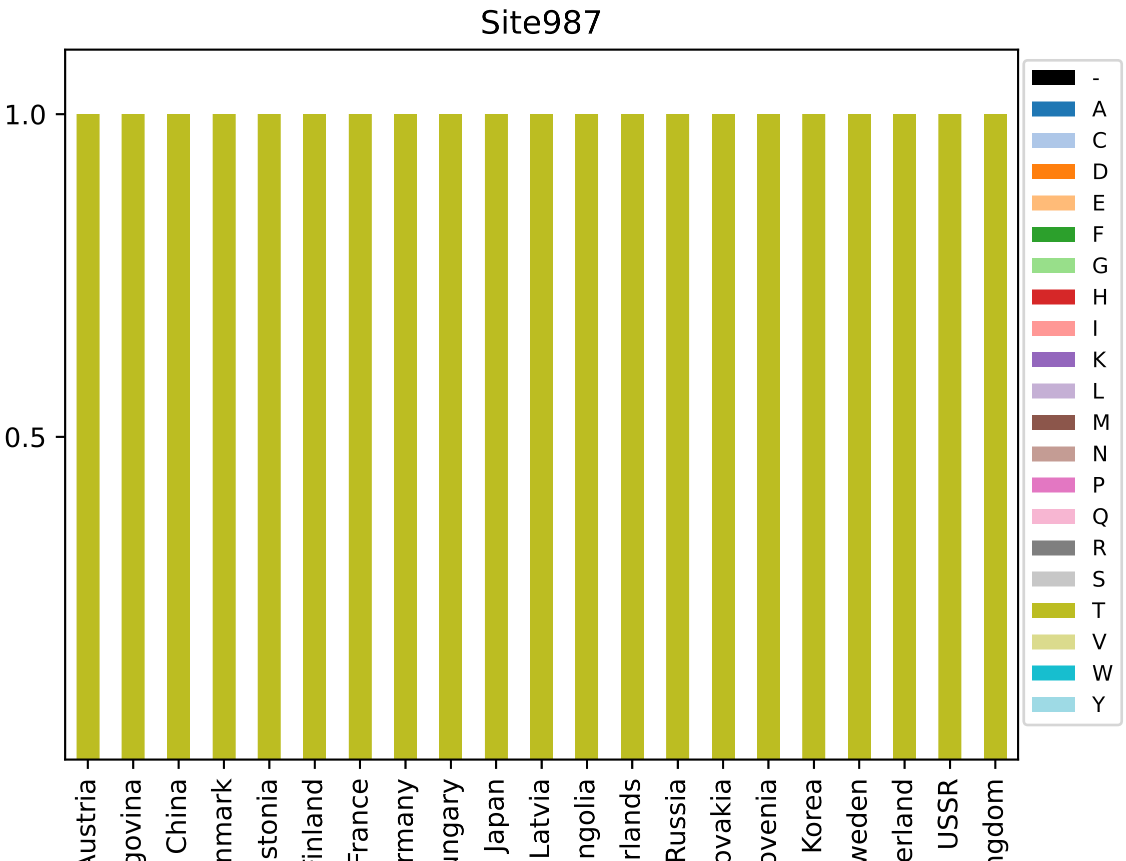

Site987
1.0
0.5
-
A
C
D
E
F
G
H
I
K
L
M
N
P
Q
R
S
T
V
W
Y
Austria
govina
China
nmark
stonia
Finland
France
rmany
ungary
Japan
Latvia
ngolia
rlands
Russia
ovakia
ovenia
Korea
weden
erland
USSR
ngdom

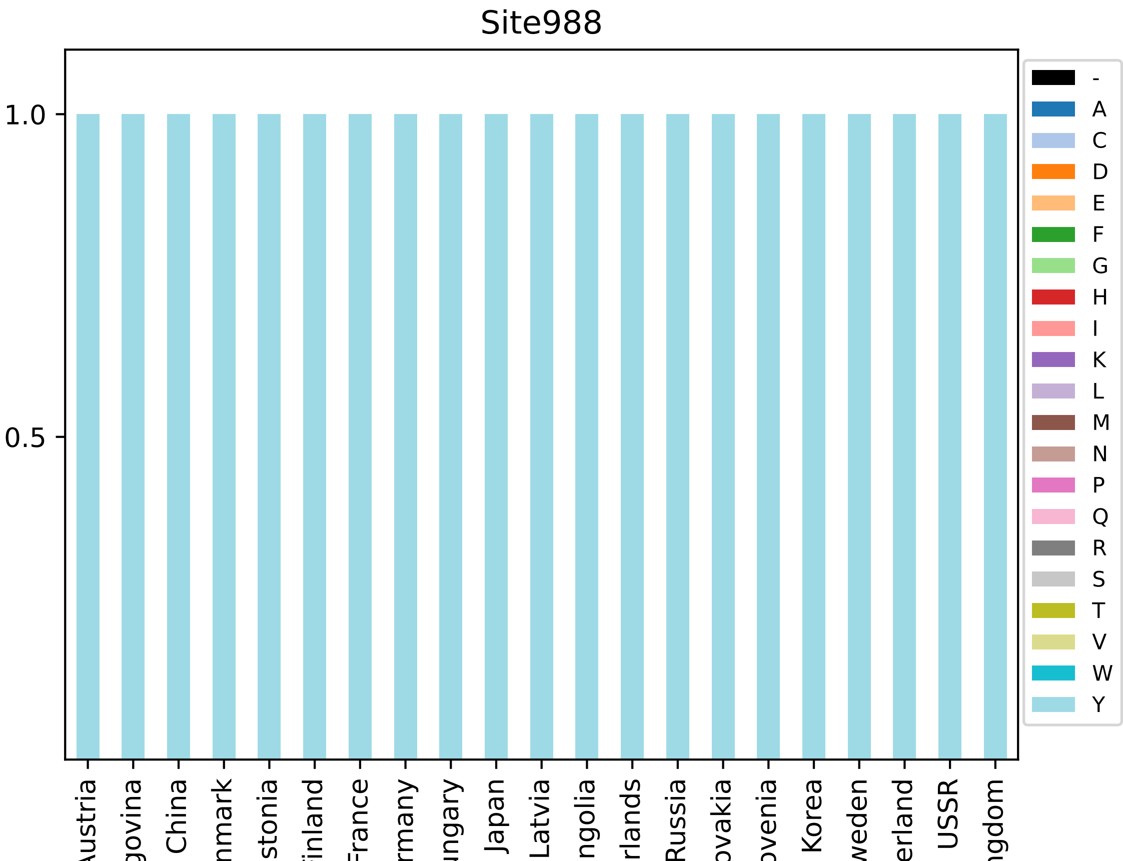
Site988
1.0
0.5
-
A
C
D
E
F
G
H
I
K
L
M
N
P
Q
R
S
T
V
W
Y
China
Japan
Latvia
Russia
Korea
USSR

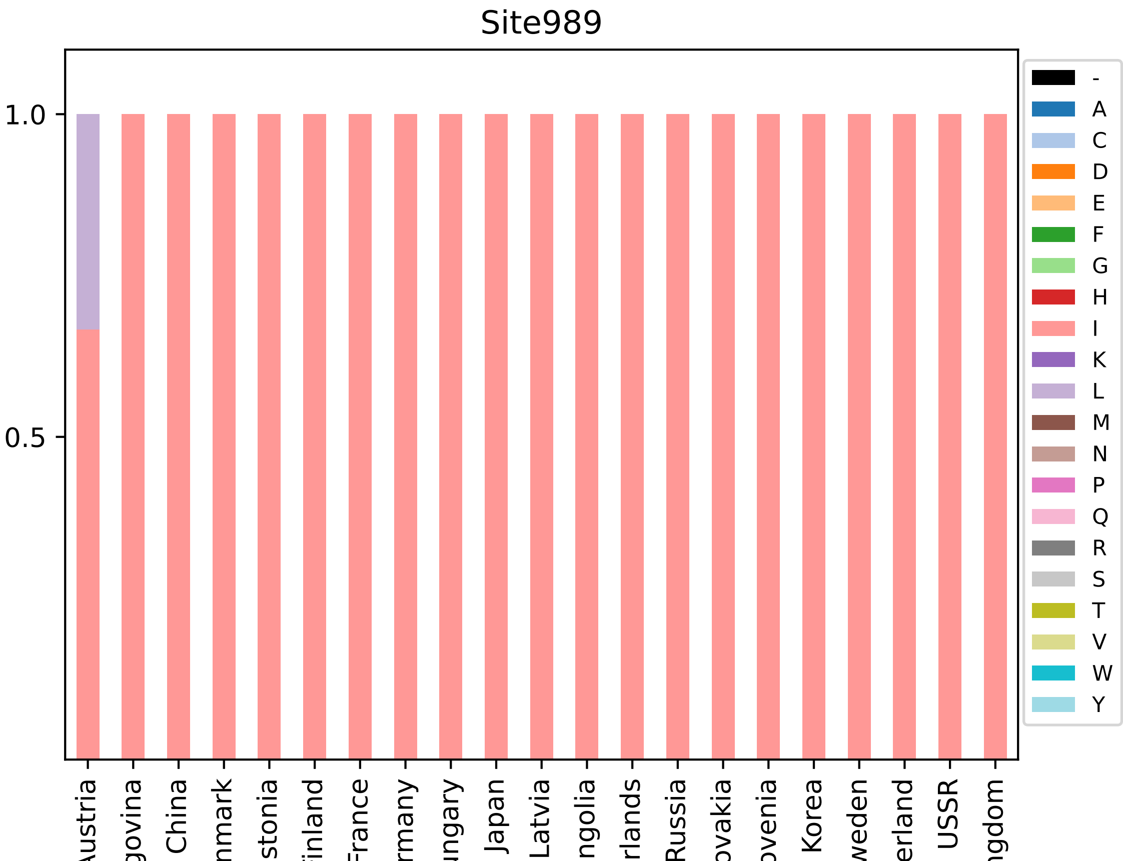

Site989
1.0
0.5
-
A
C
D
E
F
G
H
I
K
L
M
N
P
Q
R
S
T
V
W
Y
Austria
govina
China
nmark
stonia
inland
France
rmany
ungary
Japan
Latvia
ngolia
rlands
Russia
ovakia
ovenia
Korea
weden
erland
USSR
ngdom

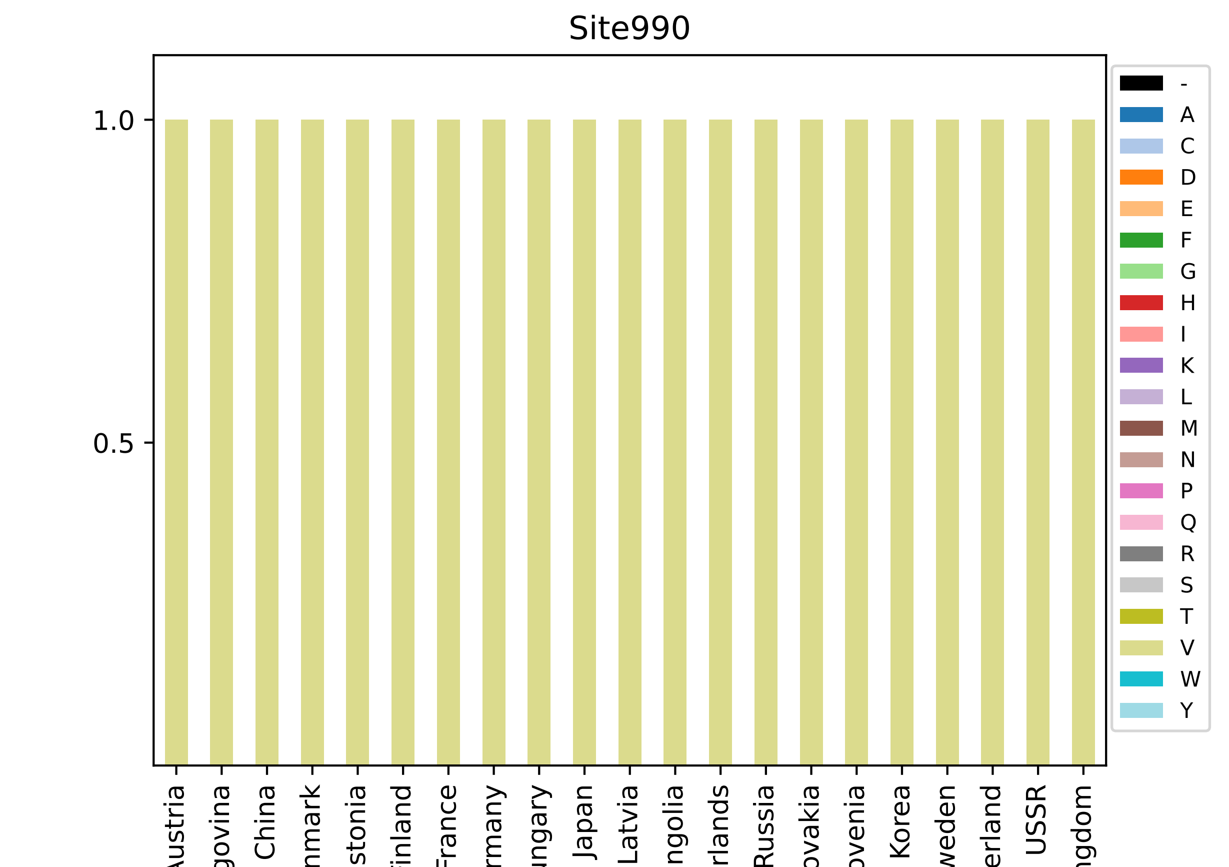

Site990
1.0
0.5
-
A
C
D
E
F
G
H
I
K
L
M
N
P
Q
R
S
T
V
W
Y
China
Japan
Latvia
Russia
Korea
USSR

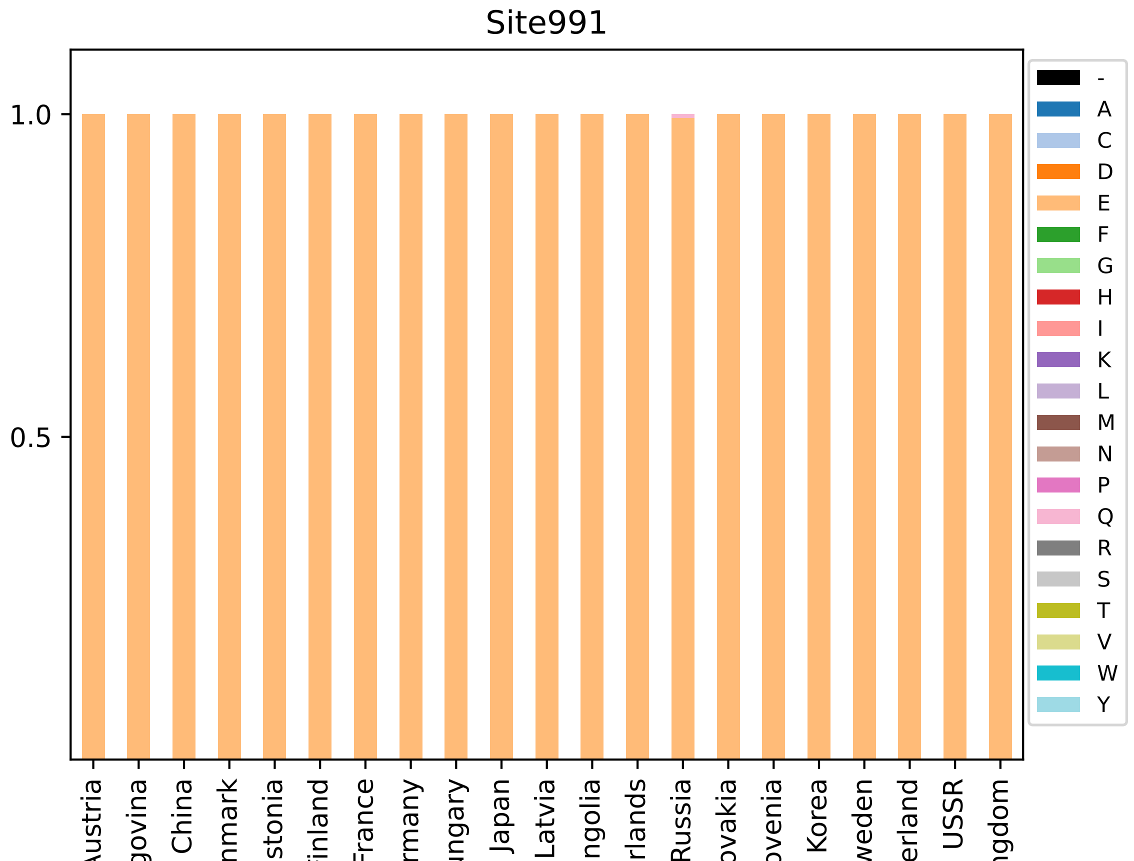

Site991
1.0
0.5
-
A
C
D
E
F
G
H
I
K
L
M
N
P
Q
R
S
T
V
W
Y
Austria
govina
China
nmark
stonia
inland
France
rmany
ungary
Japan
Latvia
ngolia
rlands
Russia
ovakia
ovenia
Korea
weden
erland
USSR
ngdom

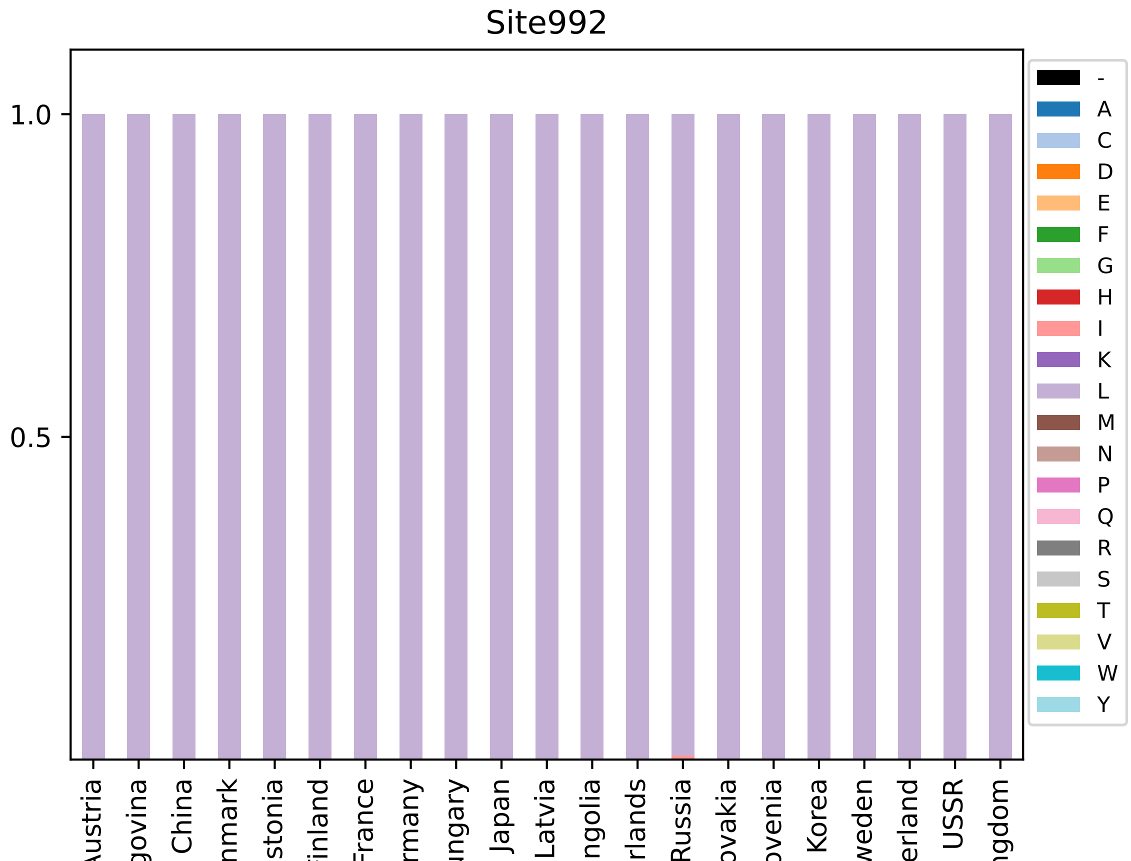

Site992
1.0
0.5
-
A
C
D
E
F
G
H
I
K
L
M
N
P
Q
R
S
T
V
W
Y
Austria
China
Finland
France
Japan
Latvia
Russia
Korea
USSR

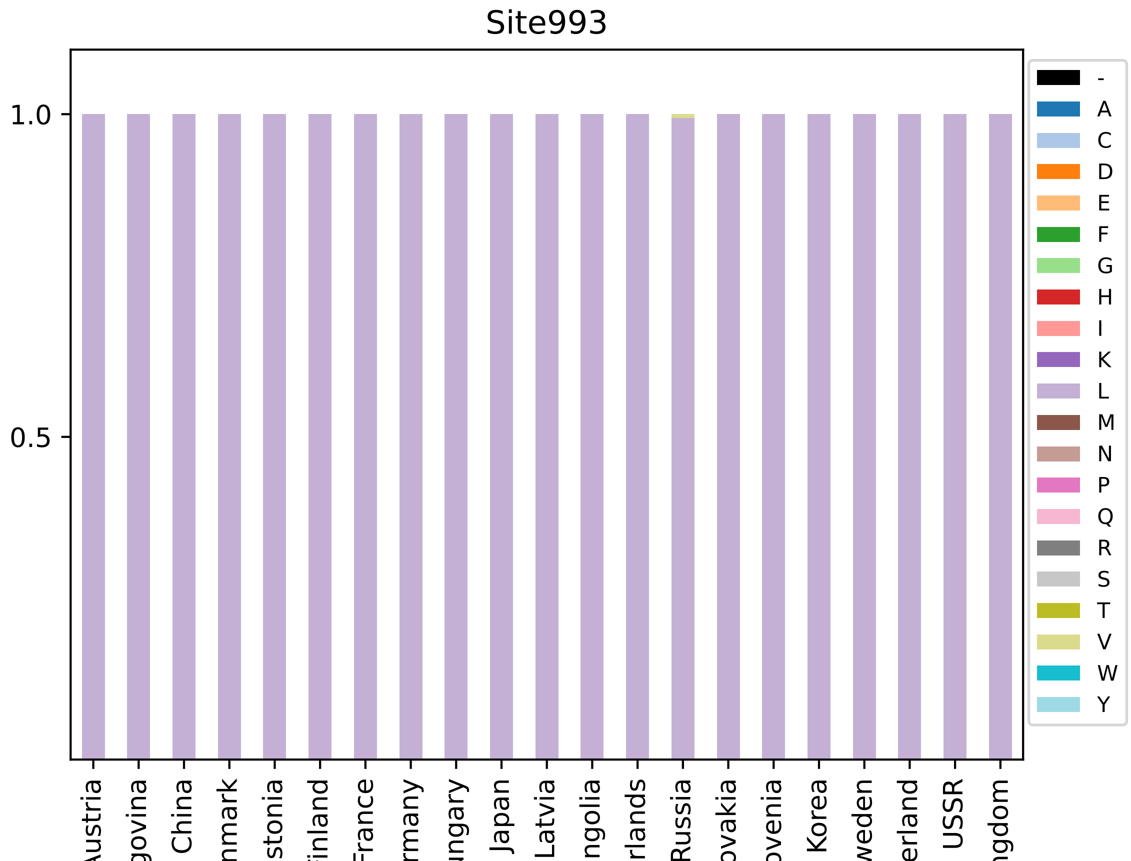
Site993
1.0
0.5
-
A
C
D
E
F
G
H
I
K
L
M
N
P
Q
R
S
T
V
W
Y
China
Japan
Latvia
Russia
Korea
USSR

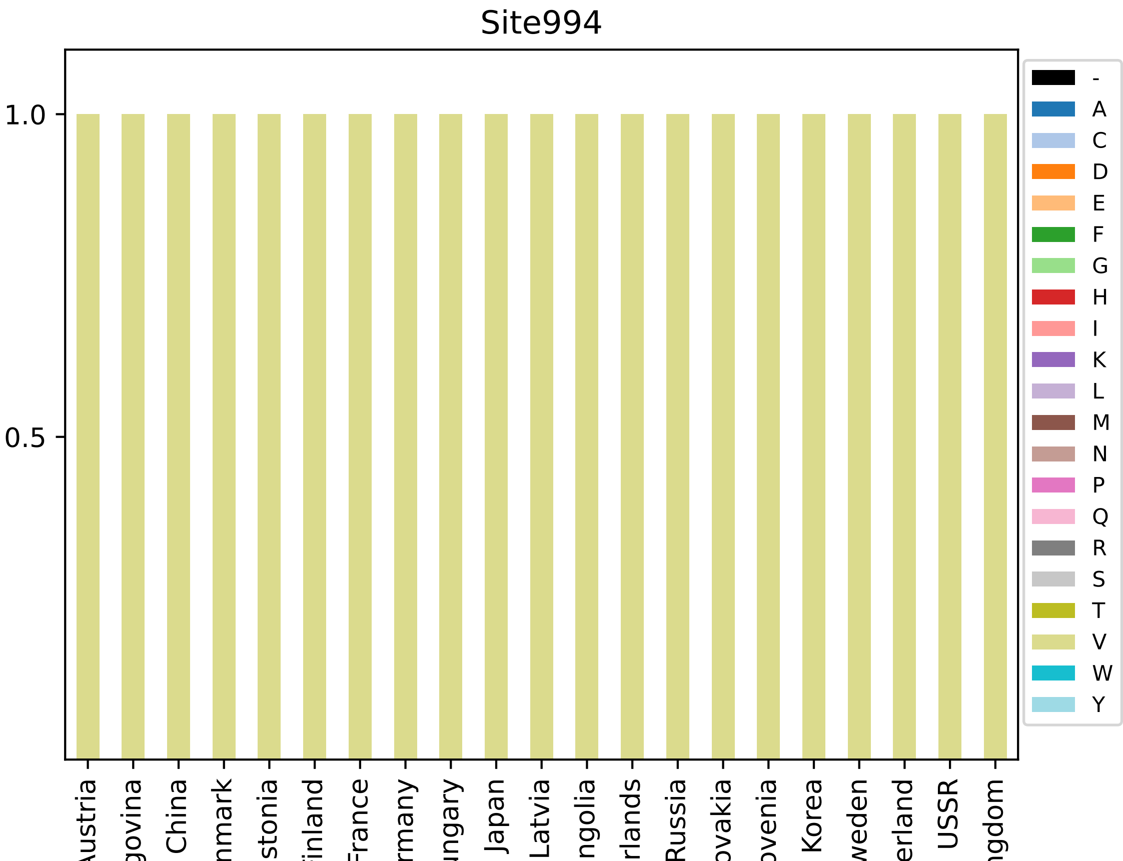

Site994
1.0
0.5
-
A
C
D
E
F
G
H
I
K
L
M
N
P
Q
R
S
T
V
W
Y
Austria
govina
China
nmark
stonia
inland
France
rmany
ungary
Japan
Latvia
ngolia
rlands
Russia
ovakia
ovenia
Korea
weden
erland
USSR
ngdom

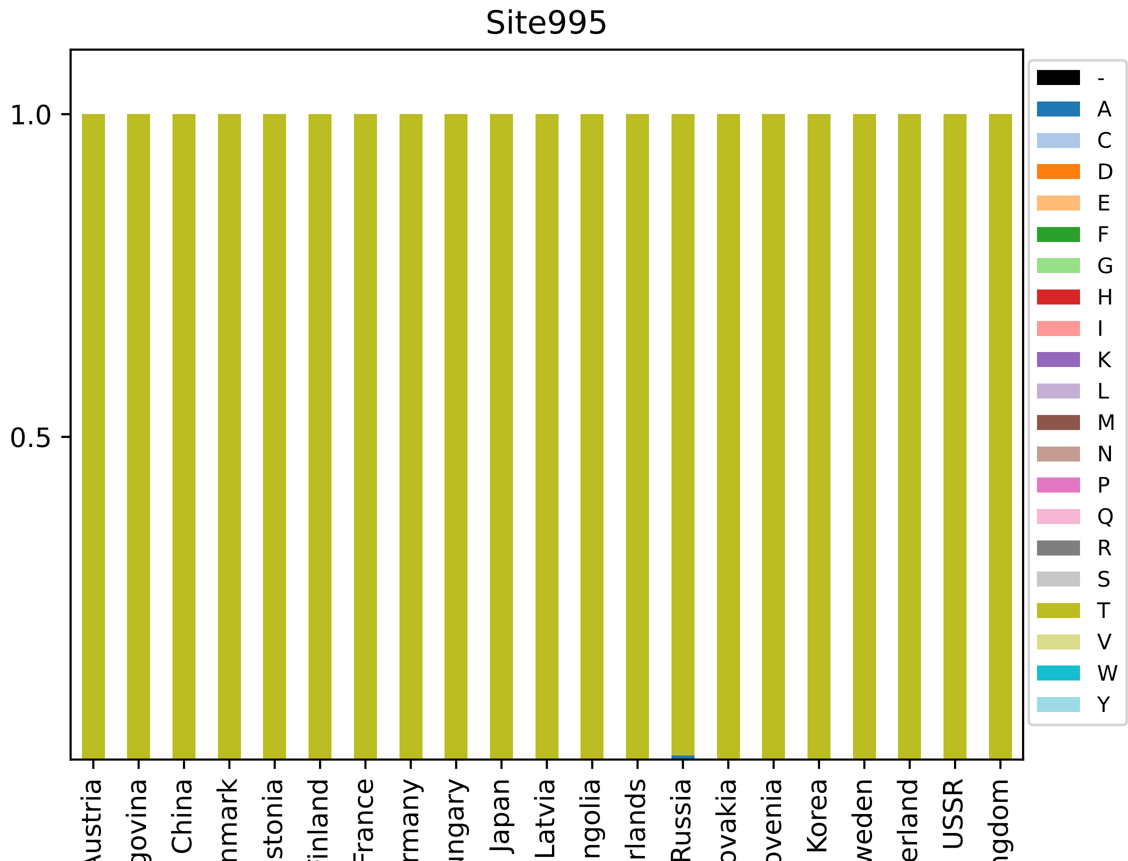

Site995
1.0
0.5
Austria
govina
China
nmark
stonia
Finland
France
rmany
ungary
Japan
Latvia
ngolia
rlands
Russia
ovakia
ovenia
Korea
weden
erland
USSR
ngdom
-
A
C
D
E
F
G
H
I
K
L
M
N
P
Q
R
S
T
V
W
Y

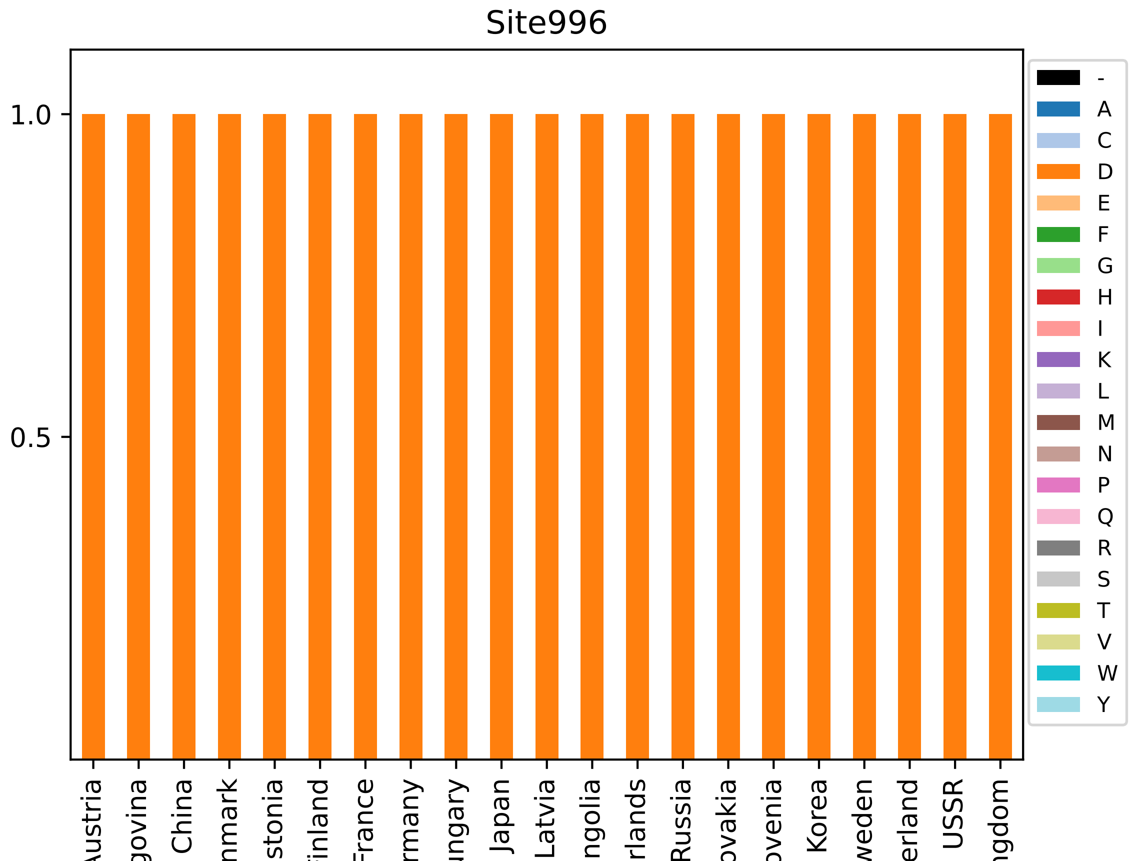
Site996
1.0
0.5
-
A
C
D
E
F
G
H
I
K
L
M
N
P
Q
R
S
T
V
W
Y
China
Japan
Latvia
Russia
Korea
USSR

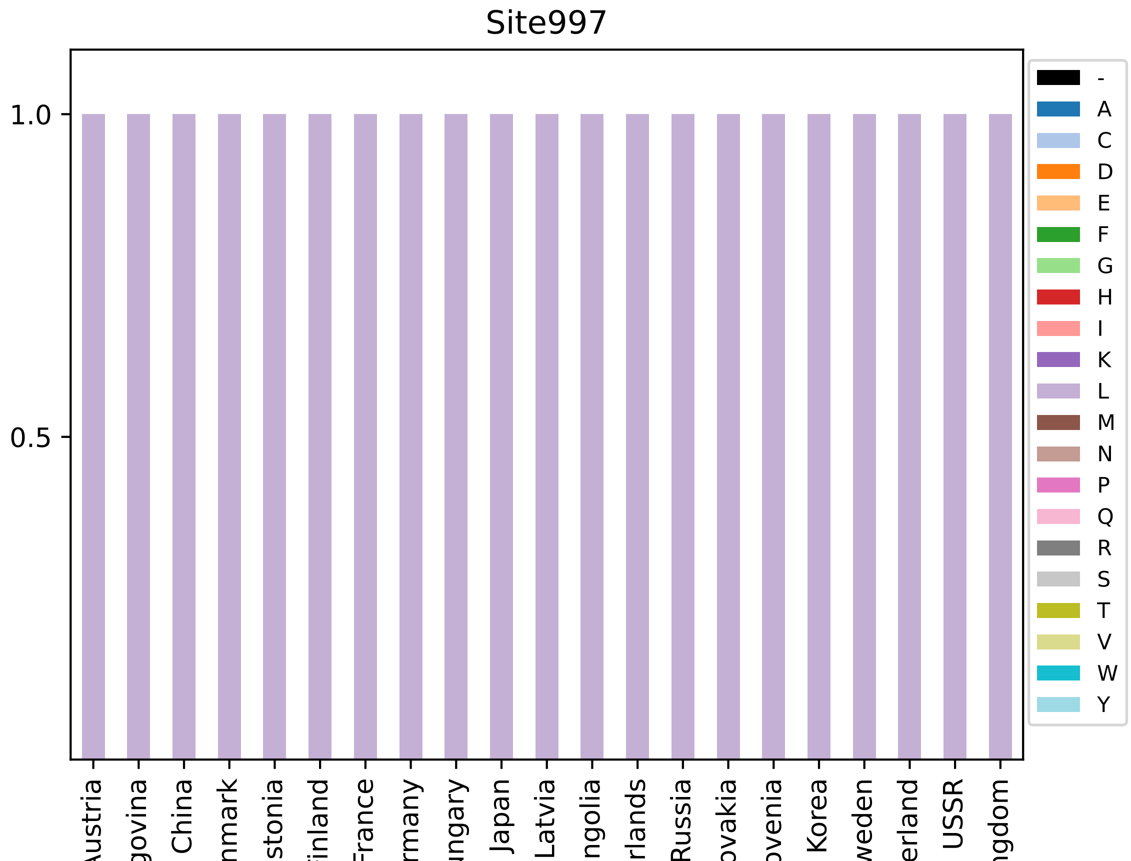

Site997
1.0
0.5
-
A
C
D
E
F
G
H
I
K
L
M
N
P
Q
R
S
T
V
W
Y
China
Japan
Latvia
Russia
Korea
USSR

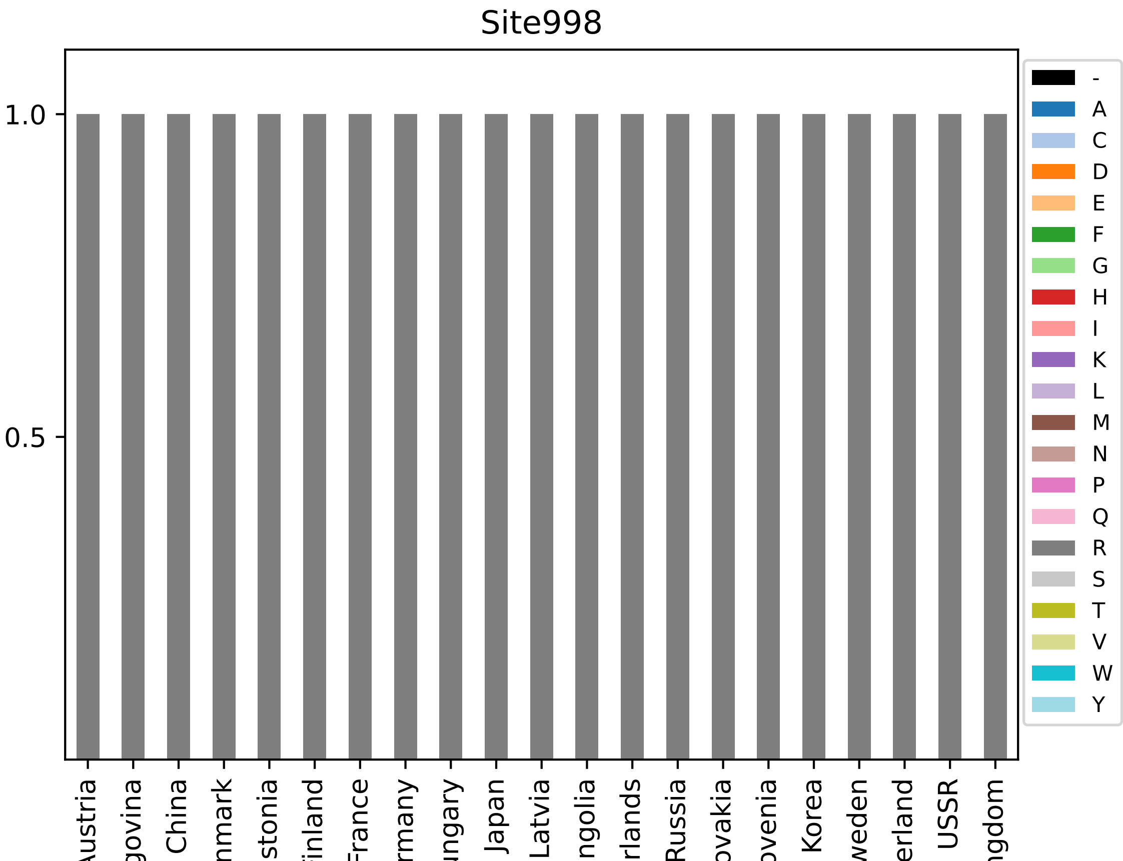

Site998
1.0
0.5
-
A
C
D
E
F
G
H
I
K
L
M
N
P
Q
R
S
T
V
W
Y
Austria
govina
China
nmark
stonia
inland
France
rmany
ungary
Japan
Latvia
ngolia
rlands
Russia
ovakia
ovenia
Korea
weden
erland
USSR
ngdom

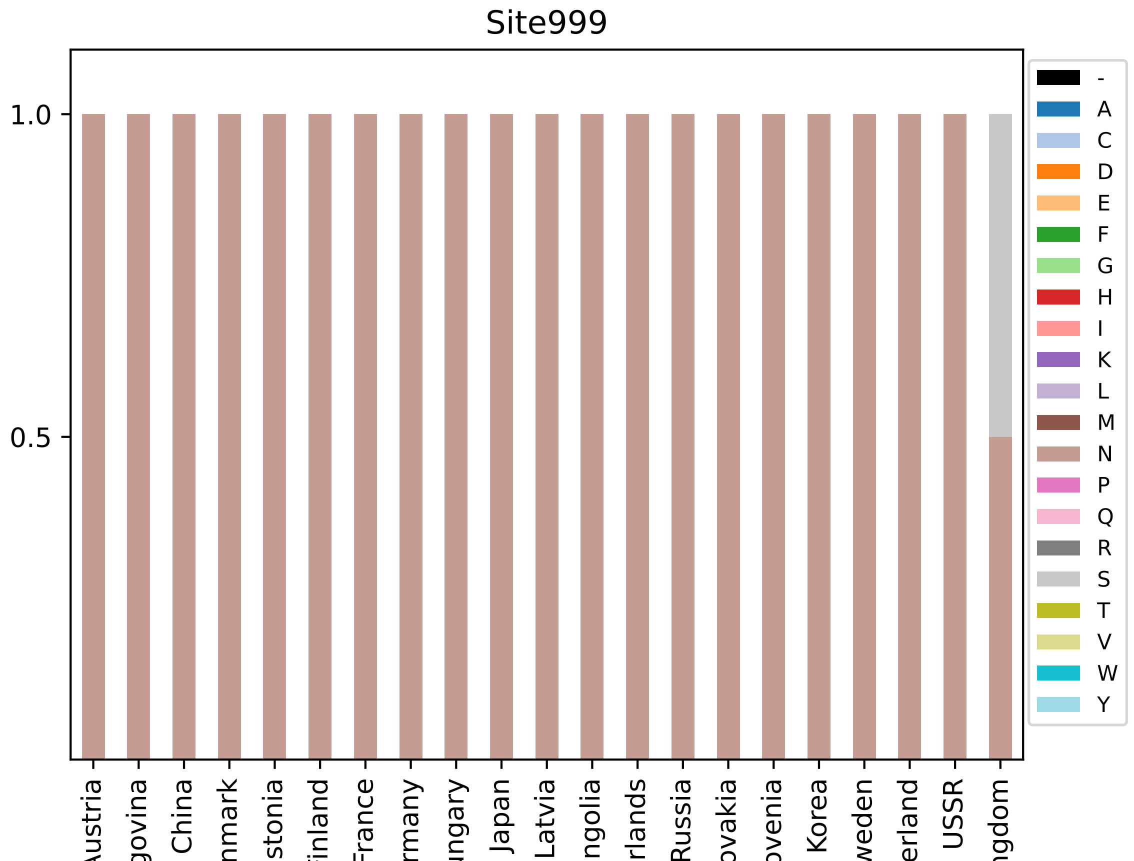

Site999
1.0
0.5
-
A
C
D
E
F
G
H
I
K
L
M
N
P
Q
R
S
T
V
W
Y
China
Japan
Latvia
Russia
Korea
USSR

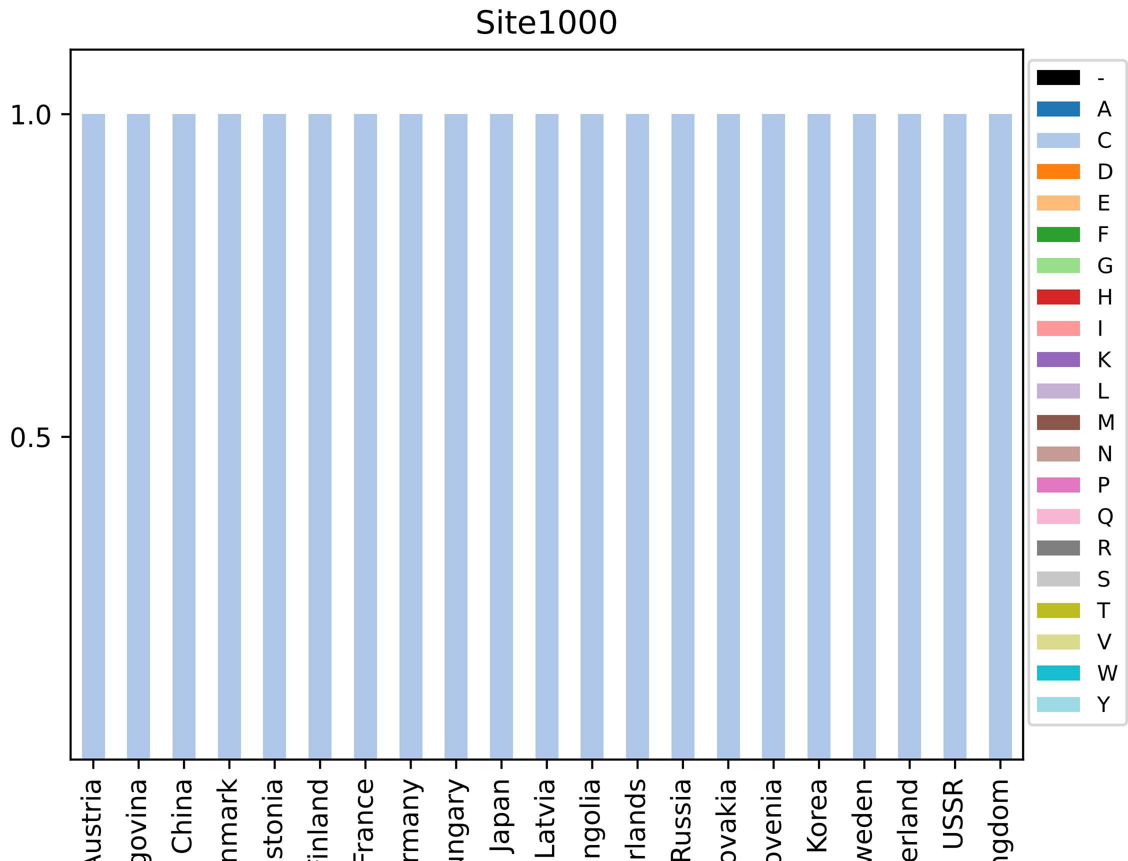
Site1000
1.0
0.5
-
A
C
D
E
F
G
H
I
K
L
M
N
P
Q
R
S
T
V
W
Y
Austria
China
Estonia
Finland
France
Hungary
Japan
Latvia
Russia
Korea
Sweden
USSR

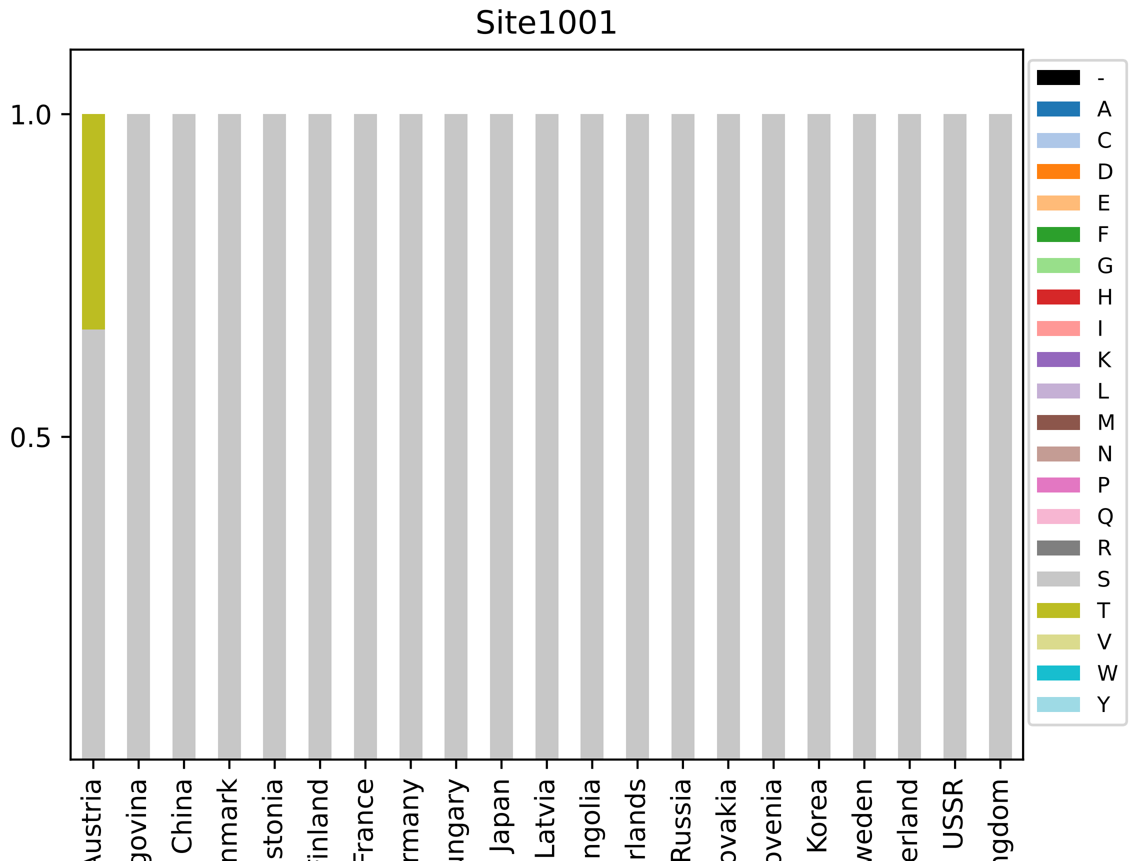

Site1001
1.0
0.5
-
A
C
D
E
F
G
H
I
K
L
M
N
P
Q
R
S
T
V
W
Y
Austria
China
Finland
France
Japan
Latvia
Russia
Korea
USSR

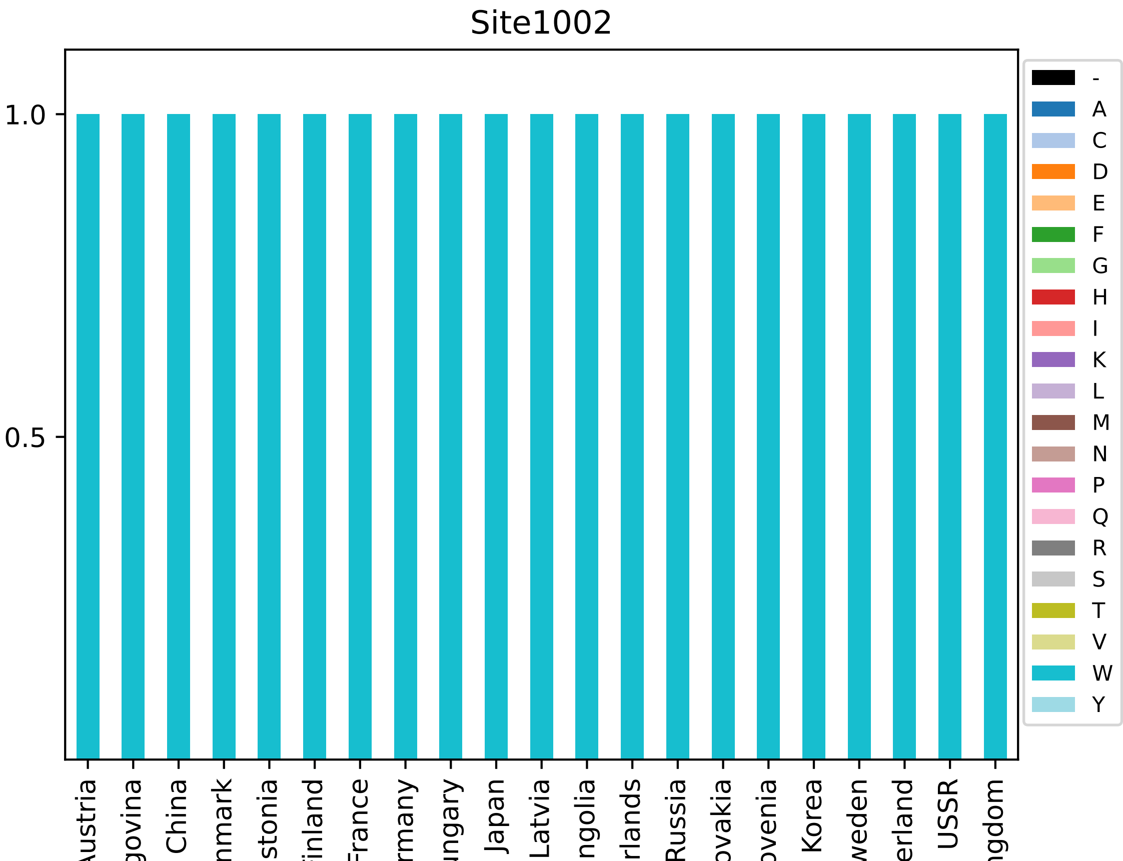

Site1002
1.0
0.5
-
A
C
D
E
F
G
H
I
K
L
M
N
P
Q
R
S
T
V
W
Y
China
Japan
Latvia
Russia
Korea
USSR

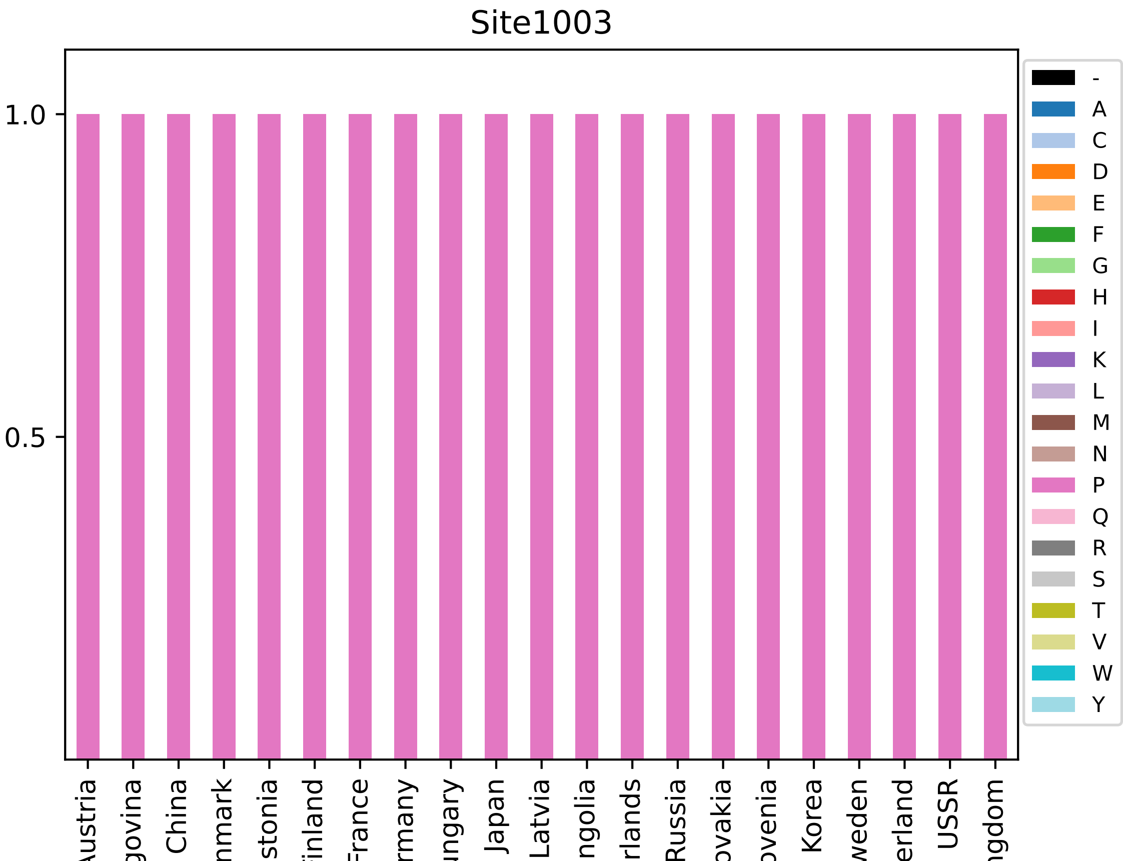

Site1003
1.0
0.5
-
A
C
D
E
F
G
H
I
K
L
M
N
P
Q
R
S
T
V
W
Y
Austria
China
Japan
Latvia
Russia
Korea
USSR

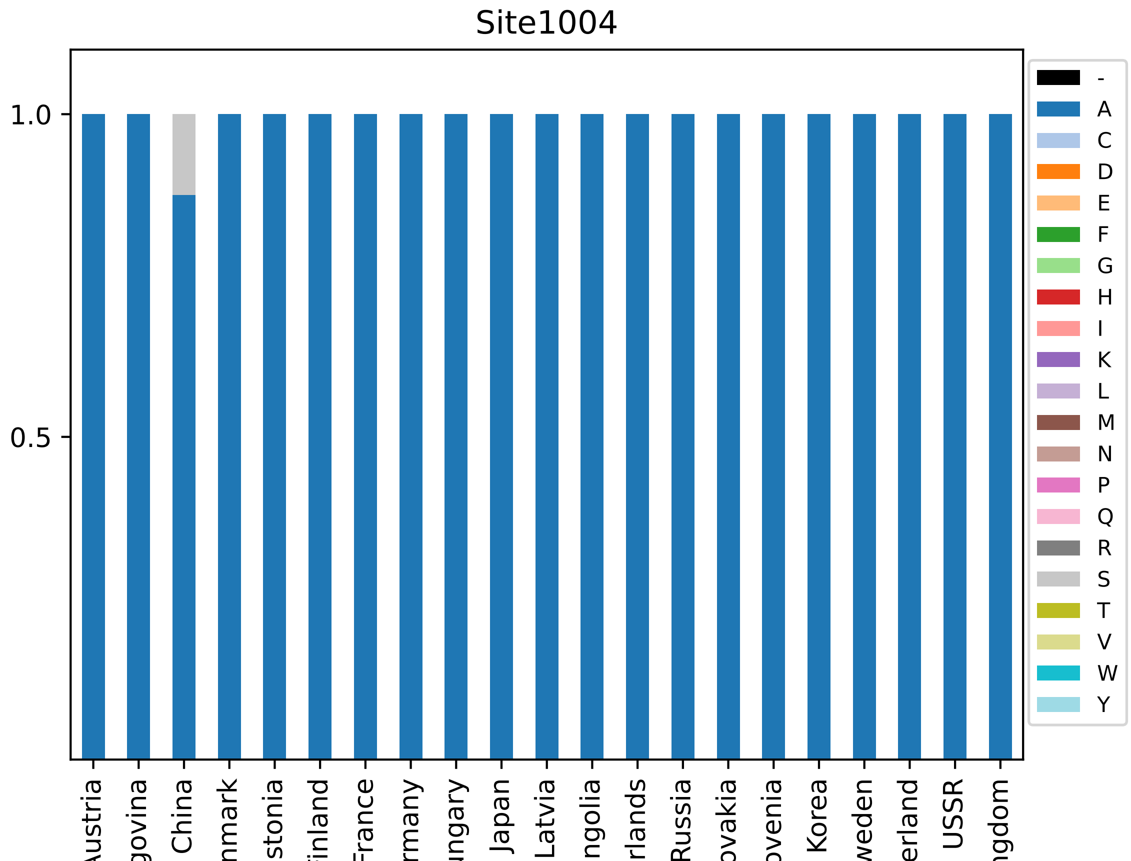
Site1004
1.0
0.5
-
A
C
D
E
F
G
H
I
K
L
M
N
P
Q
R
S
T
V
W
Y
Austria
govina
China
nmark
stonia
inland
France
rmany
ungary
Japan
Latvia
ngolia
rlands
Russia
ovakia
ovenia
Korea
weden
erland
USSR
ngdom

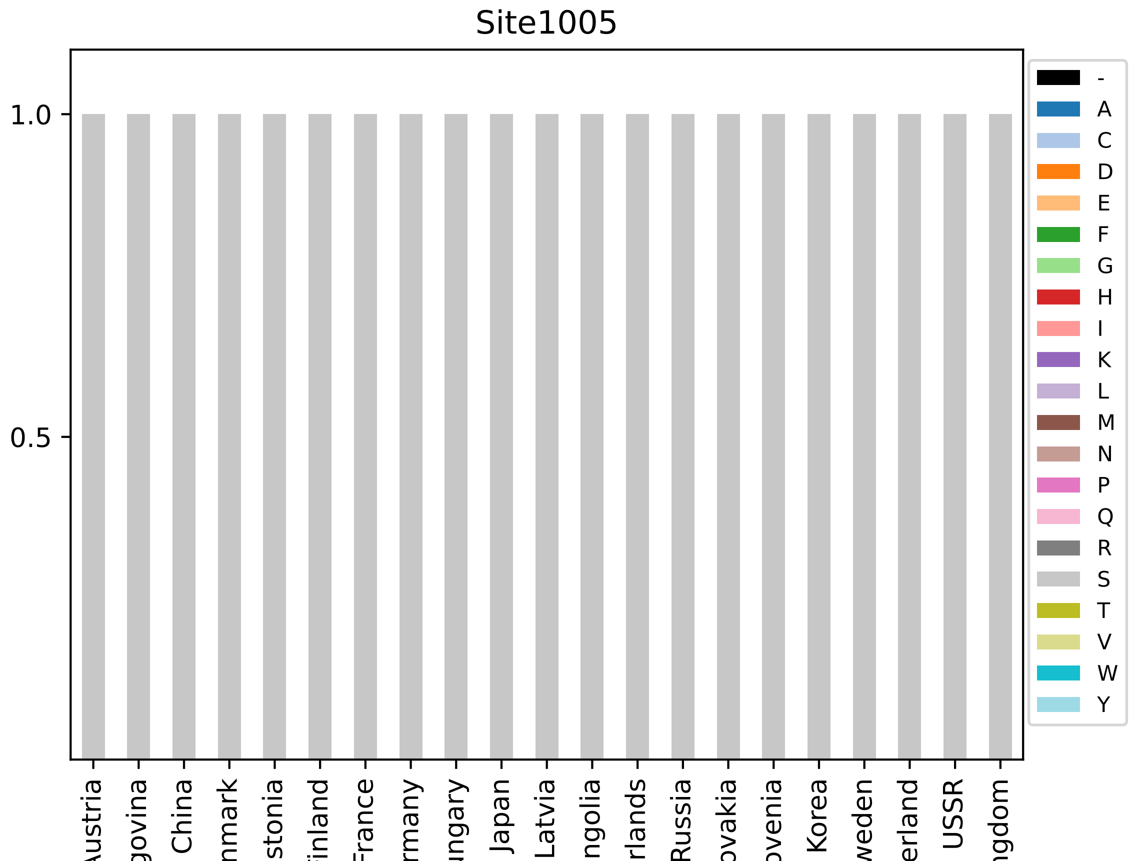

Site1005
1.0
0.5
-
A
C
D
E
F
G
H
I
K
L
M
N
P
Q
R
S
T
V
W
Y
China
Japan
Latvia
Russia
Korea
USSR

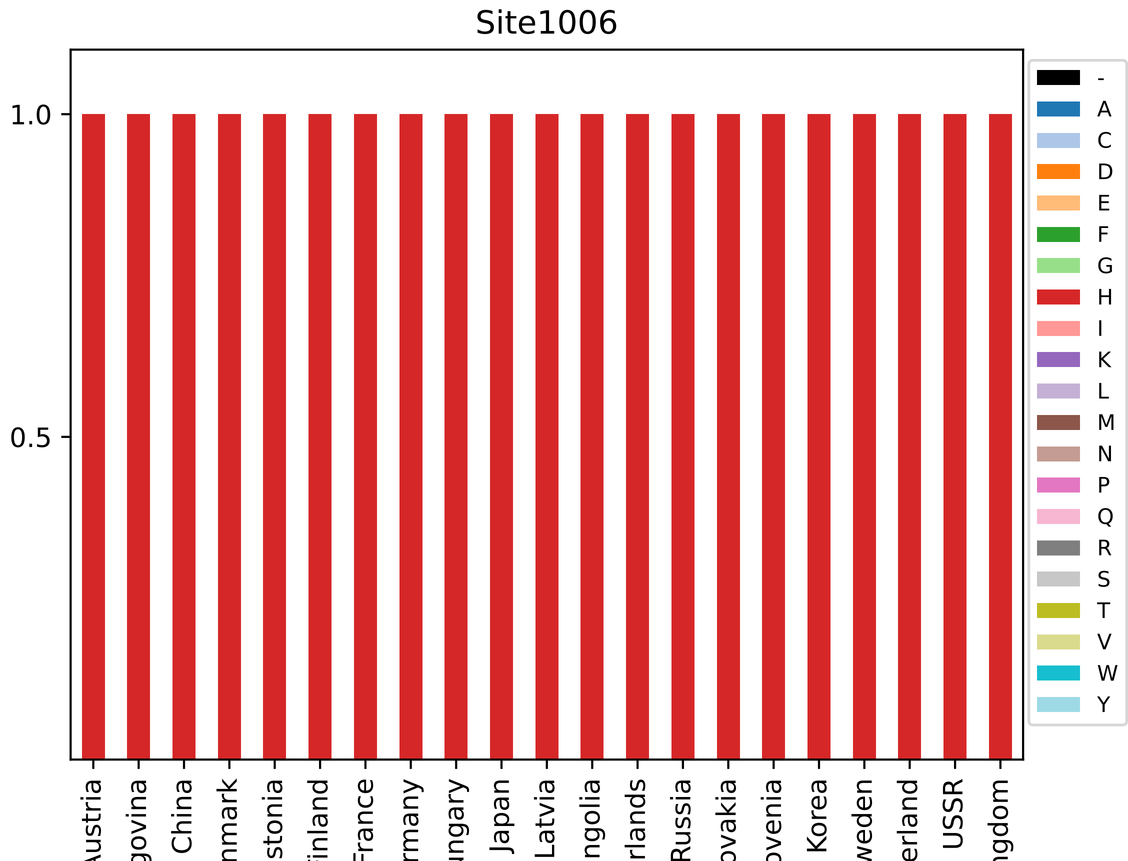
Site1006
1.0
0.5
-
A
C
D
E
F
G
H
I
K
L
M
N
P
Q
R
S
T
V
W
Y
China
Japan
Latvia
Russia
Korea
USSR

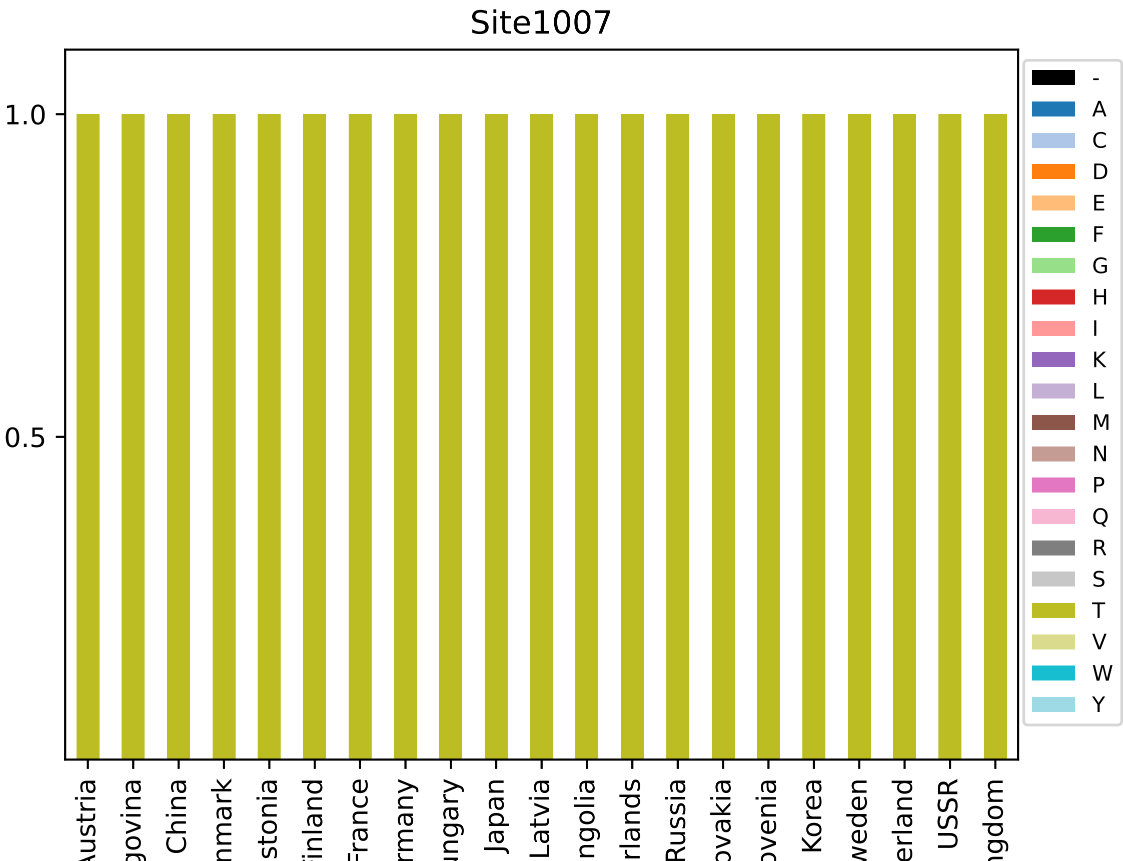

Site1007
1.0
0.5
-
A
C
D
E
F
G
H
I
K
L
M
N
P
Q
R
S
T
V
W
Y
Austria
govina
China
nmark
stonia
inland
France
rmany
ungary
Japan
Latvia
ngolia
rlands
Russia
ovakia
ovenia
Korea
weden
erland
USSR
ngdom

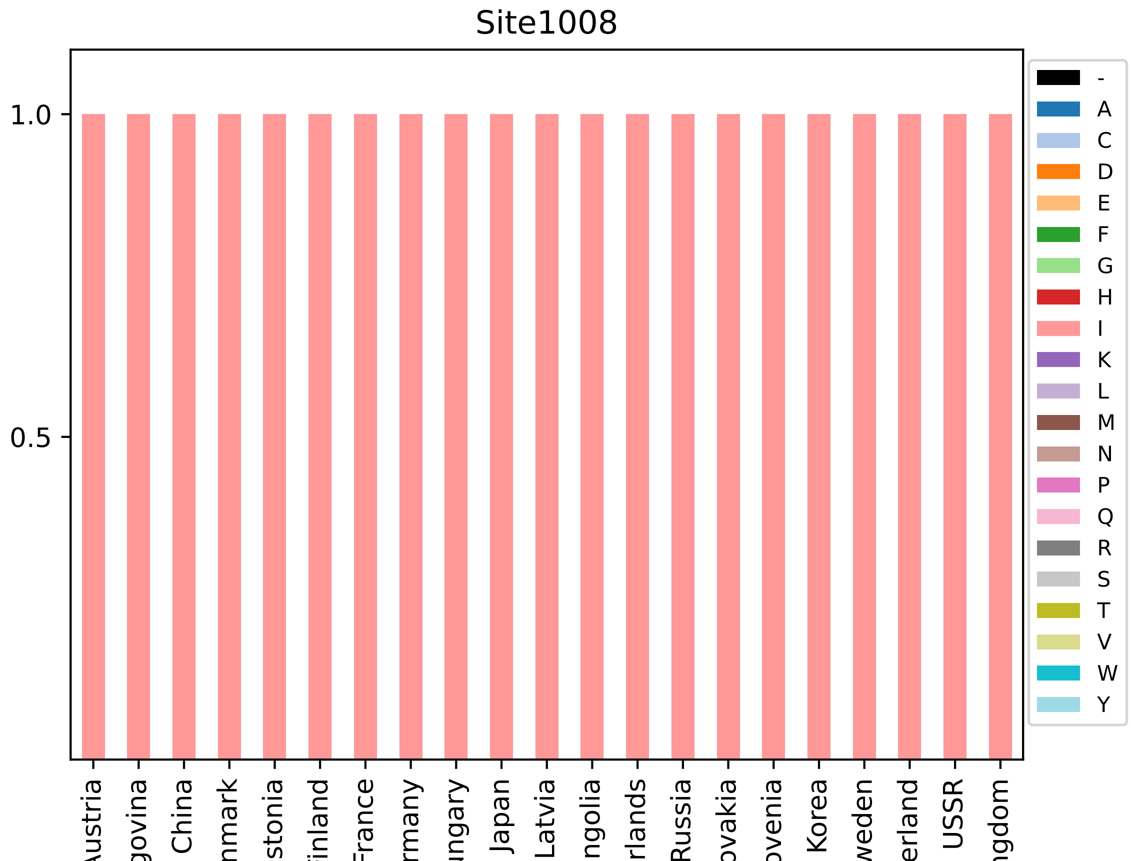
Site1008
1.0
0.5
-
A
C
D
E
F
G
H
I
K
L
M
N
P
Q
R
S
T
V
W
Y
China
Japan
Latvia
Russia
Korea
USSR

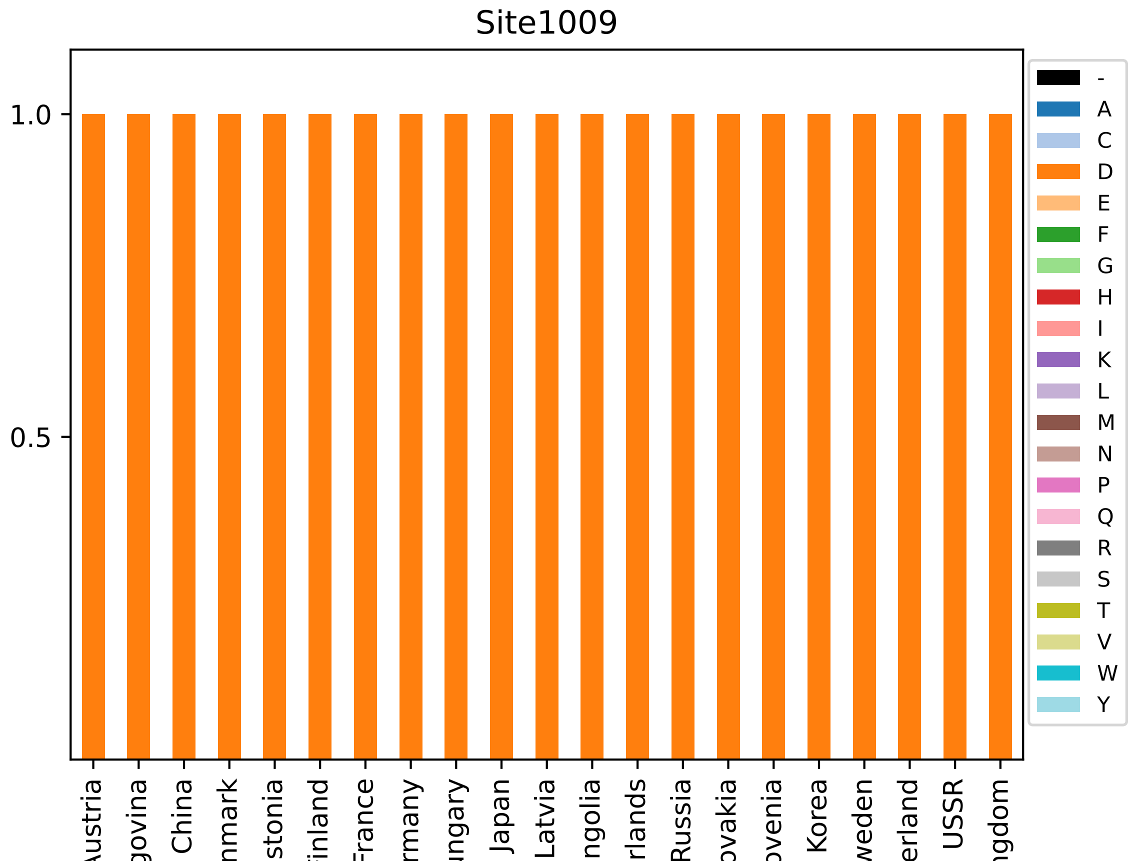

Site1009
1.0
0.5
-
A
C
D
E
F
G
H
I
K
L
M
N
P
Q
R
S
T
V
W
Y
China
Japan
Latvia
Russia
Korea
USSR

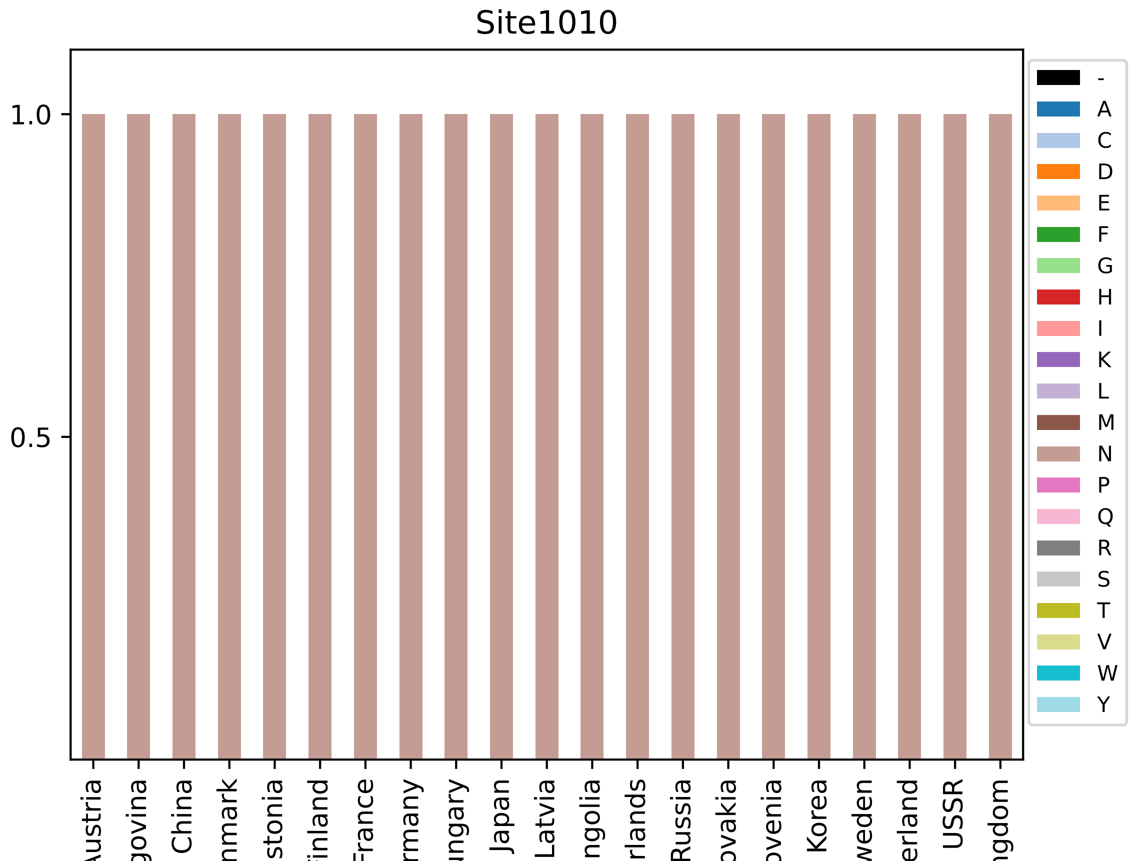

Site1010
1.0
0.5
-
A
C
D
E
F
G
H
I
K
L
M
N
P
Q
R
S
T
V
W
Y
Austria
China
Japan
Latvia
Russia
Korea
USSR

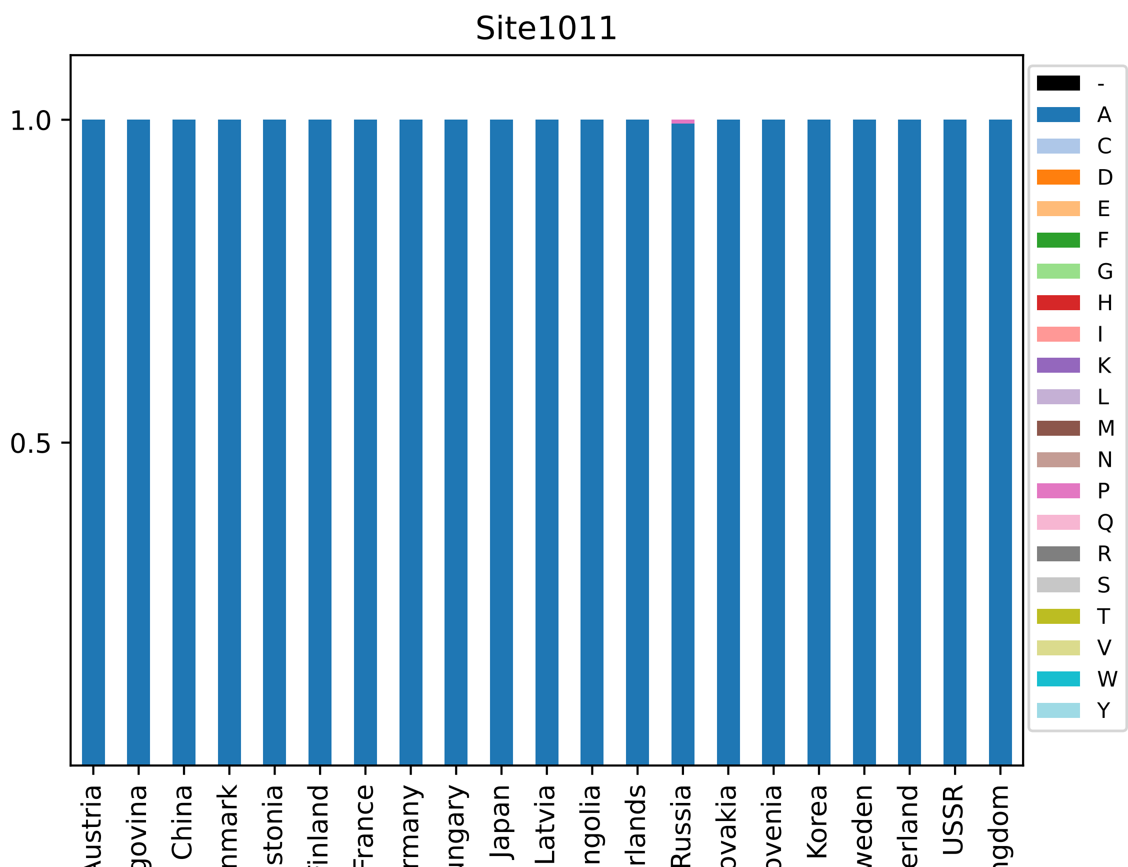

Site1011
1.0
0.5
-
A
C
D
E
F
G
H
I
K
L
M
N
P
Q
R
S
T
V
W
Y
Austria
China
Japan
Latvia
Russia
Korea
USSR

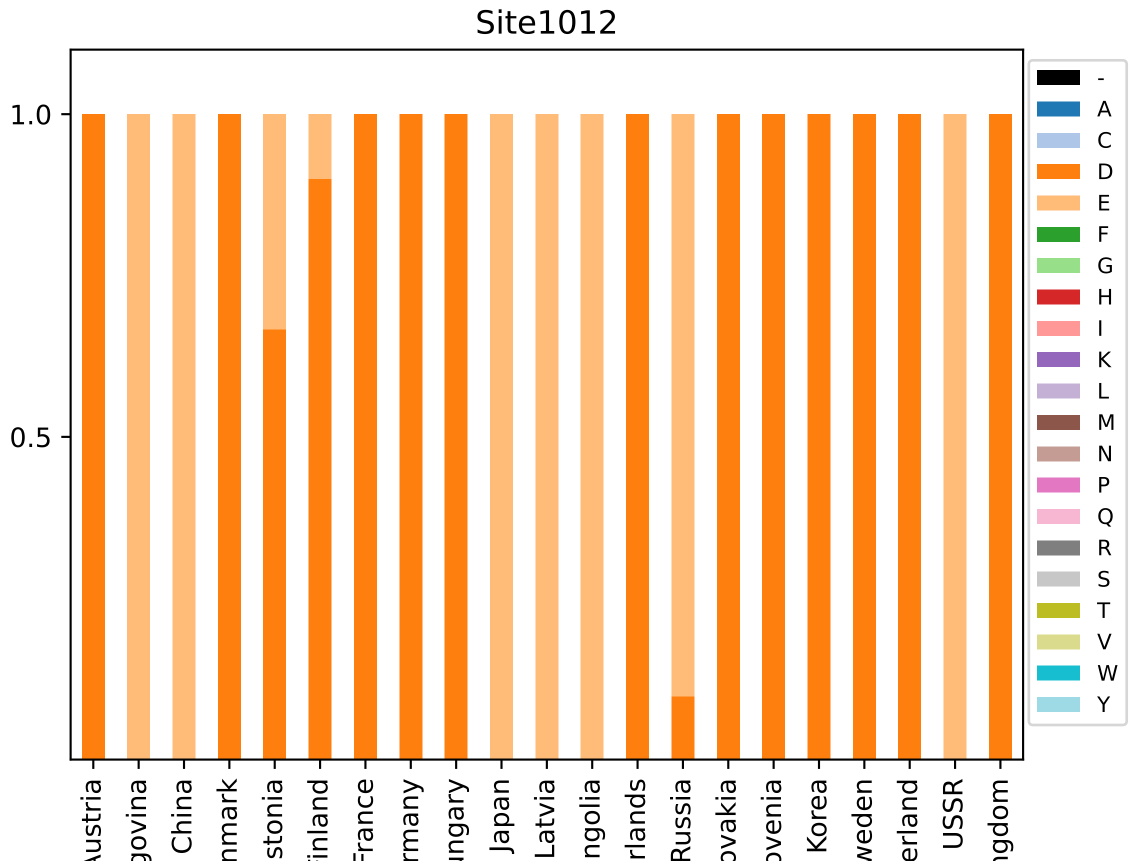
Site1012
1.0
0.5
-
A
C
D
E
F
G
H
I
K
L
M
N
P
Q
R
S
T
V
W
Y
China
Japan
Latvia
Russia
Korea
USSR

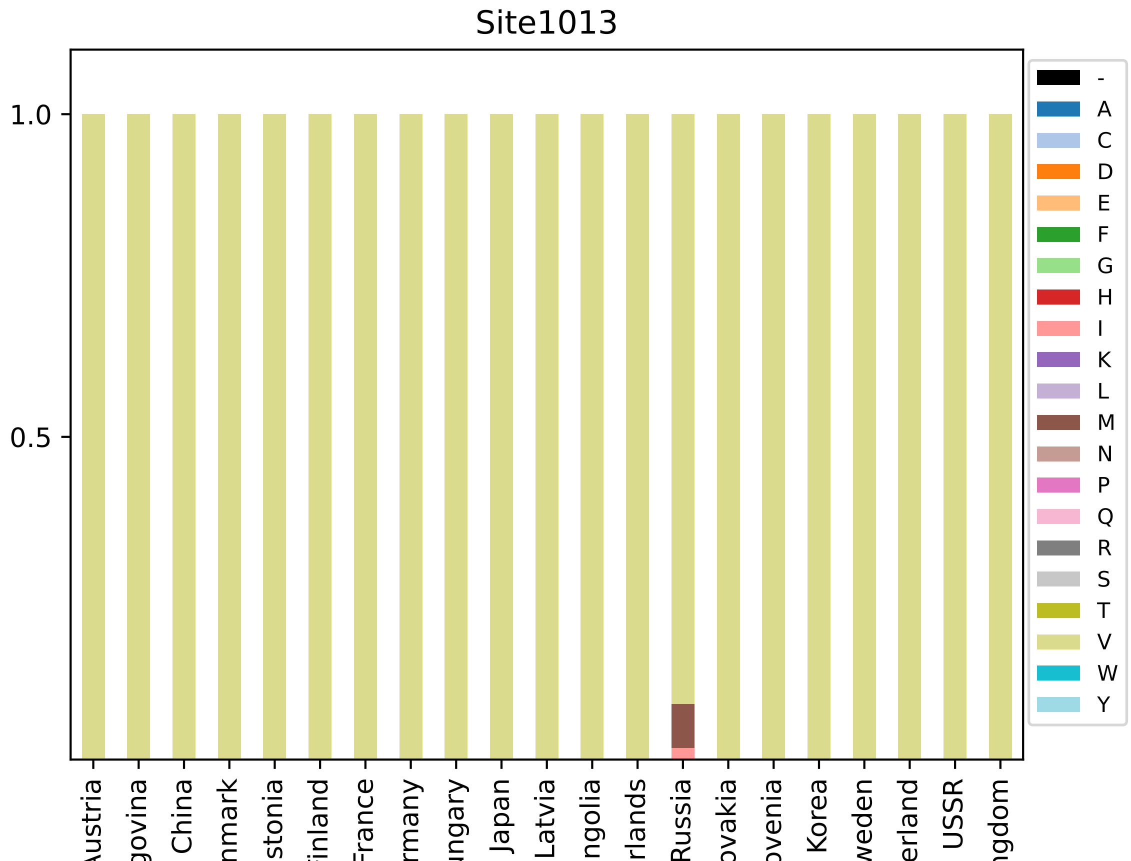

Site1013
1.0
0.5
-
A
C
D
E
F
G
H
I
K
L
M
N
P
Q
R
S
T
V
W
Y
Austria
govina
China
nmark
stonia
Finland
France
rmany
ungary
Japan
Latvia
ngolia
rlands
Russia
ovakia
ovenia
Korea
weden
erland
USSR
ngdom

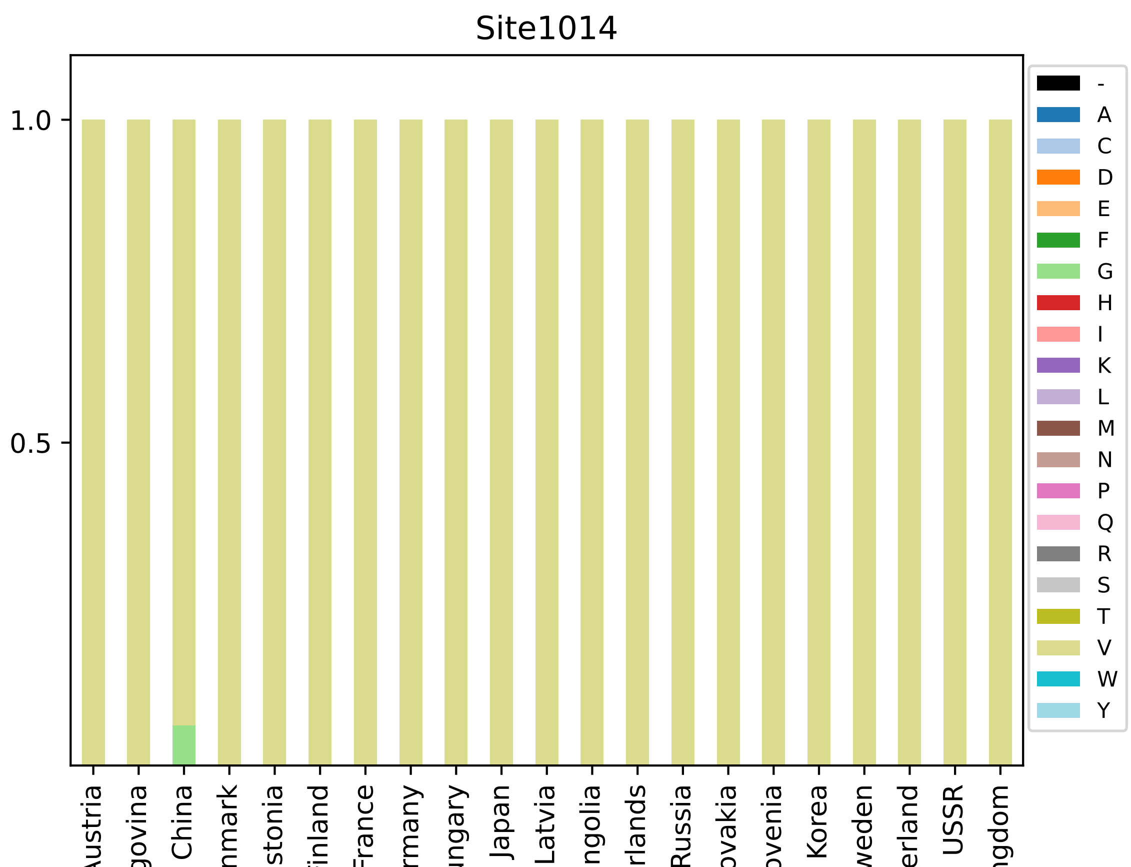

Site1014
1.0
0.5
-
A
C
D
E
F
G
H
I
K
L
M
N
P
Q
R
S
T
V
W
Y
Austria
govina
China
nmark
stonia
inland
France
rmany
ungary
Japan
Latvia
ngolia
rlands
Russia
ovakia
ovenia
Korea
weden
erland
USSR
ngdom

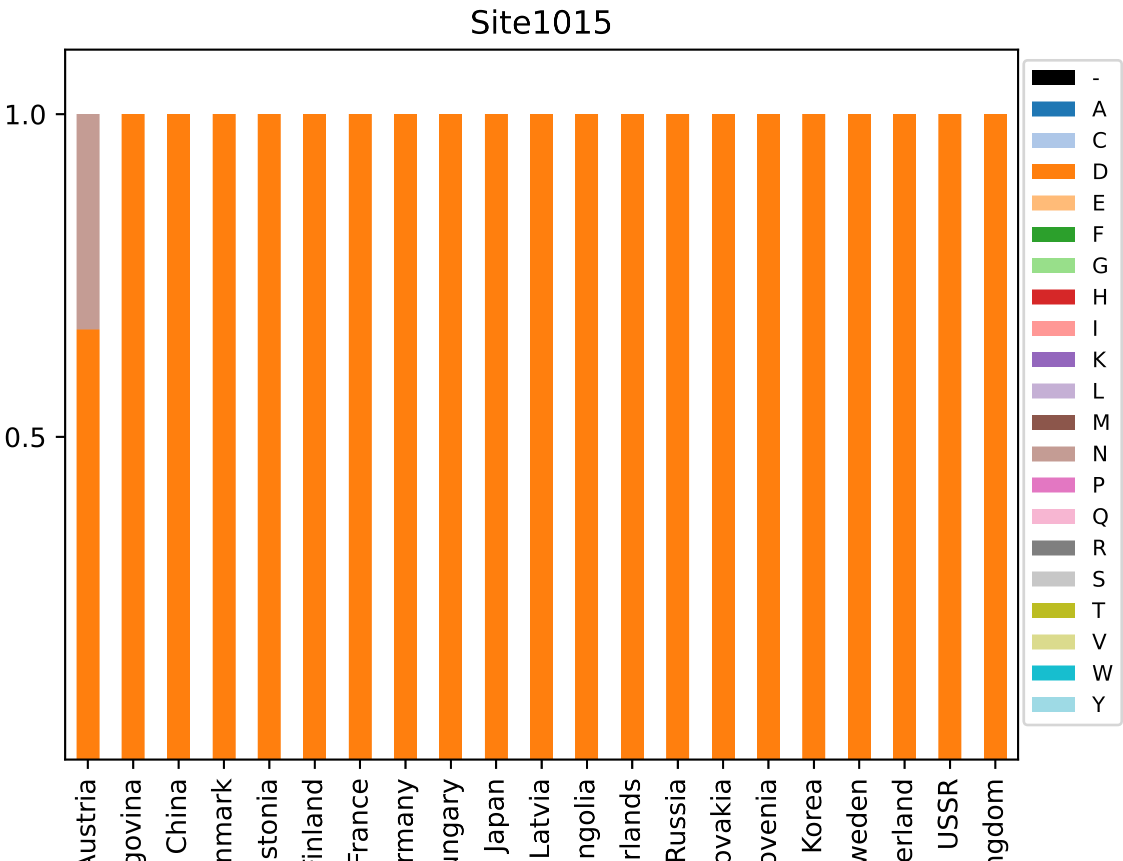

Site1015
1.0
0.5
-
A
C
D
E
F
G
H
I
K
L
M
N
P
Q
R
S
T
V
W
Y
Austria
China
Japan
Latvia
Russia
Korea
USSR

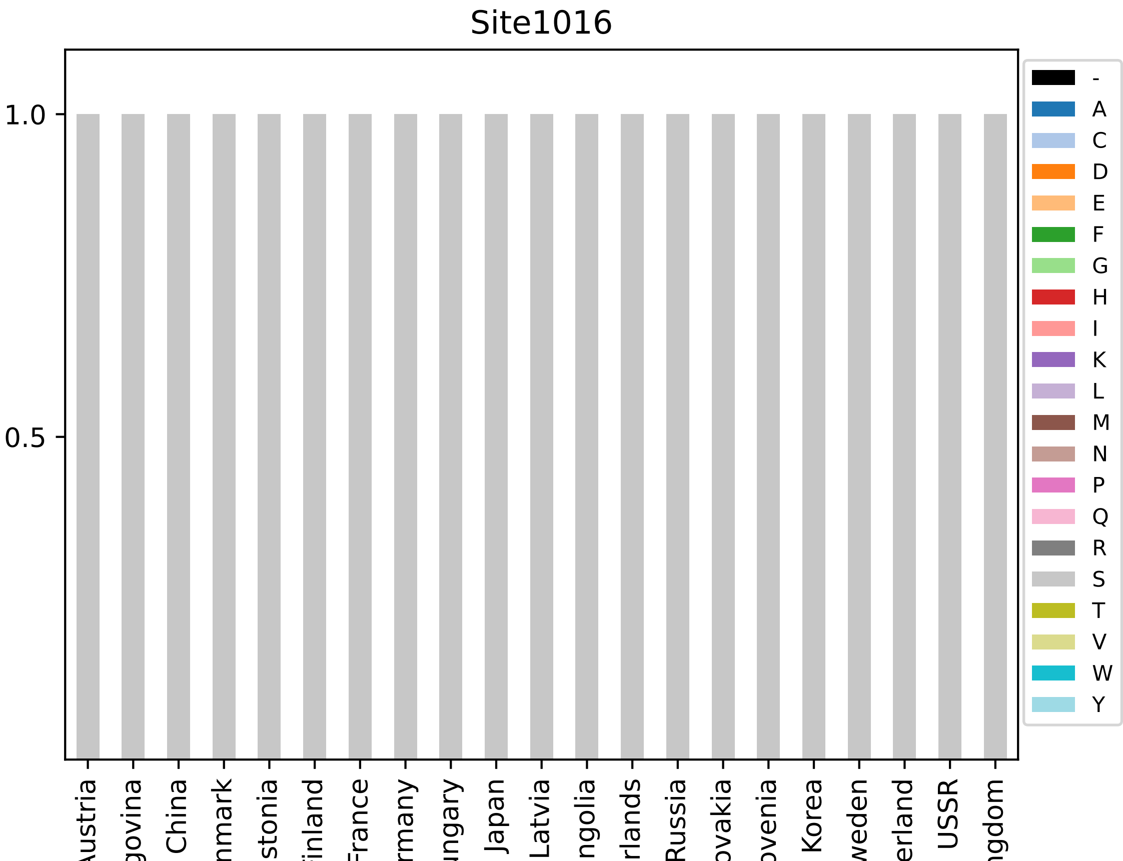
Site1016
1.0
0.5
-
A
C
D
E
F
G
H
I
K
L
M
N
P
Q
R
S
T
V
W
Y
China
Japan
Latvia
Russia
Korea
USSR

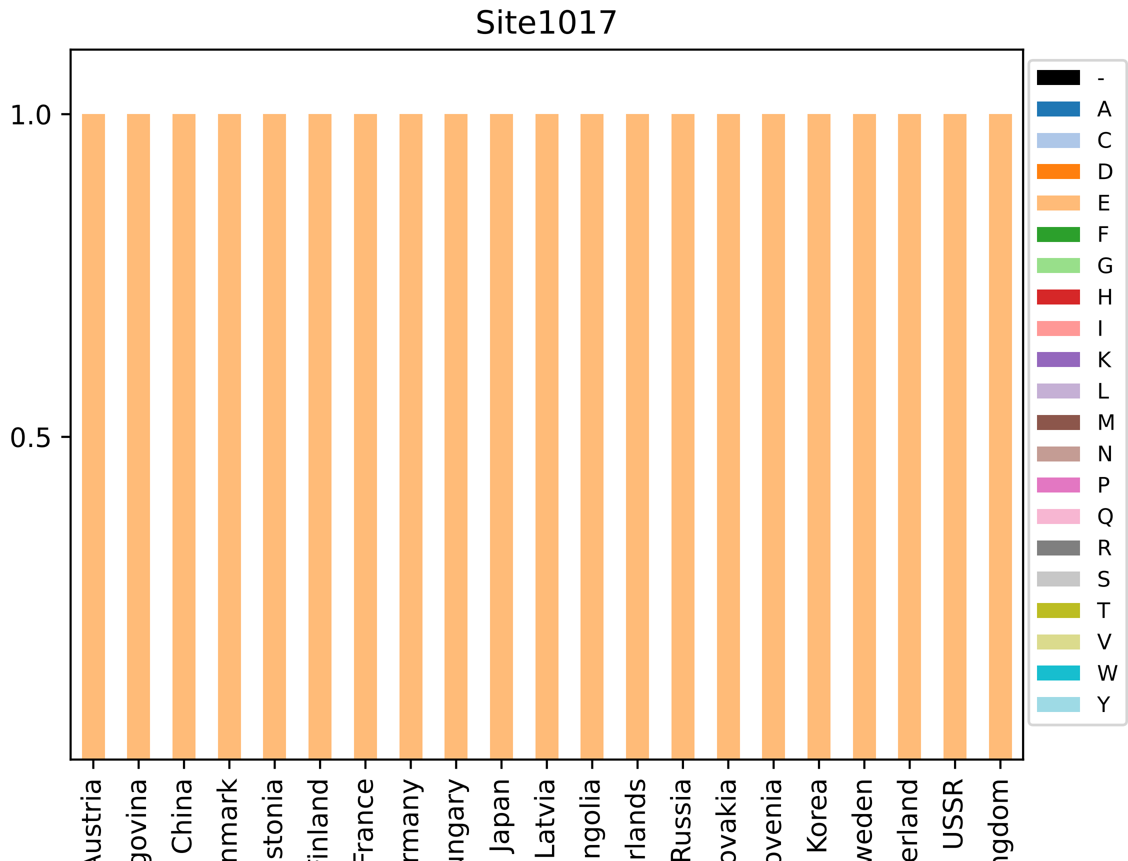
Site1017
1.0
0.5
-
A
C
D
E
F
G
H
I
K
L
M
N
P
Q
R
S
T
V
W
Y
China
Japan
Latvia
Korea
USSR

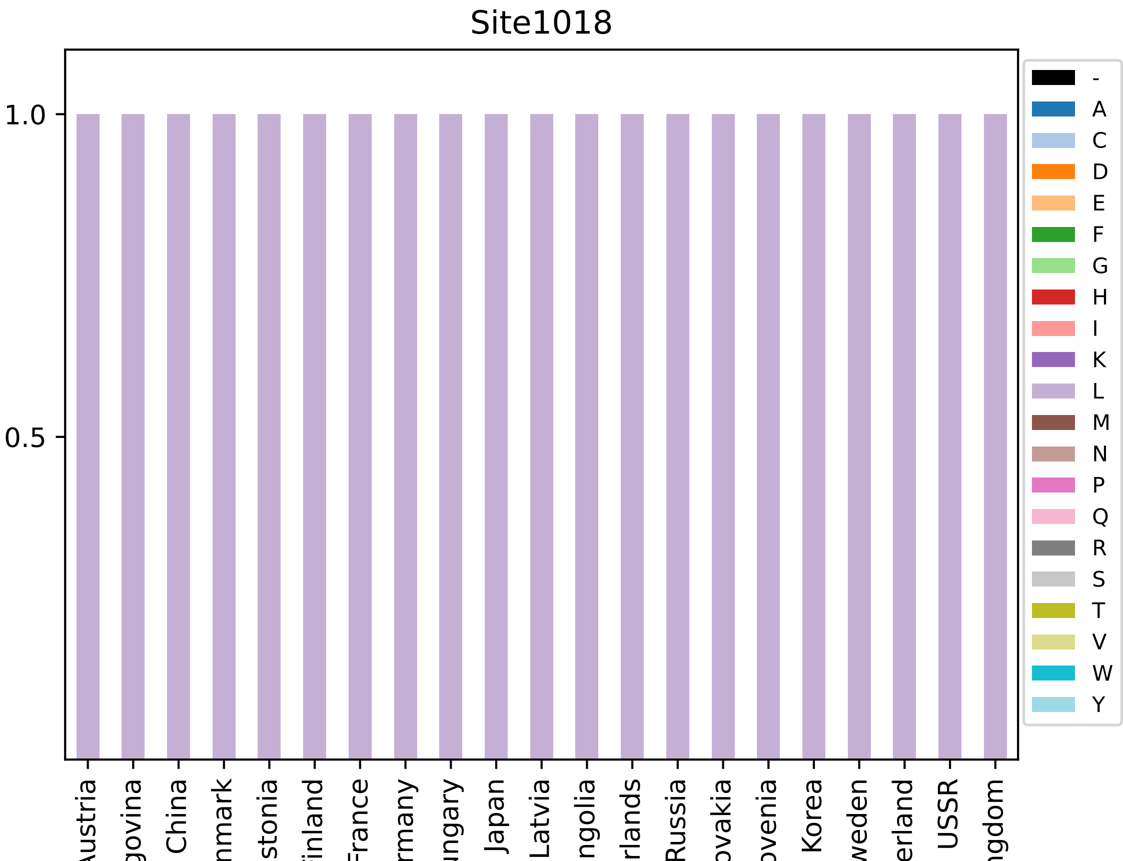

Site1018
1.0
0.5
-
A
C
D
E
F
G
H
I
K
L
M
N
P
Q
R
S
T
V
W
Y
Austria
govina
China
nmark
stonia
inland
France
rmany
ungary
Japan
Latvia
ngolia
rlands
Russia
ovakia
ovenia
Korea
weden
erland
USSR
ngdom

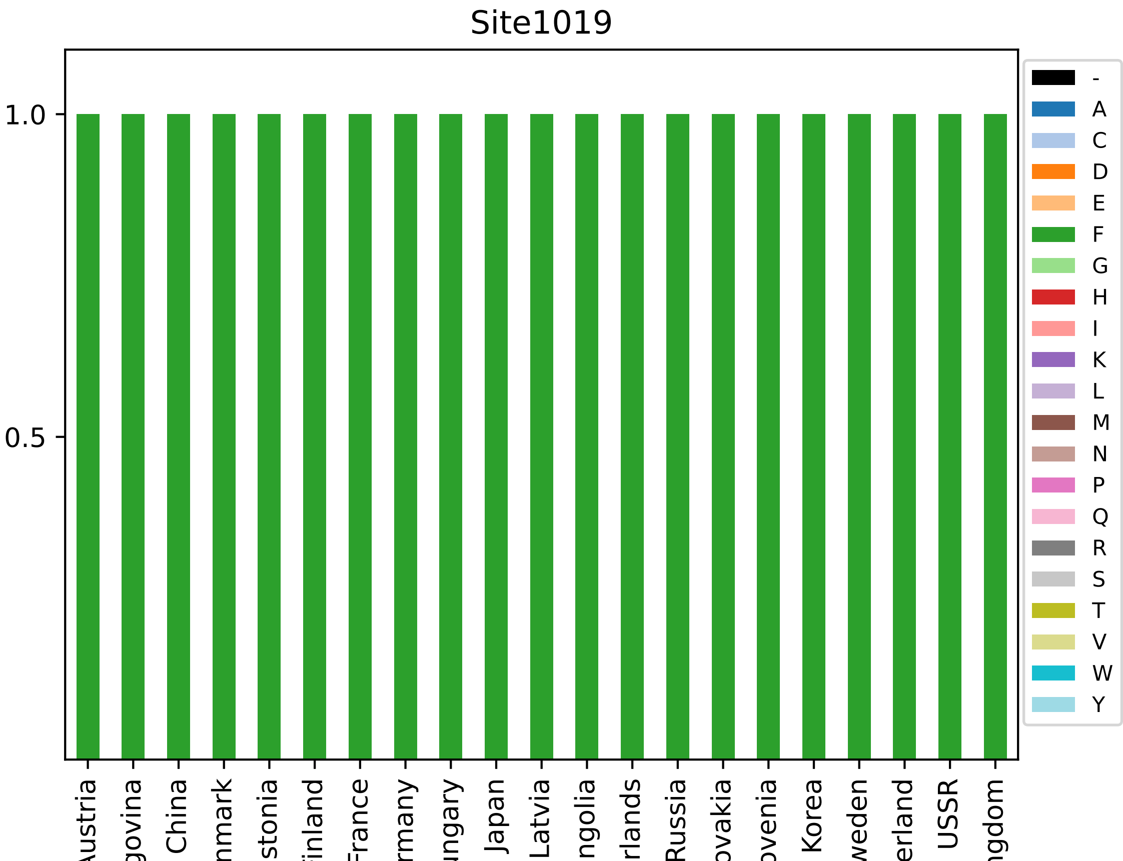
Site1019
1.0
0.5
-
A
C
D
E
F
G
H
I
K
L
M
N
P
Q
R
S
T
V
W
Y
Austria
govina
China
nmark
stonia
Finland
France
rmany
ungary
Japan
Latvia
ngolia
rlands
Russia
ovakia
ovenia
Korea
weden
erland
USSR
ngdom

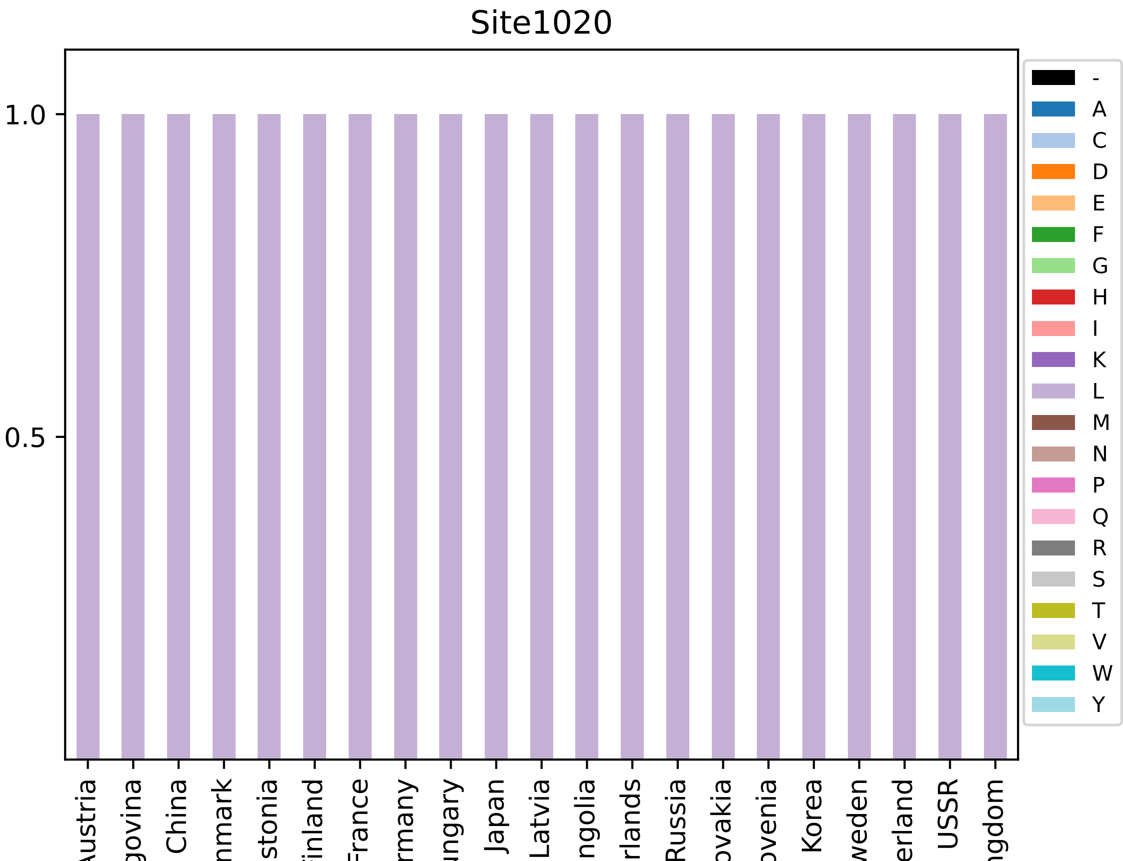
Site1020
1.0
0.5
-
A
C
D
E
F
G
H
I
K
L
M
N
P
Q
R
S
T
V
W
Y
China
stonia
Japan
Latvia
ngolia
rlands
Russia
ovakia
Korea
weden
erland
USSR
ngdom

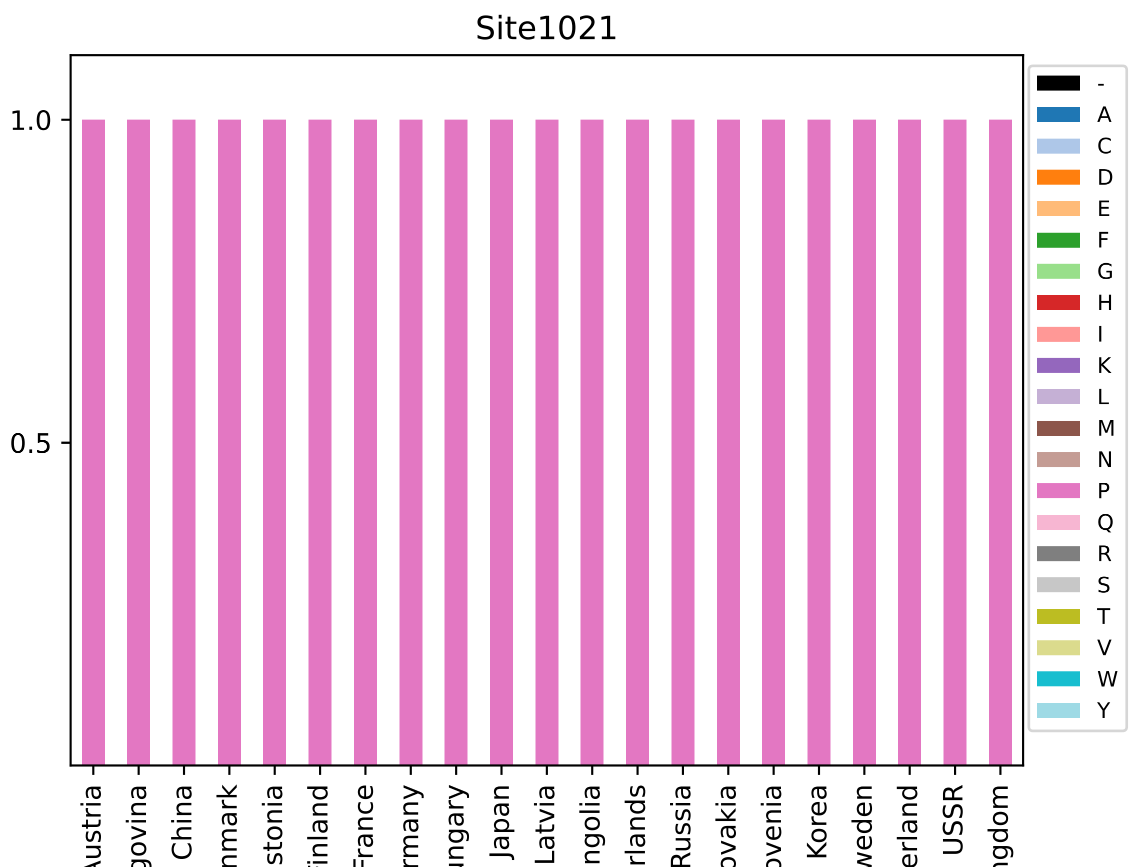
Site1021
1.0
0.5
China
Japan
Latvia
Russia
Korea
USSR
-
A
C
D
E
F
G
H
I
K
L
M
N
P
Q
R
S
T
V
W
Y

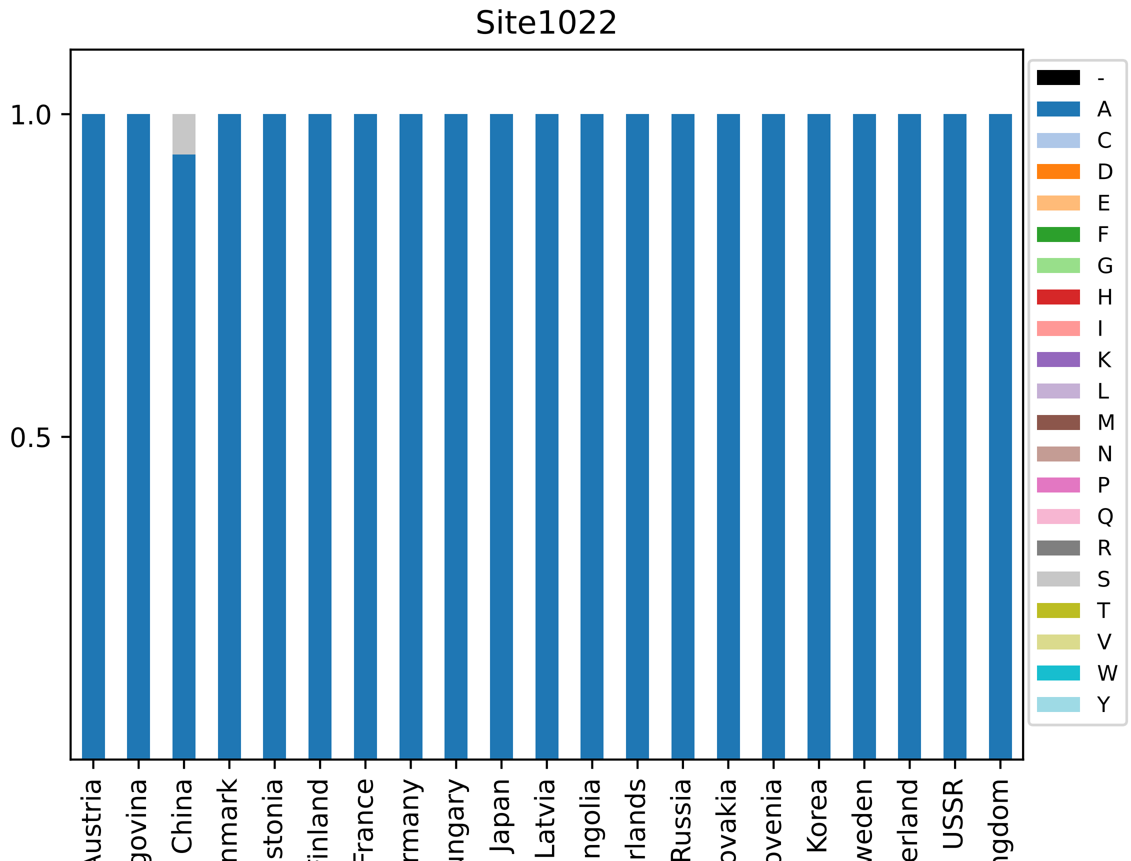

Site1022
1.0
0.5
-
A
C
D
E
F
G
H
I
K
L
M
N
P
Q
R
S
T
V
W
Y
Austria
China
Finland
France
Japan
Latvia
Russia
Korea
USSR

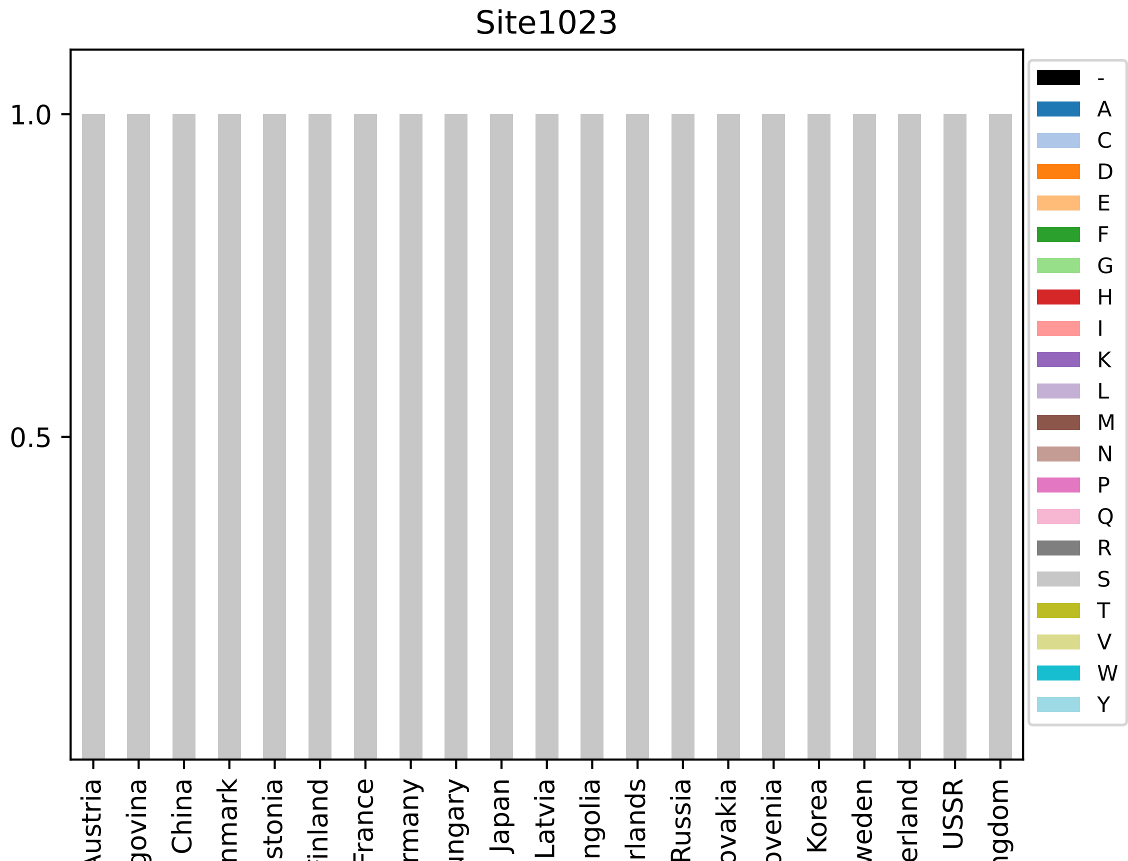
Site1023
1.0
0.5
Austria
govina
China
nmark
stonia
Finland
France
rmany
ungary
Japan
Latvia
ngolia
rlands
Russia
ovakia
ovenia
Korea
weden
erland
USSR
ngdom
-
A
C
D
E
F
G
H
I
K
L
M
N
P
Q
R
S
T
V
W
Y

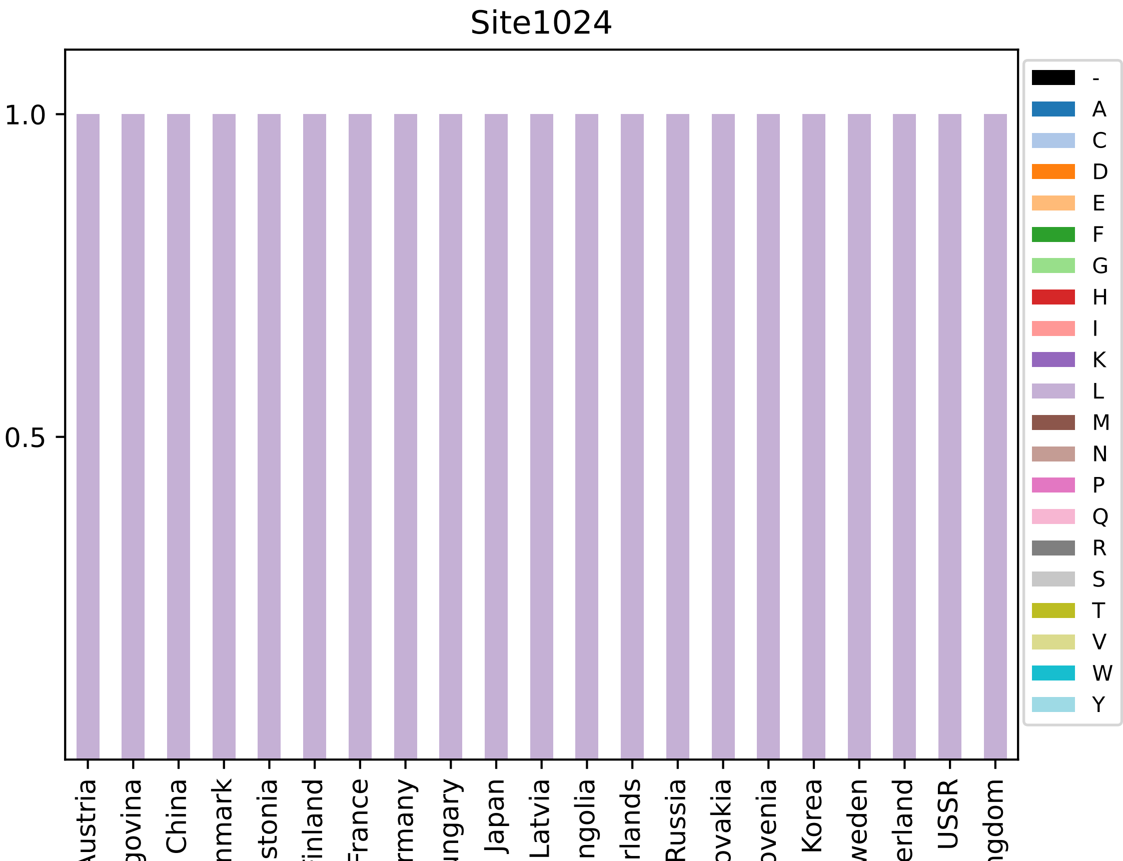

Site1024
1.0
0.5
-
A
C
D
E
F
G
H
I
K
L
M
N
P
Q
R
S
T
V
W
Y
Austria
China
Japan
Latvia
Russia
Korea
USSR

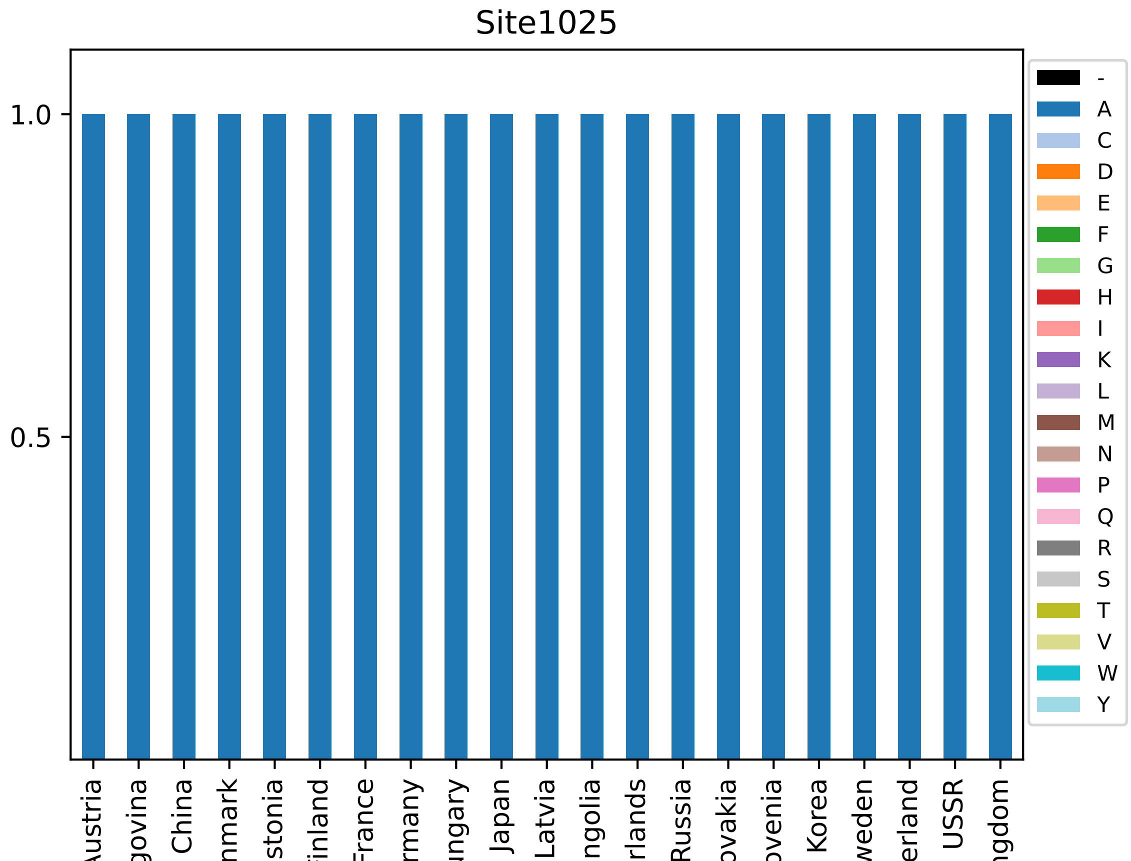

Site1025
1.0
0.5
-
A
C
D
E
F
G
H
I
K
L
M
N
P
Q
R
S
T
V
W
Y
Austria
govina
China
nmark
stonia
inland
France
rmany
ungary
Japan
Latvia
ngolia
rlands
Russia
ovakia
ovenia
Korea
weden
erland
USSR
ngdom

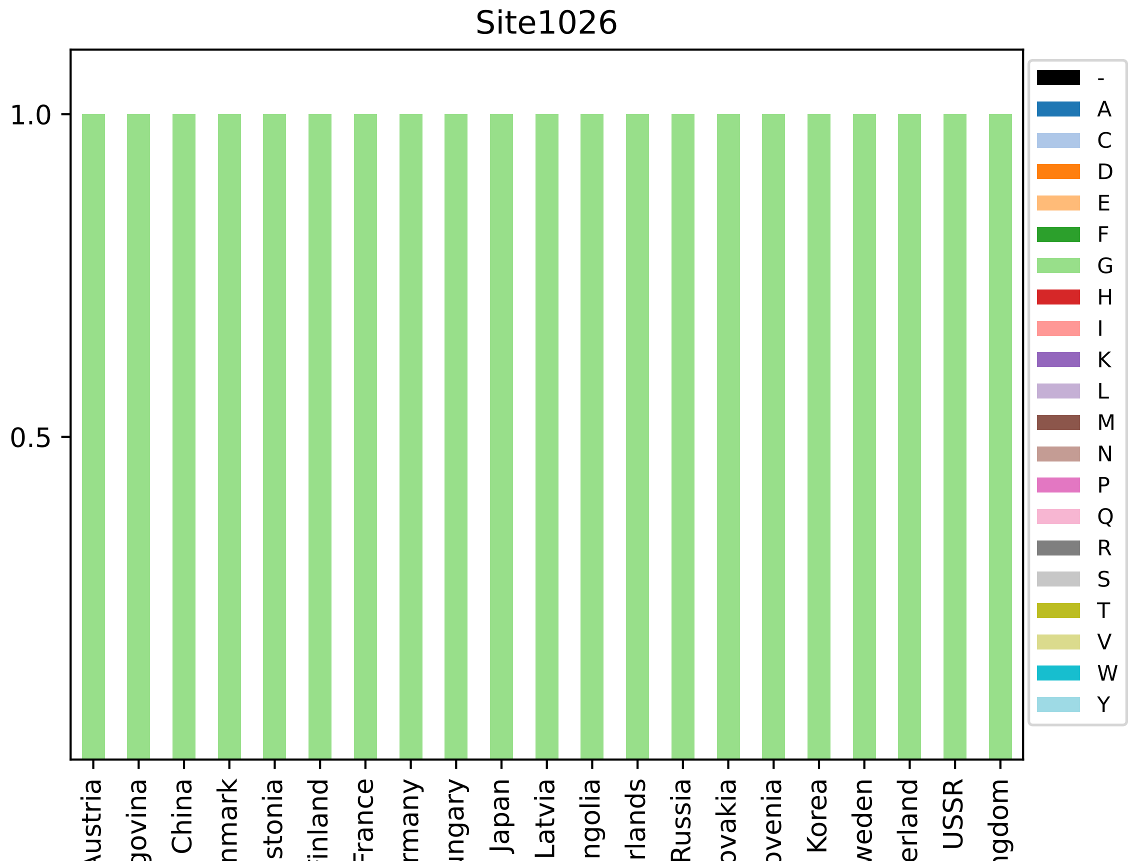
Site1026
1.0
0.5
-
A
C
D
E
F
G
H
I
K
L
M
N
P
Q
R
S
T
V
W
Y
China
Japan
Latvia
Korea
USSR

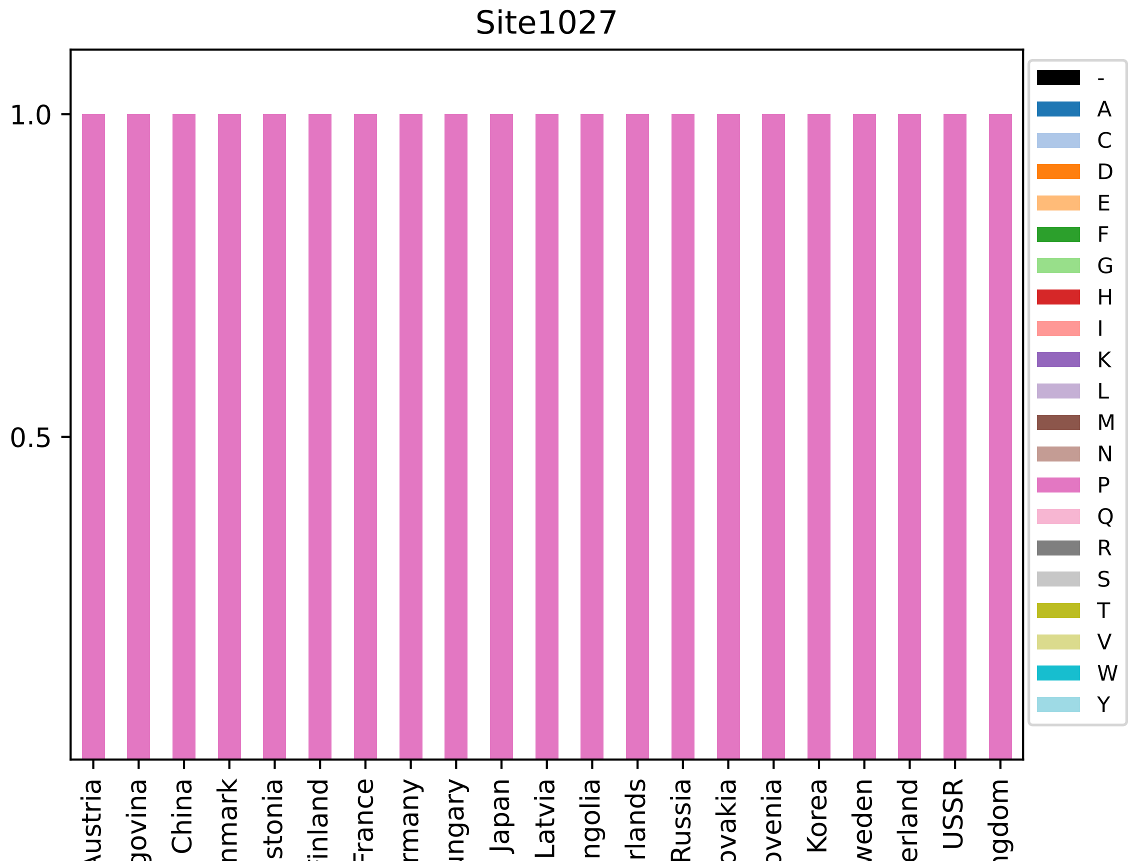

Site1027
1.0
0.5
-
A
C
D
E
F
G
H
I
K
L
M
N
P
Q
R
S
T
V
W
Y
China
Japan
Latvia
Russia
Korea
USSR

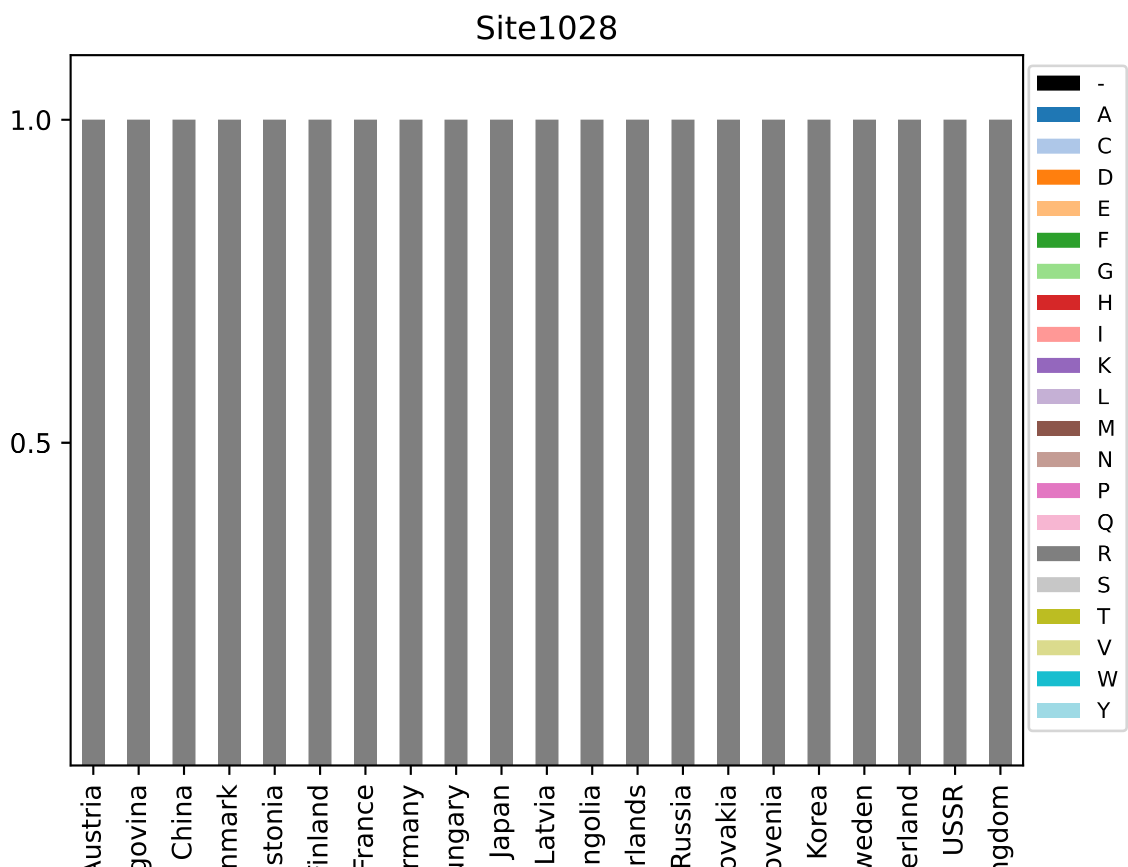

Site1028
1.0
0.5
-
A
C
D
E
F
G
H
I
K
L
M
N
P
Q
R
S
T
V
W
Y
China
Japan
Latvia
Russia
Korea
USSR

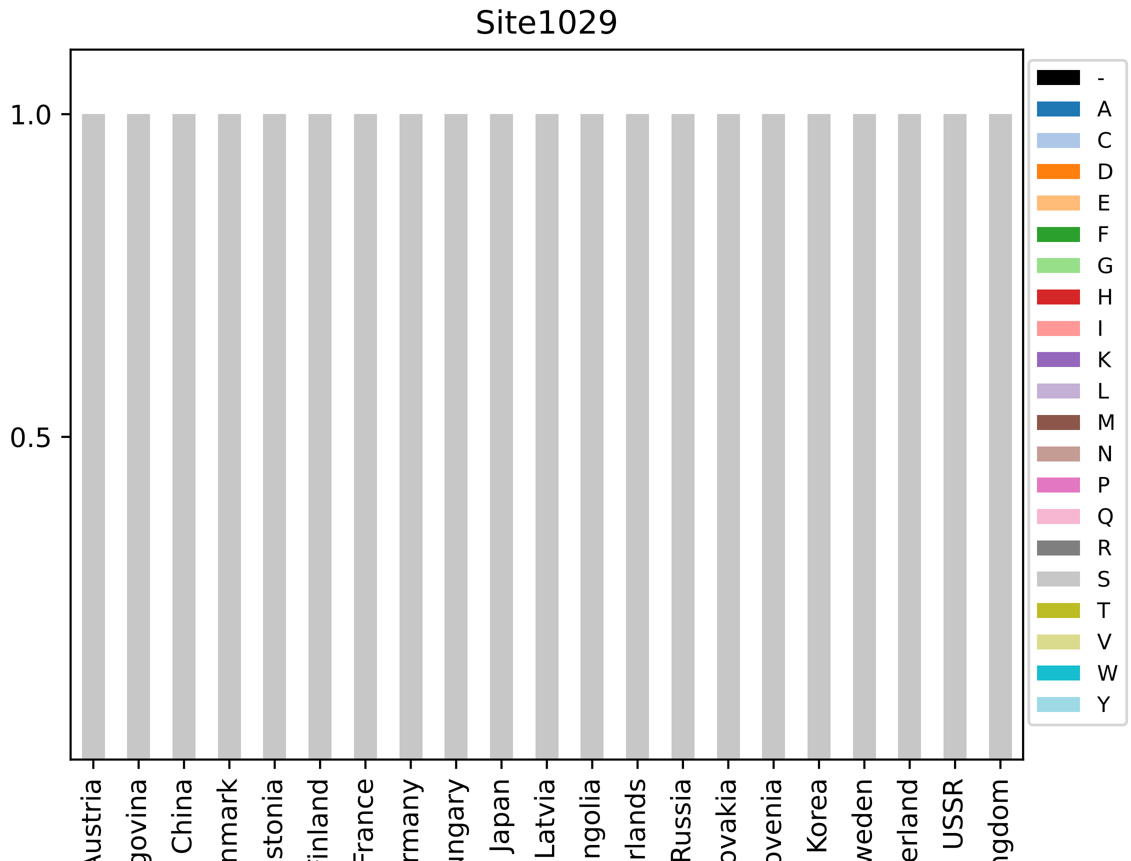
Site1029
1.0
0.5
-
A
C
D
E
F
G
H
I
K
L
M
N
P
Q
R
S
T
V
W
Y
Austria
govina
China
nmark
stonia
inland
France
rmany
ungary
Japan
Latvia
ngolia
rlands
Russia
ovakia
ovenia
Korea
weden
erland
USSR
ngdom

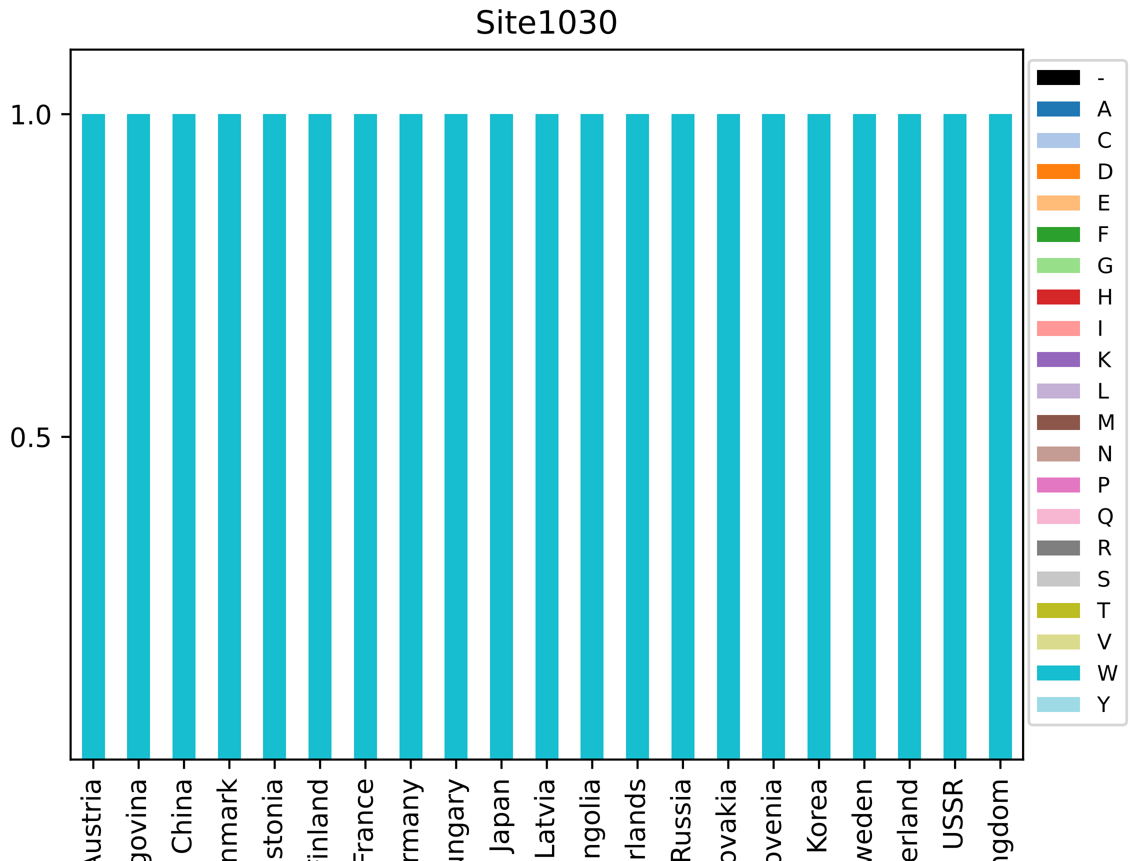
Site1030
1.0
0.5
-
A
C
D
E
F
G
H
I
K
L
M
N
P
Q
R
S
T
V
W
Y
China
Japan
Latvia
Russia
Korea
USSR

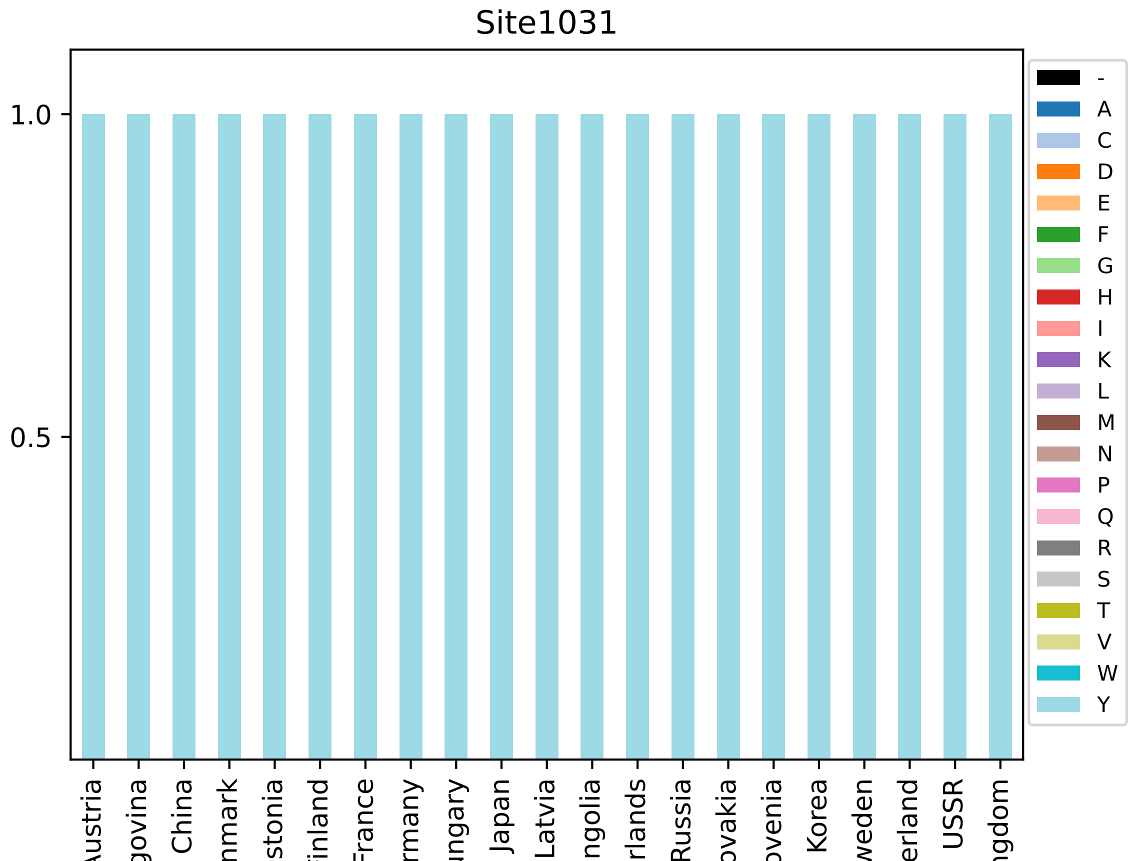
Site1031
1.0
0.5
-
A
C
D
E
F
G
H
I
K
L
M
N
P
Q
R
S
T
V
W
Y
China
Japan
Latvia
Russia
Korea
USSR

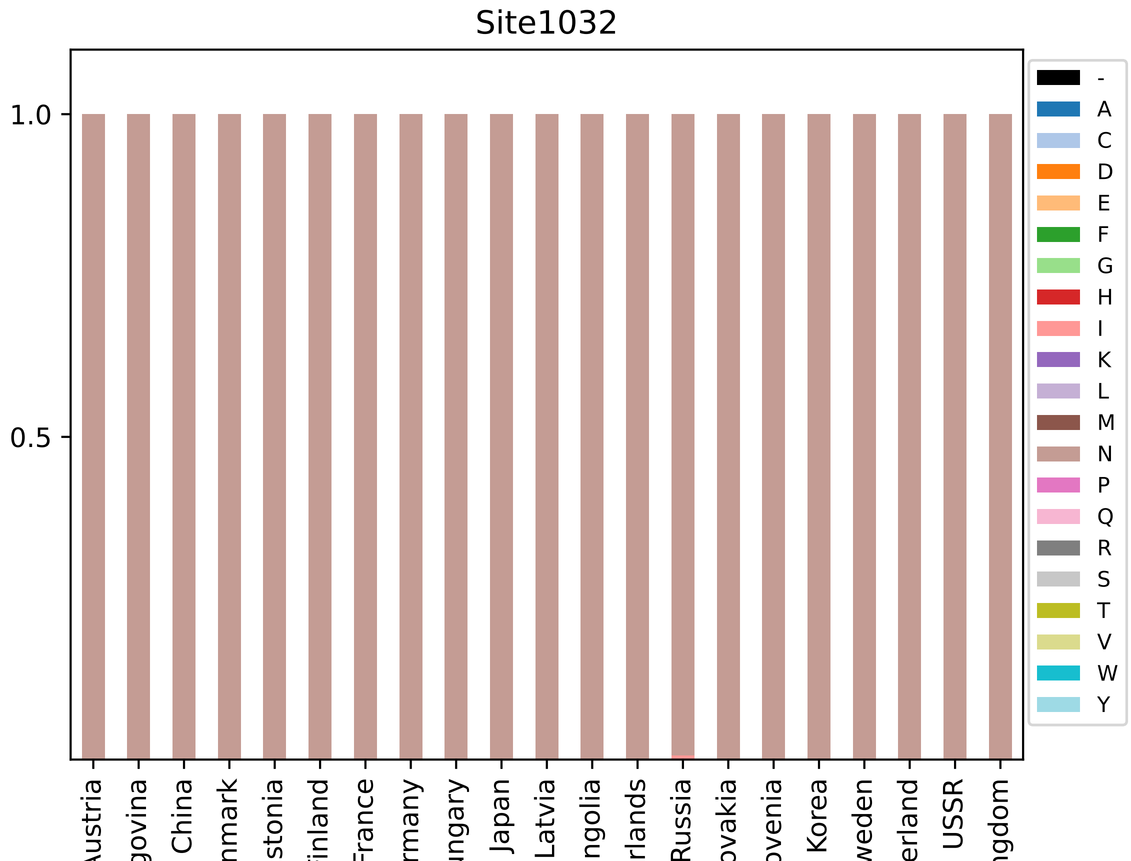
Site1032
1.0
0.5
-
A
C
D
E
F
G
H
I
K
L
M
N
P
Q
R
S
T
V
W
Y
ustria
govina
China
nmark
stonia
inland
rance
rmany
ungary
Japan
Latvia
ngolia
rlands
Russia
ovakia
ovenia
Korea
weden
erland
USSR
ngdom

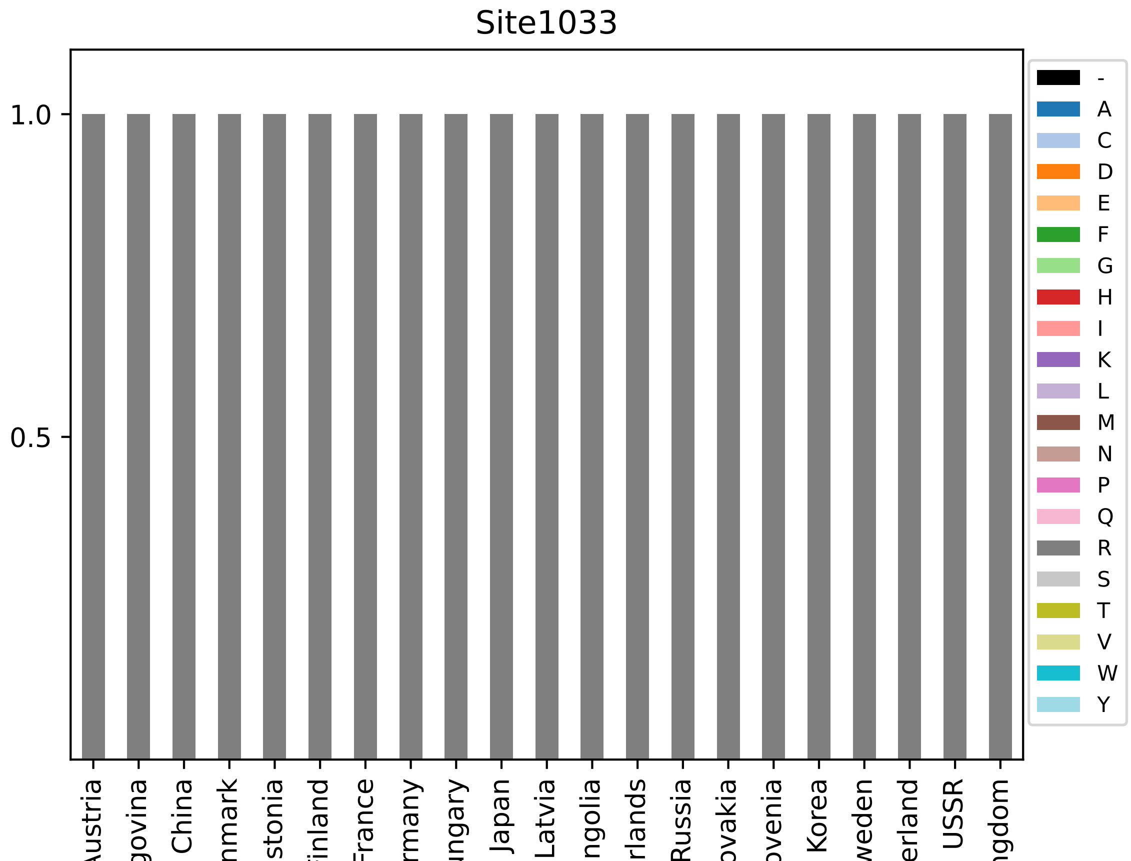

Site1033
1.0
0.5
Austria
govina
China
nmark
stonia
Finland
France
rmany
ungary
Japan
Latvia
ngolia
rlands
Russia
ovakia
ovenia
Korea
weden
erland
USSR
ngdom
-
A
C
D
E
F
G
H
I
K
L
M
N
P
Q
R
S
T
V
W
Y

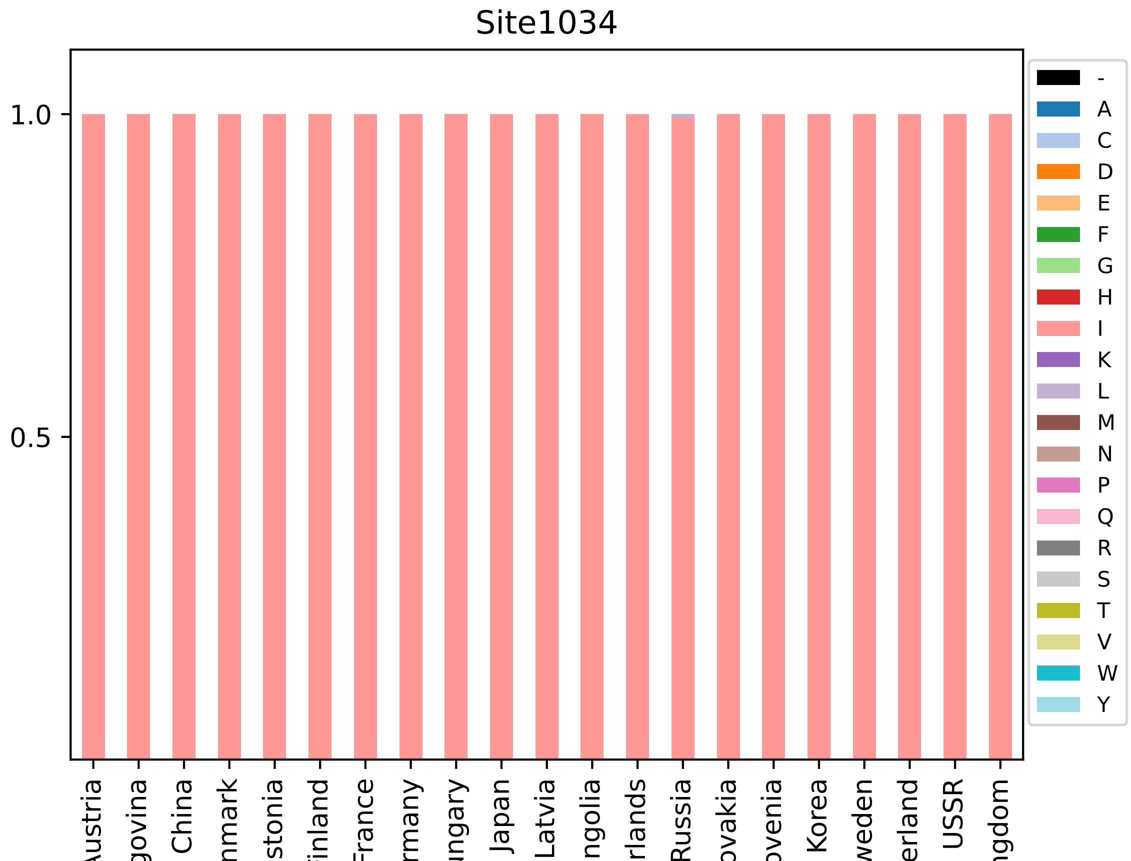
Site1034
1.0
0.5
China
Japan
Latvia
Russia
Korea
USSR
-
A
C
D
E
F
G
H
I
K
L
M
N
P
Q
R
S
T
V
W
Y

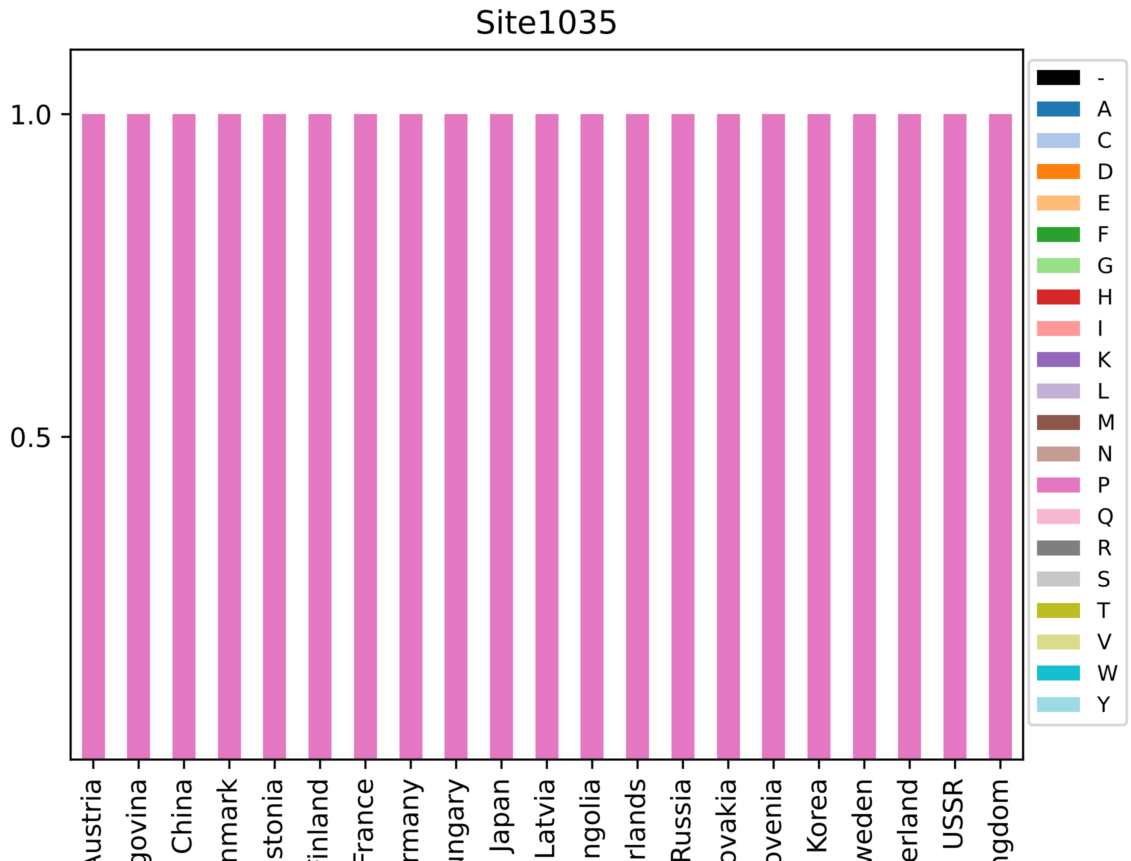

Site1035
1.0
0.5
-
A
C
D
E
F
G
H
I
K
L
M
N
P
Q
R
S
T
V
W
Y
China
Japan
Latvia
Russia
Korea
USSR

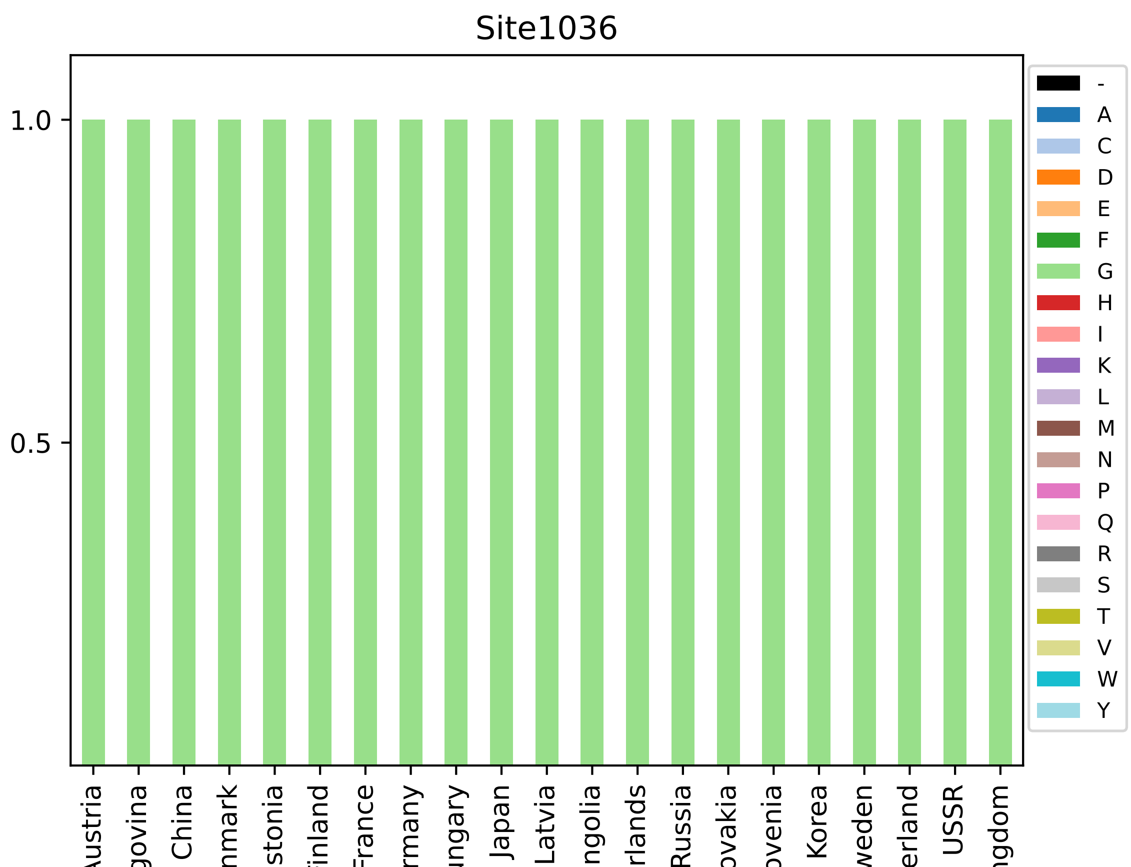

Site1036
1.0
0.5
-
A
C
D
E
F
G
H
I
K
L
M
N
P
Q
R
S
T
V
W
Y
Austria
govina
China
nmark
stonia
Finland
France
rmany
ungary
Japan
Latvia
ngolia
rlands
Russia
ovakia
ovenia
Korea
weden
erland
USSR
ngdom

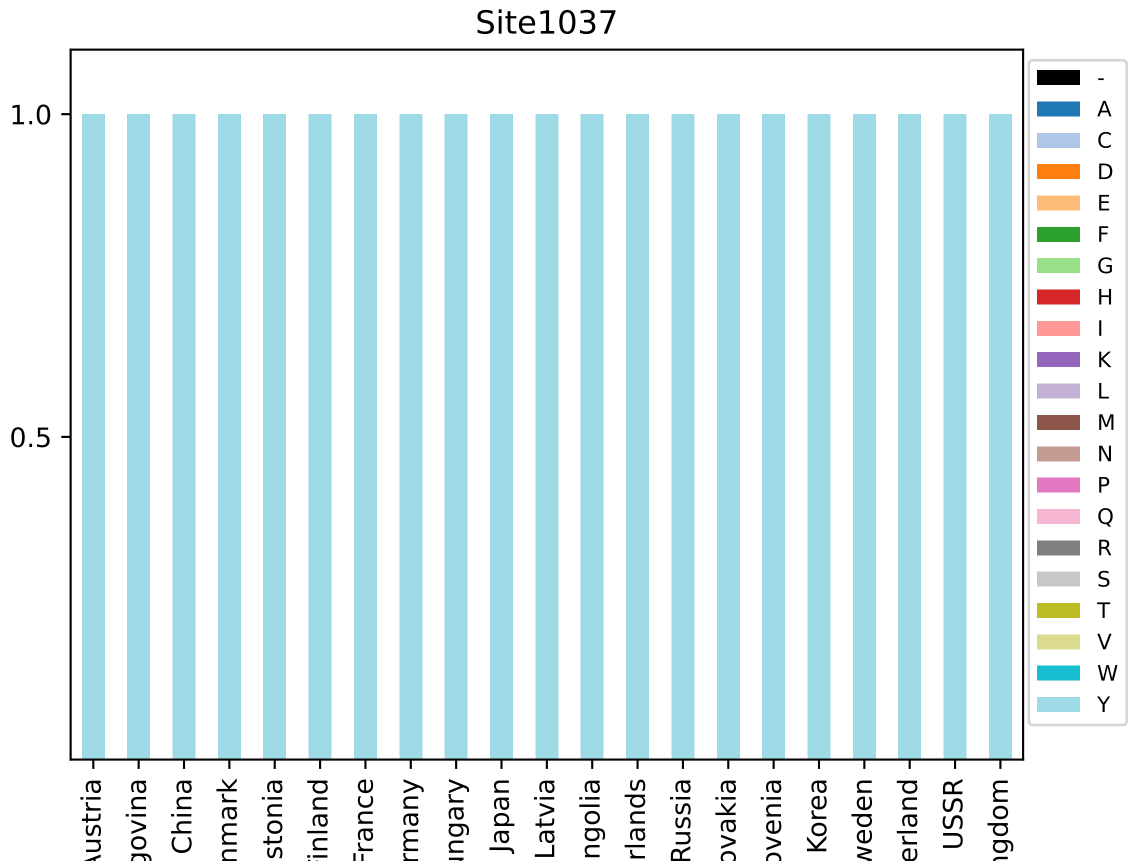
Site1037
1.0
0.5
-
A
C
D
E
F
G
H
I
K
L
M
N
P
Q
R
S
T
V
W
Y
China
Japan
Latvia
Korea
USSR

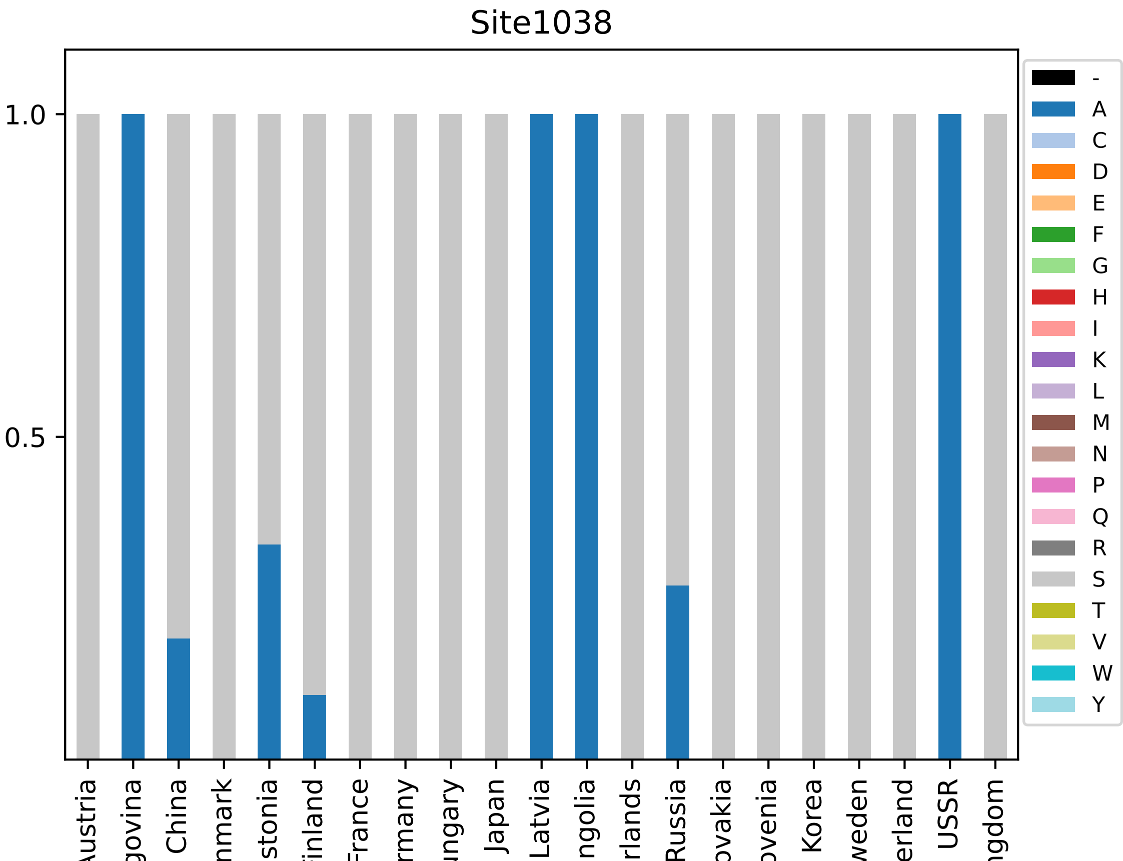

Site1038
1.0
0.5
-
A
C
D
E
F
G
H
I
K
L
M
N
P
Q
R
S
T
V
W
Y
China
Japan
Latvia
Russia
Korea
USSR

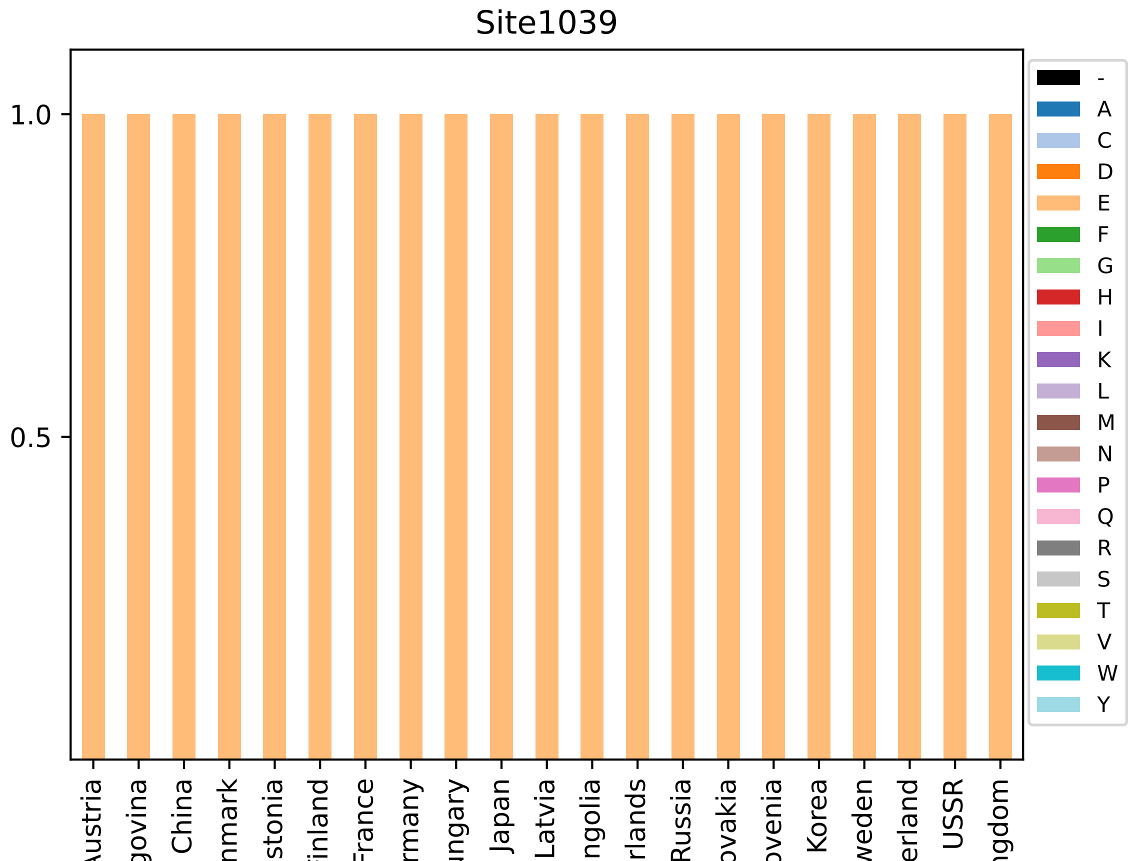

Site1039
1.0
0.5
-
A
C
D
E
F
G
H
I
K
L
M
N
P
Q
R
S
T
V
W
Y
China
Japan
Latvia
Russia
Korea
USSR

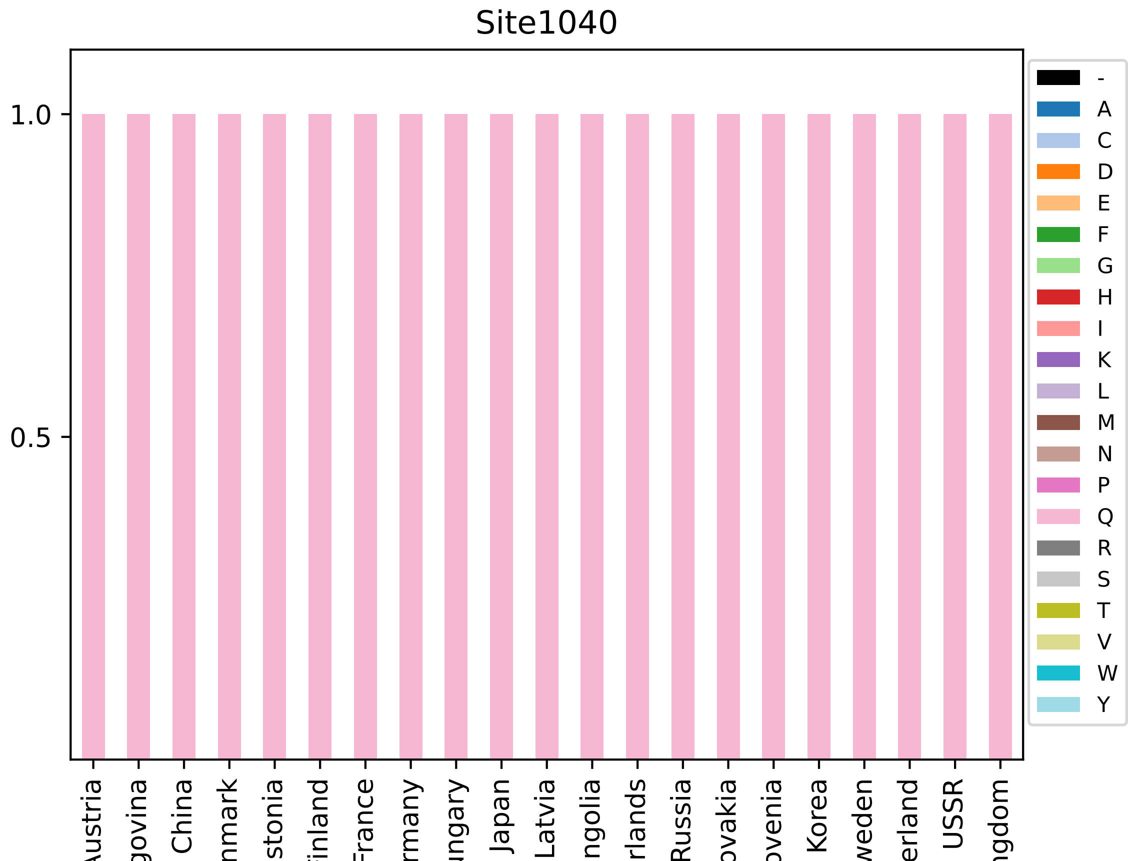
Site1040
1.0
0.5
-
A
C
D
E
F
G
H
I
K
L
M
N
P
Q
R
S
T
V
W
Y
Austria
govina
China
nmark
stonia
Finland
France
rmany
ungary
Japan
Latvia
ngolia
rlands
Russia
ovakia
ovenia
Korea
weden
erland
USSR
ngdom

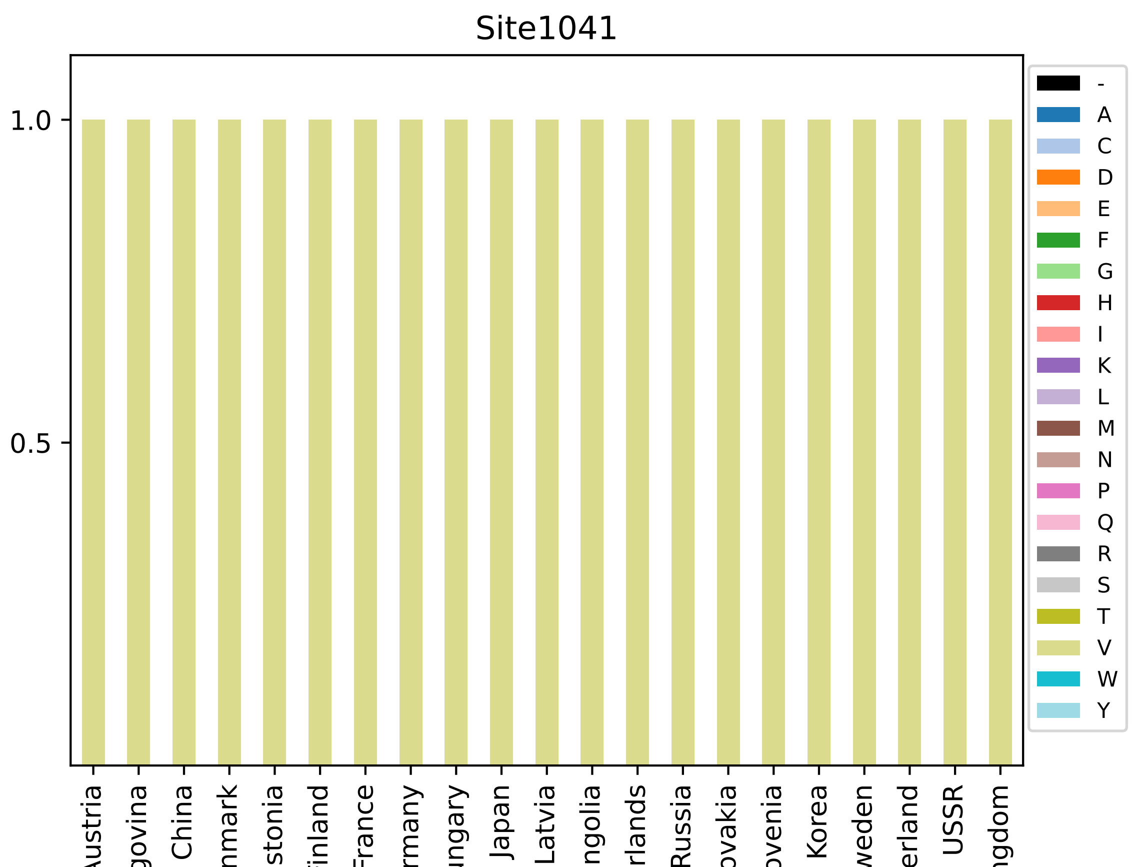

Site1041
1.0
0.5
-
A
C
D
E
F
G
H
I
K
L
M
N
P
Q
R
S
T
V
W
Y
China
Japan
Latvia
Russia
Korea
USSR

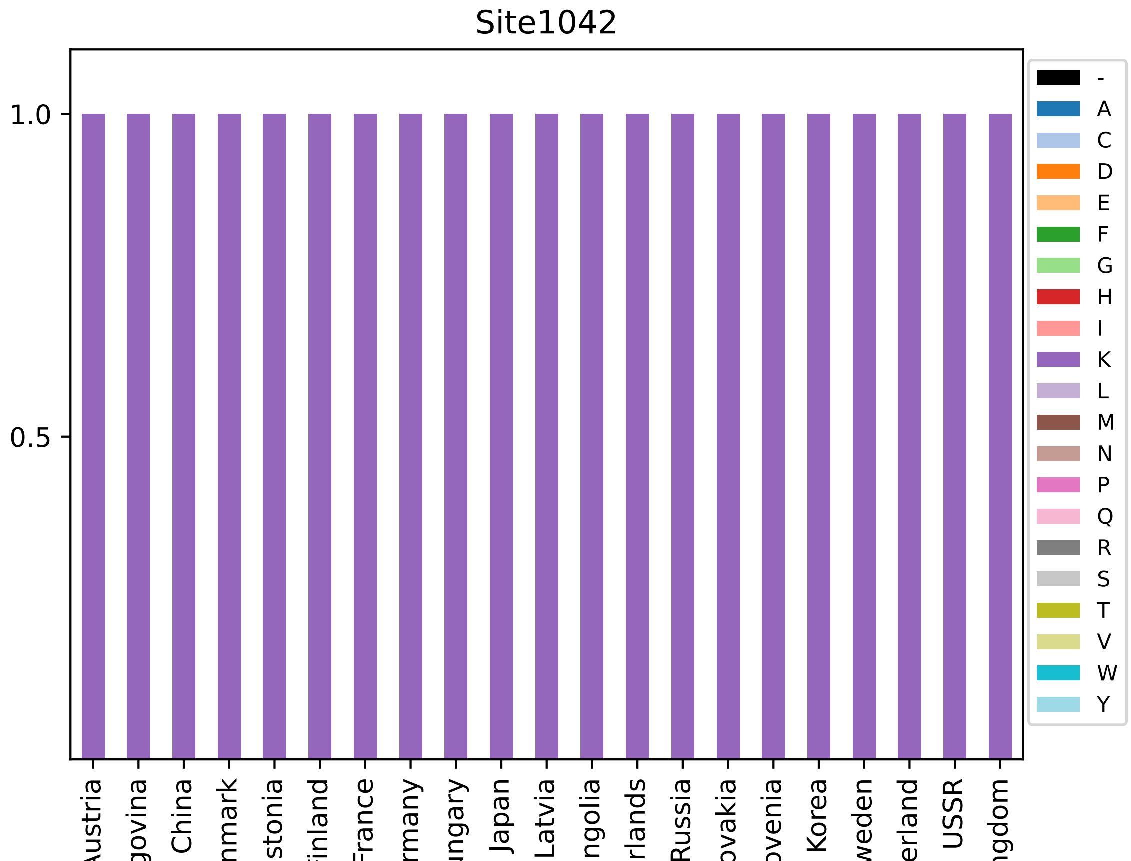

Site1042
1.0
0.5
-
A
C
D
E
F
G
H
I
K
L
M
N
P
Q
R
S
T
V
W
Y
China
Japan
Latvia
Russia
Korea
USSR

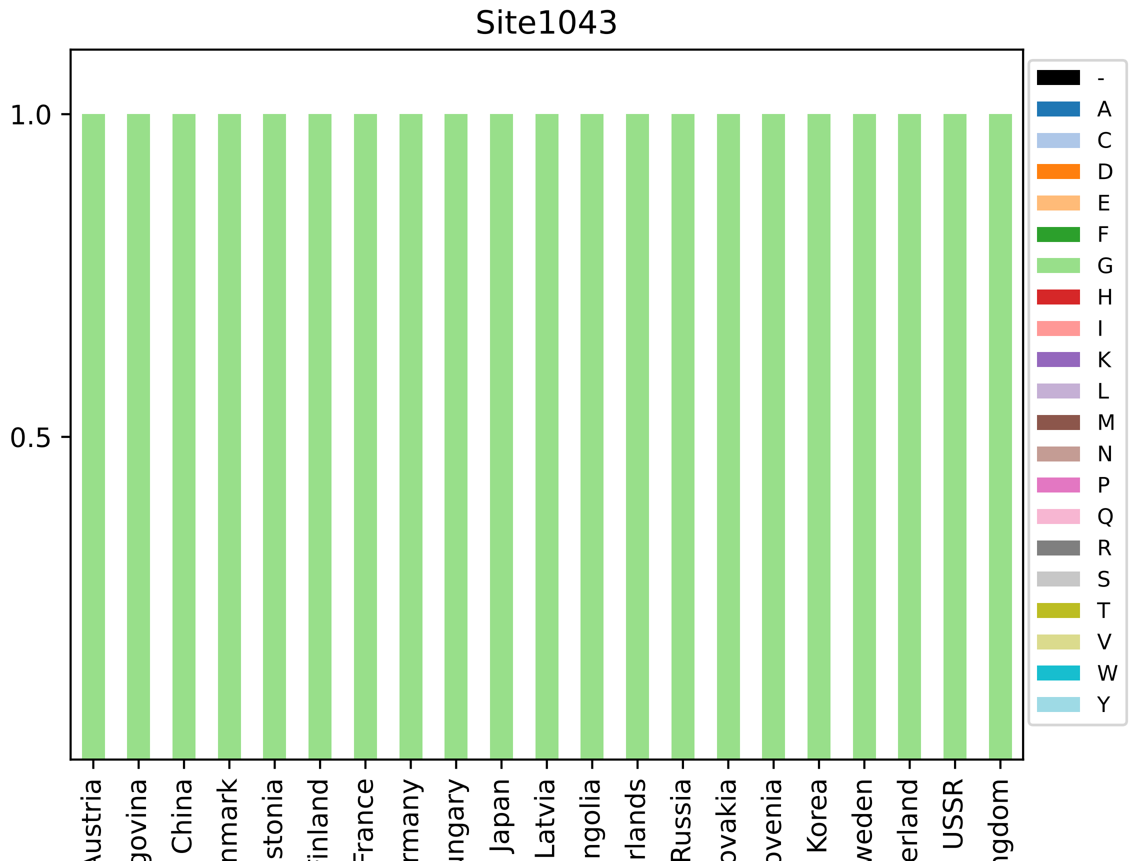

Site1043
1.0
0.5
-
A
C
D
E
F
G
H
I
K
L
M
N
P
Q
R
S
T
V
W
Y
China
Japan
Latvia
Russia
Korea
USSR

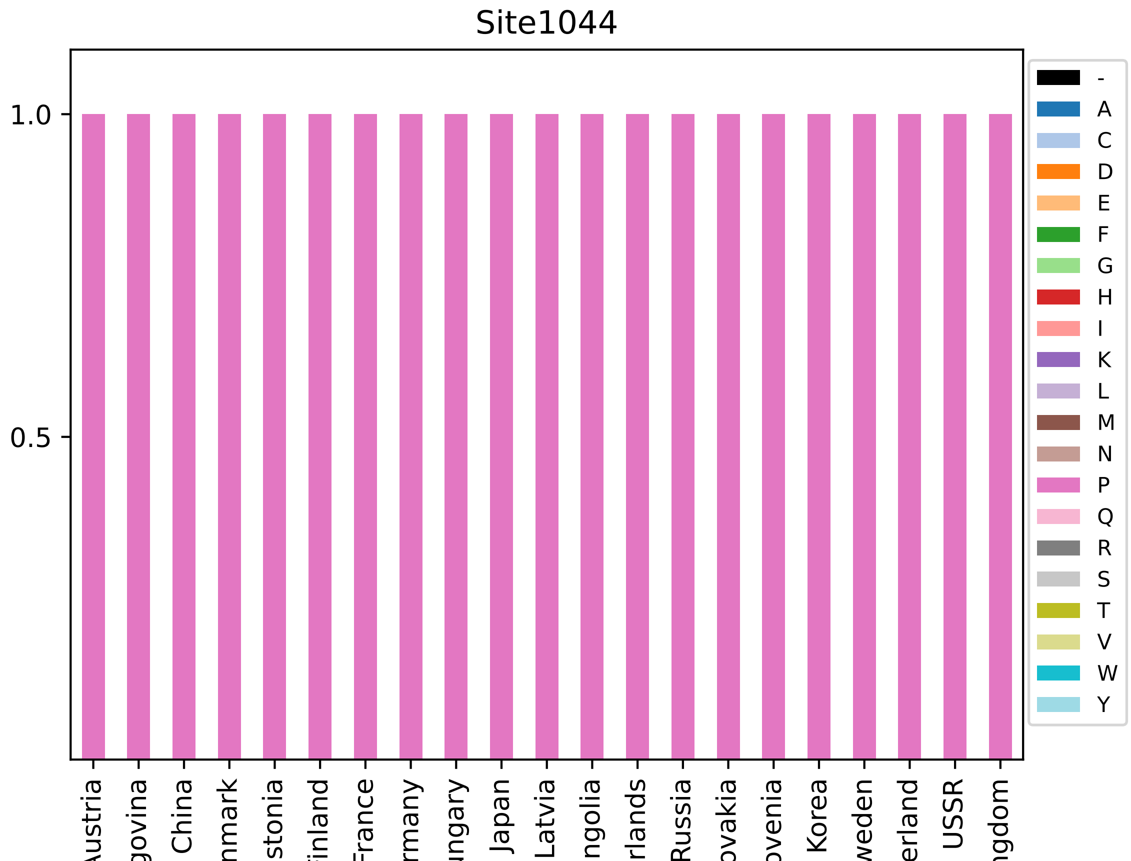
Site1044
1.0
0.5
-
A
C
D
E
F
G
H
I
K
L
M
N
P
Q
R
S
T
V
W
Y
Austria
govina
China
nmark
stonia
Finland
France
rmany
ungary
Japan
Latvia
ngolia
rlands
Russia
ovakia
ovenia
Korea
weden
erland
USSR
ngdom

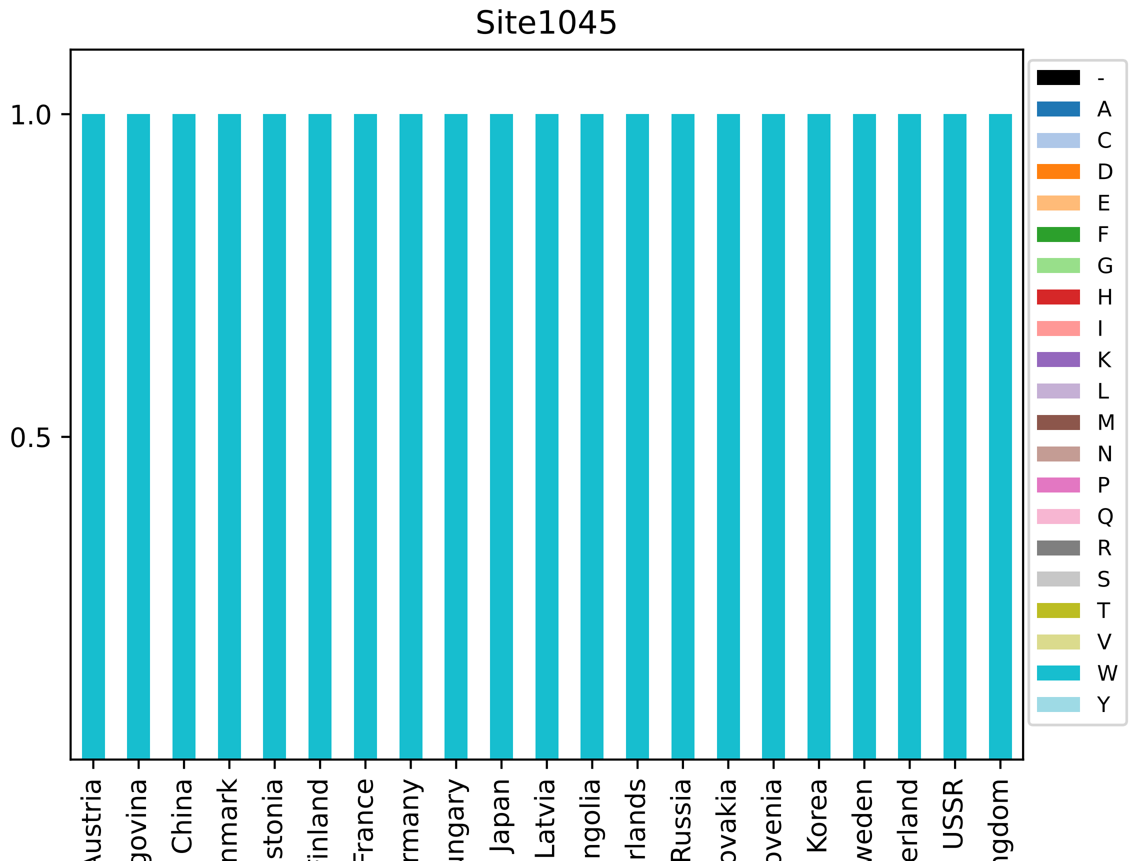

Site1045
1.0
0.5
China
Japan
Latvia
Russia
Korea
USSR
-
A
C
D
E
F
G
H
I
K
L
M
N
P
Q
R
S
T
V
W
Y

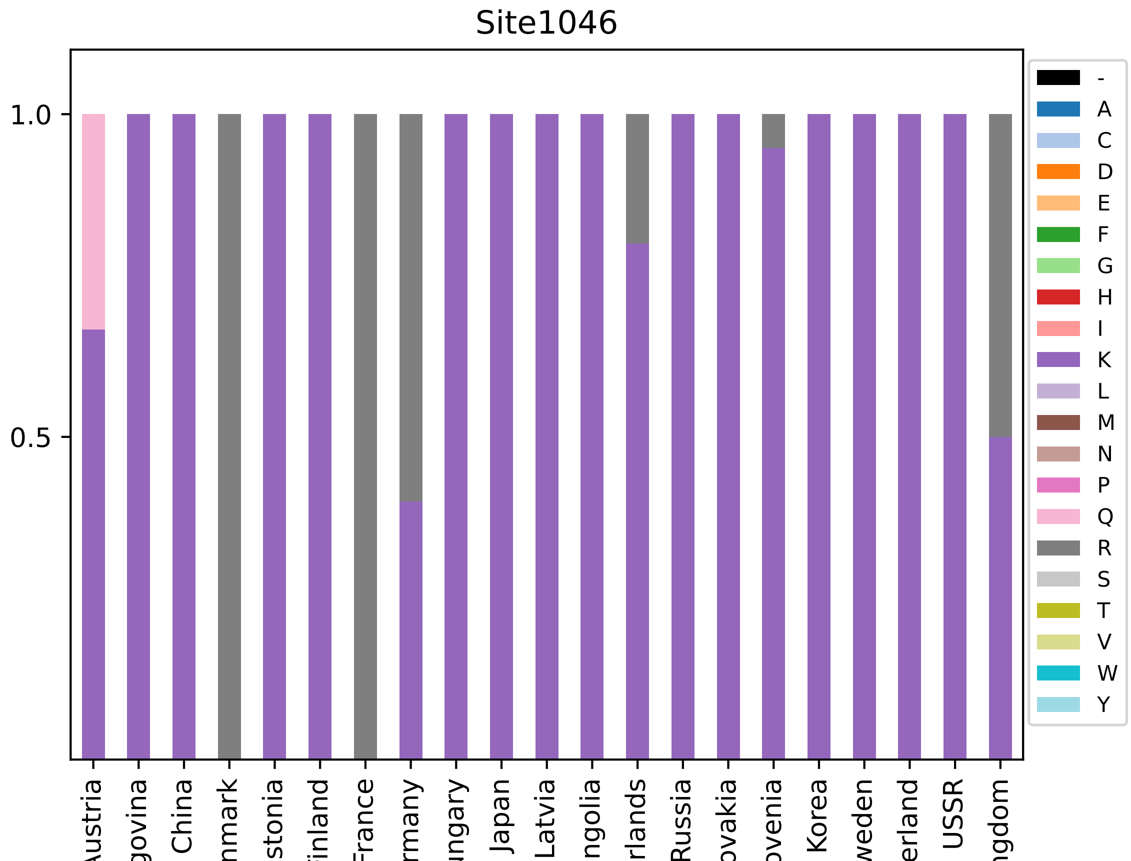
Site1046
1.0
0.5
-
A
C
D
E
F
G
H
I
K
L
M
N
P
Q
R
S
T
V
W
Y
China
Japan
Latvia
Russia
Korea
USSR

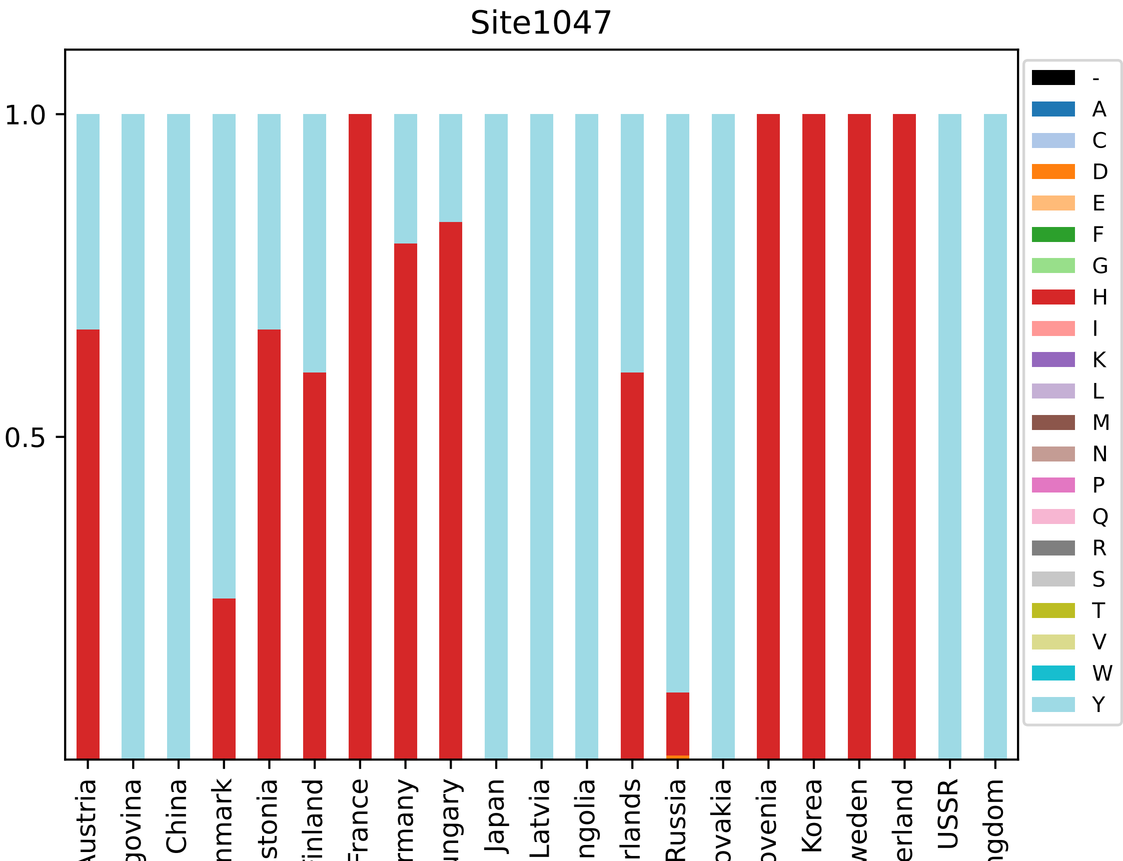

Site1047
1.0
0.5
-
A
C
D
E
F
G
H
I
K
L
M
N
P
Q
R
S
T
V
W
Y
China
France
Japan
Latvia
Russia
Korea
USSR

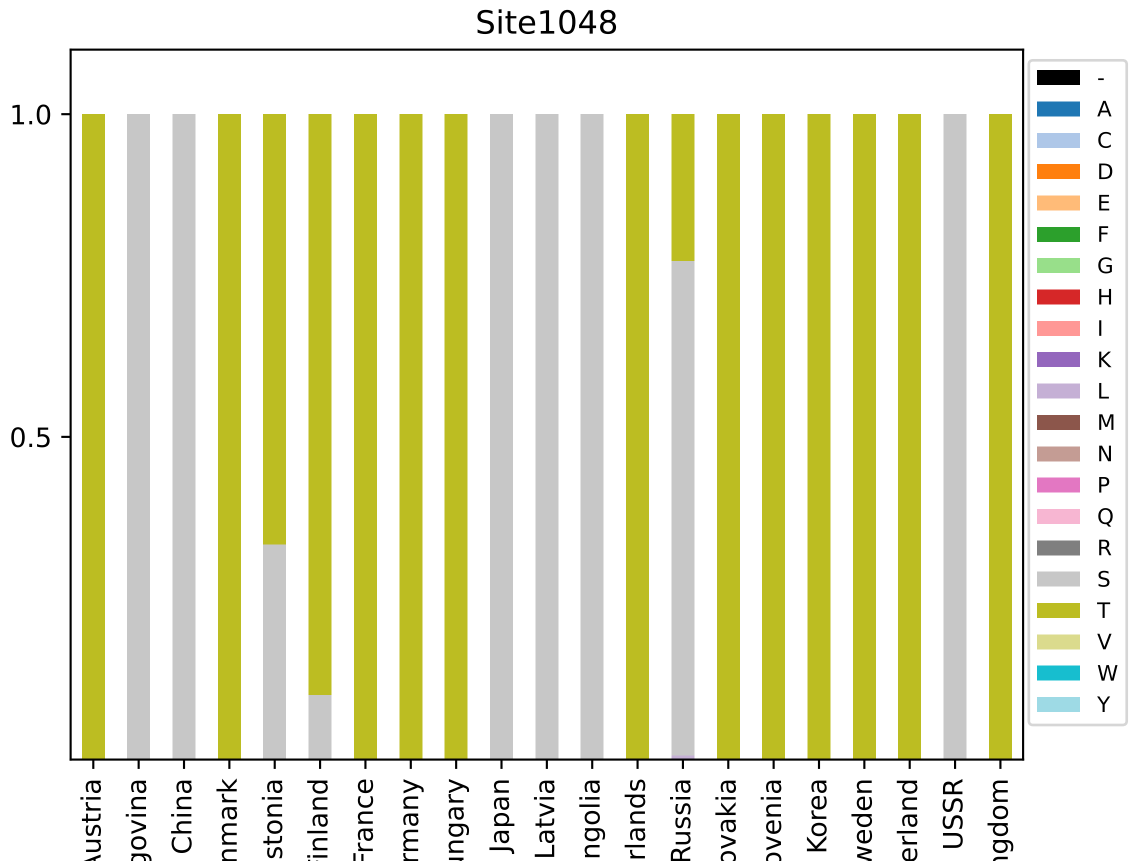
Site1048
1.0
0.5
-
A
C
D
E
F
G
H
I
K
L
M
N
P
Q
R
S
T
V
W
Y
Austria
govina
China
nmark
stonia
inland
France
rmany
ungary
Japan
Latvia
ngolia
rlands
Russia
ovakia
ovenia
Korea
weden
erland
USSR
ngdom

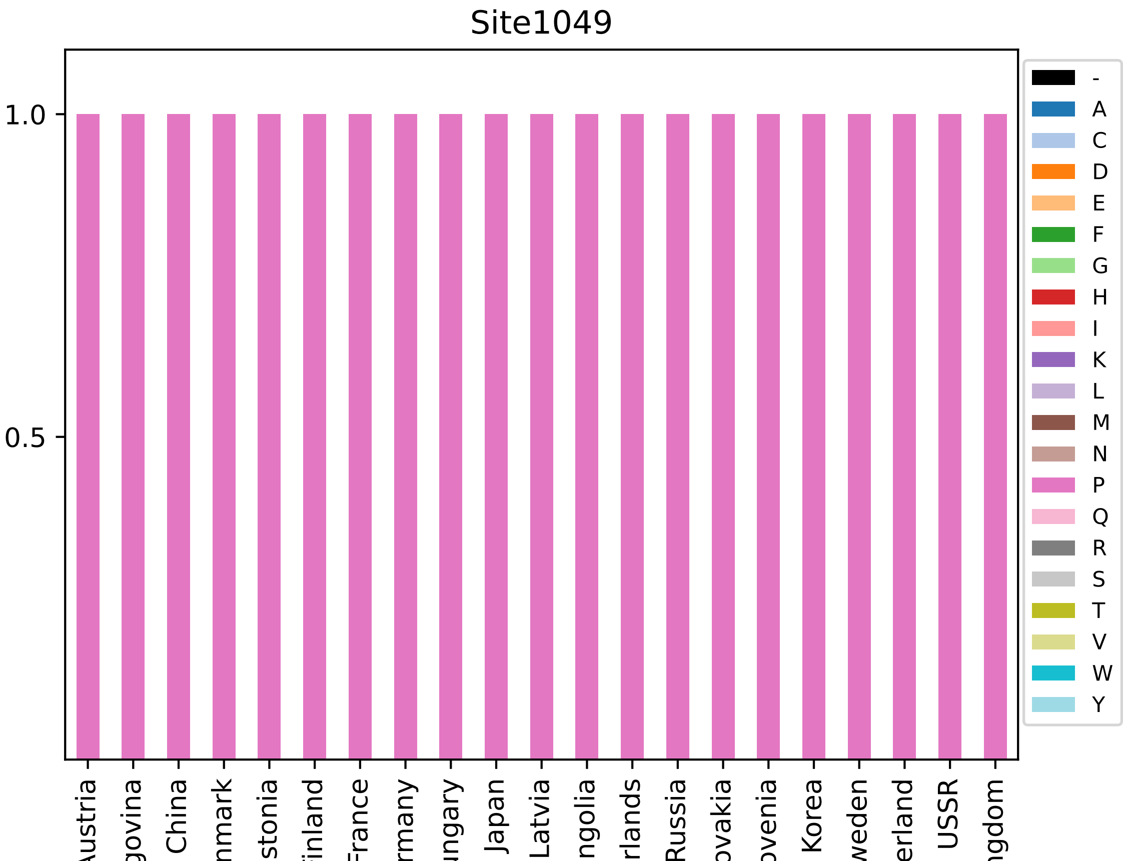
Site1049
1.0
0.5
-
A
C
D
E
F
G
H
I
K
L
M
N
P
Q
R
S
T
V
W
Y
China
Japan
Latvia
Russia
Korea
USSR

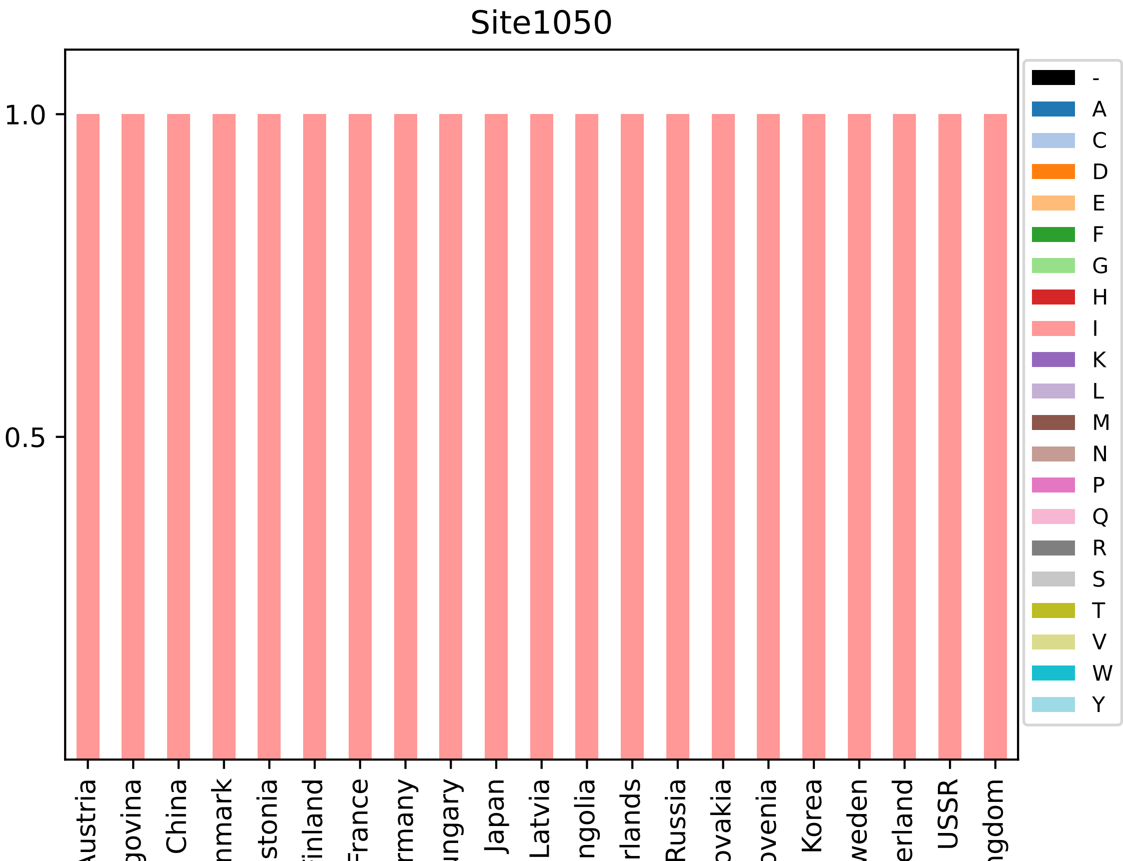
Site1050
1.0
0.5
-
A
C
D
E
F
G
H
I
K
L
M
N
P
Q
R
S
T
V
W
Y
China
Japan
Latvia
Russia
Korea
USSR

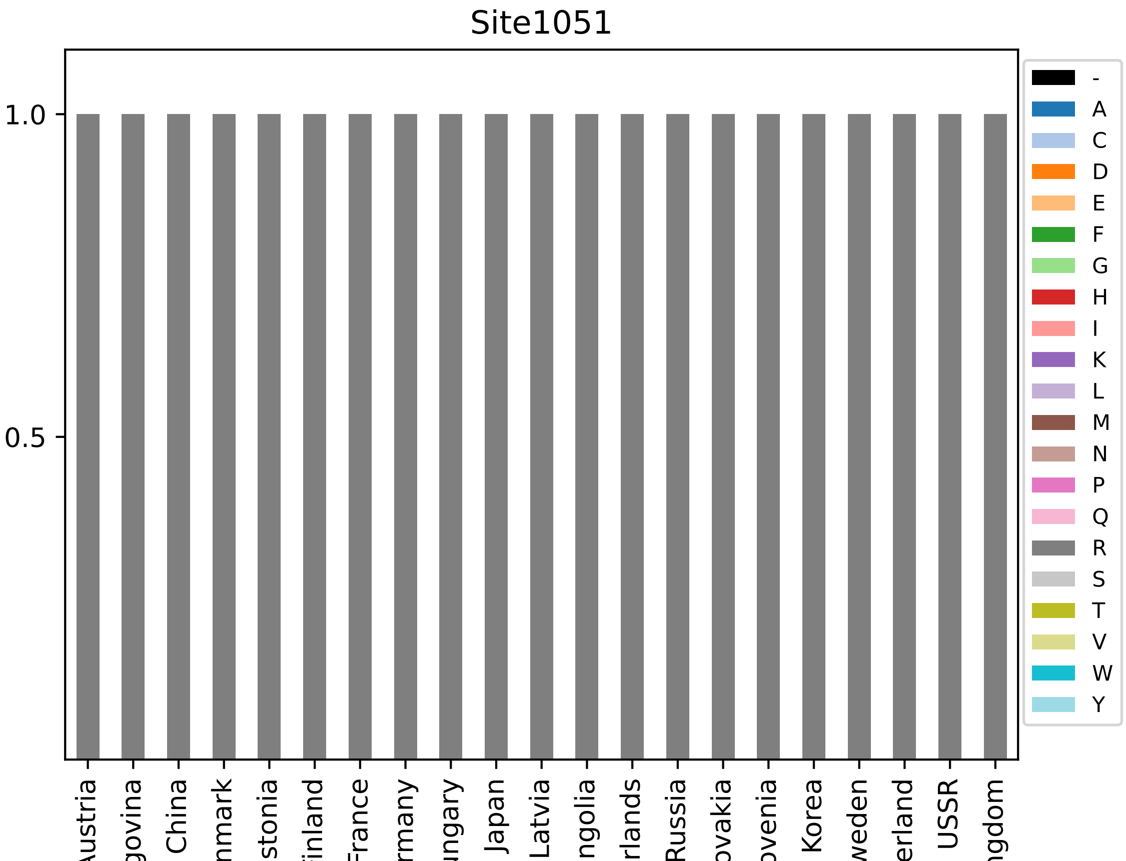

Site1051
1.0
0.5
-
A
C
D
E
F
G
H
I
K
L
M
N
P
Q
R
S
T
V
W
Y
Austria
govina
China
nmark
stonia
inland
France
rmany
ungary
Japan
Latvia
ngolia
rlands
Russia
ovakia
ovenia
Korea
weden
erland
USSR
ngdom

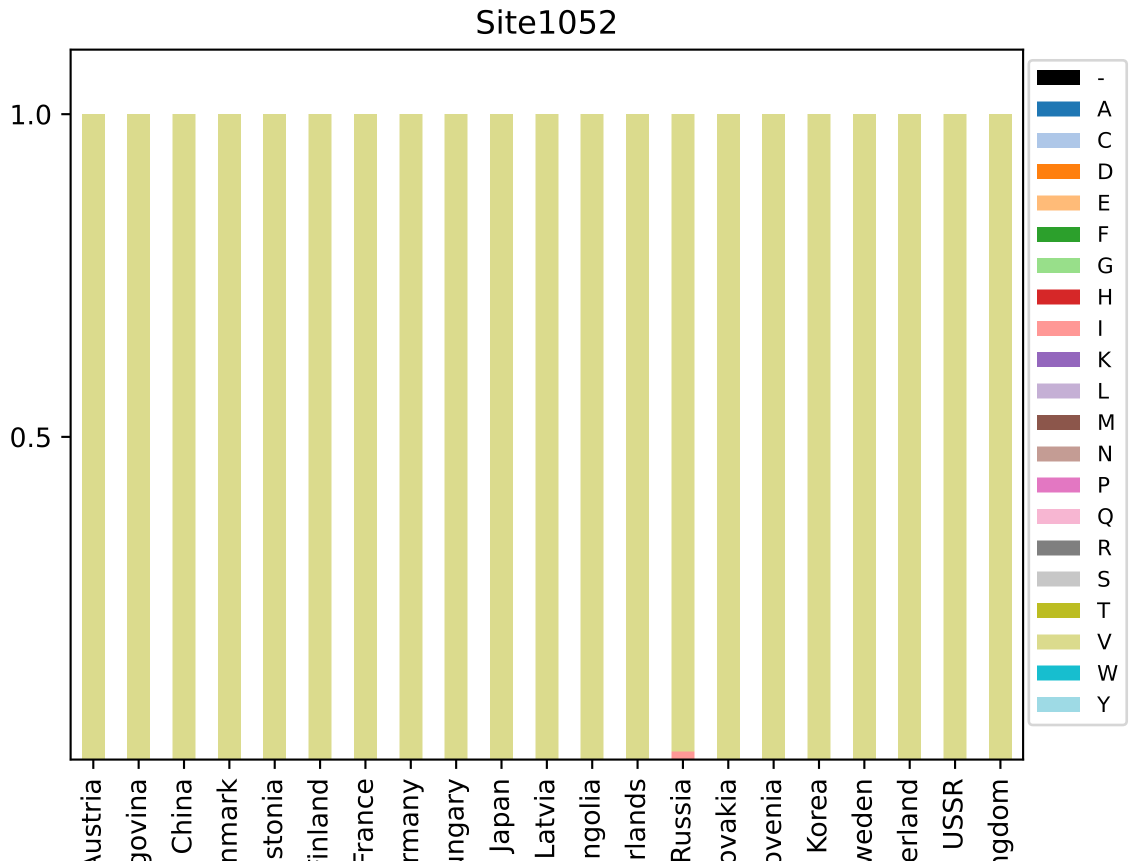

Site1052
1.0
0.5
-
A
C
D
E
F
G
H
I
K
L
M
N
P
Q
R
S
T
V
W
Y
Austria
govina
China
nmark
stonia
Finland
France
rmany
ungary
Japan
Latvia
ngolia
rlands
Russia
ovakia
ovenia
Korea
weden
erland
USSR
ngdom

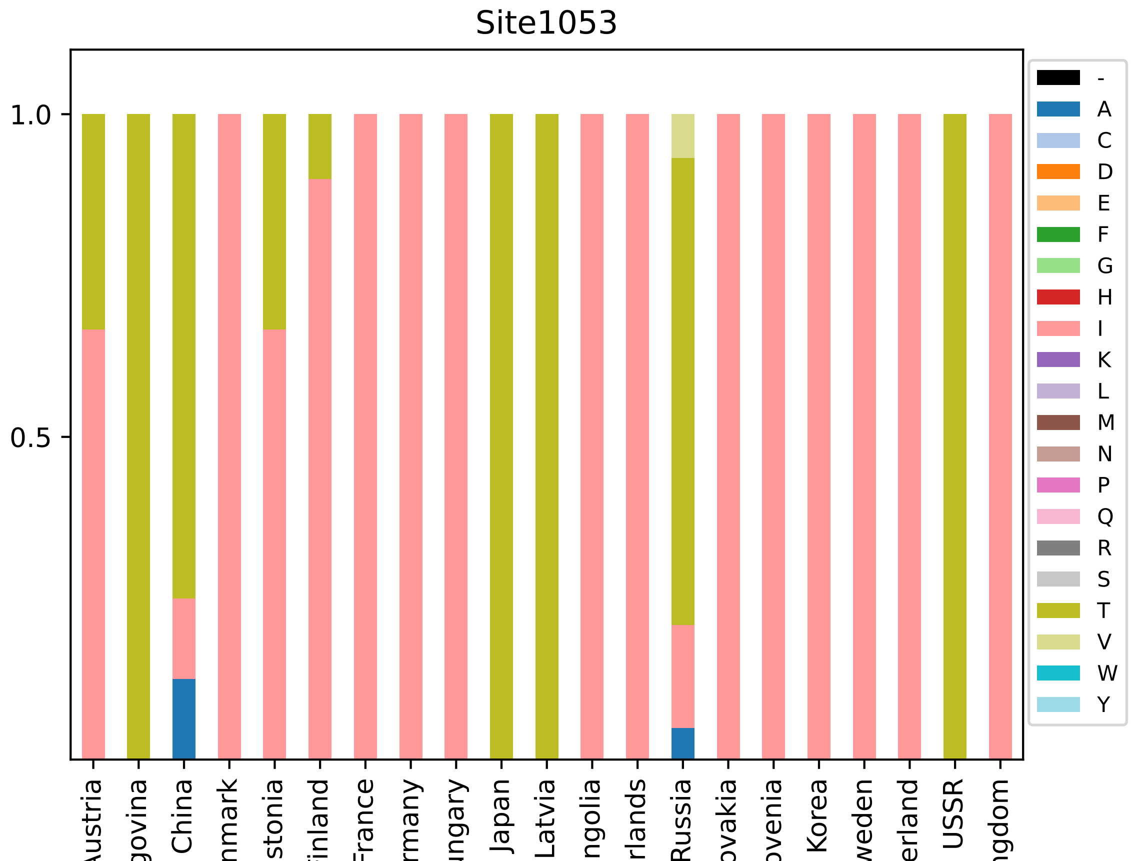

Site1053
1.0
0.5
-
A
C
D
E
F
G
H
I
K
L
M
N
P
Q
R
S
T
V
W
Y
Austria
govina
China
nmark
stonia
Finland
France
rmany
ungary
Japan
Latvia
ngolia
rlands
Russia
ovakia
ovenia
Korea
weden
erland
USSR
ngdom

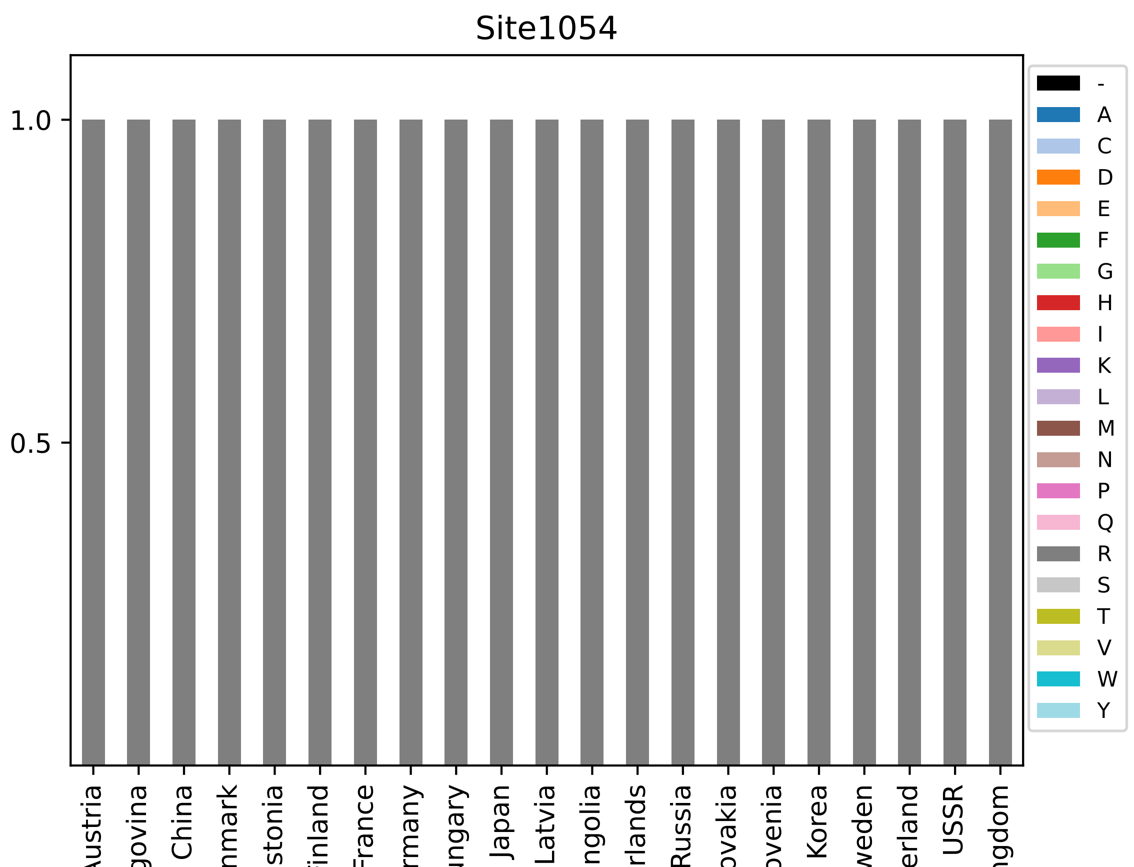

Site1054
1.0
0.5
Austria
govina
China
nmark
stonia
Finland
France
rmany
ungary
Japan
Latvia
ngolia
rlands
Russia
ovakia
ovenia
Korea
weden
erland
USSR
ngdom
-
A
C
D
E
F
G
H
I
K
L
M
N
P
Q
R
S
T
V
W
Y

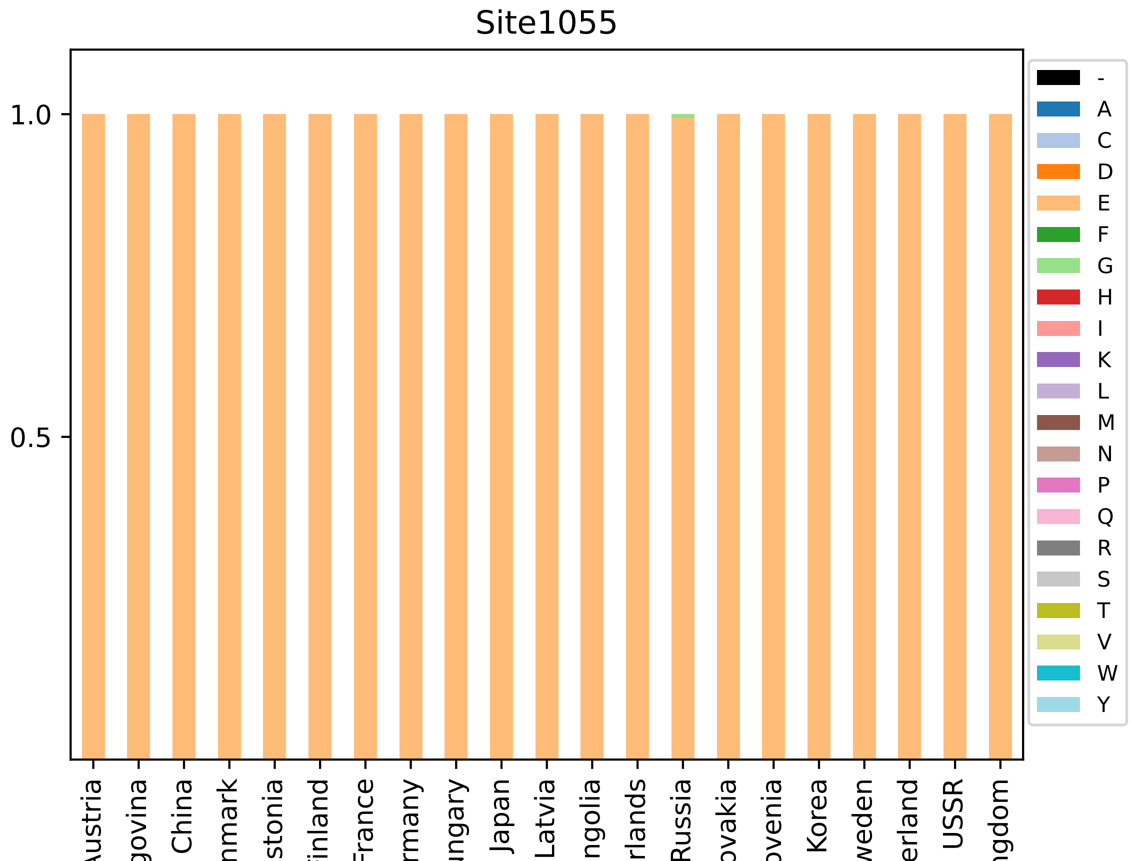

Site1055
1.0
0.5
-
A
C
D
E
F
G
H
I
K
L
M
N
P
Q
R
S
T
V
W
Y
China
Japan
Latvia
Russia
Korea
USSR

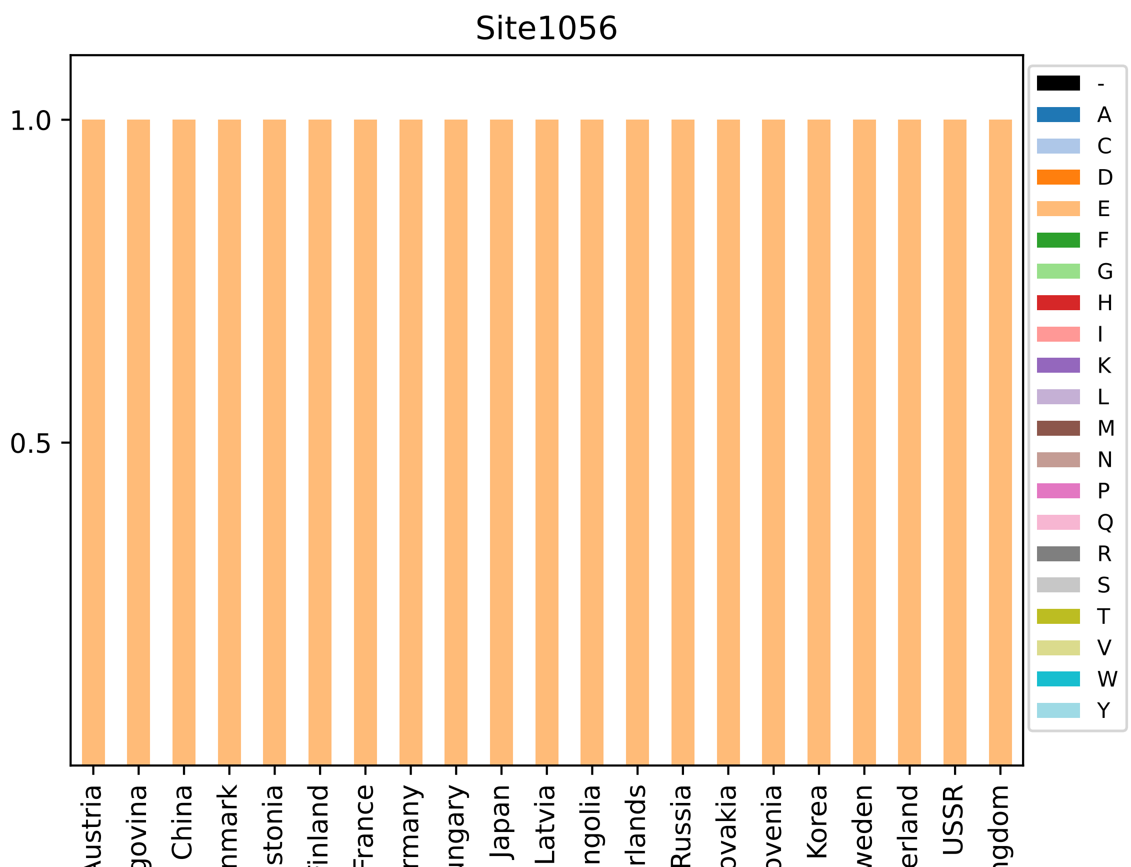

Site1056
1.0
0.5
-
A
C
D
E
F
G
H
I
K
L
M
N
P
Q
R
S
T
V
W
Y
China
Japan
Latvia
Russia
Korea
USSR

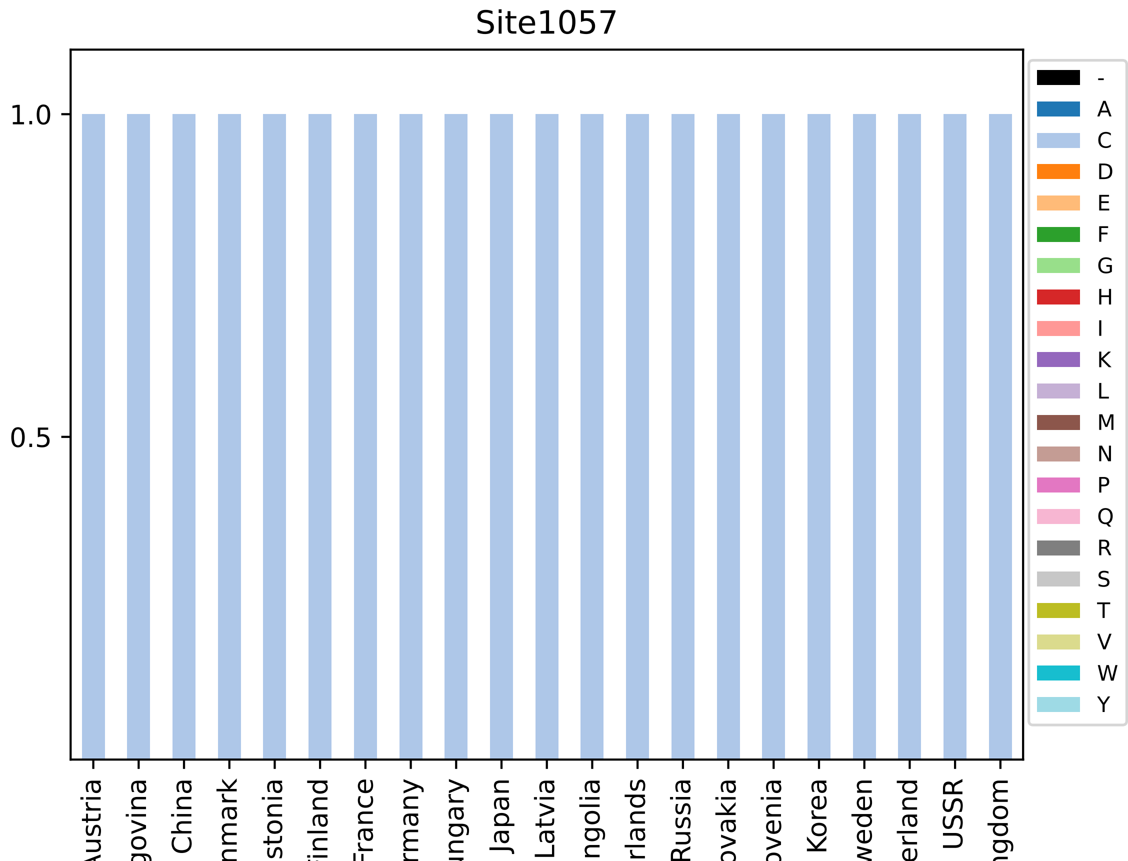

Site1057
1.0
0.5
-
A
C
D
E
F
G
H
I
K
L
M
N
P
Q
R
S
T
V
W
Y
Austria
govina
China
nmark
stonia
Finland
France
rmany
ungary
Japan
Latvia
ngolia
rlands
Russia
ovakia
ovenia
Korea
weden
erland
USSR
ngdom

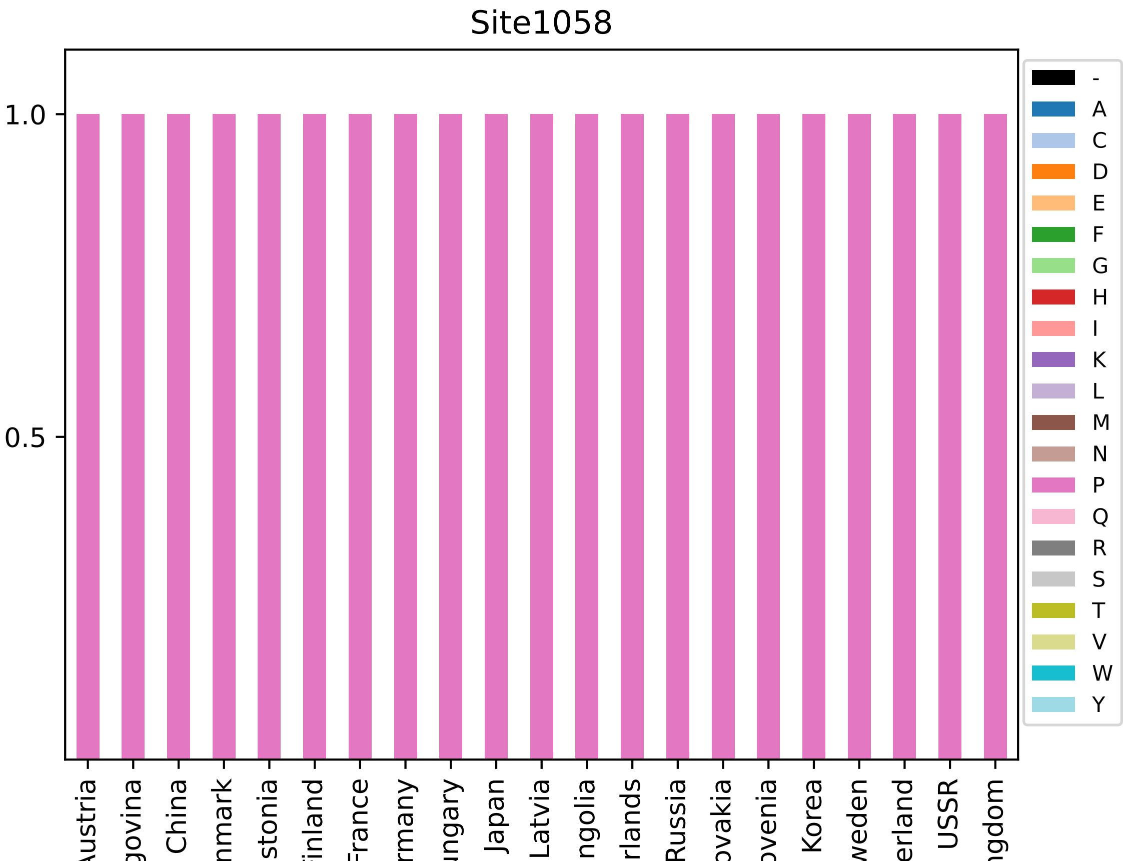

Site1058
1.0
0.5
-
A
C
D
E
F
G
H
I
K
L
M
N
P
Q
R
S
T
V
W
Y
Austria
govina
China
nmark
stonia
inland
France
rmany
ungary
Japan
Latvia
ngolia
rlands
Russia
ovakia
ovenia
Korea
weden
erland
USSR
ngdom

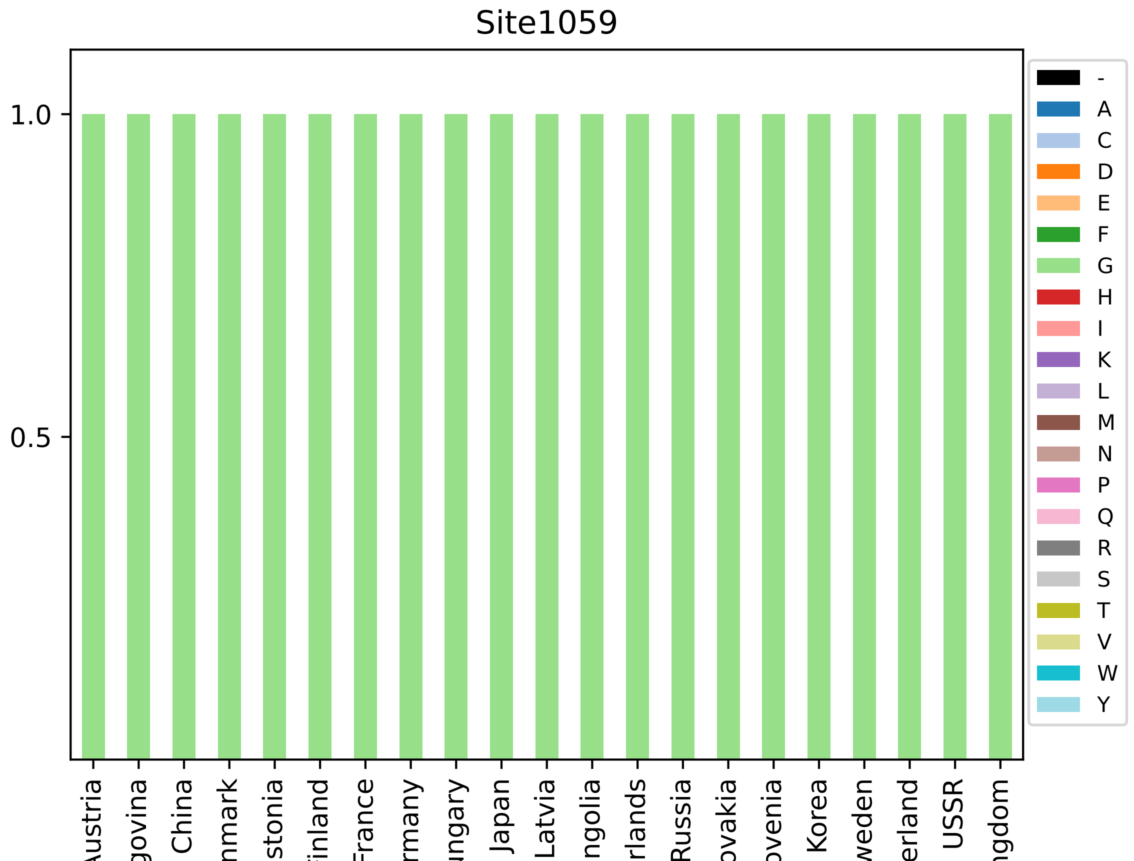

Site1059
1.0
0.5
-
A
C
D
E
F
G
H
I
K
L
M
N
P
Q
R
S
T
V
W
Y
Austria
China
Japan
Latvia
Russia
Korea
USSR

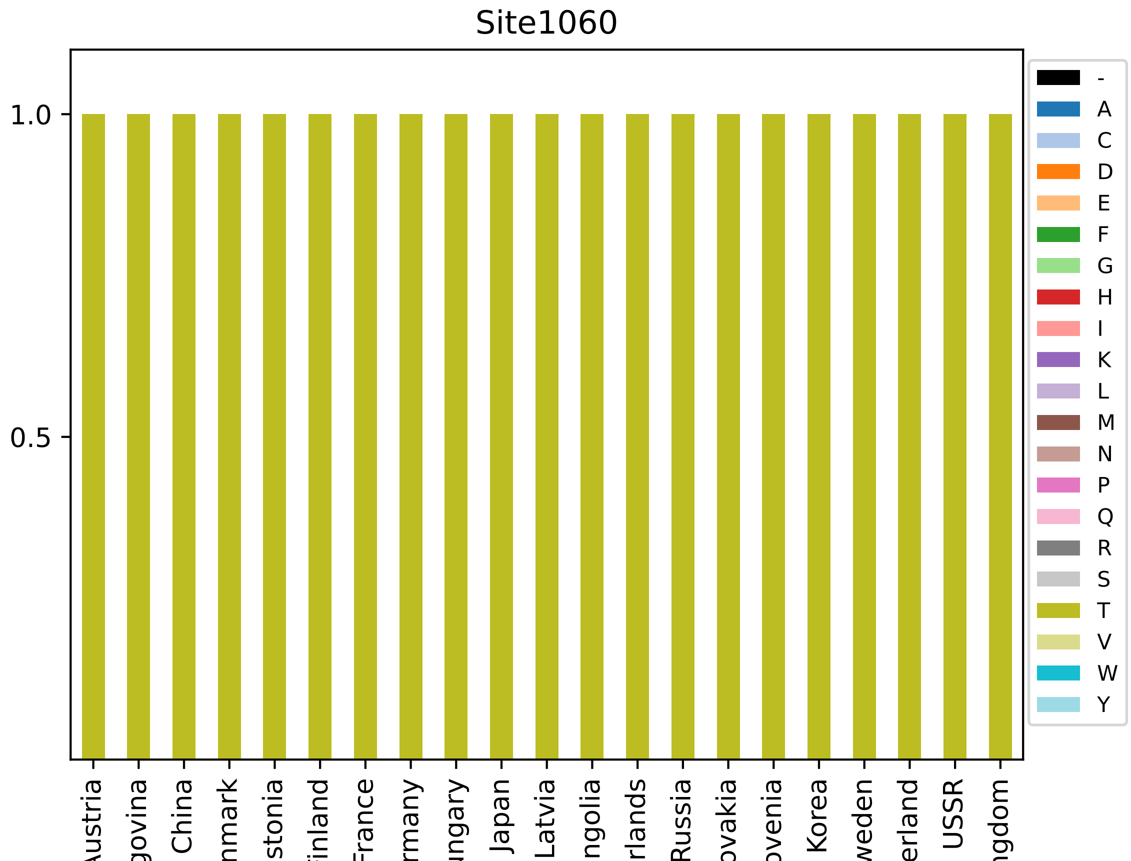

Site1060
1.0
0.5
-
A
C
D
E
F
G
H
I
K
L
M
N
P
Q
R
S
T
V
W
Y
Austria
govina
China
nmark
stonia
inland
France
rmany
ungary
Japan
Latvia
ngolia
rlands
Russia
ovakia
ovenia
Korea
weden
erland
USSR
ngdom

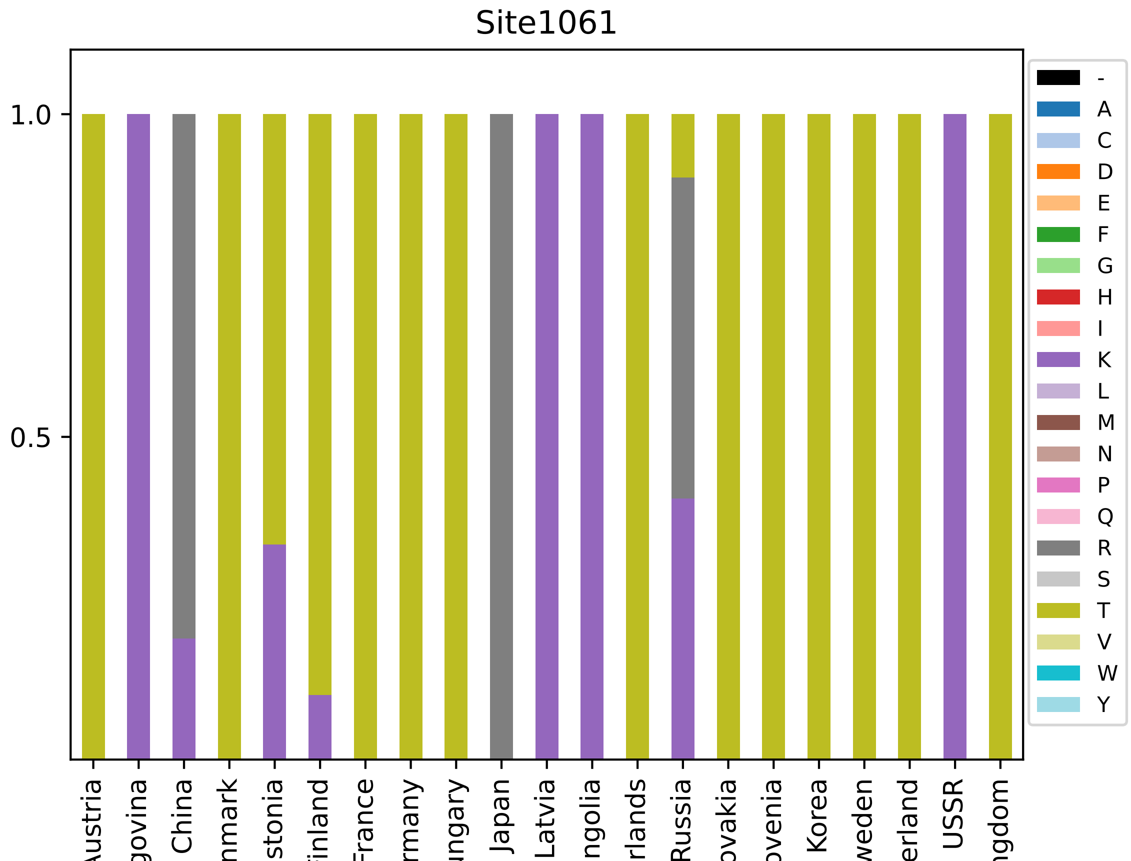

Site1061
1.0
0.5
-
A
C
D
E
F
G
H
I
K
L
M
N
P
Q
R
S
T
V
W
Y
Austria
govina
China
nmark
stonia
inland
France
rmany
ungary
Japan
Latvia
ngolia
rlands
Russia
ovakia
ovenia
Korea
weden
erland
USSR
ngdom

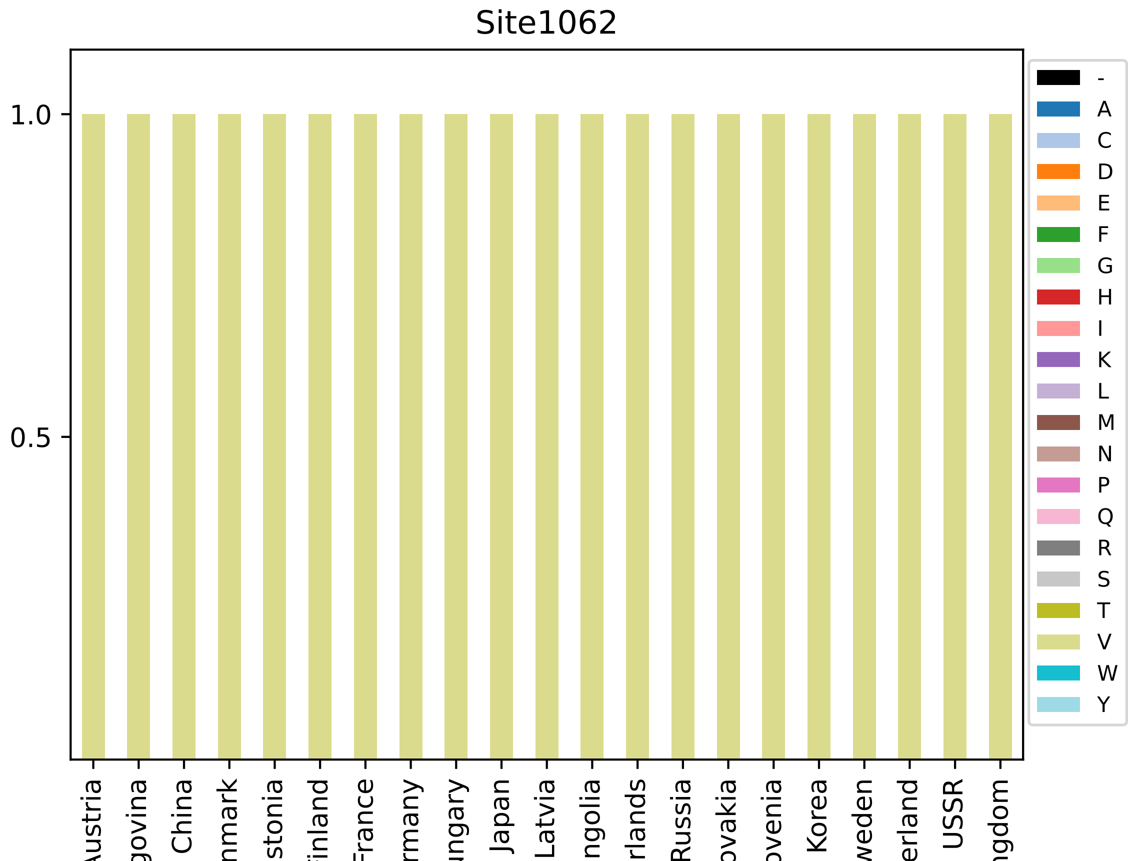

Site1062
1.0
0.5
-
A
C
D
E
F
G
H
I
K
L
M
N
P
Q
R
S
T
V
W
Y
China
Japan
Latvia
Russia
Korea
USSR

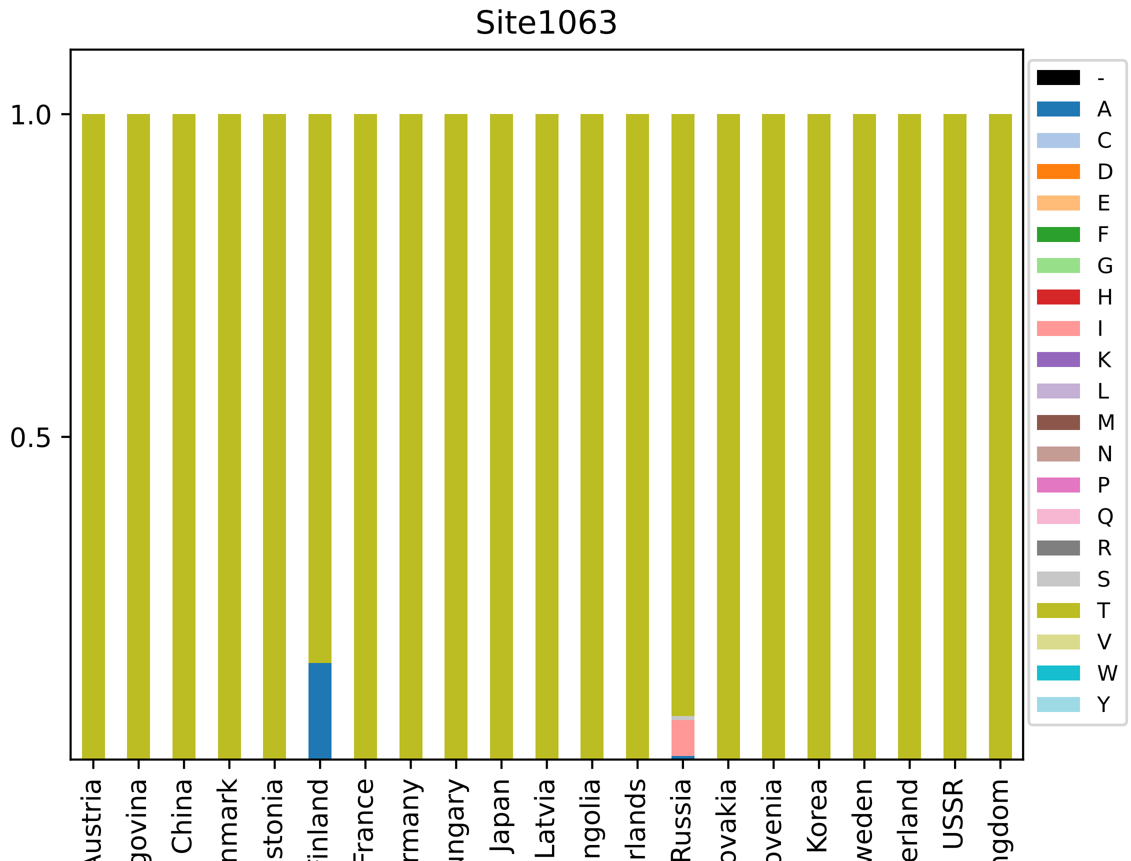
Site1063
1.0
0.5
-
A
C
D
E
F
G
H
I
K
L
M
N
P
Q
R
S
T
V
W
Y
Austria
govina
China
nmark
stonia
Finland
France
rmany
ungary
Japan
Latvia
ngolia
rlands
Russia
ovakia
ovenia
Korea
weden
erland
USSR
ngdom

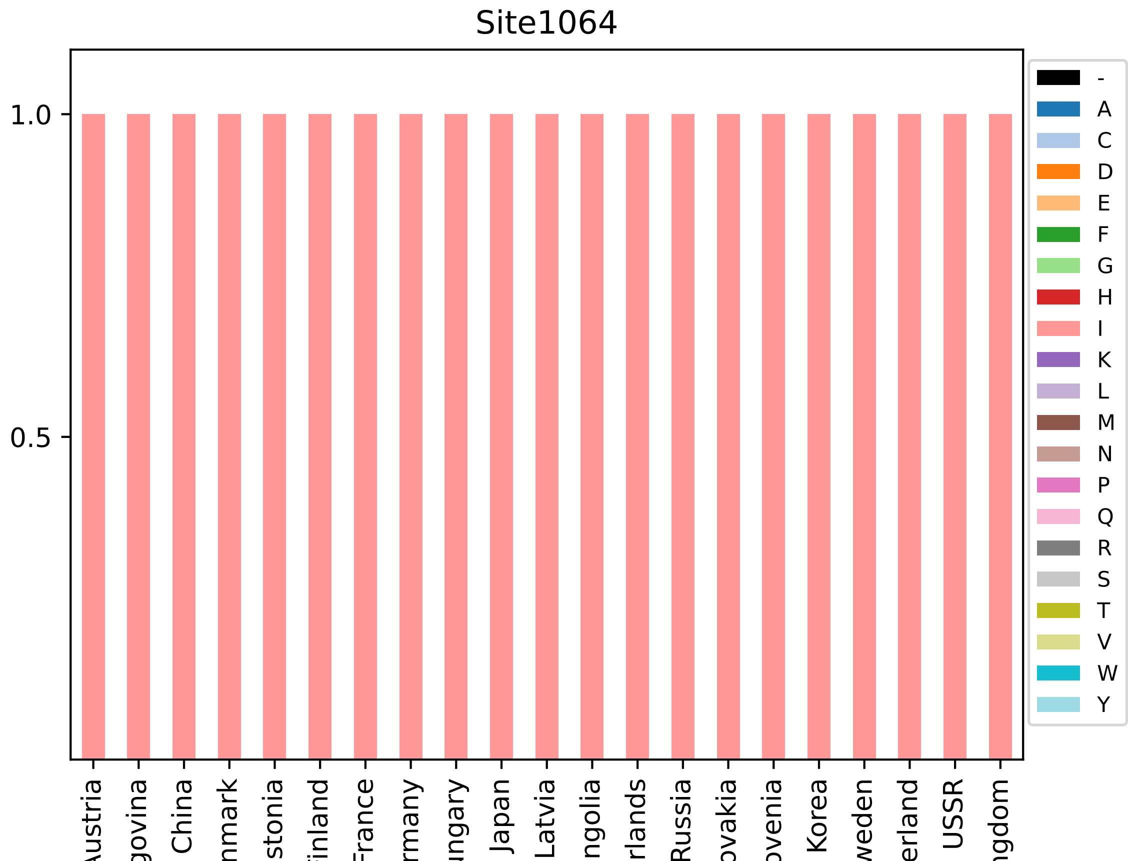
Site1064
1.0
0.5
-
A
C
D
E
F
G
H
I
K
L
M
N
P
Q
R
S
T
V
W
Y
China
Japan
Latvia
Russia
Korea
USSR

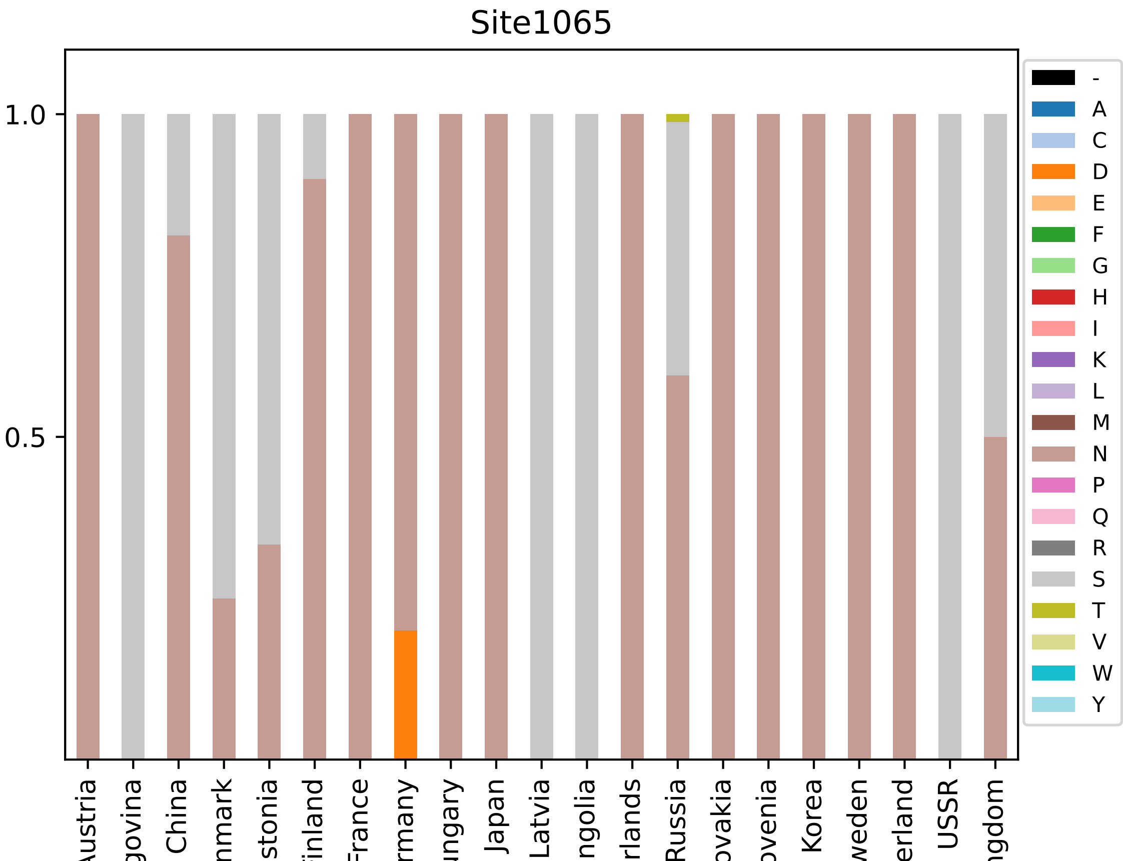
Site1065
1.0
0.5
-
A
C
D
E
F
G
H
I
K
L
M
N
P
Q
R
S
T
V
W
Y
Austria
China
Japan
Latvia
Russia
Korea
USSR

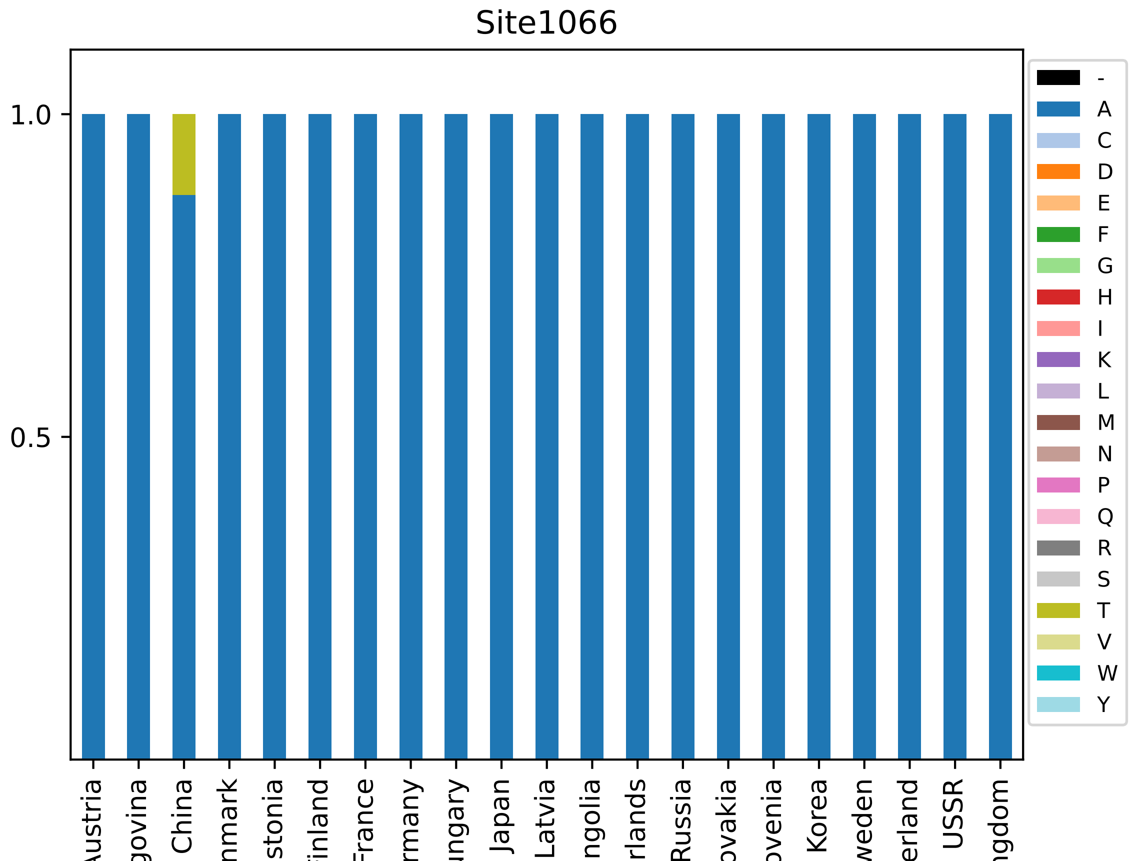
Site1066
1.0
0.5
-
A
C
D
E
F
G
H
I
K
L
M
N
P
Q
R
S
T
V
W
Y
Austria
China
Finland
France
Japan
Latvia
Russia
Korea
USSR

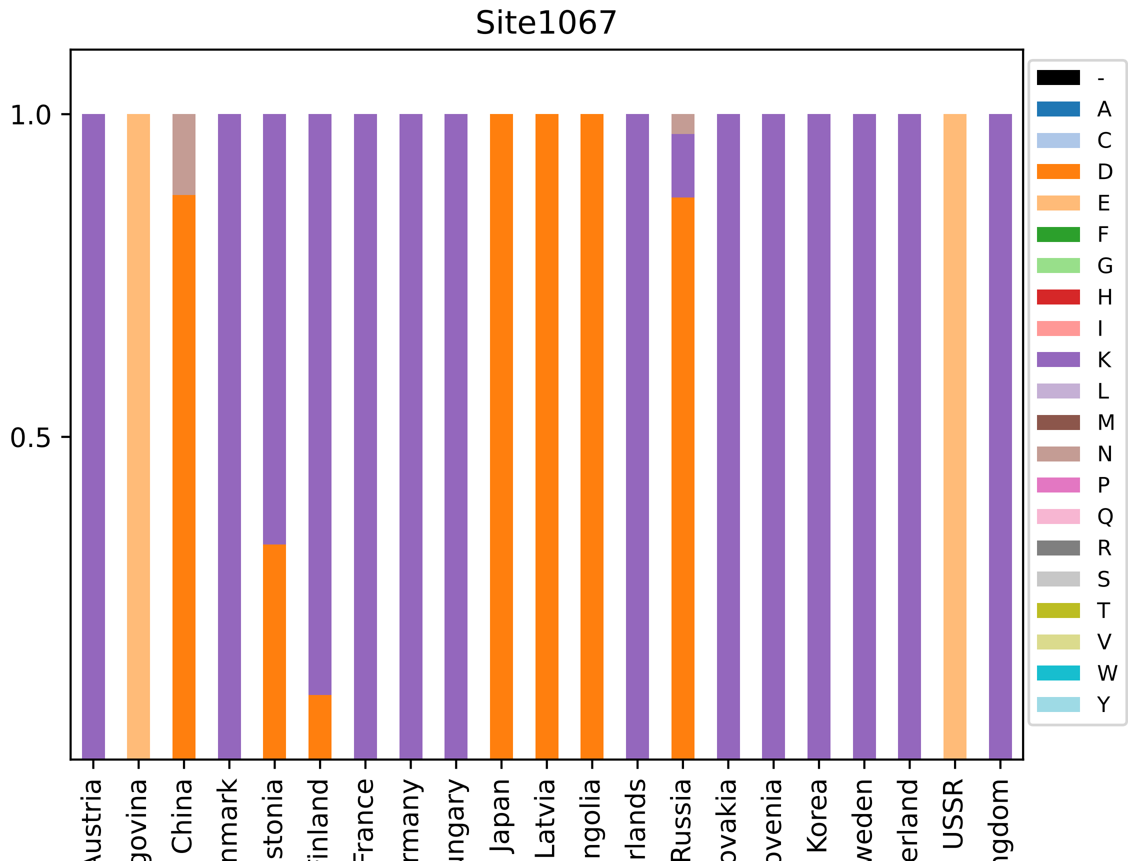
Site1067
1.0
0.5
-
A
C
D
E
F
G
H
I
K
L
M
N
P
Q
R
S
T
V
W
Y
Austria
govina
China
nmark
stonia
inland
France
rmany
ungary
Japan
Latvia
ngolia
rlands
Russia
ovakia
ovenia
Korea
weden
erland
USSR
ngdom

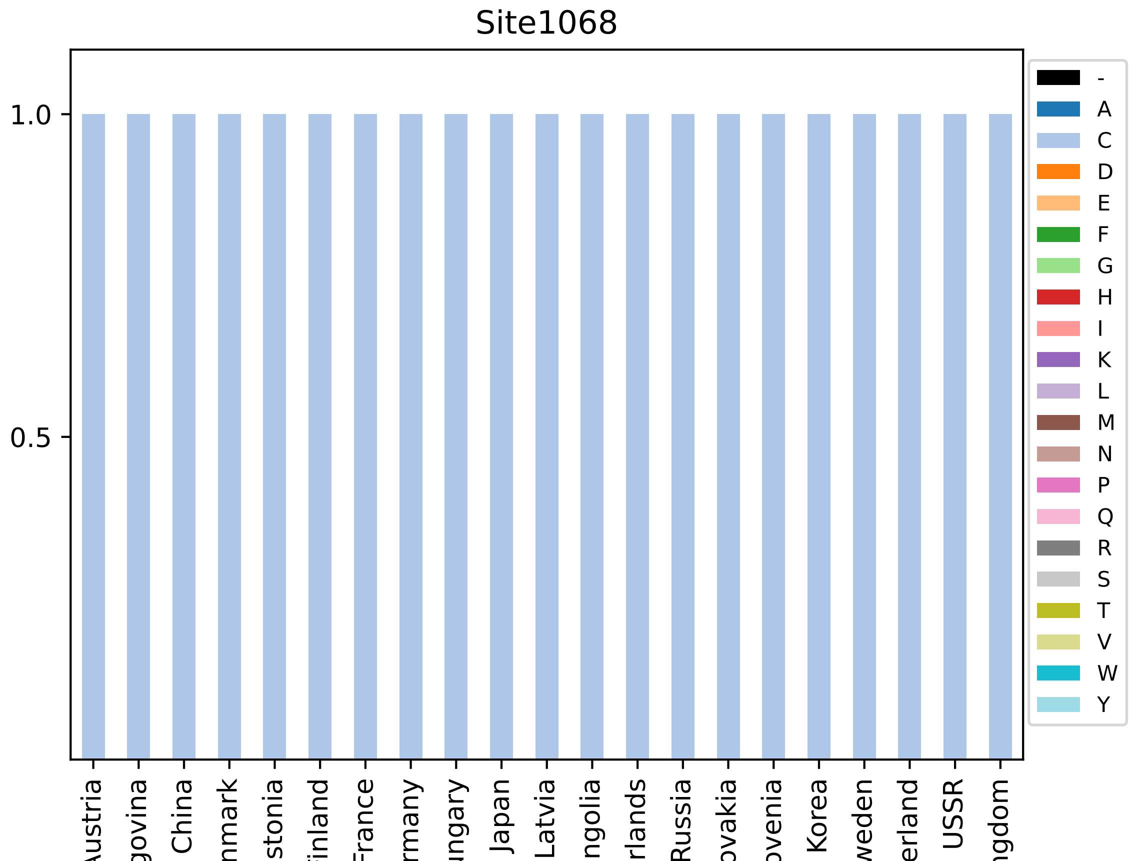

Site1068
1.0
0.5
-
A
C
D
E
F
G
H
I
K
L
M
N
P
Q
R
S
T
V
W
Y
Austria
China
Japan
Latvia
Russia
Korea
USSR

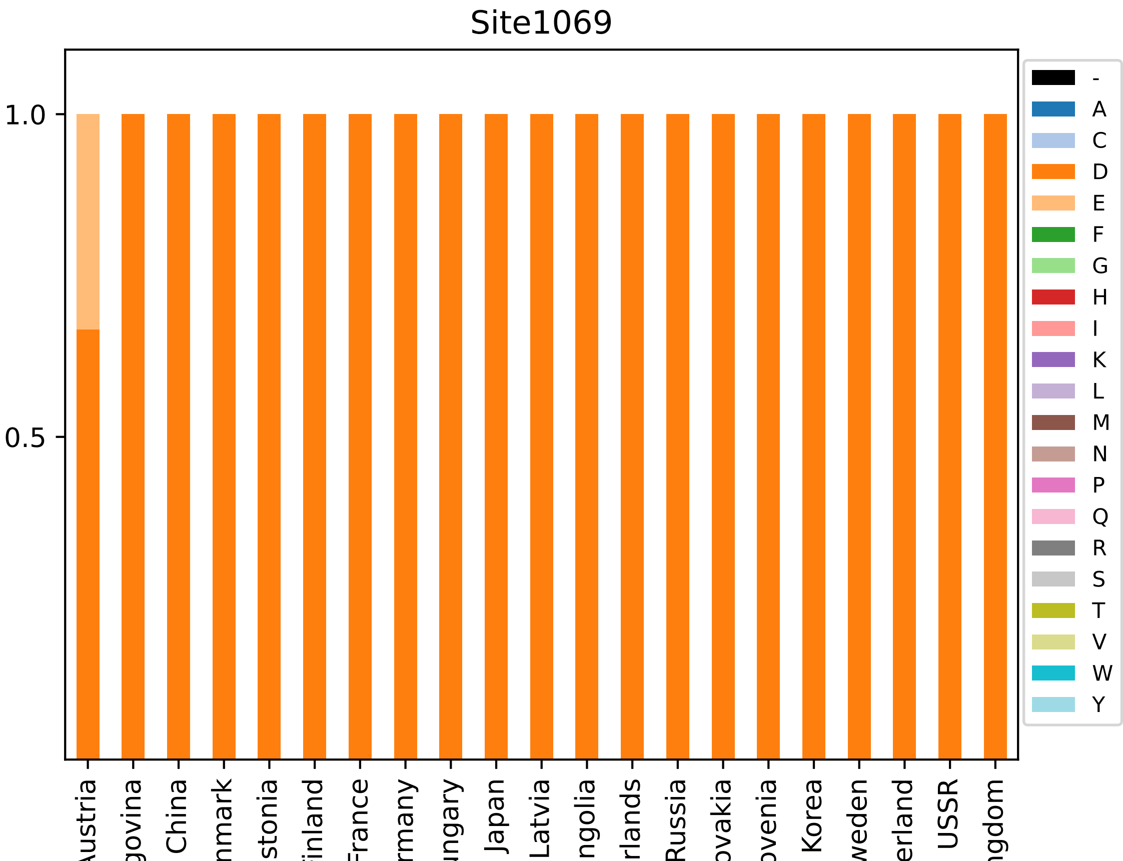
Site1069
1.0
0.5
-
A
C
D
E
F
G
H
I
K
L
M
N
P
Q
R
S
T
V
W
Y
China
Japan
Latvia
Russia
Korea
USSR

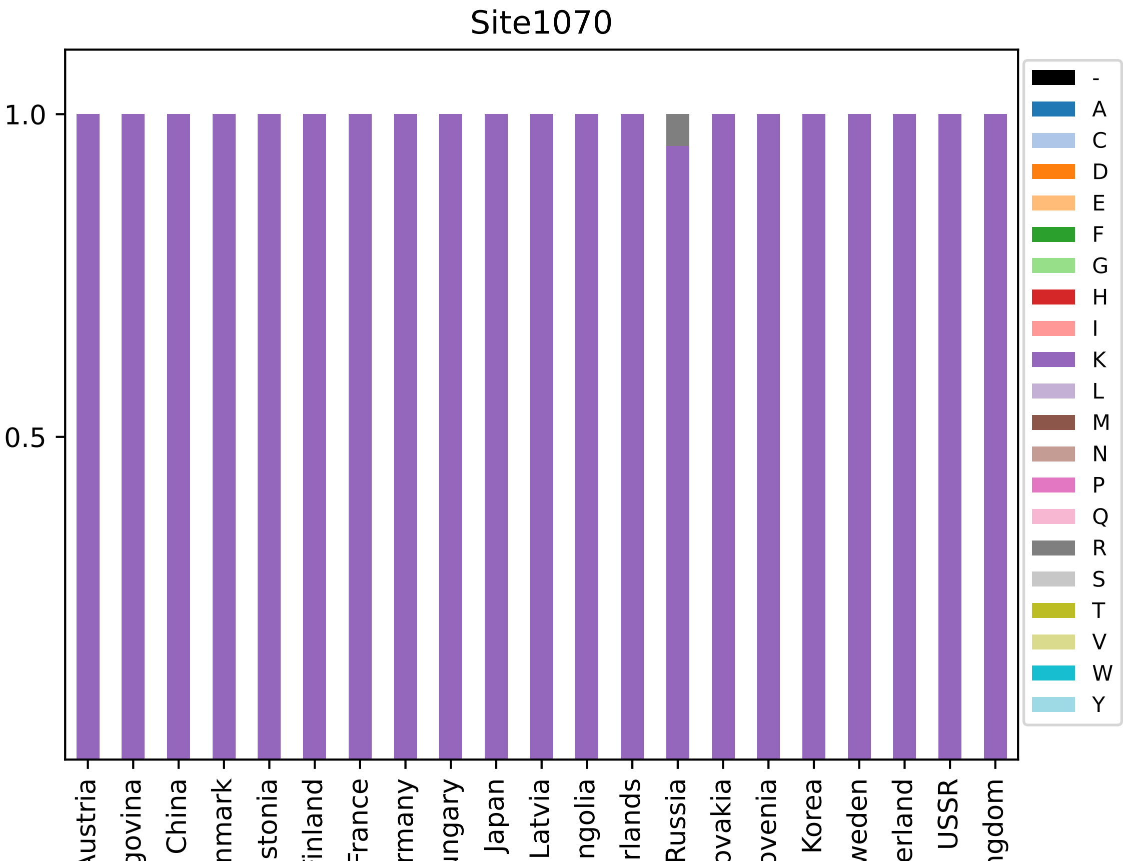

Site1070
1.0
0.5
-
A
C
D
E
F
G
H
I
K
L
M
N
P
Q
R
S
T
V
W
Y
Austria
govina
China
nmark
stonia
inland
France
rmany
ungary
Japan
Latvia
ngolia
rlands
Russia
ovakia
ovenia
Korea
weden
erland
USSR
ngdom

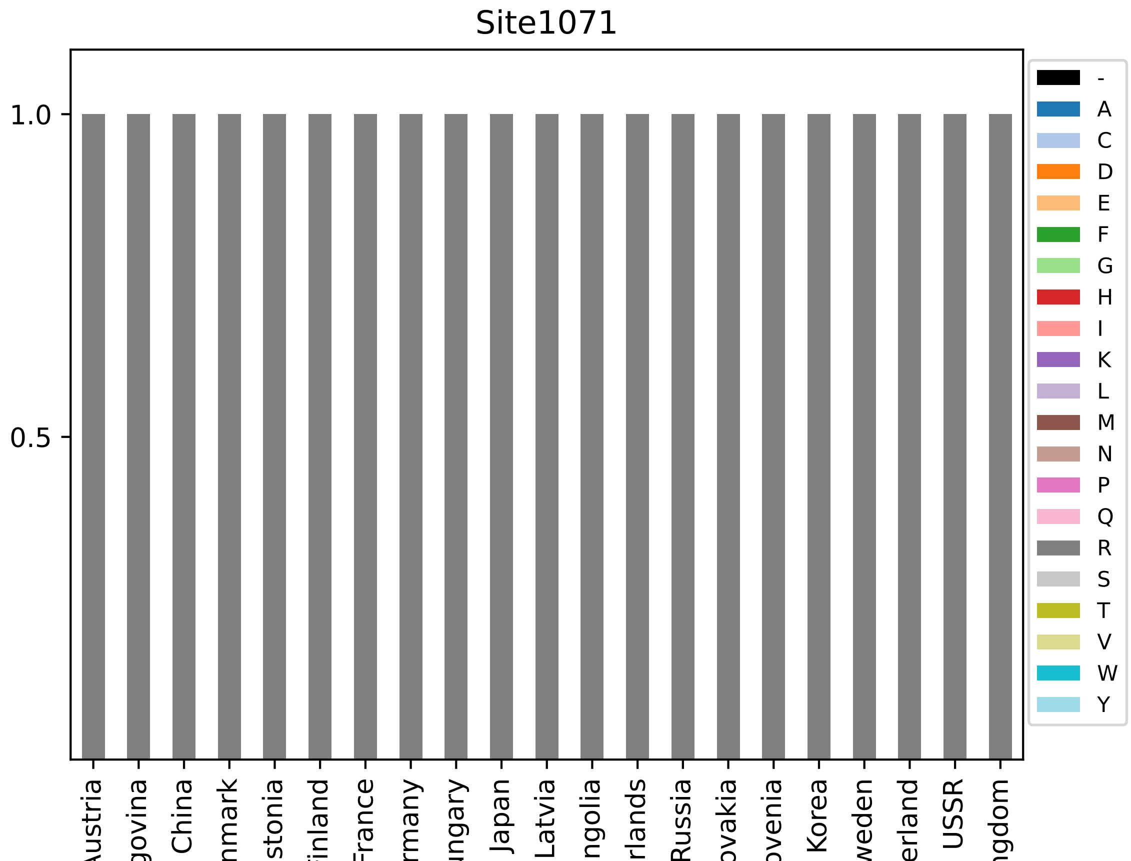

Site1071
1.0
0.5
-
A
C
D
E
F
G
H
I
K
L
M
N
P
Q
R
S
T
V
W
Y
China
Japan
Latvia
Russia
Korea
USSR

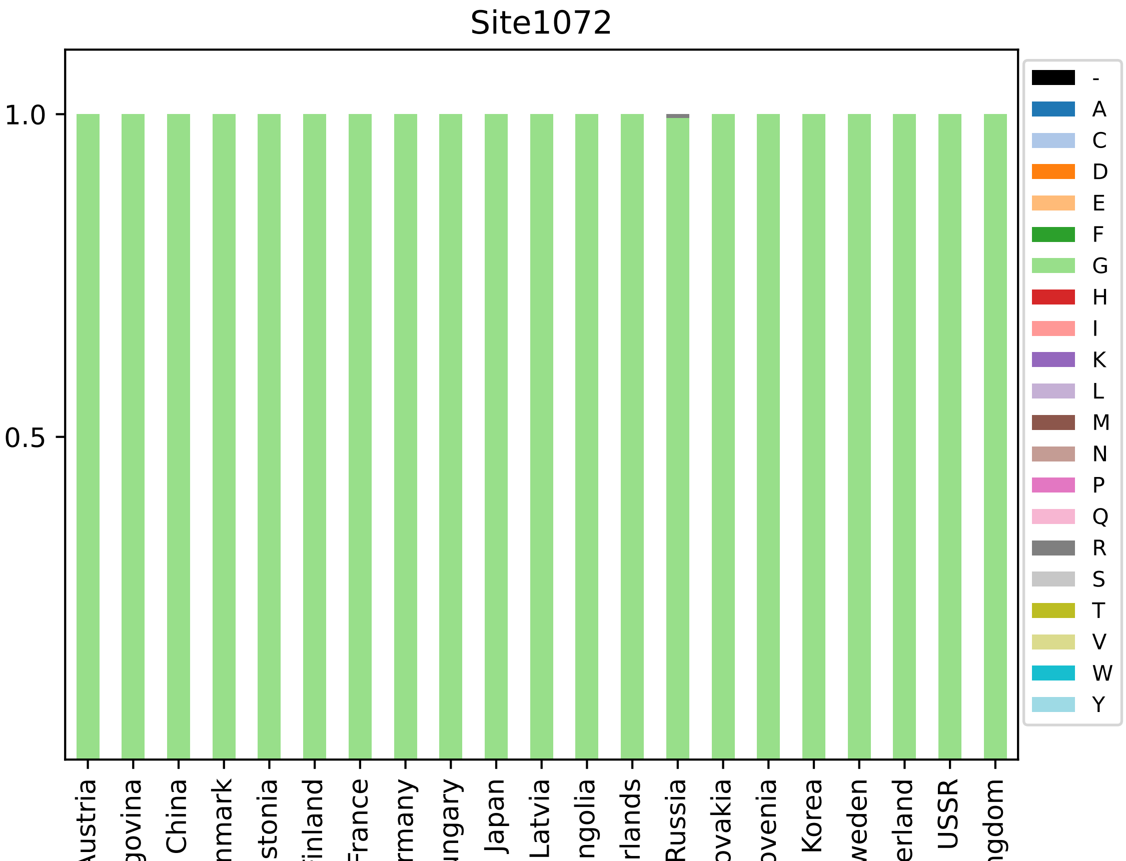
Site1072
1.0
0.5
-
A
C
D
E
F
G
H
I
K
L
M
N
P
Q
R
S
T
V
W
Y
Austria
govina
China
nmark
stonia
Finland
France
rmany
ungary
Japan
Latvia
ngolia
rlands
Russia
ovakia
ovenia
Korea
weden
erland
USSR
ngdom

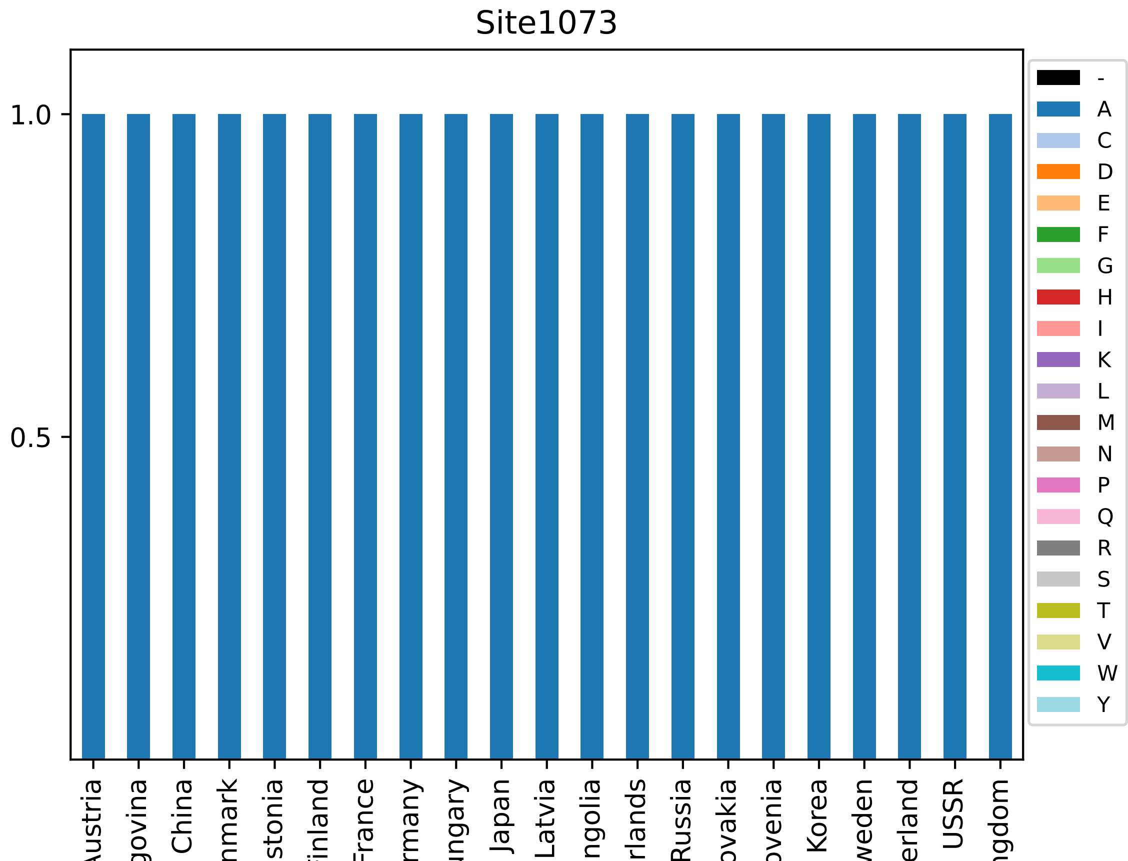

Site1073
1.0
0.5
-
A
C
D
E
F
G
H
I
K
L
M
N
P
Q
R
S
T
V
W
Y
China
Japan
Latvia
Russia
Korea
USSR

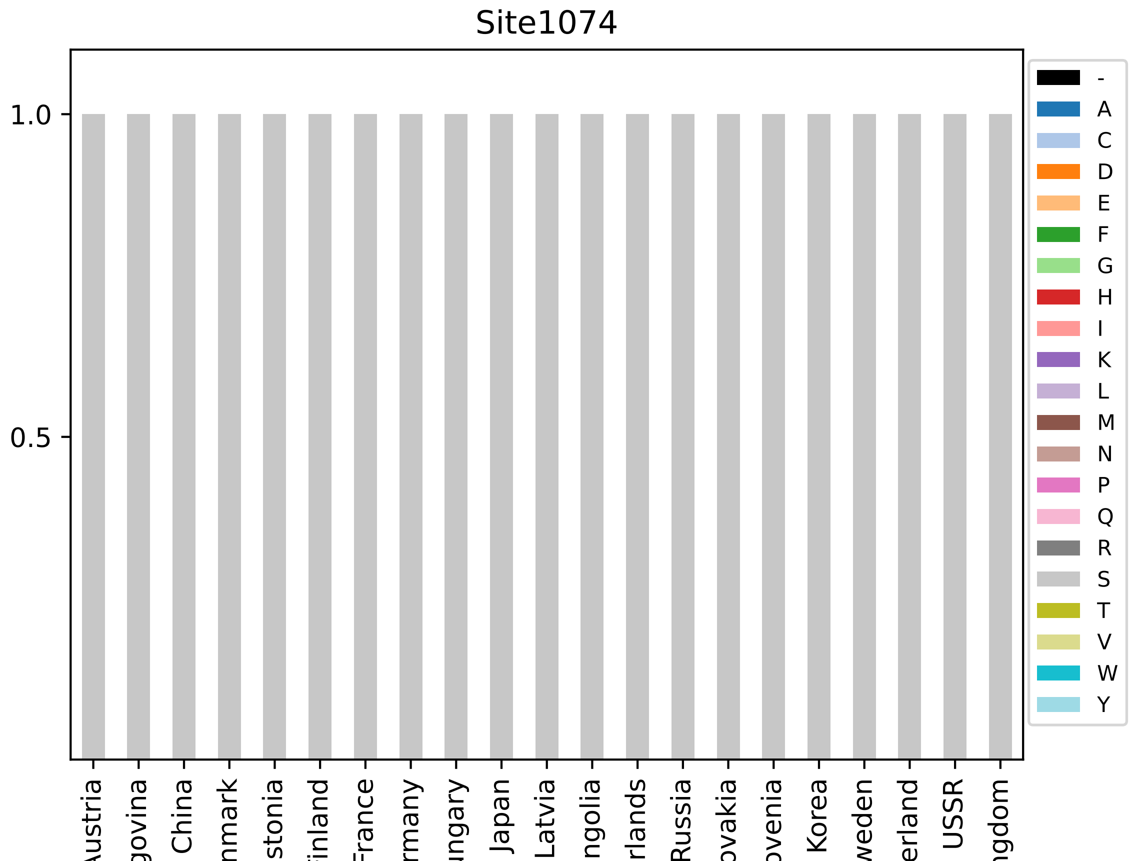
Site1074
1.0
0.5
-
A
C
D
E
F
G
H
I
K
L
M
N
P
Q
R
S
T
V
W
Y
Austria
govina
China
nmark
stonia
Finland
France
rmany
ungary
Japan
Latvia
ngolia
rlands
Russia
ovakia
ovenia
Korea
weden
erland
USSR
ngdom

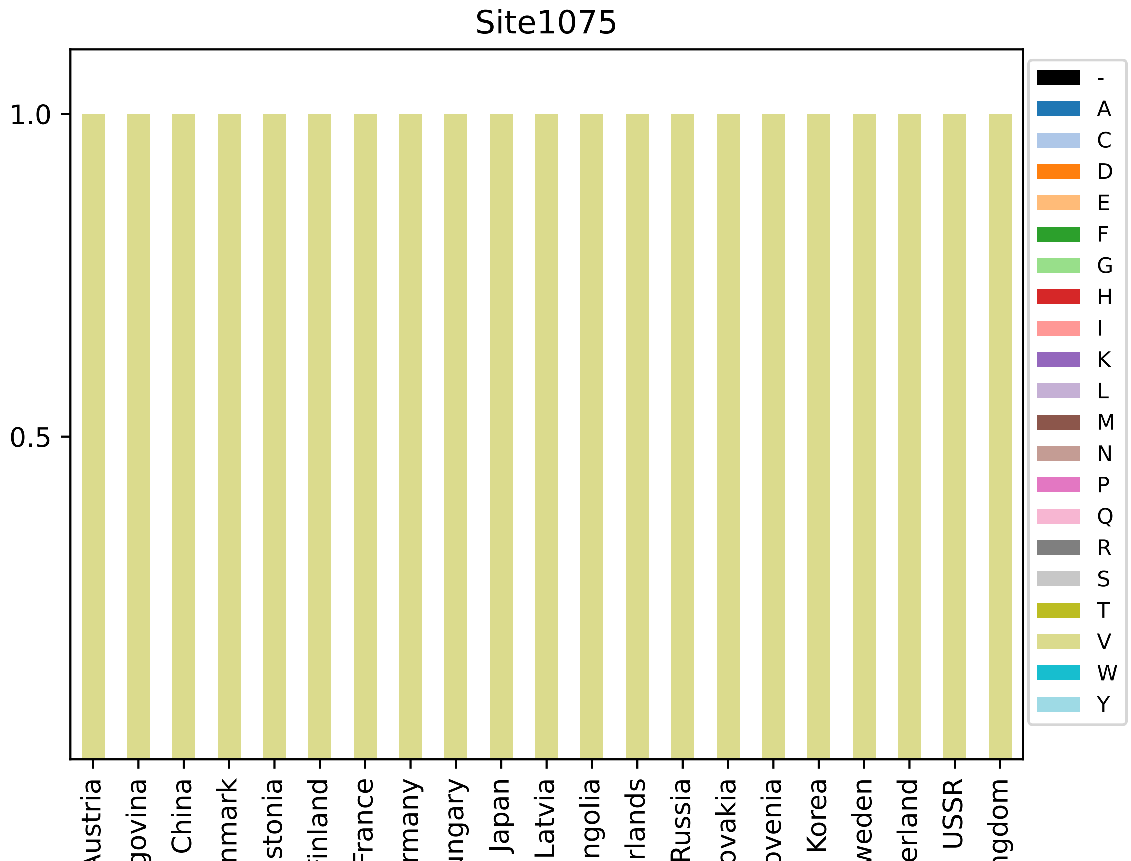
Site1075
1.0
0.5
-
A
C
D
E
F
G
H
I
K
L
M
N
P
Q
R
S
T
V
W
Y
Austria
China
Japan
Latvia
Russia
Korea
USSR

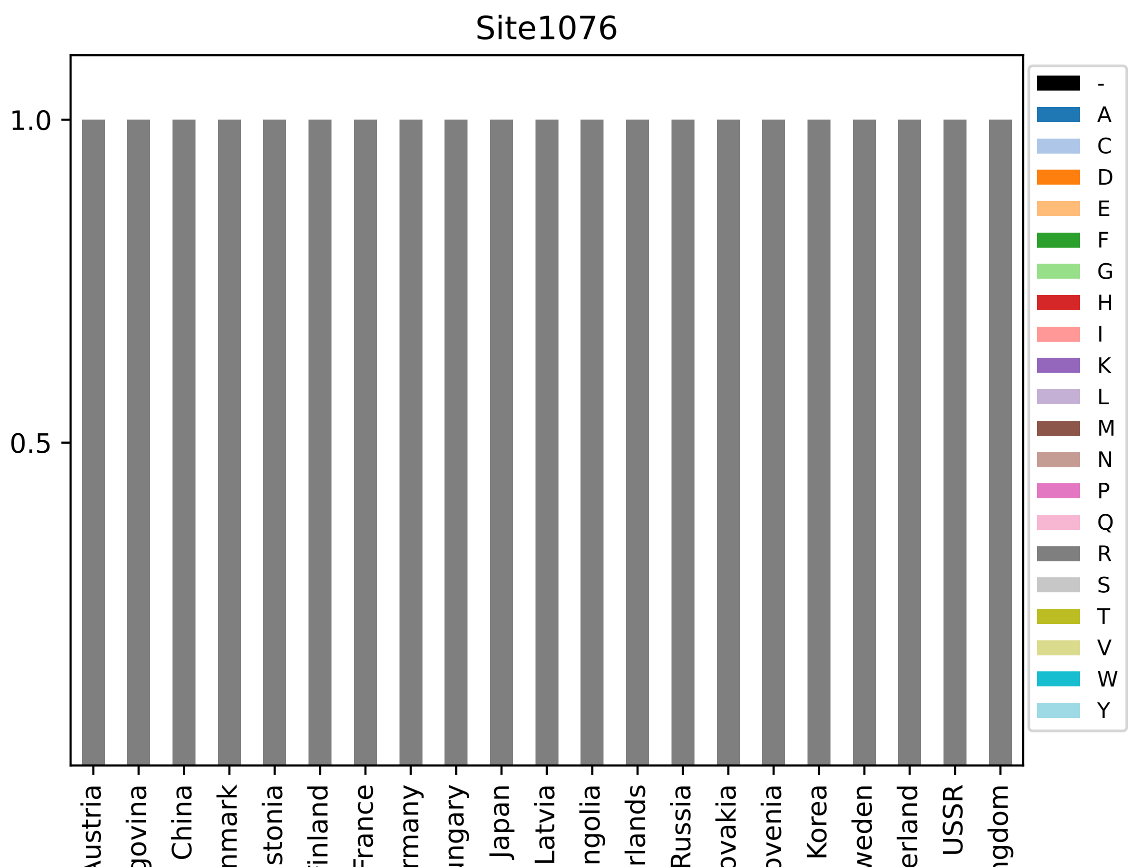
Site1076
1.0
0.5
Austria
China
Estonia
Finland
France
Hungary
Japan
Latvia
Russia
Korea
Sweden
USSR
-
A
C
D
E
F
G
H
I
K
L
M
N
P
Q
R
S
T
V
W
Y

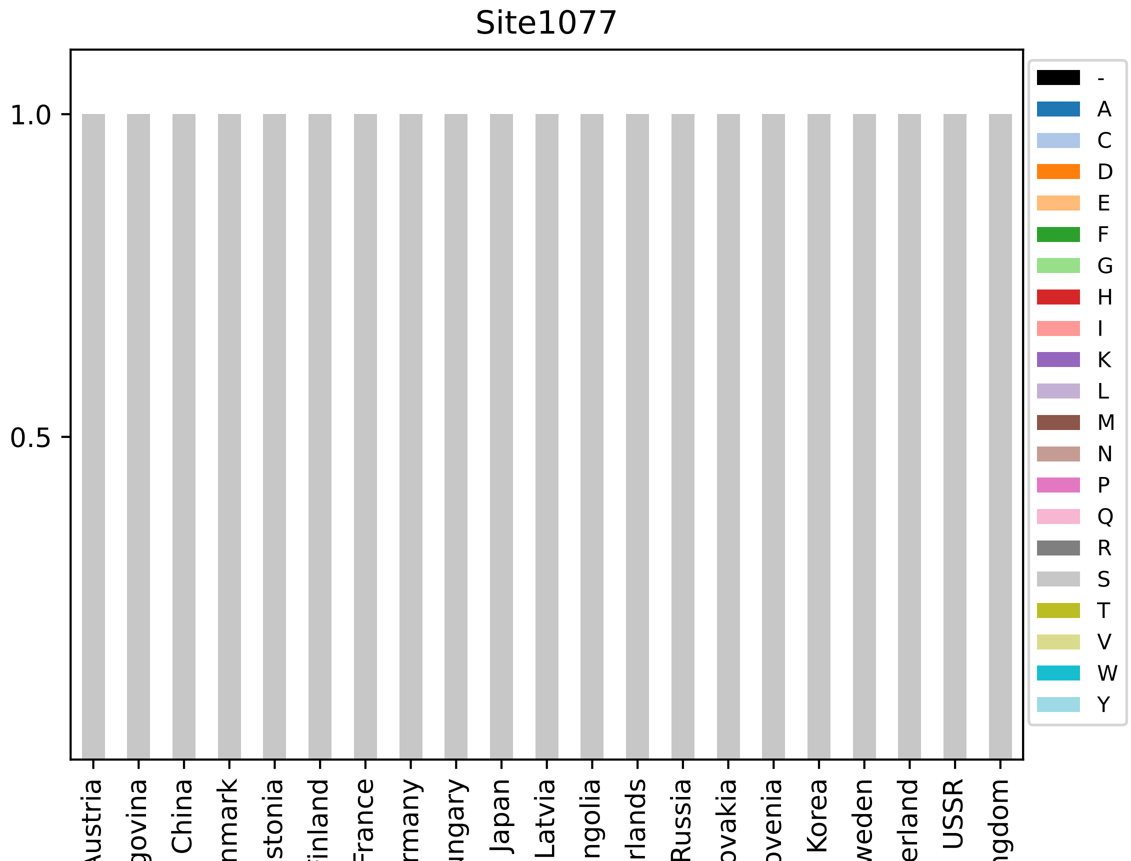

Site1077
1.0
0.5
-
A
C
D
E
F
G
H
I
K
L
M
N
P
Q
R
S
T
V
W
Y
Austria
govina
China
nmark
stonia
Finland
France
rmany
ungary
Japan
Latvia
ngolia
rlands
Russia
ovakia
ovenia
Korea
weden
erland
USSR
ngdom

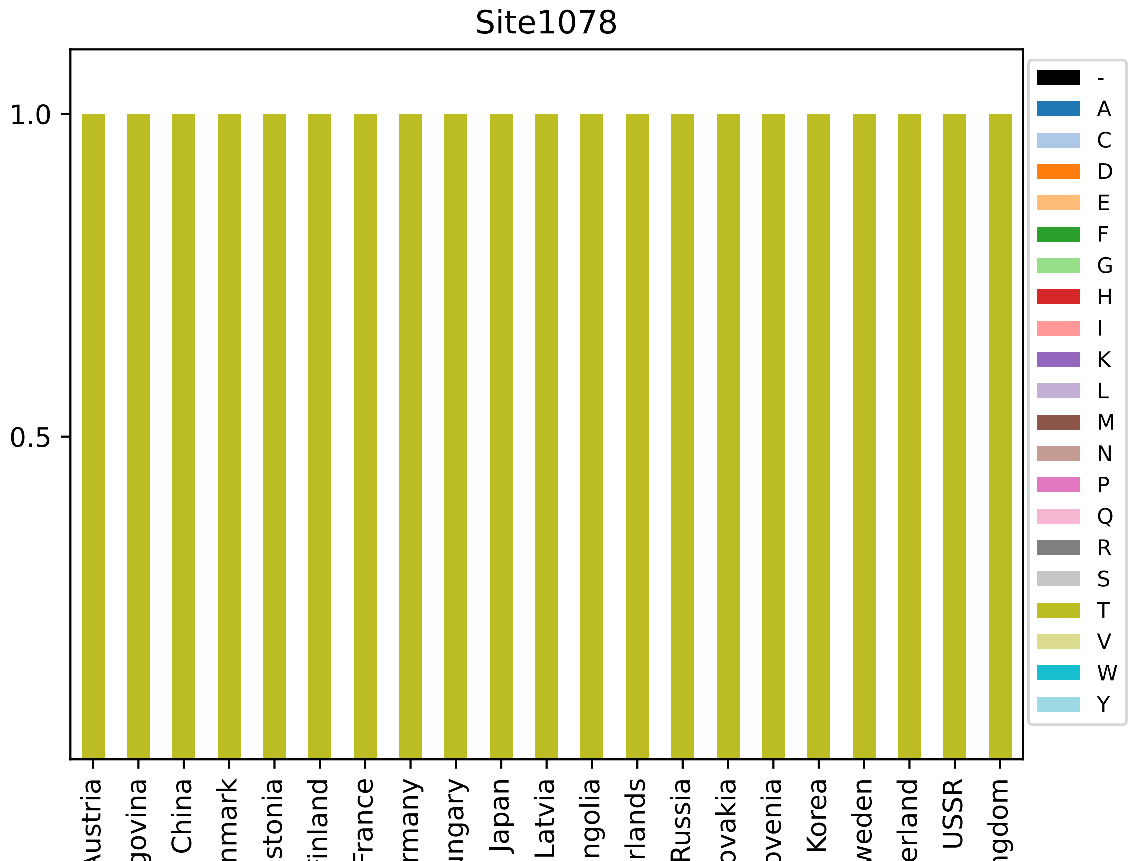

Site1078
1.0
0.5
-
A
C
D
E
F
G
H
I
K
L
M
N
P
Q
R
S
T
V
W
Y
Austria
govina
China
nmark
stonia
Finland
France
rmany
ungary
Japan
Latvia
ngolia
rlands
Russia
ovakia
ovenia
Korea
weden
erland
USSR
ngdom

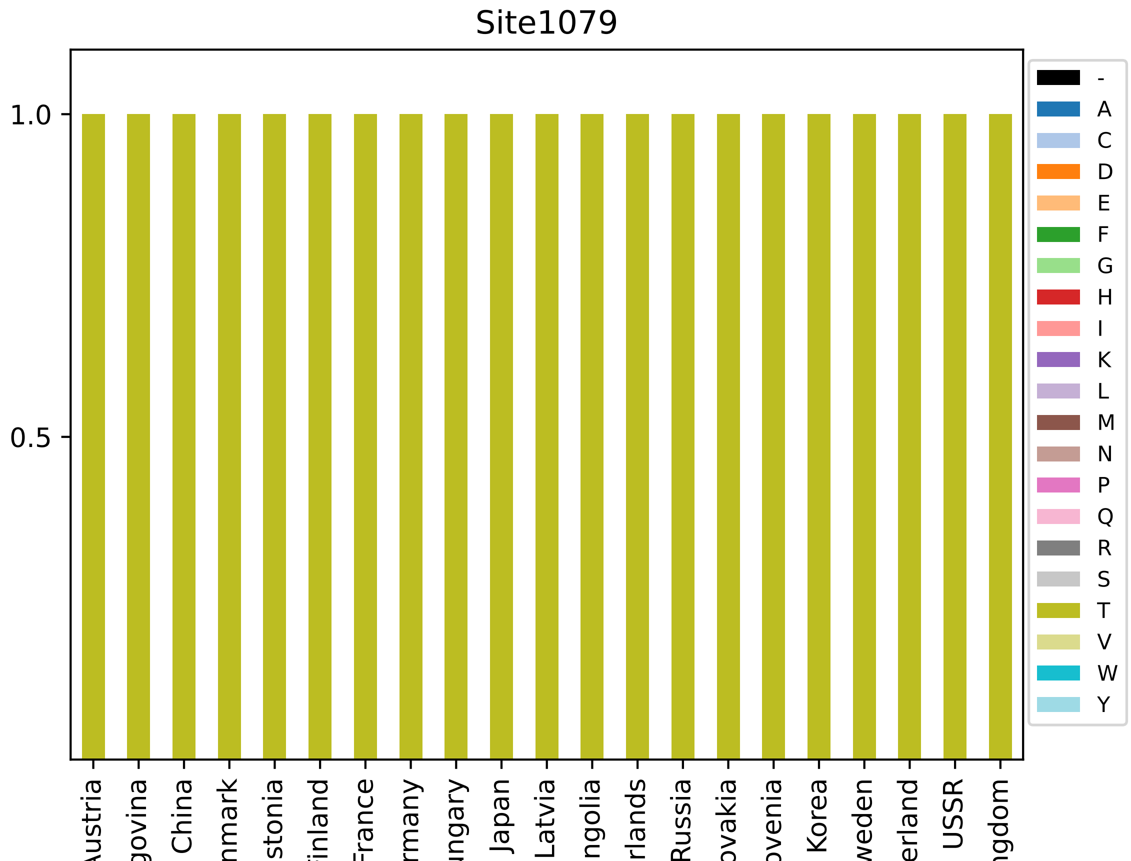

Site1079
1.0
0.5
-
A
C
D
E
F
G
H
I
K
L
M
N
P
Q
R
S
T
V
W
Y
China
Japan
Latvia
Russia
Korea
USSR

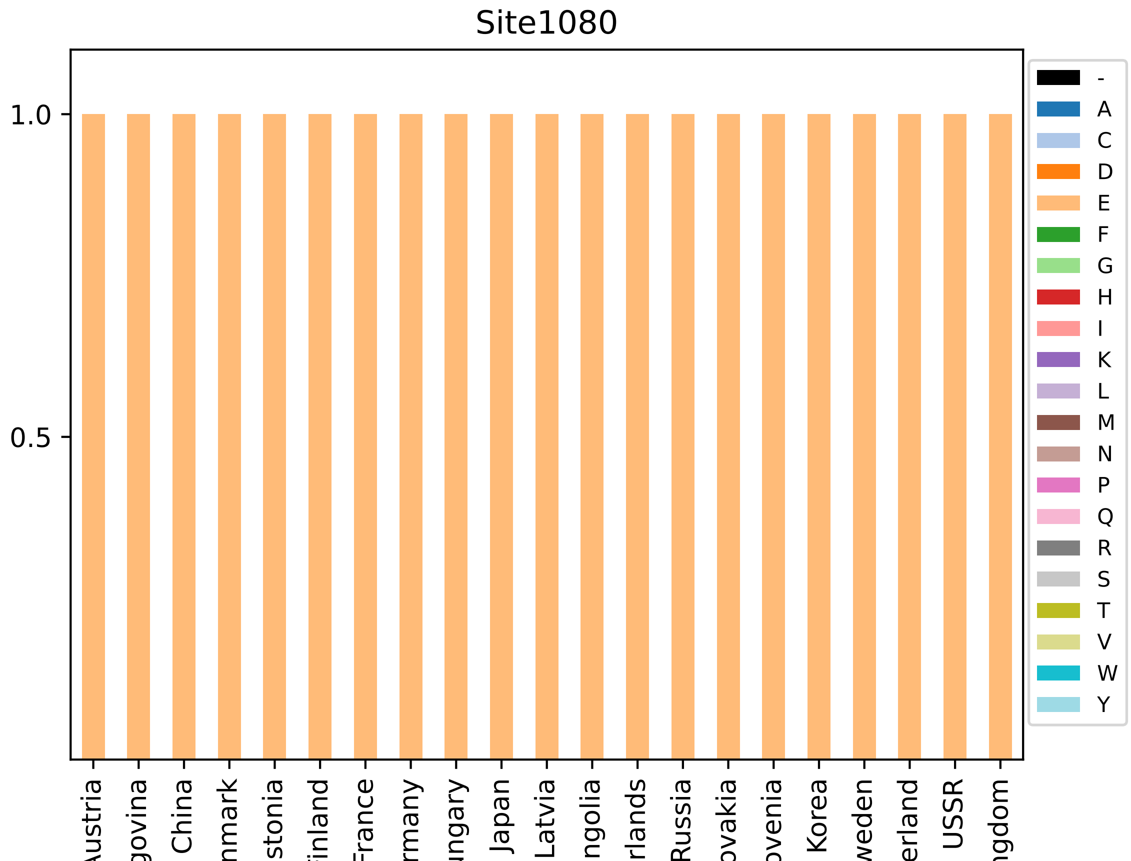
Site1080
1.0
0.5
-
A
C
D
E
F
G
H
I
K
L
M
N
P
Q
R
S
T
V
W
Y
China
Japan
Latvia
Russia
Korea
USSR

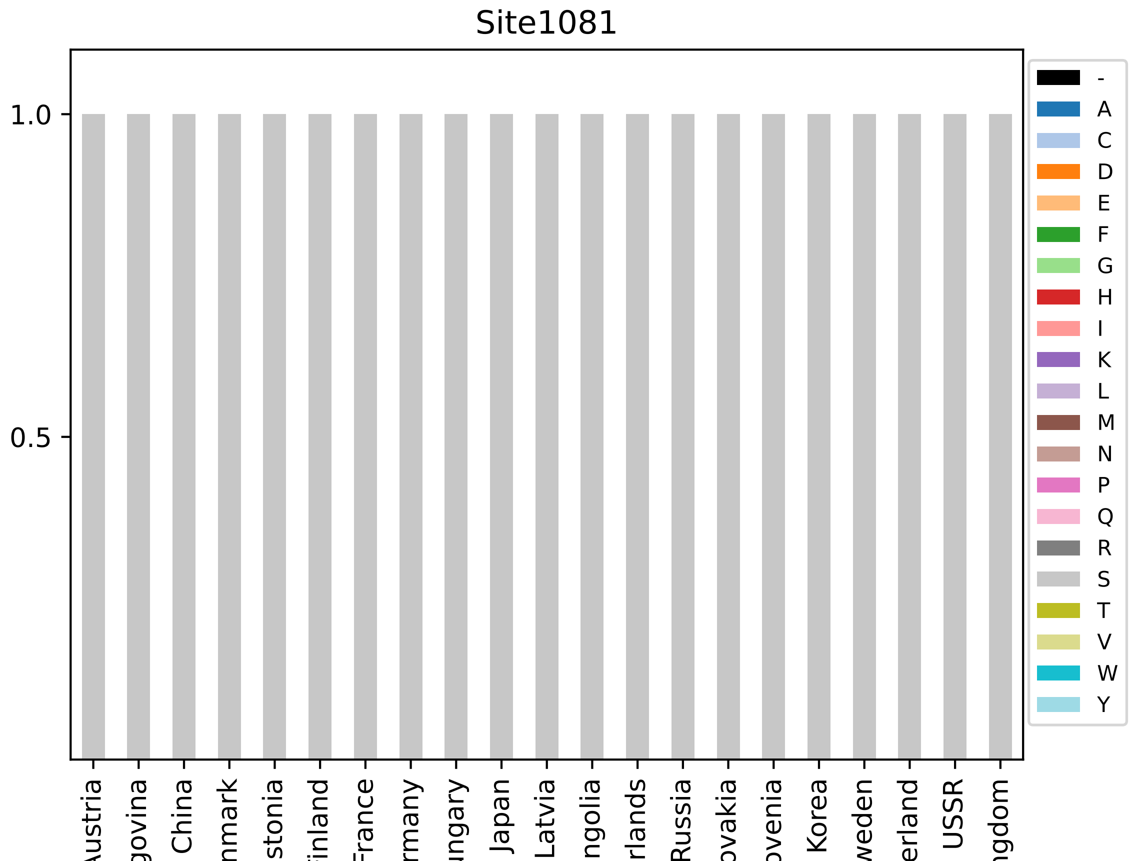

Site1081
1.0
0.5
Austria
govina
China
nmark
stonia
Finland
France
rmany
ungary
Japan
Latvia
ngolia
rlands
Russia
ovakia
ovenia
Korea
weden
erland
USSR
ngdom
-
A
C
D
E
F
G
H
I
K
L
M
N
P
Q
R
S
T
V
W
Y

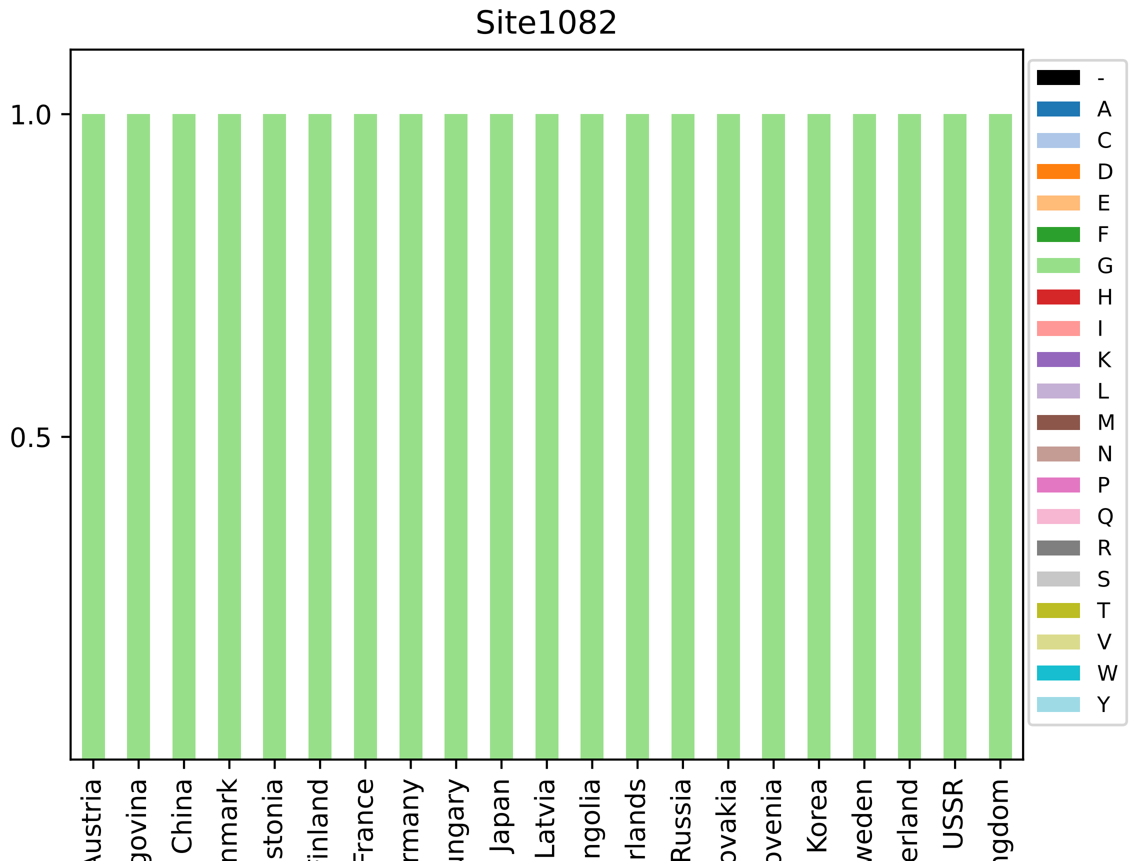
Site1082
1.0
0.5
-
A
C
D
E
F
G
H
I
K
L
M
N
P
Q
R
S
T
V
W
Y
China
Japan
Latvia
Russia
Korea
USSR

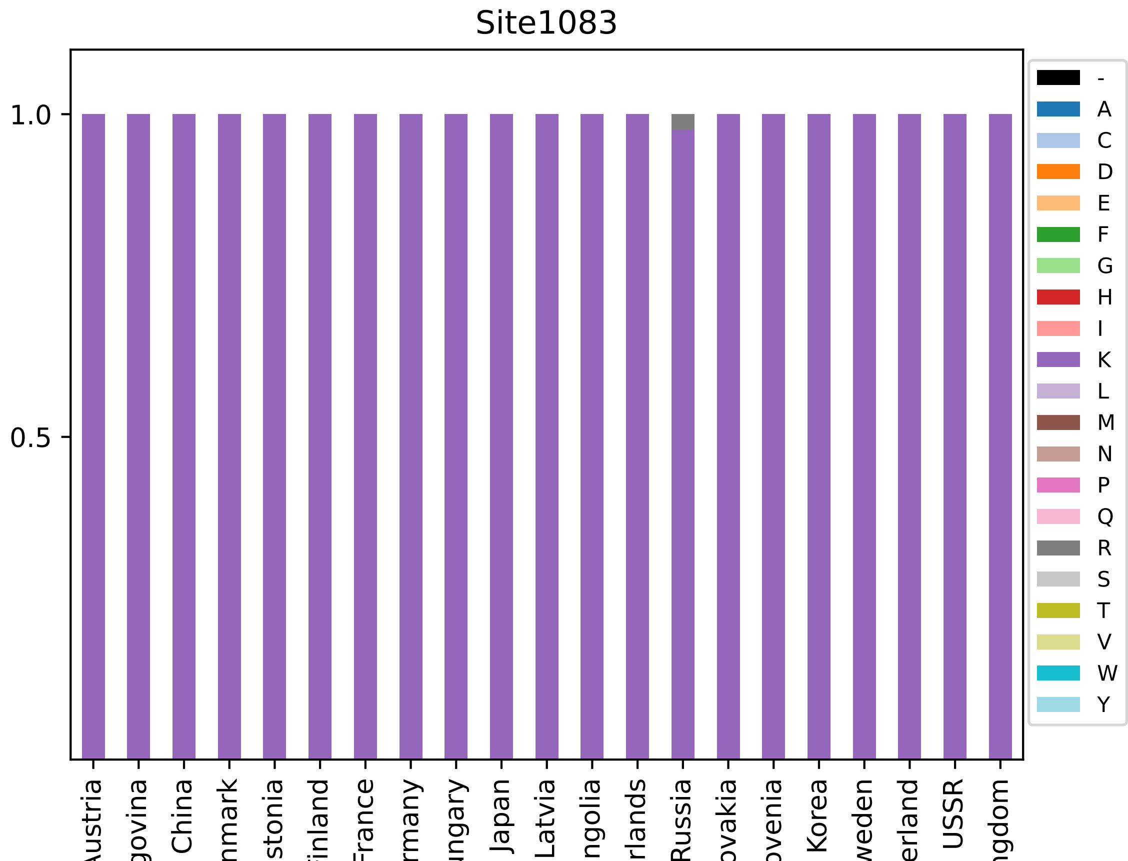
Site1083
1.0
0.5
-
A
C
D
E
F
G
H
I
K
L
M
N
P
Q
R
S
T
V
W
Y
China
Japan
Latvia
Russia
Korea
USSR

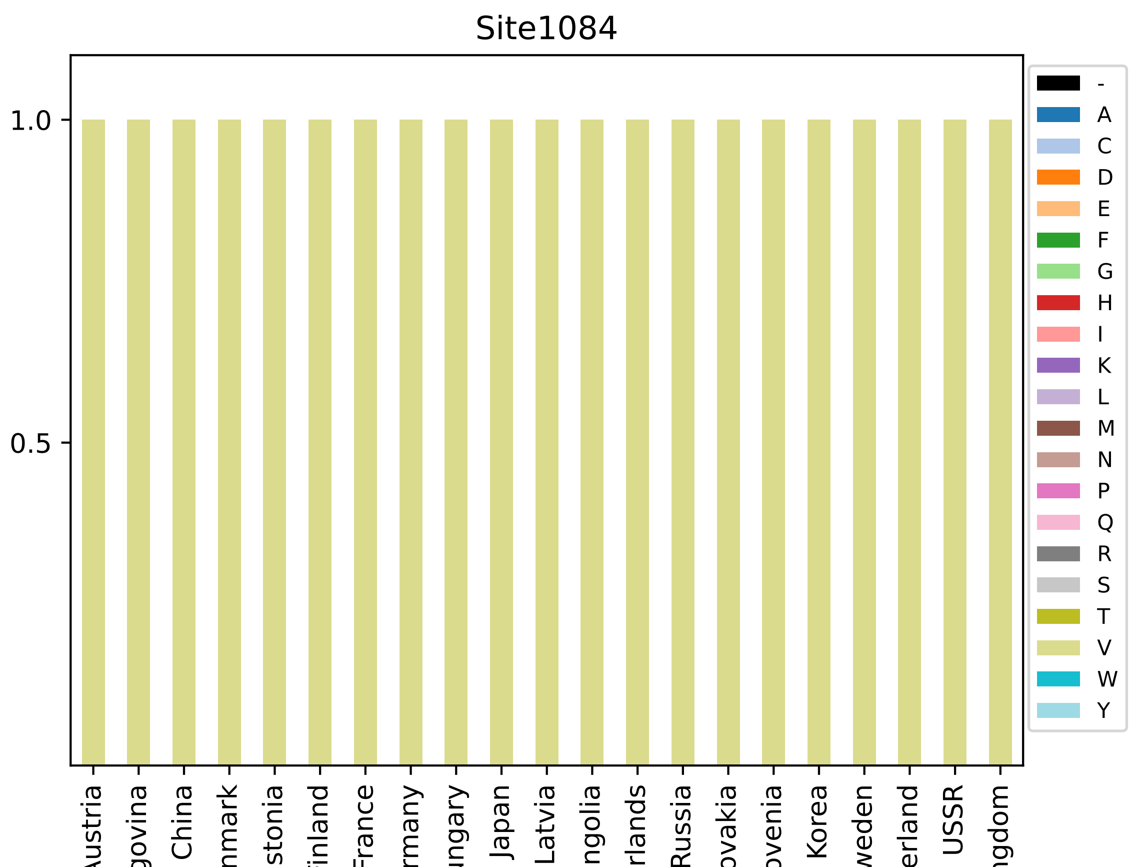
Site1084
1.0
0.5
Austria
govina
China
nmark
stonia
Finland
France
rmany
ungary
Japan
Latvia
ngolia
rlands
Russia
ovakia
ovenia
Korea
weden
erland
USSR
ngdom
-
A
C
D
E
F
G
H
I
K
L
M
N
P
Q
R
S
T
V
W
Y

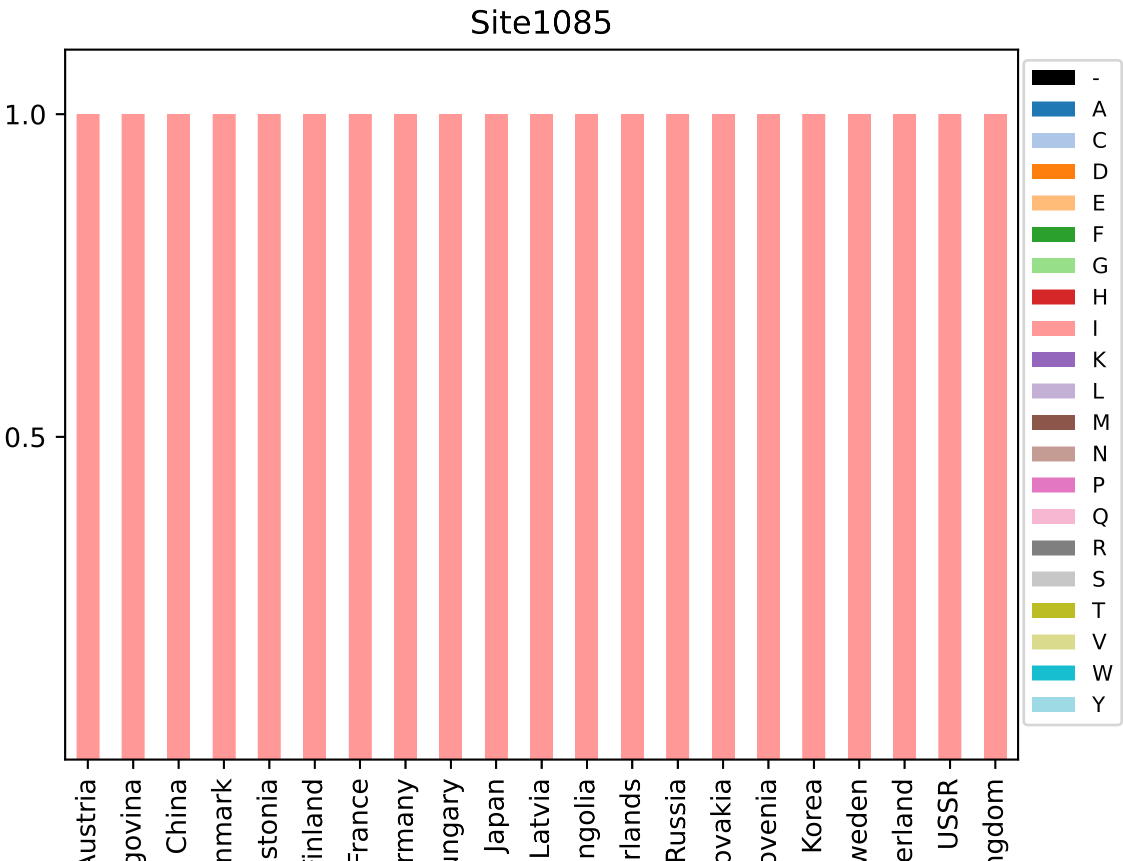

Site1085
1.0
0.5
-
A
C
D
E
F
G
H
I
K
L
M
N
P
Q
R
S
T
V
W
Y
Austria
govina
China
nmark
stonia
inland
France
rmany
ungary
Japan
Latvia
ngolia
rlands
Russia
ovakia
ovenia
Korea
weden
erland
USSR
ngdom

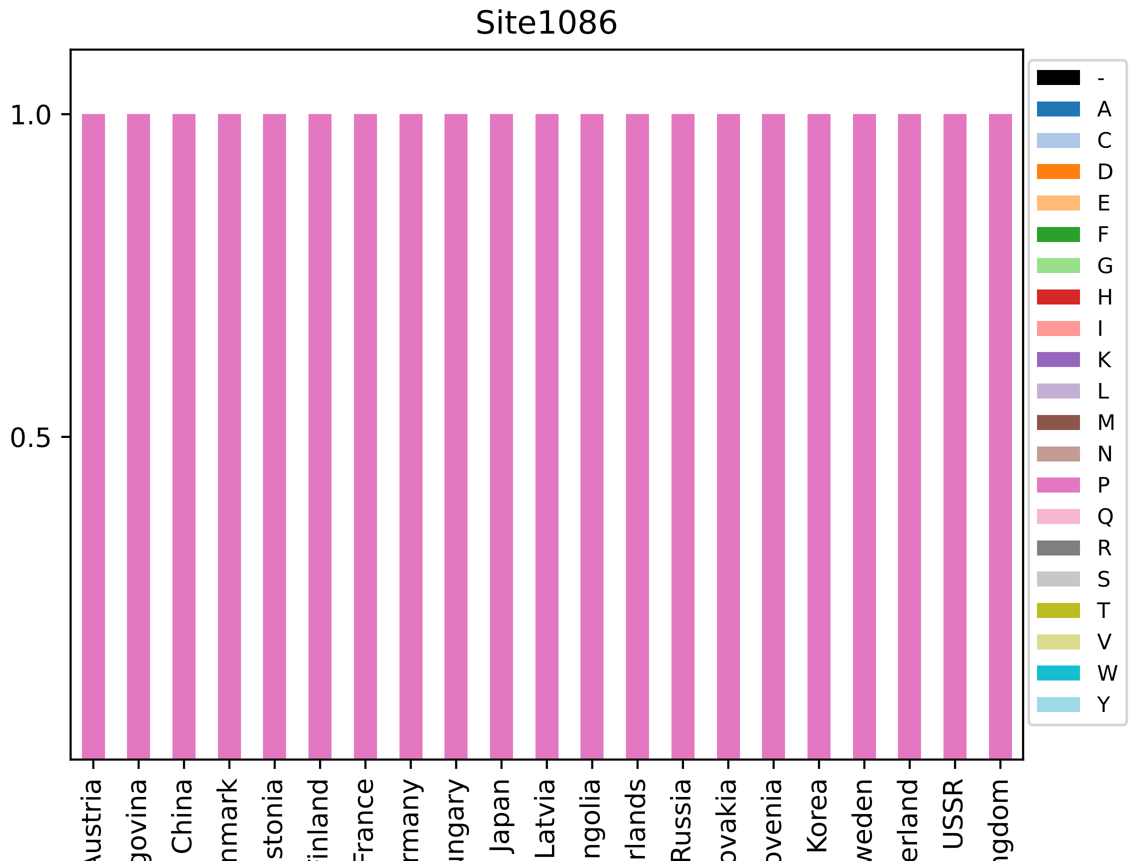
Site1086
1.0
0.5
-
A
C
D
E
F
G
H
I
K
L
M
N
P
Q
R
S
T
V
W
Y
China
Japan
Latvia
Russia
Korea
USSR

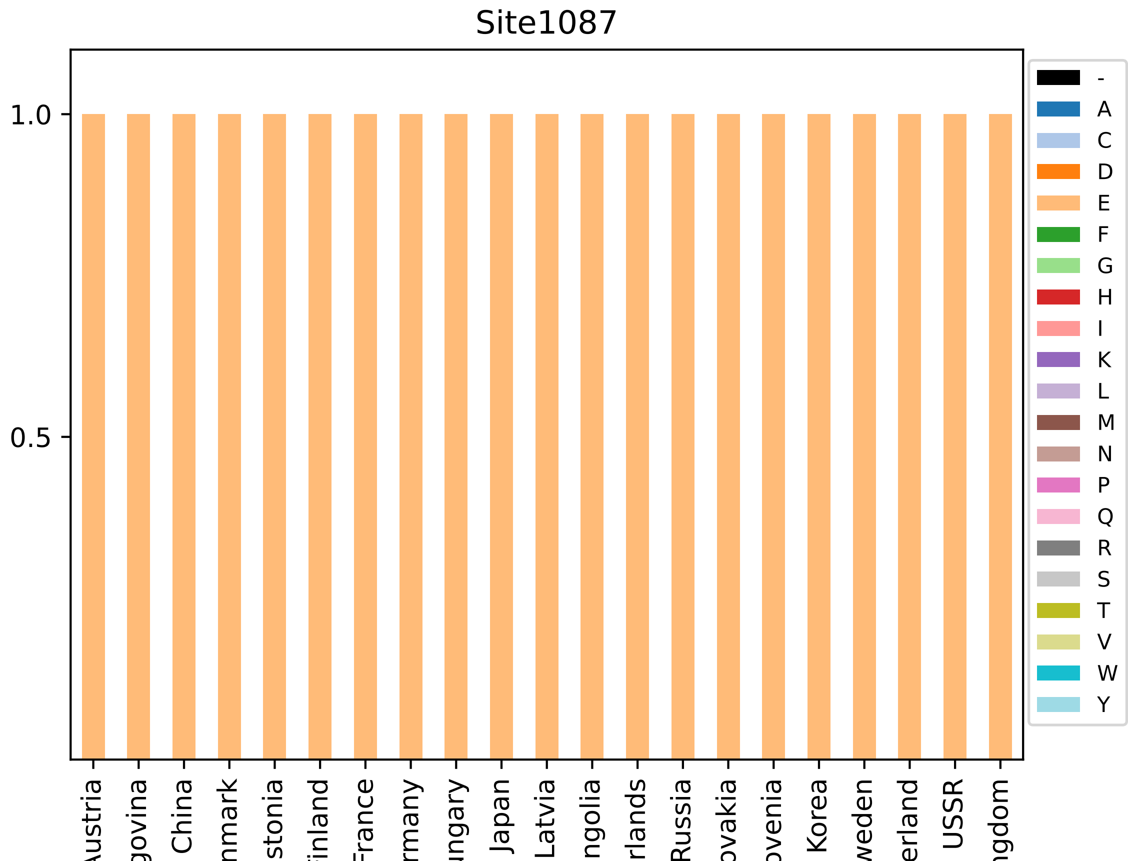

Site1087
1.0
0.5
-
A
C
D
E
F
G
H
I
K
L
M
N
P
Q
R
S
T
V
W
Y
China
Japan
Latvia
Russia
Korea
USSR

Site1088

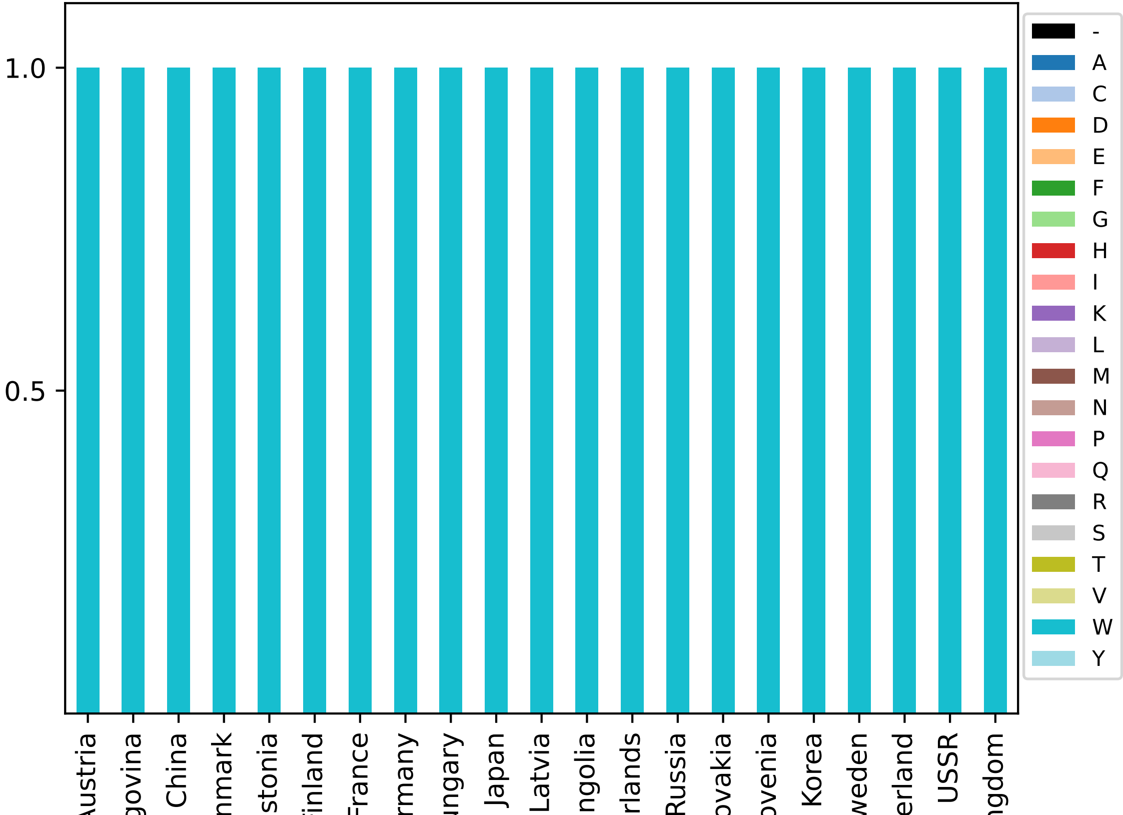

1.0
0.5
-
A
C
D
E
F
G
H
I
K
L
M
N
P
Q
R
S
T
V
W
Y
China
Japan
Latvia
Russia
Korea
USSR

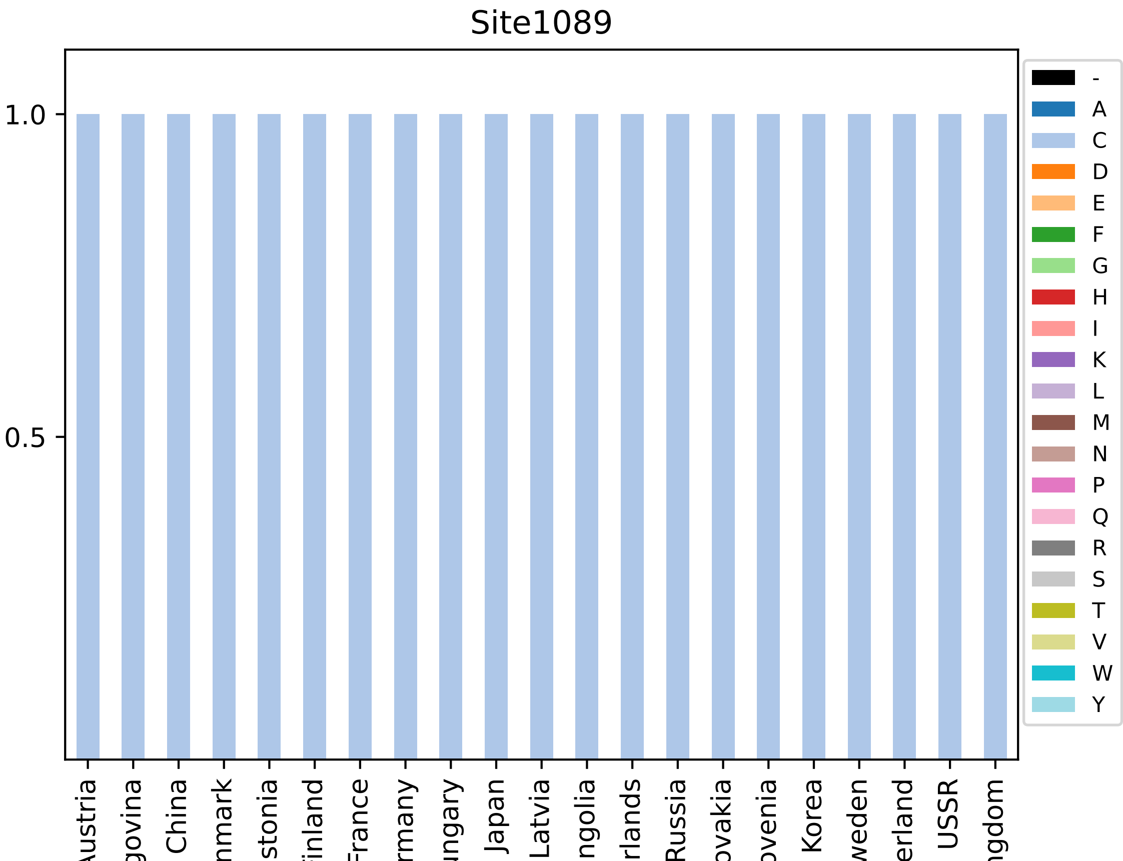
Site1089
1.0
0.5
-
A
C
D
E
F
G
H
I
K
L
M
N
P
Q
R
S
T
V
W
Y
China
Japan
Latvia
Russia
Korea
USSR

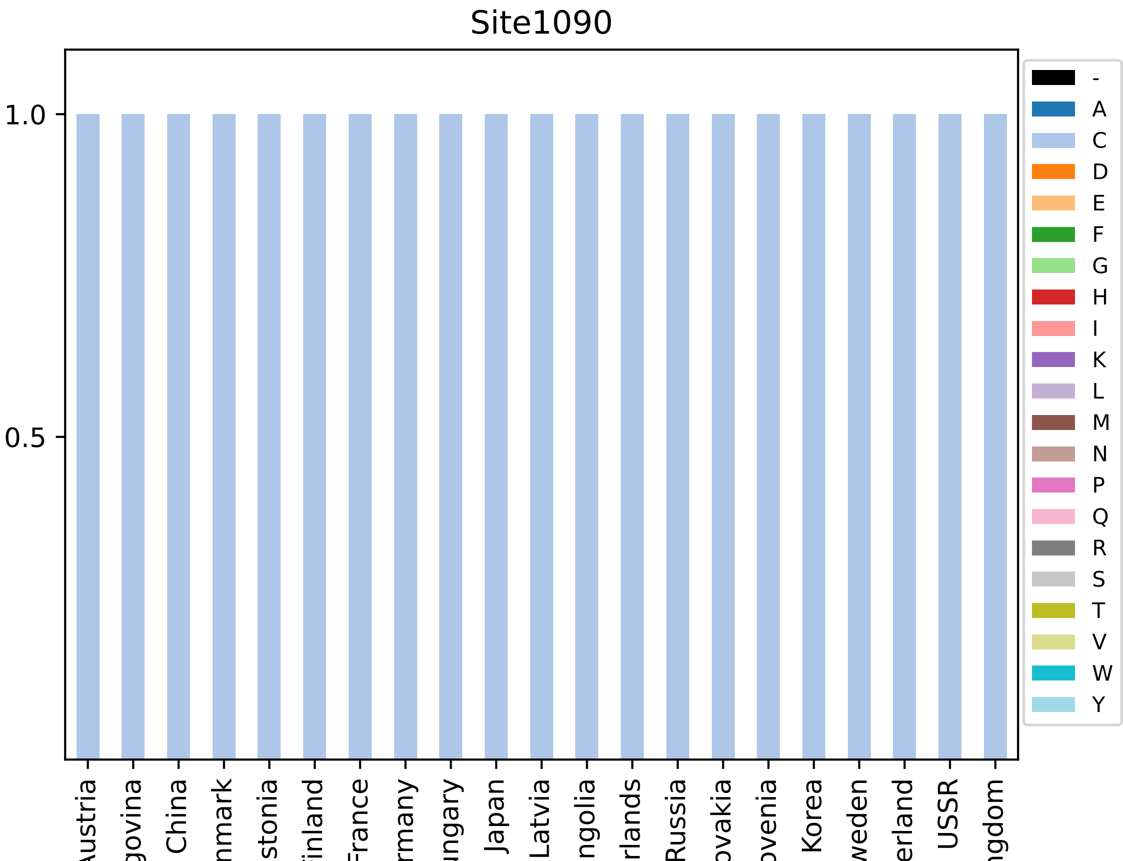
Site1090
1.0
0.5
-
A
C
D
E
F
G
H
I
K
L
M
N
P
Q
R
S
T
V
W
Y
China
Japan
Latvia
Russia
Korea
USSR

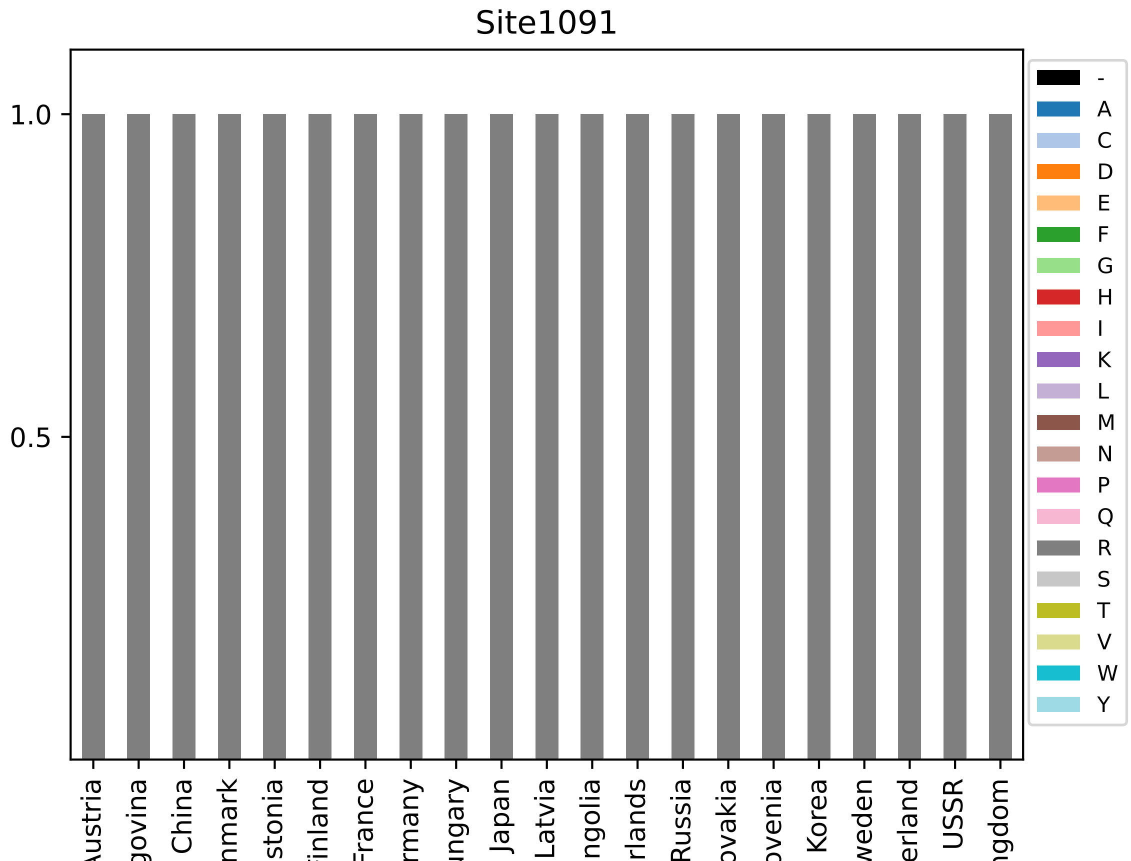
Site1091
1.0
0.5
-
A
C
D
E
F
G
H
I
K
L
M
N
P
Q
R
S
T
V
W
Y
China
Japan
Latvia
Russia
Korea
USSR

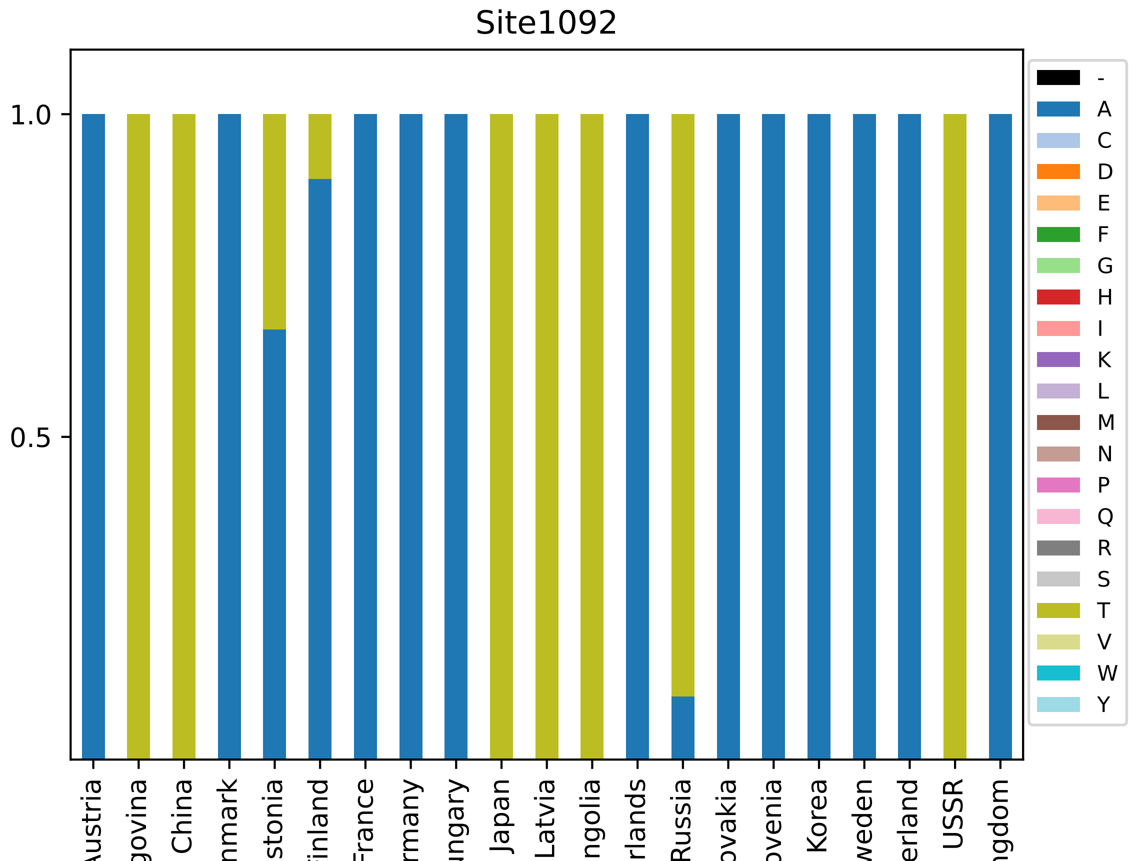

Site1092
1.0
0.5
-
A
C
D
E
F
G
H
I
K
L
M
N
P
Q
R
S
T
V
W
Y
China
Japan
Latvia
Russia
Korea
USSR

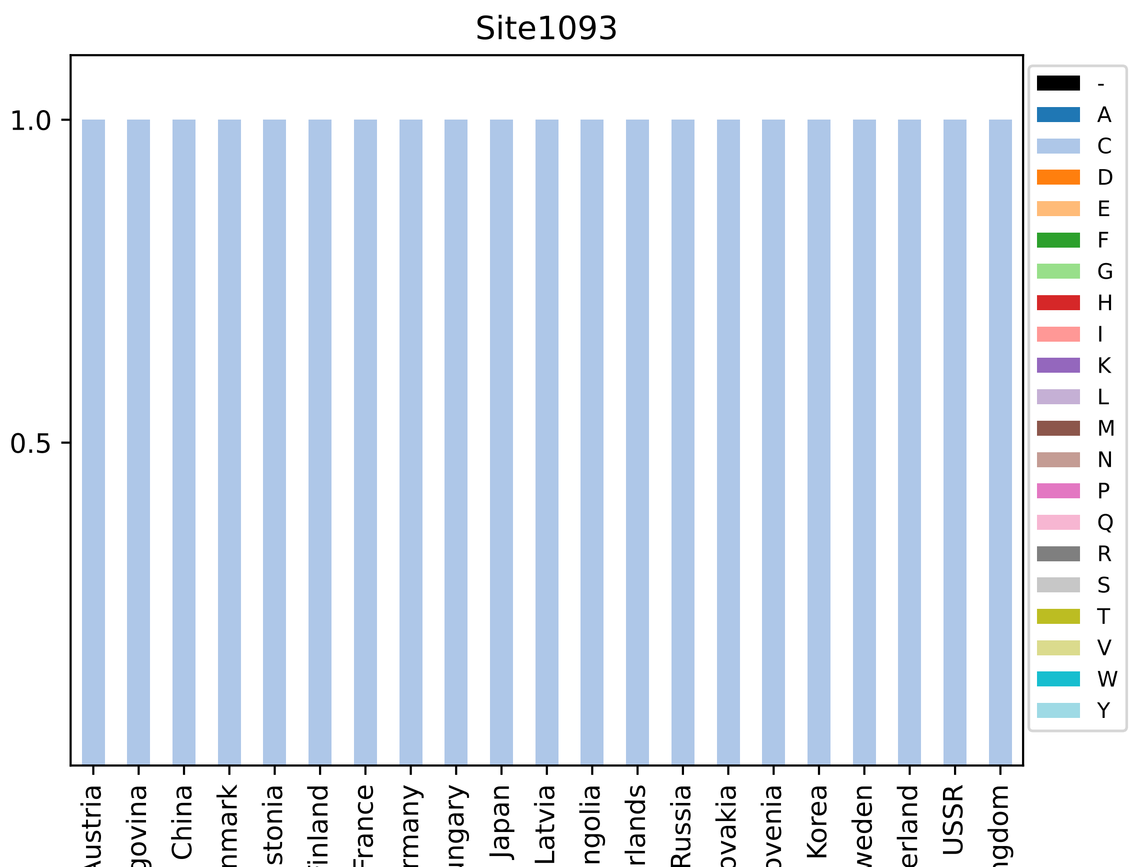

Site1093
1.0
0.5
-
A
C
D
E
F
G
H
I
K
L
M
N
P
Q
R
S
T
V
W
Y
Japan
Latvia
Russia
Korea
USSR
China

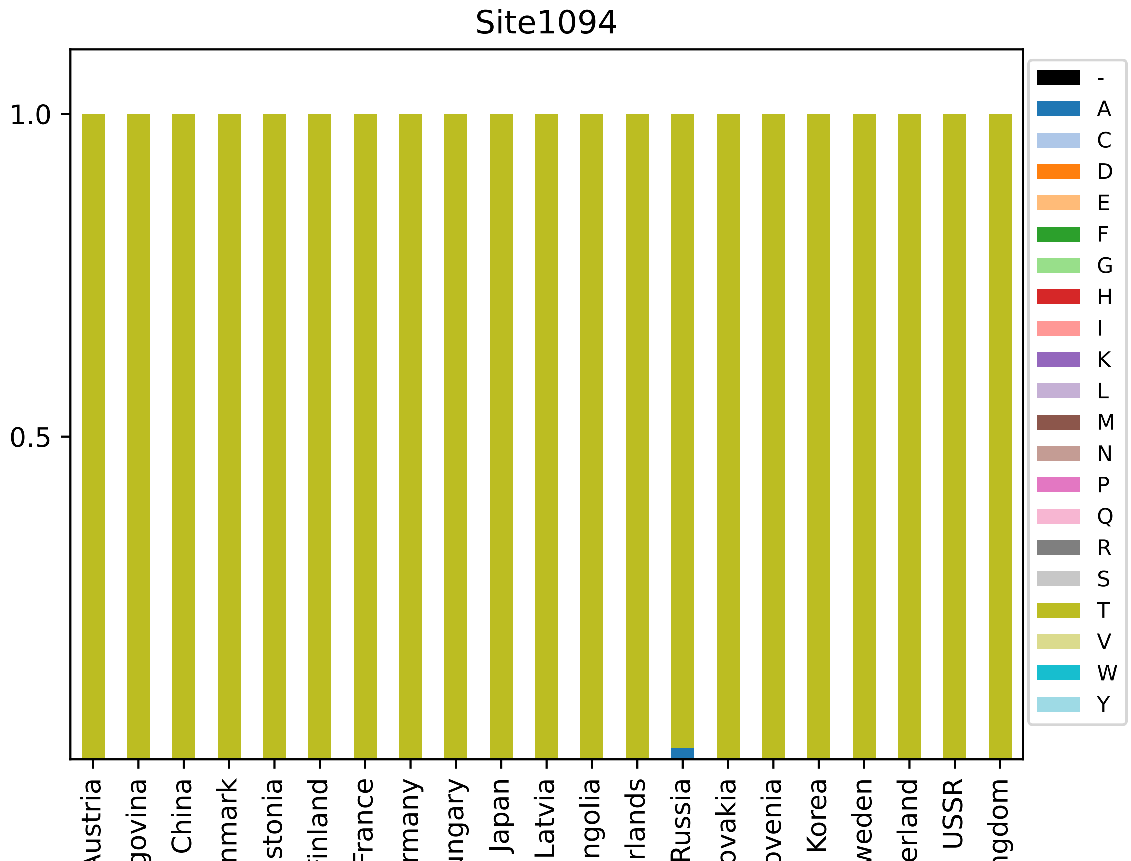

Site1094
1.0
0.5
China
Japan
Latvia
Russia
Korea
USSR
-
A
C
D
E
F
G
H
I
K
L
M
N
P
Q
R
S
T
V
W
Y

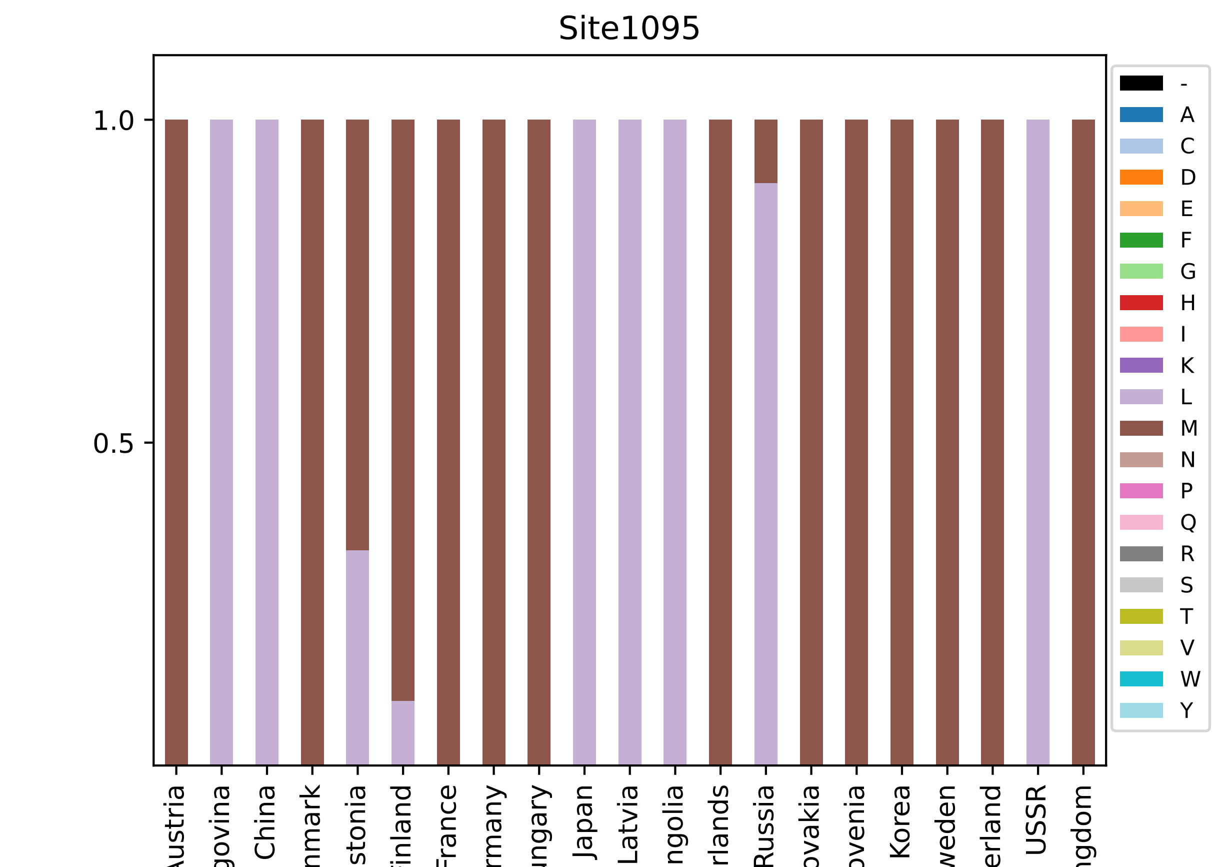

Site1095
1.0
0.5
Austria
govina
China
nmark
stonia
inland
France
rmany
ungary
Japan
Latvia
ngolia
rlands
Russia
ovakia
ovenia
Korea
weden
erland
USSR
ngdom
-
A
C
D
E
F
G
H
I
K
L
M
N
P
Q
R
S
T
V
W
Y

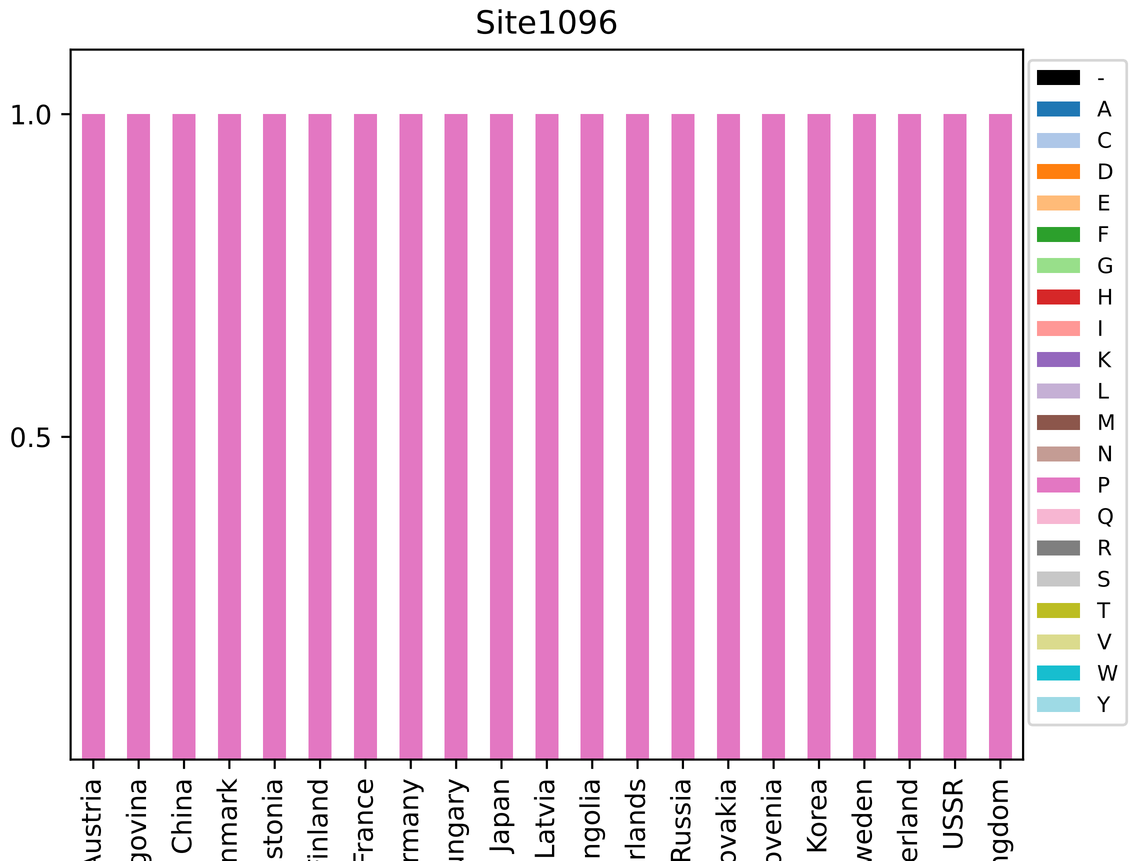
Site1096
1.0
0.5
-
A
C
D
E
F
G
H
I
K
L
M
N
P
Q
R
S
T
V
W
Y
China
Japan
Latvia
Russia
Korea
USSR

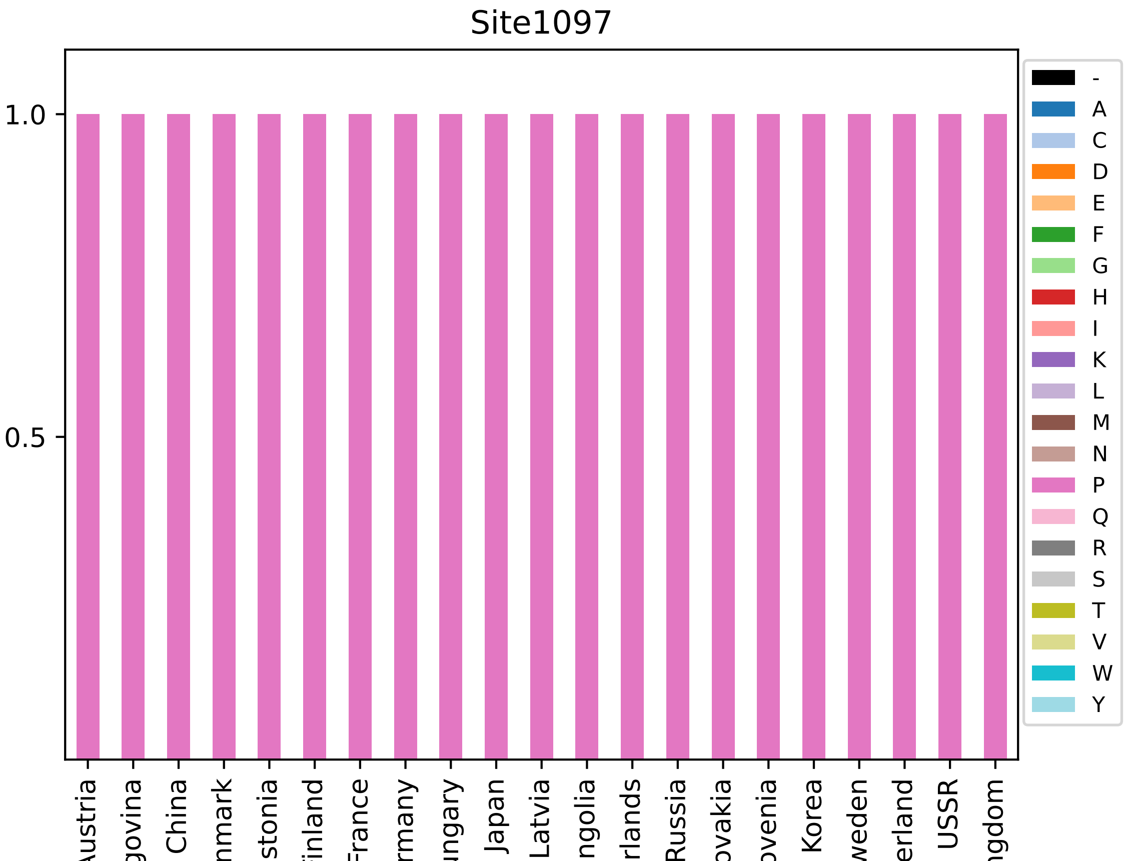

Site1097
1.0
0.5
-
A
C
D
E
F
G
H
I
K
L
M
N
P
Q
R
S
T
V
W
Y
China
Japan
Latvia
Russia
Korea
USSR

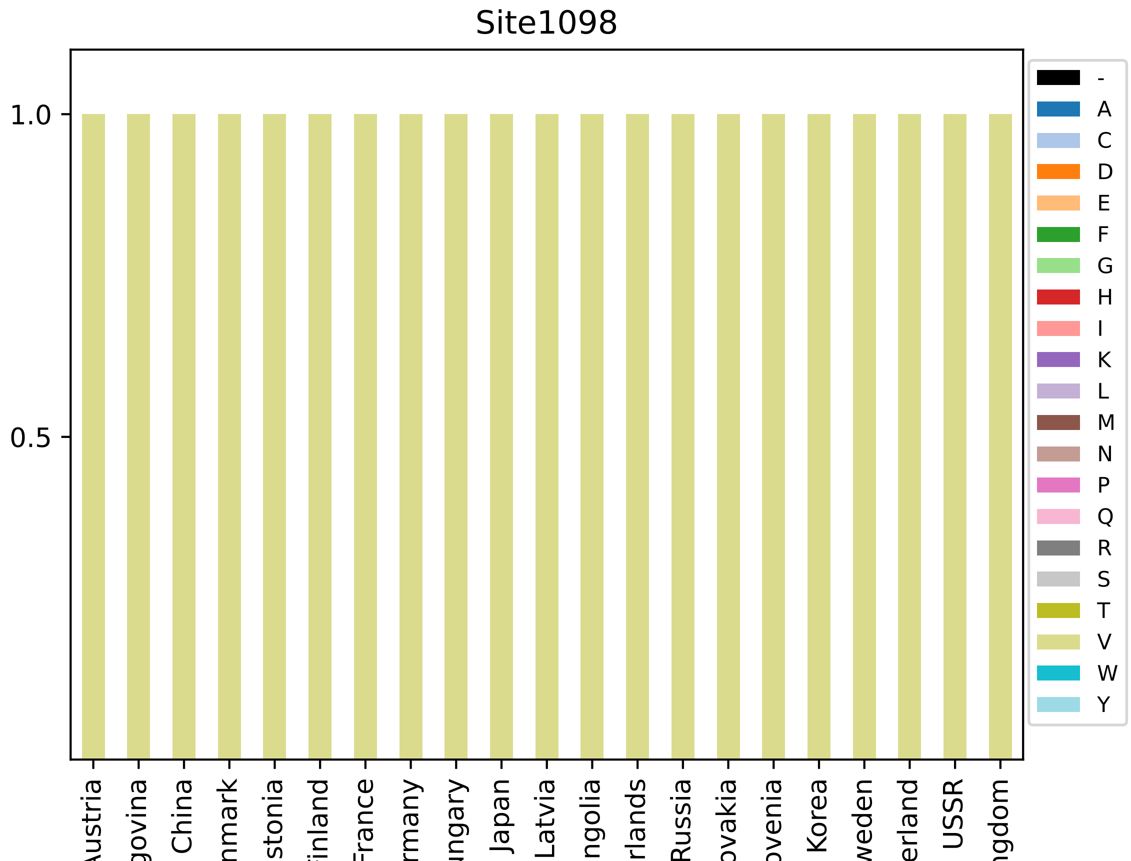
Site1098
1.0
0.5
-
A
C
D
E
F
G
H
I
K
L
M
N
P
Q
R
S
T
V
W
Y
China
Japan
Latvia
Russia
Korea
USSR

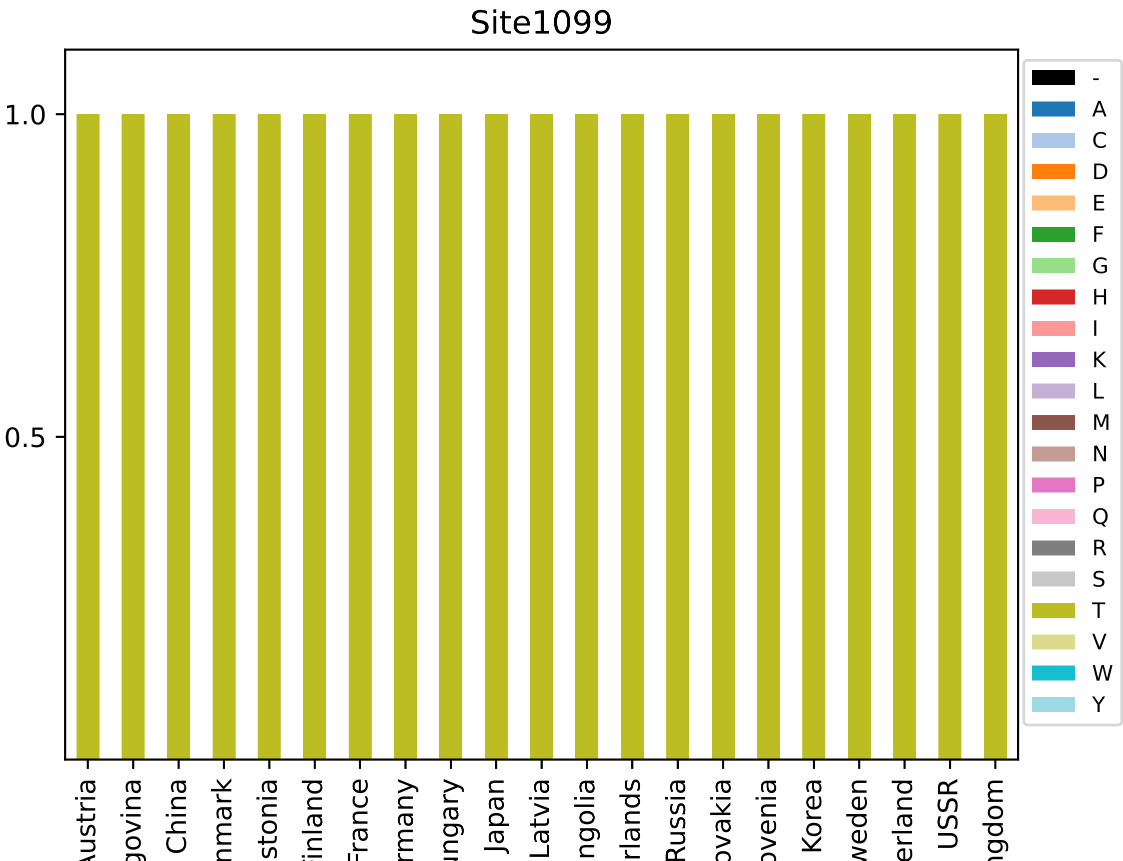
Site1099
1.0
0.5
-
A
C
D
E
F
G
H
I
K
L
M
N
P
Q
R
S
T
V
W
Y
Austria
govina
China
nmark
stonia
inland
France
rmany
ungary
Japan
Latvia
ngolia
rlands
Russia
ovakia
ovenia
Korea
weden
erland
USSR
ngdom

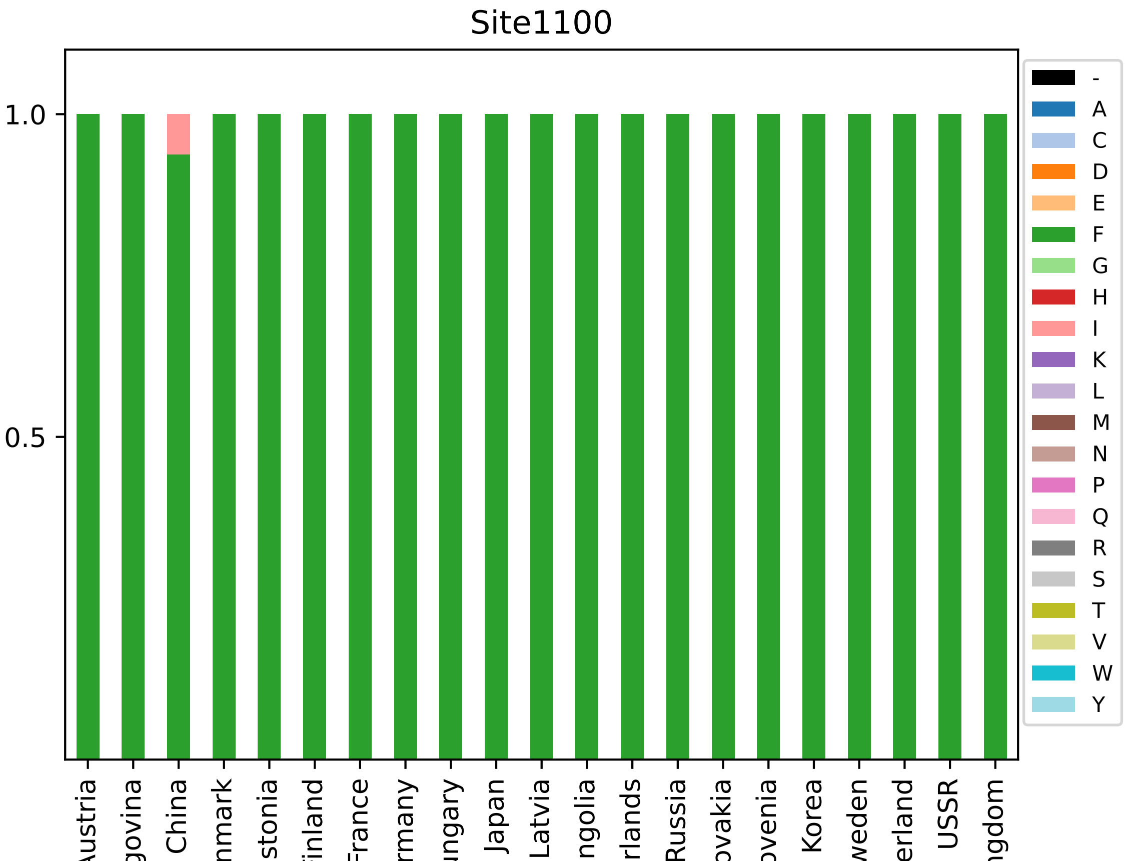
Site1100
1.0
0.5
-
A
C
D
E
F
G
H
I
K
L
M
N
P
Q
R
S
T
V
W
Y
Austria
China
Japan
Latvia
Russia
Korea
USSR

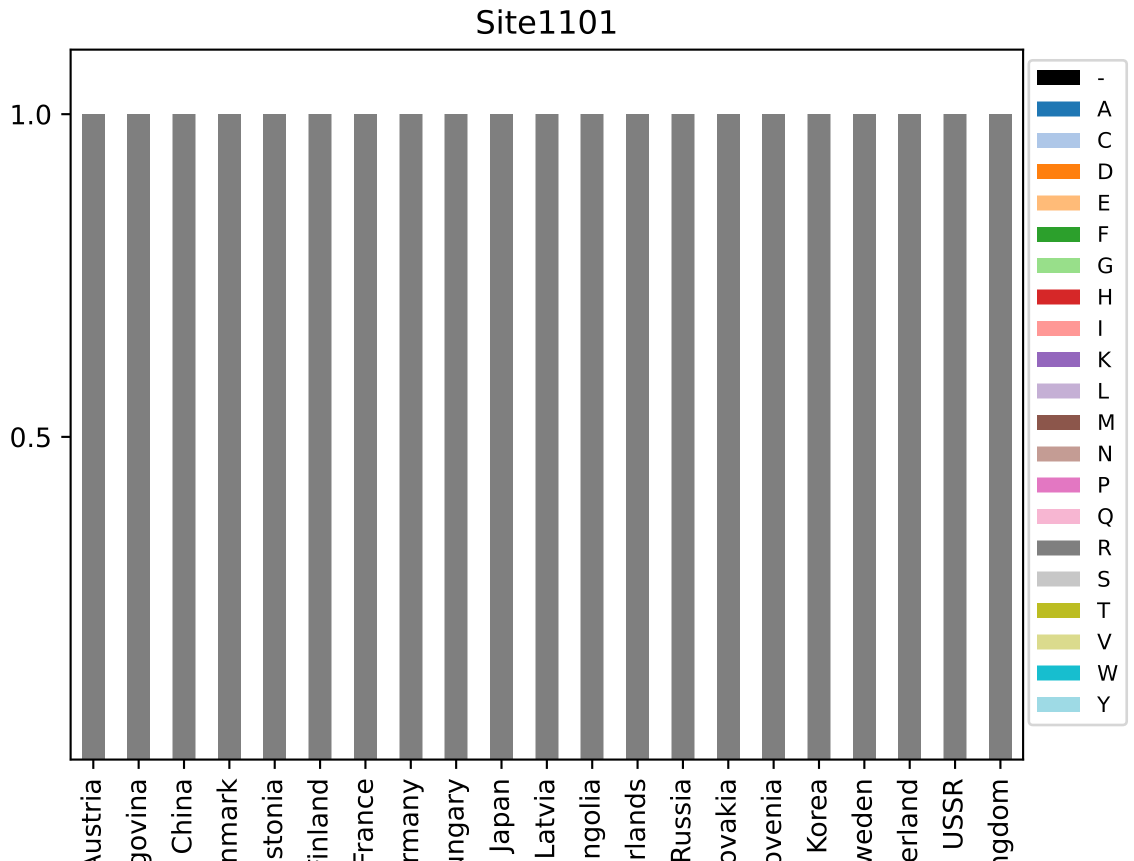
Site1101
1.0
0.5
-
A
C
D
E
F
G
H
I
K
L
M
N
P
Q
R
S
T
V
W
Y
Austria
China
Japan
Latvia
Russia
Korea
USSR

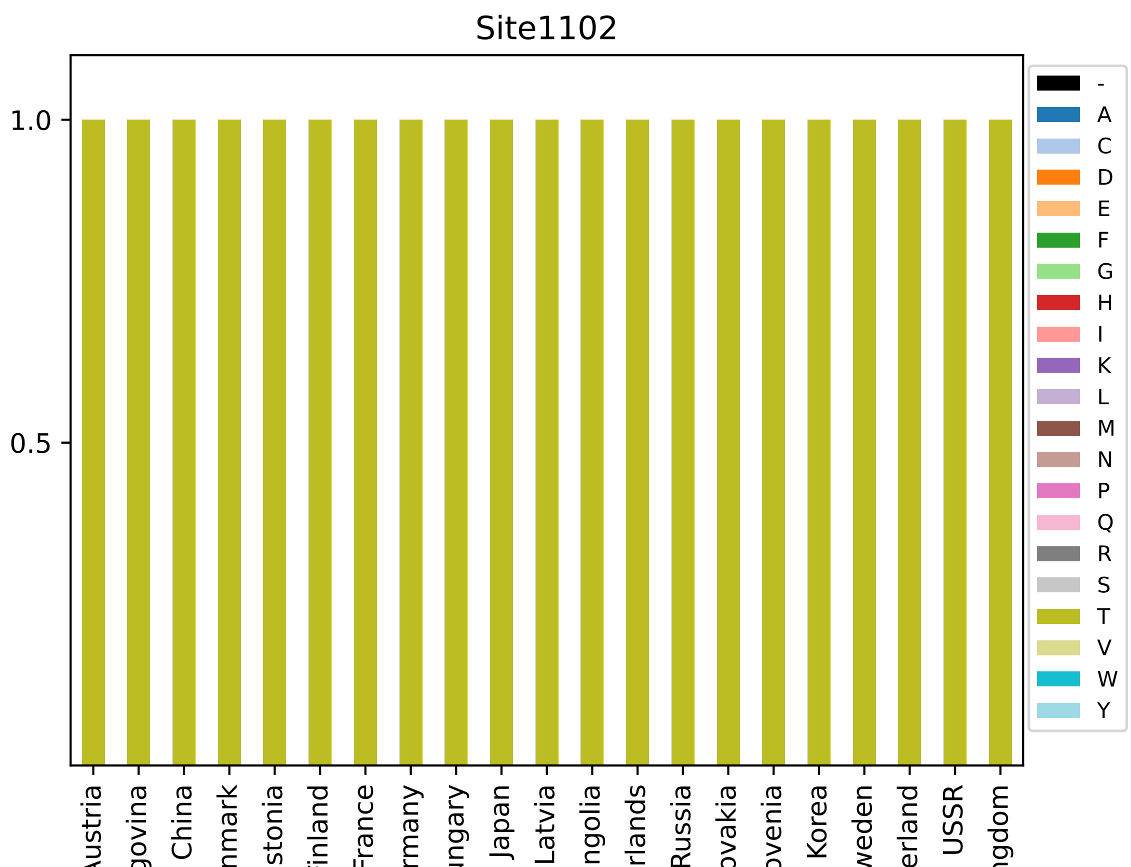

Site1102
1.0
0.5
-
A
C
D
E
F
G
H
I
K
L
M
N
P
Q
R
S
T
V
W
Y
China
Japan
Latvia
Russia
Korea
USSR

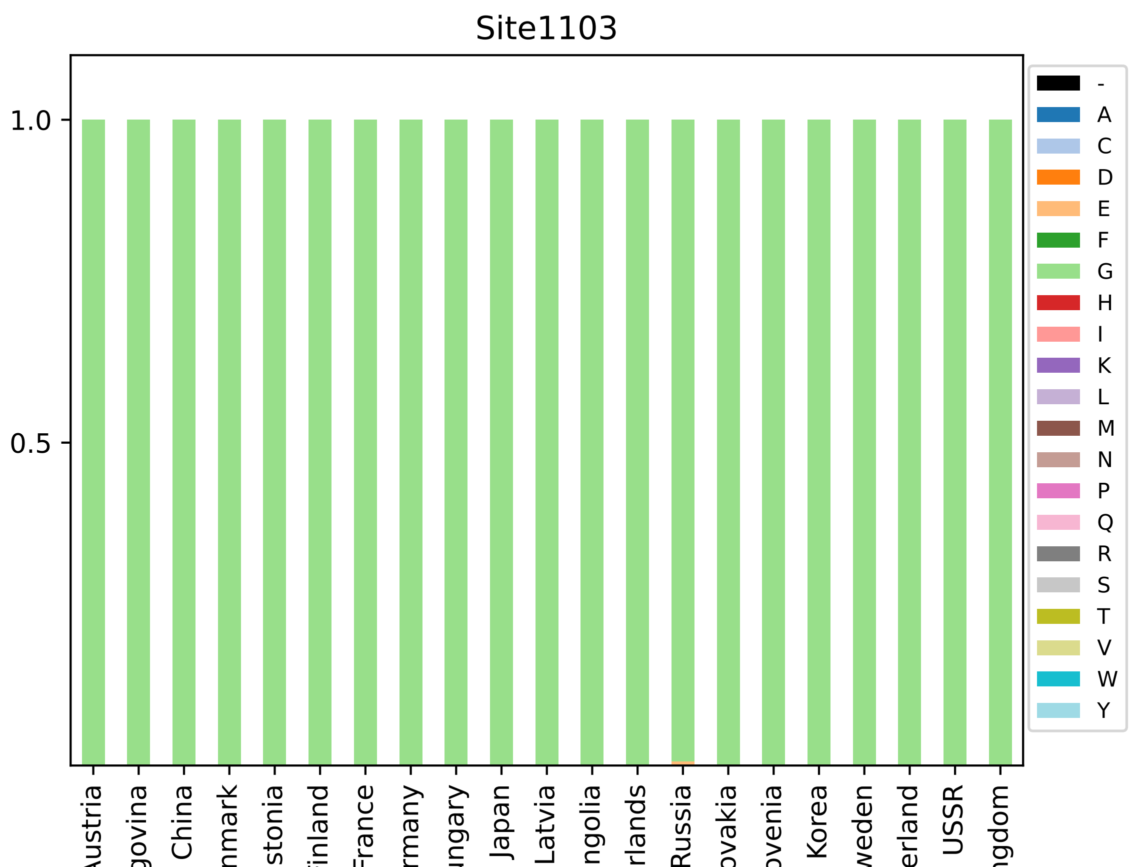
Site1103
1.0
0.5
-
A
C
D
E
F
G
H
I
K
L
M
N
P
Q
R
S
T
V
W
Y
Austria
China
Japan
Latvia
Russia
Korea
USSR

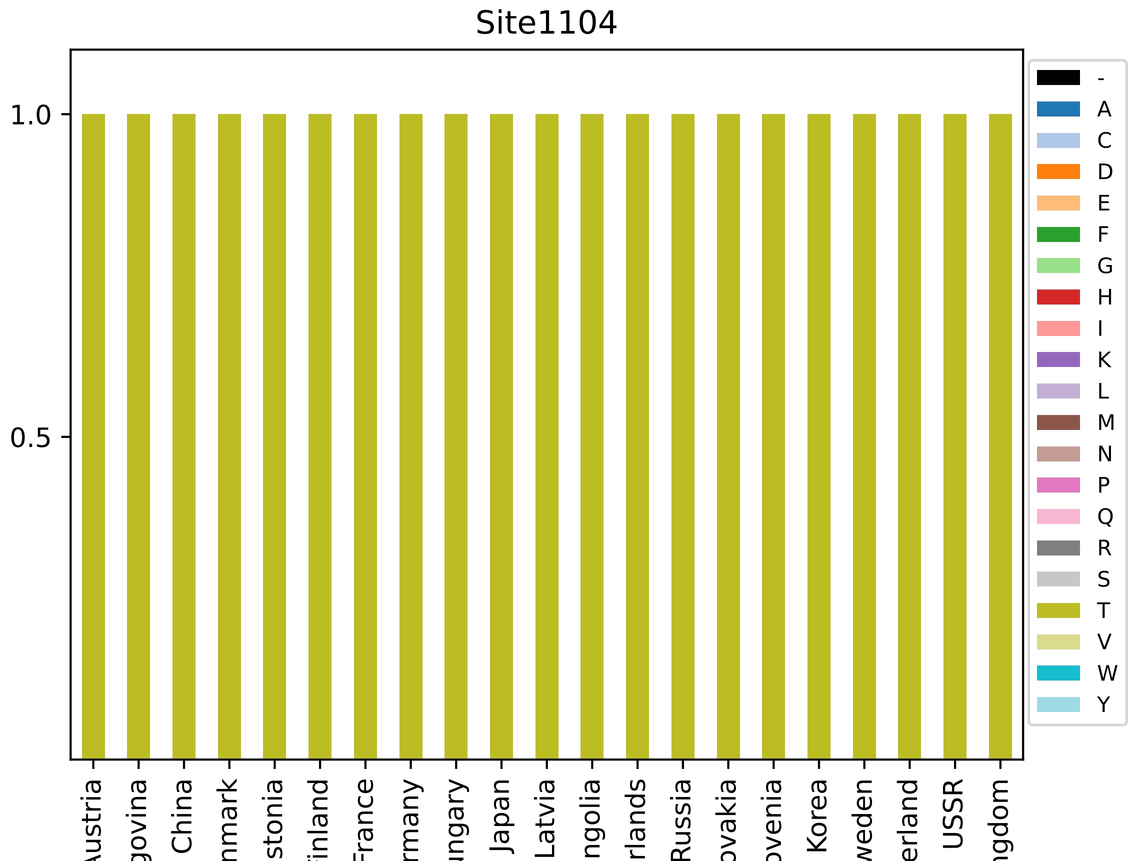
Site1104
1.0
0.5
-
A
C
D
E
F
G
H
I
K
L
M
N
P
Q
R
S
T
V
W
Y
Austria
China
Japan
Latvia
Russia
Korea
USSR

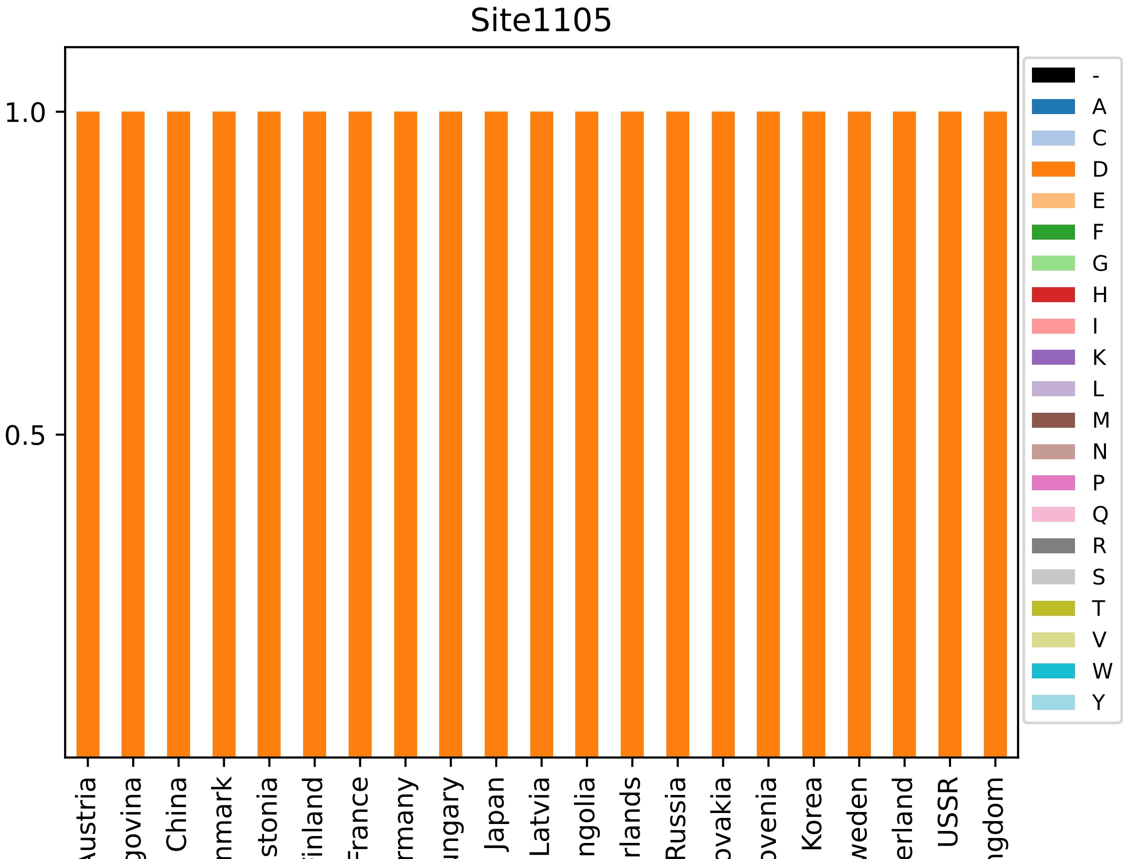

Site1105
1.0
0.5
-
A
C
D
E
F
G
H
I
K
L
M
N
P
Q
R
S
T
V
W
Y
ustria
govina
China
nmark
stonia
inland
rance
rmany
ungary
Japan
Latvia
ngolia
rlands
Russia
ovakia
ovenia
Korea
weden
erland
USSR
ngdom

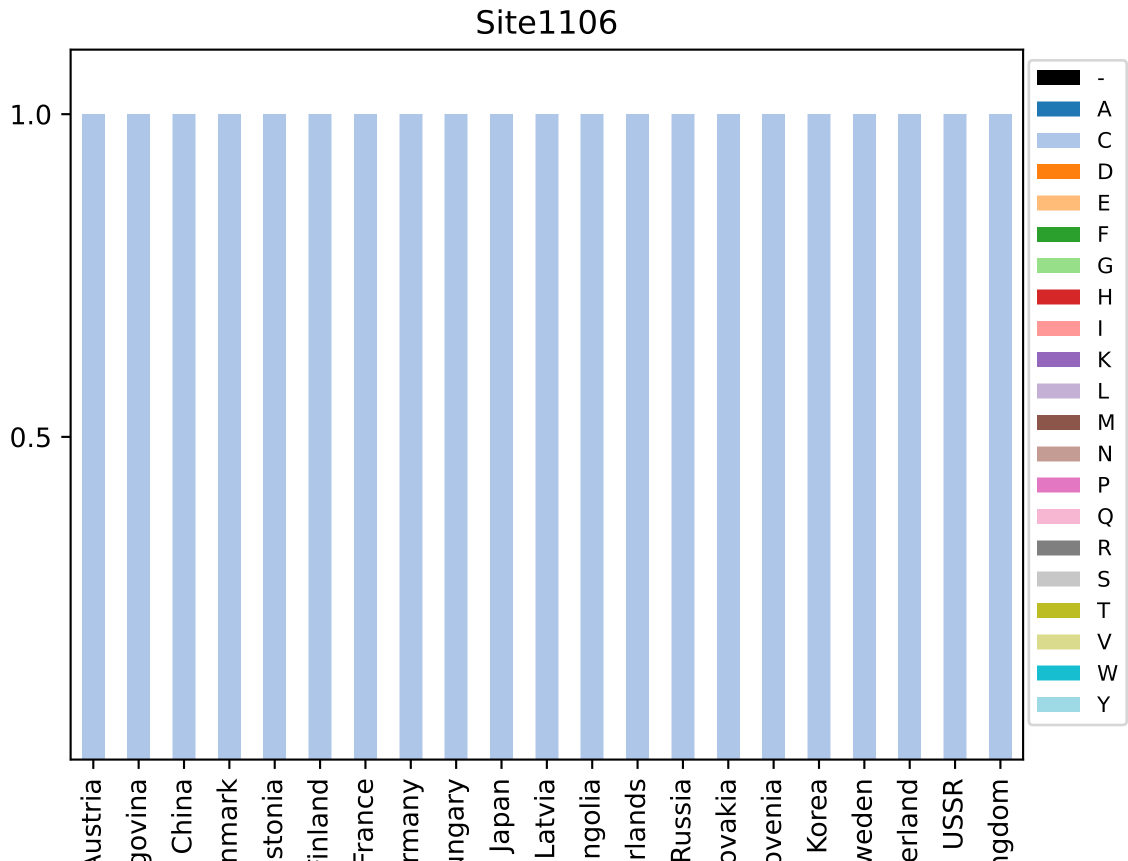

Site1106
1.0
0.5
-
A
C
D
E
F
G
H
I
K
L
M
N
P
Q
R
S
T
V
W
Y
Austria
China
Japan
Latvia
Russia
Korea
USSR

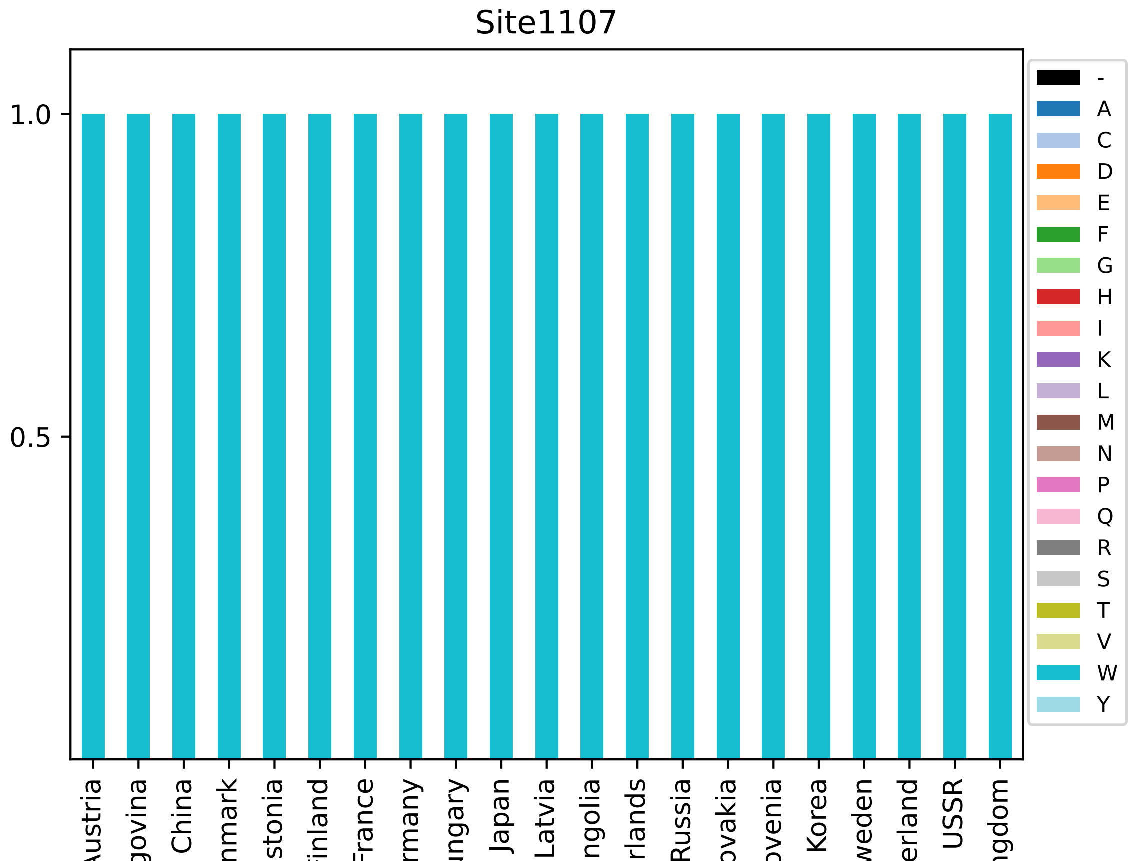
Site1107
1.0
0.5
-
A
C
D
E
F
G
H
I
K
L
M
N
P
Q
R
S
T
V
W
Y
Austria
govina
China
nmark
stonia
inland
France
rmany
ungary
Japan
Latvia
ngolia
rlands
Russia
ovakia
ovenia
Korea
weden
erland
USSR
ngdom

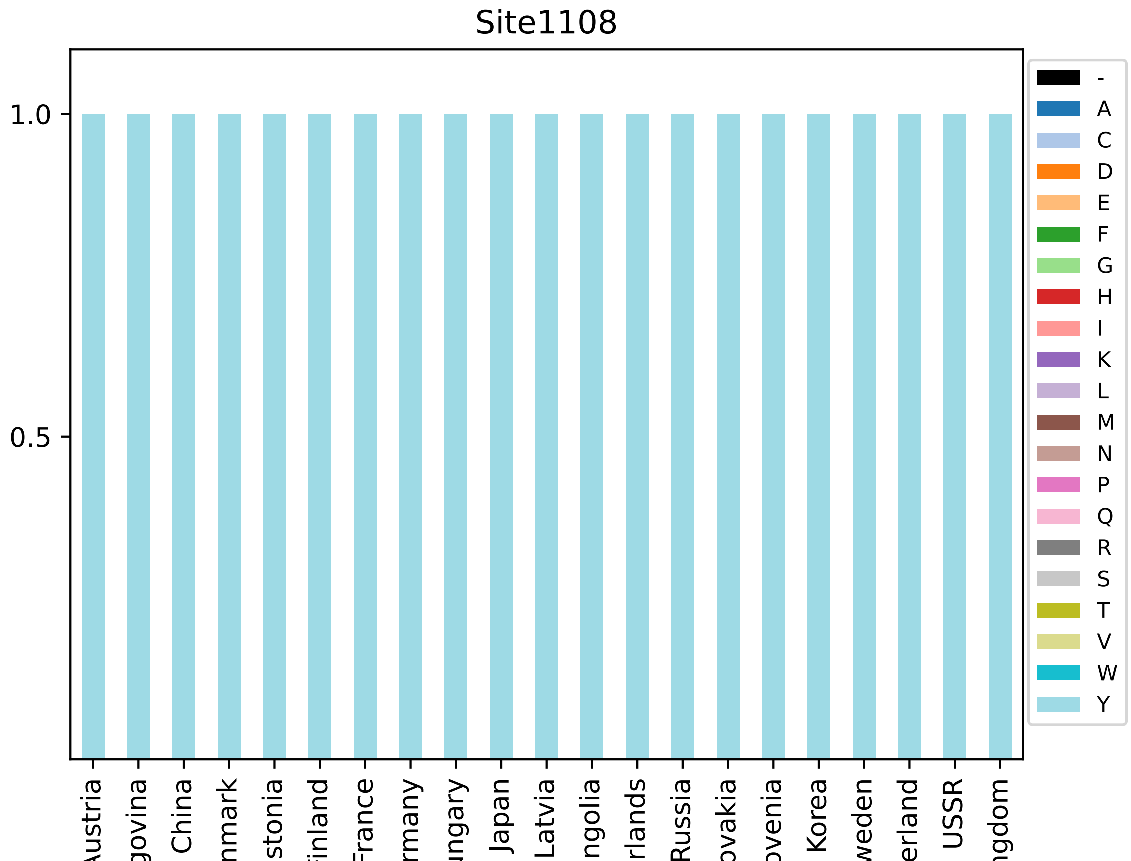
Site1108
1.0
0.5
-
A
C
D
E
F
G
H
I
K
L
M
N
P
Q
R
S
T
V
W
Y
China
Japan
Latvia
Russia
Korea
USSR

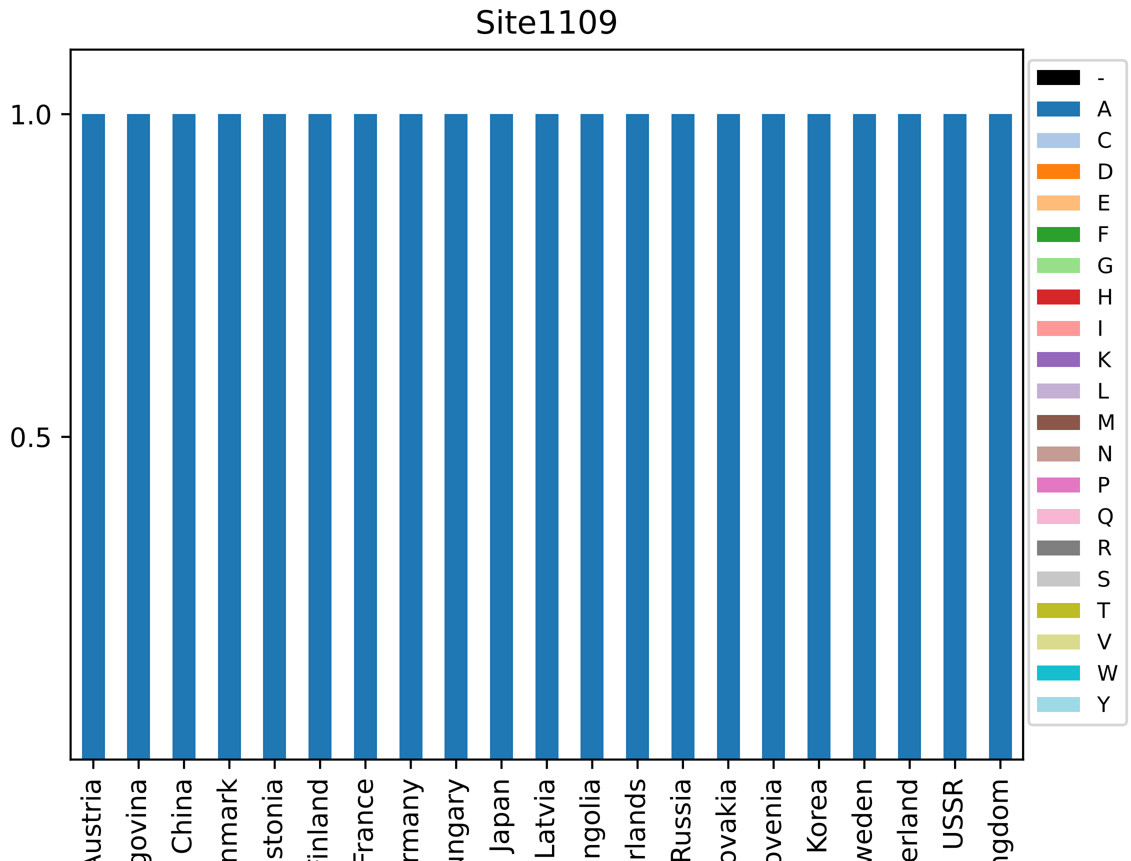

Site1109
1.0
0.5
-
A
C
D
E
F
G
H
I
K
L
M
N
P
Q
R
S
T
V
W
Y
China
Japan
Latvia
Russia
Korea
USSR

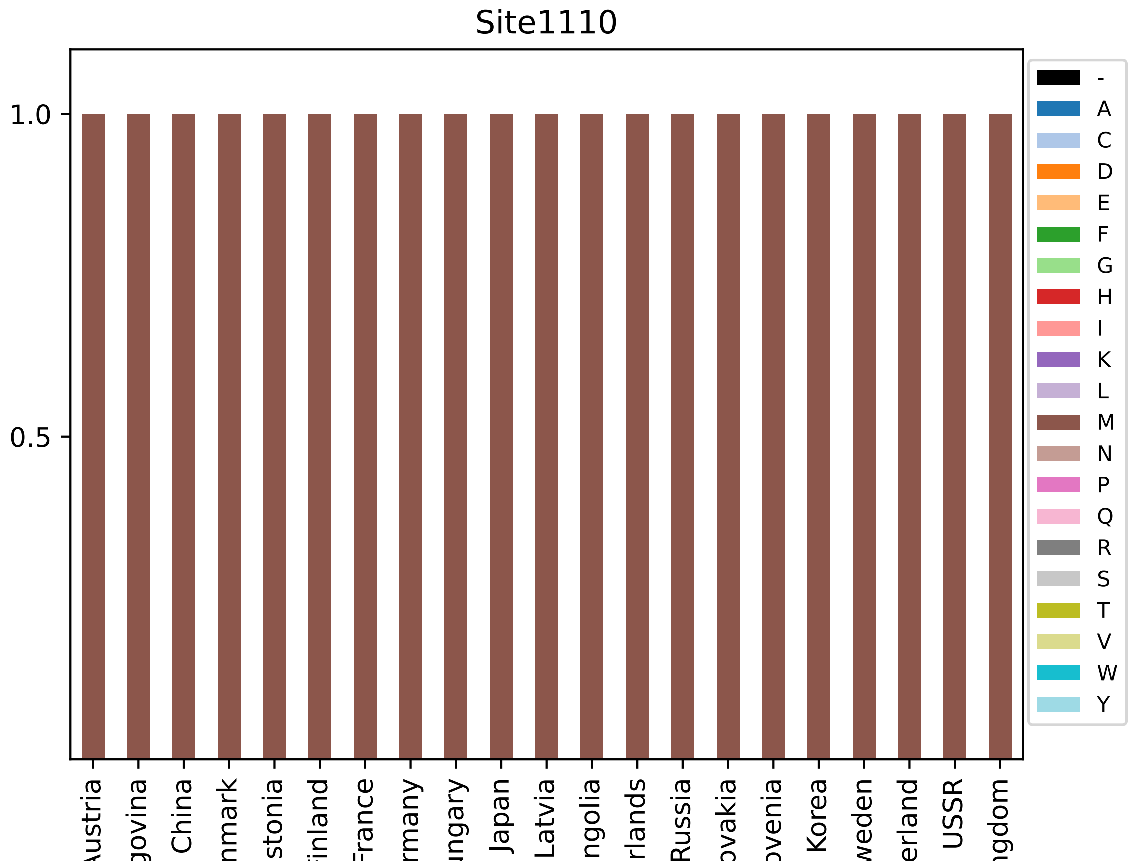
Site1110
1.0
0.5
-
A
C
D
E
F
G
H
I
K
L
M
N
P
Q
R
S
T
V
W
Y
Austria
govina
China
nmark
stonia
Finland
France
rmany
ungary
Japan
Latvia
ngolia
rlands
Russia
ovakia
ovenia
Korea
weden
erland
USSR
ngdom

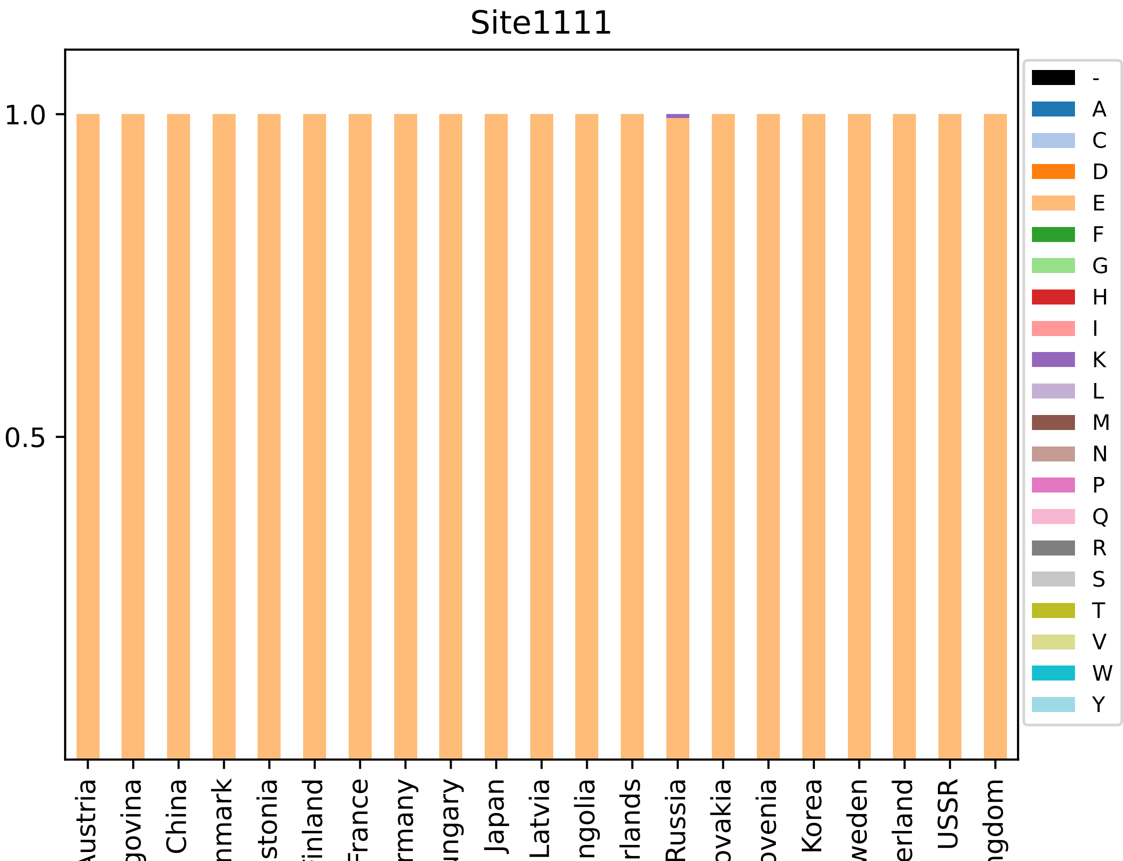

Site1111
1.0
0.5
-
A
C
D
E
F
G
H
I
K
L
M
N
P
Q
R
S
T
V
W
Y
China
Japan
Latvia
Russia
Korea
USSR

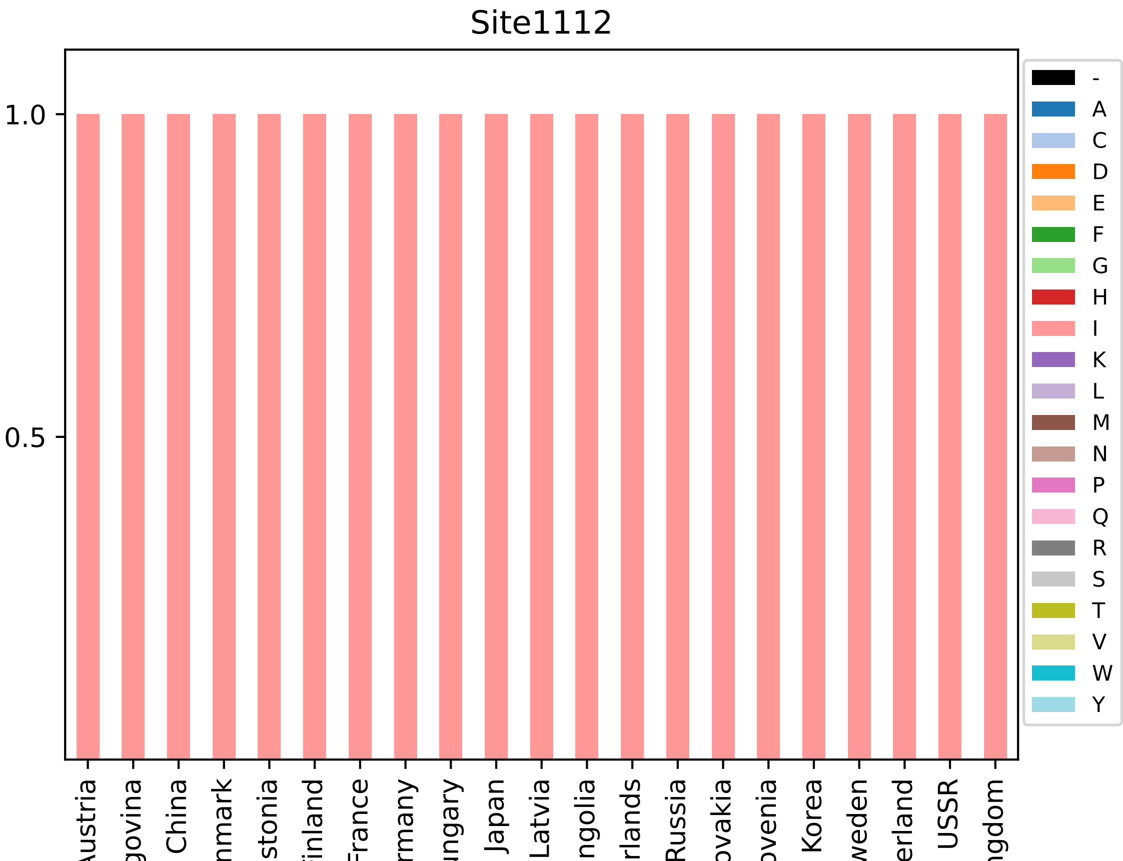
Site1112
1.0
0.5
-
A
C
D
E
F
G
H
I
K
L
M
N
P
Q
R
S
T
V
W
Y
China
Japan
Latvia
Russia
Korea
USSR

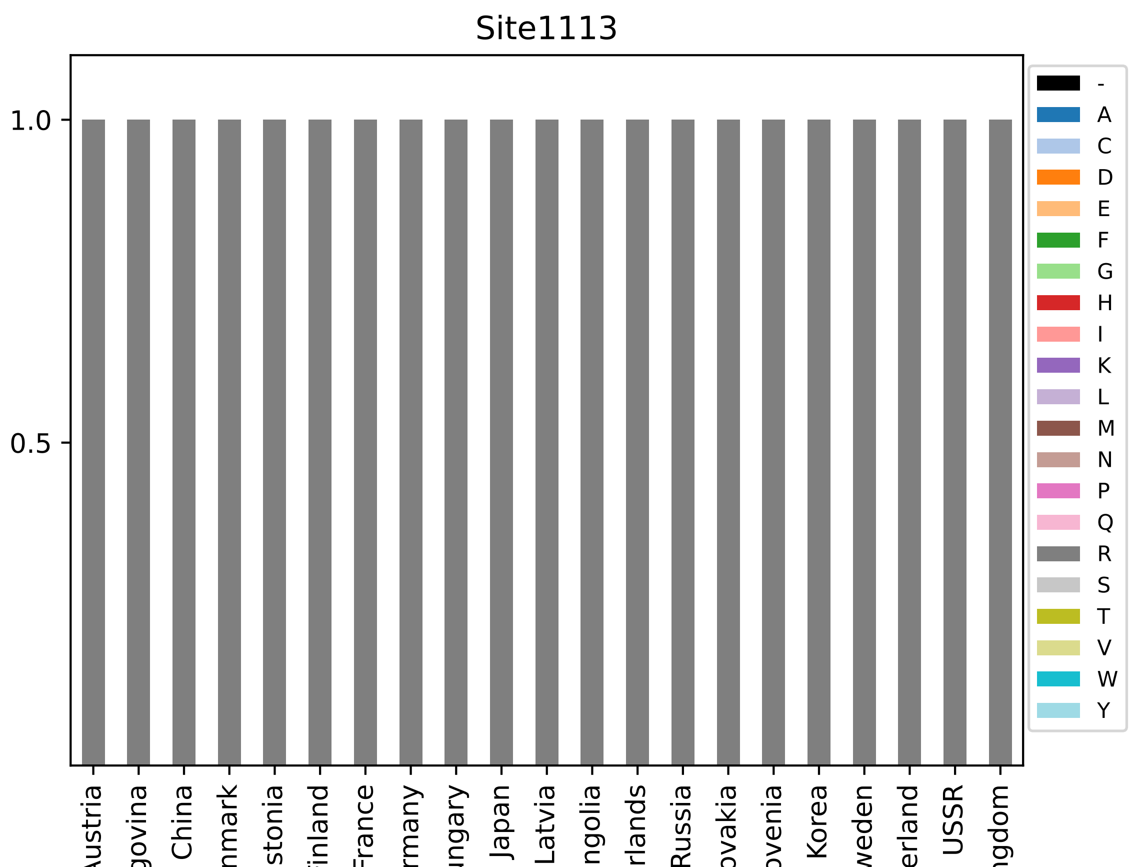

Site1113
1.0
0.5
-
A
C
D
E
F
G
H
I
K
L
M
N
P
Q
R
S
T
V
W
Y
China
Japan
Latvia
Russia
Korea
USSR

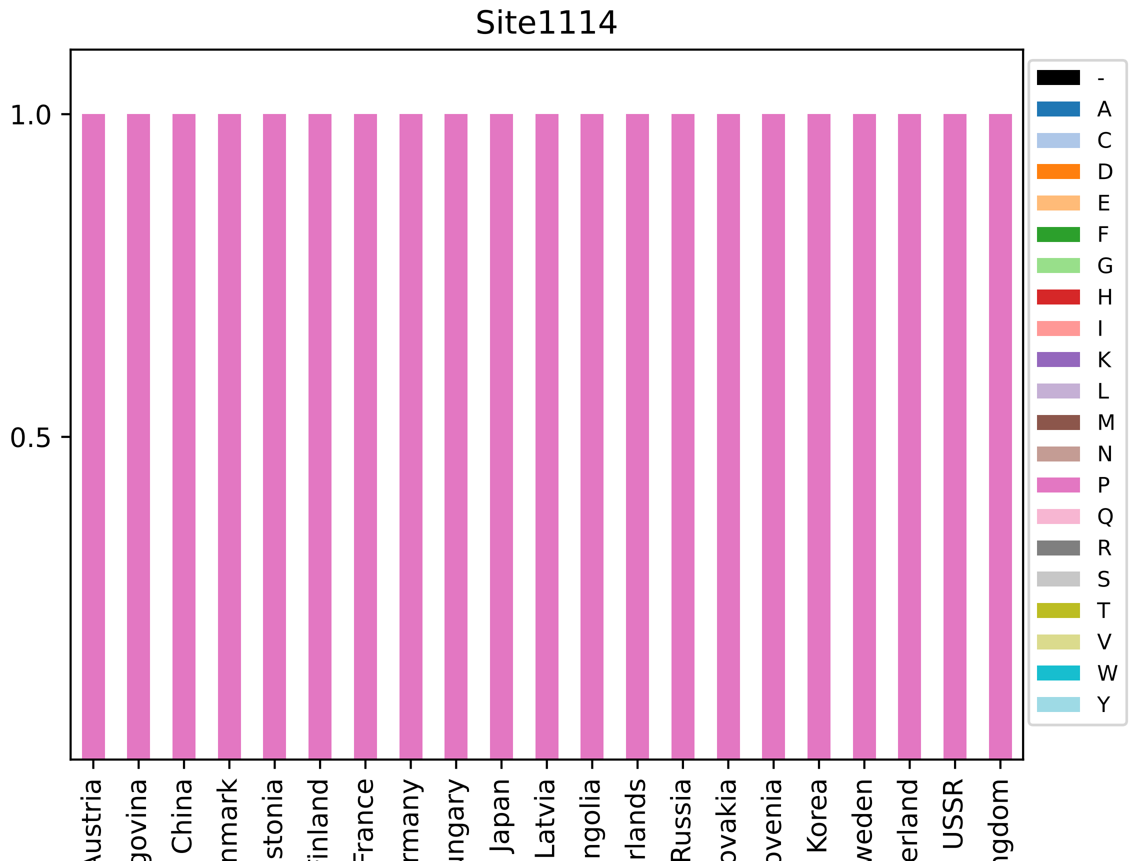
Site1114
1.0
0.5
-
A
C
D
E
F
G
H
I
K
L
M
N
P
Q
R
S
T
V
W
Y
China
Japan
Latvia
Russia
Korea
USSR

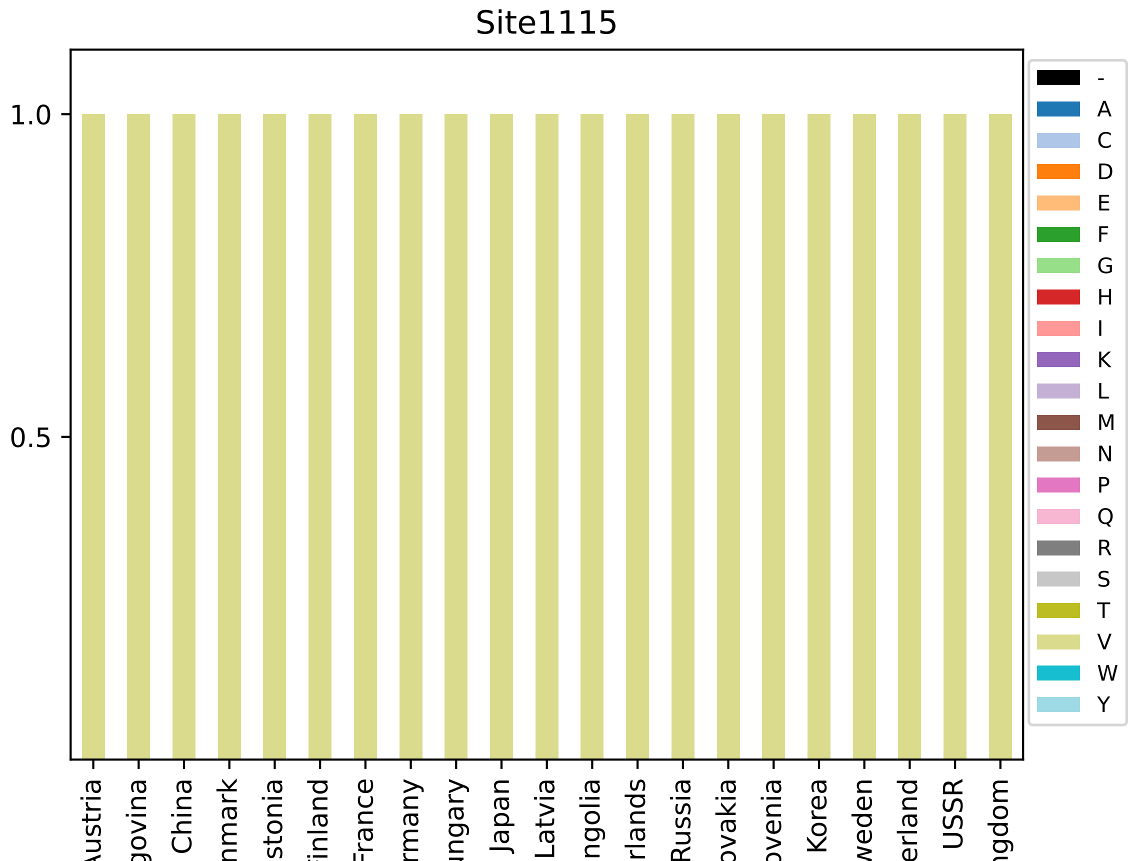
Site1115
1.0
0.5
-
A
C
D
E
F
G
H
I
K
L
M
N
P
Q
R
S
T
V
W
Y
China
Japan
Latvia
Russia
Korea
USSR

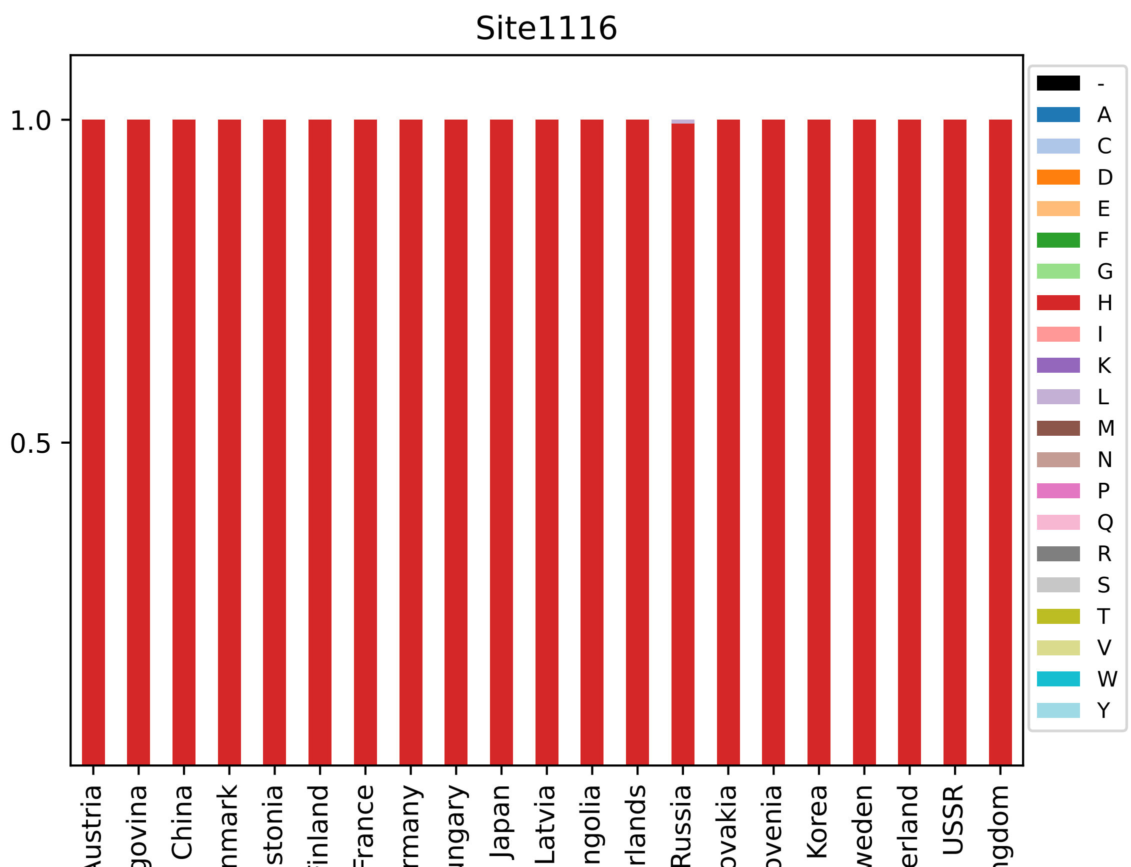

Site1116
1.0
0.5
-
A
C
D
E
F
G
H
I
K
L
M
N
P
Q
R
S
T
V
W
Y
China
Japan
Latvia
Russia
Korea
USSR

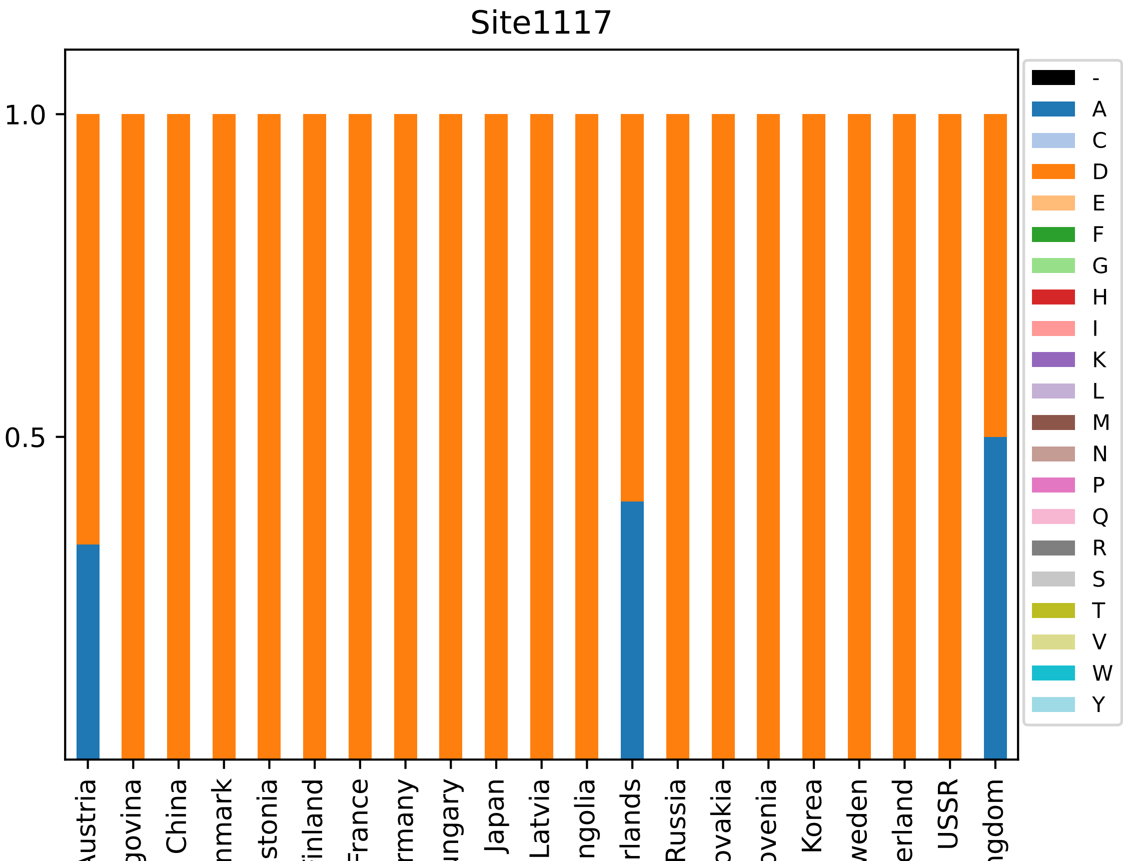
Site1117
1.0
0.5
-
A
C
D
E
F
G
H
I
K
L
M
N
P
Q
R
S
T
V
W
Y
Austria
China
Japan
Latvia
Russia
Korea
USSR

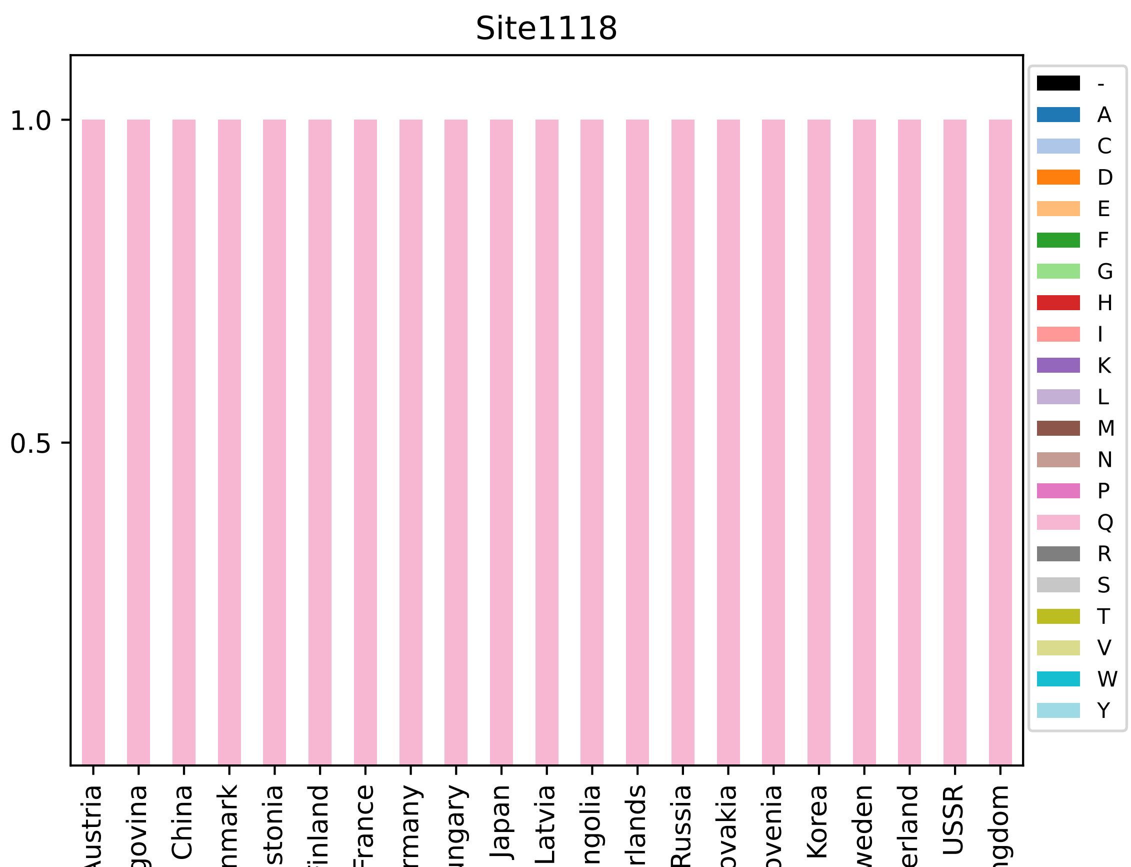
Site1118
1.0
0.5
-
A
C
D
E
F
G
H
I
K
L
M
N
P
Q
R
S
T
V
W
Y
China
Japan
Latvia
Russia
Korea
USSR

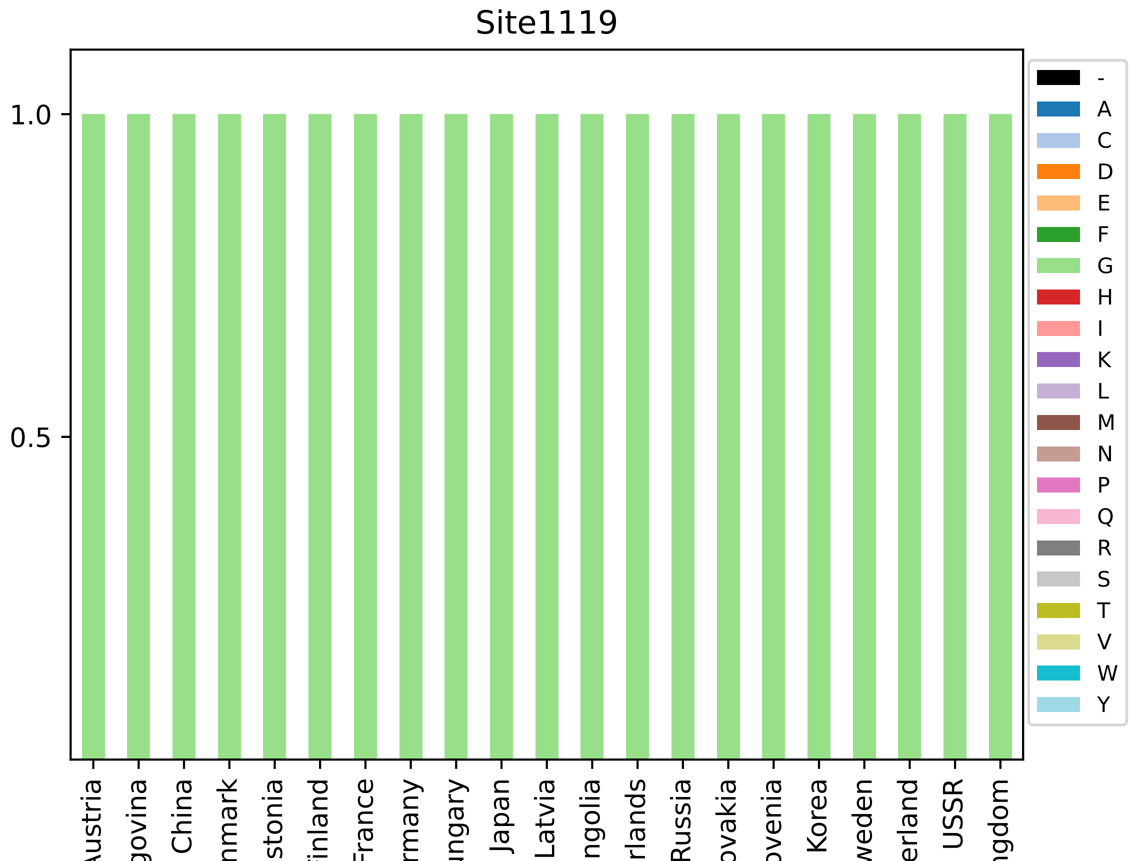

Site1119
1.0
0.5
-
A
C
D
E
F
G
H
I
K
L
M
N
P
Q
R
S
T
V
W
Y
Austria
govina
China
nmark
stonia
Finland
France
rmany
ungary
Japan
Latvia
ngolia
rlands
Russia
ovakia
ovenia
Korea
weden
erland
USSR
ngdom

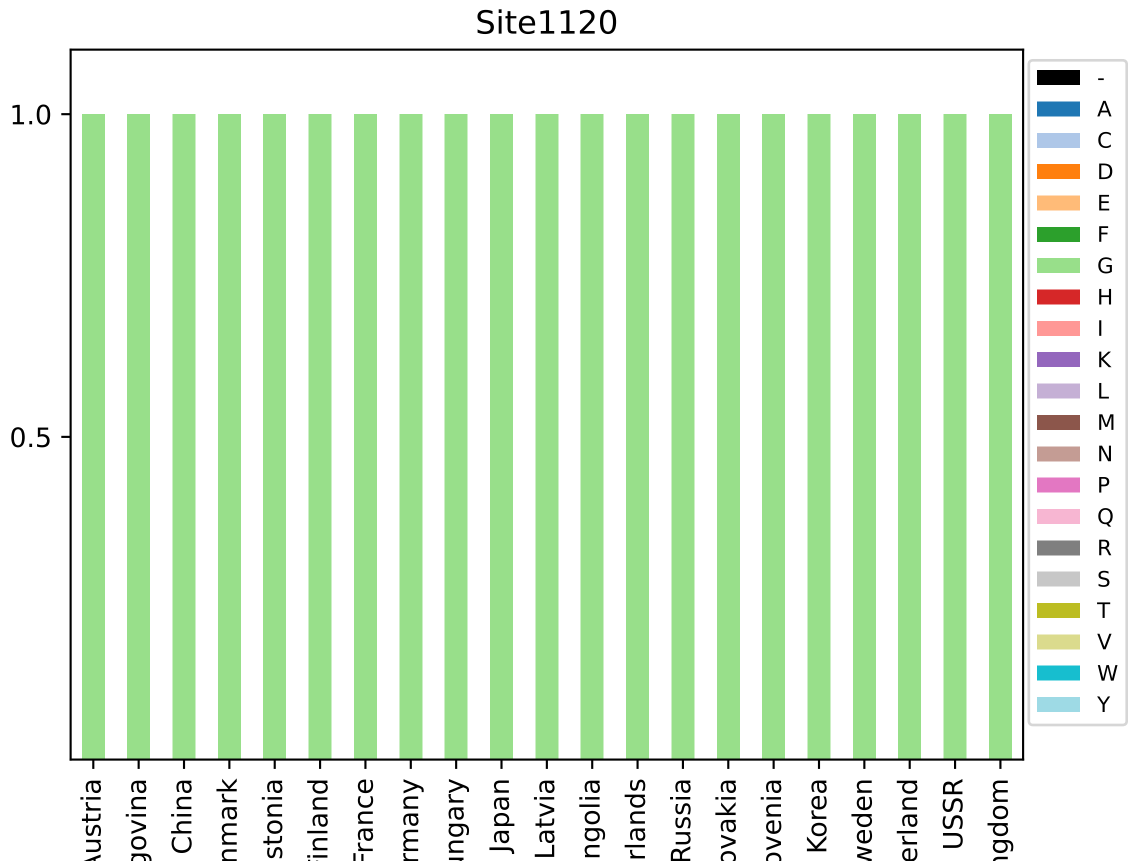

Site1120
1.0
0.5
-
A
C
D
E
F
G
H
I
K
L
M
N
P
Q
R
S
T
V
W
Y
ustria
govina
China
nmark
stonia
inland
rance
rmany
ungary
Japan
Latvia
ngolia
rlands
Russia
ovakia
ovenia
Korea
weden
erland
USSR
ngdom

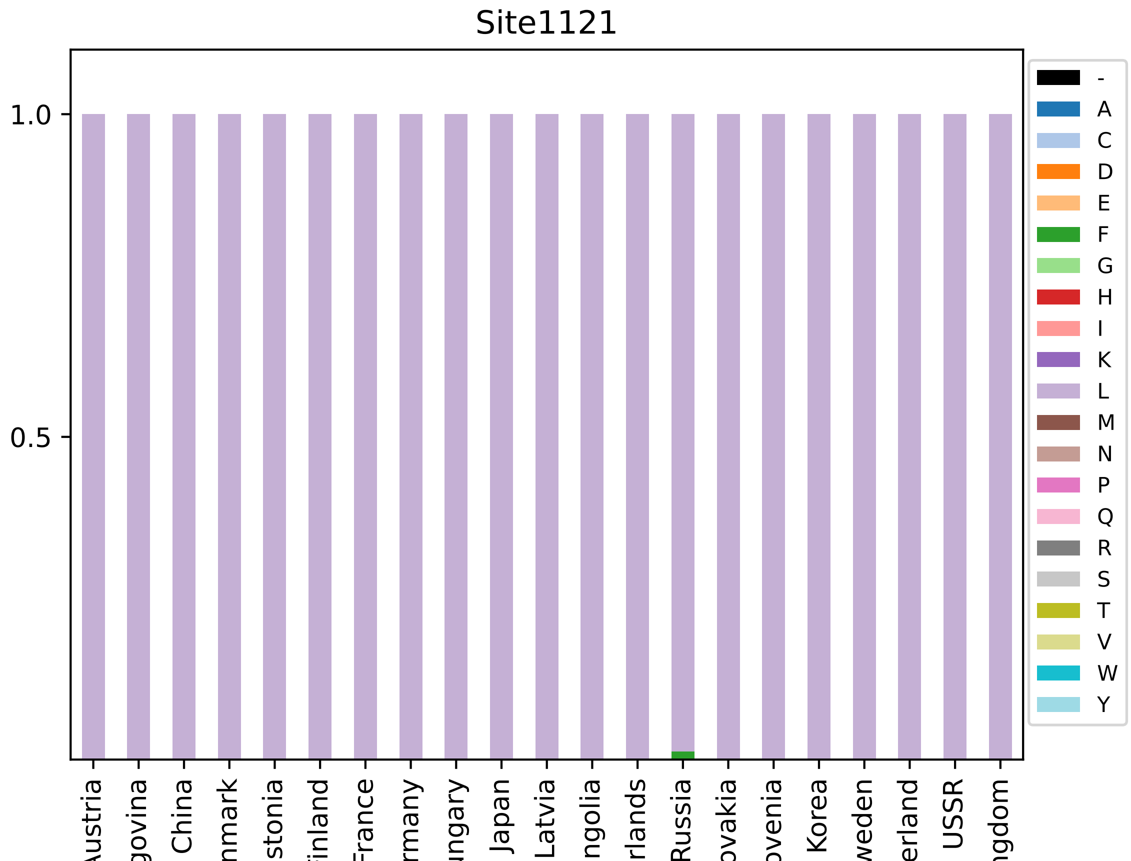
Site1121
1.0
0.5
China
Japan
Latvia
Russia
Korea
USSR
-
A
C
D
E
F
G
H
I
K
L
M
N
P
Q
R
S
T
V
W
Y

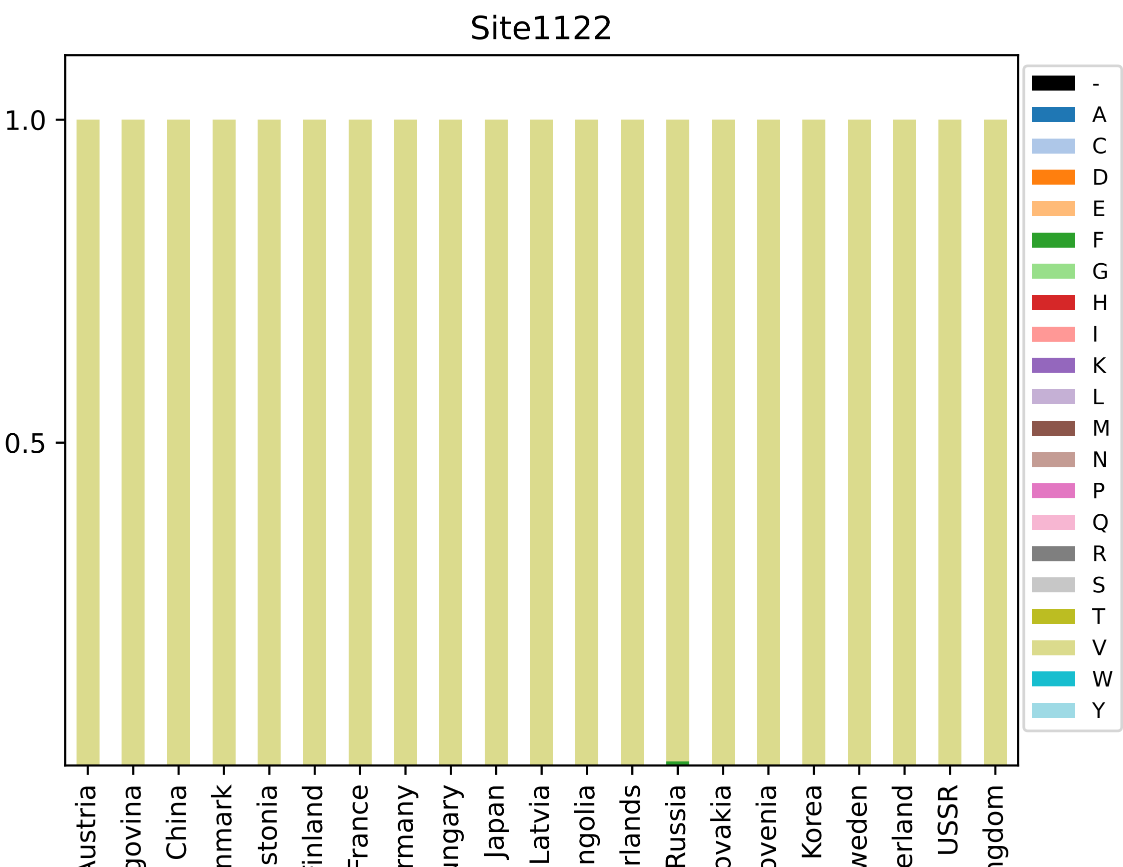
Site1122
1.0
0.5
-
A
C
D
E
F
G
H
I
K
L
M
N
P
Q
R
S
T
V
W
Y
Austria
govina
China
nmark
stonia
Finland
France
rmany
ungary
Japan
Latvia
ngolia
rlands
Russia
ovakia
ovenia
Korea
weden
erland
USSR
ngdom

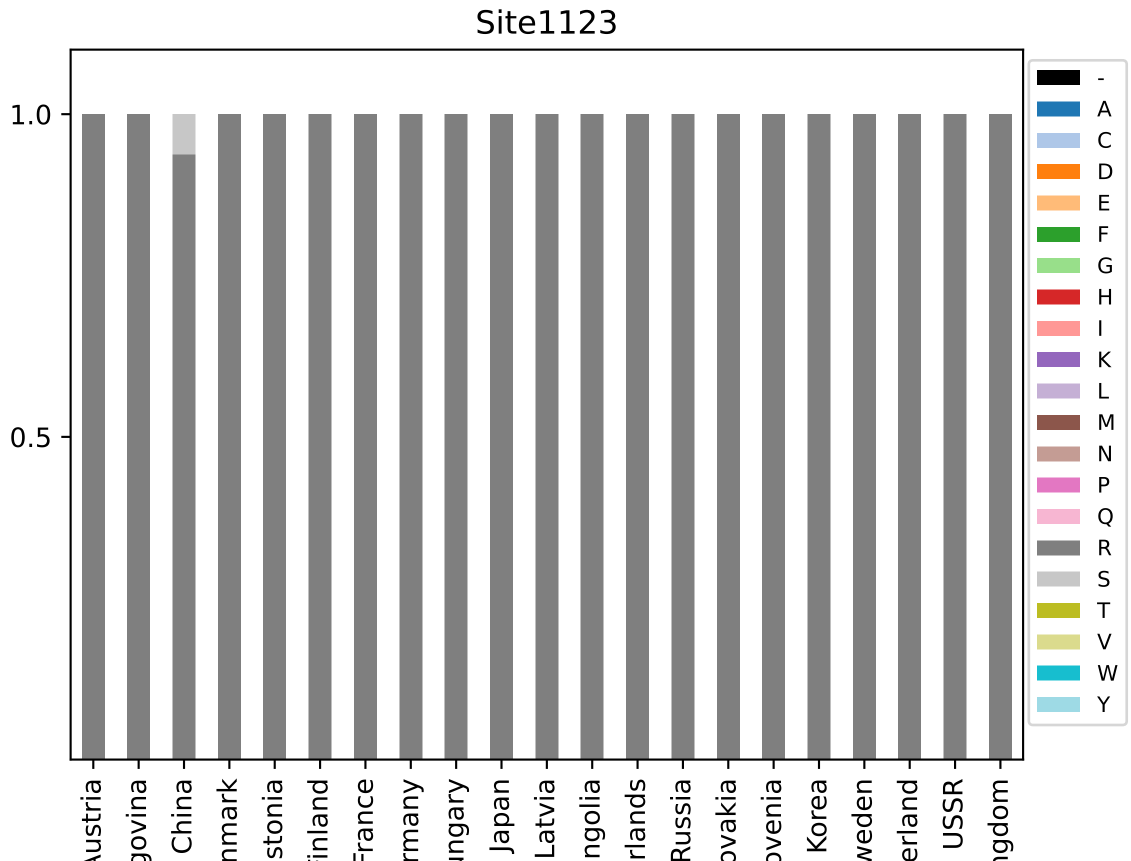

Site1123
1.0
0.5
-
A
C
D
E
F
G
H
I
K
L
M
N
P
Q
R
S
T
V
W
Y
China
Japan
Latvia
Russia
Korea
USSR

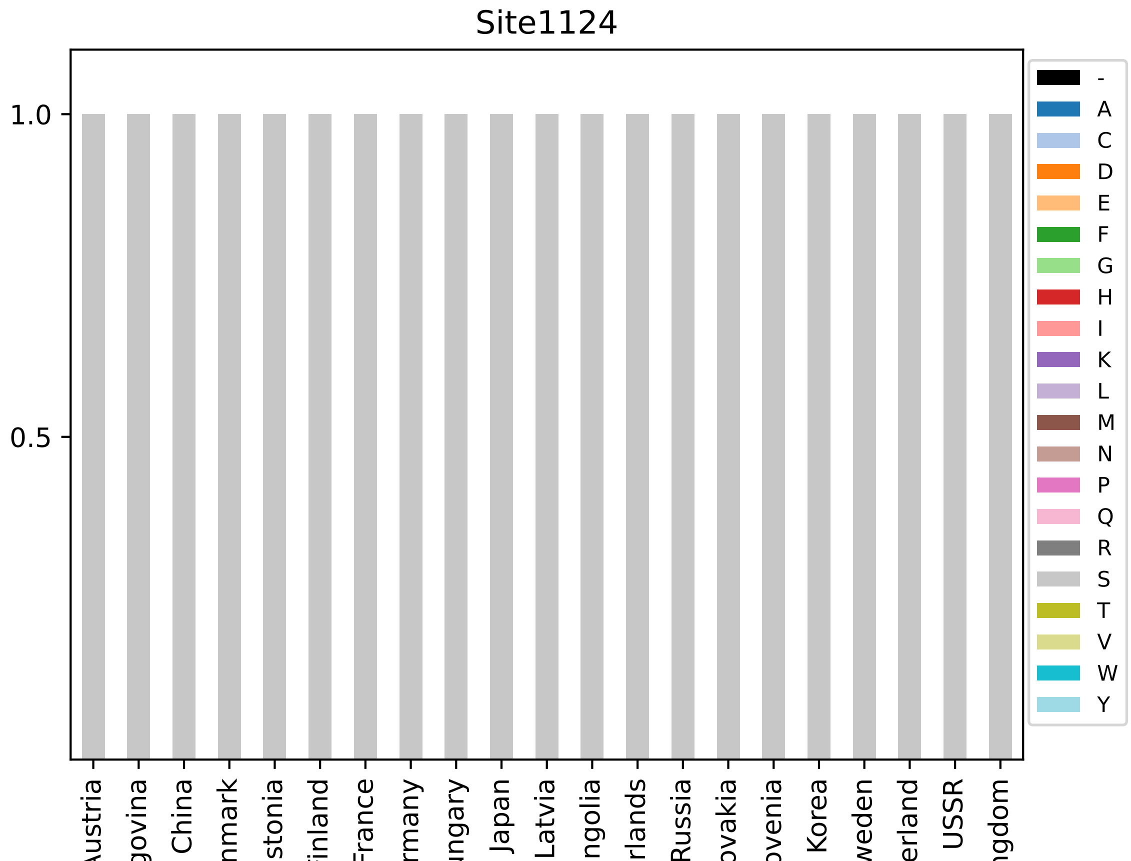

Site1124
1.0
0.5
-
A
C
D
E
F
G
H
I
K
L
M
N
P
Q
R
S
T
V
W
Y
Austria
govina
China
nmark
stonia
inland
France
rmany
ungary
Japan
Latvia
ngolia
rlands
Russia
ovakia
ovenia
Korea
weden
erland
USSR
ngdom

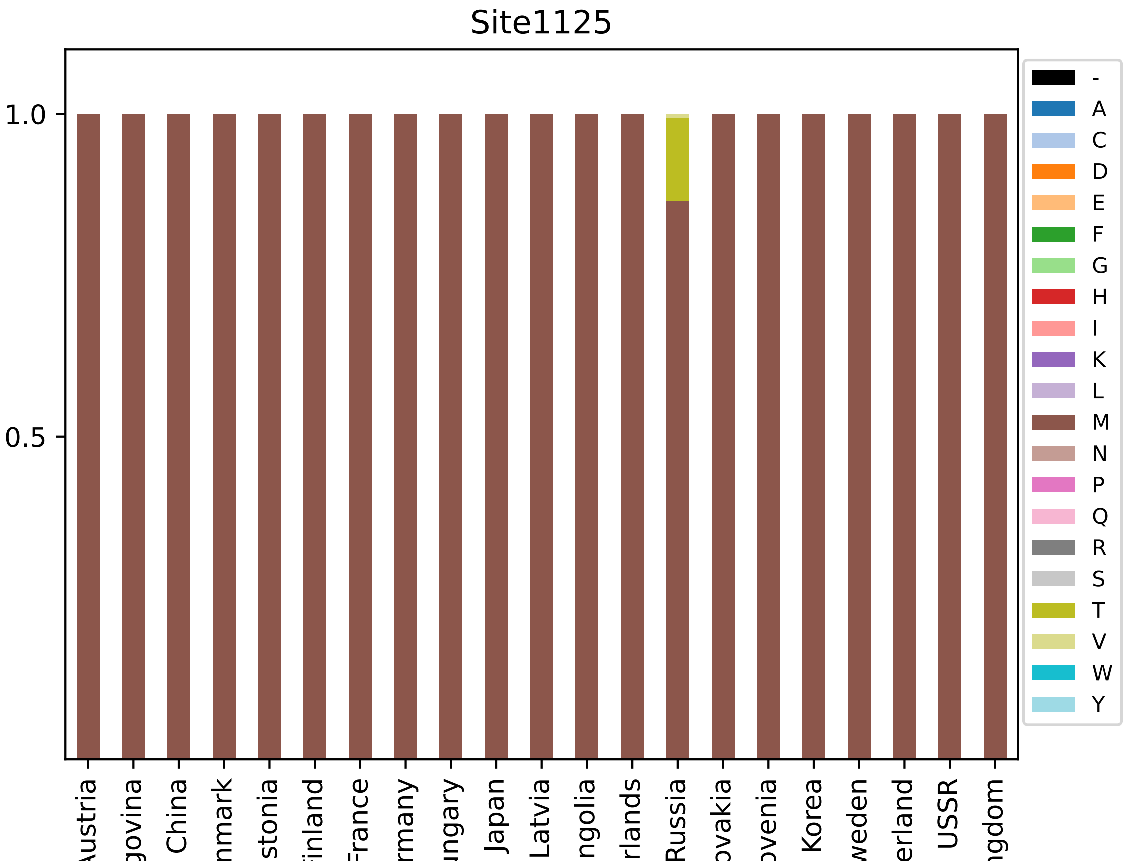
Site1125
1.0
0.5
-
A
C
D
E
F
G
H
I
K
L
M
N
P
Q
R
S
T
V
W
Y
China
Japan
Latvia
Russia
Korea
USSR

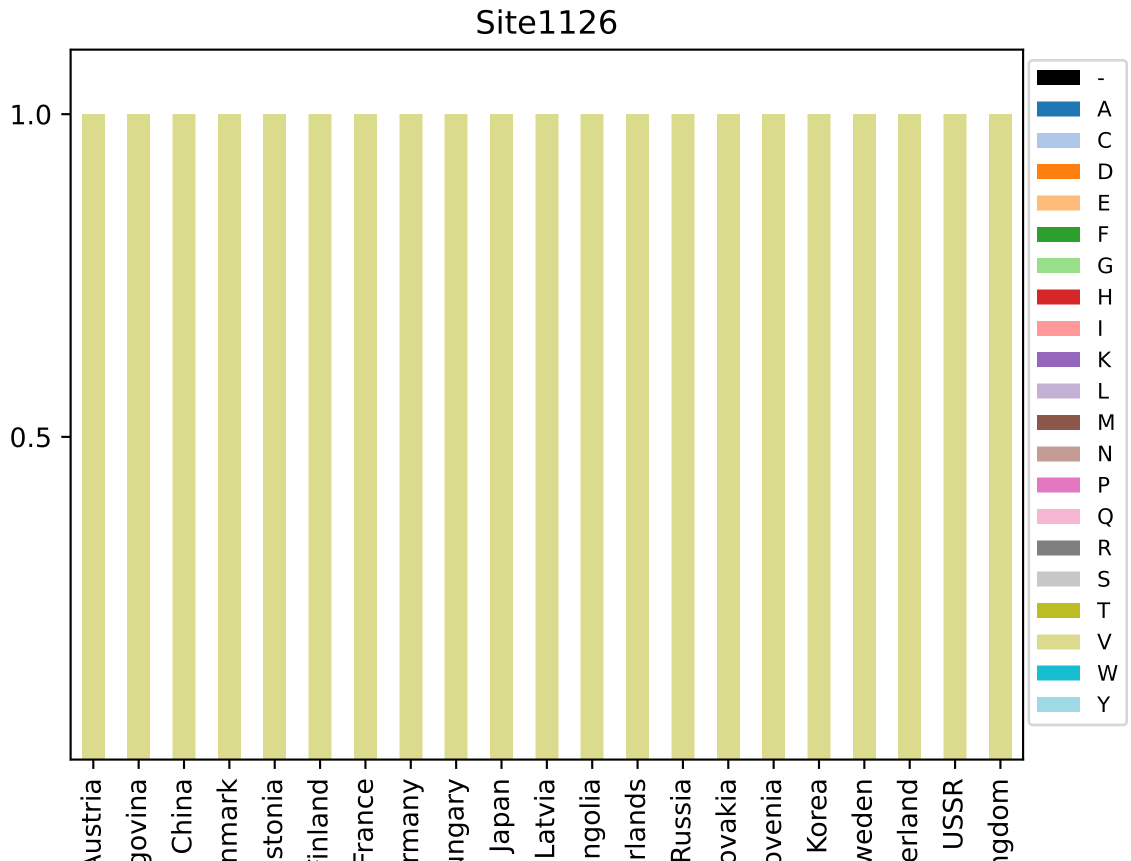
Site1126
1.0
0.5
China
Japan
Latvia
Russia
Korea
USSR
-
A
C
D
E
F
G
H
I
K
L
M
N
P
Q
R
S
T
V
W
Y

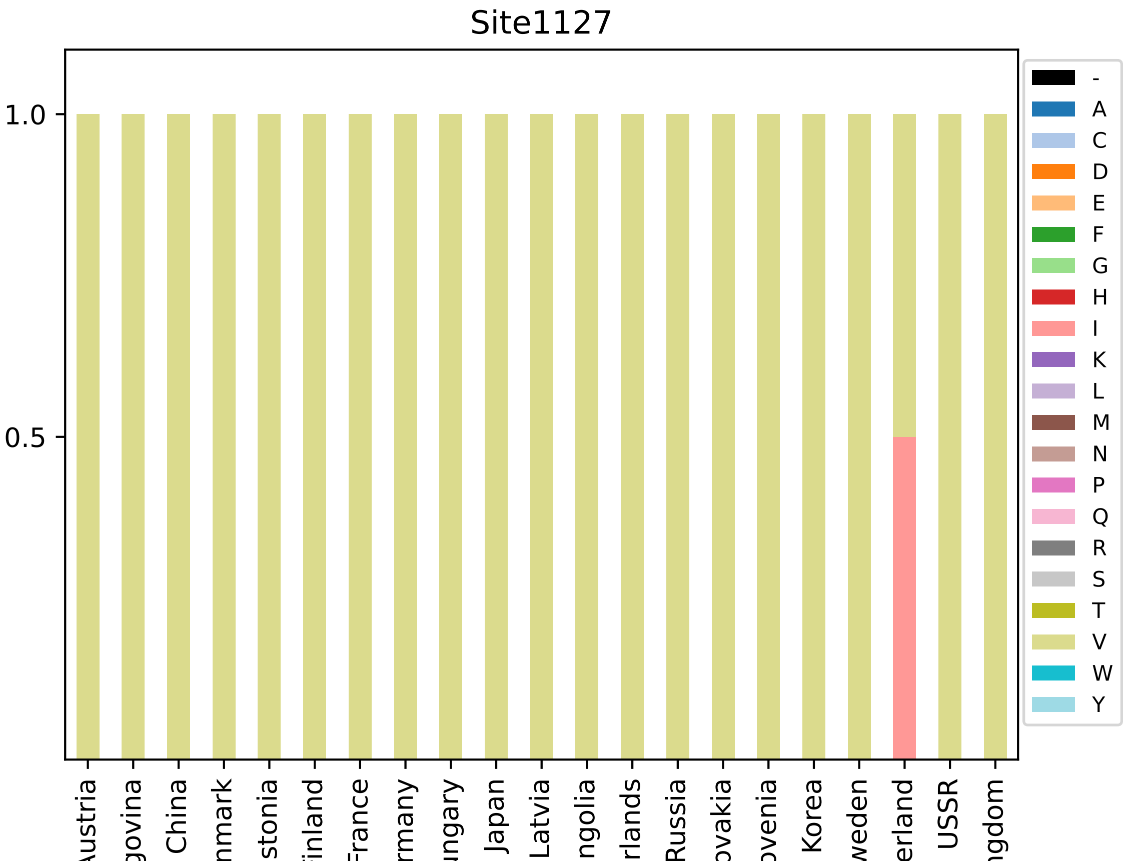

Site1127
1.0
0.5
-
A
C
D
E
F
G
H
I
K
L
M
N
P
Q
R
S
T
V
W
Y
Austria
govina
China
nmark
stonia
Finland
France
rmany
ungary
Japan
Latvia
ngolia
rlands
Russia
ovakia
ovenia
Korea
weden
erland
USSR
ngdom

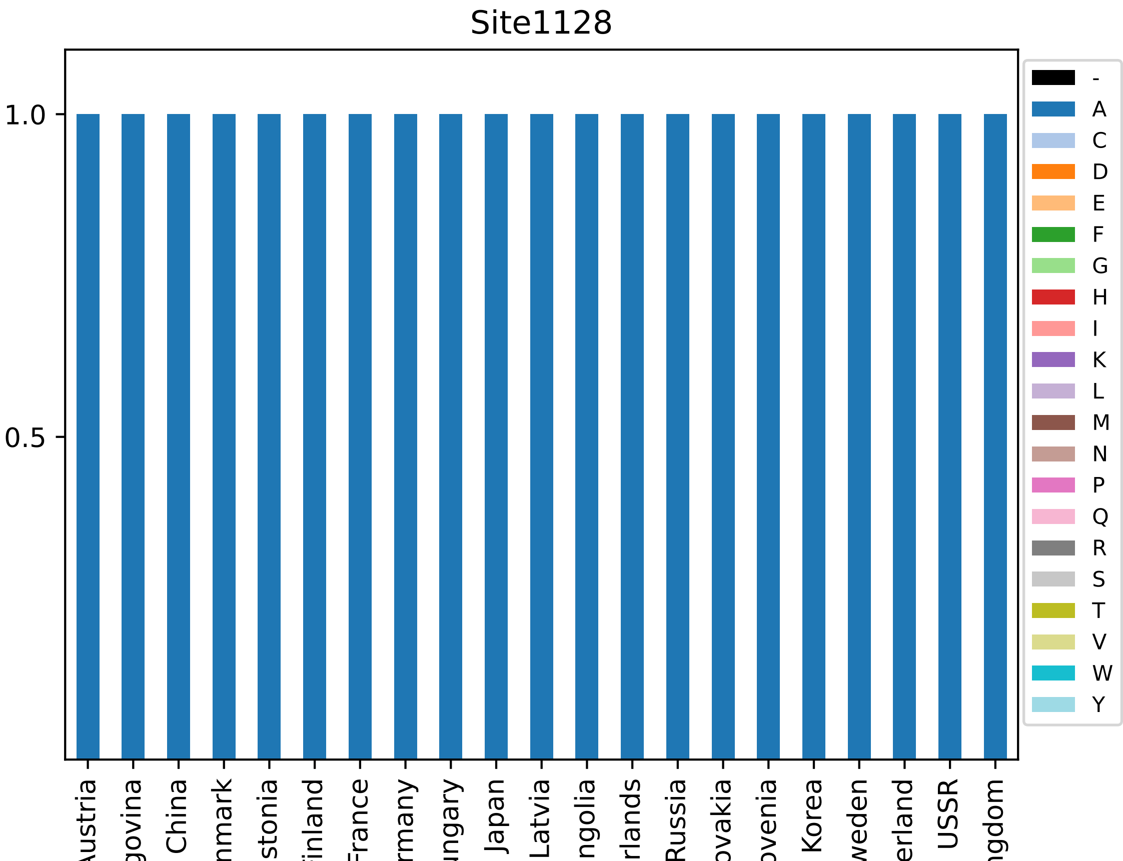
Site1128
1.0
0.5
-
A
C
D
E
F
G
H
I
K
L
M
N
P
Q
R
S
T
V
W
Y
Austria
govina
China
nmark
stonia
inland
France
rmany
ungary
Japan
Latvia
ngolia
rlands
Russia
ovakia
ovenia
Korea
weden
erland
USSR
ngdom

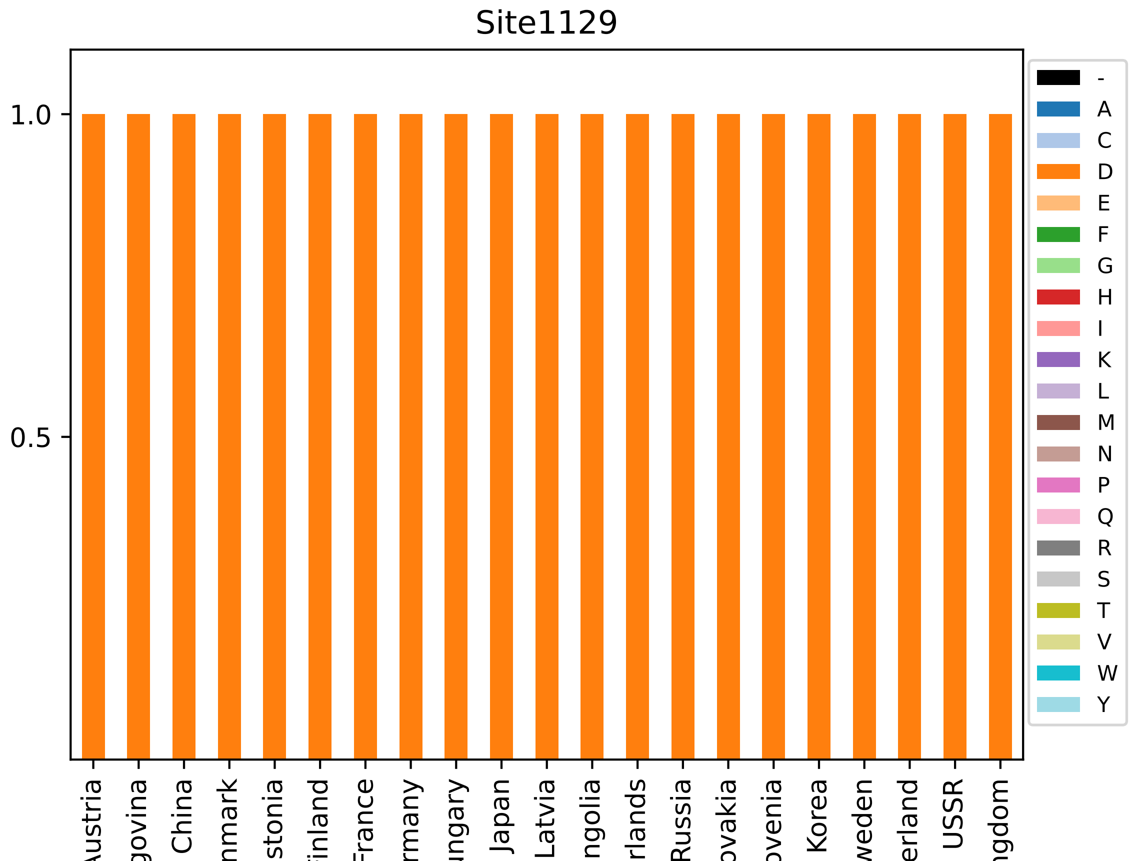
Site1129
1.0
0.5
-
A
C
D
E
F
G
H
I
K
L
M
N
P
Q
R
S
T
V
W
Y
China
Japan
Latvia
Russia
Korea
USSR

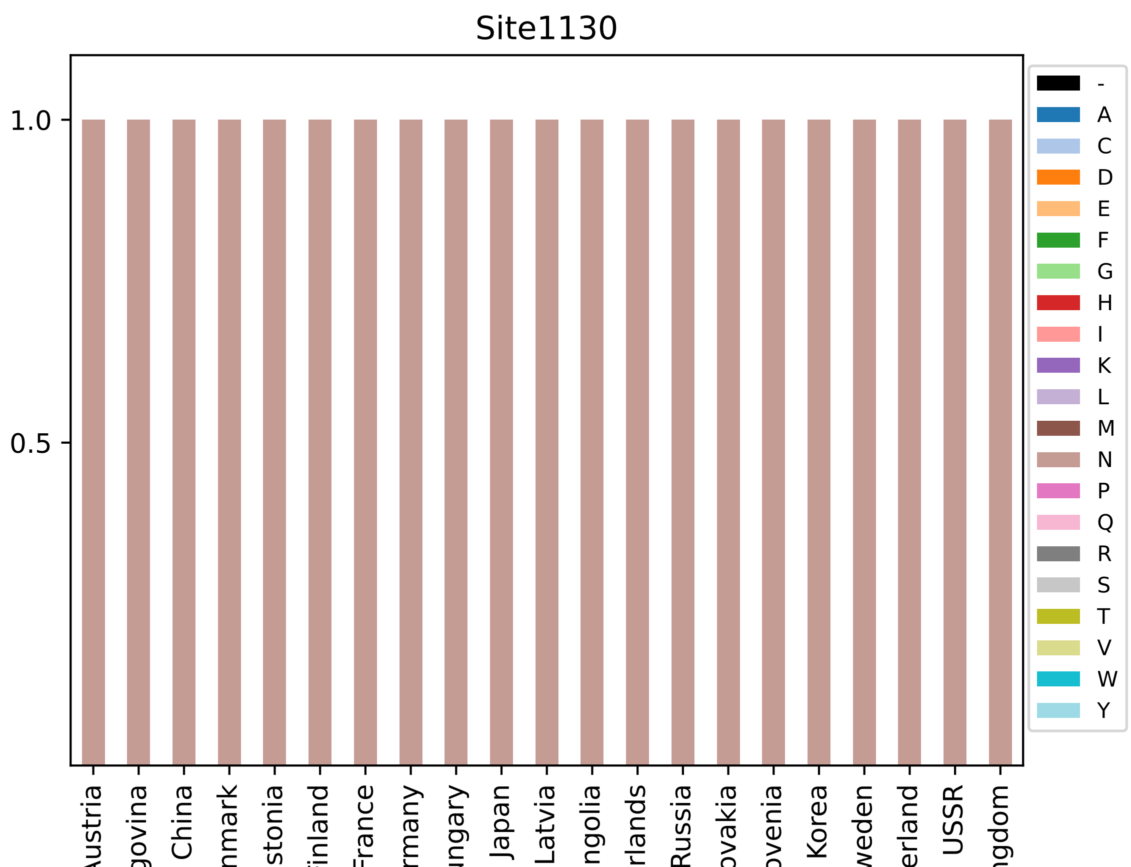

Site1130
1.0
0.5
-
A
C
D
E
F
G
H
I
K
L
M
N
P
Q
R
S
T
V
W
Y
Austria
China
Japan
Latvia
Russia
Korea
USSR

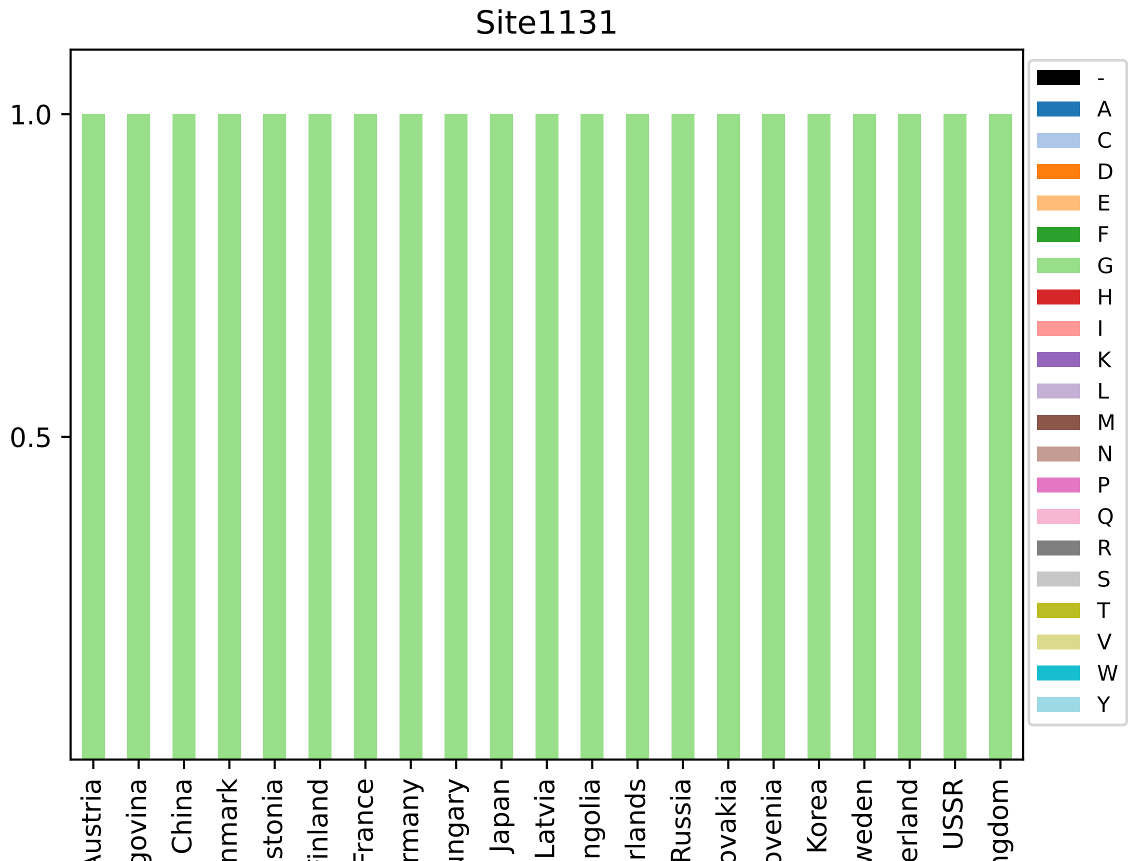
Site1131
1.0
0.5
-
A
C
D
E
F
G
H
I
K
L
M
N
P
Q
R
S
T
V
W
Y
China
Japan
Latvia
Russia
Korea
USSR

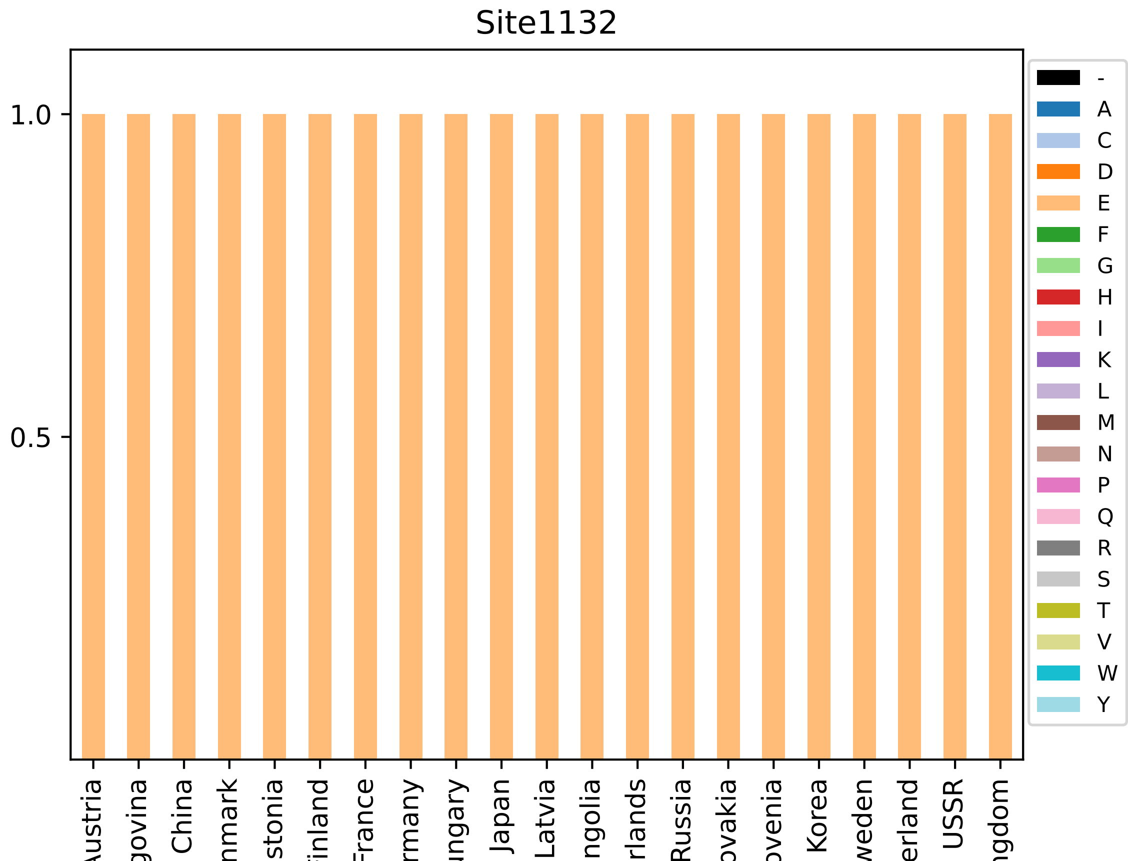
Site1132
1.0
0.5
-
A
C
D
E
F
G
H
I
K
L
M
N
P
Q
R
S
T
V
W
Y
China
Japan
Latvia
Russia
Korea
USSR

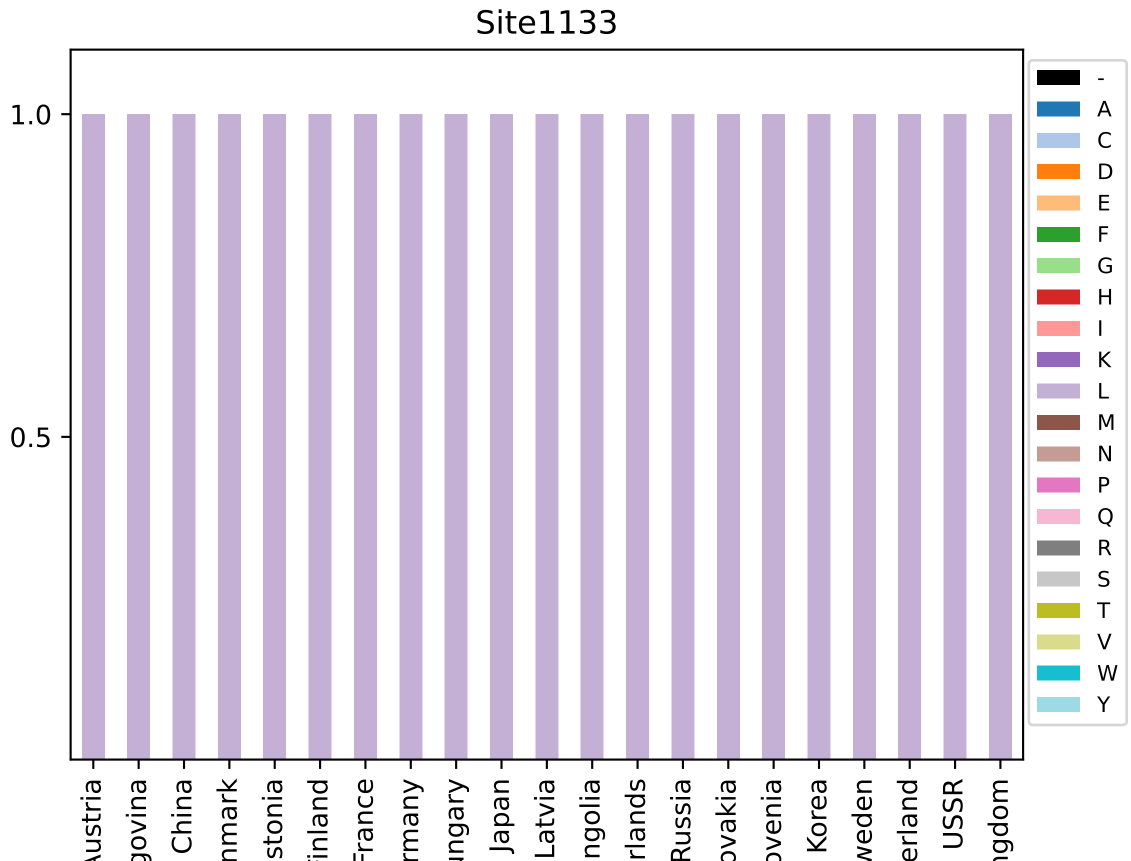

Site1133
1.0
0.5
-
A
C
D
E
F
G
H
I
K
L
M
N
P
Q
R
S
T
V
W
Y
Austria
China
Japan
Latvia
Russia
Korea
USSR

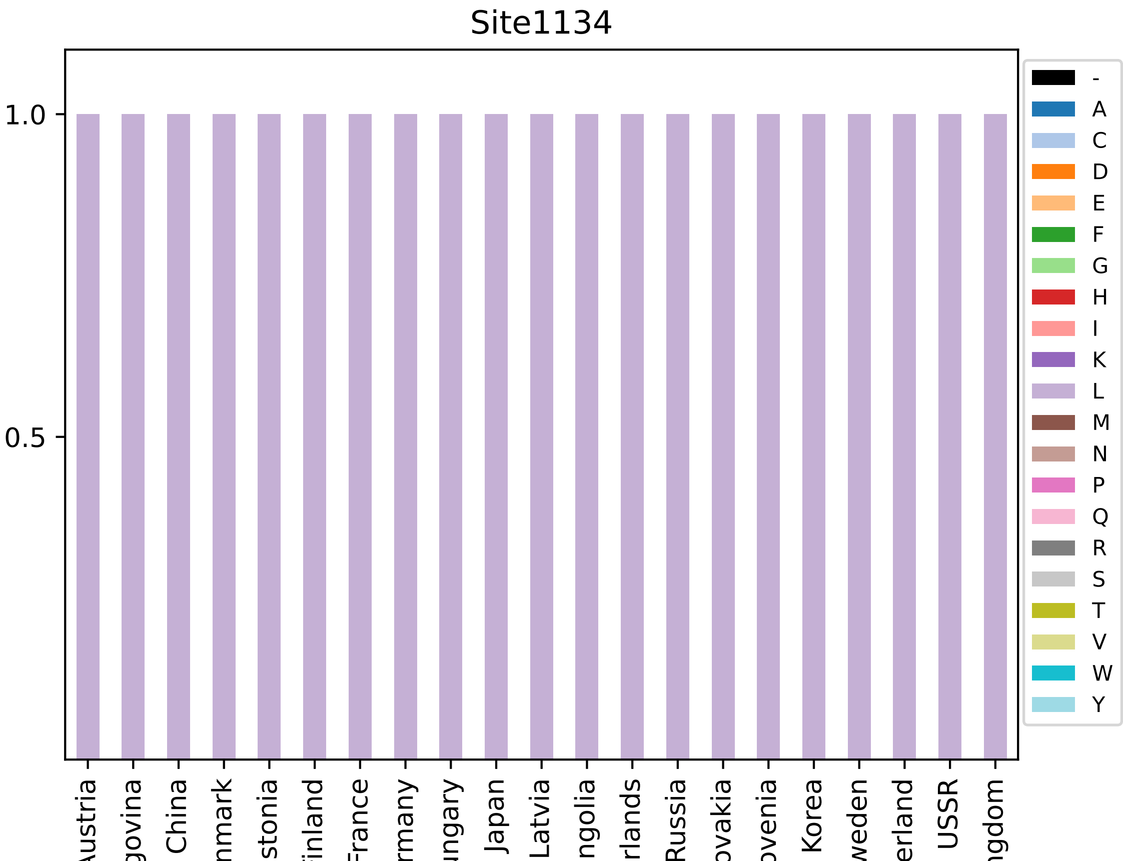

Site1134
1.0
0.5
-
A
C
D
E
F
G
H
I
K
L
M
N
P
Q
R
S
T
V
W
Y
China
Japan
Latvia
Russia
Korea
USSR

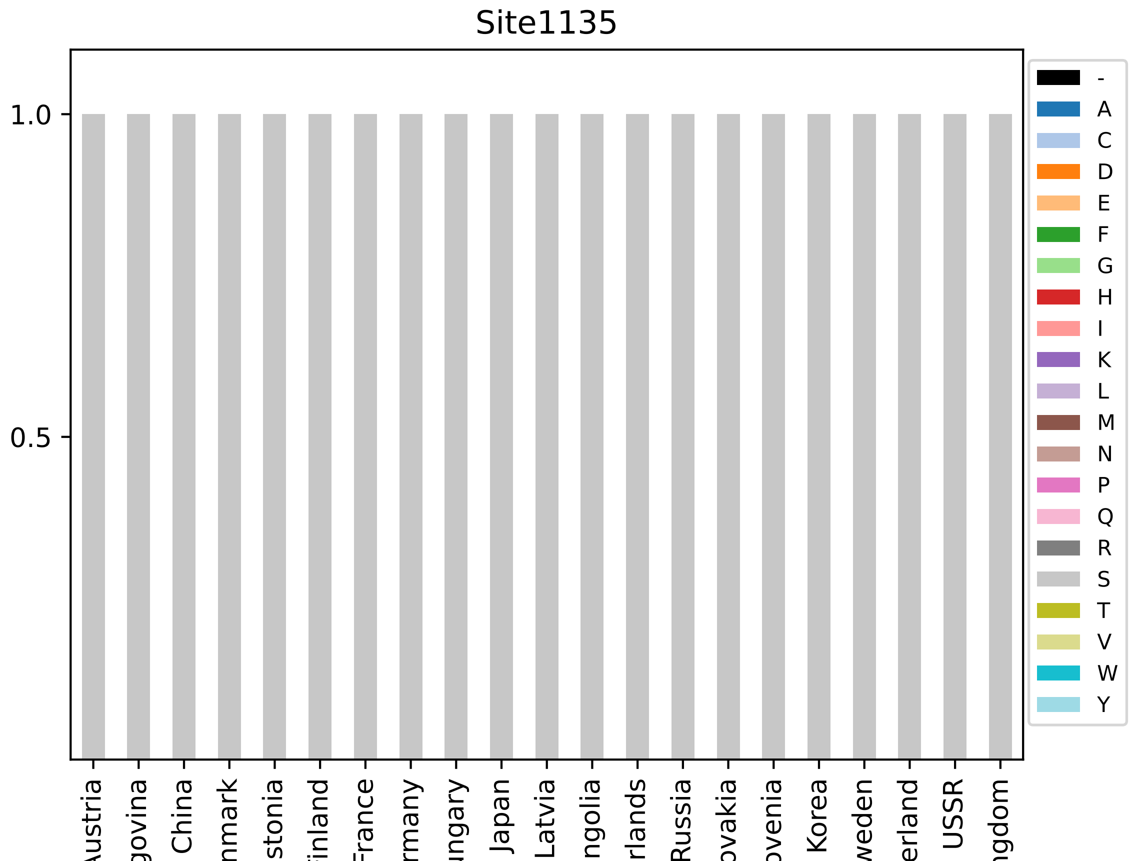

Site1135
1.0
0.5
-
A
C
D
E
F
G
H
I
K
L
M
N
P
Q
R
S
T
V
W
Y
China
Japan
Latvia
Russia
Korea
USSR

Site1136

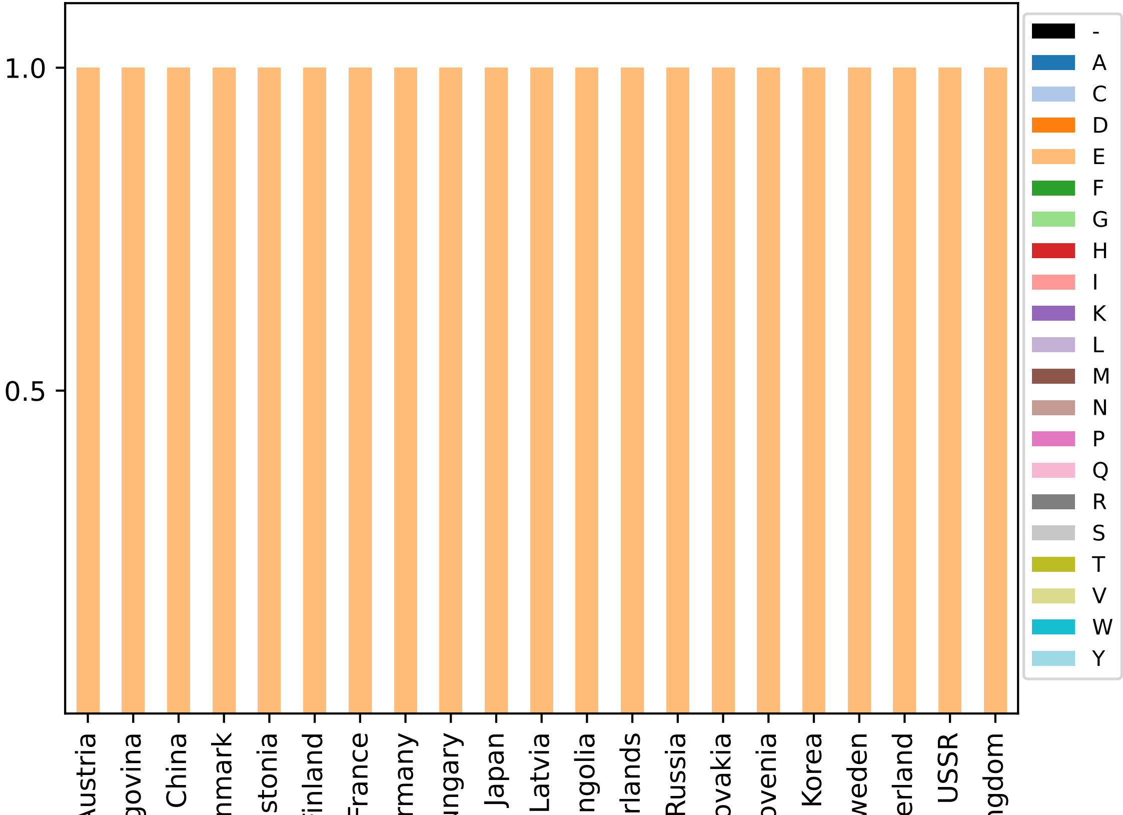
1.0
0.5
-
A
C
D
E
F
G
H
I
K
L
M
N
P
Q
R
S
T
V
W
Y
China
Japan
Latvia
Russia
Korea
USSR

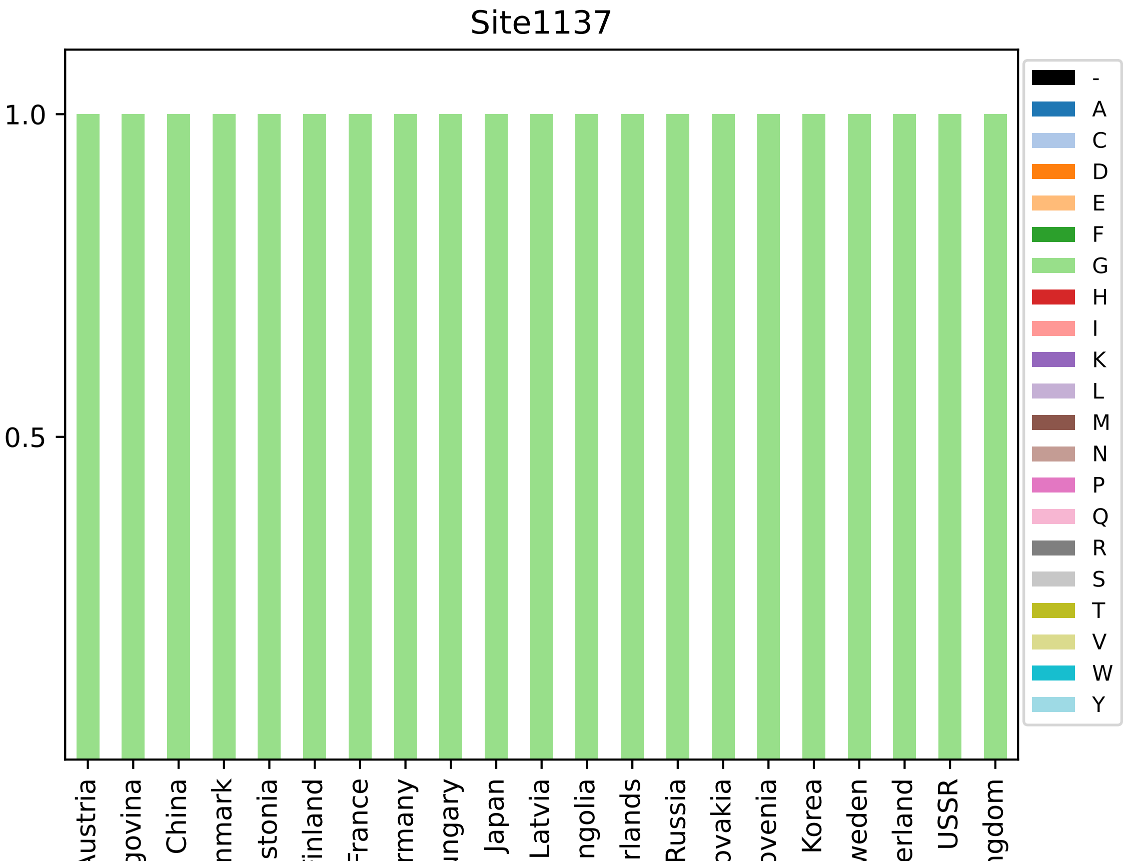
Site1137
1.0
0.5
-
A
C
D
E
F
G
H
I
K
L
M
N
P
Q
R
S
T
V
W
Y
China
Japan
Latvia
Russia
Korea
USSR

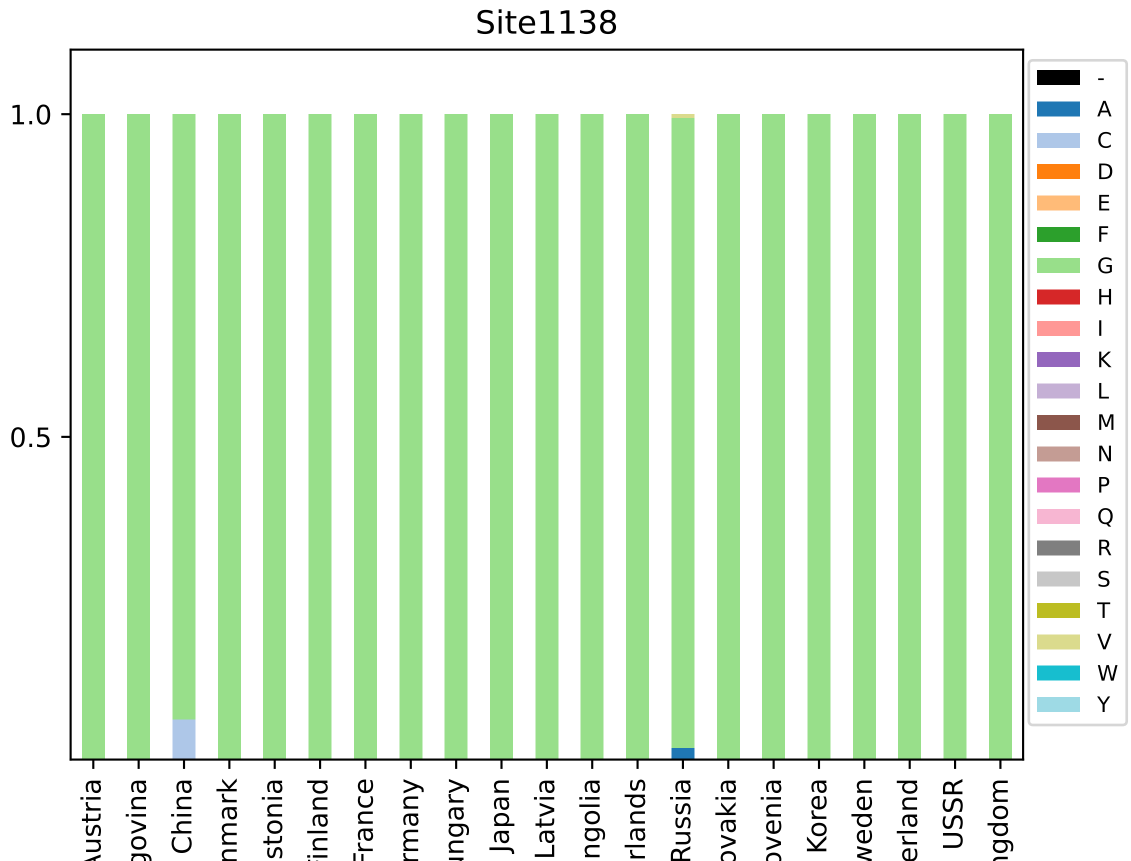
Site1138
1.0
0.5
China
Japan
Latvia
Russia
Korea
USSR
-
A
C
D
E
F
G
H
I
K
L
M
N
P
Q
R
S
T
V
W
Y

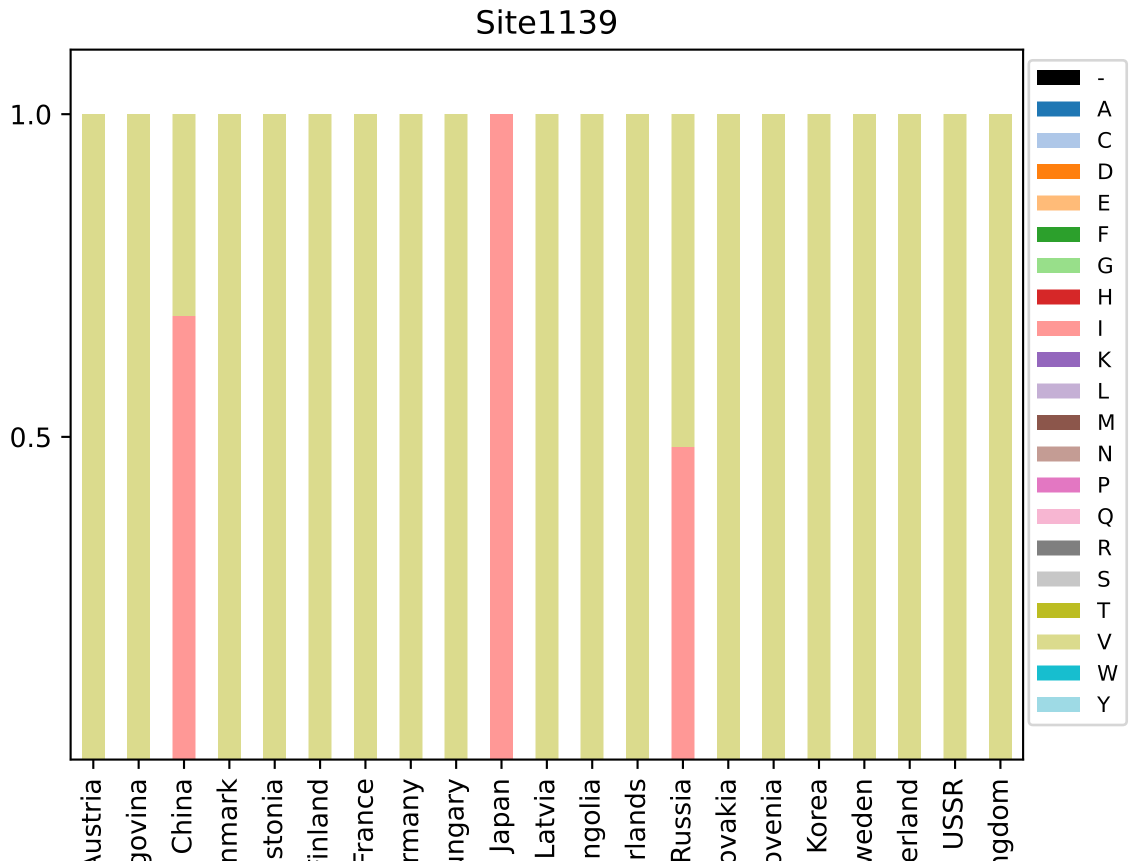
Site1139
1.0
0.5
-
A
C
D
E
F
G
H
I
K
L
M
N
P
Q
R
S
T
V
W
Y
China
Japan
Latvia
Russia
Korea
USSR

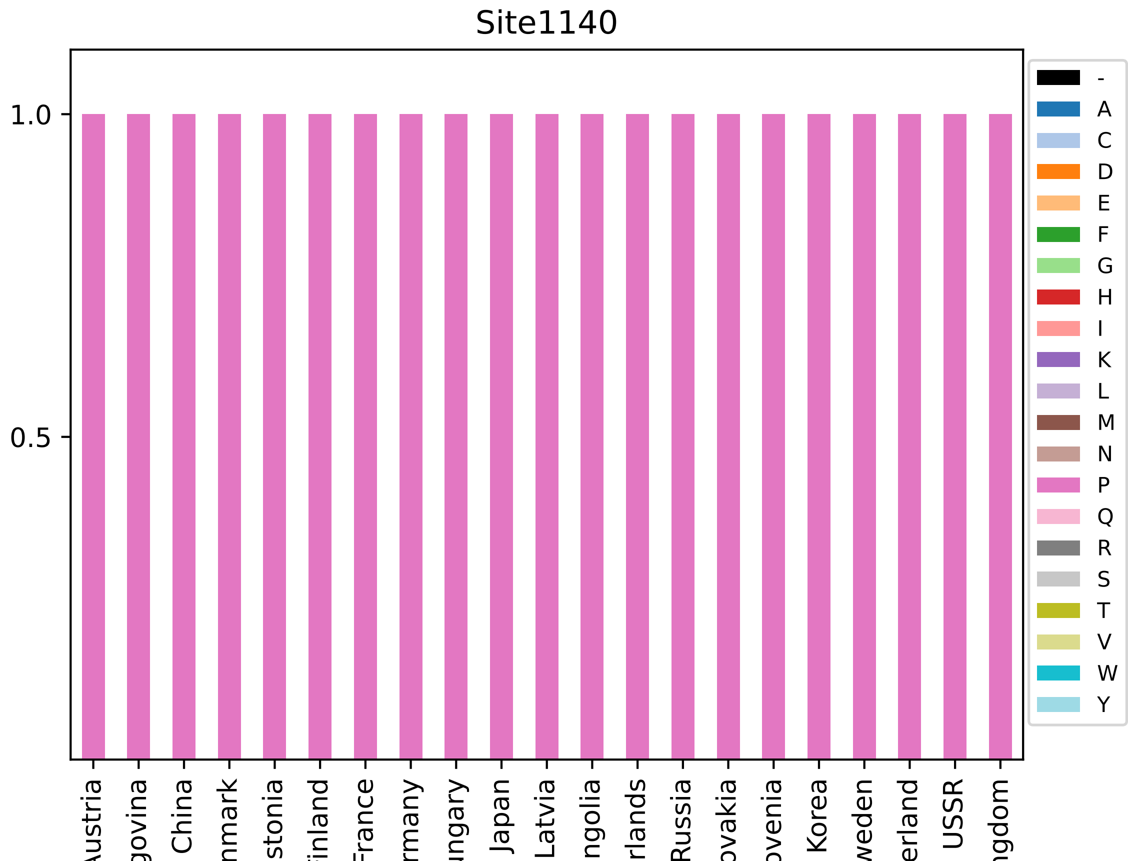

Site1140
1.0
0.5
-
A
C
D
E
F
G
H
I
K
L
M
N
P
Q
R
S
T
V
W
Y
China
Japan
Latvia
Russia
Korea
USSR

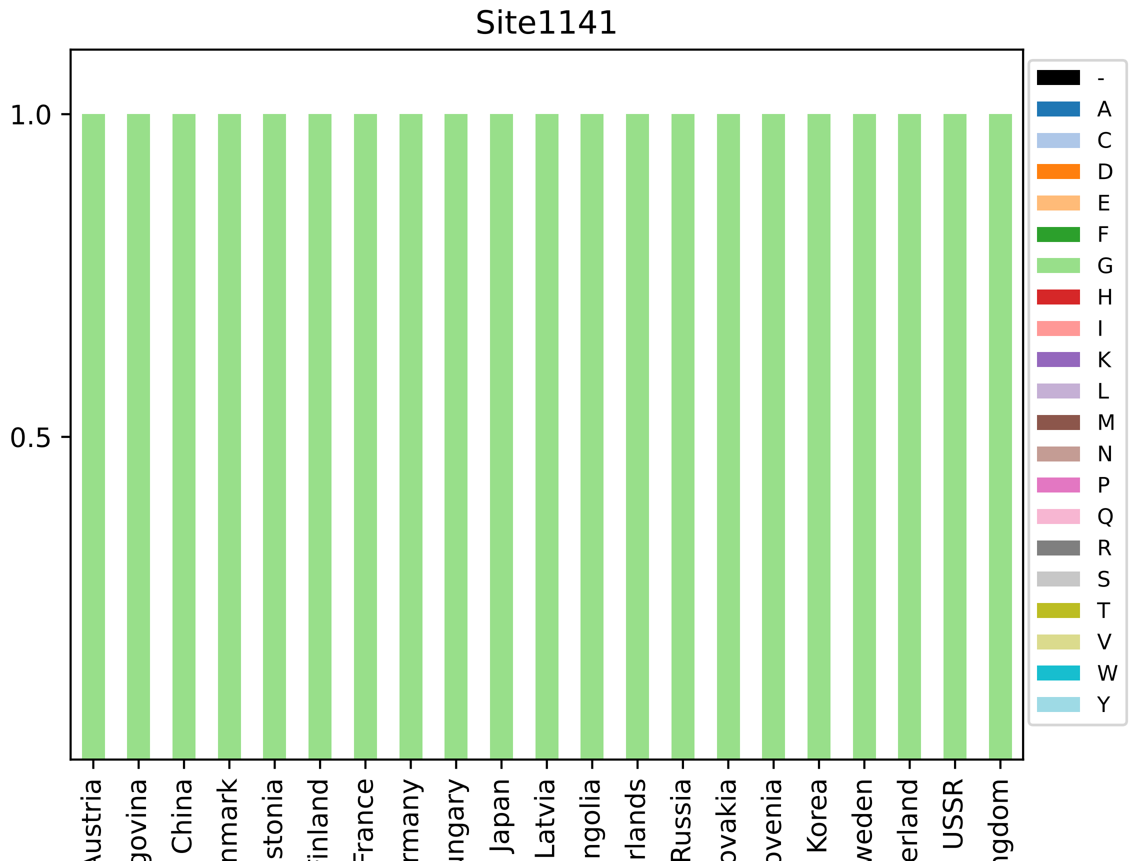

Site1141
1.0
0.5
-
A
C
D
E
F
G
H
I
K
L
M
N
P
Q
R
S
T
V
W
Y
China
Japan
Latvia
Russia
Korea
USSR

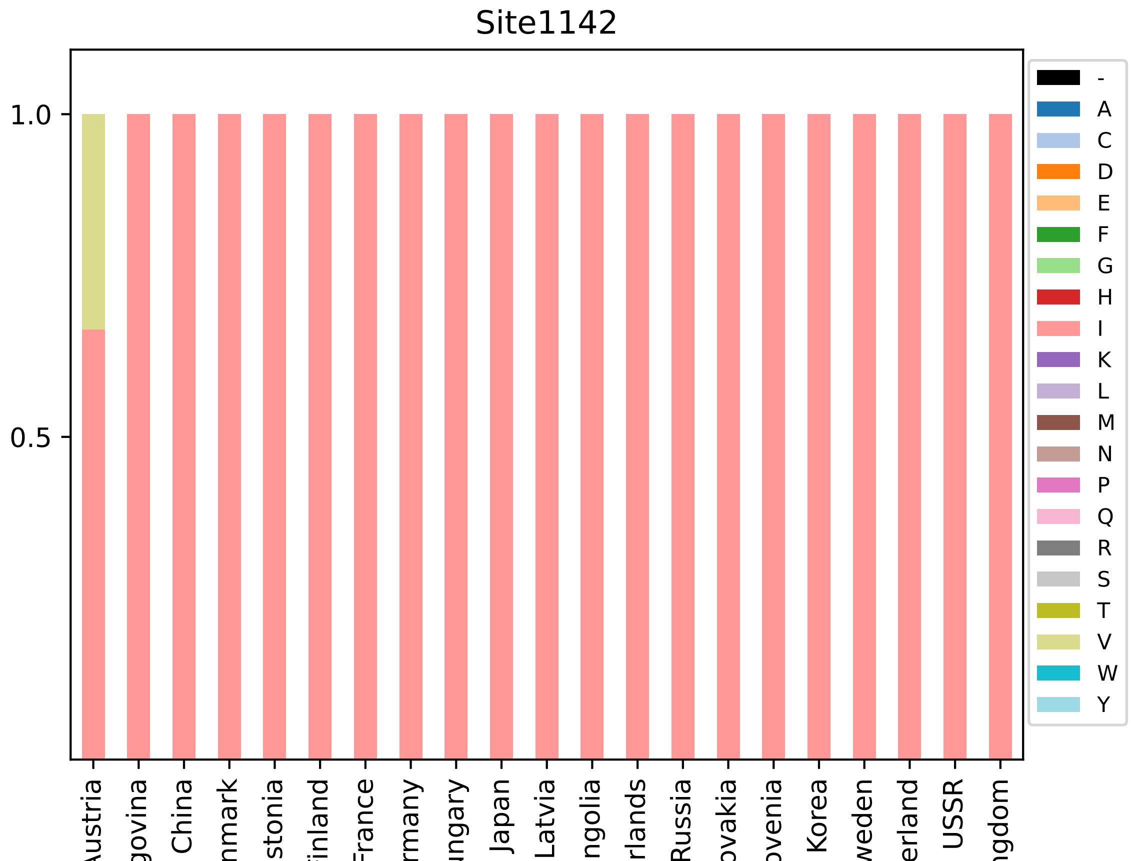
Site1142
1.0
0.5
-
A
C
D
E
F
G
H
I
K
L
M
N
P
Q
R
S
T
V
W
Y
China
Japan
Latvia
Russia
Korea
USSR

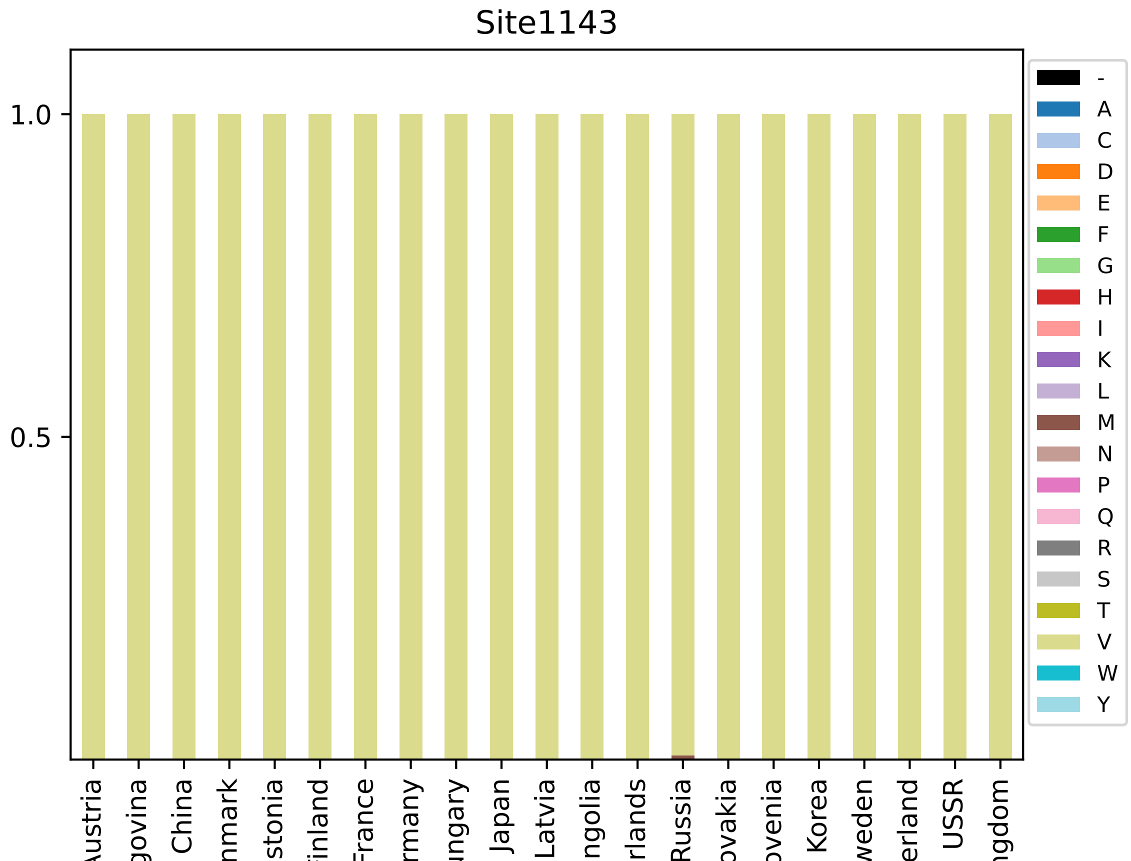

Site1143
1.0
0.5
-
A
C
D
E
F
G
H
I
K
L
M
N
P
Q
R
S
T
V
W
Y
Austria
China
Japan
Latvia
Russia
Korea
USSR

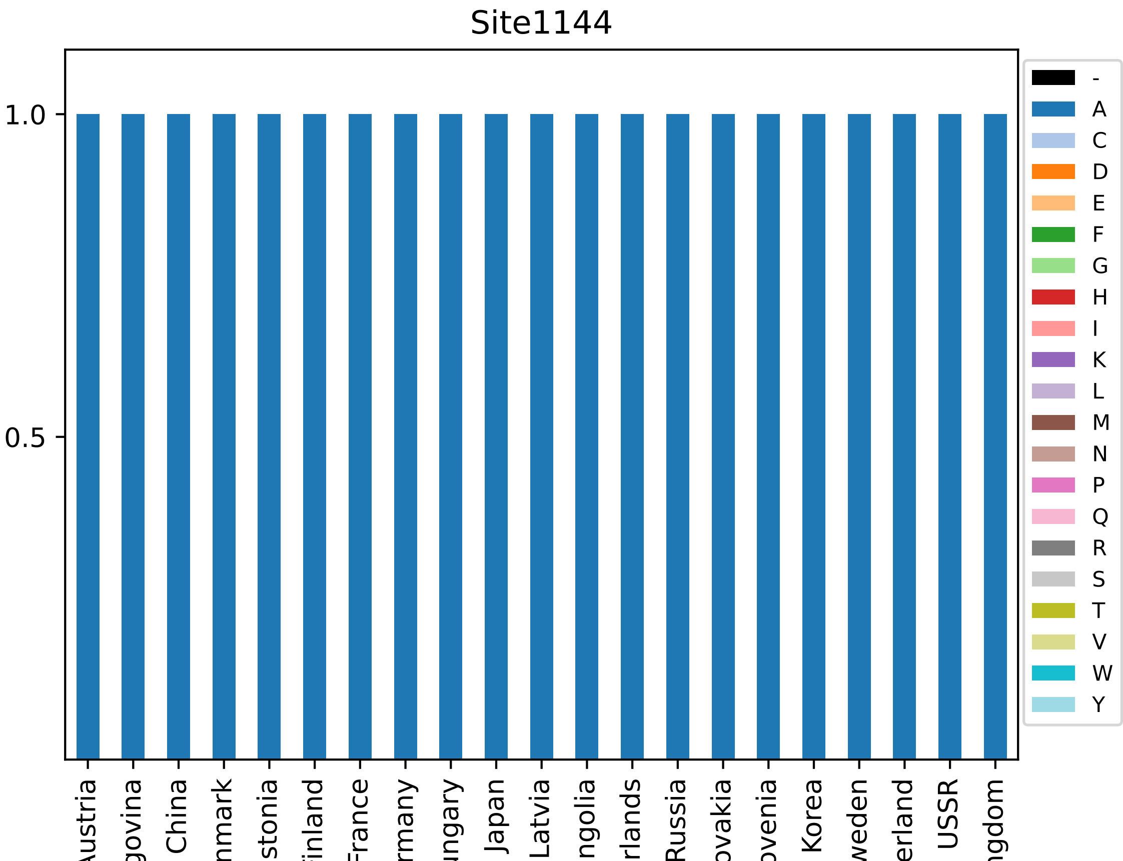
Site1144
1.0
0.5
-
A
C
D
E
F
G
H
I
K
L
M
N
P
Q
R
S
T
V
W
Y
China
Japan
Latvia
Russia
Korea
USSR

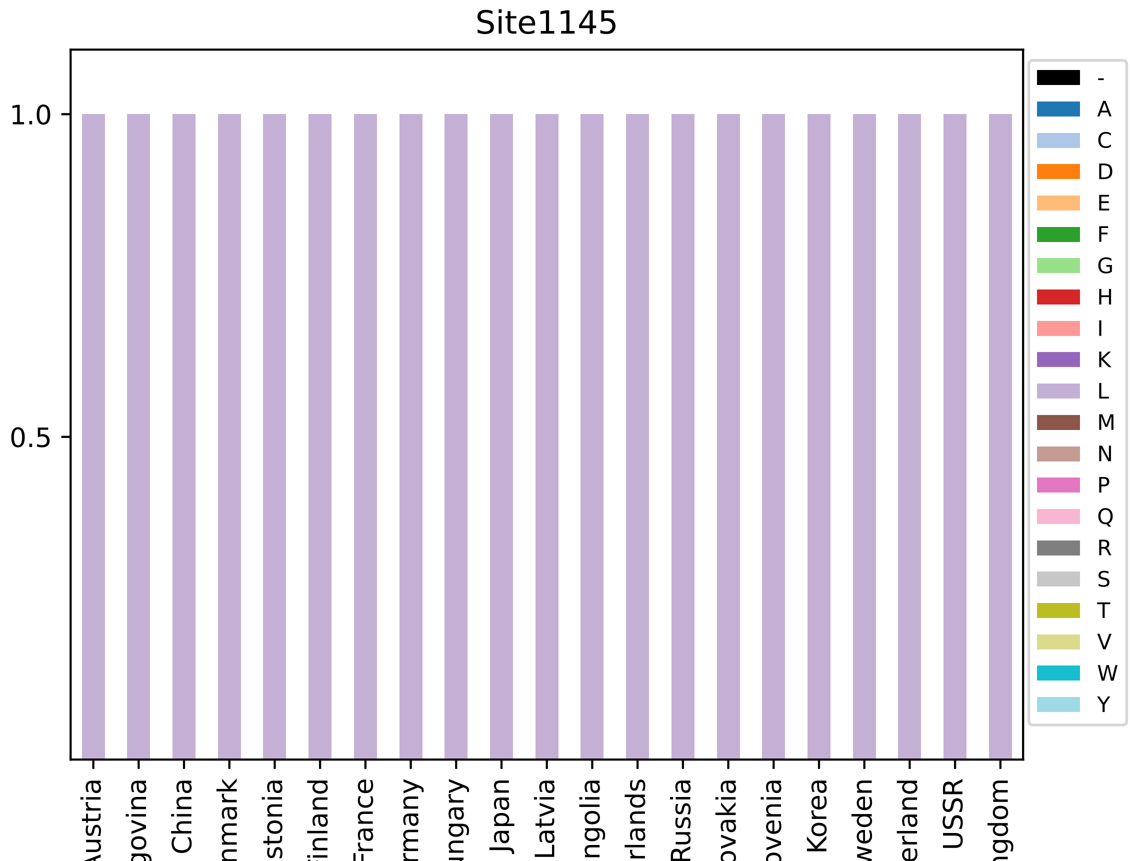

Site1145
1.0
0.5
Austria
govina
China
nmark
stonia
Finland
France
rmany
ungary
Japan
Latvia
ngolia
rlands
Russia
ovakia
ovenia
Korea
weden
erland
USSR
ngdom
-
A
C
D
E
F
G
H
I
K
L
M
N
P
Q
R
S
T
V
W
Y

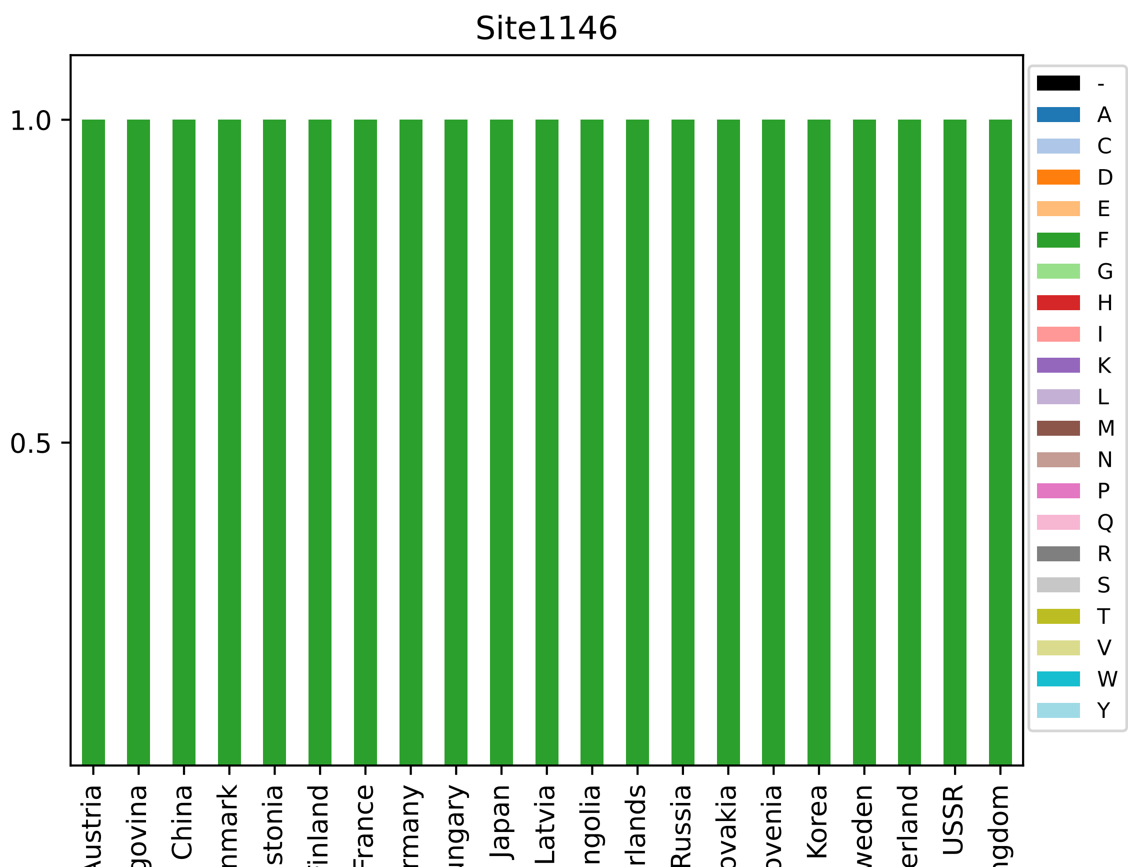

Site1146
1.0
0.5
-
A
C
D
E
F
G
H
I
K
L
M
N
P
Q
R
S
T
V
W
Y
Austria
govina
China
nmark
stonia
Finland
France
rmany
ungary
Japan
Latvia
ngolia
rlands
Russia
ovakia
ovenia
Korea
weden
erland
USSR
ngdom

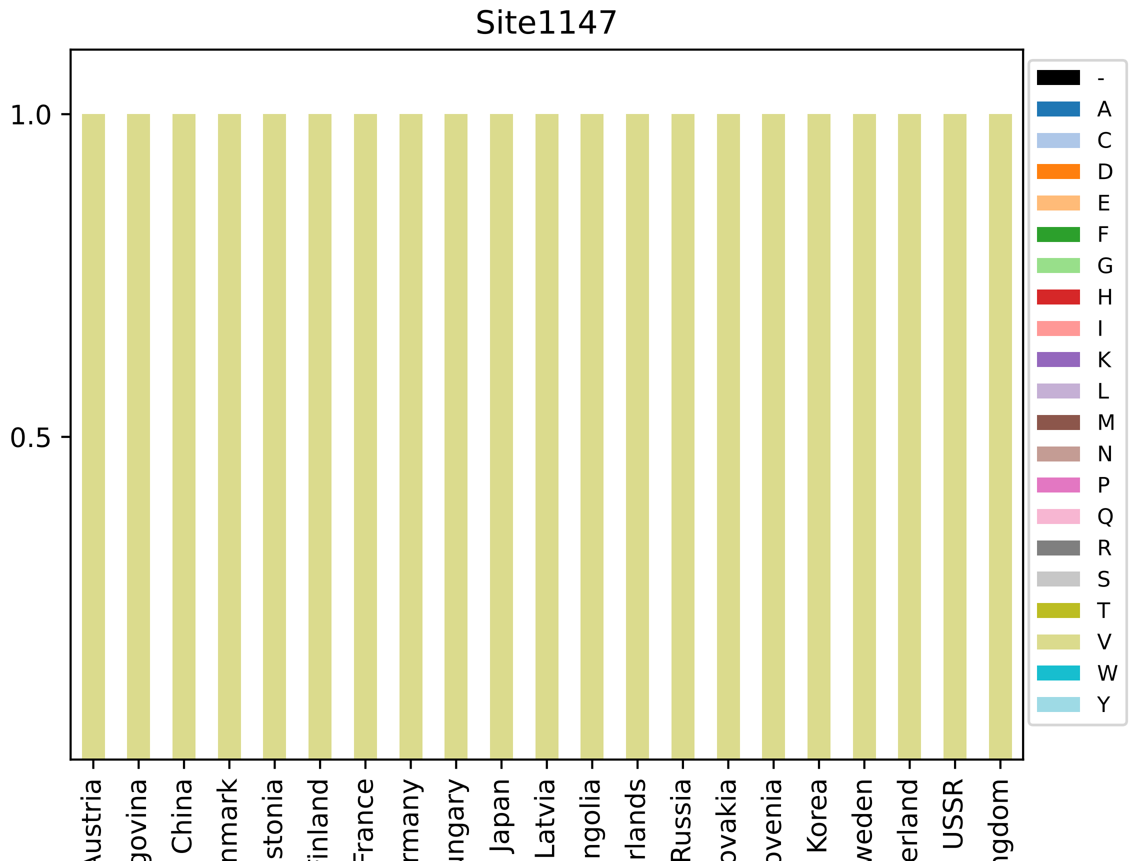

Site1147
1.0
0.5
-
A
C
D
E
F
G
H
I
K
L
M
N
P
Q
R
S
T
V
W
Y
China
Japan
Latvia
Russia
Korea
USSR

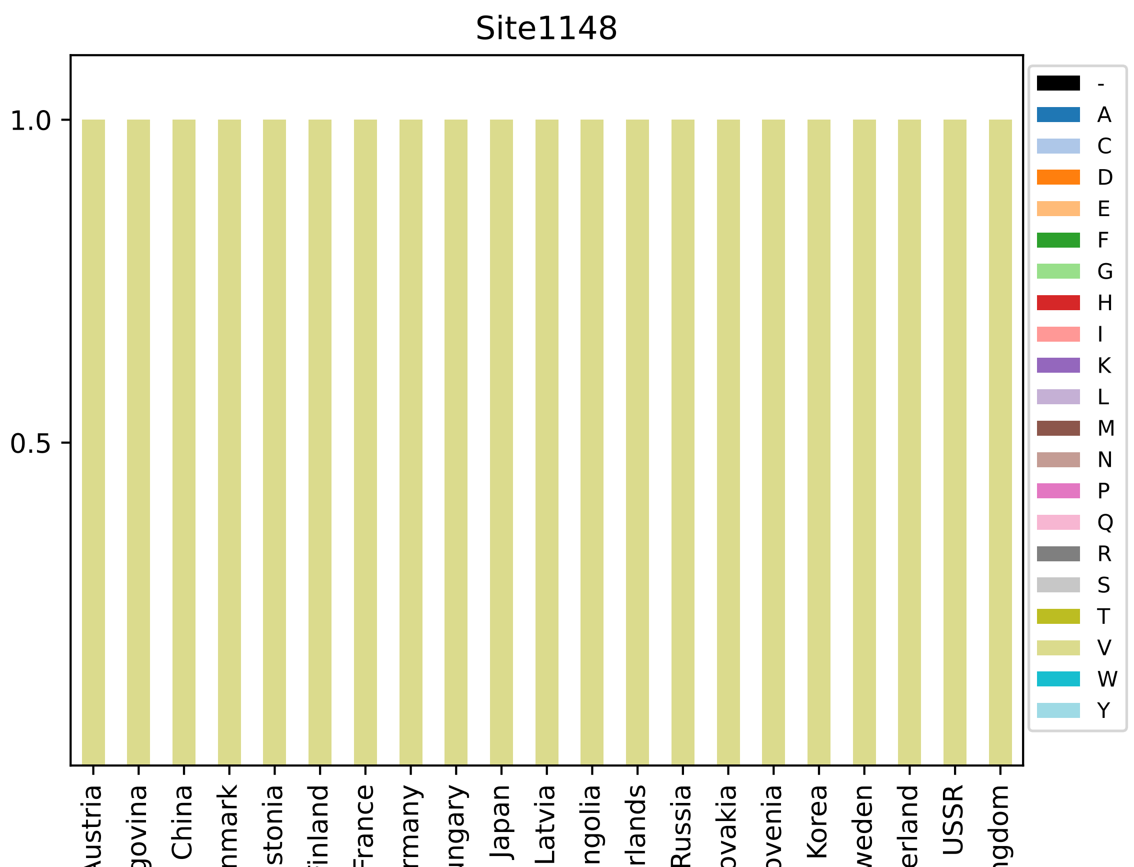

Site1148
1.0
0.5
-
A
C
D
E
F
G
H
I
K
L
M
N
P
Q
R
S
T
V
W
Y
Austria
China
Japan
Latvia
Russia
Korea
USSR

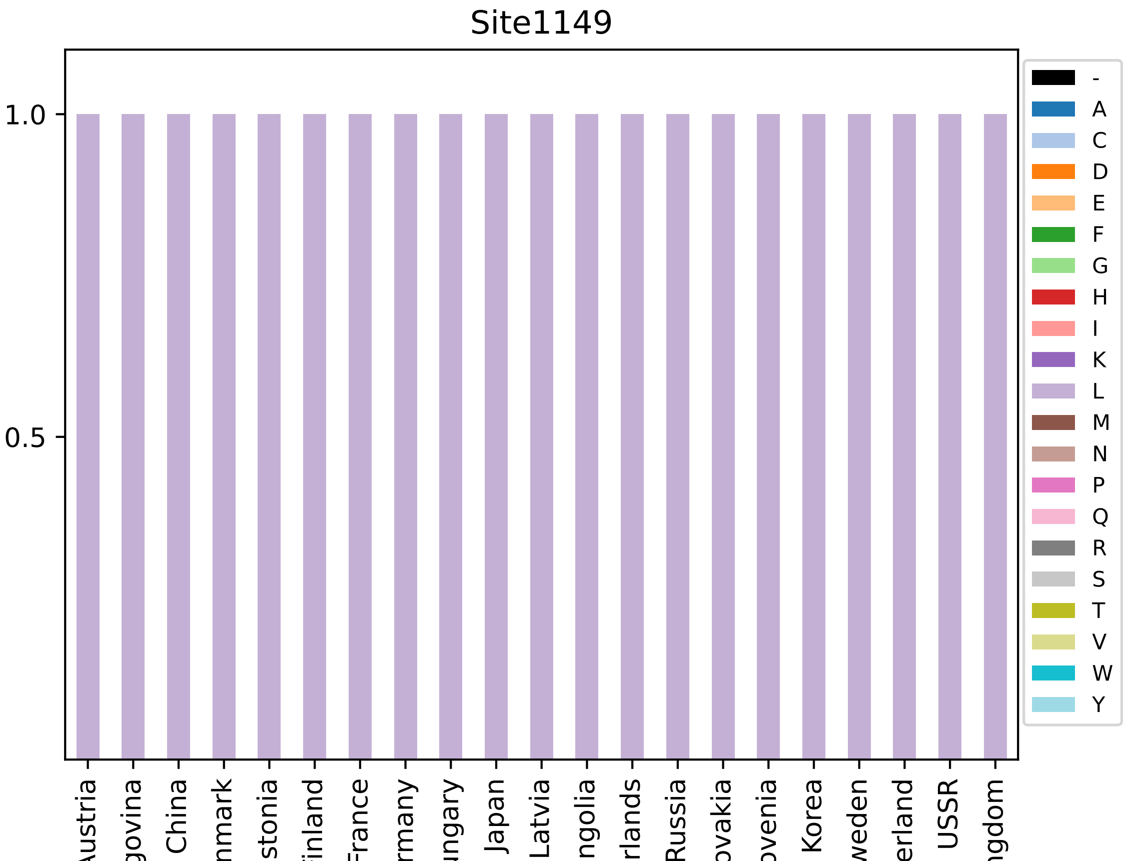
Site1149
1.0
0.5
-
A
C
D
E
F
G
H
I
K
L
M
N
P
Q
R
S
T
V
W
Y
Austria
China
Japan
Latvia
Russia
Korea
USSR

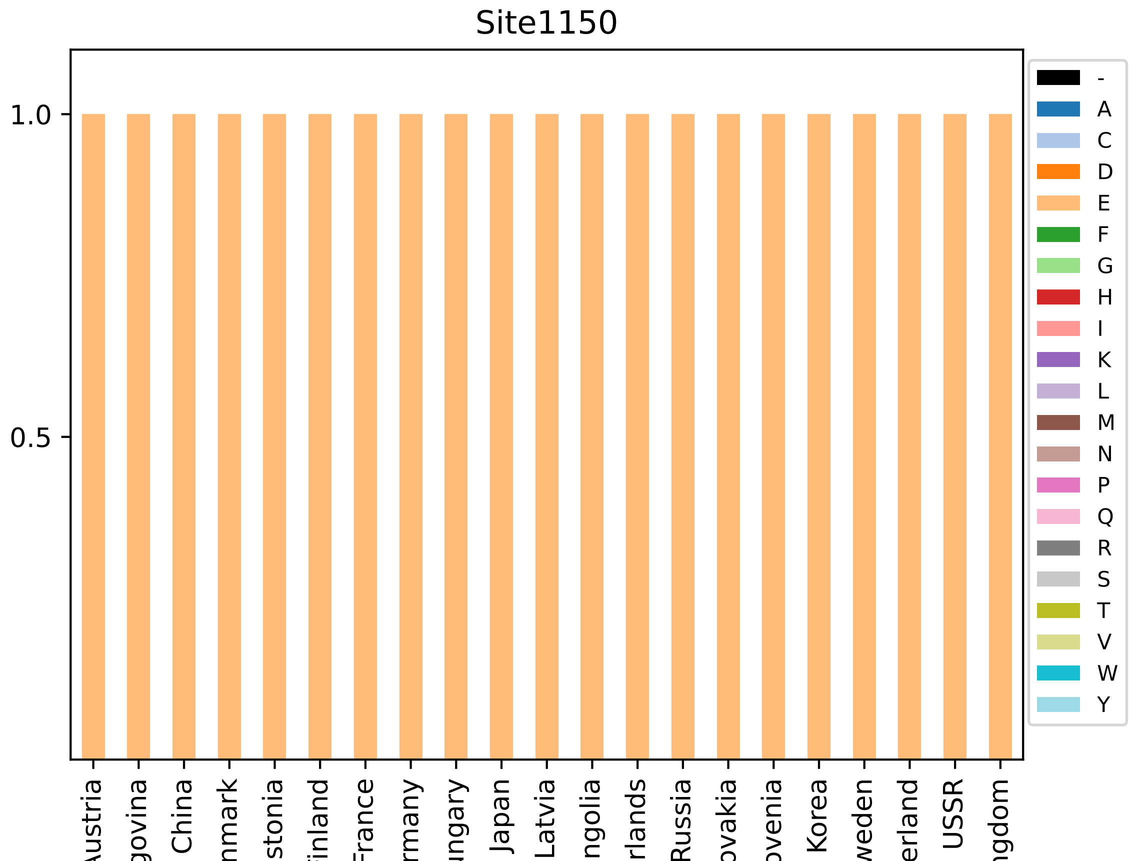

Site1150
1.0
0.5
-
A
C
D
E
F
G
H
I
K
L
M
N
P
Q
R
S
T
V
W
Y
China
Japan
Latvia
Russia
Korea
USSR

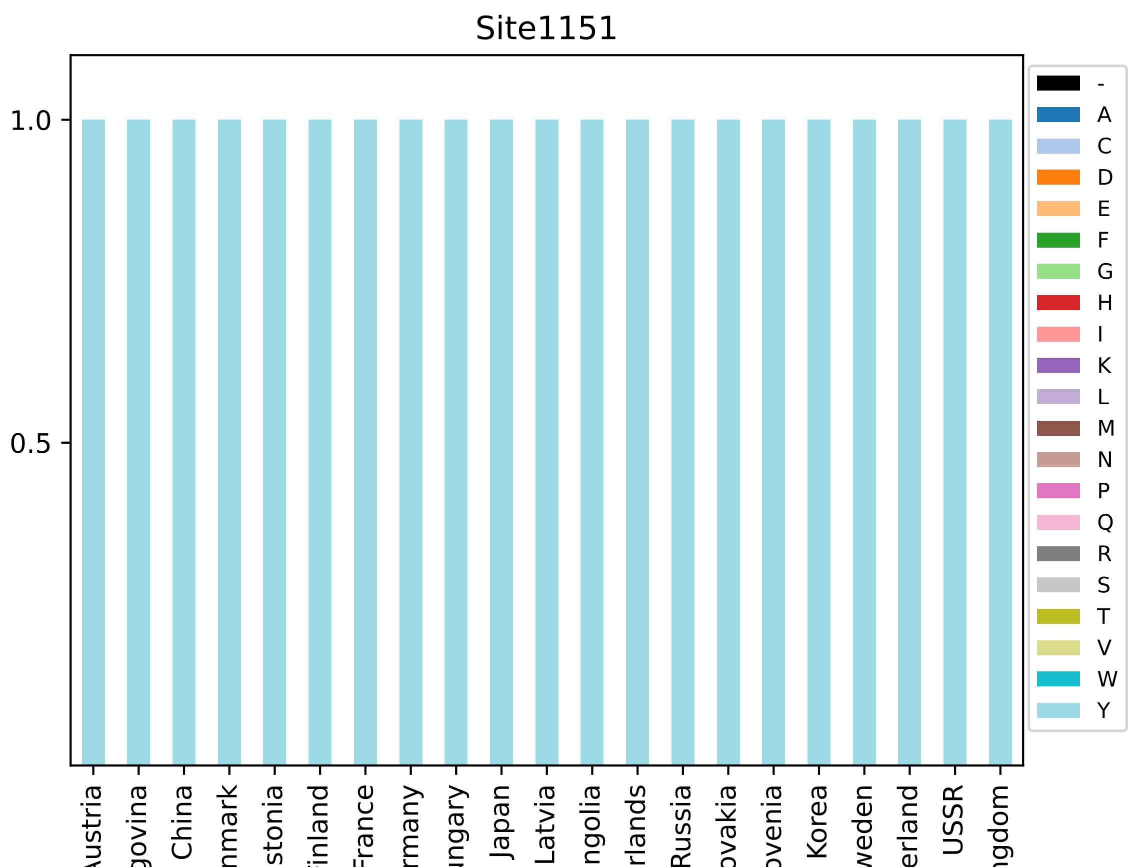
Site1151
1.0
0.5
-
A
C
D
E
F
G
H
I
K
L
M
N
P
Q
R
S
T
V
W
Y
China
Japan
Latvia
Russia
Korea
USSR

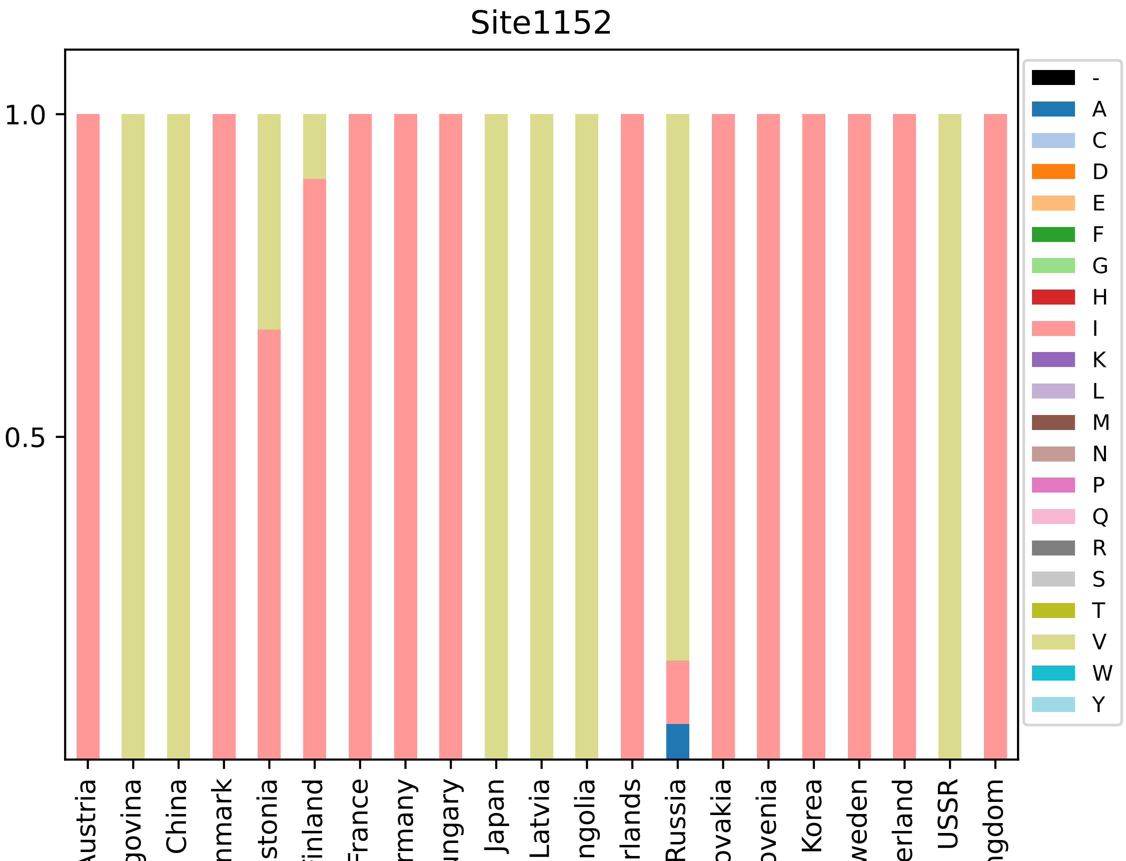

Site1152
1.0
0.5
-
A
C
D
E
F
G
H
I
K
L
M
N
P
Q
R
S
T
V
W
Y
China
Japan
Latvia
Russia
Korea
USSR

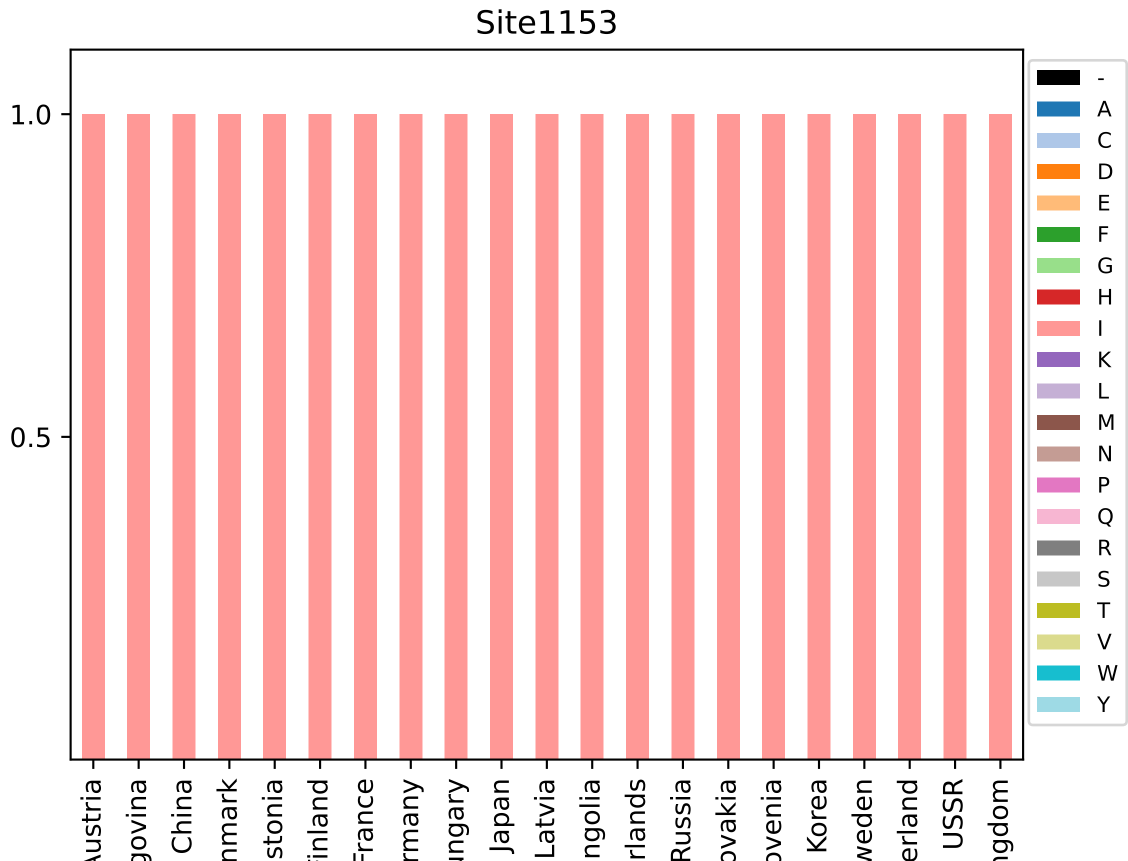

Site1153
1.0
0.5
-
A
C
D
E
F
G
H
I
K
L
M
N
P
Q
R
S
T
V
W
Y
China
Japan
Latvia
Russia
Korea
USSR

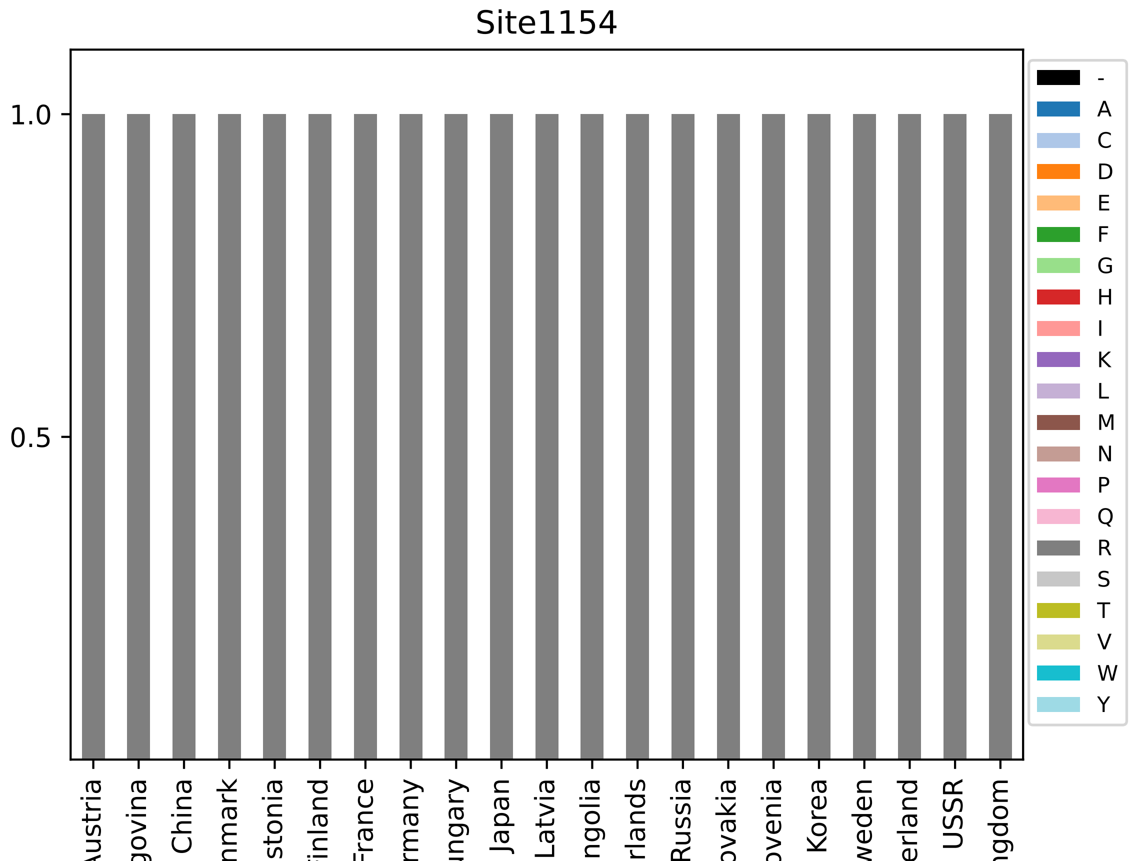
Site1154
1.0
0.5
-
A
C
D
E
F
G
H
I
K
L
M
N
P
Q
R
S
T
V
W
Y
Austria
govina
China
nmark
stonia
Finland
France
rmany
ungary
Japan
Latvia
ngolia
rlands
Russia
ovakia
ovenia
Korea
weden
erland
USSR
ngdom

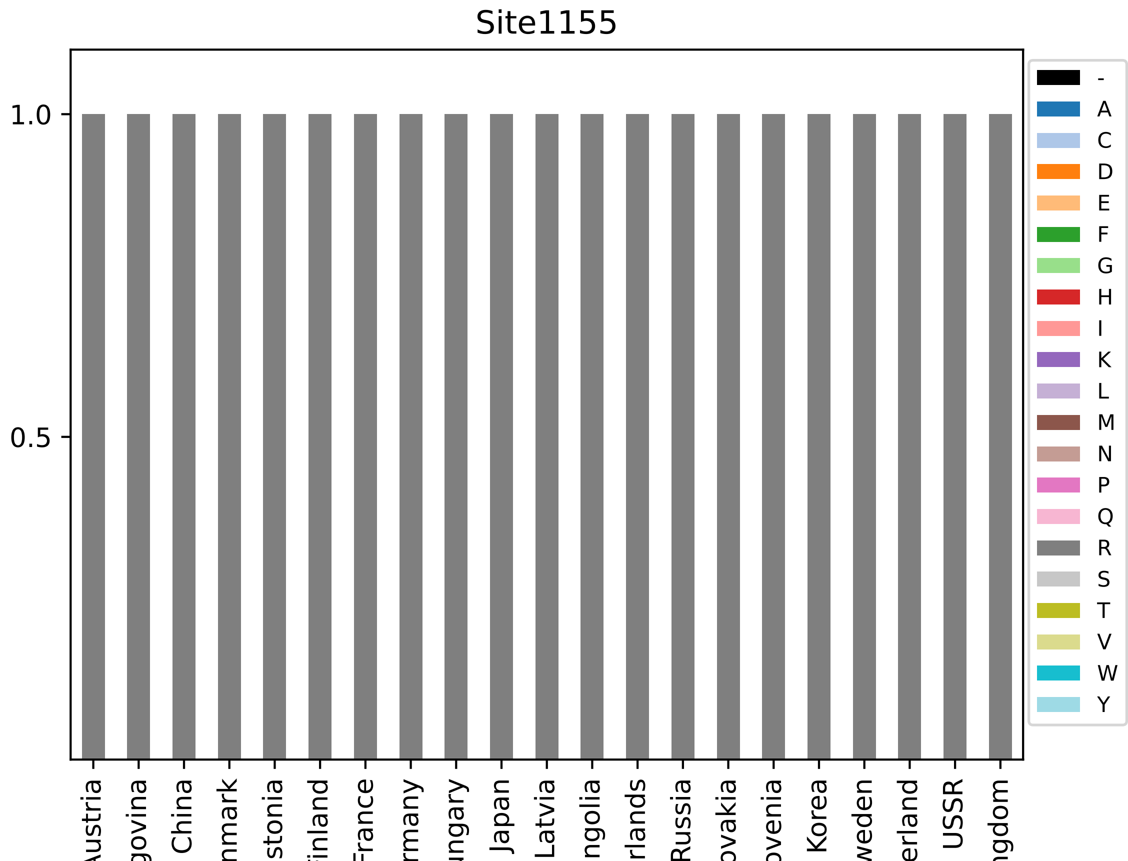
Site1155
1.0
0.5
-
A
C
D
E
F
G
H
I
K
L
M
N
P
Q
R
S
T
V
W
Y
ustria
govina
China
nmark
stonia
inland
rance
rmany
ungary
Japan
Latvia
ngolia
rlands
Russia
ovakia
ovenia
Korea
weden
erland
USSR
ngdom

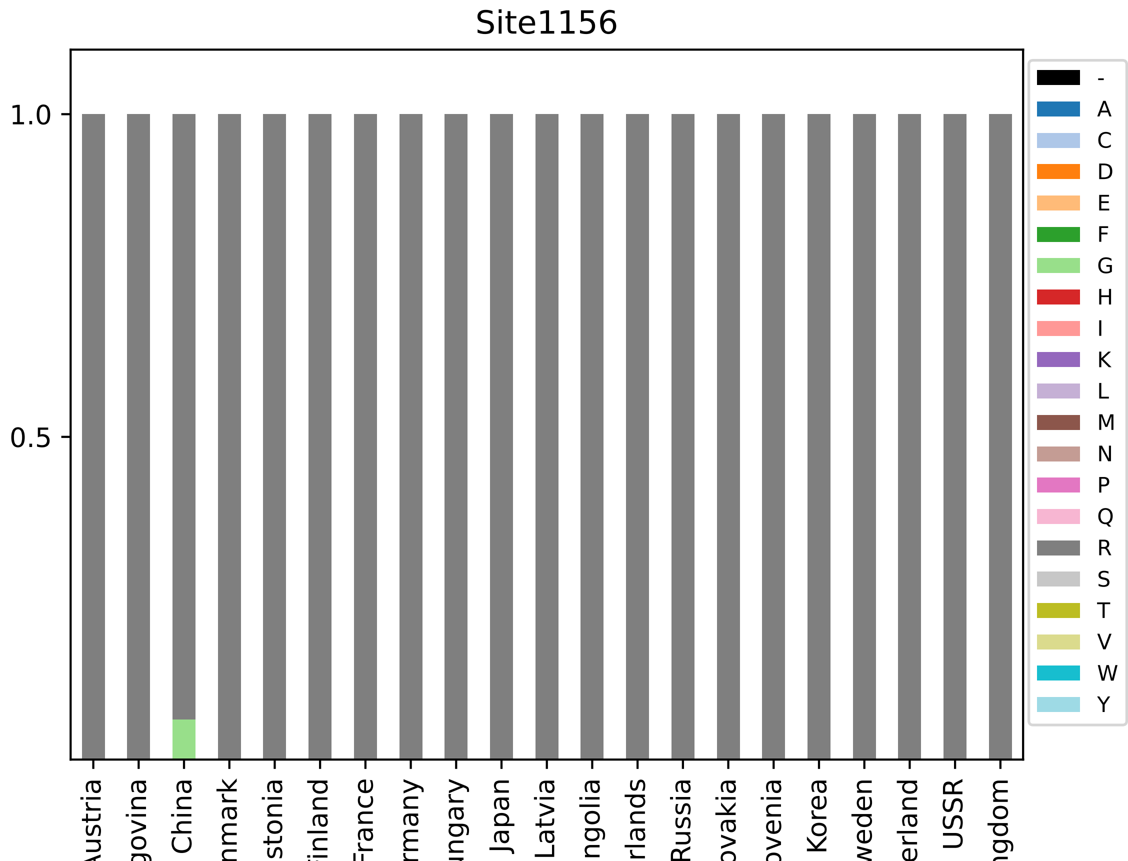
Site1156
1.0
0.5
-
A
C
D
E
F
G
H
I
K
L
M
N
P
Q
R
S
T
V
W
Y
China
Japan
Latvia
Russia
Korea
USSR

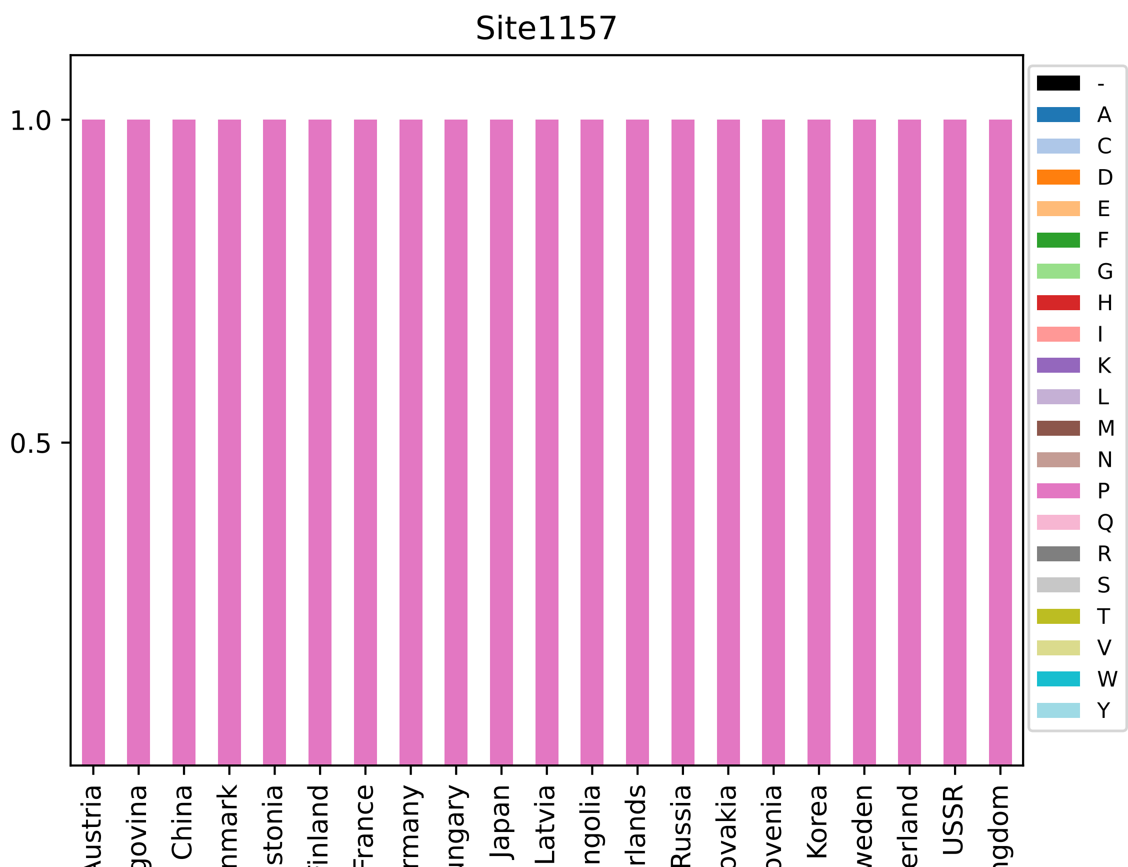

Site1157
1.0
0.5
-
A
C
D
E
F
G
H
I
K
L
M
N
P
Q
R
S
T
V
W
Y
China
Japan
Latvia
Russia
Korea
USSR

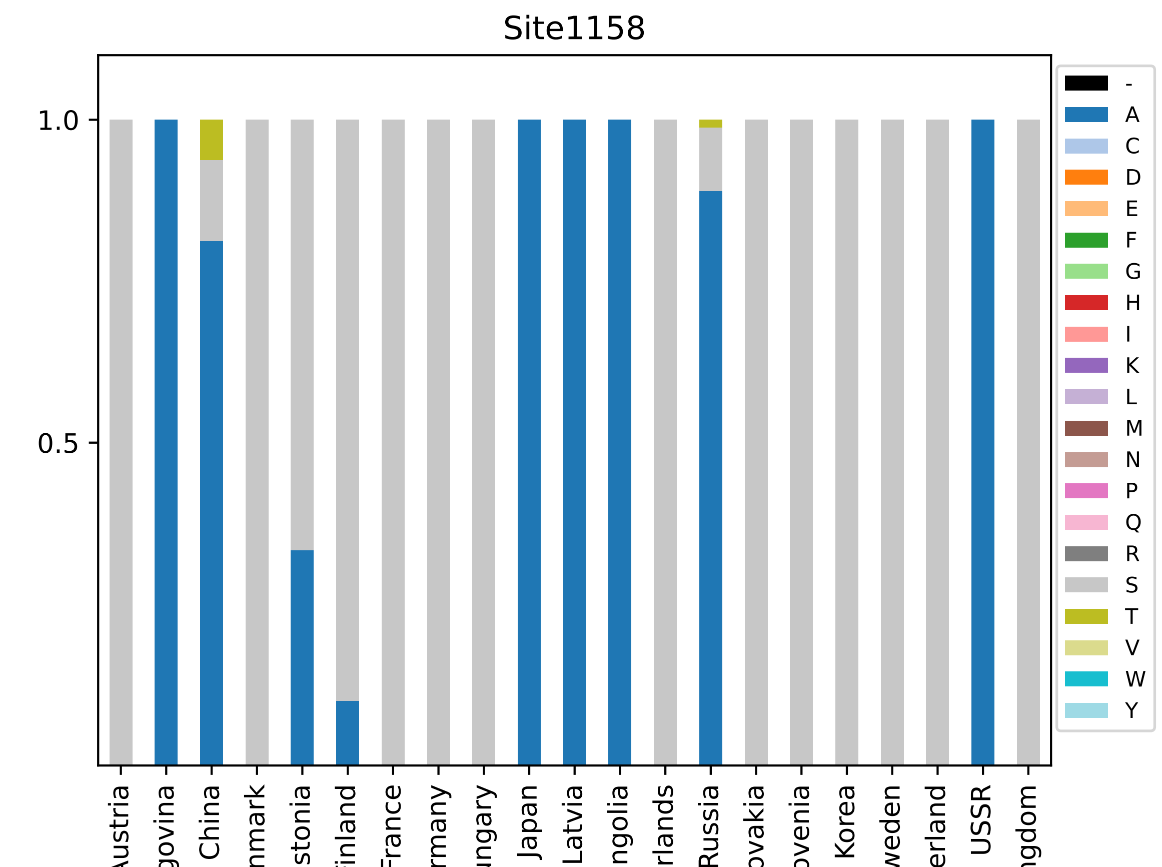

Site1158
1.0
0.5
Austria
govina
China
nmark
stonia
inland
France
rmany
ungary
Japan
Latvia
ngolia
rlands
Russia
ovakia
ovenia
Korea
weden
erland
USSR
ngdom
-
A
C
D
E
F
G
H
I
K
L
M
N
P
Q
R
S
T
V
W
Y

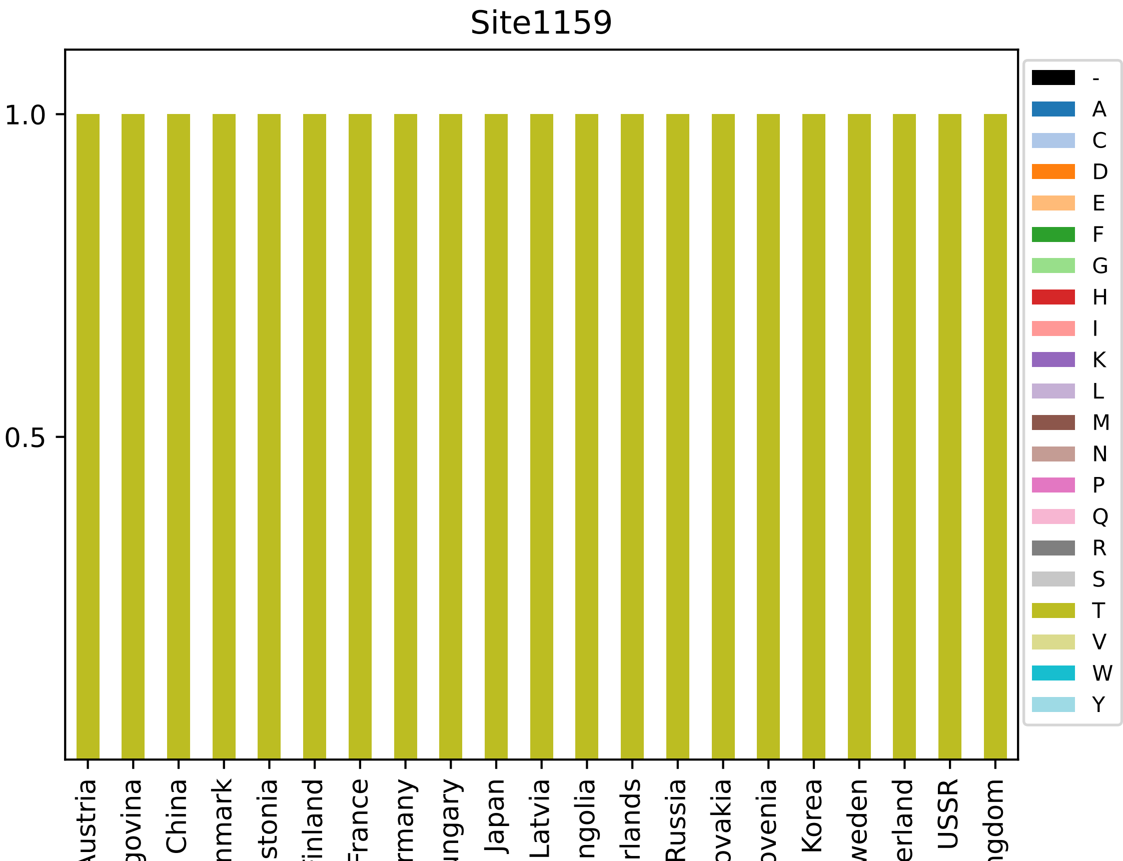
Site1159
1.0
0.5
Austria
govina
China
nmark
stonia
Finland
France
rmany
ungary
Japan
Latvia
ngolia
rlands
Russia
ovakia
ovenia
Korea
weden
erland
USSR
ngdom
-
A
C
D
E
F
G
H
I
K
L
M
N
P
Q
R
S
T
V
W
Y

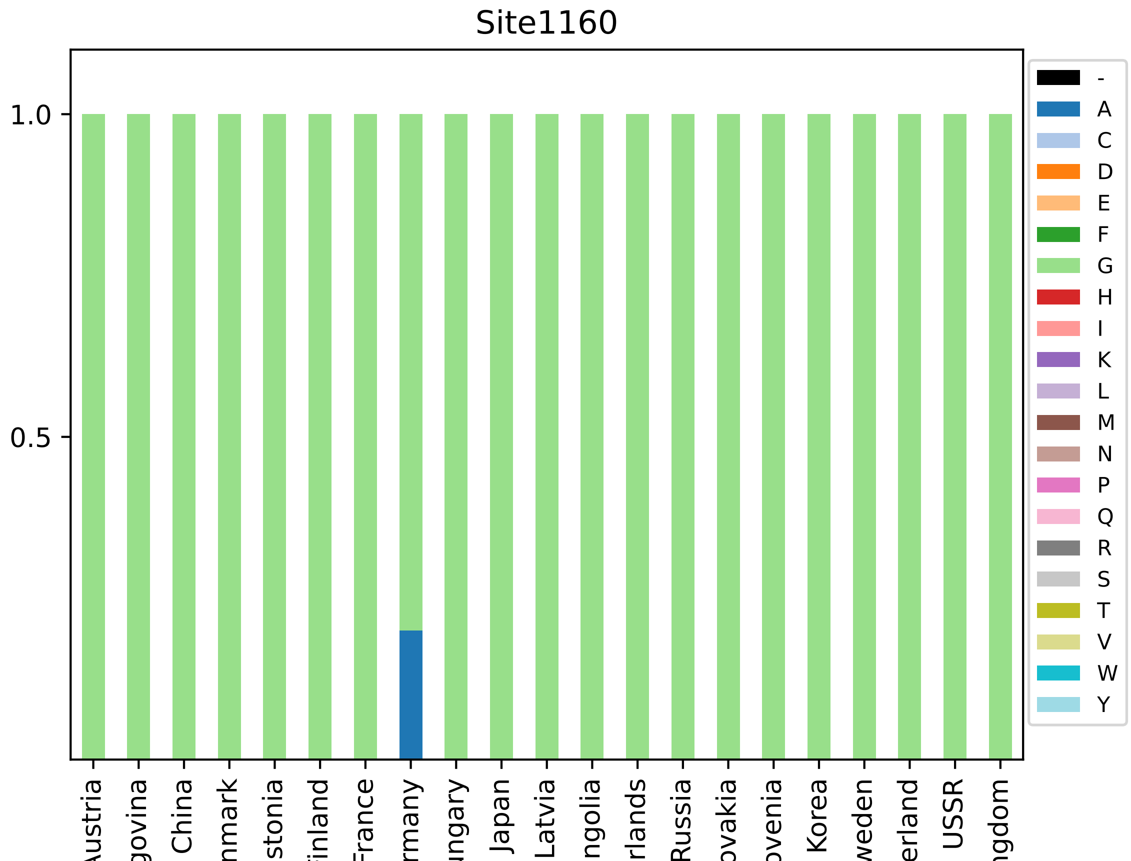
Site1160
1.0
0.5
-
A
C
D
E
F
G
H
I
K
L
M
N
P
Q
R
S
T
V
W
Y
Austria
govina
China
nmark
stonia
Finland
France
rmany
ungary
Japan
Latvia
ngolia
rlands
Russia
ovakia
ovenia
Korea
weden
erland
USSR
ngdom

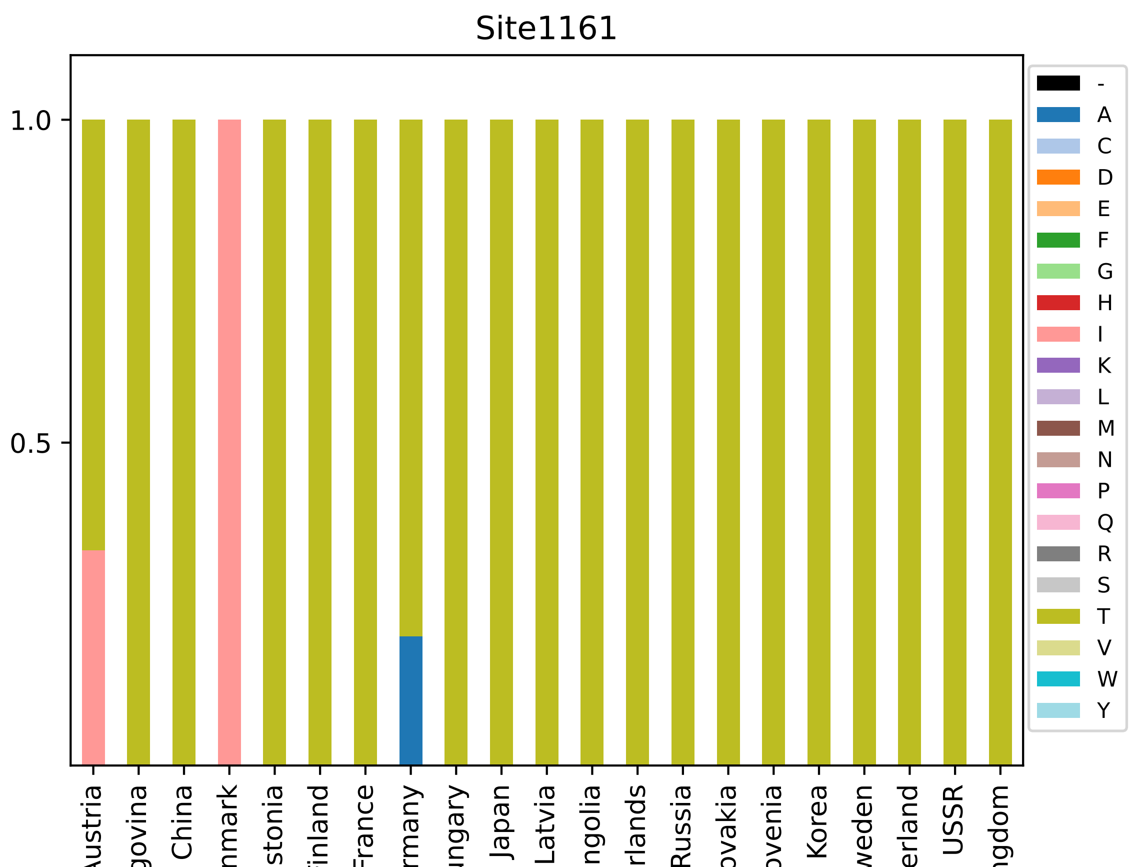
Site1161
1.0
0.5
-
A
C
D
E
F
G
H
I
K
L
M
N
P
Q
R
S
T
V
W
Y
Austria
China
Estonia
Finland
France
Japan
Latvia
Russia
Korea
USSR

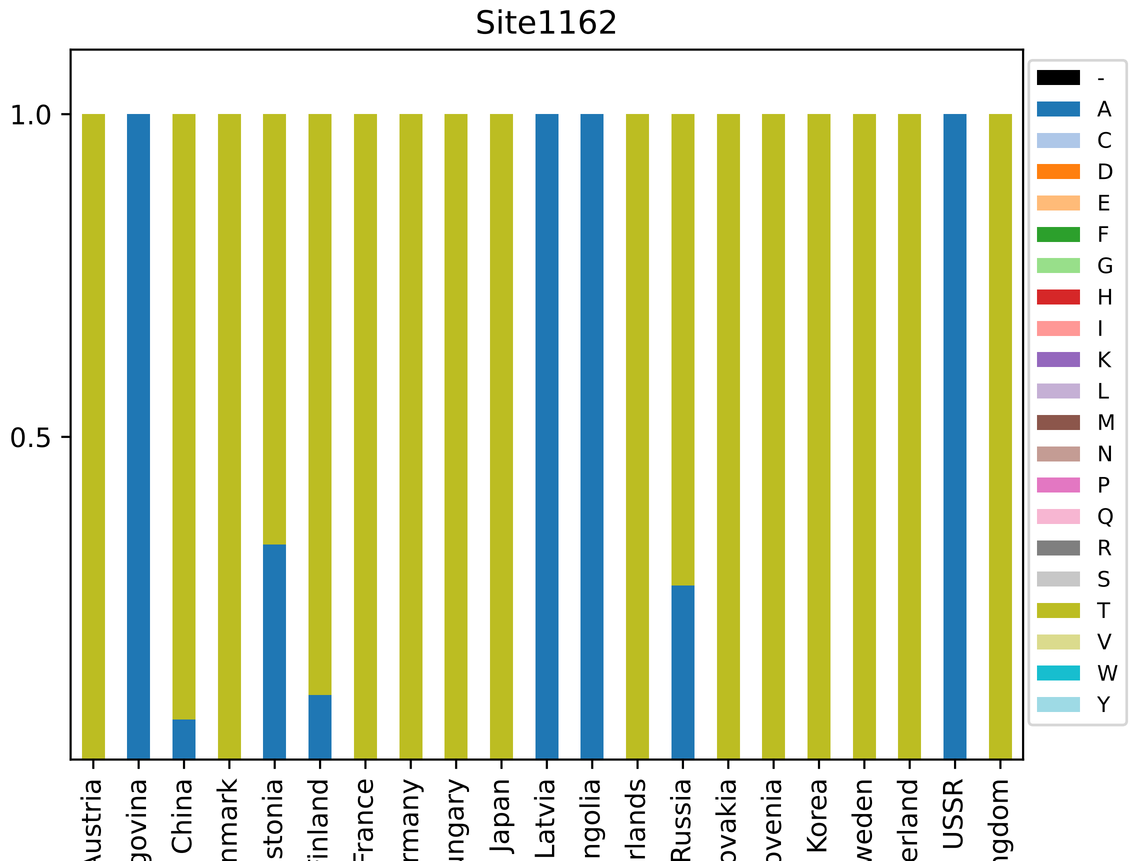

Site1162
1.0
0.5
-
A
C
D
E
F
G
H
I
K
L
M
N
P
Q
R
S
T
V
W
Y
Austria
China
Japan
Latvia
Russia
Korea
USSR

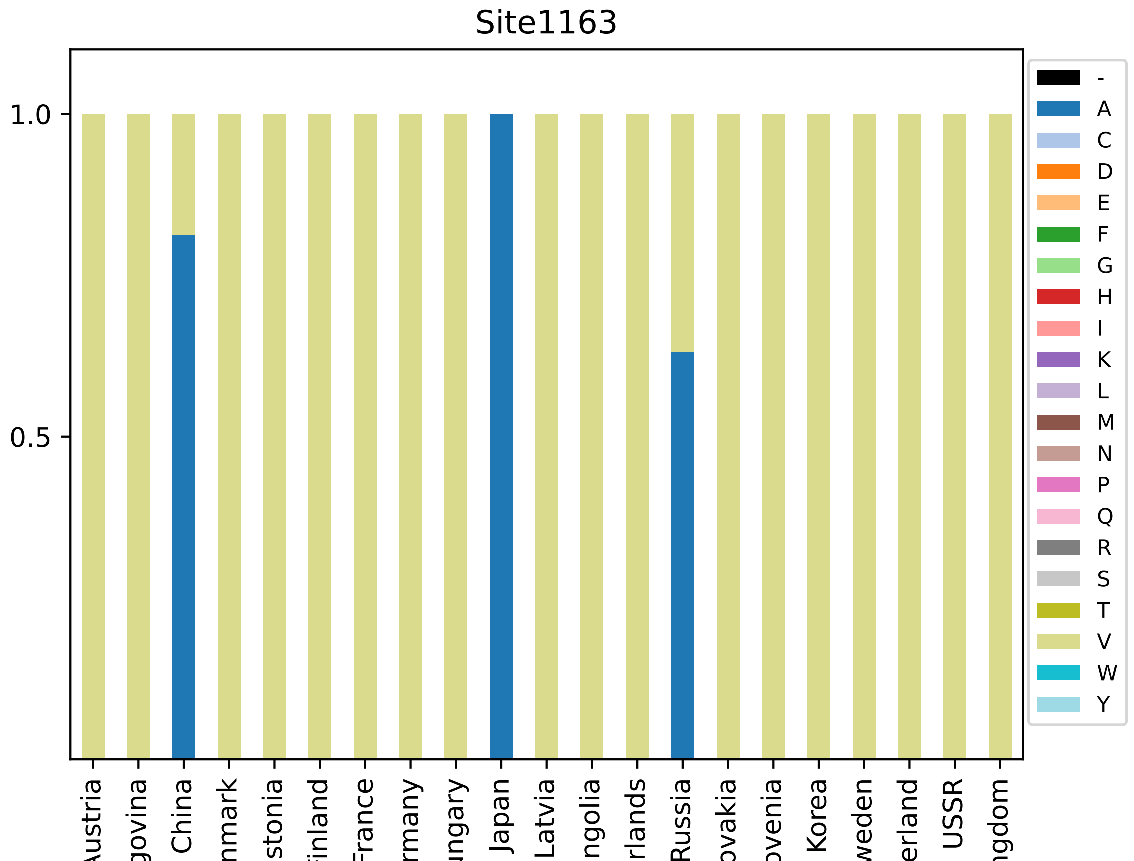
Site1163
1.0
0.5
-
A
C
D
E
F
G
H
I
K
L
M
N
P
Q
R
S
T
V
W
Y
China
Japan
Latvia
Russia
Korea
USSR

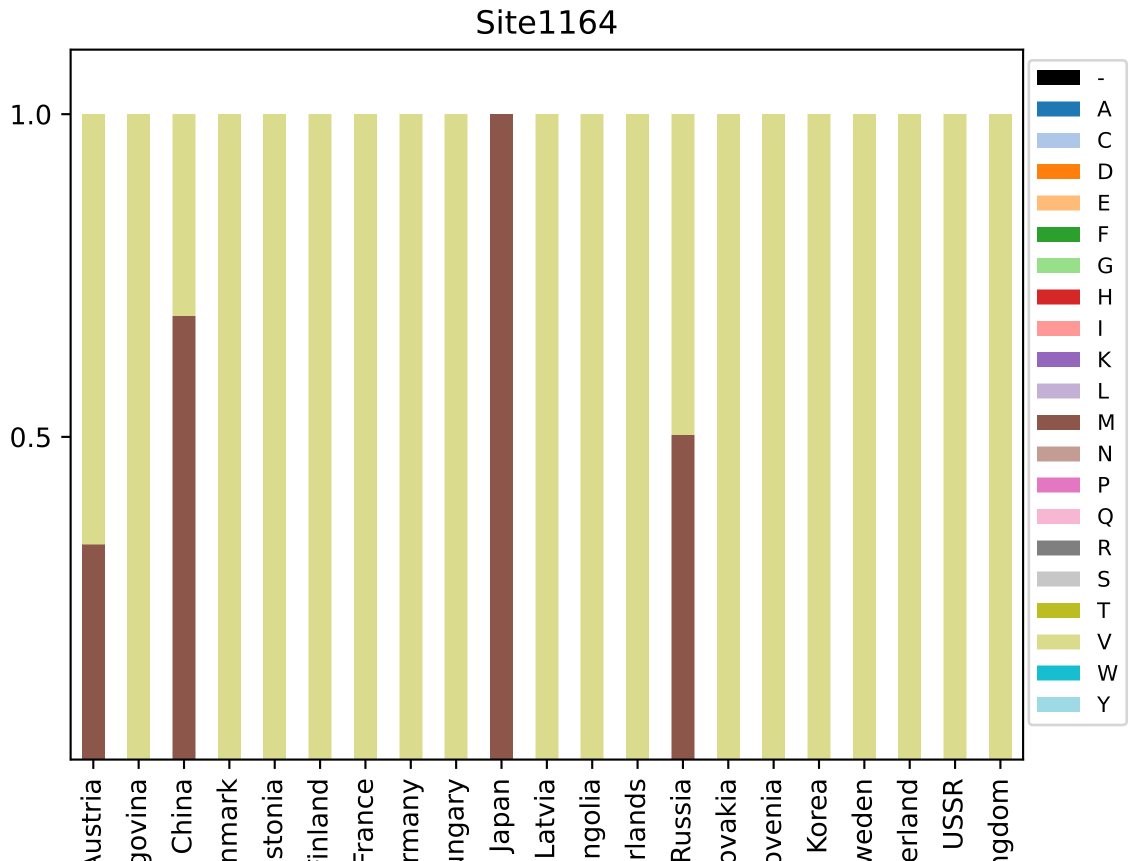

Site1164
1.0
0.5
-
A
C
D
E
F
G
H
I
K
L
M
N
P
Q
R
S
T
V
W
Y
China
Japan
Latvia
Russia
Korea
USSR

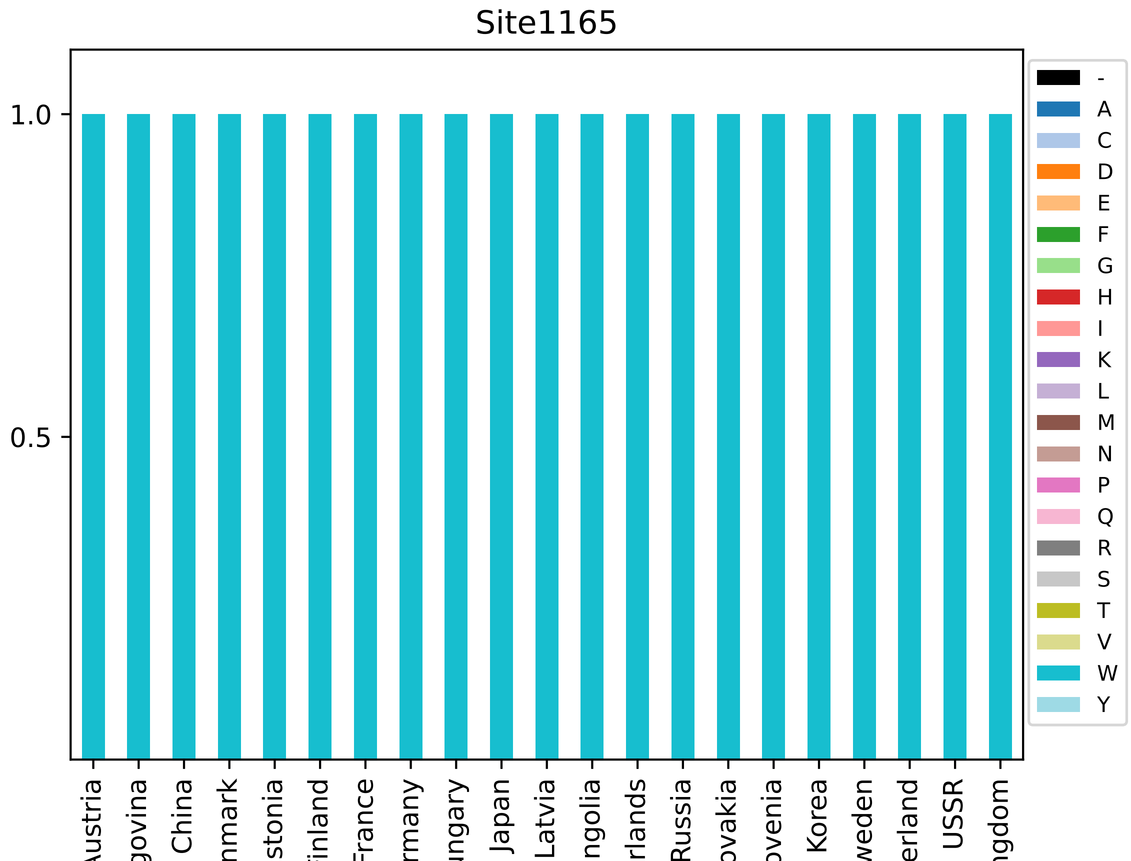
Site1165
1.0
0.5
-
A
C
D
E
F
G
H
I
K
L
M
N
P
Q
R
S
T
V
W
Y
Austria
govina
China
nmark
stonia
inland
France
rmany
ungary
Japan
Latvia
ngolia
rlands
Russia
ovakia
ovenia
Korea
weden
erland
USSR
ngdom

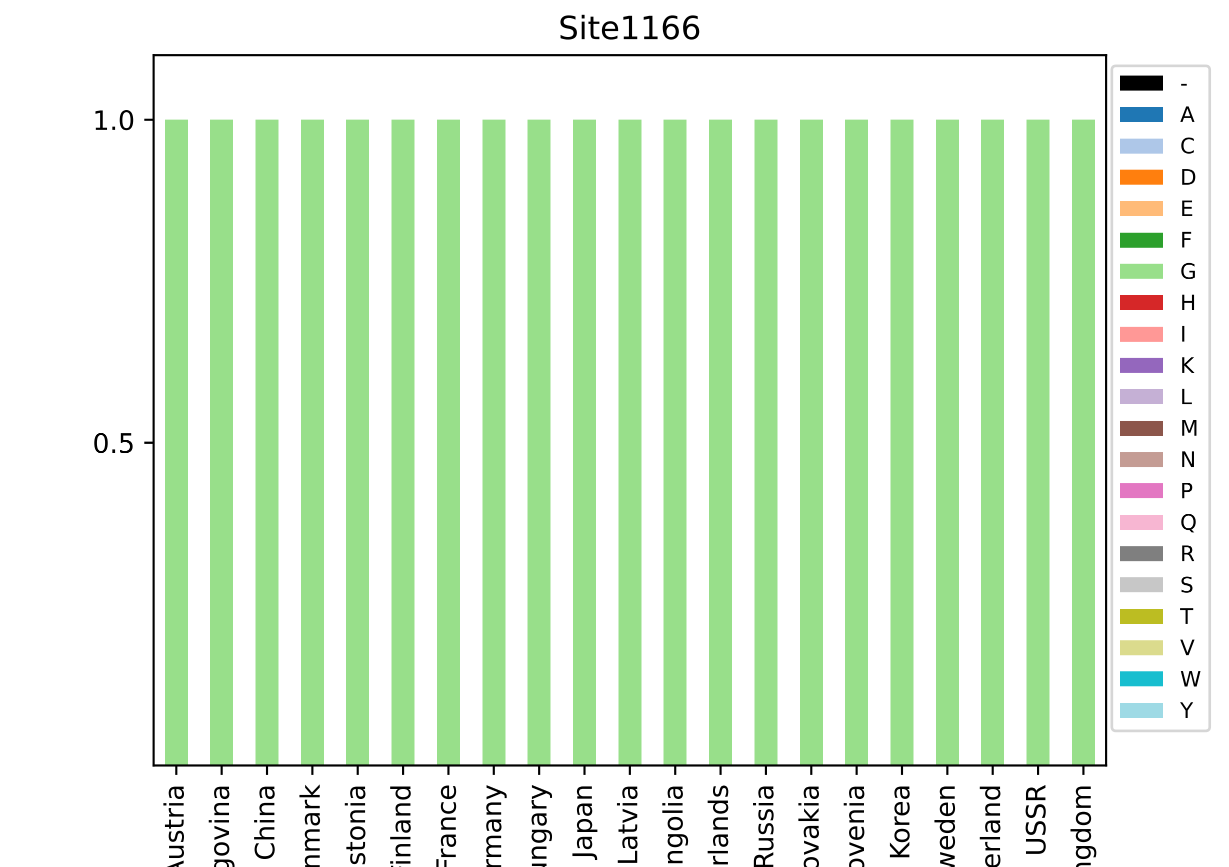

Site1166
1.0
0.5
Austria
govina
China
nmark
stonia
Finland
France
rmany
ungary
Japan
Latvia
ngolia
rlands
Russia
ovakia
ovenia
Korea
weden
erland
USSR
ngdom
-
A
C
D
E
F
G
H
I
K
L
M
N
P
Q
R
S
T
V
W
Y

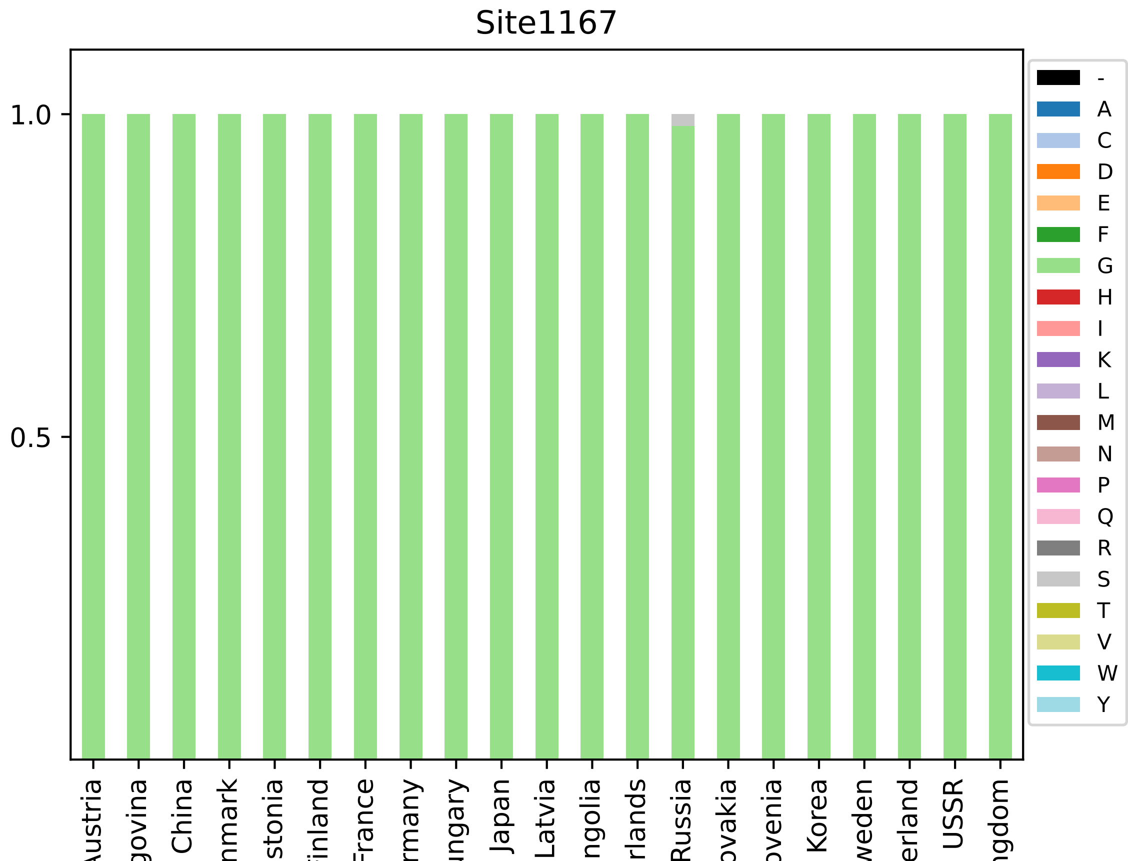
Site1167
1.0
0.5
-
A
C
D
E
F
G
H
I
K
L
M
N
P
Q
R
S
T
V
W
Y
Austria
govina
China
nmark
stonia
Finland
France
rmany
ungary
Japan
Latvia
ngolia
rlands
Russia
ovakia
ovenia
Korea
weden
erland
USSR
ngdom

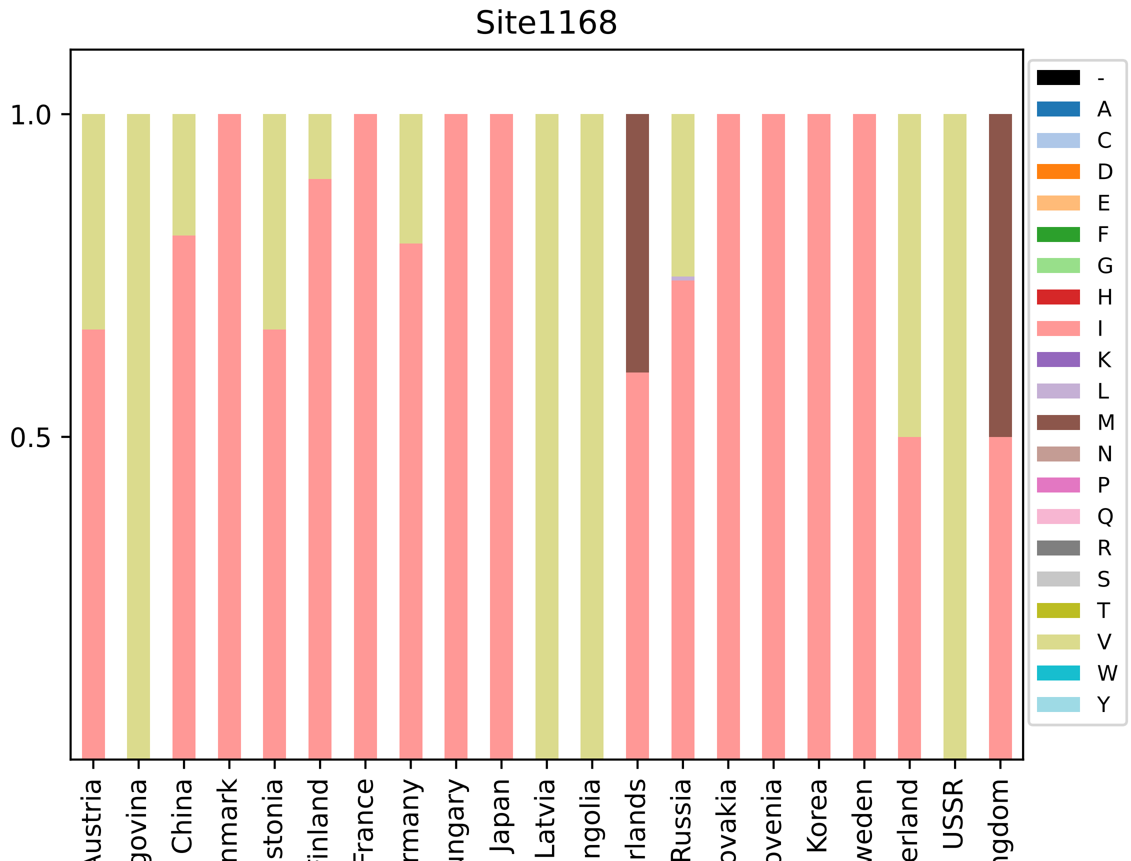
Site1168
1.0
0.5
-
A
C
D
E
F
G
H
I
K
L
M
N
P
Q
R
S
T
V
W
Y
Austria
govina
China
nmark
stonia
Finland
France
rmany
ungary
Japan
Latvia
ngolia
rlands
Russia
ovakia
ovenia
Korea
weden
erland
USSR
ngdom

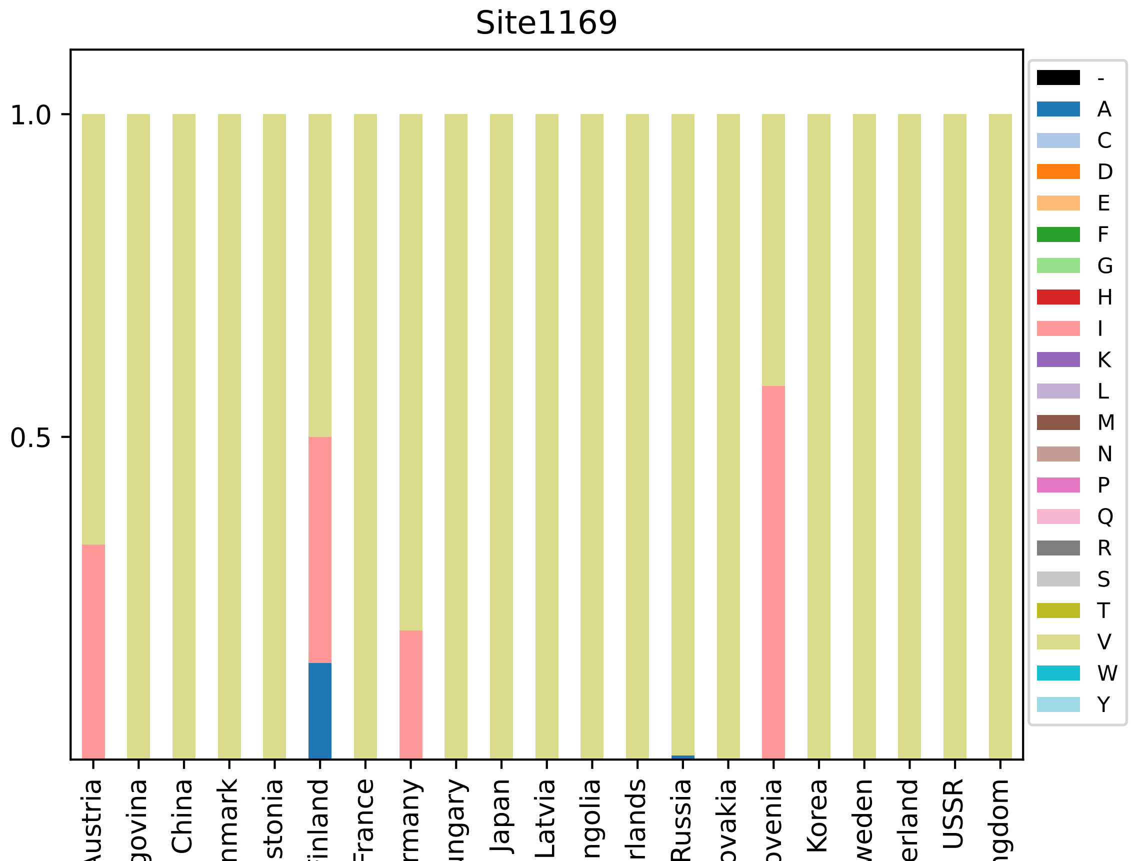

Site1169
1.0
0.5
Austria
govina
China
nmark
stonia
inland
rance
rmany
ungary
Japan
Latvia
ngolia
rlands
Russia
ovakia
ovenia
Korea
weden
erland
USSR
ngdom
-
A
C
D
E
F
G
H
I
K
L
M
N
P
Q
R
S
T
V
W
Y

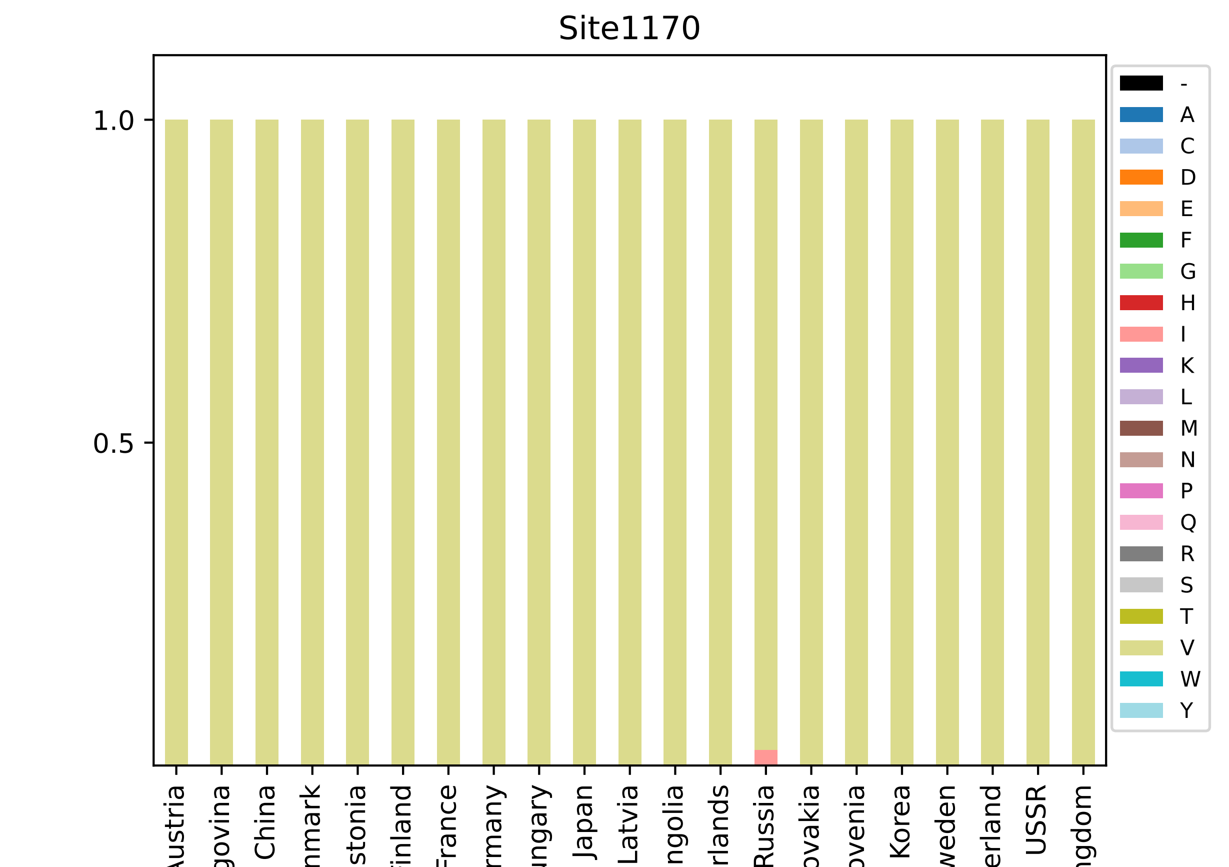
Site1170
1.0
0.5
-
A
C
D
E
F
G
H
I
K
L
M
N
P
Q
R
S
T
V
W
Y
Austria
govina
China
nmark
stonia
inland
France
rmany
ungary
Japan
Latvia
ngolia
rlands
Russia
ovakia
ovenia
Korea
weden
erland
USSR
ngdom

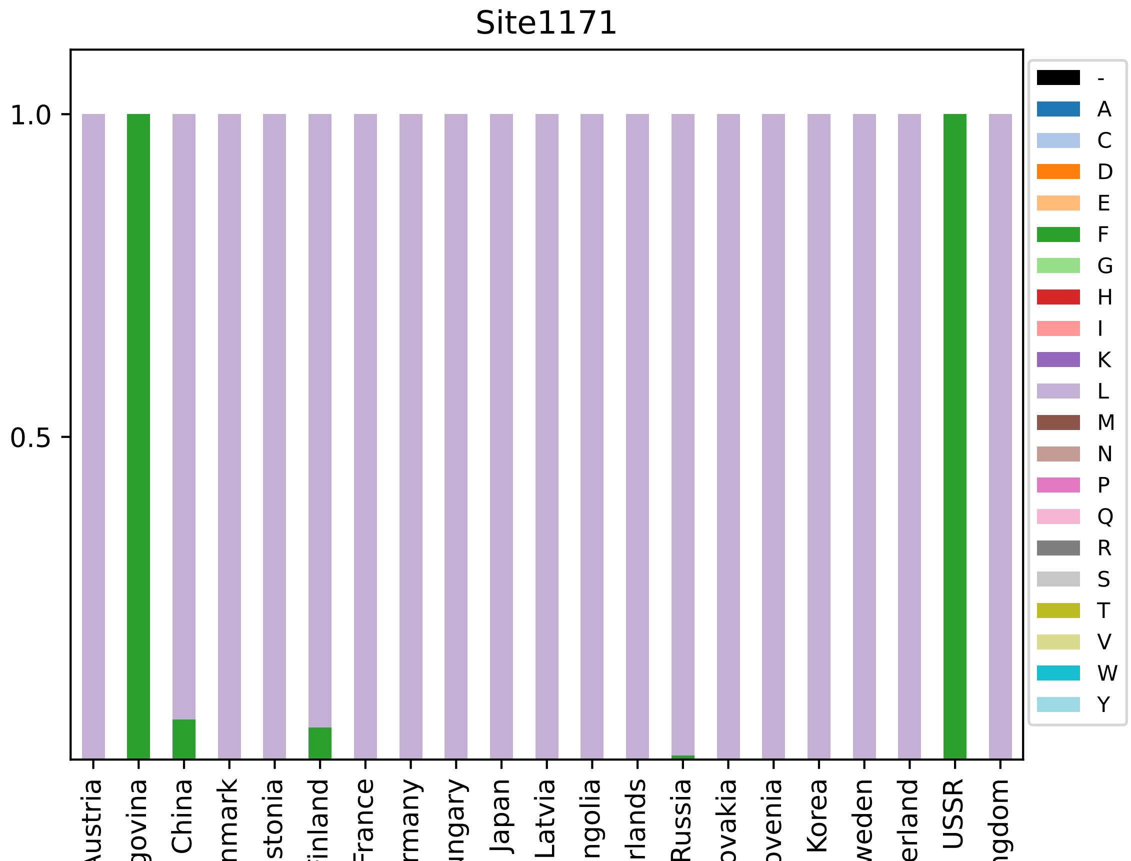
Site1171
1.0
0.5
-
A
C
D
E
F
G
H
I
K
L
M
N
P
Q
R
S
T
V
W
Y
China
Japan
Latvia
Russia
Korea
USSR

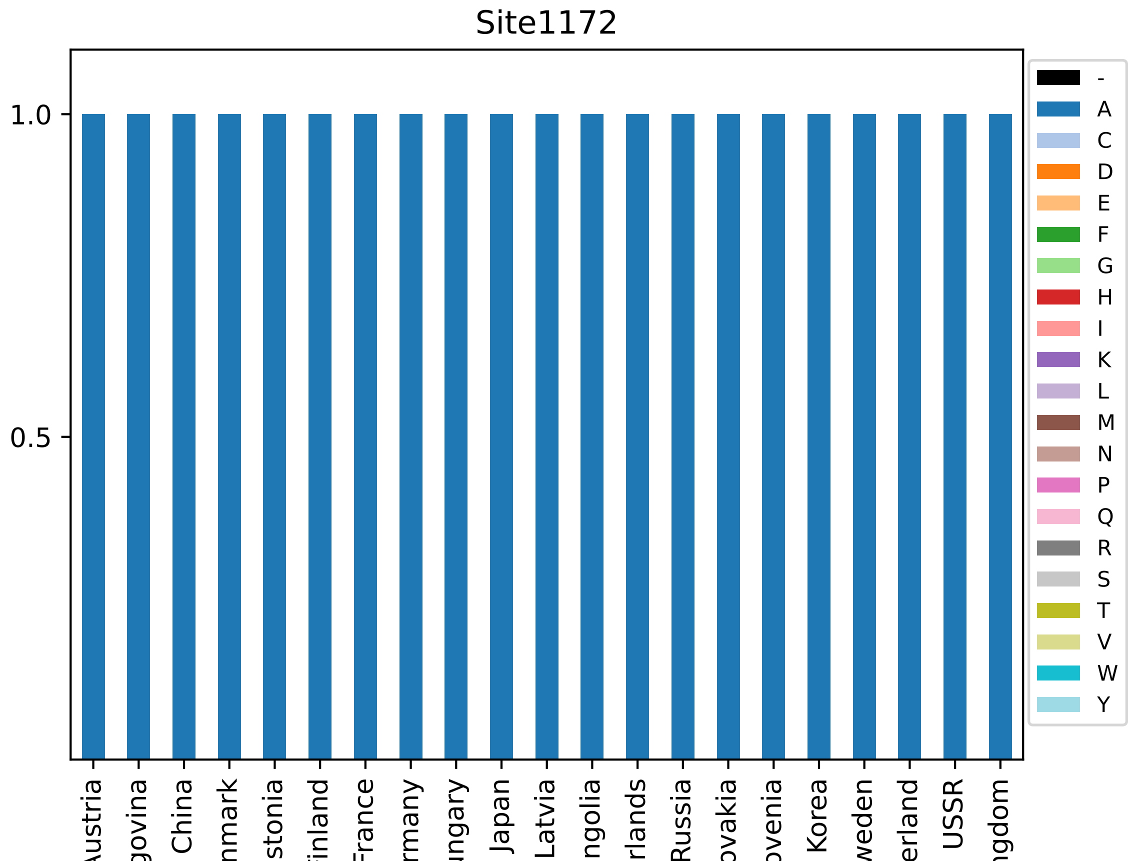

Site1172
1.0
0.5
-
A
C
D
E
F
G
H
I
K
L
M
N
P
Q
R
S
T
V
W
Y
Austria
govina
China
nmark
stonia
Finland
France
rmany
ungary
Japan
Latvia
ngolia
rlands
Russia
ovakia
ovenia
Korea
weden
erland
USSR
ngdom

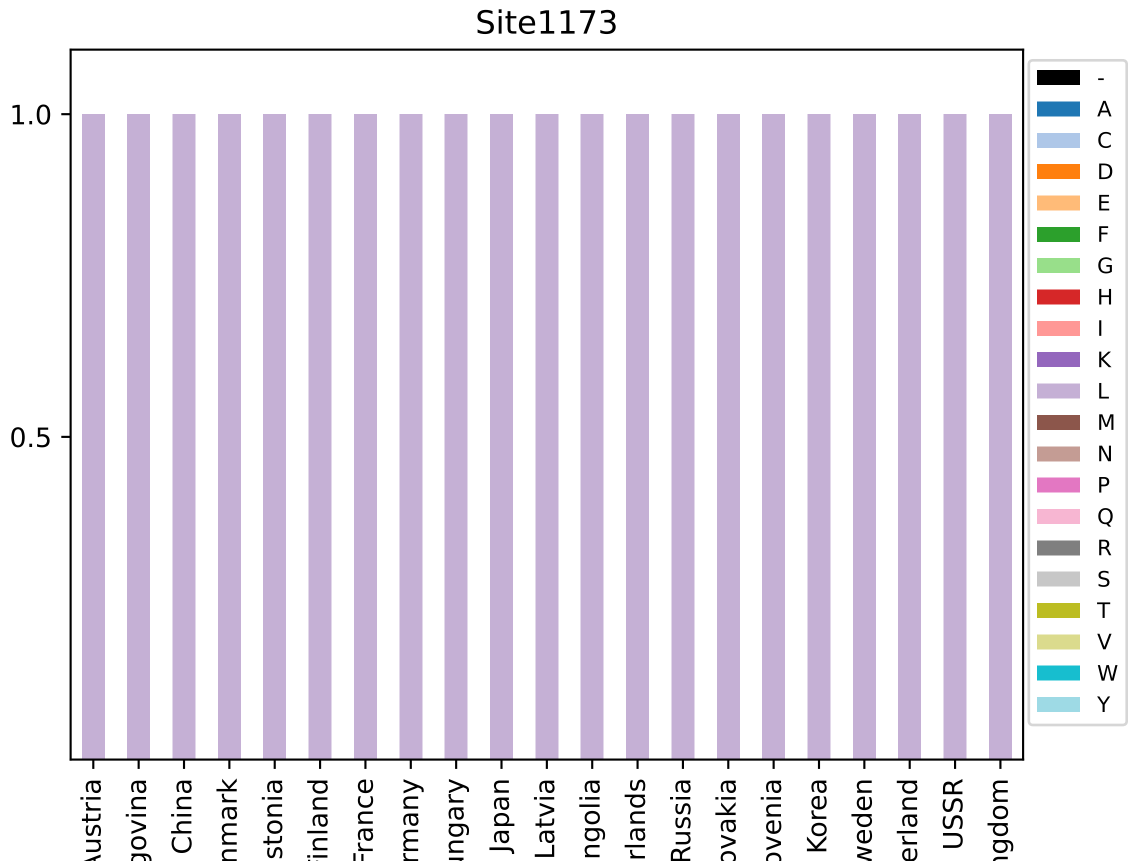

Site1173
1.0
0.5
-
A
C
D
E
F
G
H
I
K
L
M
N
P
Q
R
S
T
V
W
Y
Austria
govina
China
nmark
stonia
Finland
France
rmany
ungary
Japan
Latvia
ngolia
rlands
Russia
ovakia
ovenia
Korea
weden
erland
USSR
ngdom

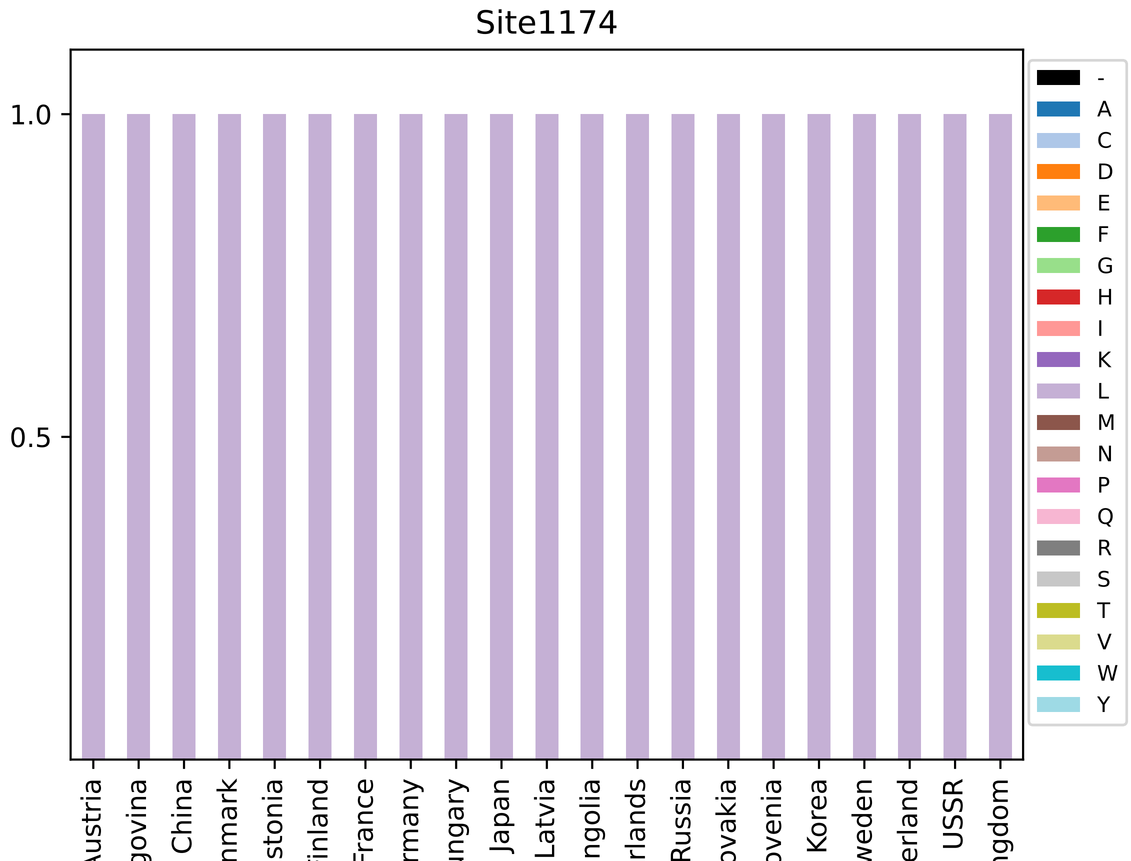

Site1174
1.0
0.5
-
A
C
D
E
F
G
H
I
K
L
M
N
P
Q
R
S
T
V
W
Y
China
Japan
Latvia
Russia
Korea
USSR

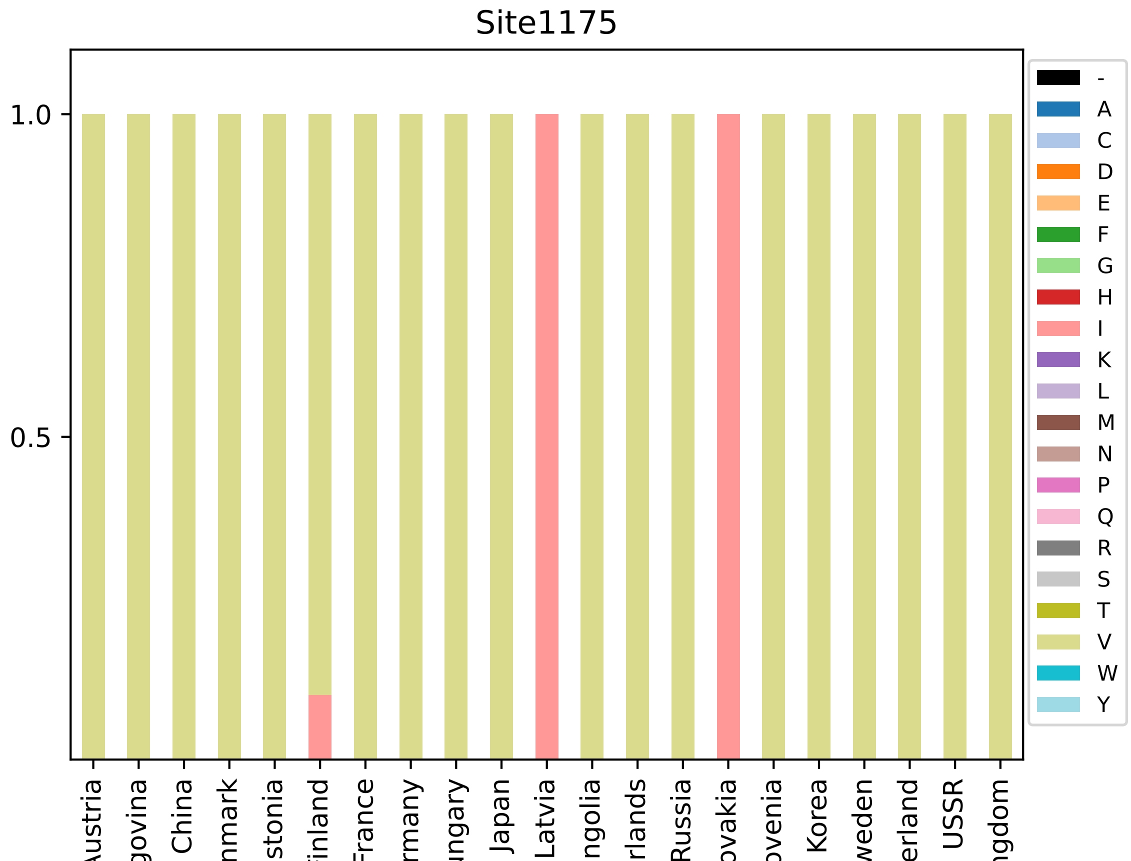
Site1175
1.0
0.5
-
A
C
D
E
F
G
H
I
K
L
M
N
P
Q
R
S
T
V
W
Y
Austria
govina
China
nmark
stonia
Finland
France
rmany
ungary
Japan
Latvia
ngolia
rlands
Russia
ovakia
ovenia
Korea
weden
erland
USSR
ngdom

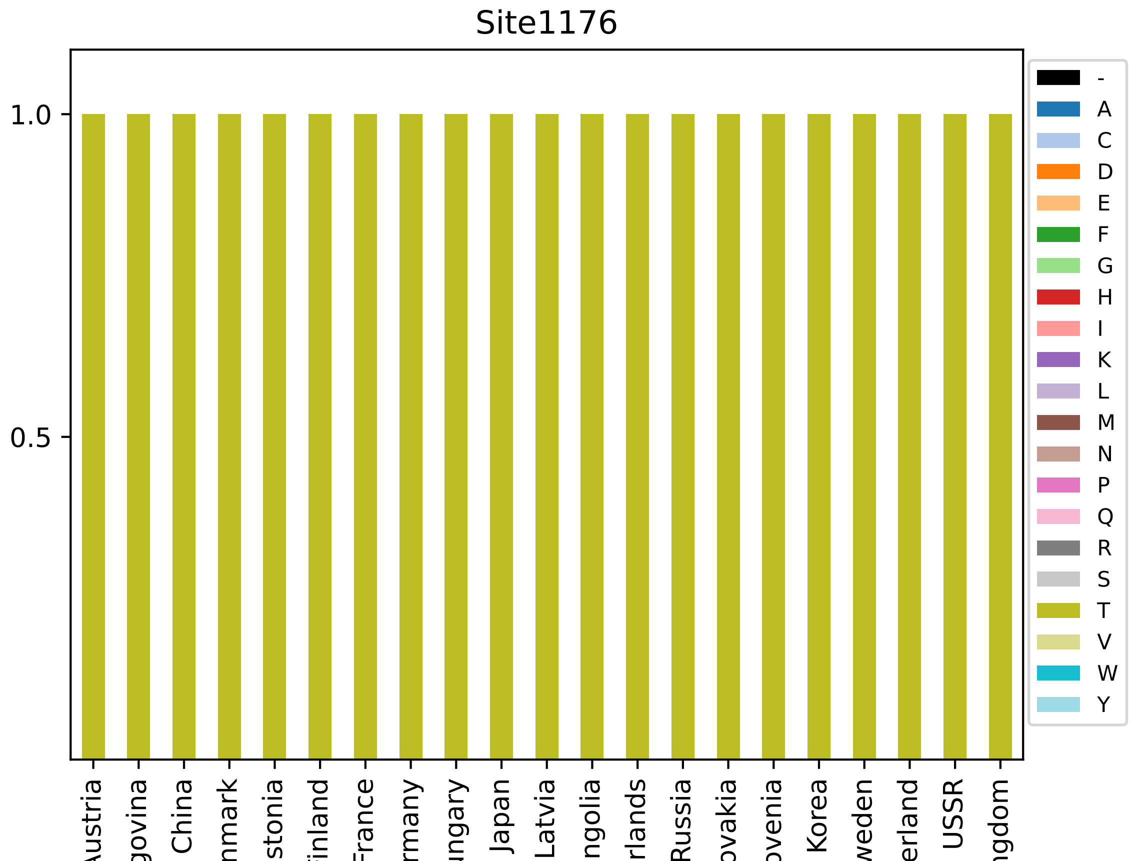
Site1176
1.0
0.5
-
A
C
D
E
F
G
H
I
K
L
M
N
P
Q
R
S
T
V
W
Y
Austria
govina
China
nmark
stonia
Finland
France
rmany
ungary
Japan
Latvia
ngolia
rlands
Russia
ovakia
ovenia
Korea
weden
erland
USSR
ngdom

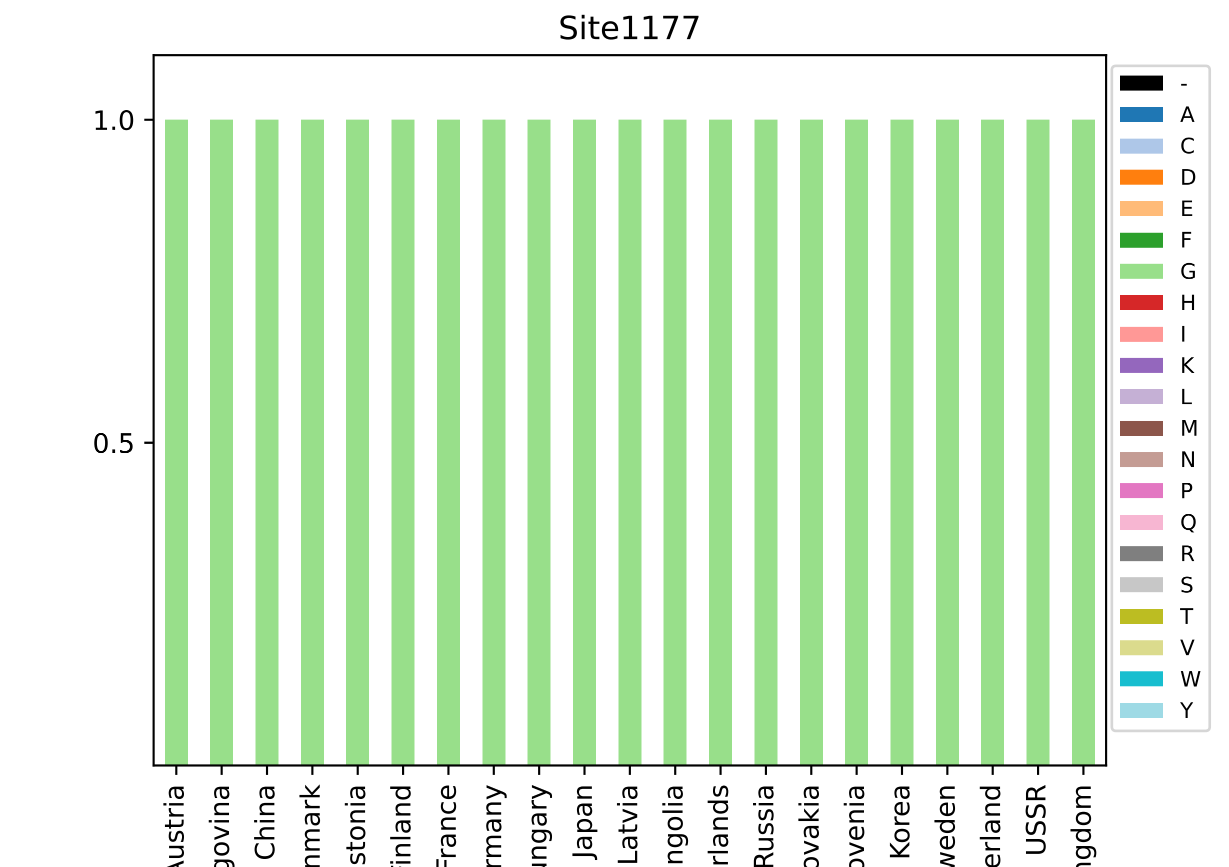
Site1177
1.0
0.5
-
A
C
D
E
F
G
H
I
K
L
M
N
P
Q
R
S
T
V
W
Y
Austria
govina
China
nmark
stonia
Finland
France
rmany
ungary
Japan
Latvia
ngolia
rlands
Russia
ovakia
ovenia
Korea
weden
erland
USSR
ngdom

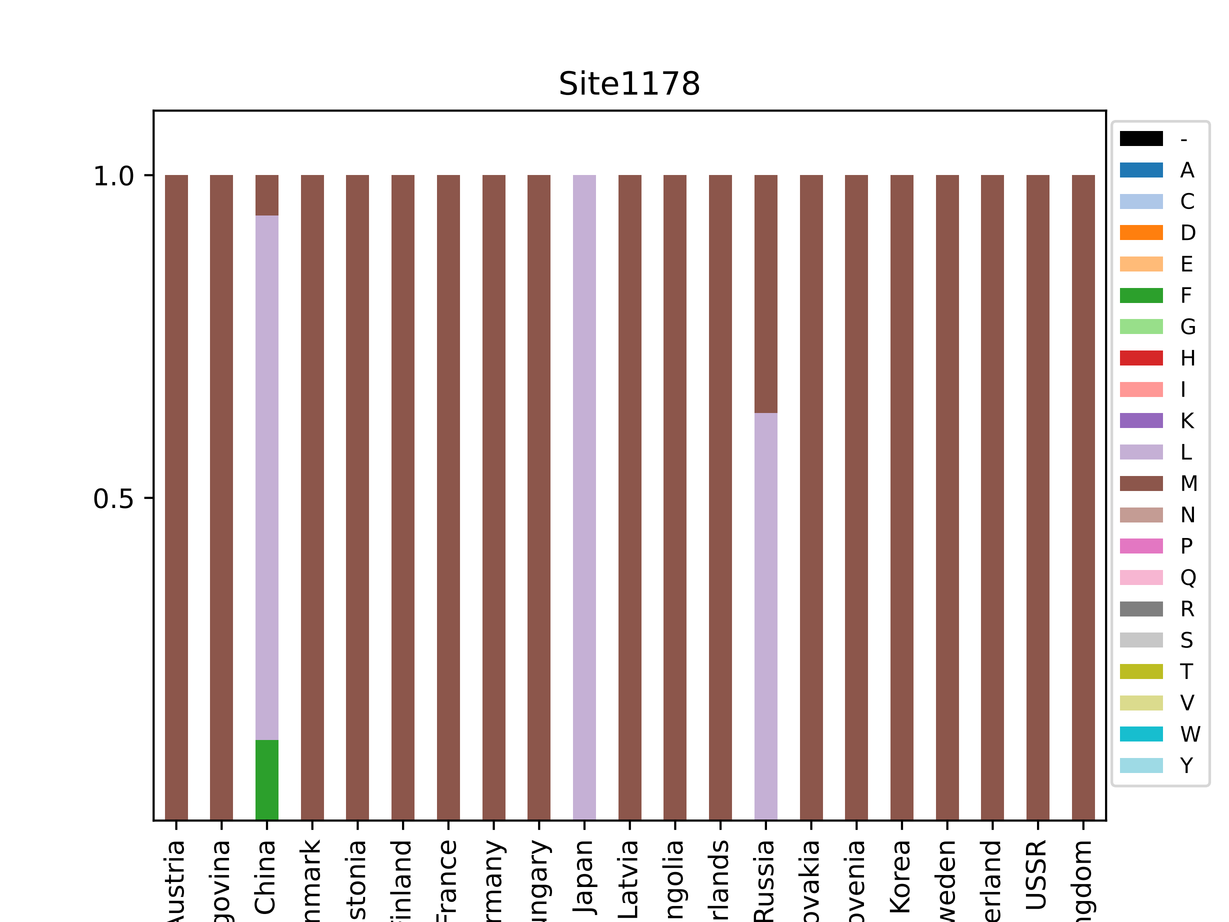

Site1178
1.0
0.5
-
A
C
D
E
F
G
H
I
K
L
M
N
P
Q
R
S
T
V
W
Y
Austria
govina
China
nmark
stonia
inland
France
rmany
ungary
Japan
Latvia
ngolia
rlands
Russia
ovakia
ovenia
Korea
weden
erland
USSR
ngdom

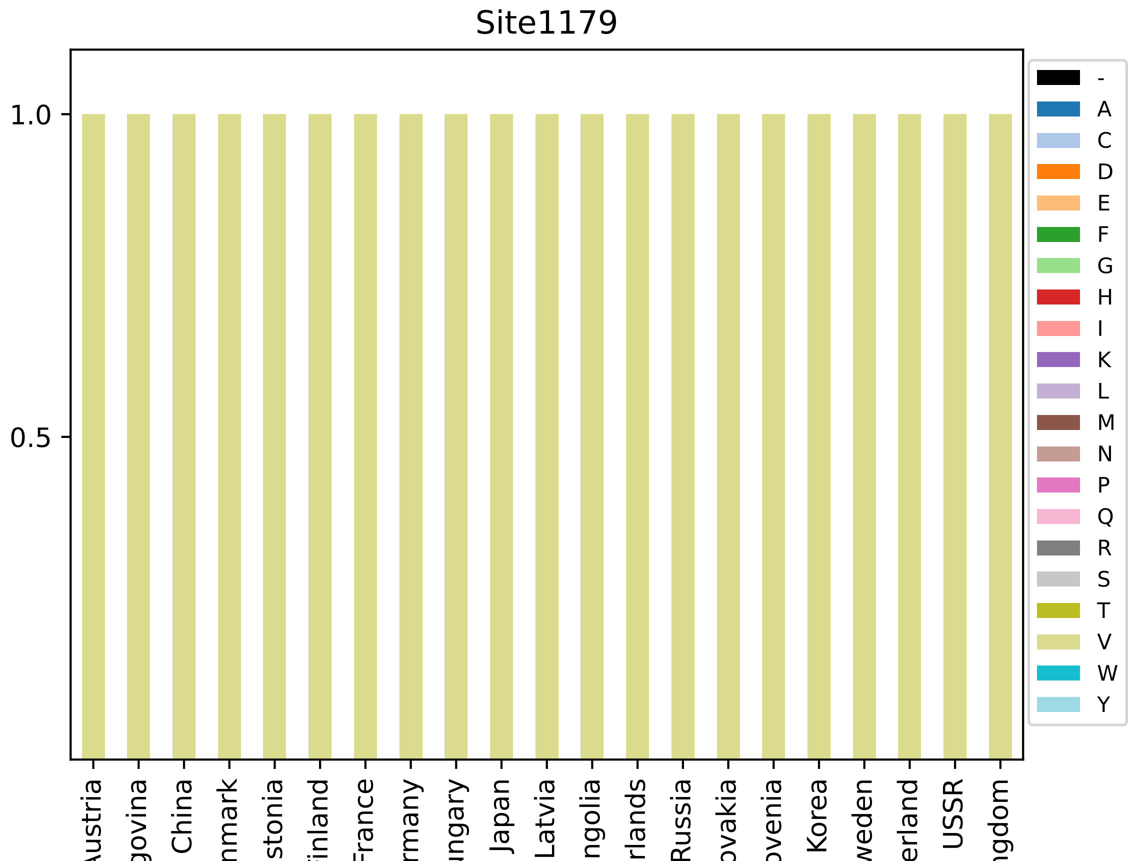
Site1179
1.0
0.5
-
A
C
D
E
F
G
H
I
K
L
M
N
P
Q
R
S
T
V
W
Y
China
Japan
Latvia
Russia
Korea
USSR

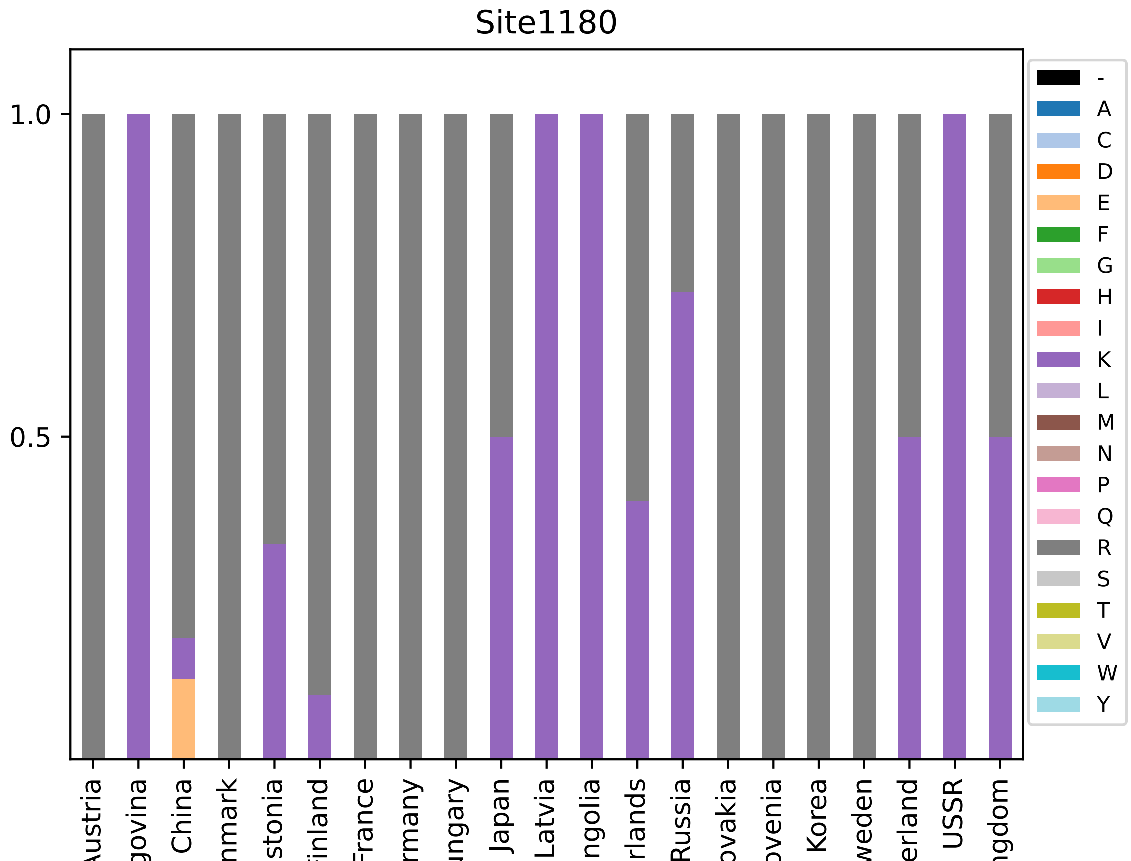

Site1180
1.0
0.5
-
A
C
D
E
F
G
H
I
K
L
M
N
P
Q
R
S
T
V
W
Y
China
Japan
Latvia
Russia
Korea
USSR

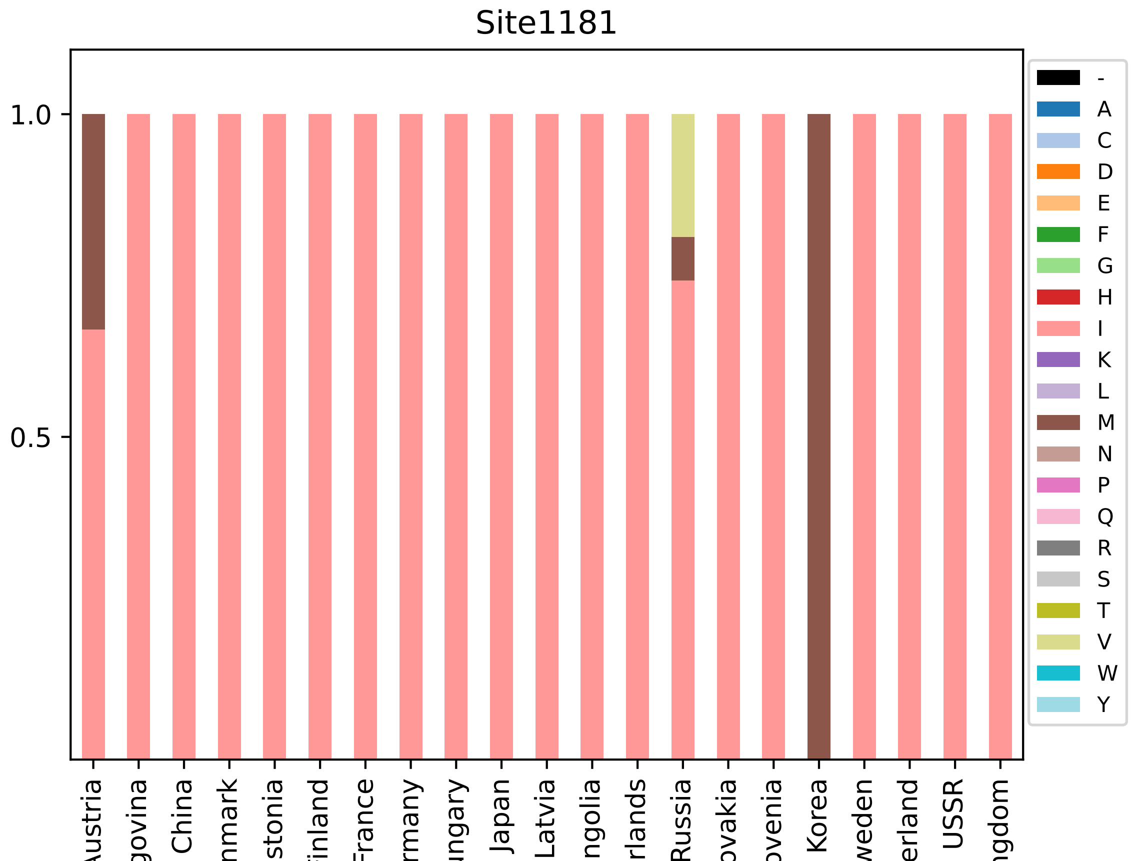
Site1181
1.0
0.5
-
A
C
D
E
F
G
H
I
K
L
M
N
P
Q
R
S
T
V
W
Y
Austria
govina
China
nmark
stonia
Finland
France
rmany
ungary
Japan
Latvia
ngolia
rlands
Russia
ovakia
ovenia
Korea
weden
erland
USSR
ngdom

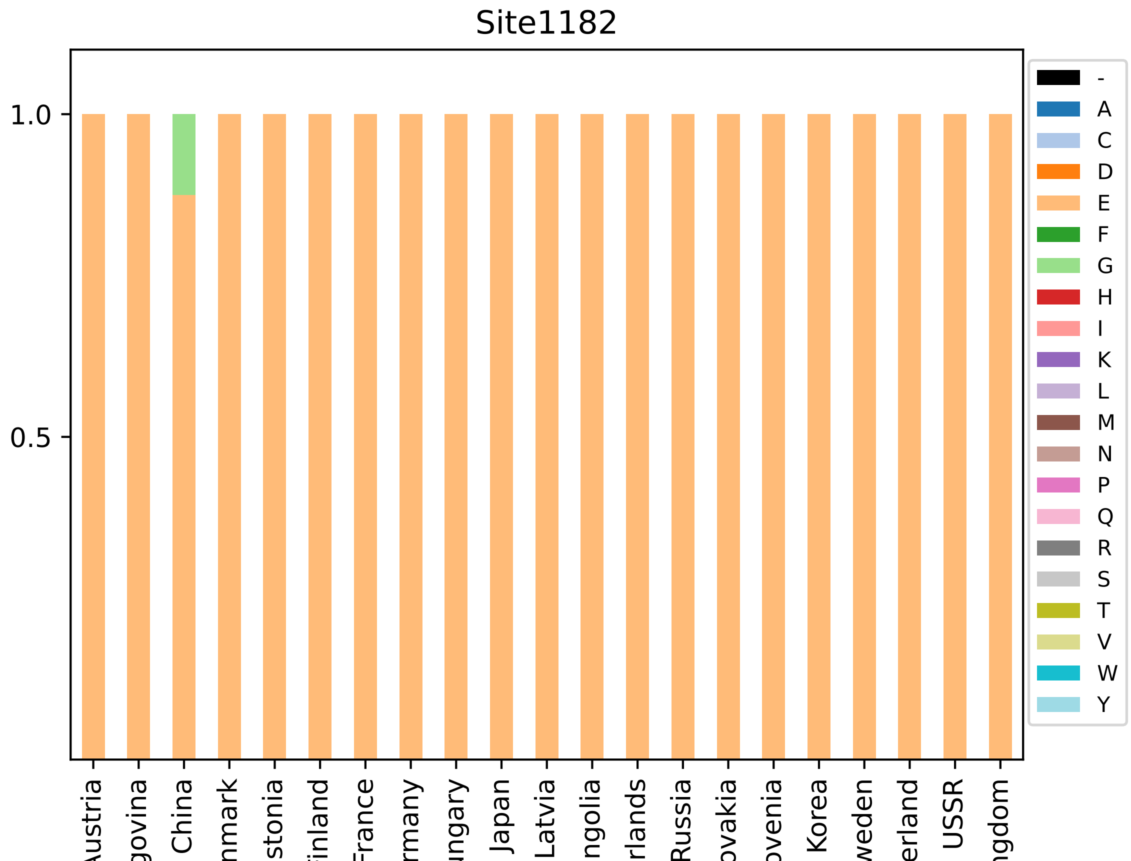
Site1182
1.0
0.5
China
Japan
Latvia
Korea
USSR
-
A
C
D
E
F
G
H
I
K
L
M
N
P
Q
R
S
T
V
W
Y

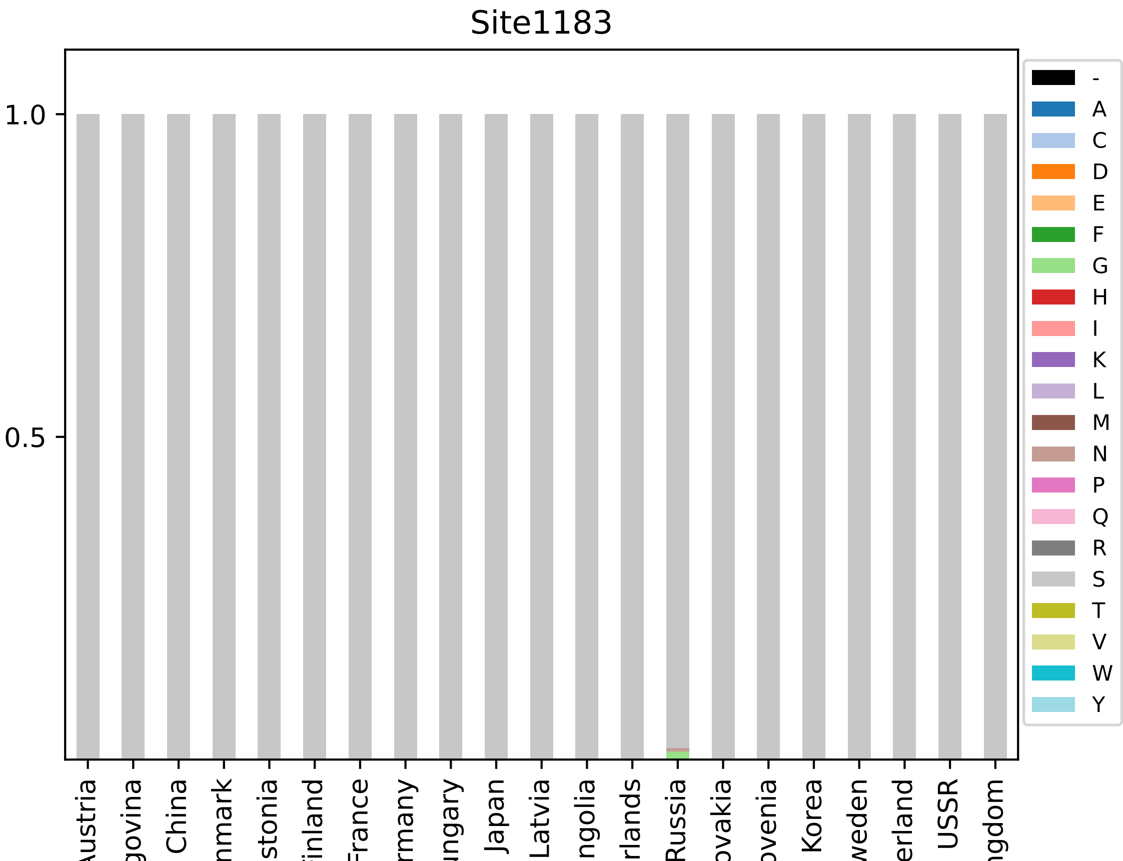
Site1183
1.0
0.5
China
Japan
Latvia
Russia
Korea
USSR
-
A
C
D
E
F
G
H
I
K
L
M
N
P
Q
R
S
T
V
W
Y

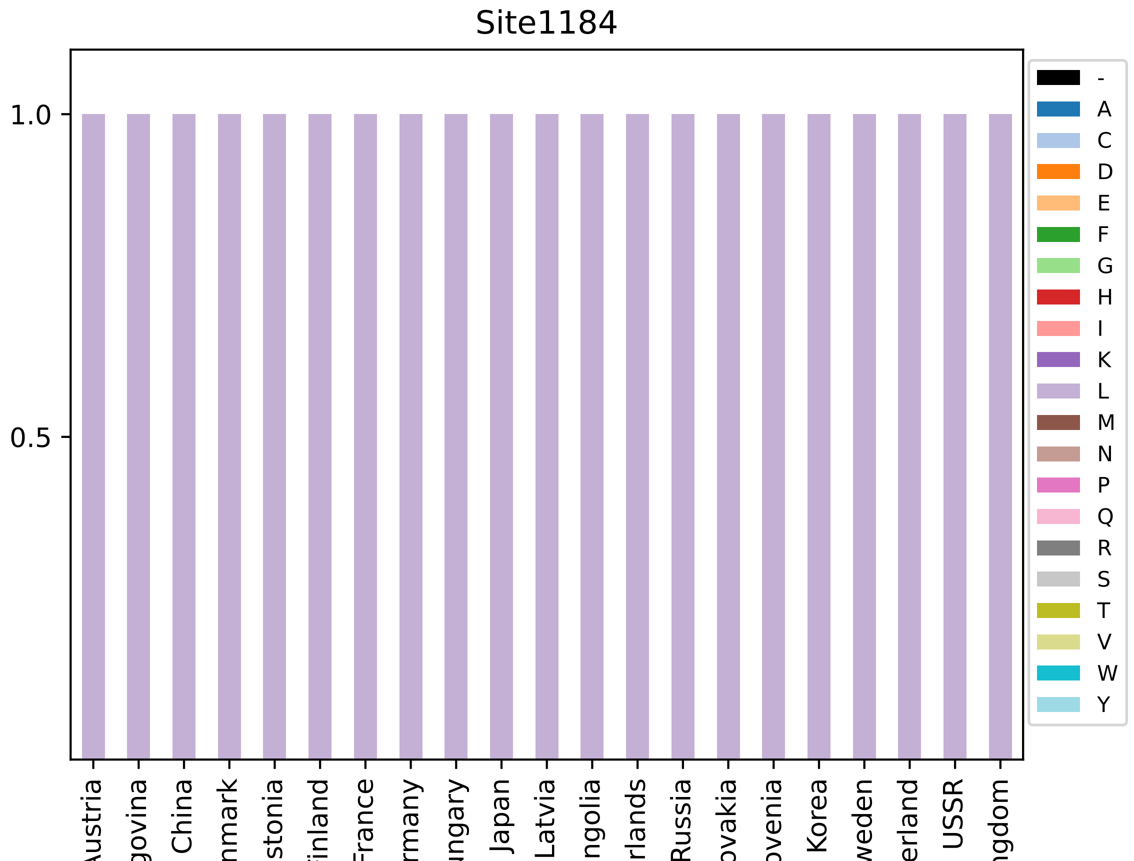

Site1184
1.0
0.5
-
A
C
D
E
F
G
H
I
K
L
M
N
P
Q
R
S
T
V
W
Y
China
Japan
Latvia
Russia
Korea
USSR

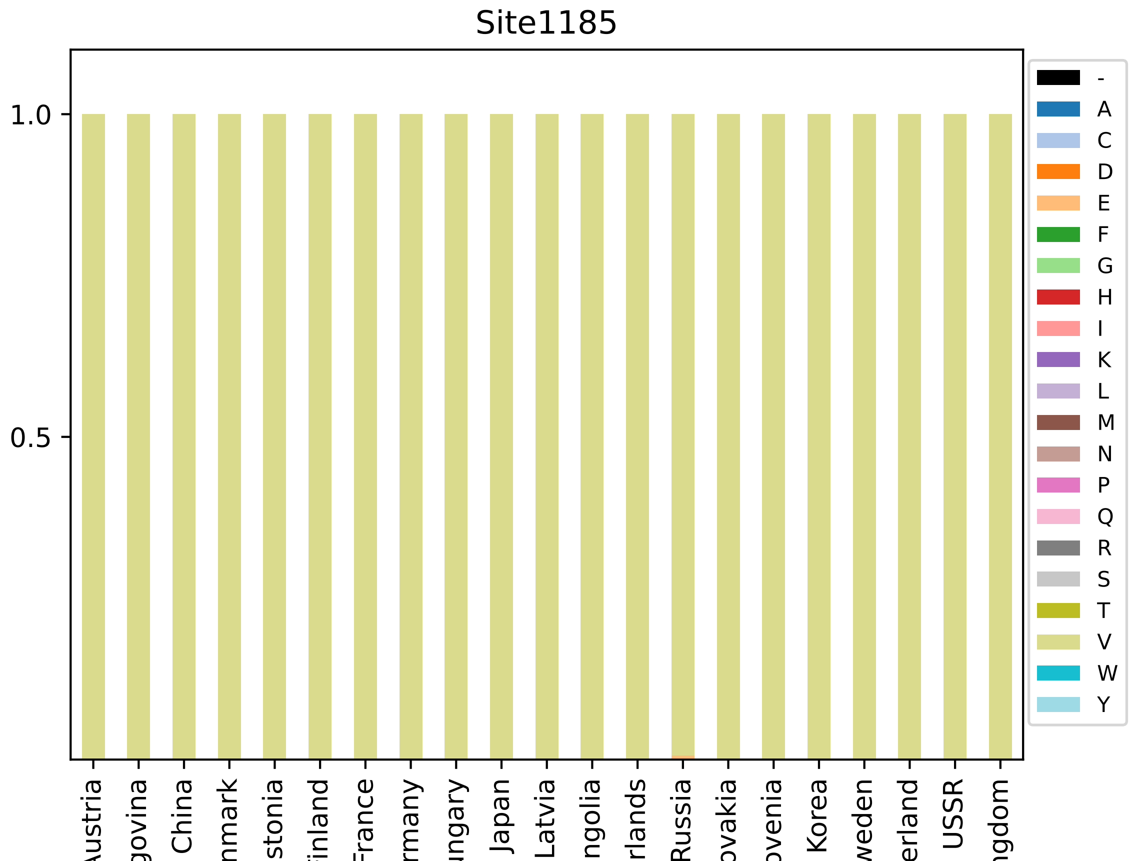
Site1185
1.0
0.5
-
A
C
D
E
F
G
H
I
K
L
M
N
P
Q
R
S
T
V
W
Y
China
Japan
Latvia
Russia
Korea
USSR

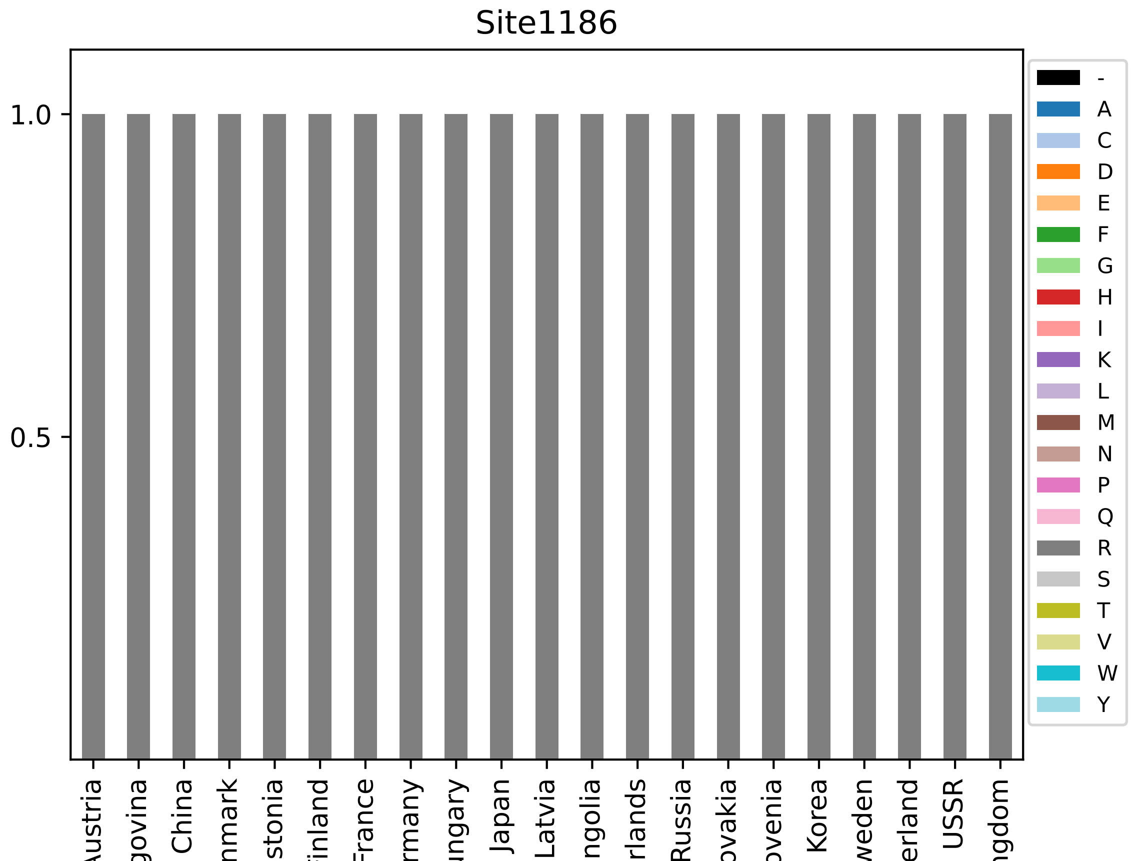

Site1186
1.0
0.5
-
A
C
D
E
F
G
H
I
K
L
M
N
P
Q
R
S
T
V
W
Y
Austria
China
Japan
Latvia
Russia
Korea
USSR

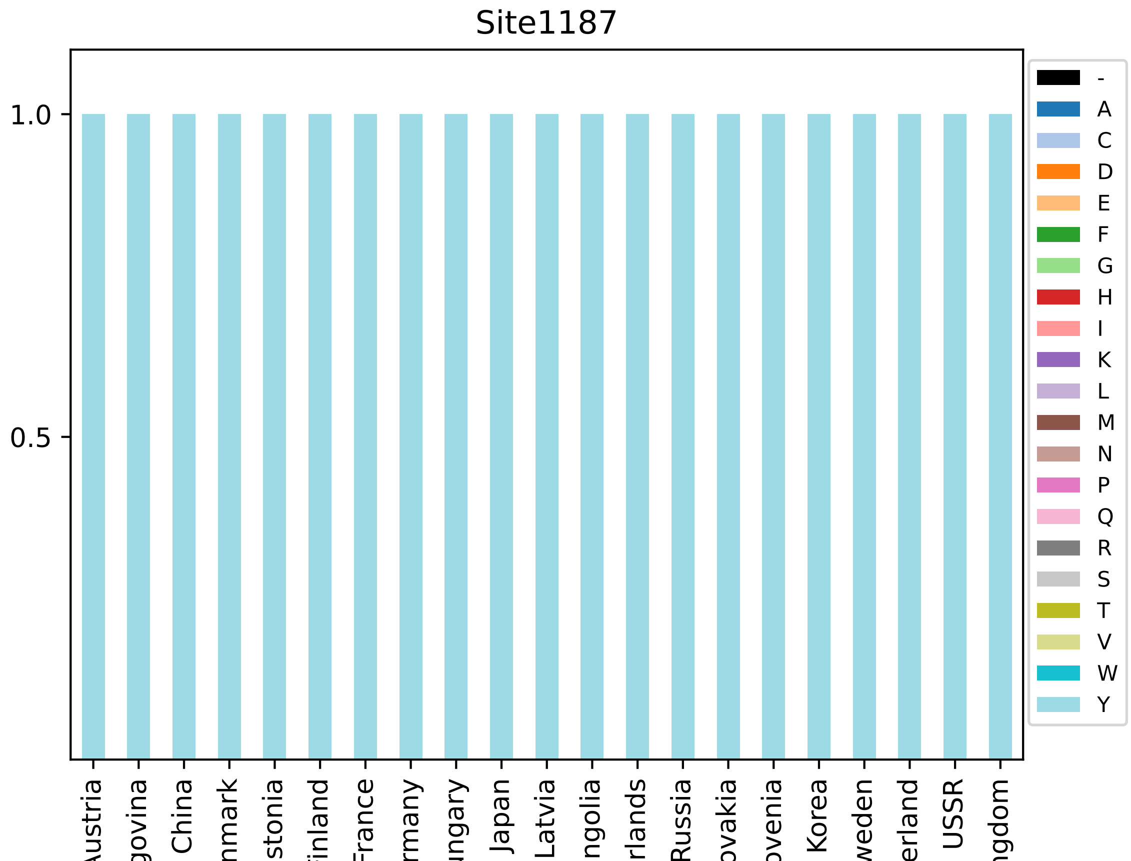
Site1187
1.0
0.5
-
A
C
D
E
F
G
H
I
K
L
M
N
P
Q
R
S
T
V
W
Y
China
Japan
Latvia
Russia
Korea
USSR

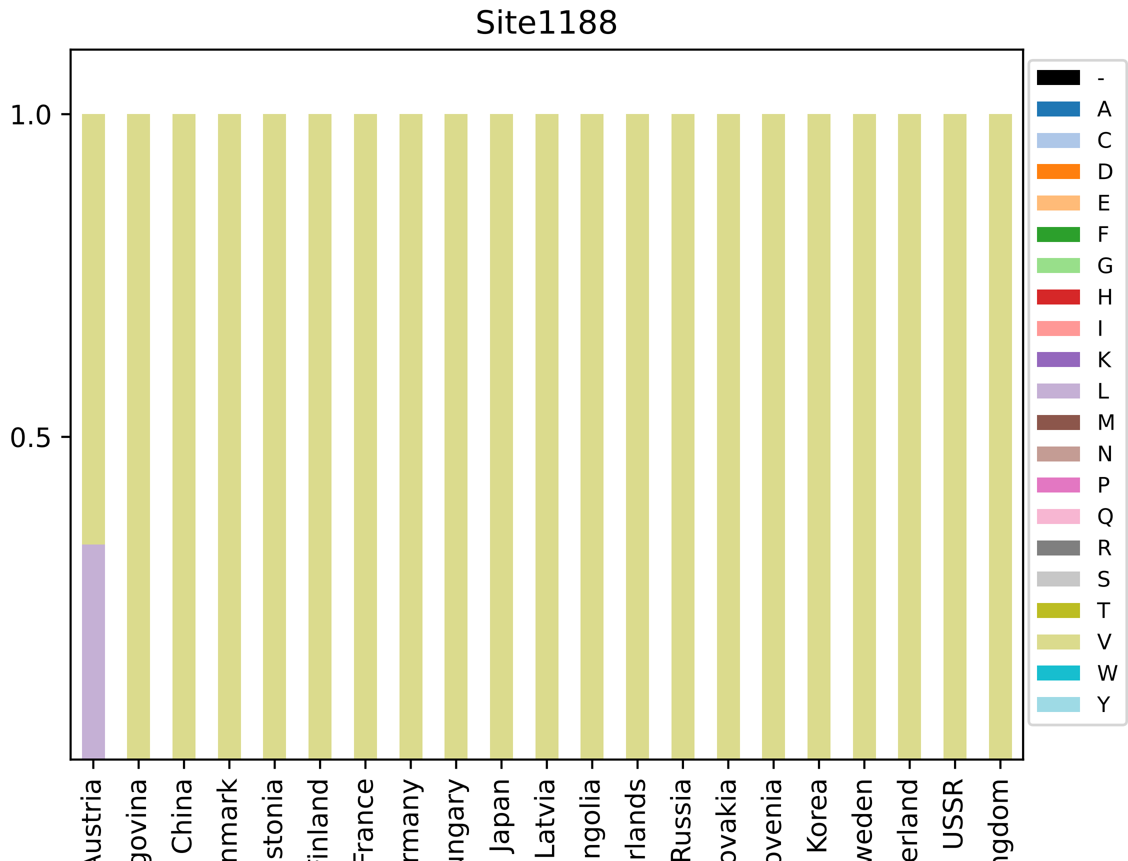

Site1188
1.0
0.5
-
A
C
D
E
F
G
H
I
K
L
M
N
P
Q
R
S
T
V
W
Y
Austria
govina
China
nmark
stonia
inland
France
rmany
ungary
Japan
Latvia
ngolia
rlands
Russia
ovakia
ovenia
Korea
weden
erland
USSR
ngdom

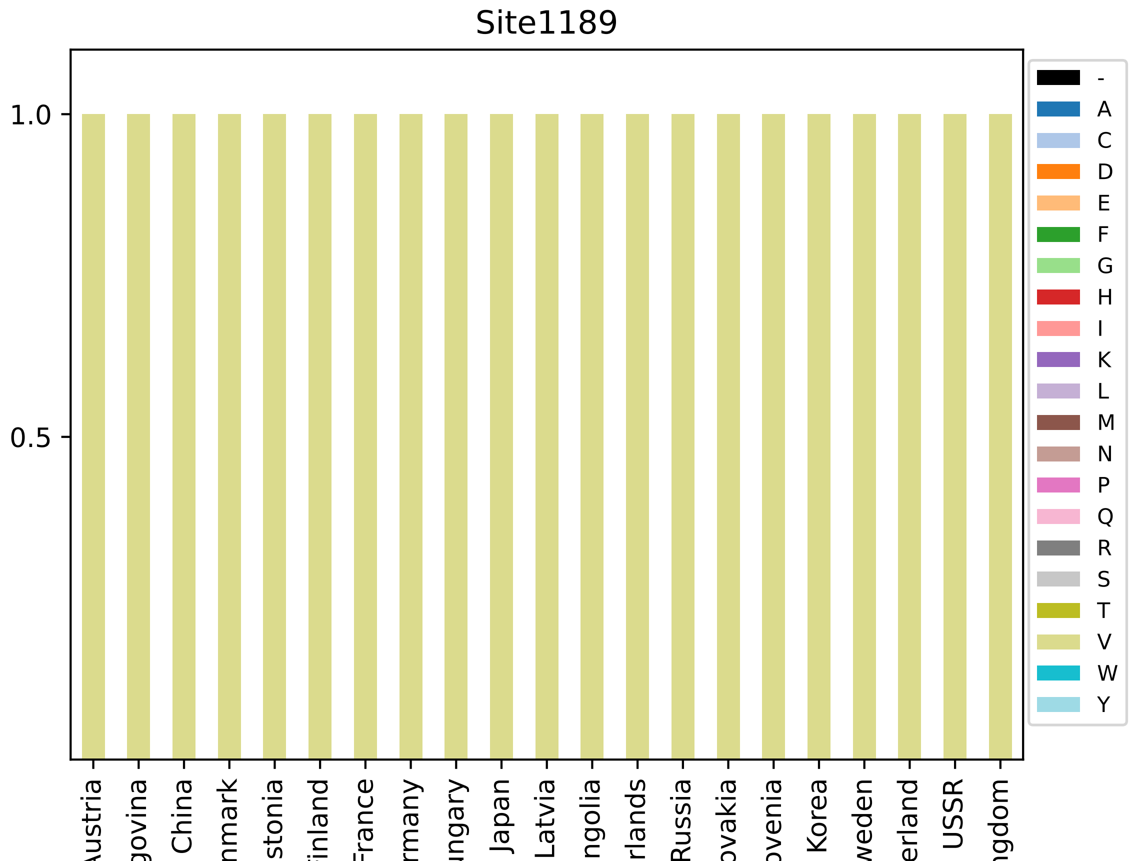

Site1189
1.0
0.5
-
A
C
D
E
F
G
H
I
K
L
M
N
P
Q
R
S
T
V
W
Y
China
Japan
Latvia
Russia
Korea
USSR

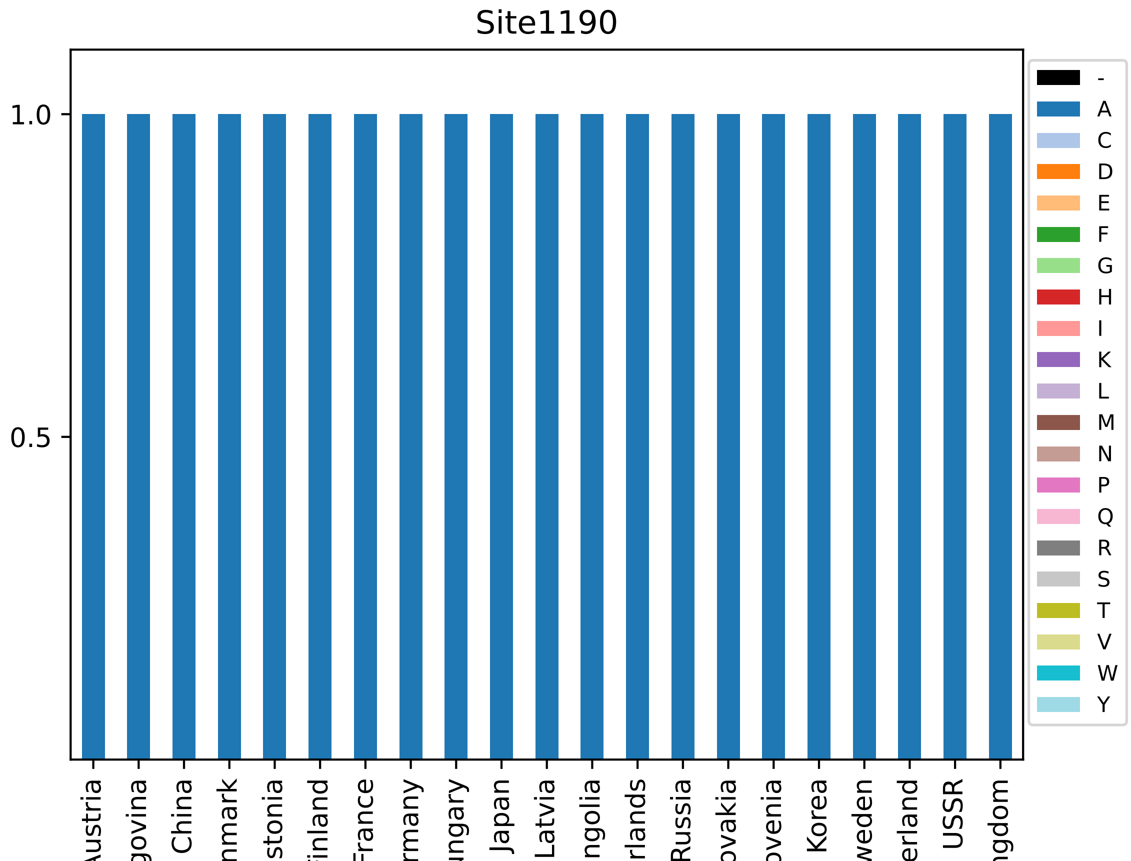

Site1190
1.0
0.5
China
Japan
Latvia
Russia
Korea
USSR
-
A
C
D
E
F
G
H
I
K
L
M
N
P
Q
R
S
T
V
W
Y

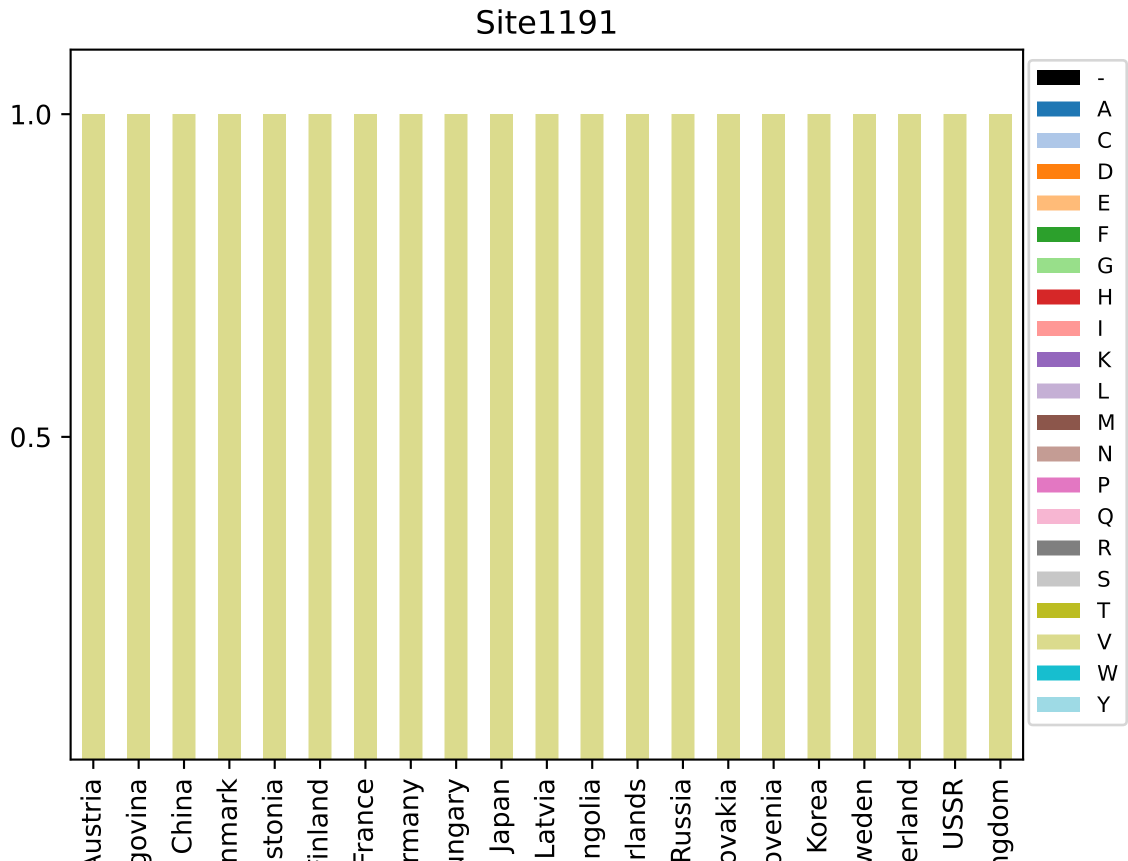

Site1191
1.0
0.5
-
A
C
D
E
F
G
H
I
K
L
M
N
P
Q
R
S
T
V
W
Y
China
Japan
Latvia
Russia
Korea
USSR

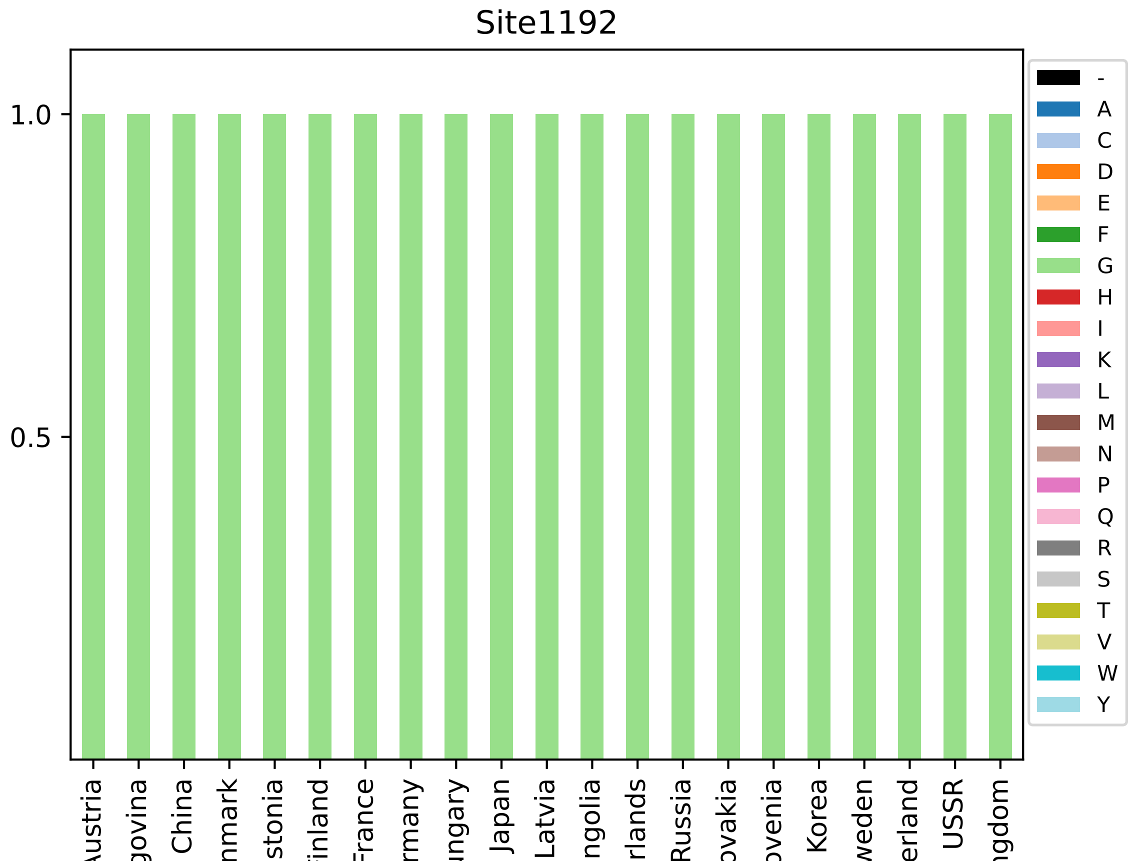

Site1192
1.0
0.5
-
A
C
D
E
F
G
H
I
K
L
M
N
P
Q
R
S
T
V
W
Y
Austria
China
Japan
Latvia
Russia
Korea
USSR

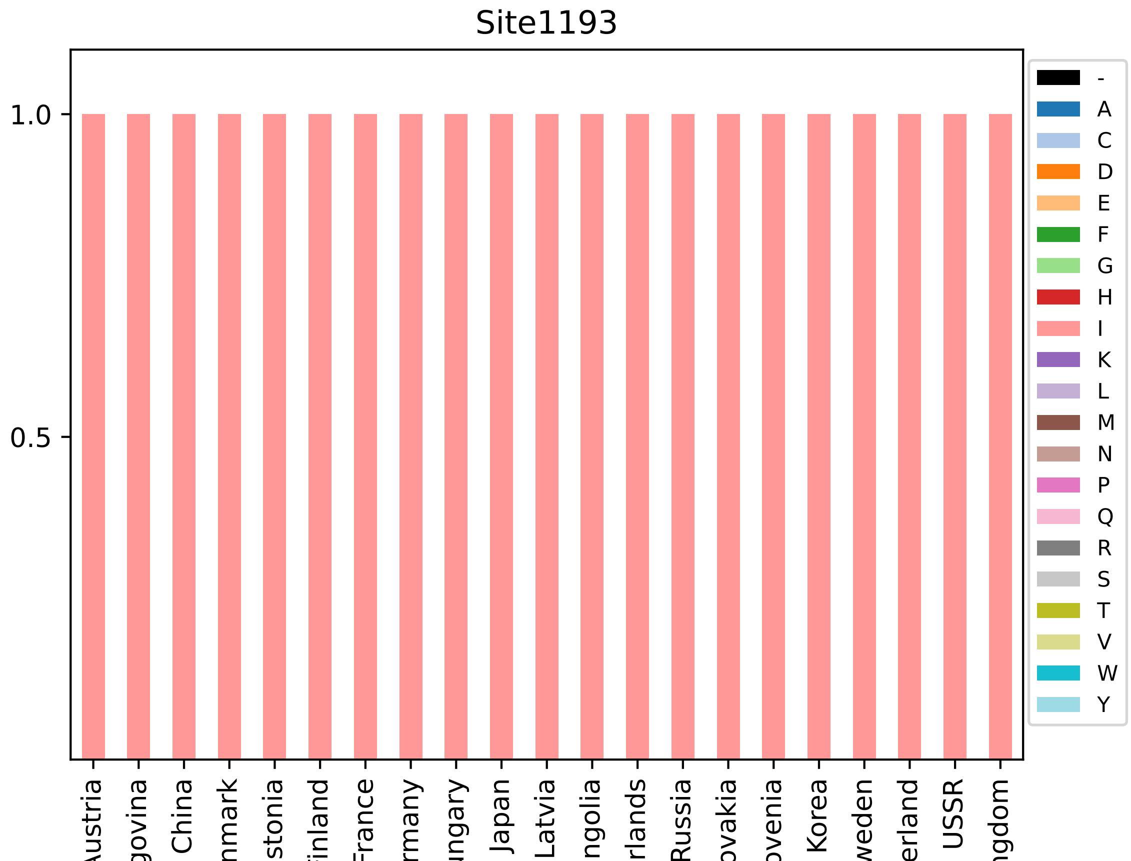

Site1193
1.0
0.5
-
A
C
D
E
F
G
H
I
K
L
M
N
P
Q
R
S
T
V
W
Y
China
Japan
Latvia
Russia
Korea
USSR

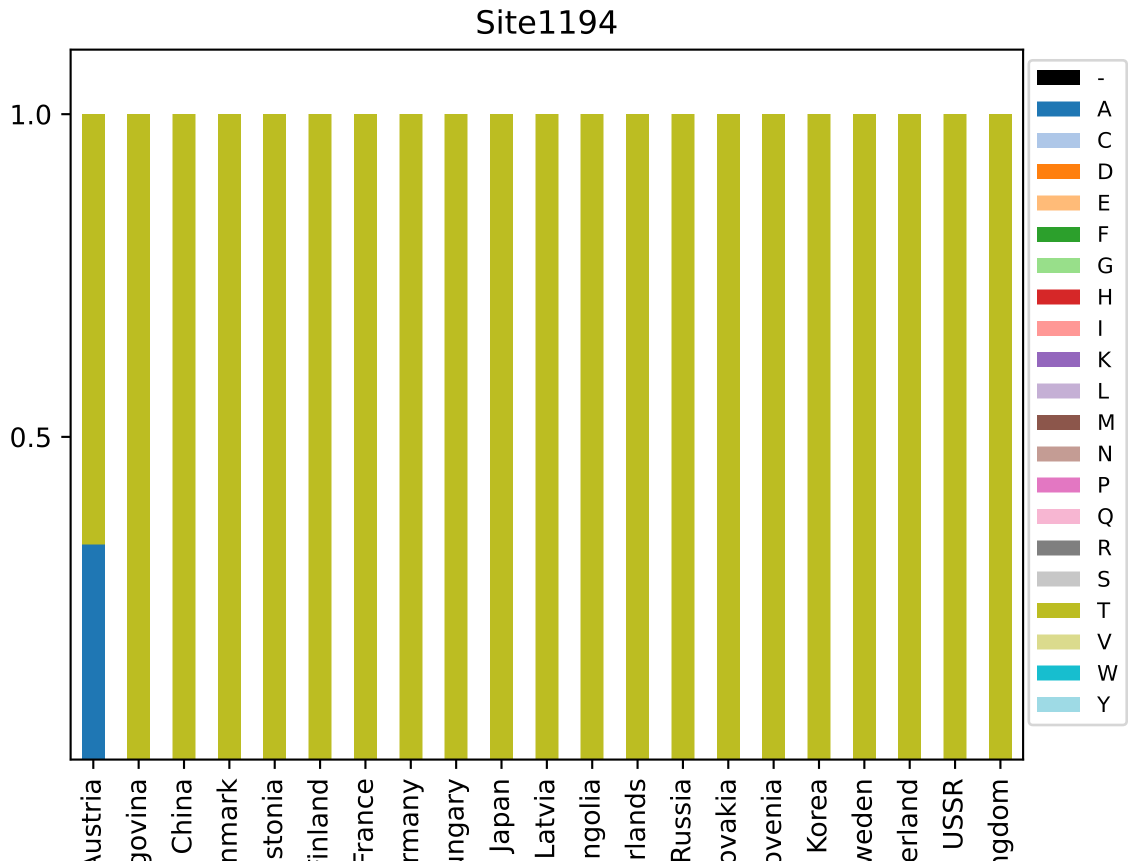
Site1194
1.0
0.5
-
A
C
D
E
F
G
H
I
K
L
M
N
P
Q
R
S
T
V
W
Y
Austria
China
Japan
Latvia
Russia
Korea
USSR

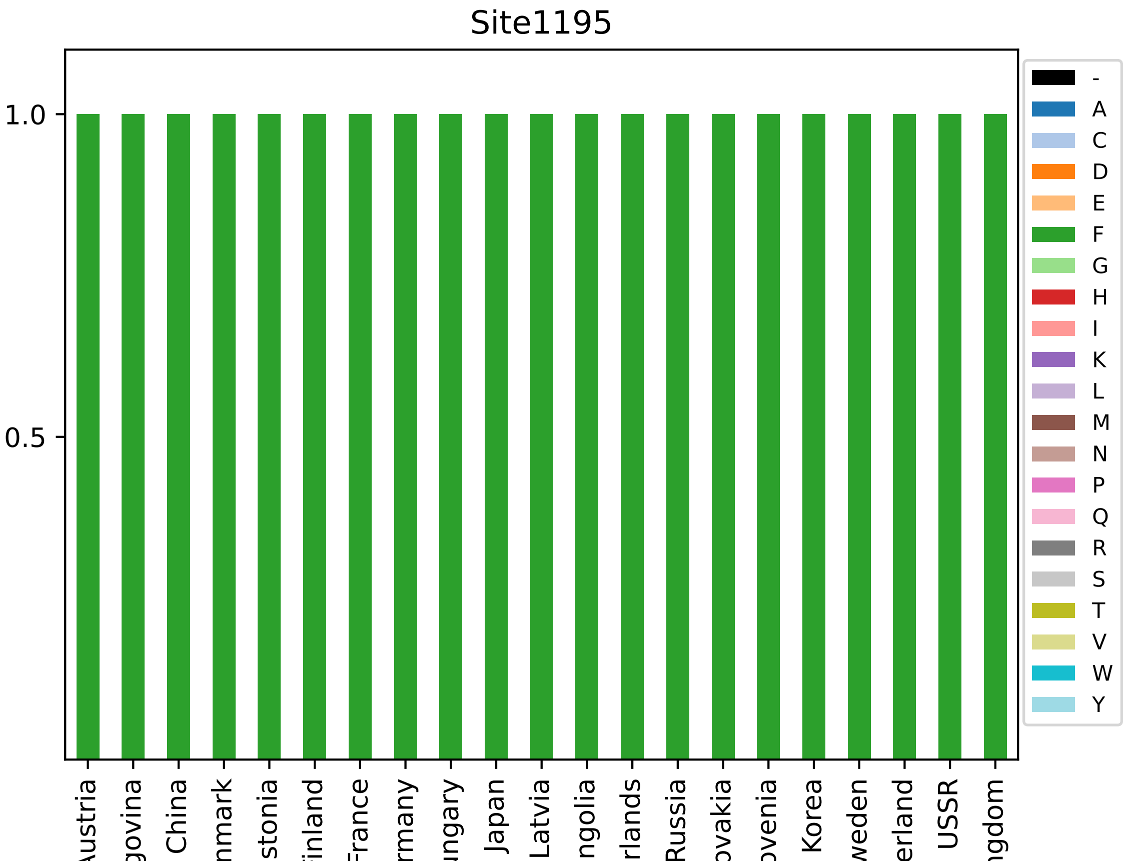
Site1195
1.0
0.5
-
A
C
D
E
F
G
H
I
K
L
M
N
P
Q
R
S
T
V
W
Y
Austria
govina
China
nmark
stonia
inland
France
rmany
ungary
Japan
Latvia
ngolia
rlands
Russia
ovakia
ovenia
Korea
weden
erland
USSR
ngdom

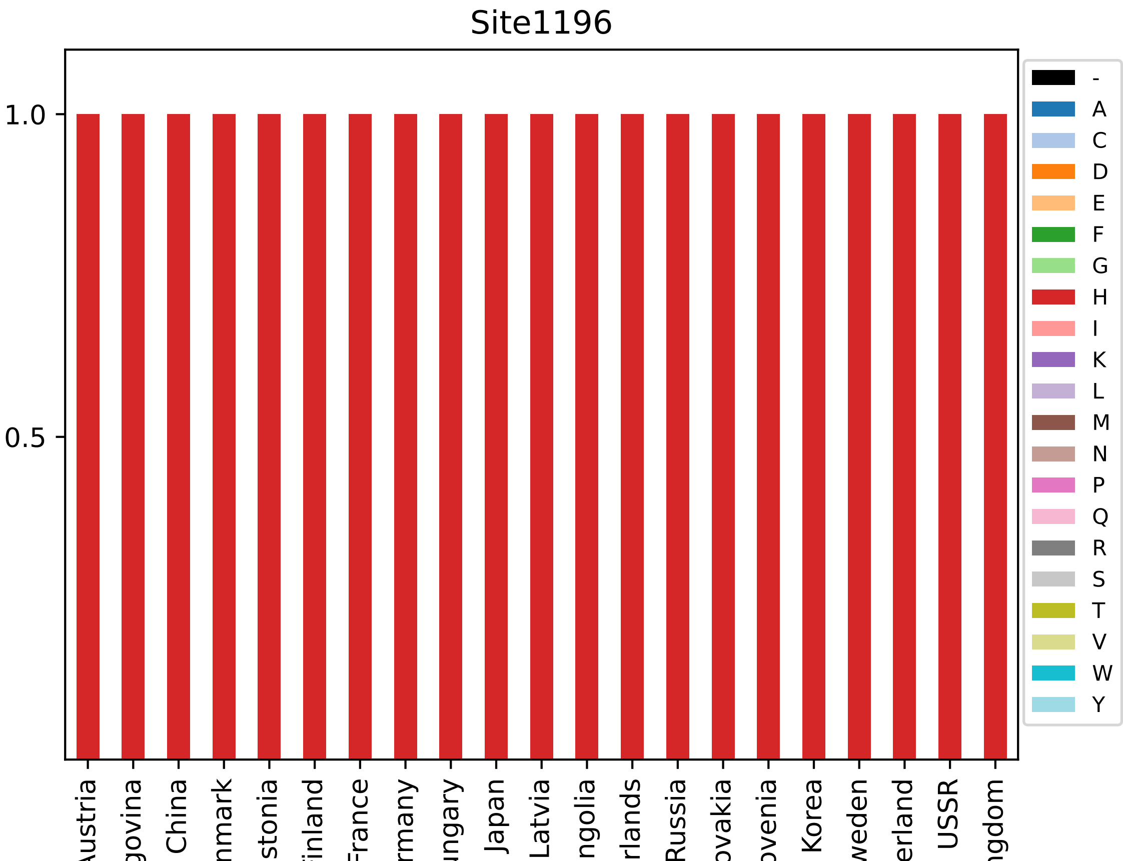
Site1196
1.0
0.5
-
A
C
D
E
F
G
H
I
K
L
M
N
P
Q
R
S
T
V
W
Y
Austria
China
Japan
Latvia
Russia
Korea
USSR

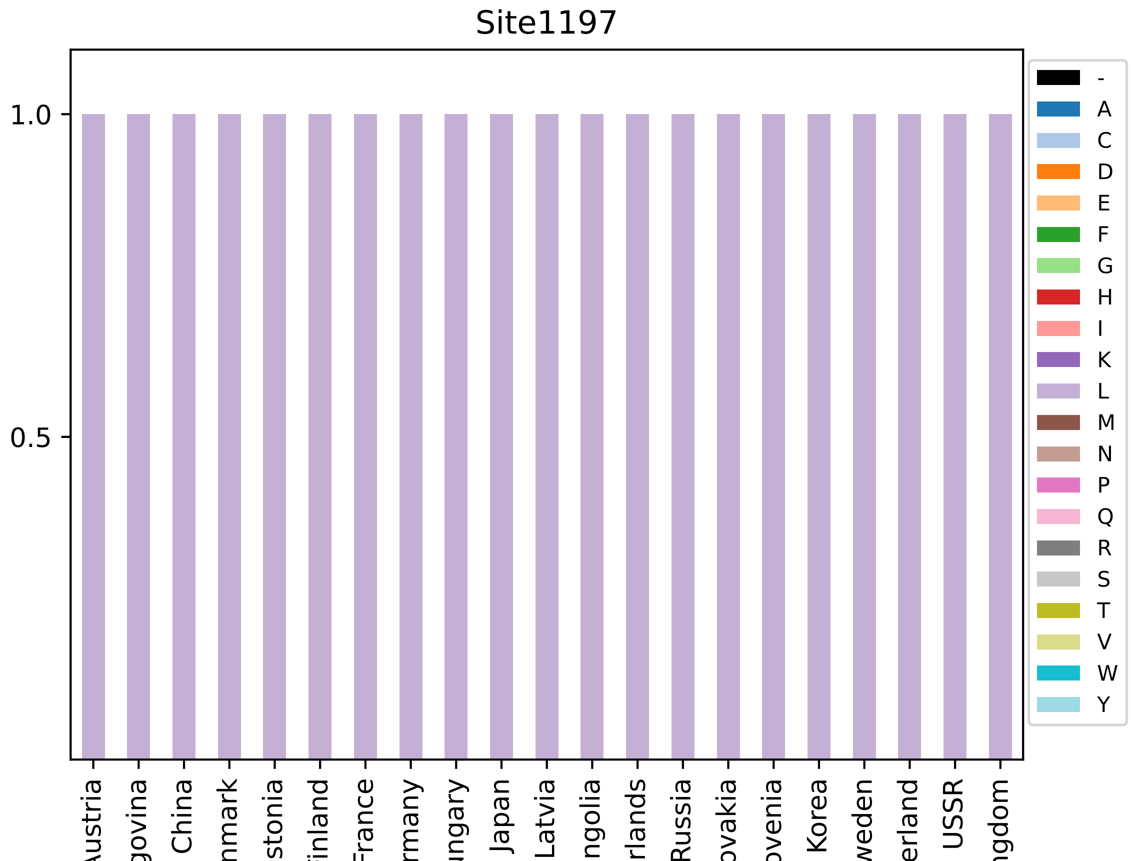
Site1197
1.0
0.5
-
A
C
D
E
F
G
H
I
K
L
M
N
P
Q
R
S
T
V
W
Y
Austria
China
Japan
Latvia
Russia
Korea
USSR

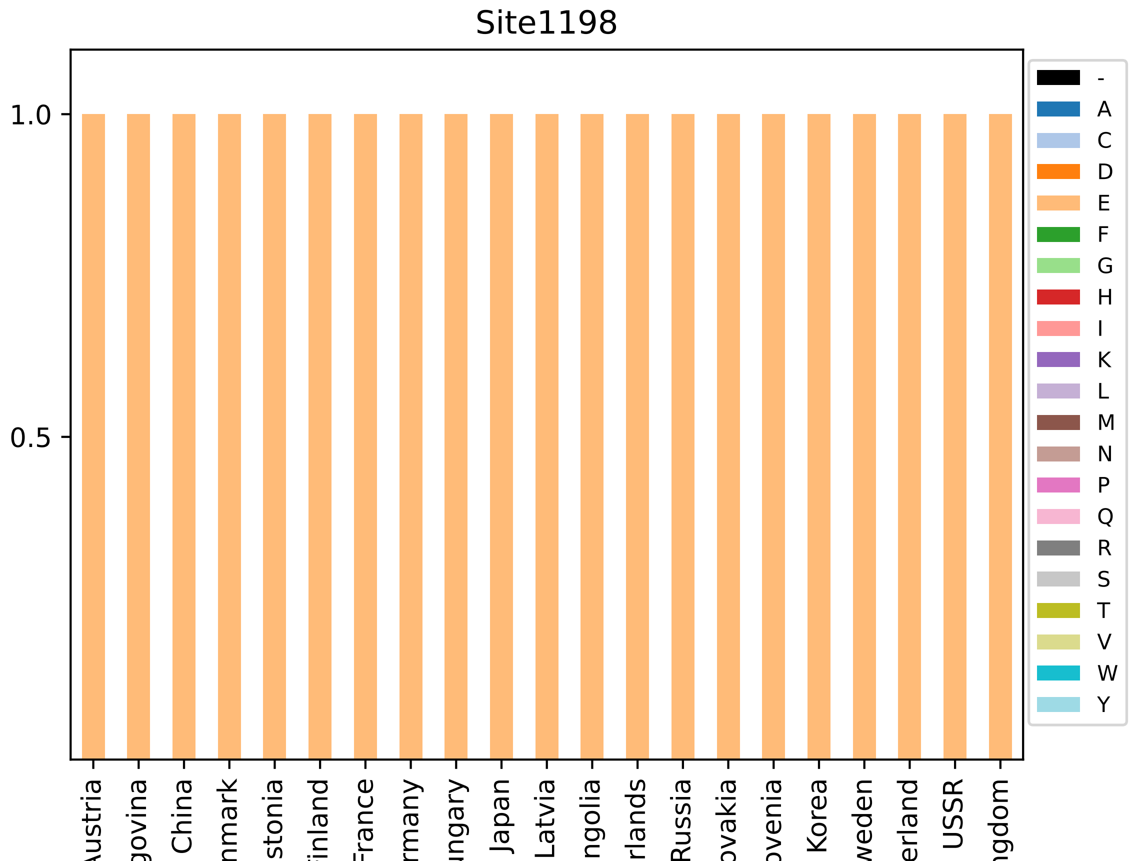
Site1198
1.0
0.5
-
A
C
D
E
F
G
H
I
K
L
M
N
P
Q
R
S
T
V
W
Y
China
Japan
Latvia
Russia
Korea
USSR

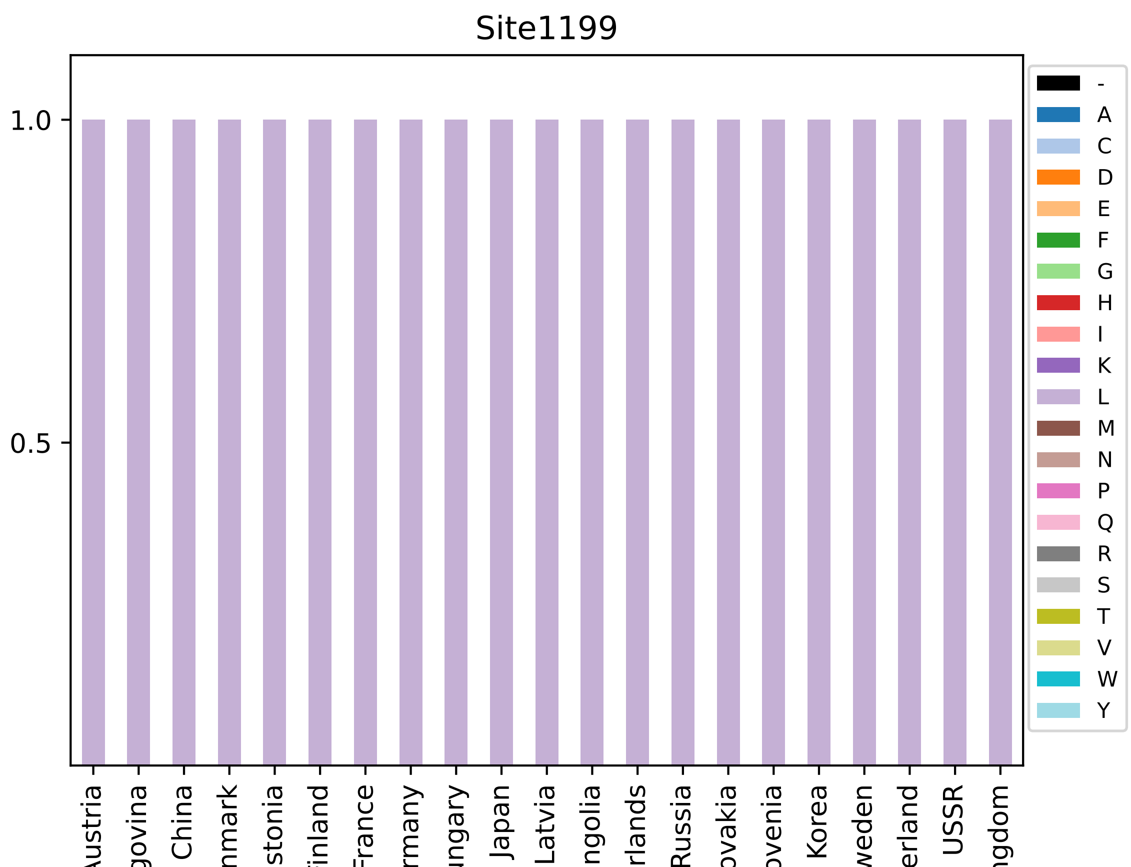

Site1199
1.0
0.5
-
A
C
D
E
F
G
H
I
K
L
M
N
P
Q
R
S
T
V
W
Y
China
Japan
Latvia
Russia
Korea
USSR

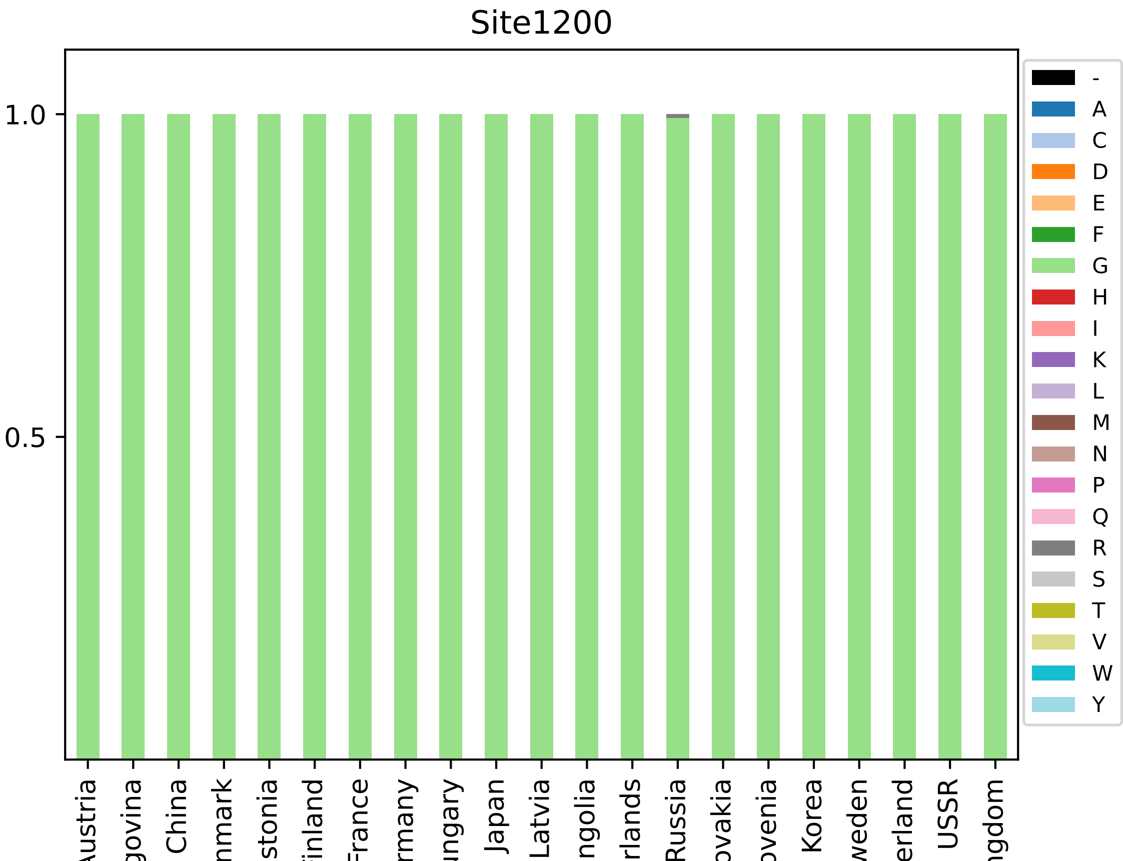

Site1200
1.0
0.5
-
A
C
D
E
F
G
H
I
K
L
M
N
P
Q
R
S
T
V
W
Y
Austria
China
Estonia
Finland
France
Germany
Hungary
Japan
Latvia
Mongolia
Russia
Korea
Sweden
USSR

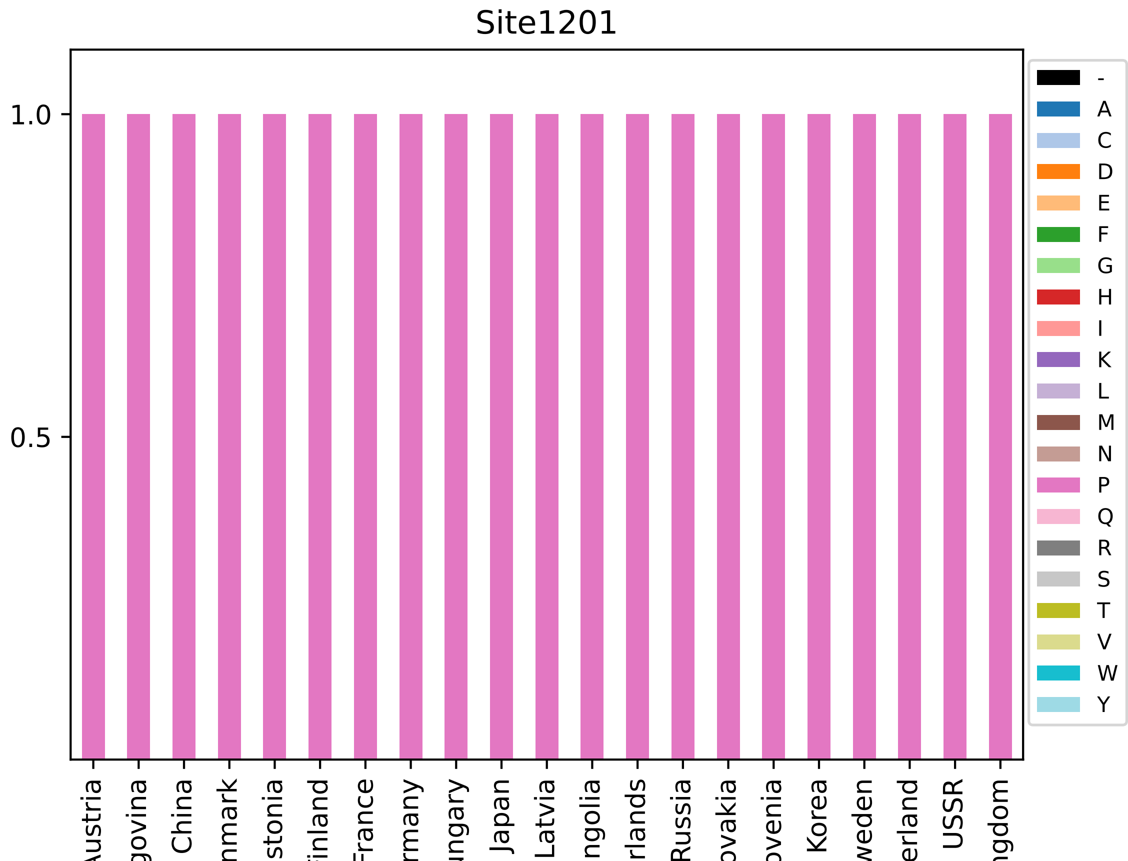
Site1201
1.0
0.5
-
A
C
D
E
F
G
H
I
K
L
M
N
P
Q
R
S
T
V
W
Y
China
Japan
Latvia
Russia
Korea
USSR

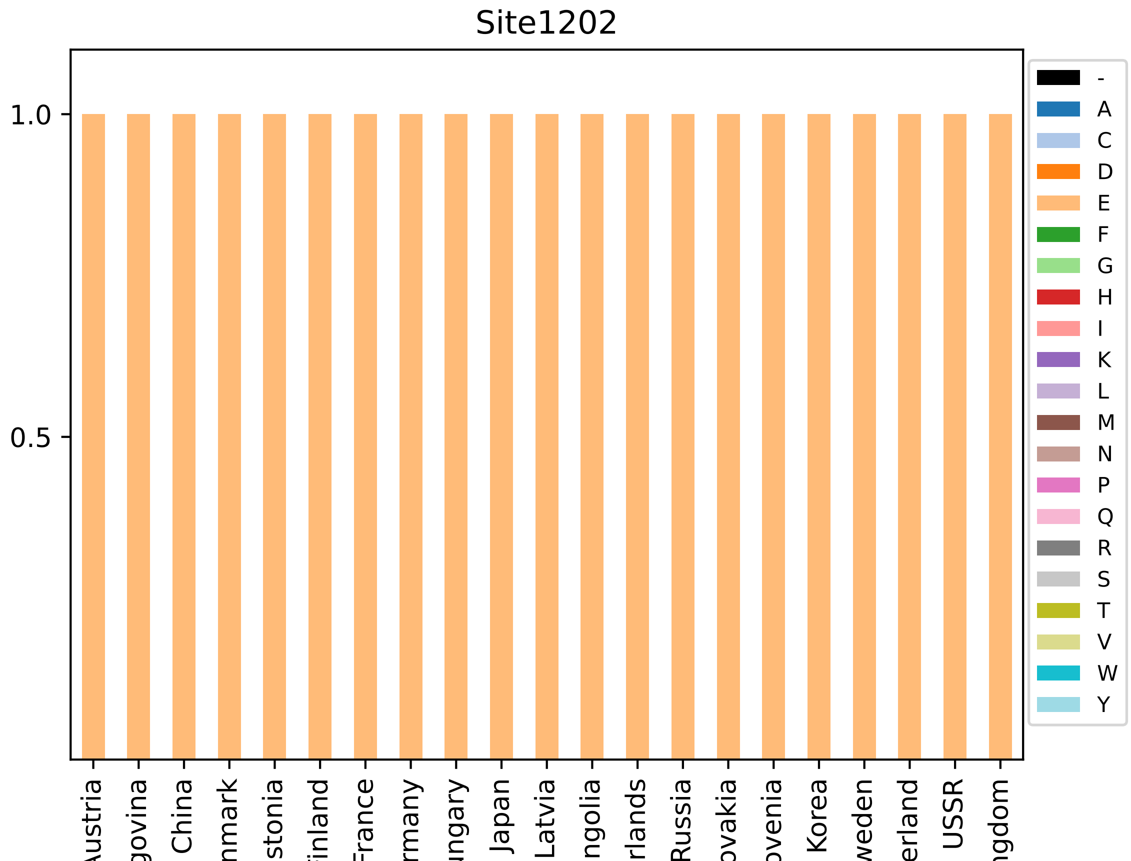

Site1202
1.0
0.5
-
A
C
D
E
F
G
H
I
K
L
M
N
P
Q
R
S
T
V
W
Y
Austria
govina
China
nmark
stonia
inland
France
rmany
ungary
Japan
Latvia
ngolia
rlands
Russia
ovakia
ovenia
Korea
weden
erland
USSR
ngdom

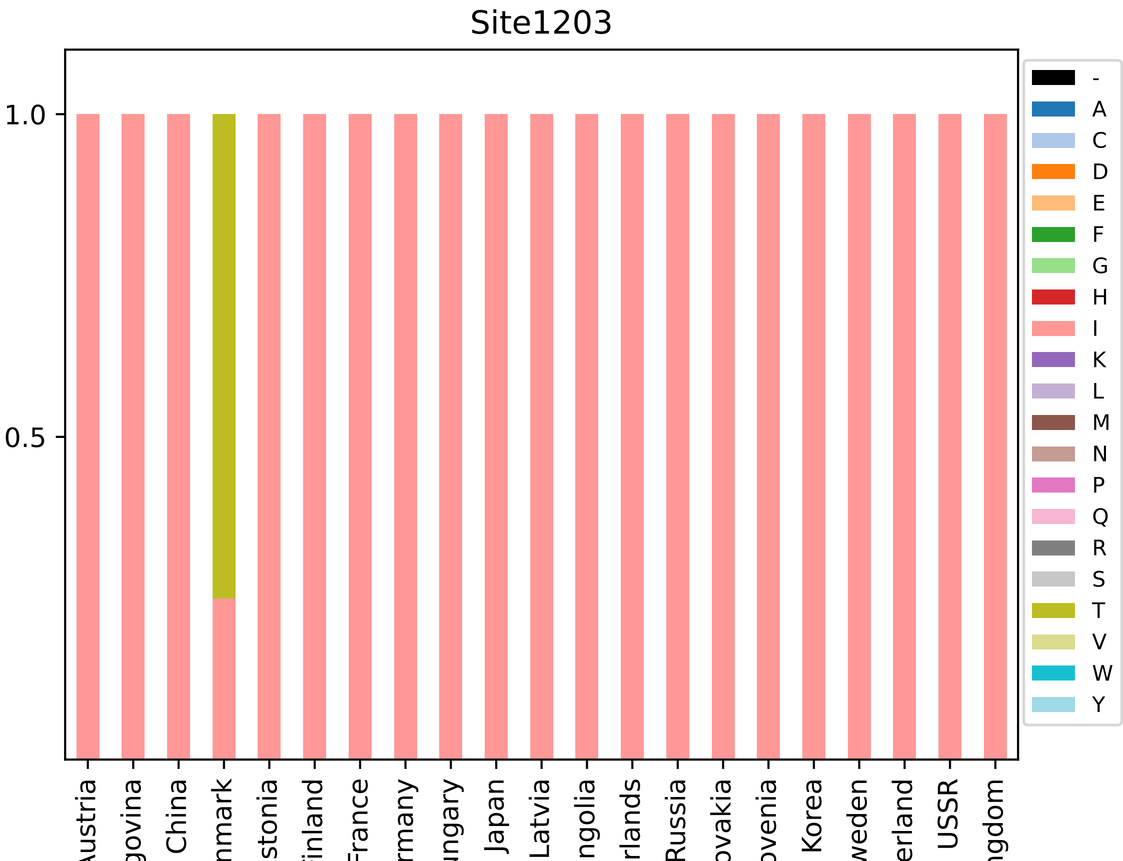
Site1203
1.0
0.5
-
A
C
D
E
F
G
H
I
K
L
M
N
P
Q
R
S
T
V
W
Y
Austria
govina
China
nmark
stonia
inland
rance
rmany
ungary
Japan
Latvia
ngolia
rlands
Russia
ovakia
ovenia
Korea
weden
erland
USSR
ngdom

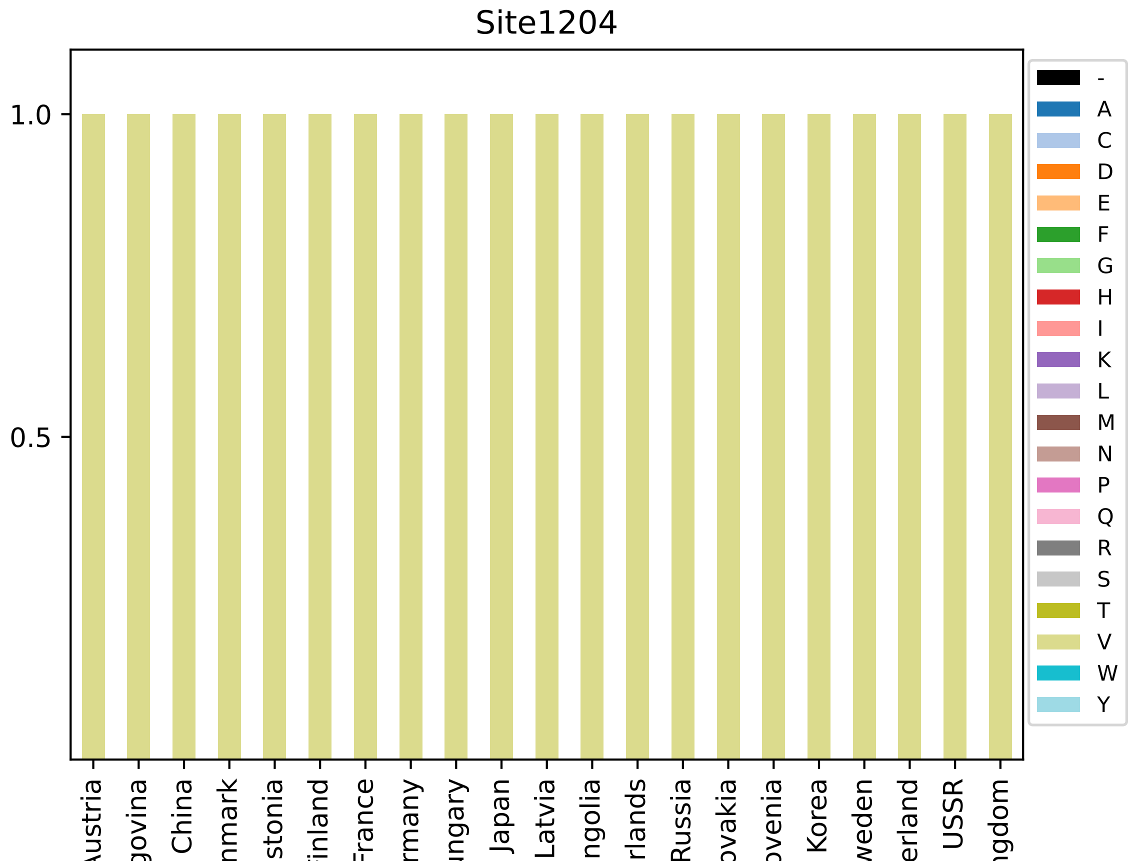
Site1204
1.0
0.5
-
A
C
D
E
F
G
H
I
K
L
M
N
P
Q
R
S
T
V
W
Y
Austria
govina
China
nmark
stonia
inland
France
rmany
ungary
Japan
Latvia
ngolia
rlands
Russia
ovakia
ovenia
Korea
weden
erland
USSR
ngdom

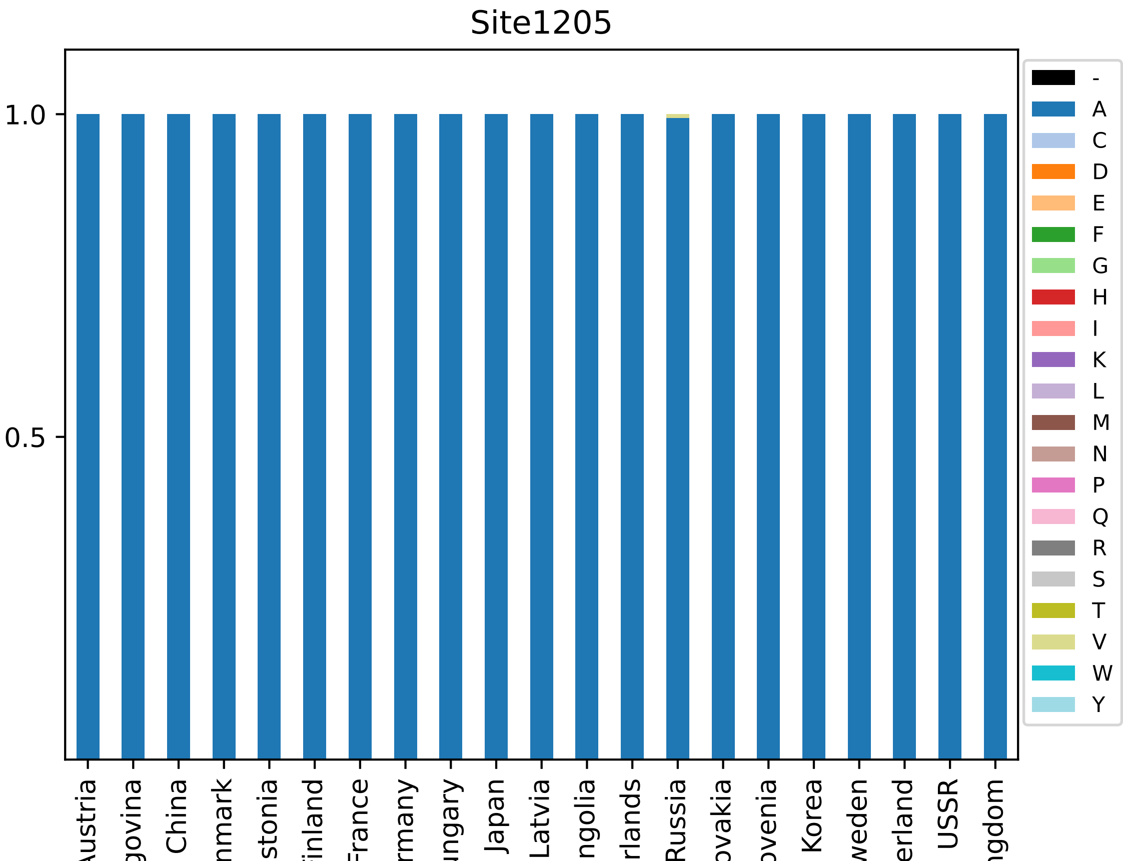

Site1205
1.0
0.5
-
A
C
D
E
F
G
H
I
K
L
M
N
P
Q
R
S
T
V
W
Y
Austria
govina
China
nmark
stonia
Finland
France
rmany
ungary
Japan
Latvia
ngolia
rlands
Russia
ovakia
ovenia
Korea
weden
erland
USSR
ngdom

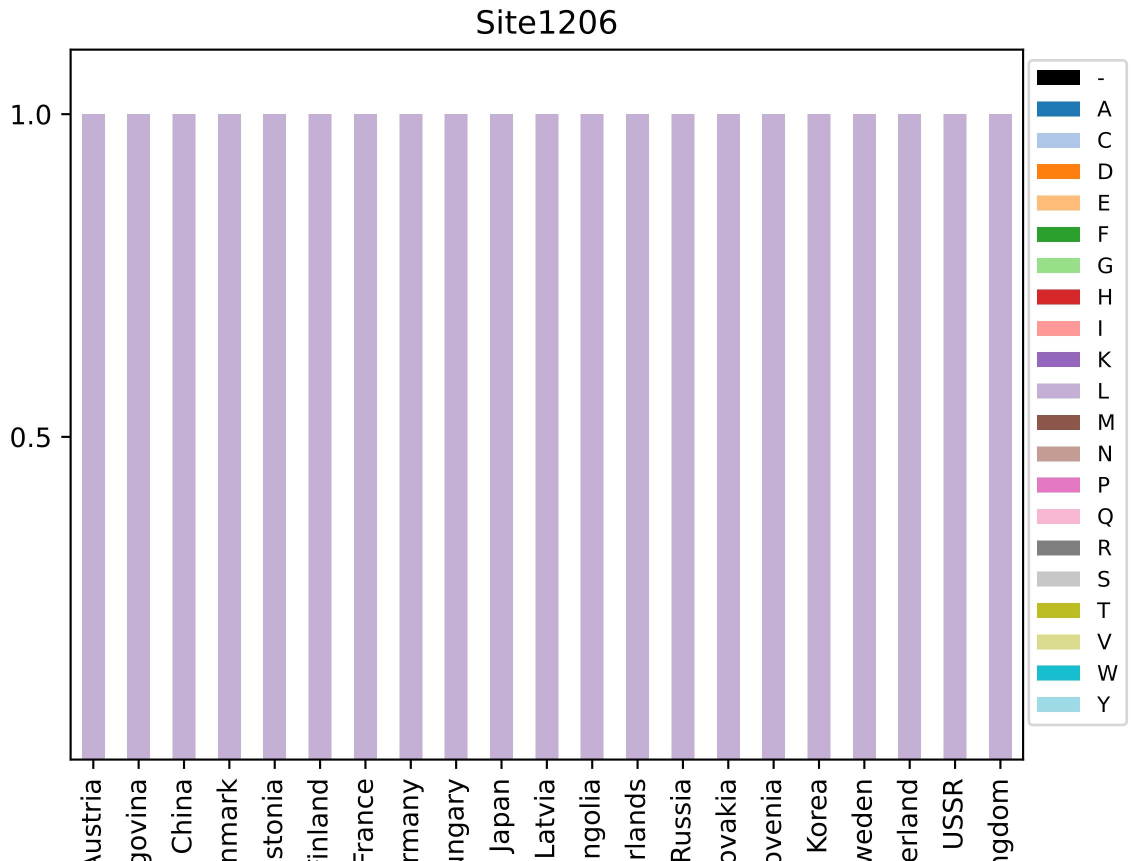

Site1206
1.0
0.5
-
A
C
D
E
F
G
H
I
K
L
M
N
P
Q
R
S
T
V
W
Y
Austria
China
Japan
Latvia
Russia
Korea
USSR

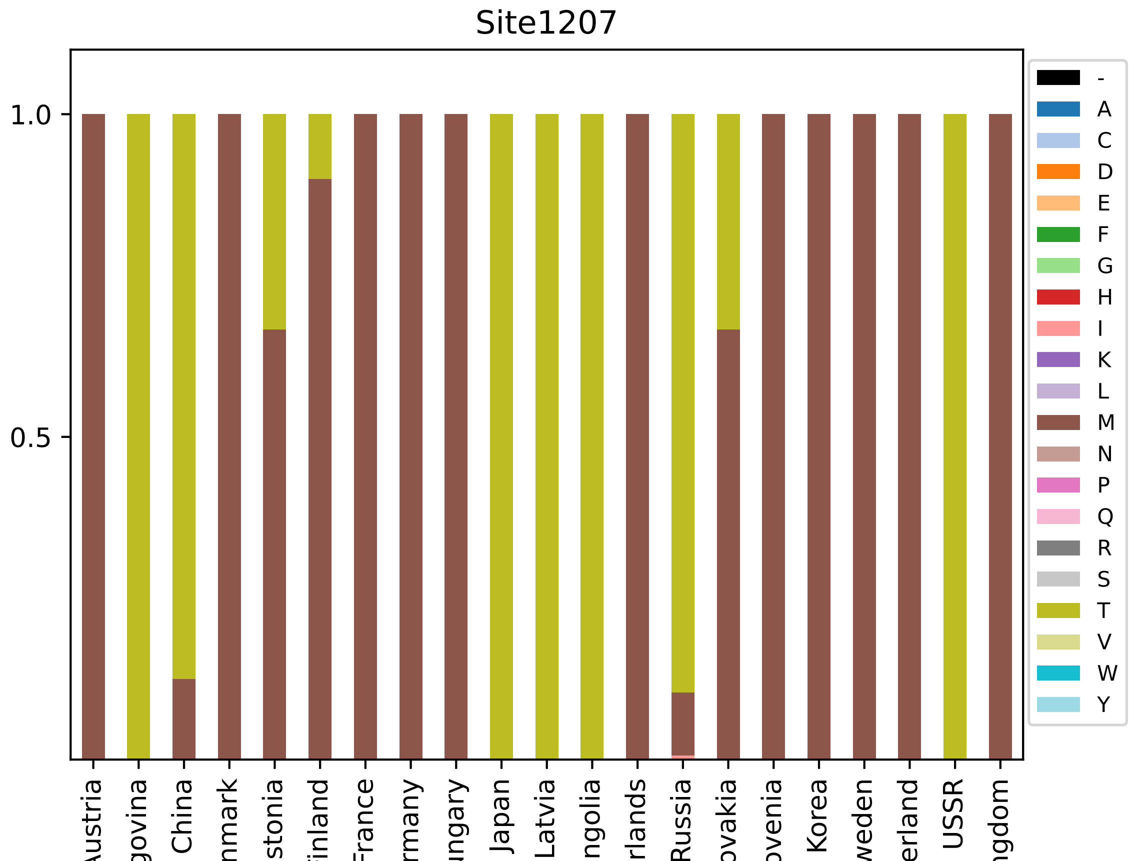

Site1207
1.0
0.5
-
A
C
D
E
F
G
H
I
K
L
M
N
P
Q
R
S
T
V
W
Y
Austria
China
Finland
France
Japan
Latvia
Russia
Korea
USSR

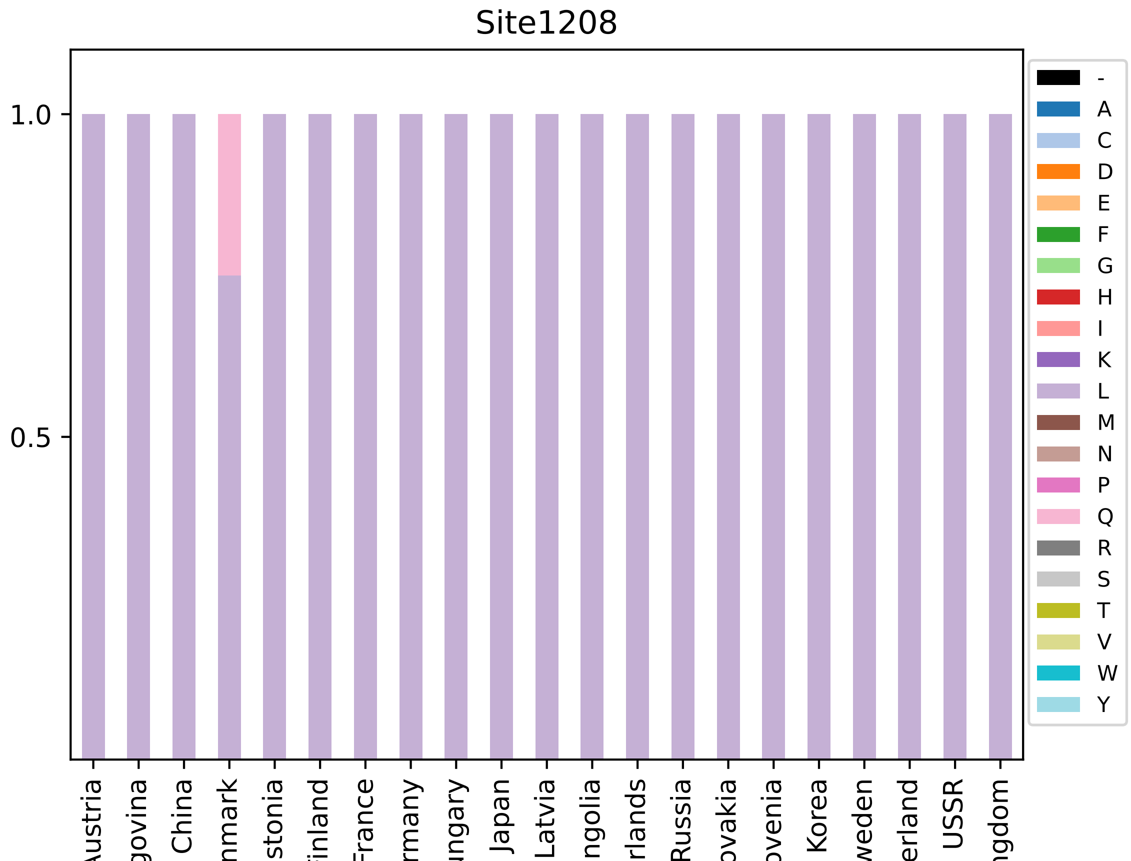
Site1208
1.0
0.5
-
A
C
D
E
F
G
H
I
K
L
M
N
P
Q
R
S
T
V
W
Y
China
Japan
Latvia
Russia
Korea
USSR

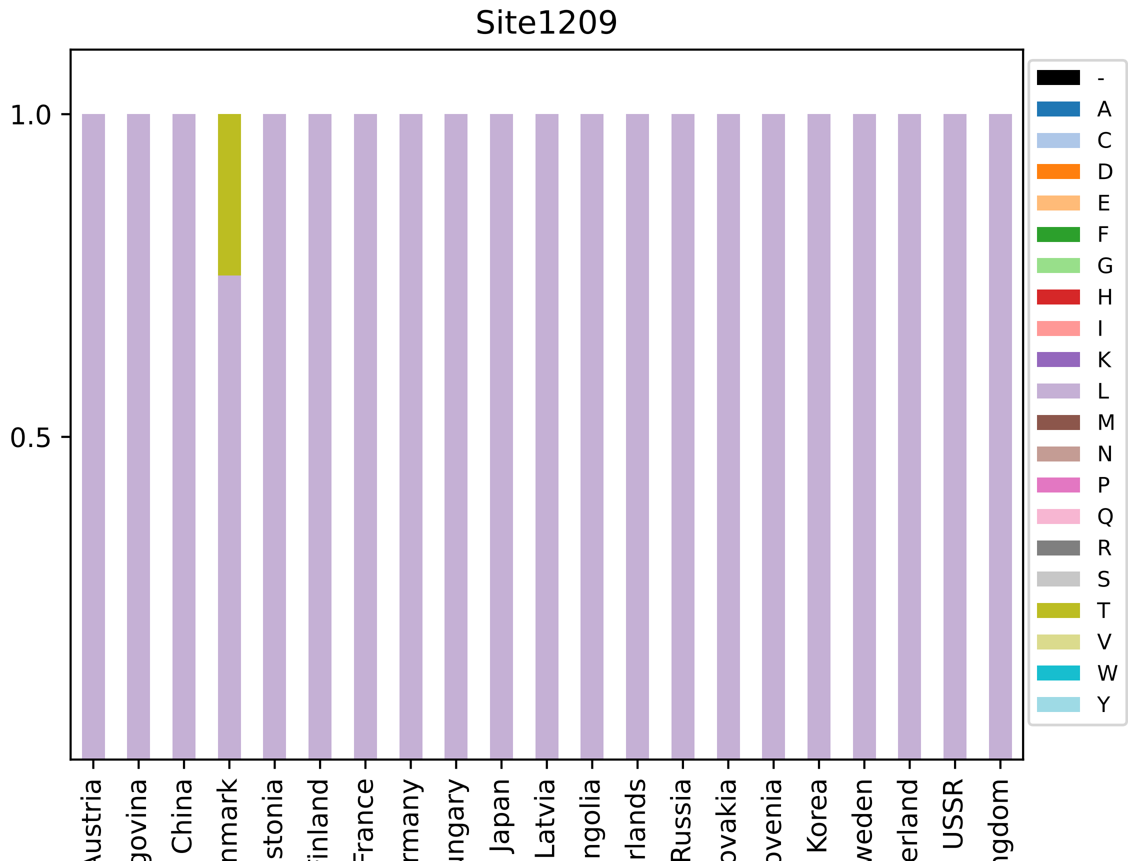

Site1209
1.0
0.5
-
A
C
D
E
F
G
H
I
K
L
M
N
P
Q
R
S
T
V
W
Y
Austria
China
Japan
Latvia
Russia
Korea
USSR

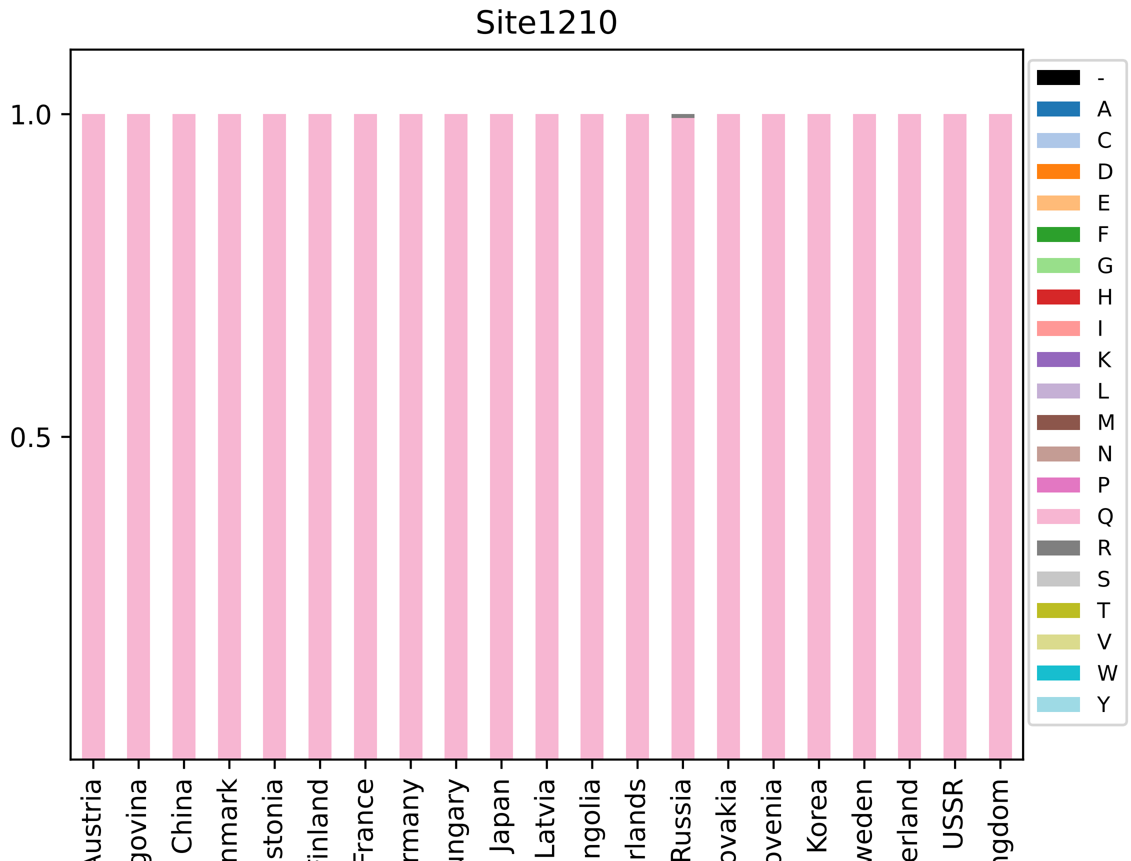
Site1210
1.0
0.5
-
A
C
D
E
F
G
H
I
K
L
M
N
P
Q
R
S
T
V
W
Y
Austria
govina
China
nmark
stonia
Finland
France
rmany
ungary
Japan
Latvia
ngolia
rlands
Russia
ovakia
ovenia
Korea
weden
erland
USSR
ngdom

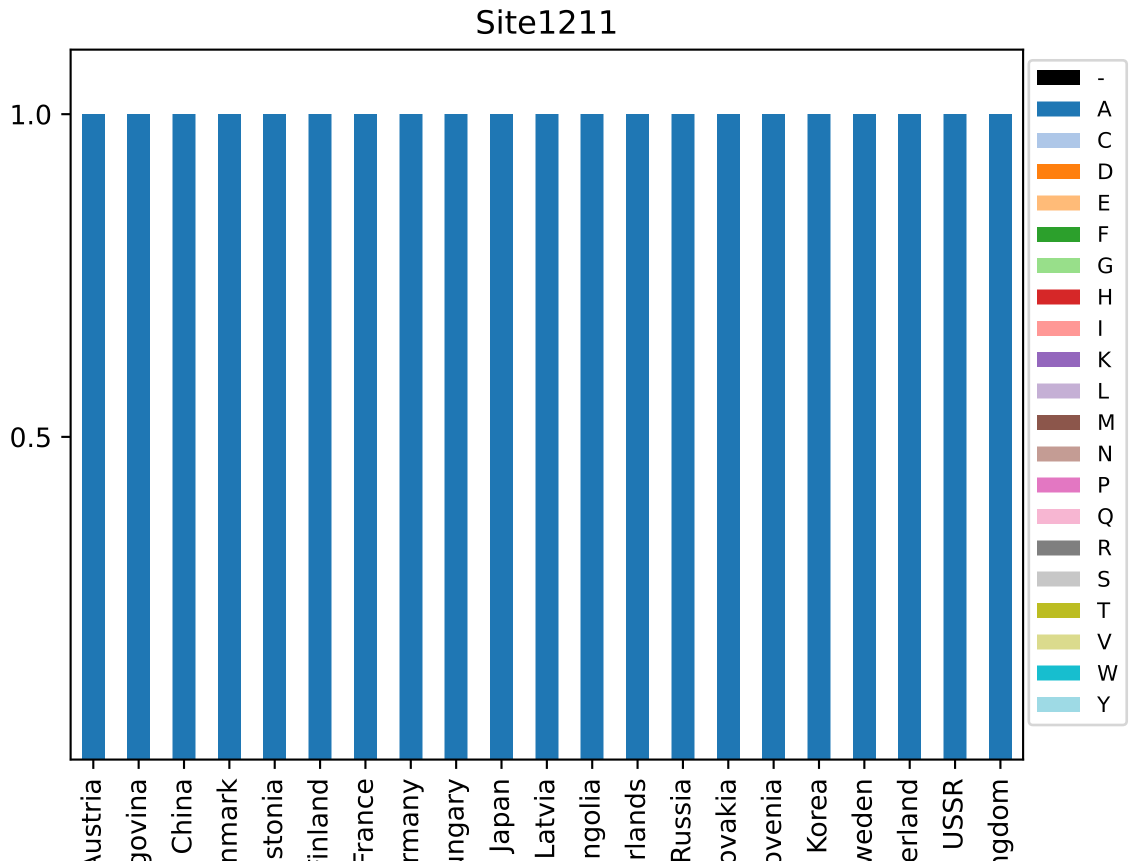
Site1211
1.0
0.5
Austria
govina
China
nmark
stonia
Finland
France
rmany
ungary
Japan
Latvia
ngolia
rlands
Russia
ovakia
ovenia
Korea
weden
erland
USSR
ngdom
-
A
C
D
E
F
G
H
I
K
L
M
N
P
Q
R
S
T
V
W
Y

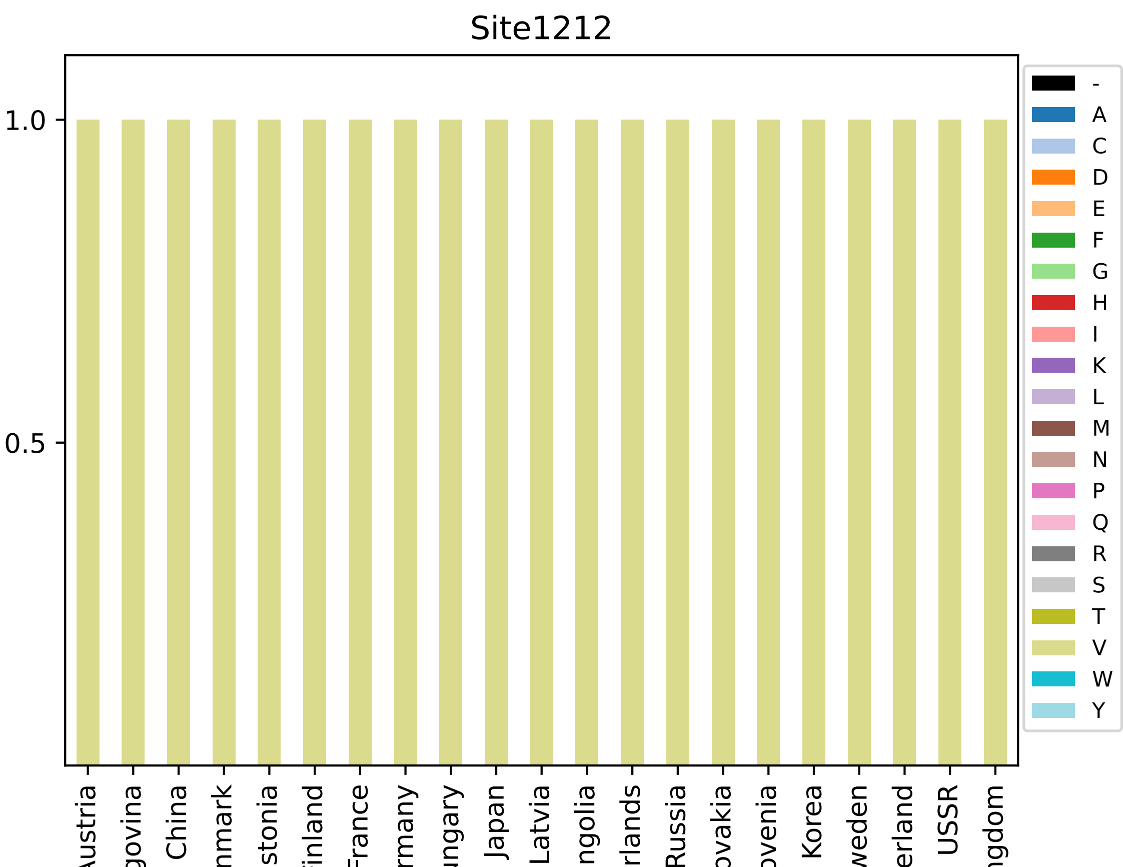

Site1212
1.0
0.5
-
A
C
D
E
F
G
H
I
K
L
M
N
P
Q
R
S
T
V
W
Y
China
Japan
Latvia
Russia
Korea
USSR

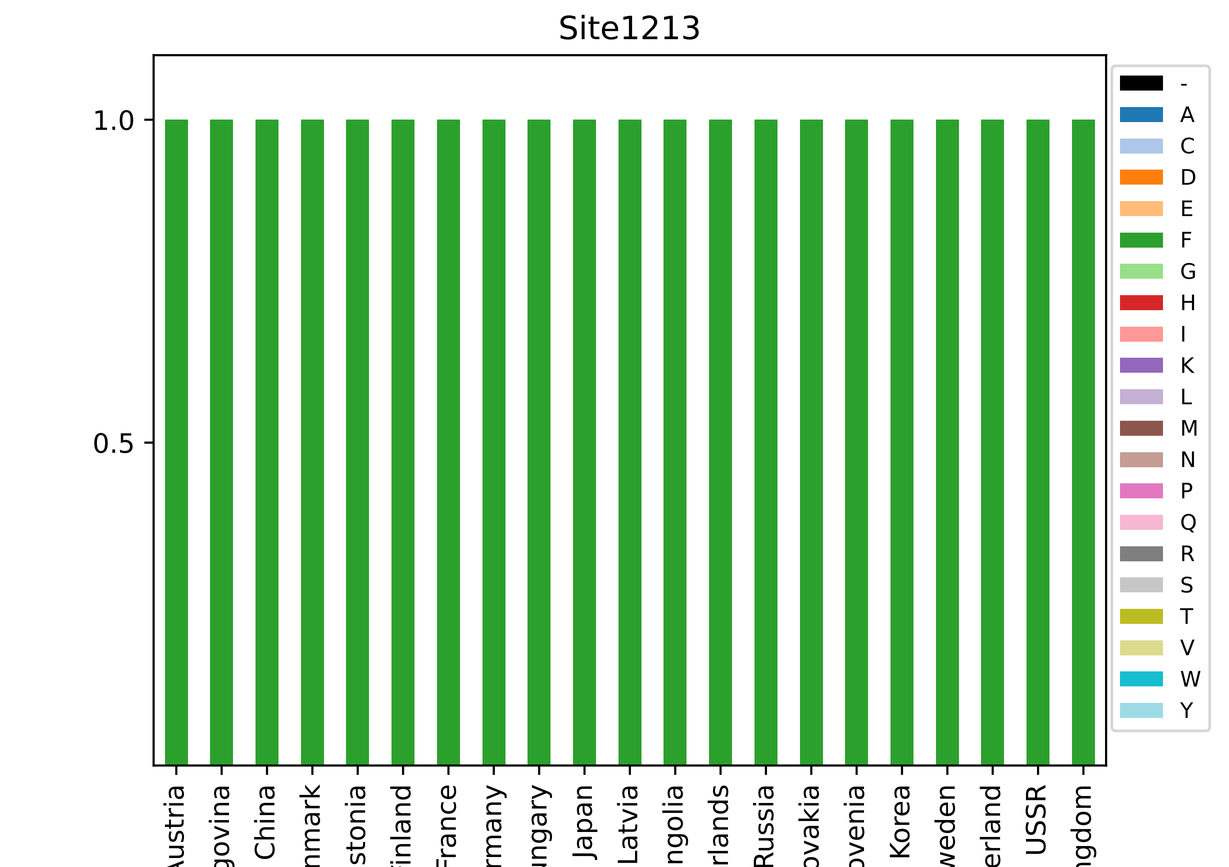
Site1213
1.0
0.5
-
A
C
D
E
F
G
H
I
K
L
M
N
P
Q
R
S
T
V
W
Y
Austria
govina
China
nmark
stonia
Finland
France
rmany
ungary
Japan
Latvia
ngolia
rlands
Russia
ovakia
ovenia
Korea
weden
erland
USSR
ngdom

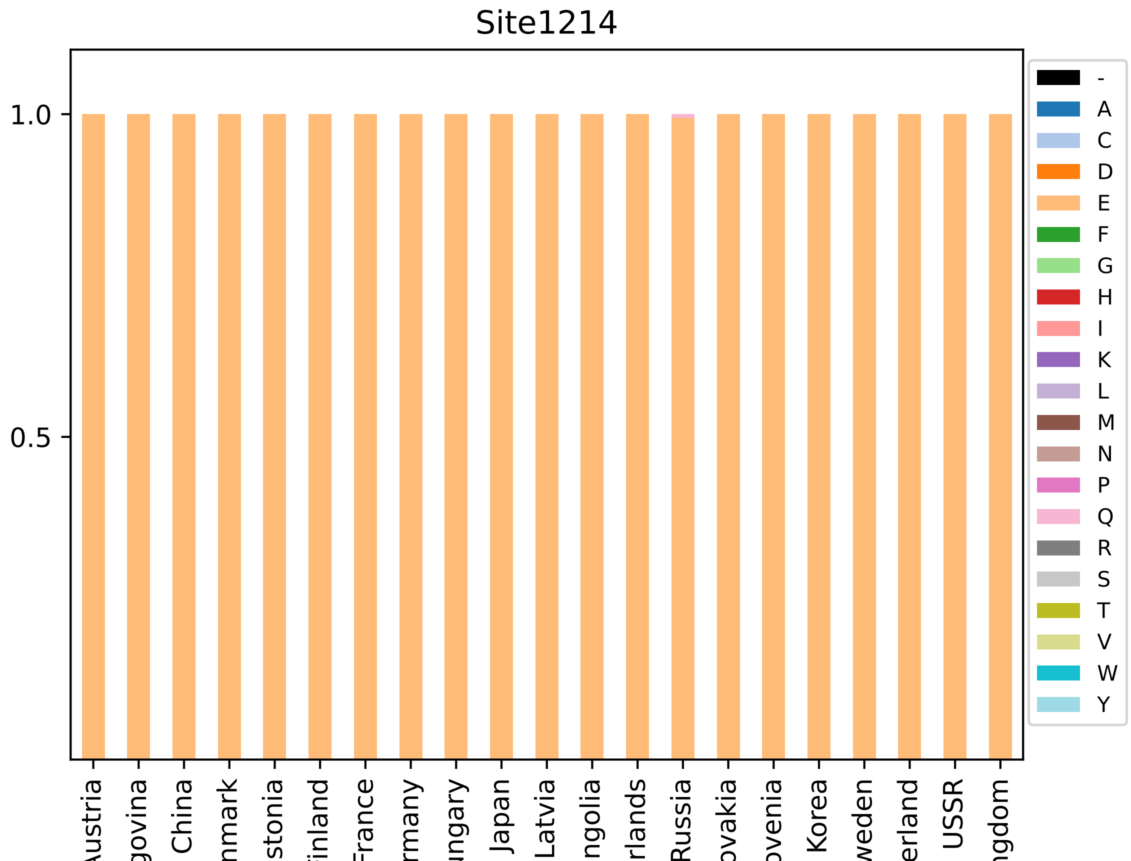

Site1214
1.0
0.5
-
A
C
D
E
F
G
H
I
K
L
M
N
P
Q
R
S
T
V
W
Y
China
Japan
Latvia
Russia
Korea
USSR

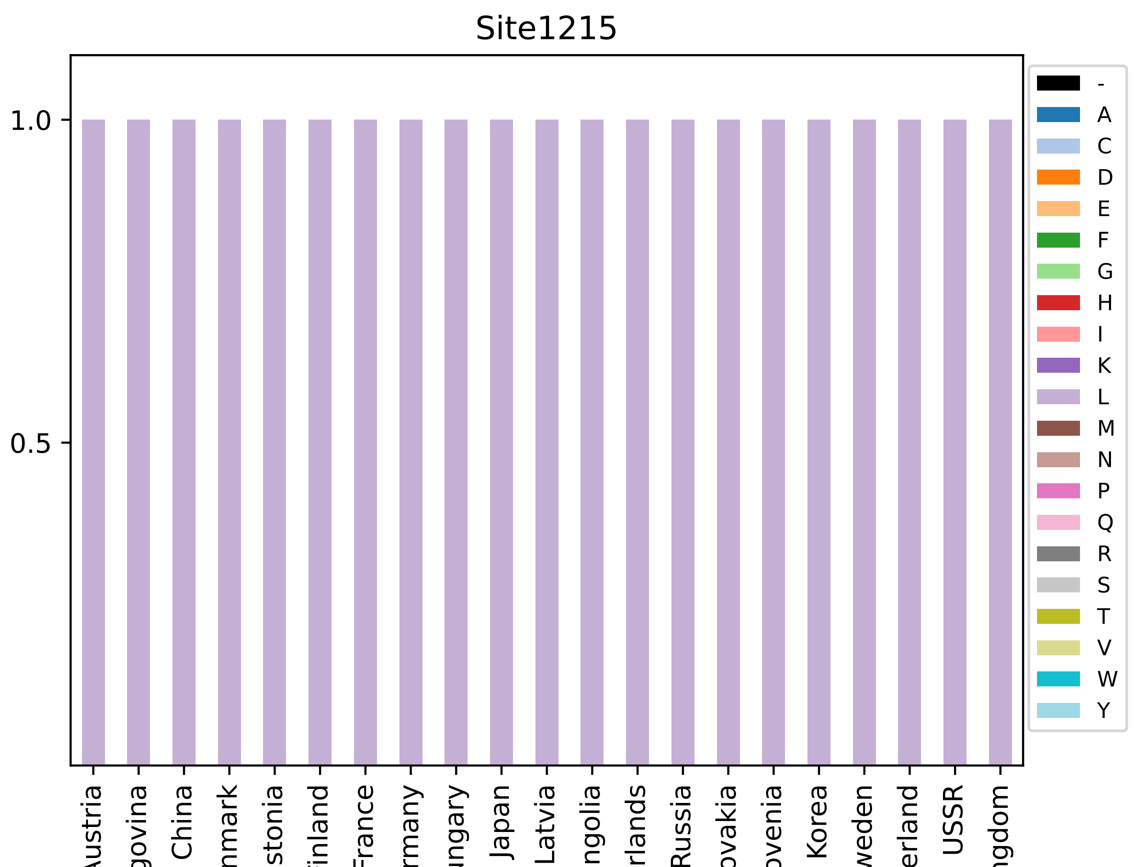
Site1215
1.0
0.5
-
A
C
D
E
F
G
H
I
K
L
M
N
P
Q
R
S
T
V
W
Y
Austria
govina
China
nmark
stonia
inland
France
rmany
ungary
Japan
Latvia
ngolia
rlands
Russia
ovakia
ovenia
Korea
weden
erland
USSR
ngdom

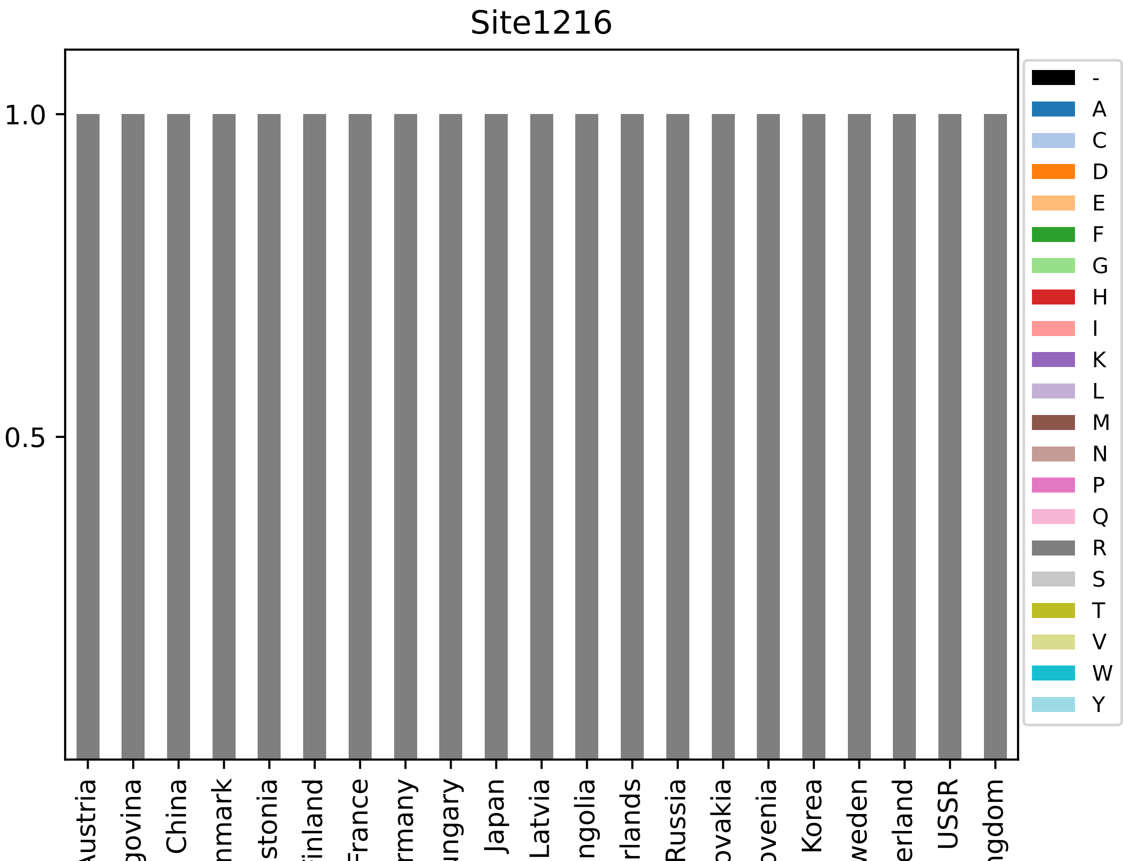

Site1216
1.0
0.5
-
A
C
D
E
F
G
H
I
K
L
M
N
P
Q
R
S
T
V
W
Y
Austria
China
Finland
France
Japan
Latvia
Russia
Korea
USSR

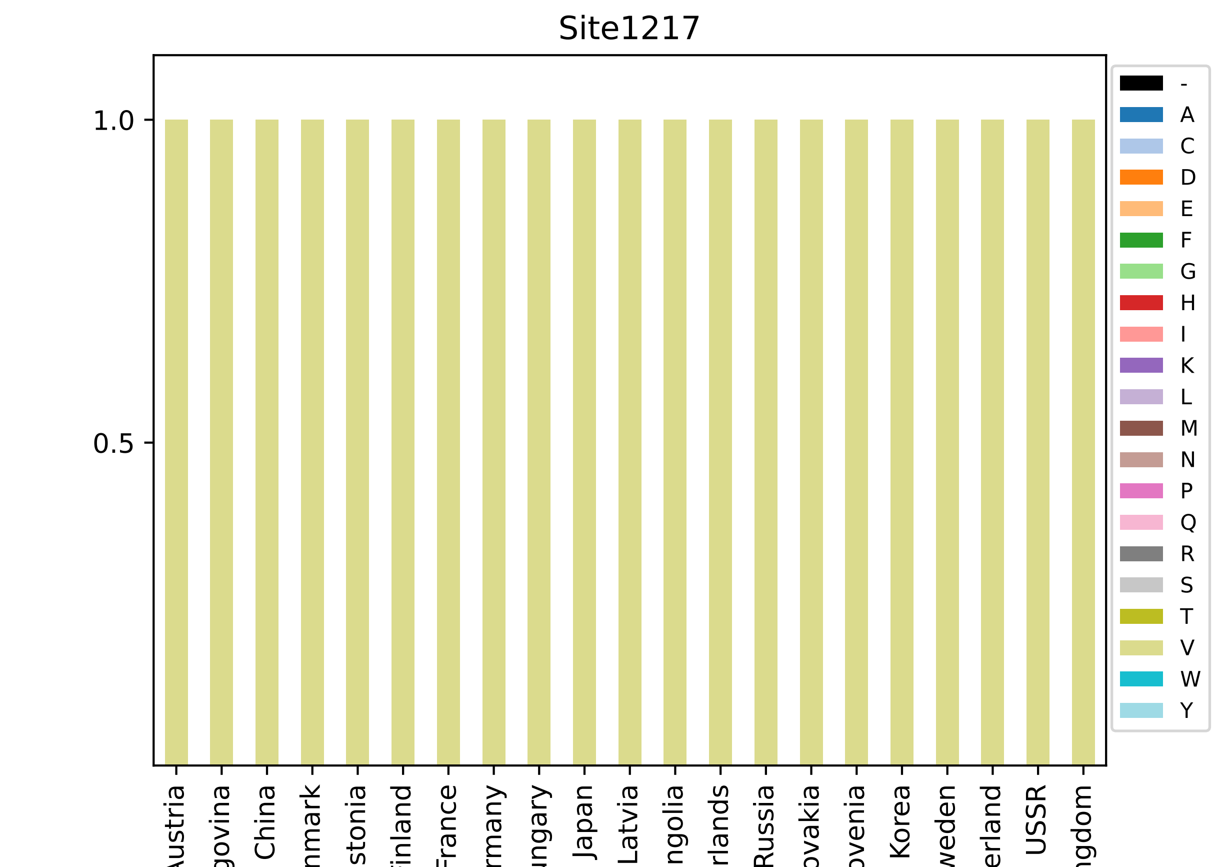

Site1217
1.0
0.5
-
A
C
D
E
F
G
H
I
K
L
M
N
P
Q
R
S
T
V
W
Y
China
Japan
Latvia
Russia
Korea
USSR

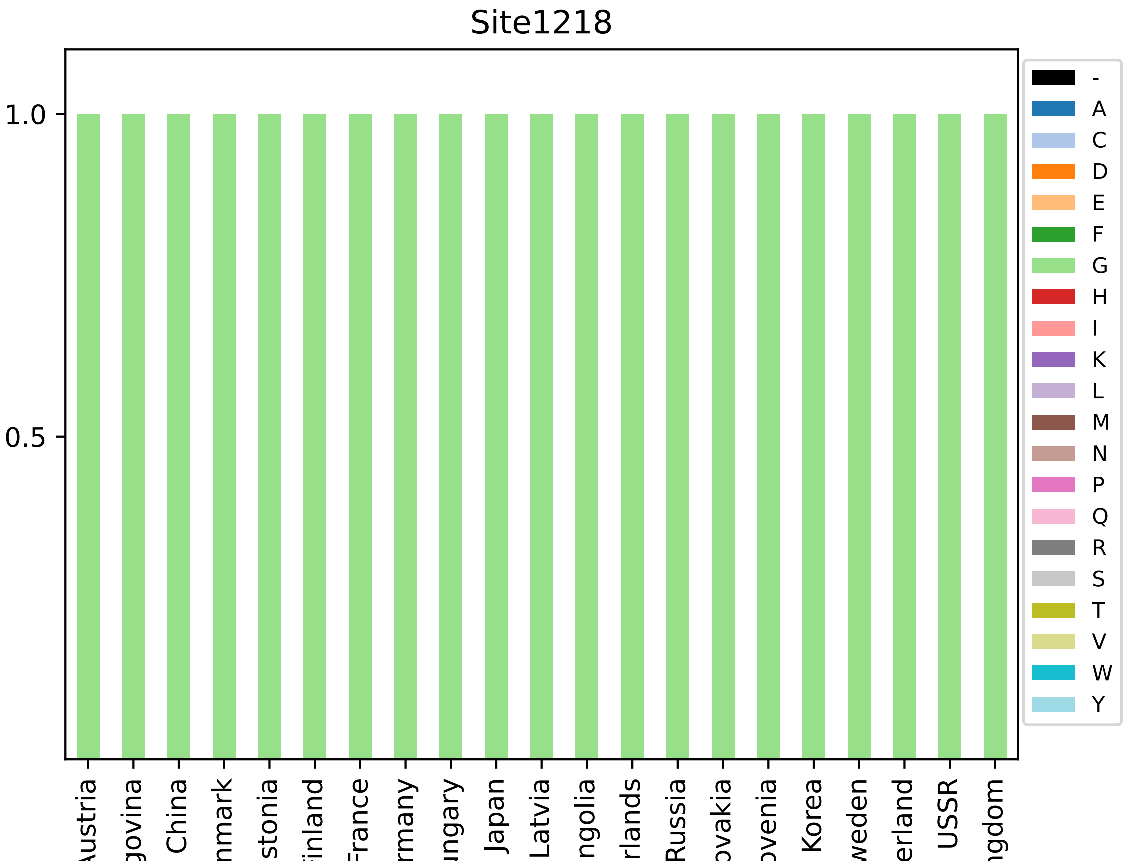
Site1218
1.0
0.5
-
A
C
D
E
F
G
H
I
K
L
M
N
P
Q
R
S
T
V
W
Y
China
Japan
Latvia
Russia
Korea
USSR

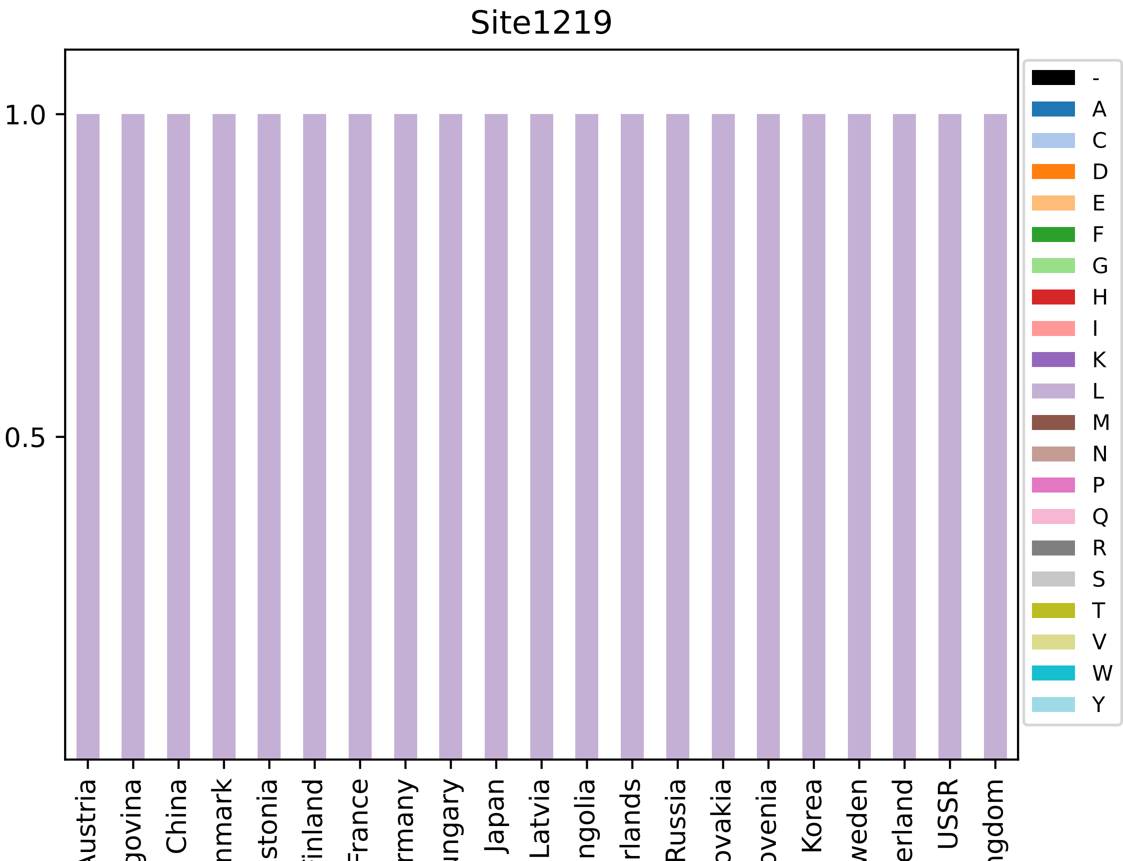

Site1219
1.0
0.5
-
A
C
D
E
F
G
H
I
K
L
M
N
P
Q
R
S
T
V
W
Y
China
Japan
Latvia
Russia
Korea
USSR

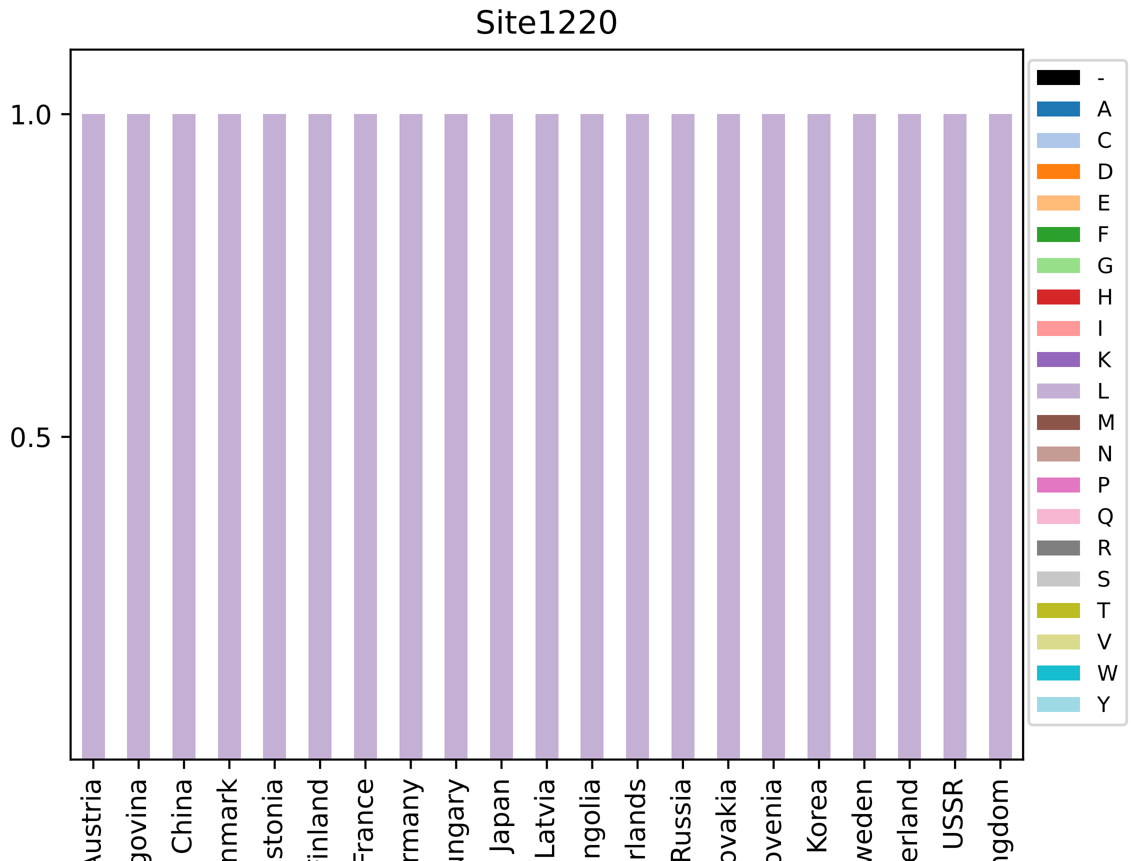
Site1220
1.0
0.5
-
A
C
D
E
F
G
H
I
K
L
M
N
P
Q
R
S
T
V
W
Y
China
Japan
Latvia
Russia
Korea
USSR

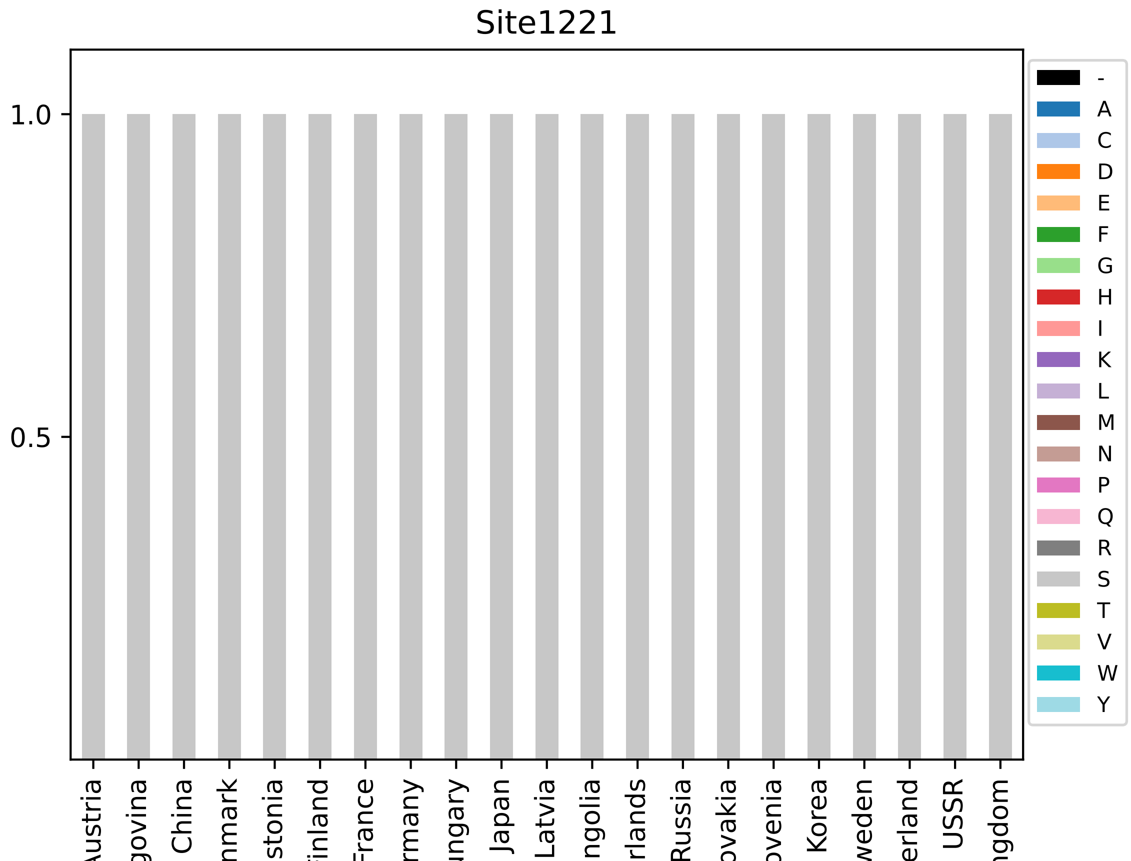

Site1221
1.0
0.5
-
A
C
D
E
F
G
H
I
K
L
M
N
P
Q
R
S
T
V
W
Y
Austria
govina
China
nmark
stonia
inland
France
rmany
ungary
Japan
Latvia
ngolia
rlands
Russia
ovakia
ovenia
Korea
weden
erland
USSR
ngdom

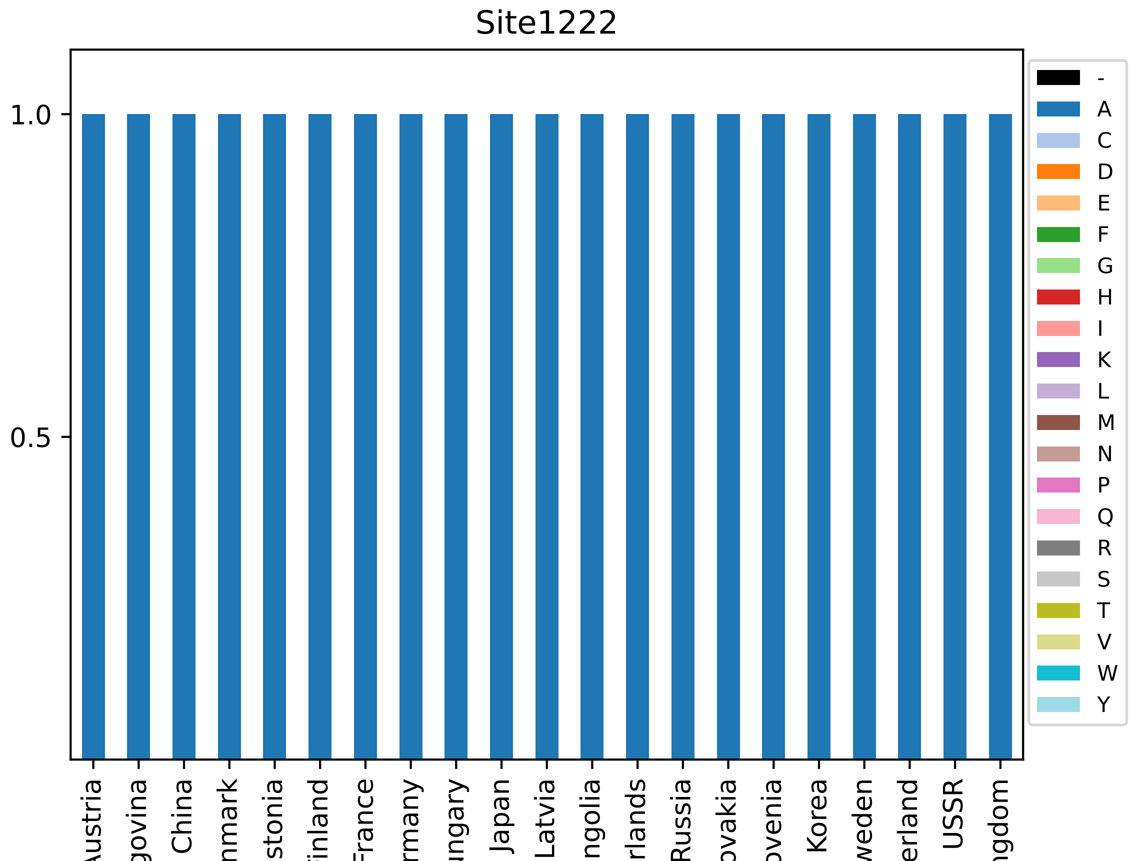

Site1222
1.0
0.5
-
A
C
D
E
F
G
H
I
K
L
M
N
P
Q
R
S
T
V
W
Y
China
Japan
Latvia
Russia
Korea
USSR

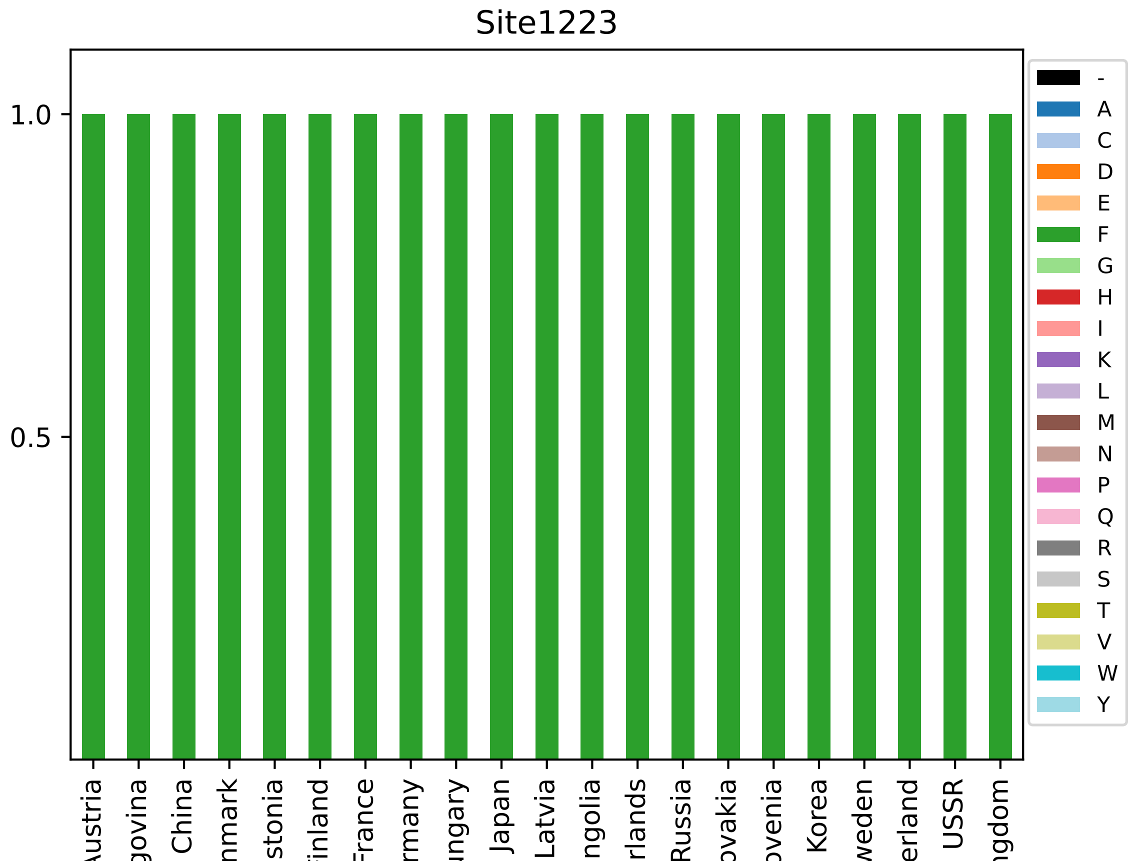
Site1223
1.0
0.5
-
A
C
D
E
F
G
H
I
K
L
M
N
P
Q
R
S
T
V
W
Y
Austria
govina
China
nmark
stonia
inland
France
rmany
ungary
Japan
Latvia
ngolia
rlands
Russia
ovakia
ovenia
Korea
weden
erland
USSR
ngdom

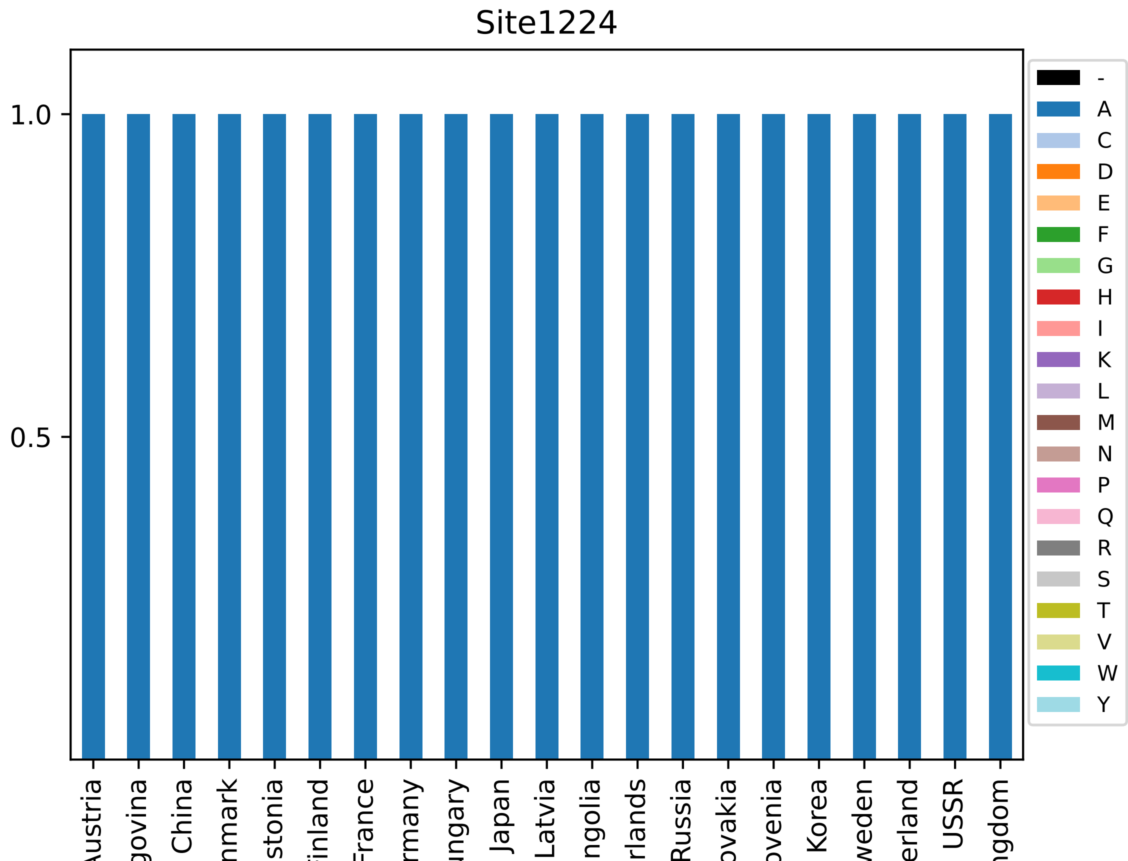

Site1224
1.0
0.5
-
A
C
D
E
F
G
H
I
K
L
M
N
P
Q
R
S
T
V
W
Y
China
Japan
Latvia
Russia
Korea
USSR

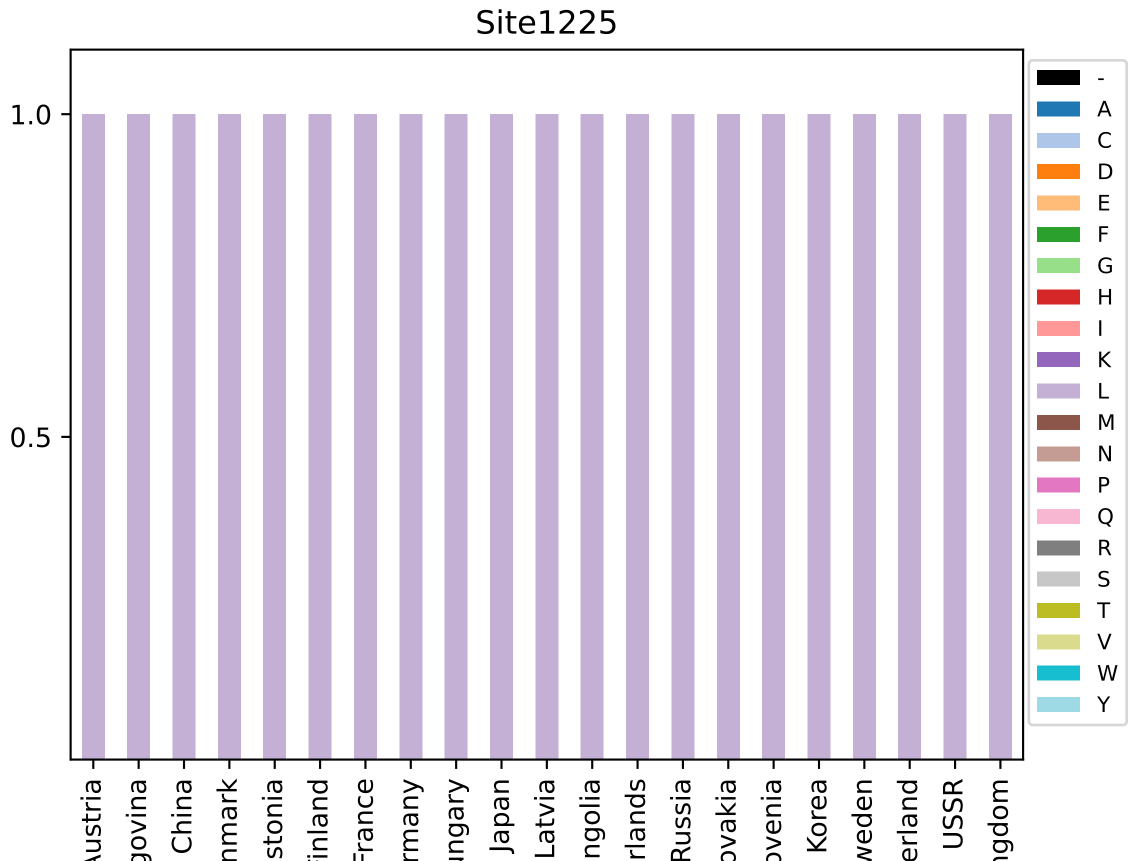
Site1225
1.0
0.5
-
A
C
D
E
F
G
H
I
K
L
M
N
P
Q
R
S
T
V
W
Y
China
Japan
Latvia
Russia
Korea
USSR

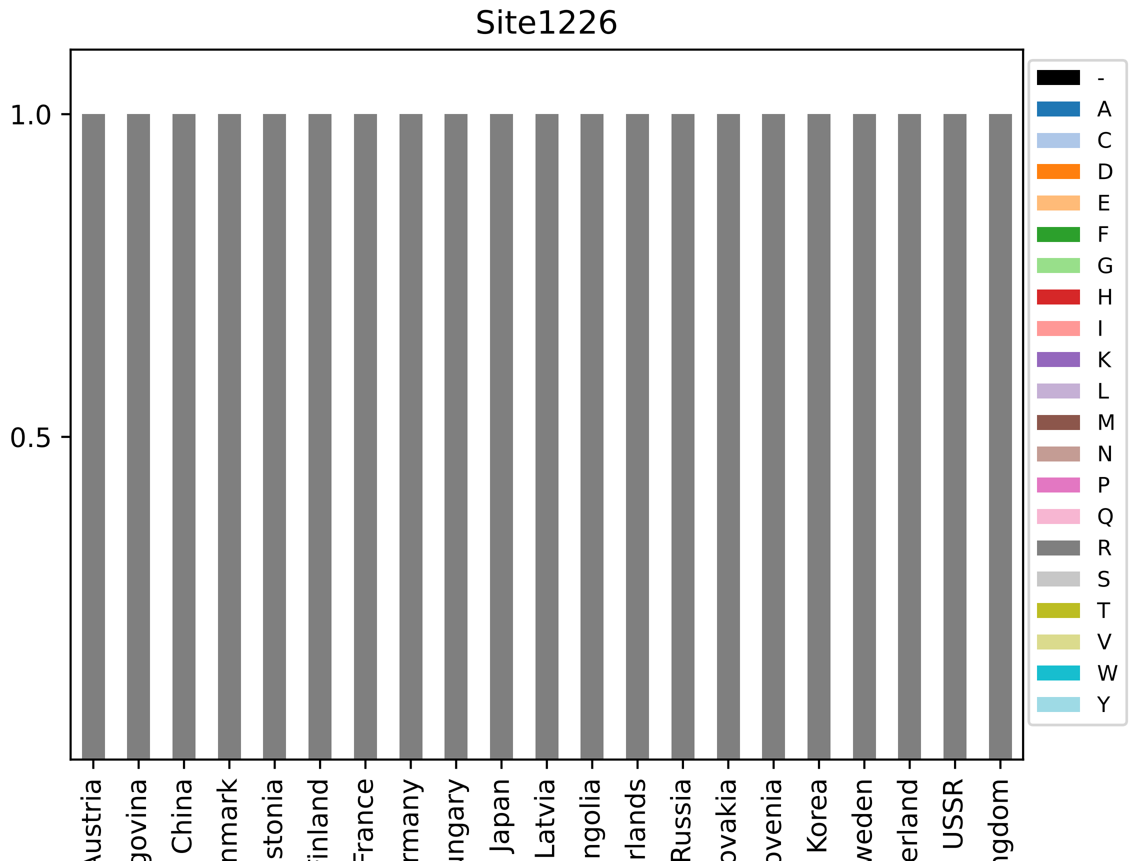
Site1226
1.0
0.5
-
A
C
D
E
F
G
H
I
K
L
M
N
P
Q
R
S
T
V
W
Y
China
Japan
Latvia
Russia
Korea
USSR

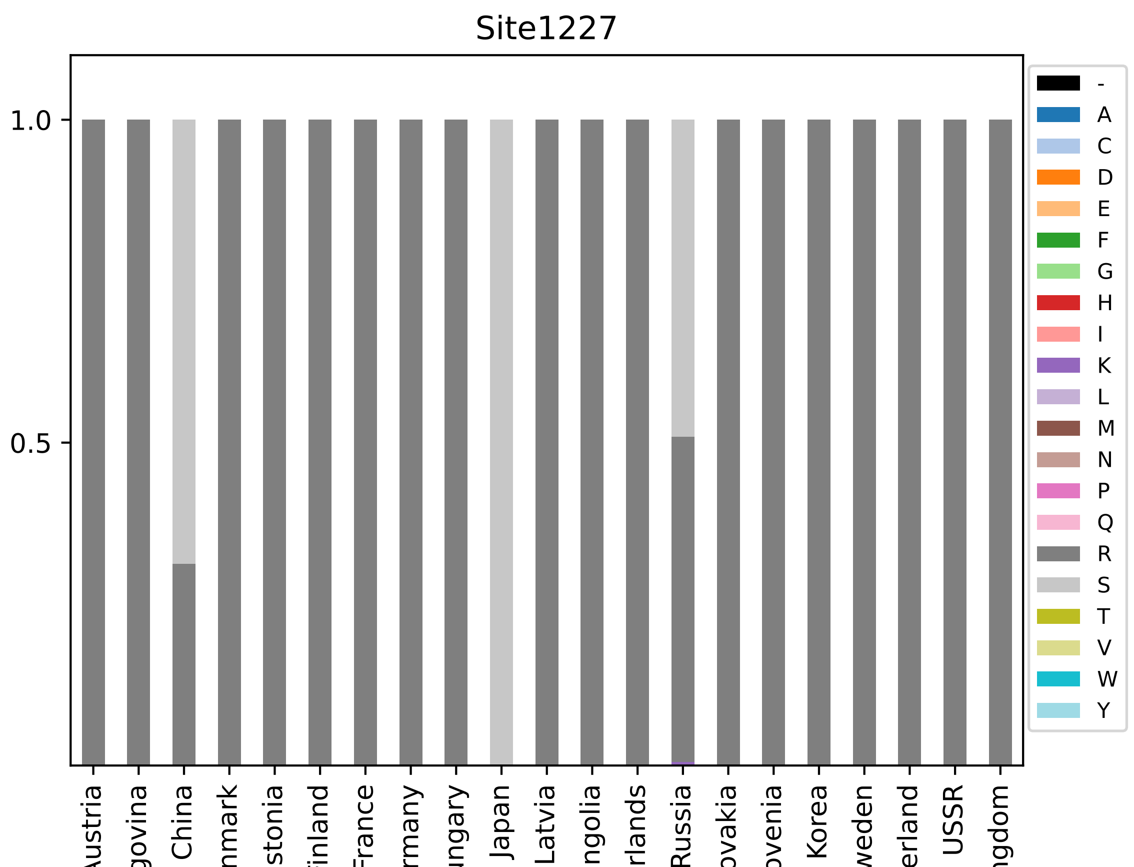

Site1227
1.0
0.5
-
A
C
D
E
F
G
H
I
K
L
M
N
P
Q
R
S
T
V
W
Y
China
Japan
Latvia
Russia
Korea
USSR

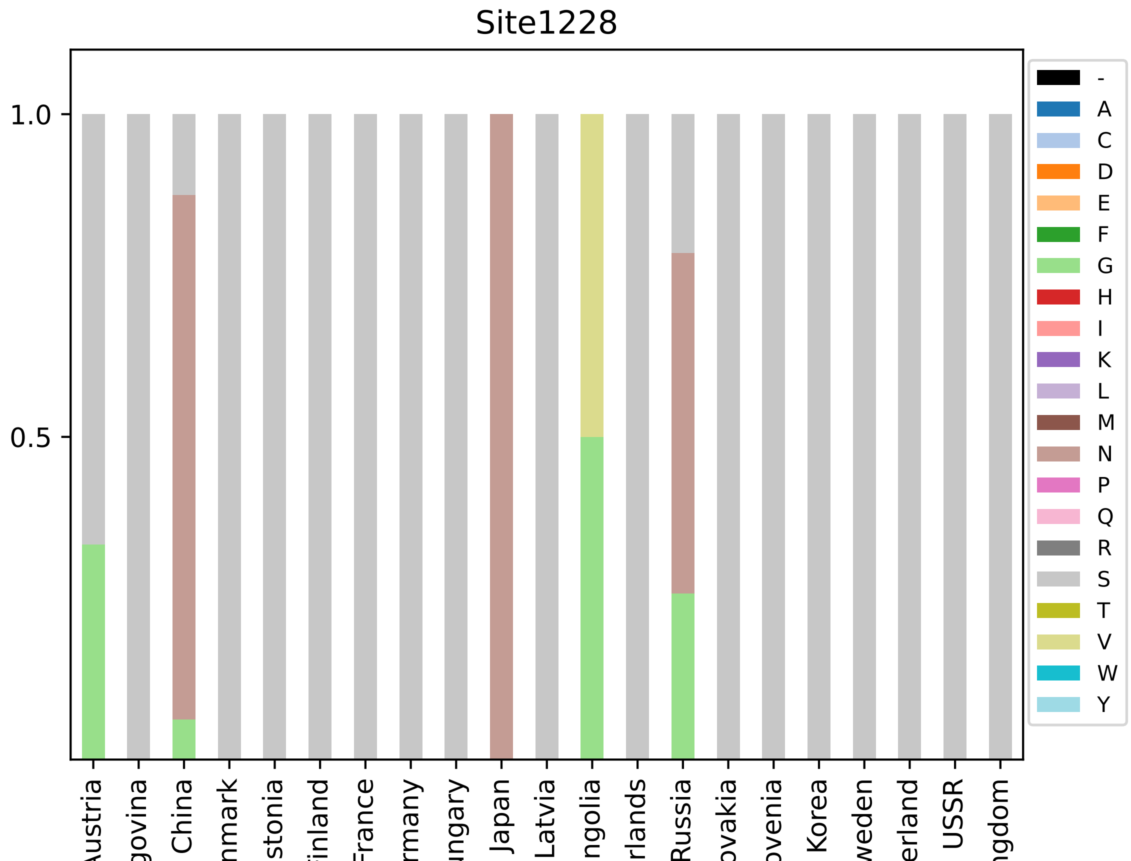

Site1228
1.0
0.5
-
A
C
D
E
F
G
H
I
K
L
M
N
P
Q
R
S
T
V
W
Y
Austria
govina
China
nmark
stonia
Finland
France
rmany
ungary
Japan
Latvia
ngolia
rlands
Russia
ovakia
ovenia
Korea
weden
erland
USSR
ngdom

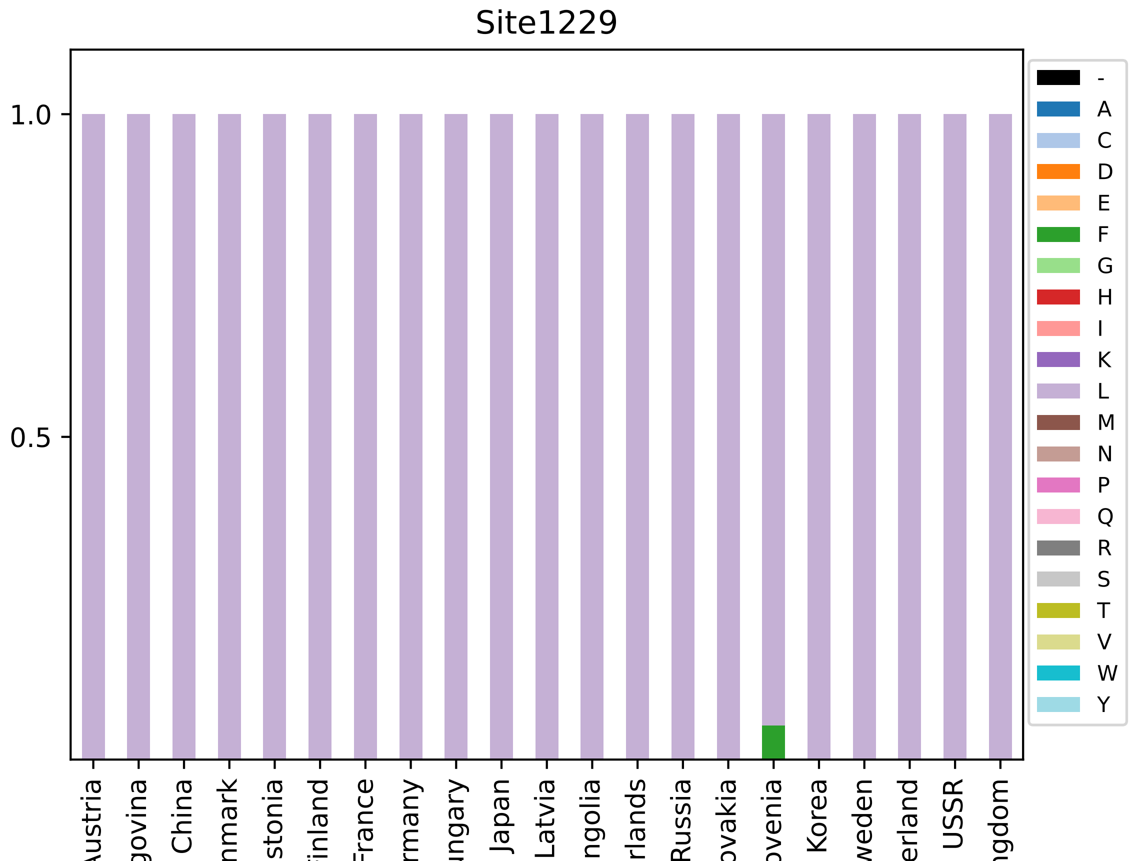

Site1229
1.0
0.5
-
A
C
D
E
F
G
H
I
K
L
M
N
P
Q
R
S
T
V
W
Y
China
Japan
Latvia
Russia
Korea
USSR

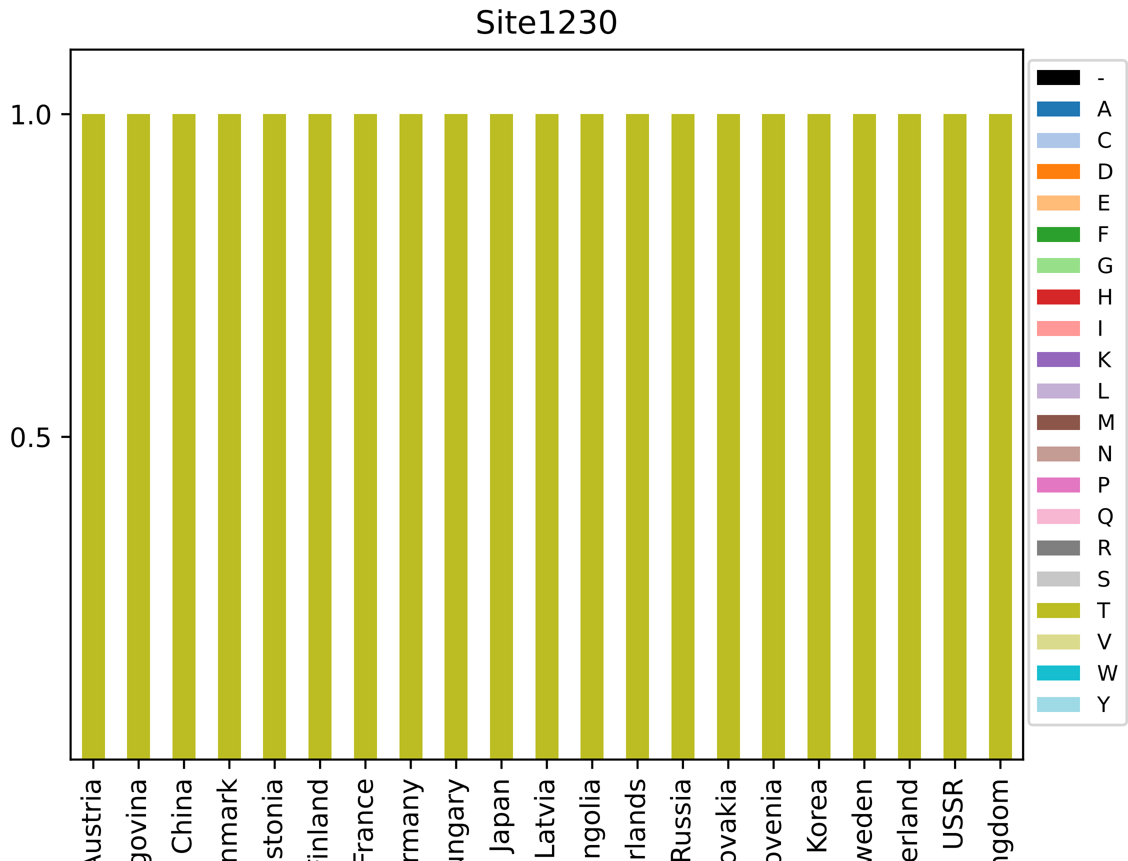

Site1230
1.0
0.5
-
A
C
D
E
F
G
H
I
K
L
M
N
P
Q
R
S
T
V
W
Y
China
Japan
Latvia
Russia
Korea
USSR

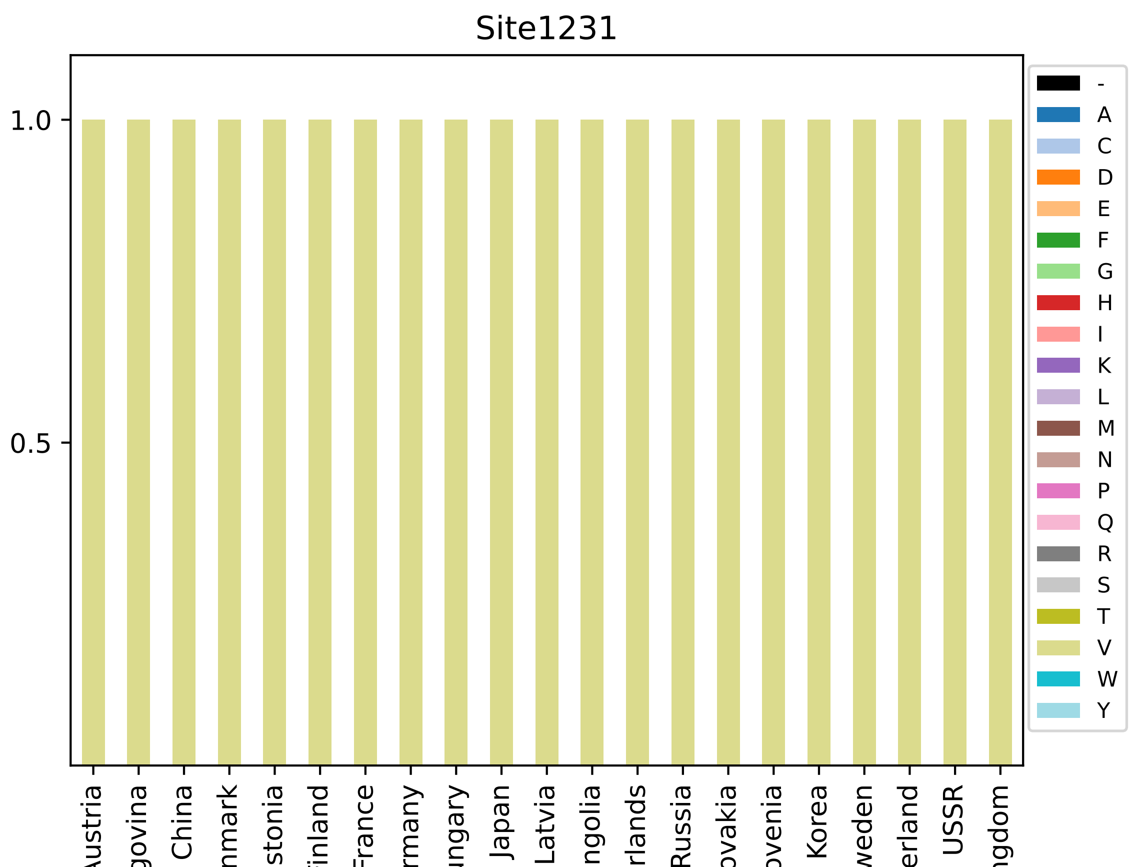
Site1231
1.0
0.5
-
A
C
D
E
F
G
H
I
K
L
M
N
P
Q
R
S
T
V
W
Y
Austria
govina
China
nmark
stonia
inland
France
rmany
ungary
Japan
Latvia
ngolia
rlands
Russia
ovakia
ovenia
Korea
weden
erland
USSR
ngdom

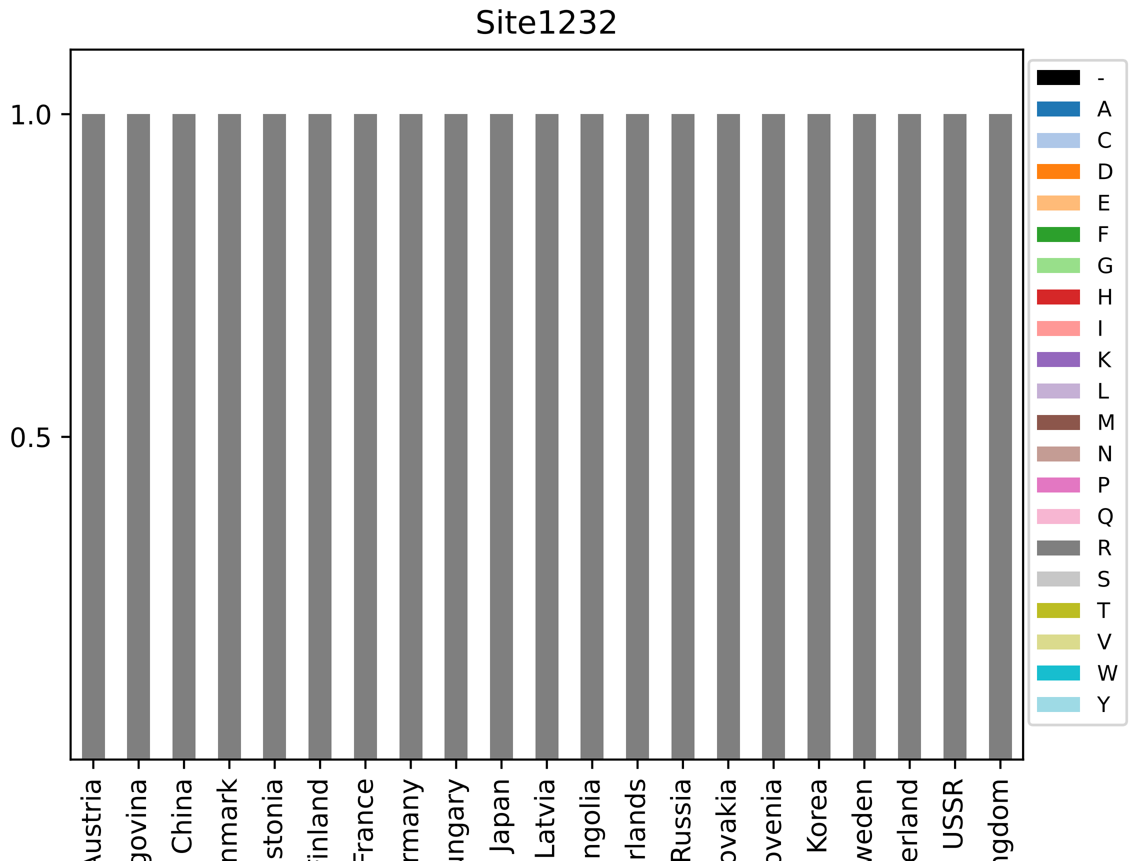

Site1232
1.0
0.5
-
A
C
D
E
F
G
H
I
K
L
M
N
P
Q
R
S
T
V
W
Y
China
Japan
Latvia
Russia
Korea
USSR

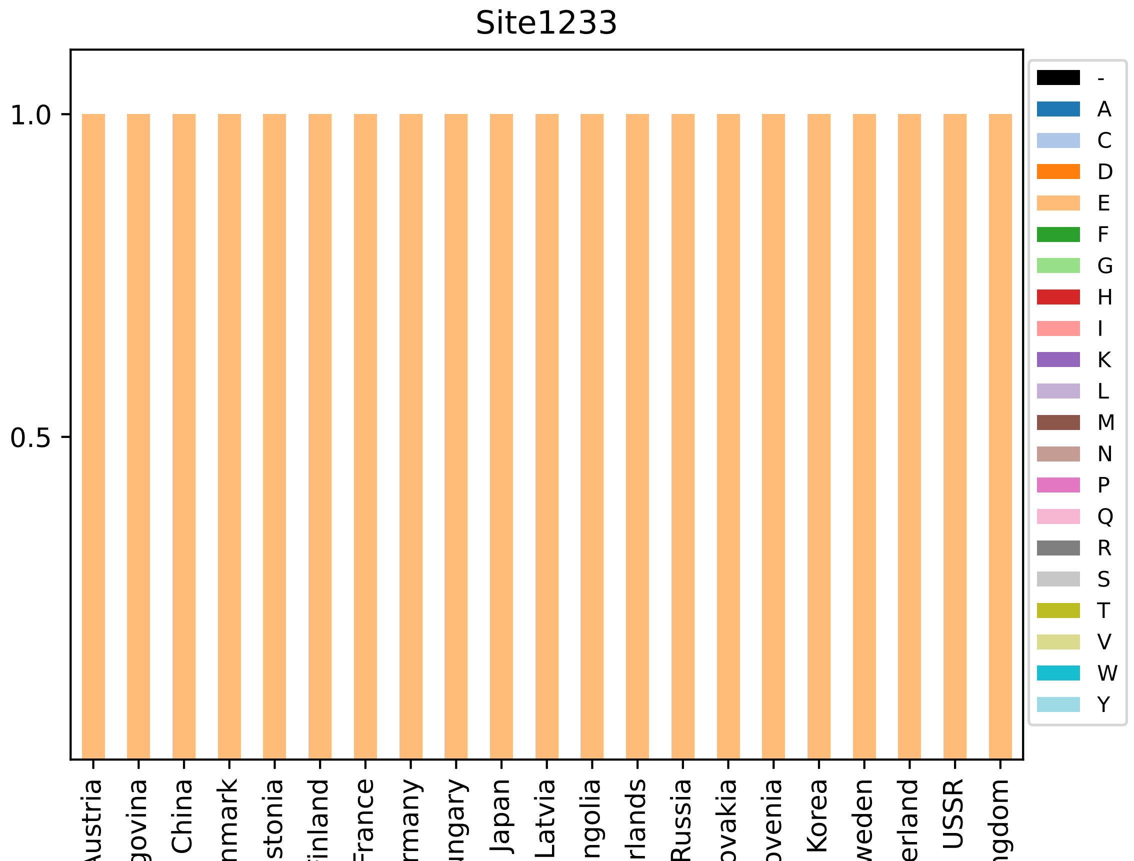

Site1233
1.0
0.5
-
A
C
D
E
F
G
H
I
K
L
M
N
P
Q
R
S
T
V
W
Y
Austria
China
Japan
Latvia
Russia
Korea
USSR

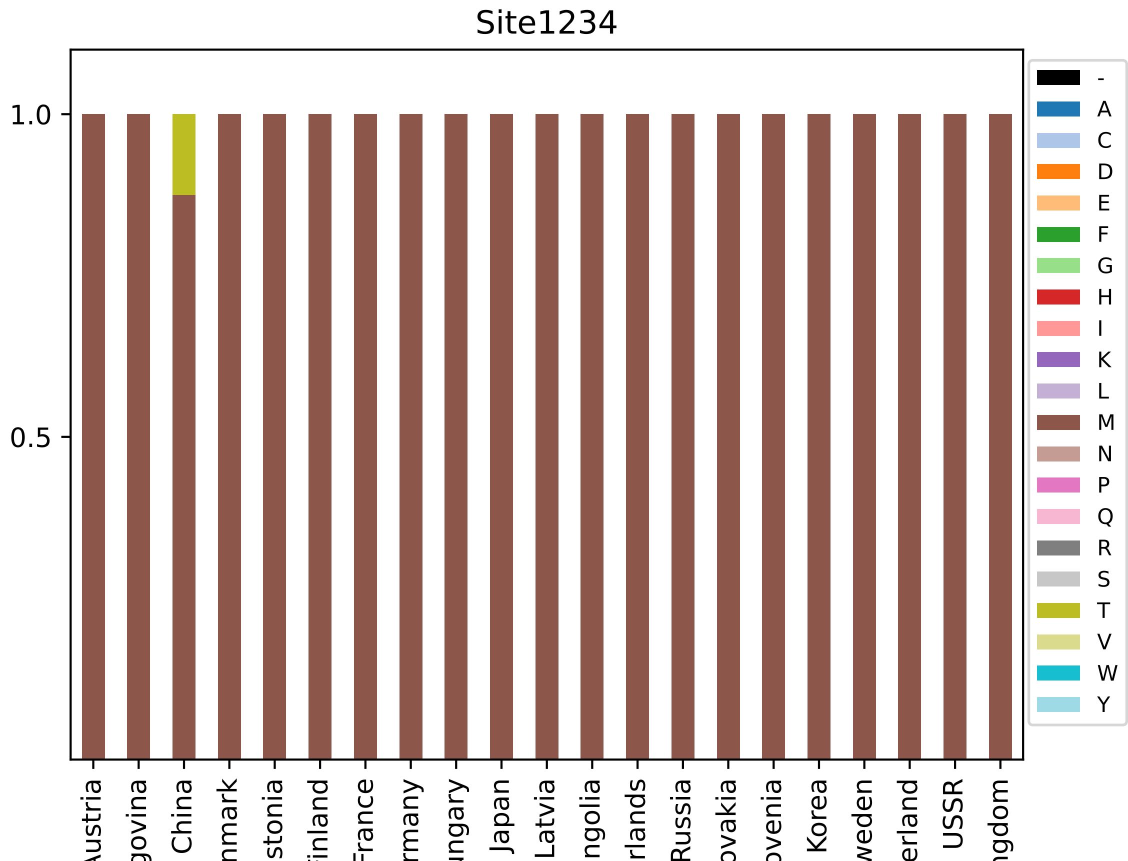
Site1234
1.0
0.5
-
A
C
D
E
F
G
H
I
K
L
M
N
P
Q
R
S
T
V
W
Y
China
Japan
Latvia
Russia
Korea
USSR

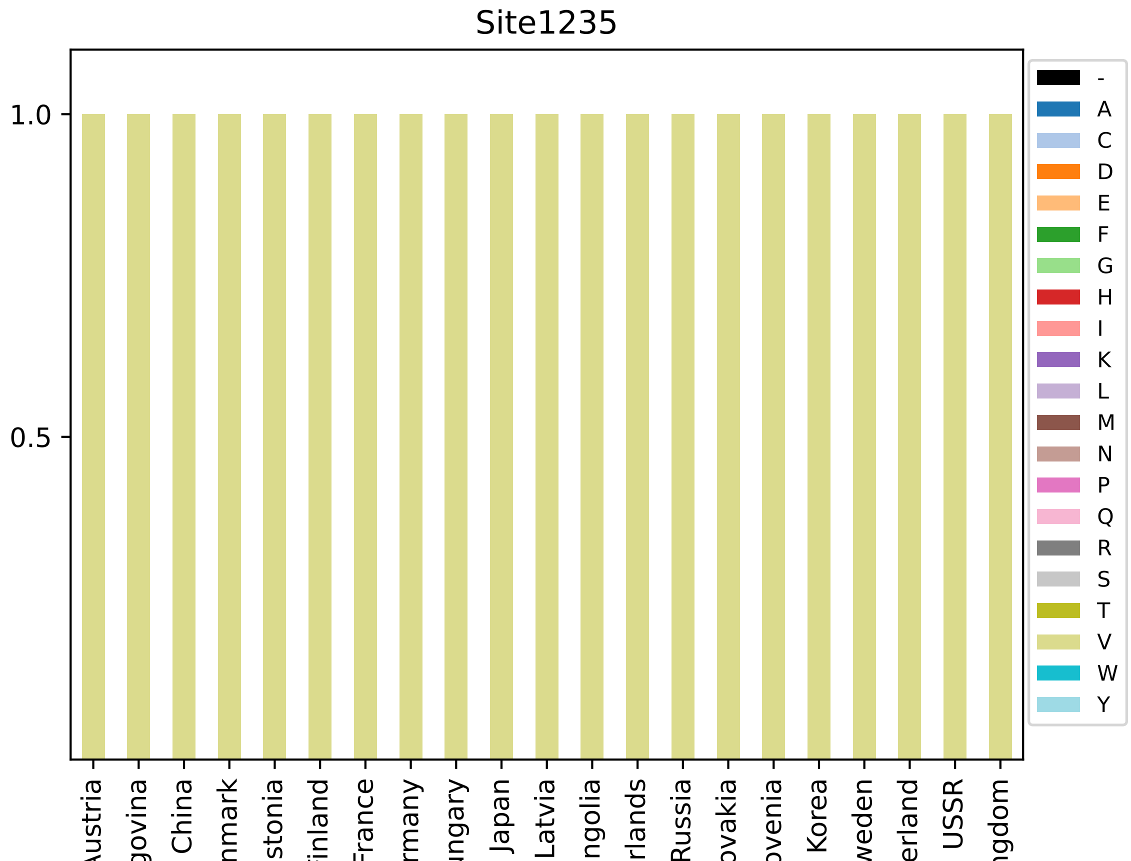
Site1235
1.0
0.5
-
A
C
D
E
F
G
H
I
K
L
M
N
P
Q
R
S
T
V
W
Y
China
Japan
Latvia
Russia
Korea
USSR

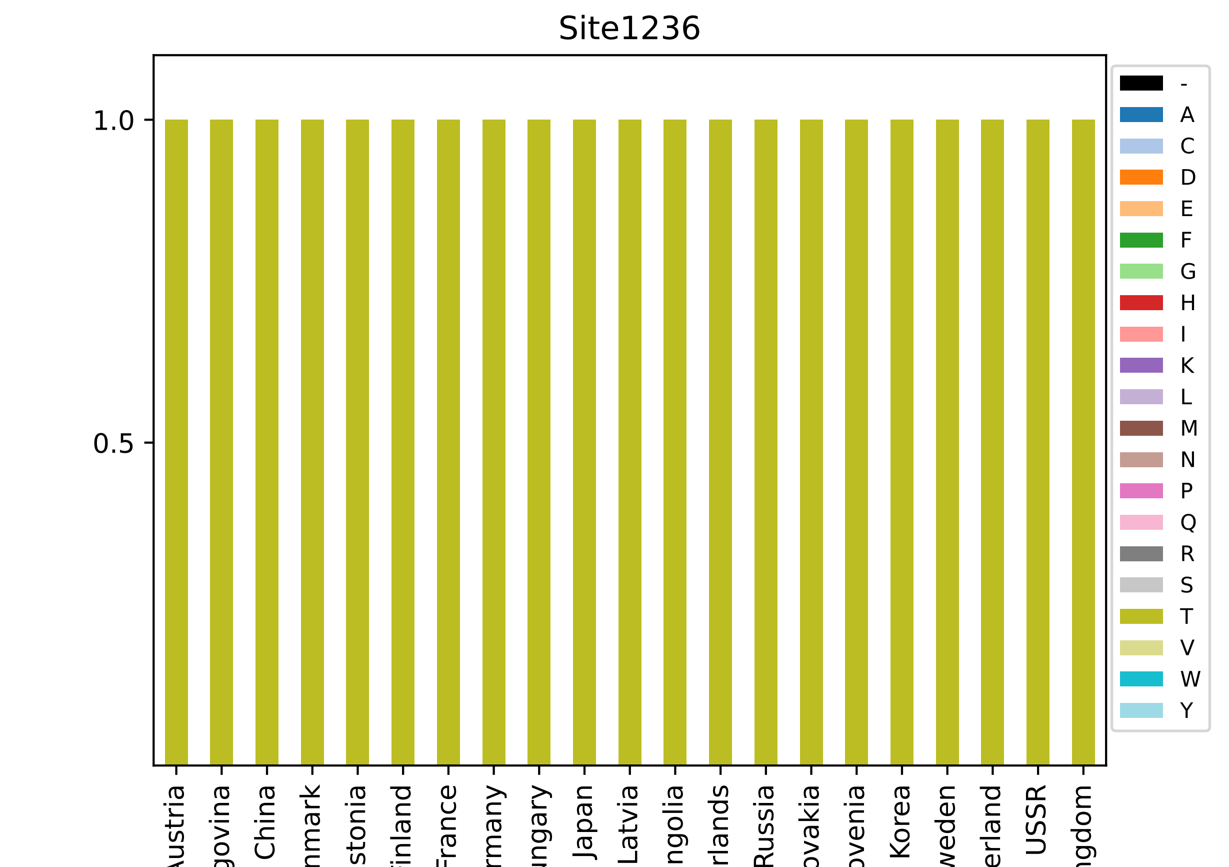
Site1236
1.0
0.5
-
A
C
D
E
F
G
H
I
K
L
M
N
P
Q
R
S
T
V
W
Y
Austria
govina
China
nmark
stonia
inland
France
rmany
ungary
Japan
Latvia
ngolia
rlands
Russia
ovakia
ovenia
Korea
weden
erland
USSR
ngdom

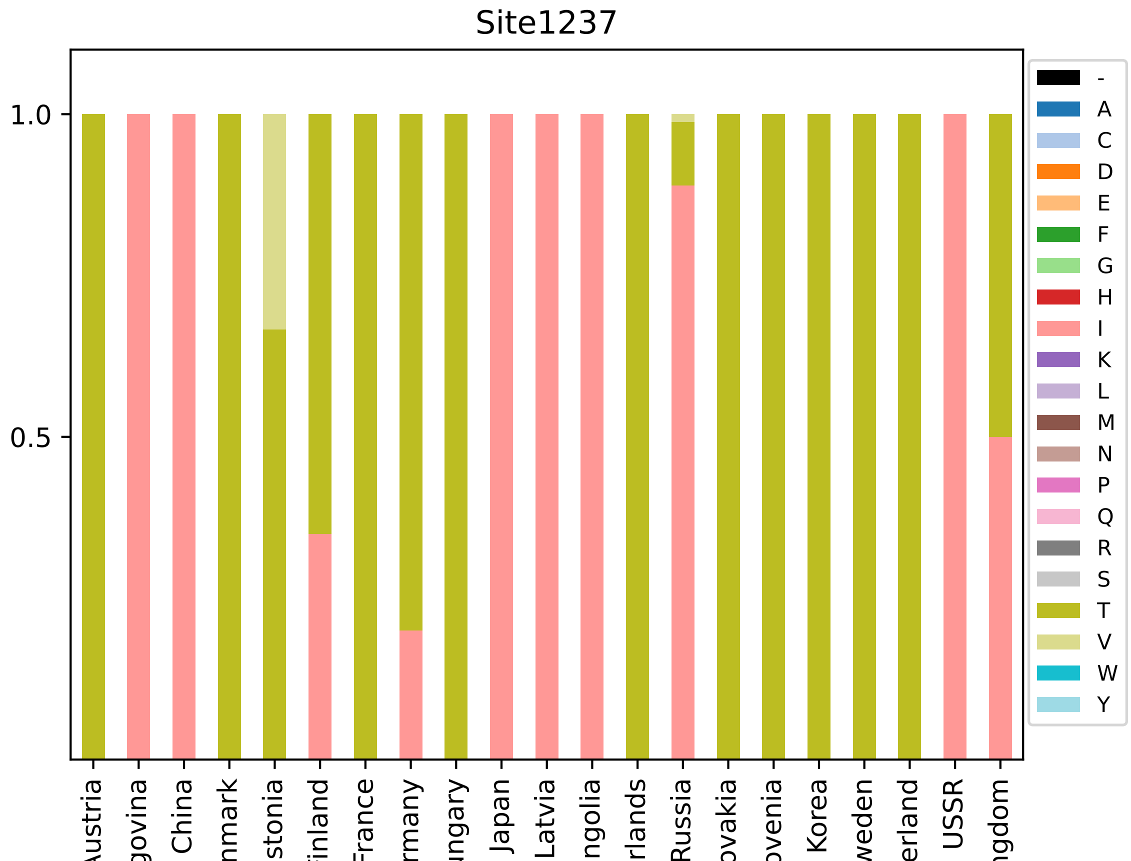

Site1237
1.0
0.5
-
A
C
D
E
F
G
H
I
K
L
M
N
P
Q
R
S
T
V
W
Y
Austria
govina
China
nmark
stonia
Finland
France
rmany
ungary
Japan
Latvia
ngolia
rlands
Russia
ovakia
ovenia
Korea
weden
erland
USSR
ngdom

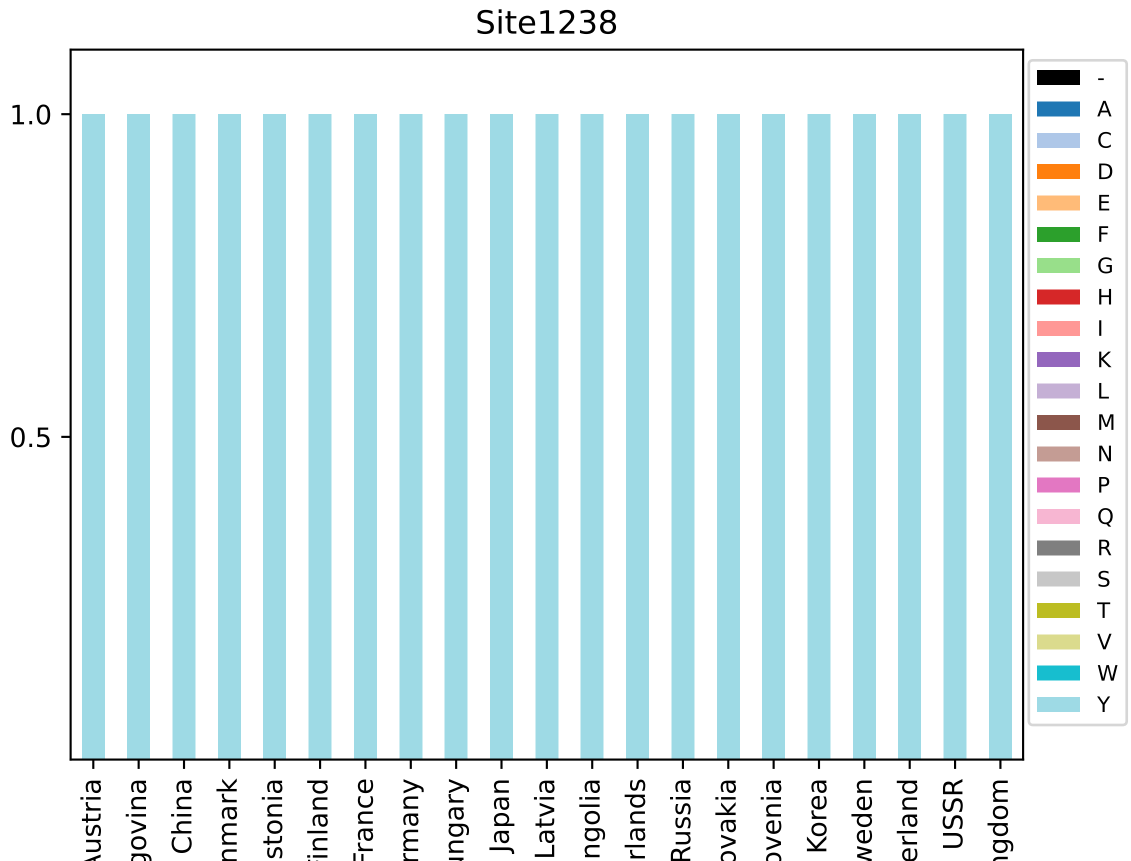

Site1238
1.0
0.5
-
A
C
D
E
F
G
H
I
K
L
M
N
P
Q
R
S
T
V
W
Y
China
Japan
Latvia
Russia
Korea
USSR

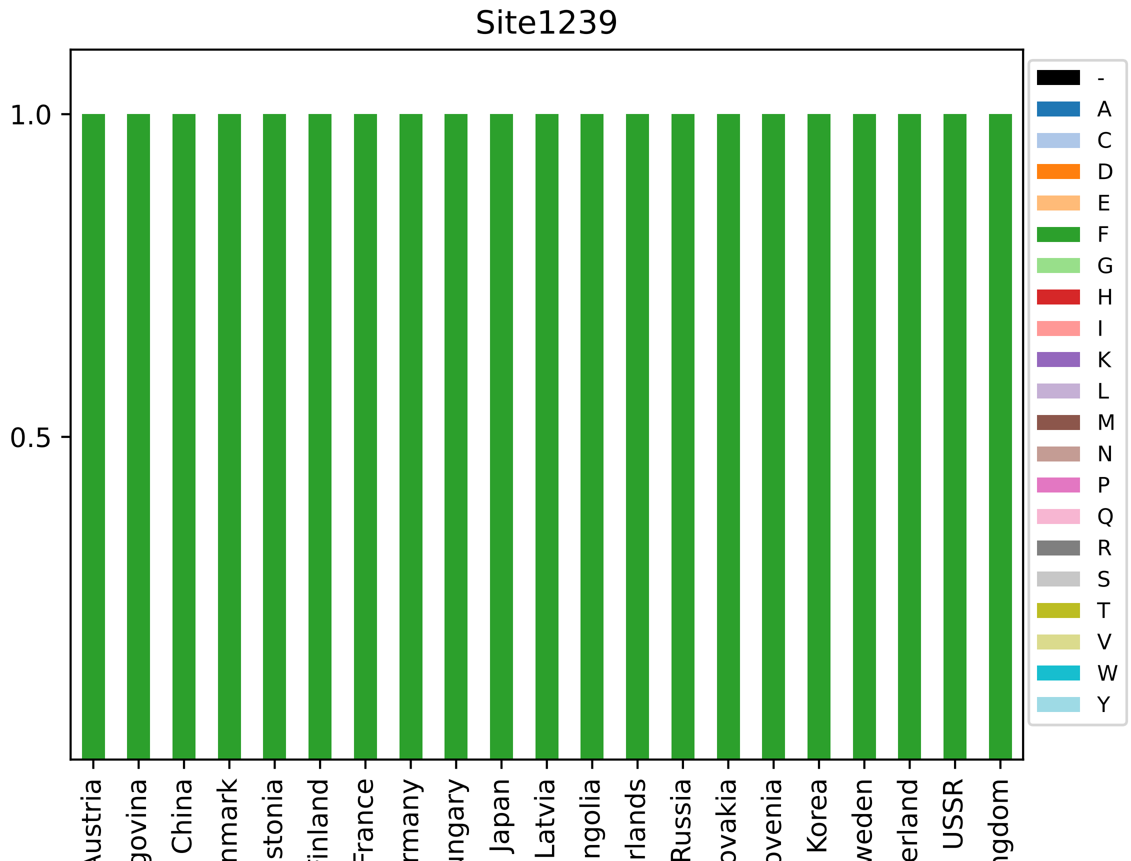

Site1239
1.0
0.5
-
A
C
D
E
F
G
H
I
K
L
M
N
P
Q
R
S
T
V
W
Y
Austria
govina
China
nmark
stonia
Finland
France
rmany
ungary
Japan
Latvia
ngolia
rlands
Russia
ovakia
ovenia
Korea
weden
erland
USSR
ngdom

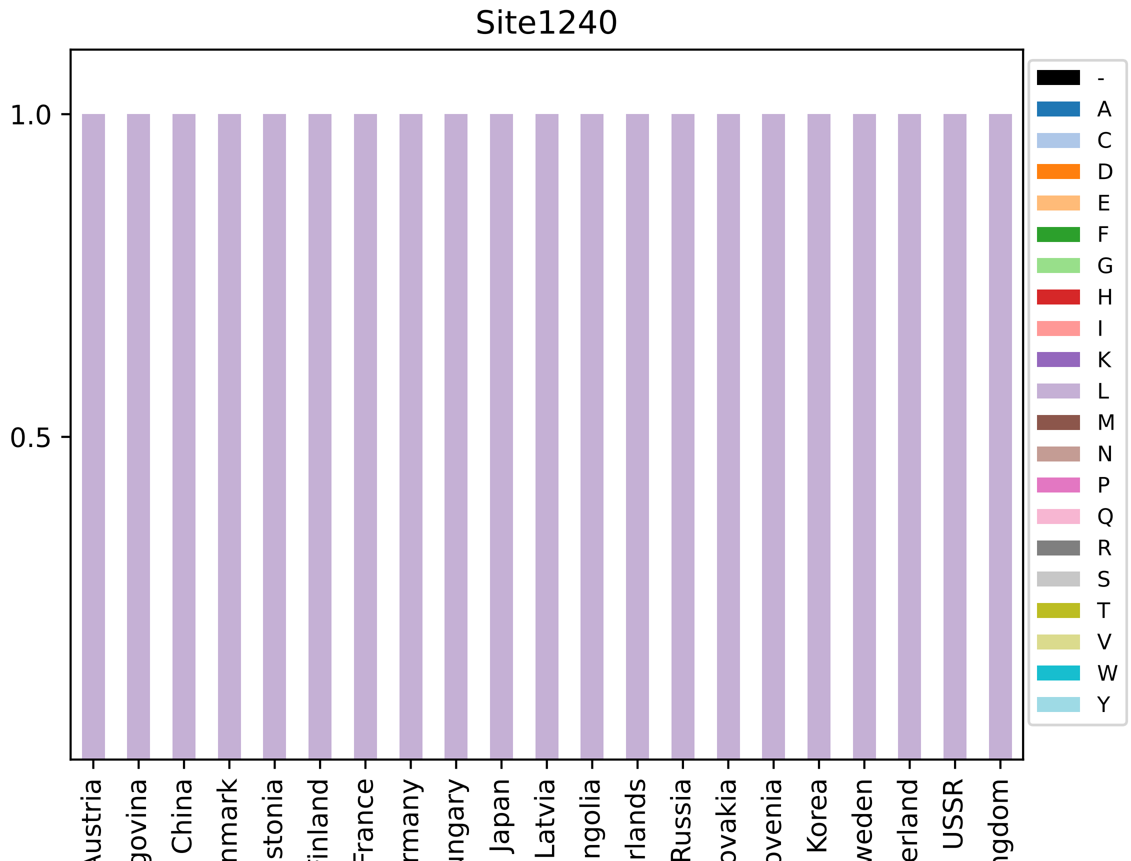
Site1240
1.0
0.5
-
A
C
D
E
F
G
H
I
K
L
M
N
P
Q
R
S
T
V
W
Y
China
Japan
Latvia
Russia
Korea
USSR

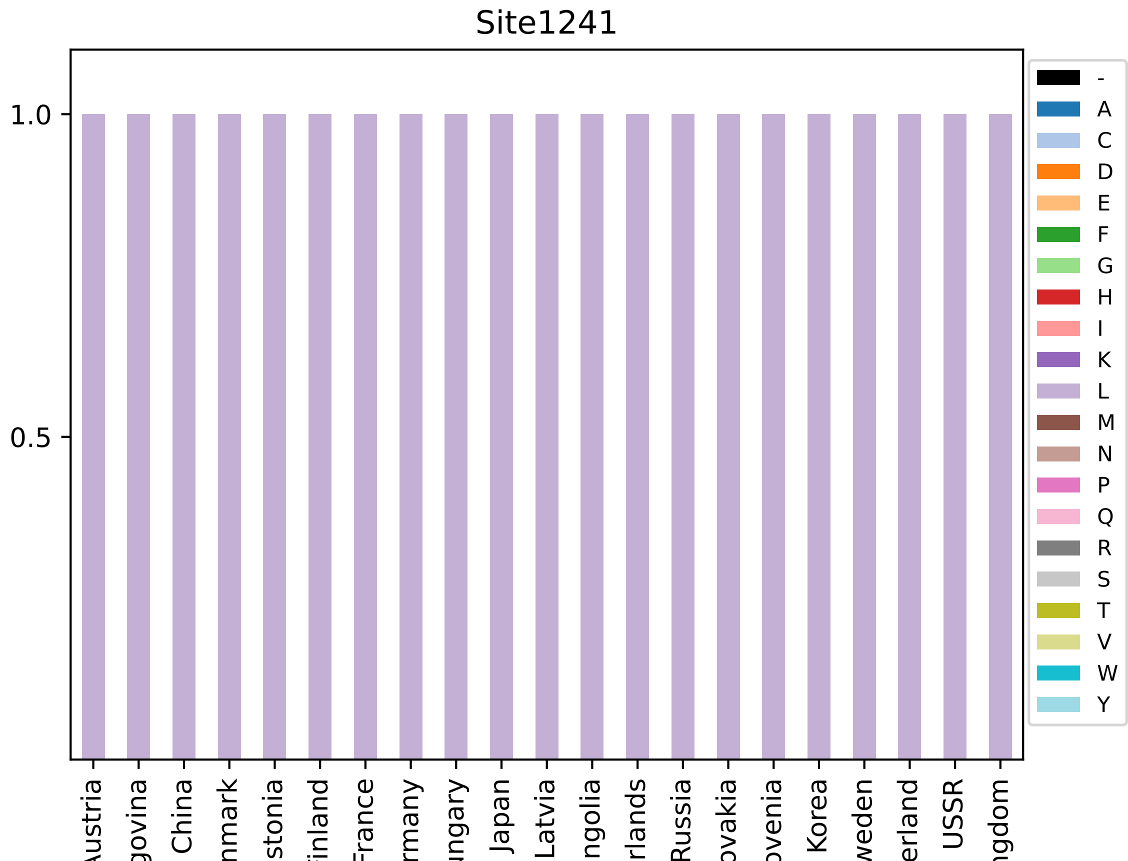

Site1241
1.0
0.5
Austria
govina
China
nmark
stonia
inland
France
rmany
ungary
Japan
Latvia
ngolia
rlands
Russia
ovakia
ovenia
Korea
weden
erland
USSR
ngdom
-
A
C
D
E
F
G
H
I
K
L
M
N
P
Q
R
S
T
V
W
Y

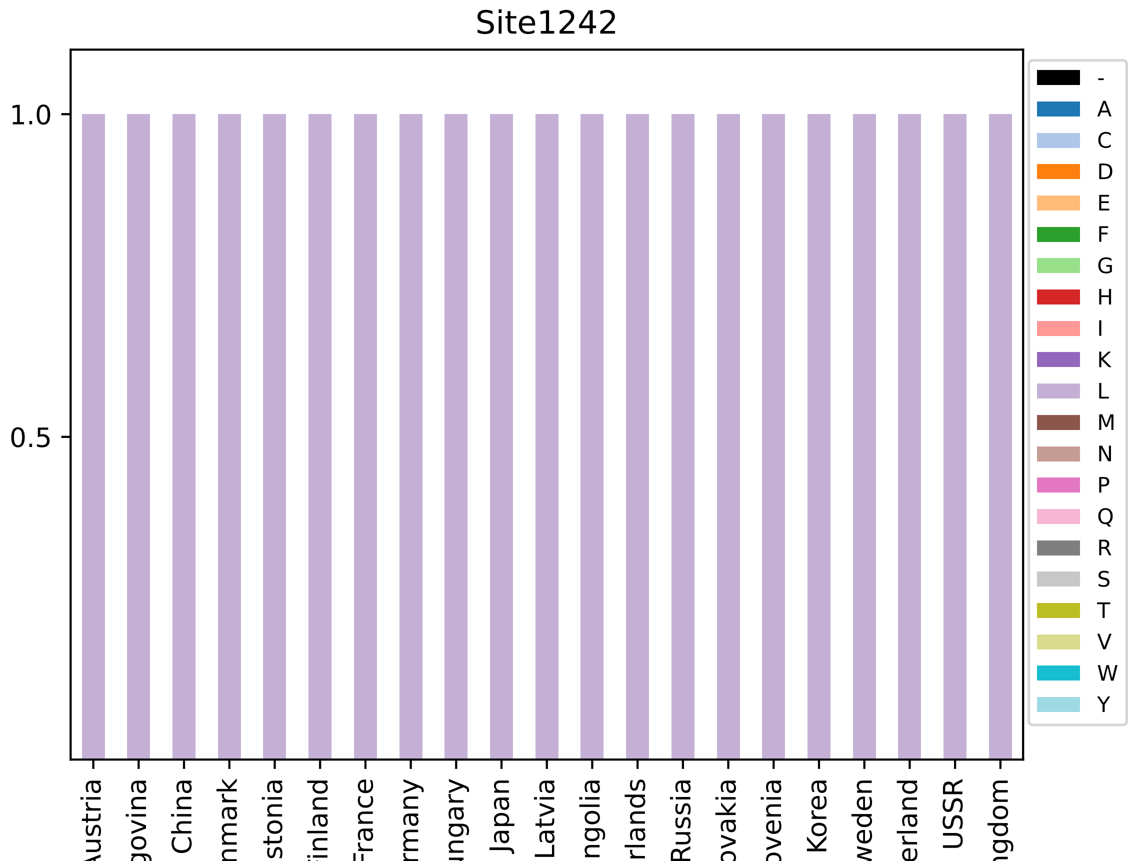

Site1242
1.0
0.5
-
A
C
D
E
F
G
H
I
K
L
M
N
P
Q
R
S
T
V
W
Y
China
Japan
Latvia
Russia
Korea
USSR

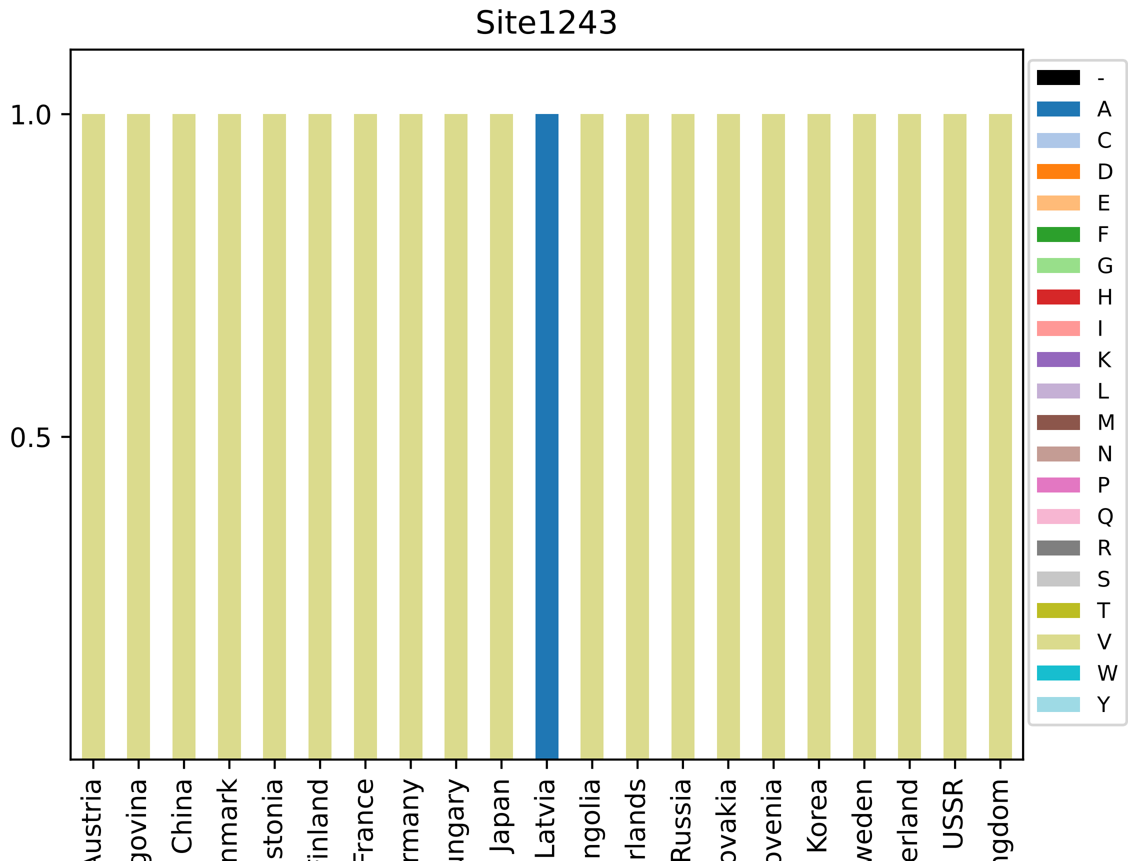

Site1243
1.0
0.5
-
A
C
D
E
F
G
H
I
K
L
M
N
P
Q
R
S
T
V
W
Y
Austria
govina
China
nmark
stonia
inland
France
rmany
ungary
Japan
Latvia
ngolia
rlands
Russia
ovakia
ovenia
Korea
weden
erland
USSR
ngdom

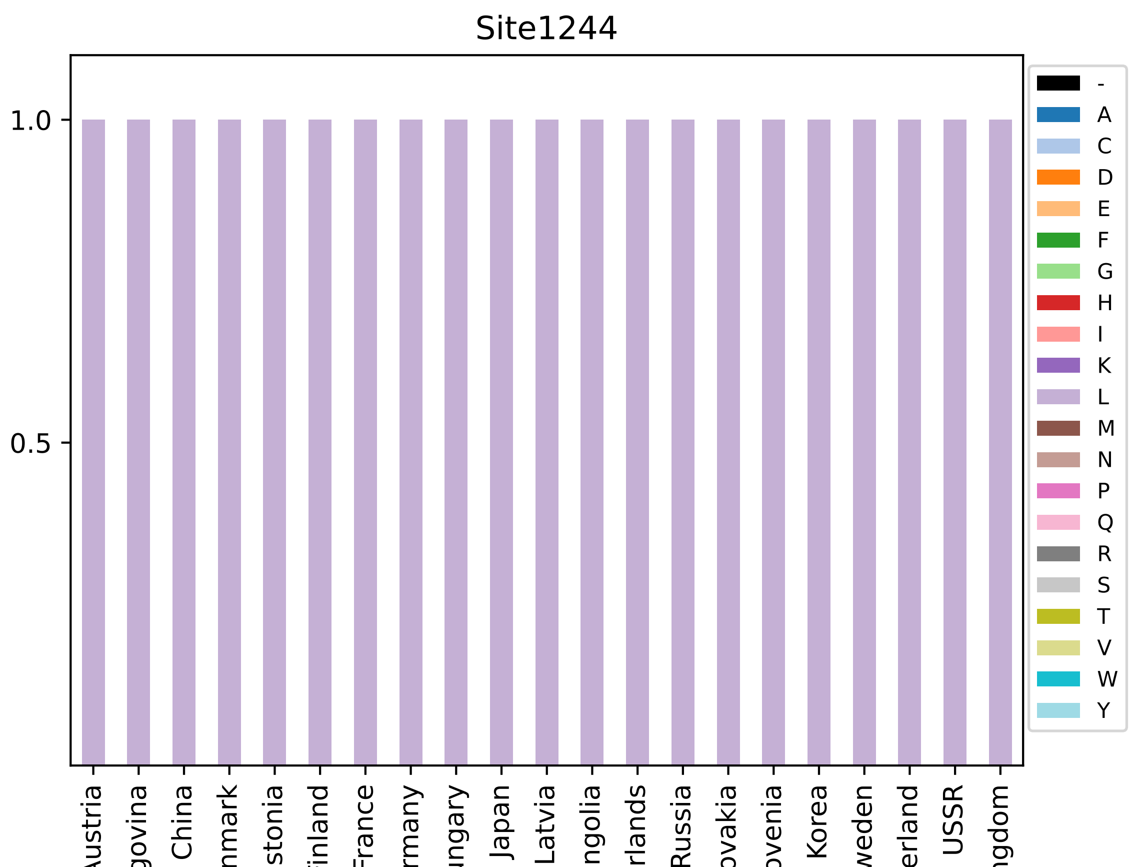
Site1244
1.0
0.5
-
A
C
D
E
F
G
H
I
K
L
M
N
P
Q
R
S
T
V
W
Y
Austria
China
Finland
France
Japan
Latvia
Russia
Korea
USSR

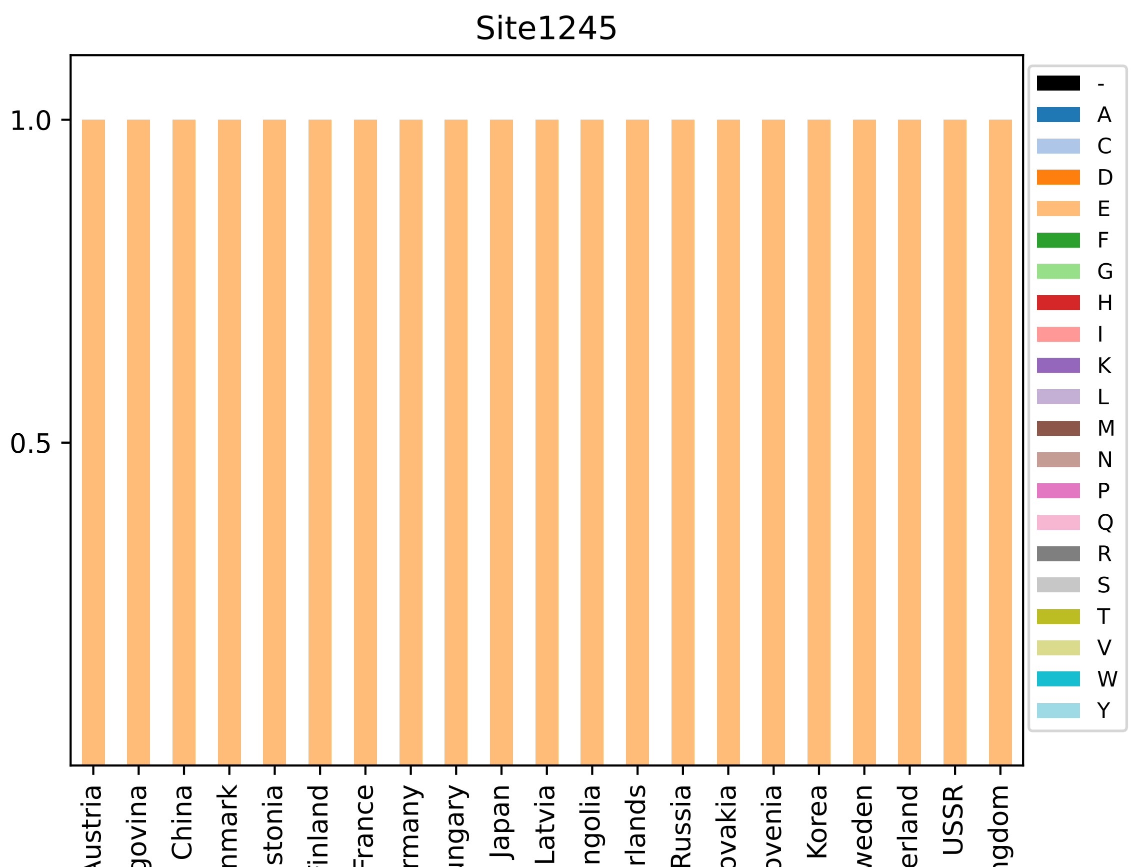

Site1245
1.0
0.5
-
A
C
D
E
F
G
H
I
K
L
M
N
P
Q
R
S
T
V
W
Y
Austria
govina
China
nmark
stonia
inland
France
rmany
ungary
Japan
Latvia
ngolia
rlands
Russia
ovakia
ovenia
Korea
weden
erland
USSR
ngdom

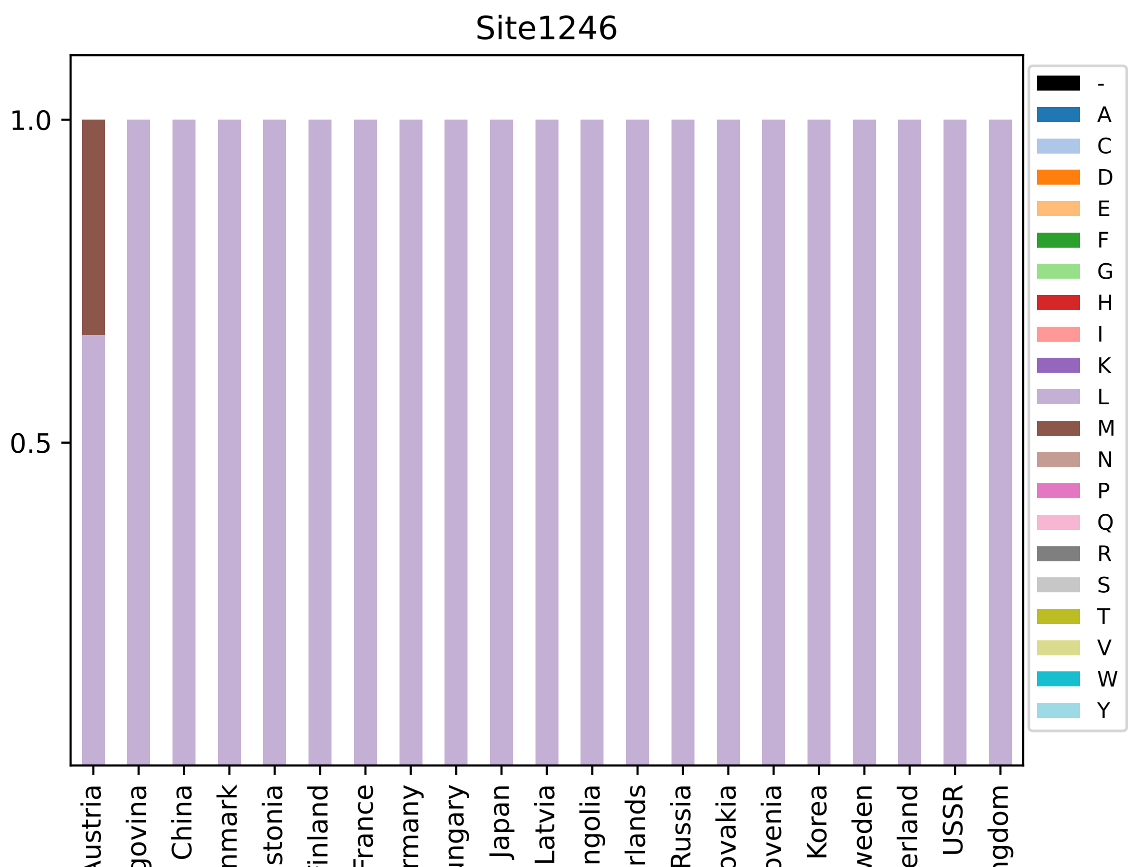
Site1246
1.0
0.5
-
A
C
D
E
F
G
H
I
K
L
M
N
P
Q
R
S
T
V
W
Y
China
Japan
Latvia
Russia
Korea
USSR

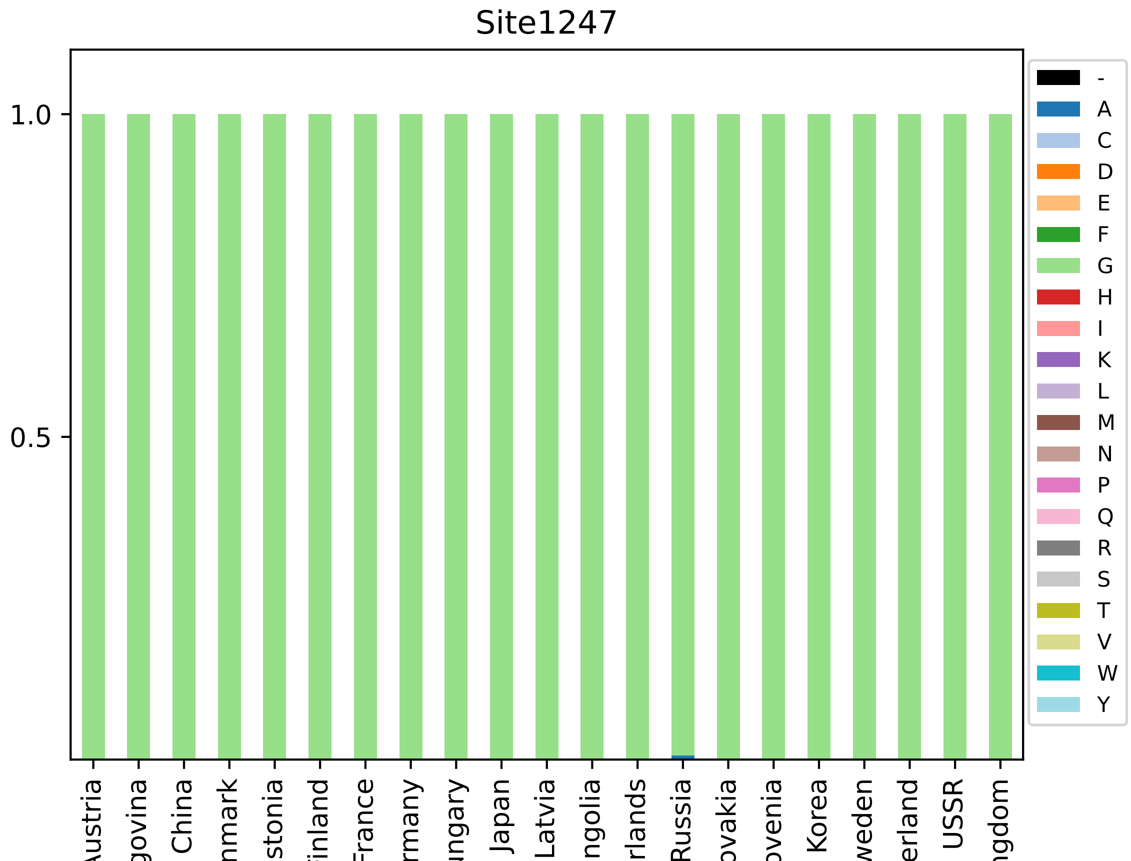

Site1247
1.0
0.5
-
A
C
D
E
F
G
H
I
K
L
M
N
P
Q
R
S
T
V
W
Y
China
Japan
Latvia
Russia
Korea
USSR

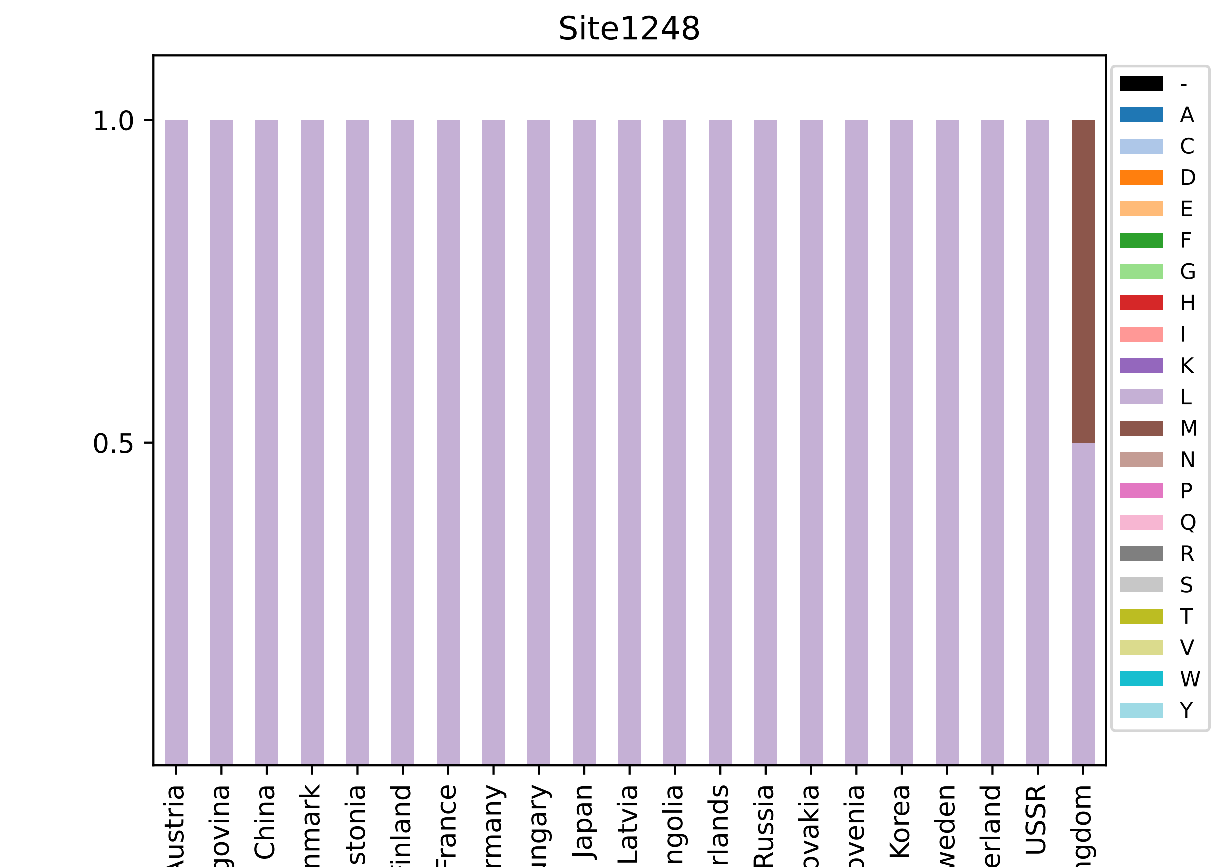
Site1248
1.0
0.5
-
A
C
D
E
F
G
H
I
K
L
M
N
P
Q
R
S
T
V
W
Y
Austria
govina
China
nmark
stonia
Finland
France
rmany
ungary
Japan
Latvia
ngolia
rlands
Russia
ovakia
ovenia
Korea
weden
erland
USSR
ngdom

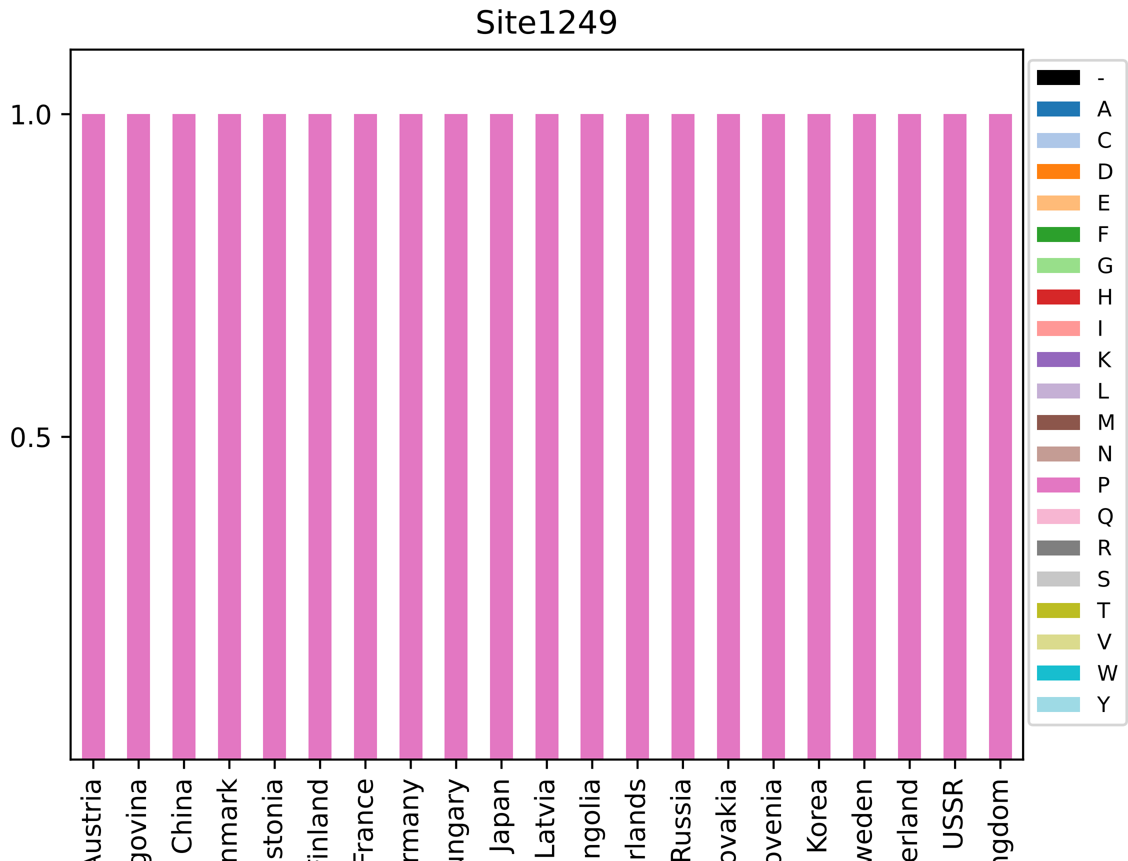

Site1249
1.0
0.5
China
Japan
Latvia
Russia
Korea
USSR
-
A
C
D
E
F
G
H
I
K
L
M
N
P
Q
R
S
T
V
W
Y

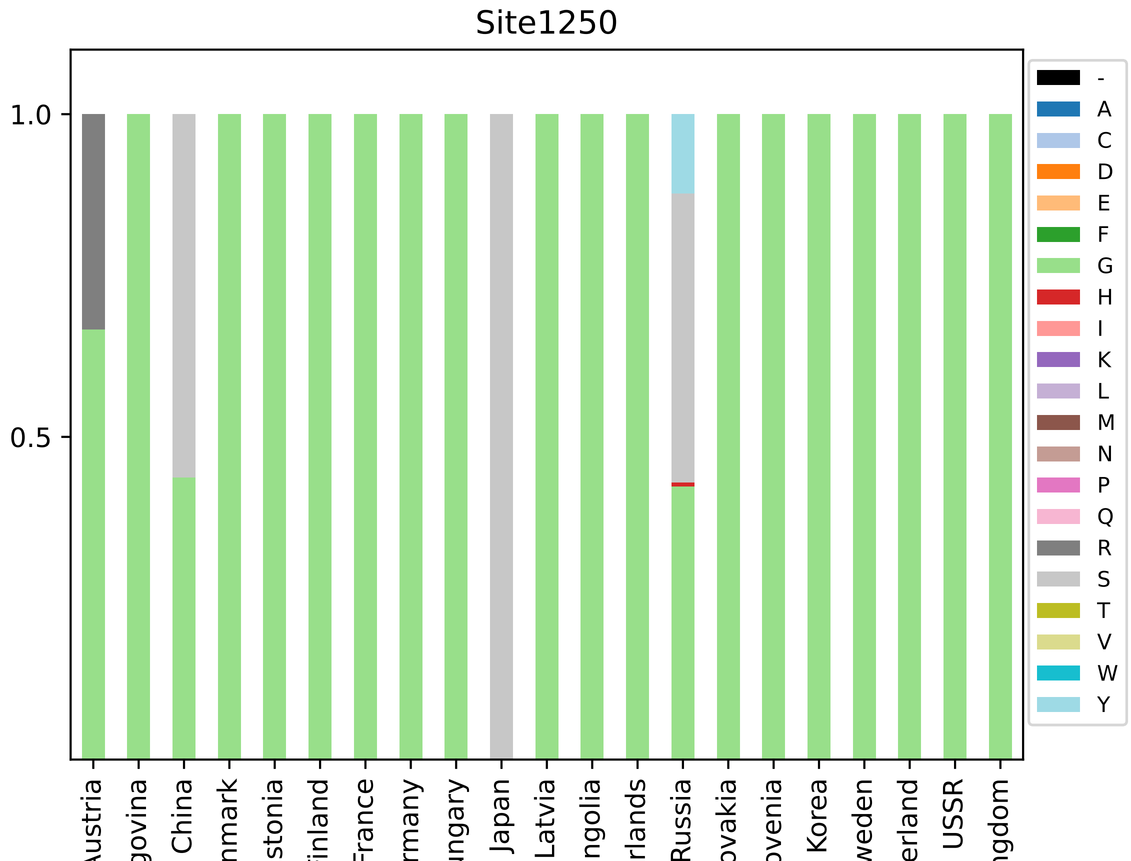

Site1250
1.0
0.5
-
A
C
D
E
F
G
H
I
K
L
M
N
P
Q
R
S
T
V
W
Y
Austria
govina
China
nmark
stonia
Finland
France
rmany
ungary
Japan
Latvia
ngolia
rlands
Russia
ovakia
ovenia
Korea
weden
erland
USSR
ngdom

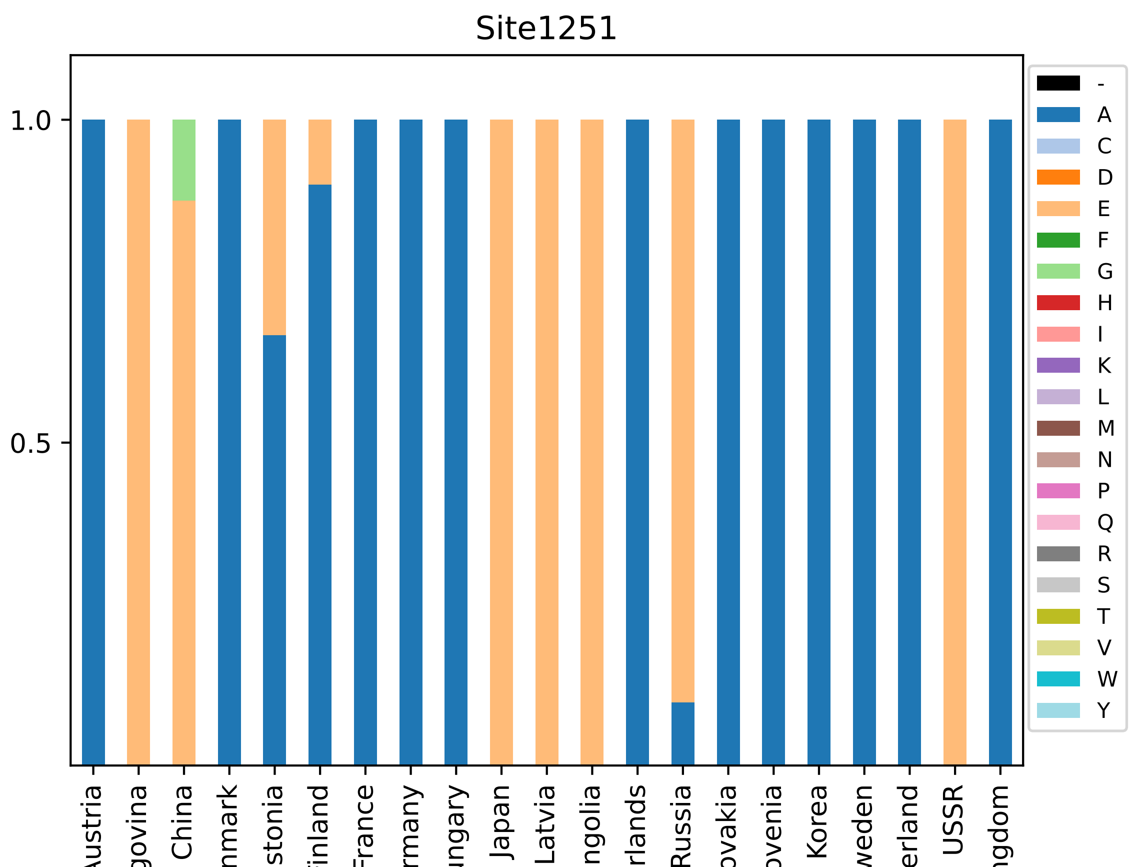

Site1251
1.0
0.5
-
A
C
D
E
F
G
H
I
K
L
M
N
P
Q
R
S
T
V
W
Y
Austria
govina
China
nmark
stonia
Finland
France
rmany
ungary
Japan
Latvia
ngolia
rlands
Russia
ovakia
ovenia
Korea
weden
erland
USSR
ngdom

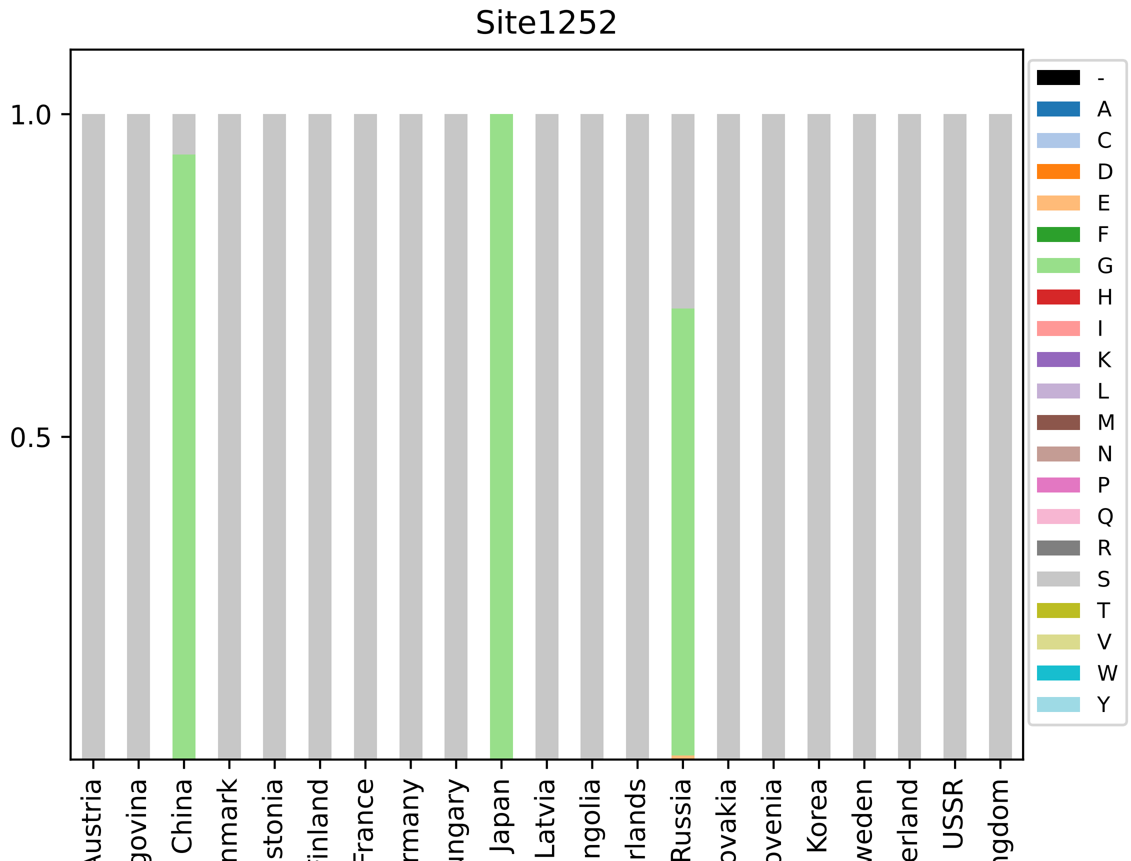
Site1252
1.0
0.5
-
A
C
D
E
F
G
H
I
K
L
M
N
P
Q
R
S
T
V
W
Y
Austria
govina
China
nmark
stonia
inland
France
rmany
ungary
Japan
Latvia
ngolia
rlands
Russia
ovakia
ovenia
Korea
weden
erland
USSR
ngdom

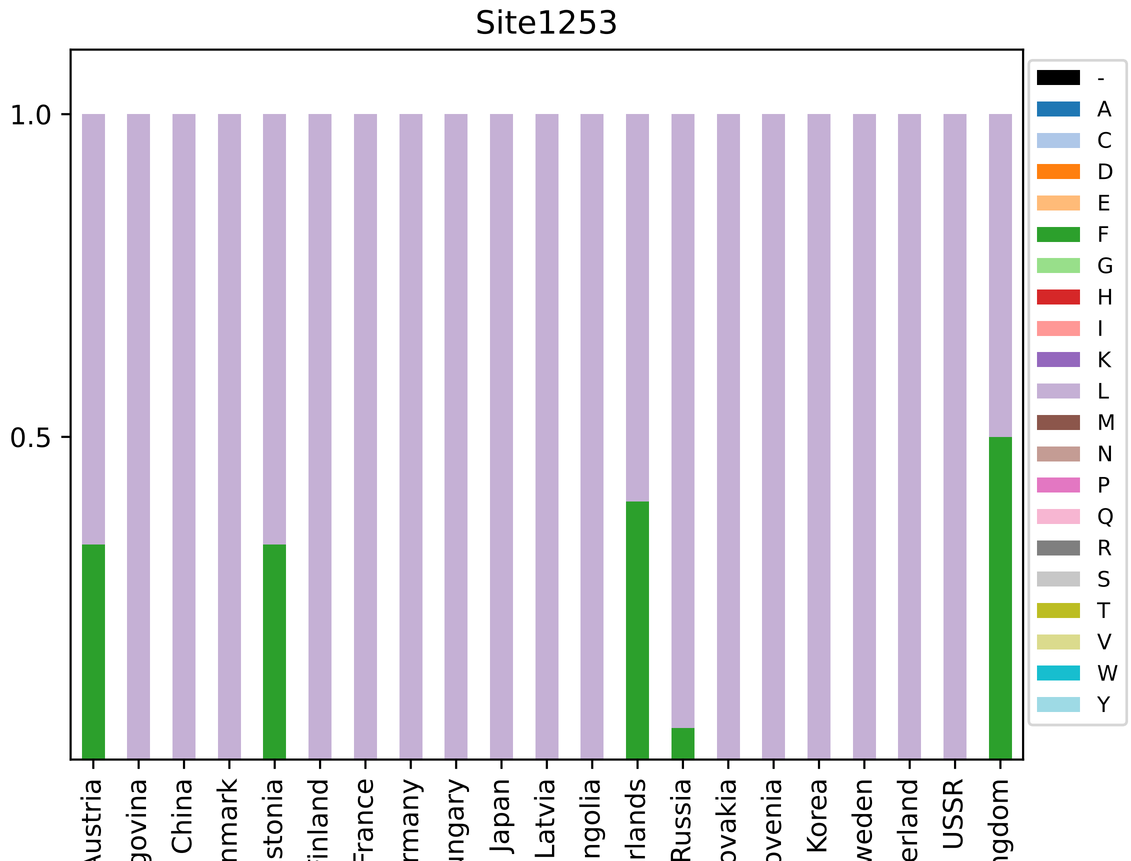

Site1253
1.0
0.5
-
A
C
D
E
F
G
H
I
K
L
M
N
P
Q
R
S
T
V
W
Y
Austria
govina
China
nmark
stonia
inland
France
rmany
ungary
Japan
Latvia
ngolia
rlands
Russia
ovakia
ovenia
Korea
weden
erland
USSR
ngdom

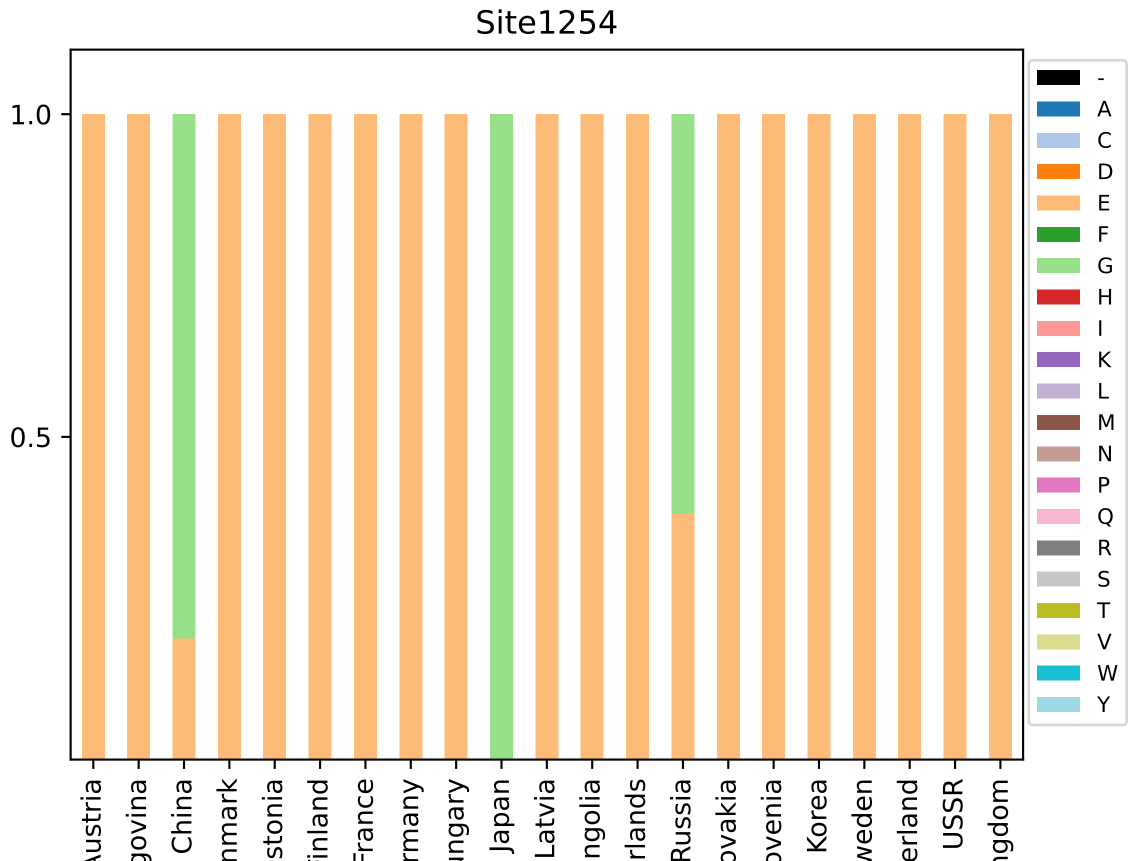
Site1254
1.0
0.5
-
A
C
D
E
F
G
H
I
K
L
M
N
P
Q
R
S
T
V
W
Y
ustria
govina
China
nmark
stonia
inland
France
rmany
ungary
Japan
Latvia
ngolia
rlands
Russia
ovakia
ovenia
Korea
weden
erland
USSR
ngdom

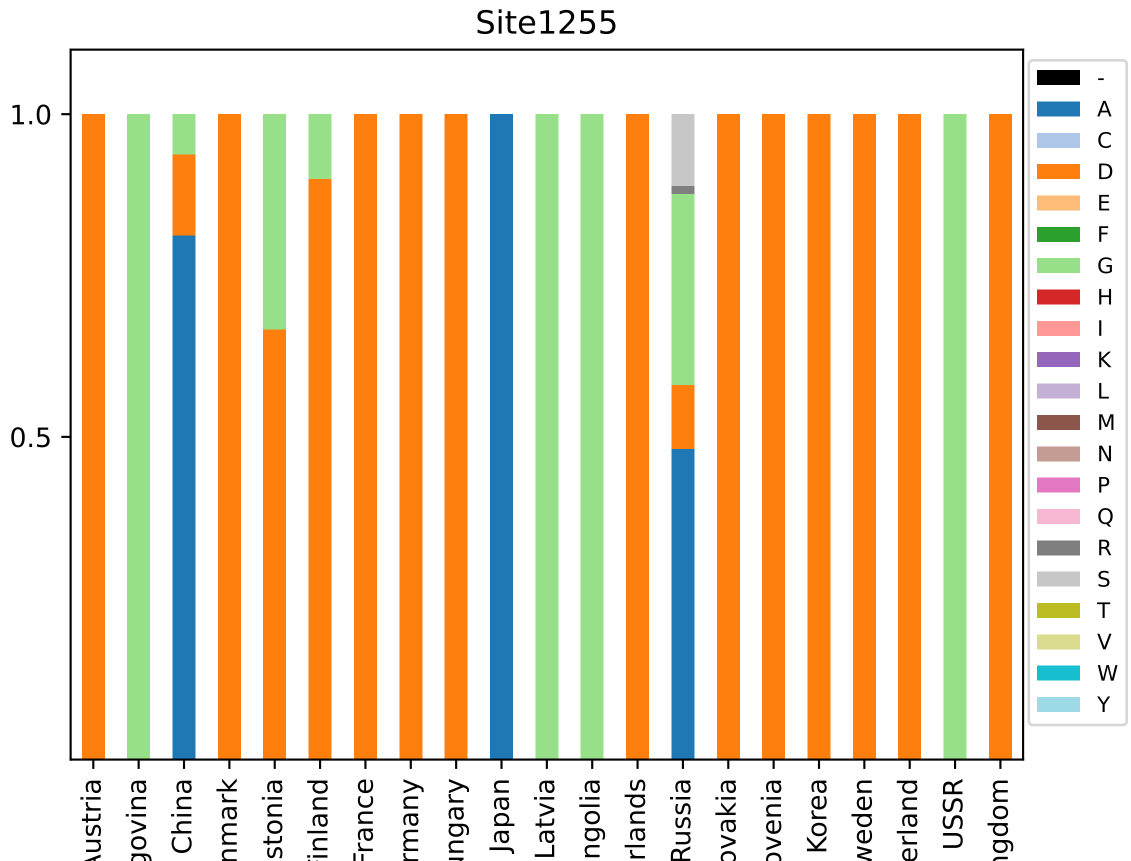
Site1255
1.0
0.5
China
Japan
Latvia
Russia
Korea
USSR
-
A
C
D
E
F
G
H
I
K
L
M
N
P
Q
R
S
T
V
W
Y

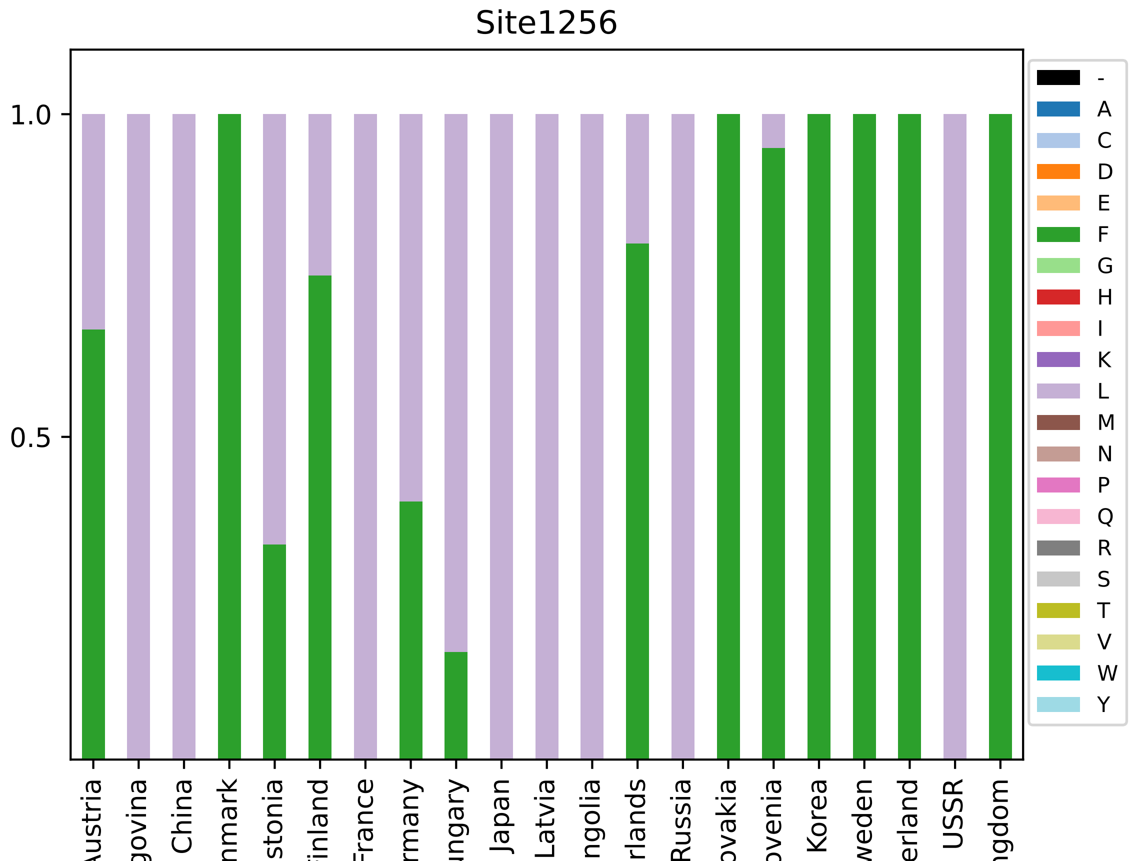

Site1256
1.0
0.5
-
A
C
D
E
F
G
H
I
K
L
M
N
P
Q
R
S
T
V
W
Y
China
Japan
Latvia
Russia
Korea
USSR

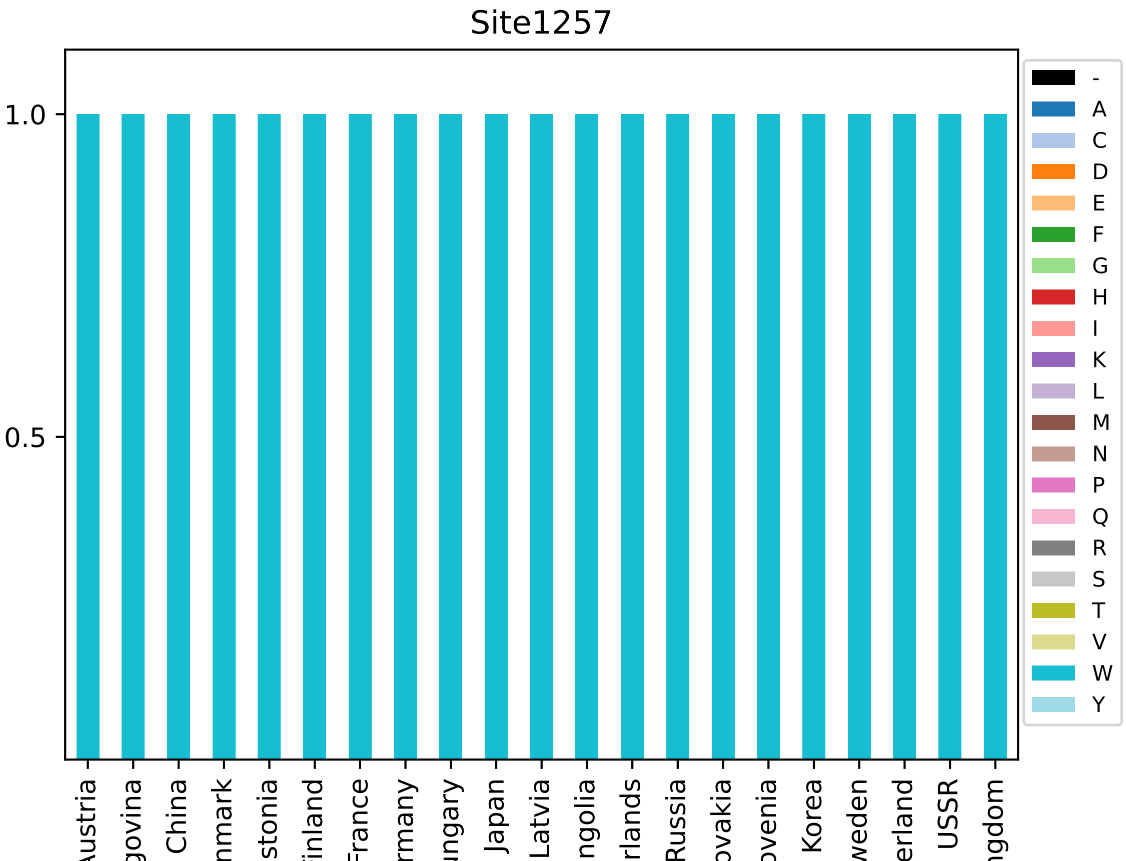

Site1257
1.0
0.5
-
A
C
D
E
F
G
H
I
K
L
M
N
P
Q
R
S
T
V
W
Y
China
Japan
Latvia
Russia
Korea
USSR

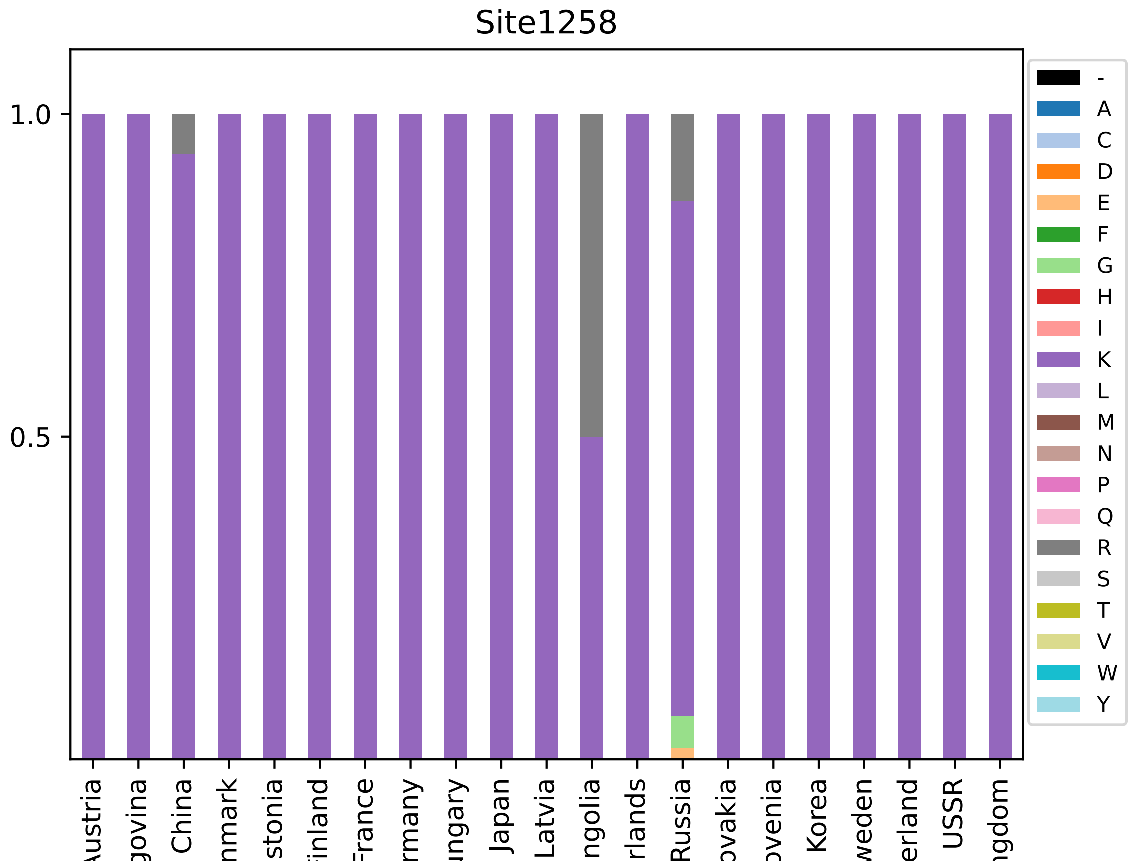

Site1258
1.0
0.5
-
A
C
D
E
F
G
H
I
K
L
M
N
P
Q
R
S
T
V
W
Y
China
Japan
Latvia
Russia
Korea
USSR

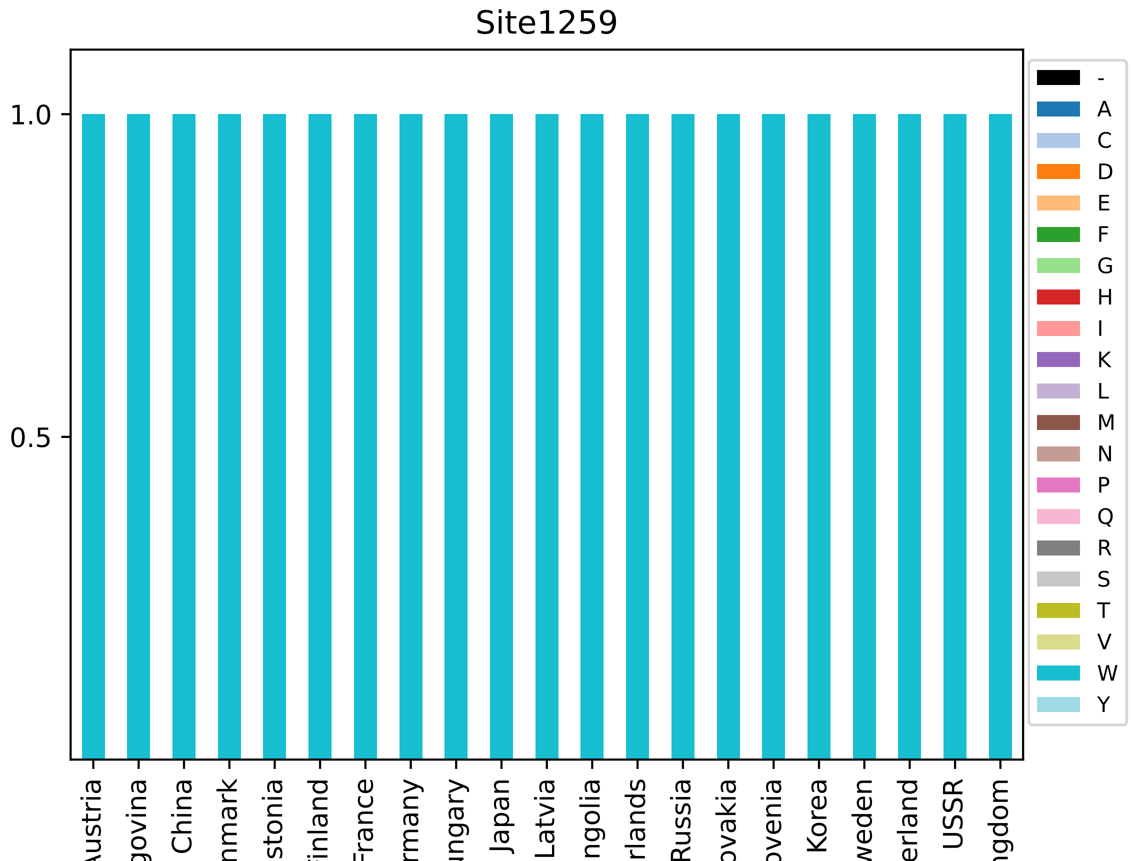
Site1259
1.0
0.5
-
A
C
D
E
F
G
H
I
K
L
M
N
P
Q
R
S
T
V
W
Y
China
Japan
Latvia
Russia
Korea
USSR

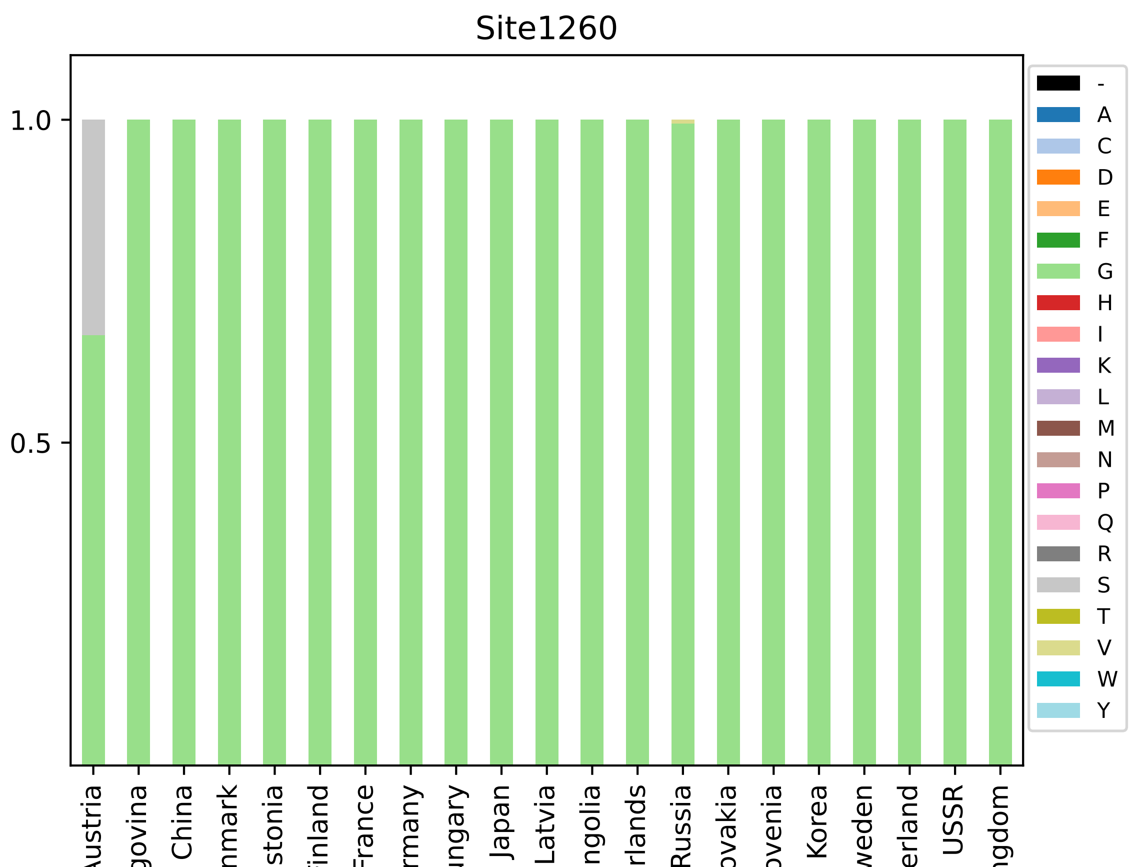

Site1260
1.0
0.5
-
A
C
D
E
F
G
H
I
K
L
M
N
P
Q
R
S
T
V
W
Y
Austria
China
Japan
Latvia
Russia
Korea
USSR

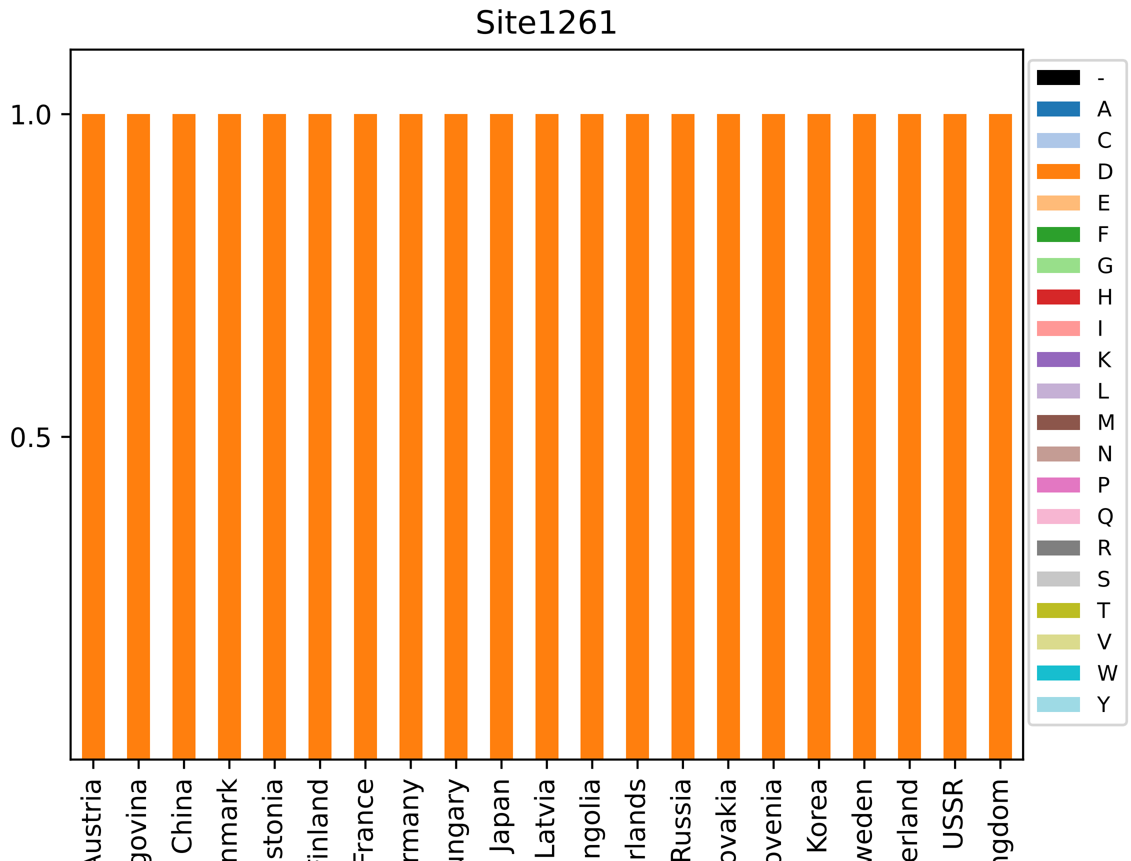

Site1261
1.0
0.5
-
A
C
D
E
F
G
H
I
K
L
M
N
P
Q
R
S
T
V
W
Y
China
Japan
Latvia
Russia
Korea
USSR

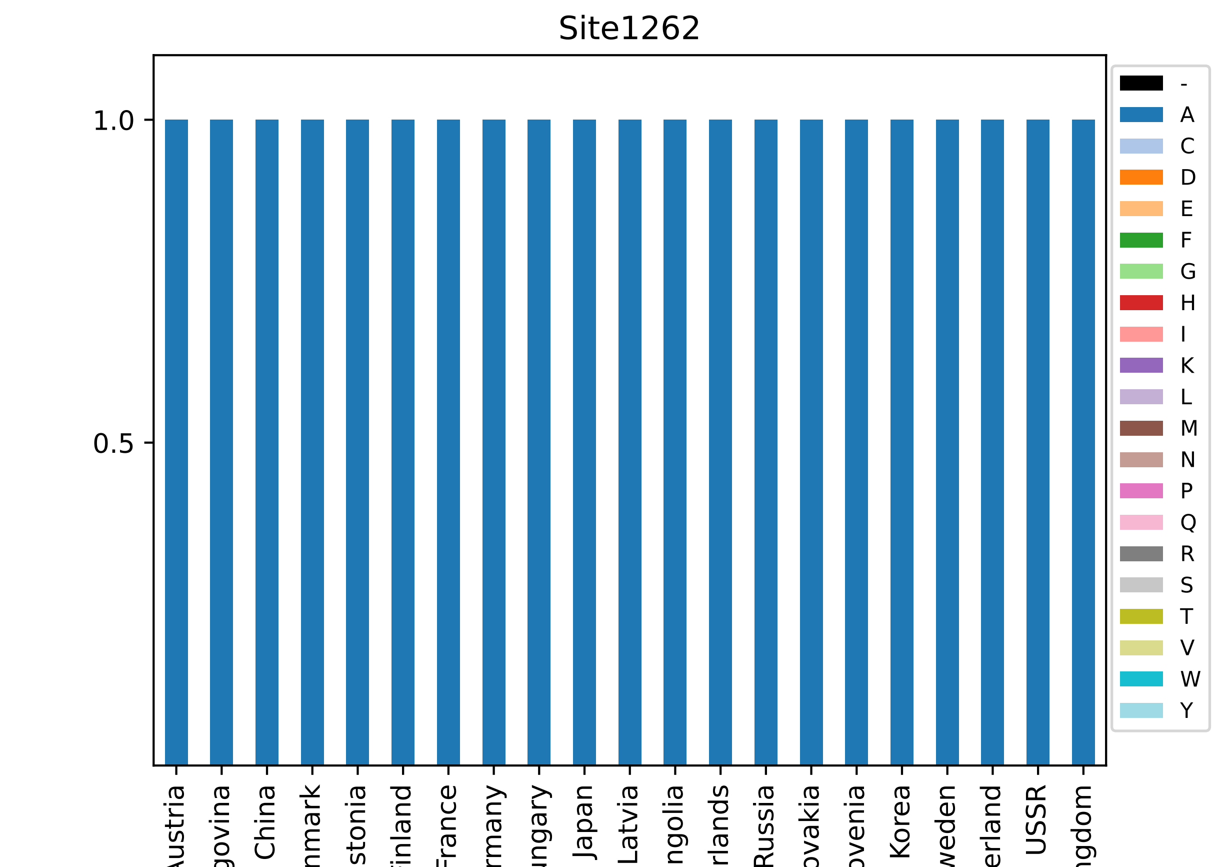

Site1262
1.0
0.5
-
A
C
D
E
F
G
H
I
K
L
M
N
P
Q
R
S
T
V
W
Y
Austria
govina
China
nmark
stonia
inland
France
rmany
ungary
Japan
Latvia
ngolia
rlands
Russia
ovakia
ovenia
Korea
weden
erland
USSR
ngdom

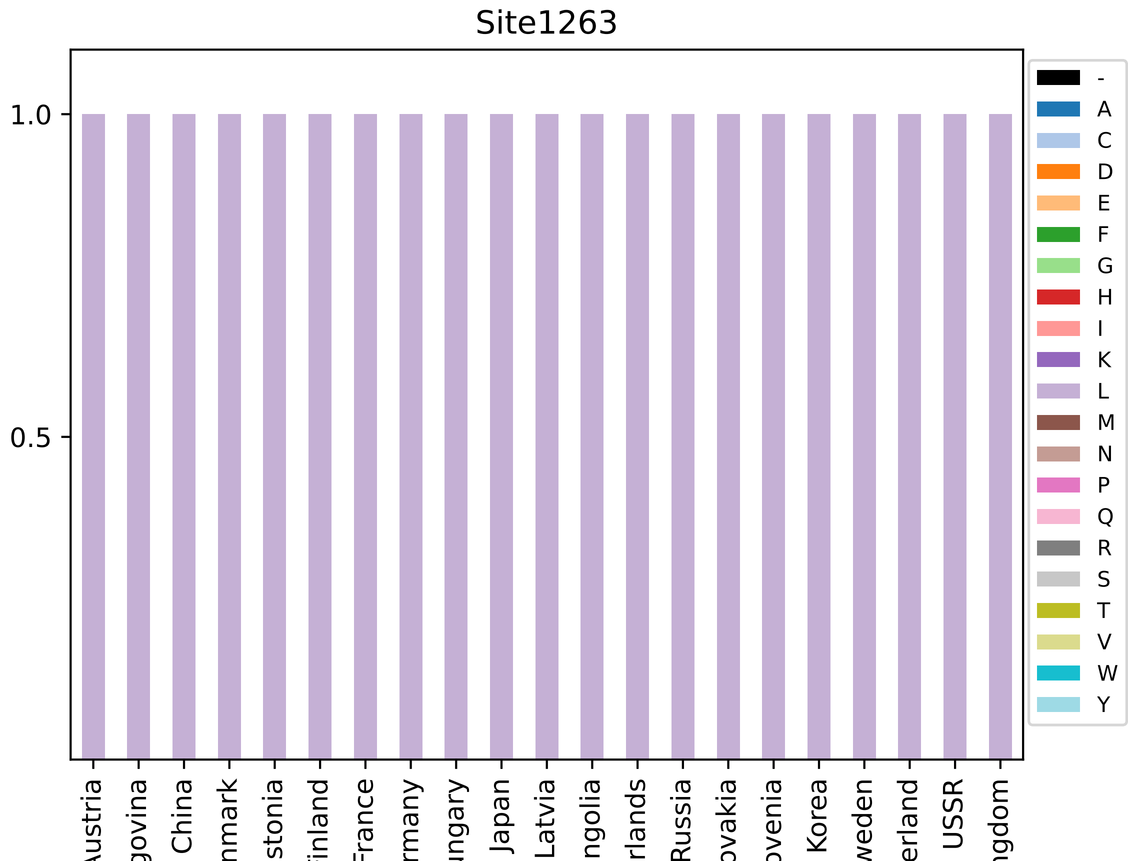

Site1263
1.0
0.5
-
A
C
D
E
F
G
H
I
K
L
M
N
P
Q
R
S
T
V
W
Y
Austria
China
Japan
Latvia
Russia
Korea
USSR

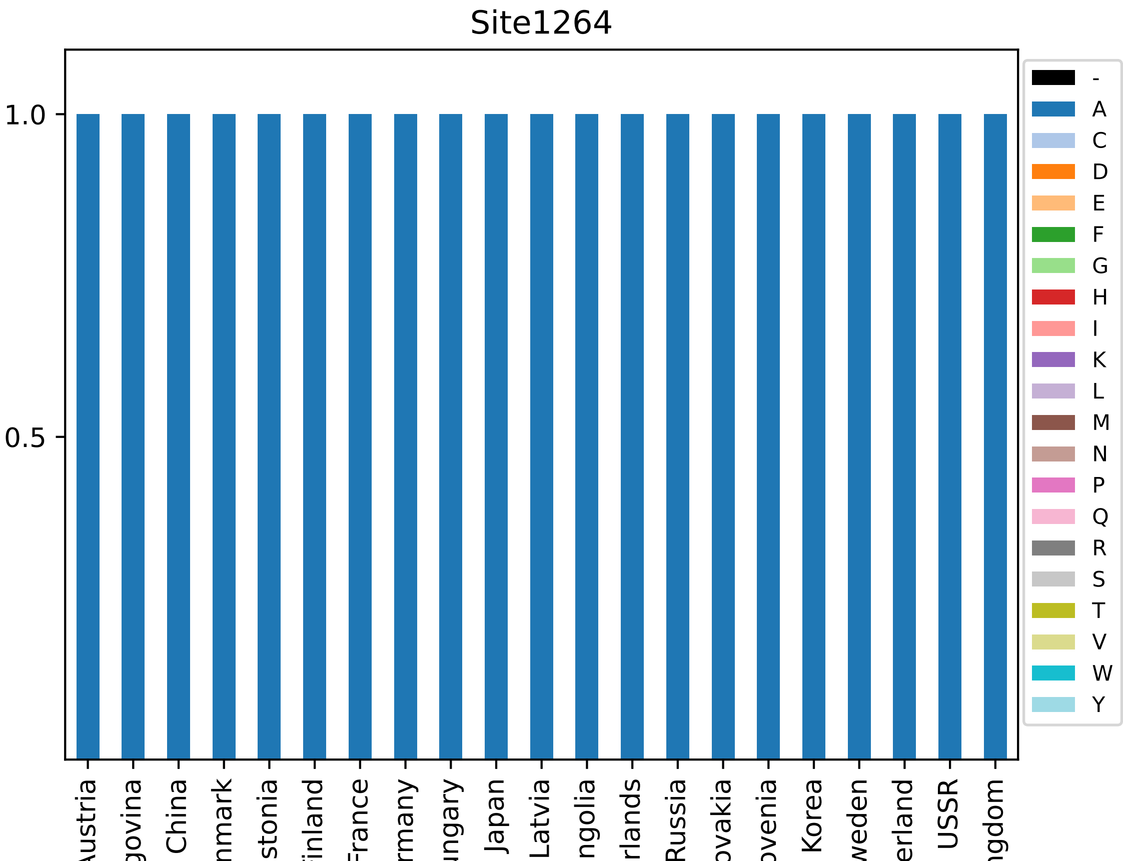

Site1264
1.0
0.5
-
A
C
D
E
F
G
H
I
K
L
M
N
P
Q
R
S
T
V
W
Y
China
Japan
Latvia
Russia
Korea
USSR

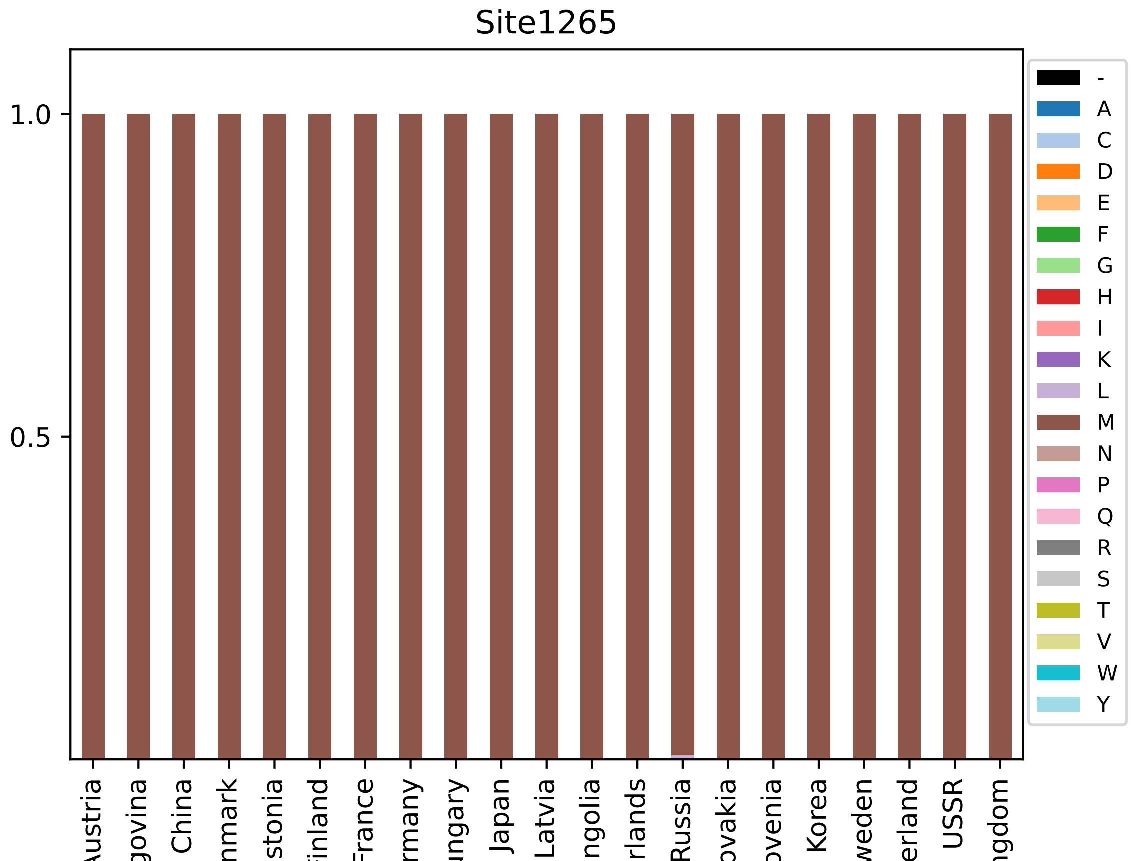

Site1265
1.0
0.5
-
A
C
D
E
F
G
H
I
K
L
M
N
P
Q
R
S
T
V
W
Y
Austria
govina
China
nmark
stonia
inland
France
rmany
ungary
Japan
Latvia
ngolia
rlands
Russia
ovakia
ovenia
Korea
weden
erland
USSR
ngdom

Site1266

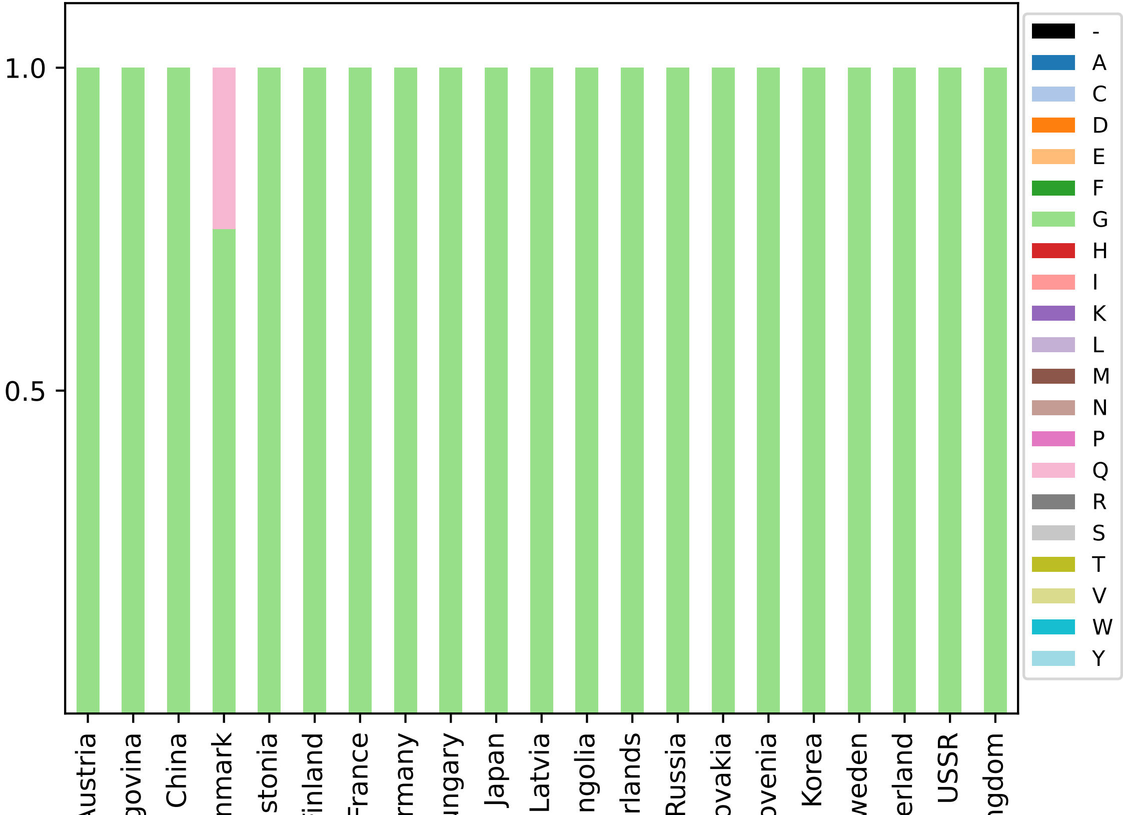

1.0
0.5
-
A
C
D
E
F
G
H
I
K
L
M
N
P
Q
R
S
T
V
W
Y
China
Japan
Latvia
Russia
Korea
USSR

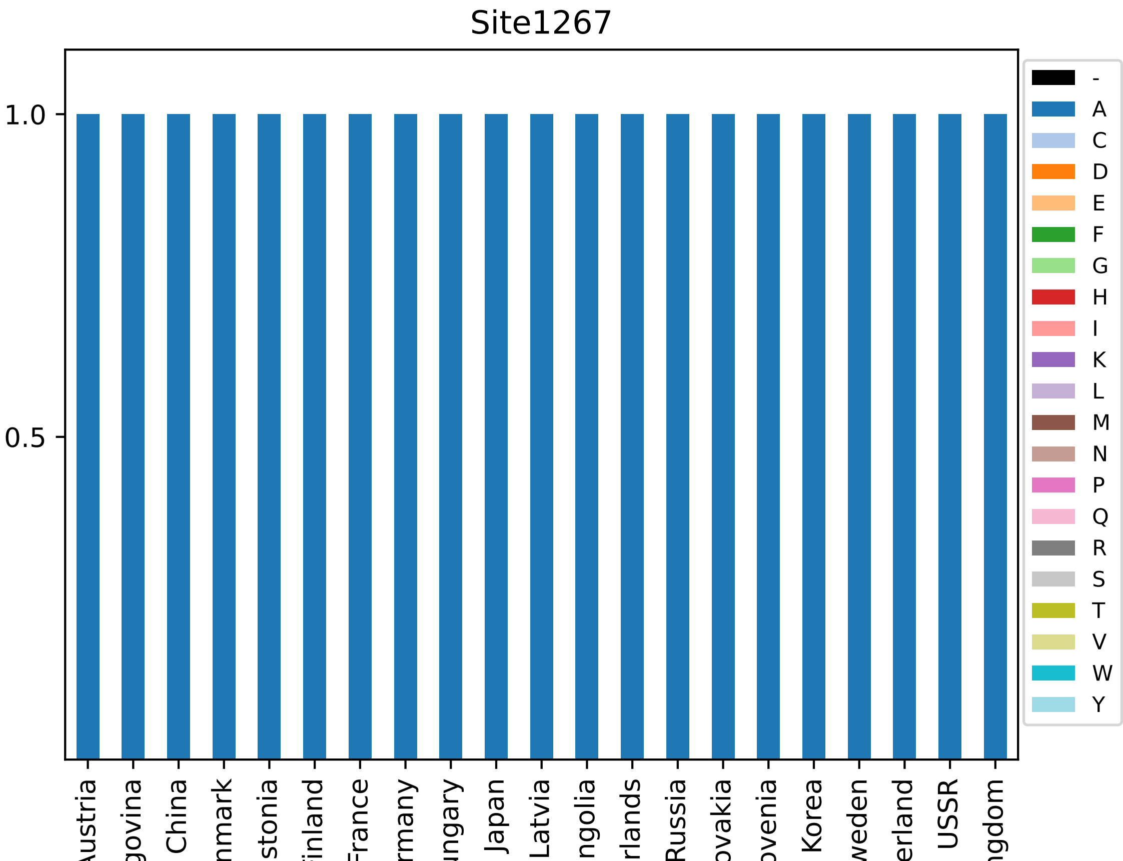

Site1267
1.0
0.5
Austria
govina
China
nmark
stonia
inland
France
rmany
ungary
Japan
Latvia
ngolia
rlands
Russia
ovakia
ovenia
Korea
weden
erland
USSR
ngdom
-
A
C
D
E
F
G
H
I
K
L
M
N
P
Q
R
S
T
V
W
Y

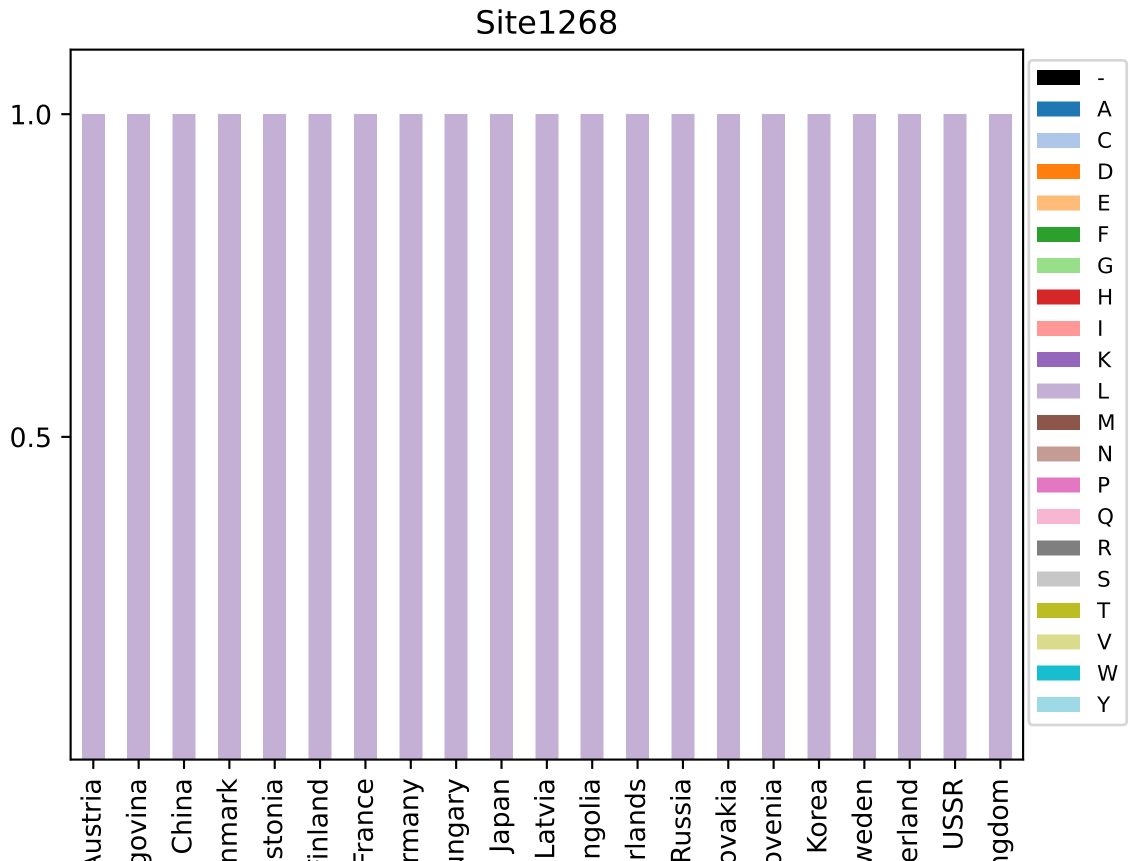

Site1268
1.0
0.5
-
A
C
D
E
F
G
H
I
K
L
M
N
P
Q
R
S
T
V
W
Y
China
Japan
Latvia
Russia
Korea
USSR

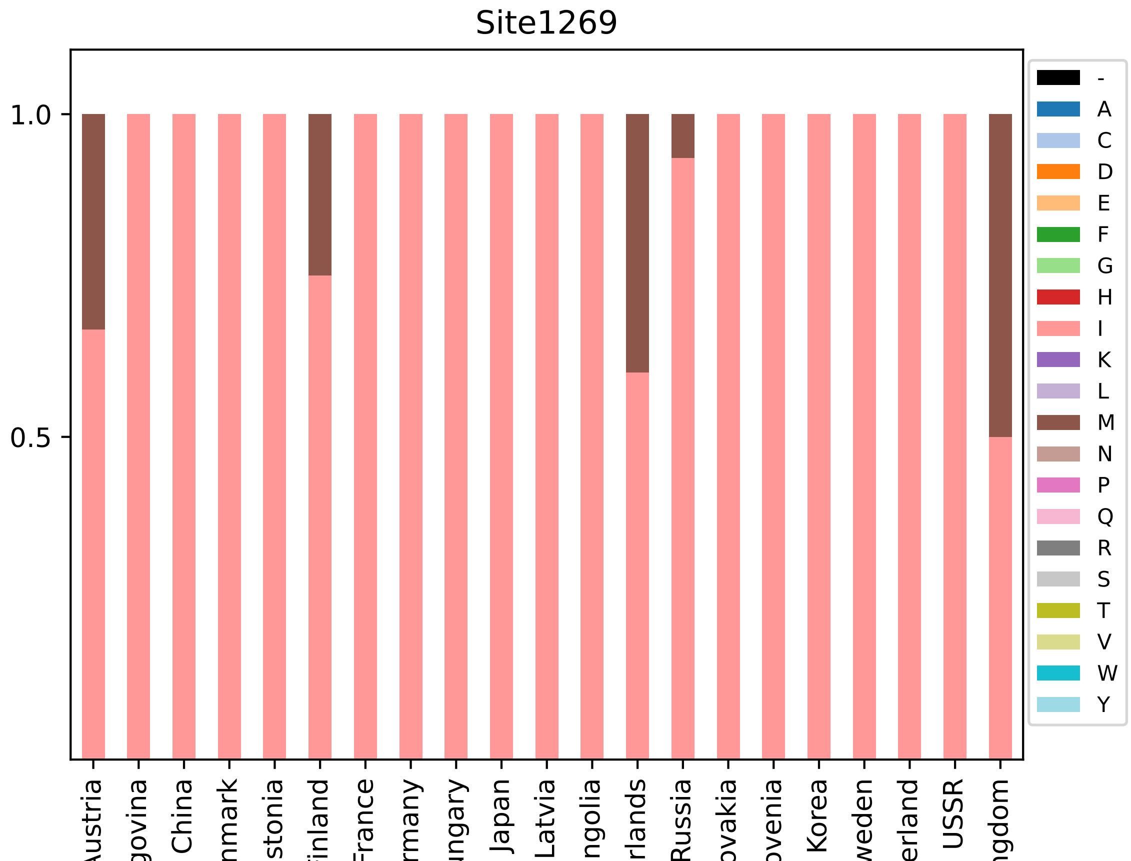

Site1269
1.0
0.5
Austria
govina
China
nmark
stonia
Finland
France
rmany
ungary
Japan
Latvia
ngolia
rlands
Russia
ovakia
ovenia
Korea
weden
erland
USSR
ngdom
-
A
C
D
E
F
G
H
I
K
L
M
N
P
Q
R
S
T
V
W
Y

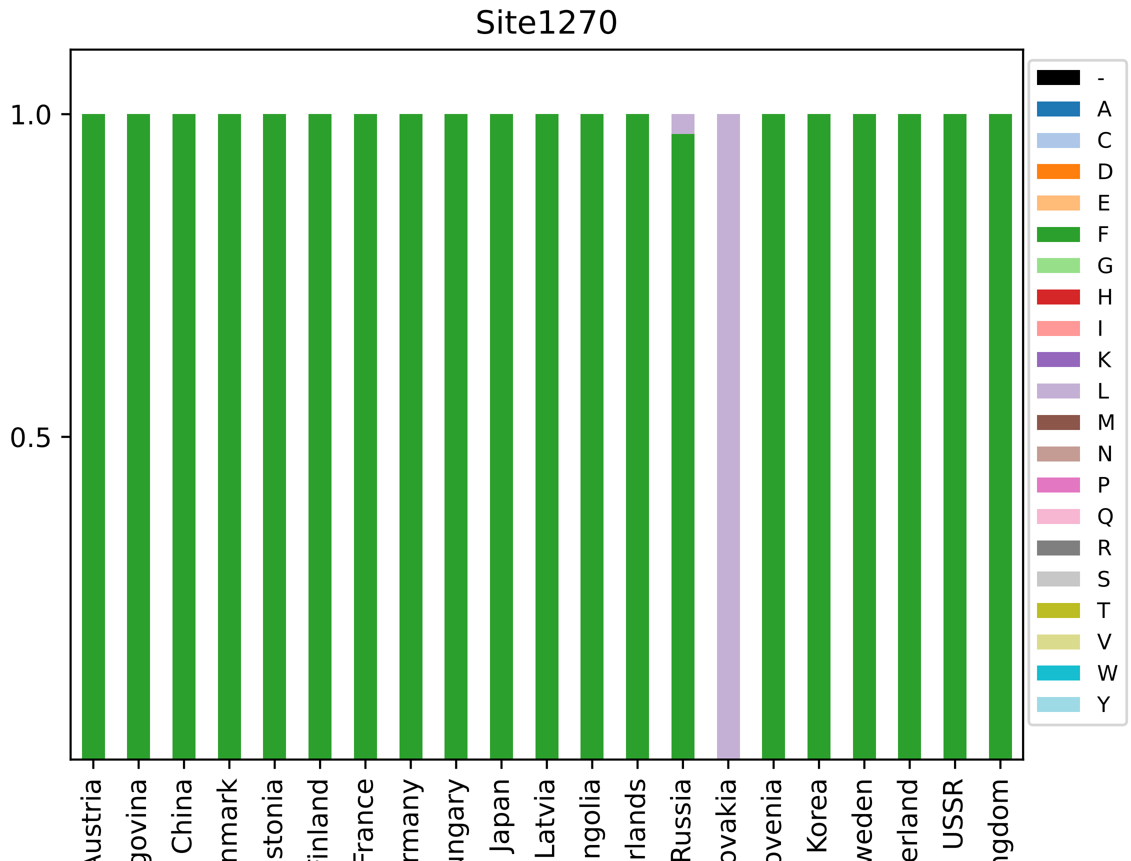

Site1270
1.0
0.5
-
A
C
D
E
F
G
H
I
K
L
M
N
P
Q
R
S
T
V
W
Y
Austria
govina
China
nmark
stonia
Finland
France
rmany
ungary
Japan
Latvia
ngolia
rlands
Russia
ovakia
ovenia
Korea
weden
erland
USSR
ngdom

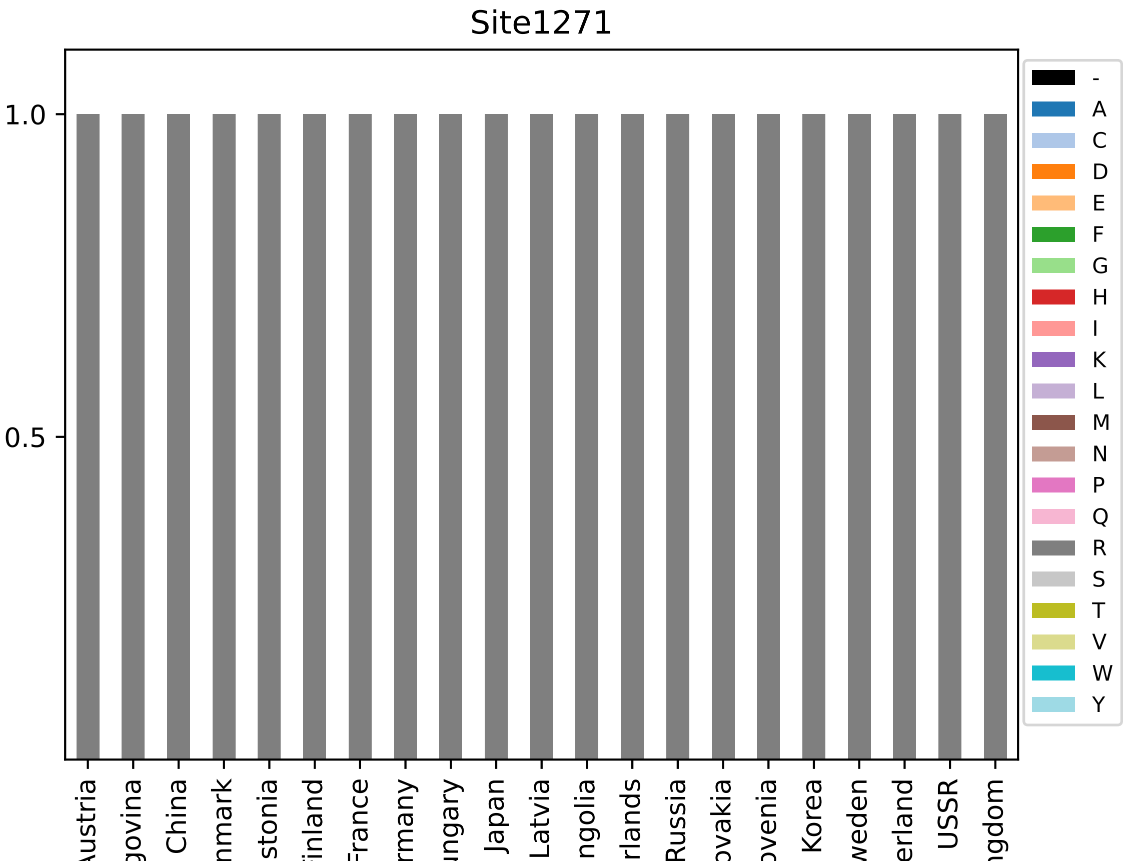

Site1271
1.0
0.5
Austria
govina
China
nmark
stonia
Finland
France
rmany
ungary
Japan
Latvia
ngolia
rlands
Russia
ovakia
ovenia
Korea
weden
erland
USSR
ngdom
-
A
C
D
E
F
G
H
I
K
L
M
N
P
Q
R
S
T
V
W
Y

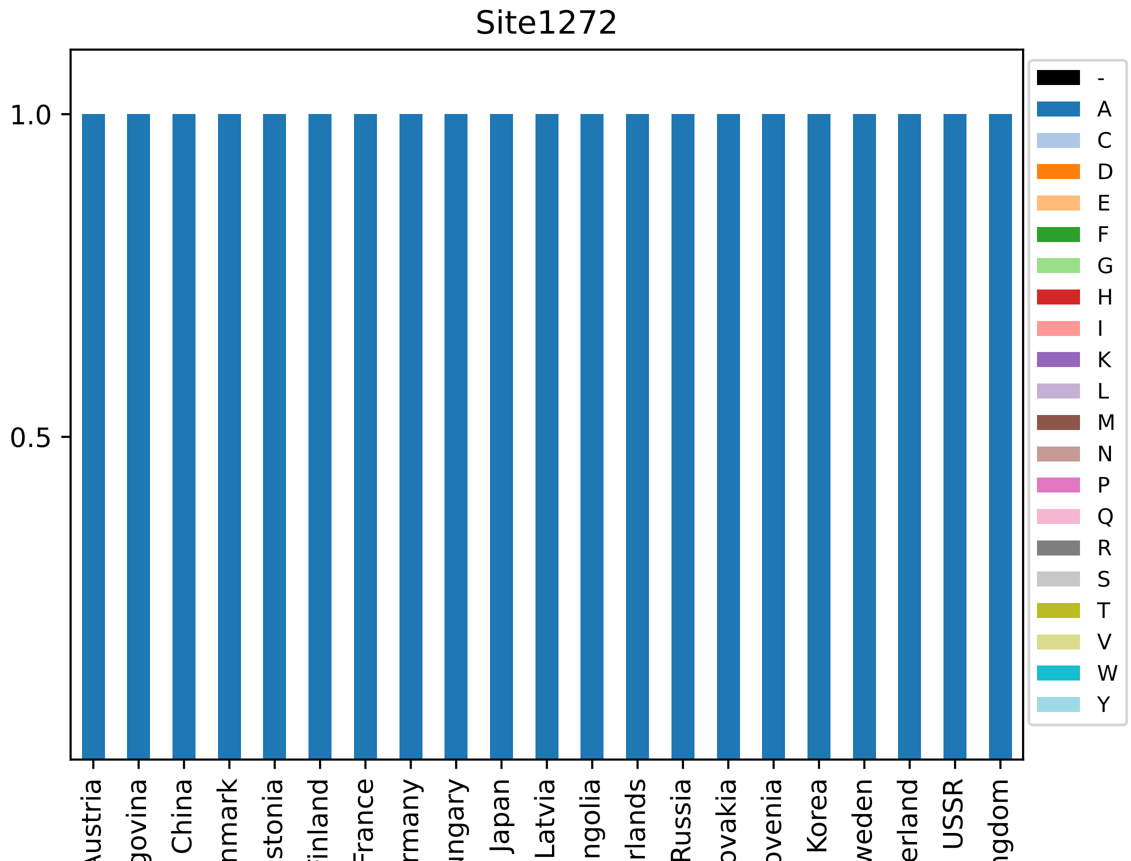
Site1272
1.0
0.5
-
A
C
D
E
F
G
H
I
K
L
M
N
P
Q
R
S
T
V
W
Y
China
Japan
Latvia
Russia
Korea
USSR

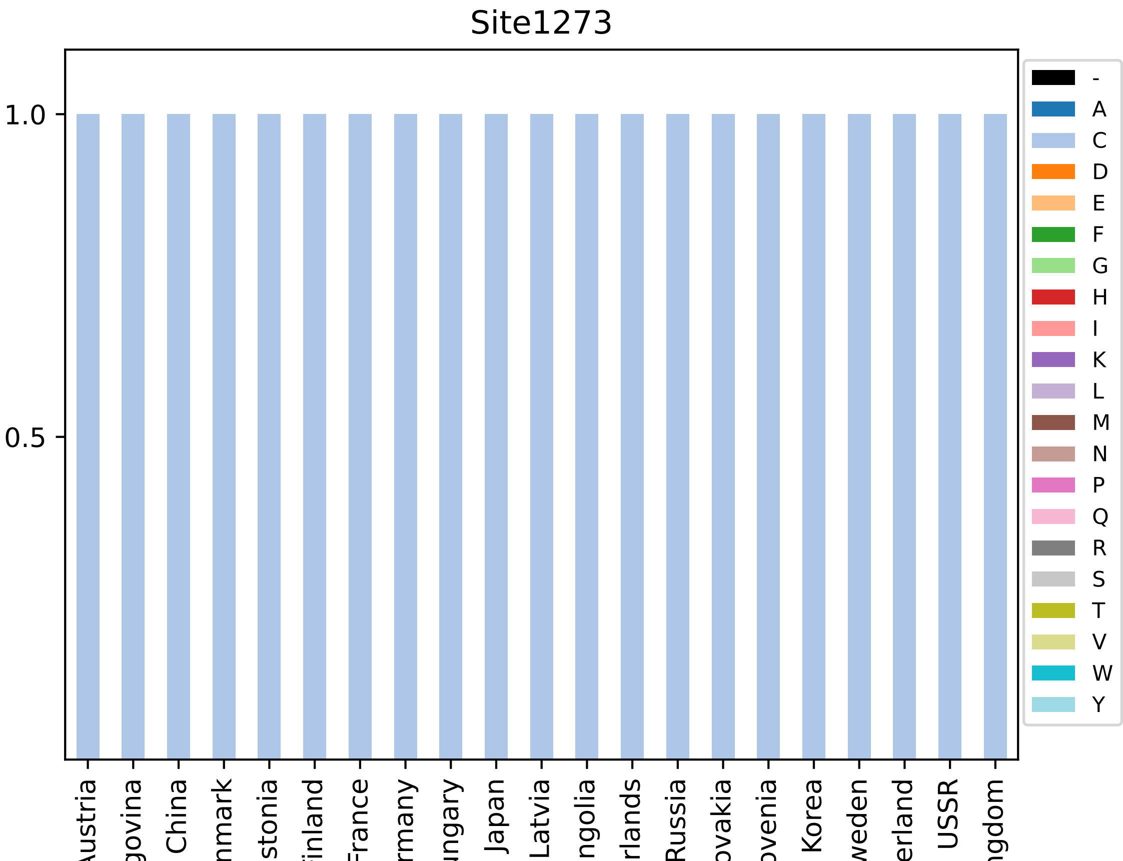
Site1273
1.0
0.5
-
A
C
D
E
F
G
H
I
K
L
M
N
P
Q
R
S
T
V
W
Y
China
Japan
Latvia
Russia
Korea
USSR

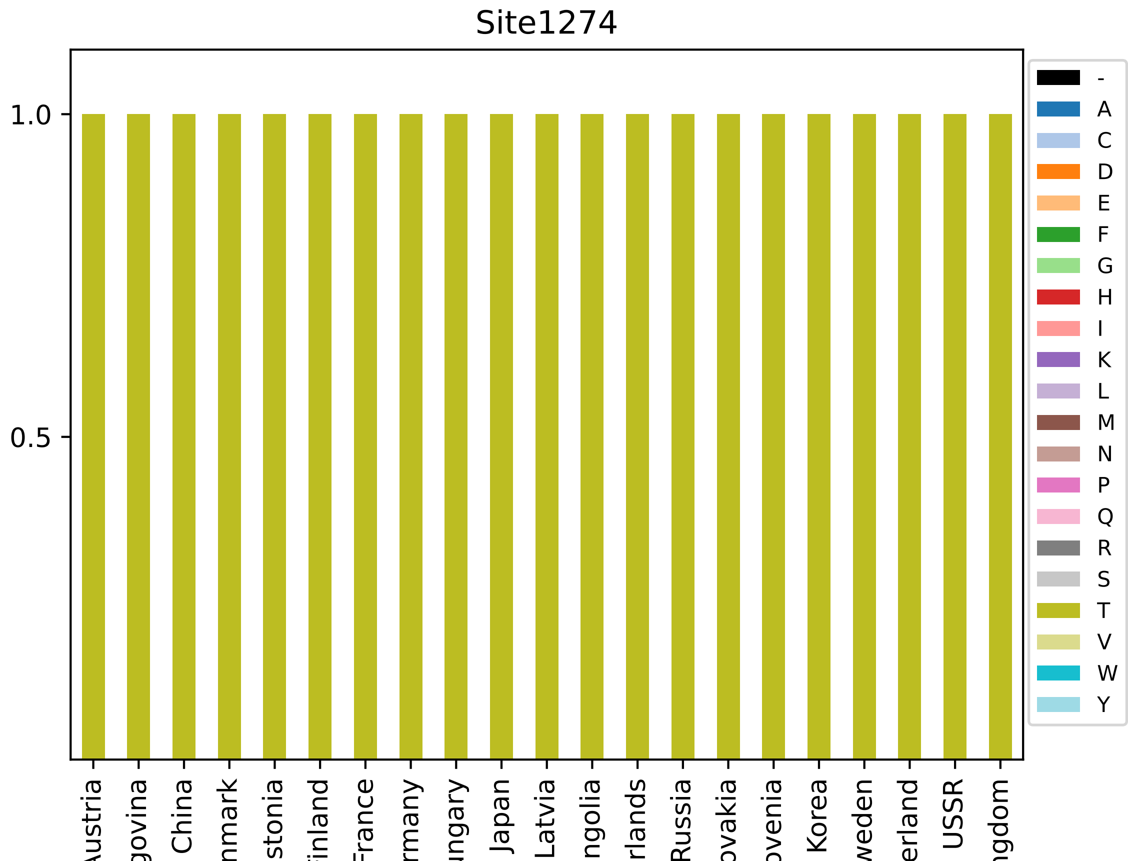
Site1274
1.0
0.5
-
A
C
D
E
F
G
H
I
K
L
M
N
P
Q
R
S
T
V
W
Y
China
Japan
Latvia
Russia
Korea
USSR

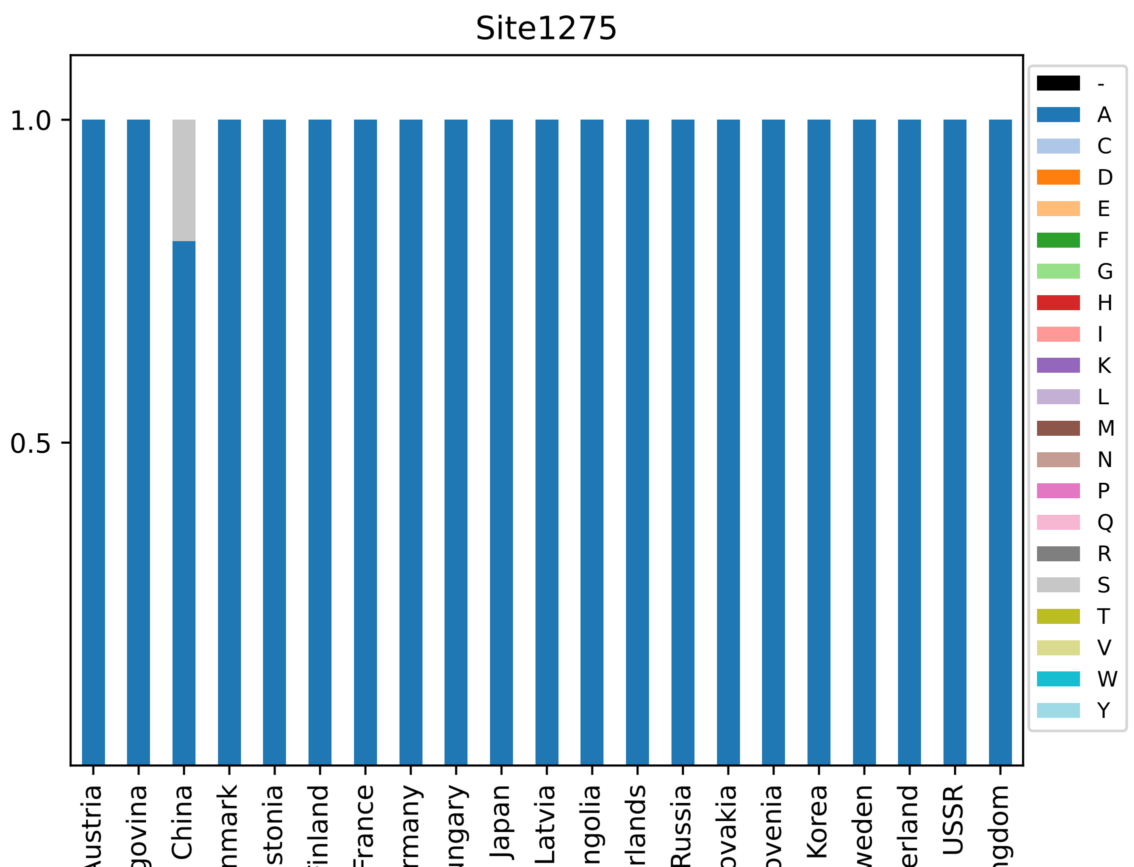

Site1275
1.0
0.5
Austria
govina
China
nmark
stonia
Finland
France
rmany
ungary
Japan
Latvia
ngolia
rlands
Russia
ovakia
ovenia
Korea
weden
erland
USSR
ngdom
-
A
C
D
E
F
G
H
I
K
L
M
N
P
Q
R
S
T
V
W
Y

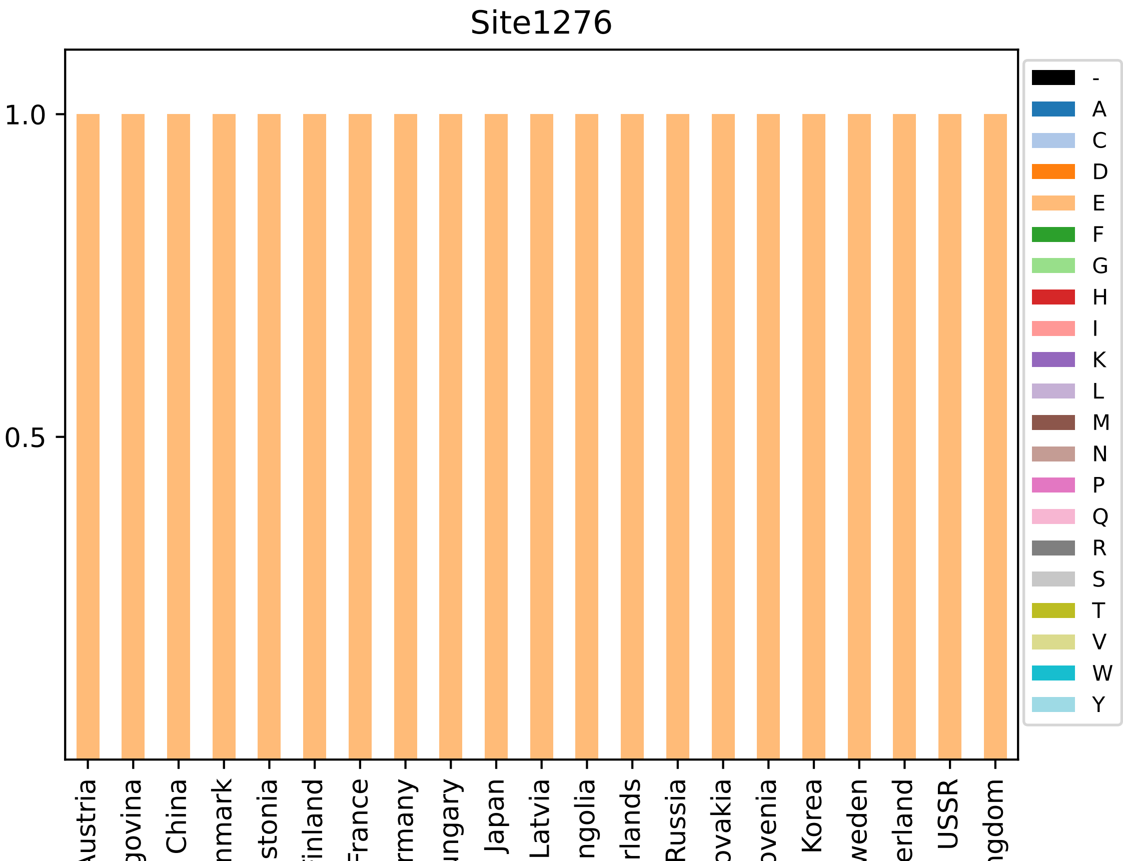
Site1276
1.0
0.5
-
A
C
D
E
F
G
H
I
K
L
M
N
P
Q
R
S
T
V
W
Y
China
Japan
Latvia
Russia
Korea
USSR

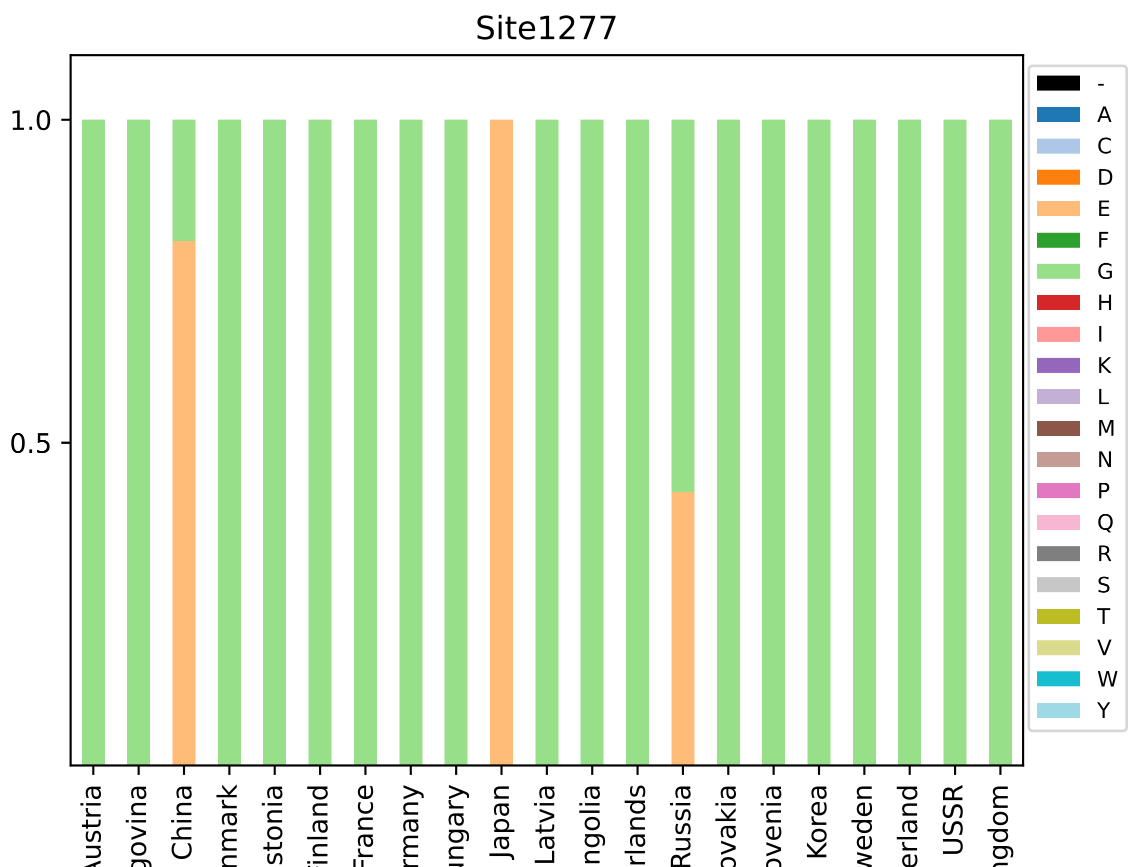
Site1277
1.0
0.5
Austria
govina
China
nmark
stonia
Finland
France
rmany
ungary
Japan
Latvia
ngolia
rlands
Russia
ovakia
ovenia
Korea
weden
erland
USSR
ngdom
-
A
C
D
E
F
G
H
I
K
L
M
N
P
Q
R
S
T
V
W
Y

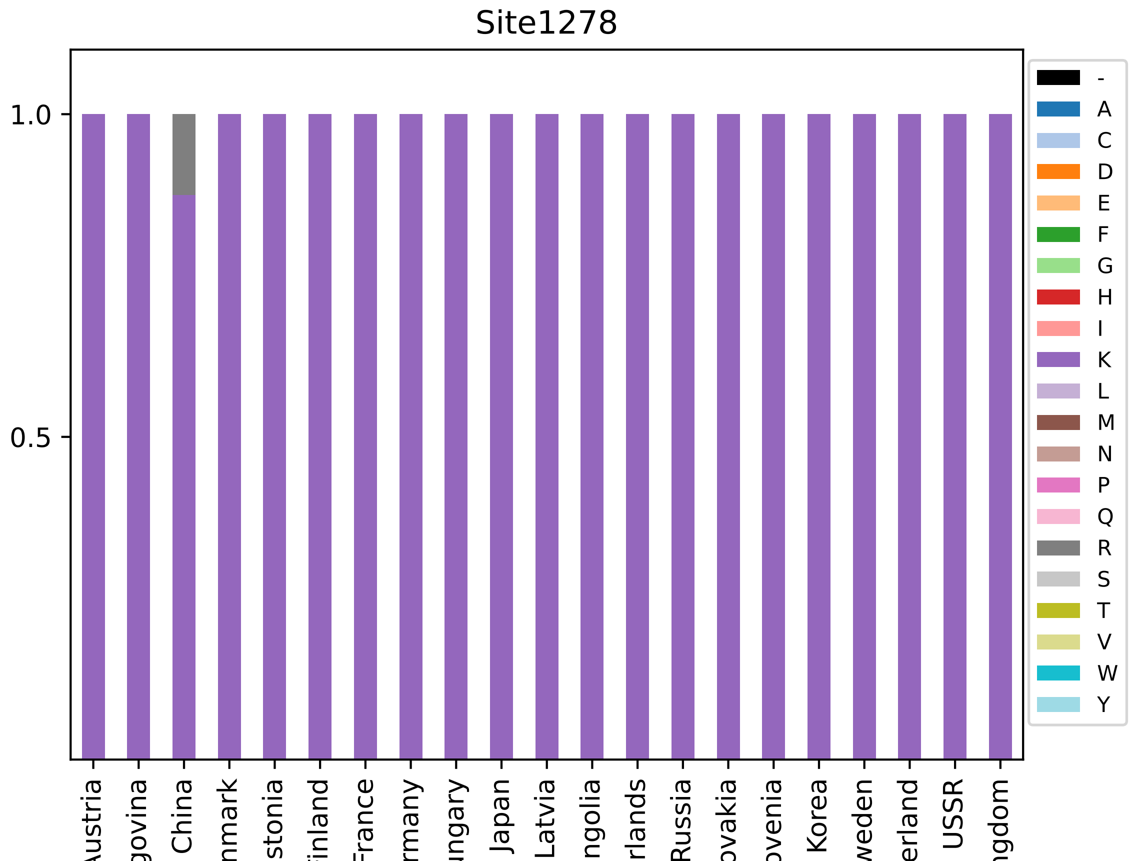
Site1278
1.0
0.5
-
A
C
D
E
F
G
H
I
K
L
M
N
P
Q
R
S
T
V
W
Y
China
Japan
Latvia
Russia
Korea
USSR

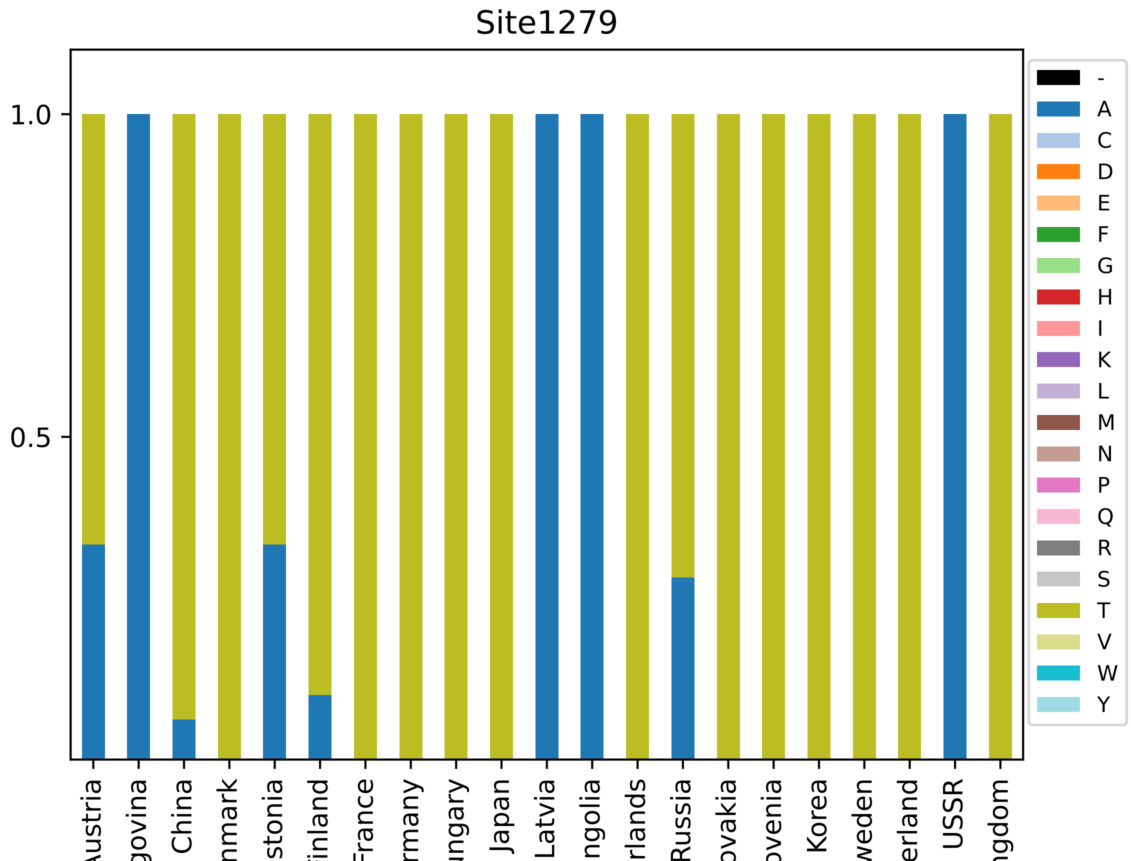
Site1279
1.0
0.5
-
A
C
D
E
F
G
H
I
K
L
M
N
P
Q
R
S
T
V
W
Y
Austria
govina
China
nmark
stonia
Finland
France
rmany
ungary
Japan
Latvia
ngolia
rlands
Russia
ovakia
ovenia
Korea
weden
erland
USSR
ngdom

Site1280

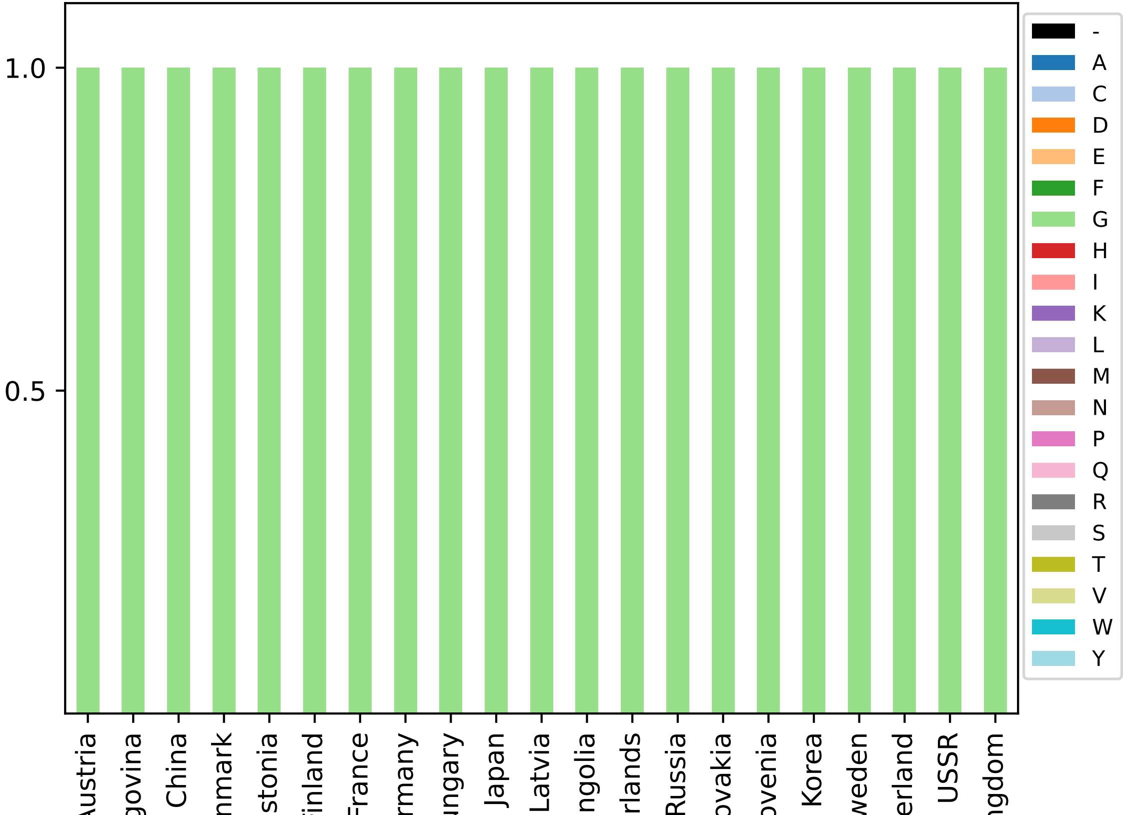
1.0
0.5
China
Japan
Latvia
Russia
Korea
USSR
-
A
C
D
E
F
G
H
I
K
L
M
N
P
Q
R
S
T
V
W
Y

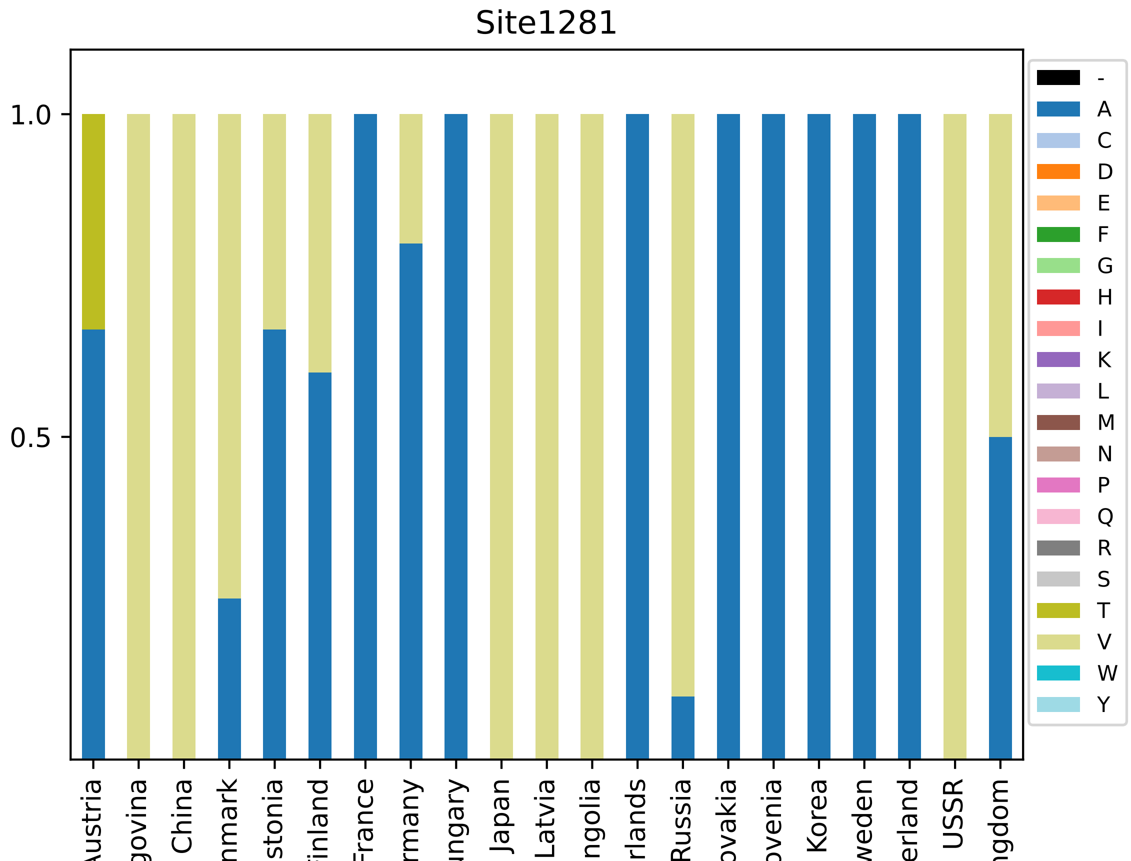
Site1281
1.0
0.5
-
A
C
D
E
F
G
H
I
K
L
M
N
P
Q
R
S
T
V
W
Y
Austria
China
Japan
Latvia
Russia
Korea
USSR

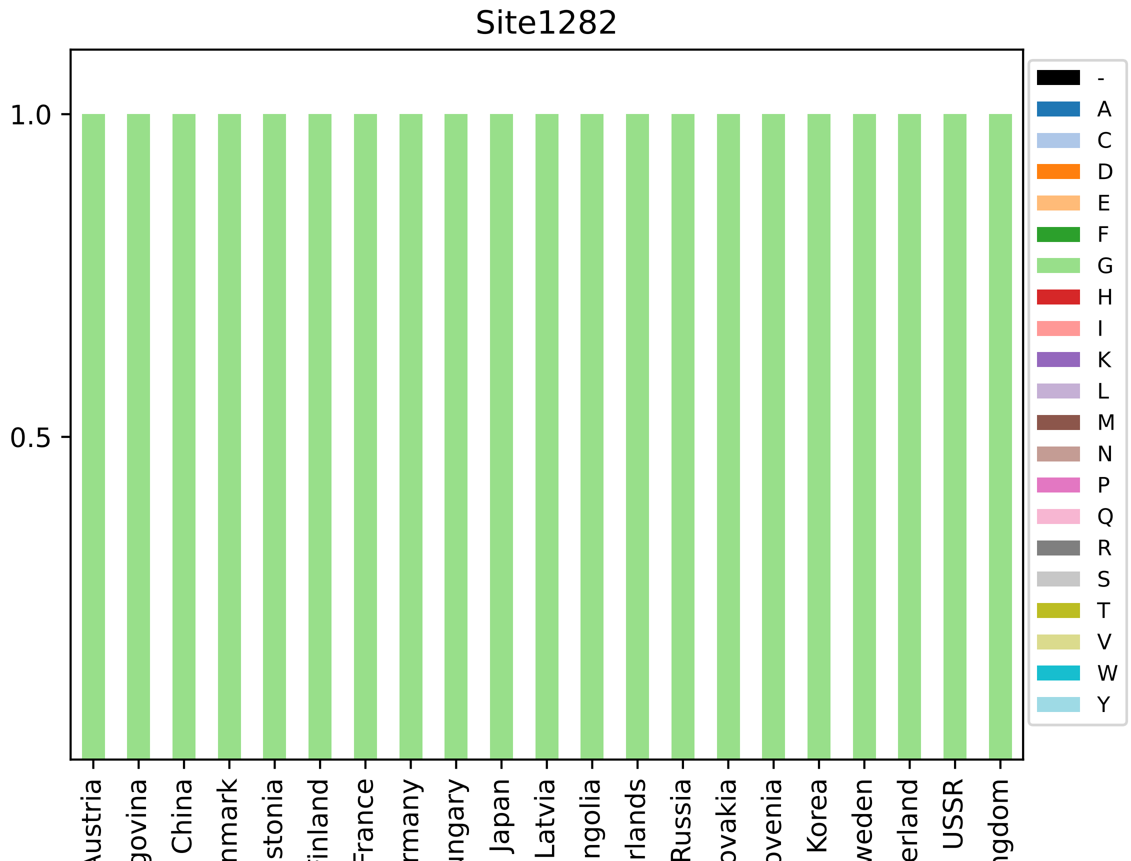

Site1282
1.0
0.5
Austria
govina
China
nmark
stonia
inland
France
rmany
ungary
Japan
Latvia
ngolia
rlands
Russia
ovakia
ovenia
Korea
weden
erland
USSR
ngdom
-
A
C
D
E
F
G
H
I
K
L
M
N
P
Q
R
S
T
V
W
Y

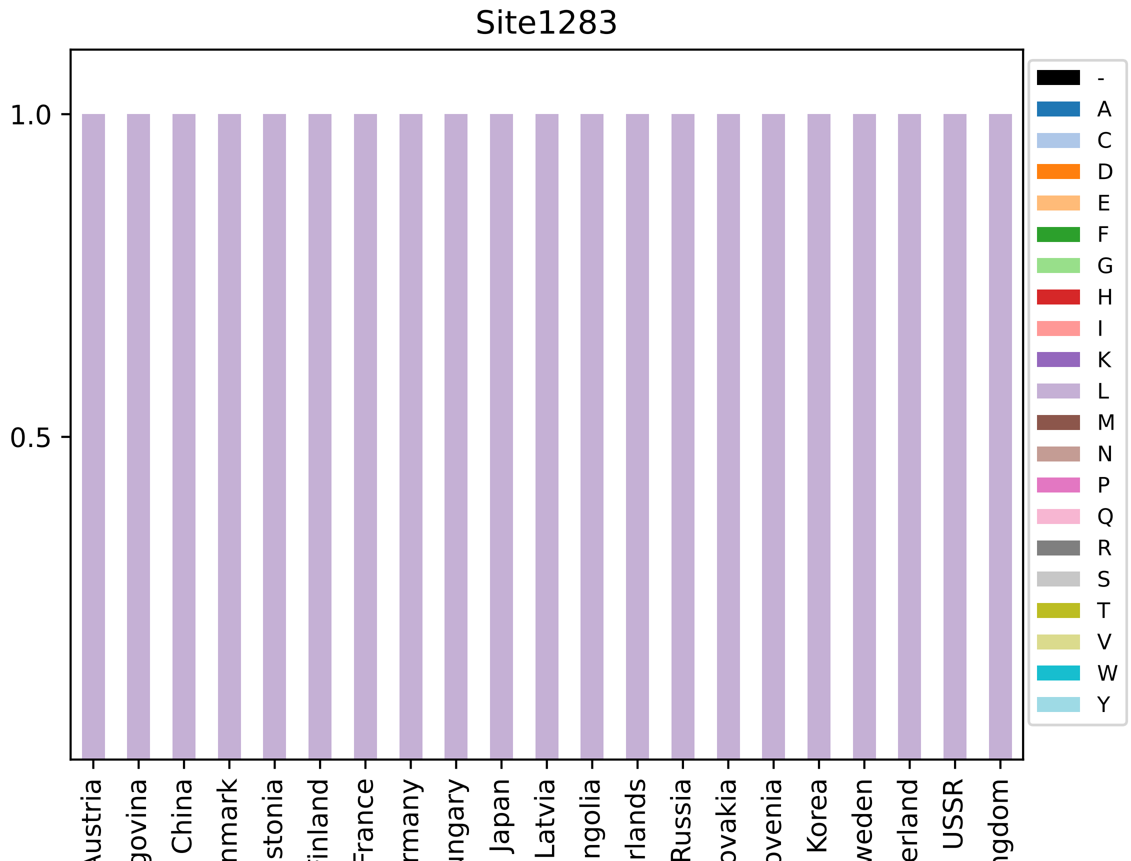
Site1283
1.0
0.5
-
A
C
D
E
F
G
H
I
K
L
M
N
P
Q
R
S
T
V
W
Y
Austria
govina
China
nmark
stonia
Finland
France
rmany
ungary
Japan
Latvia
ngolia
rlands
Russia
ovakia
ovenia
Korea
weden
erland
USSR
ngdom

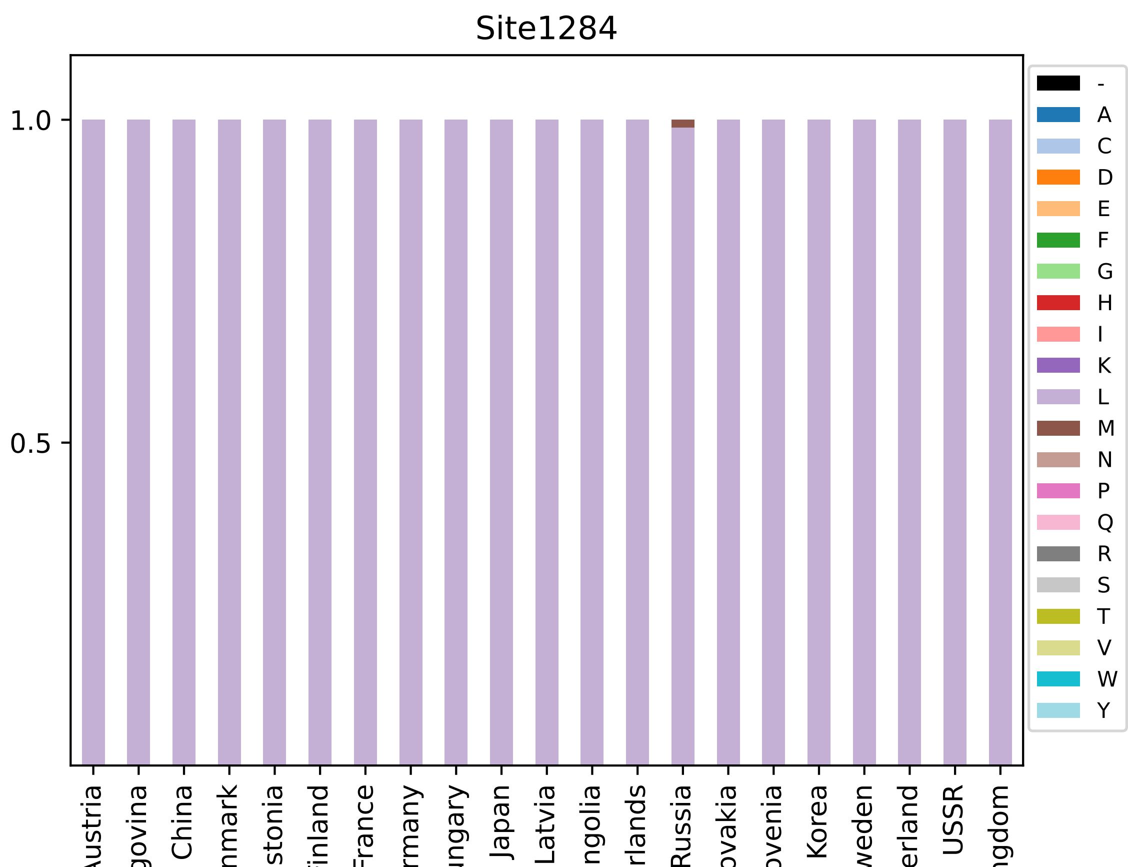
Site1284
1.0
0.5
Austria
govina
China
nmark
stonia
Finland
France
rmany
ungary
Japan
Latvia
ngolia
rlands
Russia
ovakia
ovenia
Korea
weden
erland
USSR
ngdom
-
A
C
D
E
F
G
H
I
K
L
M
N
P
Q
R
S
T
V
W
Y

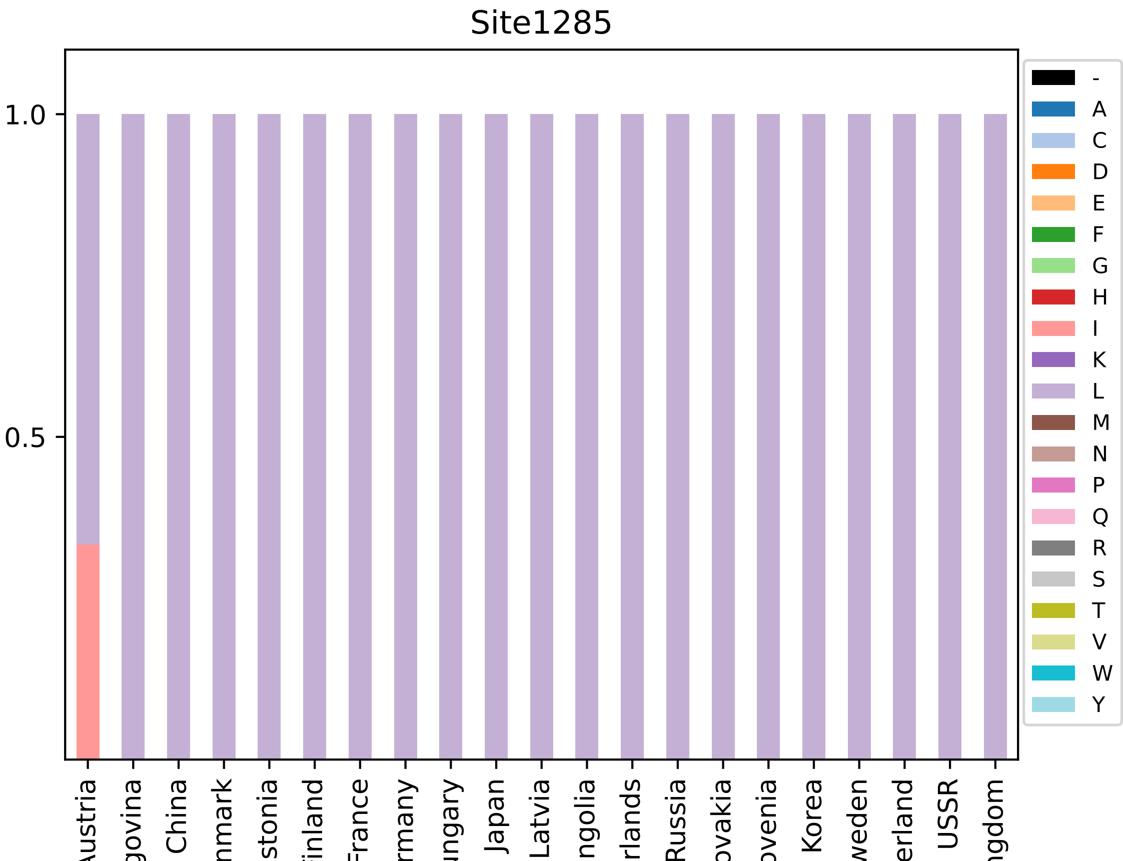

Site1285
1.0
0.5
-
A
C
D
E
F
G
H
I
K
L
M
N
P
Q
R
S
T
V
W
Y
China
Japan
Latvia
Russia
Korea
USSR

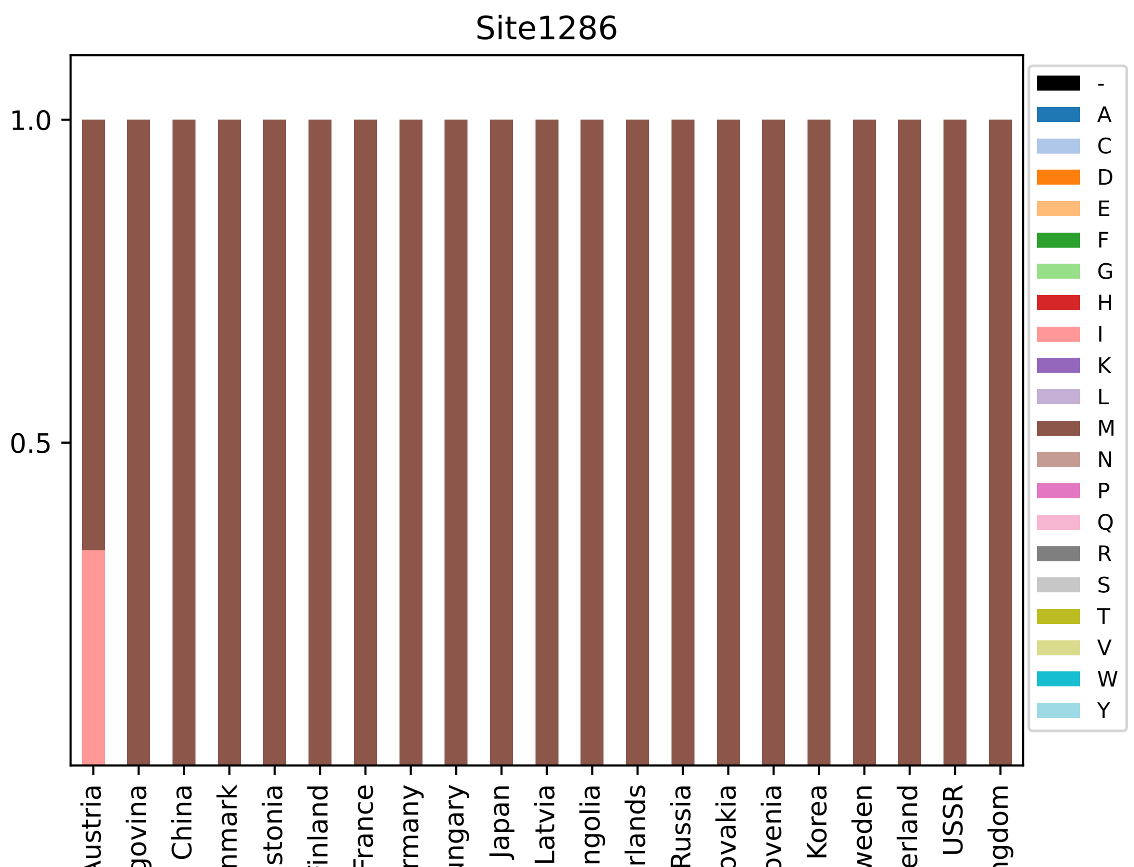

Site1286
1.0
0.5
-
A
C
D
E
F
G
H
I
K
L
M
N
P
Q
R
S
T
V
W
Y
Japan
Latvia
Russia
Korea
USSR
China

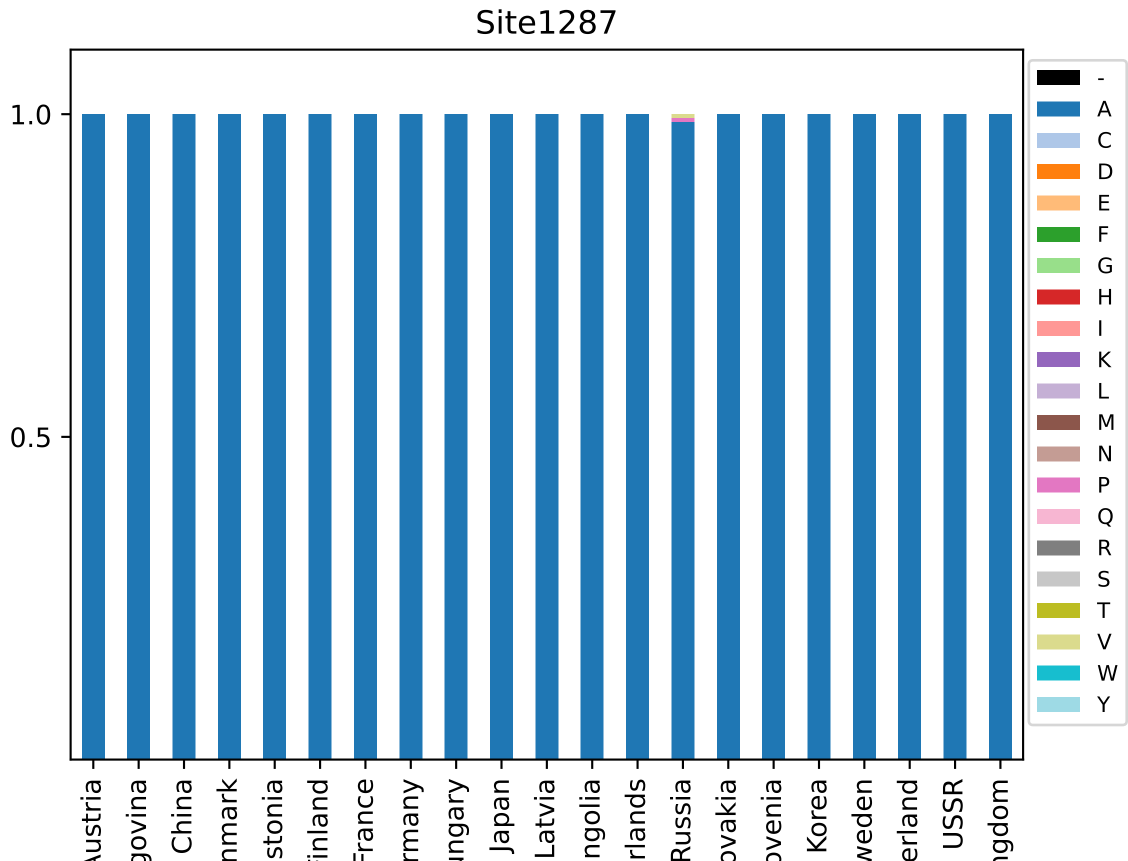

Site1287
1.0
0.5
-
A
C
D
E
F
G
H
I
K
L
M
N
P
Q
R
S
T
V
W
Y
China
Japan
Latvia
Russia
Korea
USSR

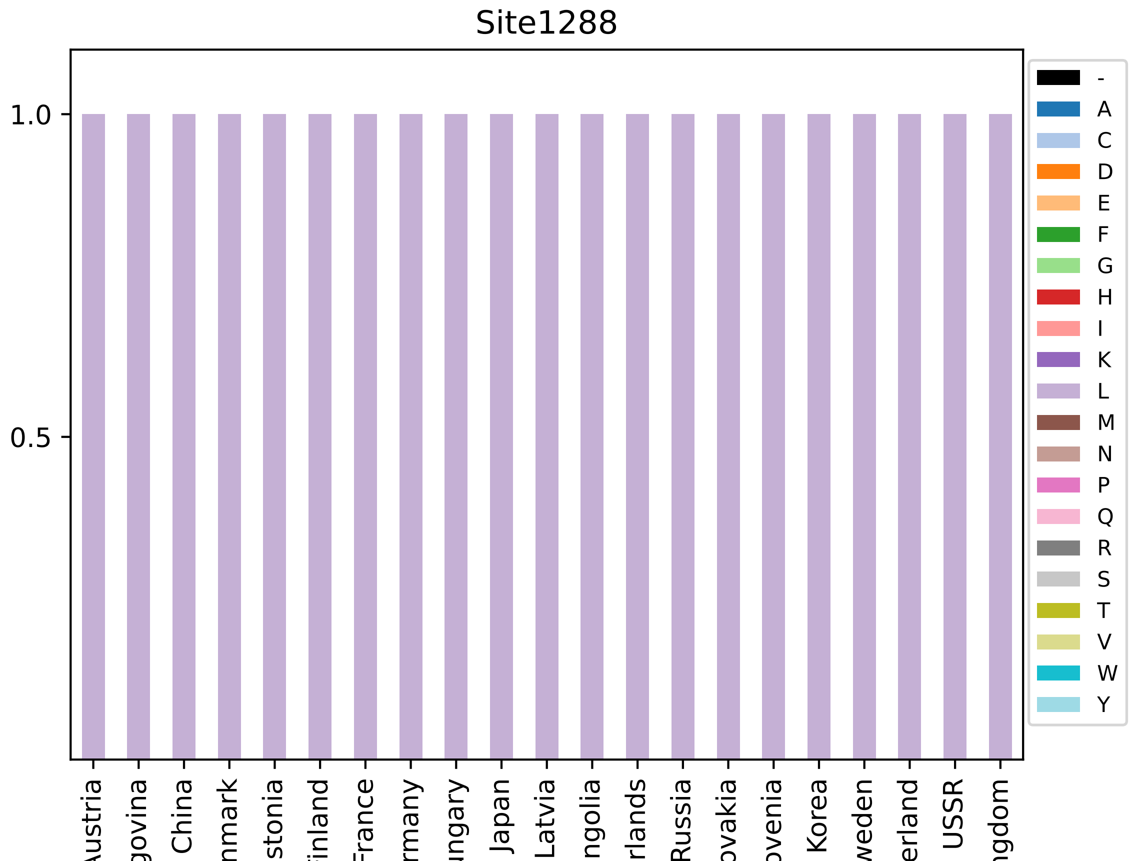
Site1288
1.0
0.5
-
A
C
D
E
F
G
H
I
K
L
M
N
P
Q
R
S
T
V
W
Y
Austria
China
Japan
Latvia
Russia
Korea
USSR

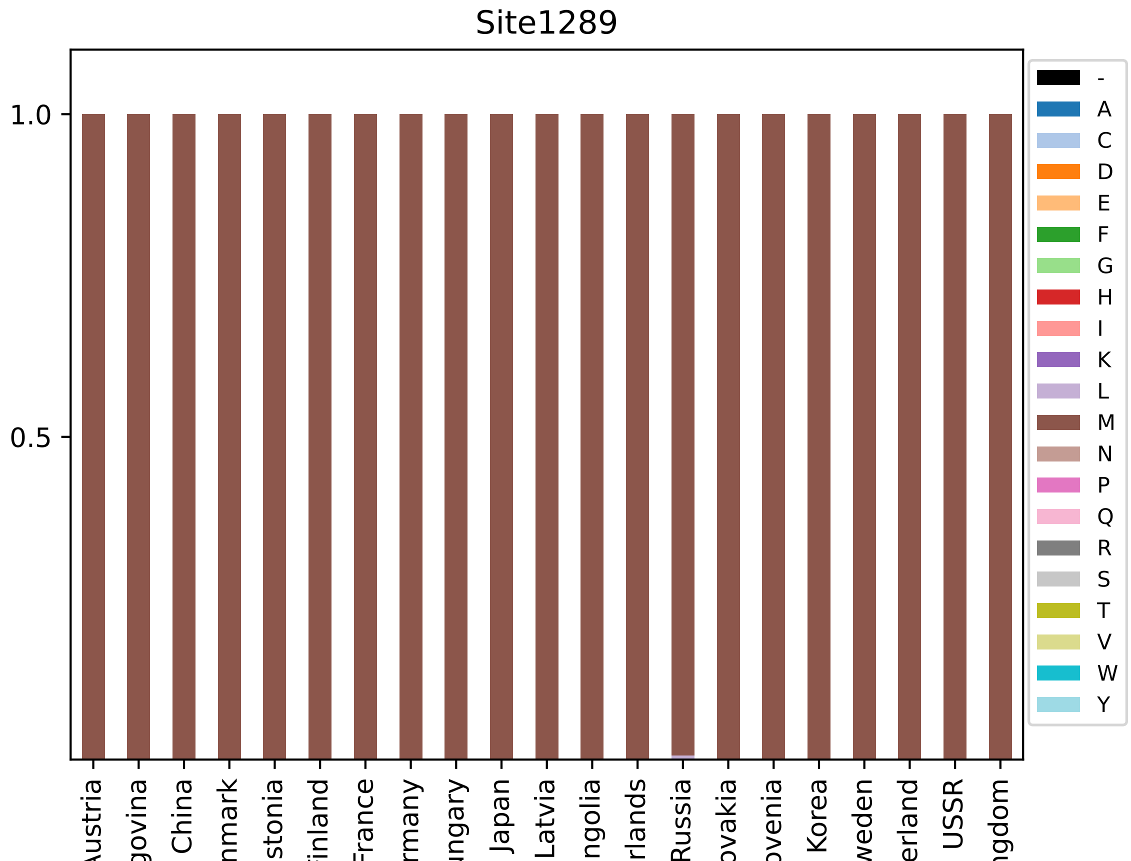
Site1289
1.0
0.5
-
A
C
D
E
F
G
H
I
K
L
M
N
P
Q
R
S
T
V
W
Y
Austria
govina
China
nmark
stonia
inland
France
rmany
ungary
Japan
Latvia
ngolia
rlands
Russia
ovakia
ovenia
Korea
weden
erland
USSR
ngdom

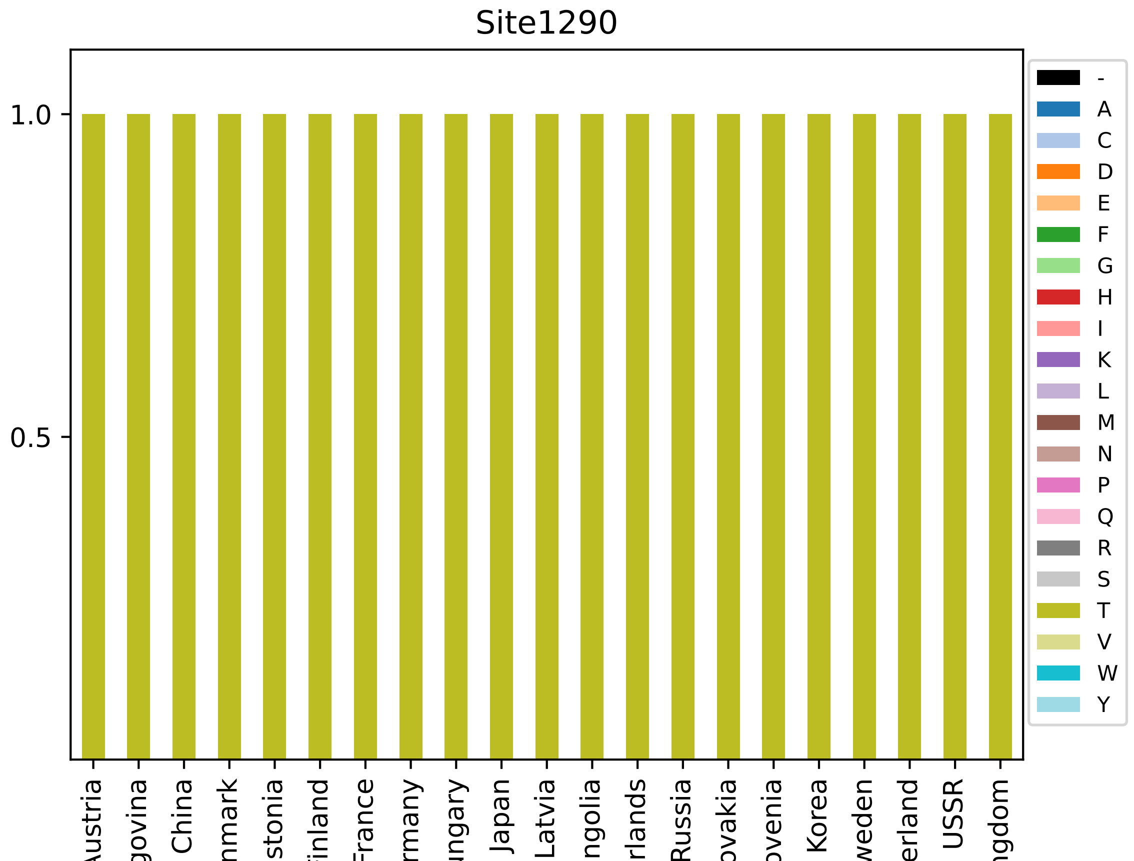

Site1290
1.0
0.5
-
A
C
D
E
F
G
H
I
K
L
M
N
P
Q
R
S
T
V
W
Y
Austria
govina
China
nmark
stonia
inland
France
rmany
ungary
Japan
Latvia
ngolia
rlands
Russia
ovakia
ovenia
Korea
weden
erland
USSR
ngdom

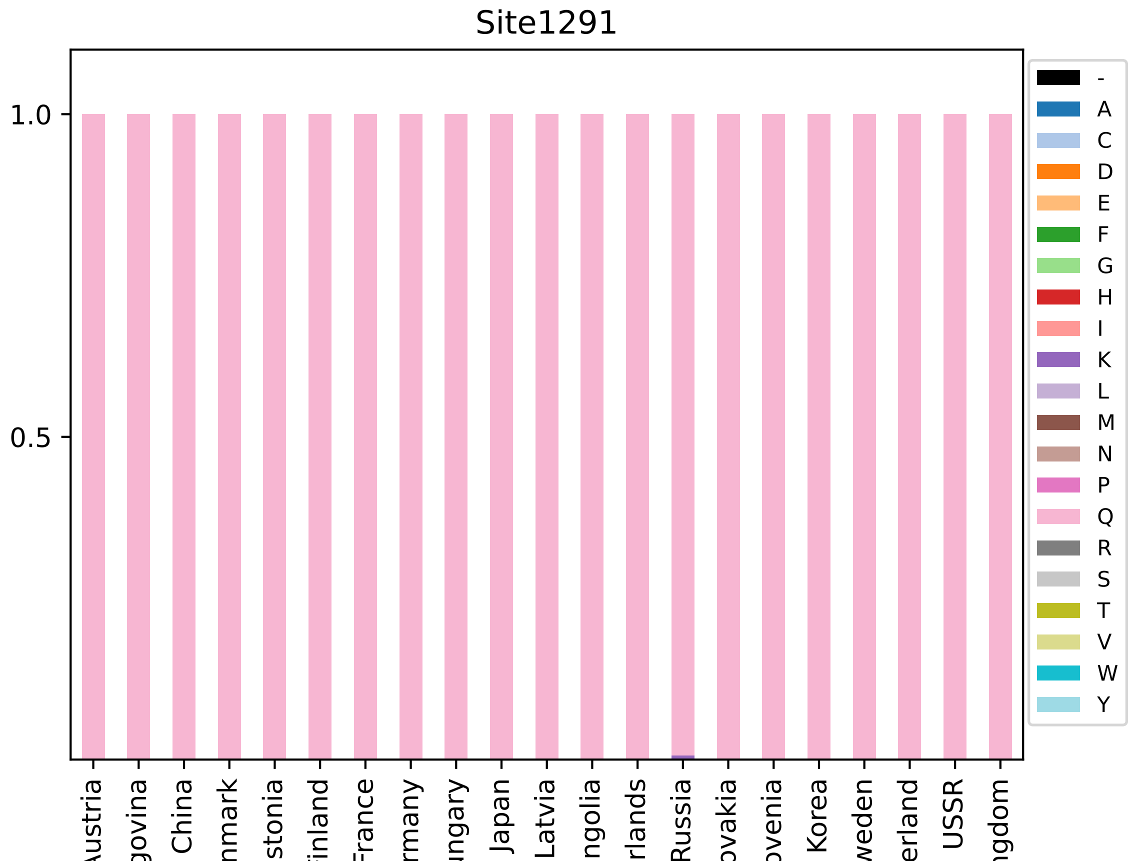
Site1291
1.0
0.5
-
A
C
D
E
F
G
H
I
K
L
M
N
P
Q
R
S
T
V
W
Y
China
Japan
Latvia
Russia
Korea
USSR

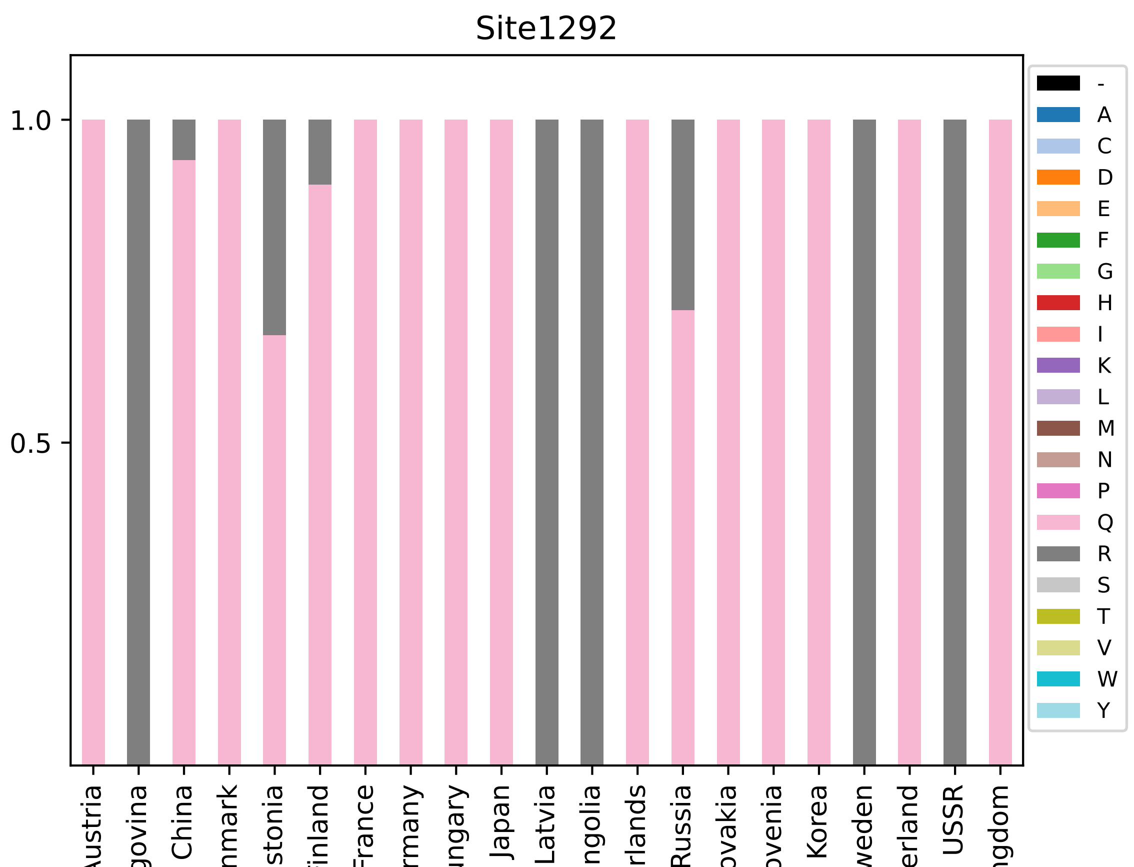

Site1292
1.0
0.5
-
A
C
D
E
F
G
H
I
K
L
M
N
P
Q
R
S
T
V
W
Y
Austria
govina
China
nmark
stonia
inland
France
rmany
ungary
Japan
Latvia
ngolia
rlands
Russia
ovakia
ovenia
Korea
weden
erland
USSR
ngdom

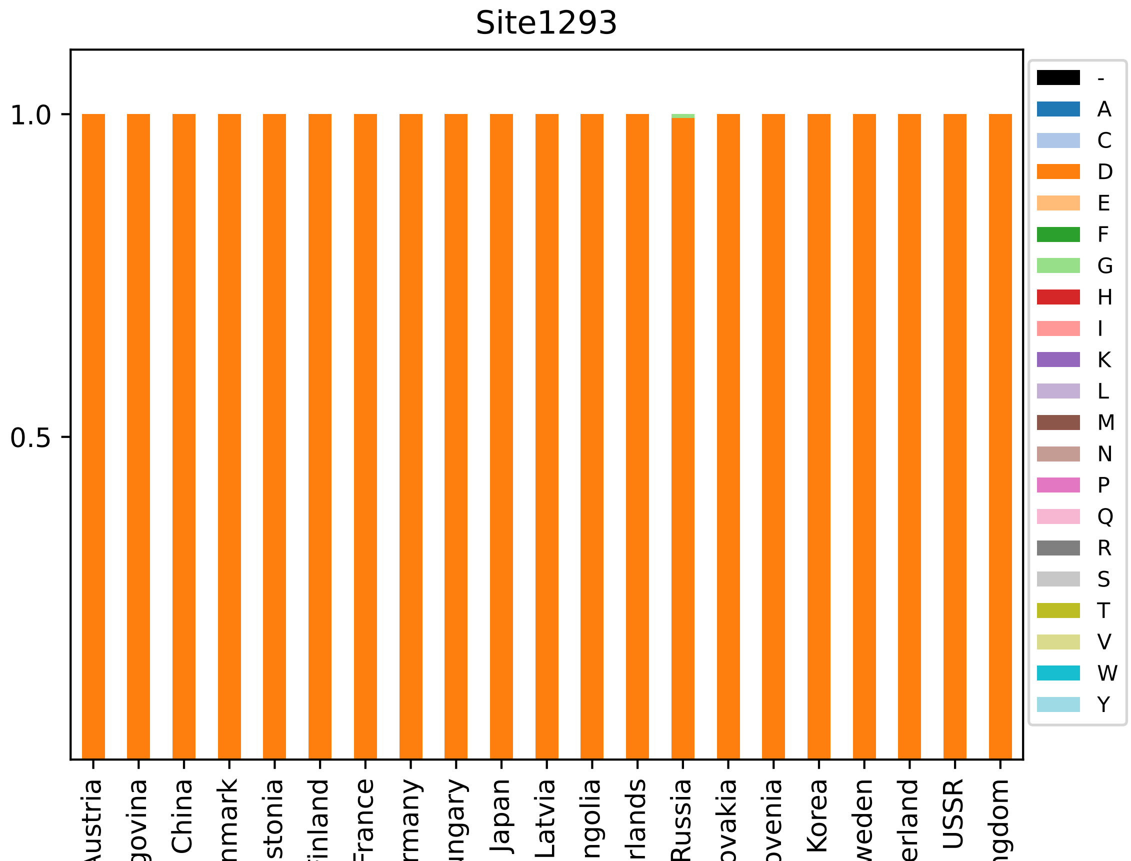

Site1293
1.0
0.5
-
A
C
D
E
F
G
H
I
K
L
M
N
P
Q
R
S
T
V
W
Y
Austria
govina
China
nmark
stonia
Finland
France
rmany
ungary
Japan
Latvia
ngolia
rlands
Russia
ovakia
ovenia
Korea
weden
erland
USSR
ngdom

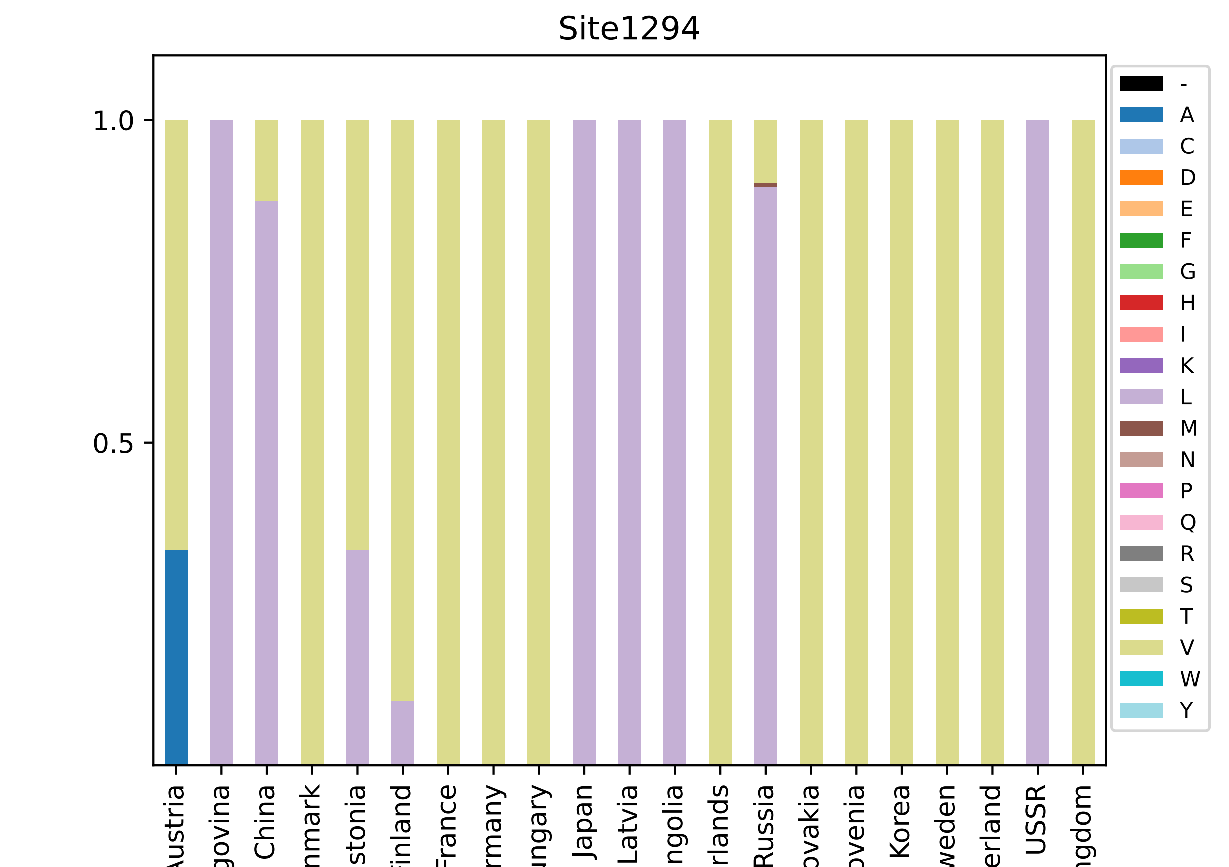

Site1294
1.0
0.5
-
A
C
D
E
F
G
H
I
K
L
M
N
P
Q
R
S
T
V
W
Y
Austria
govina
China
nmark
stonia
inland
France
rmany
ungary
Japan
Latvia
ngolia
rlands
Russia
ovakia
ovenia
Korea
weden
erland
USSR
ngdom

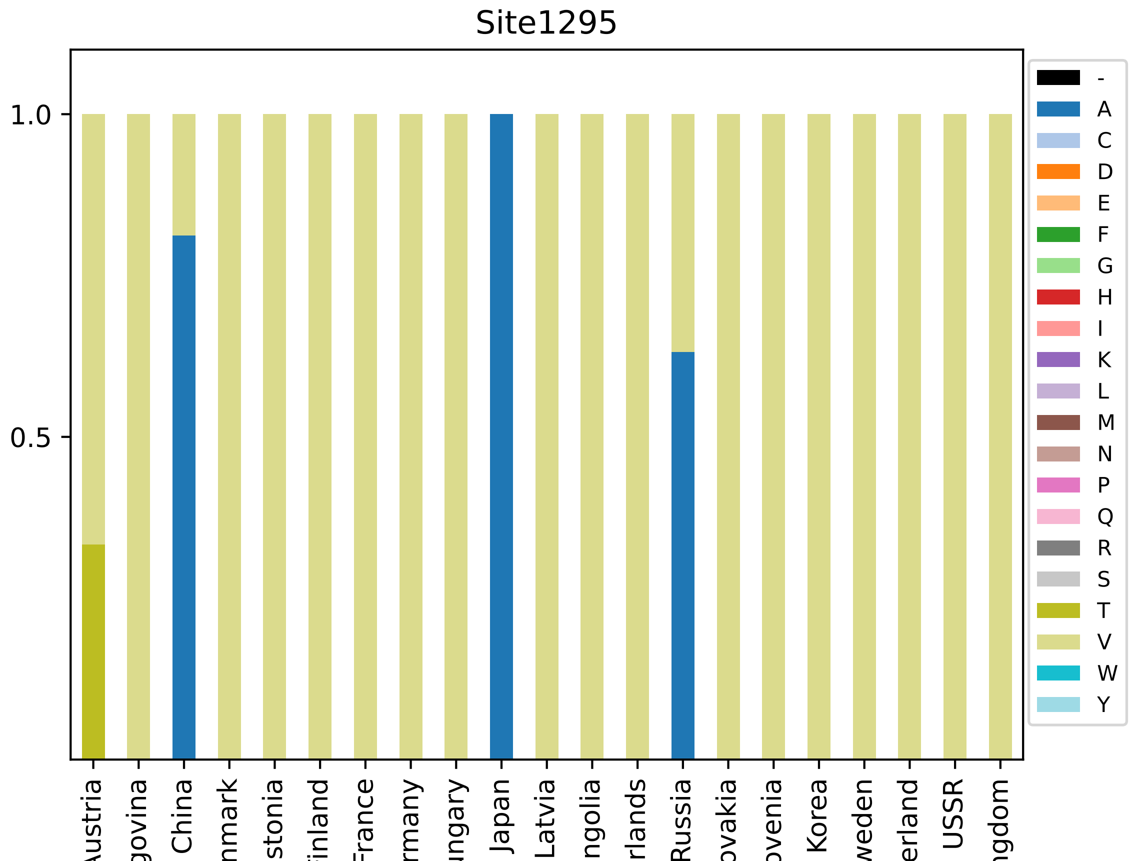

Site1295
1.0
0.5
-
A
C
D
E
F
G
H
I
K
L
M
N
P
Q
R
S
T
V
W
Y
Austria
govina
China
nmark
stonia
Finland
France
rmany
ungary
Japan
Latvia
ngolia
rlands
Russia
ovakia
ovenia
Korea
weden
erland
USSR
ngdom

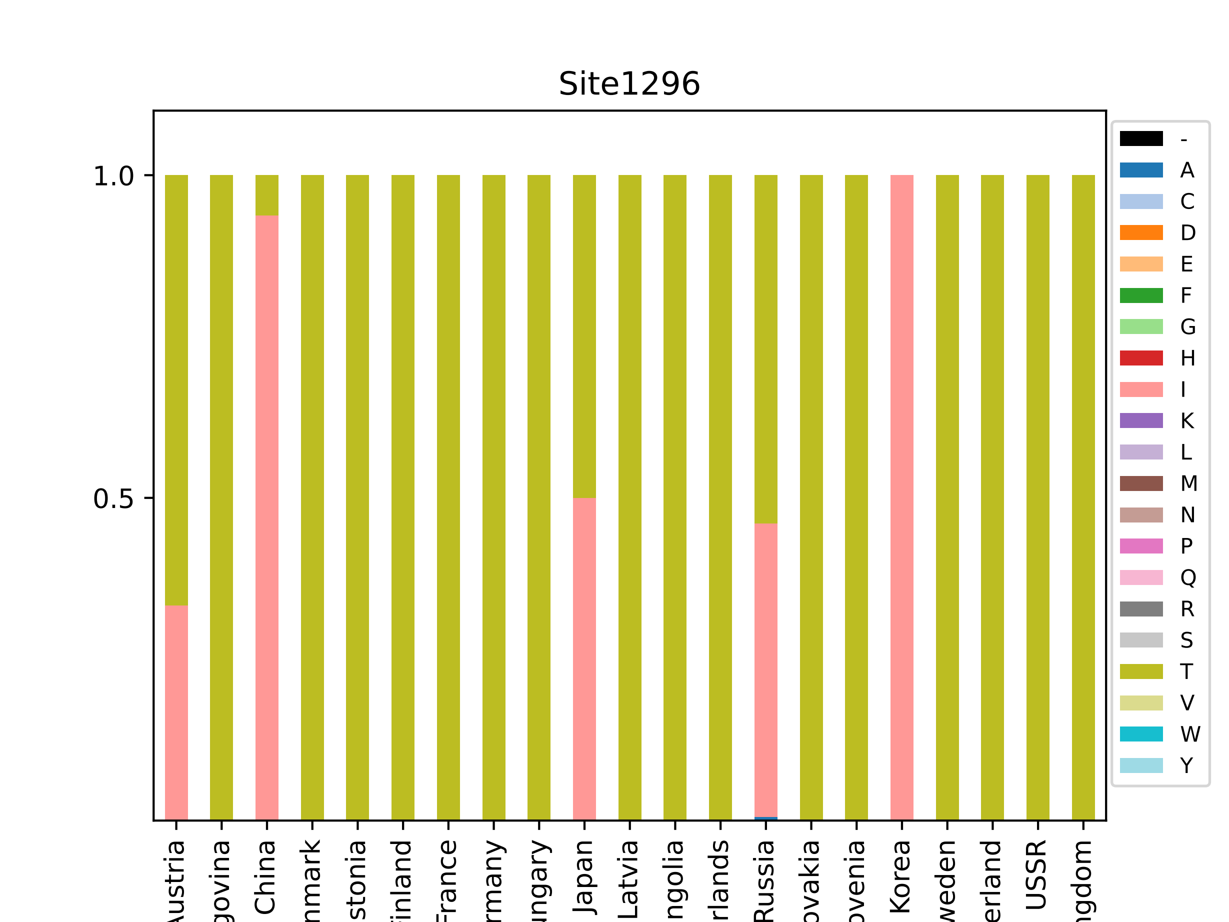

Site1296
1.0
0.5
-
A
C
D
E
F
G
H
I
K
L
M
N
P
Q
R
S
T
V
W
Y
Austria
govina
China
nmark
stonia
Finland
France
rmany
ungary
Japan
Latvia
ngolia
rlands
Russia
ovakia
ovenia
Korea
weden
erland
USSR
ngdom

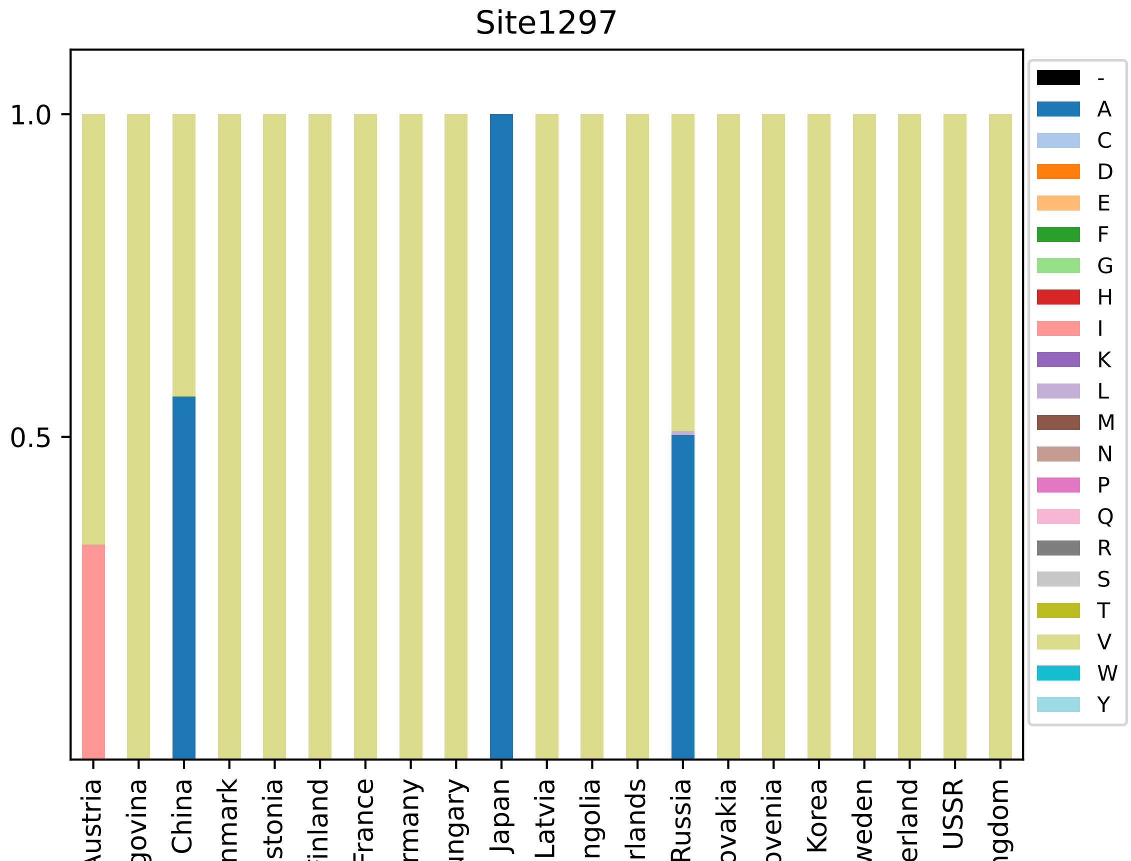
Site1297
1.0
0.5
-
A
C
D
E
F
G
H
I
K
L
M
N
P
Q
R
S
T
V
W
Y
China
Japan
Latvia
Russia
Korea
USSR

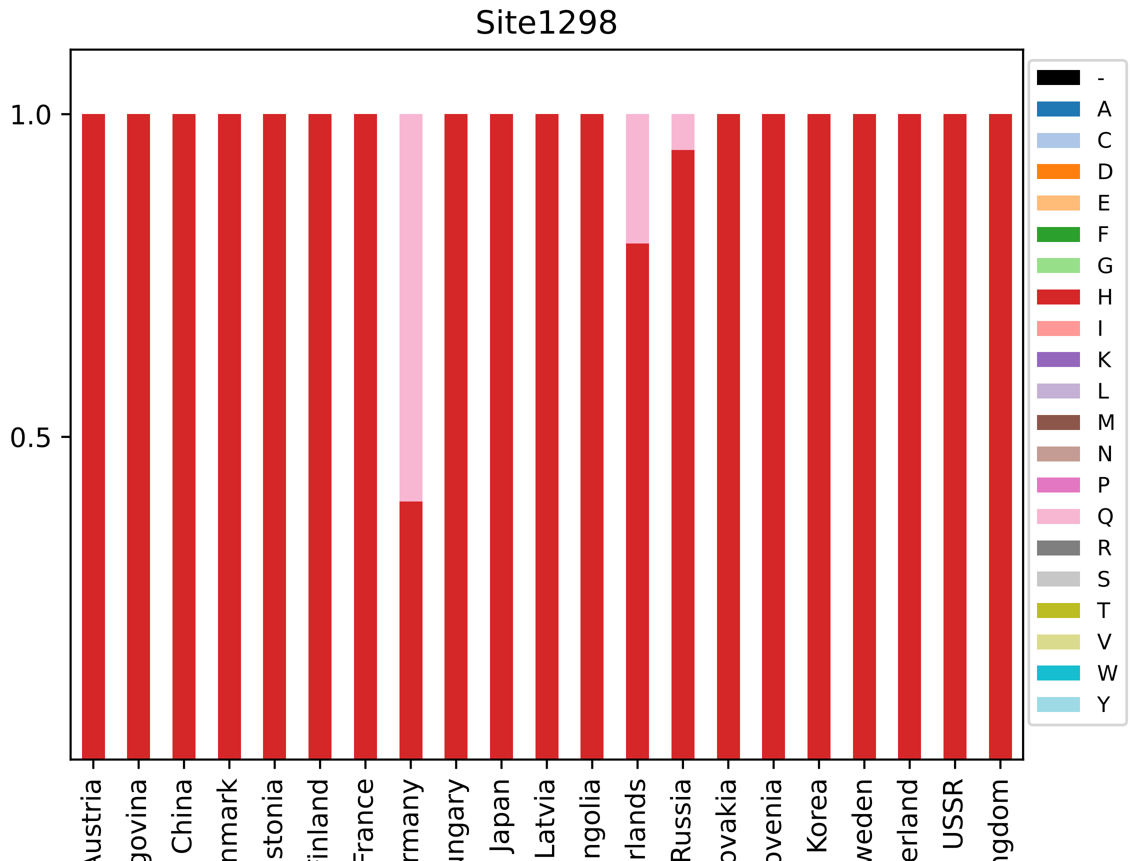
Site1298
1.0
0.5
-
A
C
D
E
F
G
H
I
K
L
M
N
P
Q
R
S
T
V
W
Y
Austria
China
Finland
France
Japan
Latvia
Russia
Korea
USSR

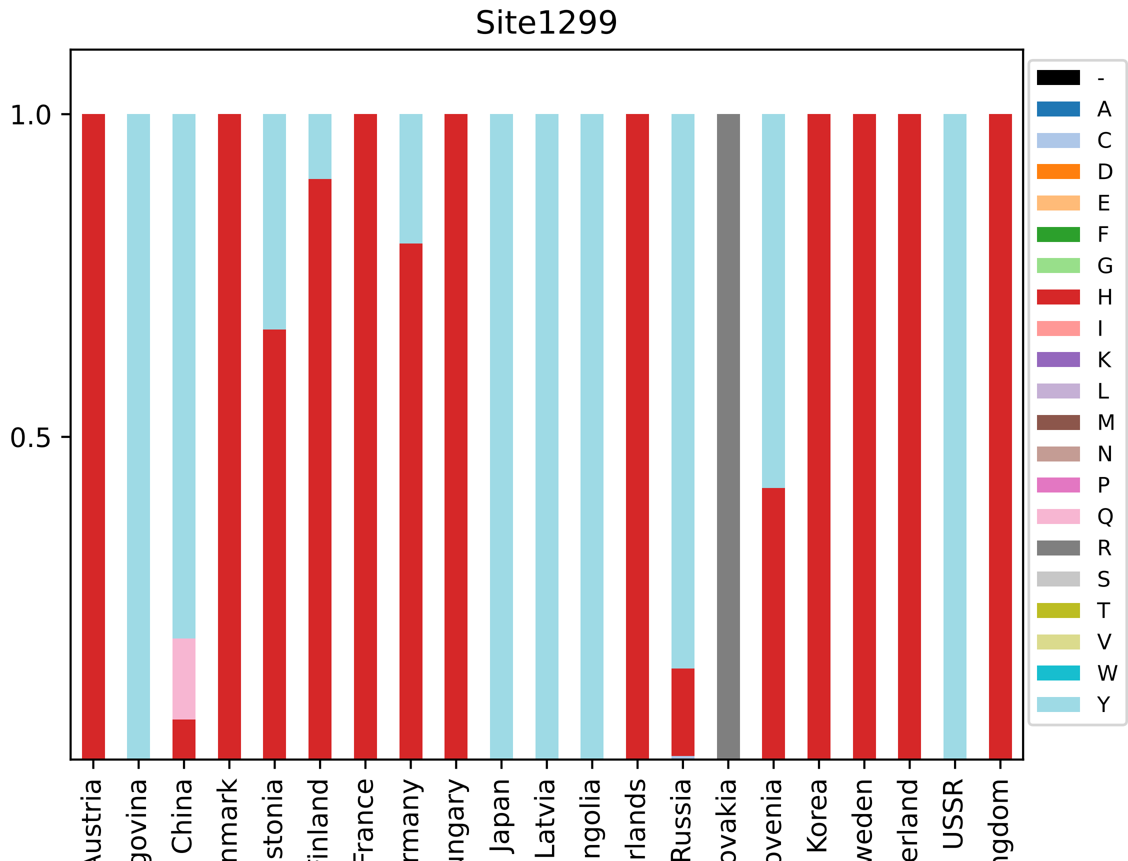

Site1299
1.0
0.5
-
A
C
D
E
F
G
H
I
K
L
M
N
P
Q
R
S
T
V
W
Y
Austria
govina
China
nmark
stonia
Finland
France
rmany
ungary
Japan
Latvia
ngolia
rlands
Russia
ovakia
ovenia
Korea
weden
erland
USSR
ngdom

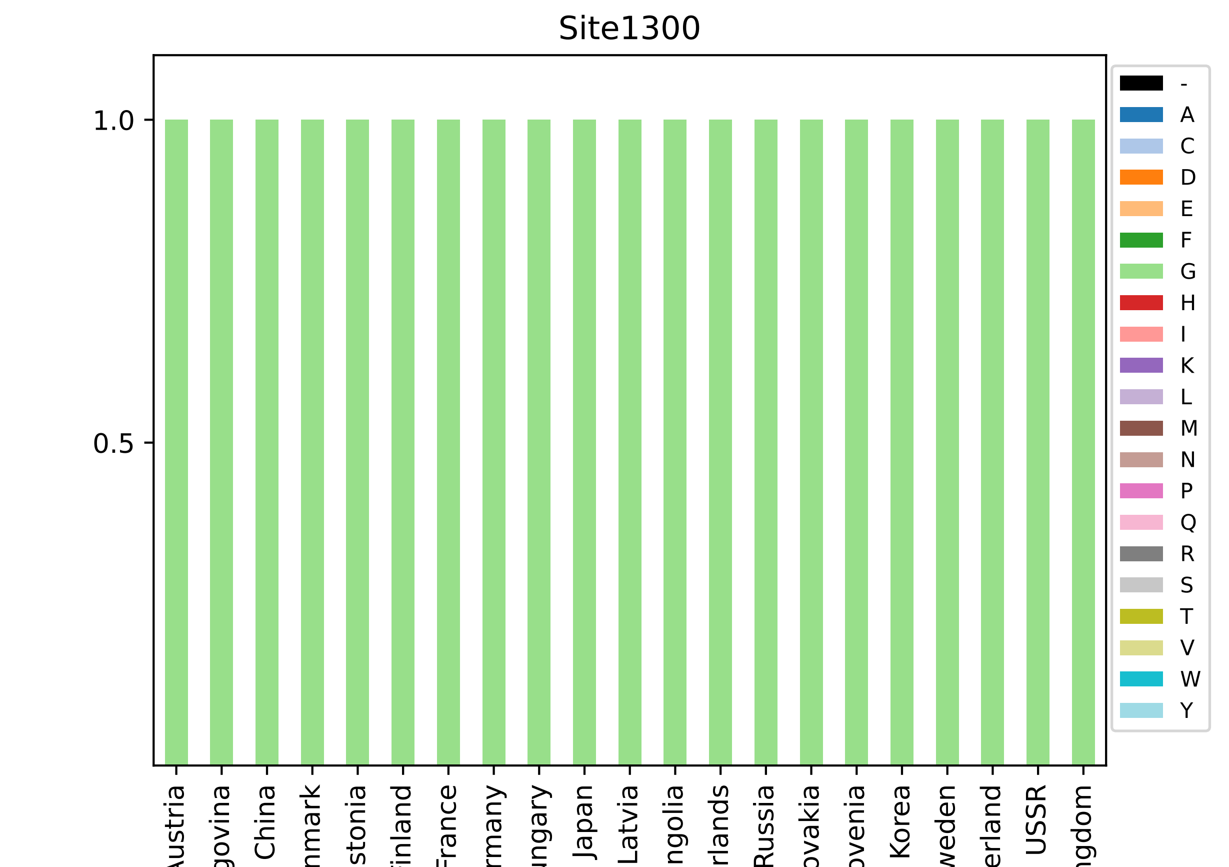

Site1300
1.0
0.5
-
A
C
D
E
F
G
H
I
K
L
M
N
P
Q
R
S
T
V
W
Y
China
Japan
Latvia
Russia
Korea
USSR

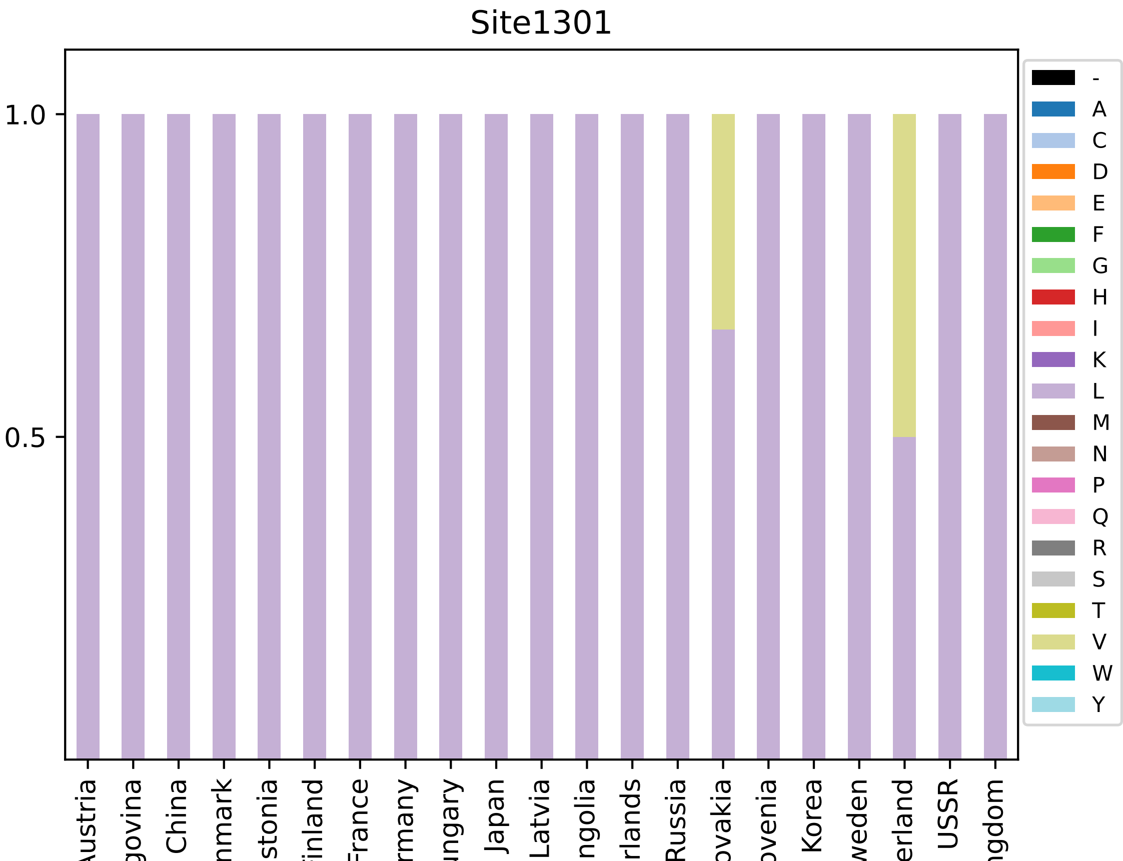

Site1301
1.0
0.5
-
A
C
D
E
F
G
H
I
K
L
M
N
P
Q
R
S
T
V
W
Y
Austria
govina
China
nmark
stonia
Finland
France
rmany
ungary
Japan
Latvia
ngolia
rlands
Russia
ovakia
ovenia
Korea
weden
erland
USSR
ngdom

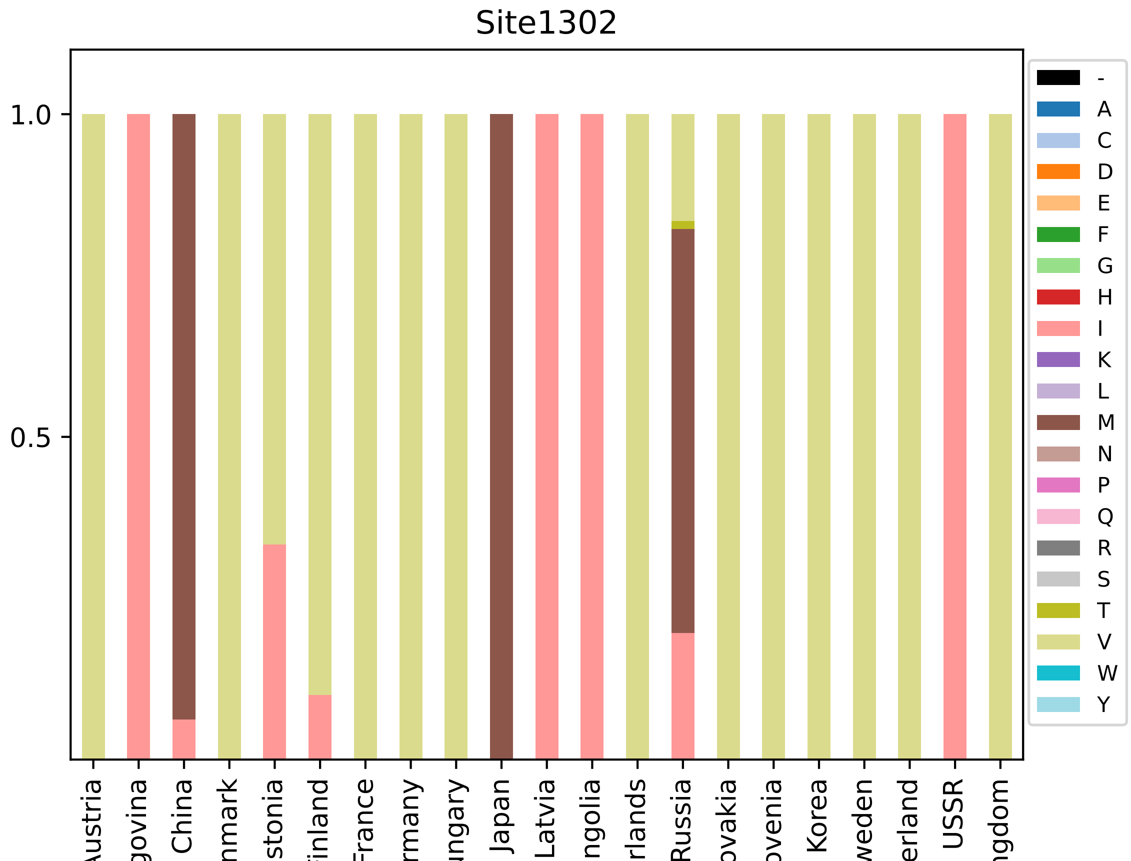

Site1302
1.0
0.5
-
A
C
D
E
F
G
H
I
K
L
M
N
P
Q
R
S
T
V
W
Y
China
Japan
Latvia
Russia
Korea
USSR

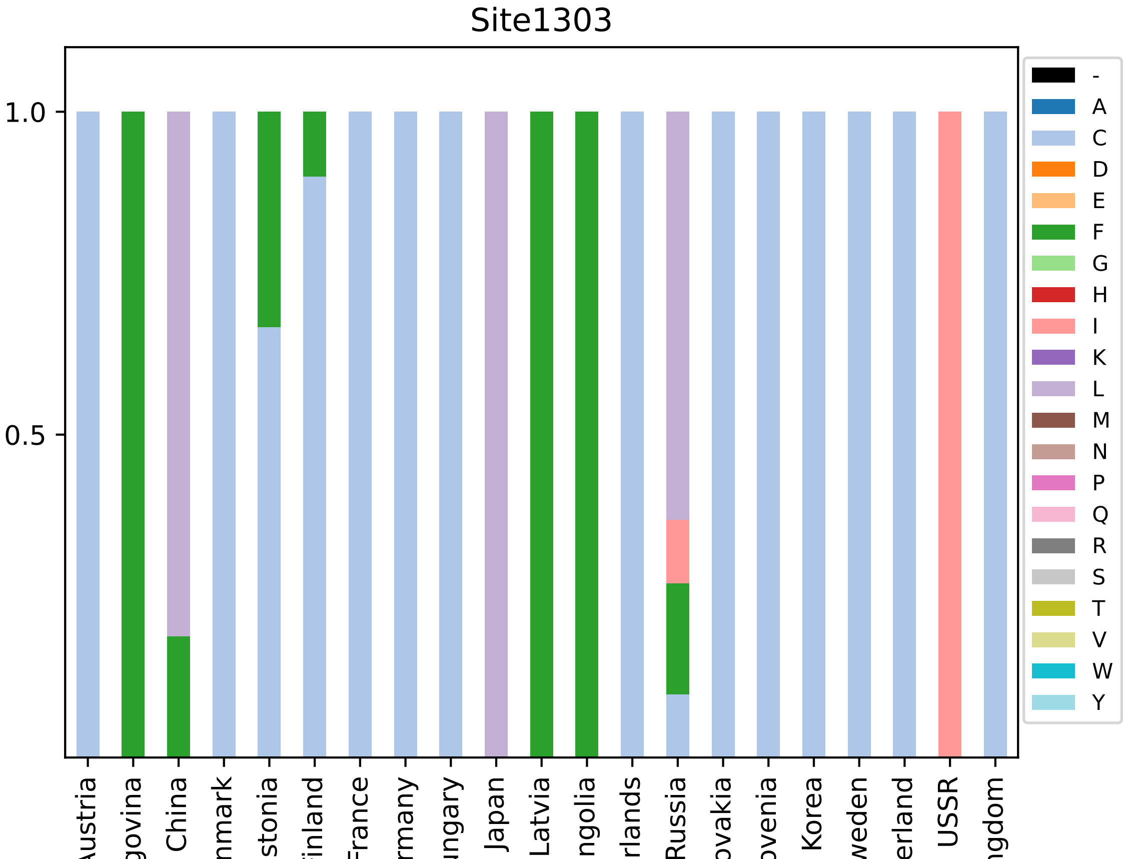
Site1303
1.0
0.5
-
A
C
D
E
F
G
H
I
K
L
M
N
P
Q
R
S
T
V
W
Y
China
Japan
Latvia
Russia
Korea
USSR

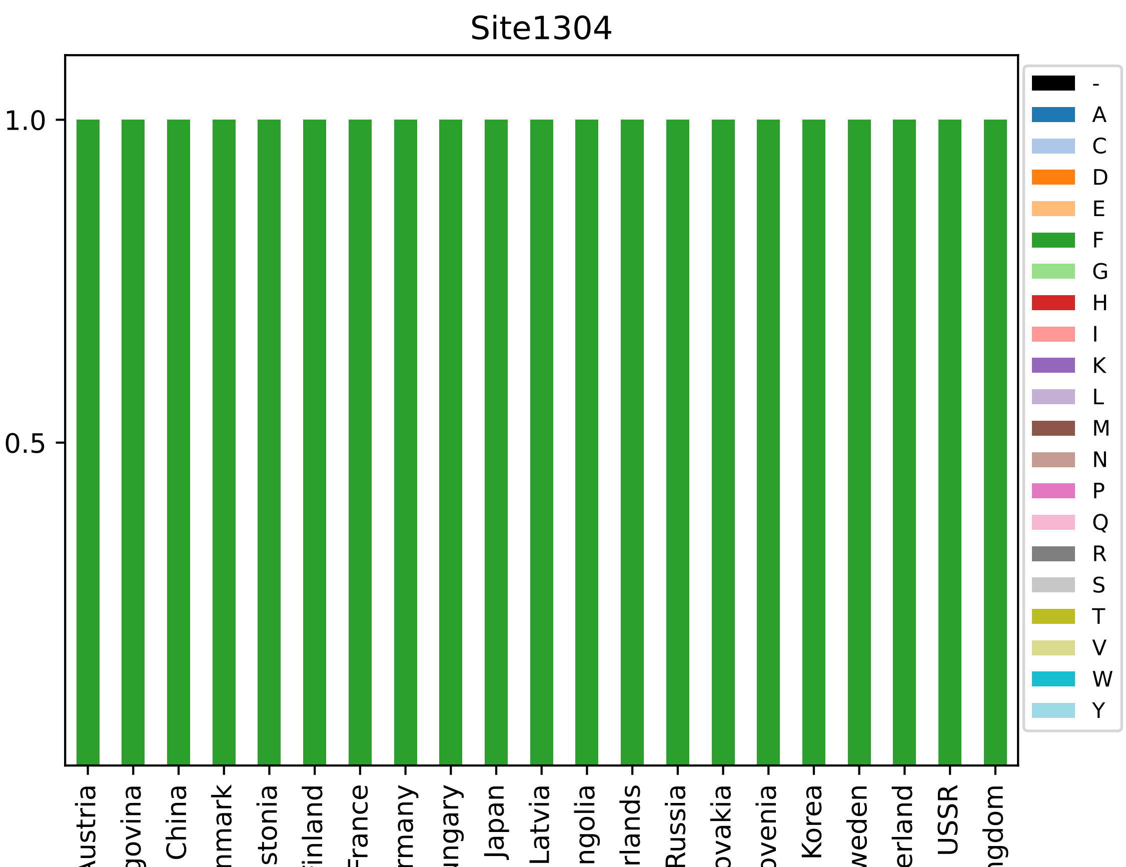

Site1304
1.0
0.5
-
A
C
D
E
F
G
H
I
K
L
M
N
P
Q
R
S
T
V
W
Y
Austria
China
Japan
Latvia
Russia
Korea
USSR

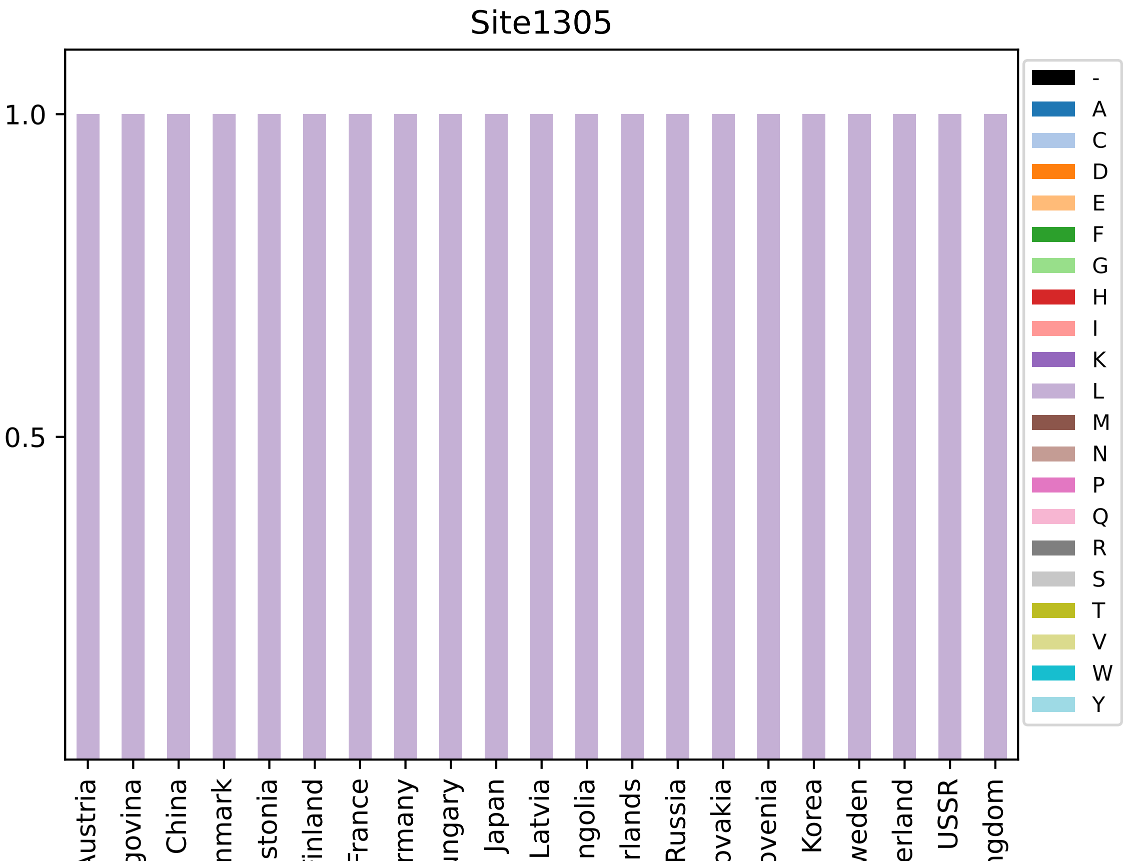
Site1305
1.0
0.5
-
A
C
D
E
F
G
H
I
K
L
M
N
P
Q
R
S
T
V
W
Y
China
Japan
Latvia
Russia
Korea
USSR

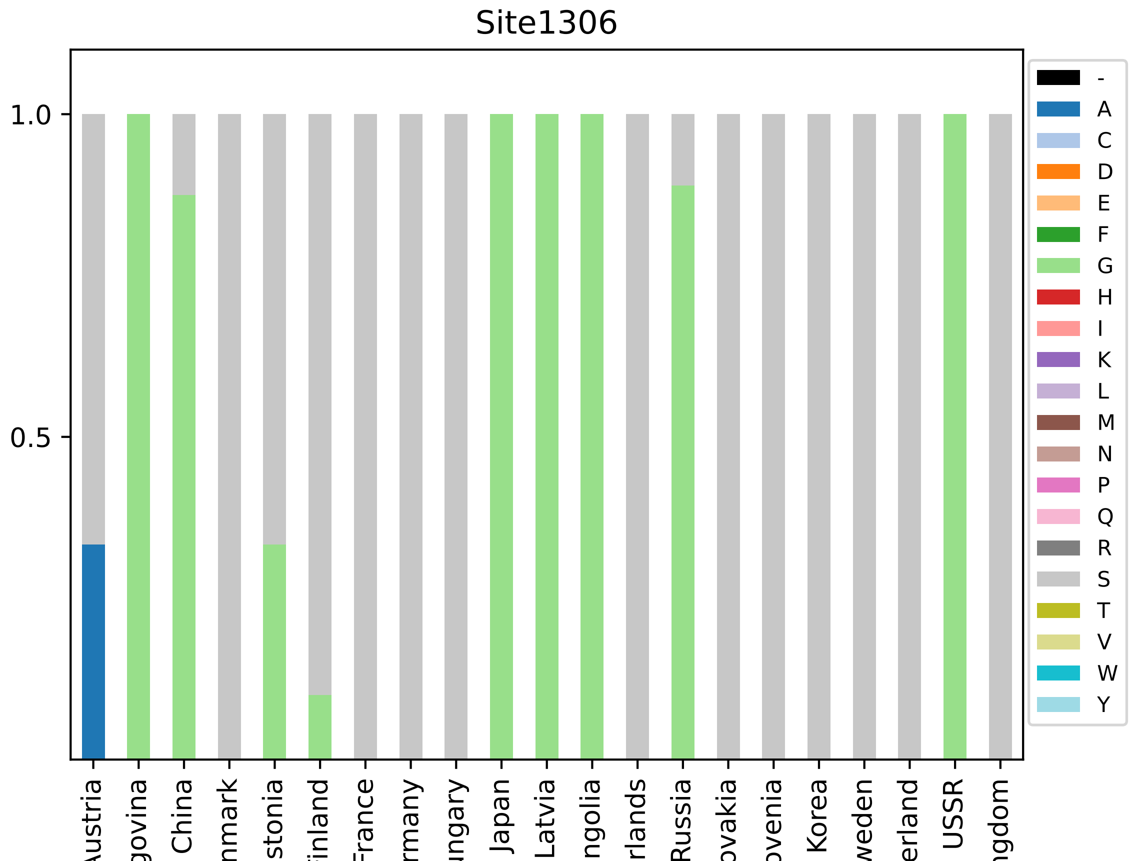

Site1306
1.0
0.5
-
A
C
D
E
F
G
H
I
K
L
M
N
P
Q
R
S
T
V
W
Y
Austria
govina
China
nmark
stonia
inland
France
rmany
ungary
Japan
Latvia
ngolia
rlands
Russia
ovakia
ovenia
Korea
weden
erland
USSR
ngdom

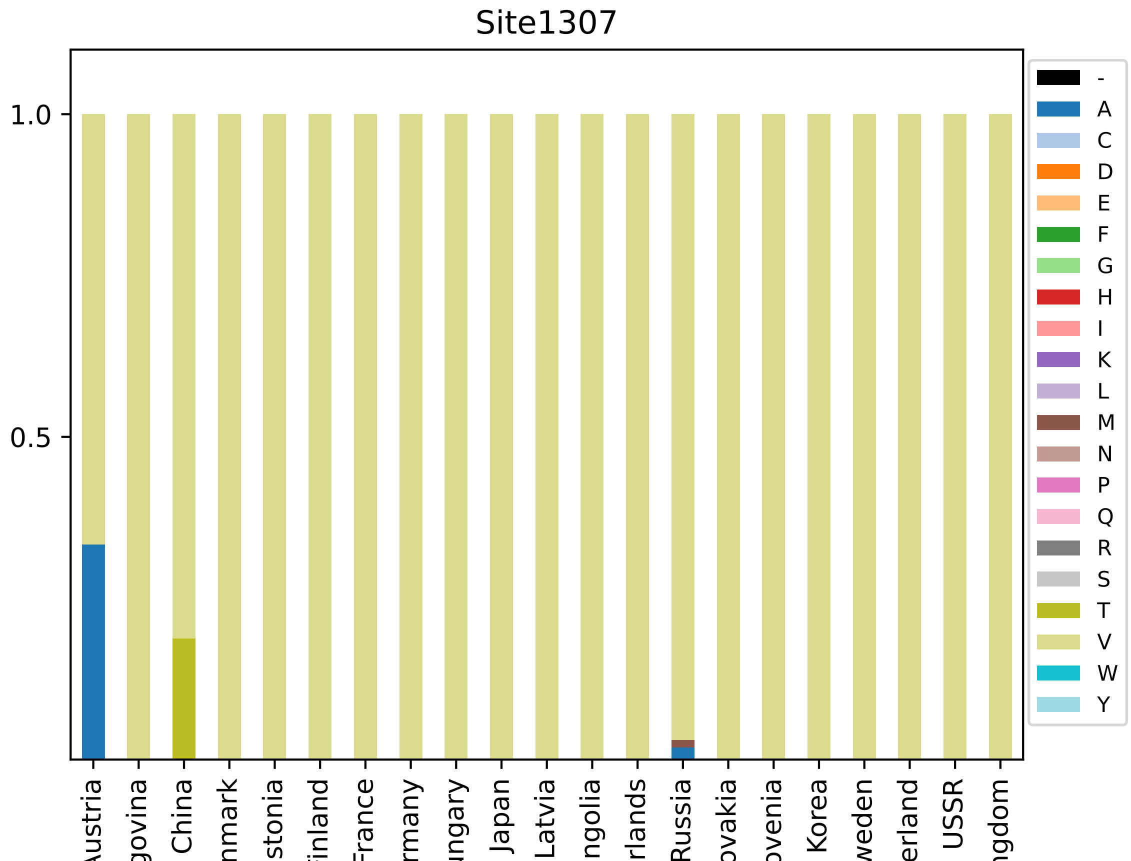
Site1307
1.0
0.5
-
A
C
D
E
F
G
H
I
K
L
M
N
P
Q
R
S
T
V
W
Y
China
Japan
Latvia
Russia
Korea
USSR

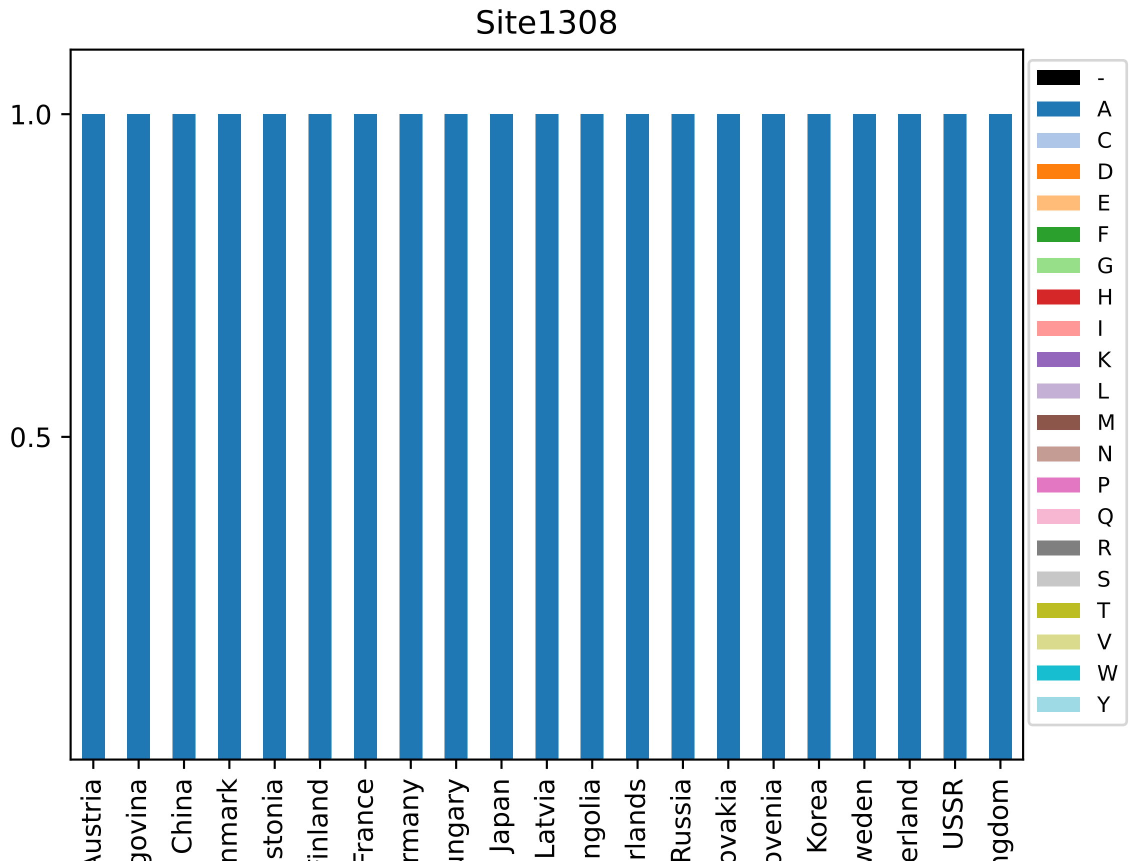
Site1308
1.0
0.5
-
A
C
D
E
F
G
H
I
K
L
M
N
P
Q
R
S
T
V
W
Y
Austria
govina
China
nmark
stonia
inland
France
rmany
ungary
Japan
Latvia
ngolia
rlands
Russia
ovakia
ovenia
Korea
weden
erland
USSR
ngdom

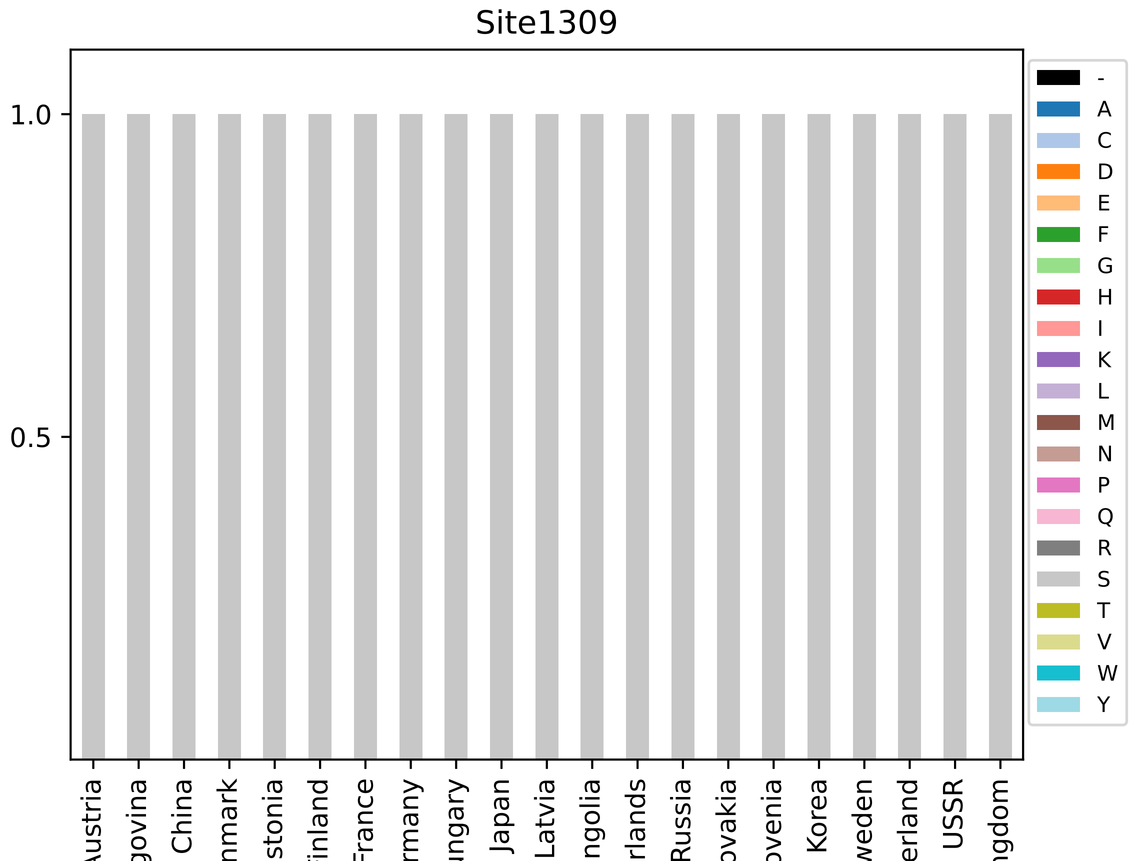

Site1309
1.0
0.5
-
A
C
D
E
F
G
H
I
K
L
M
N
P
Q
R
S
T
V
W
Y
China
Japan
Latvia
Russia
Korea
USSR

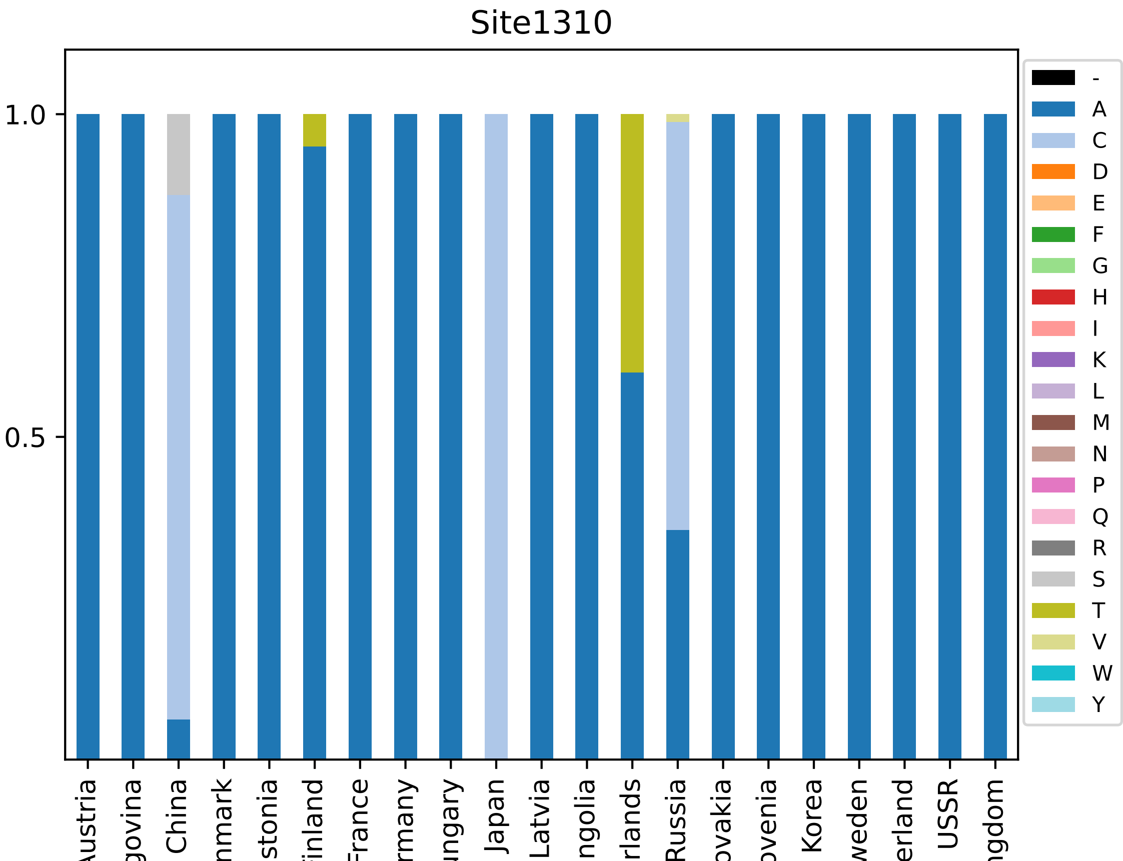

Site1310
1.0
0.5
-
A
C
D
E
F
G
H
I
K
L
M
N
P
Q
R
S
T
V
W
Y
China
Japan
Latvia
Russia
Korea
USSR

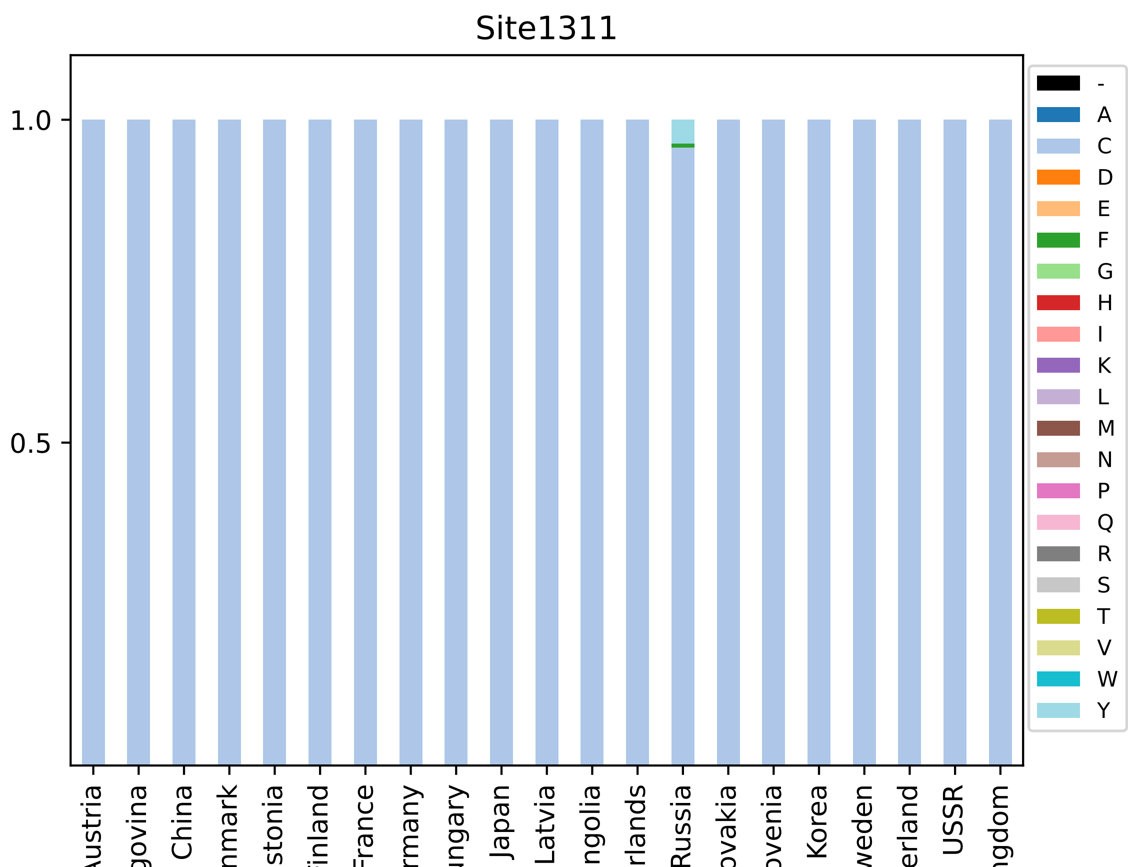

Site1311
1.0
0.5
-
A
C
D
E
F
G
H
I
K
L
M
N
P
Q
R
S
T
V
W
Y
China
Japan
Latvia
Russia
Korea
USSR

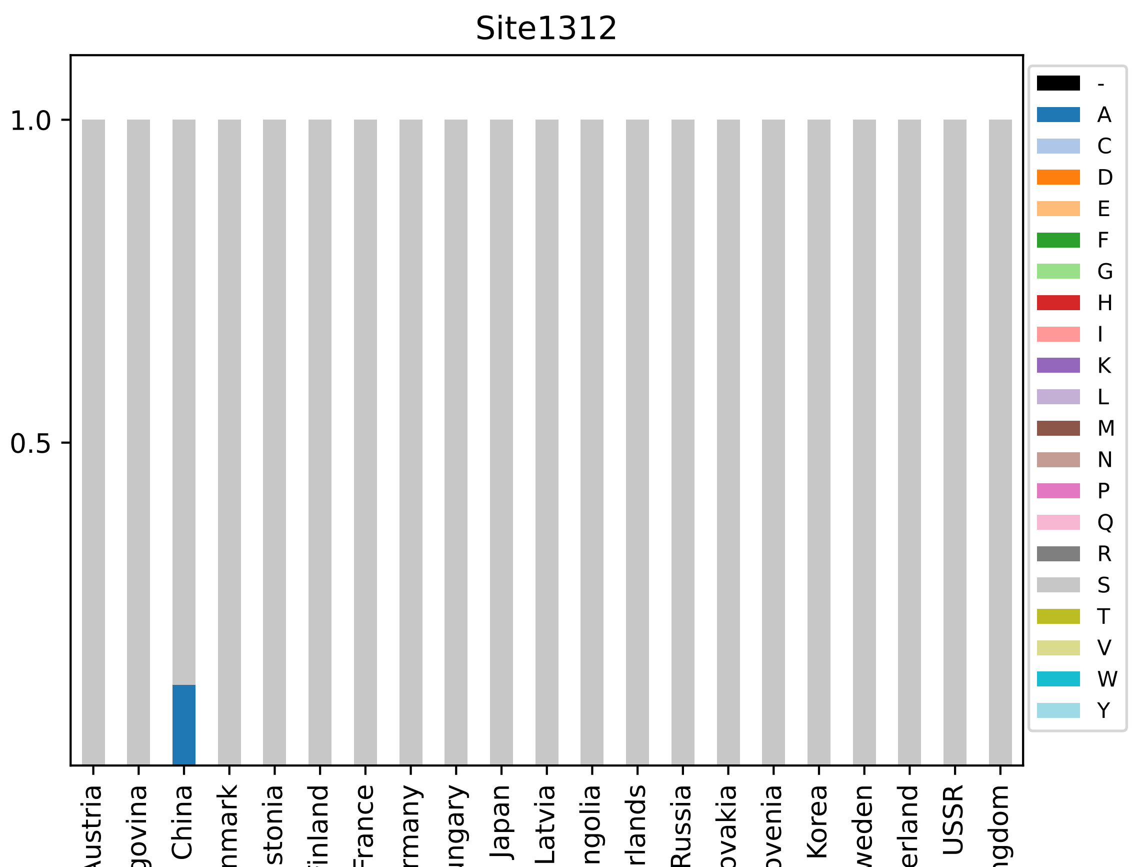

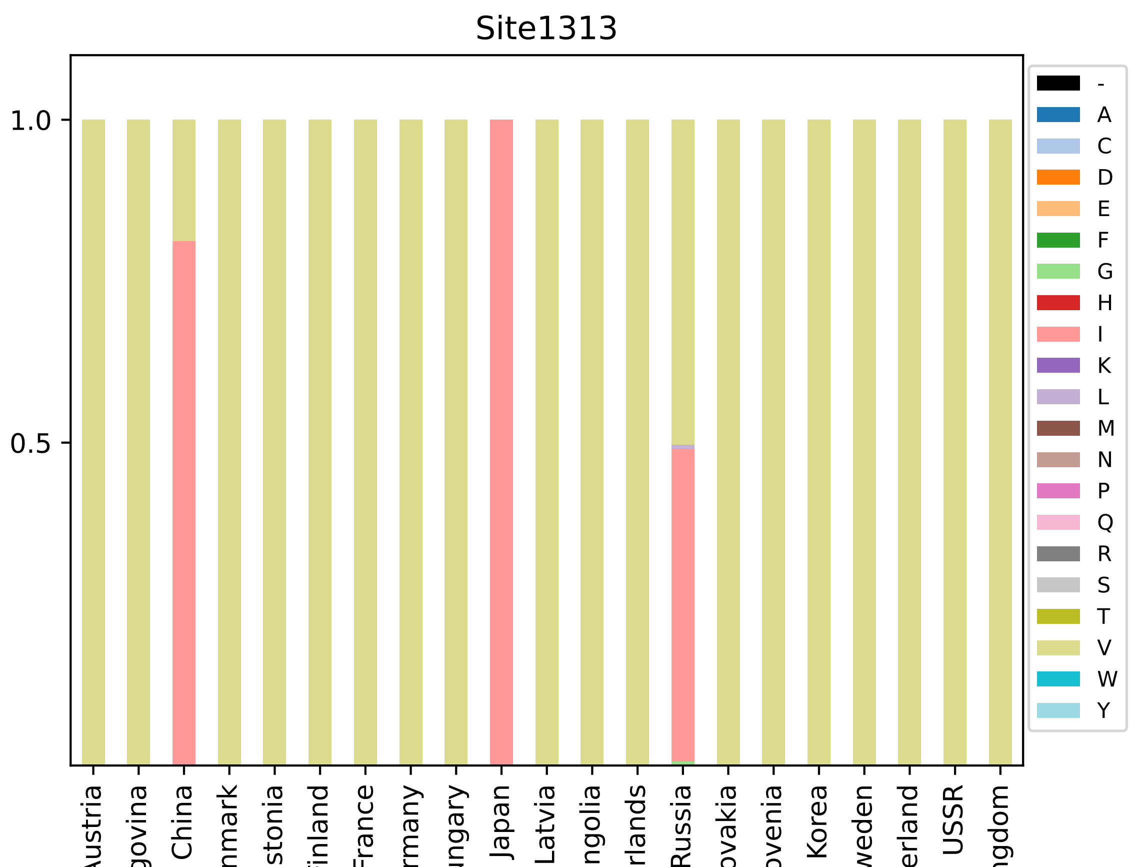

Site1313
1.0
0.5
-
A
C
D
E
F
G
H
I
K
L
M
N
P
Q
R
S
T
V
W
Y
Austria
govina
China
nmark
stonia
Finland
France
rmany
ungary
Japan
Latvia
ngolia
rlands
Russia
ovakia
ovenia
Korea
weden
erland
USSR
ngdom

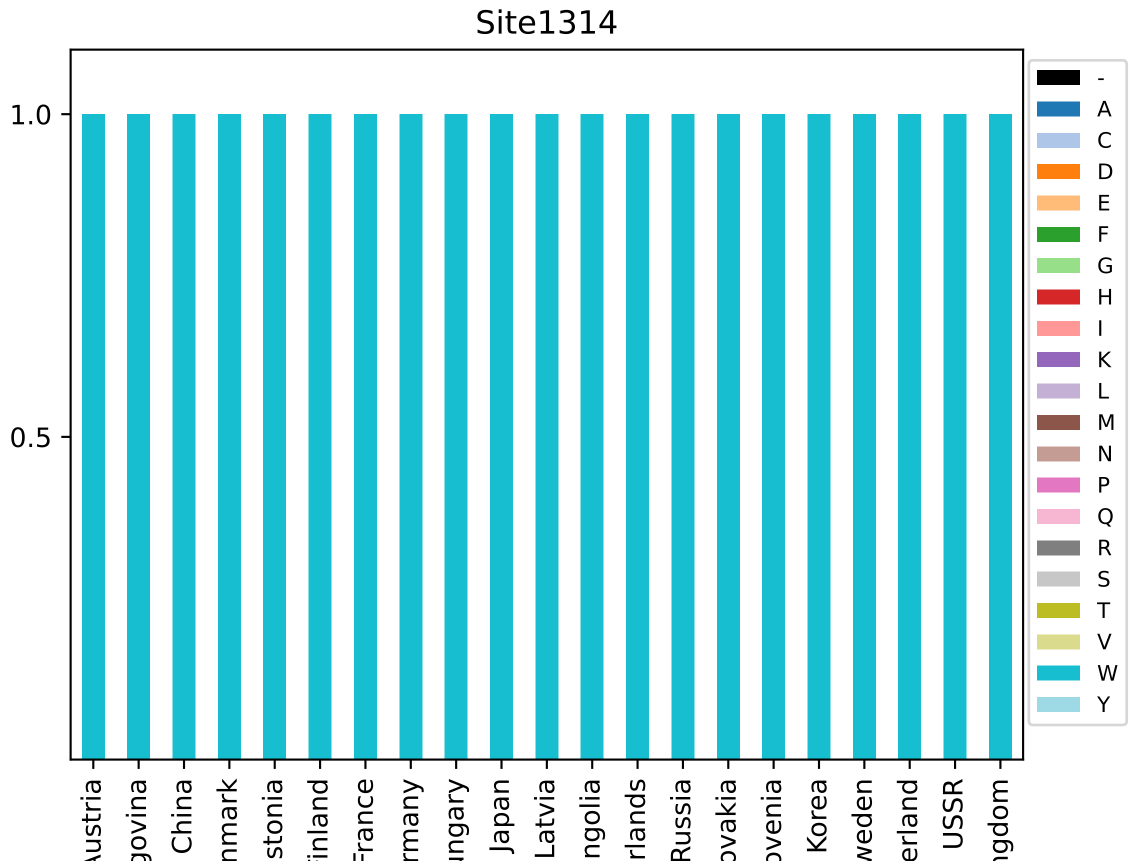

Site1314
1.0
0.5
-
A
C
D
E
F
G
H
I
K
L
M
N
P
Q
R
S
T
V
W
Y
China
Japan
Latvia
Russia
Korea
USSR

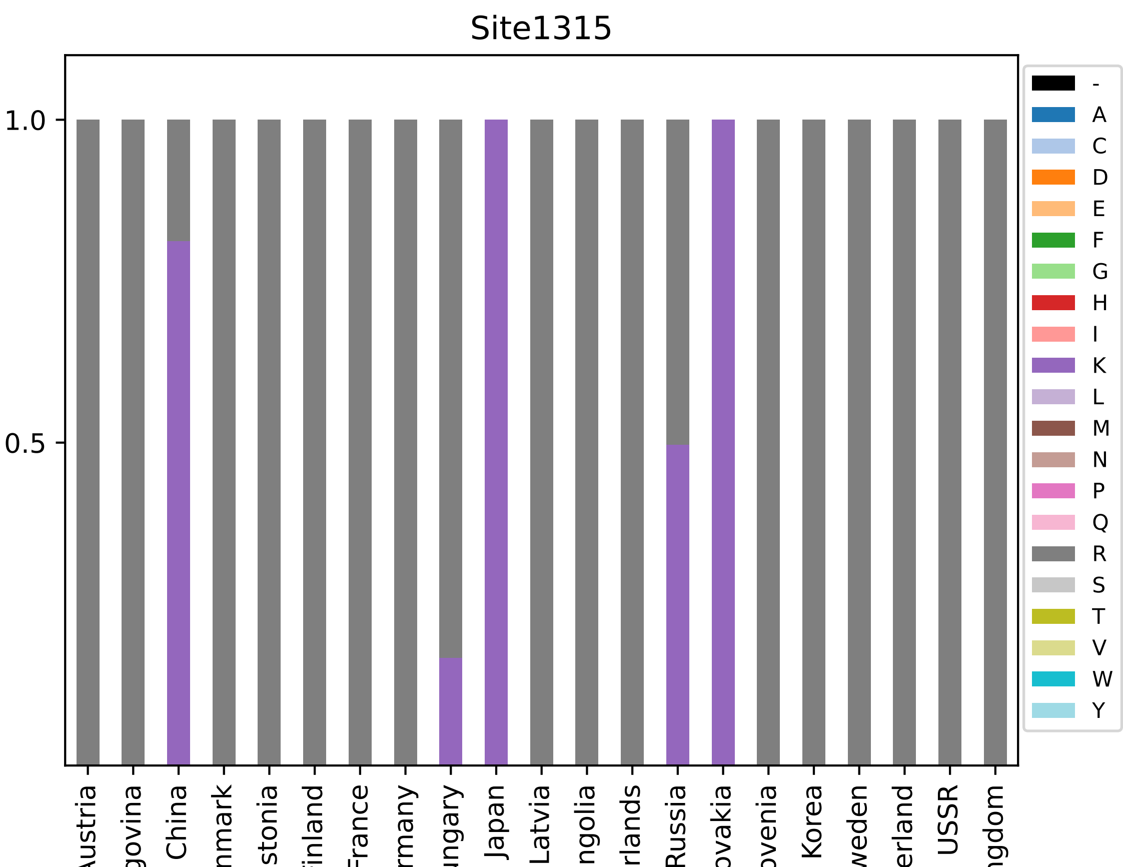
Site1315
1.0
0.5
-
A
C
D
E
F
G
H
I
K
L
M
N
P
Q
R
S
T
V
W
Y
ustria
govina
China
nmark
stonia
inland
France
rmany
ungary
Japan
Latvia
ngolia
rlands
Russia
ovakia
ovenia
Korea
weden
erland
USSR
ngdom

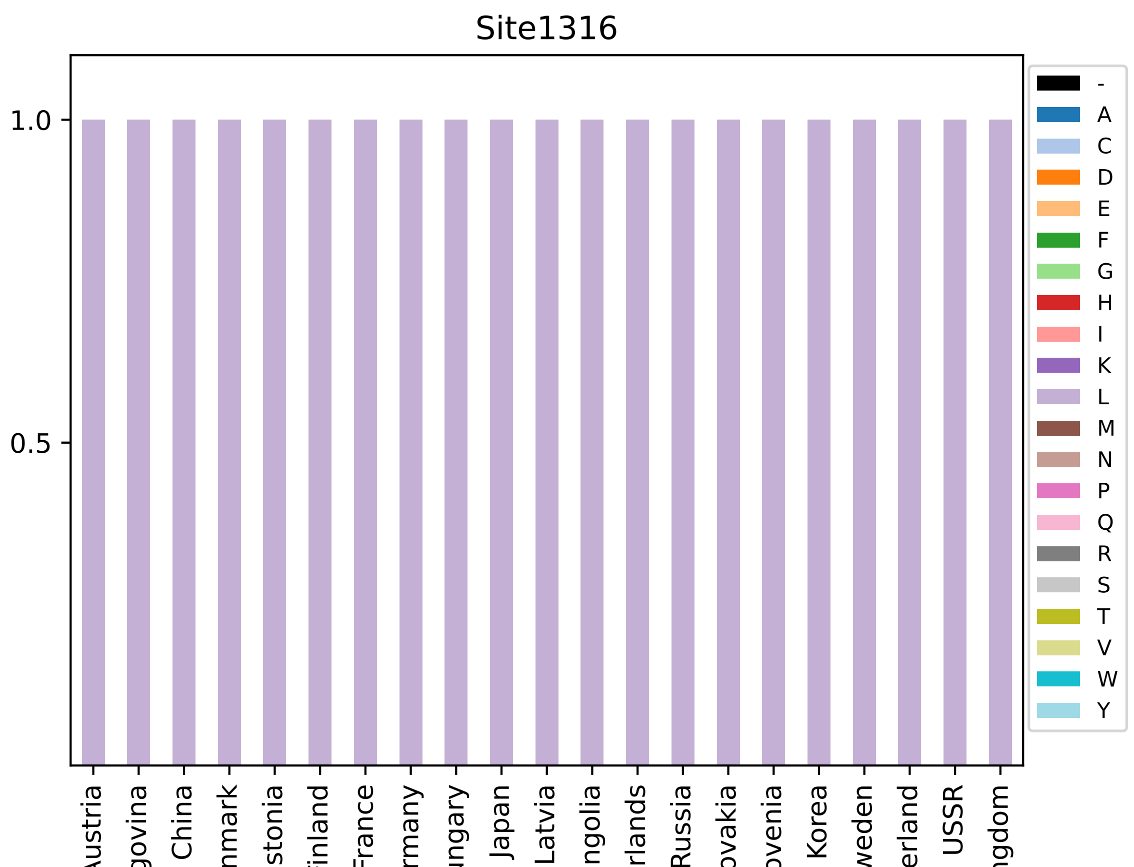
Site1316
1.0
0.5
-
A
C
D
E
F
G
H
I
K
L
M
N
P
Q
R
S
T
V
W
Y
Austria
govina
China
nmark
stonia
inland
France
rmany
ungary
Japan
Latvia
ngolia
rlands
Russia
ovakia
ovenia
Korea
weden
erland
USSR
ngdom

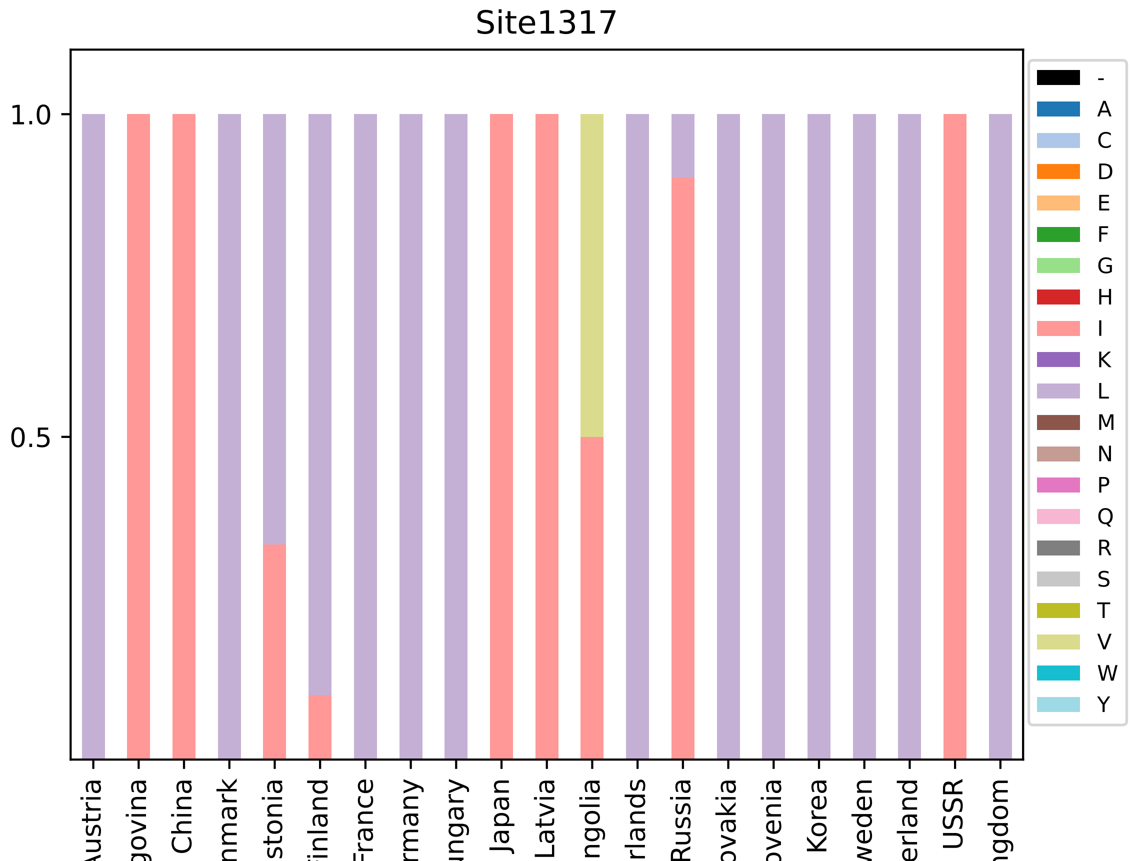
Site1317
1.0
0.5
-
A
C
D
E
F
G
H
I
K
L
M
N
P
Q
R
S
T
V
W
Y
China
Japan
Latvia
Russia
Korea
USSR

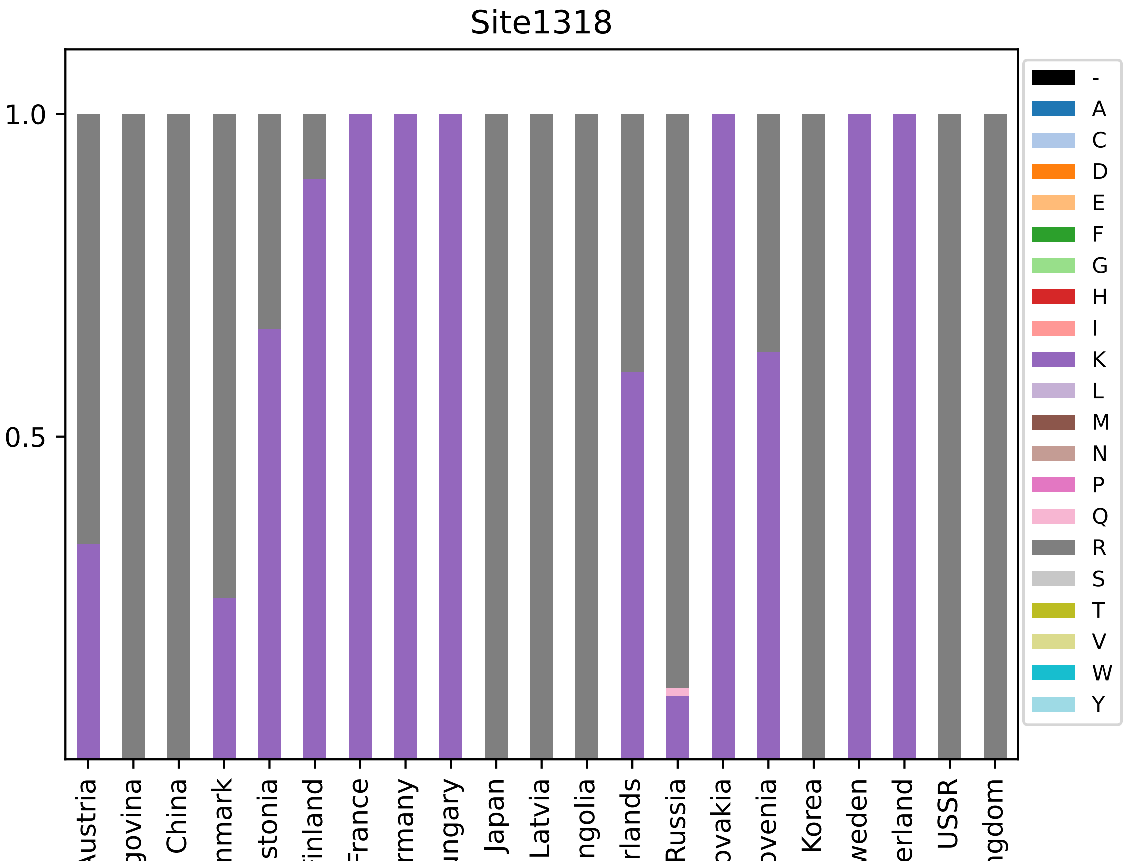
Site1318
1.0
0.5
-
A
C
D
E
F
G
H
I
K
L
M
N
P
Q
R
S
T
V
W
Y
Austria
govina
China
nmark
stonia
inland
France
rmany
ungary
Japan
Latvia
ngolia
rlands
Russia
ovakia
ovenia
Korea
weden
erland
USSR
ngdom

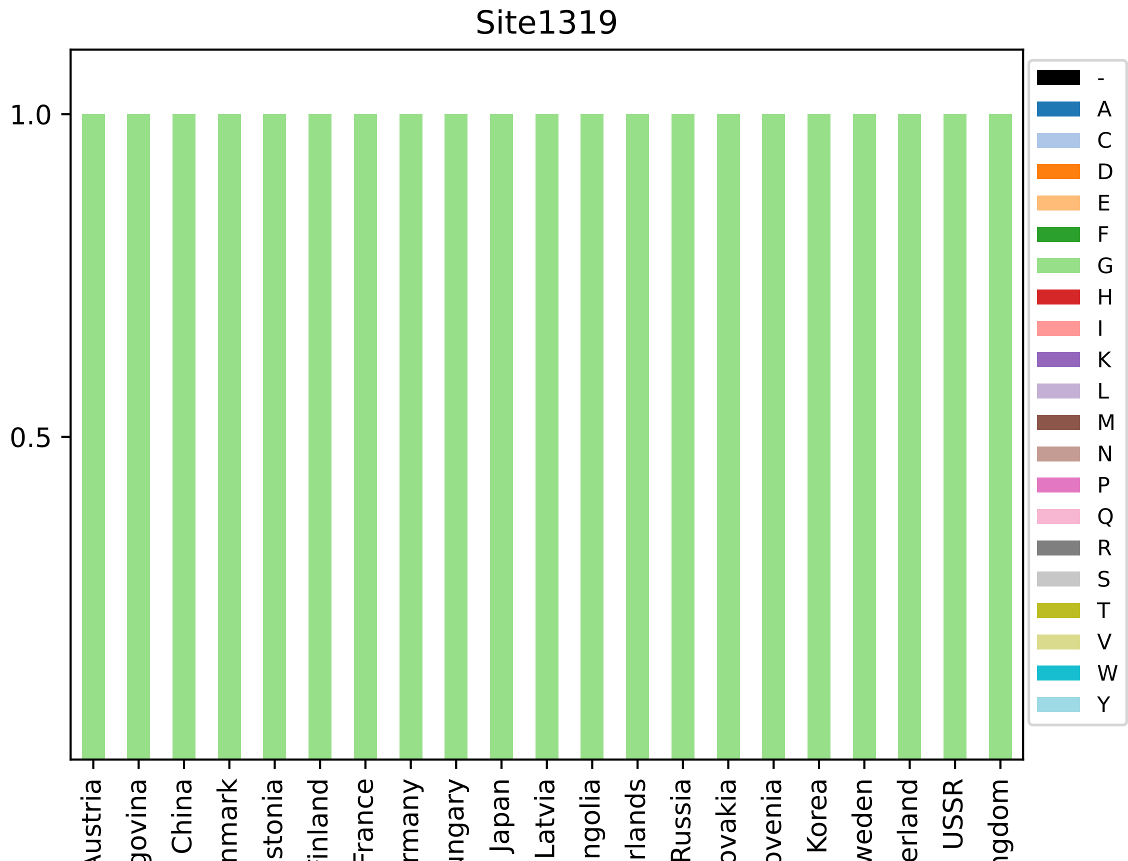

Site1319
1.0
0.5
-
A
C
D
E
F
G
H
I
K
L
M
N
P
Q
R
S
T
V
W
Y
Austria
China
Japan
Latvia
Russia
Korea
USSR

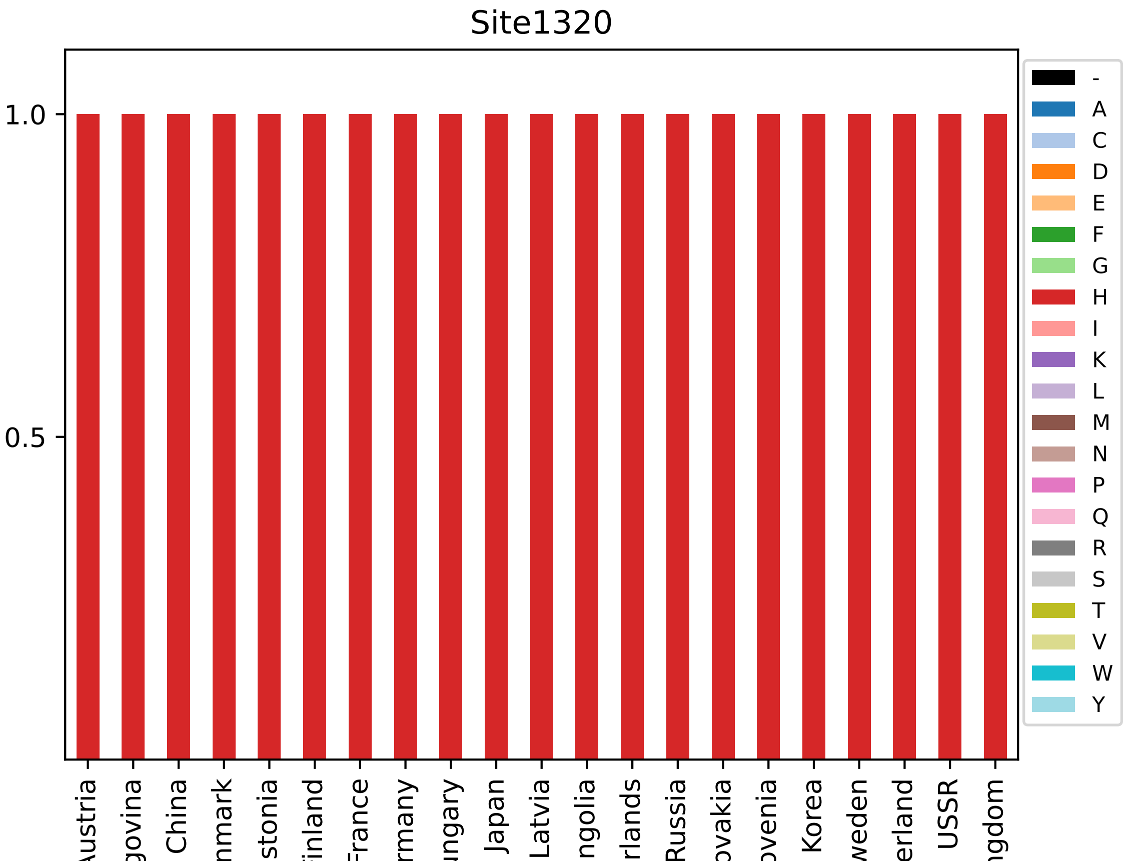
Site1320
1.0
0.5
-
A
C
D
E
F
G
H
I
K
L
M
N
P
Q
R
S
T
V
W
Y
China
Japan
Latvia
Russia
Korea
USSR

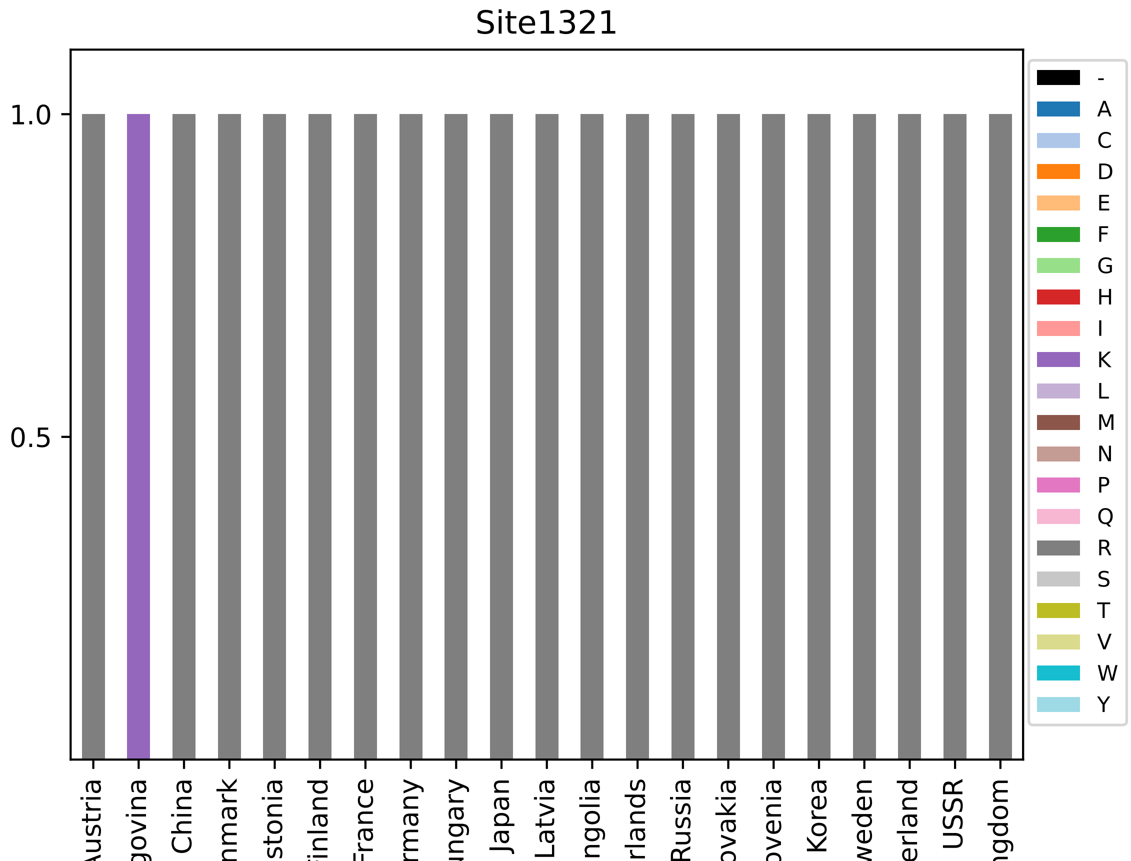

Site1321
1.0
0.5
-
A
C
D
E
F
G
H
I
K
L
M
N
P
Q
R
S
T
V
W
Y
China
Japan
Latvia
Russia
Korea
USSR

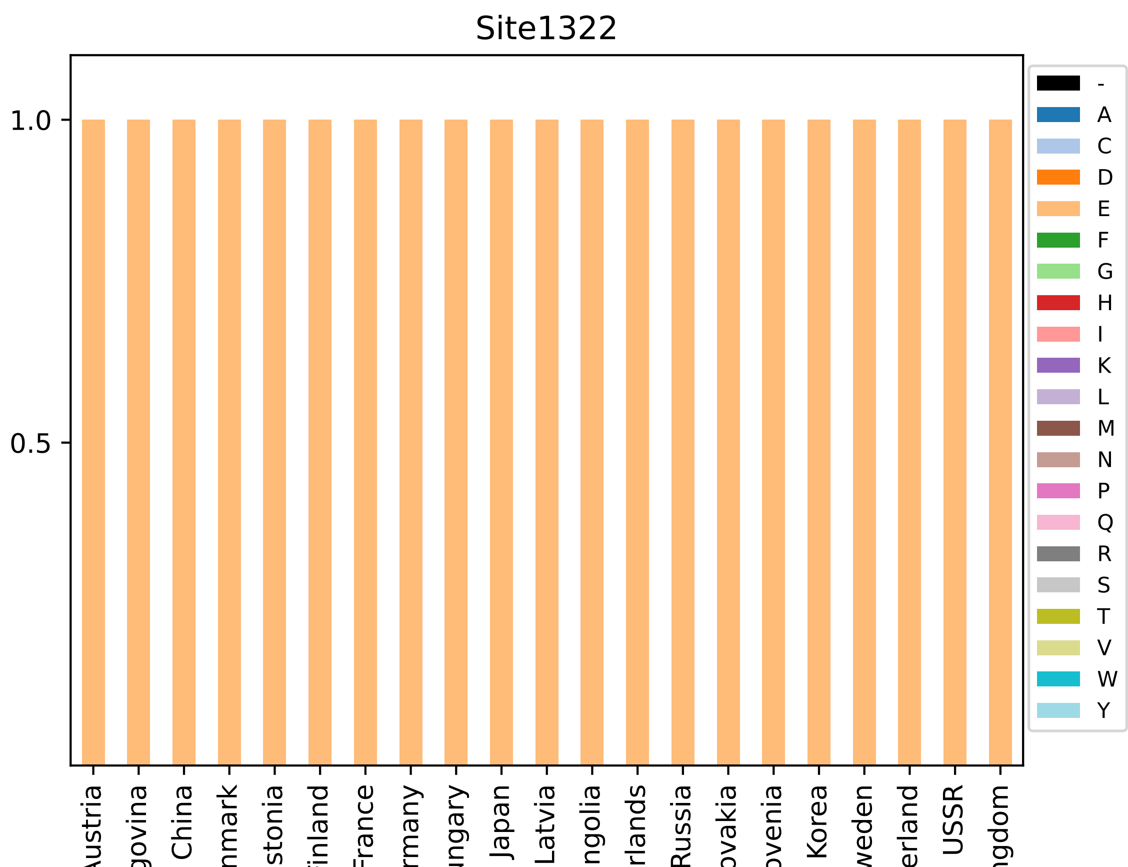
Site1322
1.0
0.5
-
A
C
D
E
F
G
H
I
K
L
M
N
P
Q
R
S
T
V
W
Y
China
Japan
Latvia
Russia
Korea
USSR

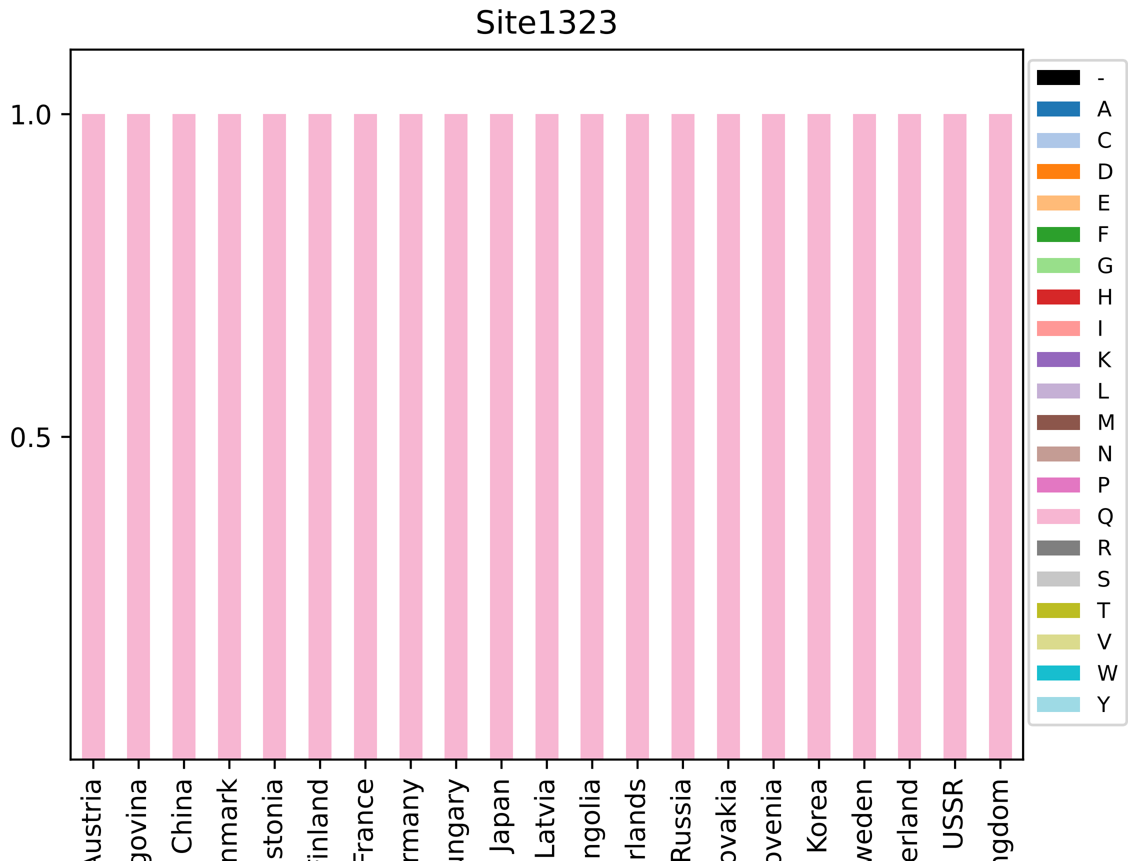

Site1323
1.0
0.5
-
A
C
D
E
F
G
H
I
K
L
M
N
P
Q
R
S
T
V
W
Y
Austria
China
Japan
Latvia
Russia
Korea
USSR

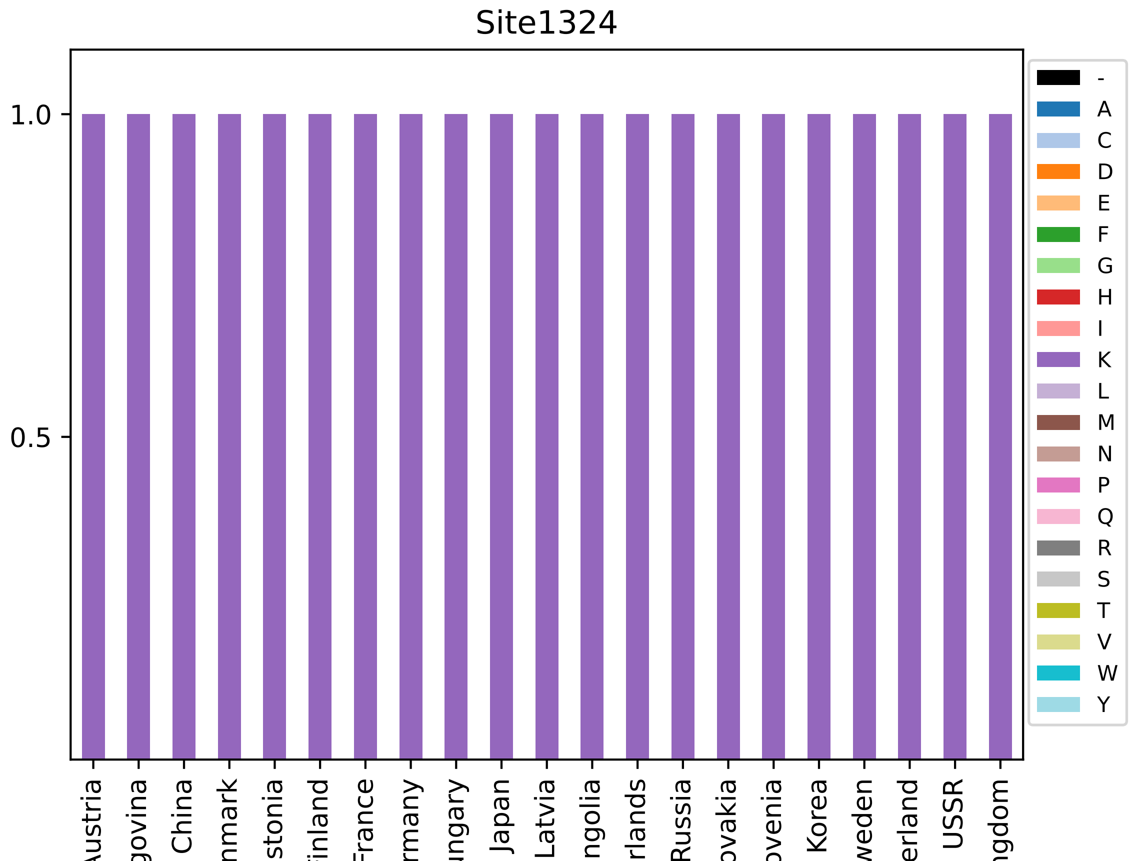

Site1324
1.0
0.5
-
A
C
D
E
F
G
H
I
K
L
M
N
P
Q
R
S
T
V
W
Y
China
Japan
Latvia
Russia
Korea
USSR

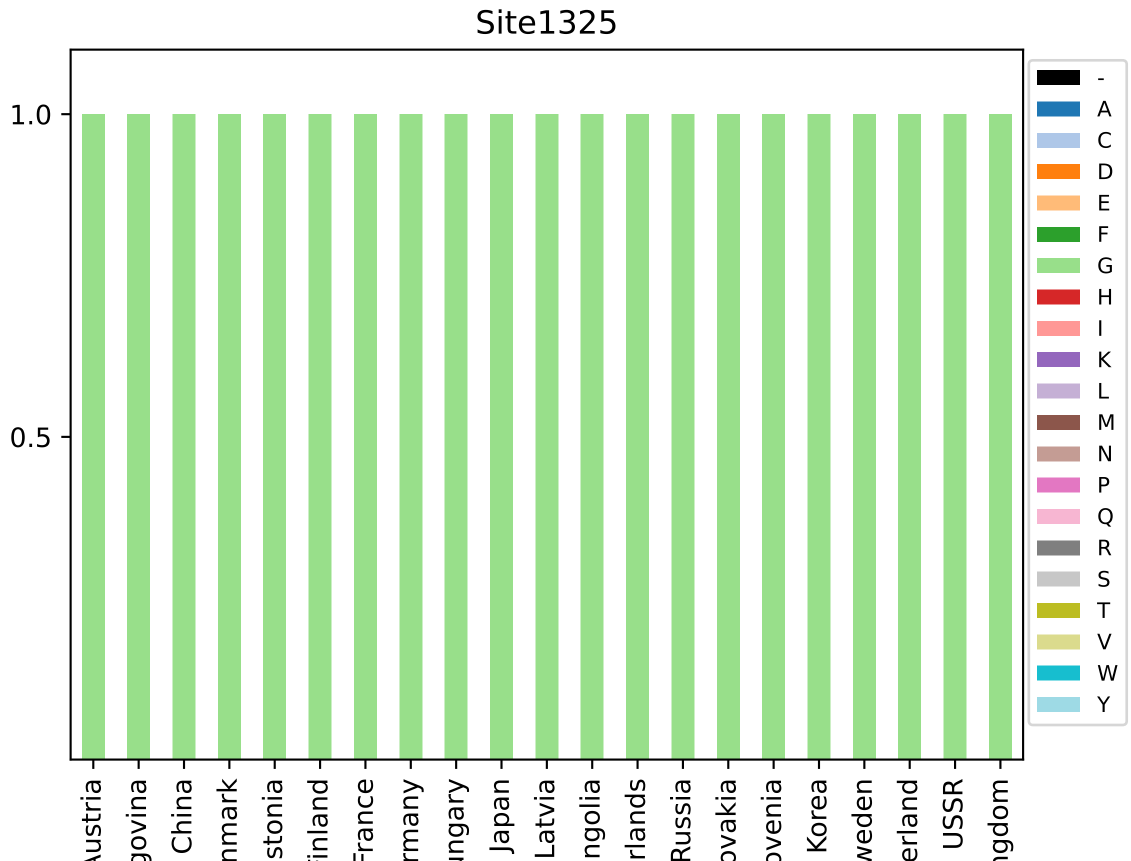

Site1325
1.0
0.5
-
A
C
D
E
F
G
H
I
K
L
M
N
P
Q
R
S
T
V
W
Y
Austria
China
Finland
France
Japan
Latvia
Russia
Korea
USSR

Site1326

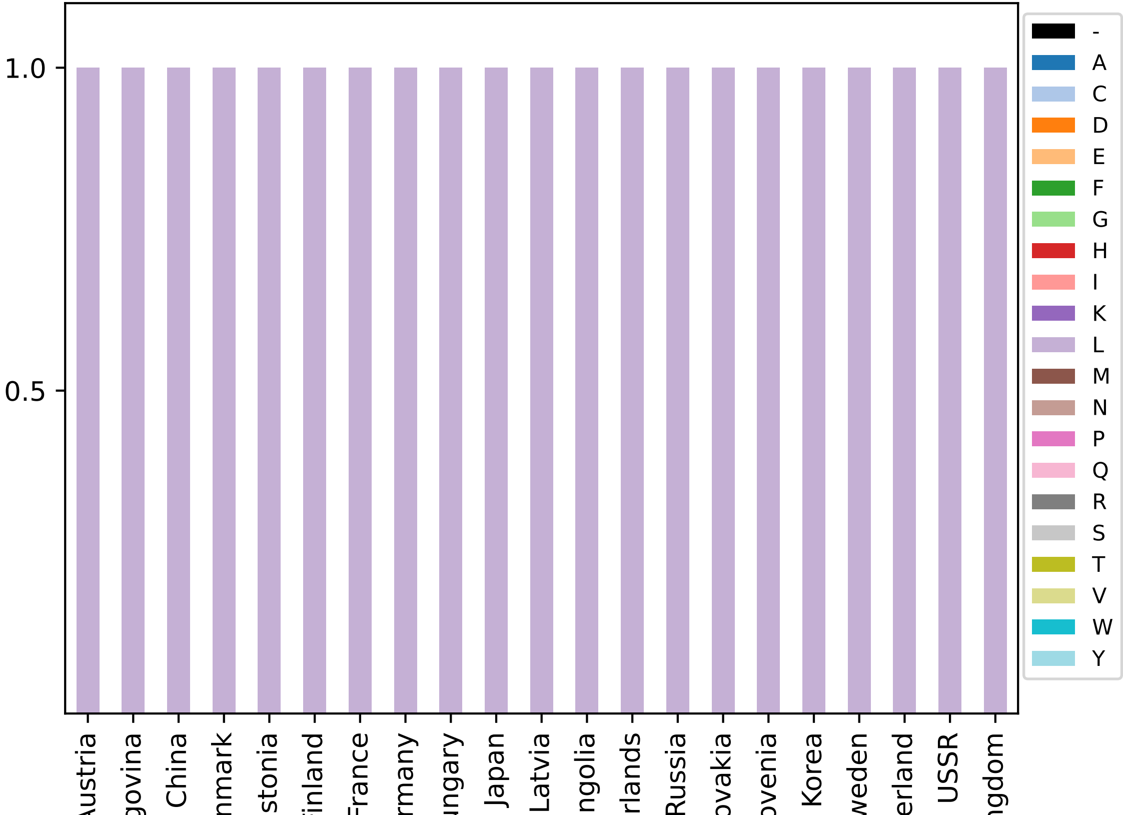
1.0
0.5
-
A
C
D
E
F
G
H
I
K
L
M
N
P
Q
R
S
T
V
W
Y
China
Japan
Latvia
Russia
Korea
USSR

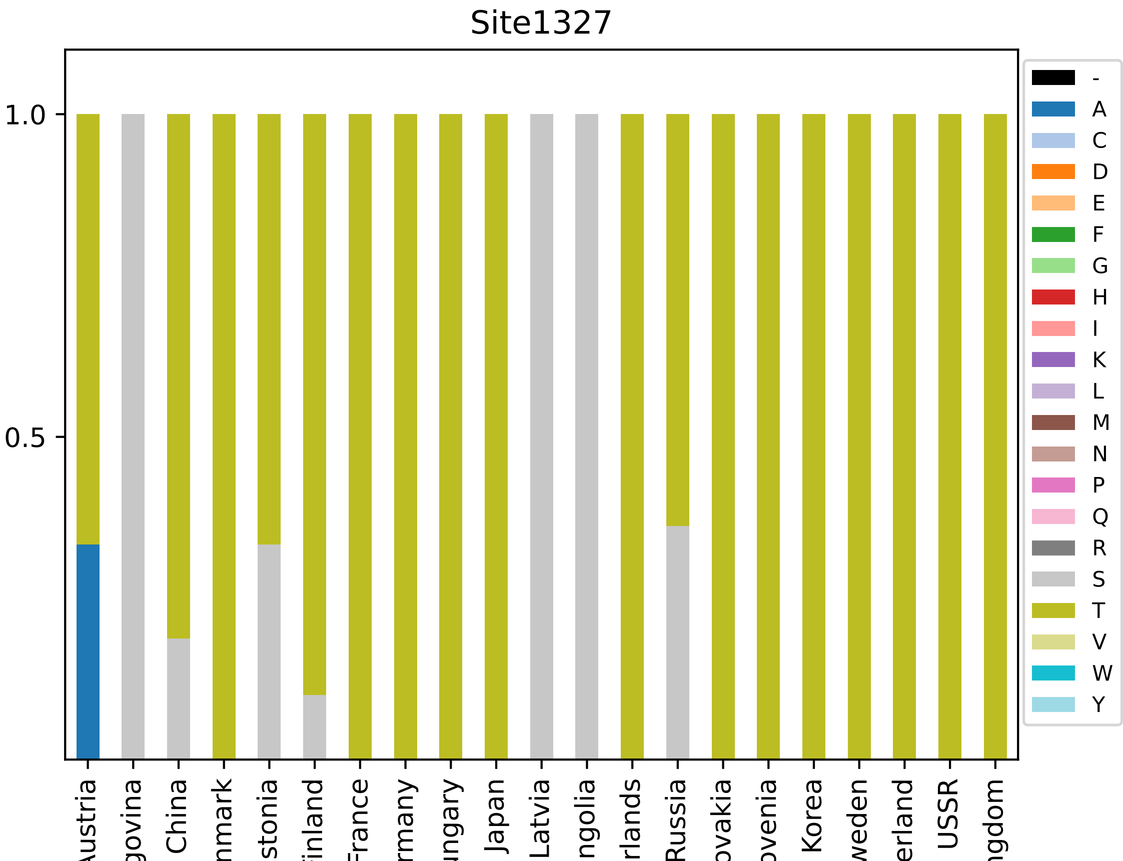
Site1327
1.0
0.5
-
A
C
D
E
F
G
H
I
K
L
M
N
P
Q
R
S
T
V
W
Y
Austria
China
Japan
Latvia
Russia
Korea
USSR

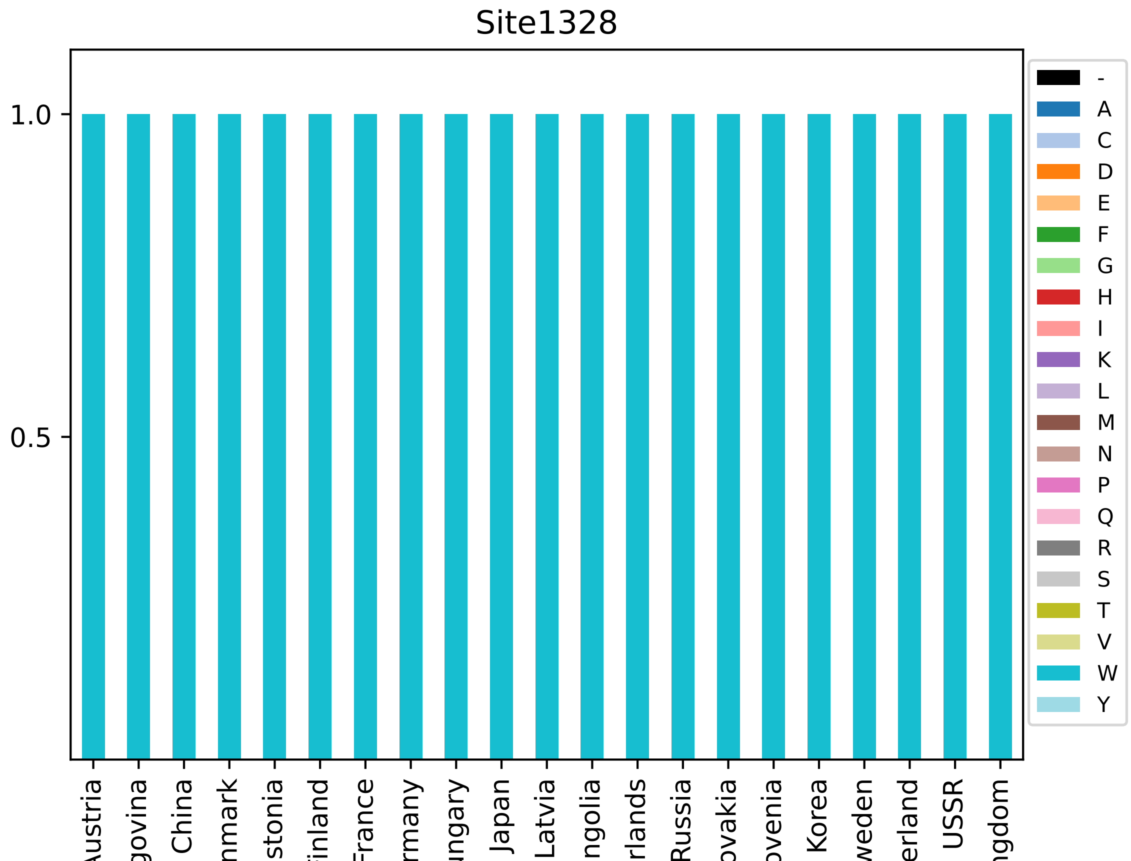

Site1328
1.0
0.5
-
A
C
D
E
F
G
H
I
K
L
M
N
P
Q
R
S
T
V
W
Y
China
Japan
Latvia
Russia
Korea
USSR

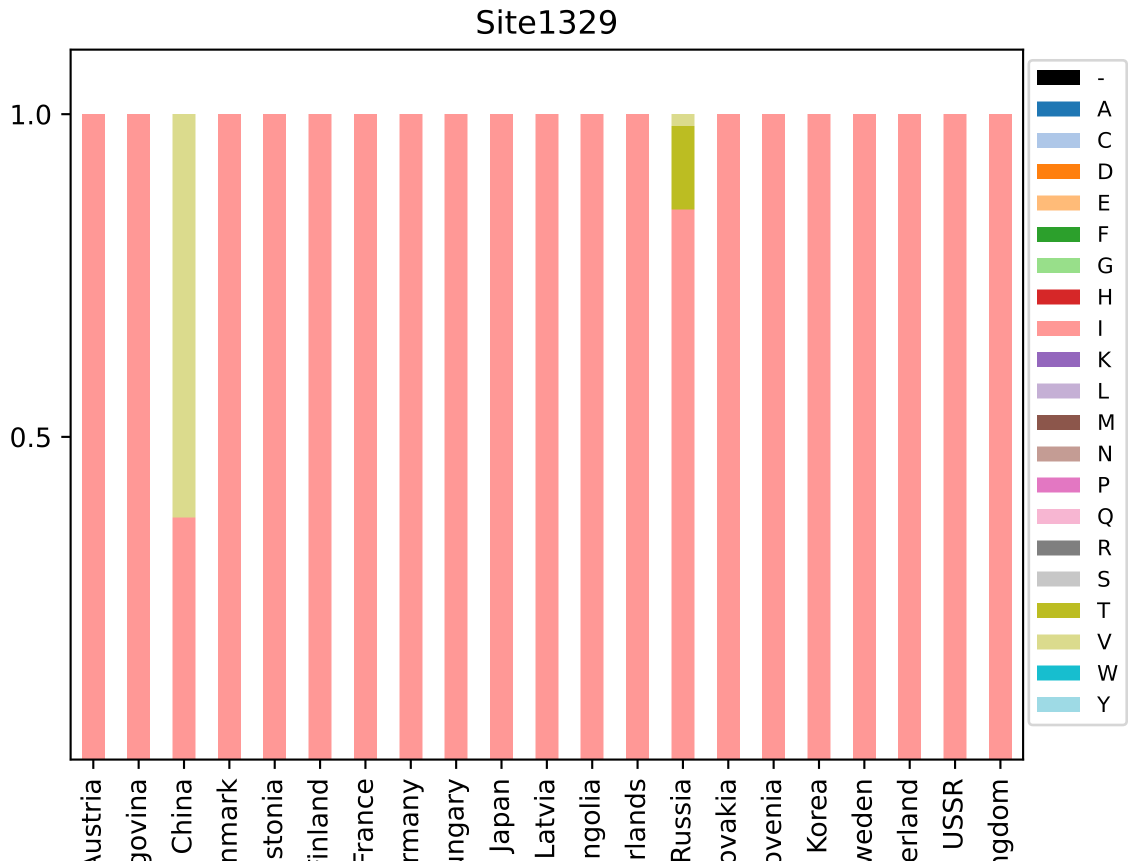
Site1329
1.0
0.5
-
A
C
D
E
F
G
H
I
K
L
M
N
P
Q
R
S
T
V
W
Y
Austria
China
Japan
Latvia
Russia
Korea
USSR

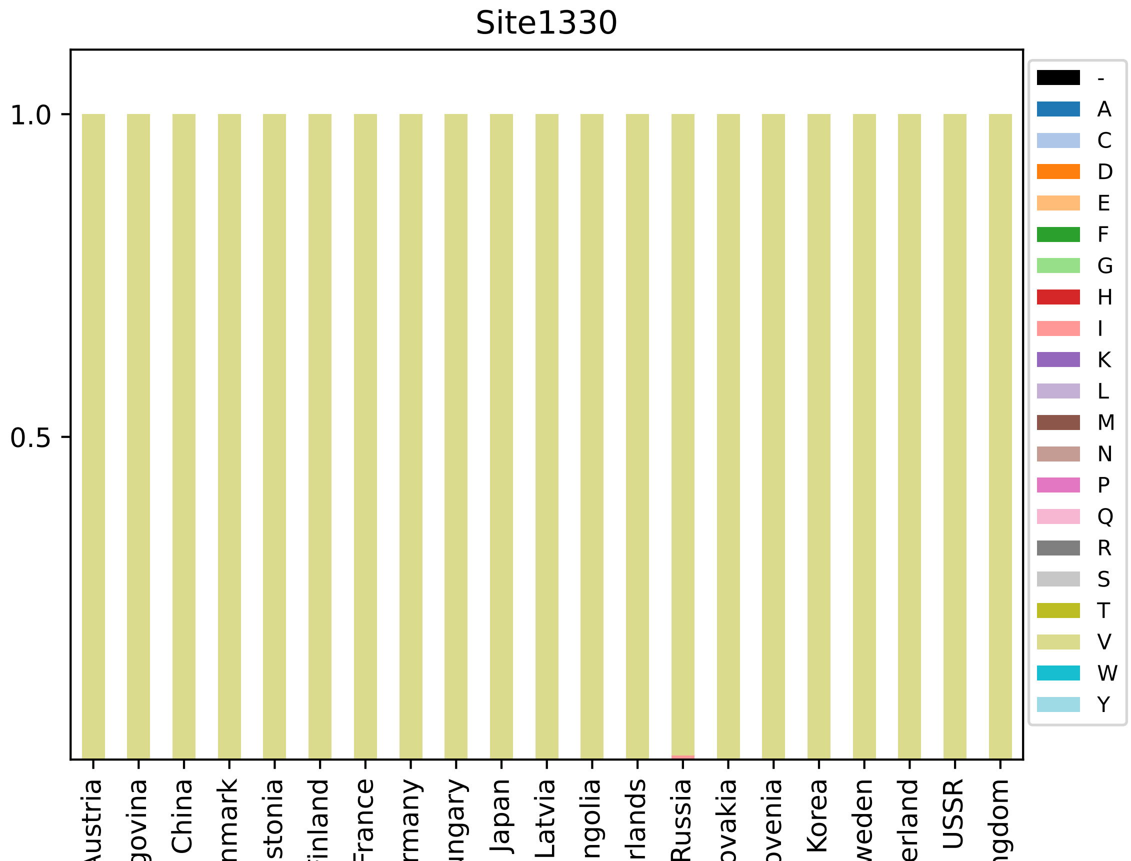

Site1330
1.0
0.5
-
A
C
D
E
F
G
H
I
K
L
M
N
P
Q
R
S
T
V
W
Y
Austria
govina
China
nmark
stonia
Finland
France
rmany
ungary
Japan
Latvia
ngolia
rlands
Russia
ovakia
ovenia
Korea
weden
erland
USSR
ngdom

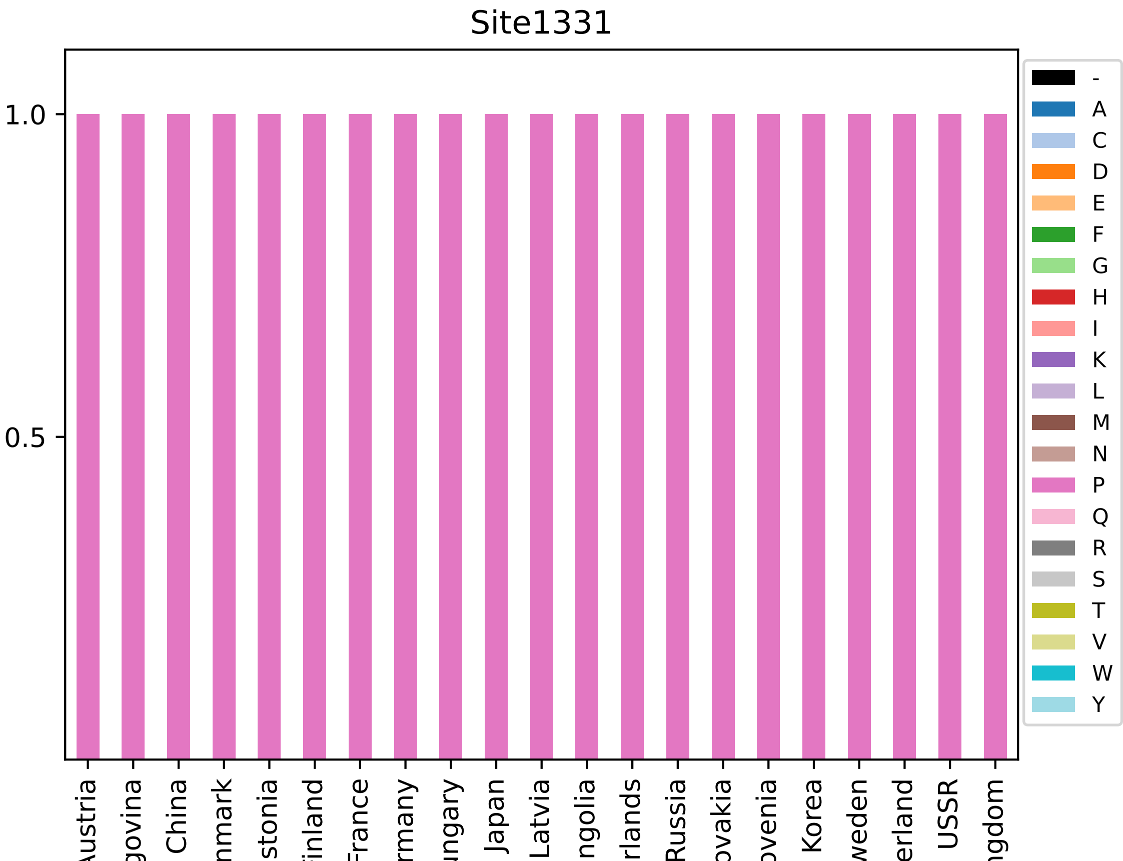
Site1331
1.0
0.5
-
A
C
D
E
F
G
H
I
K
L
M
N
P
Q
R
S
T
V
W
Y
China
Japan
Latvia
Russia
Korea
USSR

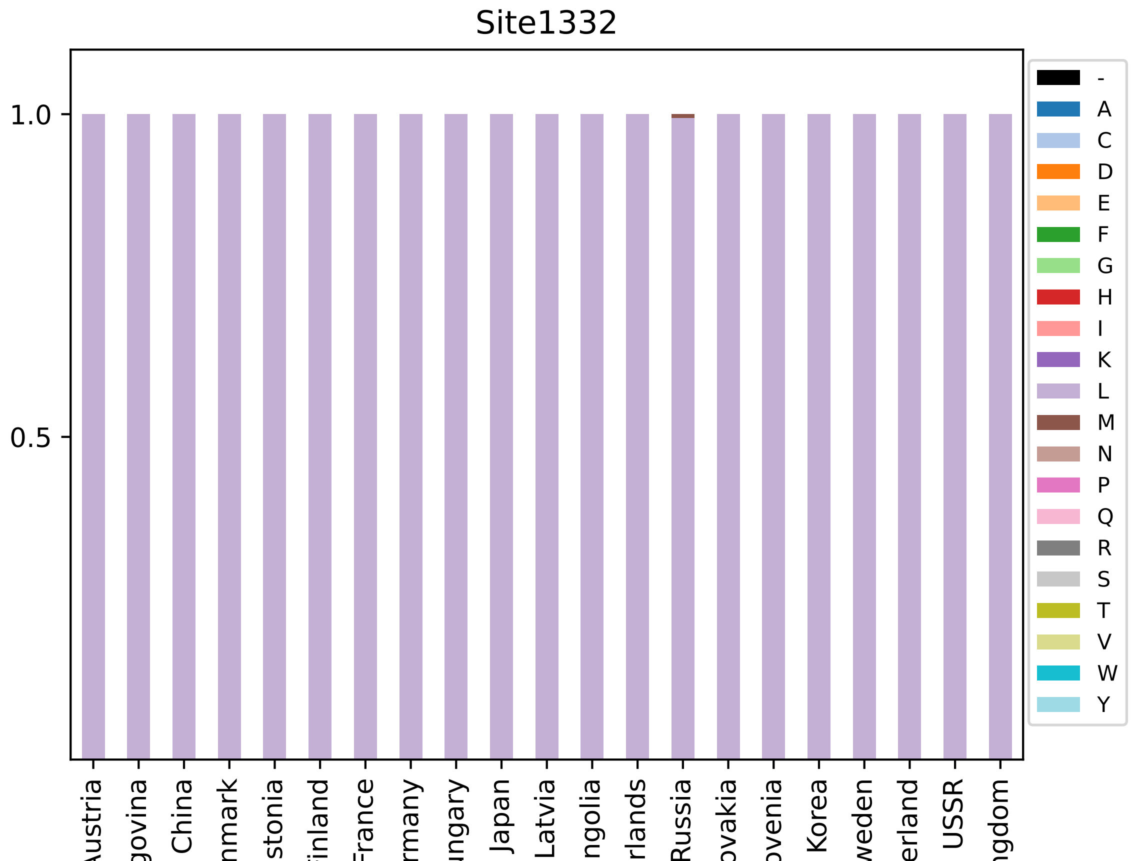

Site1332
1.0
0.5
-
A
C
D
E
F
G
H
I
K
L
M
N
P
Q
R
S
T
V
W
Y
Austria
China
Japan
Latvia
Russia
Korea
USSR

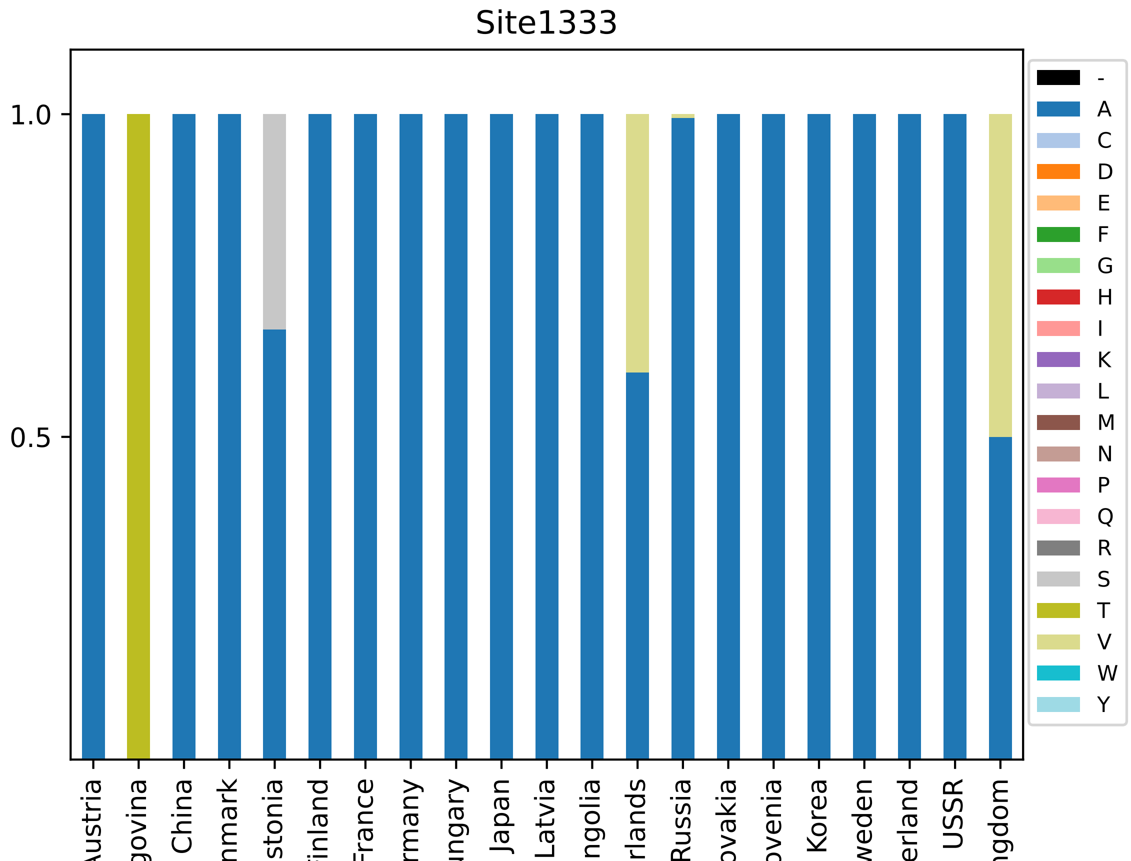

Site1333
1.0
0.5
-
A
C
D
E
F
G
H
I
K
L
M
N
P
Q
R
S
T
V
W
Y
Austria
govina
China
nmark
stonia
inland
France
rmany
ungary
Japan
Latvia
ngolia
rlands
Russia
ovakia
ovenia
Korea
weden
erland
USSR
ngdom

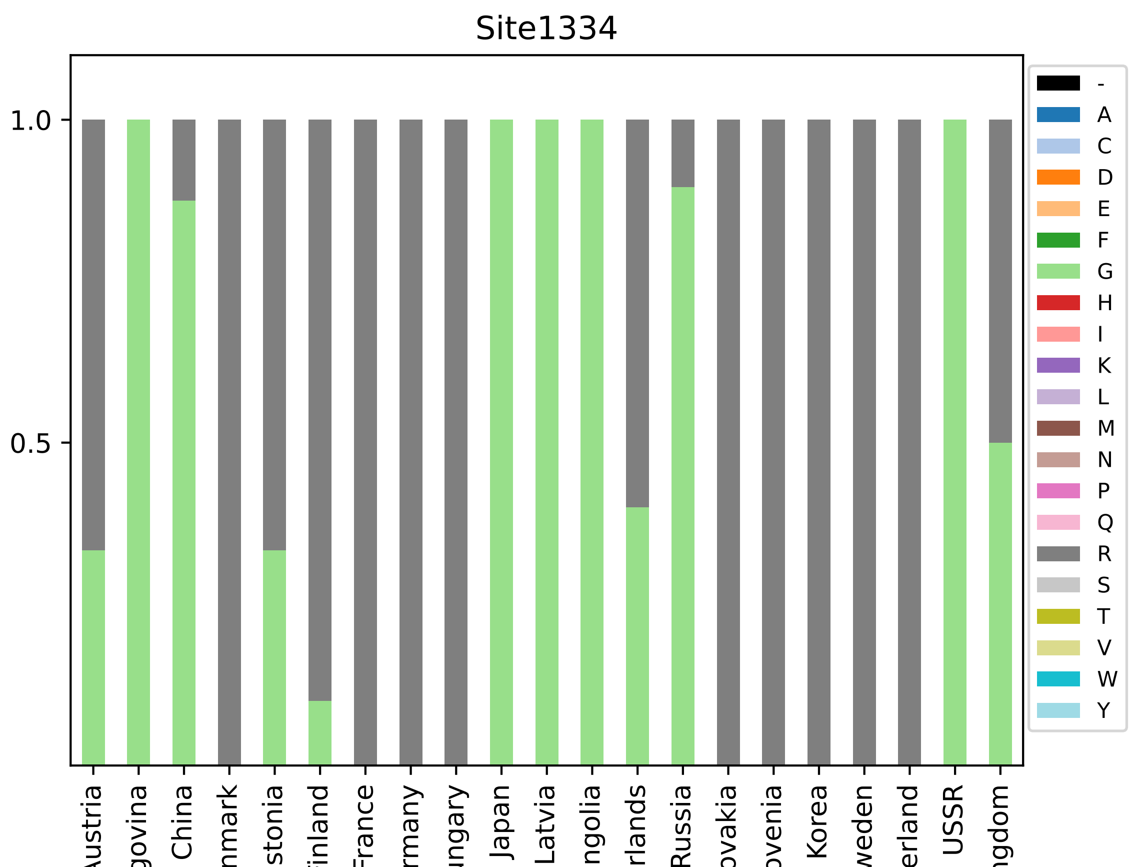

Site1334
1.0
0.5
-
A
C
D
E
F
G
H
I
K
L
M
N
P
Q
R
S
T
V
W
Y
Austria
China
Japan
Latvia
Russia
Korea
USSR

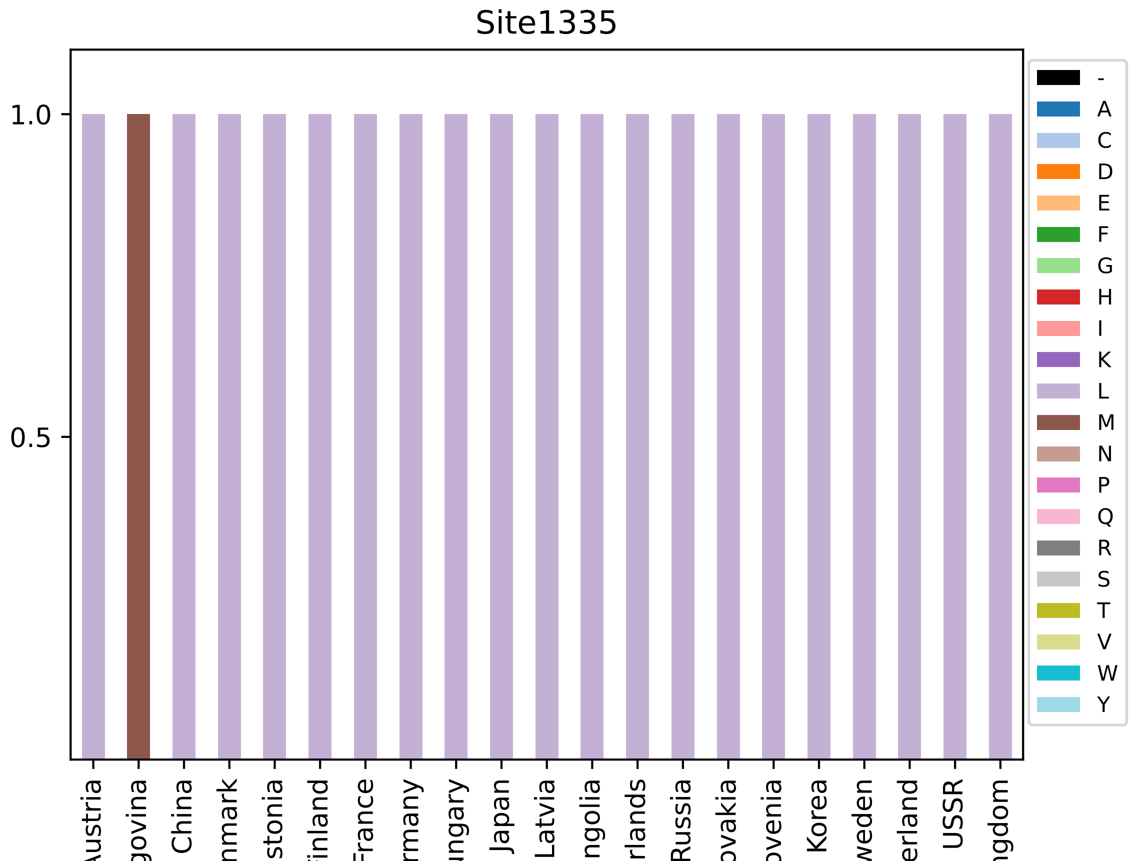

Site1335
1.0
0.5
-
A
C
D
E
F
G
H
I
K
L
M
N
P
Q
R
S
T
V
W
Y
China
Japan
Latvia
Russia
Korea
USSR

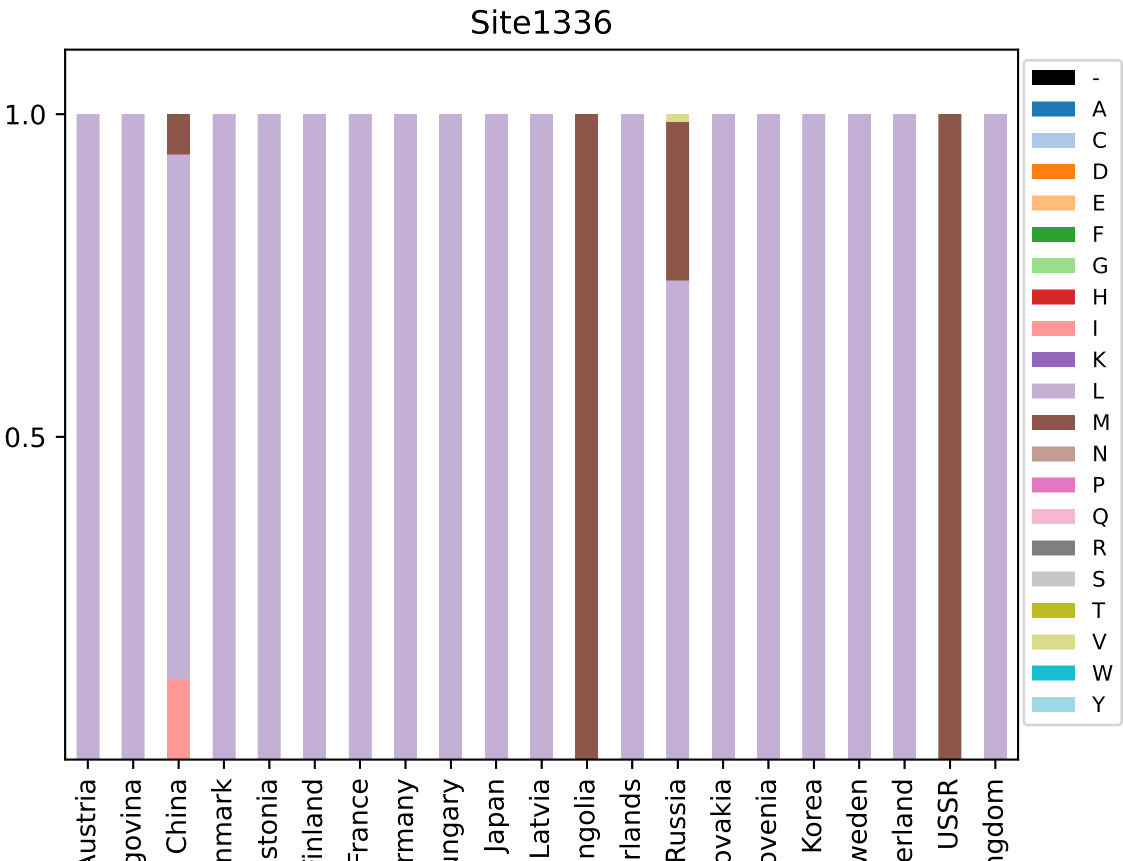

Site1336
1.0
0.5
-
A
C
D
E
F
G
H
I
K
L
M
N
P
Q
R
S
T
V
W
Y
Austria
govina
China
nmark
stonia
Finland
France
rmany
ungary
Japan
Latvia
ngolia
rlands
Russia
ovakia
ovenia
Korea
weden
erland
USSR
ngdom

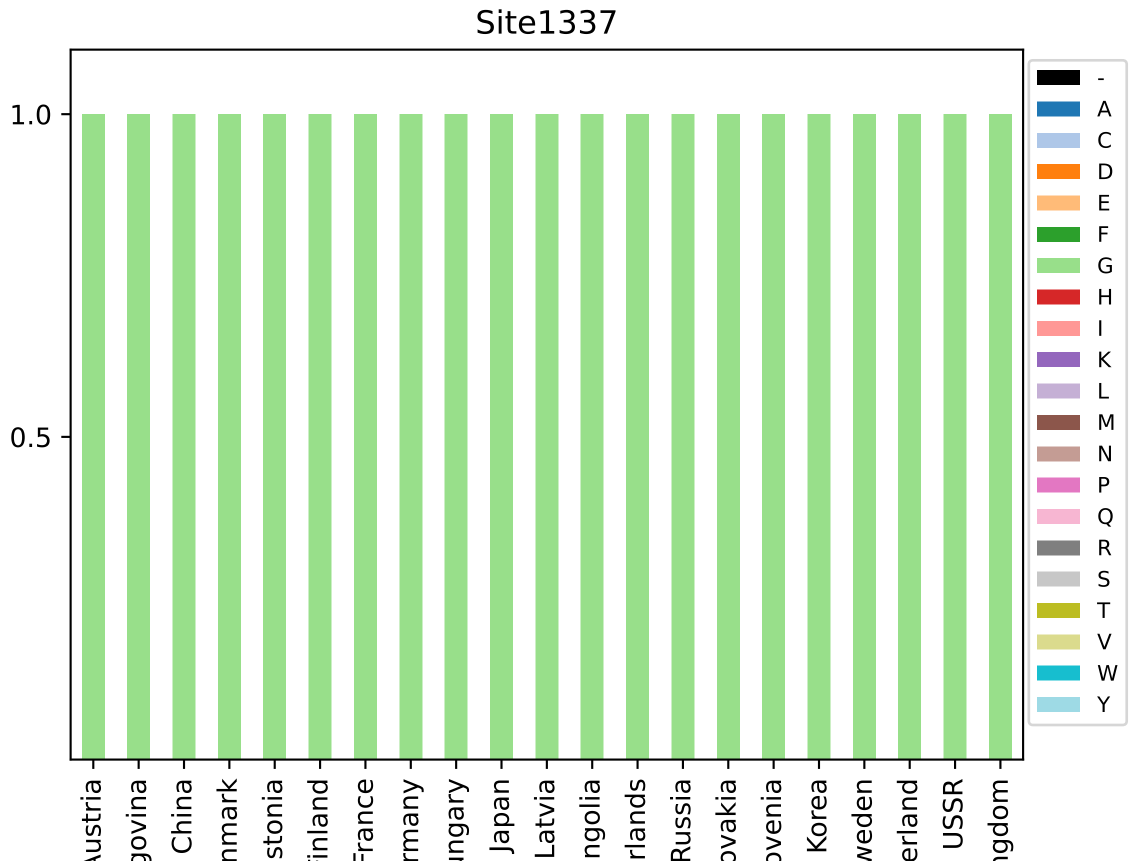

Site1337
1.0
0.5
Austria
govina
China
nmark
stonia
Finland
France
rmany
ungary
Japan
Latvia
ngolia
rlands
Russia
ovakia
ovenia
Korea
weden
erland
USSR
ngdom
-
A
C
D
E
F
G
H
I
K
L
M
N
P
Q
R
S
T
V
W
Y

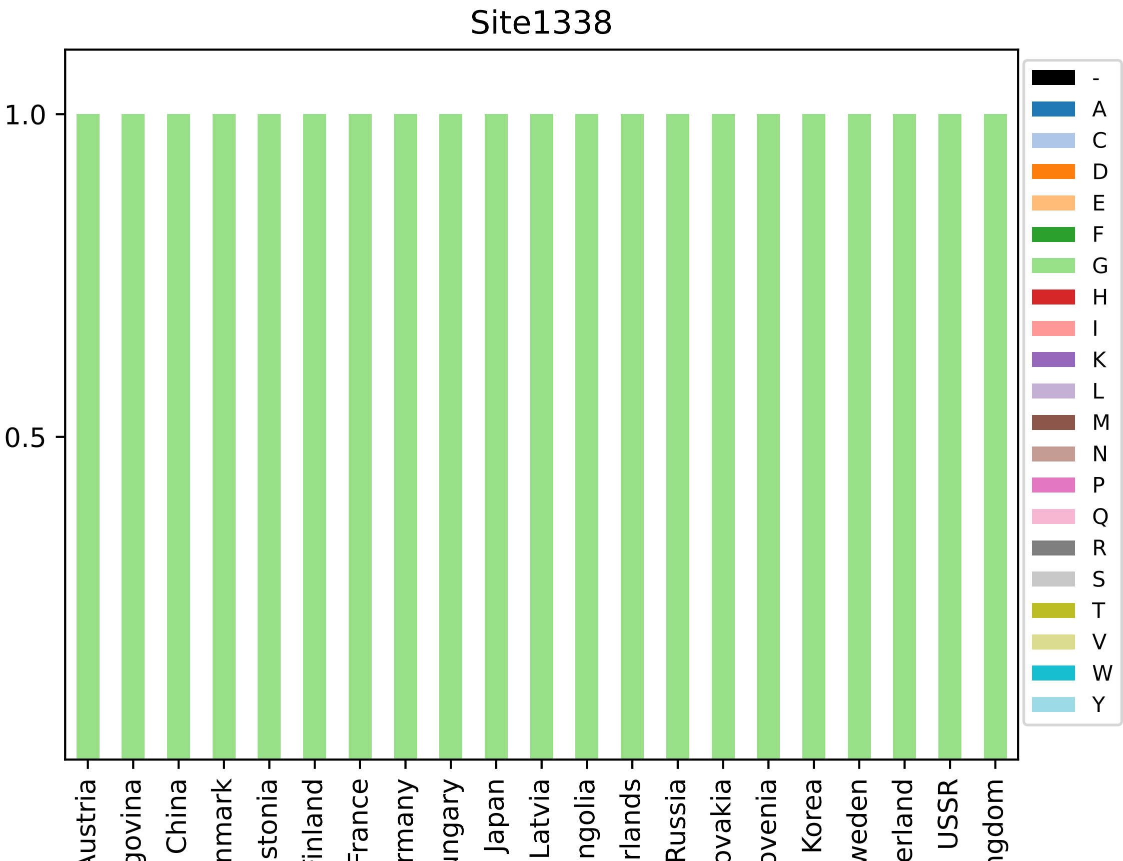
Site1338
1.0
0.5
-
A
C
D
E
F
G
H
I
K
L
M
N
P
Q
R
S
T
V
W
Y
China
Japan
Latvia
Russia
Korea
USSR

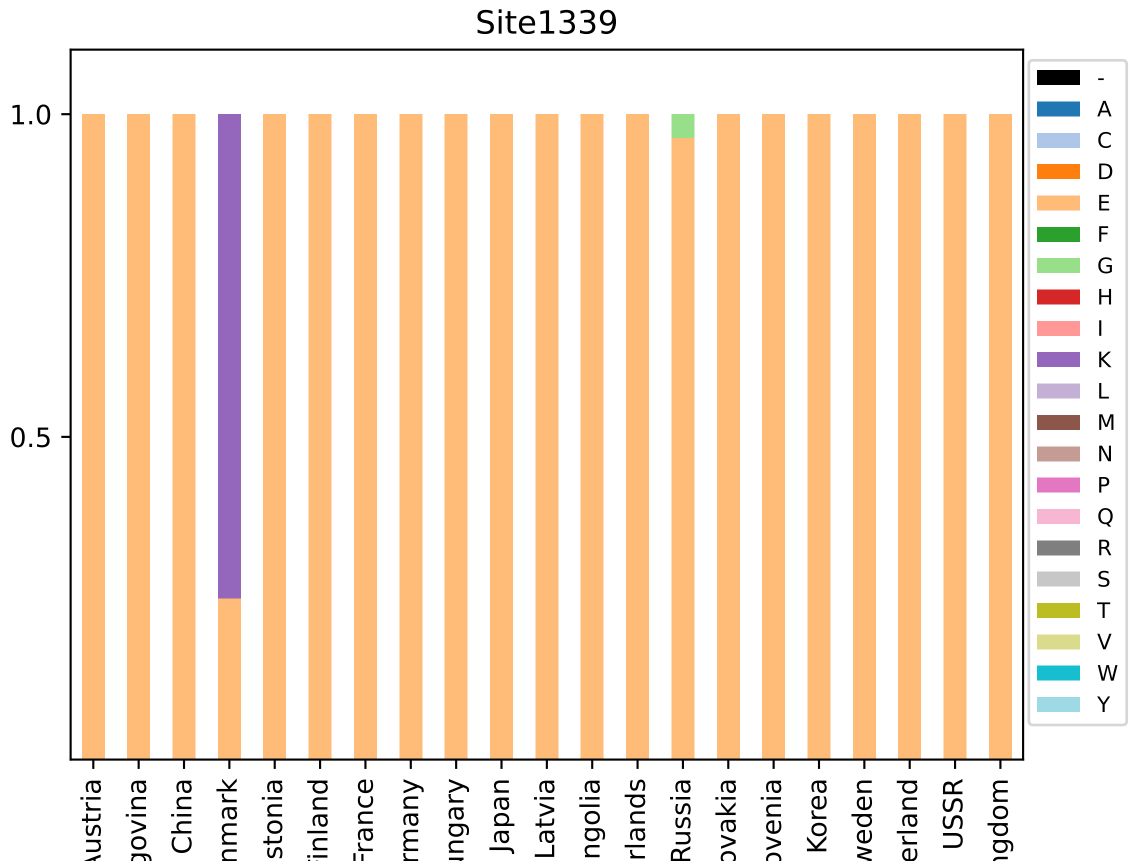
Site1339
1.0
0.5
-
A
C
D
E
F
G
H
I
K
L
M
N
P
Q
R
S
T
V
W
Y
Austria
China
Estonia
Finland
France
Germany
Hungary
Japan
Latvia
Russia
Korea
Sweden
USSR

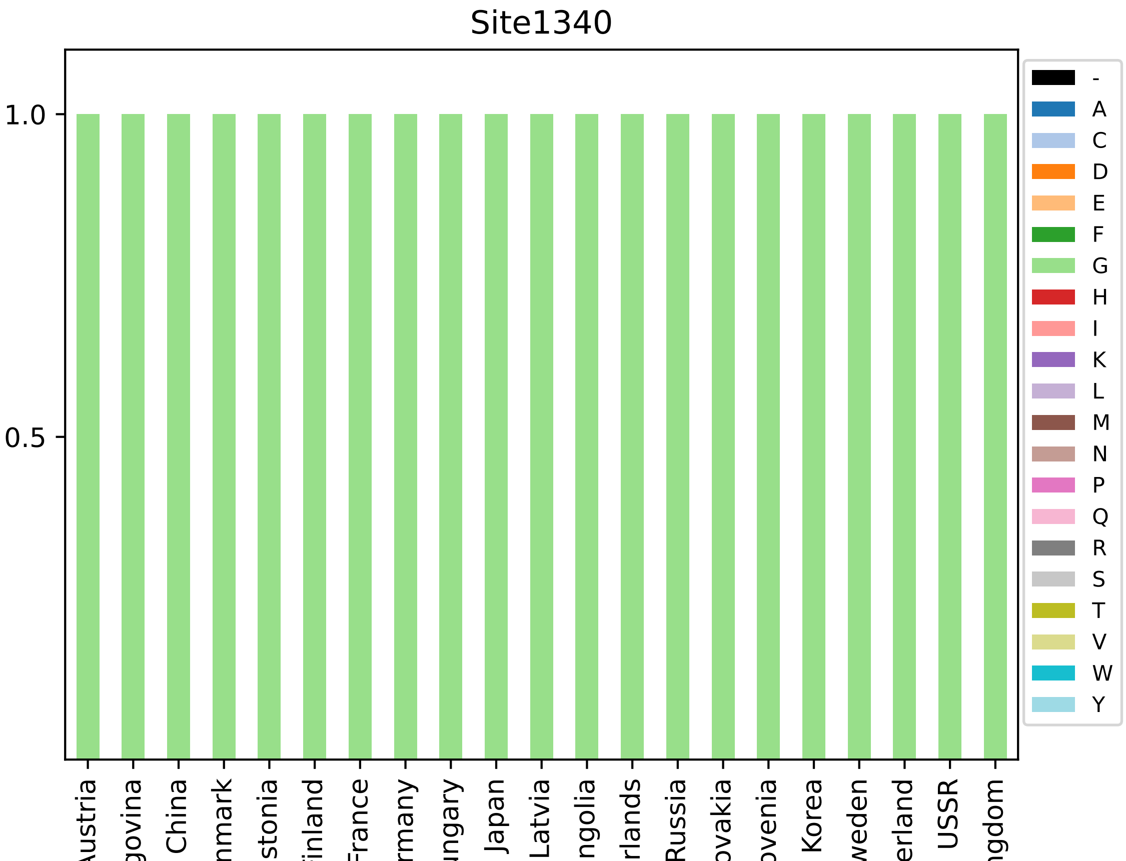

Site1340
1.0
0.5
-
A
C
D
E
F
G
H
I
K
L
M
N
P
Q
R
S
T
V
W
Y
Japan
Latvia
Russia
Korea
USSR
China

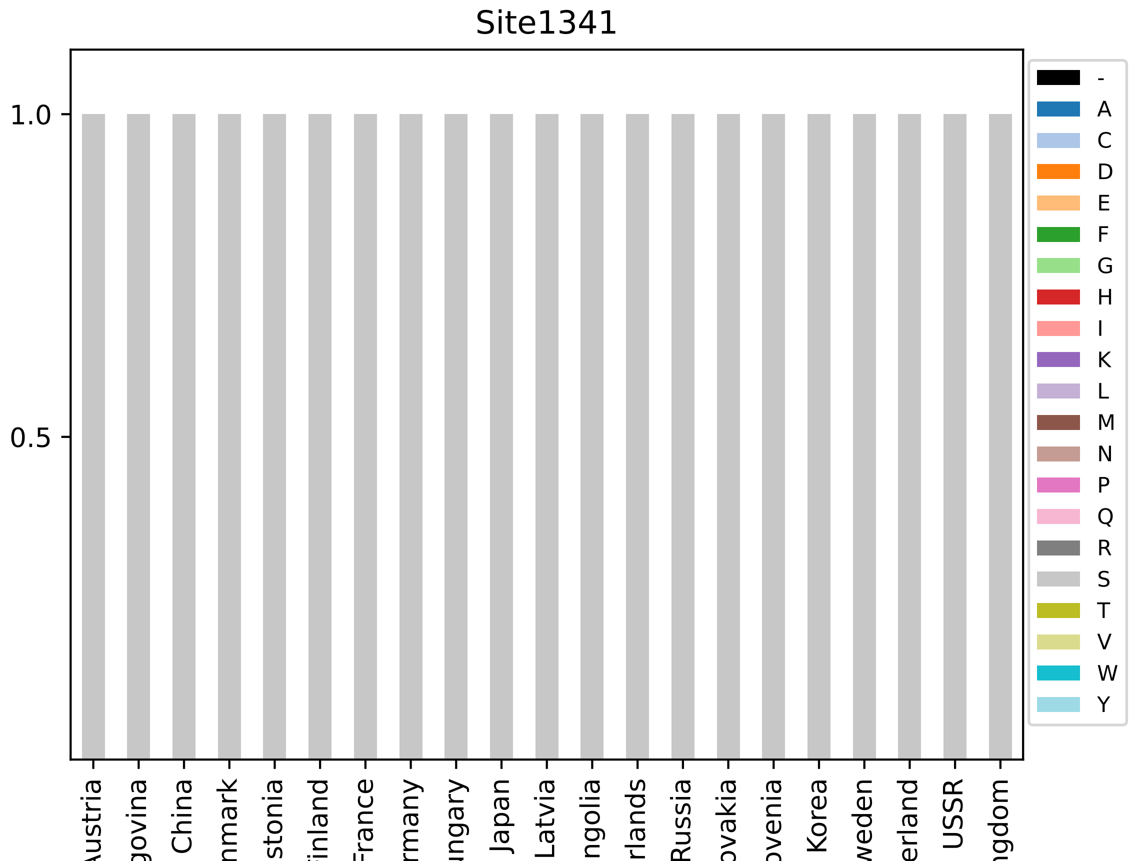
Site1341
1.0
0.5
-
A
C
D
E
F
G
H
I
K
L
M
N
P
Q
R
S
T
V
W
Y
Austria
China
Finland
Japan
Latvia
Russia
Korea
USSR

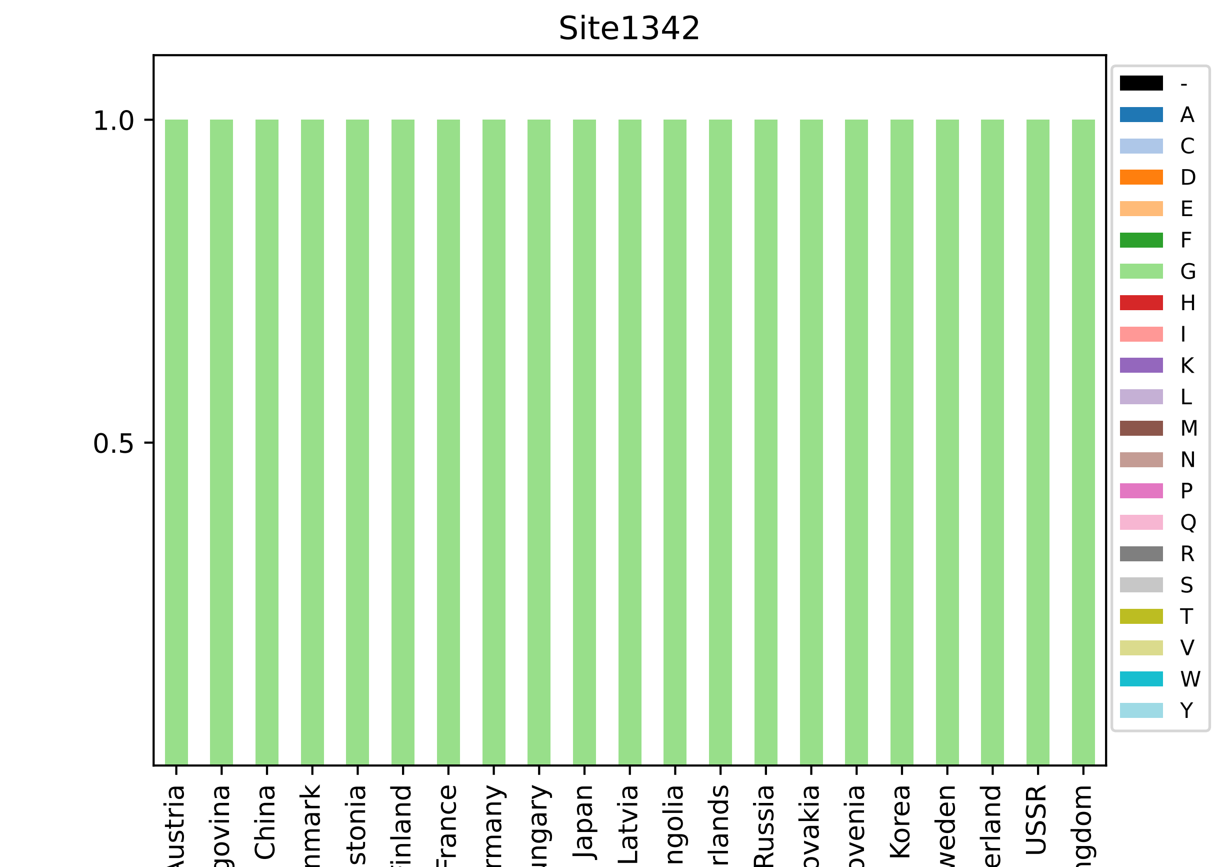

Site1342
1.0
0.5
-
A
C
D
E
F
G
H
I
K
L
M
N
P
Q
R
S
T
V
W
Y
Austria
China
Japan
Latvia
Russia
Korea
USSR

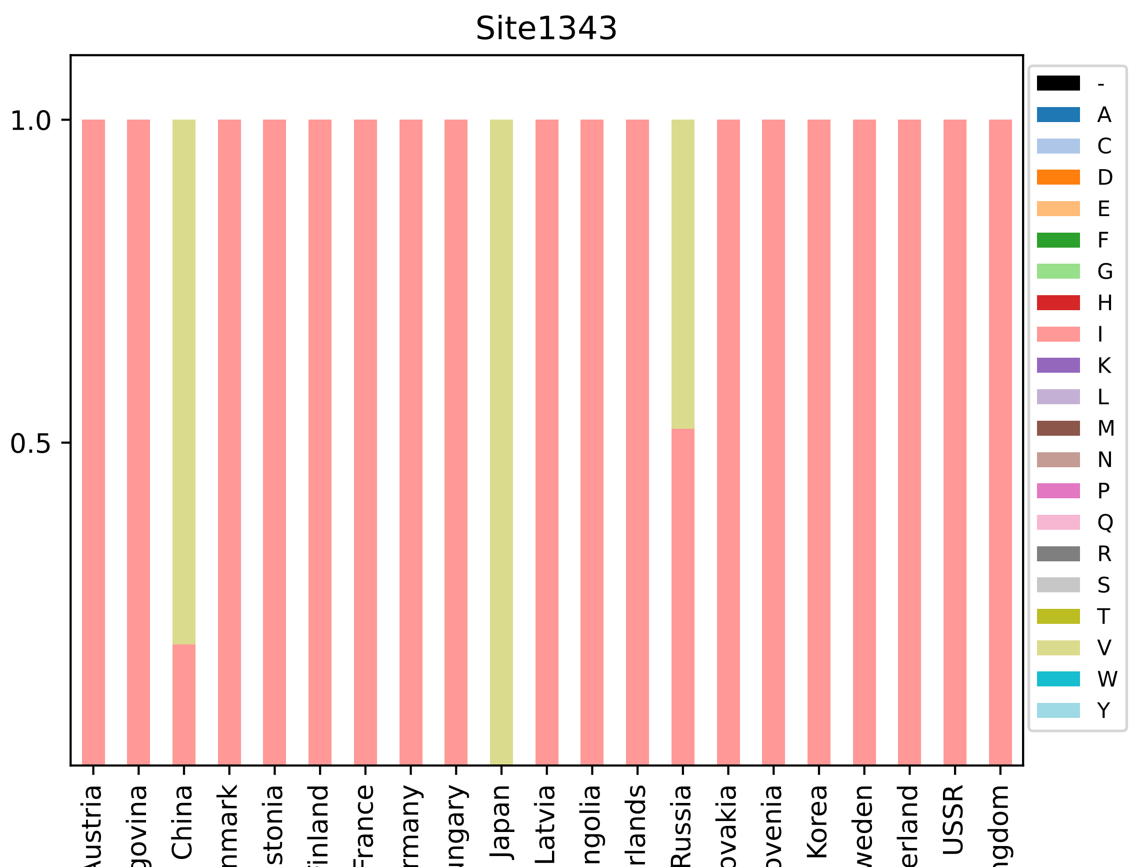

Site1343
1.0
0.5
-
A
C
D
E
F
G
H
I
K
L
M
N
P
Q
R
S
T
V
W
Y
China
Japan
Latvia
Russia
Korea
USSR

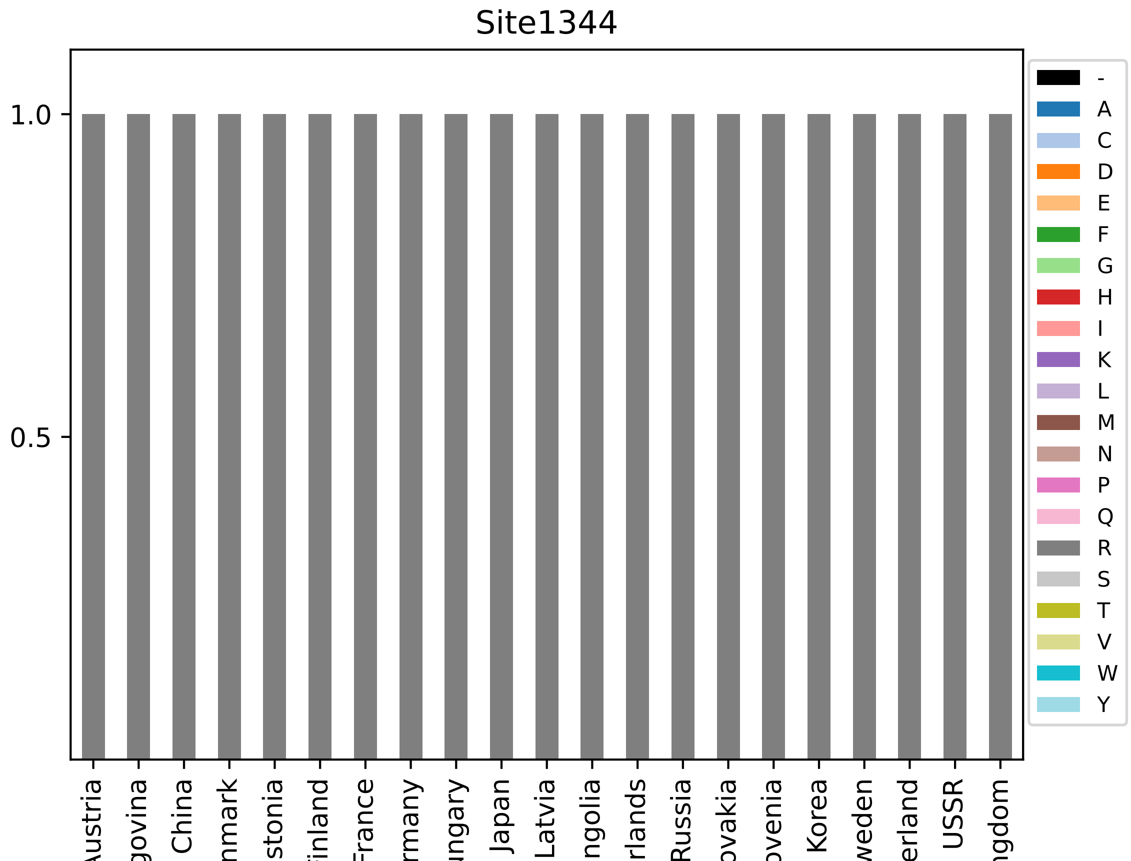
Site1344
1.0
0.5
Austria
govina
China
nmark
stonia
Finland
France
rmany
ungary
Japan
Latvia
ngolia
rlands
Russia
ovakia
ovenia
Korea
weden
erland
USSR
ngdom
-
A
C
D
E
F
G
H
I
K
L
M
N
P
Q
R
S
T
V
W
Y

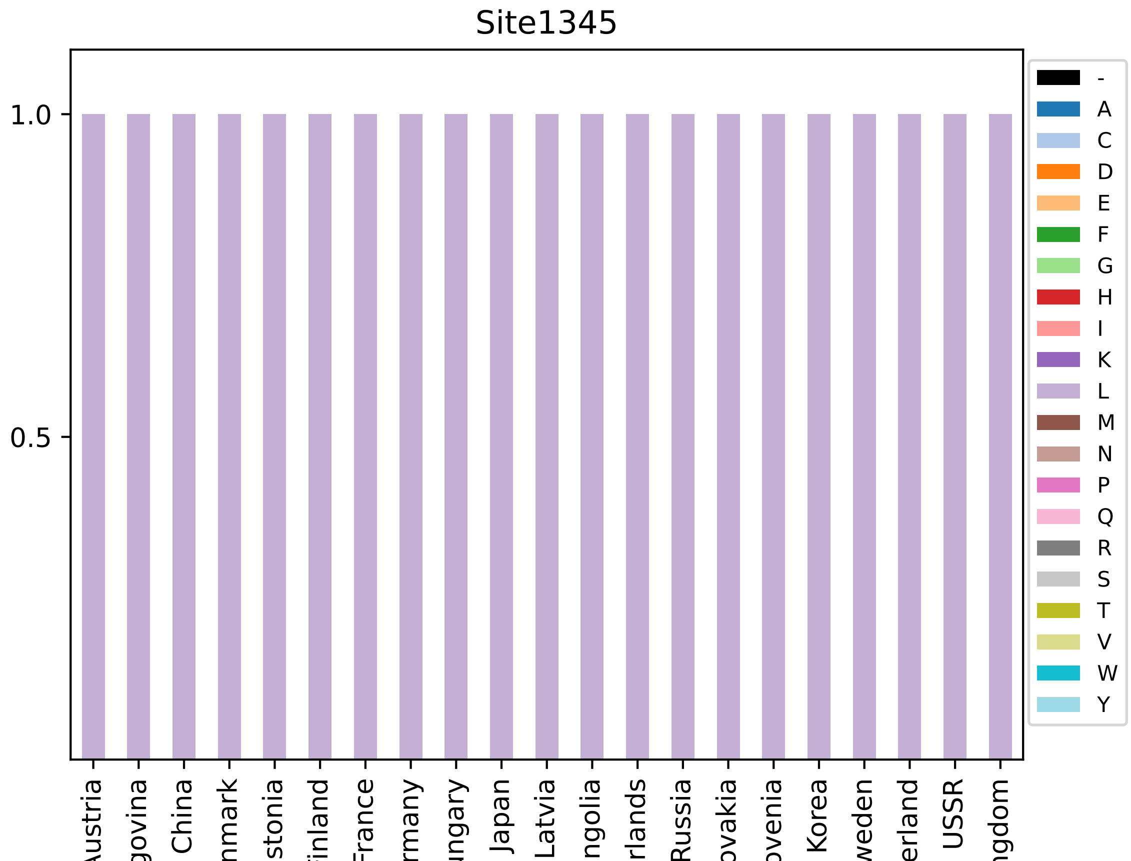
Site1345
1.0
0.5
-
A
C
D
E
F
G
H
I
K
L
M
N
P
Q
R
S
T
V
W
Y
ustria
govina
China
nmark
stonia
inland
rance
rmany
ungary
Japan
Latvia
ngolia
rlands
Russia
ovakia
ovenia
Korea
weden
erland
USSR
ngdom

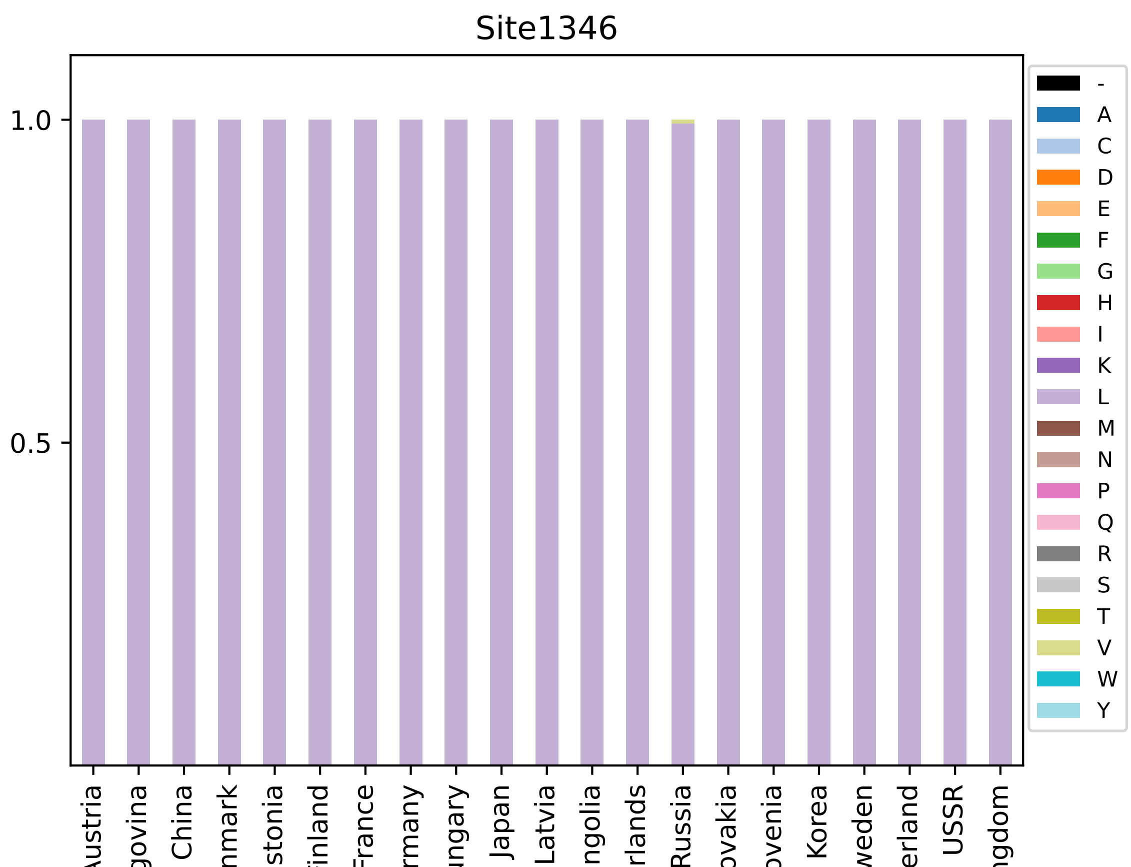
Site1346
1.0
0.5
-
A
C
D
E
F
G
H
I
K
L
M
N
P
Q
R
S
T
V
W
Y
Austria
govina
China
nmark
stonia
inland
France
rmany
ungary
Japan
Latvia
ngolia
rlands
Russia
ovakia
ovenia
Korea
weden
erland
USSR
ngdom

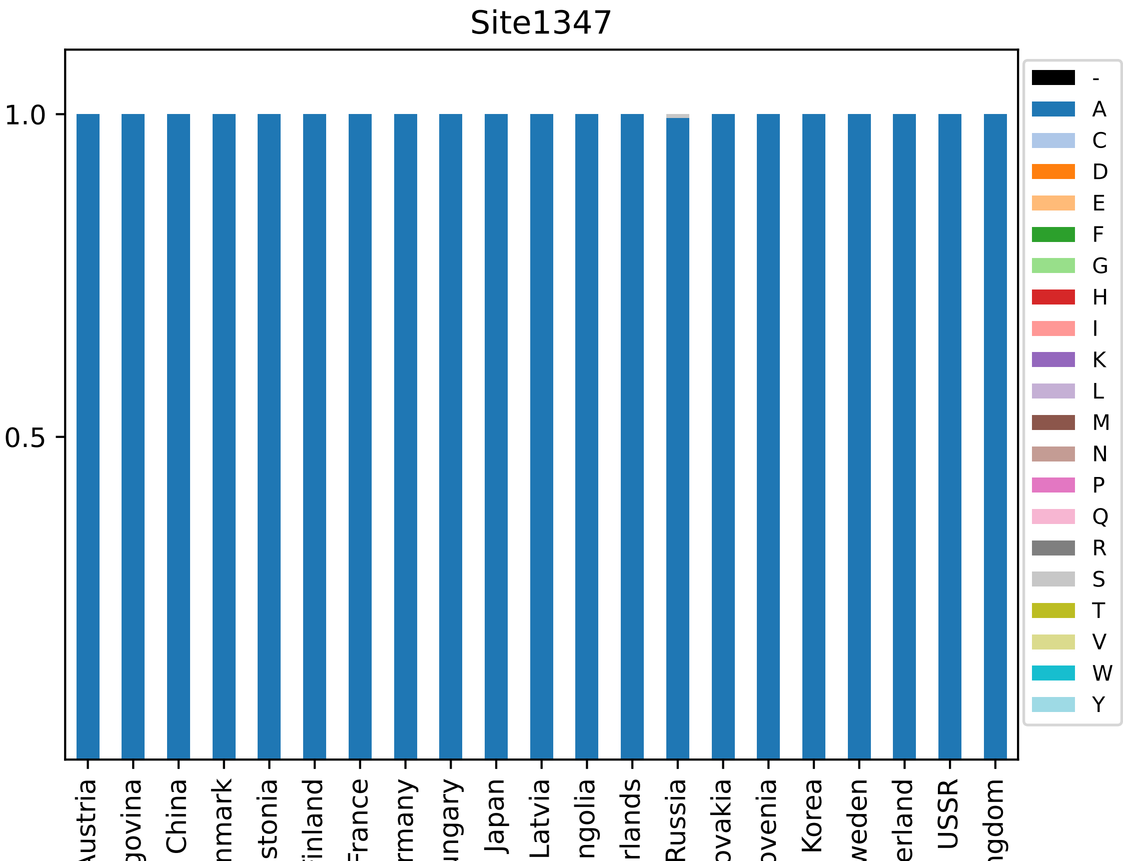

Site1347
1.0
0.5
-
A
C
D
E
F
G
H
I
K
L
M
N
P
Q
R
S
T
V
W
Y
China
Japan
Latvia
Russia
Korea
USSR

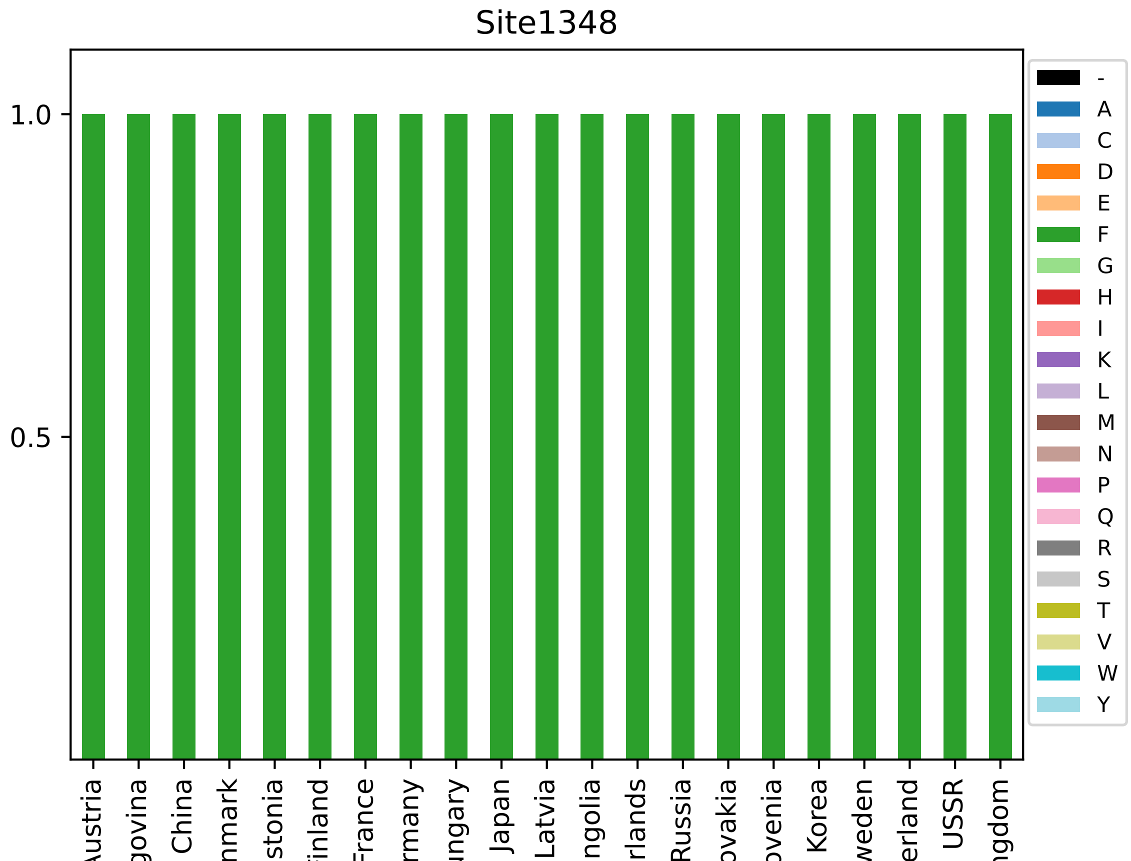
Site1348
1.0
0.5
-
A
C
D
E
F
G
H
I
K
L
M
N
P
Q
R
S
T
V
W
Y
China
Japan
Latvia
Russia
Korea
USSR

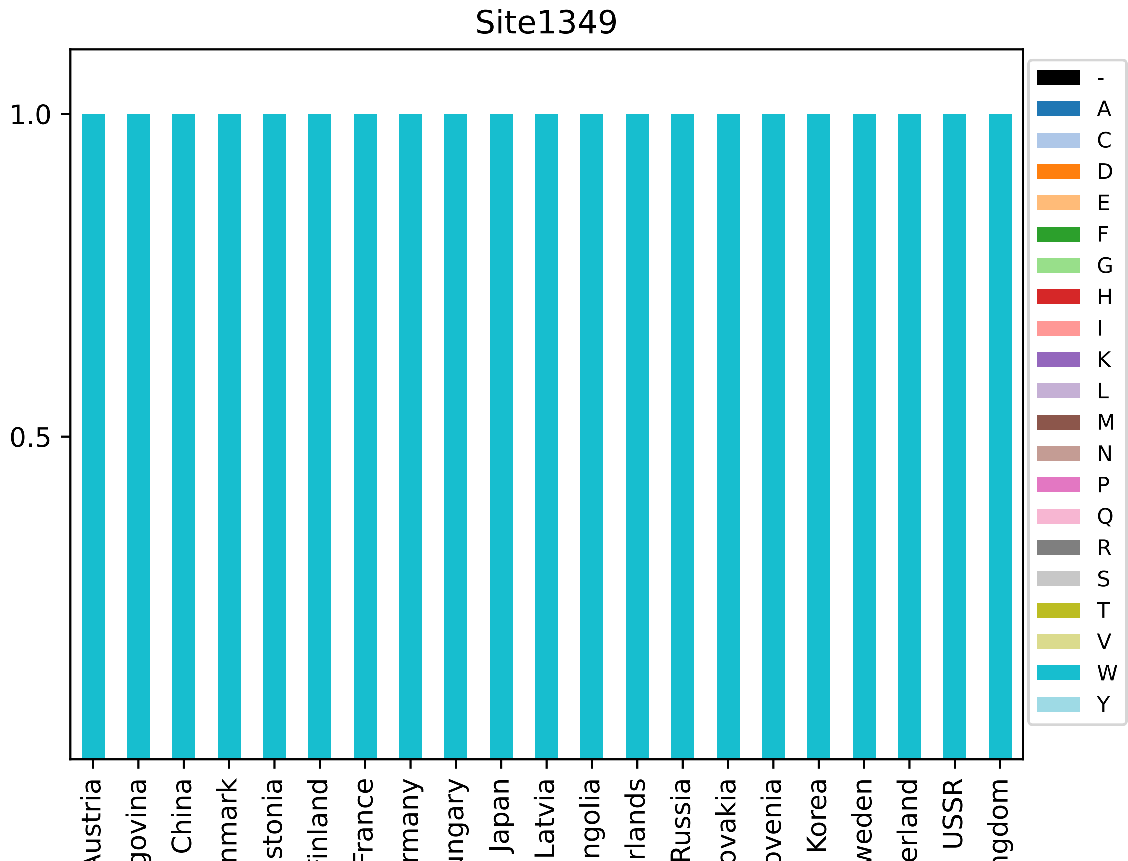
Site1349
1.0
0.5
-
A
C
D
E
F
G
H
I
K
L
M
N
P
Q
R
S
T
V
W
Y
Austria
govina
China
nmark
stonia
inland
France
rmany
ungary
Japan
Latvia
ngolia
rlands
Russia
ovakia
ovenia
Korea
weden
erland
USSR
ngdom

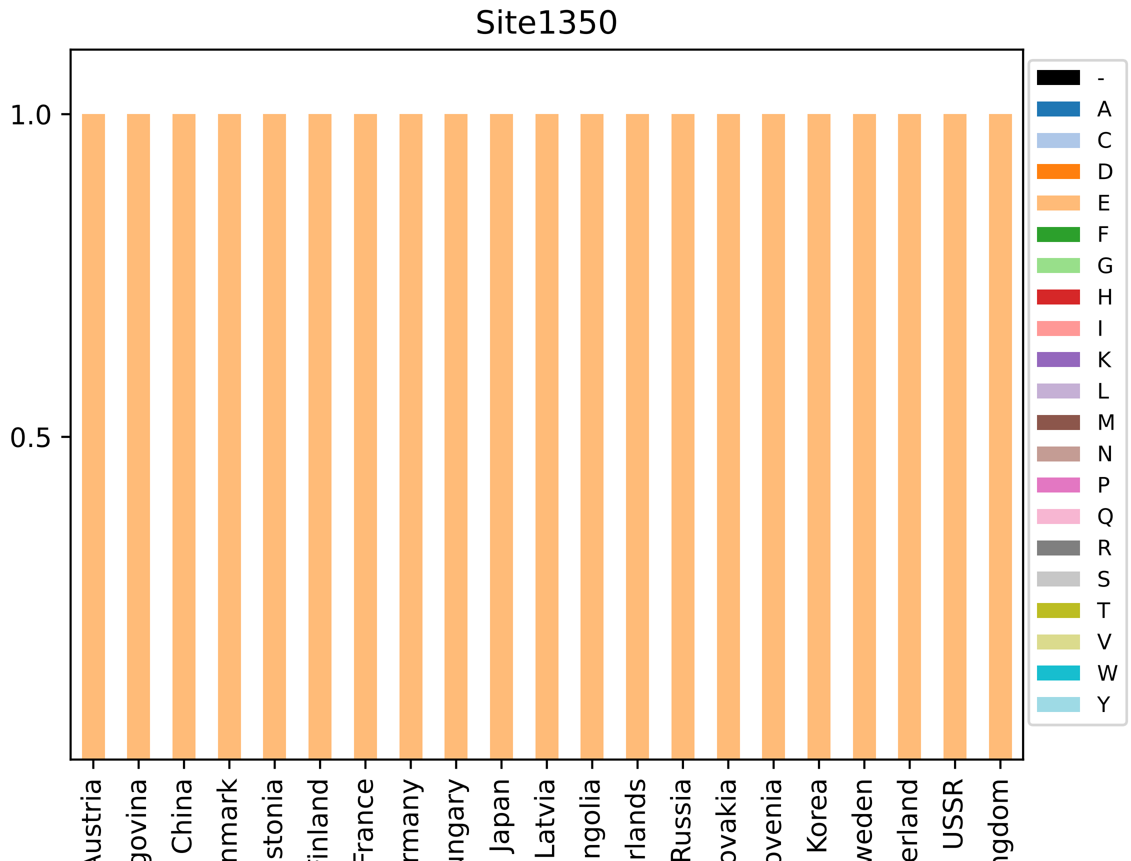

Site1350
1.0
0.5
-
A
C
D
E
F
G
H
I
K
L
M
N
P
Q
R
S
T
V
W
Y
China
Japan
Latvia
Russia
Korea
USSR

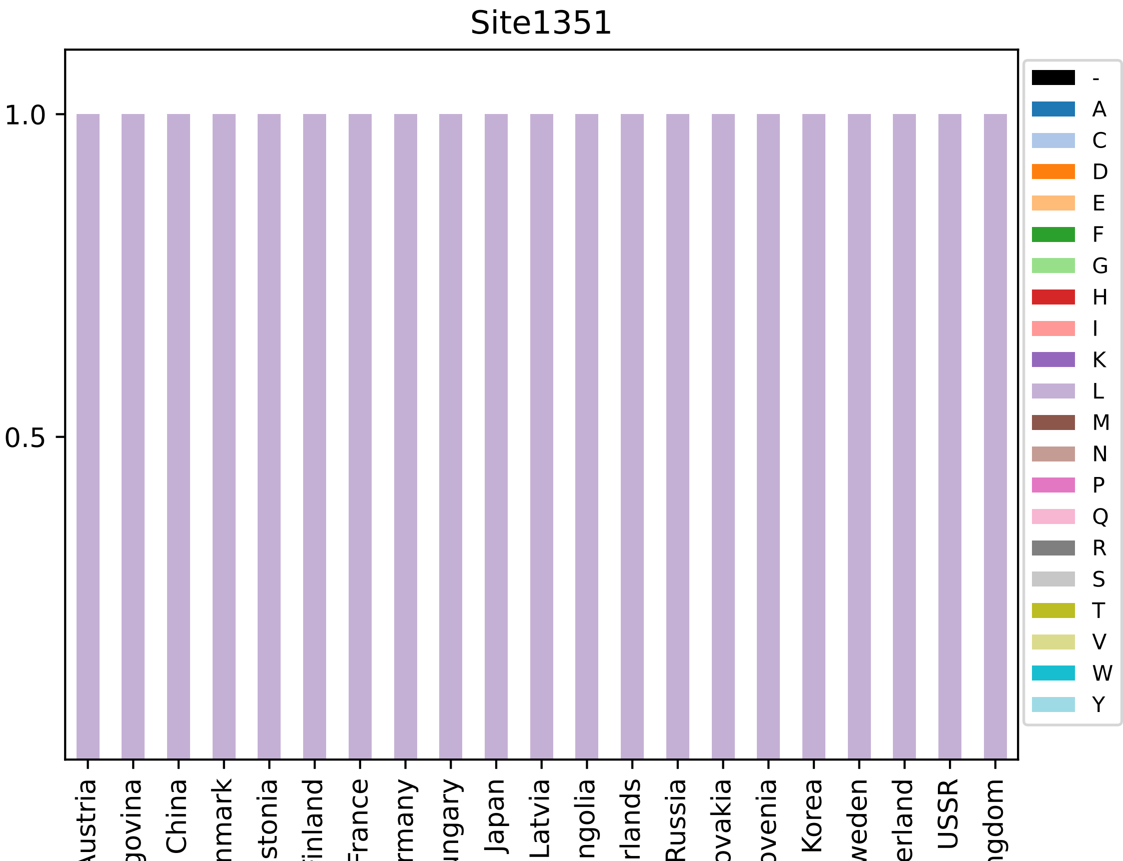

Site1351
1.0
0.5
-
A
C
D
E
F
G
H
I
K
L
M
N
P
Q
R
S
T
V
W
Y
Austria
govina
China
nmark
stonia
Finland
France
rmany
ungary
Japan
Latvia
ngolia
rlands
Russia
ovakia
ovenia
Korea
weden
erland
USSR
ngdom

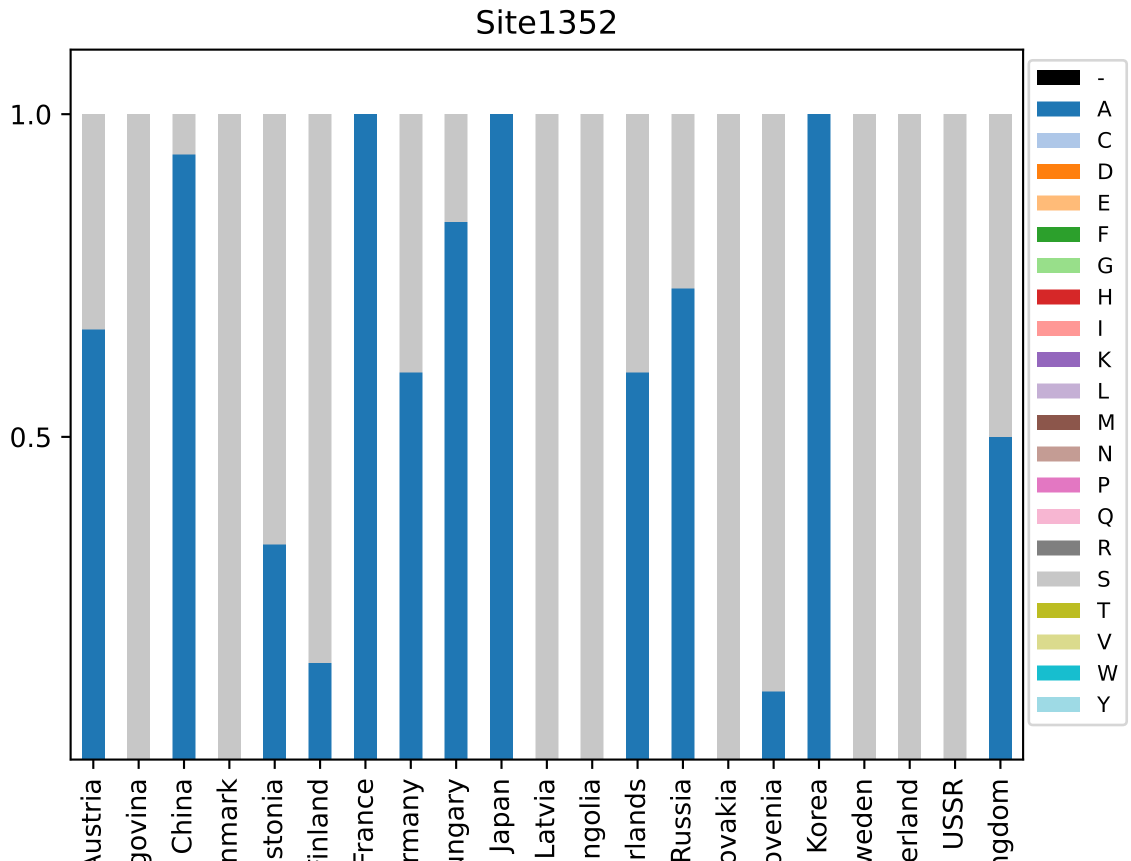
Site1352
1.0
0.5
-
A
C
D
E
F
G
H
I
K
L
M
N
P
Q
R
S
T
V
W
Y
Austria
China
Estonia
Finland
France
Japan
Latvia
Russia
Korea
USSR

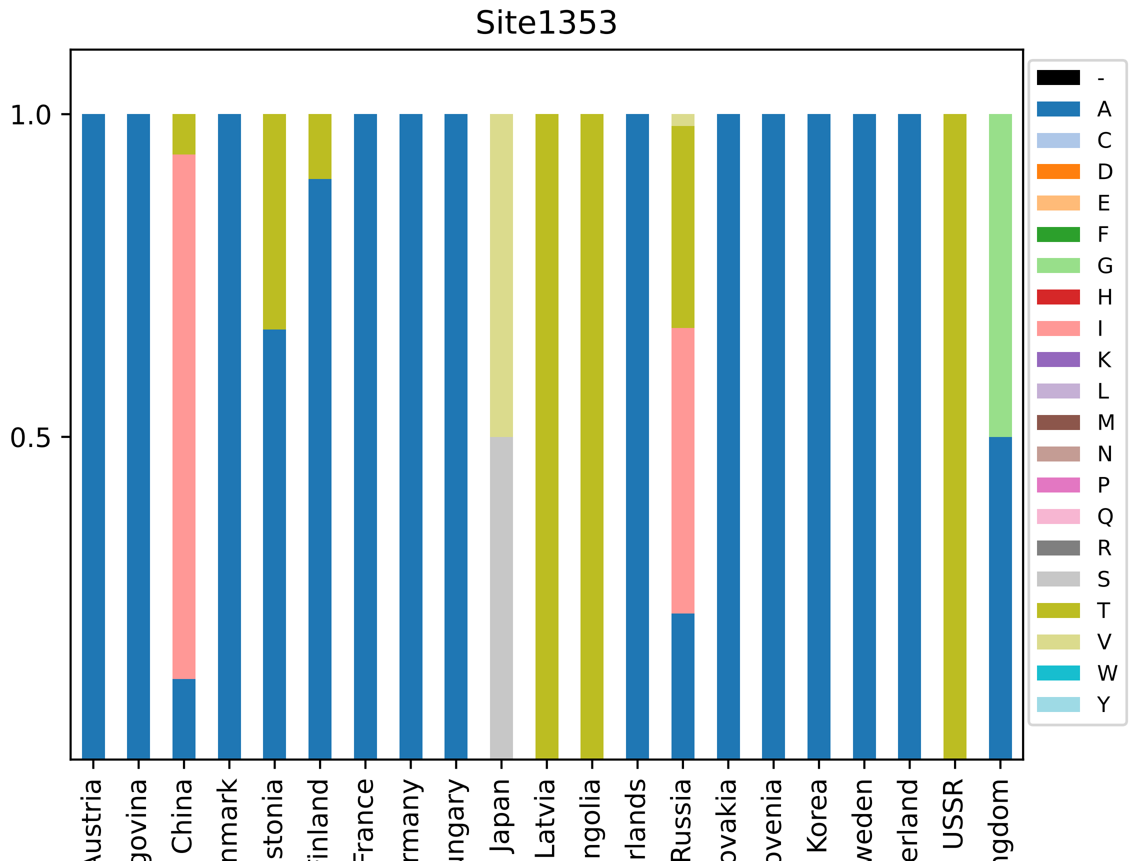
Site1353
1.0
0.5
-
A
C
D
E
F
G
H
I
K
L
M
N
P
Q
R
S
T
V
W
Y
Austria
govina
China
nmark
stonia
Finland
France
rmany
ungary
Japan
Latvia
ngolia
rlands
Russia
ovakia
ovenia
Korea
weden
erland
USSR
ngdom

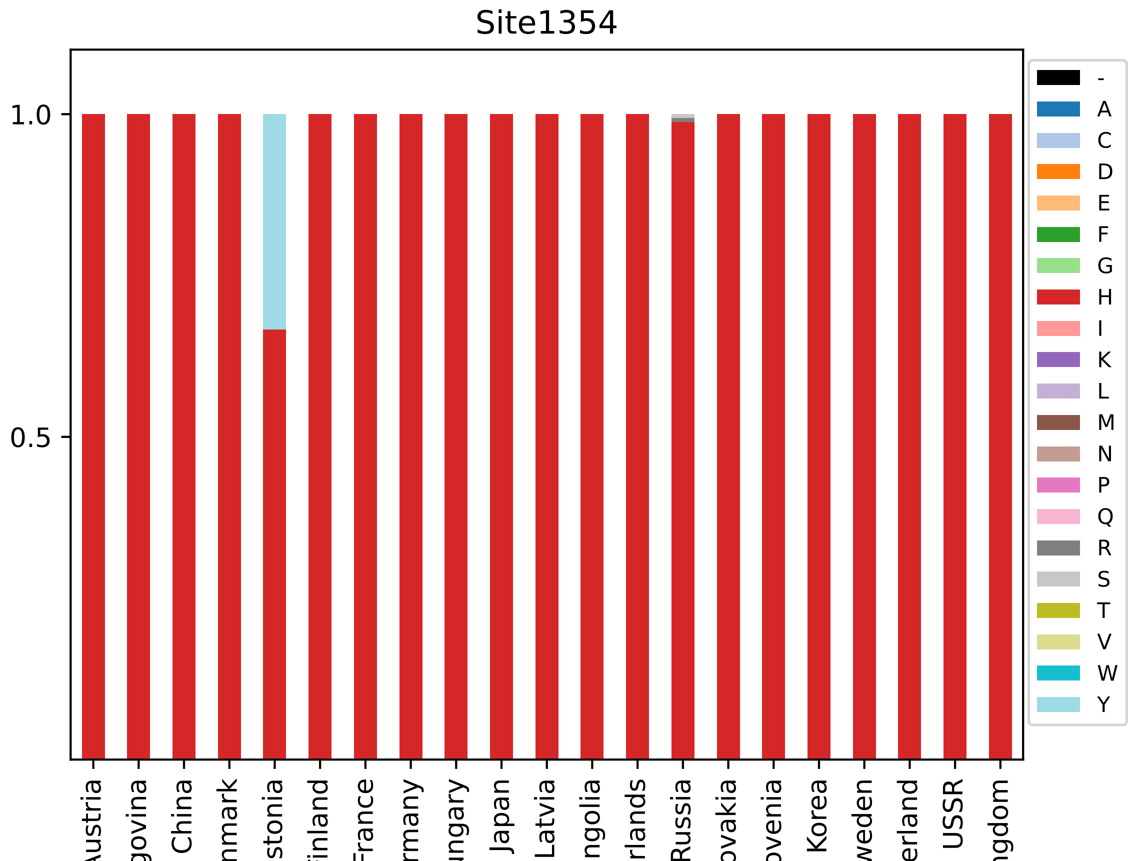

Site1354
1.0
0.5
-
A
C
D
E
F
G
H
I
K
L
M
N
P
Q
R
S
T
V
W
Y
Austria
govina
China
nmark
stonia
inland
France
rmany
ungary
Japan
Latvia
ngolia
rlands
Russia
ovakia
ovenia
Korea
weden
erland
USSR
ngdom

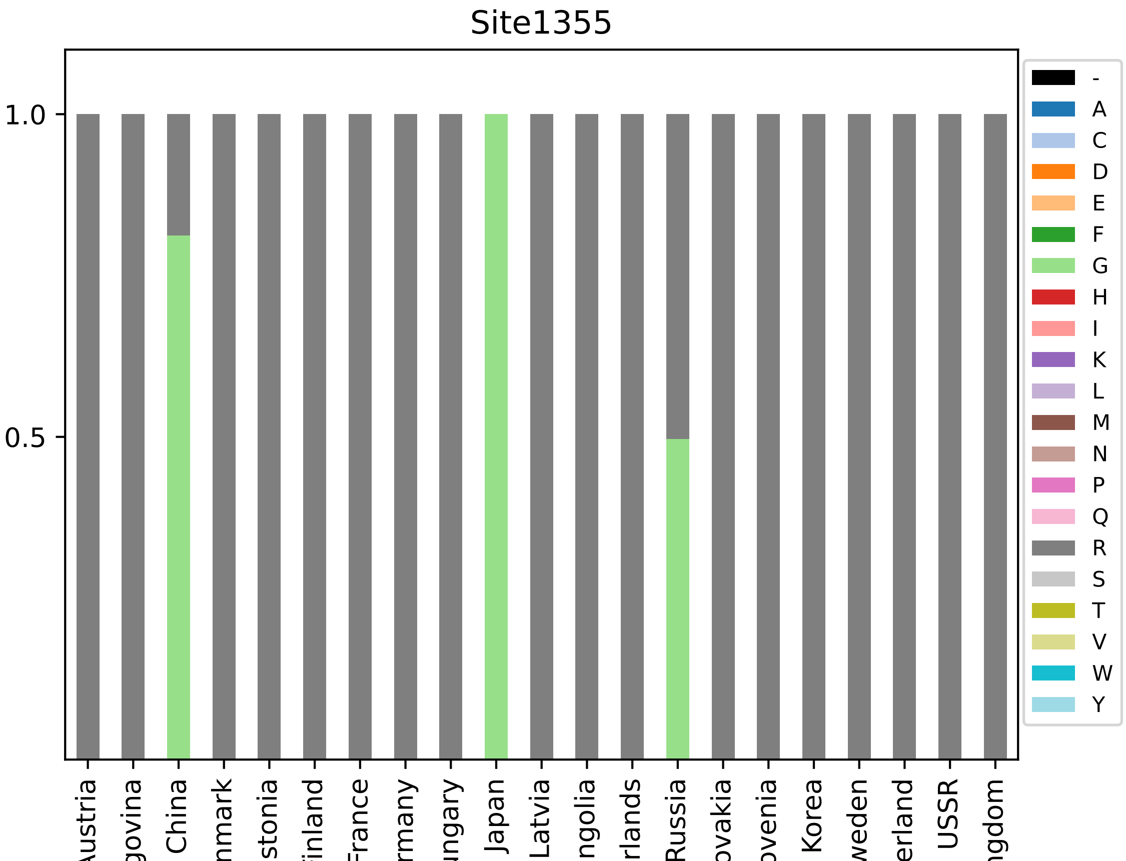
Site1355
1.0
0.5
-
A
C
D
E
F
G
H
I
K
L
M
N
P
Q
R
S
T
V
W
Y
China
Japan
Latvia
Russia
Korea
USSR

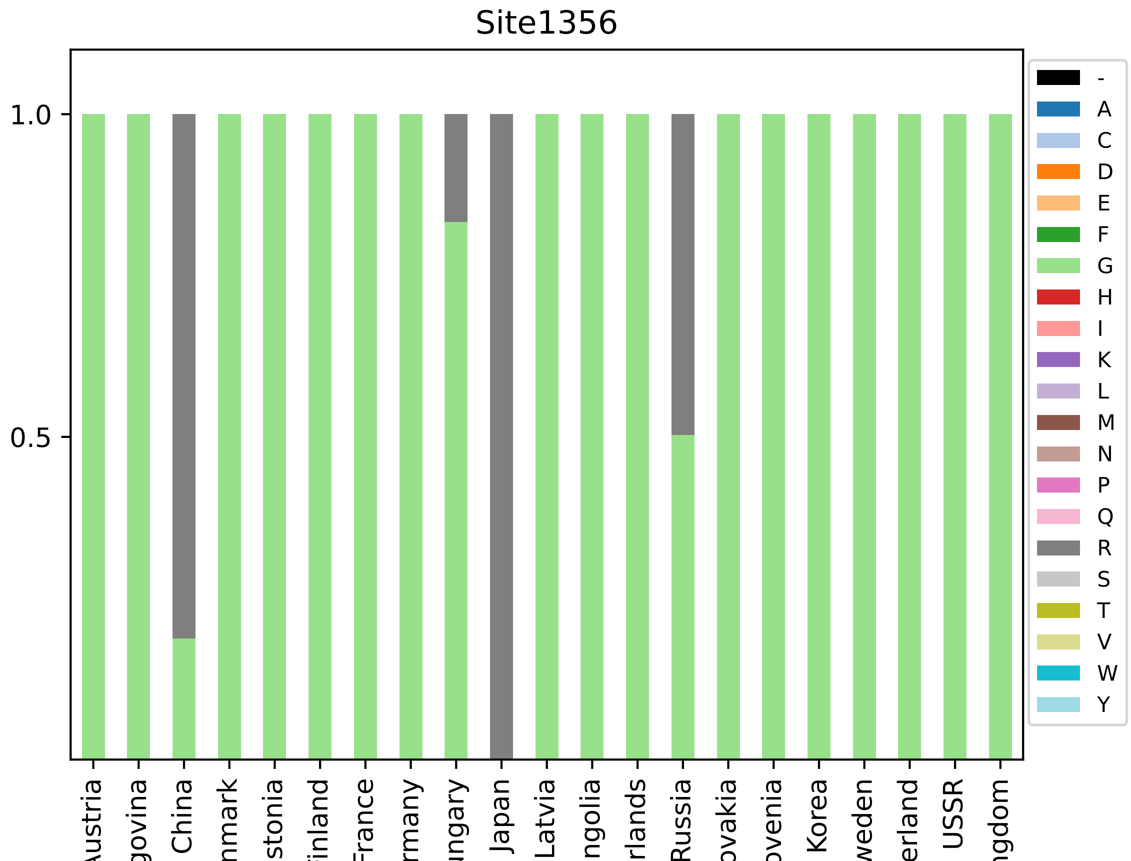

Site1356
1.0
0.5
-
A
C
D
E
F
G
H
I
K
L
M
N
P
Q
R
S
T
V
W
Y
Austria
govina
China
nmark
stonia
Finland
France
rmany
ungary
Japan
Latvia
ngolia
rlands
Russia
ovakia
ovenia
Korea
weden
erland
USSR
ngdom

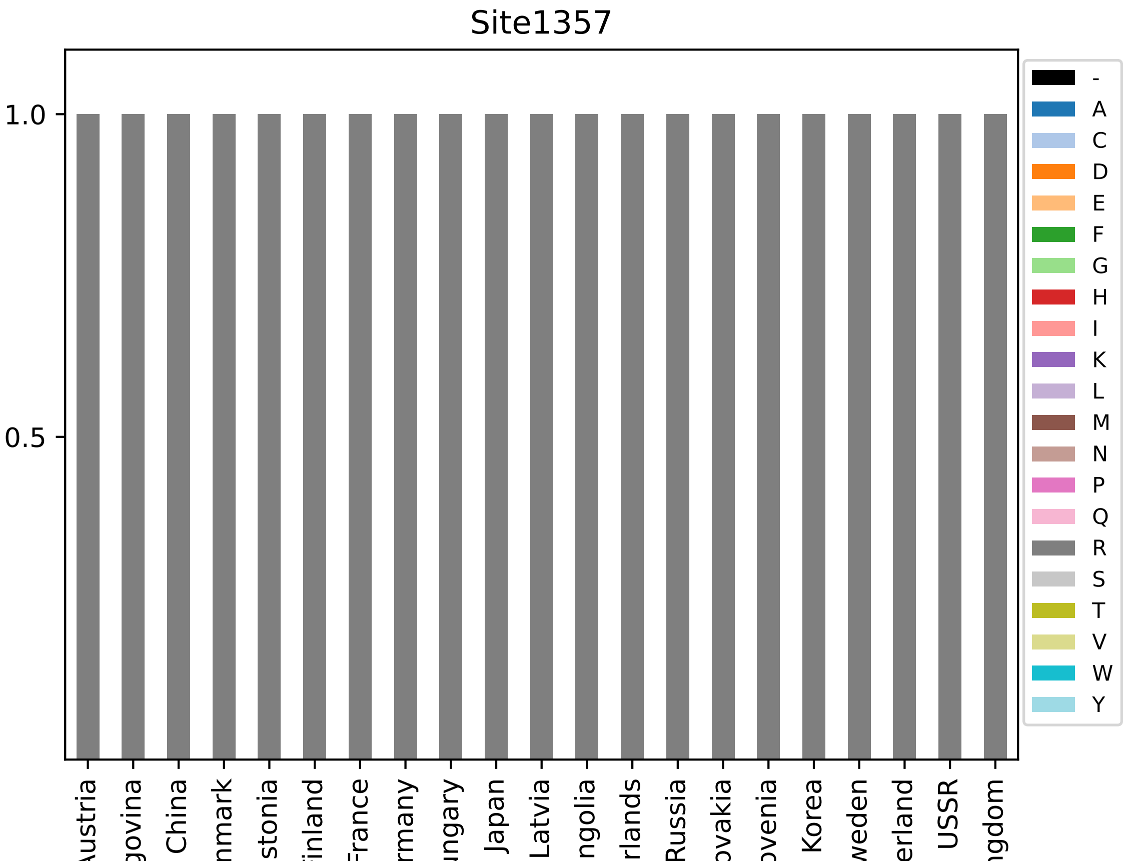
Site1357
1.0
0.5
-
A
C
D
E
F
G
H
I
K
L
M
N
P
Q
R
S
T
V
W
Y
Austria
China
Finland
France
Japan
Latvia
Russia
Korea
USSR

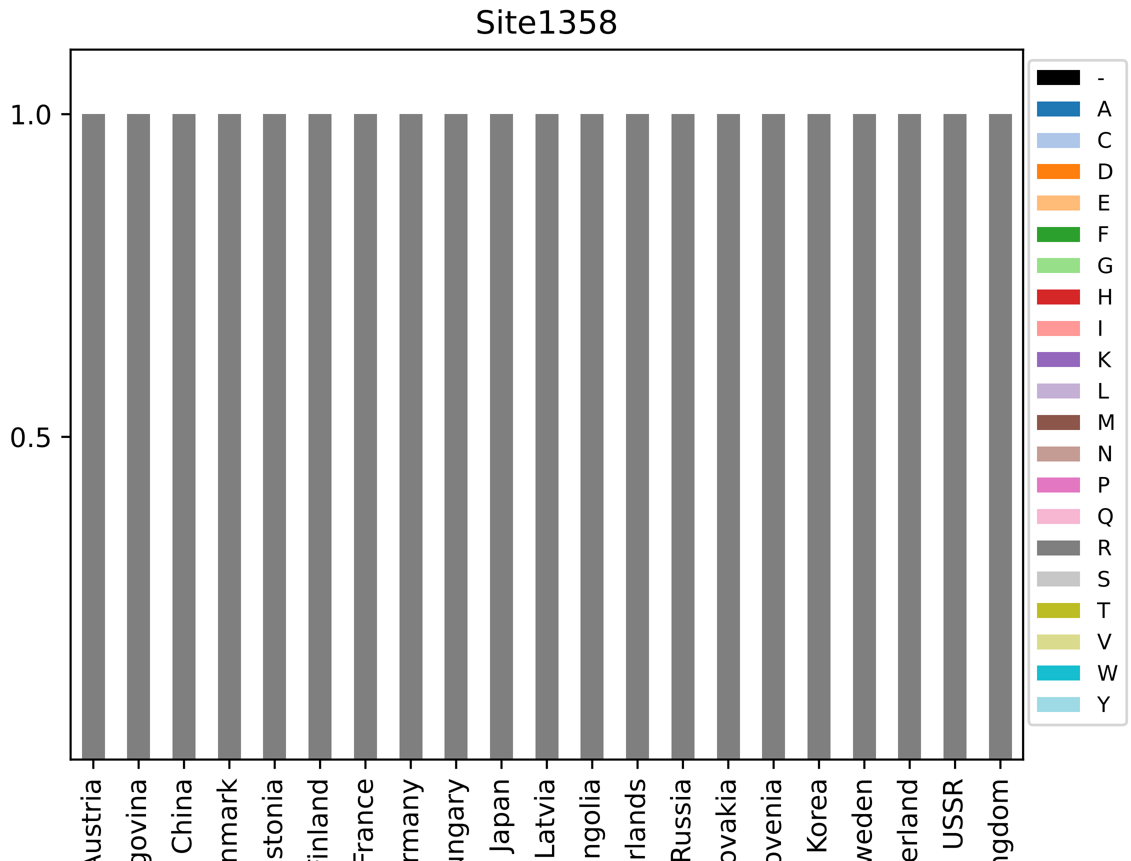

Site1358
1.0
0.5
-
A
C
D
E
F
G
H
I
K
L
M
N
P
Q
R
S
T
V
W
Y
China
Japan
Latvia
Russia
Korea
USSR

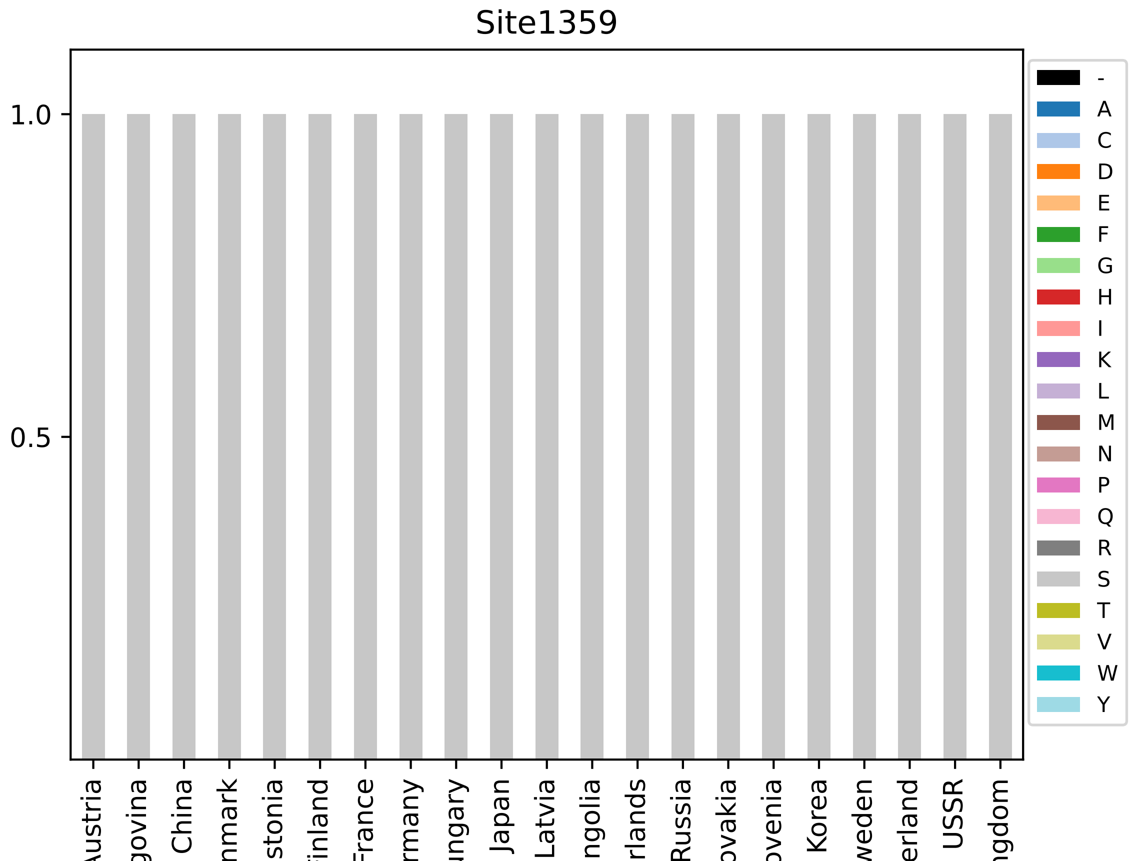

Site1359
1.0
0.5
-
A
C
D
E
F
G
H
I
K
L
M
N
P
Q
R
S
T
V
W
Y
Austria
govina
China
nmark
stonia
Finland
France
rmany
ungary
Japan
Latvia
ngolia
rlands
Russia
ovakia
ovenia
Korea
weden
erland
USSR
ngdom

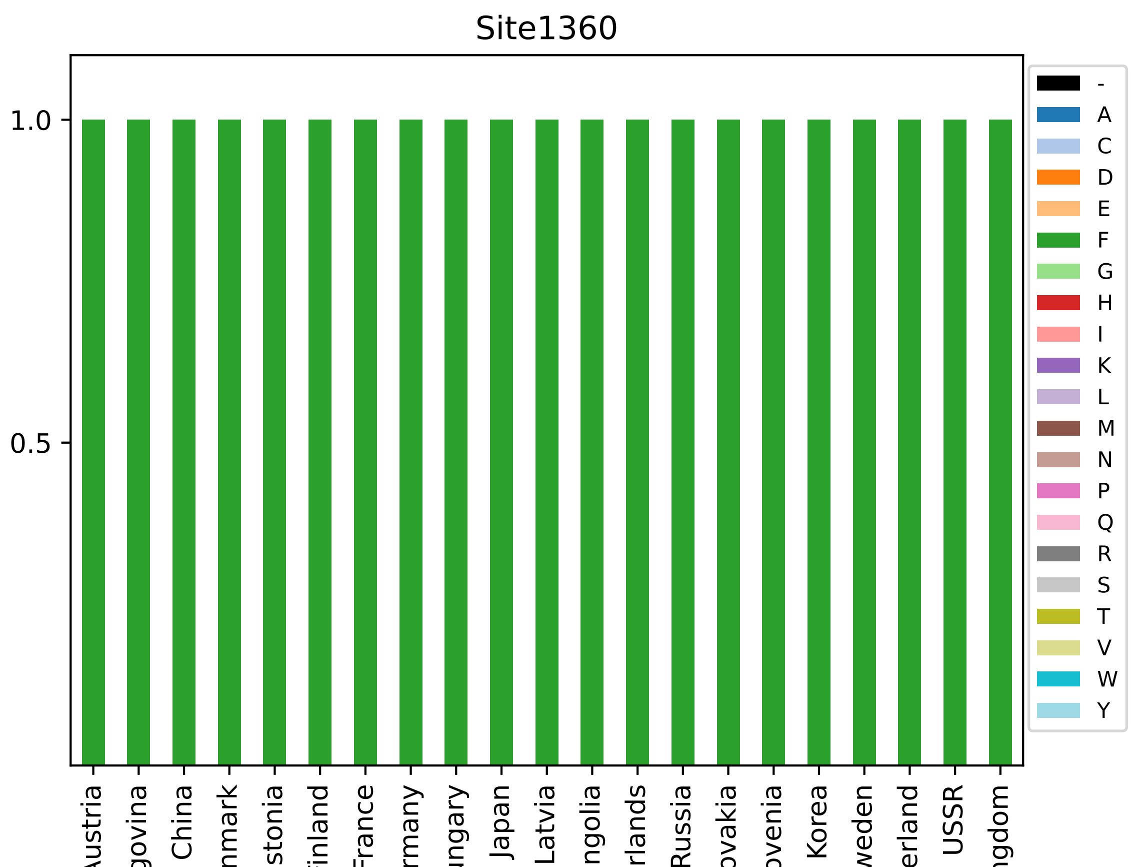
Site1360
1.0
0.5
Austria
govina
China
nmark
stonia
Finland
France
rmany
ungary
Japan
Latvia
ngolia
rlands
Russia
ovakia
ovenia
Korea
weden
erland
USSR
ngdom
-
A
C
D
E
F
G
H
I
K
L
M
N
P
Q
R
S
T
V
W
Y

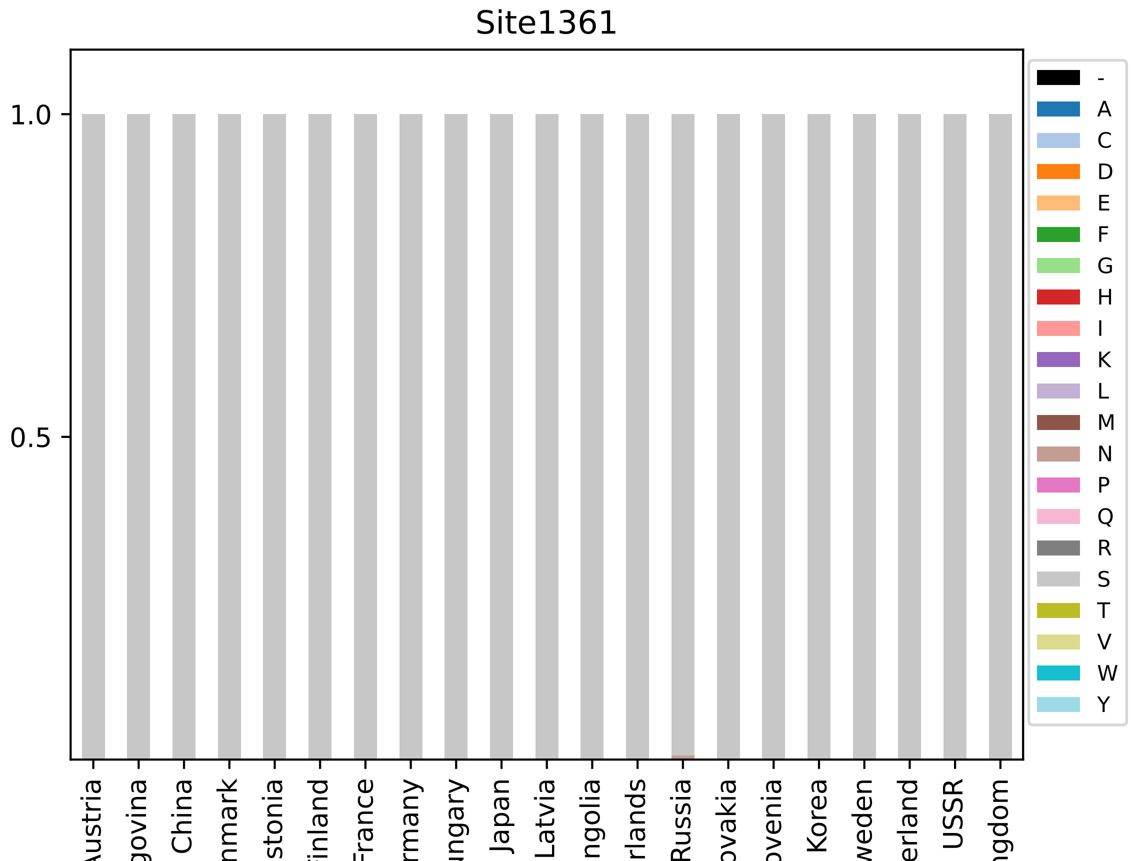

Site1361
1.0
0.5
-
A
C
D
E
F
G
H
I
K
L
M
N
P
Q
R
S
T
V
W
Y
Austria
govina
China
nmark
stonia
Finland
France
rmany
ungary
Japan
Latvia
ngolia
rlands
Russia
ovakia
ovenia
Korea
weden
erland
USSR
ngdom

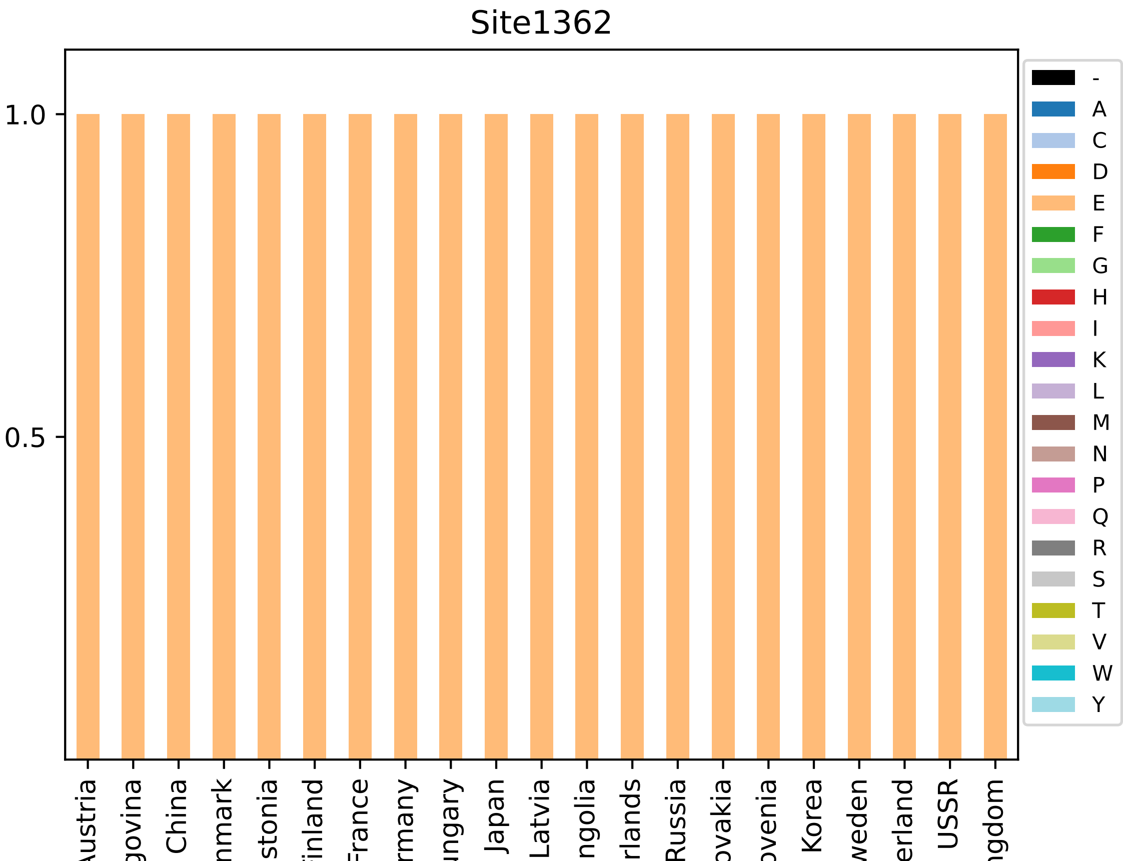
Site1362
1.0
0.5
-
A
C
D
E
F
G
H
I
K
L
M
N
P
Q
R
S
T
V
W
Y
China
Japan
Latvia
Russia
Korea
USSR

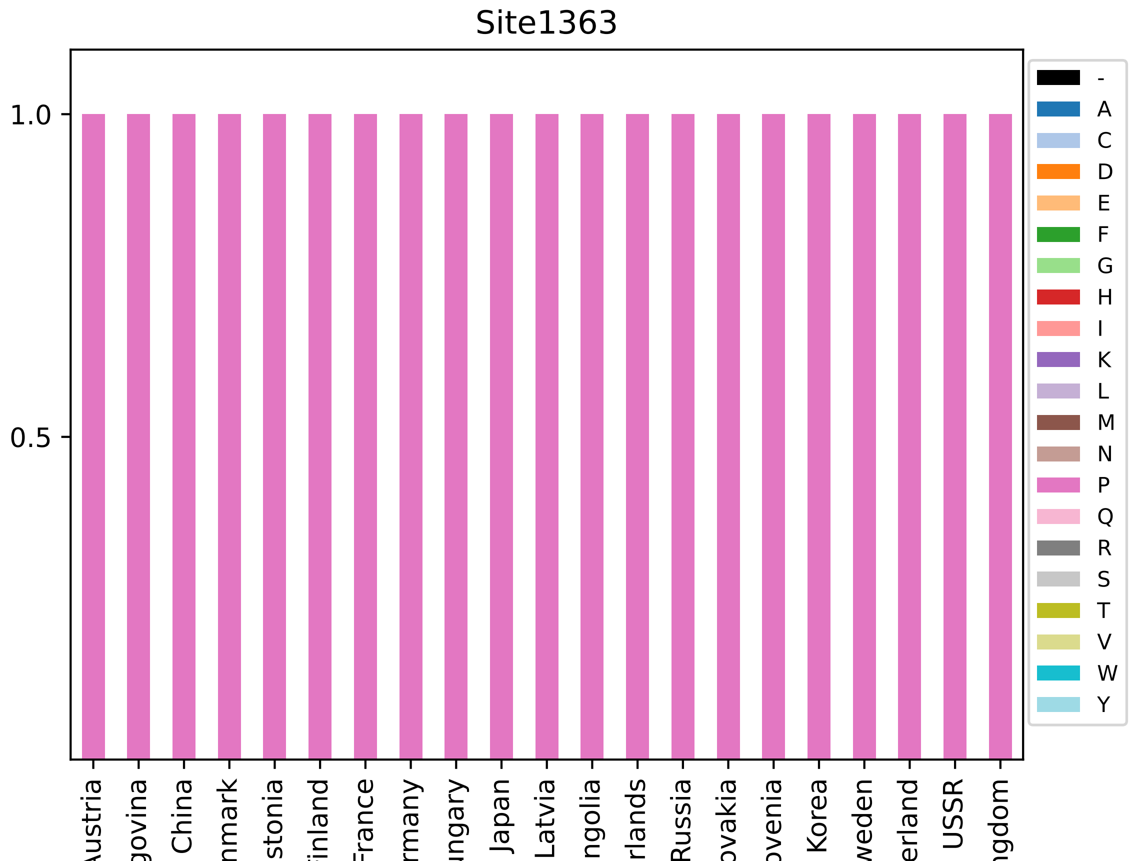

Site1363
1.0
0.5
Austria
govina
China
nmark
stonia
Finland
France
rmany
ungary
Japan
Latvia
ngolia
rlands
Russia
ovakia
ovenia
Korea
weden
erland
USSR
ngdom
-
A
C
D
E
F
G
H
I
K
L
M
N
P
Q
R
S
T
V
W
Y

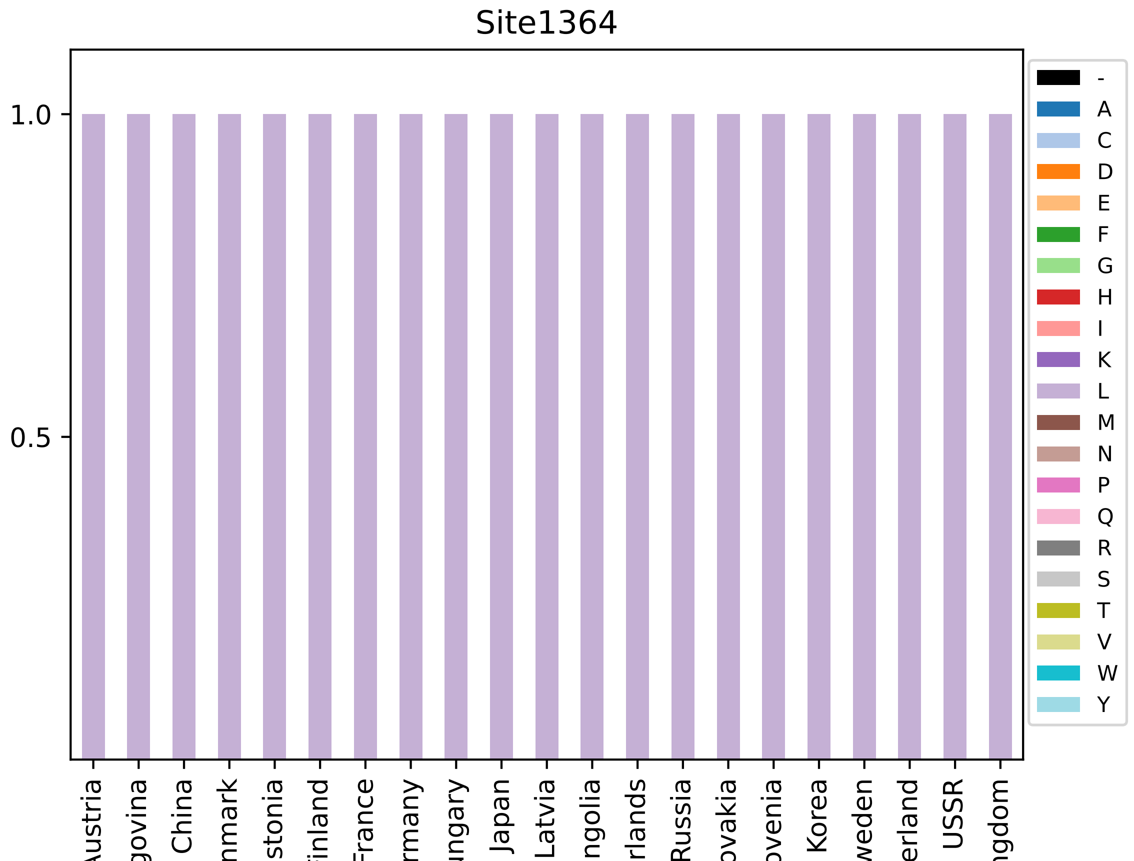

Site1364
1.0
0.5
-
A
C
D
E
F
G
H
I
K
L
M
N
P
Q
R
S
T
V
W
Y
China
Japan
Latvia
Russia
Korea
USSR

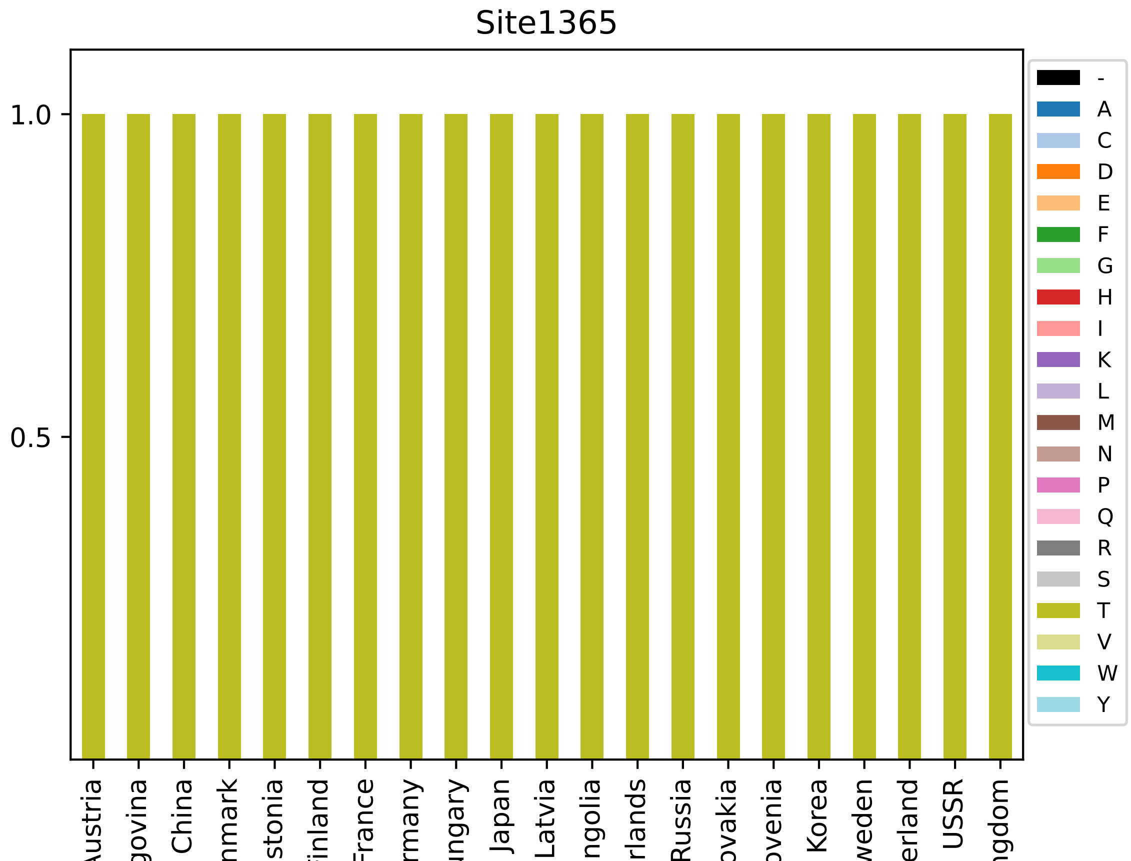

Site1365
1.0
0.5
-
A
C
D
E
F
G
H
I
K
L
M
N
P
Q
R
S
T
V
W
Y
China
Japan
Latvia
Russia
Korea
USSR

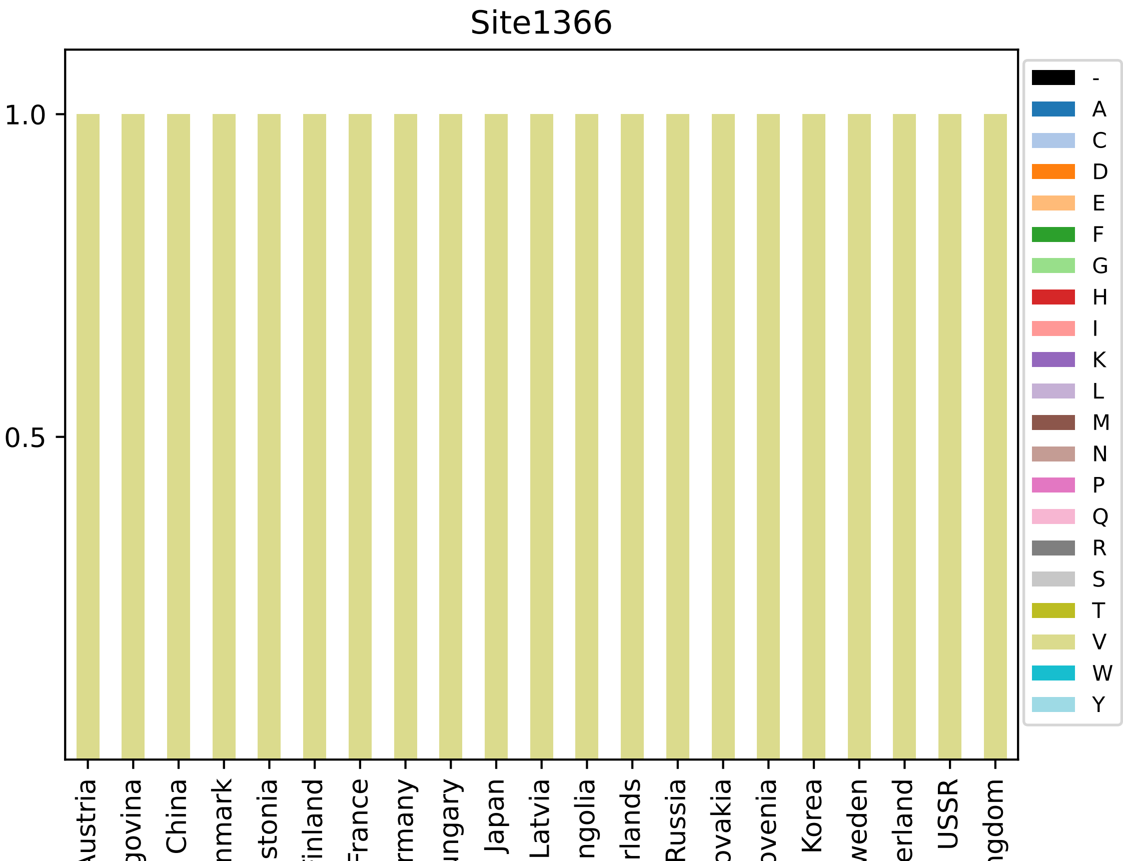

Site1366
1.0
0.5
-
A
C
D
E
F
G
H
I
K
L
M
N
P
Q
R
S
T
V
W
Y
China
Japan
Latvia
Russia
Korea
USSR

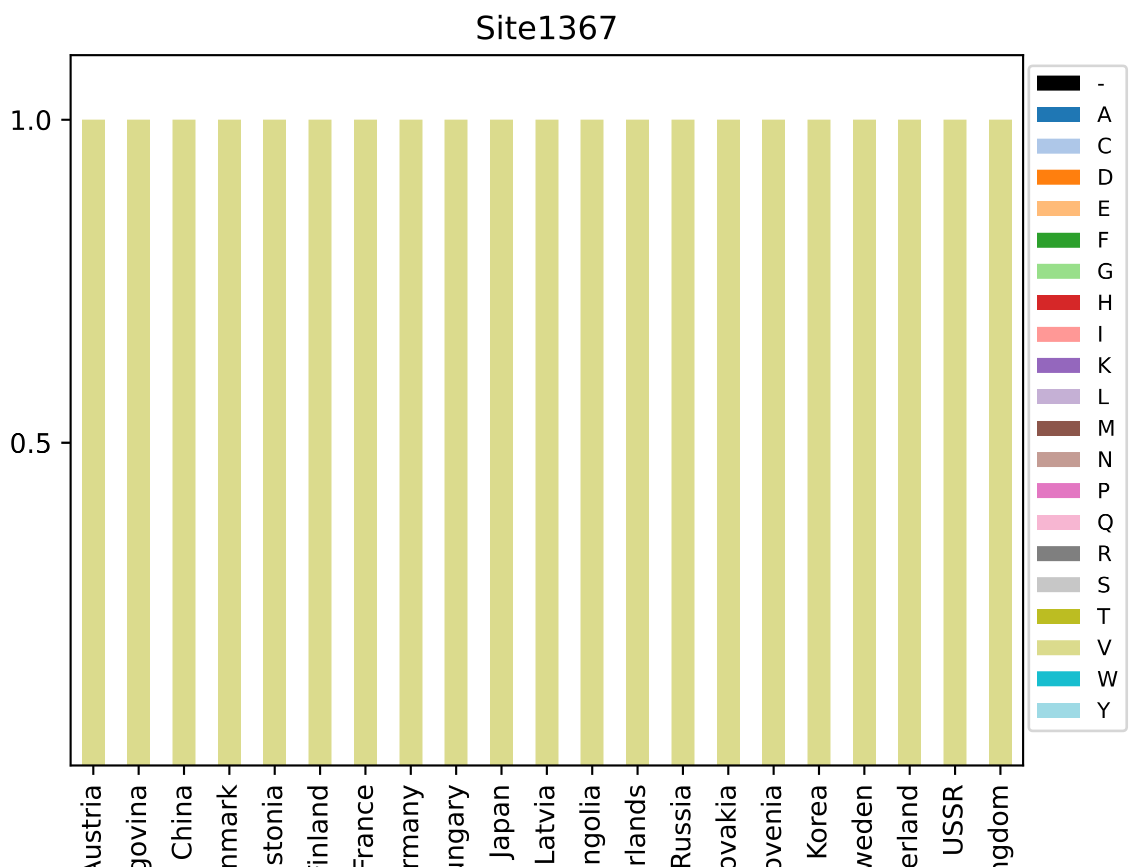

Site1367
1.0
0.5
-
A
C
D
E
F
G
H
I
K
L
M
N
P
Q
R
S
T
V
W
Y
China
Japan
Latvia
Russia
Korea
USSR

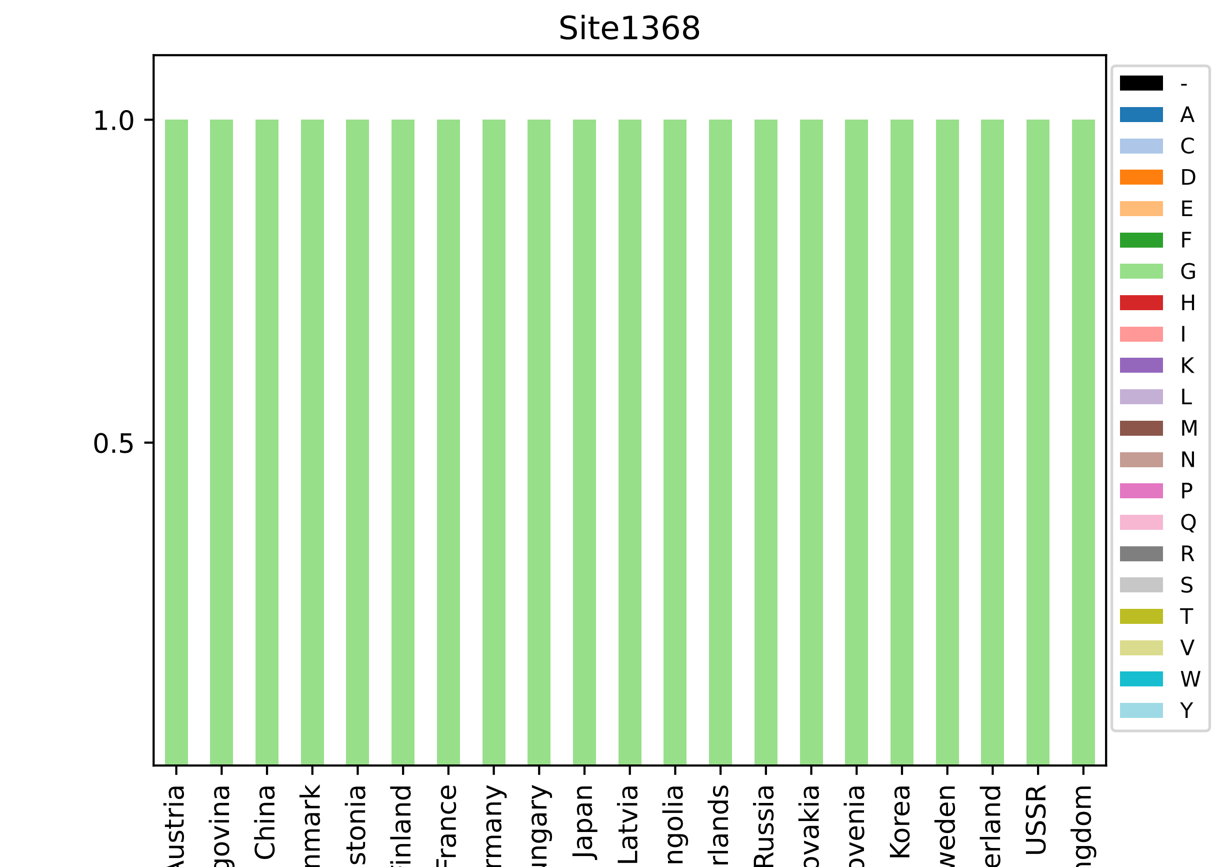

Site1368
1.0
0.5
-
A
C
D
E
F
G
H
I
K
L
M
N
P
Q
R
S
T
V
W
Y
Austria
govina
China
nmark
stonia
Finland
France
rmany
ungary
Japan
Latvia
ngolia
rlands
Russia
ovakia
ovenia
Korea
weden
erland
USSR
ngdom

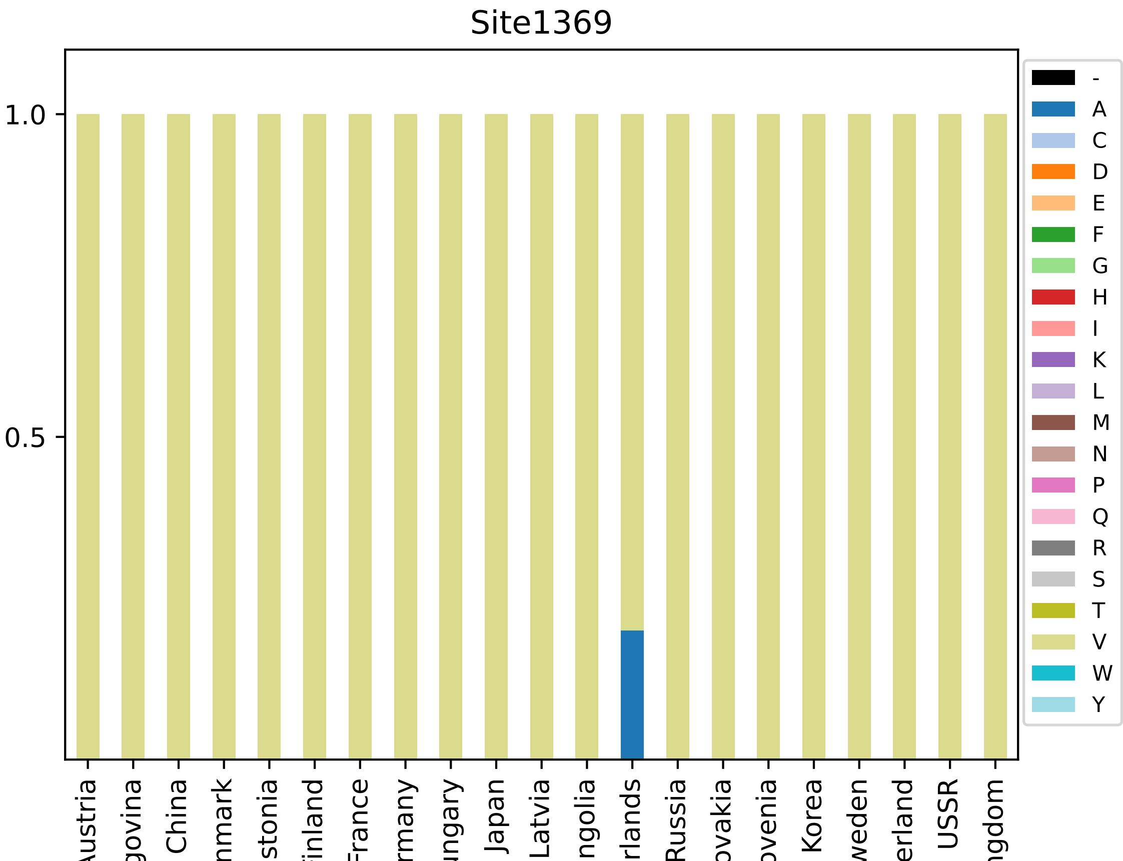
Site1369
1.0
0.5
-
A
C
D
E
F
G
H
I
K
L
M
N
P
Q
R
S
T
V
W
Y
Austria
govina
China
nmark
stonia
Finland
France
rmany
ungary
Japan
Latvia
ngolia
rlands
Russia
ovakia
ovenia
Korea
weden
erland
USSR
ngdom

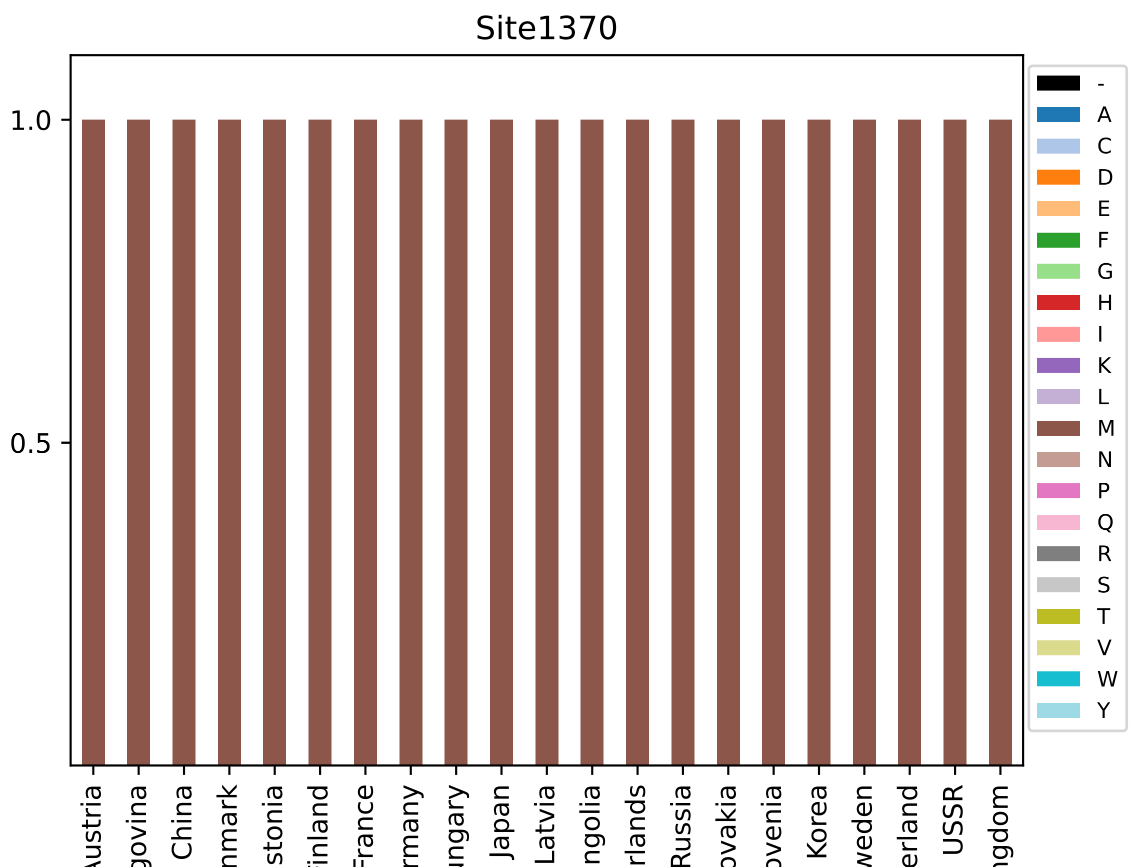
Site1370
1.0
0.5
-
A
C
D
E
F
G
H
I
K
L
M
N
P
Q
R
S
T
V
W
Y
Austria
China
Japan
Latvia
Russia
Korea
USSR

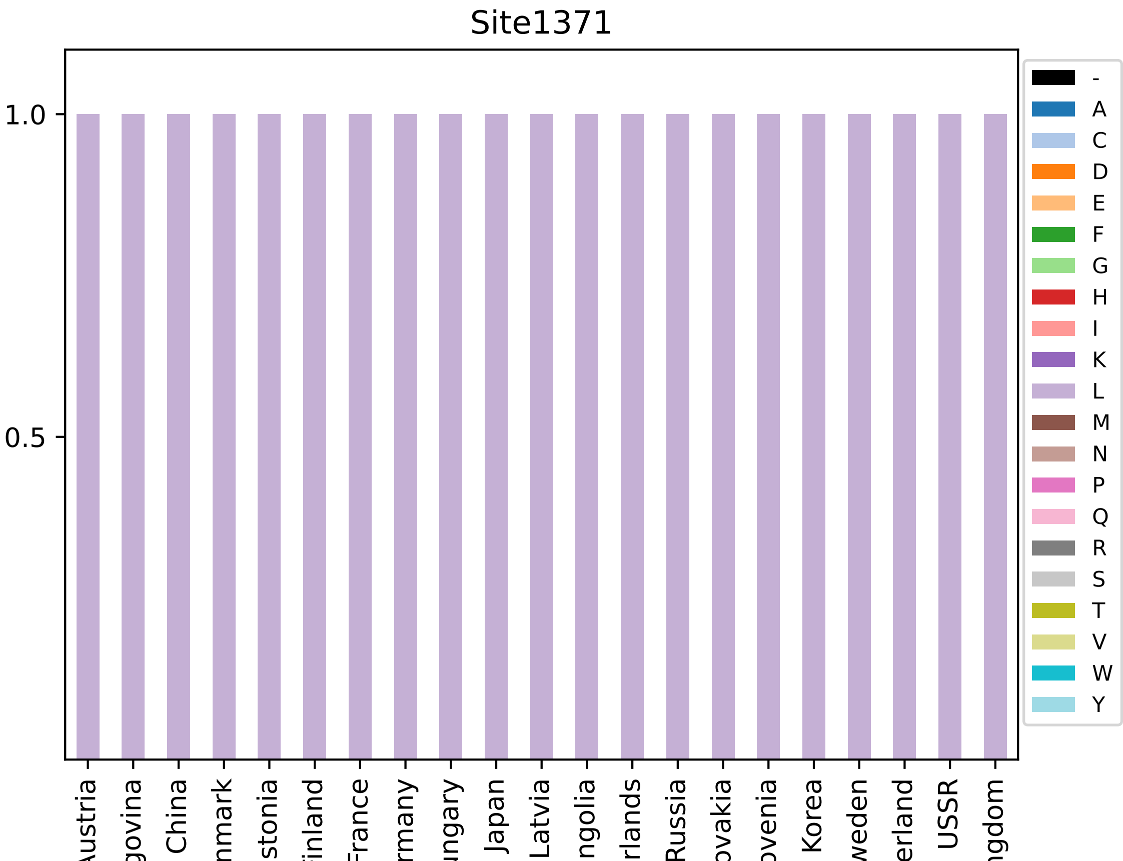
Site1371
1.0
0.5
-
A
C
D
E
F
G
H
I
K
L
M
N
P
Q
R
S
T
V
W
Y
Austria
govina
China
nmark
stonia
inland
France
rmany
ungary
Japan
Latvia
ngolia
rlands
Russia
ovakia
ovenia
Korea
weden
erland
USSR
ngdom

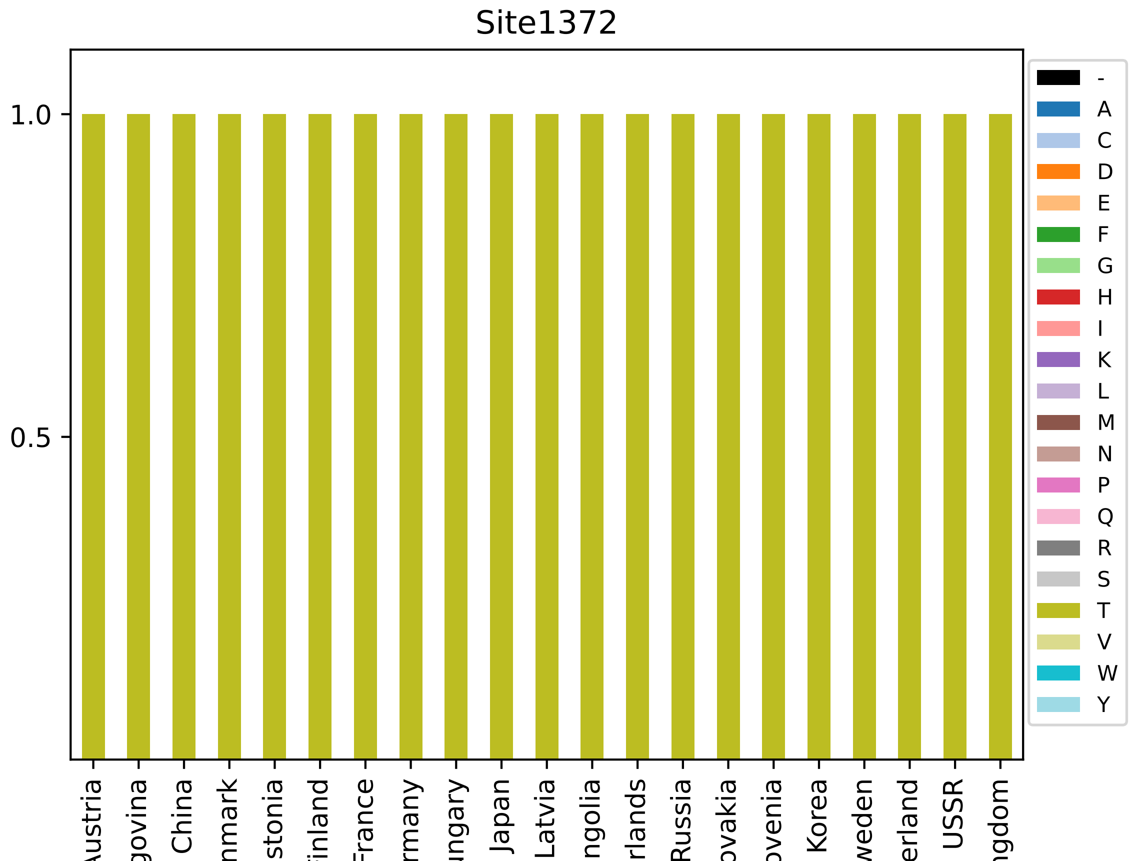

Site1372
1.0
0.5
-
A
C
D
E
F
G
H
I
K
L
M
N
P
Q
R
S
T
V
W
Y
Austria
China
Finland
France
Japan
Latvia
Russia
Korea
USSR

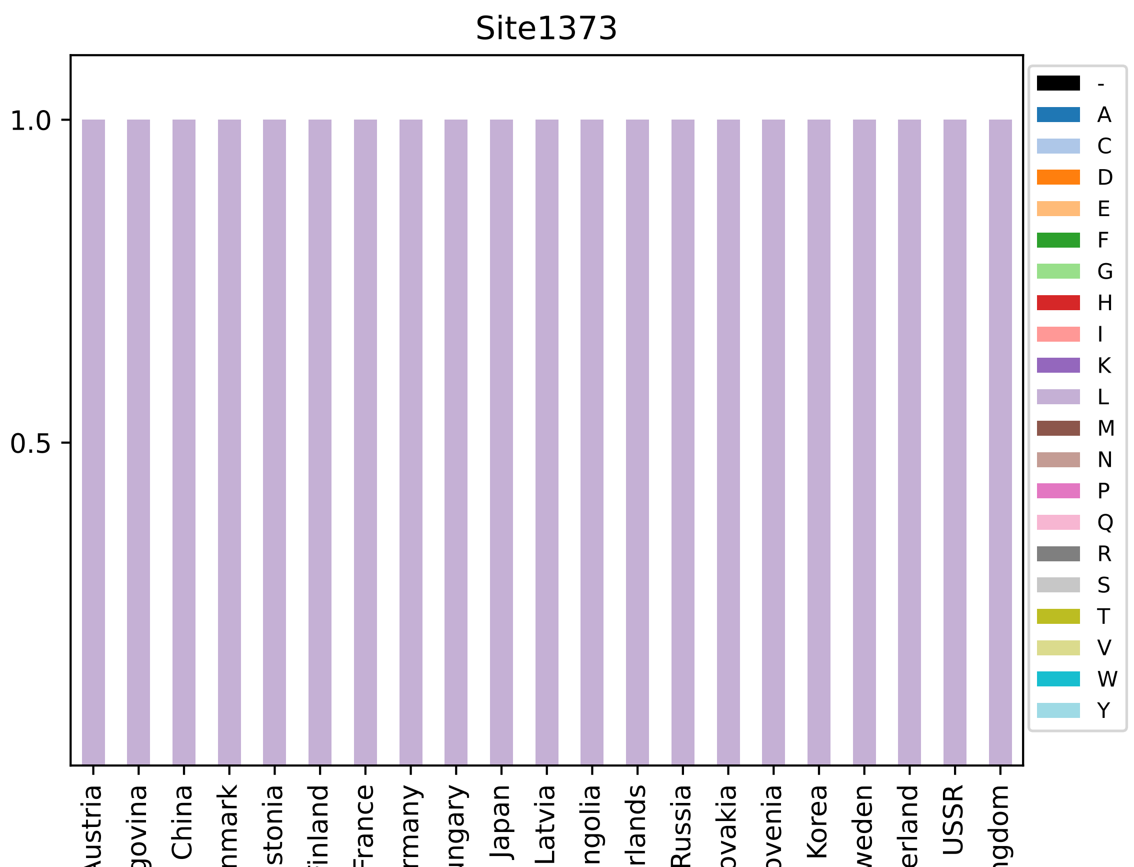
Site1373
1.0
0.5
-
A
C
D
E
F
G
H
I
K
L
M
N
P
Q
R
S
T
V
W
Y
China
Japan
Latvia
Russia
Korea
USSR

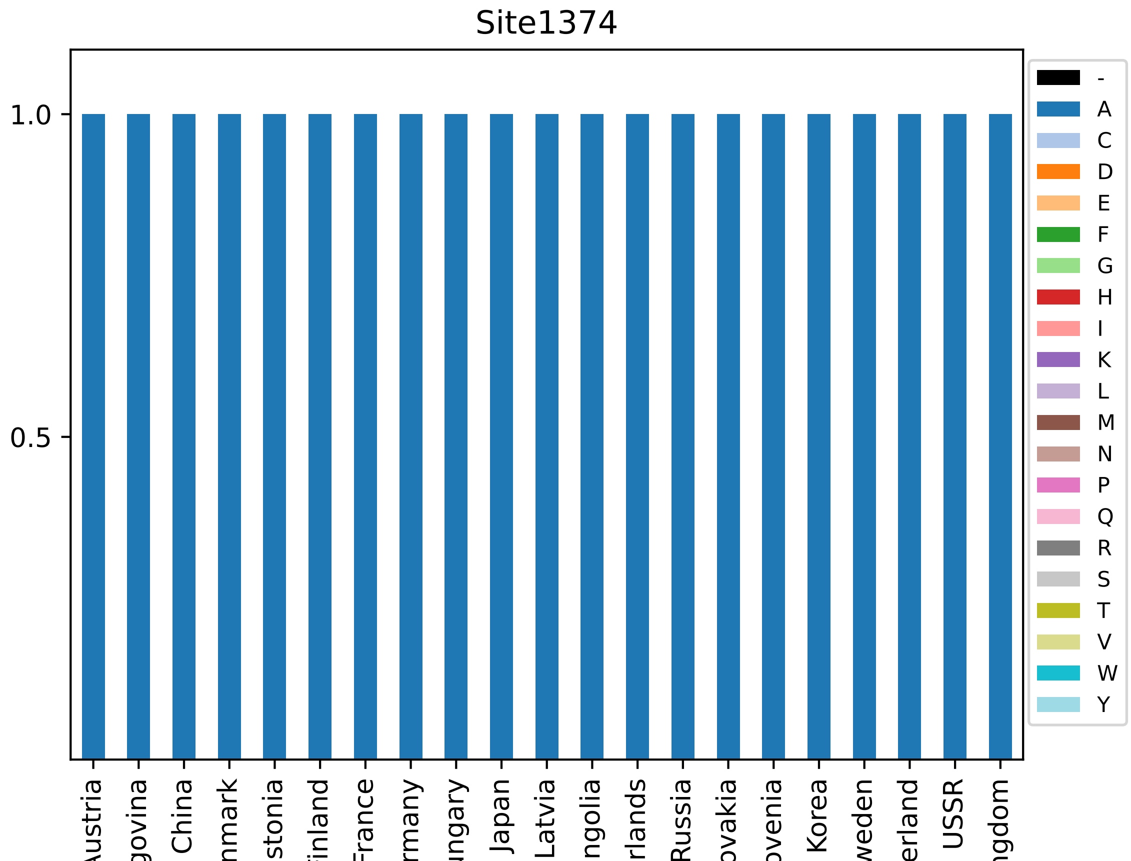
Site1374
1.0
0.5
Austria
govina
China
nmark
stonia
inland
France
rmany
ungary
Japan
Latvia
ngolia
rlands
Russia
ovakia
ovenia
Korea
weden
erland
USSR
ngdom
-
A
C
D
E
F
G
H
I
K
L
M
N
P
Q
R
S
T
V
W
Y

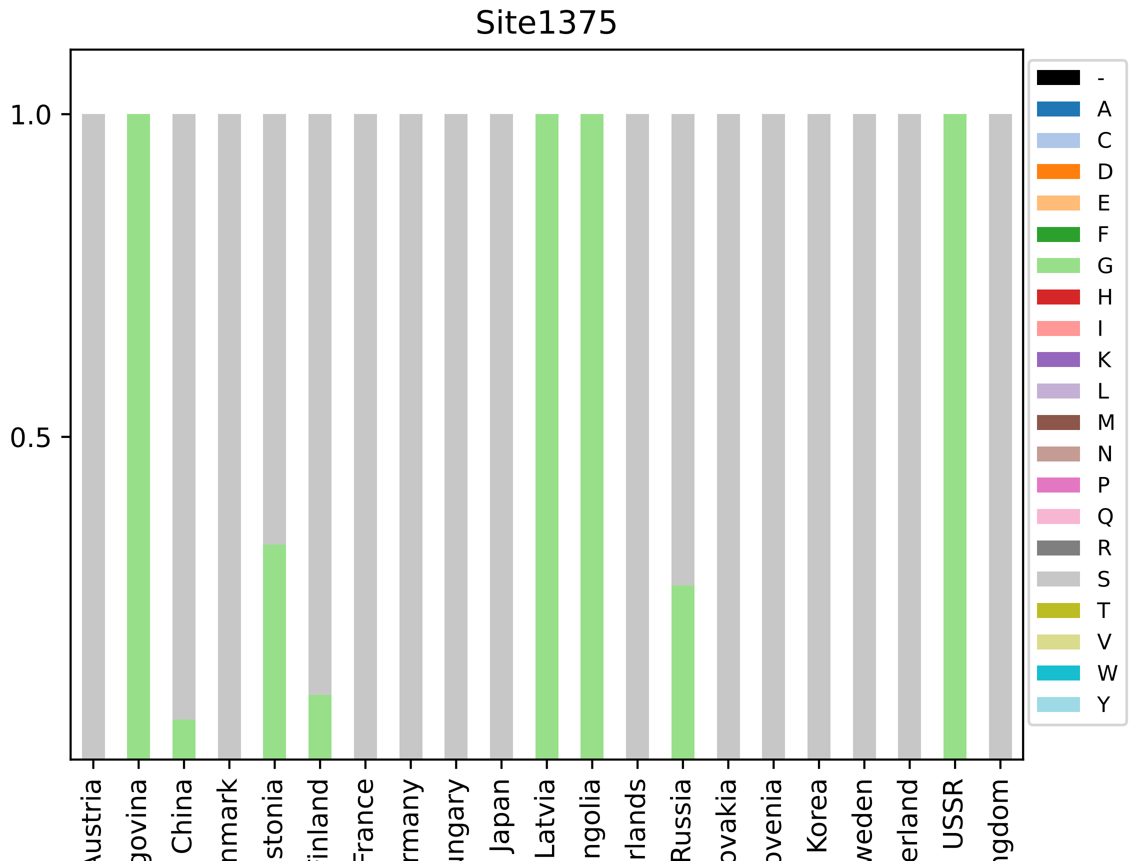

Site1375
1.0
0.5
-
A
C
D
E
F
G
H
I
K
L
M
N
P
Q
R
S
T
V
W
Y
China
Latvia
Russia
Korea
USSR
Japan

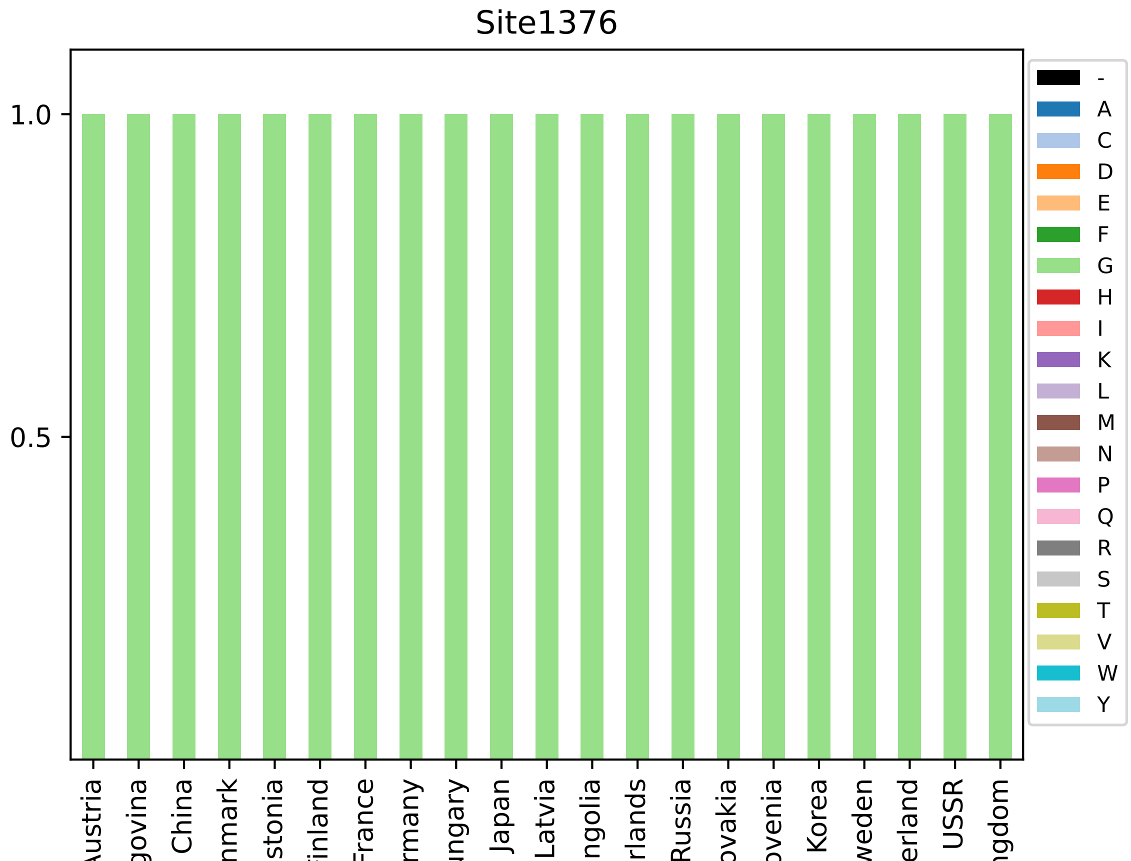

Site1376
1.0
0.5
-
A
C
D
E
F
G
H
I
K
L
M
N
P
Q
R
S
T
V
W
Y
Austria
China
Finland
France
Japan
Latvia
Russia
Korea
USSR

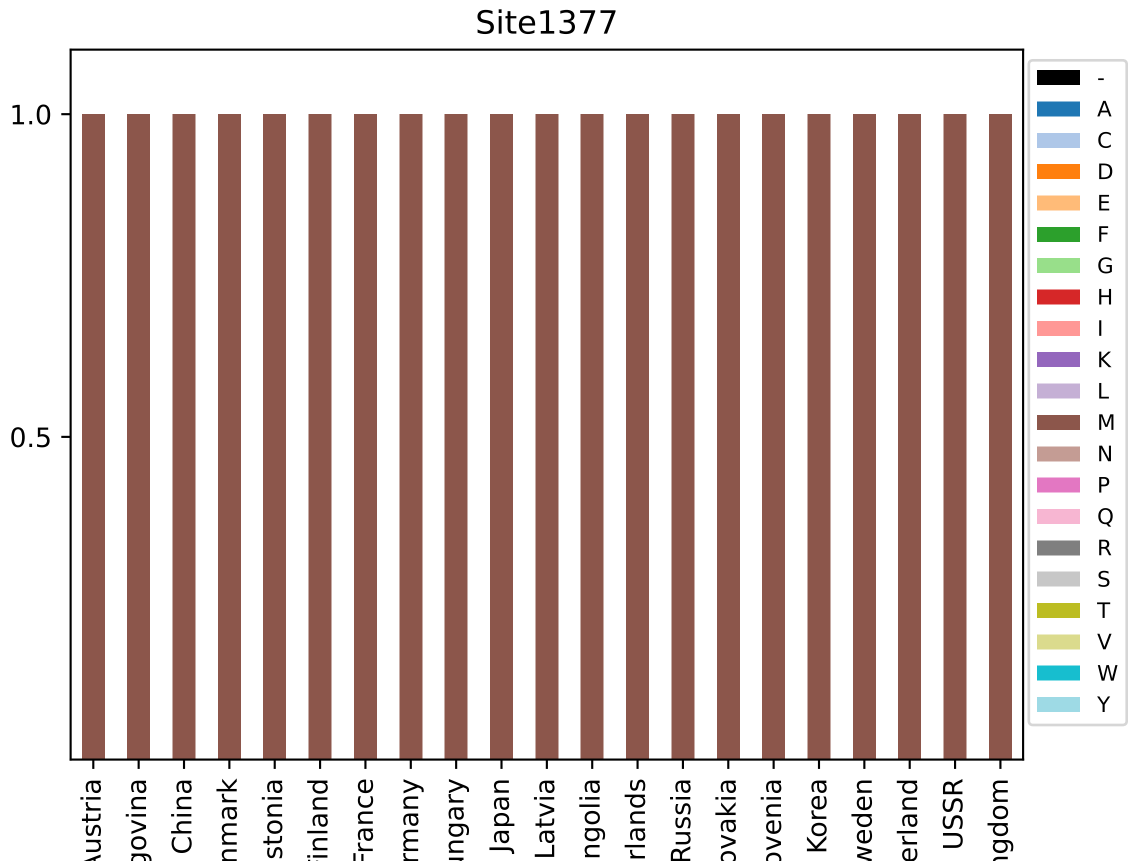

Site1377
1.0
0.5
Austria
govina
China
nmark
stonia
inland
France
rmany
ungary
Japan
Latvia
ngolia
rlands
Russia
ovakia
ovenia
Korea
weden
erland
USSR
ngdom
-
A
C
D
E
F
G
H
I
K
L
M
N
P
Q
R
S
T
V
W
Y

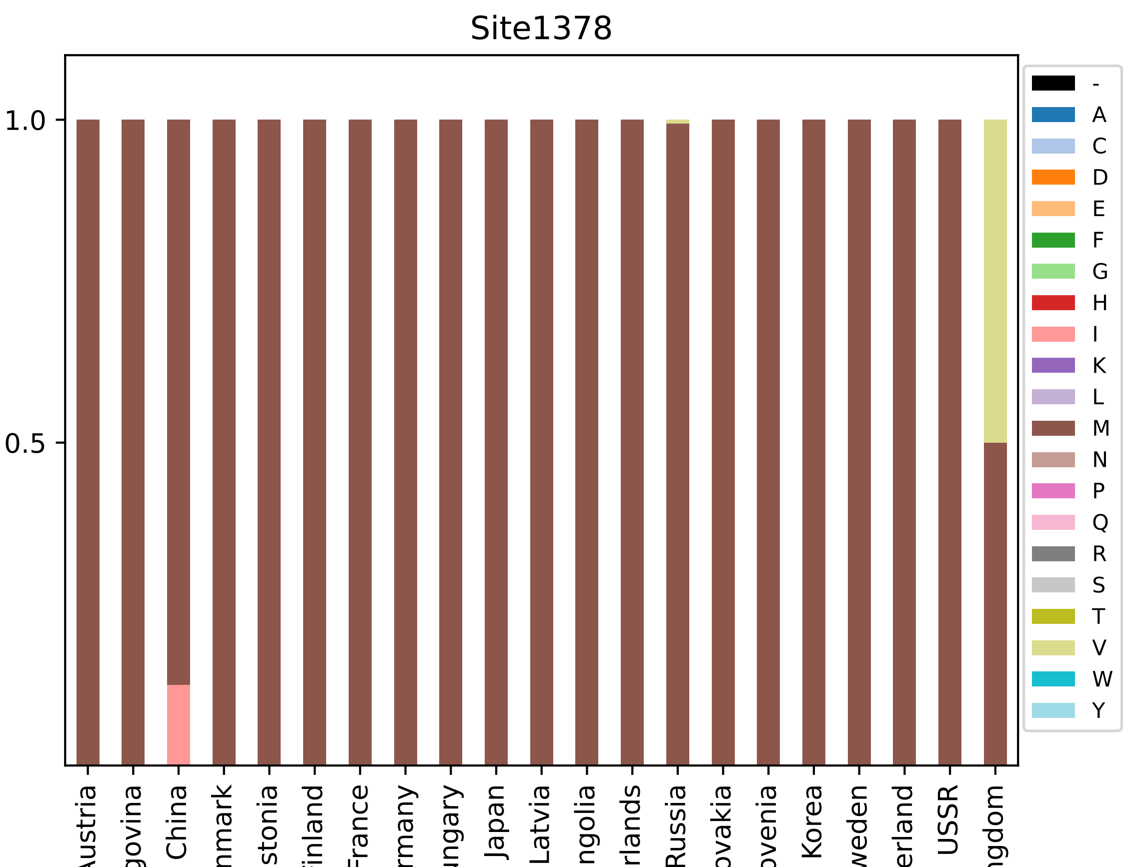

Site1378
1.0
0.5
-
A
C
D
E
F
G
H
I
K
L
M
N
P
Q
R
S
T
V
W
Y
China
Japan
Latvia
Russia
Korea
USSR

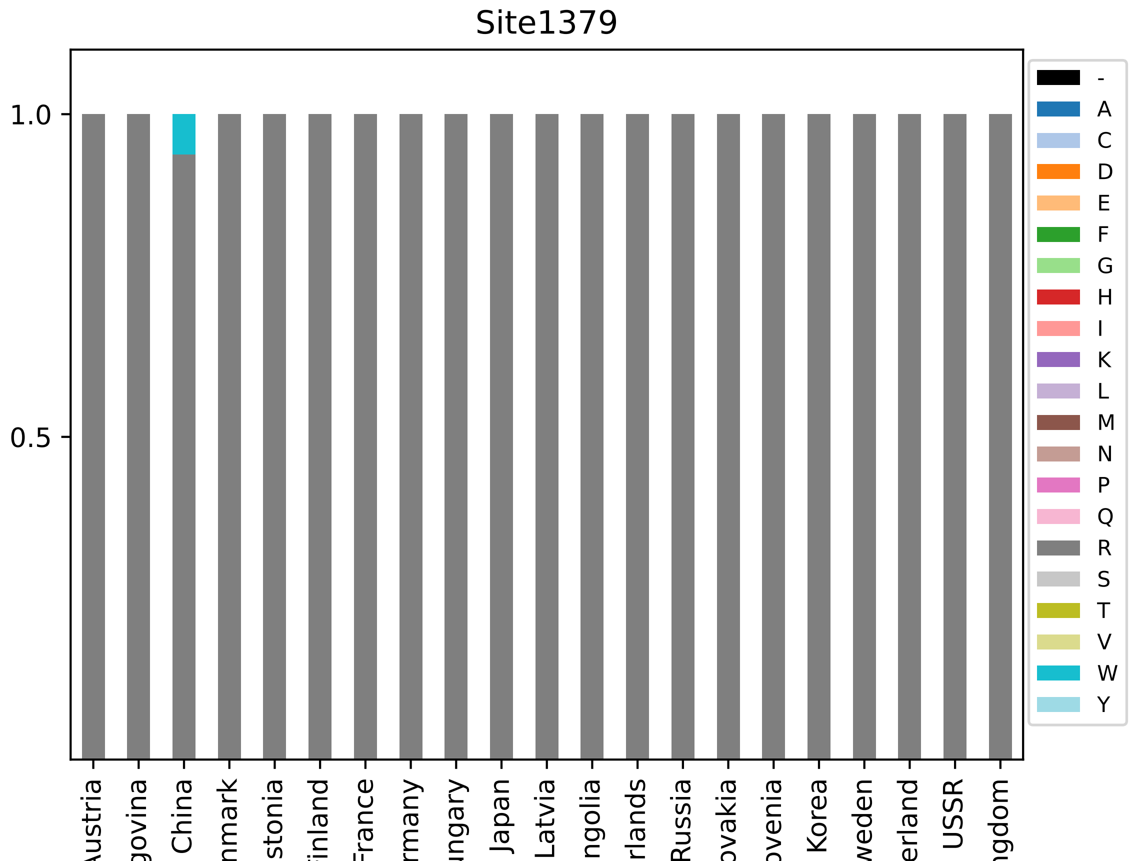

Site1379
1.0
0.5
-
A
C
D
E
F
G
H
I
K
L
M
N
P
Q
R
S
T
V
W
Y
China
Japan
Latvia
Russia
Korea
USSR

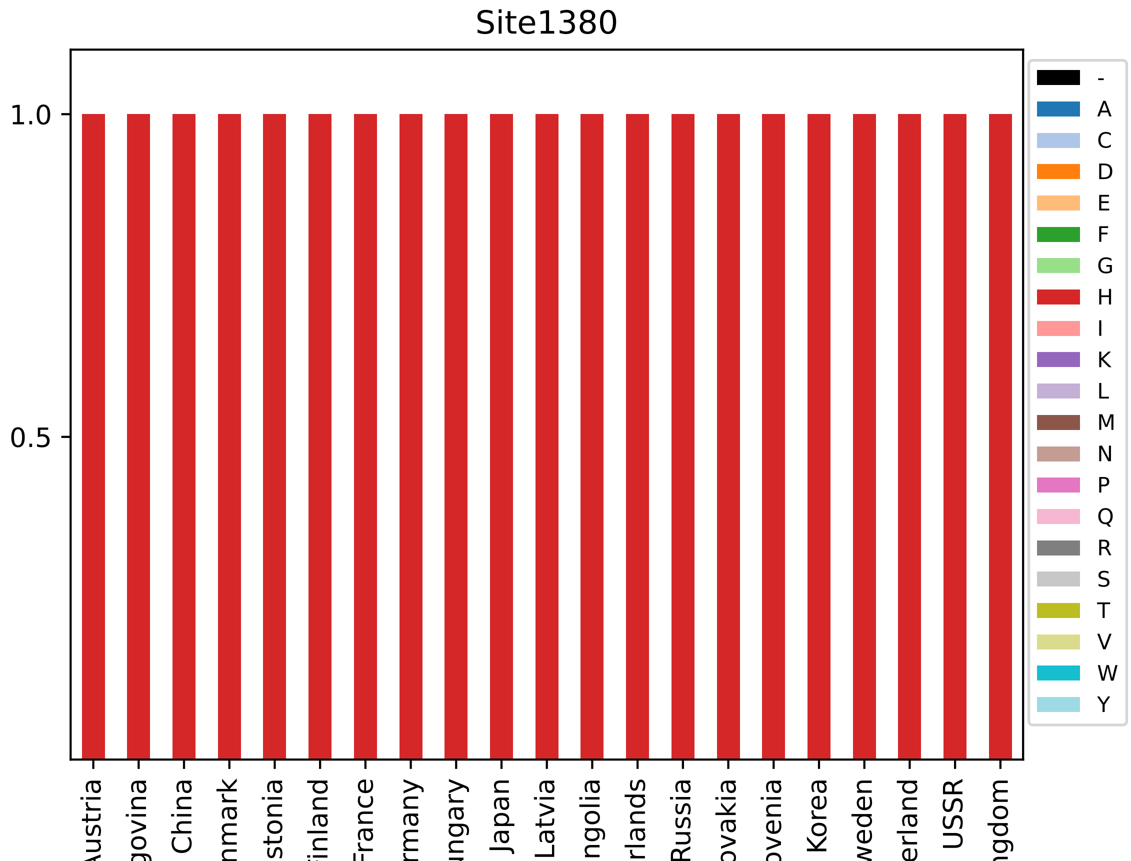
Site1380
1.0
0.5
-
A
C
D
E
F
G
H
I
K
L
M
N
P
Q
R
S
T
V
W
Y
China
Japan
Latvia
Russia
Korea
USSR

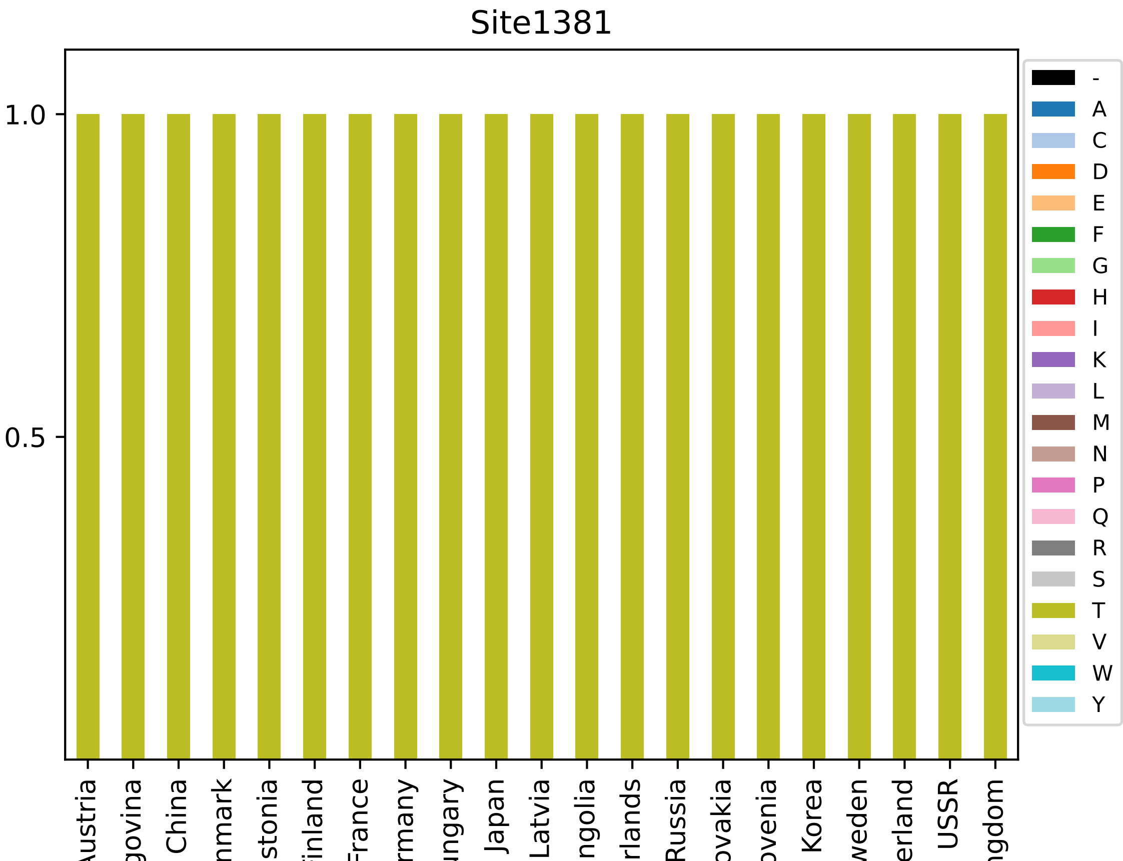

Site1381
1.0
0.5
-
A
C
D
E
F
G
H
I
K
L
M
N
P
Q
R
S
T
V
W
Y
China
Japan
Latvia
Russia
Korea
USSR

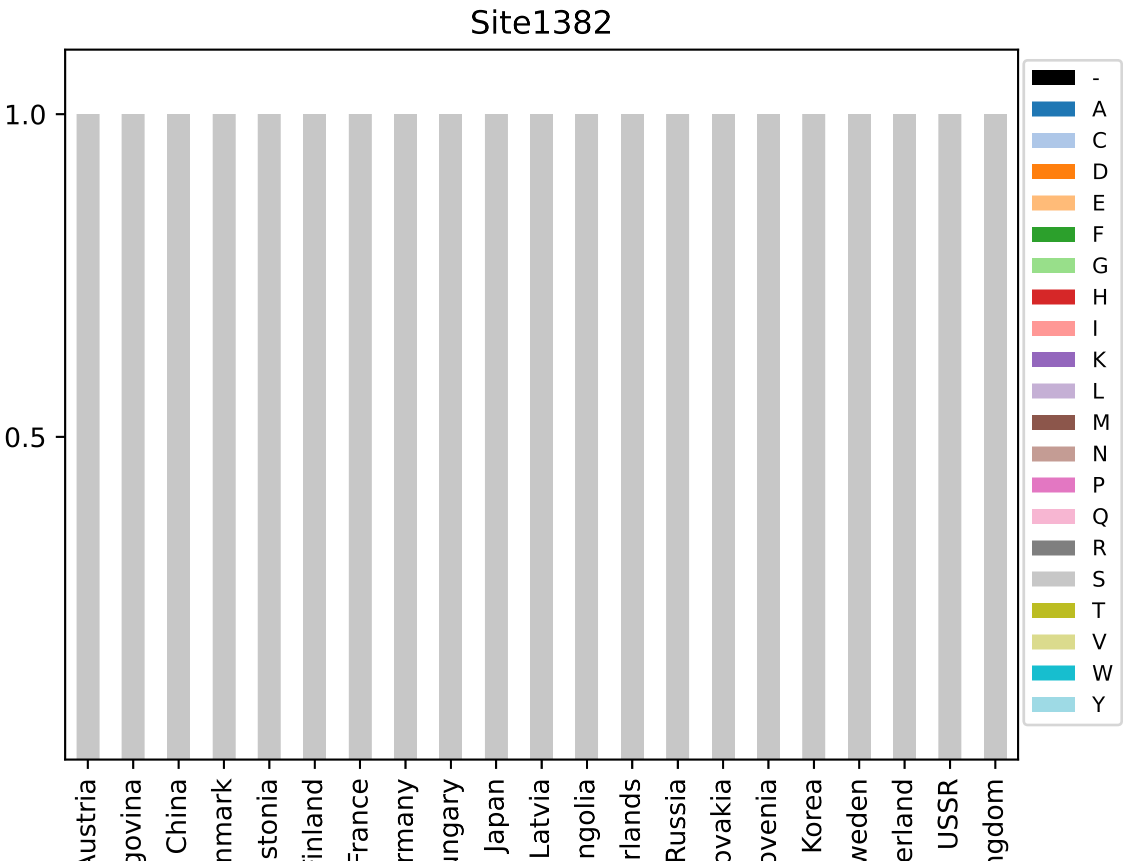
Site1382
1.0
0.5
-
A
C
D
E
F
G
H
I
K
L
M
N
P
Q
R
S
T
V
W
Y
China
Japan
Latvia
Russia
Korea
USSR

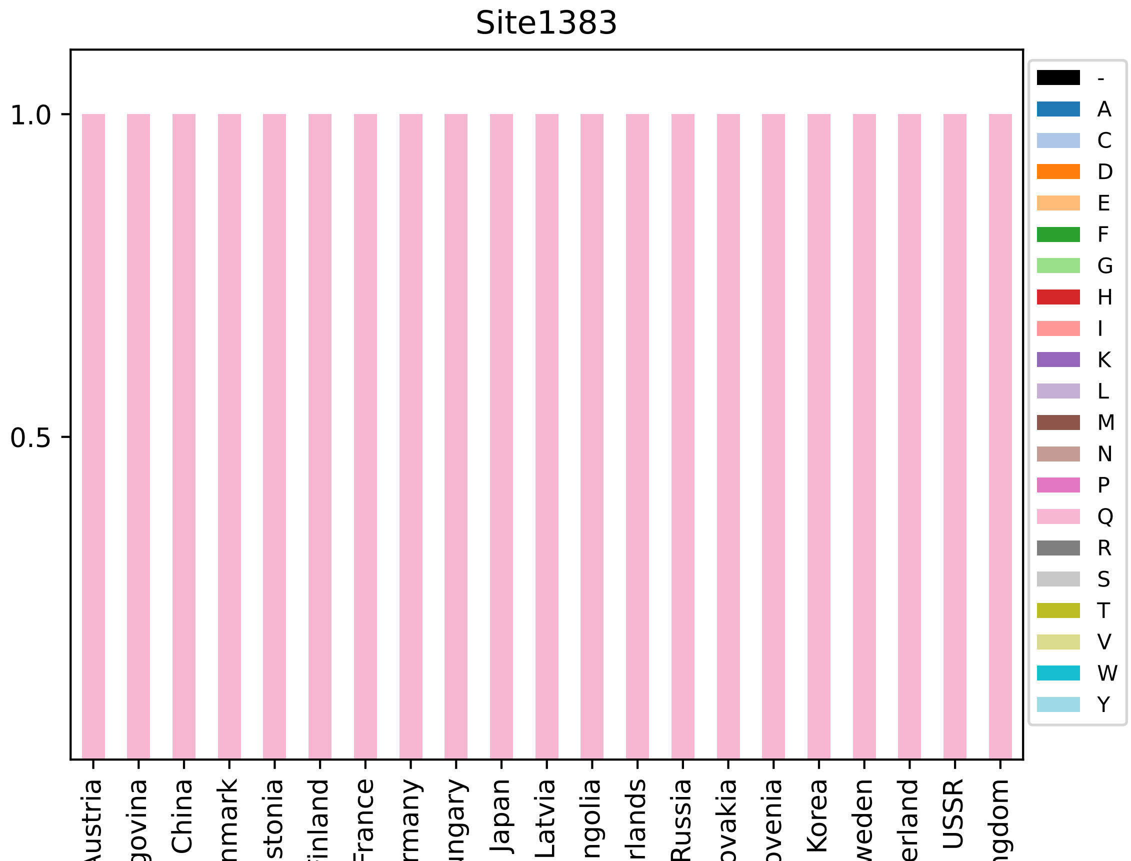

Site1383
1.0
0.5
-
A
C
D
E
F
G
H
I
K
L
M
N
P
Q
R
S
T
V
W
Y
China
Japan
Latvia
Russia
Korea
USSR

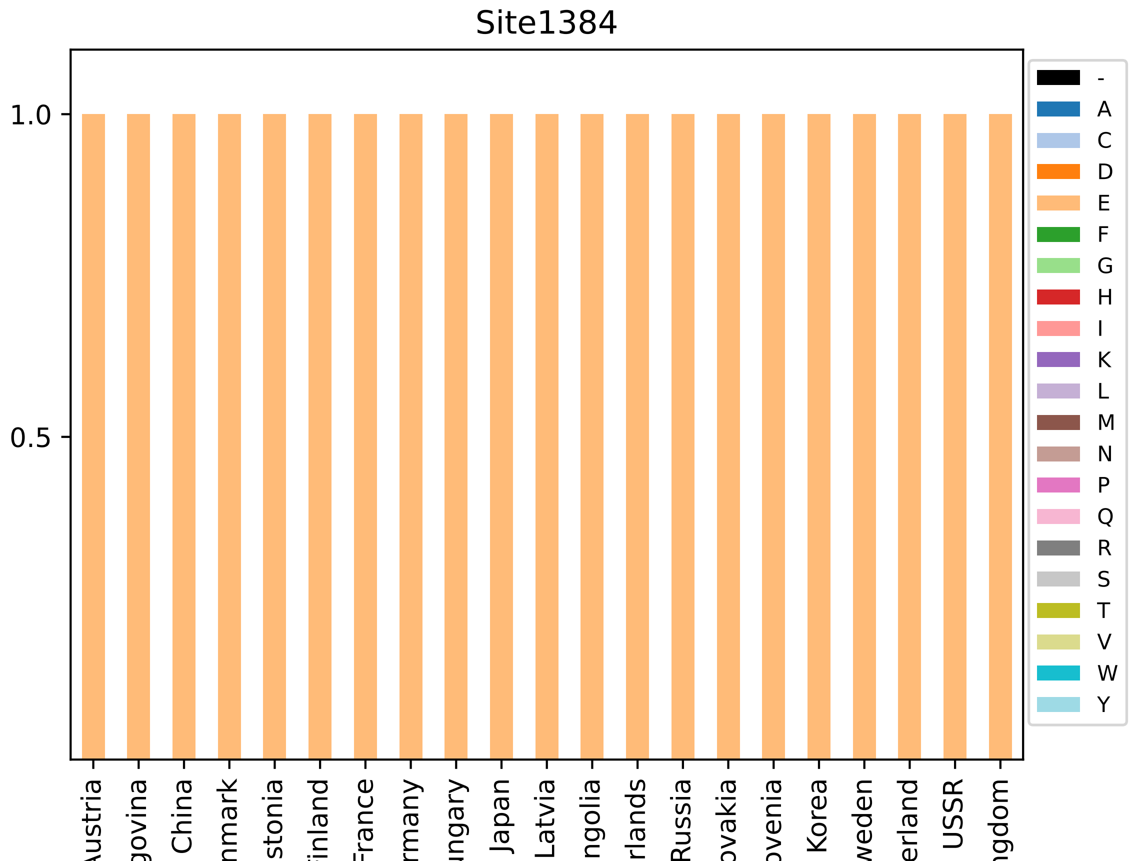

Site1384
1.0
0.5
-
A
C
D
E
F
G
H
I
K
L
M
N
P
Q
R
S
T
V
W
Y
Austria
China
Japan
Latvia
Russia
Korea
USSR

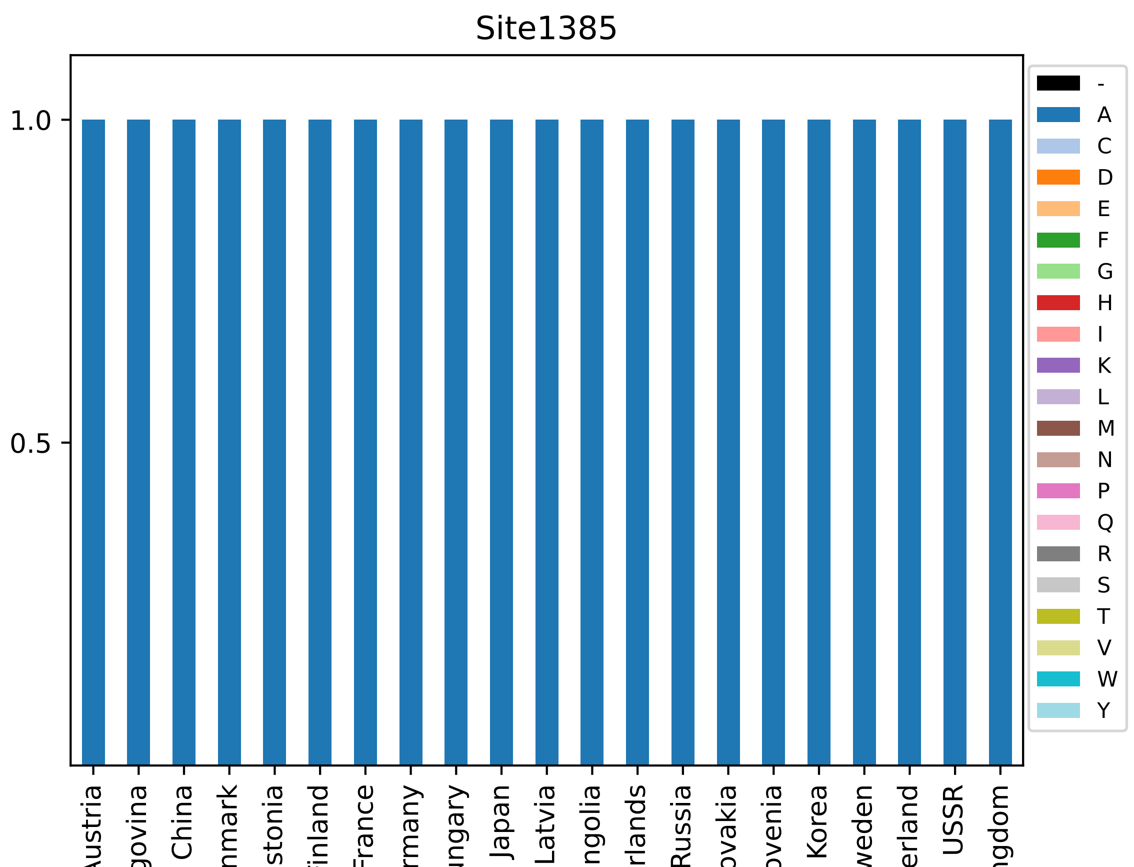
Site1385
1.0
0.5
-
A
C
D
E
F
G
H
I
K
L
M
N
P
Q
R
S
T
V
W
Y
Austria
govina
China
nmark
stonia
Finland
France
rmany
ungary
Japan
Latvia
ngolia
rlands
Russia
ovakia
ovenia
Korea
weden
erland
USSR
ngdom

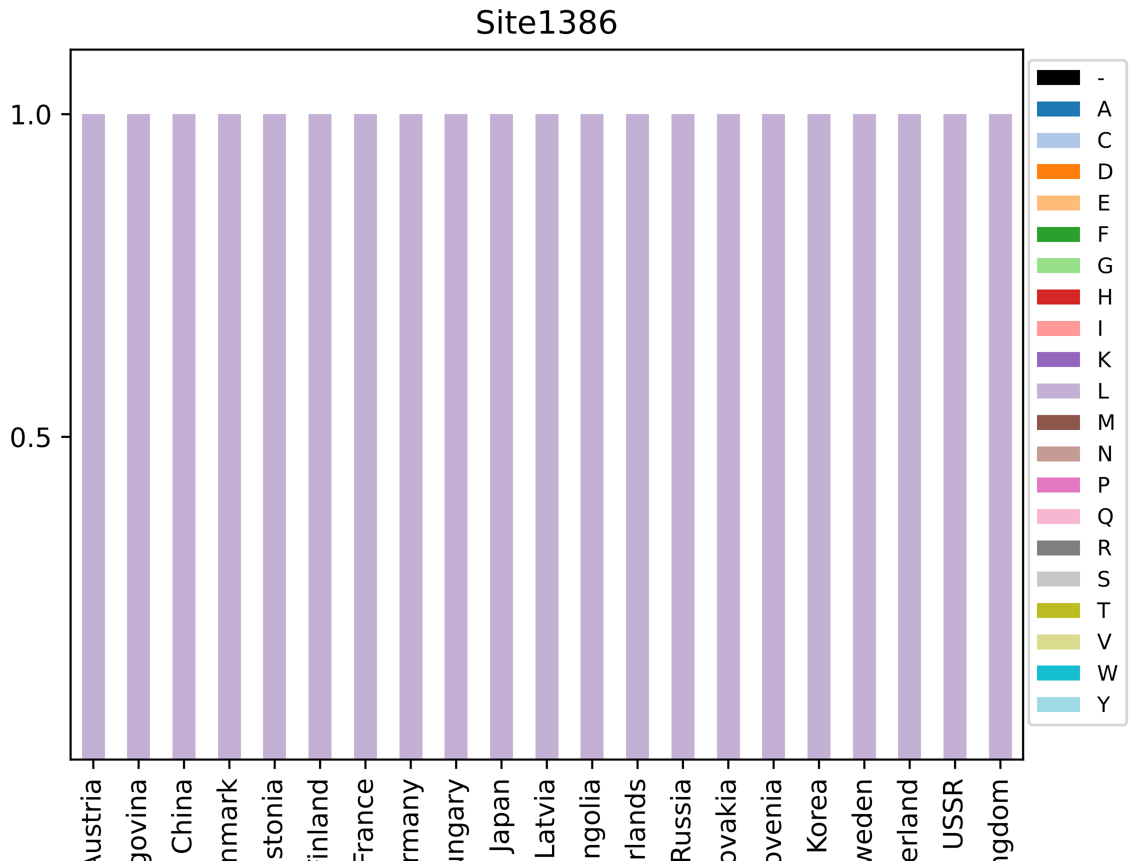

Site1386
1.0
0.5
-
A
C
D
E
F
G
H
I
K
L
M
N
P
Q
R
S
T
V
W
Y
China
Japan
Latvia
Russia
Korea
USSR

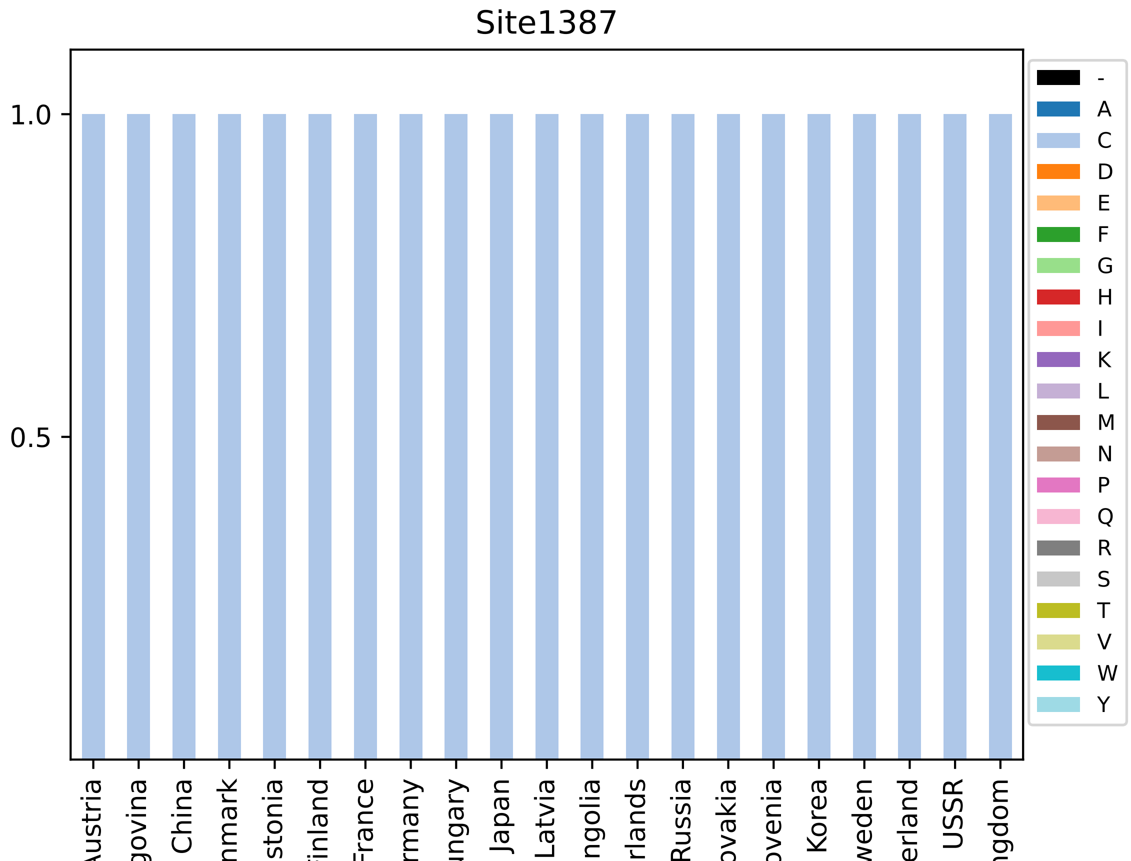

Site1387
1.0
0.5
-
A
C
D
E
F
G
H
I
K
L
M
N
P
Q
R
S
T
V
W
Y
Austria
China
Japan
Latvia
Russia
Korea
USSR

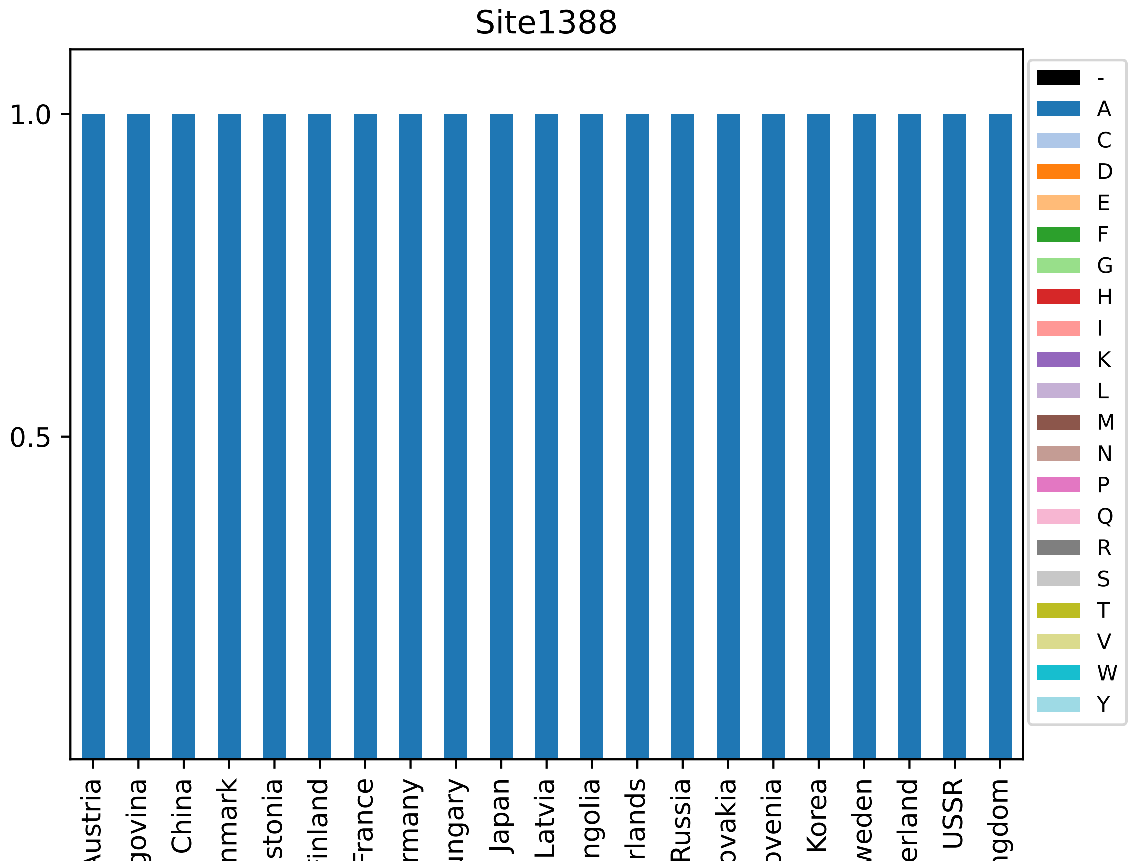

Site1388
1.0
0.5
-
A
C
D
E
F
G
H
I
K
L
M
N
P
Q
R
S
T
V
W
Y
Austria
govina
China
nmark
stonia
Finland
France
rmany
ungary
Japan
Latvia
ngolia
rlands
Russia
ovakia
ovenia
Korea
weden
erland
USSR
ngdom

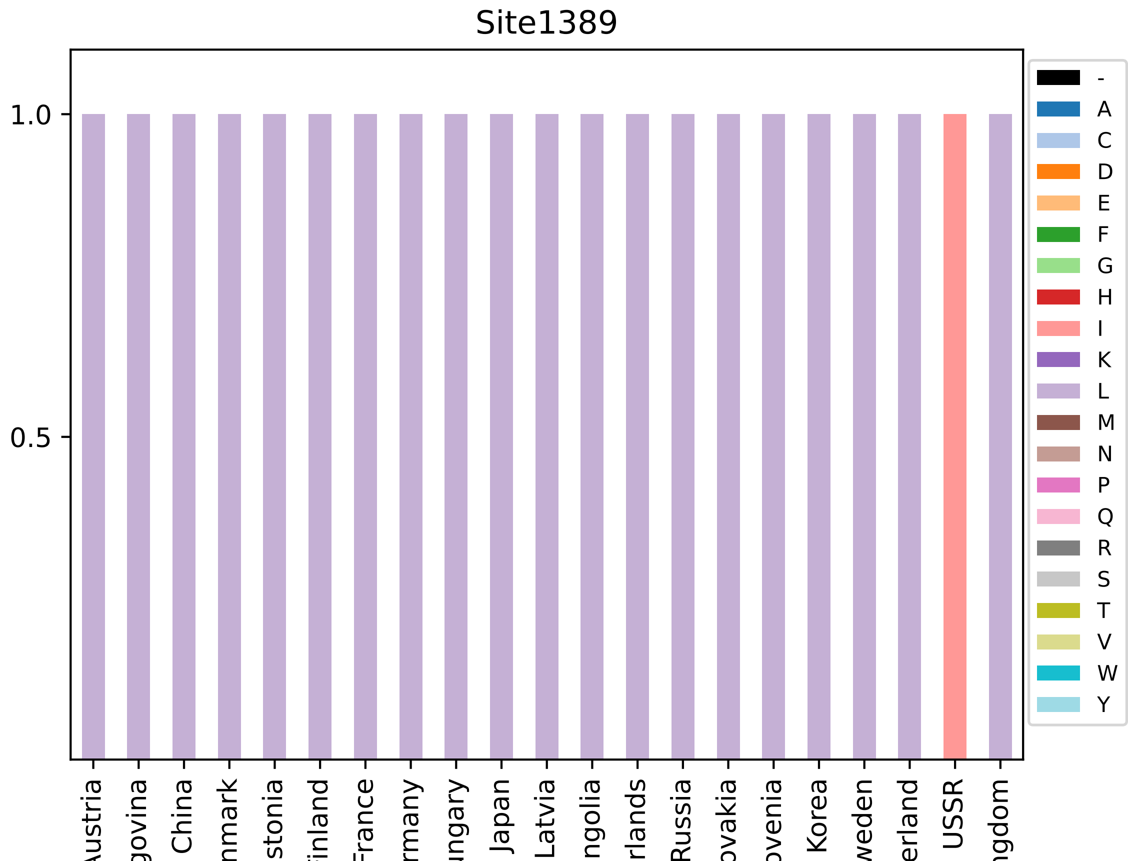
Site1389
1.0
0.5
-
A
C
D
E
F
G
H
I
K
L
M
N
P
Q
R
S
T
V
W
Y
China
Japan
Latvia
Russia
Korea
USSR

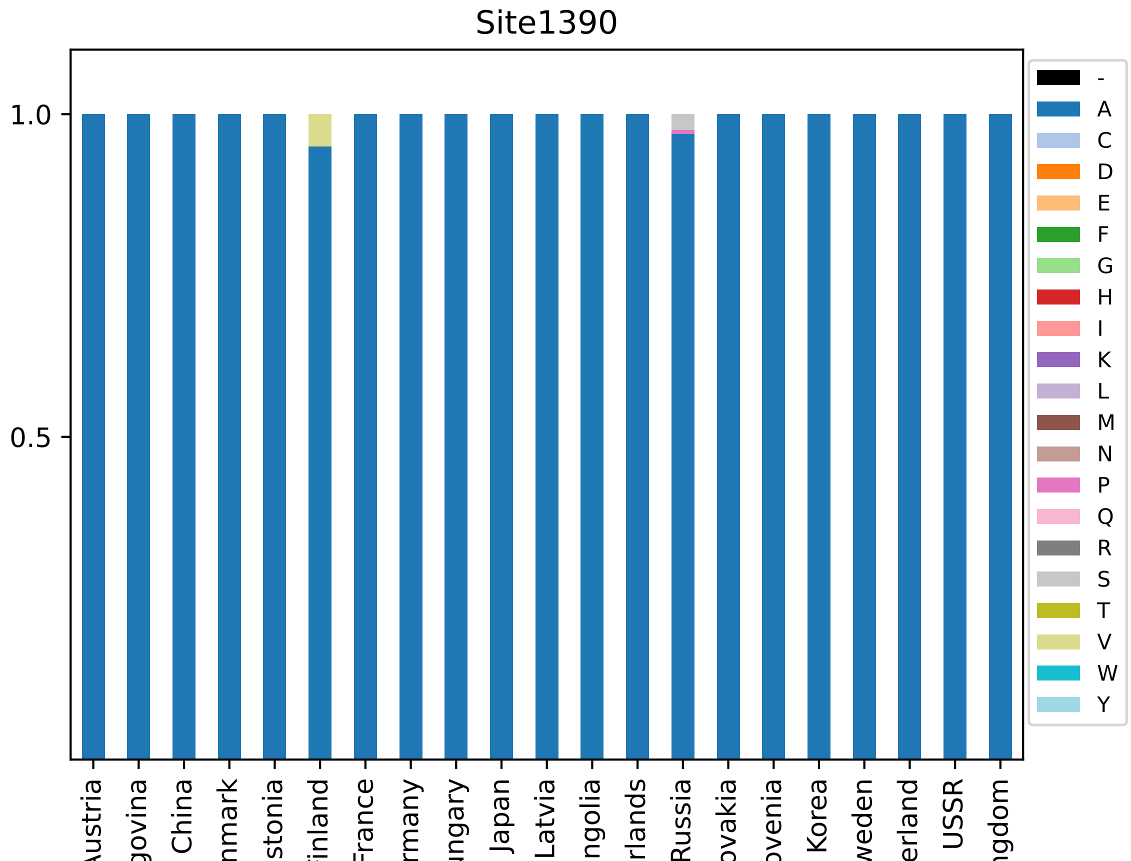

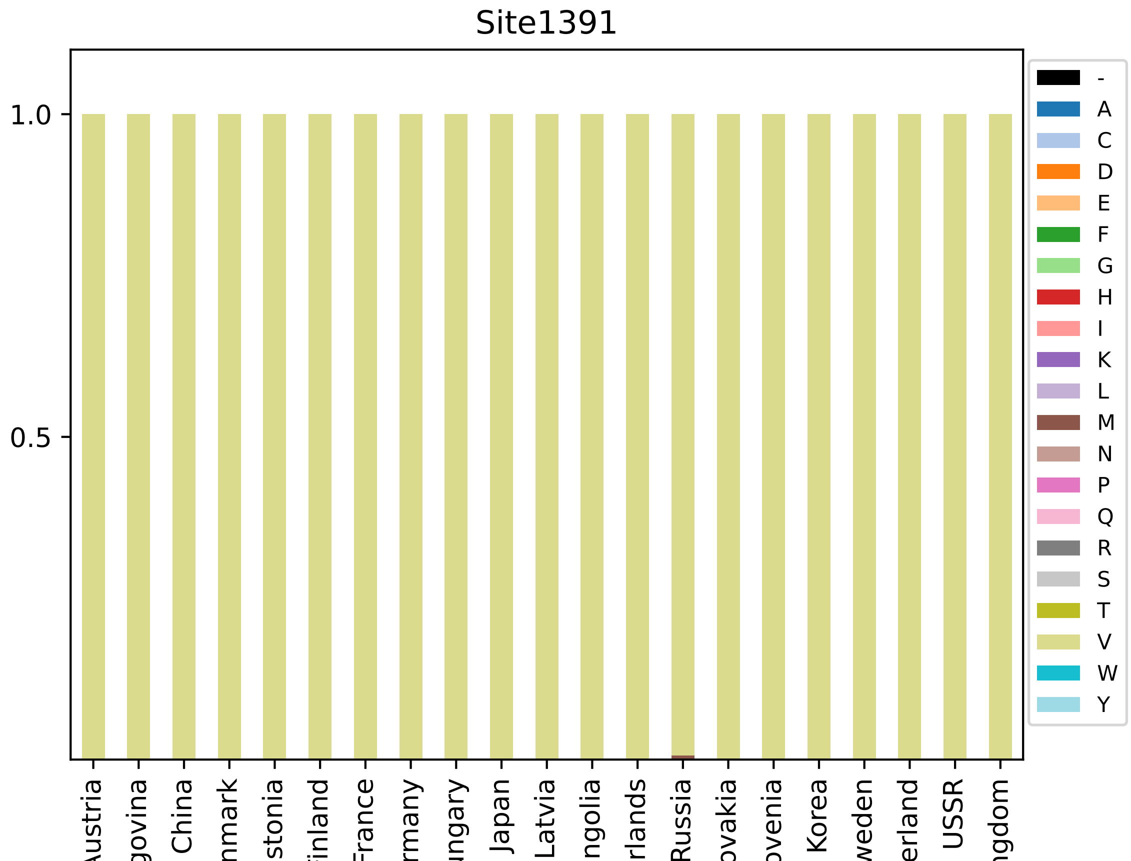

Site1391
1.0
0.5
-
A
C
D
E
F
G
H
I
K
L
M
N
P
Q
R
S
T
V
W
Y
Austria
govina
China
nmark
stonia
inland
France
rmany
ungary
Japan
Latvia
ngolia
rlands
Russia
ovakia
ovenia
Korea
weden
erland
USSR
ngdom

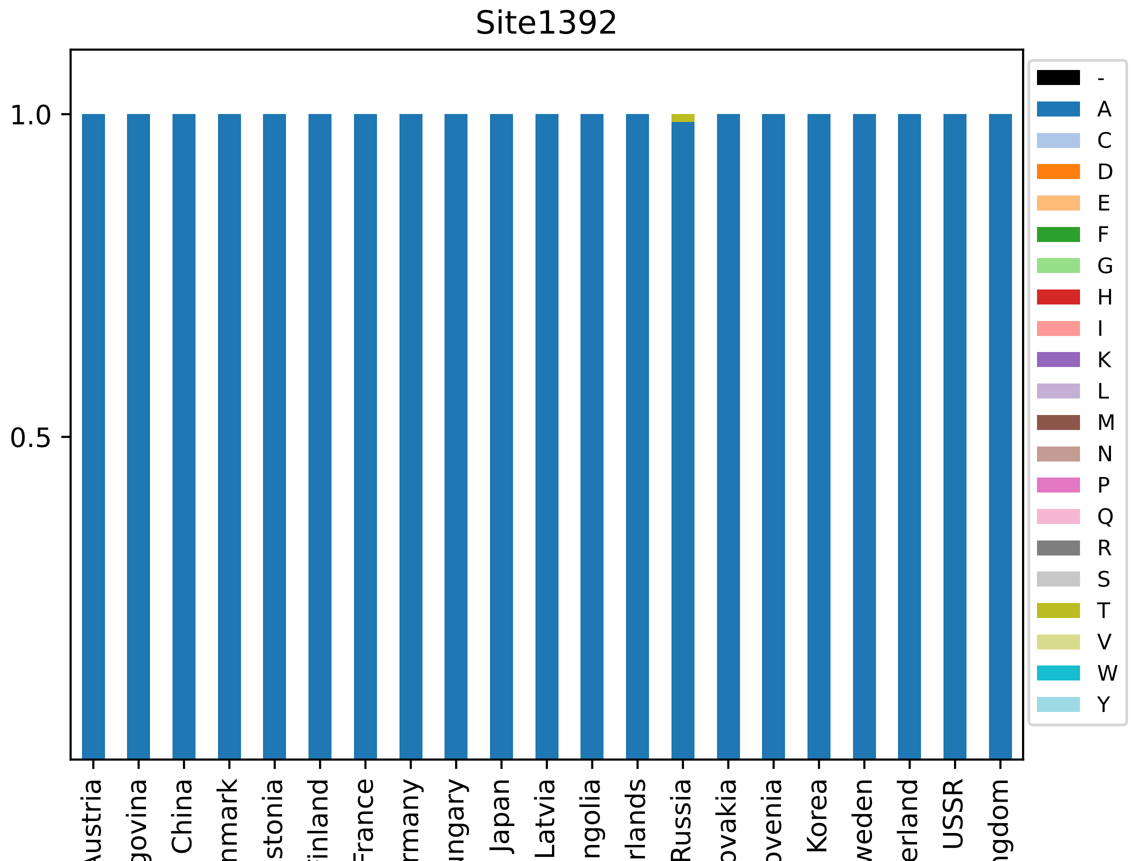

Site1392
1.0
0.5
-
A
C
D
E
F
G
H
I
K
L
M
N
P
Q
R
S
T
V
W
Y
Austria
govina
China
nmark
stonia
inland
France
rmany
ungary
Japan
Latvia
ngolia
rlands
Russia
ovakia
ovenia
Korea
weden
erland
USSR
ngdom

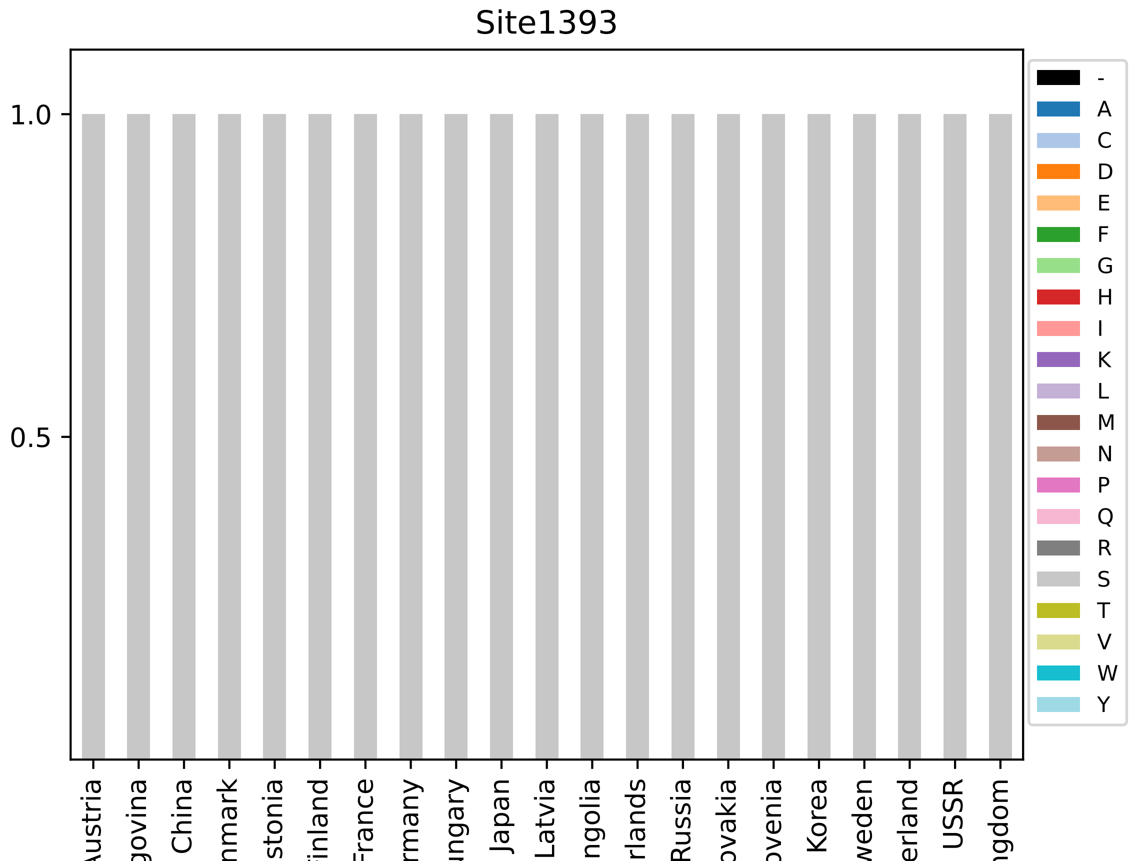
Site1393
1.0
0.5
-
A
C
D
E
F
G
H
I
K
L
M
N
P
Q
R
S
T
V
W
Y
Austria
govina
China
nmark
stonia
inland
France
rmany
ungary
Japan
Latvia
ngolia
rlands
Russia
ovakia
ovenia
Korea
weden
erland
USSR
ngdom

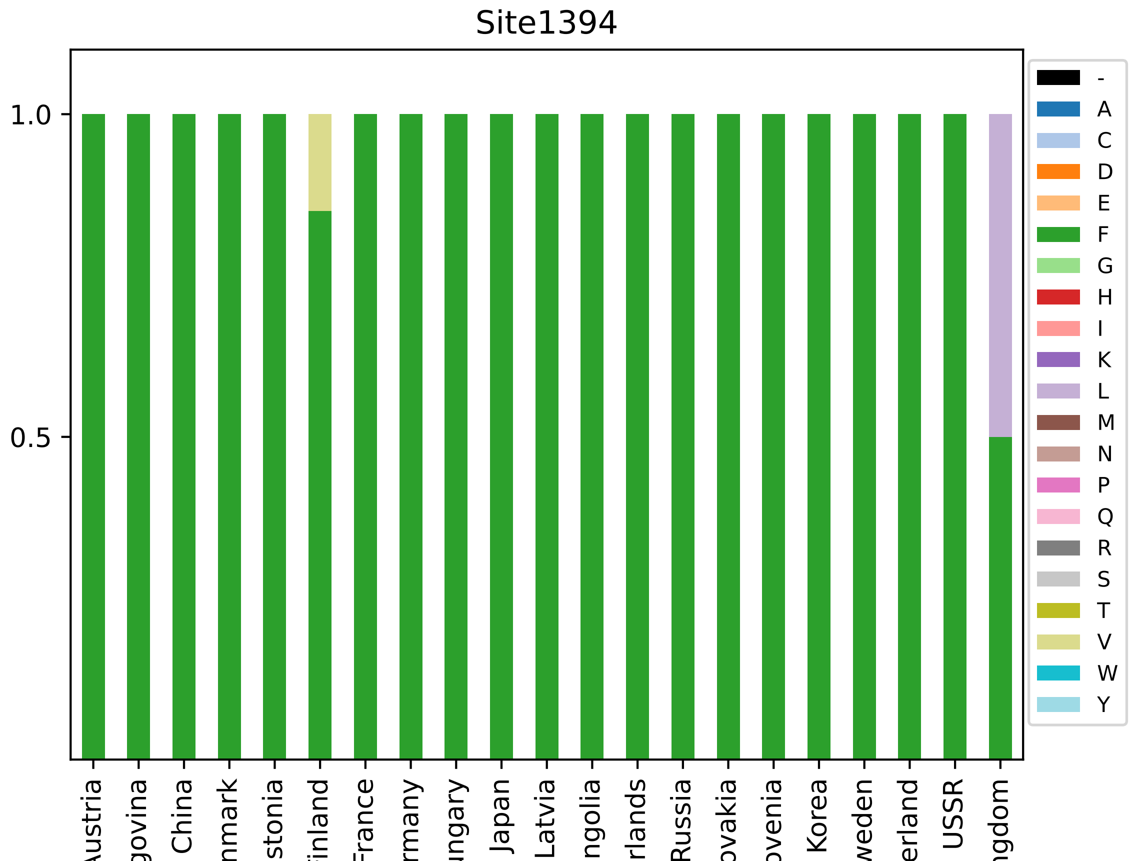

Site1394
1.0
0.5
-
A
C
D
E
F
G
H
I
K
L
M
N
P
Q
R
S
T
V
W
Y
Austria
govina
China
nmark
stonia
Finland
France
rmany
ungary
Japan
Latvia
ngolia
rlands
Russia
ovakia
ovenia
Korea
weden
erland
USSR
ngdom

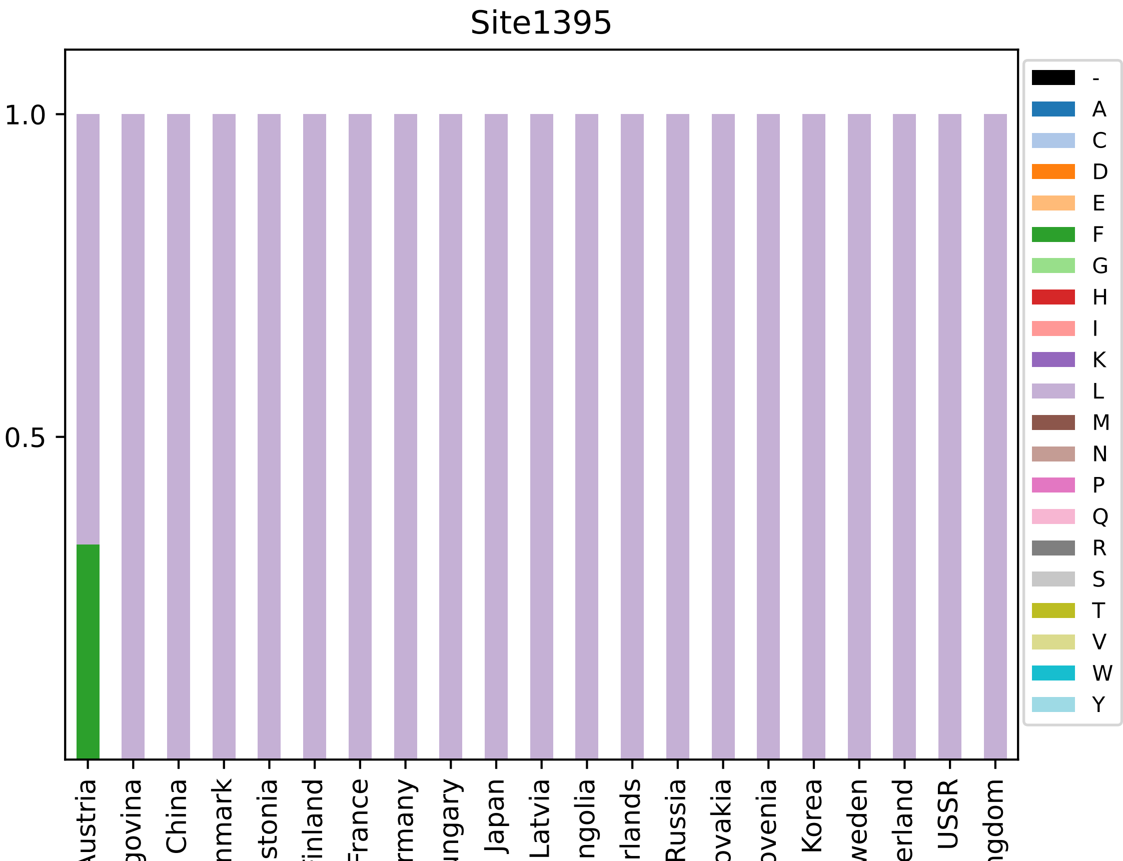

Site1395
1.0
0.5
Austria
govina
China
nmark
stonia
inland
France
rmany
ungary
Japan
Latvia
ngolia
rlands
Russia
ovakia
ovenia
Korea
weden
erland
USSR
ngdom
-
A
C
D
E
F
G
H
I
K
L
M
N
P
Q
R
S
T
V
W
Y

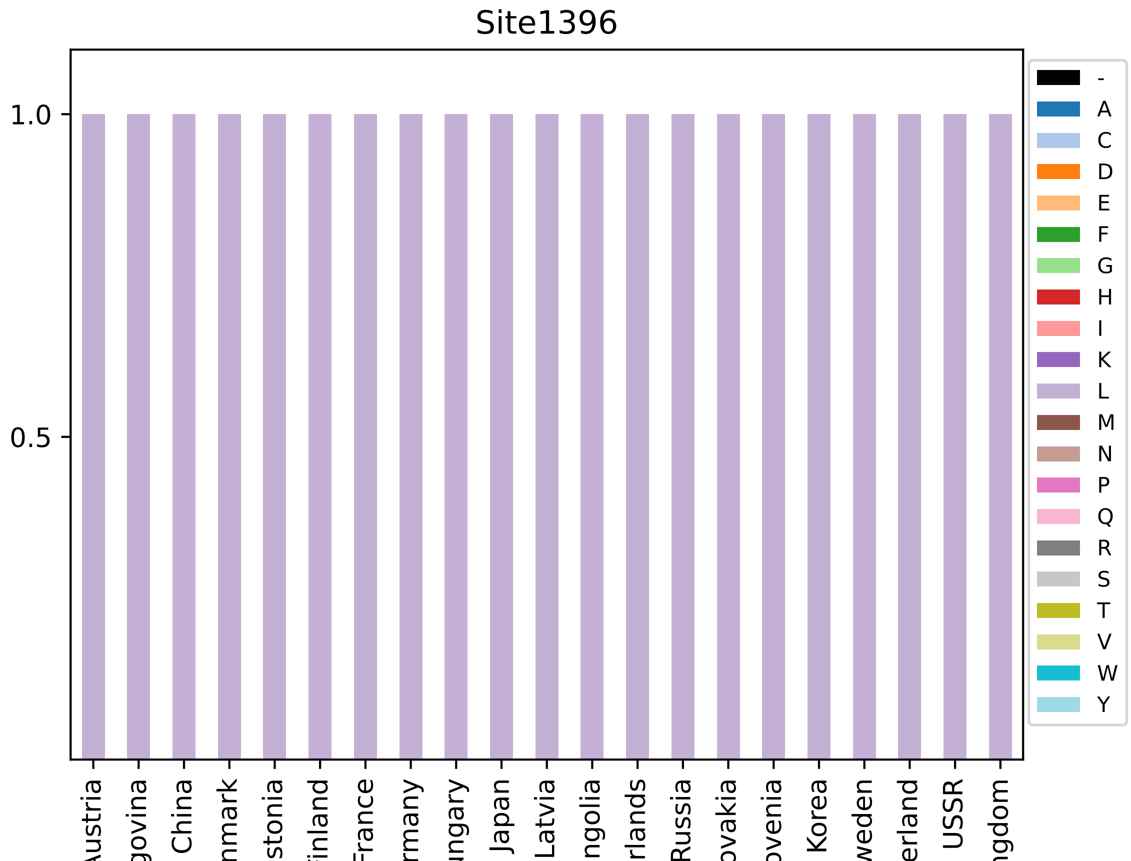

Site1396
1.0
0.5
-
A
C
D
E
F
G
H
I
K
L
M
N
P
Q
R
S
T
V
W
Y
Austria
China
Japan
Latvia
Russia
Korea
USSR

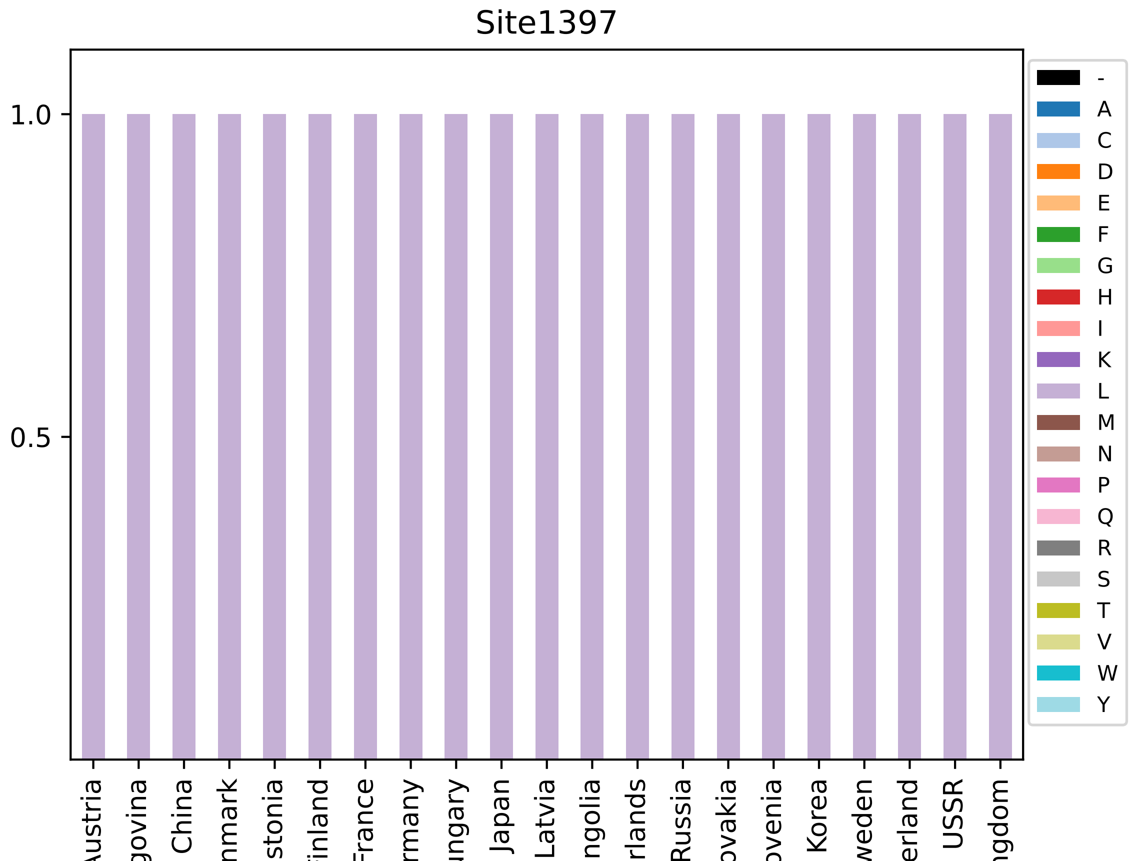
Site1397
1.0
0.5
-
A
C
D
E
F
G
H
I
K
L
M
N
P
Q
R
S
T
V
W
Y
Japan
Latvia
Russia
Korea
USSR
China

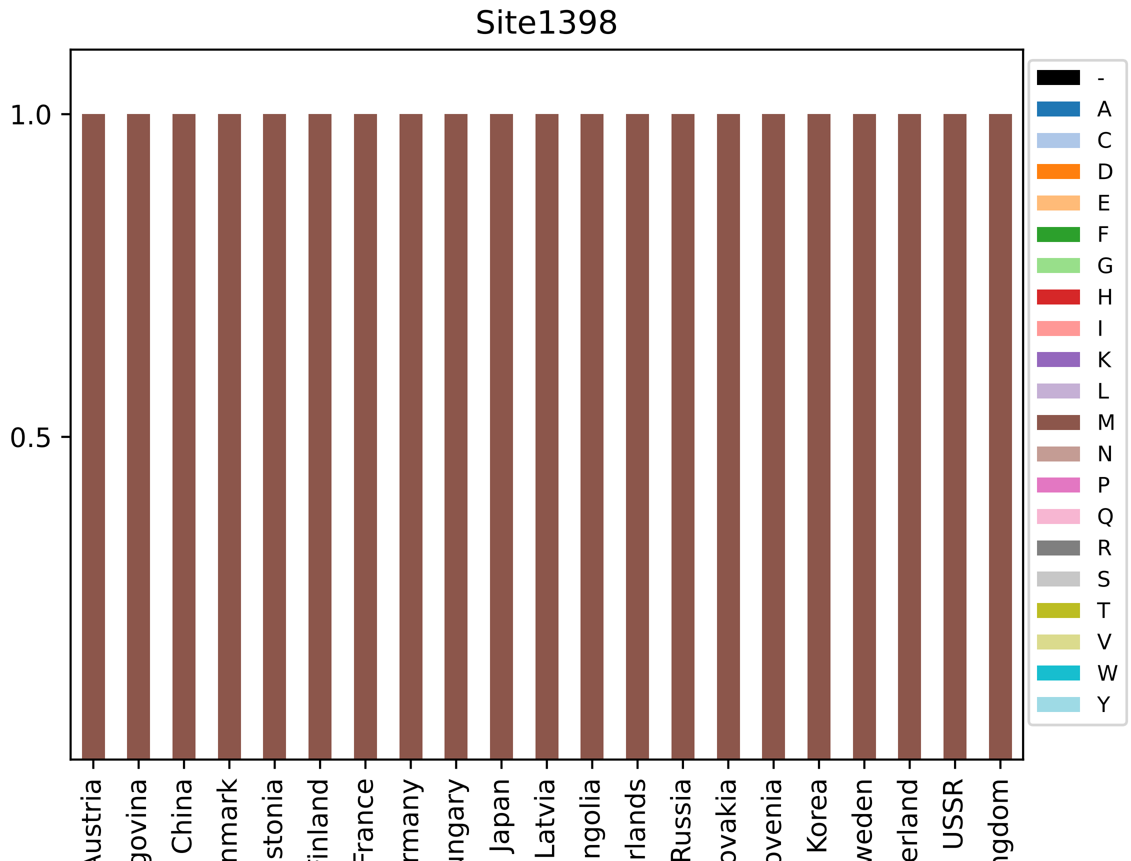
Site1398
1.0
0.5
-
A
C
D
E
F
G
H
I
K
L
M
N
P
Q
R
S
T
V
W
Y
Austria
govina
China
nmark
stonia
Finland
France
rmany
ungary
Japan
Latvia
ngolia
rlands
Russia
ovakia
ovenia
Korea
weden
erland
USSR
ngdom

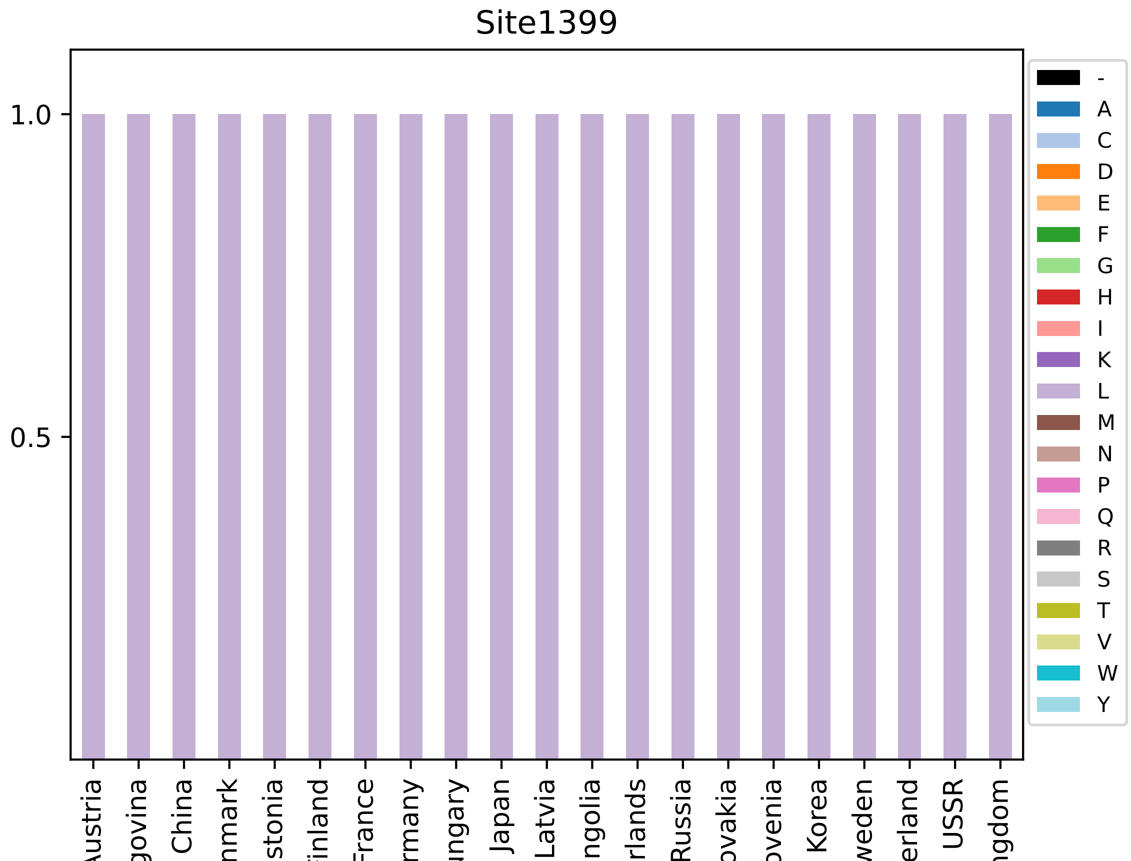
Site1399
1.0
0.5
Austria
govina
China
nmark
stonia
Finland
France
rmany
ungary
Japan
Latvia
ngolia
rlands
Russia
ovakia
ovenia
Korea
weden
erland
USSR
ngdom
-
A
C
D
E
F
G
H
I
K
L
M
N
P
Q
R
S
T
V
W
Y

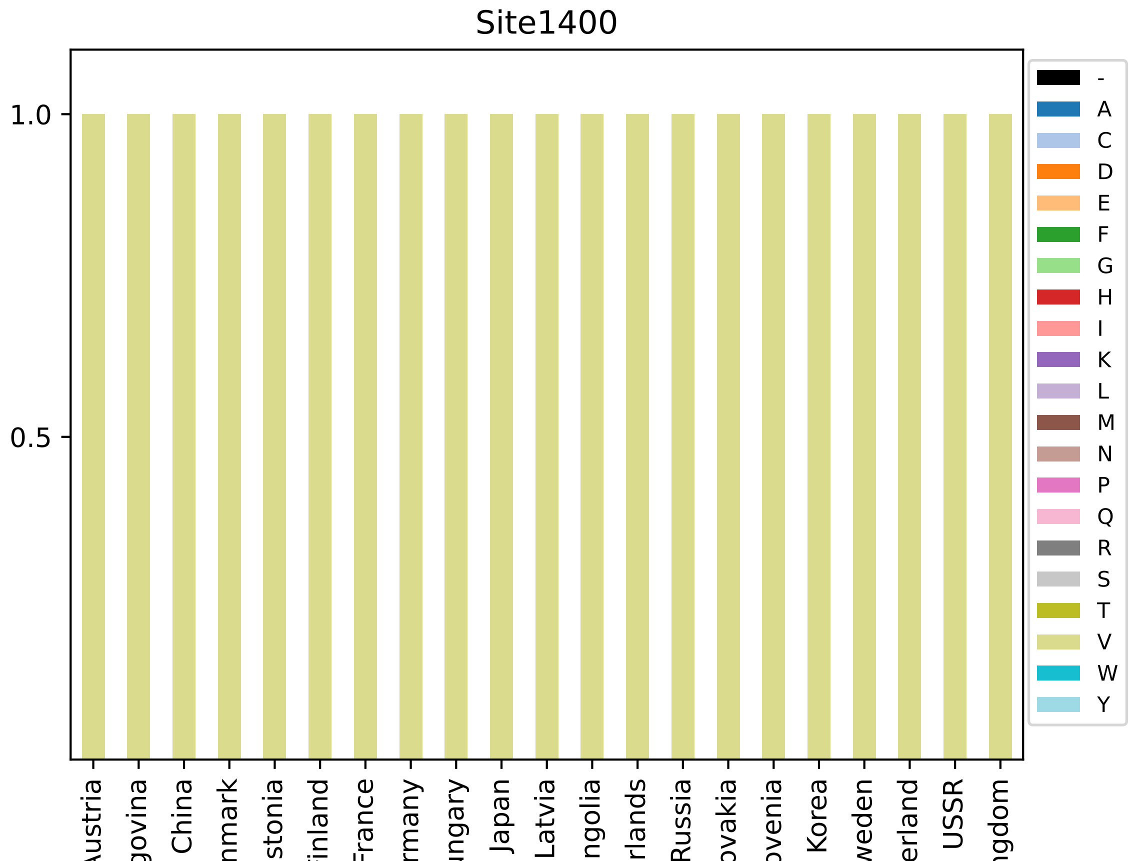
Site1400
1.0
0.5
-
A
C
D
E
F
G
H
I
K
L
M
N
P
Q
R
S
T
V
W
Y
Austria
govina
China
nmark
stonia
inland
France
rmany
ungary
Japan
Latvia
ngolia
rlands
Russia
ovakia
ovenia
Korea
weden
erland
USSR
ngdom

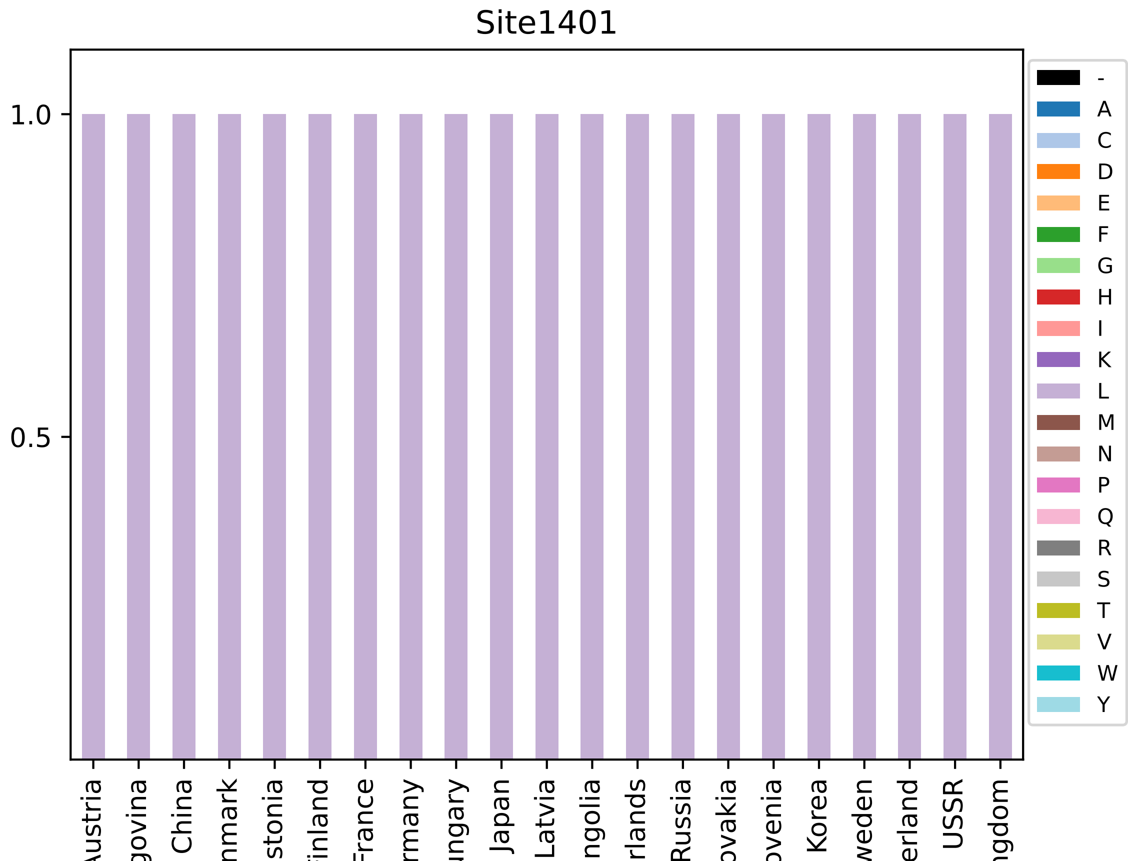

Site1401
1.0
0.5
China
Japan
Latvia
Russia
Korea
USSR
-
A
C
D
E
F
G
H
I
K
L
M
N
P
Q
R
S
T
V
W
Y

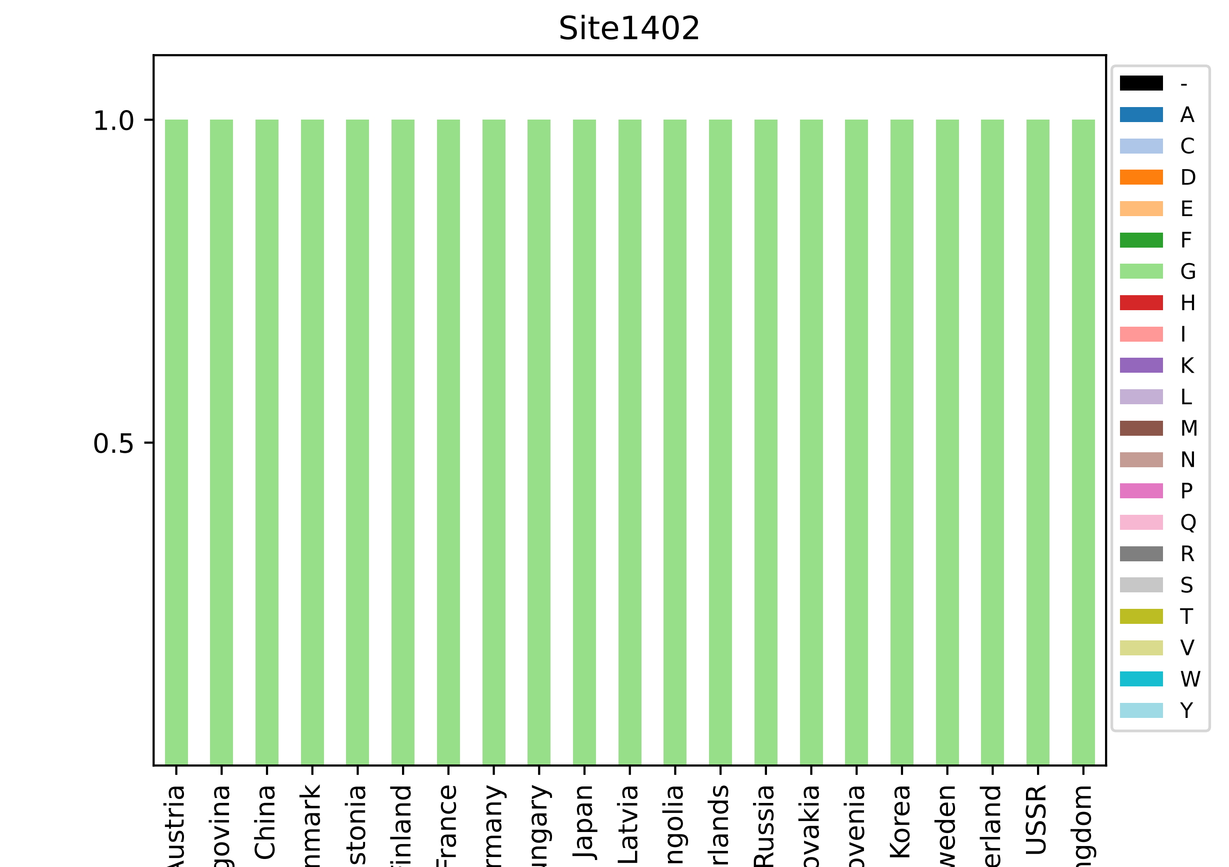

Site1402
1.0
0.5
-
A
C
D
E
F
G
H
I
K
L
M
N
P
Q
R
S
T
V
W
Y
Austria
govina
China
nmark
stonia
inland
France
rmany
ungary
Japan
Latvia
ngolia
rlands
Russia
ovakia
ovenia
Korea
weden
erland
USSR
ngdom

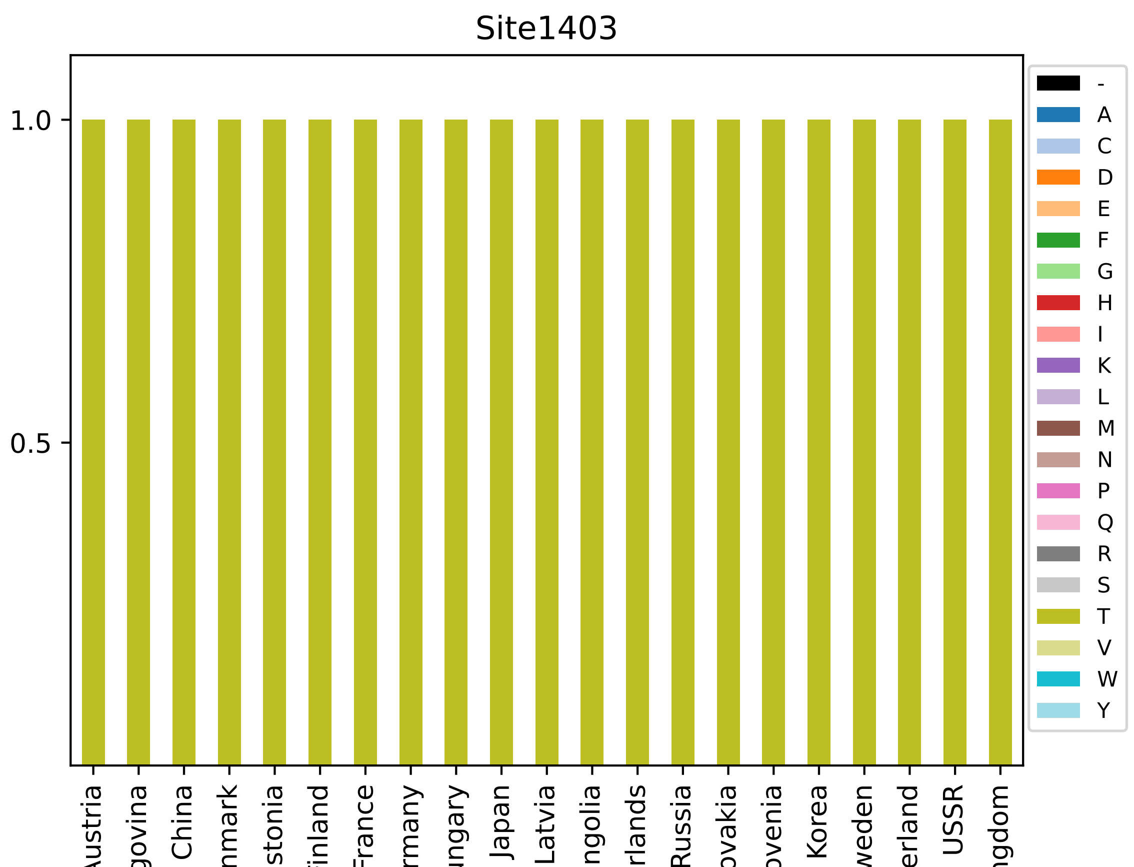

Site1403
1.0
0.5
Austria
govina
China
nmark
stonia
inland
France
rmany
ungary
Japan
Latvia
ngolia
rlands
Russia
ovakia
ovenia
Korea
weden
erland
USSR
ngdom
-
A
C
D
E
F
G
H
I
K
L
M
N
P
Q
R
S
T
V
W
Y

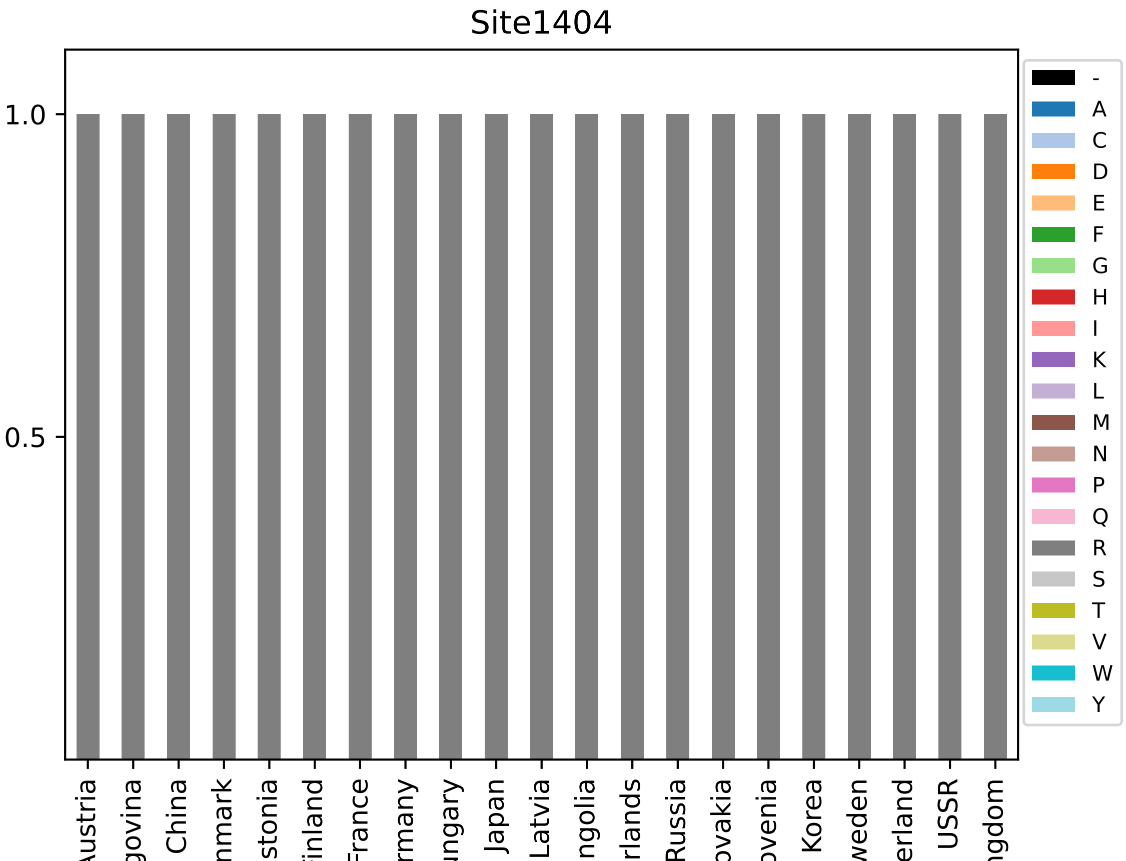

Site1404
1.0
0.5
-
A
C
D
E
F
G
H
I
K
L
M
N
P
Q
R
S
T
V
W
Y
Austria
China
Japan
Latvia
Russia
Korea
USSR

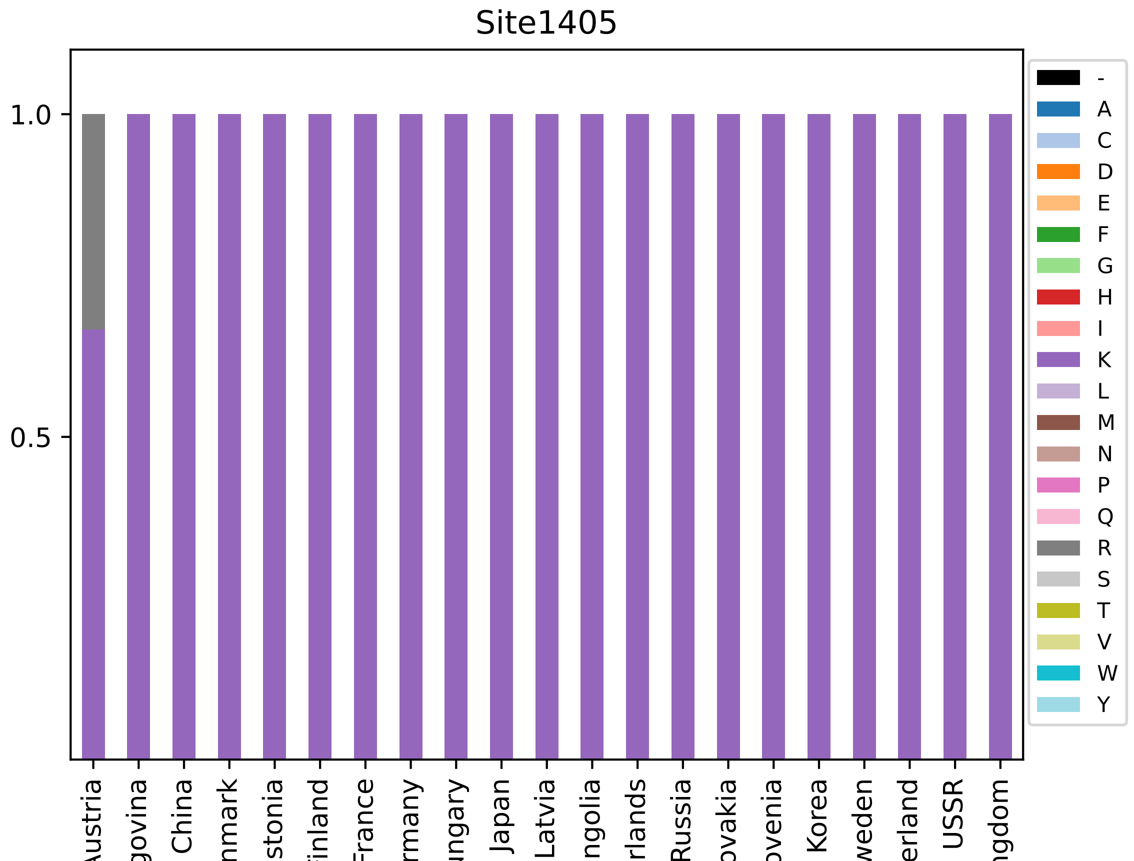
Site1405
1.0
0.5
-
A
C
D
E
F
G
H
I
K
L
M
N
P
Q
R
S
T
V
W
Y
China
Japan
Latvia
Russia
Korea
USSR

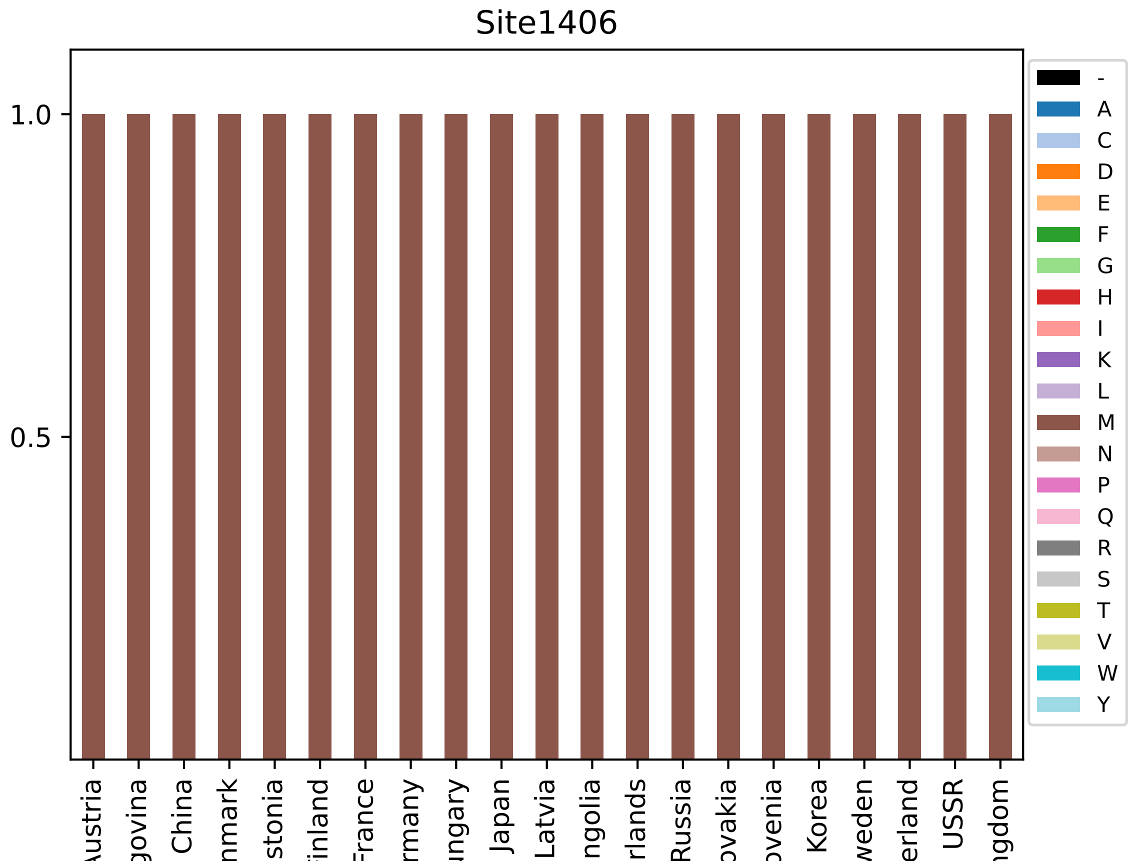

Site1406
1.0
0.5
-
A
C
D
E
F
G
H
I
K
L
M
N
P
Q
R
S
T
V
W
Y
China
Japan
Latvia
Russia
Korea
USSR

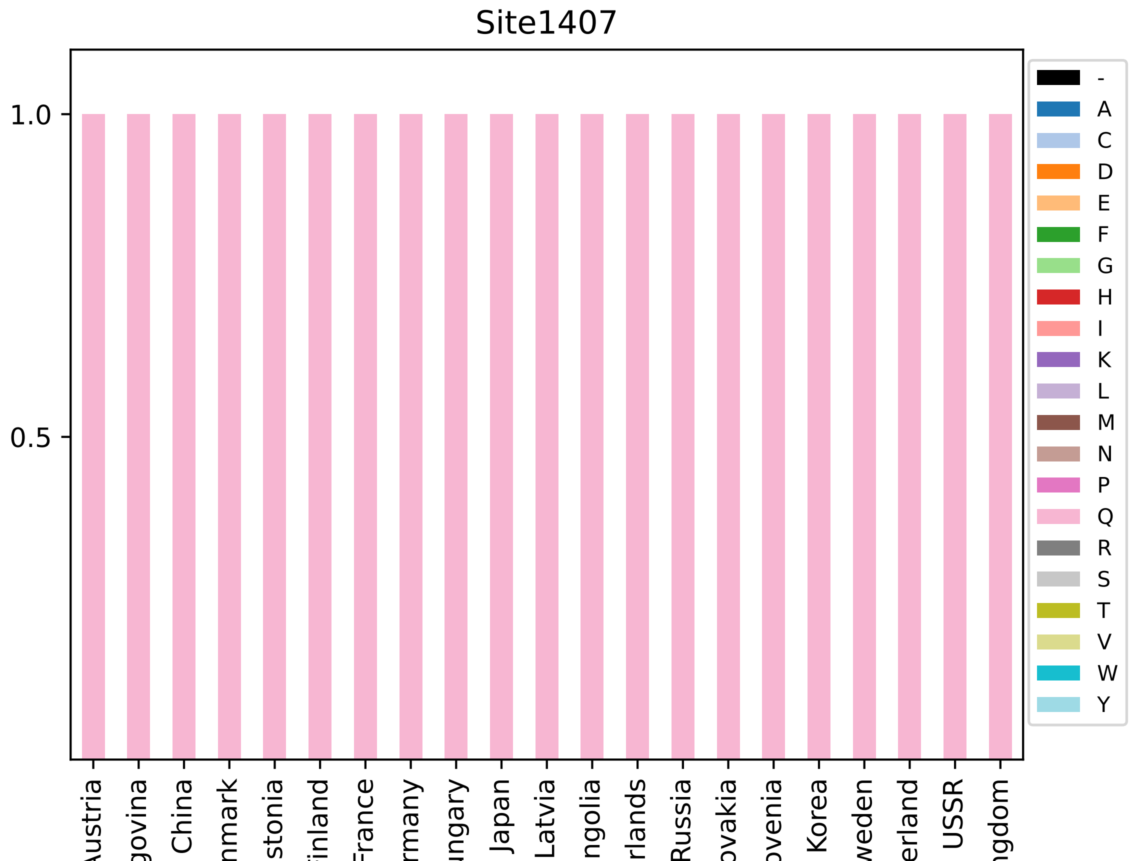
Site1407
1.0
0.5
-
A
C
D
E
F
G
H
I
K
L
M
N
P
Q
R
S
T
V
W
Y
China
Japan
Latvia
Russia
Korea
USSR

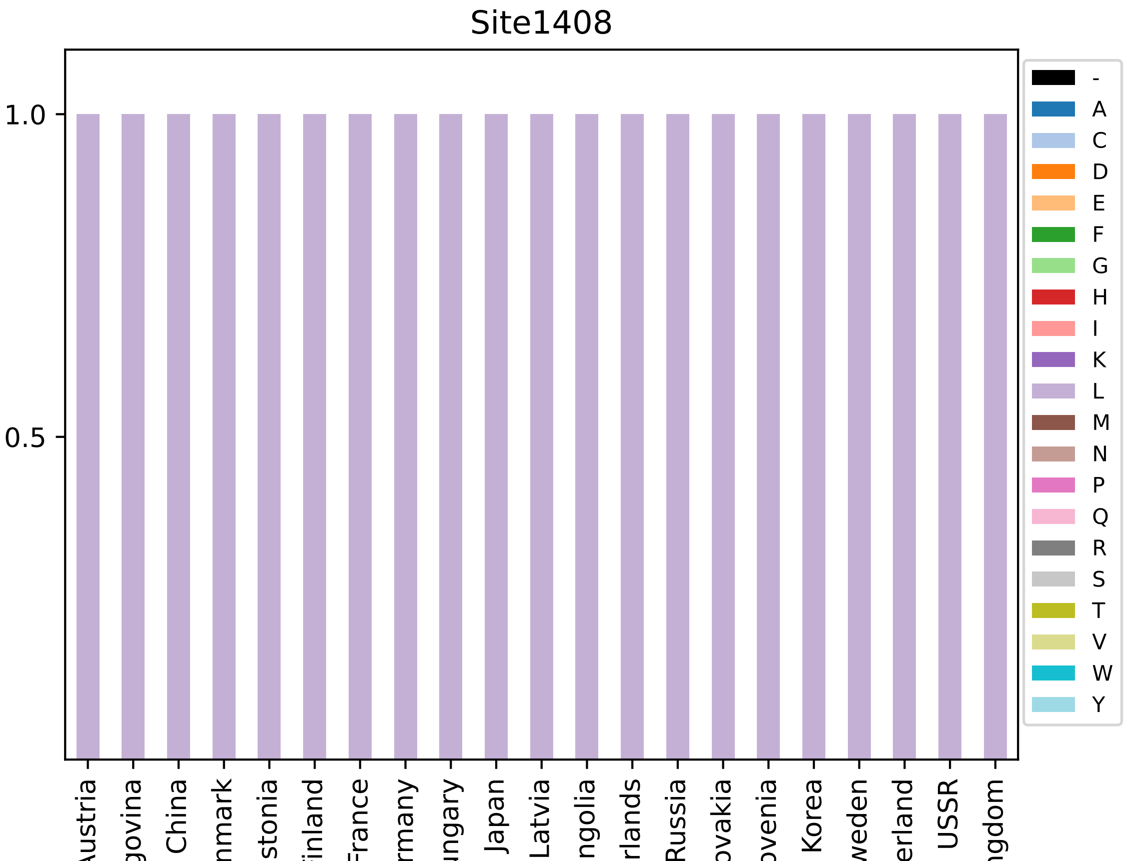
Site1408
1.0
0.5
China
Japan
Latvia
Russia
Korea
USSR
-
A
C
D
E
F
G
H
I
K
L
M
N
P
Q
R
S
T
V
W
Y

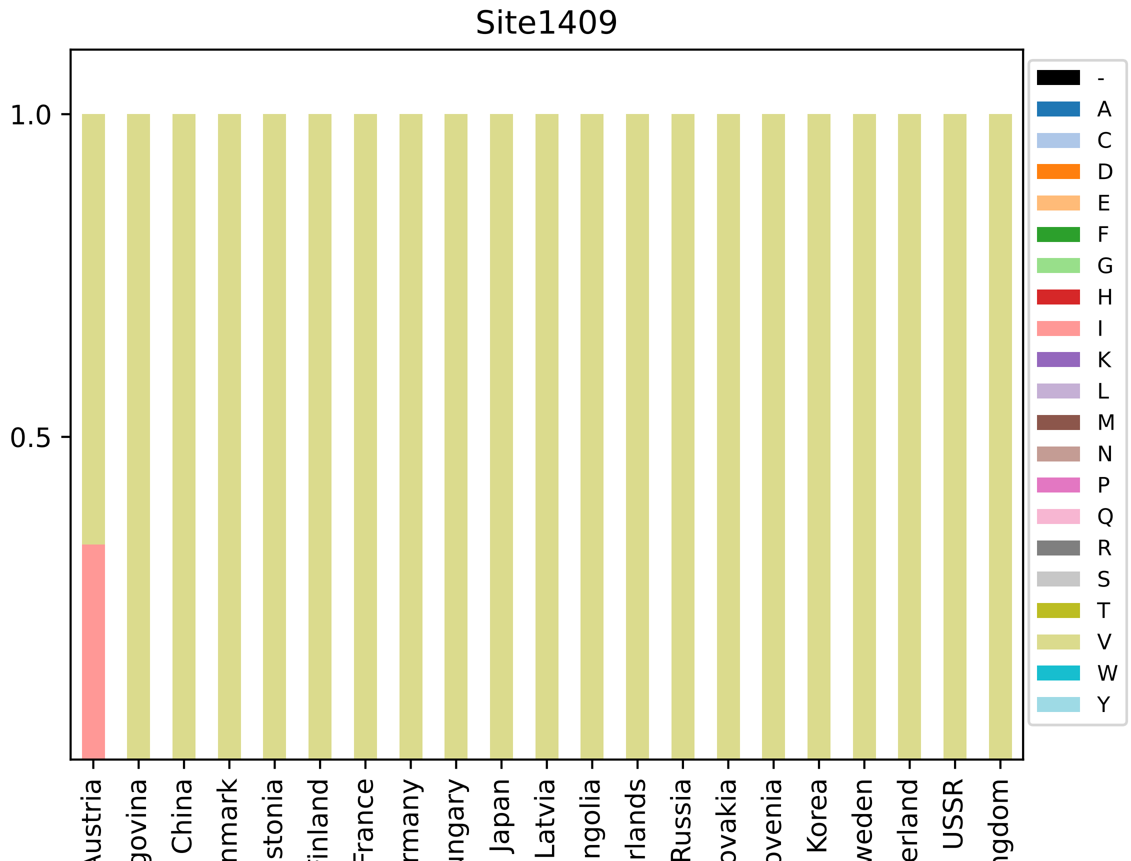

Site1409
1.0
0.5
-
A
C
D
E
F
G
H
I
K
L
M
N
P
Q
R
S
T
V
W
Y
Austria
govina
China
nmark
stonia
Finland
France
rmany
ungary
Japan
Latvia
ngolia
rlands
Russia
ovakia
ovenia
Korea
weden
erland
USSR
ngdom

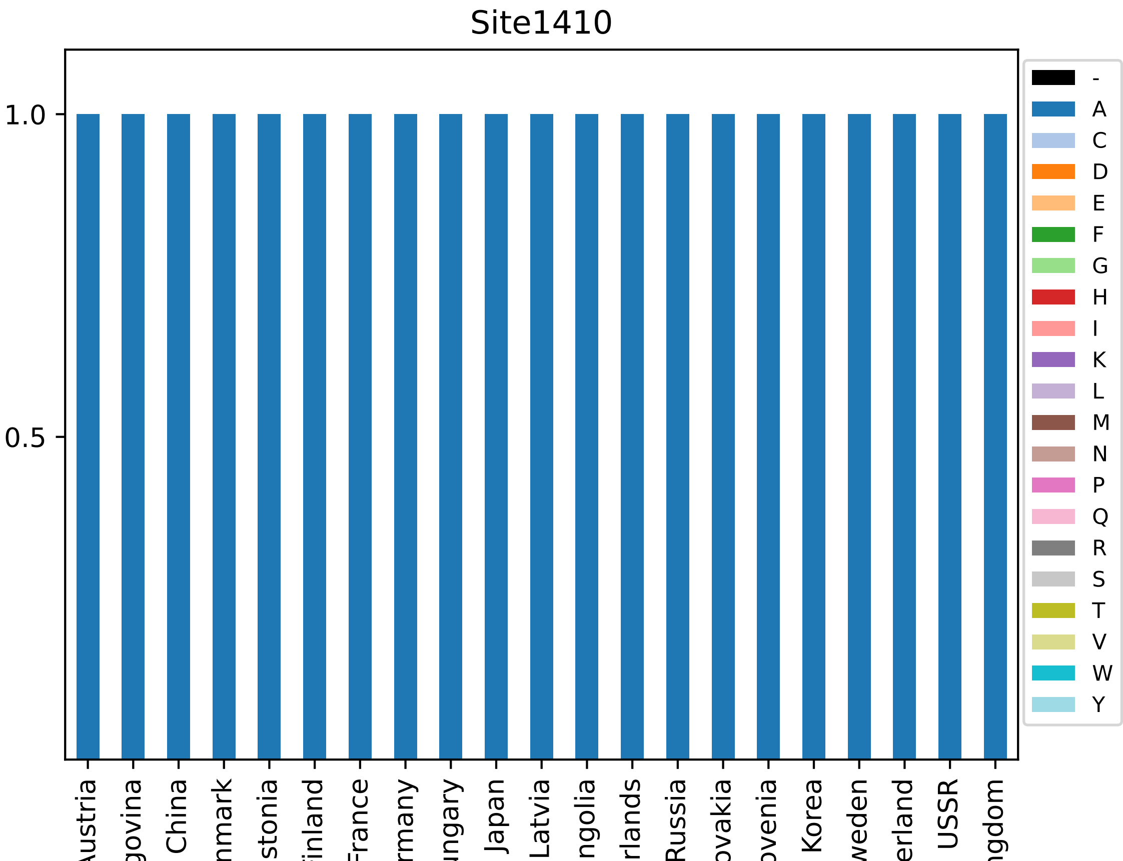

Site1410
1.0
0.5
-
A
C
D
E
F
G
H
I
K
L
M
N
P
Q
R
S
T
V
W
Y
Austria
China
Japan
Latvia
Russia
Korea
USSR

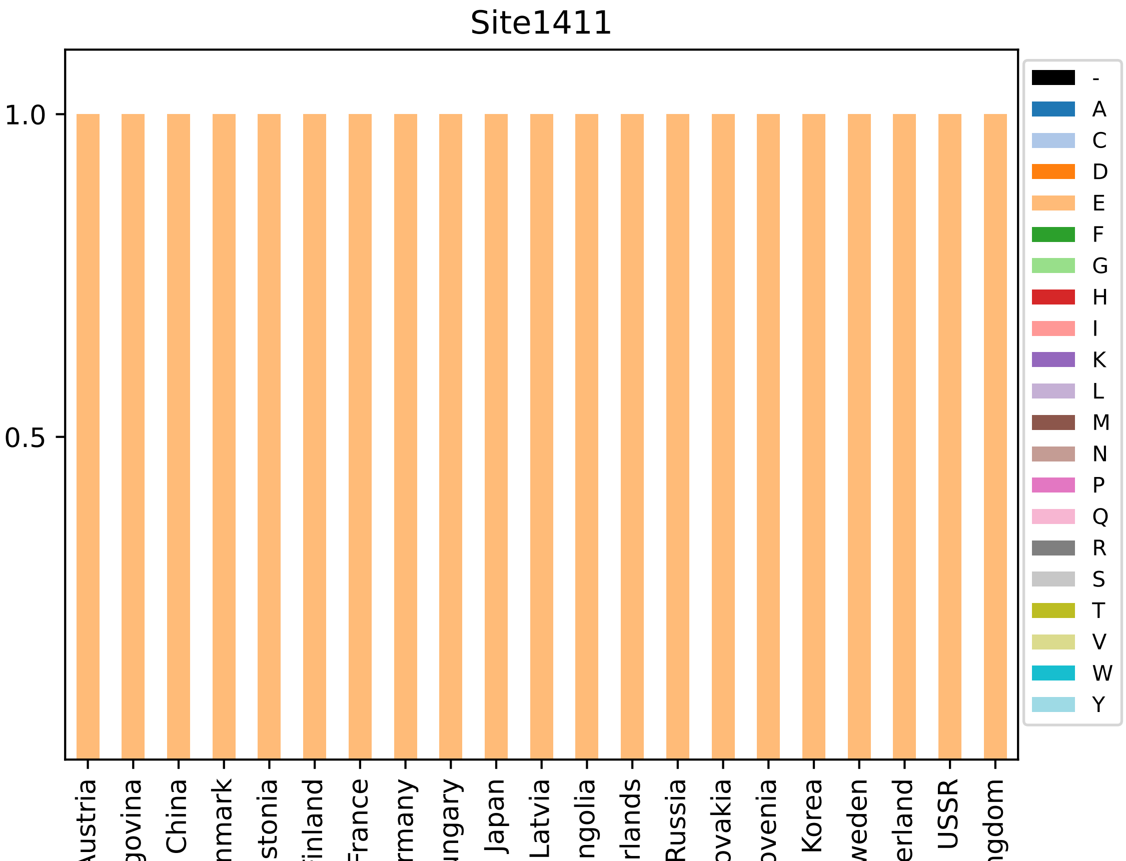

Site1411
1.0
0.5
-
A
C
D
E
F
G
H
I
K
L
M
N
P
Q
R
S
T
V
W
Y
Austria
govina
China
nmark
stonia
Finland
France
rmany
ungary
Japan
Latvia
ngolia
rlands
Russia
ovakia
ovenia
Korea
weden
erland
USSR
ngdom

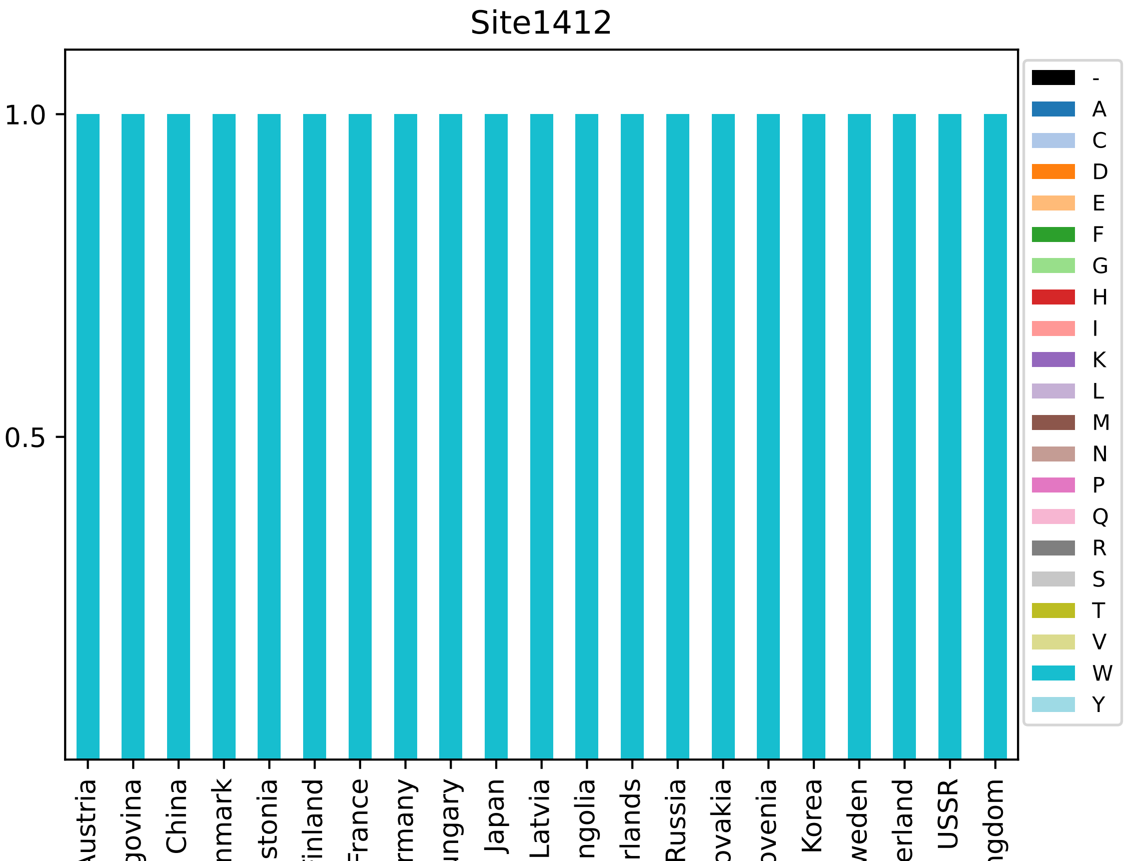

Site1412
1.0
0.5
China
Japan
Latvia
Russia
Korea
USSR
-
A
C
D
E
F
G
H
I
K
L
M
N
P
Q
R
S
T
V
W
Y

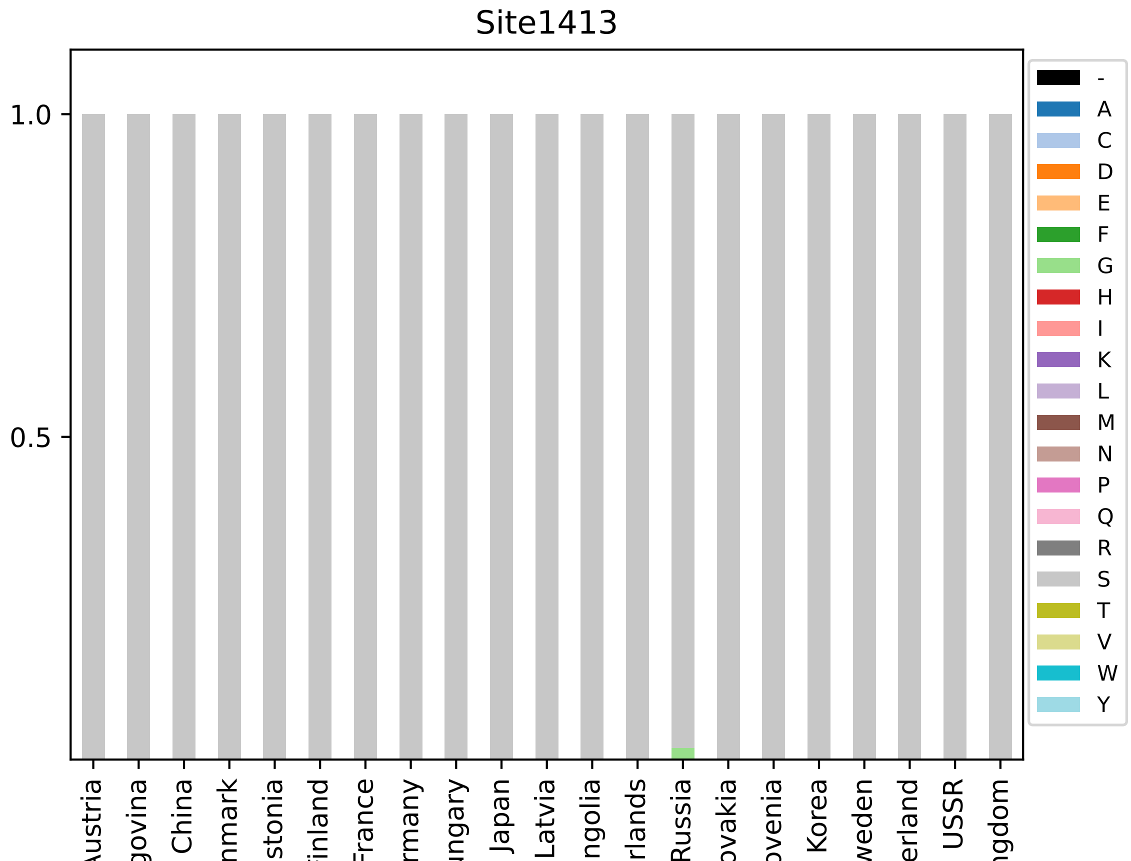
Site1413
1.0
0.5
-
A
C
D
E
F
G
H
I
K
L
M
N
P
Q
R
S
T
V
W
Y
China
Japan
Latvia
Russia
Korea
USSR

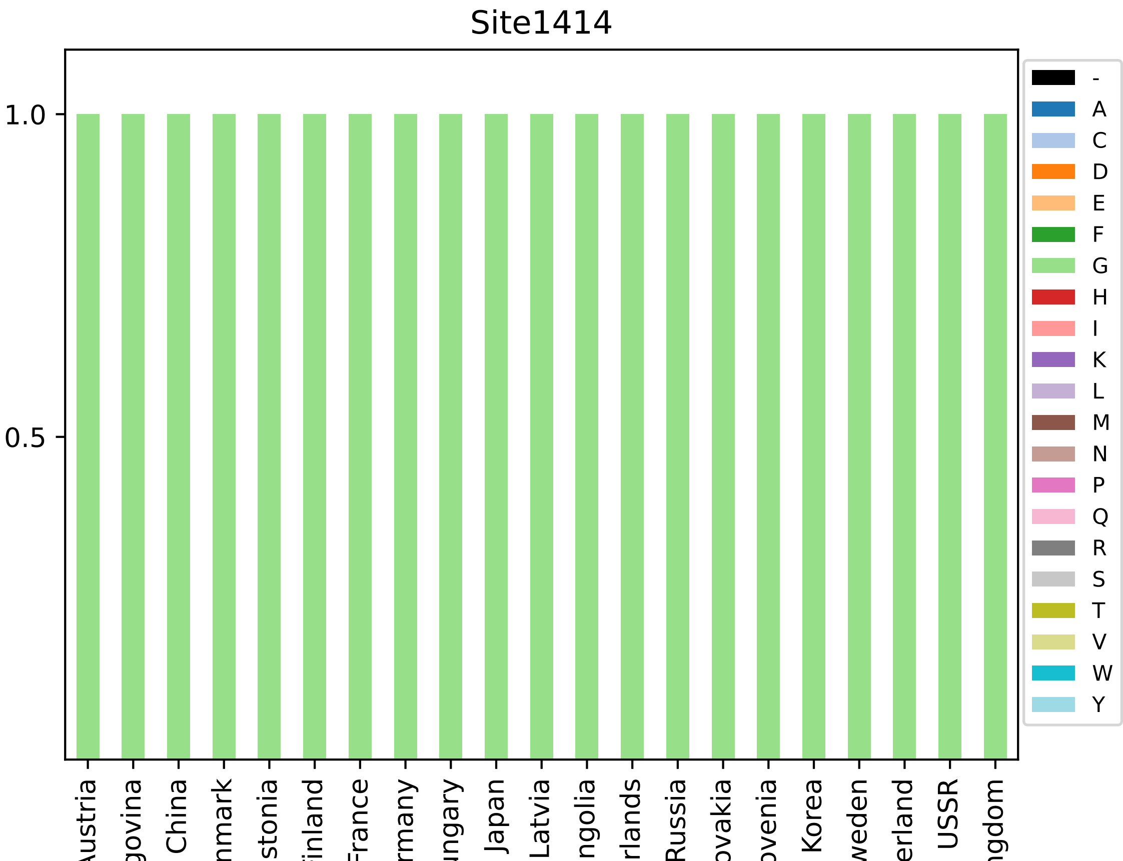

Site1414
1.0
0.5
-
A
C
D
E
F
G
H
I
K
L
M
N
P
Q
R
S
T
V
W
Y
China
Japan
Latvia
Russia
Korea
USSR

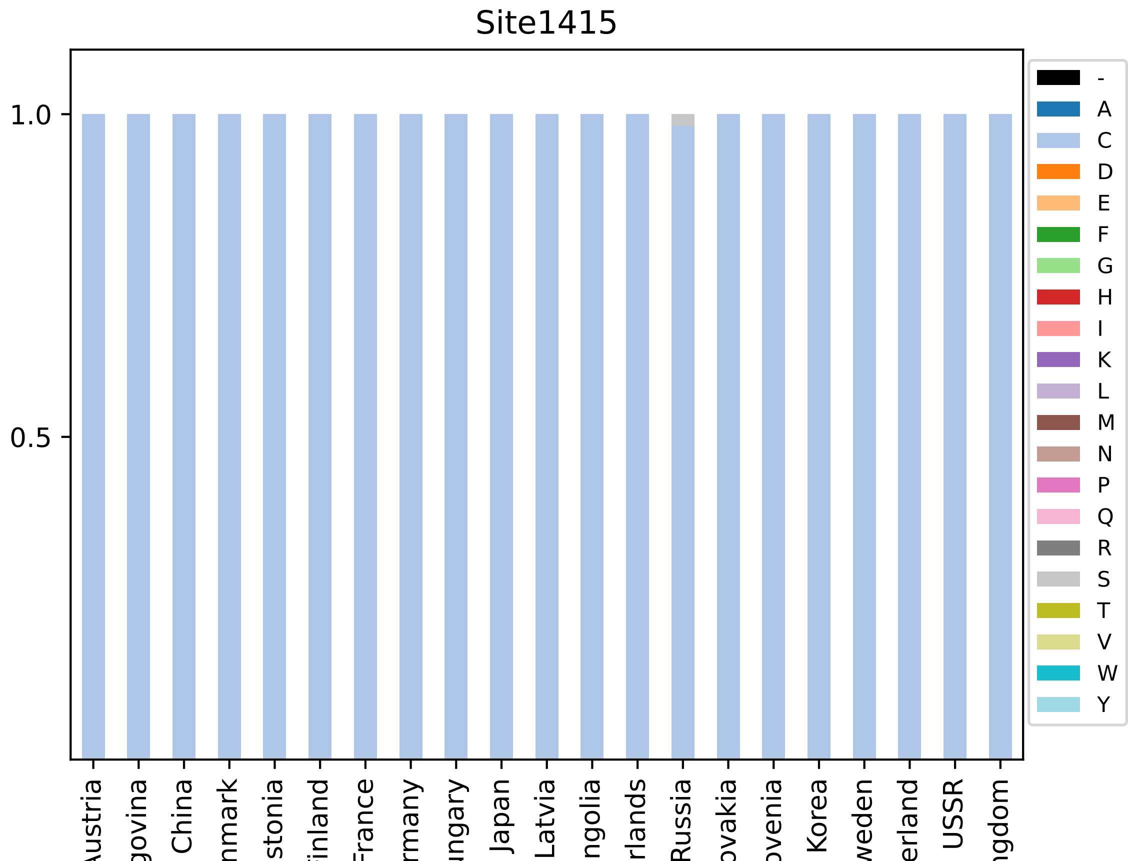
Site1415
1.0
0.5
-
A
C
D
E
F
G
H
I
K
L
M
N
P
Q
R
S
T
V
W
Y
China
Japan
Latvia
Russia
Korea
USSR

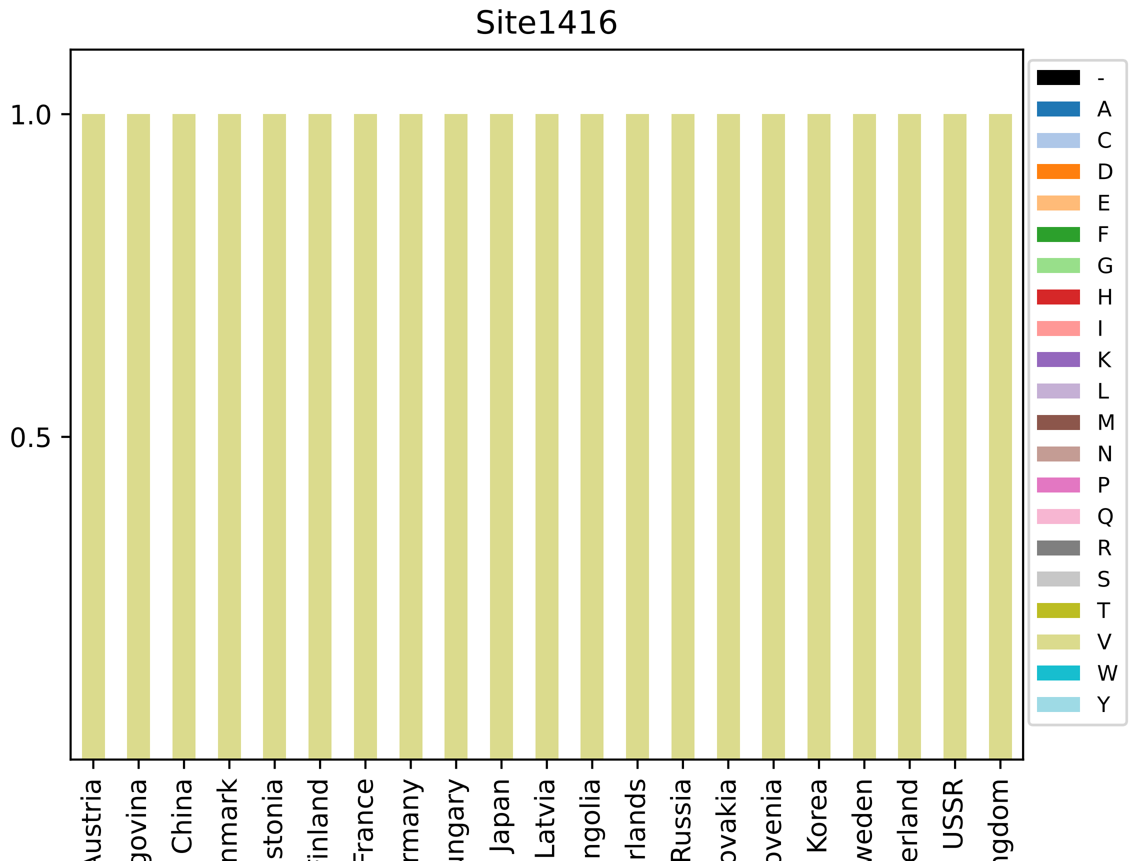

Site1416
1.0
0.5
Austria
China
Japan
Latvia
Russia
Korea
USSR
-
A
C
D
E
F
G
H
I
K
L
M
N
P
Q
R
S
T
V
W
Y

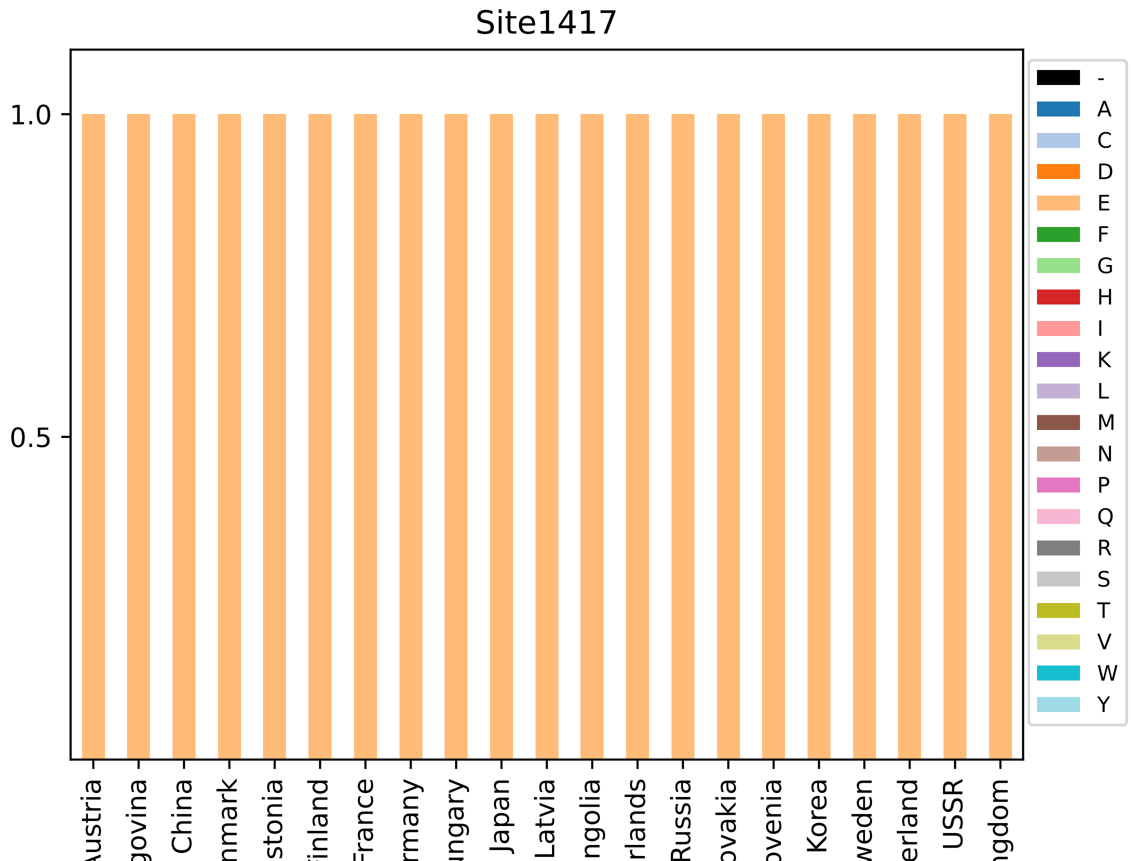

Site1417
1.0
0.5
-
A
C
D
E
F
G
H
I
K
L
M
N
P
Q
R
S
T
V
W
Y
Austria
govina
China
nmark
stonia
Finland
France
rmany
ungary
Japan
Latvia
ngolia
rlands
Russia
ovakia
ovenia
Korea
weden
erland
USSR
ngdom

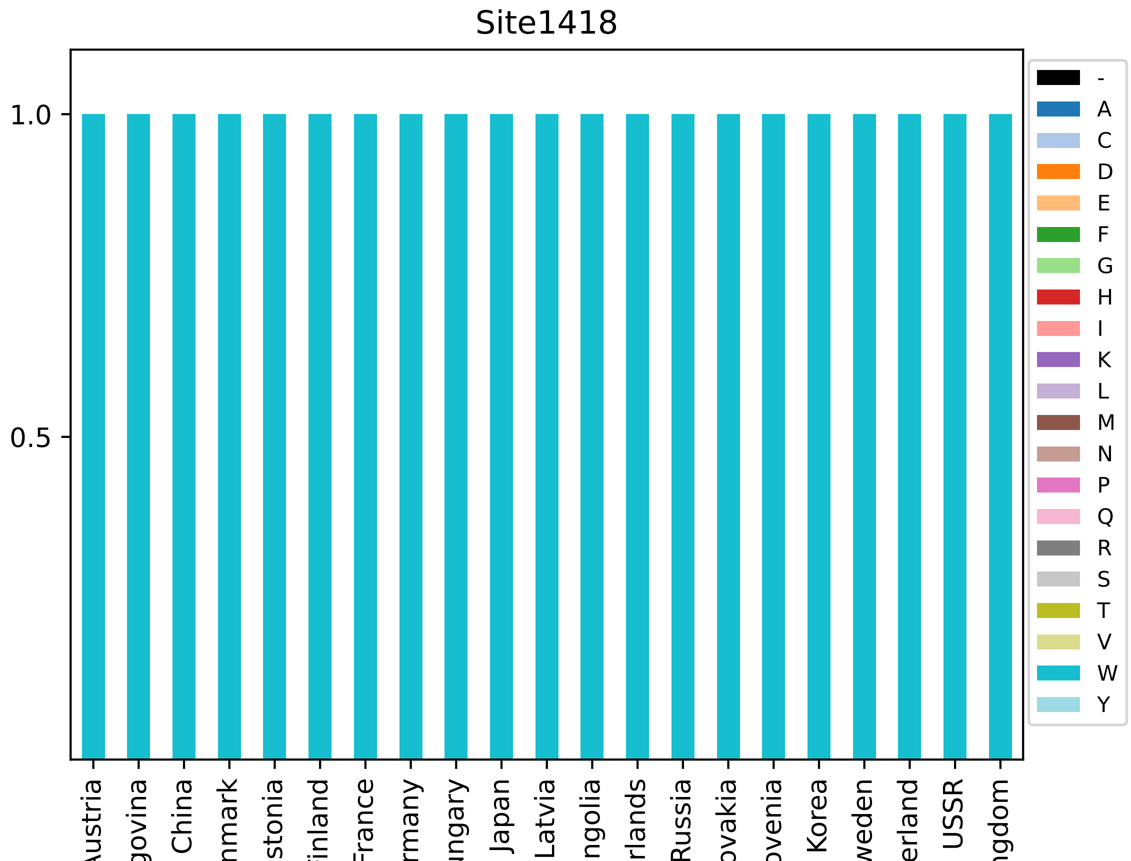

Site1418
1.0
0.5
Austria
govina
China
nmark
stonia
inland
France
rmany
ungary
Japan
Latvia
ngolia
rlands
Russia
ovakia
ovenia
Korea
weden
erland
USSR
ngdom
-
A
C
D
E
F
G
H
I
K
L
M
N
P
Q
R
S
T
V
W
Y

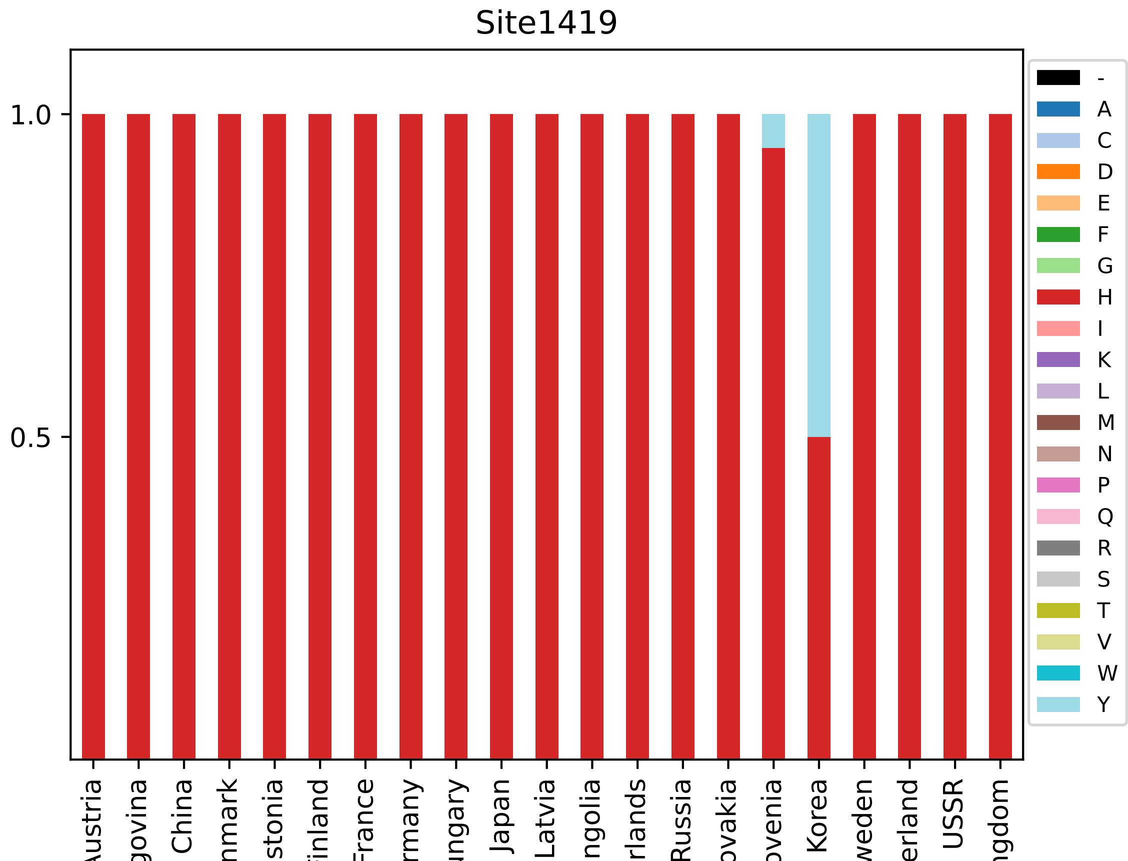

Site1419
1.0
0.5
-
A
C
D
E
F
G
H
I
K
L
M
N
P
Q
R
S
T
V
W
Y
China
Japan
Latvia
Russia
Korea
USSR

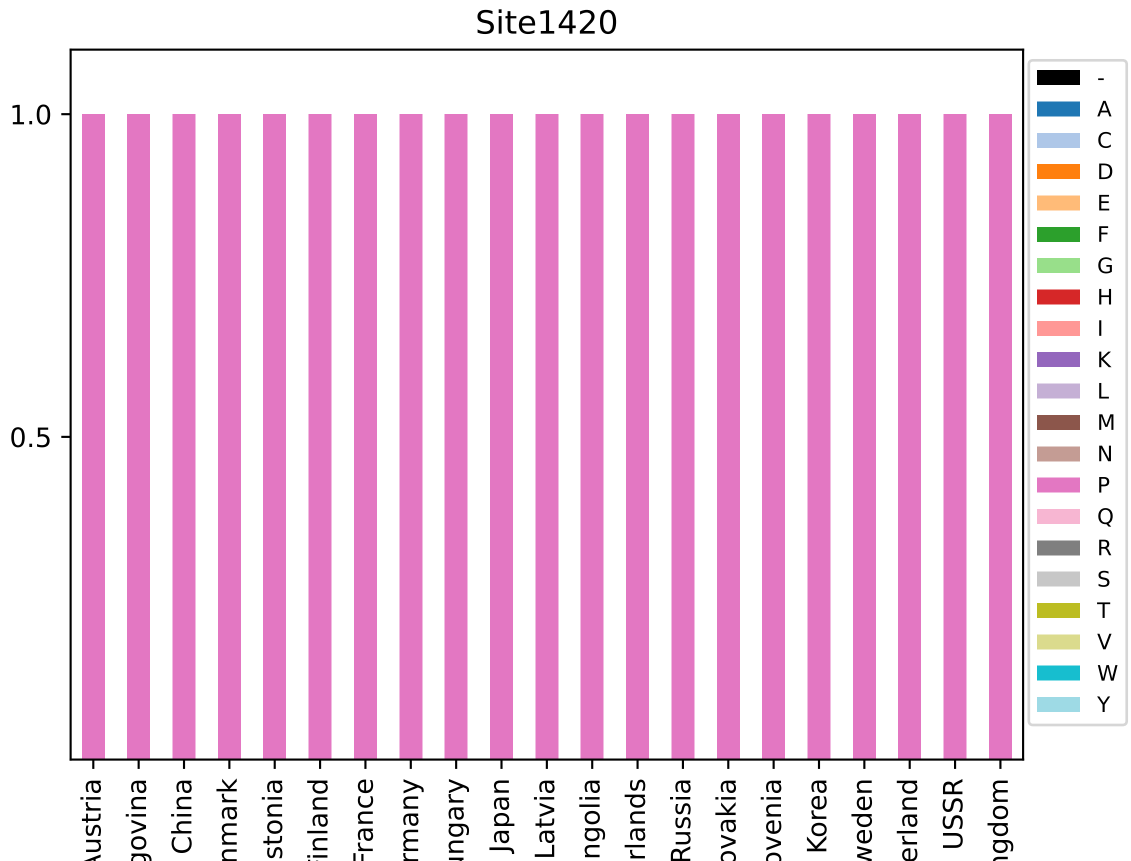
Site1420
1.0
0.5
-
A
C
D
E
F
G
H
I
K
L
M
N
P
Q
R
S
T
V
W
Y
China
Japan
Latvia
Russia
Korea
USSR

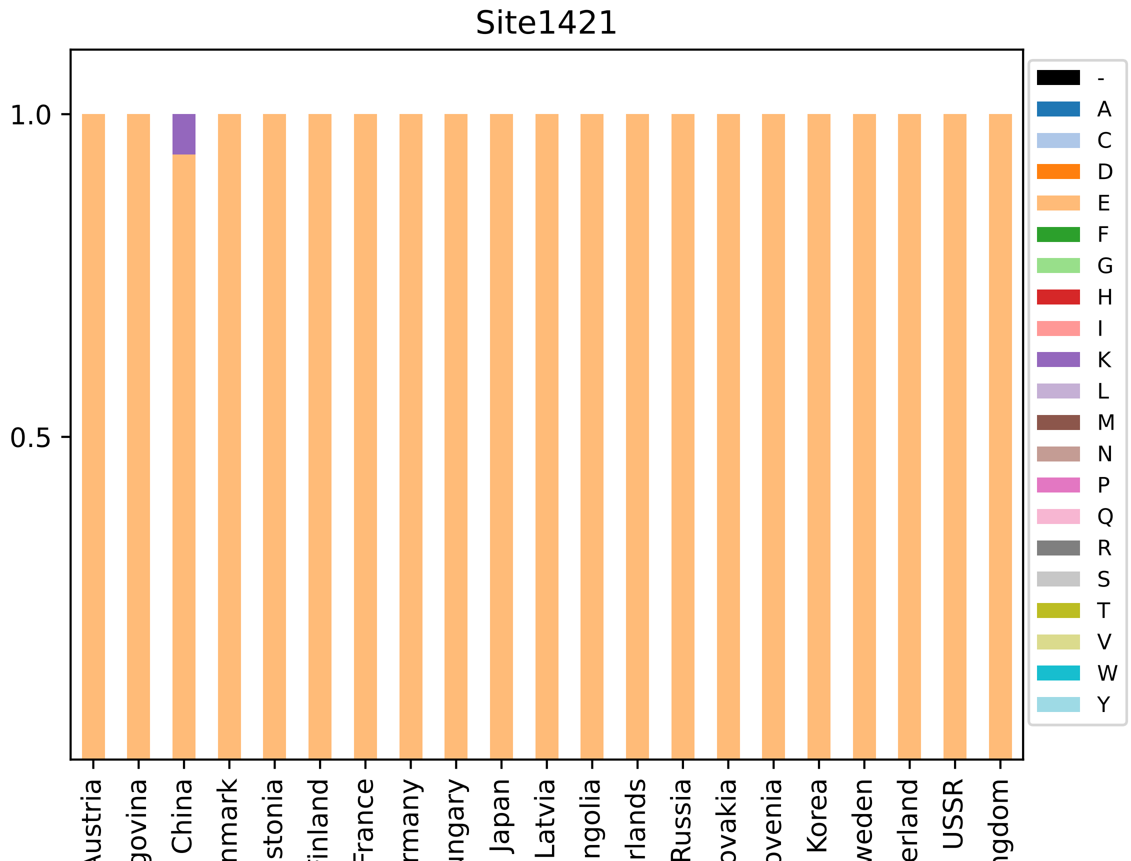

Site1421
1.0
0.5
-
A
C
D
E
F
G
H
I
K
L
M
N
P
Q
R
S
T
V
W
Y
China
Japan
Latvia
Russia
Korea
USSR

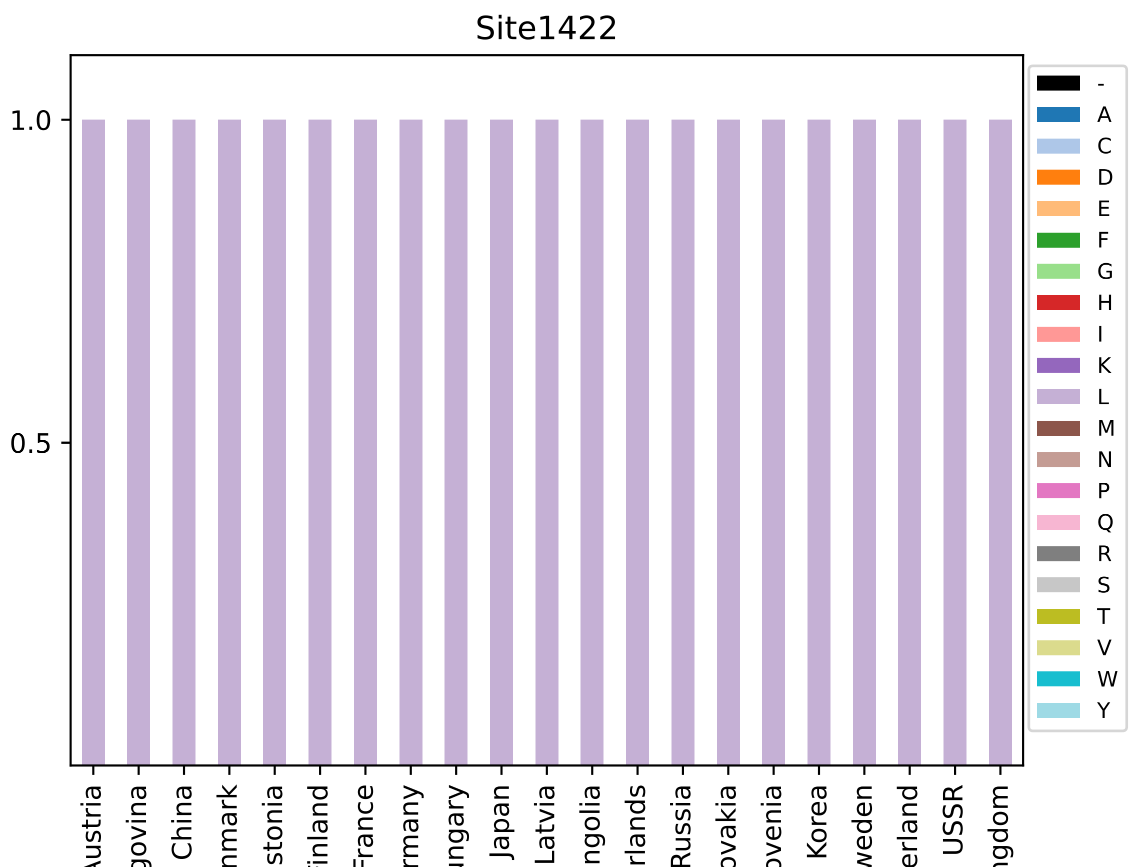

Site1422
1.0
0.5
-
A
C
D
E
F
G
H
I
K
L
M
N
P
Q
R
S
T
V
W
Y
Austria
China
Japan
Latvia
Russia
Korea
USSR

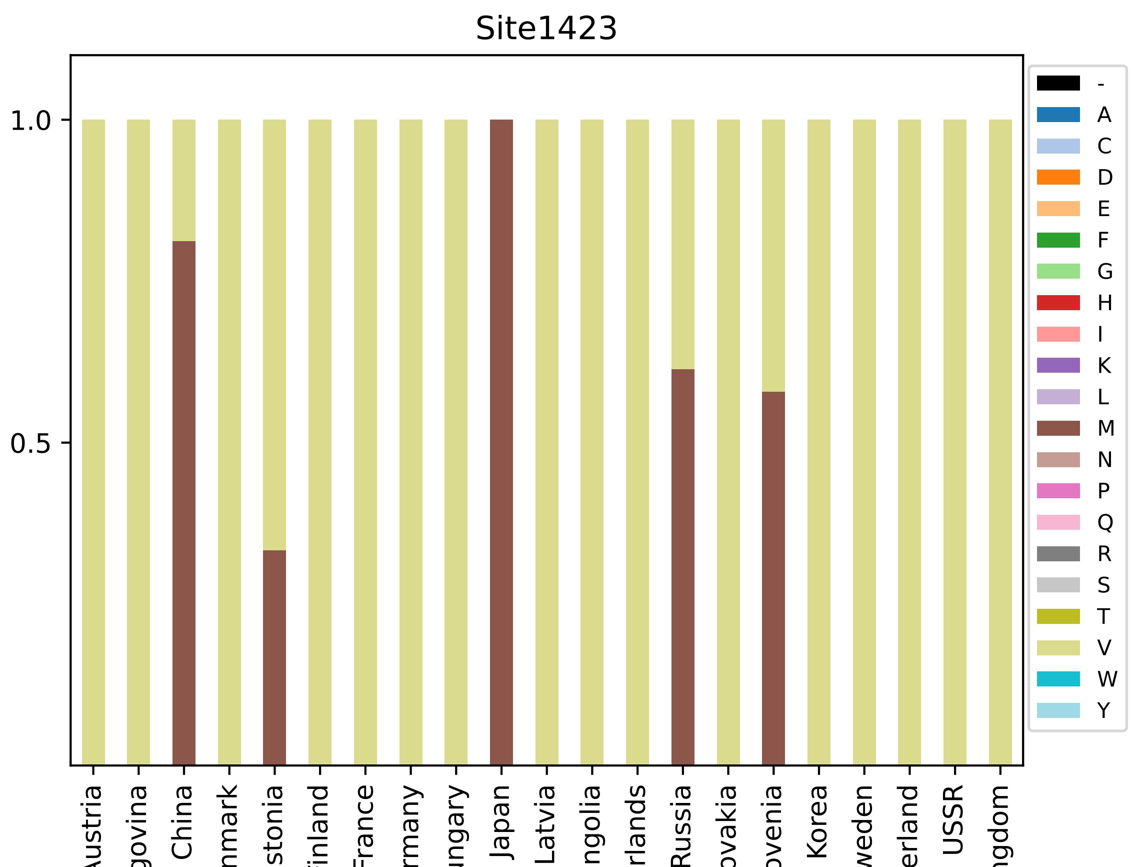

Site1423
1.0
0.5
-
A
C
D
E
F
G
H
I
K
L
M
N
P
Q
R
S
T
V
W
Y
China
Japan
Latvia
Russia
Korea
USSR

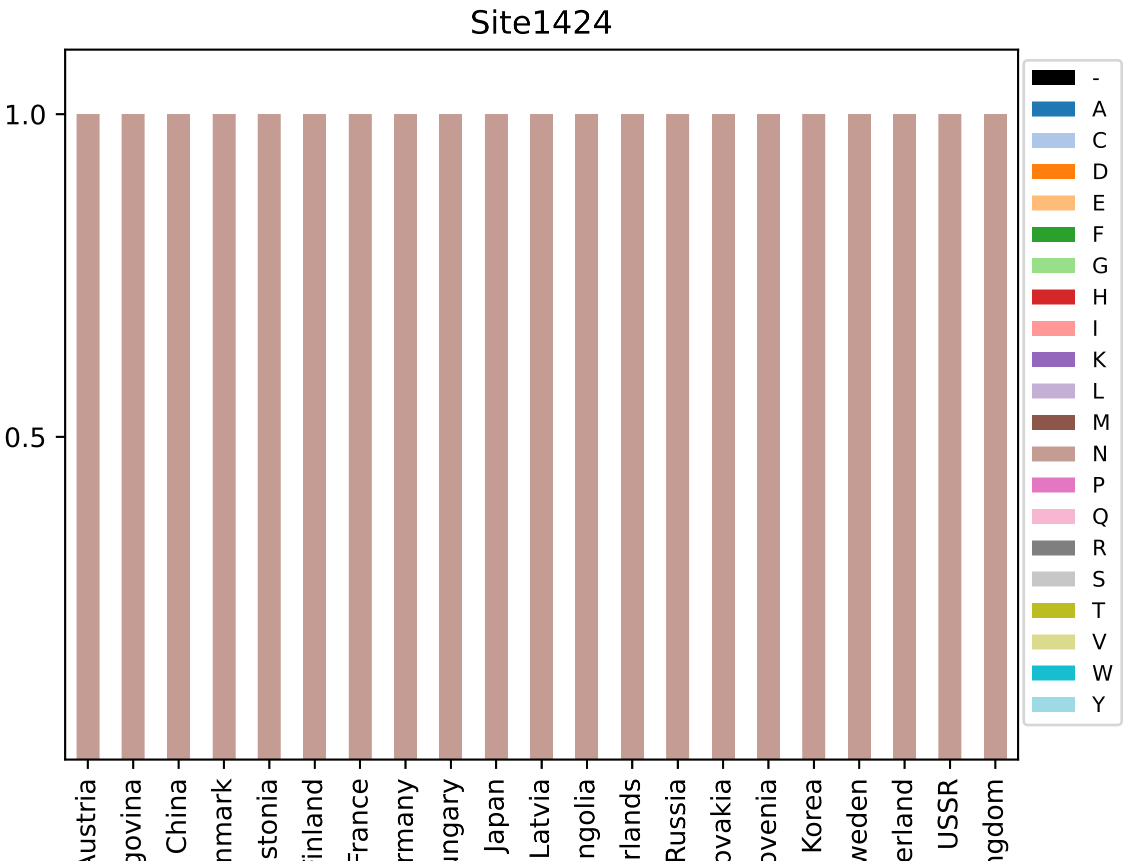
Site1424
1.0
0.5
-
A
C
D
E
F
G
H
I
K
L
M
N
P
Q
R
S
T
V
W
Y
Austria
China
Japan
Latvia
Russia
Korea
USSR

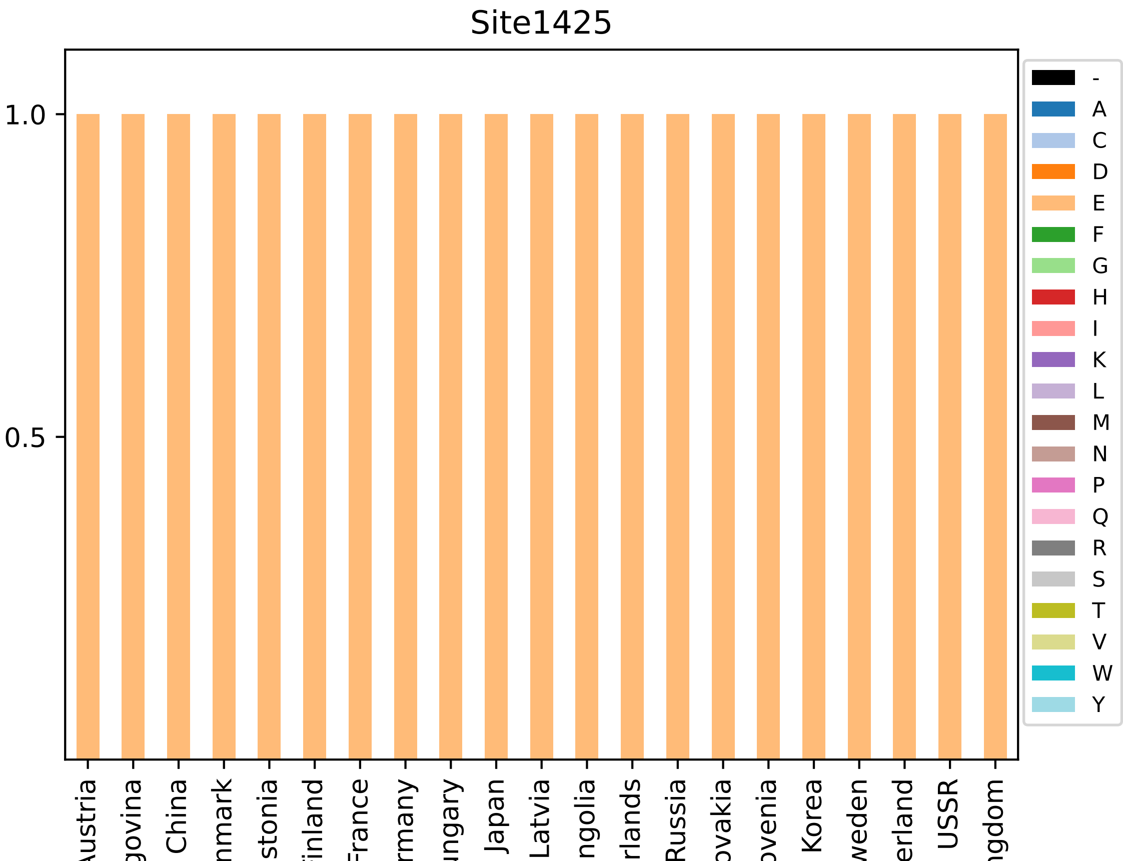
Site1425
1.0
0.5
-
A
C
D
E
F
G
H
I
K
L
M
N
P
Q
R
S
T
V
W
Y
China
Japan
Latvia
Russia
Korea
USSR

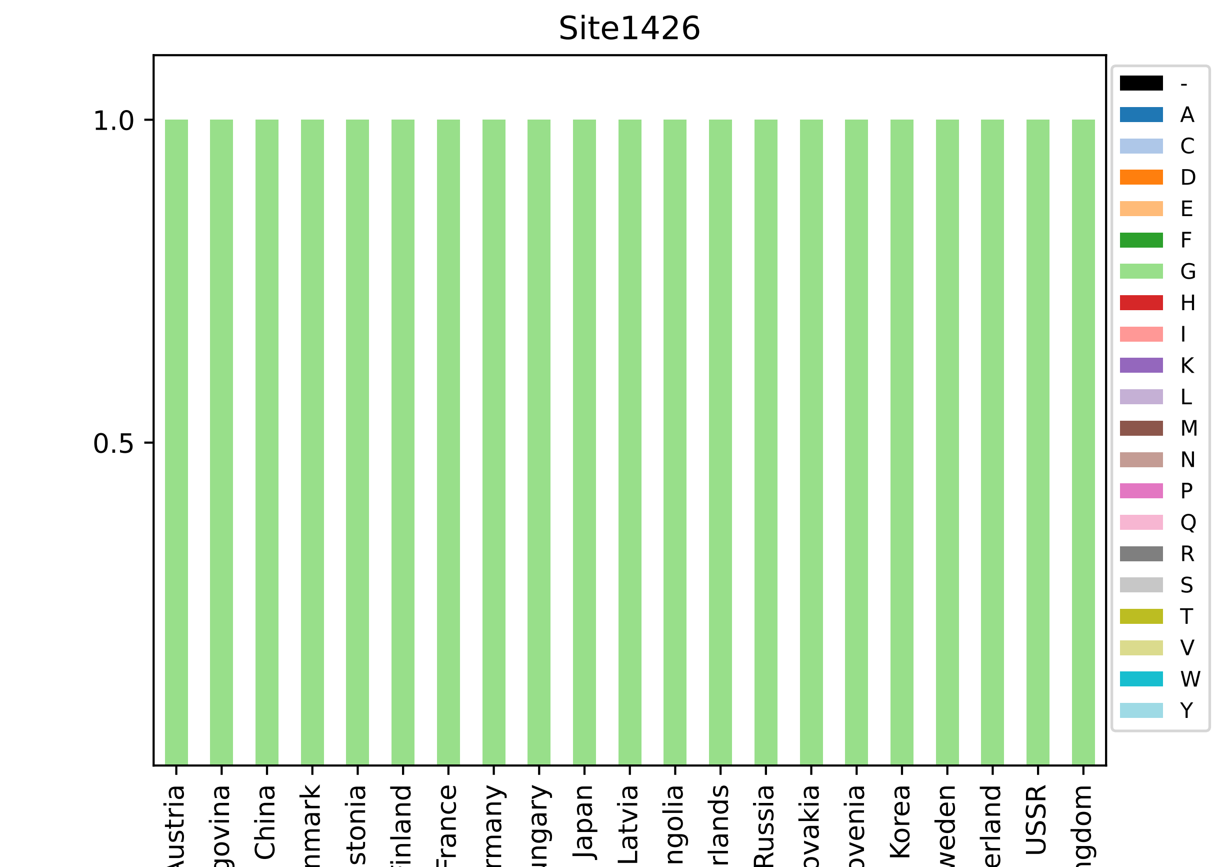

Site1426
1.0
0.5
Austria
govina
China
nmark
stonia
Finland
France
rmany
ungary
Japan
Latvia
ngolia
rlands
Russia
ovakia
ovenia
Korea
weden
erland
USSR
ngdom
-
A
C
D
E
F
G
H
I
K
L
M
N
P
Q
R
S
T
V
W
Y

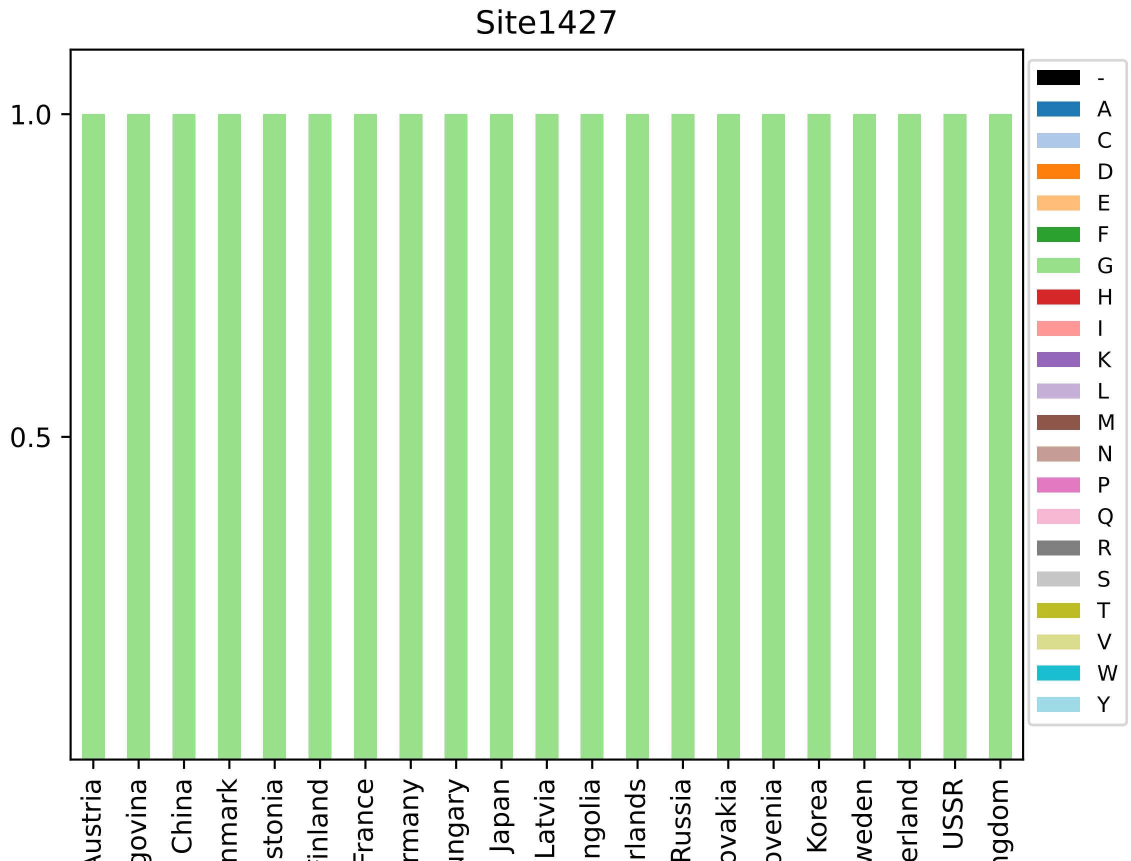

Site1427
1.0
0.5
-
A
C
D
E
F
G
H
I
K
L
M
N
P
Q
R
S
T
V
W
Y
China
Japan
Latvia
Russia
Korea
USSR

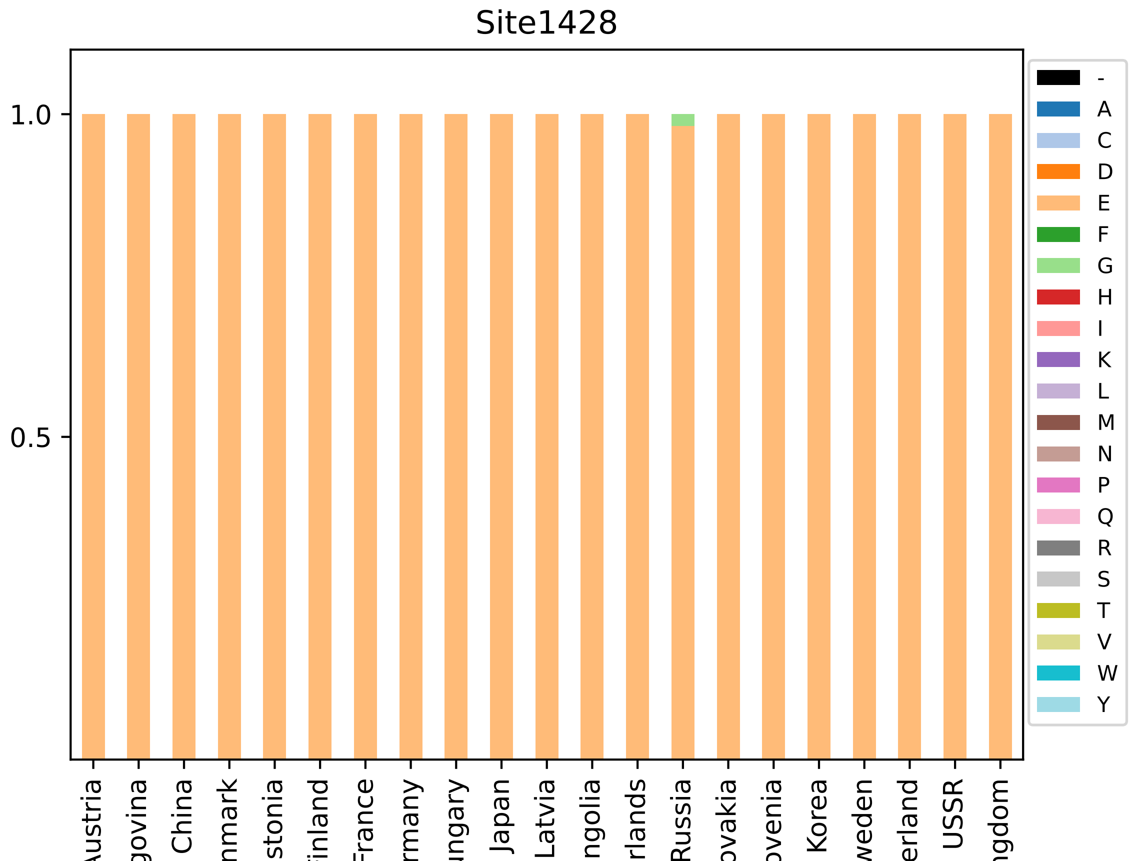

Site1428
1.0
0.5
-
A
C
D
E
F
G
H
I
K
L
M
N
P
Q
R
S
T
V
W
Y
Austria
China
Finland
France
Japan
Latvia
Russia
Korea
USSR

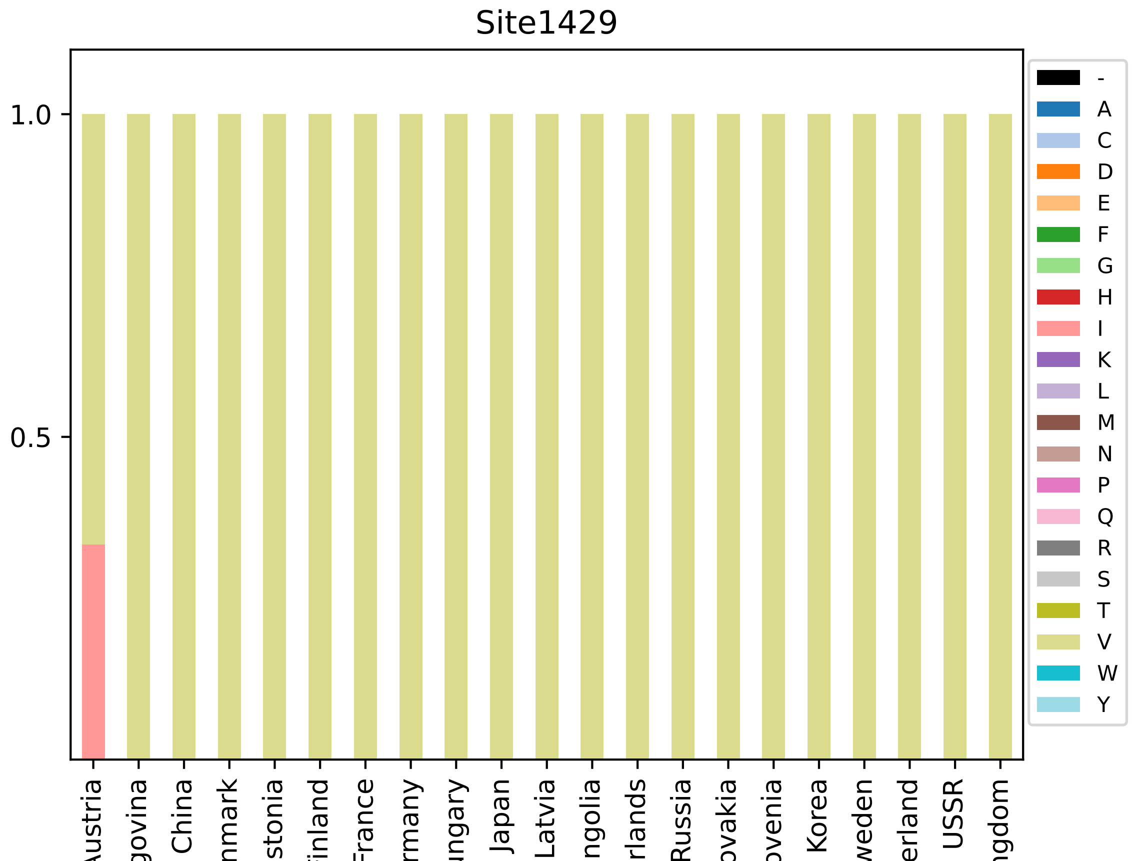

Site1429
1.0
0.5
-
A
C
D
E
F
G
H
I
K
L
M
N
P
Q
R
S
T
V
W
Y
China
Japan
Latvia
Russia
Korea
USSR

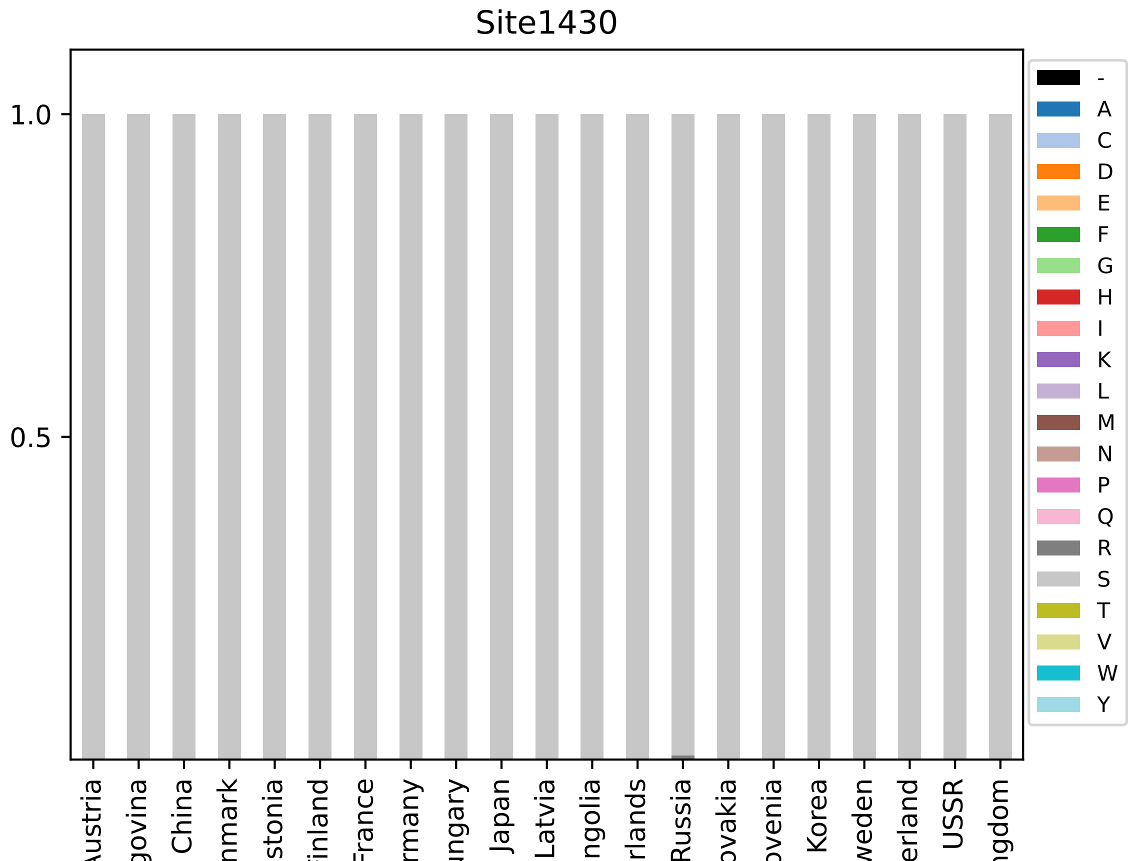

Site1430
1.0
0.5
-
A
C
D
E
F
G
H
I
K
L
M
N
P
Q
R
S
T
V
W
Y
Austria
China
Japan
Latvia
Russia
Korea
USSR

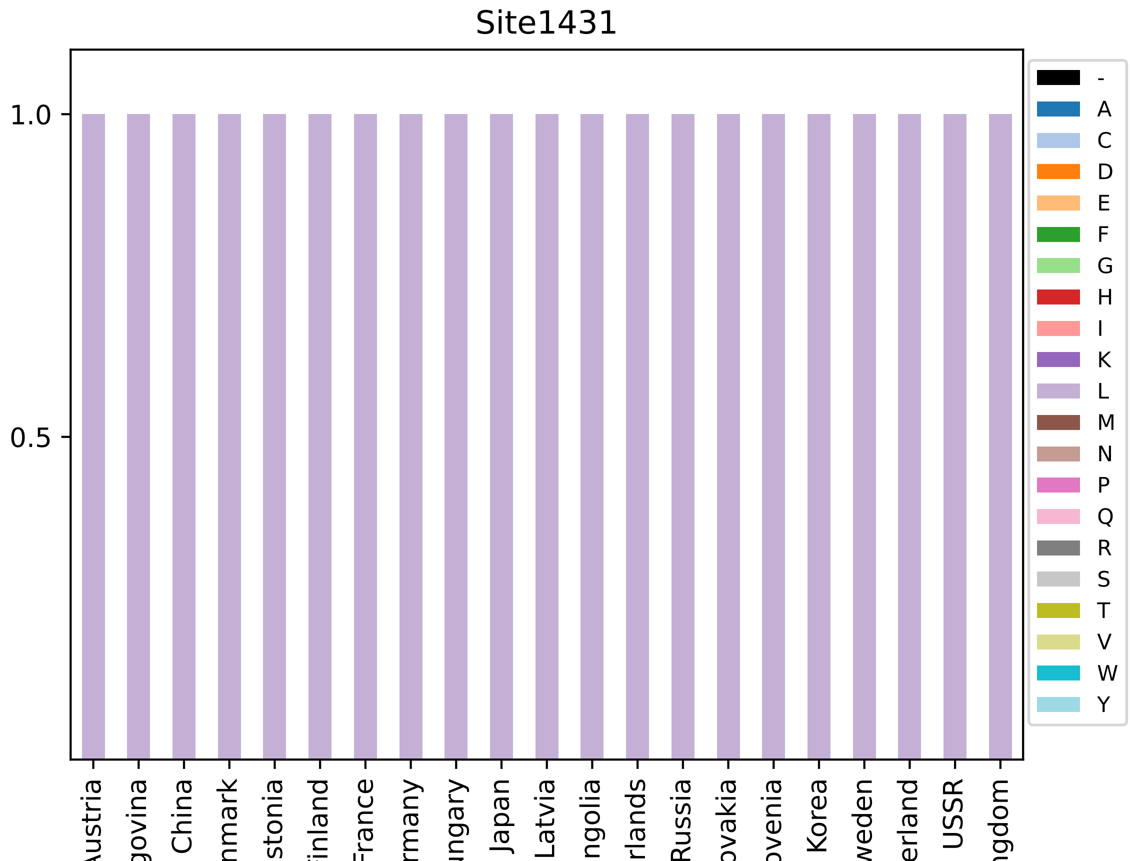

Site1431
1.0
0.5
-
A
C
D
E
F
G
H
I
K
L
M
N
P
Q
R
S
T
V
W
Y
Austria
China
Finland
France
Japan
Latvia
Russia
Korea
USSR

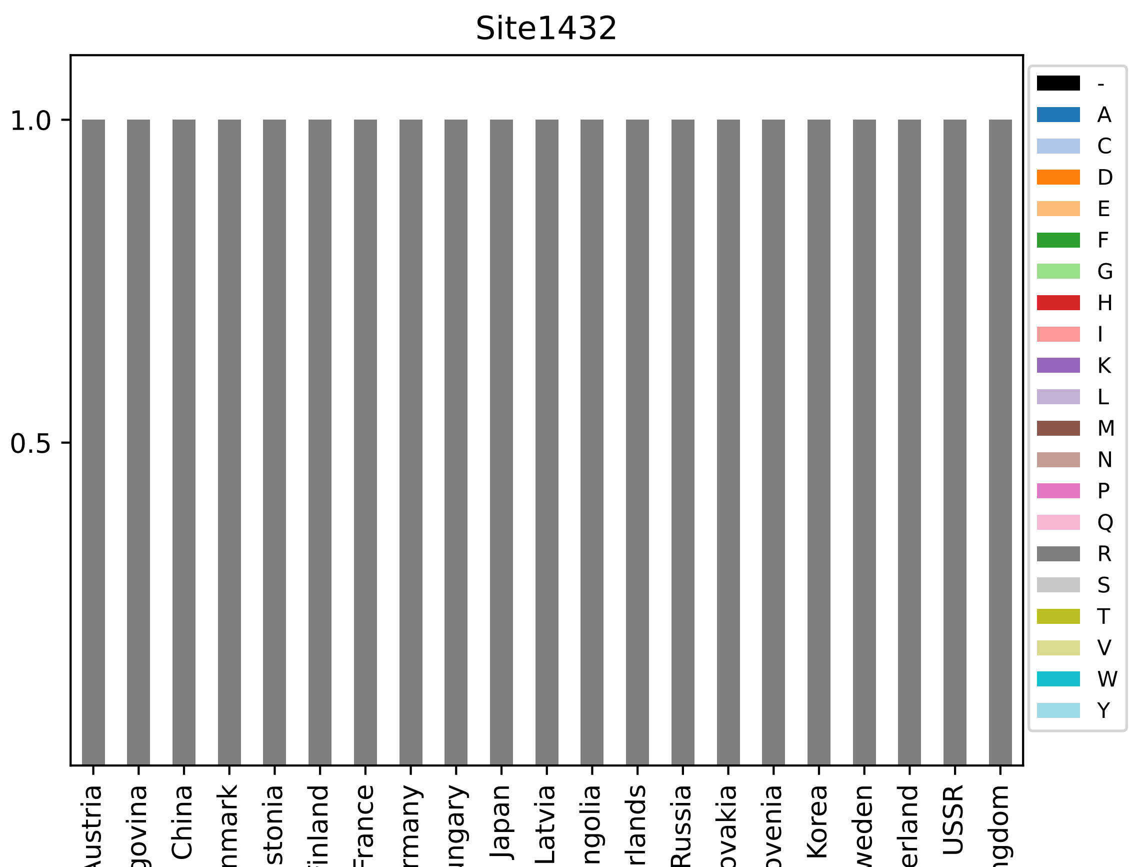

Site1432
1.0
0.5
-
A
C
D
E
F
G
H
I
K
L
M
N
P
Q
R
S
T
V
W
Y
Austria
China
Japan
Latvia
Russia
Korea
USSR

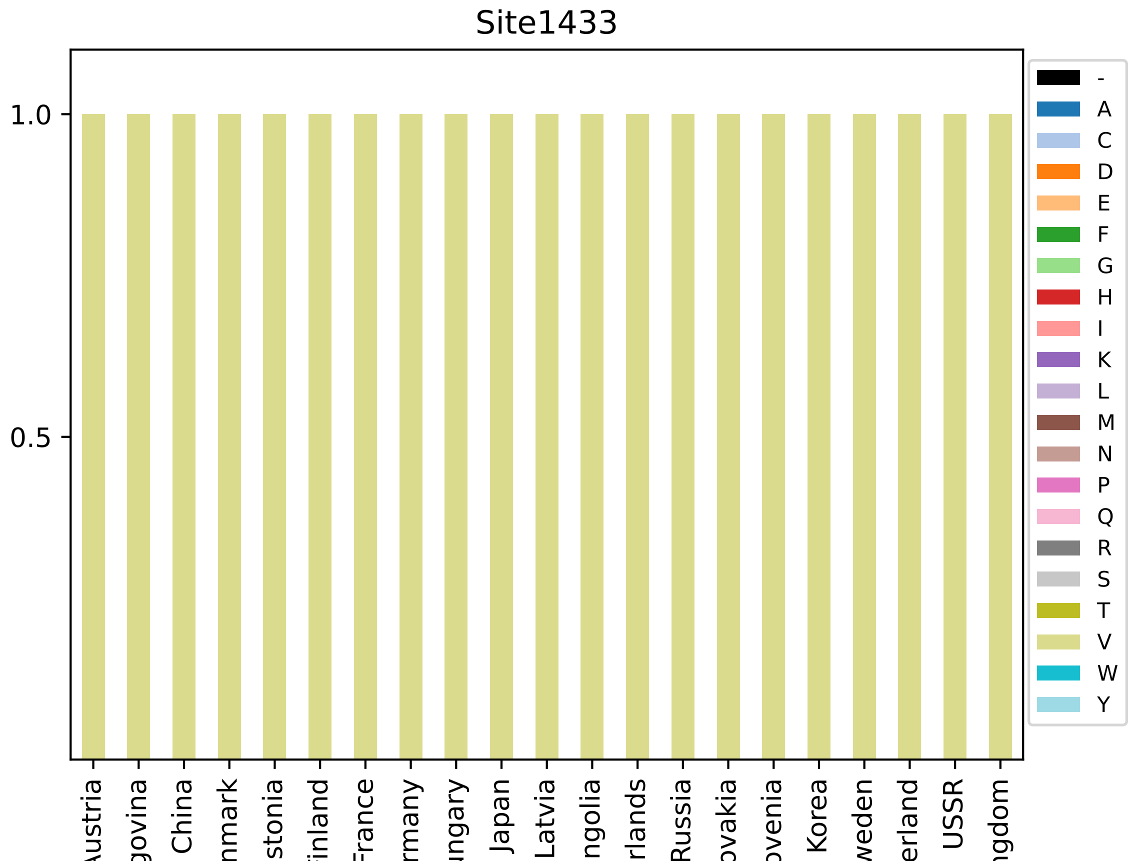
Site1433
1.0
0.5
-
A
C
D
E
F
G
H
I
K
L
M
N
P
Q
R
S
T
V
W
Y
China
Japan
Latvia
Russia
Korea
USSR

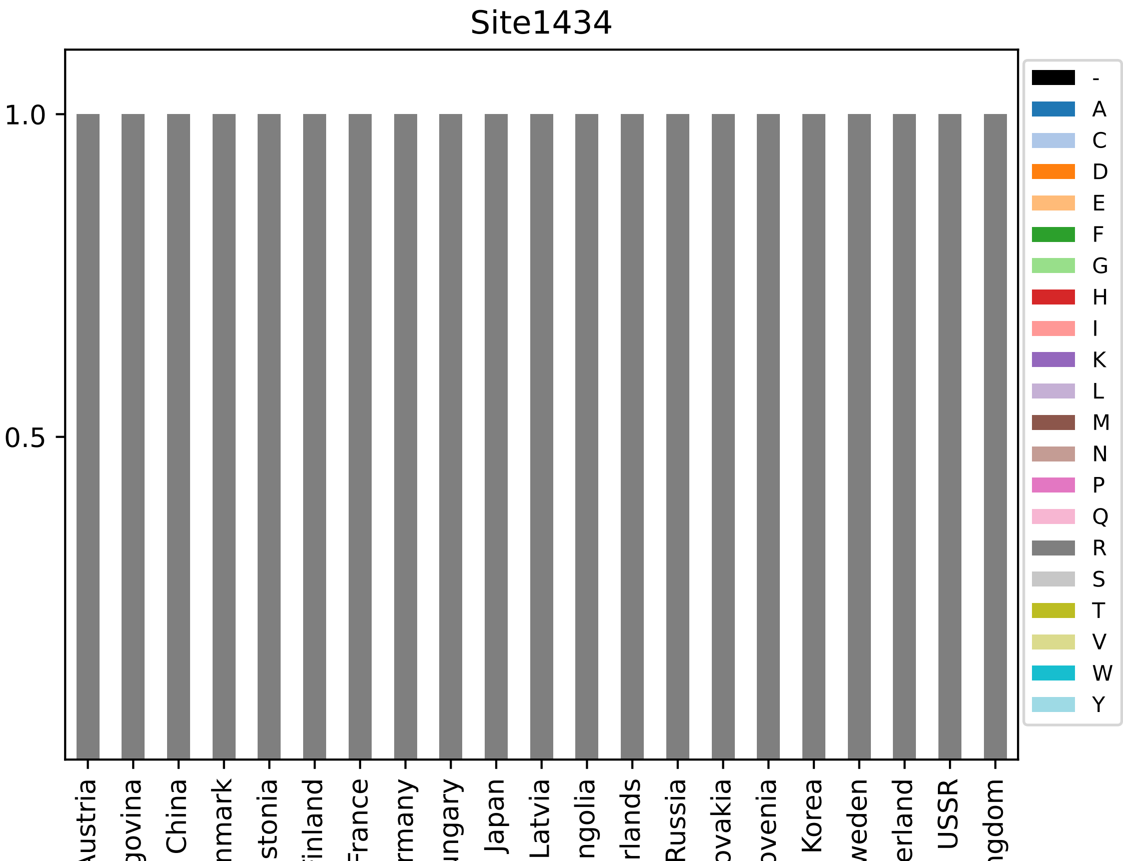

Site1434
1.0
0.5
-
A
C
D
E
F
G
H
I
K
L
M
N
P
Q
R
S
T
V
W
Y
Austria
govina
China
nmark
stonia
inland
France
rmany
ungary
Japan
Latvia
ngolia
rlands
Russia
ovakia
ovenia
Korea
weden
erland
USSR
ngdom

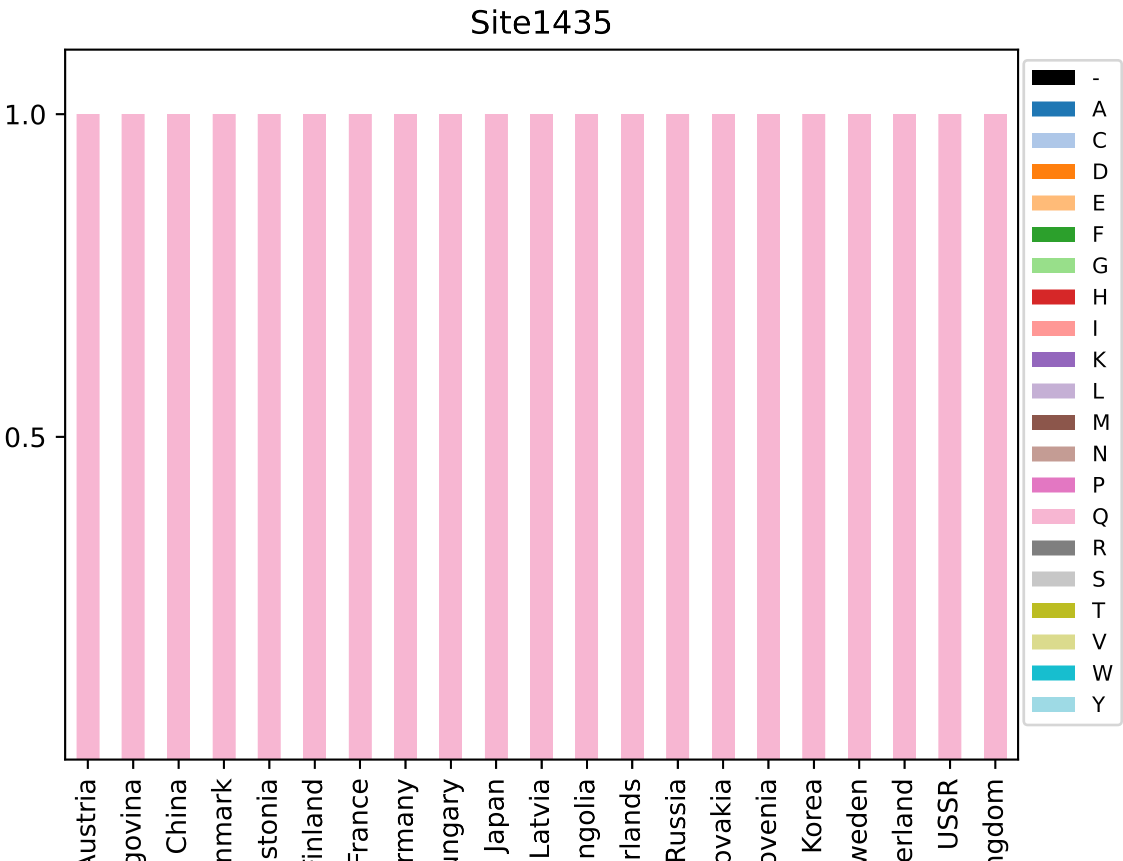
Site1435
1.0
0.5
China
Japan
Latvia
Russia
Korea
USSR
-
A
C
D
E
F
G
H
I
K
L
M
N
P
Q
R
S
T
V
W
Y

Site1436

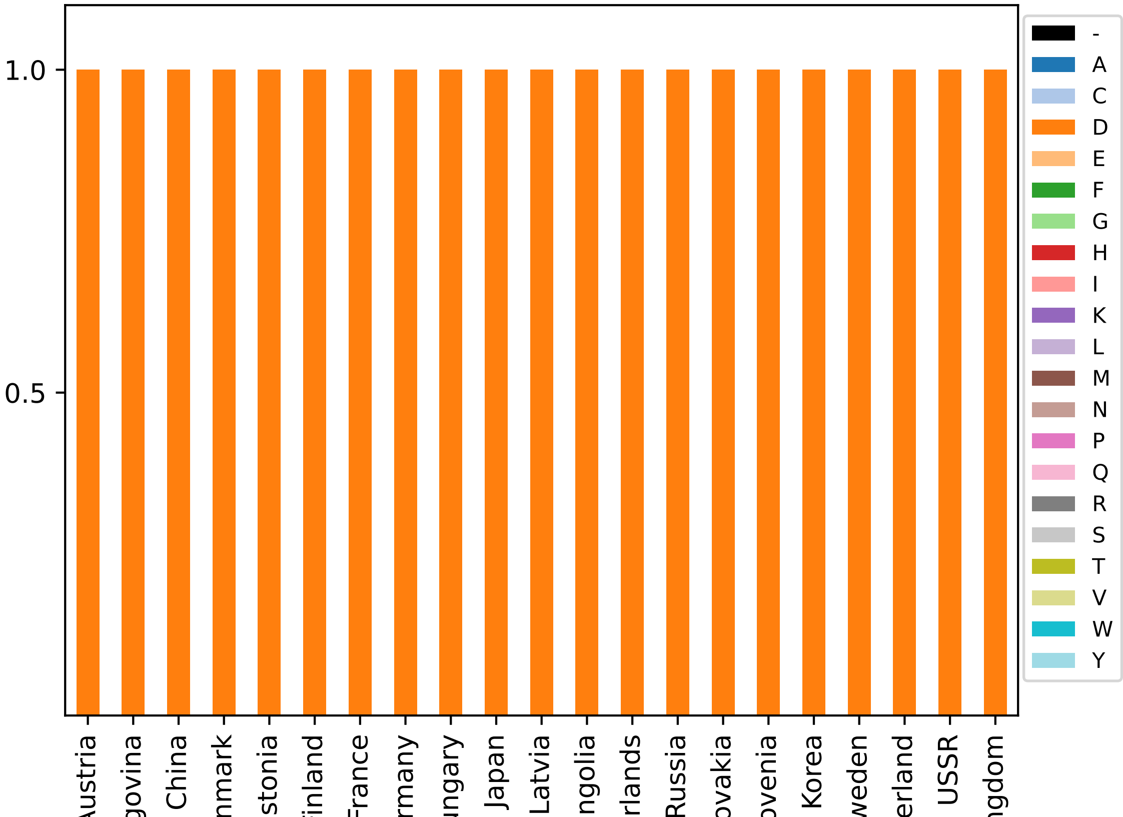

Site1436
1.0
0.5
Austria
govina
China
nmark
stonia
Finland
France
rmany
ungary
Japan
Latvia
ngolia
rlands
Russia
ovakia
ovenia
Korea
weden
erland
USSR
ngdom
-
A
C
D
E
F
G
H
I
K
L
M
N
P
Q
R
S
T
V
W
Y

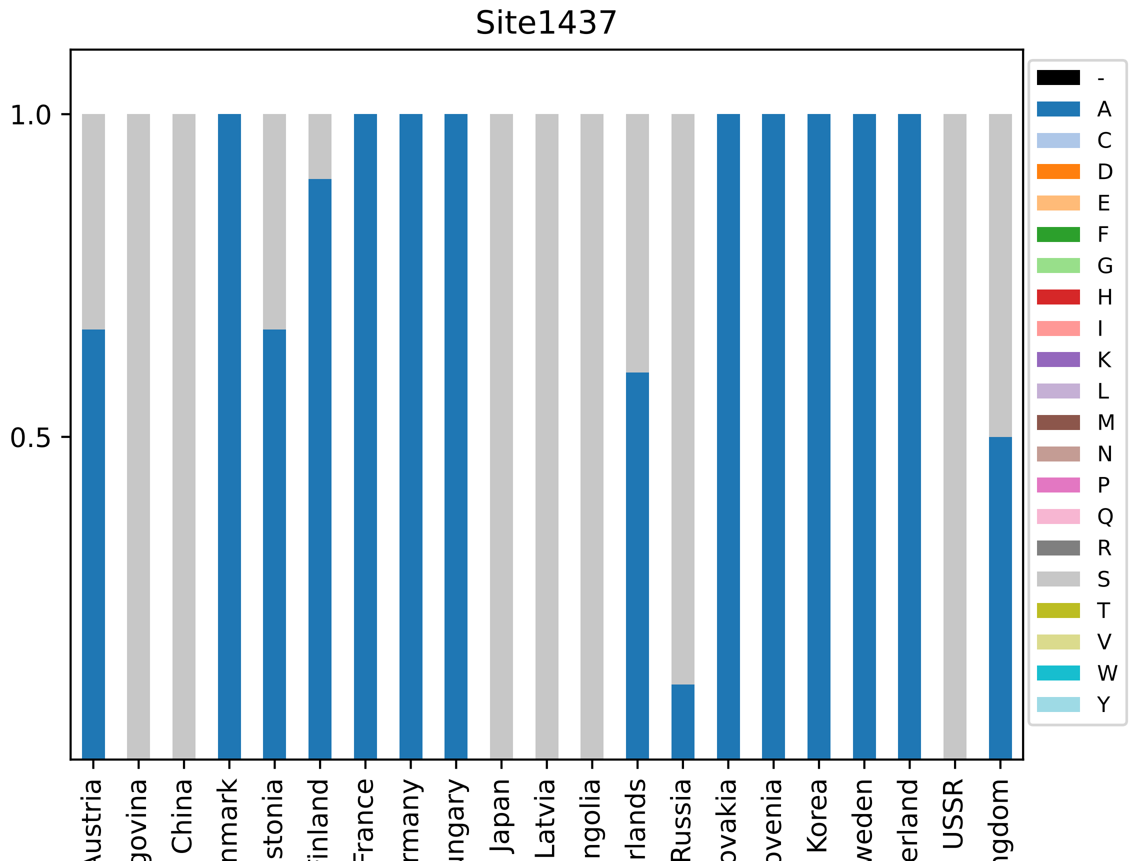

Site1437
1.0
0.5
-
A
C
D
E
F
G
H
I
K
L
M
N
P
Q
R
S
T
V
W
Y
Austria
govina
China
nmark
stonia
Finland
France
rmany
ungary
Japan
Latvia
ngolia
rlands
Russia
ovakia
ovenia
Korea
weden
erland
USSR
ngdom

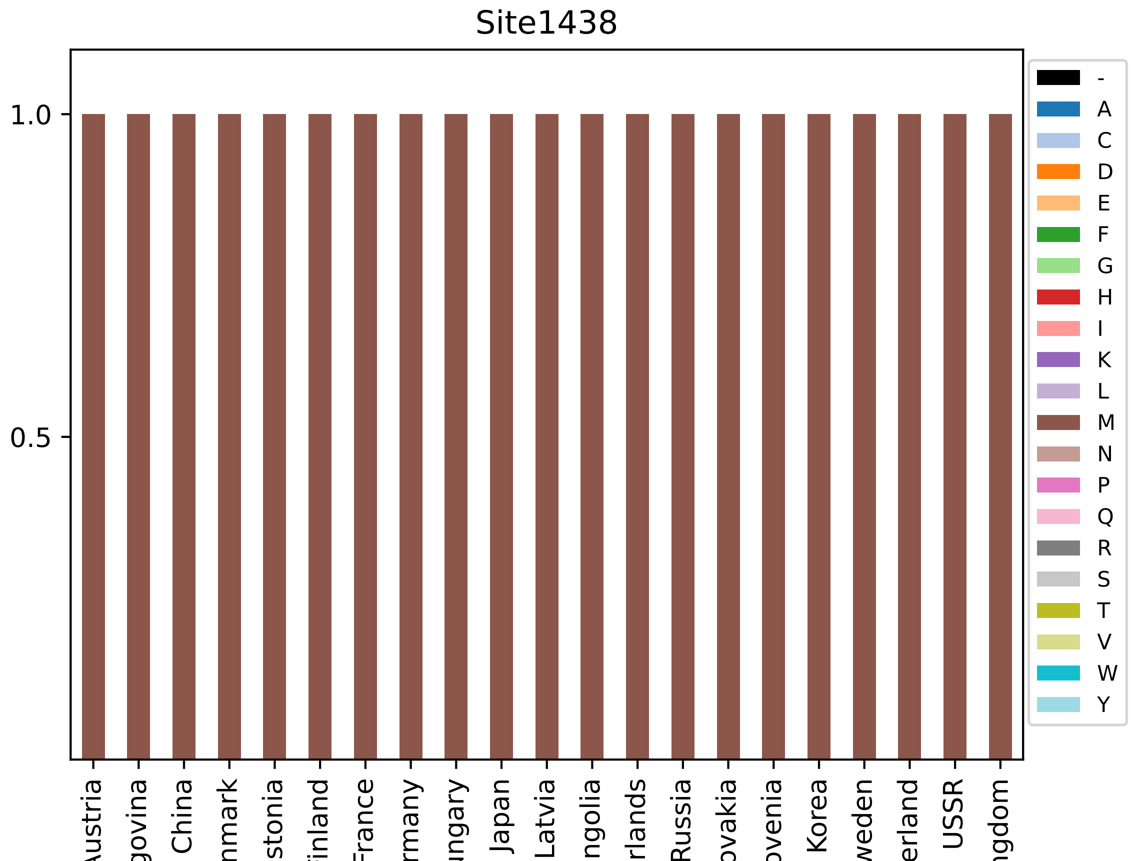
Site1438
1.0
0.5
Austria
govina
China
nmark
stonia
inland
France
rmany
ungary
Japan
Latvia
ngolia
rlands
Russia
ovakia
ovenia
Korea
weden
erland
USSR
ngdom
-
A
C
D
E
F
G
H
I
K
L
M
N
P
Q
R
S
T
V
W
Y

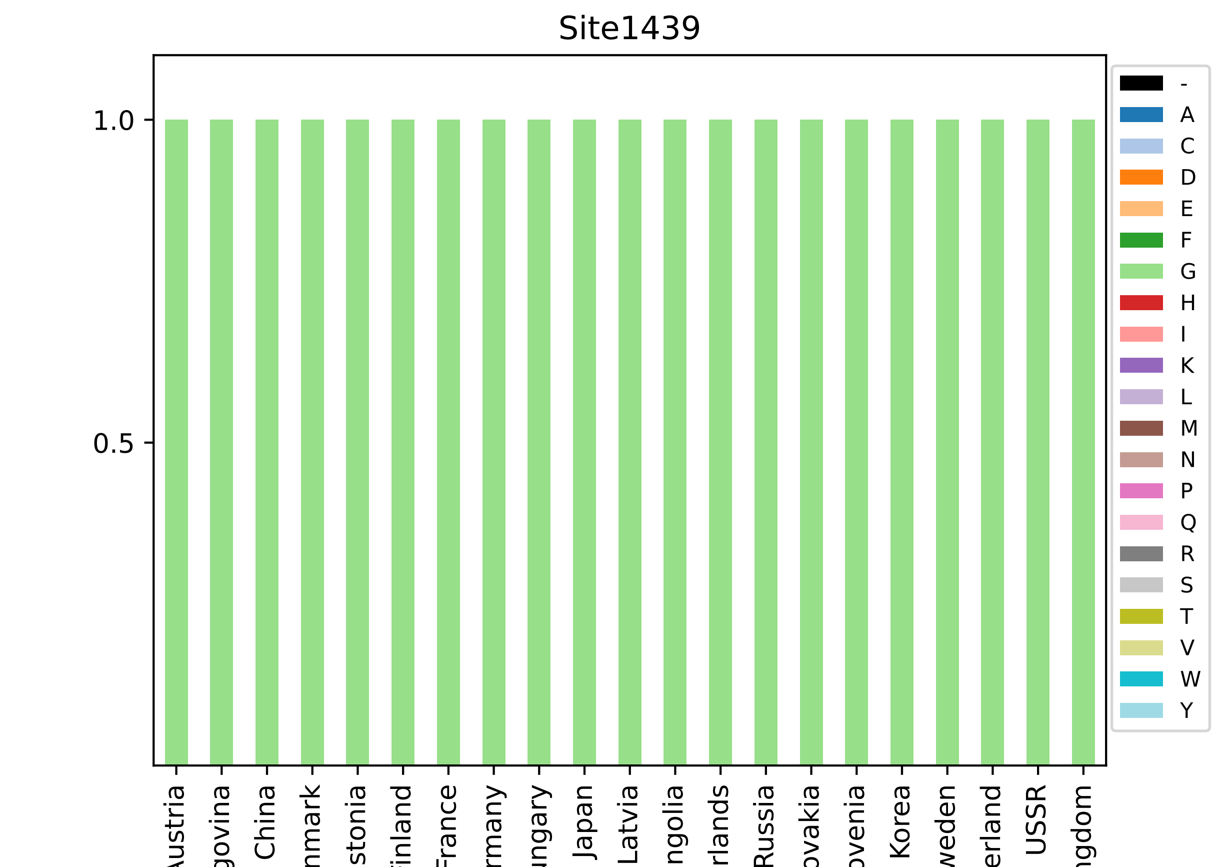

Site1439
1.0
0.5
-
A
C
D
E
F
G
H
I
K
L
M
N
P
Q
R
S
T
V
W
Y
China
Japan
Latvia
Russia
Korea
USSR

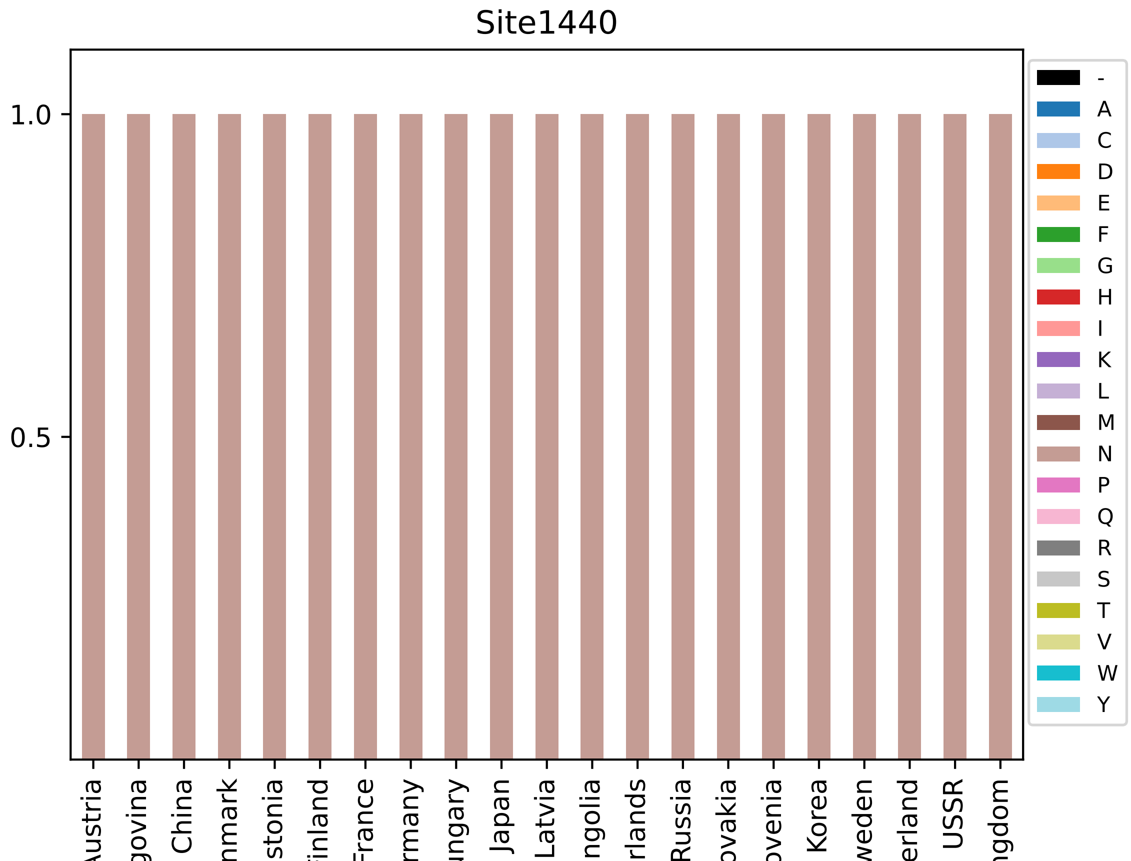

Site1440
1.0
0.5
-
A
C
D
E
F
G
H
I
K
L
M
N
P
Q
R
S
T
V
W
Y
Austria
China
Finland
France
Japan
Latvia
Russia
Korea
USSR

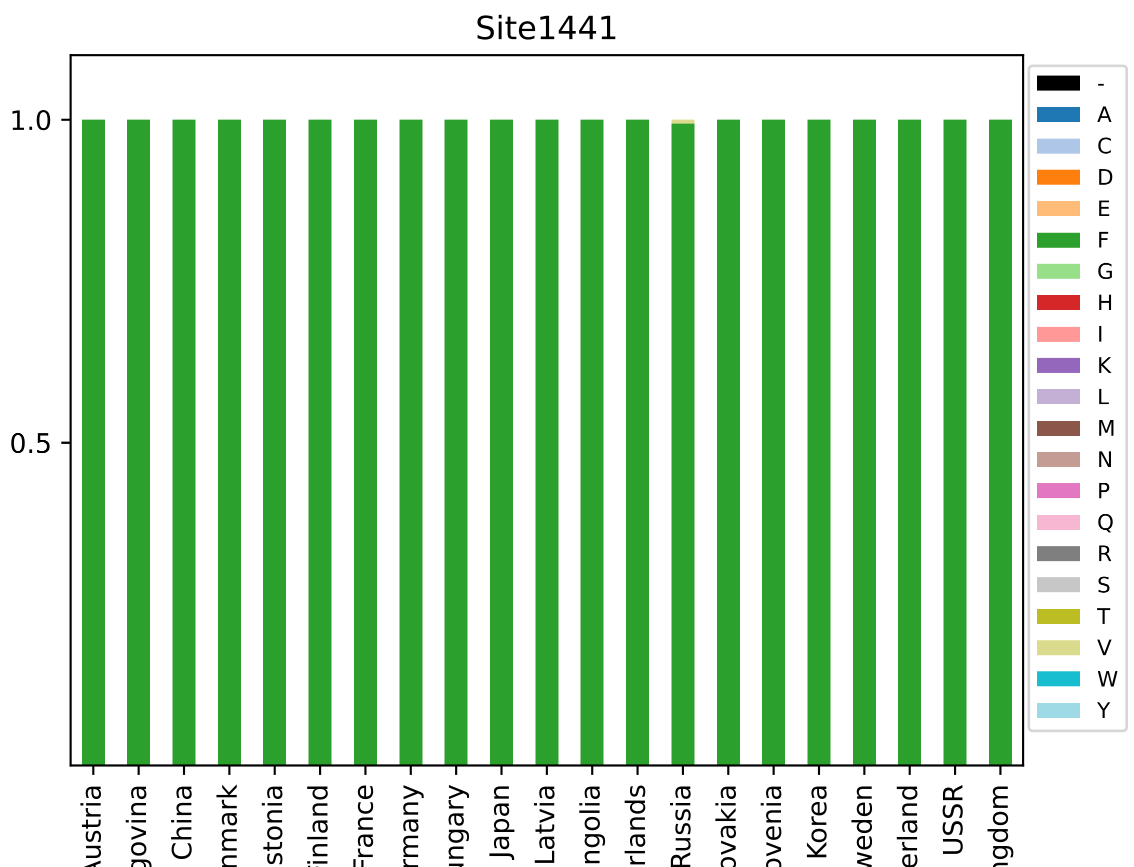

Site1441
1.0
0.5
-
A
C
D
E
F
G
H
I
K
L
M
N
P
Q
R
S
T
V
W
Y
China
Japan
Latvia
Russia
Korea
USSR

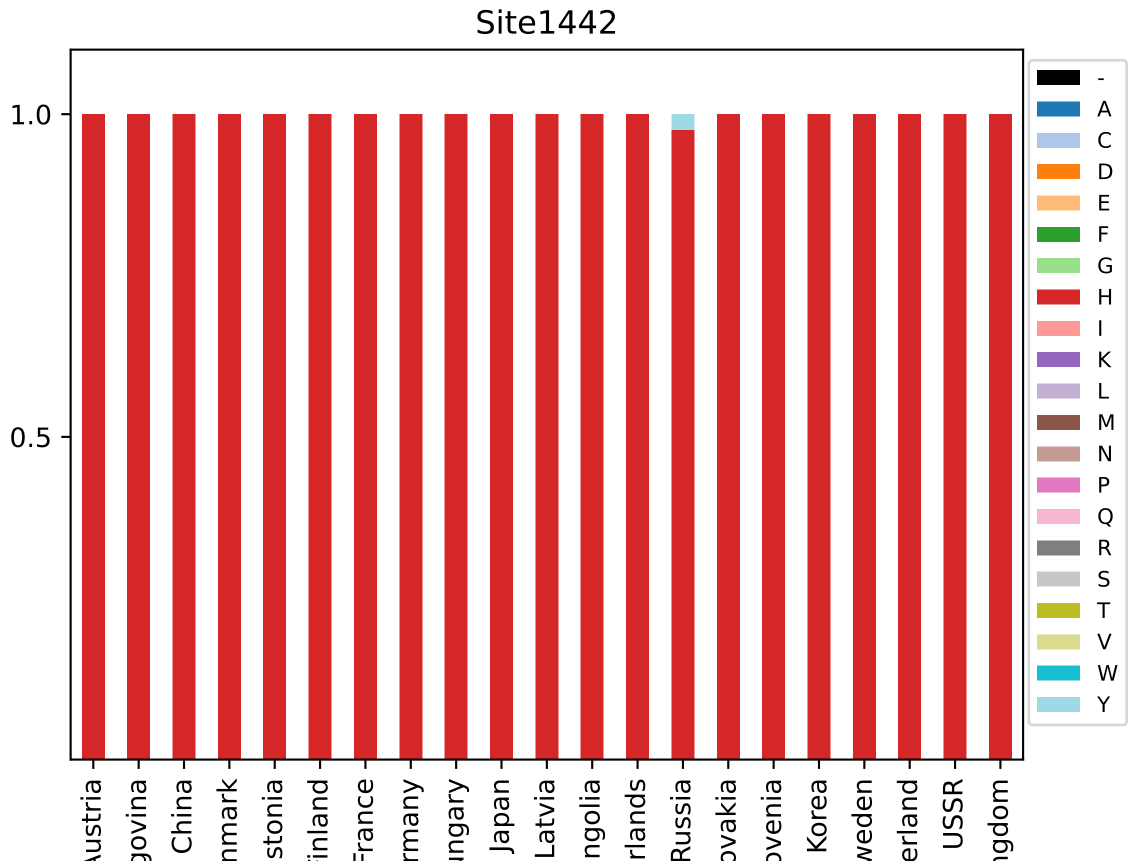
Site1442
1.0
0.5
-
A
C
D
E
F
G
H
I
K
L
M
N
P
Q
R
S
T
V
W
Y
China
Japan
Latvia
Russia
Korea
USSR

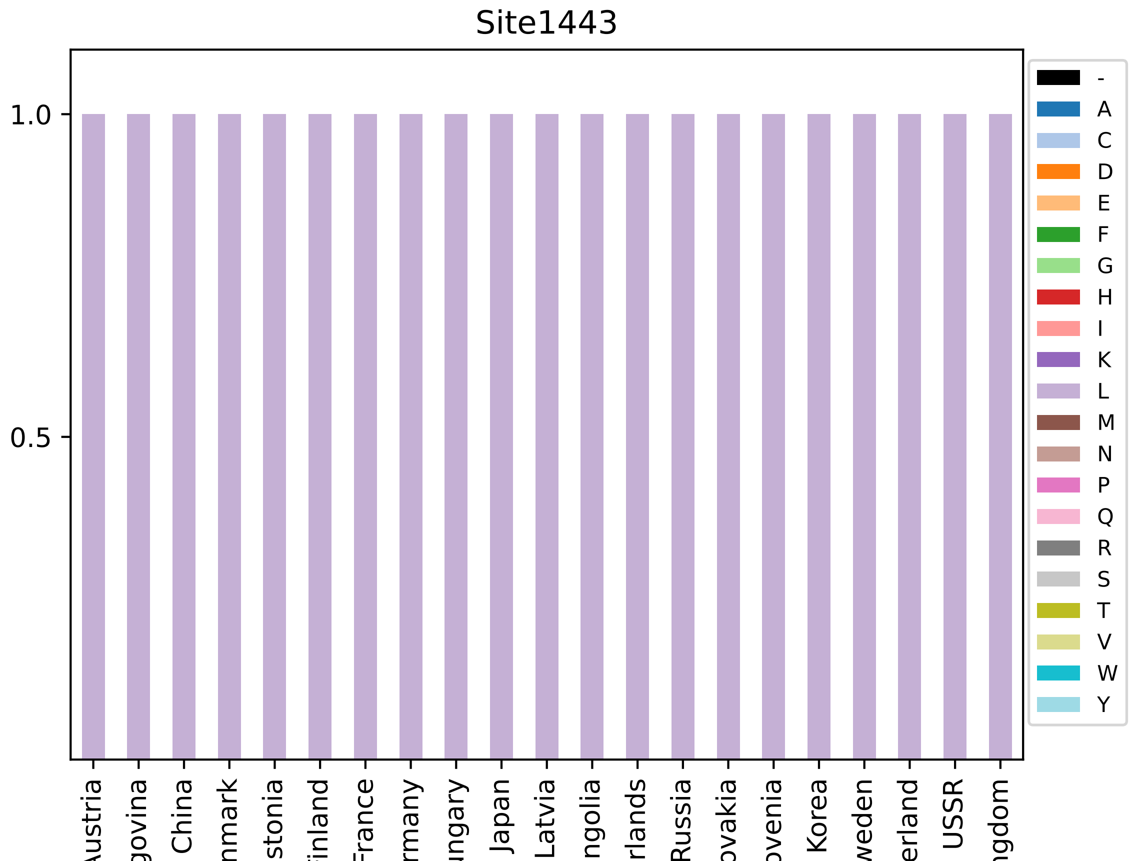
Site1443
1.0
0.5
China
Japan
Latvia
Russia
Korea
USSR
-
A
C
D
E
F
G
H
I
K
L
M
N
P
Q
R
S
T
V
W
Y

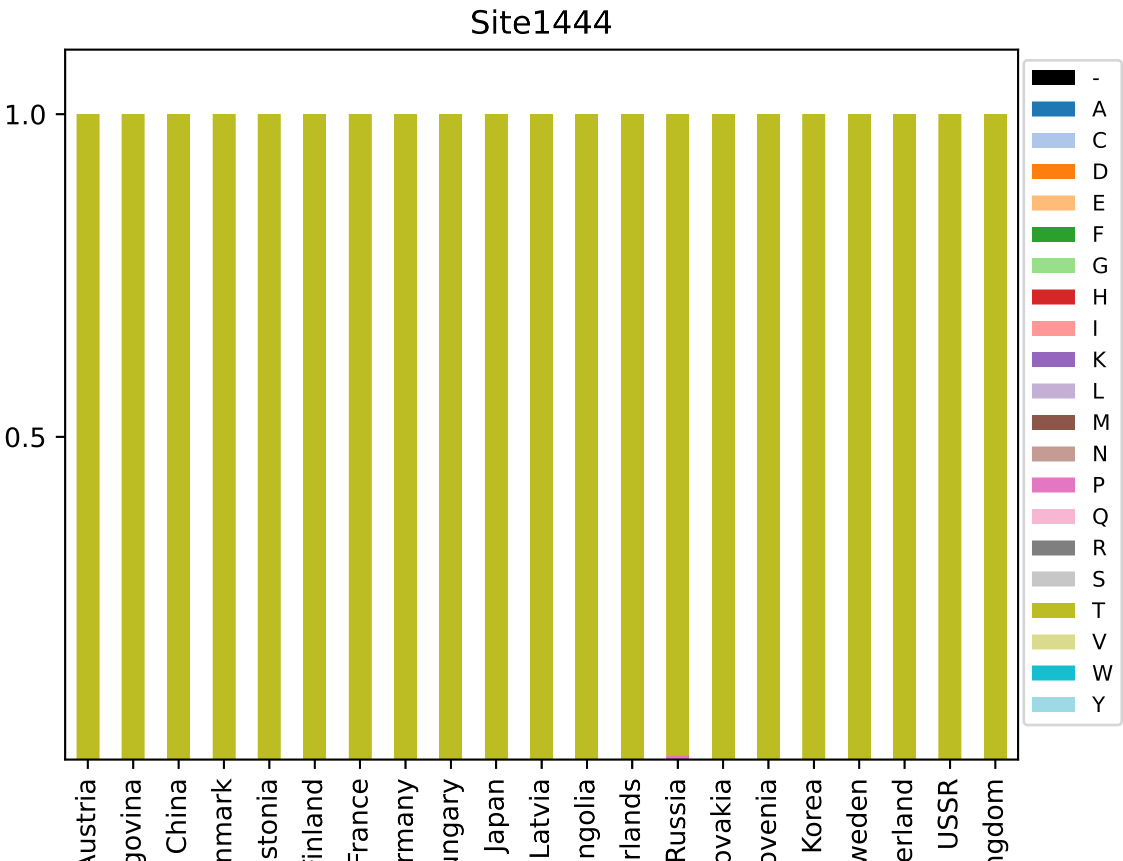

Site1444
1.0
0.5
-
A
C
D
E
F
G
H
I
K
L
M
N
P
Q
R
S
T
V
W
Y
Austria
govina
China
nmark
stonia
Finland
France
rmany
ungary
Japan
Latvia
ngolia
rlands
Russia
ovakia
ovenia
Korea
weden
erland
USSR
ngdom

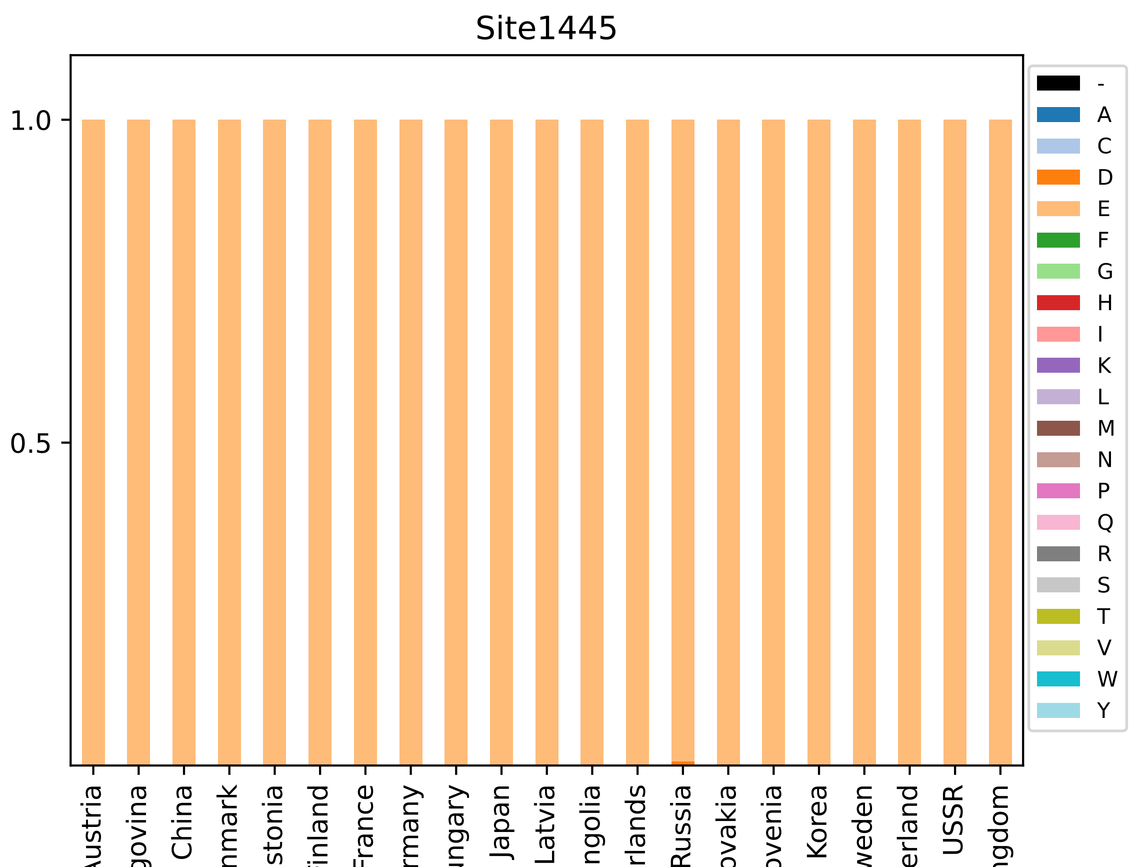
Site1445
1.0
0.5
-
A
C
D
E
F
G
H
I
K
L
M
N
P
Q
R
S
T
V
W
Y
Austria
China
Estonia
Finland
France
Hungary
Japan
Latvia
Russia
Korea
Sweden
USSR

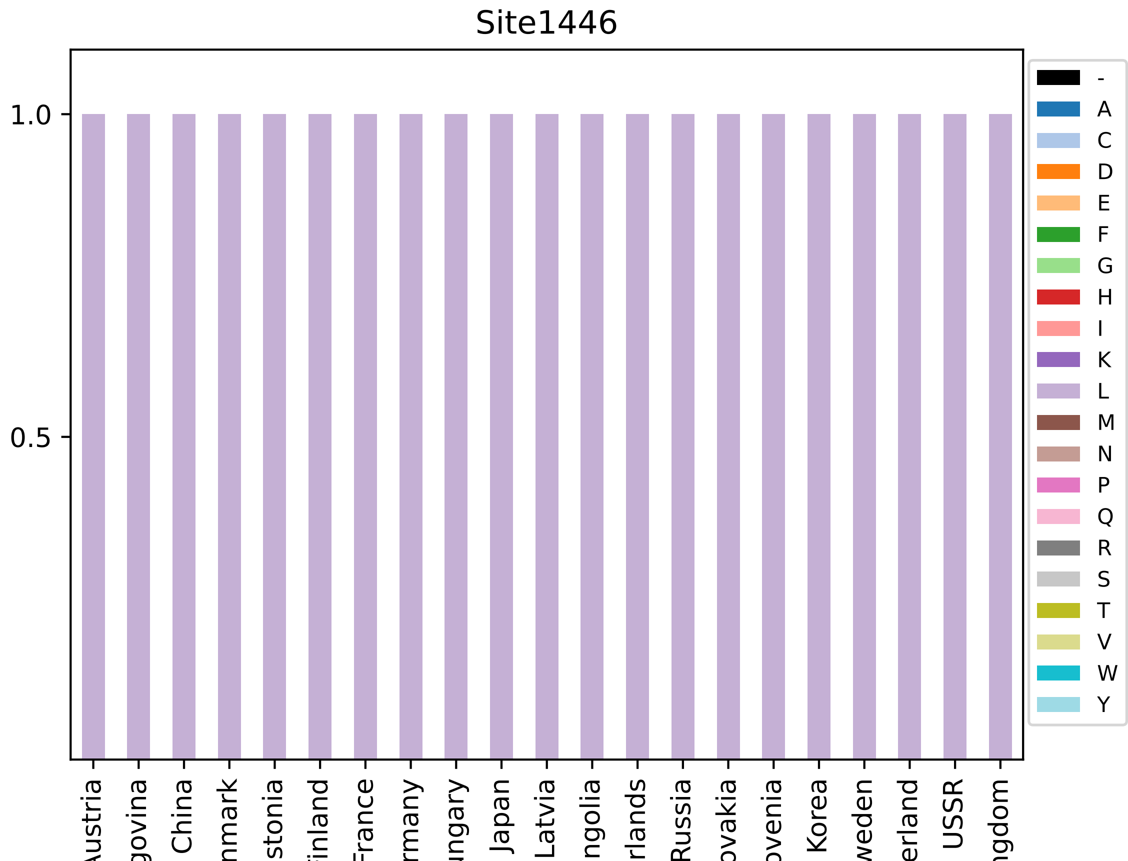
Site1446
1.0
0.5
-
A
C
D
E
F
G
H
I
K
L
M
N
P
Q
R
S
T
V
W
Y
China
Japan
Latvia
Russia
Korea
USSR

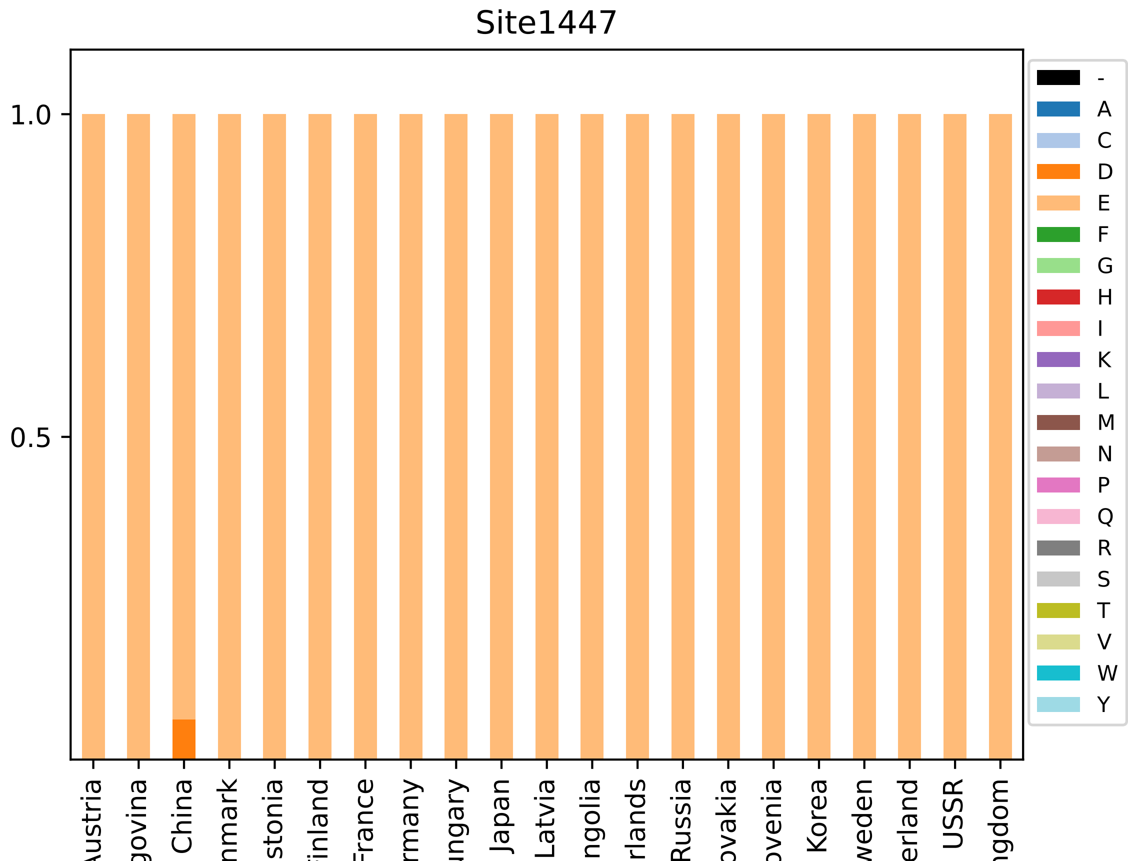
Site1447
1.0
0.5
-
A
C
D
E
F
G
H
I
K
L
M
N
P
Q
R
S
T
V
W
Y
China
Japan
Latvia
Russia
Korea
USSR

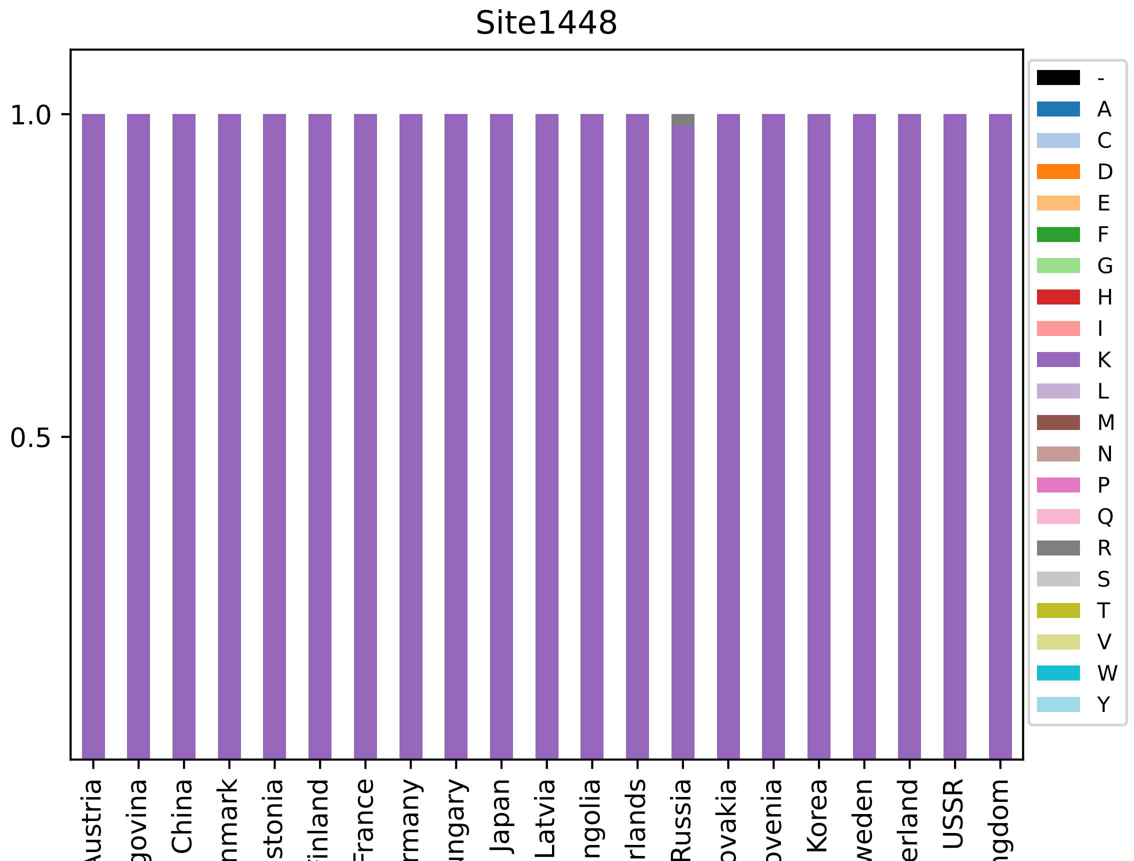

Site1448
1.0
0.5
-
A
C
D
E
F
G
H
I
K
L
M
N
P
Q
R
S
T
V
W
Y
Austria
China
Japan
Latvia
Russia
Korea
USSR

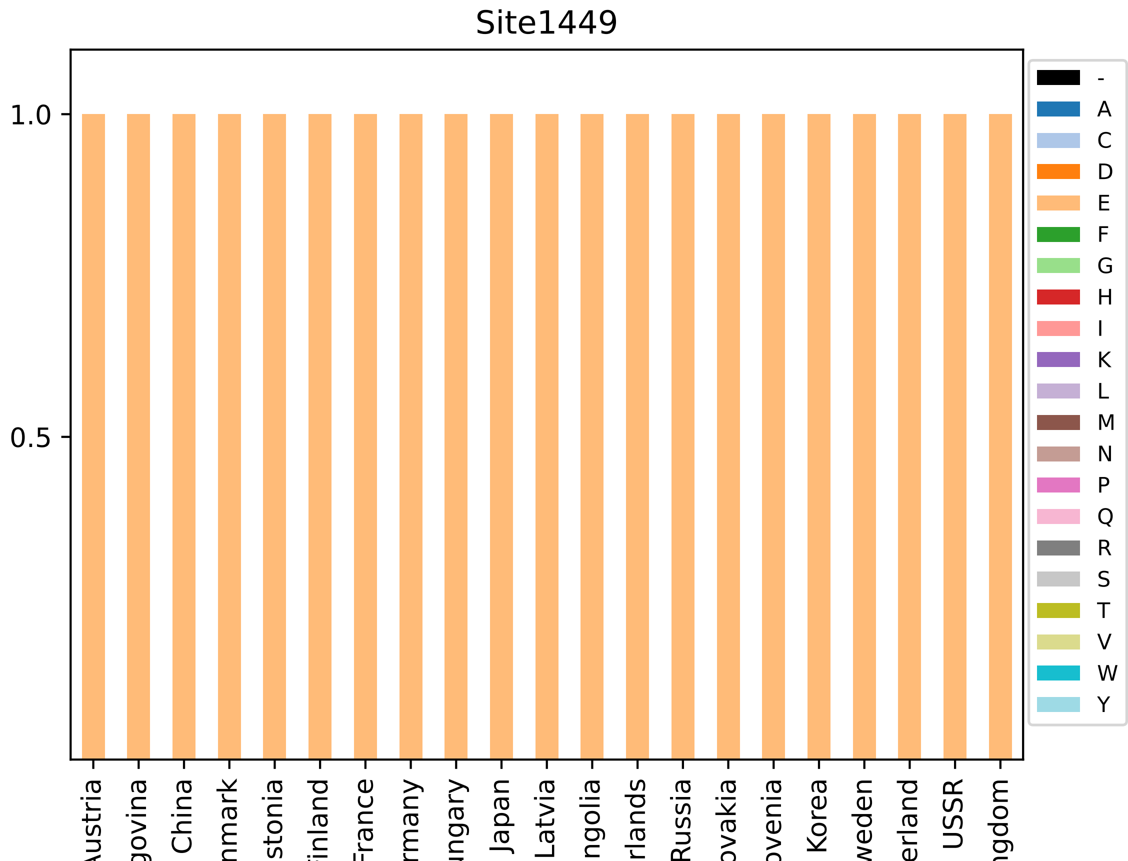
Site1449
1.0
0.5
Austria
govina
China
nmark
stonia
Finland
France
rmany
ungary
Japan
Latvia
ngolia
rlands
Russia
ovakia
ovenia
Korea
weden
erland
USSR
ngdom
-
A
C
D
E
F
G
H
I
K
L
M
N
P
Q
R
S
T
V
W
Y

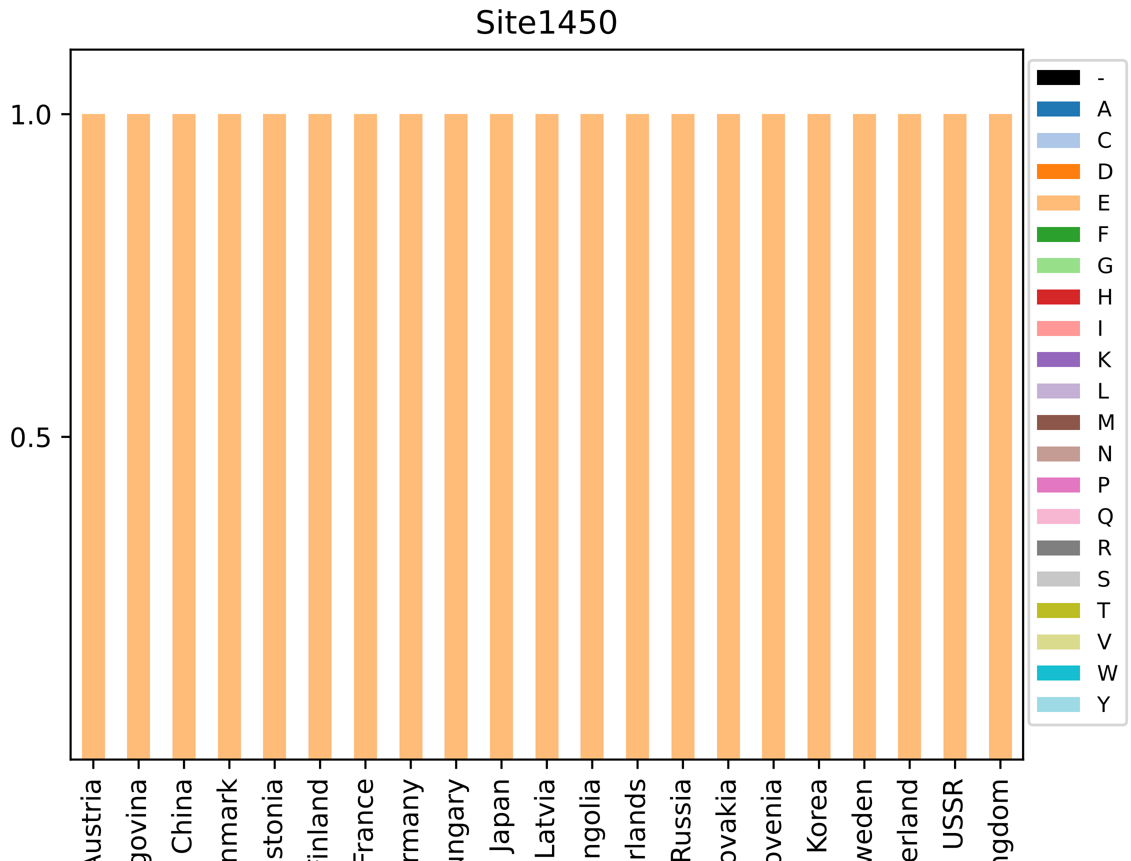
Site1450
1.0
0.5
Austria
govina
China
nmark
stonia
Finland
France
rmany
ungary
Japan
Latvia
ngolia
rlands
Russia
ovakia
ovenia
Korea
weden
erland
USSR
ngdom
-
A
C
D
E
F
G
H
I
K
L
M
N
P
Q
R
S
T
V
W
Y

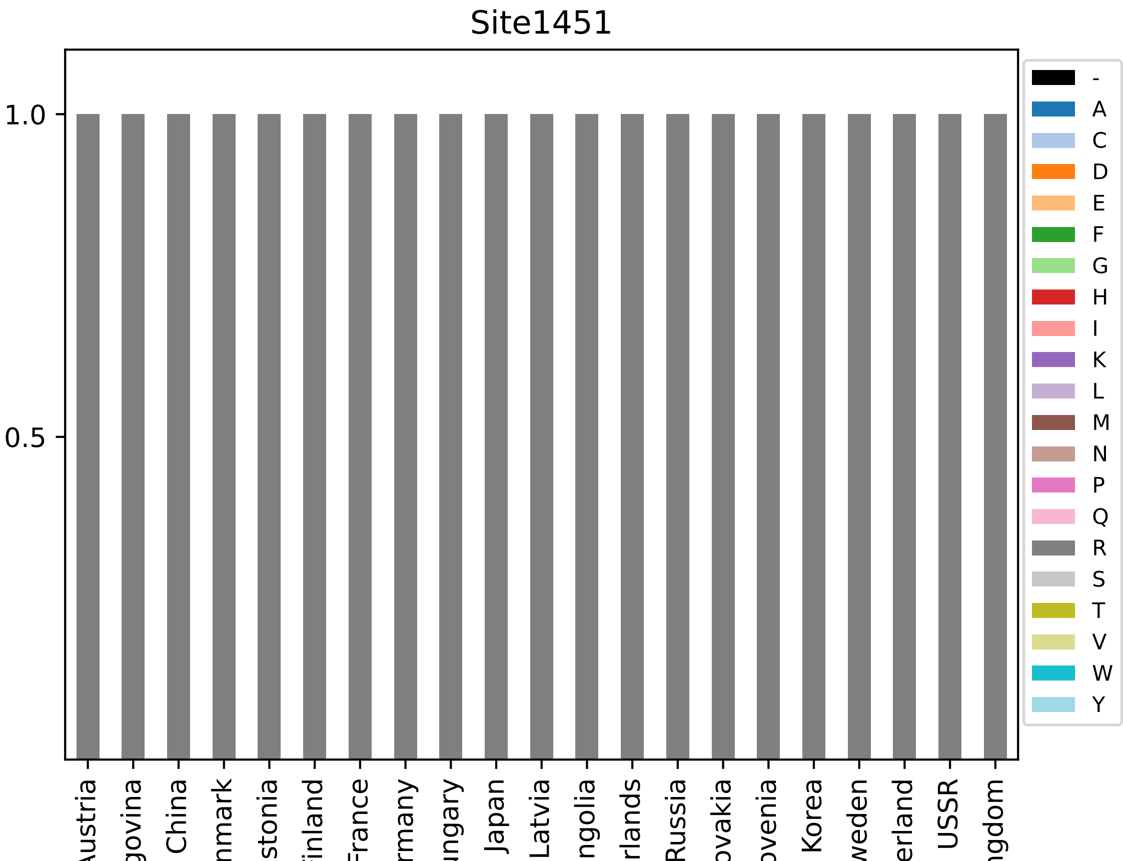

Site1451
1.0
0.5
-
A
C
D
E
F
G
H
I
K
L
M
N
P
Q
R
S
T
V
W
Y
Austria
China
Japan
Latvia
Russia
Korea
USSR

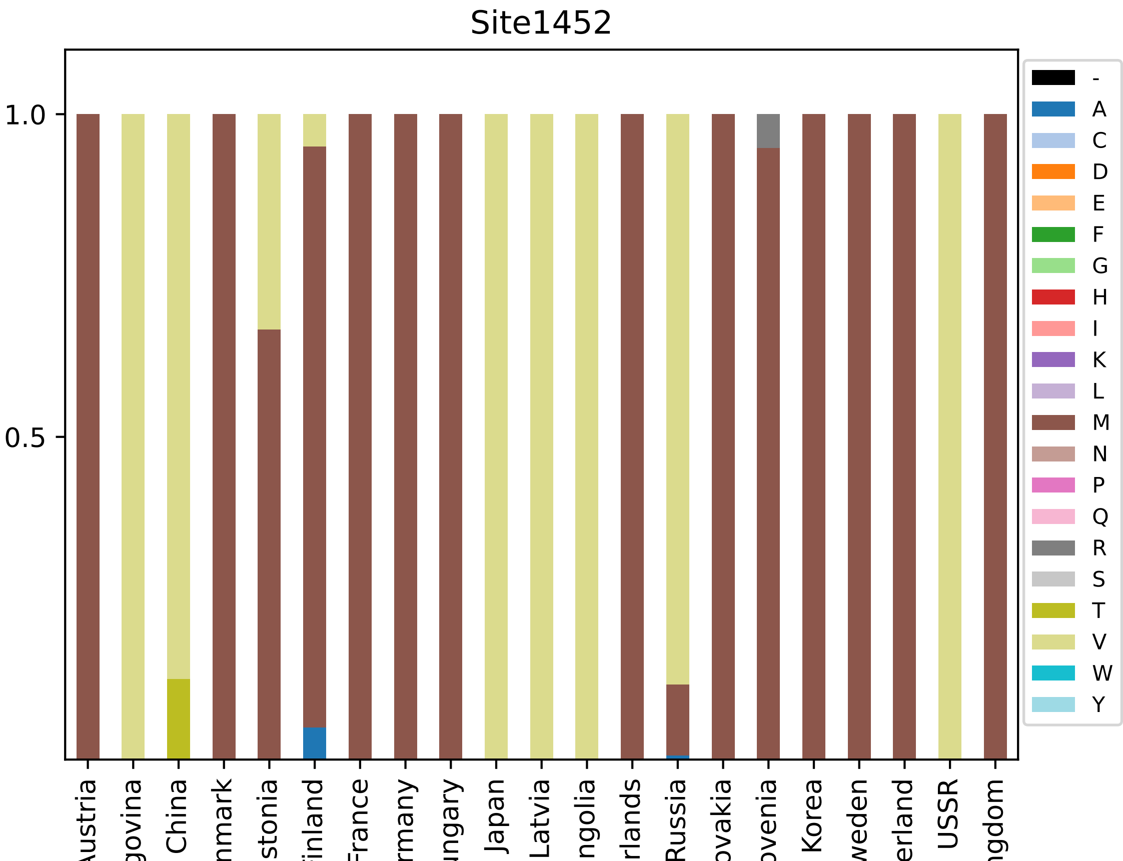

Site1452
1.0
0.5
-
A
C
D
E
F
G
H
I
K
L
M
N
P
Q
R
S
T
V
W
Y
Austria
China
Finland
France
Japan
Latvia
Russia
Korea
USSR

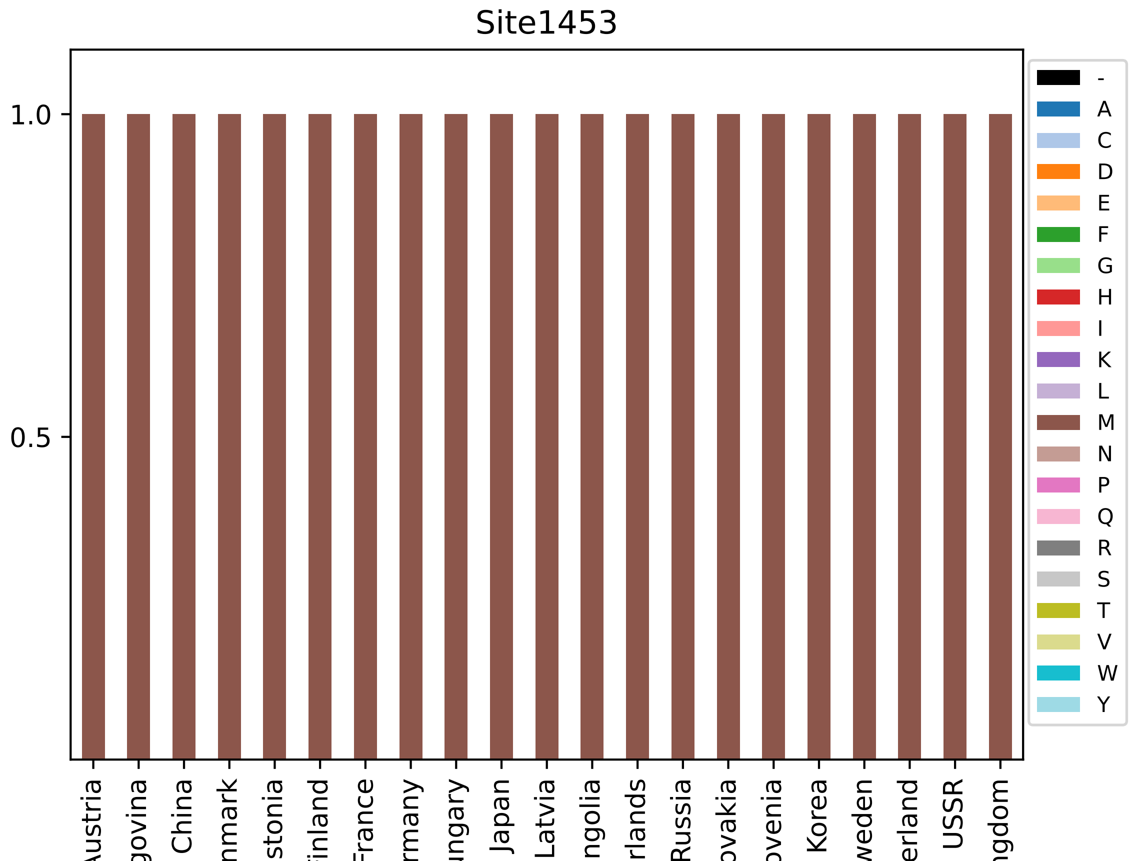

Site1453
1.0
0.5
-
A
C
D
E
F
G
H
I
K
L
M
N
P
Q
R
S
T
V
W
Y
China
Japan
Latvia
Russia
Korea
USSR

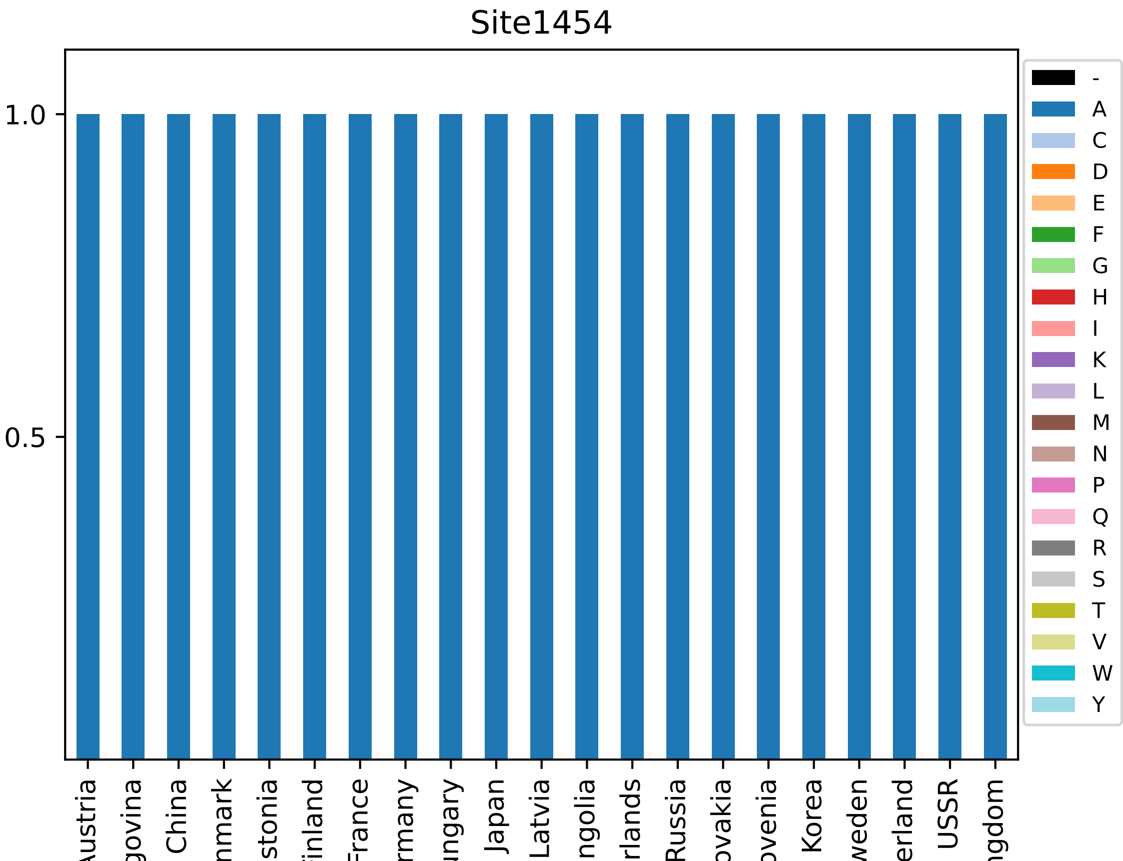

Site1454
1.0
0.5
-
A
C
D
E
F
G
H
I
K
L
M
N
P
Q
R
S
T
V
W
Y
Austria
govina
China
nmark
stonia
Finland
France
rmany
ungary
Japan
Latvia
ngolia
rlands
Russia
ovakia
ovenia
Korea
weden
erland
USSR
ngdom

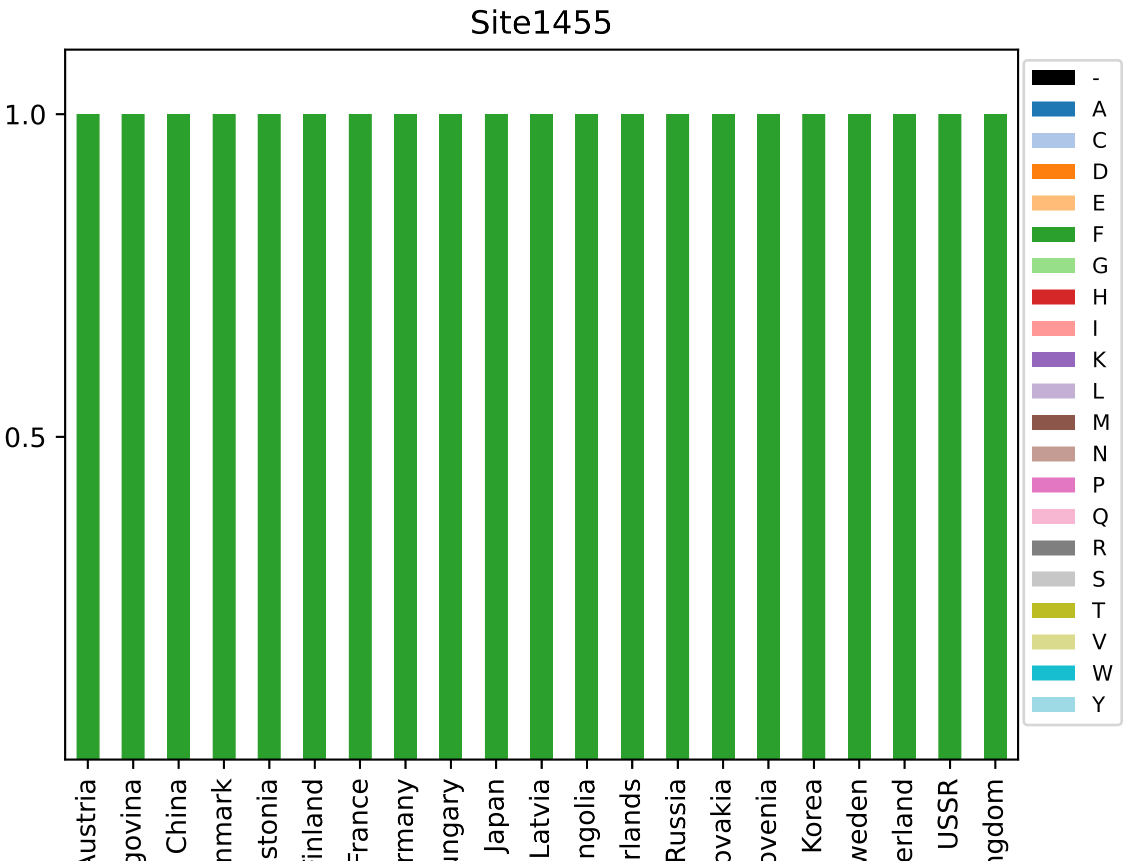

Site1455
1.0
0.5
-
A
C
D
E
F
G
H
I
K
L
M
N
P
Q
R
S
T
V
W
Y
Austria
China
Japan
Latvia
Russia
Korea
USSR

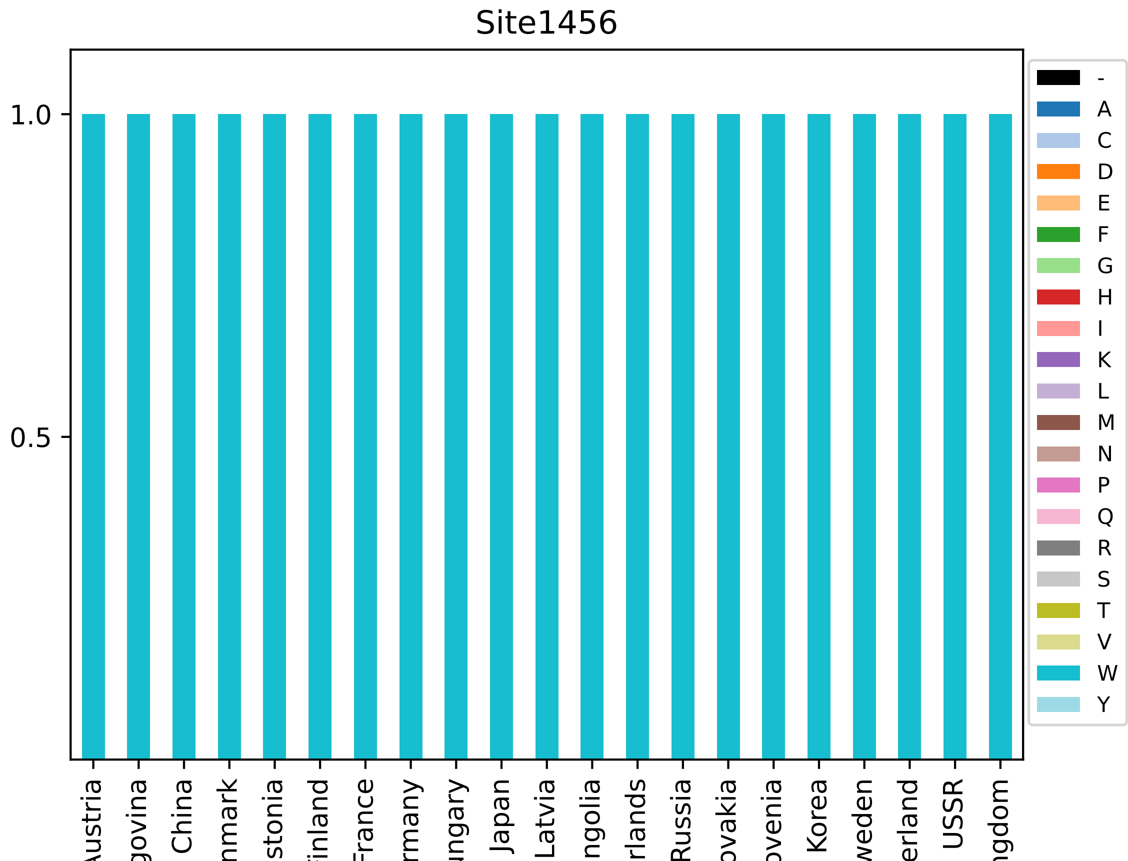

Site1456
1.0
0.5
-
A
C
D
E
F
G
H
I
K
L
M
N
P
Q
R
S
T
V
W
Y
China
Japan
Latvia
Russia
Korea
USSR

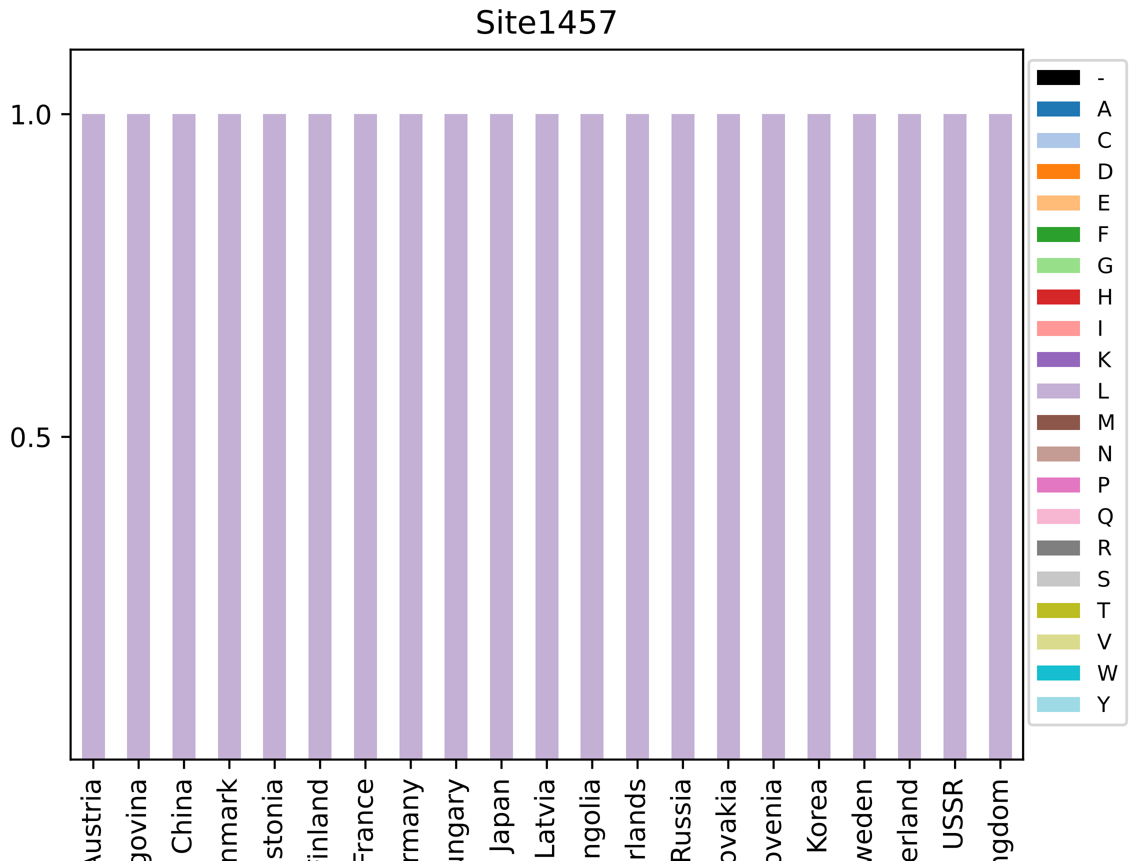

Site1457
1.0
0.5
-
A
C
D
E
F
G
H
I
K
L
M
N
P
Q
R
S
T
V
W
Y
Austria
China
Estonia
Finland
France
Hungary
Japan
Latvia
Russia
Korea
Sweden
USSR

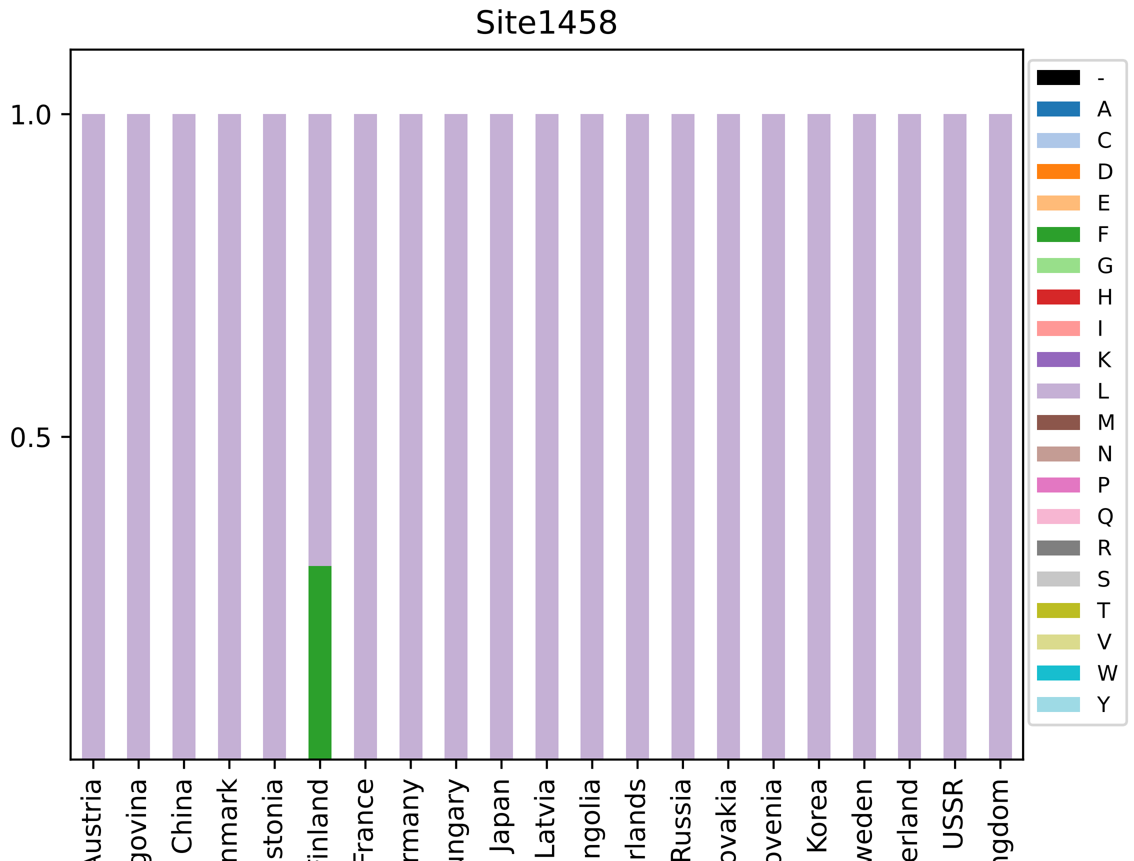

Site1458
1.0
0.5
-
A
C
D
E
F
G
H
I
K
L
M
N
P
Q
R
S
T
V
W
Y
China
Japan
Latvia
Russia
Korea
USSR

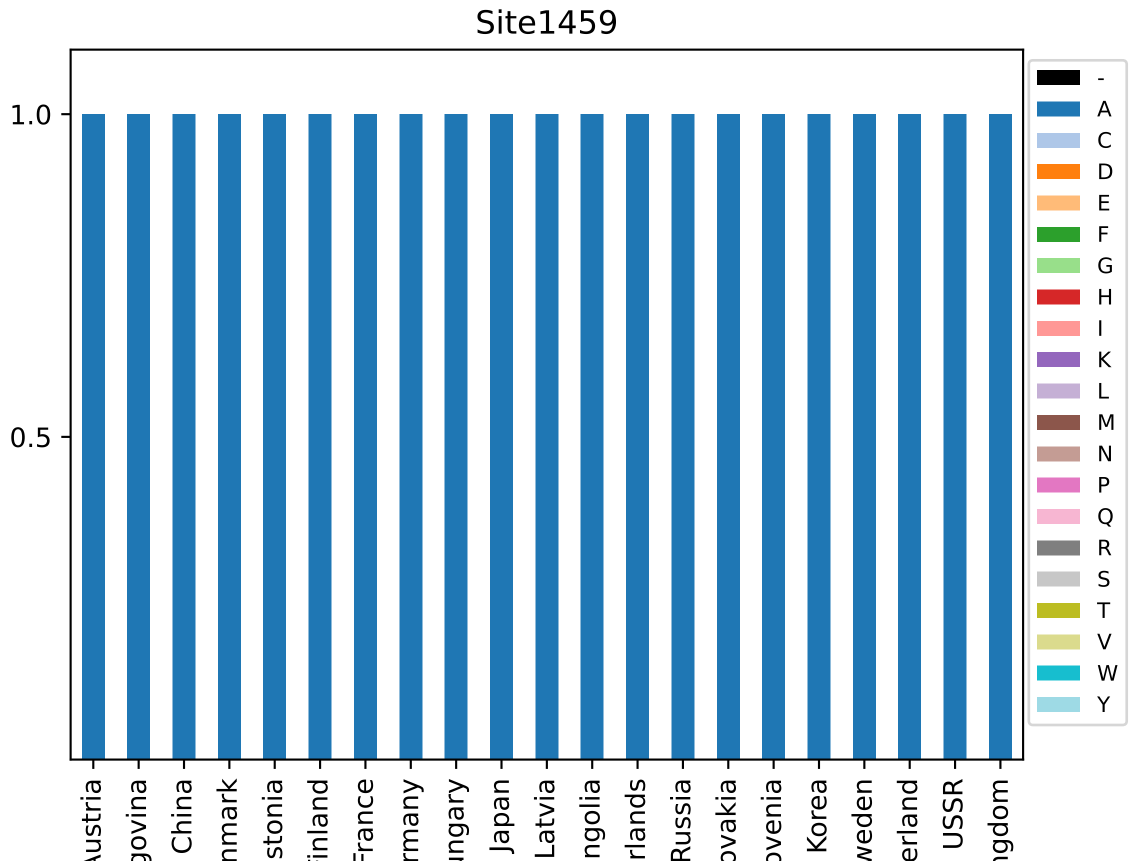
Site1459
1.0
0.5
China
Japan
Latvia
Russia
Korea
USSR
-
A
C
D
E
F
G
H
I
K
L
M
N
P
Q
R
S
T
V
W
Y

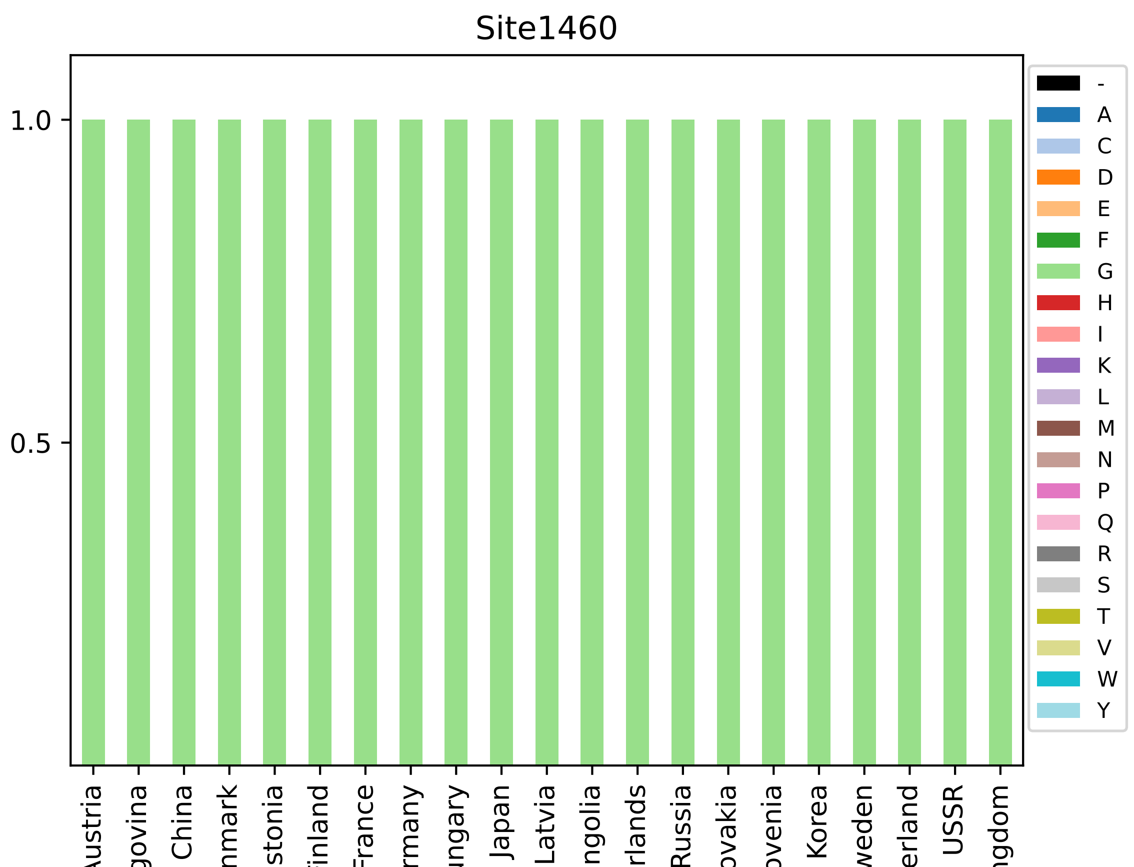

Site1460
1.0
0.5
-
A
C
D
E
F
G
H
I
K
L
M
N
P
Q
R
S
T
V
W
Y
Austria
govina
China
nmark
stonia
Finland
France
rmany
ungary
Japan
Latvia
ngolia
rlands
Russia
ovakia
ovenia
Korea
weden
erland
USSR
ngdom

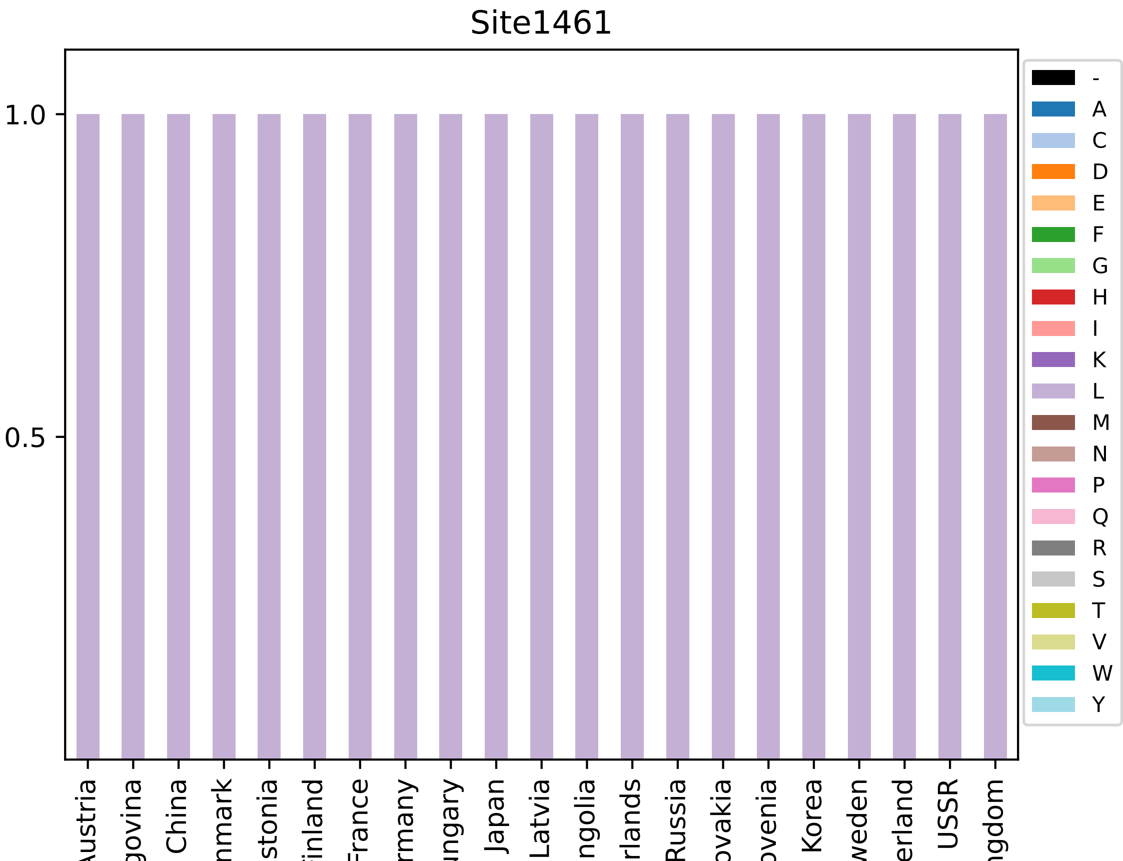

Site1461
1.0
0.5
-
A
C
D
E
F
G
H
I
K
L
M
N
P
Q
R
S
T
V
W
Y
China
Japan
Latvia
Russia
Korea
USSR

Site1462

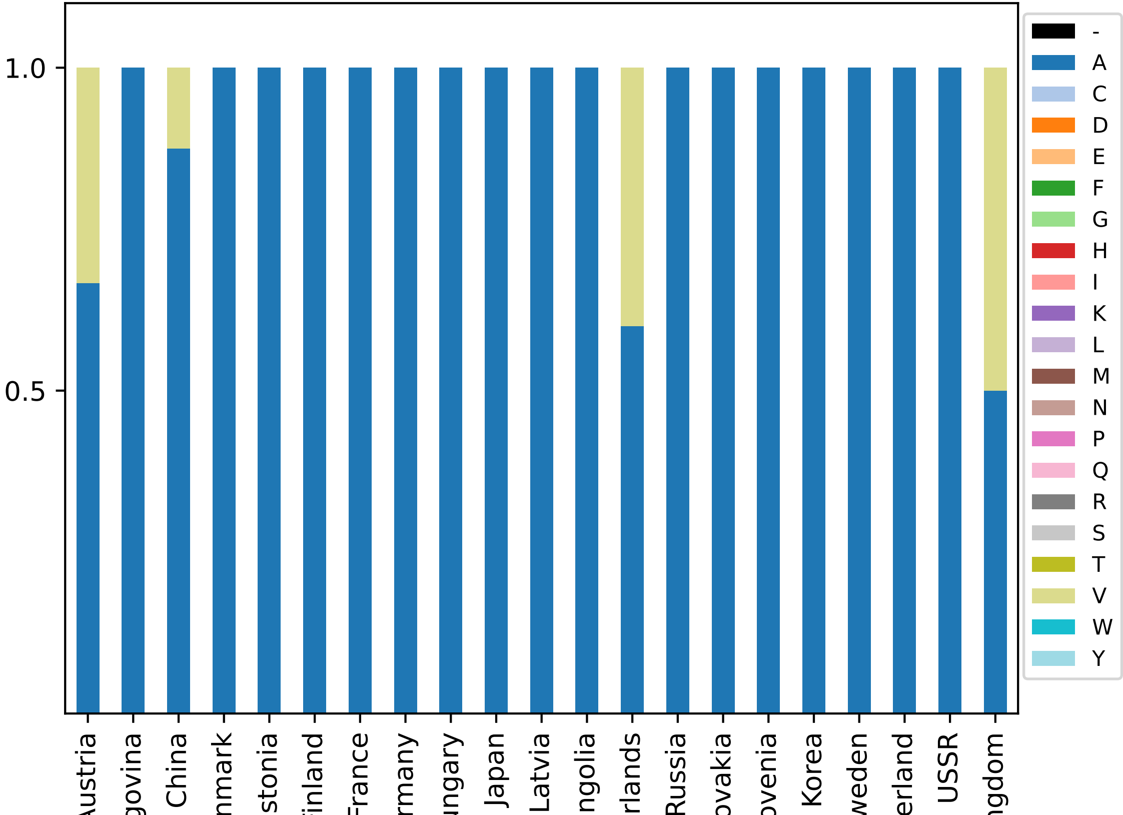

1.0
0.5
-
A
C
D
E
F
G
H
I
K
L
M
N
P
Q
R
S
T
V
W
Y
China
Japan
Latvia
Russia
Korea
USSR

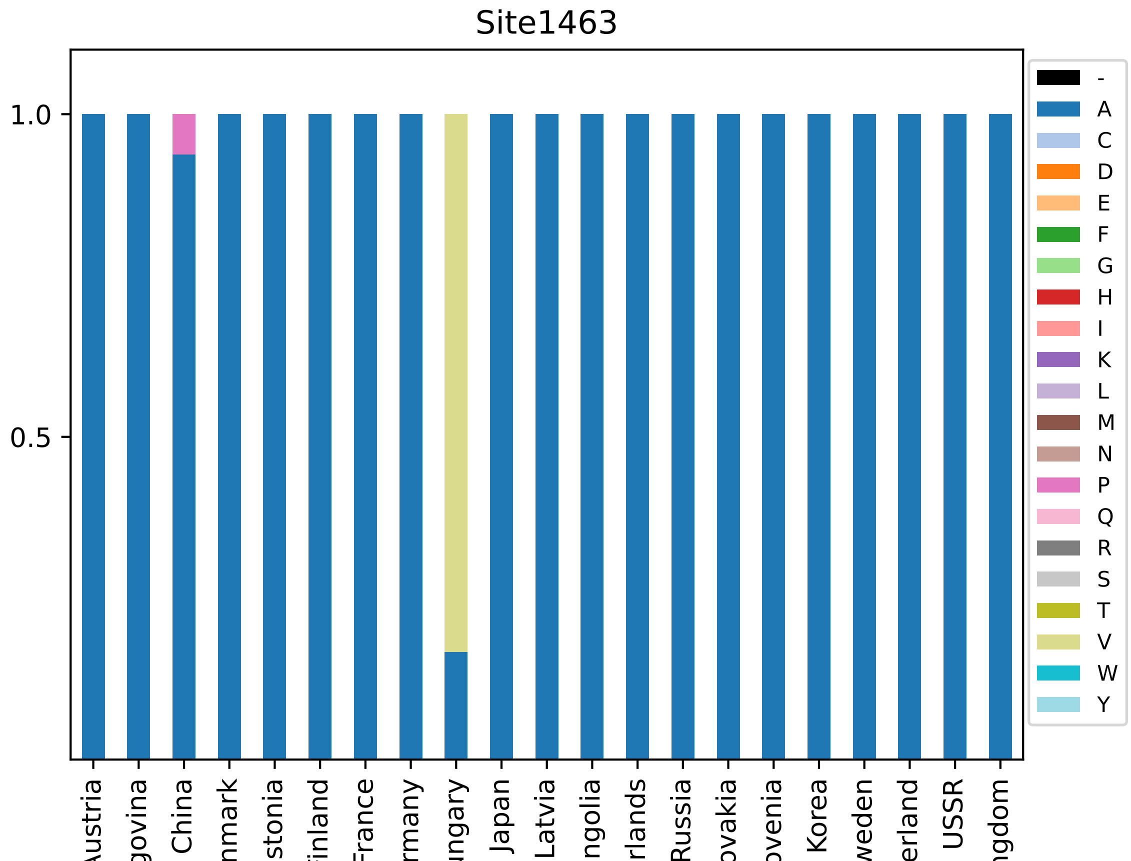
Site1463
1.0
0.5
-
A
C
D
E
F
G
H
I
K
L
M
N
P
Q
R
S
T
V
W
Y
Austria
govina
China
nmark
stonia
inland
France
rmany
ungary
Japan
Latvia
ngolia
rlands
Russia
ovakia
ovenia
Korea
weden
erland
USSR
ngdom

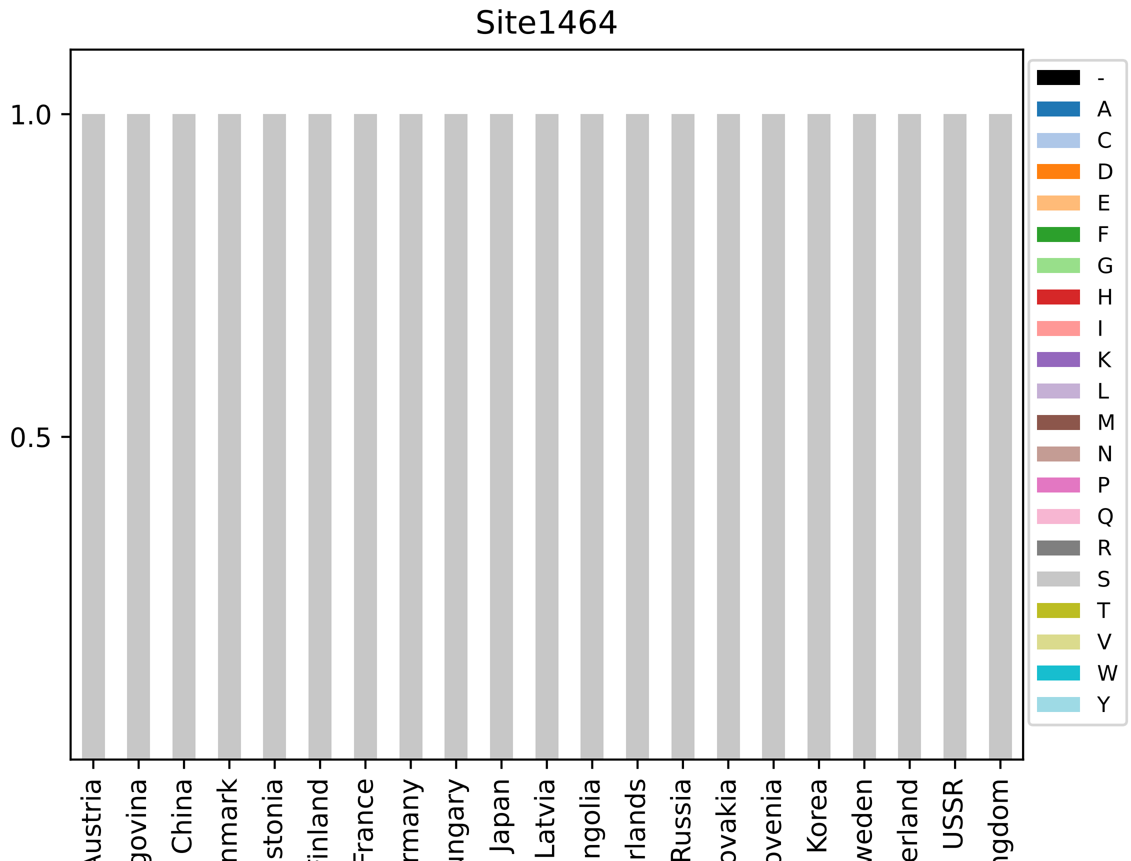
Site1464
1.0
0.5
Austria
govina
China
nmark
stonia
Finland
France
rmany
ungary
Japan
Latvia
ngolia
rlands
Russia
ovakia
ovenia
Korea
weden
erland
USSR
ngdom
-
A
C
D
E
F
G
H
I
K
L
M
N
P
Q
R
S
T
V
W
Y

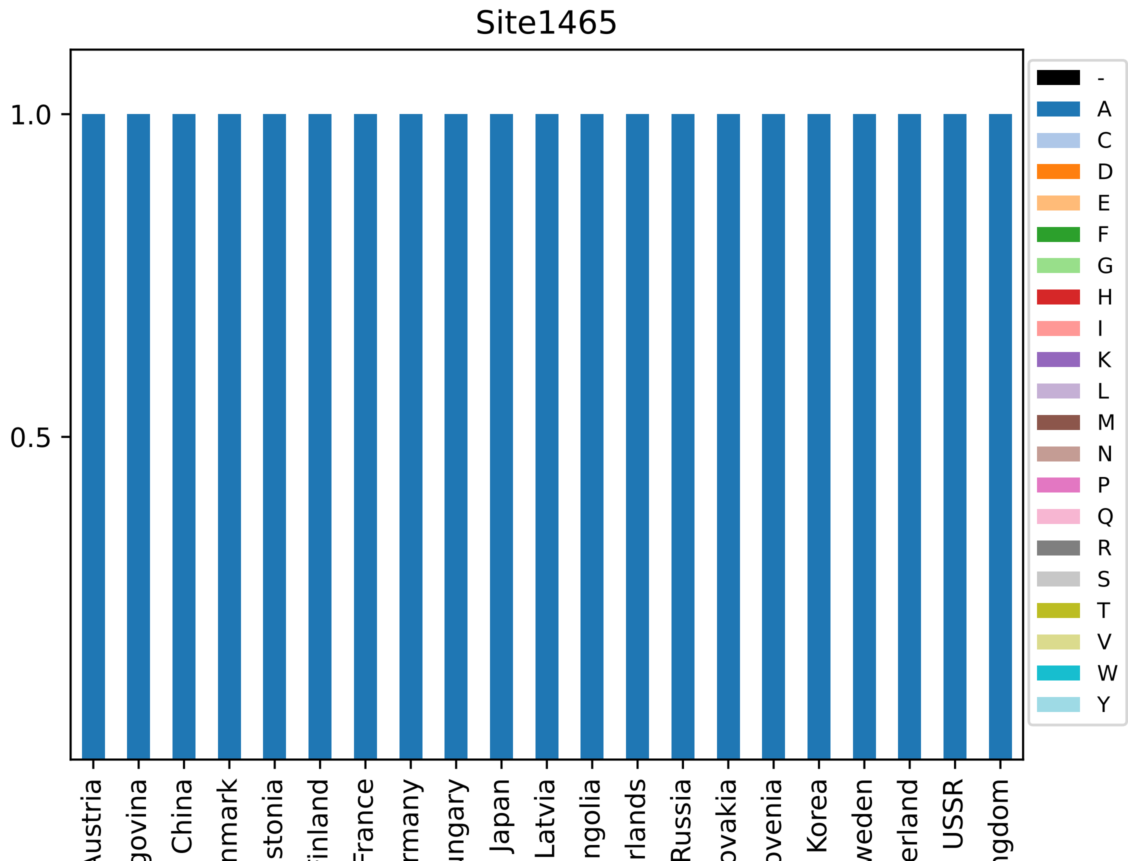

Site1465
1.0
0.5
-
A
C
D
E
F
G
H
I
K
L
M
N
P
Q
R
S
T
V
W
Y
Austria
govina
China
nmark
stonia
inland
France
rmany
ungary
Japan
Latvia
ngolia
rlands
Russia
ovakia
ovenia
Korea
weden
erland
USSR
ngdom

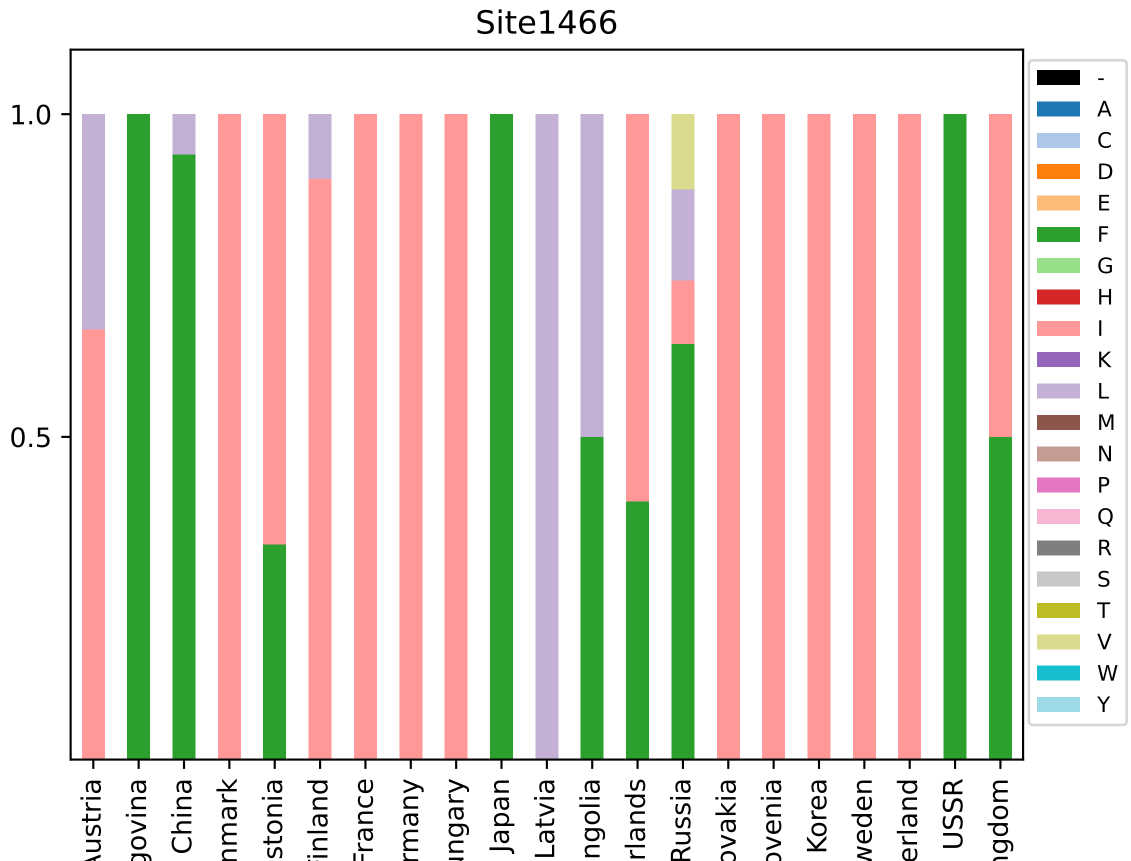

Site1466
1.0
0.5
-
A
C
D
E
F
G
H
I
K
L
M
N
P
Q
R
S
T
V
W
Y
Austria
govina
China
nmark
stonia
inland
France
rmany
ungary
Japan
Latvia
ngolia
rlands
Russia
ovakia
ovenia
Korea
weden
erland
USSR
ngdom

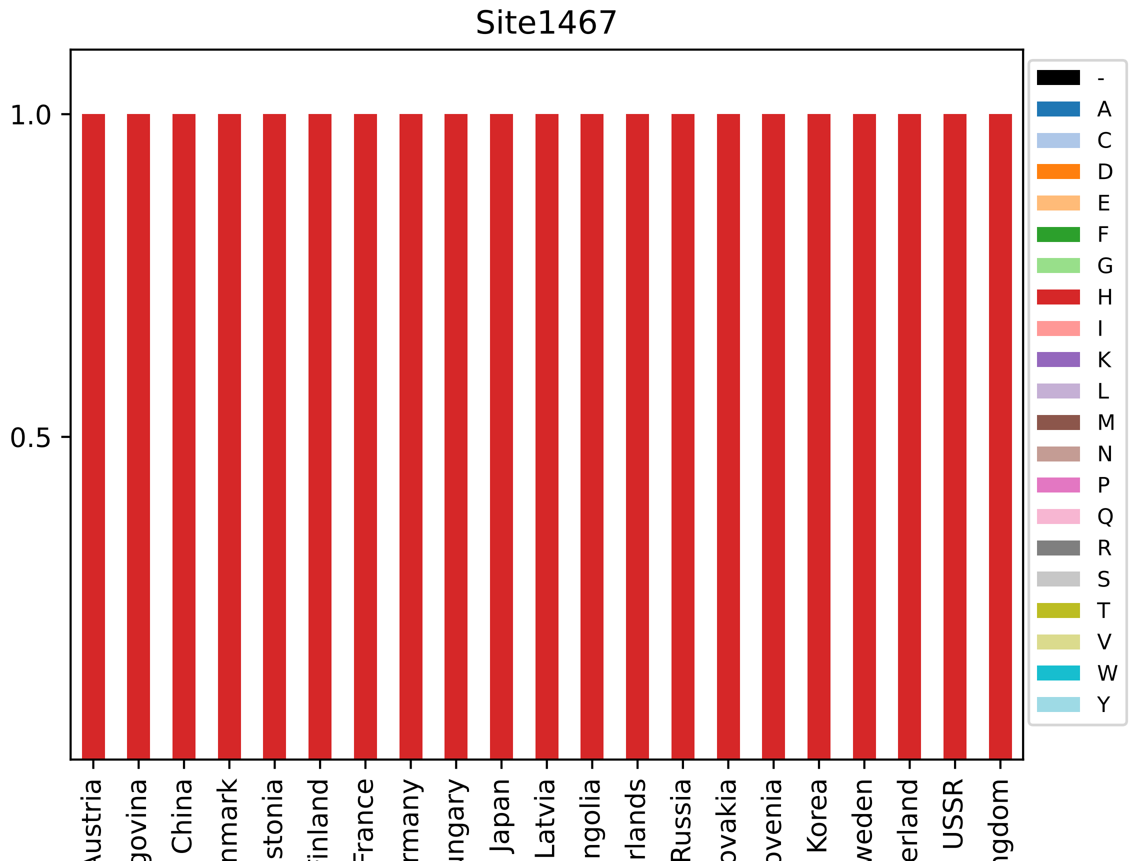

Site1467
1.0
0.5
-
A
C
D
E
F
G
H
I
K
L
M
N
P
Q
R
S
T
V
W
Y
Austria
China
Japan
Latvia
Russia
Korea
USSR

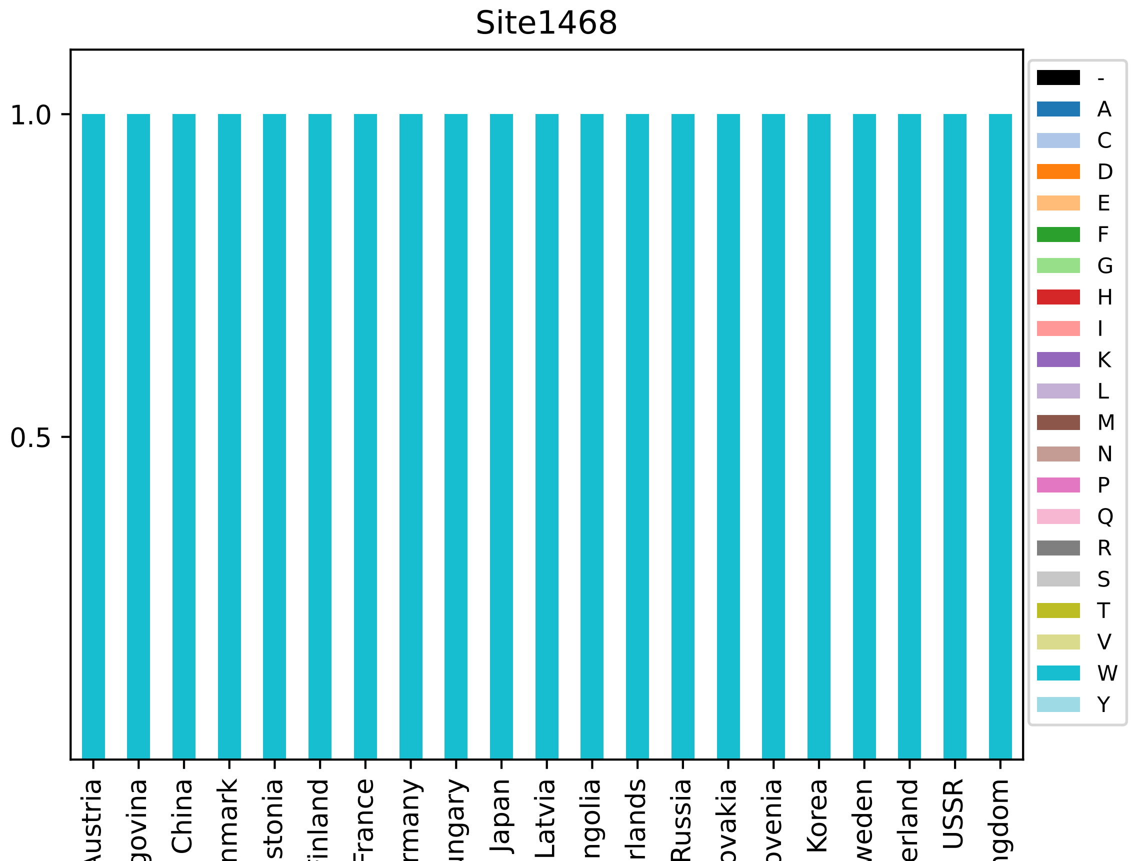

Site1468
1.0
0.5
-
A
C
D
E
F
G
H
I
K
L
M
N
P
Q
R
S
T
V
W
Y
China
Japan
Latvia
Russia
Korea
USSR

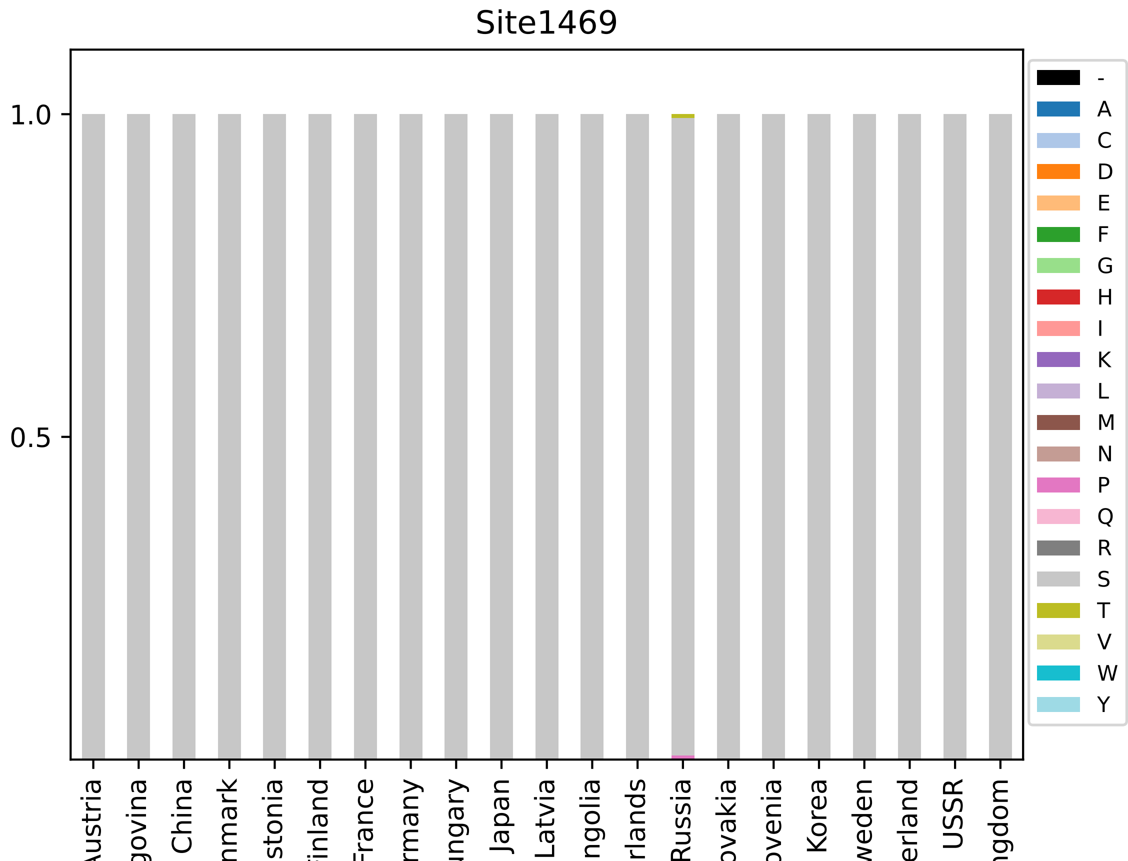
Site1469
1.0
0.5
-
A
C
D
E
F
G
H
I
K
L
M
N
P
Q
R
S
T
V
W
Y
Austria
govina
China
nmark
stonia
inland
France
rmany
ungary
Japan
Latvia
ngolia
rlands
Russia
ovakia
ovenia
Korea
weden
erland
USSR
ngdom

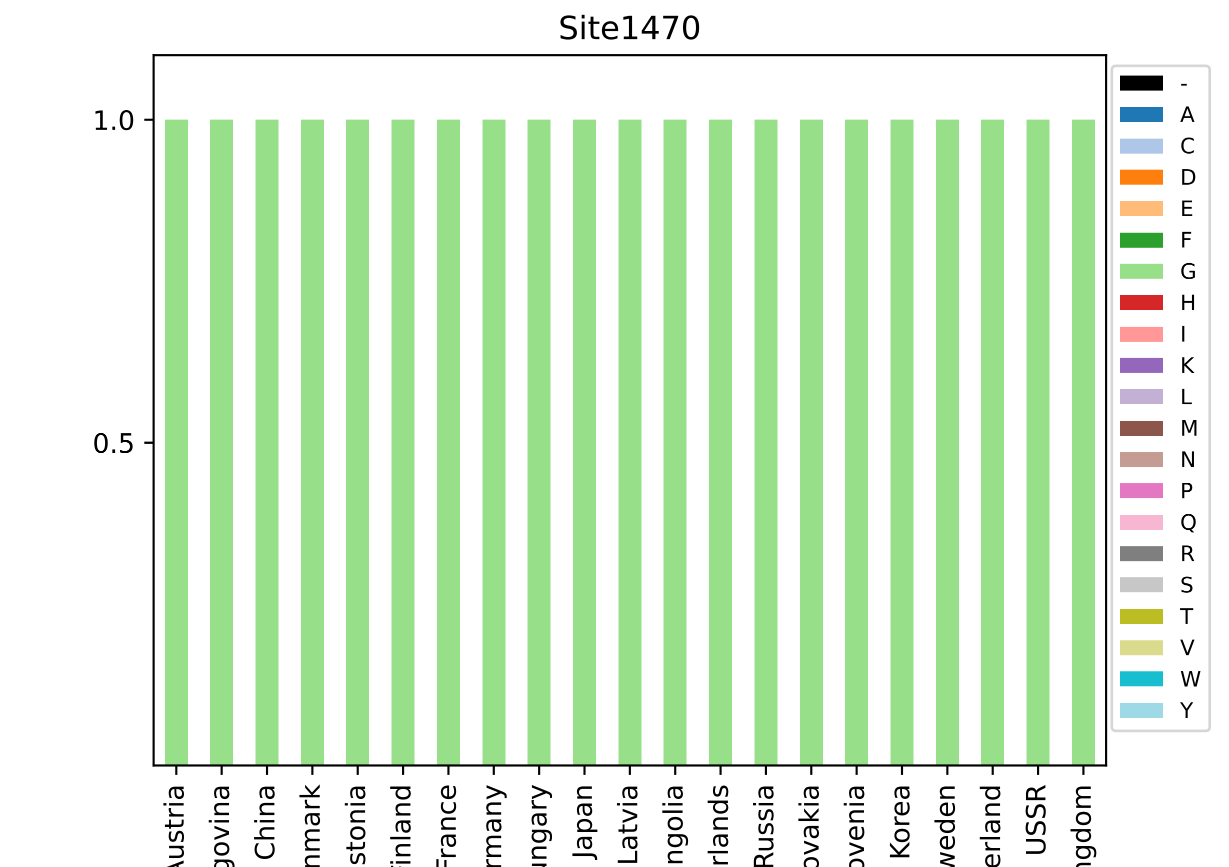

Site1470
1.0
0.5
-
A
C
D
E
F
G
H
I
K
L
M
N
P
Q
R
S
T
V
W
Y
China
Japan
Latvia
Russia
Korea
USSR

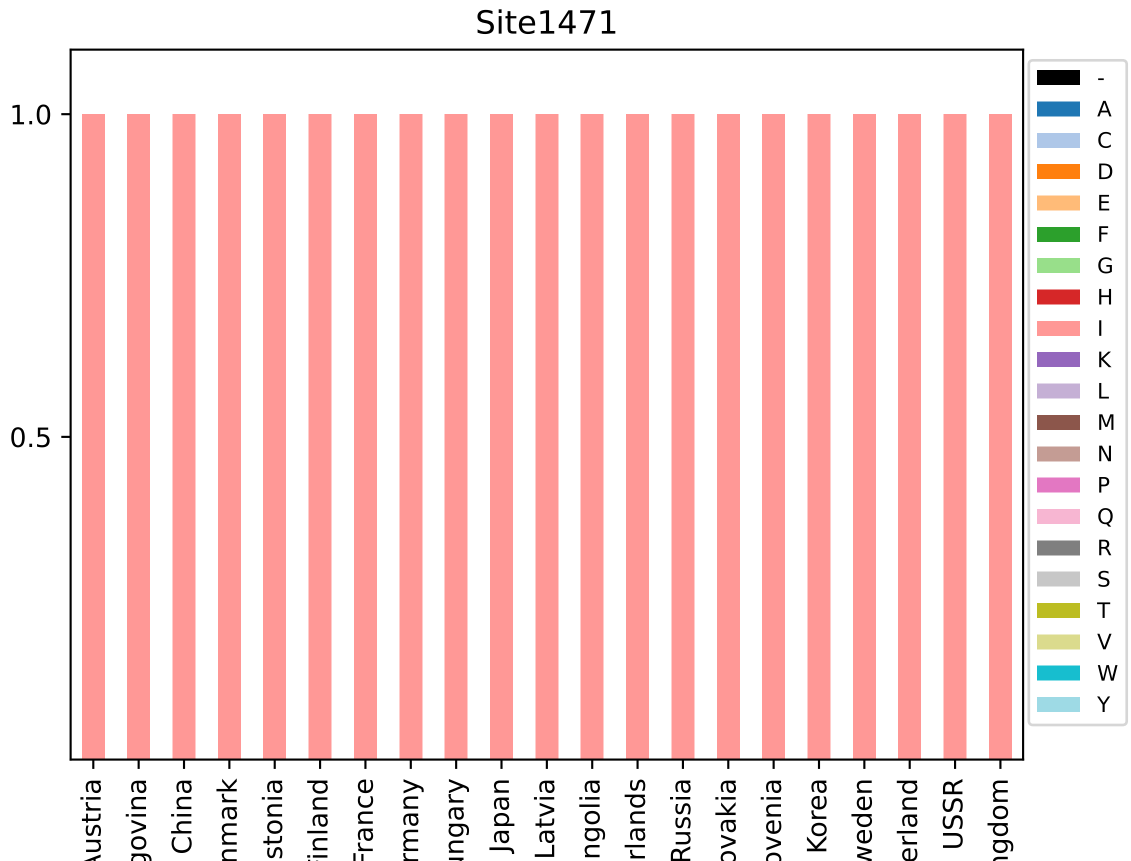

Site1471
1.0
0.5
Austria
govina
China
nmark
stonia
Finland
France
rmany
ungary
Japan
Latvia
ngolia
rlands
Russia
ovakia
ovenia
Korea
weden
erland
USSR
ngdom
-
A
C
D
E
F
G
H
I
K
L
M
N
P
Q
R
S
T
V
W
Y

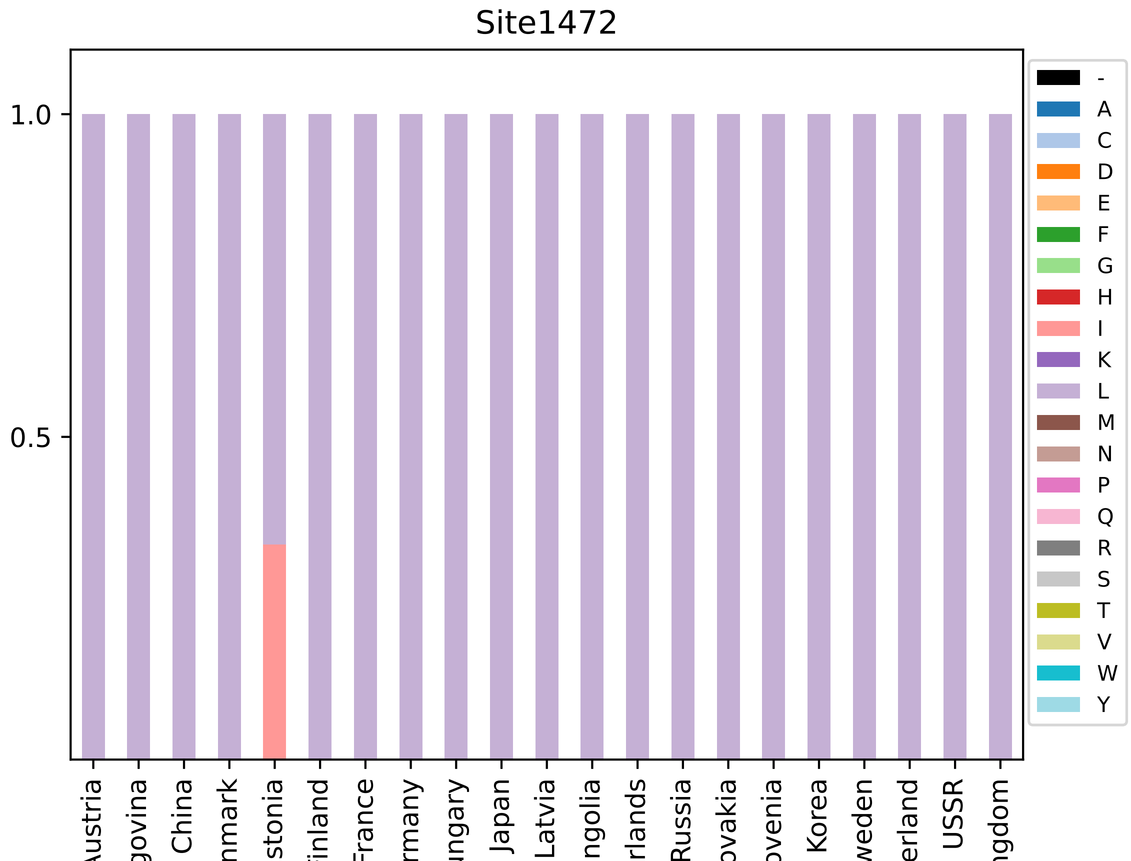

Site1472
1.0
0.5
-
A
C
D
E
F
G
H
I
K
L
M
N
P
Q
R
S
T
V
W
Y
China
Japan
Latvia
Russia
Korea
USSR

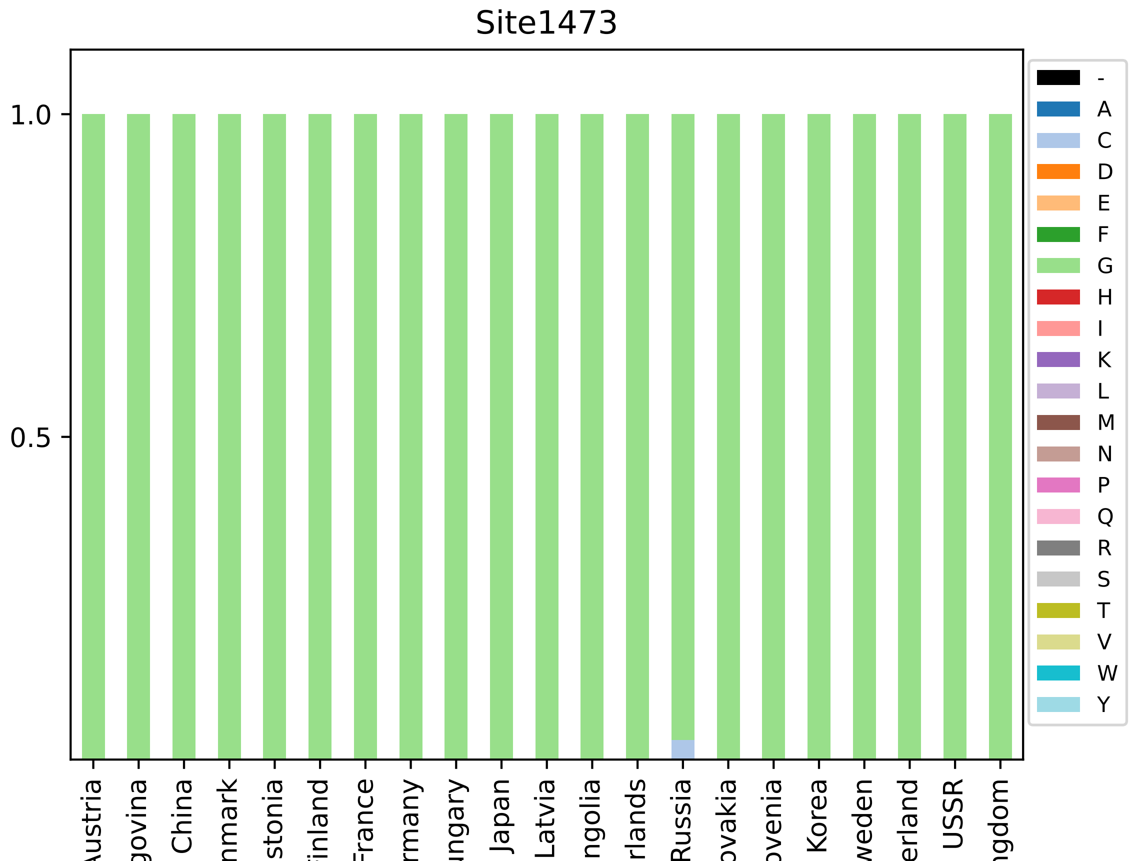
Site1473
1.0
0.5
-
A
C
D
E
F
G
H
I
K
L
M
N
P
Q
R
S
T
V
W
Y
China
Japan
Latvia
Russia
Korea
USSR

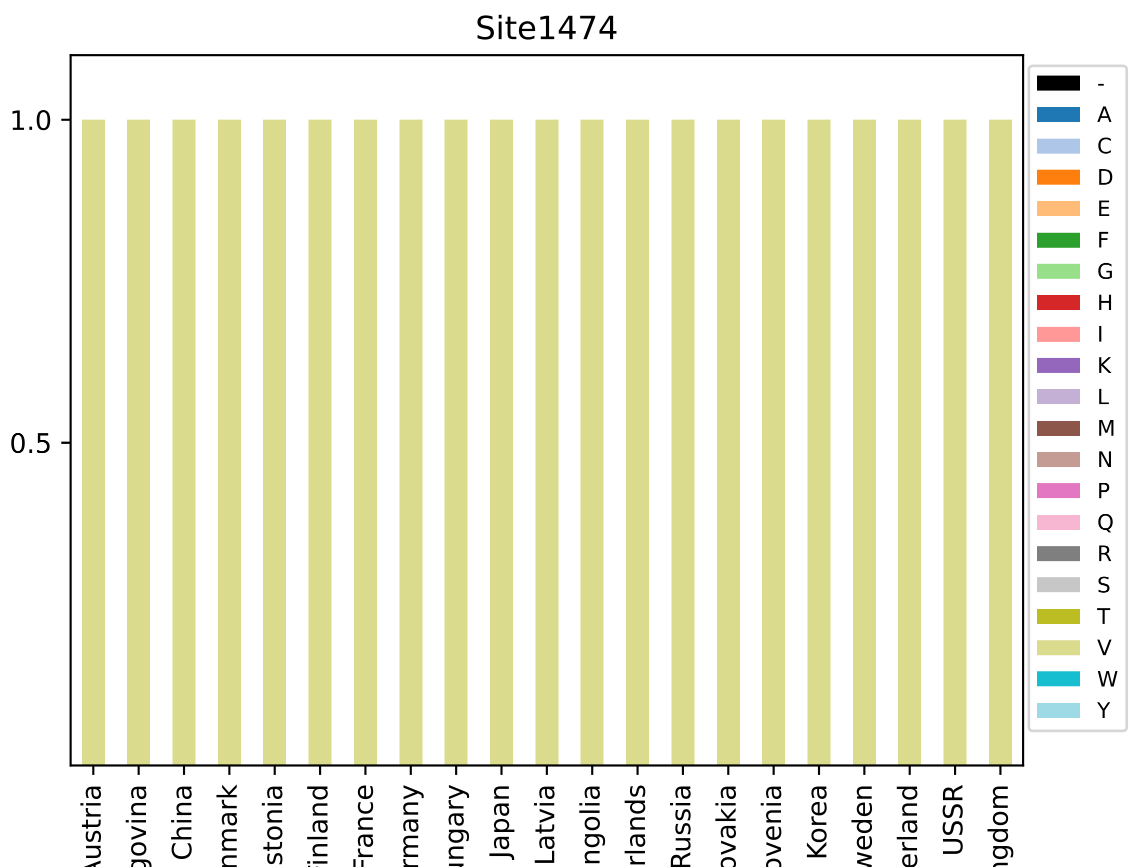

Site1474
1.0
0.5
-
A
C
D
E
F
G
H
I
K
L
M
N
P
Q
R
S
T
V
W
Y
China
Japan
Latvia
Russia
Korea
USSR

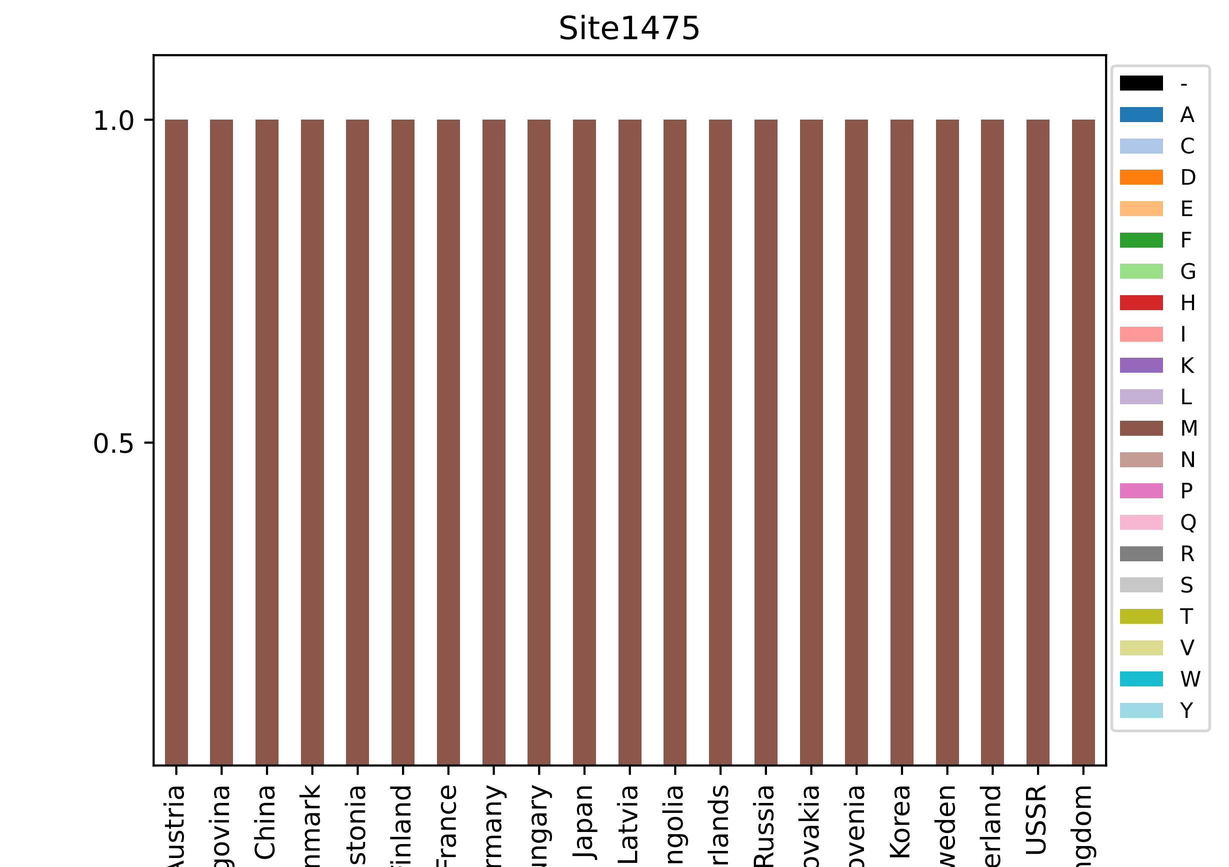

Site1475
1.0
0.5
-
A
C
D
E
F
G
H
I
K
L
M
N
P
Q
R
S
T
V
W
Y
Austria
govina
China
nmark
stonia
inland
France
rmany
ungary
Japan
Latvia
ngolia
rlands
Russia
ovakia
ovenia
Korea
weden
erland
USSR
ngdom

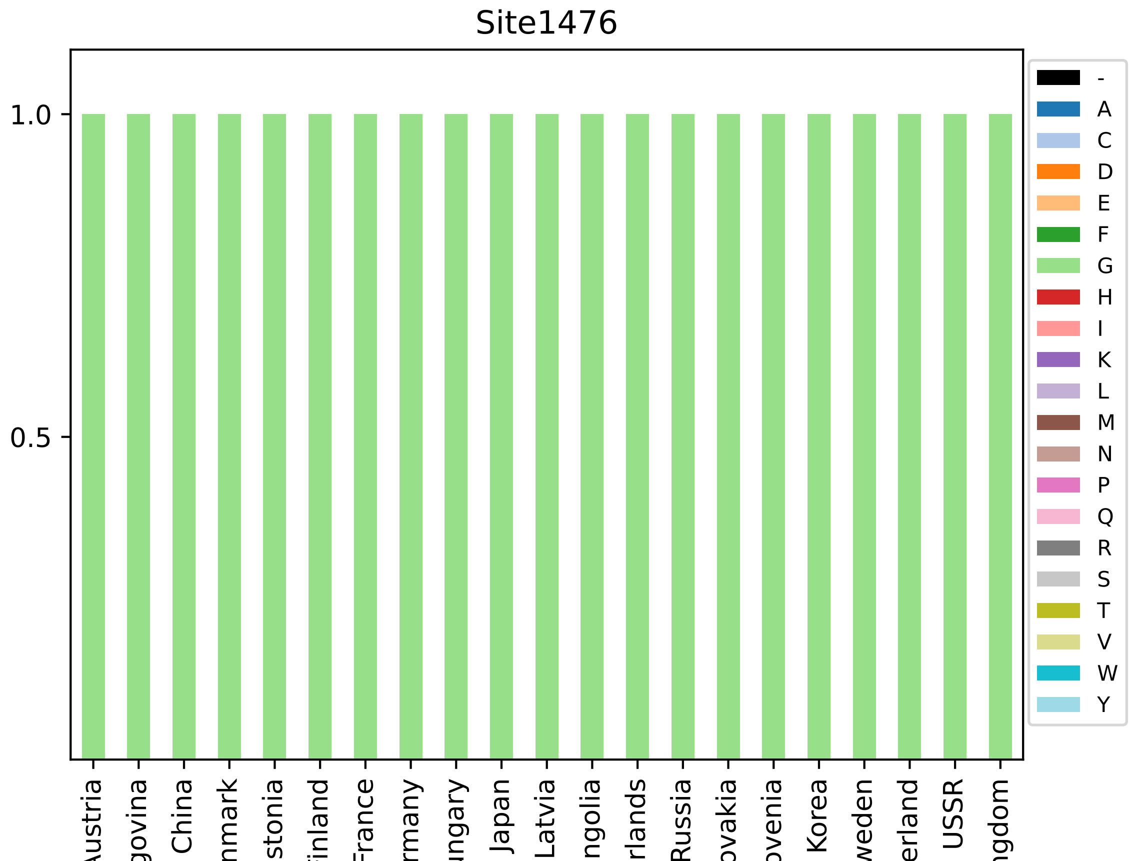
Site1476
1.0
0.5
-
A
C
D
E
F
G
H
I
K
L
M
N
P
Q
R
S
T
V
W
Y
China
Japan
Latvia
Russia
Korea
USSR

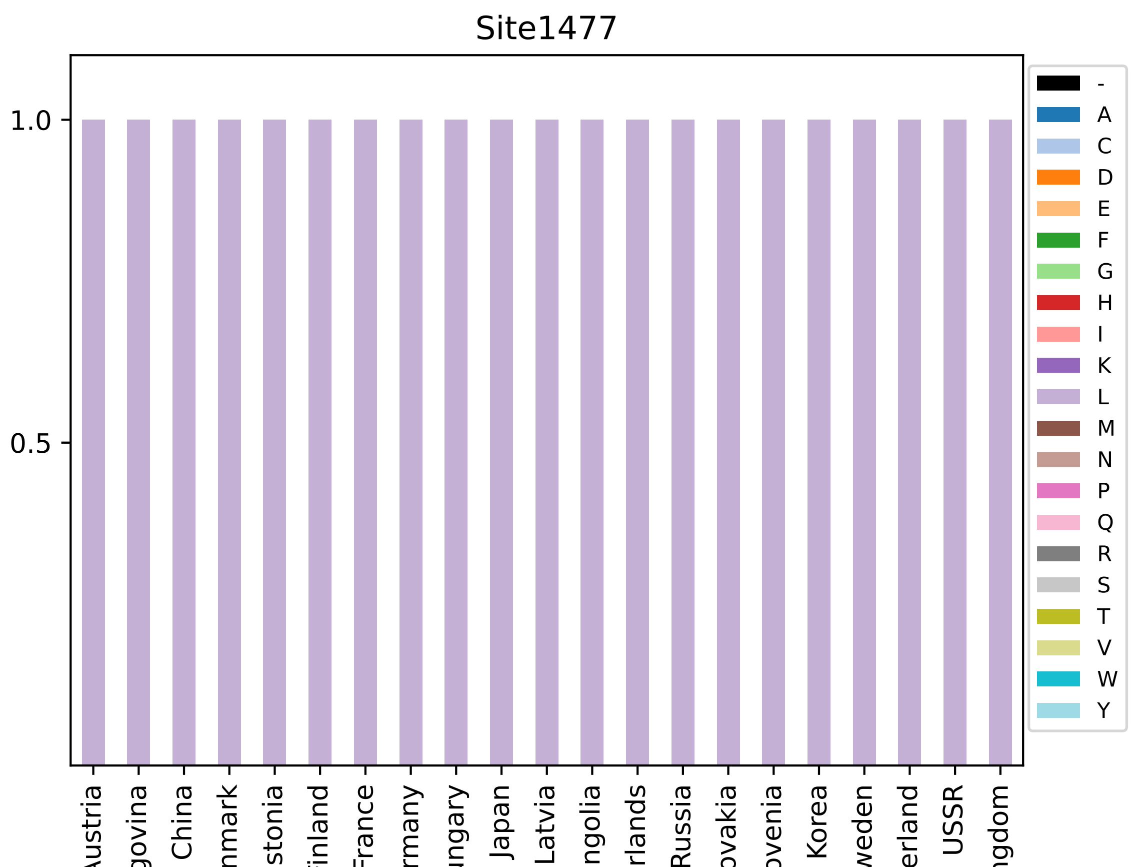
Site1477
1.0
0.5
-
A
C
D
E
F
G
H
I
K
L
M
N
P
Q
R
S
T
V
W
Y
China
Japan
Latvia
Russia
Korea
USSR

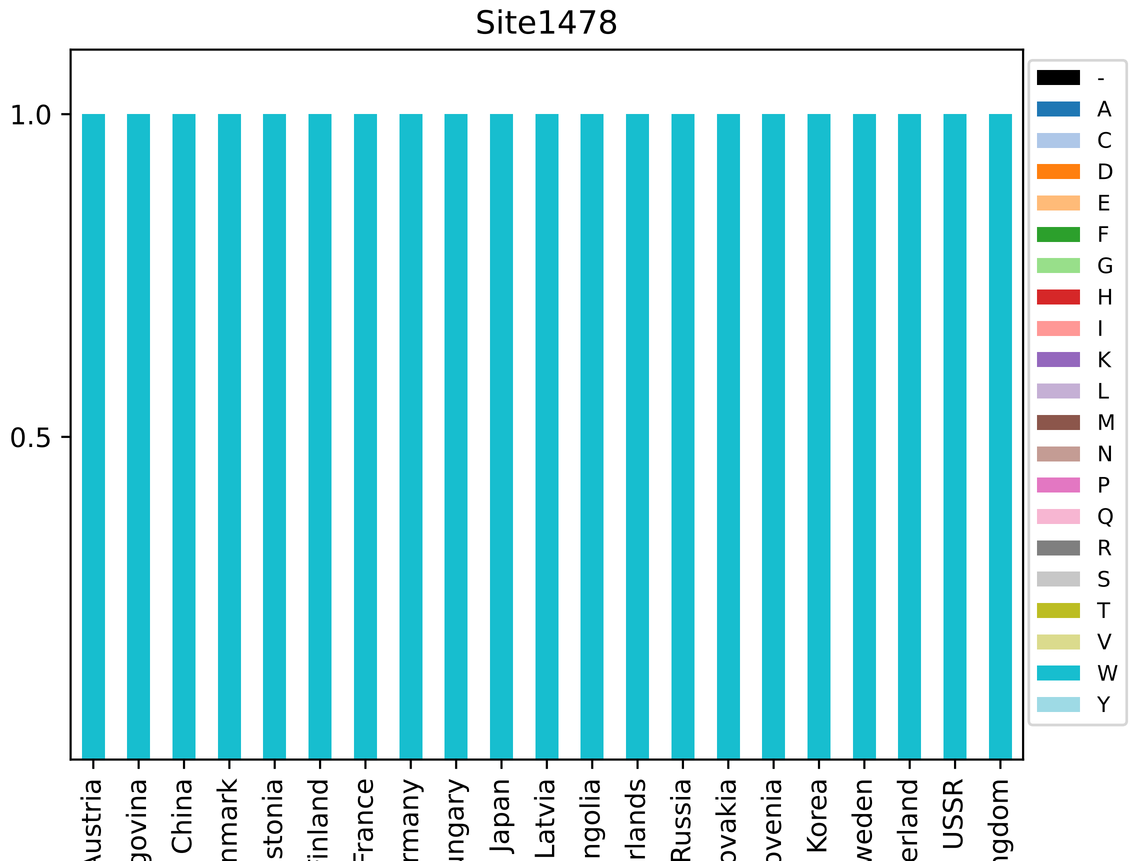

Site1478
1.0
0.5
-
A
C
D
E
F
G
H
I
K
L
M
N
P
Q
R
S
T
V
W
Y
China
Japan
Latvia
Russia
Korea
USSR

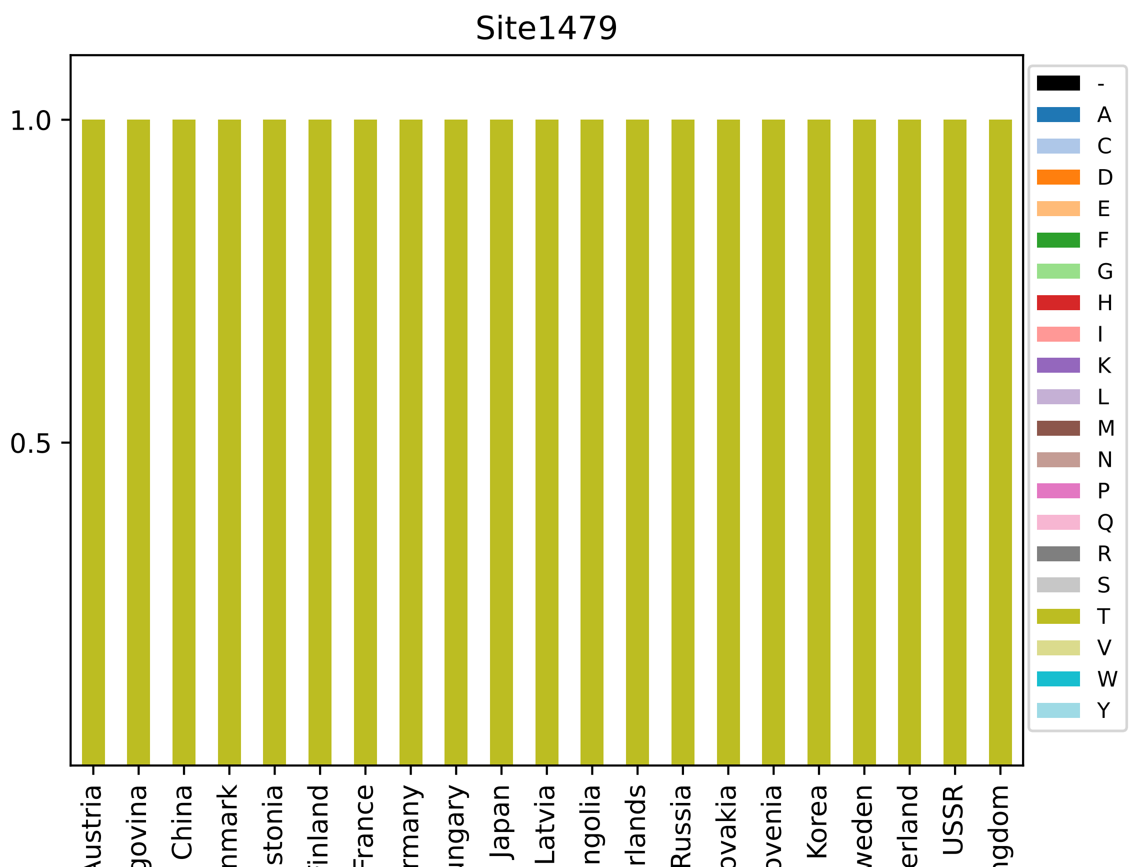

Site1479
1.0
0.5
-
A
C
D
E
F
G
H
I
K
L
M
N
P
Q
R
S
T
V
W
Y
Austria
govina
China
nmark
stonia
inland
France
rmany
ungary
Japan
Latvia
ngolia
rlands
Russia
ovakia
ovenia
Korea
weden
erland
USSR
ngdom

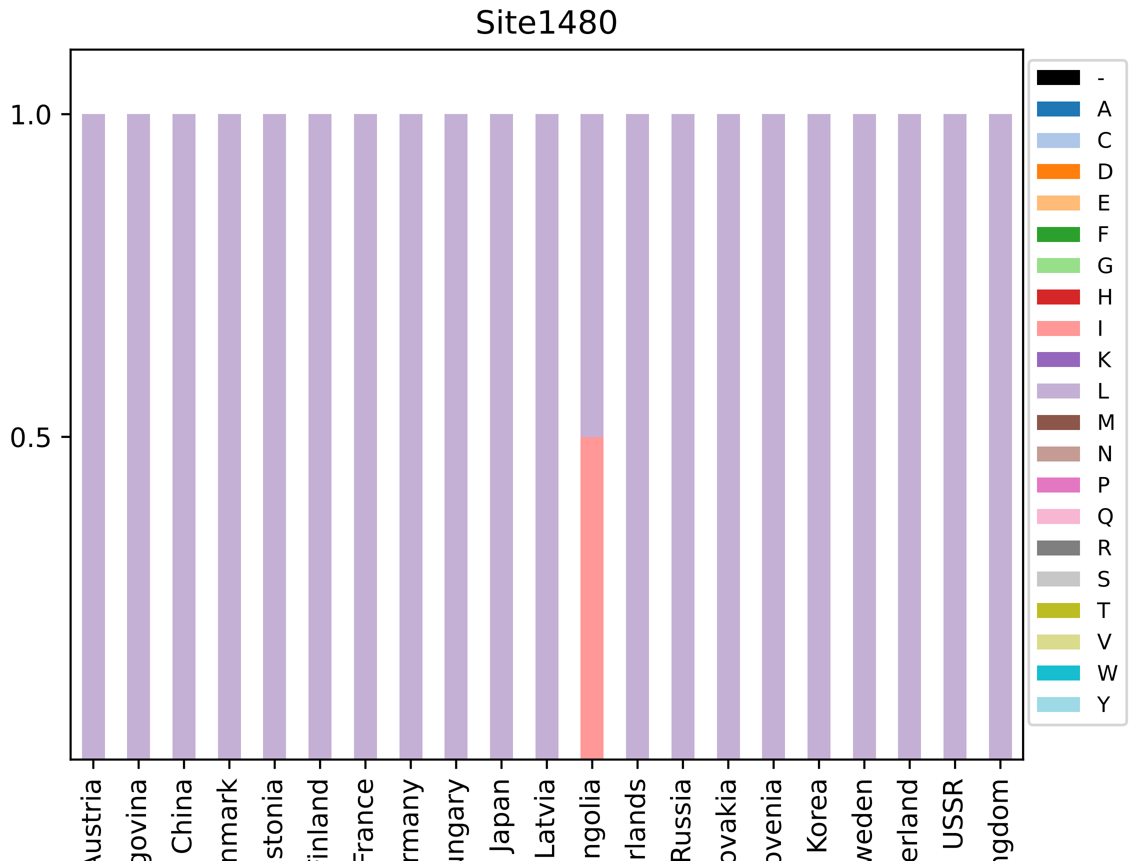

Site1480
1.0
0.5
-
A
C
D
E
F
G
H
I
K
L
M
N
P
Q
R
S
T
V
W
Y
Austria
govina
China
nmark
stonia
inland
France
rmany
ungary
Japan
Latvia
ngolia
rlands
Russia
ovakia
ovenia
Korea
weden
erland
USSR
ngdom

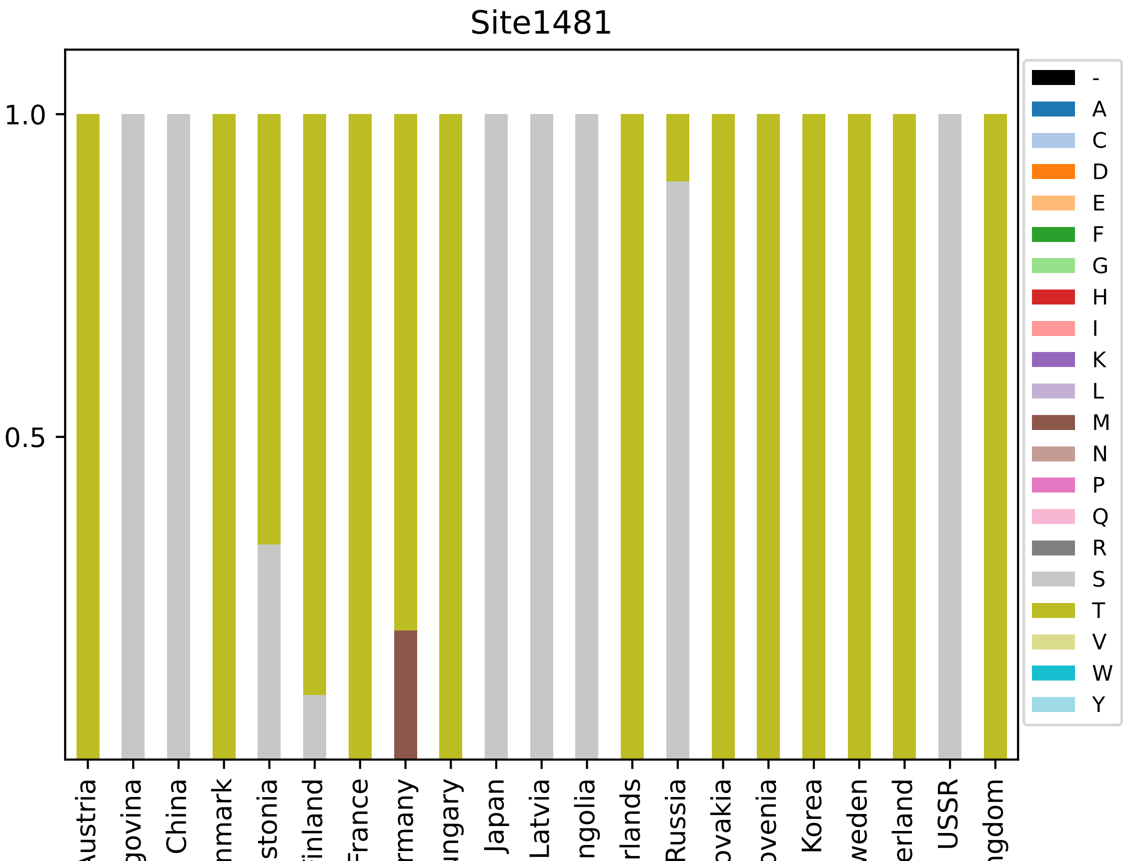
Site1481
1.0
0.5
-
A
C
D
E
F
G
H
I
K
L
M
N
P
Q
R
S
T
V
W
Y
Austria
China
Japan
Latvia
Russia
Korea
USSR

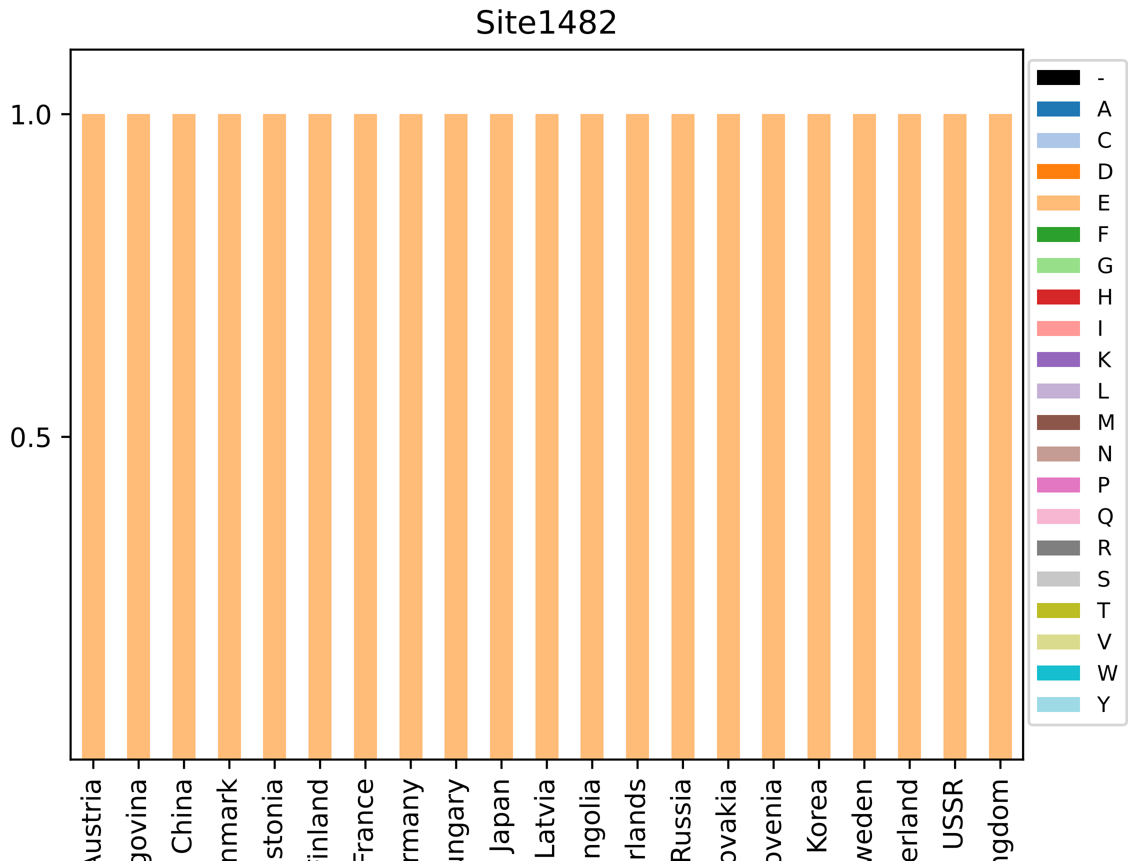

Site1482
1.0
0.5
-
A
C
D
E
F
G
H
I
K
L
M
N
P
Q
R
S
T
V
W
Y
China
Japan
Latvia
Russia
Korea
USSR

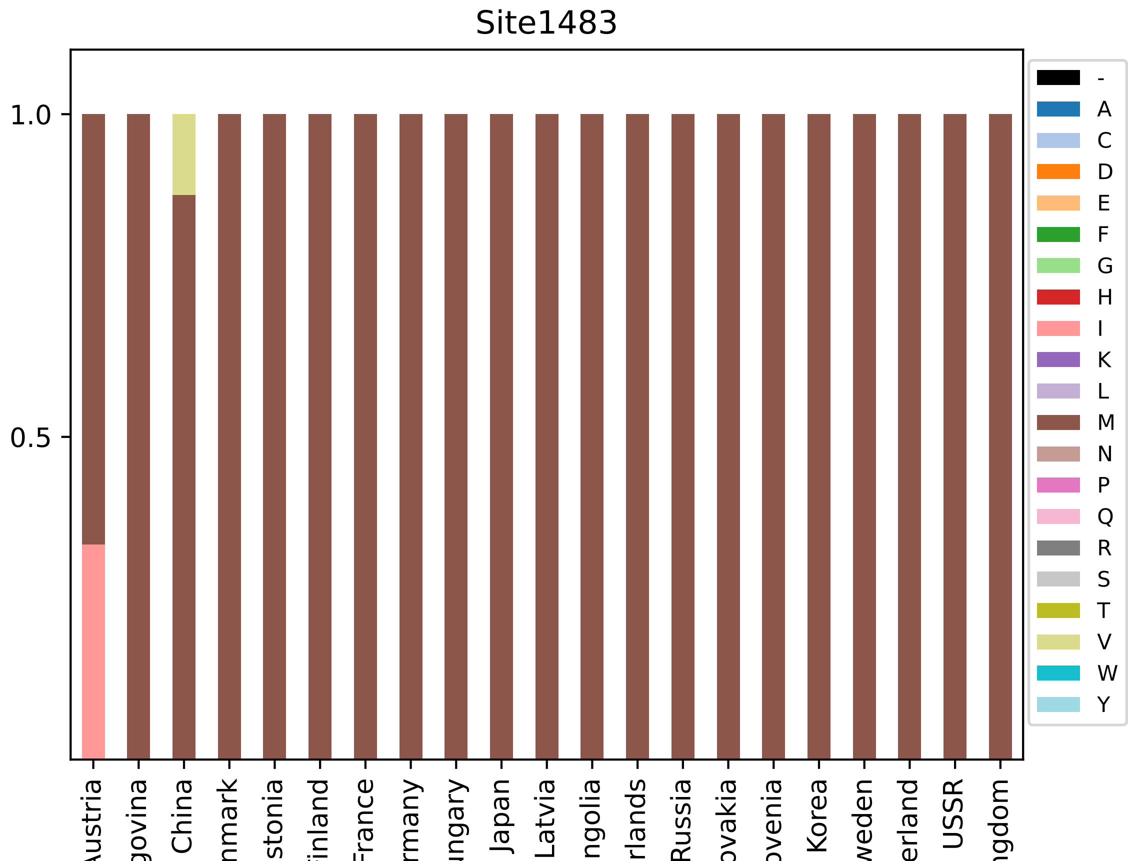

Site1483
1.0
0.5
-
A
C
D
E
F
G
H
I
K
L
M
N
P
Q
R
S
T
V
W
Y
China
Japan
Latvia
Russia
Korea
USSR

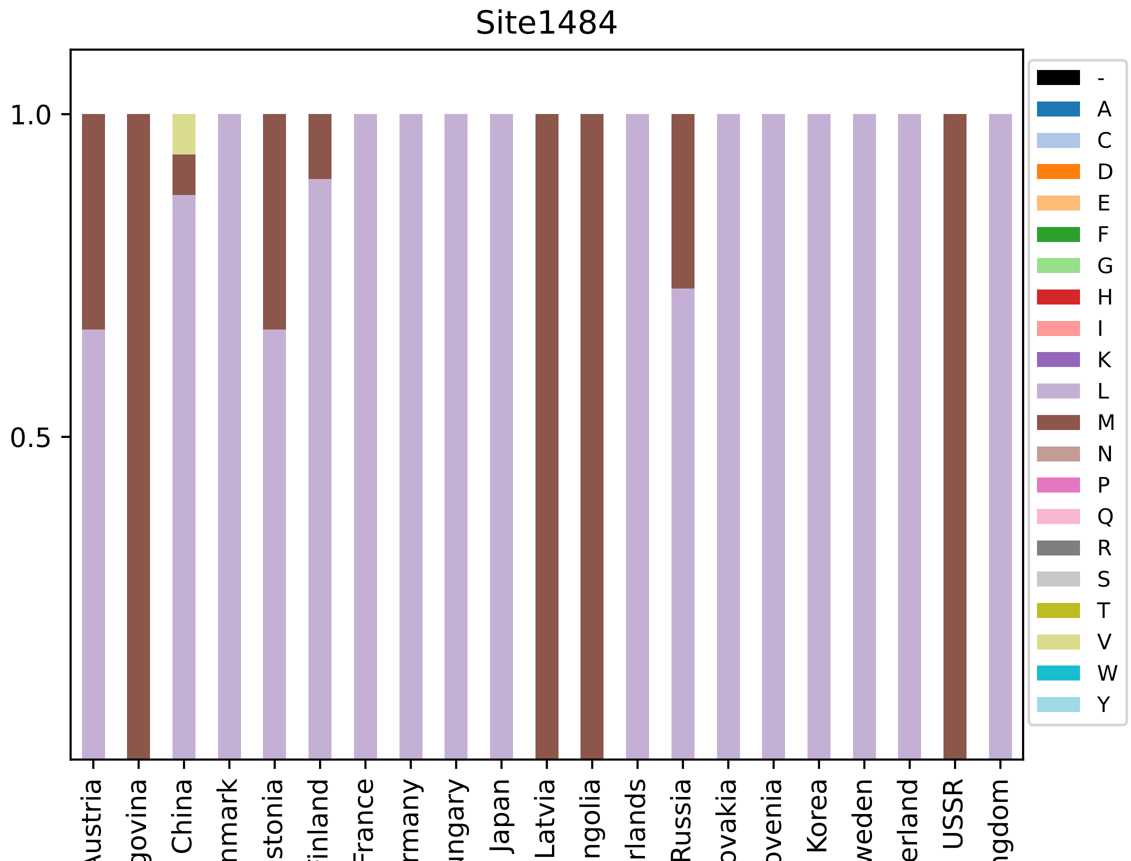

Site1484
1.0
0.5
-
A
C
D
E
F
G
H
I
K
L
M
N
P
Q
R
S
T
V
W
Y
Austria
govina
China
nmark
stonia
Finland
France
rmany
ungary
Japan
Latvia
ngolia
rlands
Russia
ovakia
ovenia
Korea
weden
erland
USSR
ngdom

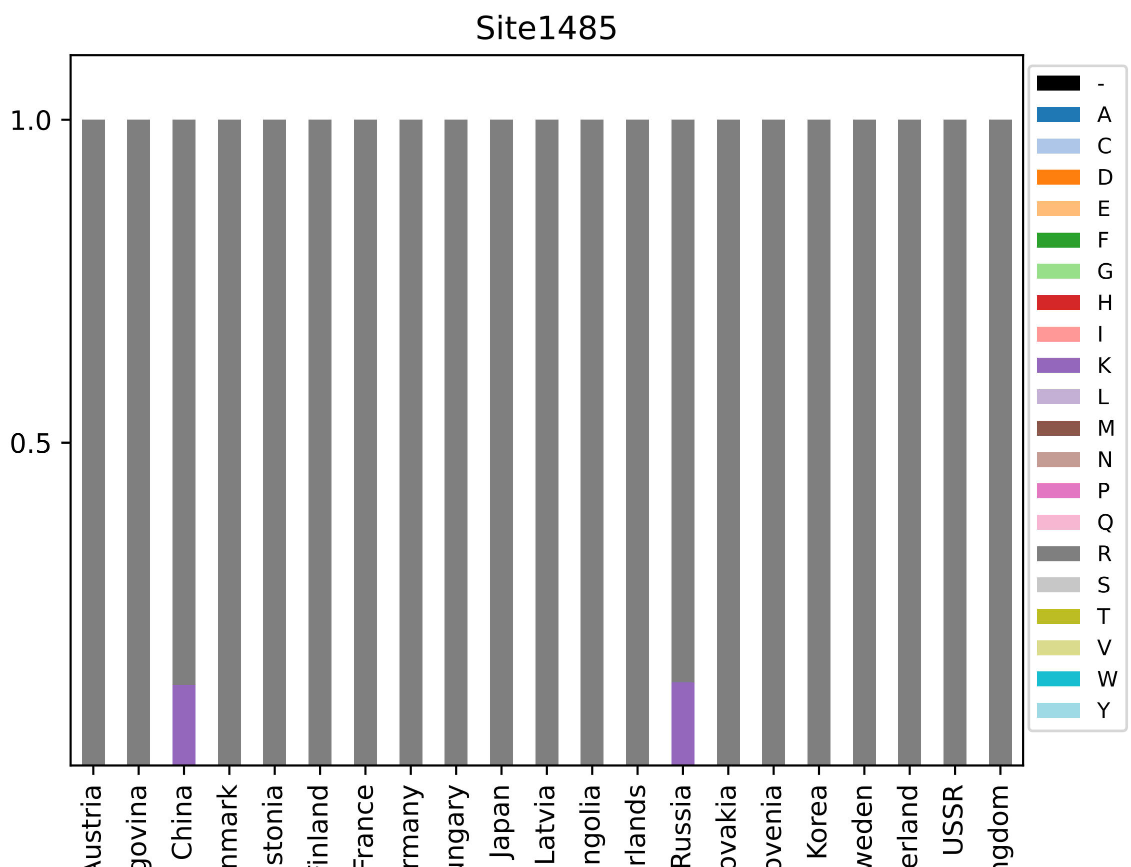

Site1485
1.0
0.5
-
A
C
D
E
F
G
H
I
K
L
M
N
P
Q
R
S
T
V
W
Y
China
Japan
Latvia
Russia
Korea
USSR

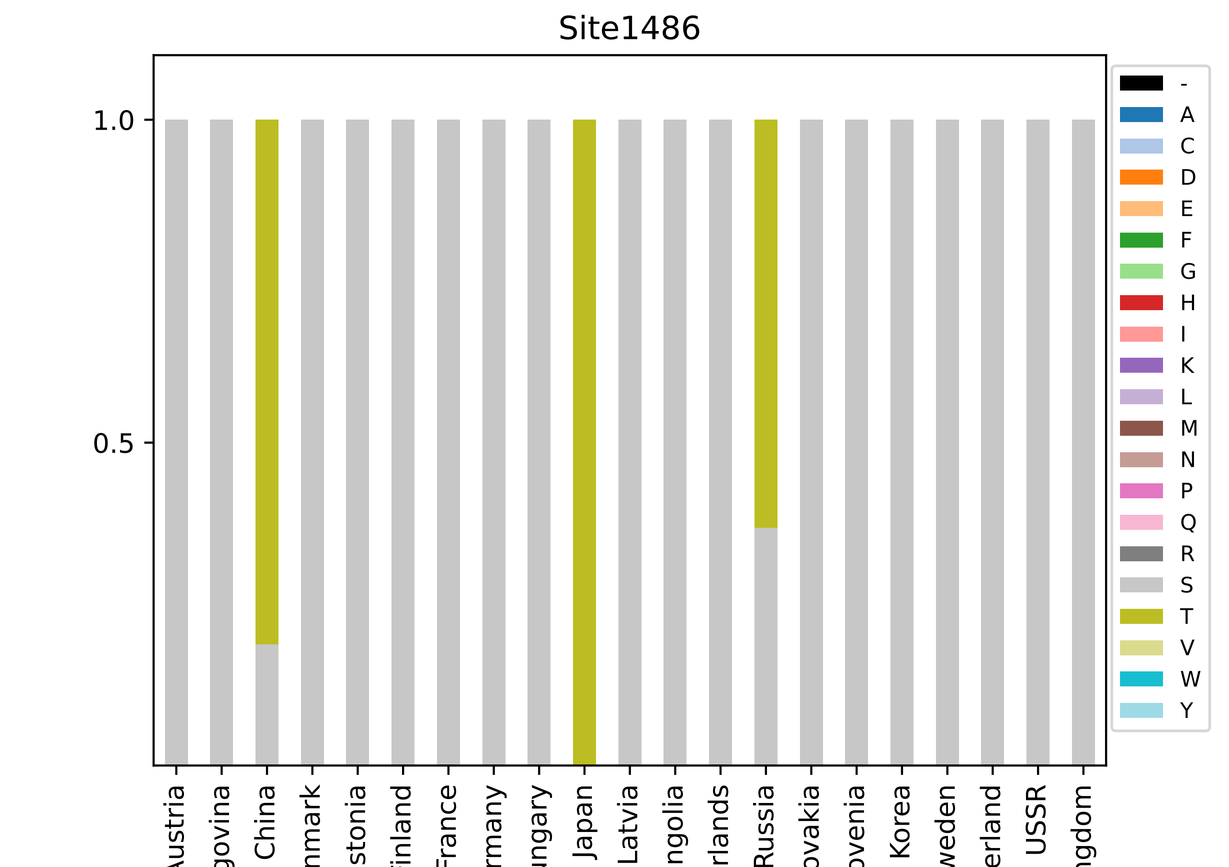

Site1486
1.0
0.5
-
A
C
D
E
F
G
H
I
K
L
M
N
P
Q
R
S
T
V
W
Y
Austria
govina
China
nmark
stonia
Finland
France
rmany
ungary
Japan
Latvia
ngolia
rlands
Russia
ovakia
ovenia
Korea
weden
erland
USSR
ngdom

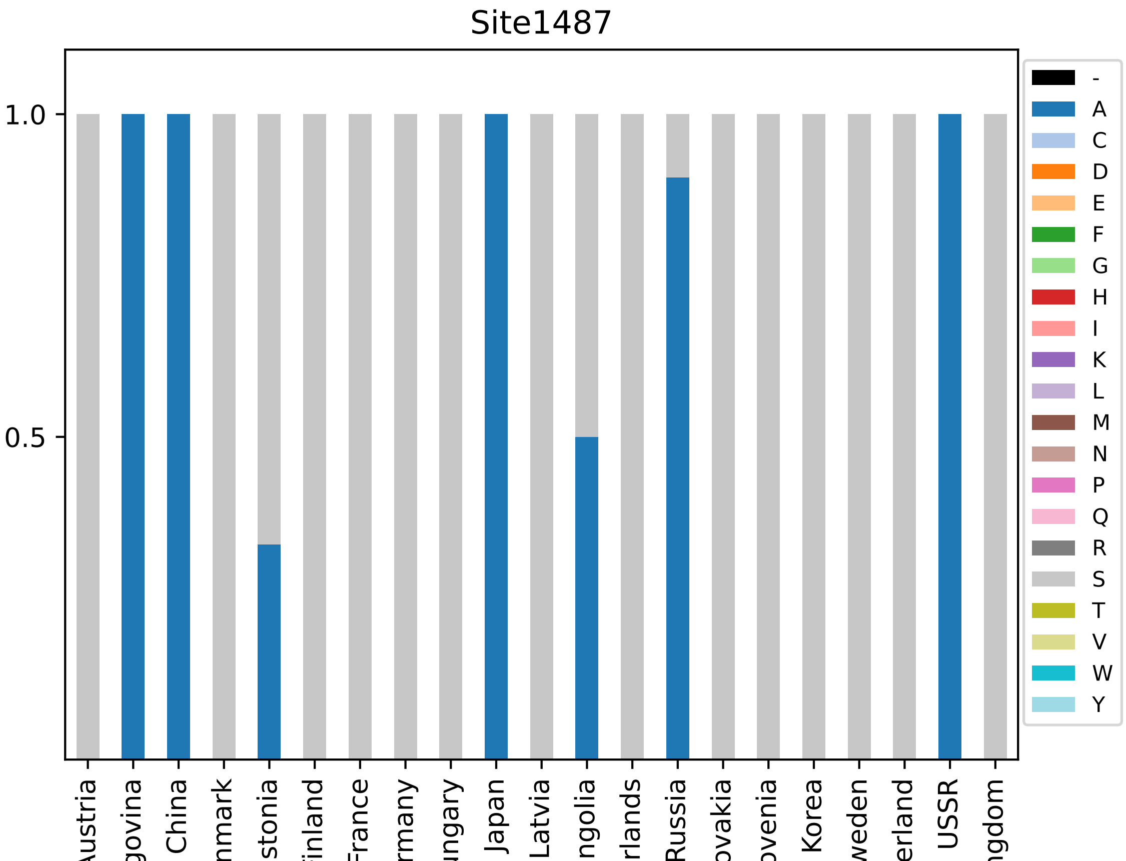
Site1487
1.0
0.5
-
A
C
D
E
F
G
H
I
K
L
M
N
P
Q
R
S
T
V
W
Y
Austria
govina
China
nmark
stonia
inland
France
rmany
ungary
Japan
Latvia
ngolia
rlands
Russia
ovakia
ovenia
Korea
weden
erland
USSR
ngdom

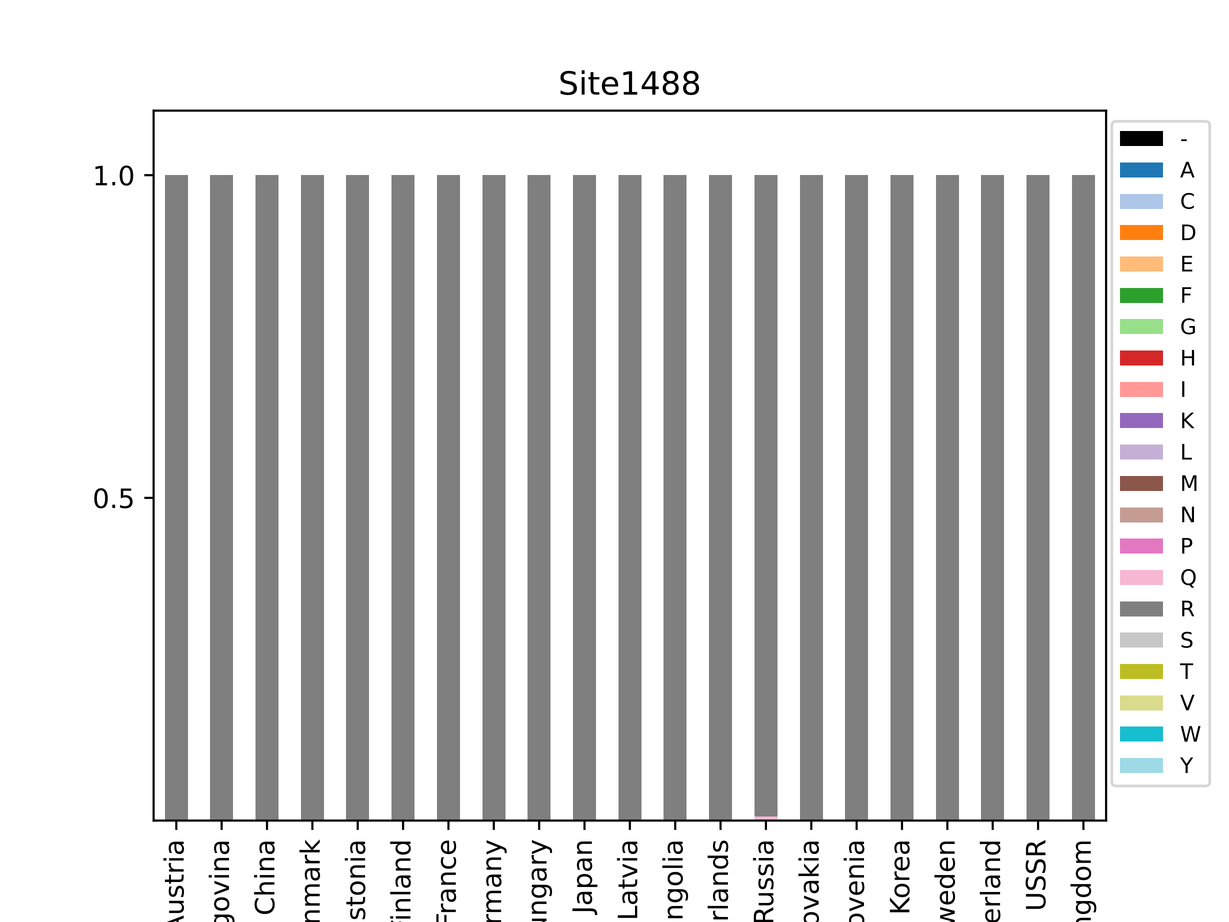

Site1488
1.0
0.5
-
A
C
D
E
F
G
H
I
K
L
M
N
P
Q
R
S
T
V
W
Y
Austria
govina
China
nmark
stonia
Finland
France
rmany
ungary
Japan
Latvia
ngolia
rlands
Russia
ovakia
ovenia
Korea
weden
erland
USSR
ngdom

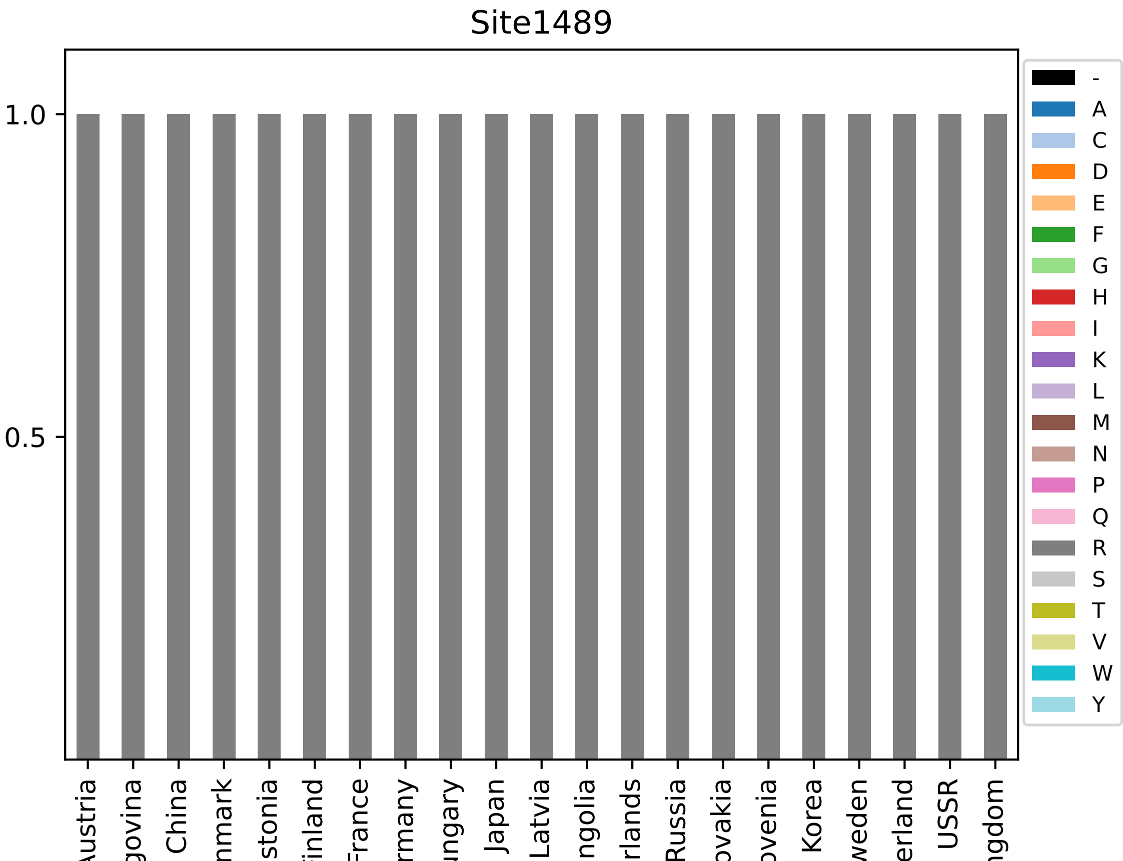
Site1489
1.0
0.5
Austria
govina
China
nmark
stonia
inland
France
rmany
ungary
Japan
Latvia
ngolia
rlands
Russia
ovakia
ovenia
Korea
weden
erland
USSR
ngdom
-
A
C
D
E
F
G
H
I
K
L
M
N
P
Q
R
S
T
V
W
Y

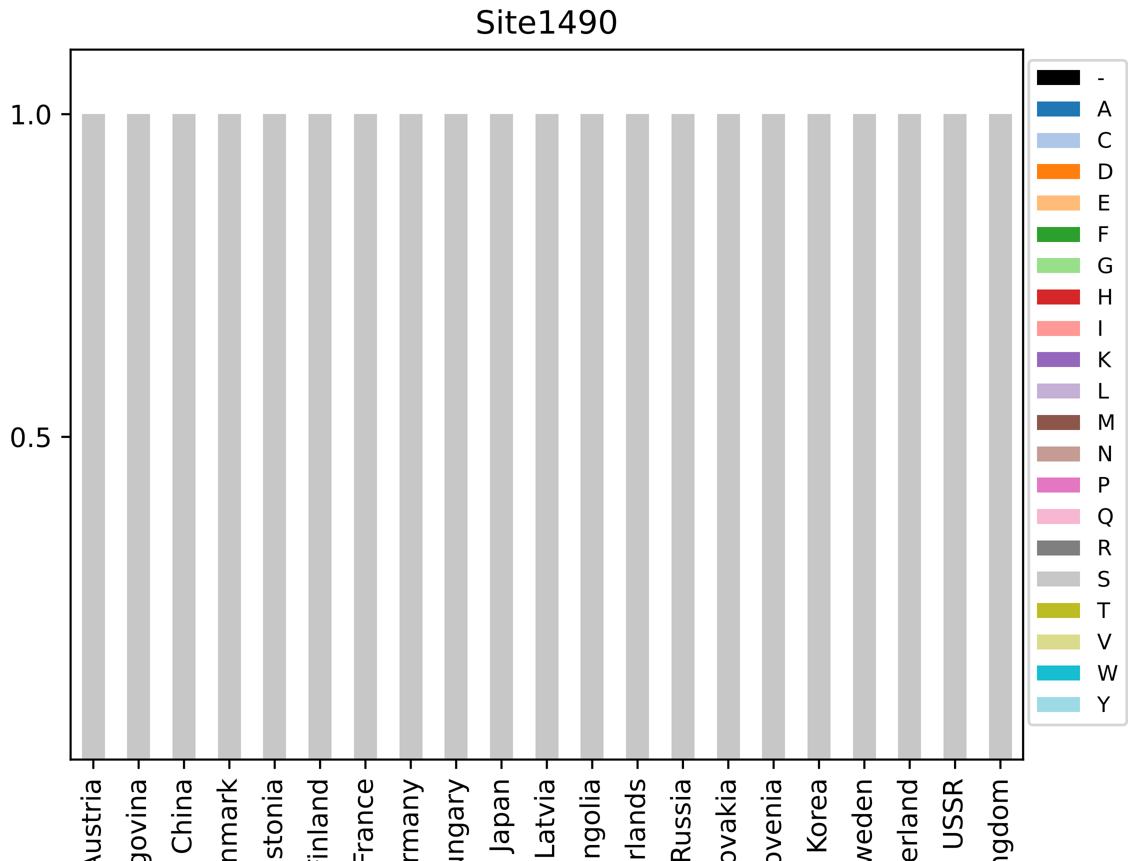

Site1490
1.0
0.5
-
A
C
D
E
F
G
H
I
K
L
M
N
P
Q
R
S
T
V
W
Y
Austria
China
Japan
Latvia
Russia
Korea
USSR

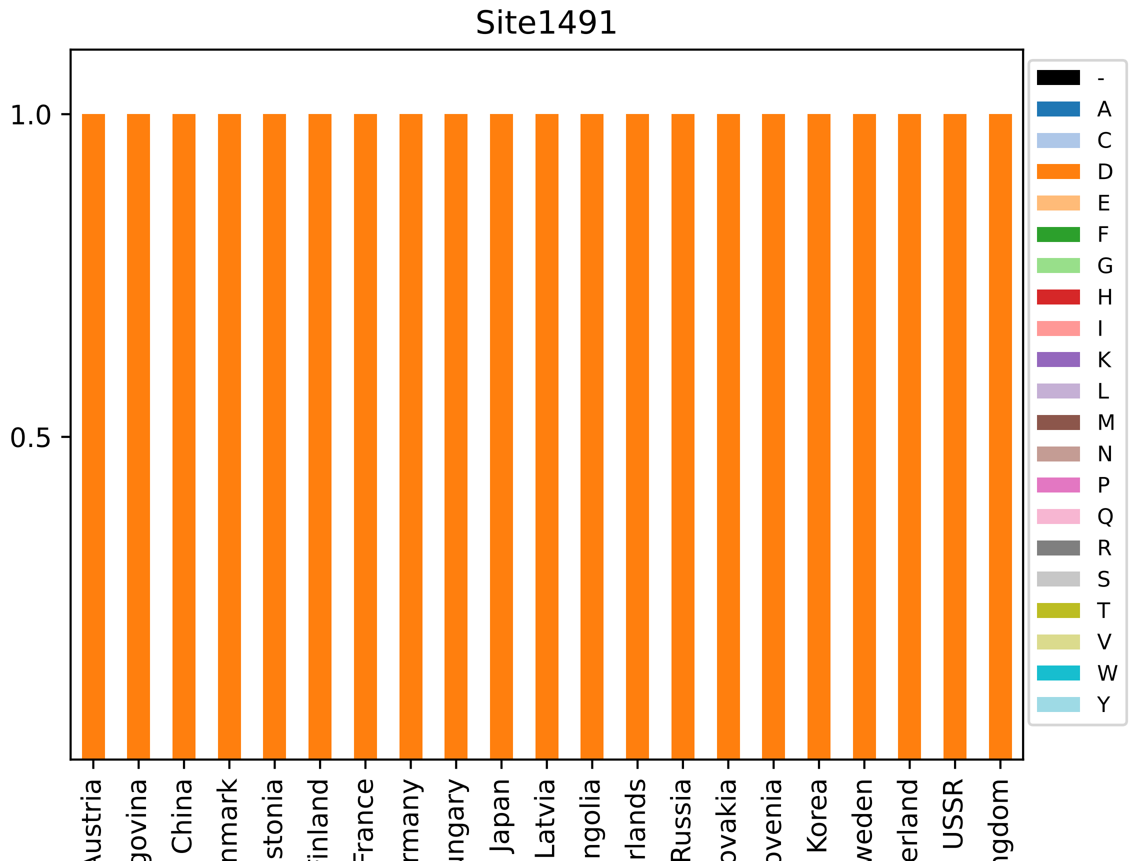
Site1491
1.0
0.5
-
A
C
D
E
F
G
H
I
K
L
M
N
P
Q
R
S
T
V
W
Y
China
Japan
Latvia
Russia
Korea
USSR

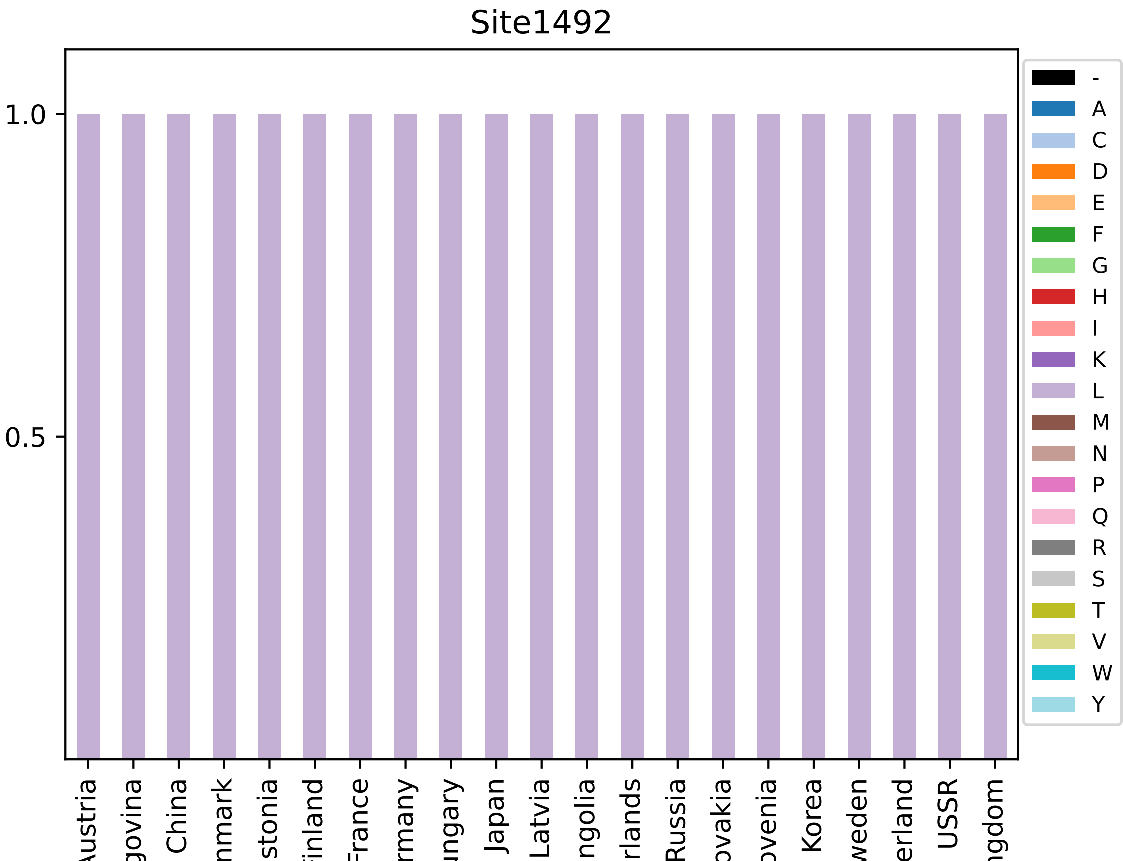

Site1492
1.0
0.5
-
A
C
D
E
F
G
H
I
K
L
M
N
P
Q
R
S
T
V
W
Y
China
Japan
Latvia
Russia
Korea
USSR

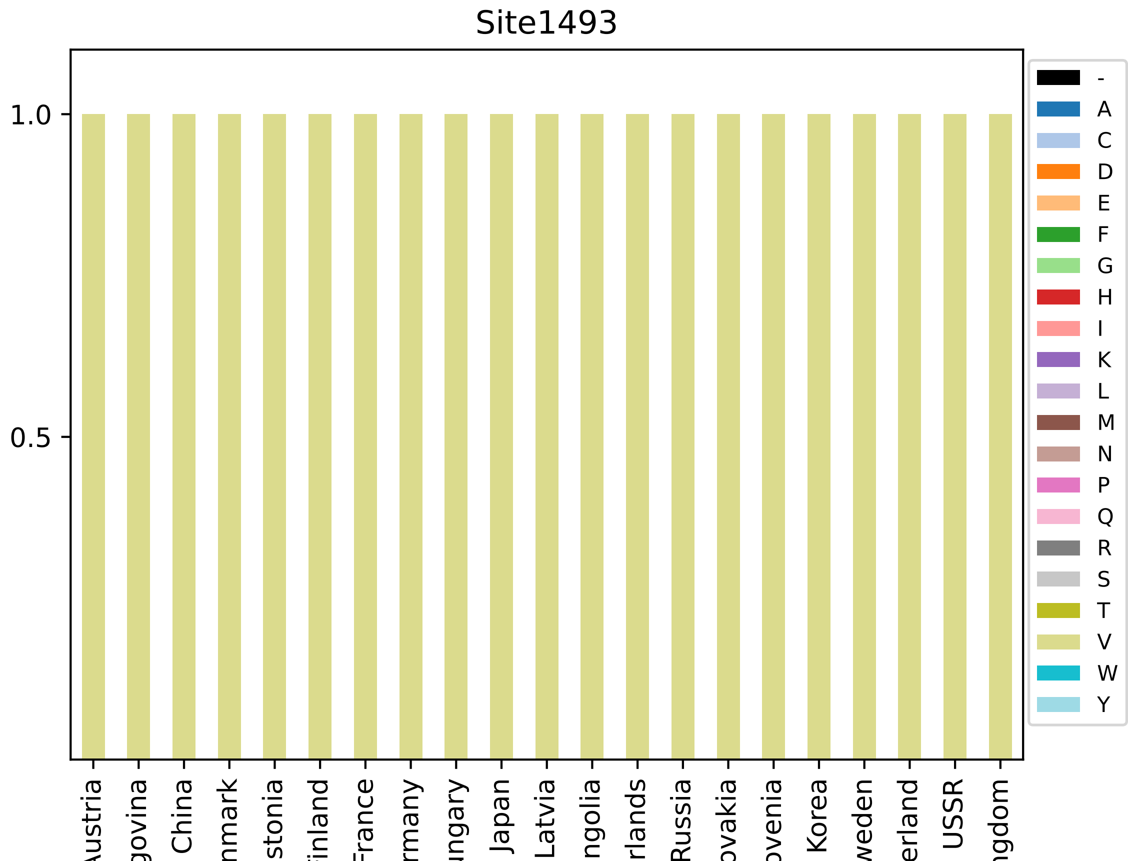
Site1493
1.0
0.5
-
A
C
D
E
F
G
H
I
K
L
M
N
P
Q
R
S
T
V
W
Y
China
Japan
Latvia
Russia
Korea
USSR

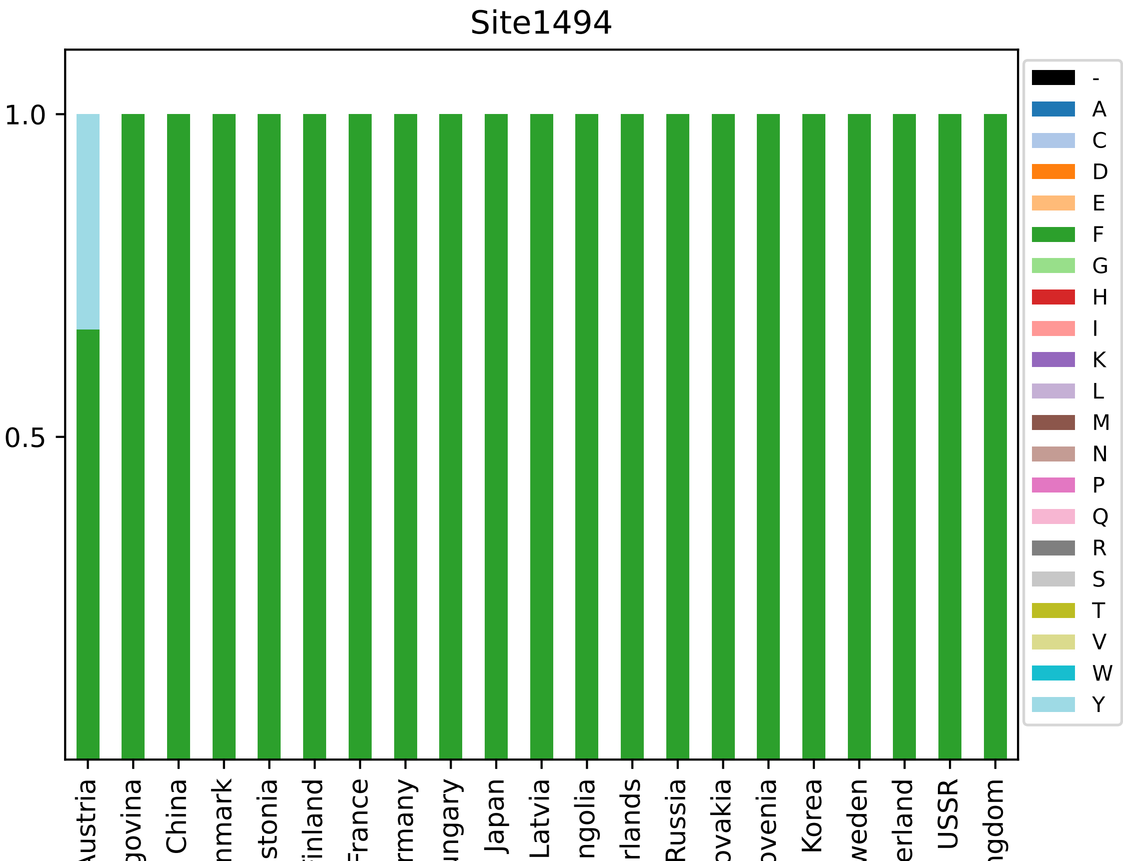

Site1494
1.0
0.5
-
A
C
D
E
F
G
H
I
K
L
M
N
P
Q
R
S
T
V
W
Y
China
Japan
Latvia
Russia
Korea
USSR

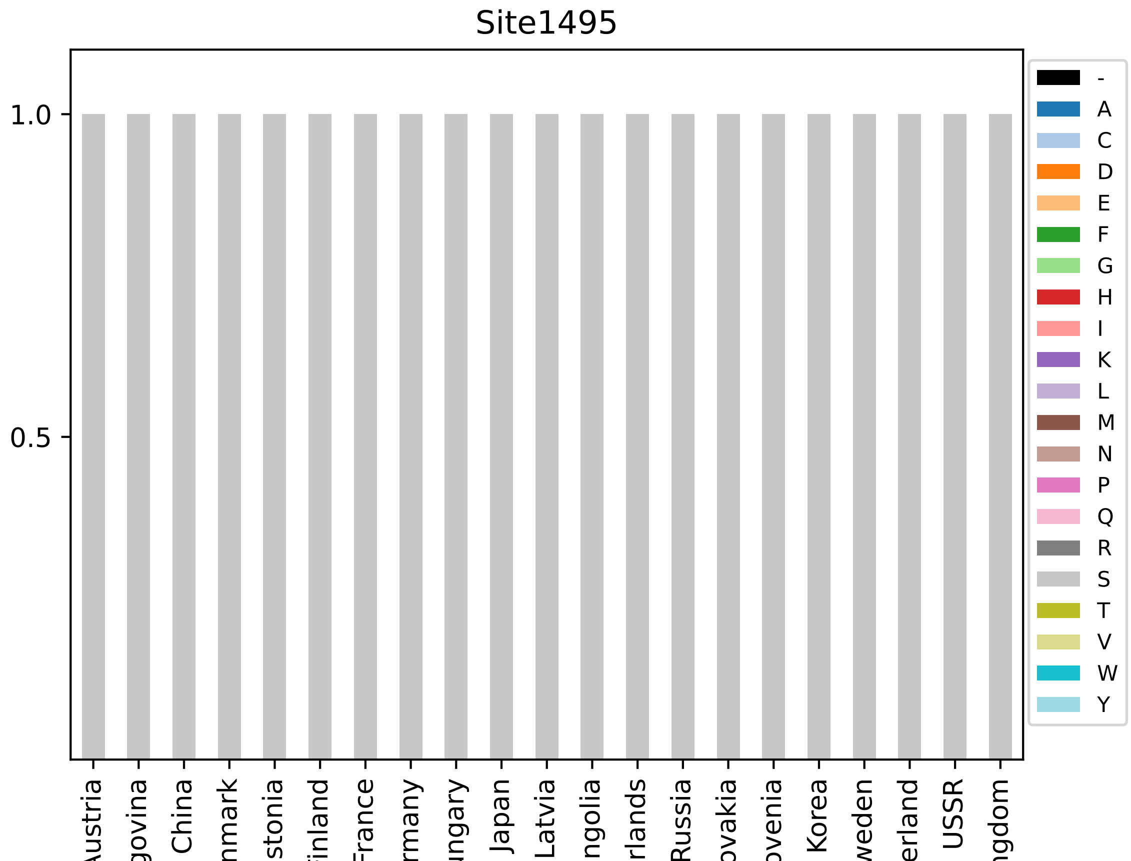
Site1495
1.0
0.5
Austria
govina
China
nmark
stonia
Finland
France
rmany
ungary
Japan
Latvia
ngolia
rlands
Russia
ovakia
ovenia
Korea
weden
erland
USSR
ngdom
-
A
C
D
E
F
G
H
I
K
L
M
N
P
Q
R
S
T
V
W
Y

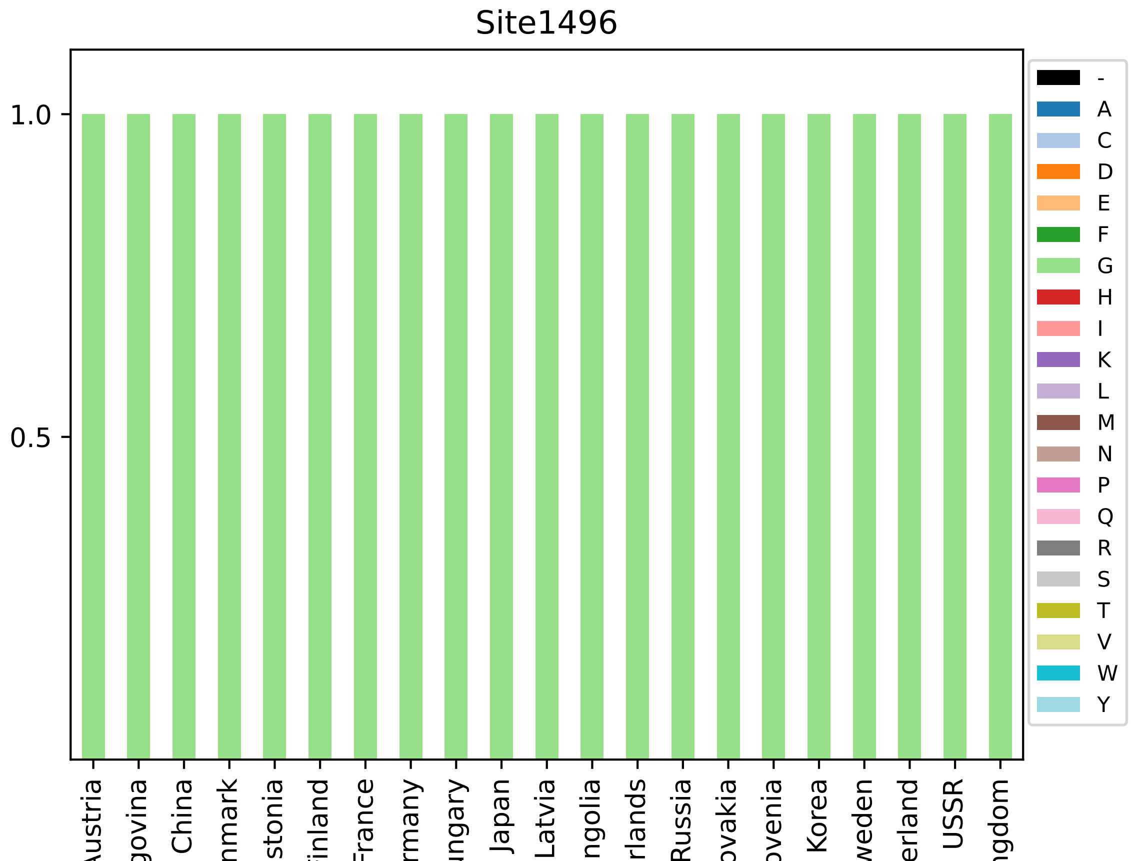

Site1496
1.0
0.5
-
A
C
D
E
F
G
H
I
K
L
M
N
P
Q
R
S
T
V
W
Y
Austria
govina
China
nmark
stonia
Finland
France
rmany
ungary
Japan
Latvia
ngolia
rlands
Russia
ovakia
ovenia
Korea
weden
erland
USSR
ngdom

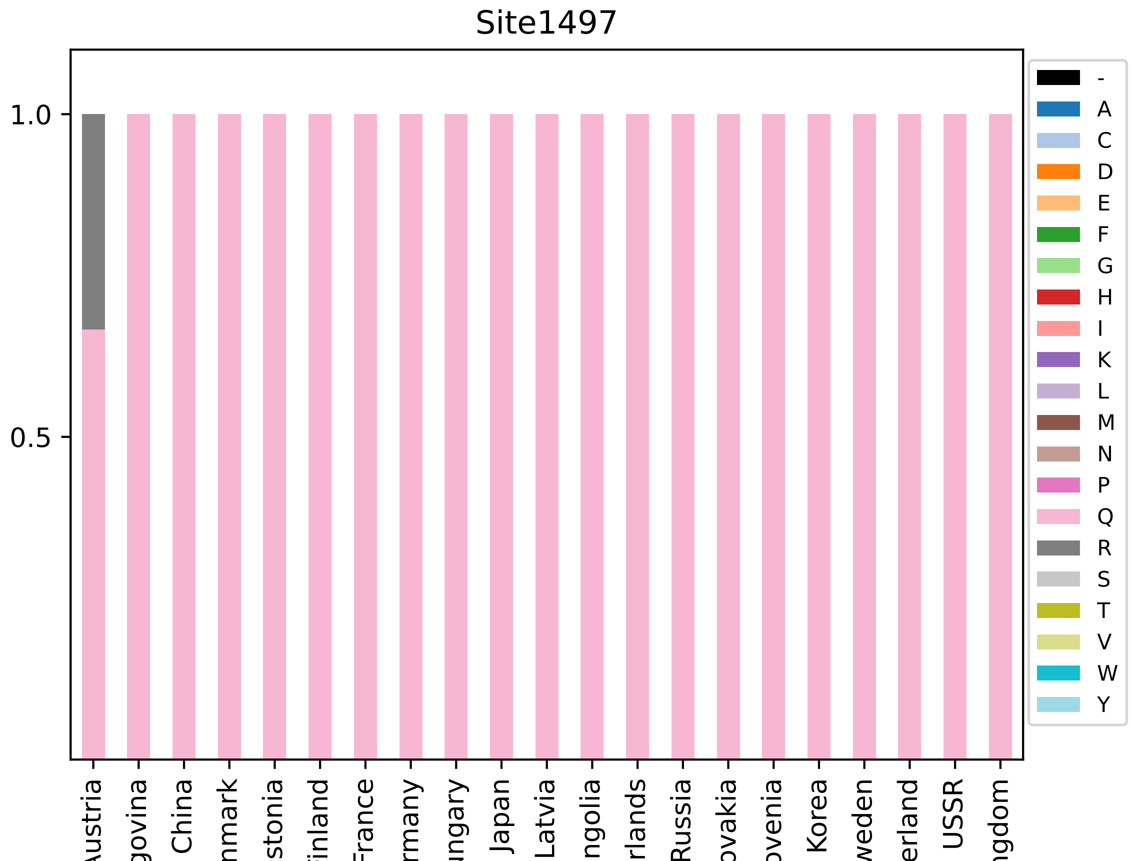
Site1497
1.0
0.5
-
A
C
D
E
F
G
H
I
K
L
M
N
P
Q
R
S
T
V
W
Y
Austria
China
Japan
Latvia
Russia
Korea
USSR

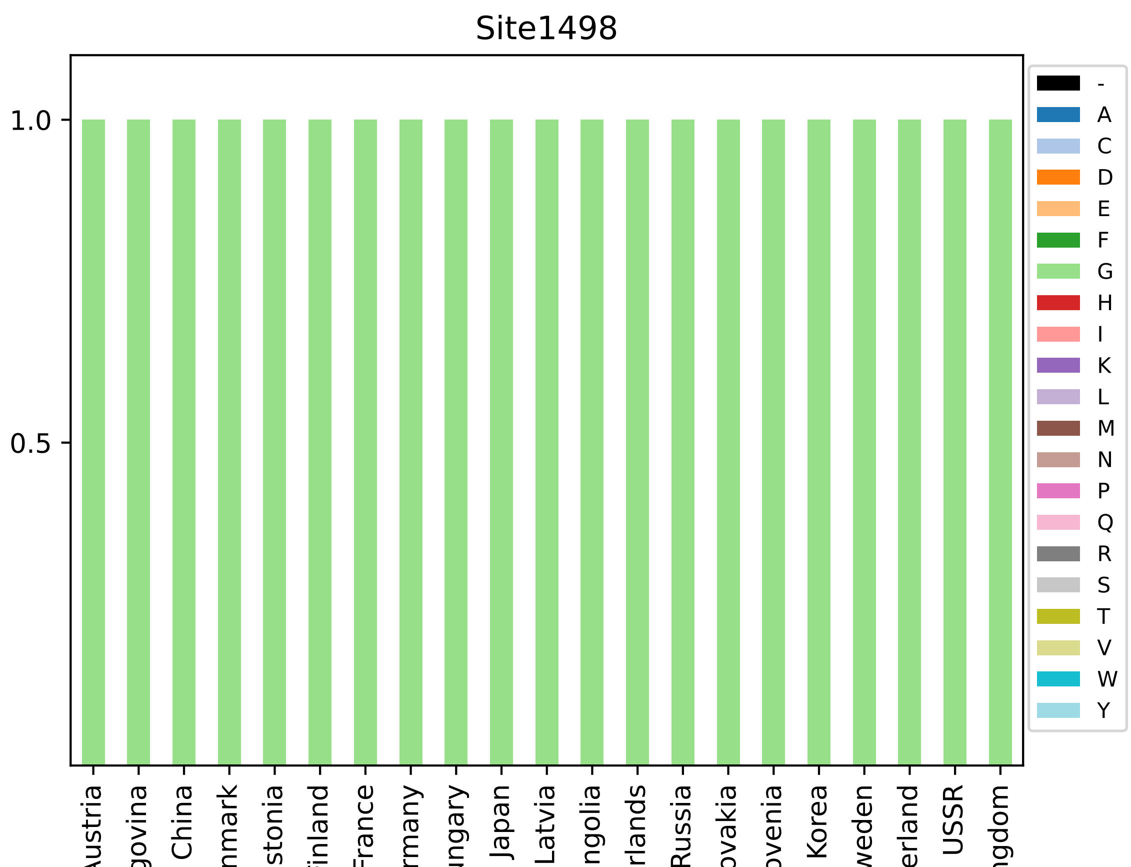
Site1498
1.0
0.5
-
A
C
D
E
F
G
H
I
K
L
M
N
P
Q
R
S
T
V
W
Y
China
Japan
Latvia
Russia
Korea
USSR

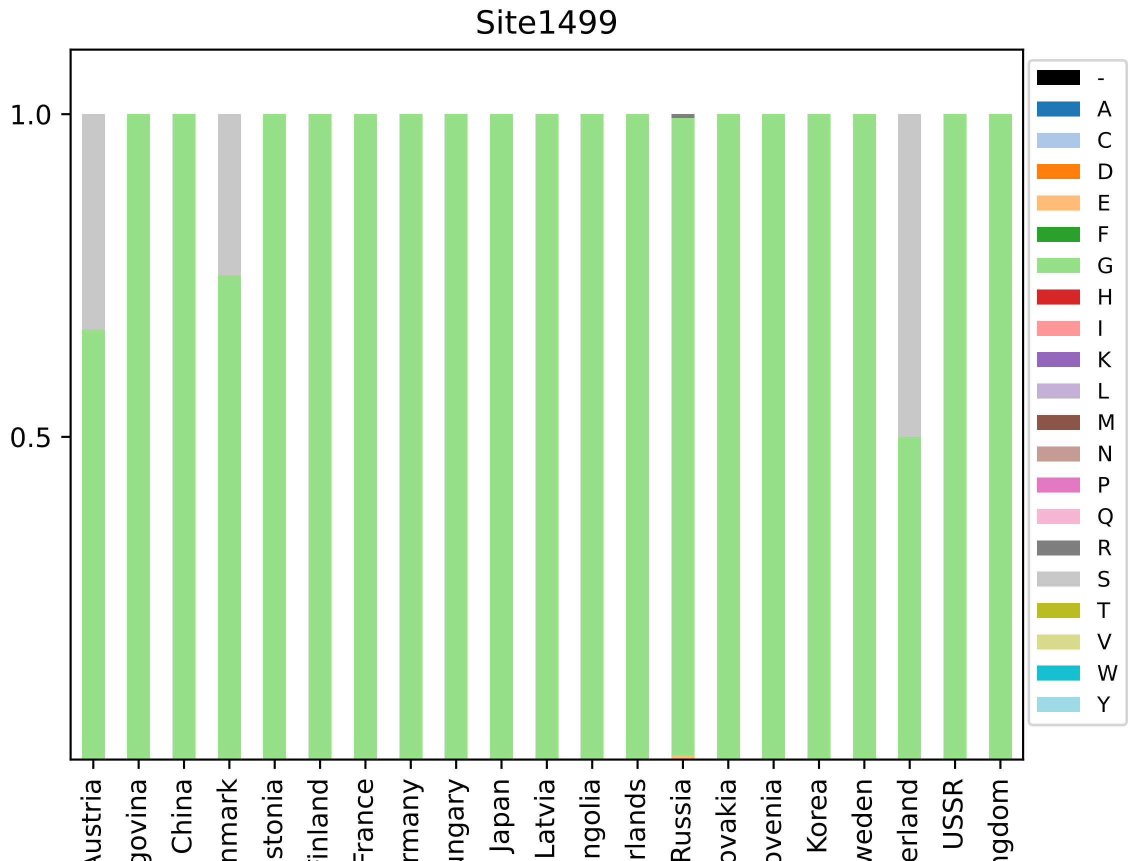
Site1499
1.0
0.5
-
A
C
D
E
F
G
H
I
K
L
M
N
P
Q
R
S
T
V
W
Y
China
Japan
Latvia
Russia
Korea
USSR

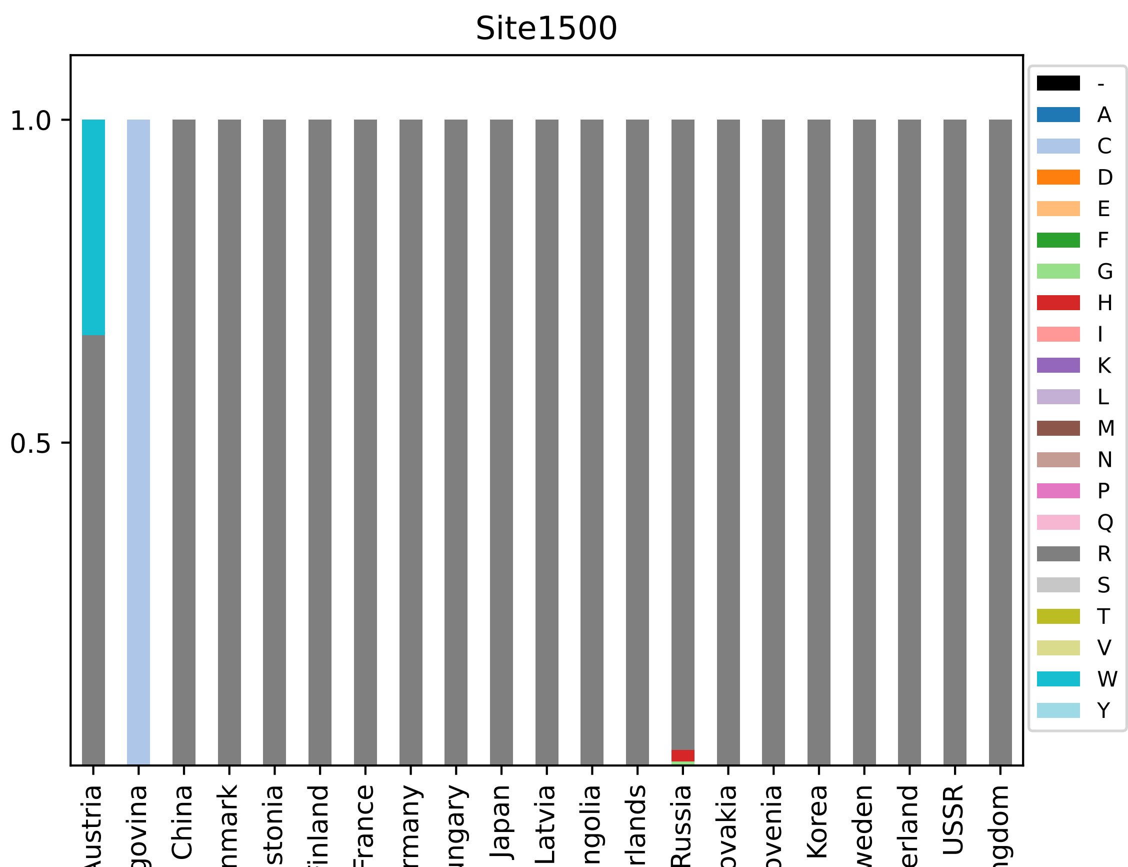

Site1500
1.0
0.5
-
A
C
D
E
F
G
H
I
K
L
M
N
P
Q
R
S
T
V
W
Y
Austria
China
Japan
Latvia
Russia
Korea
USSR

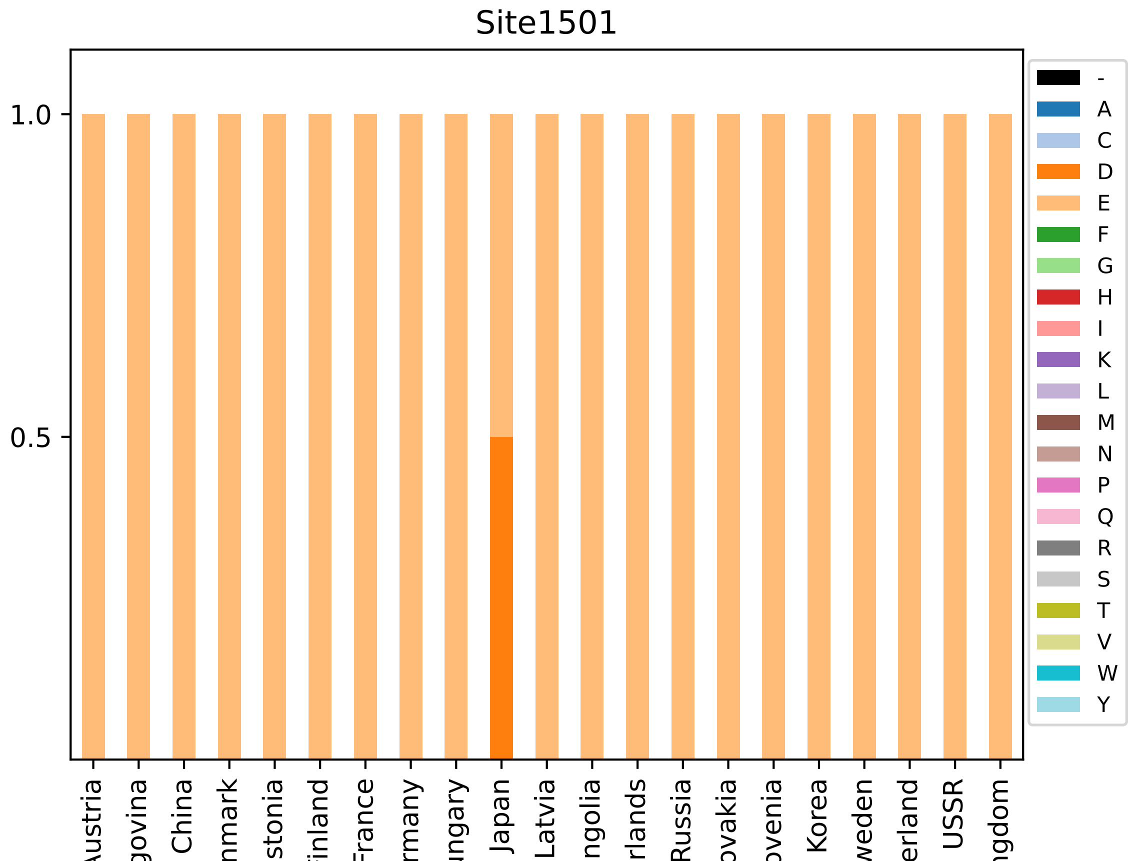

Site1501
1.0
0.5
-
A
C
D
E
F
G
H
I
K
L
M
N
P
Q
R
S
T
V
W
Y
China
Japan
Latvia
Russia
Korea
USSR

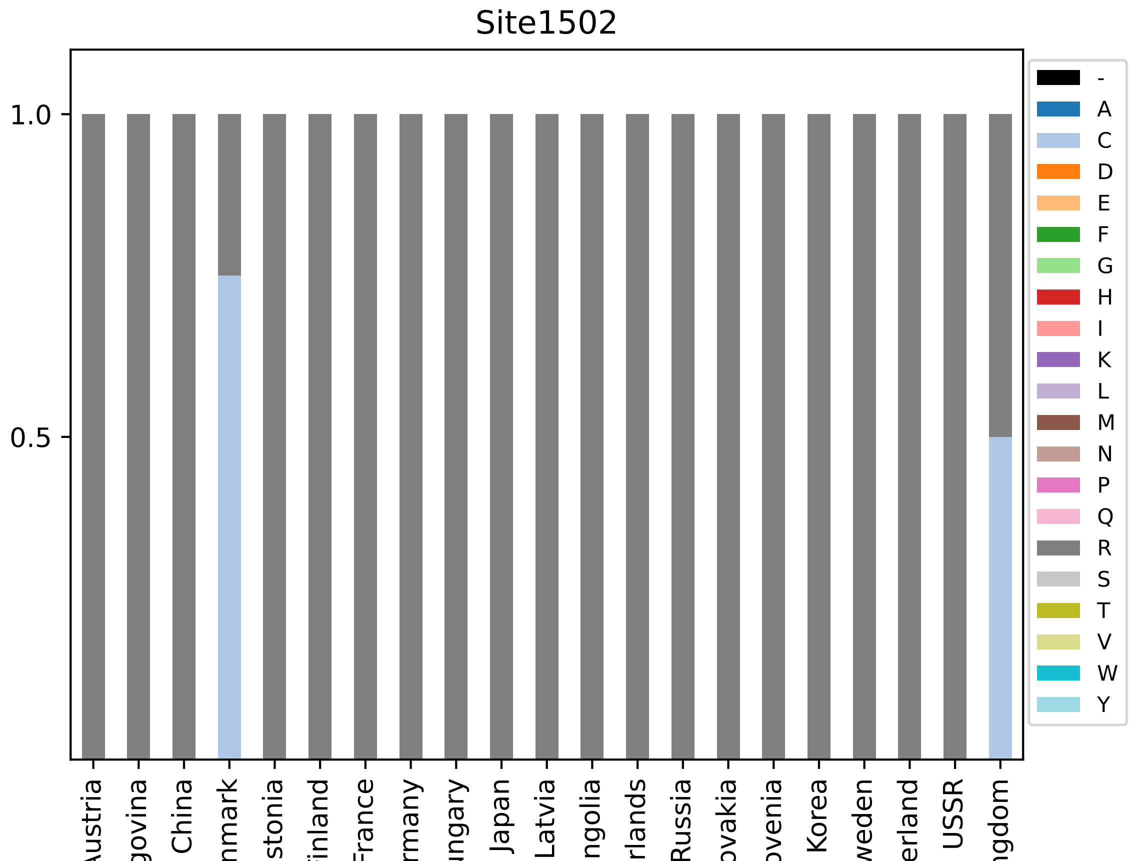
Site1502
1.0
0.5
-
A
C
D
E
F
G
H
I
K
L
M
N
P
Q
R
S
T
V
W
Y
China
Japan
Latvia
Russia
Korea
USSR

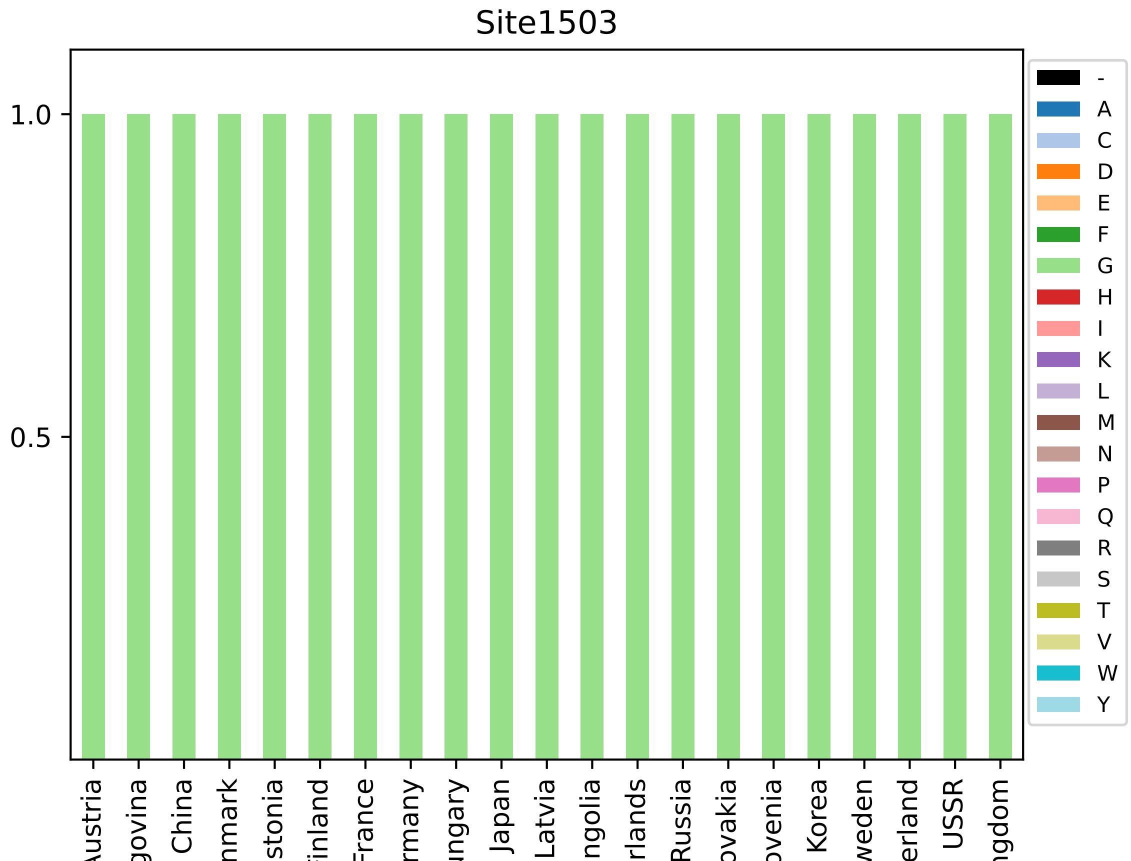

Site1503
1.0
0.5
-
A
C
D
E
F
G
H
I
K
L
M
N
P
Q
R
S
T
V
W
Y
China
Japan
Latvia
Russia
Korea
USSR

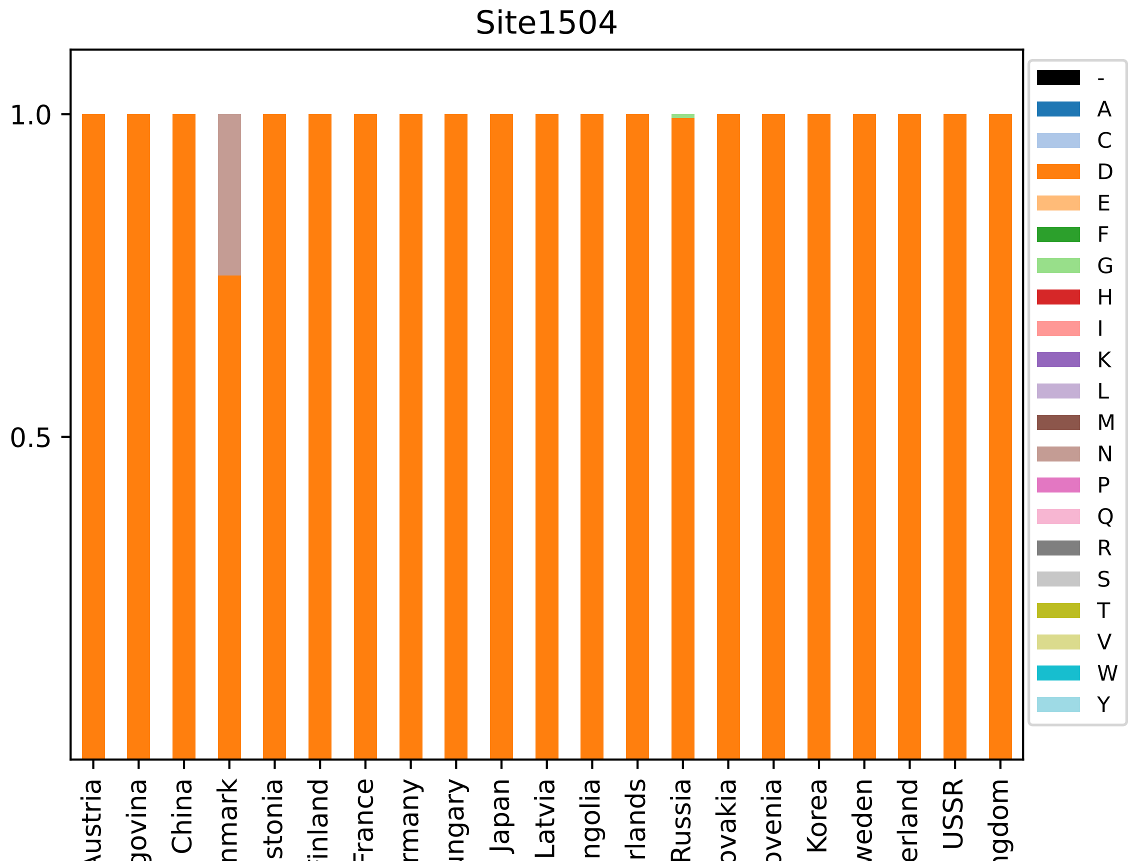

Site1504
1.0
0.5
-
A
C
D
E
F
G
H
I
K
L
M
N
P
Q
R
S
T
V
W
Y
Austria
govina
China
nmark
stonia
Finland
France
rmany
ungary
Japan
Latvia
ngolia
rlands
Russia
ovakia
ovenia
Korea
weden
erland
USSR
ngdom

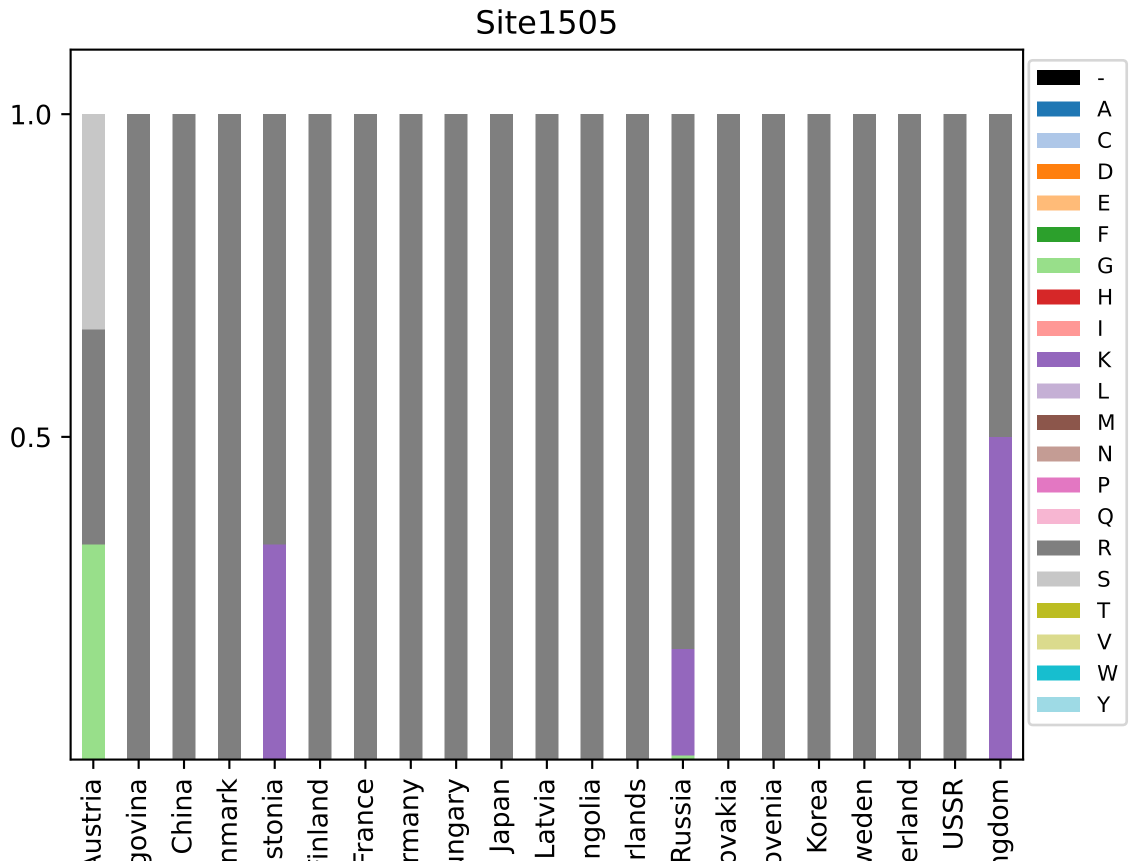

Site1505
1.0
0.5
-
A
C
D
E
F
G
H
I
K
L
M
N
P
Q
R
S
T
V
W
Y
Austria
govina
China
nmark
stonia
inland
France
rmany
ungary
Japan
Latvia
ngolia
rlands
Russia
ovakia
ovenia
Korea
weden
erland
USSR
ngdom

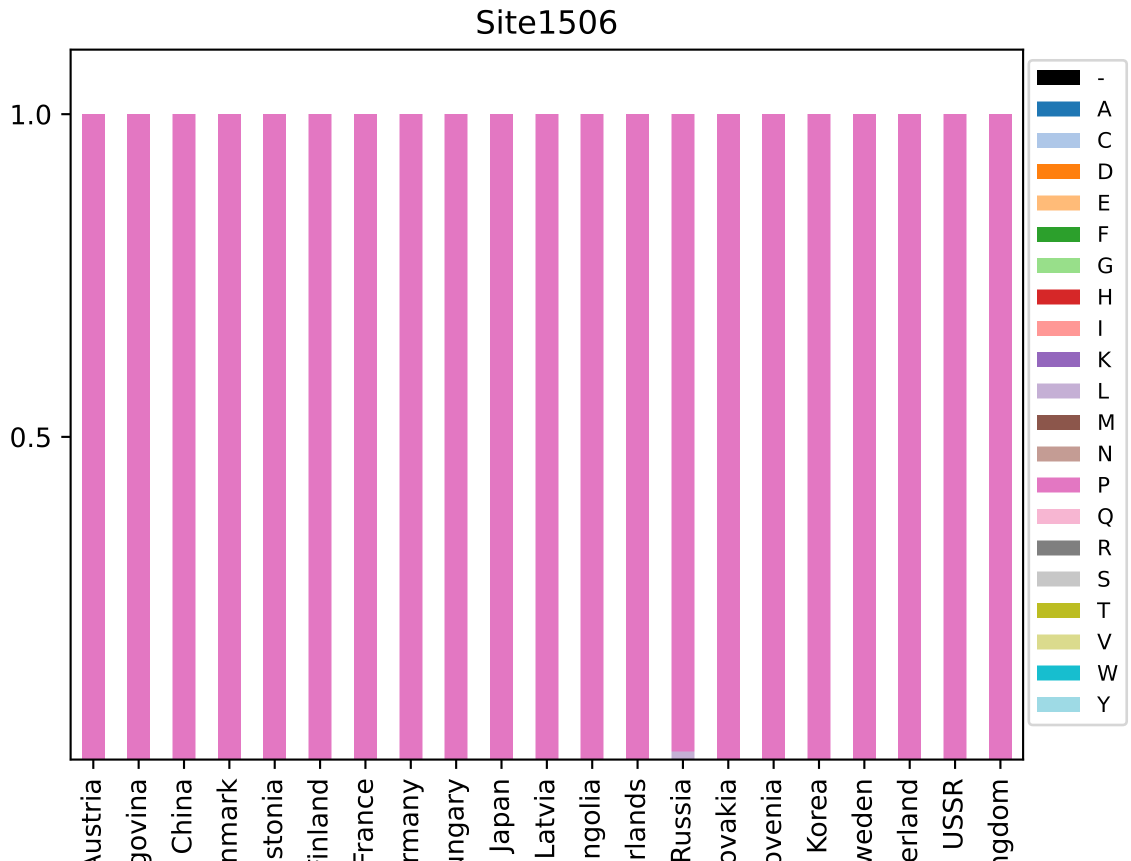
Site1506
1.0
0.5
-
A
C
D
E
F
G
H
I
K
L
M
N
P
Q
R
S
T
V
W
Y
China
Japan
Latvia
Russia
Korea
USSR

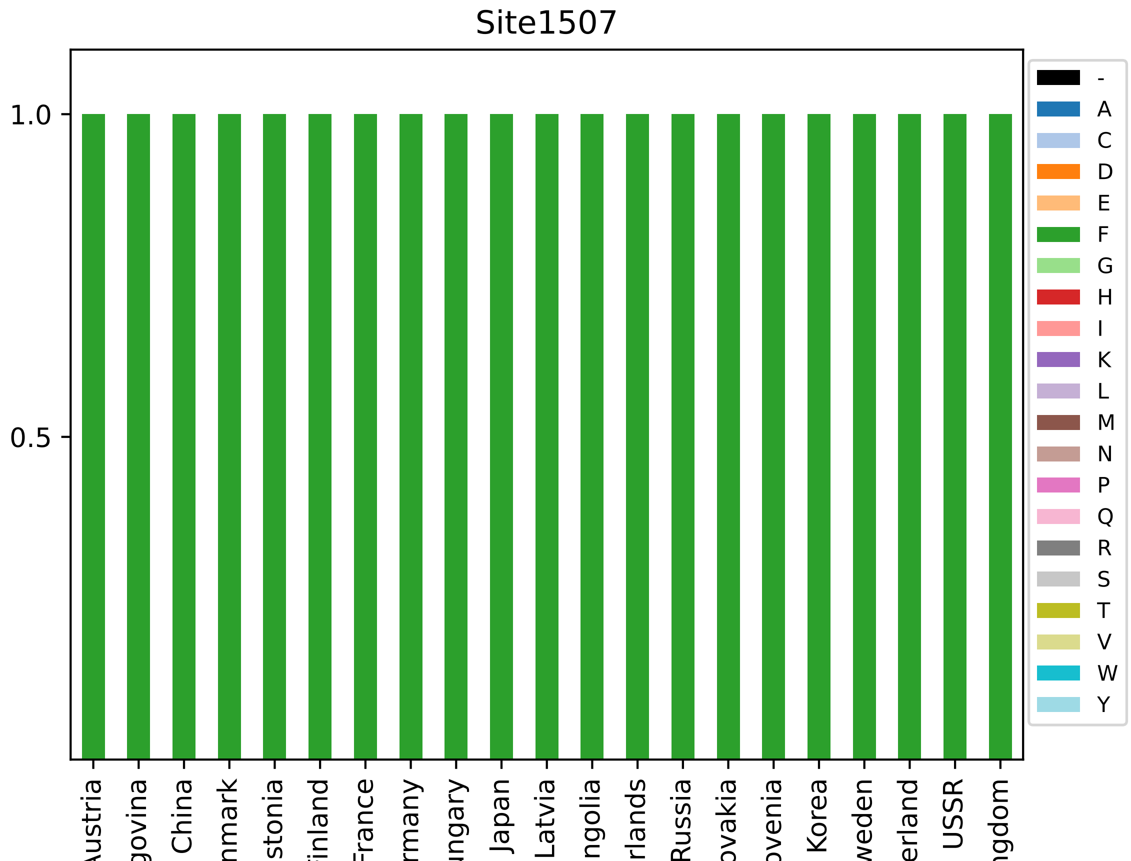
Site1507
1.0
0.5
-
A
C
D
E
F
G
H
I
K
L
M
N
P
Q
R
S
T
V
W
Y
Austria
govina
China
nmark
stonia
Finland
France
rmany
ungary
Japan
Latvia
ngolia
rlands
Russia
ovakia
ovenia
Korea
weden
erland
USSR
ngdom

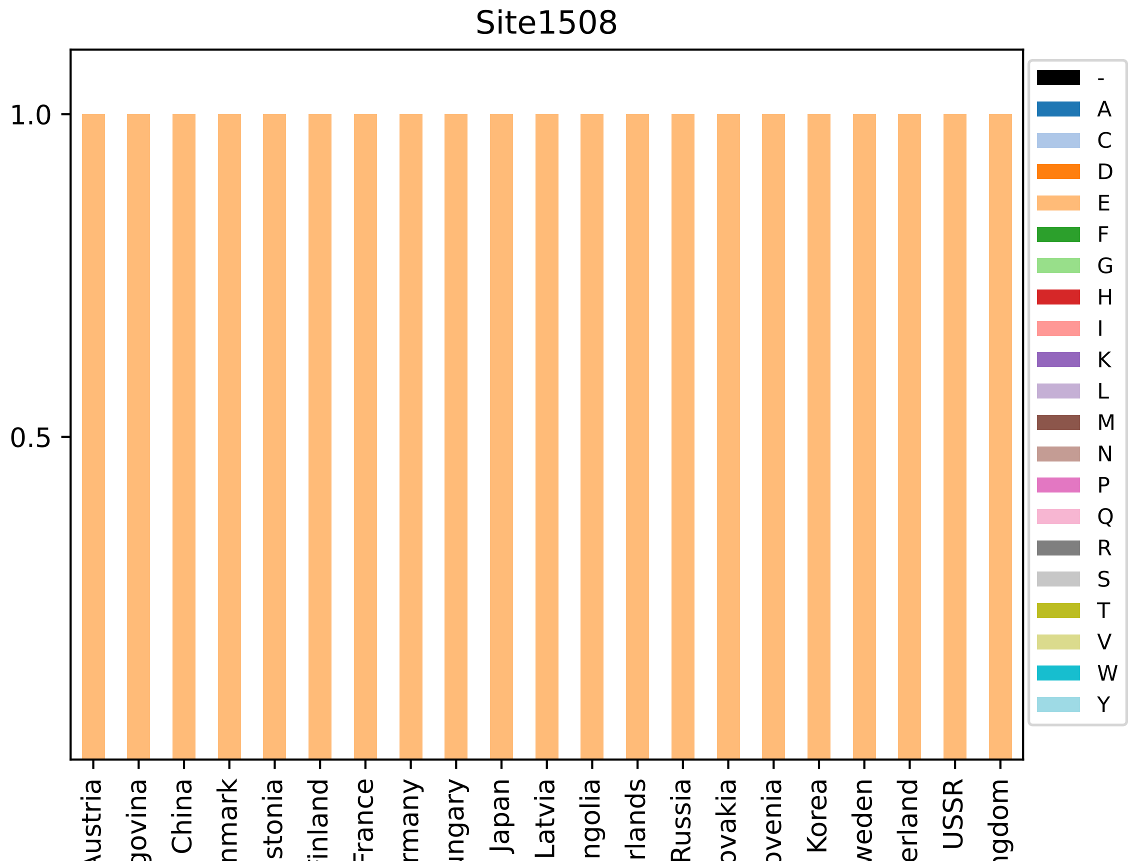
Site1508
1.0
0.5
-
A
C
D
E
F
G
H
I
K
L
M
N
P
Q
R
S
T
V
W
Y
China
Japan
Latvia
Russia
Korea
USSR

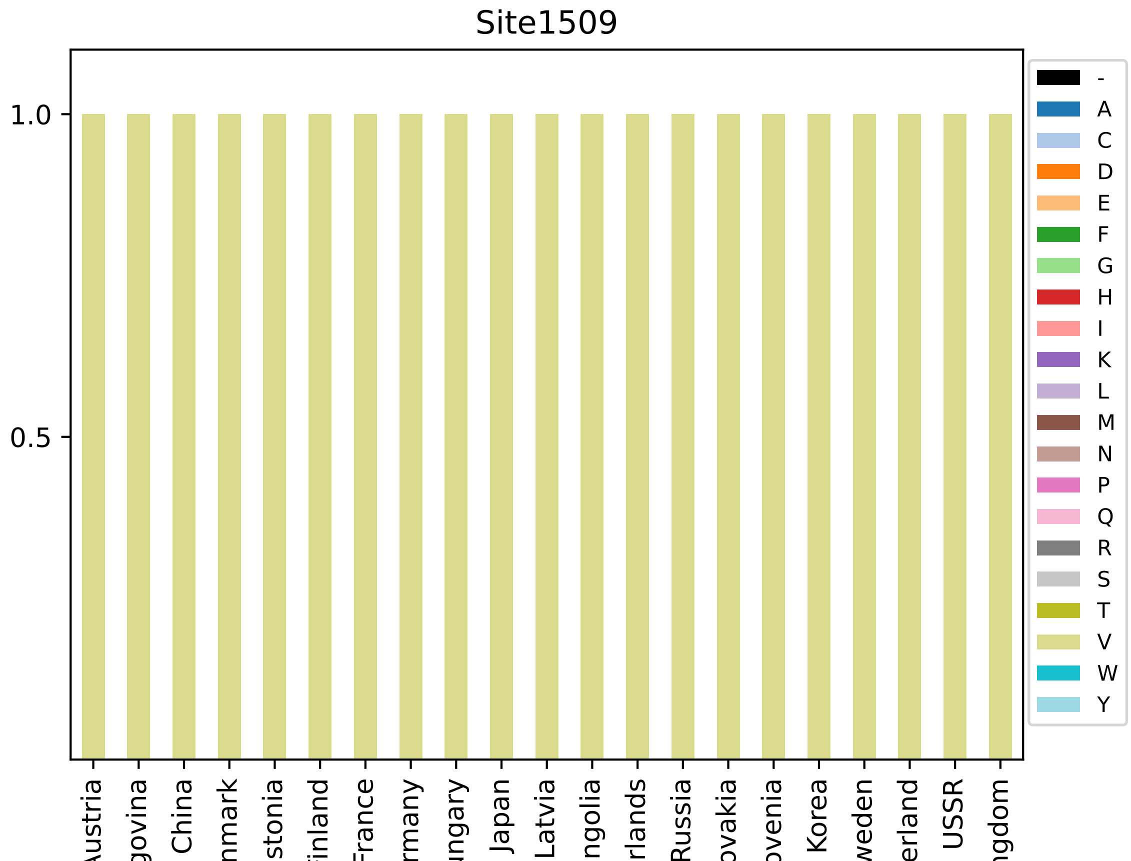
Site1509
1.0
0.5
-
A
C
D
E
F
G
H
I
K
L
M
N
P
Q
R
S
T
V
W
Y
China
Japan
Latvia
Russia
Korea
USSR

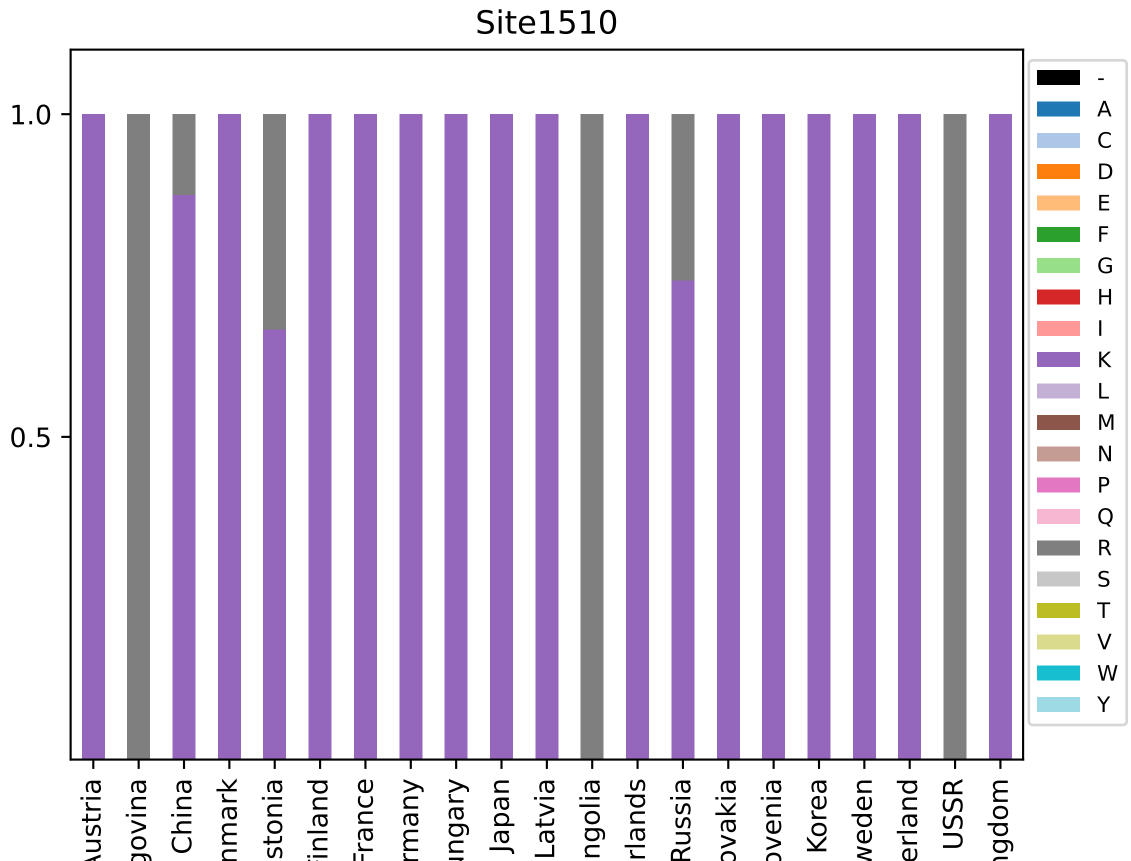

Site1510
1.0
0.5
-
A
C
D
E
F
G
H
I
K
L
M
N
P
Q
R
S
T
V
W
Y
China
Latvia
Japan
Russia
Korea
USSR

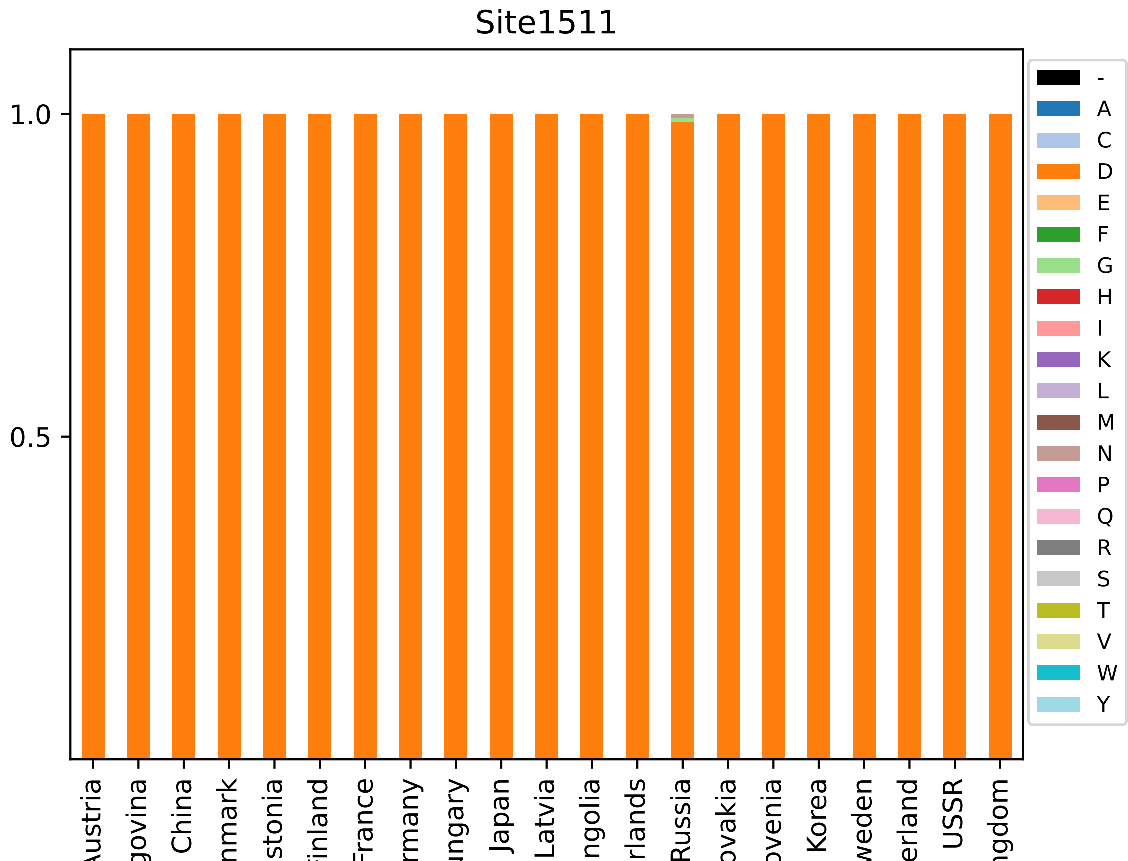
Site1511
1.0
0.5
-
A
C
D
E
F
G
H
I
K
L
M
N
P
Q
R
S
T
V
W
Y
Austria
China
Japan
Latvia
Russia
Korea
USSR

Site1512

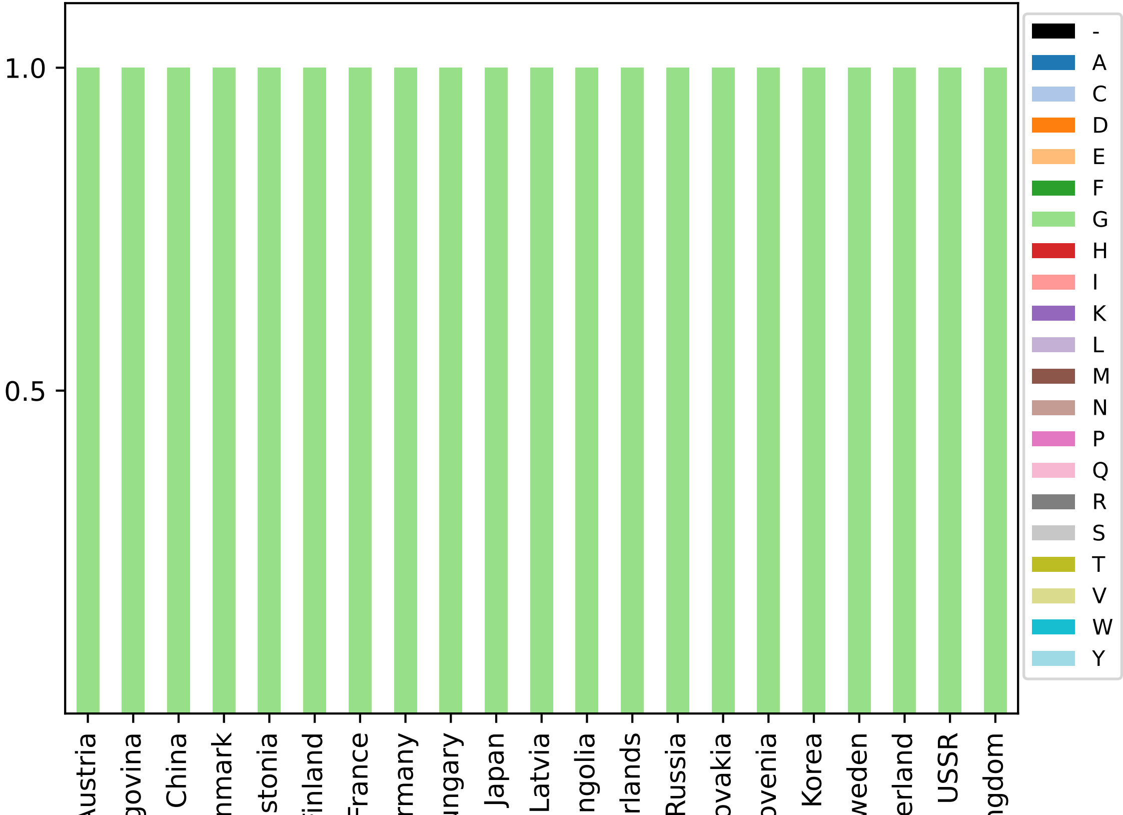

1.0
0.5
-
A
C
D
E
F
G
H
I
K
L
M
N
P
Q
R
S
T
V
W
Y
China
Japan
Latvia
Russia
Korea
USSR

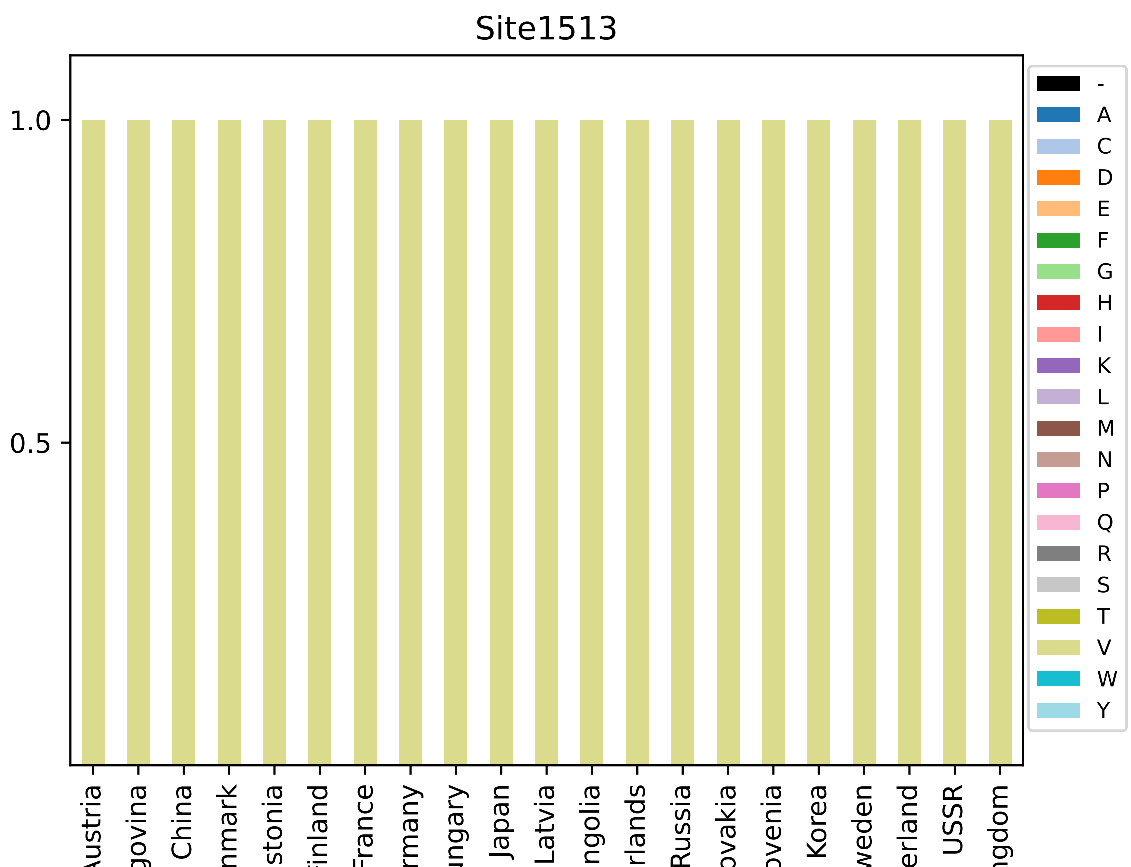

Site1513
1.0
0.5
-
A
C
D
E
F
G
H
I
K
L
M
N
P
Q
R
S
T
V
W
Y
Austria
China
Japan
Latvia
Russia
Korea
USSR

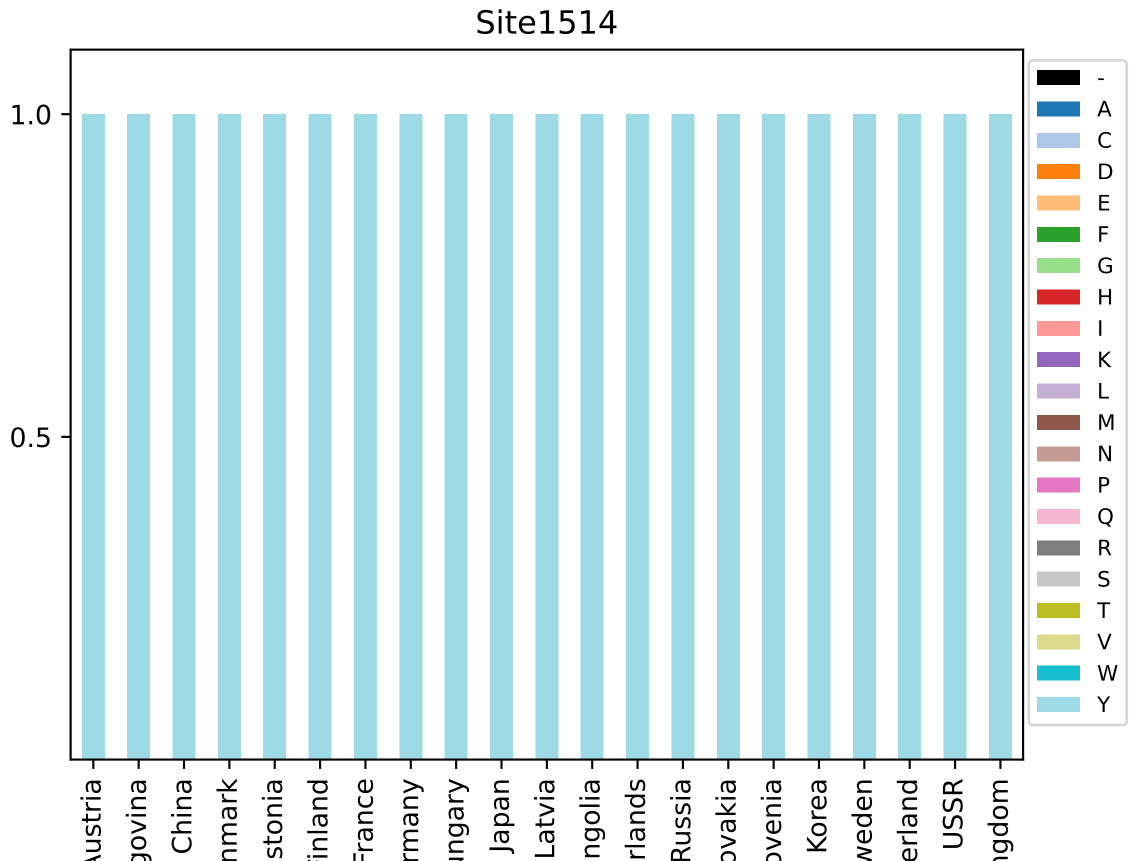

Site1514
1.0
0.5
-
A
C
D
E
F
G
H
I
K
L
M
N
P
Q
R
S
T
V
W
Y
China
Japan
Latvia
Russia
Korea
USSR

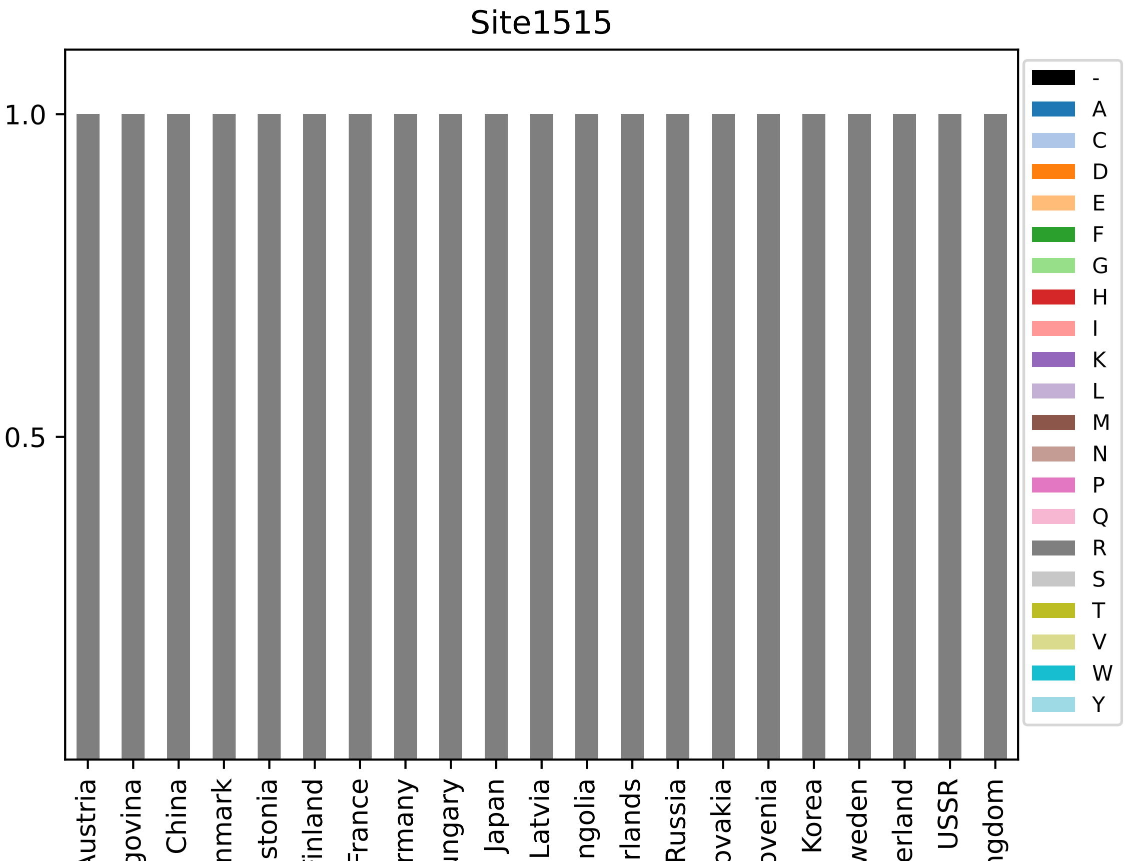

Site1515
1.0
0.5
-
A
C
D
E
F
G
H
I
K
L
M
N
P
Q
R
S
T
V
W
Y
Austria
govina
China
nmark
stonia
inland
France
rmany
ungary
Japan
Latvia
ngolia
rlands
Russia
ovakia
ovenia
Korea
weden
erland
USSR
ngdom

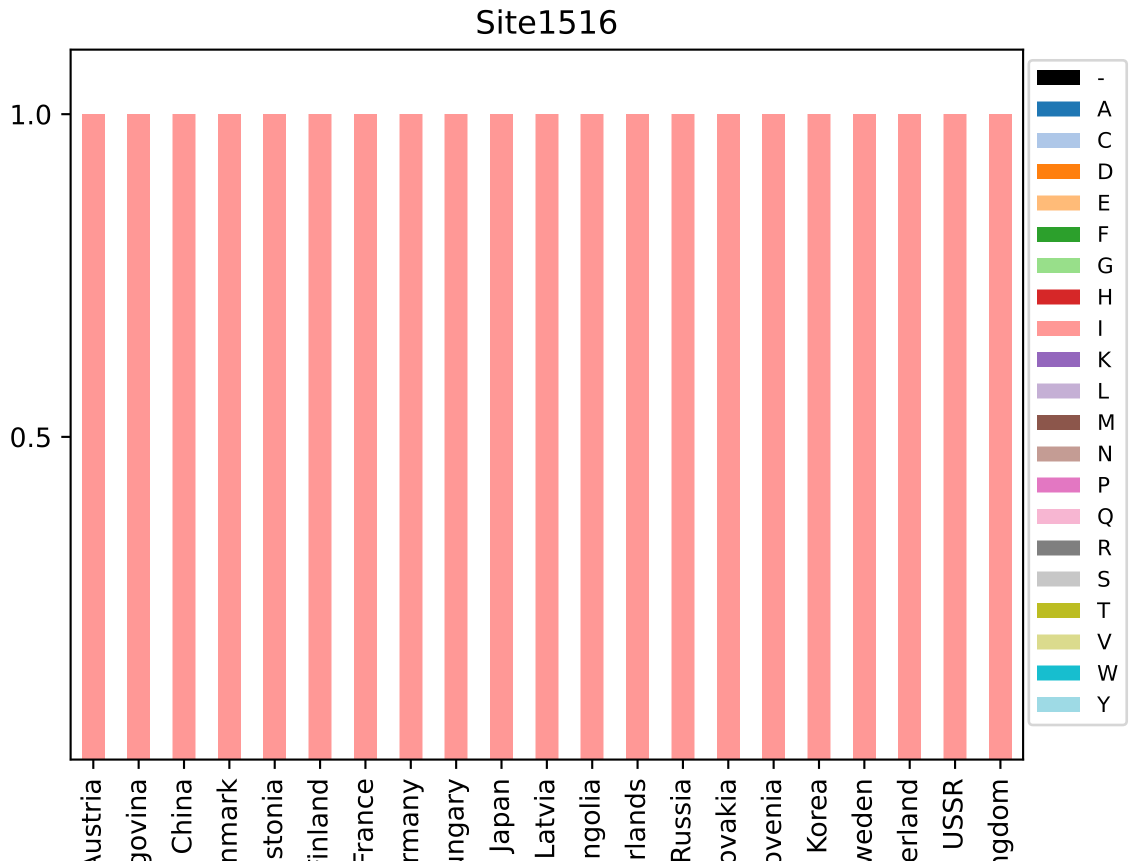

Site1516
1.0
0.5
-
A
C
D
E
F
G
H
I
K
L
M
N
P
Q
R
S
T
V
W
Y
China
Japan
Latvia
Russia
Korea
USSR

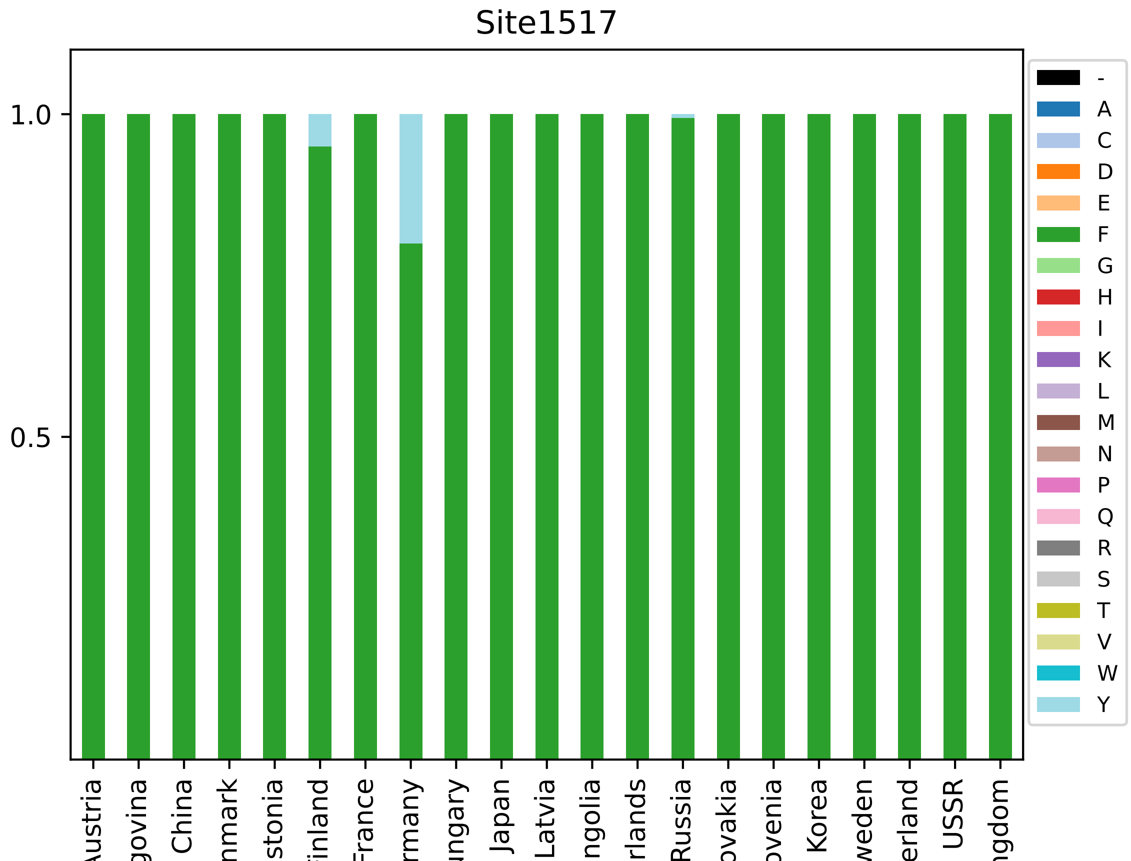
Site1517
1.0
0.5
-
A
C
D
E
F
G
H
I
K
L
M
N
P
Q
R
S
T
V
W
Y
China
Japan
Latvia
Russia
Korea
USSR

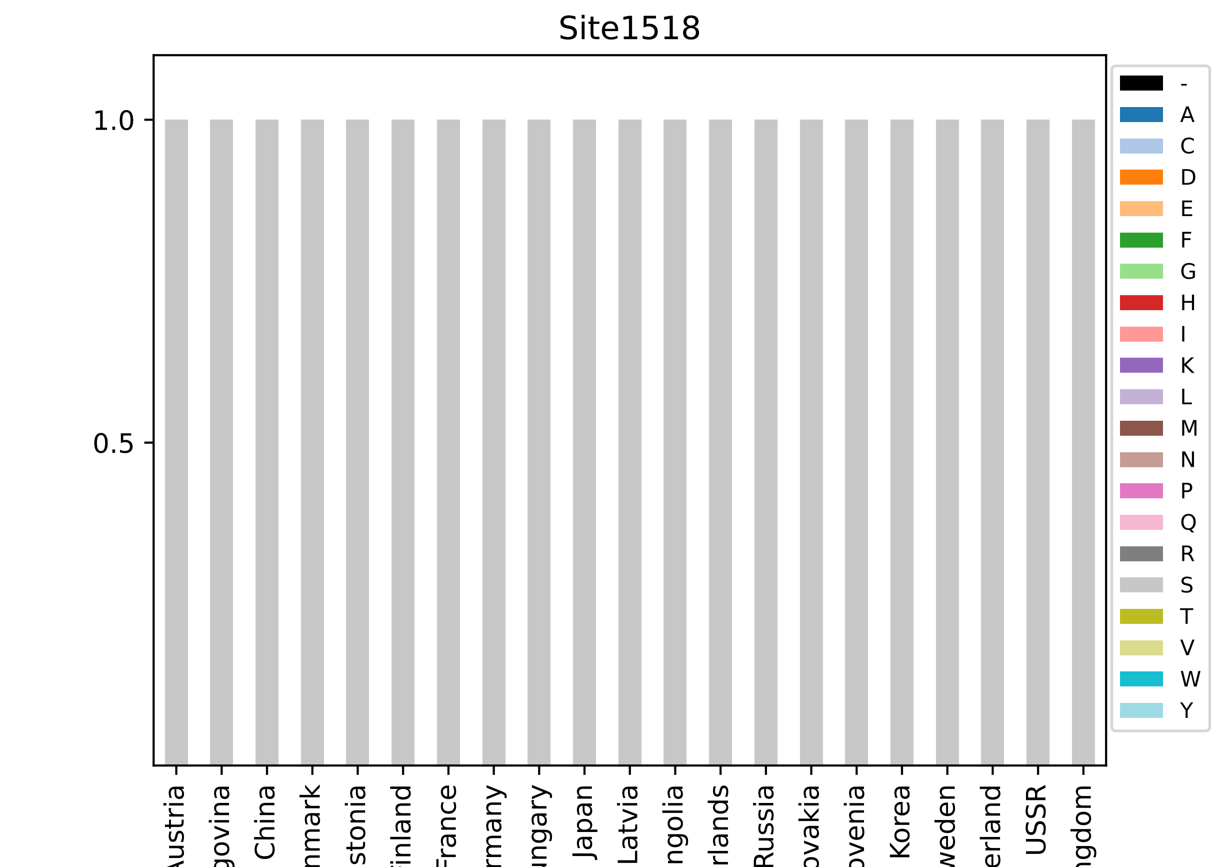
Site1518
1.0
0.5
Austria
govina
China
nmark
stonia
Finland
France
rmany
ungary
Japan
Latvia
ngolia
rlands
Russia
ovakia
ovenia
Korea
weden
erland
USSR
ngdom
-
A
C
D
E
F
G
H
I
K
L
M
N
P
Q
R
S
T
V
W
Y

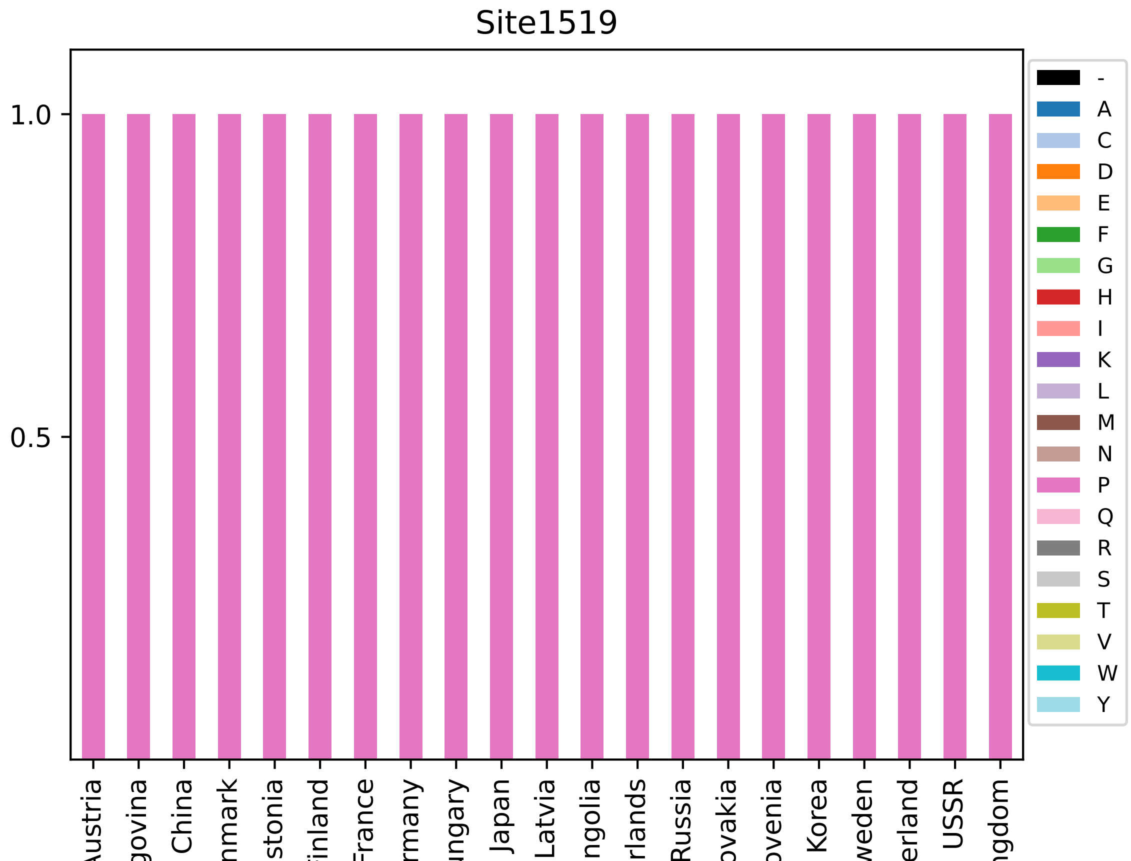

Site1519
1.0
0.5
-
A
C
D
E
F
G
H
I
K
L
M
N
P
Q
R
S
T
V
W
Y
Austria
China
Japan
Latvia
Russia
Korea
USSR

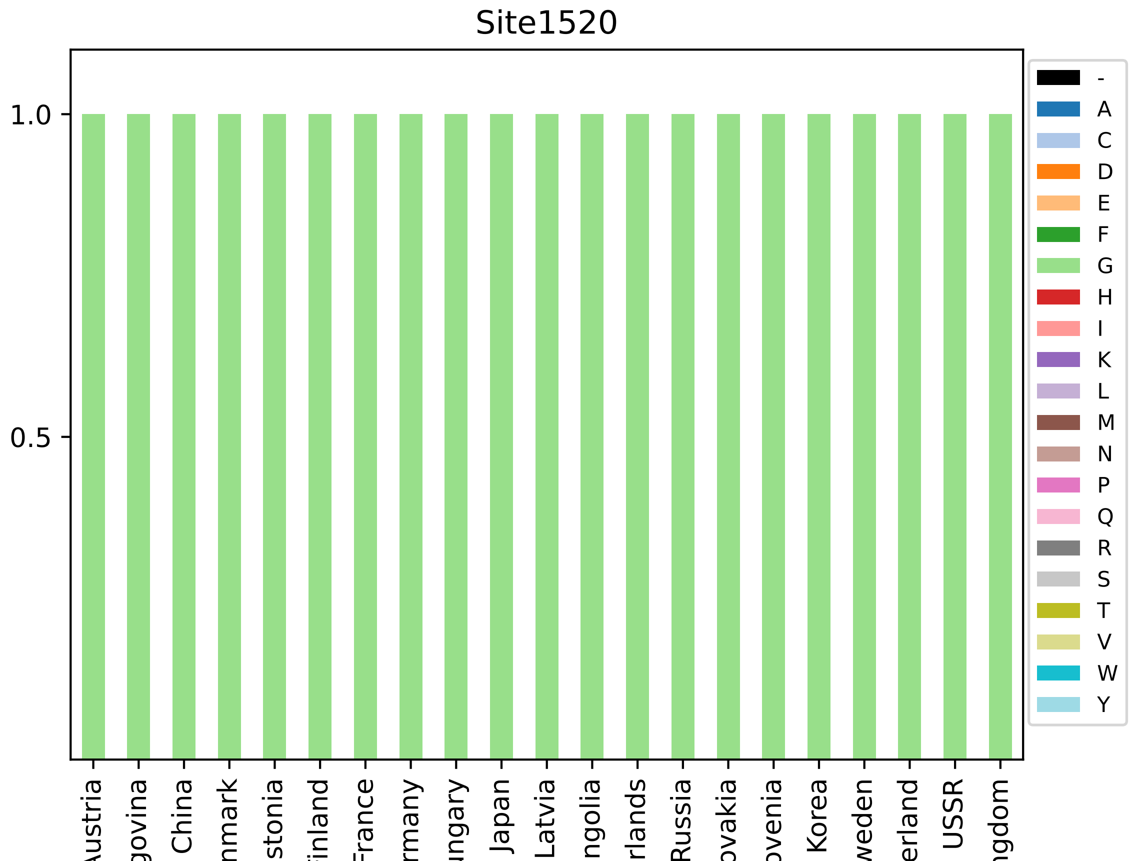
Site1520
1.0
0.5
-
A
C
D
E
F
G
H
I
K
L
M
N
P
Q
R
S
T
V
W
Y
Austria
govina
China
nmark
stonia
inland
France
rmany
ungary
Japan
Latvia
ngolia
rlands
Russia
ovakia
ovenia
Korea
weden
erland
USSR
ngdom

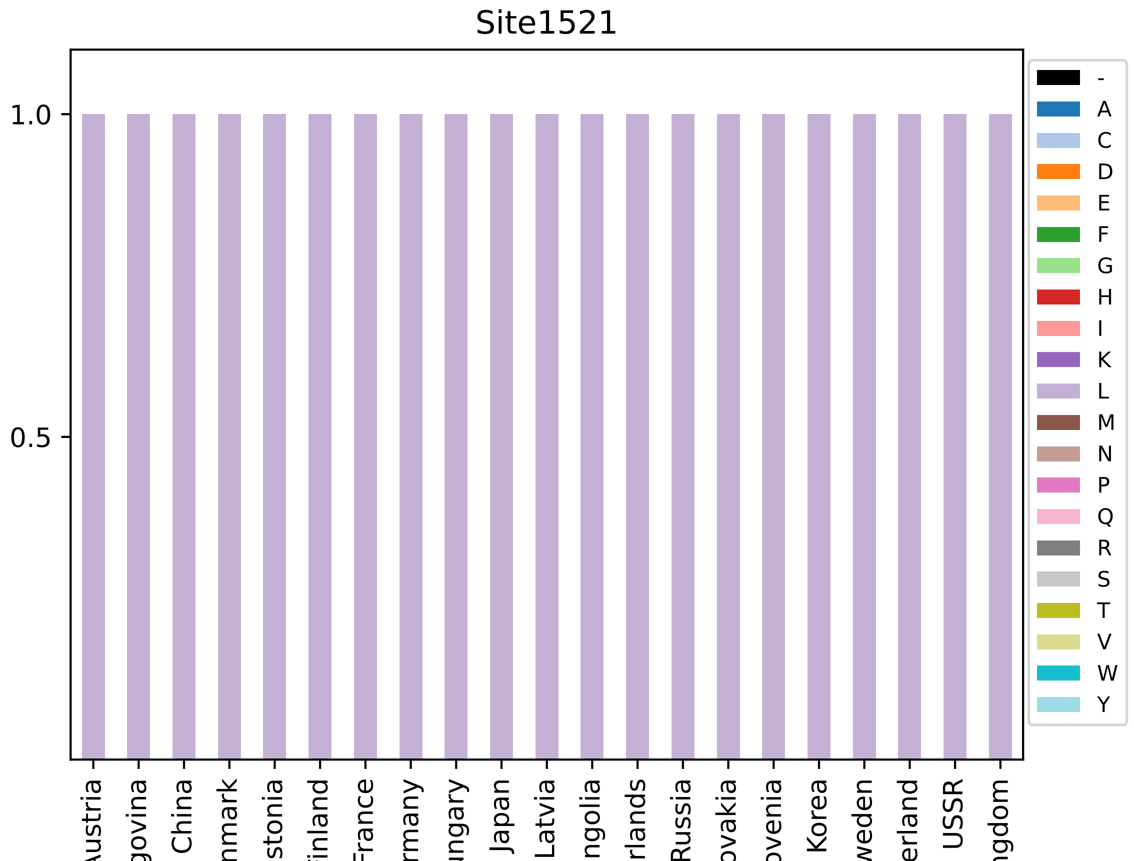
Site1521
1.0
0.5
Austria
govina
China
nmark
stonia
Finland
France
rmany
ungary
Japan
Latvia
ngolia
rlands
Russia
ovakia
ovenia
Korea
weden
erland
USSR
ngdom
-
A
C
D
E
F
G
H
I
K
L
M
N
P
Q
R
S
T
V
W
Y

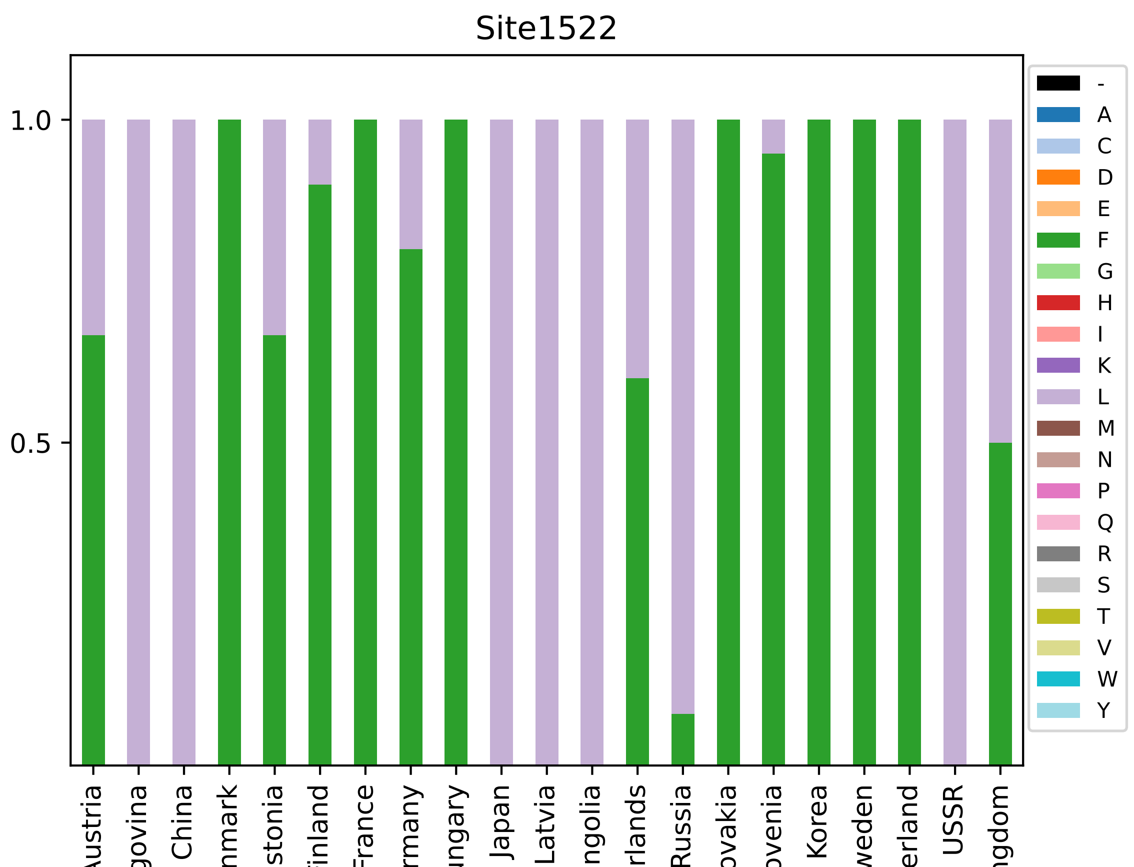
Site1522
1.0
0.5
-
A
C
D
E
F
G
H
I
K
L
M
N
P
Q
R
S
T
V
W
Y
Austria
govina
China
nmark
stonia
inland
France
rmany
ungary
Japan
Latvia
ngolia
rlands
Russia
ovakia
ovenia
Korea
weden
erland
USSR
ngdom

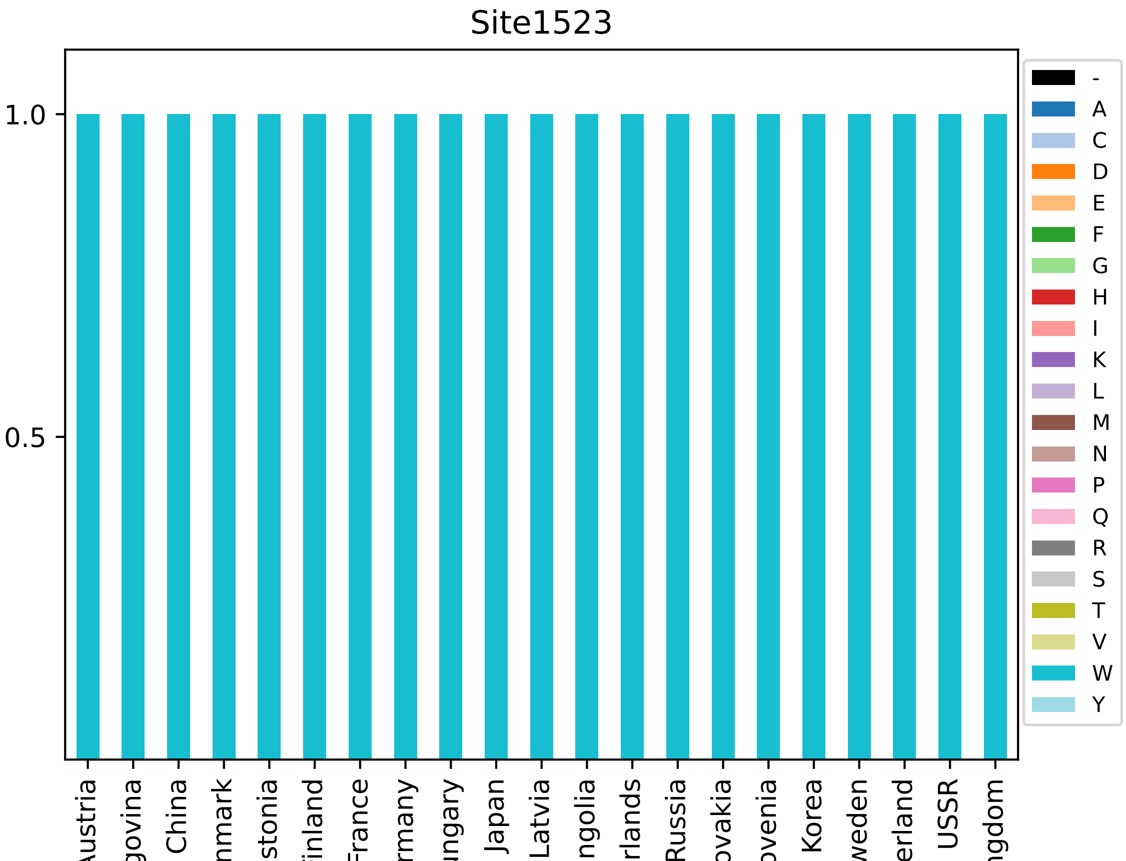
Site1523
1.0
0.5
-
A
C
D
E
F
G
H
I
K
L
M
N
P
Q
R
S
T
V
W
Y
Austria
China
Japan
Latvia
Russia
Korea
USSR

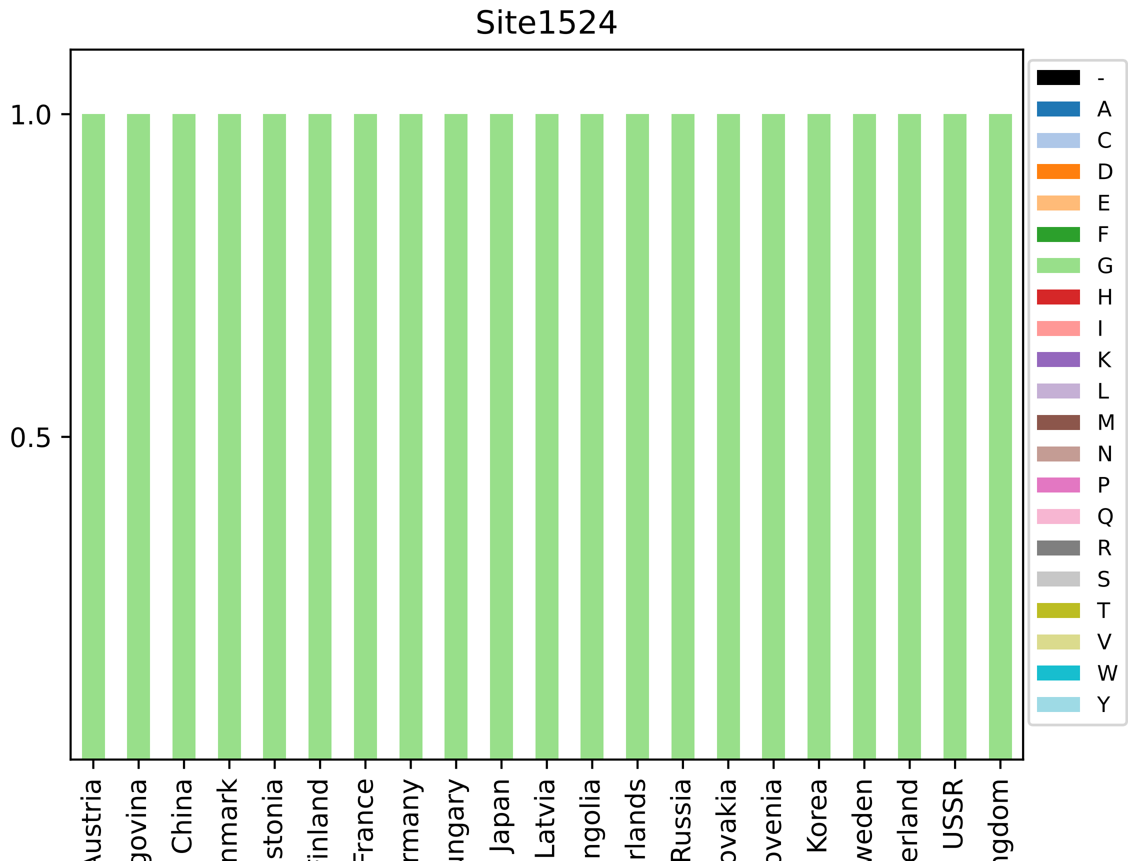
Site1524
1.0
0.5
-
A
C
D
E
F
G
H
I
K
L
M
N
P
Q
R
S
T
V
W
Y
China
Japan
Latvia
Russia
Korea
USSR

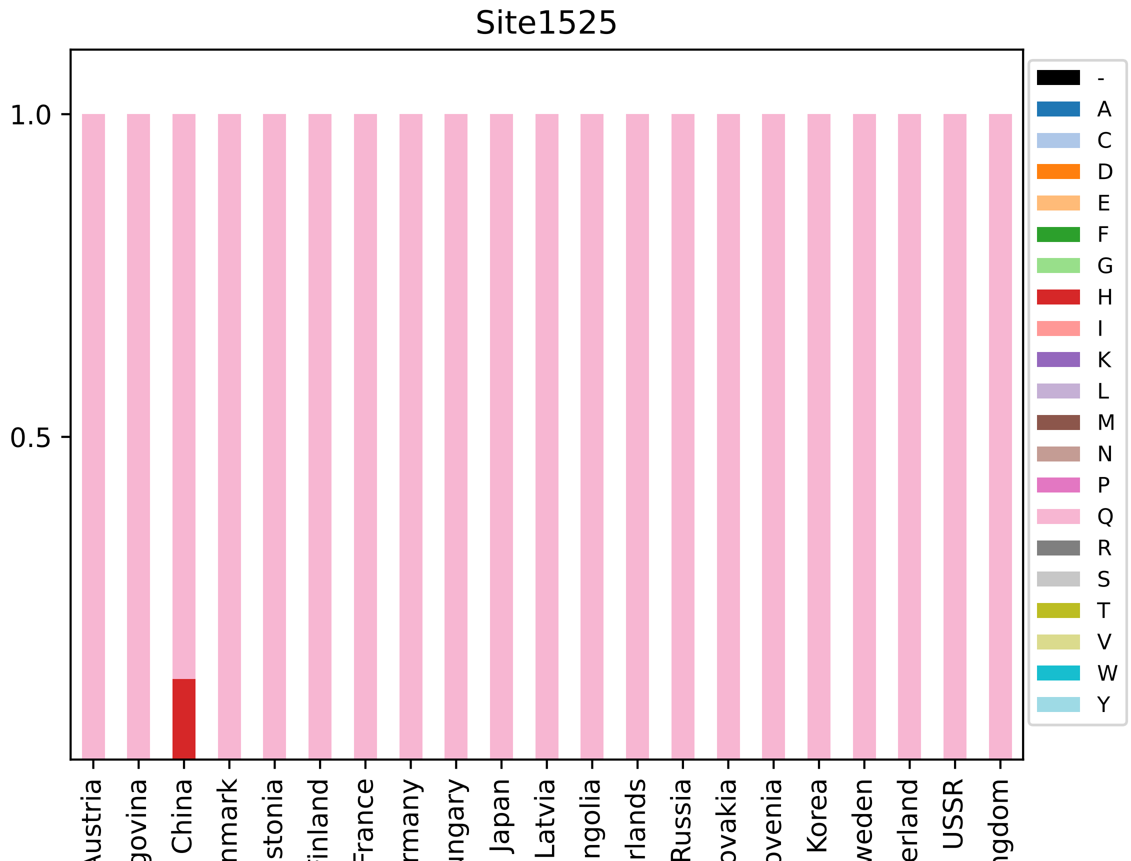

Site1525
1.0
0.5
-
A
C
D
E
F
G
H
I
K
L
M
N
P
Q
R
S
T
V
W
Y
China
Japan
Latvia
Russia
Korea
USSR

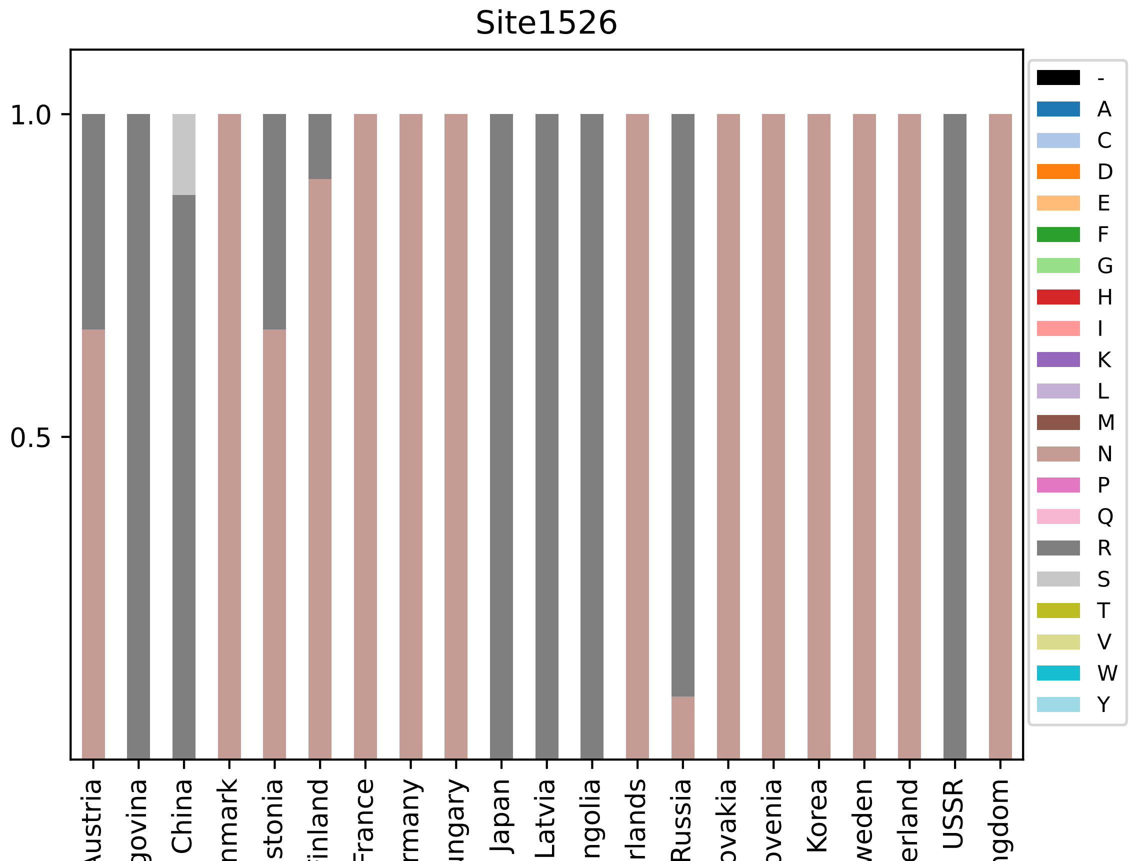
Site1526
1.0
0.5
-
A
C
D
E
F
G
H
I
K
L
M
N
P
Q
R
S
T
V
W
Y
Austria
govina
China
nmark
stonia
inland
France
rmany
ungary
Japan
Latvia
ngolia
rlands
Russia
ovakia
ovenia
Korea
weden
erland
USSR
ngdom

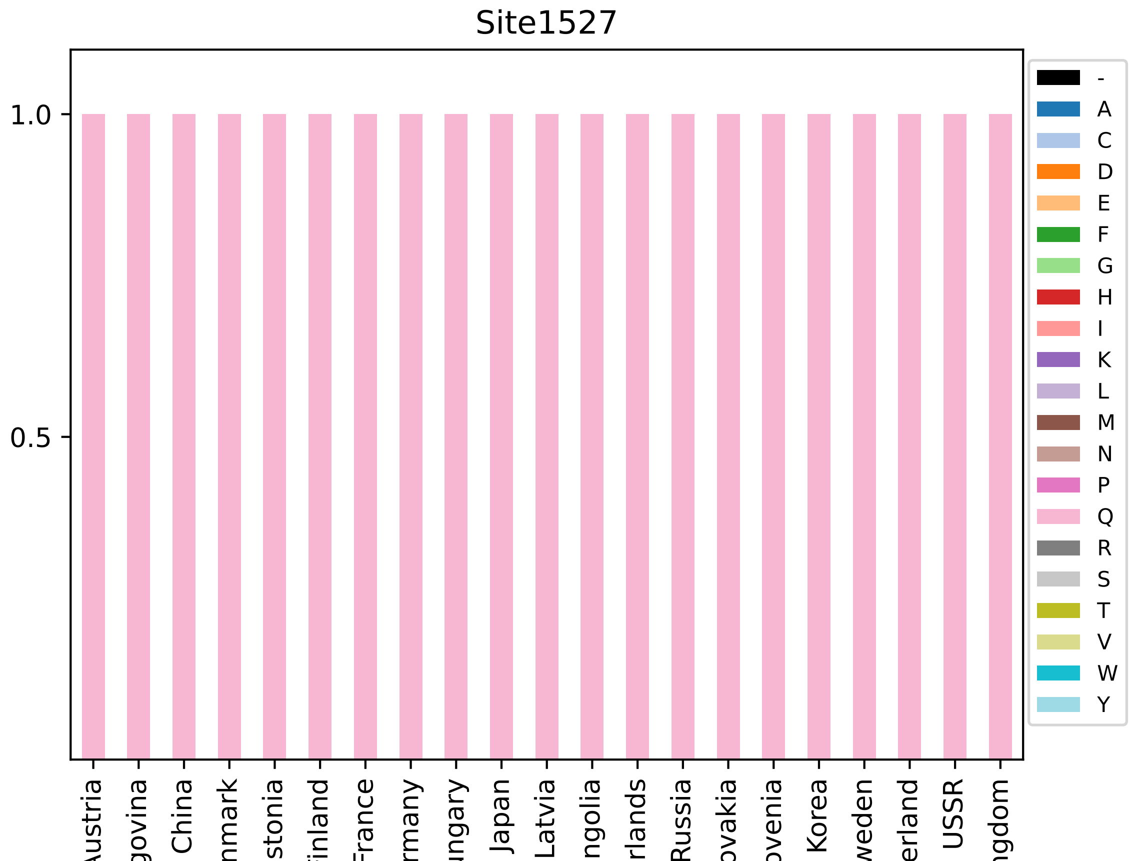
Site1527
1.0
0.5
-
A
C
D
E
F
G
H
I
K
L
M
N
P
Q
R
S
T
V
W
Y
Austria
China
Estonia
Finland
France
Hungary
Japan
Latvia
Russia
Korea
Sweden
USSR

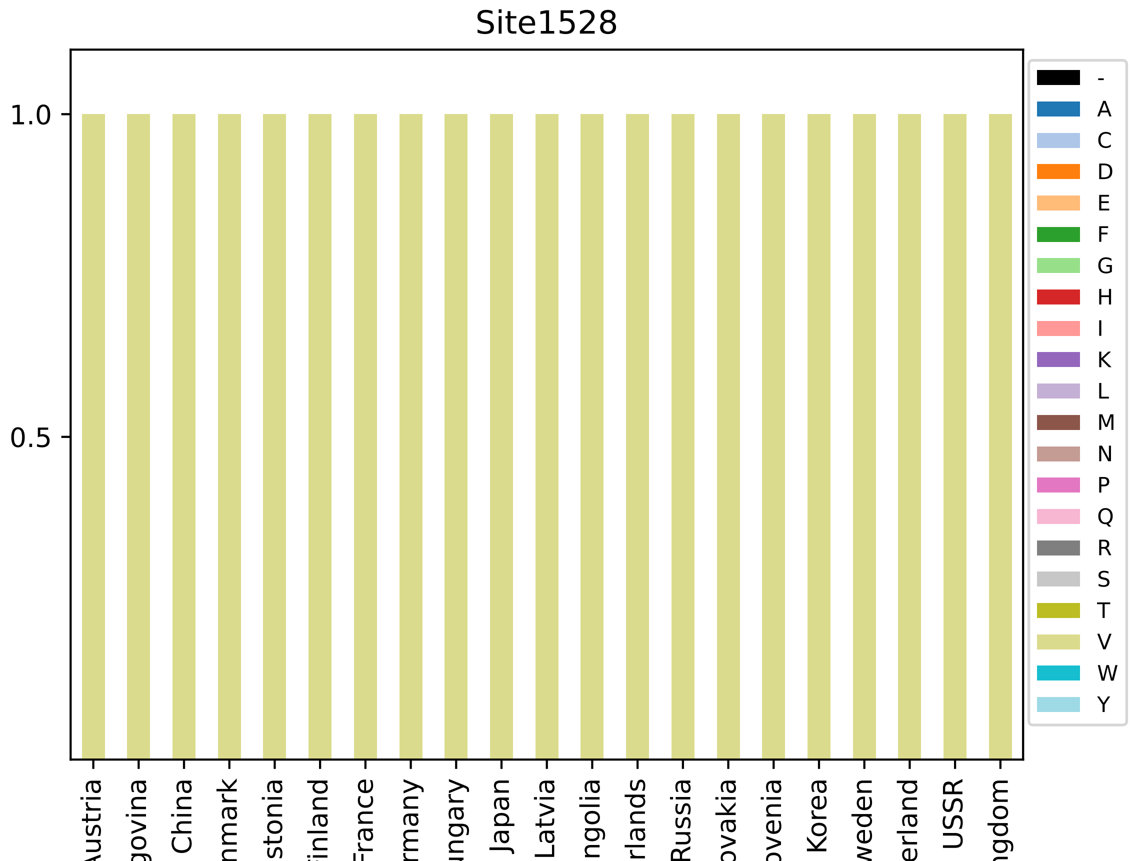

Site1528
1.0
0.5
Austria
govina
China
nmark
stonia
Finland
France
rmany
ungary
Japan
Latvia
ngolia
rlands
Russia
ovakia
ovenia
Korea
weden
erland
USSR
ngdom
-
A
C
D
E
F
G
H
I
K
L
M
N
P
Q
R
S
T
V
W
Y

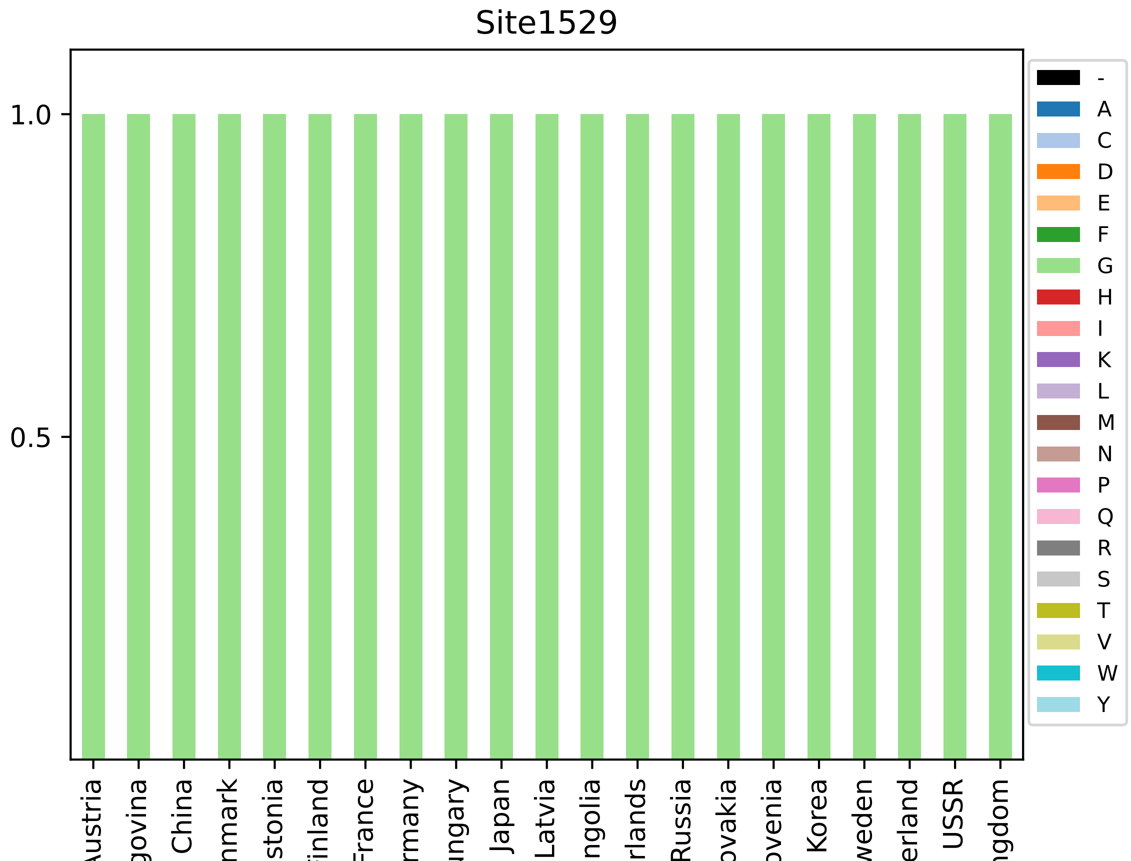

Site1529
1.0
0.5
-
A
C
D
E
F
G
H
I
K
L
M
N
P
Q
R
S
T
V
W
Y
China
Japan
Latvia
Russia
Korea
USSR

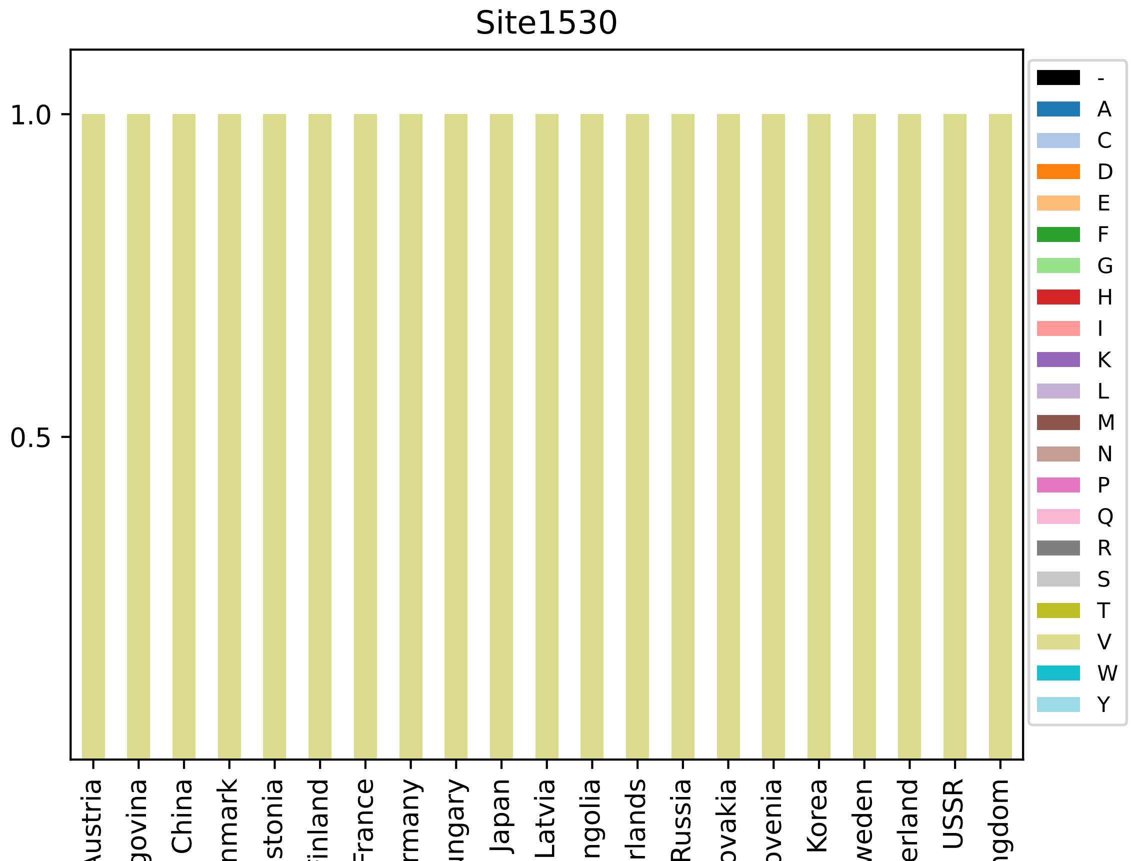
Site1530
1.0
0.5
China
Japan
Latvia
Russia
Korea
USSR
-
A
C
D
E
F
G
H
I
K
L
M
N
P
Q
R
S
T
V
W
Y

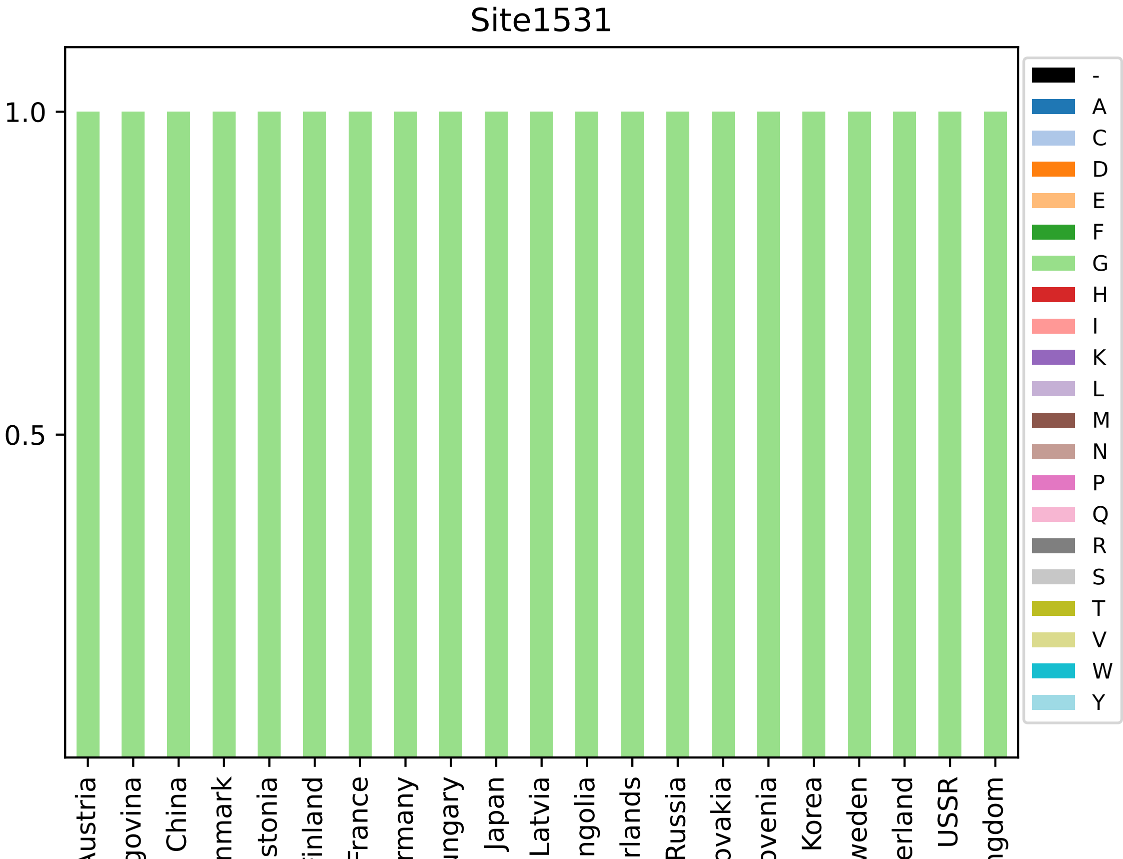
Site1531
1.0
0.5
-
A
C
D
E
F
G
H
I
K
L
M
N
P
Q
R
S
T
V
W
Y
China
Japan
Latvia
Korea
USSR

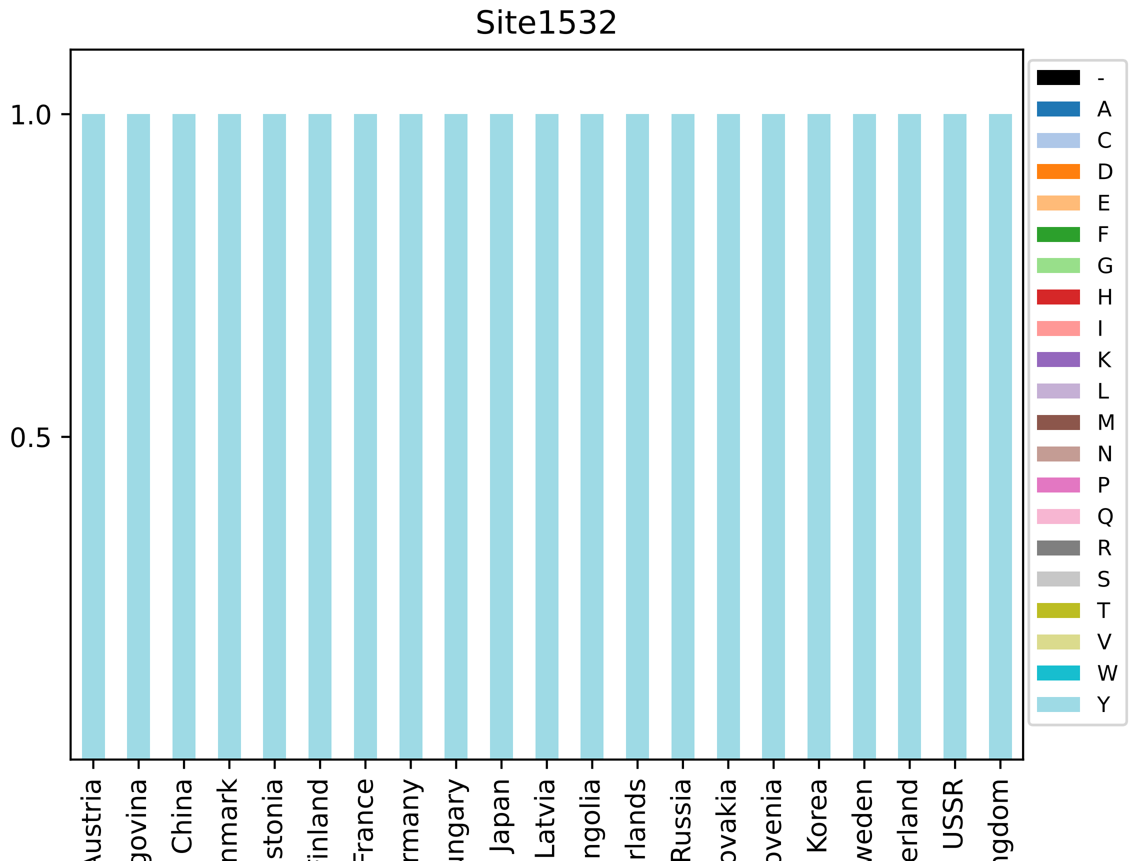

Site1532
1.0
0.5
-
A
C
D
E
F
G
H
I
K
L
M
N
P
Q
R
S
T
V
W
Y
China
Japan
Latvia
Russia
Korea
USSR

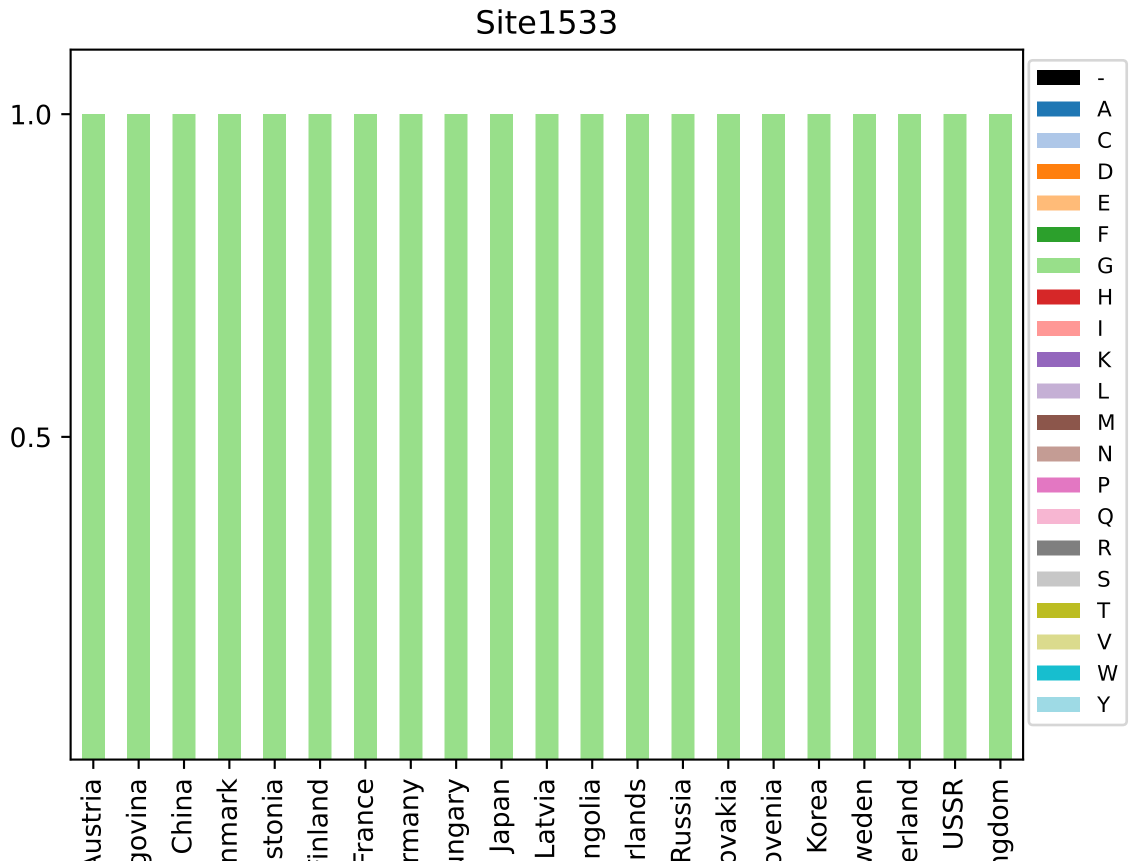

Site1533
1.0
0.5
-
A
C
D
E
F
G
H
I
K
L
M
N
P
Q
R
S
T
V
W
Y
Austria
govina
China
nmark
stonia
Finland
France
rmany
ungary
Japan
Latvia
ngolia
rlands
Russia
ovakia
ovenia
Korea
weden
erland
USSR
ngdom

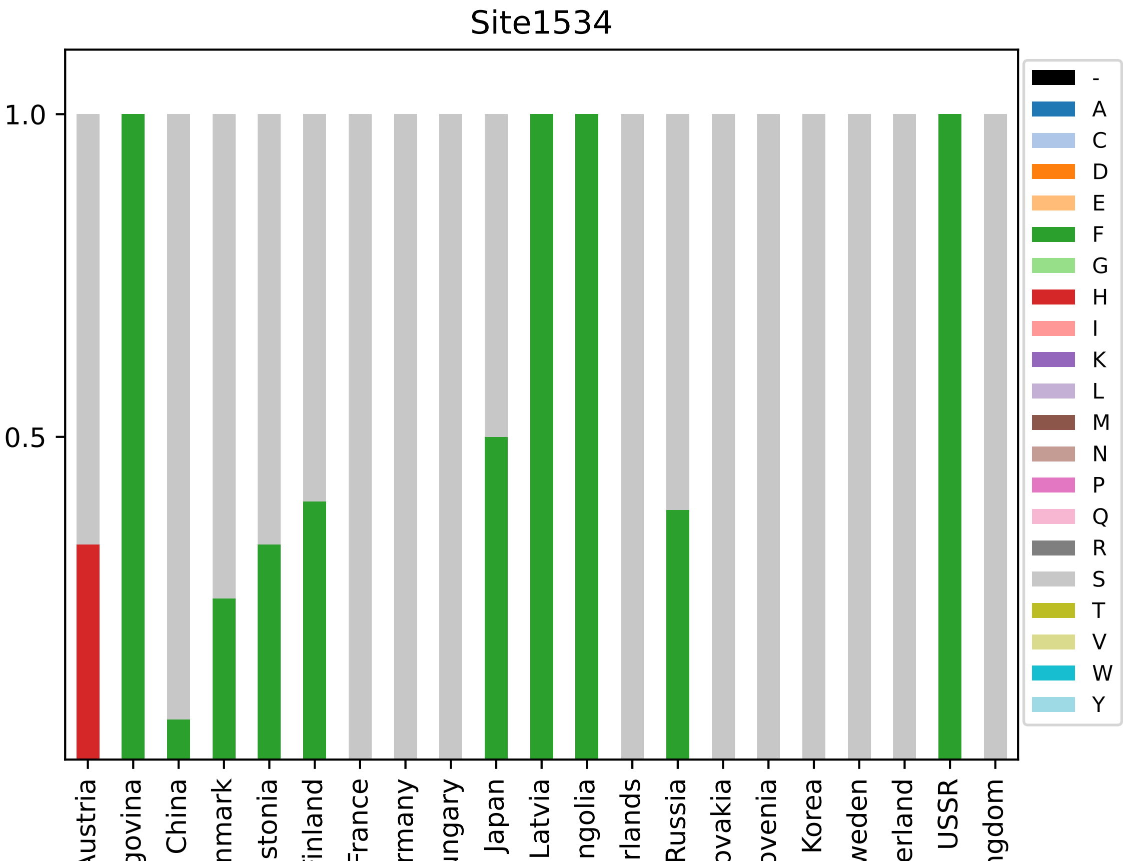

Site1534
1.0
0.5
-
A
C
D
E
F
G
H
I
K
L
M
N
P
Q
R
S
T
V
W
Y
Austria
govina
China
nmark
stonia
inland
France
rmany
ungary
Japan
Latvia
ngolia
rlands
Russia
ovakia
ovenia
Korea
weden
erland
USSR
ngdom

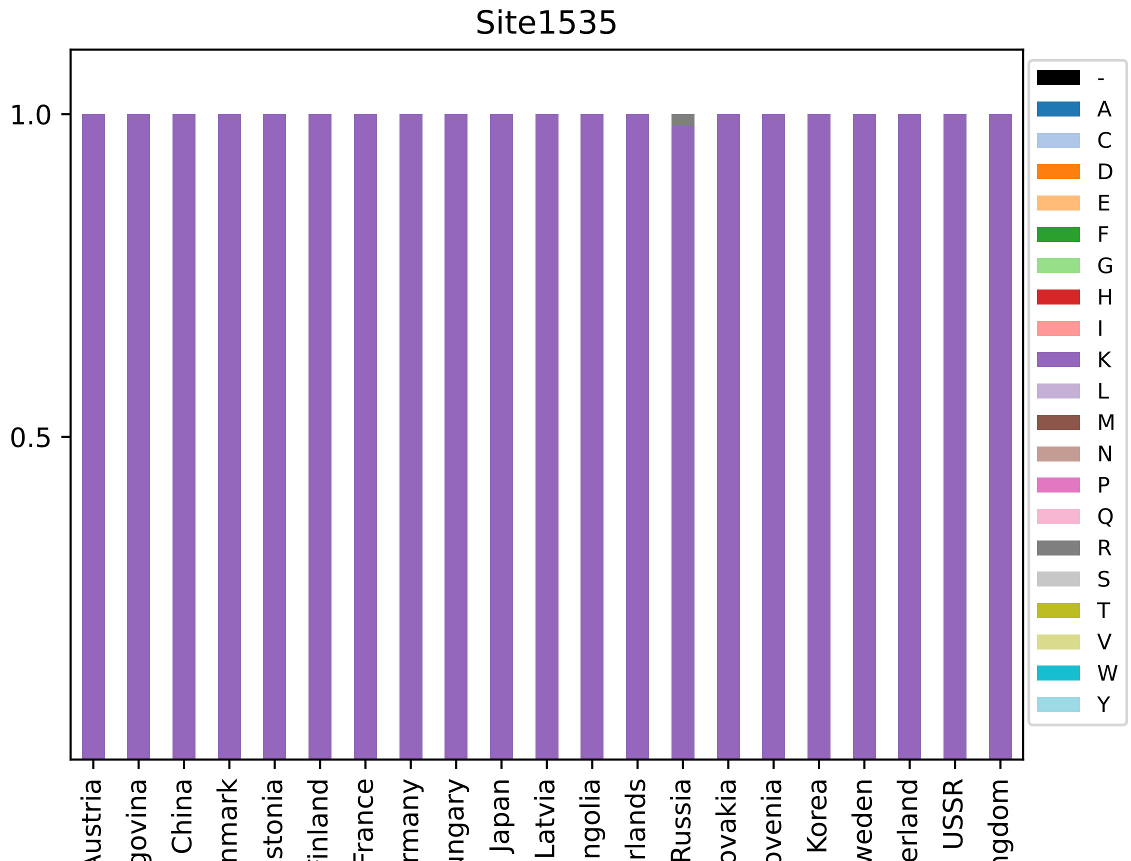

Site1535
1.0
0.5
-
A
C
D
E
F
G
H
I
K
L
M
N
P
Q
R
S
T
V
W
Y
Austria
govina
China
nmark
stonia
Finland
France
rmany
ungary
Japan
Latvia
ngolia
rlands
Russia
ovakia
ovenia
Korea
weden
erland
USSR
ngdom

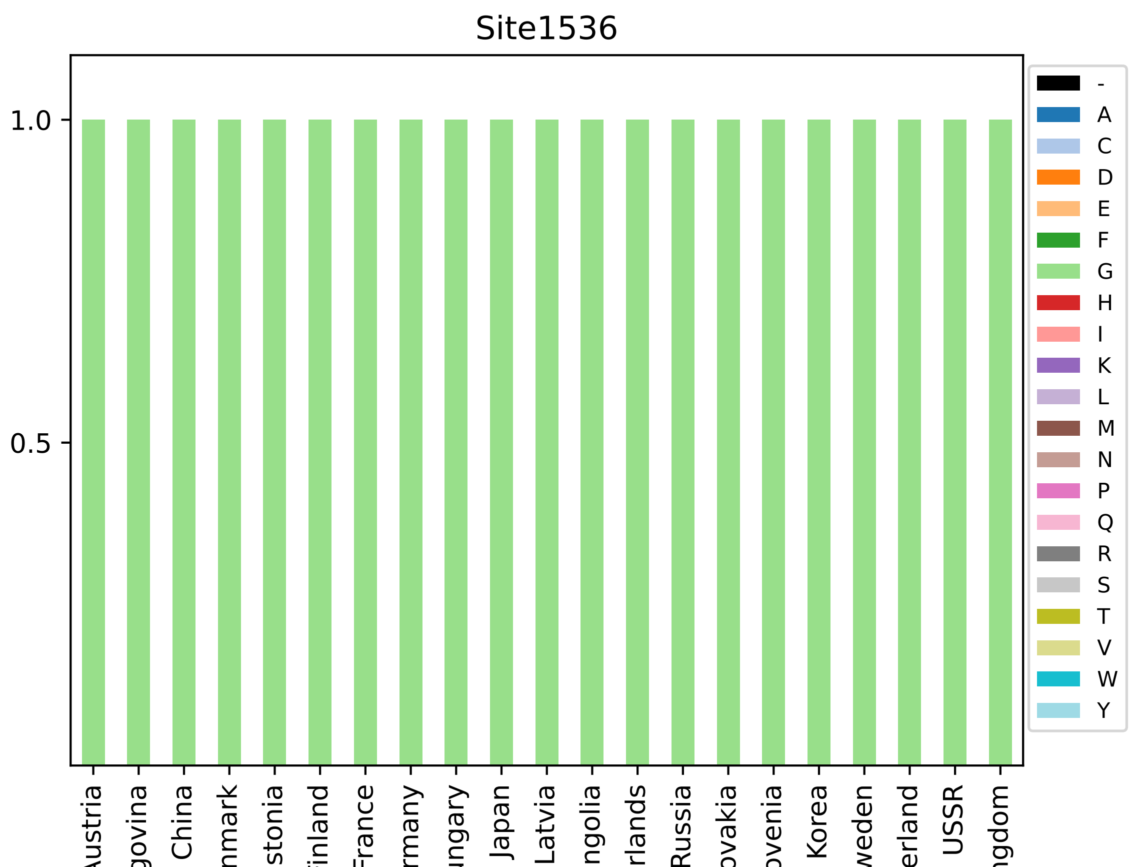

Site1536
1.0
0.5
-
A
C
D
E
F
G
H
I
K
L
M
N
P
Q
R
S
T
V
W
Y
Austria
China
Finland
France
Japan
Latvia
Russia
Korea
USSR

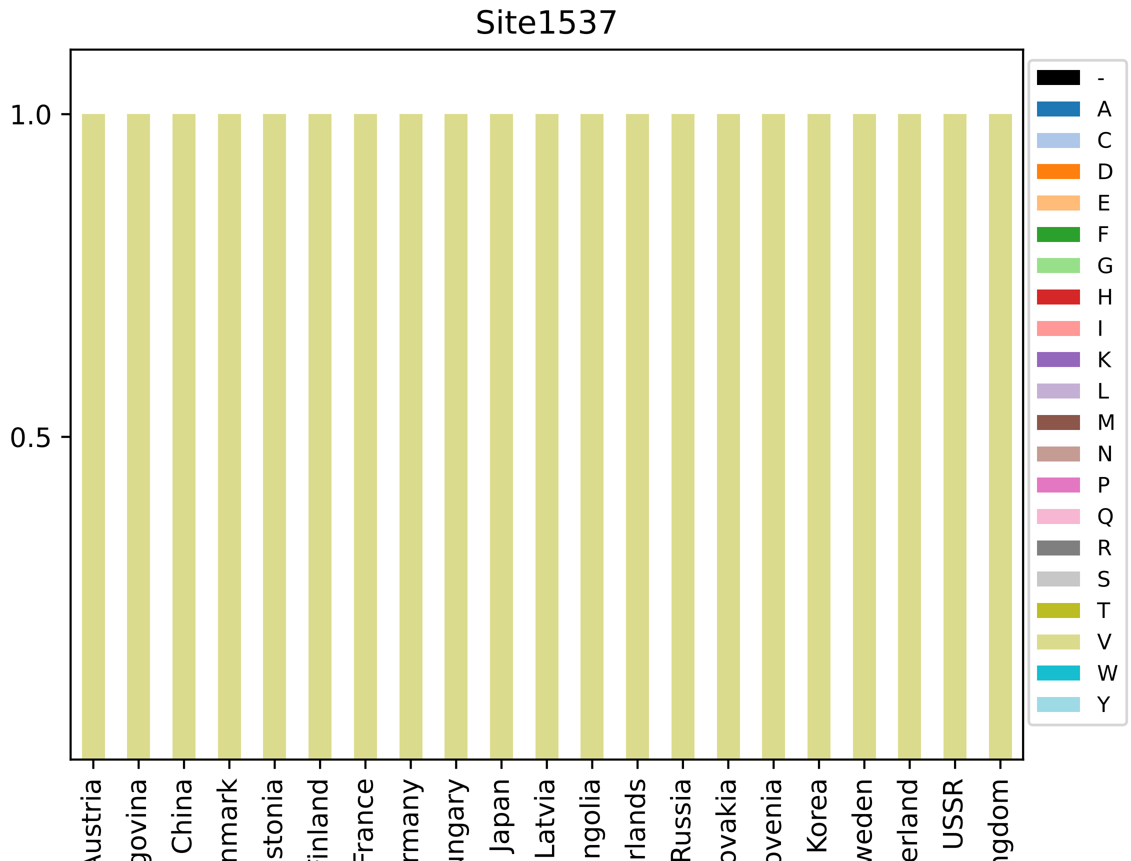

Site1537
1.0
0.5
Austria
govina
China
nmark
stonia
Finland
France
rmany
ungary
Japan
Latvia
ngolia
rlands
Russia
ovakia
ovenia
Korea
weden
erland
USSR
ngdom
-
A
C
D
E
F
G
H
I
K
L
M
N
P
Q
R
S
T
V
W
Y

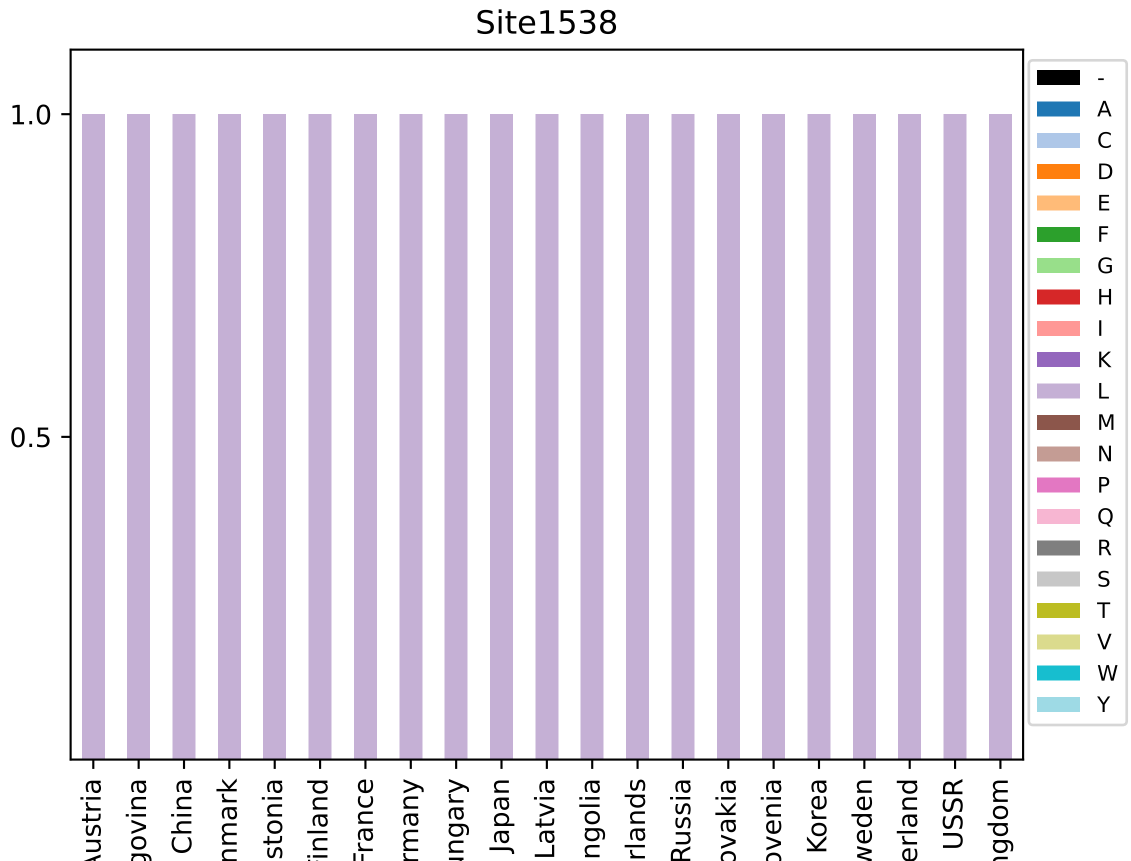
Site1538
1.0
0.5
Austria
govina
China
nmark
stonia
inland
France
rmany
ungary
Japan
Latvia
ngolia
rlands
Russia
ovakia
ovenia
Korea
weden
erland
USSR
ngdom
-
A
C
D
E
F
G
H
I
K
L
M
N
P
Q
R
S
T
V
W
Y

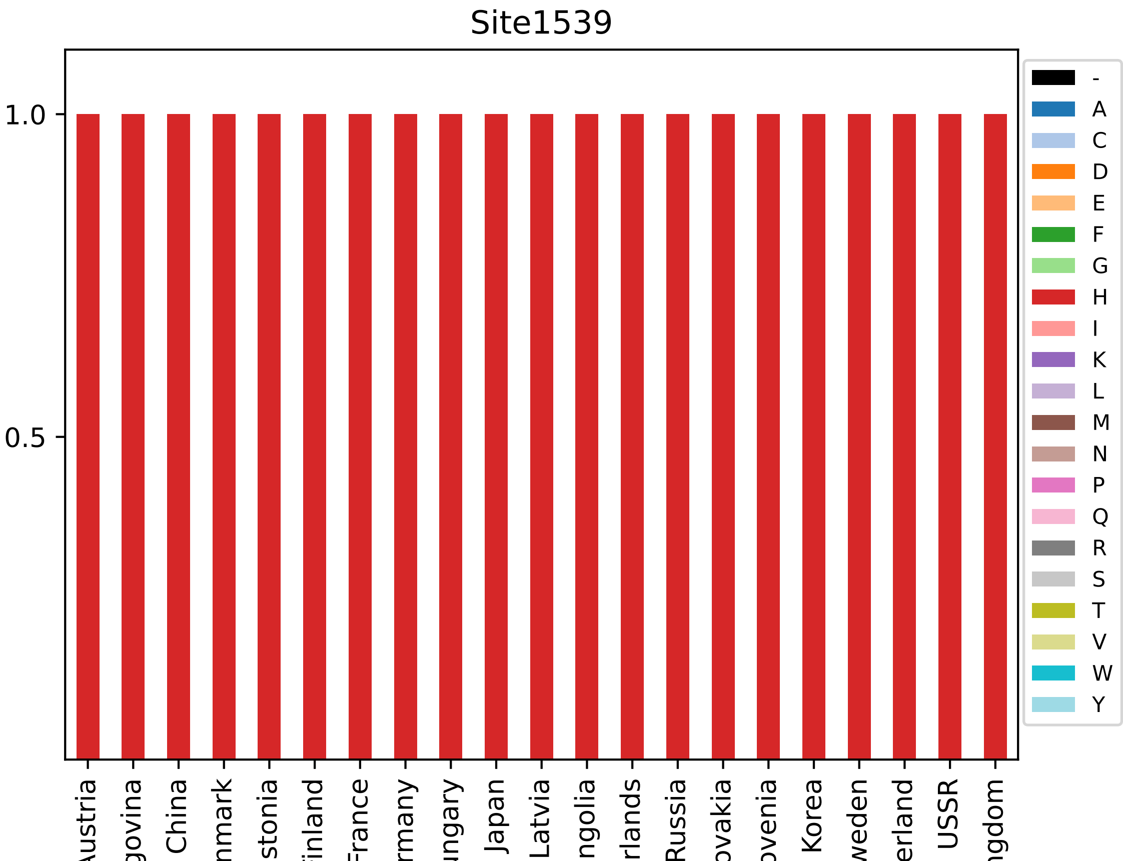
Site1539
1.0
0.5
-
A
C
D
E
F
G
H
I
K
L
M
N
P
Q
R
S
T
V
W
Y
China
Japan
Latvia
Russia
Korea
USSR

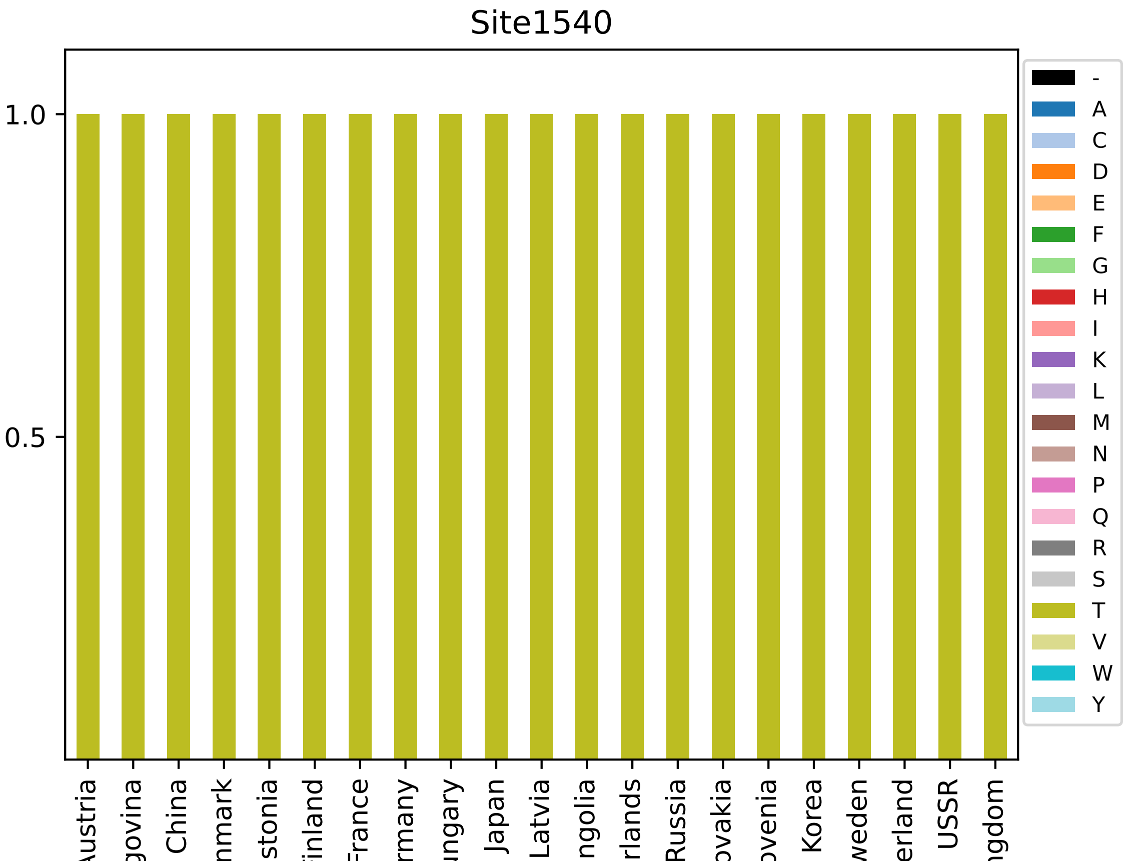
Site1540
1.0
0.5
-
A
C
D
E
F
G
H
I
K
L
M
N
P
Q
R
S
T
V
W
Y
China
Japan
Latvia
Russia
Korea
USSR

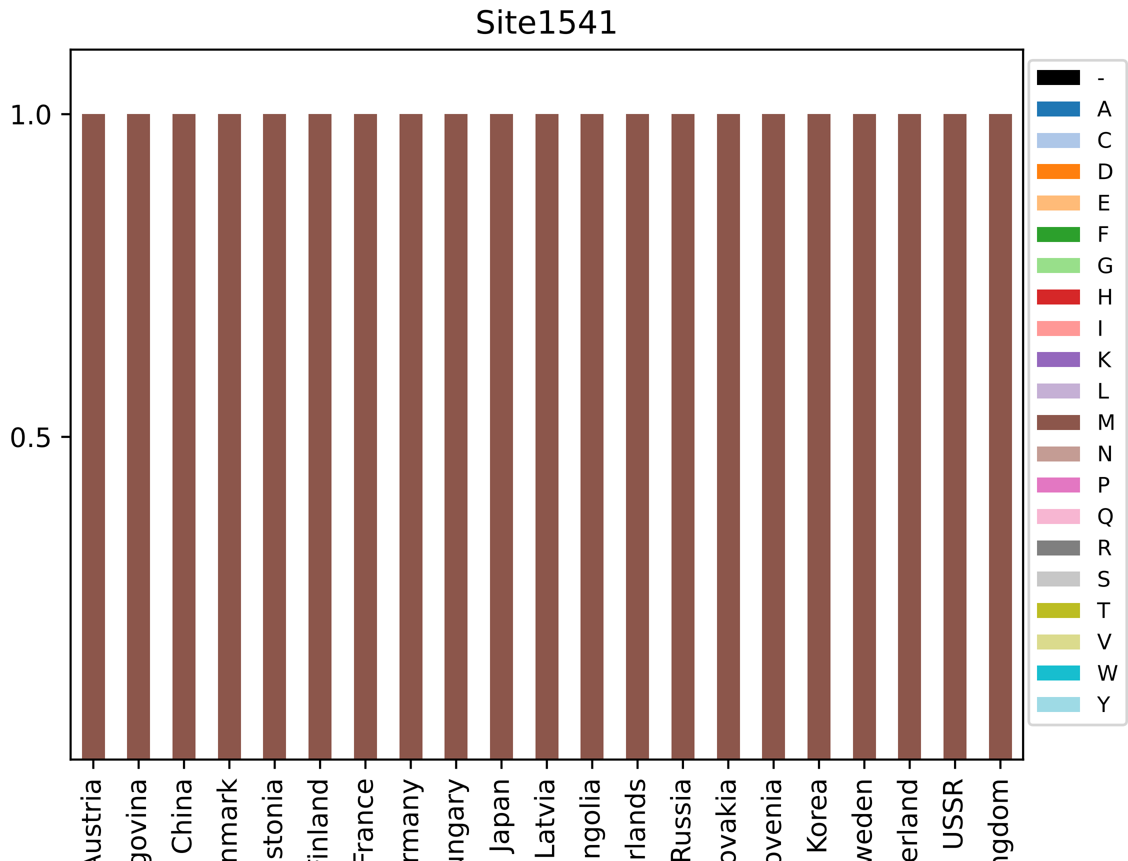

Site1541
1.0
0.5
-
A
C
D
E
F
G
H
I
K
L
M
N
P
Q
R
S
T
V
W
Y
China
Japan
Latvia
Russia
Korea
USSR

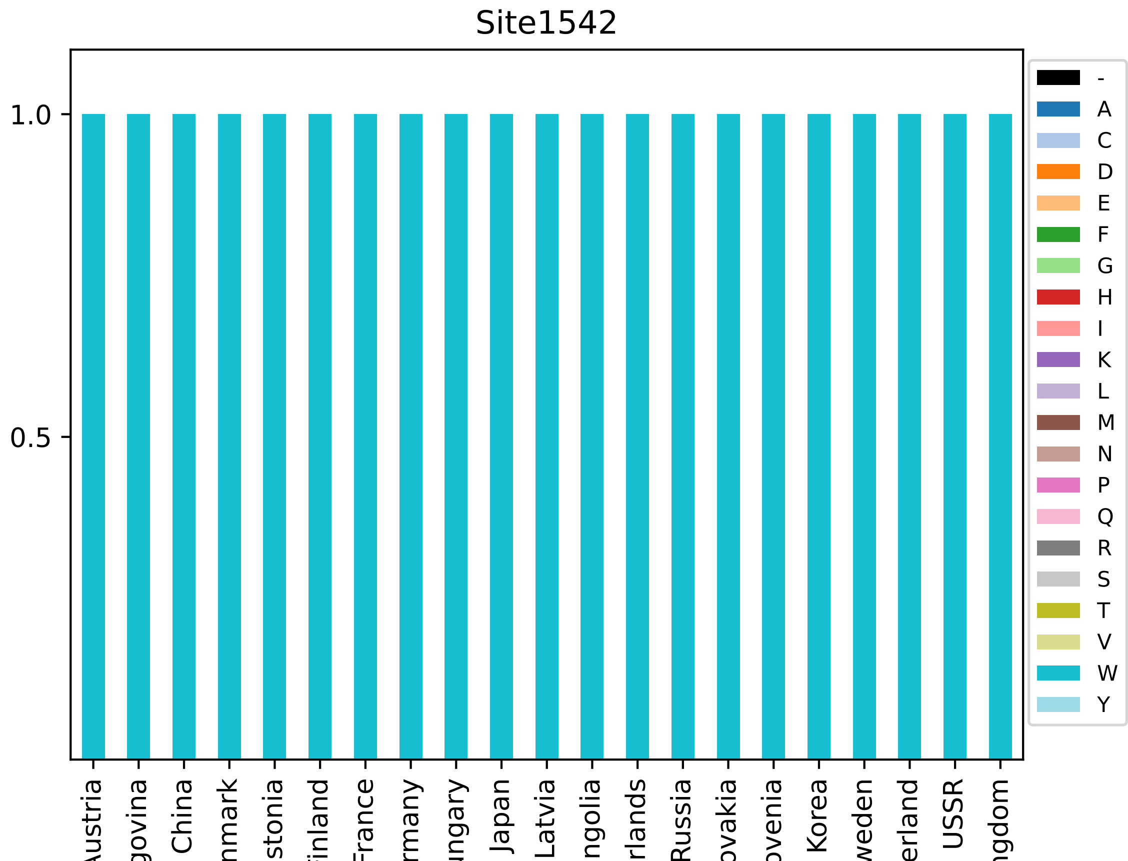

Site1542
1.0
0.5
-
A
C
D
E
F
G
H
I
K
L
M
N
P
Q
R
S
T
V
W
Y
China
Japan
Latvia
Russia
Korea
USSR

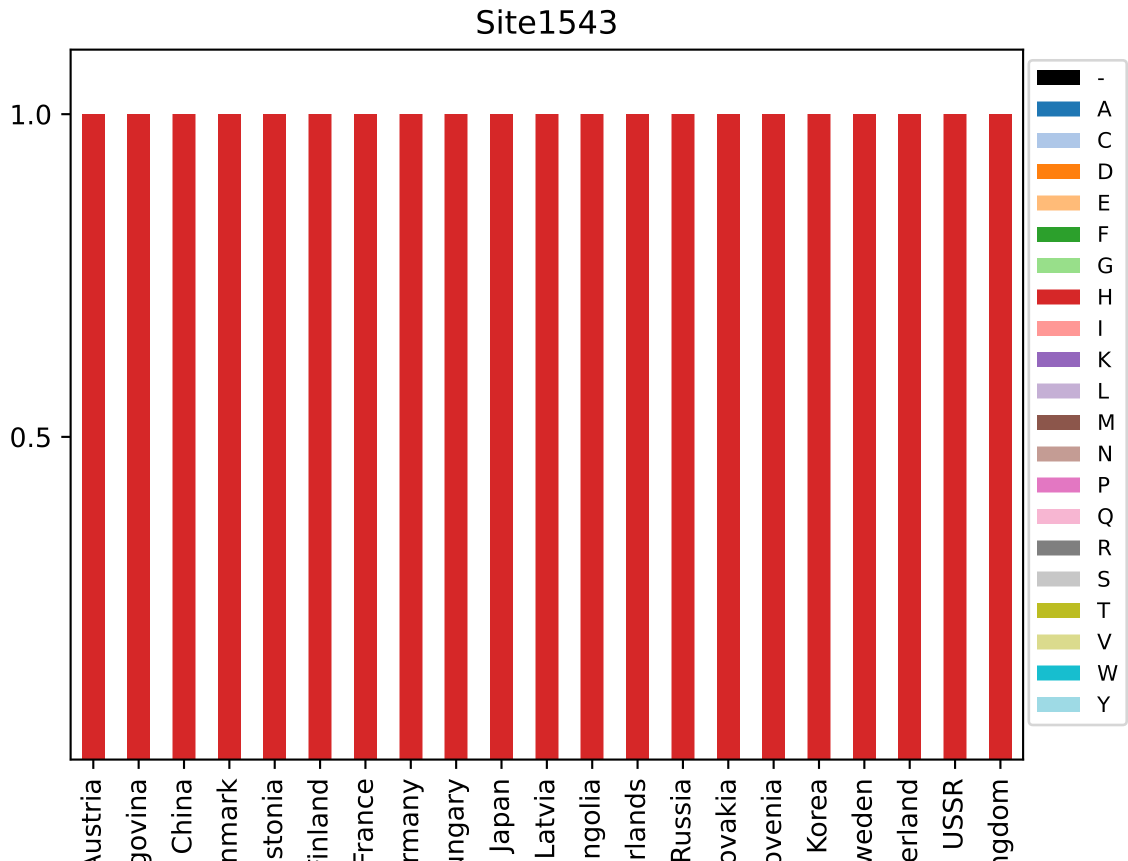

Site1543
1.0
0.5
-
A
C
D
E
F
G
H
I
K
L
M
N
P
Q
R
S
T
V
W
Y
China
Japan
Latvia
Russia
Korea
USSR

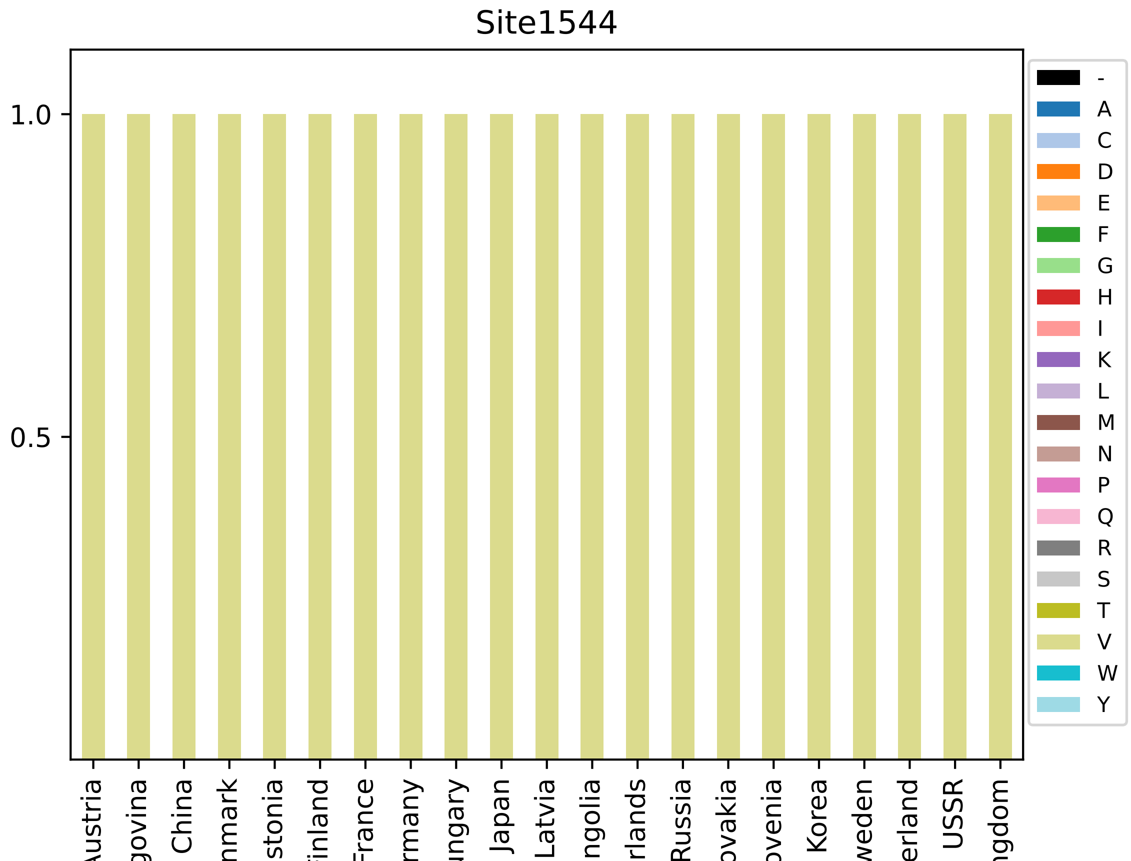

Site1544
1.0
0.5
Austria
govina
China
nmark
stonia
Finland
France
rmany
ungary
Japan
Latvia
ngolia
rlands
Russia
ovakia
ovenia
Korea
weden
erland
USSR
ngdom
-
A
C
D
E
F
G
H
I
K
L
M
N
P
Q
R
S
T
V
W
Y

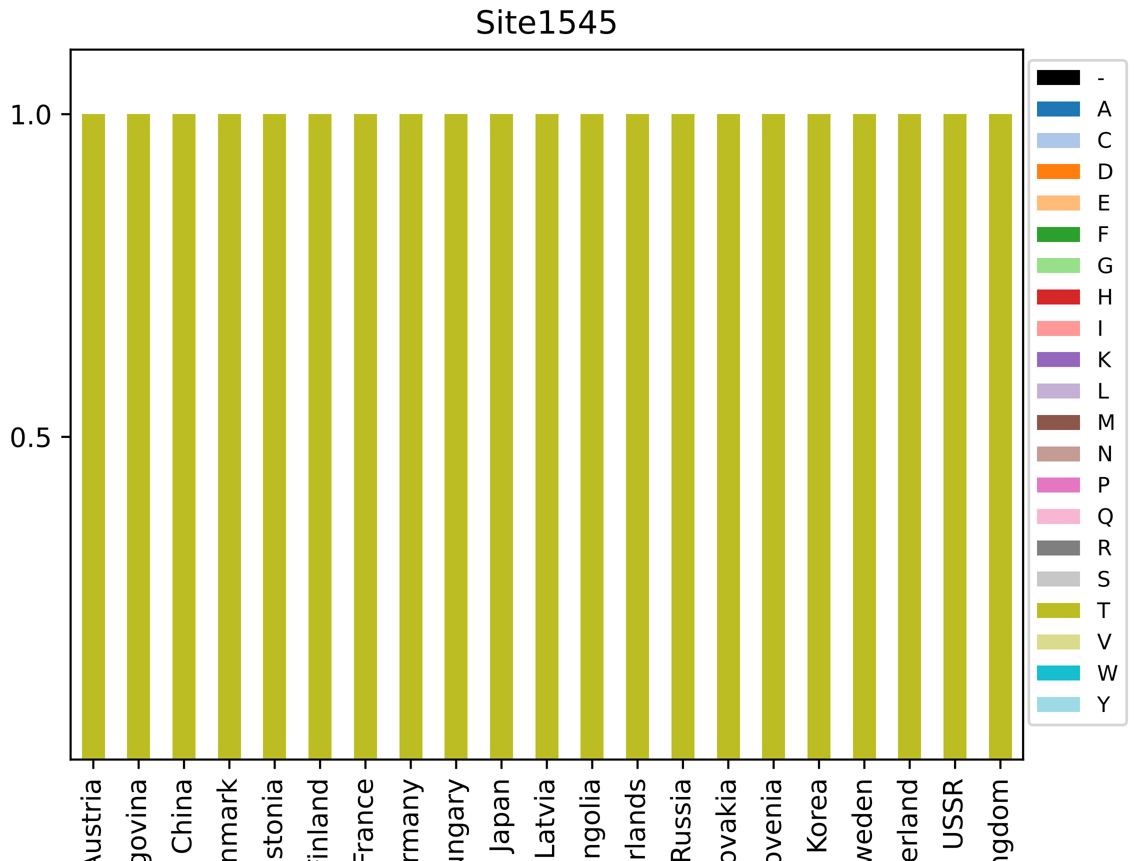

Site1545
1.0
0.5
-
A
C
D
E
F
G
H
I
K
L
M
N
P
Q
R
S
T
V
W
Y
Austria
govina
China
nmark
stonia
inland
France
rmany
ungary
Japan
Latvia
ngolia
rlands
Russia
ovakia
ovenia
Korea
weden
erland
USSR
ngdom

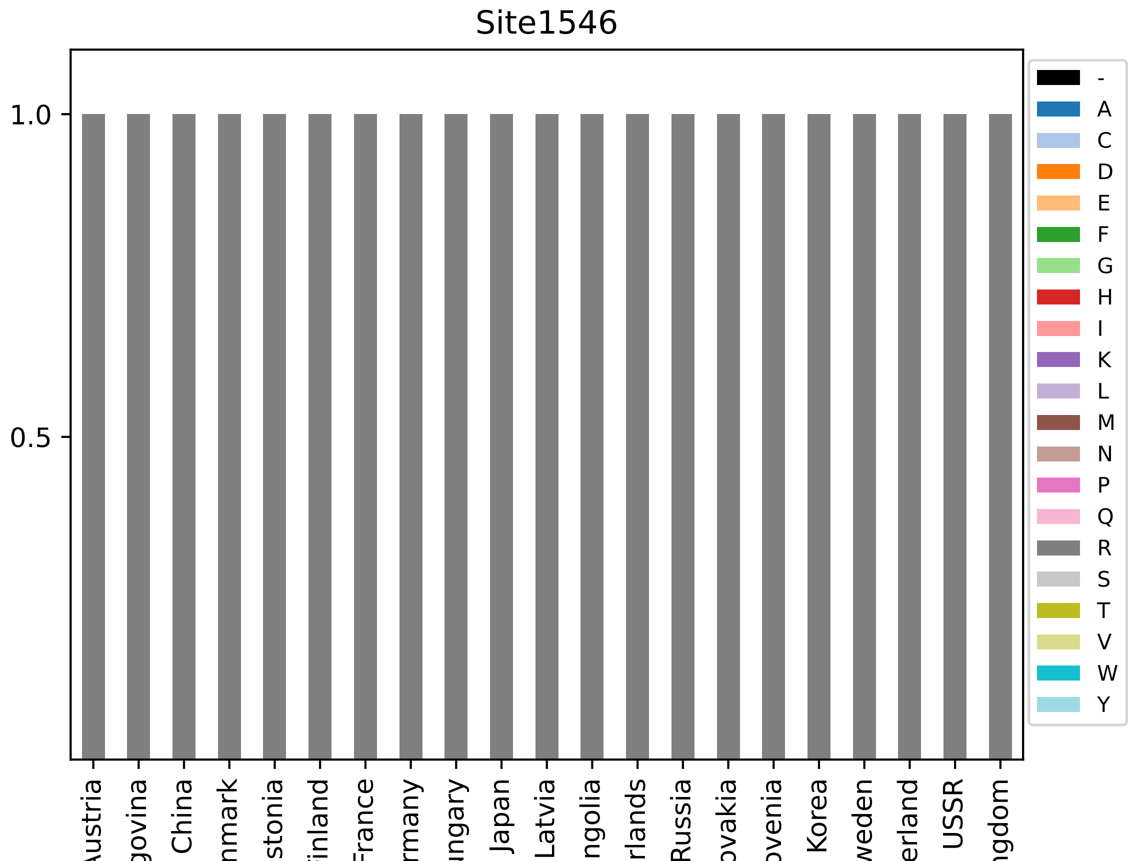

Site1546
1.0
0.5
-
A
C
D
E
F
G
H
I
K
L
M
N
P
Q
R
S
T
V
W
Y
Austria
China
Japan
Latvia
Russia
Korea
USSR

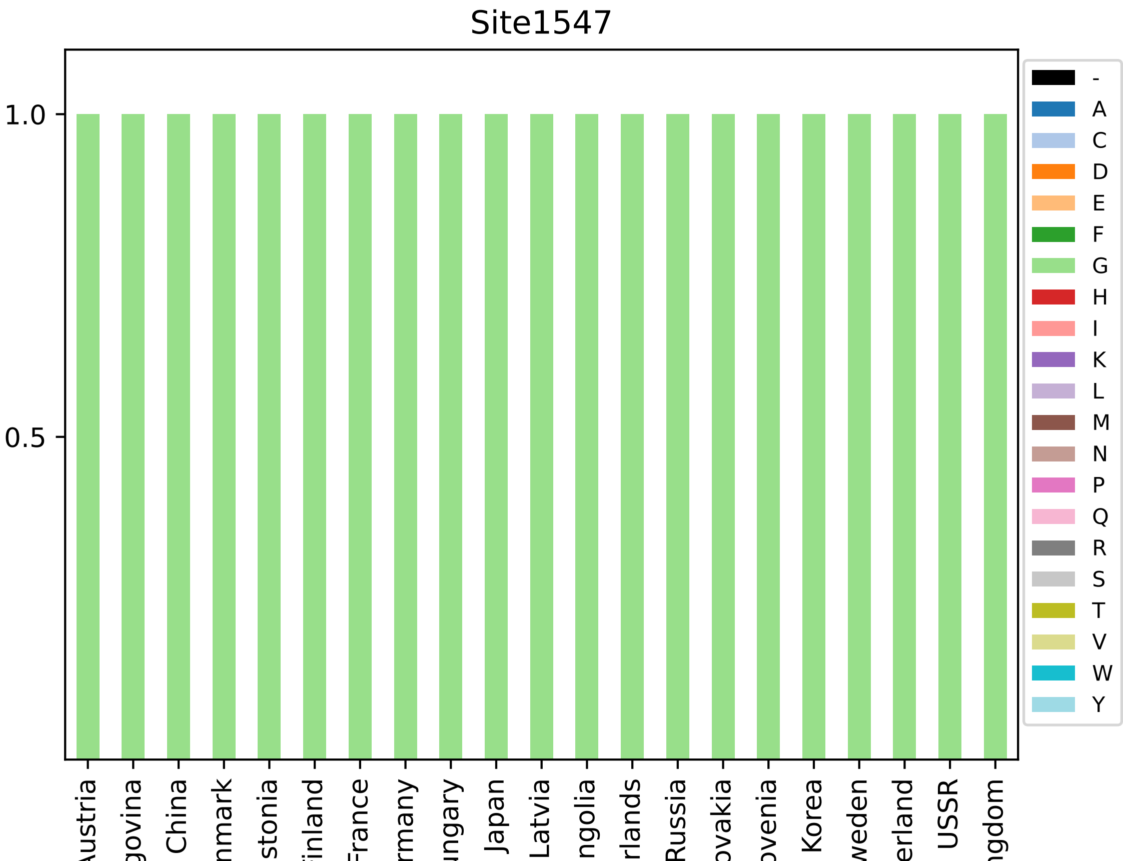

Site1547
1.0
0.5
-
A
C
D
E
F
G
H
I
K
L
M
N
P
Q
R
S
T
V
W
Y
China
Japan
Latvia
Russia
Korea
USSR

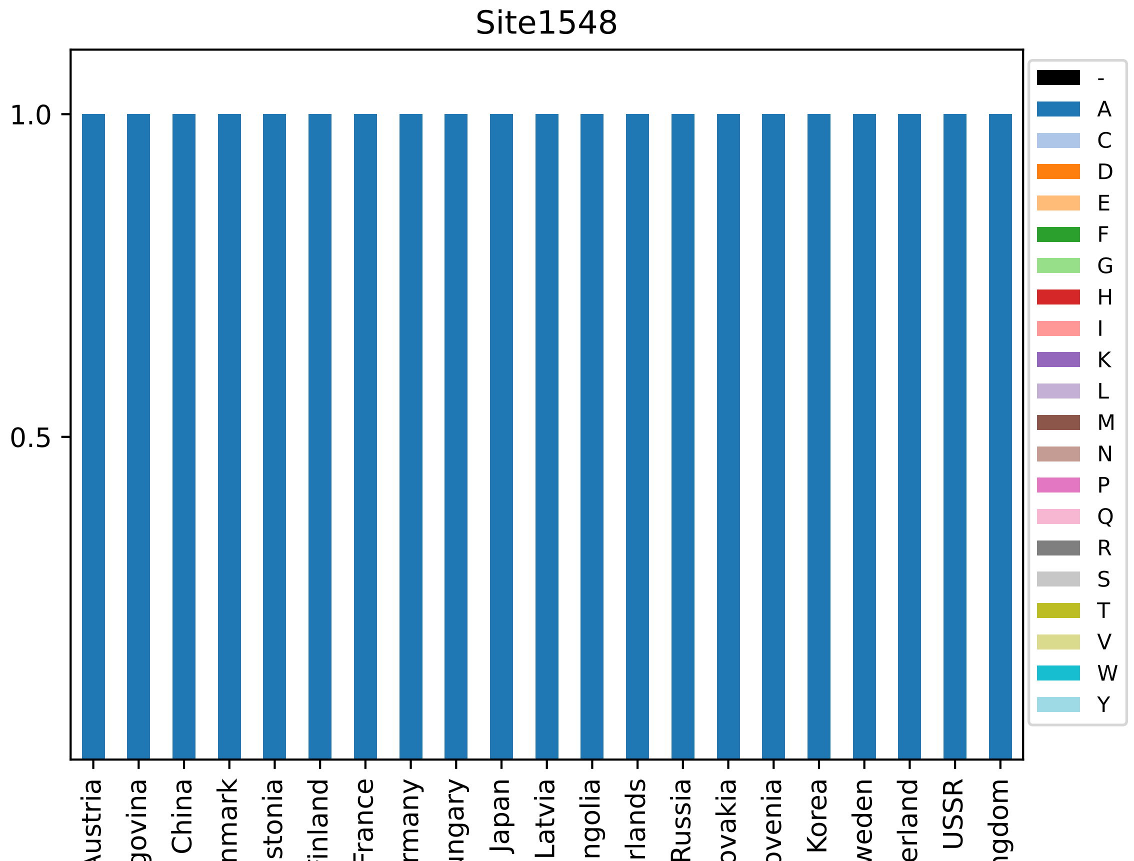
Site1548
1.0
0.5
Austria
govina
China
nmark
stonia
Finland
France
rmany
ungary
Japan
Latvia
ngolia
rlands
Russia
ovakia
ovenia
Korea
weden
erland
USSR
ngdom
-
A
C
D
E
F
G
H
I
K
L
M
N
P
Q
R
S
T
V
W
Y

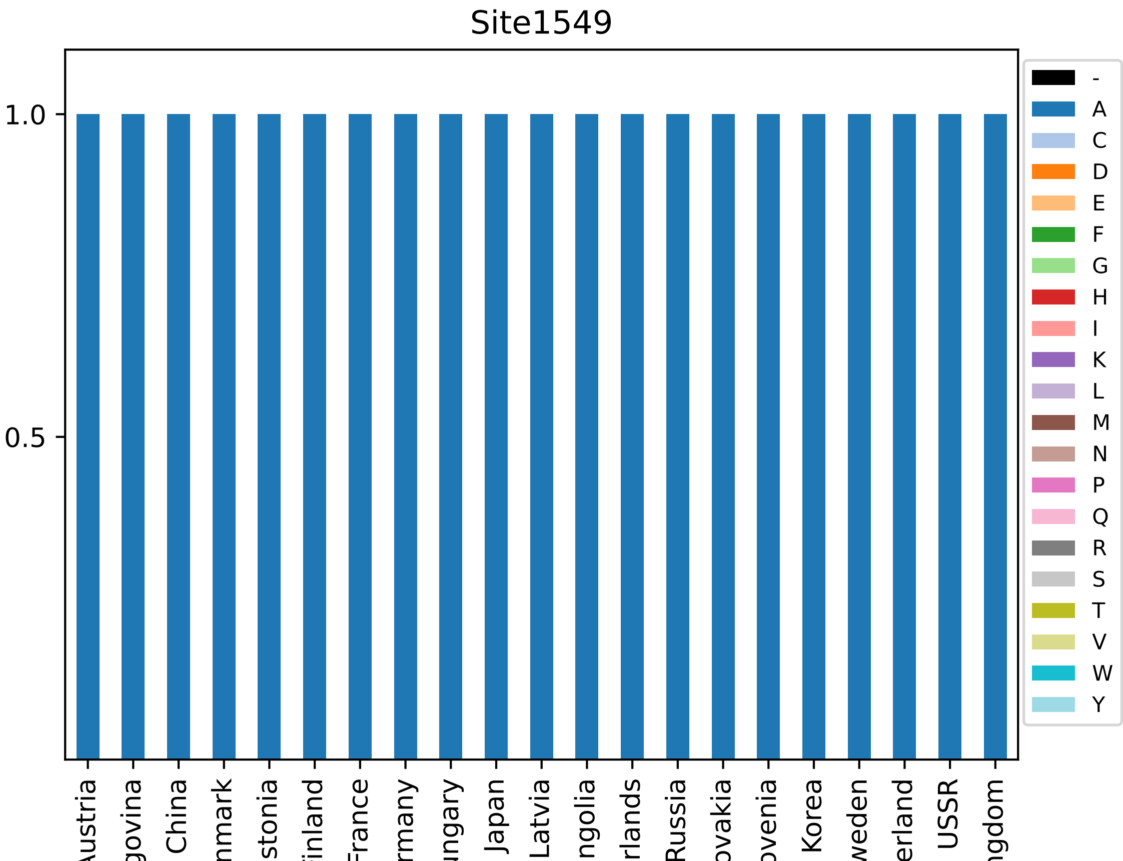

Site1549
1.0
0.5
-
A
C
D
E
F
G
H
I
K
L
M
N
P
Q
R
S
T
V
W
Y
China
Japan
Latvia
Russia
Korea
USSR

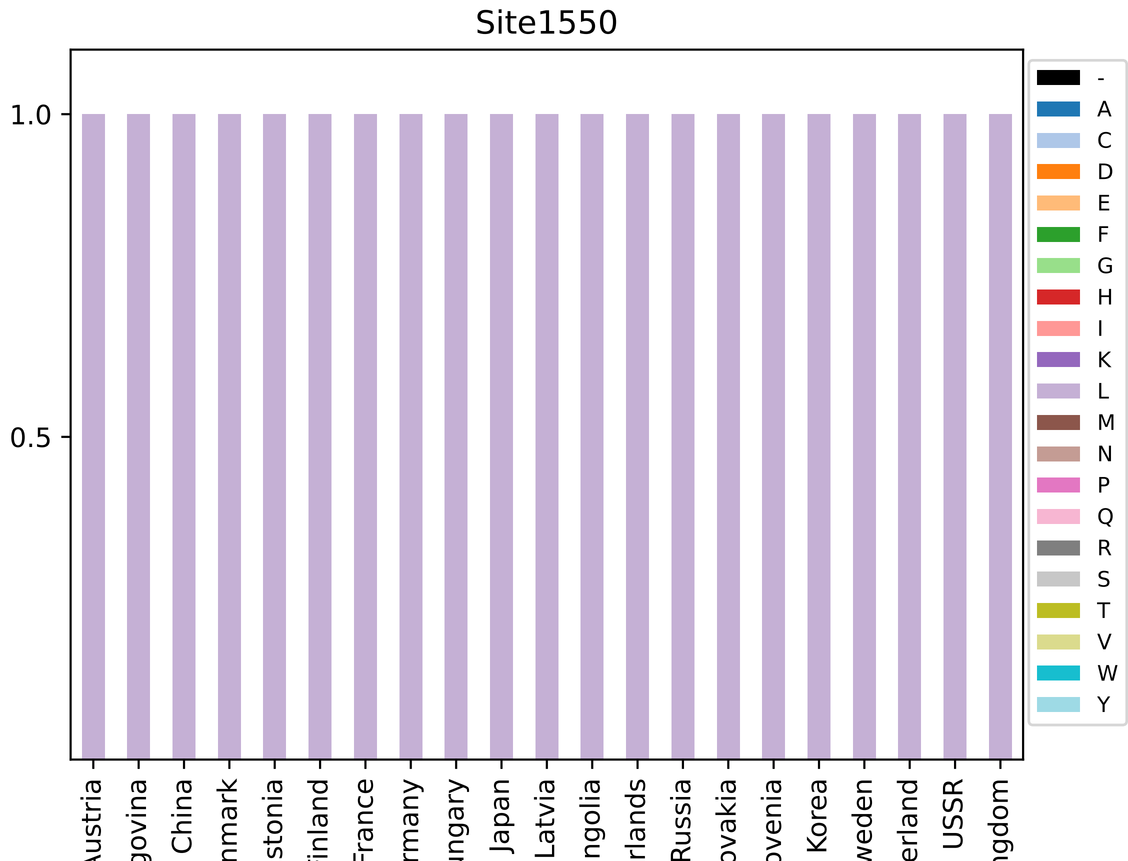
Site1550
1.0
0.5
-
A
C
D
E
F
G
H
I
K
L
M
N
P
Q
R
S
T
V
W
Y
Austria
China
Japan
Latvia
Russia
Korea
USSR

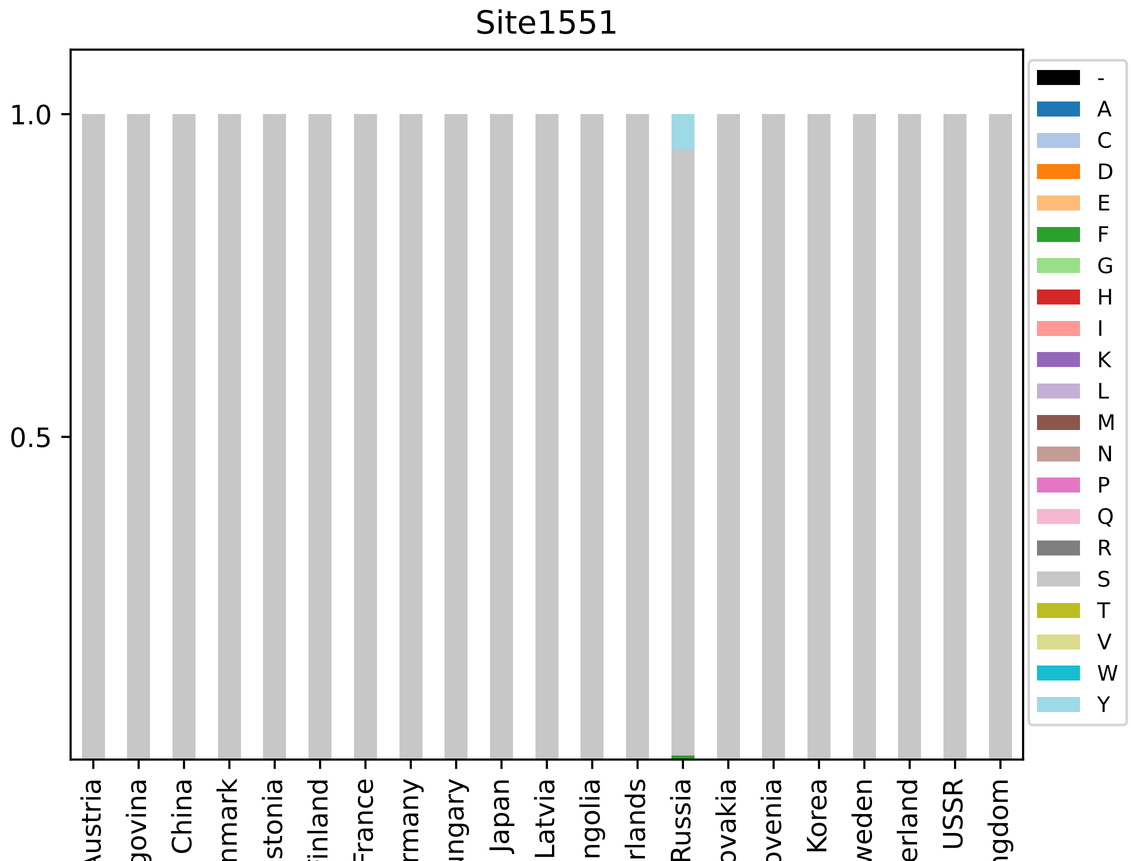
Site1551
1.0
0.5
-
A
C
D
E
F
G
H
I
K
L
M
N
P
Q
R
S
T
V
W
Y
Austria
govina
China
nmark
stonia
Finland
France
rmany
ungary
Japan
Latvia
ngolia
rlands
Russia
ovakia
ovenia
Korea
weden
erland
USSR
ngdom

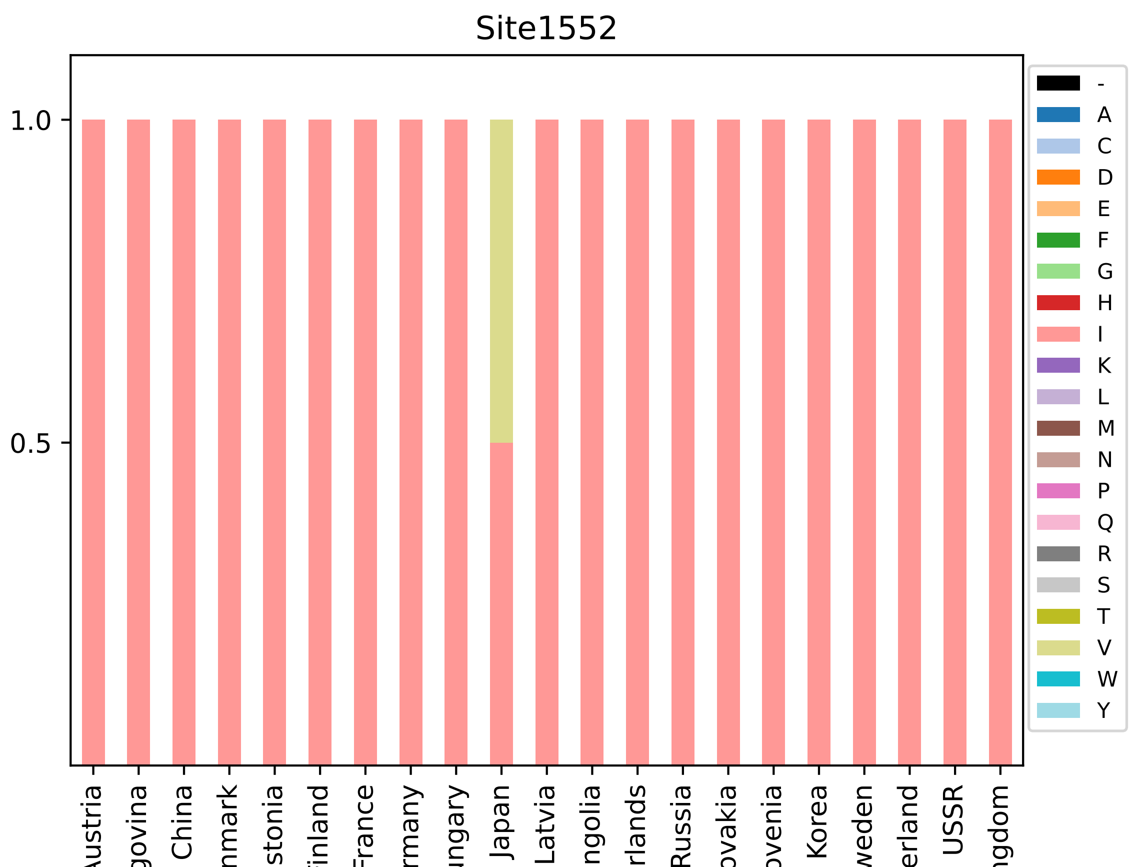

Site1552
1.0
0.5
-
A
C
D
E
F
G
H
I
K
L
M
N
P
Q
R
S
T
V
W
Y
Austria
govina
China
nmark
stonia
Finland
France
rmany
ungary
Japan
Latvia
ngolia
rlands
Russia
ovakia
ovenia
Korea
weden
erland
USSR
ngdom

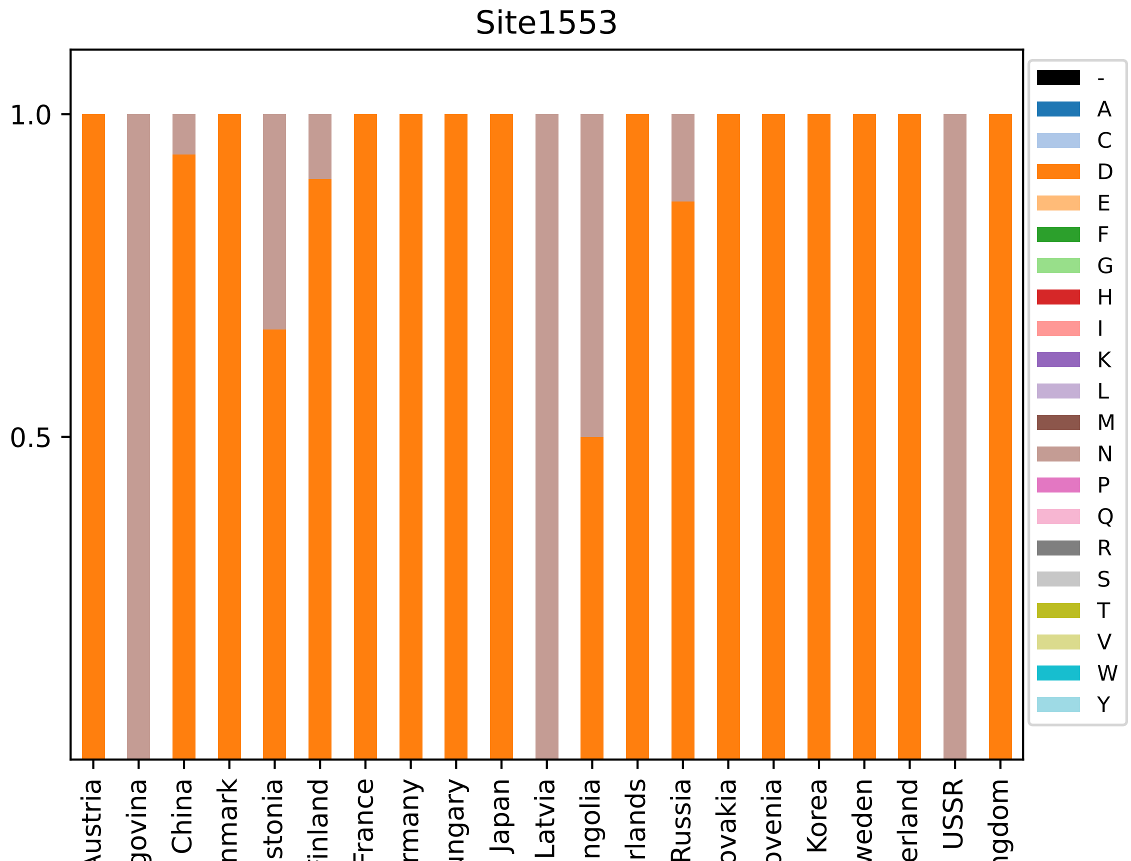

Site1553
1.0
0.5
-
A
C
D
E
F
G
H
I
K
L
M
N
P
Q
R
S
T
V
W
Y
Austria
China
Latvia
Japan
Russia
Korea
USSR

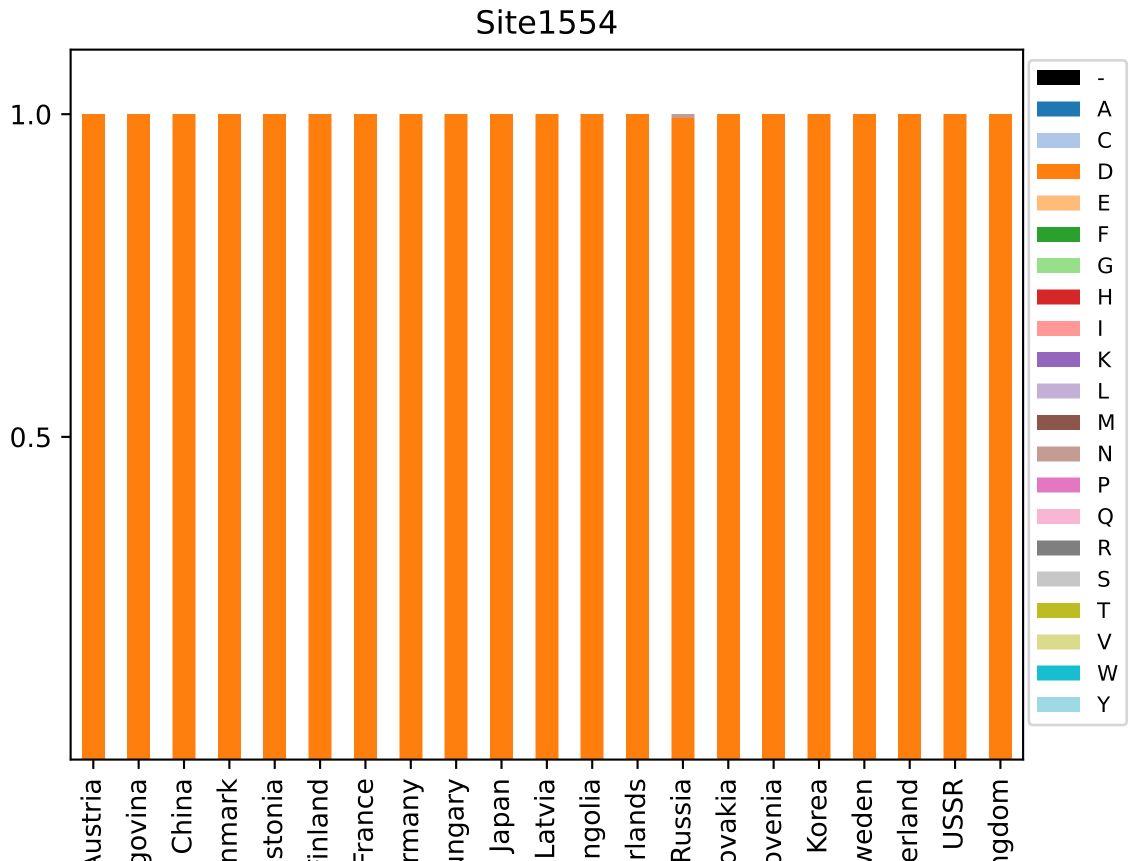

Site1554
1.0
0.5
-
A
C
D
E
F
G
H
I
K
L
M
N
P
Q
R
S
T
V
W
Y
China
Japan
Latvia
Russia
Korea
USSR

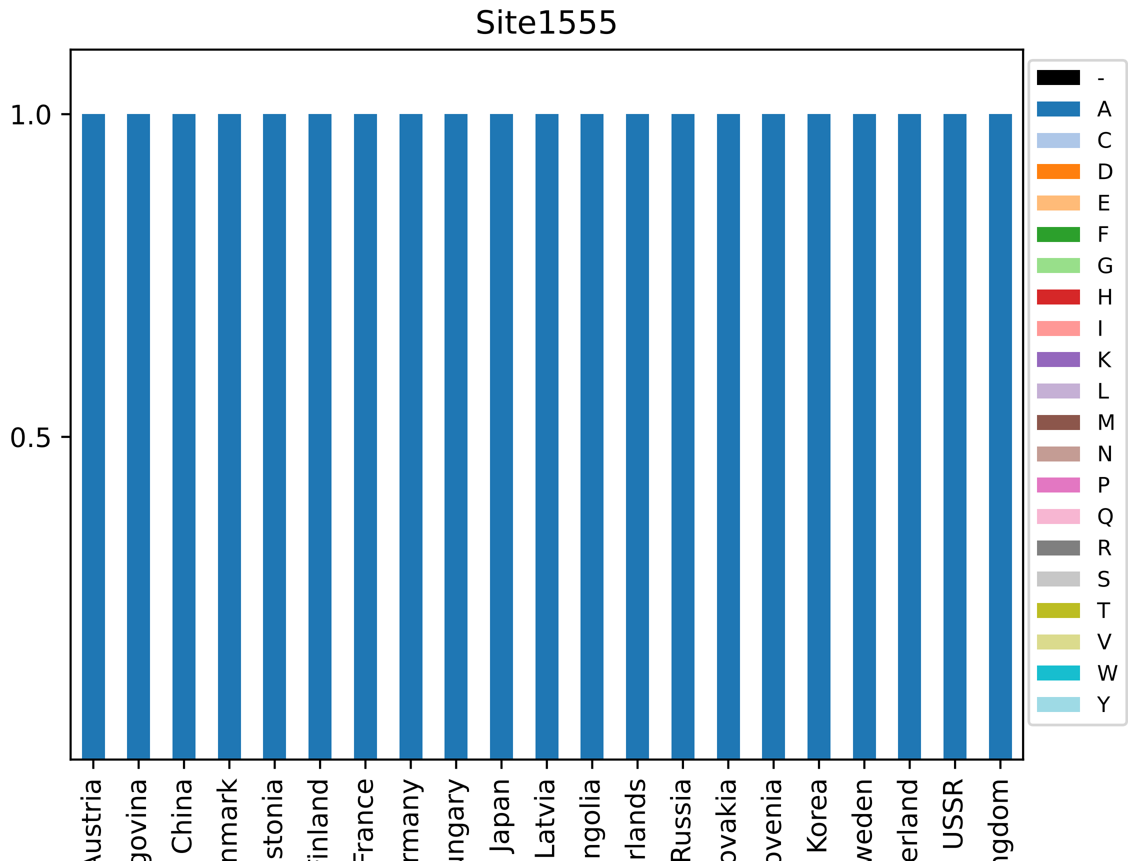

Site1555
1.0
0.5
-
A
C
D
E
F
G
H
I
K
L
M
N
P
Q
R
S
T
V
W
Y
Austria
China
Japan
Latvia
Russia
Korea
USSR

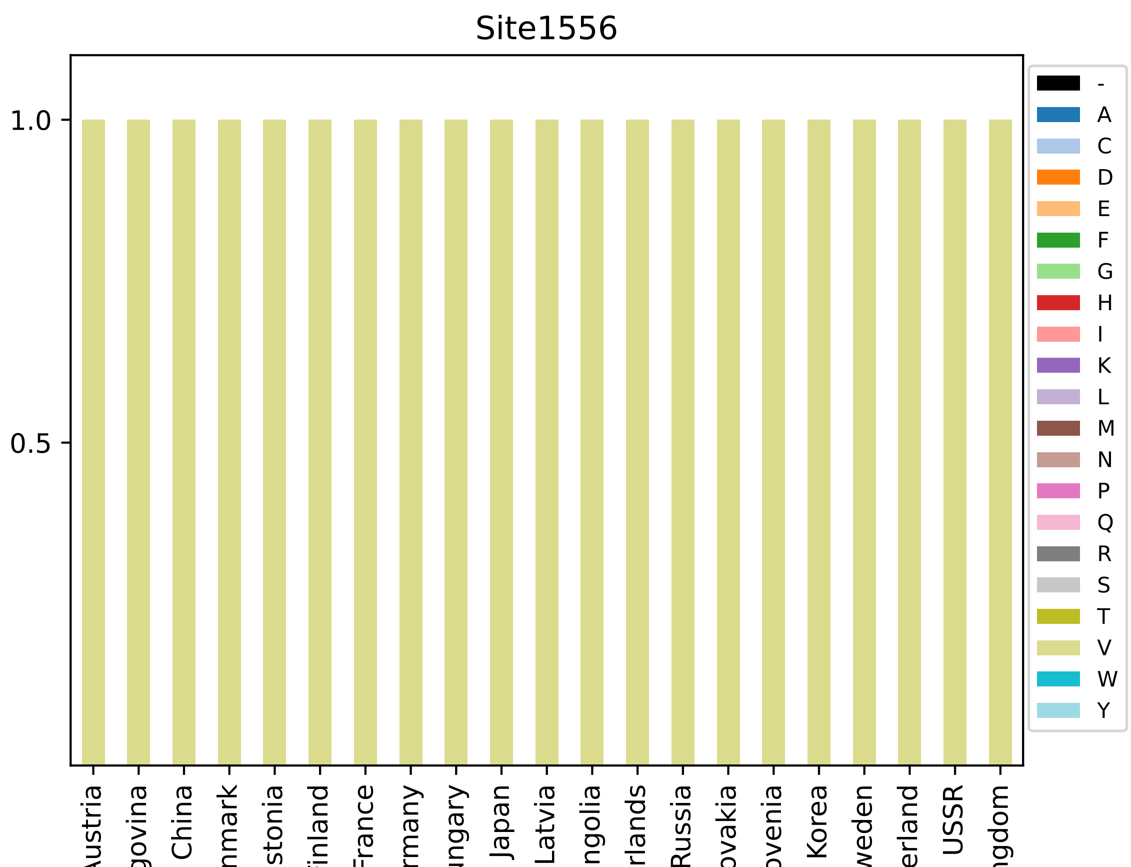
Site1556
1.0
0.5
-
A
C
D
E
F
G
H
I
K
L
M
N
P
Q
R
S
T
V
W
Y
China
Japan
Latvia
Russia
Korea
USSR

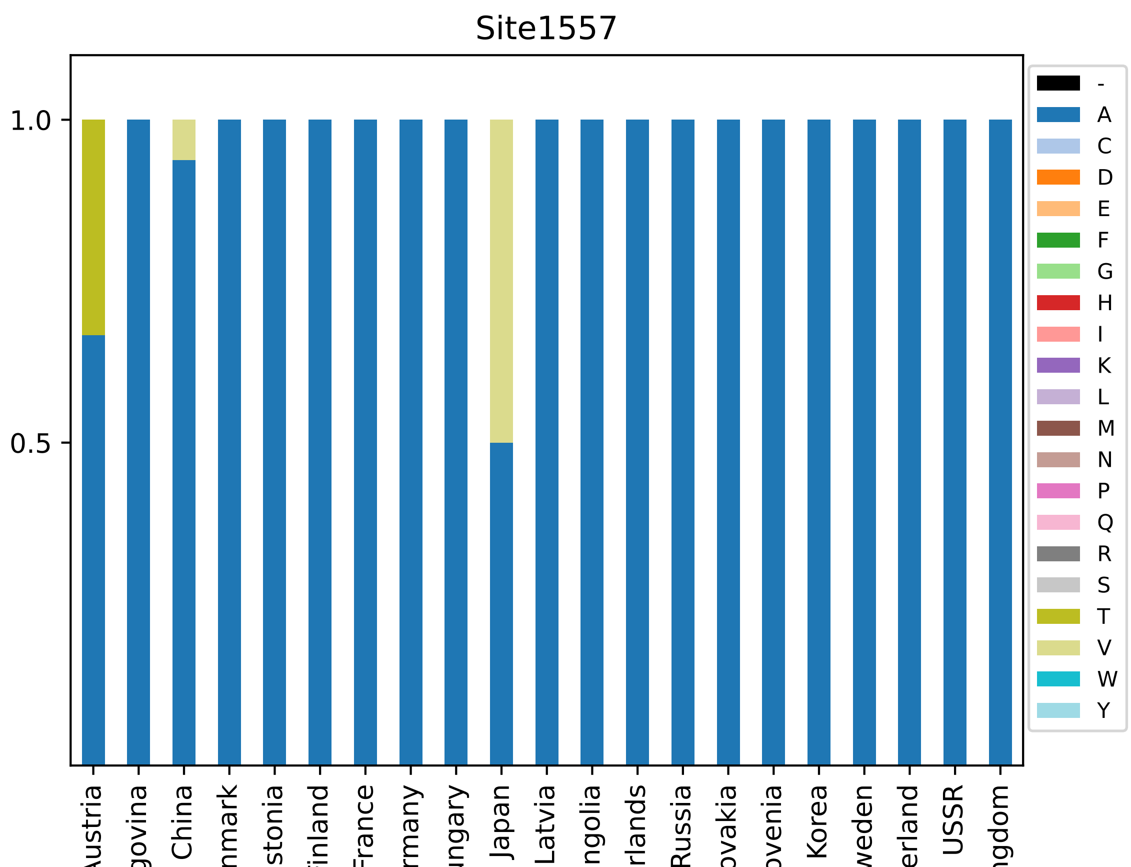

Site1557
1.0
0.5
-
A
C
D
E
F
G
H
I
K
L
M
N
P
Q
R
S
T
V
W
Y
Austria
China
Japan
Latvia
Russia
Korea
USSR

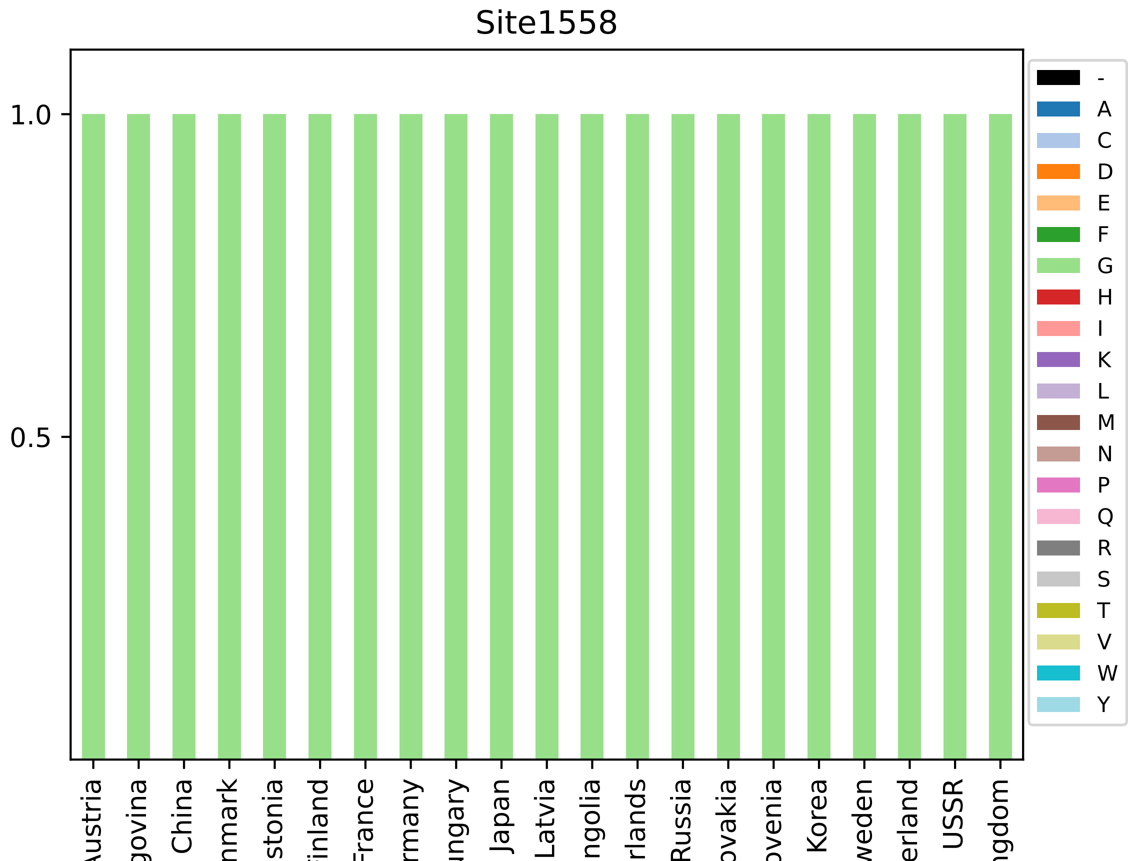

Site1558
1.0
0.5
-
A
C
D
E
F
G
H
I
K
L
M
N
P
Q
R
S
T
V
W
Y
China
Japan
Latvia
Russia
Korea
USSR

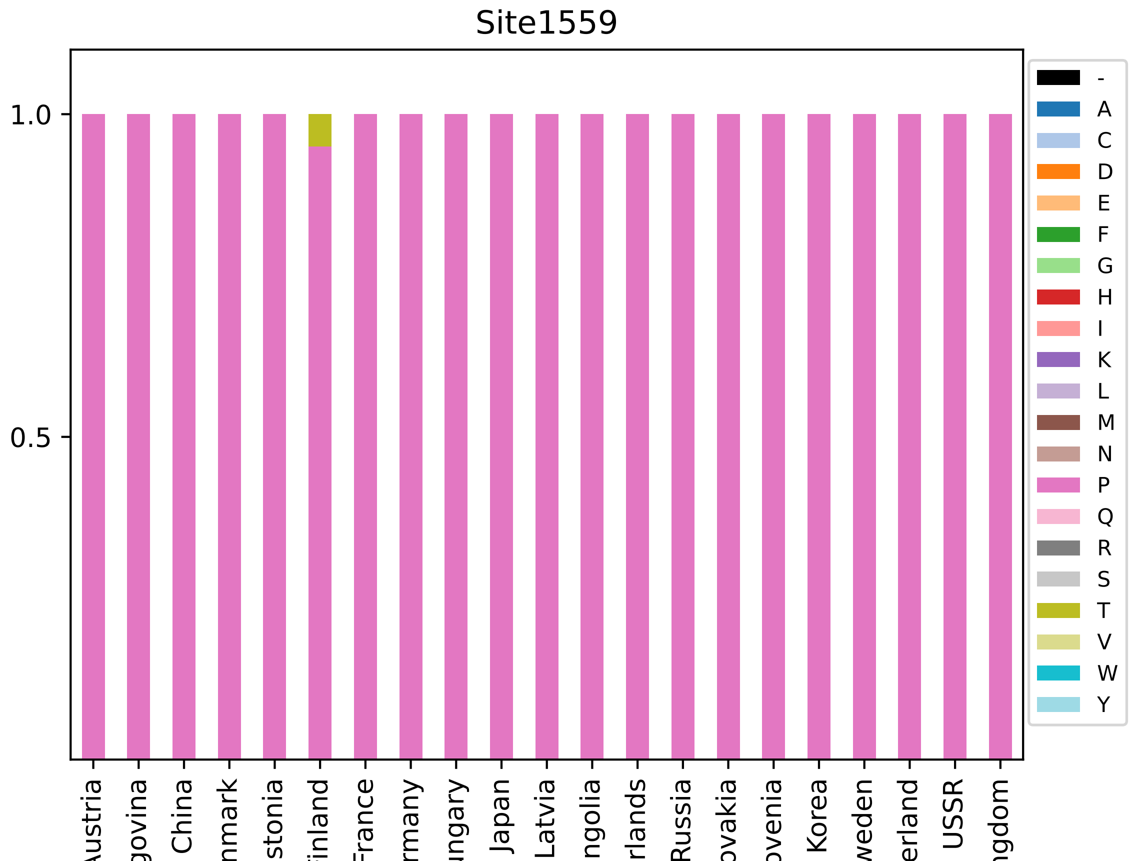
Site1559
1.0
0.5
-
A
C
D
E
F
G
H
I
K
L
M
N
P
Q
R
S
T
V
W
Y
China
Japan
Latvia
Russia
Korea
USSR

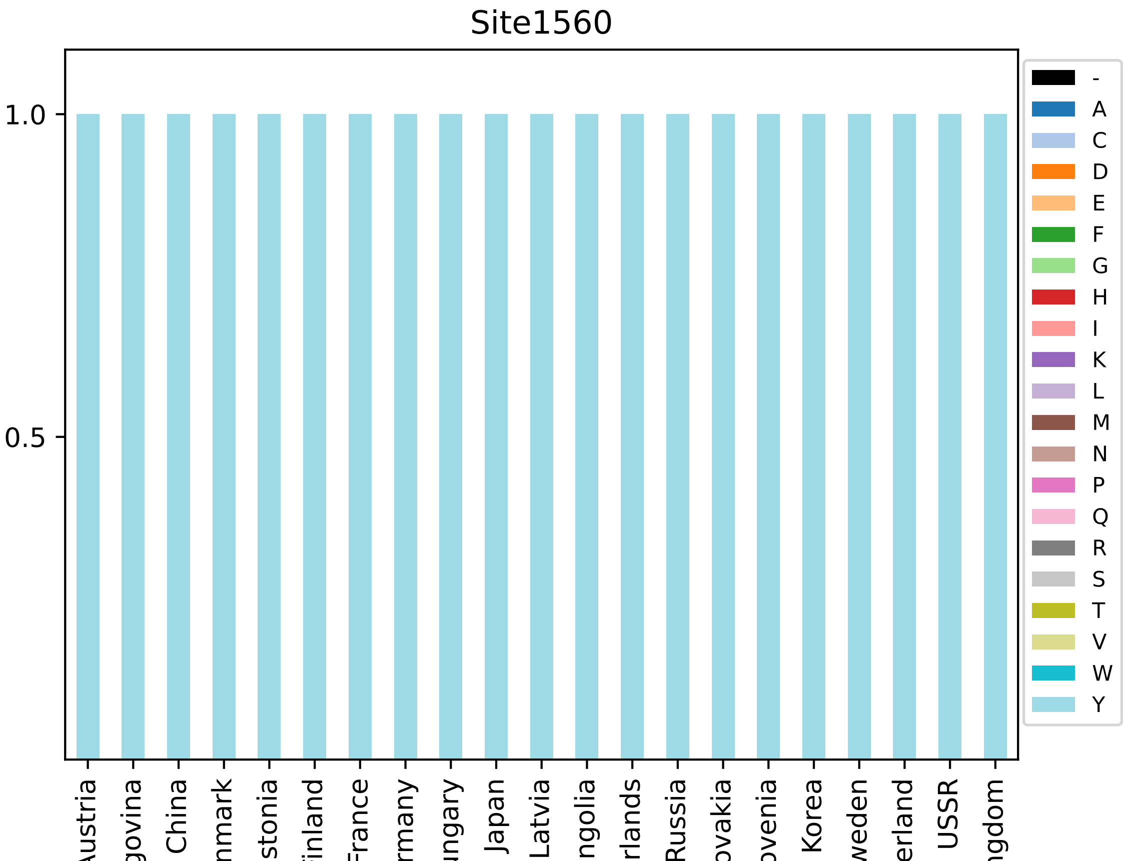

Site1560
1.0
0.5
China
Japan
Latvia
Russia
Korea
USSR
-
A
C
D
E
F
G
H
I
K
L
M
N
P
Q
R
S
T
V
W
Y

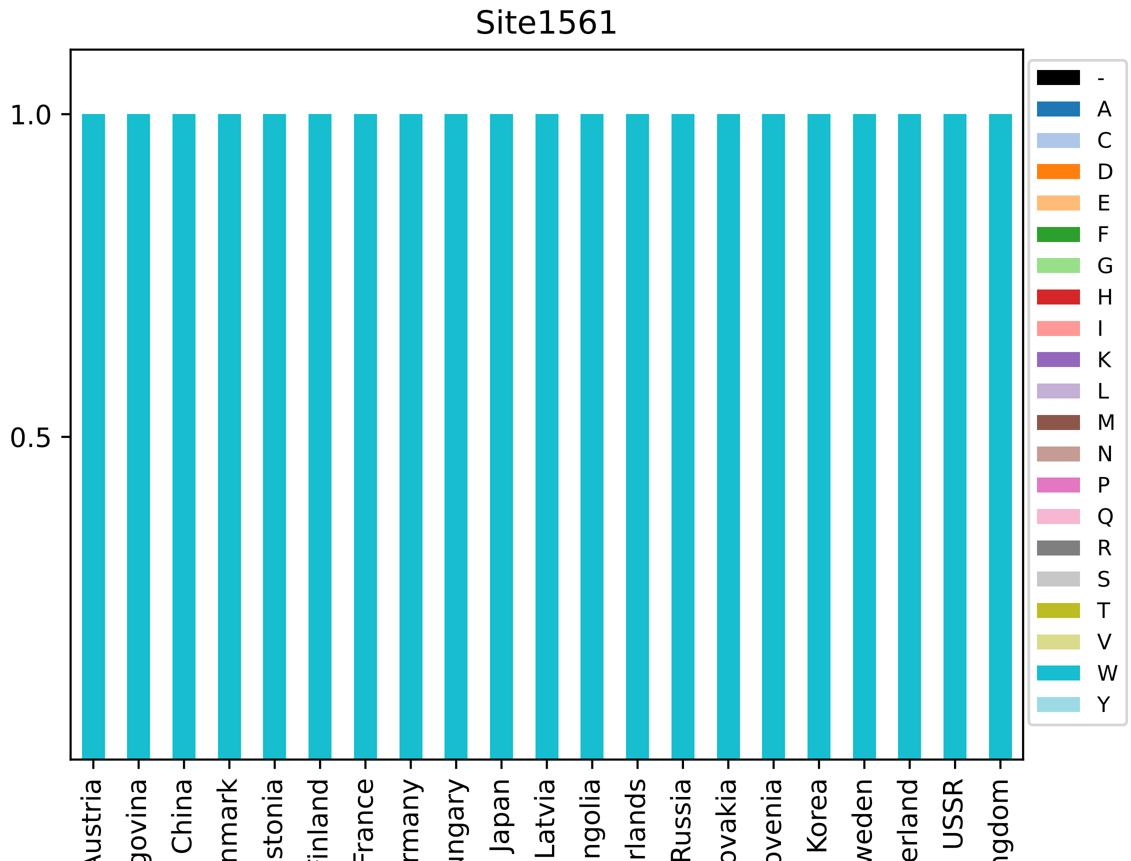
Site1561
1.0
0.5
-
A
C
D
E
F
G
H
I
K
L
M
N
P
Q
R
S
T
V
W
Y
China
Japan
Latvia
Russia
Korea
USSR

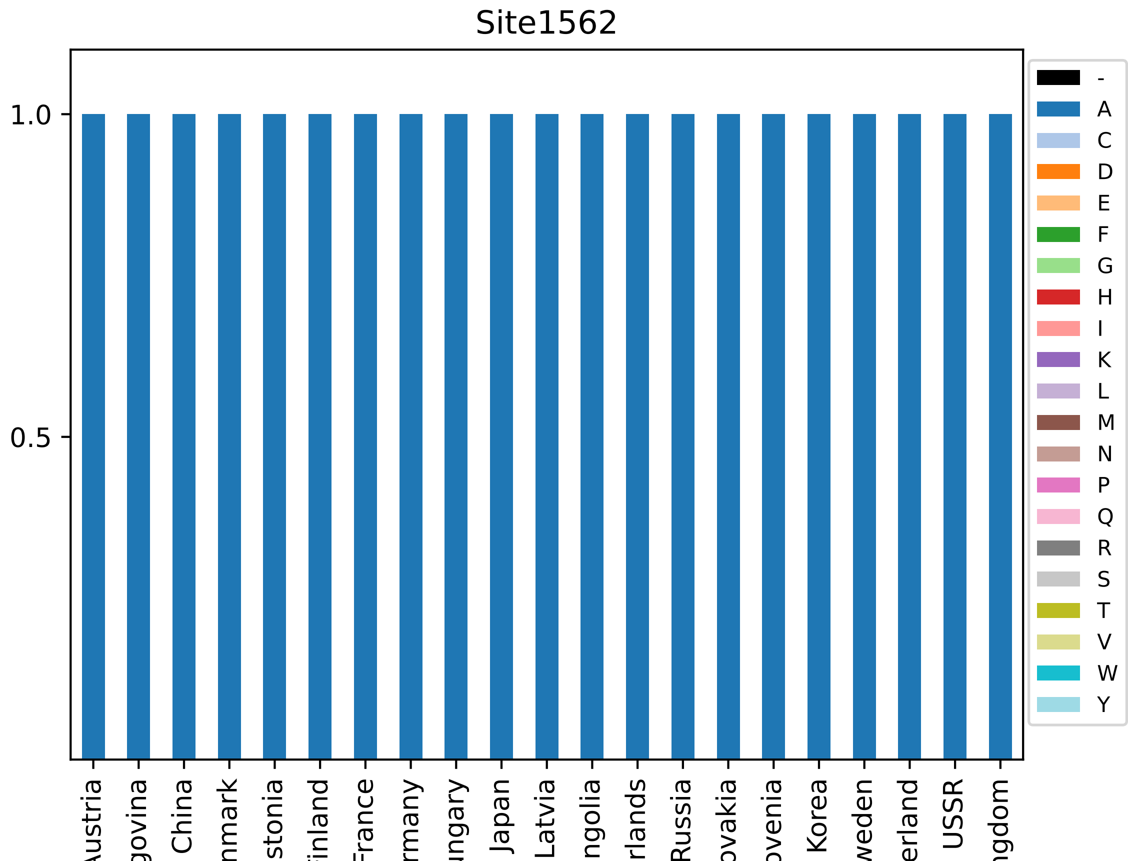

Site1562
1.0
0.5
Austria
govina
China
nmark
stonia
Finland
France
rmany
ungary
Japan
Latvia
ngolia
rlands
Russia
ovakia
ovenia
Korea
weden
erland
USSR
ngdom
-
A
C
D
E
F
G
H
I
K
L
M
N
P
Q
R
S
T
V
W
Y

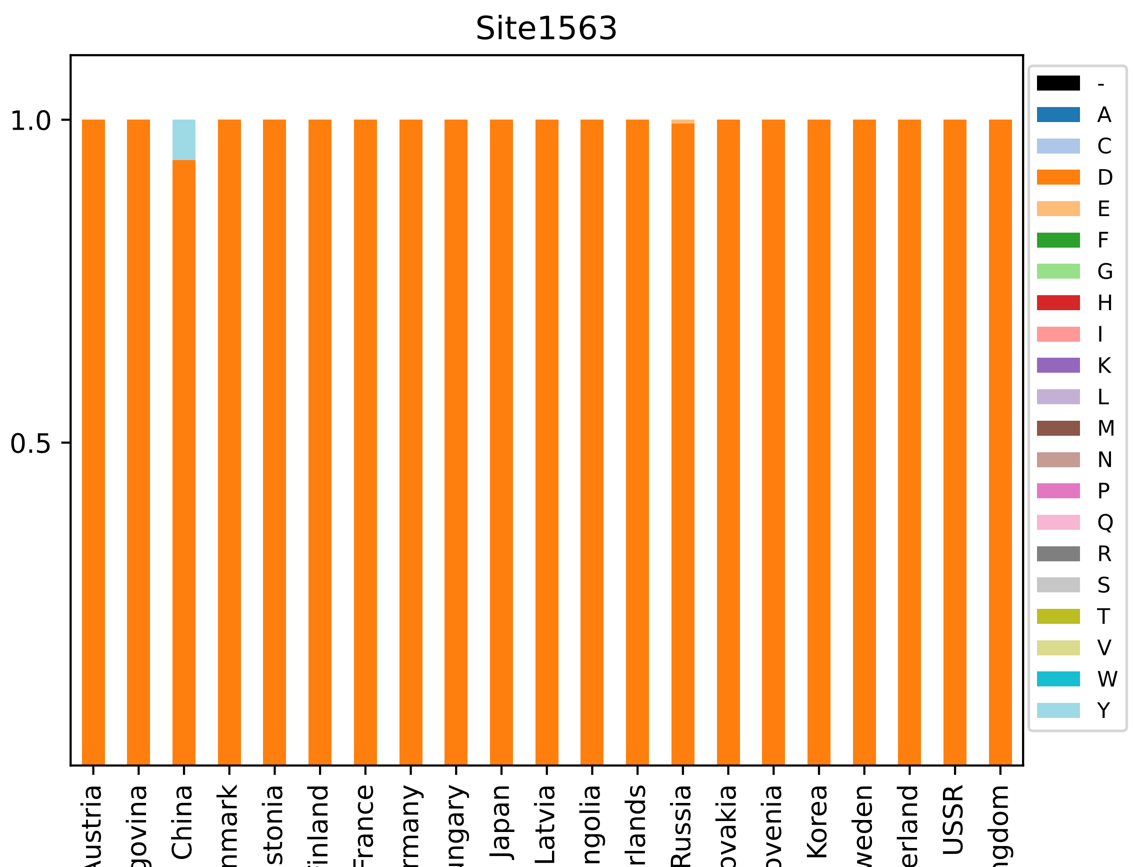

Site1563
1.0
0.5
Austria
govina
China
nmark
stonia
Finland
France
rmany
ungary
Japan
Latvia
ngolia
rlands
Russia
ovakia
ovenia
Korea
weden
erland
USSR
ngdom
-
A
C
D
E
F
G
H
I
K
L
M
N
P
Q
R
S
T
V
W
Y

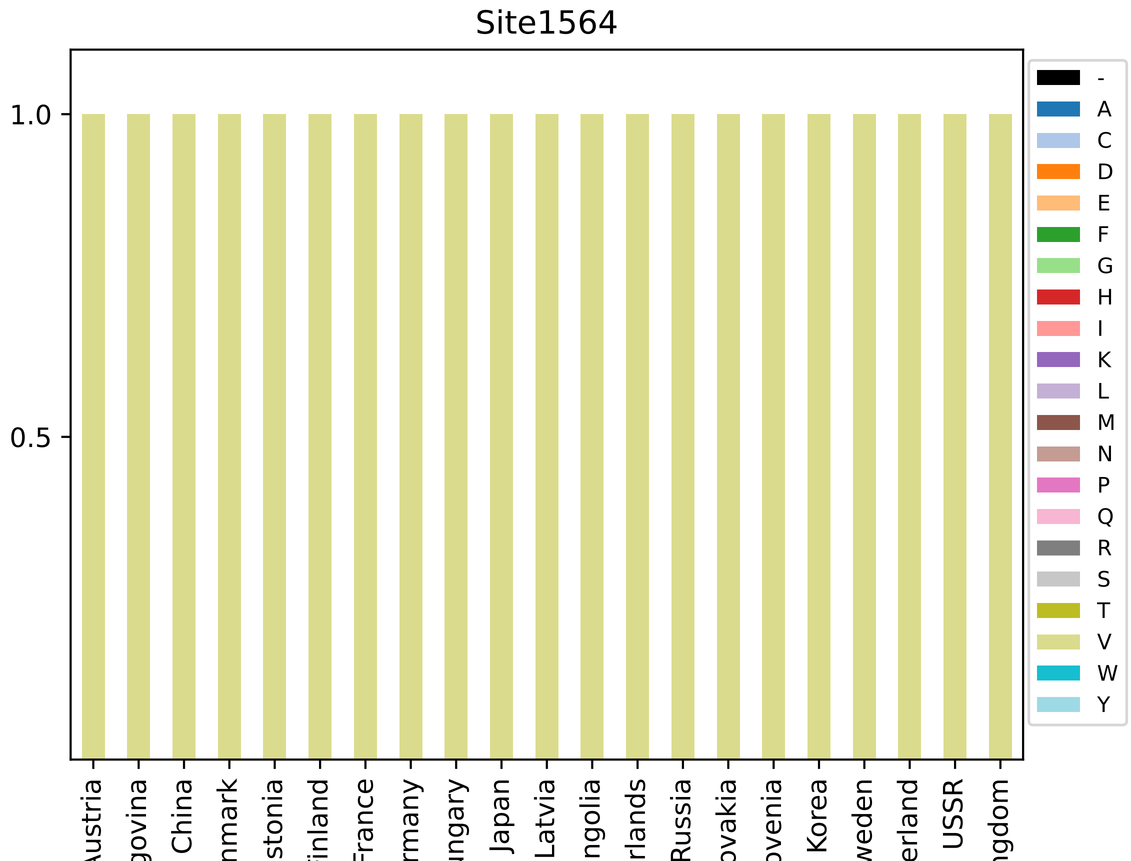

Site1564
1.0
0.5
-
A
C
D
E
F
G
H
I
K
L
M
N
P
Q
R
S
T
V
W
Y
China
Japan
Latvia
Russia
Korea
USSR

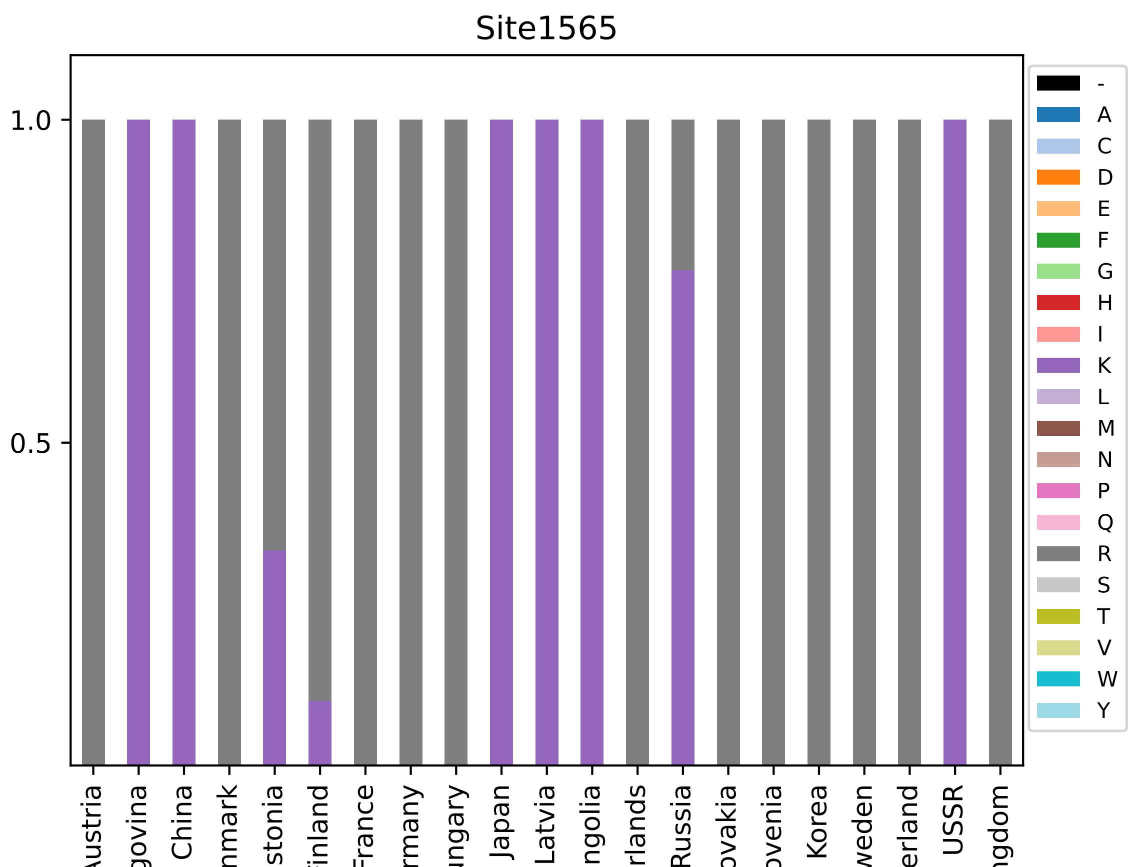

Site1565
1.0
0.5
-
A
C
D
E
F
G
H
I
K
L
M
N
P
Q
R
S
T
V
W
Y
China
Japan
Latvia
Russia
Korea
USSR

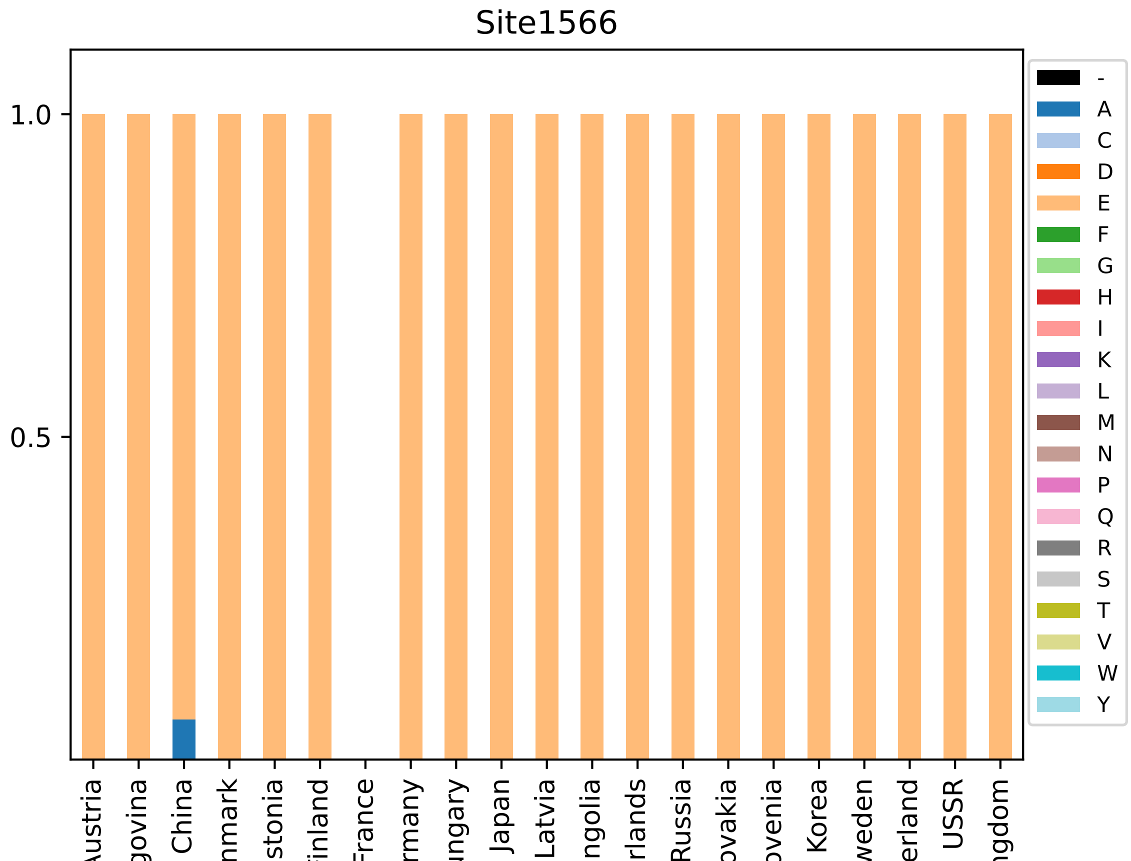
Site1566
1.0
0.5
-
A
C
D
E
F
G
H
I
K
L
M
N
P
Q
R
S
T
V
W
Y
Austria
govina
China
nmark
stonia
Finland
France
rmany
ungary
Japan
Latvia
ngolia
rlands
Russia
ovakia
ovenia
Korea
weden
erland
USSR
ngdom

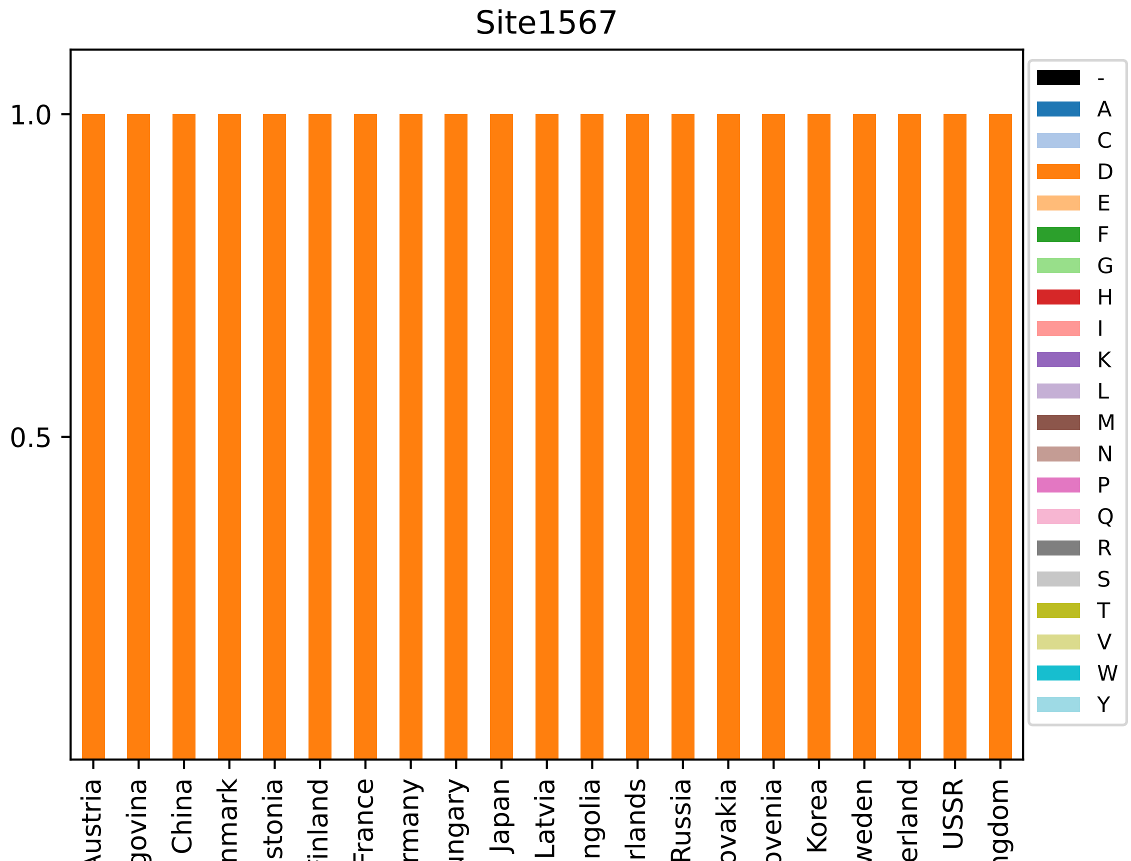
Site1567
1.0
0.5
-
A
C
D
E
F
G
H
I
K
L
M
N
P
Q
R
S
T
V
W
Y
China
Japan
Latvia
Russia
Korea
USSR

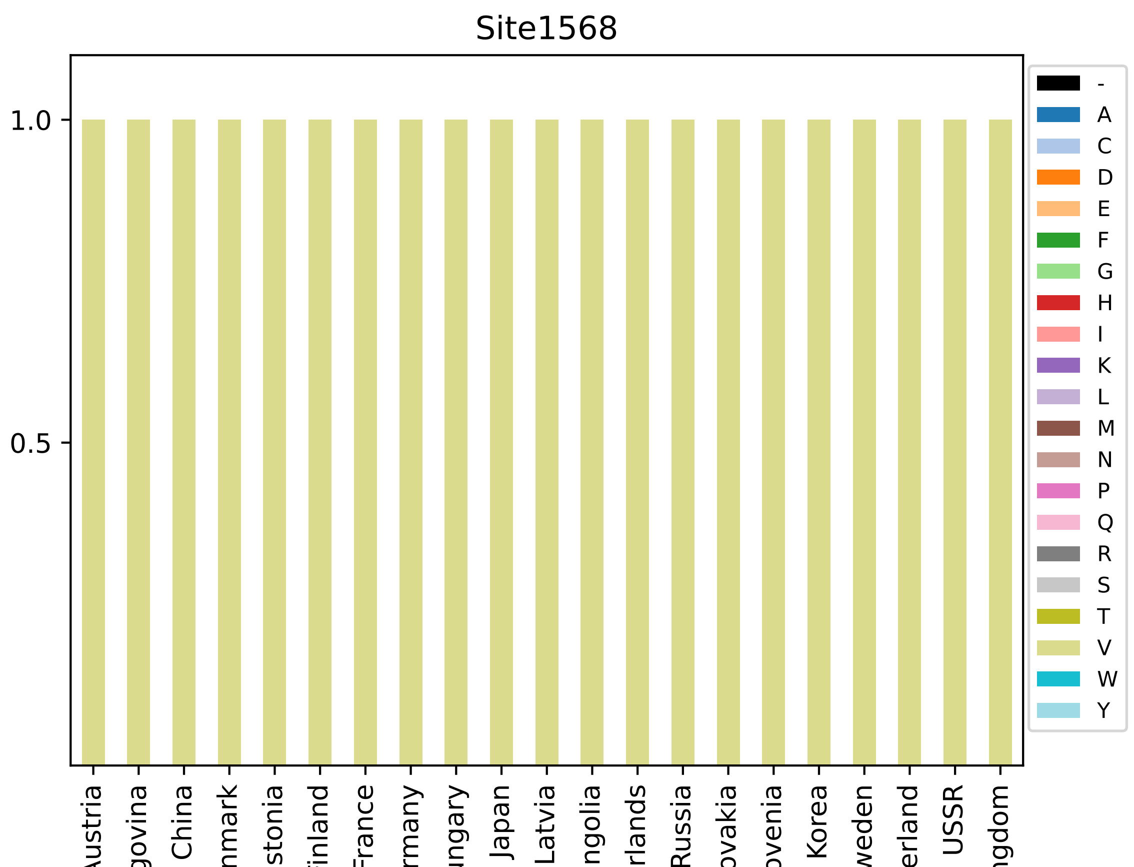

Site1568
1.0
0.5
-
A
C
D
E
F
G
H
I
K
L
M
N
P
Q
R
S
T
V
W
Y
Austria
govina
China
nmark
stonia
Finland
France
rmany
ungary
Japan
Latvia
ngolia
rlands
Russia
ovakia
ovenia
Korea
weden
erland
USSR
ngdom

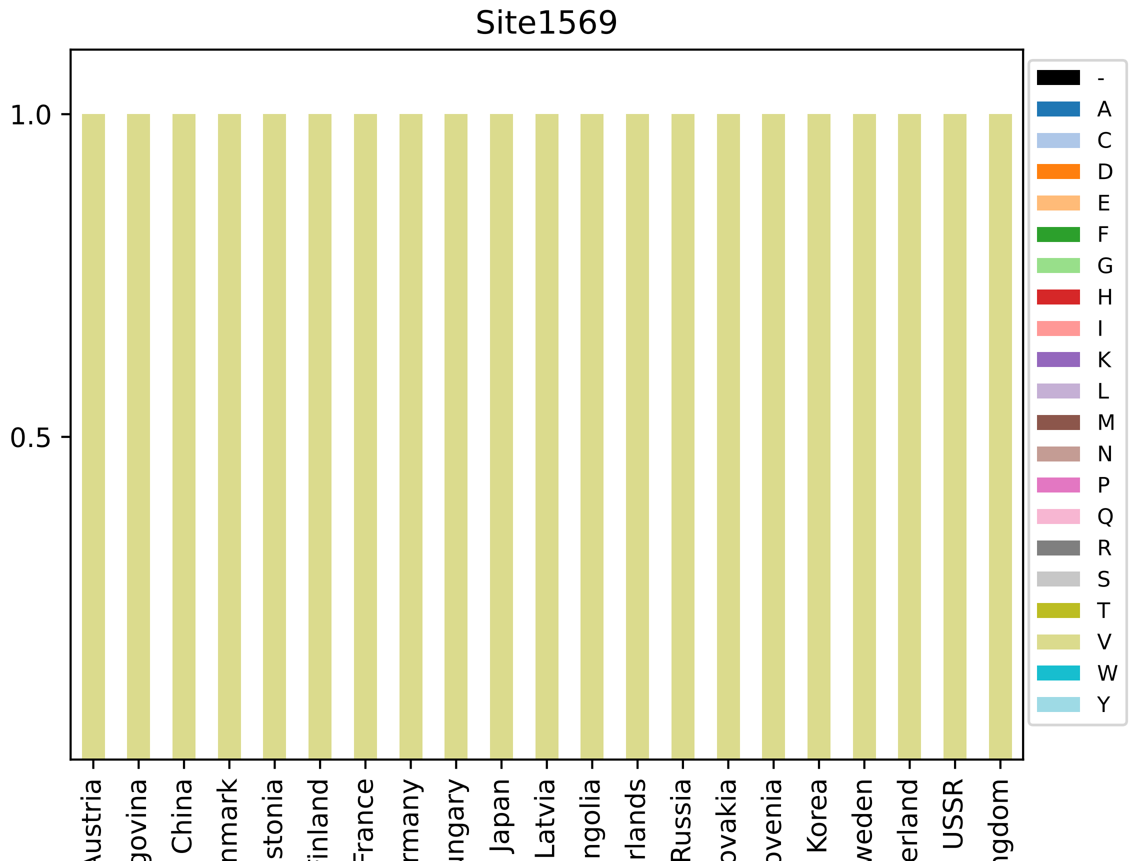
Site1569
1.0
0.5
-
A
C
D
E
F
G
H
I
K
L
M
N
P
Q
R
S
T
V
W
Y
China
Japan
Latvia
Russia
Korea
USSR

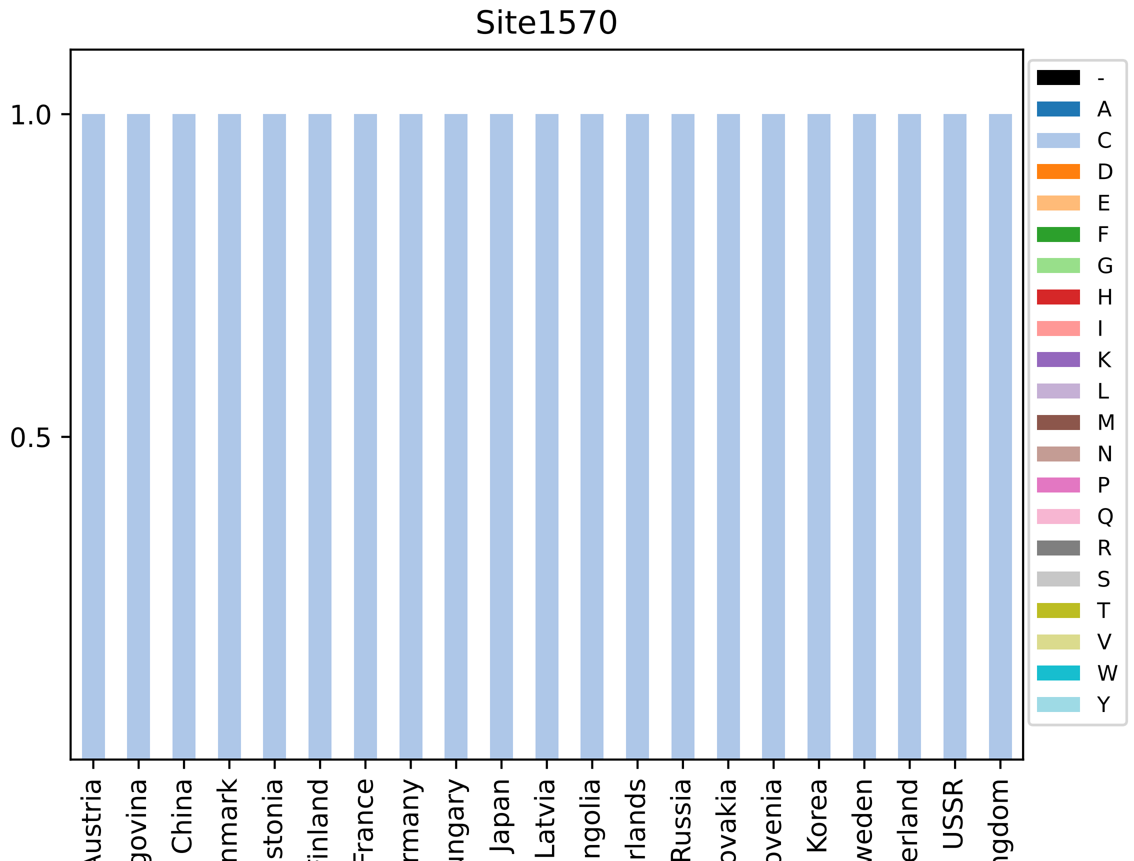

Site1570
1.0
0.5
-
A
C
D
E
F
G
H
I
K
L
M
N
P
Q
R
S
T
V
W
Y
China
Japan
Latvia
Russia
Korea
USSR

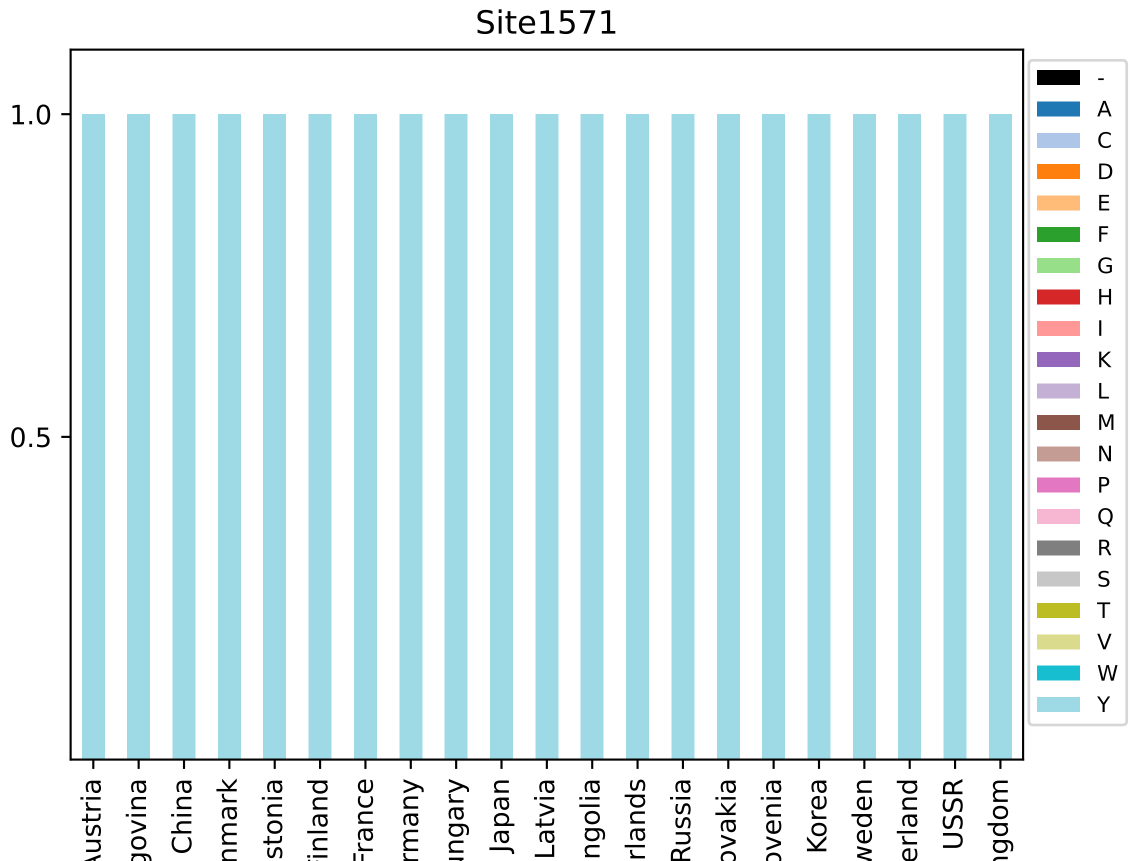

Site1571
1.0
0.5
-
A
C
D
E
F
G
H
I
K
L
M
N
P
Q
R
S
T
V
W
Y
Austria
China
Japan
Latvia
Russia
Korea
USSR

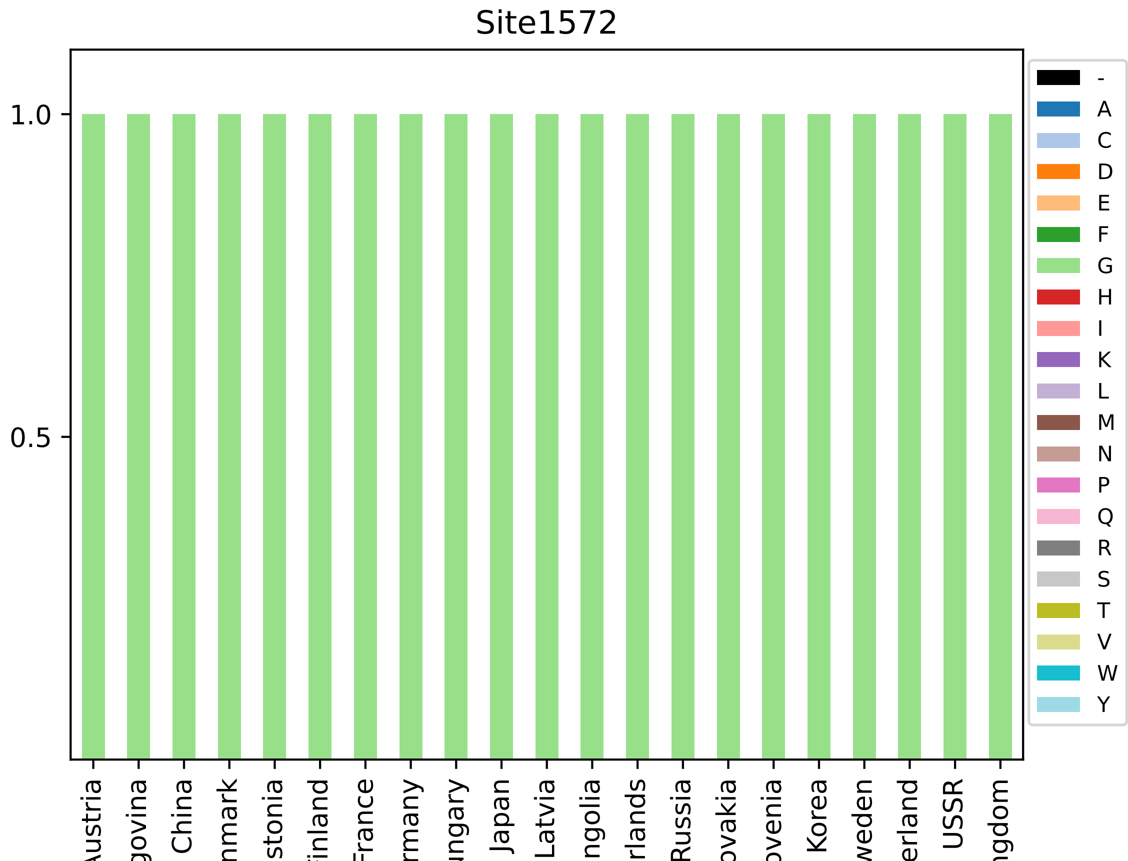
Site1572
1.0
0.5
-
A
C
D
E
F
G
H
I
K
L
M
N
P
Q
R
S
T
V
W
Y
Austria
govina
China
nmark
stonia
Finland
France
rmany
ungary
Japan
Latvia
ngolia
rlands
Russia
ovakia
ovenia
Korea
weden
erland
USSR
ngdom

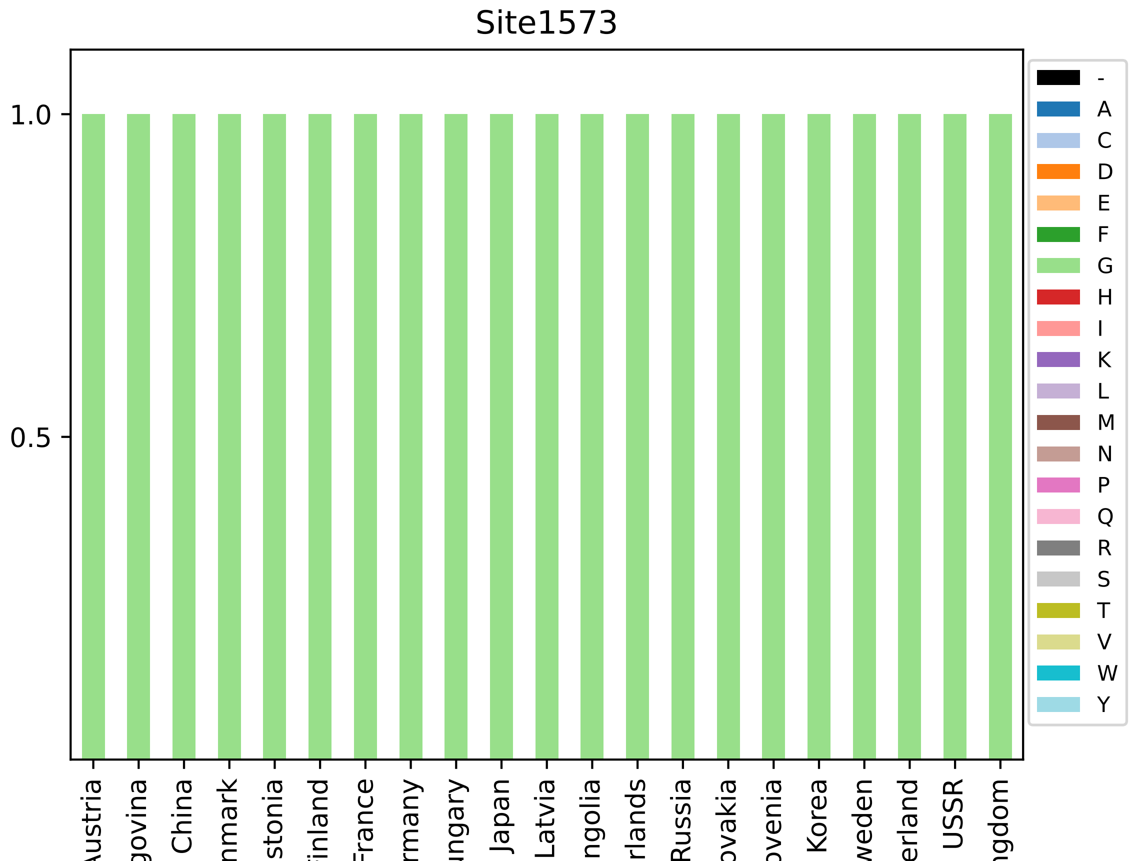

Site1573
1.0
0.5
-
A
C
D
E
F
G
H
I
K
L
M
N
P
Q
R
S
T
V
W
Y
China
Japan
Latvia
Russia
Korea
USSR

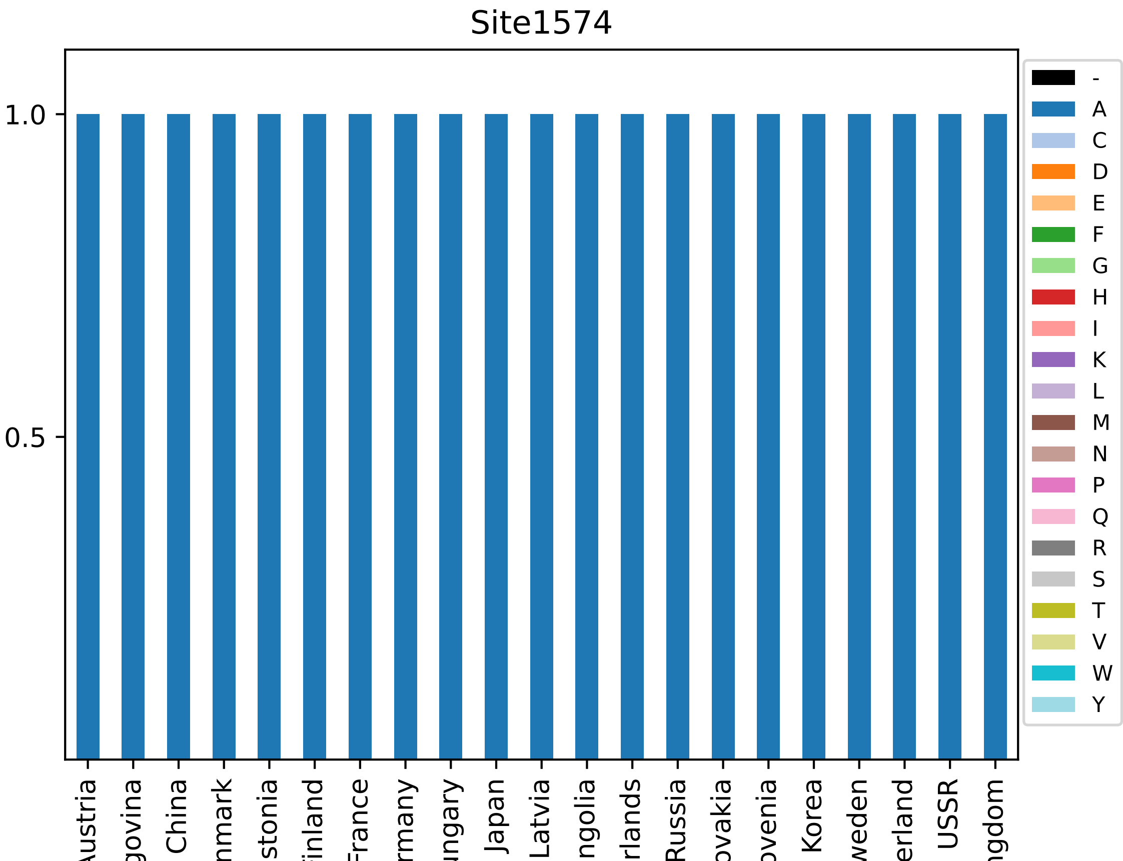
Site1574
1.0
0.5
-
A
C
D
E
F
G
H
I
K
L
M
N
P
Q
R
S
T
V
W
Y
China
Japan
Latvia
Russia
Korea
USSR

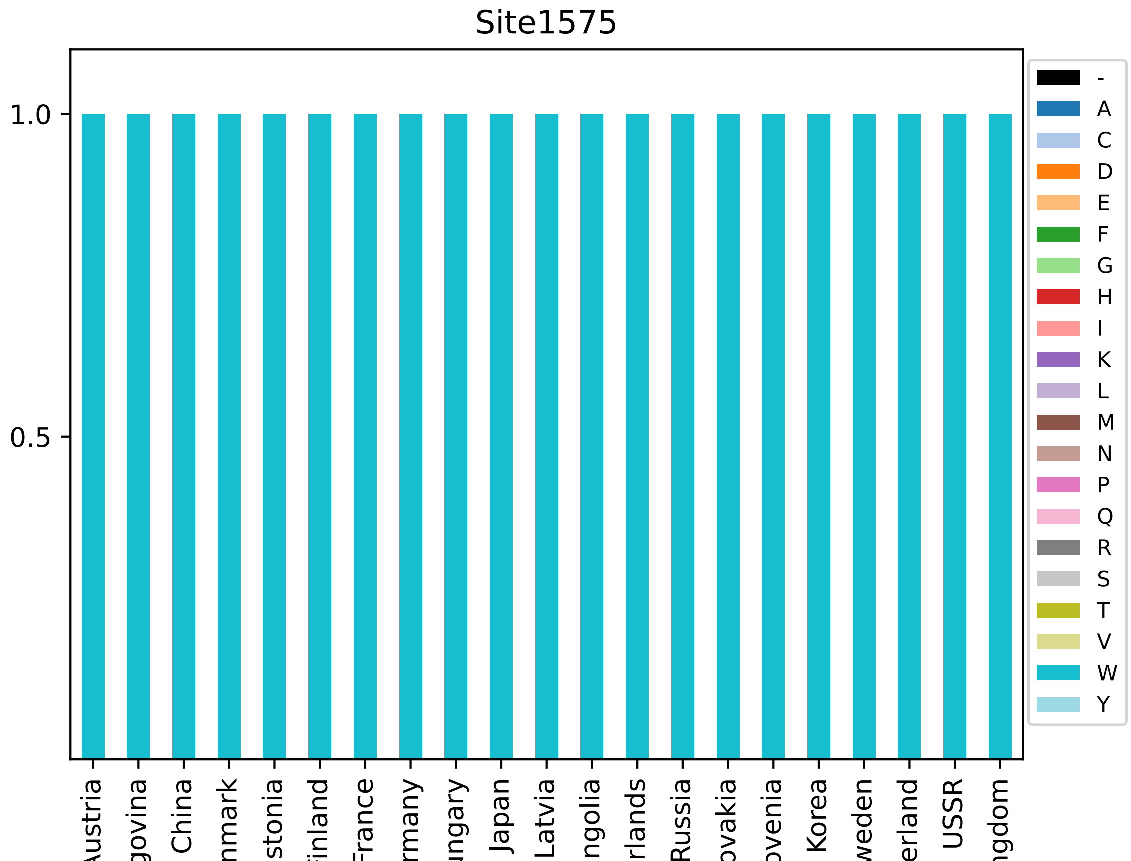
Site1575
1.0
0.5
-
A
C
D
E
F
G
H
I
K
L
M
N
P
Q
R
S
T
V
W
Y
China
Japan
Latvia
Russia
Korea
USSR

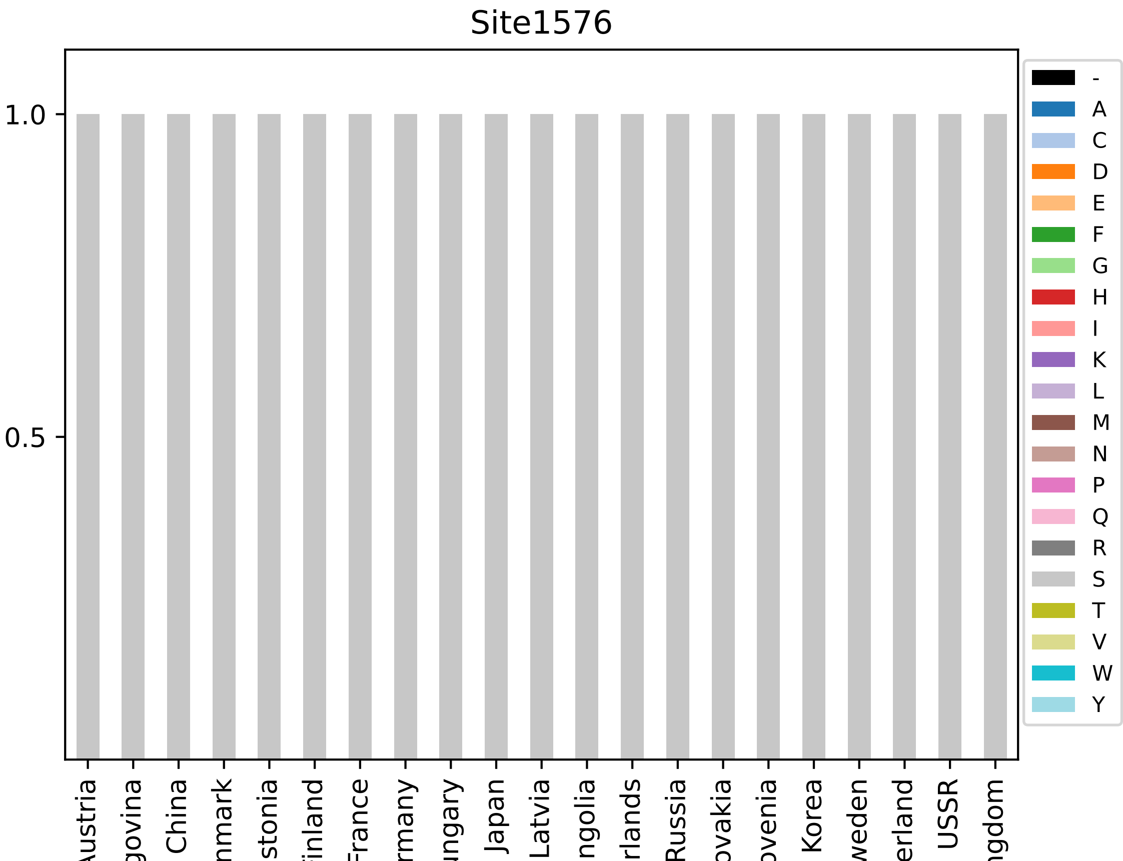
Site1576
1.0
0.5
-
A
C
D
E
F
G
H
I
K
L
M
N
P
Q
R
S
T
V
W
Y
Austria
govina
China
nmark
stonia
inland
France
rmany
ungary
Japan
Latvia
ngolia
rlands
Russia
ovakia
ovenia
Korea
weden
erland
USSR
ngdom

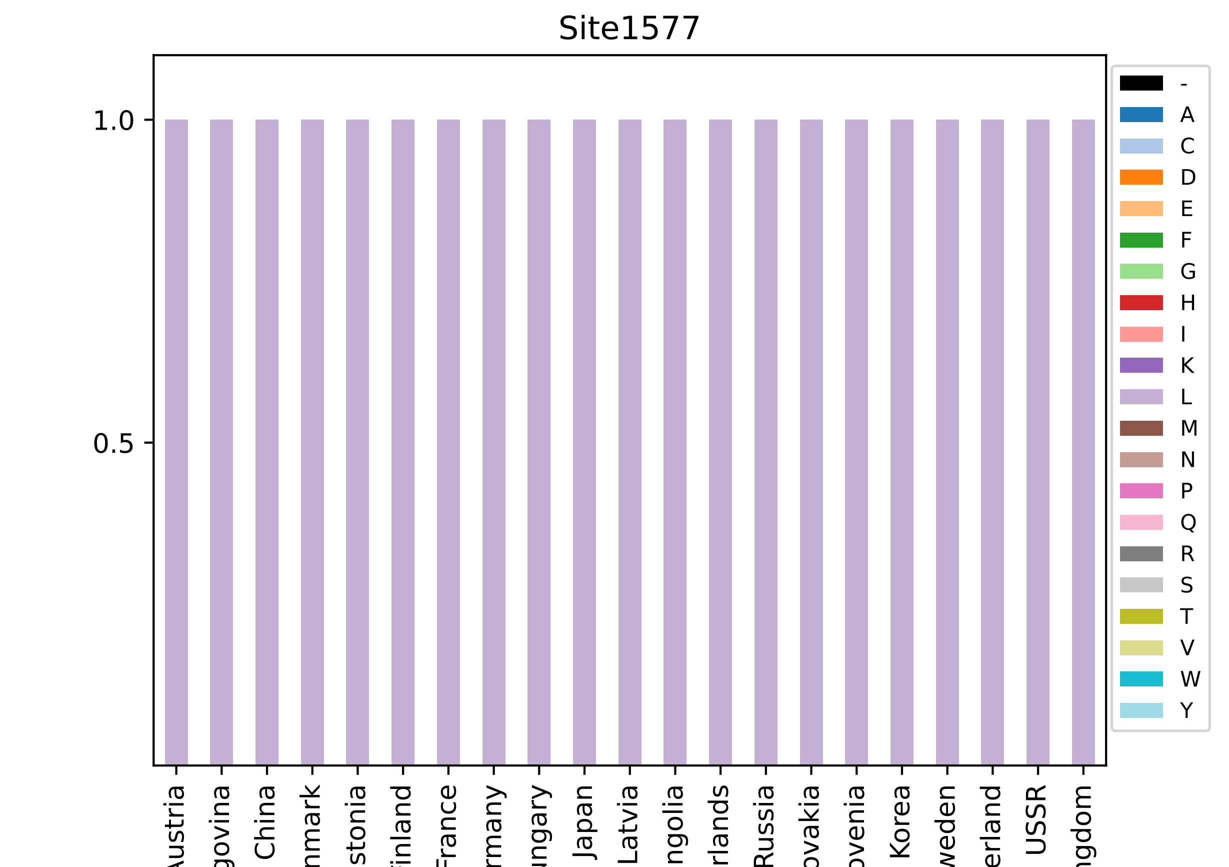

Site1577
1.0
0.5
-
A
C
D
E
F
G
H
I
K
L
M
N
P
Q
R
S
T
V
W
Y
Austria
govina
China
nmark
stonia
Finland
France
rmany
ungary
Japan
Latvia
ngolia
rlands
Russia
ovakia
ovenia
Korea
weden
erland
USSR
ngdom

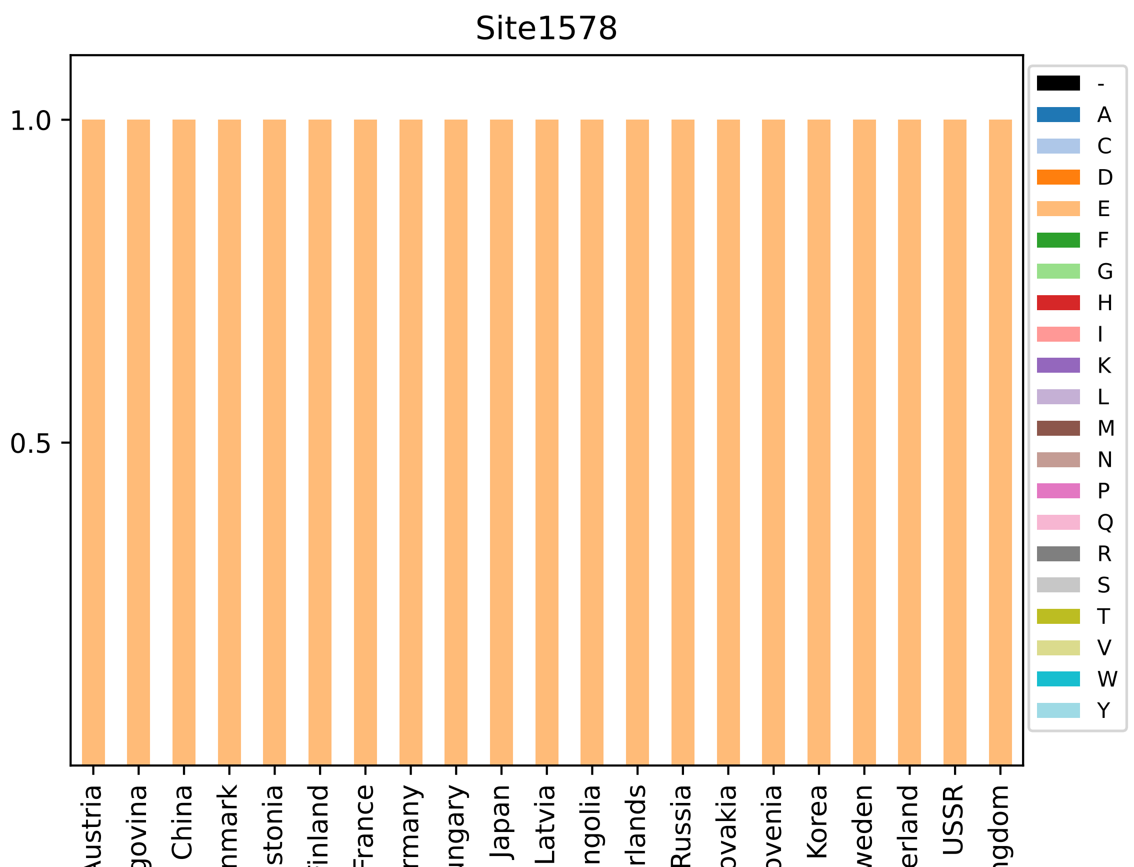

Site1578
1.0
0.5
-
A
C
D
E
F
G
H
I
K
L
M
N
P
Q
R
S
T
V
W
Y
China
Japan
Latvia
Russia
Korea
USSR

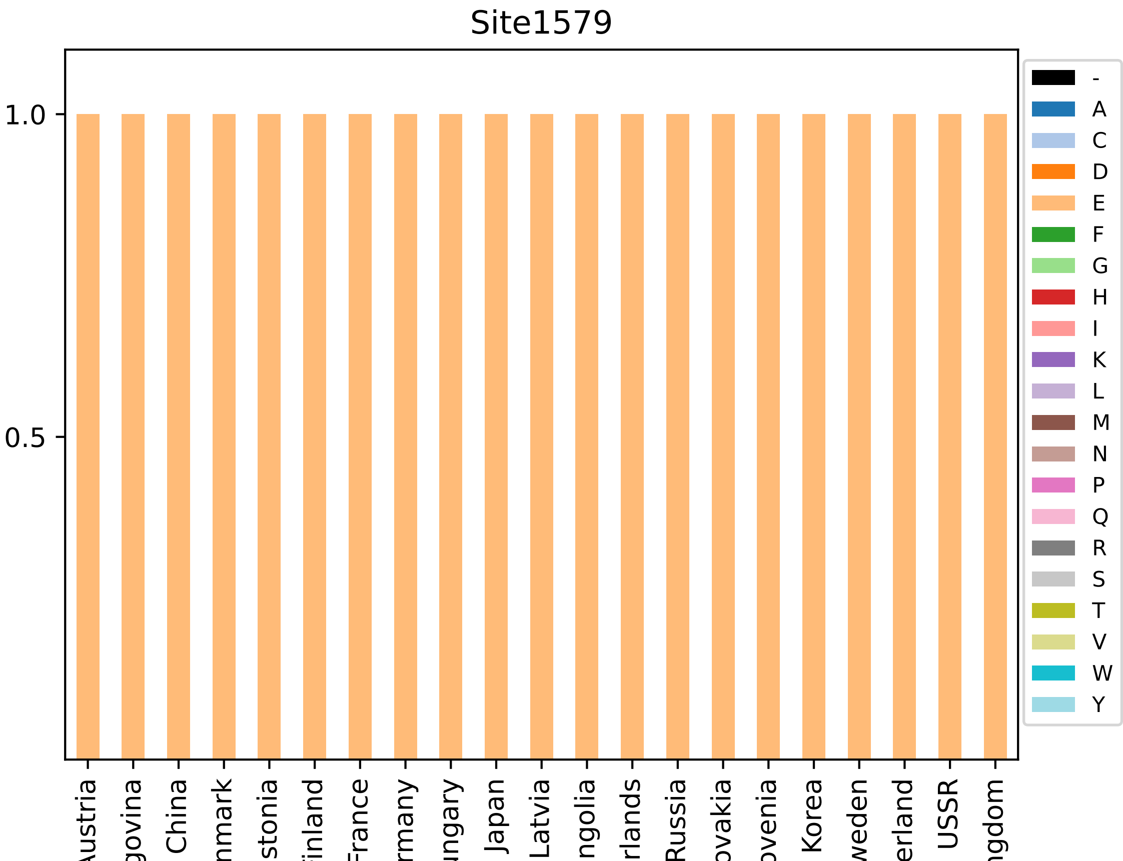
Site1579
1.0
0.5
-
A
C
D
E
F
G
H
I
K
L
M
N
P
Q
R
S
T
V
W
Y
China
Japan
Latvia
Russia
Korea
USSR

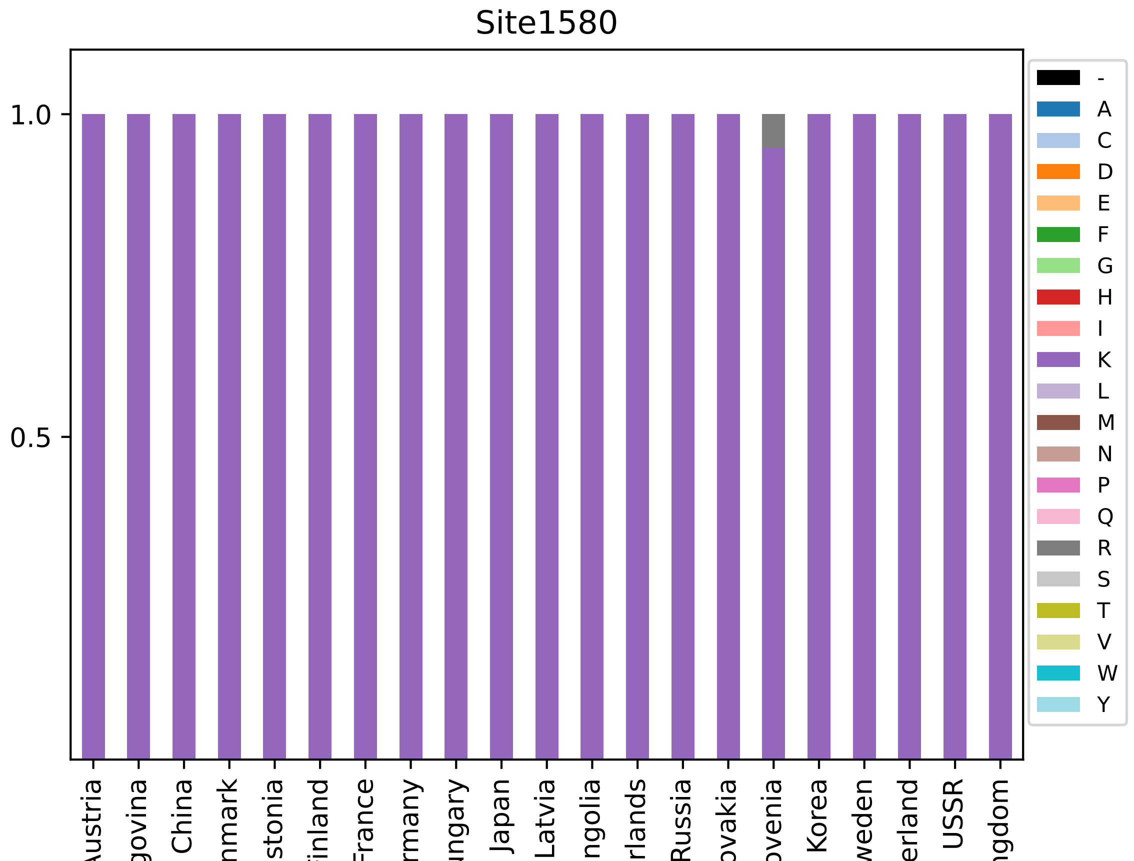

Site1580
1.0
0.5
-
A
C
D
E
F
G
H
I
K
L
M
N
P
Q
R
S
T
V
W
Y
Austria
govina
China
nmark
stonia
inland
France
rmany
ungary
Japan
Latvia
ngolia
rlands
Russia
ovakia
ovenia
Korea
weden
erland
USSR
ngdom

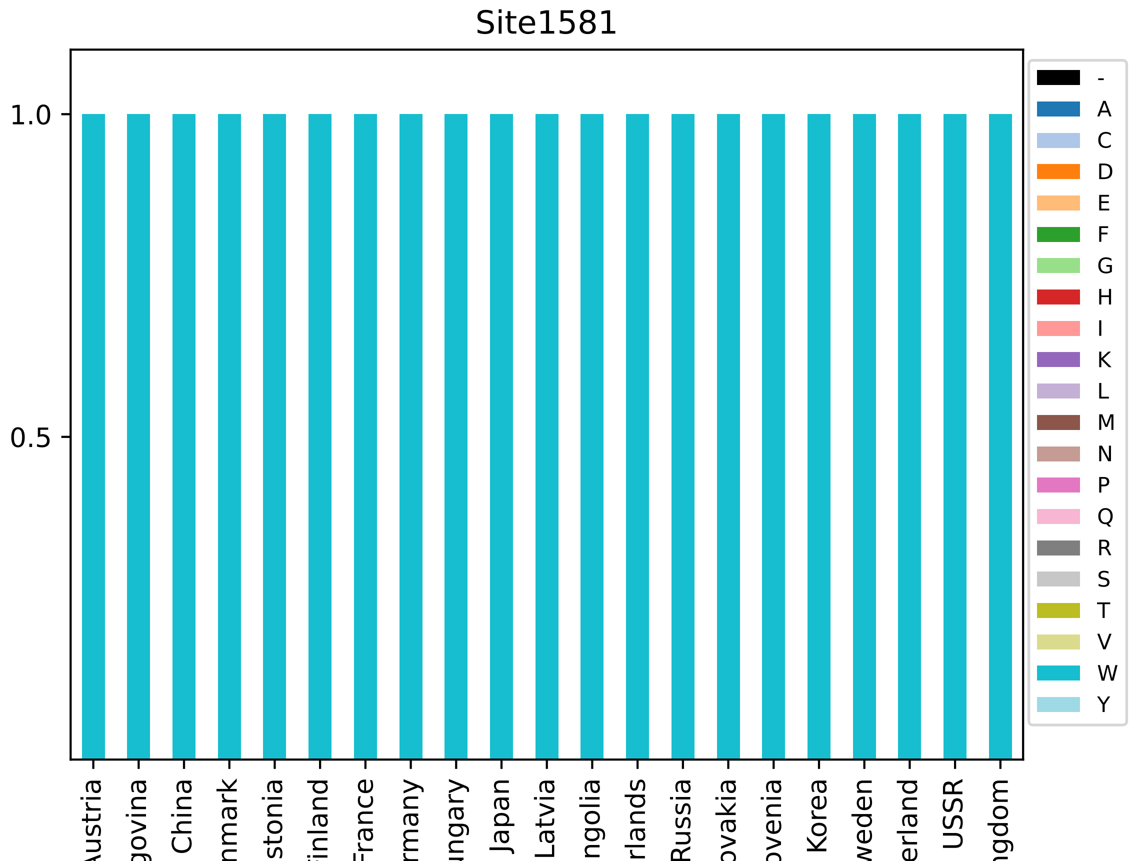

Site1581
1.0
0.5
-
A
C
D
E
F
G
H
I
K
L
M
N
P
Q
R
S
T
V
W
Y
China
Japan
Latvia
Russia
Korea
USSR

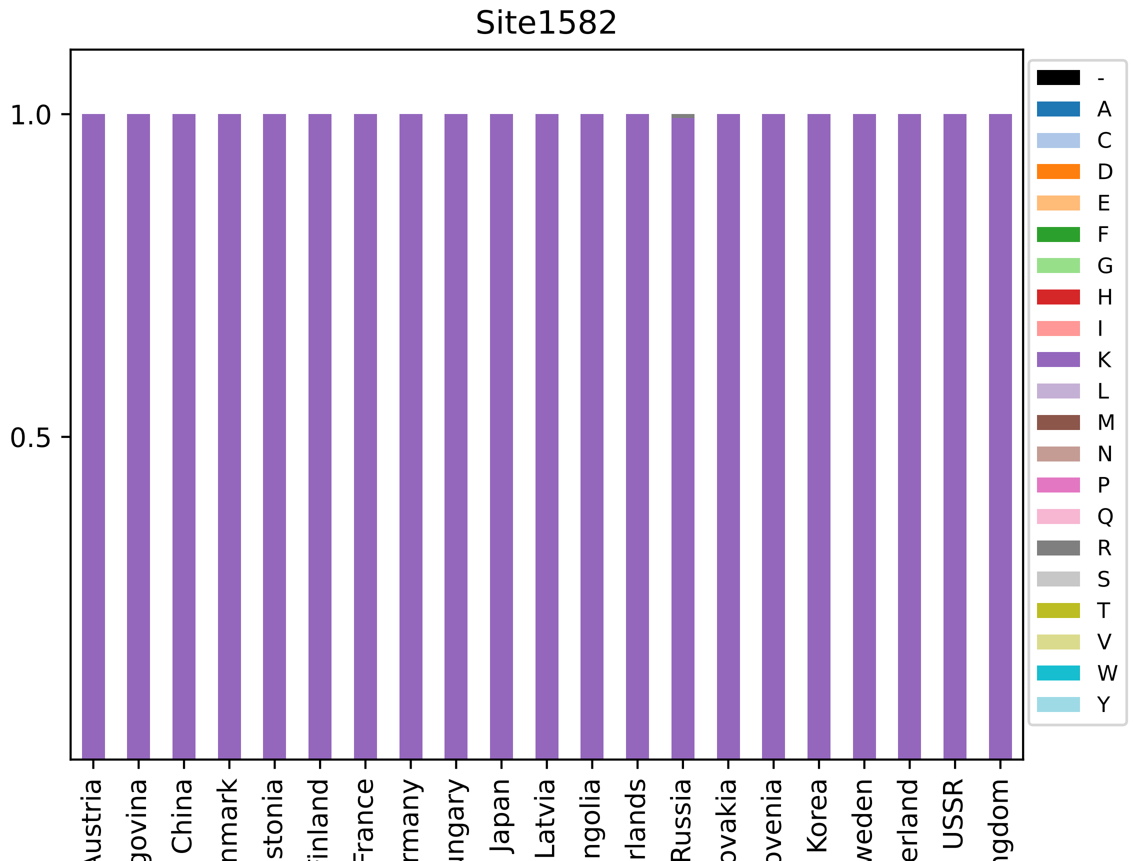
Site1582
1.0
0.5
-
A
C
D
E
F
G
H
I
K
L
M
N
P
Q
R
S
T
V
W
Y
China
Finland
France
Japan
Latvia
Russia
Korea
USSR

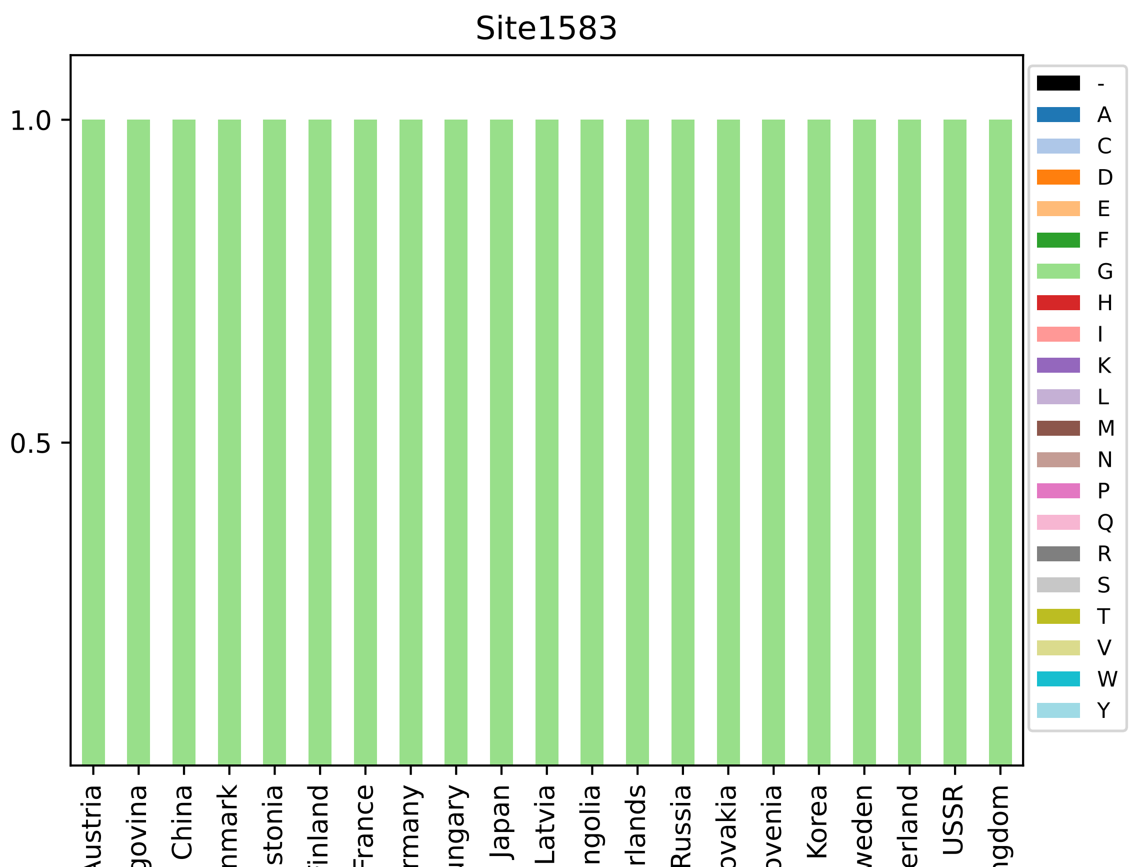
Site1583
1.0
0.5
-
A
C
D
E
F
G
H
I
K
L
M
N
P
Q
R
S
T
V
W
Y
China
Japan
Latvia
Russia
Korea
USSR

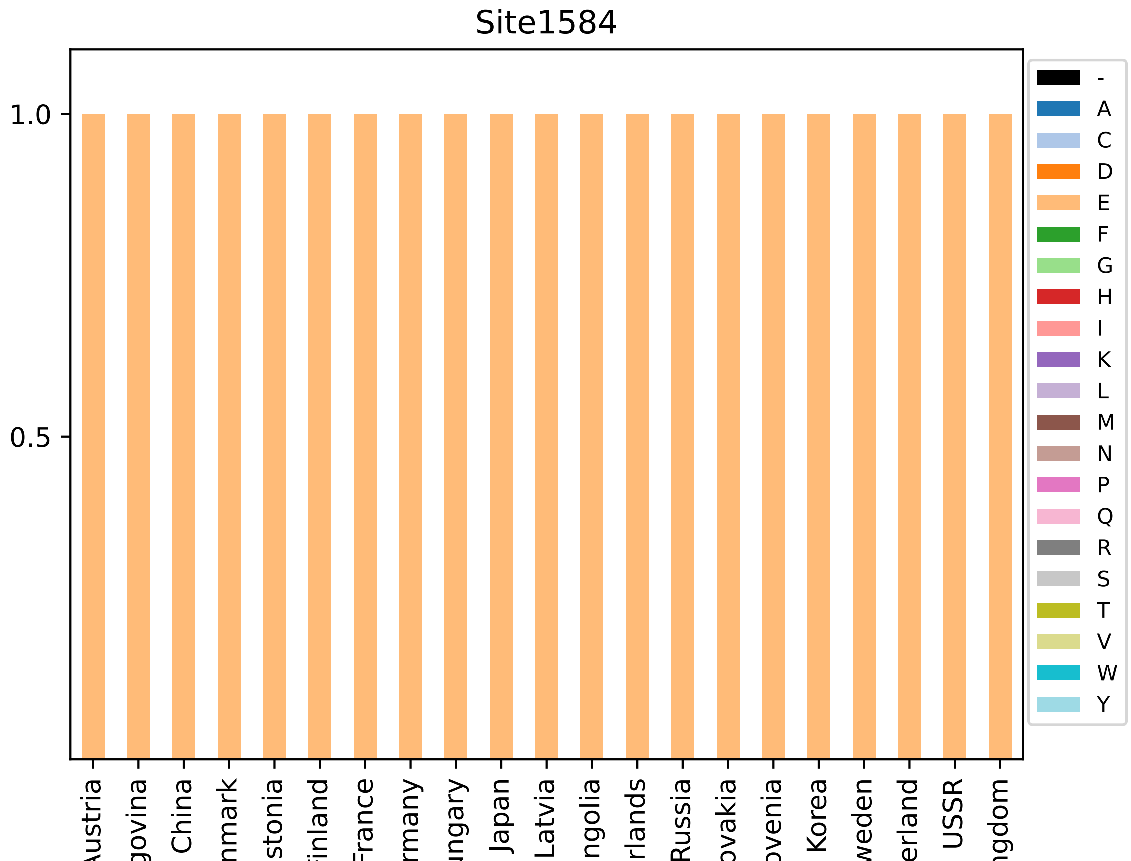

Site1584
1.0
0.5
-
A
C
D
E
F
G
H
I
K
L
M
N
P
Q
R
S
T
V
W
Y
China
Japan
Latvia
Russia
Korea
USSR

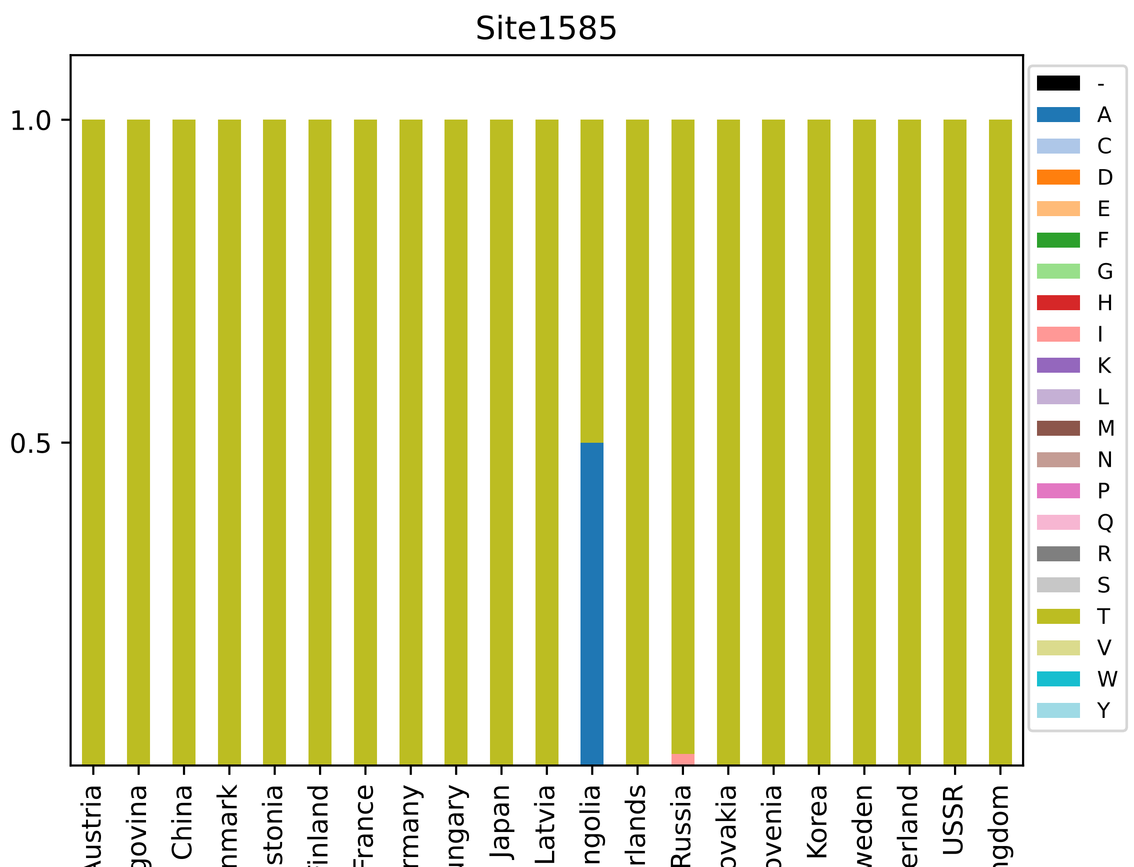

Site1585
1.0
0.5
-
A
C
D
E
F
G
H
I
K
L
M
N
P
Q
R
S
T
V
W
Y
Austria
govina
China
nmark
stonia
inland
France
rmany
ungary
Japan
Latvia
ngolia
rlands
Russia
ovakia
ovenia
Korea
weden
erland
USSR
ngdom

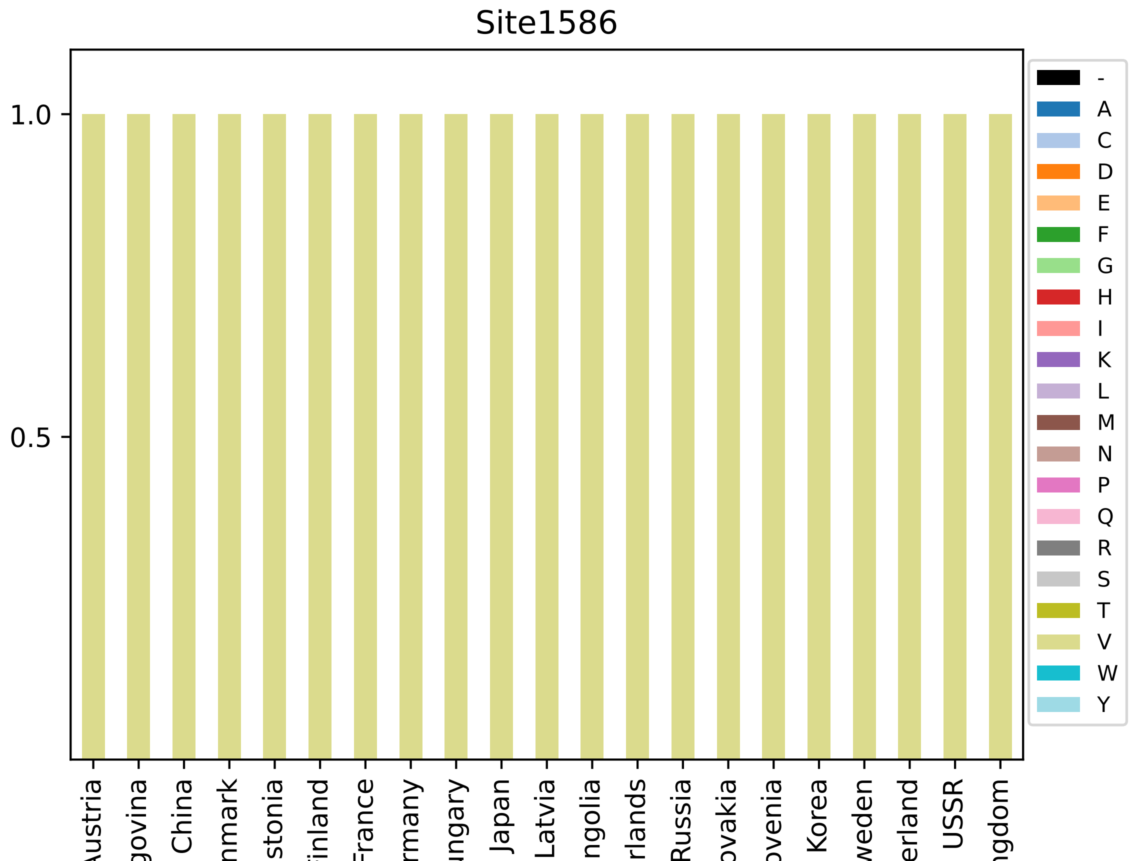
Site1586
1.0
0.5
-
A
C
D
E
F
G
H
I
K
L
M
N
P
Q
R
S
T
V
W
Y
Austria
China
Japan
Latvia
Russia
Korea
USSR

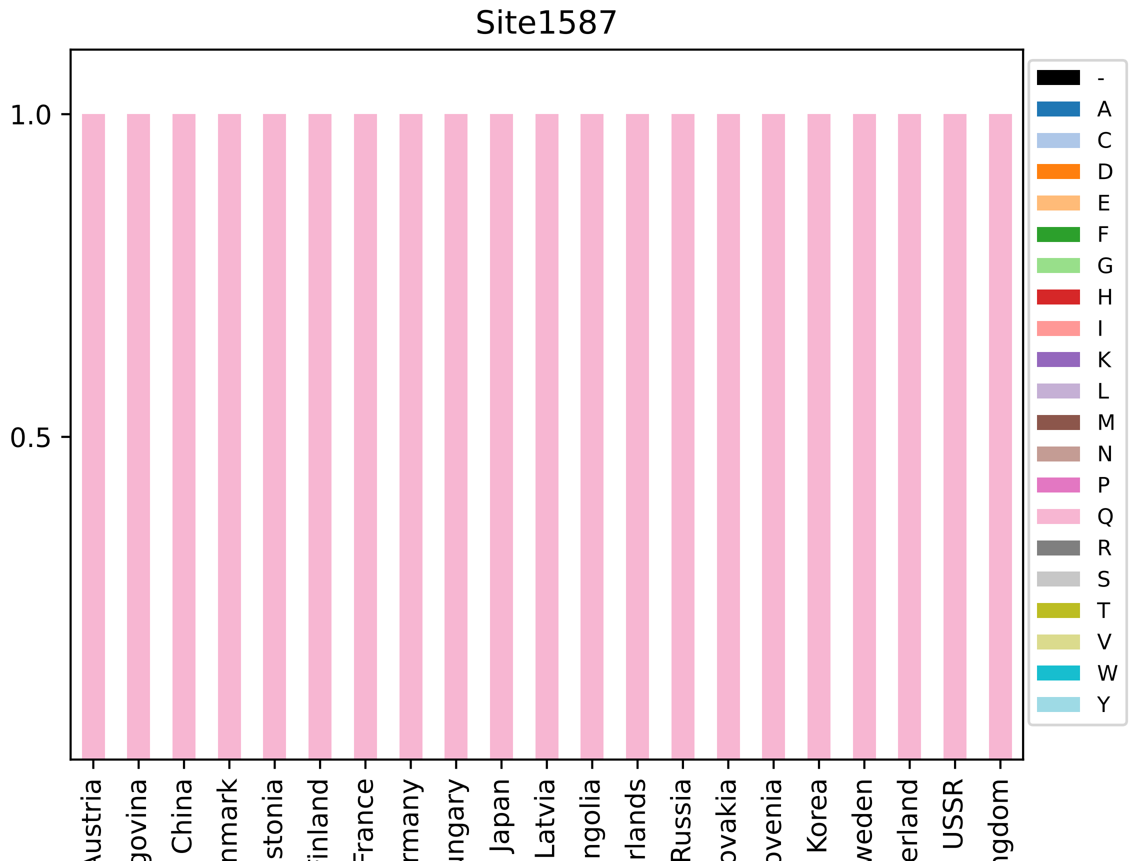
Site1587
1.0
0.5
Austria
govina
China
nmark
stonia
Finland
France
rmany
ungary
Japan
Latvia
ngolia
rlands
Russia
ovakia
ovenia
Korea
weden
erland
USSR
ngdom
-
A
C
D
E
F
G
H
I
K
L
M
N
P
Q
R
S
T
V
W
Y

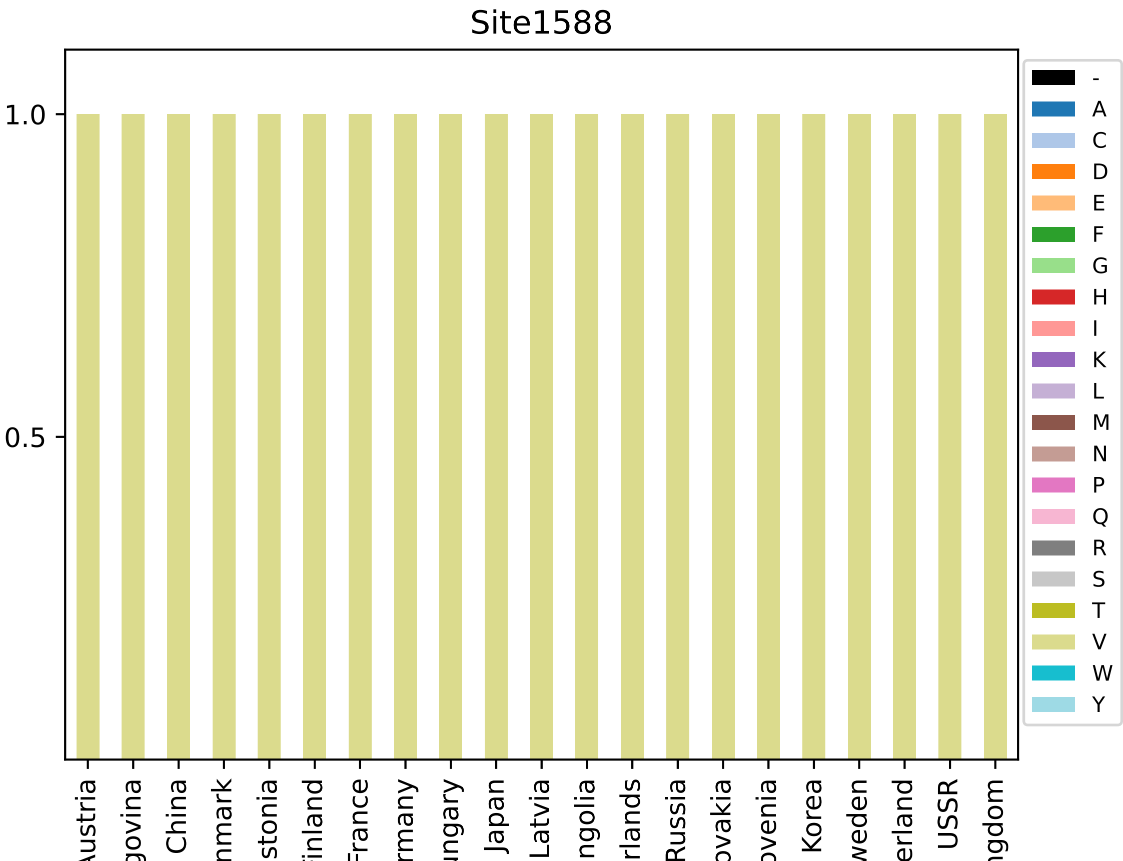

Site1588
1.0
0.5
-
A
C
D
E
F
G
H
I
K
L
M
N
P
Q
R
S
T
V
W
Y
China
Japan
Latvia
Russia
Korea
USSR

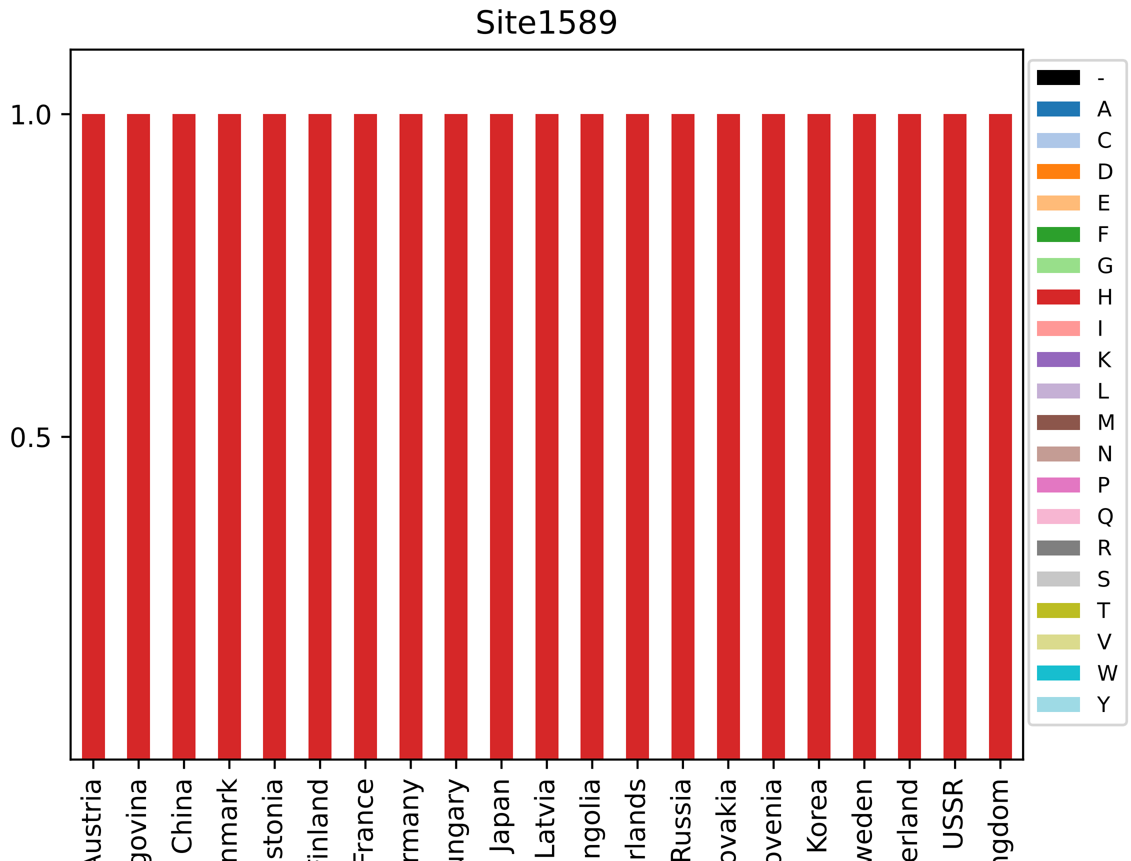

Site1589
1.0
0.5
-
A
C
D
E
F
G
H
I
K
L
M
N
P
Q
R
S
T
V
W
Y
ustria
govina
China
nmark
stonia
inland
rance
rmany
ungary
Japan
Latvia
ngolia
rlands
Russia
ovakia
ovenia
Korea
weden
erland
USSR
ngdom

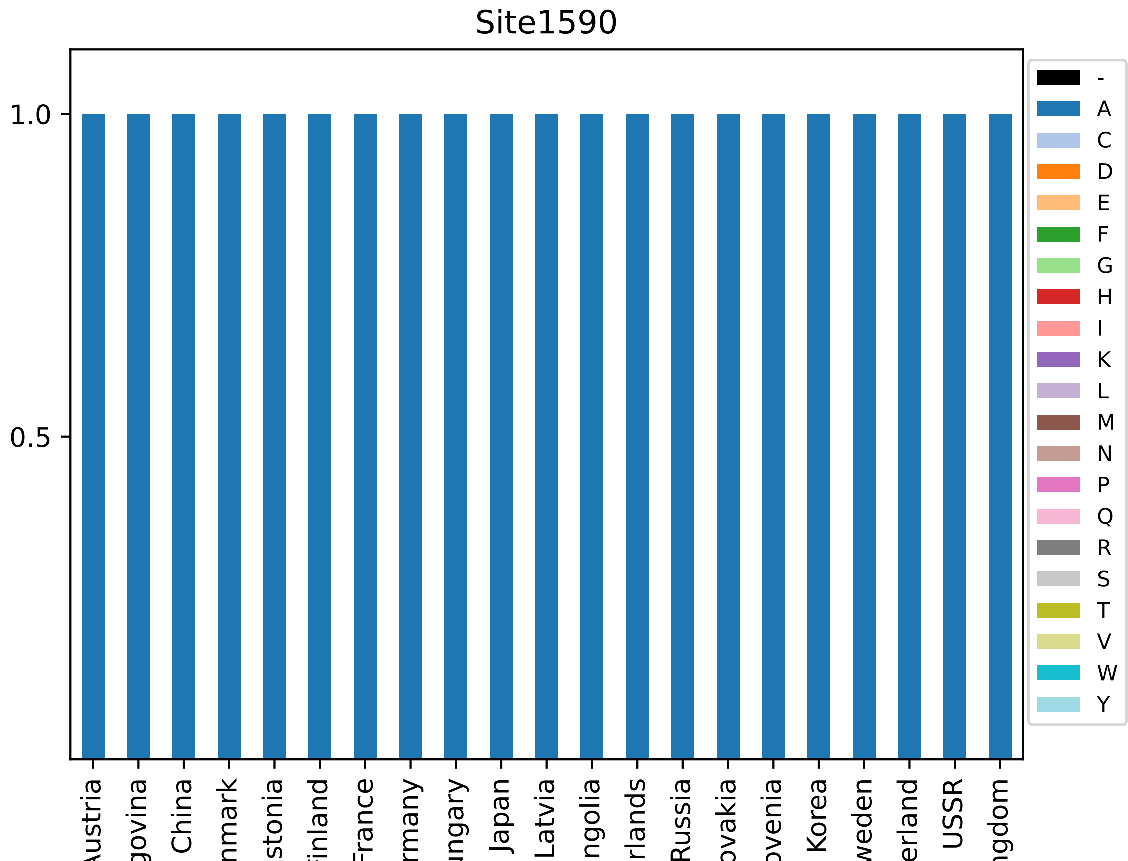

Site1590
1.0
0.5
-
A
C
D
E
F
G
H
I
K
L
M
N
P
Q
R
S
T
V
W
Y
Austria
govina
China
nmark
stonia
inland
France
rmany
ungary
Japan
Latvia
ngolia
rlands
Russia
ovakia
ovenia
Korea
weden
erland
USSR
ngdom

Site1591

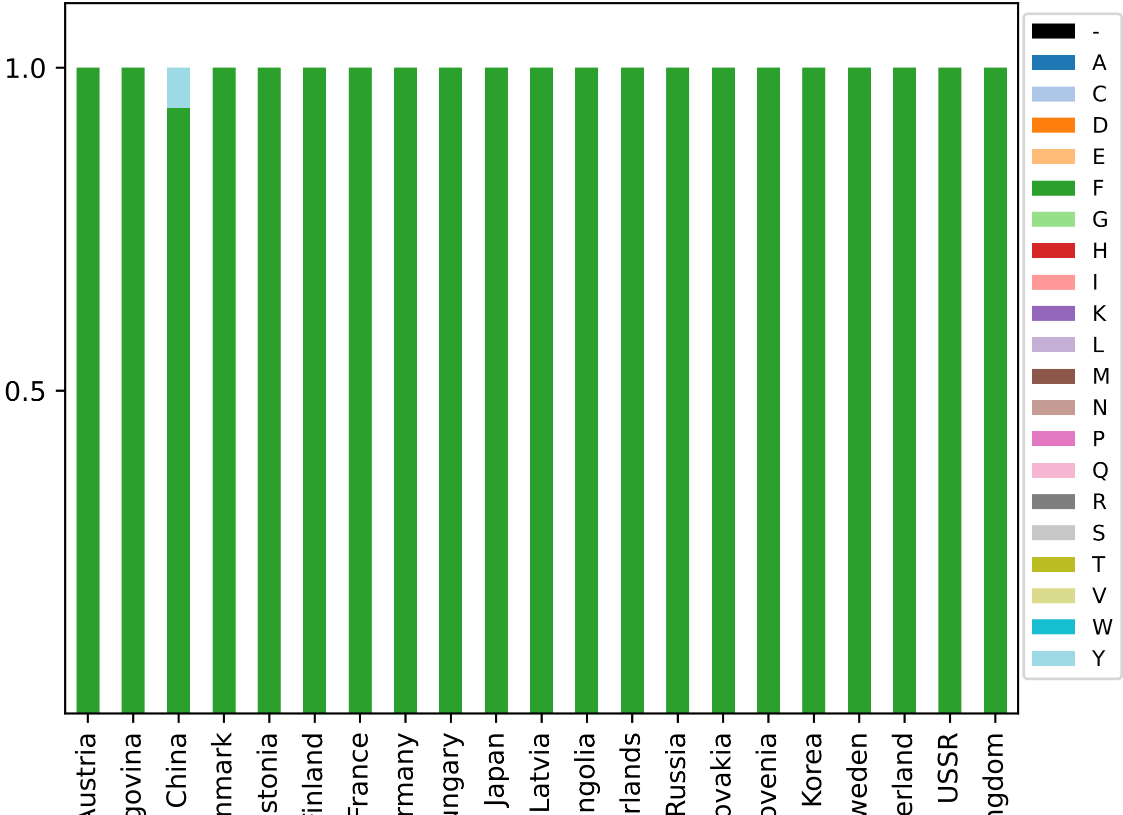

1.0
0.5
China
Japan
Latvia
Russia
Korea
USSR
-
A
C
D
E
F
G
H
I
K
L
M
N
P
Q
R
S
T
V
W
Y

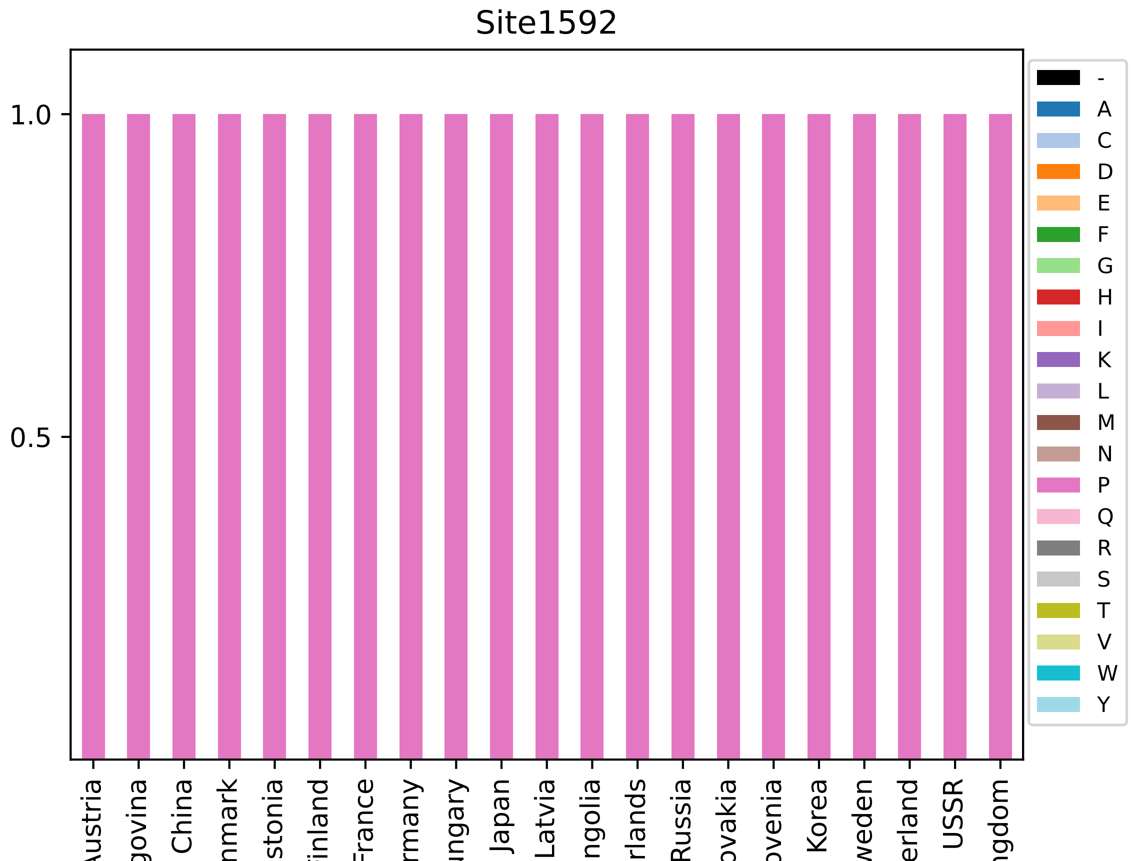

Site1592
1.0
0.5
-
A
C
D
E
F
G
H
I
K
L
M
N
P
Q
R
S
T
V
W
Y
China
Japan
Latvia
Russia
Korea
USSR

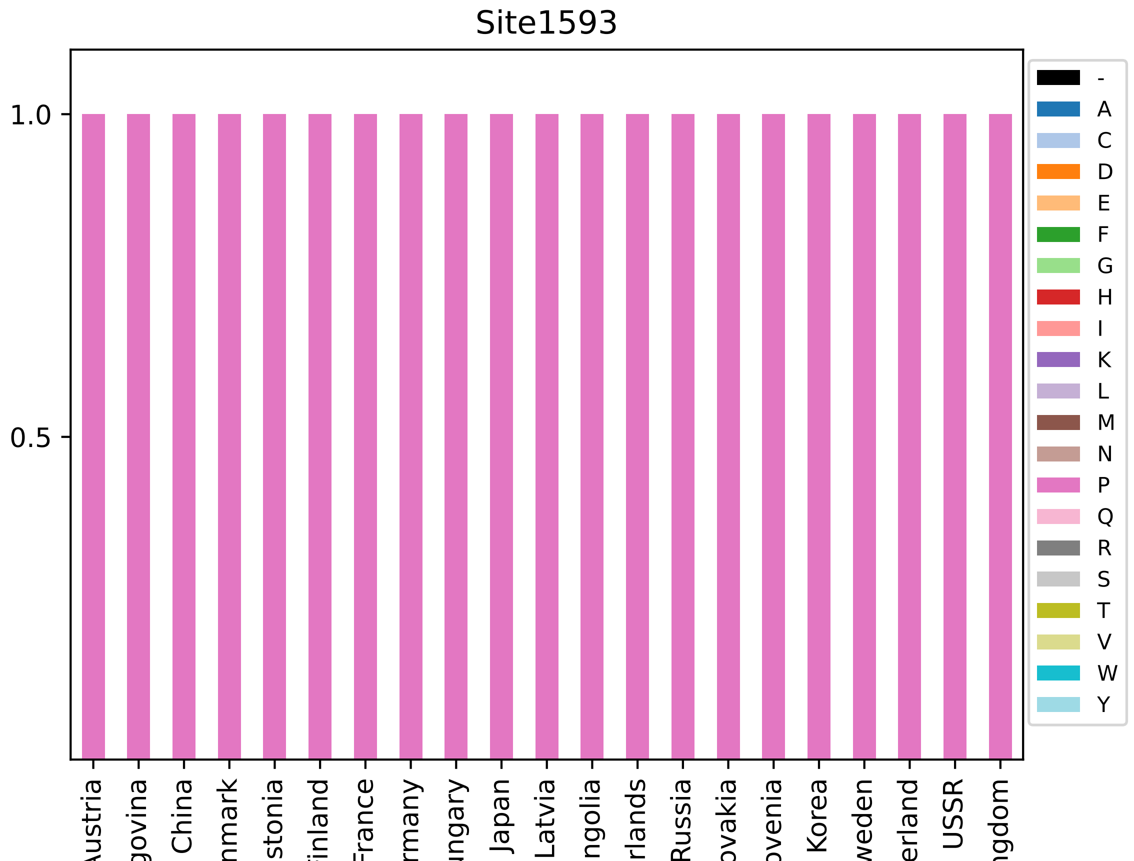
Site1593
1.0
0.5
Austria
govina
China
nmark
stonia
Finland
France
rmany
ungary
Japan
Latvia
ngolia
rlands
Russia
ovakia
ovenia
Korea
weden
erland
USSR
ngdom
-
A
C
D
E
F
G
H
I
K
L
M
N
P
Q
R
S
T
V
W
Y

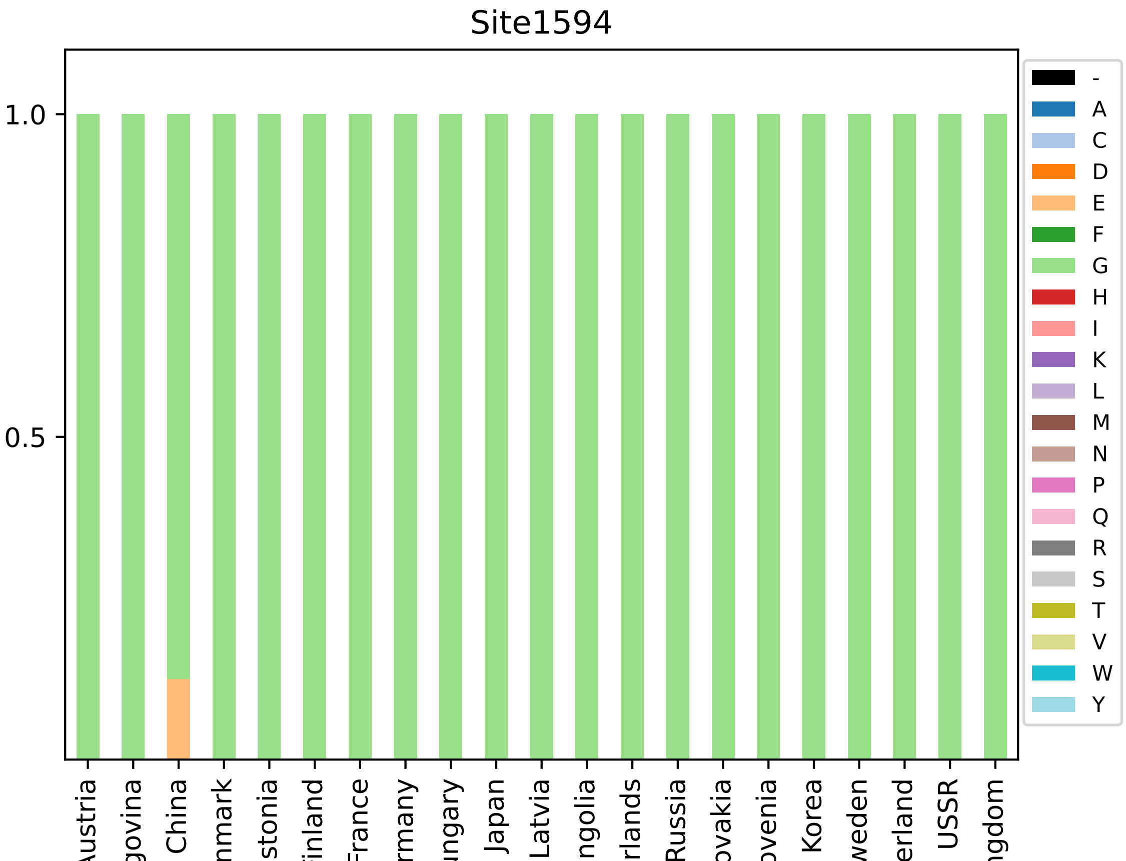
Site1594
1.0
0.5
-
A
C
D
E
F
G
H
I
K
L
M
N
P
Q
R
S
T
V
W
Y
China
Japan
Latvia
Russia
Korea
USSR

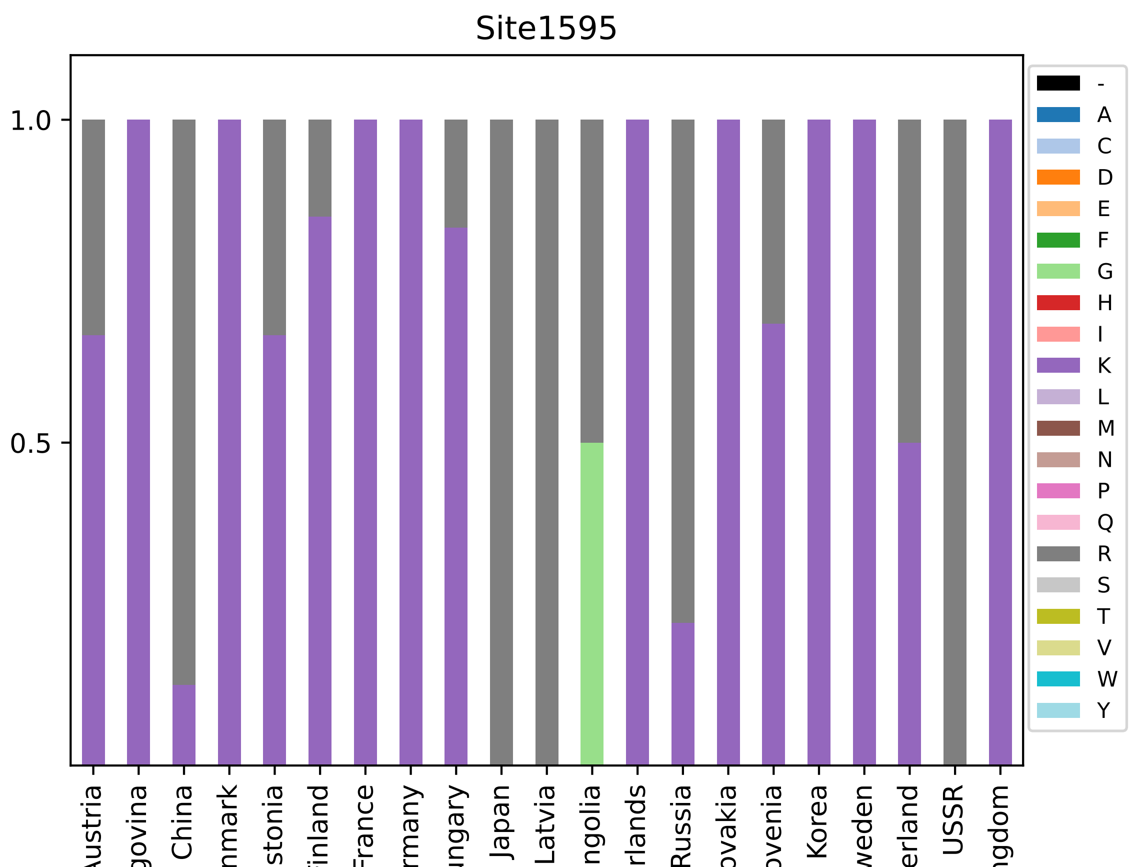
Site1595
1.0
0.5
-
A
C
D
E
F
G
H
I
K
L
M
N
P
Q
R
S
T
V
W
Y
Austria
China
Finland
France
Japan
Latvia
Russia
Korea
USSR

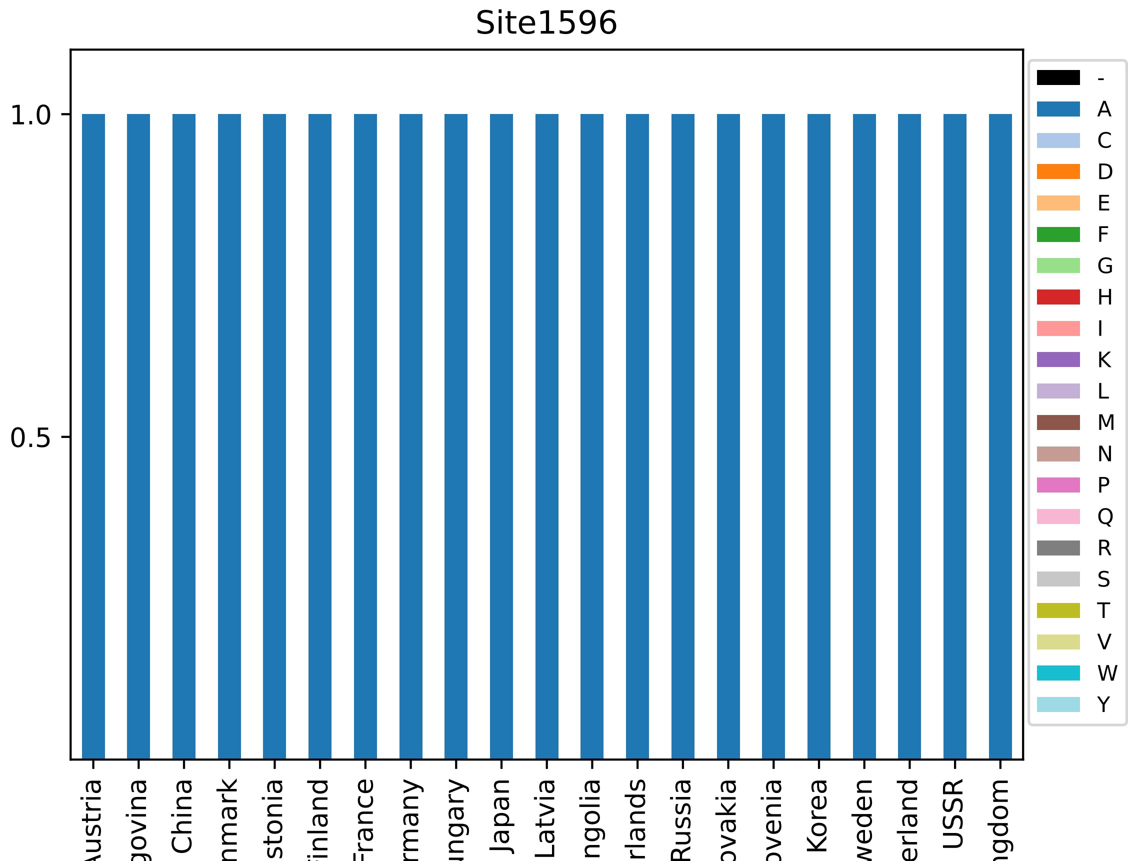
Site1596
1.0
0.5
-
A
C
D
E
F
G
H
I
K
L
M
N
P
Q
R
S
T
V
W
Y
China
Japan
Latvia
Russia
Korea
USSR

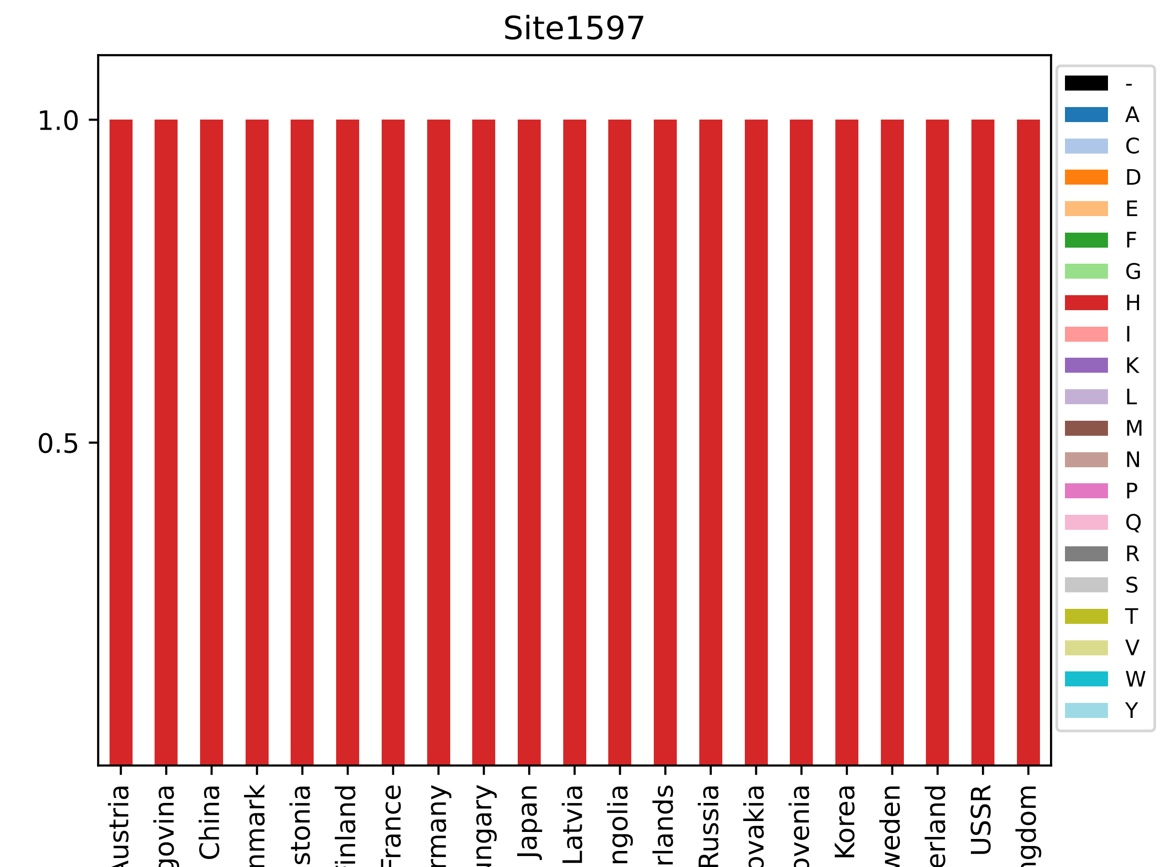

Site1597
1.0
0.5
-
A
C
D
E
F
G
H
I
K
L
M
N
P
Q
R
S
T
V
W
Y
Austria
govina
China
nmark
stonia
inland
France
rmany
ungary
Japan
Latvia
ngolia
rlands
Russia
ovakia
ovenia
Korea
weden
erland
USSR
ngdom

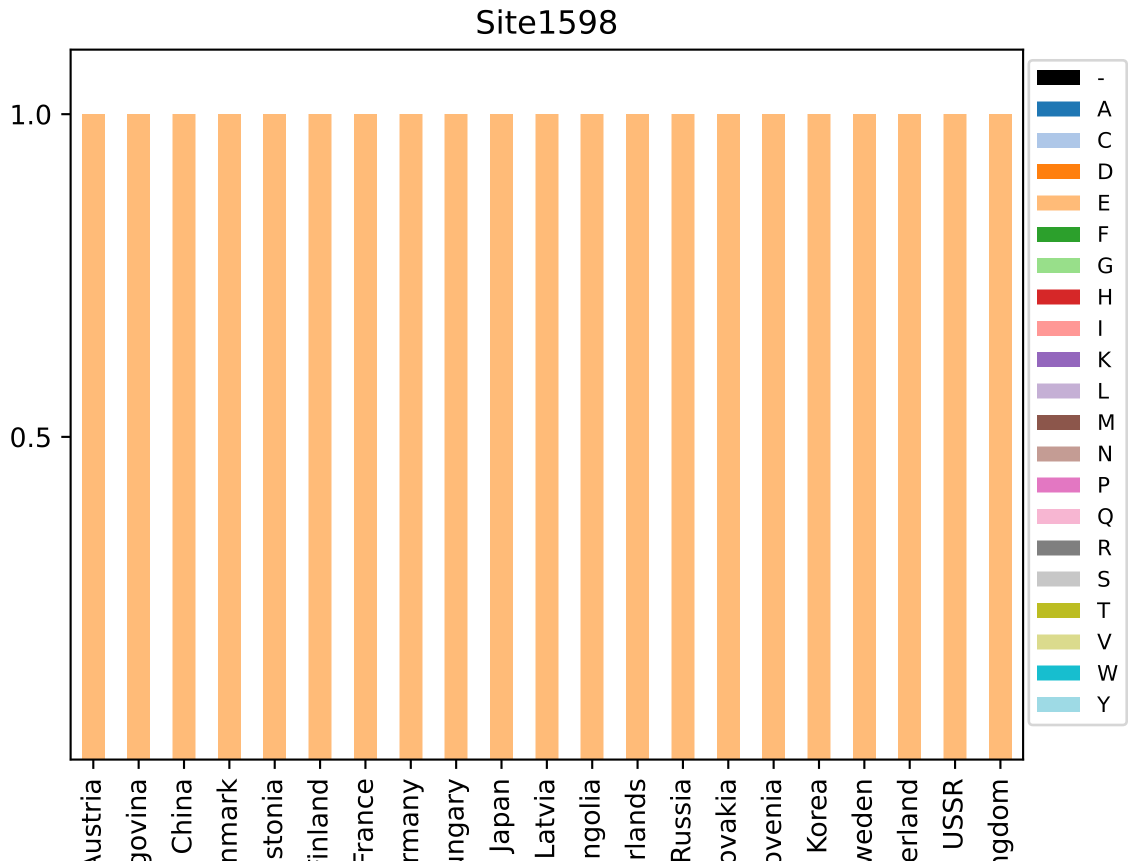

Site1598
1.0
0.5
-
A
C
D
E
F
G
H
I
K
L
M
N
P
Q
R
S
T
V
W
Y
China
Japan
Latvia
Russia
Korea
USSR

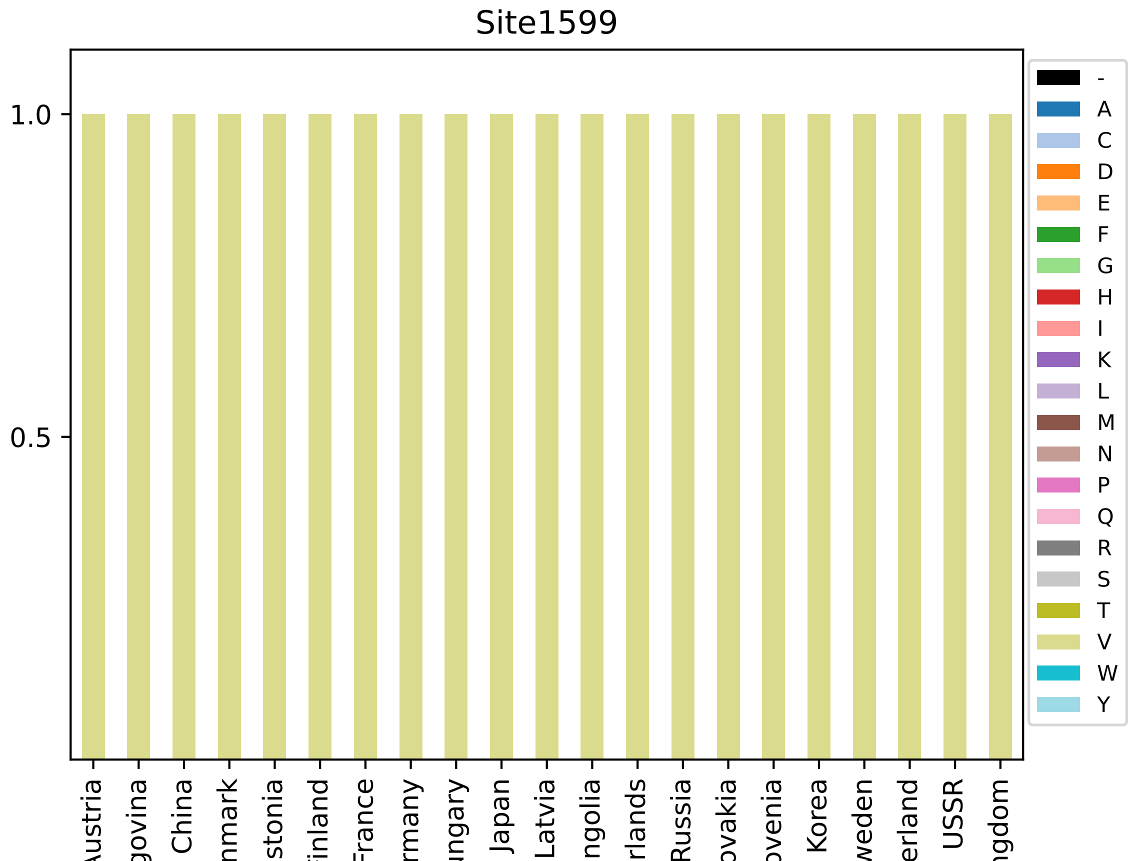
Site1599
1.0
0.5
-
A
C
D
E
F
G
H
I
K
L
M
N
P
Q
R
S
T
V
W
Y
Austria
China
Japan
Latvia
Russia
Korea
USSR

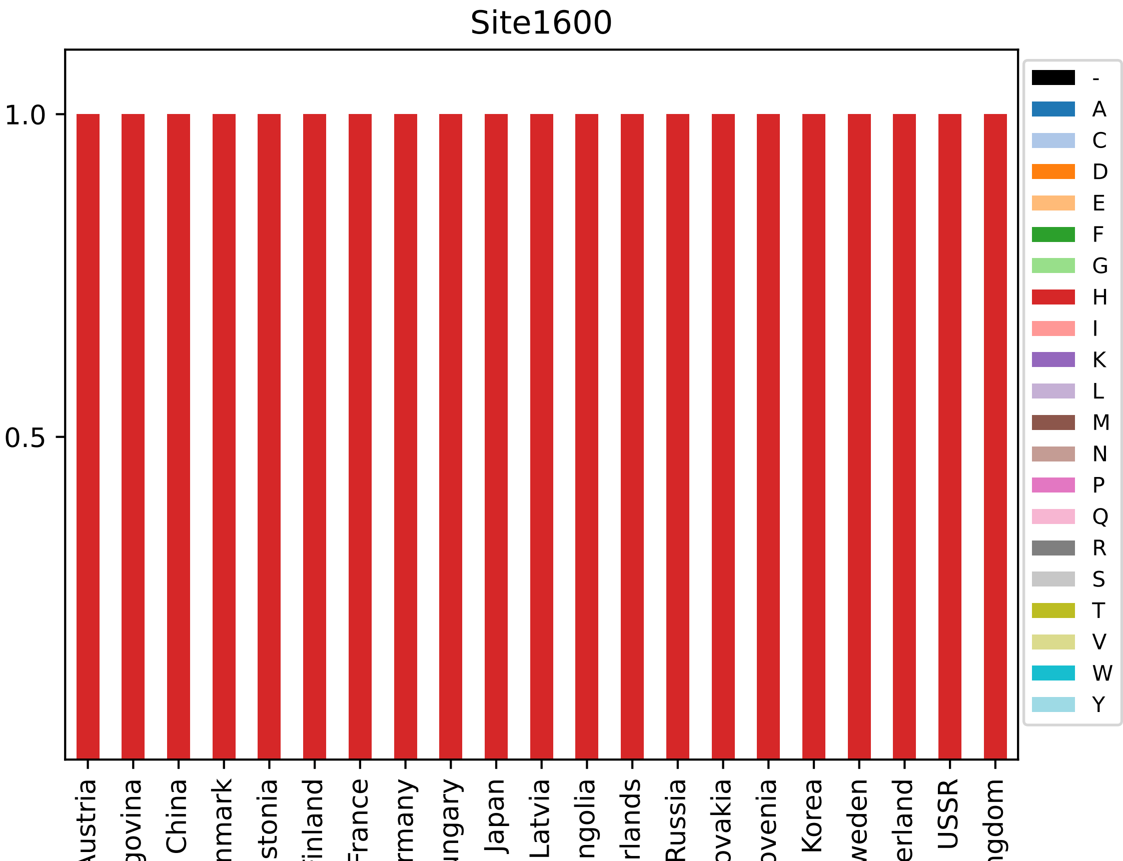
Site1600
1.0
0.5
-
A
C
D
E
F
G
H
I
K
L
M
N
P
Q
R
S
T
V
W
Y
China
Japan
Latvia
Russia
Korea
USSR

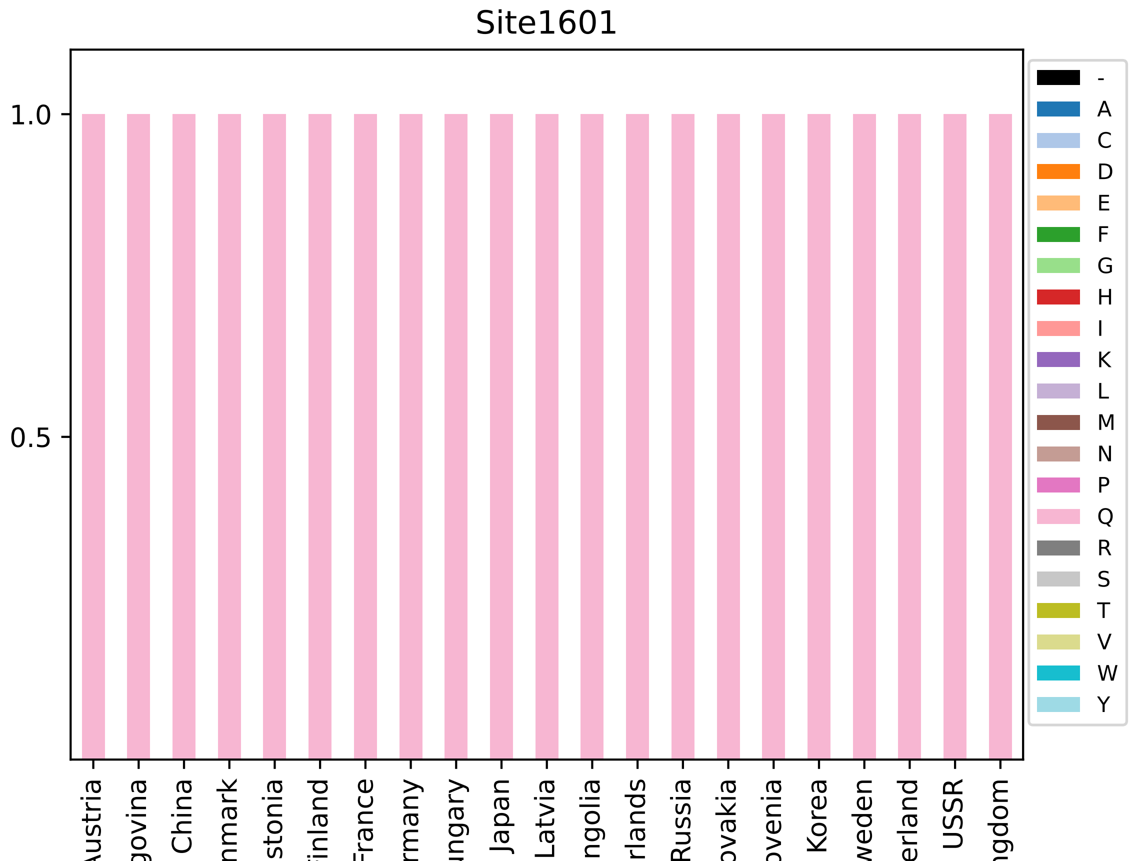

Site1601
1.0
0.5
-
A
C
D
E
F
G
H
I
K
L
M
N
P
Q
R
S
T
V
W
Y
Austria
govina
China
nmark
stonia
Finland
France
rmany
ungary
Japan
Latvia
ngolia
rlands
Russia
ovakia
ovenia
Korea
weden
erland
USSR
ngdom

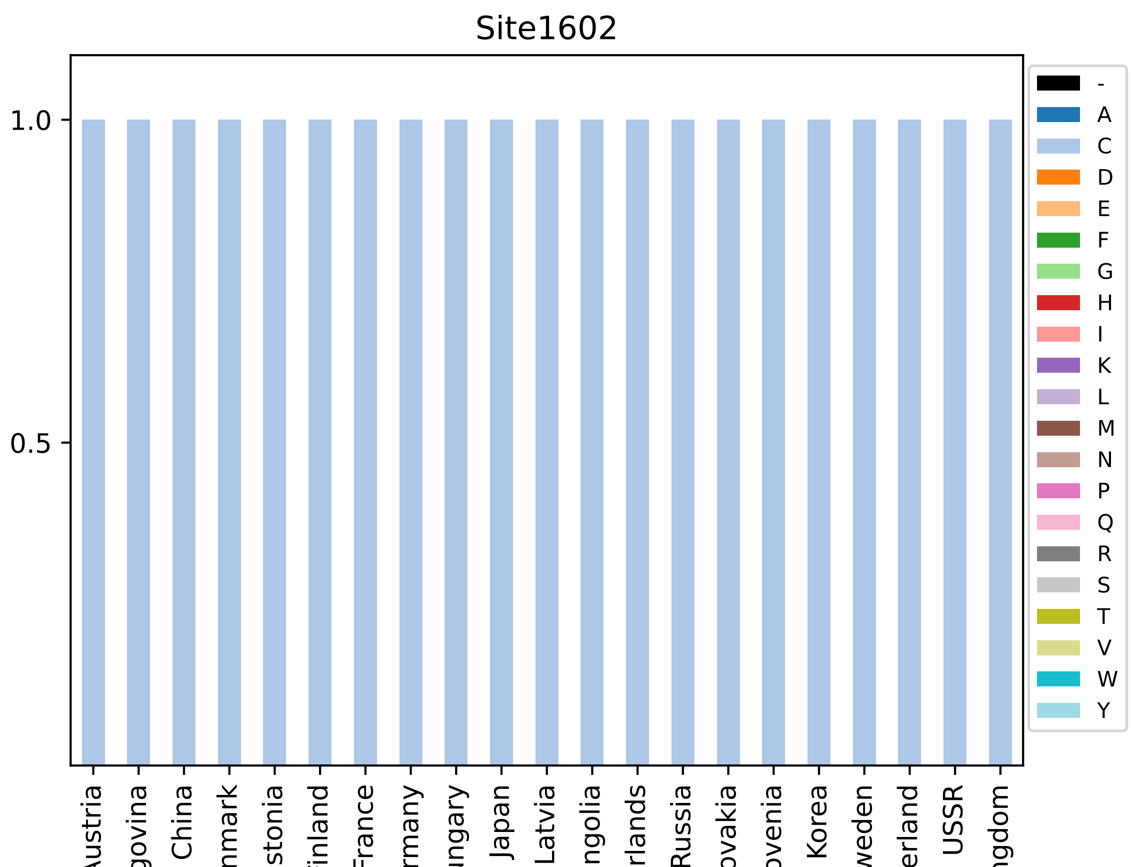
Site1602
1.0
0.5
-
A
C
D
E
F
G
H
I
K
L
M
N
P
Q
R
S
T
V
W
Y
China
Japan
Latvia
Russia
Korea
USSR

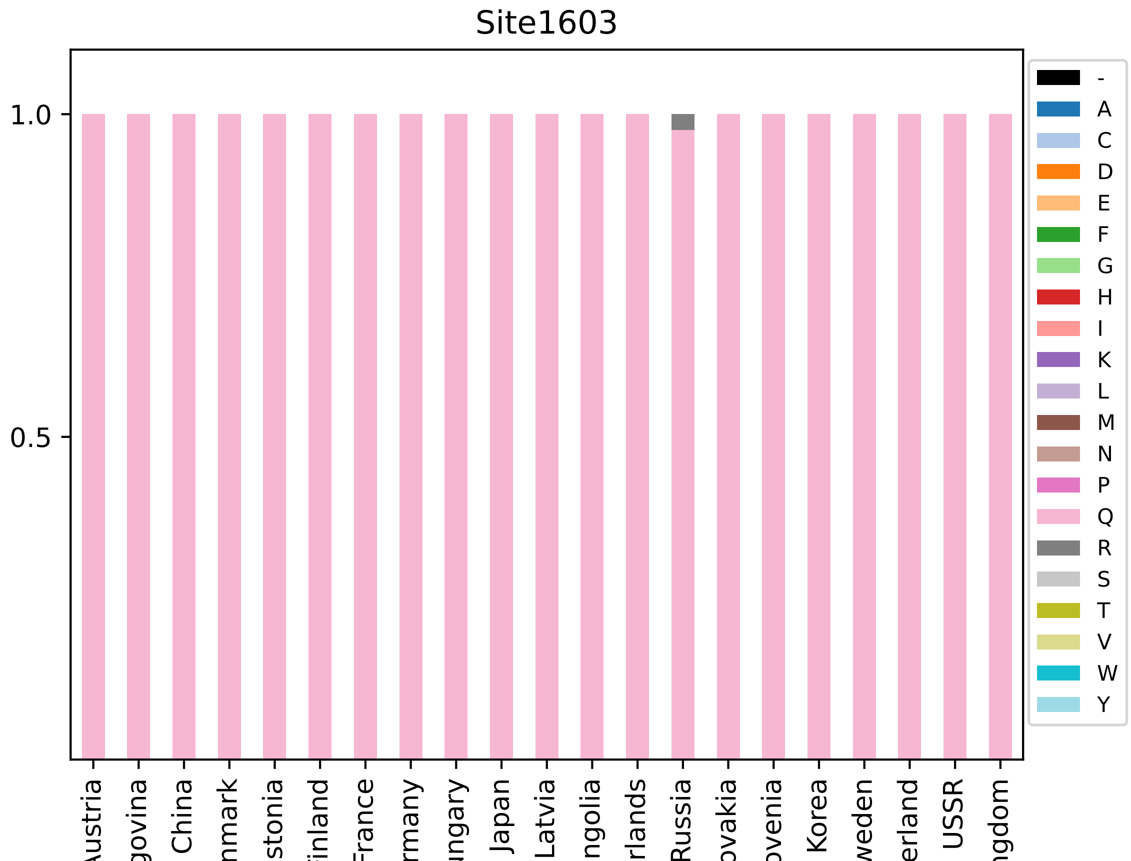

Site1603
1.0
0.5
-
A
C
D
E
F
G
H
I
K
L
M
N
P
Q
R
S
T
V
W
Y
Austria
China
Estonia
Finland
France
Hungary
Japan
Latvia
Russia
Korea
Sweden
USSR

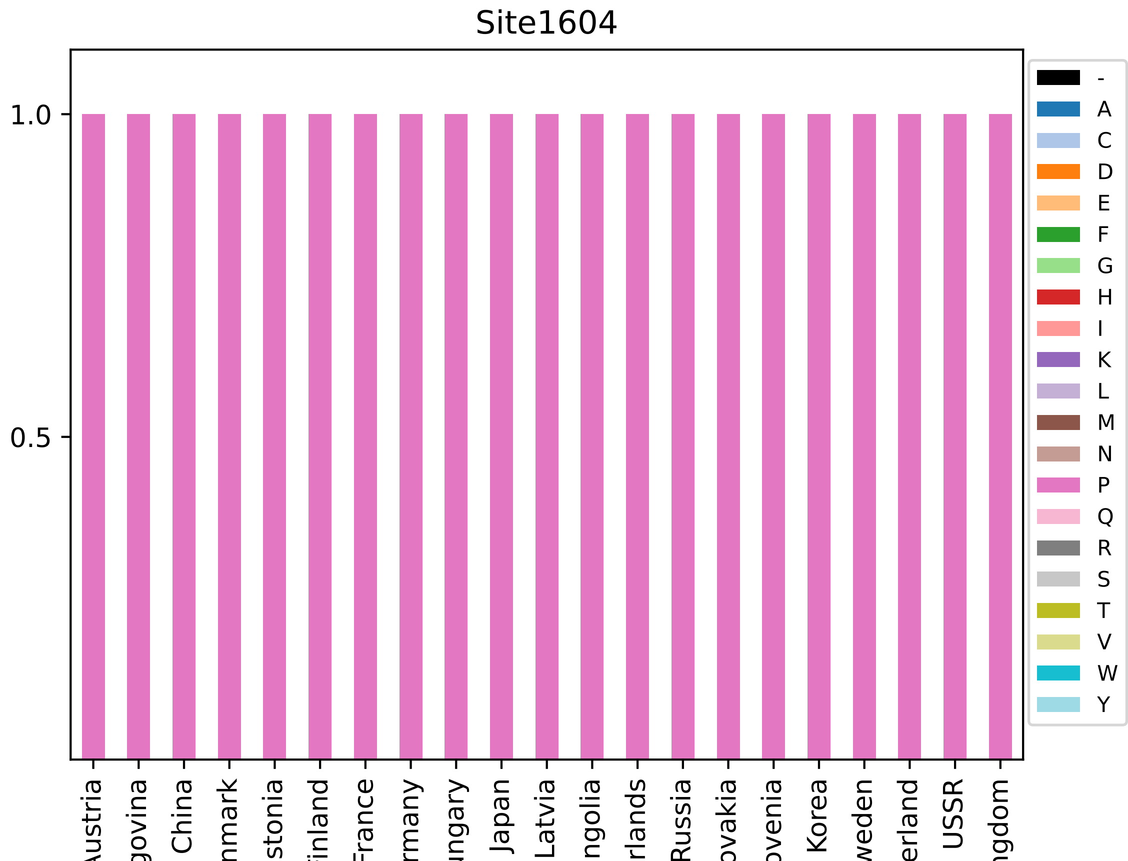

Site1604
1.0
0.5
-
A
C
D
E
F
G
H
I
K
L
M
N
P
Q
R
S
T
V
W
Y
China
Japan
Latvia
Russia
Korea
USSR

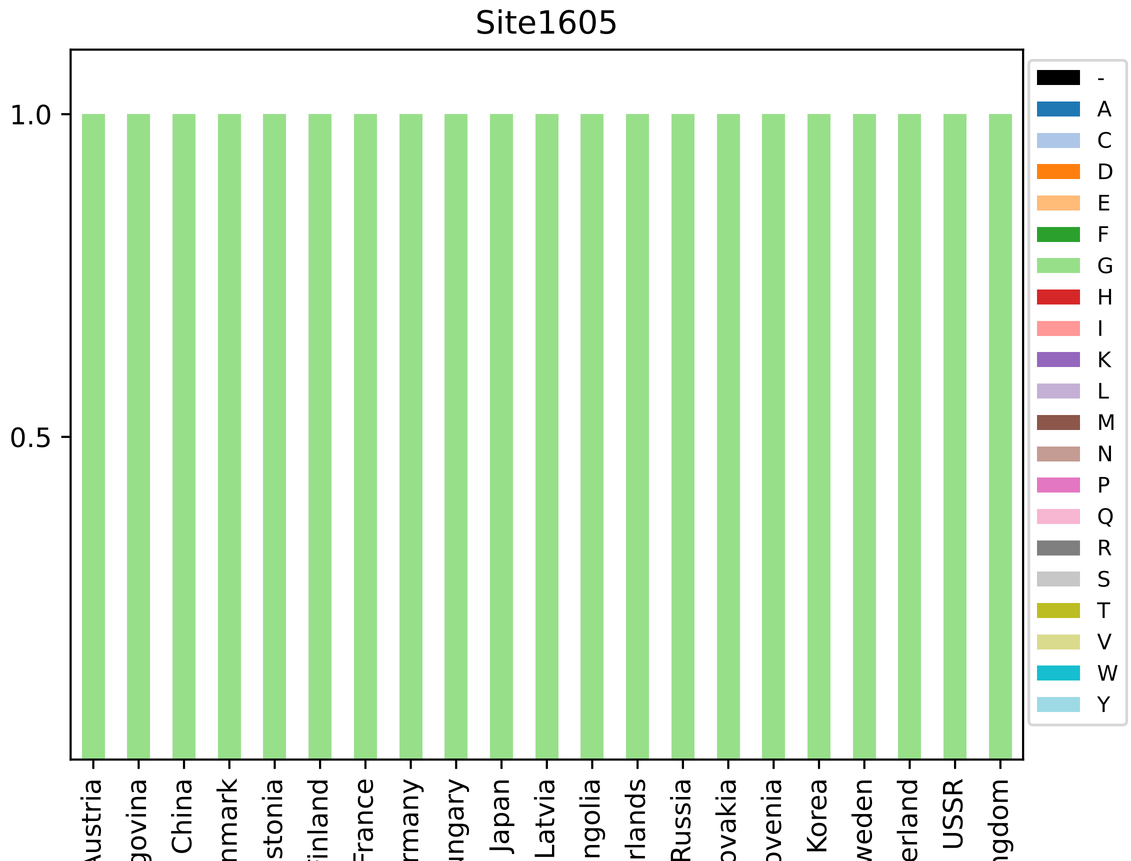
Site1605
1.0
0.5
-
A
C
D
E
F
G
H
I
K
L
M
N
P
Q
R
S
T
V
W
Y
Austria
govina
China
nmark
stonia
inland
France
rmany
ungary
Japan
Latvia
ngolia
rlands
Russia
ovakia
ovenia
Korea
weden
erland
USSR
ngdom

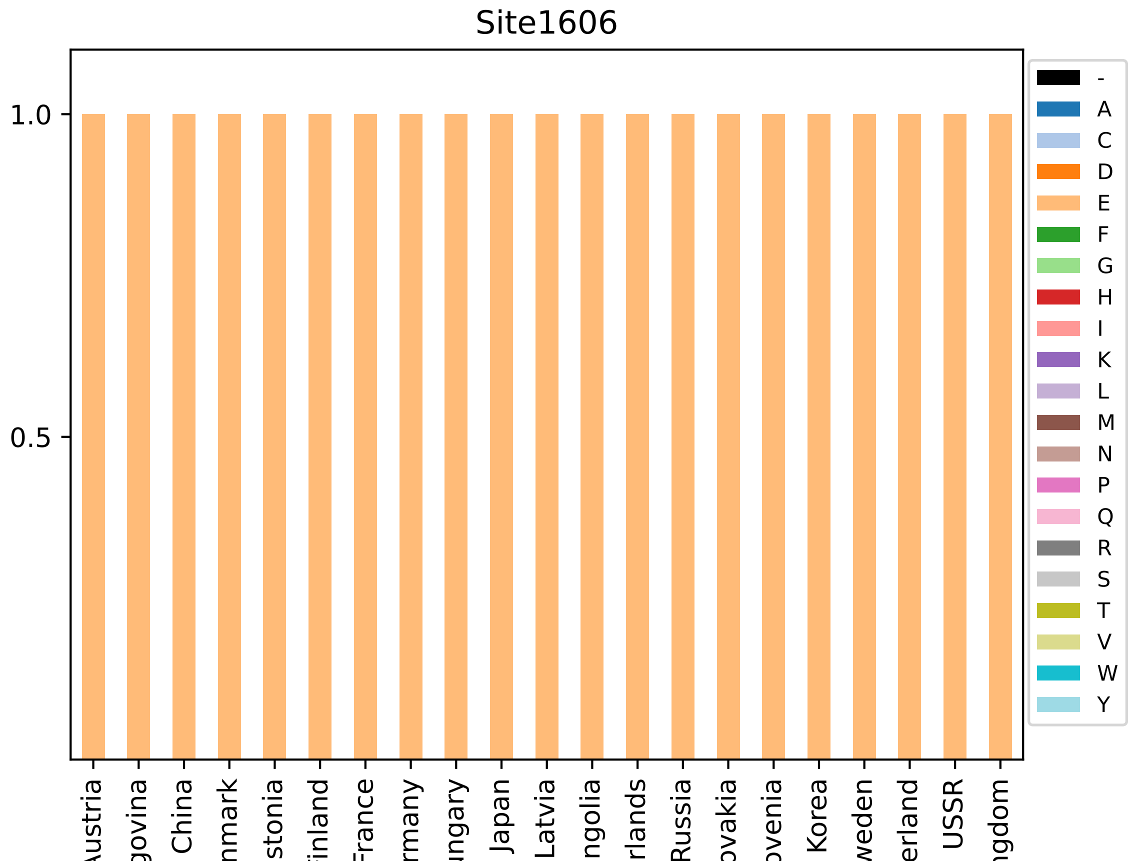

Site1606
1.0
0.5
-
A
C
D
E
F
G
H
I
K
L
M
N
P
Q
R
S
T
V
W
Y
Austria
China
Japan
Latvia
Russia
Korea
USSR

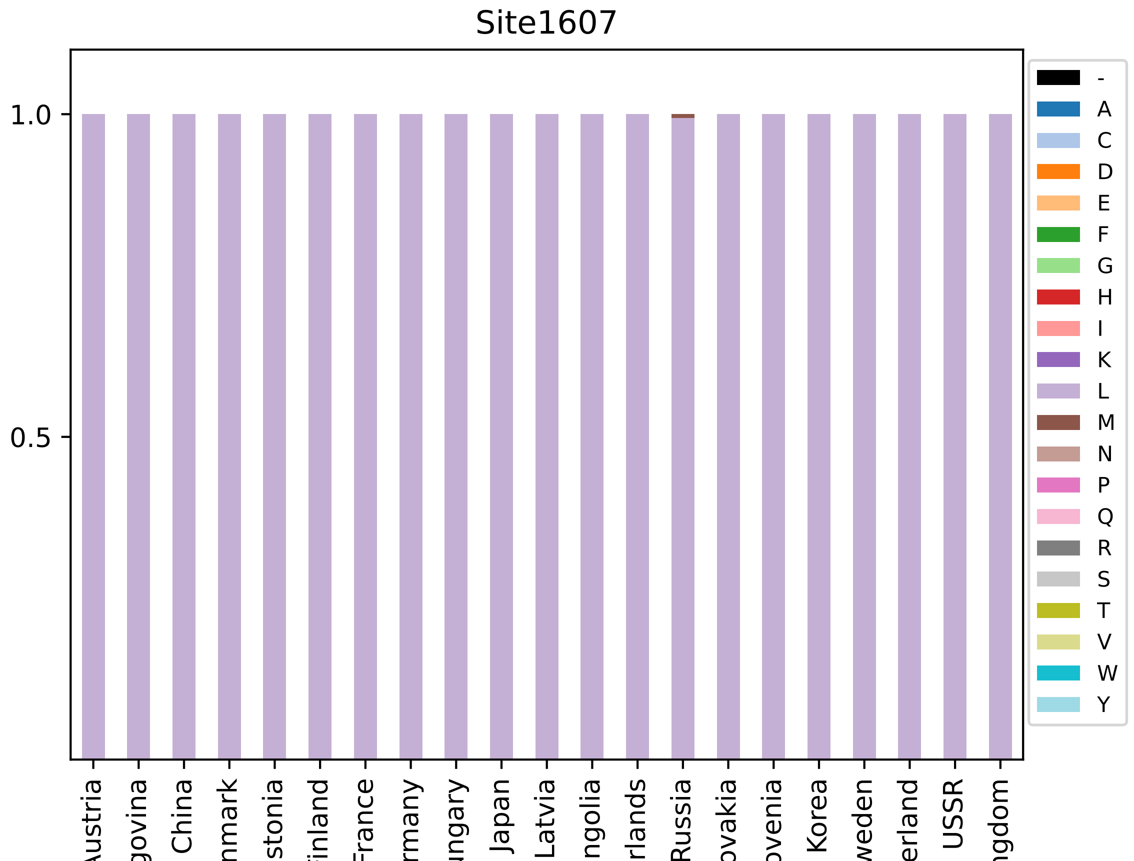
Site1607
1.0
0.5
-
A
C
D
E
F
G
H
I
K
L
M
N
P
Q
R
S
T
V
W
Y
China
Japan
Latvia
Russia
Korea
USSR

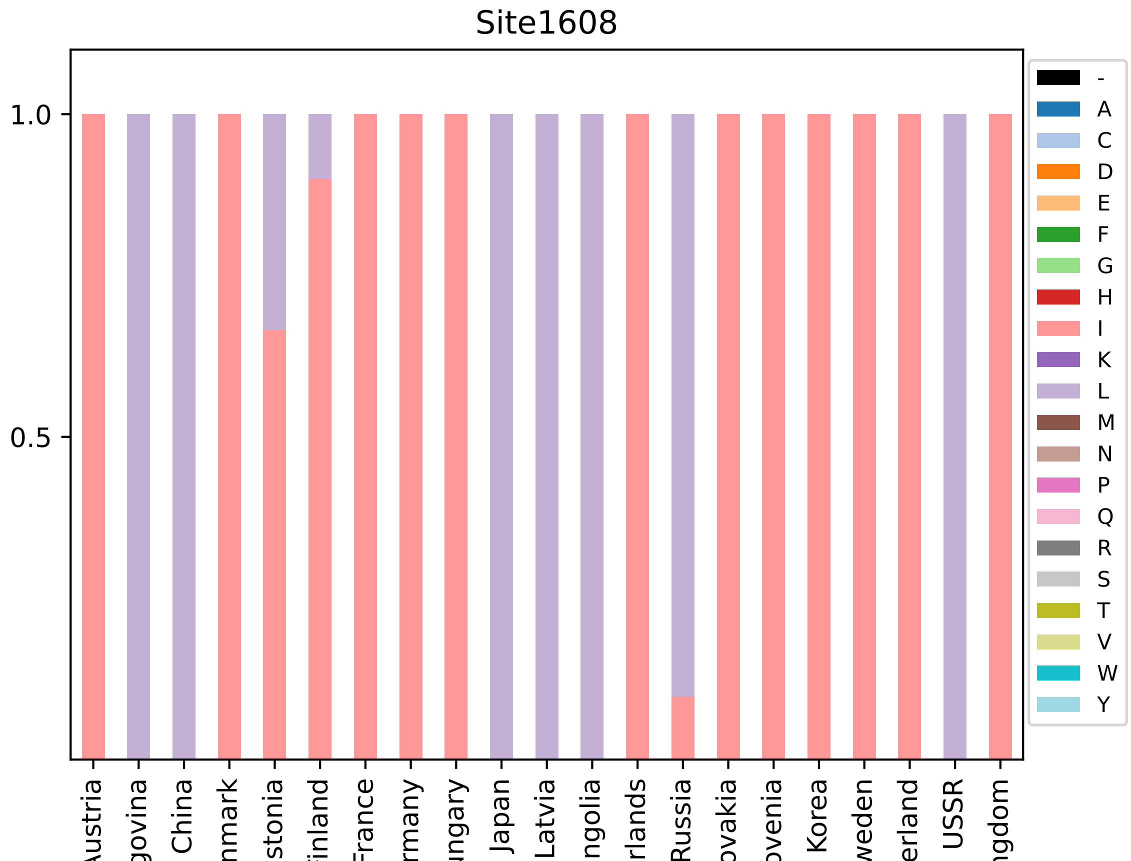

Site1608
1.0
0.5
-
A
C
D
E
F
G
H
I
K
L
M
N
P
Q
R
S
T
V
W
Y
Austria
govina
China
nmark
stonia
Finland
France
rmany
ungary
Japan
Latvia
ngolia
rlands
Russia
ovakia
ovenia
Korea
weden
erland
USSR
ngdom

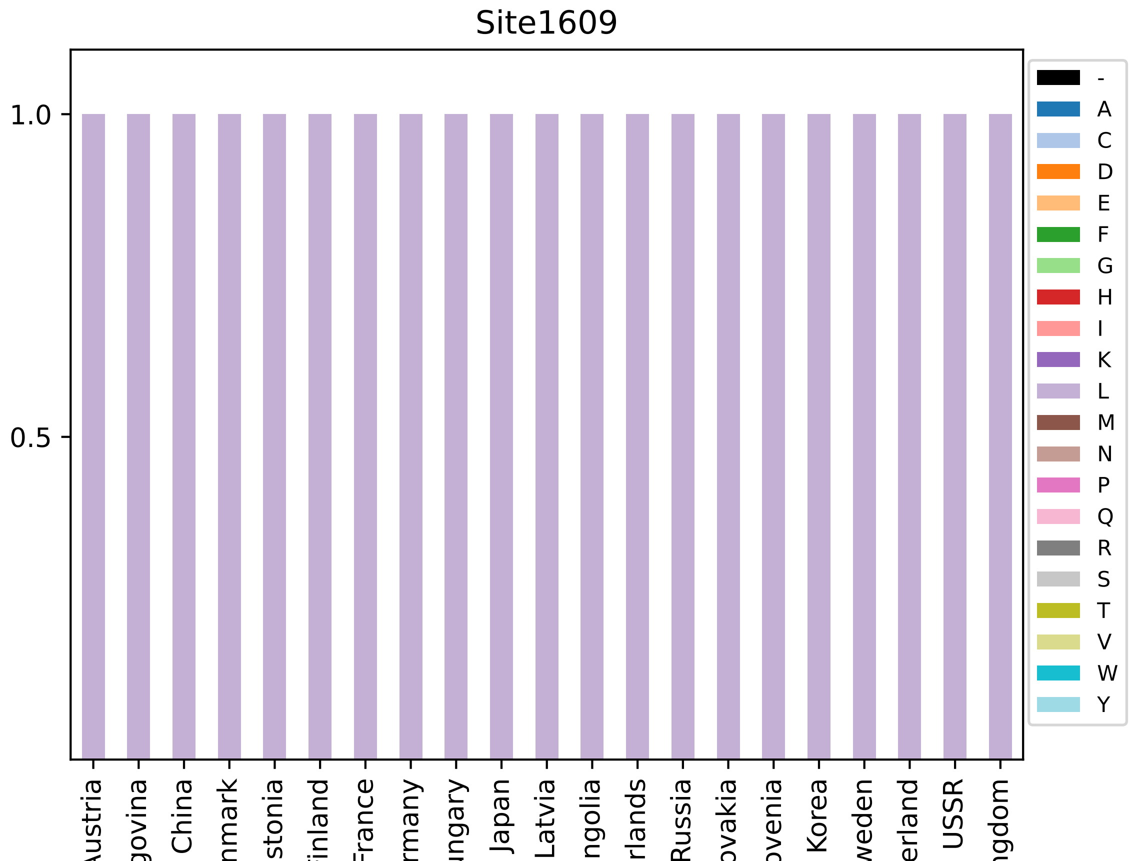
Site1609
1.0
0.5
-
A
C
D
E
F
G
H
I
K
L
M
N
P
Q
R
S
T
V
W
Y
Austria
govina
China
nmark
stonia
Finland
France
rmany
ungary
Japan
Latvia
ngolia
rlands
Russia
ovakia
ovenia
Korea
weden
erland
USSR
ngdom

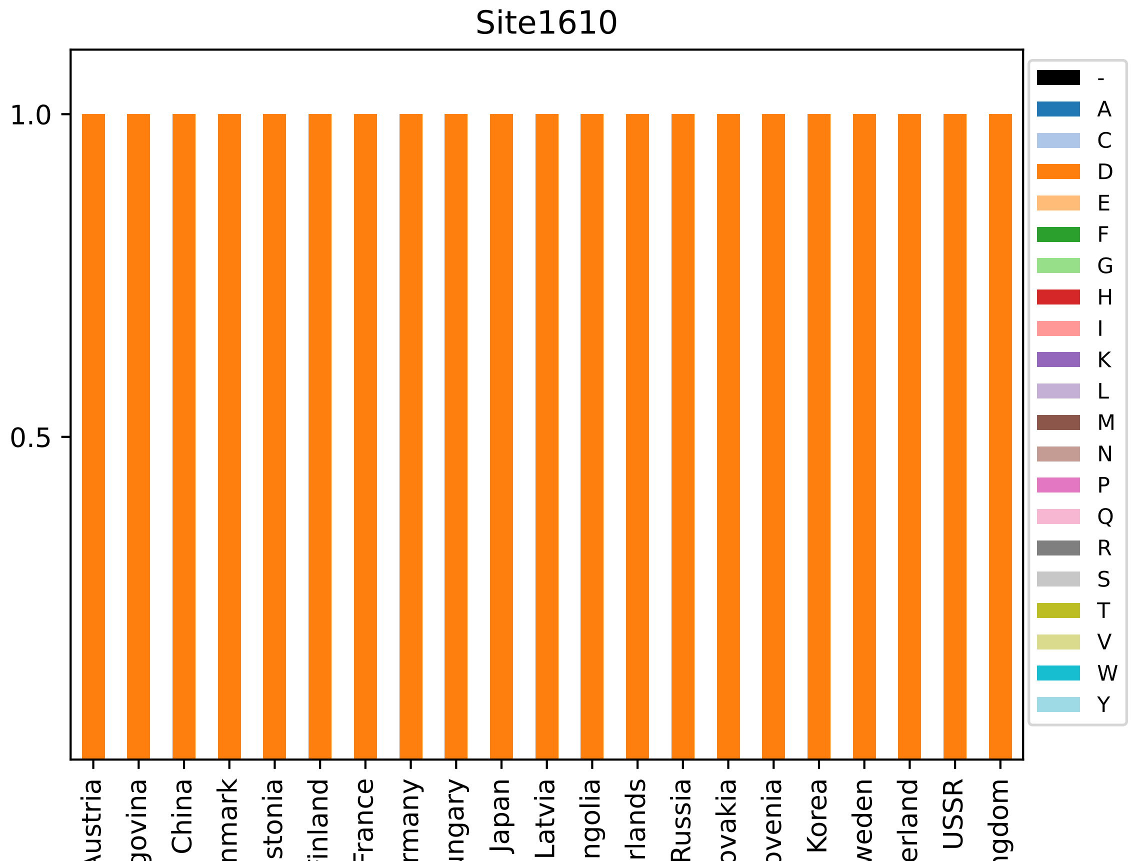

Site1610
1.0
0.5
-
A
C
D
E
F
G
H
I
K
L
M
N
P
Q
R
S
T
V
W
Y
Austria
govina
China
nmark
stonia
Finland
France
rmany
ungary
Japan
Latvia
ngolia
rlands
Russia
ovakia
ovenia
Korea
weden
erland
USSR
ngdom

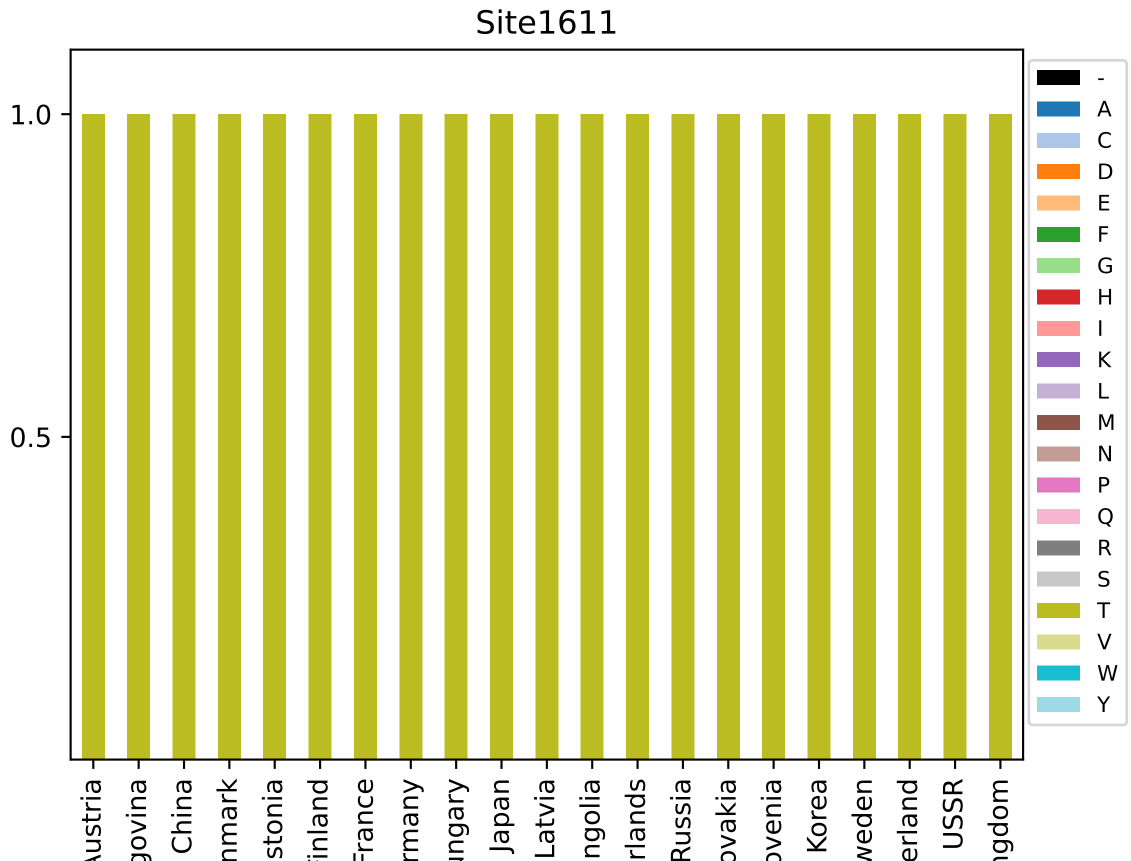

Site1611
1.0
0.5
-
A
C
D
E
F
G
H
I
K
L
M
N
P
Q
R
S
T
V
W
Y
Austria
China
Japan
Latvia
Russia
Korea
USSR

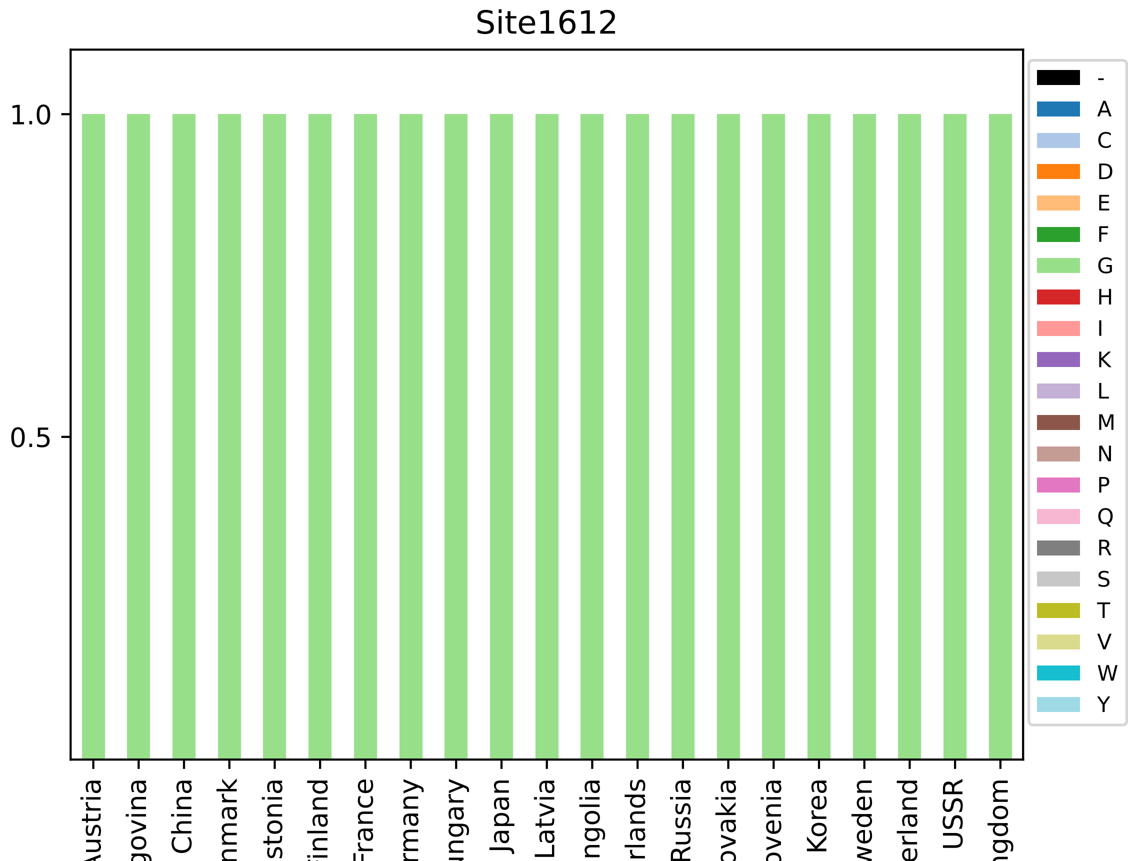
Site1612
1.0
0.5
-
A
C
D
E
F
G
H
I
K
L
M
N
P
Q
R
S
T
V
W
Y
Austria
China
Japan
Latvia
Russia
Korea
USSR

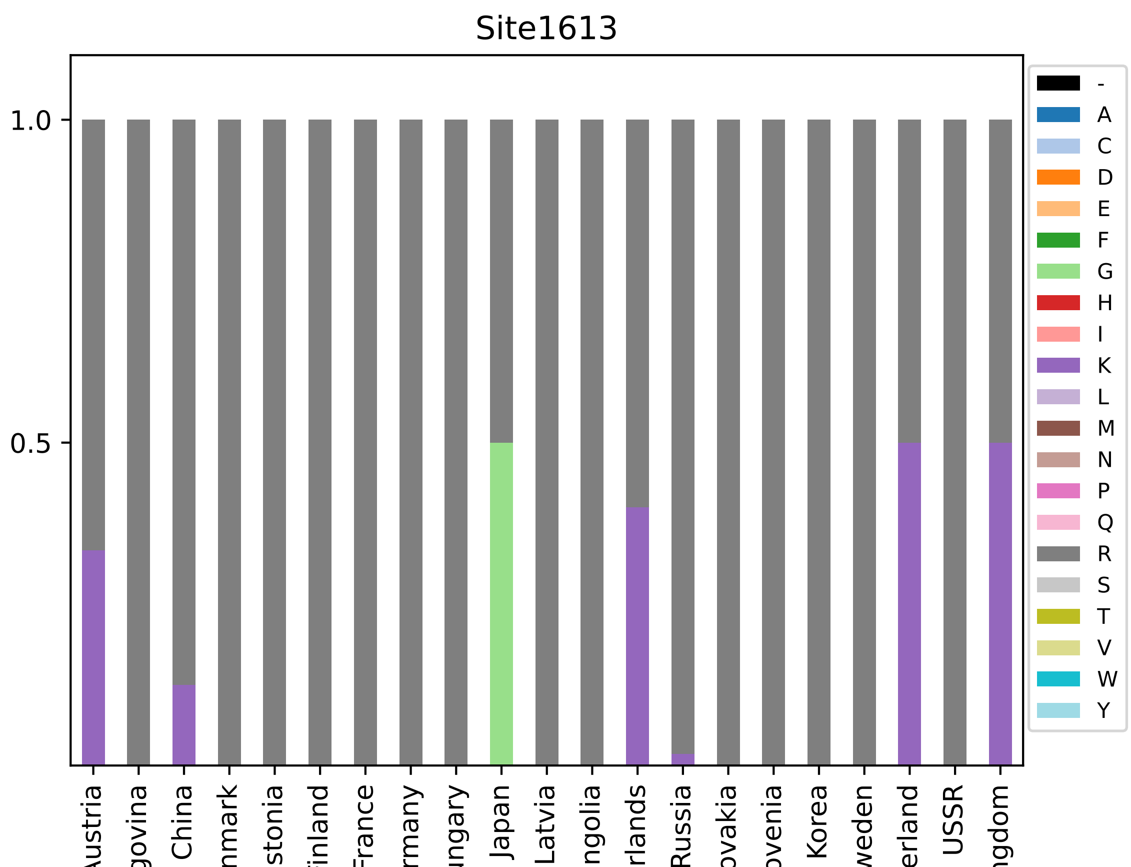

Site1613
1.0
0.5
-
A
C
D
E
F
G
H
I
K
L
M
N
P
Q
R
S
T
V
W
Y
Austria
govina
China
nmark
stonia
inland
France
rmany
ungary
Japan
Latvia
ngolia
rlands
Russia
ovakia
ovenia
Korea
weden
erland
USSR
ngdom

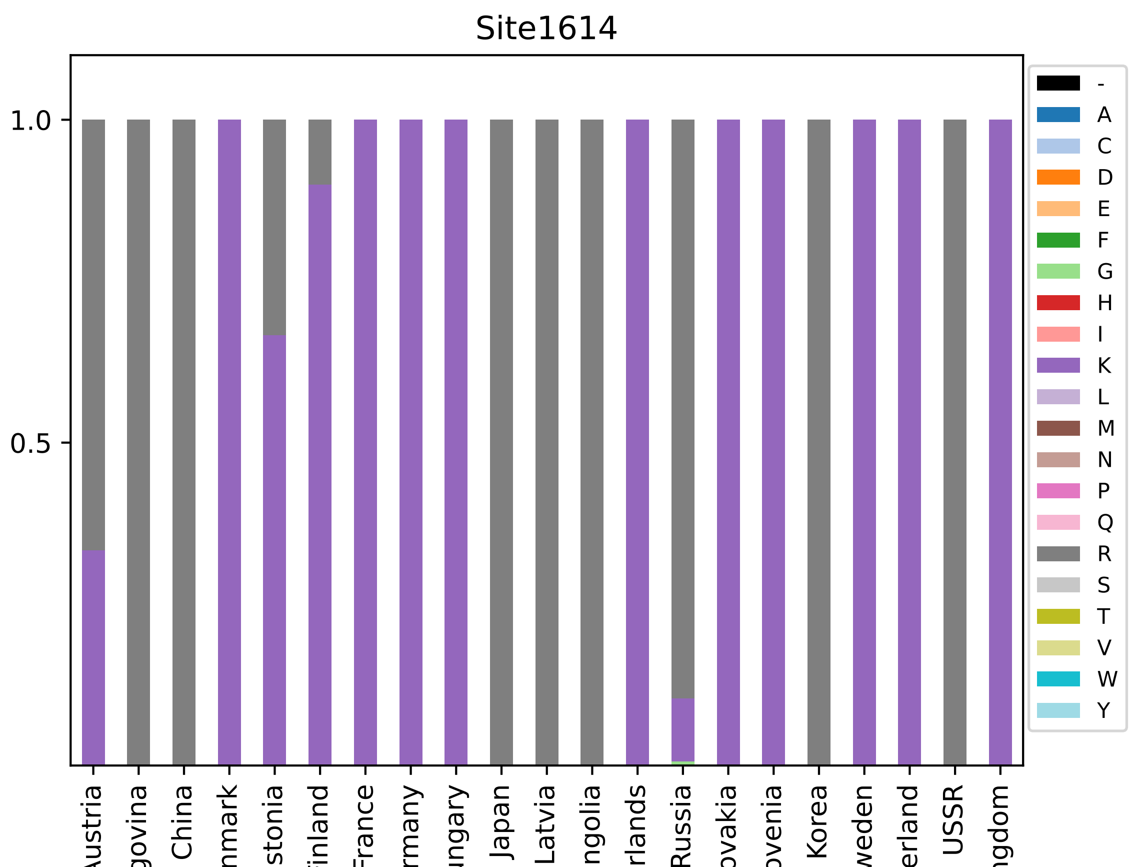

Site1614
1.0
0.5
-
A
C
D
E
F
G
H
I
K
L
M
N
P
Q
R
S
T
V
W
Y
Austria
govina
China
nmark
stonia
Finland
France
rmany
ungary
Japan
Latvia
ngolia
rlands
Russia
ovakia
ovenia
Korea
weden
erland
USSR
ngdom

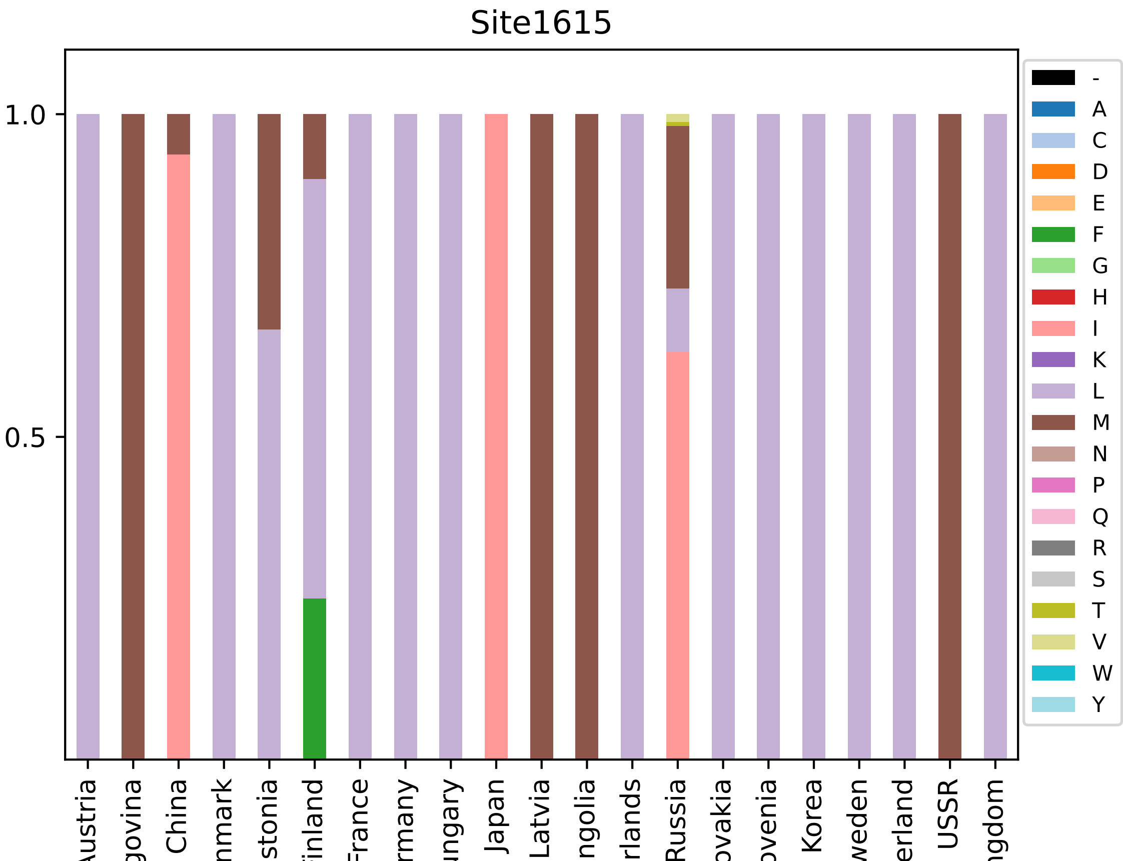

Site1615
1.0
0.5
-
A
C
D
E
F
G
H
I
K
L
M
N
P
Q
R
S
T
V
W
Y
Austria
govina
China
nmark
stonia
Finland
France
rmany
ungary
Japan
Latvia
ngolia
rlands
Russia
ovakia
ovenia
Korea
weden
erland
USSR
ngdom

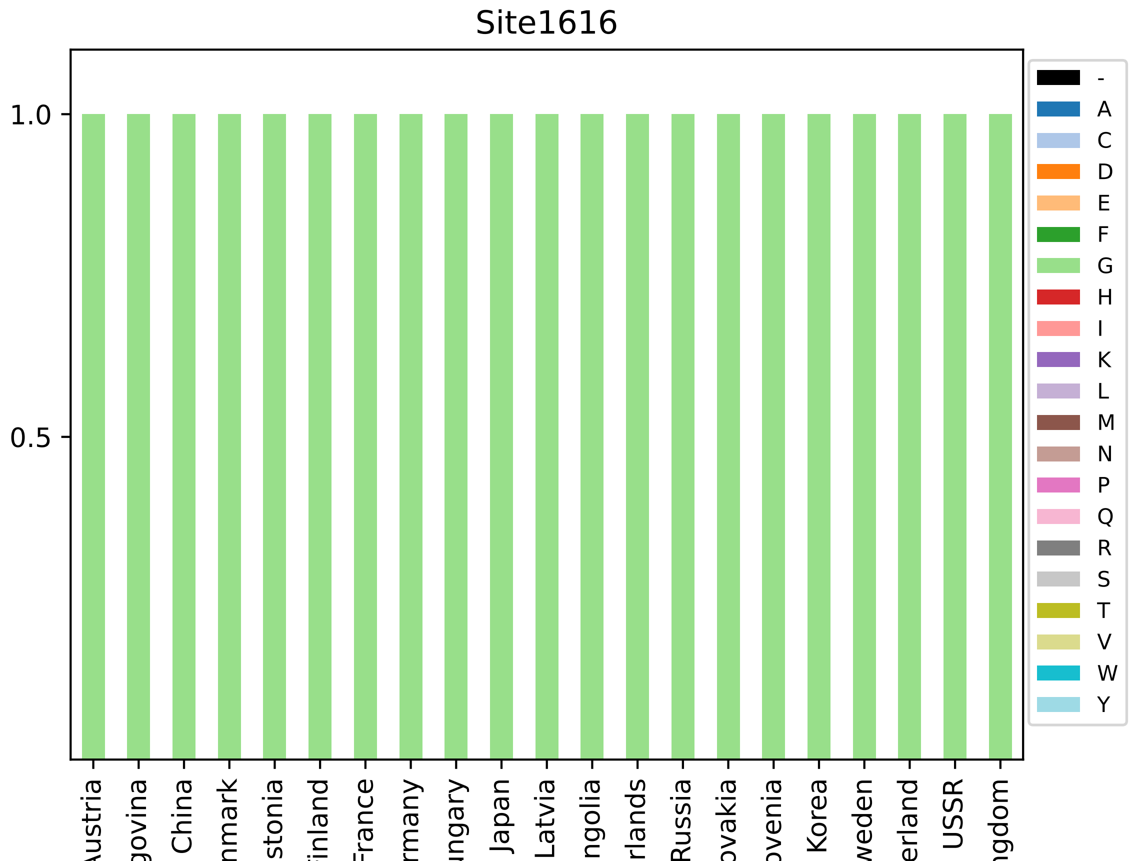
Site1616
1.0
0.5
-
A
C
D
E
F
G
H
I
K
L
M
N
P
Q
R
S
T
V
W
Y
Austria
govina
China
nmark
stonia
Finland
France
rmany
ungary
Japan
Latvia
ngolia
rlands
Russia
ovakia
ovenia
Korea
weden
erland
USSR
ngdom

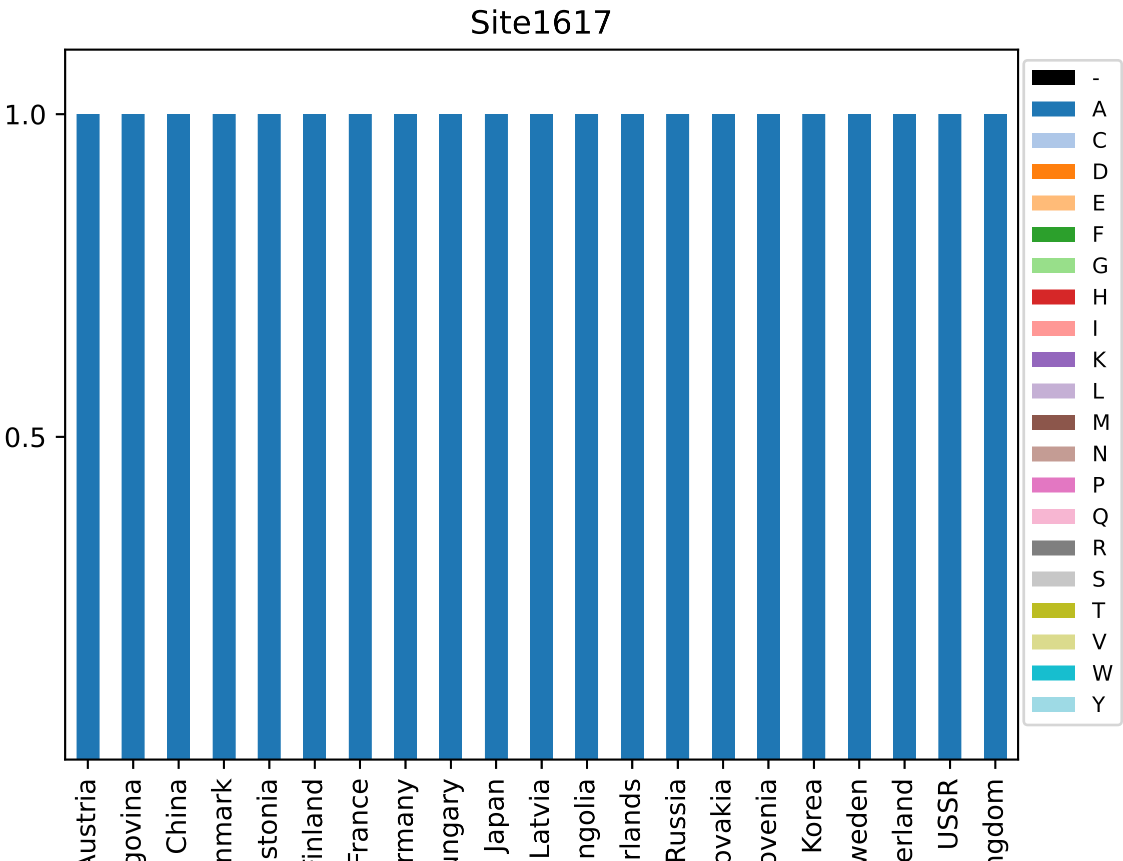
Site1617
1.0
0.5
-
A
C
D
E
F
G
H
I
K
L
M
N
P
Q
R
S
T
V
W
Y
China
Japan
Latvia
Russia
Korea
USSR

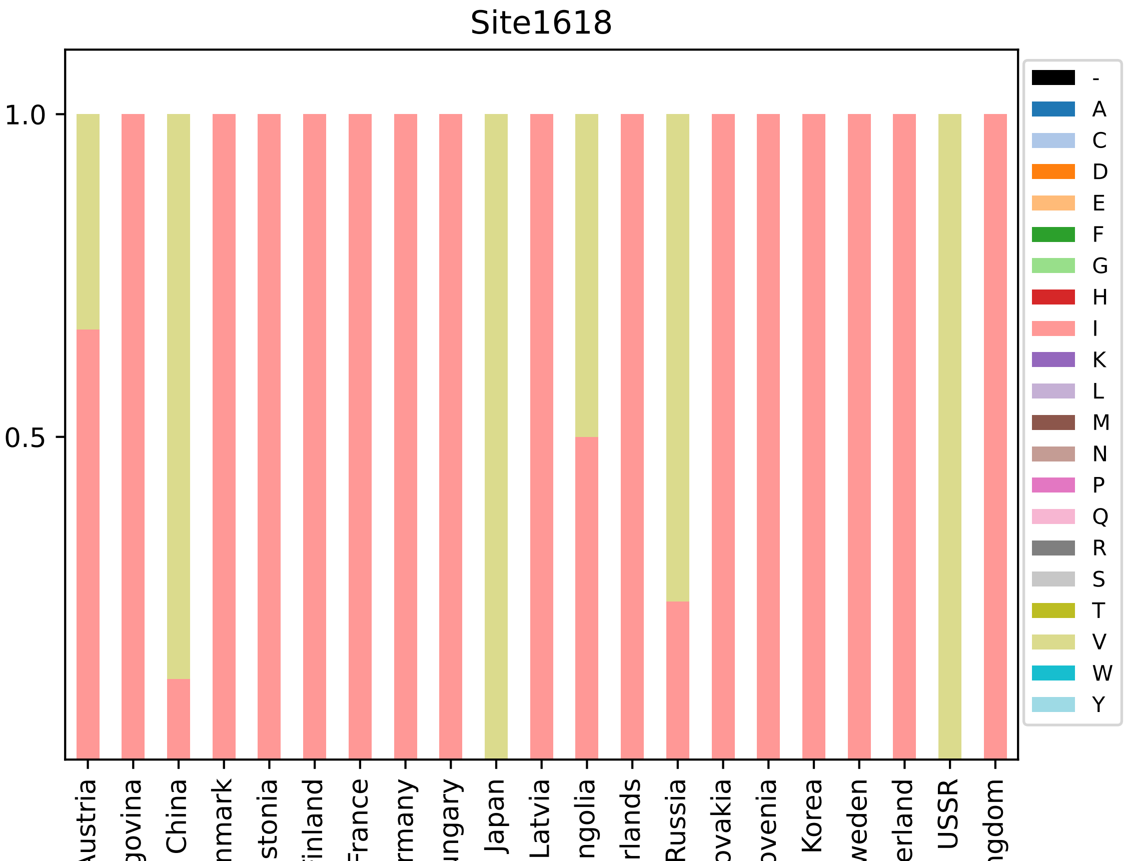

Site1618
1.0
0.5
-
A
C
D
E
F
G
H
I
K
L
M
N
P
Q
R
S
T
V
W
Y
Austria
govina
China
nmark
stonia
inland
France
rmany
ungary
Japan
Latvia
ngolia
rlands
Russia
ovakia
ovenia
Korea
weden
erland
USSR
ngdom

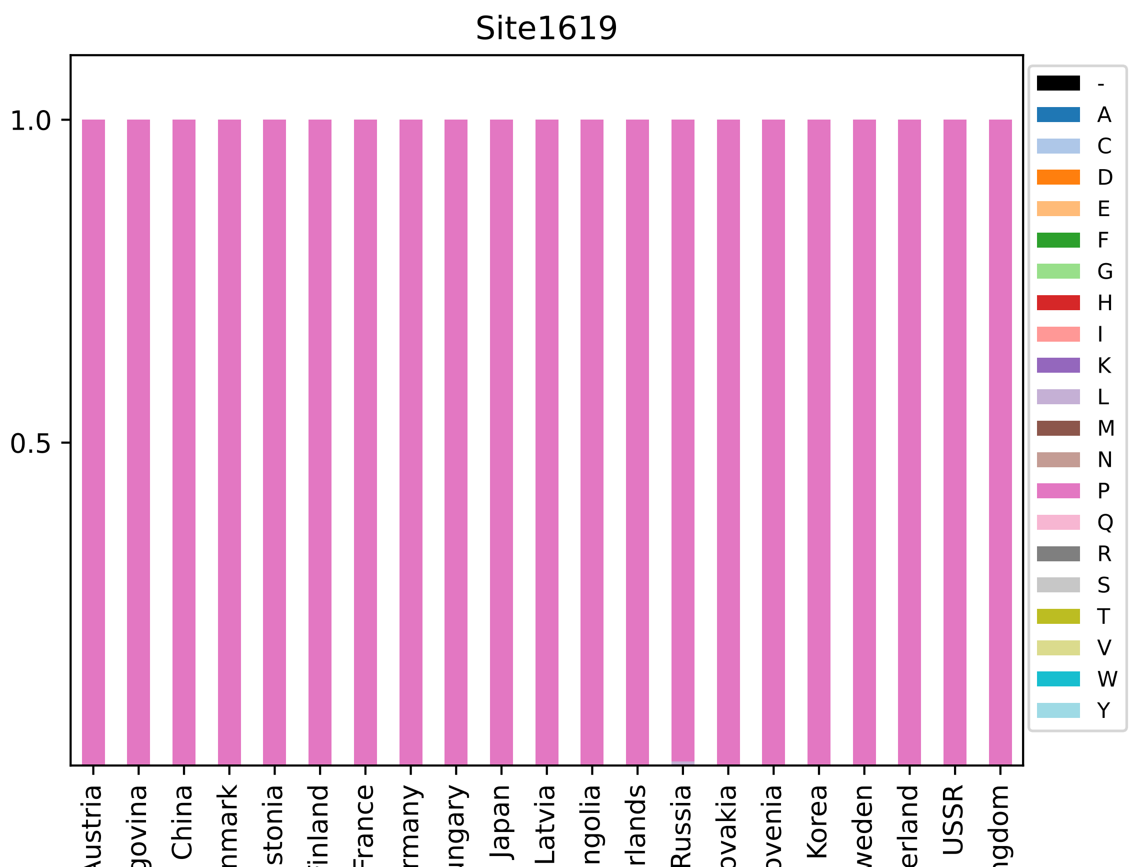

Site1619
1.0
0.5
-
A
C
D
E
F
G
H
I
K
L
M
N
P
Q
R
S
T
V
W
Y
China
Japan
Latvia
Russia
Korea
USSR

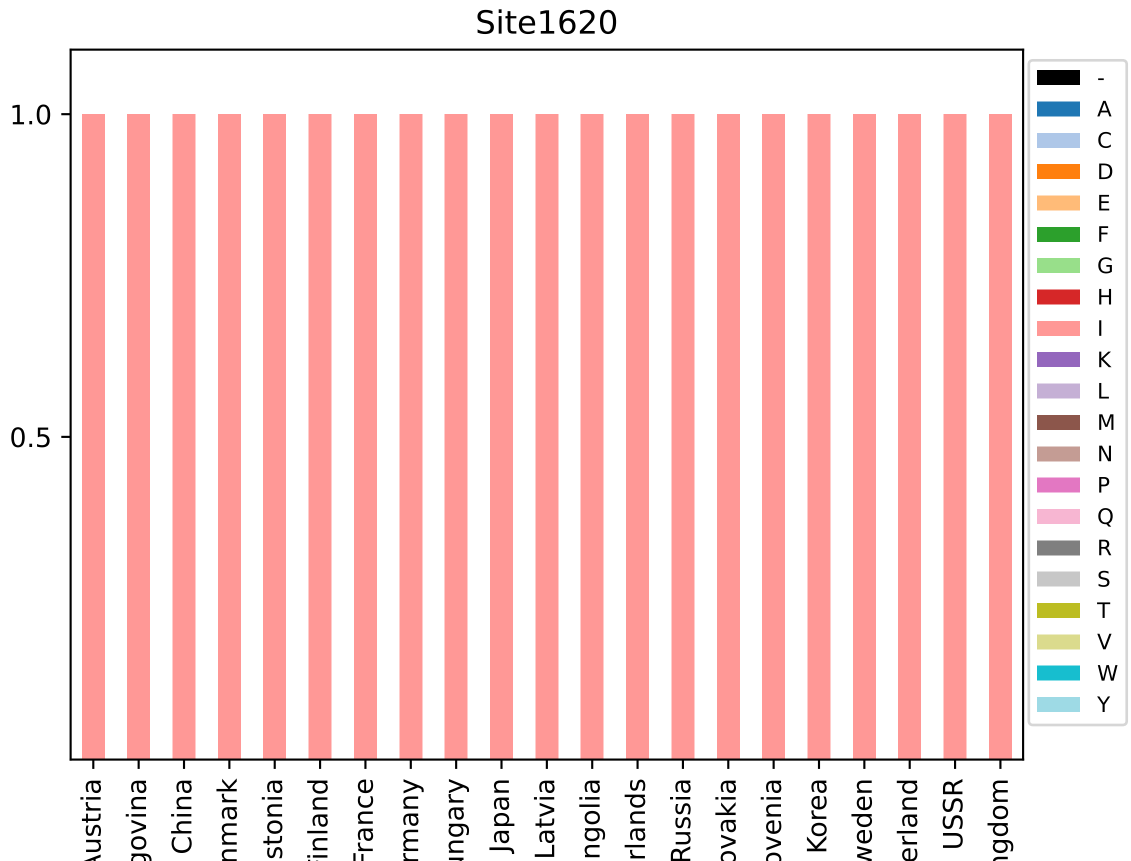
Site1620
1.0
0.5
-
A
C
D
E
F
G
H
I
K
L
M
N
P
Q
R
S
T
V
W
Y
Austria
China
Japan
Latvia
Russia
Korea
USSR

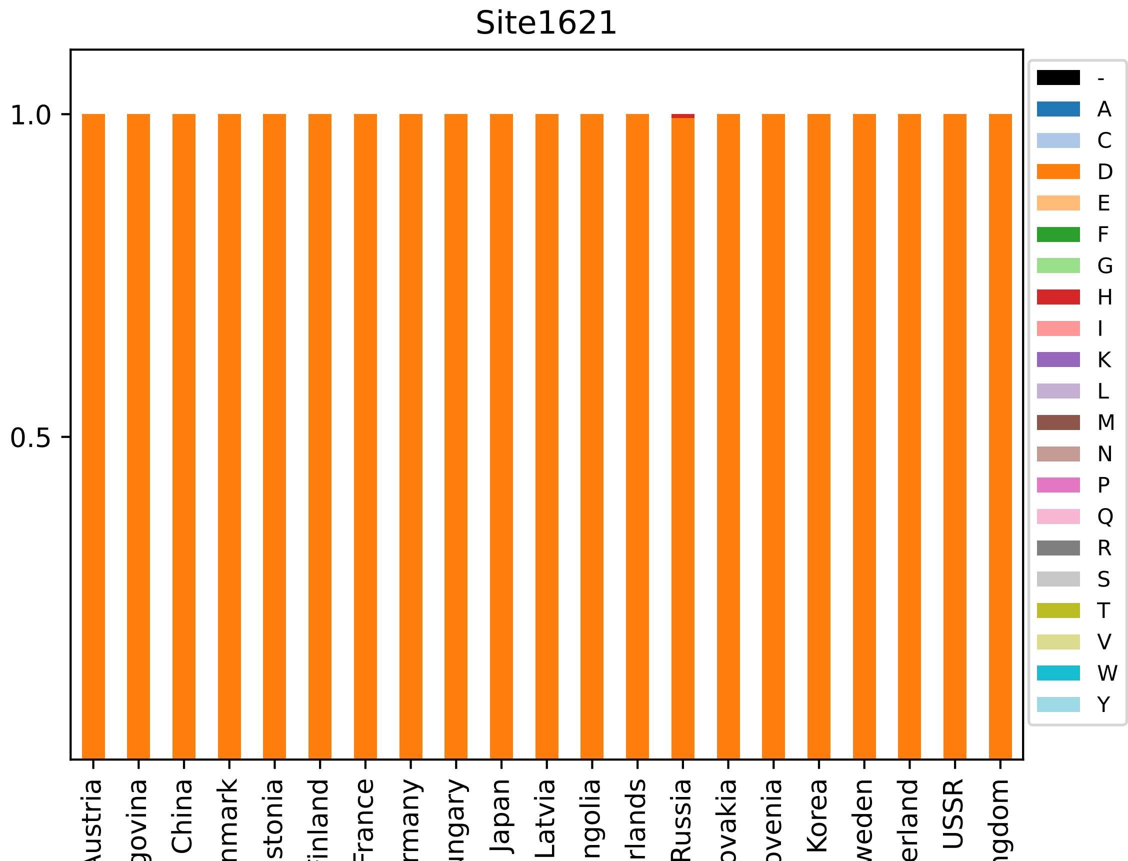

Site1621
1.0
0.5
-
A
C
D
E
F
G
H
I
K
L
M
N
P
Q
R
S
T
V
W
Y
Austria
govina
China
nmark
stonia
inland
France
rmany
ungary
Japan
Latvia
ngolia
rlands
Russia
ovakia
ovenia
Korea
weden
erland
USSR
ngdom

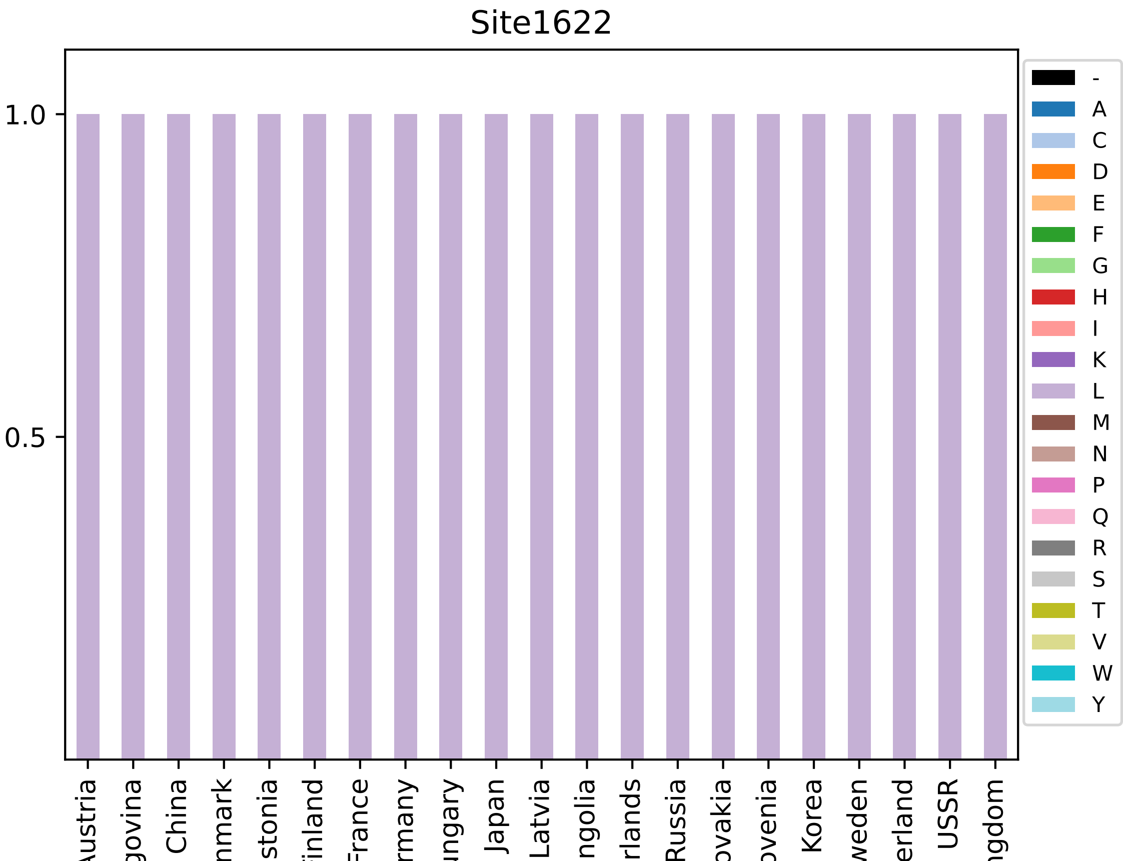
Site1622
1.0
0.5
-
A
C
D
E
F
G
H
I
K
L
M
N
P
Q
R
S
T
V
W
Y
China
Japan
Latvia
Russia
Korea
USSR

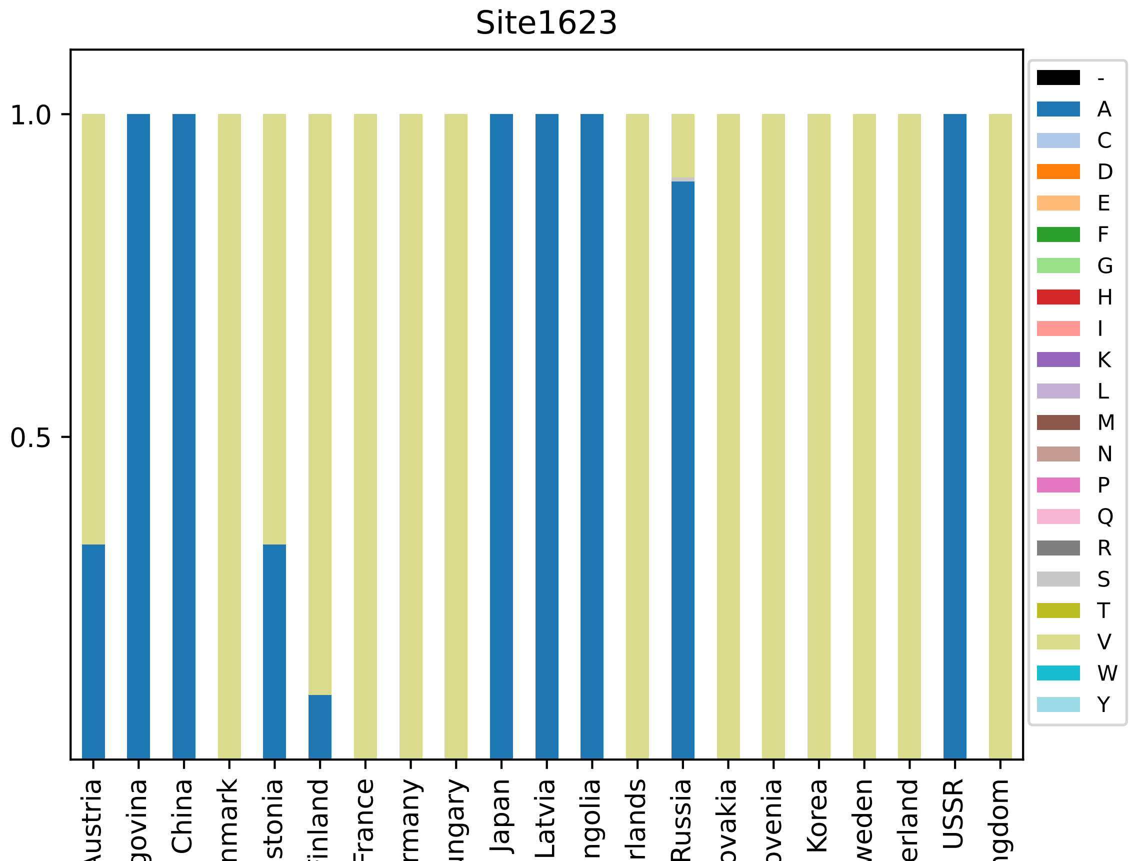

Site1623
1.0
0.5
Austria
govina
China
nmark
stonia
inland
France
rmany
ungary
Japan
Latvia
ngolia
rlands
Russia
ovakia
ovenia
Korea
weden
erland
USSR
ngdom
-
A
C
D
E
F
G
H
I
K
L
M
N
P
Q
R
S
T
V
W
Y

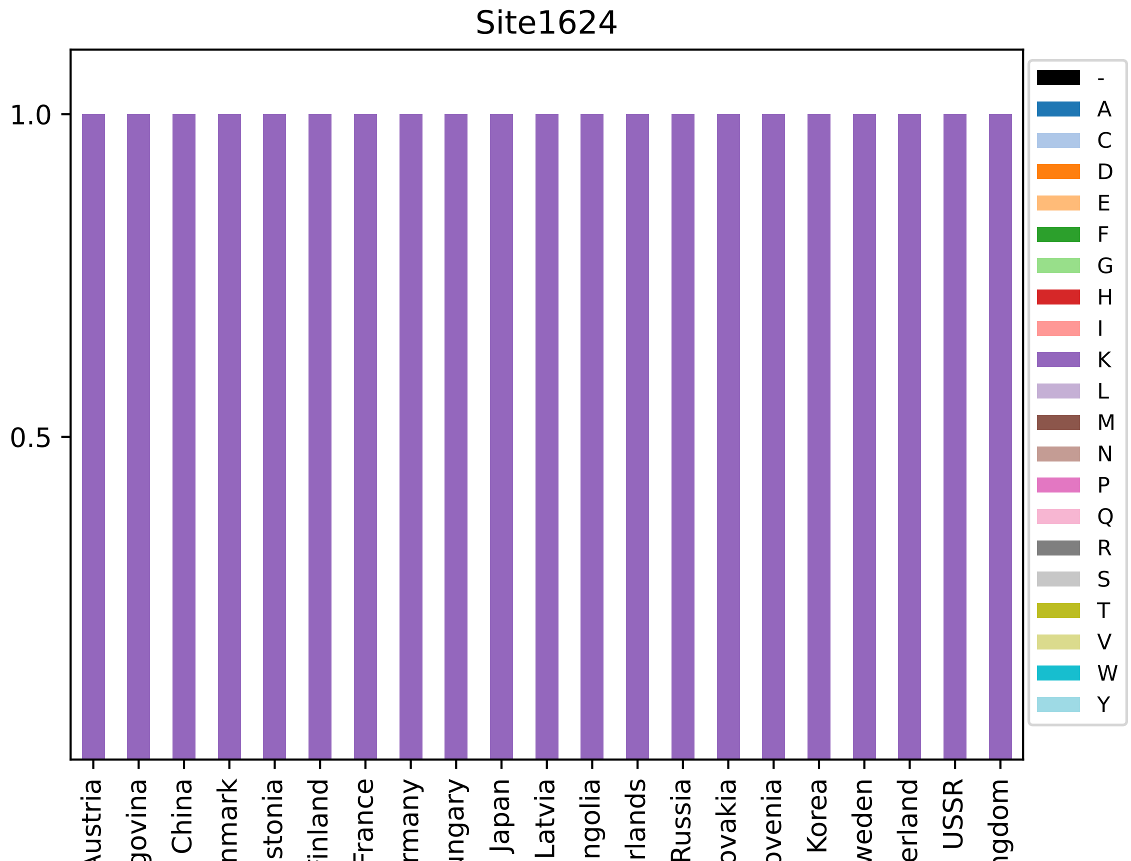

Site1624
1.0
0.5
-
A
C
D
E
F
G
H
I
K
L
M
N
P
Q
R
S
T
V
W
Y
Austria
govina
China
nmark
stonia
inland
France
rmany
ungary
Japan
Latvia
ngolia
rlands
Russia
ovakia
ovenia
Korea
weden
erland
USSR
ngdom

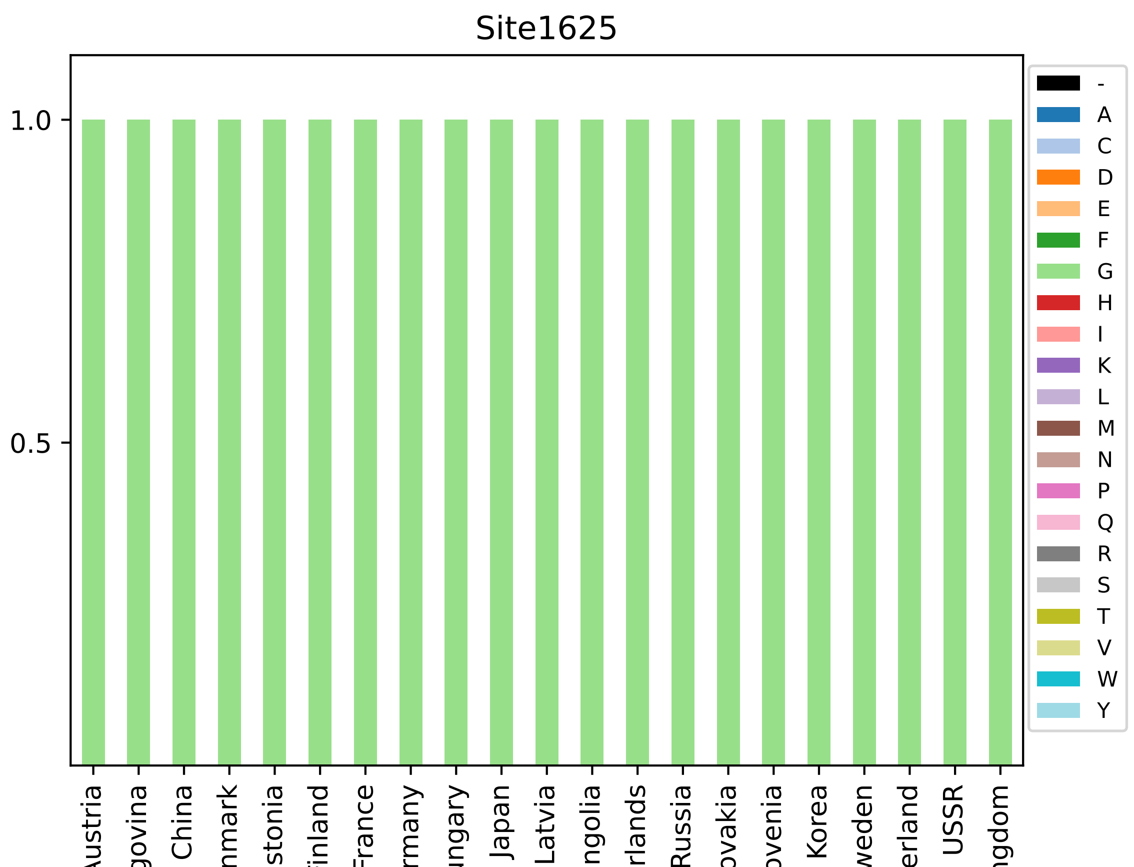
Site1625
1.0
0.5
-
A
C
D
E
F
G
H
I
K
L
M
N
P
Q
R
S
T
V
W
Y
China
Japan
Latvia
Russia
Korea
USSR

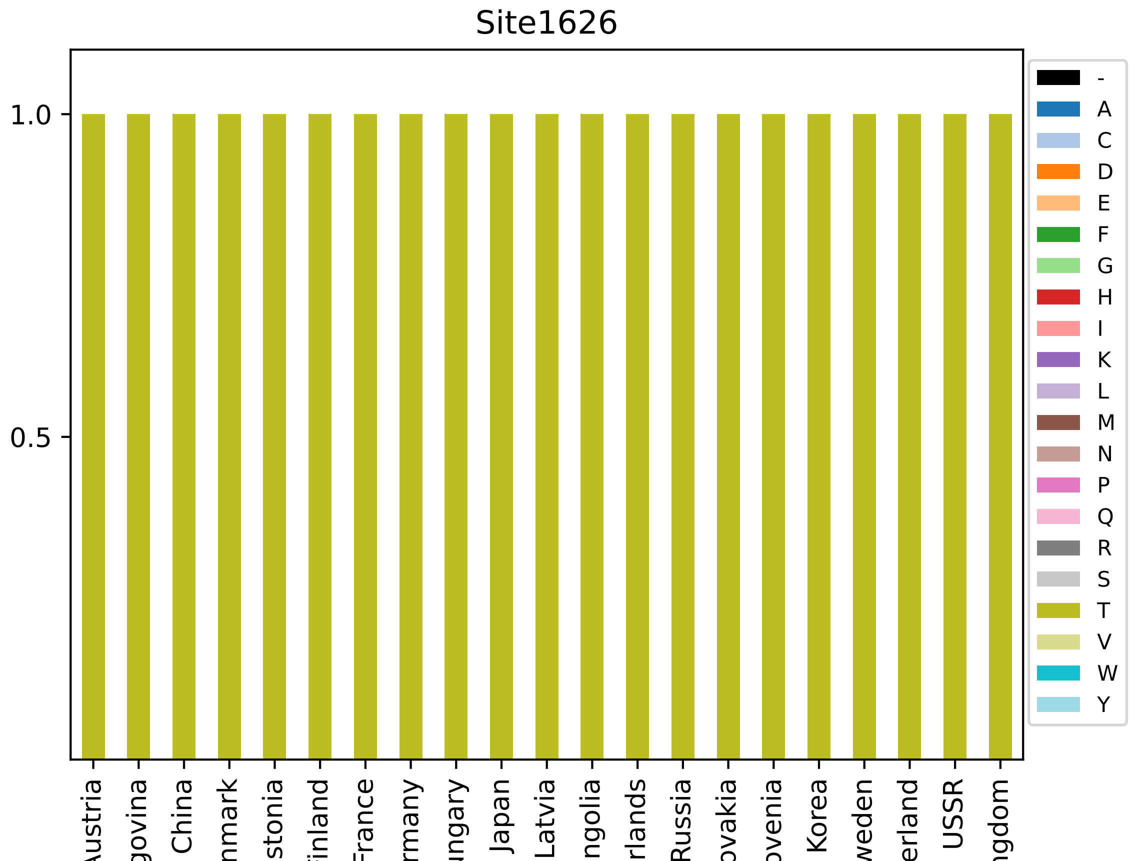

Site1626
1.0
0.5
-
A
C
D
E
F
G
H
I
K
L
M
N
P
Q
R
S
T
V
W
Y
China
Japan
Latvia
Russia
Korea
USSR

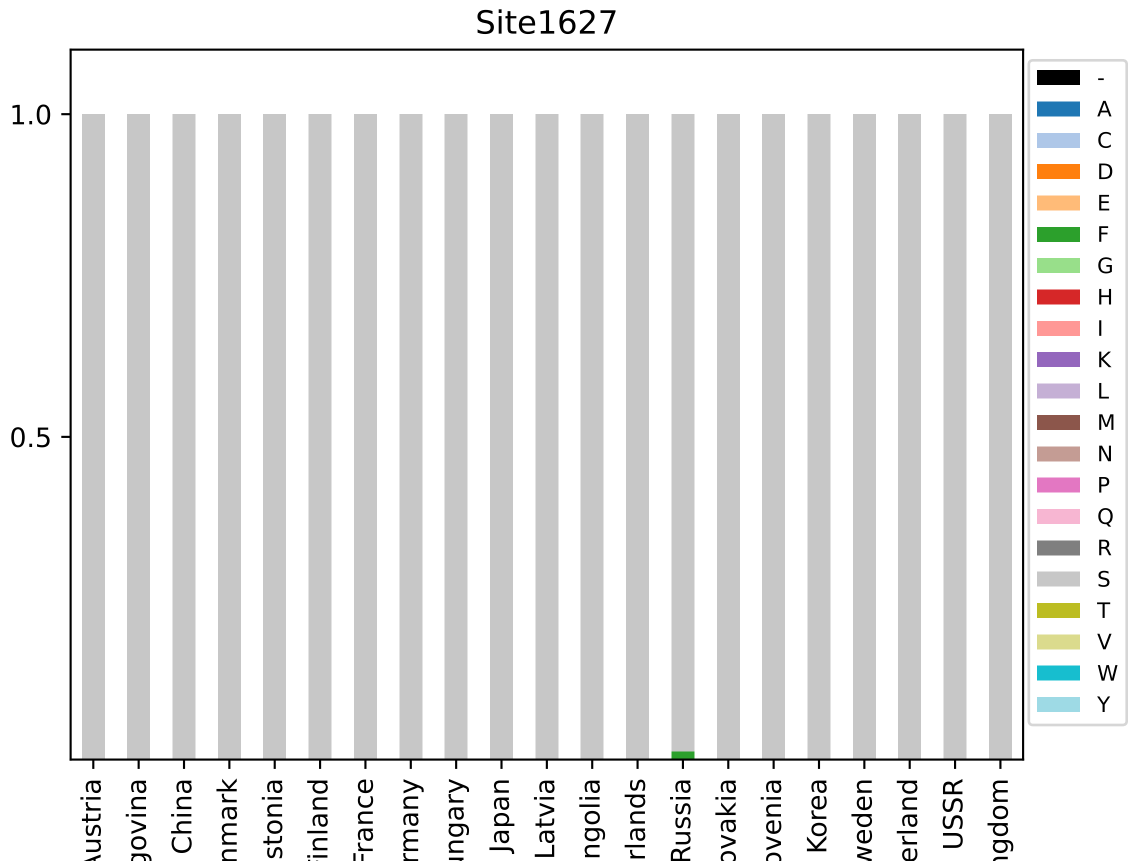

Site1627
1.0
0.5
-
A
C
D
E
F
G
H
I
K
L
M
N
P
Q
R
S
T
V
W
Y
Austria
govina
China
nmark
stonia
Finland
France
rmany
ungary
Japan
Latvia
ngolia
rlands
Russia
ovakia
ovenia
Korea
weden
erland
USSR
ngdom

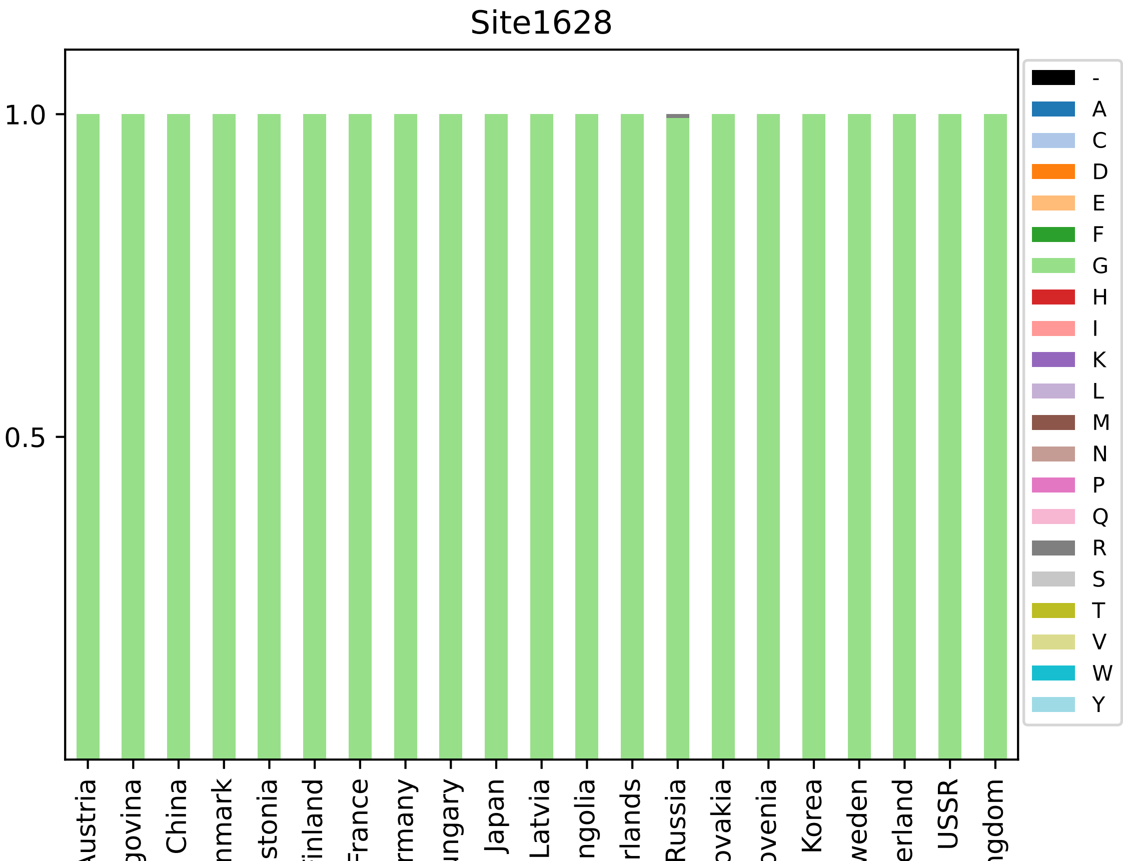

Site1628
1.0
0.5
-
A
C
D
E
F
G
H
I
K
L
M
N
P
Q
R
S
T
V
W
Y
China
Japan
Latvia
Russia
Korea
USSR

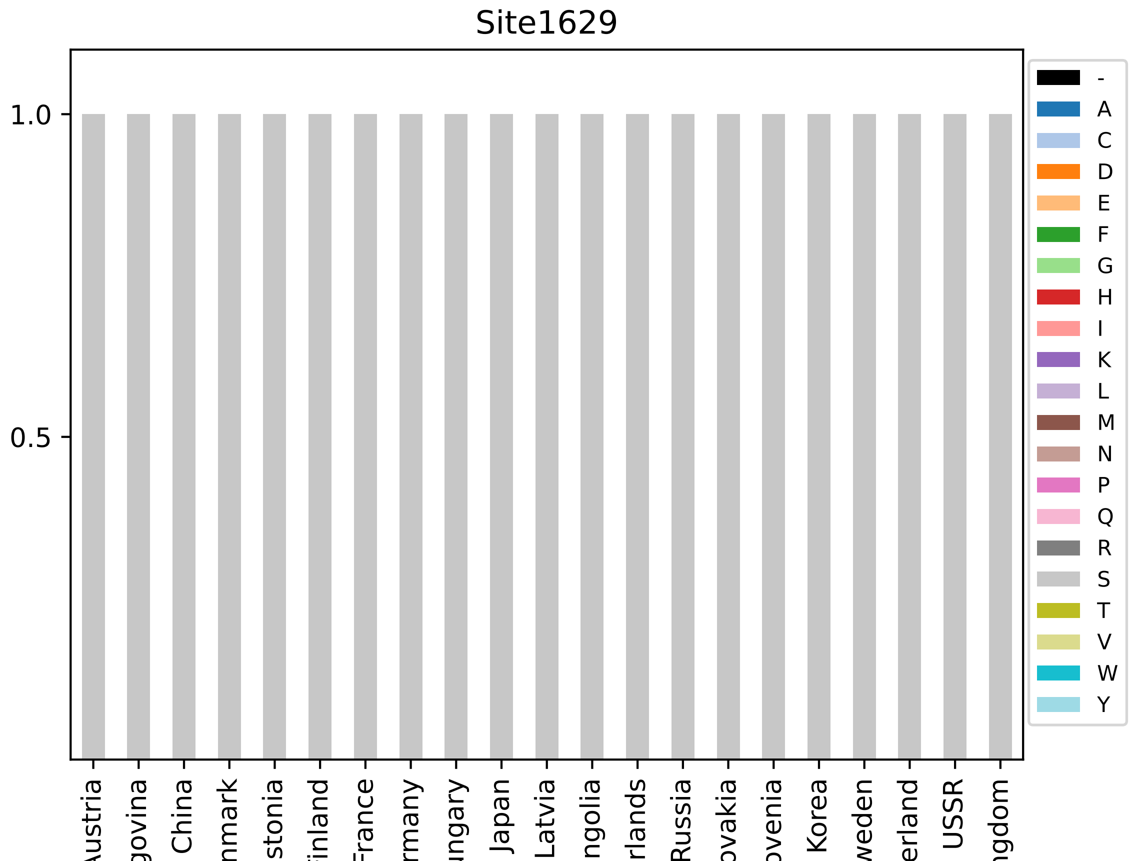

Site1629
1.0
0.5
-
A
C
D
E
F
G
H
I
K
L
M
N
P
Q
R
S
T
V
W
Y
China
Japan
Latvia
Russia
Korea
USSR

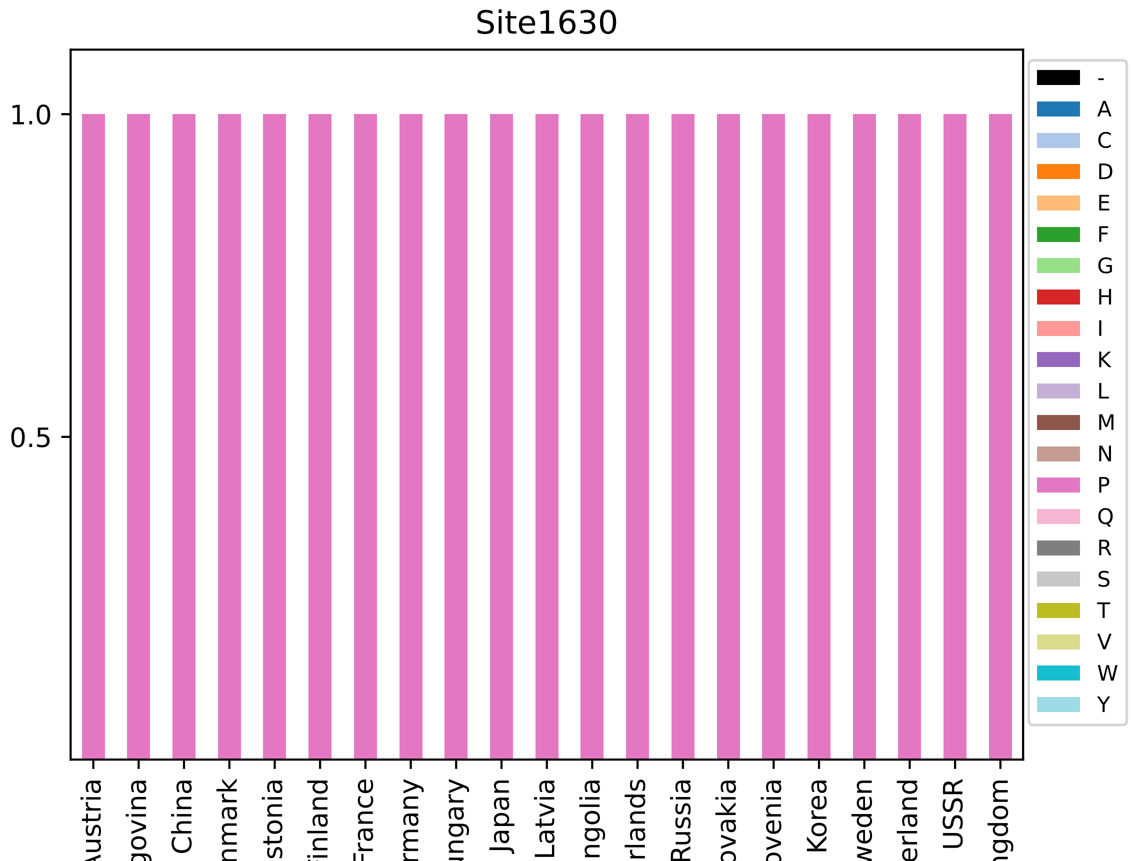
Site1630
1.0
0.5
-
A
C
D
E
F
G
H
I
K
L
M
N
P
Q
R
S
T
V
W
Y
China
Japan
Latvia
Russia
Korea
USSR

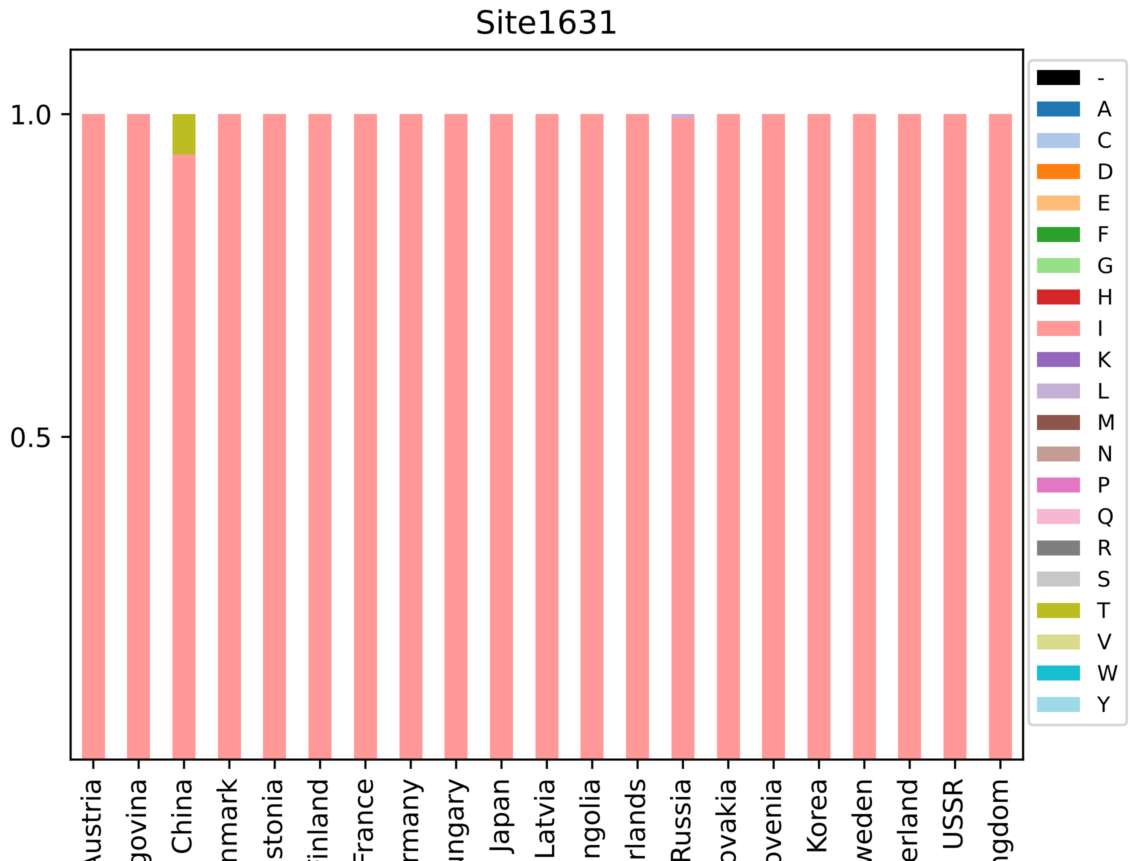

Site1631
1.0
0.5
-
A
C
D
E
F
G
H
I
K
L
M
N
P
Q
R
S
T
V
W
Y
Austria
govina
China
nmark
stonia
inland
France
rmany
ungary
Japan
Latvia
ngolia
rlands
Russia
ovakia
ovenia
Korea
weden
erland
USSR
ngdom

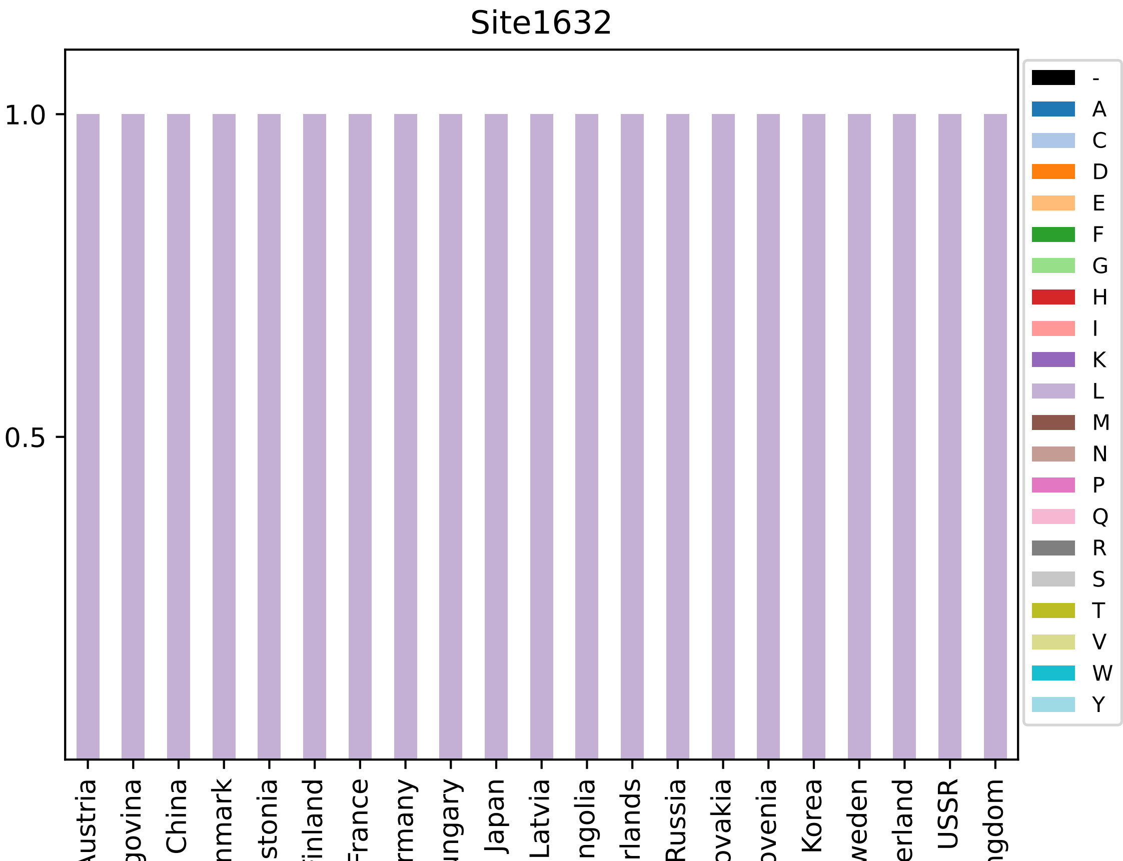

Site1632
1.0
0.5
-
A
C
D
E
F
G
H
I
K
L
M
N
P
Q
R
S
T
V
W
Y
Austria
China
Japan
Latvia
Russia
Korea
USSR

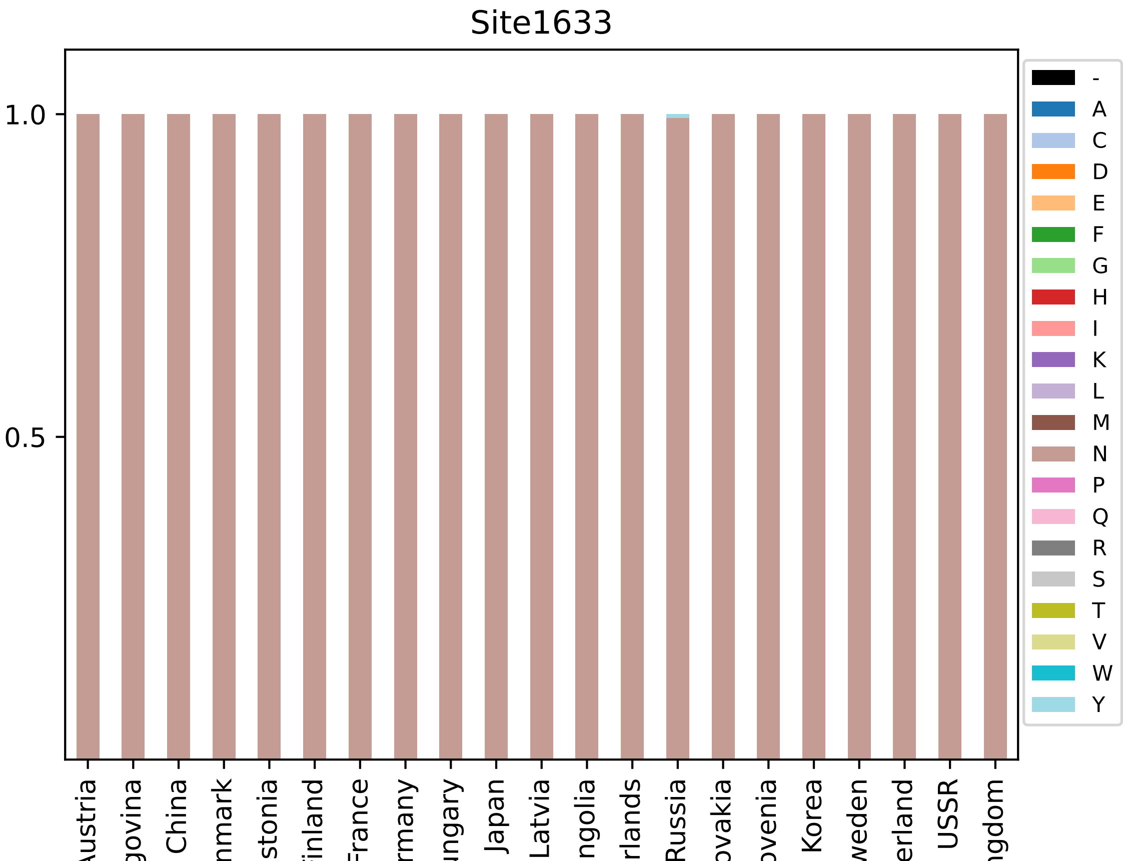
Site1633
1.0
0.5
-
A
C
D
E
F
G
H
I
K
L
M
N
P
Q
R
S
T
V
W
Y
Austria
govina
China
nmark
stonia
inland
France
rmany
ungary
Japan
Latvia
ngolia
rlands
Russia
ovakia
ovenia
Korea
weden
erland
USSR
ngdom

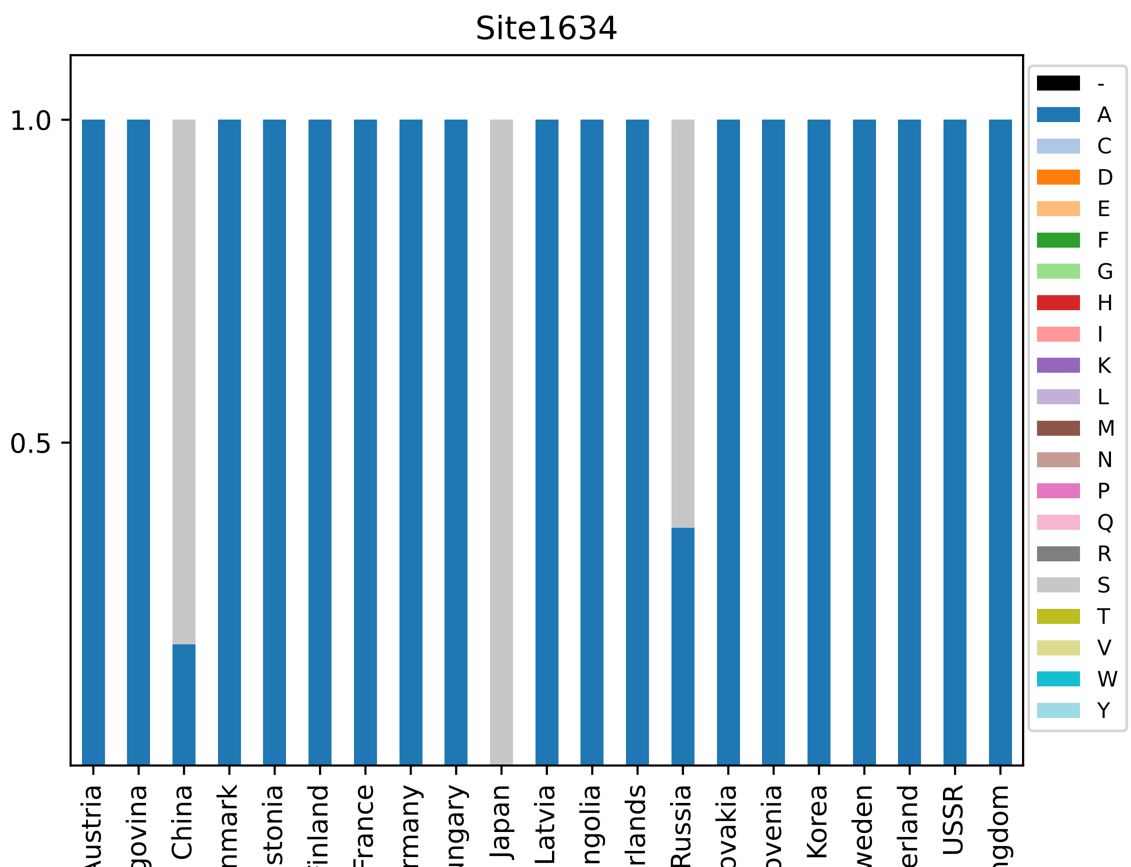
Site1634
1.0
0.5
-
A
C
D
E
F
G
H
I
K
L
M
N
P
Q
R
S
T
V
W
Y
China
Japan
Latvia
Russia
Korea
USSR

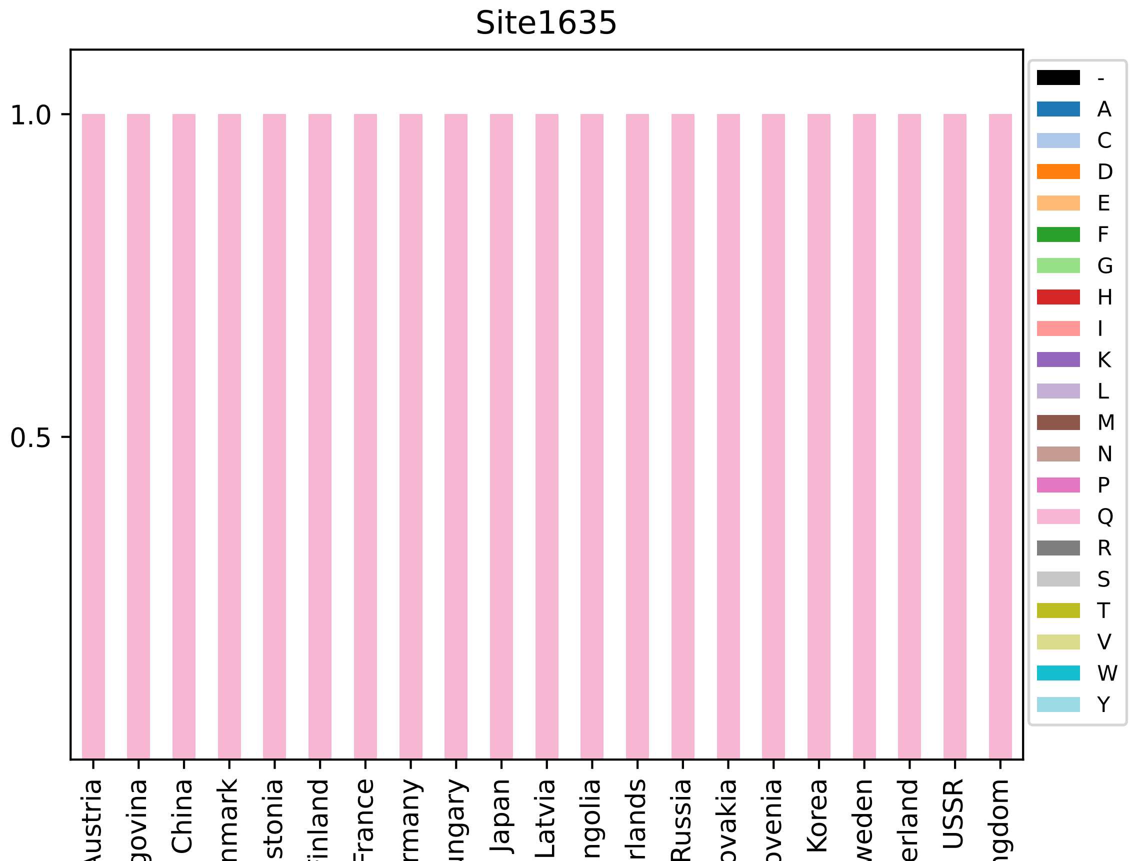
Site1635
1.0
0.5
-
A
C
D
E
F
G
H
I
K
L
M
N
P
Q
R
S
T
V
W
Y
China
Japan
Latvia
Russia
Korea
USSR

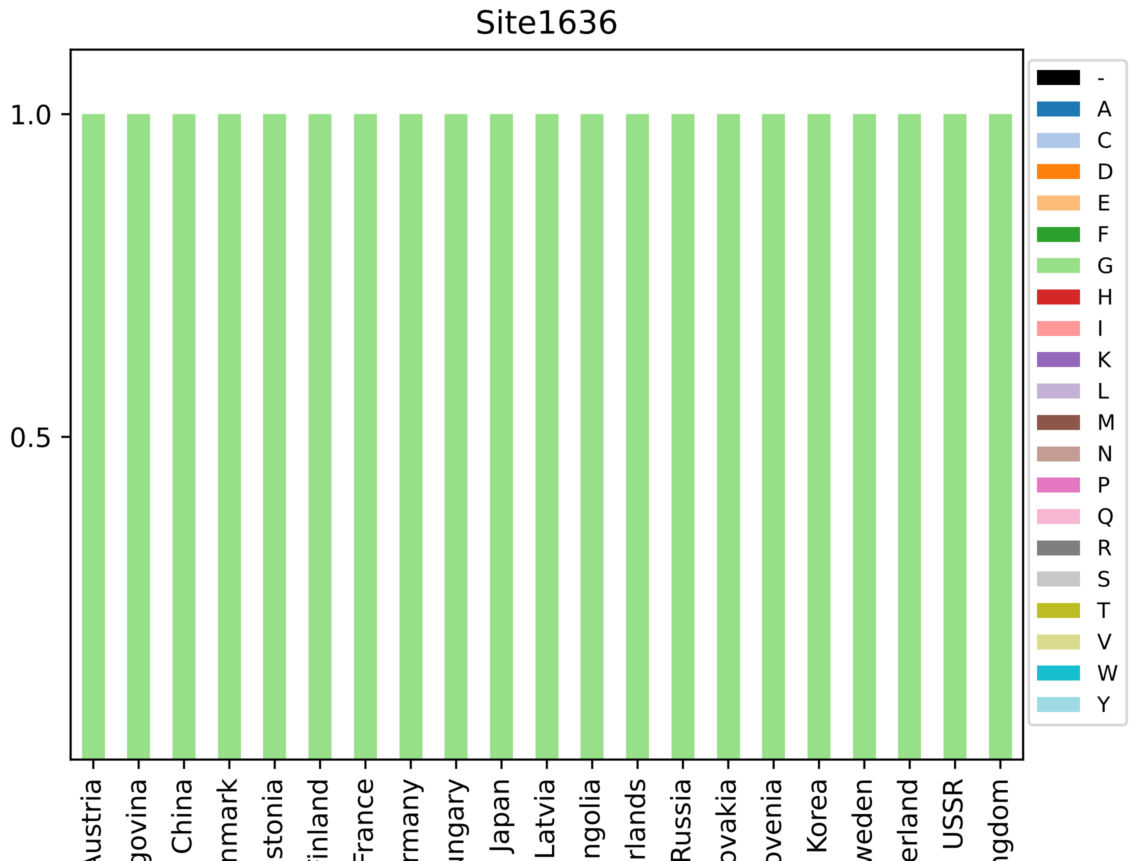

Site1636
1.0
0.5
-
A
C
D
E
F
G
H
I
K
L
M
N
P
Q
R
S
T
V
W
Y
Austria
China
Japan
Latvia
Russia
Korea
USSR

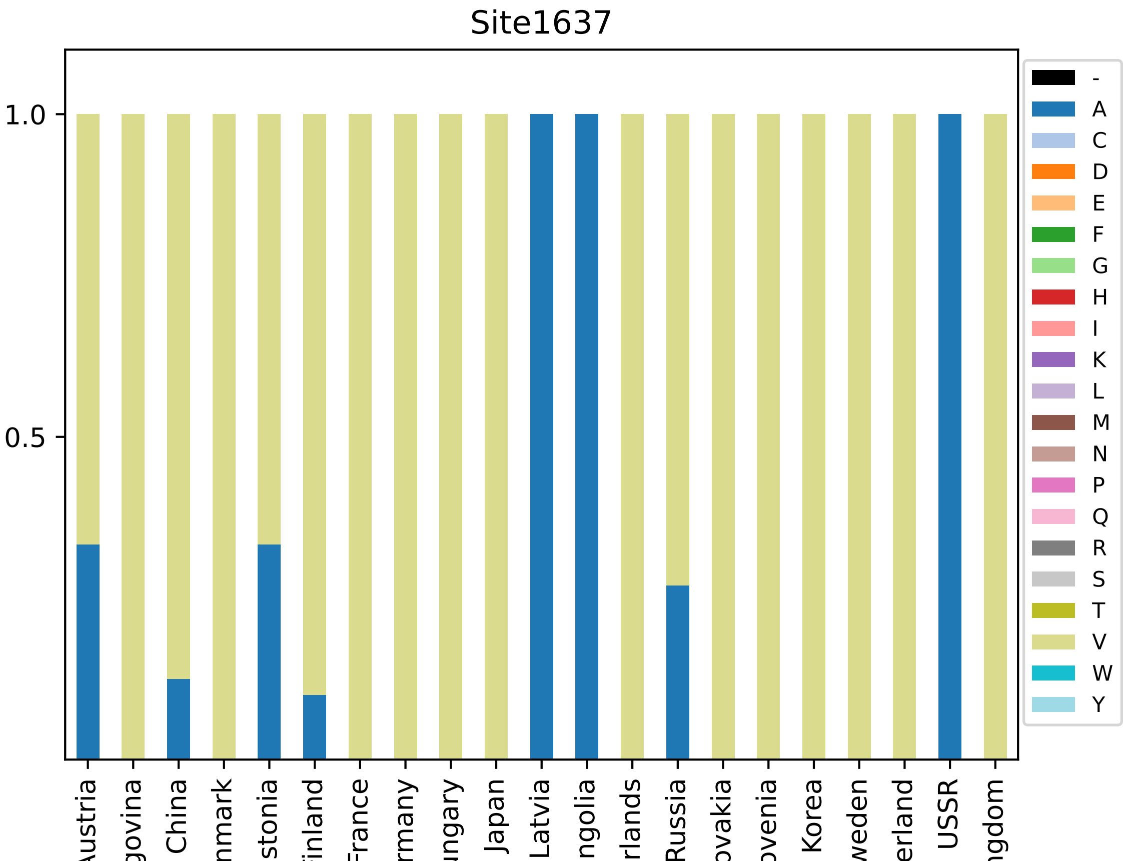

Site1637
1.0
0.5
Austria
govina
China
nmark
stonia
Finland
France
rmany
ungary
Japan
Latvia
ngolia
rlands
Russia
ovakia
ovenia
Korea
weden
erland
USSR
ngdom
-
A
C
D
E
F
G
H
I
K
L
M
N
P
Q
R
S
T
V
W
Y

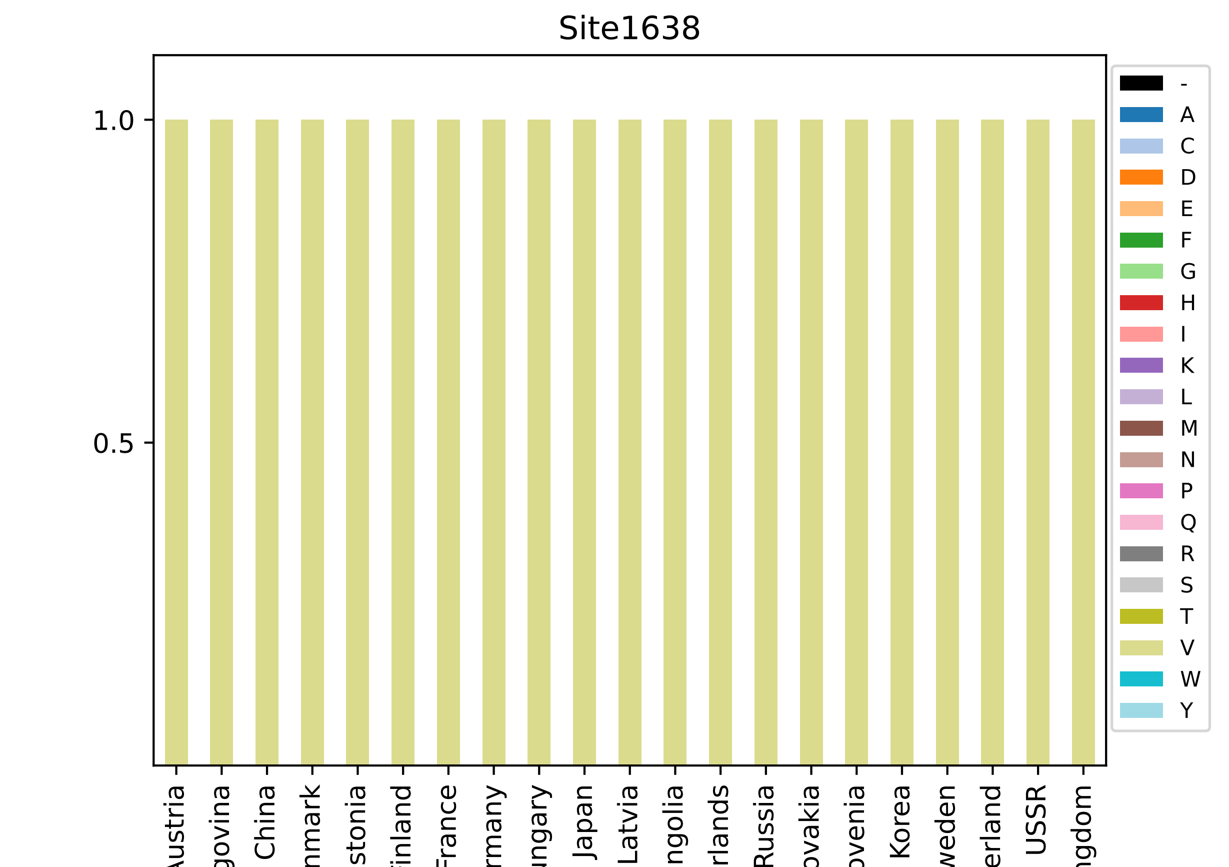

Site1638
1.0
0.5
-
A
C
D
E
F
G
H
I
K
L
M
N
P
Q
R
S
T
V
W
Y
Austria
govina
China
nmark
stonia
Finland
France
rmany
ungary
Japan
Latvia
ngolia
rlands
Russia
ovakia
ovenia
Korea
weden
erland
USSR
ngdom

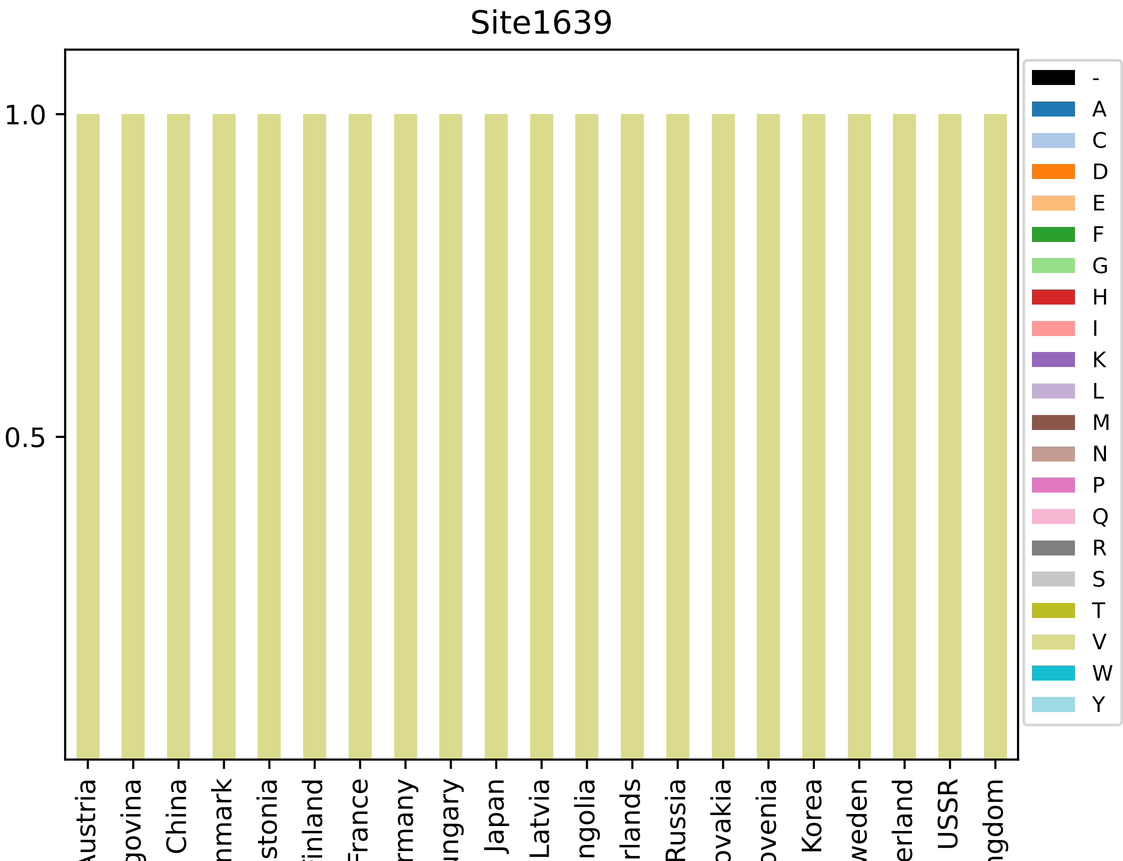
Site1639
1.0
0.5
China
Japan
Latvia
Russia
Korea
USSR
-
A
C
D
E
F
G
H
I
K
L
M
N
P
Q
R
S
T
V
W
Y

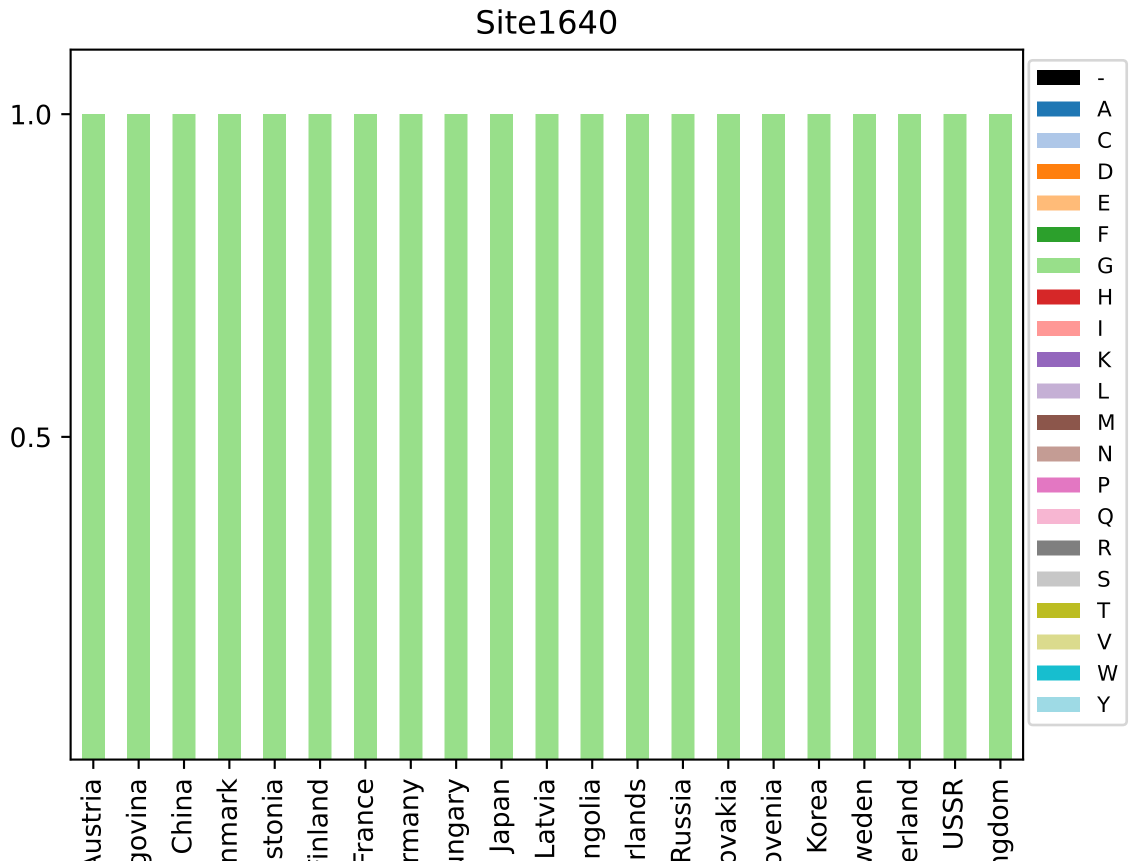
Site1640
1.0
0.5
-
A
C
D
E
F
G
H
I
K
L
M
N
P
Q
R
S
T
V
W
Y
China
Japan
Latvia
Russia
Korea
USSR

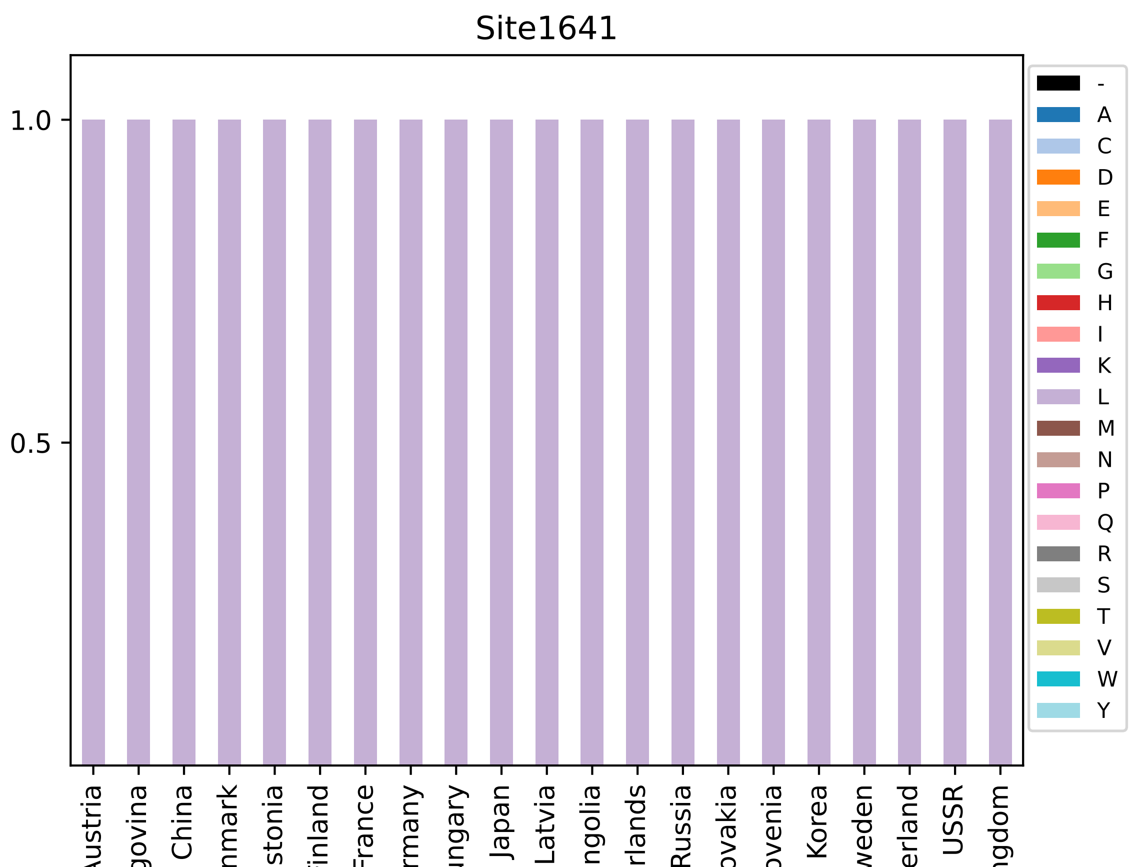
Site1641
1.0
0.5
-
A
C
D
E
F
G
H
I
K
L
M
N
P
Q
R
S
T
V
W
Y
Austria
China
Japan
Latvia
Russia
Korea
USSR

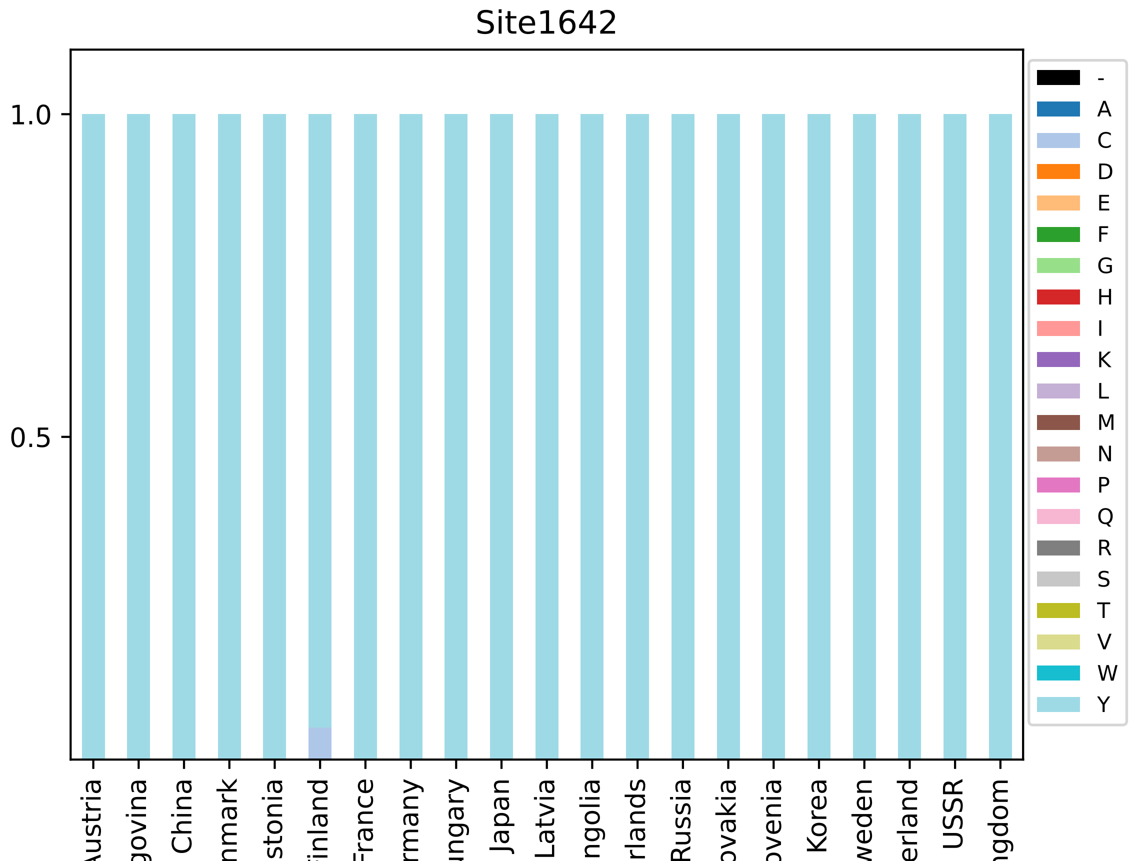
Site1642
1.0
0.5
-
A
C
D
E
F
G
H
I
K
L
M
N
P
Q
R
S
T
V
W
Y
China
Finland
France
Japan
Latvia
Russia
Korea
USSR

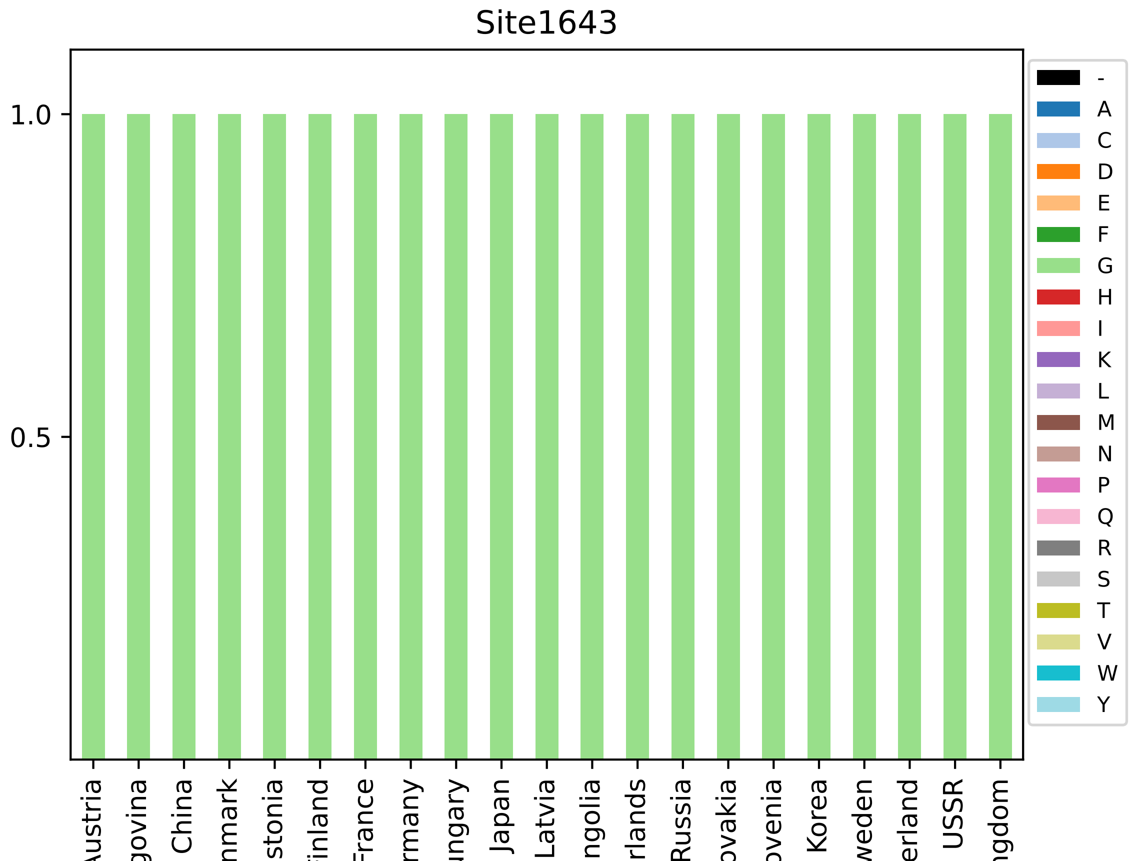

Site1643
1.0
0.5
-
A
C
D
E
F
G
H
I
K
L
M
N
P
Q
R
S
T
V
W
Y
China
Japan
Latvia
Russia
Korea
USSR

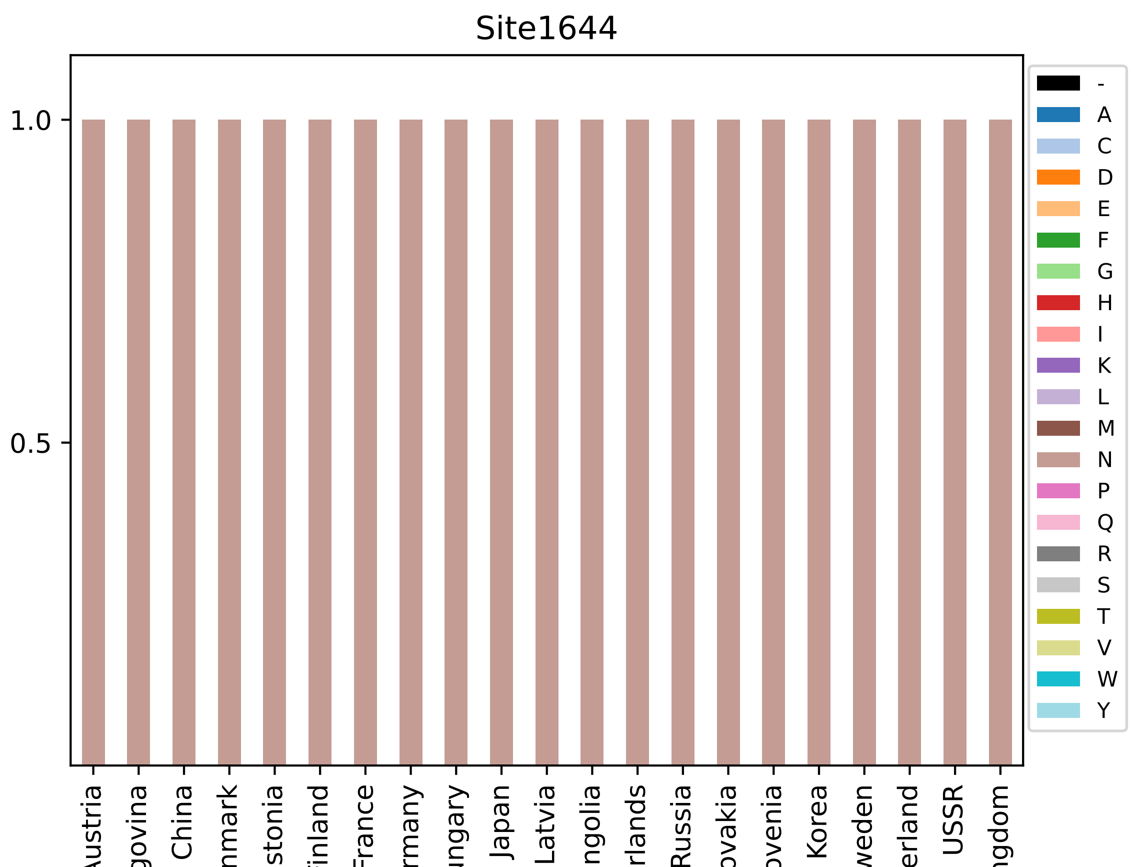

Site1644
1.0
0.5
-
A
C
D
E
F
G
H
I
K
L
M
N
P
Q
R
S
T
V
W
Y
Austria
govina
China
nmark
stonia
Finland
France
rmany
ungary
Japan
Latvia
ngolia
rlands
Russia
ovakia
ovenia
Korea
weden
erland
USSR
ngdom

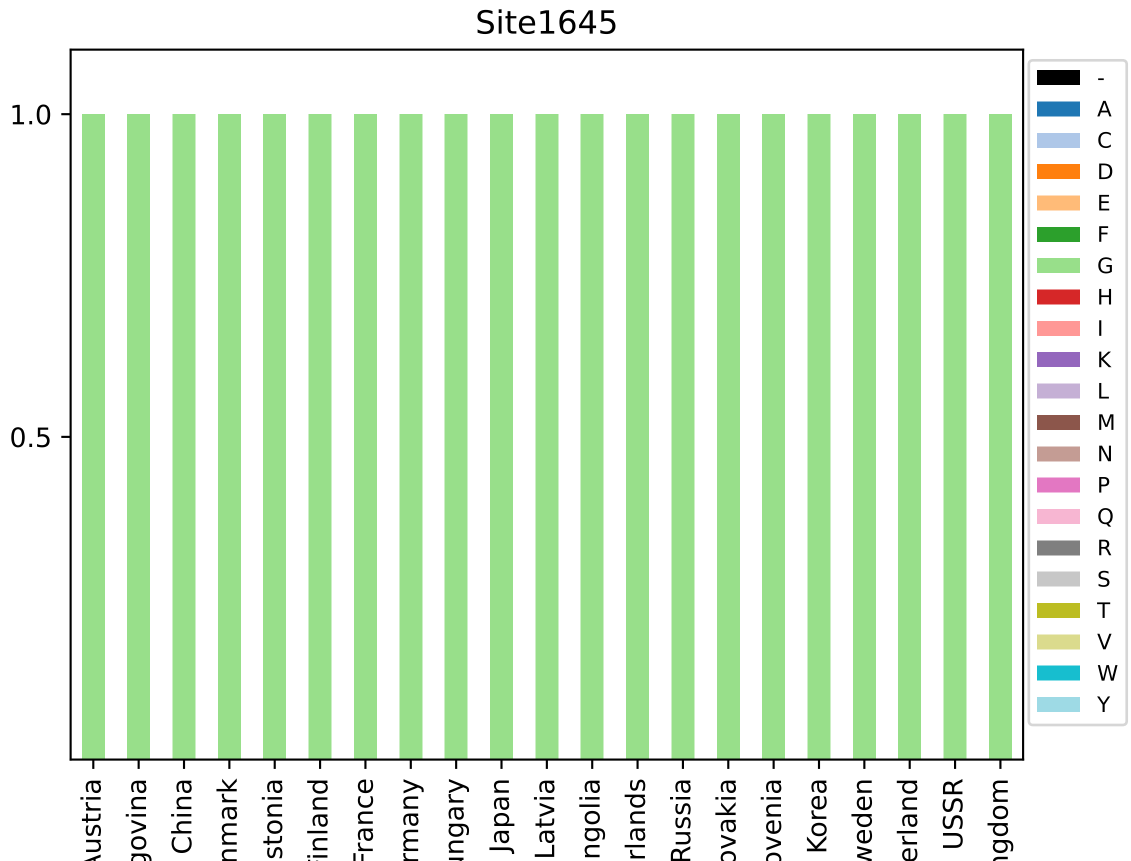
Site1645
1.0
0.5
-
A
C
D
E
F
G
H
I
K
L
M
N
P
Q
R
S
T
V
W
Y
China
Japan
Latvia
Russia
Korea
USSR

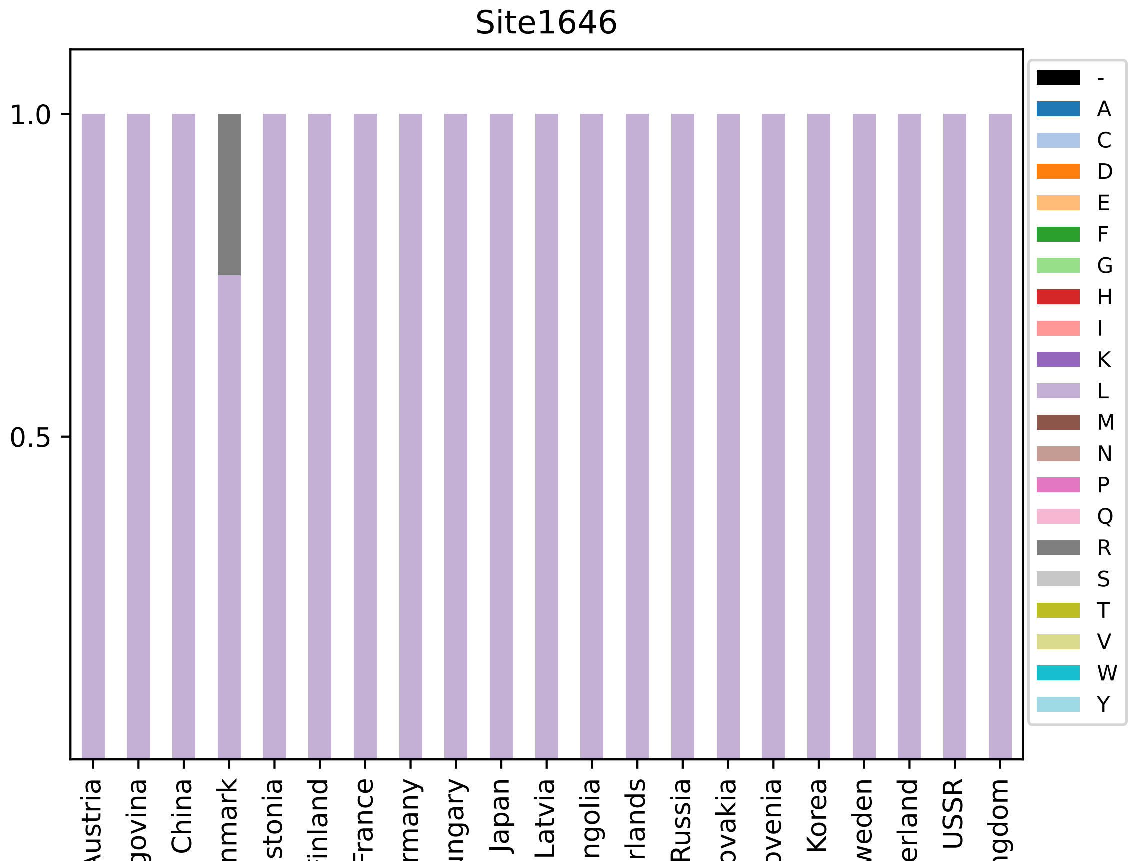

Site1646
1.0
0.5
-
A
C
D
E
F
G
H
I
K
L
M
N
P
Q
R
S
T
V
W
Y
Austria
govina
China
nmark
stonia
Finland
France
rmany
ungary
Japan
Latvia
ngolia
rlands
Russia
ovakia
ovenia
Korea
weden
erland
USSR
ngdom

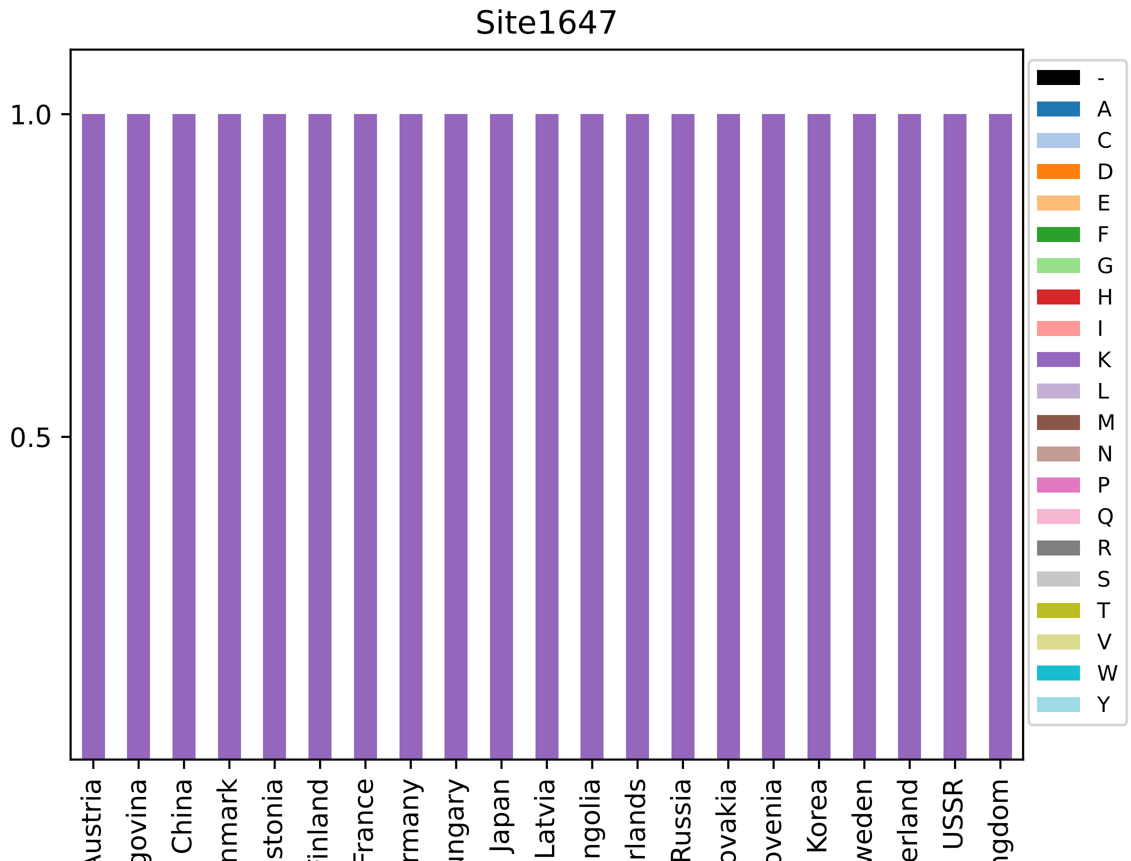
Site1647
1.0
0.5
-
A
C
D
E
F
G
H
I
K
L
M
N
P
Q
R
S
T
V
W
Y
China
Japan
Latvia
Russia
Korea
USSR

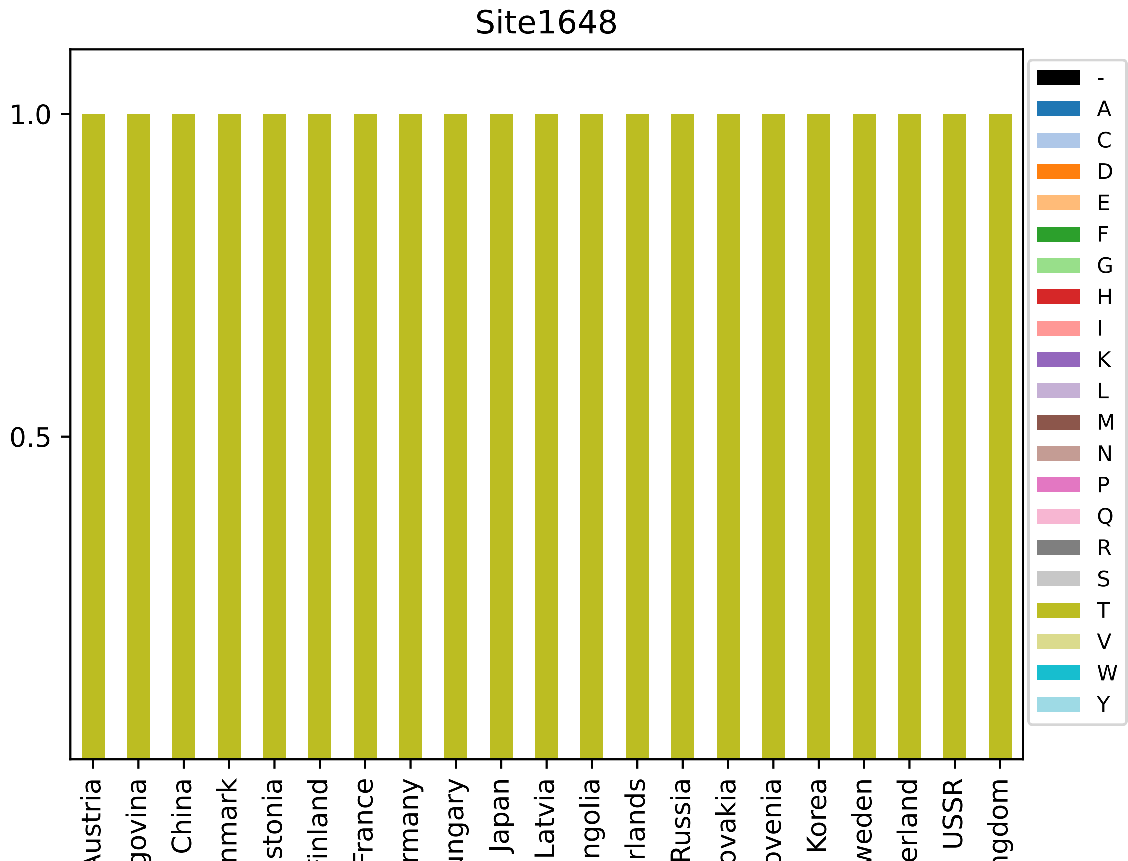
Site1648
1.0
0.5
-
A
C
D
E
F
G
H
I
K
L
M
N
P
Q
R
S
T
V
W
Y
China
Japan
Latvia
Russia
Korea
USSR

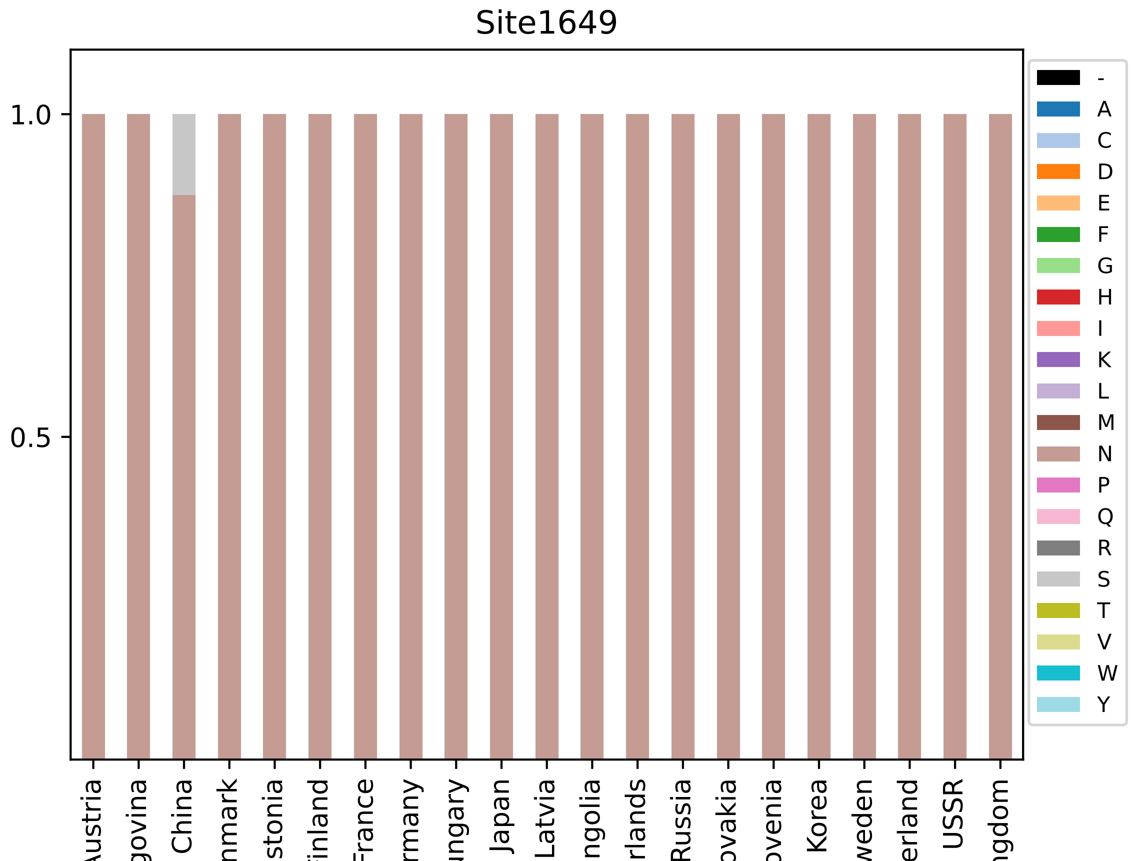
Site1649
1.0
0.5
-
A
C
D
E
F
G
H
I
K
L
M
N
P
Q
R
S
T
V
W
Y
Austria
govina
China
nmark
stonia
inland
France
rmany
ungary
Japan
Latvia
ngolia
rlands
Russia
ovakia
ovenia
Korea
weden
erland
USSR
ngdom

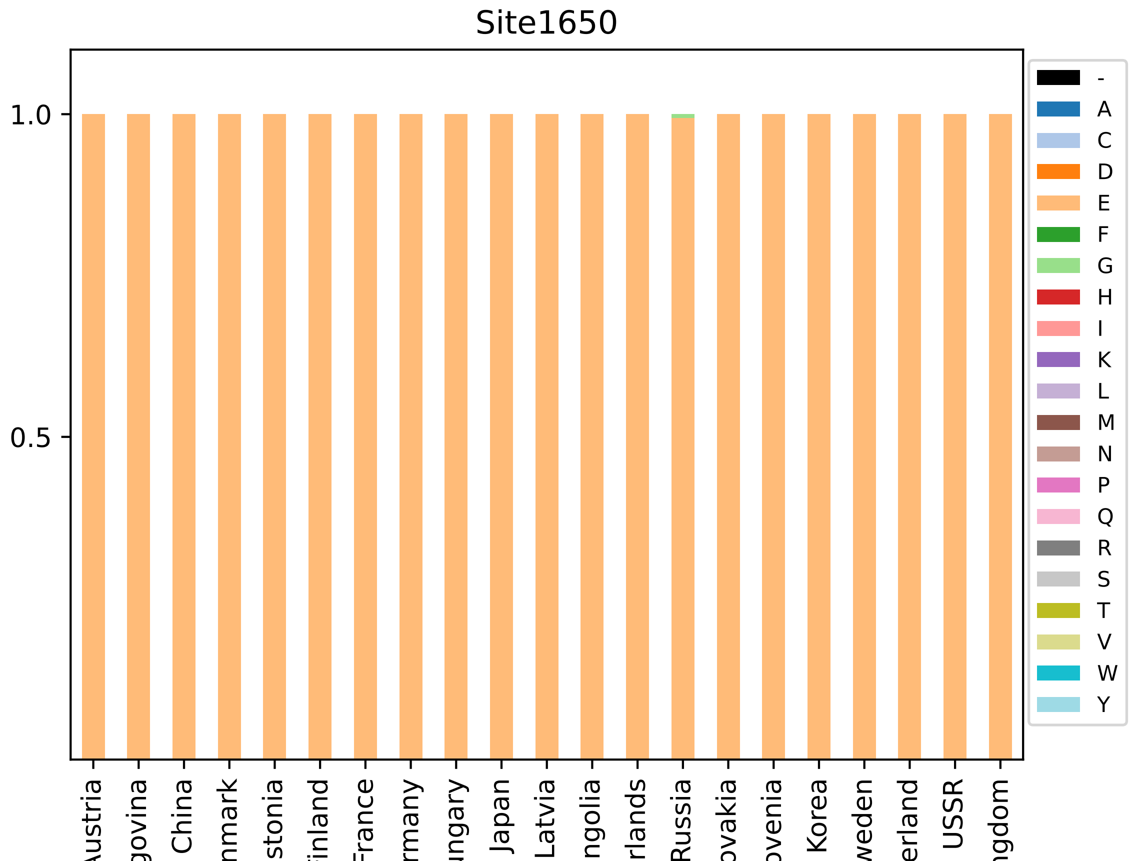
Site1650
1.0
0.5
-
A
C
D
E
F
G
H
I
K
L
M
N
P
Q
R
S
T
V
W
Y
China
Japan
Latvia
Russia
Korea
USSR

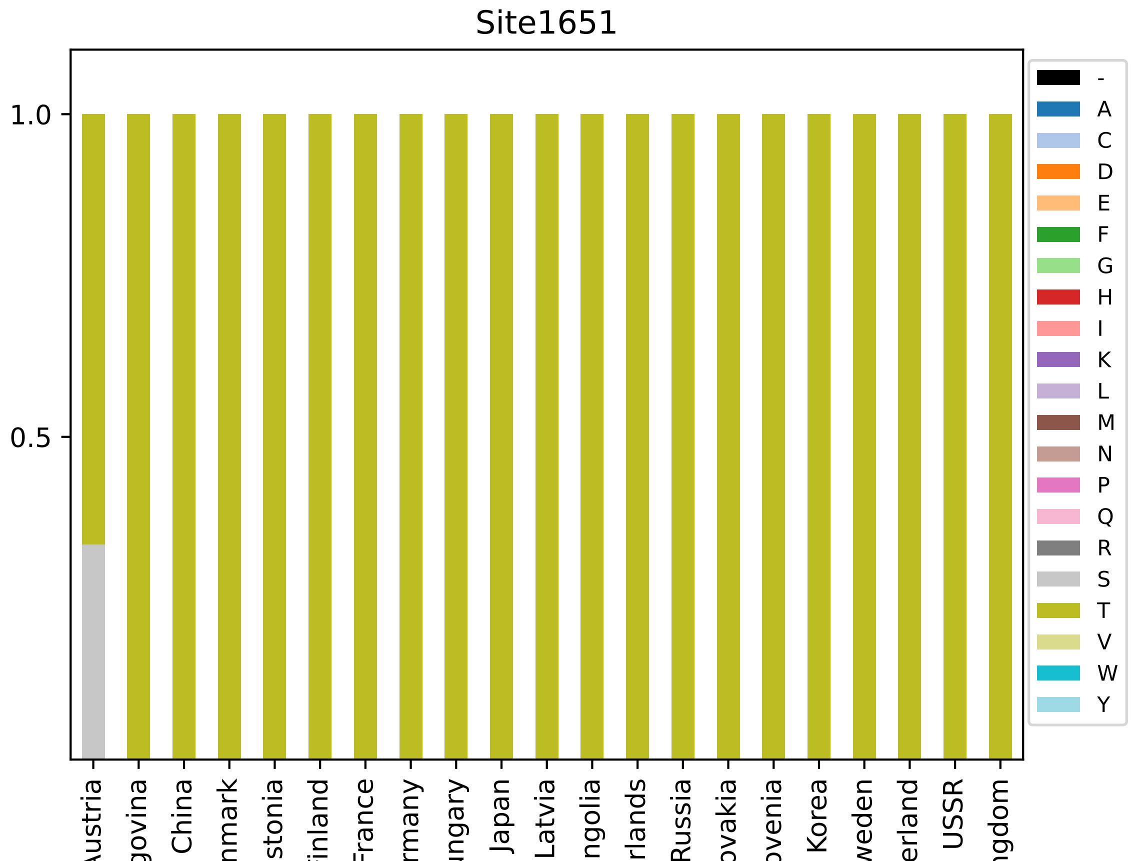

Site1651
1.0
0.5
-
A
C
D
E
F
G
H
I
K
L
M
N
P
Q
R
S
T
V
W
Y
China
Japan
Latvia
Russia
Korea
USSR

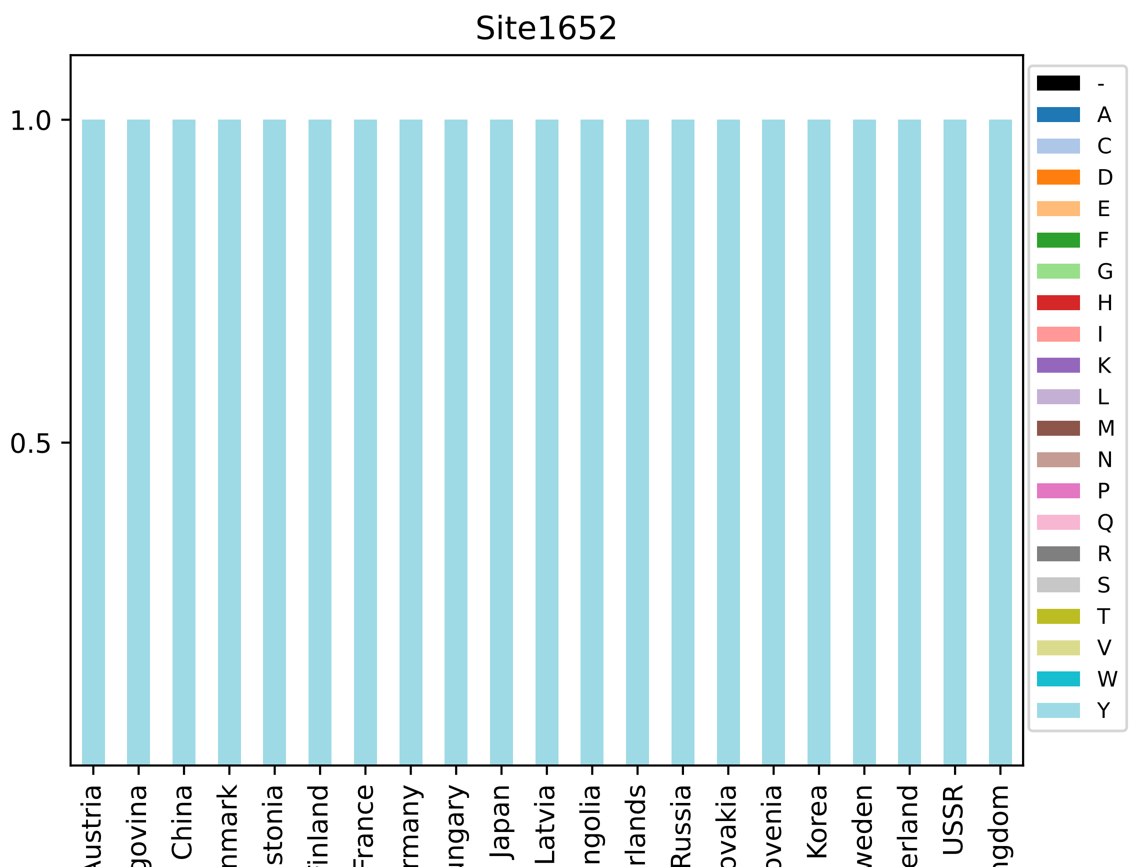

Site1652
1.0
0.5
China
Japan
Latvia
Russia
Korea
USSR
-
A
C
D
E
F
G
H
I
K
L
M
N
P
Q
R
S
T
V
W
Y

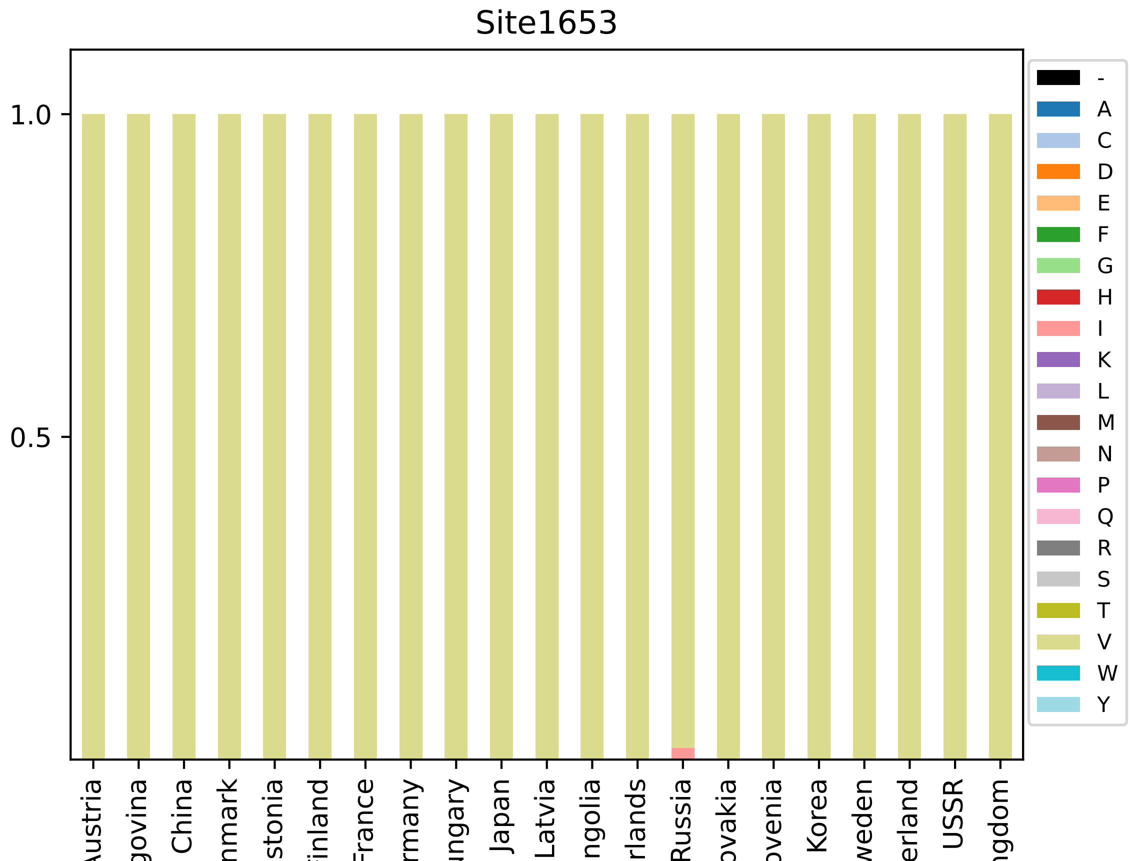

Site1653
1.0
0.5
-
A
C
D
E
F
G
H
I
K
L
M
N
P
Q
R
S
T
V
W
Y
Austria
China
Finland
France
Japan
Latvia
Russia
Korea
USSR

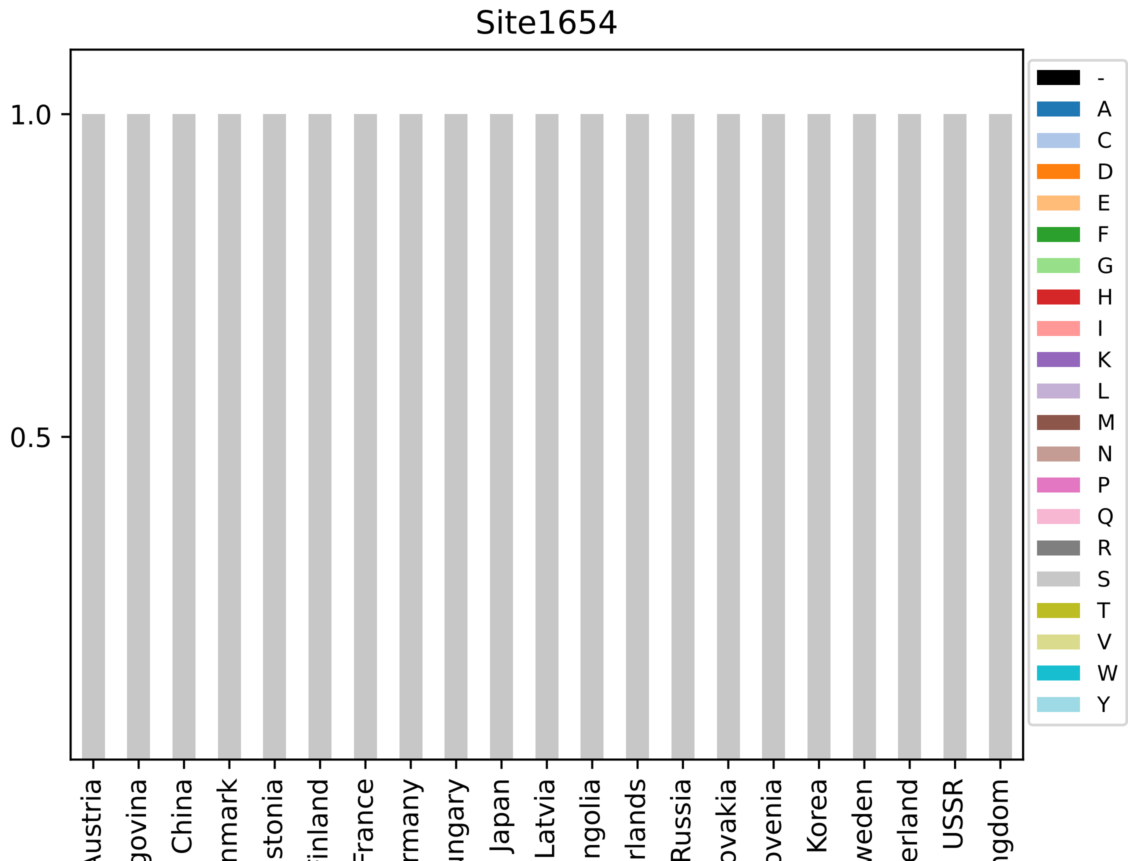

Site1654
1.0
0.5
-
A
C
D
E
F
G
H
I
K
L
M
N
P
Q
R
S
T
V
W
Y
Austria
govina
China
nmark
stonia
Finland
France
rmany
ungary
Japan
Latvia
ngolia
rlands
Russia
ovakia
ovenia
Korea
weden
erland
USSR
ngdom

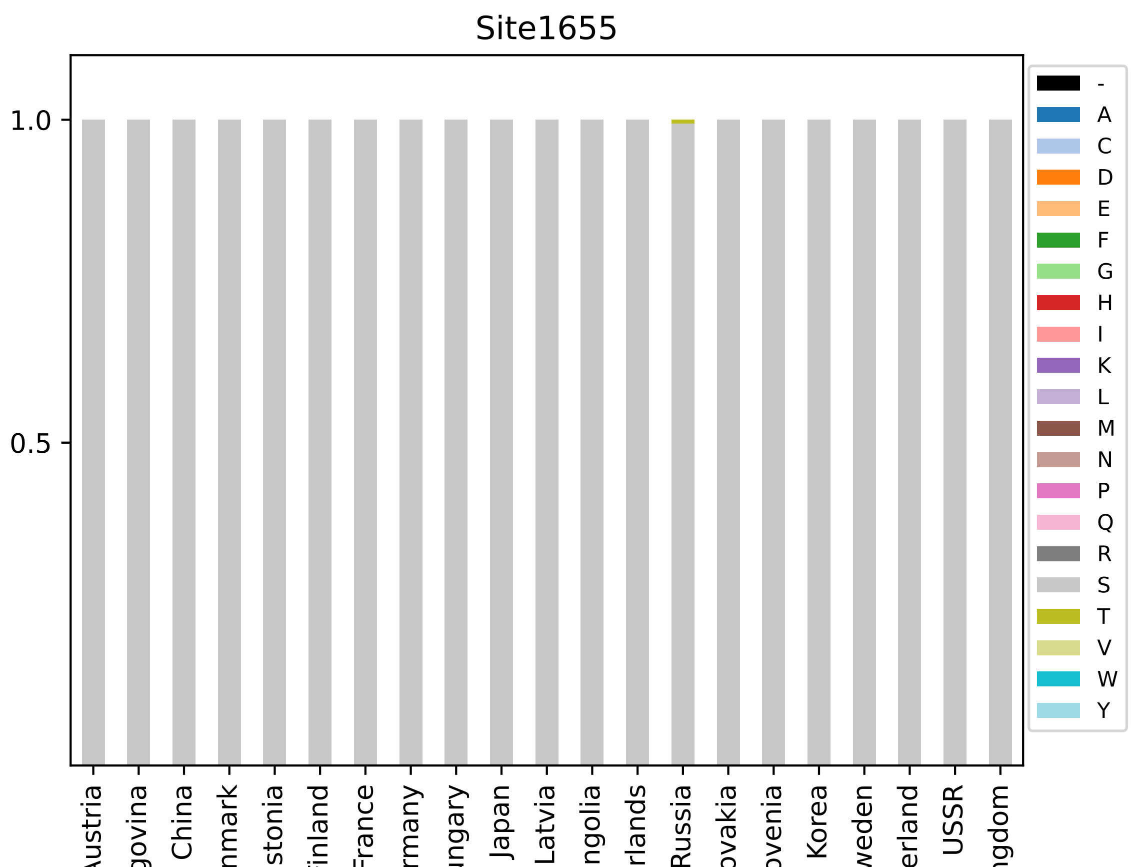

Site1655
1.0
0.5
-
A
C
D
E
F
G
H
I
K
L
M
N
P
Q
R
S
T
V
W
Y
Austria
govina
China
nmark
stonia
Finland
France
rmany
ungary
Japan
Latvia
ngolia
rlands
Russia
ovakia
ovenia
Korea
weden
erland
USSR
ngdom

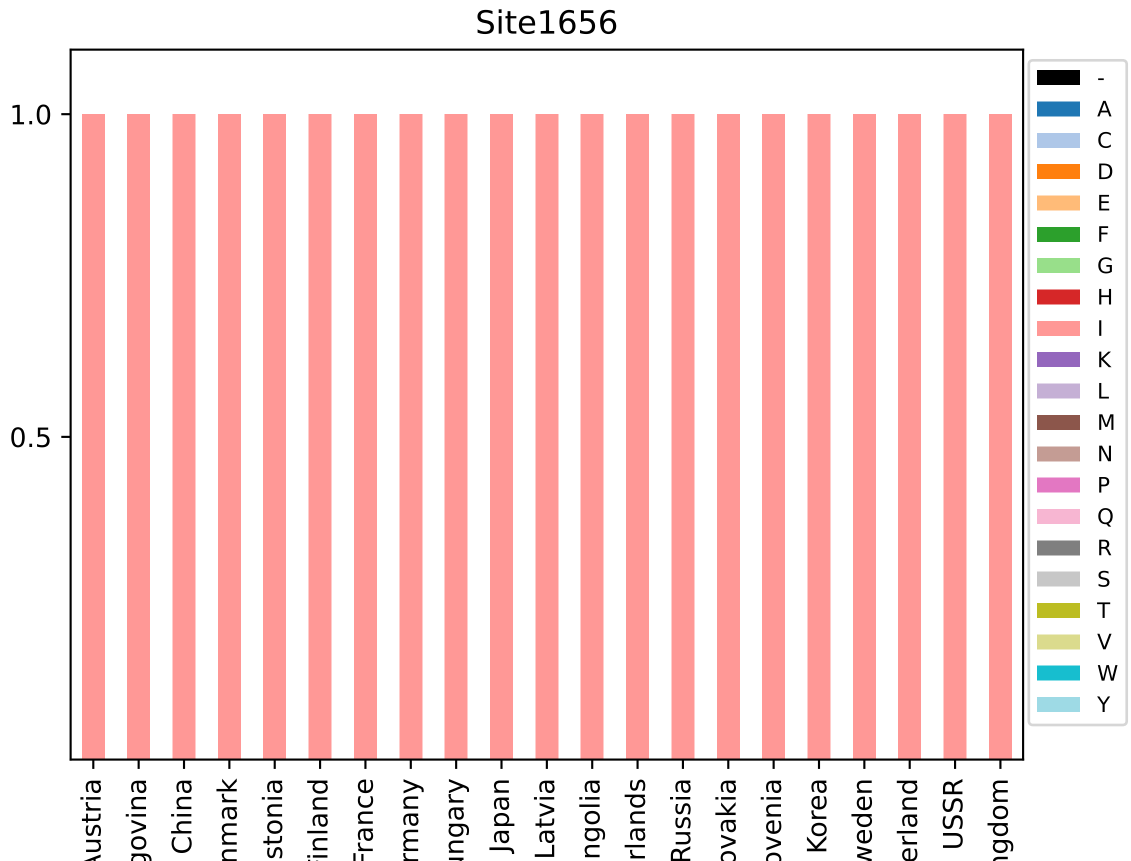
Site1656
1.0
0.5
-
A
C
D
E
F
G
H
I
K
L
M
N
P
Q
R
S
T
V
W
Y
Austria
govina
China
nmark
stonia
inland
France
rmany
ungary
Japan
Latvia
ngolia
rlands
Russia
ovakia
ovenia
Korea
weden
erland
USSR
ngdom

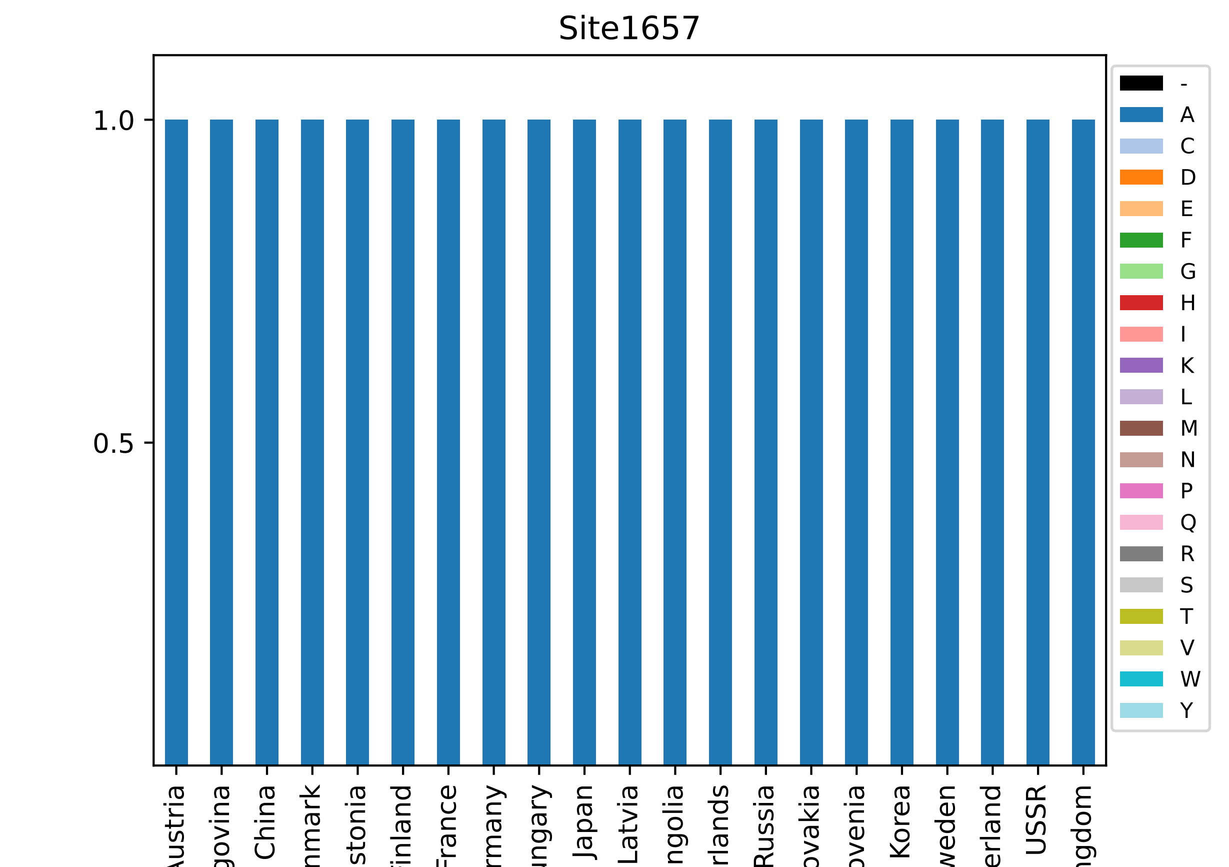
Site1657
1.0
0.5
Austria
govina
China
nmark
stonia
Finland
France
rmany
ungary
Japan
Latvia
ngolia
rlands
Russia
ovakia
ovenia
Korea
weden
erland
USSR
ngdom
-
A
C
D
E
F
G
H
I
K
L
M
N
P
Q
R
S
T
V
W
Y

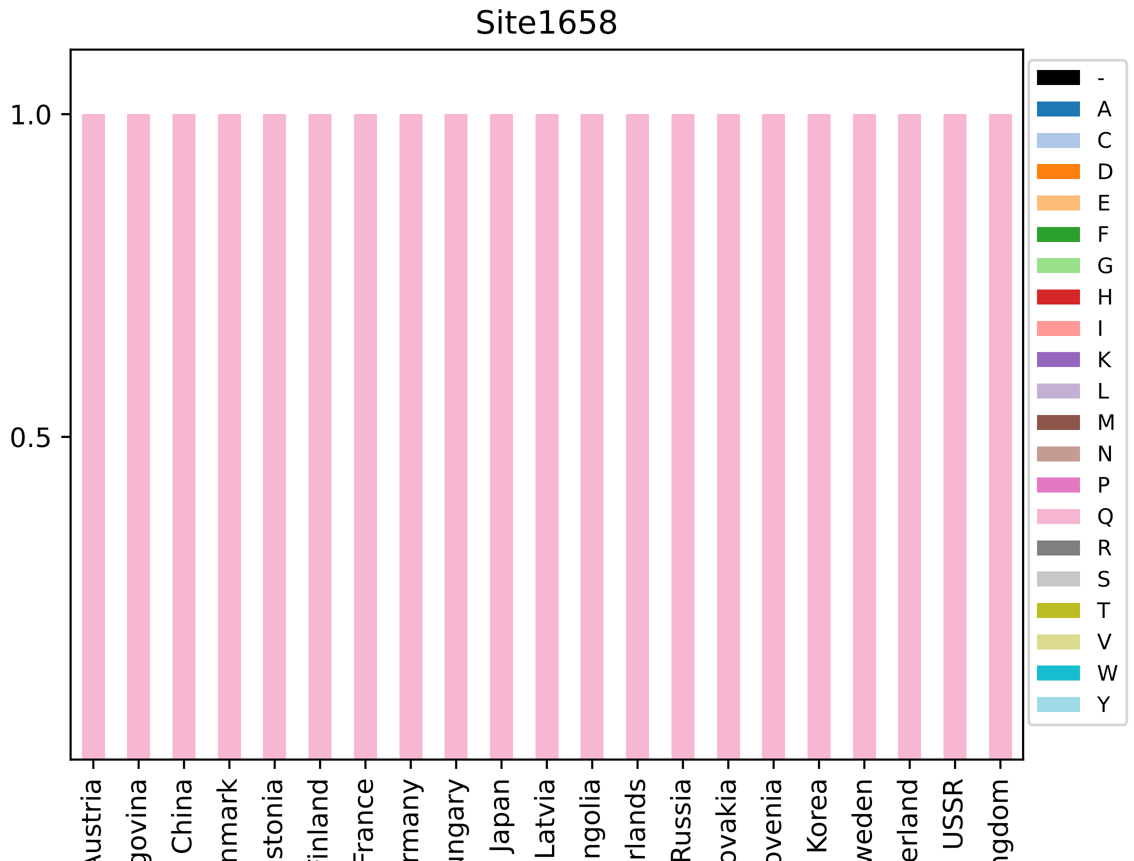

Site1658
1.0
0.5
-
A
C
D
E
F
G
H
I
K
L
M
N
P
Q
R
S
T
V
W
Y
China
Japan
Latvia
Russia
Korea
USSR

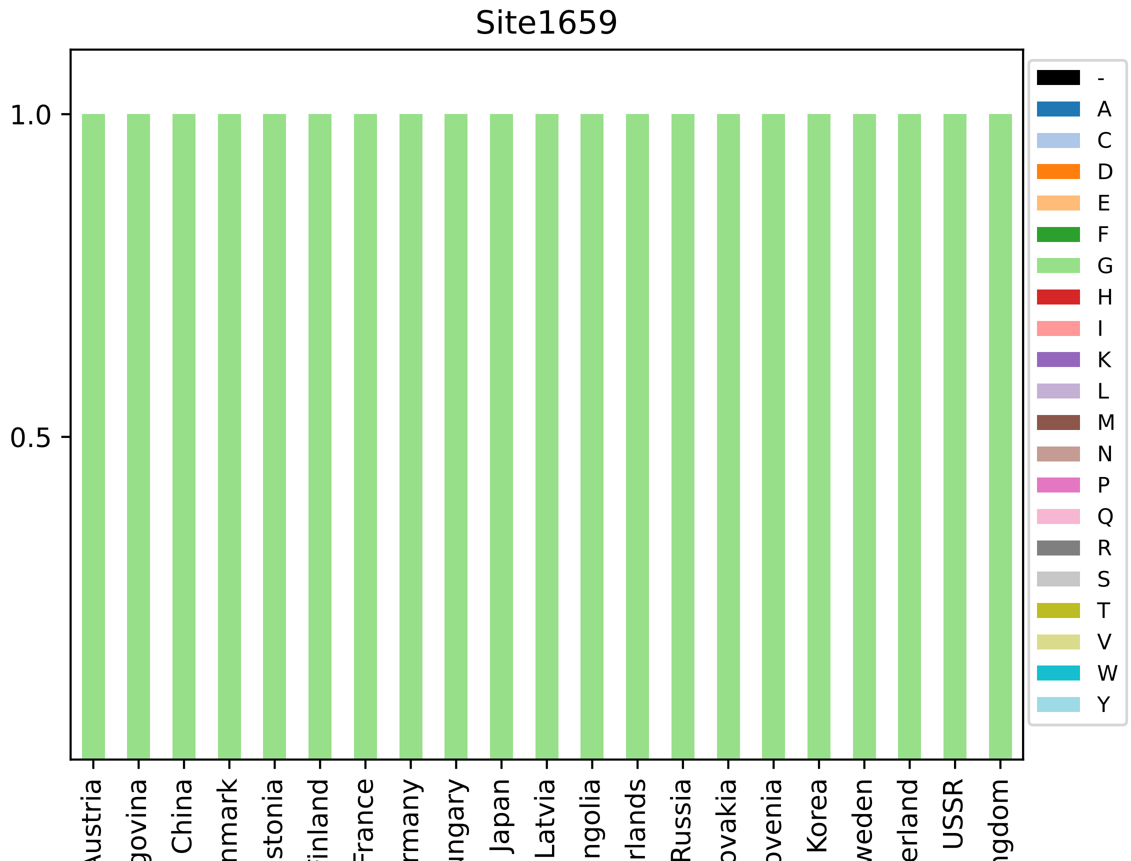

Site1659
1.0
0.5
Austria
govina
China
nmark
stonia
inland
France
rmany
ungary
Japan
Latvia
ngolia
rlands
Russia
ovakia
ovenia
Korea
weden
erland
USSR
ngdom
-
A
C
D
E
F
G
H
I
K
L
M
N
P
Q
R
S
T
V
W
Y

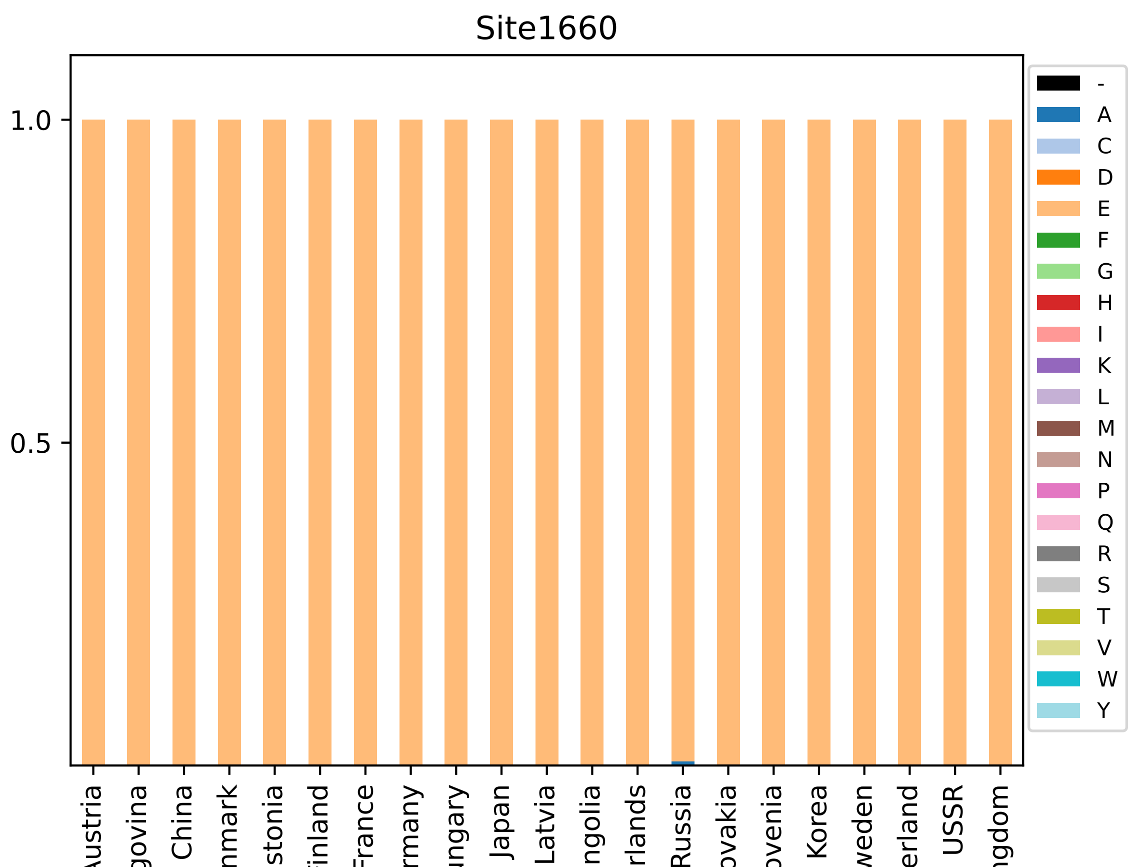

Site1660
1.0
0.5
-
A
C
D
E
F
G
H
I
K
L
M
N
P
Q
R
S
T
V
W
Y
China
Japan
Latvia
Russia
Korea
USSR

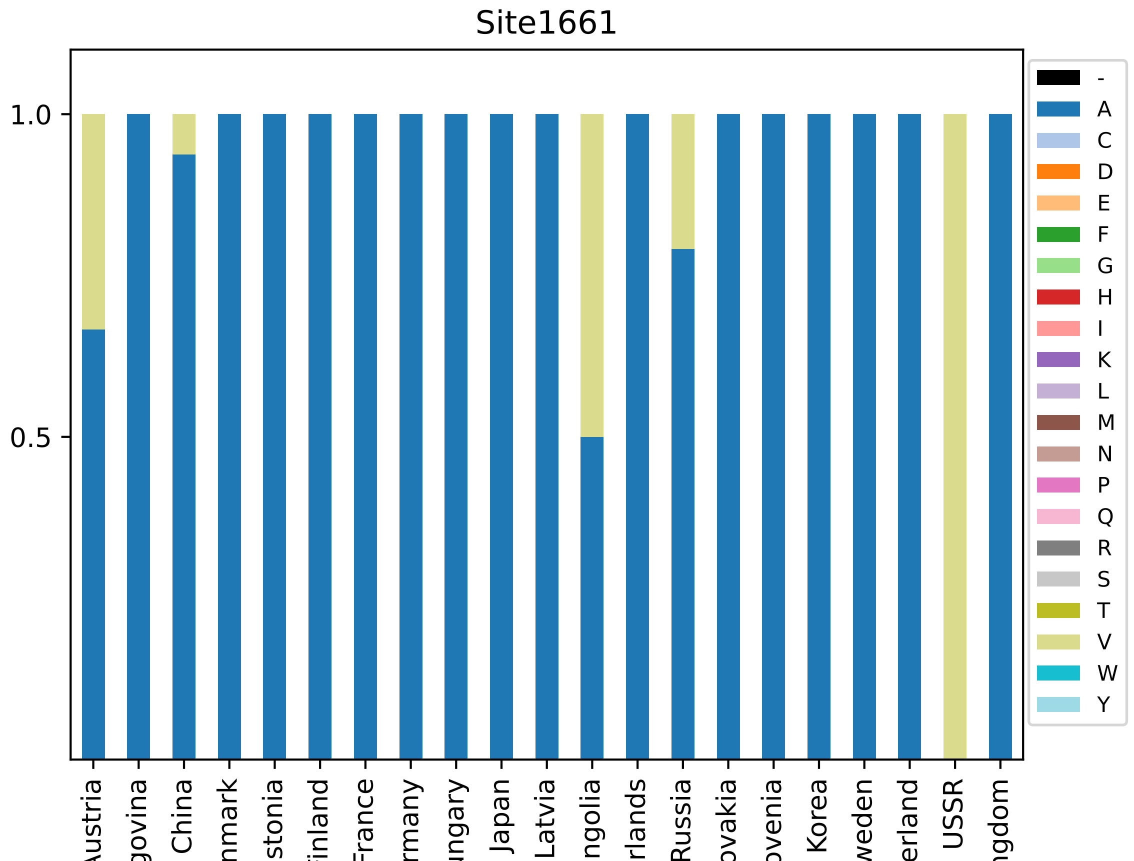

Site1661
1.0
0.5
-
A
C
D
E
F
G
H
I
K
L
M
N
P
Q
R
S
T
V
W
Y
Austria
govina
China
nmark
stonia
inland
France
rmany
ungary
Japan
Latvia
ngolia
rlands
Russia
ovakia
ovenia
Korea
weden
erland
USSR
ngdom

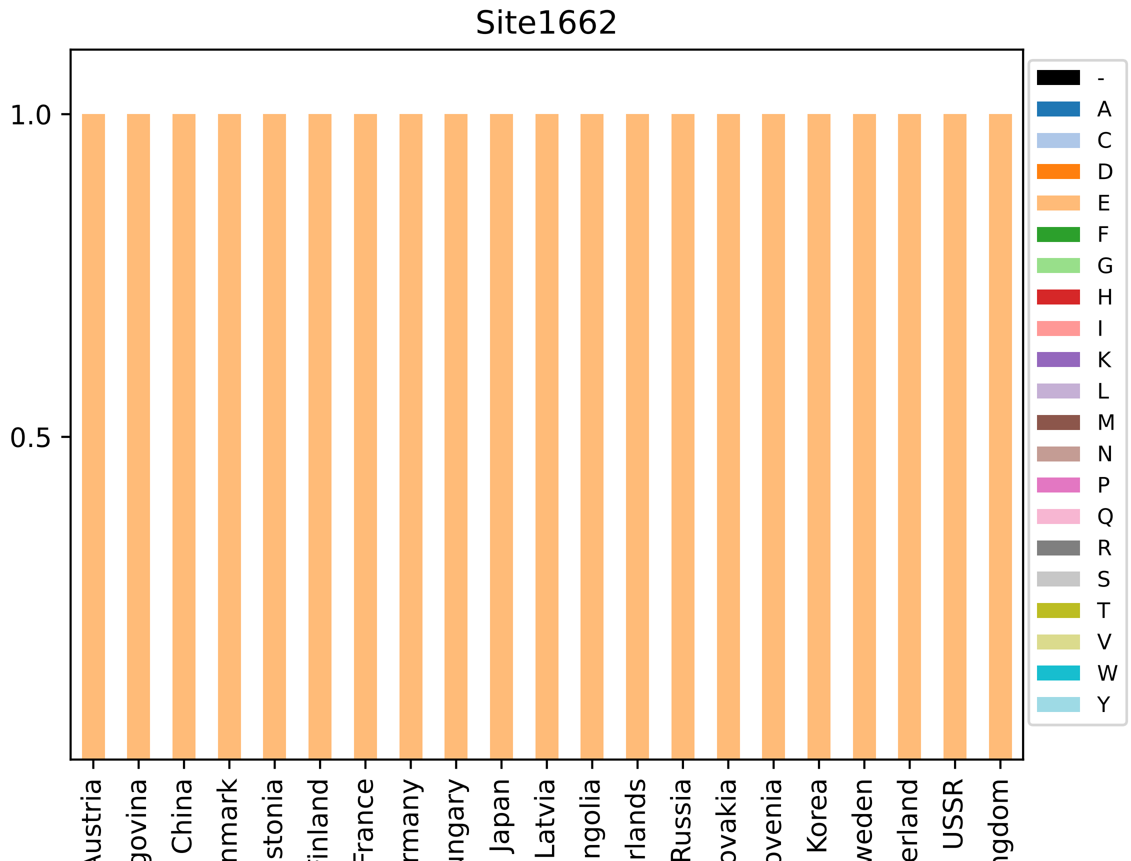

Site1662
1.0
0.5
-
A
C
D
E
F
G
H
I
K
L
M
N
P
Q
R
S
T
V
W
Y
Austria
China
Japan
Latvia
Russia
Korea
USSR

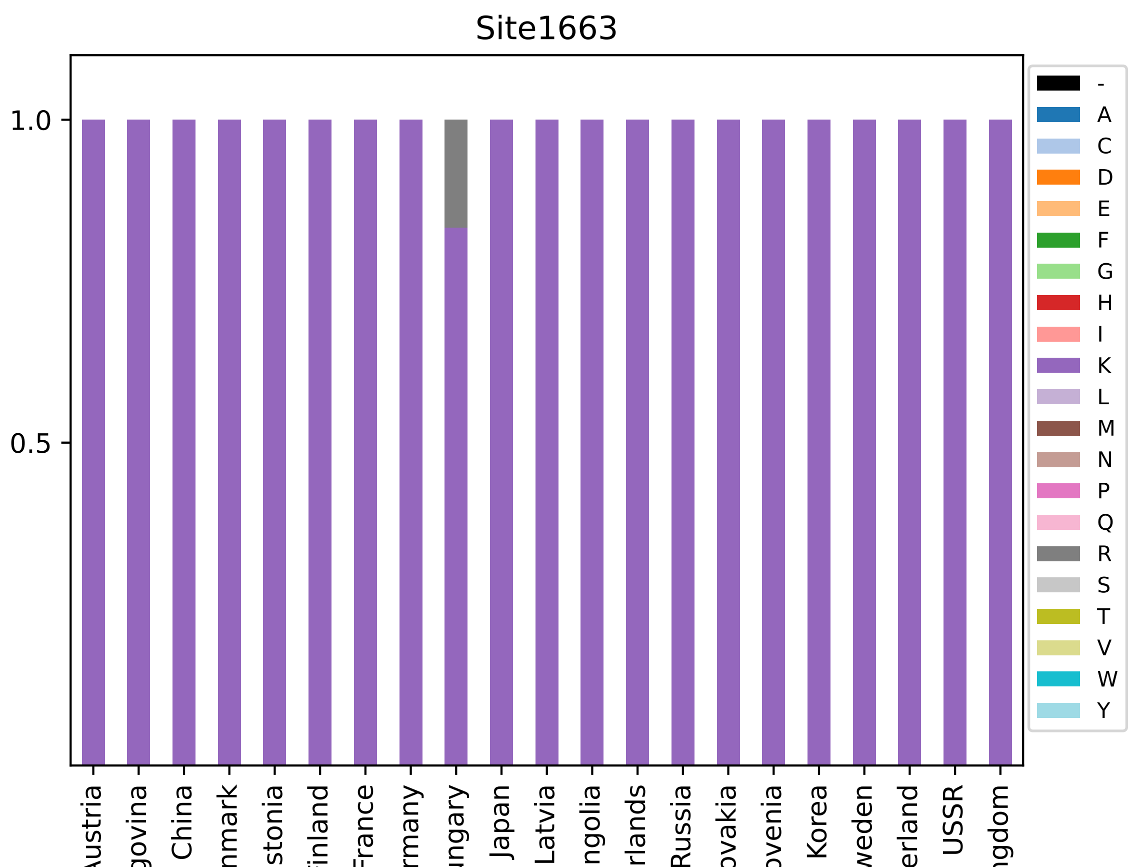

Site1663
1.0
0.5
-
A
C
D
E
F
G
H
I
K
L
M
N
P
Q
R
S
T
V
W
Y
China
France
Japan
Latvia
Russia
Korea
USSR

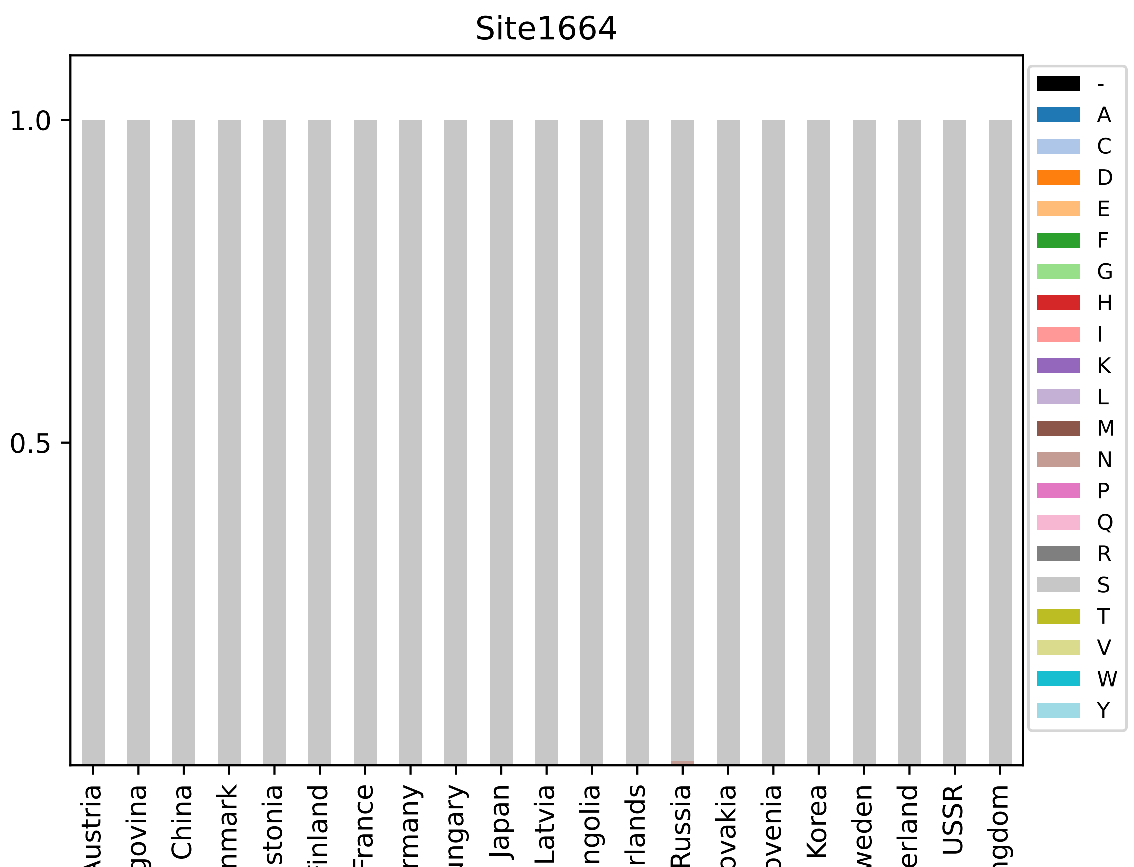

Site1664
1.0
0.5
-
A
C
D
E
F
G
H
I
K
L
M
N
P
Q
R
S
T
V
W
Y
Austria
govina
China
nmark
stonia
Finland
France
rmany
ungary
Japan
Latvia
ngolia
rlands
Russia
ovakia
ovenia
Korea
weden
erland
USSR
ngdom

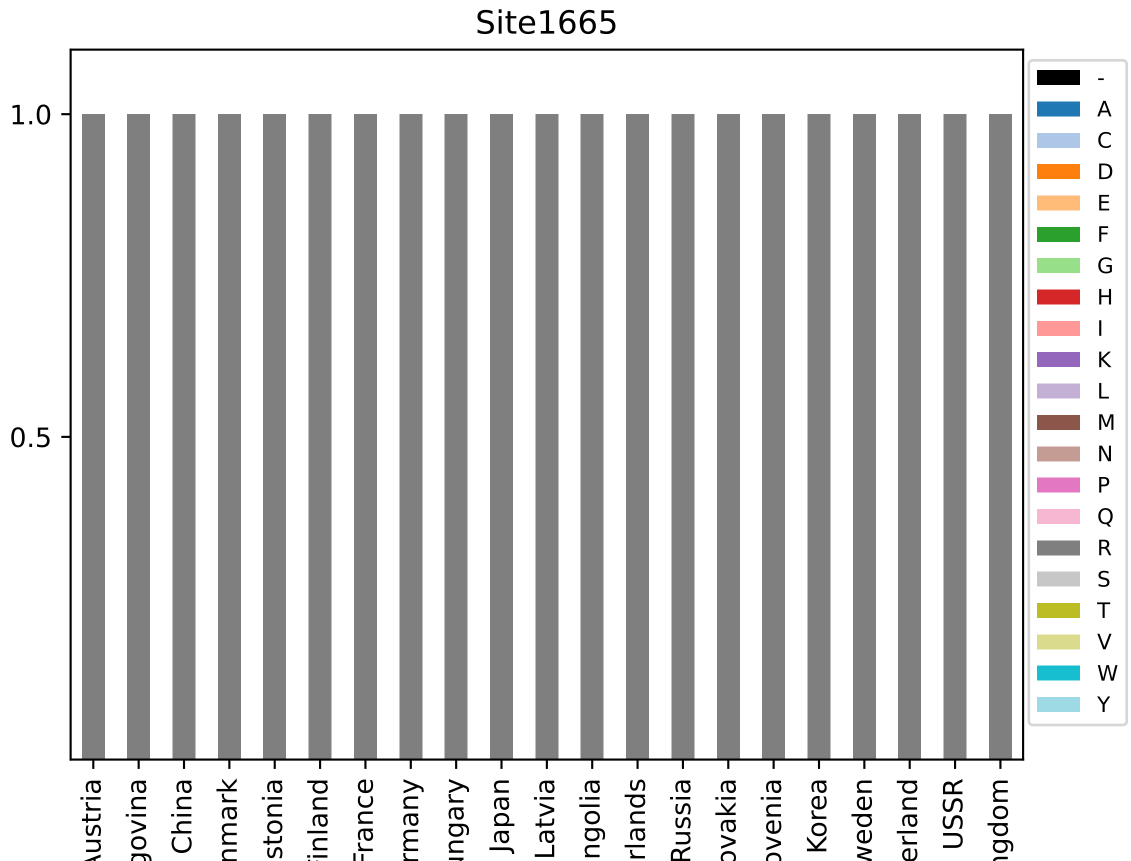
Site1665
1.0
0.5
-
A
C
D
E
F
G
H
I
K
L
M
N
P
Q
R
S
T
V
W
Y
China
Japan
Latvia
Russia
Korea
USSR

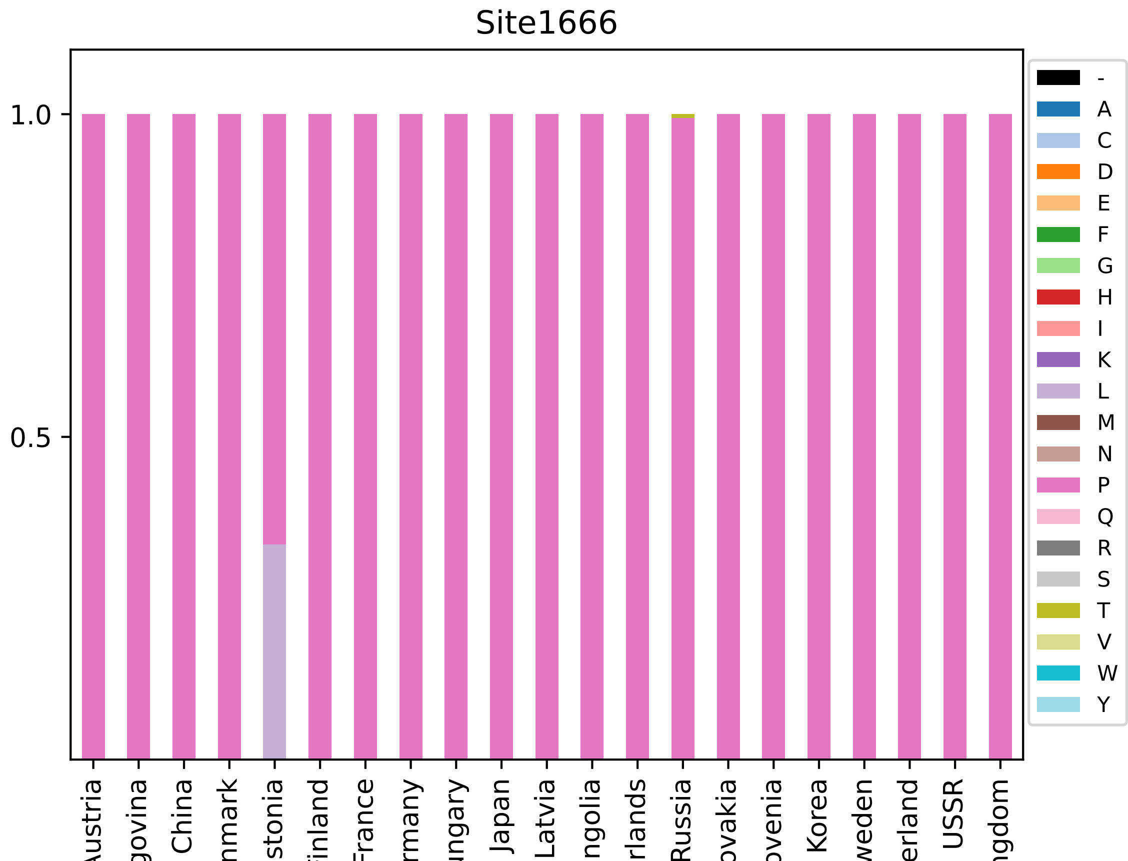

Site1666
1.0
0.5
-
A
C
D
E
F
G
H
I
K
L
M
N
P
Q
R
S
T
V
W
Y
China
Japan
Latvia
Russia
Korea
USSR

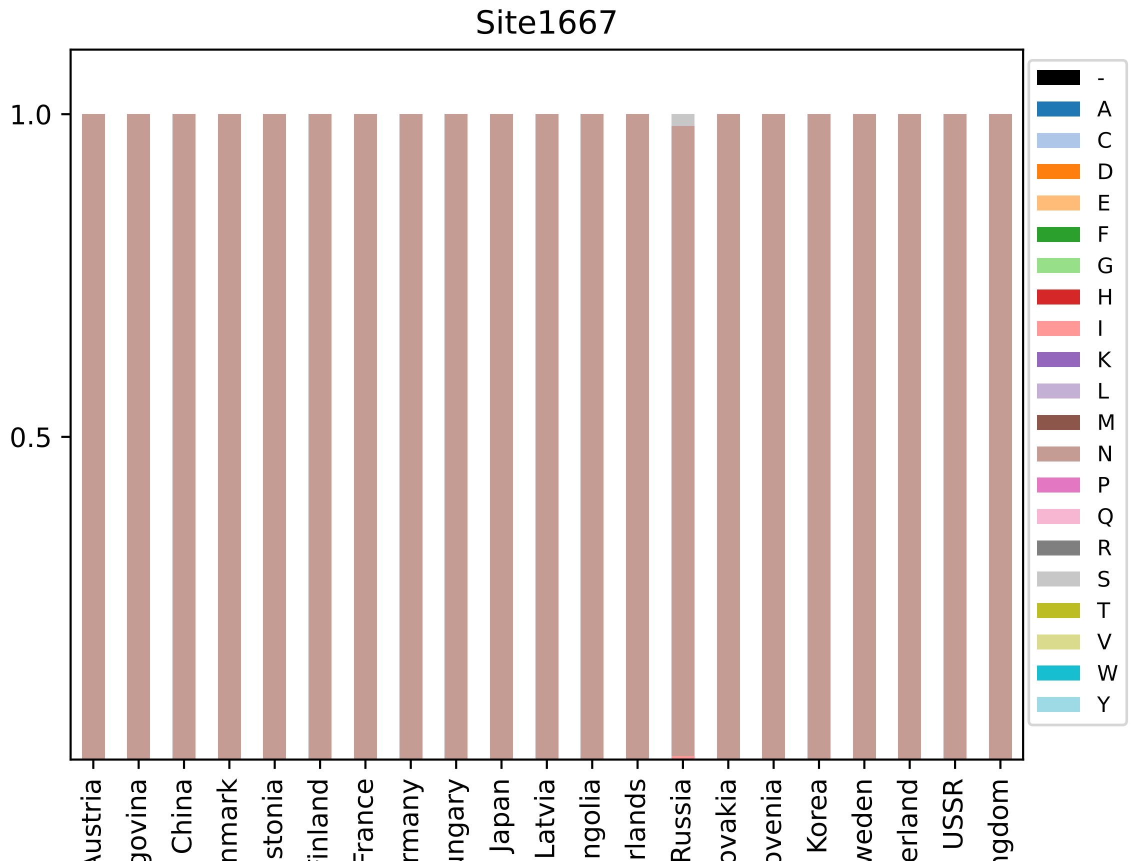

Site1667
1.0
0.5
-
A
C
D
E
F
G
H
I
K
L
M
N
P
Q
R
S
T
V
W
Y
Austria
China
Japan
Latvia
Russia
Korea
USSR

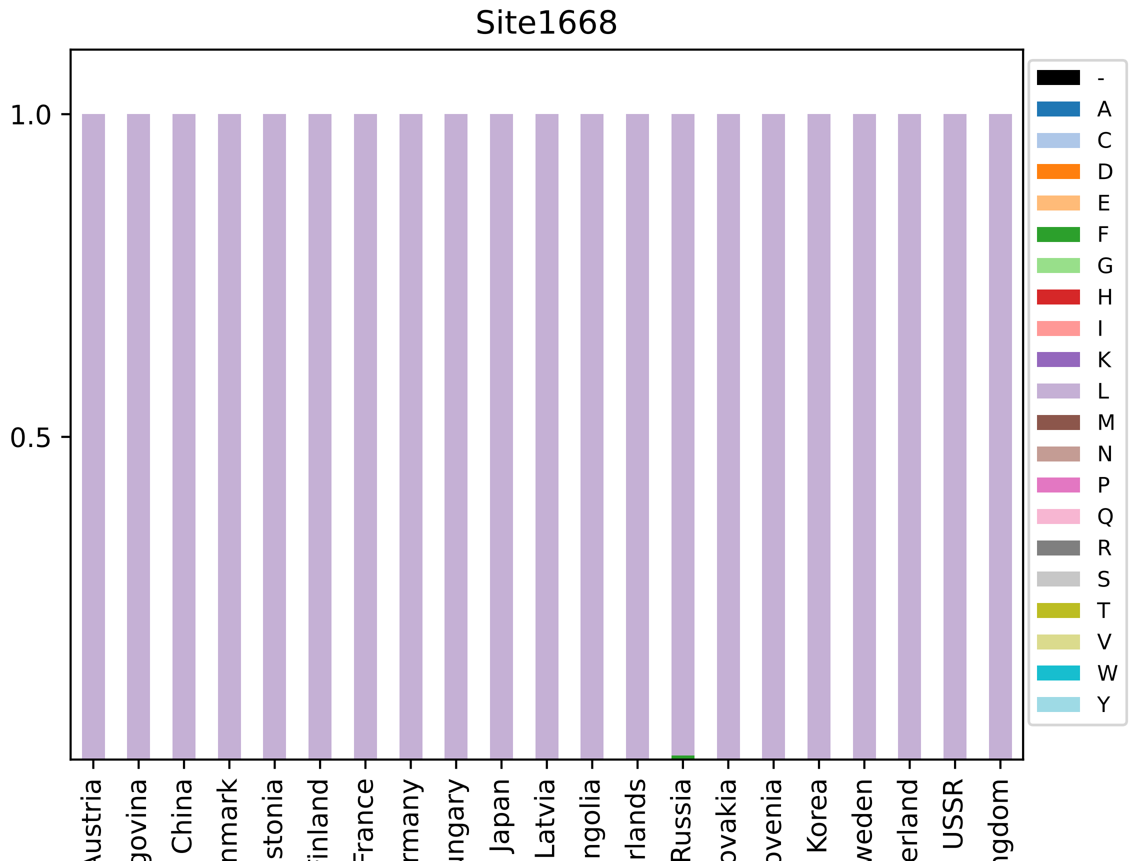

Site1668
1.0
0.5
Austria
govina
China
nmark
stonia
inland
France
rmany
ungary
Japan
Latvia
ngolia
rlands
Russia
ovakia
ovenia
Korea
weden
erland
USSR
ngdom
-
A
C
D
E
F
G
H
I
K
L
M
N
P
Q
R
S
T
V
W
Y

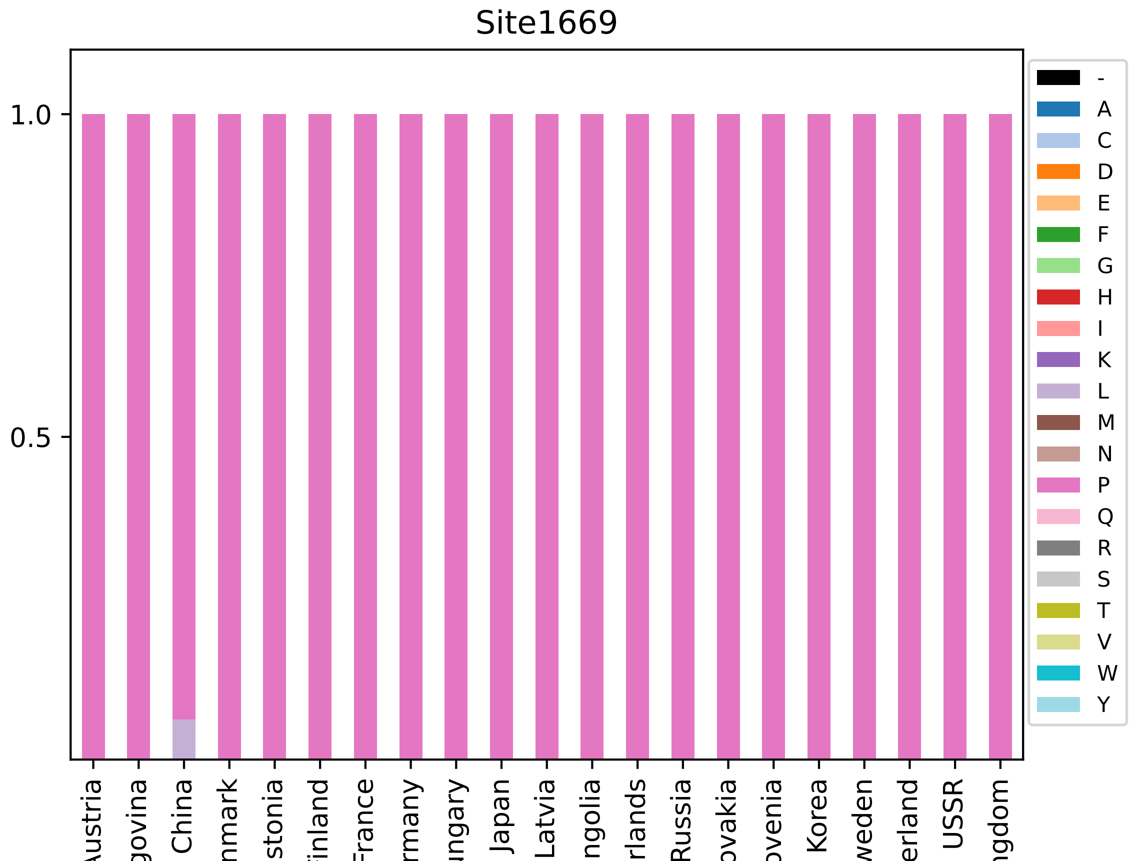

Site1669
1.0
0.5
China
Japan
Latvia
Russia
Korea
USSR
-
A
C
D
E
F
G
H
I
K
L
M
N
P
Q
R
S
T
V
W
Y

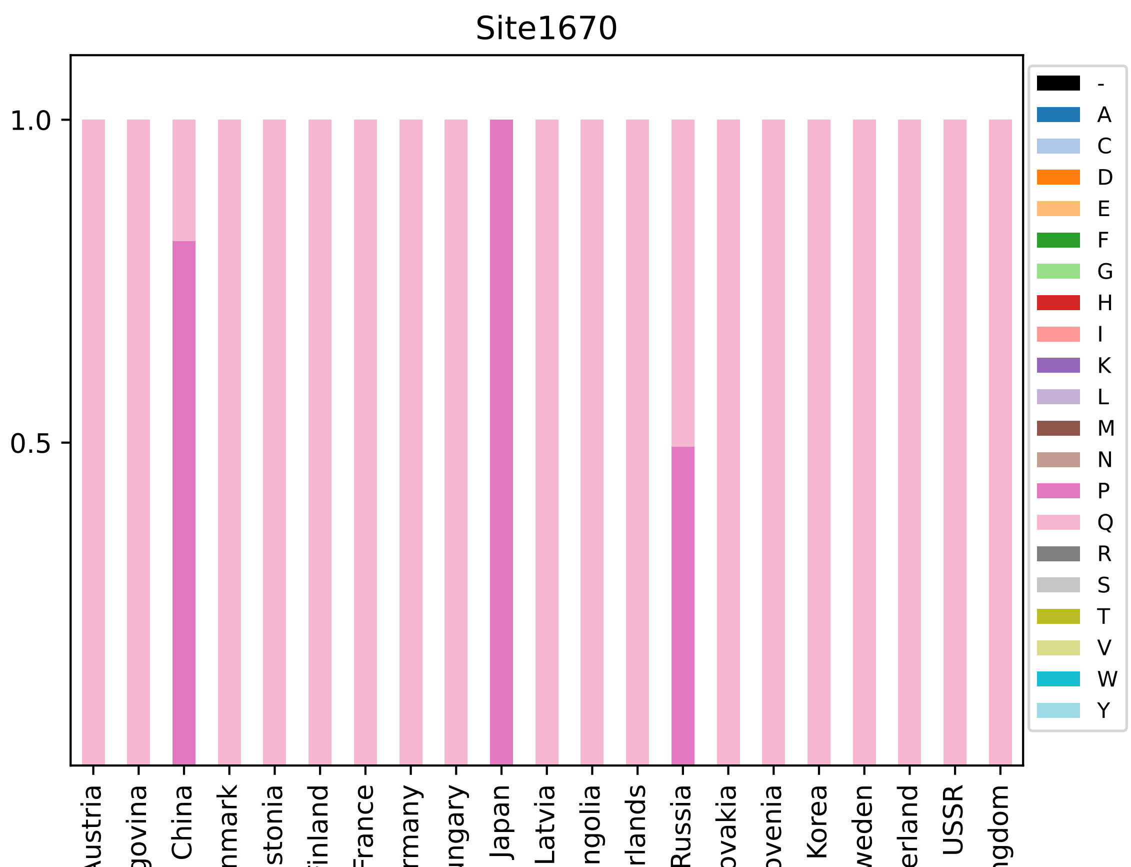

Site1670
1.0
0.5
-
A
C
D
E
F
G
H
I
K
L
M
N
P
Q
R
S
T
V
W
Y
China
Japan
Latvia
Russia
Korea
USSR

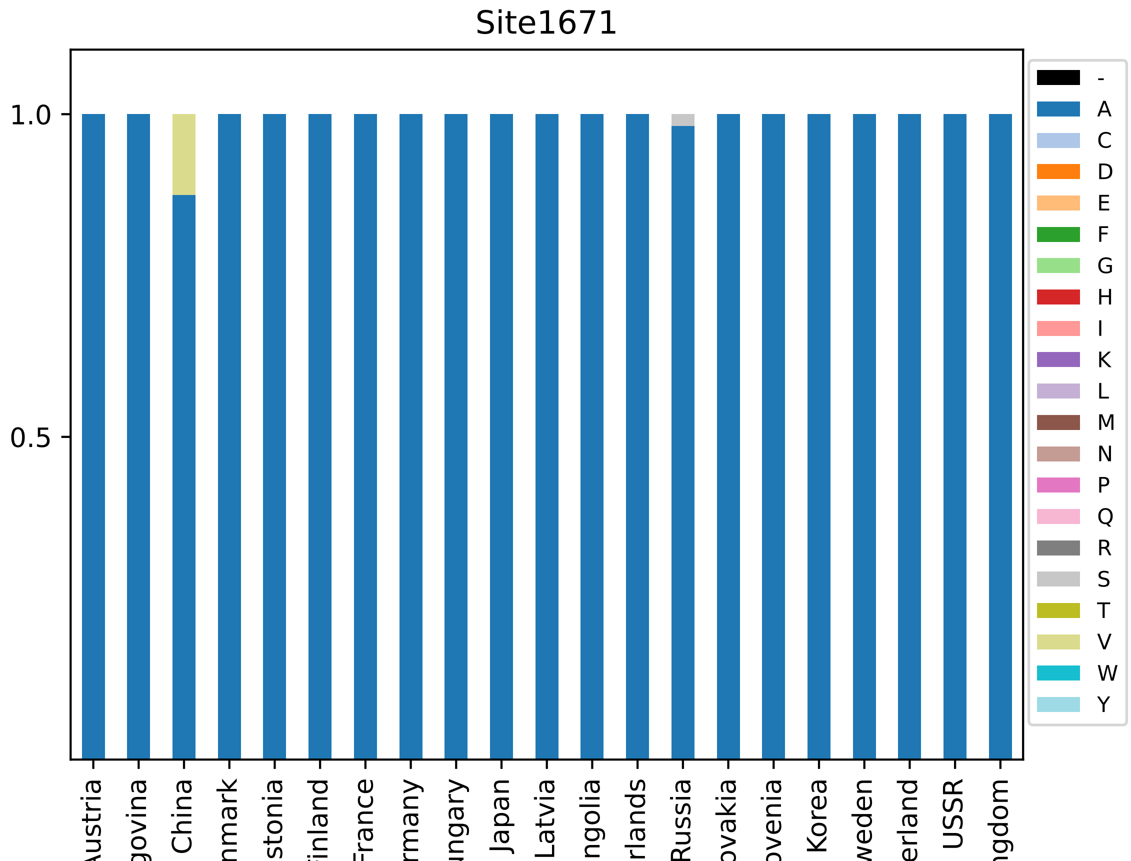
Site1671
1.0
0.5
-
A
C
D
E
F
G
H
I
K
L
M
N
P
Q
R
S
T
V
W
Y
China
Japan
Latvia
Russia
Korea
USSR

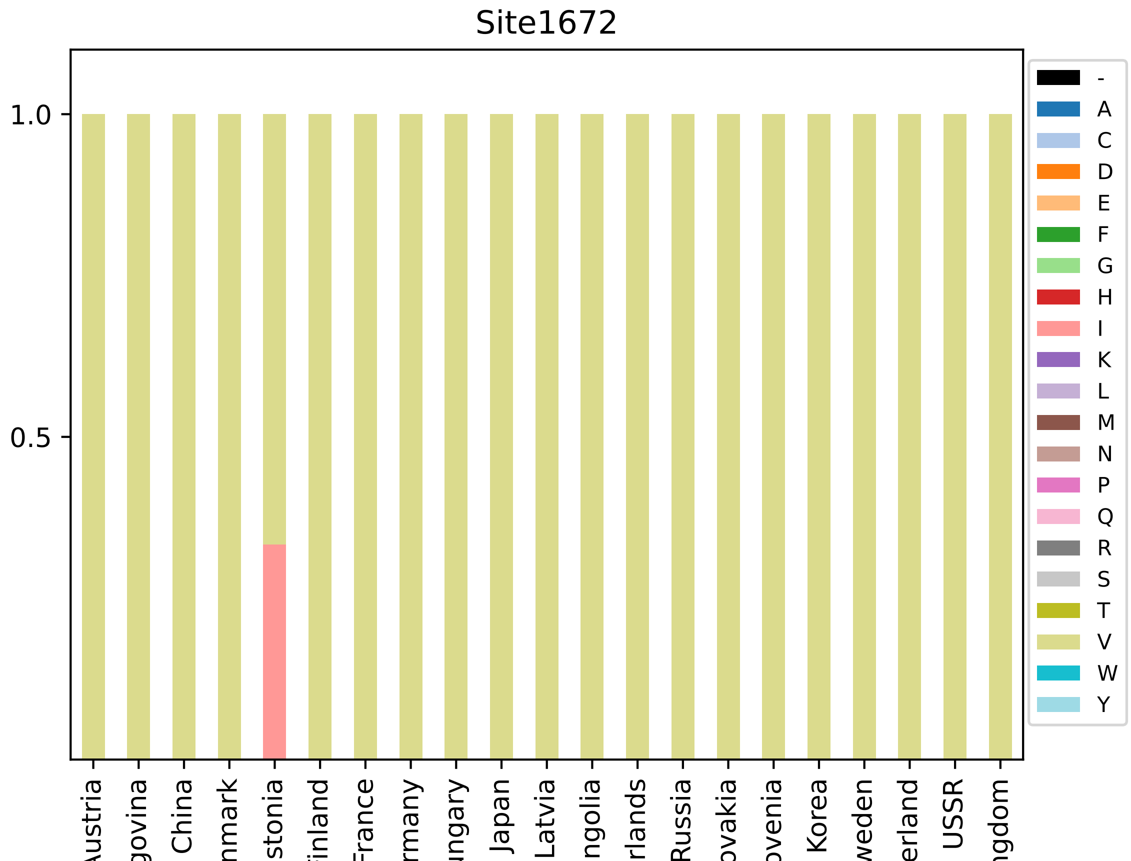
Site1672
1.0
0.5
Austria
govina
China
nmark
stonia
Finland
France
rmany
ungary
Japan
Latvia
ngolia
rlands
Russia
ovakia
ovenia
Korea
weden
erland
USSR
ngdom
-
A
C
D
E
F
G
H
I
K
L
M
N
P
Q
R
S
T
V
W
Y

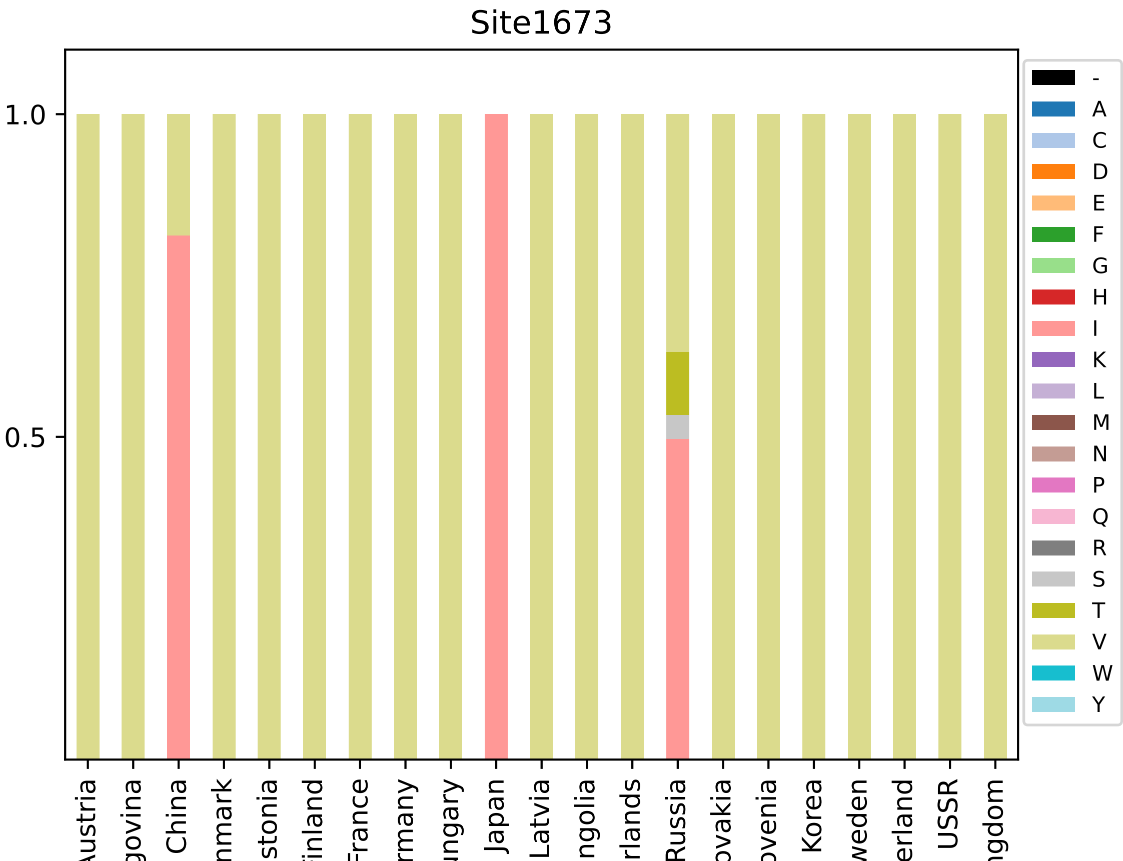
Site1673
1.0
0.5
-
A
C
D
E
F
G
H
I
K
L
M
N
P
Q
R
S
T
V
W
Y
China
Japan
Latvia
Russia
Korea
USSR

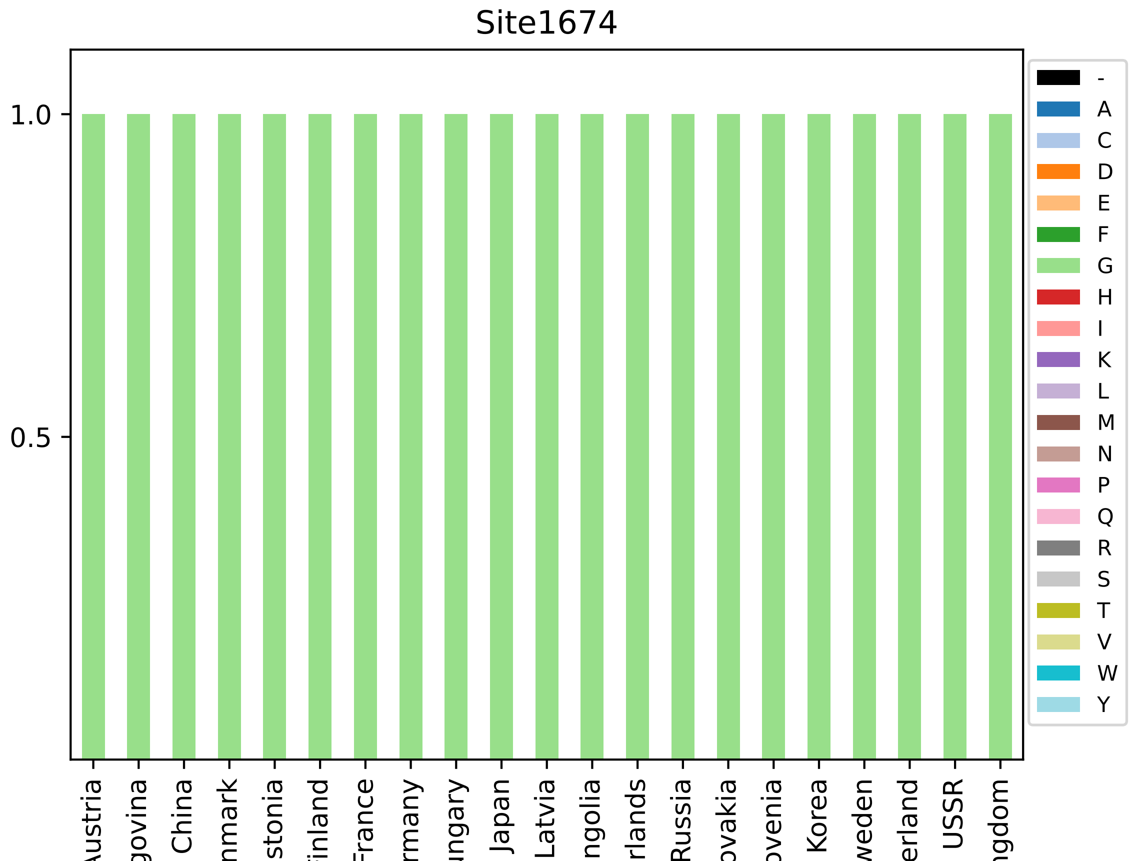
Site1674
1.0
0.5
-
A
C
D
E
F
G
H
I
K
L
M
N
P
Q
R
S
T
V
W
Y
Austria
govina
China
nmark
stonia
inland
France
rmany
ungary
Japan
Latvia
ngolia
rlands
Russia
ovakia
ovenia
Korea
weden
erland
USSR
ngdom

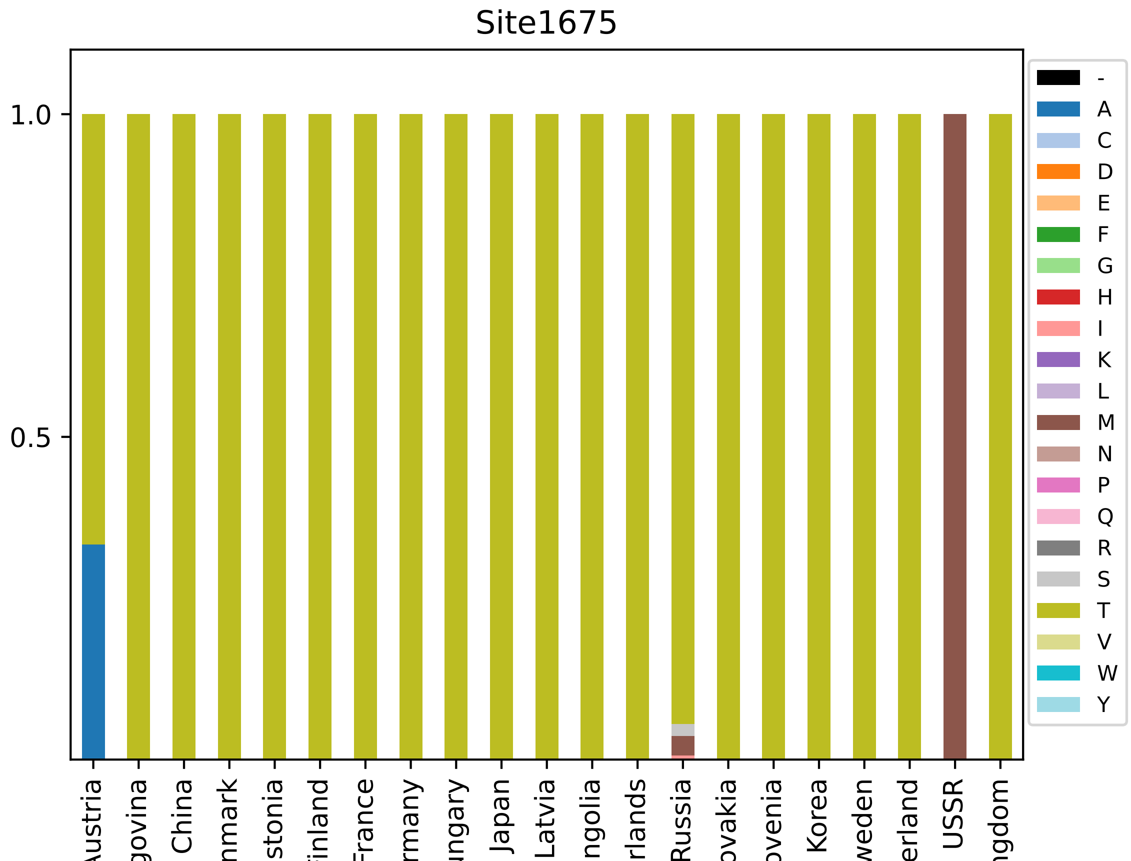
Site1675
1.0
0.5
-
A
C
D
E
F
G
H
I
K
L
M
N
P
Q
R
S
T
V
W
Y
Austria
China
Japan
Latvia
Russia
Korea
USSR

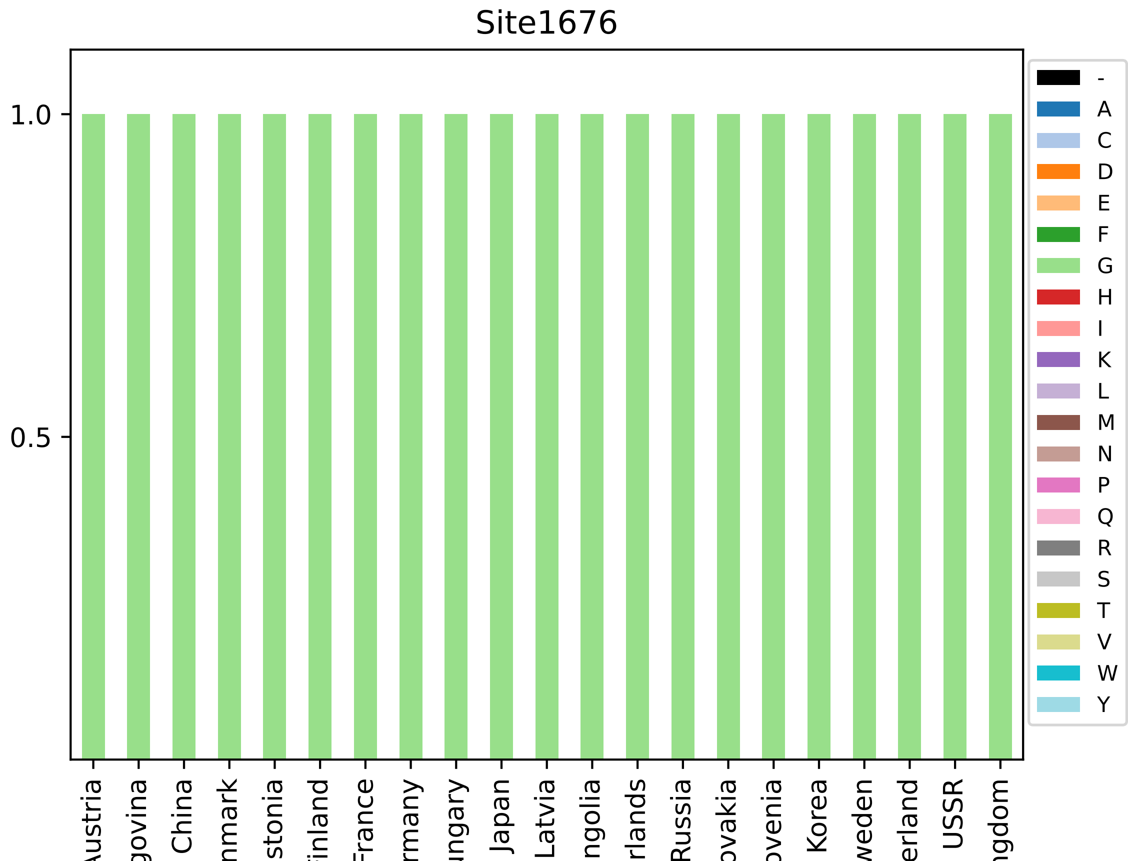
Site1676
1.0
0.5
-
A
C
D
E
F
G
H
I
K
L
M
N
P
Q
R
S
T
V
W
Y
Austria
govina
China
nmark
stonia
inland
France
rmany
ungary
Japan
Latvia
ngolia
rlands
Russia
ovakia
ovenia
Korea
weden
erland
USSR
ngdom

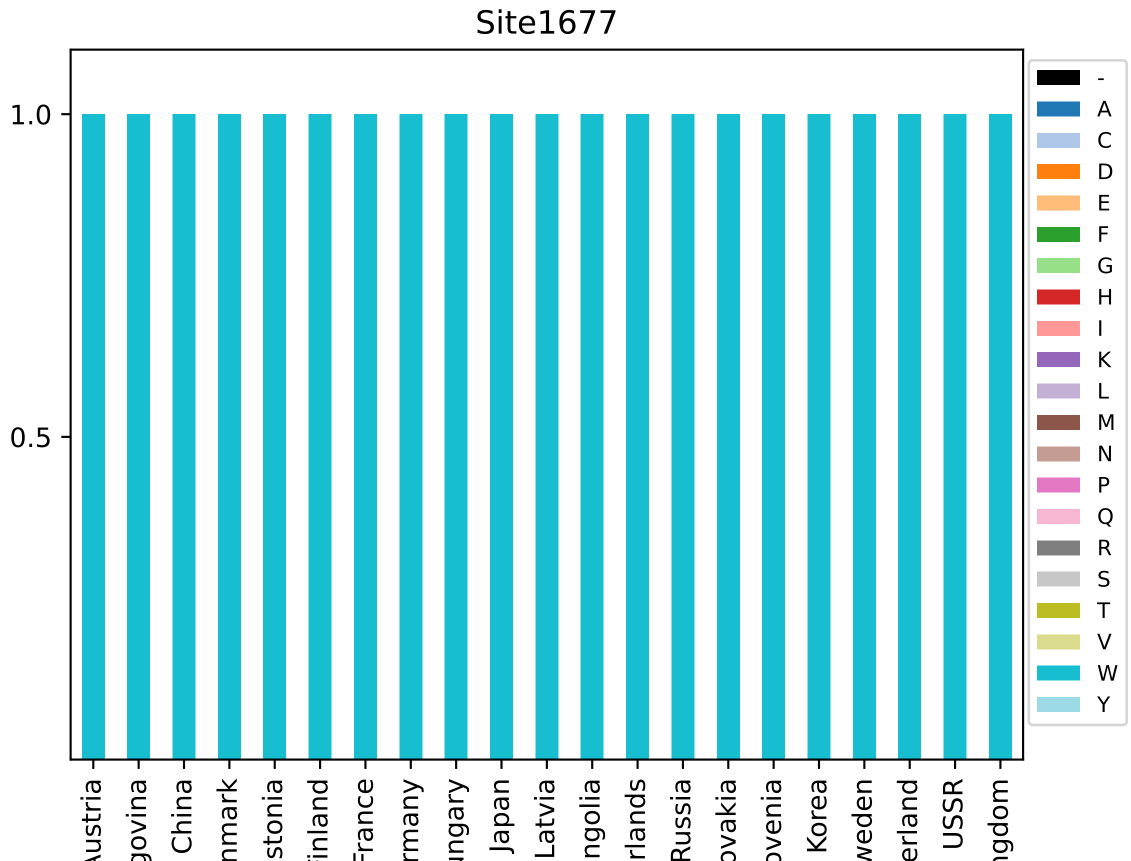
Site1677
1.0
0.5
-
A
C
D
E
F
G
H
I
K
L
M
N
P
Q
R
S
T
V
W
Y
Austria
China
Japan
Latvia
Russia
Korea
USSR

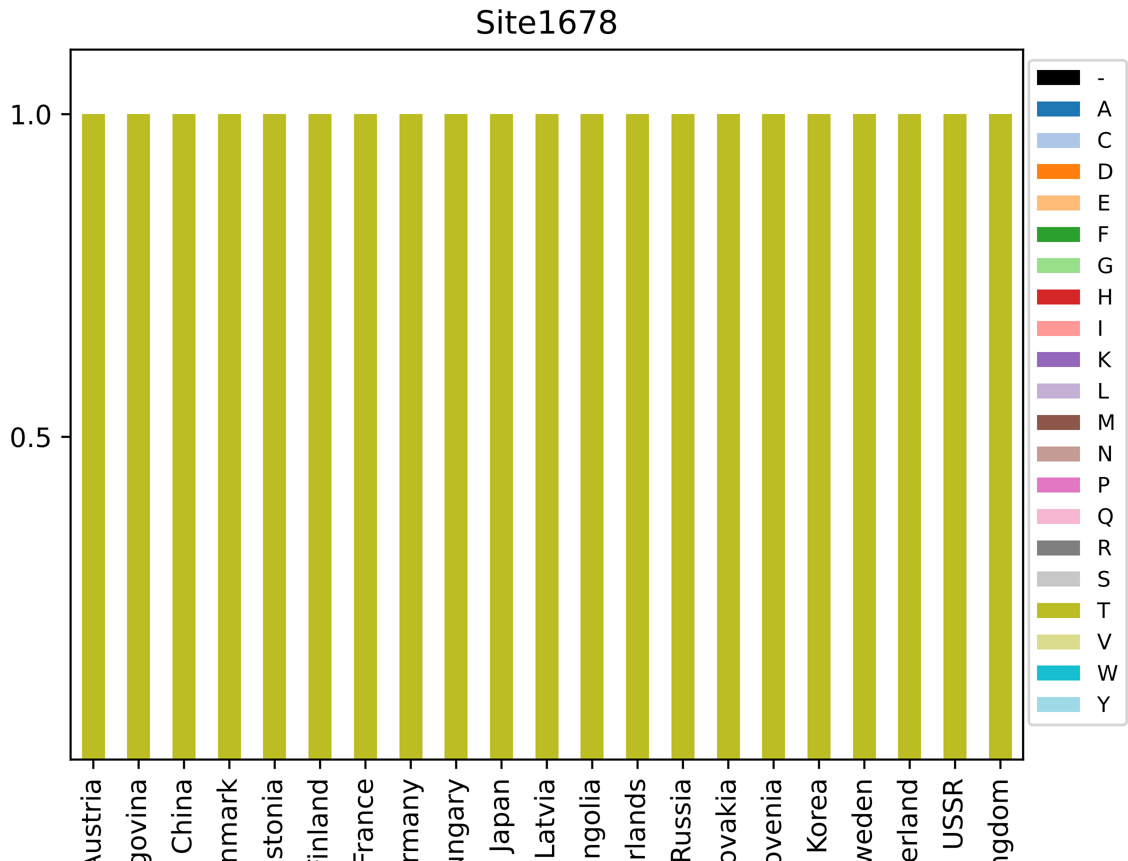
Site1678
1.0
0.5
-
A
C
D
E
F
G
H
I
K
L
M
N
P
Q
R
S
T
V
W
Y
Austria
govina
China
nmark
stonia
inland
France
rmany
ungary
Japan
Latvia
ngolia
rlands
Russia
ovakia
ovenia
Korea
weden
erland
USSR
ngdom

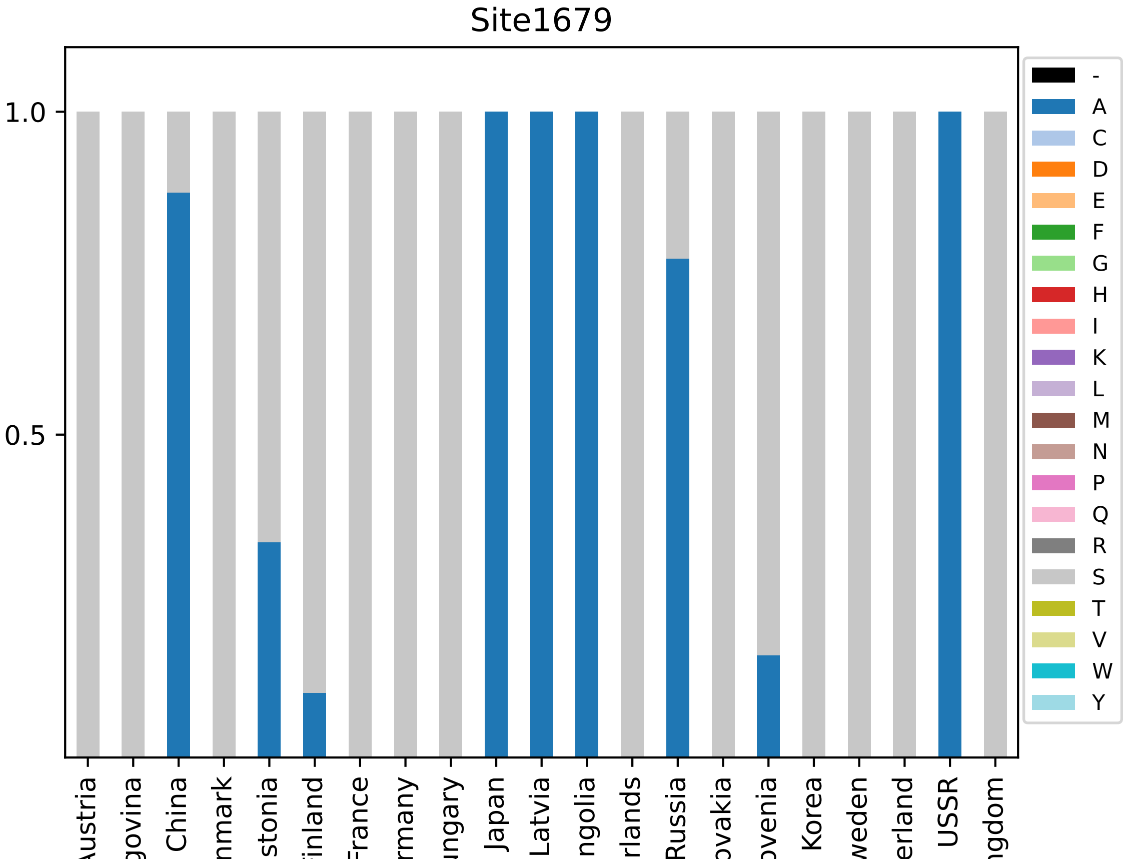

Site1679
1.0
0.5
-
A
C
D
E
F
G
H
I
K
L
M
N
P
Q
R
S
T
V
W
Y
China
Japan
Latvia
Russia
Korea
USSR

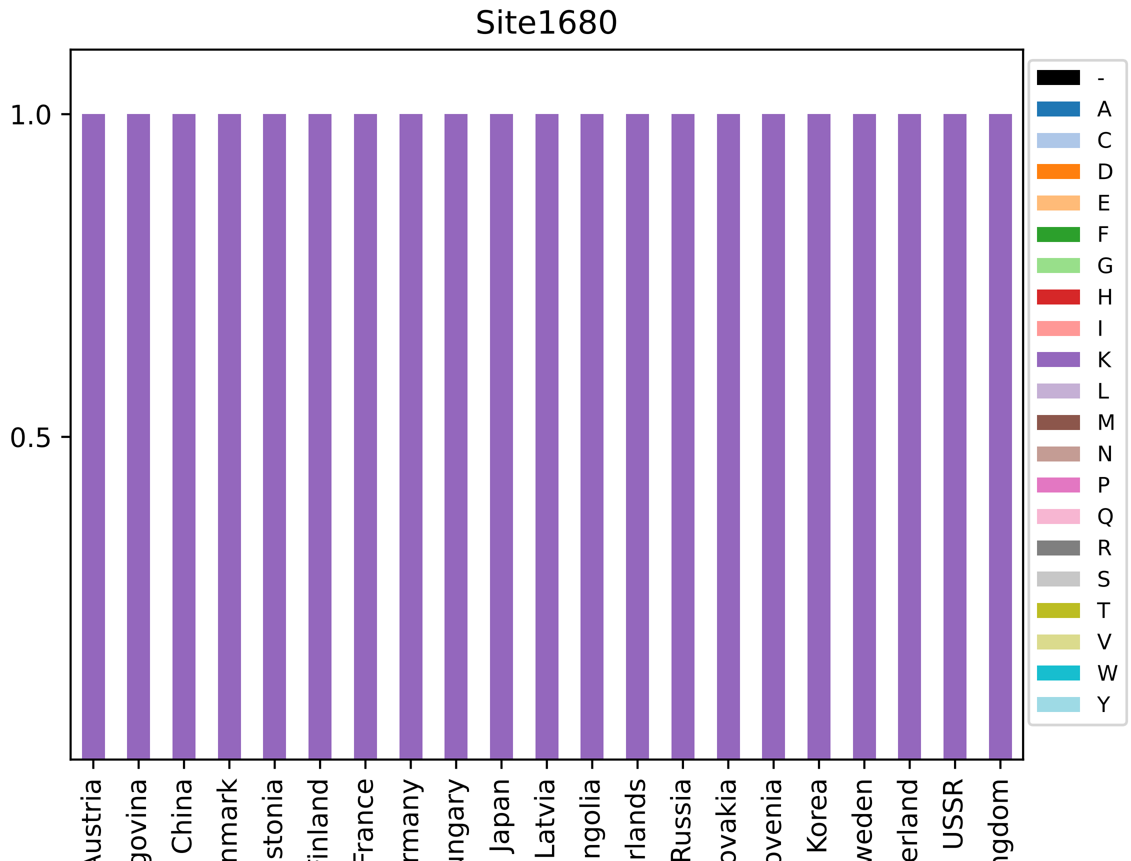
Site1680
1.0
0.5
-
A
C
D
E
F
G
H
I
K
L
M
N
P
Q
R
S
T
V
W
Y
Austria
govina
China
nmark
stonia
inland
France
rmany
ungary
Japan
Latvia
ngolia
rlands
Russia
ovakia
ovenia
Korea
weden
erland
USSR
ngdom

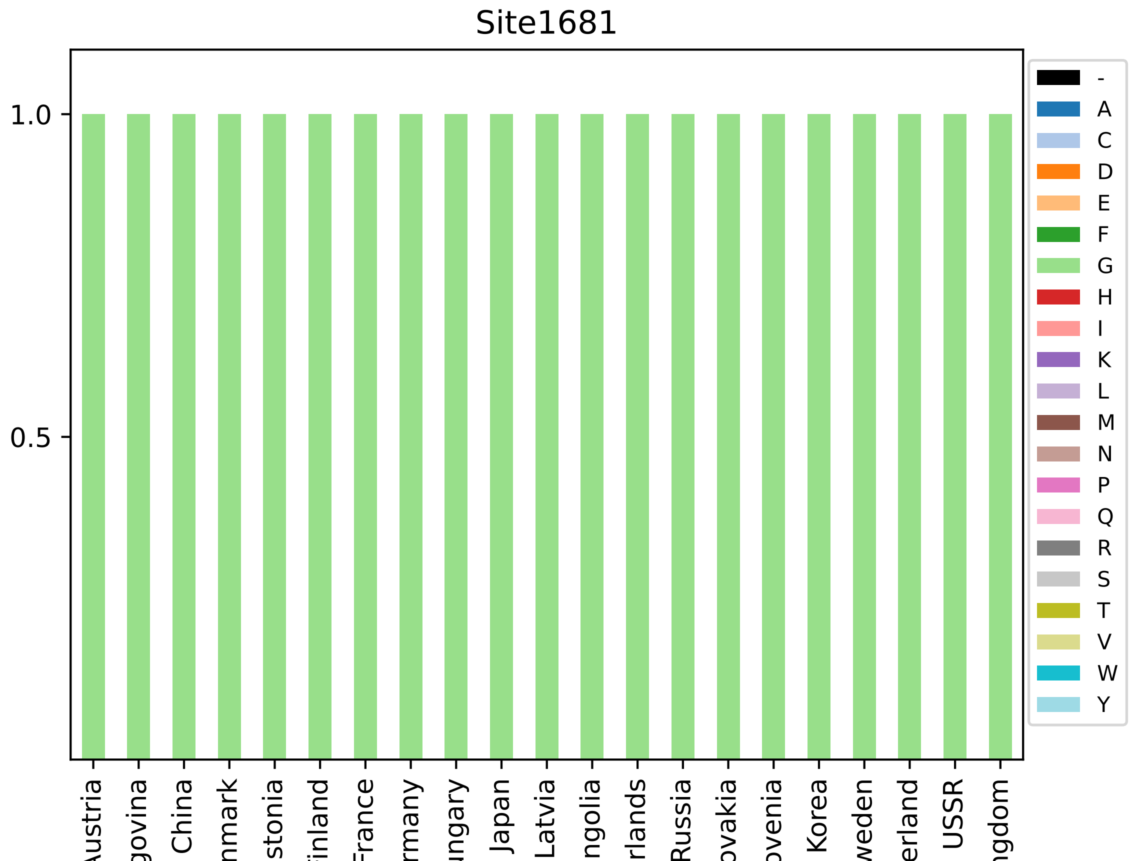
Site1681
1.0
0.5
-
A
C
D
E
F
G
H
I
K
L
M
N
P
Q
R
S
T
V
W
Y
ustria
govina
China
nmark
stonia
inland
rance
rmany
ungary
Japan
Latvia
ngolia
rlands
Russia
ovakia
ovenia
Korea
weden
erland
USSR
ngdom

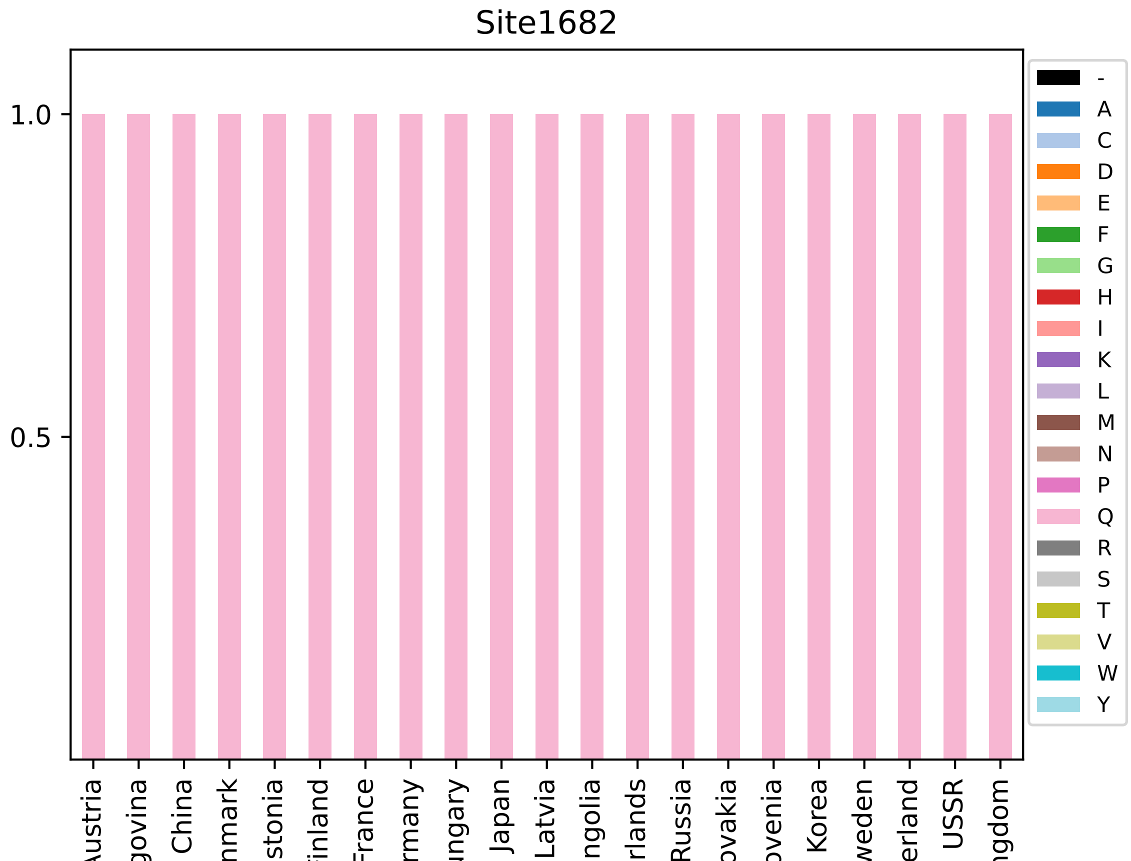

Site1682
1.0
0.5
-
A
C
D
E
F
G
H
I
K
L
M
N
P
Q
R
S
T
V
W
Y
Austria
China
Japan
Latvia
Russia
Korea
USSR

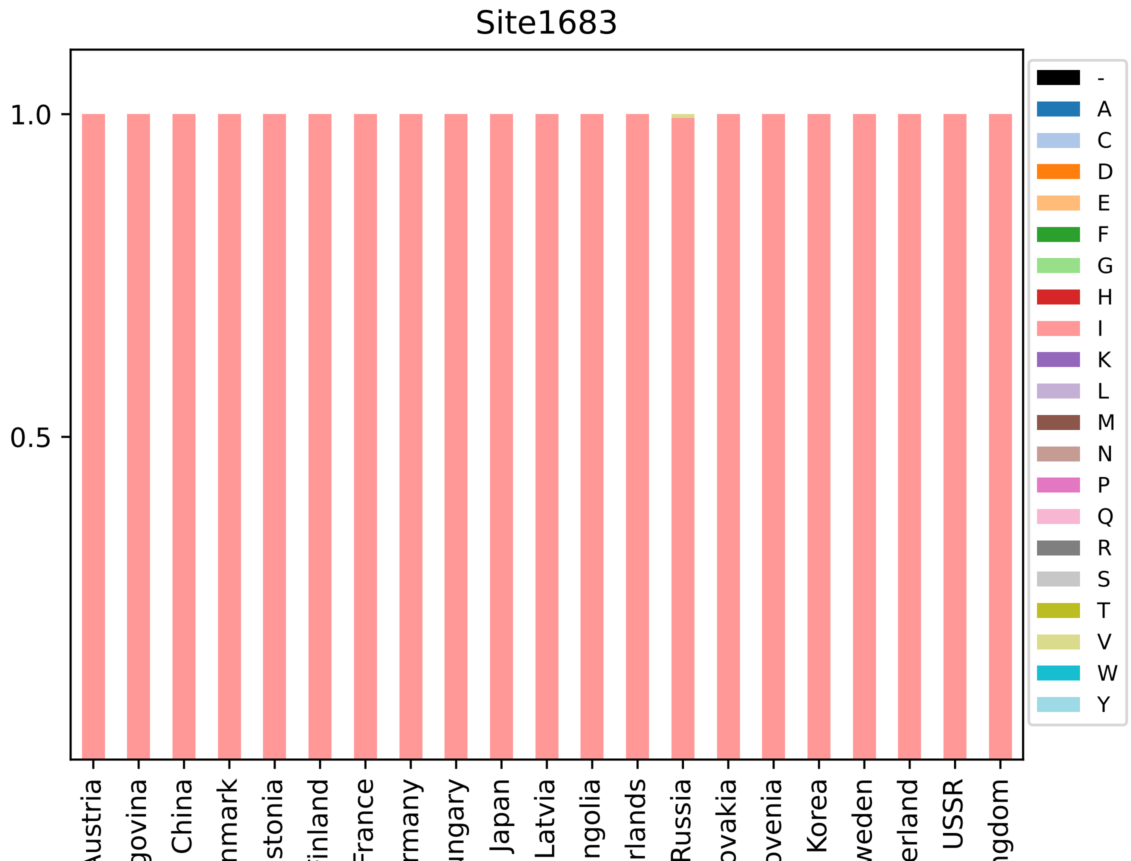

Site1683
1.0
0.5
-
A
C
D
E
F
G
H
I
K
L
M
N
P
Q
R
S
T
V
W
Y
Austria
govina
China
nmark
stonia
inland
France
rmany
ungary
Japan
Latvia
ngolia
rlands
Russia
ovakia
ovenia
Korea
weden
erland
USSR
ngdom

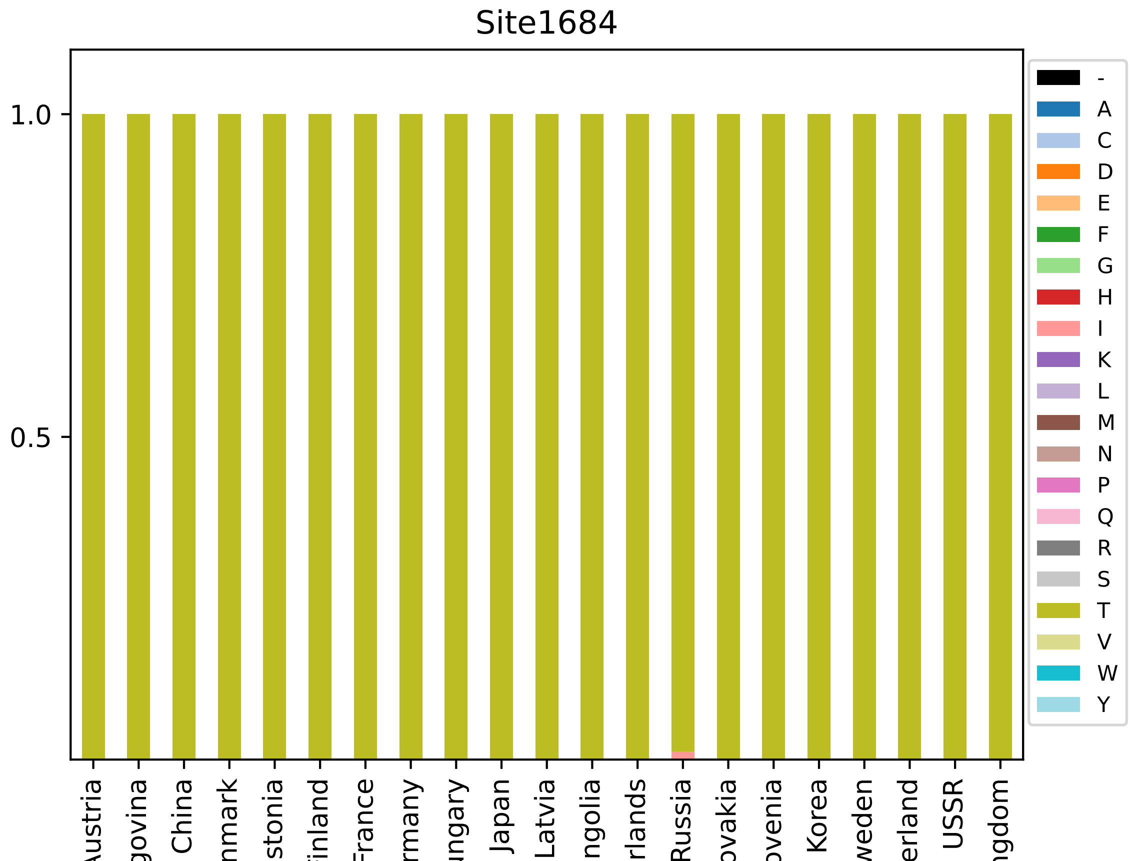
Site1684
1.0
0.5
-
A
C
D
E
F
G
H
I
K
L
M
N
P
Q
R
S
T
V
W
Y
China
Japan
Latvia
Russia
Korea
USSR

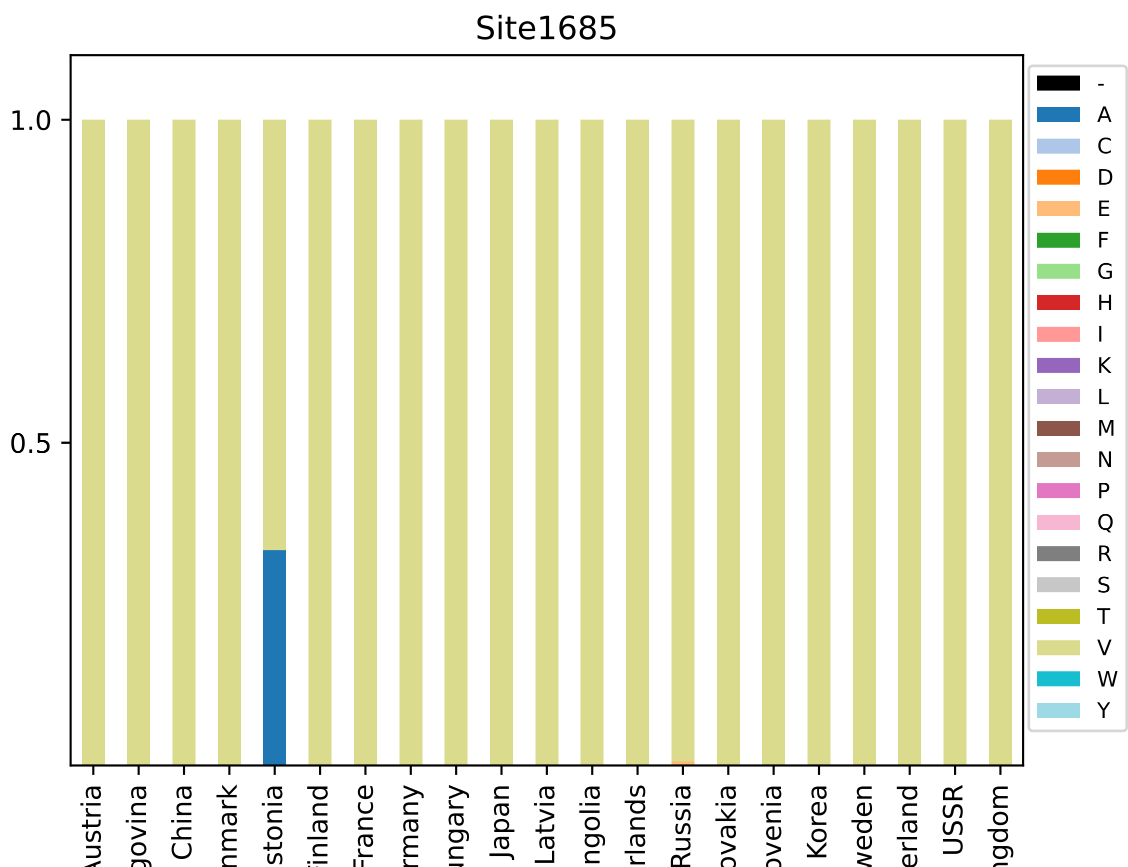

Site1685
1.0
0.5
-
A
C
D
E
F
G
H
I
K
L
M
N
P
Q
R
S
T
V
W
Y
Austria
govina
China
nmark
stonia
Finland
France
rmany
ungary
Japan
Latvia
ngolia
rlands
Russia
ovakia
ovenia
Korea
weden
erland
USSR
ngdom

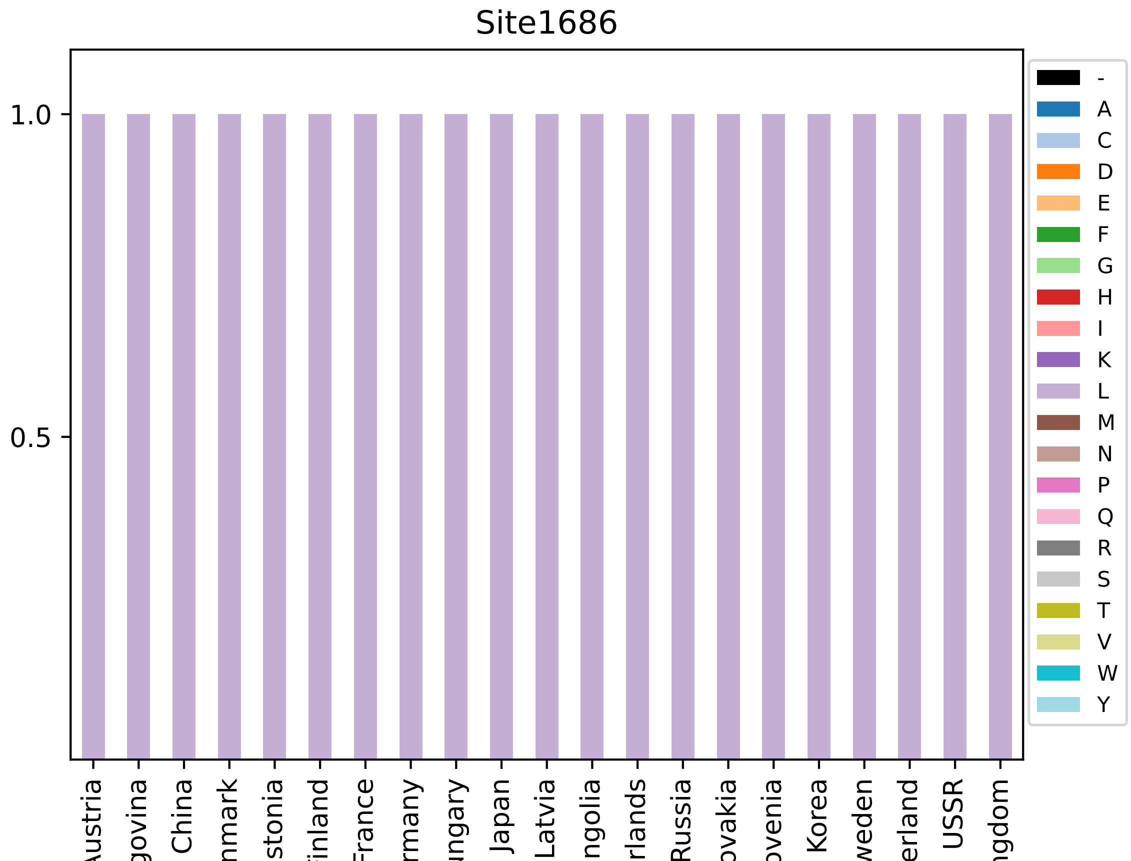
Site1686
1.0
0.5
-
A
C
D
E
F
G
H
I
K
L
M
N
P
Q
R
S
T
V
W
Y
Austria
govina
China
nmark
stonia
Finland
France
rmany
ungary
Japan
Latvia
ngolia
rlands
Russia
ovakia
ovenia
Korea
weden
erland
USSR
ngdom

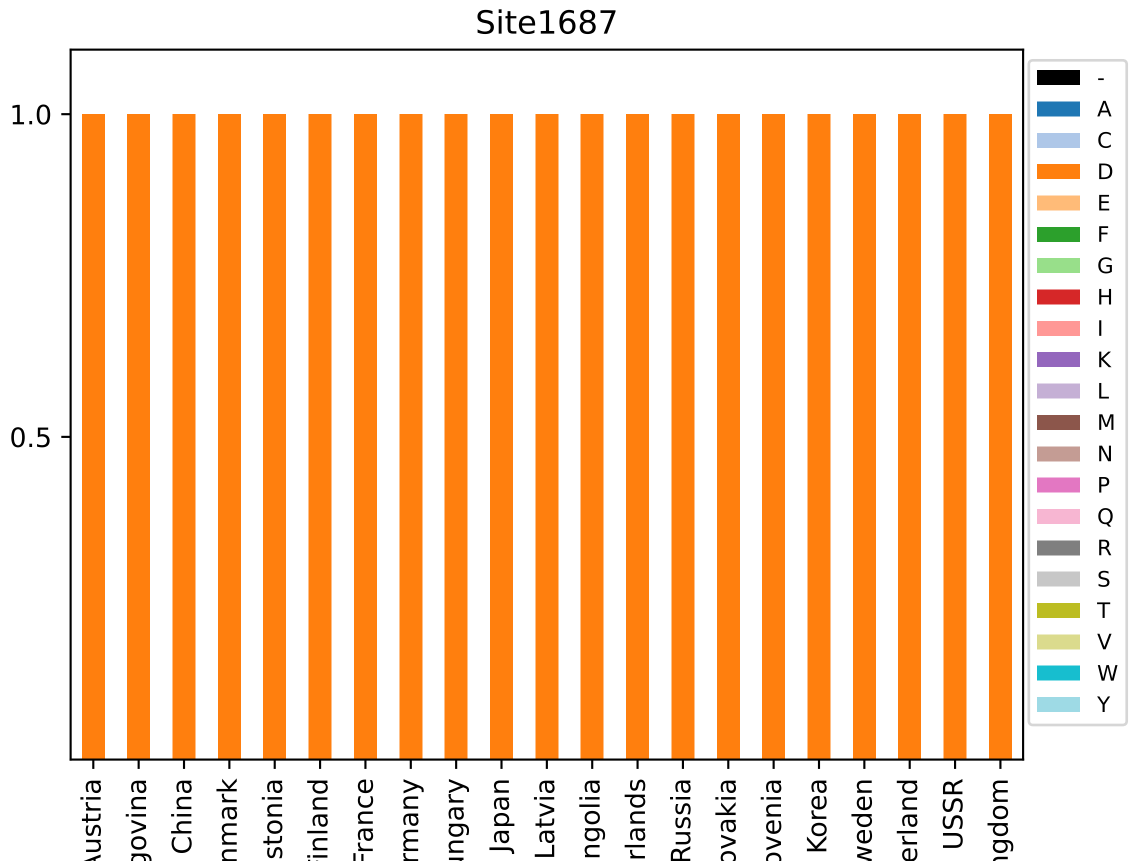
Site1687
1.0
0.5
-
A
C
D
E
F
G
H
I
K
L
M
N
P
Q
R
S
T
V
W
Y
China
Japan
Latvia
Russia
Korea
USSR

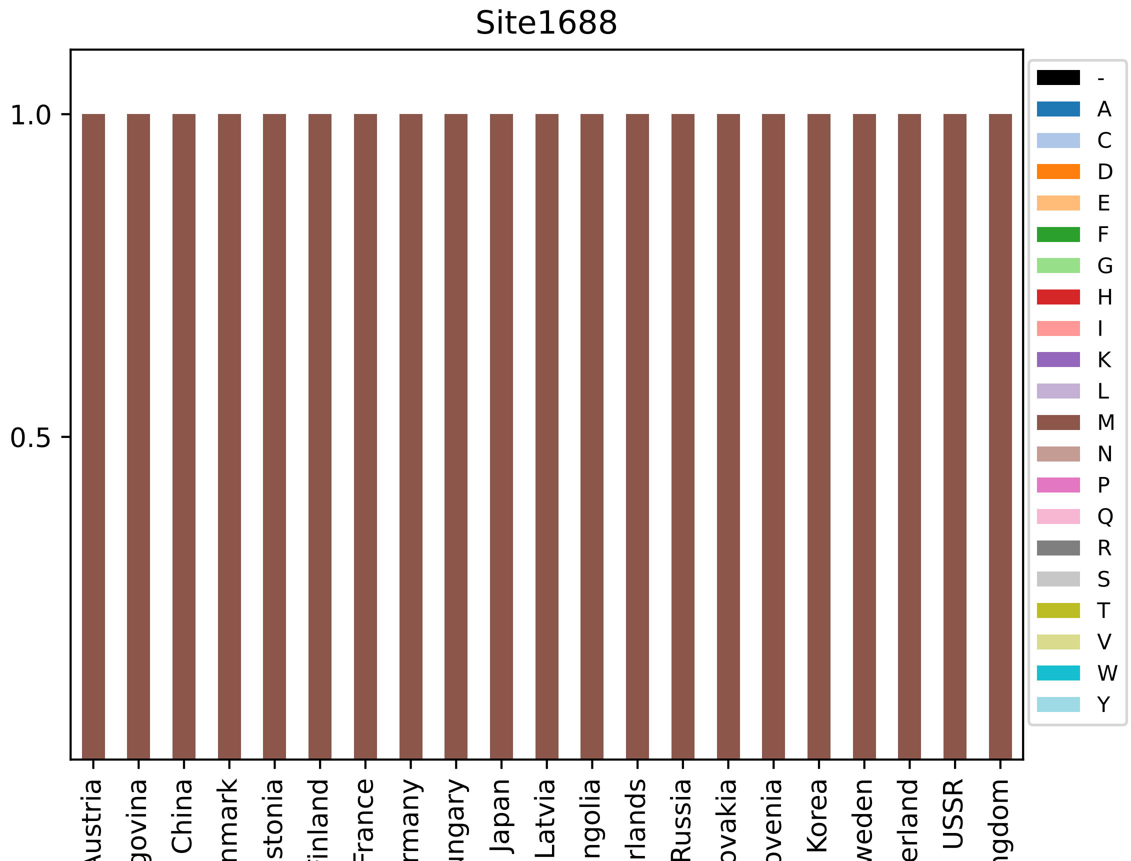
Site1688
1.0
0.5
-
A
C
D
E
F
G
H
I
K
L
M
N
P
Q
R
S
T
V
W
Y
Austria
govina
China
nmark
stonia
inland
France
rmany
ungary
Japan
Latvia
ngolia
rlands
Russia
ovakia
ovenia
Korea
weden
erland
USSR
ngdom

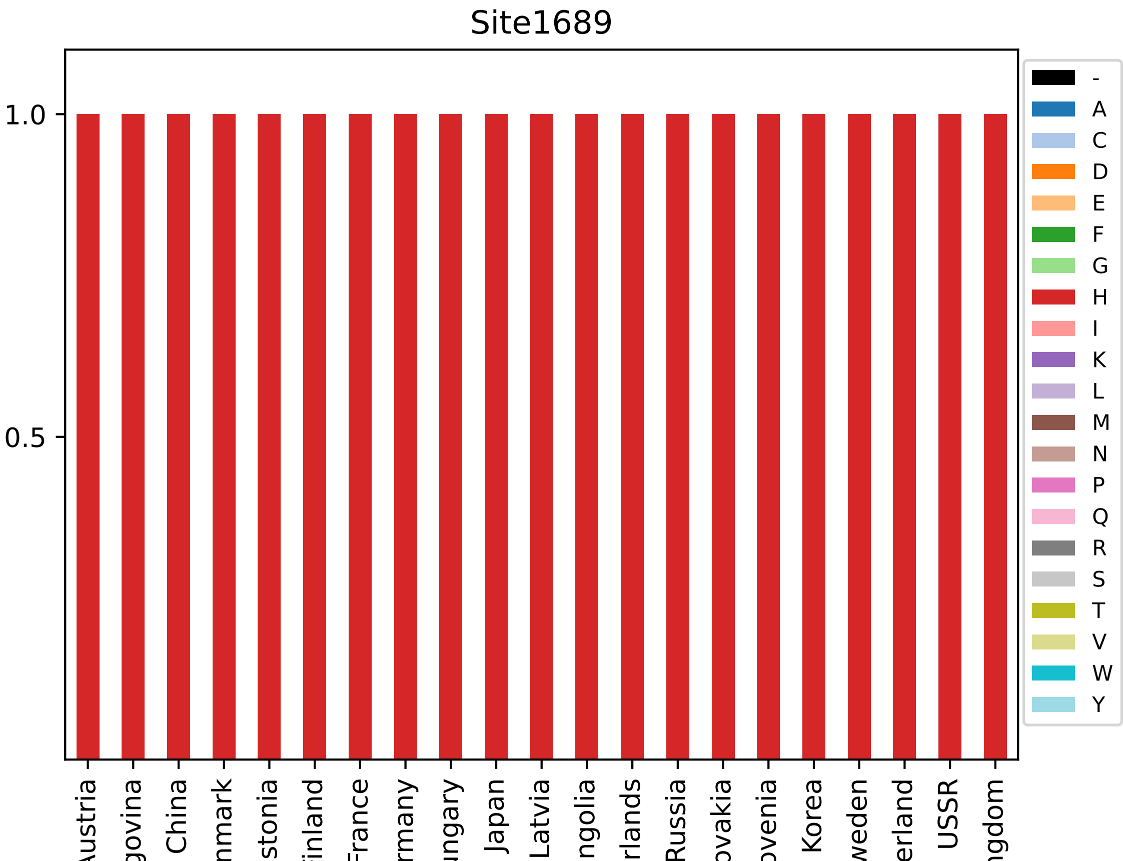
Site1689
1.0
0.5
-
A
C
D
E
F
G
H
I
K
L
M
N
P
Q
R
S
T
V
W
Y
China
Japan
Latvia
Russia
Korea
USSR

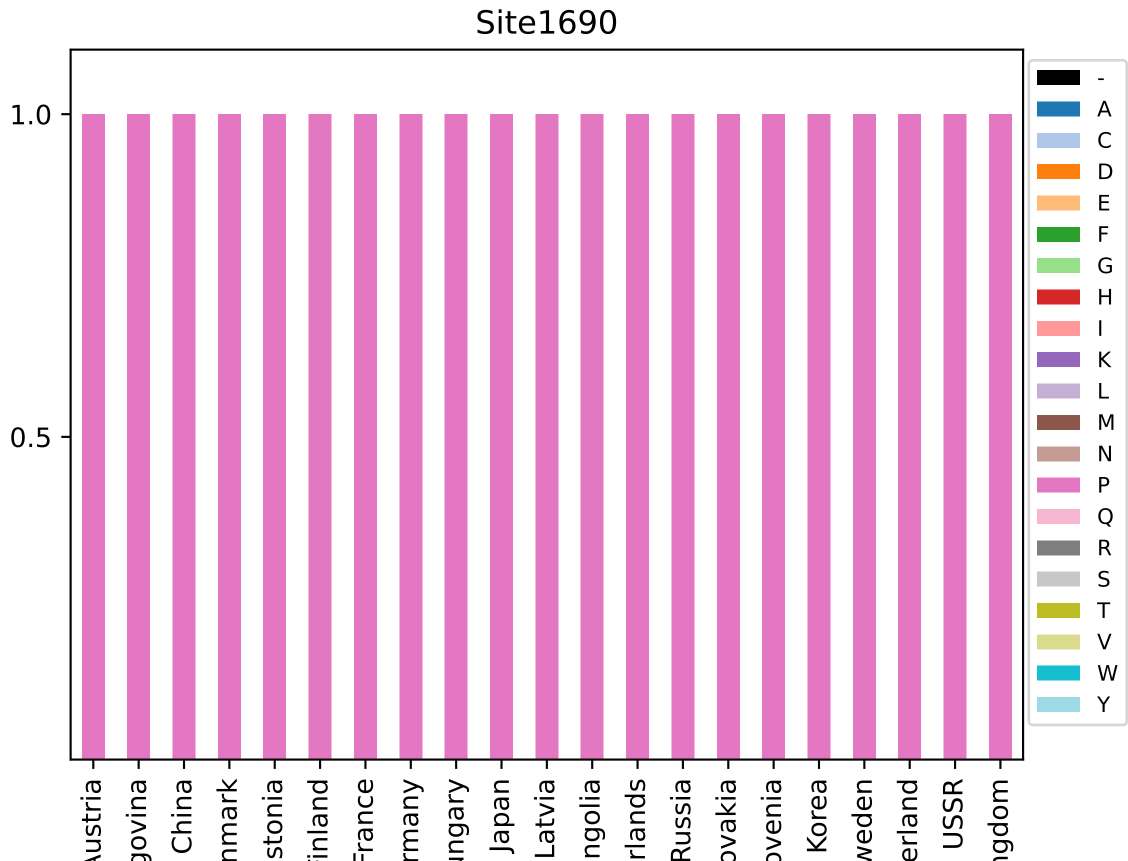

Site1690
1.0
0.5
China
Japan
Latvia
Russia
Korea
USSR
-
A
C
D
E
F
G
H
I
K
L
M
N
P
Q
R
S
T
V
W
Y

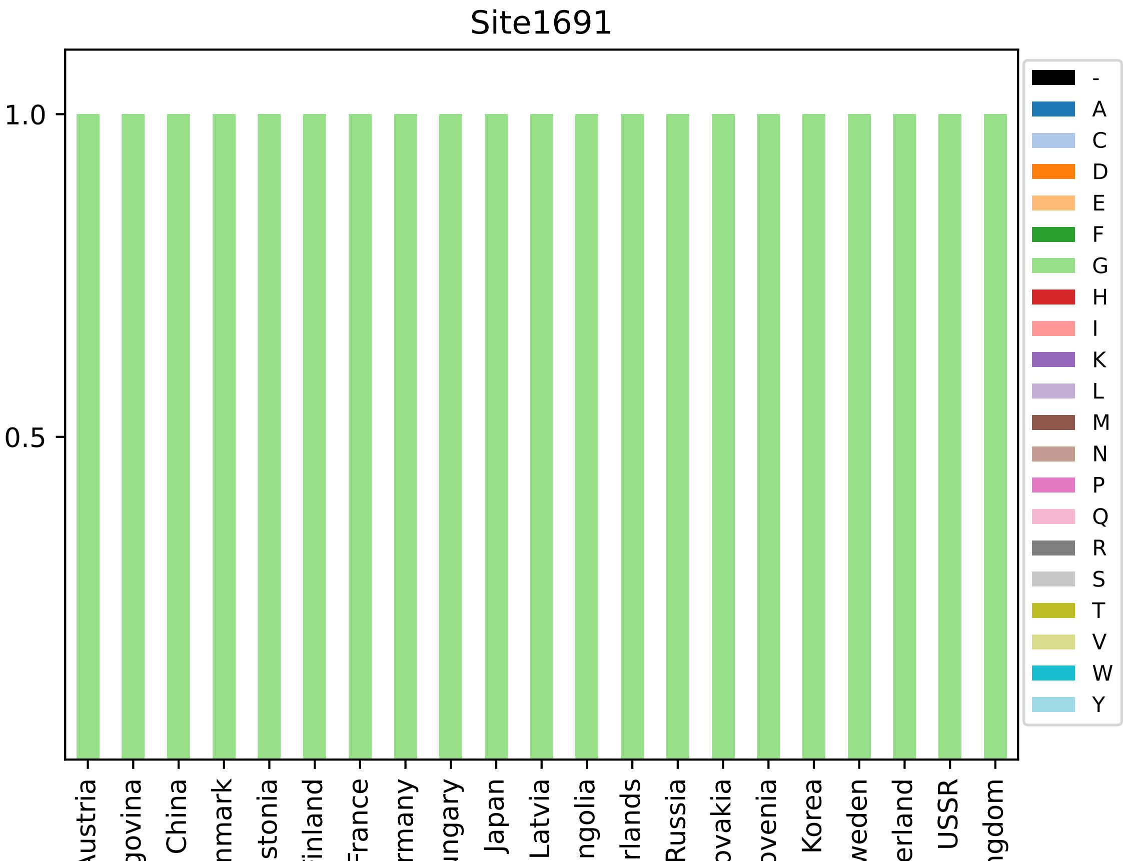

Site1691
1.0
0.5
-
A
C
D
E
F
G
H
I
K
L
M
N
P
Q
R
S
T
V
W
Y
China
Japan
Latvia
Russia
Korea
USSR

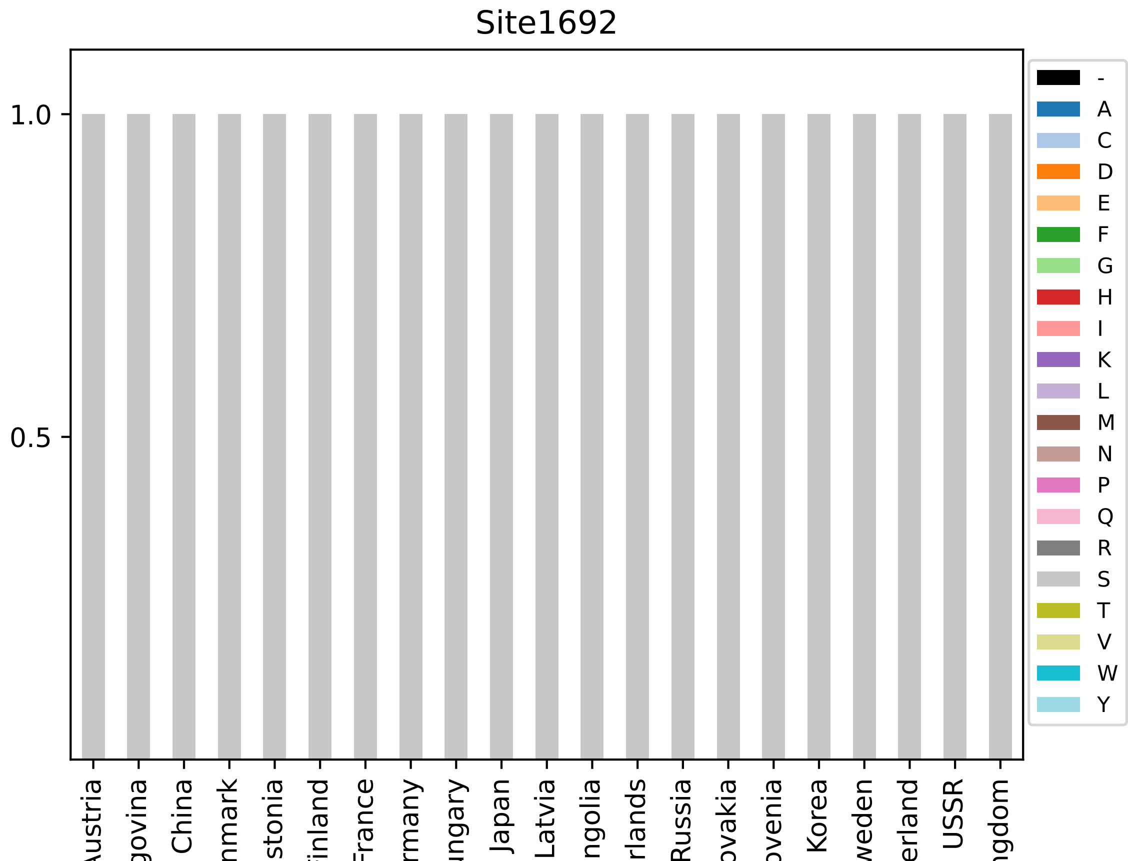

Site1692
1.0
0.5
-
A
C
D
E
F
G
H
I
K
L
M
N
P
Q
R
S
T
V
W
Y
Austria
govina
China
nmark
stonia
inland
France
rmany
ungary
Japan
Latvia
ngolia
rlands
Russia
ovakia
ovenia
Korea
weden
erland
USSR
ngdom

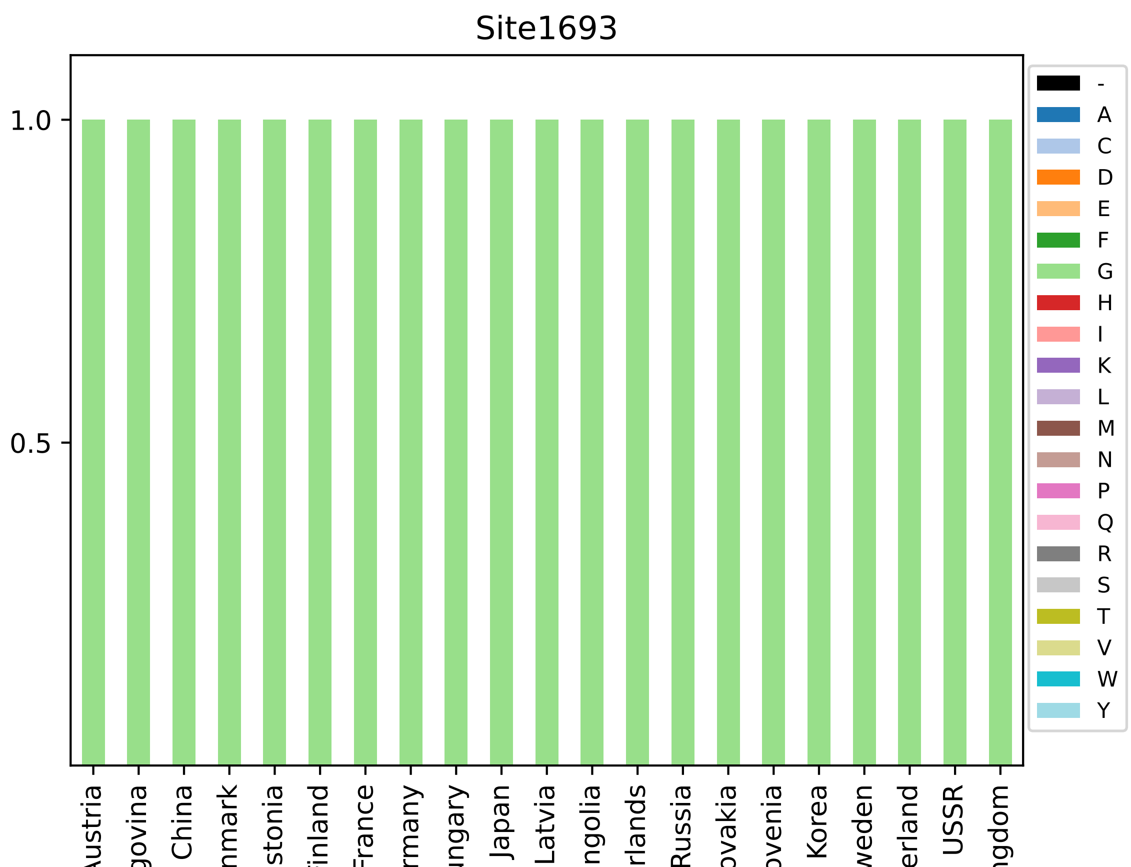
Site1693
1.0
0.5
-
A
C
D
E
F
G
H
I
K
L
M
N
P
Q
R
S
T
V
W
Y
China
Japan
Latvia
Russia
Korea
USSR

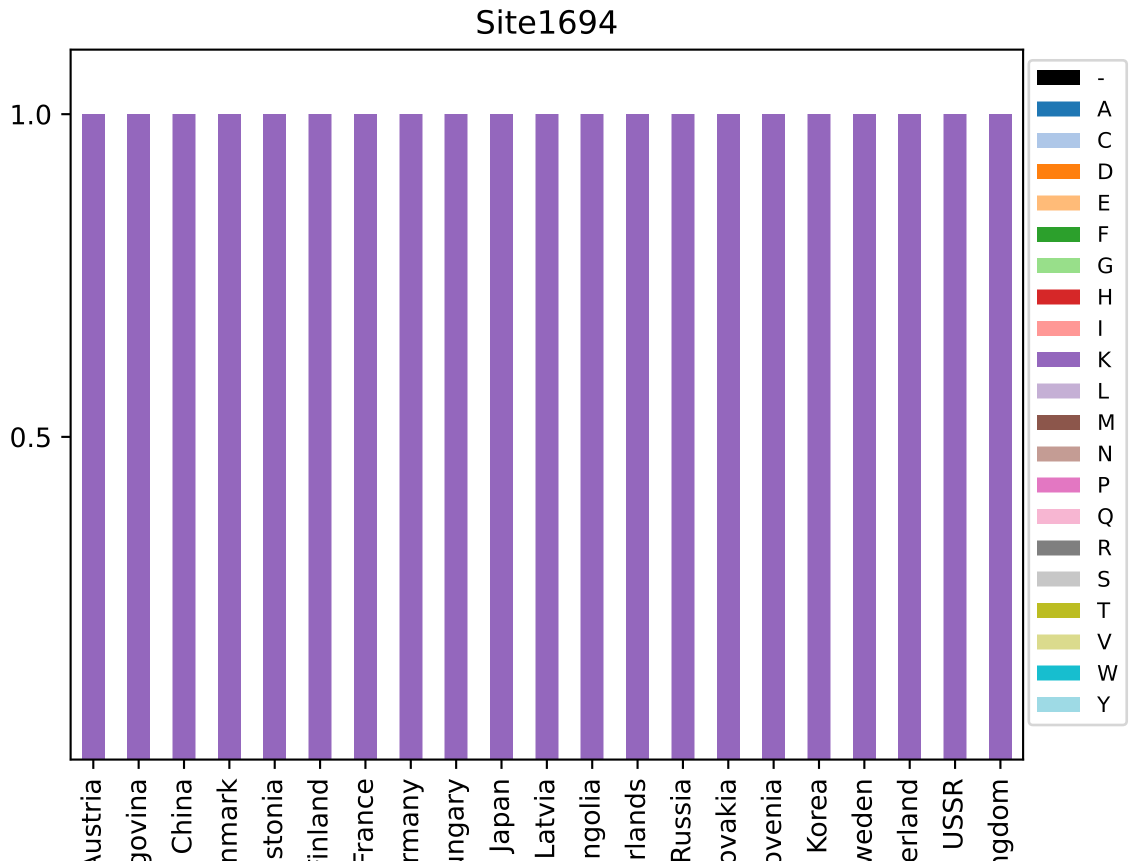

Site1694
1.0
0.5
-
A
C
D
E
F
G
H
I
K
L
M
N
P
Q
R
S
T
V
W
Y
Austria
govina
China
nmark
stonia
Finland
France
rmany
ungary
Japan
Latvia
ngolia
rlands
Russia
ovakia
ovenia
Korea
weden
erland
USSR
ngdom

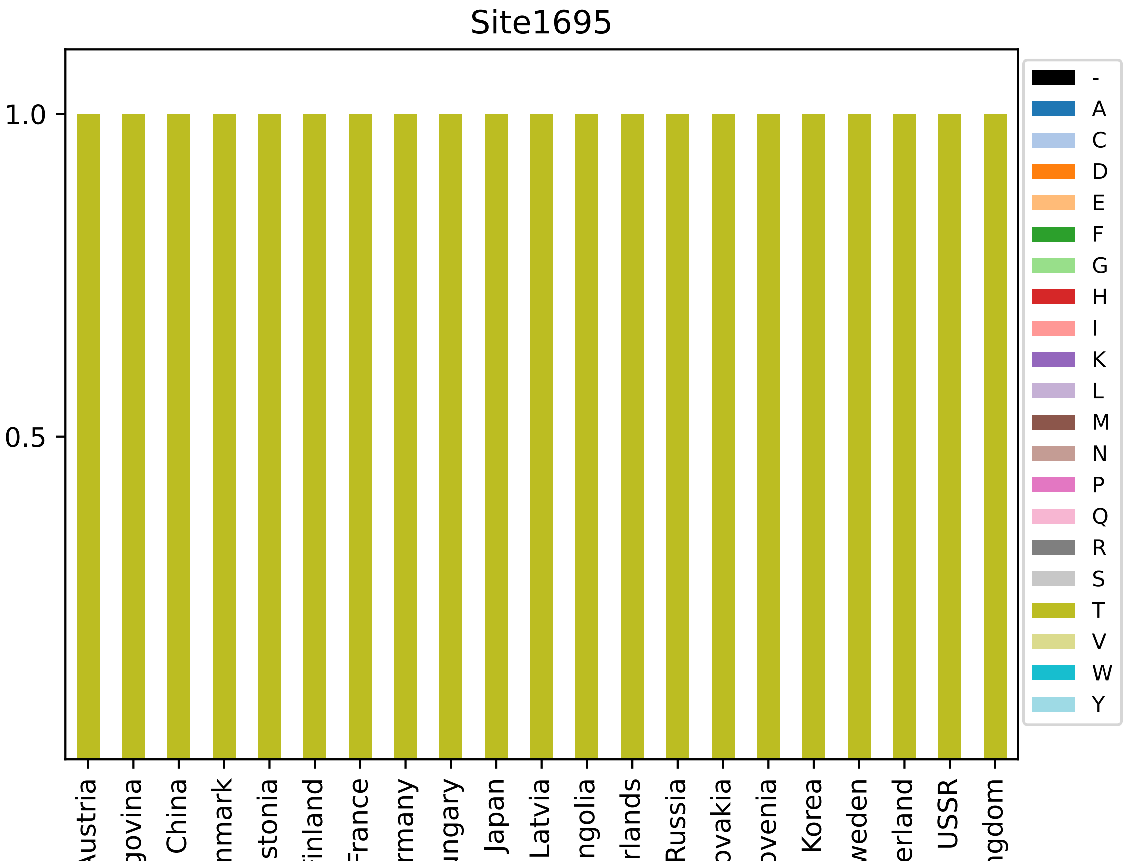

Site1695
1.0
0.5
-
A
C
D
E
F
G
H
I
K
L
M
N
P
Q
R
S
T
V
W
Y
China
Japan
Latvia
Russia
Korea
USSR

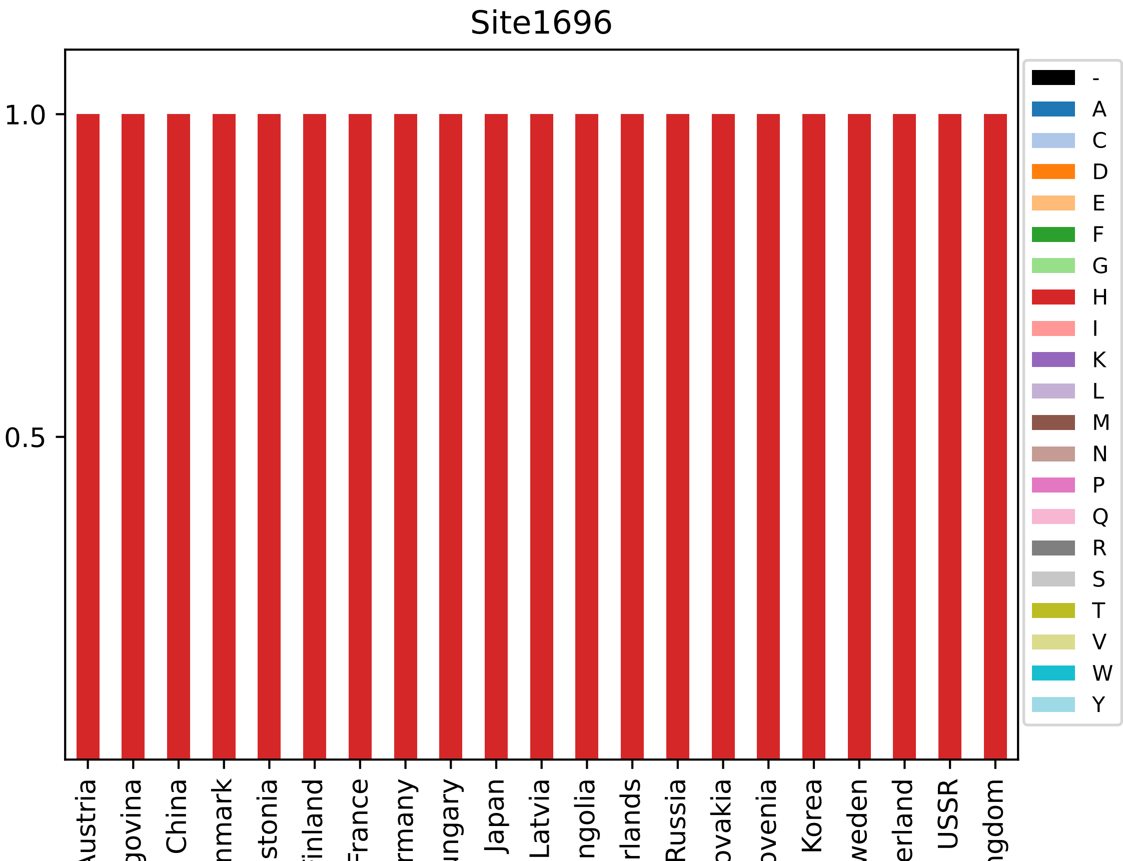
Site1696
1.0
0.5
-
A
C
D
E
F
G
H
I
K
L
M
N
P
Q
R
S
T
V
W
Y
China
Japan
Latvia
Russia
Korea
USSR

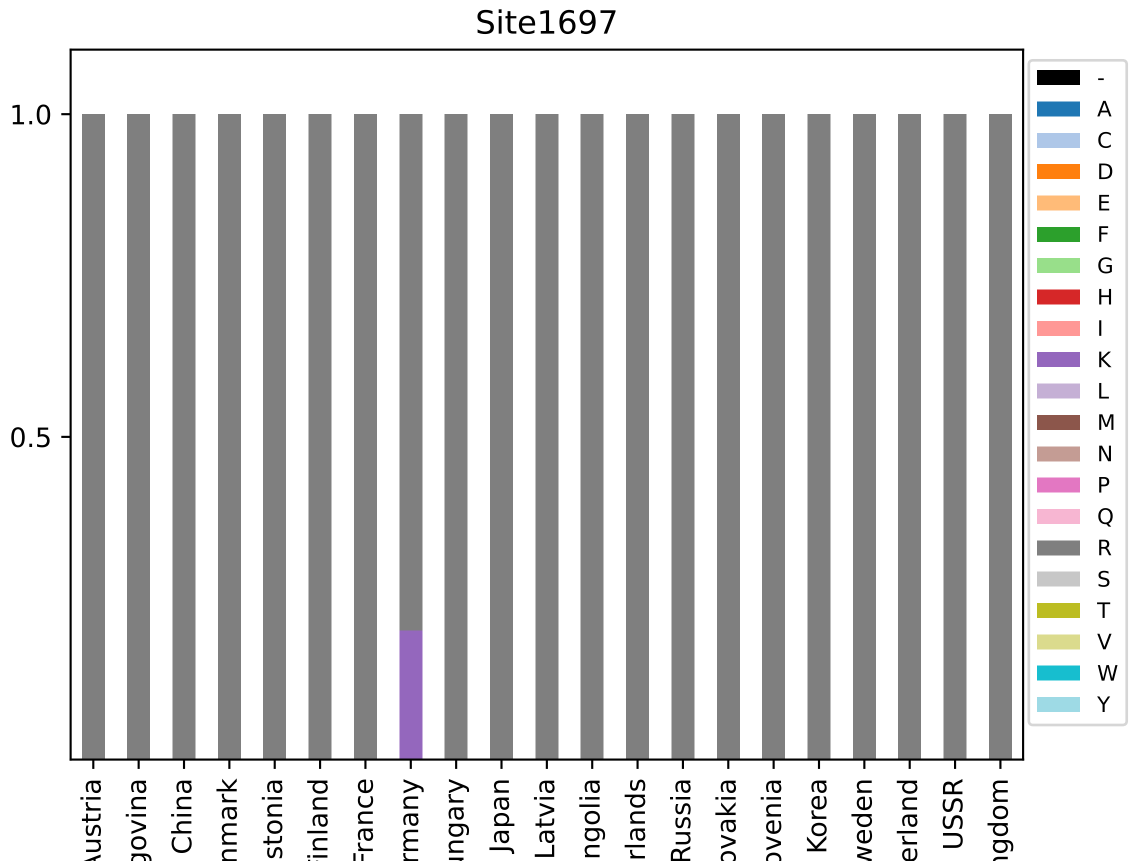

Site1697
1.0
0.5
-
A
C
D
E
F
G
H
I
K
L
M
N
P
Q
R
S
T
V
W
Y
Austria
govina
China
nmark
stonia
Finland
France
rmany
ungary
Japan
Latvia
ngolia
rlands
Russia
ovakia
ovenia
Korea
weden
erland
USSR
ngdom

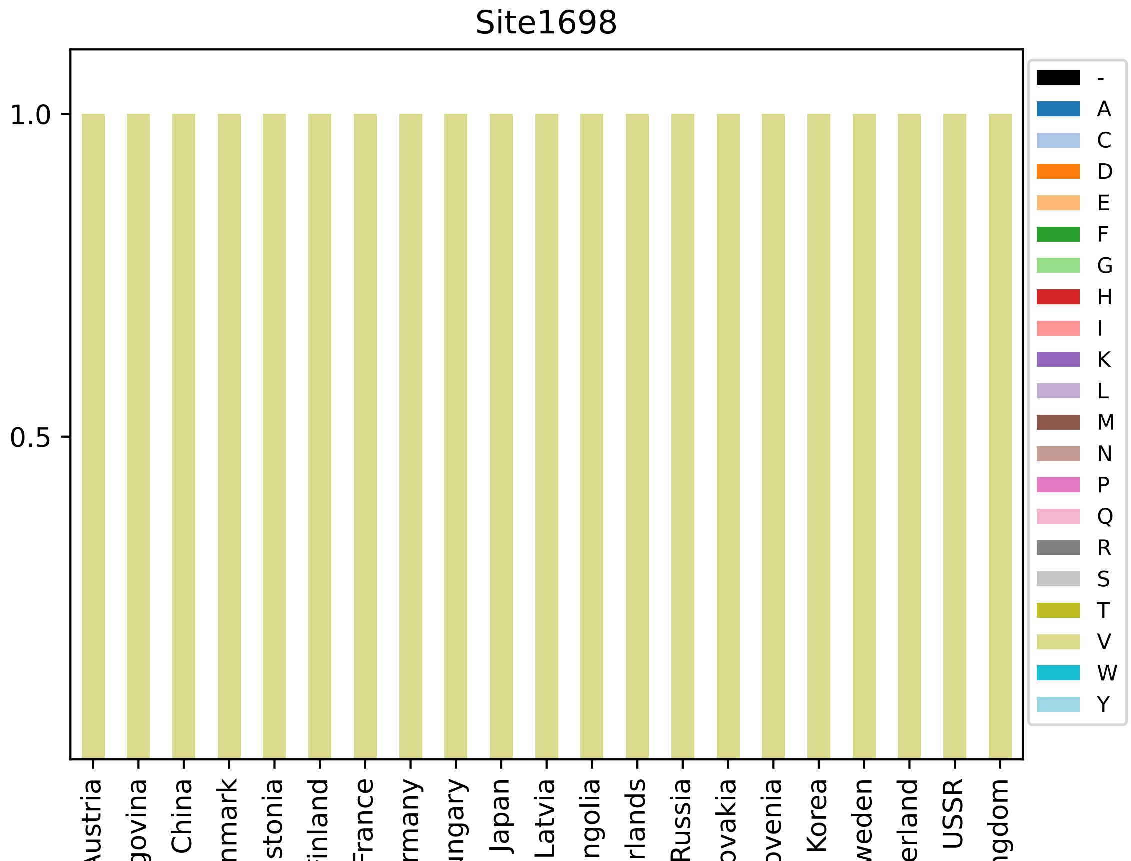

Site1698
1.0
0.5
-
A
C
D
E
F
G
H
I
K
L
M
N
P
Q
R
S
T
V
W
Y
China
France
Japan
Latvia
Russia
Korea
USSR

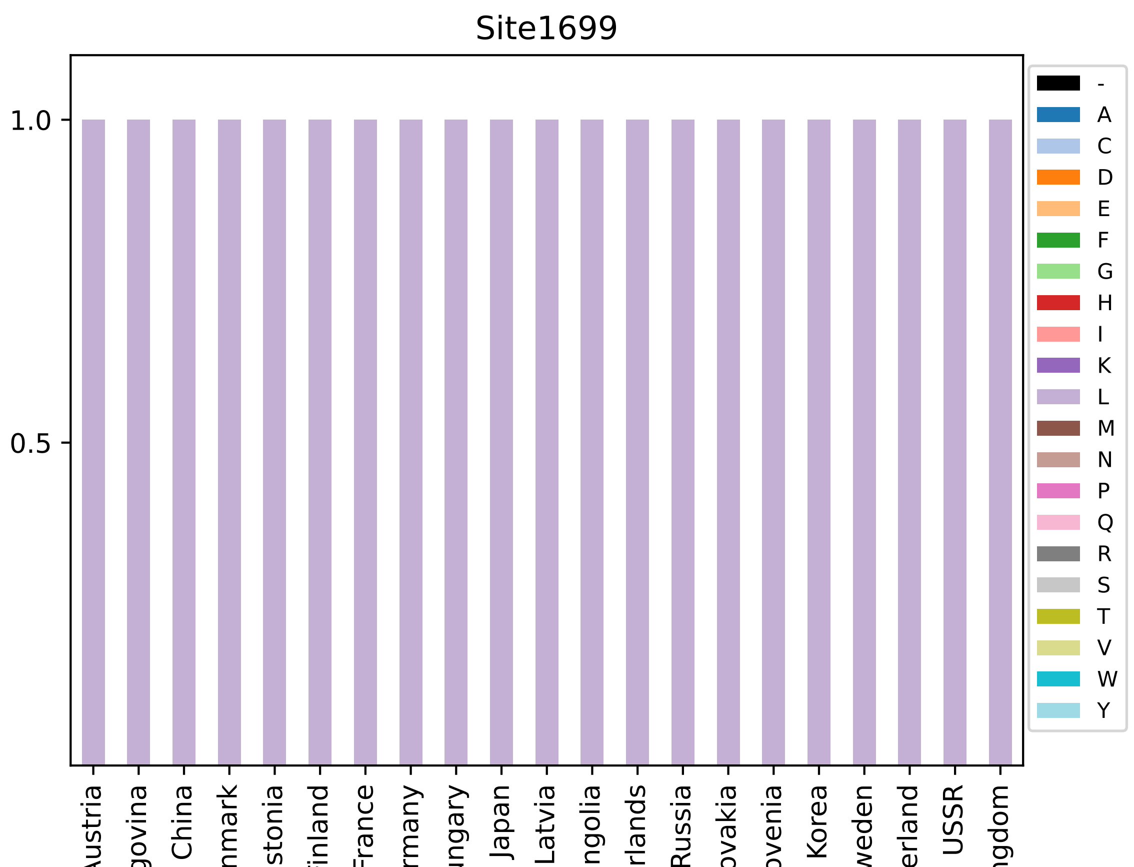

Site1699
1.0
0.5
-
A
C
D
E
F
G
H
I
K
L
M
N
P
Q
R
S
T
V
W
Y
Austria
China
Japan
Latvia
Russia
Korea
USSR

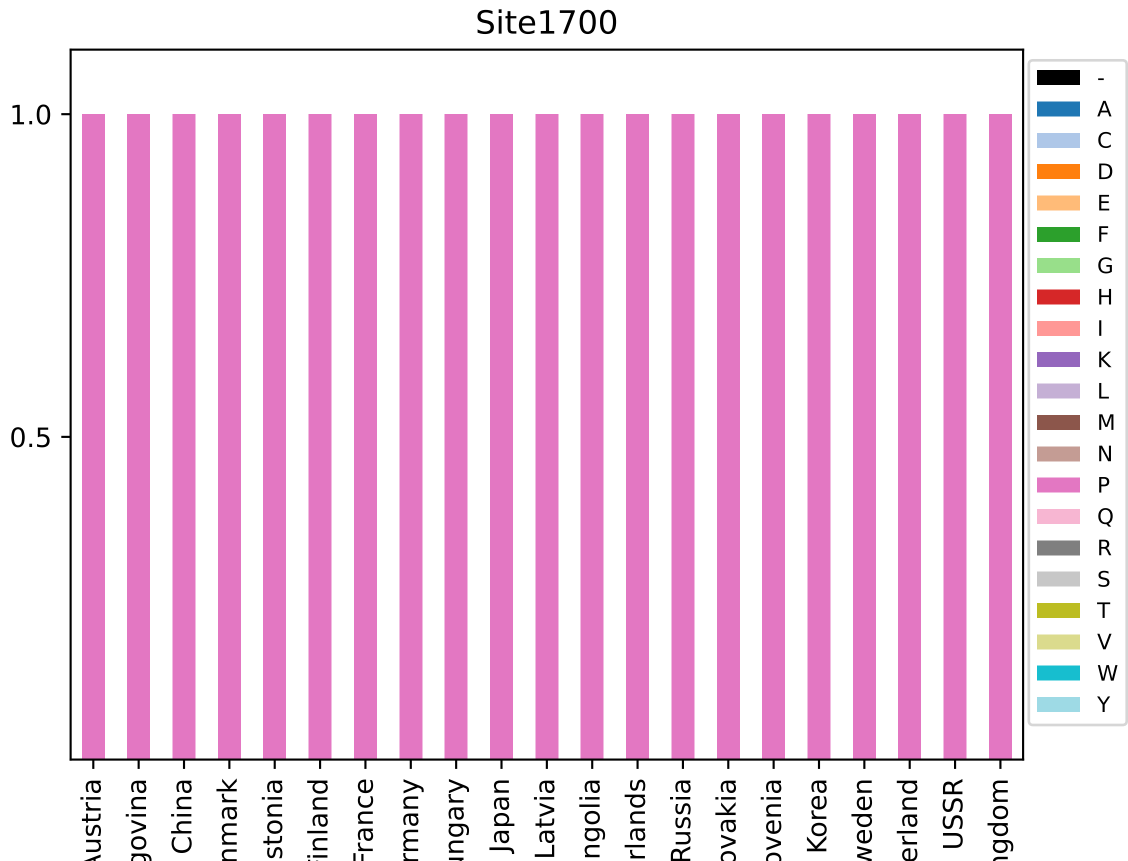
Site1700
1.0
0.5
-
A
C
D
E
F
G
H
I
K
L
M
N
P
Q
R
S
T
V
W
Y
China
Japan
Latvia
Russia
Korea
USSR

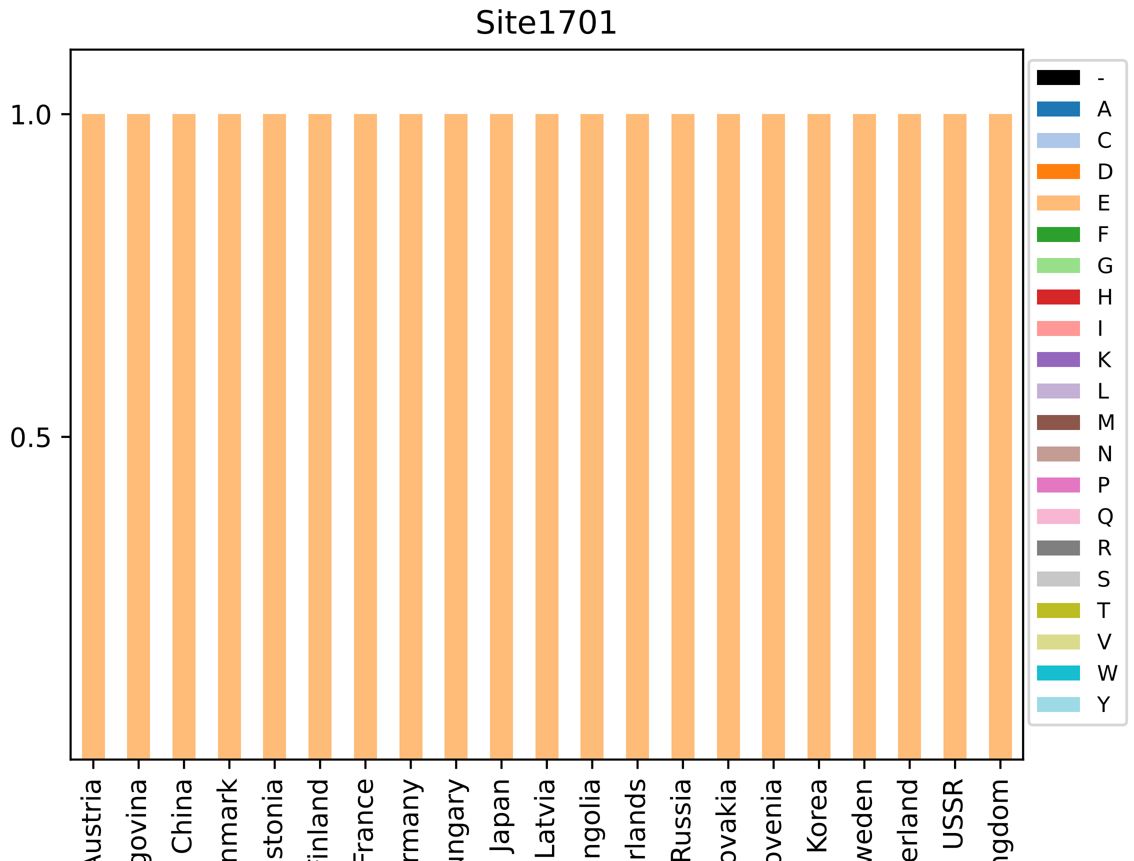
Site1701
1.0
0.5
-
A
C
D
E
F
G
H
I
K
L
M
N
P
Q
R
S
T
V
W
Y
China
Japan
Latvia
Russia
Korea
USSR

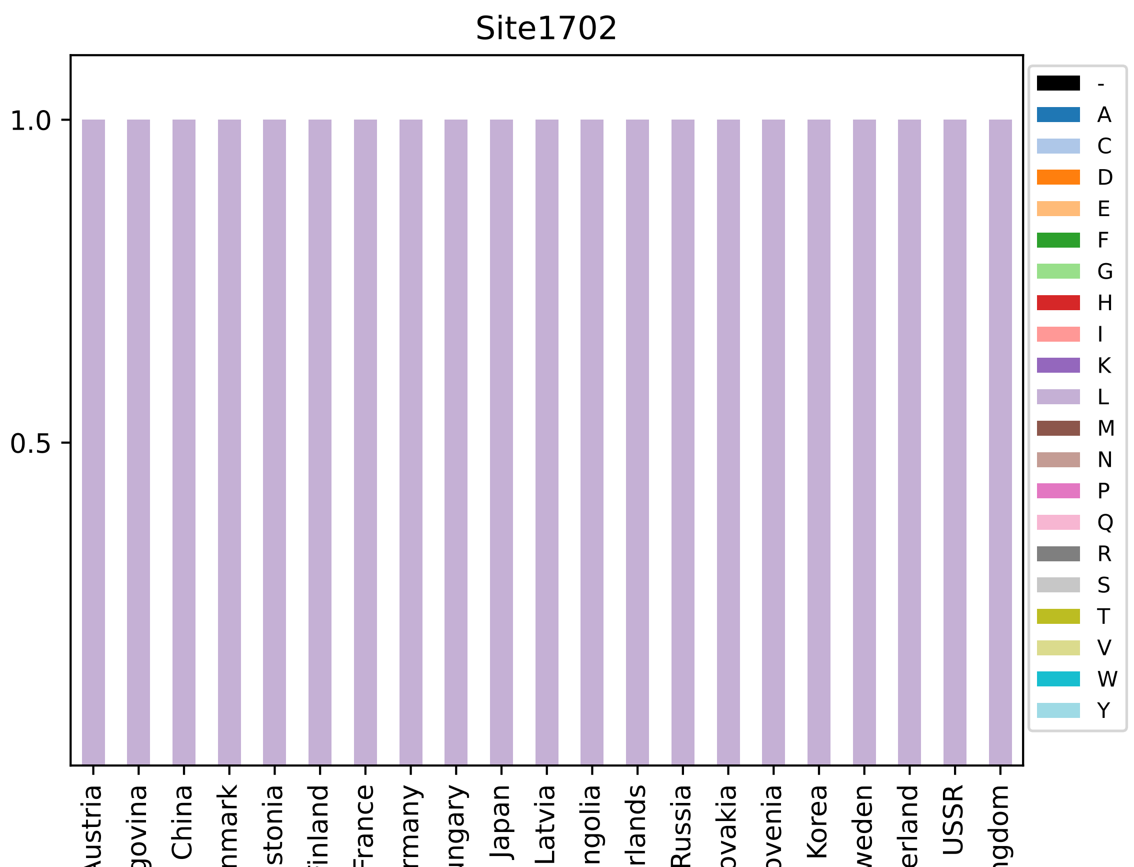
Site1702
1.0
0.5
-
A
C
D
E
F
G
H
I
K
L
M
N
P
Q
R
S
T
V
W
Y
China
Japan
Latvia
Russia
Korea
USSR

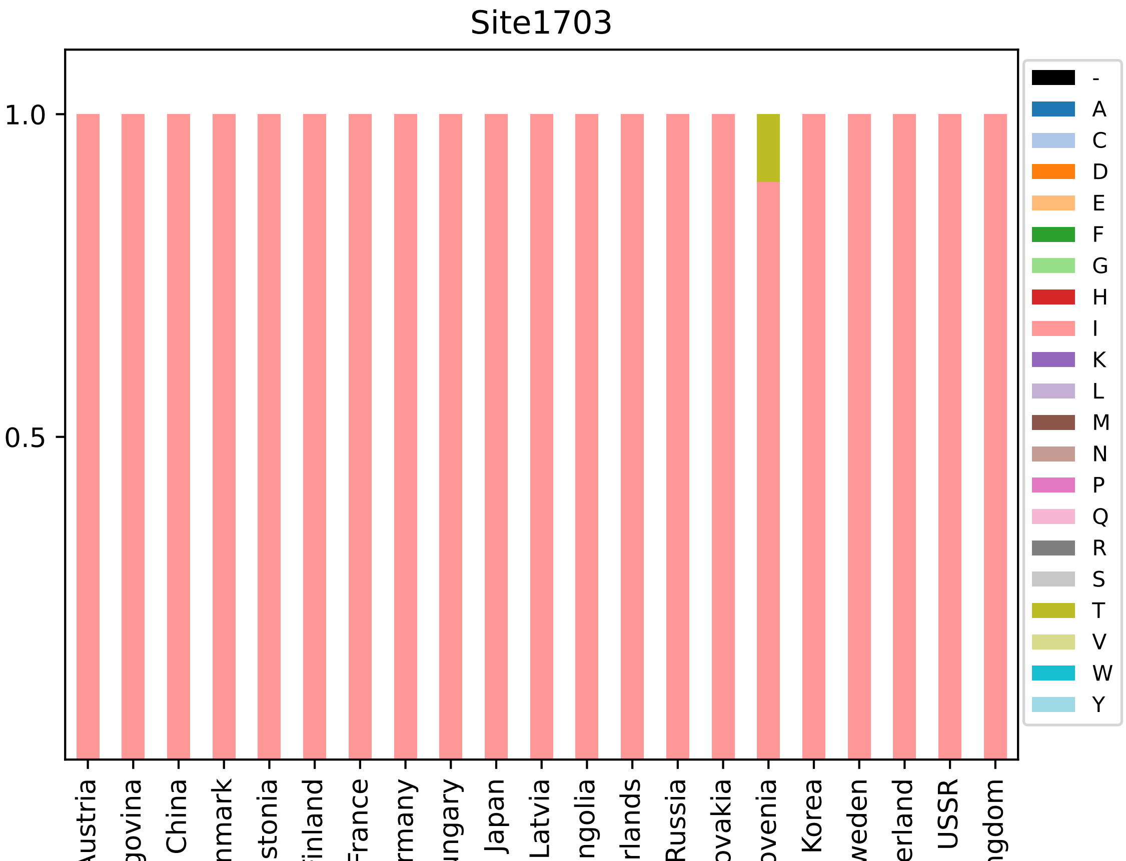

Site1703
1.0
0.5
-
A
C
D
E
F
G
H
I
K
L
M
N
P
Q
R
S
T
V
W
Y
Austria
China
Japan
Latvia
Russia
Korea
USSR

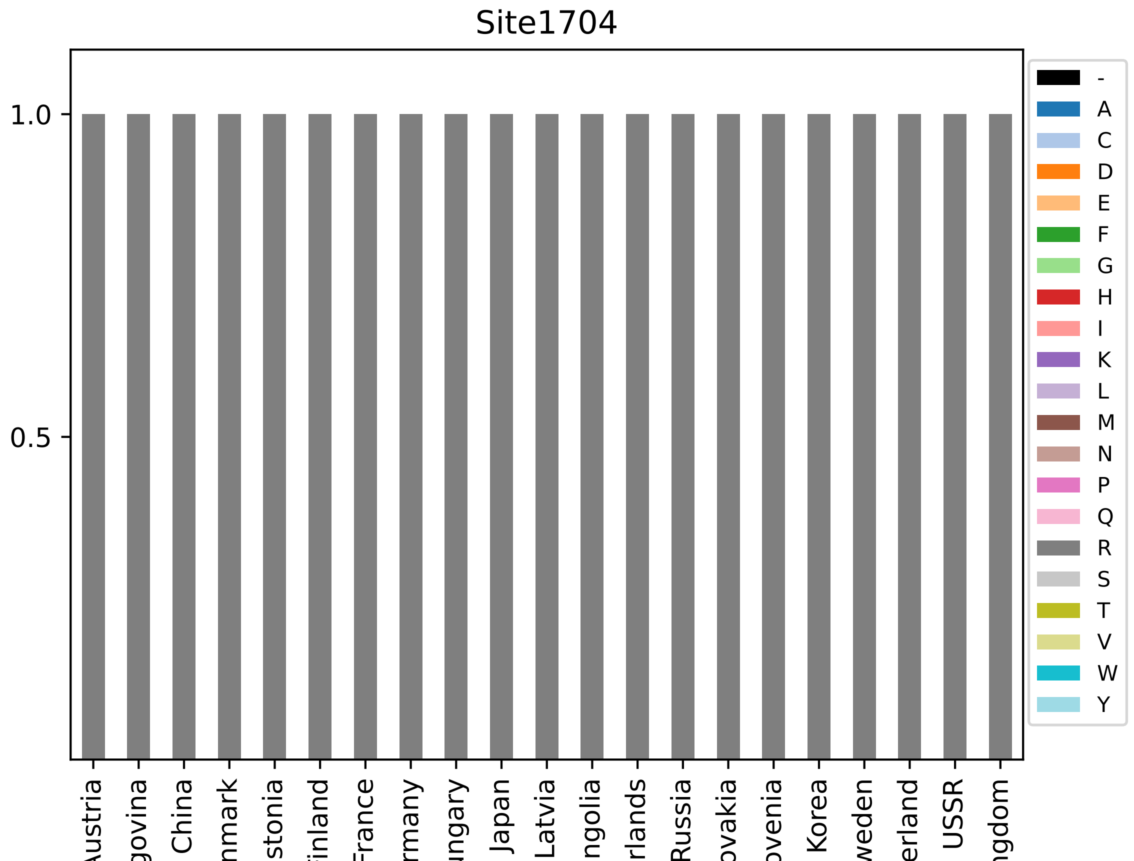
Site1704
1.0
0.5
Austria
govina
China
nmark
stonia
Finland
France
rmany
ungary
Japan
Latvia
ngolia
rlands
Russia
ovakia
ovenia
Korea
weden
erland
USSR
ngdom
-
A
C
D
E
F
G
H
I
K
L
M
N
P
Q
R
S
T
V
W
Y

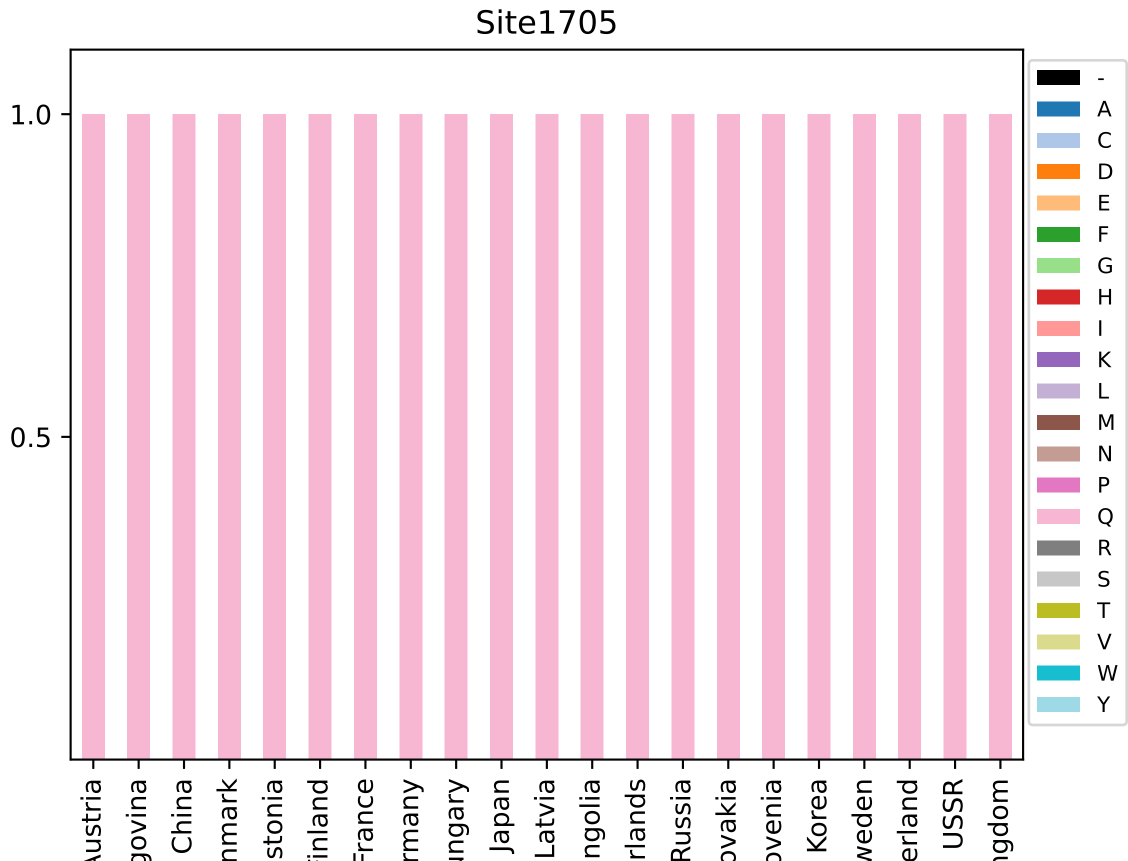

Site1705
1.0
0.5
-
A
C
D
E
F
G
H
I
K
L
M
N
P
Q
R
S
T
V
W
Y
China
Japan
Latvia
Russia
Korea
USSR

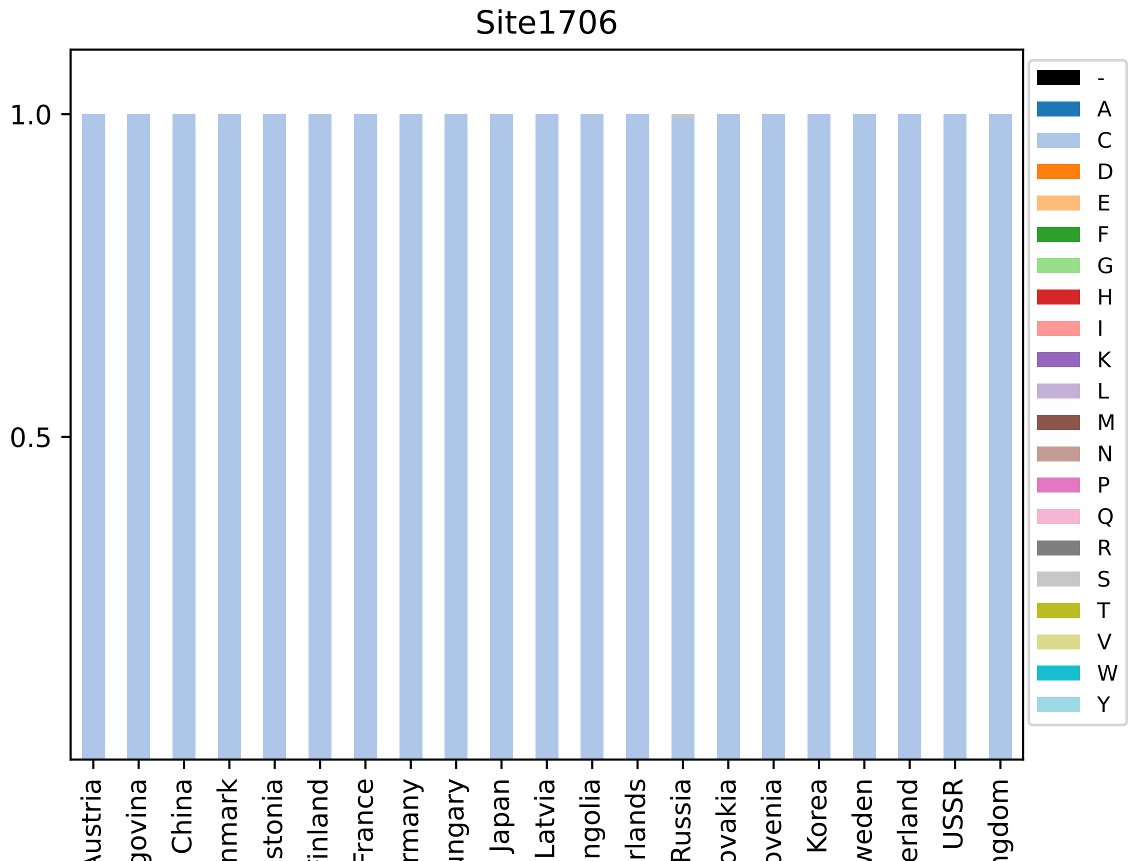
Site1706
1.0
0.5
-
A
C
D
E
F
G
H
I
K
L
M
N
P
Q
R
S
T
V
W
Y
Austria
govina
China
nmark
stonia
Finland
France
rmany
ungary
Japan
Latvia
ngolia
rlands
Russia
ovakia
ovenia
Korea
weden
erland
USSR
ngdom

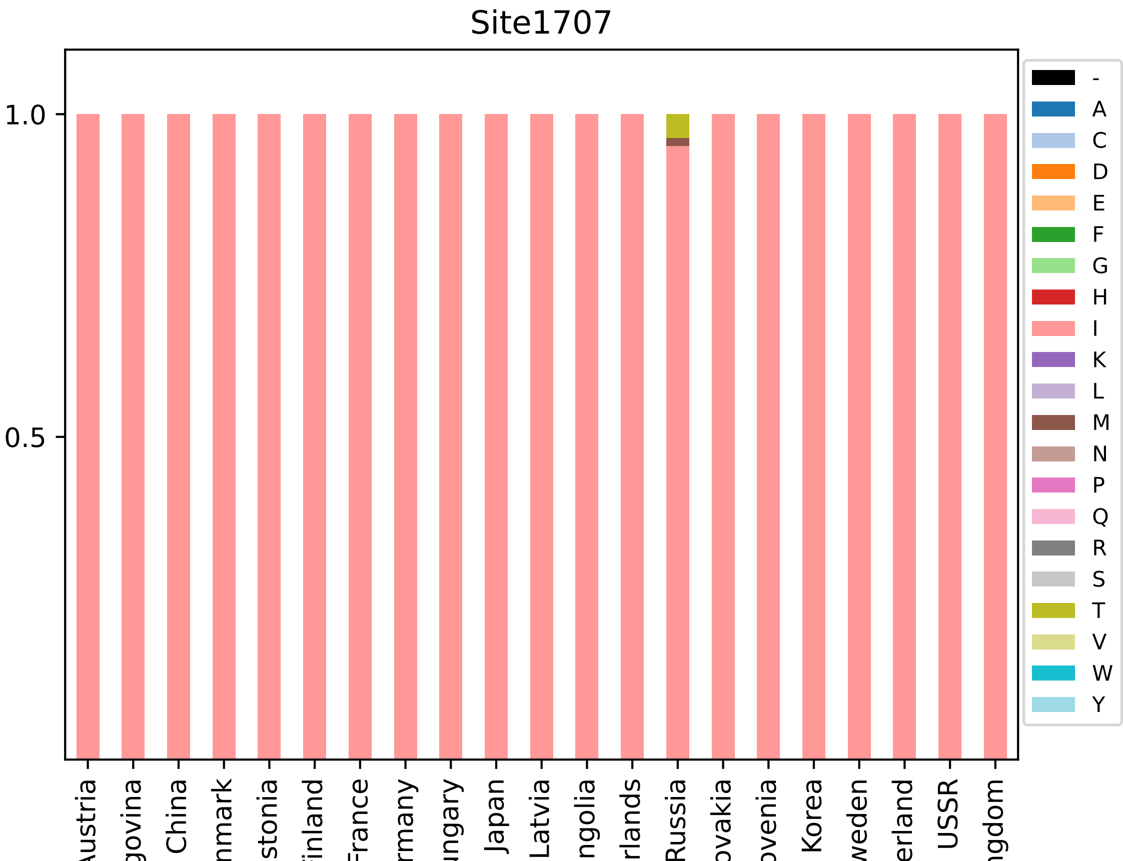
Site1707
1.0
0.5
-
A
C
D
E
F
G
H
I
K
L
M
N
P
Q
R
S
T
V
W
Y
Austria
China
Japan
Latvia
Russia
Korea
USSR

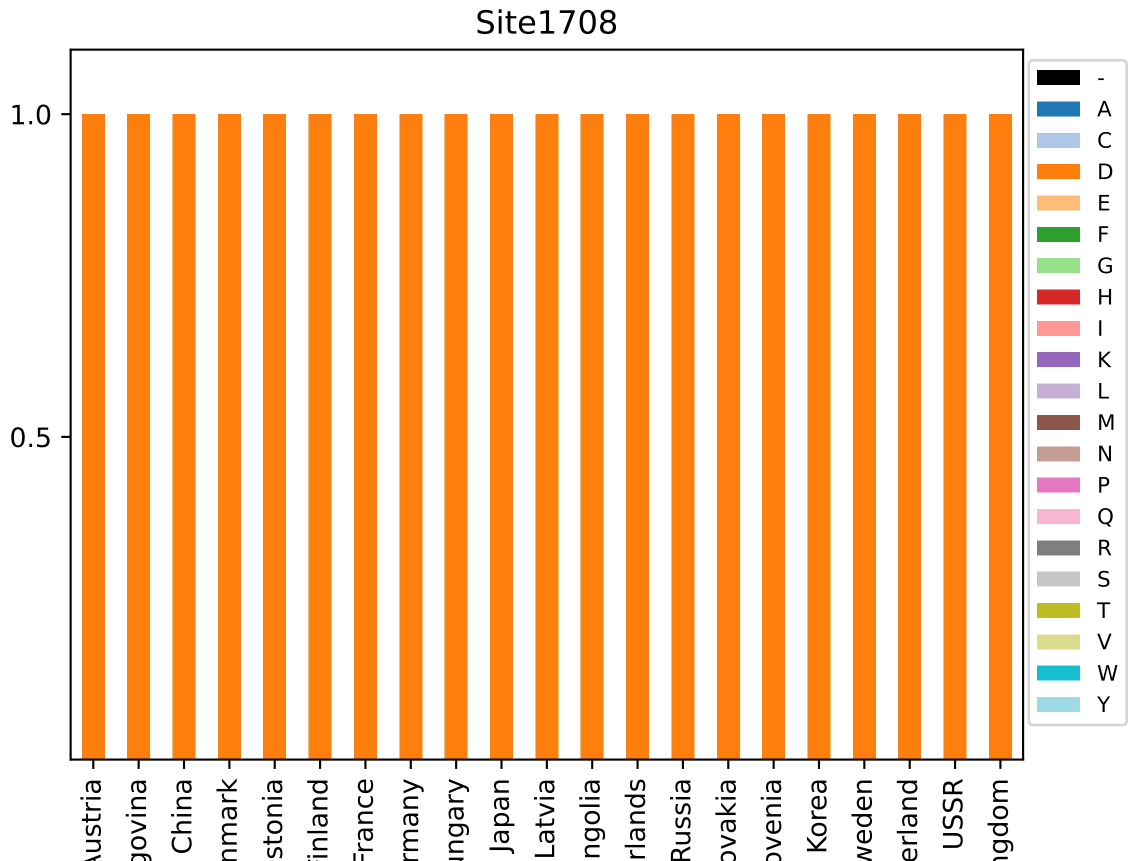
Site1708
1.0
0.5
-
A
C
D
E
F
G
H
I
K
L
M
N
P
Q
R
S
T
V
W
Y
Austria
govina
China
nmark
stonia
Finland
France
rmany
ungary
Japan
Latvia
ngolia
rlands
Russia
ovakia
ovenia
Korea
weden
erland
USSR
ngdom

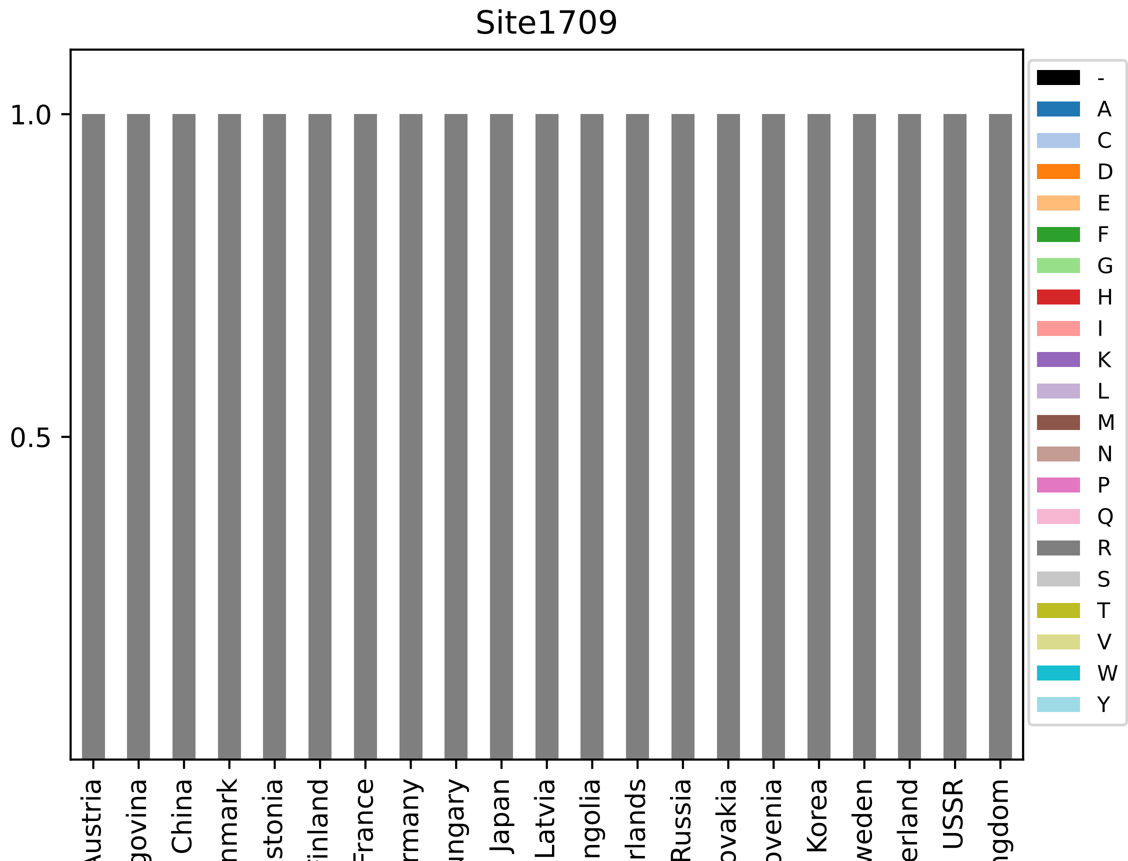

Site1709
1.0
0.5
-
A
C
D
E
F
G
H
I
K
L
M
N
P
Q
R
S
T
V
W
Y
China
Japan
Latvia
Russia
Korea
USSR

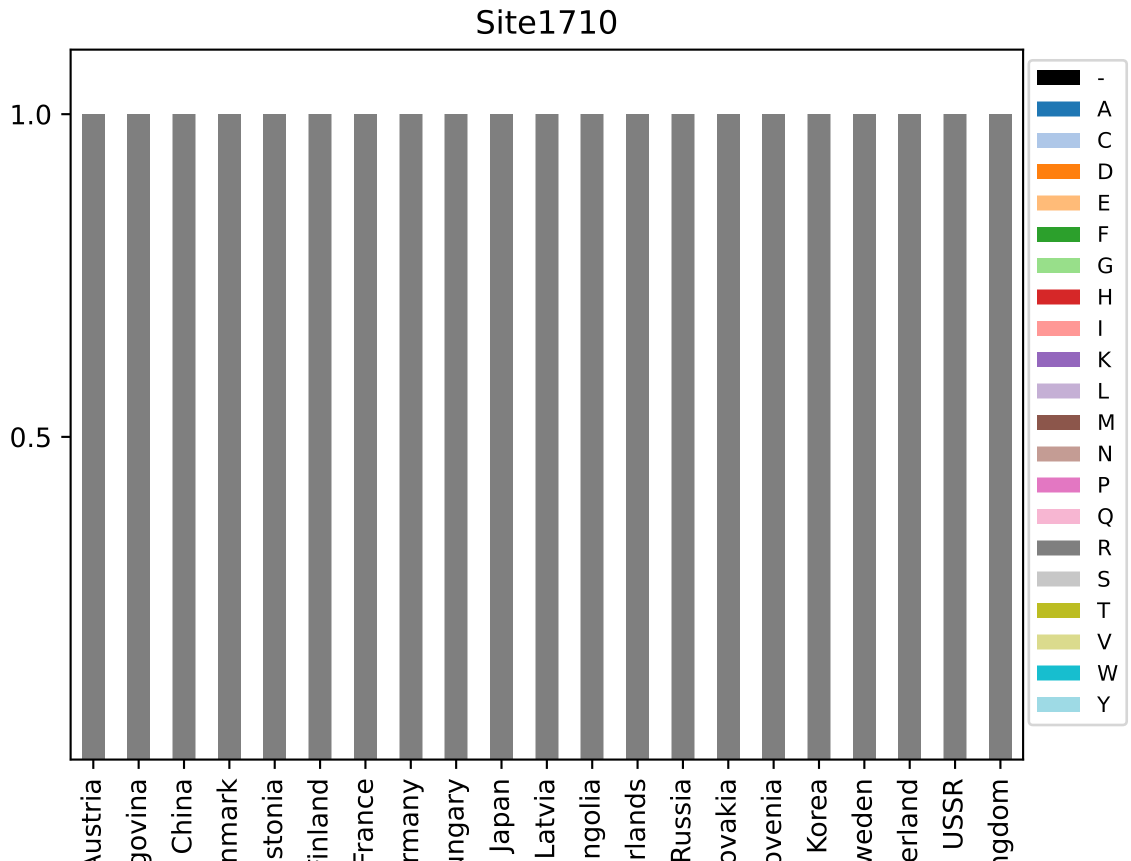
Site1710
1.0
0.5
-
A
C
D
E
F
G
H
I
K
L
M
N
P
Q
R
S
T
V
W
Y
Austria
govina
China
nmark
stonia
inland
France
rmany
ungary
Japan
Latvia
ngolia
rlands
Russia
ovakia
ovenia
Korea
weden
erland
USSR
ngdom

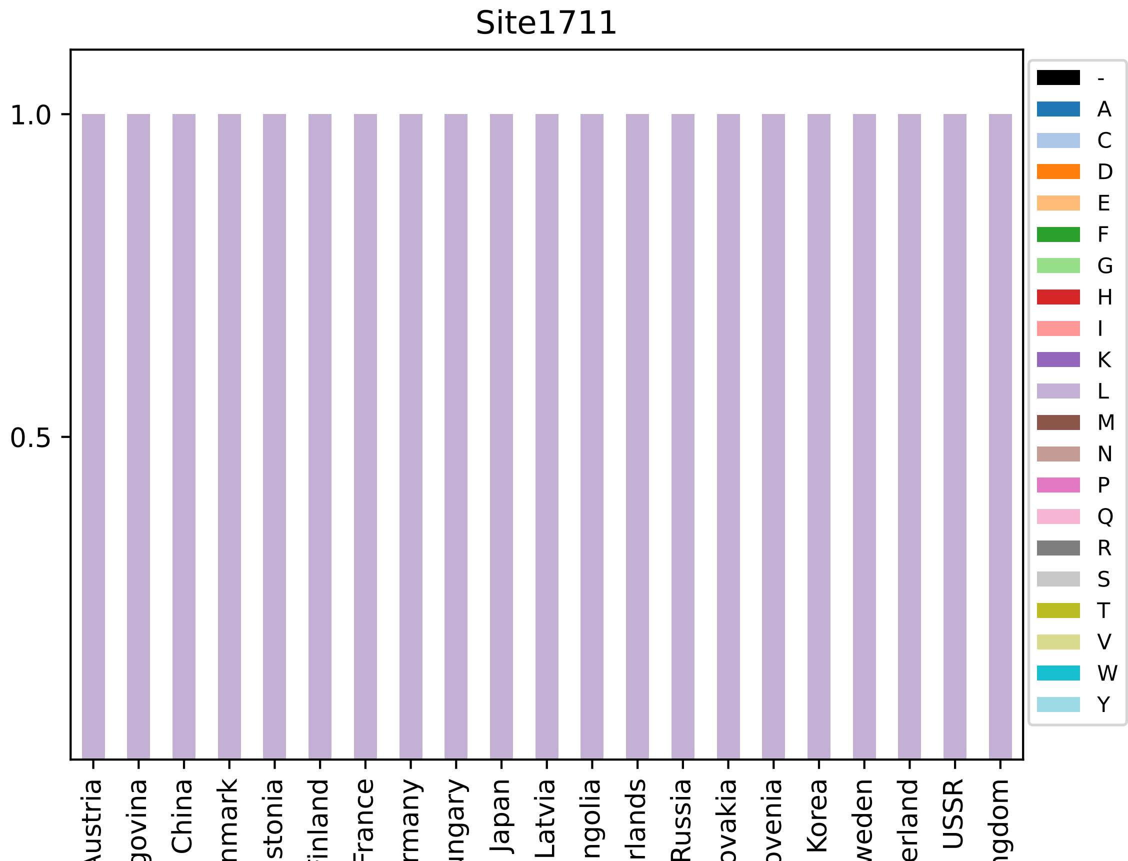

Site1711
1.0
0.5
-
A
C
D
E
F
G
H
I
K
L
M
N
P
Q
R
S
T
V
W
Y
Austria
govina
China
nmark
stonia
inland
France
rmany
ungary
Japan
Latvia
ngolia
rlands
Russia
ovakia
ovenia
Korea
weden
erland
USSR
ngdom

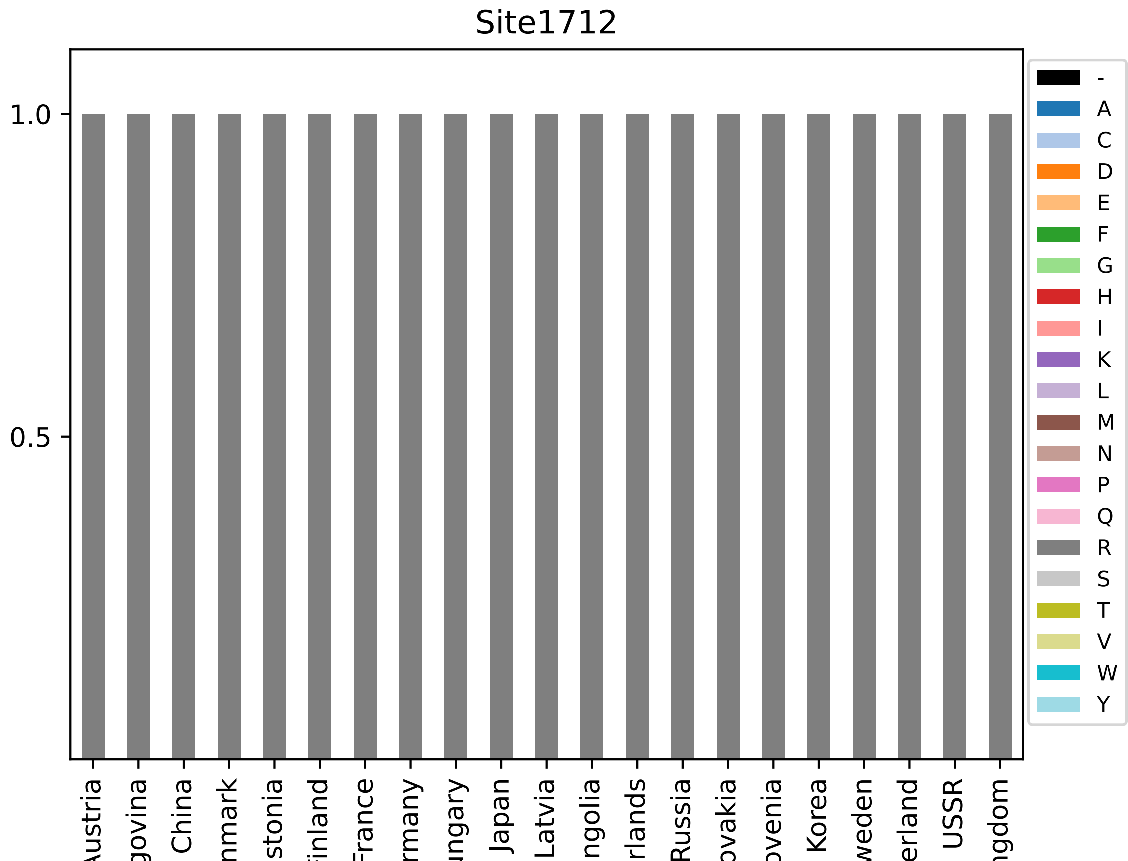
Site1712
1.0
0.5
-
A
C
D
E
F
G
H
I
K
L
M
N
P
Q
R
S
T
V
W
Y
China
Japan
Latvia
Russia
Korea
USSR

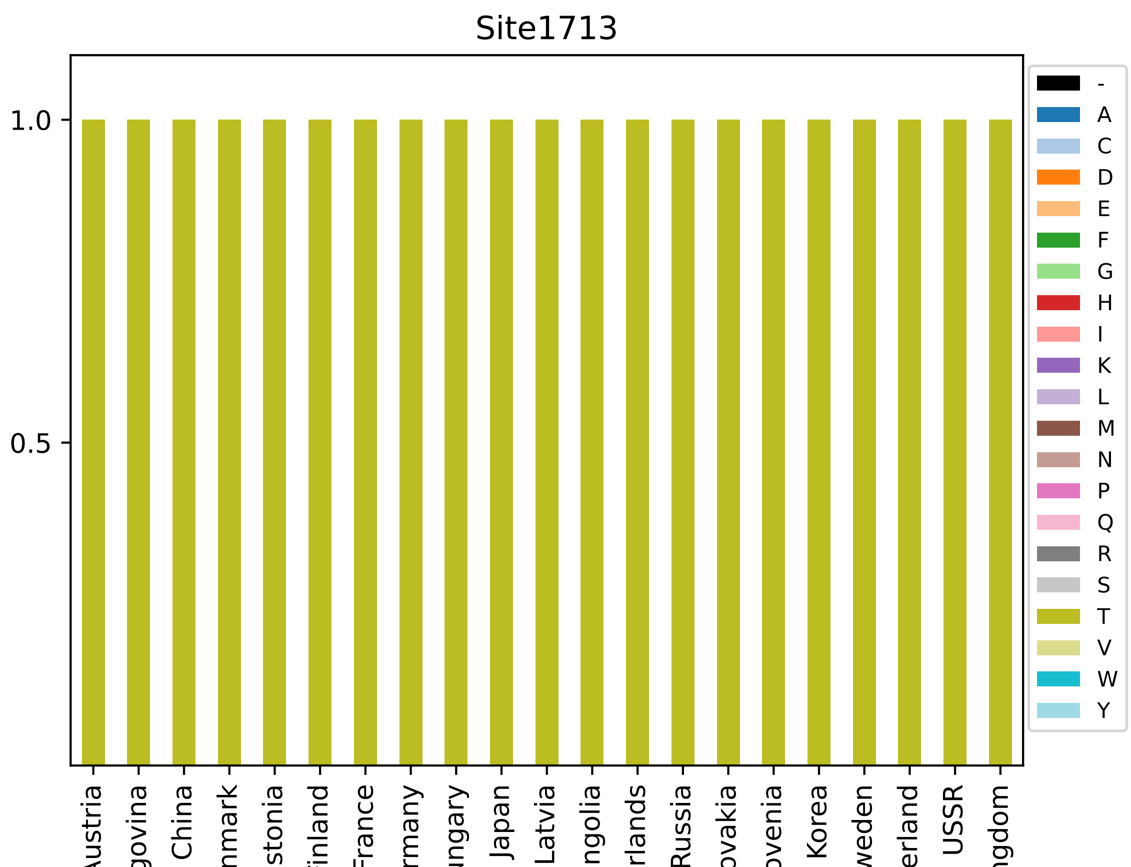

Site1713
1.0
0.5
-
A
C
D
E
F
G
H
I
K
L
M
N
P
Q
R
S
T
V
W
Y
China
Japan
Latvia
Russia
Korea
USSR

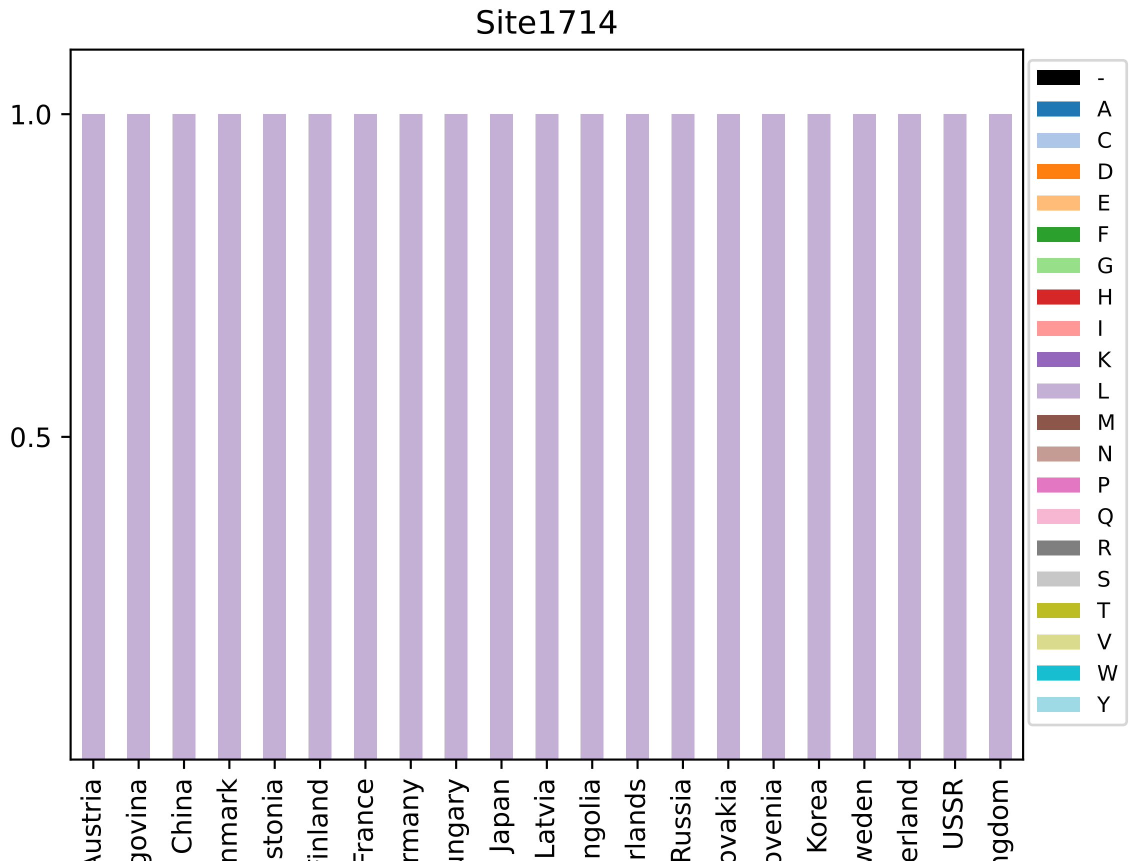

Site1714
1.0
0.5
-
A
C
D
E
F
G
H
I
K
L
M
N
P
Q
R
S
T
V
W
Y
China
Japan
Latvia
Russia
Korea
USSR

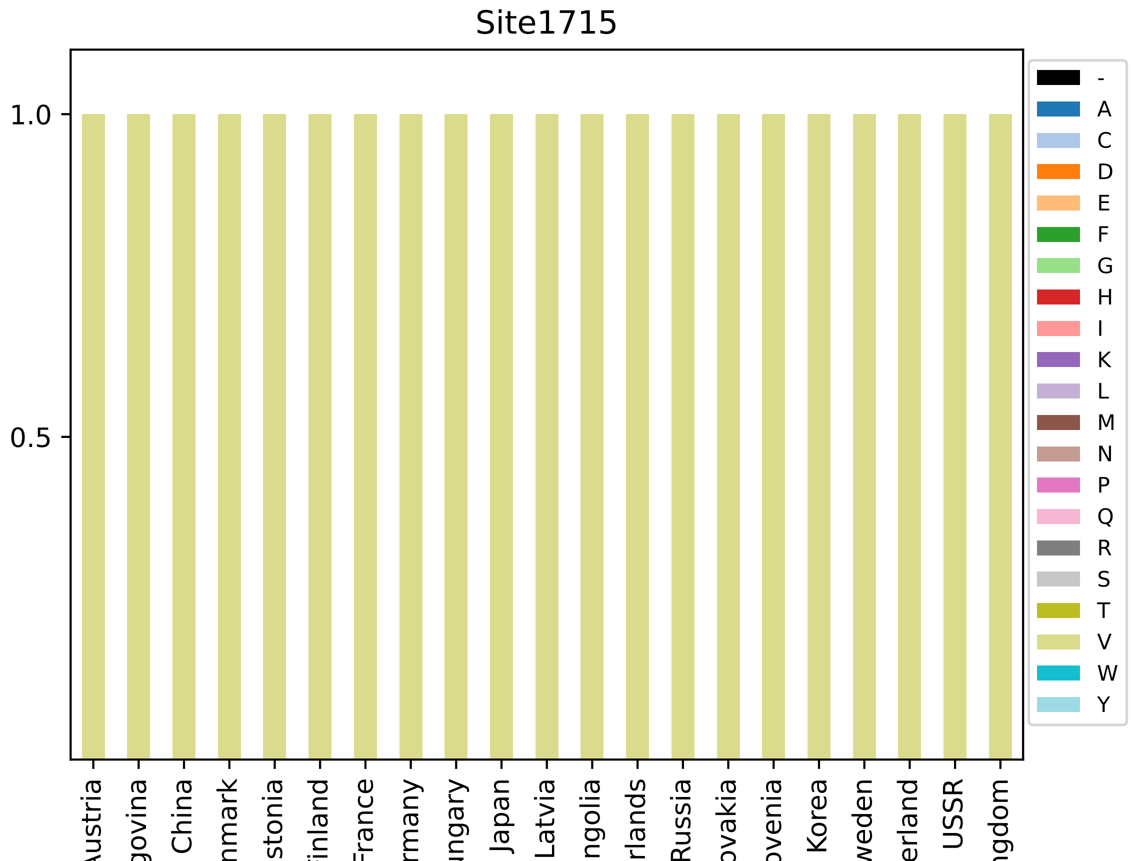
Site1715
1.0
0.5
-
A
C
D
E
F
G
H
I
K
L
M
N
P
Q
R
S
T
V
W
Y
Austria
govina
China
nmark
stonia
Finland
France
rmany
ungary
Japan
Latvia
ngolia
rlands
Russia
ovakia
ovenia
Korea
weden
erland
USSR
ngdom

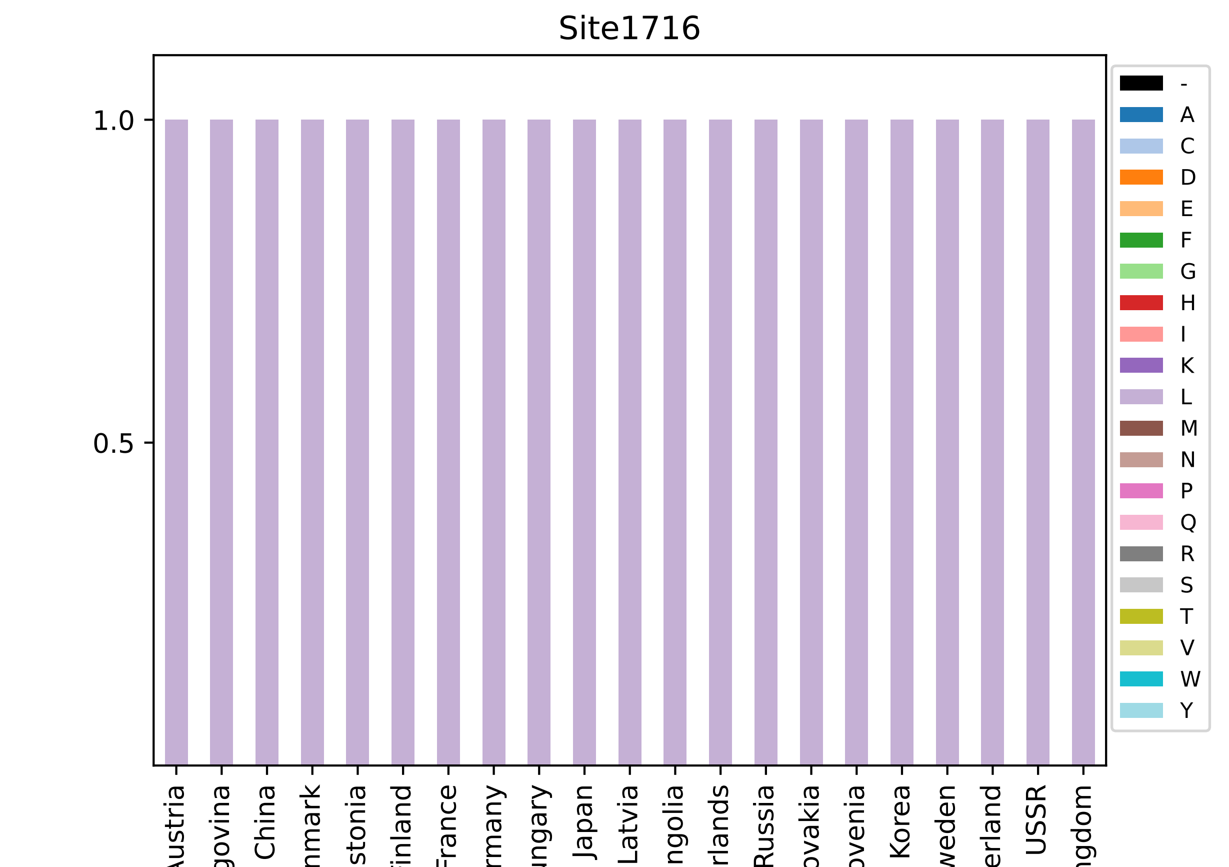

Site1716
1.0
0.5
-
A
C
D
E
F
G
H
I
K
L
M
N
P
Q
R
S
T
V
W
Y
China
Japan
Latvia
Russia
Korea
USSR

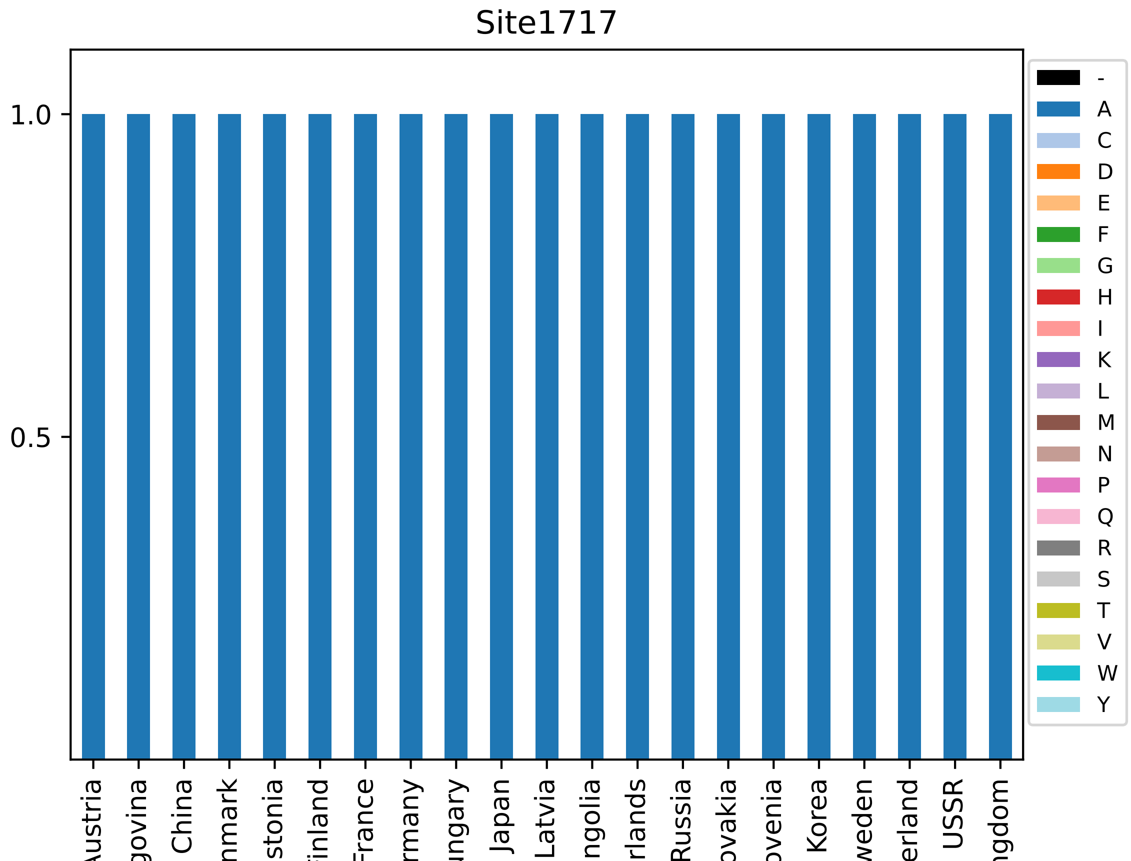
Site1717
1.0
0.5
-
A
C
D
E
F
G
H
I
K
L
M
N
P
Q
R
S
T
V
W
Y
China
Japan
Latvia
Russia
Korea
USSR

Site1718

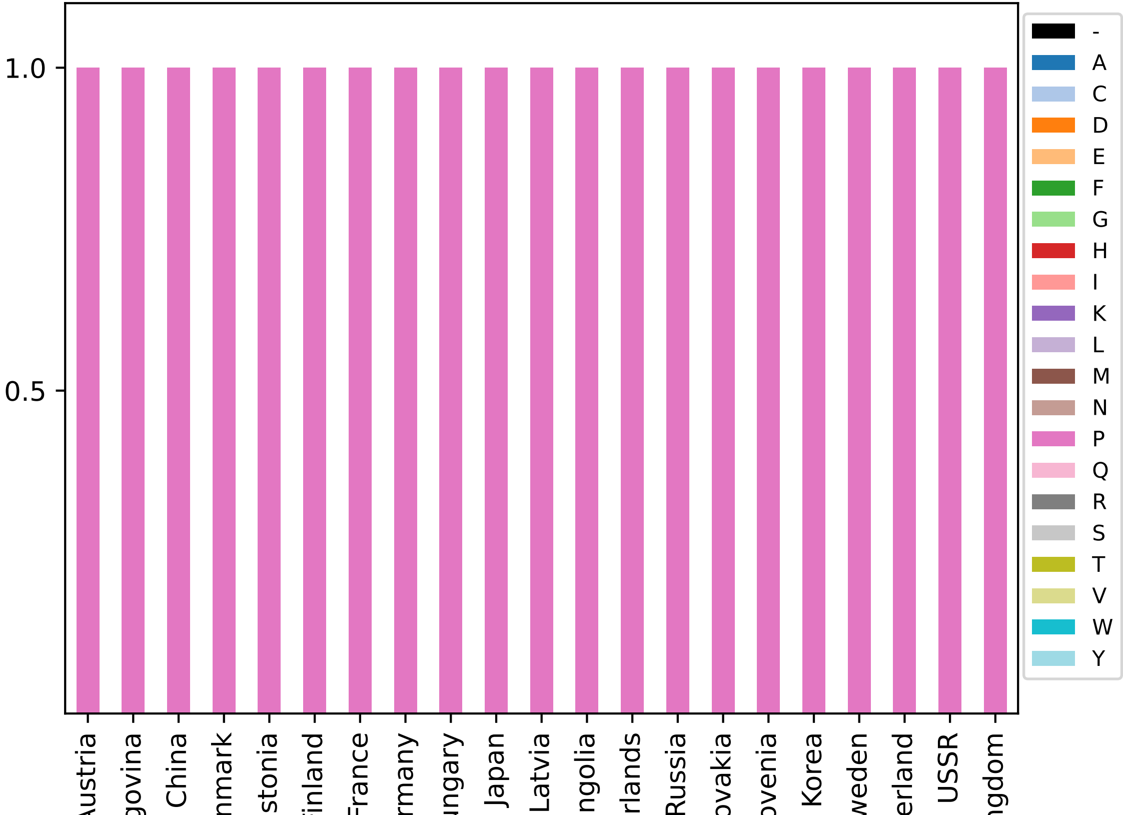

1.0
0.5
China
Japan
Latvia
Russia
Korea
USSR
-
A
C
D
E
F
G
H
I
K
L
M
N
P
Q
R
S
T
V
W
Y

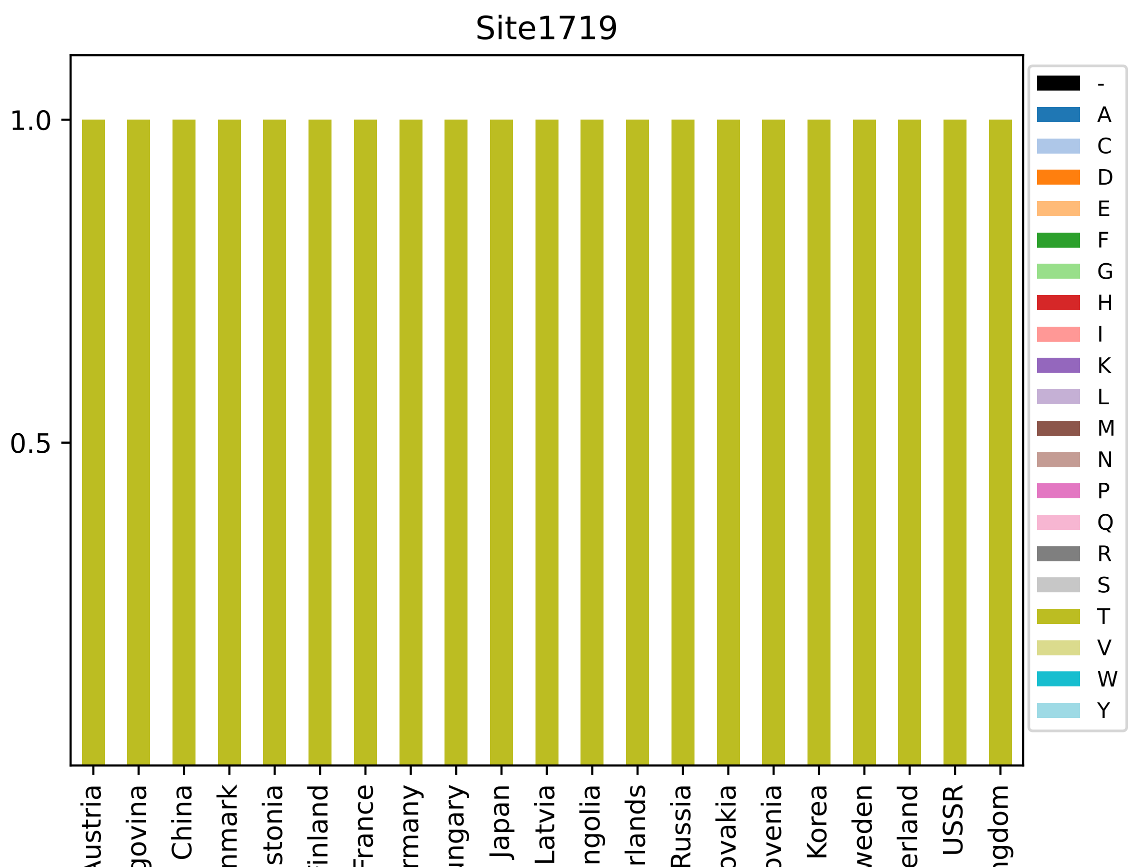

Site1719
1.0
0.5
-
A
C
D
E
F
G
H
I
K
L
M
N
P
Q
R
S
T
V
W
Y
China
Japan
Latvia
Russia
Korea
USSR

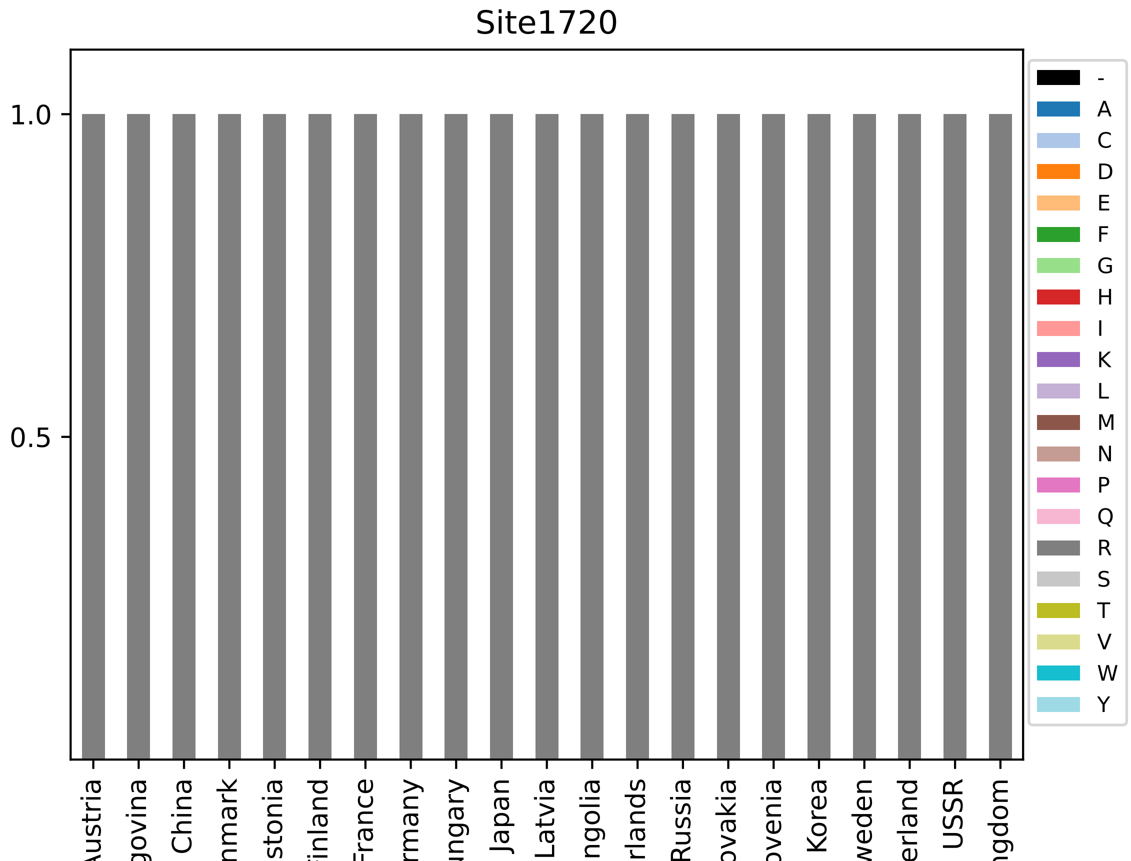
Site1720
1.0
0.5
-
A
C
D
E
F
G
H
I
K
L
M
N
P
Q
R
S
T
V
W
Y
Austria
govina
China
nmark
stonia
Finland
France
rmany
ungary
Japan
Latvia
ngolia
rlands
Russia
ovakia
ovenia
Korea
weden
erland
USSR
ngdom

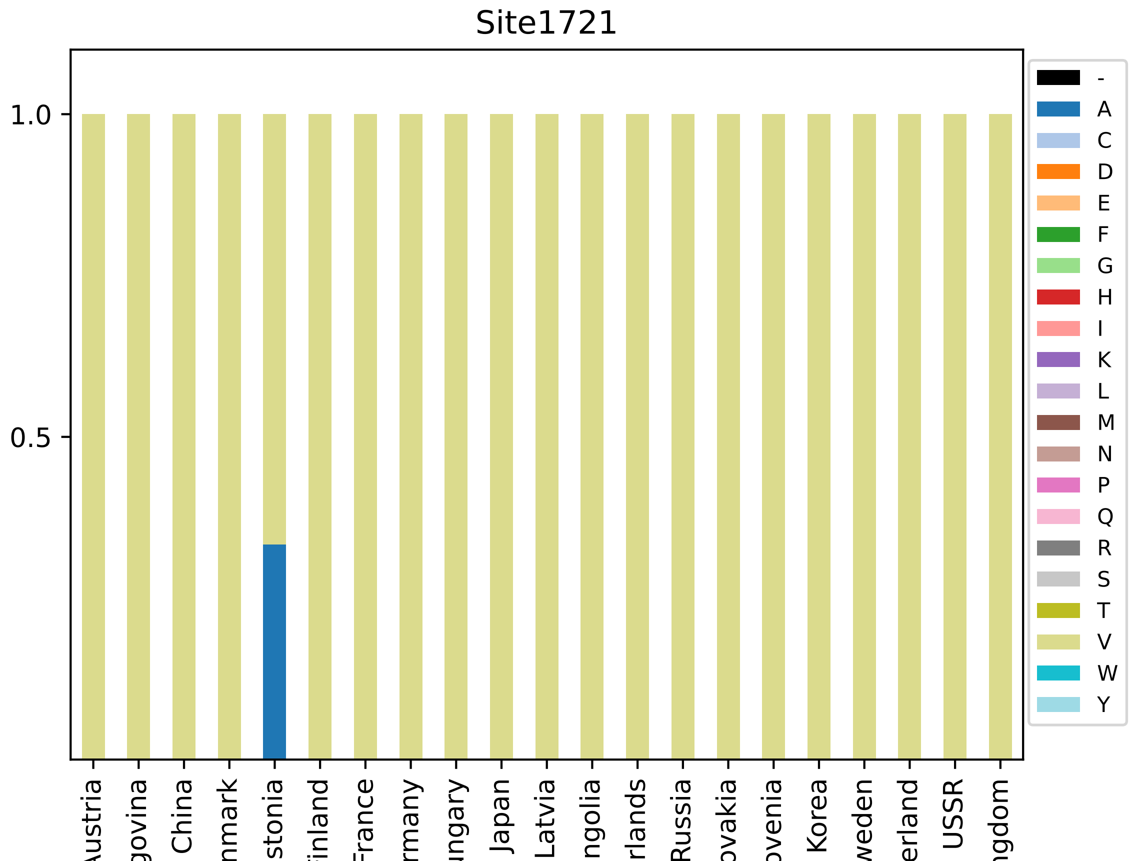

Site1721
1.0
0.5
-
A
C
D
E
F
G
H
I
K
L
M
N
P
Q
R
S
T
V
W
Y
Austria
govina
China
nmark
stonia
Finland
France
rmany
ungary
Japan
Latvia
ngolia
rlands
Russia
ovakia
ovenia
Korea
weden
erland
USSR
ngdom

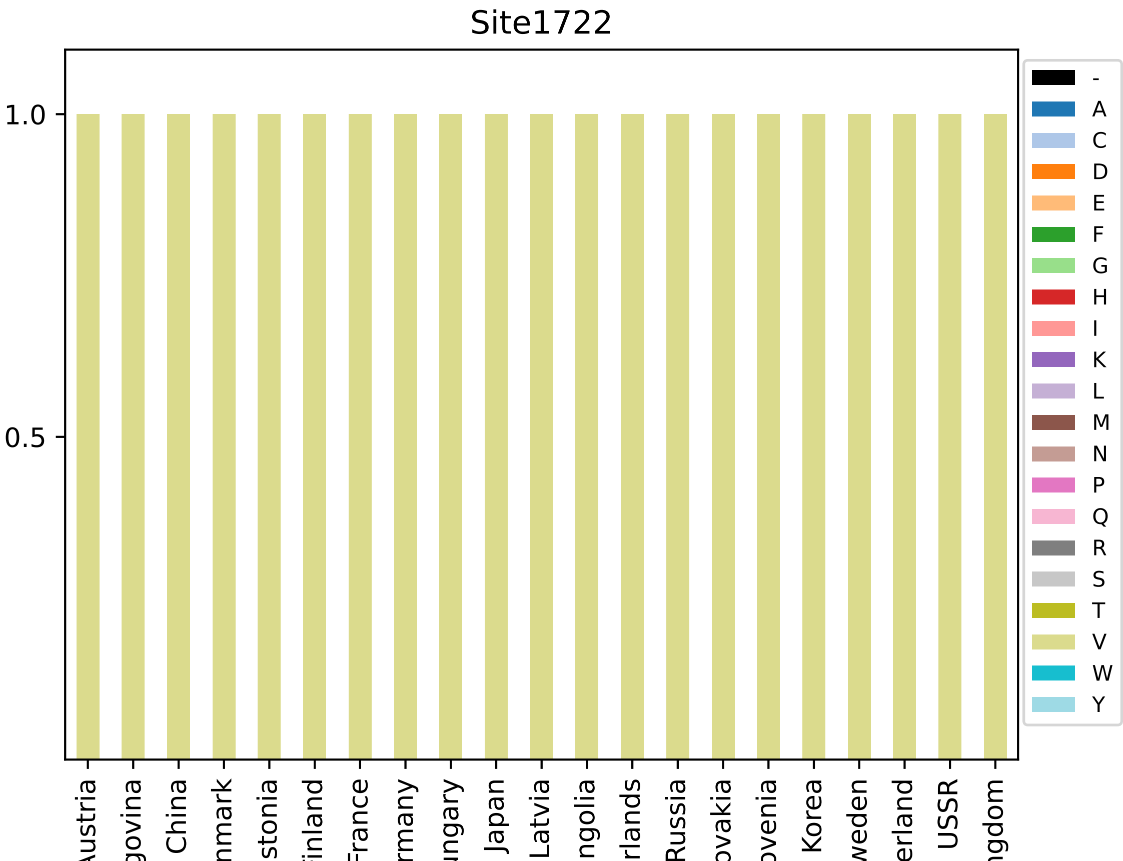
Site1722
1.0
0.5
-
A
C
D
E
F
G
H
I
K
L
M
N
P
Q
R
S
T
V
W
Y
China
Japan
Latvia
Russia
Korea
USSR

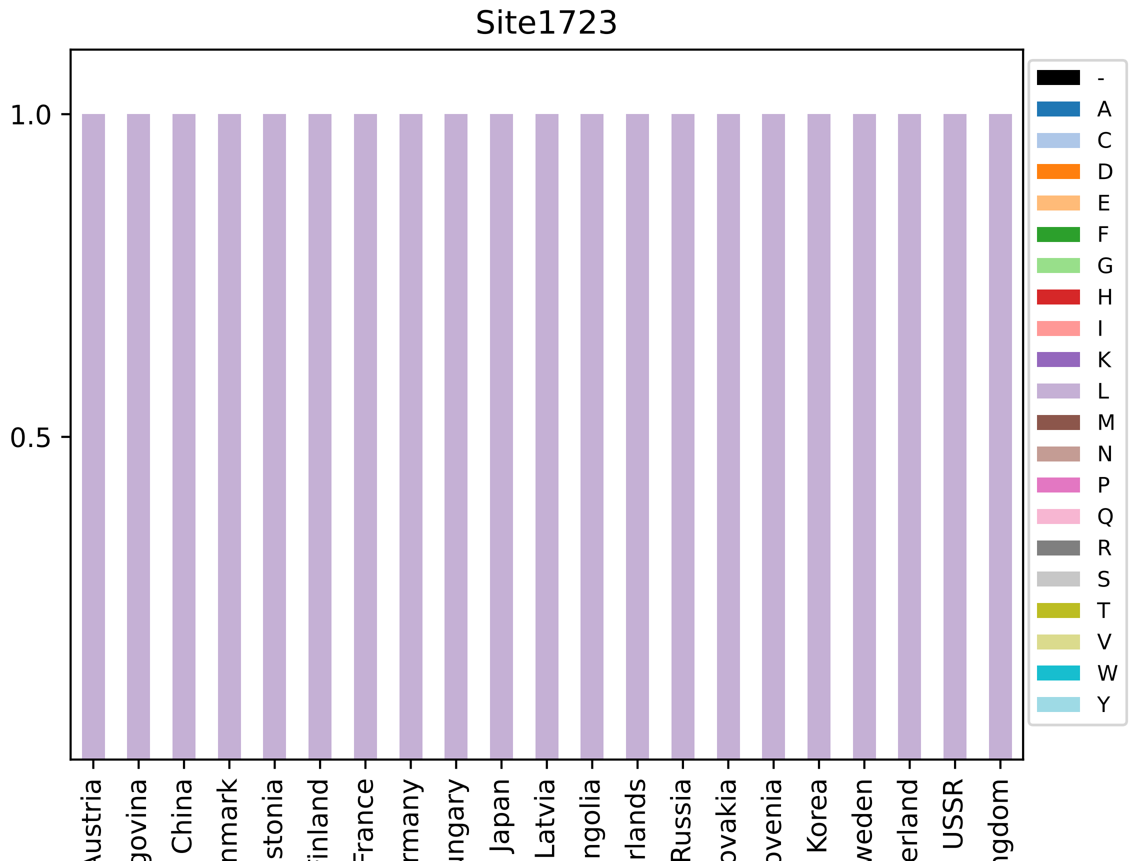
Site1723
1.0
0.5
-
A
C
D
E
F
G
H
I
K
L
M
N
P
Q
R
S
T
V
W
Y
China
Japan
Latvia
Russia
Korea
USSR

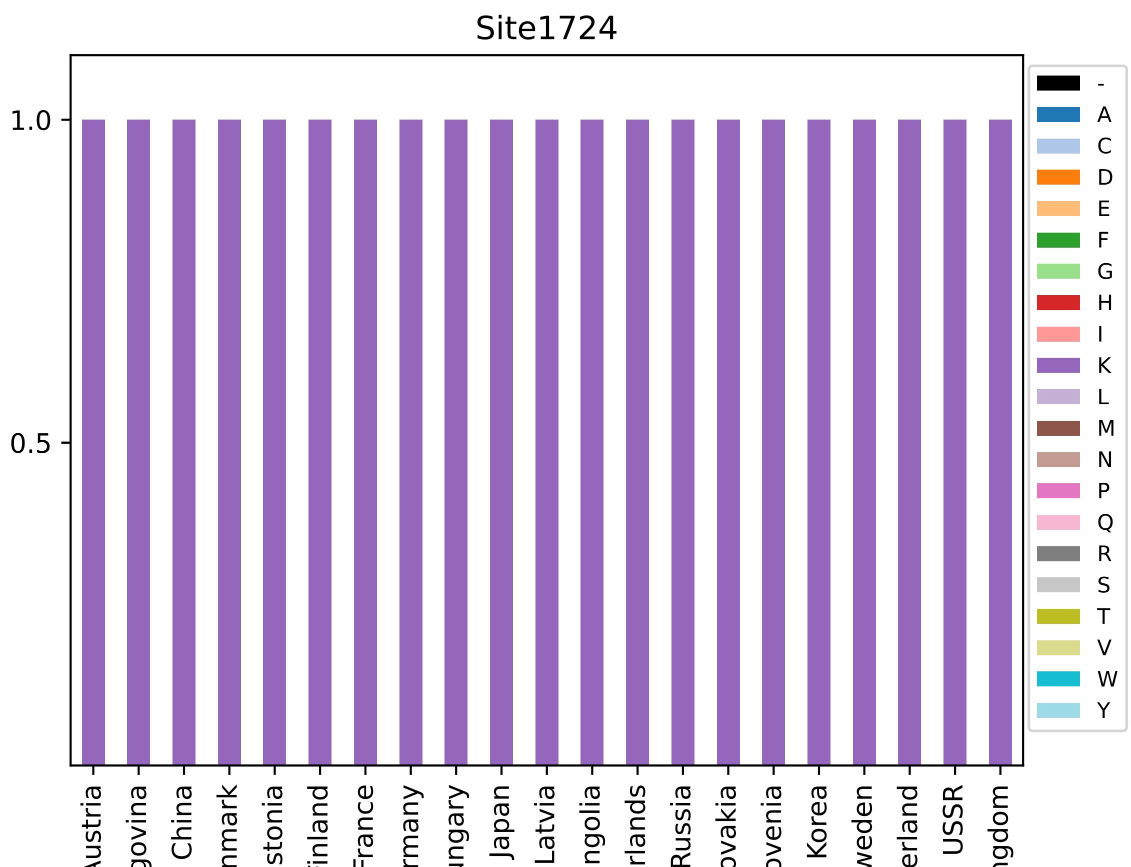

Site1724
1.0
0.5
-
A
C
D
E
F
G
H
I
K
L
M
N
P
Q
R
S
T
V
W
Y
China
Japan
Latvia
Russia
Korea
USSR

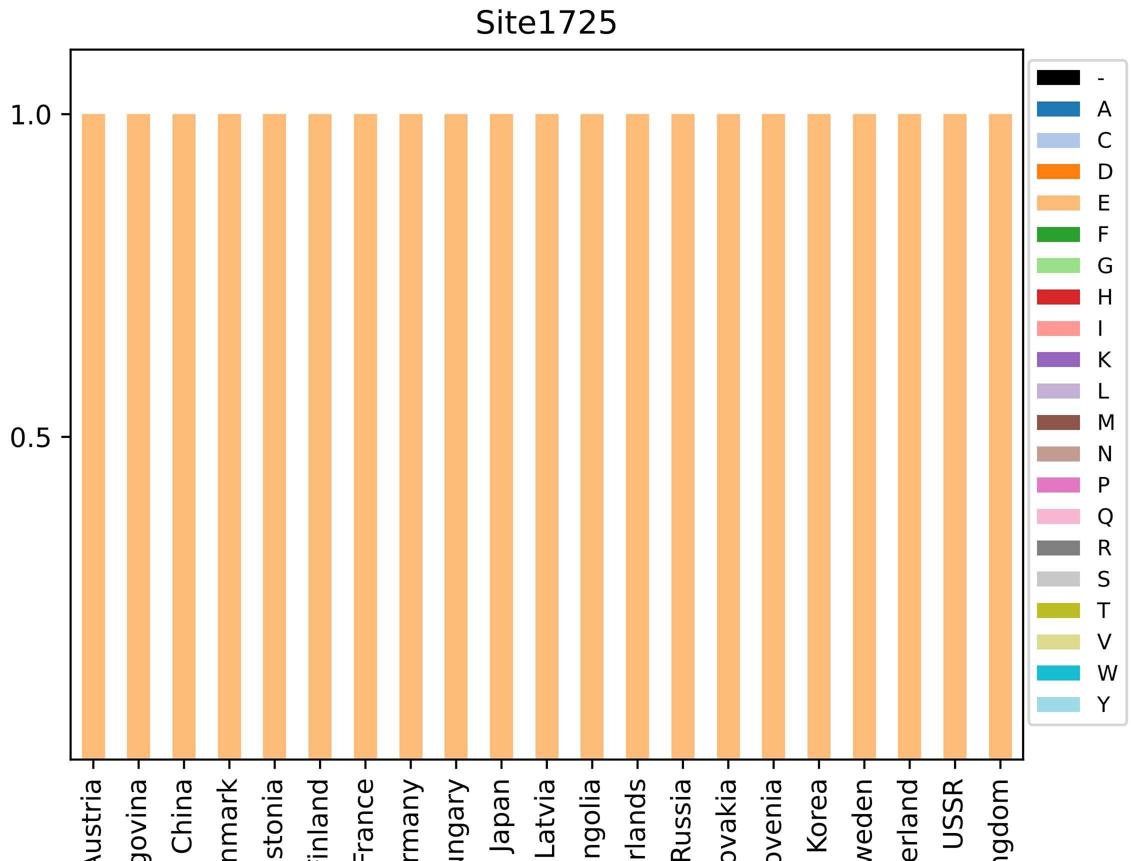
Site1725
1.0
0.5
-
A
C
D
E
F
G
H
I
K
L
M
N
P
Q
R
S
T
V
W
Y
China
Latvia
Russia
Korea
USSR

Site1726

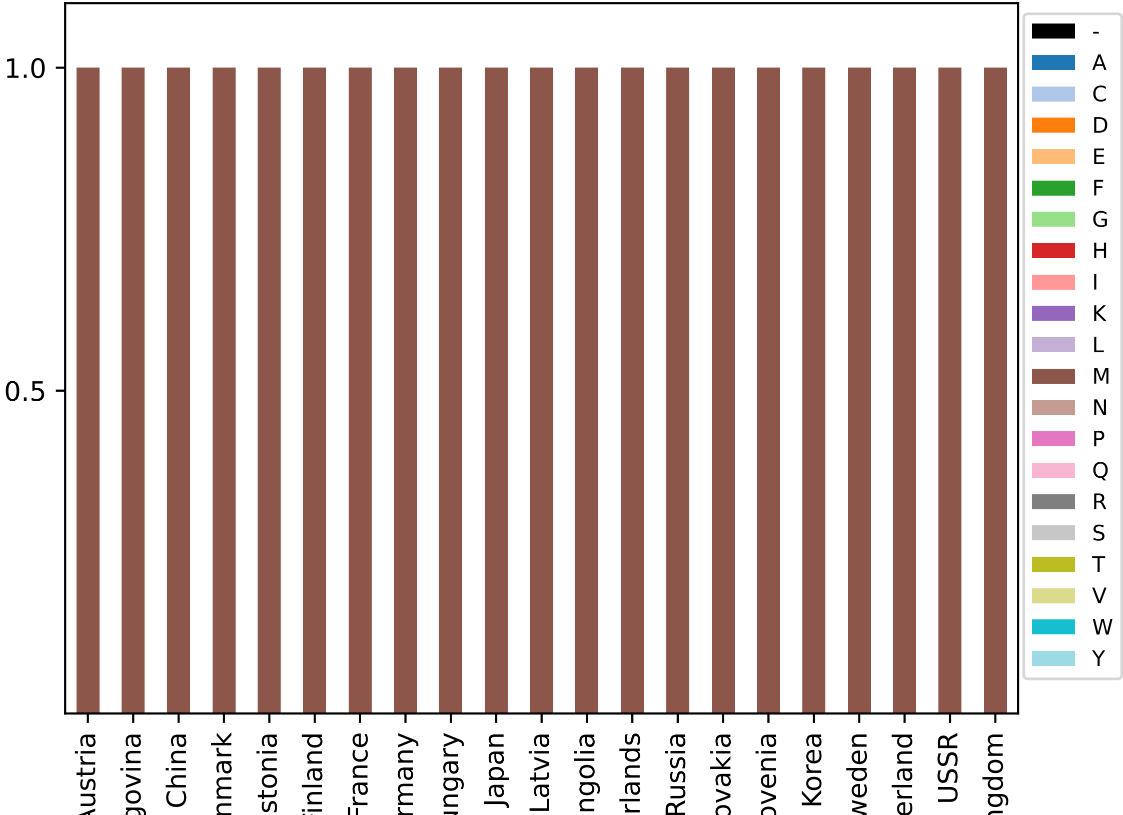
1.0
0.5
-
A
C
D
E
F
G
H
I
K
L
M
N
P
Q
R
S
T
V
W
Y
Austria
govina
China
nmark
stonia
Finland
France
rmany
ungary
Japan
Latvia
ngolia
rlands
Russia
ovakia
ovenia
Korea
weden
erland
USSR
ngdom

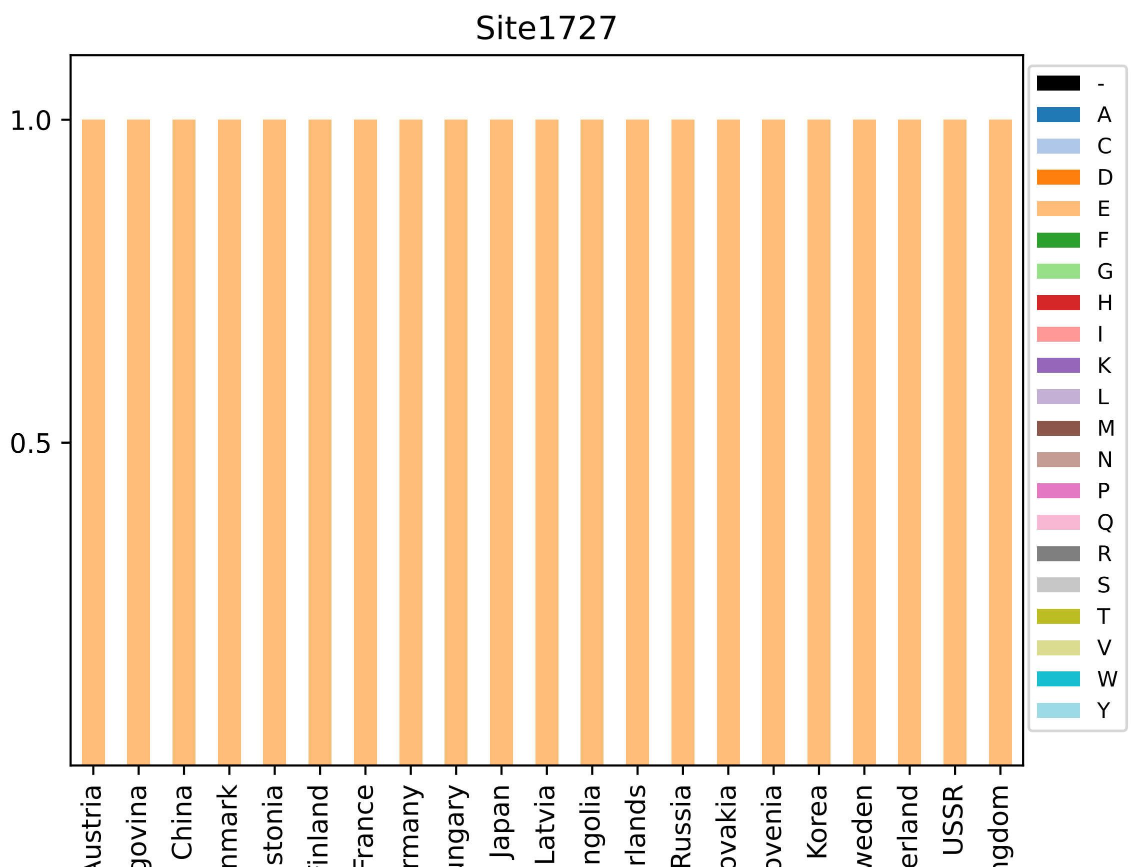

Site1727
1.0
0.5
-
A
C
D
E
F
G
H
I
K
L
M
N
P
Q
R
S
T
V
W
Y
Austria
govina
China
nmark
stonia
Finland
France
rmany
ungary
Japan
Latvia
ngolia
rlands
Russia
ovakia
ovenia
Korea
weden
erland
USSR
ngdom

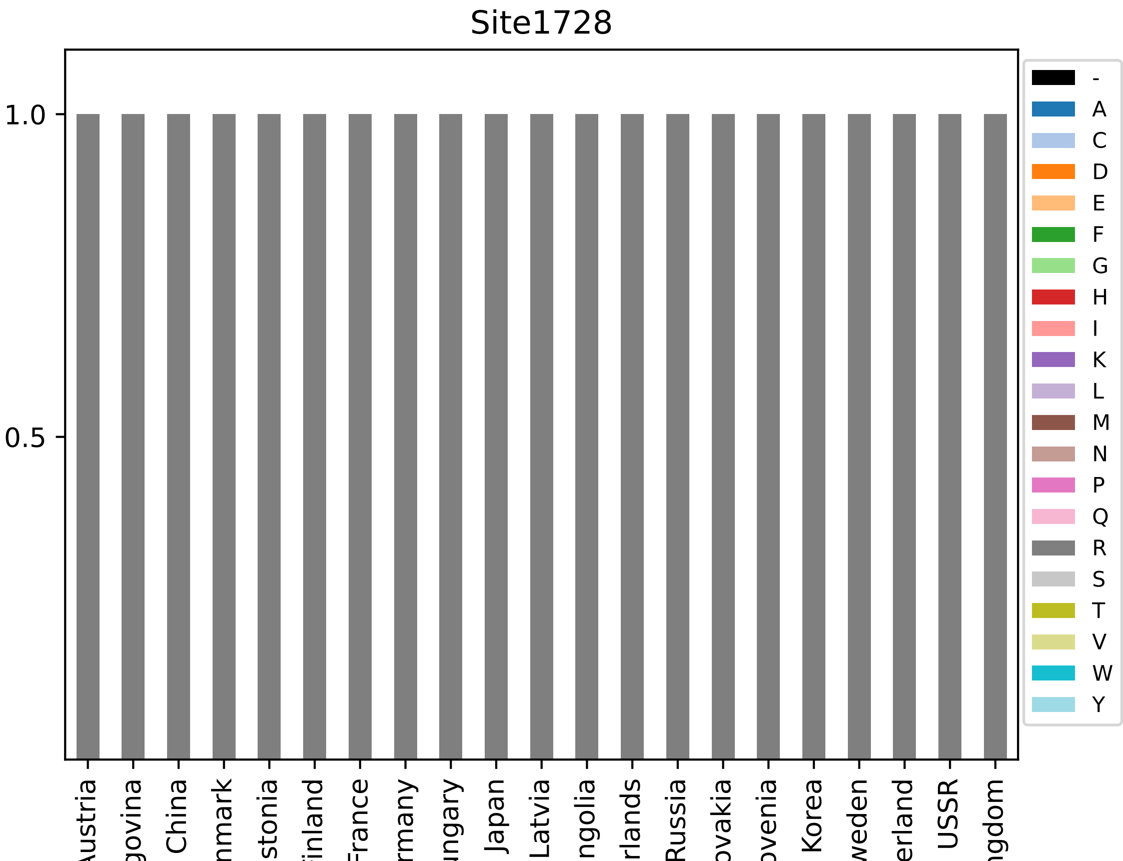
Site1728
1.0
0.5
-
A
C
D
E
F
G
H
I
K
L
M
N
P
Q
R
S
T
V
W
Y
China
Japan
Latvia
Russia
Korea
USSR

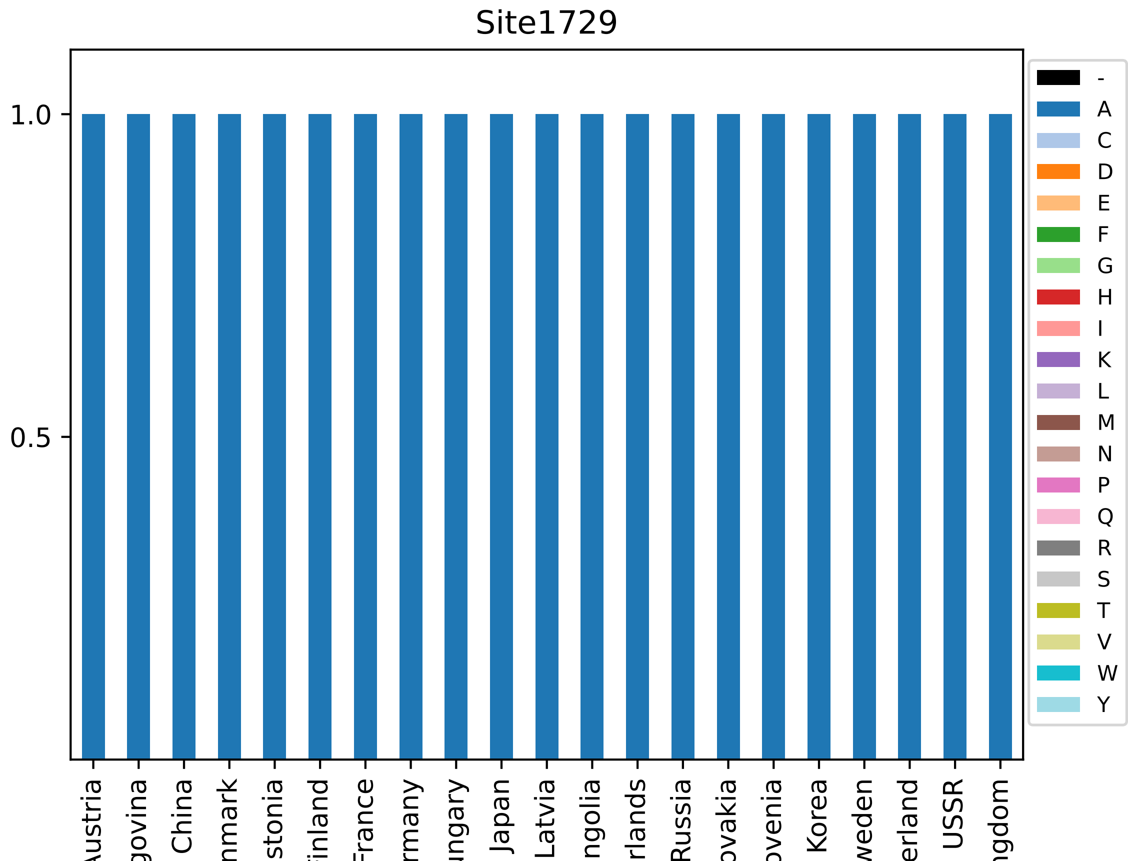
Site1729
1.0
0.5
-
A
C
D
E
F
G
H
I
K
L
M
N
P
Q
R
S
T
V
W
Y
China
Japan
Latvia
Russia
Korea
USSR

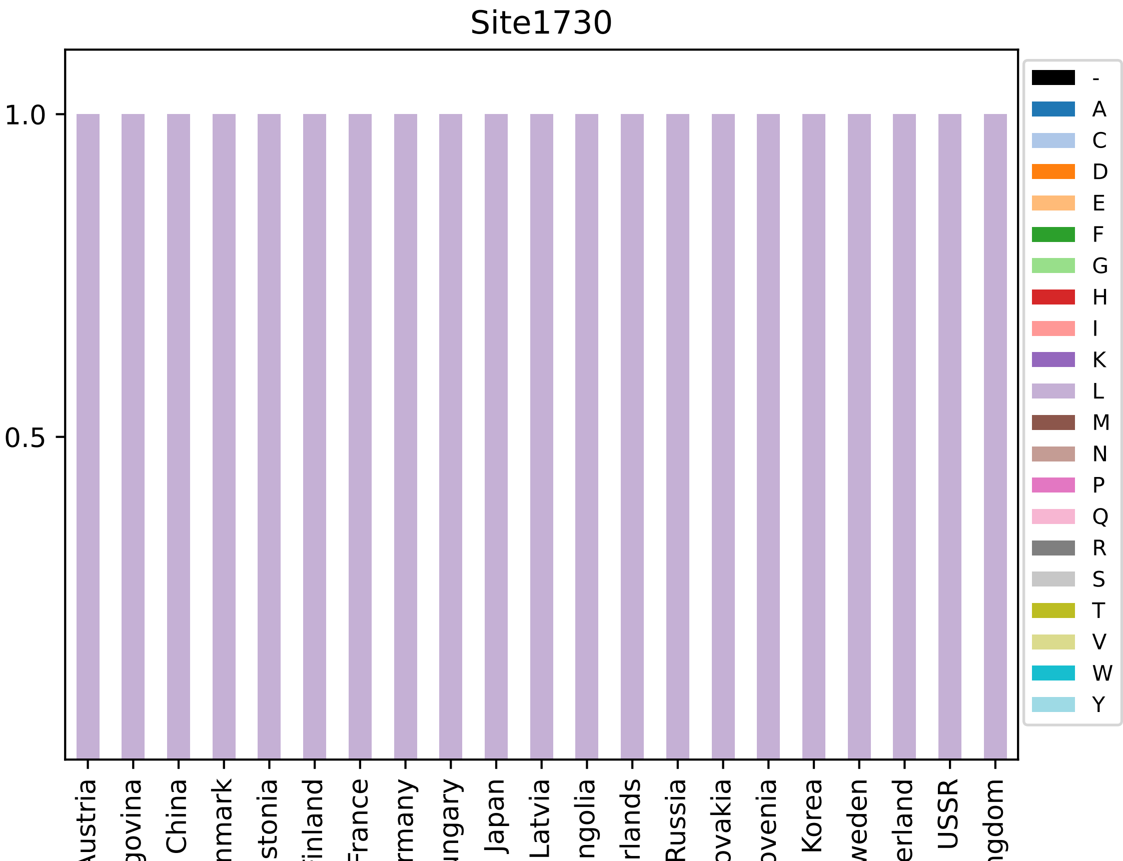
Site1730
1.0
0.5
-
A
C
D
E
F
G
H
I
K
L
M
N
P
Q
R
S
T
V
W
Y
Austria
China
Japan
Latvia
Russia
Korea
USSR

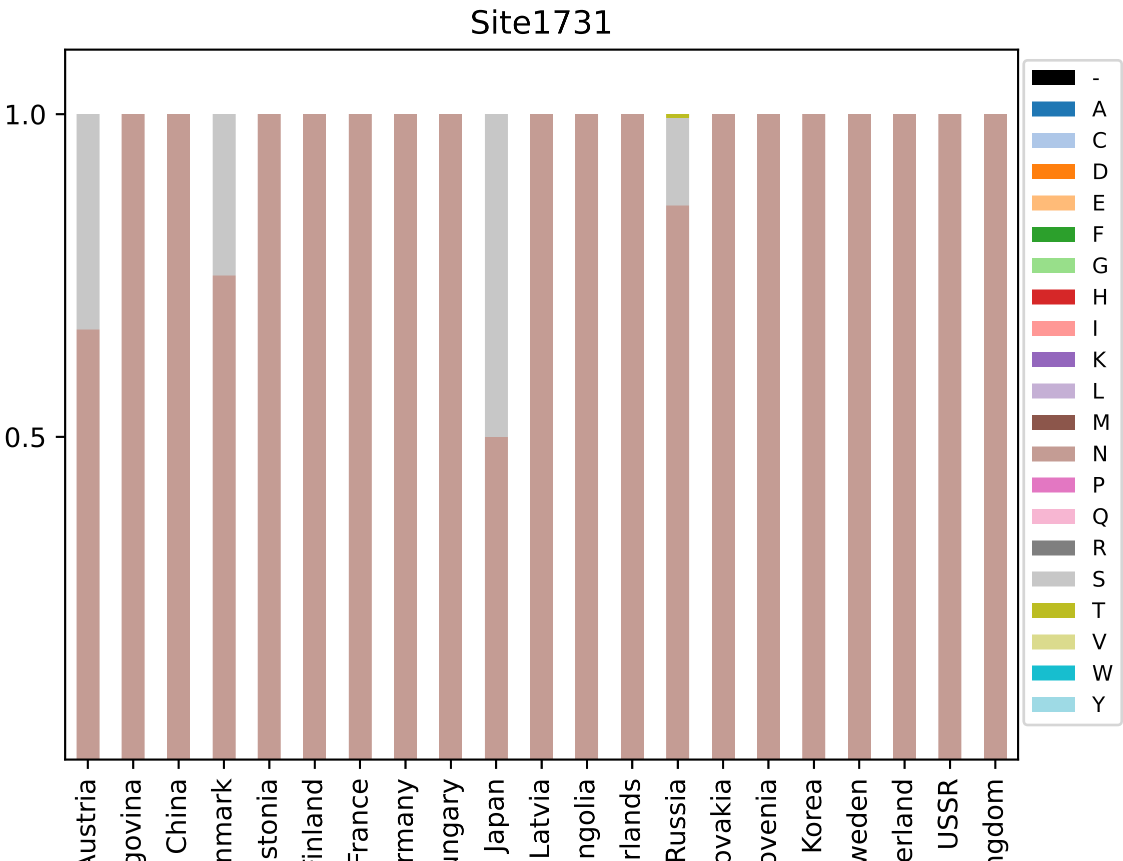

Site1731
1.0
0.5
-
A
C
D
E
F
G
H
I
K
L
M
N
P
Q
R
S
T
V
W
Y
Austria
govina
China
nmark
stonia
Finland
France
rmany
ungary
Japan
Latvia
ngolia
rlands
Russia
ovakia
ovenia
Korea
weden
erland
USSR
ngdom

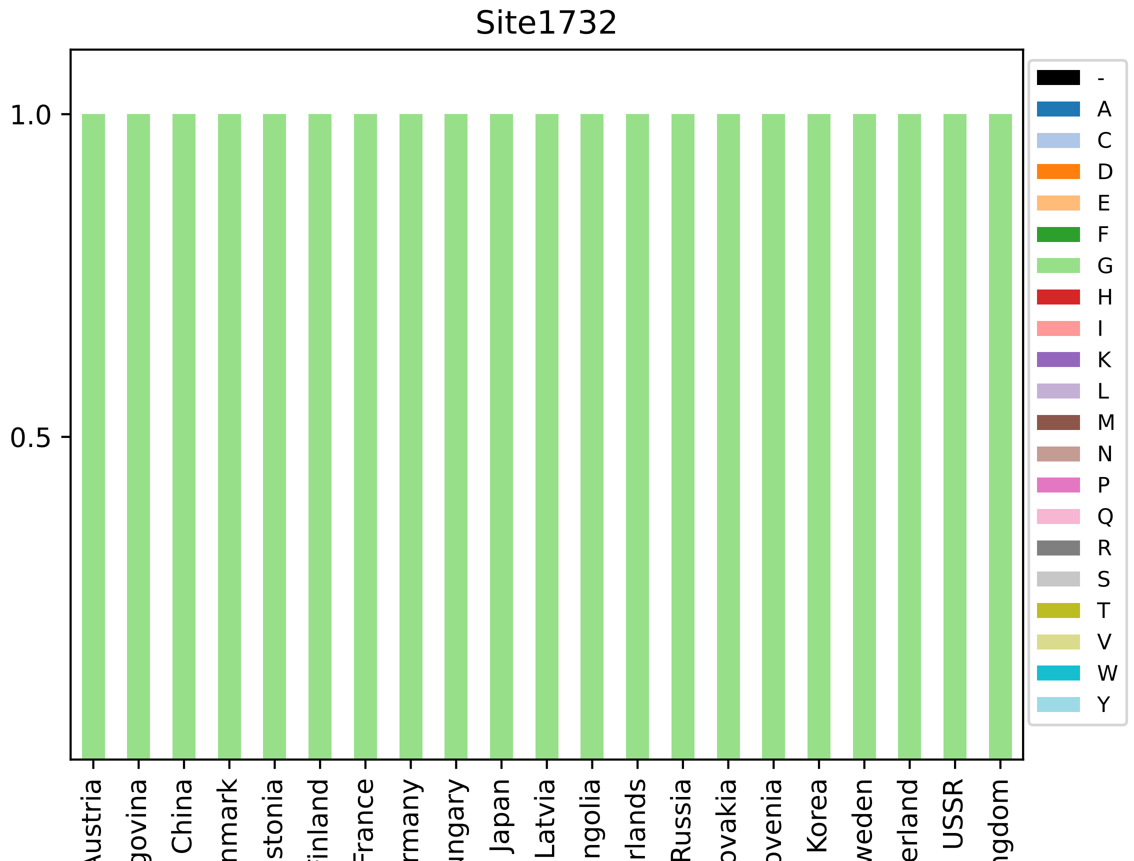
Site1732
1.0
0.5
-
A
C
D
E
F
G
H
I
K
L
M
N
P
Q
R
S
T
V
W
Y
Austria
China
Japan
Latvia
Russia
Korea
USSR

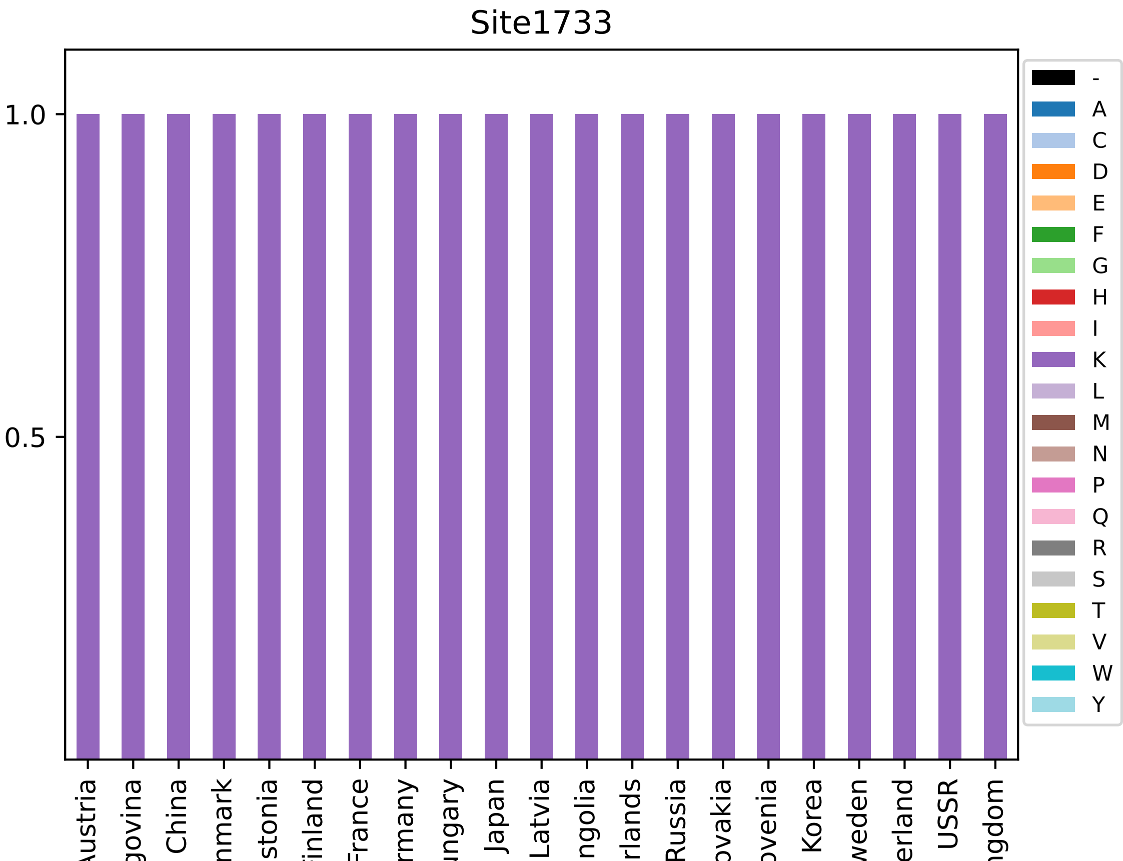

Site1733
1.0
0.5
-
A
C
D
E
F
G
H
I
K
L
M
N
P
Q
R
S
T
V
W
Y
Austria
China
Finland
France
Japan
Latvia
Russia
Korea
USSR

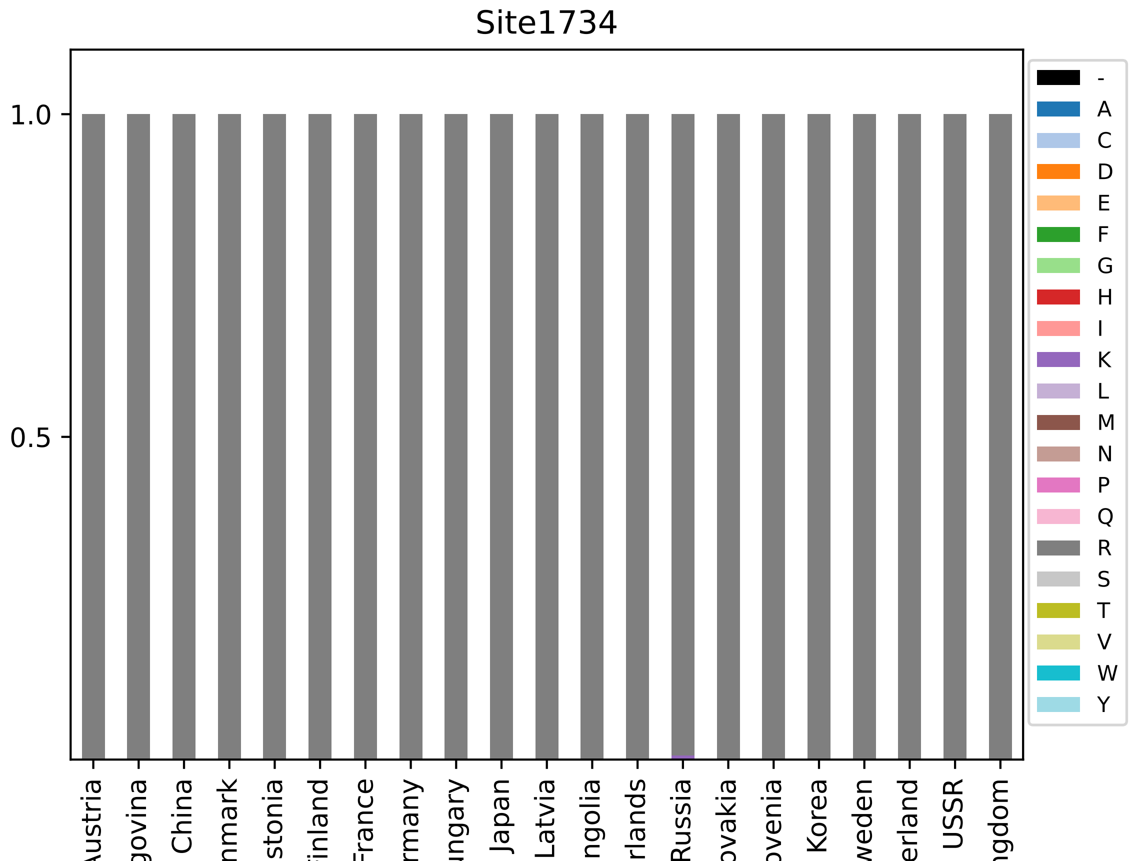
Site1734
1.0
0.5
-
A
C
D
E
F
G
H
I
K
L
M
N
P
Q
R
S
T
V
W
Y
Austria
China
Japan
Latvia
Russia
Korea
USSR

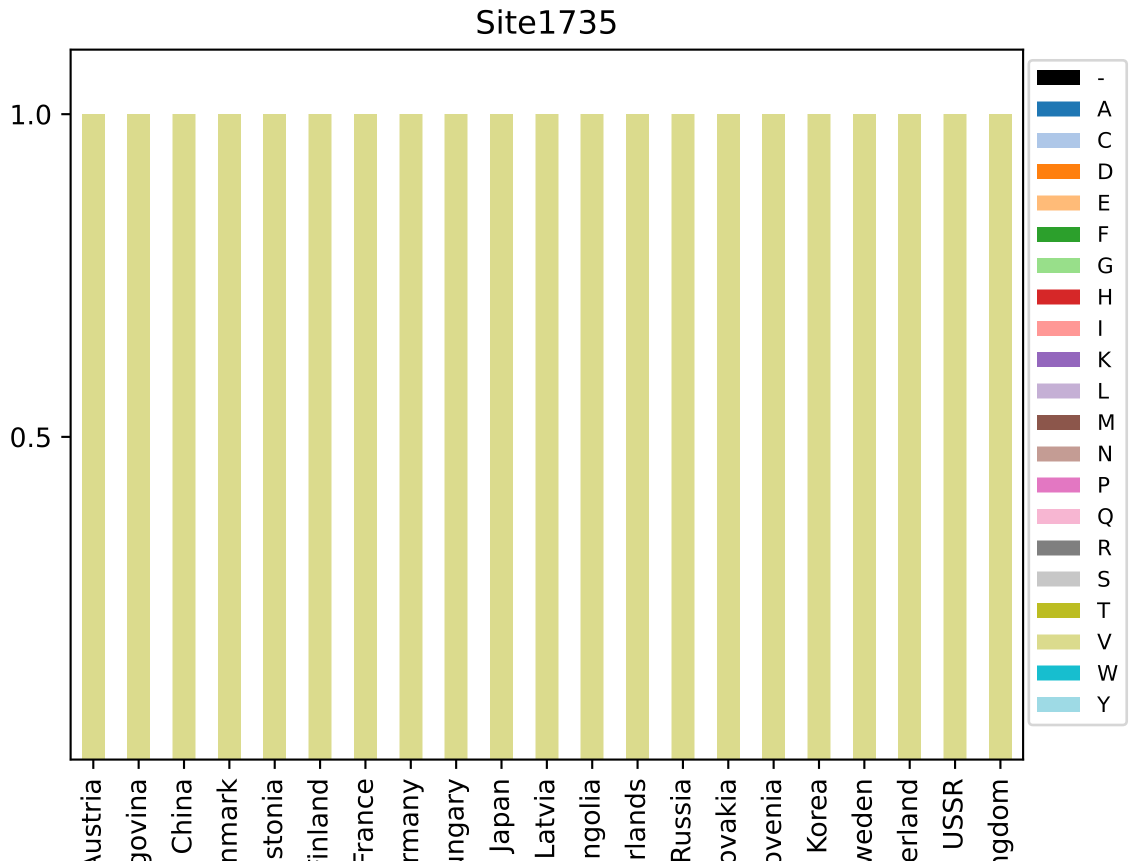

Site1735
1.0
0.5
China
Japan
Latvia
Russia
Korea
USSR
-
A
C
D
E
F
G
H
I
K
L
M
N
P
Q
R
S
T
V
W
Y

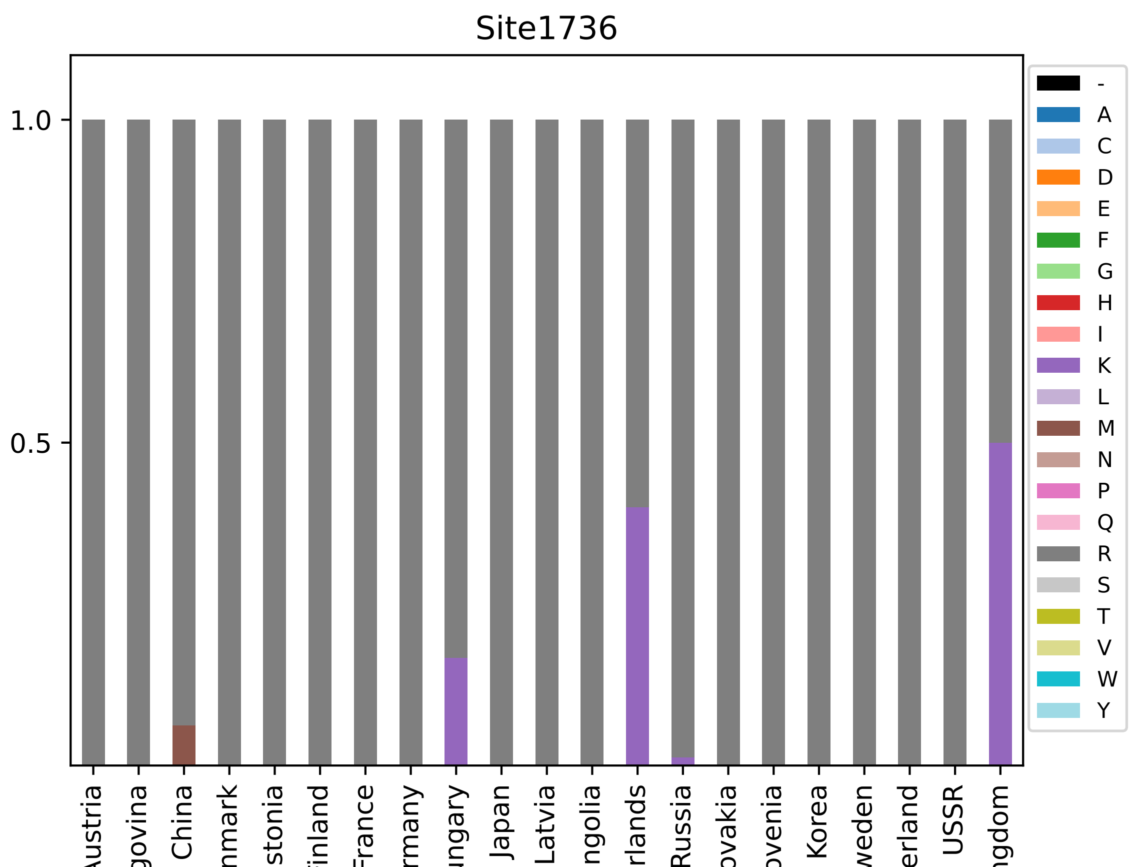

Site1736
1.0
0.5
-
A
C
D
E
F
G
H
I
K
L
M
N
P
Q
R
S
T
V
W
Y
China
Japan
Latvia
Russia
Korea
USSR

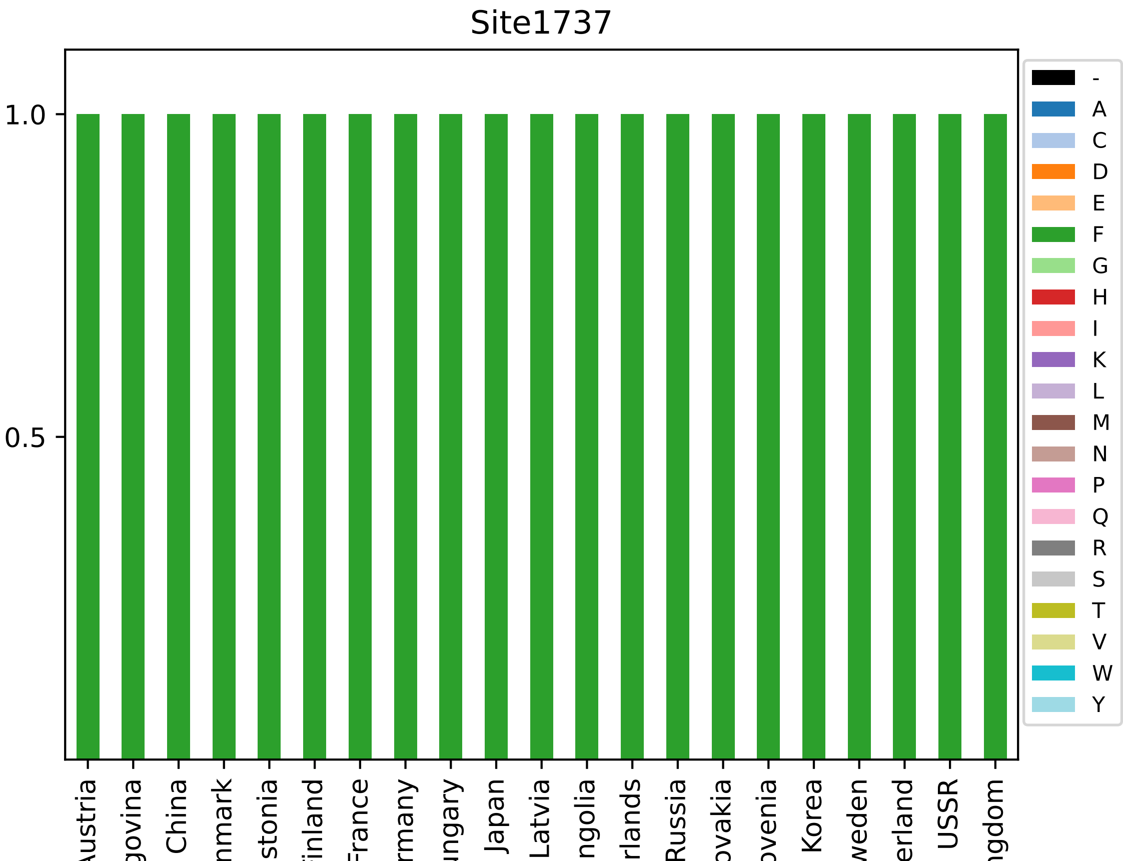

Site1737
1.0
0.5
-
A
C
D
E
F
G
H
I
K
L
M
N
P
Q
R
S
T
V
W
Y
Austria
China
Japan
Latvia
Russia
Korea
USSR

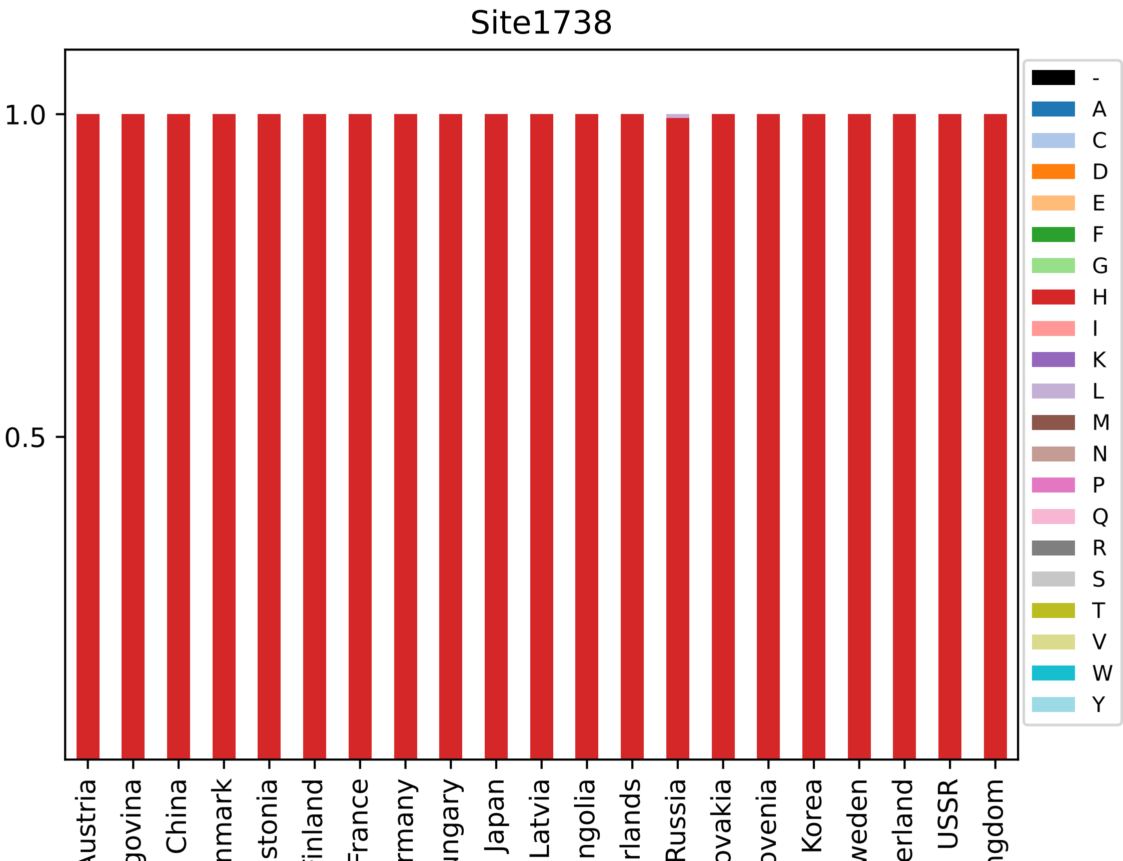
Site1738
1.0
0.5
-
A
C
D
E
F
G
H
I
K
L
M
N
P
Q
R
S
T
V
W
Y
Austria
govina
China
nmark
stonia
Finland
France
rmany
ungary
Japan
Latvia
ngolia
rlands
Russia
ovakia
ovenia
Korea
weden
erland
USSR
ngdom

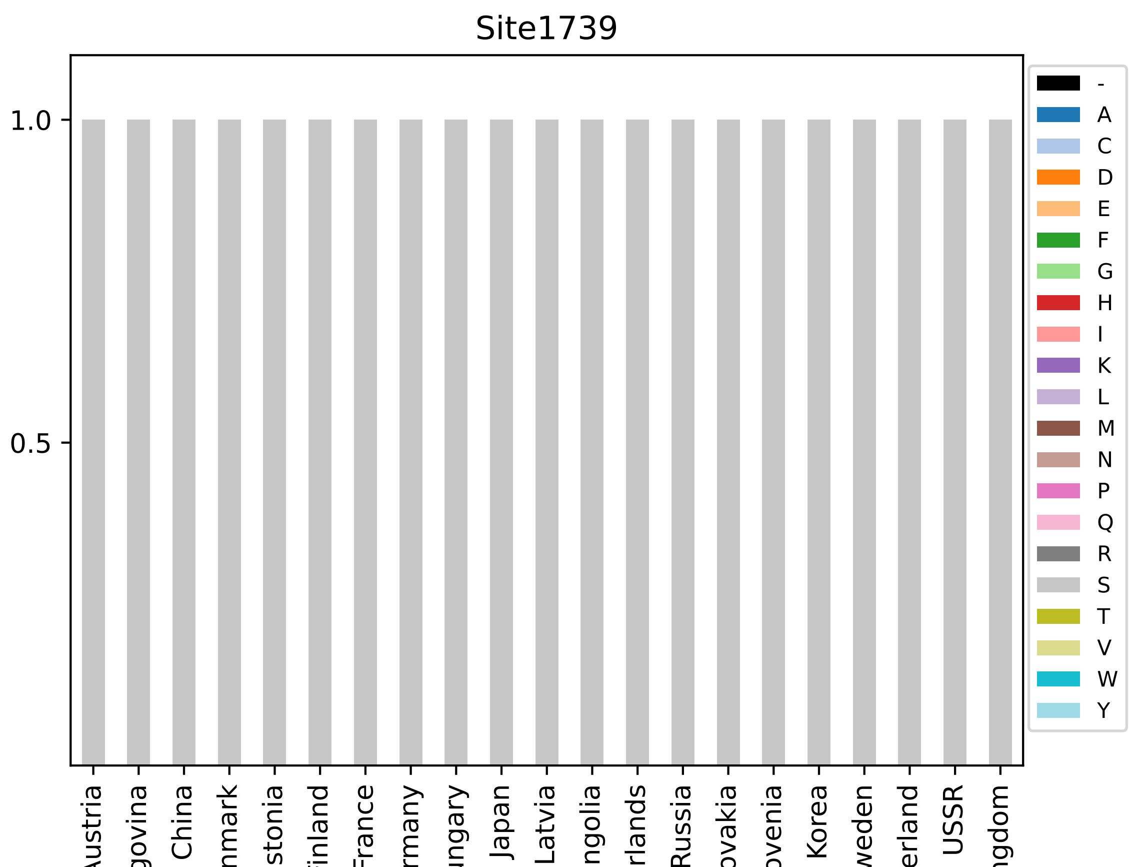
Site1739
1.0
0.5
-
A
C
D
E
F
G
H
I
K
L
M
N
P
Q
R
S
T
V
W
Y
Austria
govina
China
nmark
stonia
Finland
France
rmany
ungary
Japan
Latvia
ngolia
rlands
Russia
ovakia
ovenia
Korea
weden
erland
USSR
ngdom

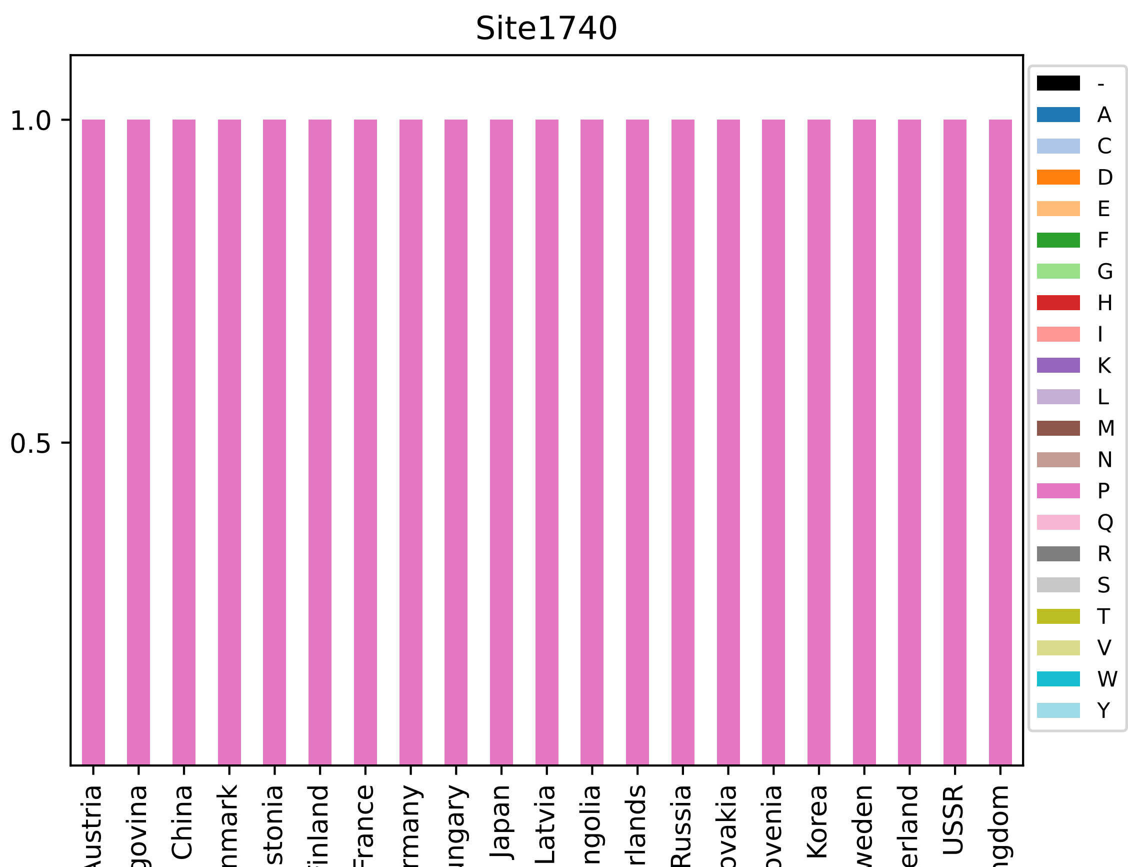

Site1740
1.0
0.5
-
A
C
D
E
F
G
H
I
K
L
M
N
P
Q
R
S
T
V
W
Y
China
nmark
stonia
Japan
Latvia
ngolia
rlands
Russia
ovakia
ovenia
Korea
weden
erland
USSR
ngdom

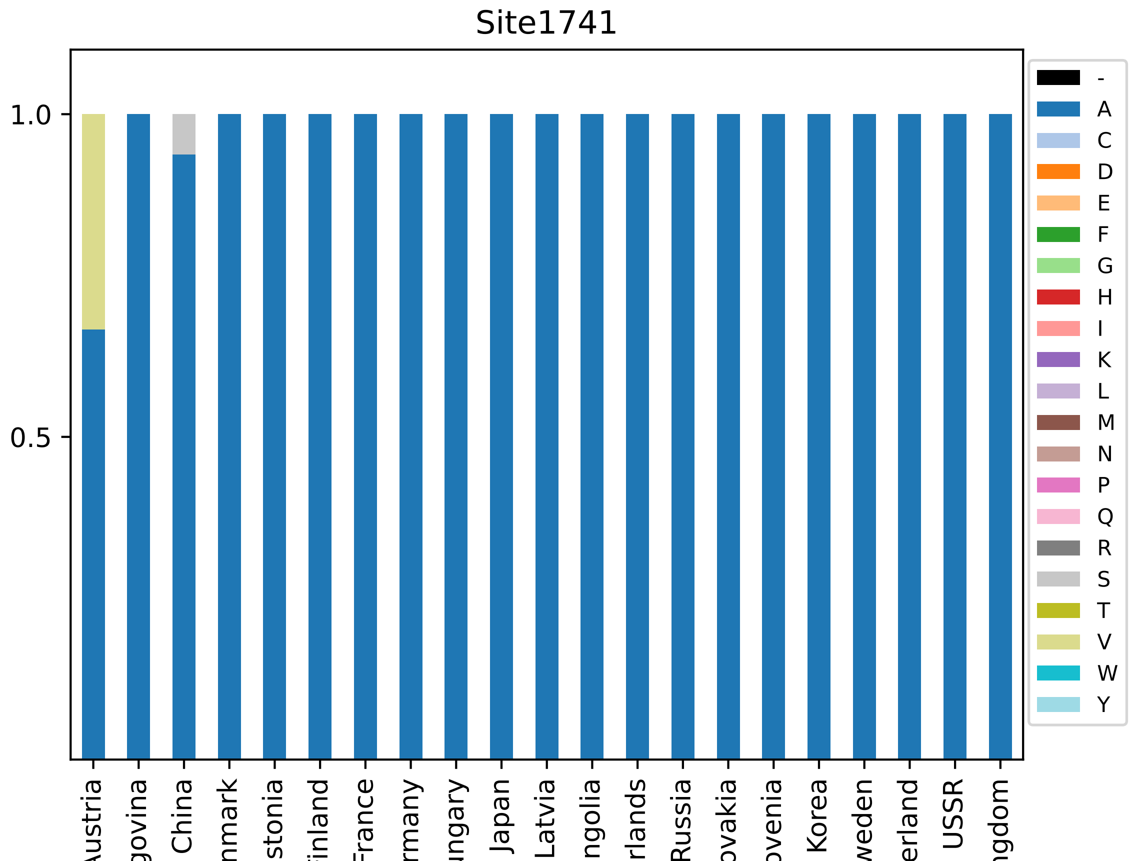

Site1741
1.0
0.5
Austria
govina
China
nmark
stonia
inland
France
rmany
ungary
Japan
Latvia
ngolia
rlands
Russia
ovakia
ovenia
Korea
weden
erland
USSR
ngdom
-
A
C
D
E
F
G
H
I
K
L
M
N
P
Q
R
S
T
V
W
Y

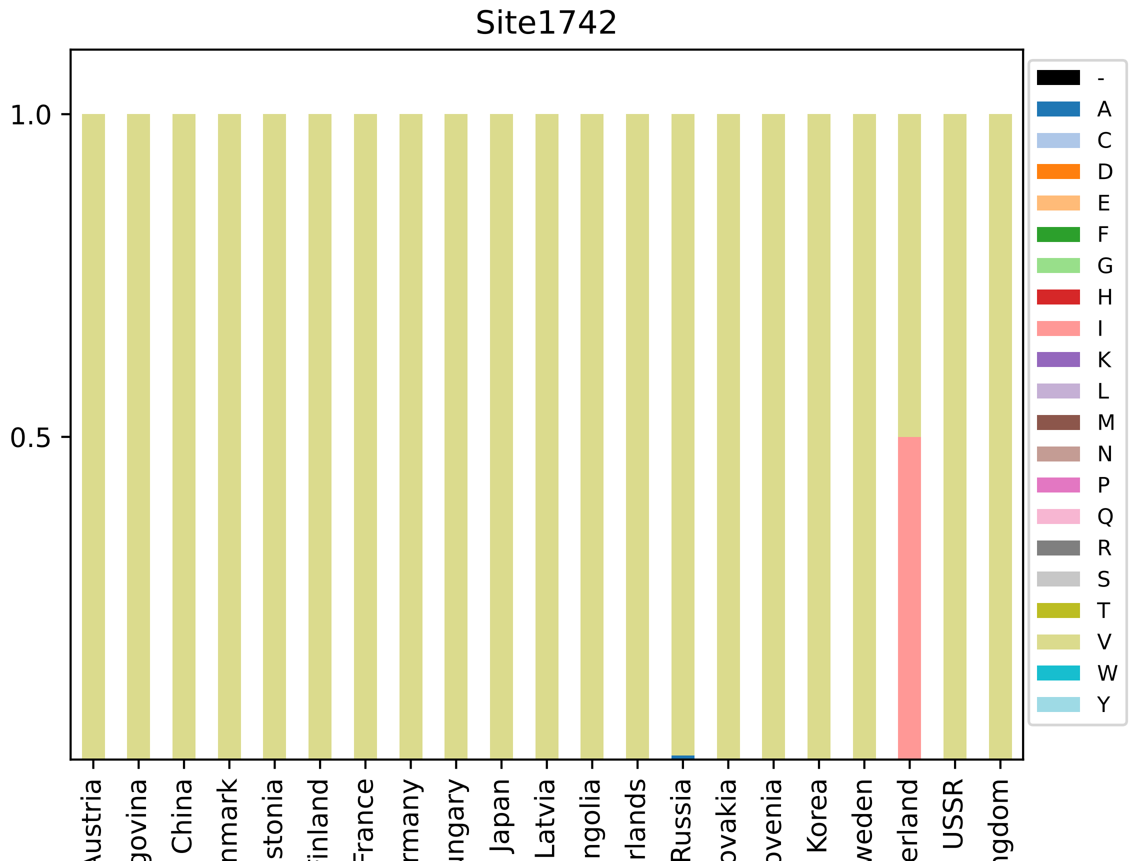

Site1742
1.0
0.5
Austria
govina
China
nmark
stonia
Finland
France
rmany
ungary
Japan
Latvia
ngolia
rlands
Russia
ovakia
ovenia
Korea
weden
erland
USSR
ngdom
-
A
C
D
E
F
G
H
I
K
L
M
N
P
Q
R
S
T
V
W
Y

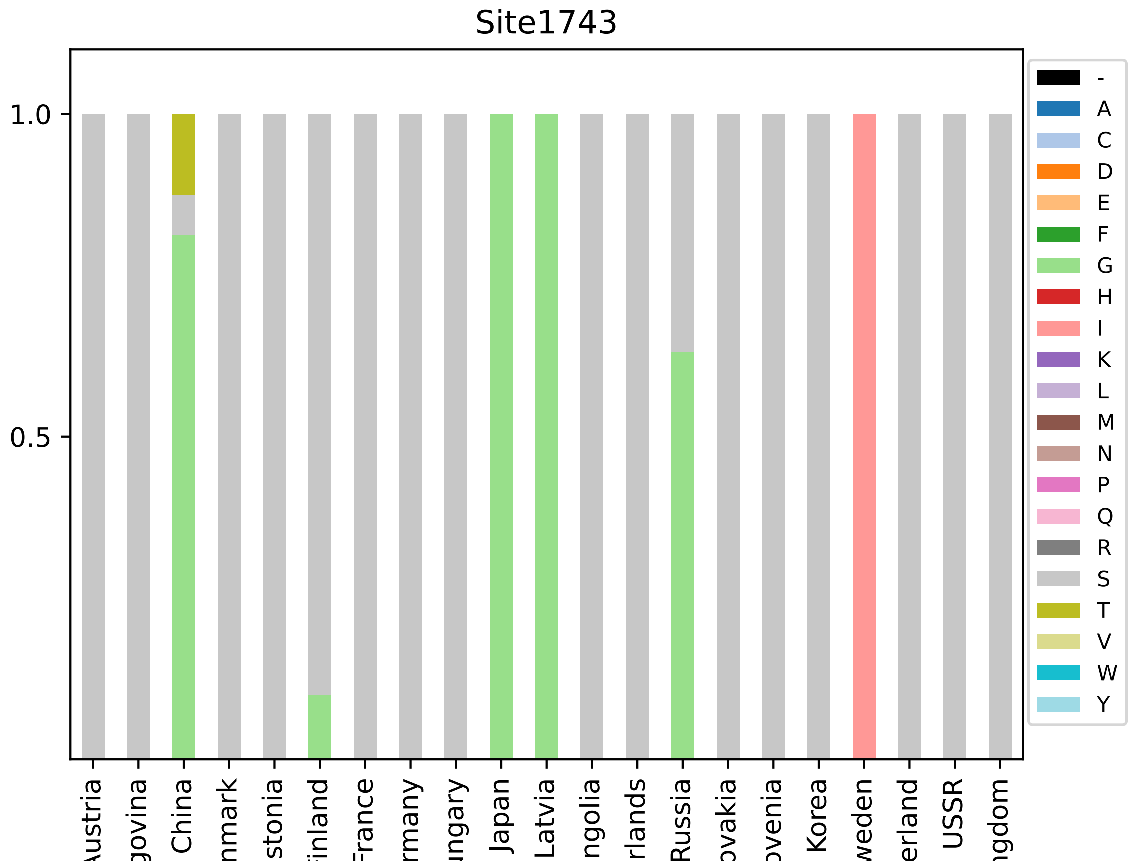
Site1743
1.0
0.5
Austria
govina
China
nmark
stonia
inland
France
rmany
ungary
Japan
Latvia
ngolia
rlands
Russia
ovakia
ovenia
Korea
weden
erland
USSR
ngdom
-
A
C
D
E
F
G
H
I
K
L
M
N
P
Q
R
S
T
V
W
Y

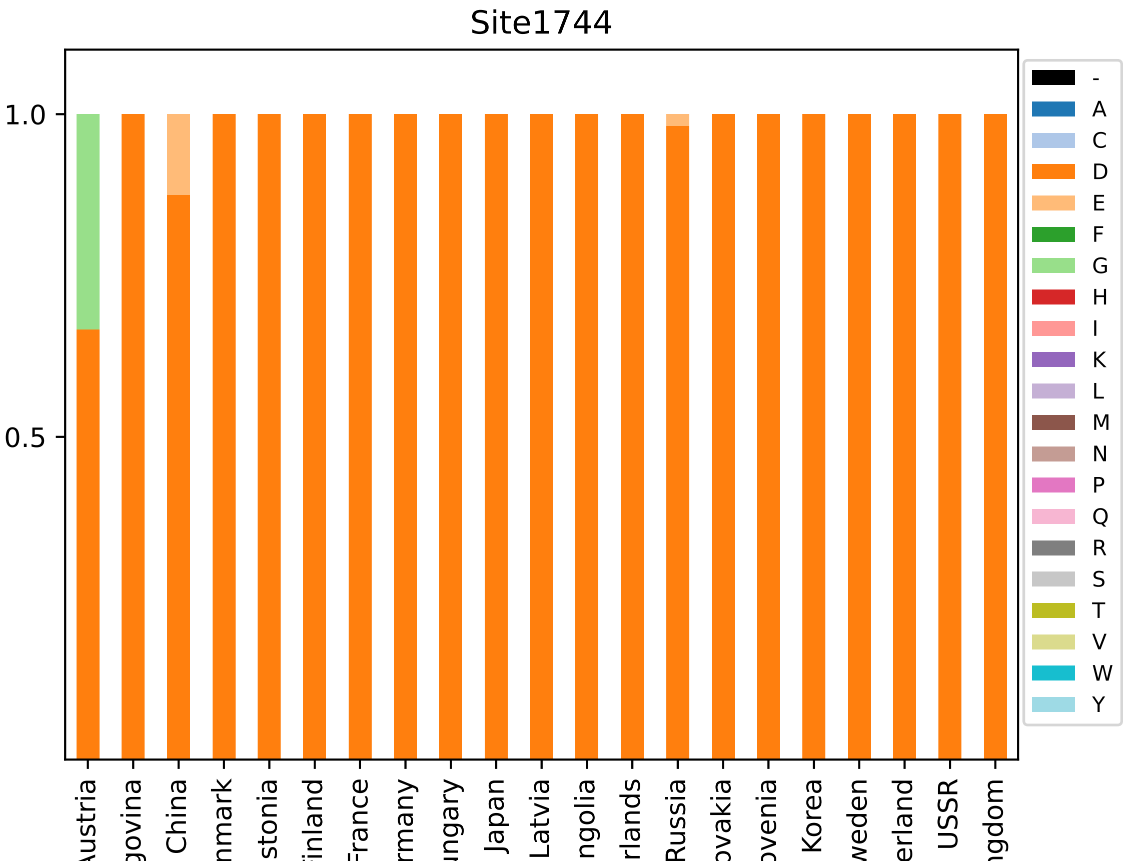
Site1744
1.0
0.5
-
A
C
D
E
F
G
H
I
K
L
M
N
P
Q
R
S
T
V
W
Y
China
Japan
Latvia
Russia
Korea
USSR

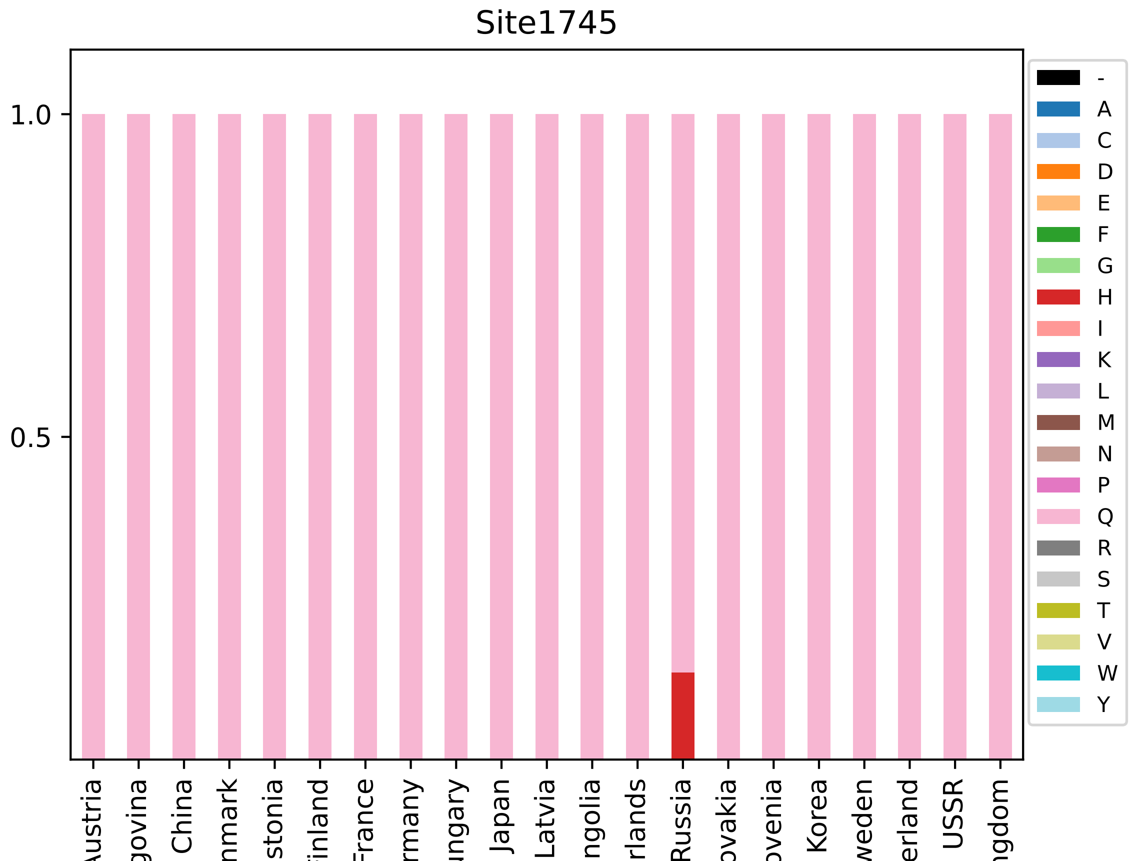
Site1745
1.0
0.5
-
A
C
D
E
F
G
H
I
K
L
M
N
P
Q
R
S
T
V
W
Y
China
Japan
Latvia
Russia
Korea
USSR

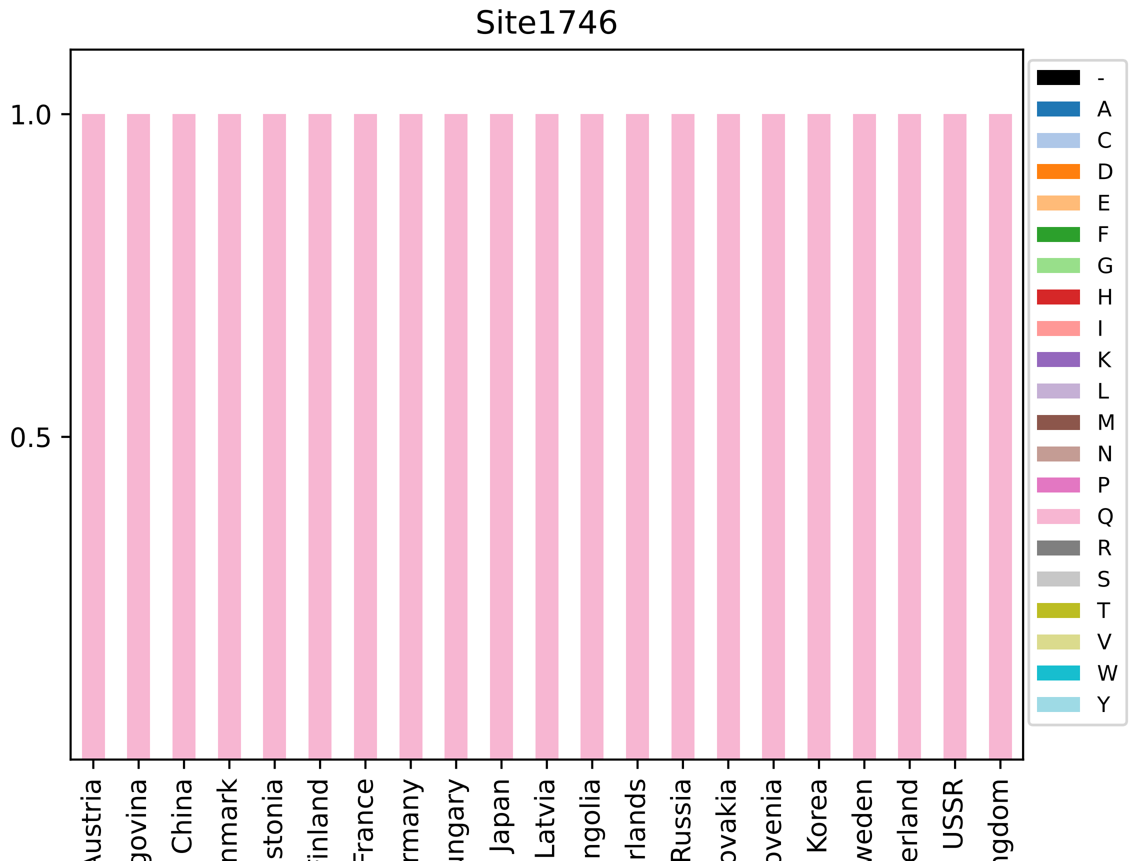
Site1746
1.0
0.5
-
A
C
D
E
F
G
H
I
K
L
M
N
P
Q
R
S
T
V
W
Y
China
Japan
Latvia
Russia
Korea
USSR

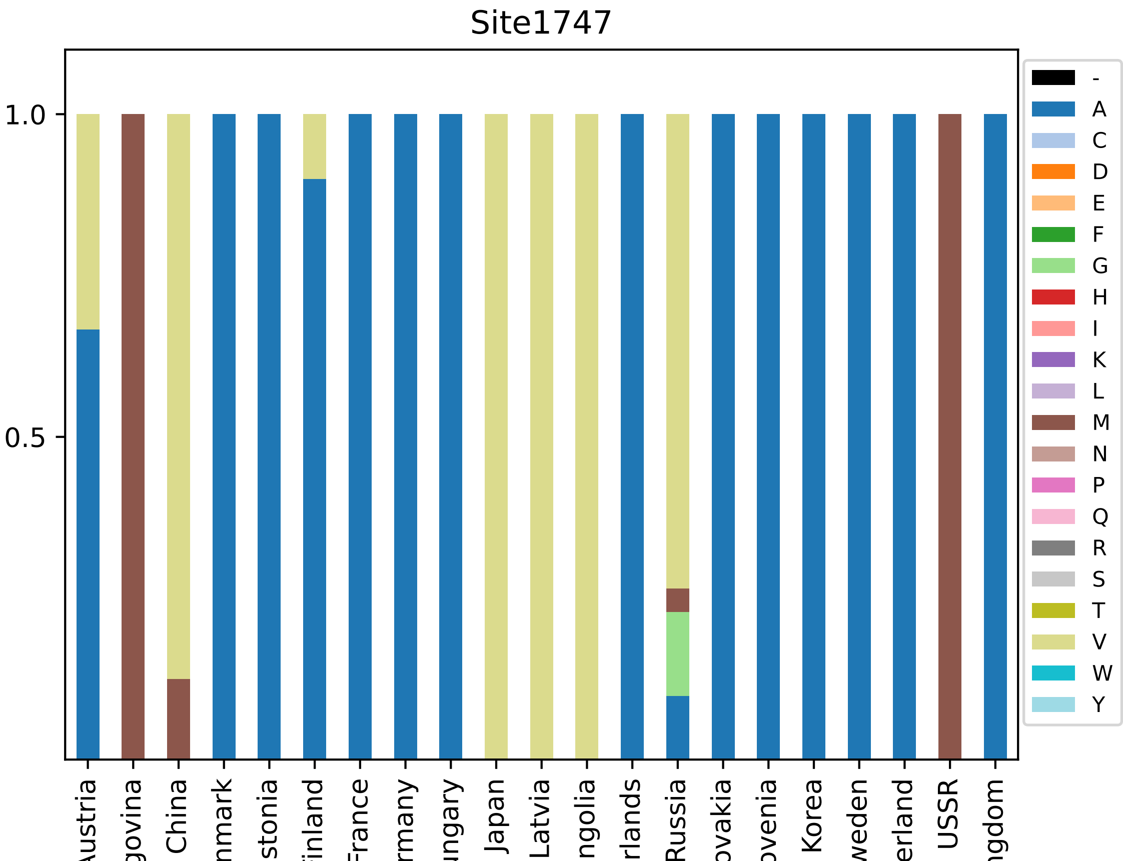
Site1747
1.0
0.5
-
A
C
D
E
F
G
H
I
K
L
M
N
P
Q
R
S
T
V
W
Y
China
Japan
Latvia
Russia
Korea
USSR

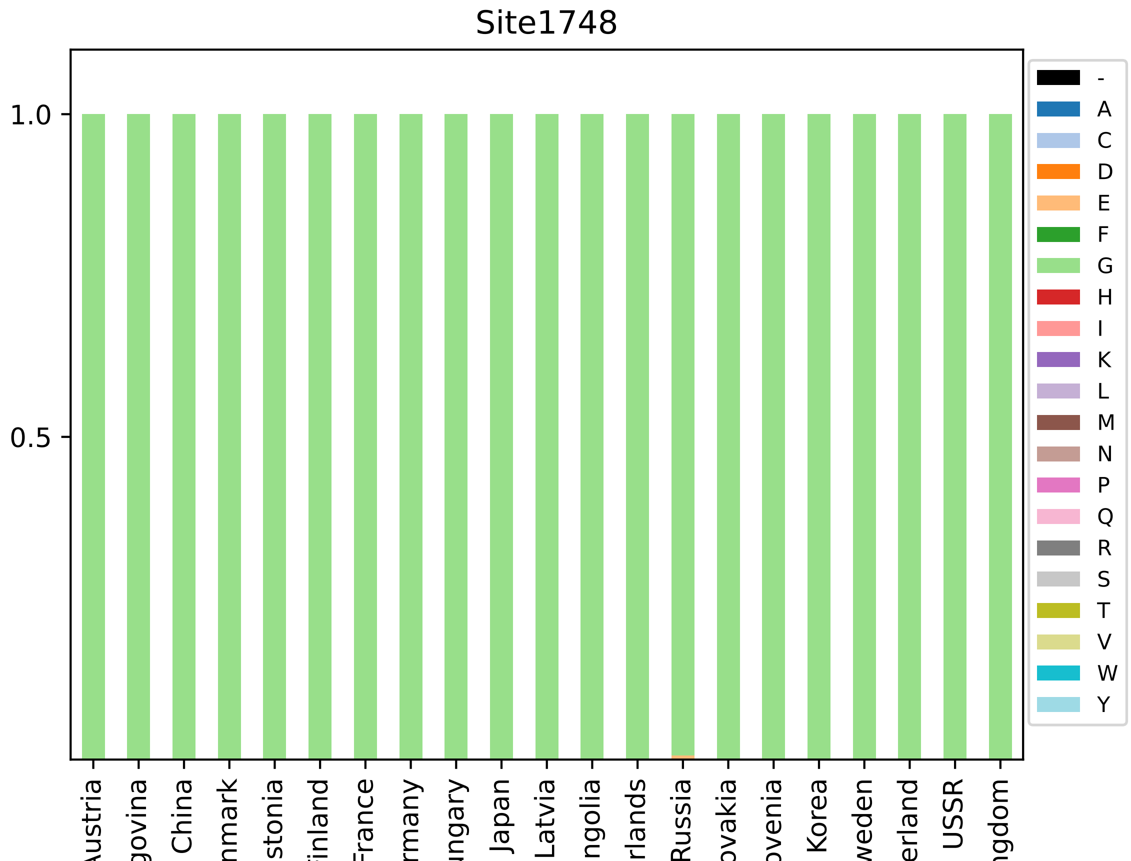
Site1748
1.0
0.5
-
A
C
D
E
F
G
H
I
K
L
M
N
P
Q
R
S
T
V
W
Y
Austria
China
Japan
Latvia
Russia
Korea
USSR

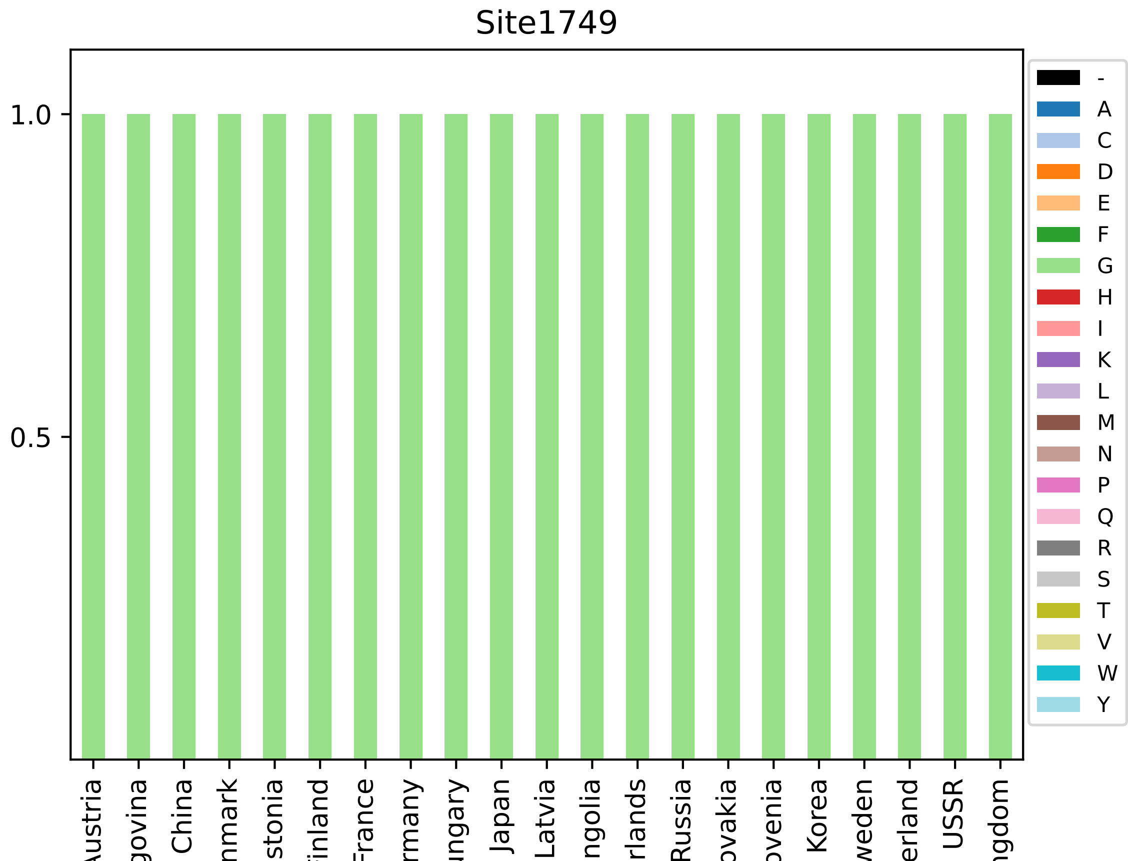
Site1749
1.0
0.5
-
A
C
D
E
F
G
H
I
K
L
M
N
P
Q
R
S
T
V
W
Y
China
Japan
Latvia
Russia
Korea
USSR

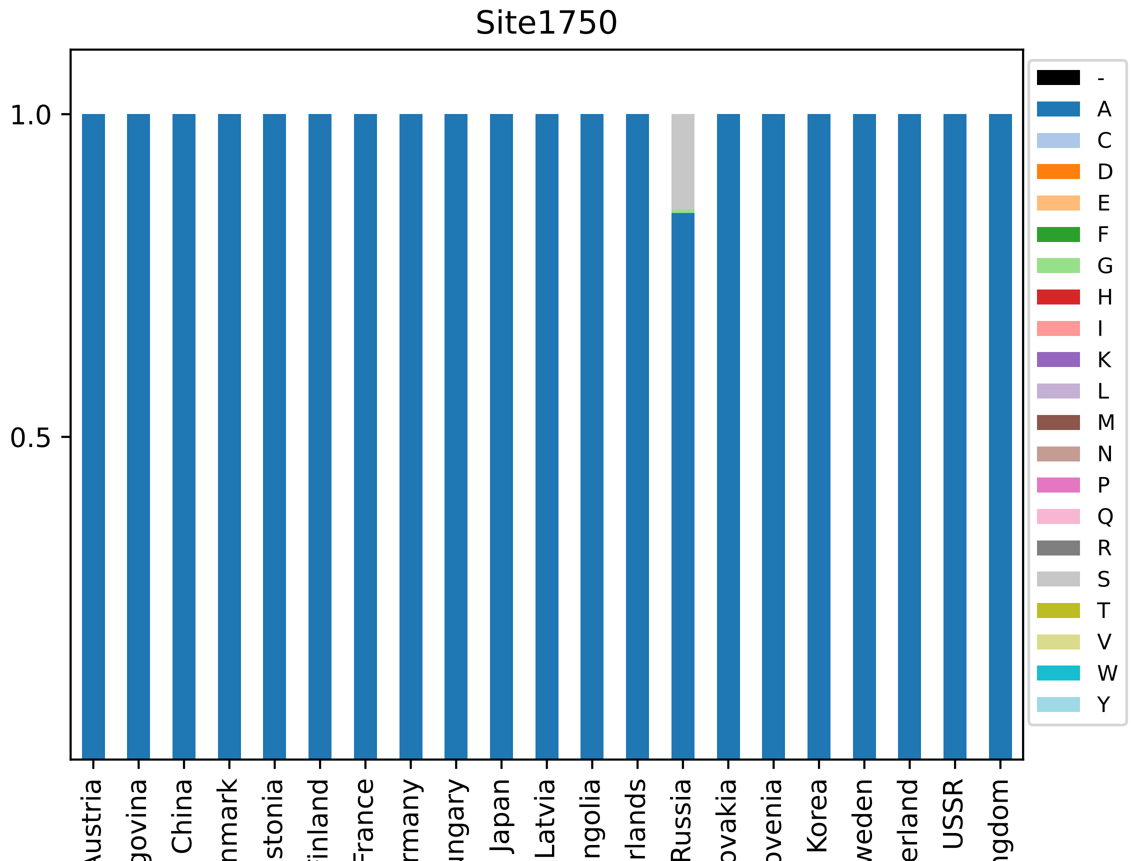

Site1750
1.0
0.5
-
A
C
D
E
F
G
H
I
K
L
M
N
P
Q
R
S
T
V
W
Y
Austria
govina
China
nmark
stonia
inland
France
rmany
ungary
Japan
Latvia
ngolia
rlands
Russia
ovakia
ovenia
Korea
weden
erland
USSR
ngdom

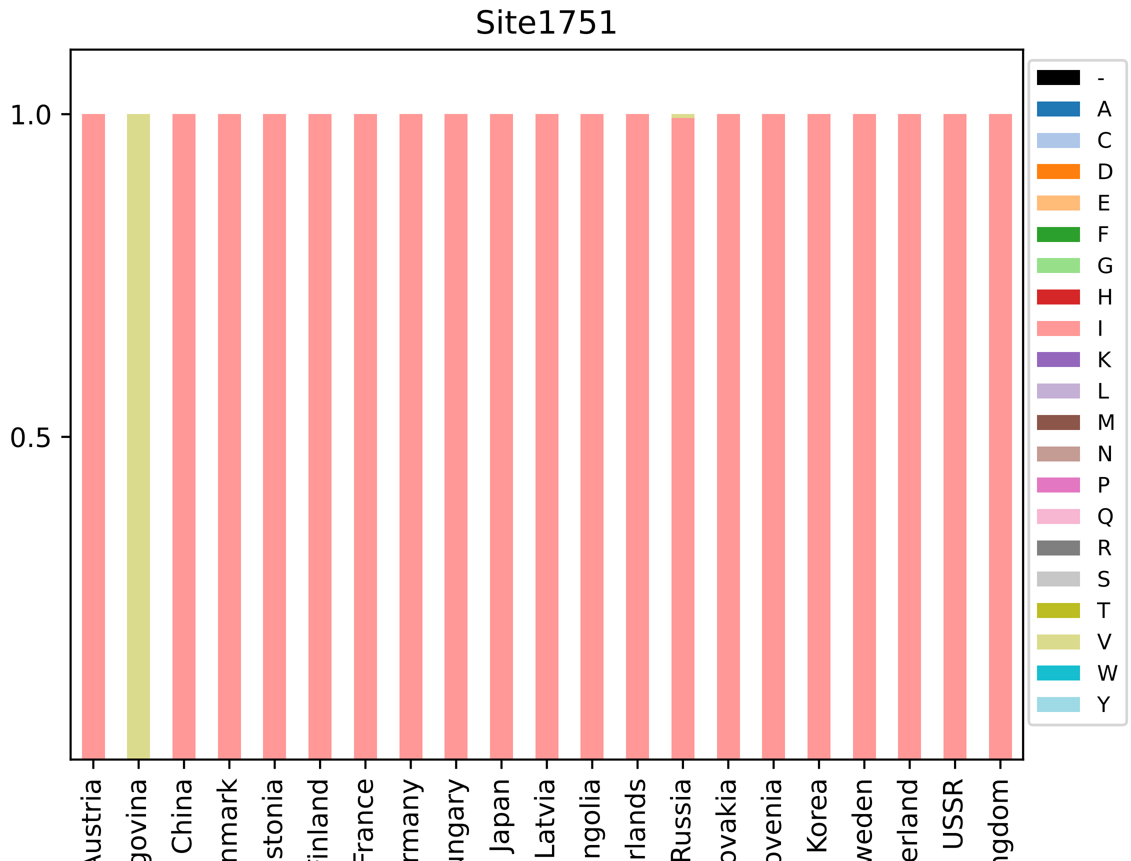

Site1751
1.0
0.5
-
A
C
D
E
F
G
H
I
K
L
M
N
P
Q
R
S
T
V
W
Y
Austria
govina
China
nmark
stonia
Finland
France
rmany
ungary
Japan
Latvia
ngolia
rlands
Russia
ovakia
ovenia
Korea
weden
erland
USSR
ngdom

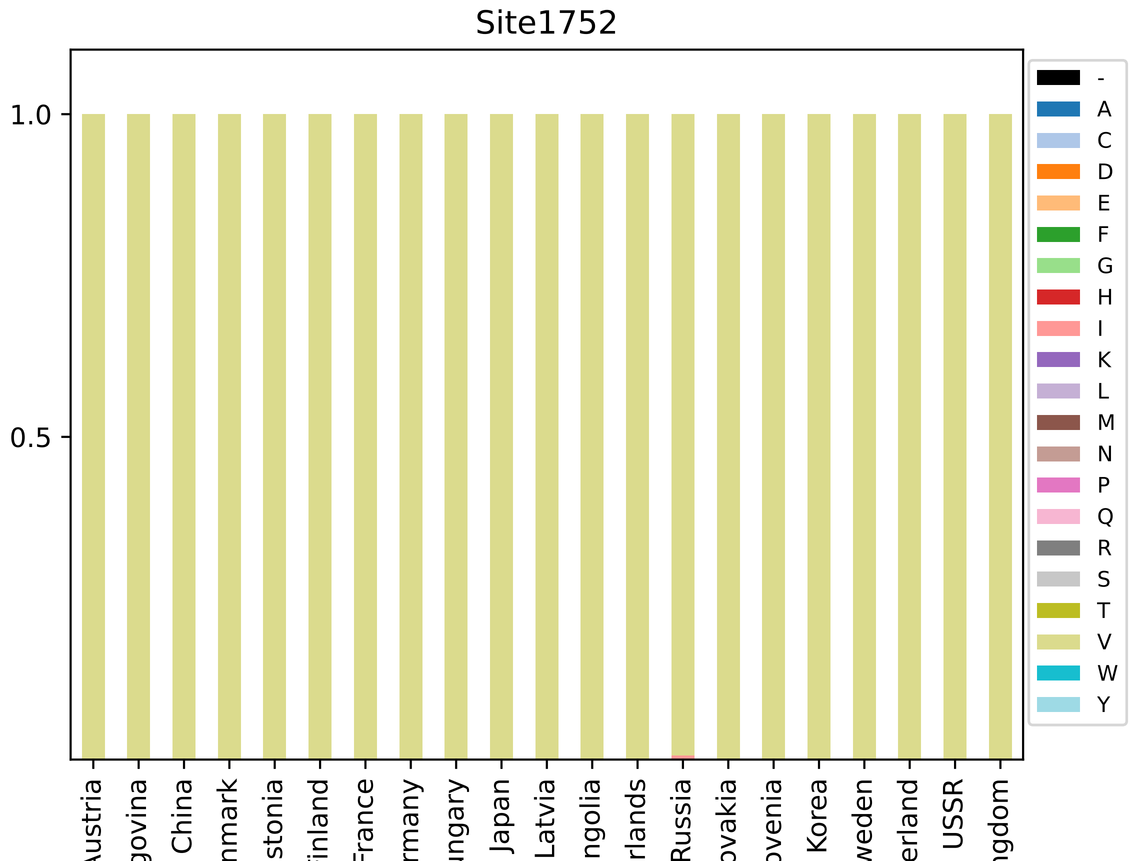
Site1752
1.0
0.5
-
A
C
D
E
F
G
H
I
K
L
M
N
P
Q
R
S
T
V
W
Y
Austria
govina
China
nmark
stonia
Finland
France
rmany
ungary
Japan
Latvia
ngolia
rlands
Russia
ovakia
ovenia
Korea
weden
erland
USSR
ngdom

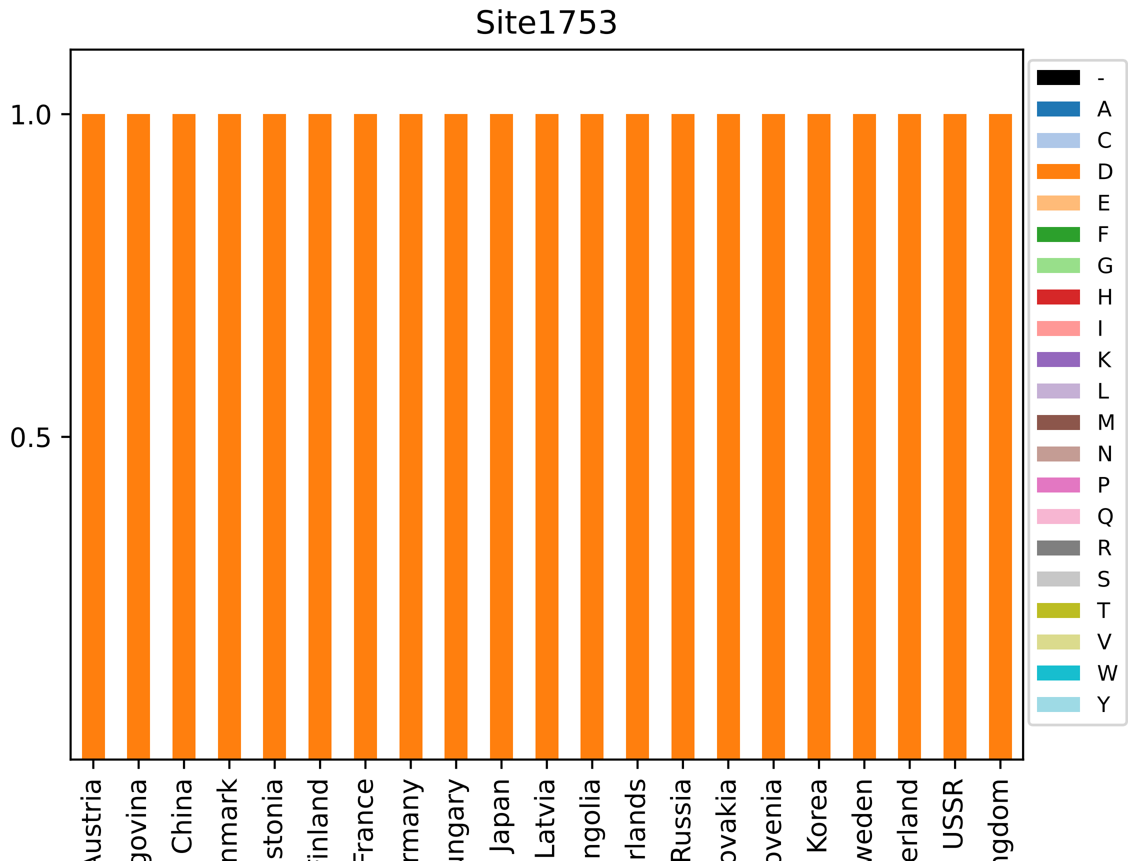
Site1753
1.0
0.5
-
A
C
D
E
F
G
H
I
K
L
M
N
P
Q
R
S
T
V
W
Y
China
Japan
Latvia
Russia
Korea
USSR

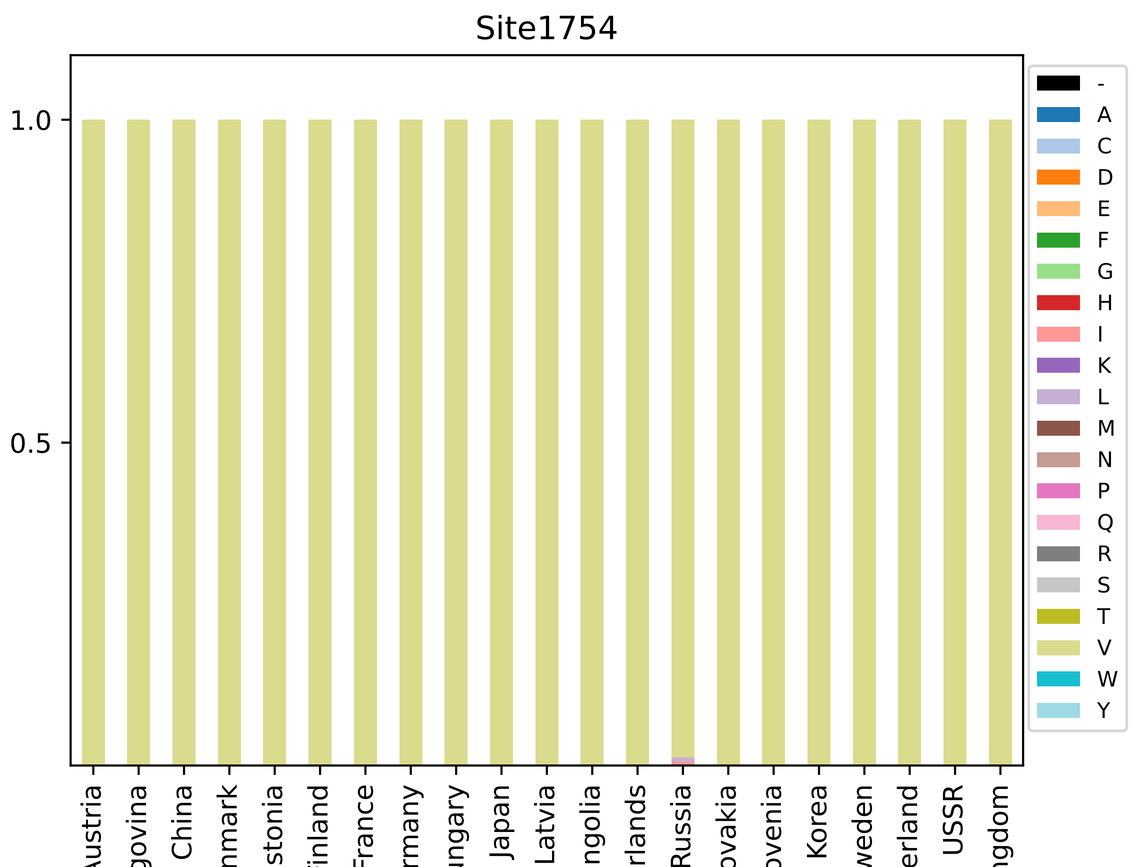

Site1754
1.0
0.5
-
A
C
D
E
F
G
H
I
K
L
M
N
P
Q
R
S
T
V
W
Y
Austria
govina
China
nmark
stonia
Finland
France
rmany
ungary
Japan
Latvia
ngolia
rlands
Russia
ovakia
ovenia
Korea
weden
erland
USSR
ngdom

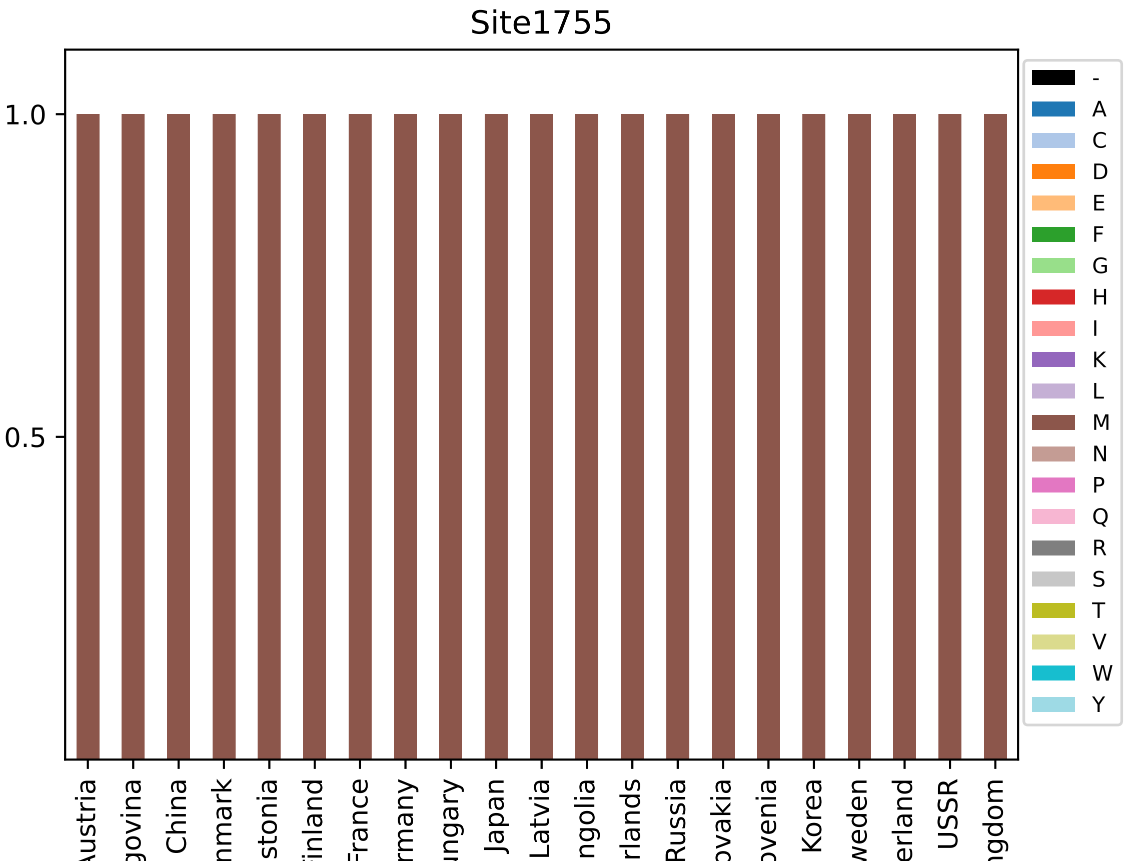
Site1755
1.0
0.5
-
A
C
D
E
F
G
H
I
K
L
M
N
P
Q
R
S
T
V
W
Y
China
Japan
Latvia
Russia
Korea
USSR

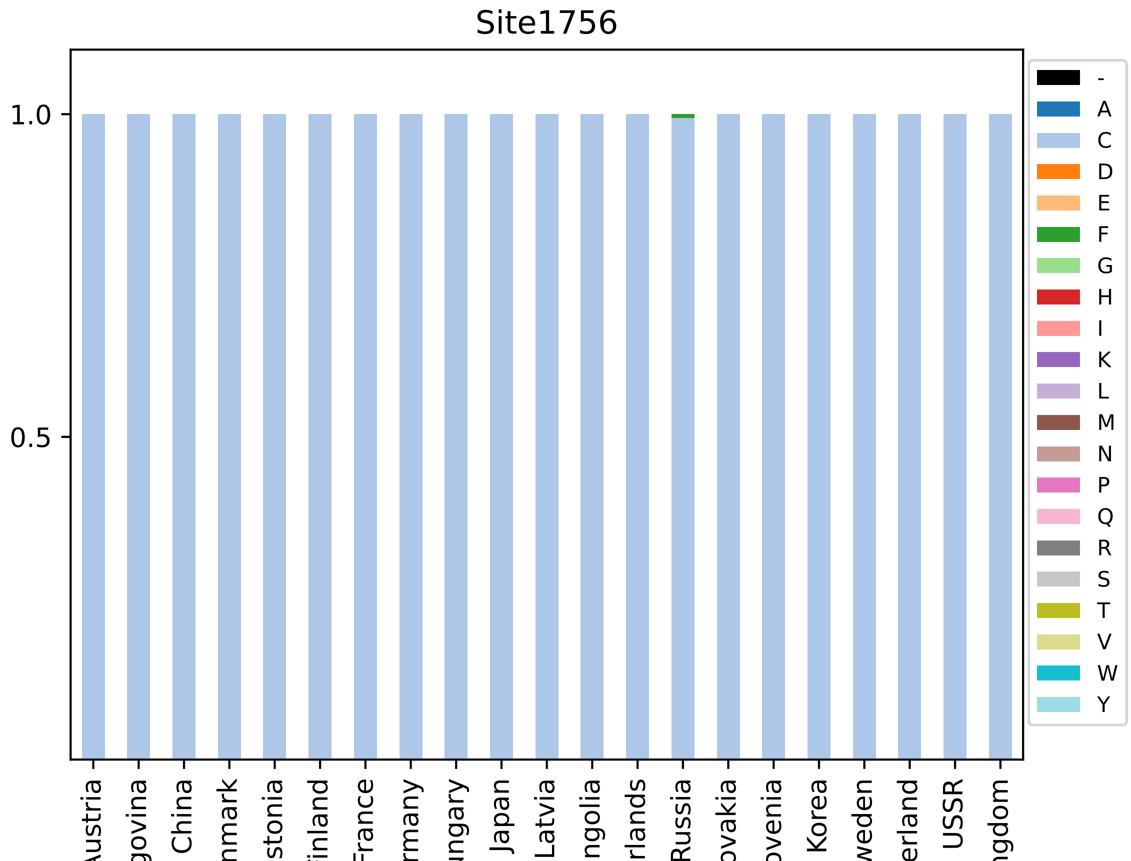

Site1756
1.0
0.5
-
A
C
D
E
F
G
H
I
K
L
M
N
P
Q
R
S
T
V
W
Y
China
Japan
Latvia
Russia
Korea
USSR

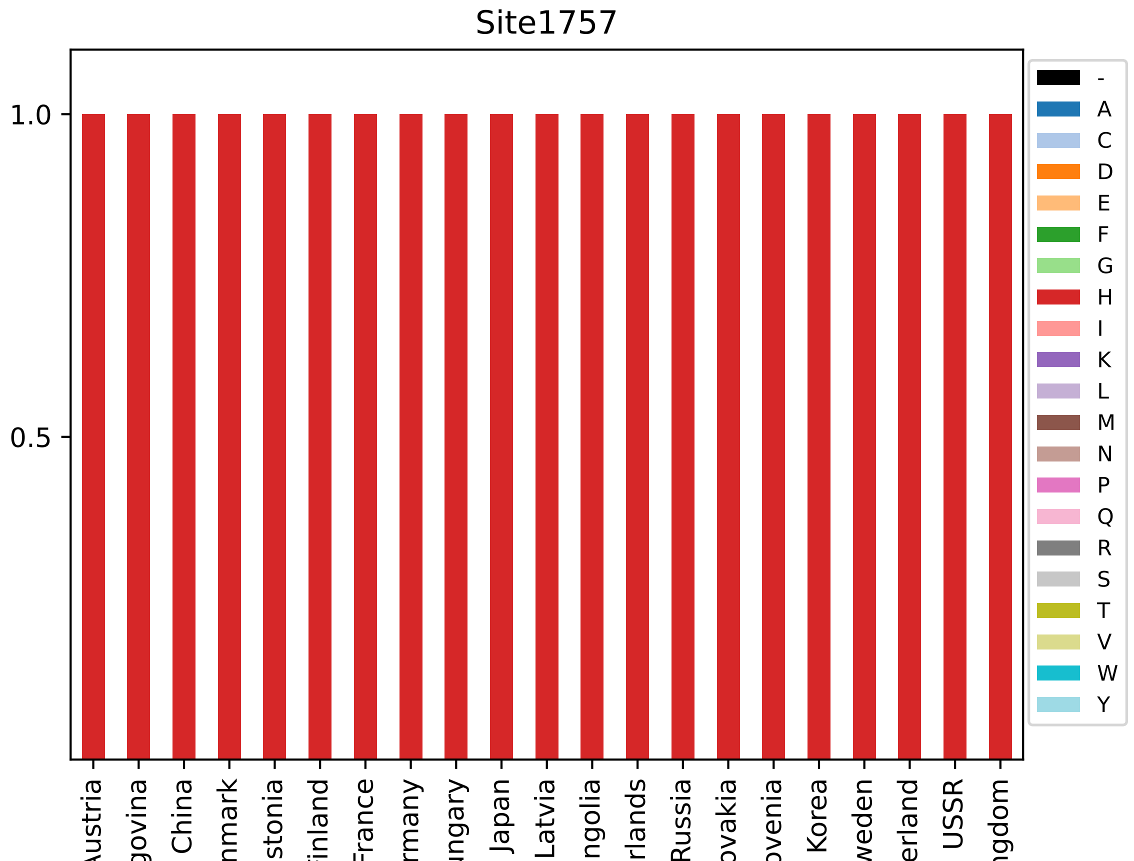

Site1757
1.0
0.5
-
A
C
D
E
F
G
H
I
K
L
M
N
P
Q
R
S
T
V
W
Y
China
Japan
Latvia
Russia
Korea
USSR

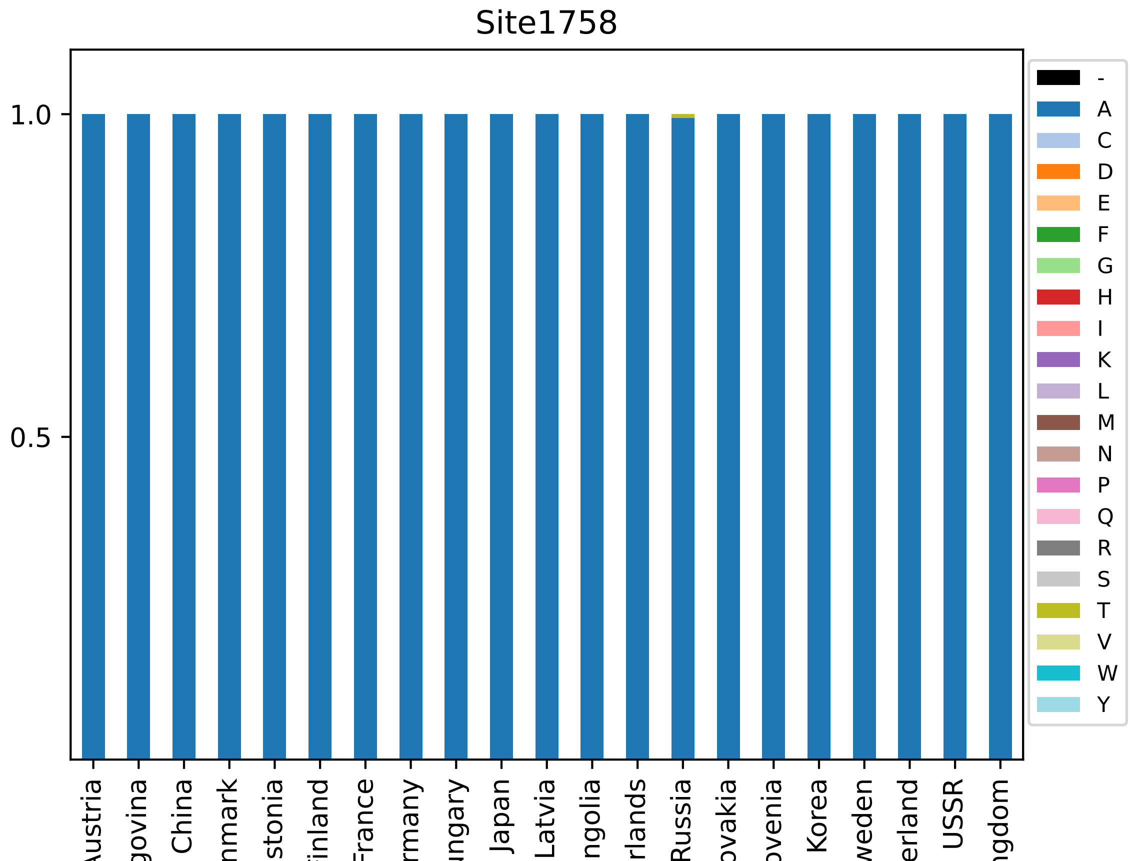

Site1758
1.0
0.5
-
A
C
D
E
F
G
H
I
K
L
M
N
P
Q
R
S
T
V
W
Y
Austria
govina
China
nmark
stonia
inland
France
rmany
ungary
Japan
Latvia
ngolia
rlands
Russia
ovakia
ovenia
Korea
weden
erland
USSR
ngdom

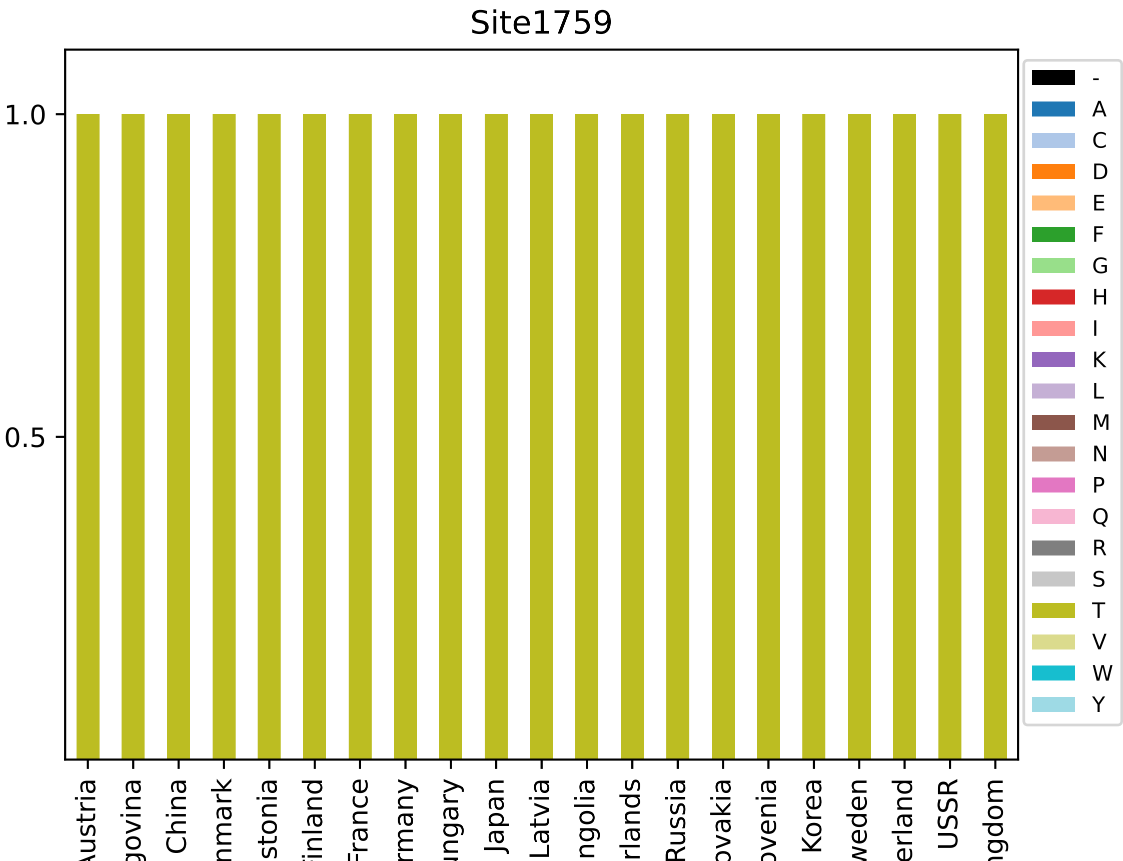

Site1759
1.0
0.5
-
A
C
D
E
F
G
H
I
K
L
M
N
P
Q
R
S
T
V
W
Y
Austria
China
Japan
Latvia
Russia
Korea
USSR

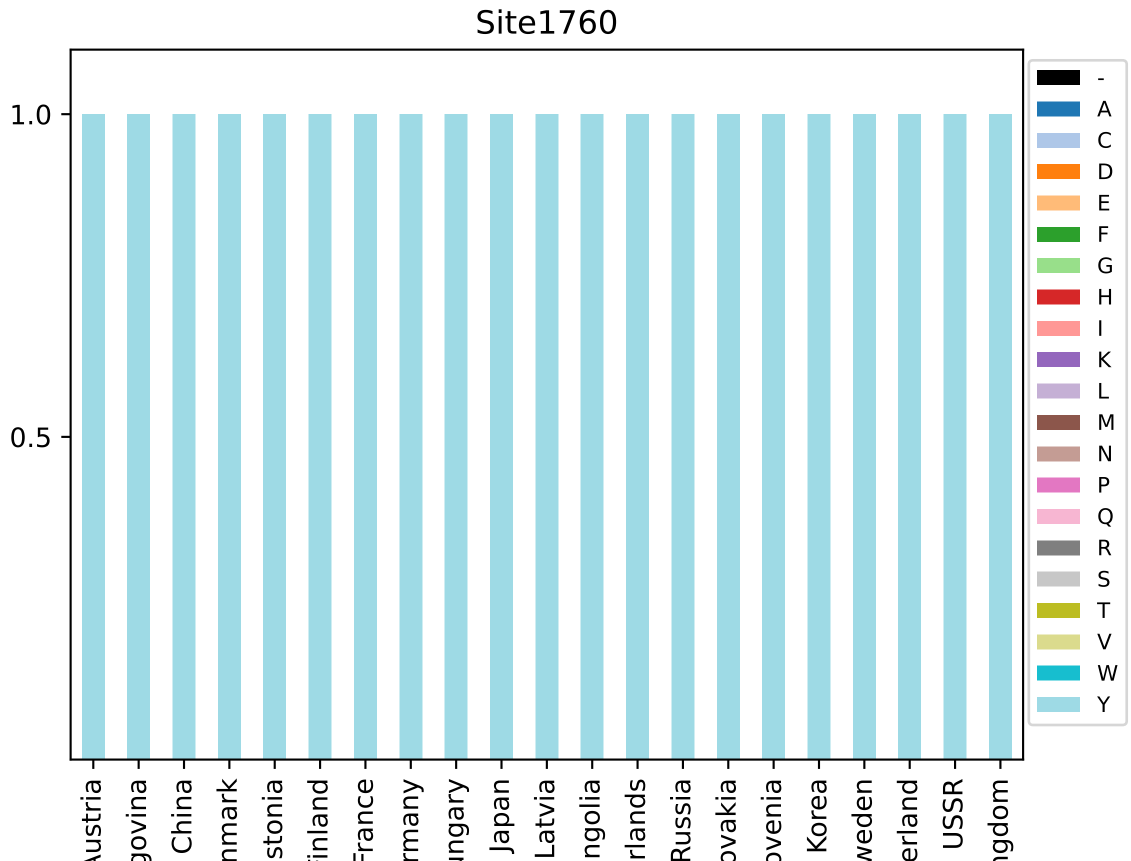
Site1760
1.0
0.5
-
A
C
D
E
F
G
H
I
K
L
M
N
P
Q
R
S
T
V
W
Y
Austria
govina
China
nmark
stonia
inland
France
rmany
ungary
Japan
Latvia
ngolia
rlands
Russia
ovakia
ovenia
Korea
weden
erland
USSR
ngdom

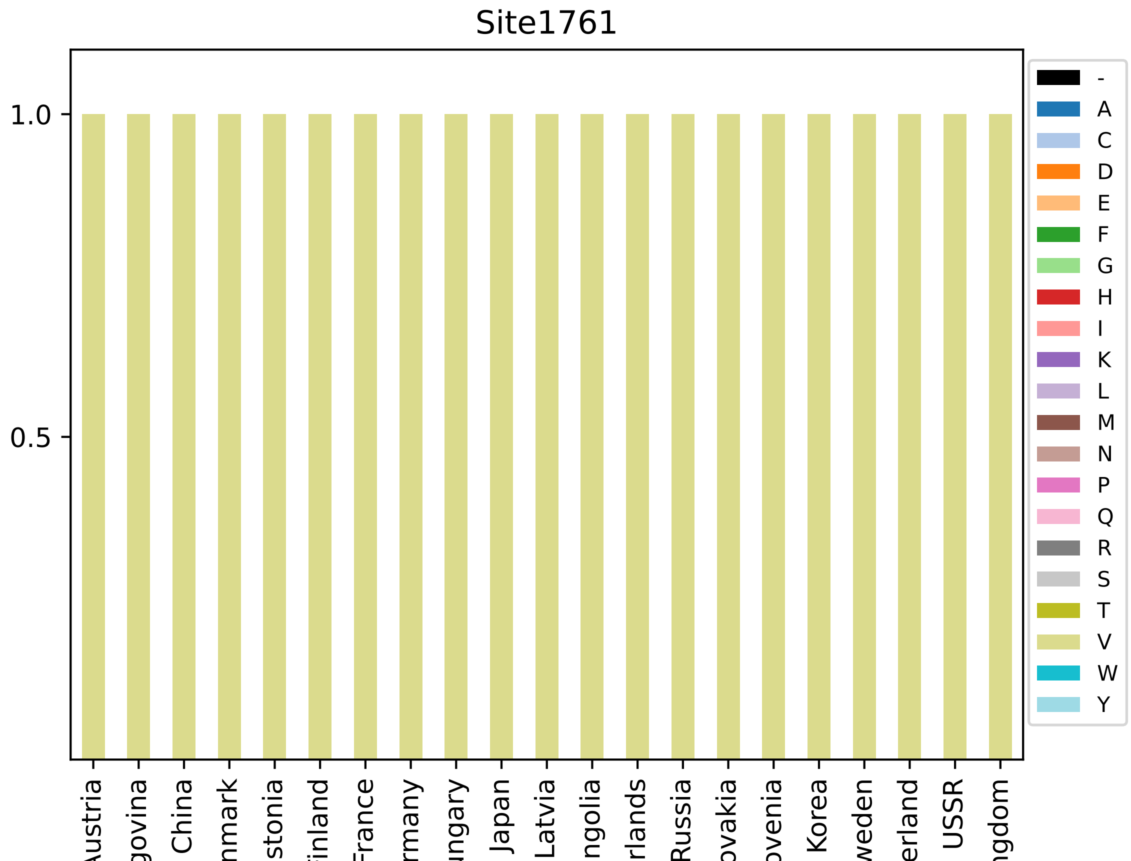

Site1761
1.0
0.5
-
A
C
D
E
F
G
H
I
K
L
M
N
P
Q
R
S
T
V
W
Y
China
Japan
Latvia
Russia
Korea
USSR

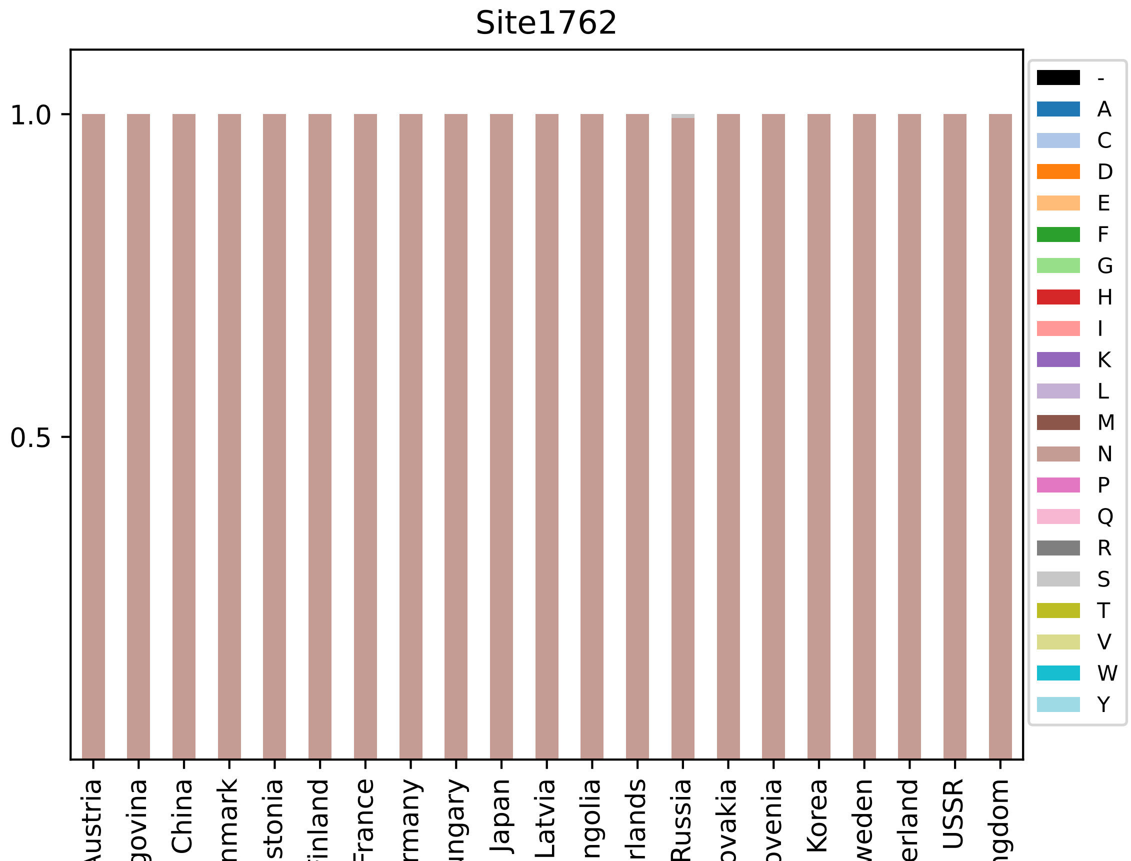

Site1762
1.0
0.5
-
A
C
D
E
F
G
H
I
K
L
M
N
P
Q
R
S
T
V
W
Y
Austria
China
Japan
Latvia
Russia
Korea
USSR

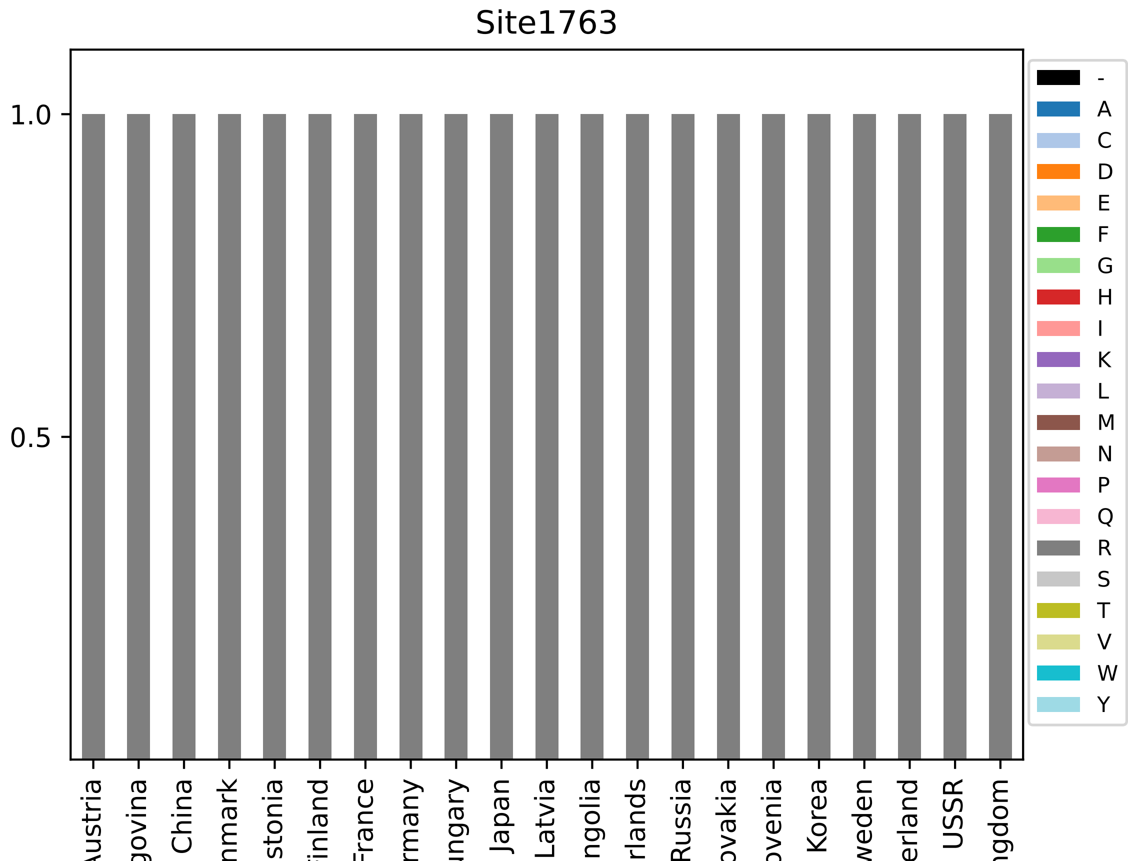

Site1763
1.0
0.5
-
A
C
D
E
F
G
H
I
K
L
M
N
P
Q
R
S
T
V
W
Y
China
Japan
Latvia
Russia
Korea
USSR

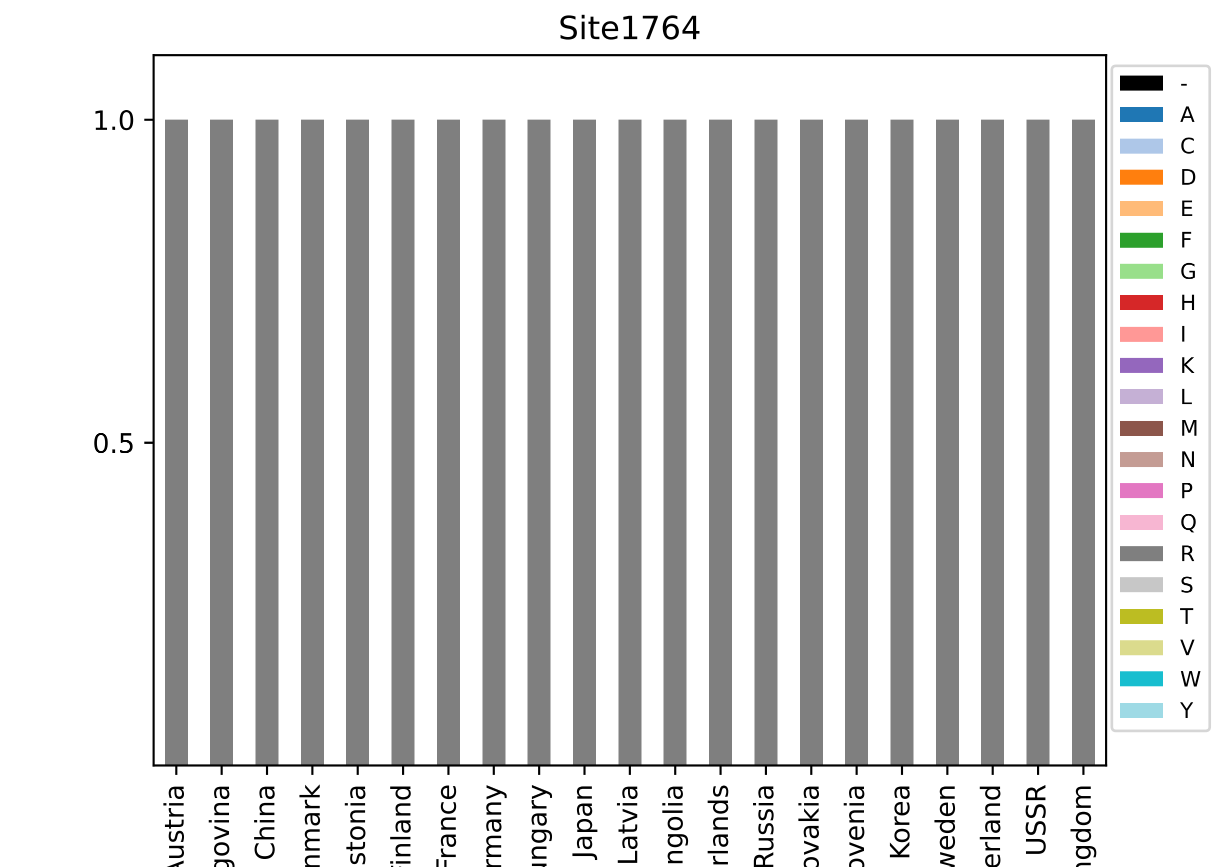
Site1764
1.0
0.5
Austria
govina
China
nmark
stonia
Finland
France
rmany
ungary
Japan
Latvia
ngolia
rlands
Russia
ovakia
ovenia
Korea
weden
erland
USSR
ngdom
-
A
C
D
E
F
G
H
I
K
L
M
N
P
Q
R
S
T
V
W
Y

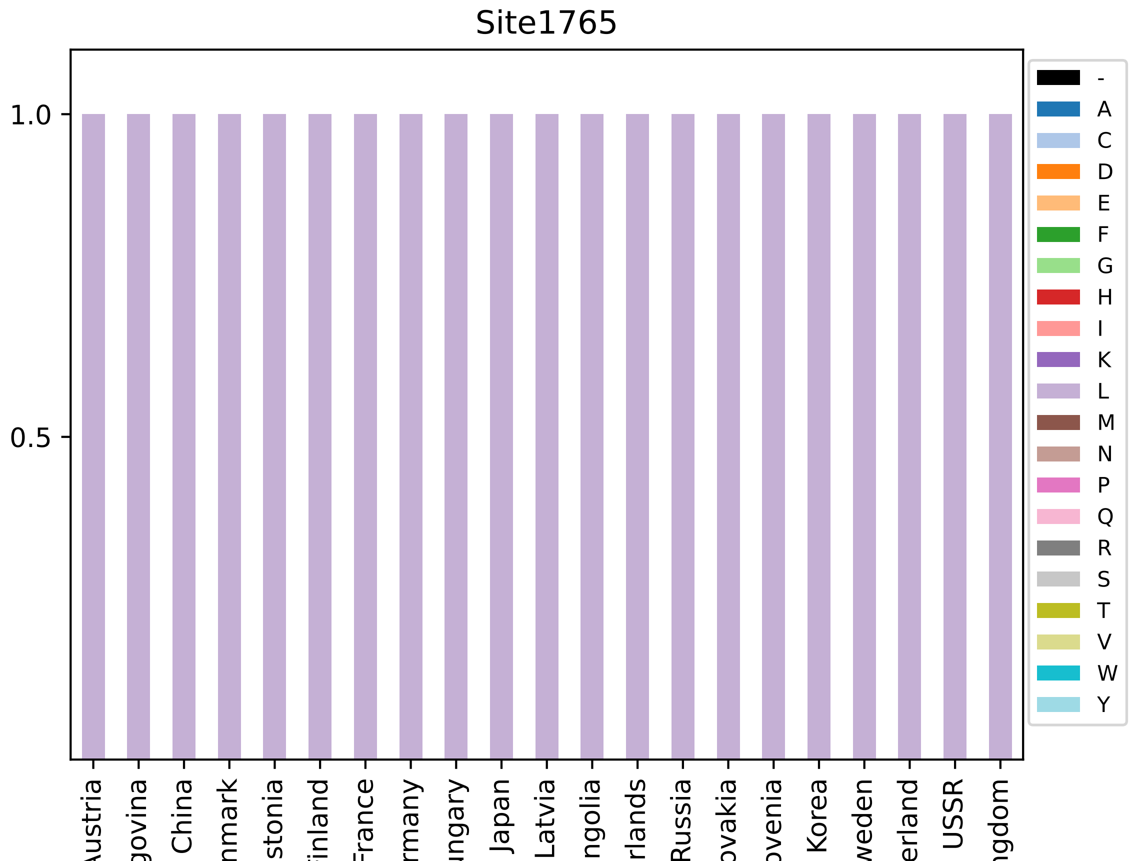

Site1765
1.0
0.5
-
A
C
D
E
F
G
H
I
K
L
M
N
P
Q
R
S
T
V
W
Y
Austria
govina
China
nmark
stonia
inland
France
rmany
ungary
Japan
Latvia
ngolia
rlands
Russia
ovakia
ovenia
Korea
weden
erland
USSR
ngdom

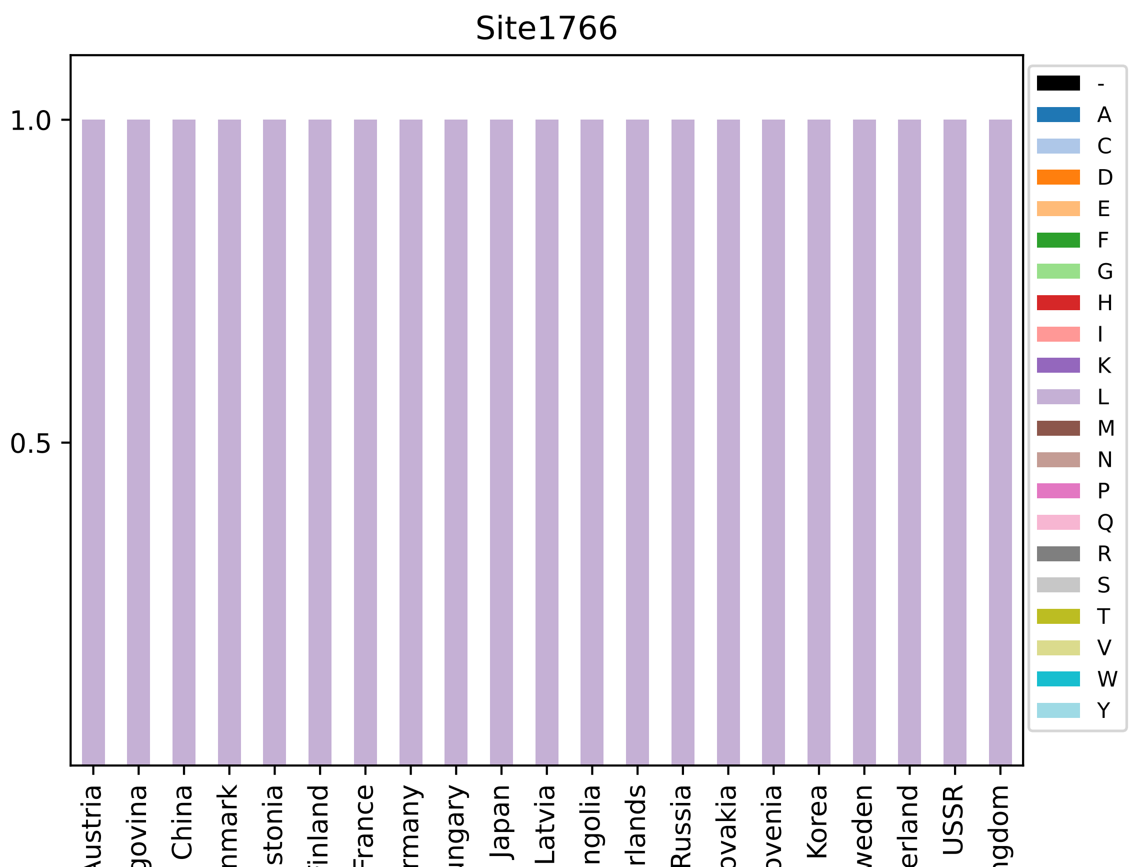
Site1766
1.0
0.5
-
A
C
D
E
F
G
H
I
K
L
M
N
P
Q
R
S
T
V
W
Y
Austria
China
Japan
Latvia
Russia
Korea
USSR

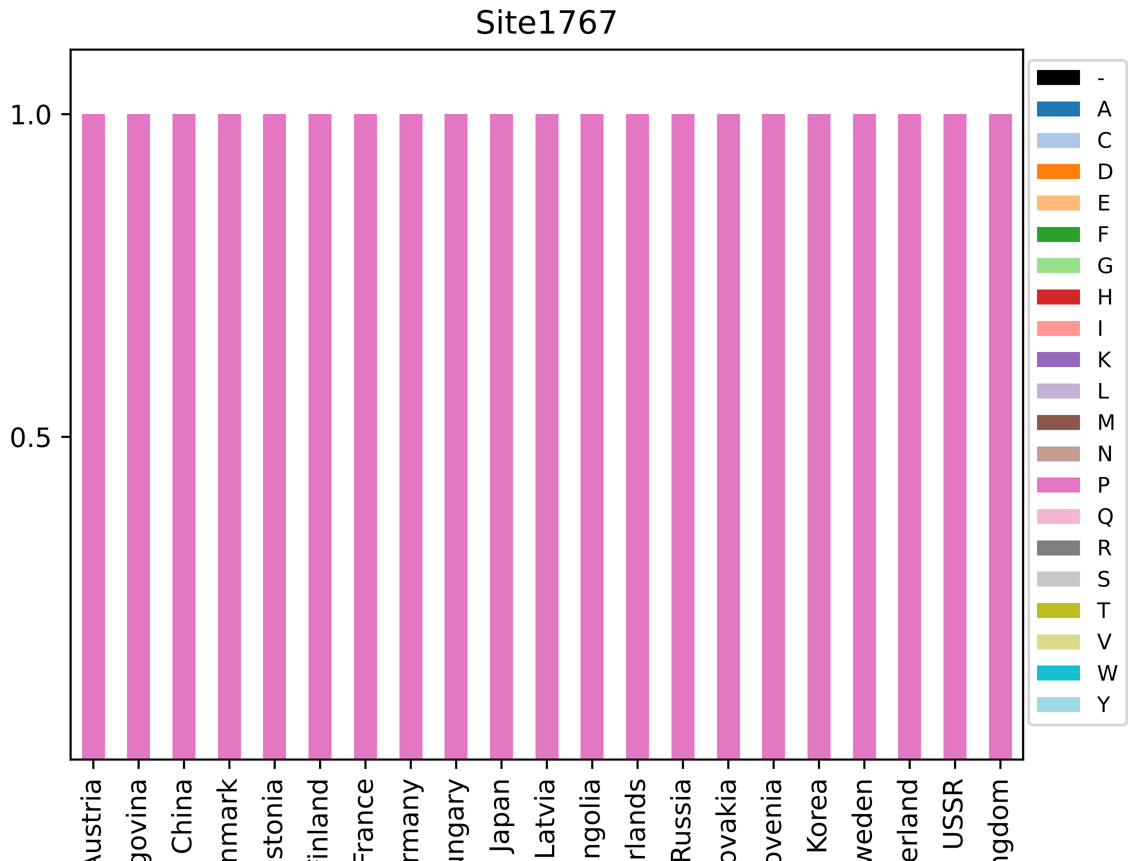
Site1767
1.0
0.5
China
Japan
Latvia
Russia
Korea
USSR
-
A
C
D
E
F
G
H
I
K
L
M
N
P
Q
R
S
T
V
W
Y

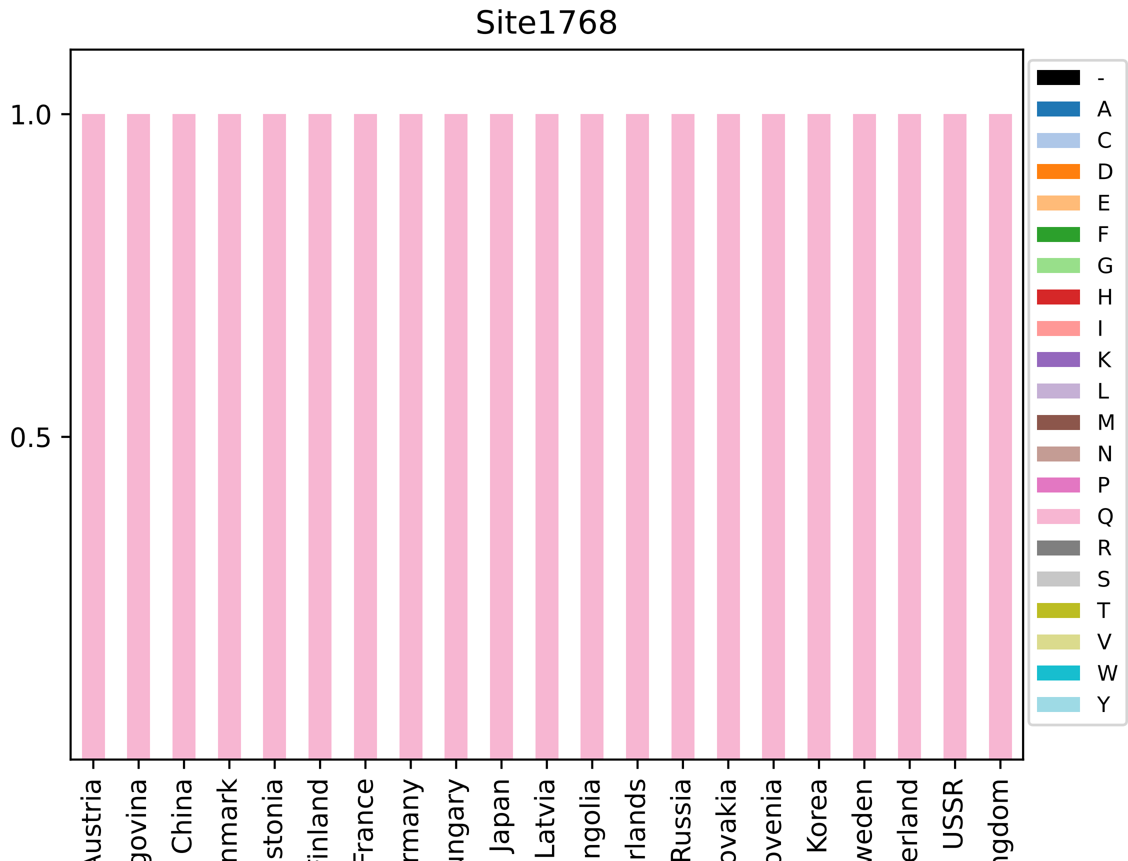

Site1768
1.0
0.5
-
A
C
D
E
F
G
H
I
K
L
M
N
P
Q
R
S
T
V
W
Y
China
Japan
Latvia
Russia
Korea
USSR

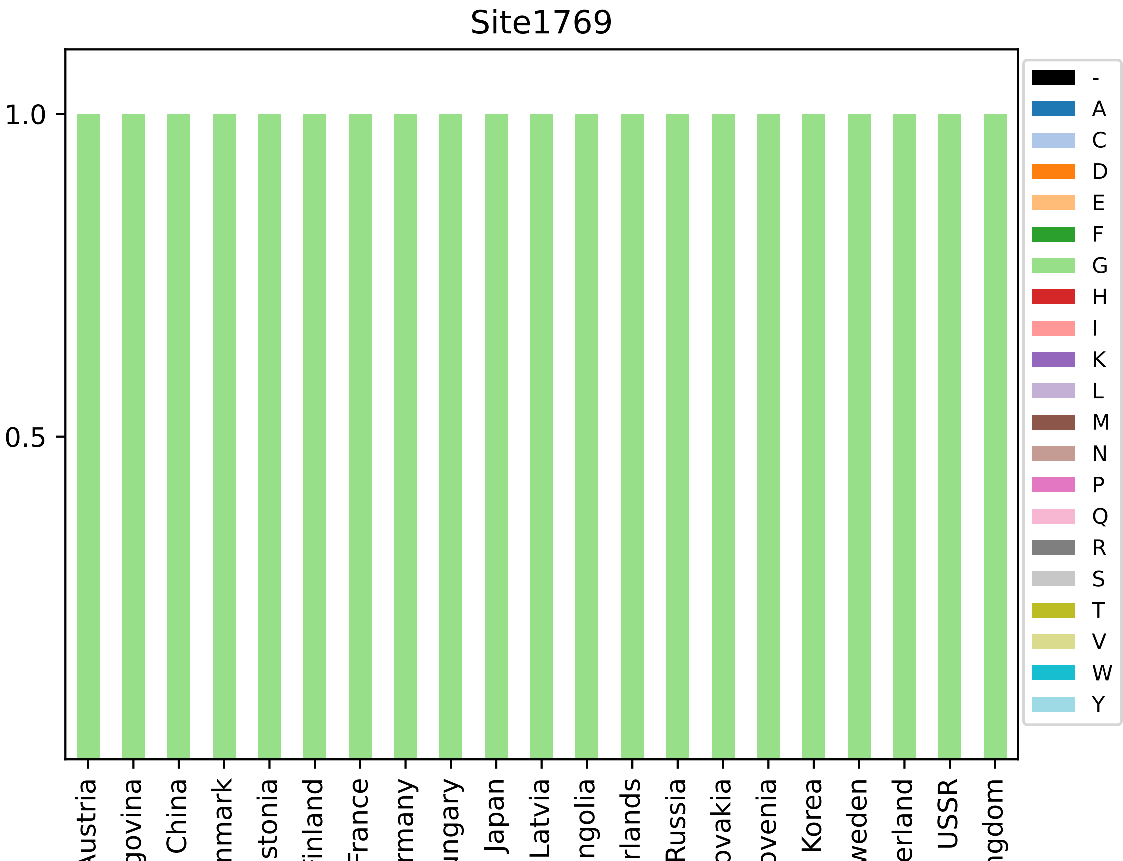
Site1769
1.0
0.5
-
A
C
D
E
F
G
H
I
K
L
M
N
P
Q
R
S
T
V
W
Y
China
Japan
Latvia
Russia
Korea
USSR

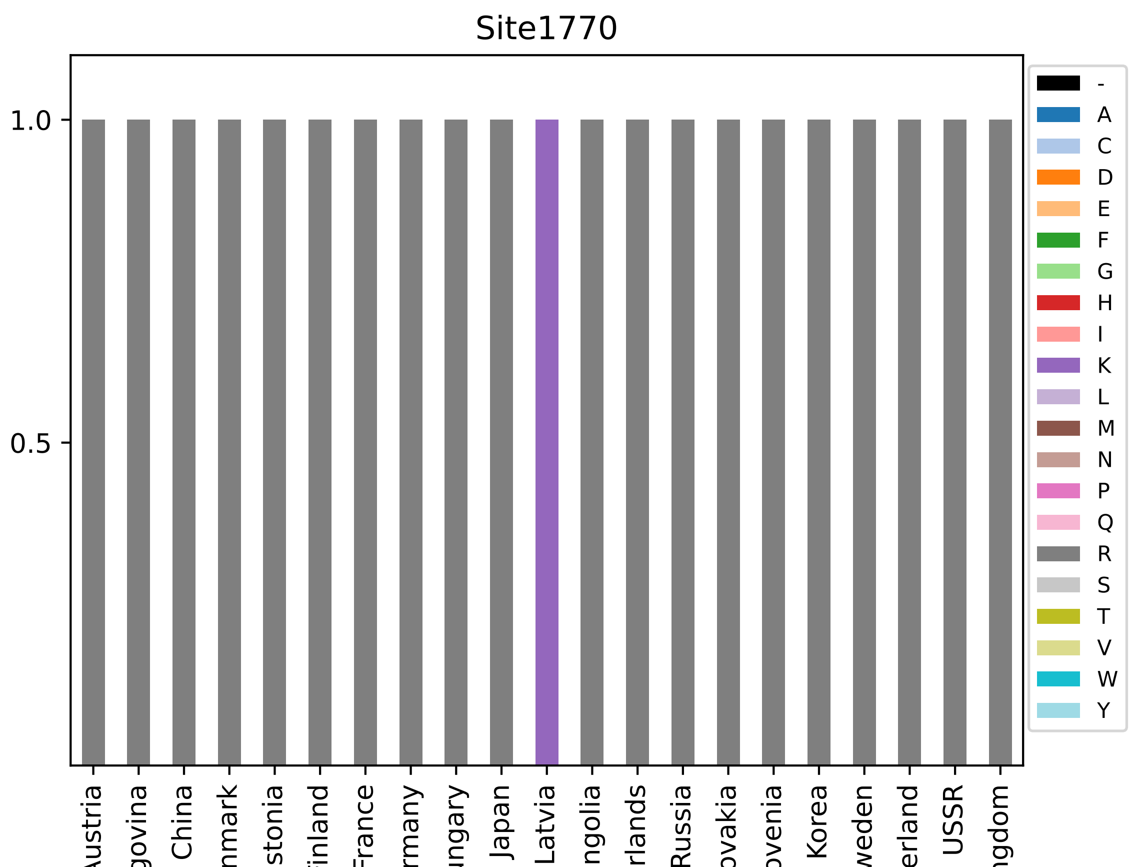

Site1770
1.0
0.5
-
A
C
D
E
F
G
H
I
K
L
M
N
P
Q
R
S
T
V
W
Y
Austria
China
Japan
Latvia
Russia
Korea
USSR

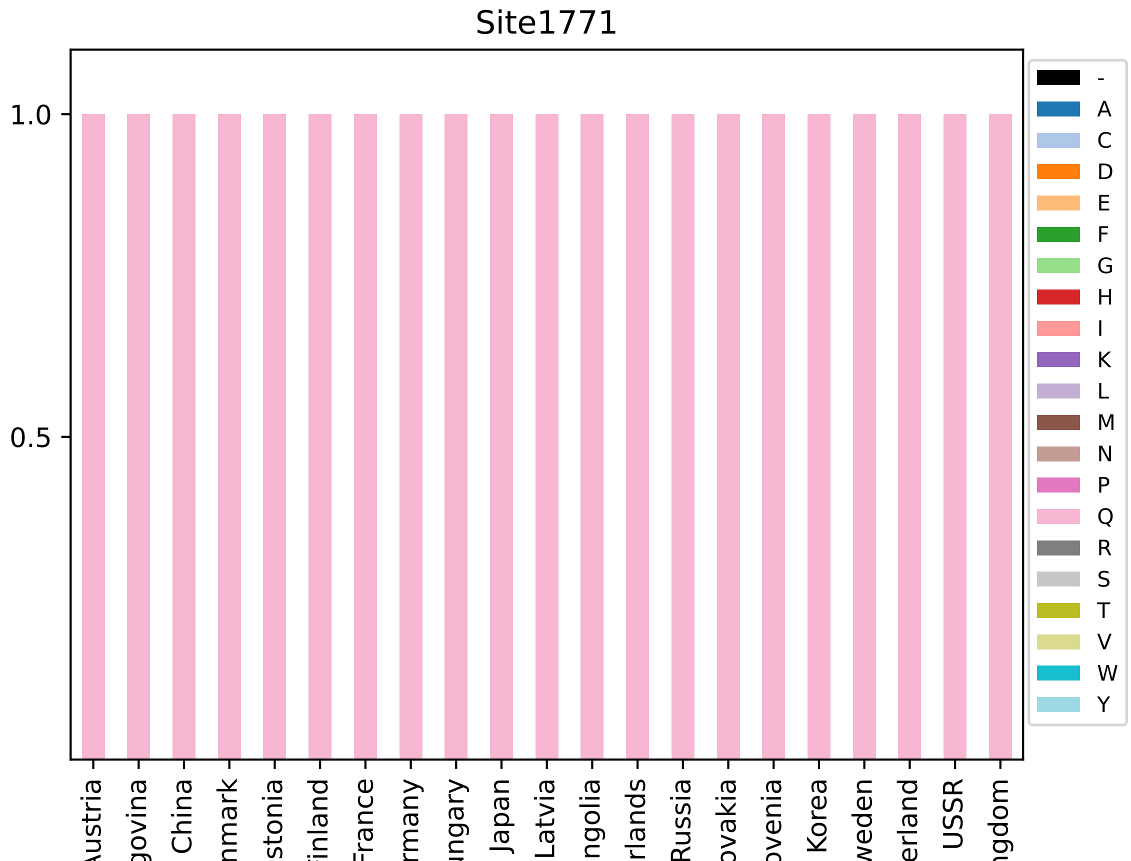
Site1771
1.0
0.5
Austria
govina
China
nmark
stonia
Finland
France
rmany
ungary
Japan
Latvia
ngolia
rlands
Russia
ovakia
ovenia
Korea
weden
erland
USSR
ngdom
-
A
C
D
E
F
G
H
I
K
L
M
N
P
Q
R
S
T
V
W
Y

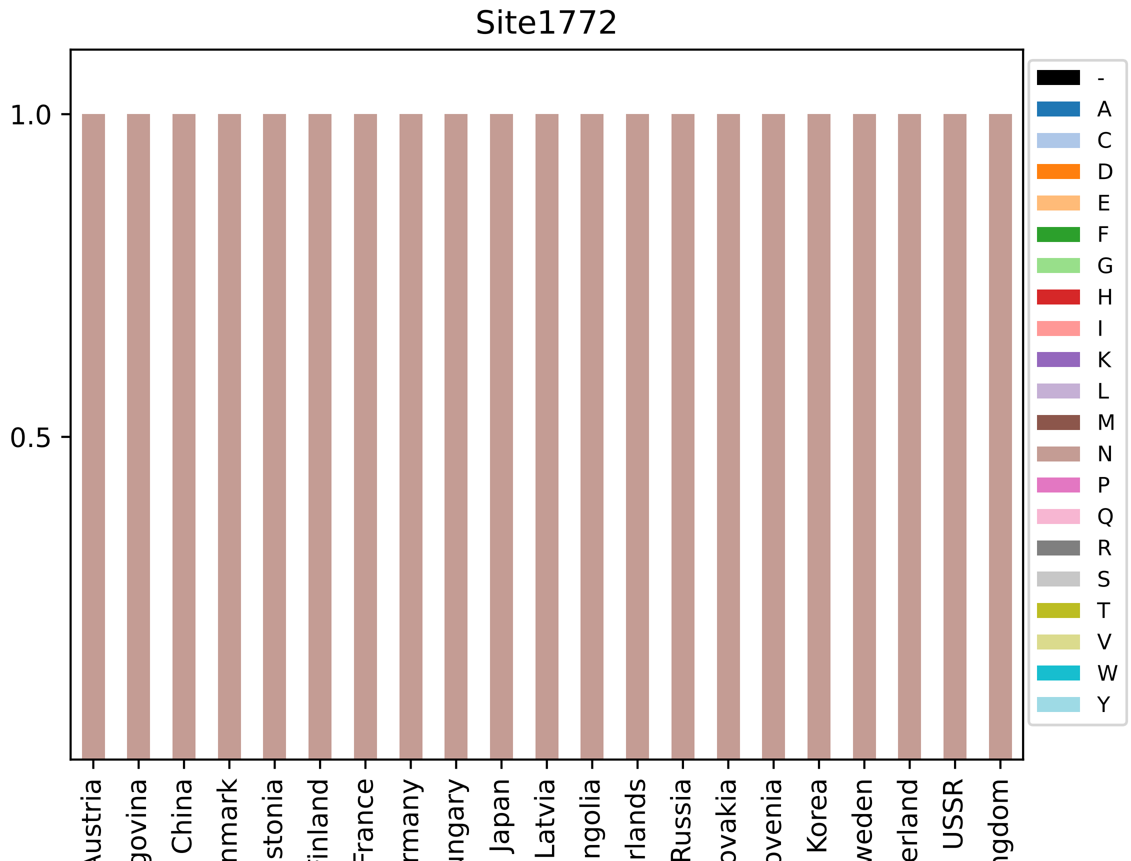

Site1772
1.0
0.5
-
A
C
D
E
F
G
H
I
K
L
M
N
P
Q
R
S
T
V
W
Y
China
Japan
Latvia
Russia
Korea
USSR

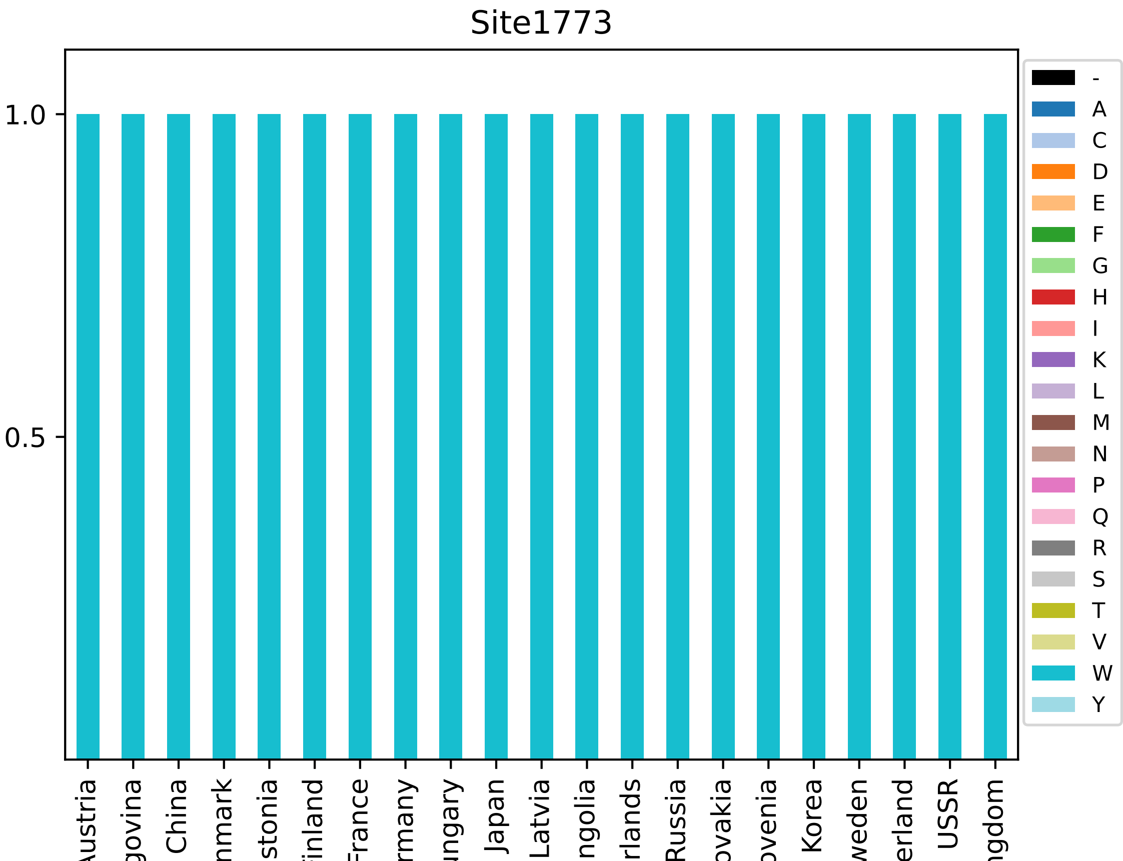

Site1773
1.0
0.5
-
A
C
D
E
F
G
H
I
K
L
M
N
P
Q
R
S
T
V
W
Y
Austria
govina
China
nmark
stonia
inland
France
rmany
ungary
Japan
Latvia
ngolia
rlands
Russia
ovakia
ovenia
Korea
weden
erland
USSR
ngdom

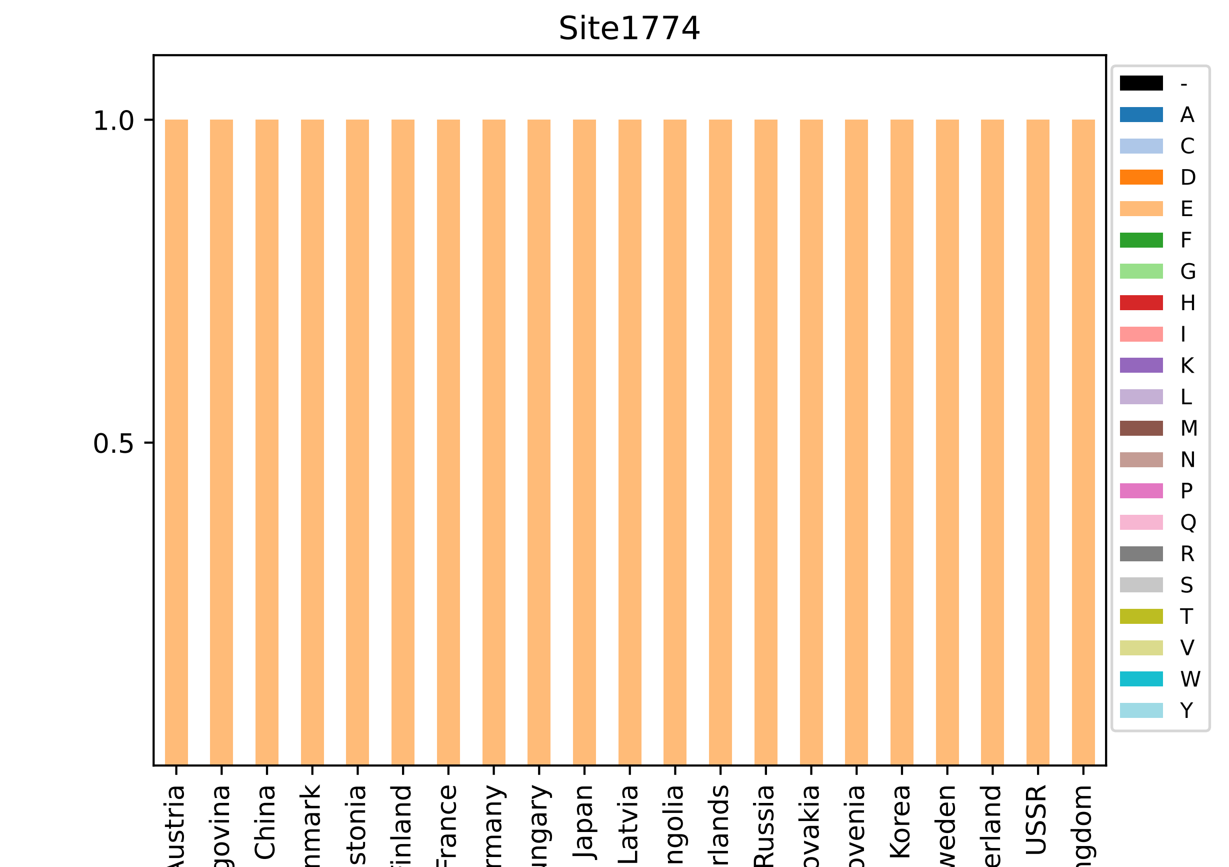

Site1774
1.0
0.5
-
A
C
D
E
F
G
H
I
K
L
M
N
P
Q
R
S
T
V
W
Y
Austria
govina
China
nmark
stonia
Finland
France
rmany
ungary
Japan
Latvia
ngolia
rlands
Russia
ovakia
ovenia
Korea
weden
erland
USSR
ngdom

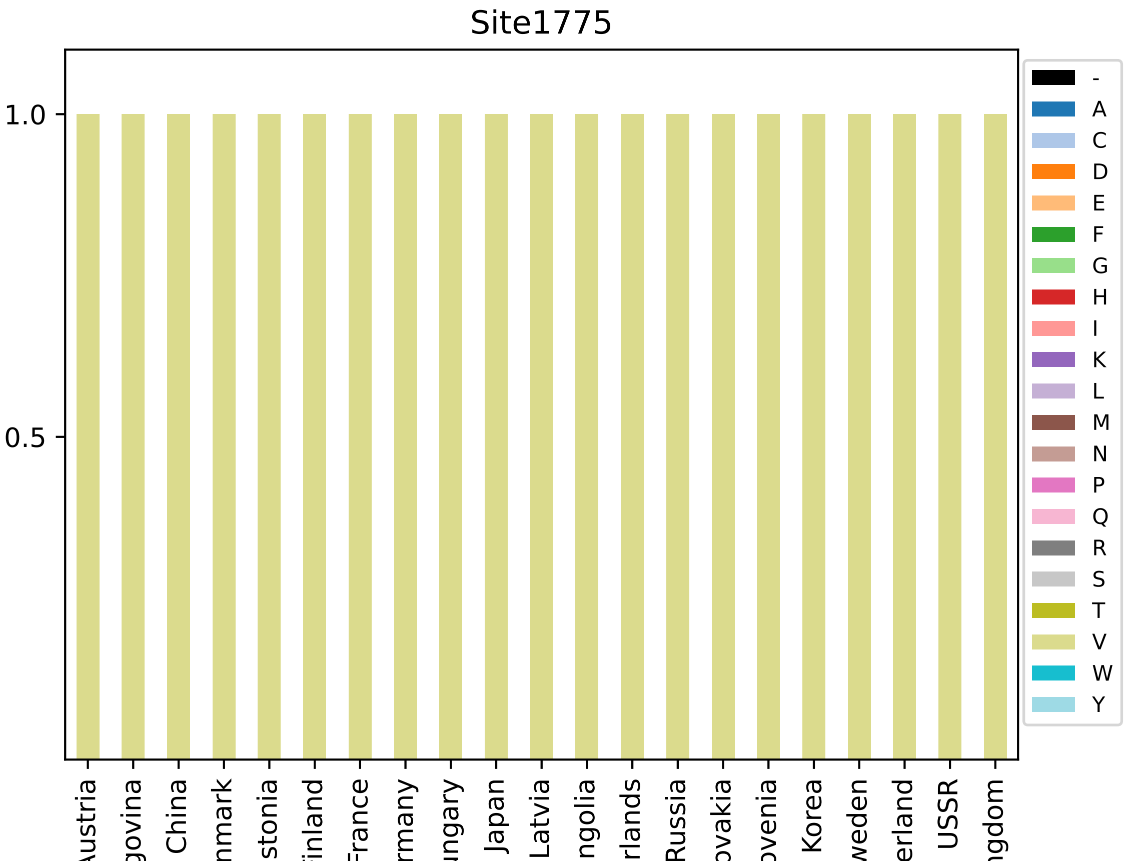

Site1775
1.0
0.5
-
A
C
D
E
F
G
H
I
K
L
M
N
P
Q
R
S
T
V
W
Y
Austria
China
Japan
Latvia
Russia
Korea
USSR

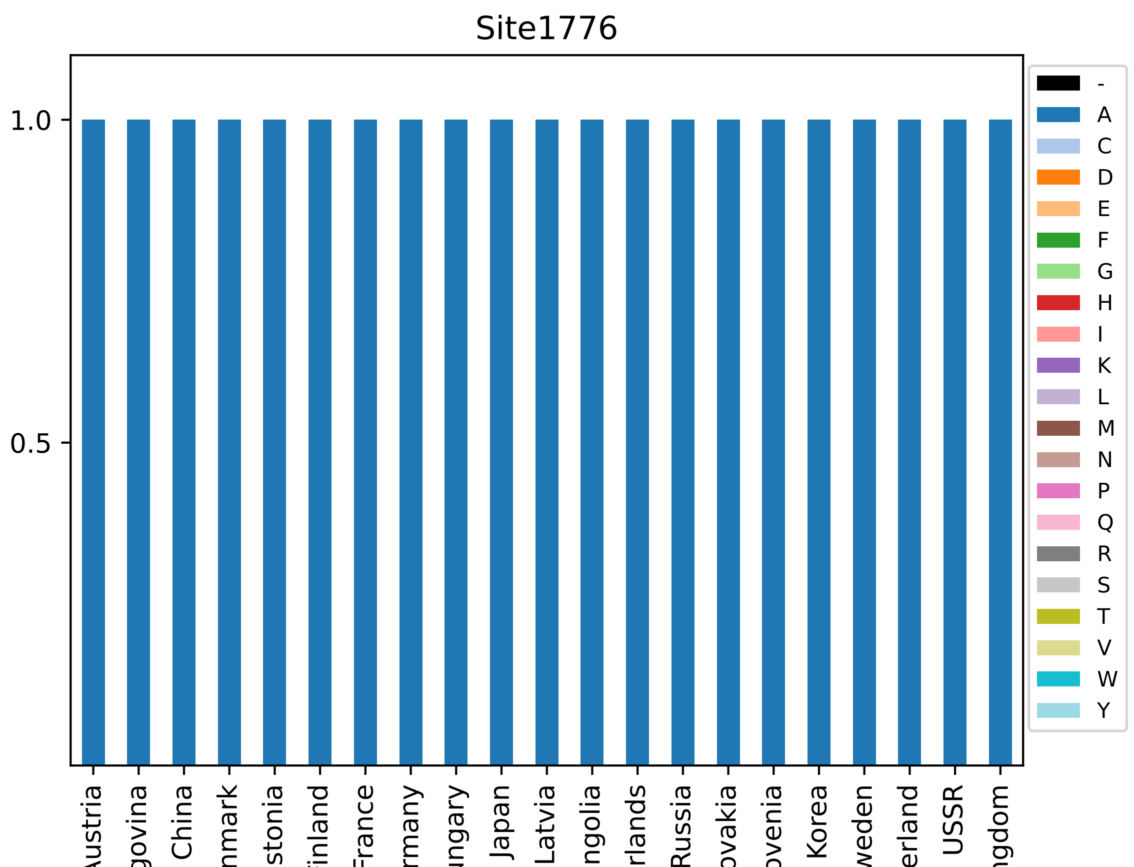

Site1776
1.0
0.5
-
A
C
D
E
F
G
H
I
K
L
M
N
P
Q
R
S
T
V
W
Y
Austria
China
Japan
Latvia
Russia
Korea
USSR

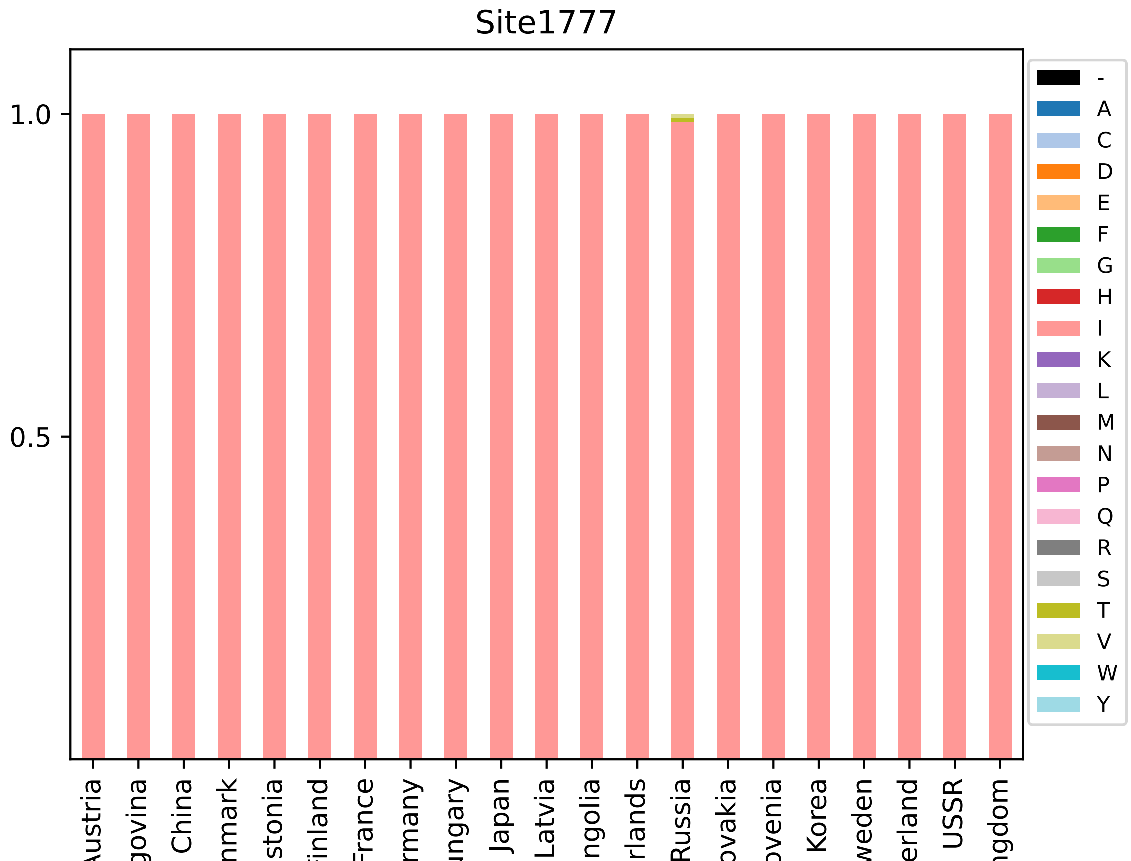
Site1777
1.0
0.5
-
A
C
D
E
F
G
H
I
K
L
M
N
P
Q
R
S
T
V
W
Y
China
Japan
Latvia
Russia
Korea
USSR

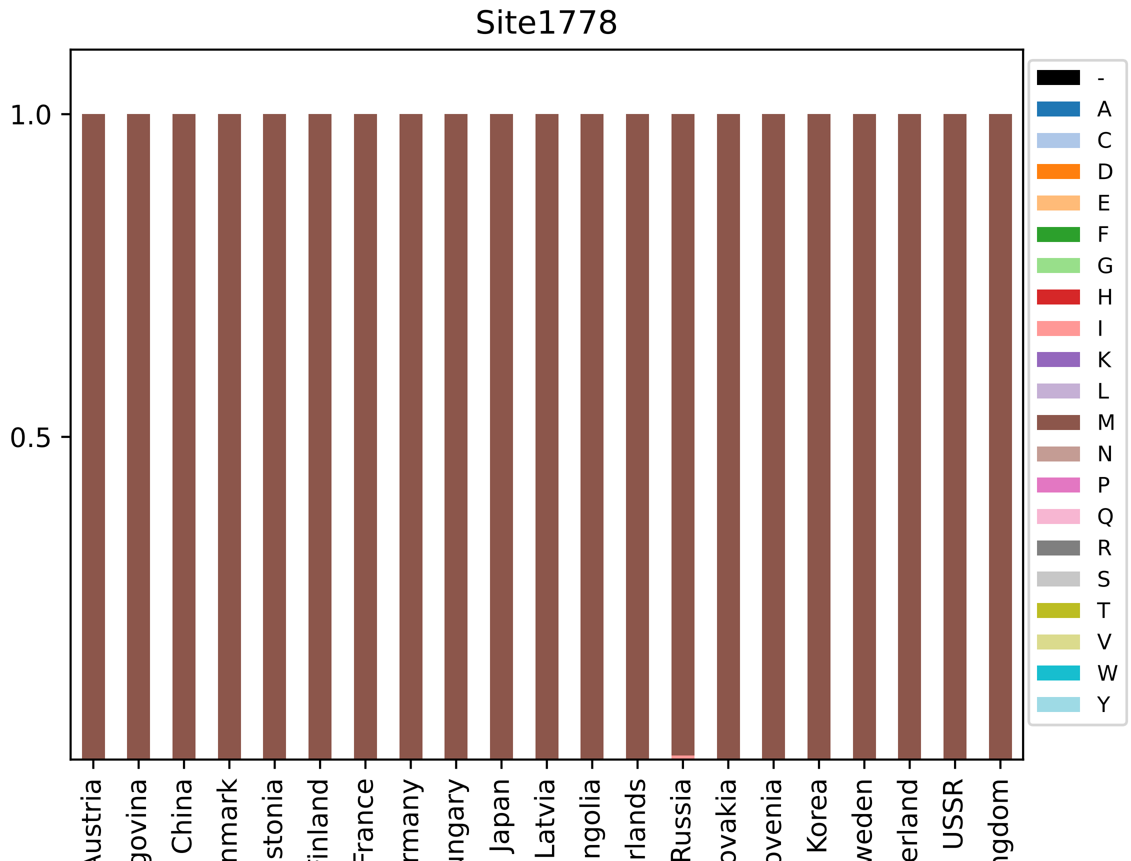
Site1778
1.0
0.5
-
A
C
D
E
F
G
H
I
K
L
M
N
P
Q
R
S
T
V
W
Y
ustria
govina
China
nmark
stonia
inland
rance
rmany
ungary
Japan
Latvia
ngolia
rlands
Russia
ovakia
ovenia
Korea
weden
erland
USSR
ngdom

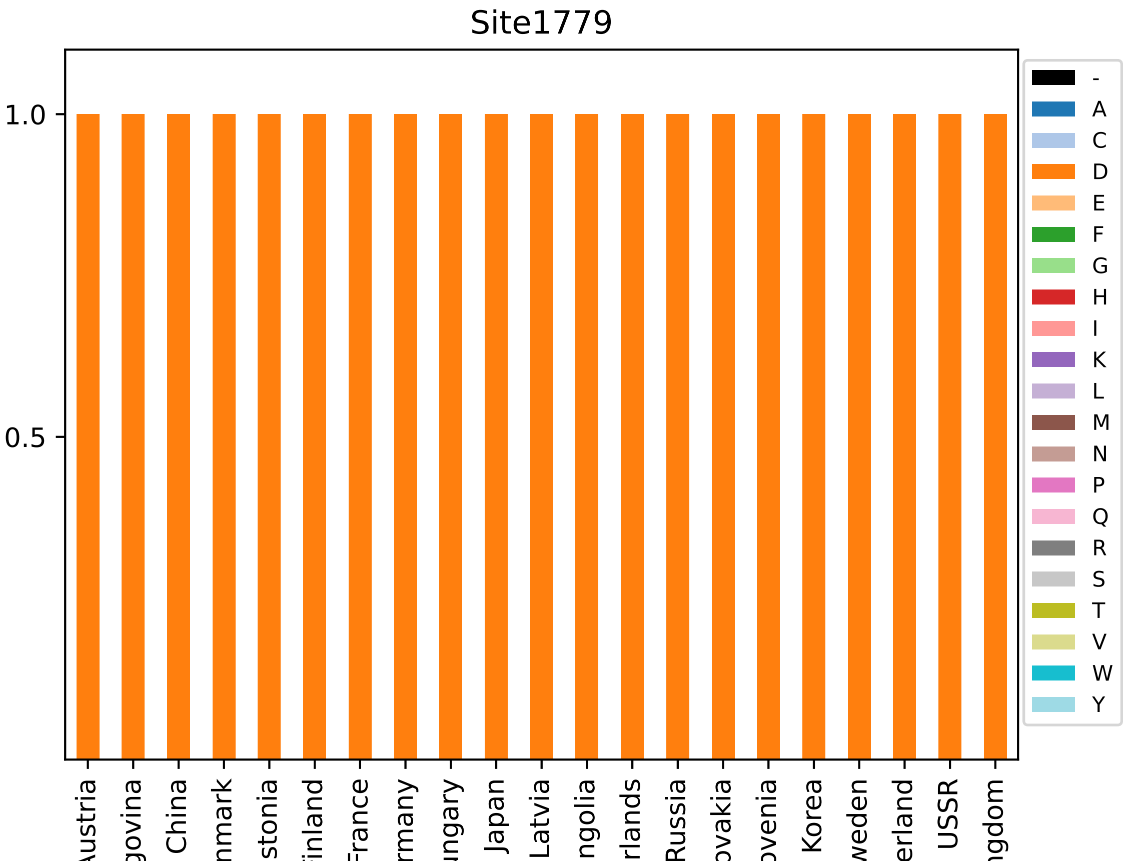
Site1779
1.0
0.5
-
A
C
D
E
F
G
H
I
K
L
M
N
P
Q
R
S
T
V
W
Y
Austria
govina
China
nmark
stonia
inland
France
rmany
ungary
Japan
Latvia
ngolia
rlands
Russia
ovakia
ovenia
Korea
weden
erland
USSR
ngdom

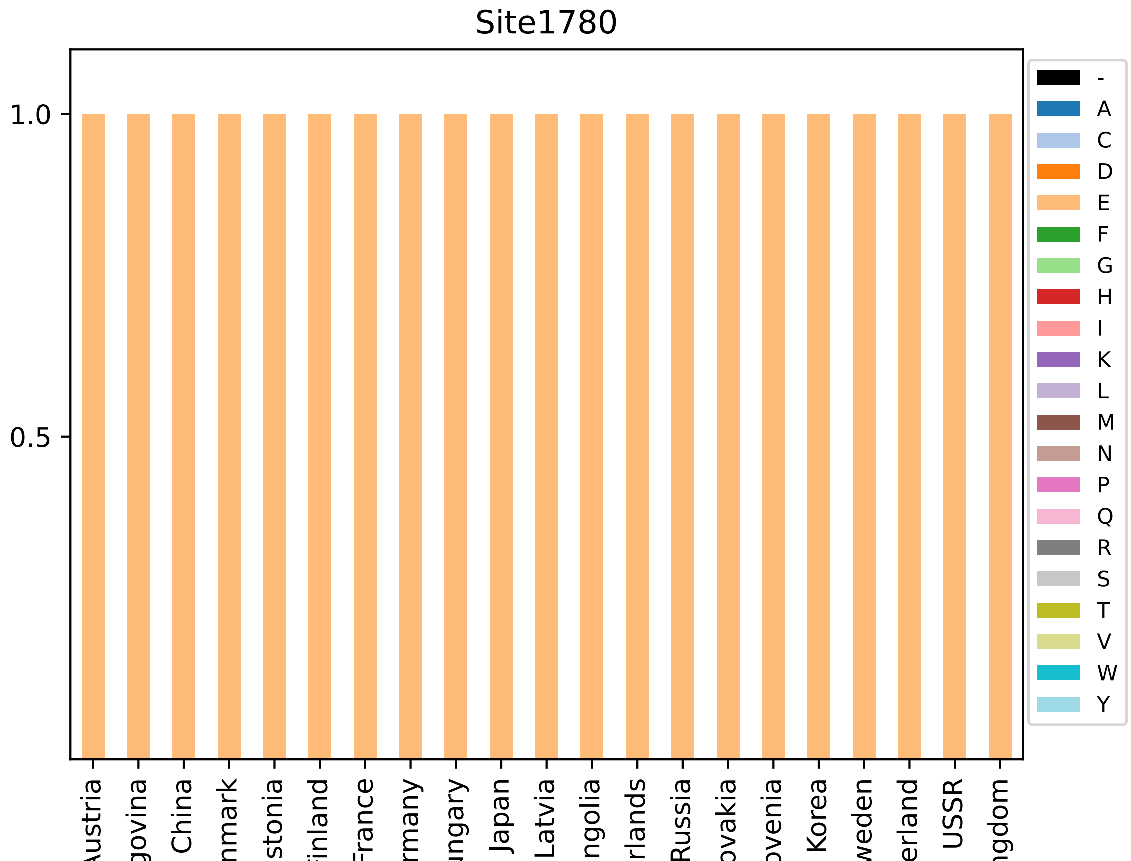
Site1780
1.0
0.5
Austria
govina
China
nmark
stonia
Finland
France
rmany
ungary
Japan
Latvia
ngolia
rlands
Russia
ovakia
ovenia
Korea
weden
erland
USSR
ngdom
-
A
C
D
E
F
G
H
I
K
L
M
N
P
Q
R
S
T
V
W
Y

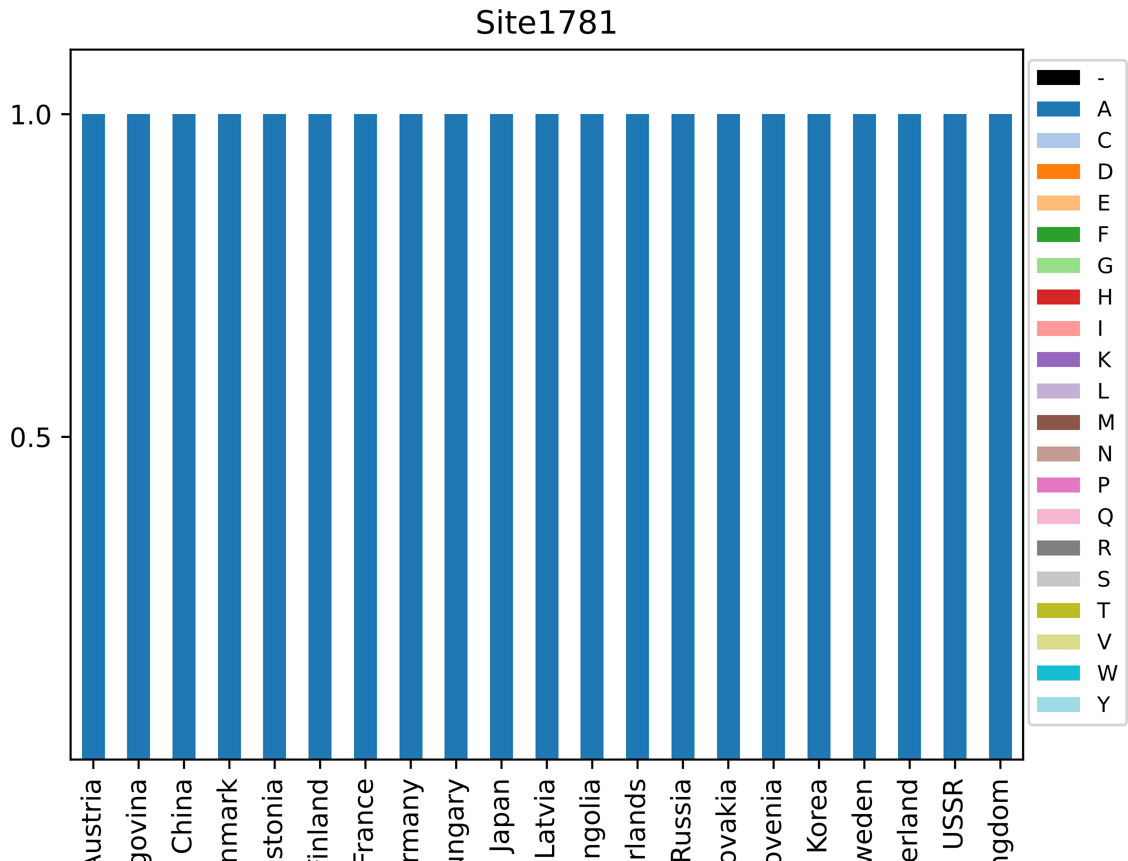

Site1781
1.0
0.5
-
A
C
D
E
F
G
H
I
K
L
M
N
P
Q
R
S
T
V
W
Y
China
Japan
Latvia
Russia
Korea
USSR

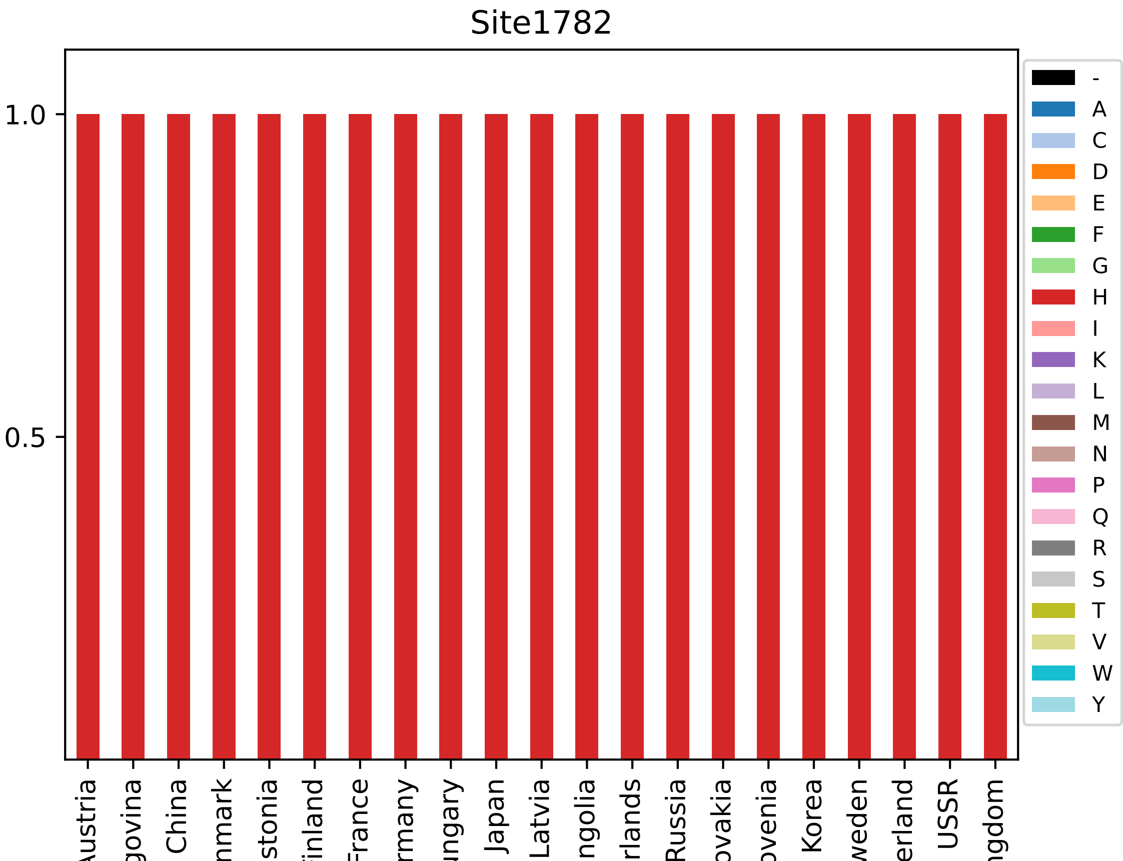

Site1782
1.0
0.5
-
A
C
D
E
F
G
H
I
K
L
M
N
P
Q
R
S
T
V
W
Y
China
Japan
Latvia
Russia
Korea
USSR

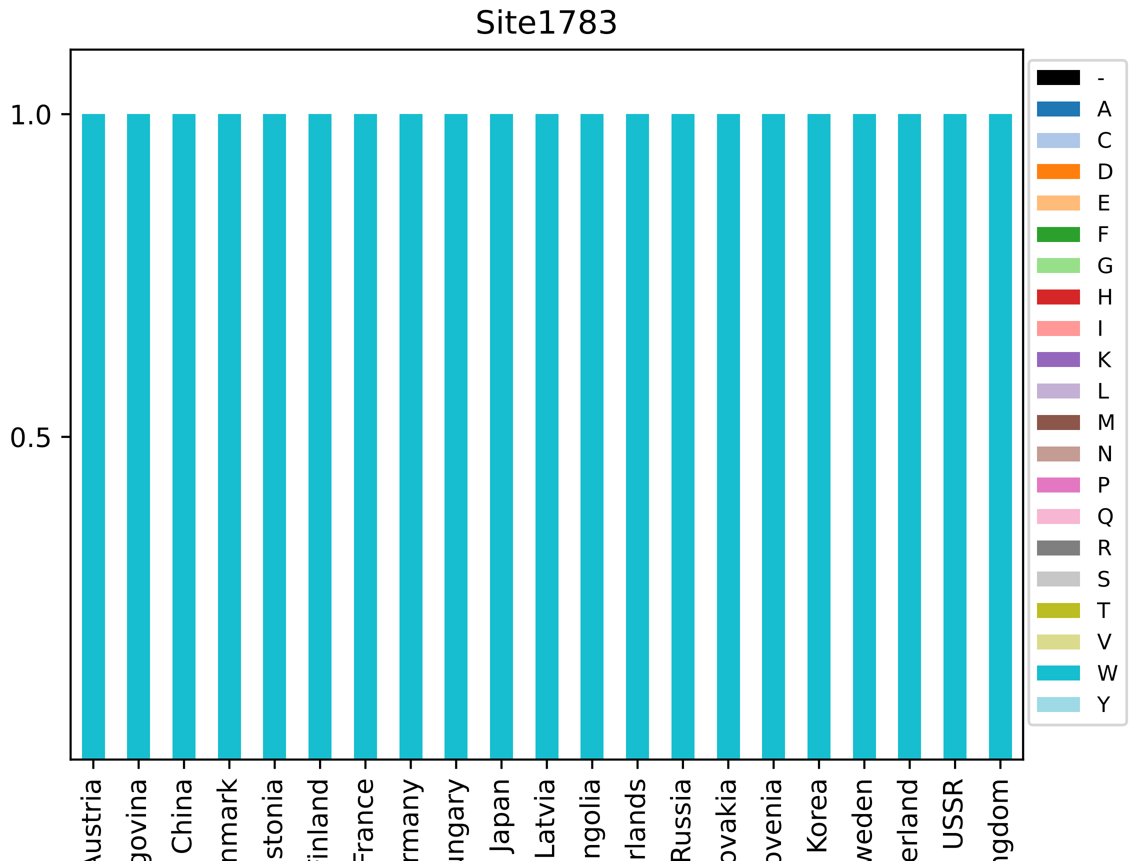

Site1783
1.0
0.5
Austria
govina
China
nmark
stonia
Finland
France
rmany
ungary
Japan
Latvia
ngolia
rlands
Russia
ovakia
ovenia
Korea
weden
erland
USSR
ngdom
-
A
C
D
E
F
G
H
I
K
L
M
N
P
Q
R
S
T
V
W
Y

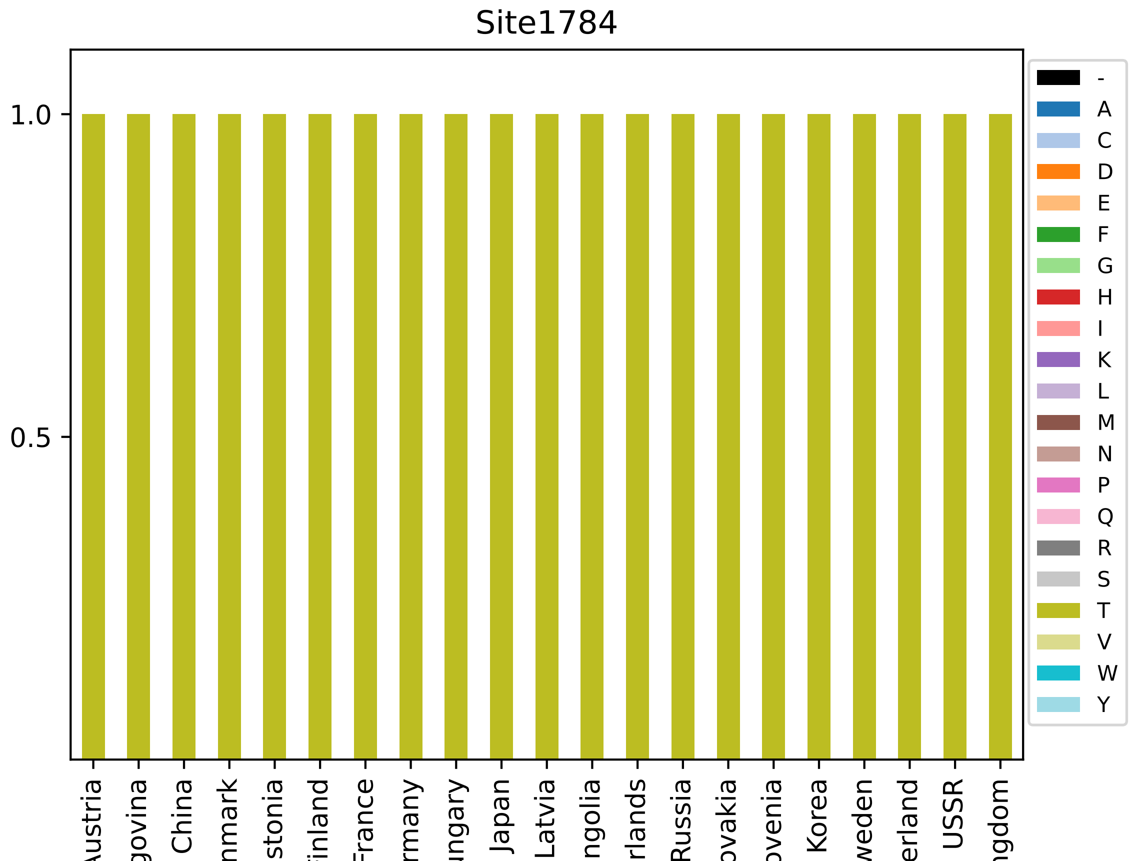

Site1784
1.0
0.5
-
A
C
D
E
F
G
H
I
K
L
M
N
P
Q
R
S
T
V
W
Y
China
Japan
Latvia
Russia
Korea
USSR

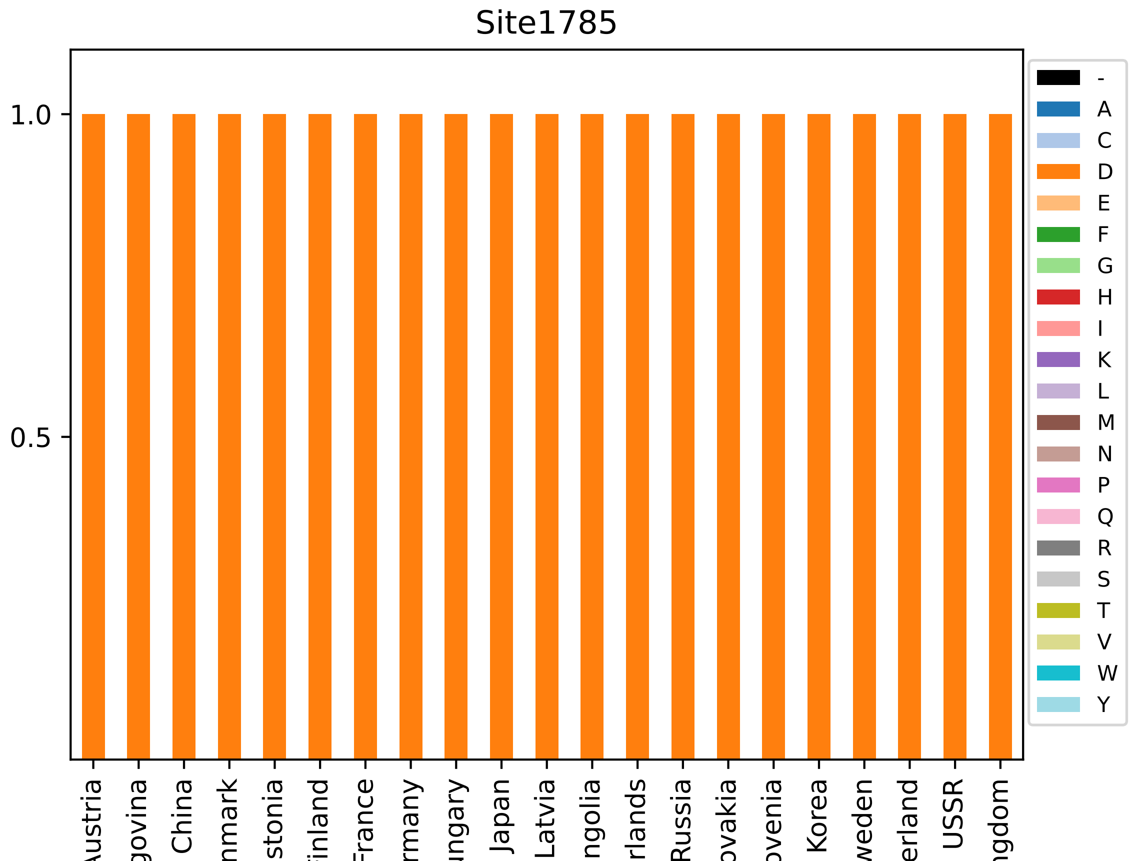

Site1785
1.0
0.5
-
A
C
D
E
F
G
H
I
K
L
M
N
P
Q
R
S
T
V
W
Y
Austria
govina
China
nmark
stonia
Finland
France
rmany
ungary
Japan
Latvia
ngolia
rlands
Russia
ovakia
ovenia
Korea
weden
erland
USSR
ngdom

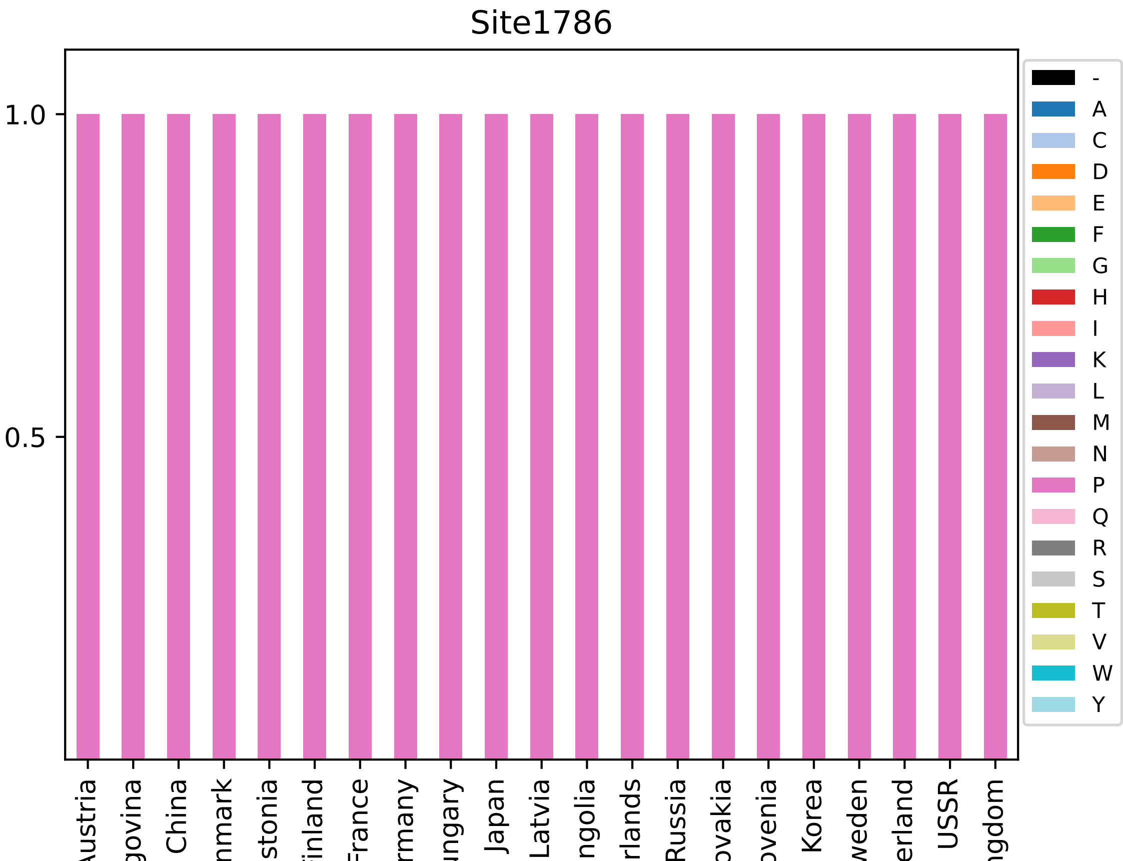
Site1786
1.0
0.5
-
A
C
D
E
F
G
H
I
K
L
M
N
P
Q
R
S
T
V
W
Y
China
Japan
Latvia
Russia
Korea
USSR

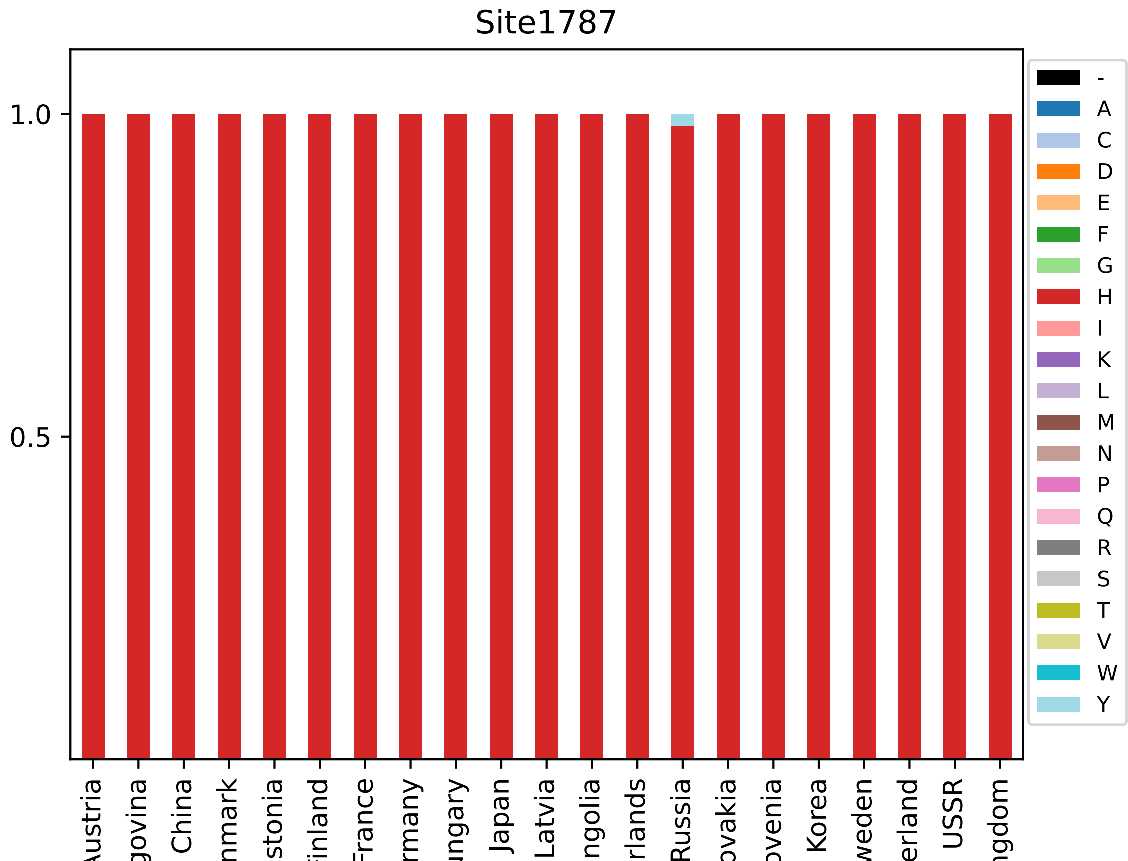
Site1787
1.0
0.5
-
A
C
D
E
F
G
H
I
K
L
M
N
P
Q
R
S
T
V
W
Y
China
Japan
Latvia
Russia
Korea
USSR

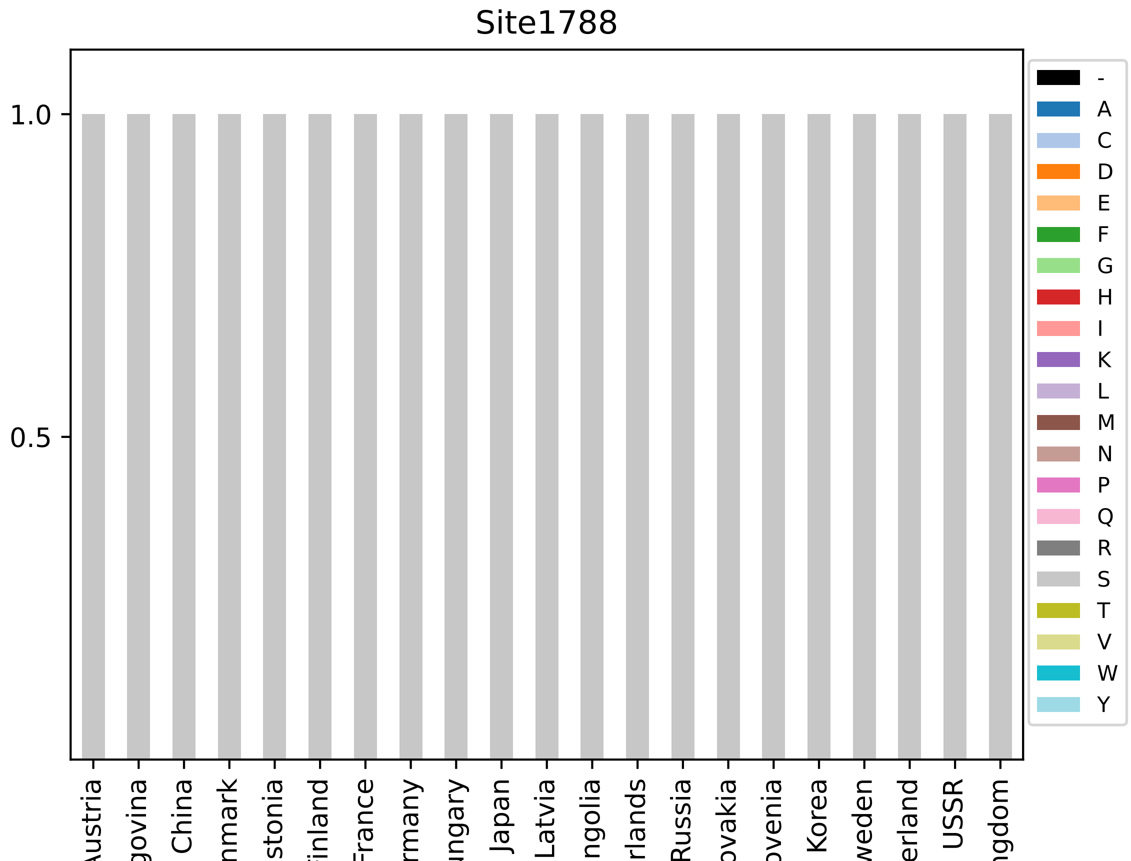
Site1788
1.0
0.5
-
A
C
D
E
F
G
H
I
K
L
M
N
P
Q
R
S
T
V
W
Y
Austria
govina
China
nmark
stonia
Finland
France
rmany
ungary
Japan
Latvia
ngolia
rlands
Russia
ovakia
ovenia
Korea
weden
erland
USSR
ngdom

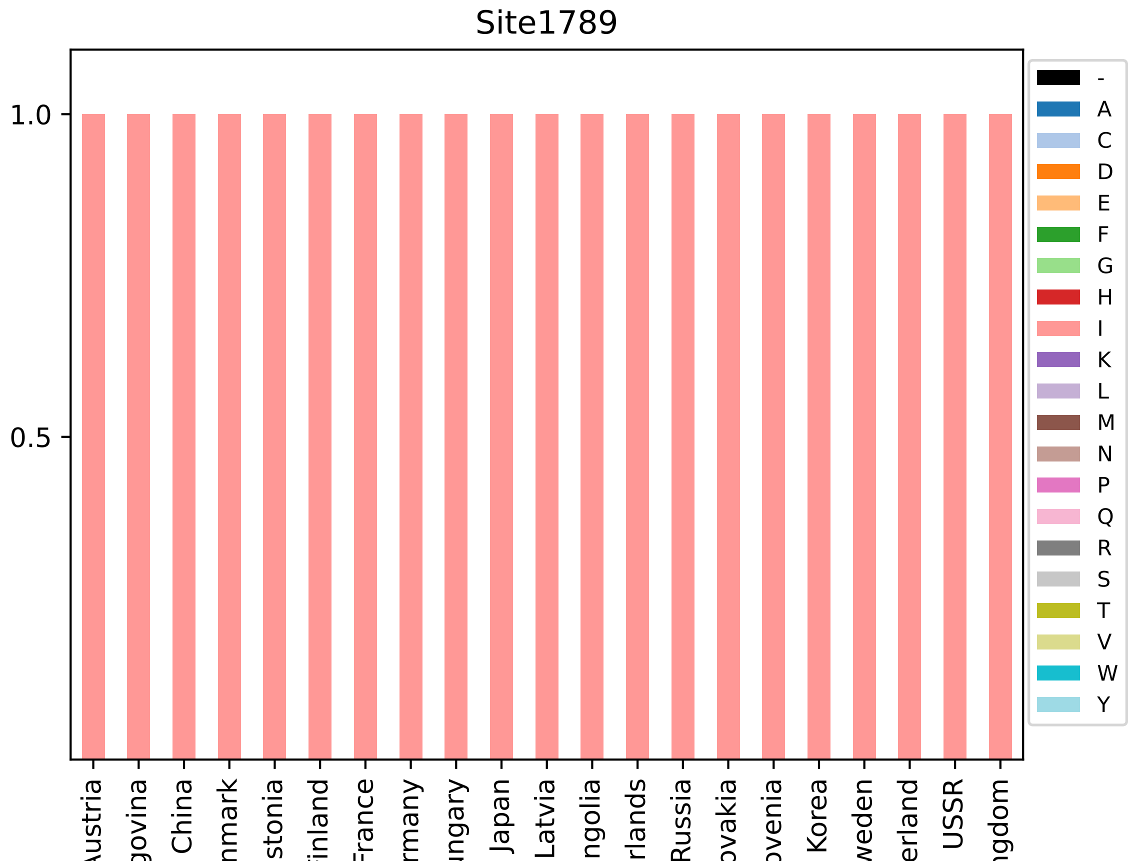

Site1789
1.0
0.5
-
A
C
D
E
F
G
H
I
K
L
M
N
P
Q
R
S
T
V
W
Y
China
Japan
Latvia
Russia
Korea
USSR

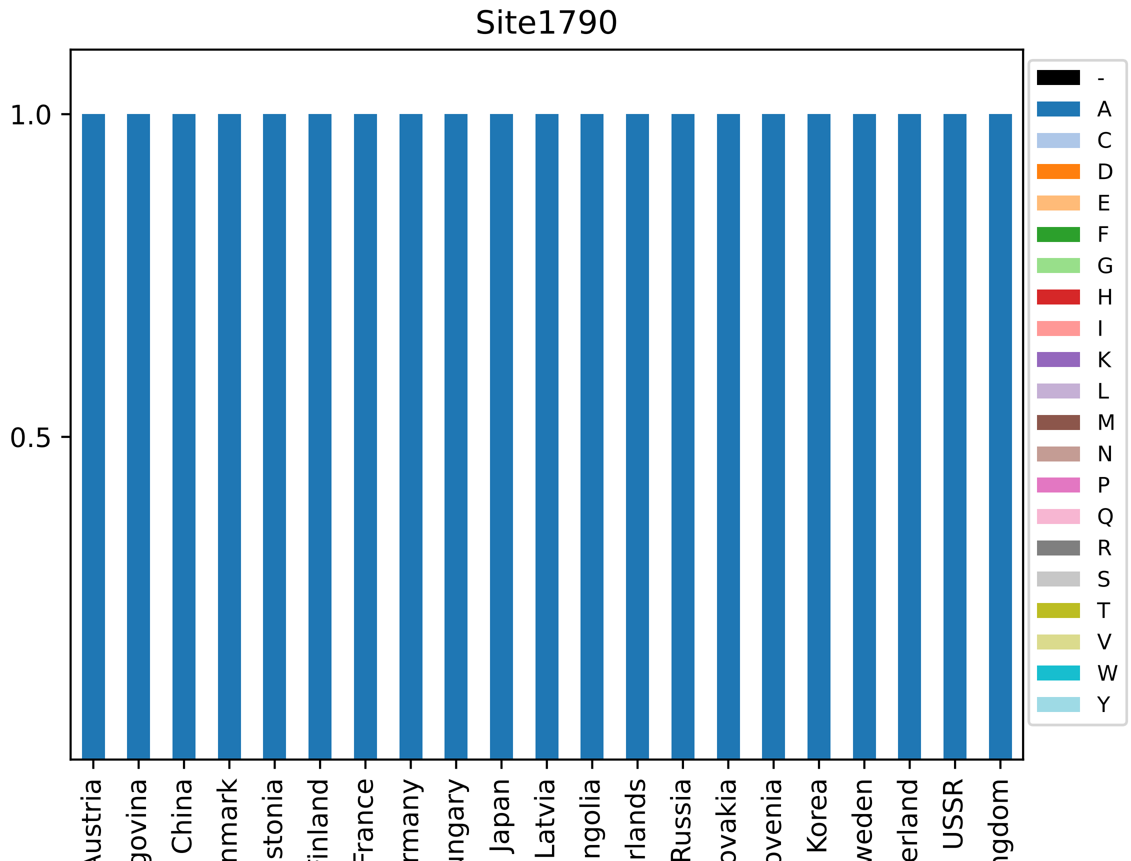

Site1790
1.0
0.5
-
A
C
D
E
F
G
H
I
K
L
M
N
P
Q
R
S
T
V
W
Y
China
Japan
Latvia
Russia
Korea
USSR

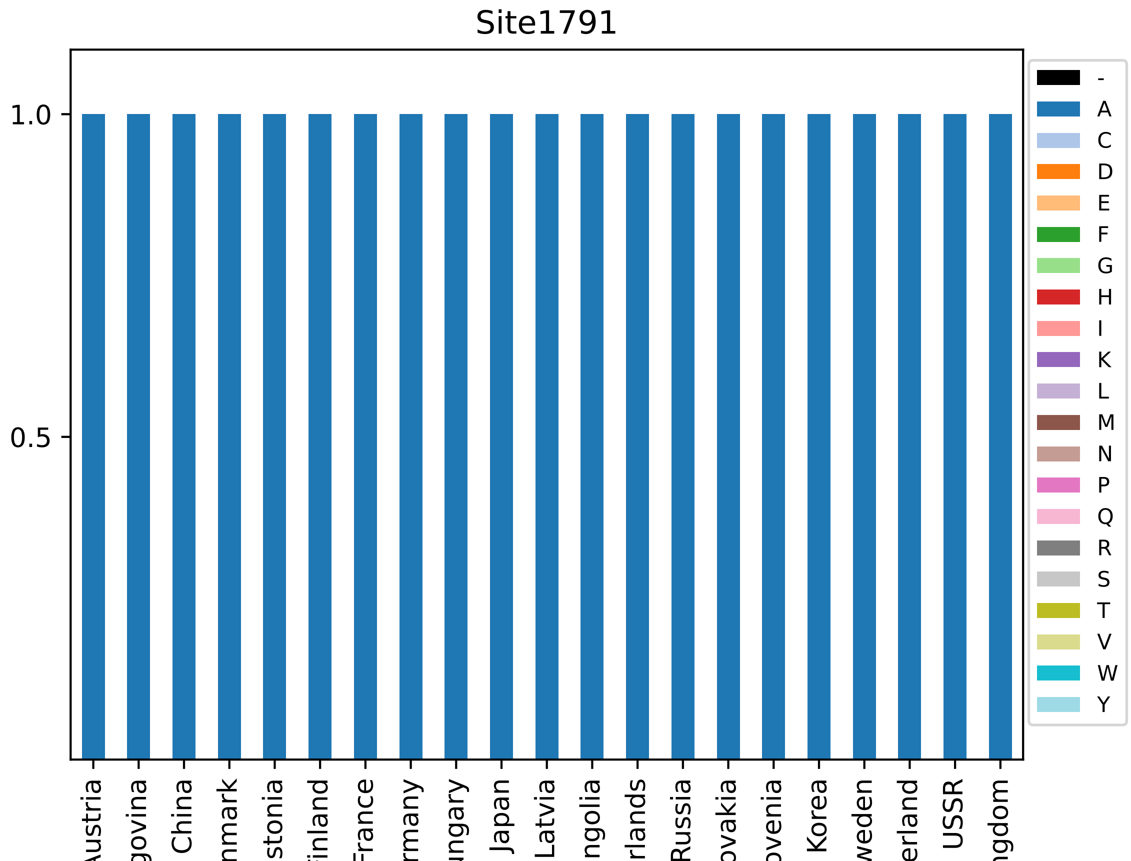
Site1791
1.0
0.5
Austria
govina
China
nmark
stonia
Finland
France
rmany
ungary
Japan
Latvia
ngolia
rlands
Russia
ovakia
ovenia
Korea
weden
erland
USSR
ngdom
-
A
C
D
E
F
G
H
I
K
L
M
N
P
Q
R
S
T
V
W
Y

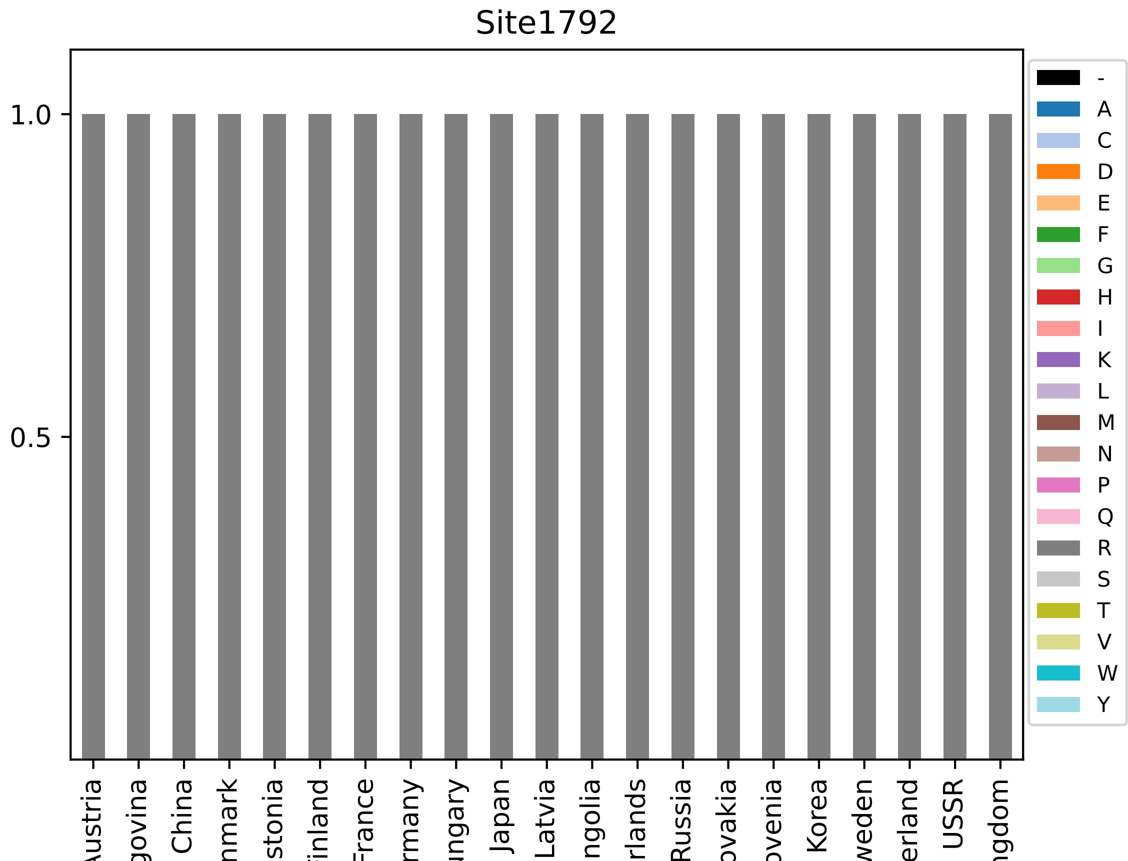

Site1792
1.0
0.5
-
A
C
D
E
F
G
H
I
K
L
M
N
P
Q
R
S
T
V
W
Y
China
Japan
Latvia
Russia
Korea
USSR

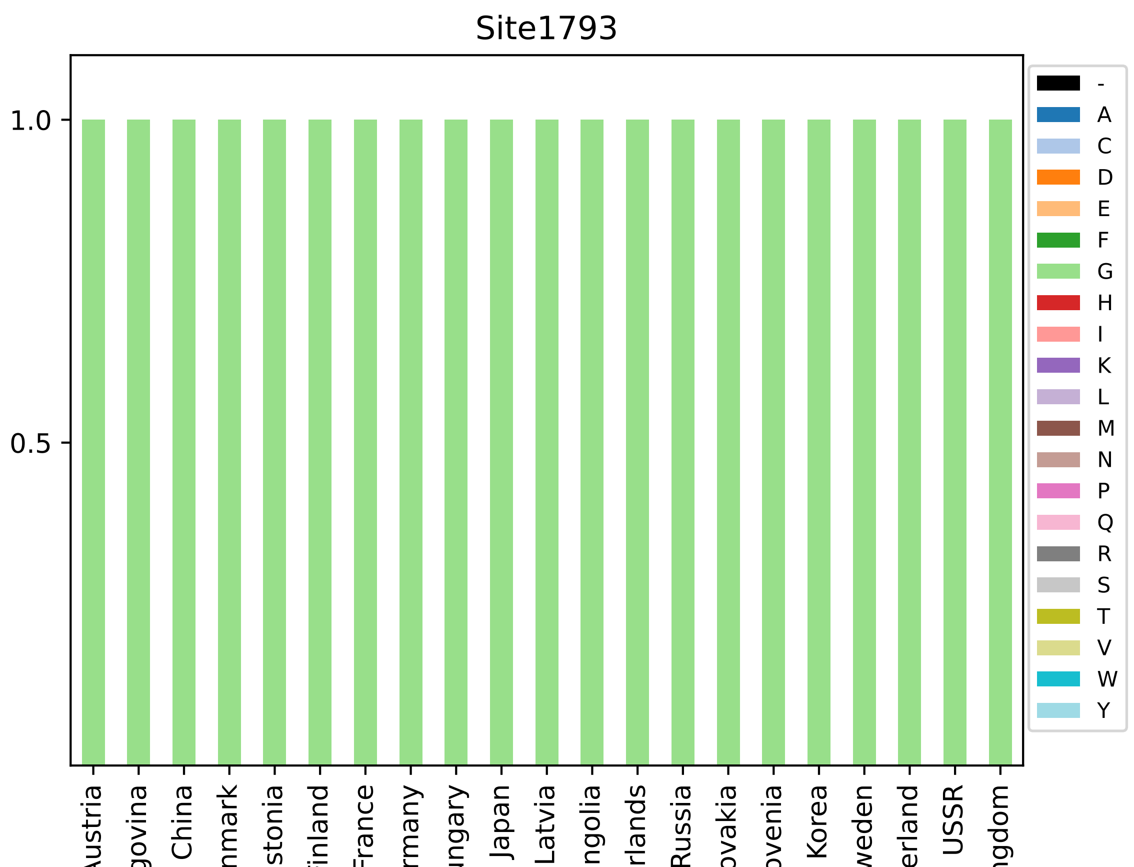

Site1793
1.0
0.5
-
A
C
D
E
F
G
H
I
K
L
M
N
P
Q
R
S
T
V
W
Y
China
Japan
Latvia
Russia
Korea
USSR

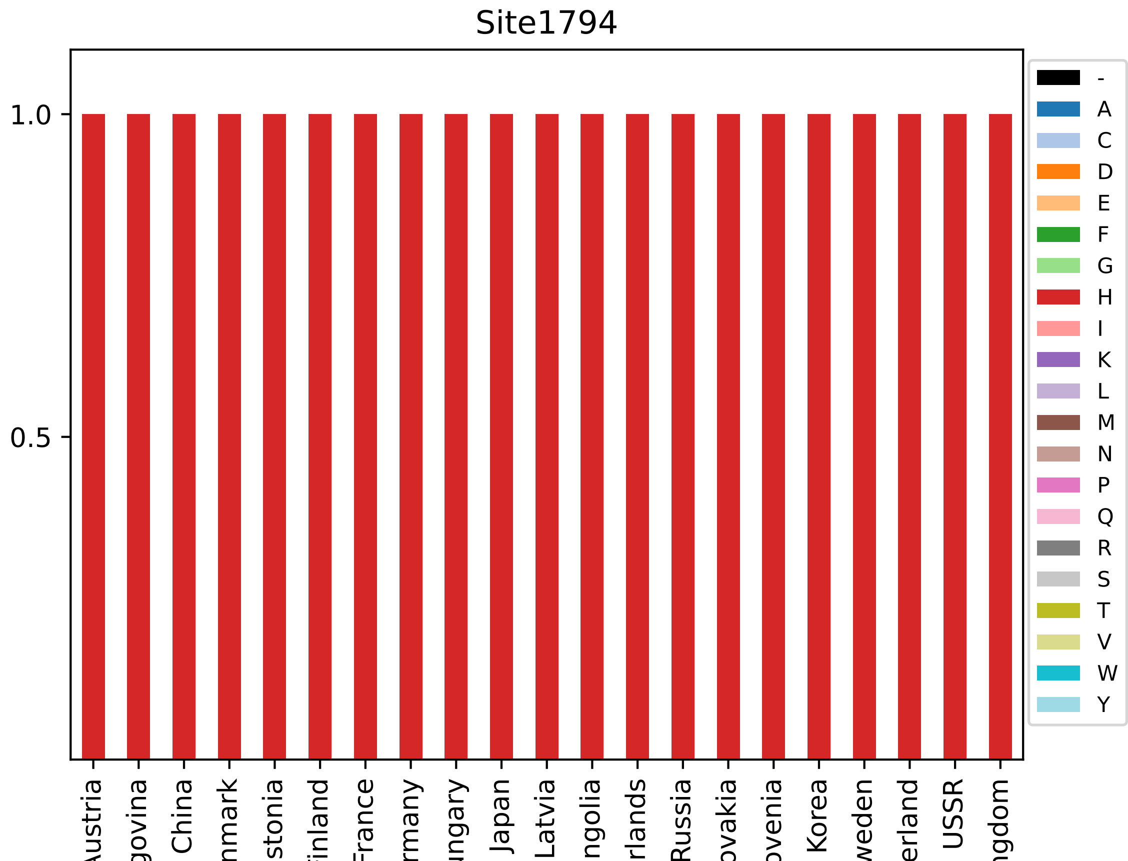

Site1794
1.0
0.5
-
A
C
D
E
F
G
H
I
K
L
M
N
P
Q
R
S
T
V
W
Y
China
Japan
Latvia
Russia
Korea
USSR

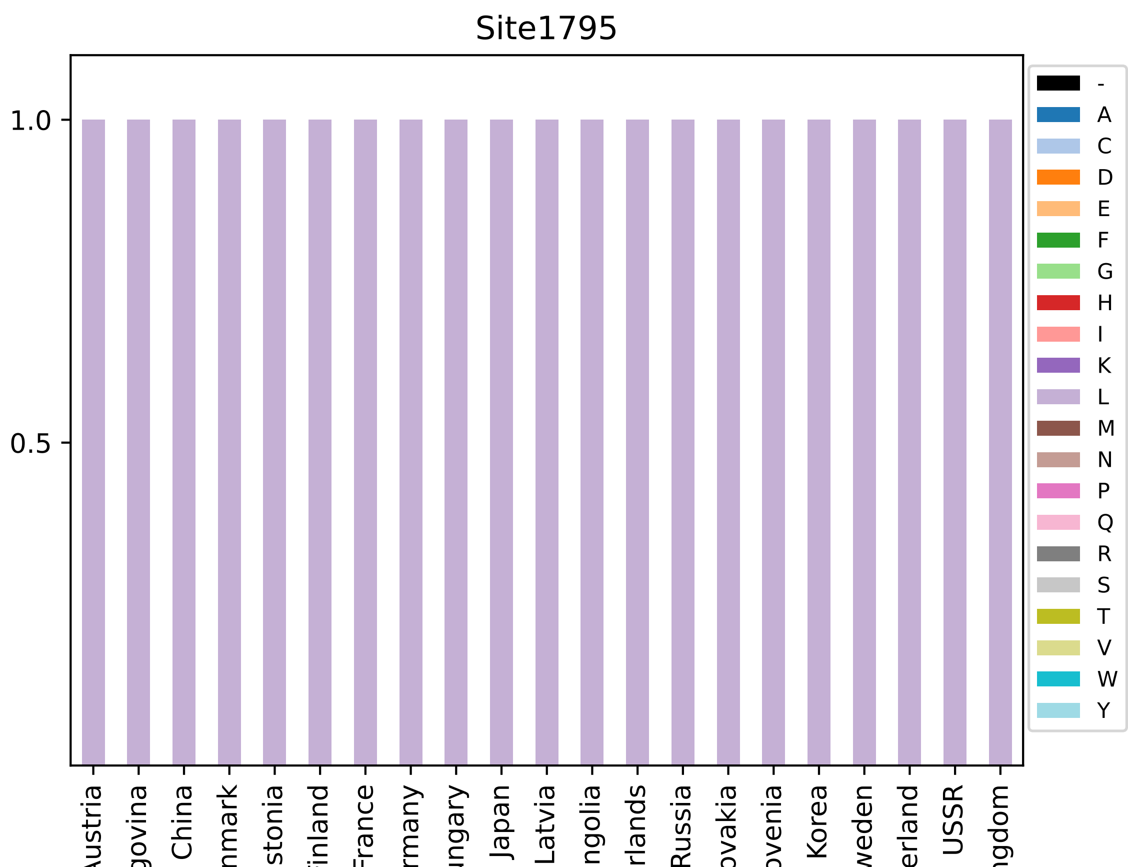
Site1795
1.0
0.5
-
A
C
D
E
F
G
H
I
K
L
M
N
P
Q
R
S
T
V
W
Y
China
Japan
Latvia
Russia
Korea
USSR

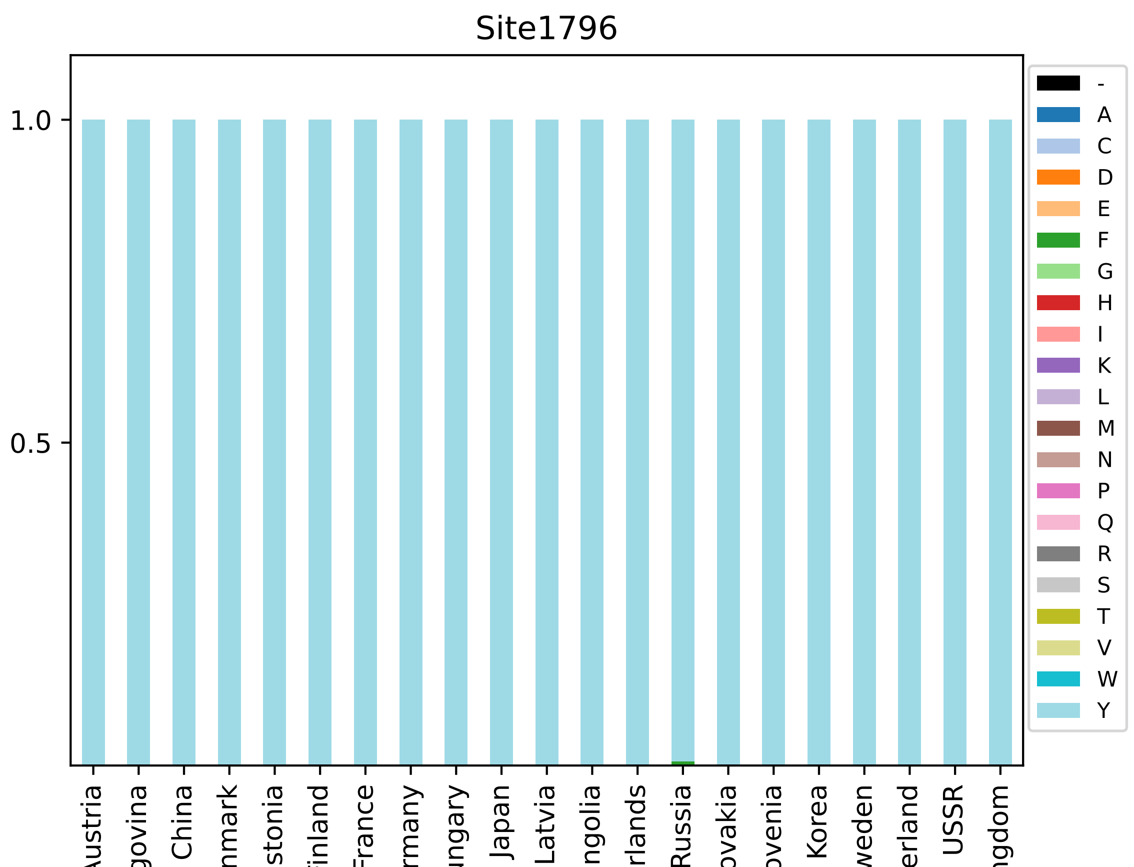
Site1796
1.0
0.5
-
A
C
D
E
F
G
H
I
K
L
M
N
P
Q
R
S
T
V
W
Y
China
Japan
Latvia
Russia
Korea
USSR

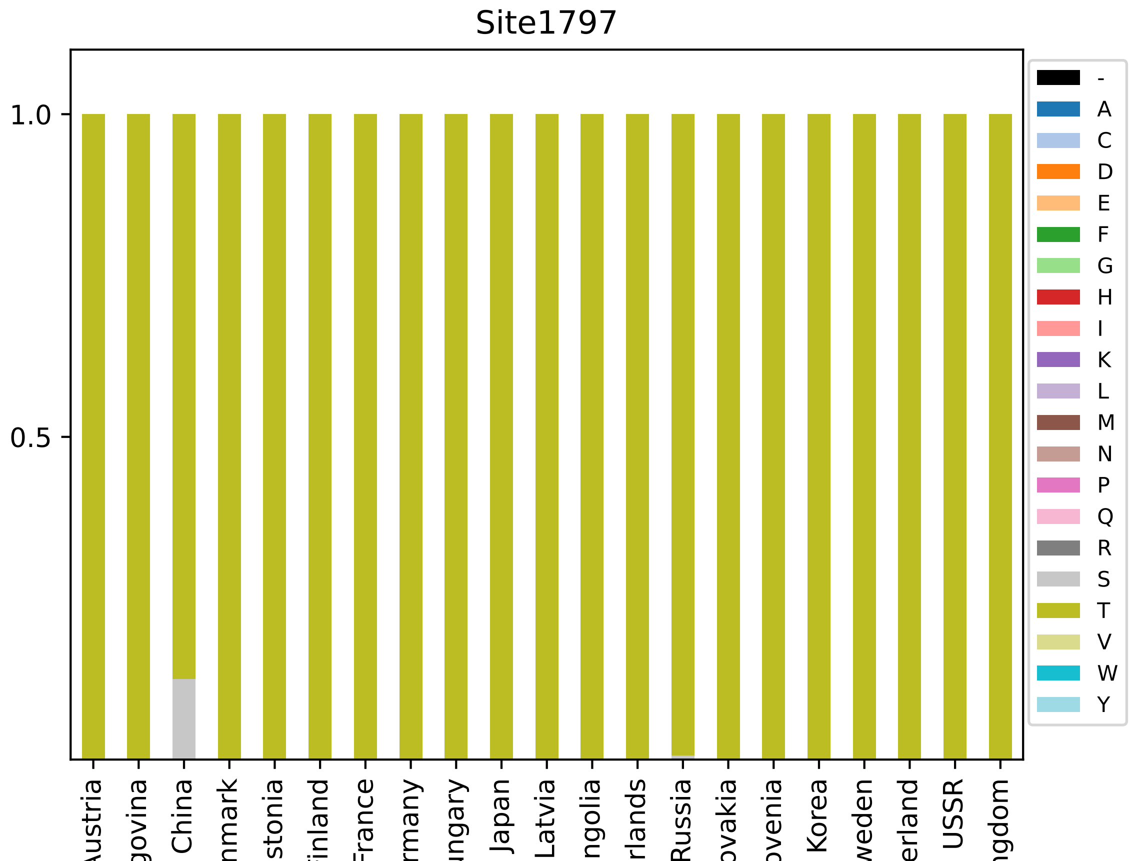

Site1797
1.0
0.5
-
A
C
D
E
F
G
H
I
K
L
M
N
P
Q
R
S
T
V
W
Y
Austria
govina
China
nmark
stonia
Finland
France
rmany
ungary
Japan
Latvia
ngolia
rlands
Russia
ovakia
ovenia
Korea
weden
erland
USSR
ngdom

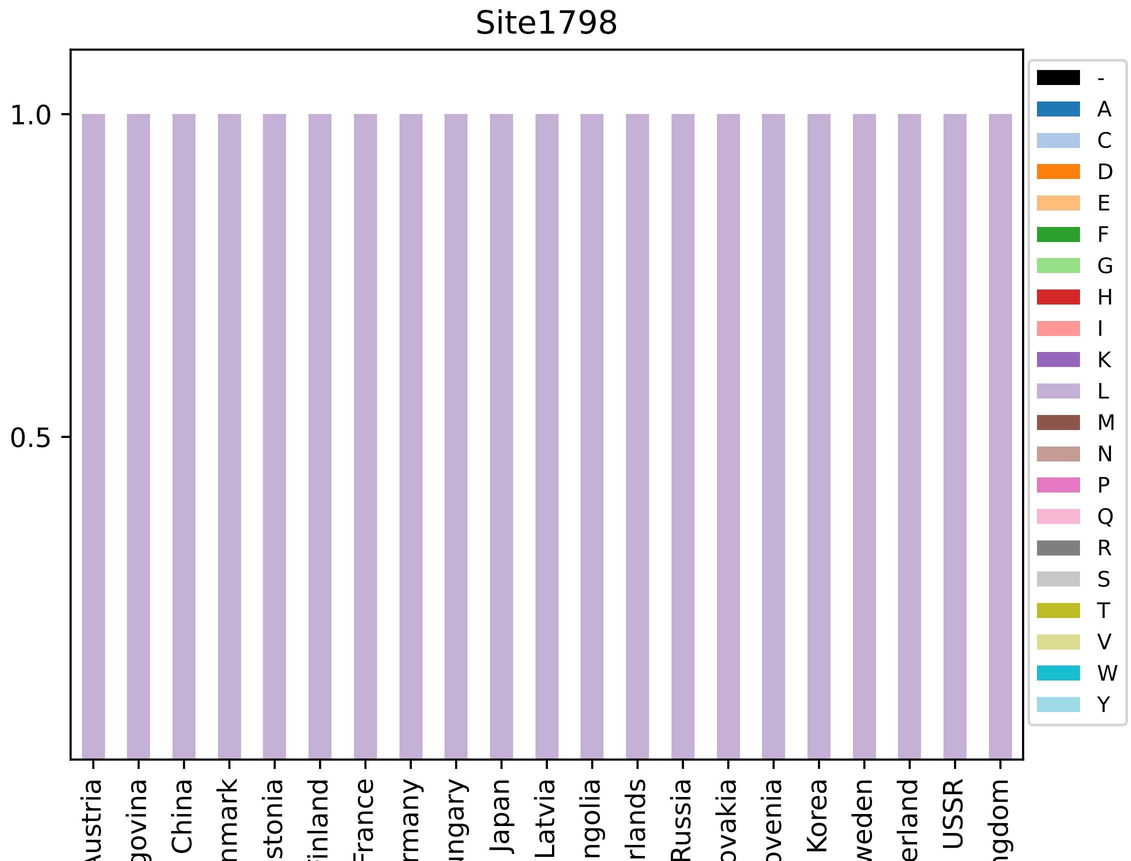
Site1798
1.0
0.5
-
A
C
D
E
F
G
H
I
K
L
M
N
P
Q
R
S
T
V
W
Y
Austria
China
Finland
France
Japan
Latvia
Russia
Korea
USSR

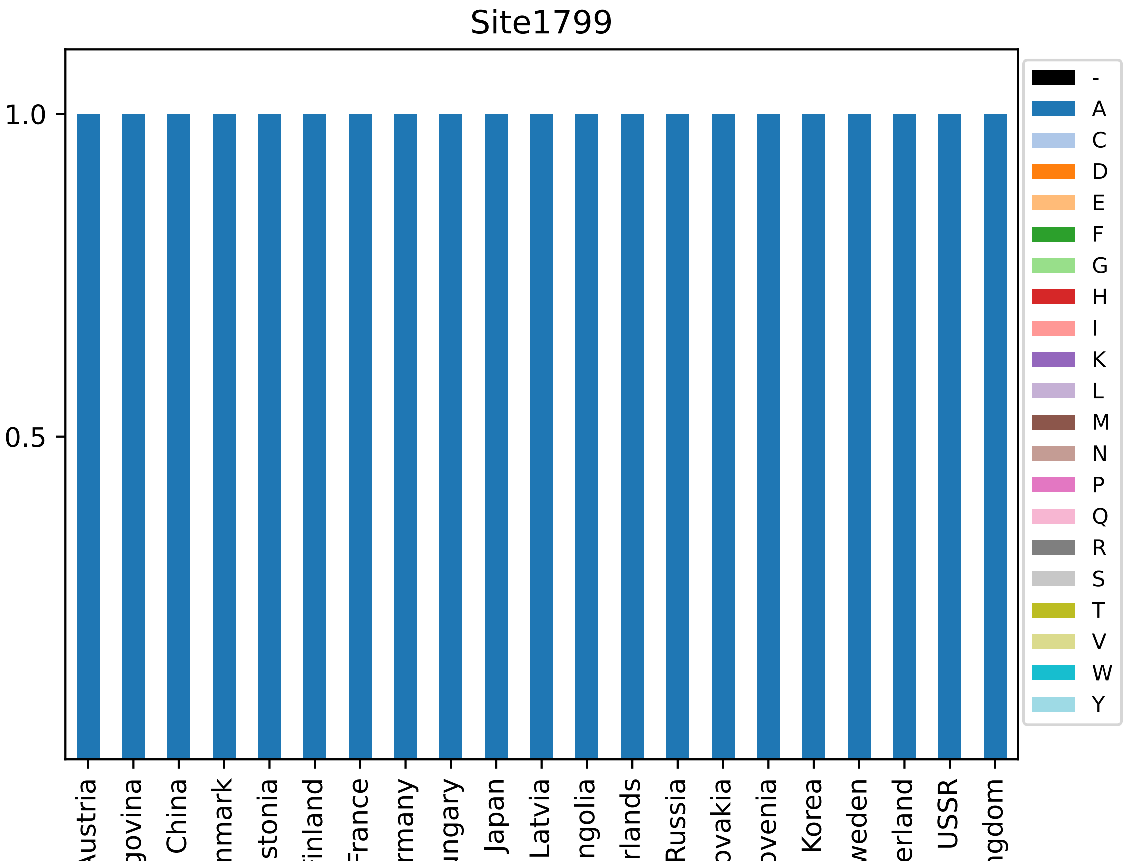

Site1799
1.0
0.5
Austria
govina
China
nmark
stonia
inland
France
rmany
ungary
Japan
Latvia
ngolia
rlands
Russia
ovakia
ovenia
Korea
weden
erland
USSR
ngdom
-
A
C
D
E
F
G
H
I
K
L
M
N
P
Q
R
S
T
V
W
Y

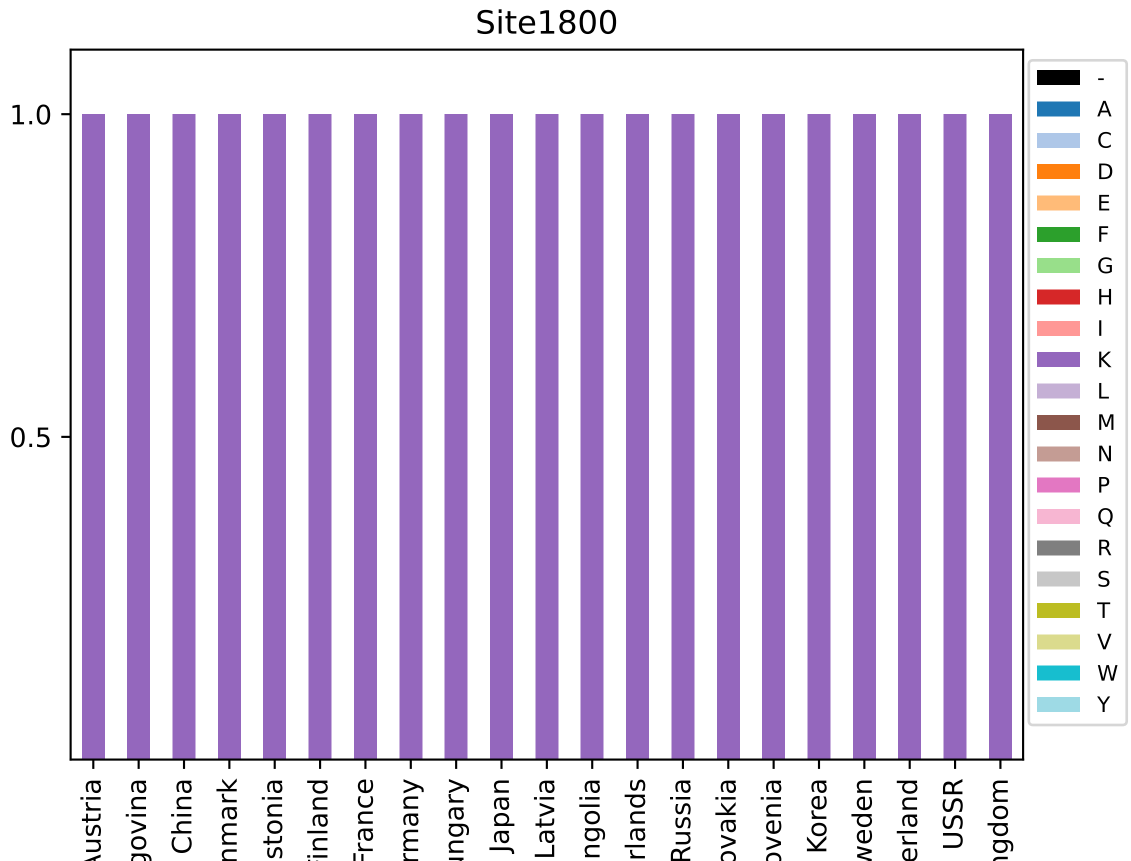
Site1800
1.0
0.5
-
A
C
D
E
F
G
H
I
K
L
M
N
P
Q
R
S
T
V
W
Y
Austria
govina
China
nmark
stonia
inland
France
rmany
ungary
Japan
Latvia
ngolia
rlands
Russia
ovakia
ovenia
Korea
weden
erland
USSR
ngdom

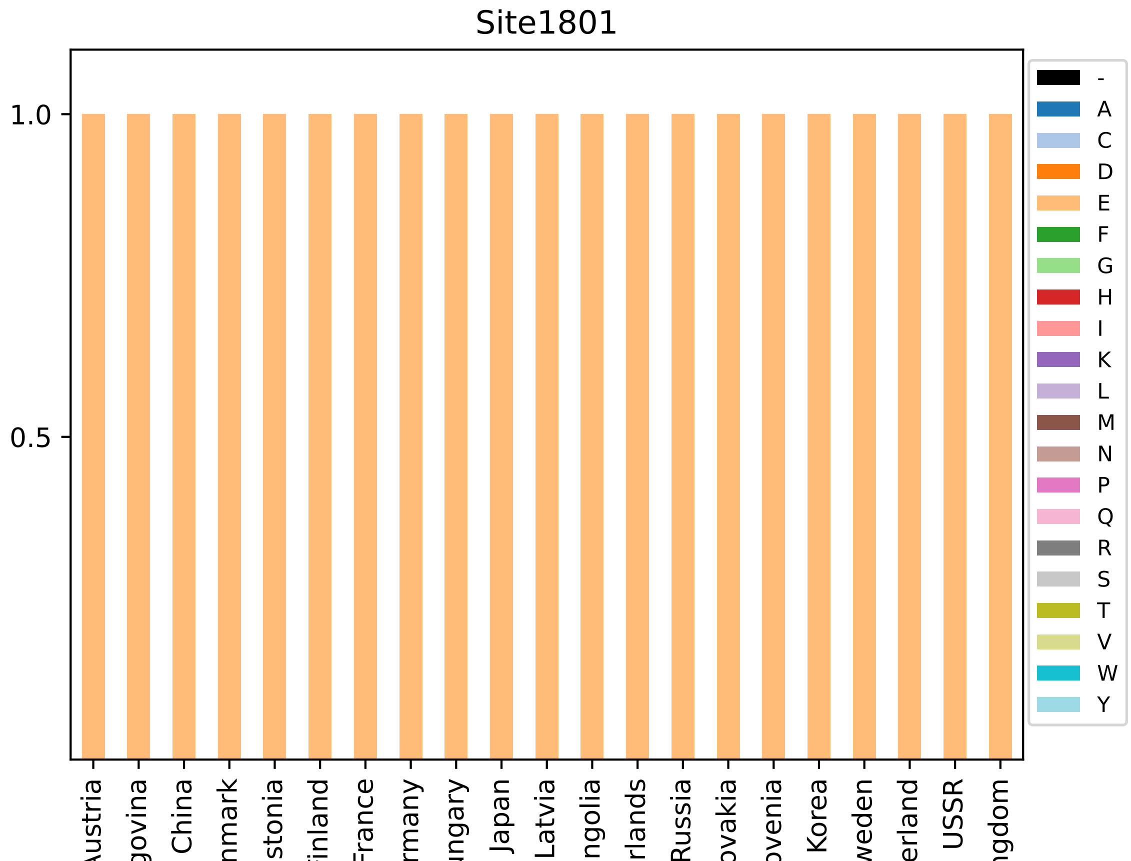

Site1801
1.0
0.5
-
A
C
D
E
F
G
H
I
K
L
M
N
P
Q
R
S
T
V
W
Y
China
Japan
Latvia
Russia
Korea
USSR

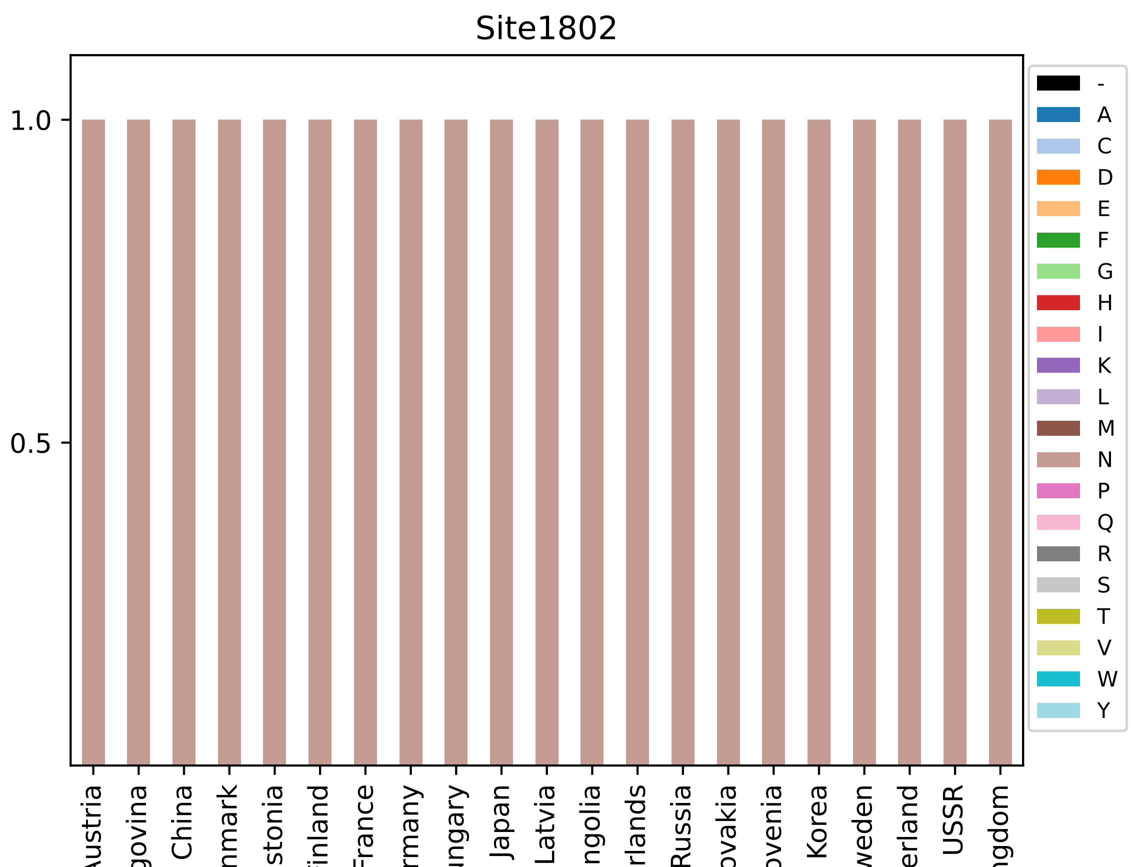

Site1802
1.0
0.5
-
A
C
D
E
F
G
H
I
K
L
M
N
P
Q
R
S
T
V
W
Y
Austria
govina
China
nmark
stonia
Finland
France
rmany
ungary
Japan
Latvia
ngolia
rlands
Russia
ovakia
ovenia
Korea
weden
erland
USSR
ngdom

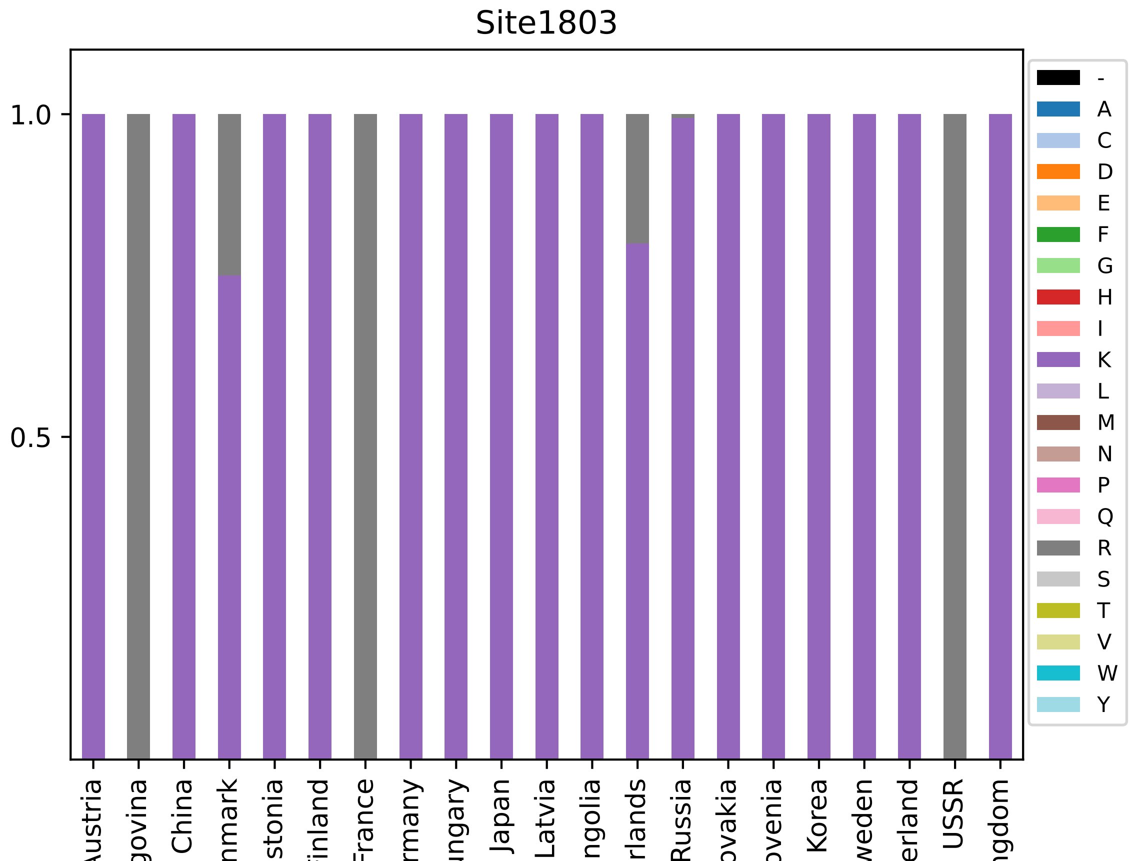
Site1803
1.0
0.5
-
A
C
D
E
F
G
H
I
K
L
M
N
P
Q
R
S
T
V
W
Y
China
Japan
Latvia
Russia
Korea
USSR

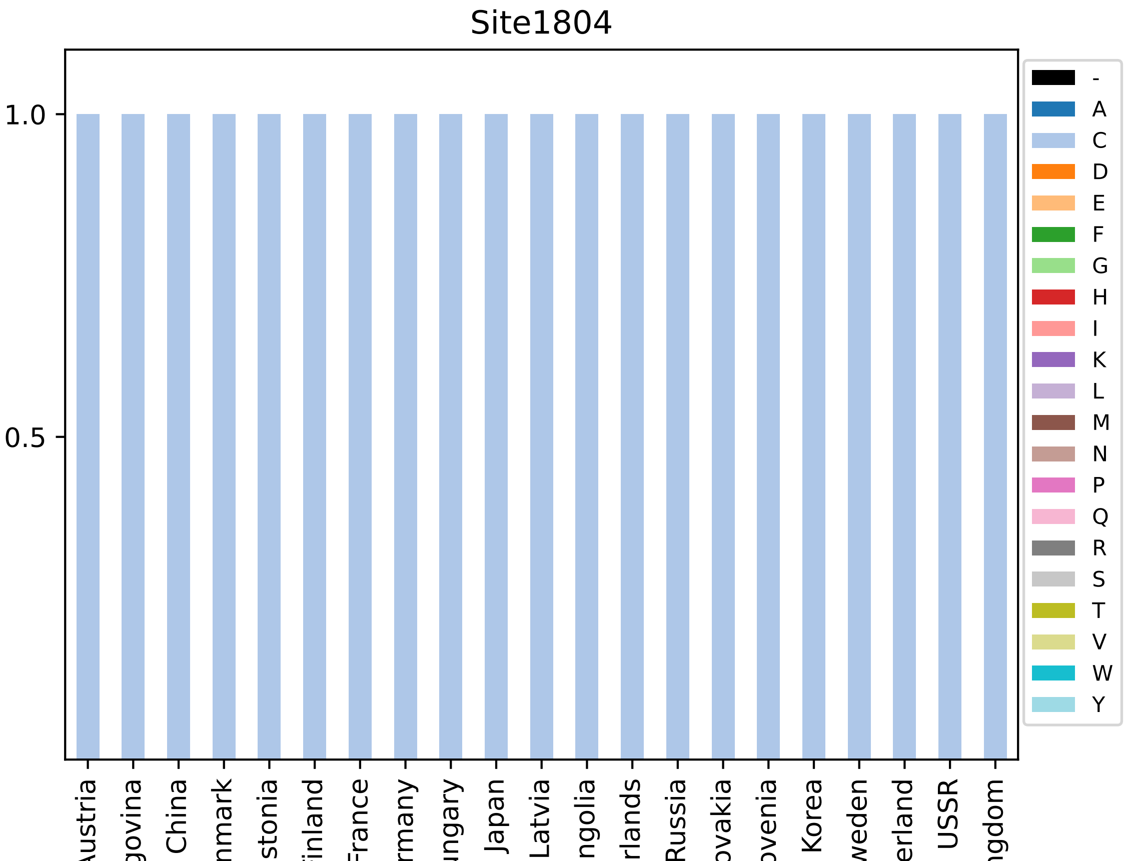
Site1804
1.0
0.5
-
A
C
D
E
F
G
H
I
K
L
M
N
P
Q
R
S
T
V
W
Y
China
Japan
Latvia
Russia
Korea
USSR

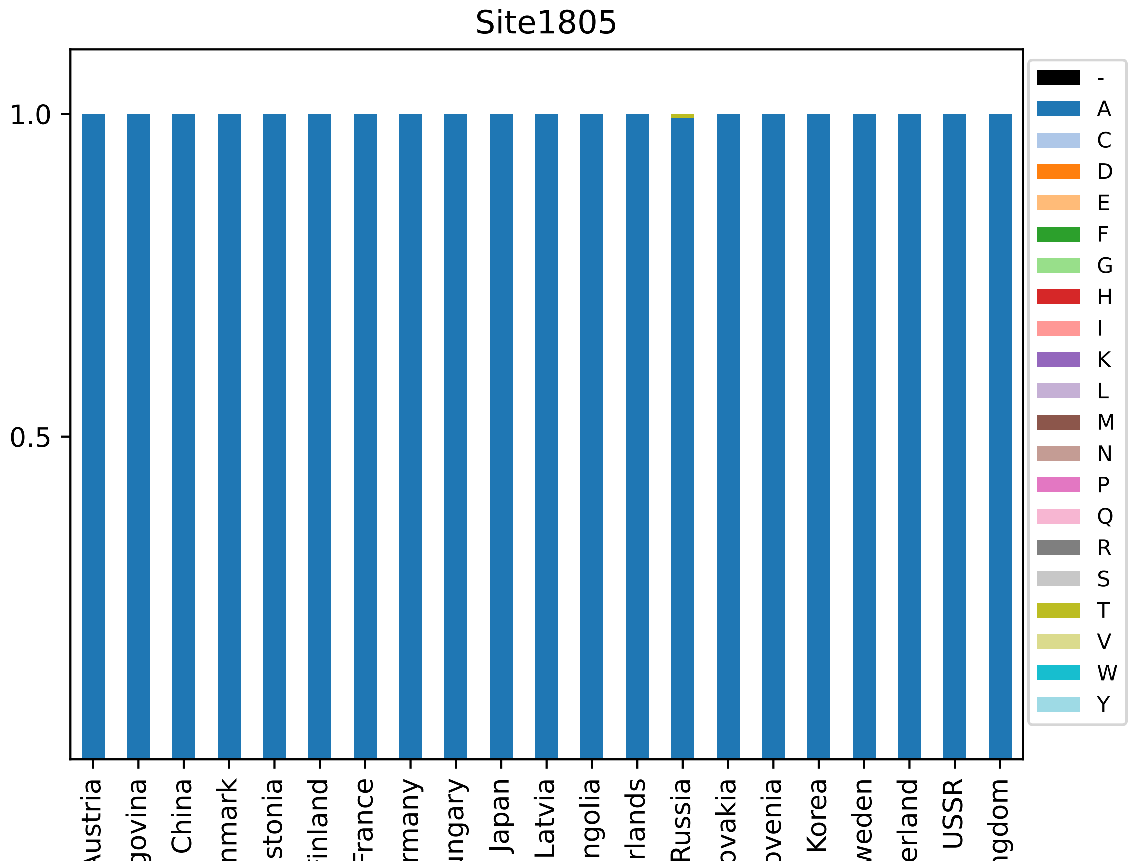

Site1805
1.0
0.5
-
A
C
D
E
F
G
H
I
K
L
M
N
P
Q
R
S
T
V
W
Y
Austria
govina
China
nmark
stonia
inland
France
rmany
ungary
Japan
Latvia
ngolia
rlands
Russia
ovakia
ovenia
Korea
weden
erland
USSR
ngdom

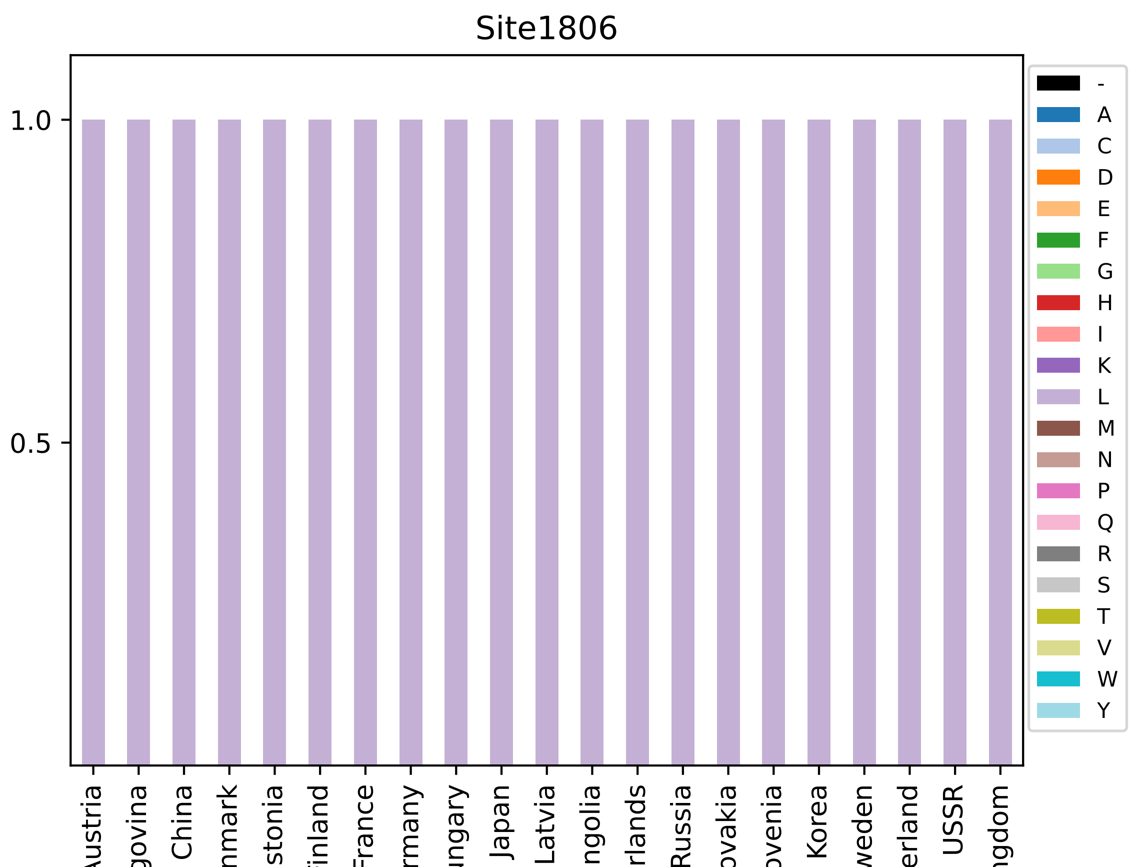

Site1806
1.0
0.5
-
A
C
D
E
F
G
H
I
K
L
M
N
P
Q
R
S
T
V
W
Y
China
Japan
Latvia
Russia
Korea
USSR

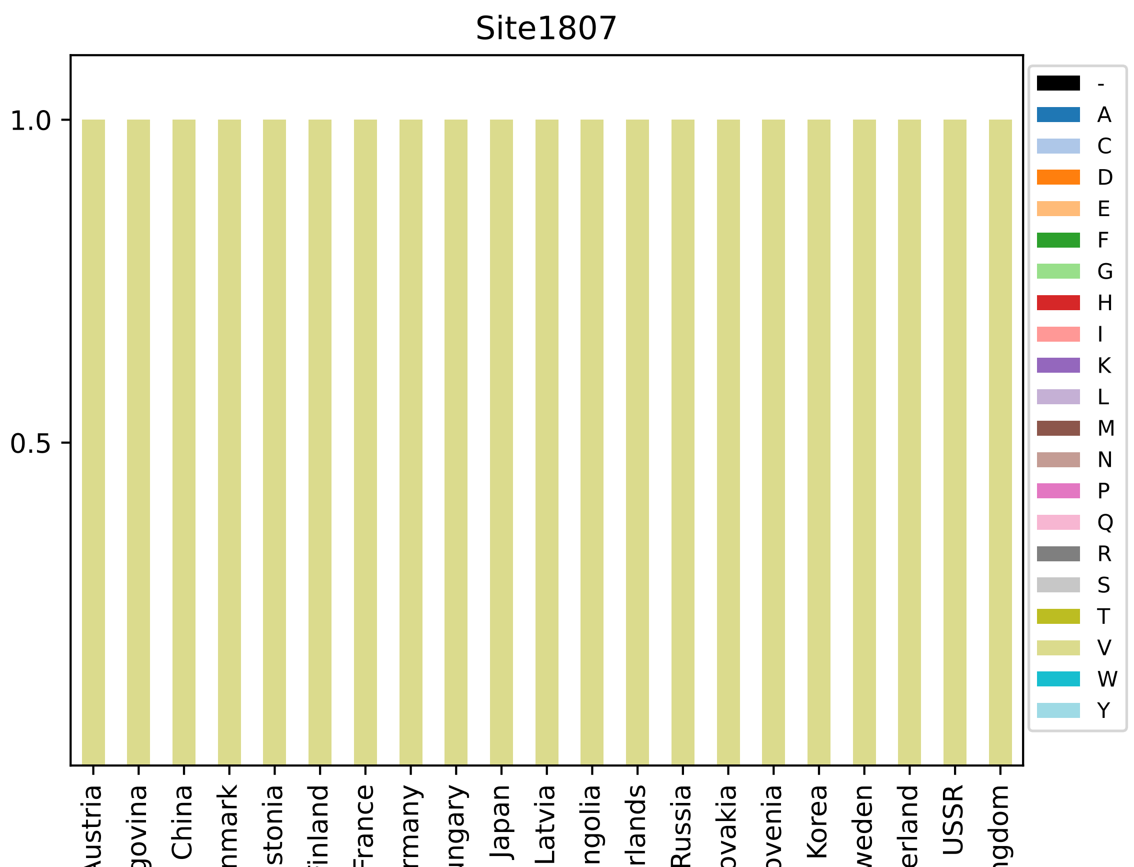
Site1807
1.0
0.5
China
Japan
Latvia
Russia
Korea
USSR
-
A
C
D
E
F
G
H
I
K
L
M
N
P
Q
R
S
T
V
W
Y

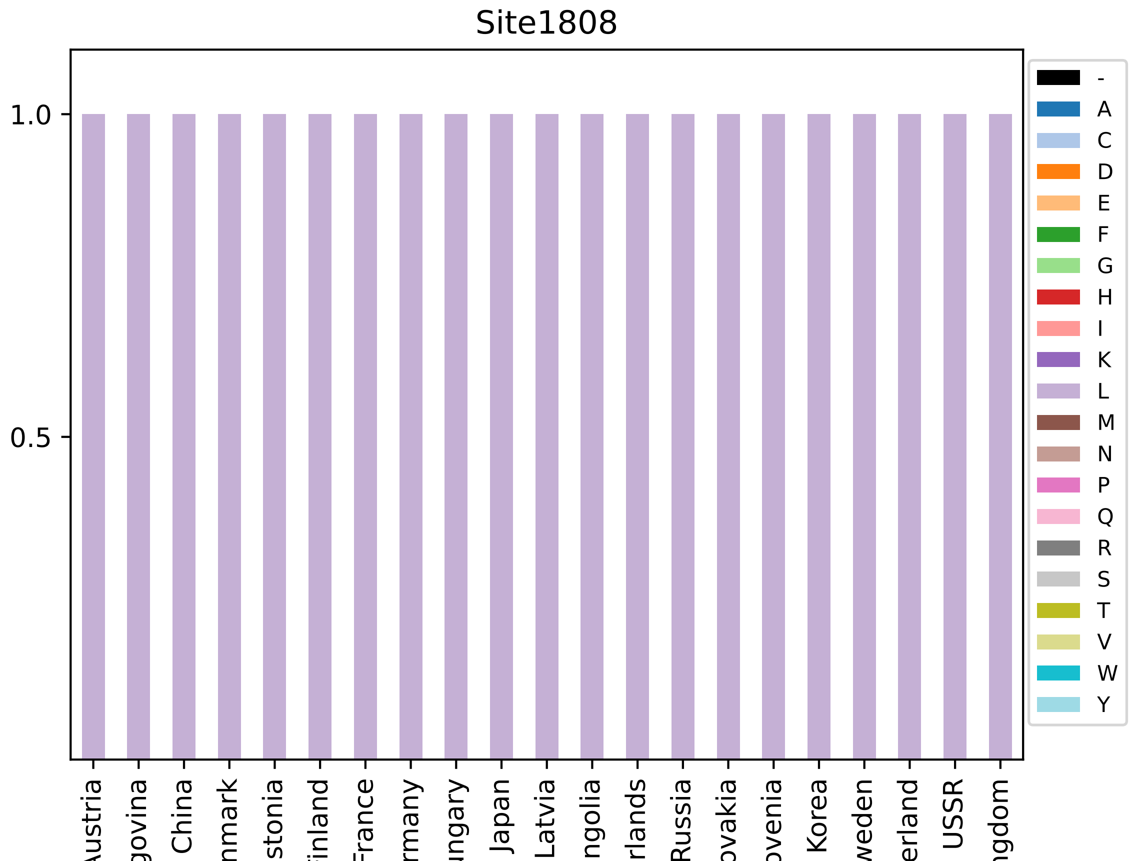
Site1808
1.0
0.5
-
A
C
D
E
F
G
H
I
K
L
M
N
P
Q
R
S
T
V
W
Y
China
Japan
Latvia
Russia
Korea
USSR

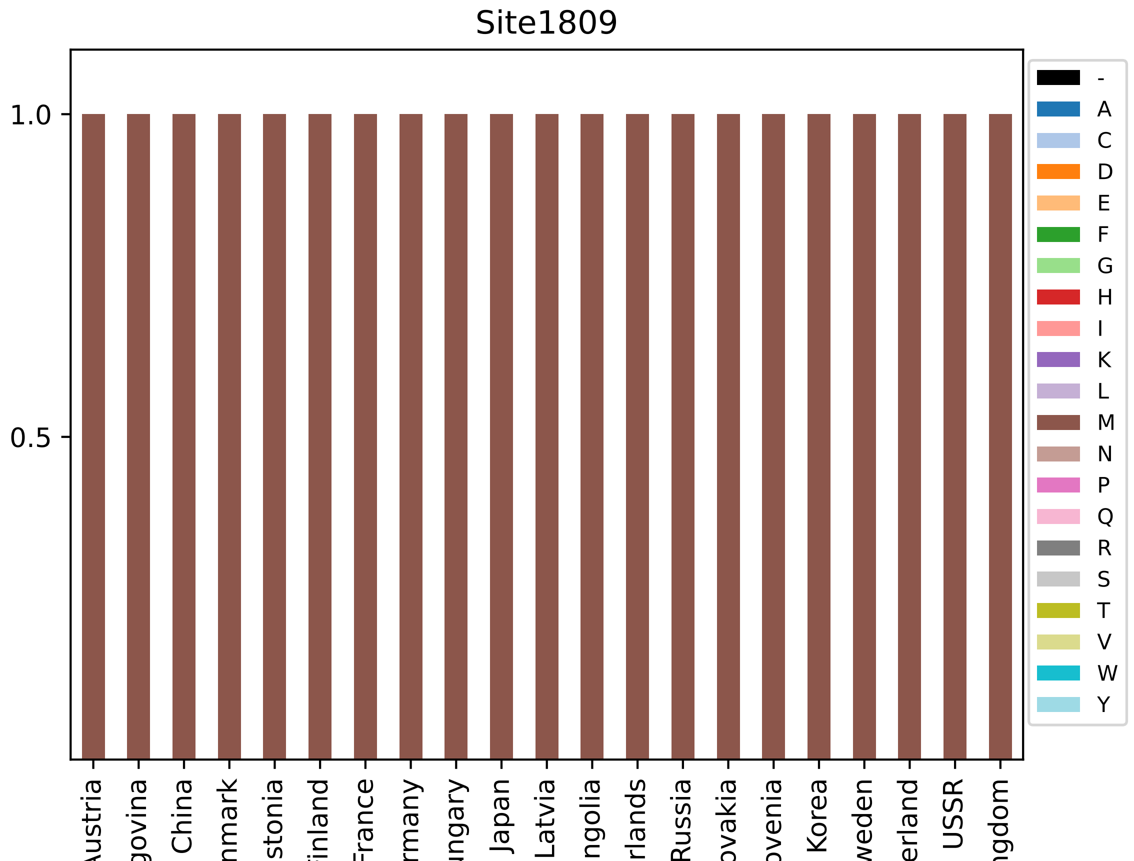

Site1809
1.0
0.5
-
A
C
D
E
F
G
H
I
K
L
M
N
P
Q
R
S
T
V
W
Y
Austria
govina
China
nmark
stonia
inland
France
rmany
ungary
Japan
Latvia
ngolia
rlands
Russia
ovakia
ovenia
Korea
weden
erland
USSR
ngdom

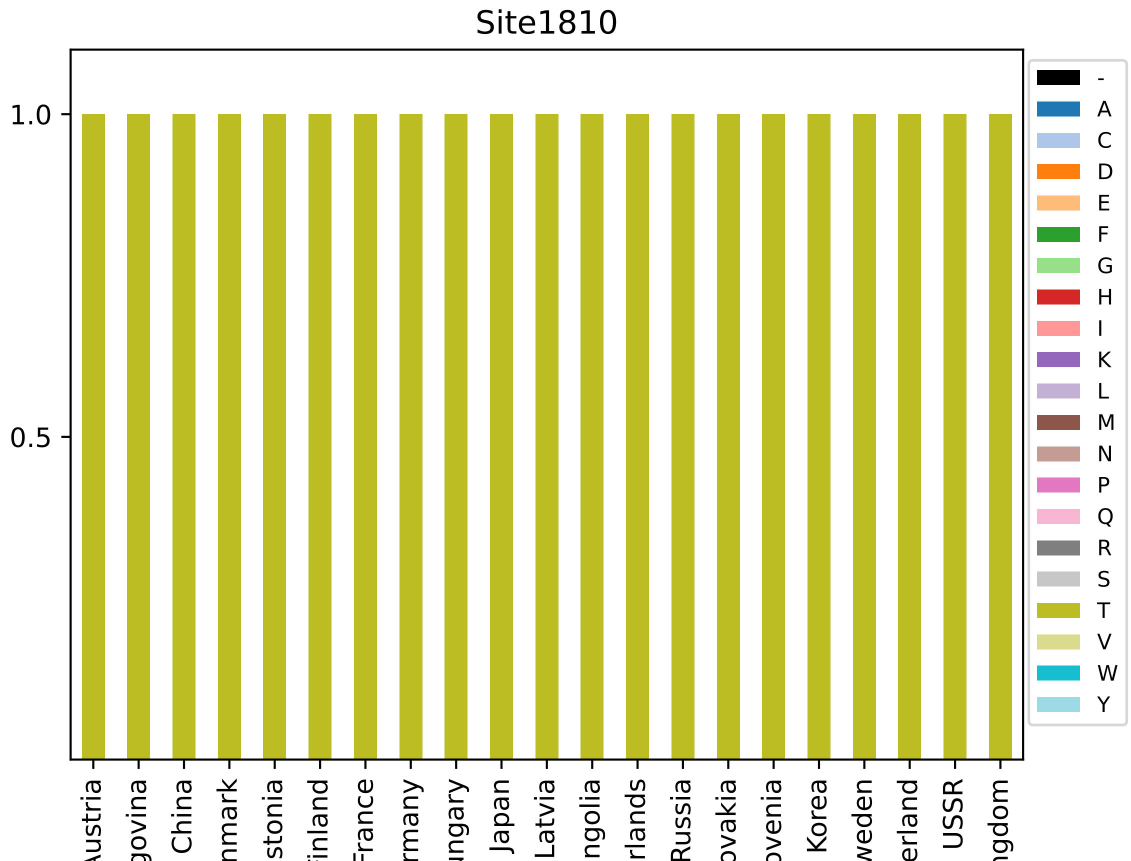

Site1810
1.0
0.5
-
A
C
D
E
F
G
H
I
K
L
M
N
P
Q
R
S
T
V
W
Y
China
Japan
Latvia
Russia
Korea
USSR

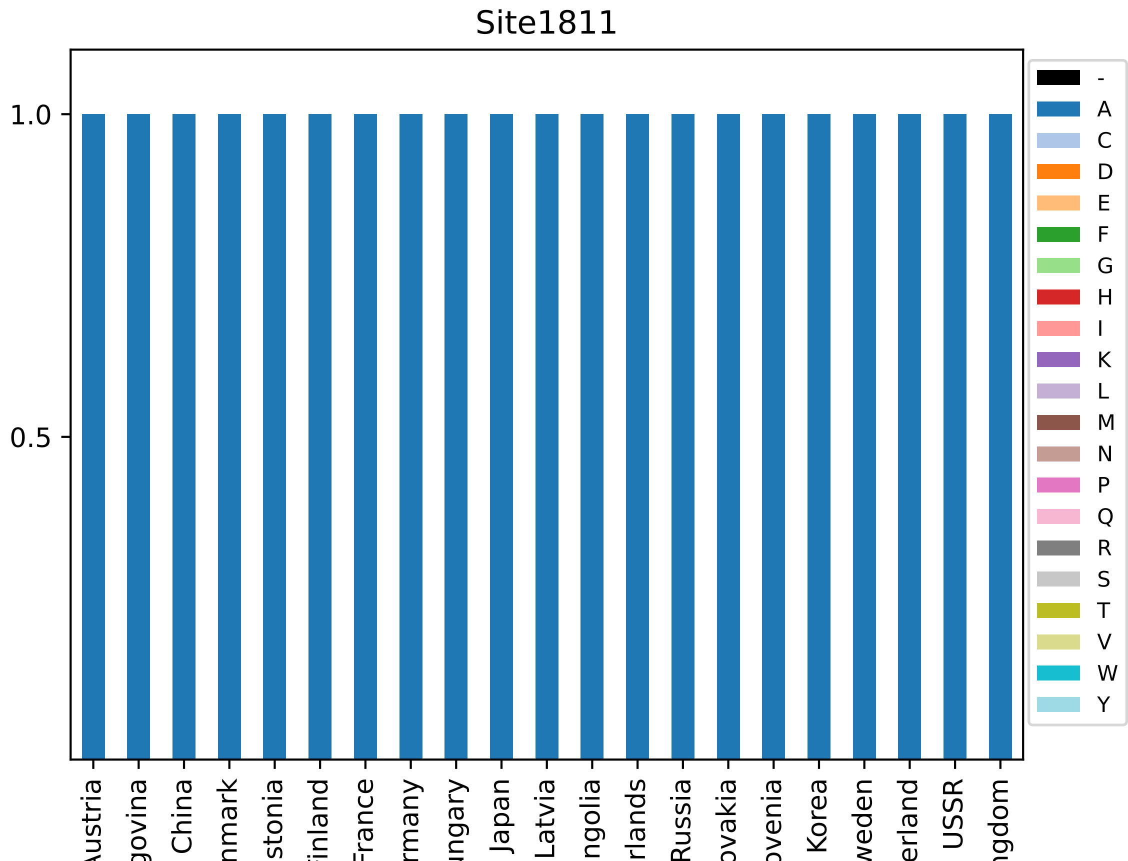
Site1811
1.0
0.5
-
A
C
D
E
F
G
H
I
K
L
M
N
P
Q
R
S
T
V
W
Y
Austria
China
Japan
Latvia
Russia
Korea
USSR

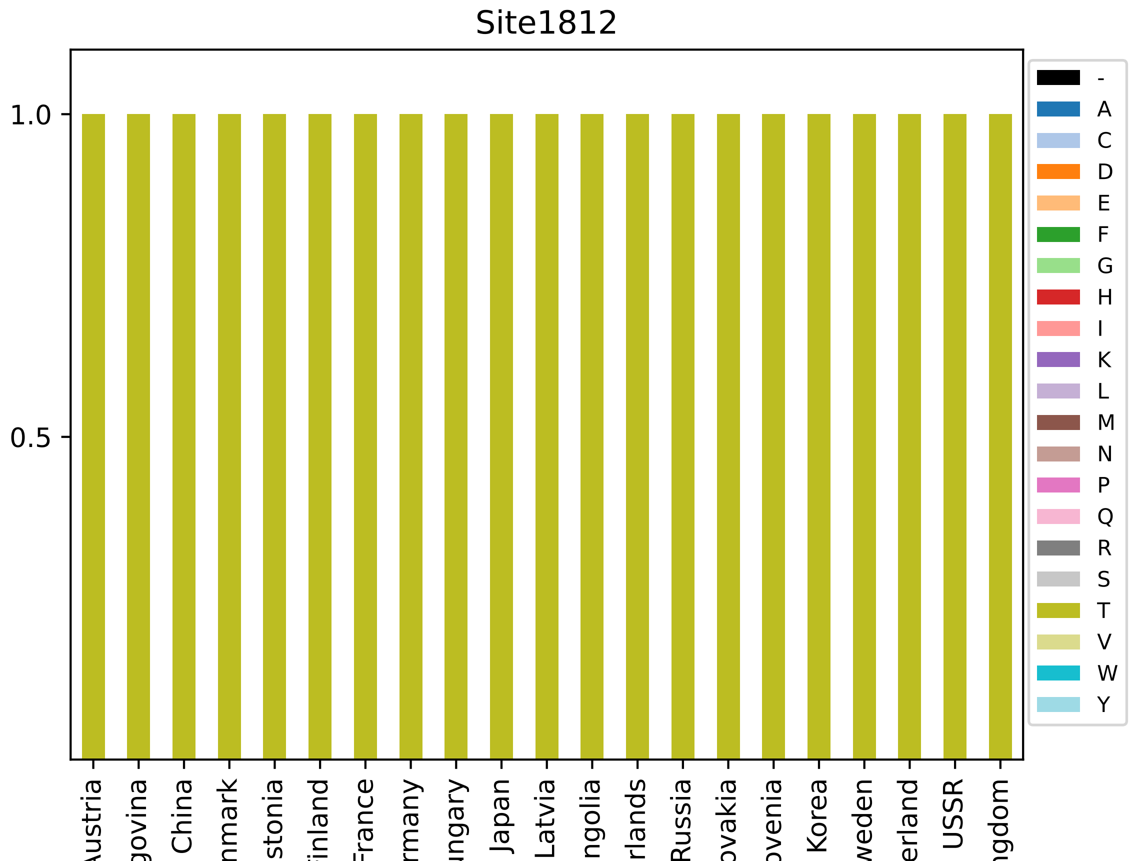

Site1812
1.0
0.5
-
A
C
D
E
F
G
H
I
K
L
M
N
P
Q
R
S
T
V
W
Y
Austria
govina
China
nmark
stonia
Finland
France
rmany
ungary
Japan
Latvia
ngolia
rlands
Russia
ovakia
ovenia
Korea
weden
erland
USSR
ngdom

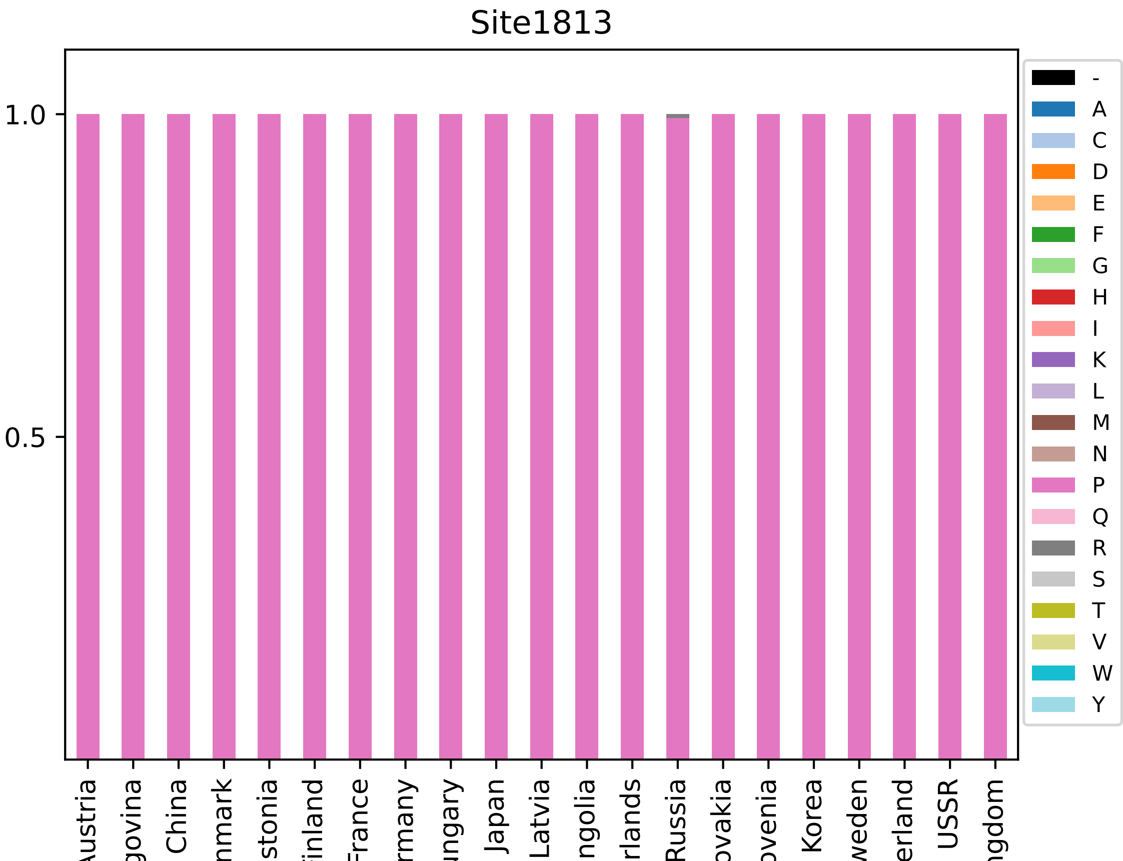

Site1813
1.0
0.5
-
A
C
D
E
F
G
H
I
K
L
M
N
P
Q
R
S
T
V
W
Y
China
Japan
Latvia
Russia
Korea
USSR

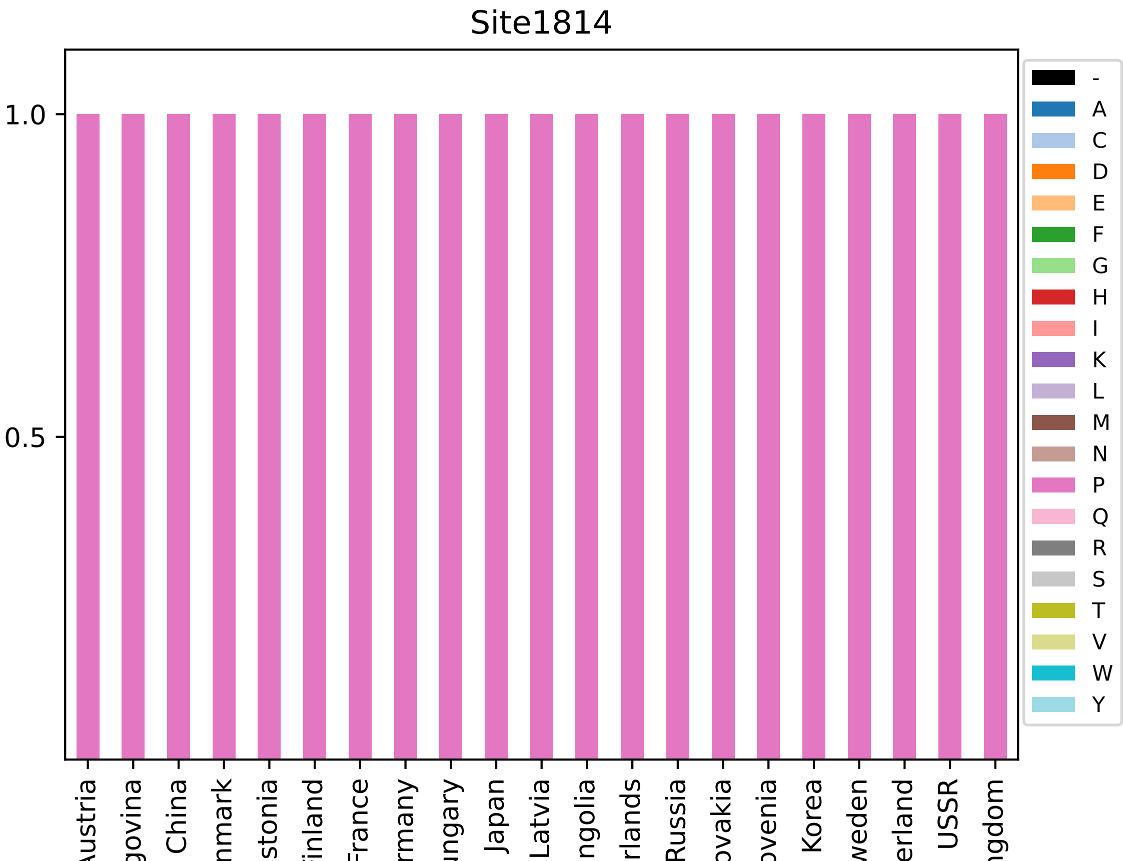
Site1814
1.0
0.5
-
A
C
D
E
F
G
H
I
K
L
M
N
P
Q
R
S
T
V
W
Y
China
Japan
Latvia
Russia
Korea
USSR

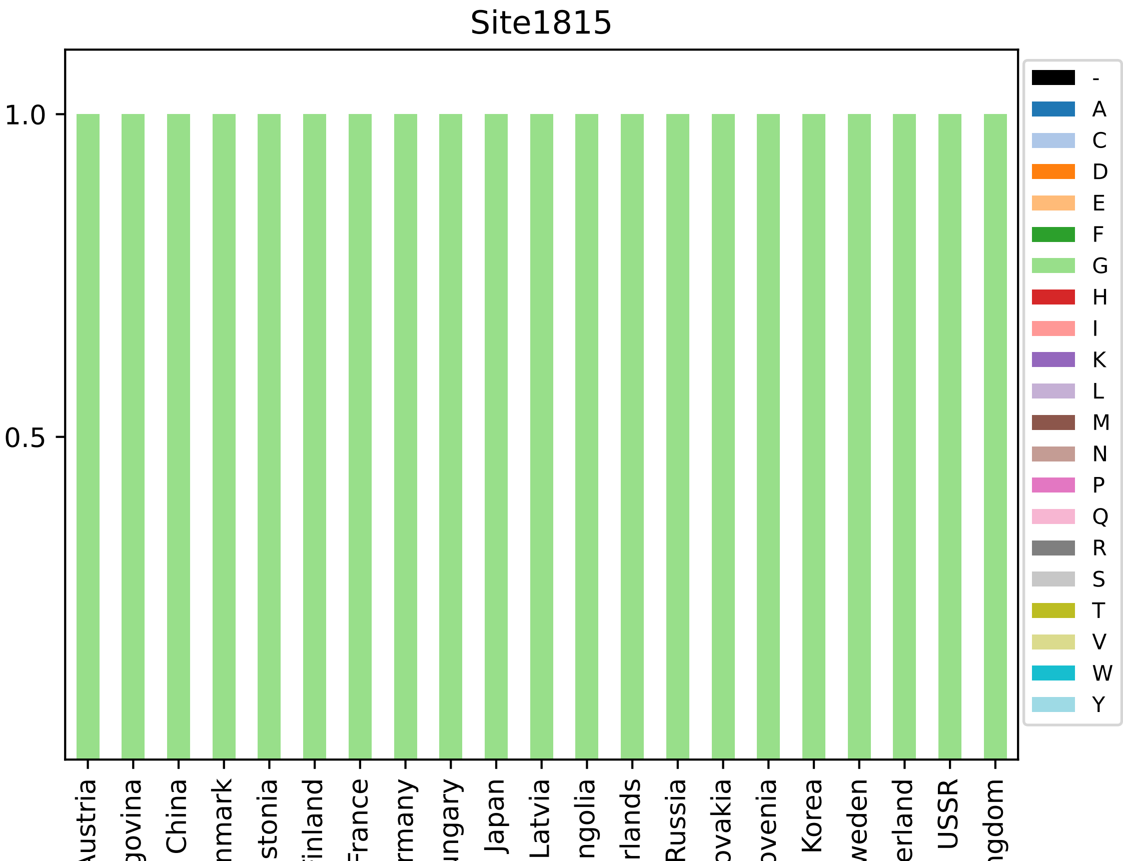
Site1815
1.0
0.5
-
A
C
D
E
F
G
H
I
K
L
M
N
P
Q
R
S
T
V
W
Y
China
Japan
Latvia
Russia
Korea
USSR

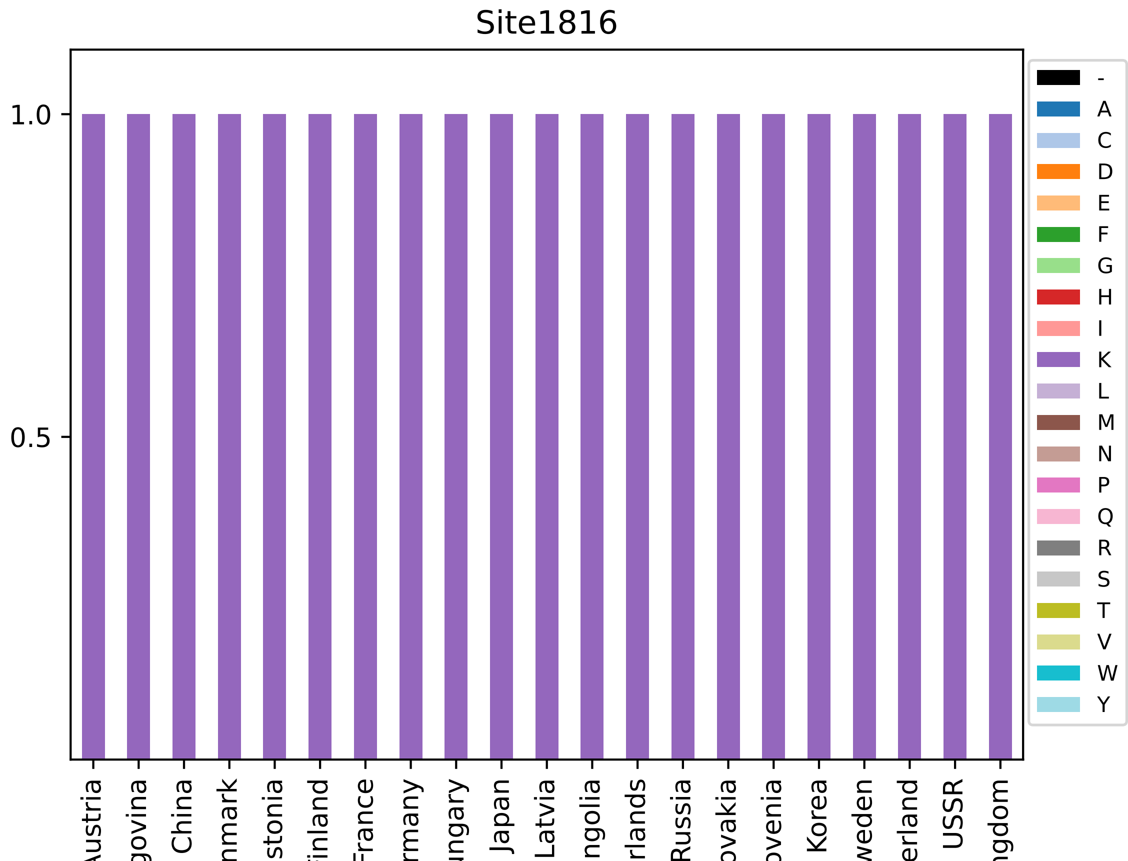

Site1816
1.0
0.5
-
A
C
D
E
F
G
H
I
K
L
M
N
P
Q
R
S
T
V
W
Y
Austria
China
Japan
Latvia
Russia
Korea
USSR

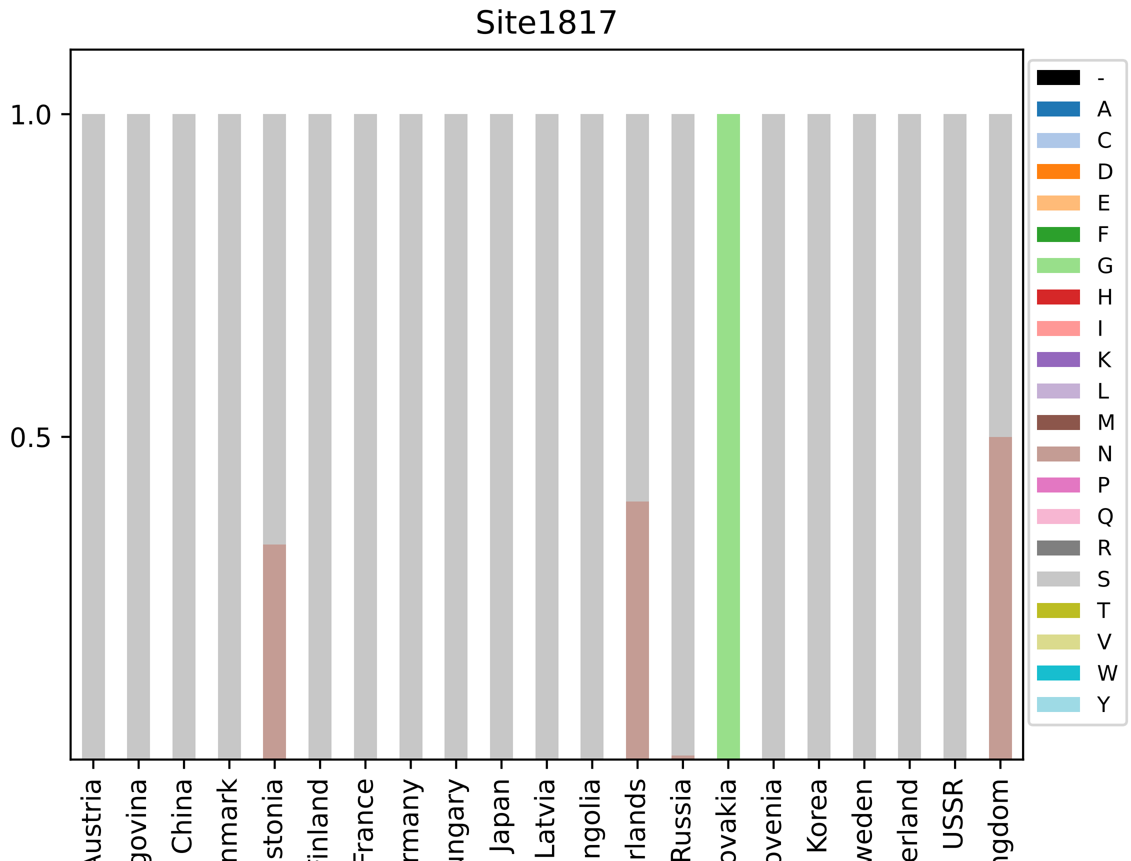

Site1817
1.0
0.5
China
Japan
Latvia
USSR
-
A
C
D
E
F
G
H
I
K
L
M
N
P
Q
R
S
T
V
W
Y

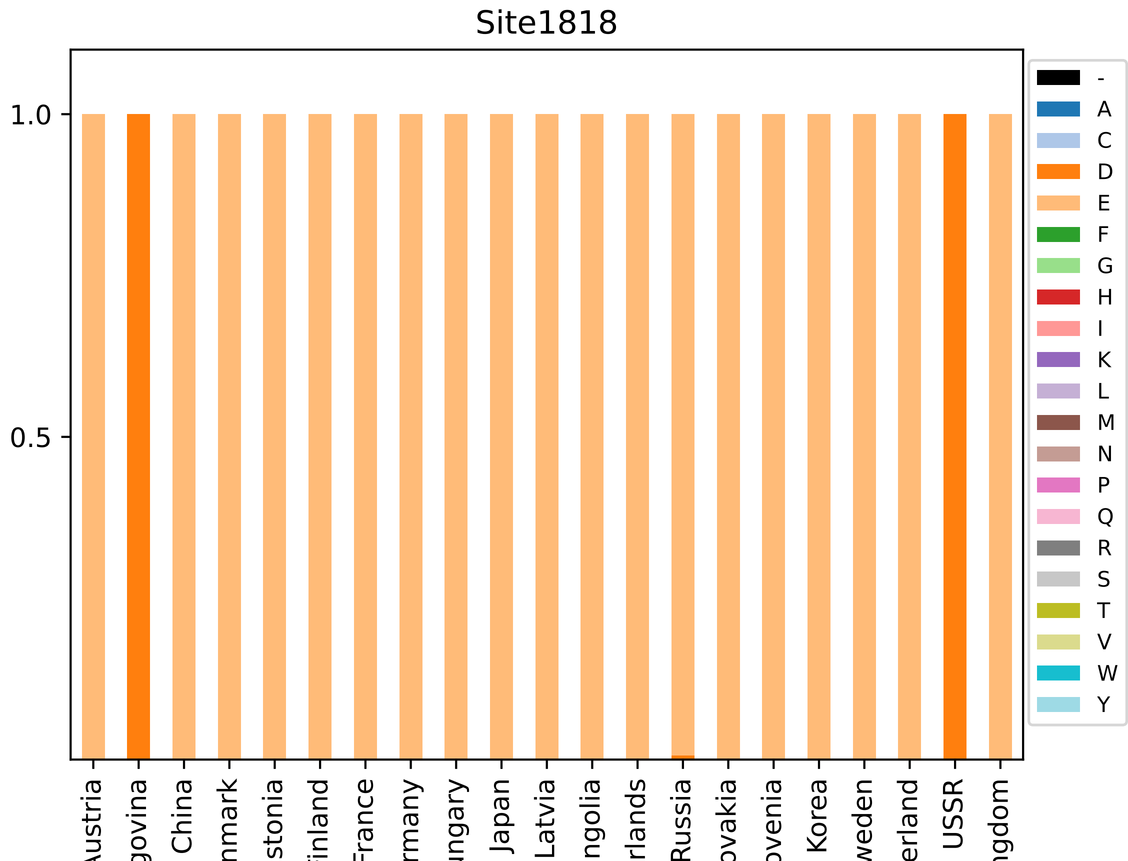

Site1818
1.0
0.5
Austria
govina
China
nmark
stonia
inland
France
rmany
ungary
Japan
Latvia
ngolia
rlands
Russia
ovakia
ovenia
Korea
weden
erland
USSR
ngdom
-
A
C
D
E
F
G
H
I
K
L
M
N
P
Q
R
S
T
V
W
Y

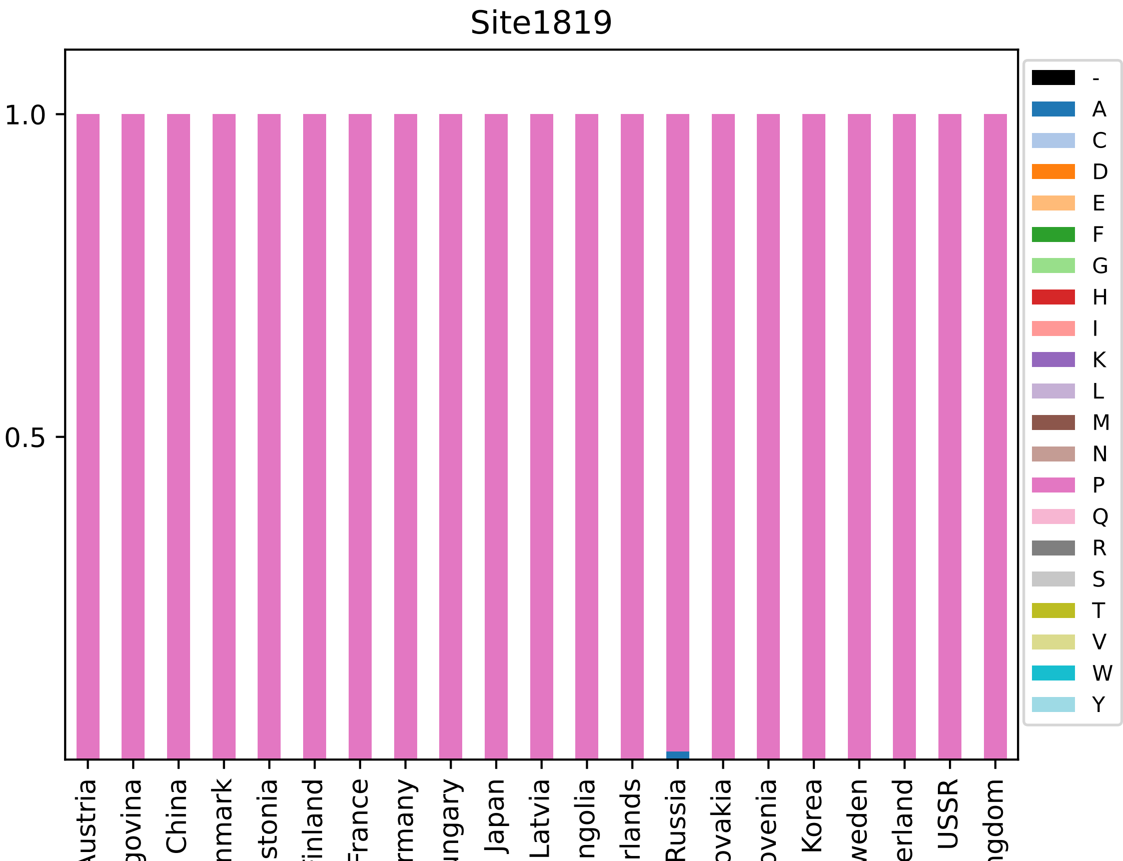
Site1819
1.0
0.5
-
A
C
D
E
F
G
H
I
K
L
M
N
P
Q
R
S
T
V
W
Y
China
Japan
Latvia
Russia
Korea
USSR

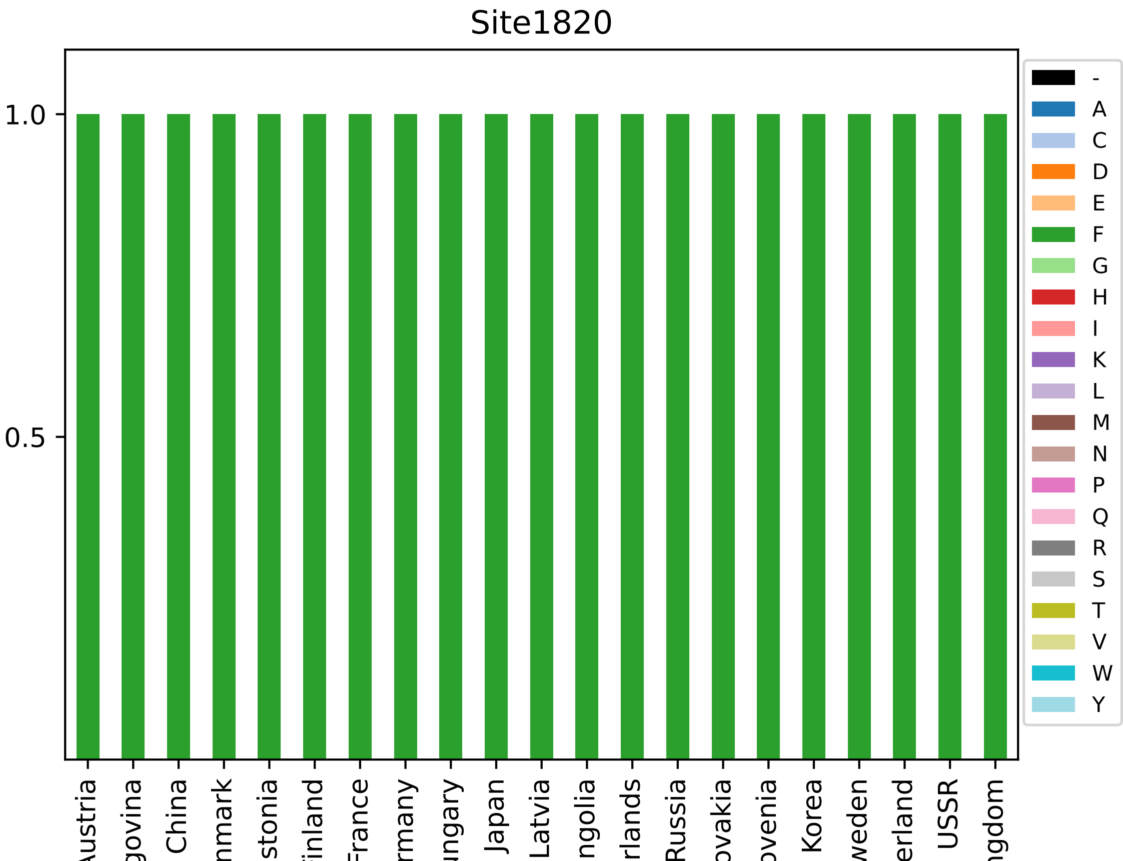
Site1820
1.0
0.5
-
A
C
D
E
F
G
H
I
K
L
M
N
P
Q
R
S
T
V
W
Y
Austria
govina
China
nmark
stonia
Finland
France
rmany
ungary
Japan
Latvia
ngolia
rlands
Russia
ovakia
ovenia
Korea
weden
erland
USSR
ngdom

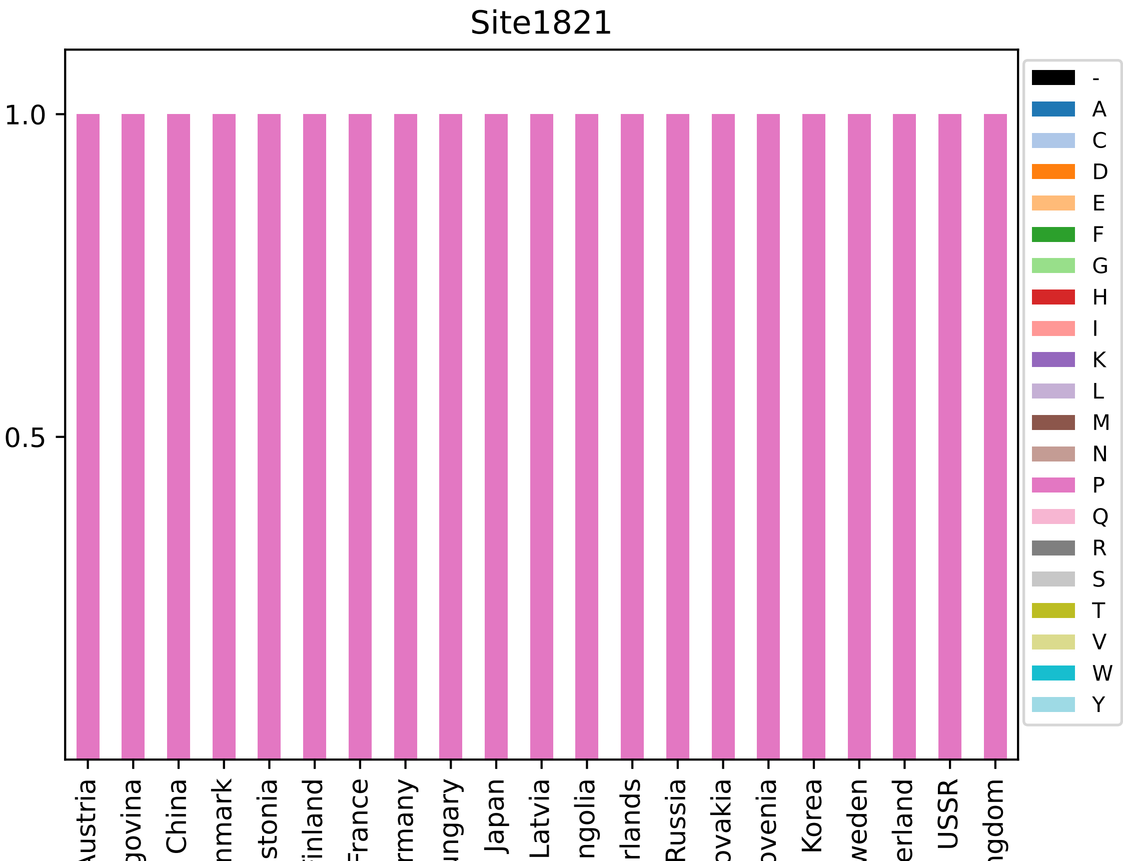

Site1821
1.0
0.5
-
A
C
D
E
F
G
H
I
K
L
M
N
P
Q
R
S
T
V
W
Y
China
Japan
Latvia
Russia
Korea
USSR

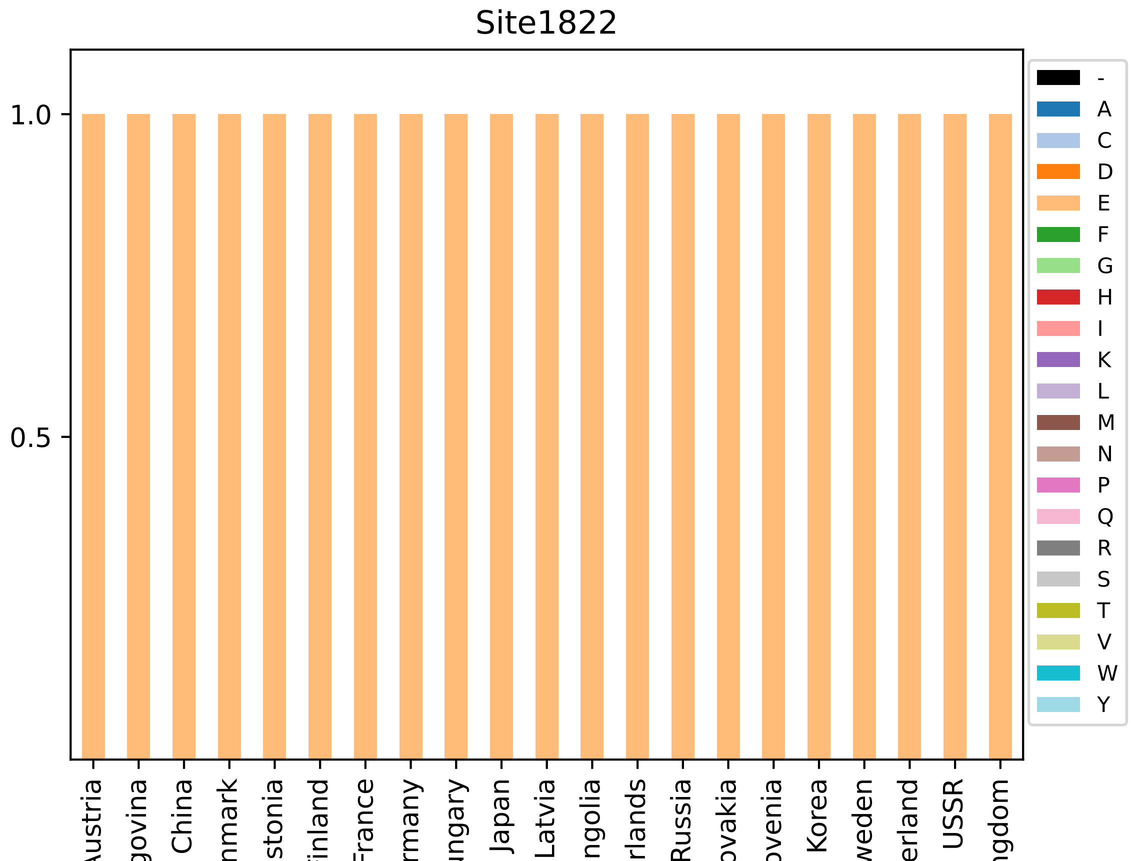
Site1822
1.0
0.5
-
A
C
D
E
F
G
H
I
K
L
M
N
P
Q
R
S
T
V
W
Y
Austria
China
Japan
Latvia
Russia
Korea
USSR

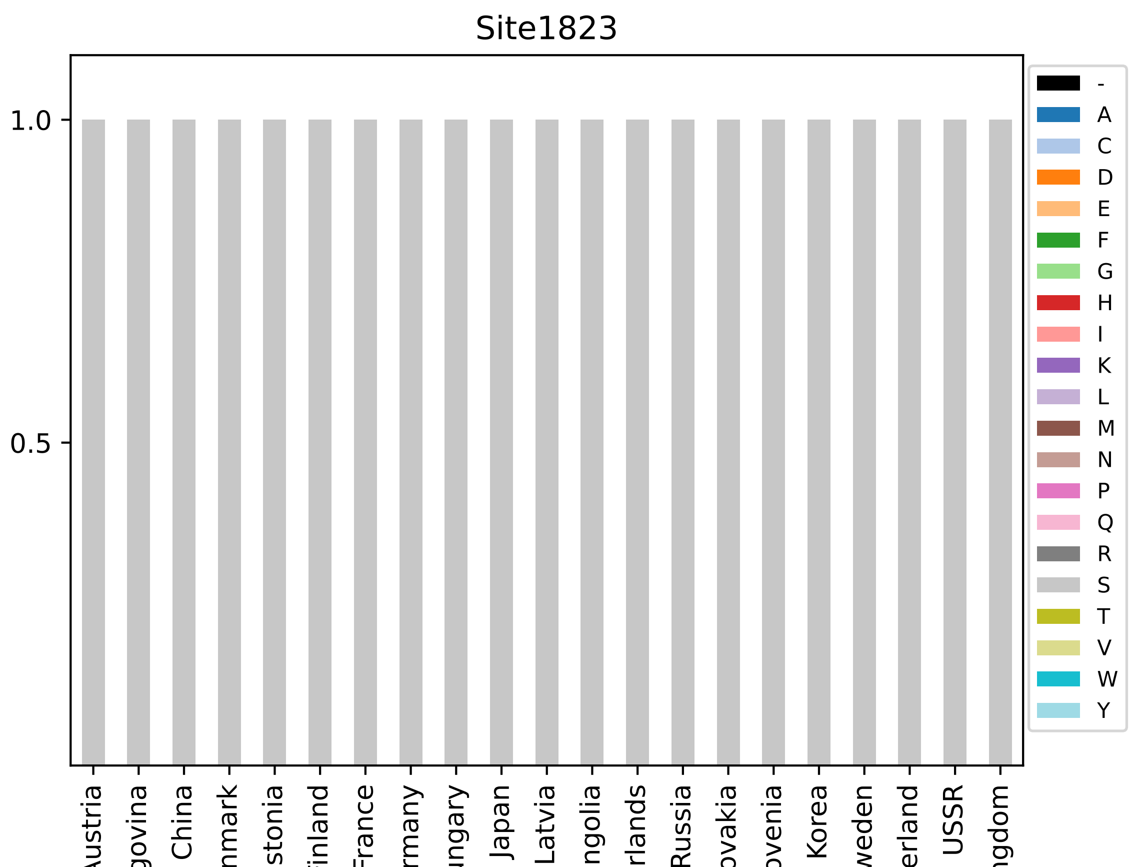
Site1823
1.0
0.5
-
A
C
D
E
F
G
H
I
K
L
M
N
P
Q
R
S
T
V
W
Y
China
Japan
Latvia
Russia
Korea
USSR

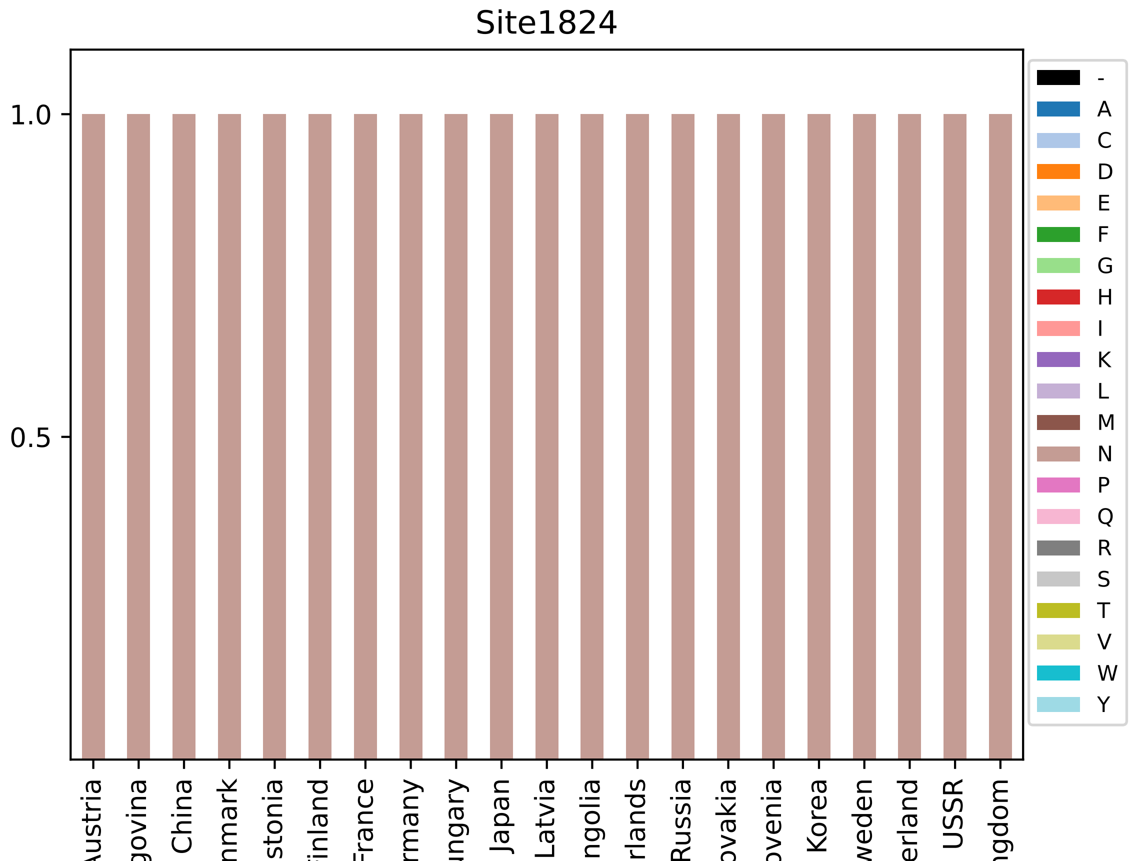

Site1824
1.0
0.5
-
A
C
D
E
F
G
H
I
K
L
M
N
P
Q
R
S
T
V
W
Y
Austria
China
Japan
Latvia
Russia
Korea
USSR

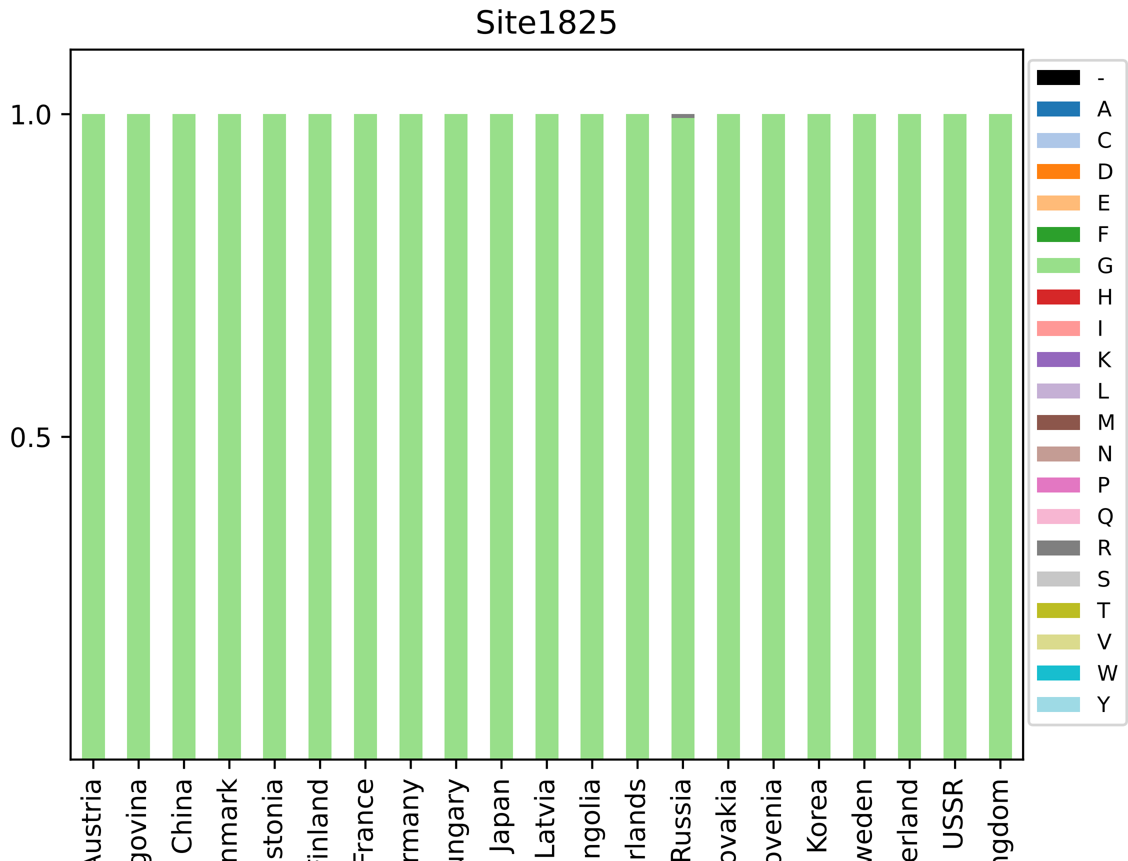

Site1825
1.0
0.5
-
A
C
D
E
F
G
H
I
K
L
M
N
P
Q
R
S
T
V
W
Y
Austria
govina
China
nmark
stonia
Finland
France
rmany
ungary
Japan
Latvia
ngolia
rlands
Russia
ovakia
ovenia
Korea
weden
erland
USSR
ngdom

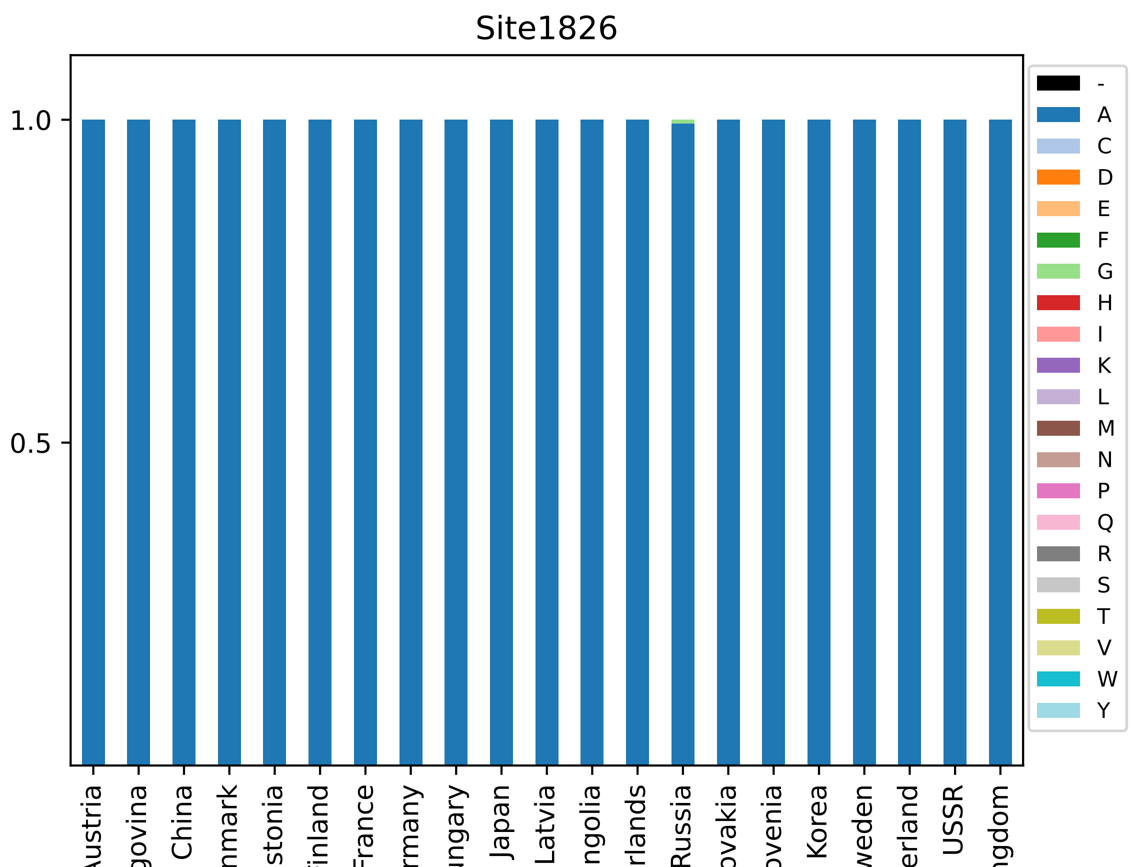
Site1826
1.0
0.5
-
A
C
D
E
F
G
H
I
K
L
M
N
P
Q
R
S
T
V
W
Y
China
Japan
Latvia
Russia
Korea
USSR

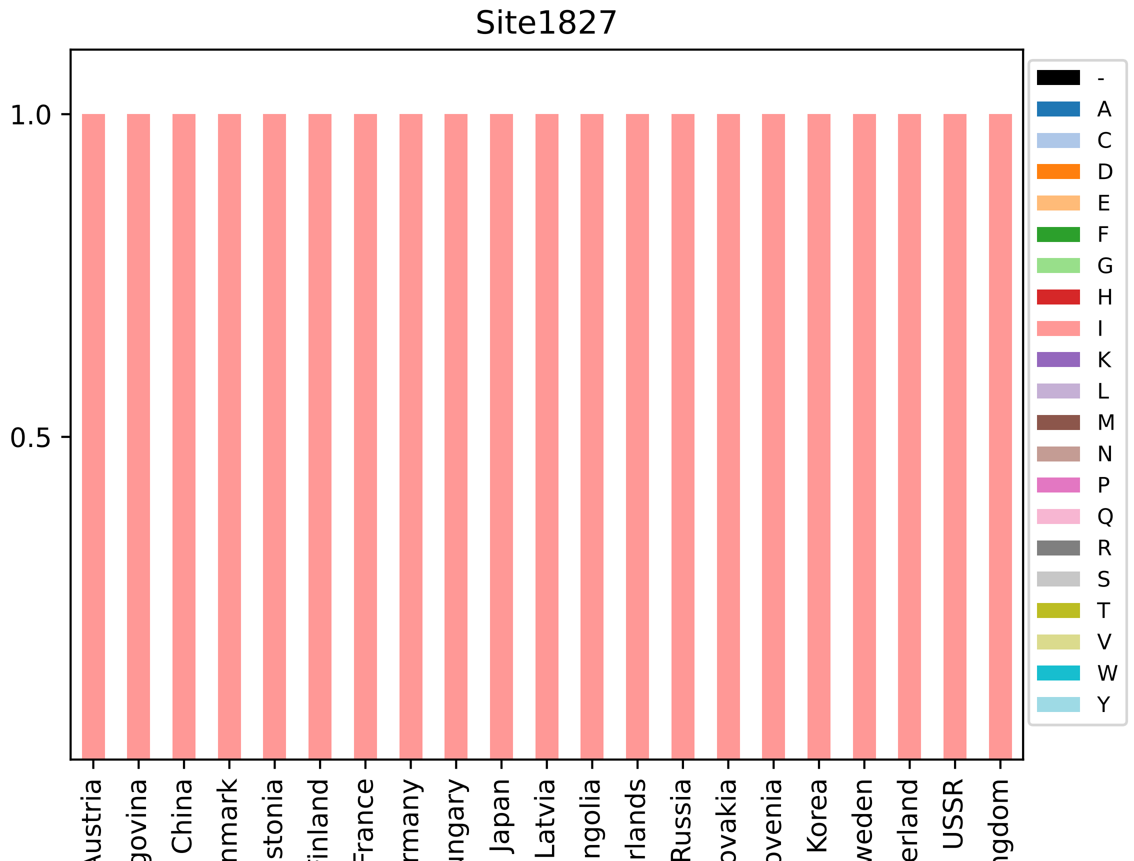

Site1827
1.0
0.5
-
A
C
D
E
F
G
H
I
K
L
M
N
P
Q
R
S
T
V
W
Y
China
Japan
Latvia
Russia
Korea
USSR

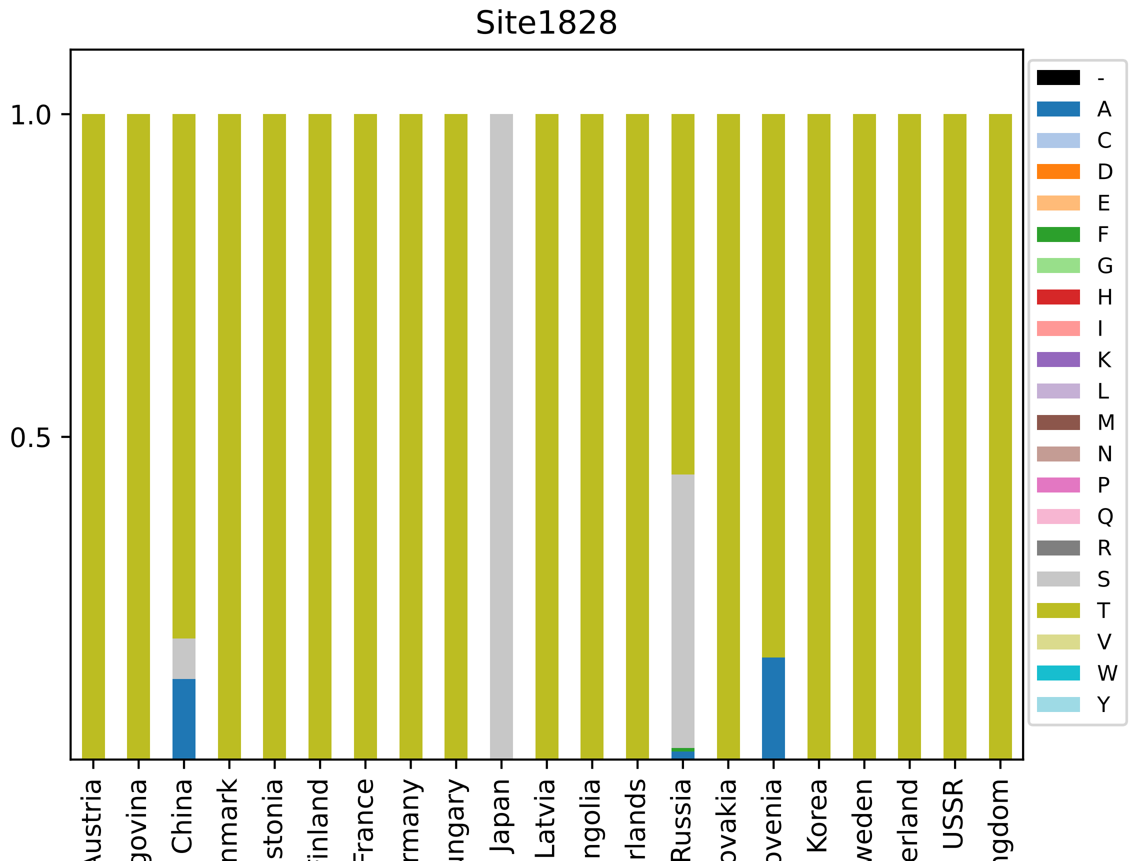

Site1828
1.0
0.5
-
A
C
D
E
F
G
H
I
K
L
M
N
P
Q
R
S
T
V
W
Y
Austria
govina
China
nmark
stonia
inland
France
rmany
ungary
Japan
Latvia
ngolia
rlands
Russia
ovakia
ovenia
Korea
weden
erland
USSR
ngdom

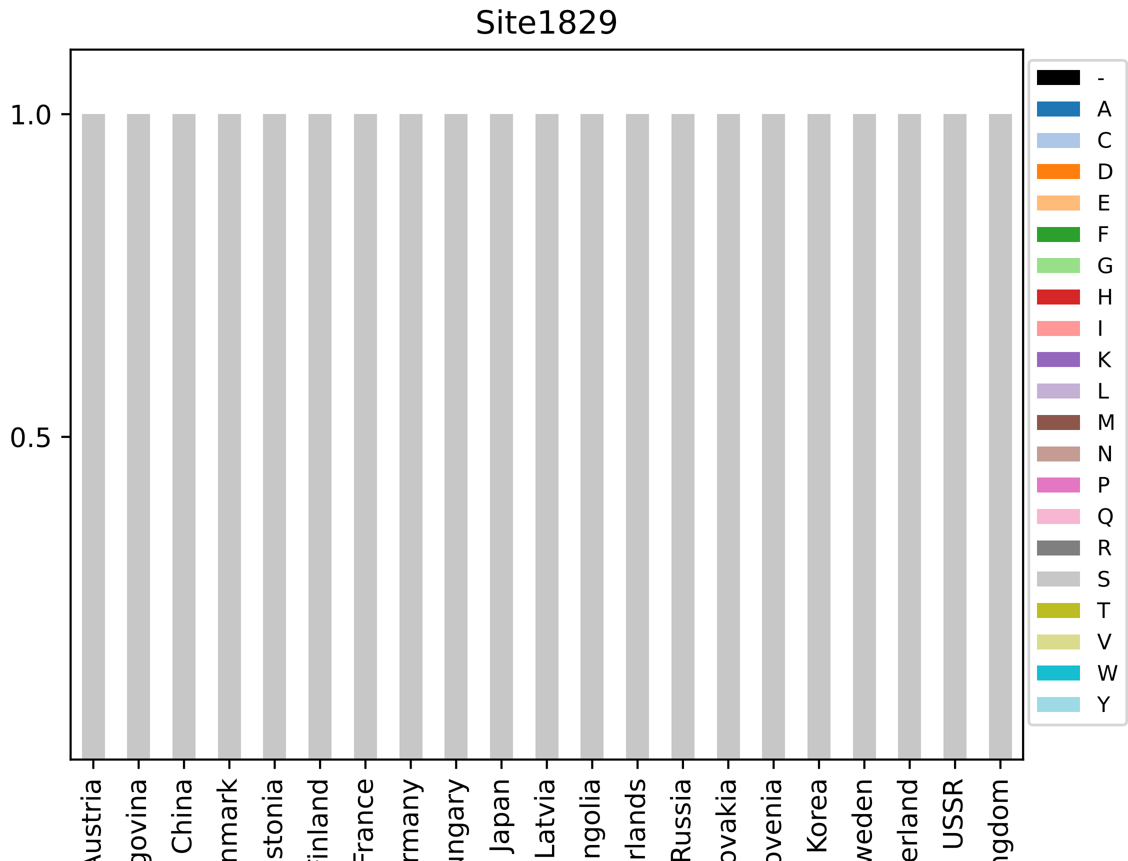
Site1829
1.0
0.5
-
A
C
D
E
F
G
H
I
K
L
M
N
P
Q
R
S
T
V
W
Y
Austria
China
Japan
Latvia
Russia
Korea
USSR

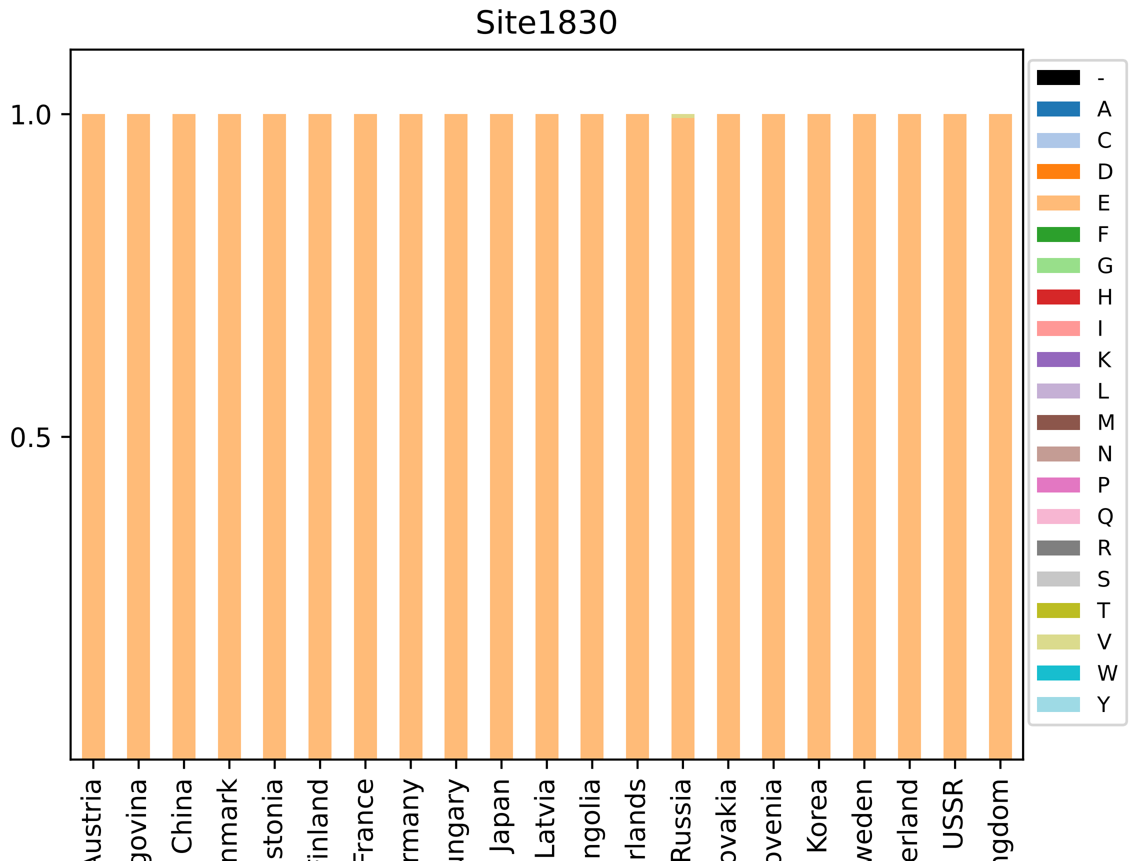
Site1830
1.0
0.5
-
A
C
D
E
F
G
H
I
K
L
M
N
P
Q
R
S
T
V
W
Y
Austria
China
Japan
Latvia
Russia
Korea
USSR

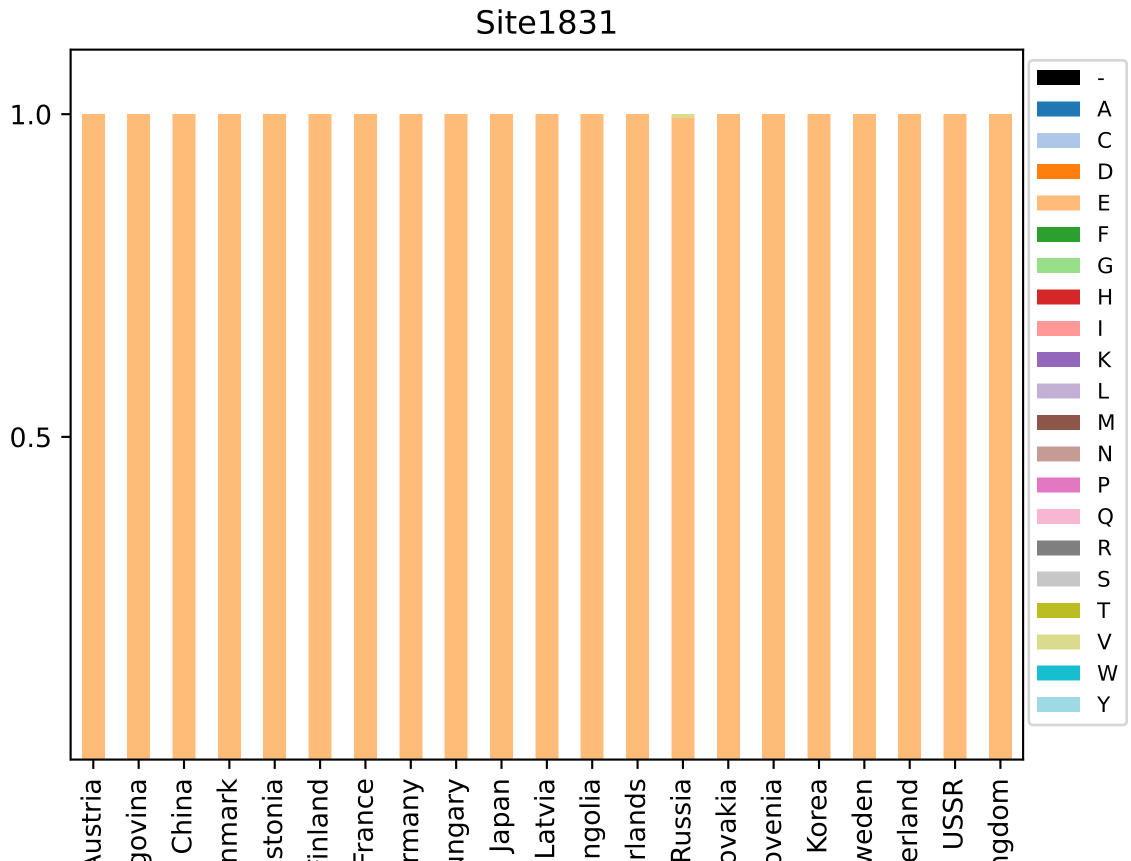
Site1831
1.0
0.5
-
A
C
D
E
F
G
H
I
K
L
M
N
P
Q
R
S
T
V
W
Y
China
Japan
Latvia
Russia
Korea
USSR

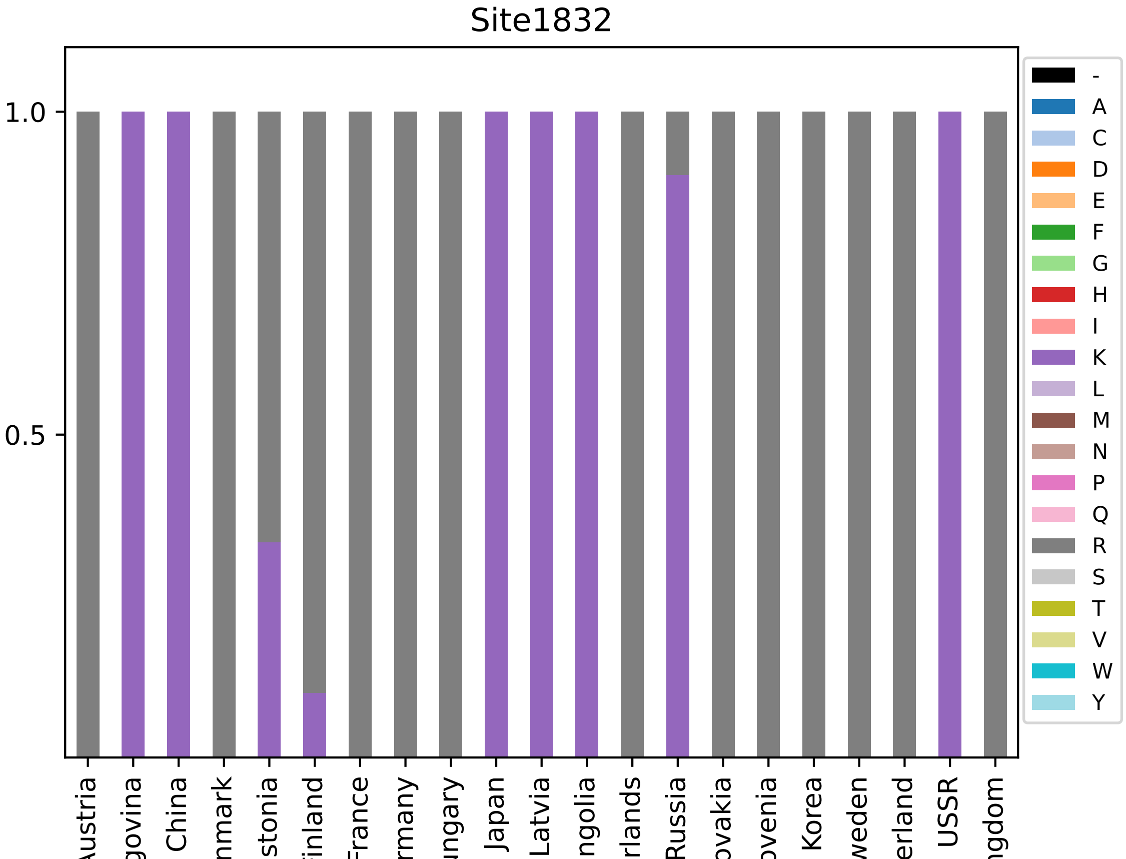

Site1832
1.0
0.5
-
A
C
D
E
F
G
H
I
K
L
M
N
P
Q
R
S
T
V
W
Y
China
Japan
Latvia
Russia
Korea
USSR

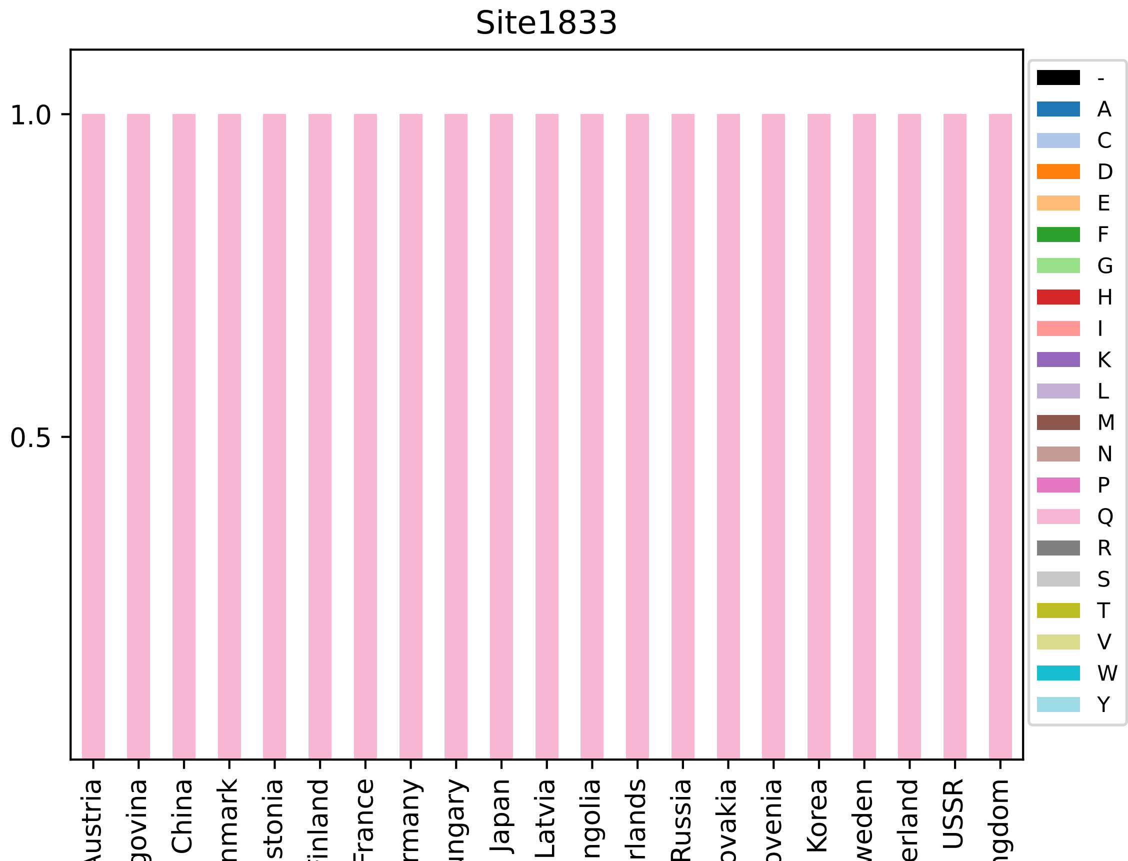

Site1833
1.0
0.5
-
A
C
D
E
F
G
H
I
K
L
M
N
P
Q
R
S
T
V
W
Y
China
Japan
Latvia
Russia
Korea
USSR

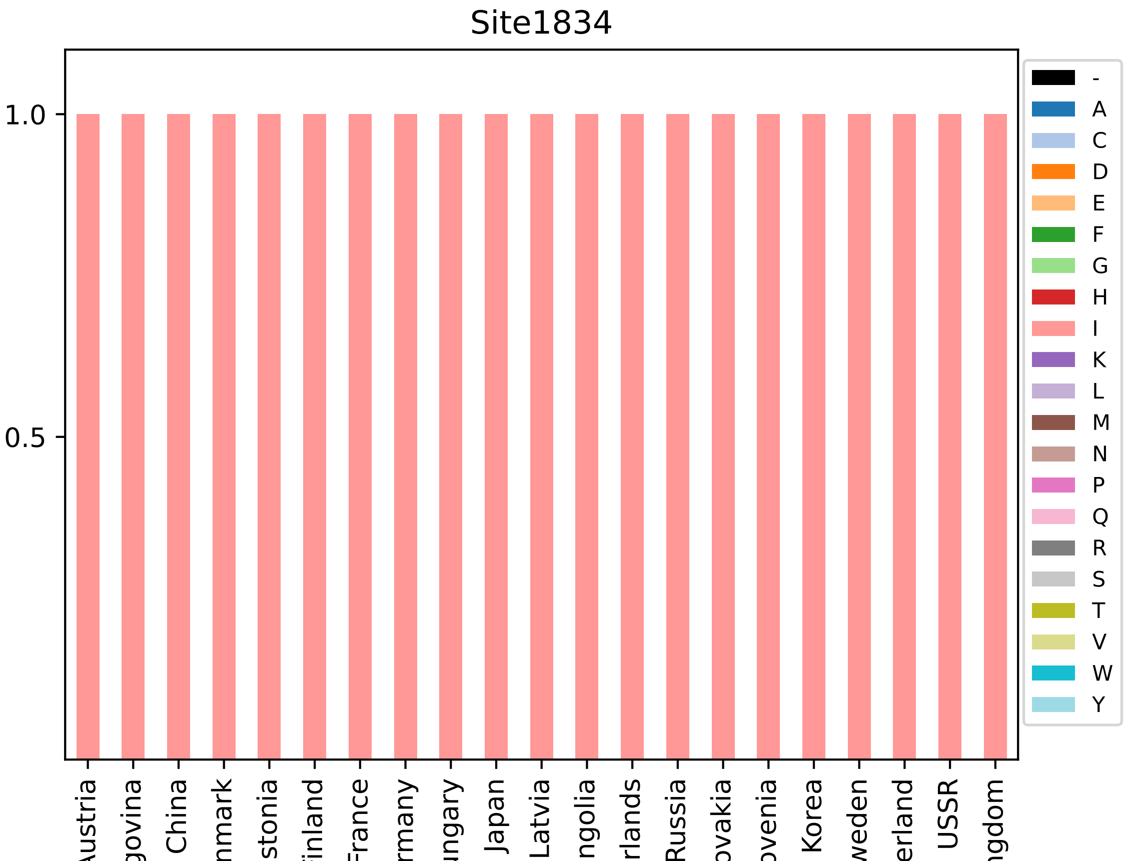
Site1834
1.0
0.5
-
A
C
D
E
F
G
H
I
K
L
M
N
P
Q
R
S
T
V
W
Y
China
Japan
Latvia
Russia
Korea
USSR

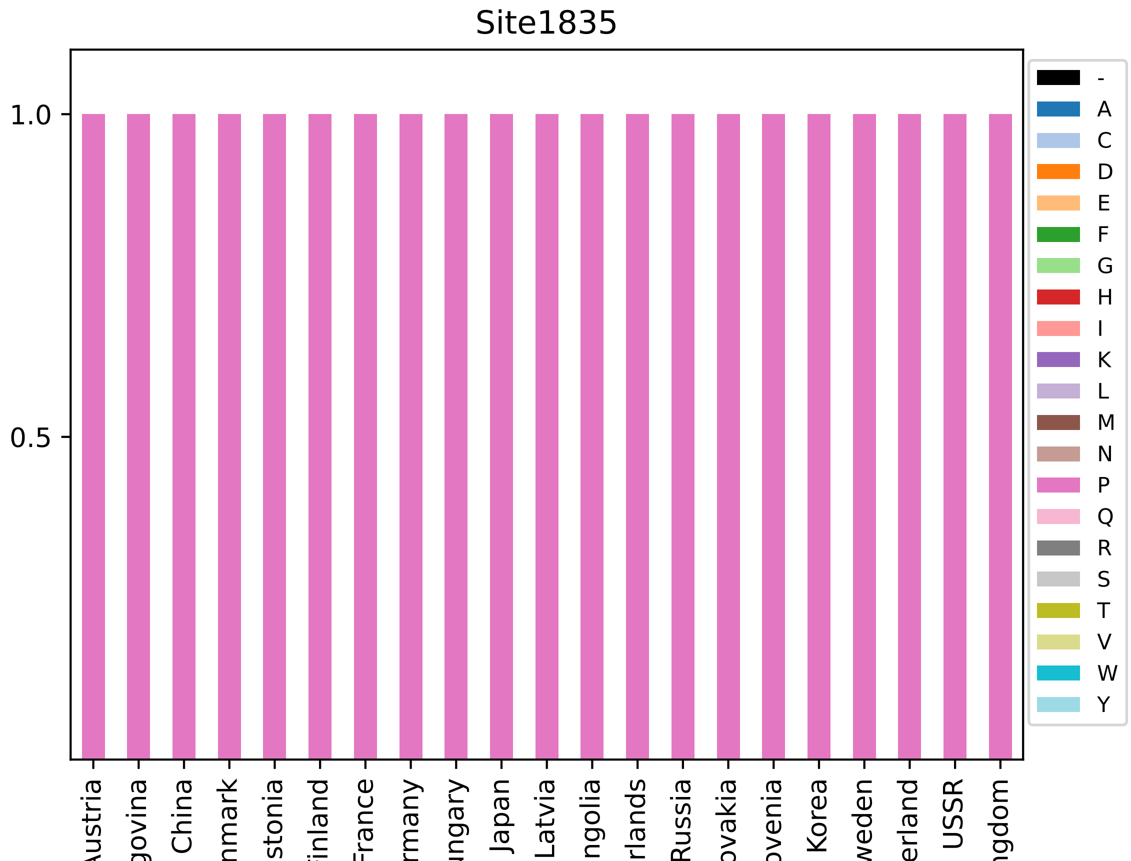

Site1835
1.0
0.5
-
A
C
D
E
F
G
H
I
K
L
M
N
P
Q
R
S
T
V
W
Y
China
Japan
Latvia
Russia
Korea
USSR

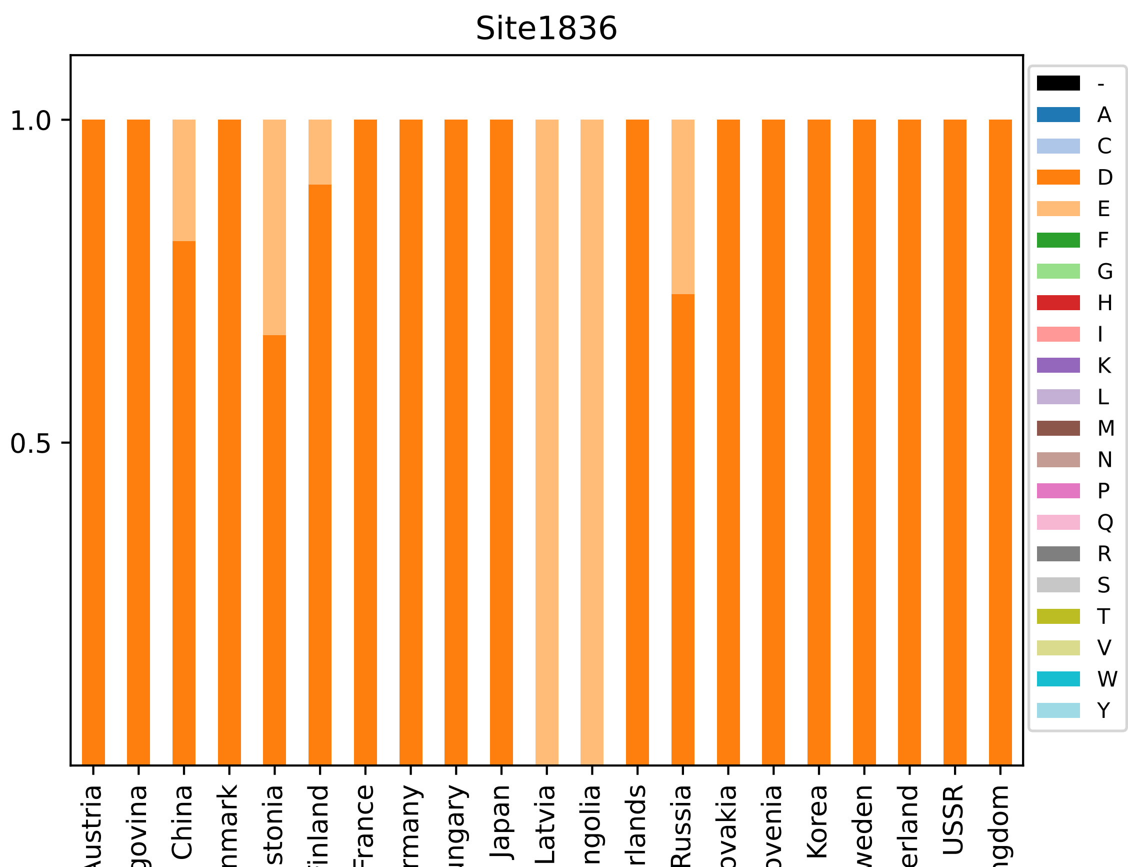
Site1836
1.0
0.5
-
A
C
D
E
F
G
H
I
K
L
M
N
P
Q
R
S
T
V
W
Y
China
Japan
Latvia
Russia
Korea
USSR

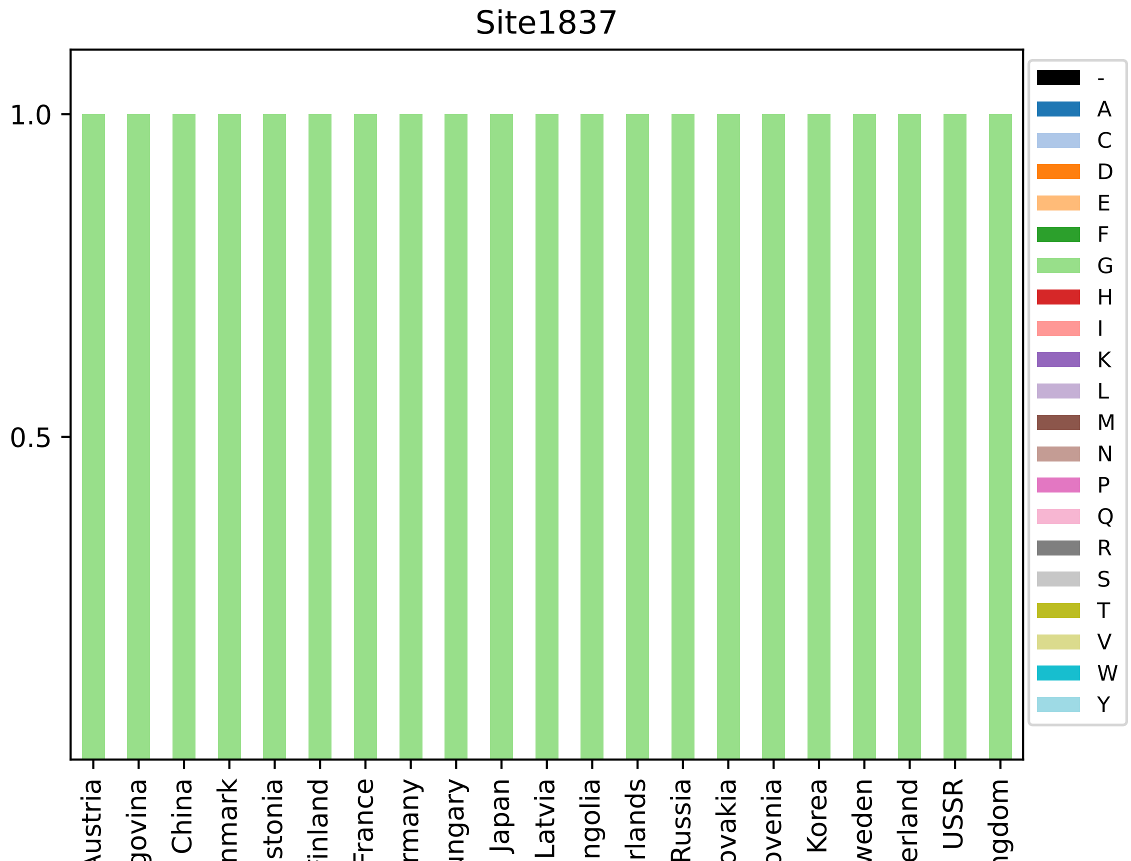
Site1837
1.0
0.5
-
A
C
D
E
F
G
H
I
K
L
M
N
P
Q
R
S
T
V
W
Y
Austria
govina
China
nmark
stonia
Finland
France
rmany
ungary
Japan
Latvia
ngolia
rlands
Russia
ovakia
ovenia
Korea
weden
erland
USSR
ngdom

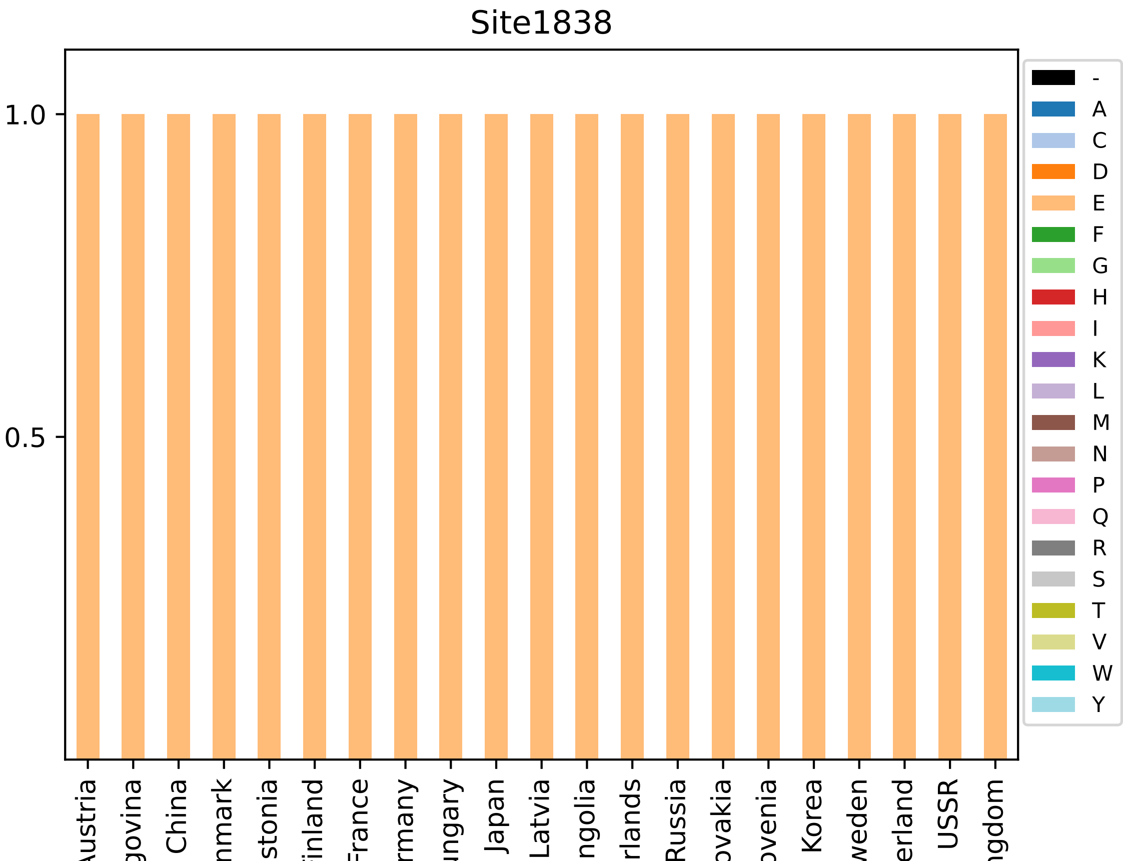
Site1838
1.0
0.5
-
A
C
D
E
F
G
H
I
K
L
M
N
P
Q
R
S
T
V
W
Y
Austria
govina
China
nmark
stonia
inland
France
rmany
ungary
Japan
Latvia
ngolia
rlands
Russia
ovakia
ovenia
Korea
weden
erland
USSR
ngdom

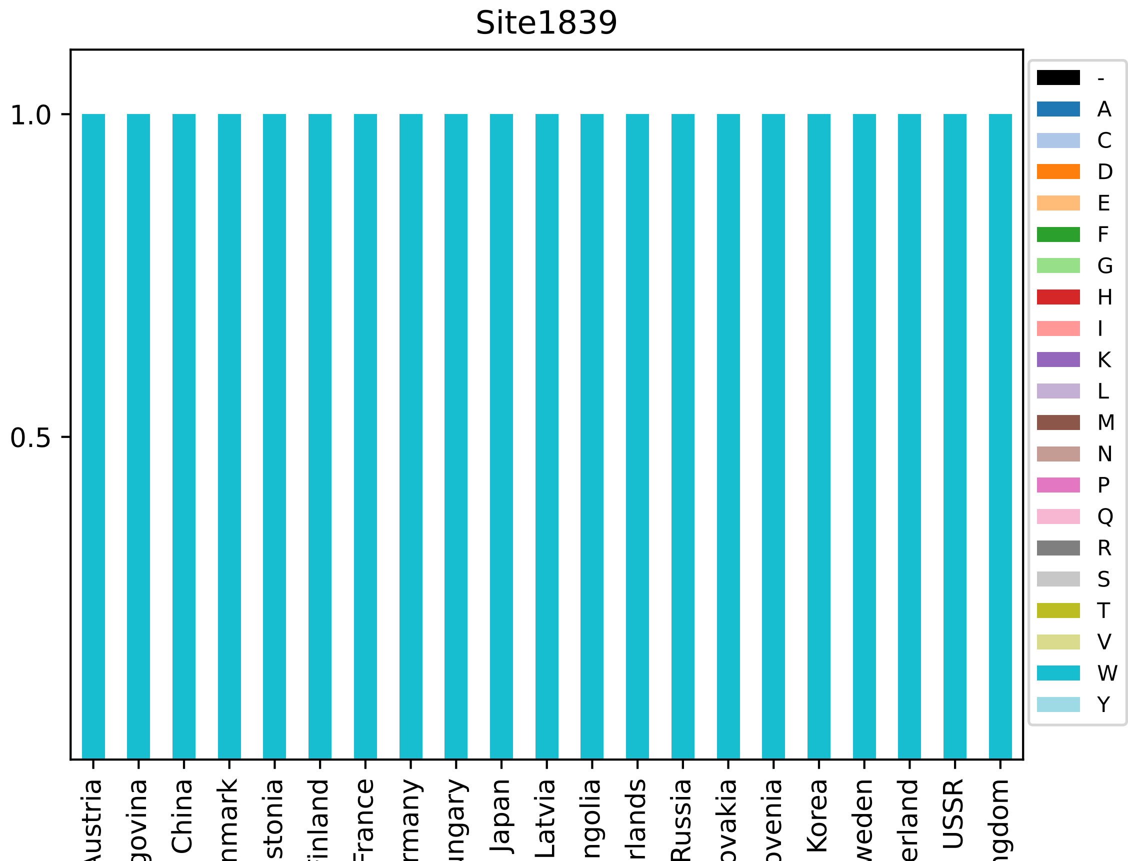
Site1839
1.0
0.5
-
A
C
D
E
F
G
H
I
K
L
M
N
P
Q
R
S
T
V
W
Y
China
Japan
Latvia
Russia
Korea
USSR

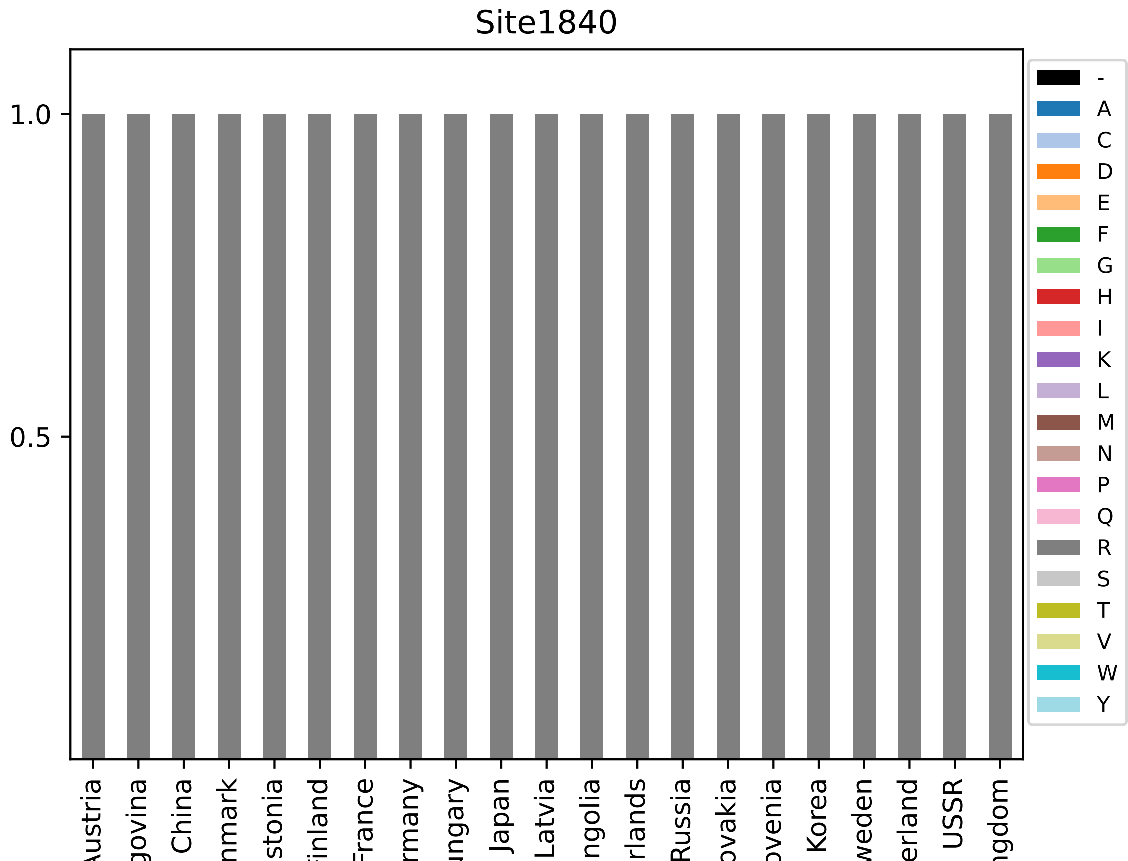

Site1840
1.0
0.5
-
A
C
D
E
F
G
H
I
K
L
M
N
P
Q
R
S
T
V
W
Y
Austria
China
Japan
Latvia
Russia
Korea
USSR

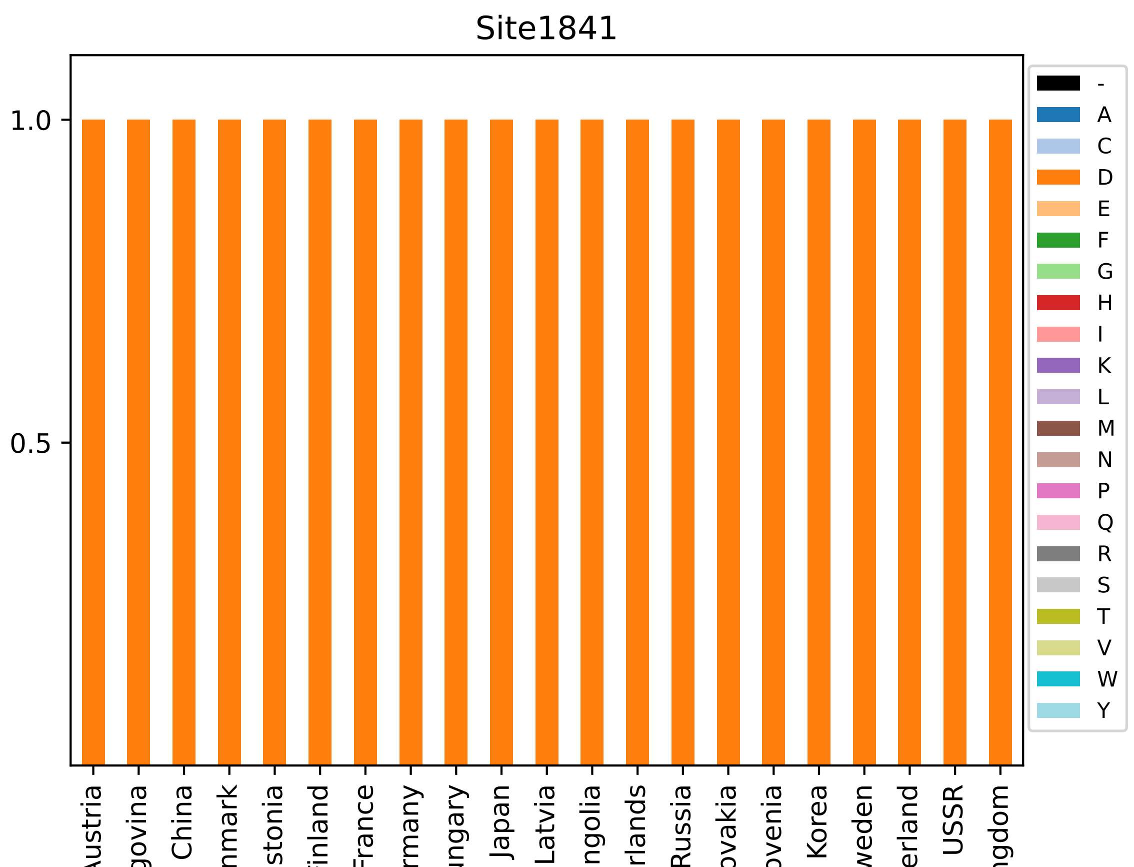
Site1841
1.0
0.5
-
A
C
D
E
F
G
H
I
K
L
M
N
P
Q
R
S
T
V
W
Y
Austria
govina
China
nmark
stonia
inland
France
rmany
ungary
Japan
Latvia
ngolia
rlands
Russia
ovakia
ovenia
Korea
weden
erland
USSR
ngdom

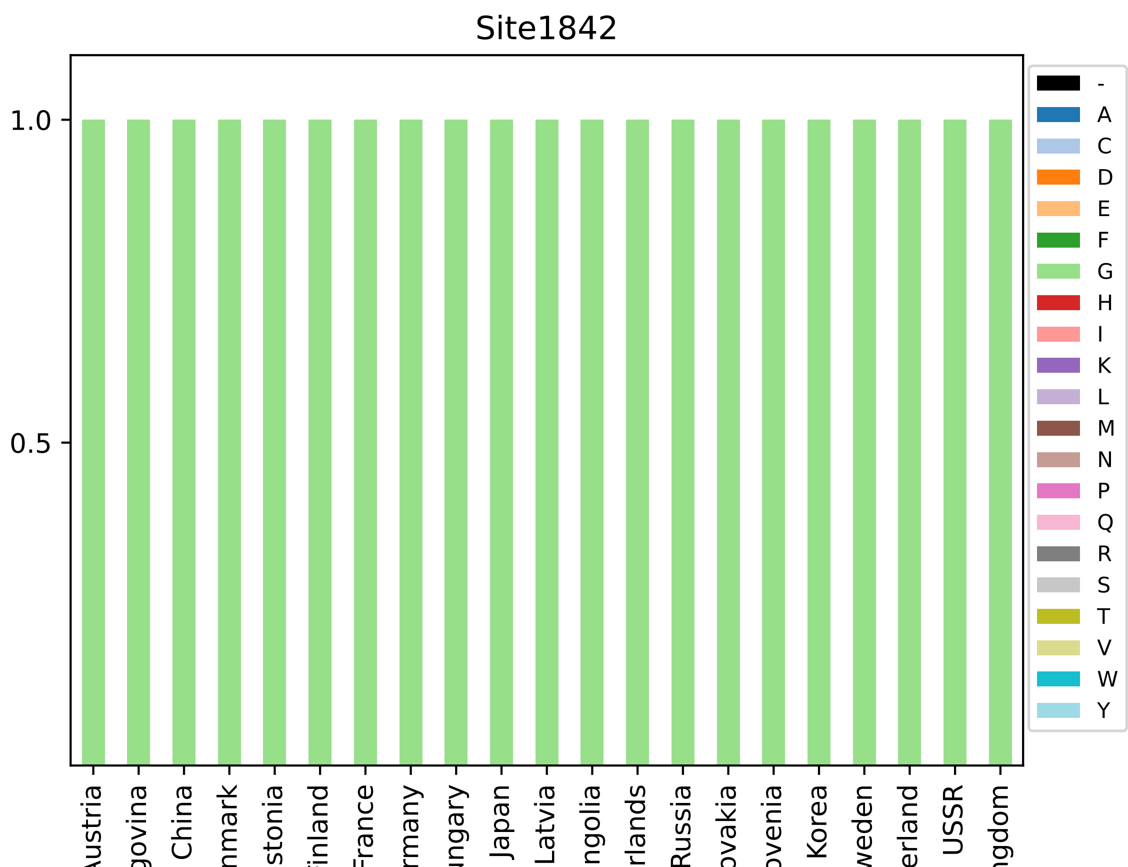
Site1842
1.0
0.5
-
A
C
D
E
F
G
H
I
K
L
M
N
P
Q
R
S
T
V
W
Y
China
Japan
Latvia
Russia
Korea
USSR

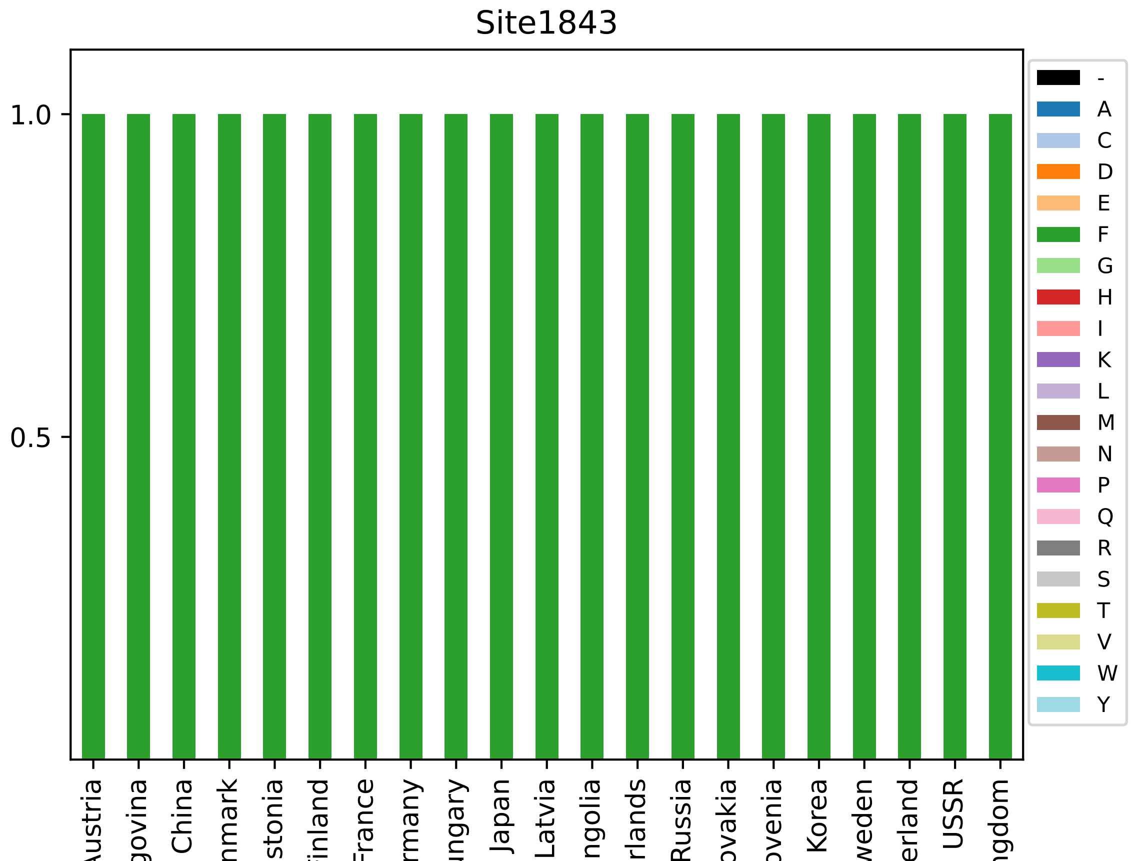
Site1843
1.0
0.5
-
A
C
D
E
F
G
H
I
K
L
M
N
P
Q
R
S
T
V
W
Y
Austria
govina
China
nmark
stonia
inland
France
rmany
ungary
Japan
Latvia
ngolia
rlands
Russia
ovakia
ovenia
Korea
weden
erland
USSR
ngdom

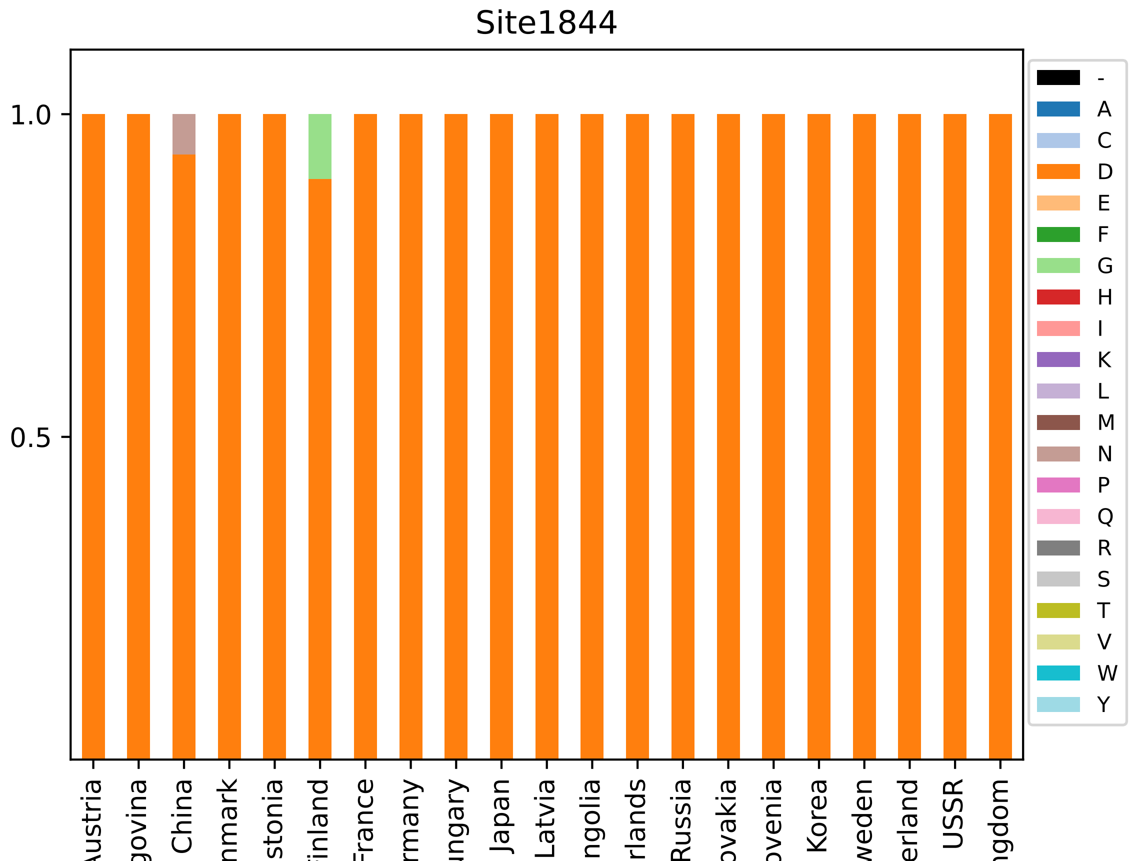

Site1844
1.0
0.5
-
A
C
D
E
F
G
H
I
K
L
M
N
P
Q
R
S
T
V
W
Y
China
Japan
Latvia
Russia
Korea
USSR

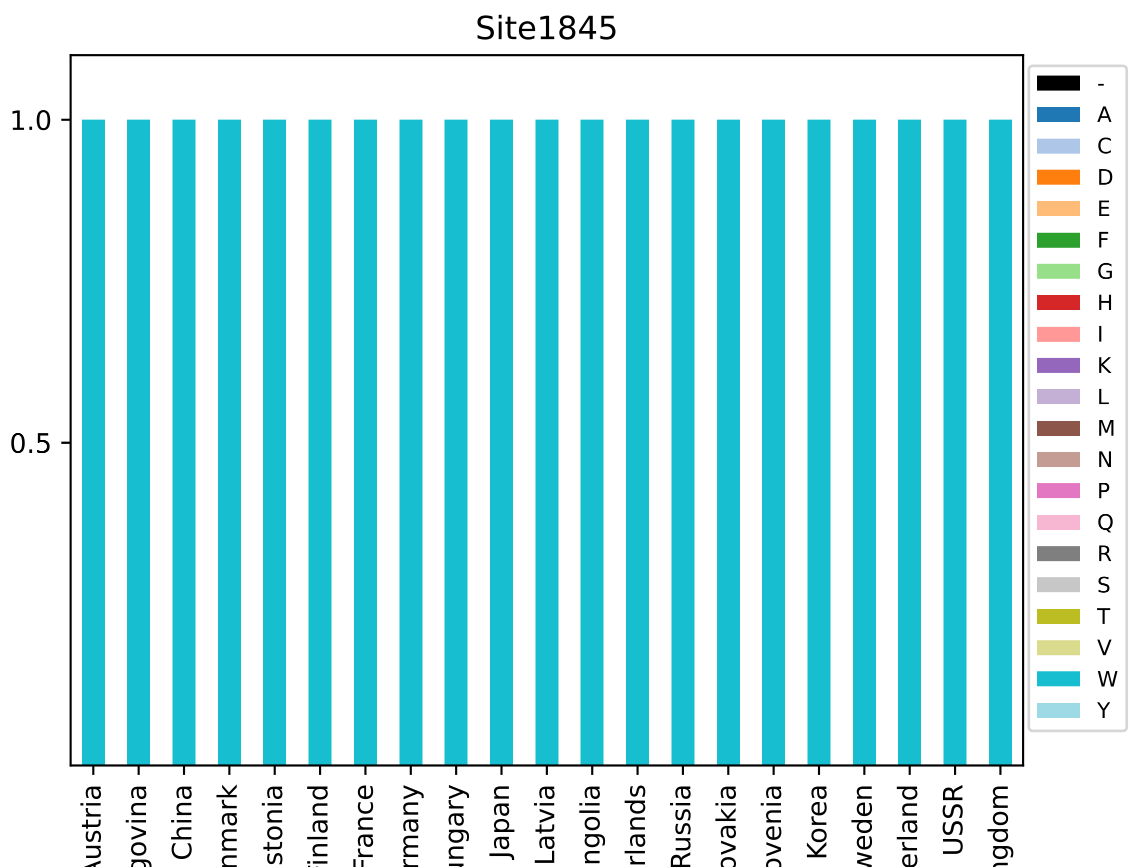
Site1845
1.0
0.5
-
A
C
D
E
F
G
H
I
K
L
M
N
P
Q
R
S
T
V
W
Y
China
Japan
Latvia
Russia
Korea
USSR

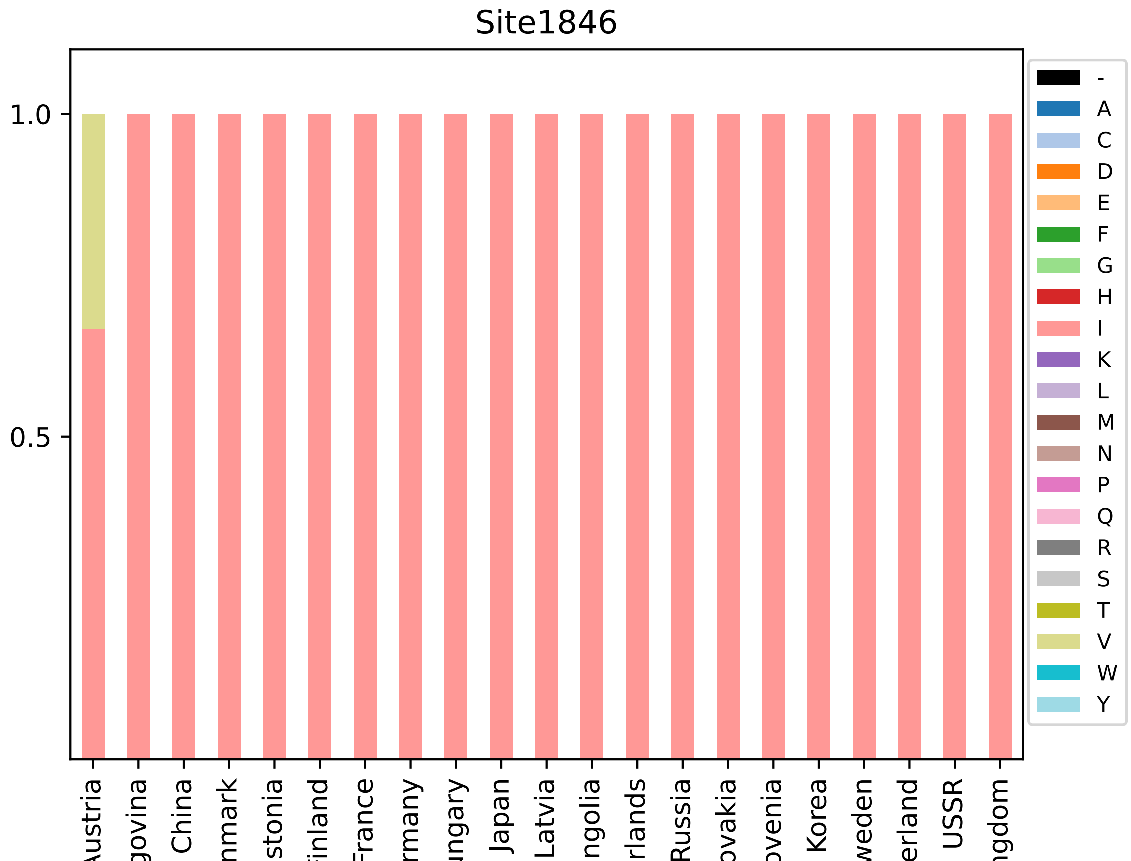

Site1846
1.0
0.5
-
A
C
D
E
F
G
H
I
K
L
M
N
P
Q
R
S
T
V
W
Y
Austria
China
Japan
Latvia
Russia
Korea
USSR

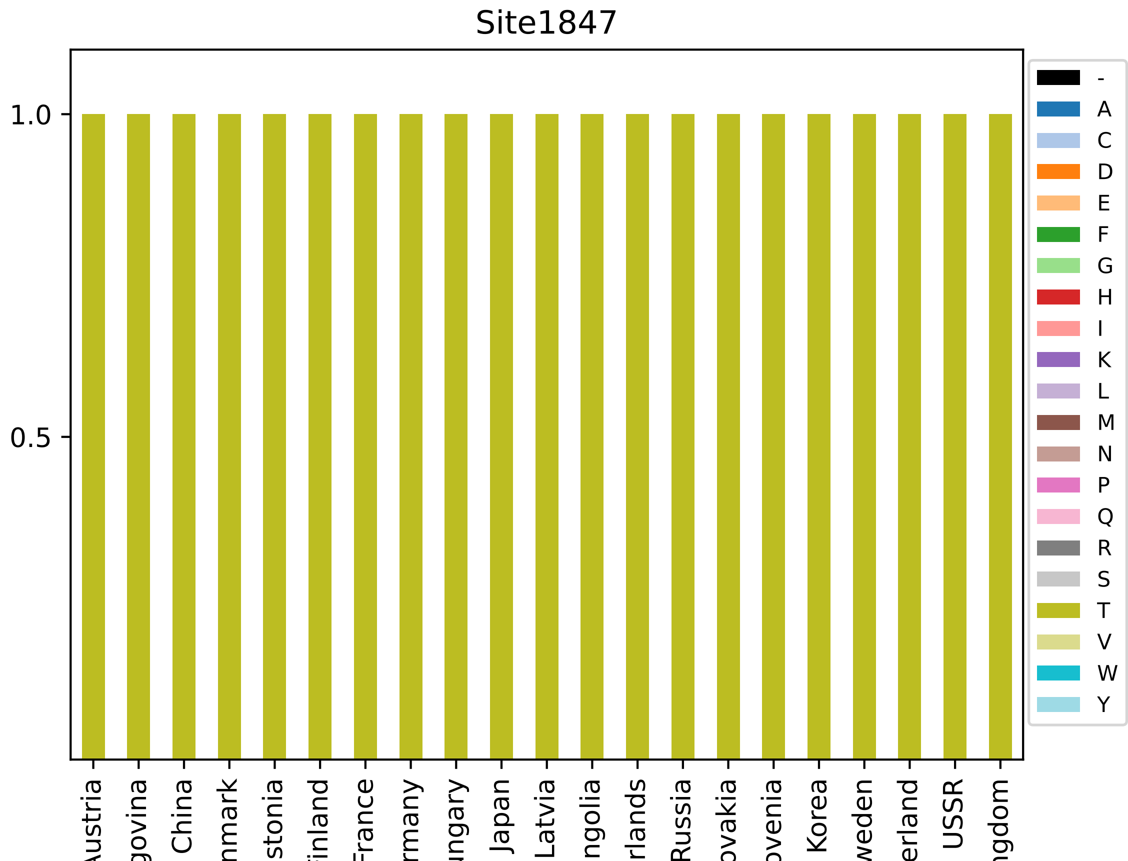

Site1847
1.0
0.5
Austria
govina
China
nmark
stonia
Finland
France
rmany
ungary
Japan
Latvia
ngolia
rlands
Russia
ovakia
ovenia
Korea
weden
erland
USSR
ngdom
-
A
C
D
E
F
G
H
I
K
L
M
N
P
Q
R
S
T
V
W
Y

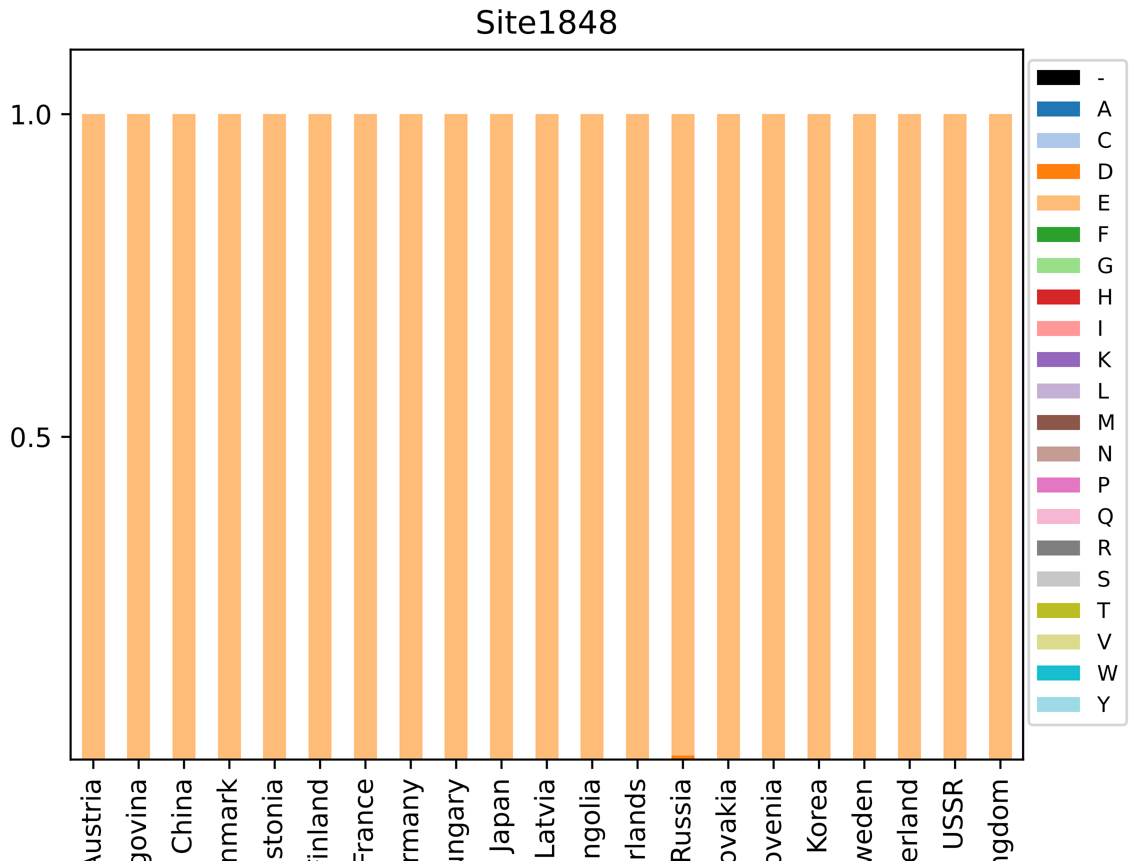

Site1848
1.0
0.5
-
A
C
D
E
F
G
H
I
K
L
M
N
P
Q
R
S
T
V
W
Y
Austria
China
Japan
Latvia
Russia
Korea
USSR

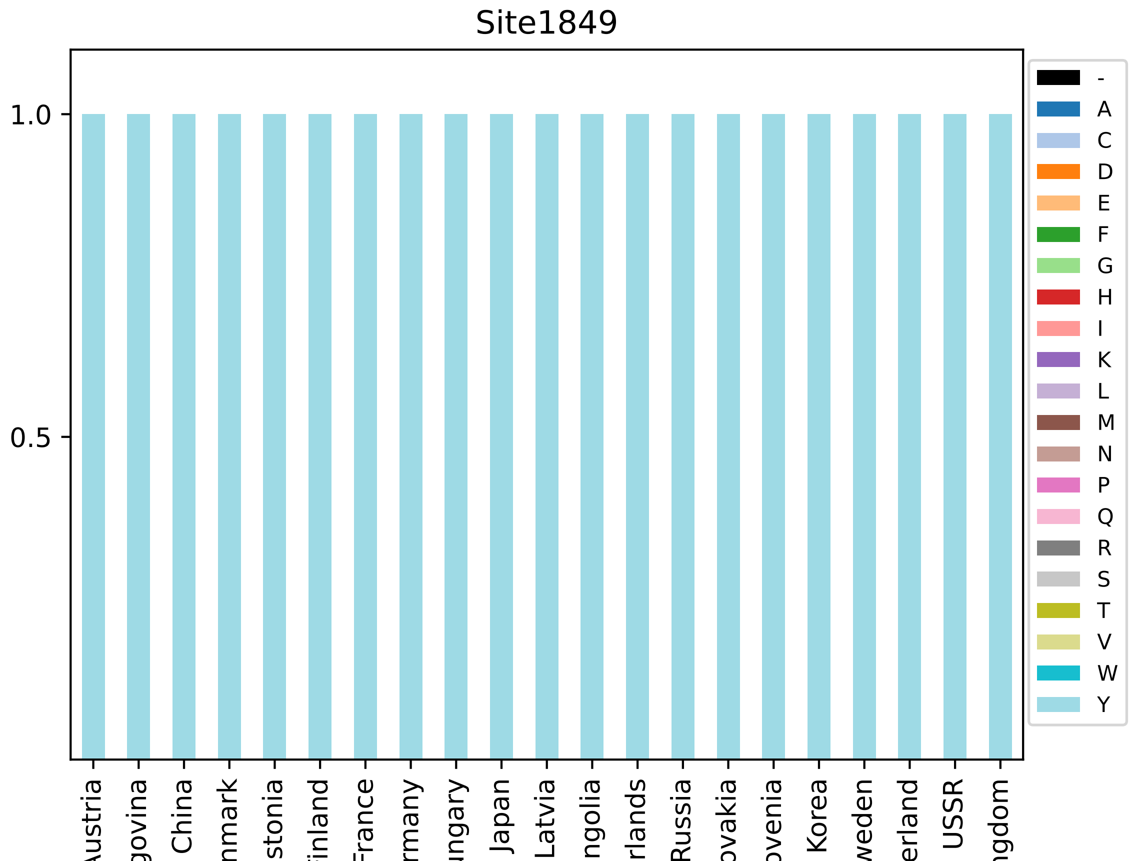

Site1849
1.0
0.5
-
A
C
D
E
F
G
H
I
K
L
M
N
P
Q
R
S
T
V
W
Y
China
Japan
Latvia
Russia
Korea
USSR

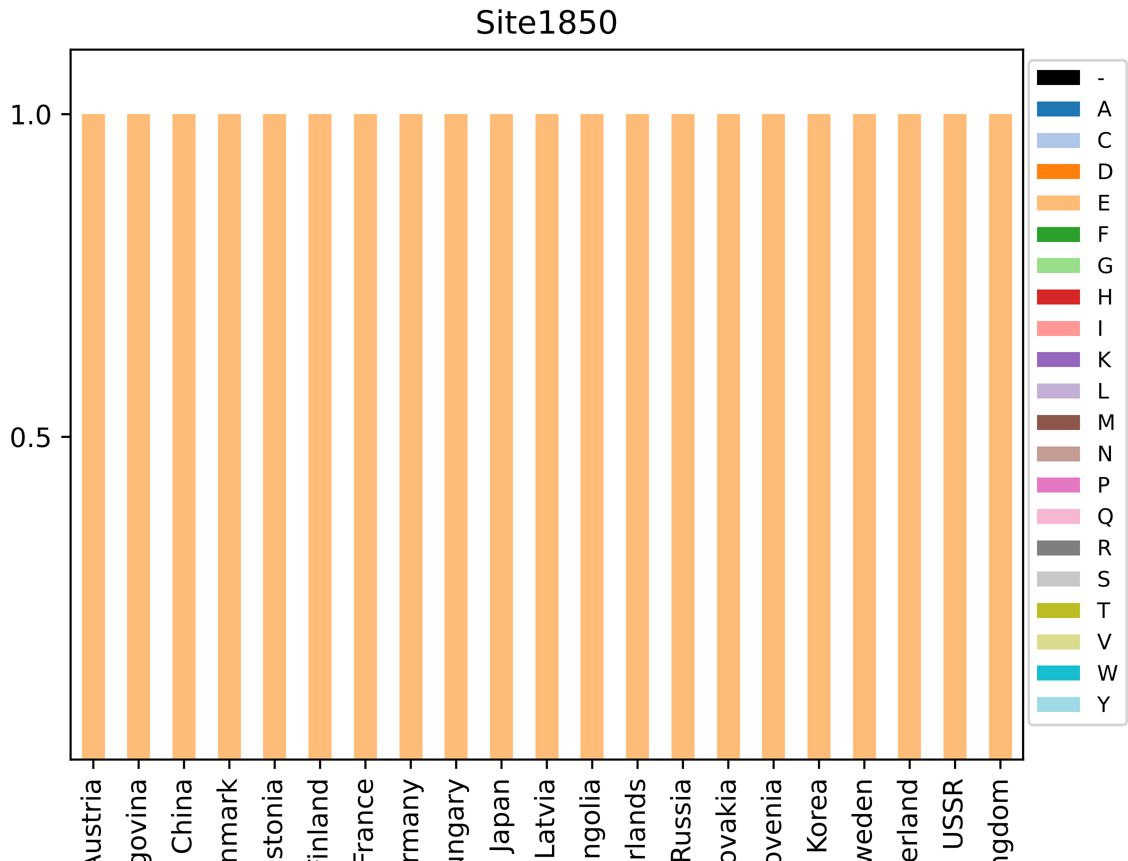

Site1850
1.0
0.5
China
Japan
Latvia
Russia
Korea
USSR
-
A
C
D
E
F
G
H
I
K
L
M
N
P
Q
R
S
T
V
W
Y

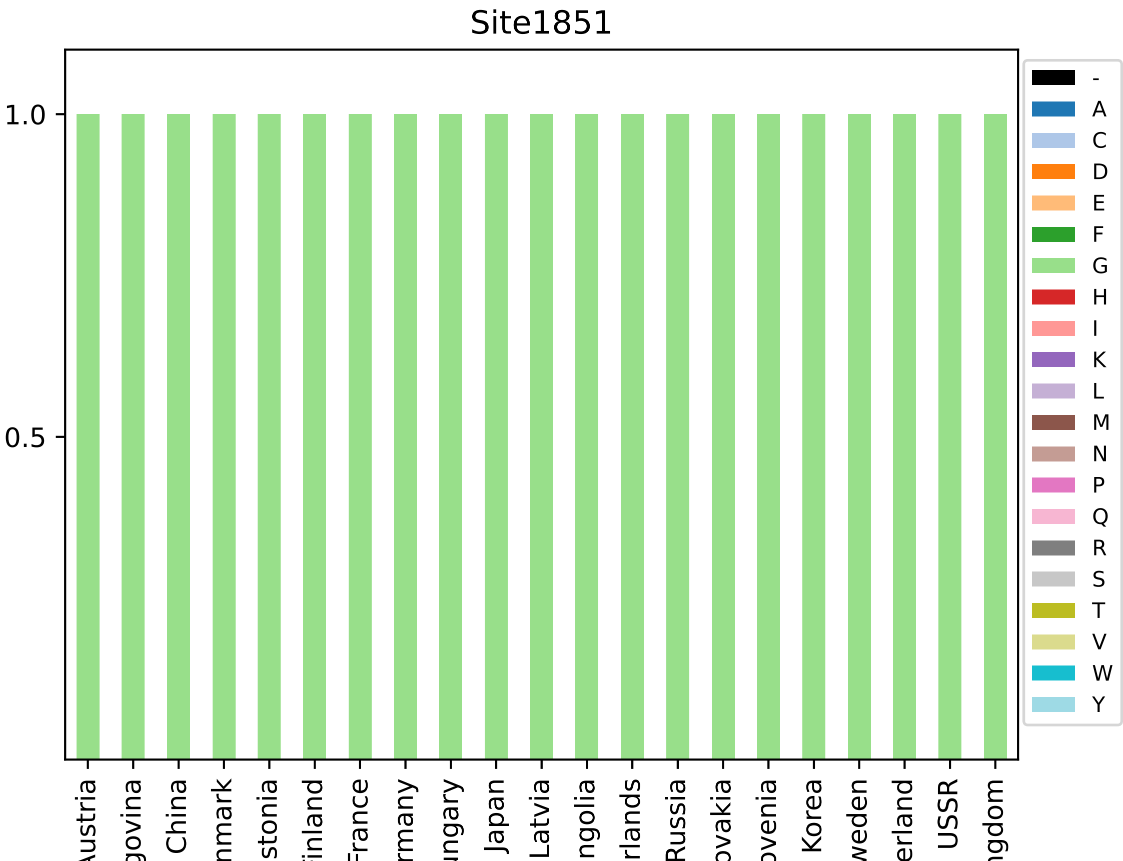

Site1851
1.0
0.5
-
A
C
D
E
F
G
H
I
K
L
M
N
P
Q
R
S
T
V
W
Y
China
Japan
Latvia
Russia
Korea
USSR

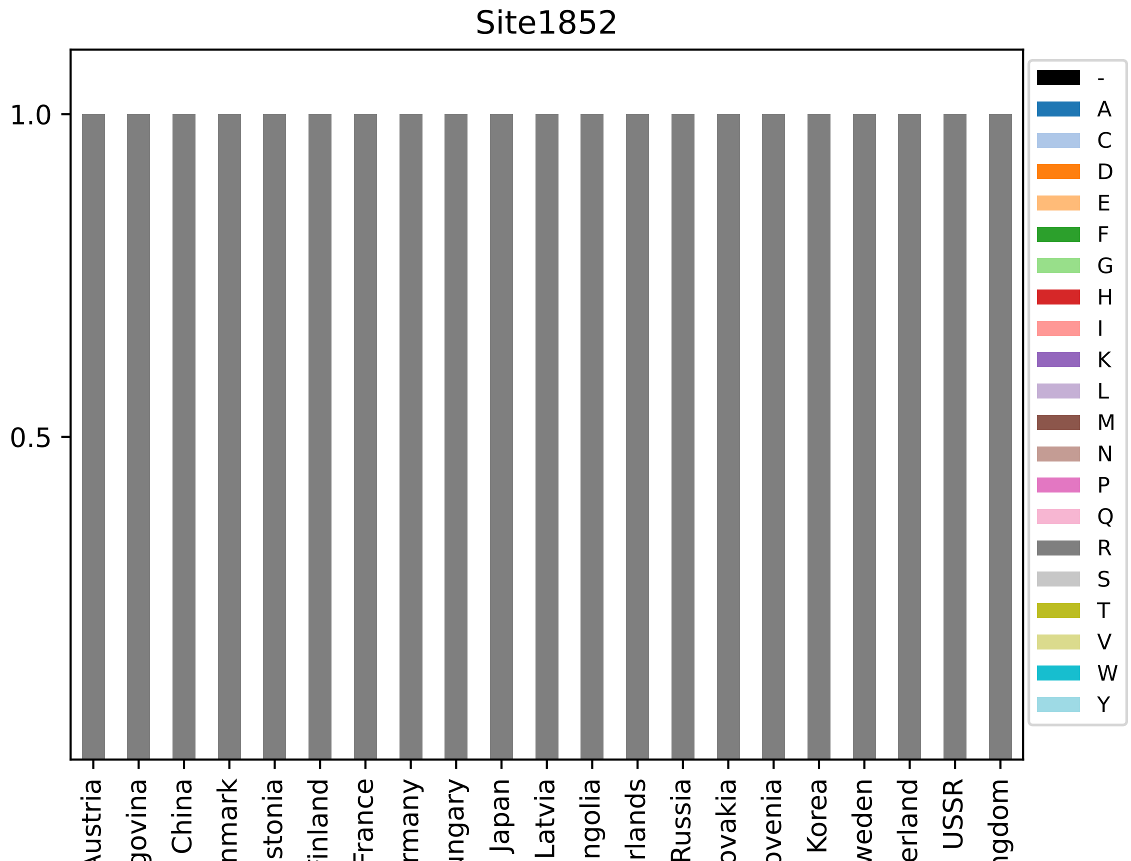
Site1852
1.0
0.5
-
A
C
D
E
F
G
H
I
K
L
M
N
P
Q
R
S
T
V
W
Y
China
Japan
Latvia
Russia
Korea
USSR

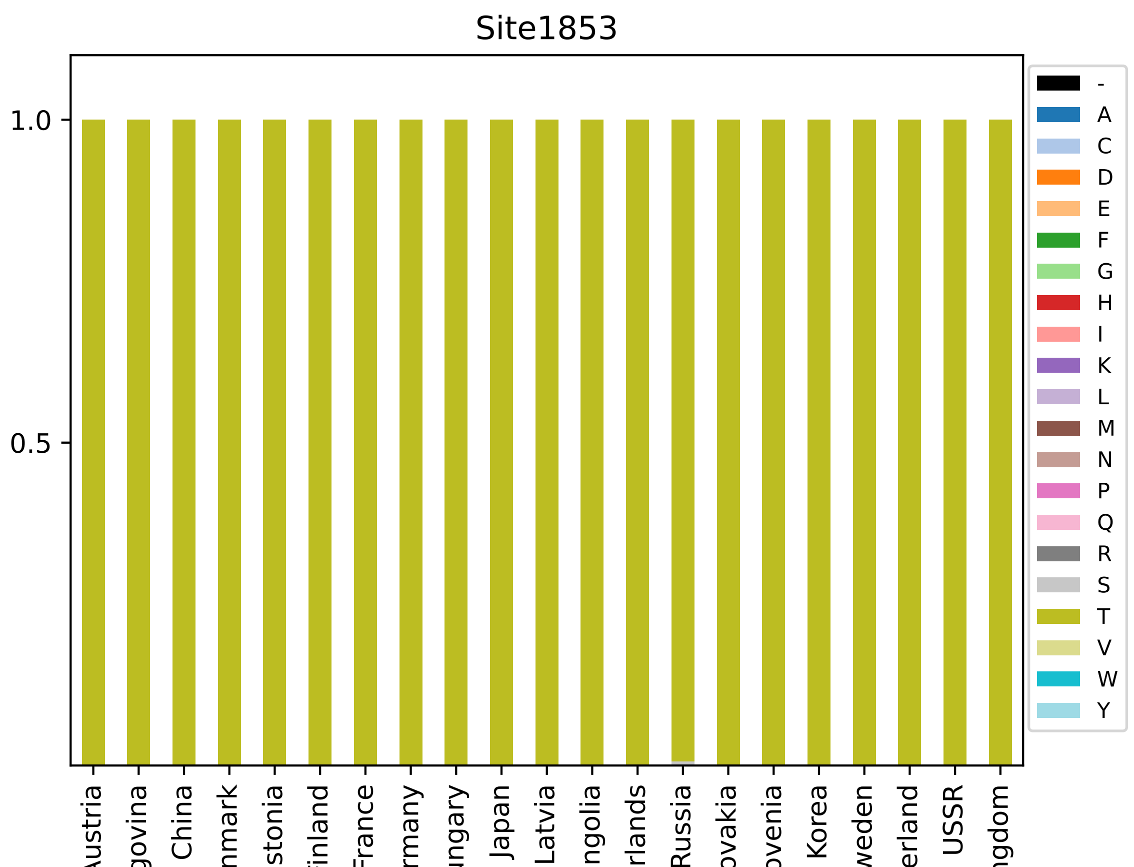

Site1853
1.0
0.5
-
A
C
D
E
F
G
H
I
K
L
M
N
P
Q
R
S
T
V
W
Y
Austria
govina
China
nmark
stonia
Finland
France
rmany
ungary
Japan
Latvia
ngolia
rlands
Russia
ovakia
ovenia
Korea
weden
erland
USSR
ngdom

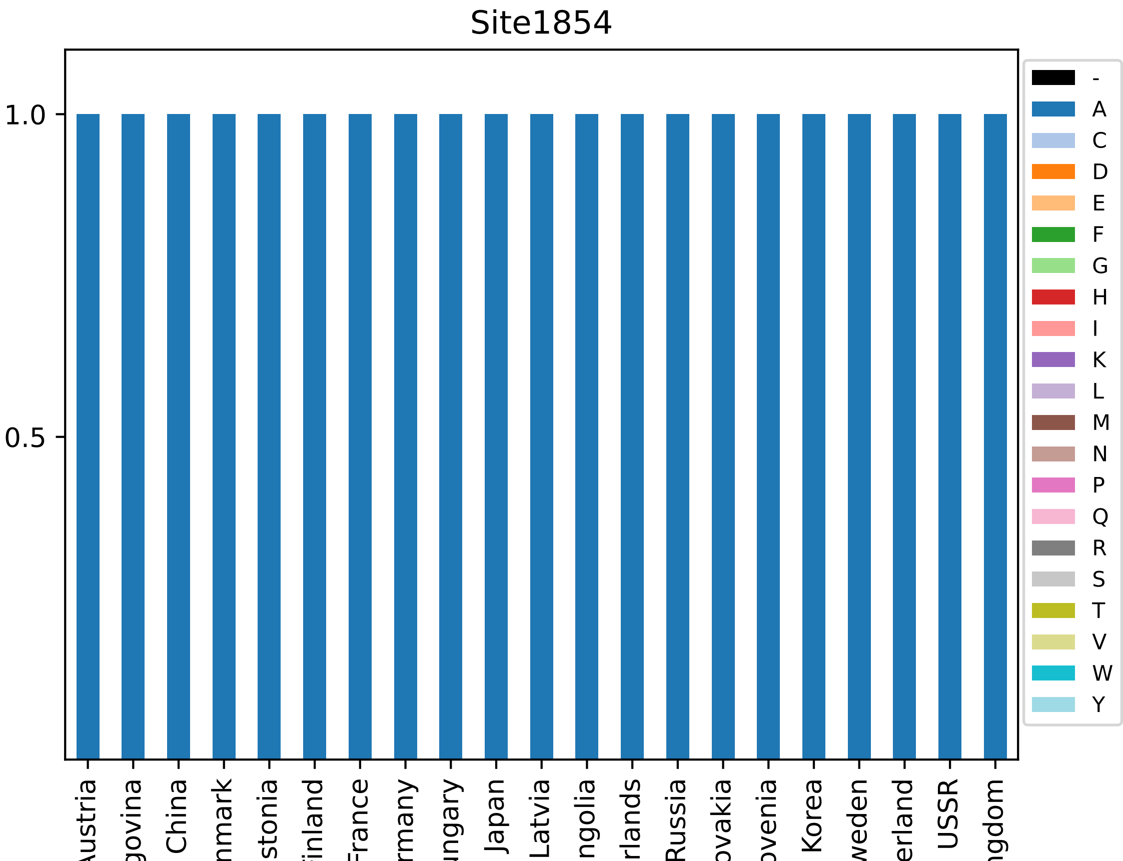

Site1854
1.0
0.5
-
A
C
D
E
F
G
H
I
K
L
M
N
P
Q
R
S
T
V
W
Y
China
Japan
Latvia
Russia
Korea
USSR

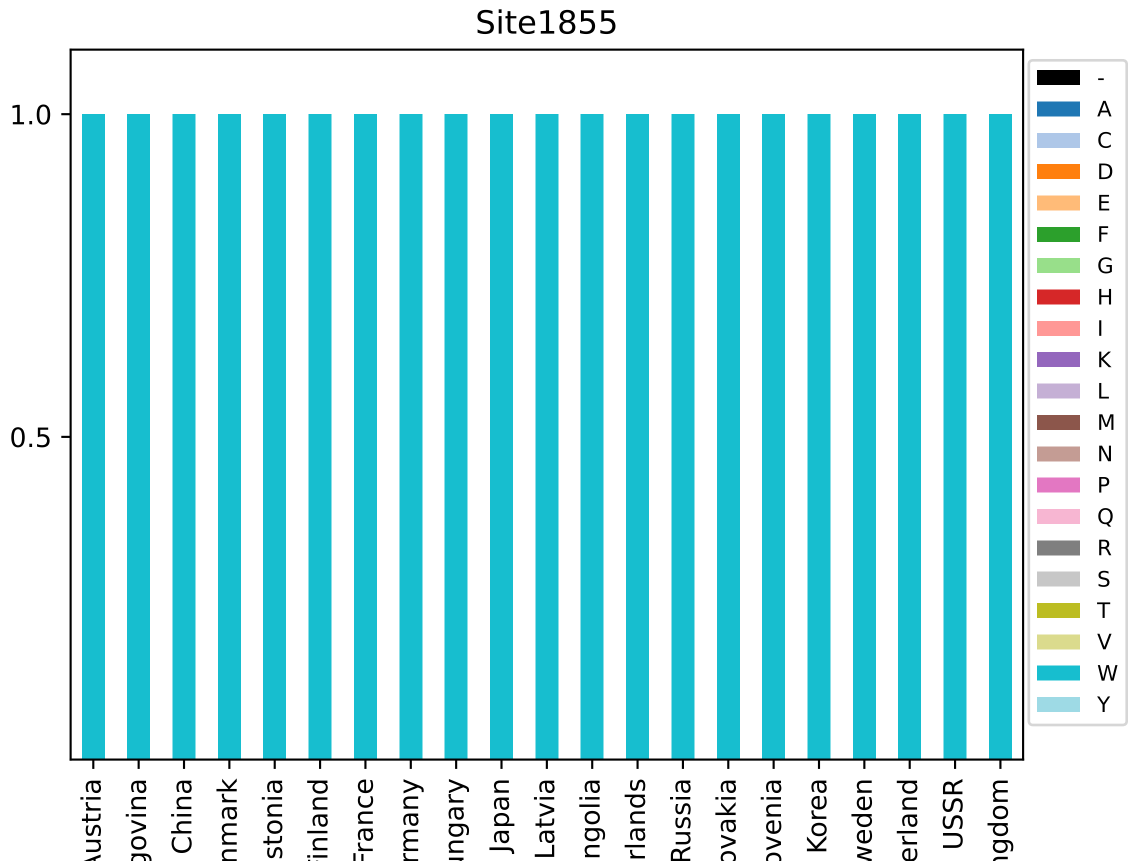

Site1855
1.0
0.5
-
A
C
D
E
F
G
H
I
K
L
M
N
P
Q
R
S
T
V
W
Y
China
Japan
Latvia
Russia
Korea
USSR

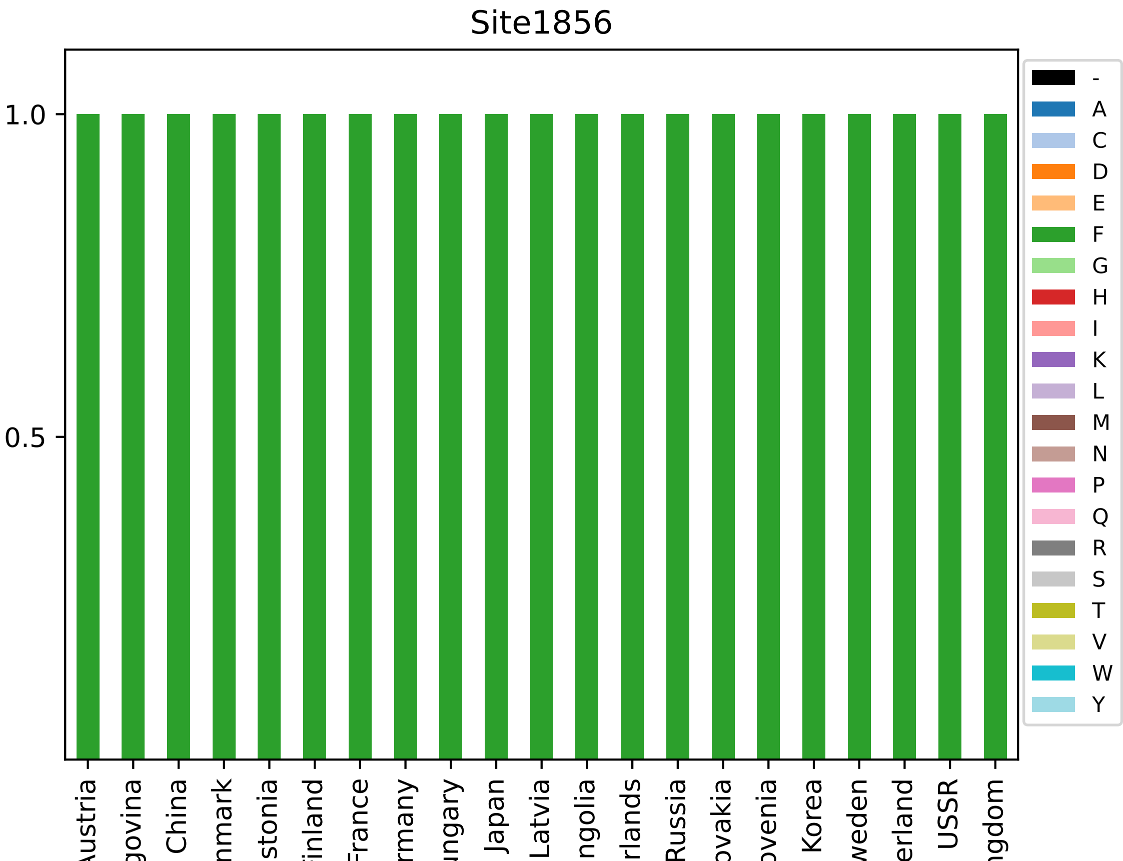

Site1856
1.0
0.5
-
A
C
D
E
F
G
H
I
K
L
M
N
P
Q
R
S
T
V
W
Y
China
Japan
Latvia
Russia
Korea
USSR

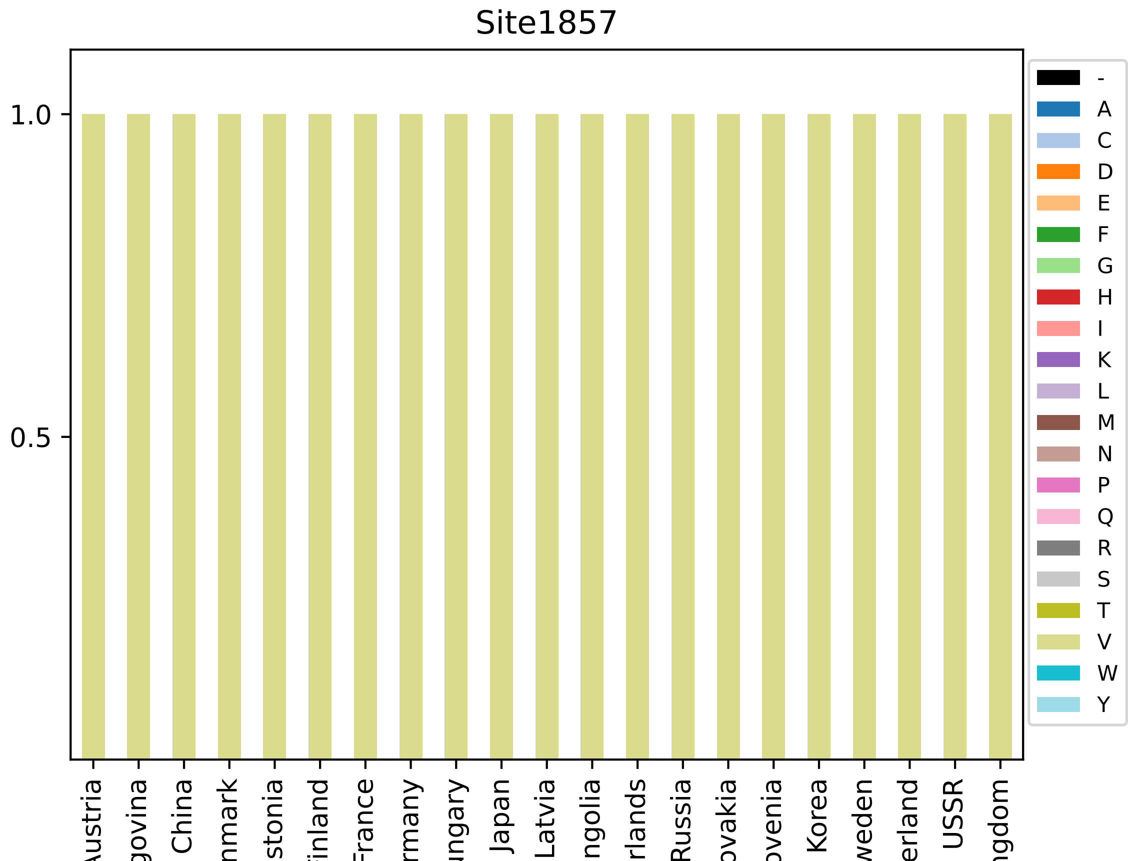
Site1857
1.0
0.5
-
A
C
D
E
F
G
H
I
K
L
M
N
P
Q
R
S
T
V
W
Y
Austria
govina
China
nmark
stonia
inland
France
rmany
ungary
Japan
Latvia
ngolia
rlands
Russia
ovakia
ovenia
Korea
weden
erland
USSR
ngdom

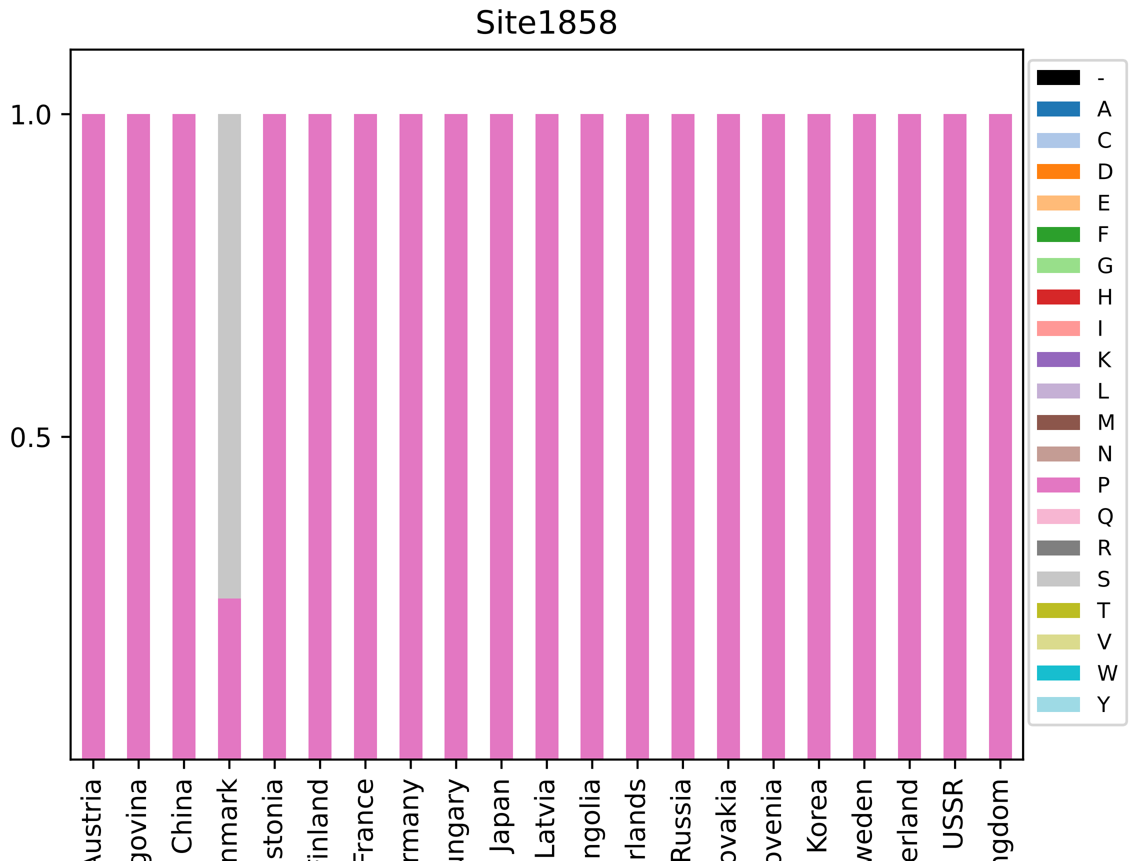

Site1858
1.0
0.5
-
A
C
D
E
F
G
H
I
K
L
M
N
P
Q
R
S
T
V
W
Y
China
Japan
Latvia
Russia
Korea
USSR

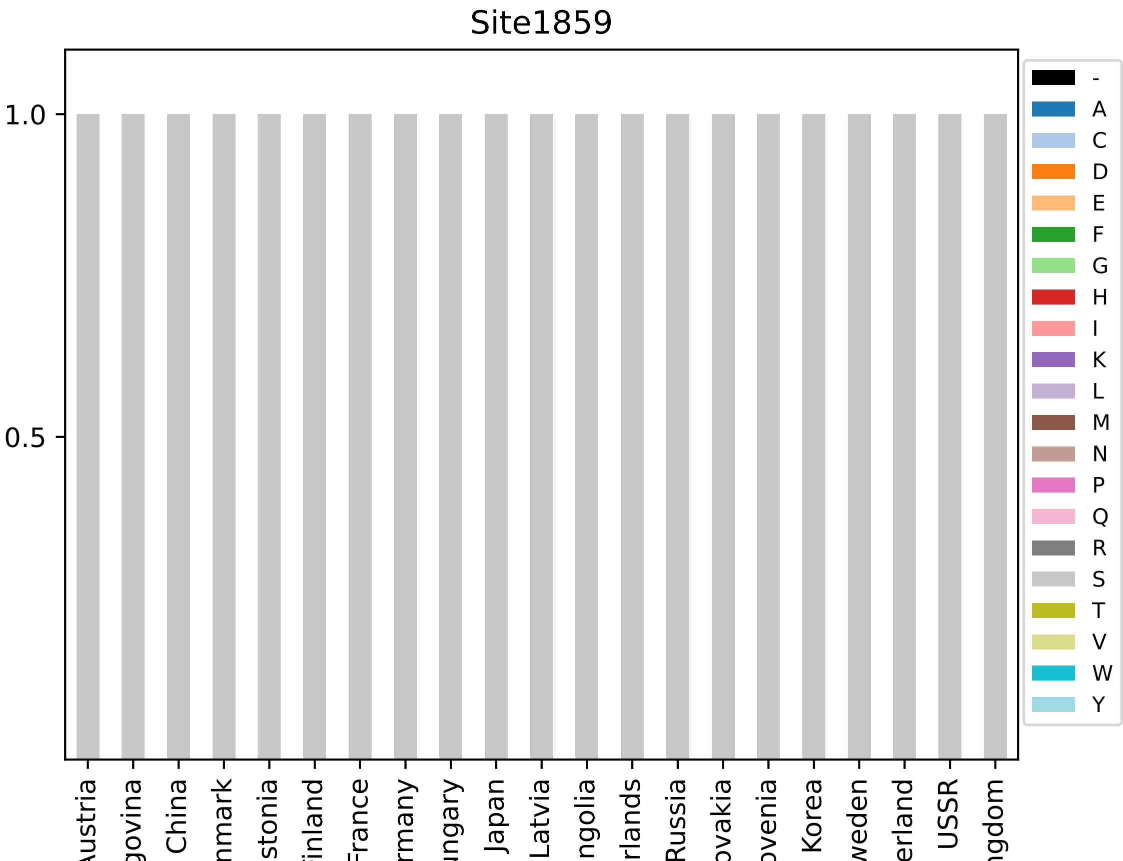
Site1859
1.0
0.5
-
A
C
D
E
F
G
H
I
K
L
M
N
P
Q
R
S
T
V
W
Y
Austria
govina
China
nmark
stonia
inland
France
rmany
ungary
Japan
Latvia
ngolia
rlands
Russia
ovakia
ovenia
Korea
weden
erland
USSR
ngdom

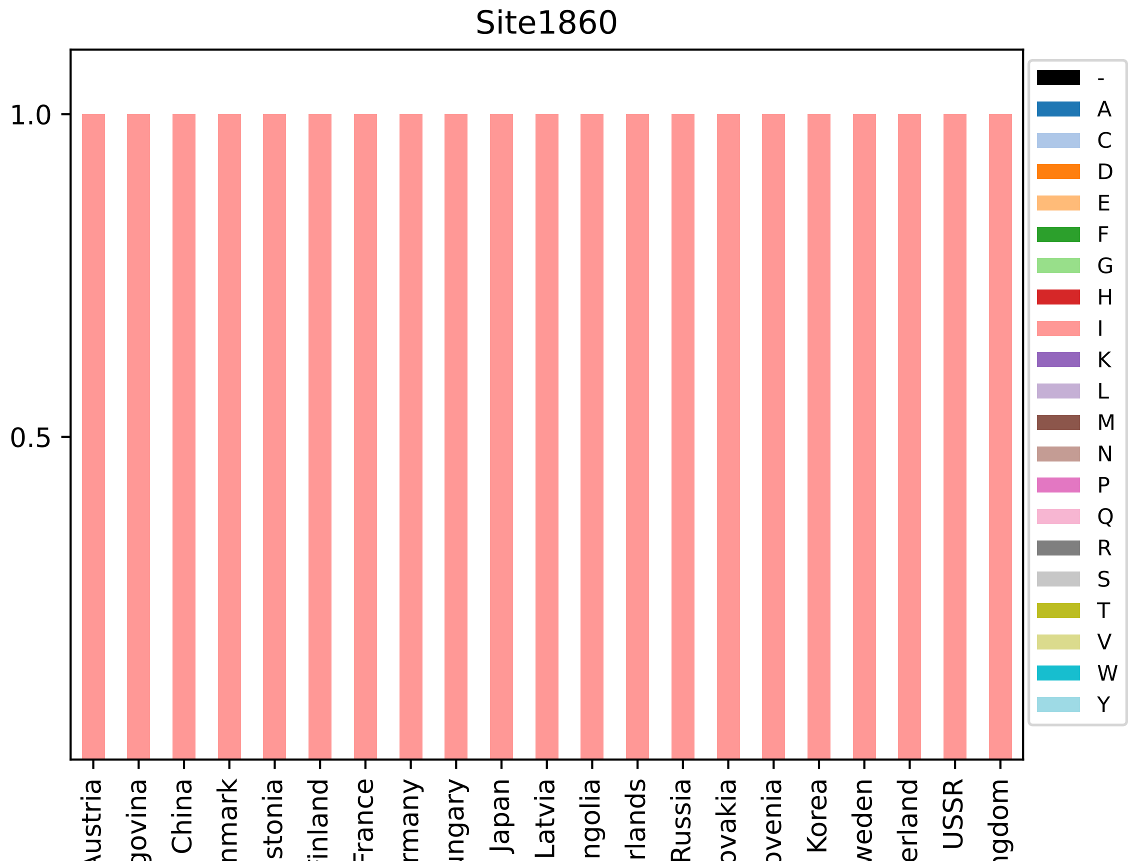

Site1860
1.0
0.5
China
Japan
Latvia
Russia
Korea
USSR
-
A
C
D
E
F
G
H
I
K
L
M
N
P
Q
R
S
T
V
W
Y

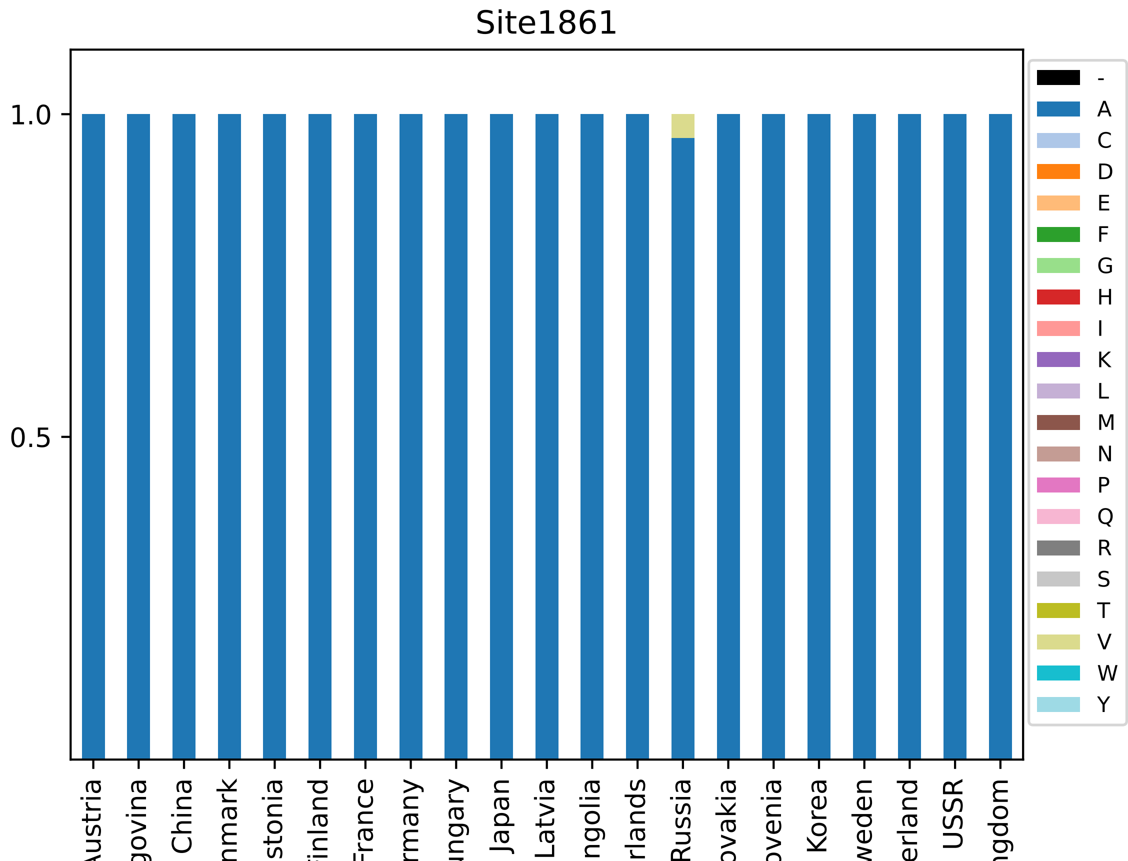

Site1861
1.0
0.5
-
A
C
D
E
F
G
H
I
K
L
M
N
P
Q
R
S
T
V
W
Y
China
Japan
Latvia
Russia
Korea
USSR

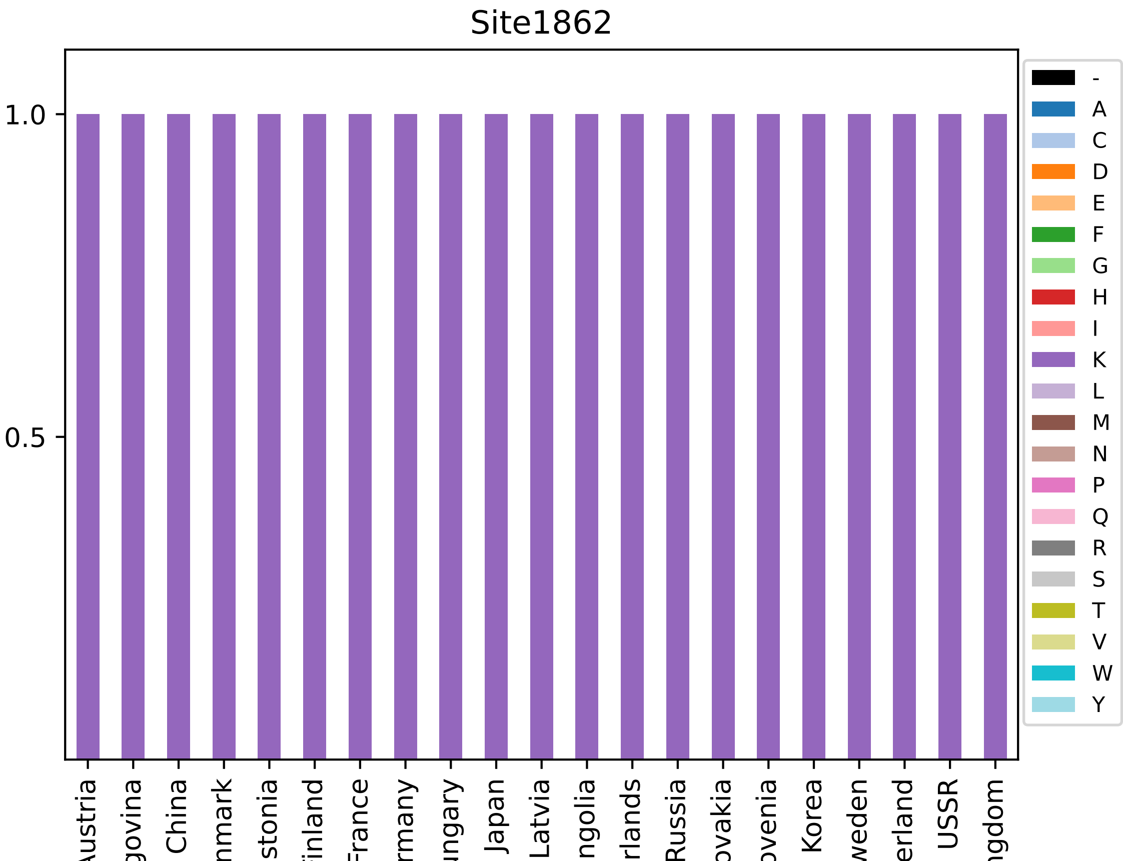

Site1862
1.0
0.5
-
A
C
D
E
F
G
H
I
K
L
M
N
P
Q
R
S
T
V
W
Y
China
Japan
Latvia
Russia
Korea
USSR

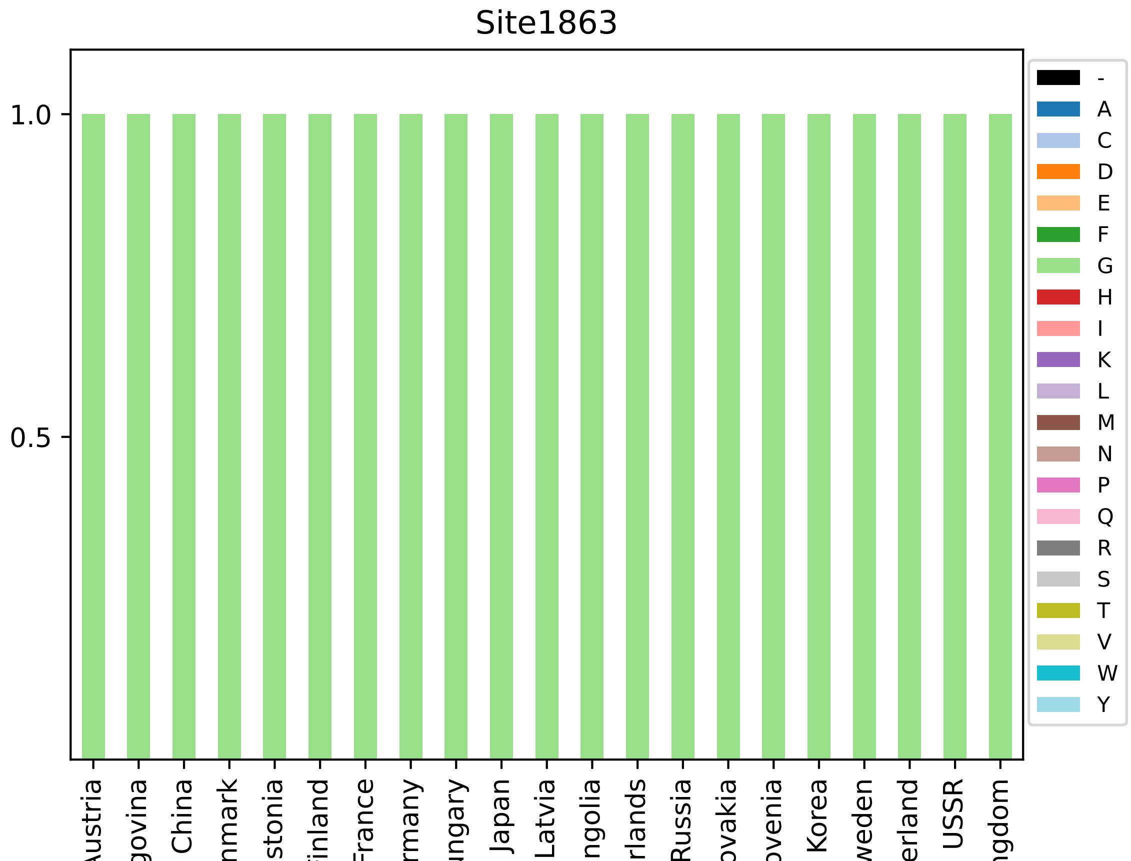

Site1863
1.0
0.5
-
A
C
D
E
F
G
H
I
K
L
M
N
P
Q
R
S
T
V
W
Y
China
Japan
Latvia
Russia
Korea
USSR

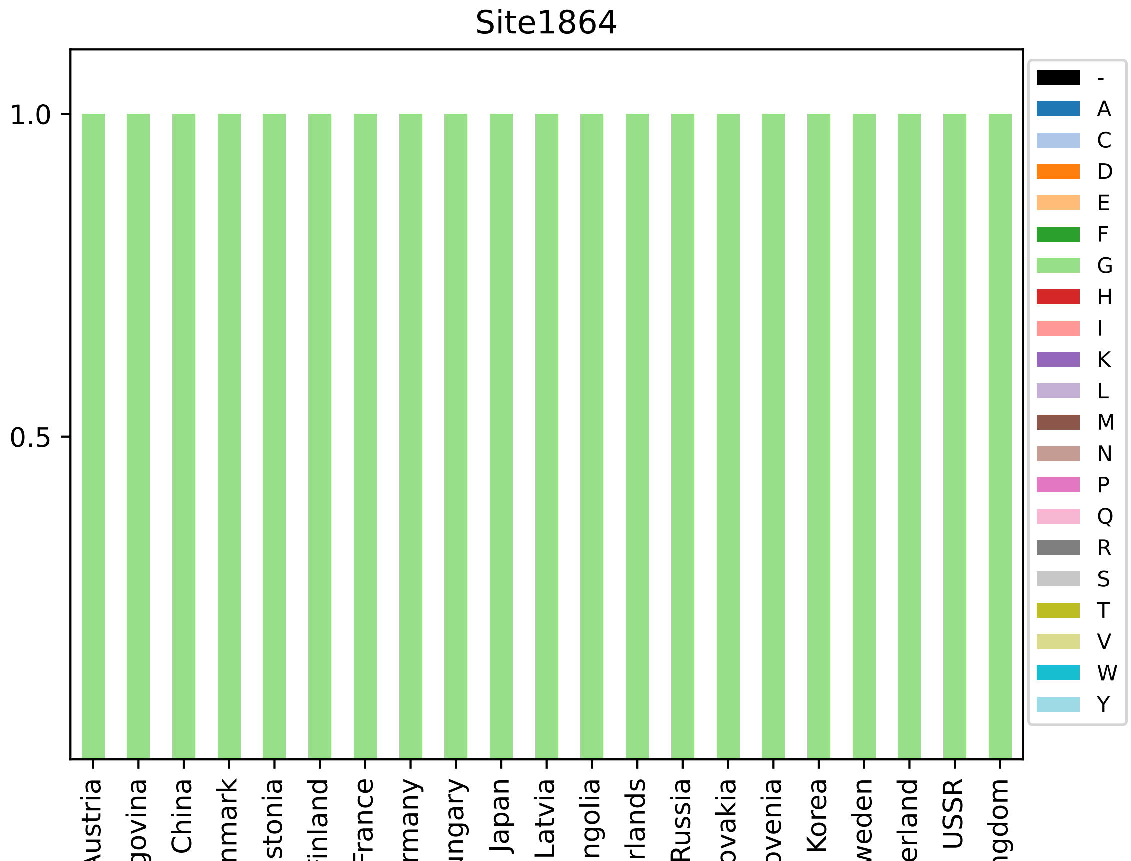

Site1864
1.0
0.5
-
A
C
D
E
F
G
H
I
K
L
M
N
P
Q
R
S
T
V
W
Y
Austria
China
Japan
Latvia
Russia
Korea
USSR

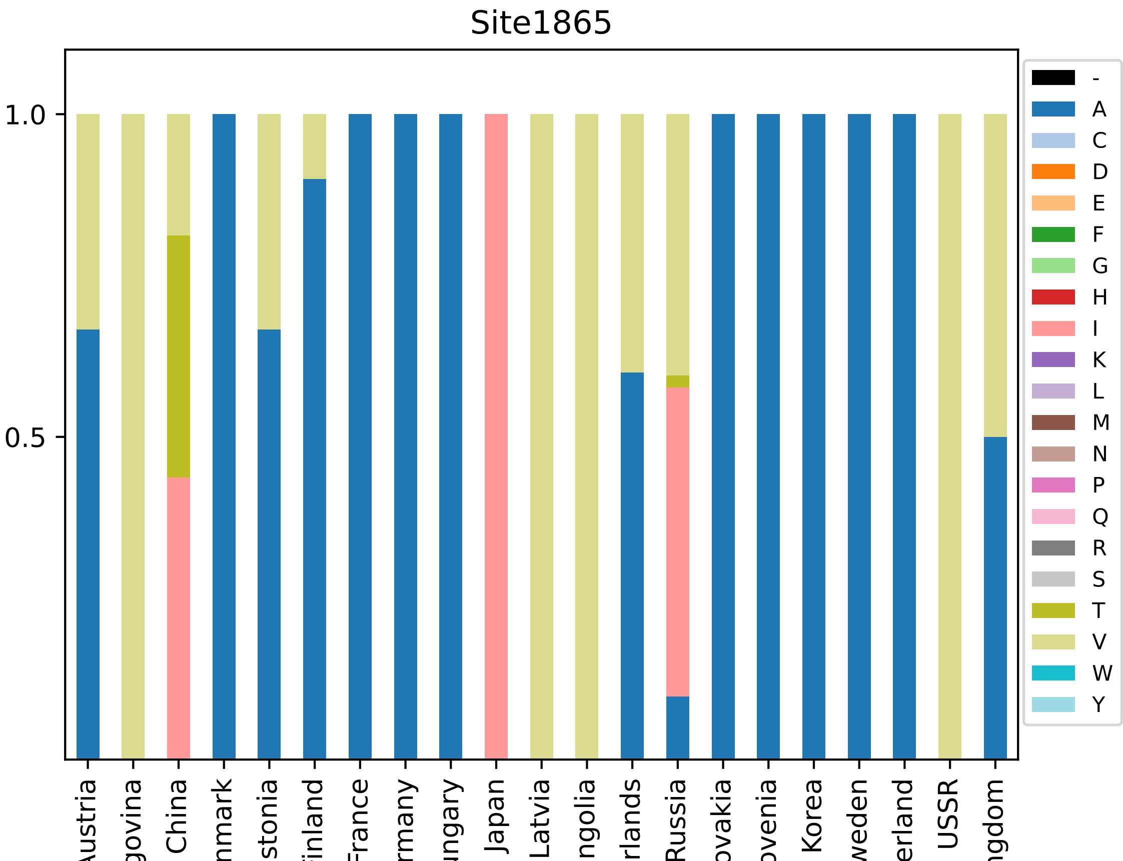

Site1865
1.0
0.5
-
A
C
D
E
F
G
H
I
K
L
M
N
P
Q
R
S
T
V
W
Y
China
Japan
Latvia
Russia
Korea
USSR

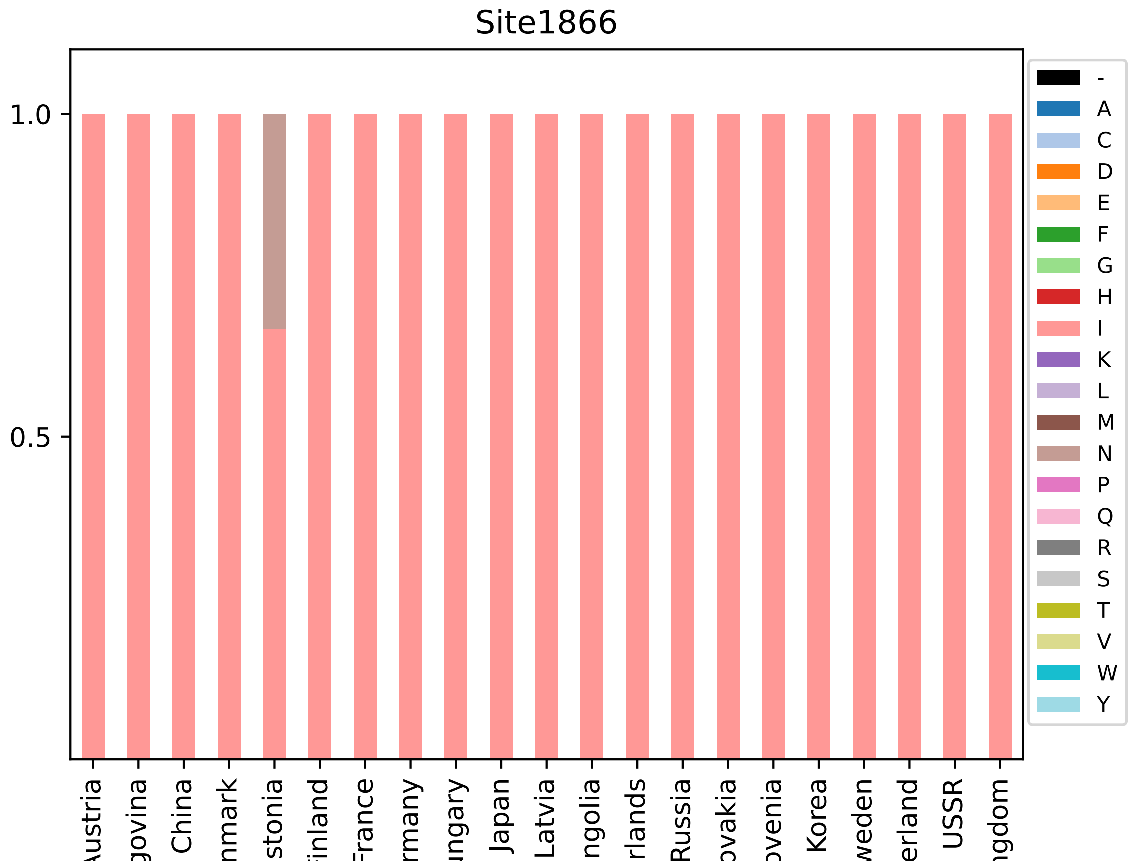
Site1866
1.0
0.5
-
A
C
D
E
F
G
H
I
K
L
M
N
P
Q
R
S
T
V
W
Y
Austria
govina
China
nmark
stonia
Finland
France
rmany
ungary
Japan
Latvia
ngolia
rlands
Russia
ovakia
ovenia
Korea
weden
erland
USSR
ngdom

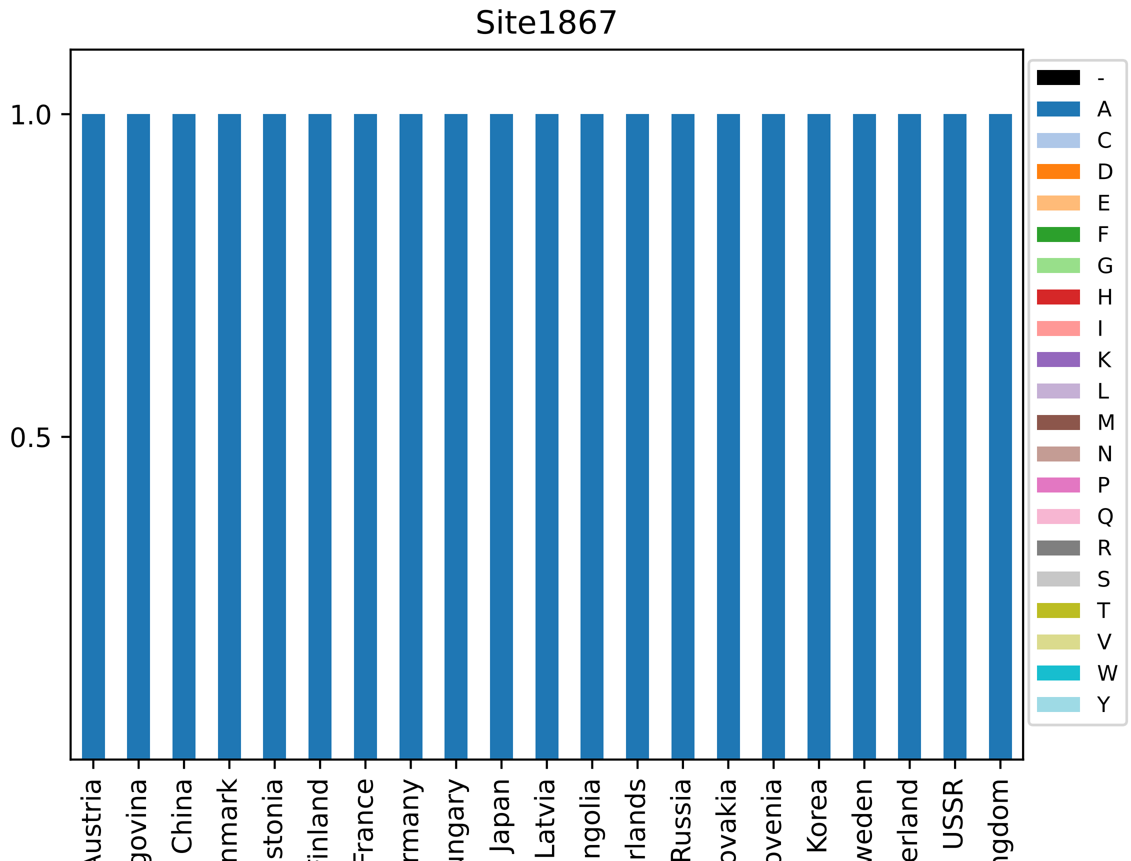

Site1867
1.0
0.5
-
A
C
D
E
F
G
H
I
K
L
M
N
P
Q
R
S
T
V
W
Y
China
Japan
Latvia
Russia
Korea
USSR

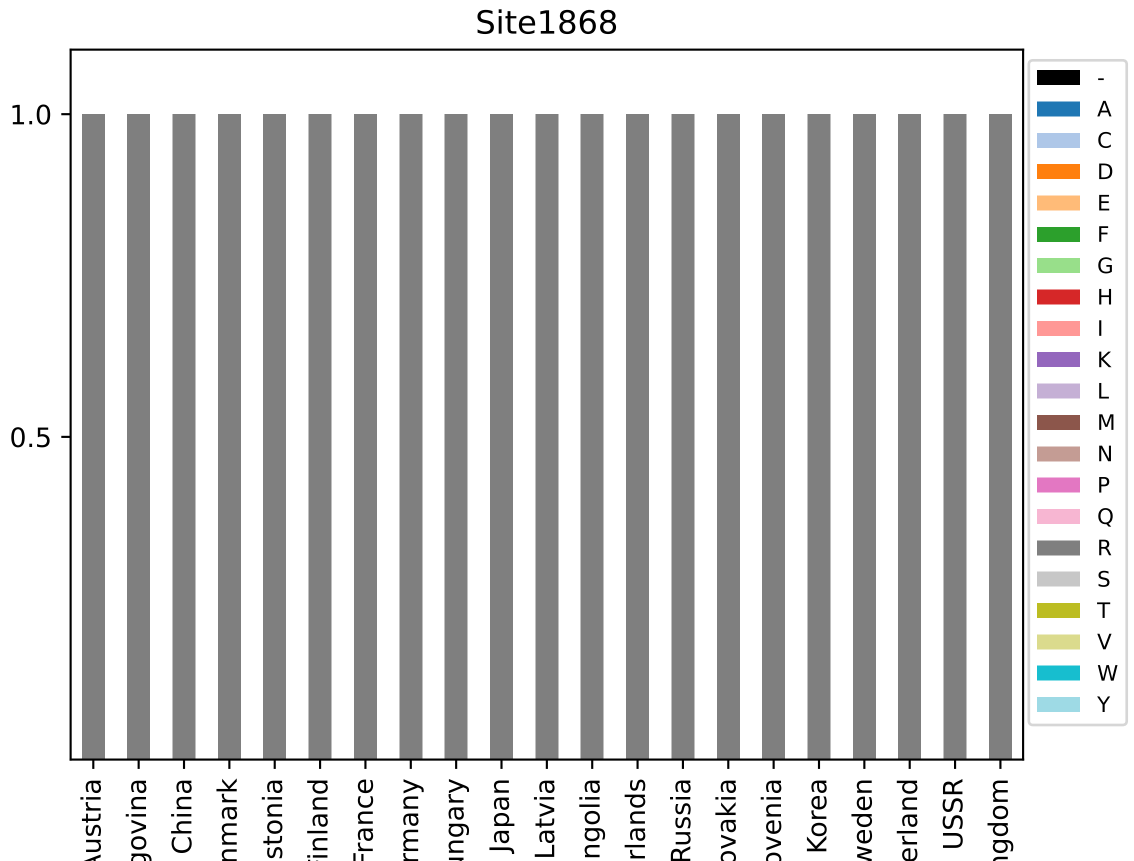
Site1868
1.0
0.5
-
A
C
D
E
F
G
H
I
K
L
M
N
P
Q
R
S
T
V
W
Y
China
Japan
Latvia
Russia
Korea
USSR

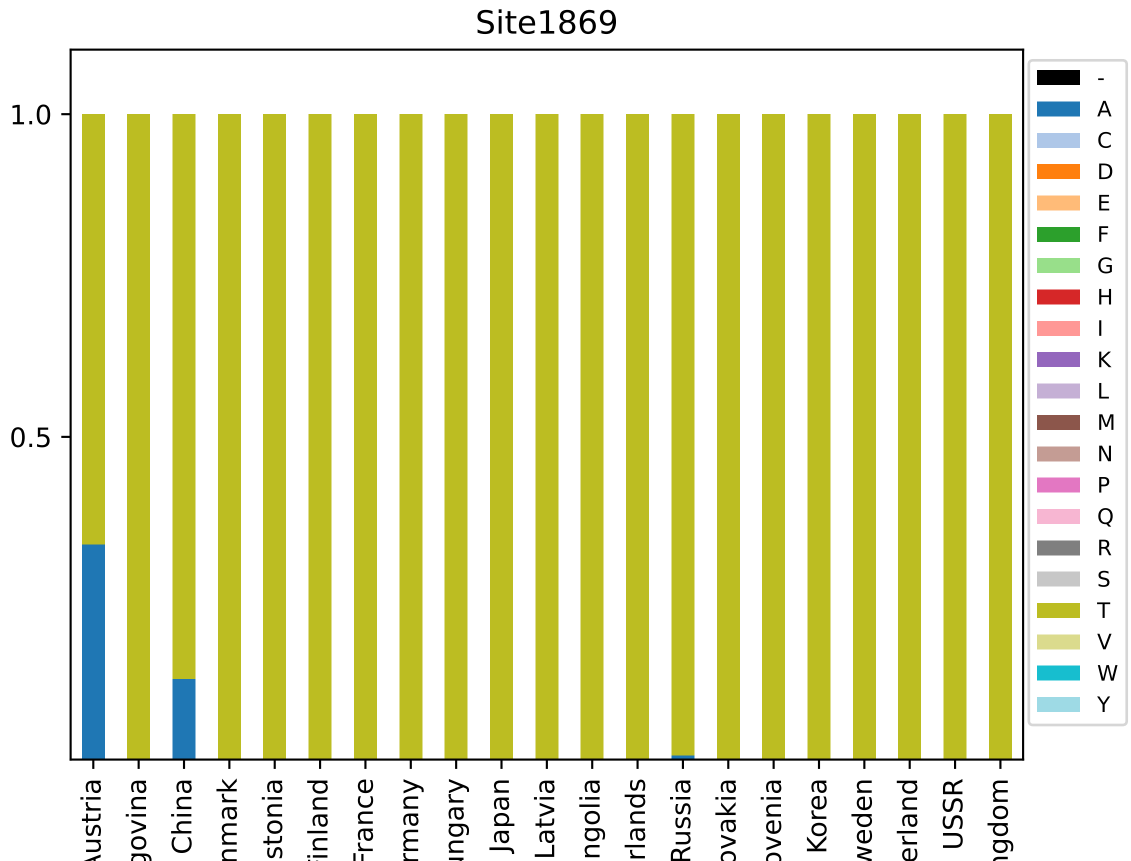

Site1869
1.0
0.5
-
A
C
D
E
F
G
H
I
K
L
M
N
P
Q
R
S
T
V
W
Y
China
Japan
Latvia
Russia
Korea
USSR

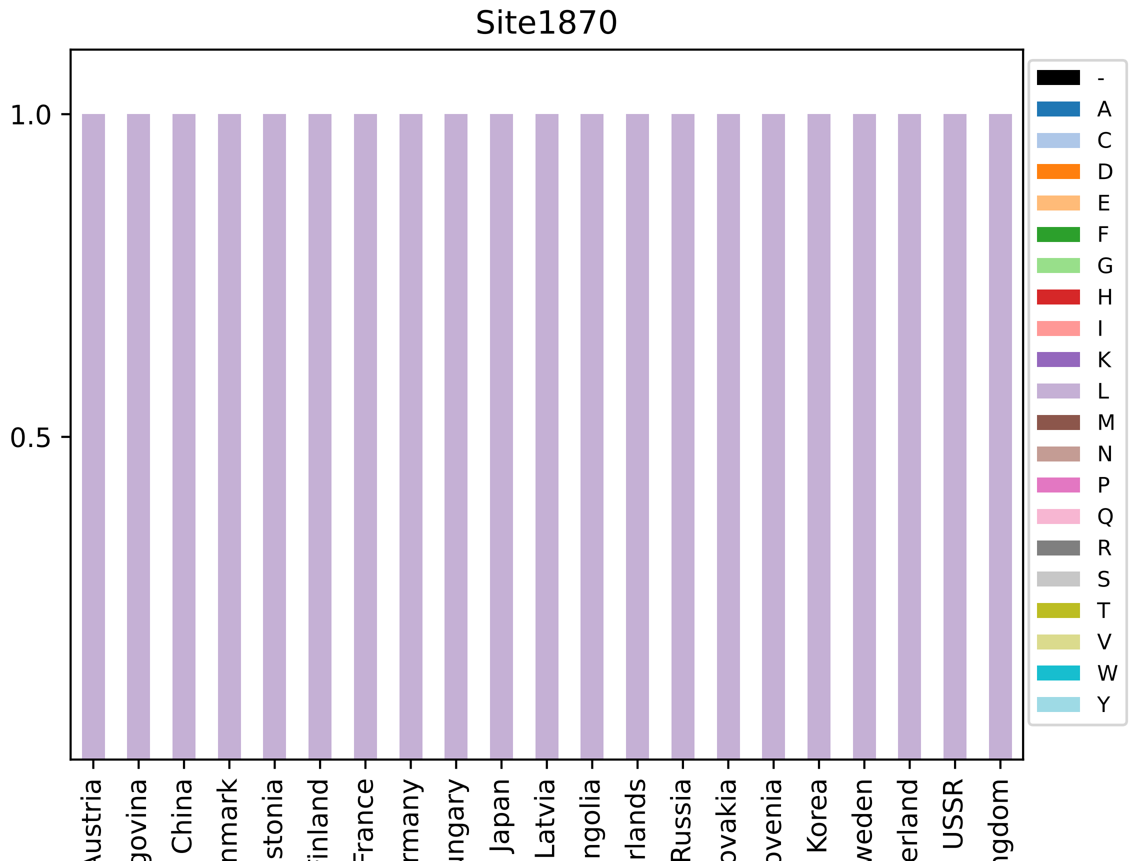

Site1870
1.0
0.5
-
A
C
D
E
F
G
H
I
K
L
M
N
P
Q
R
S
T
V
W
Y
Austria
China
Finland
France
Japan
Latvia
Russia
Korea
USSR

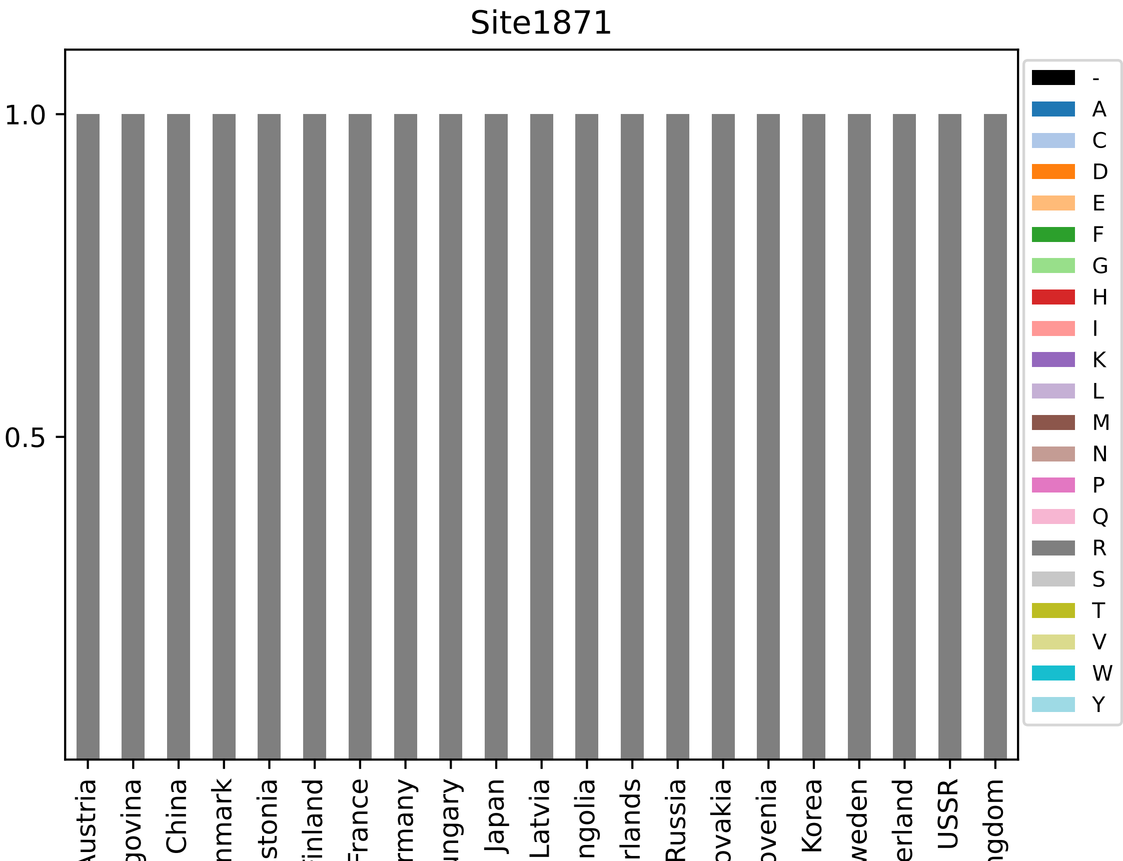

Site1871
1.0
0.5
-
A
C
D
E
F
G
H
I
K
L
M
N
P
Q
R
S
T
V
W
Y
China
Japan
Latvia
Russia
Korea
USSR

Site1872

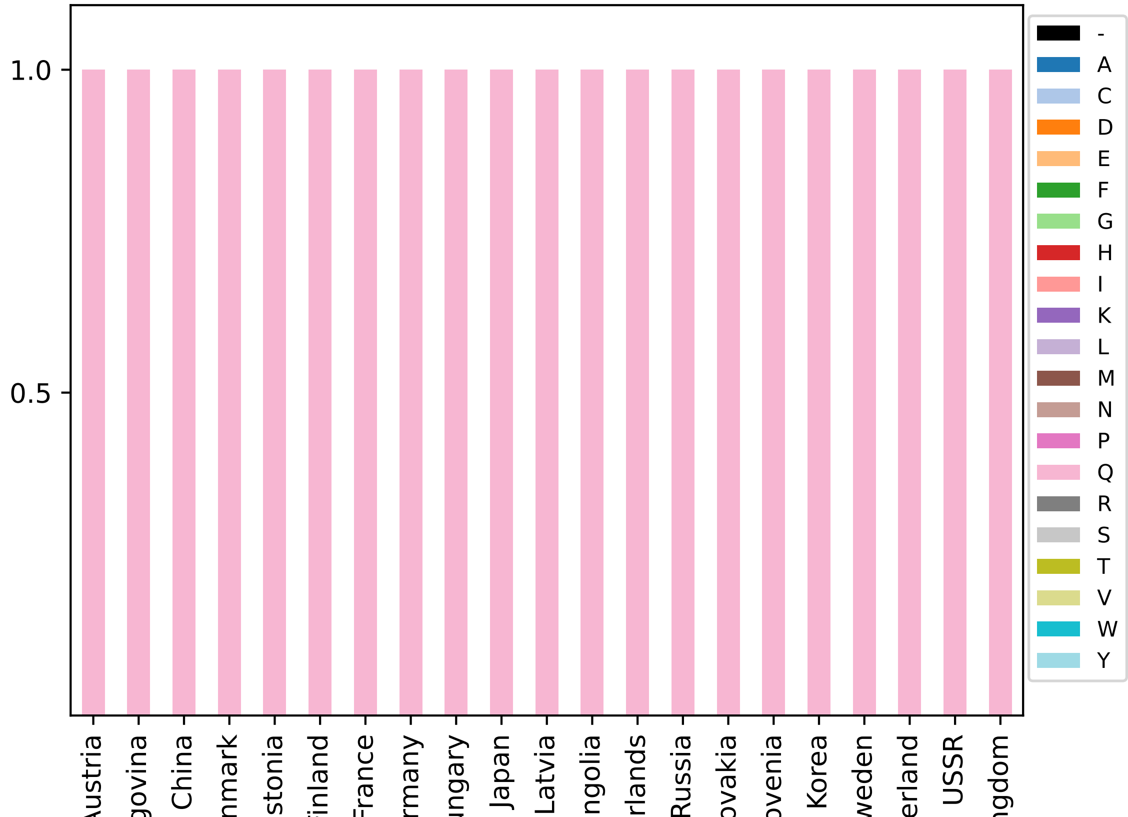
1.0
0.5
-
A
C
D
E
F
G
H
I
K
L
M
N
P
Q
R
S
T
V
W
Y
China
Japan
Latvia
Russia
Korea
USSR

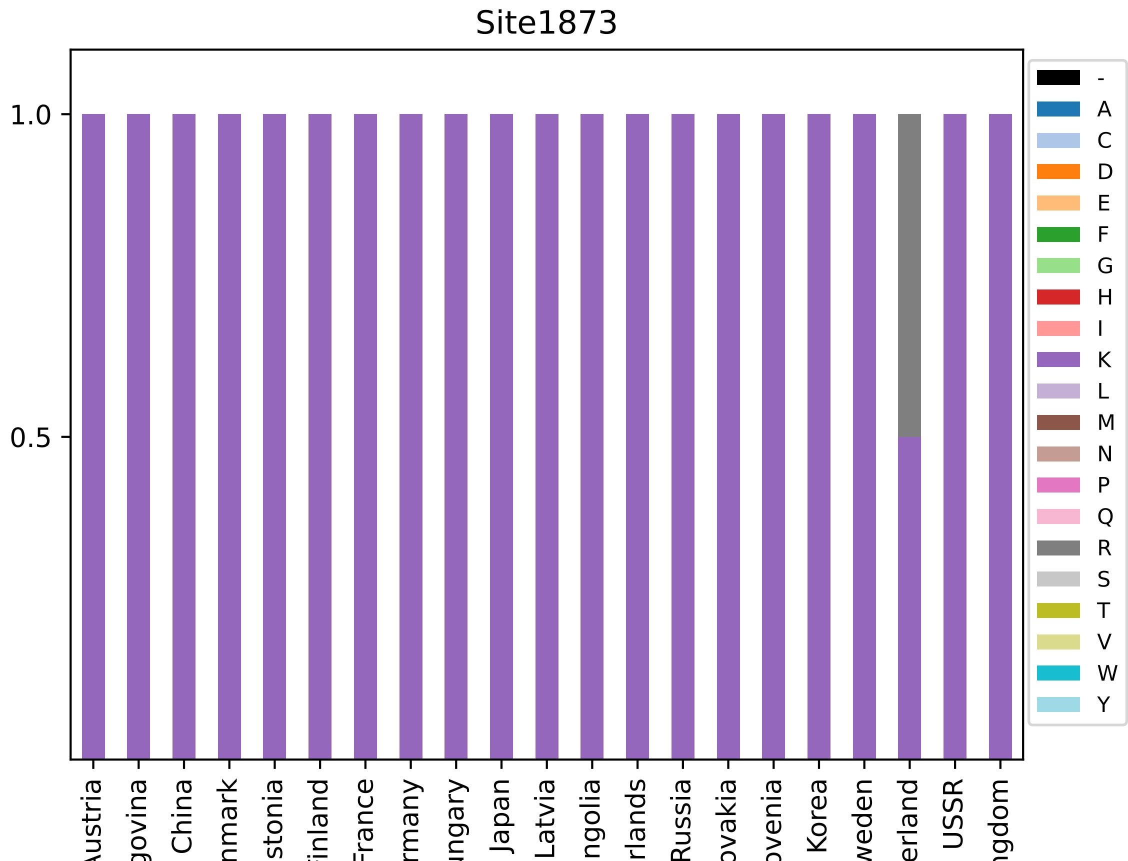

Site1873
1.0
0.5
-
A
C
D
E
F
G
H
I
K
L
M
N
P
Q
R
S
T
V
W
Y
Austria
govina
China
nmark
stonia
Finland
France
rmany
ungary
Japan
Latvia
ngolia
rlands
Russia
ovakia
ovenia
Korea
weden
erland
USSR
ngdom

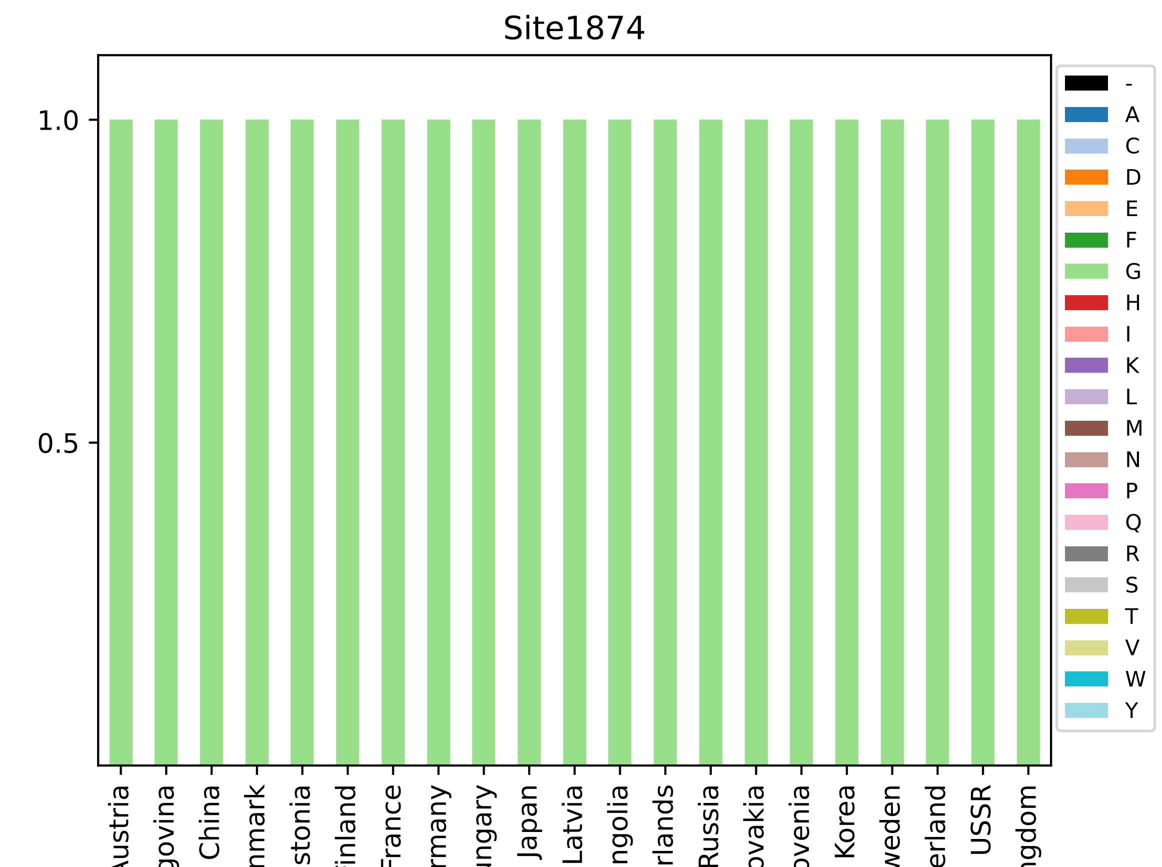
Site1874
1.0
0.5
Austria
govina
China
nmark
stonia
Finland
France
rmany
ungary
Japan
Latvia
ngolia
rlands
Russia
ovakia
ovenia
Korea
weden
erland
USSR
ngdom
-
A
C
D
E
F
G
H
I
K
L
M
N
P
Q
R
S
T
V
W
Y

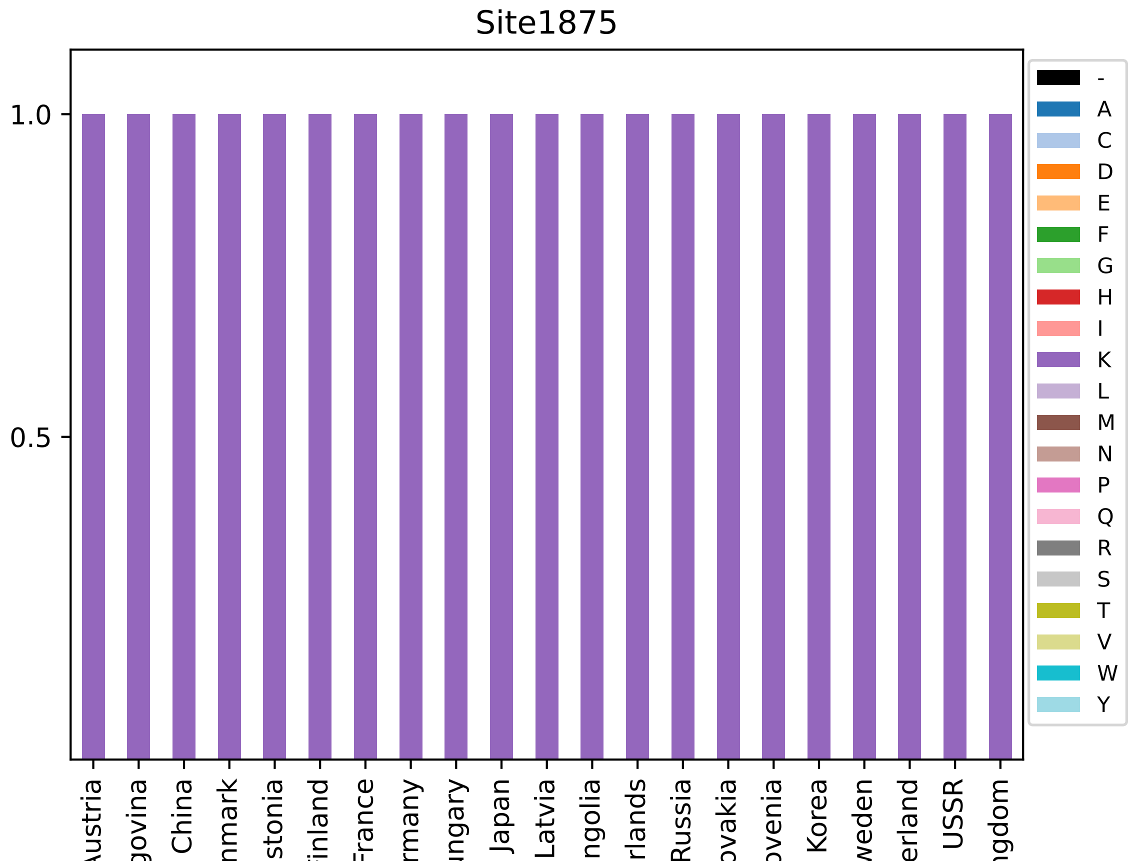
Site1875
1.0
0.5
-
A
C
D
E
F
G
H
I
K
L
M
N
P
Q
R
S
T
V
W
Y
China
Japan
Latvia
Russia
Korea
USSR

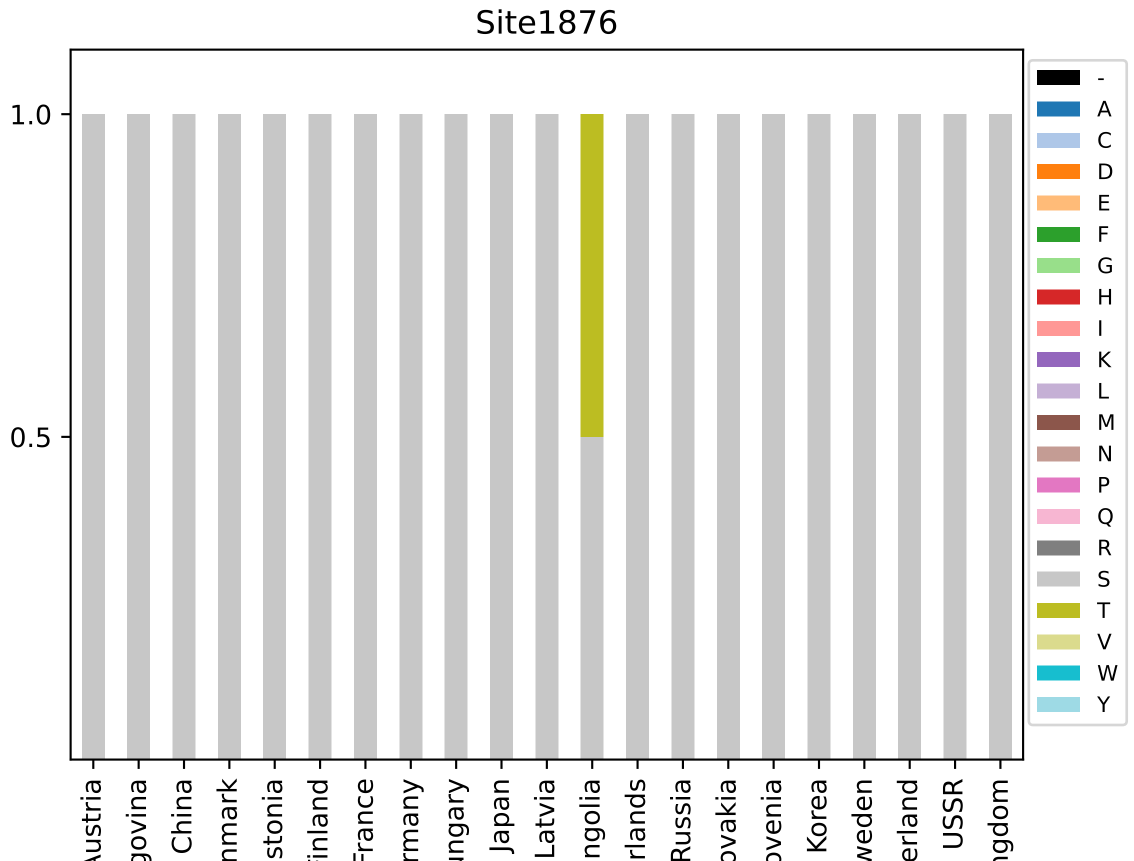
Site1876
1.0
0.5
-
A
C
D
E
F
G
H
I
K
L
M
N
P
Q
R
S
T
V
W
Y
Austria
govina
China
nmark
stonia
Finland
France
rmany
ungary
Japan
Latvia
ngolia
rlands
Russia
ovakia
ovenia
Korea
weden
erland
USSR
ngdom

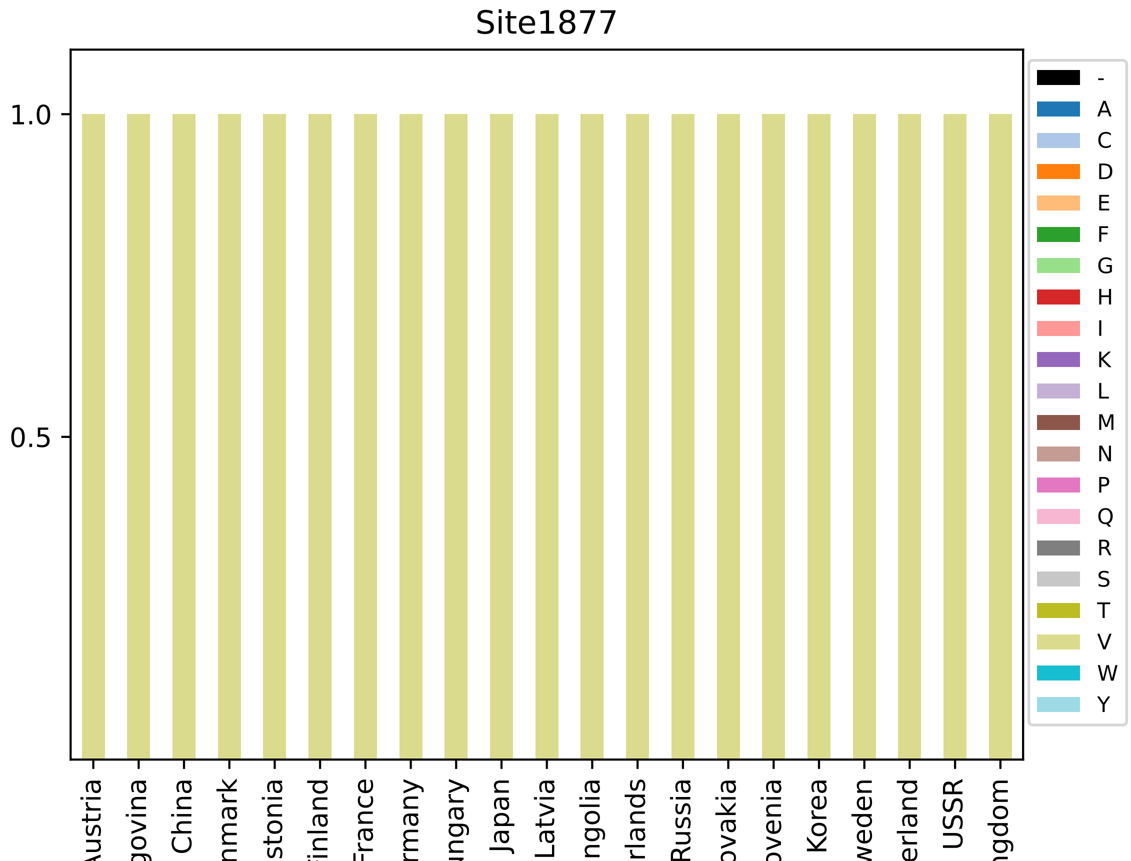

Site1877
1.0
0.5
-
A
C
D
E
F
G
H
I
K
L
M
N
P
Q
R
S
T
V
W
Y
Austria
govina
China
nmark
stonia
inland
France
rmany
ungary
Japan
Latvia
ngolia
rlands
Russia
ovakia
ovenia
Korea
weden
erland
USSR
ngdom

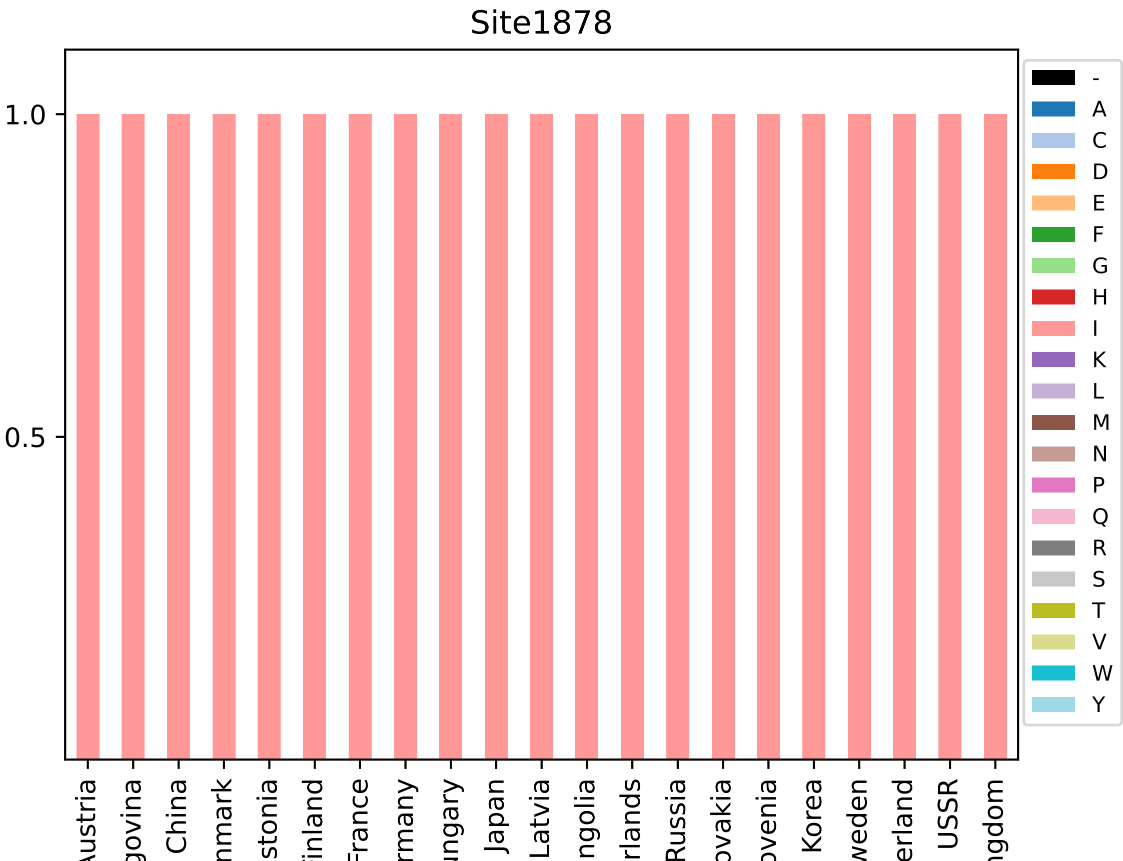

Site1878
1.0
0.5
Austria
govina
China
nmark
stonia
Finland
France
rmany
ungary
Japan
Latvia
ngolia
rlands
Russia
ovakia
ovenia
Korea
weden
erland
USSR
ngdom
-
A
C
D
E
F
G
H
I
K
L
M
N
P
Q
R
S
T
V
W
Y

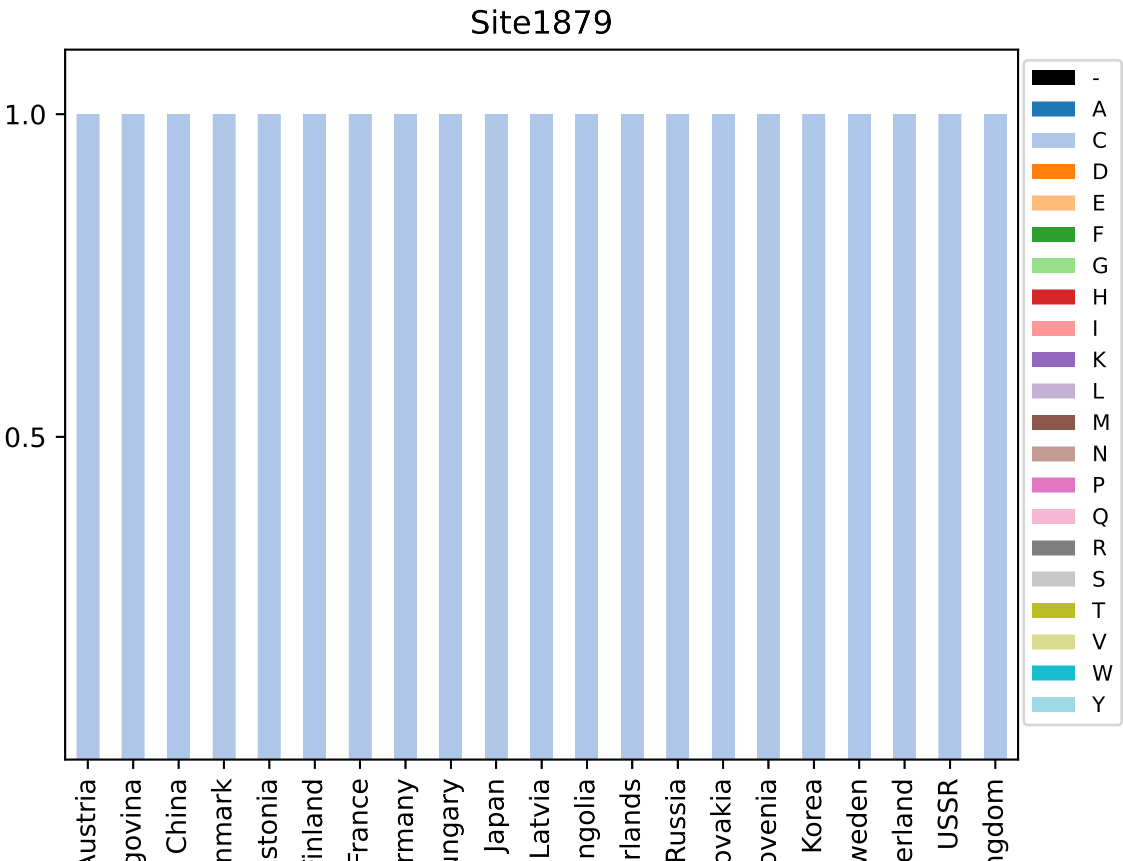
Site1879
1.0
0.5
-
A
C
D
E
F
G
H
I
K
L
M
N
P
Q
R
S
T
V
W
Y
Austria
China
Japan
Latvia
Russia
Korea
USSR

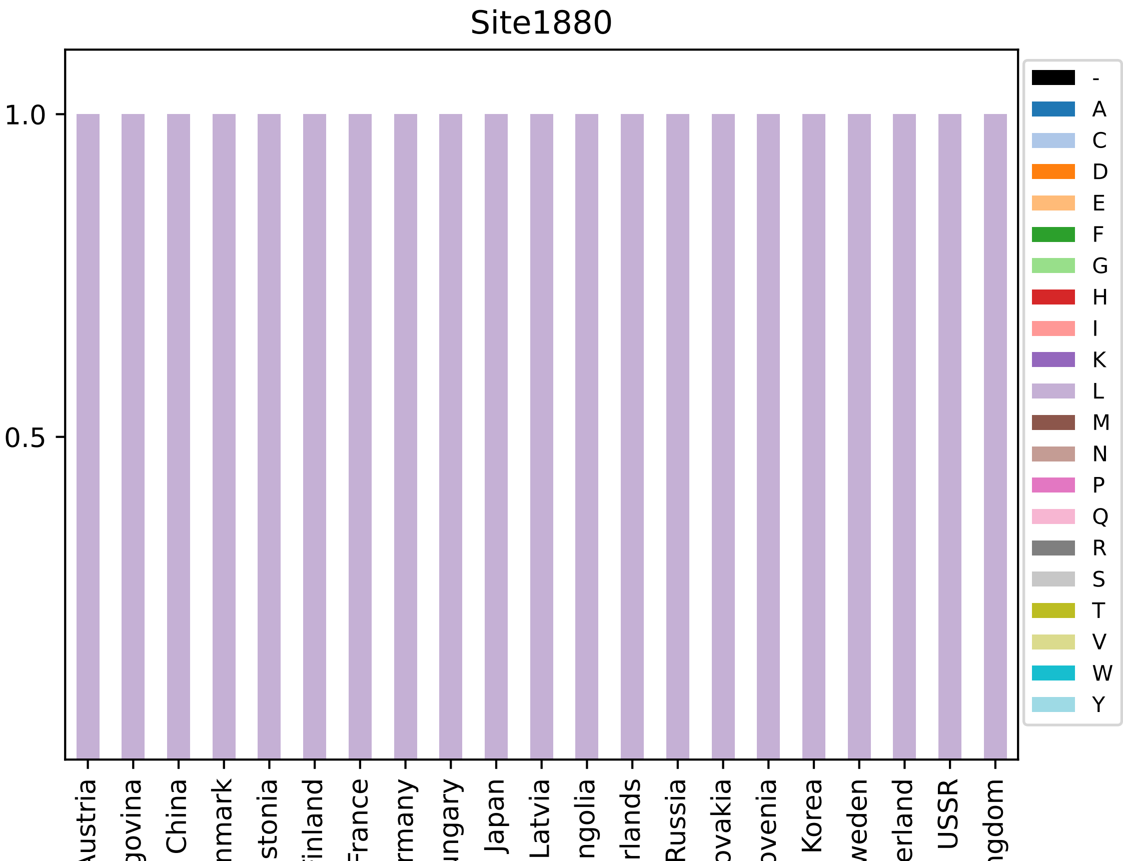

Site1880
1.0
0.5
-
A
C
D
E
F
G
H
I
K
L
M
N
P
Q
R
S
T
V
W
Y
Austria
China
Japan
Latvia
Russia
Korea
USSR

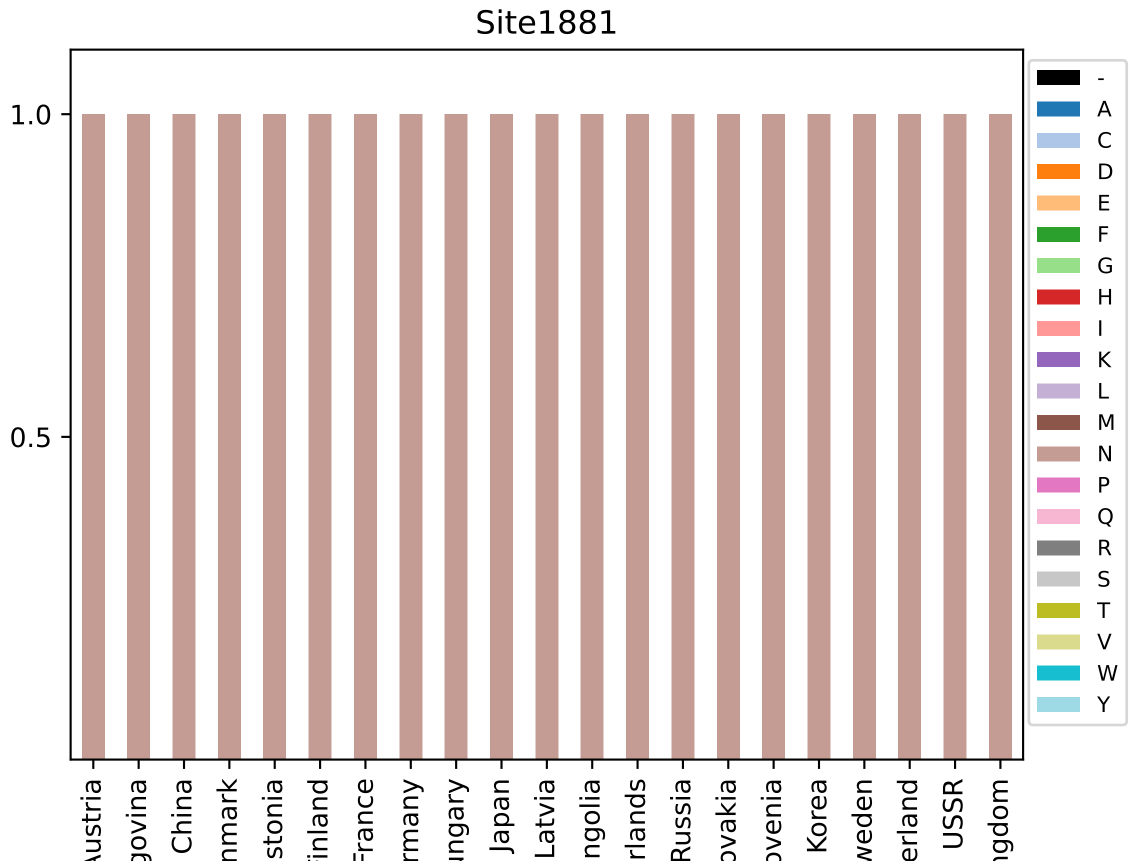

Site1881
1.0
0.5
-
A
C
D
E
F
G
H
I
K
L
M
N
P
Q
R
S
T
V
W
Y
Austria
govina
China
nmark
stonia
inland
France
rmany
ungary
Japan
Latvia
ngolia
rlands
Russia
ovakia
ovenia
Korea
weden
erland
USSR
ngdom

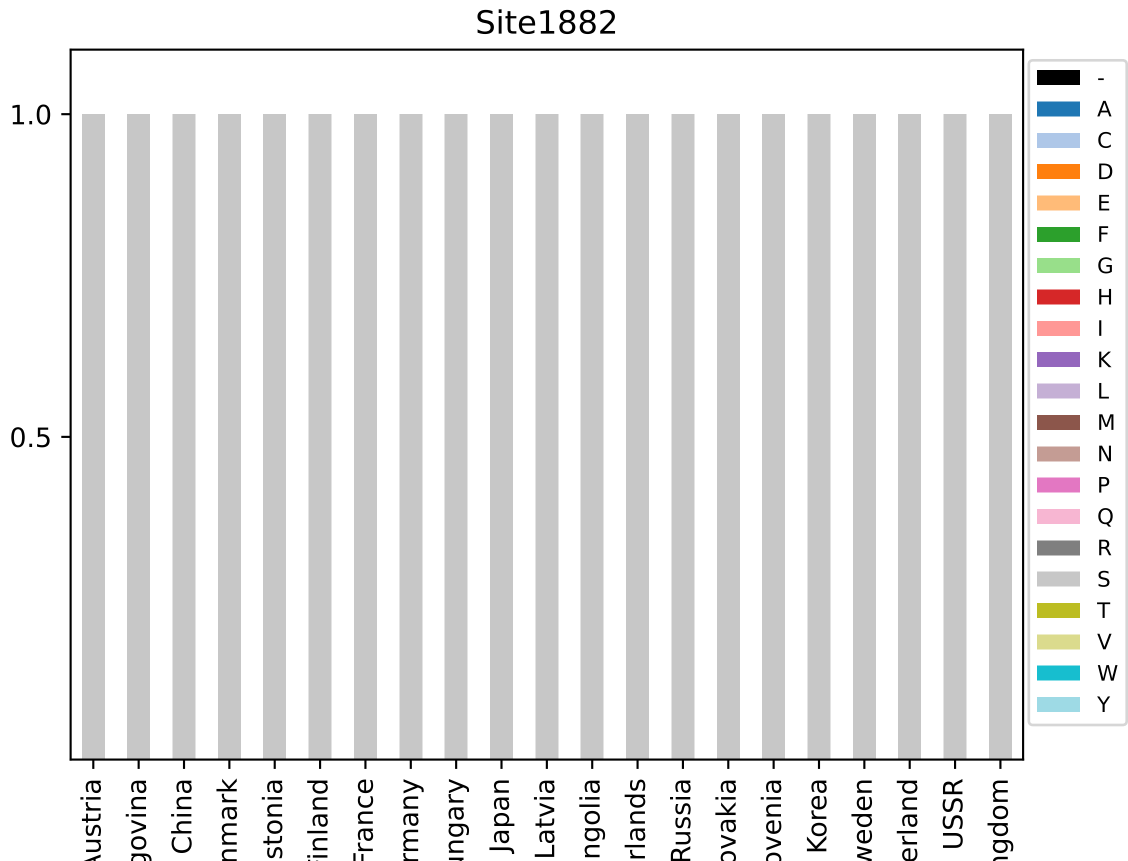

Site1882
1.0
0.5
-
A
C
D
E
F
G
H
I
K
L
M
N
P
Q
R
S
T
V
W
Y
Austria
govina
China
nmark
stonia
Finland
France
rmany
ungary
Japan
Latvia
ngolia
rlands
Russia
ovakia
ovenia
Korea
weden
erland
USSR
ngdom

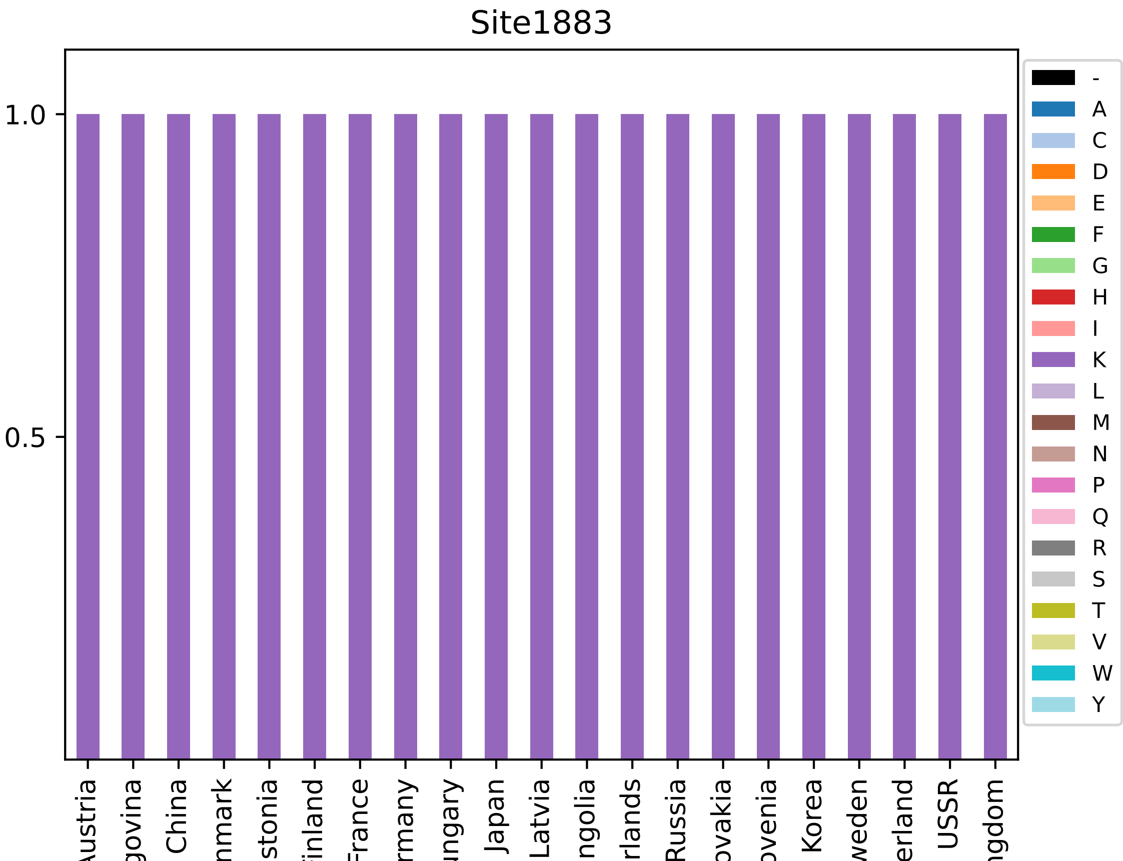
Site1883
1.0
0.5
-
A
C
D
E
F
G
H
I
K
L
M
N
P
Q
R
S
T
V
W
Y
Austria
govina
China
nmark
stonia
inland
France
rmany
ungary
Japan
Latvia
ngolia
rlands
Russia
ovakia
ovenia
Korea
weden
erland
USSR
ngdom

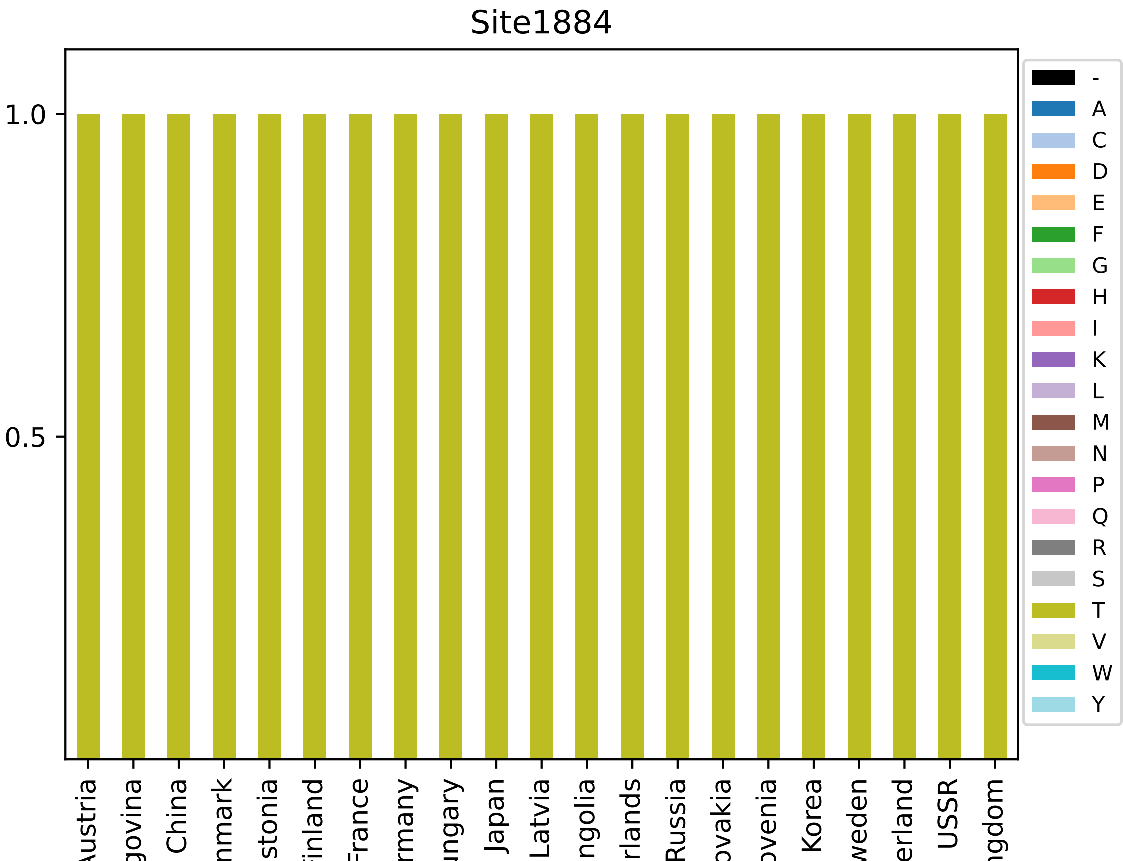
Site1884
1.0
0.5
-
A
C
D
E
F
G
H
I
K
L
M
N
P
Q
R
S
T
V
W
Y
China
Japan
Latvia
Russia
Korea
USSR

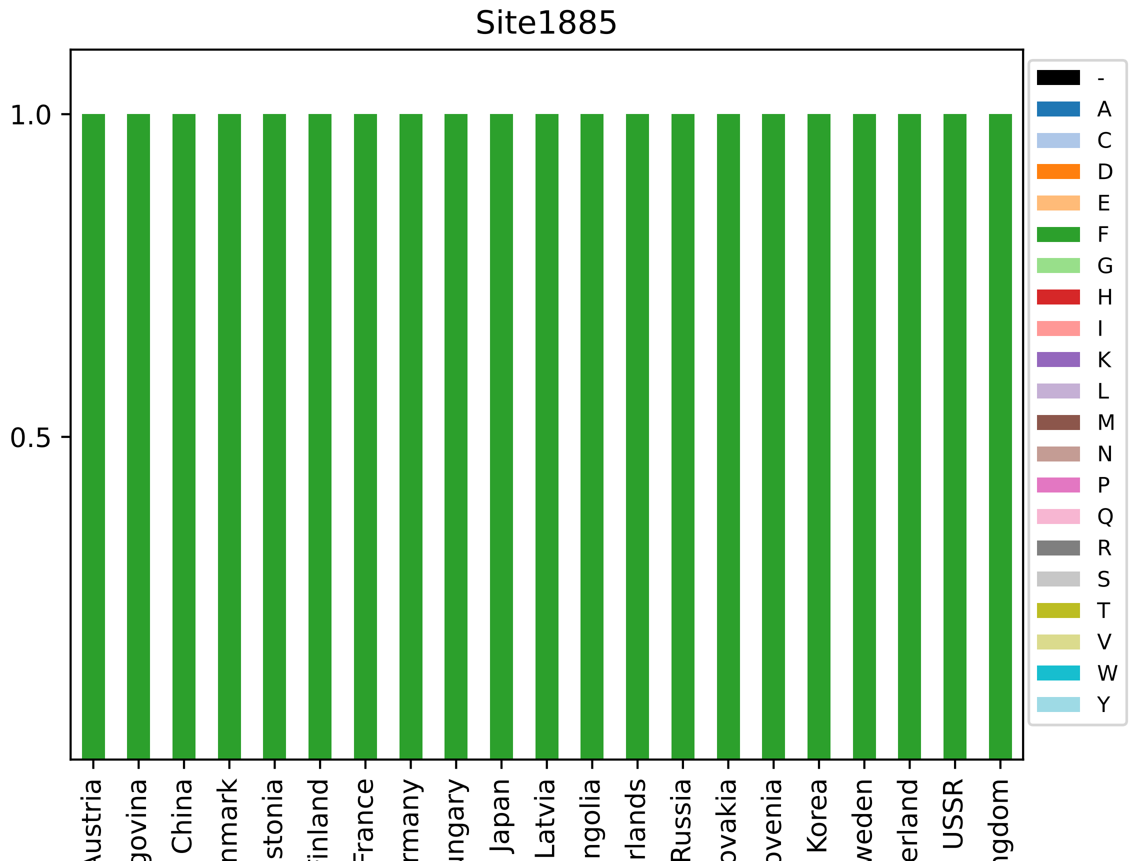
Site1885
1.0
0.5
-
A
C
D
E
F
G
H
I
K
L
M
N
P
Q
R
S
T
V
W
Y
China
Japan
Latvia
Russia
Korea
USSR

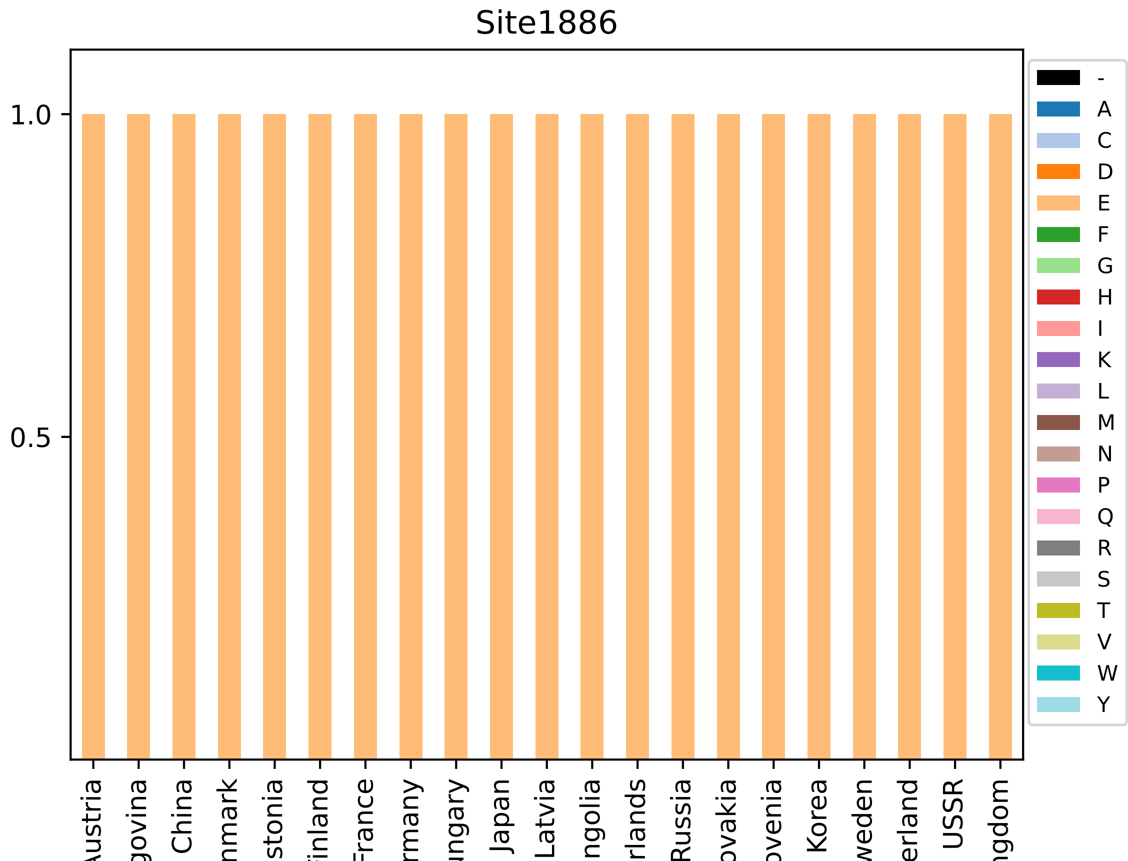

Site1886
1.0
0.5
-
A
C
D
E
F
G
H
I
K
L
M
N
P
Q
R
S
T
V
W
Y
China
Japan
Latvia
Russia
Korea
USSR

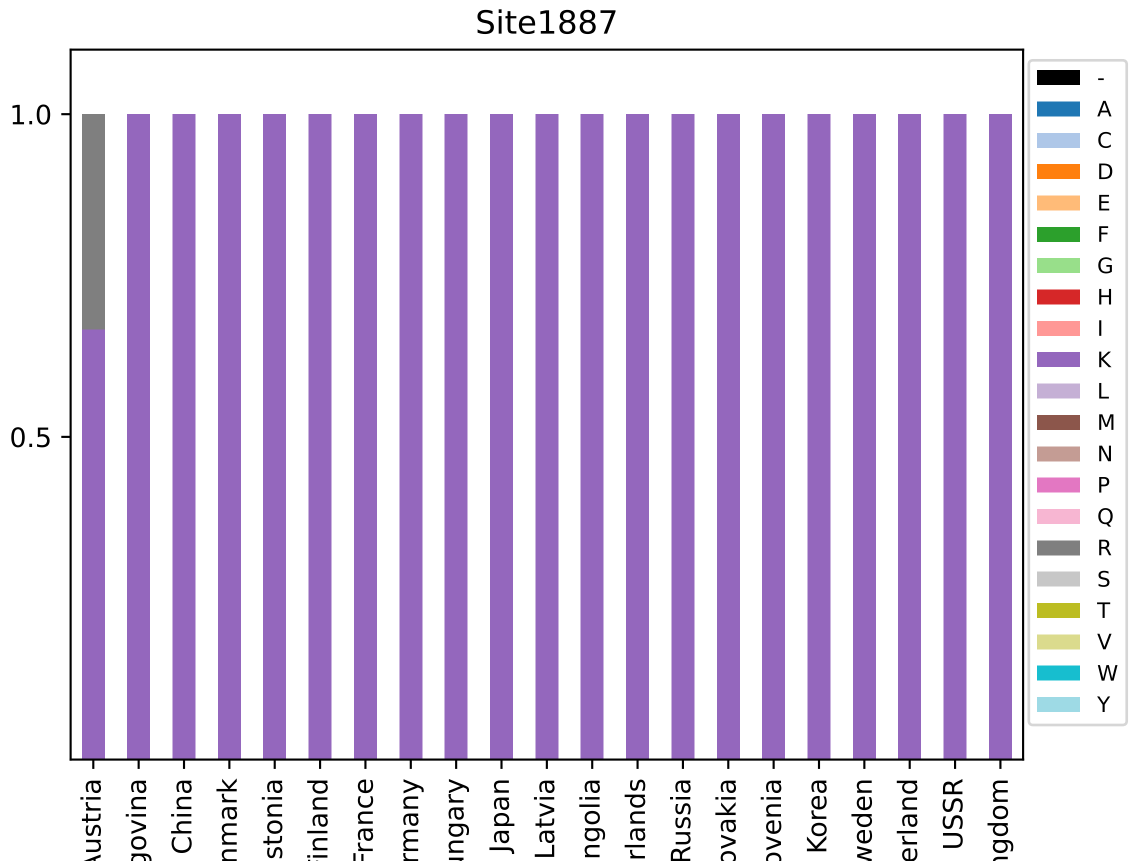
Site1887
1.0
0.5
-
A
C
D
E
F
G
H
I
K
L
M
N
P
Q
R
S
T
V
W
Y
China
Japan
Latvia
Russia
Korea
USSR

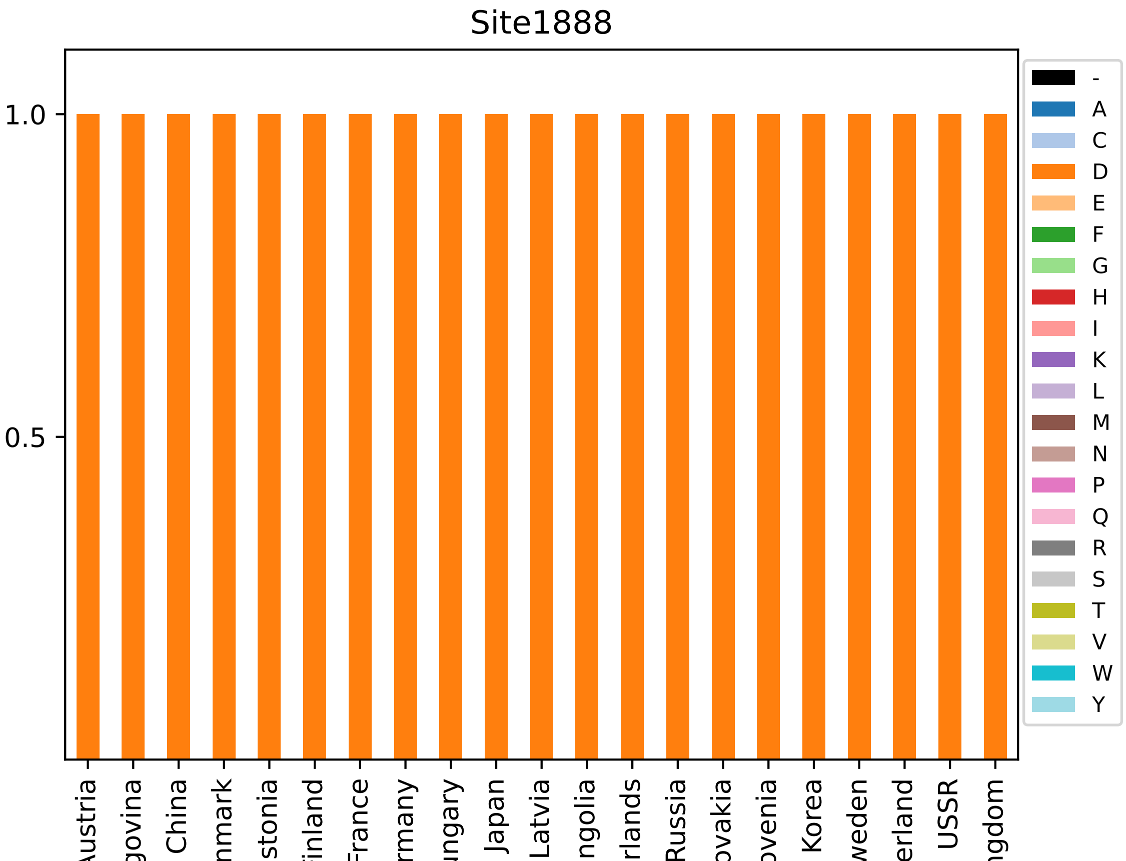
Site1888
1.0
0.5
-
A
C
D
E
F
G
H
I
K
L
M
N
P
Q
R
S
T
V
W
Y
China
Japan
Latvia
Russia
Korea
USSR

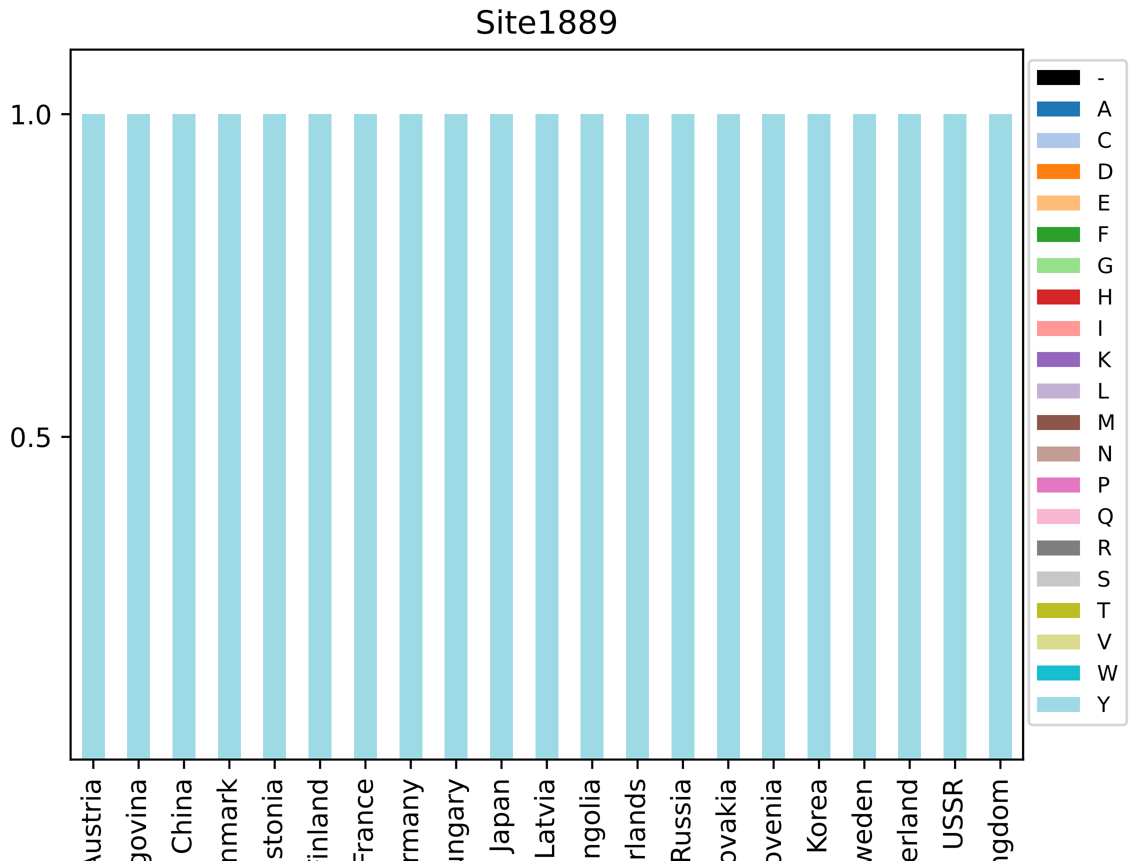

Site1889
1.0
0.5
Austria
govina
China
nmark
stonia
inland
France
rmany
ungary
Japan
Latvia
ngolia
rlands
Russia
ovakia
ovenia
Korea
weden
erland
USSR
ngdom
-
A
C
D
E
F
G
H
I
K
L
M
N
P
Q
R
S
T
V
W
Y

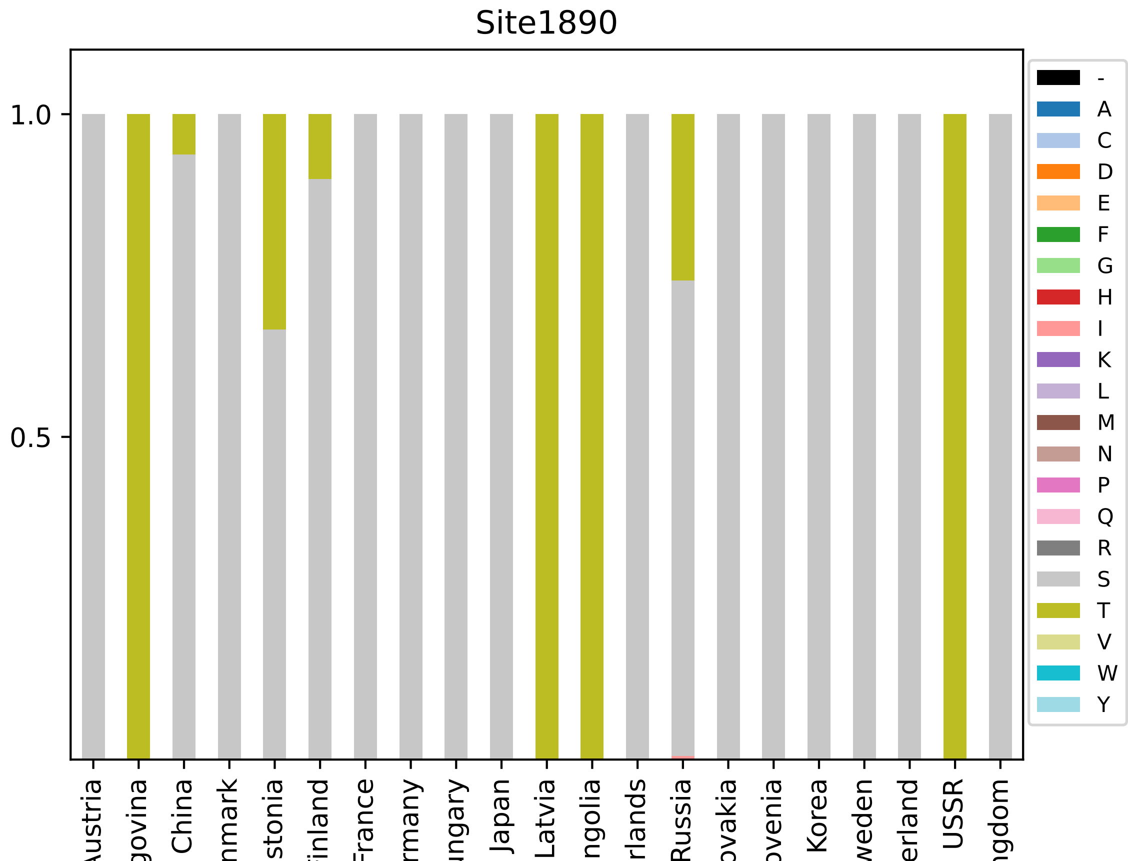
Site1890
1.0
0.5
Austria
govina
China
nmark
stonia
Finland
France
rmany
ungary
Japan
Latvia
ngolia
rlands
Russia
ovakia
ovenia
Korea
weden
erland
USSR
ngdom
-
A
C
D
E
F
G
H
I
K
L
M
N
P
Q
R
S
T
V
W
Y

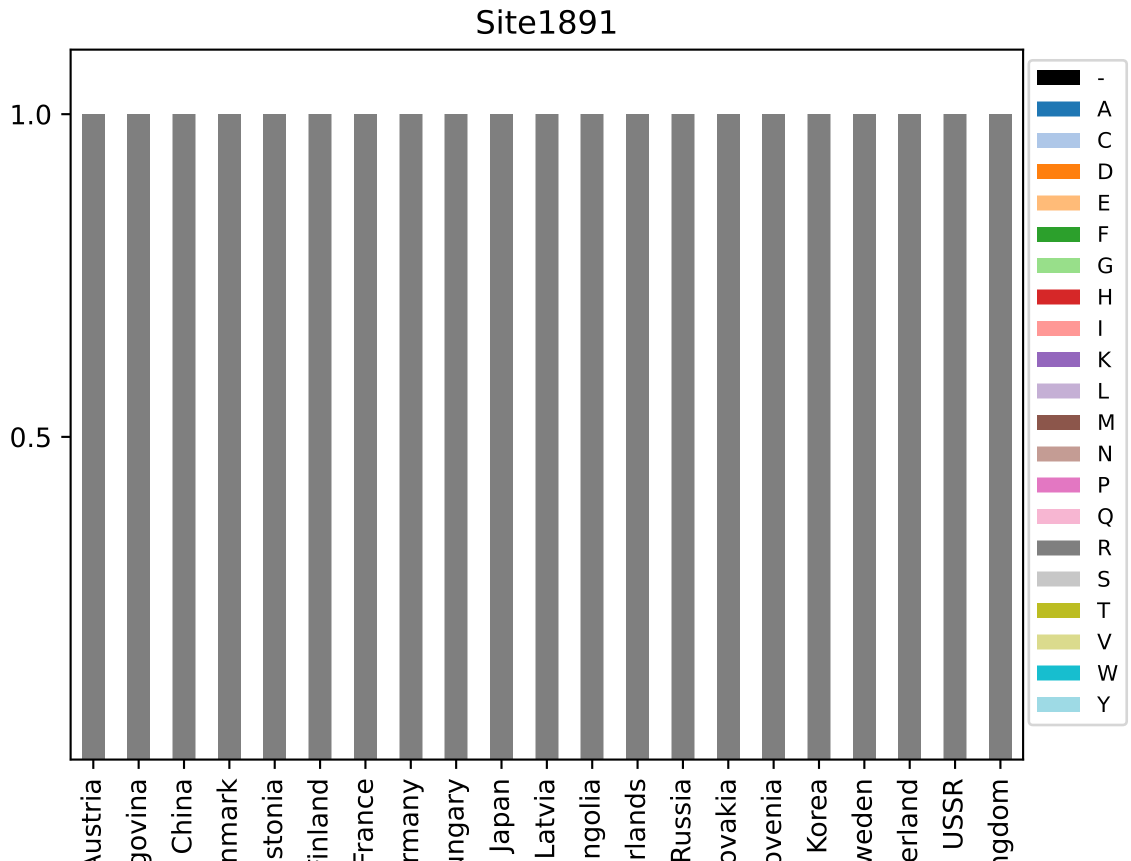

Site1891
1.0
0.5
-
A
C
D
E
F
G
H
I
K
L
M
N
P
Q
R
S
T
V
W
Y
Austria
China
Finland
Japan
Latvia
Russia
Korea
USSR

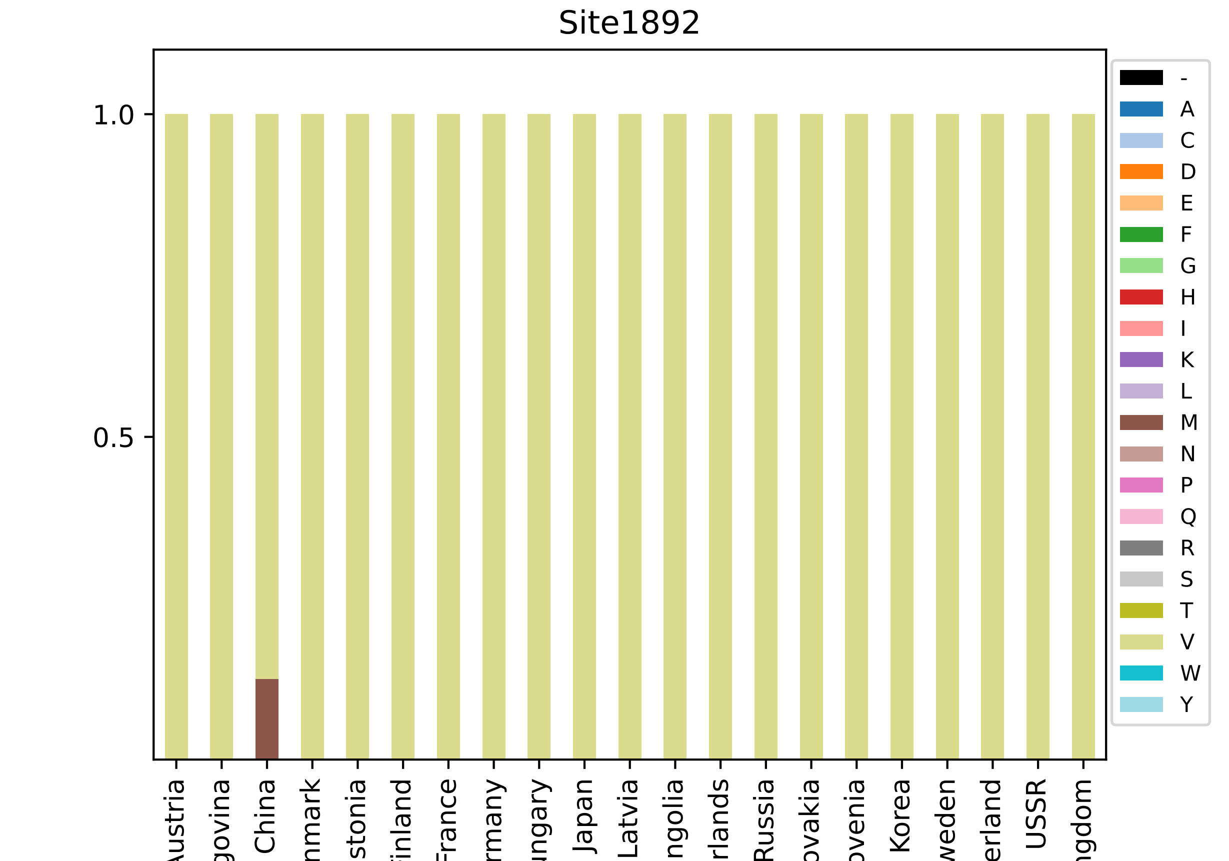
Site1892
1.0
0.5
-
A
C
D
E
F
G
H
I
K
L
M
N
P
Q
R
S
T
V
W
Y
Austria
govina
China
nmark
stonia
inland
France
rmany
ungary
Japan
Latvia
ngolia
rlands
Russia
ovakia
ovenia
Korea
weden
erland
USSR
ngdom

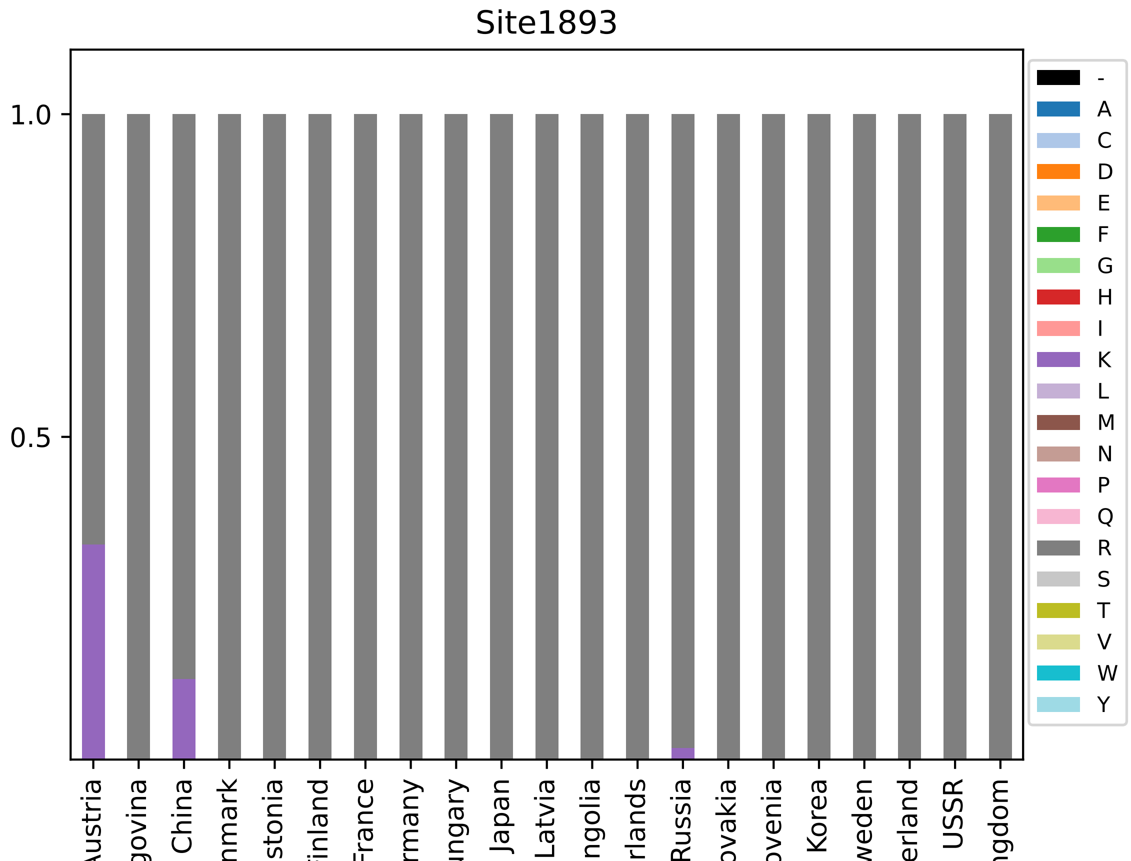
Site1893
1.0
0.5
-
A
C
D
E
F
G
H
I
K
L
M
N
P
Q
R
S
T
V
W
Y
Austria
govina
China
nmark
stonia
Finland
France
rmany
ungary
Japan
Latvia
ngolia
rlands
Russia
ovakia
ovenia
Korea
weden
erland
USSR
ngdom

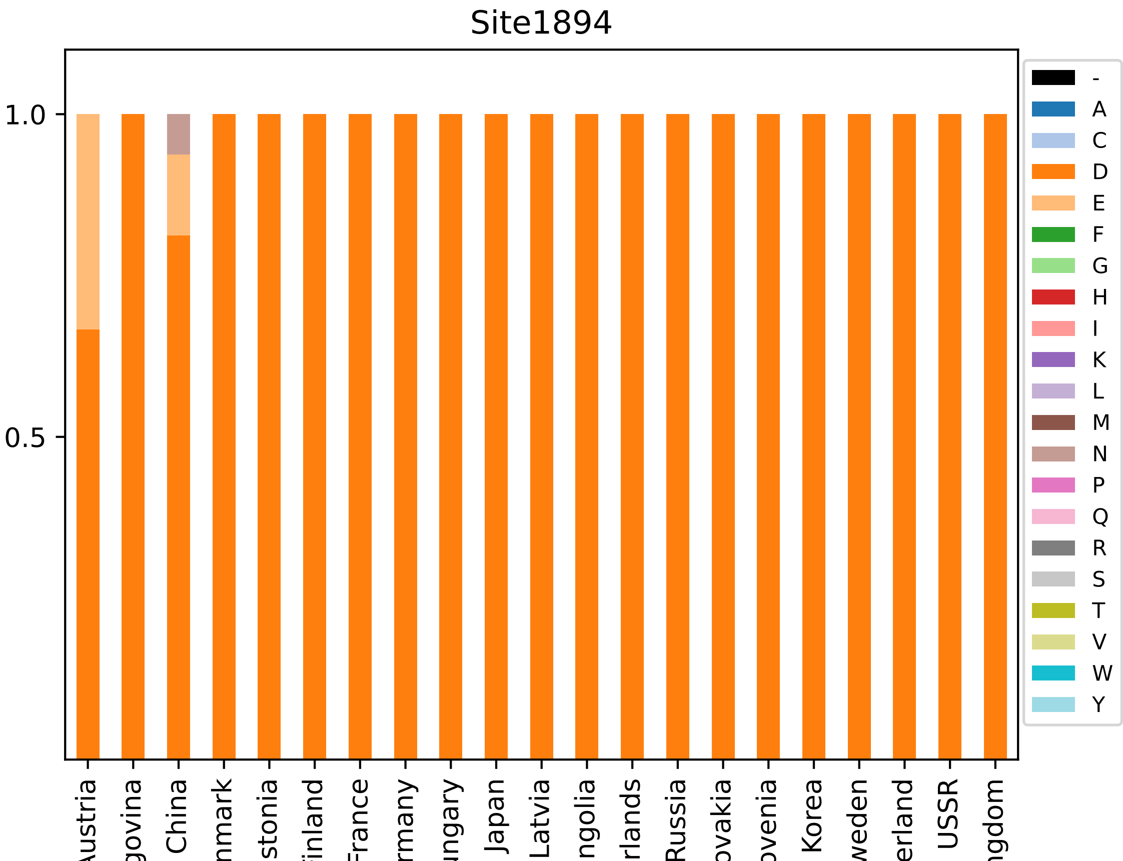

Site1894
1.0
0.5
-
A
C
D
E
F
G
H
I
K
L
M
N
P
Q
R
S
T
V
W
Y
Austria
govina
China
nmark
stonia
inland
France
rmany
ungary
Japan
Latvia
ngolia
rlands
Russia
ovakia
ovenia
Korea
weden
erland
USSR
ngdom

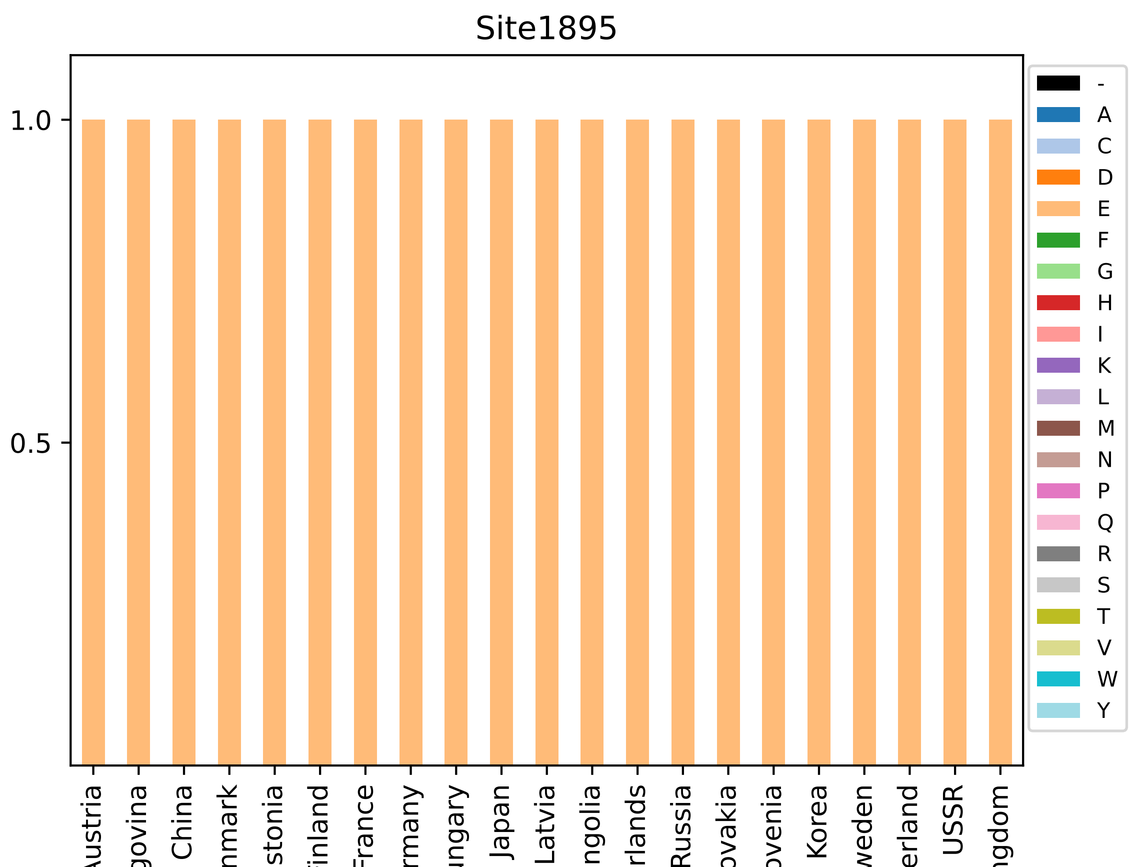

Site1895
1.0
0.5
-
A
C
D
E
F
G
H
I
K
L
M
N
P
Q
R
S
T
V
W
Y
China
Japan
Latvia
Russia
Korea
USSR

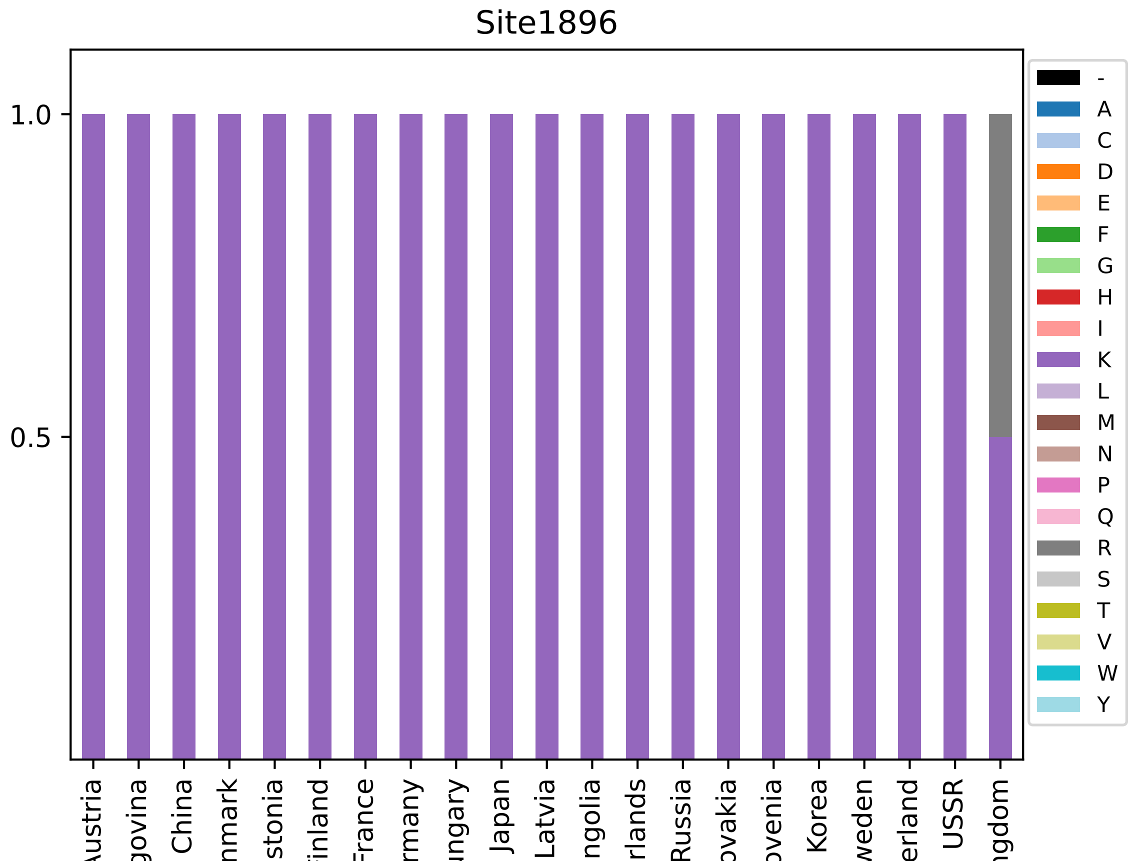

Site1896
1.0
0.5
-
A
C
D
E
F
G
H
I
K
L
M
N
P
Q
R
S
T
V
W
Y
Austria
China
Finland
France
Japan
Latvia
Russia
Korea
USSR

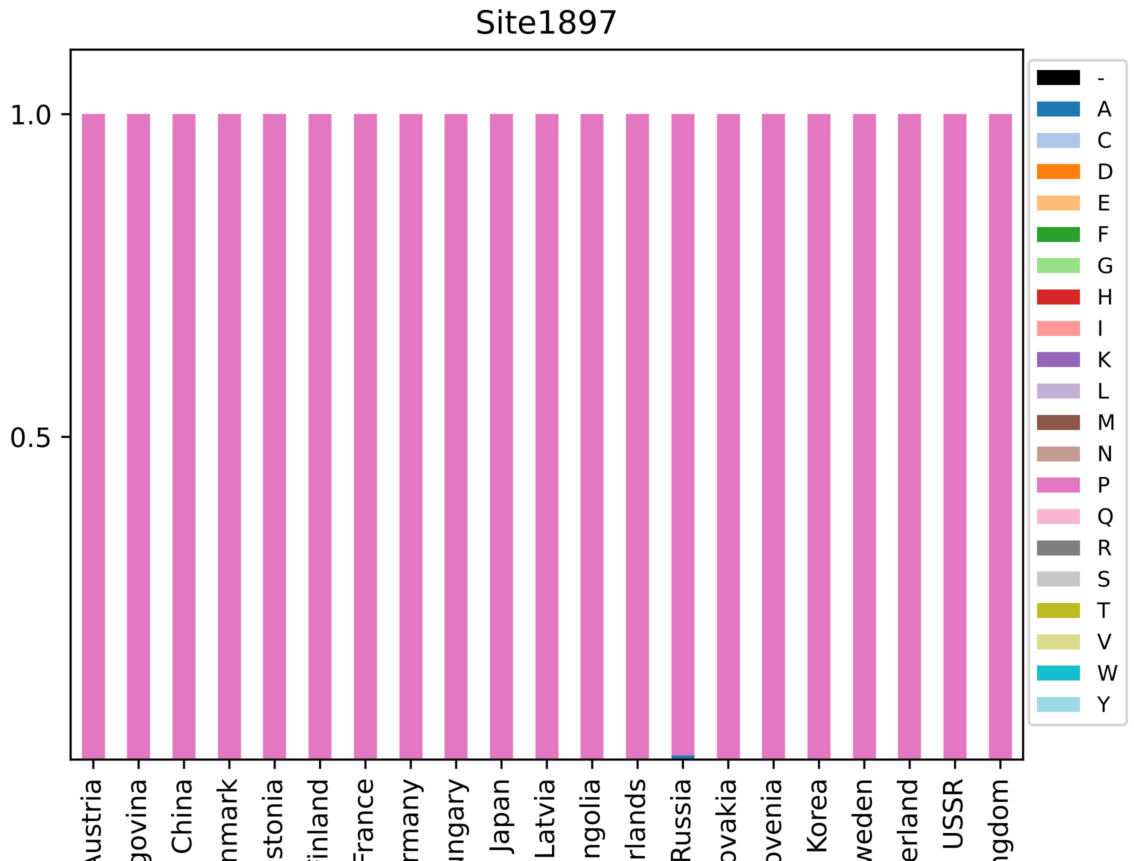
Site1897
1.0
0.5
-
A
C
D
E
F
G
H
I
K
L
M
N
P
Q
R
S
T
V
W
Y
Austria
govina
China
nmark
stonia
Finland
France
rmany
ungary
Japan
Latvia
ngolia
rlands
Russia
ovakia
ovenia
Korea
weden
erland
USSR
ngdom

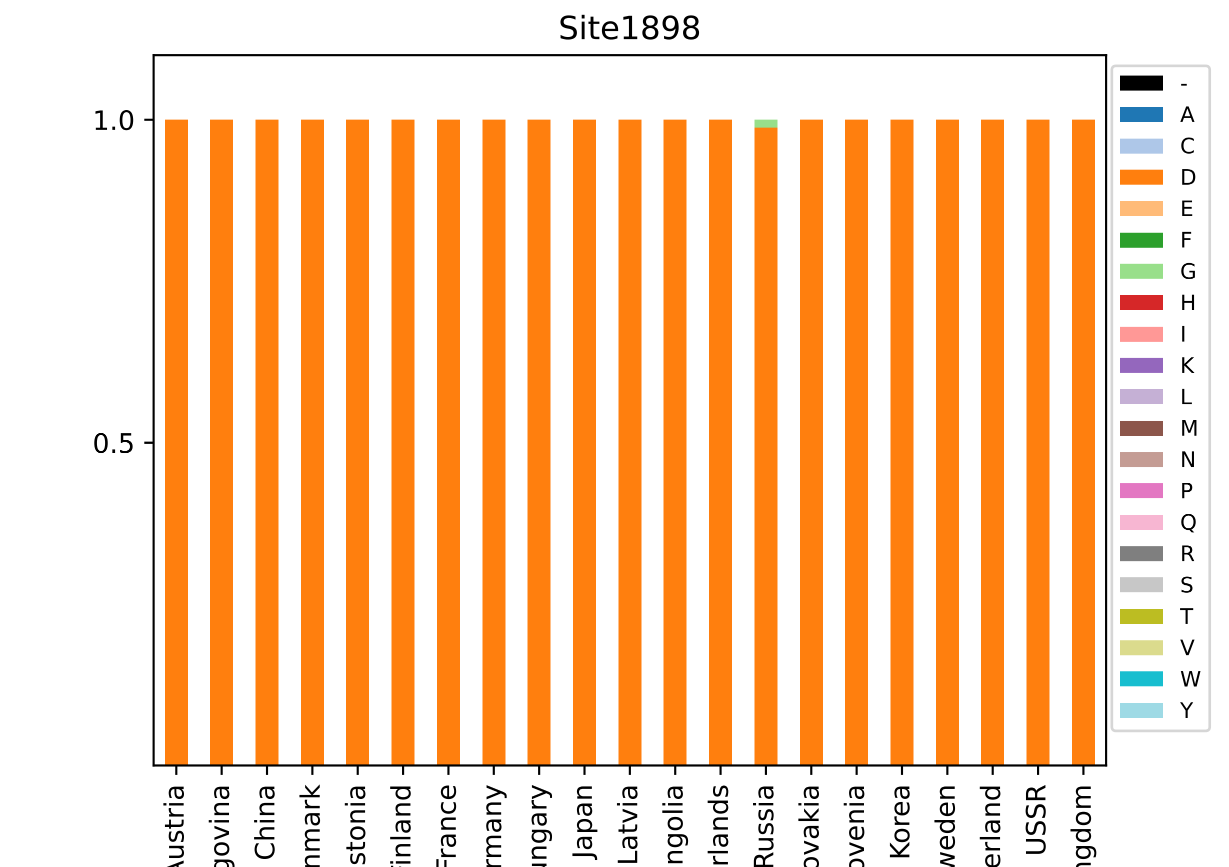
Site1898
1.0
0.5
-
A
C
D
E
F
G
H
I
K
L
M
N
P
Q
R
S
T
V
W
Y
Austria
govina
China
nmark
stonia
Finland
France
rmany
ungary
Japan
Latvia
ngolia
rlands
Russia
ovakia
ovenia
Korea
weden
erland
USSR
ngdom

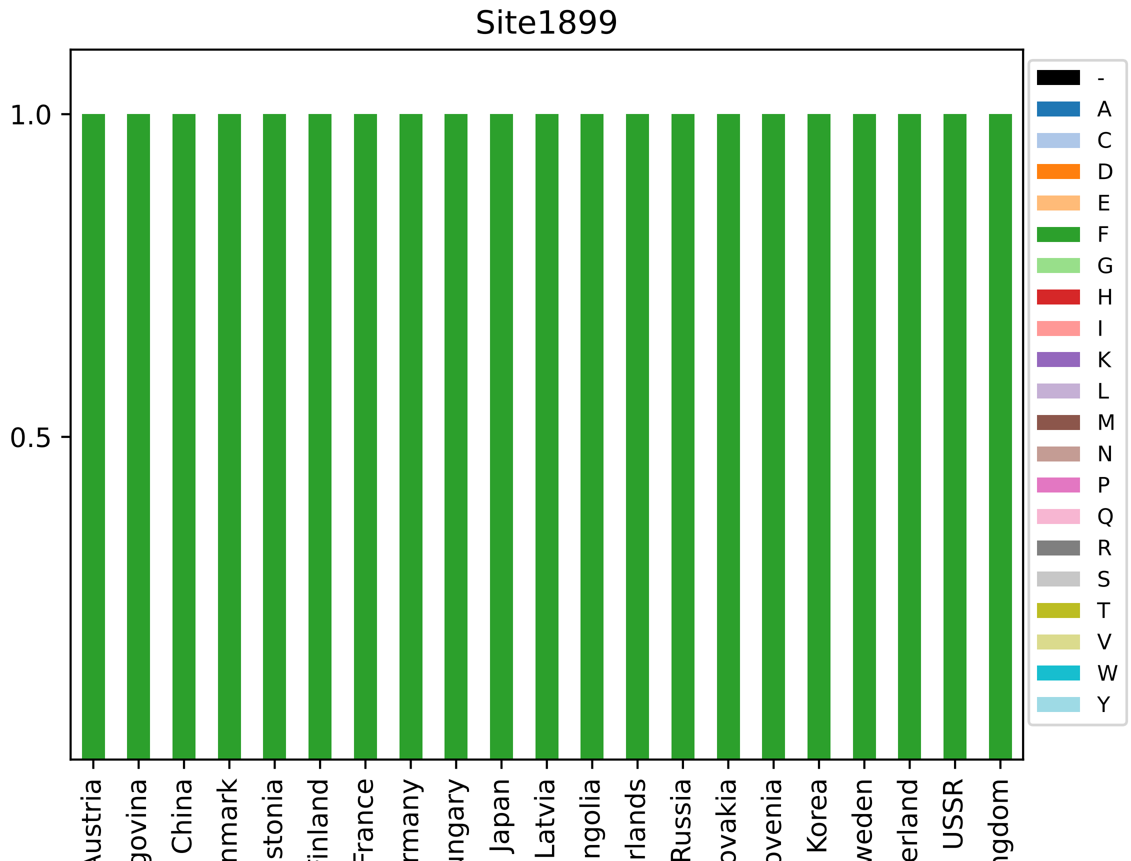
Site1899
1.0
0.5
-
A
C
D
E
F
G
H
I
K
L
M
N
P
Q
R
S
T
V
W
Y
Austria
govina
China
nmark
stonia
Finland
France
rmany
ungary
Japan
Latvia
ngolia
rlands
Russia
ovakia
ovenia
Korea
weden
erland
USSR
ngdom

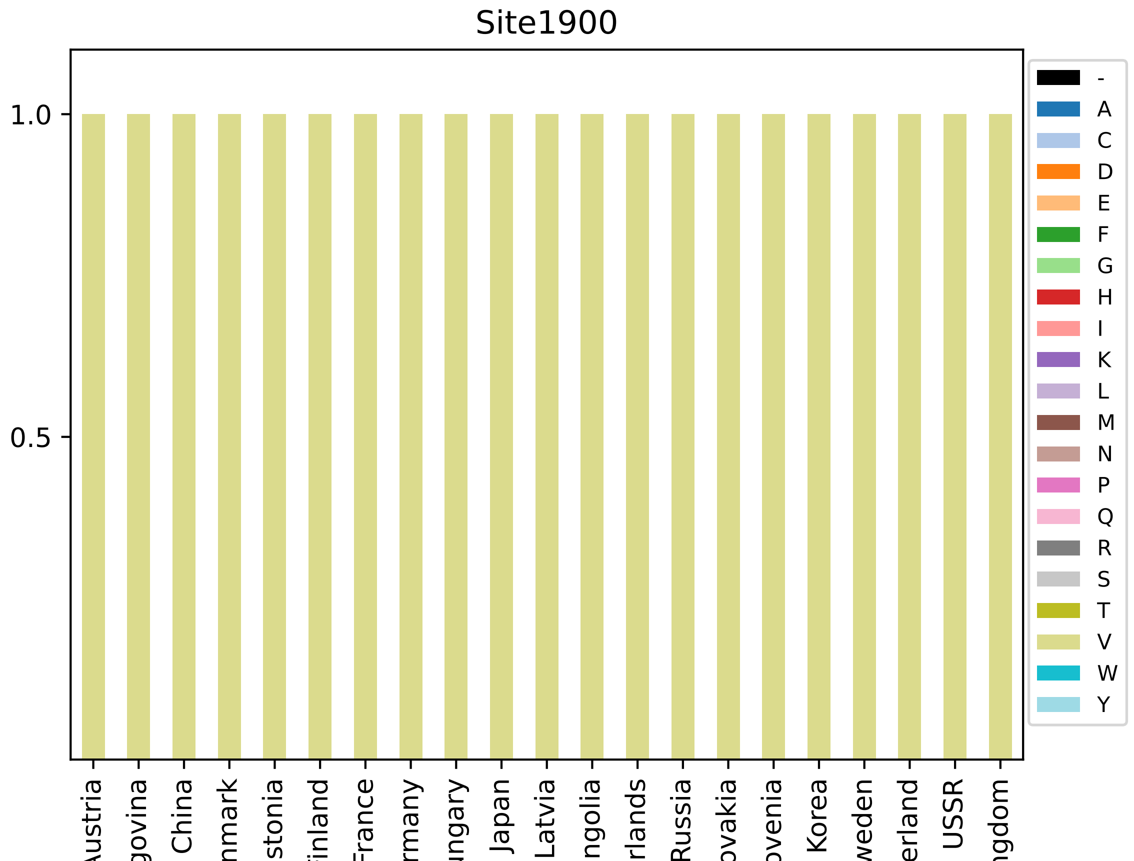
Site1900
1.0
0.5
China
Japan
Latvia
Korea
USSR
-
A
C
D
E
F
G
H
I
K
L
M
N
P
Q
R
S
T
V
W
Y

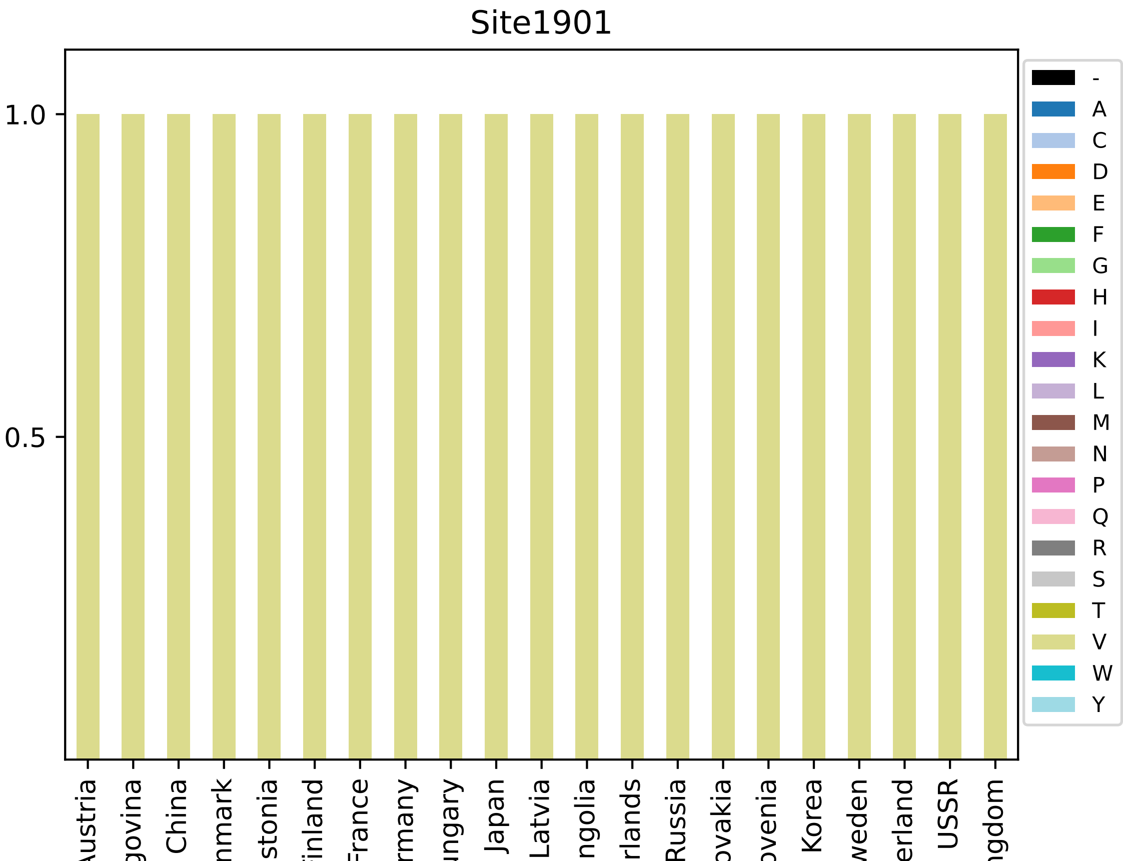

Site1901
1.0
0.5
-
A
C
D
E
F
G
H
I
K
L
M
N
P
Q
R
S
T
V
W
Y
Austria
China
Finland
France
Japan
Latvia
Russia
Korea
USSR

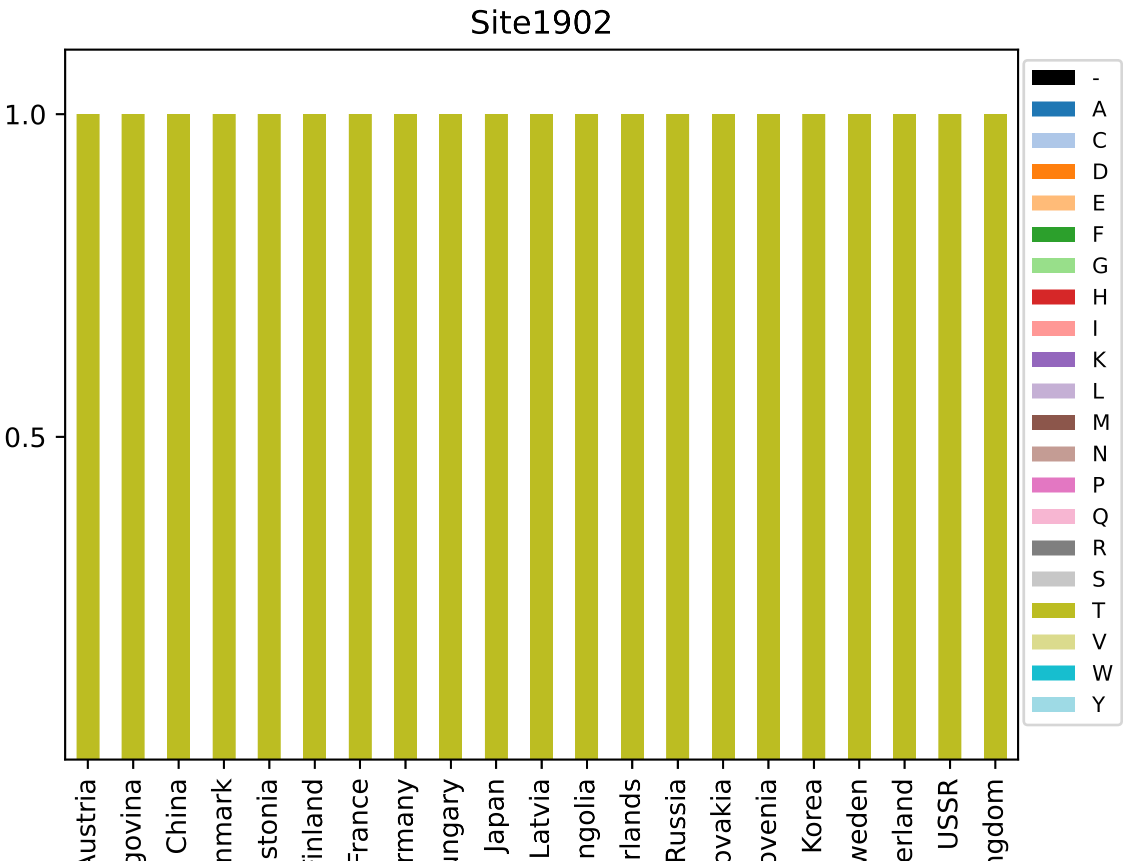

Site1902
1.0
0.5
-
A
C
D
E
F
G
H
I
K
L
M
N
P
Q
R
S
T
V
W
Y
China
Japan
Latvia
Russia
Korea
USSR

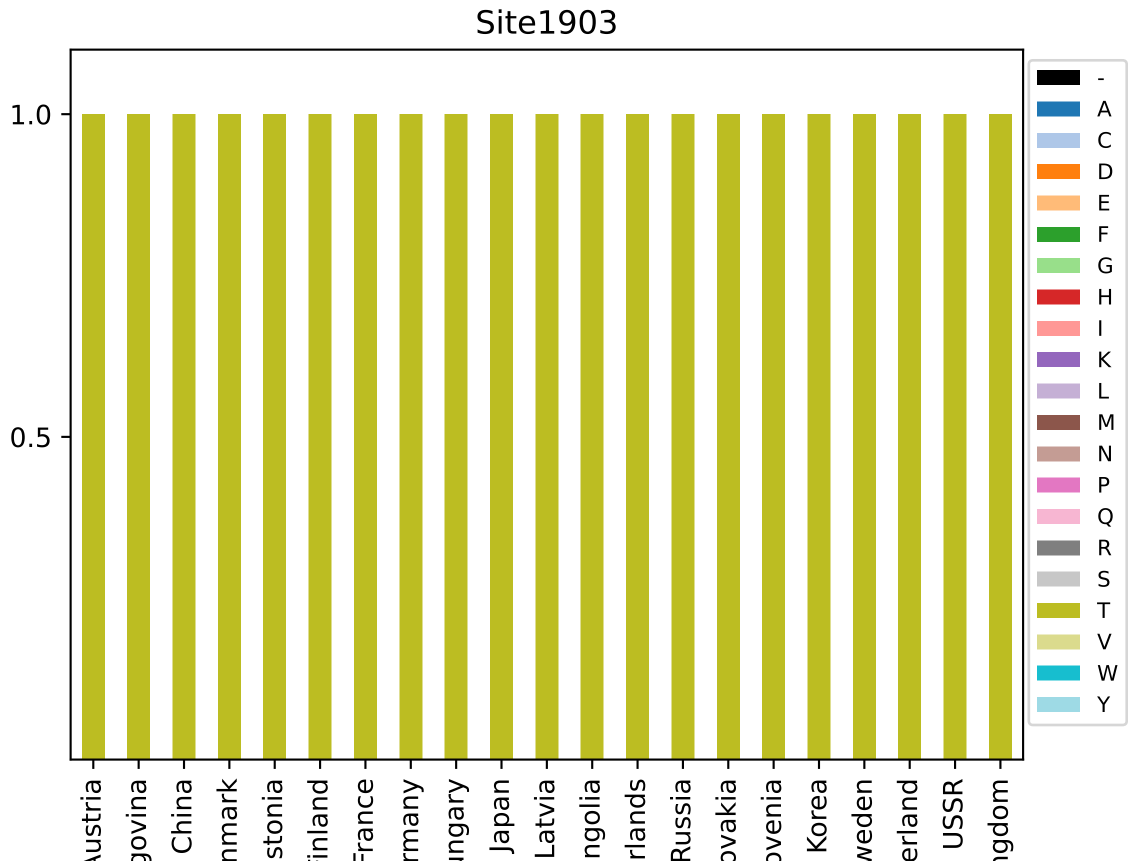
Site1903
1.0
0.5
-
A
C
D
E
F
G
H
I
K
L
M
N
P
Q
R
S
T
V
W
Y
China
Japan
Latvia
Russia
Korea
USSR

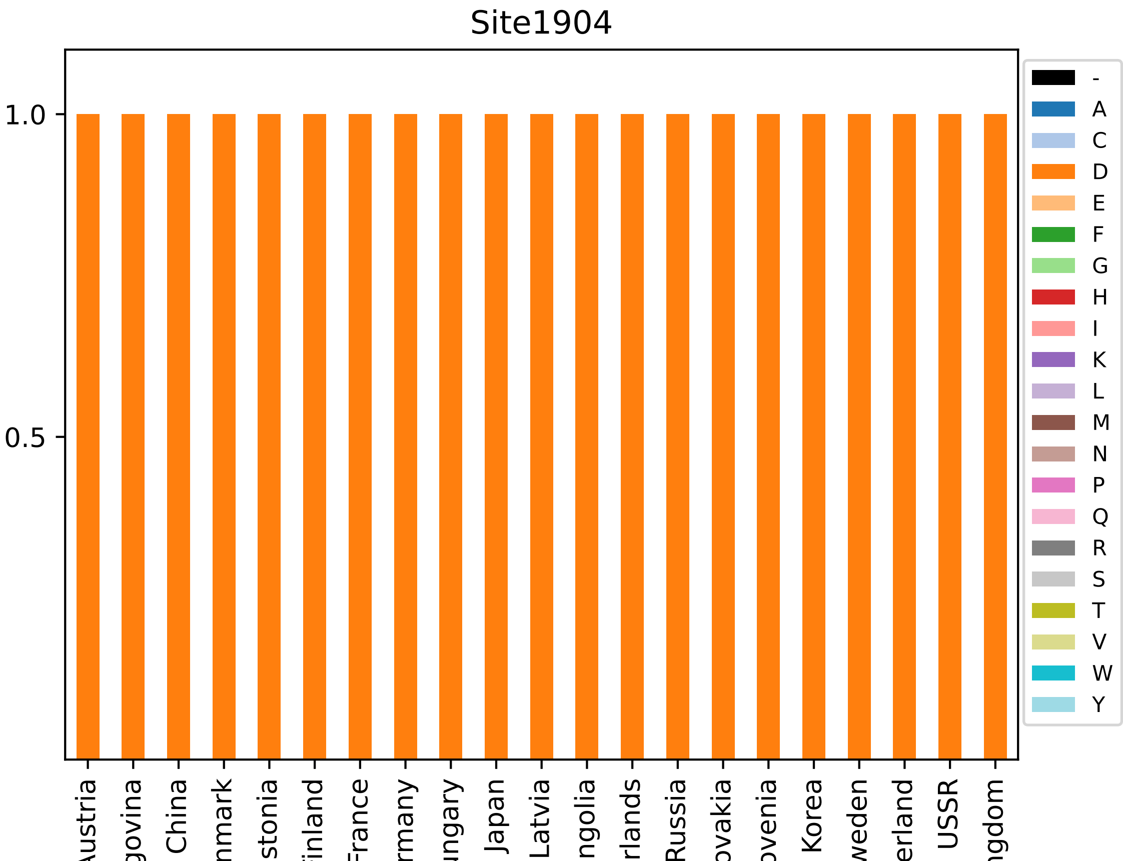

Site1904
1.0
0.5
-
A
C
D
E
F
G
H
I
K
L
M
N
P
Q
R
S
T
V
W
Y
Austria
govina
China
nmark
stonia
Finland
France
rmany
ungary
Japan
Latvia
ngolia
rlands
Russia
ovakia
ovenia
Korea
weden
erland
USSR
ngdom

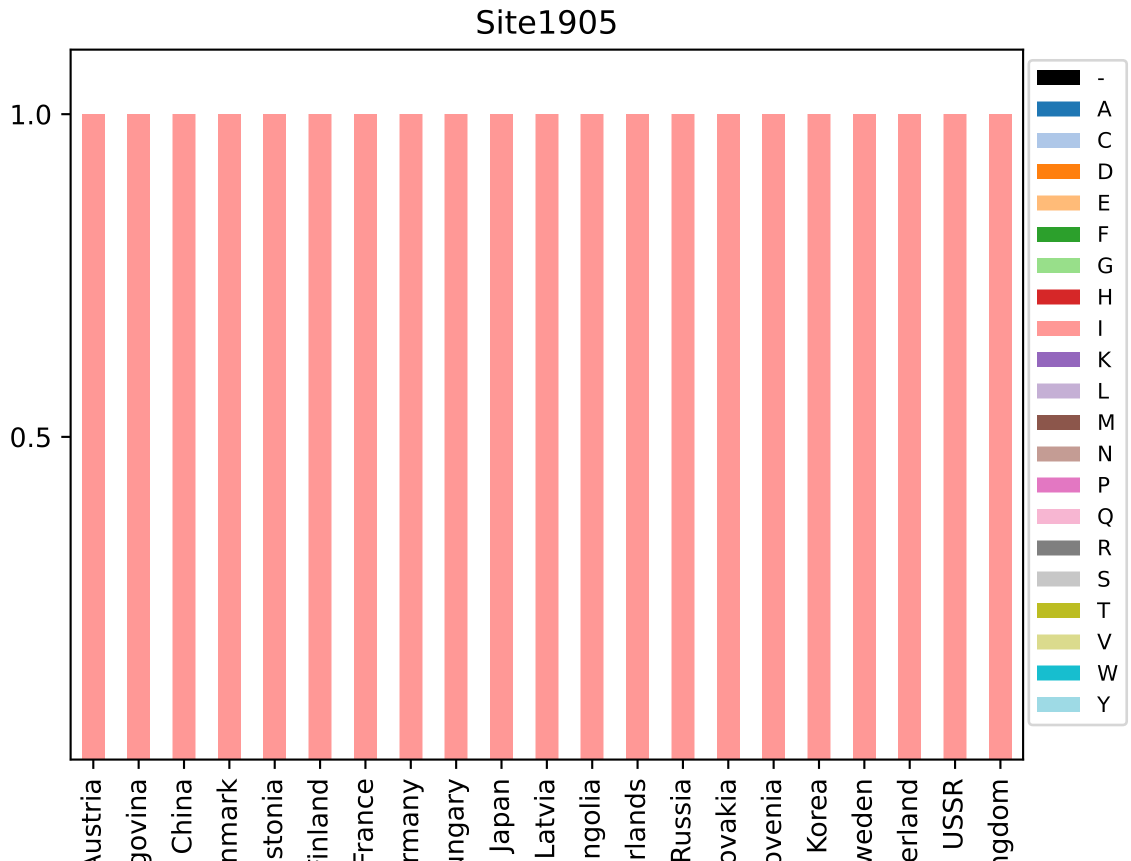
Site1905
1.0
0.5
-
A
C
D
E
F
G
H
I
K
L
M
N
P
Q
R
S
T
V
W
Y
China
Japan
Latvia
Russia
Korea
USSR

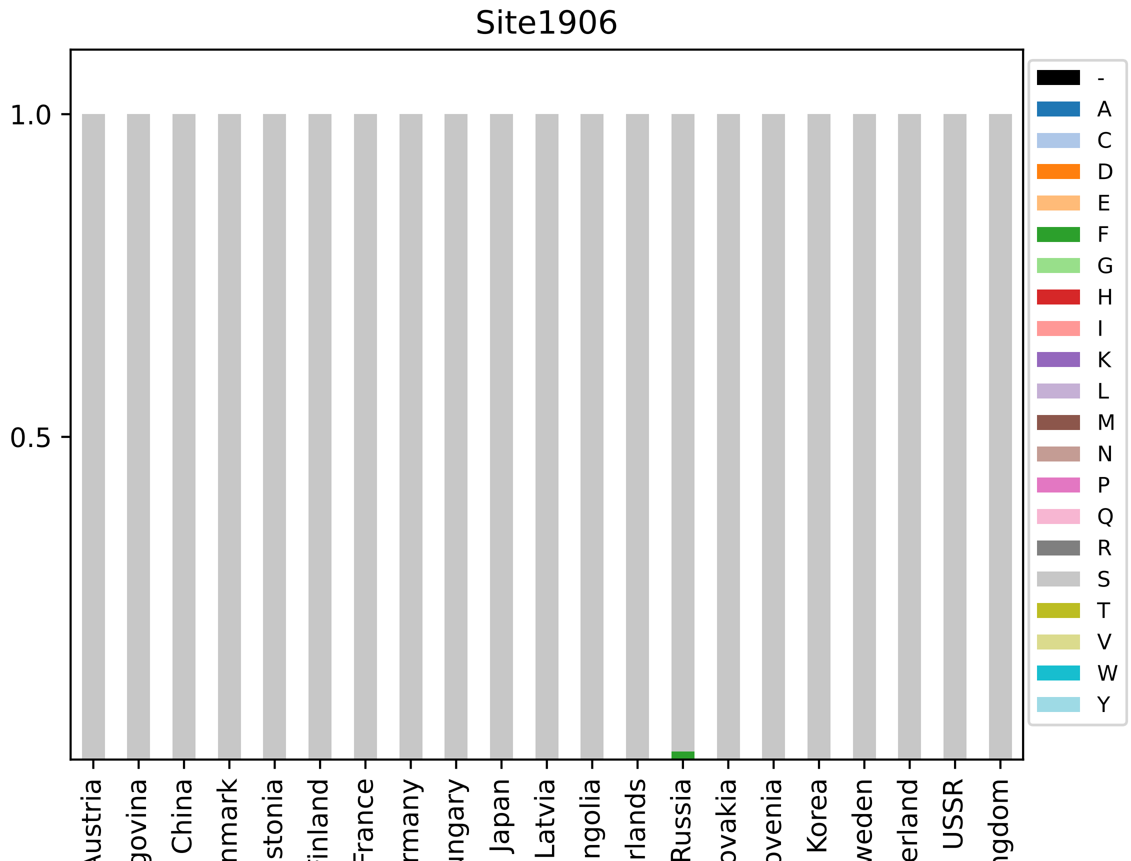

Site1906
1.0
0.5
-
A
C
D
E
F
G
H
I
K
L
M
N
P
Q
R
S
T
V
W
Y
Austria
govina
China
nmark
stonia
Finland
France
rmany
ungary
Japan
Latvia
ngolia
rlands
Russia
ovakia
ovenia
Korea
weden
erland
USSR
ngdom

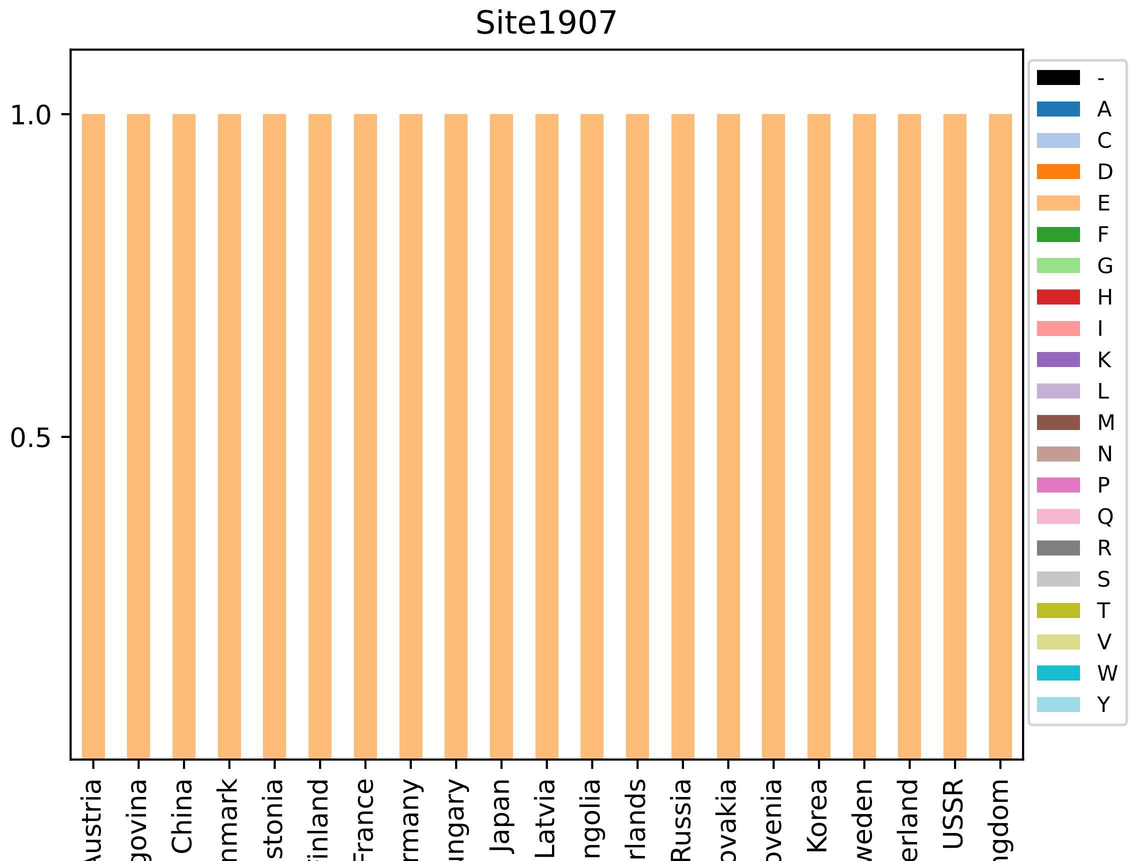
Site1907
1.0
0.5
-
A
C
D
E
F
G
H
I
K
L
M
N
P
Q
R
S
T
V
W
Y
China
Japan
Latvia
Russia
Korea
USSR

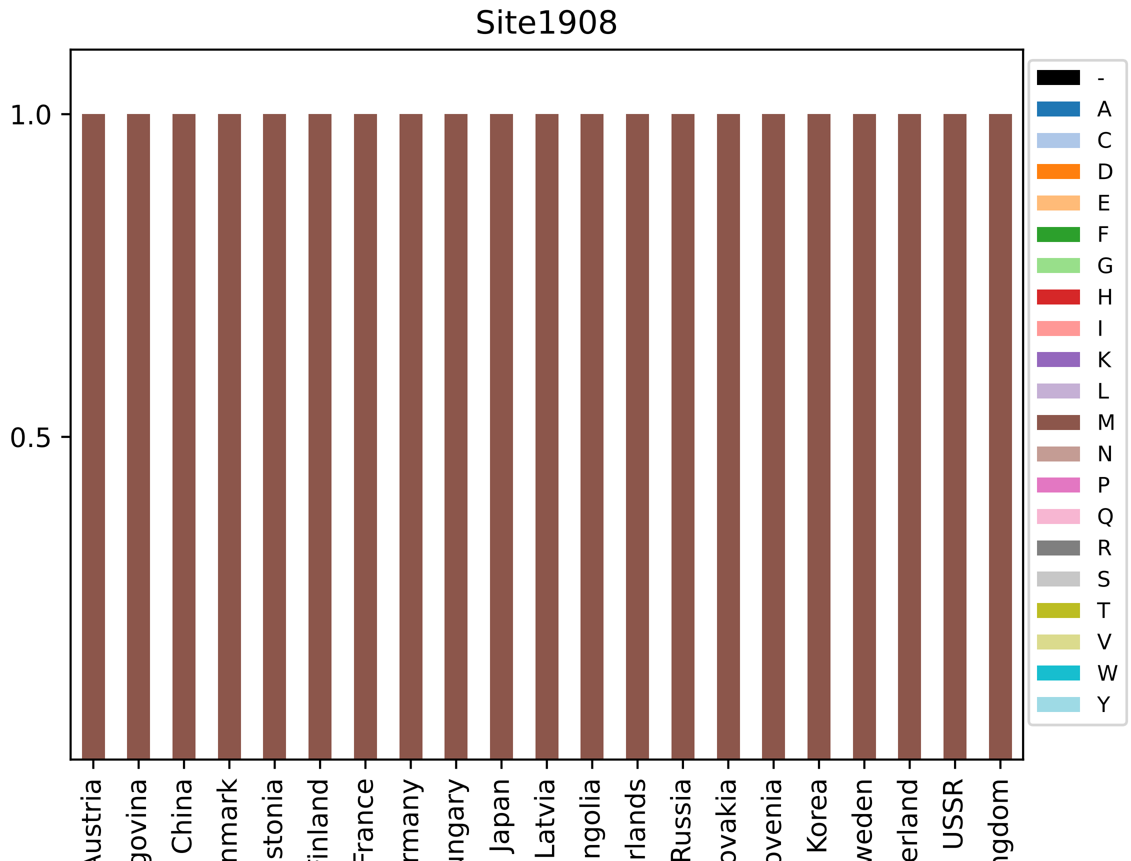

Site1908
1.0
0.5
-
A
C
D
E
F
G
H
I
K
L
M
N
P
Q
R
S
T
V
W
Y
Austria
China
Japan
Latvia
Russia
Korea
USSR

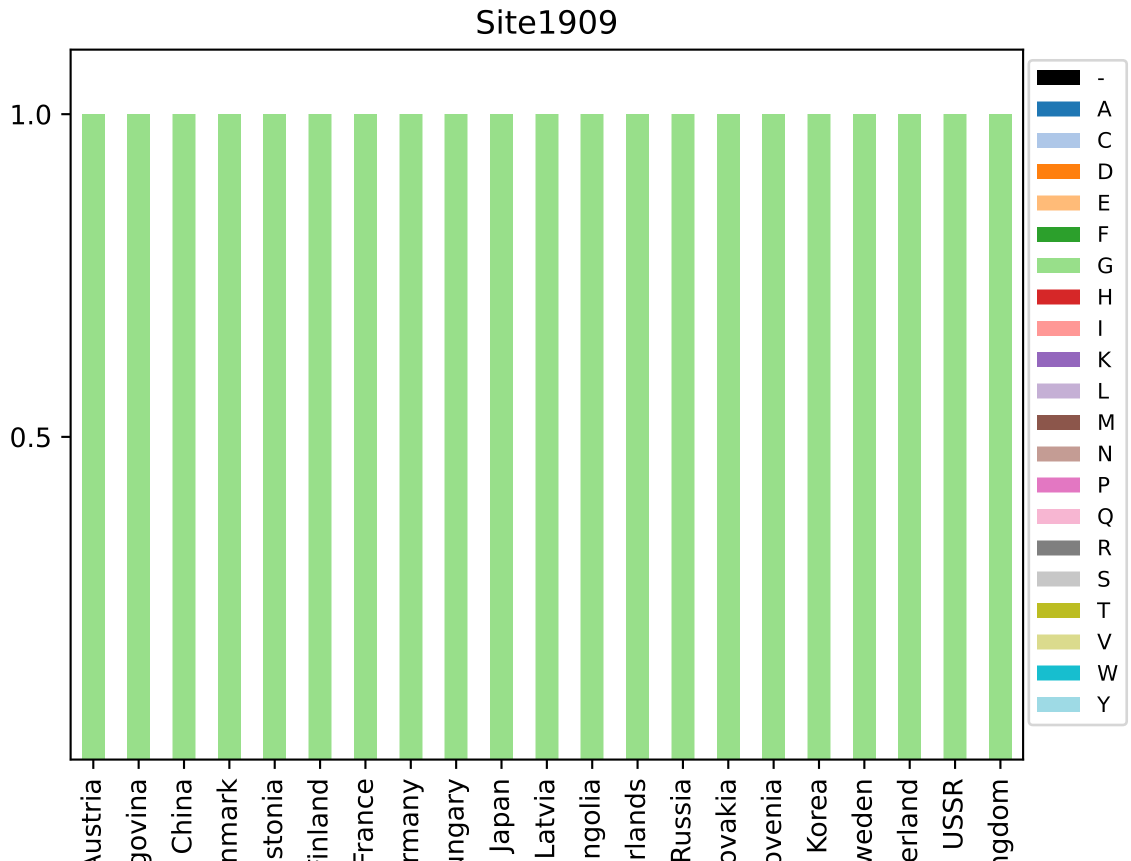

Site1909
1.0
0.5
-
A
C
D
E
F
G
H
I
K
L
M
N
P
Q
R
S
T
V
W
Y
Austria
govina
China
nmark
stonia
Finland
France
rmany
ungary
Japan
Latvia
ngolia
rlands
Russia
ovakia
ovenia
Korea
weden
erland
USSR
ngdom

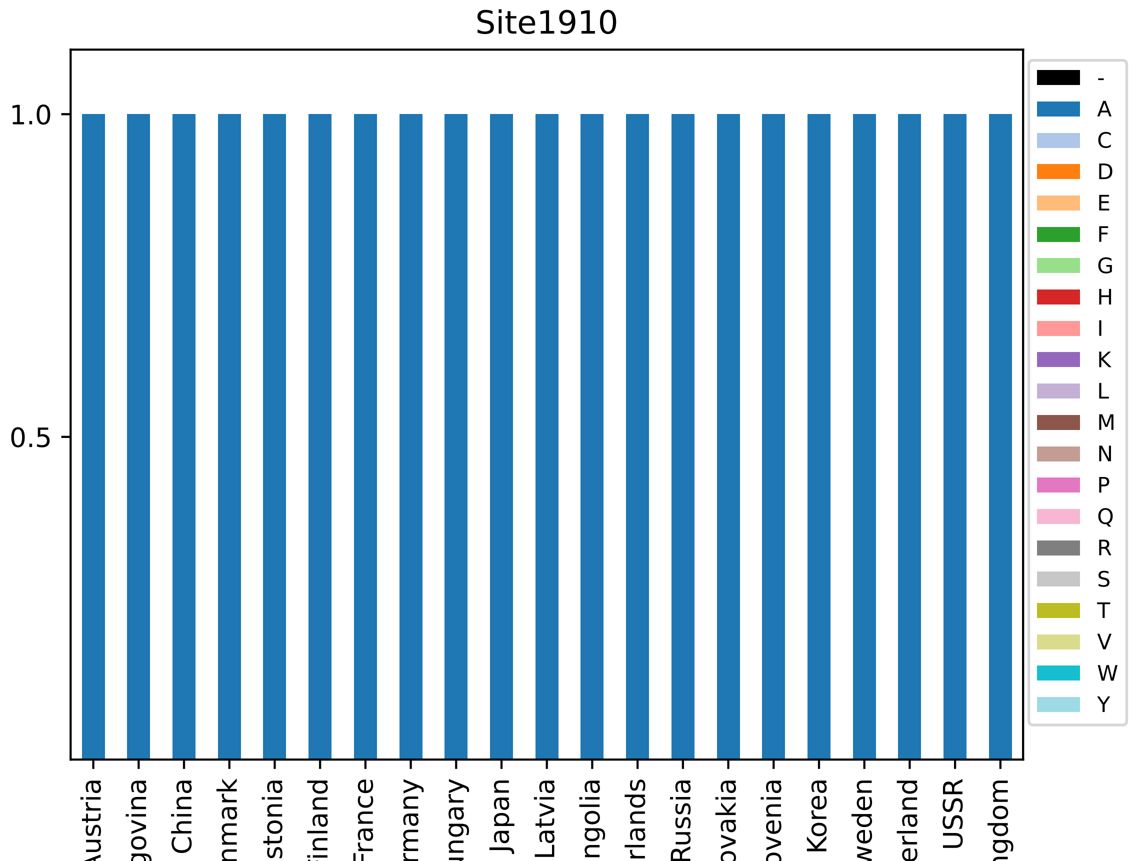

Site1910
1.0
0.5
-
A
C
D
E
F
G
H
I
K
L
M
N
P
Q
R
S
T
V
W
Y
Austria
China
Finland
Japan
Latvia
Russia
Korea
USSR

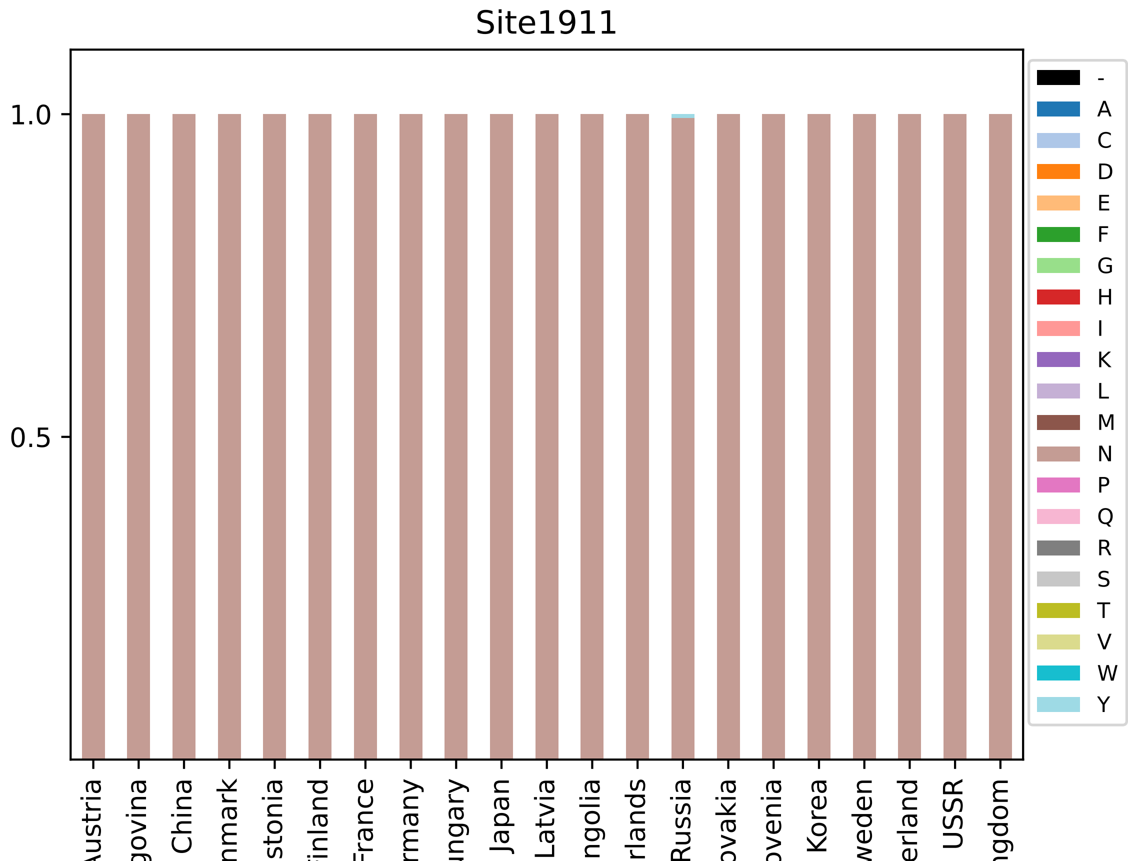
Site1911
1.0
0.5
-
A
C
D
E
F
G
H
I
K
L
M
N
P
Q
R
S
T
V
W
Y
Austria
govina
China
nmark
stonia
inland
France
rmany
ungary
Japan
Latvia
ngolia
rlands
Russia
ovakia
ovenia
Korea
weden
erland
USSR
ngdom

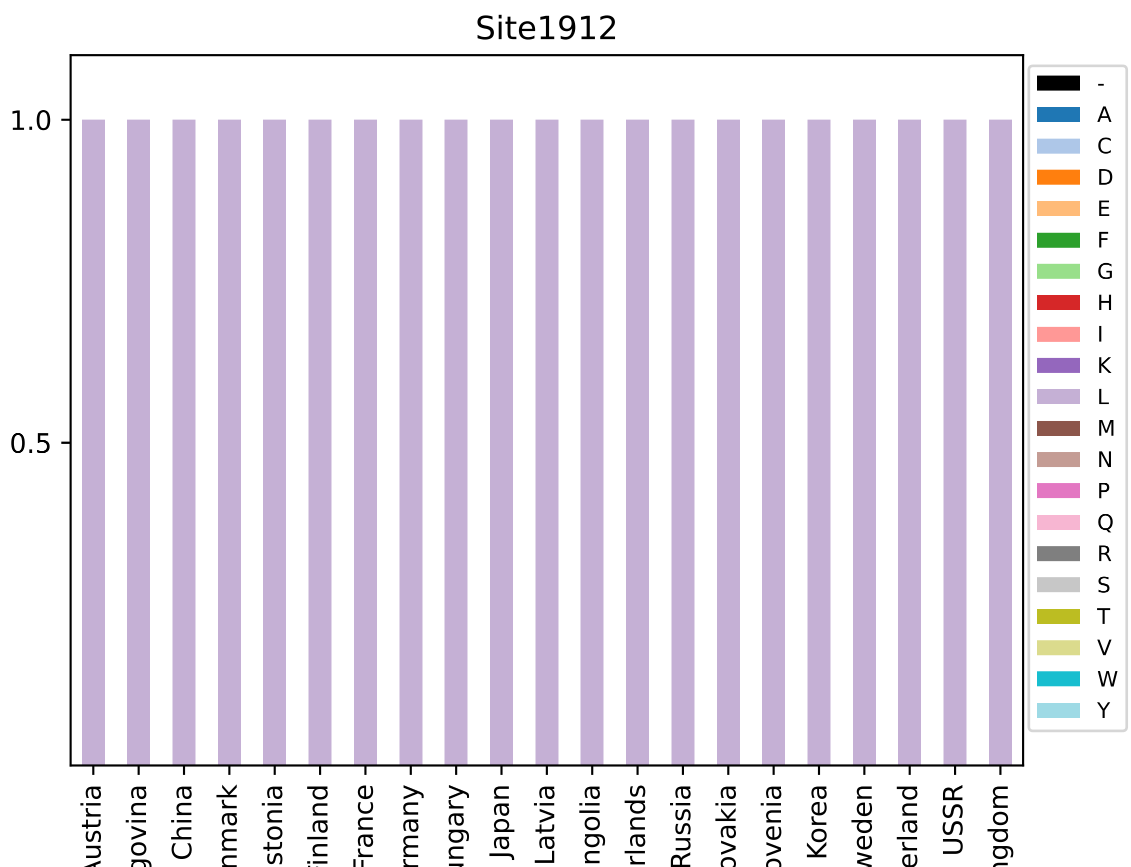

Site1912
1.0
0.5
-
A
C
D
E
F
G
H
I
K
L
M
N
P
Q
R
S
T
V
W
Y
Austria
govina
China
nmark
stonia
Finland
France
rmany
ungary
Japan
Latvia
ngolia
rlands
Russia
ovakia
ovenia
Korea
weden
erland
USSR
ngdom

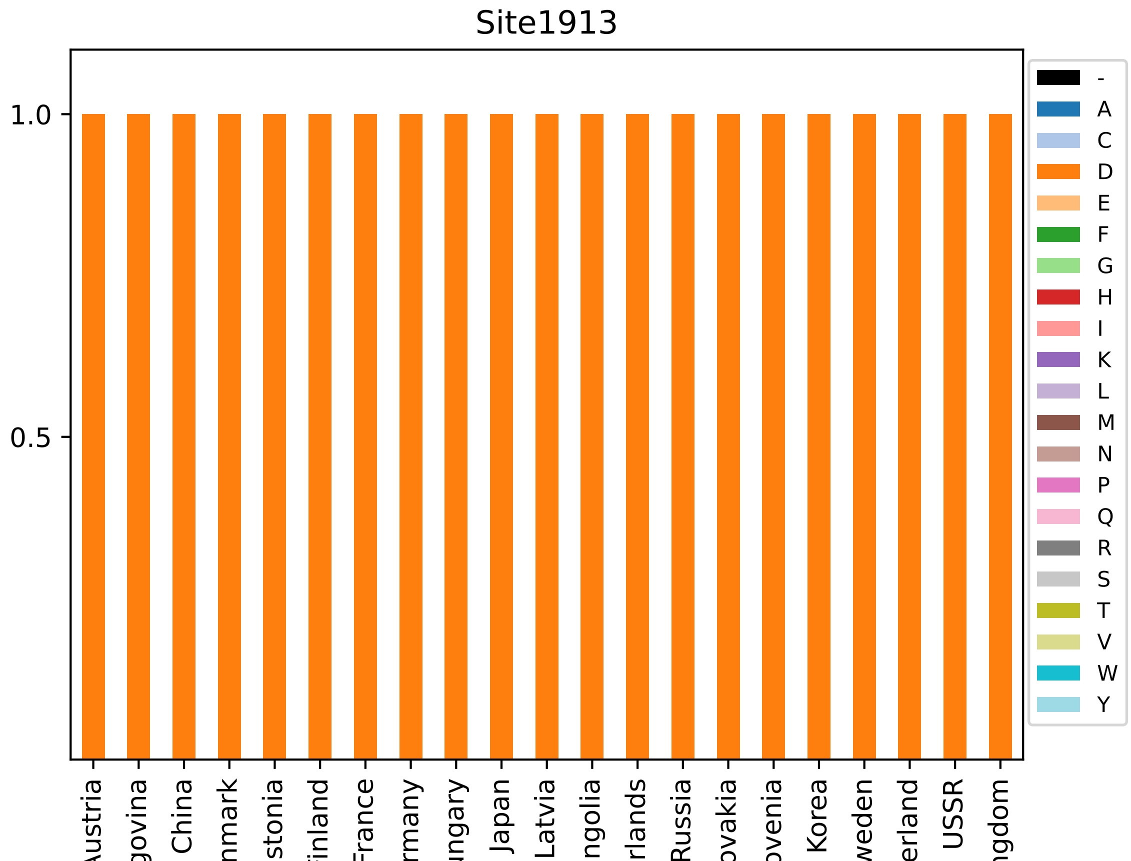

Site1913
1.0
0.5
Austria
govina
China
nmark
stonia
inland
France
rmany
ungary
Japan
Latvia
ngolia
rlands
Russia
ovakia
ovenia
Korea
weden
erland
USSR
ngdom
-
A
C
D
E
F
G
H
I
K
L
M
N
P
Q
R
S
T
V
W
Y

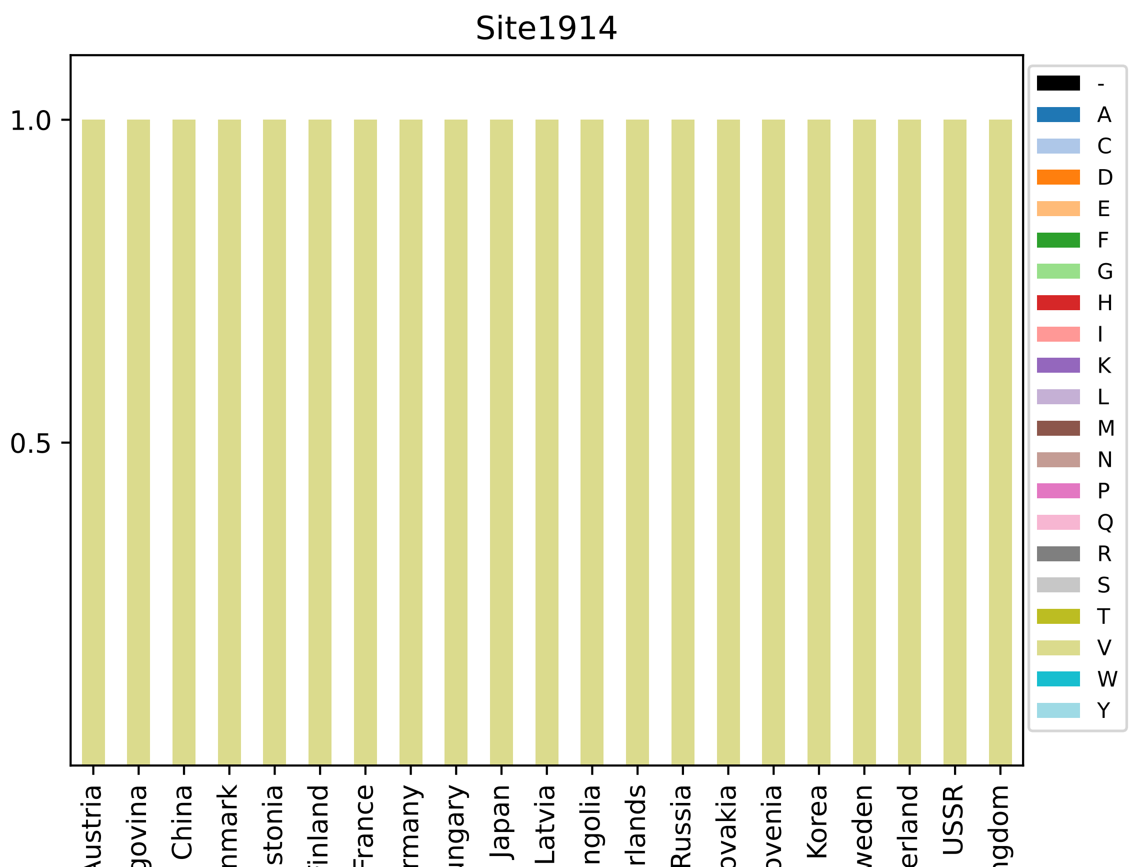
Site1914
1.0
0.5
-
A
C
D
E
F
G
H
I
K
L
M
N
P
Q
R
S
T
V
W
Y
China
Japan
Latvia
Russia
Korea
USSR

Site1915

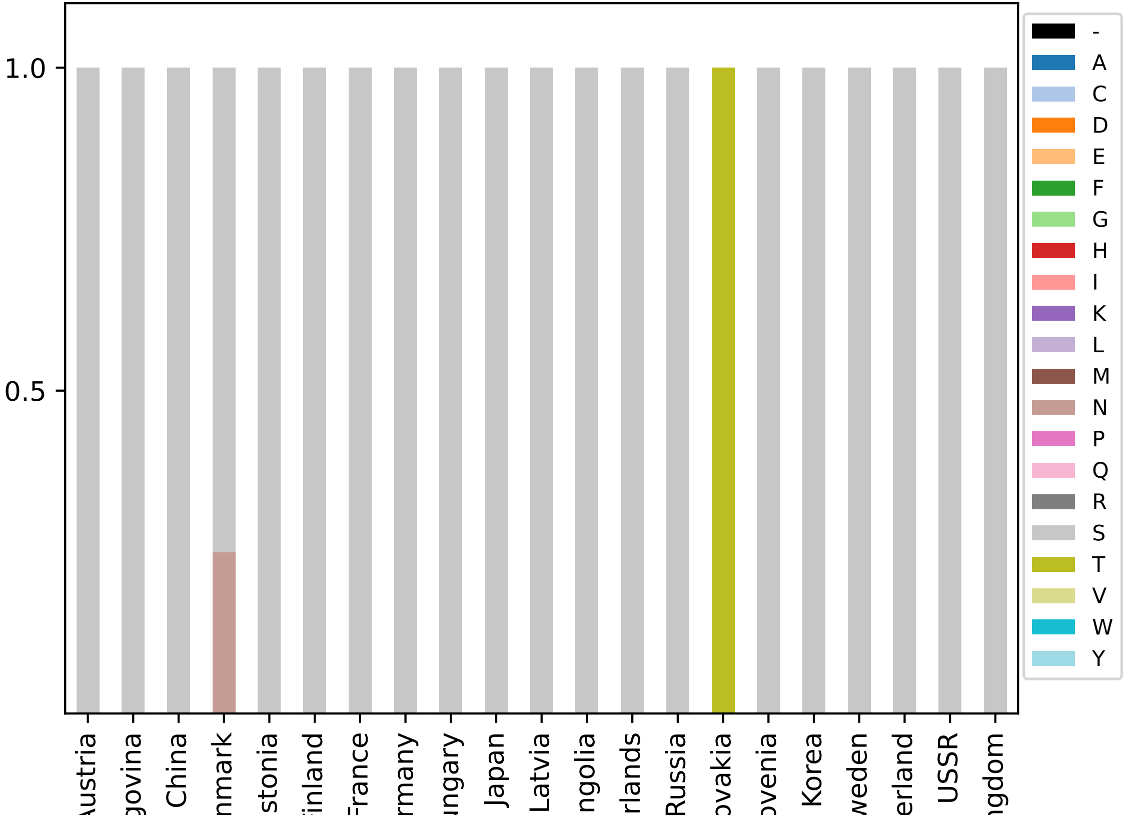

1.0
0.5
-
A
C
D
E
F
G
H
I
K
L
M
N
P
Q
R
S
T
V
W
Y
China
Japan
Latvia
Russia
Korea
USSR

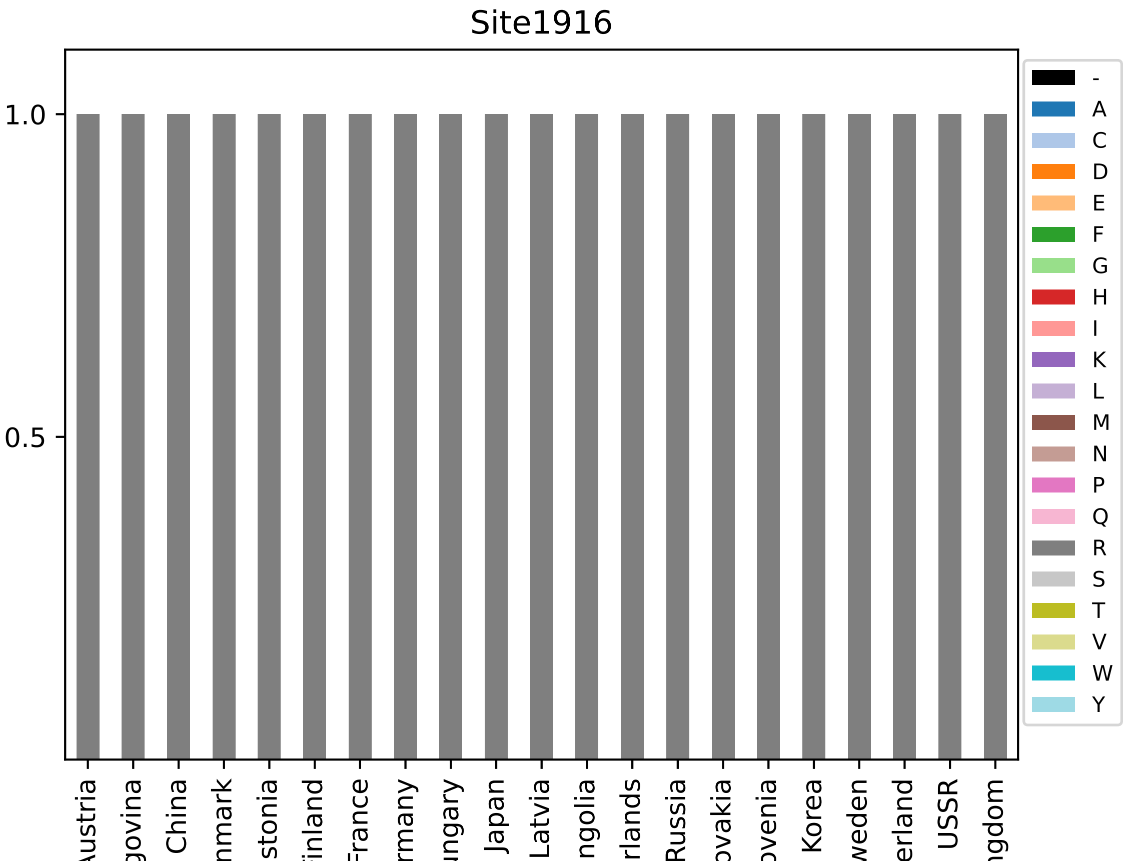
Site1916
1.0
0.5
-
A
C
D
E
F
G
H
I
K
L
M
N
P
Q
R
S
T
V
W
Y
China
Japan
Latvia
Russia
Korea
USSR

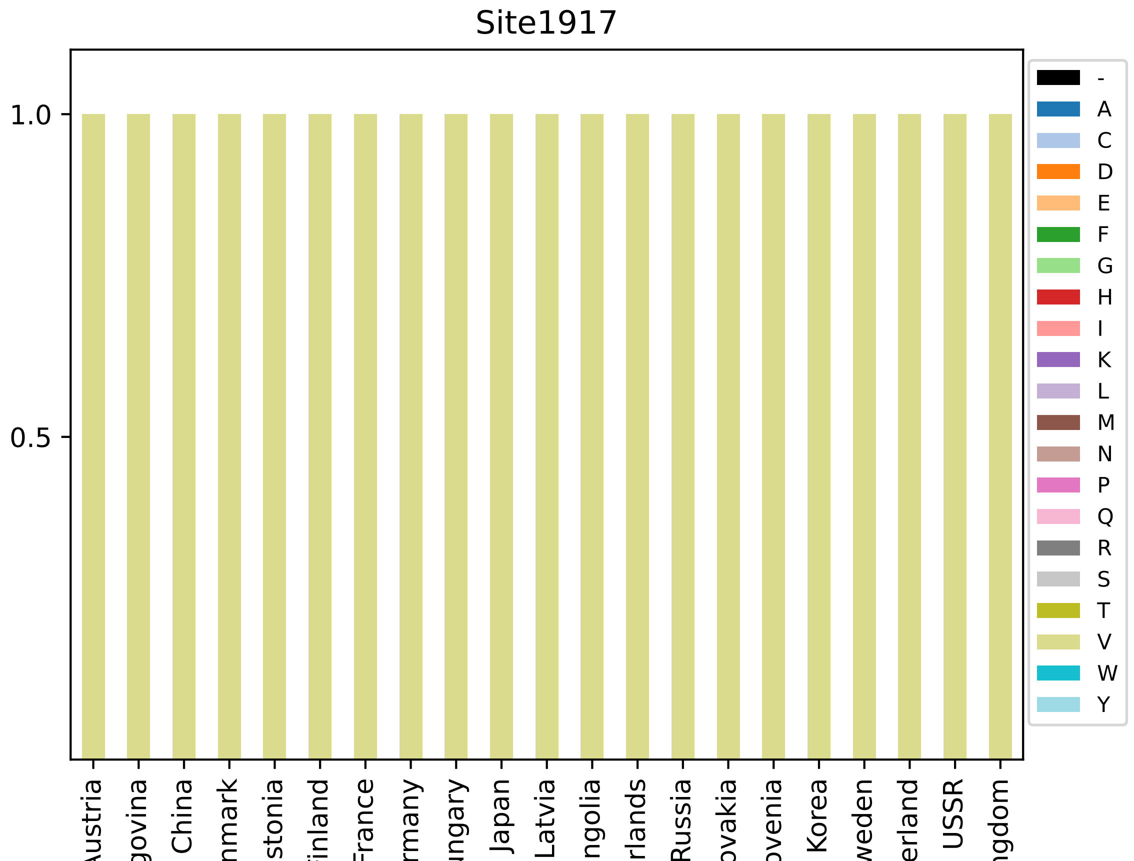

Site1917
1.0
0.5
-
A
C
D
E
F
G
H
I
K
L
M
N
P
Q
R
S
T
V
W
Y
Austria
China
Japan
Latvia
Russia
Korea
USSR

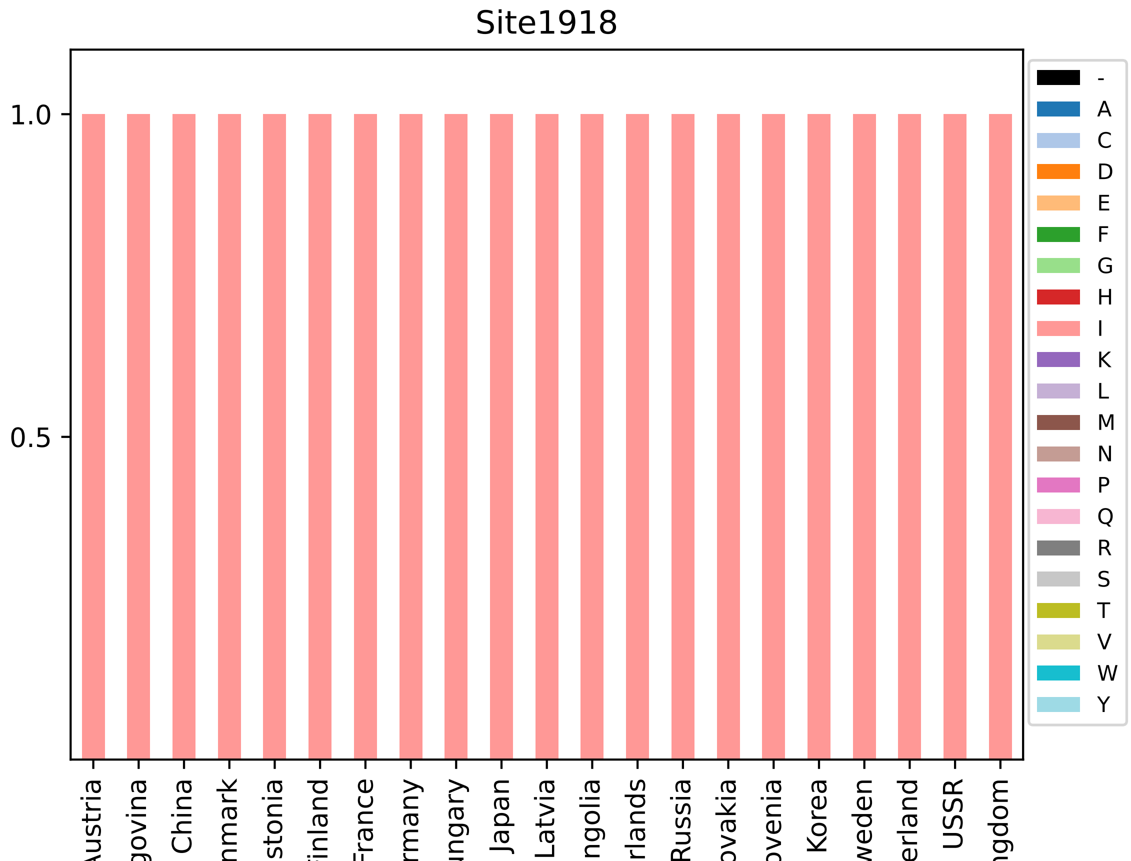
Site1918
1.0
0.5
-
A
C
D
E
F
G
H
I
K
L
M
N
P
Q
R
S
T
V
W
Y
China
Japan
Latvia
Russia
Korea
USSR

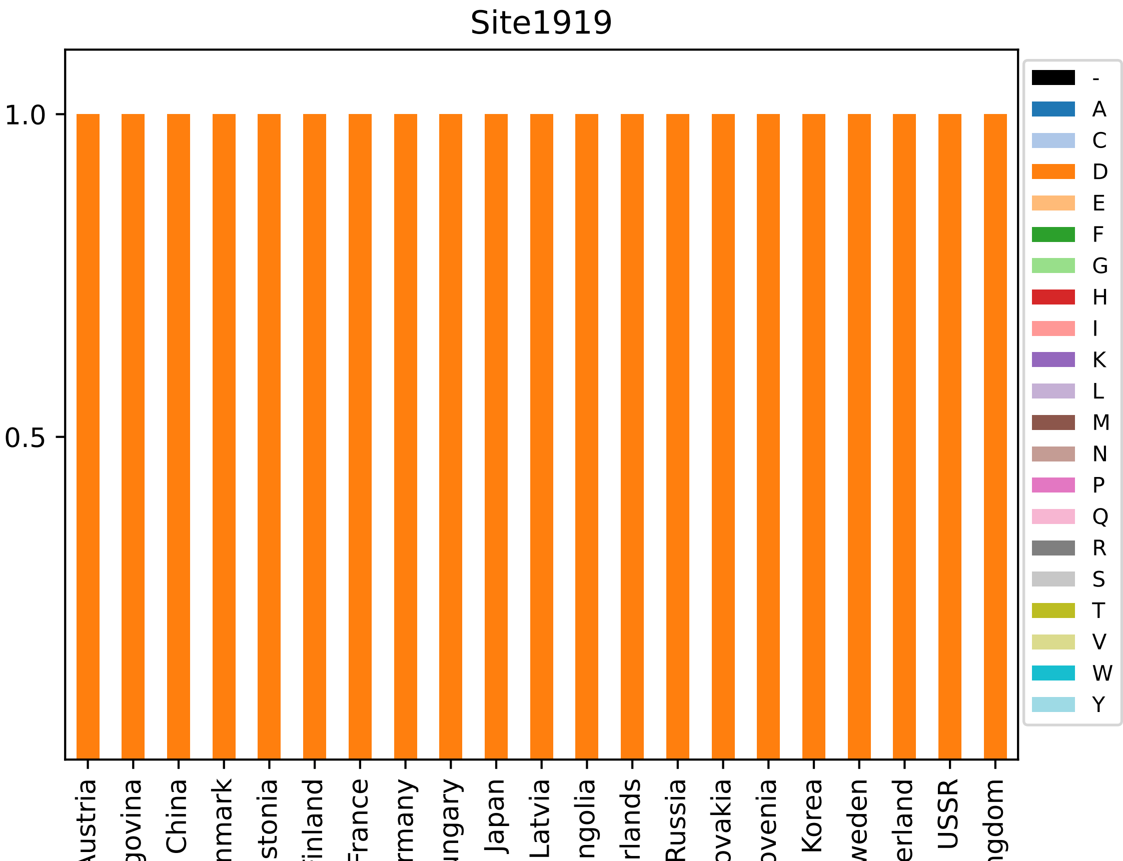
Site1919
1.0
0.5
-
A
C
D
E
F
G
H
I
K
L
M
N
P
Q
R
S
T
V
W
Y
China
Japan
Latvia
Russia
Korea
USSR

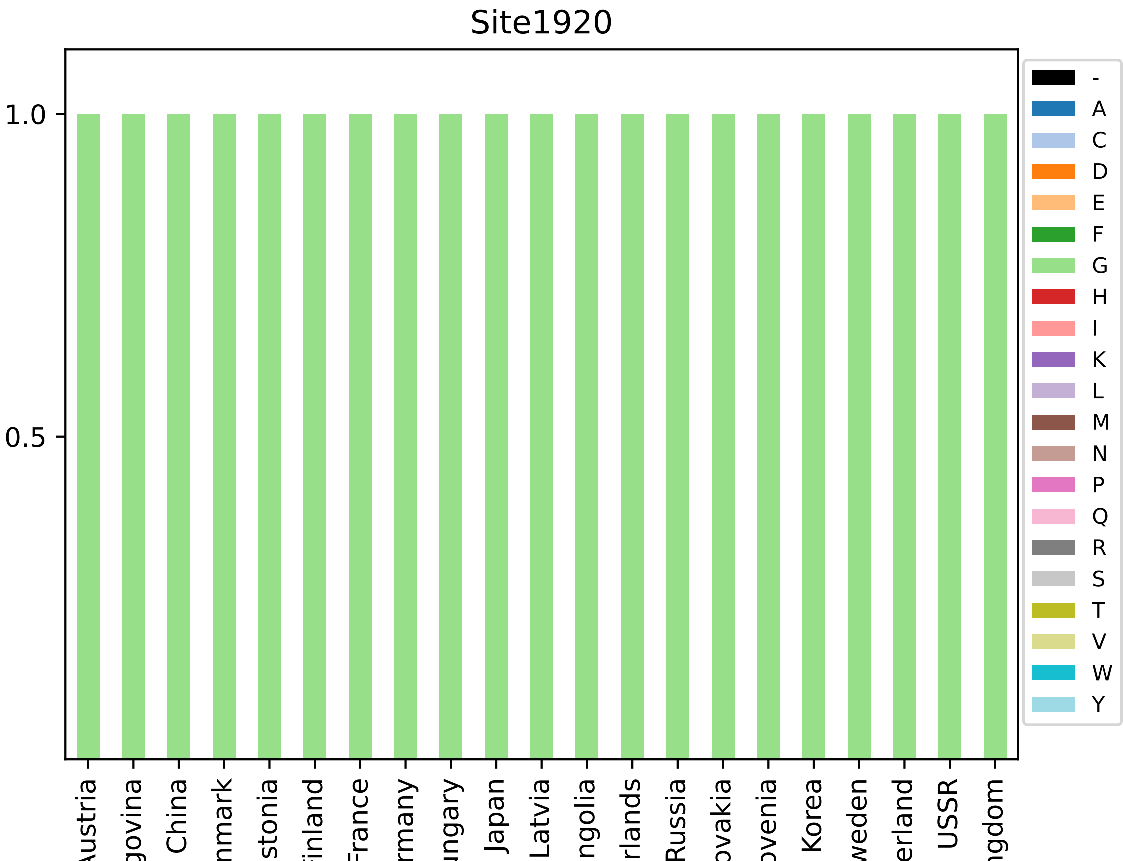
Site1920
1.0
0.5
China
Japan
Latvia
Russia
Korea
USSR
-
A
C
D
E
F
G
H
I
K
L
M
N
P
Q
R
S
T
V
W
Y

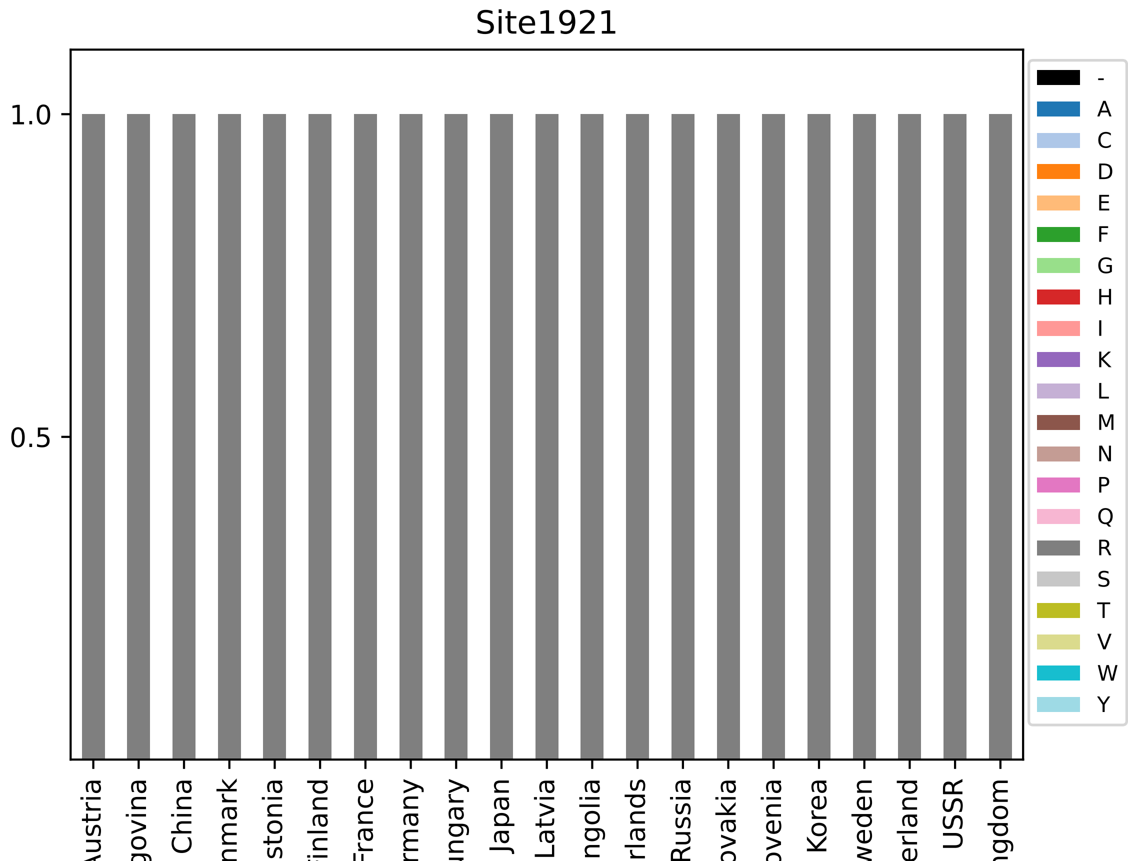
Site1921
1.0
0.5
-
A
C
D
E
F
G
H
I
K
L
M
N
P
Q
R
S
T
V
W
Y
China
Japan
Latvia
Russia
Korea
USSR

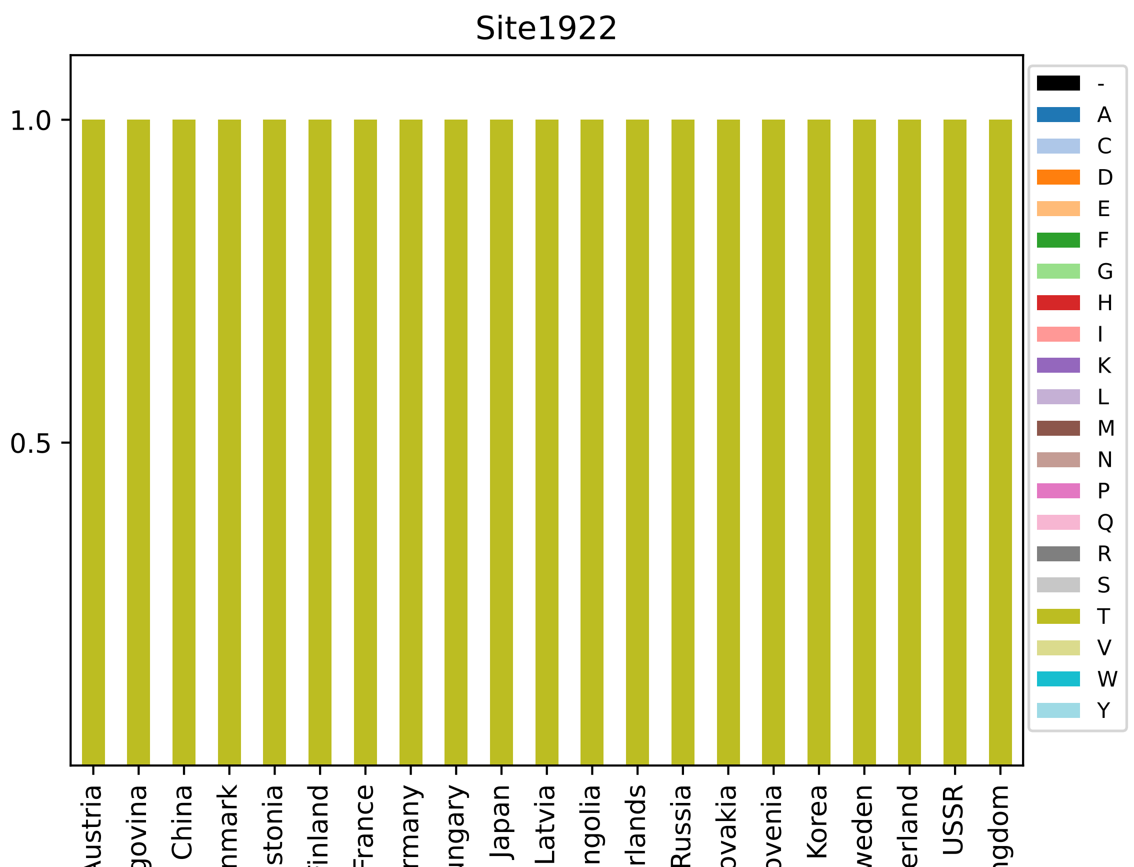

Site1922
1.0
0.5
-
A
C
D
E
F
G
H
I
K
L
M
N
P
Q
R
S
T
V
W
Y
China
Japan
Latvia
Russia
Korea
USSR

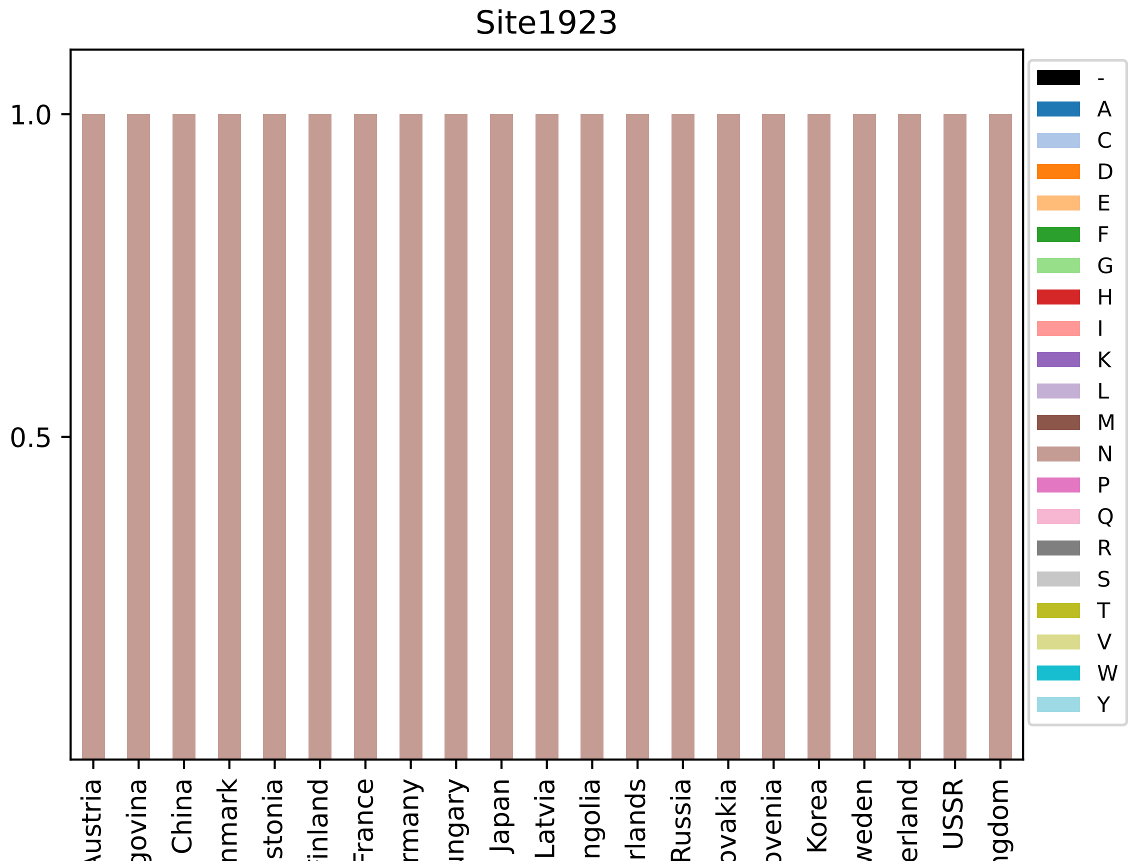

Site1923
1.0
0.5
-
A
C
D
E
F
G
H
I
K
L
M
N
P
Q
R
S
T
V
W
Y
Austria
govina
China
nmark
stonia
Finland
France
rmany
ungary
Japan
Latvia
ngolia
rlands
Russia
ovakia
ovenia
Korea
weden
erland
USSR
ngdom

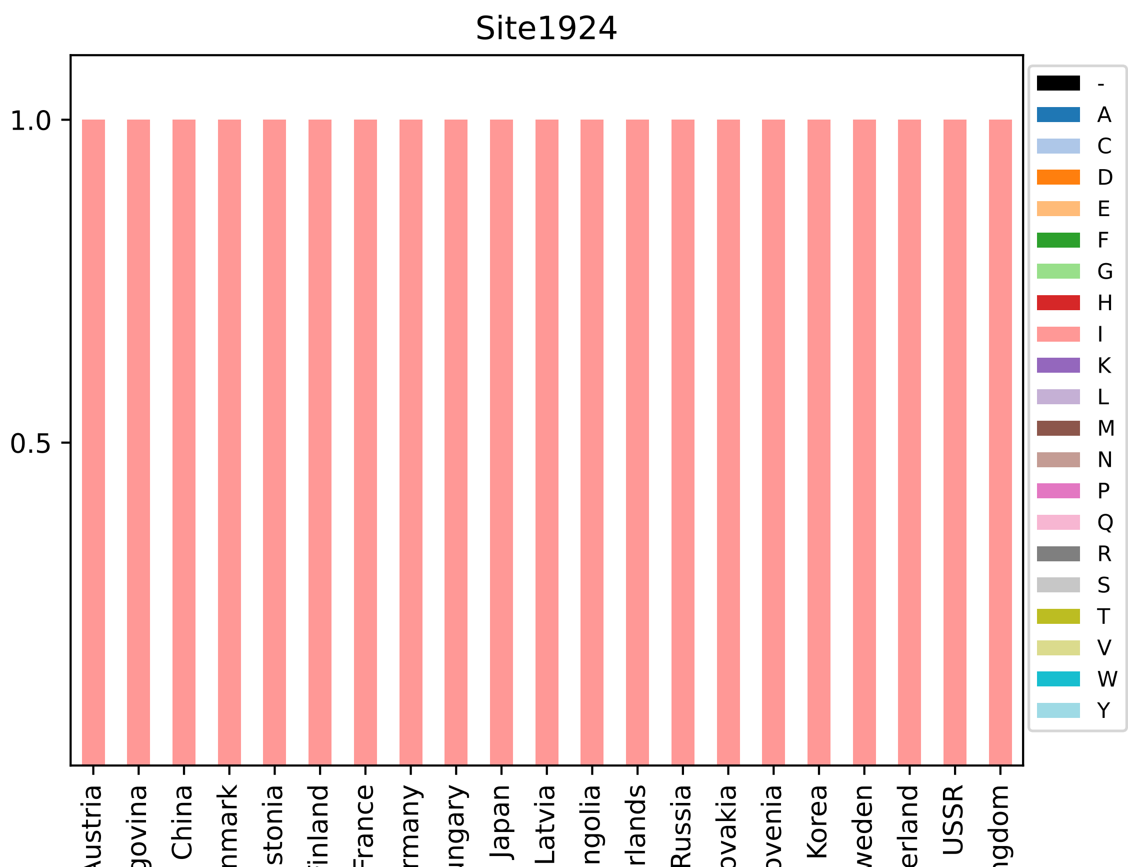
Site1924
1.0
0.5
-
A
C
D
E
F
G
H
I
K
L
M
N
P
Q
R
S
T
V
W
Y
China
Japan
Latvia
Russia
Korea
USSR

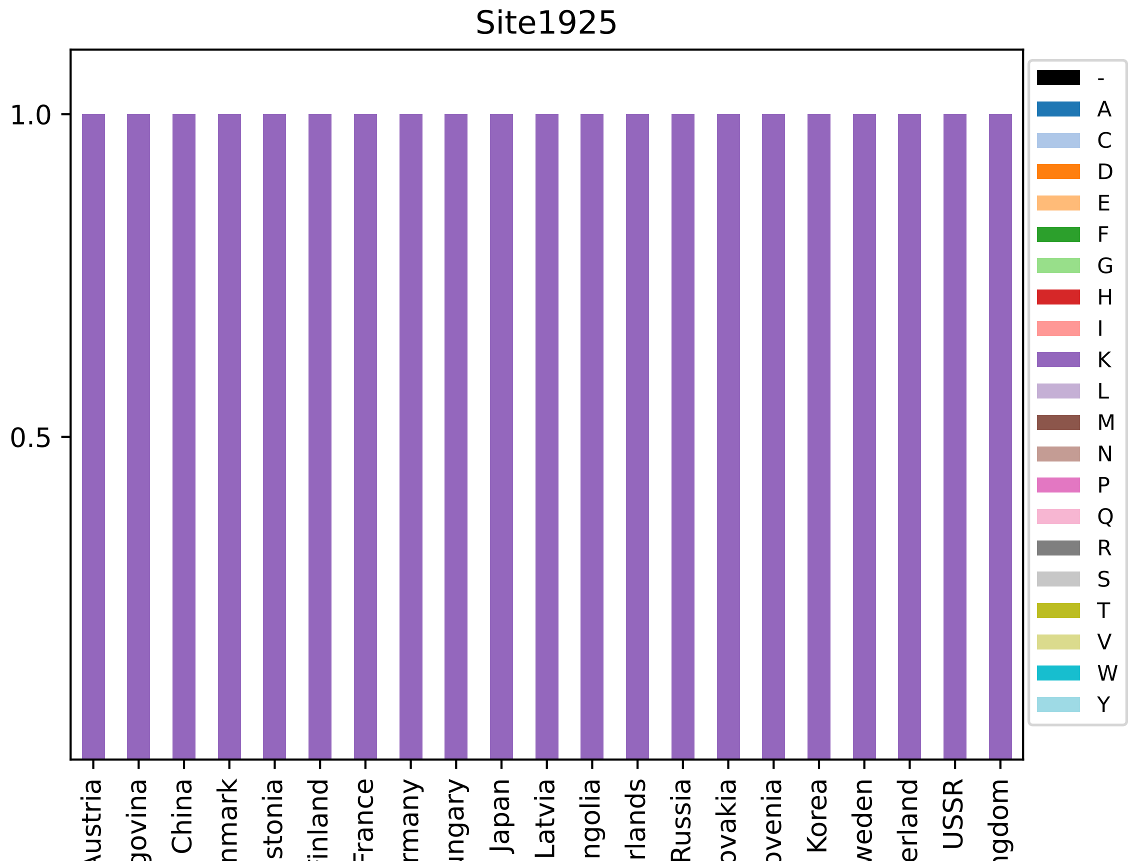

Site1925
1.0
0.5
Austria
govina
China
nmark
stonia
Finland
France
rmany
ungary
Japan
Latvia
ngolia
rlands
Russia
ovakia
ovenia
Korea
weden
erland
USSR
ngdom
-
A
C
D
E
F
G
H
I
K
L
M
N
P
Q
R
S
T
V
W
Y

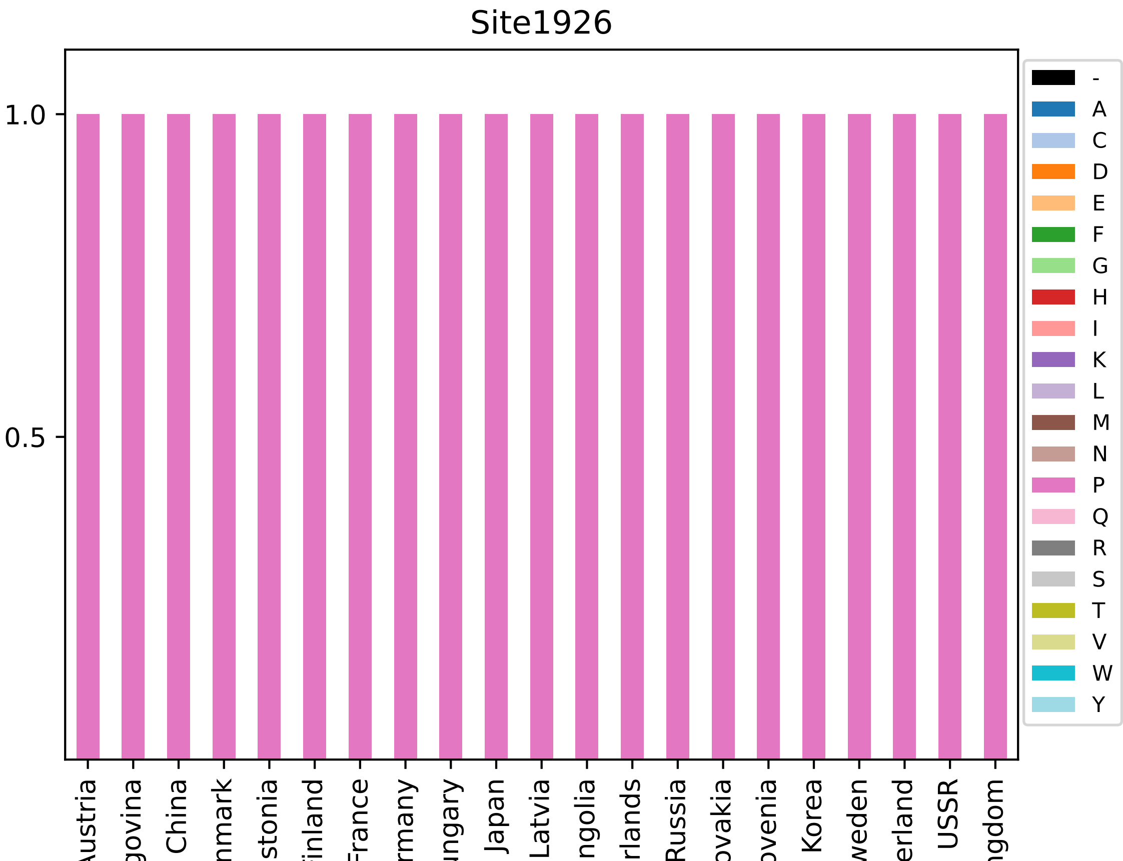

Site1926
1.0
0.5
-
A
C
D
E
F
G
H
I
K
L
M
N
P
Q
R
S
T
V
W
Y
Austria
govina
China
nmark
stonia
Finland
France
rmany
ungary
Japan
Latvia
ngolia
rlands
Russia
ovakia
ovenia
Korea
weden
erland
USSR
ngdom

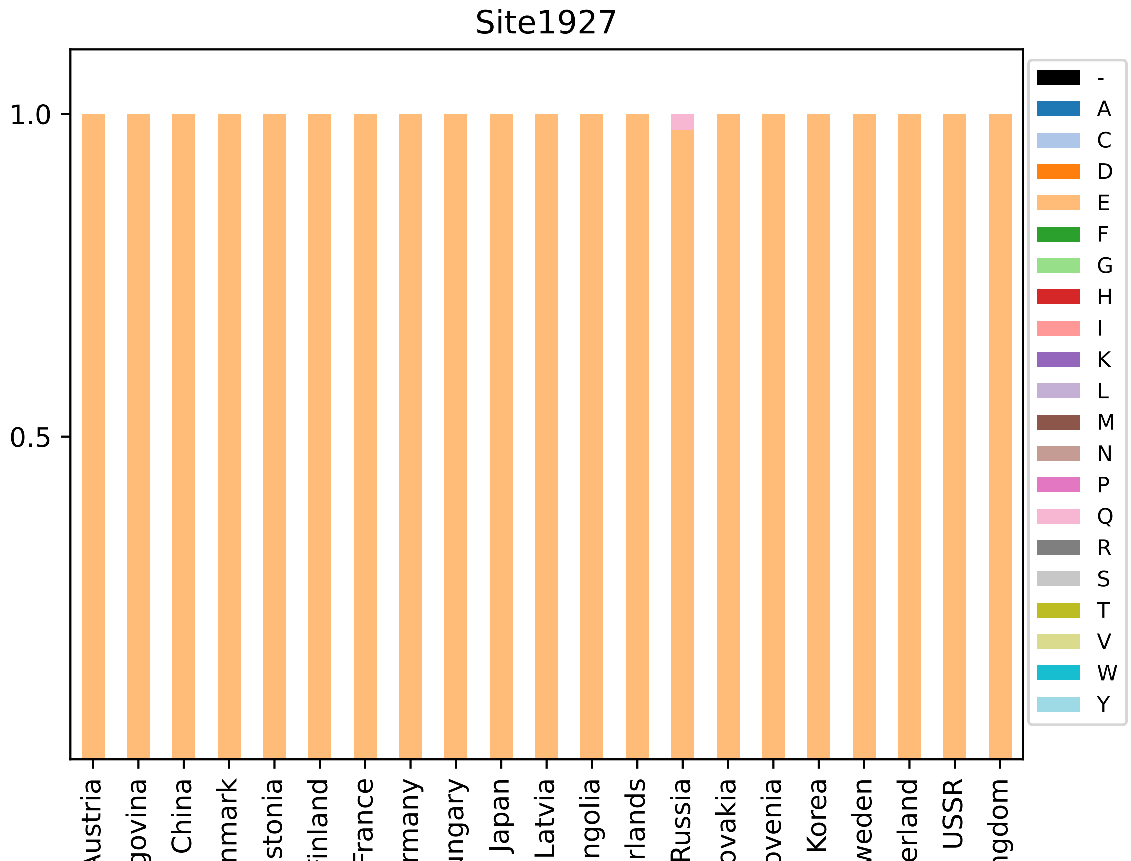
Site1927
1.0
0.5
-
A
C
D
E
F
G
H
I
K
L
M
N
P
Q
R
S
T
V
W
Y
China
Japan
Latvia
Russia
Korea
USSR

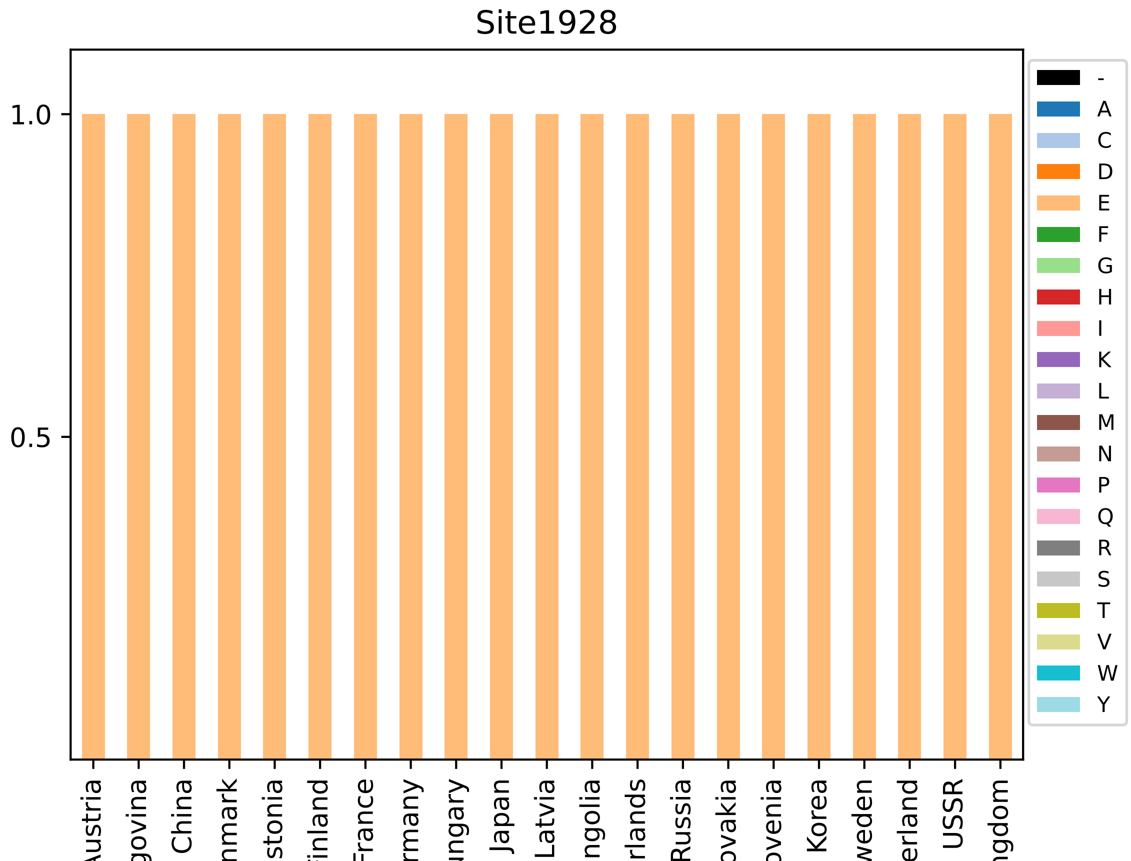
Site1928
1.0
0.5
-
A
C
D
E
F
G
H
I
K
L
M
N
P
Q
R
S
T
V
W
Y
China
Japan
Latvia
Russia
Korea
USSR

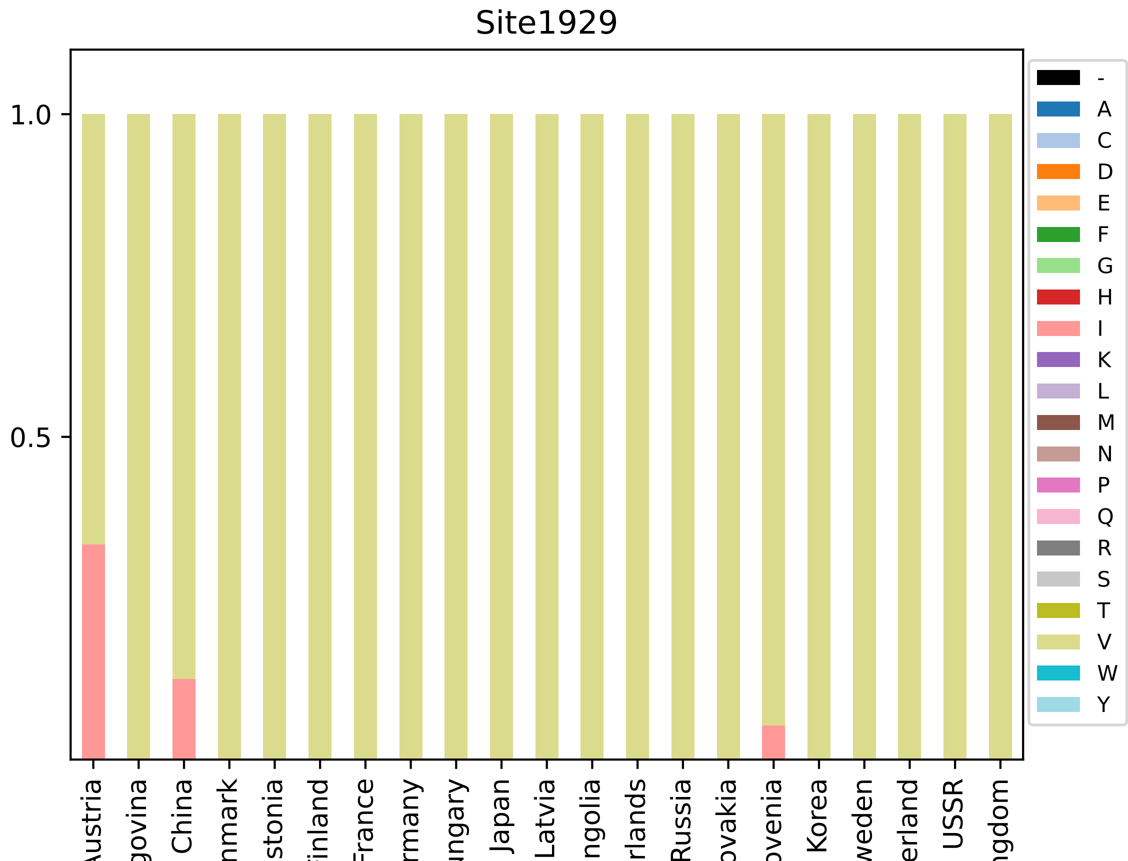

Site1929
1.0
0.5
-
A
C
D
E
F
G
H
I
K
L
M
N
P
Q
R
S
T
V
W
Y
China
Japan
Latvia
Korea
USSR

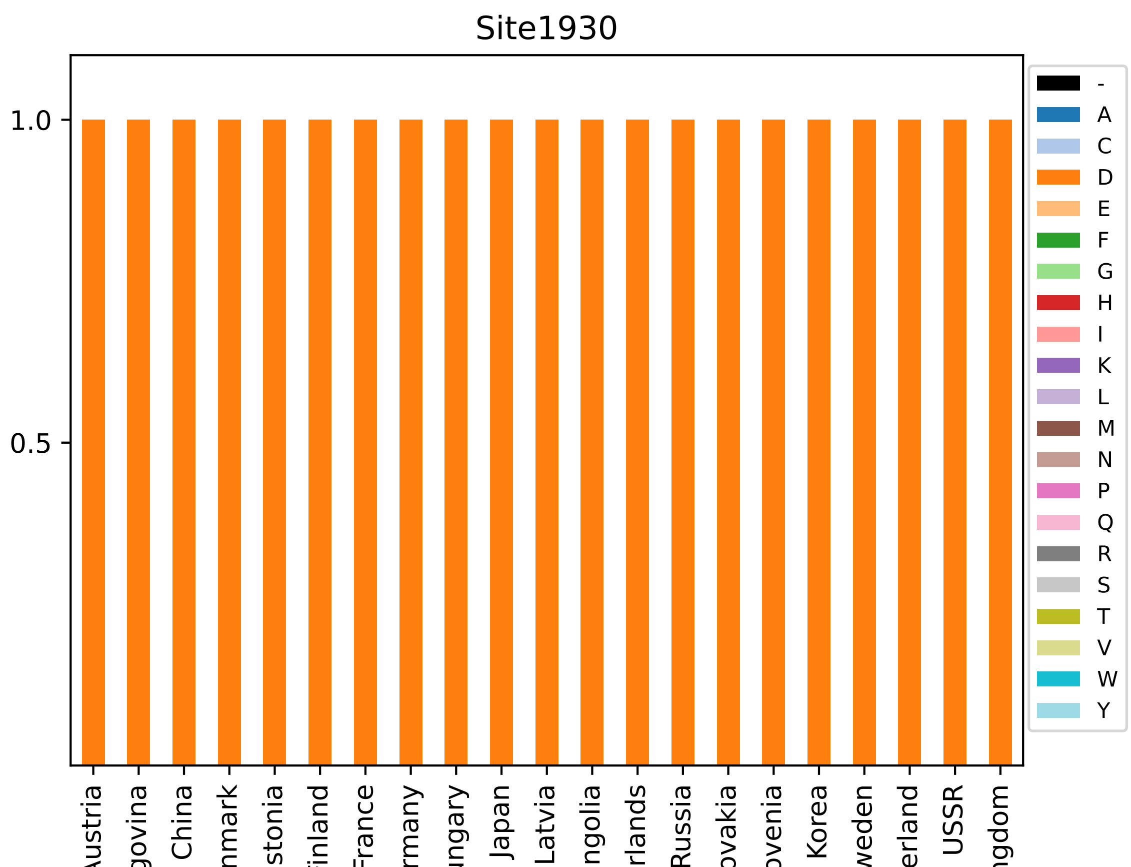
Site1930
1.0
0.5
-
A
C
D
E
F
G
H
I
K
L
M
N
P
Q
R
S
T
V
W
Y
China
Japan
Latvia
Russia
Korea
USSR

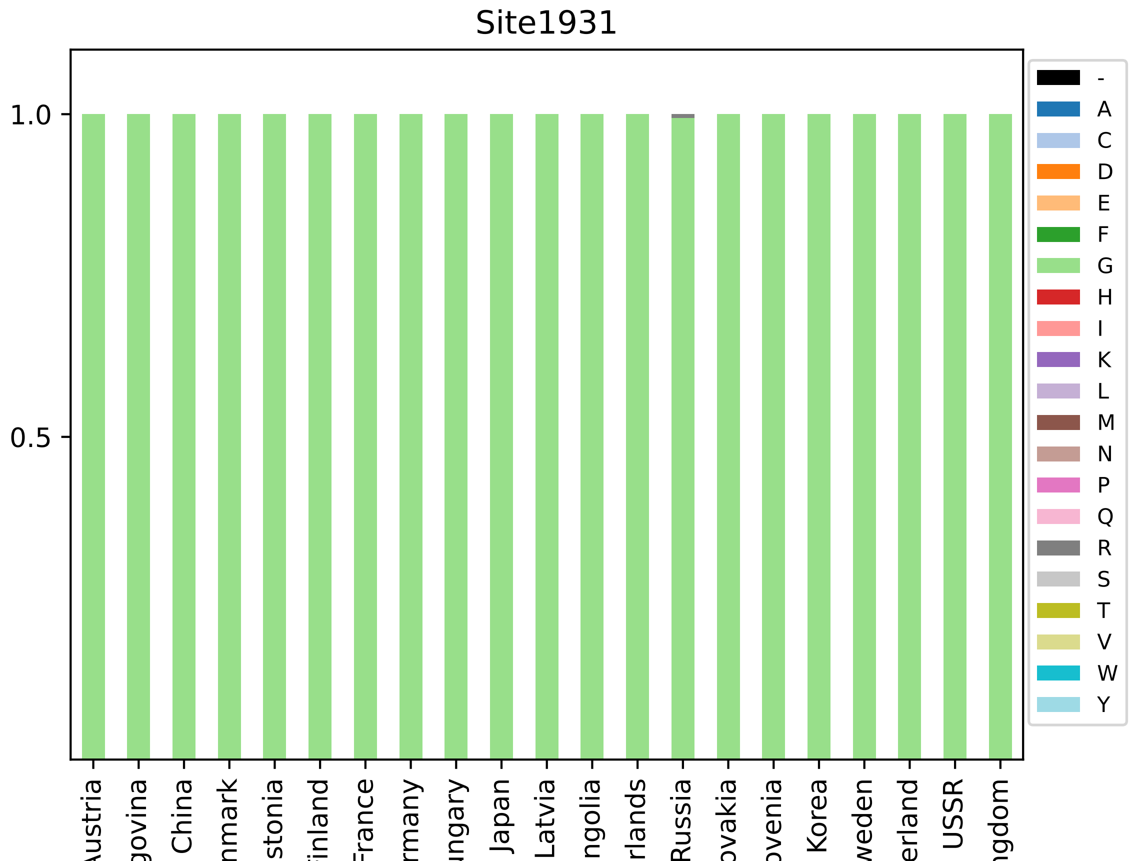
Site1931
1.0
0.5
-
A
C
D
E
F
G
H
I
K
L
M
N
P
Q
R
S
T
V
W
Y
Austria
govina
China
nmark
stonia
inland
France
rmany
ungary
Japan
Latvia
ngolia
rlands
Russia
ovakia
ovenia
Korea
weden
erland
USSR
ngdom

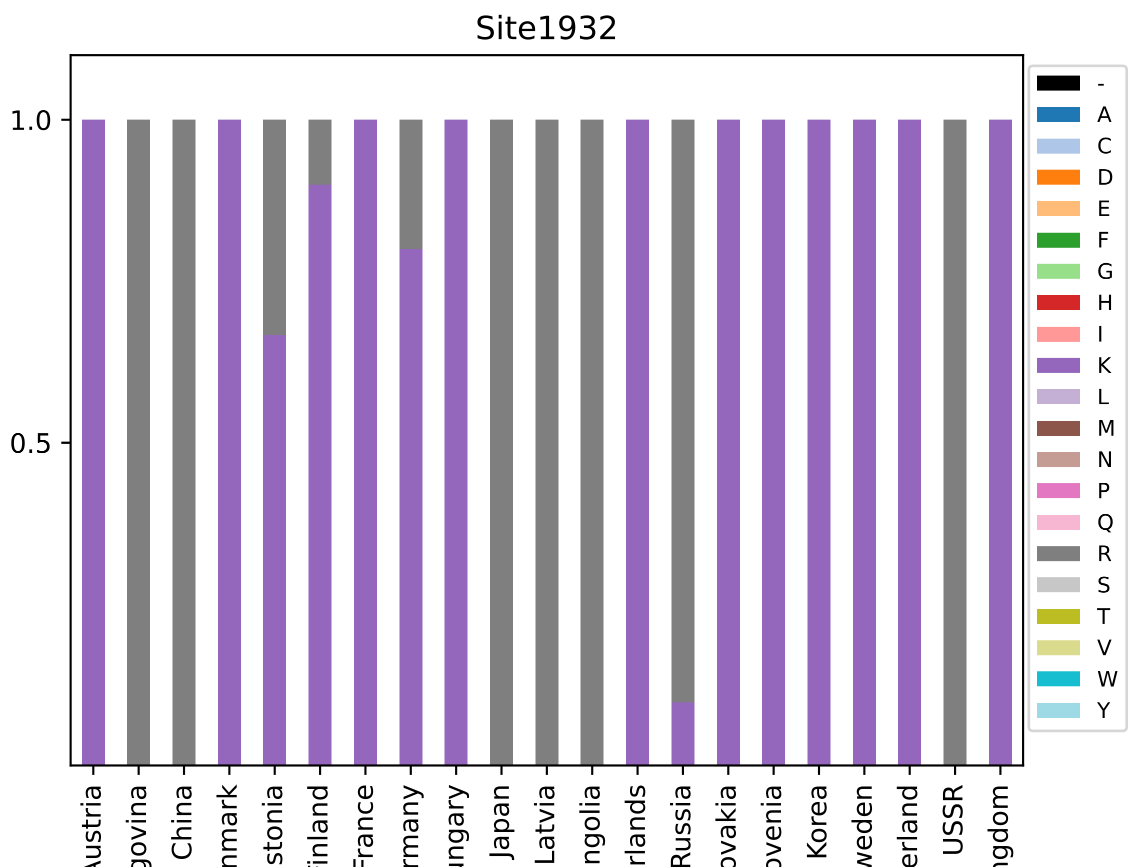
Site1932
1.0
0.5
-
A
C
D
E
F
G
H
I
K
L
M
N
P
Q
R
S
T
V
W
Y
China
Japan
Latvia
Russia
Korea
USSR

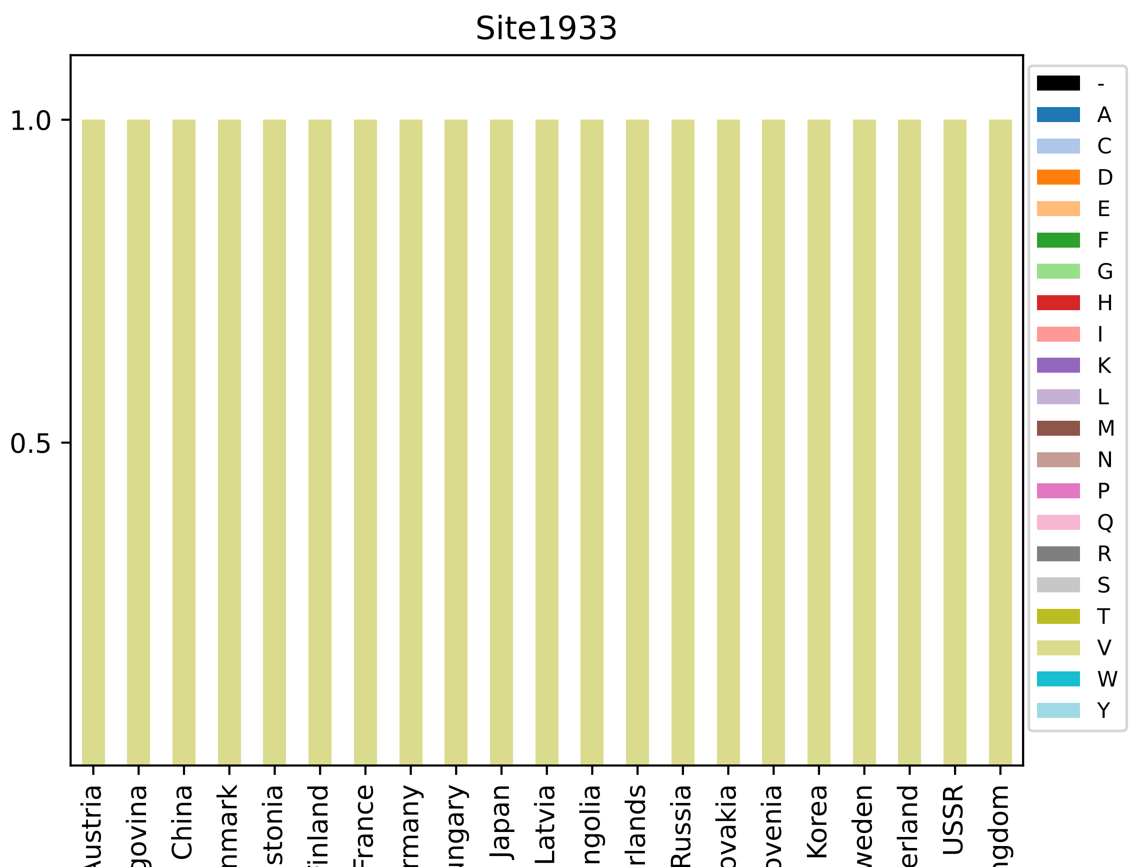
Site1933
1.0
0.5
-
A
C
D
E
F
G
H
I
K
L
M
N
P
Q
R
S
T
V
W
Y
China
Japan
Latvia
Russia
Korea
USSR

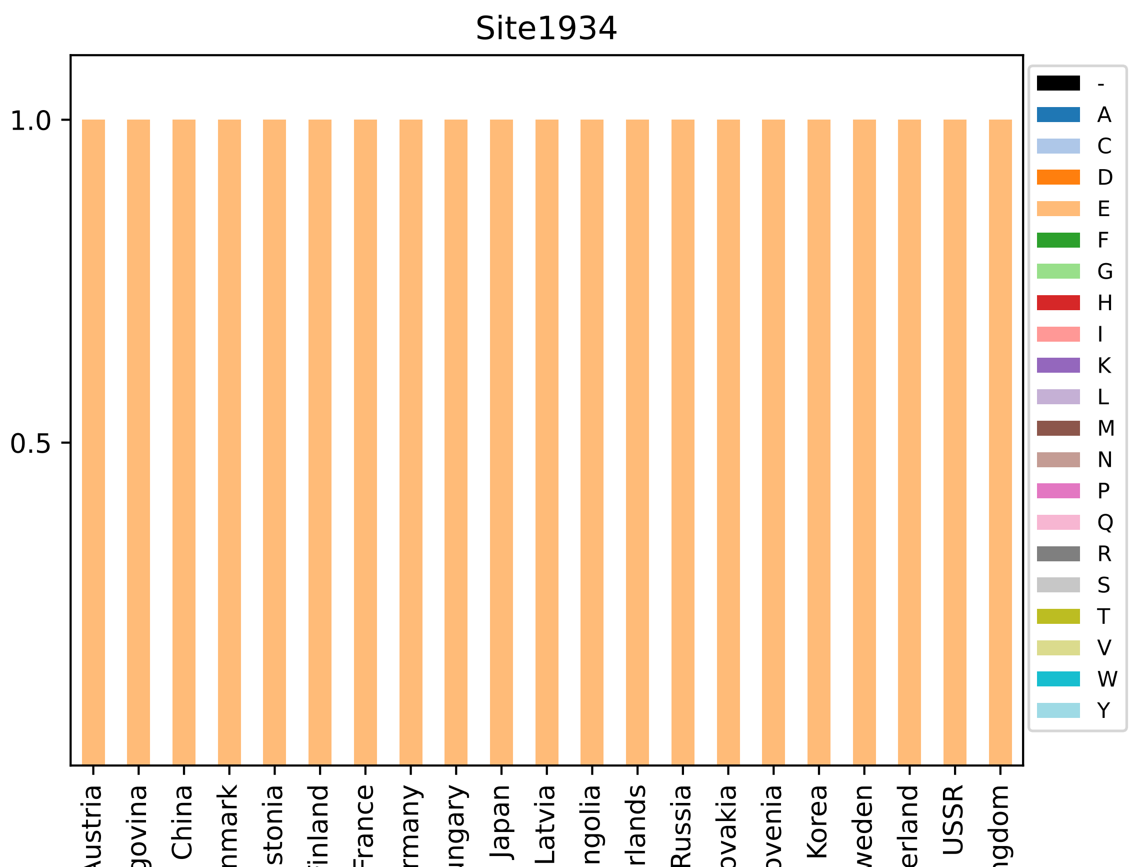

Site1934
1.0
0.5
-
A
C
D
E
F
G
H
I
K
L
M
N
P
Q
R
S
T
V
W
Y
China
Japan
Latvia
Russia
Korea
USSR

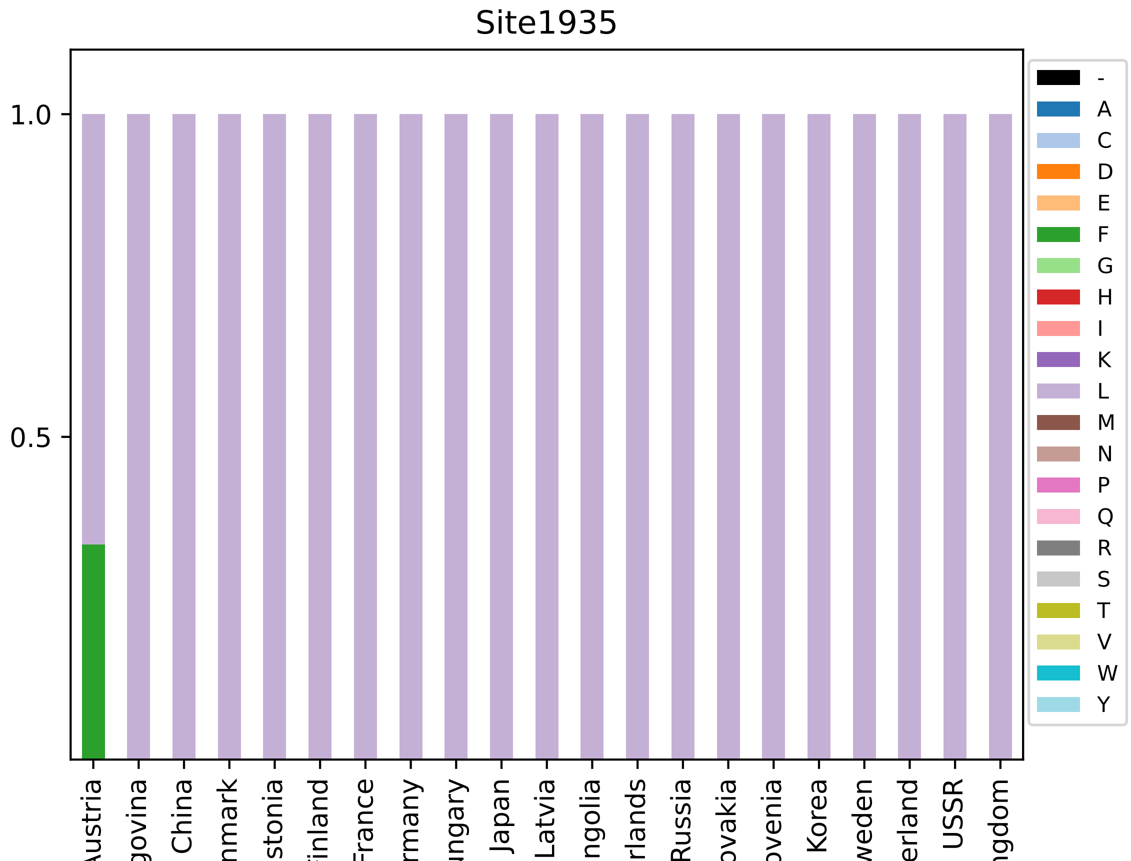

Site1935
1.0
0.5
-
A
C
D
E
F
G
H
I
K
L
M
N
P
Q
R
S
T
V
W
Y
Austria
govina
China
nmark
stonia
inland
France
rmany
ungary
Japan
Latvia
ngolia
rlands
Russia
ovakia
ovenia
Korea
weden
erland
USSR
ngdom

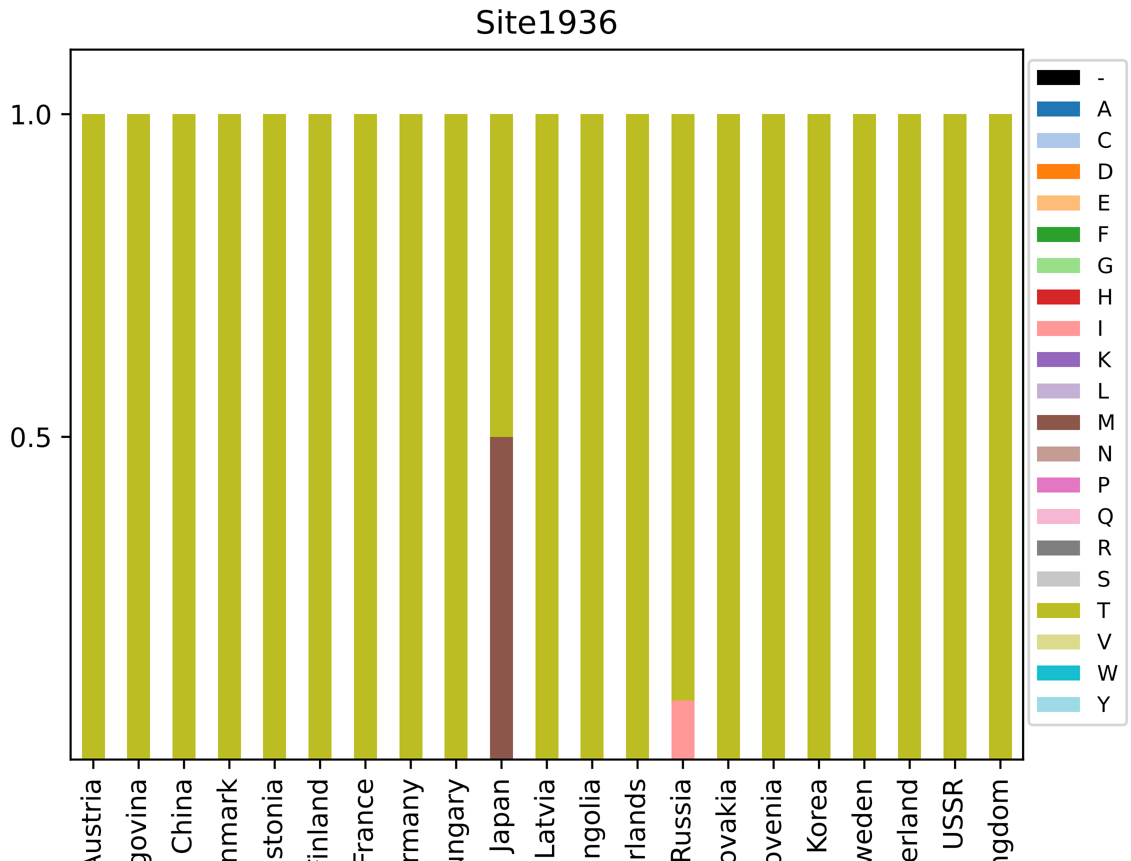
Site1936
1.0
0.5
-
A
C
D
E
F
G
H
I
K
L
M
N
P
Q
R
S
T
V
W
Y
China
Japan
Latvia
Russia
Korea
USSR

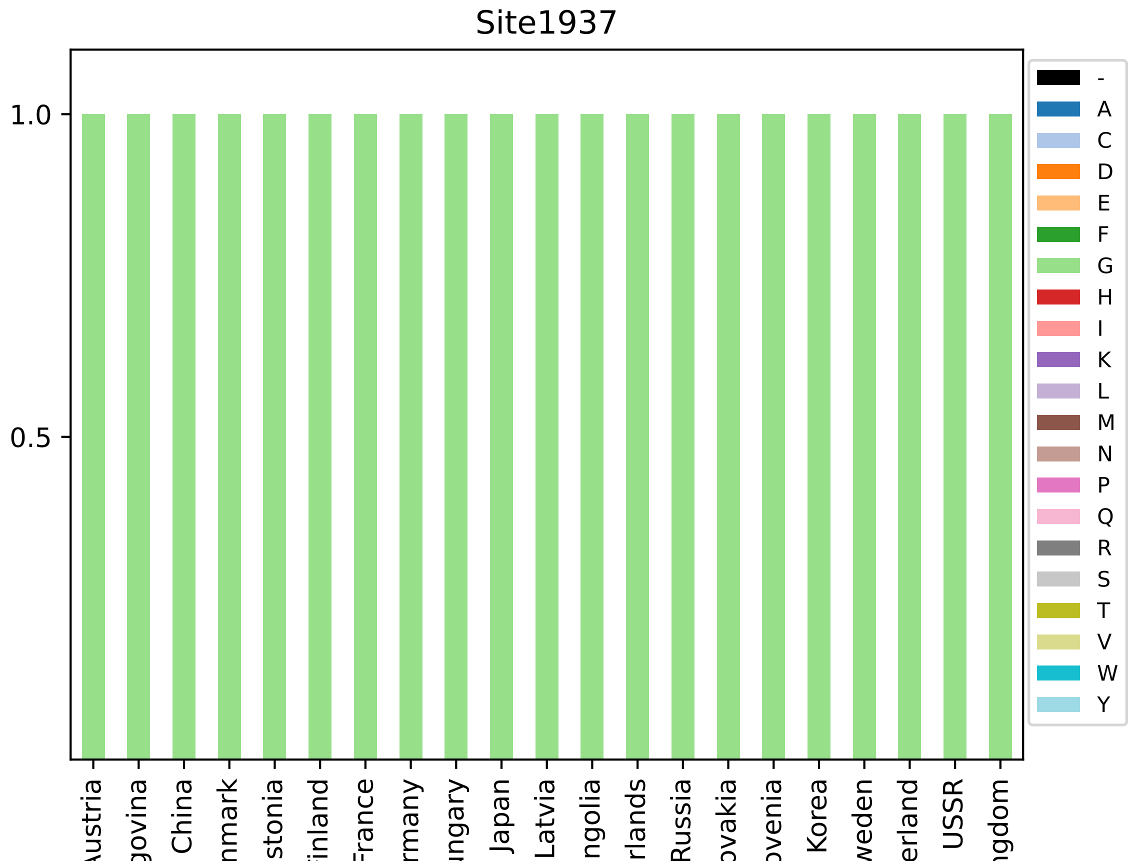

Site1937
1.0
0.5
-
A
C
D
E
F
G
H
I
K
L
M
N
P
Q
R
S
T
V
W
Y
China
Japan
Latvia
Russia
Korea
USSR

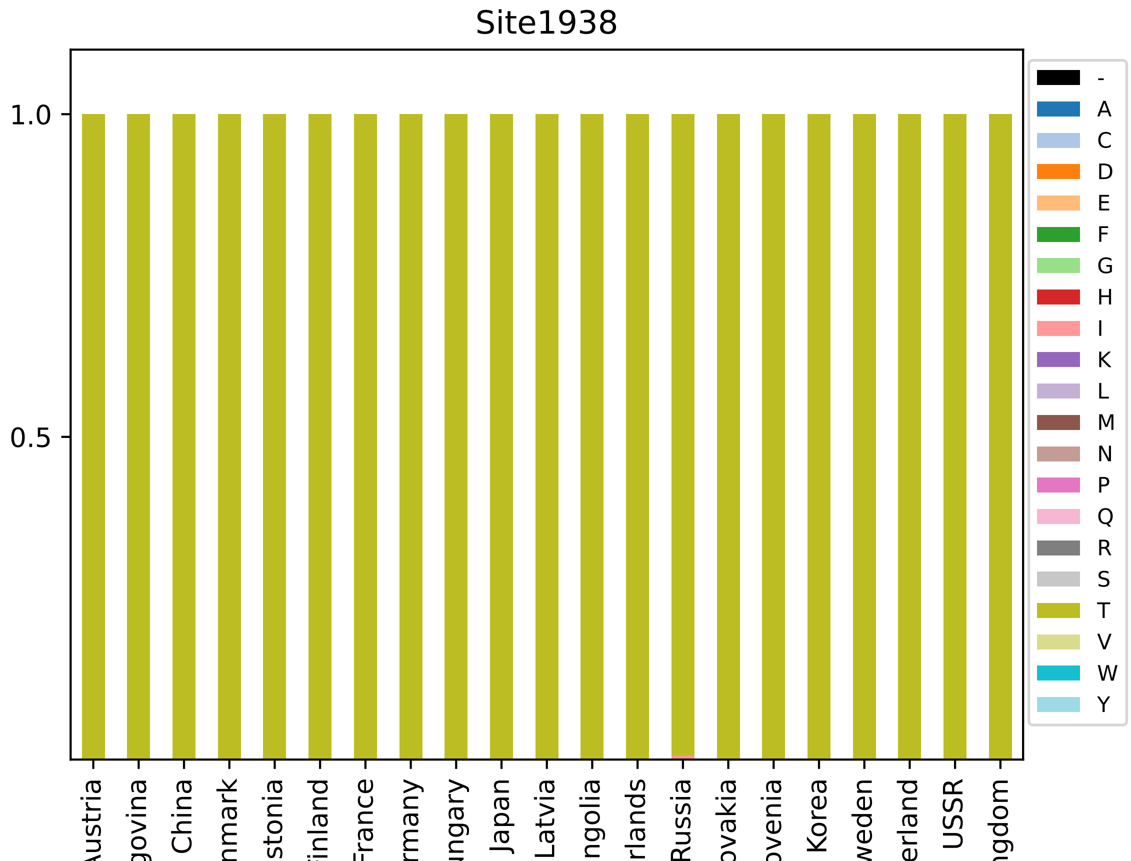

Site1938
1.0
0.5
-
A
C
D
E
F
G
H
I
K
L
M
N
P
Q
R
S
T
V
W
Y
Austria
govina
China
nmark
stonia
Finland
France
rmany
ungary
Japan
Latvia
ngolia
rlands
Russia
ovakia
ovenia
Korea
weden
erland
USSR
ngdom

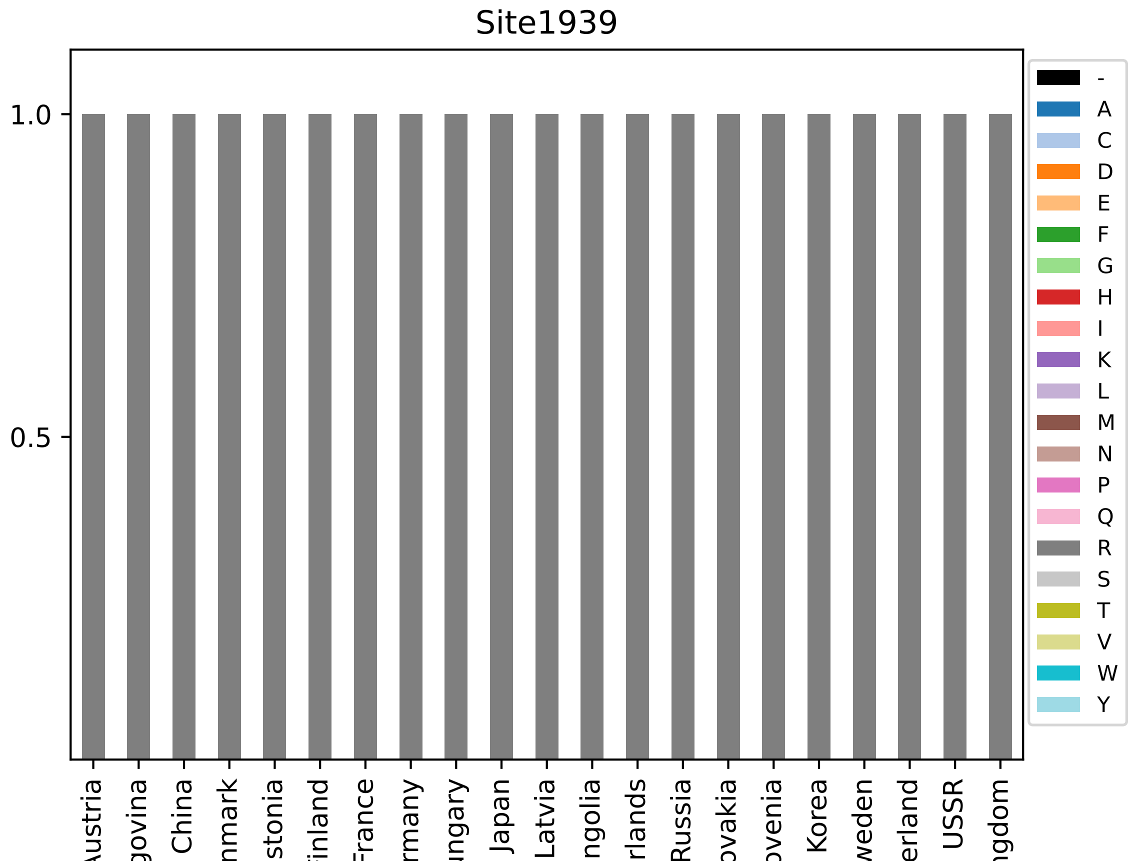

Site1939
1.0
0.5
-
A
C
D
E
F
G
H
I
K
L
M
N
P
Q
R
S
T
V
W
Y
Austria
China
Finland
France
Japan
Latvia
Russia
Korea
USSR

Site1940

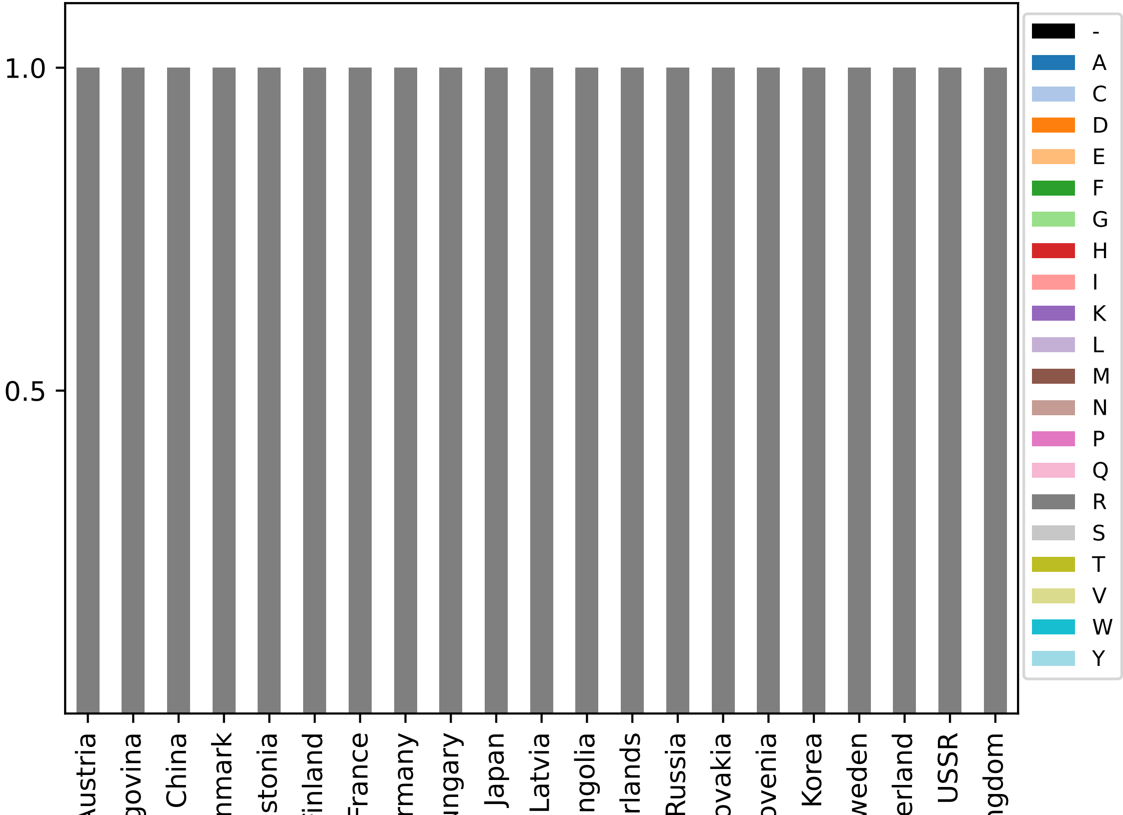

1.0
0.5
China
Japan
Latvia
Russia
Korea
USSR
-
A
C
D
E
F
G
H
I
K
L
M
N
P
Q
R
S
T
V
W
Y

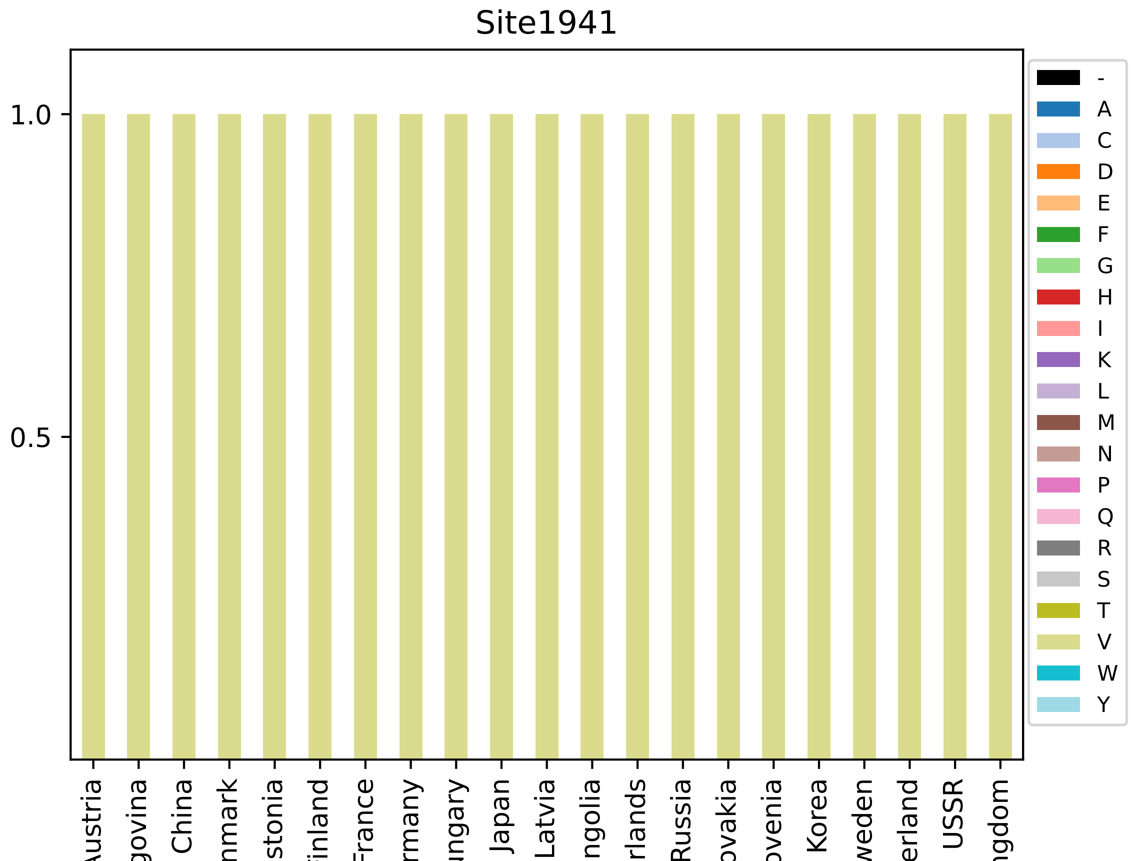

Site1941
1.0
0.5
-
A
C
D
E
F
G
H
I
K
L
M
N
P
Q
R
S
T
V
W
Y
China
Japan
Latvia
Russia
Korea
USSR

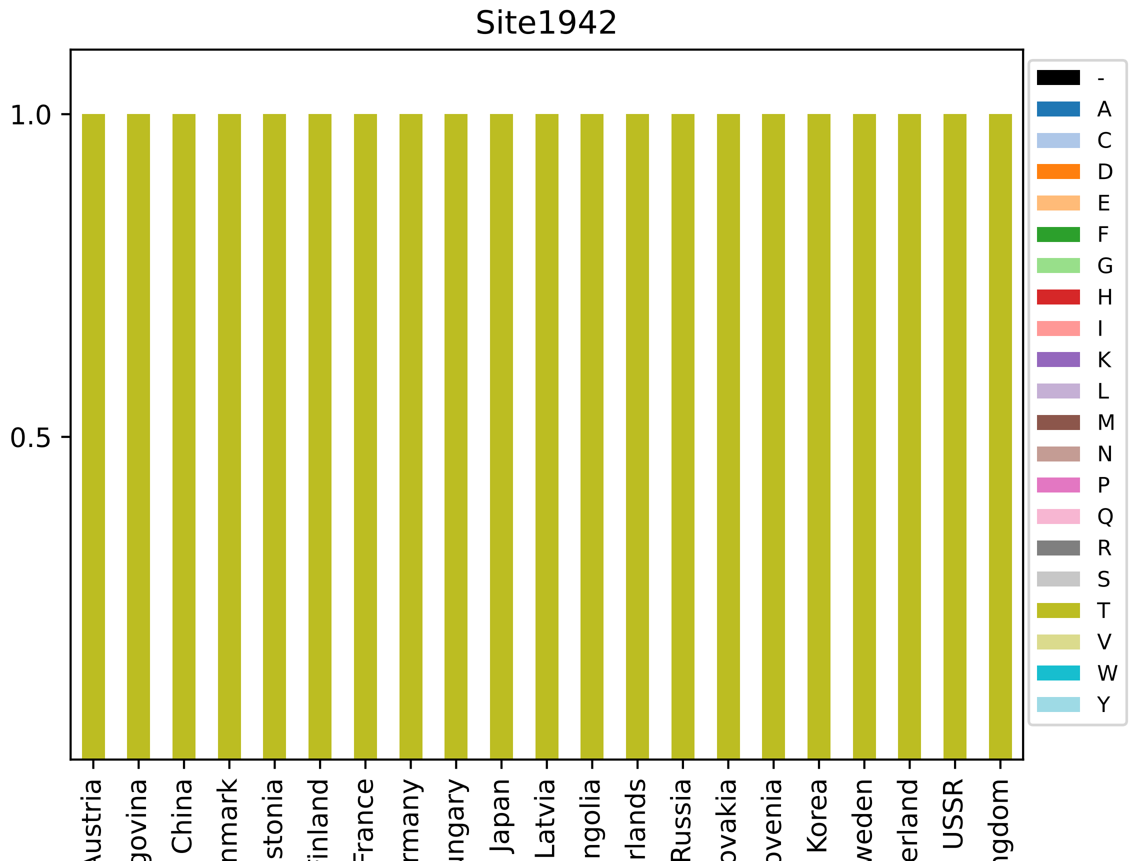
Site1942
1.0
0.5
-
A
C
D
E
F
G
H
I
K
L
M
N
P
Q
R
S
T
V
W
Y
Austria
China
Japan
Latvia
Russia
Korea
USSR

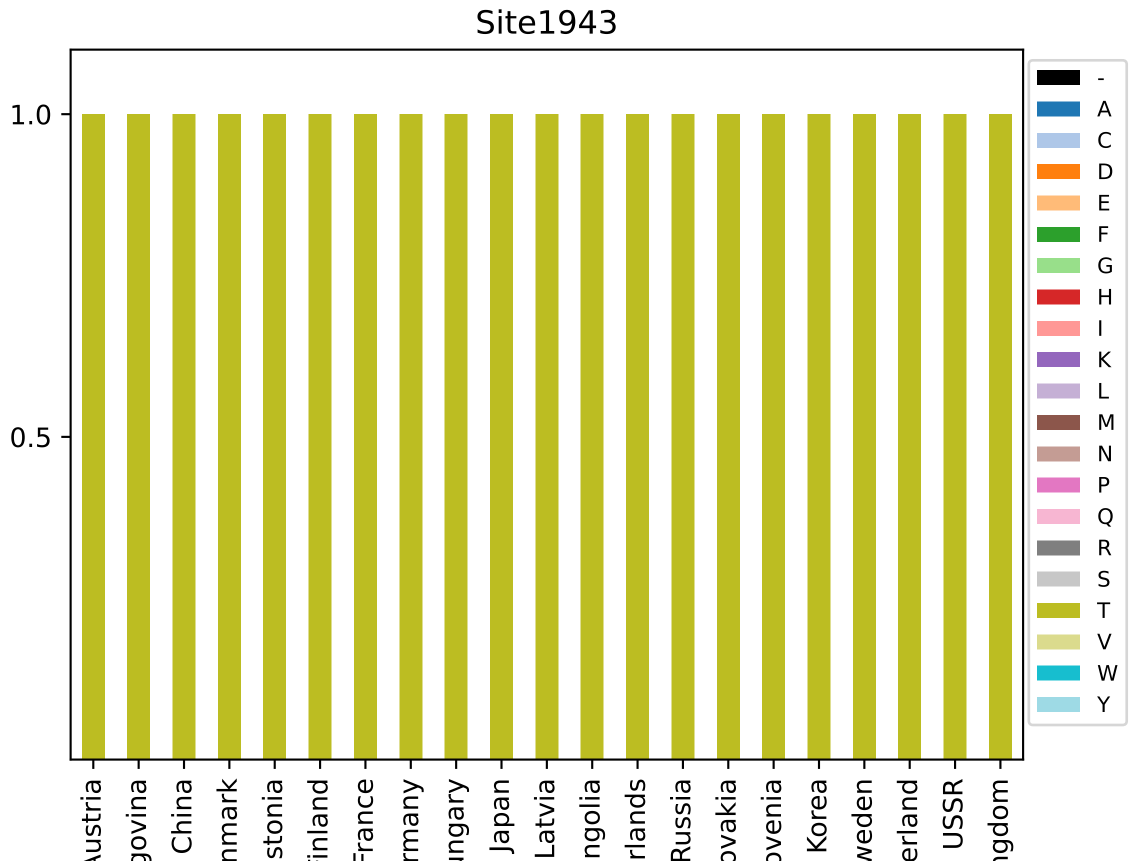
Site1943
1.0
0.5
-
A
C
D
E
F
G
H
I
K
L
M
N
P
Q
R
S
T
V
W
Y
China
Japan
Latvia
Russia
Korea
USSR

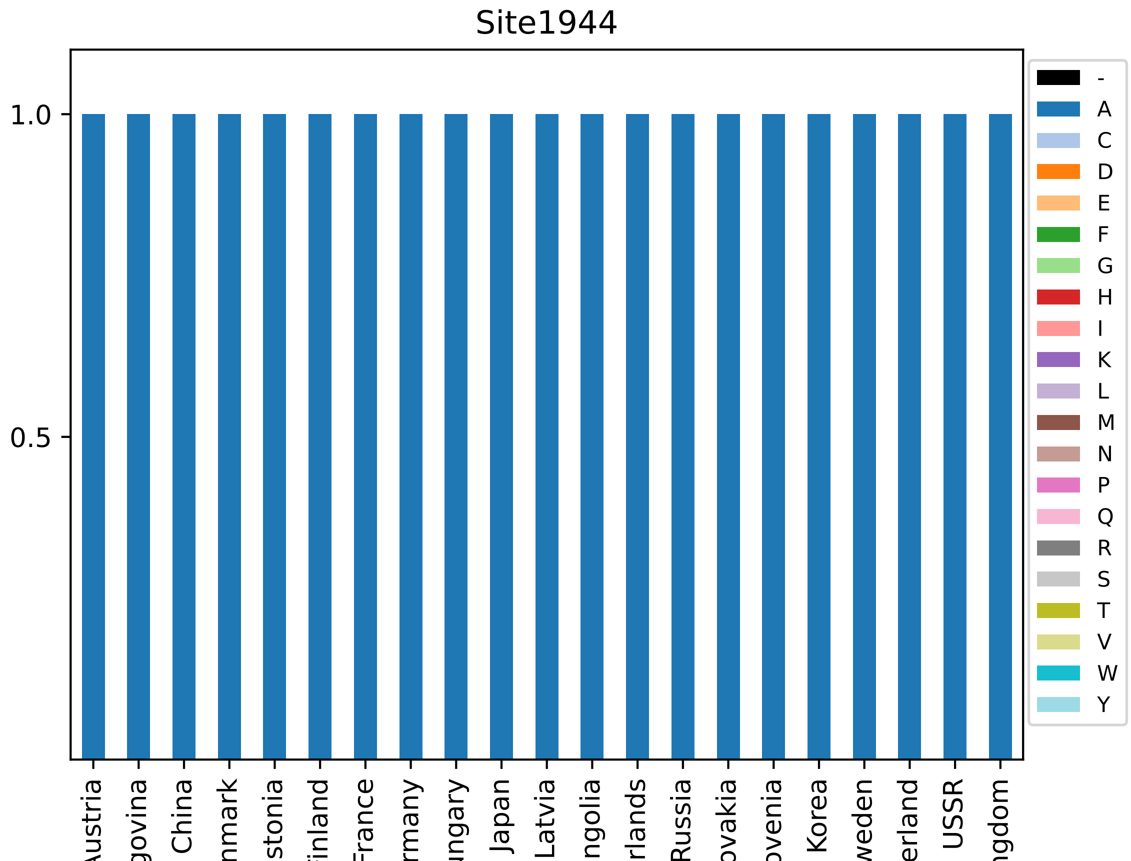
Site1944
1.0
0.5
-
A
C
D
E
F
G
H
I
K
L
M
N
P
Q
R
S
T
V
W
Y
Austria
govina
China
nmark
stonia
inland
France
rmany
ungary
Japan
Latvia
ngolia
rlands
Russia
ovakia
ovenia
Korea
weden
erland
USSR
ngdom

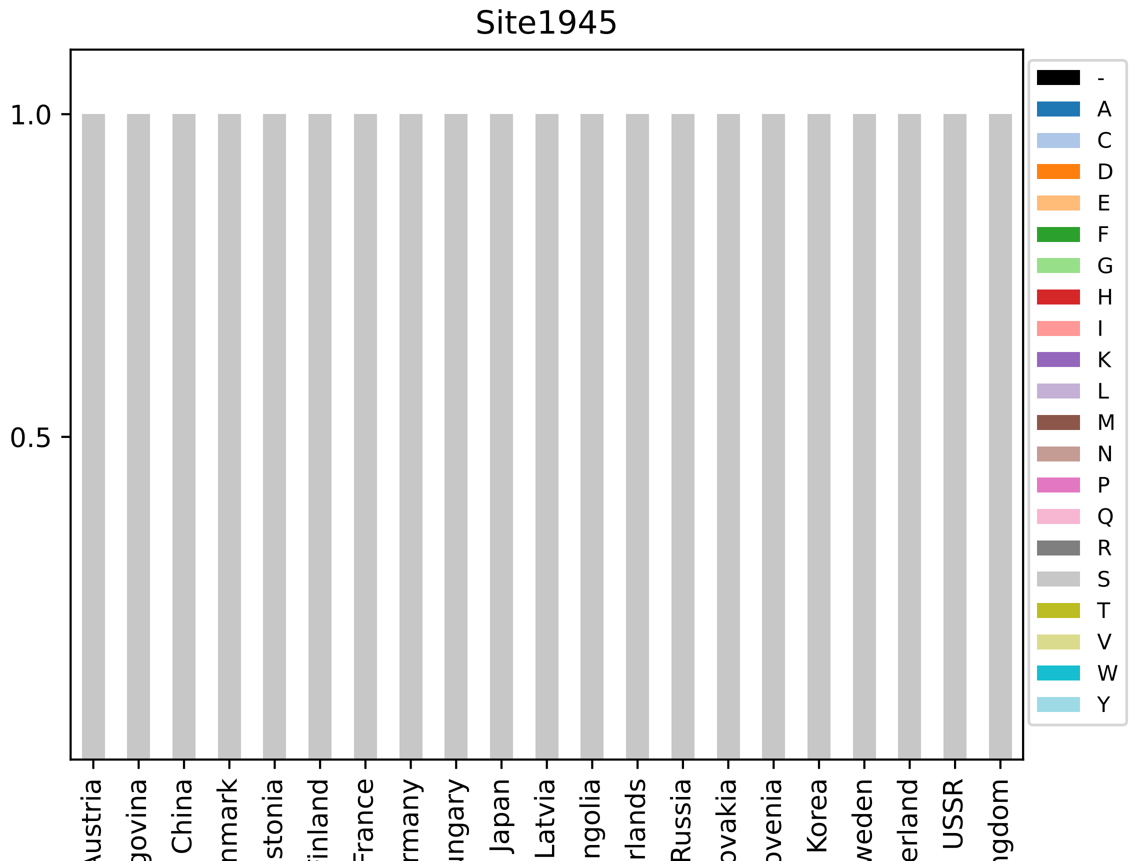
Site1945
1.0
0.5
-
A
C
D
E
F
G
H
I
K
L
M
N
P
Q
R
S
T
V
W
Y
China
Japan
Latvia
Russia
Korea
USSR

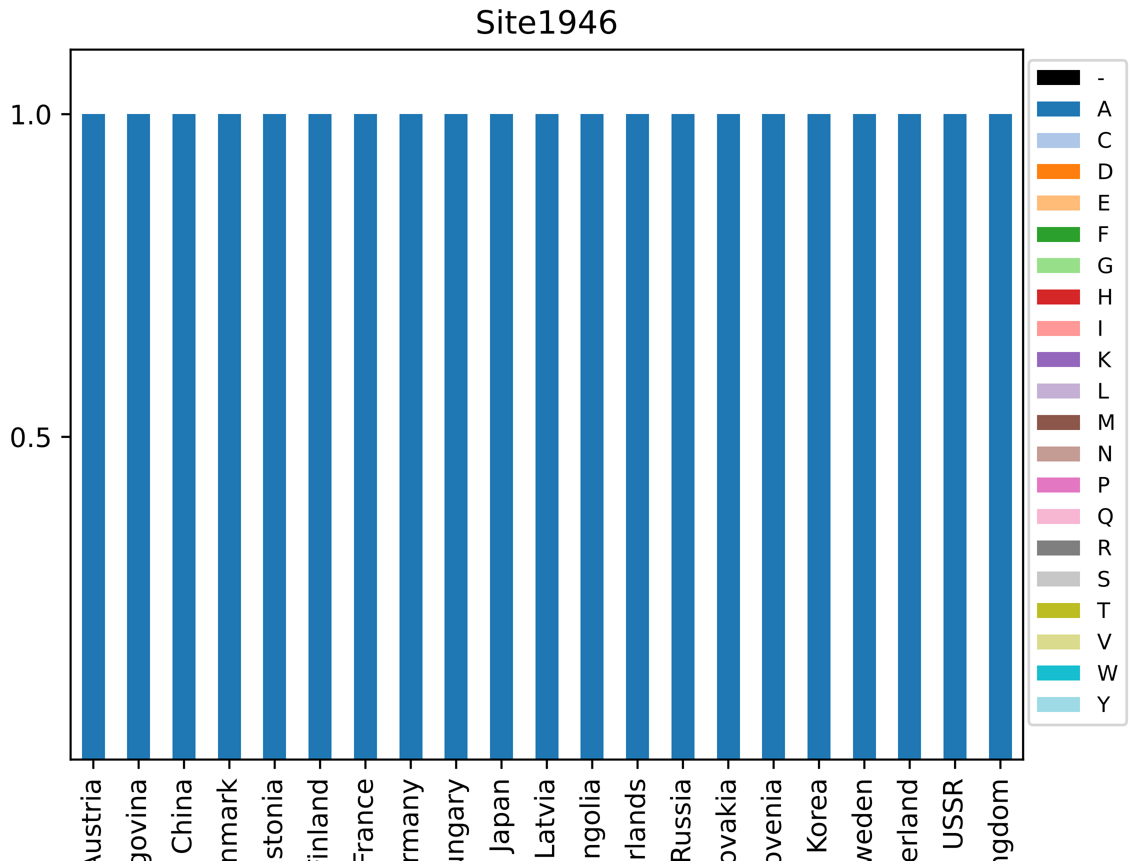

Site1946
1.0
0.5
-
A
C
D
E
F
G
H
I
K
L
M
N
P
Q
R
S
T
V
W
Y
China
Japan
Latvia
Russia
Korea
USSR

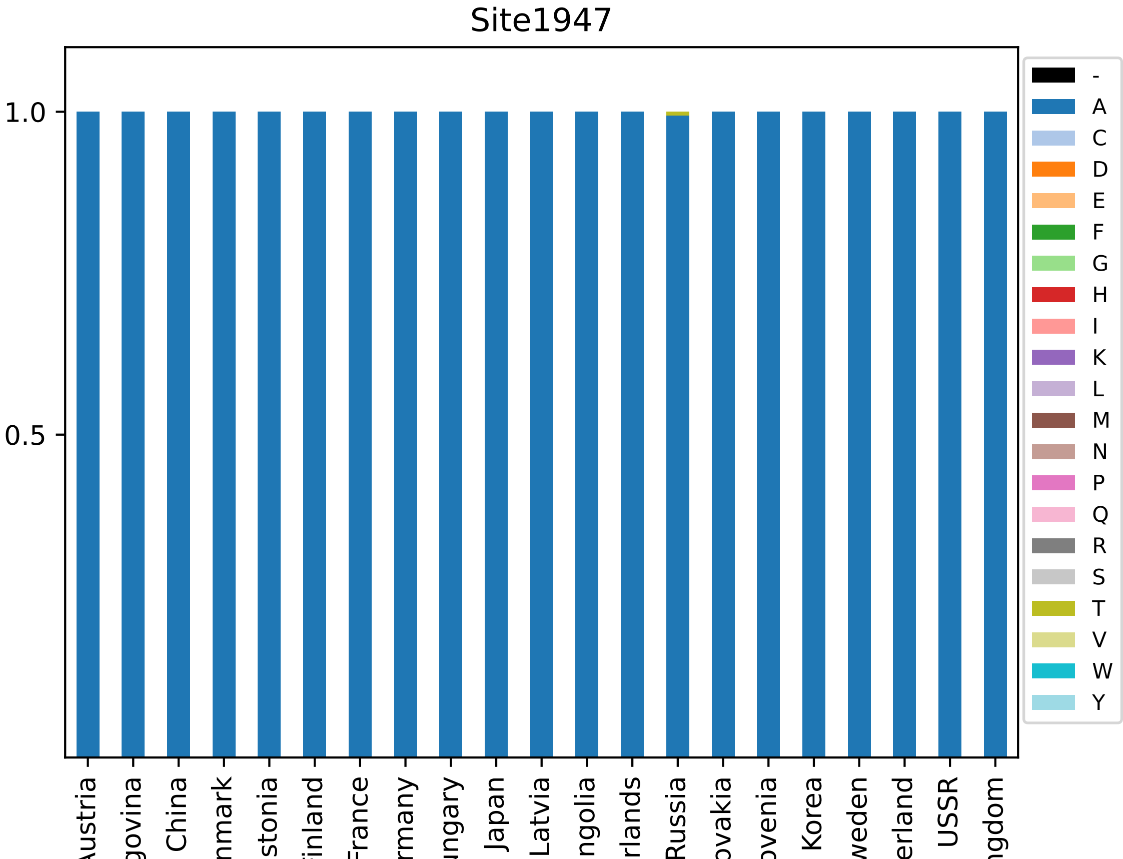
Site1947
1.0
0.5
-
A
C
D
E
F
G
H
I
K
L
M
N
P
Q
R
S
T
V
W
Y
ustria
govina
China
nmark
stonia
inland
rance
rmany
ungary
Japan
Latvia
ngolia
rlands
Russia
ovakia
ovenia
Korea
weden
erland
USSR
ngdom

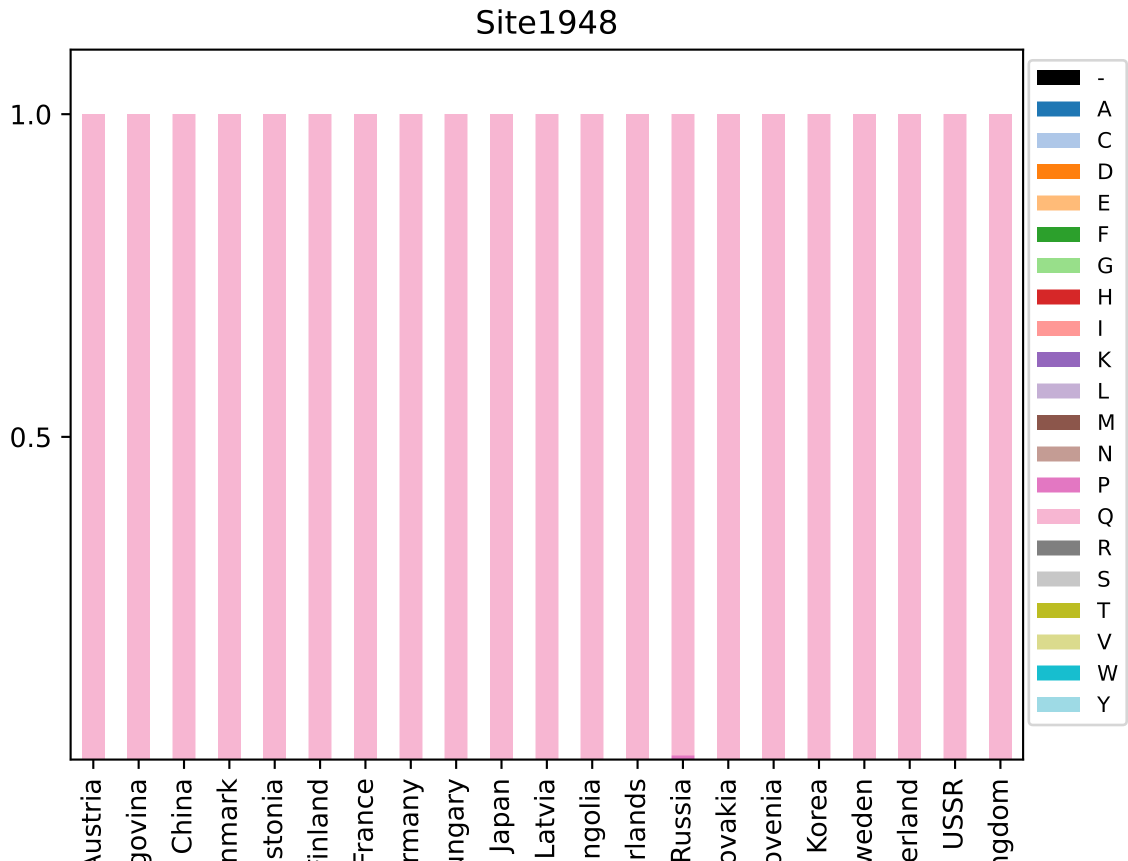
Site1948
1.0
0.5
-
A
C
D
E
F
G
H
I
K
L
M
N
P
Q
R
S
T
V
W
Y
Austria
China
Finland
France
Japan
Latvia
Russia
Korea
USSR

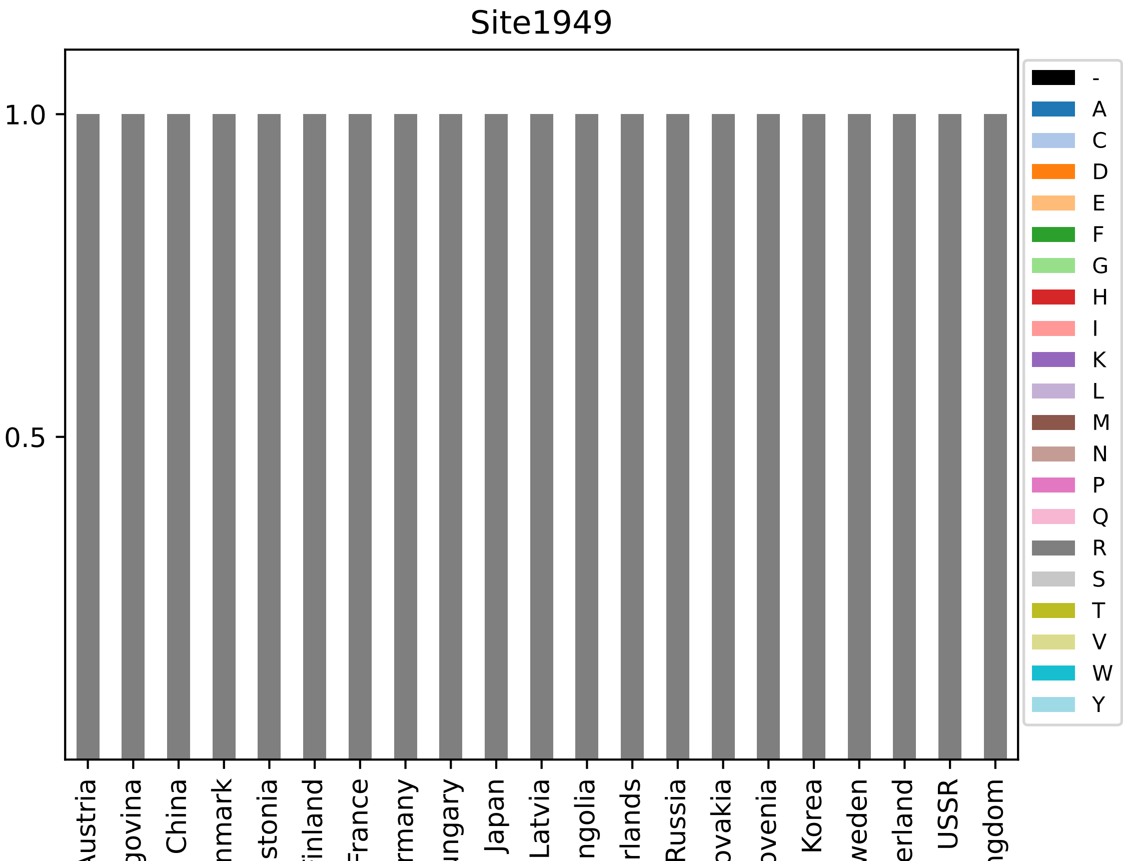

Site1949
1.0
0.5
-
A
C
D
E
F
G
H
I
K
L
M
N
P
Q
R
S
T
V
W
Y
China
Japan
Latvia
Russia
Korea
USSR

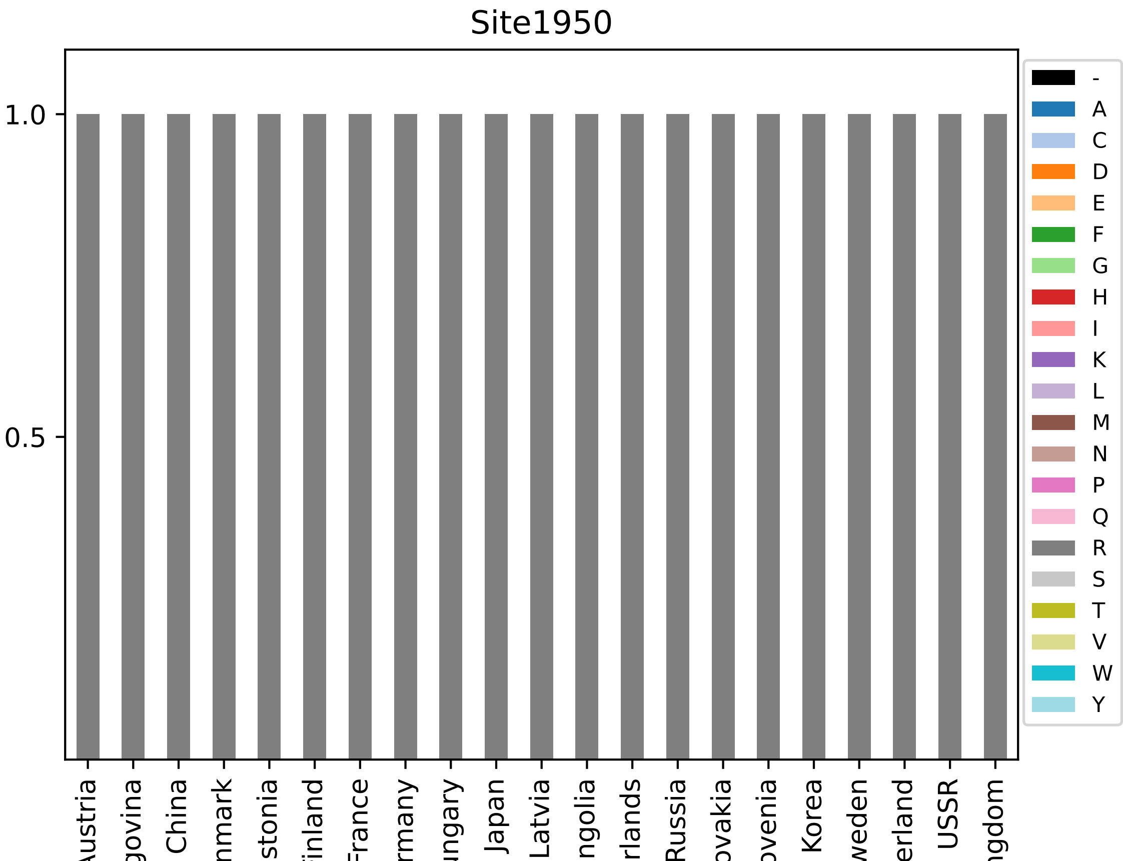
Site1950
1.0
0.5
-
A
C
D
E
F
G
H
I
K
L
M
N
P
Q
R
S
T
V
W
Y
China
Japan
Latvia
Russia
Korea
USSR

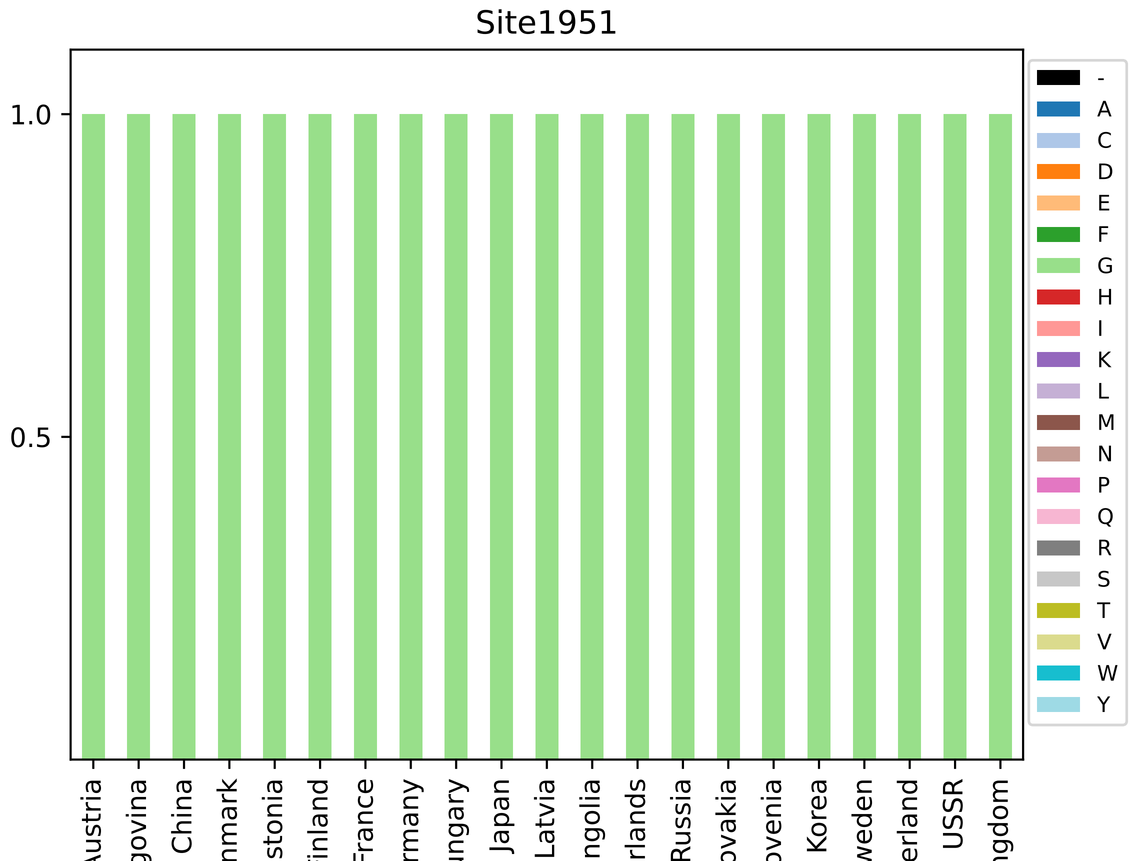
Site1951
1.0
0.5
-
A
C
D
E
F
G
H
I
K
L
M
N
P
Q
R
S
T
V
W
Y
China
Japan
Latvia
Russia
Korea
USSR

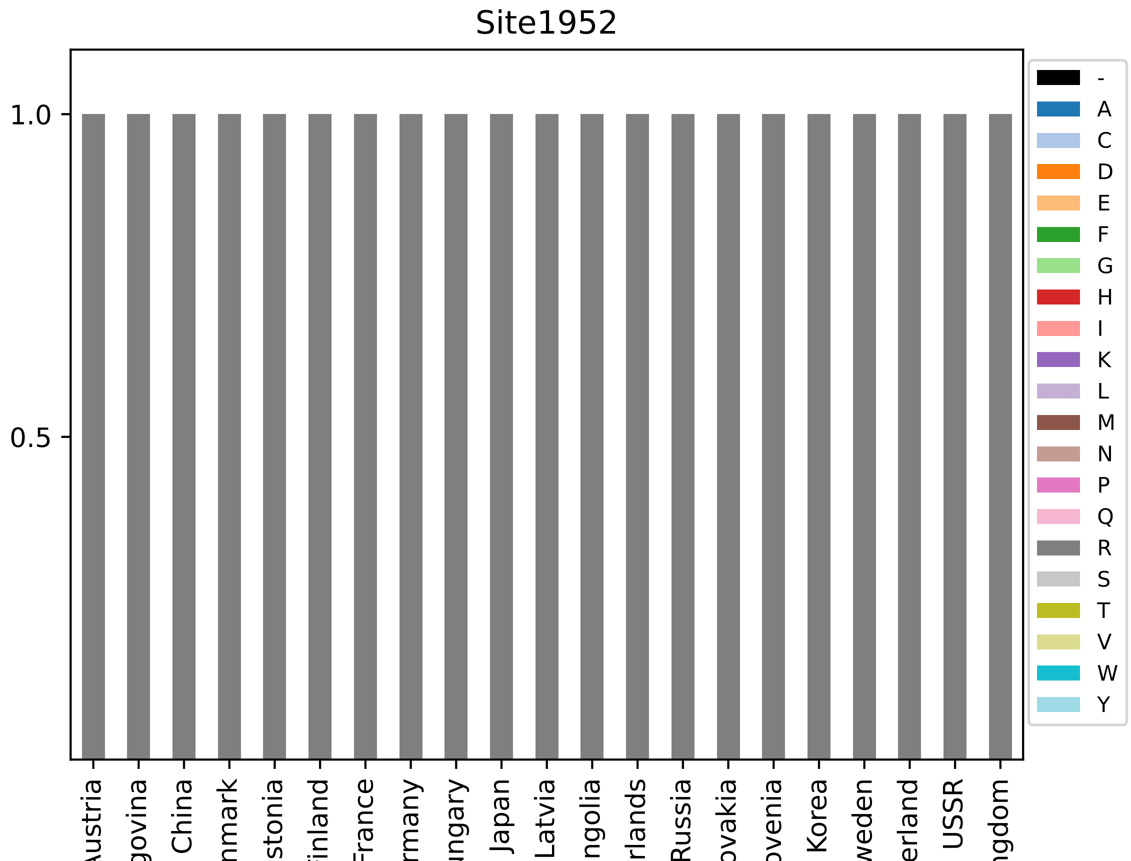

Site1952
1.0
0.5
-
A
C
D
E
F
G
H
I
K
L
M
N
P
Q
R
S
T
V
W
Y
Austria
China
Japan
Latvia
Russia
Korea
USSR

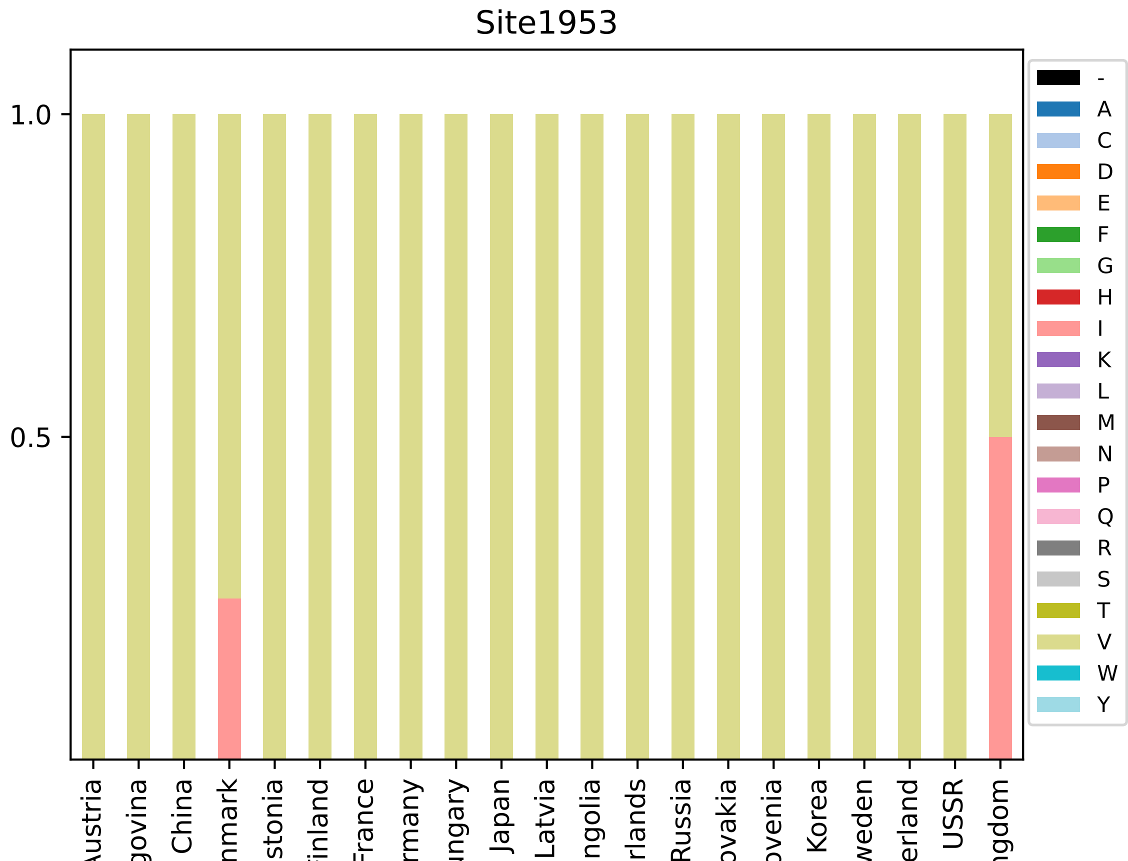

Site1953
1.0
0.5
-
A
C
D
E
F
G
H
I
K
L
M
N
P
Q
R
S
T
V
W
Y
Austria
govina
China
nmark
stonia
Finland
France
rmany
ungary
Japan
Latvia
ngolia
rlands
Russia
ovakia
ovenia
Korea
weden
erland
USSR
ngdom

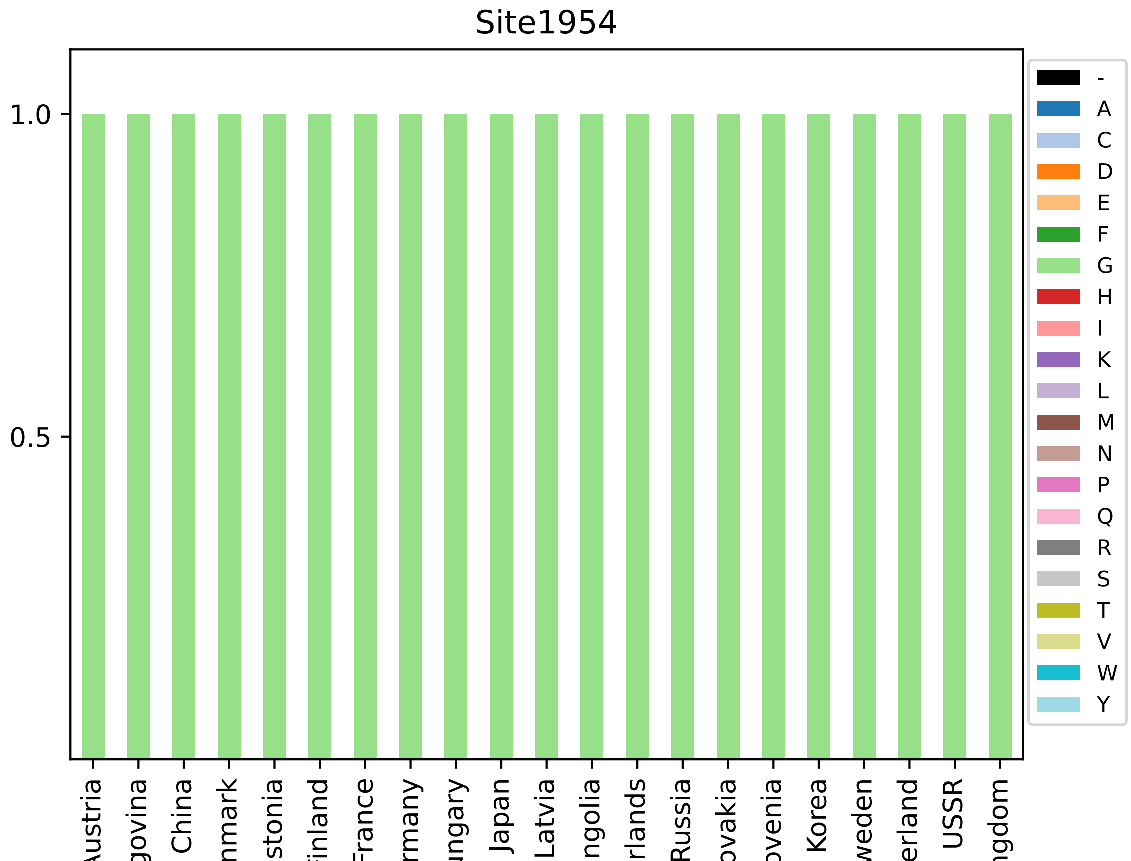
Site1954
1.0
0.5
-
A
C
D
E
F
G
H
I
K
L
M
N
P
Q
R
S
T
V
W
Y
Austria
govina
China
nmark
stonia
Finland
France
rmany
ungary
Japan
Latvia
ngolia
rlands
Russia
ovakia
ovenia
Korea
weden
erland
USSR
ngdom

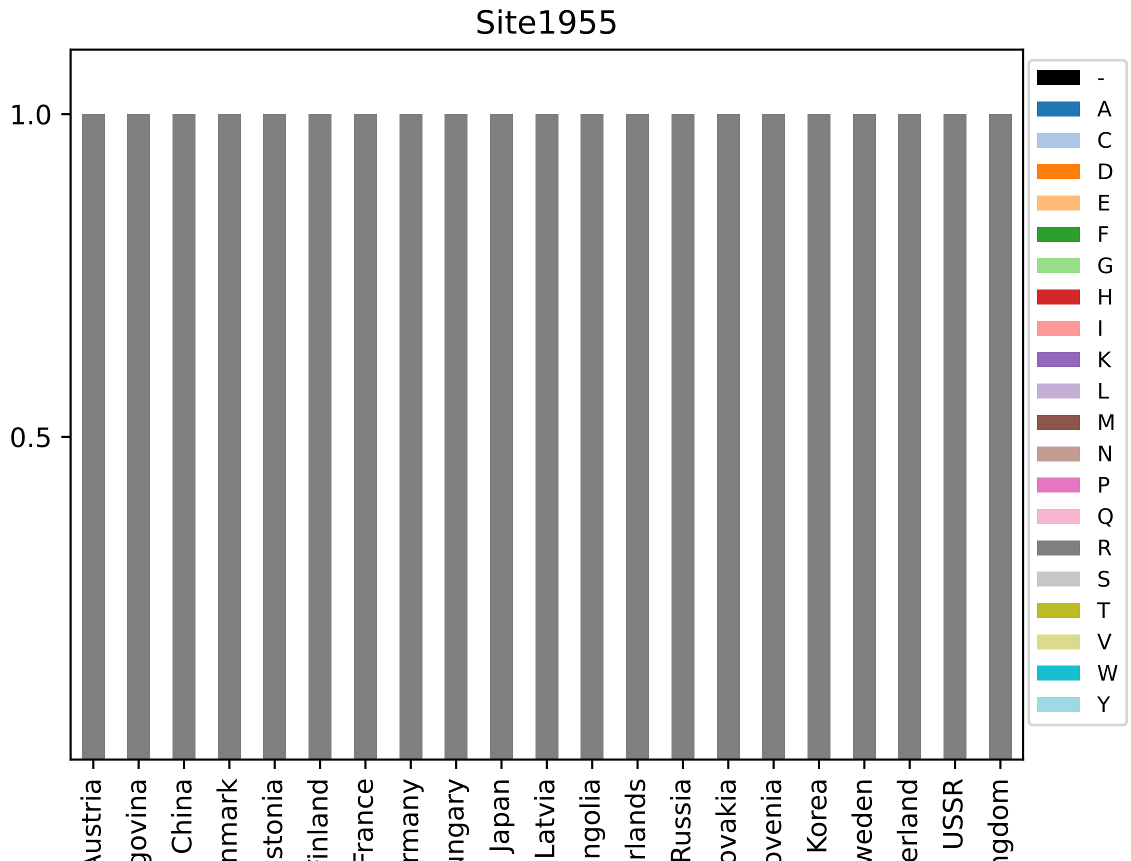

Site1955
1.0
0.5
-
A
C
D
E
F
G
H
I
K
L
M
N
P
Q
R
S
T
V
W
Y
Austria
govina
China
nmark
stonia
Finland
France
rmany
ungary
Japan
Latvia
ngolia
rlands
Russia
ovakia
ovenia
Korea
weden
erland
USSR
ngdom

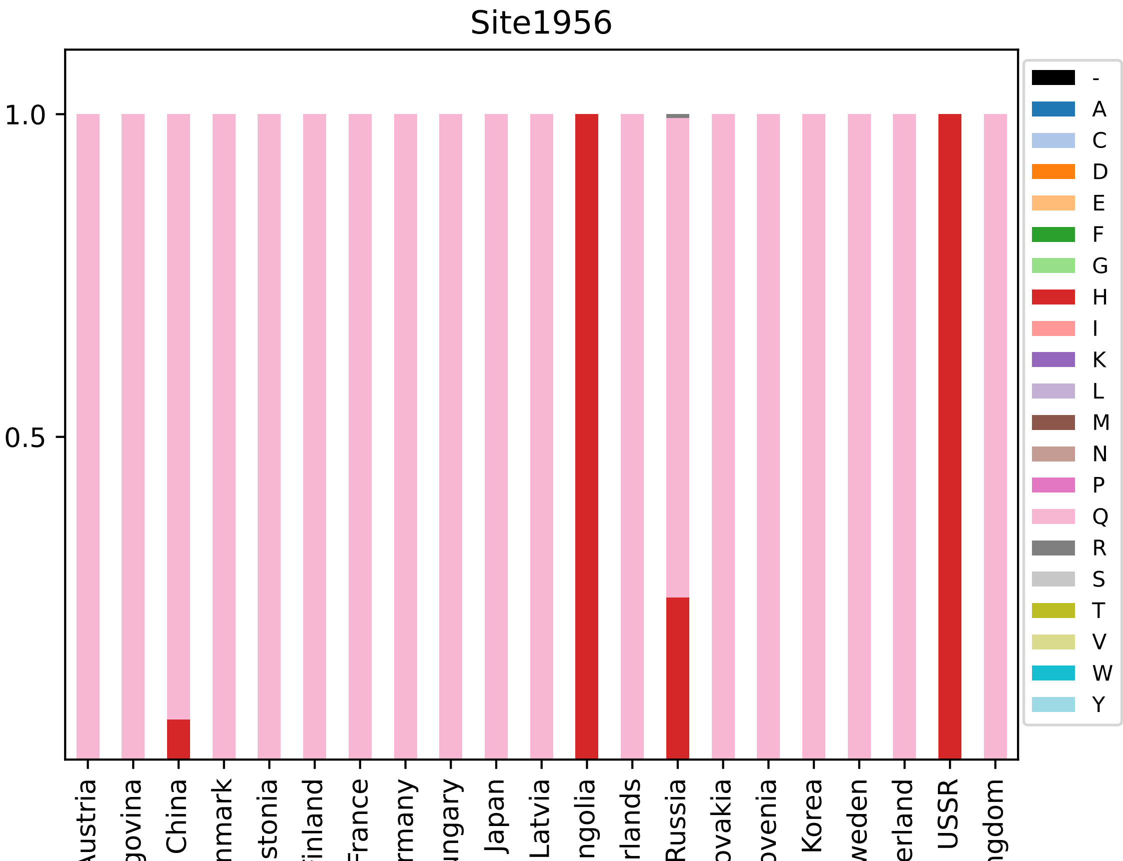

Site1956
1.0
0.5
-
A
C
D
E
F
G
H
I
K
L
M
N
P
Q
R
S
T
V
W
Y
ustria
govina
China
nmark
stonia
inland
rance
rmany
ungary
Japan
Latvia
ngolia
rlands
Russia
ovakia
ovenia
Korea
weden
erland
USSR
ngdom

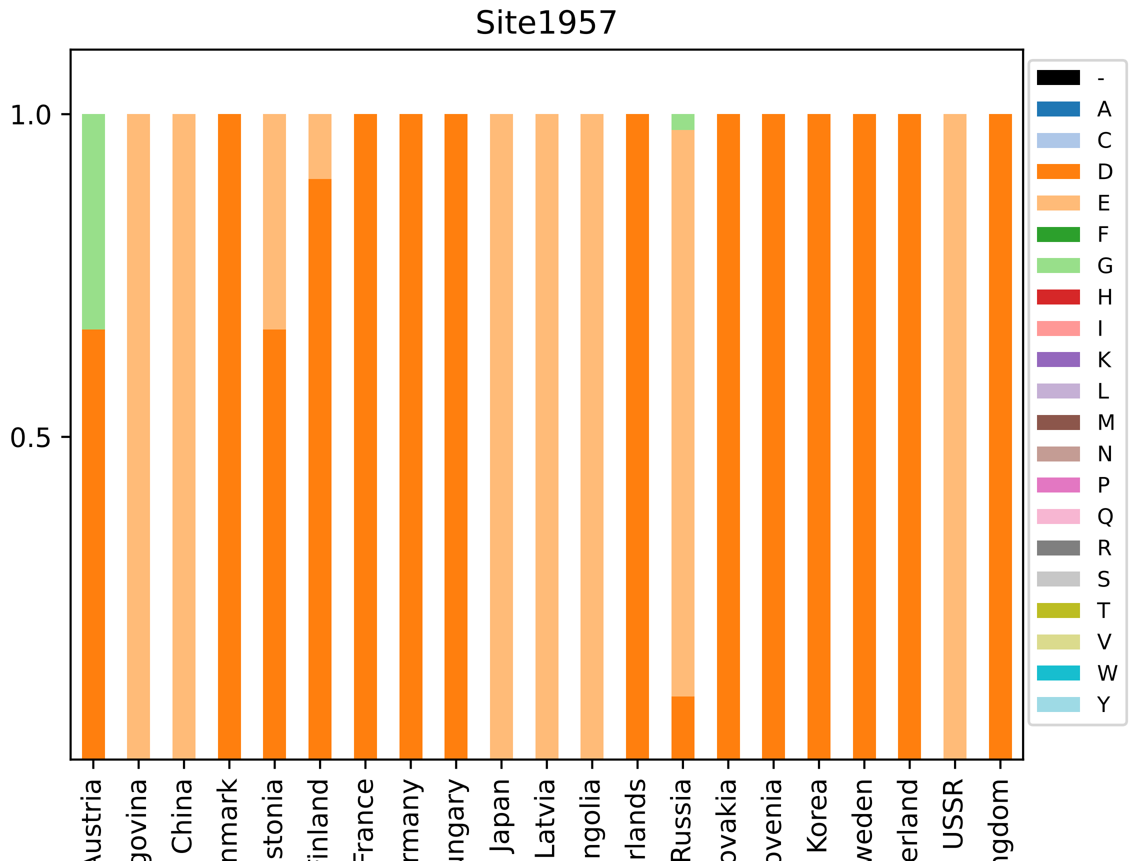
Site1957
1.0
0.5
-
A
C
D
E
F
G
H
I
K
L
M
N
P
Q
R
S
T
V
W
Y
China
Japan
Latvia
Russia
Korea
USSR

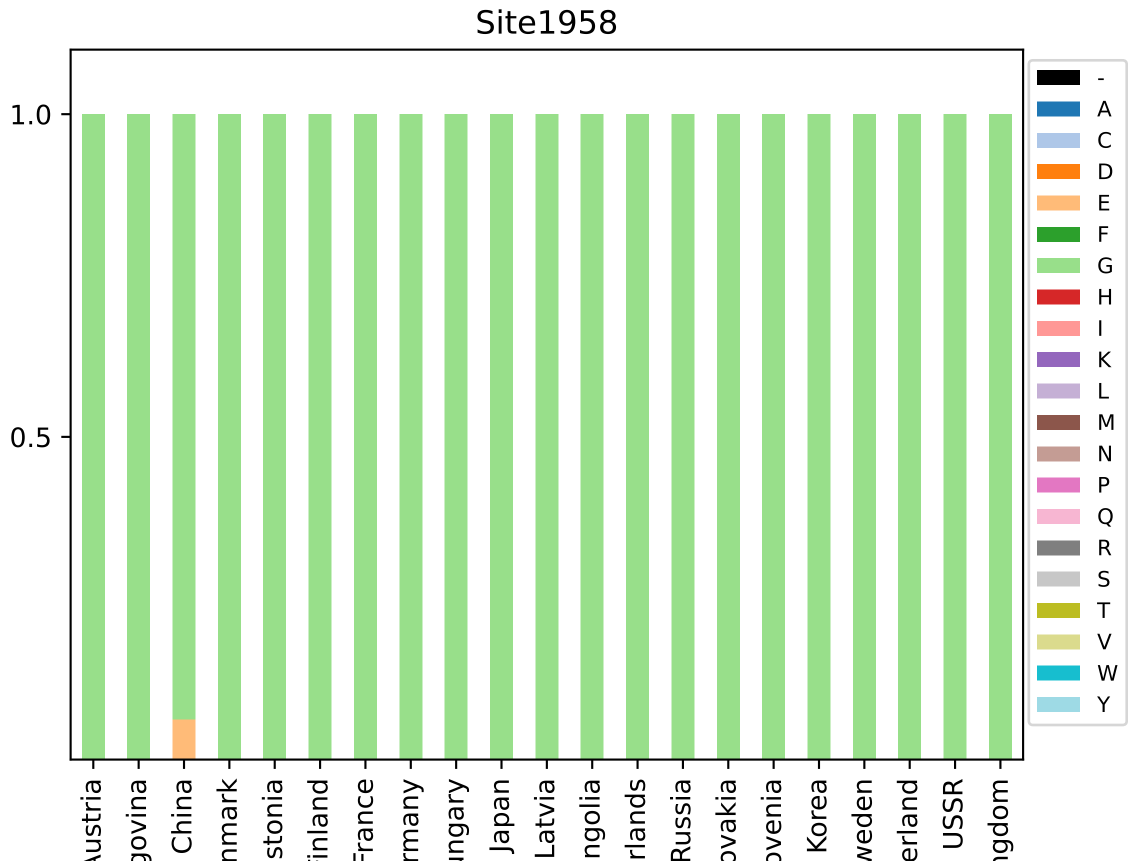

Site1958
1.0
0.5
-
A
C
D
E
F
G
H
I
K
L
M
N
P
Q
R
S
T
V
W
Y
China
Japan
Latvia
Russia
Korea
USSR

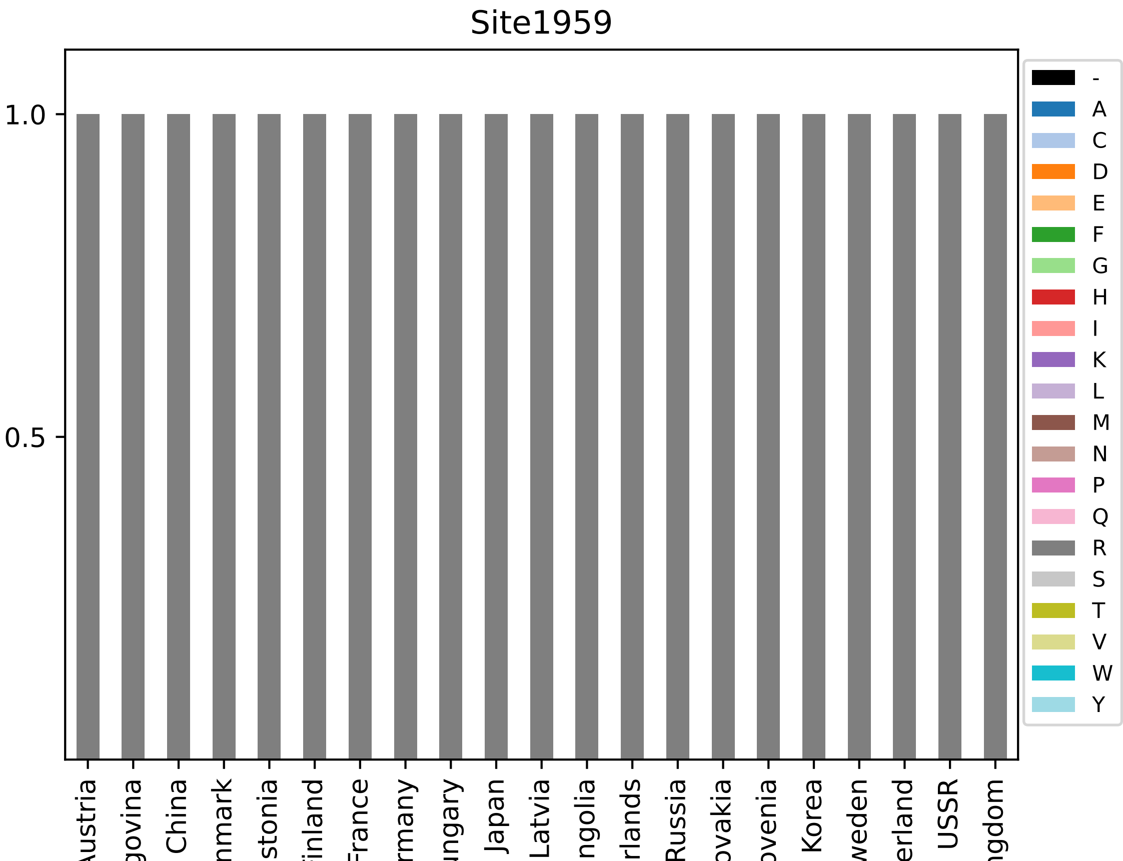
Site1959
1.0
0.5
-
A
C
D
E
F
G
H
I
K
L
M
N
P
Q
R
S
T
V
W
Y
China
Japan
Latvia
Russia
Korea
USSR

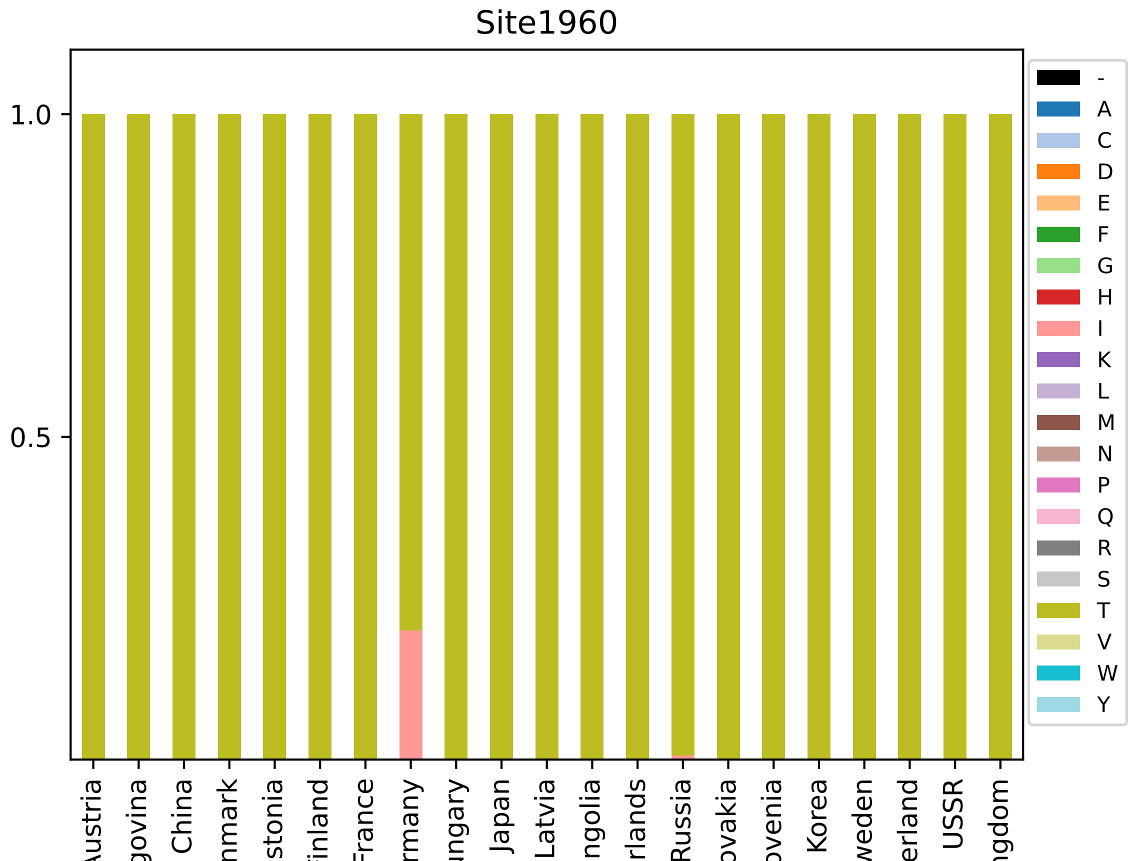
Site1960
1.0
0.5
-
A
C
D
E
F
G
H
I
K
L
M
N
P
Q
R
S
T
V
W
Y
China
Japan
Latvia
Russia
Korea
USSR

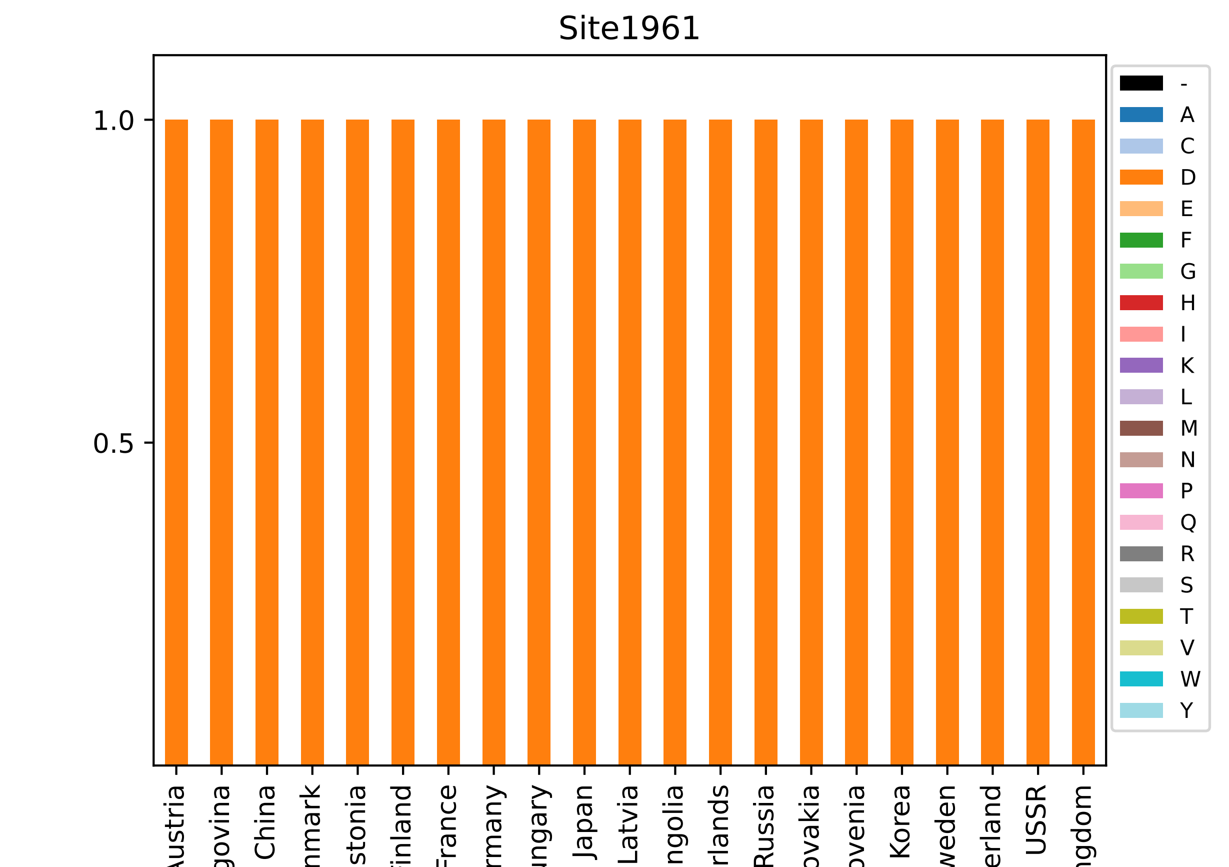

Site1961
1.0
0.5
-
A
C
D
E
F
G
H
I
K
L
M
N
P
Q
R
S
T
V
W
Y
Austria
govina
China
nmark
stonia
Finland
France
rmany
ungary
Japan
Latvia
ngolia
rlands
Russia
ovakia
ovenia
Korea
weden
erland
USSR
ngdom

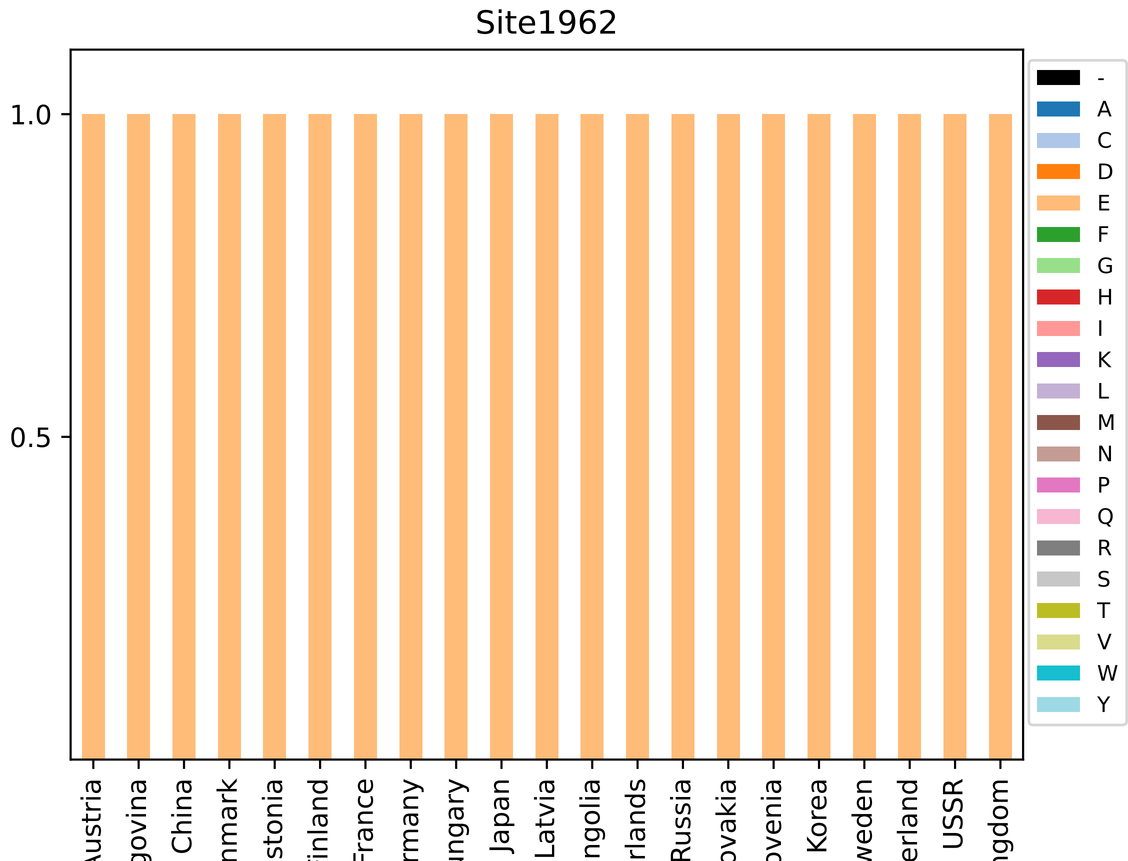
Site1962
1.0
0.5
-
A
C
D
E
F
G
H
I
K
L
M
N
P
Q
R
S
T
V
W
Y
China
Japan
Latvia
Russia
Korea
USSR

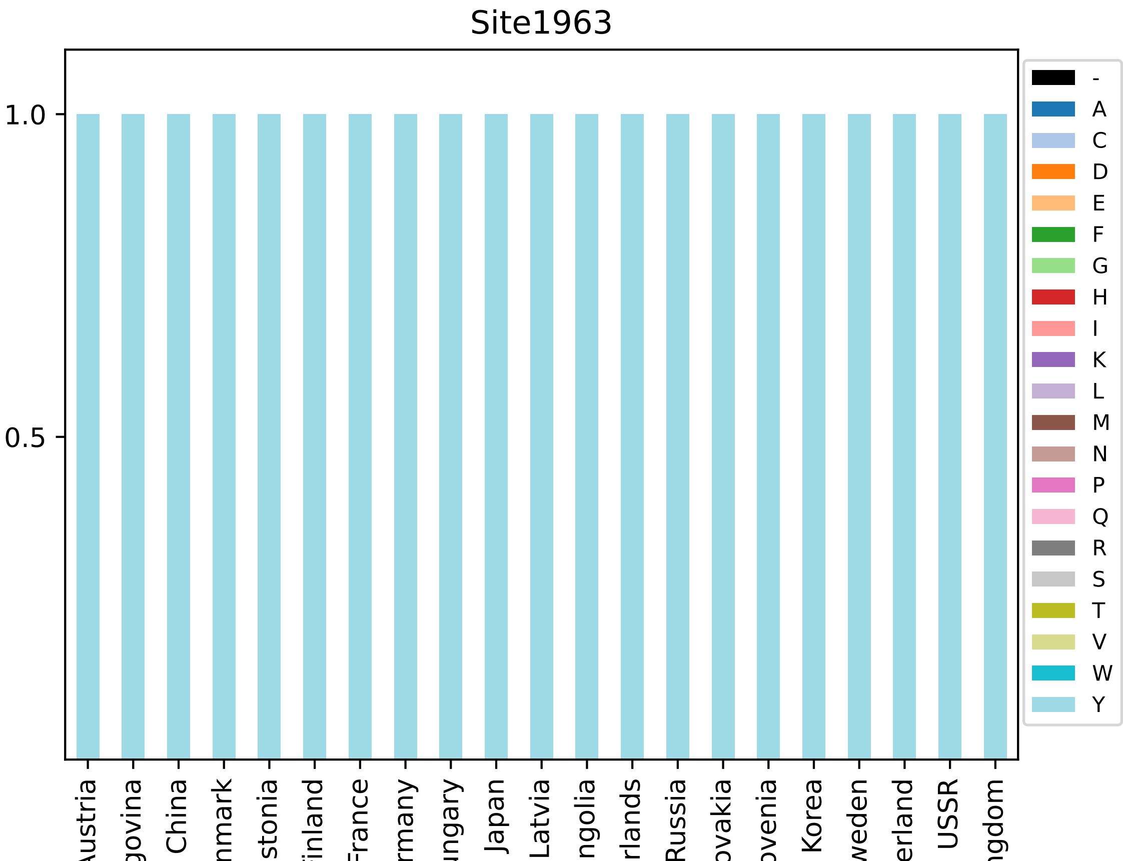
Site1963
1.0
0.5
-
A
C
D
E
F
G
H
I
K
L
M
N
P
Q
R
S
T
V
W
Y
Austria
govina
China
nmark
stonia
Finland
France
rmany
ungary
Japan
Latvia
ngolia
rlands
Russia
ovakia
ovenia
Korea
weden
erland
USSR
ngdom

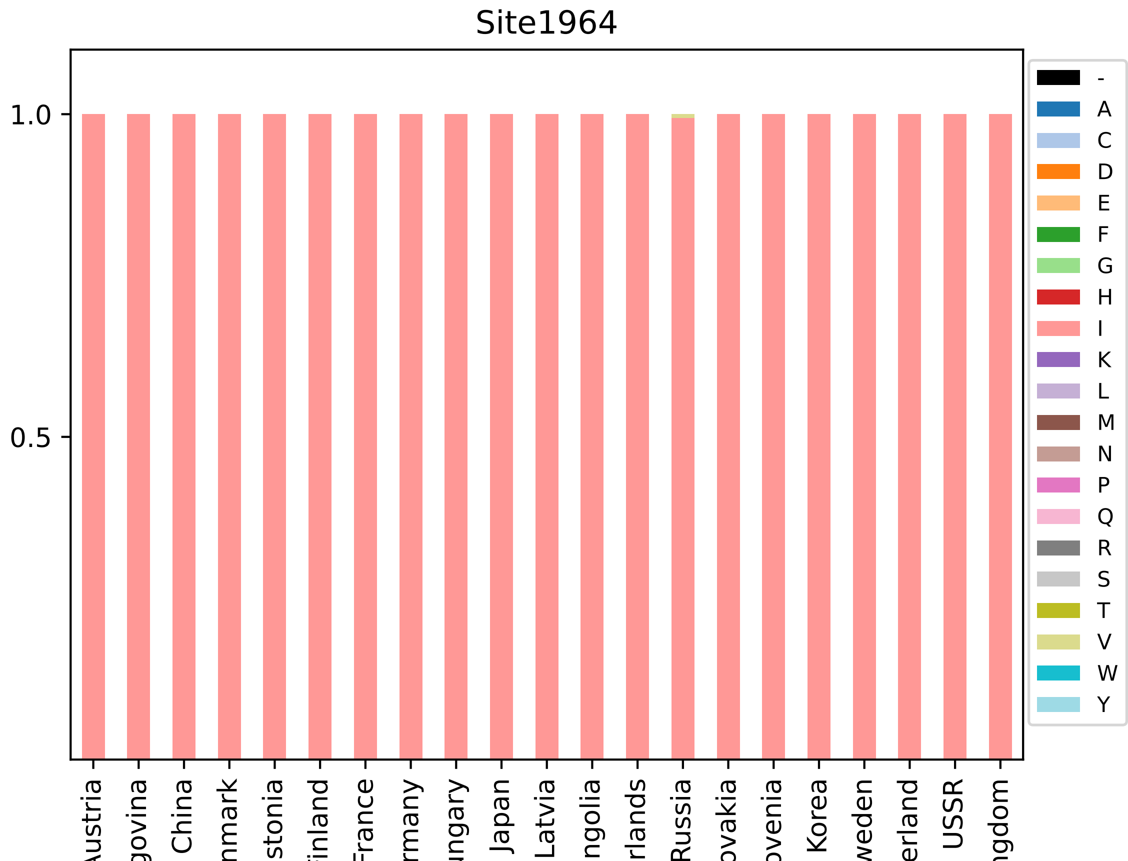

Site1964
1.0
0.5
-
A
C
D
E
F
G
H
I
K
L
M
N
P
Q
R
S
T
V
W
Y
Austria
China
Japan
Latvia
Russia
Korea
USSR

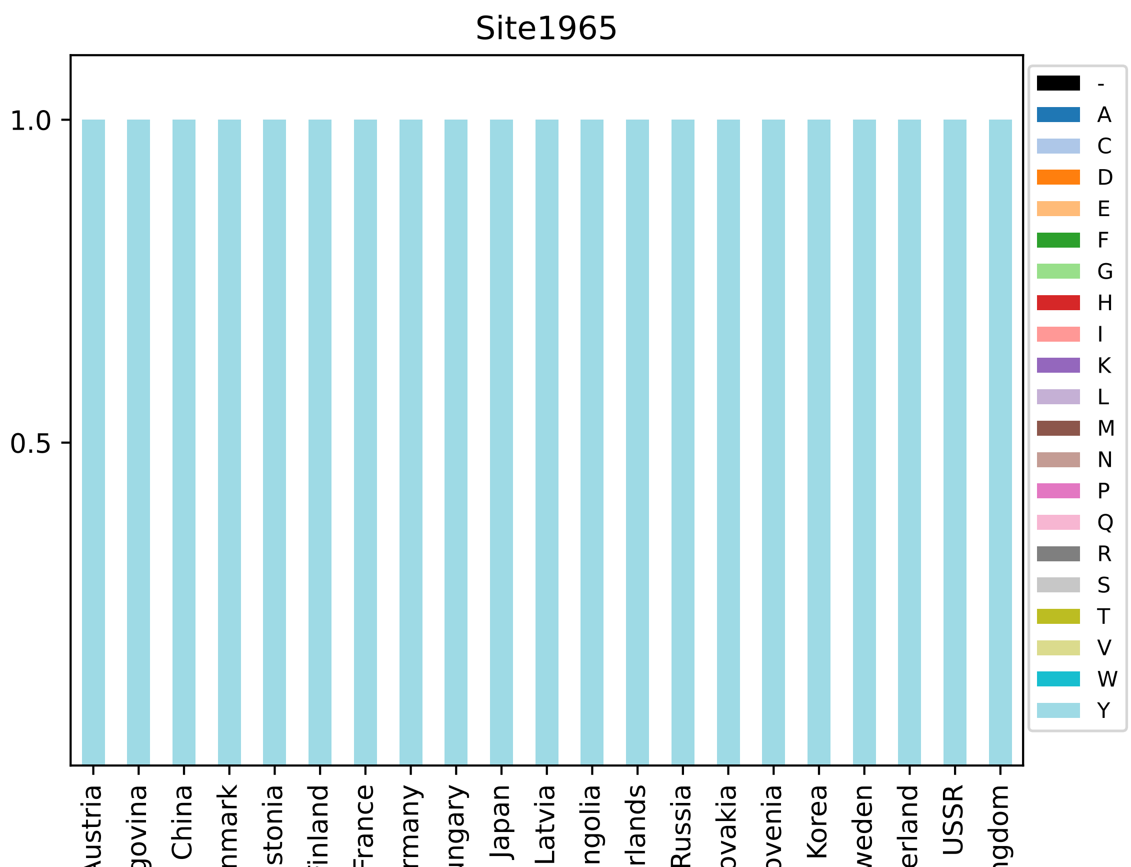
Site1965
1.0
0.5
-
A
C
D
E
F
G
H
I
K
L
M
N
P
Q
R
S
T
V
W
Y
Austria
govina
China
nmark
stonia
inland
France
rmany
ungary
Japan
Latvia
ngolia
rlands
Russia
ovakia
ovenia
Korea
weden
erland
USSR
ngdom

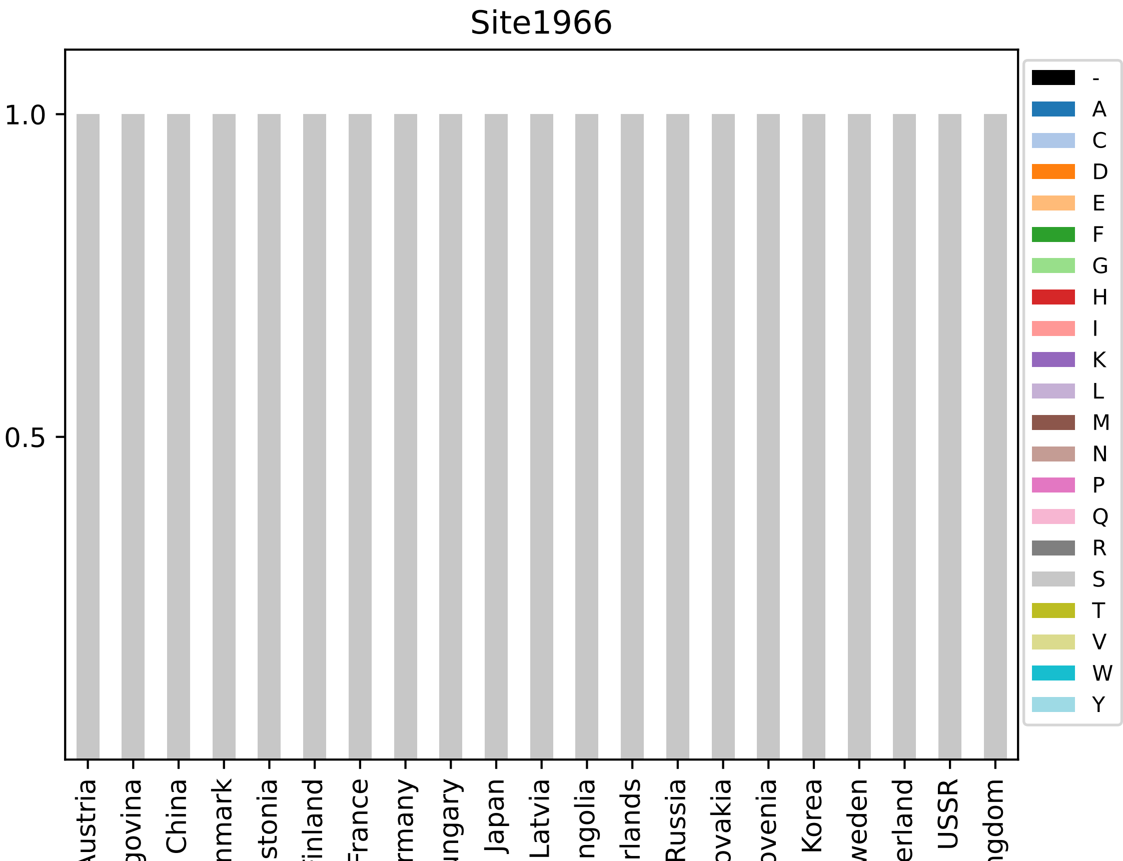
Site1966
1.0
0.5
-
A
C
D
E
F
G
H
I
K
L
M
N
P
Q
R
S
T
V
W
Y
Austria
China
Japan
Latvia
Russia
Korea
USSR

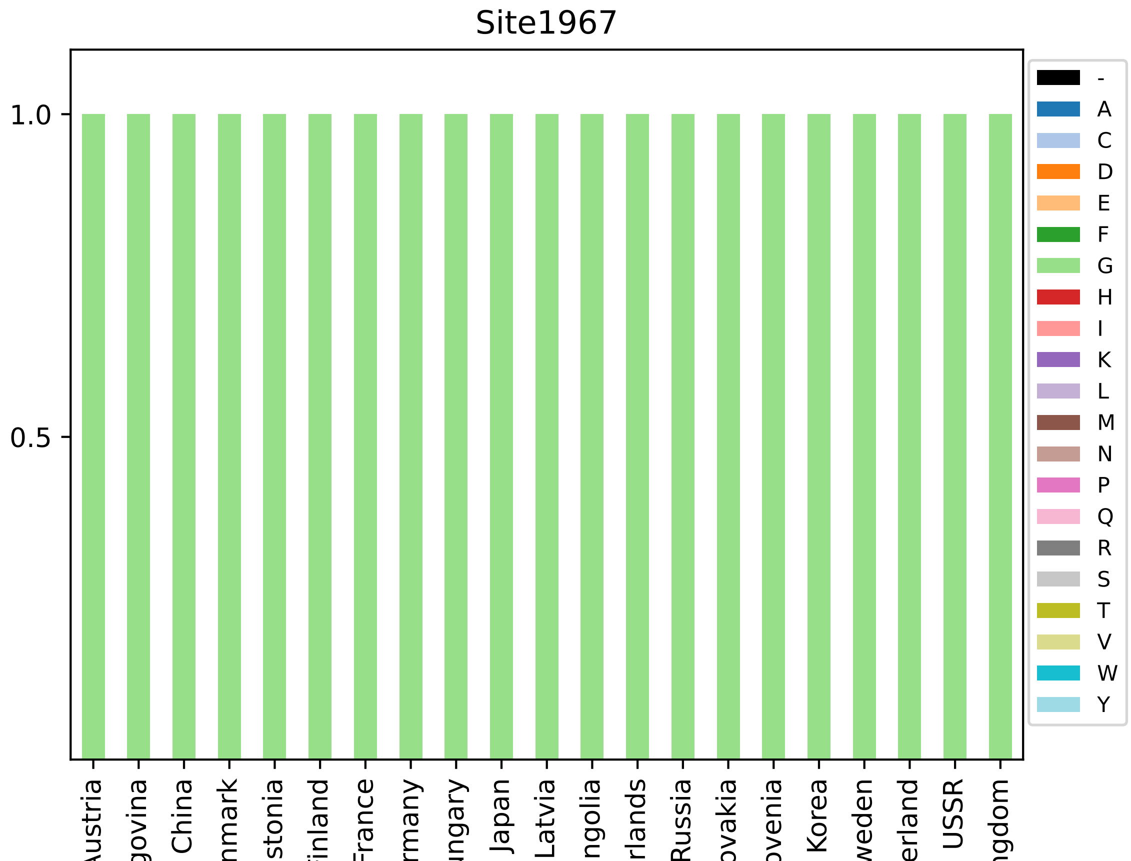
Site1967
1.0
0.5
-
A
C
D
E
F
G
H
I
K
L
M
N
P
Q
R
S
T
V
W
Y
Austria
govina
China
nmark
stonia
inland
France
rmany
ungary
Japan
Latvia
ngolia
rlands
Russia
ovakia
ovenia
Korea
weden
erland
USSR
ngdom

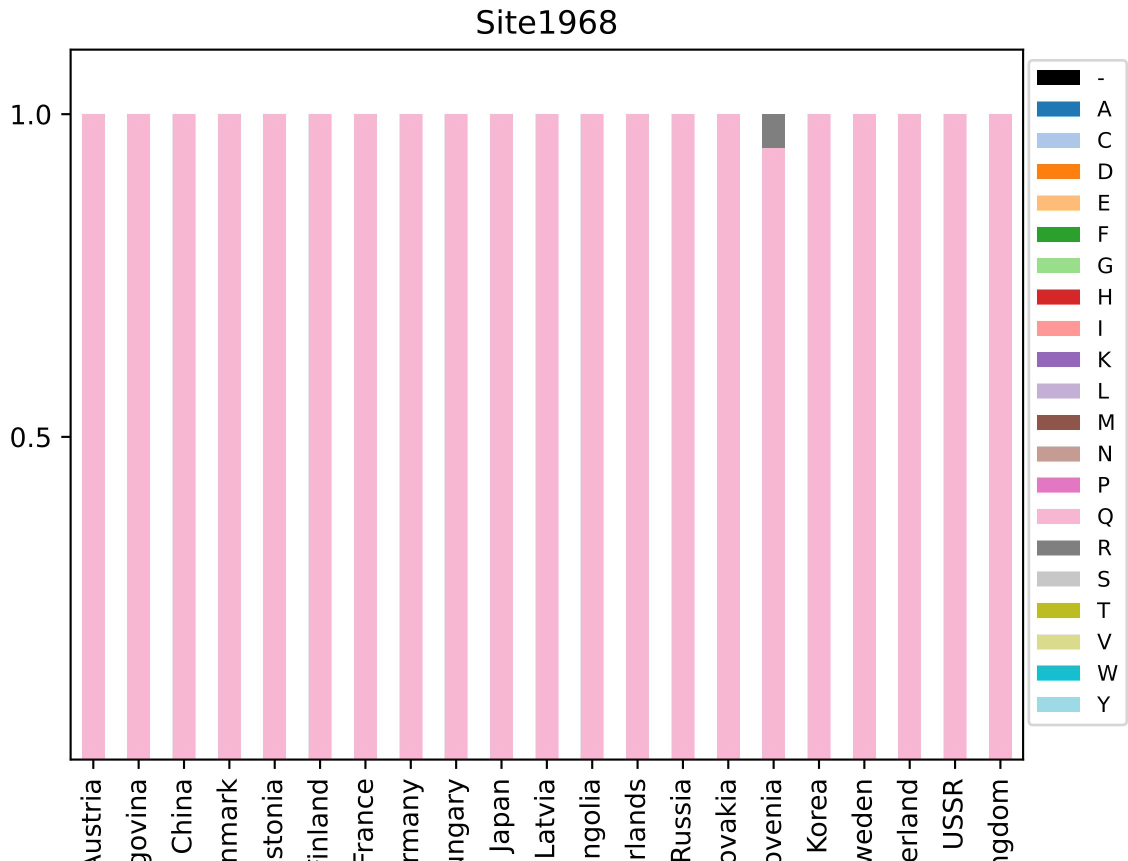
Site1968
1.0
0.5
-
A
C
D
E
F
G
H
I
K
L
M
N
P
Q
R
S
T
V
W
Y
Austria
govina
China
nmark
stonia
Finland
France
rmany
ungary
Japan
Latvia
ngolia
rlands
Russia
ovakia
ovenia
Korea
weden
erland
USSR
ngdom

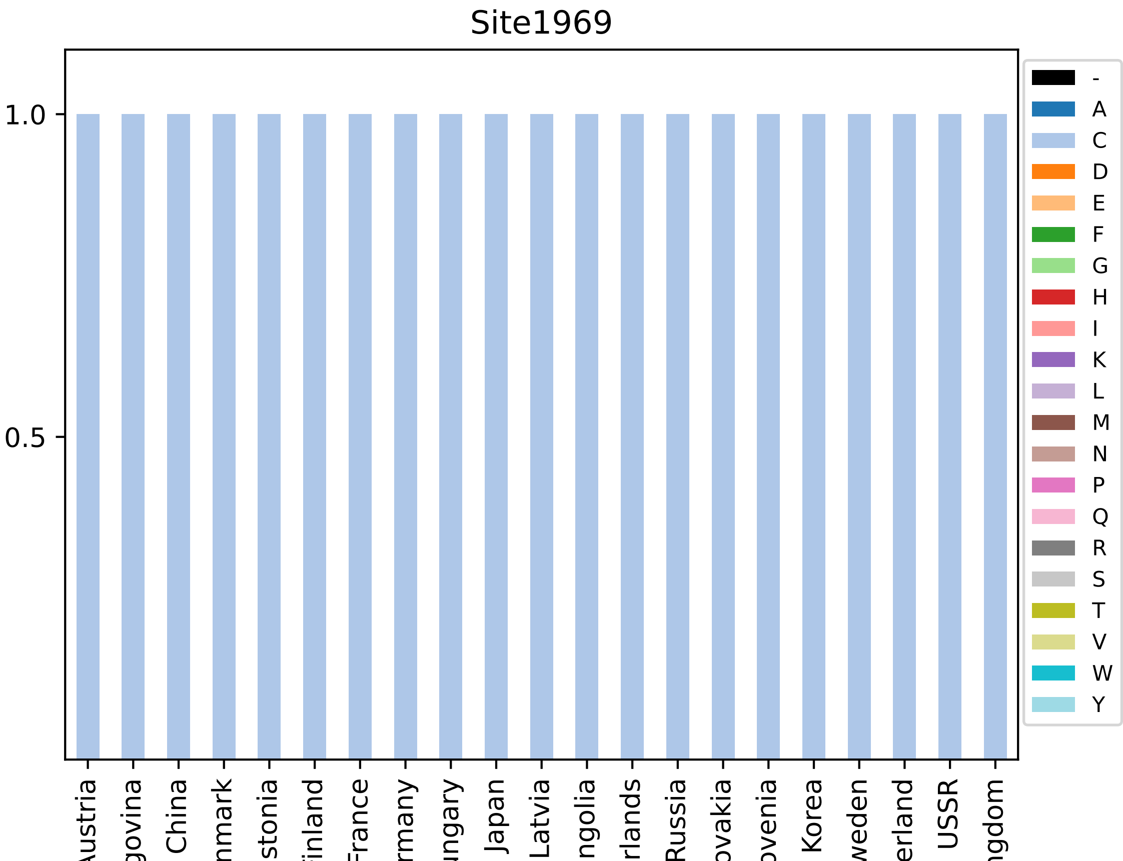

Site1969
1.0
0.5
China
Japan
Latvia
Russia
Korea
USSR
-
A
C
D
E
F
G
H
I
K
L
M
N
P
Q
R
S
T
V
W
Y

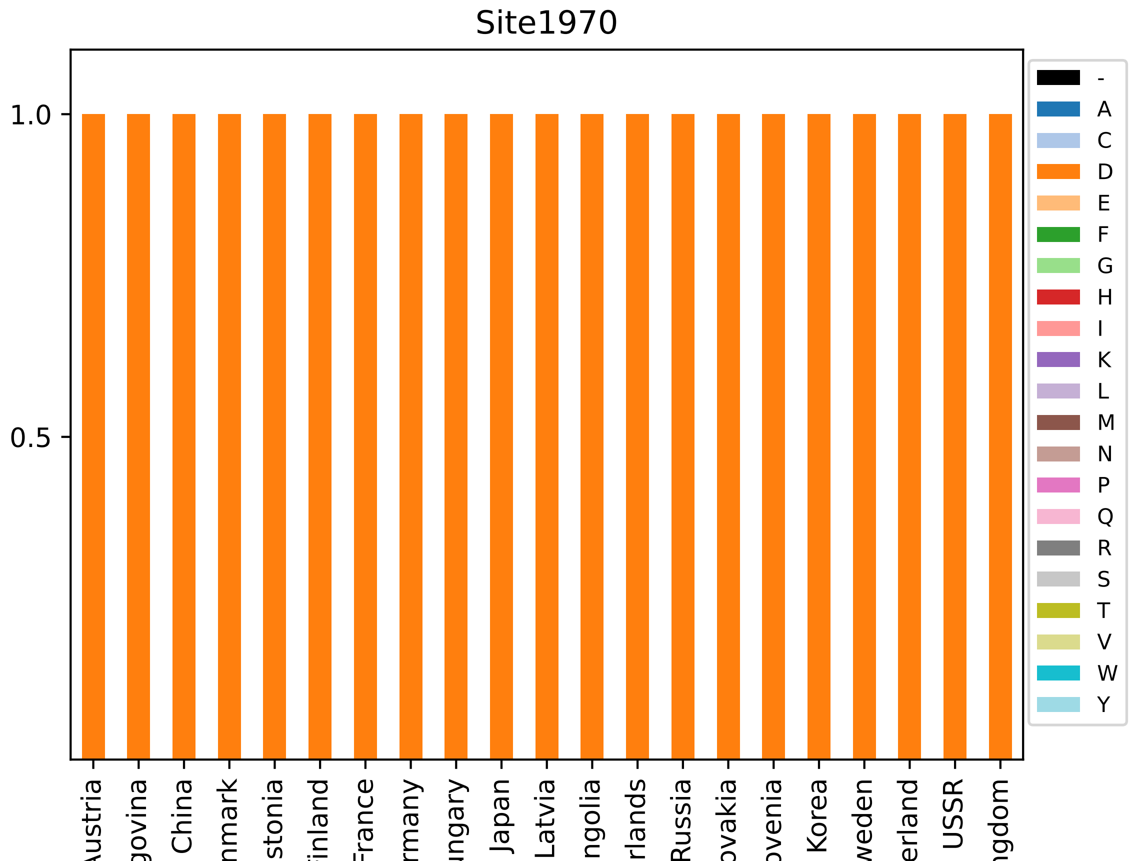

Site1970
1.0
0.5
-
A
C
D
E
F
G
H
I
K
L
M
N
P
Q
R
S
T
V
W
Y
China
Japan
Latvia
Russia
Korea
USSR

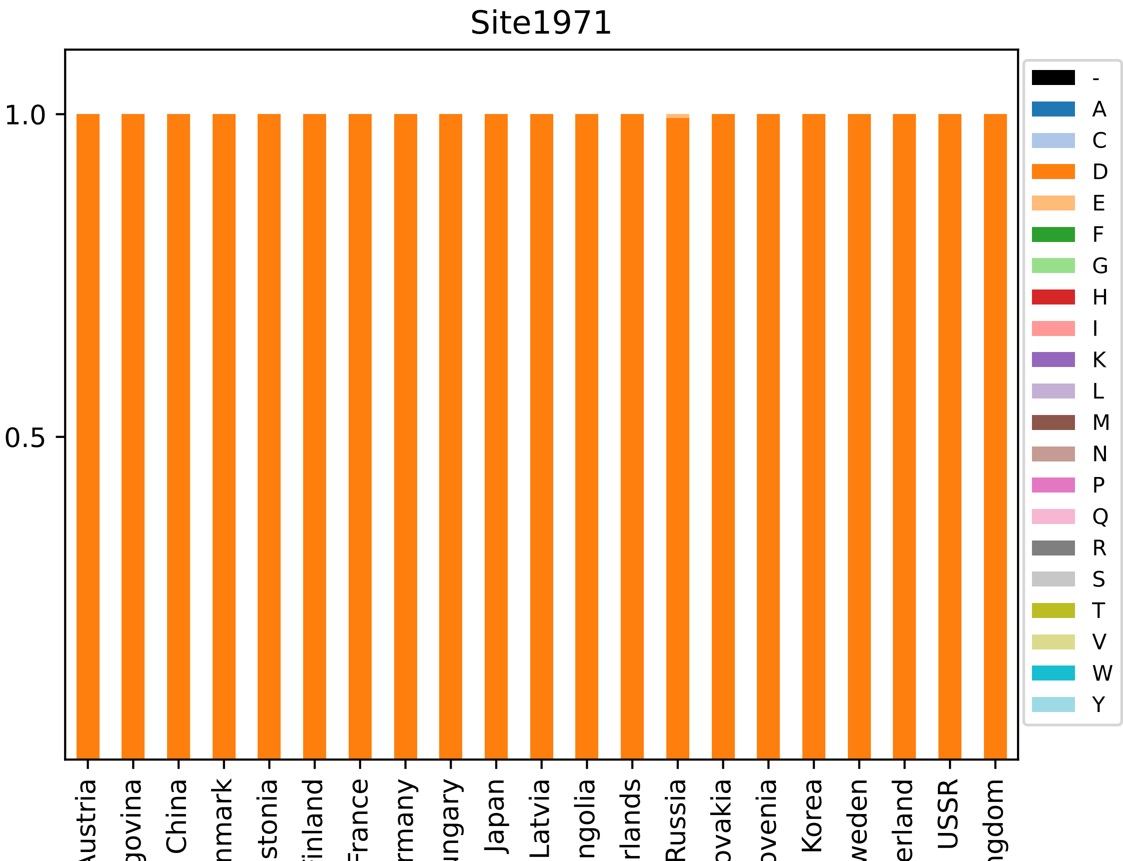
Site1971
1.0
0.5
-
A
C
D
E
F
G
H
I
K
L
M
N
P
Q
R
S
T
V
W
Y
China
Japan
Latvia
Russia
Korea
USSR

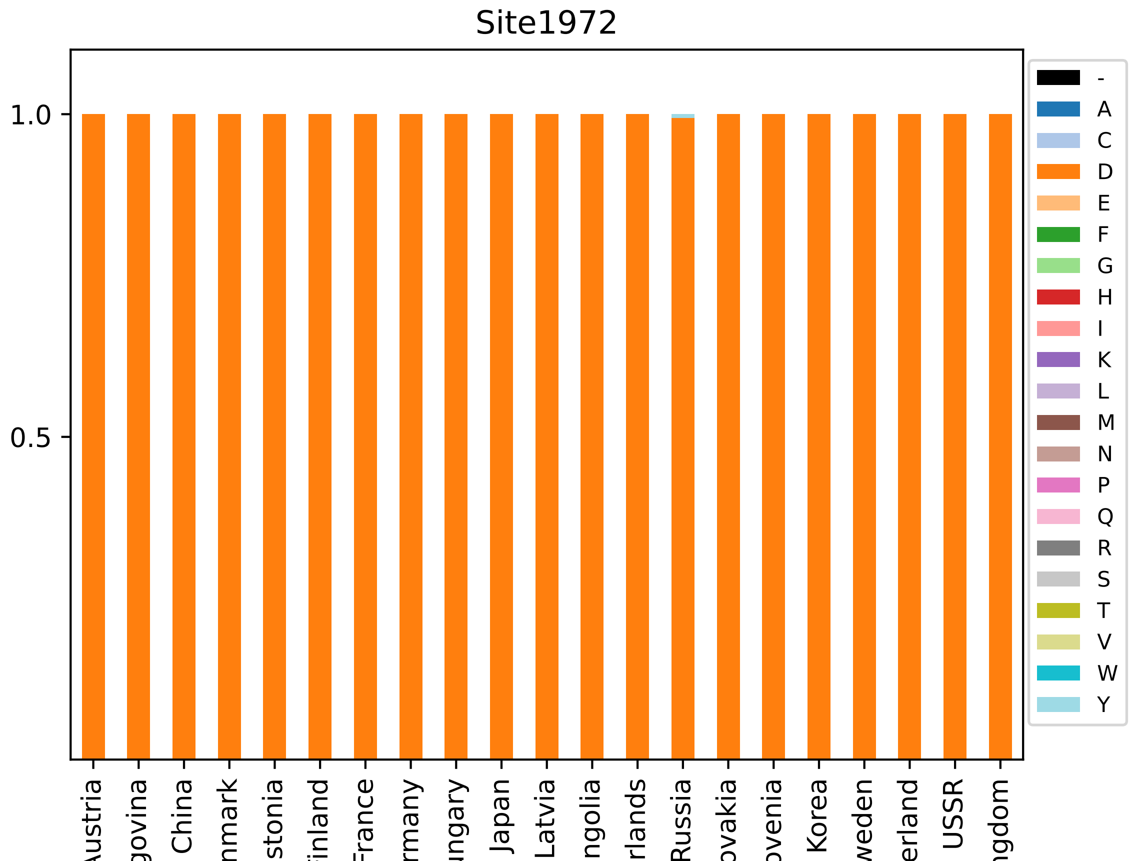

Site1972
1.0
0.5
-
A
C
D
E
F
G
H
I
K
L
M
N
P
Q
R
S
T
V
W
Y
Austria
govina
China
nmark
stonia
inland
France
rmany
ungary
Japan
Latvia
ngolia
rlands
Russia
ovakia
ovenia
Korea
weden
erland
USSR
ngdom

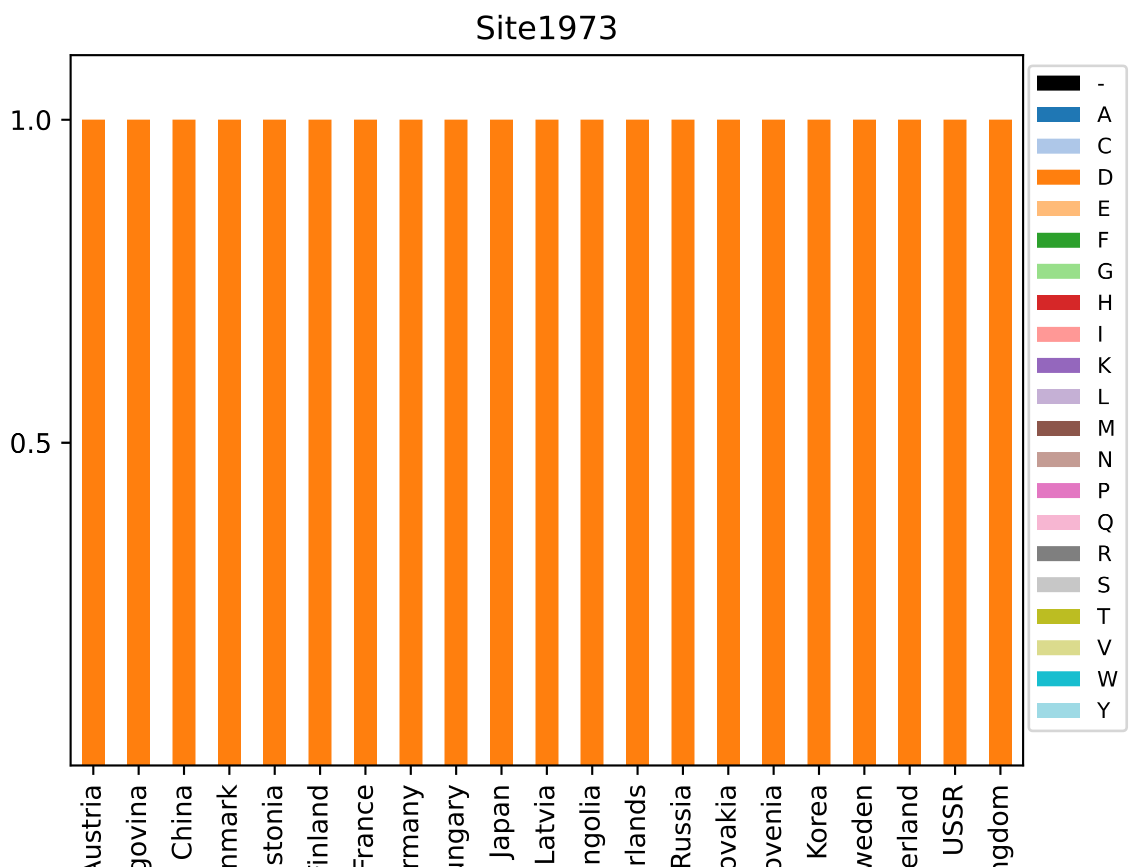
Site1973
1.0
0.5
-
A
C
D
E
F
G
H
I
K
L
M
N
P
Q
R
S
T
V
W
Y
China
Japan
Latvia
Russia
Korea
USSR

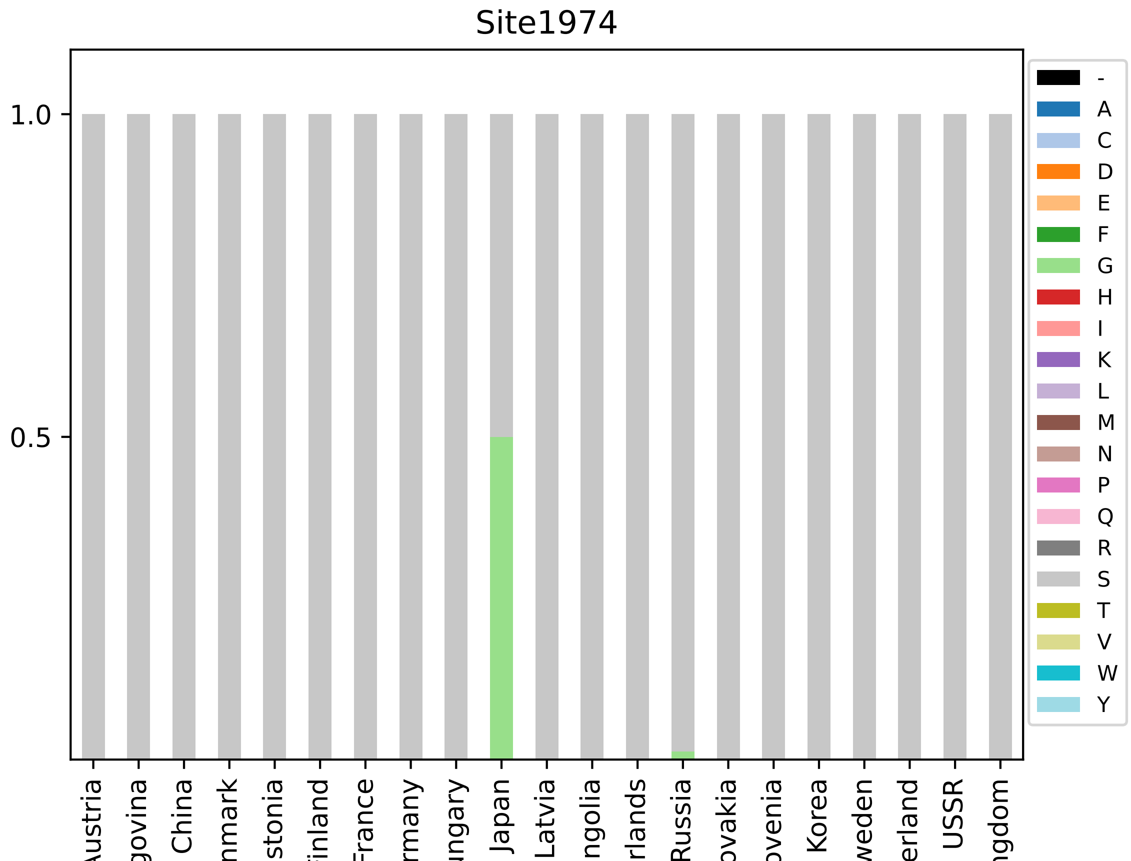
Site1974
1.0
0.5
-
A
C
D
E
F
G
H
I
K
L
M
N
P
Q
R
S
T
V
W
Y
China
Japan
Latvia
Russia
Korea
USSR

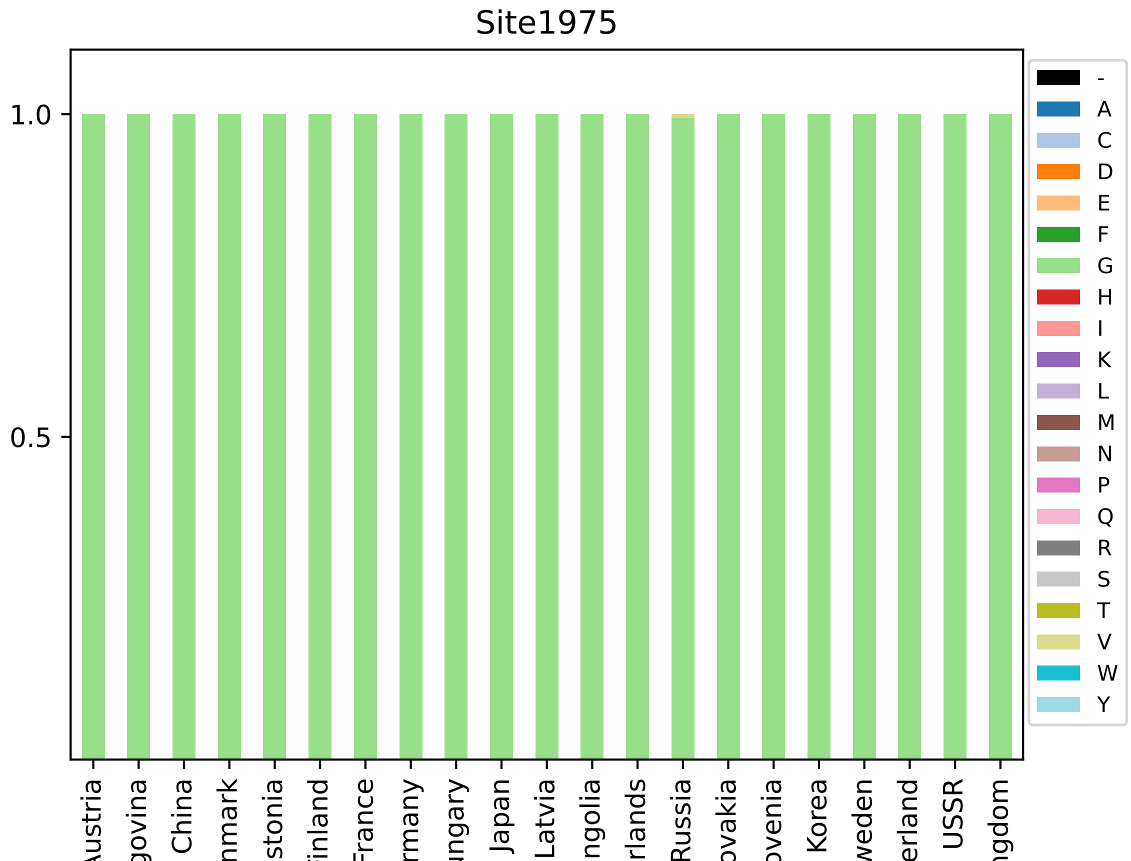

Site1975
1.0
0.5
-
A
C
D
E
F
G
H
I
K
L
M
N
P
Q
R
S
T
V
W
Y
Austria
govina
China
nmark
stonia
Finland
France
rmany
ungary
Japan
Latvia
ngolia
rlands
Russia
ovakia
ovenia
Korea
weden
erland
USSR
ngdom

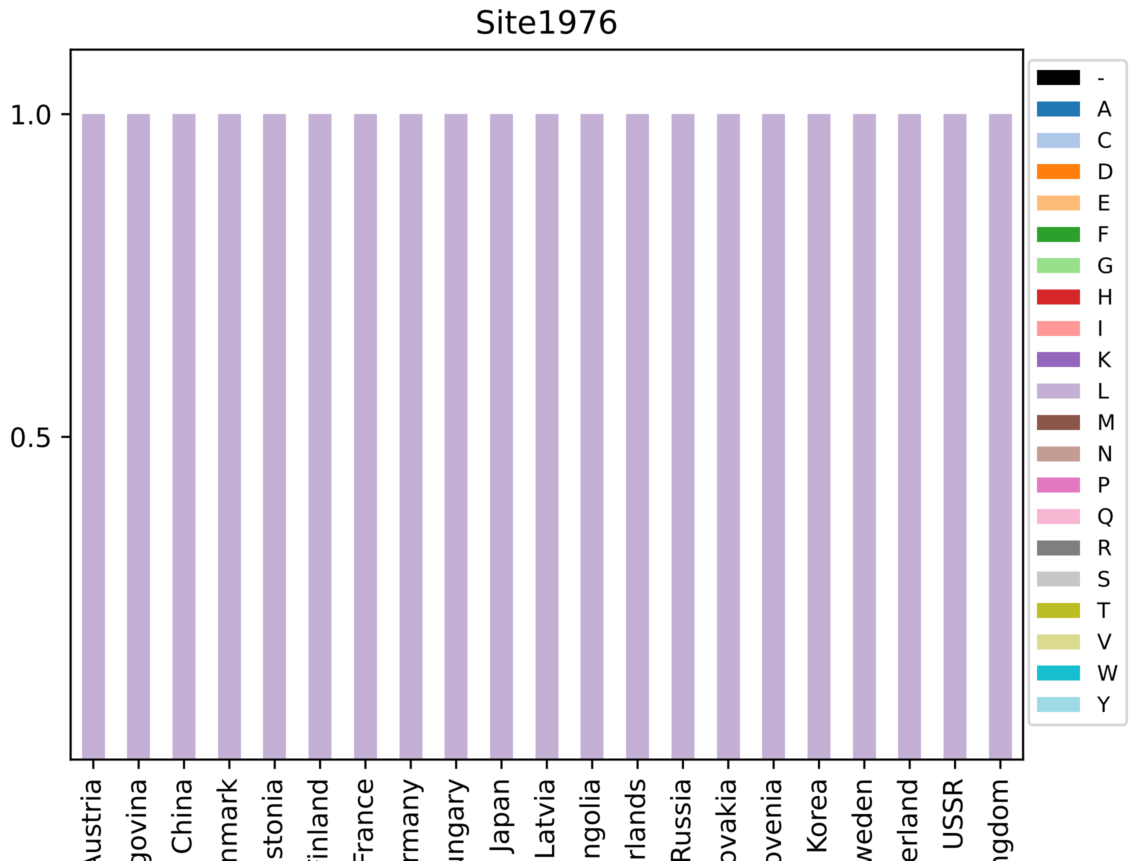
Site1976
1.0
0.5
China
Japan
Latvia
Russia
Korea
USSR
-
A
C
D
E
F
G
H
I
K
L
M
N
P
Q
R
S
T
V
W
Y

Site1977

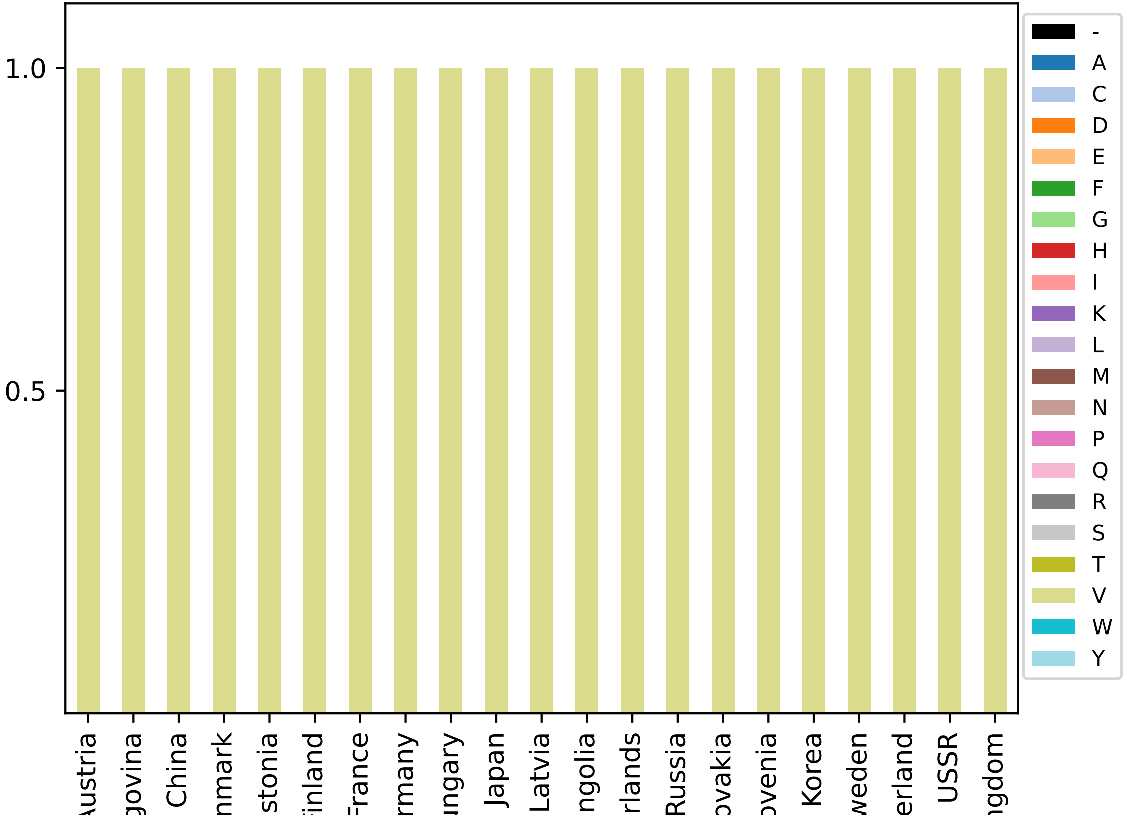
1.0
0.5
China
Japan
Latvia
Russia
Korea
USSR
-
A
C
D
E
F
G
H
I
K
L
M
N
P
Q
R
S
T
V
W
Y

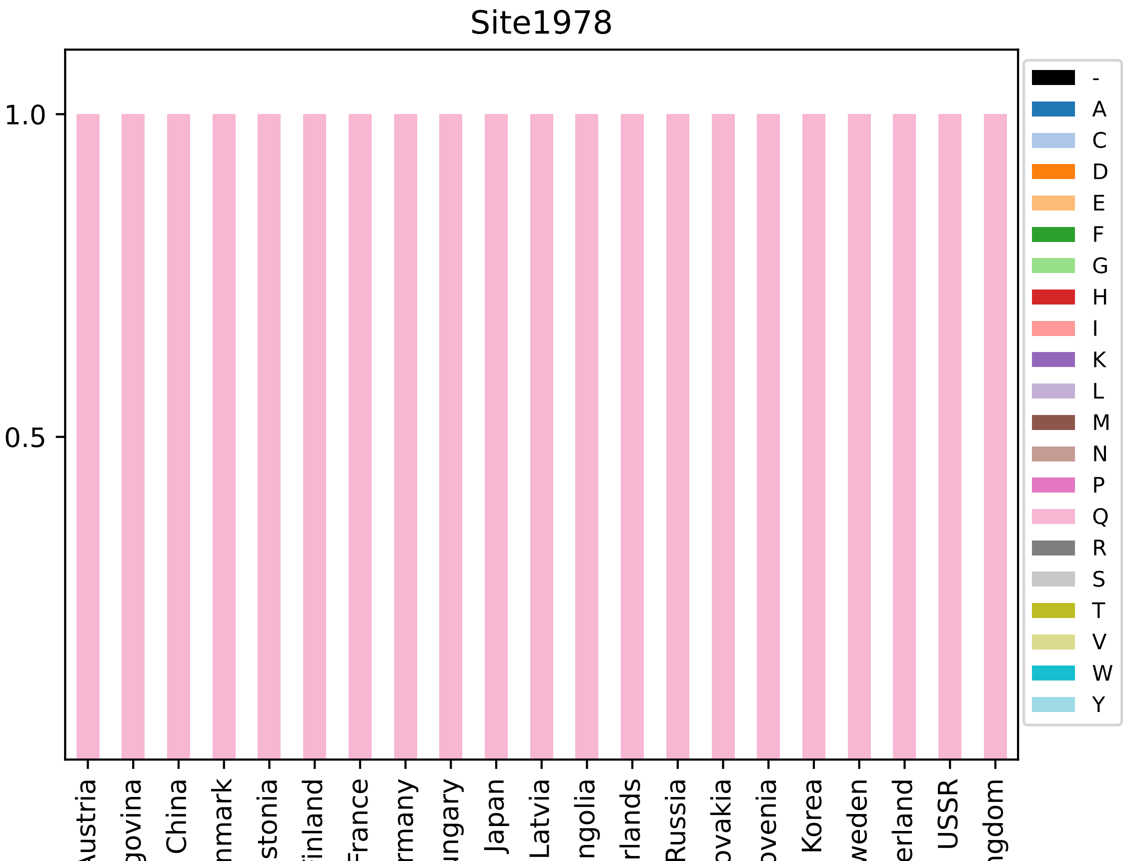

Site1978
1.0
0.5
-
A
C
D
E
F
G
H
I
K
L
M
N
P
Q
R
S
T
V
W
Y
China
Japan
Latvia
Russia
Korea
USSR

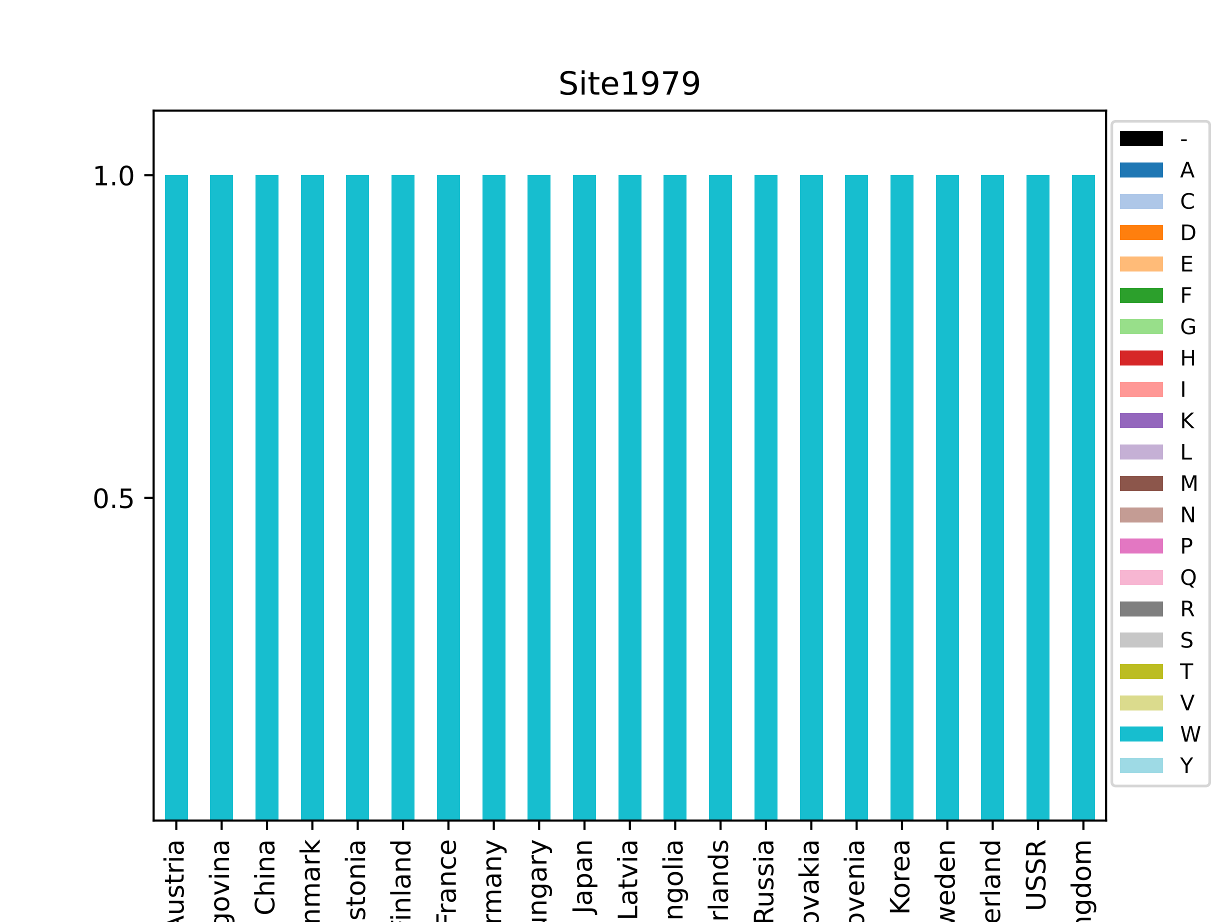

Site1979
1.0
0.5
-
A
C
D
E
F
G
H
I
K
L
M
N
P
Q
R
S
T
V
W
Y
Austria
govina
China
nmark
stonia
inland
France
rmany
ungary
Japan
Latvia
ngolia
rlands
Russia
ovakia
ovenia
Korea
weden
erland
USSR
ngdom

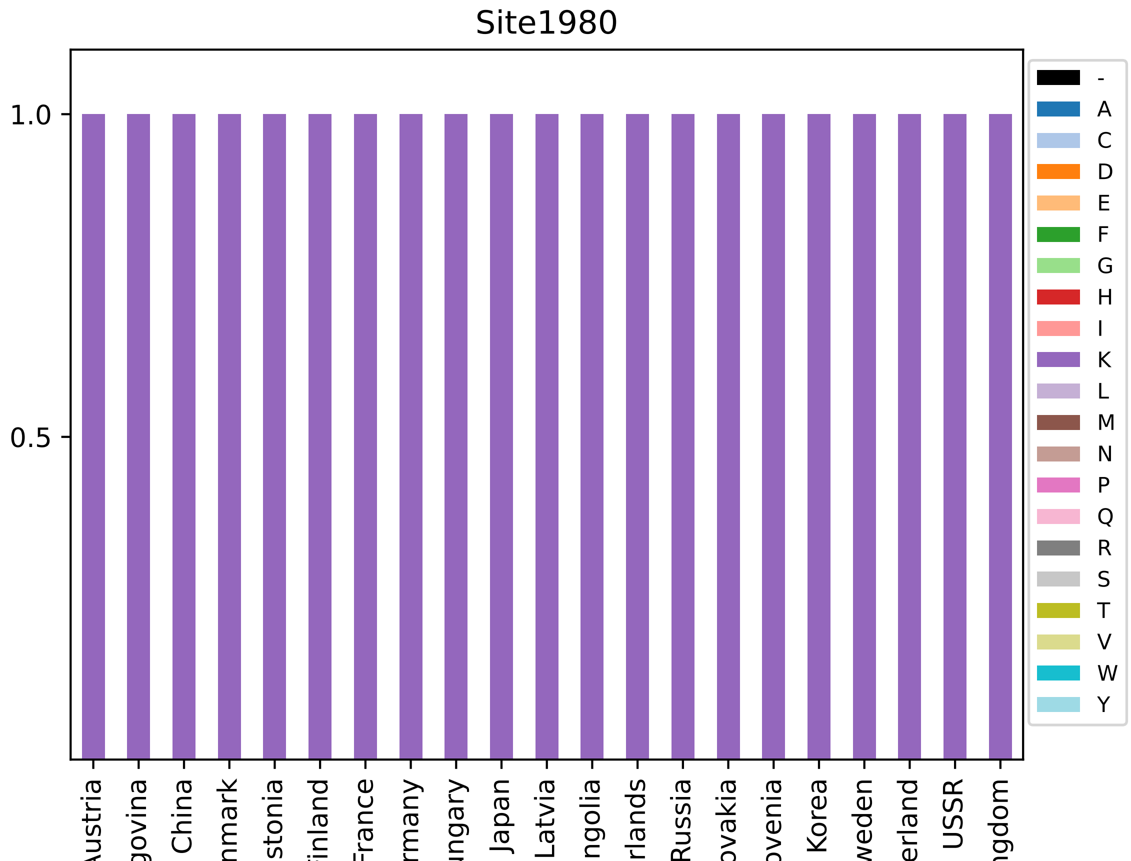
Site1980
1.0
0.5
-
A
C
D
E
F
G
H
I
K
L
M
N
P
Q
R
S
T
V
W
Y
Austria
govina
China
nmark
stonia
inland
France
rmany
ungary
Japan
Latvia
ngolia
rlands
Russia
ovakia
ovenia
Korea
weden
erland
USSR
ngdom

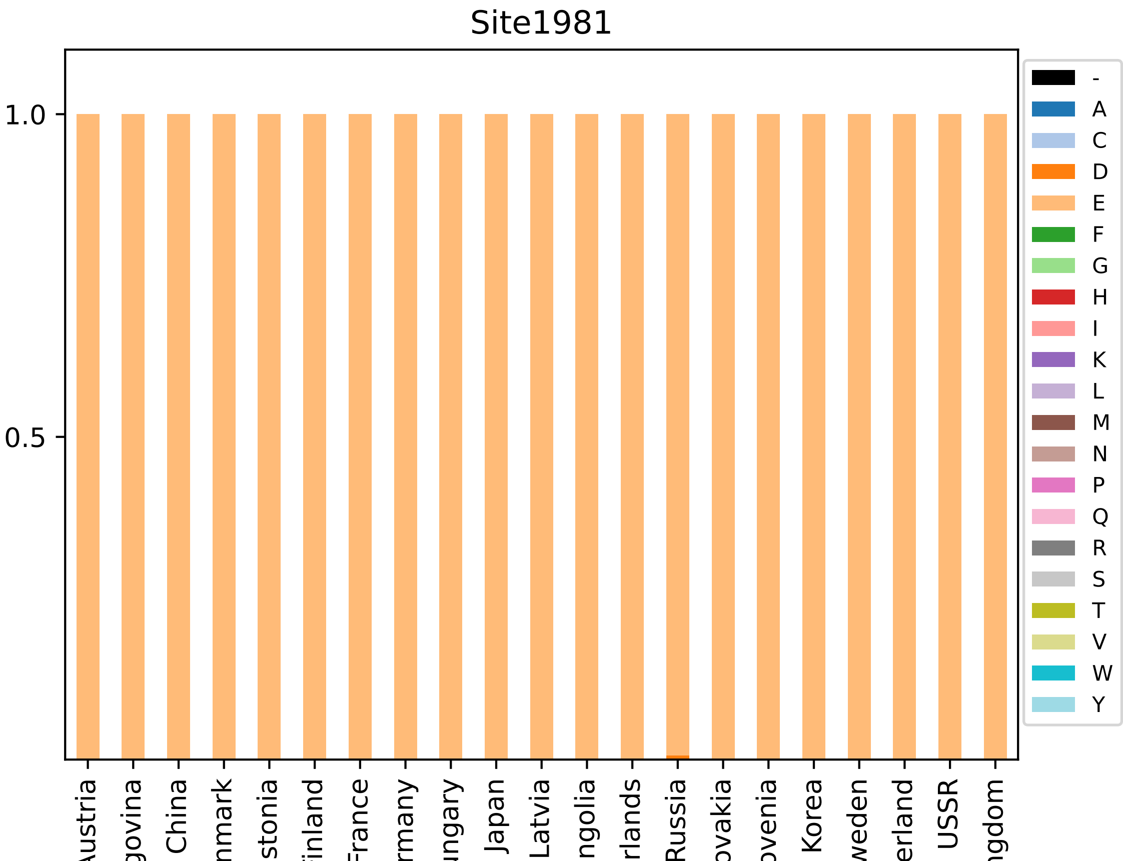

Site1981
1.0
0.5
-
A
C
D
E
F
G
H
I
K
L
M
N
P
Q
R
S
T
V
W
Y
China
Japan
Latvia
Russia
Korea
USSR

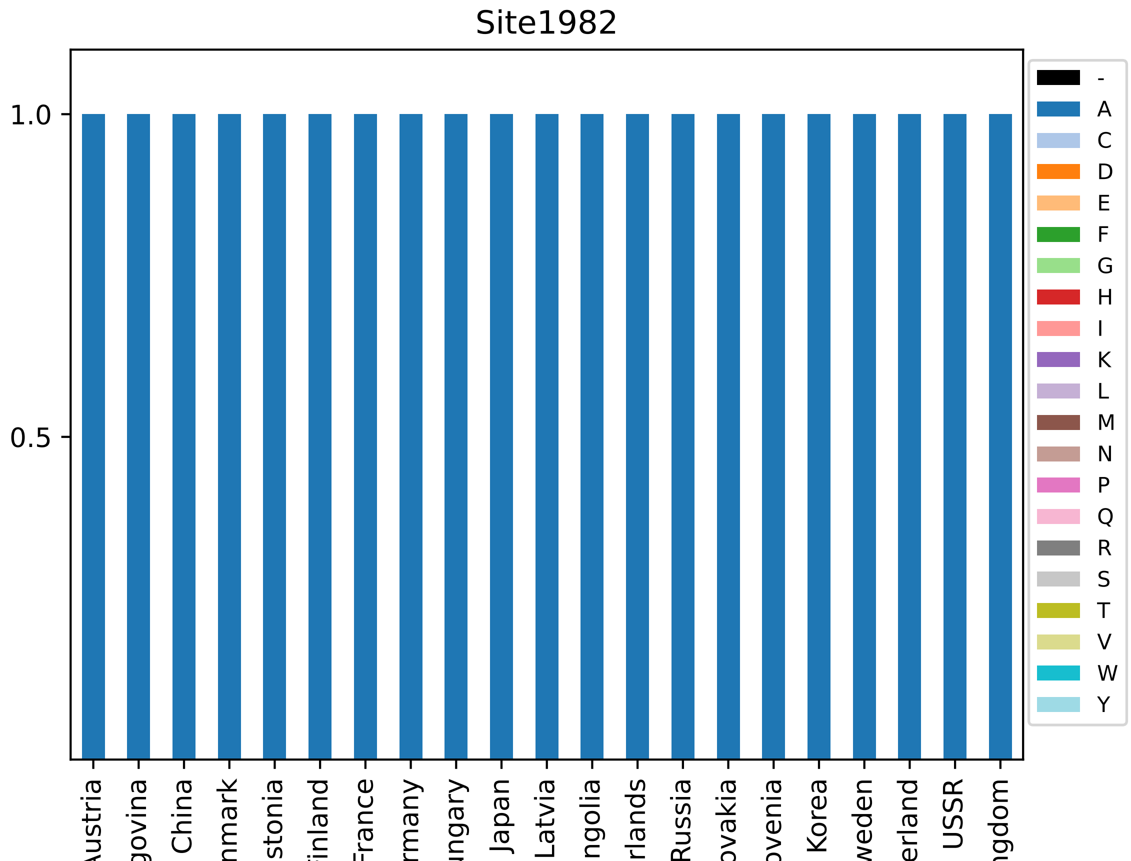

Site1982
1.0
0.5
-
A
C
D
E
F
G
H
I
K
L
M
N
P
Q
R
S
T
V
W
Y
Austria
govina
China
nmark
stonia
inland
France
rmany
ungary
Japan
Latvia
ngolia
rlands
Russia
ovakia
ovenia
Korea
weden
erland
USSR
ngdom

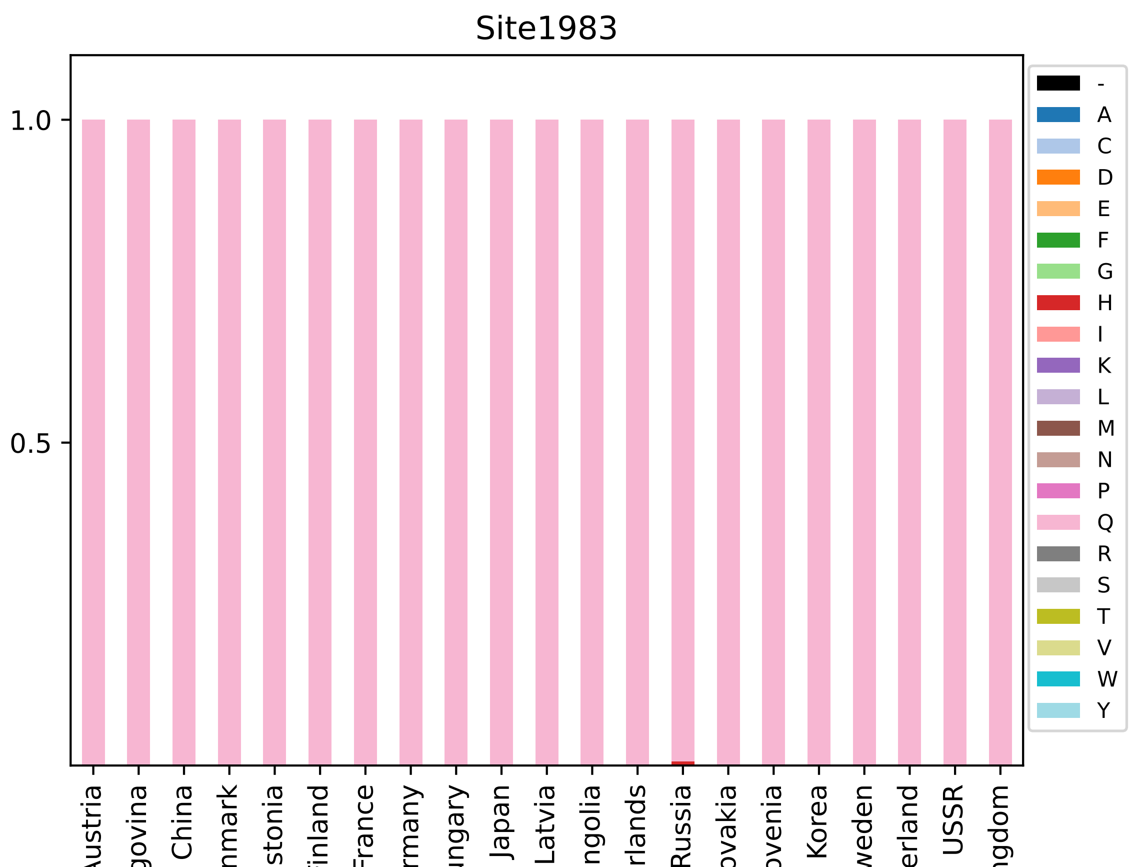
Site1983
1.0
0.5
-
A
C
D
E
F
G
H
I
K
L
M
N
P
Q
R
S
T
V
W
Y
Austria
China
Japan
Latvia
Russia
Korea
USSR

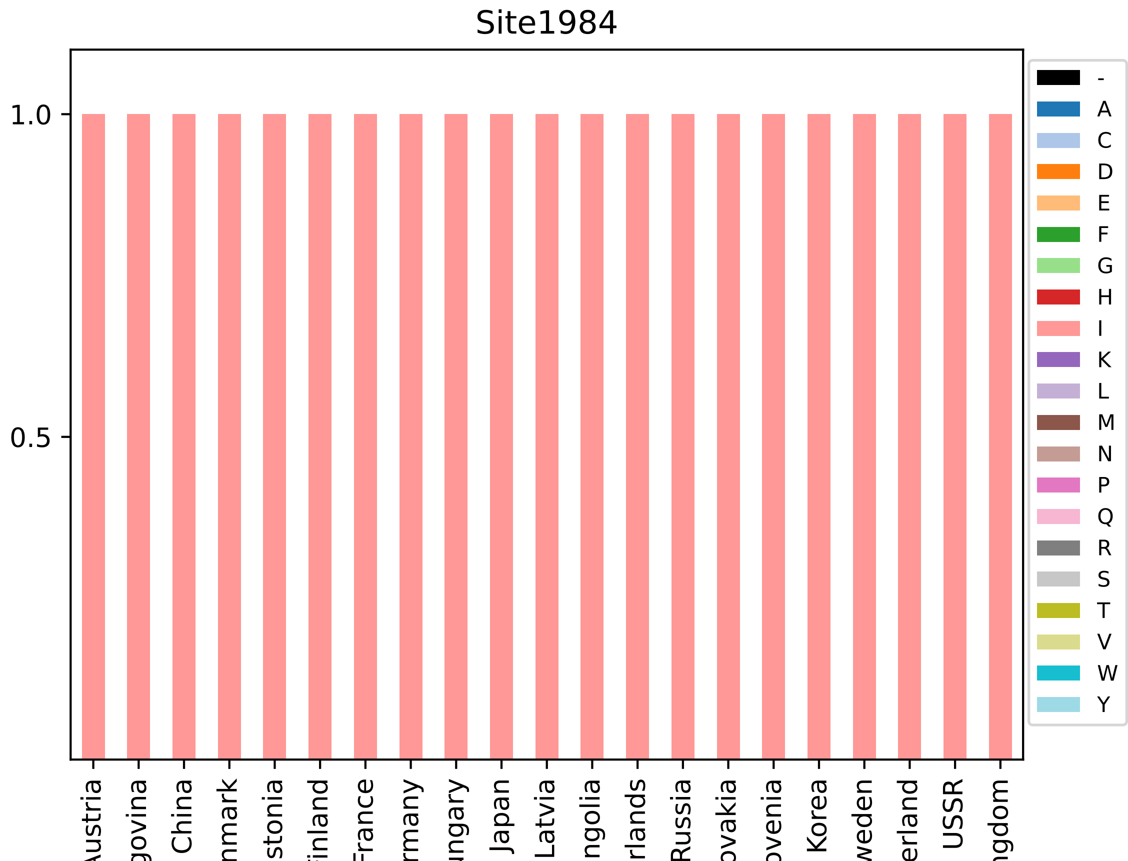
Site1984
1.0
0.5
-
A
C
D
E
F
G
H
I
K
L
M
N
P
Q
R
S
T
V
W
Y
ustria
govina
China
nmark
stonia
inland
rance
rmany
ungary
Japan
Latvia
ngolia
rlands
Russia
ovakia
ovenia
Korea
weden
erland
USSR
ngdom

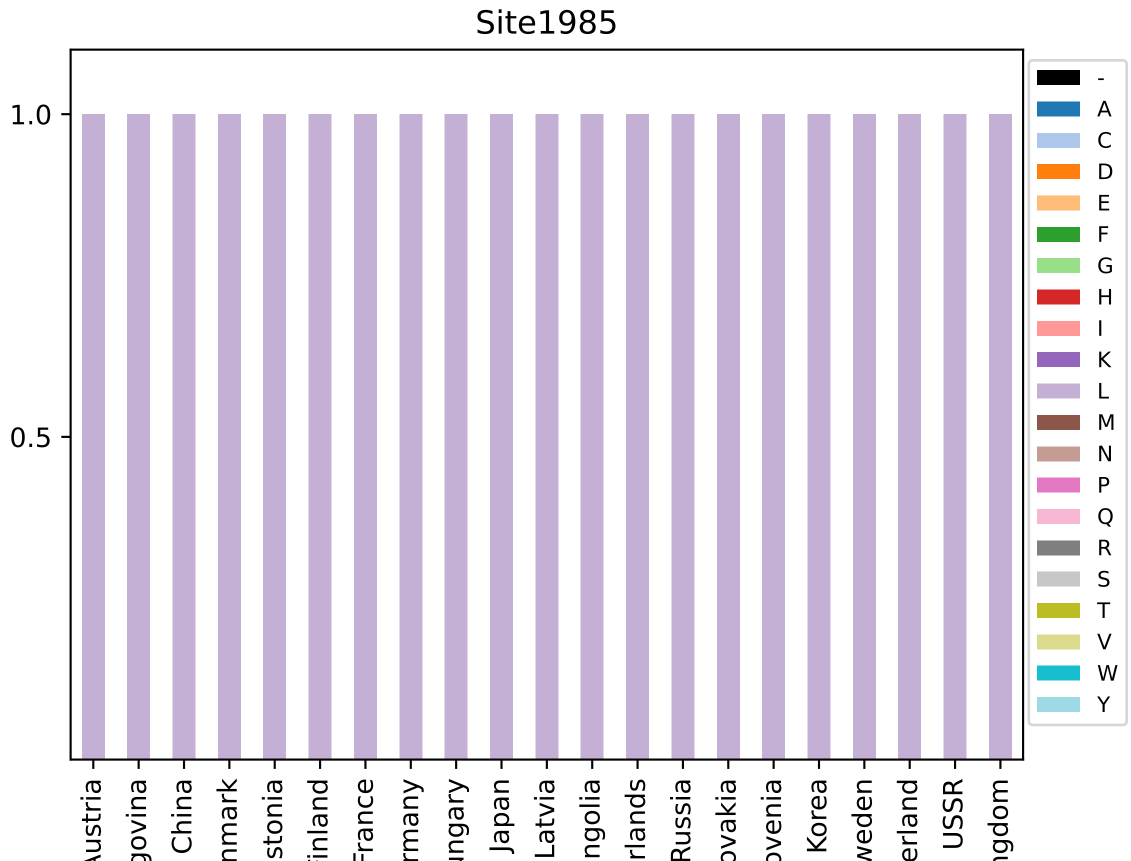
Site1985
1.0
0.5
-
A
C
D
E
F
G
H
I
K
L
M
N
P
Q
R
S
T
V
W
Y
China
Japan
Latvia
Russia
Korea
USSR

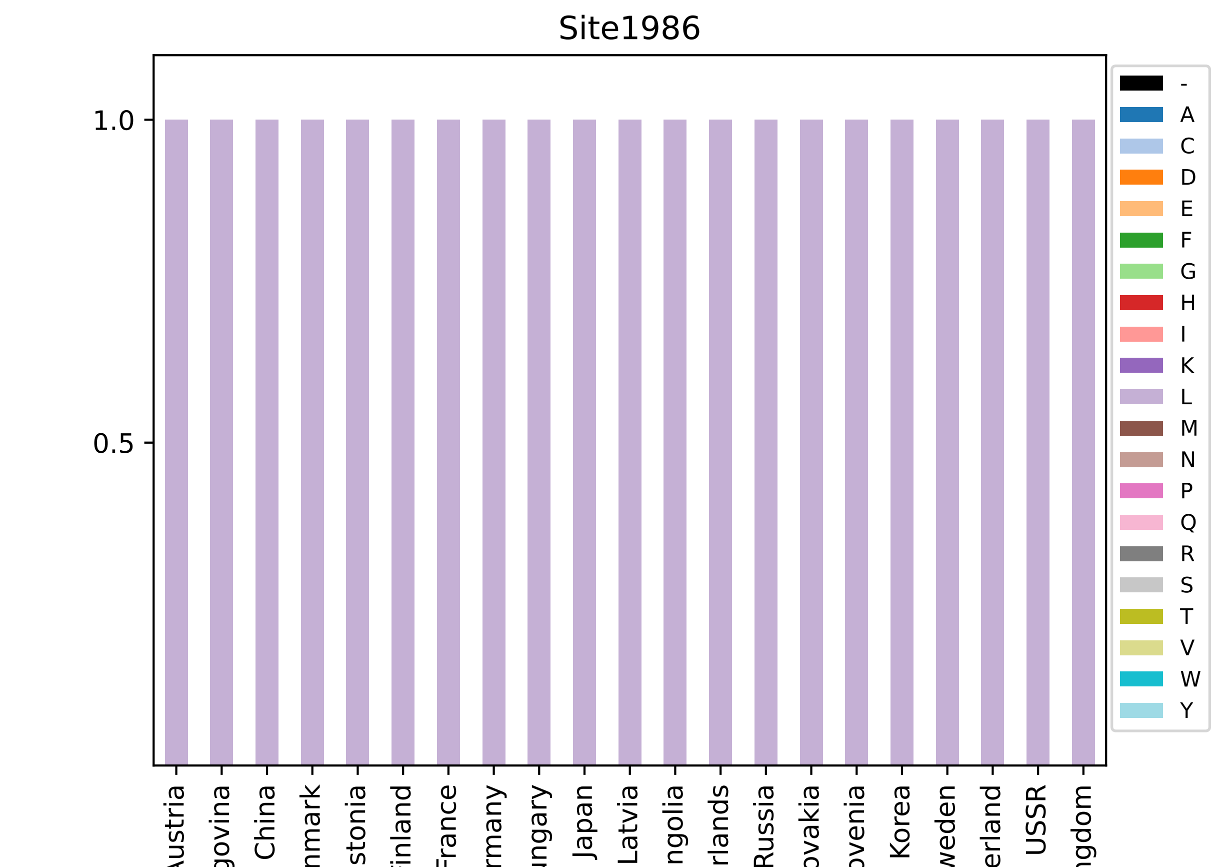
Site1986
1.0
0.5
-
A
C
D
E
F
G
H
I
K
L
M
N
P
Q
R
S
T
V
W
Y
Austria
govina
China
nmark
stonia
inland
France
rmany
ungary
Japan
Latvia
ngolia
rlands
Russia
ovakia
ovenia
Korea
weden
erland
USSR
ngdom

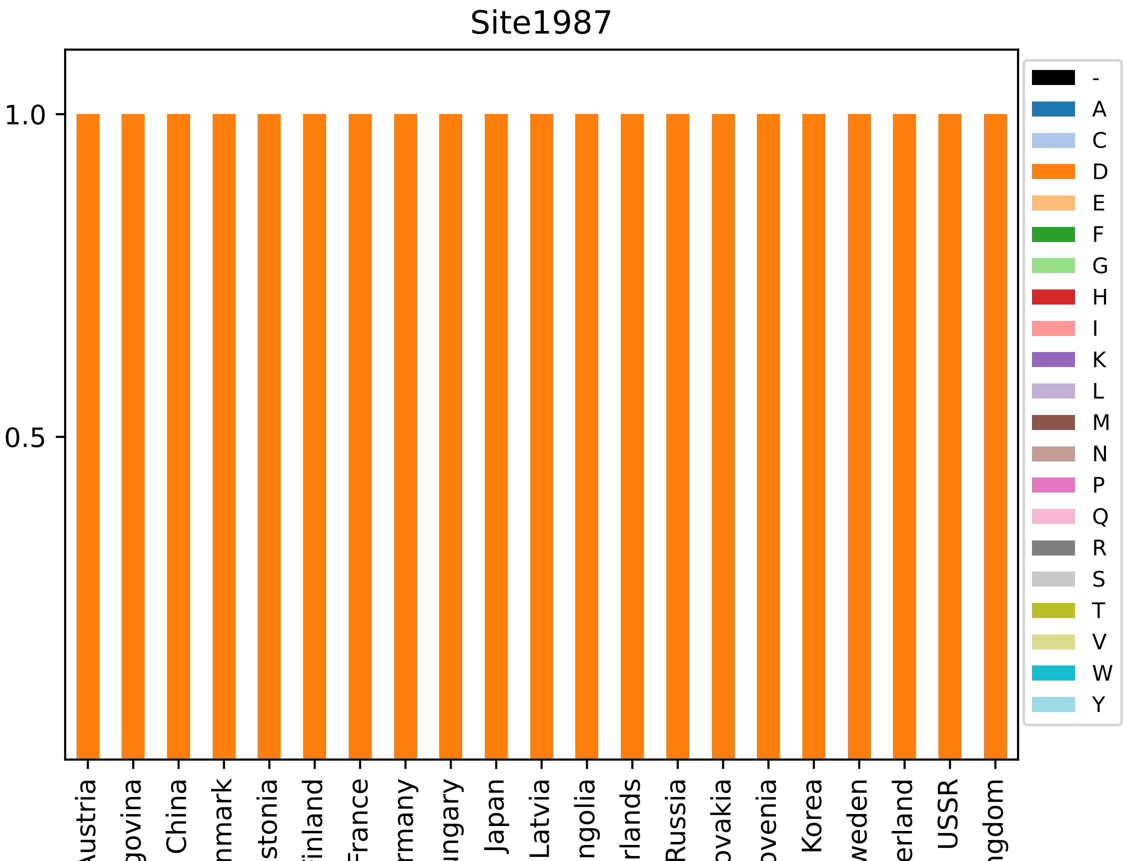
Site1987
1.0
0.5
-
A
C
D
E
F
G
H
I
K
L
M
N
P
Q
R
S
T
V
W
Y
Austria
govina
China
nmark
stonia
Finland
France
rmany
ungary
Japan
Latvia
ngolia
rlands
Russia
ovakia
ovenia
Korea
weden
erland
USSR
ngdom

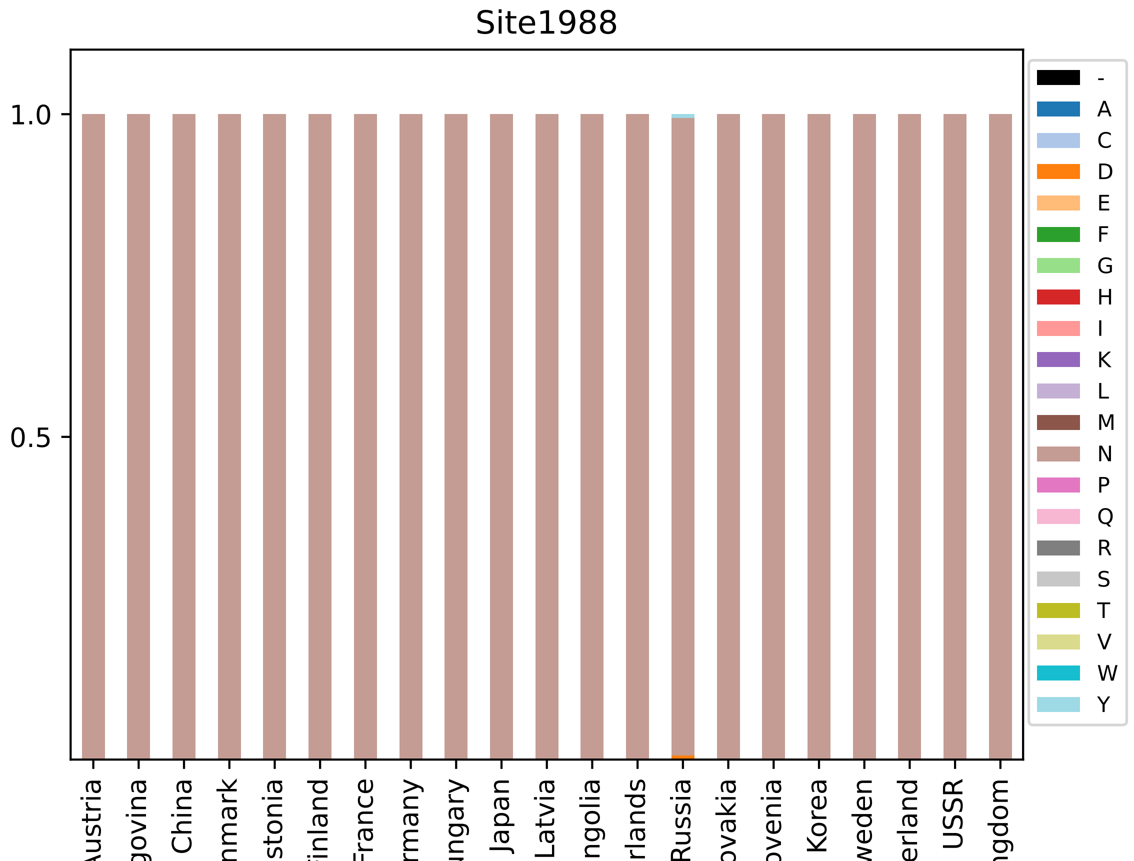
Site1988
1.0
0.5
-
A
C
D
E
F
G
H
I
K
L
M
N
P
Q
R
S
T
V
W
Y
Austria
China
Finland
France
Japan
Latvia
Russia
Korea
USSR

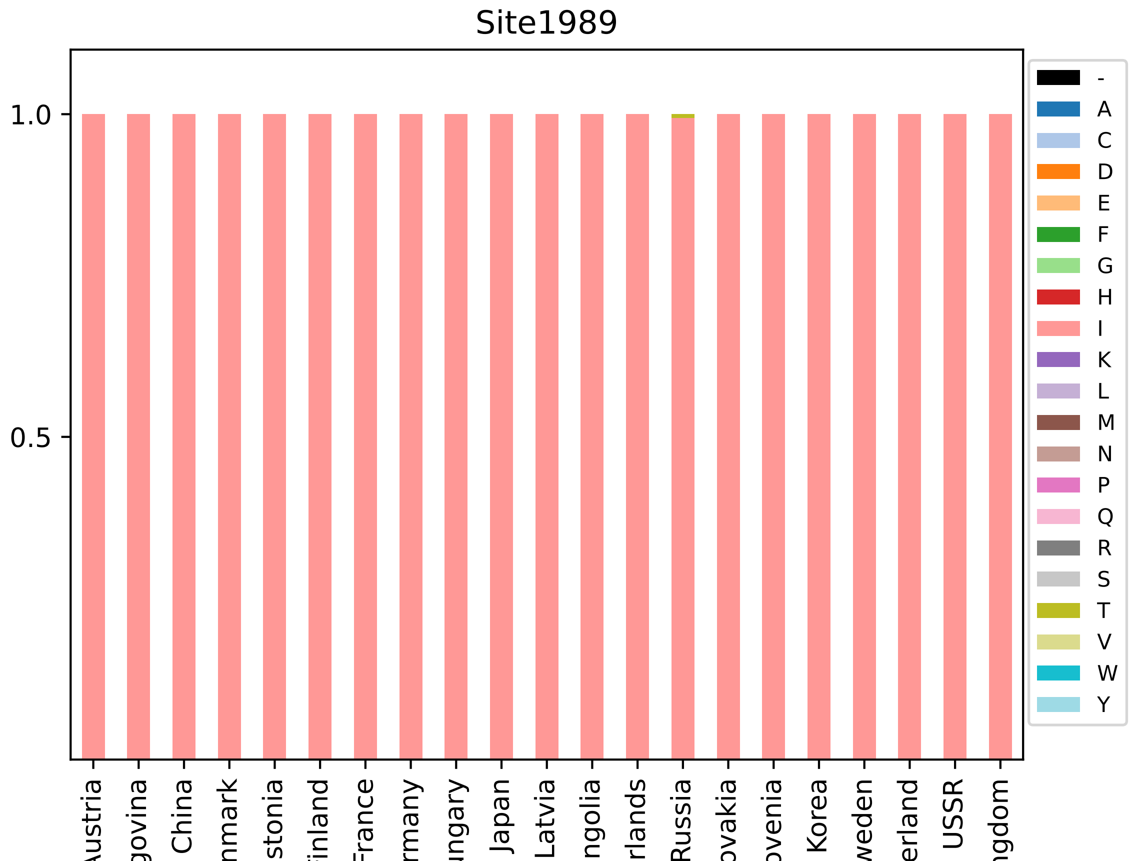

Site1989
1.0
0.5
-
A
C
D
E
F
G
H
I
K
L
M
N
P
Q
R
S
T
V
W
Y
Austria
govina
China
nmark
stonia
Finland
France
rmany
ungary
Japan
Latvia
ngolia
rlands
Russia
ovakia
ovenia
Korea
weden
erland
USSR
ngdom

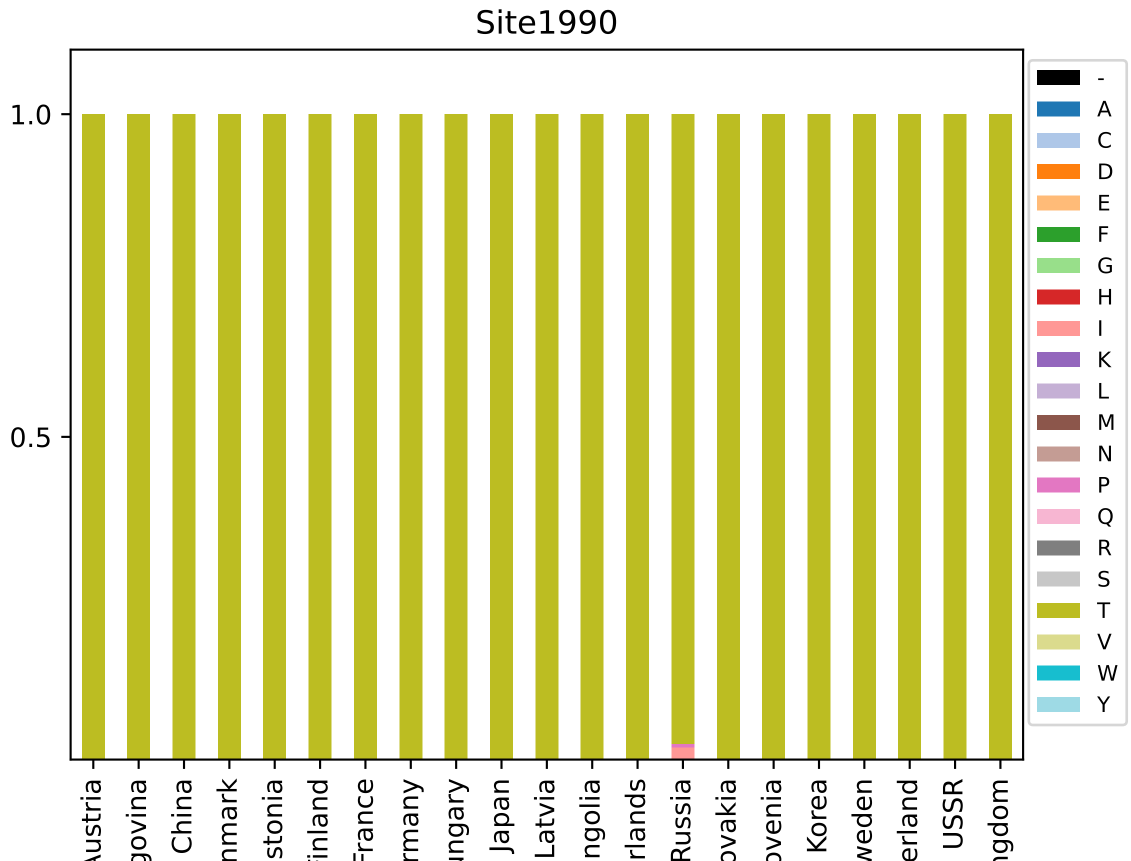

Site1990
1.0
0.5
-
A
C
D
E
F
G
H
I
K
L
M
N
P
Q
R
S
T
V
W
Y
Austria
govina
China
nmark
stonia
Finland
France
rmany
ungary
Japan
Latvia
ngolia
rlands
Russia
ovakia
ovenia
Korea
weden
erland
USSR
ngdom

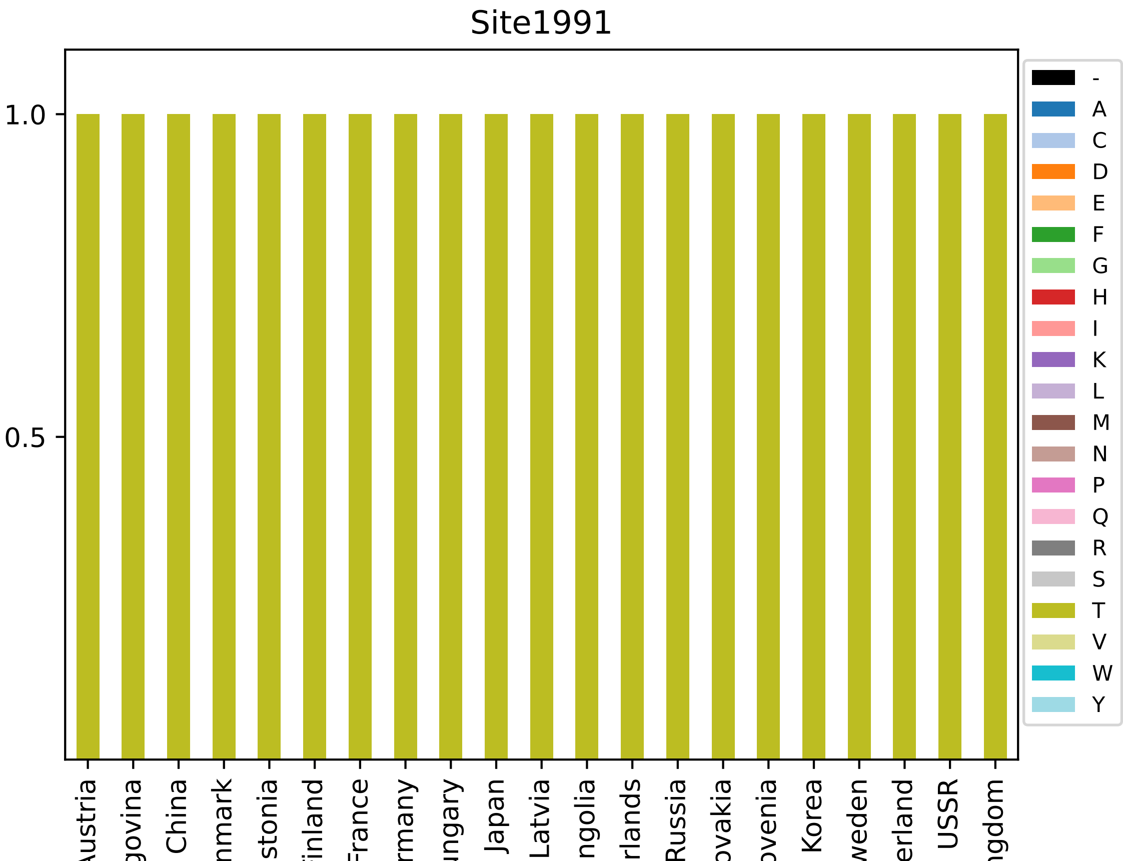

Site1991
1.0
0.5
-
A
C
D
E
F
G
H
I
K
L
M
N
P
Q
R
S
T
V
W
Y
China
Japan
Latvia
Russia
Korea
USSR

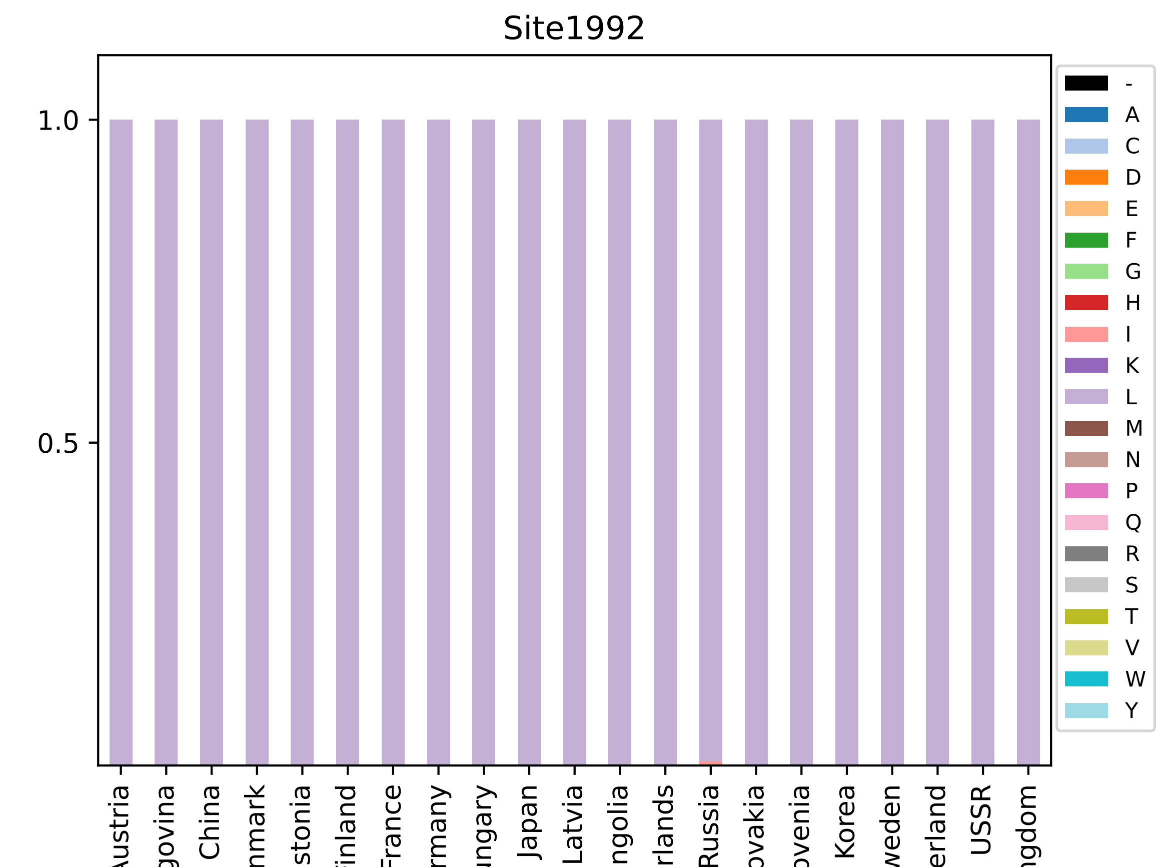
Site1992
1.0
0.5
-
A
C
D
E
F
G
H
I
K
L
M
N
P
Q
R
S
T
V
W
Y
Austria
govina
China
nmark
stonia
inland
France
rmany
ungary
Japan
Latvia
ngolia
rlands
Russia
ovakia
ovenia
Korea
weden
erland
USSR
ngdom

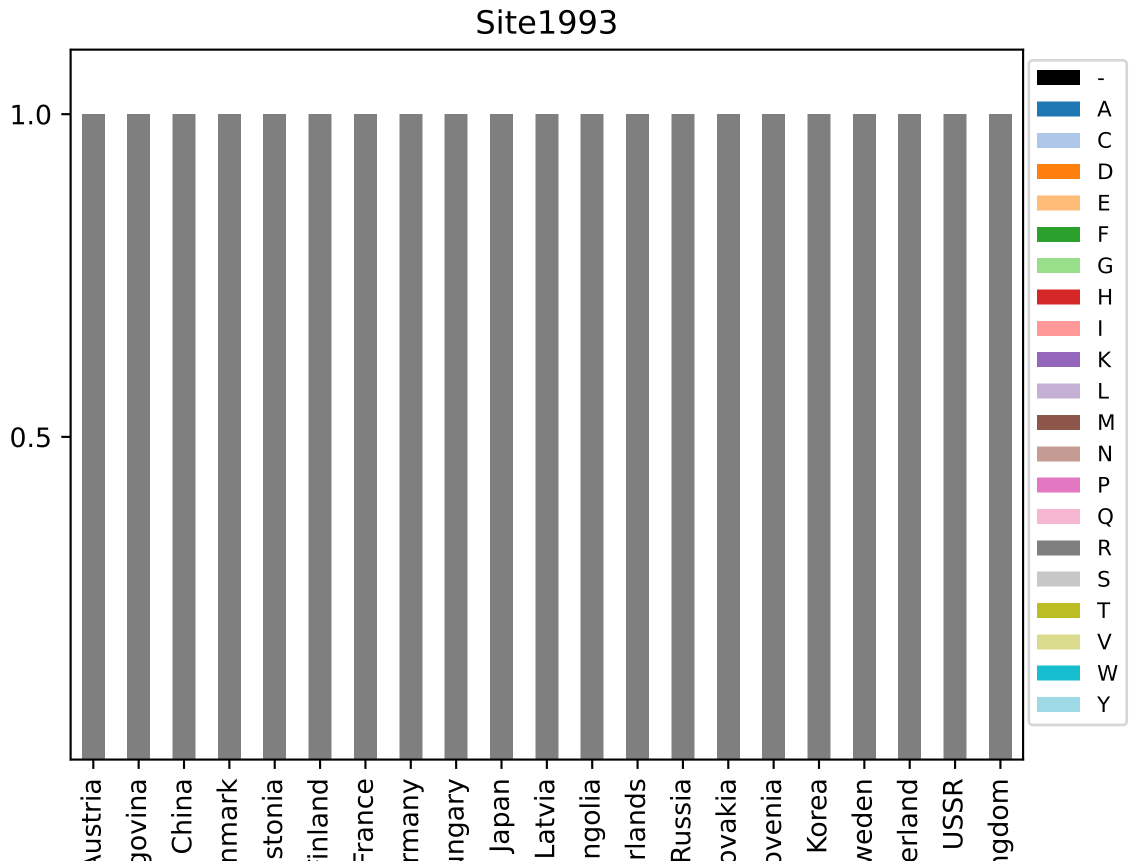

Site1993
1.0
0.5
-
A
C
D
E
F
G
H
I
K
L
M
N
P
Q
R
S
T
V
W
Y
China
Japan
Latvia
Russia
Korea
USSR

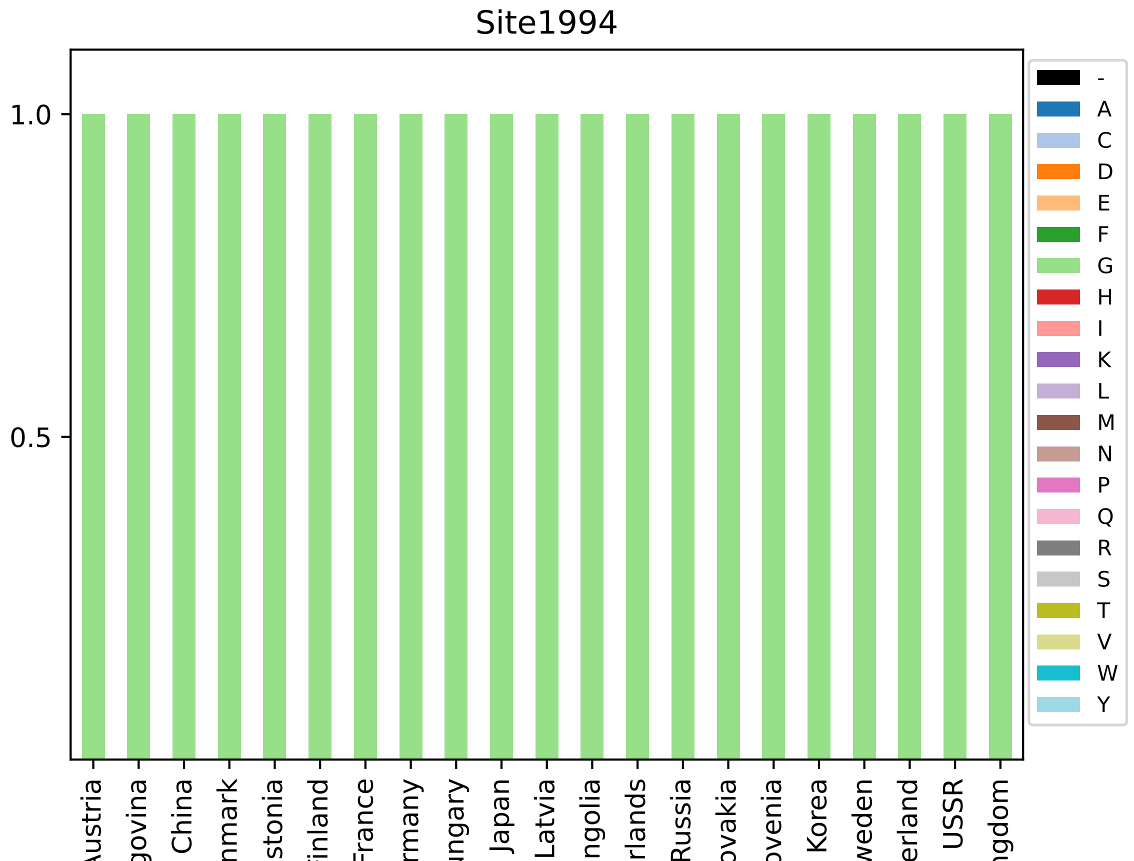
Site1994
1.0
0.5
-
A
C
D
E
F
G
H
I
K
L
M
N
P
Q
R
S
T
V
W
Y
China
Japan
Latvia
Russia
Korea
USSR

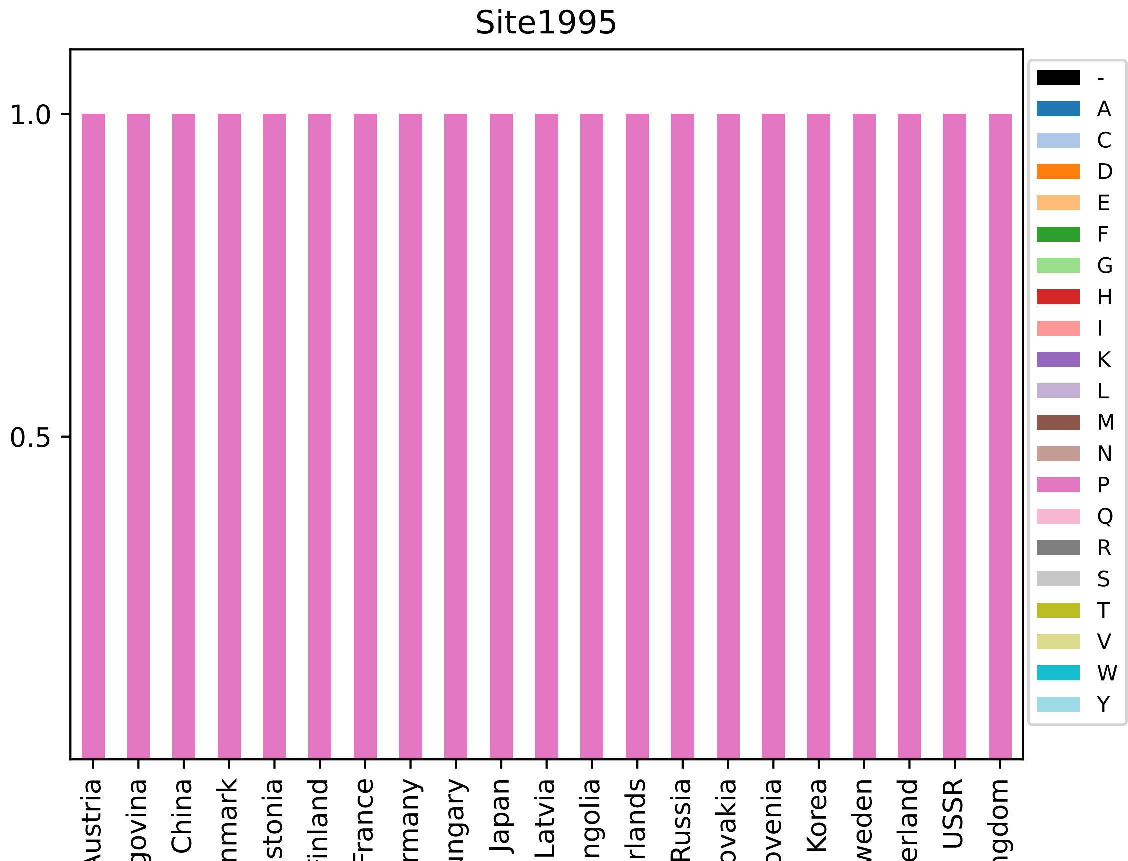

Site1995
1.0
0.5
-
A
C
D
E
F
G
H
I
K
L
M
N
P
Q
R
S
T
V
W
Y
China
Japan
Latvia
Russia
Korea
USSR

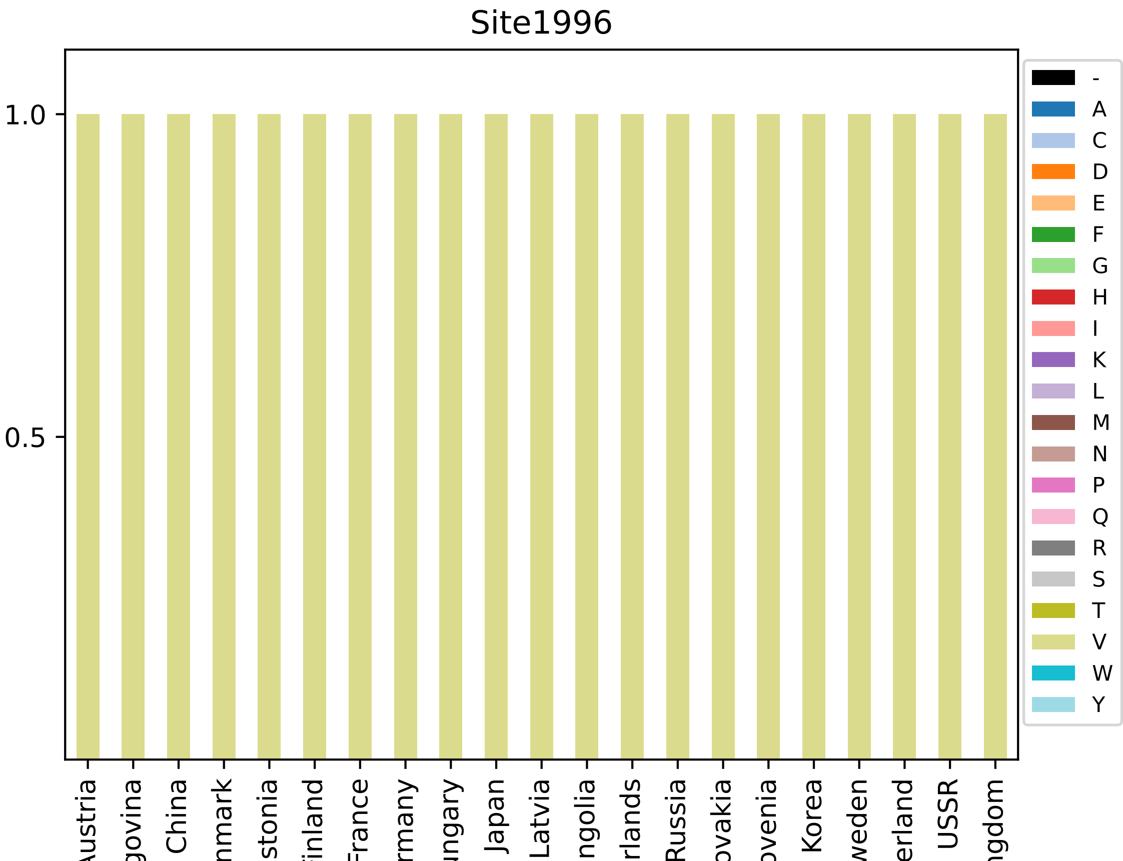

Site1996
1.0
0.5
-
A
C
D
E
F
G
H
I
K
L
M
N
P
Q
R
S
T
V
W
Y
Austria
govina
China
nmark
stonia
Finland
France
rmany
ungary
Japan
Latvia
ngolia
rlands
Russia
ovakia
ovenia
Korea
weden
erland
USSR
ngdom

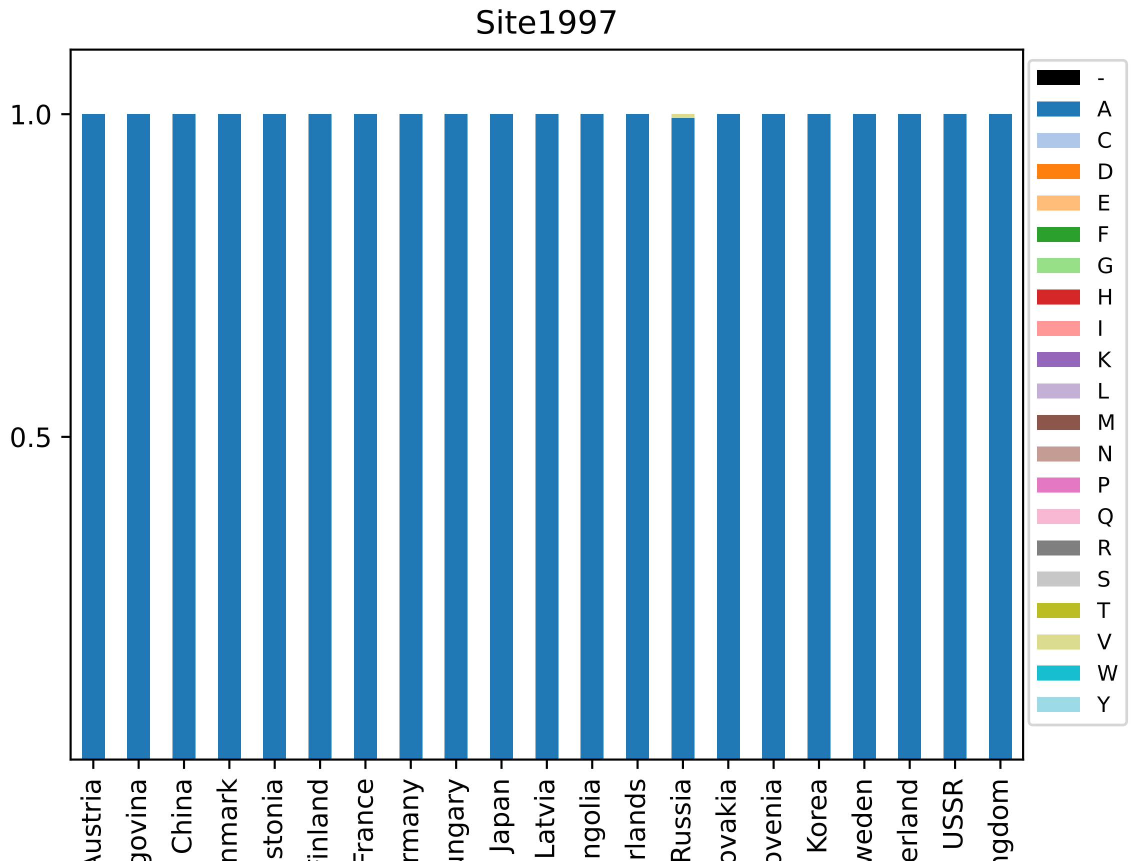
Site1997
1.0
0.5
-
A
C
D
E
F
G
H
I
K
L
M
N
P
Q
R
S
T
V
W
Y
China
Japan
Latvia
Russia
Korea
USSR

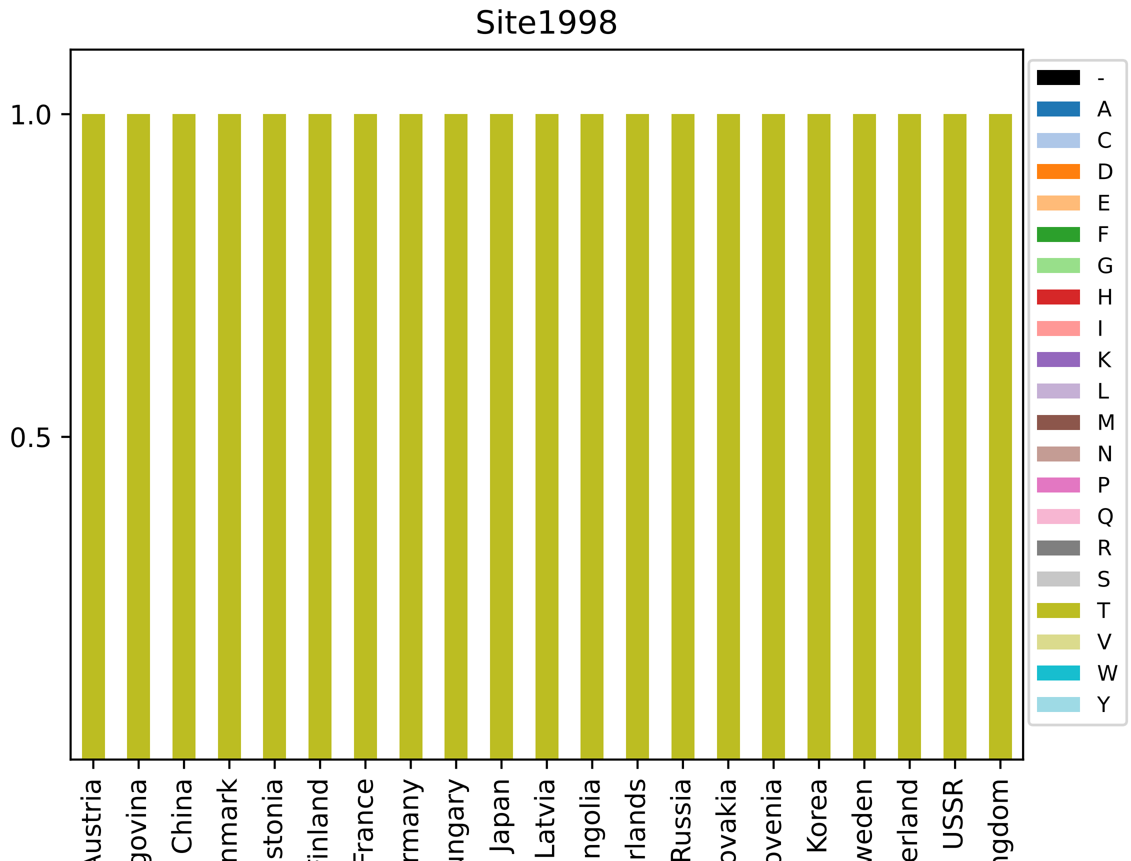
Site1998
1.0
0.5
-
A
C
D
E
F
G
H
I
K
L
M
N
P
Q
R
S
T
V
W
Y
Austria
govina
China
nmark
stonia
inland
France
rmany
ungary
Japan
Latvia
ngolia
rlands
Russia
ovakia
ovenia
Korea
weden
erland
USSR
ngdom

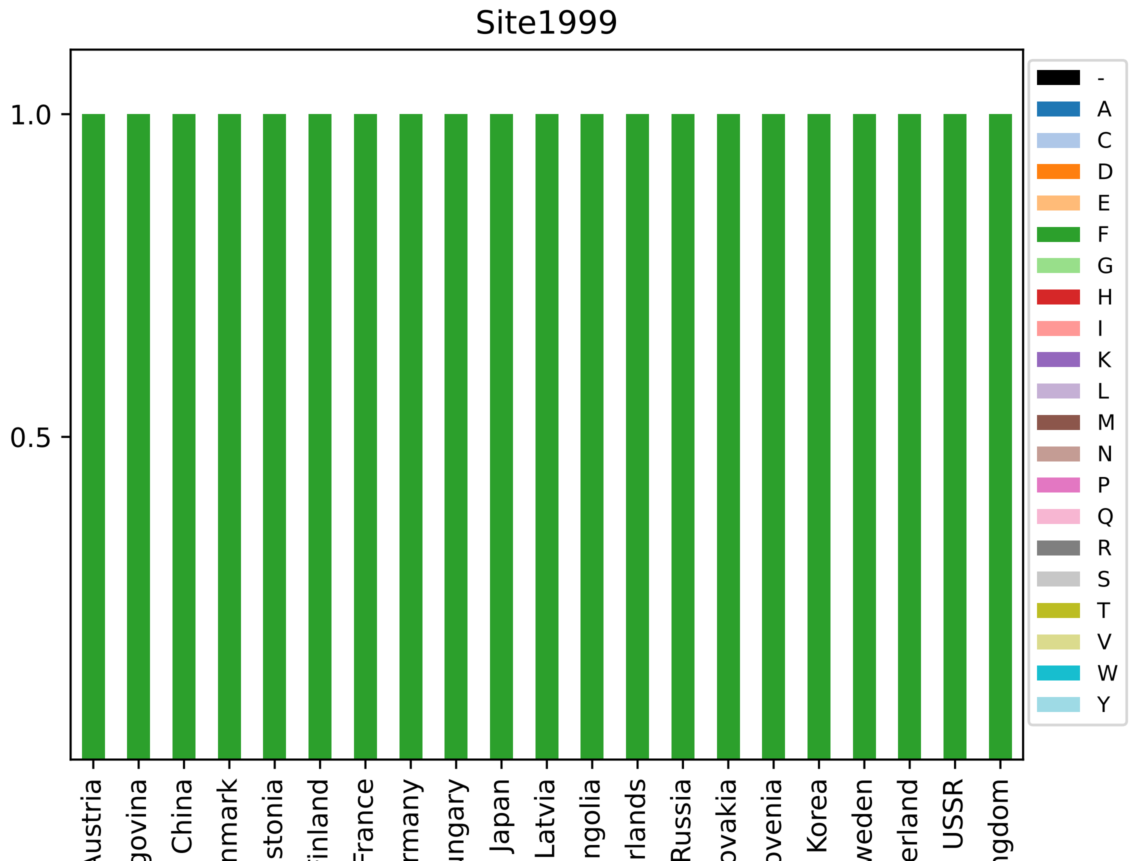
Site1999
1.0
0.5
-
A
C
D
E
F
G
H
I
K
L
M
N
P
Q
R
S
T
V
W
Y
Austria
govina
China
nmark
stonia
inland
France
rmany
ungary
Japan
Latvia
ngolia
rlands
Russia
ovakia
ovenia
Korea
weden
erland
USSR
ngdom

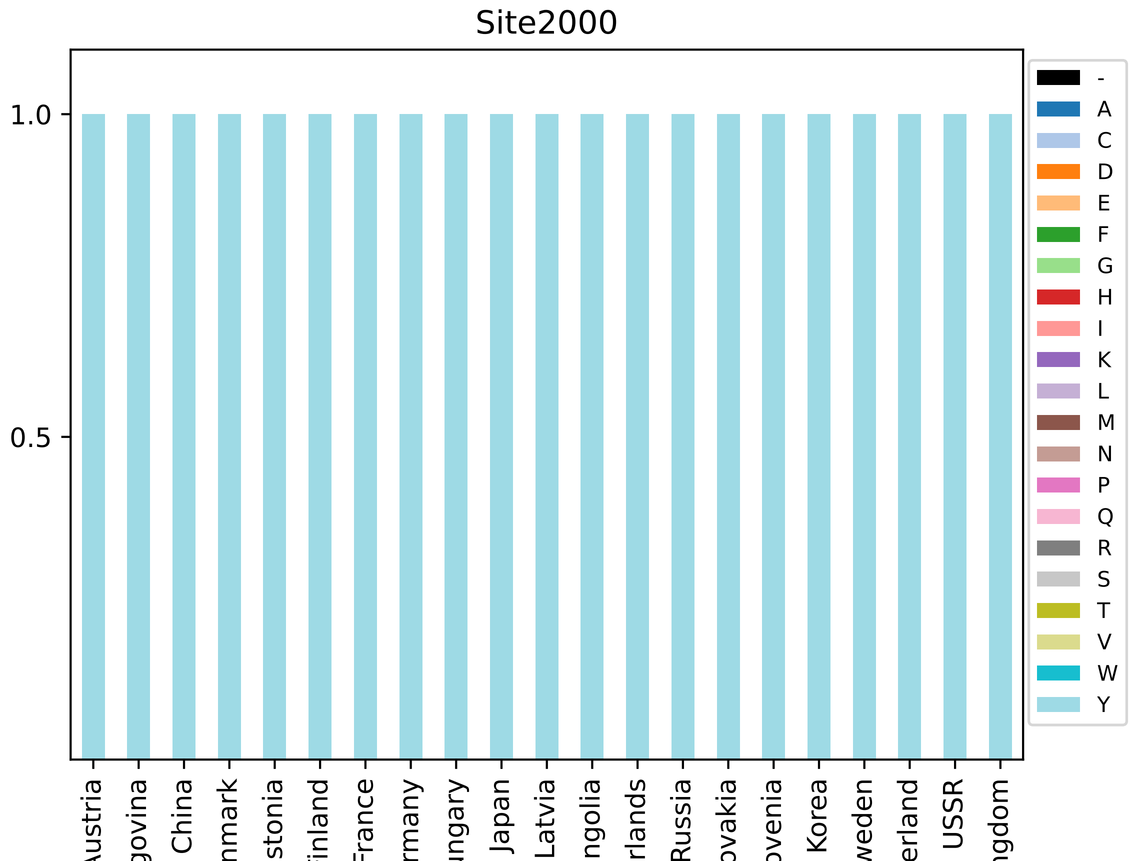
Site2000
1.0
0.5
-
A
C
D
E
F
G
H
I
K
L
M
N
P
Q
R
S
T
V
W
Y
Austria
govina
China
nmark
stonia
Finland
France
rmany
ungary
Japan
Latvia
ngolia
rlands
Russia
ovakia
ovenia
Korea
weden
erland
USSR
ngdom

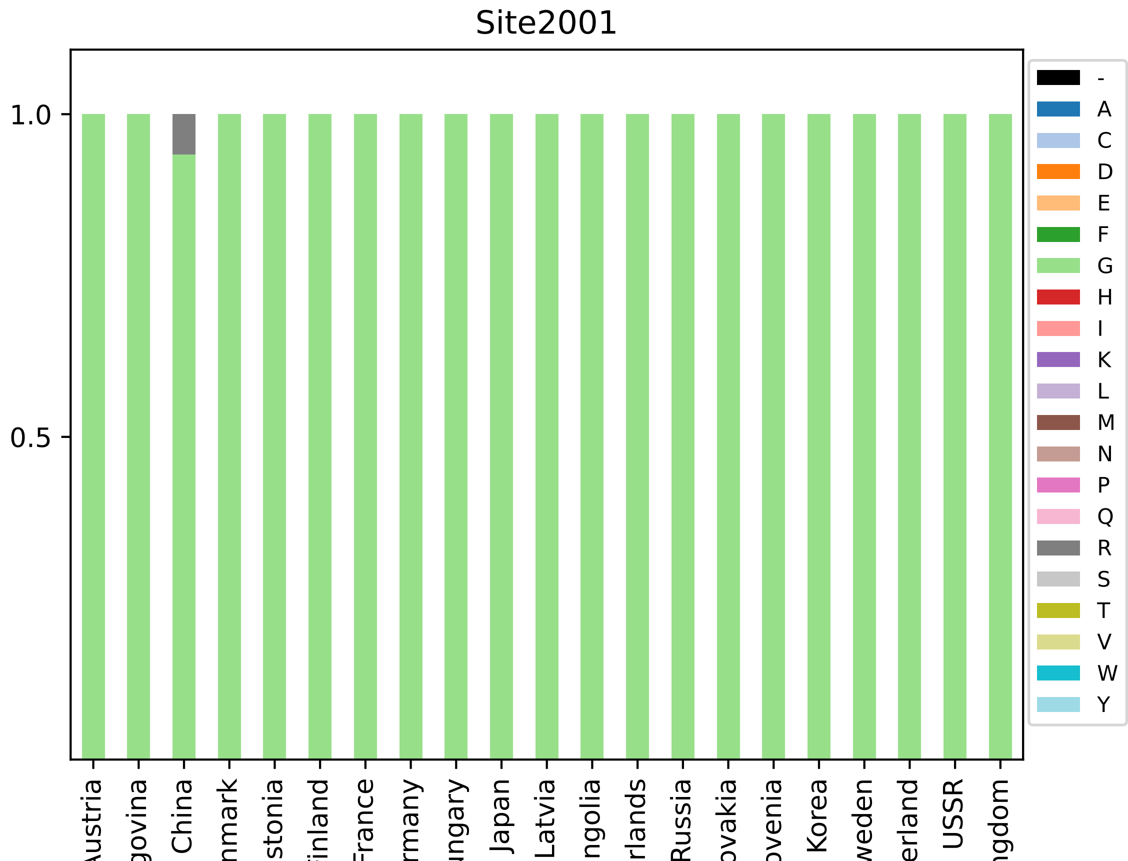

Site2001
1.0
0.5
-
A
C
D
E
F
G
H
I
K
L
M
N
P
Q
R
S
T
V
W
Y
Austria
govina
China
nmark
stonia
inland
France
rmany
ungary
Japan
Latvia
ngolia
rlands
Russia
ovakia
ovenia
Korea
weden
erland
USSR
ngdom

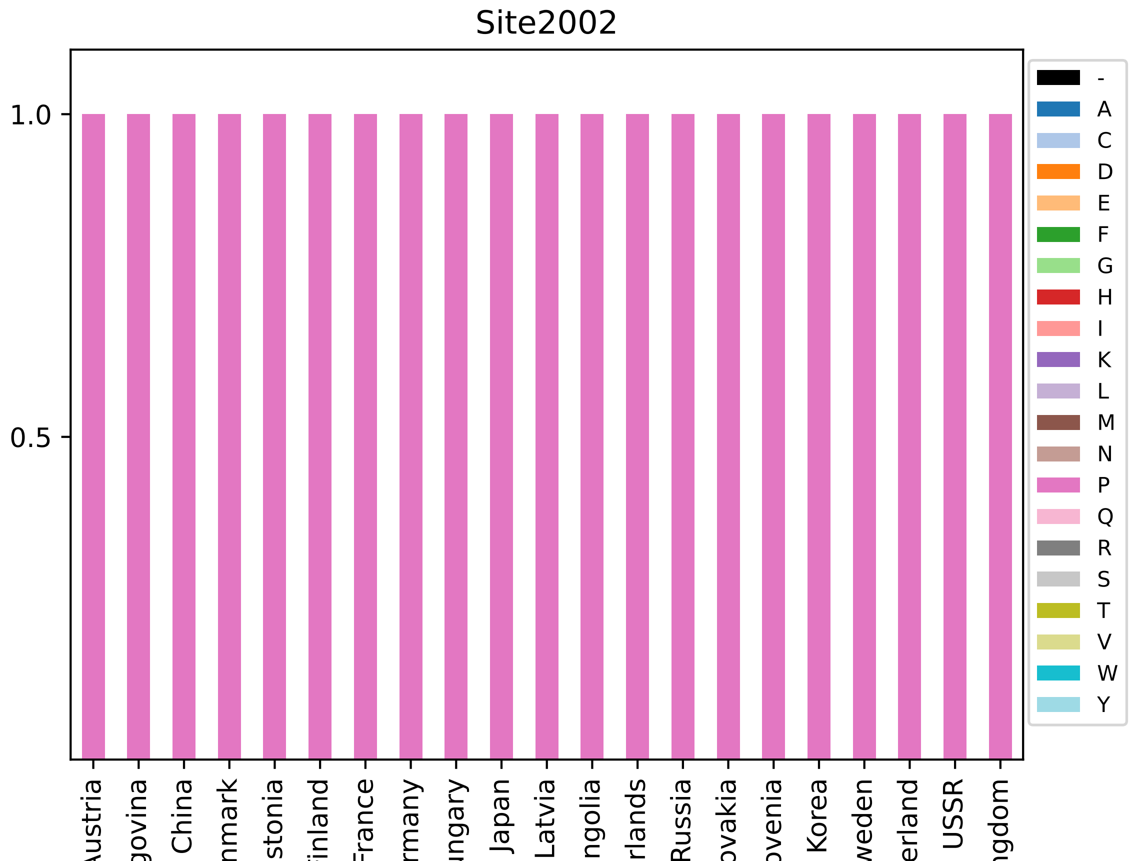
Site2002
1.0
0.5
-
A
C
D
E
F
G
H
I
K
L
M
N
P
Q
R
S
T
V
W
Y
China
Japan
Latvia
Russia
Korea
USSR

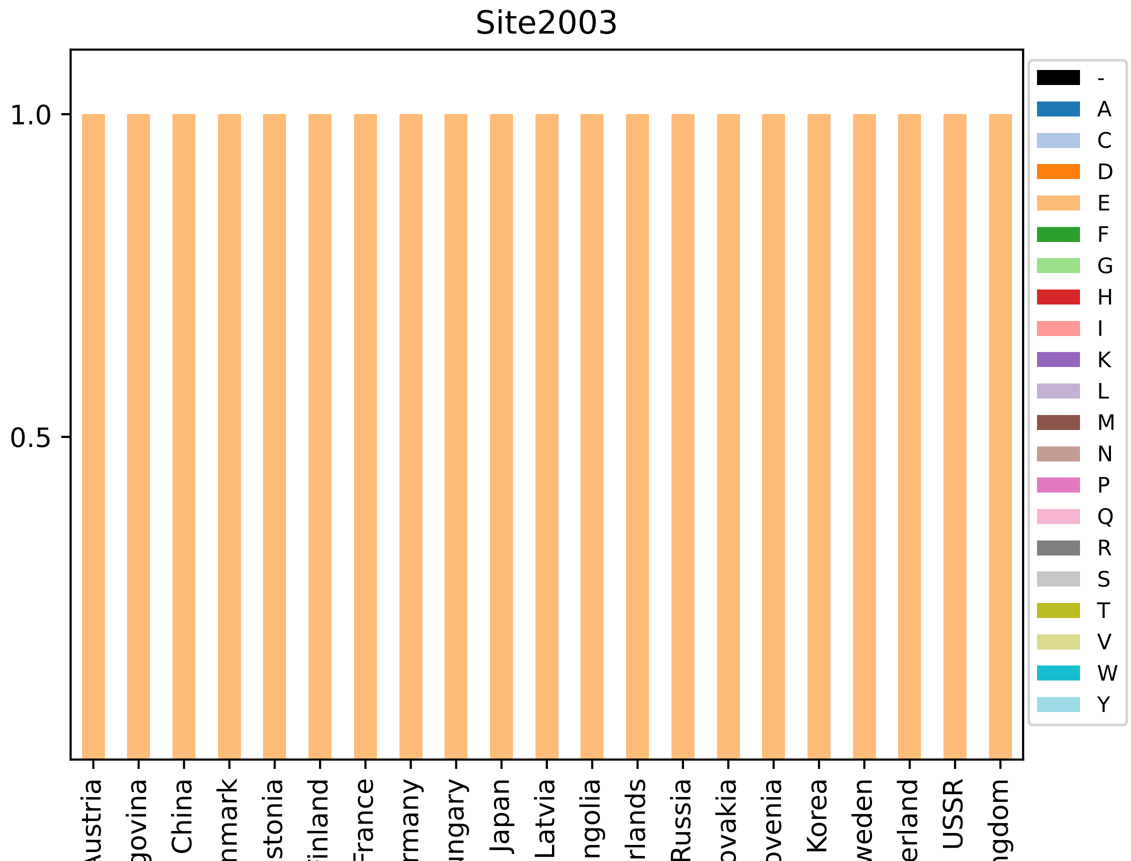
Site2003
1.0
0.5
-
A
C
D
E
F
G
H
I
K
L
M
N
P
Q
R
S
T
V
W
Y
China
Japan
Latvia
Russia
Korea
USSR

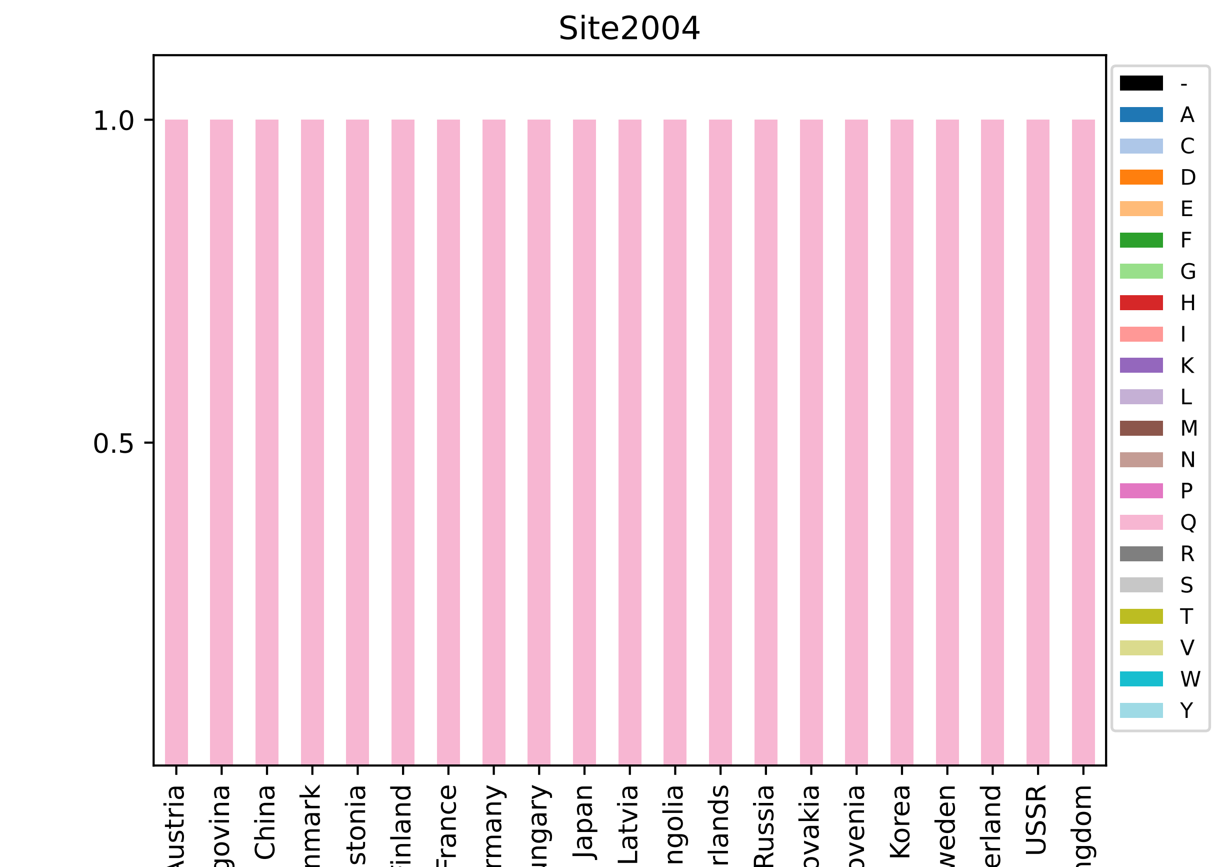

Site2004
1.0
0.5
-
A
C
D
E
F
G
H
I
K
L
M
N
P
Q
R
S
T
V
W
Y
Austria
govina
China
nmark
stonia
Finland
France
rmany
ungary
Japan
Latvia
ngolia
rlands
Russia
ovakia
ovenia
Korea
weden
erland
USSR
ngdom

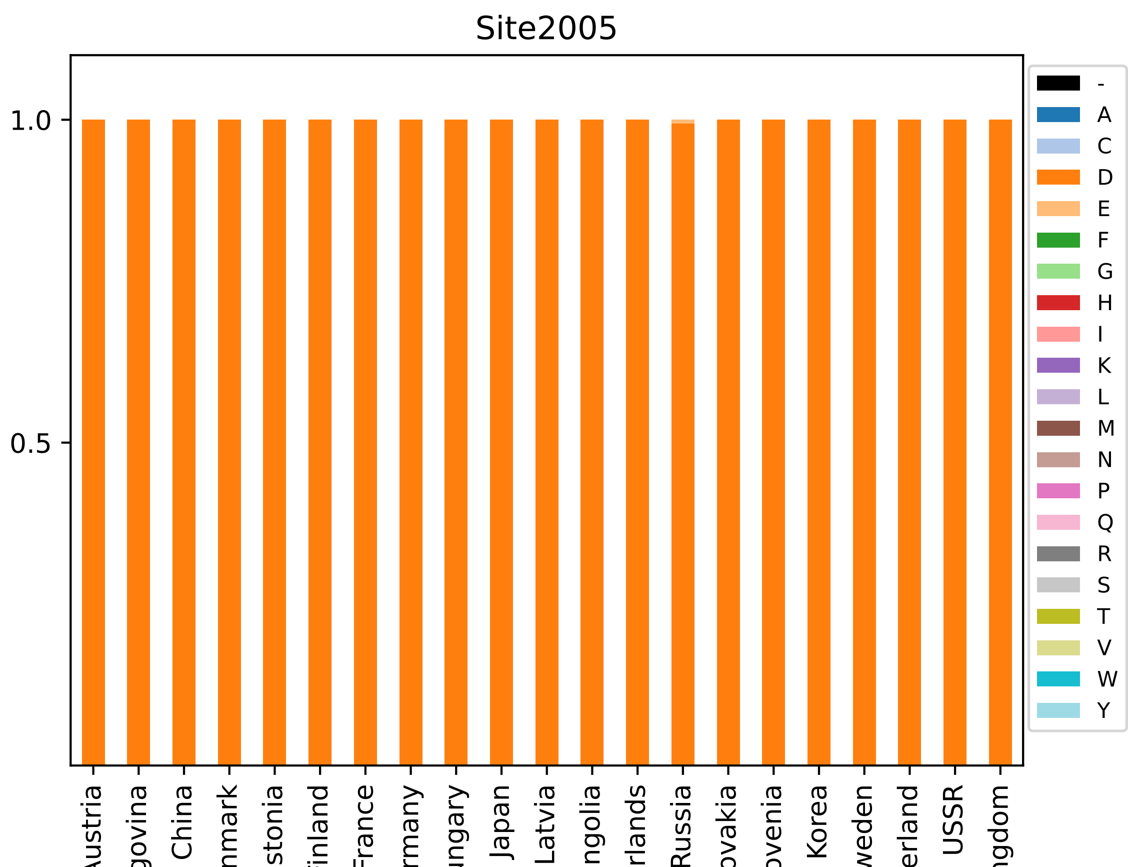
Site2005
1.0
0.5
-
A
C
D
E
F
G
H
I
K
L
M
N
P
Q
R
S
T
V
W
Y
China
Japan
Latvia
Russia
Korea
USSR

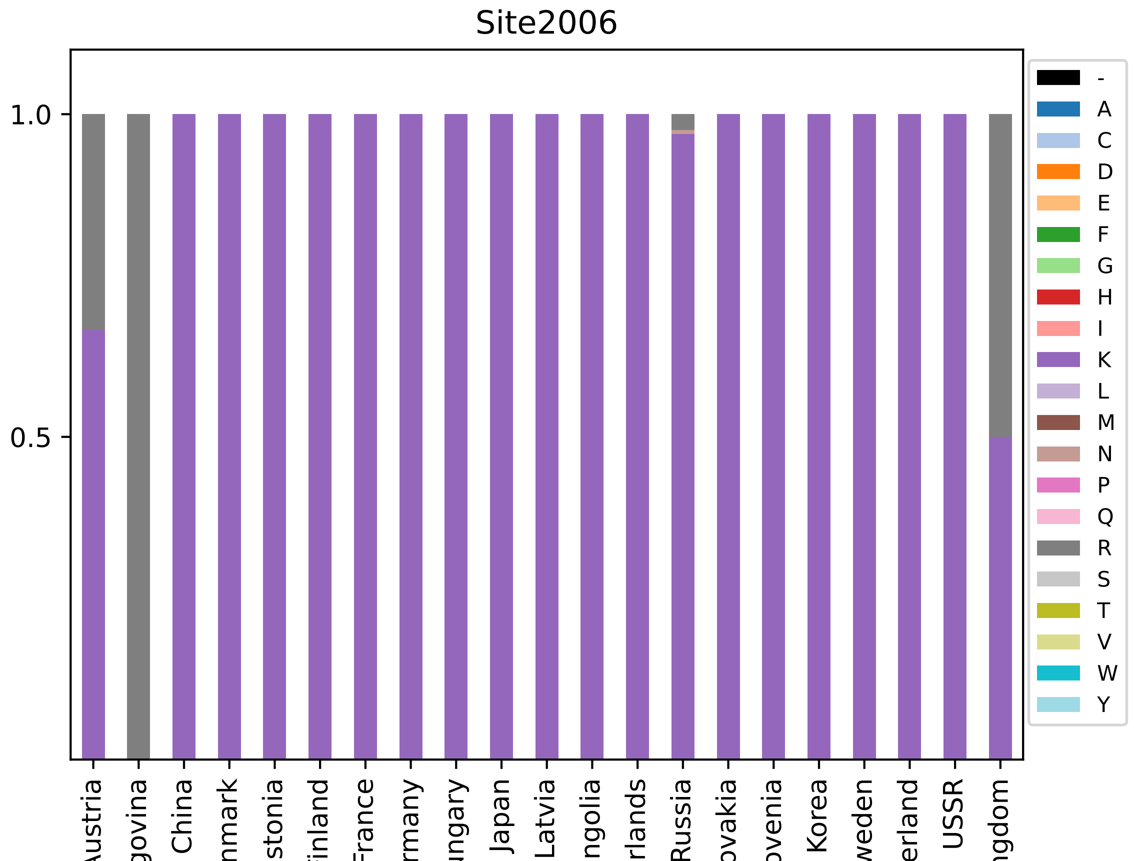

Site2006
1.0
0.5
-
A
C
D
E
F
G
H
I
K
L
M
N
P
Q
R
S
T
V
W
Y
Austria
govina
China
nmark
stonia
inland
France
rmany
ungary
Japan
Latvia
ngolia
rlands
Russia
ovakia
ovenia
Korea
weden
erland
USSR
ngdom

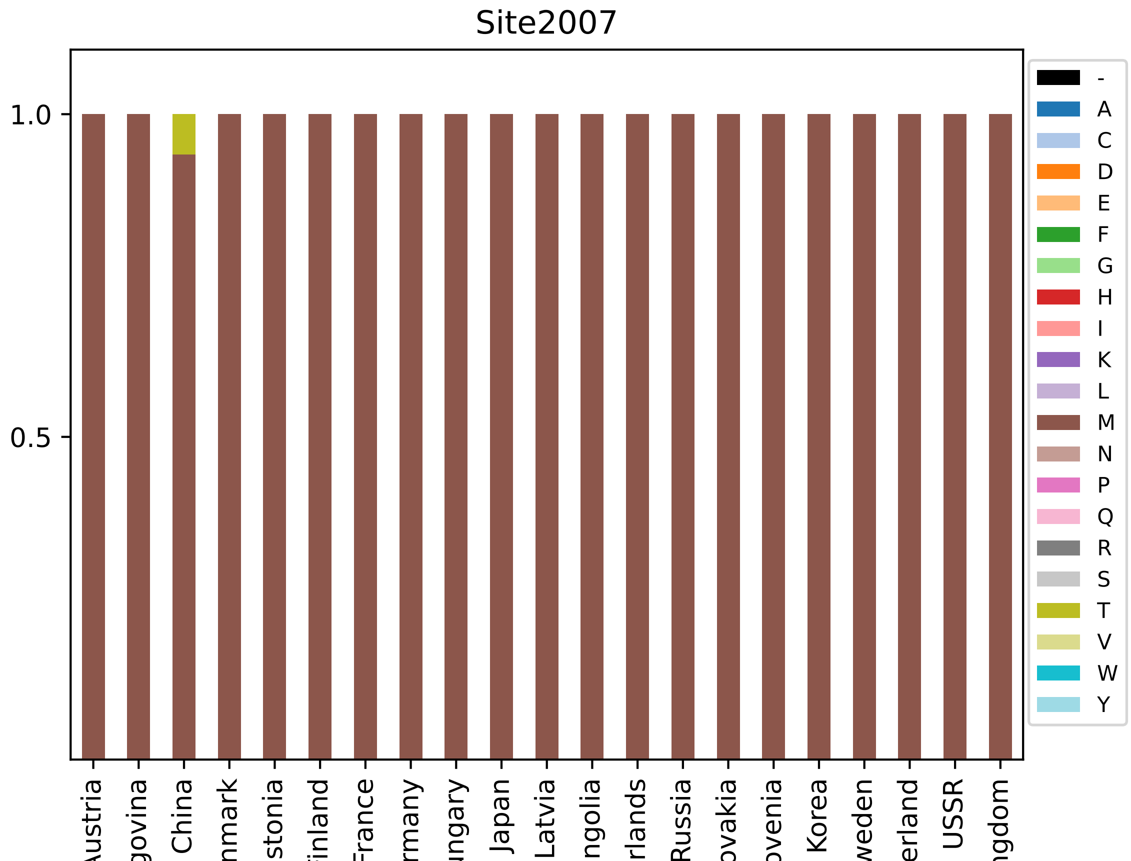

Site2007
1.0
0.5
-
A
C
D
E
F
G
H
I
K
L
M
N
P
Q
R
S
T
V
W
Y
China
Japan
Latvia
Russia
Korea
USSR

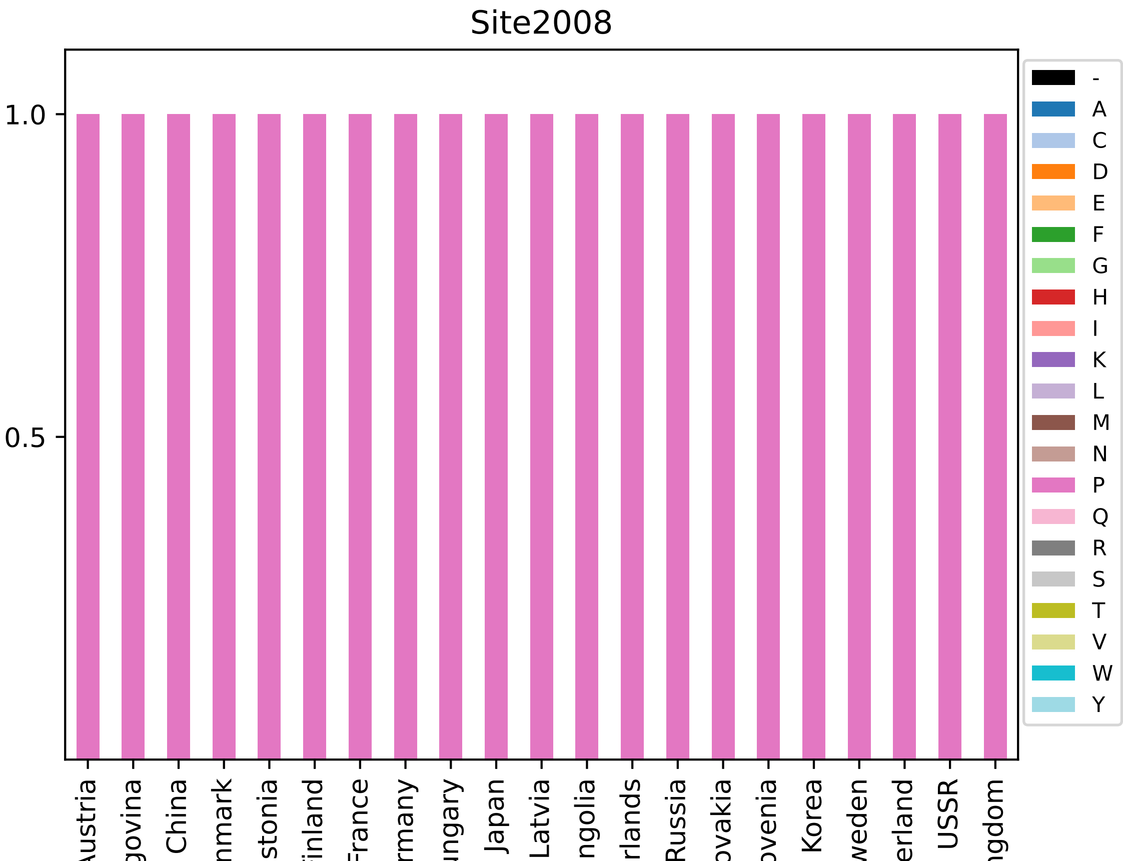

Site2008
1.0
0.5
-
A
C
D
E
F
G
H
I
K
L
M
N
P
Q
R
S
T
V
W
Y
China
Japan
Latvia
Russia
Korea
USSR

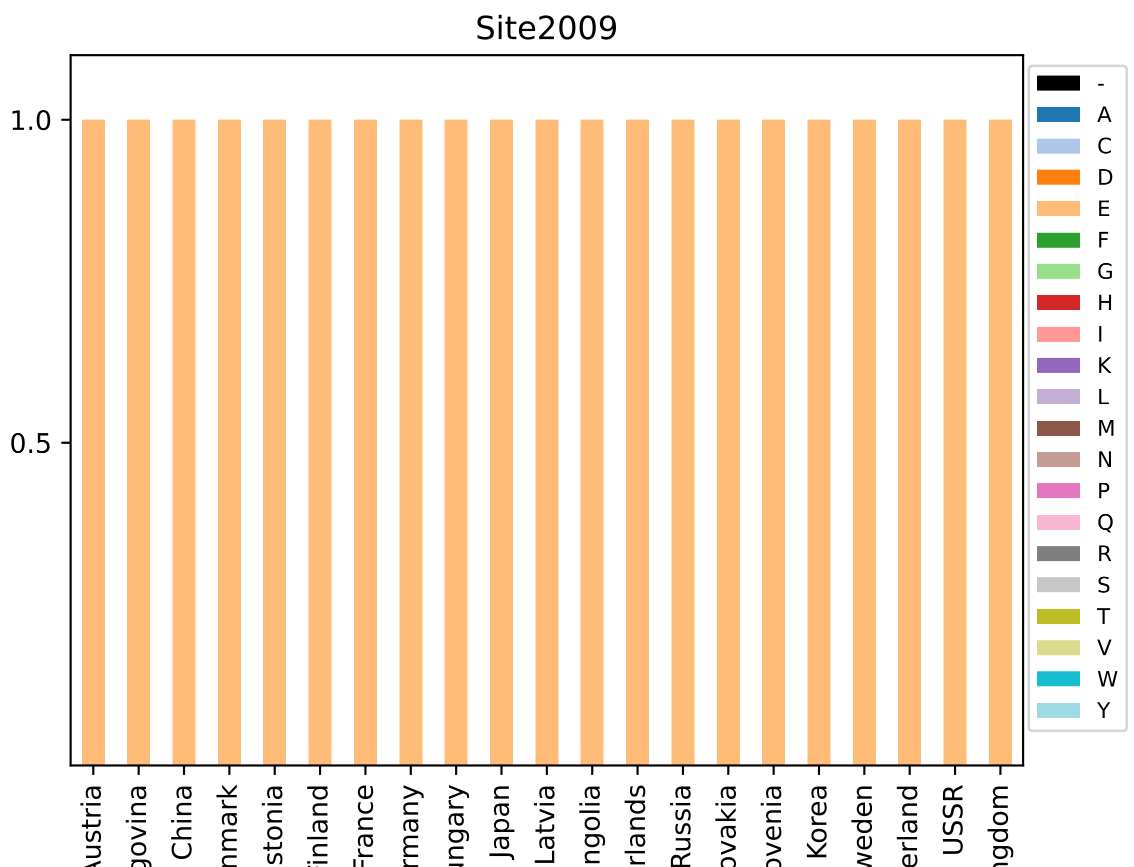
Site2009
1.0
0.5
-
A
C
D
E
F
G
H
I
K
L
M
N
P
Q
R
S
T
V
W
Y
China
Japan
Latvia
Russia
Korea
USSR

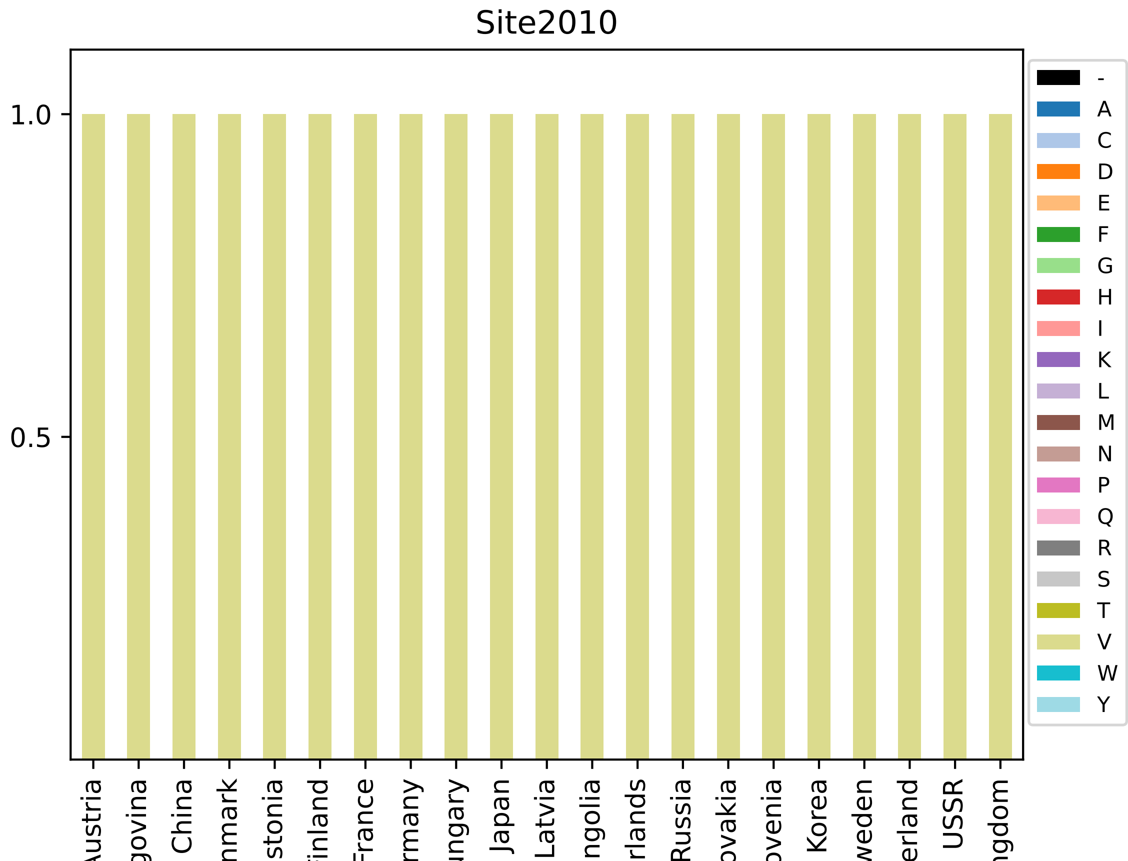

Site2010
1.0
0.5
-
A
C
D
E
F
G
H
I
K
L
M
N
P
Q
R
S
T
V
W
Y
Austria
China
Finland
France
Japan
Latvia
Russia
Korea
USSR

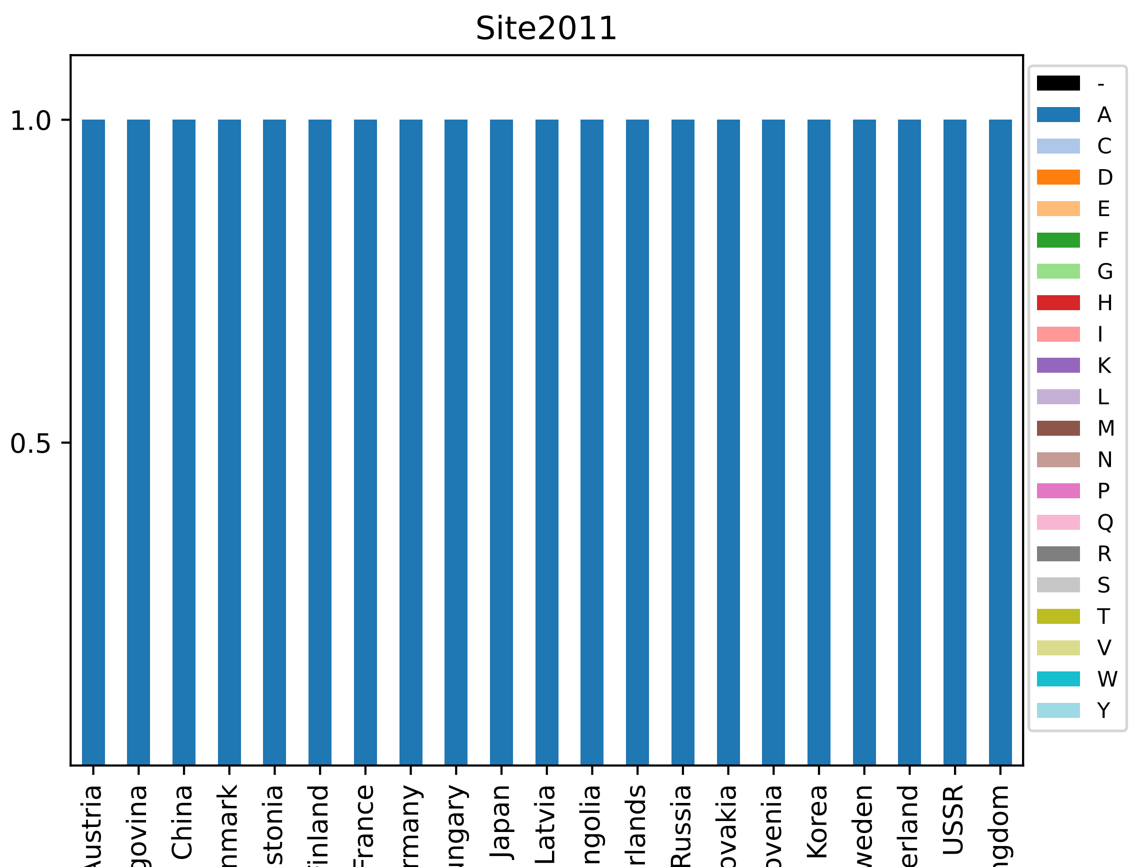

Site2011
1.0
0.5
-
A
C
D
E
F
G
H
I
K
L
M
N
P
Q
R
S
T
V
W
Y
Austria
govina
China
nmark
stonia
inland
France
rmany
ungary
Japan
Latvia
ngolia
rlands
Russia
ovakia
ovenia
Korea
weden
erland
USSR
ngdom

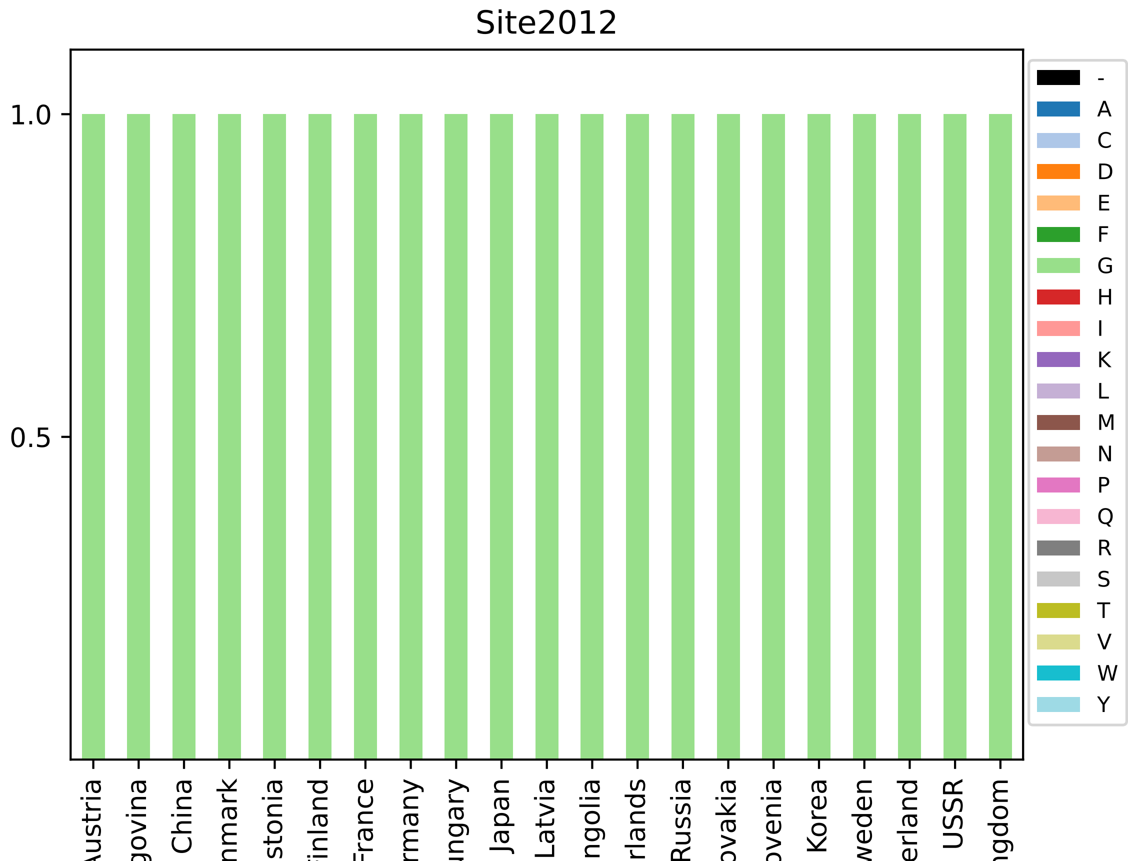
Site2012
1.0
0.5
-
A
C
D
E
F
G
H
I
K
L
M
N
P
Q
R
S
T
V
W
Y
Austria
govina
China
nmark
stonia
inland
France
rmany
ungary
Japan
Latvia
ngolia
rlands
Russia
ovakia
ovenia
Korea
weden
erland
USSR
ngdom

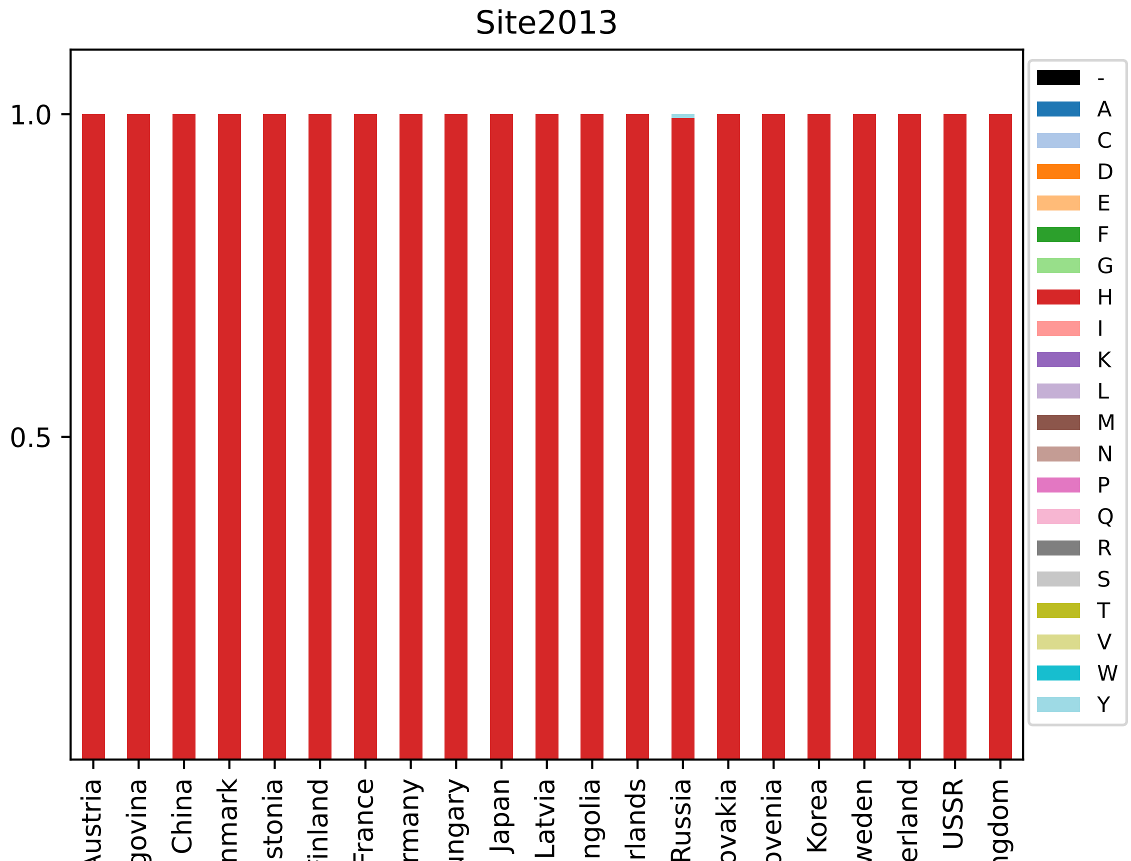
Site2013
1.0
0.5
-
A
C
D
E
F
G
H
I
K
L
M
N
P
Q
R
S
T
V
W
Y
Austria
govina
China
nmark
stonia
Finland
France
rmany
ungary
Japan
Latvia
ngolia
rlands
Russia
ovakia
ovenia
Korea
weden
erland
USSR
ngdom

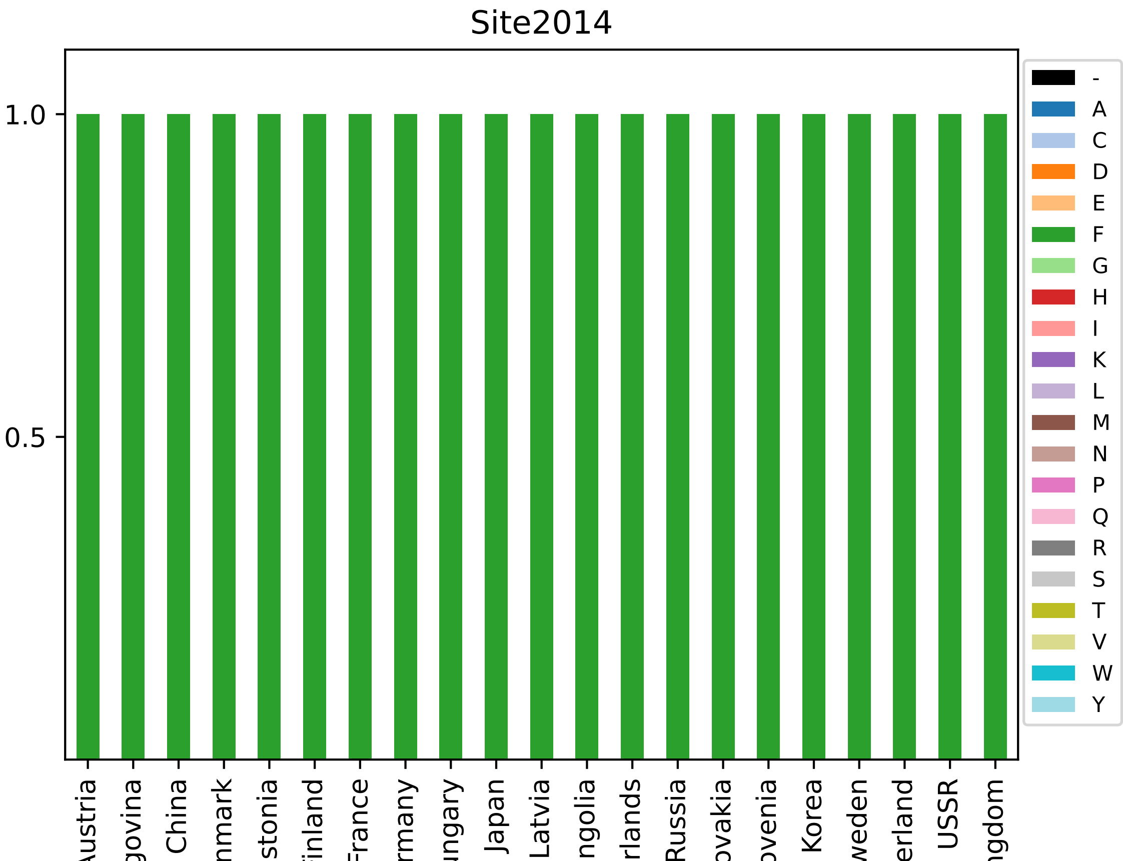

Site2014
1.0
0.5
-
A
C
D
E
F
G
H
I
K
L
M
N
P
Q
R
S
T
V
W
Y
China
Japan
Latvia
Korea
USSR

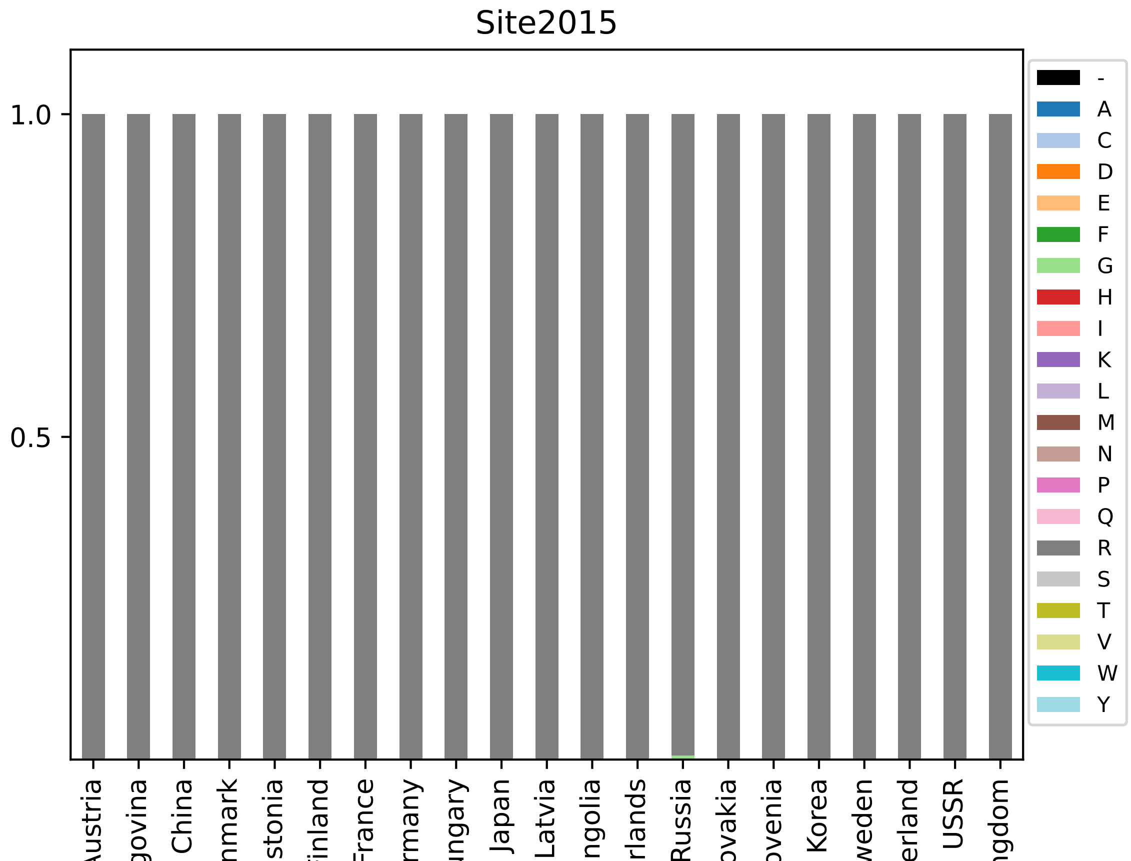

Site2015
1.0
0.5
-
A
C
D
E
F
G
H
I
K
L
M
N
P
Q
R
S
T
V
W
Y
China
Japan
Latvia
Russia
Korea
USSR

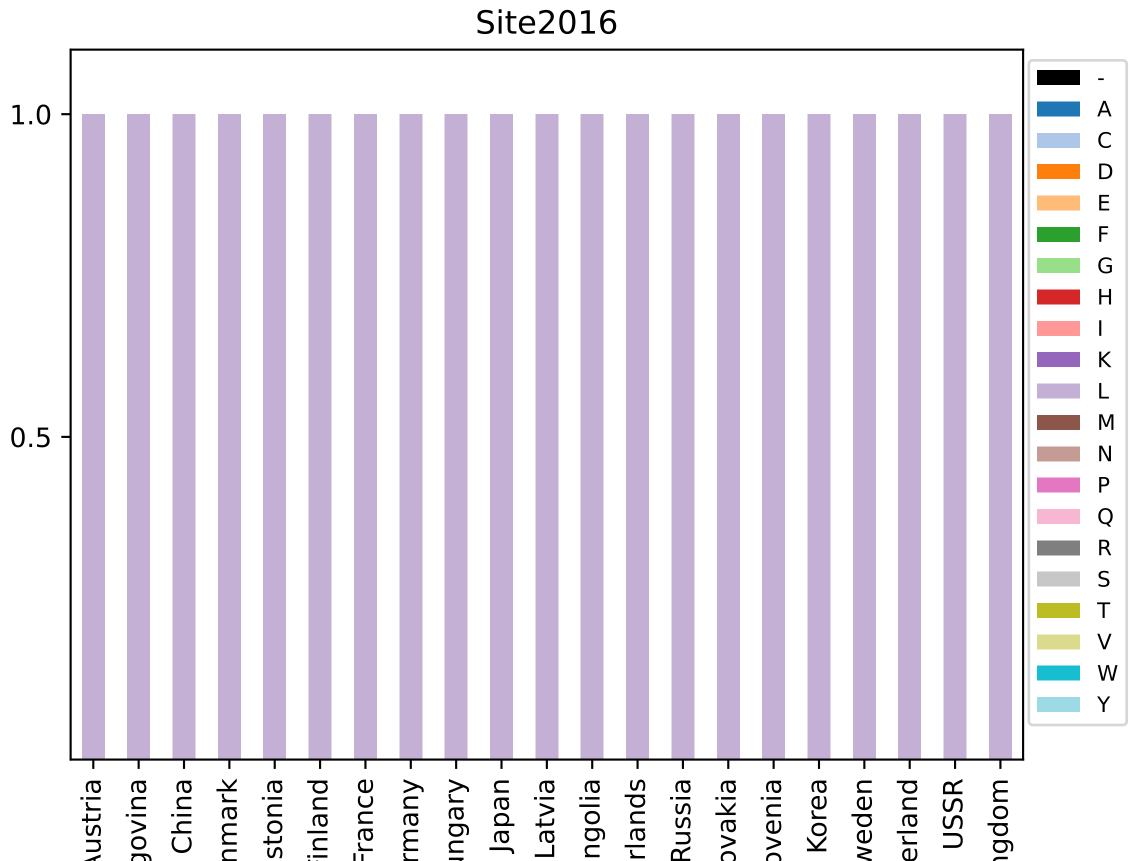

Site2016
1.0
0.5
-
A
C
D
E
F
G
H
I
K
L
M
N
P
Q
R
S
T
V
W
Y
Austria
China
Japan
Latvia
Russia
Korea
USSR

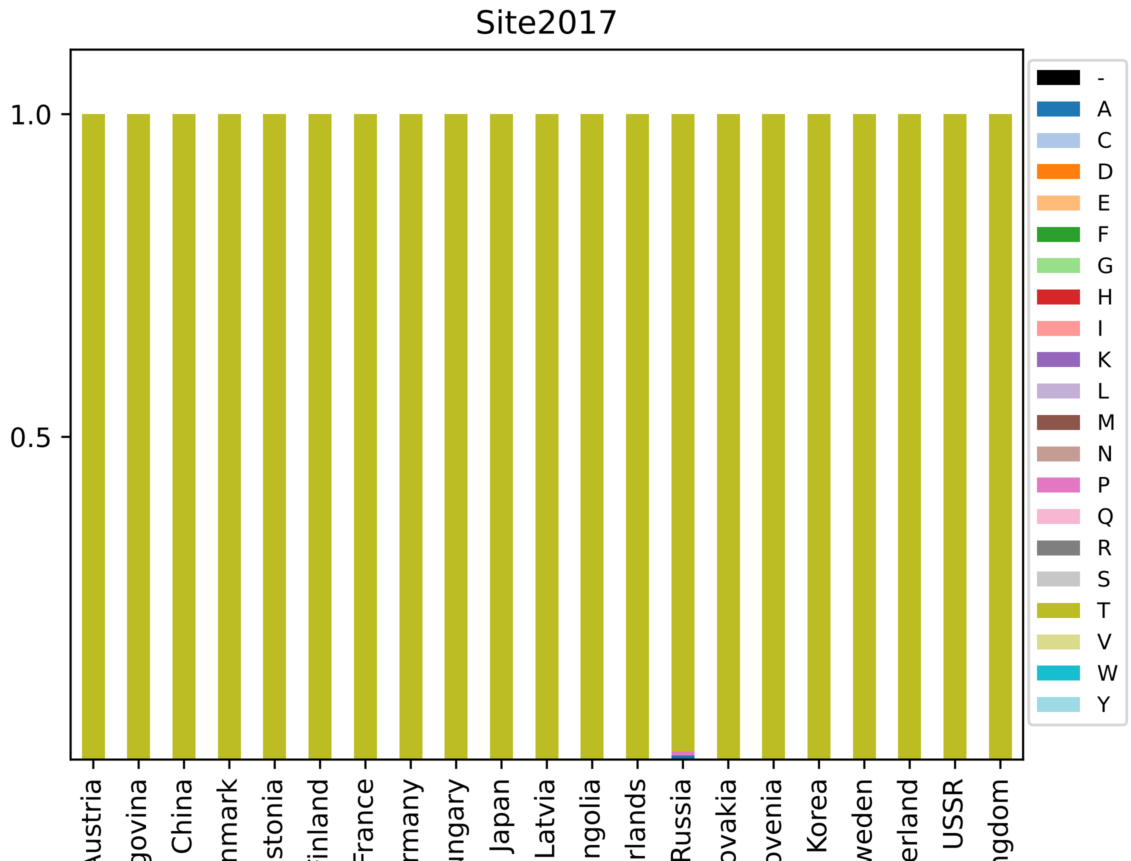

Site2017
1.0
0.5
-
A
C
D
E
F
G
H
I
K
L
M
N
P
Q
R
S
T
V
W
Y
Austria
govina
China
nmark
stonia
inland
France
rmany
ungary
Japan
Latvia
ngolia
rlands
Russia
ovakia
ovenia
Korea
weden
erland
USSR
ngdom

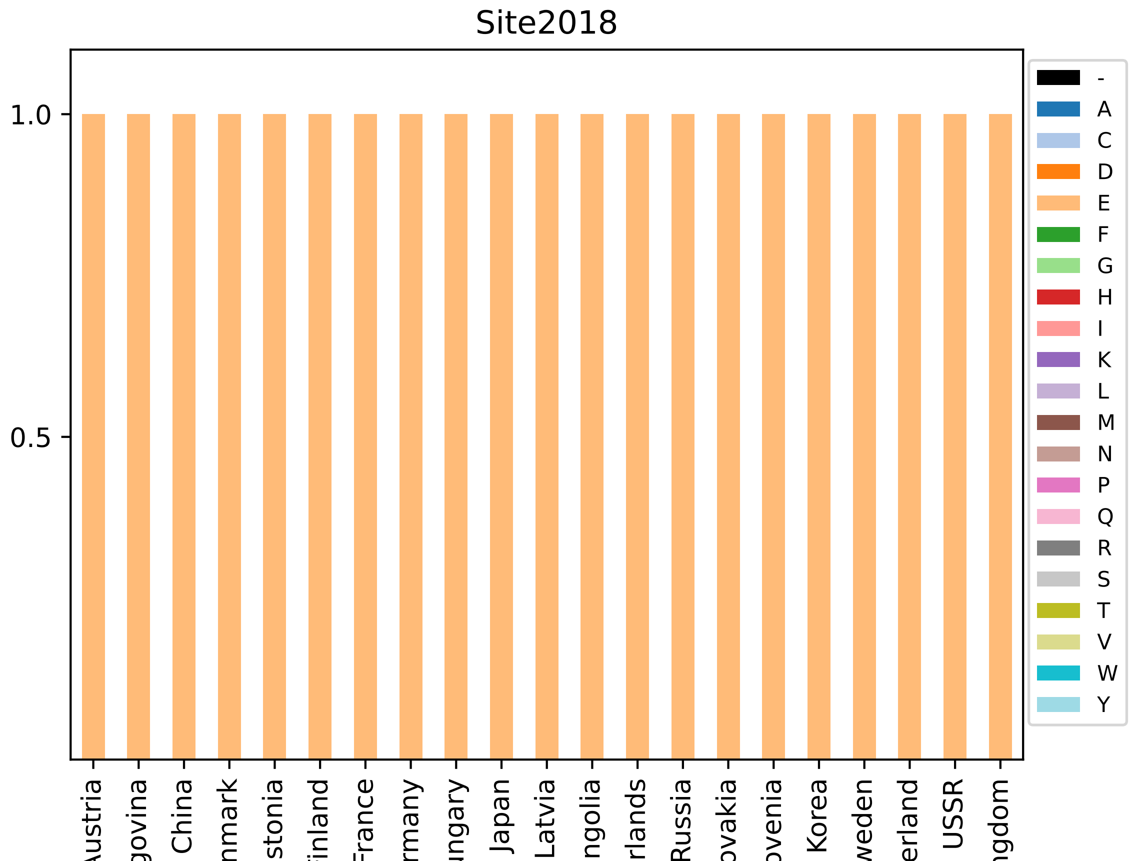
Site2018
1.0
0.5
Austria
govina
China
nmark
stonia
Finland
France
rmany
ungary
Japan
Latvia
ngolia
rlands
Russia
ovakia
ovenia
Korea
weden
erland
USSR
ngdom
-
A
C
D
E
F
G
H
I
K
L
M
N
P
Q
R
S
T
V
W
Y

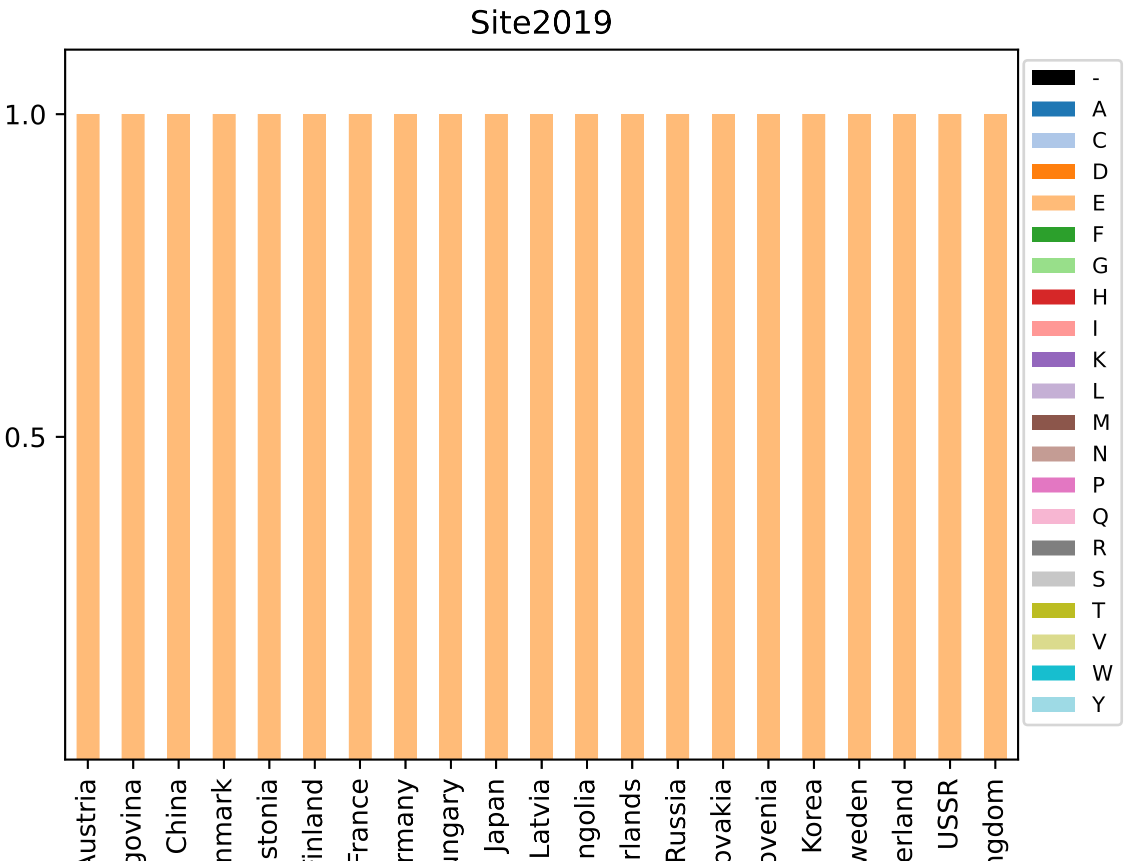
Site2019
1.0
0.5
China
Japan
Latvia
Russia
Korea
USSR
-
A
C
D
E
F
G
H
I
K
L
M
N
P
Q
R
S
T
V
W
Y

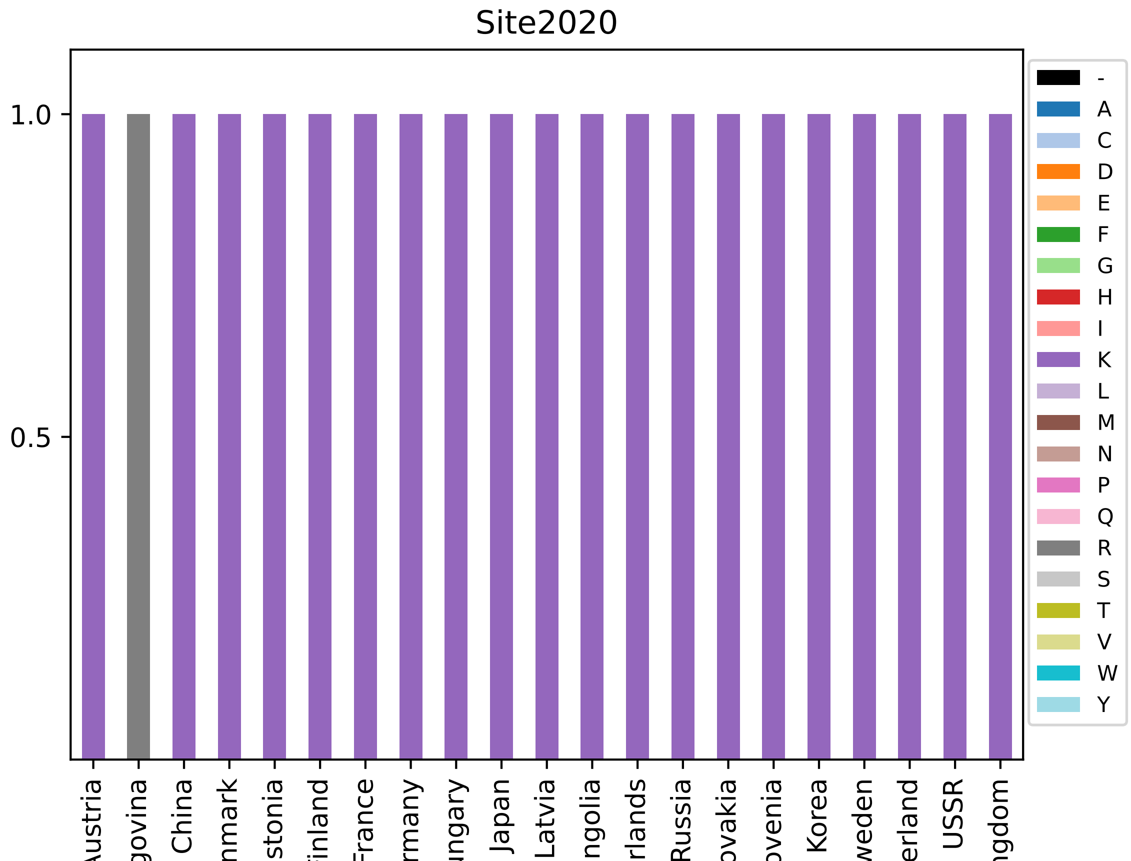
Site2020
1.0
0.5
China
Japan
Latvia
Russia
Korea
USSR
-
A
C
D
E
F
G
H
I
K
L
M
N
P
Q
R
S
T
V
W
Y

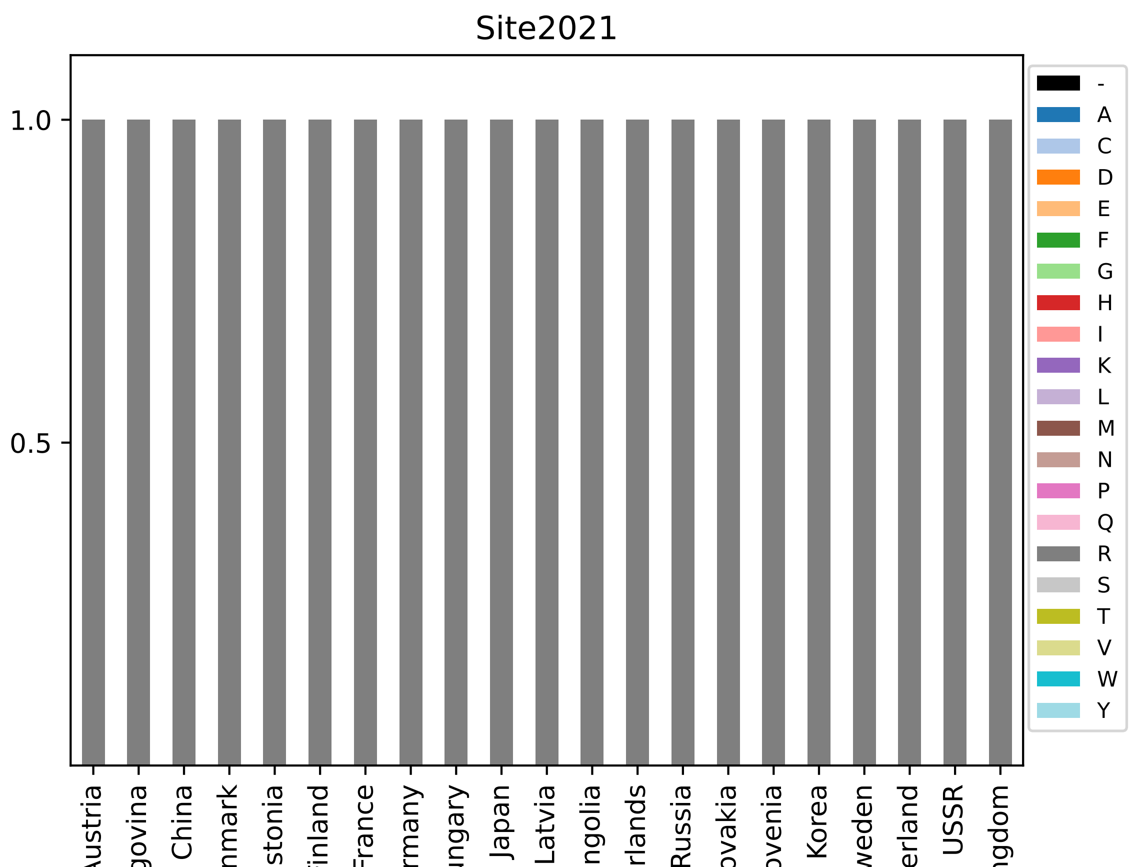

Site2021
1.0
0.5
-
A
C
D
E
F
G
H
I
K
L
M
N
P
Q
R
S
T
V
W
Y
China
Japan
Latvia
Russia
Korea
USSR

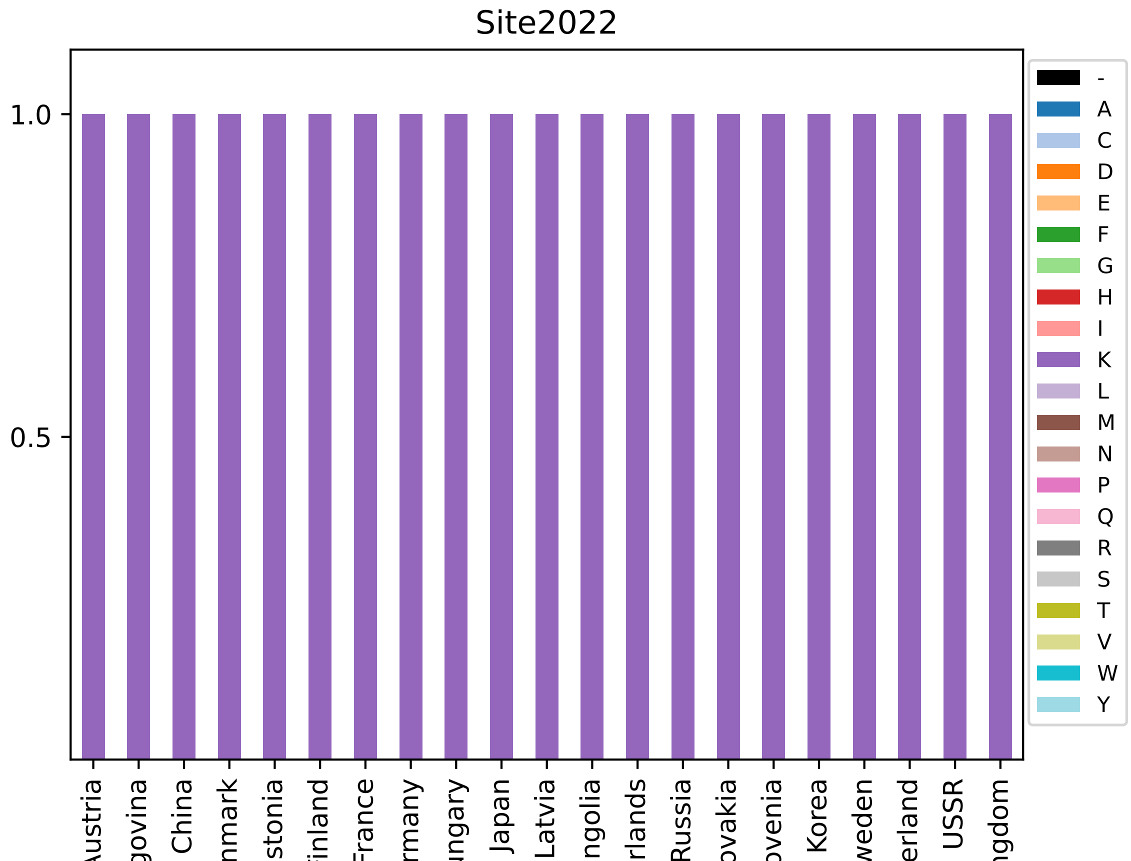
Site2022
1.0
0.5
-
A
C
D
E
F
G
H
I
K
L
M
N
P
Q
R
S
T
V
W
Y
Austria
China
Japan
Latvia
Russia
Korea
USSR

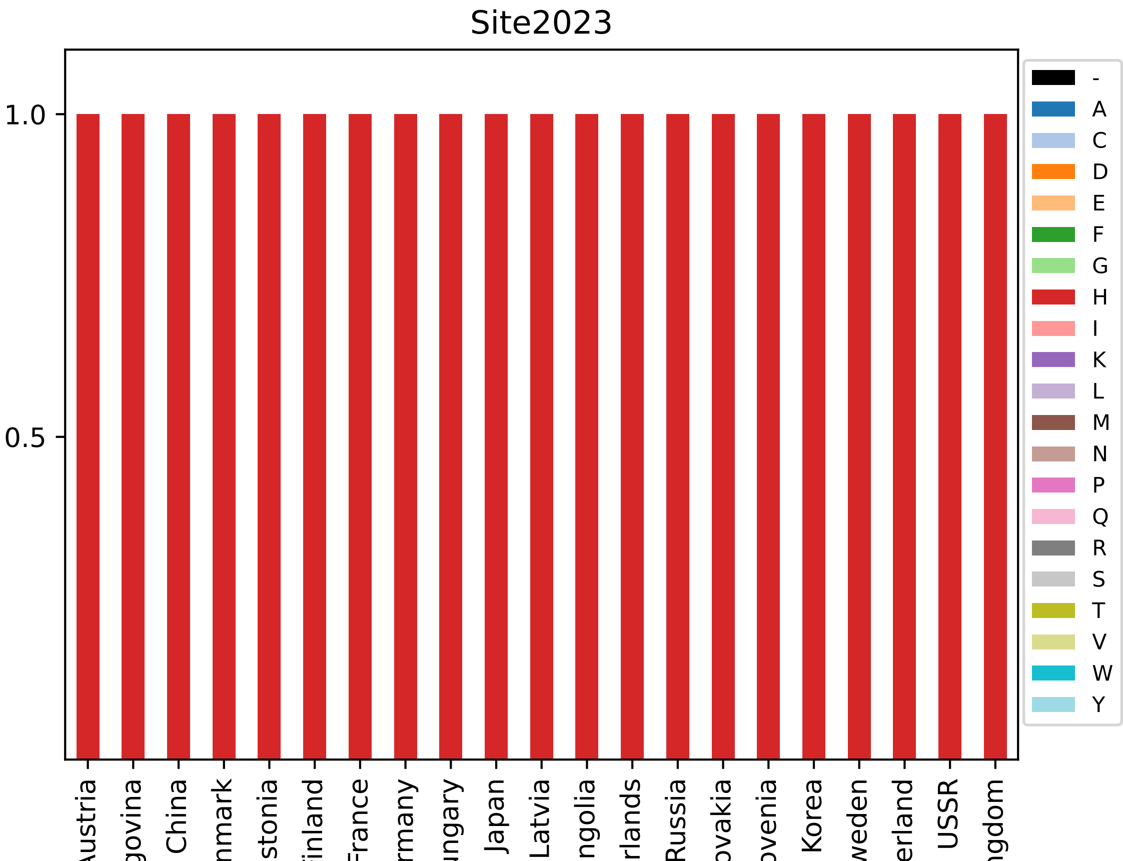
Site2023
1.0
0.5
-
A
C
D
E
F
G
H
I
K
L
M
N
P
Q
R
S
T
V
W
Y
China
Japan
Latvia
Russia
Korea
USSR

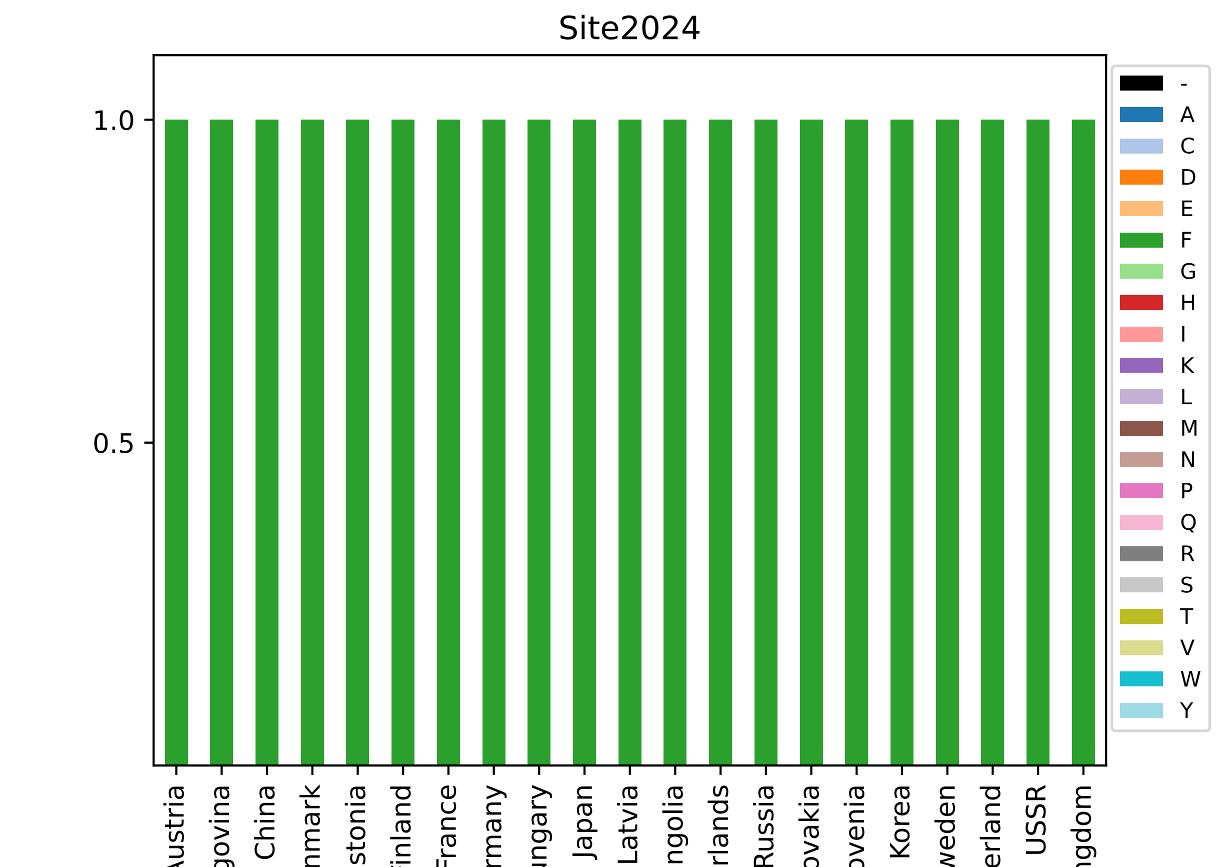
Site2024
1.0
0.5
-
A
C
D
E
F
G
H
I
K
L
M
N
P
Q
R
S
T
V
W
Y
Austria
China
Japan
Latvia
Russia
Korea
USSR

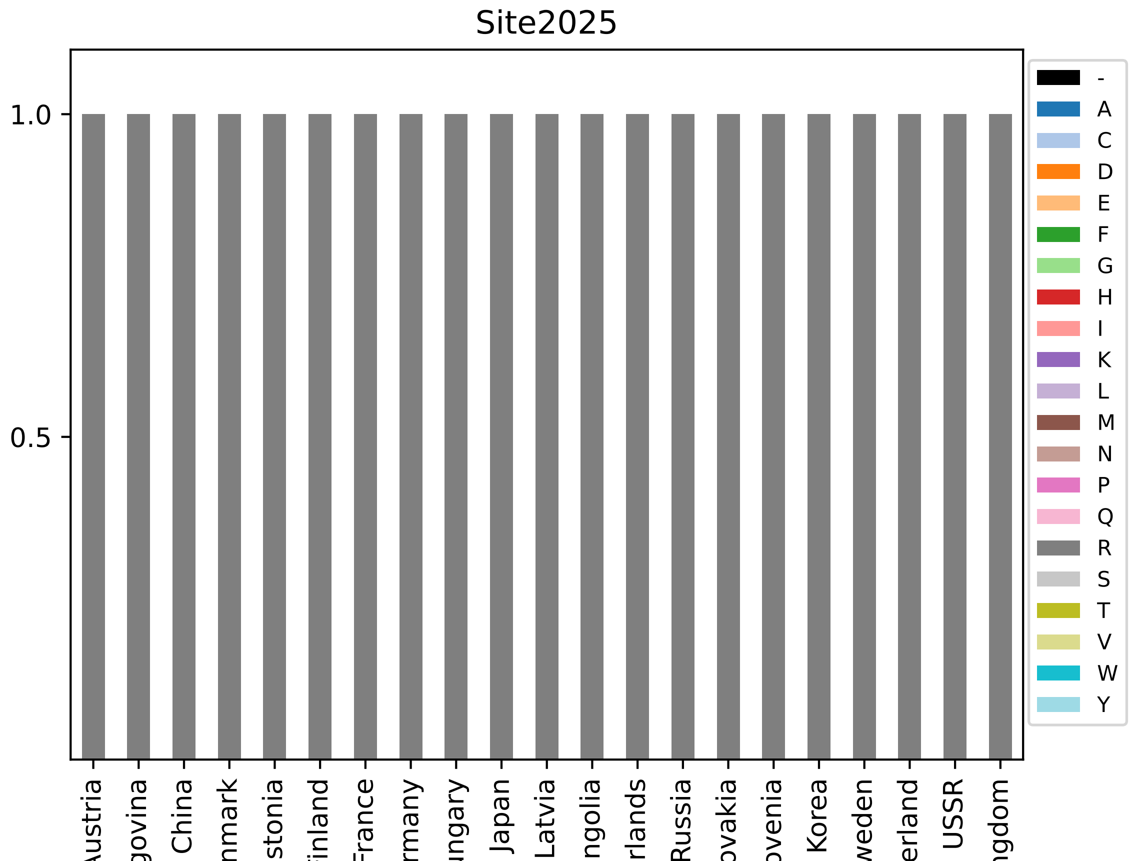

Site2025
1.0
0.5
-
A
C
D
E
F
G
H
I
K
L
M
N
P
Q
R
S
T
V
W
Y
China
Japan
Latvia
Russia
Korea
USSR

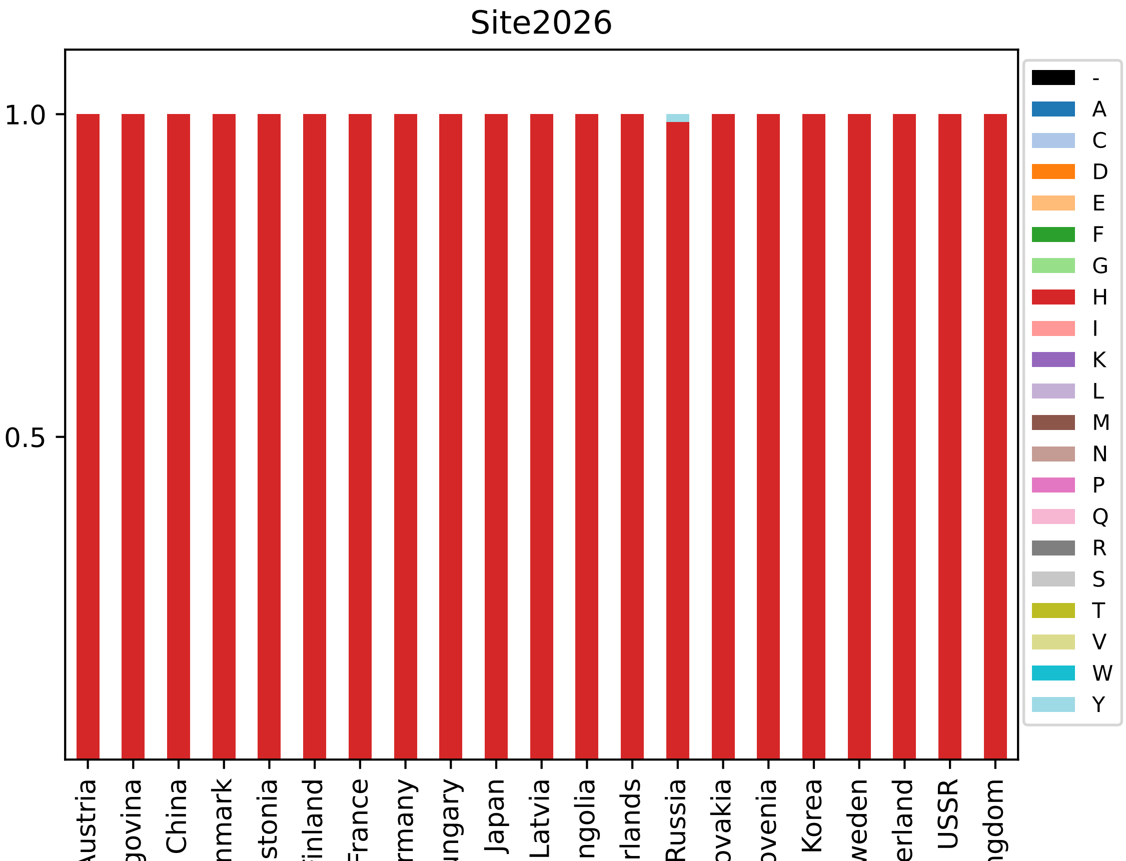

Site2026
1.0
0.5
-
A
C
D
E
F
G
H
I
K
L
M
N
P
Q
R
S
T
V
W
Y
Austria
China
Japan
Latvia
Russia
Korea
USSR

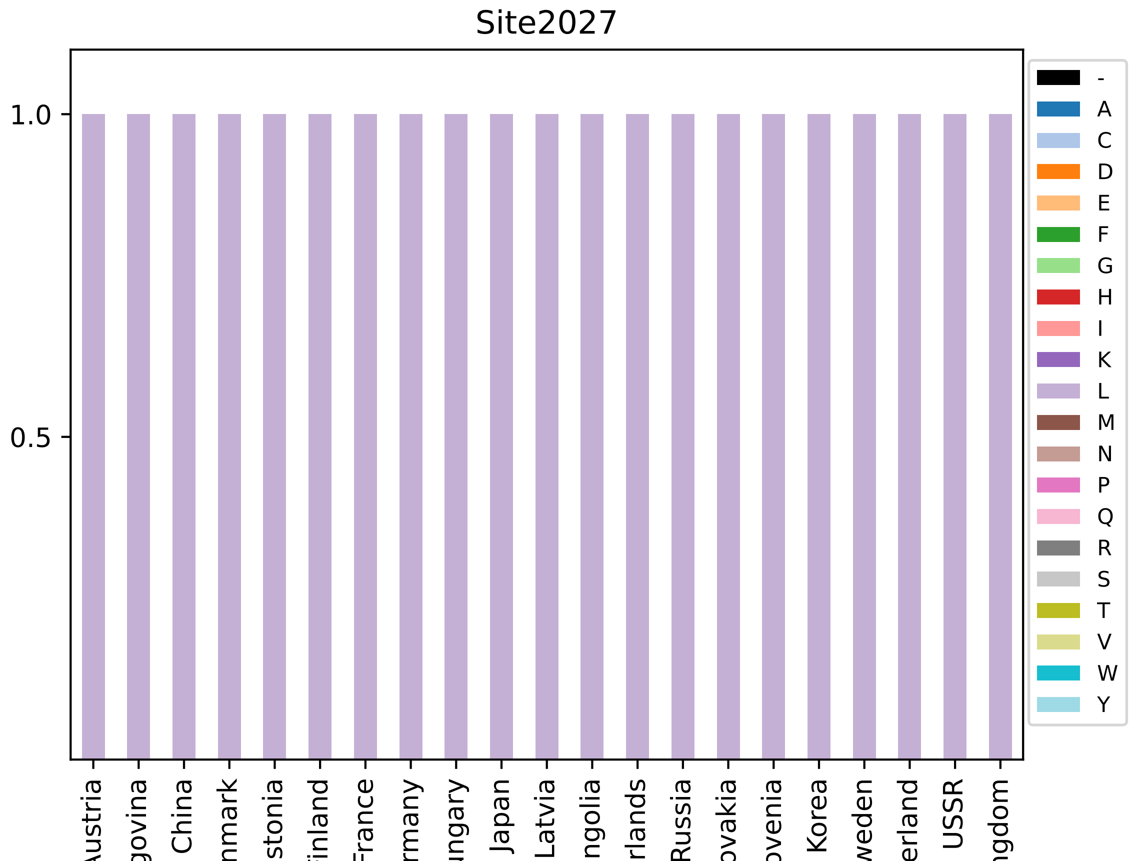

Site2027
1.0
0.5
-
A
C
D
E
F
G
H
I
K
L
M
N
P
Q
R
S
T
V
W
Y
China
Japan
Latvia
Russia
Korea
USSR

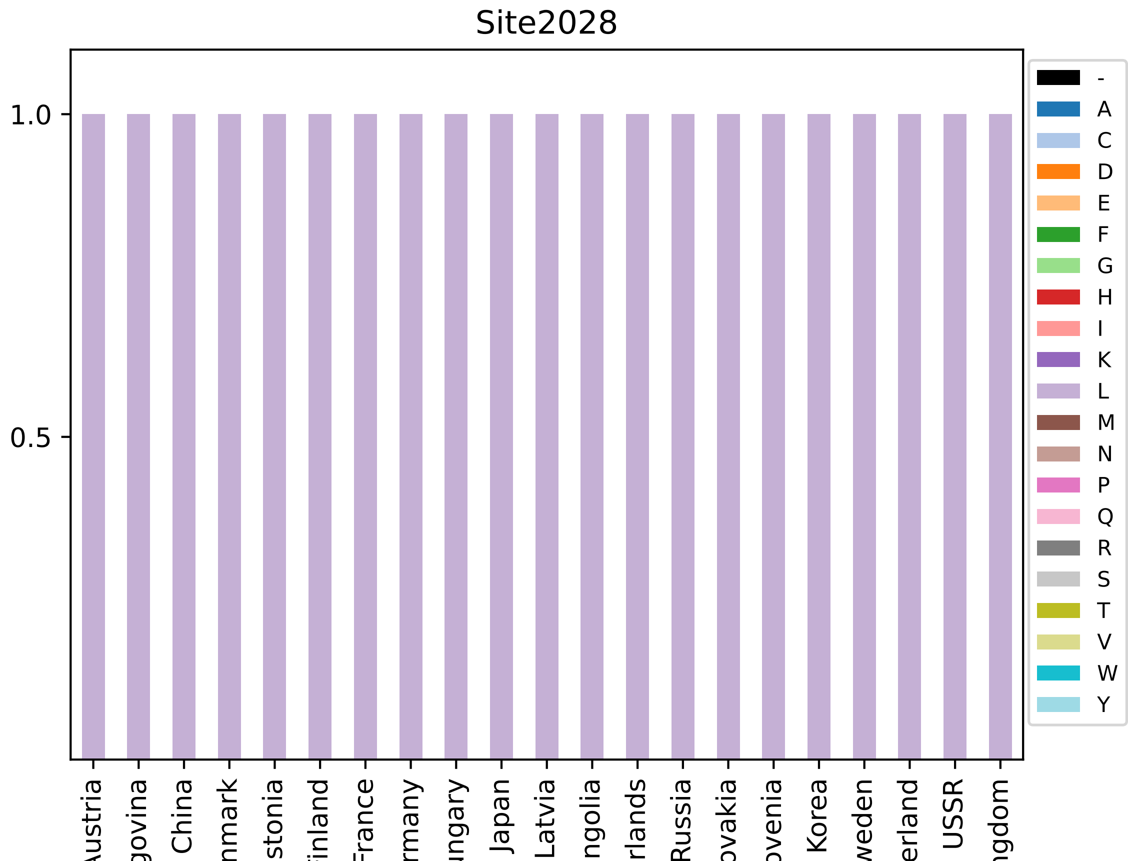

Site2028
1.0
0.5
China
Japan
Latvia
Russia
Korea
USSR
-
A
C
D
E
F
G
H
I
K
L
M
N
P
Q
R
S
T
V
W
Y

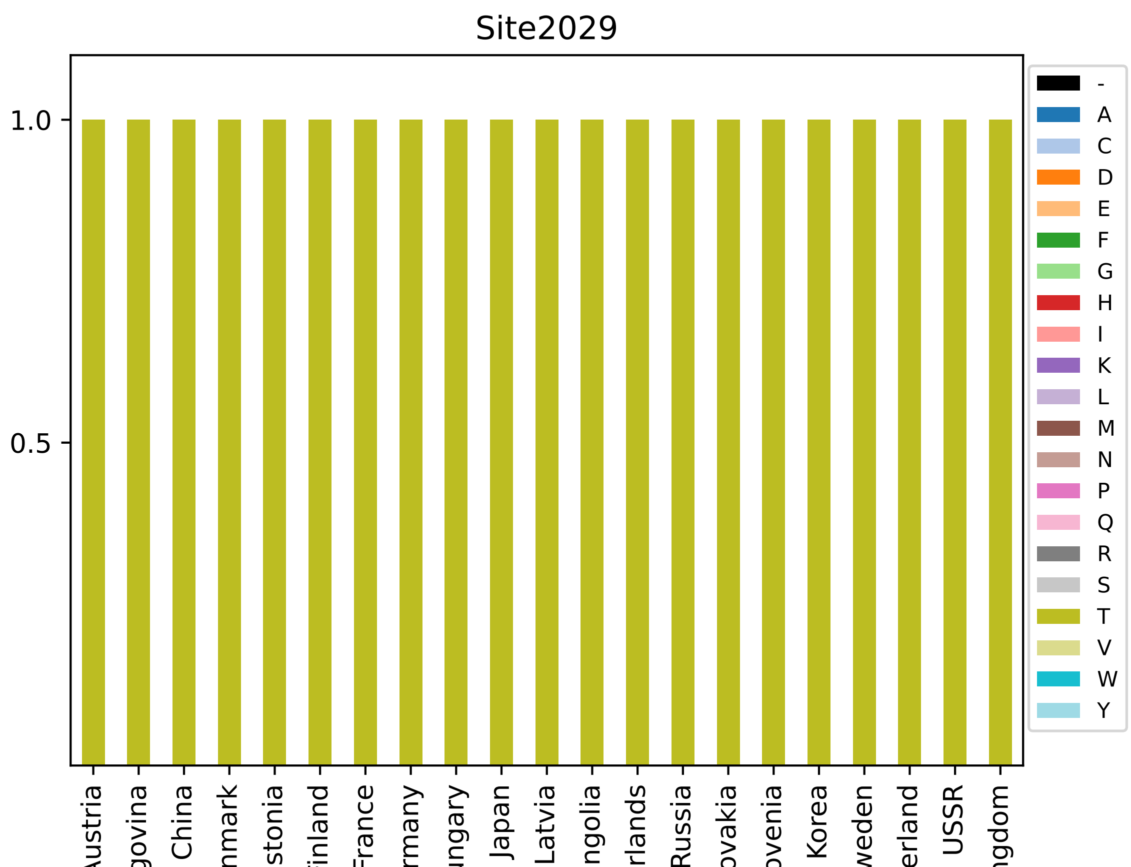

Site2029
1.0
0.5
-
A
C
D
E
F
G
H
I
K
L
M
N
P
Q
R
S
T
V
W
Y
Austria
govina
China
nmark
stonia
Finland
France
rmany
ungary
Japan
Latvia
ngolia
rlands
Russia
ovakia
ovenia
Korea
weden
erland
USSR
ngdom

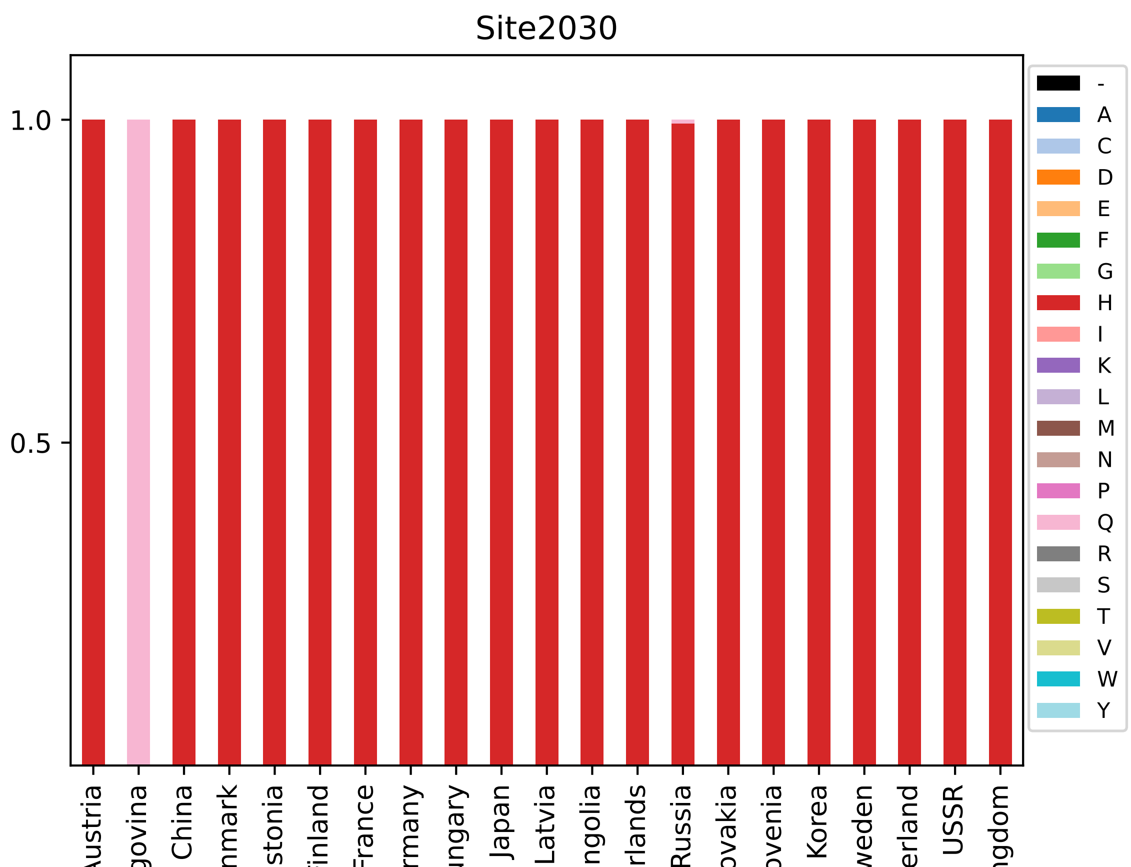
Site2030
1.0
0.5
-
A
C
D
E
F
G
H
I
K
L
M
N
P
Q
R
S
T
V
W
Y
Austria
govina
China
nmark
stonia
Finland
France
rmany
ungary
Japan
Latvia
ngolia
rlands
Russia
ovakia
ovenia
Korea
weden
erland
USSR
ngdom

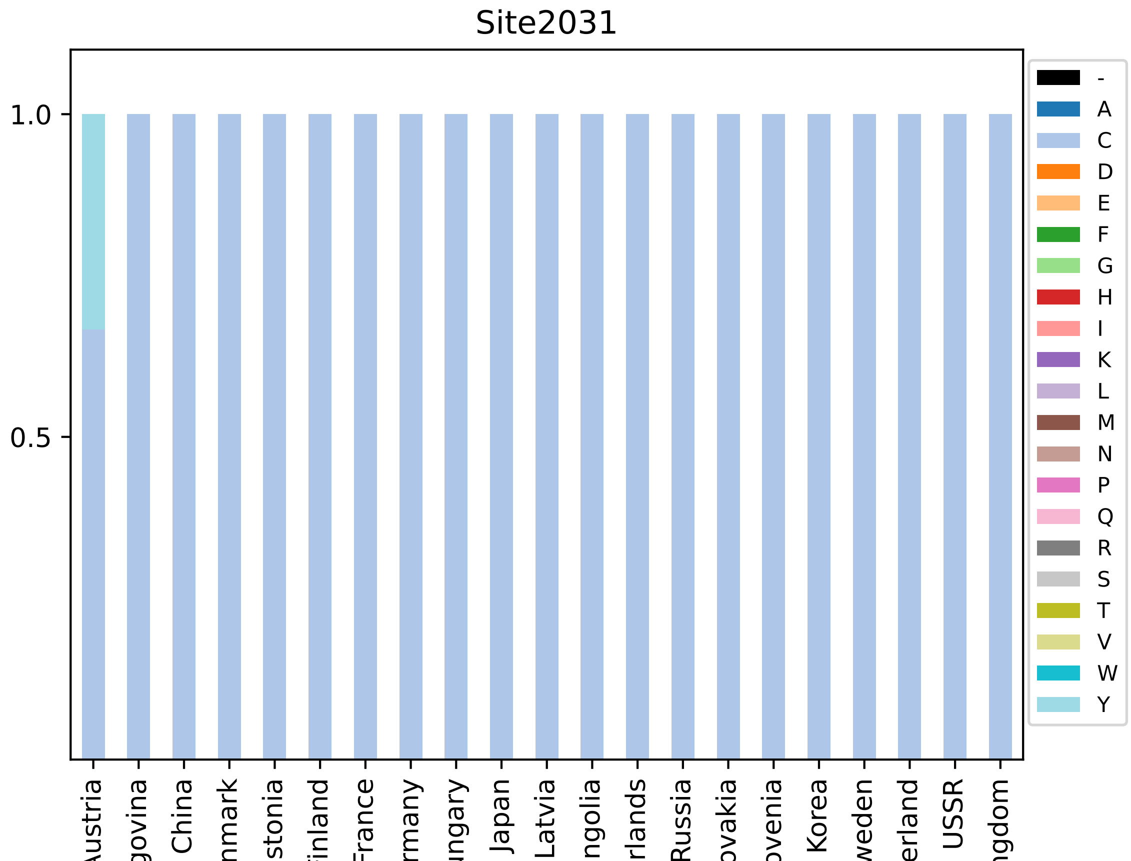

Site2031
1.0
0.5
-
A
C
D
E
F
G
H
I
K
L
M
N
P
Q
R
S
T
V
W
Y
China
Finland
France
Japan
Latvia
Russia
Korea
USSR

Site2032

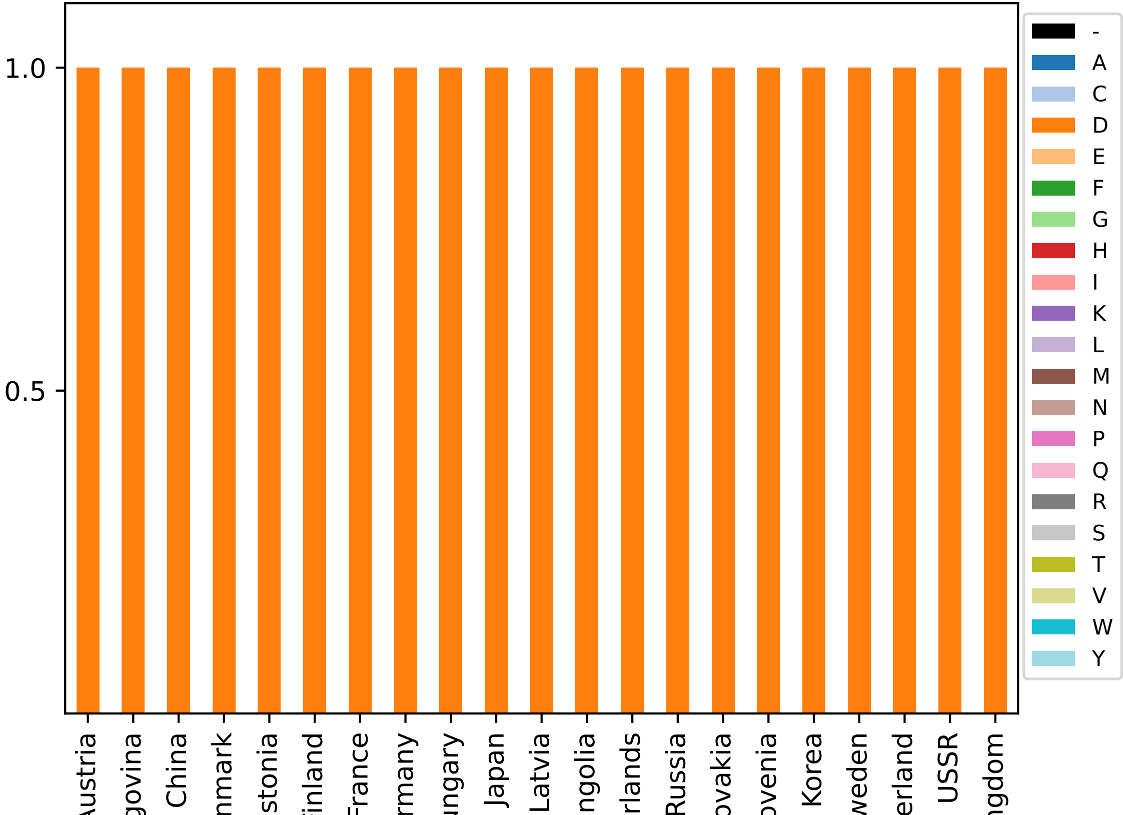

1.0
0.5
-
A
C
D
E
F
G
H
I
K
L
M
N
P
Q
R
S
T
V
W
Y
China
Japan
Latvia
Russia
Korea
USSR

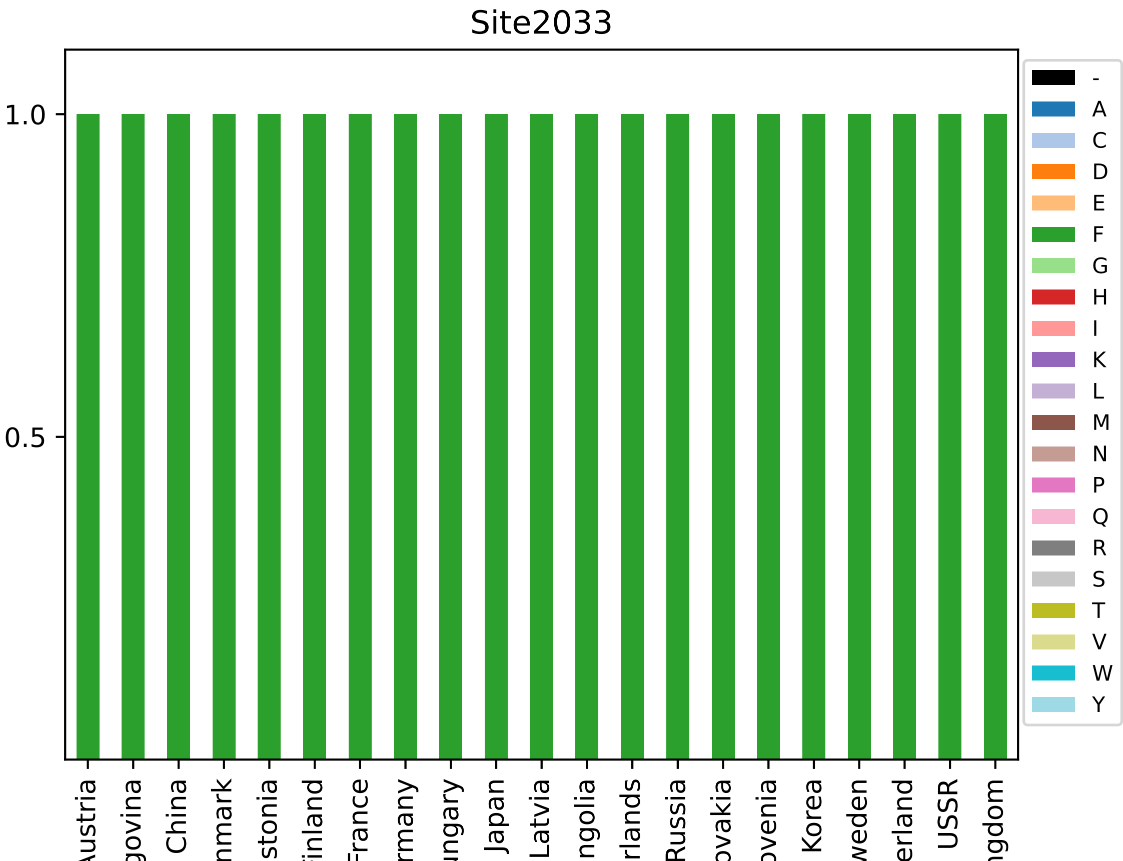

Site2033
1.0
0.5
-
A
C
D
E
F
G
H
I
K
L
M
N
P
Q
R
S
T
V
W
Y
Austria
govina
China
nmark
stonia
inland
France
rmany
ungary
Japan
Latvia
ngolia
rlands
Russia
ovakia
ovenia
Korea
weden
erland
USSR
ngdom

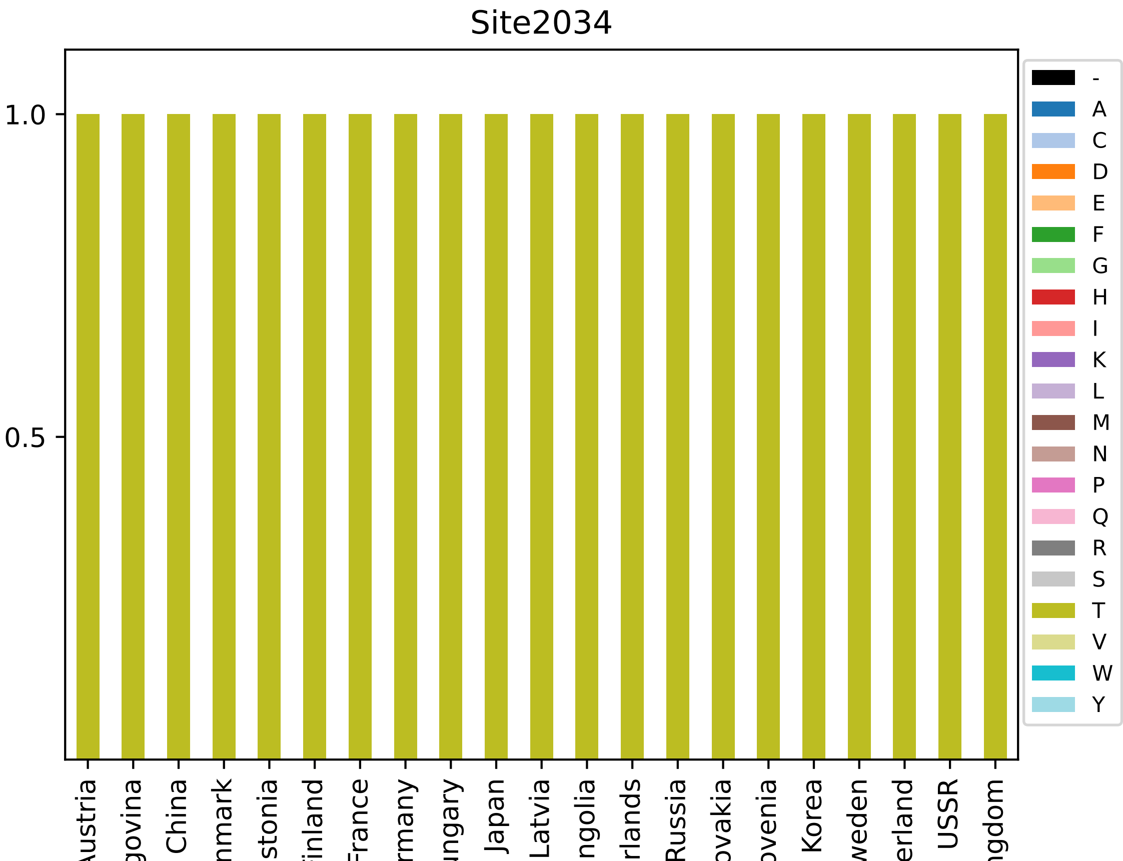

Site2034
1.0
0.5
-
A
C
D
E
F
G
H
I
K
L
M
N
P
Q
R
S
T
V
W
Y
Austria
China
Japan
Latvia
Russia
Korea
USSR

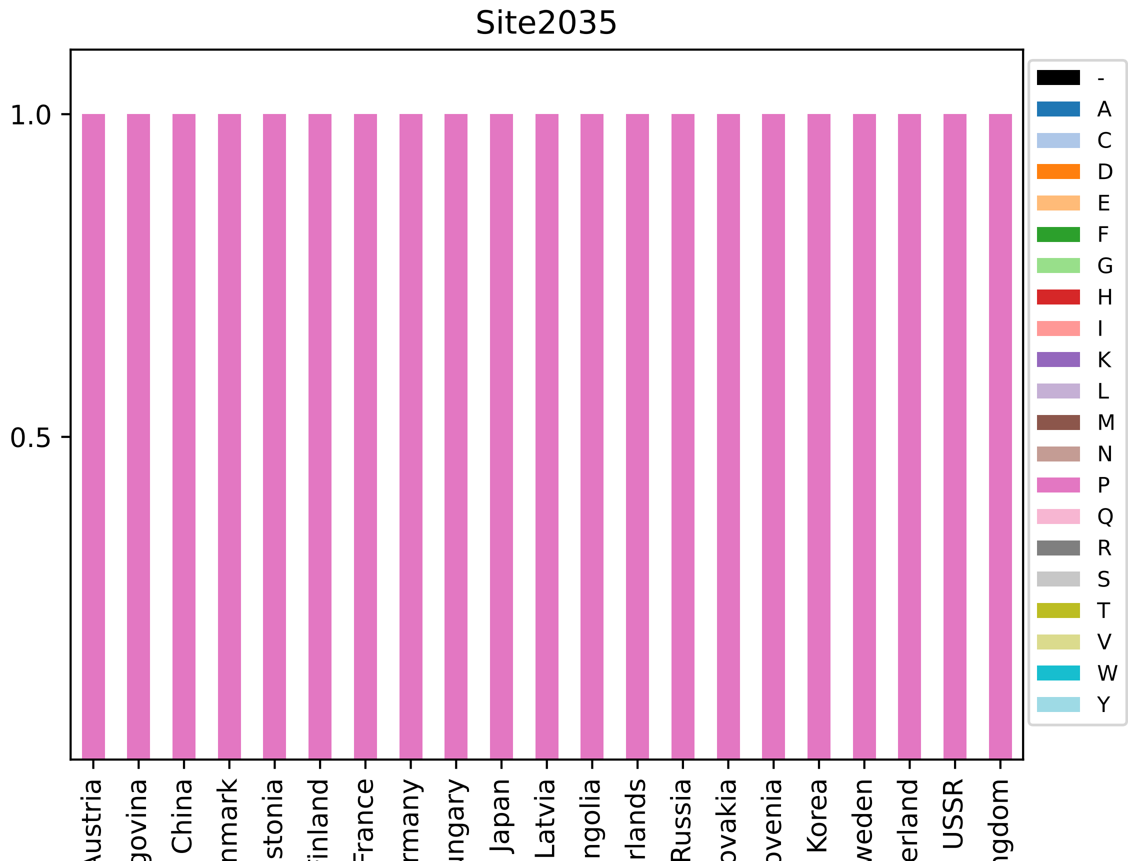

Site2035
1.0
0.5
-
A
C
D
E
F
G
H
I
K
L
M
N
P
Q
R
S
T
V
W
Y
China
Japan
Latvia
Russia
Korea
USSR

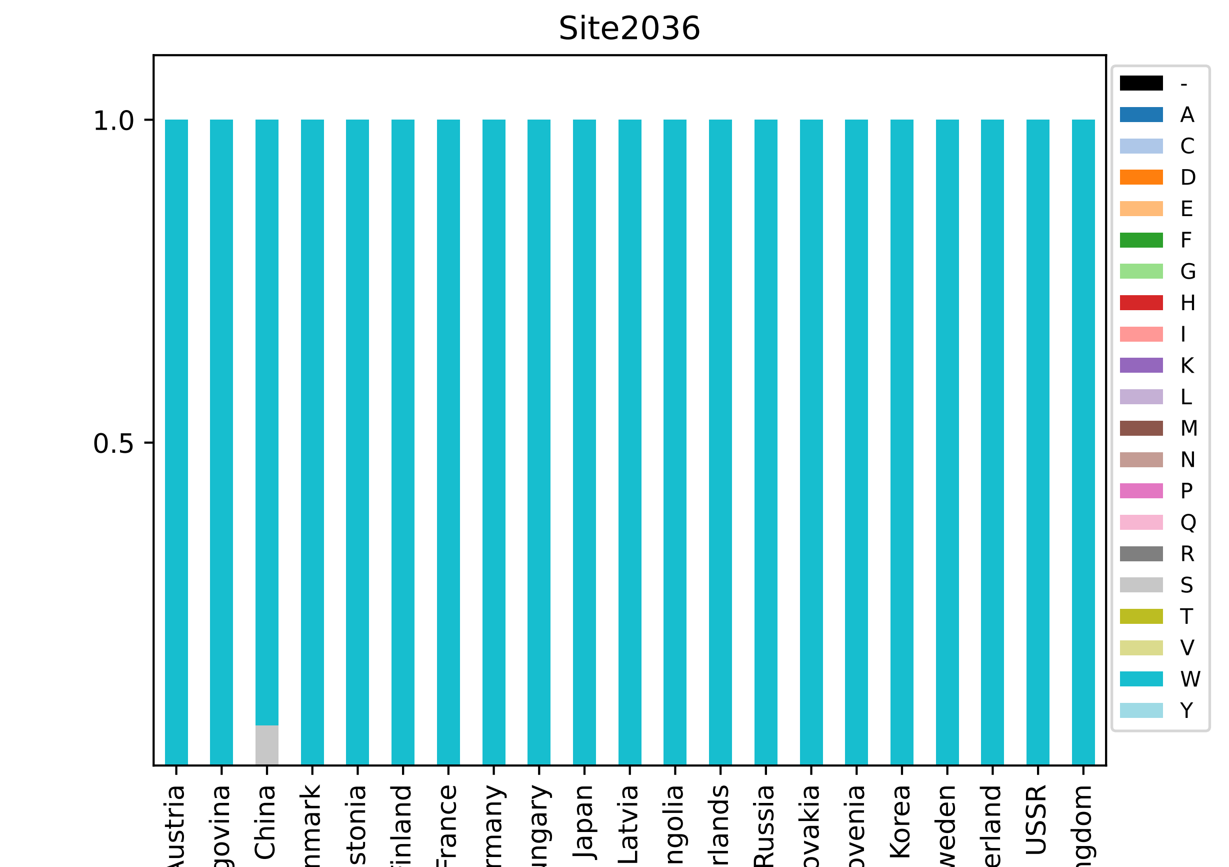
Site2036
1.0
0.5
-
A
C
D
E
F
G
H
I
K
L
M
N
P
Q
R
S
T
V
W
Y
Austria
govina
China
nmark
stonia
inland
France
rmany
ungary
Japan
Latvia
ngolia
rlands
Russia
ovakia
ovenia
Korea
weden
erland
USSR
ngdom

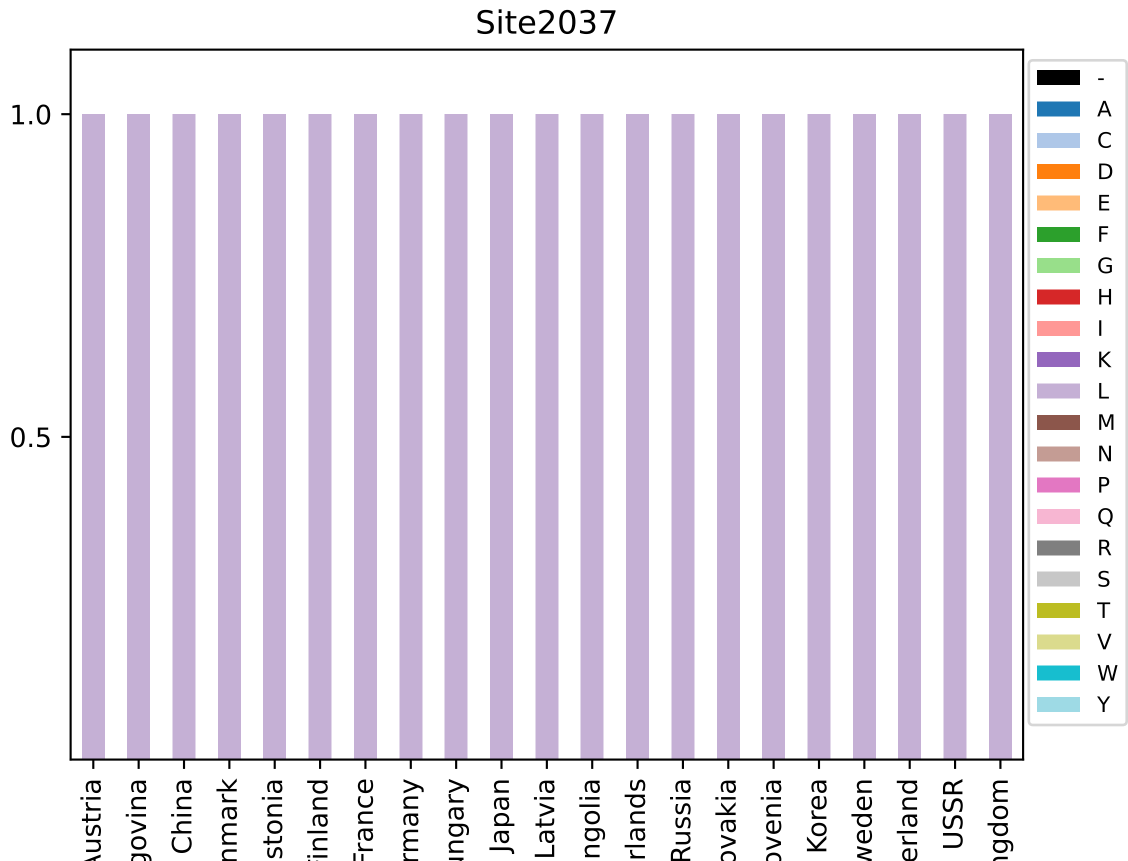

Site2037
1.0
0.5
-
A
C
D
E
F
G
H
I
K
L
M
N
P
Q
R
S
T
V
W
Y
China
Japan
Latvia
Russia
Korea
USSR

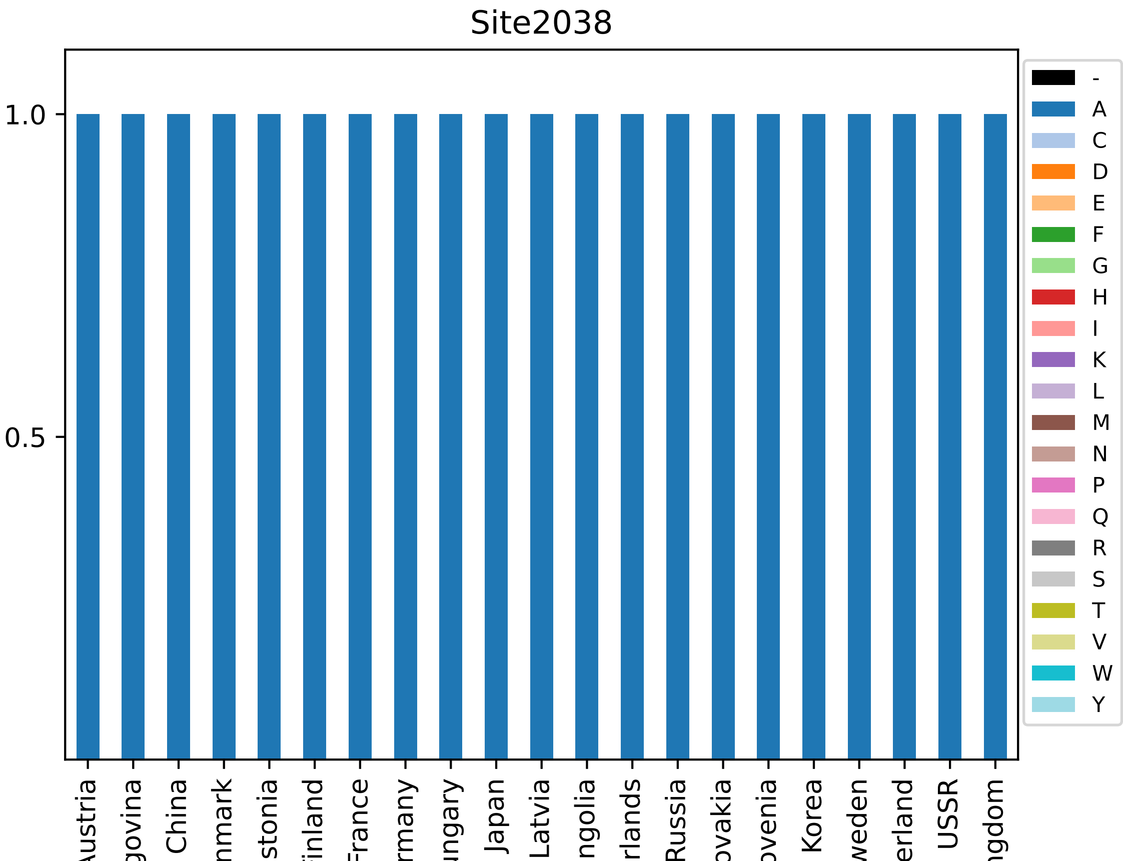
Site2038
1.0
0.5
-
A
C
D
E
F
G
H
I
K
L
M
N
P
Q
R
S
T
V
W
Y
China
Japan
Latvia
Russia
Korea
USSR

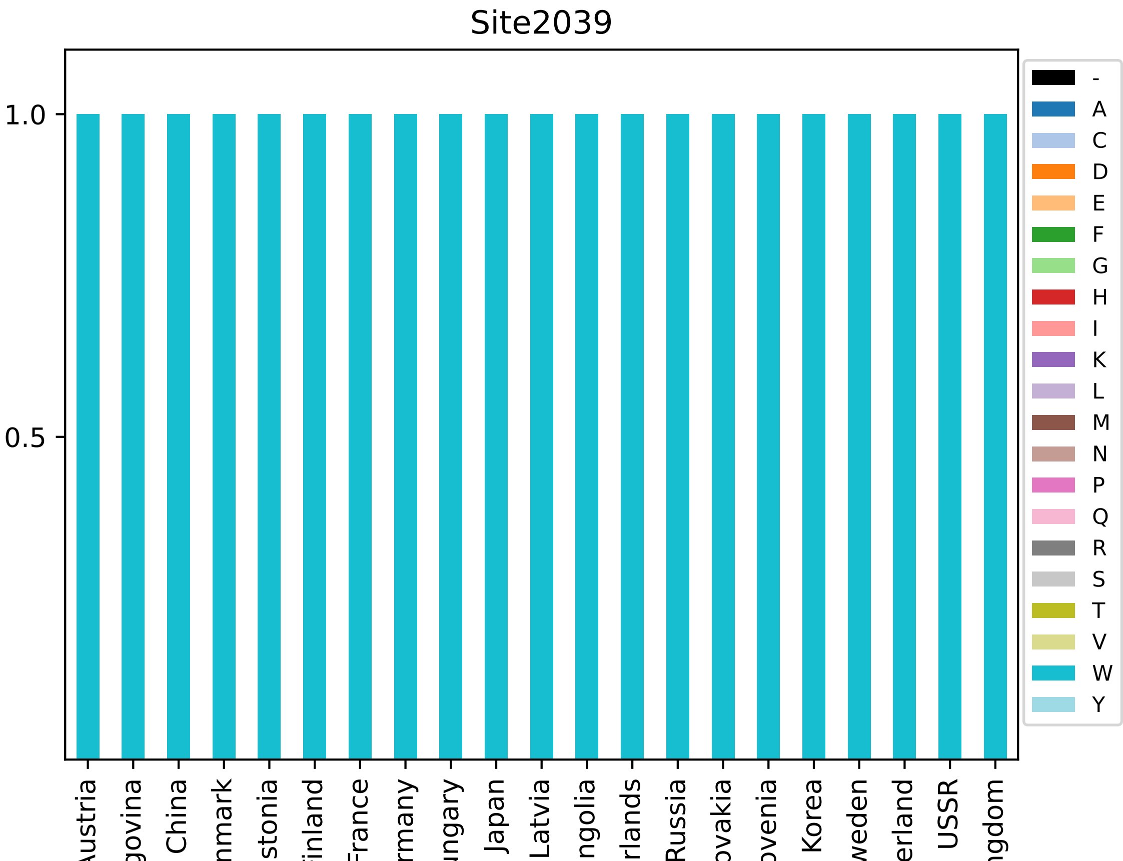

Site2039
1.0
0.5
-
A
C
D
E
F
G
H
I
K
L
M
N
P
Q
R
S
T
V
W
Y
China
Japan
Latvia
Russia
Korea
USSR

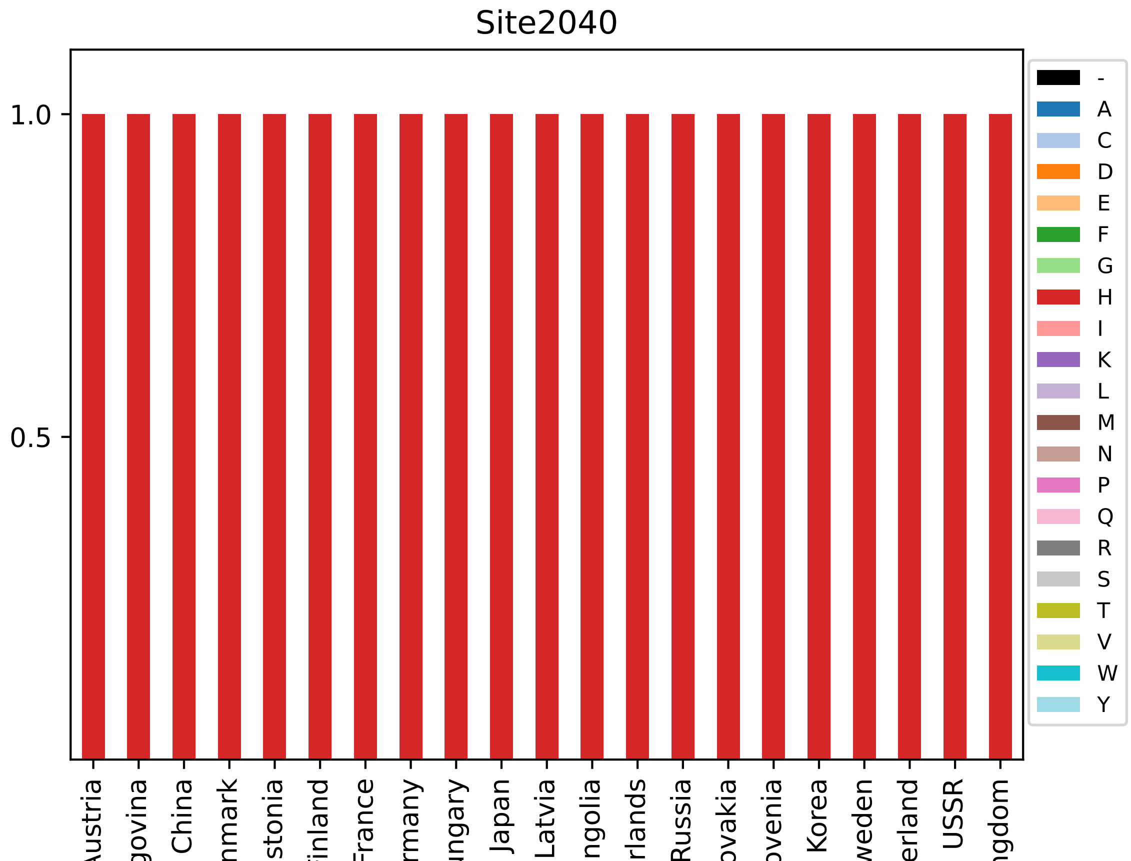
Site2040
1.0
0.5
-
A
C
D
E
F
G
H
I
K
L
M
N
P
Q
R
S
T
V
W
Y
China
Japan
Latvia
Russia
Korea
USSR

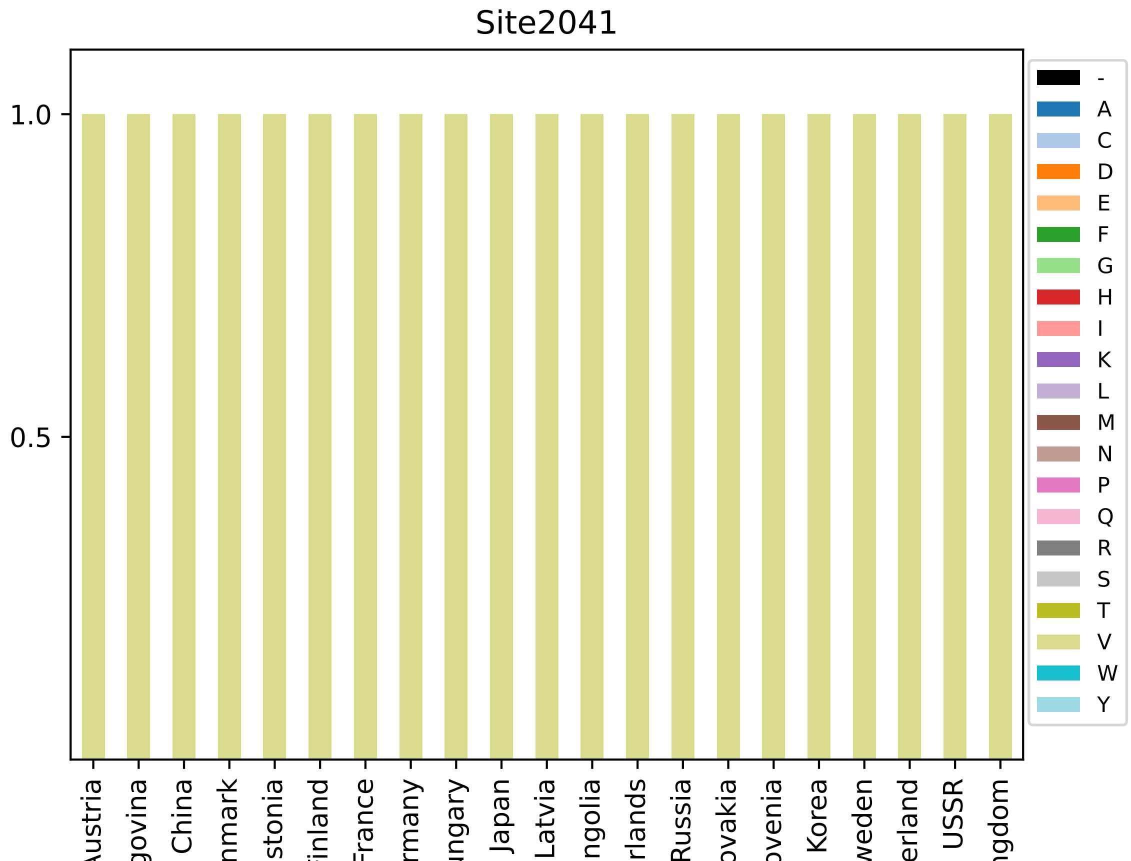

Site2041
1.0
0.5
-
A
C
D
E
F
G
H
I
K
L
M
N
P
Q
R
S
T
V
W
Y
China
Japan
Latvia
Russia
Korea
USSR

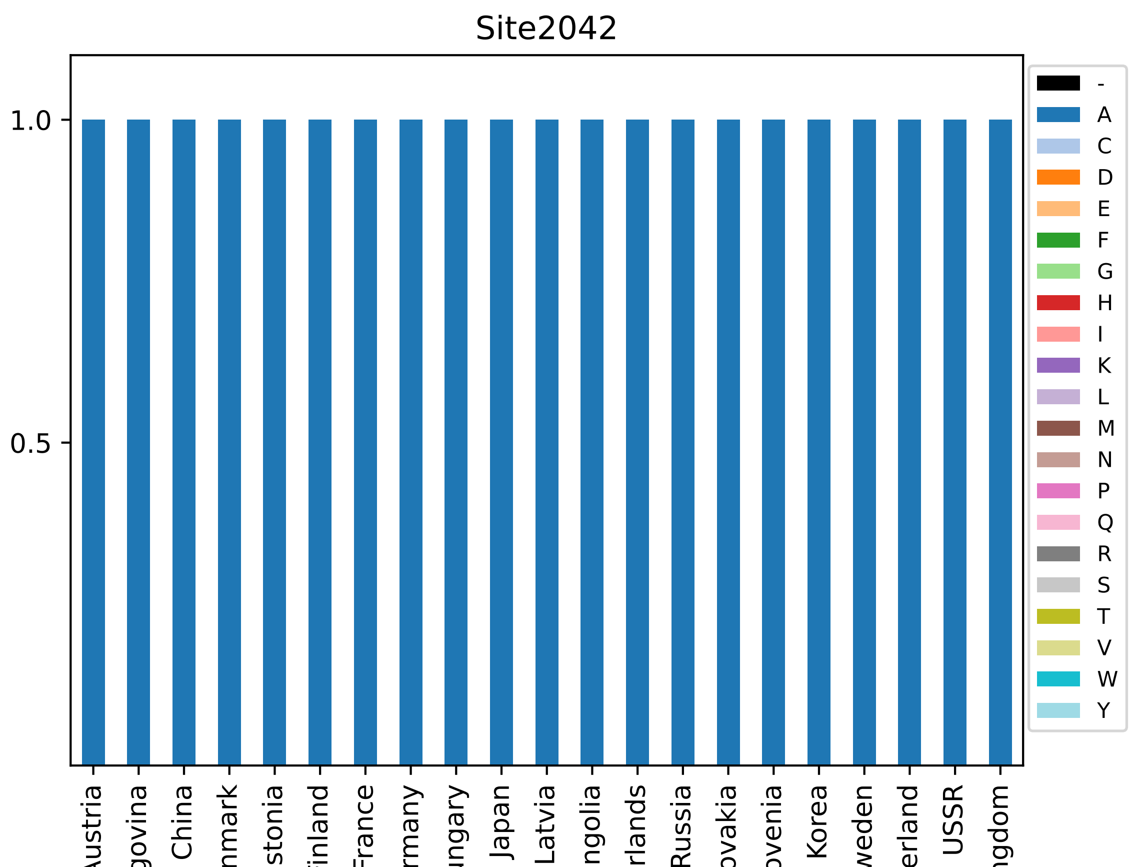

Site2042
1.0
0.5
-
A
C
D
E
F
G
H
I
K
L
M
N
P
Q
R
S
T
V
W
Y
China
Japan
Latvia
Russia
Korea
USSR

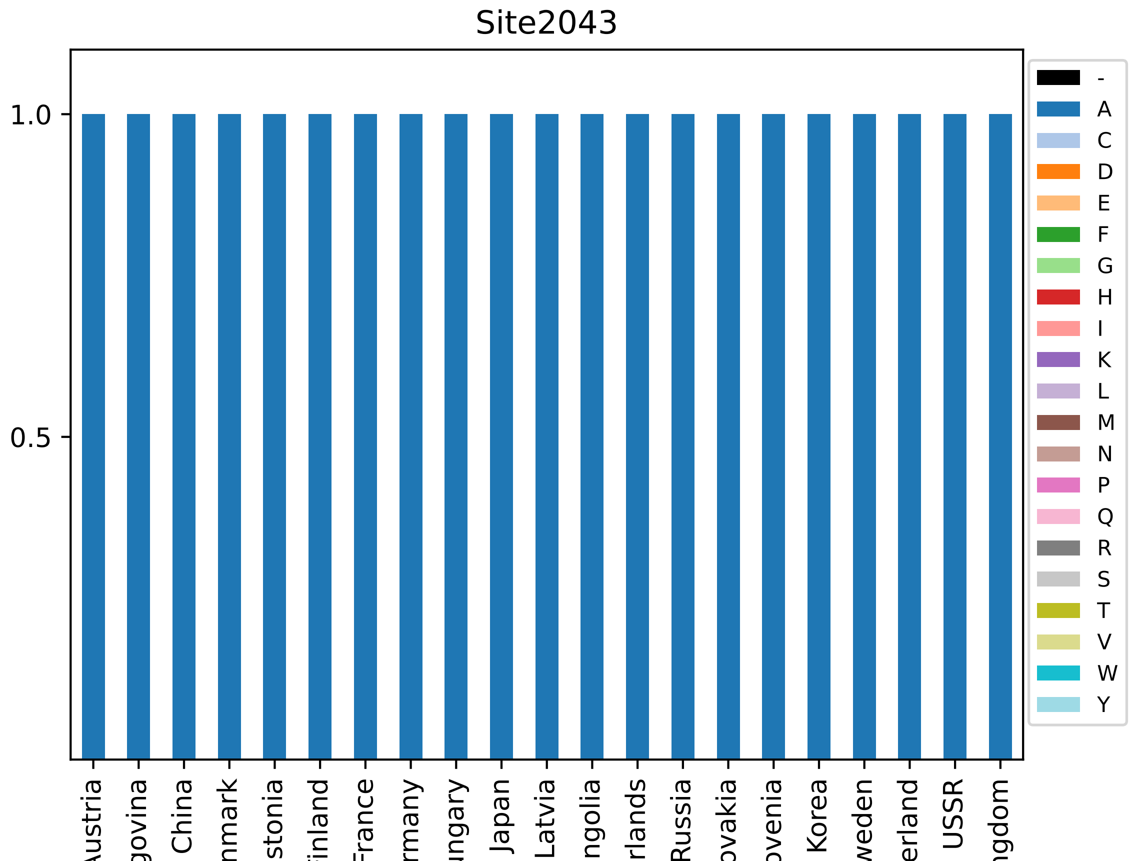

Site2043
1.0
0.5
-
A
C
D
E
F
G
H
I
K
L
M
N
P
Q
R
S
T
V
W
Y
Austria
China
Japan
Latvia
Russia
Korea
USSR

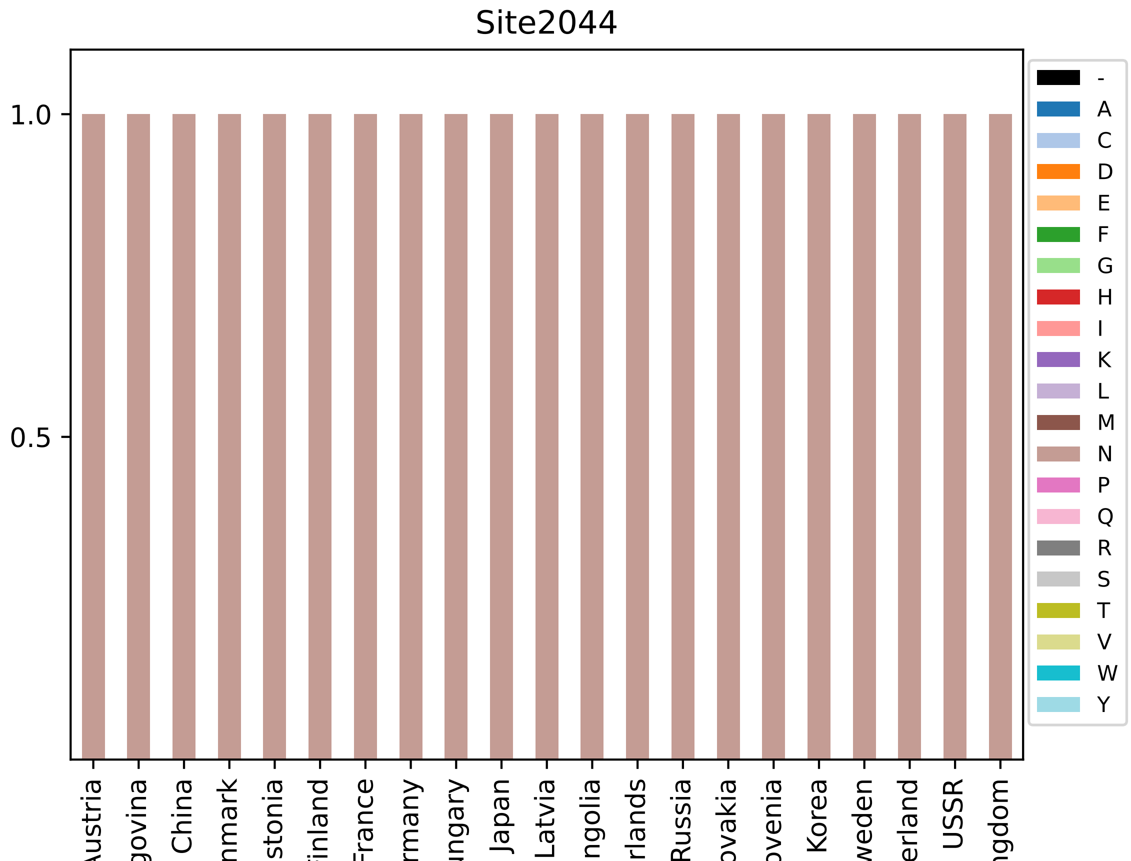
Site2044
1.0
0.5
-
A
C
D
E
F
G
H
I
K
L
M
N
P
Q
R
S
T
V
W
Y
Austria
govina
China
nmark
stonia
Finland
France
rmany
ungary
Japan
Latvia
ngolia
rlands
Russia
ovakia
ovenia
Korea
weden
erland
USSR
ngdom

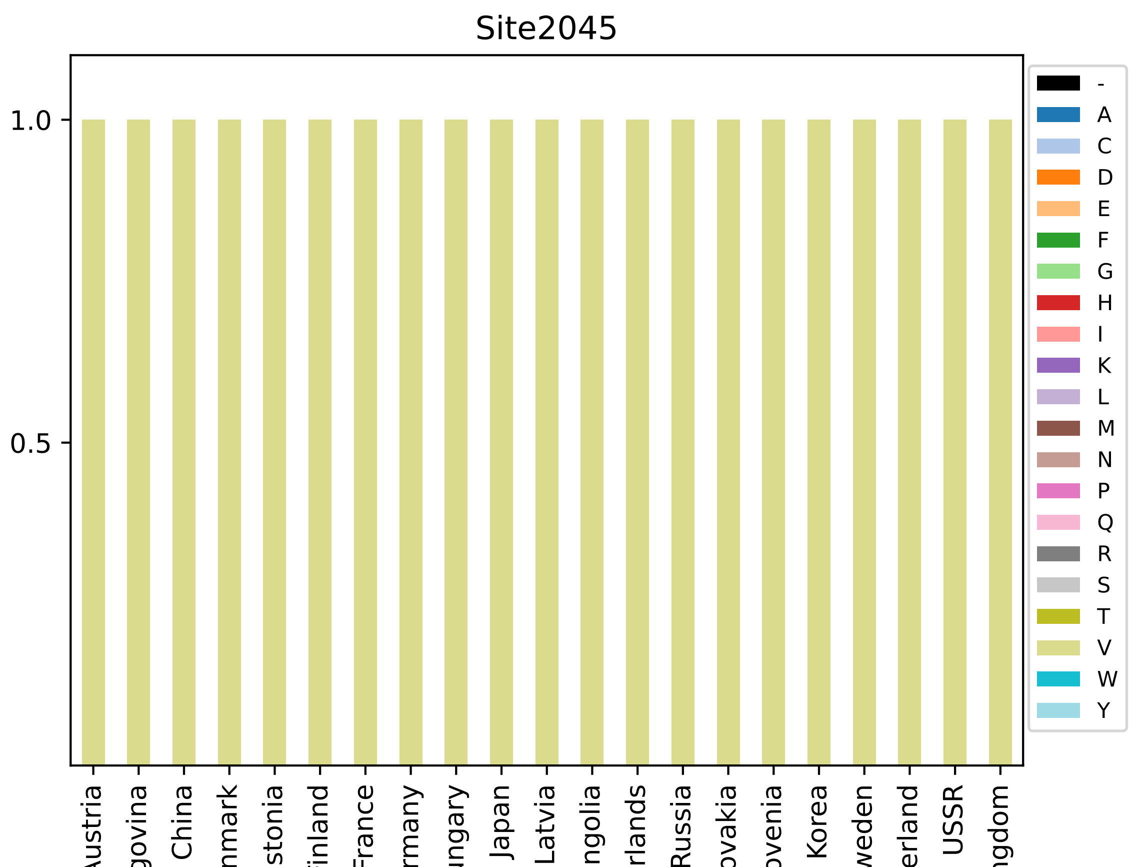

Site2045
1.0
0.5
-
A
C
D
E
F
G
H
I
K
L
M
N
P
Q
R
S
T
V
W
Y
China
Japan
Latvia
Russia
Korea
USSR

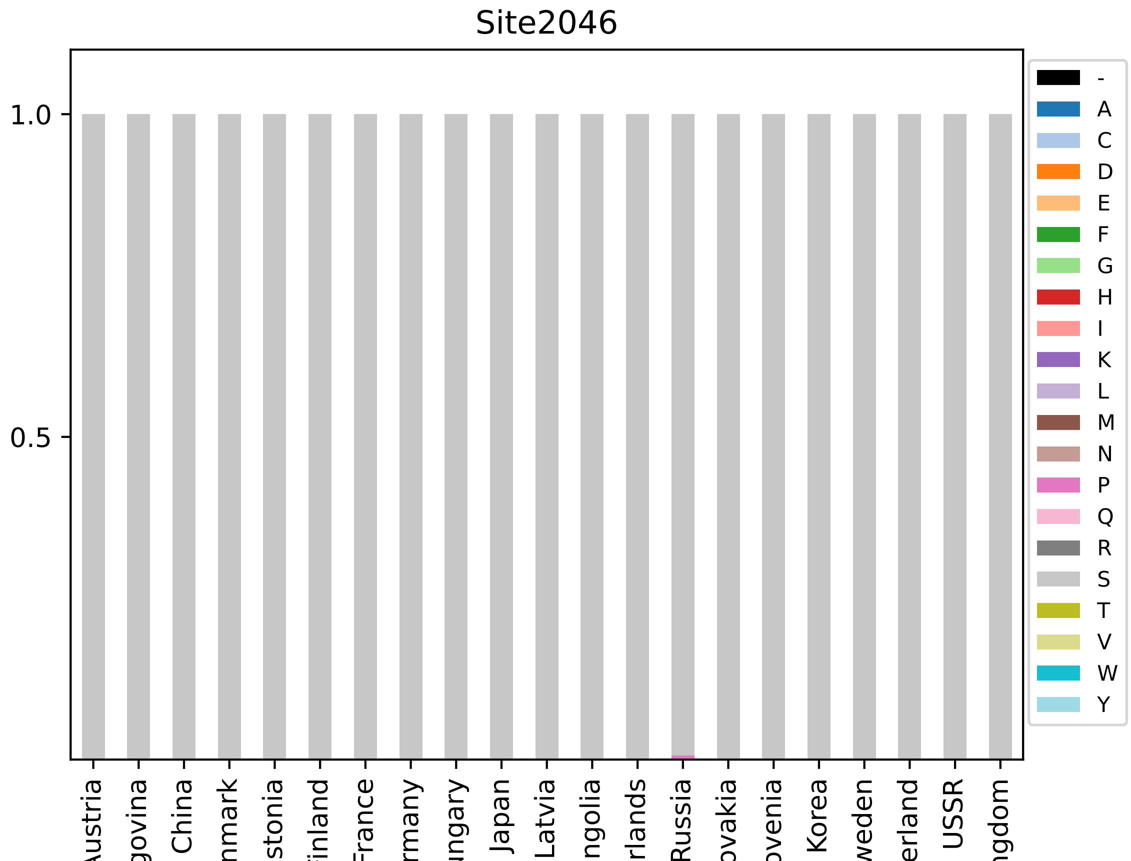

Site2046
1.0
0.5
Austria
govina
China
nmark
stonia
Finland
France
rmany
ungary
Japan
Latvia
ngolia
rlands
Russia
ovakia
ovenia
Korea
weden
erland
USSR
ngdom
-
A
C
D
E
F
G
H
I
K
L
M
N
P
Q
R
S
T
V
W
Y

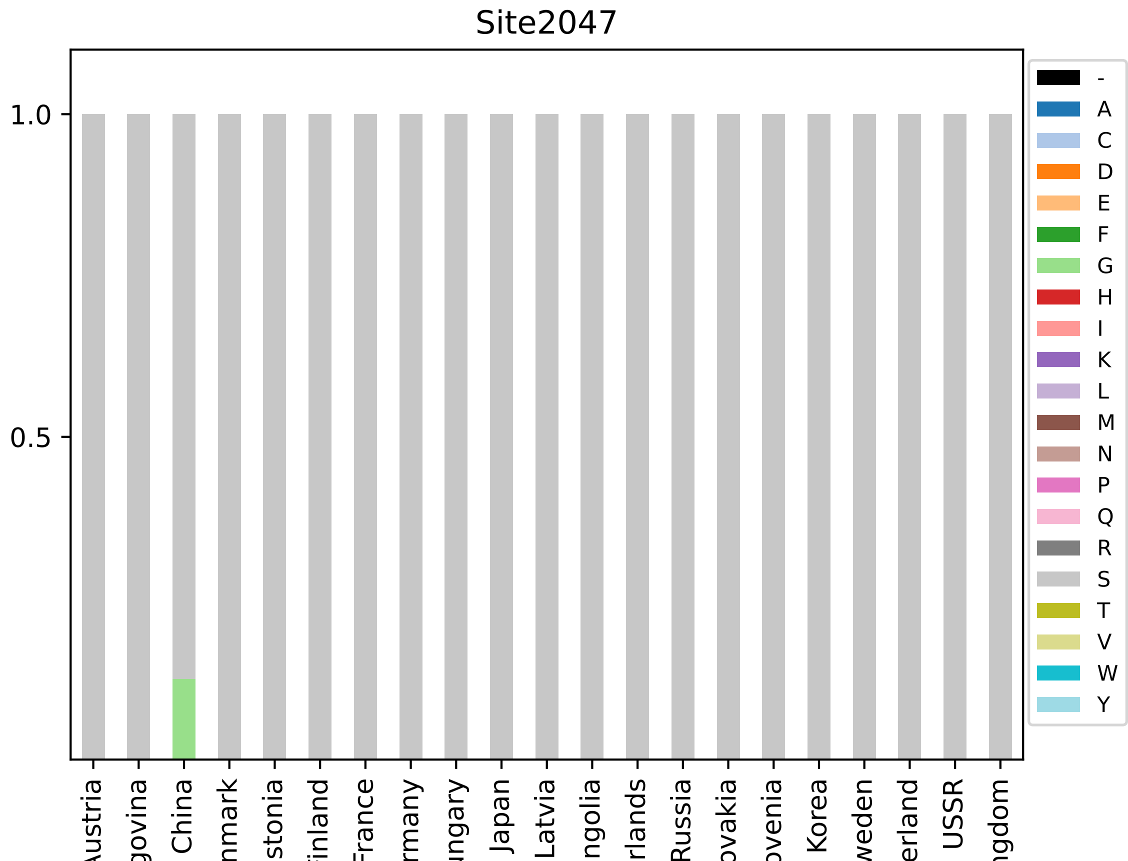

Site2047
1.0
0.5
-
A
C
D
E
F
G
H
I
K
L
M
N
P
Q
R
S
T
V
W
Y
Austria
govina
China
nmark
stonia
inland
France
rmany
ungary
Japan
Latvia
ngolia
rlands
Russia
ovakia
ovenia
Korea
weden
erland
USSR
ngdom

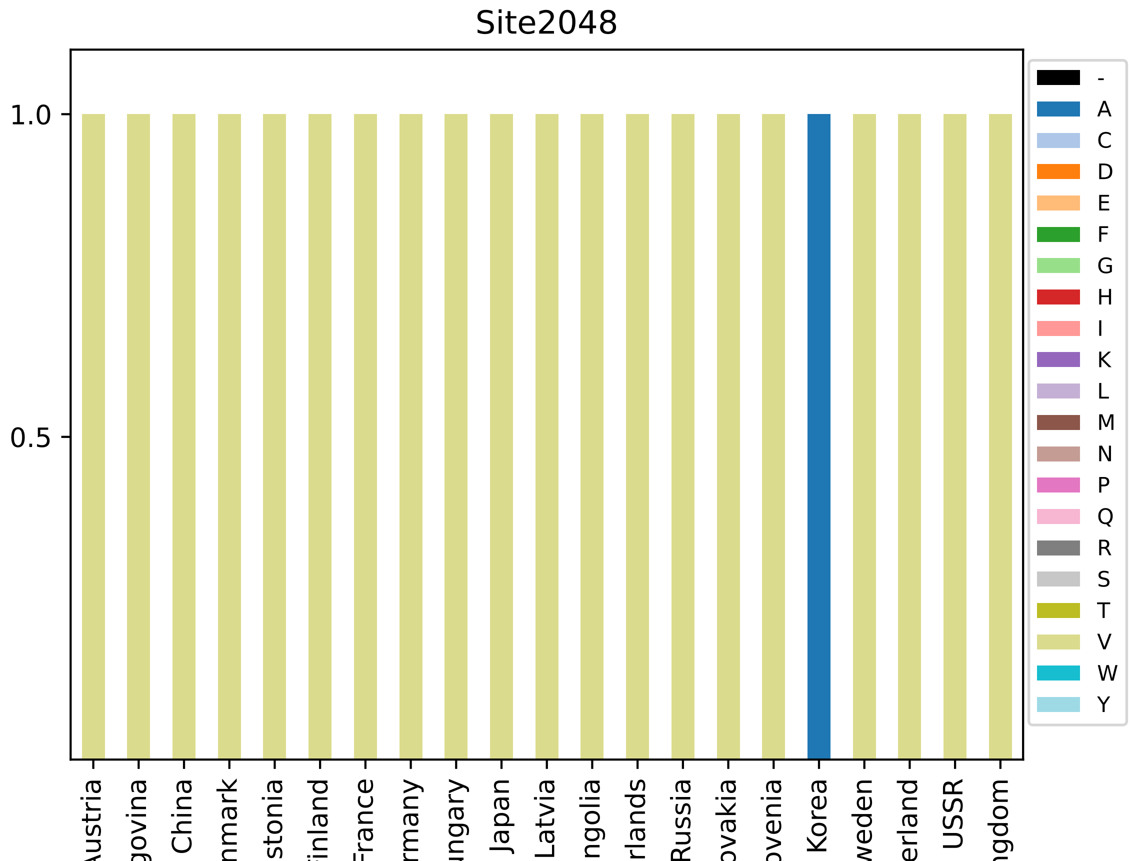

Site2048
1.0
0.5
-
A
C
D
E
F
G
H
I
K
L
M
N
P
Q
R
S
T
V
W
Y
Austria
China
Japan
Latvia
Russia
Korea
USSR

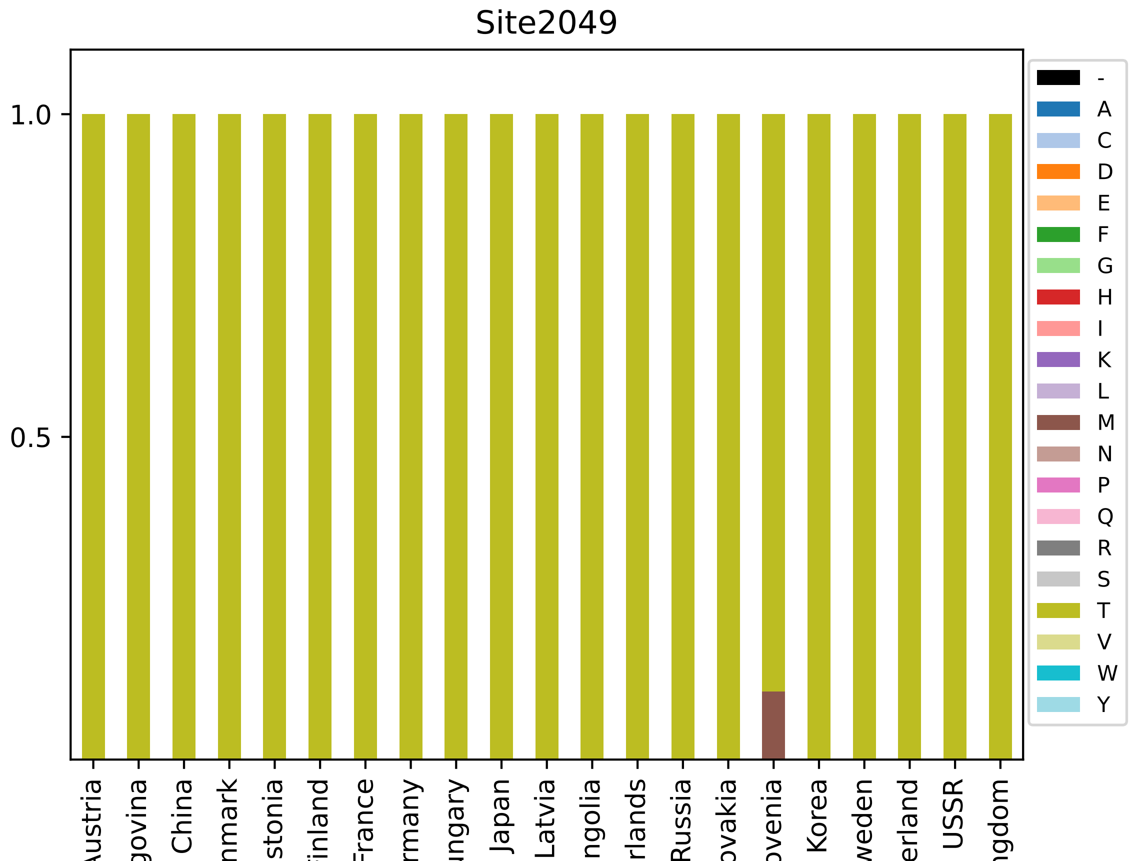

Site2049
1.0
0.5
-
A
C
D
E
F
G
H
I
K
L
M
N
P
Q
R
S
T
V
W
Y
China
Japan
Latvia
Russia
Korea
USSR

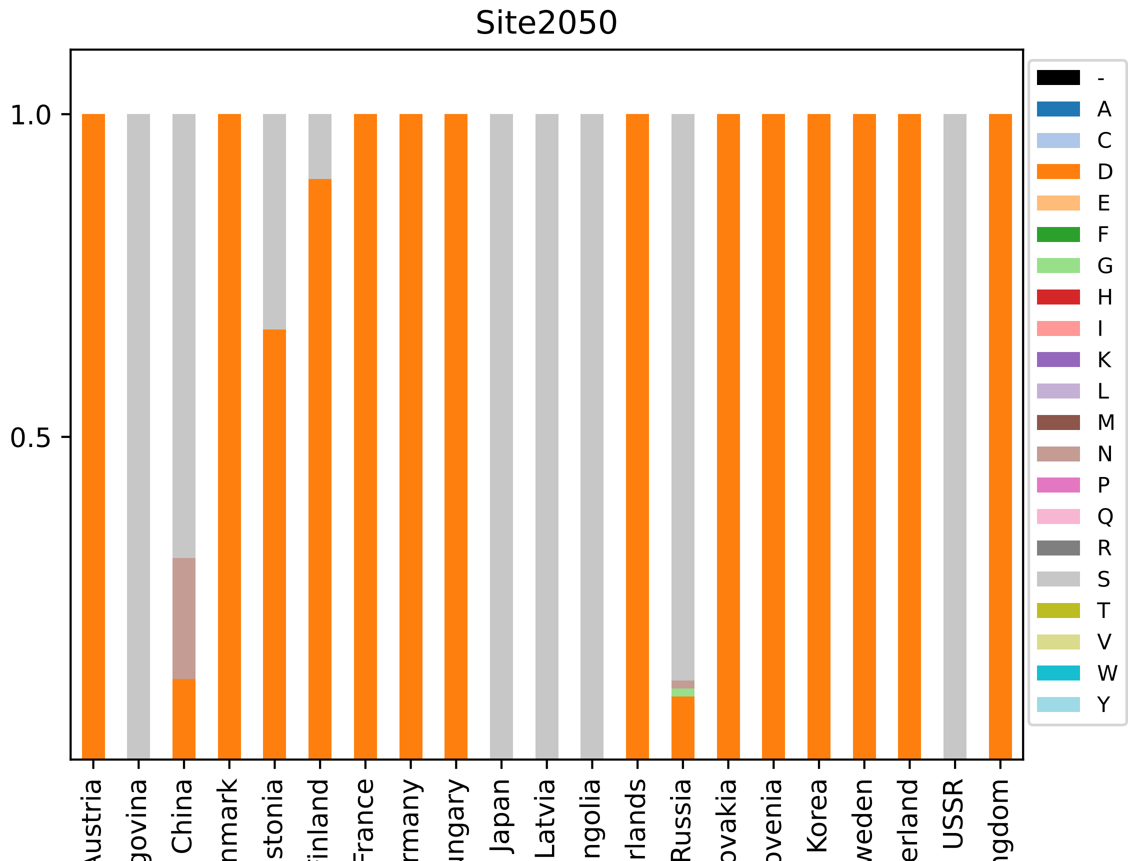

Site2050
1.0
0.5
China
Japan
Latvia
Russia
Korea
USSR
-
A
C
D
E
F
G
H
I
K
L
M
N
P
Q
R
S
T
V
W
Y

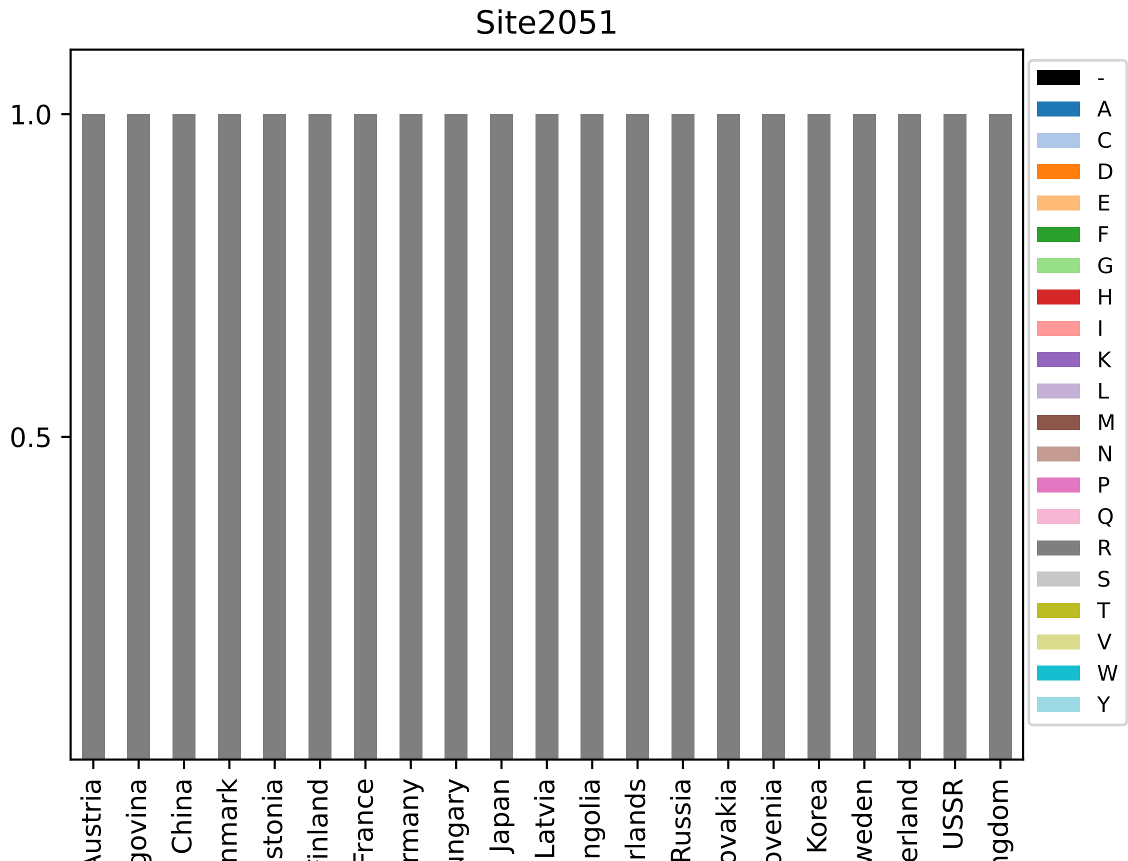
Site2051
1.0
0.5
-
A
C
D
E
F
G
H
I
K
L
M
N
P
Q
R
S
T
V
W
Y
China
Japan
Latvia
Russia
Korea
USSR

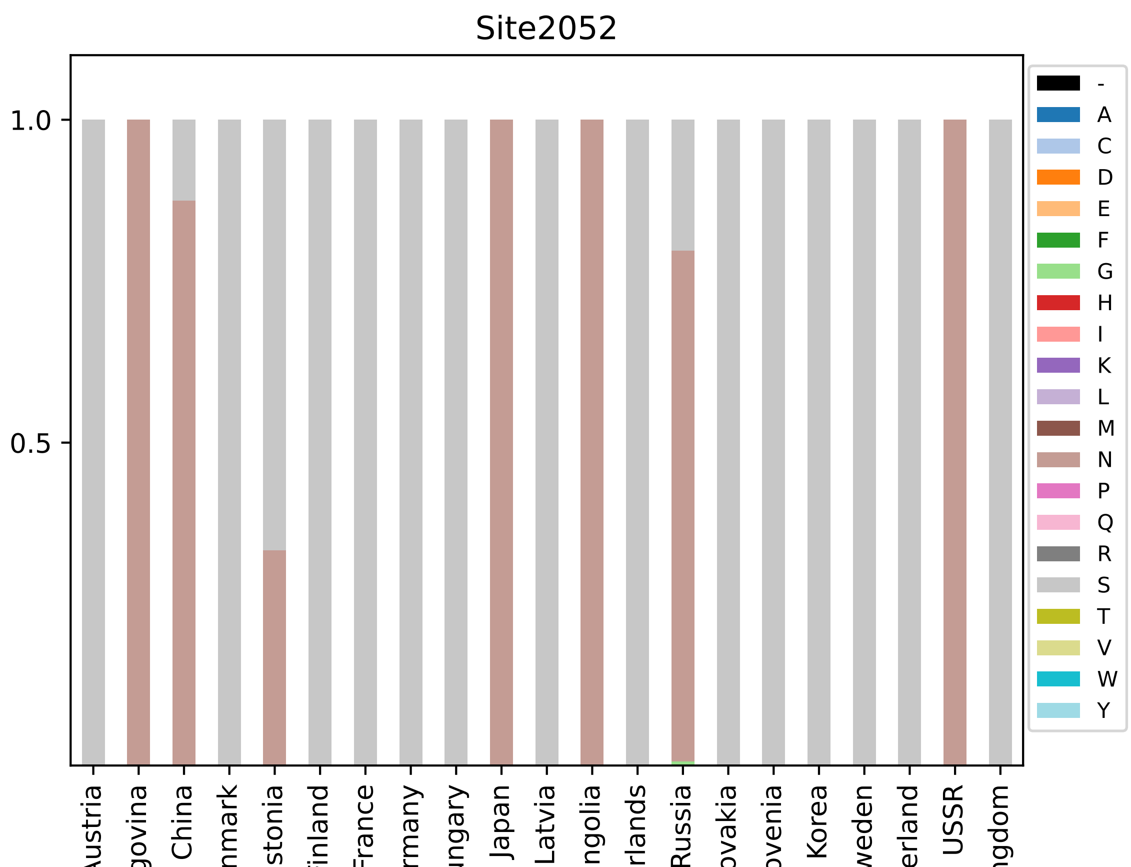

Site2052
1.0
0.5
Austria
govina
China
nmark
stonia
inland
France
rmany
ungary
Japan
Latvia
ngolia
rlands
Russia
ovakia
ovenia
Korea
weden
erland
USSR
ngdom
-
A
C
D
E
F
G
H
I
K
L
M
N
P
Q
R
S
T
V
W
Y

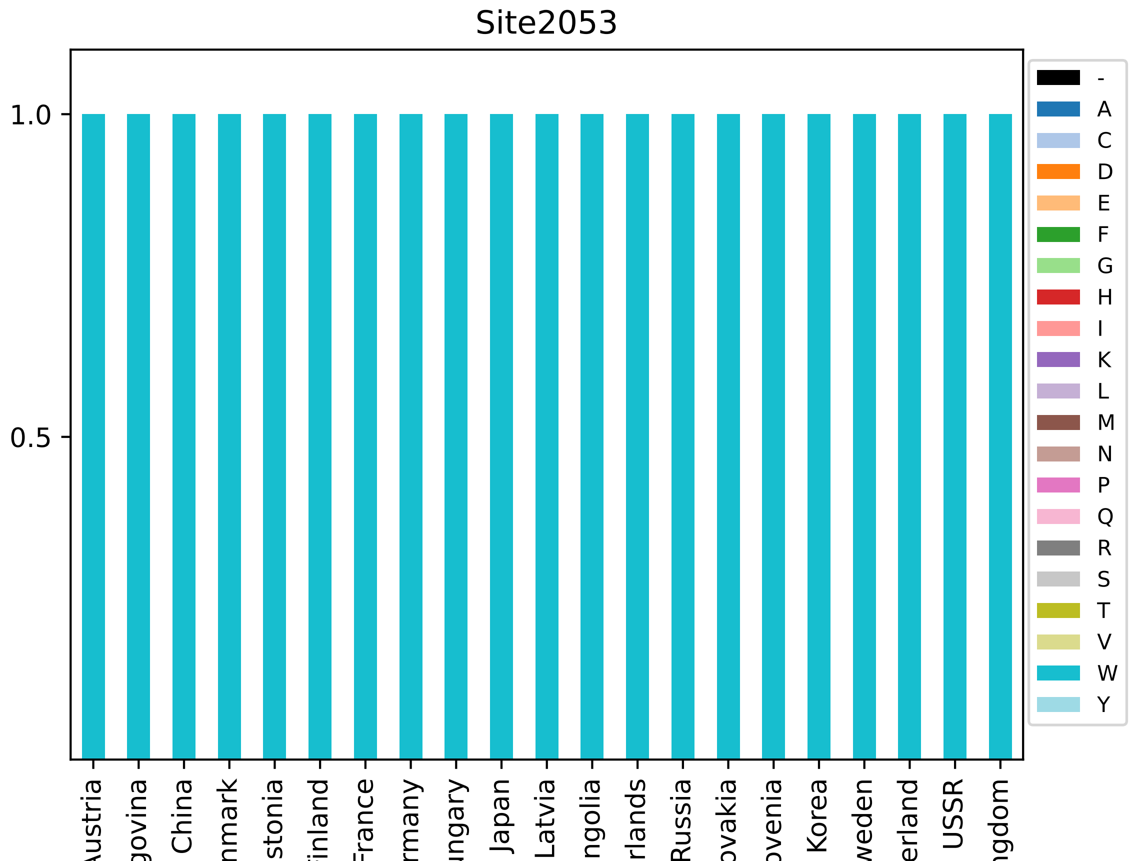

Site2053
1.0
0.5
-
A
C
D
E
F
G
H
I
K
L
M
N
P
Q
R
S
T
V
W
Y
China
Japan
Latvia
Russia
Korea
USSR

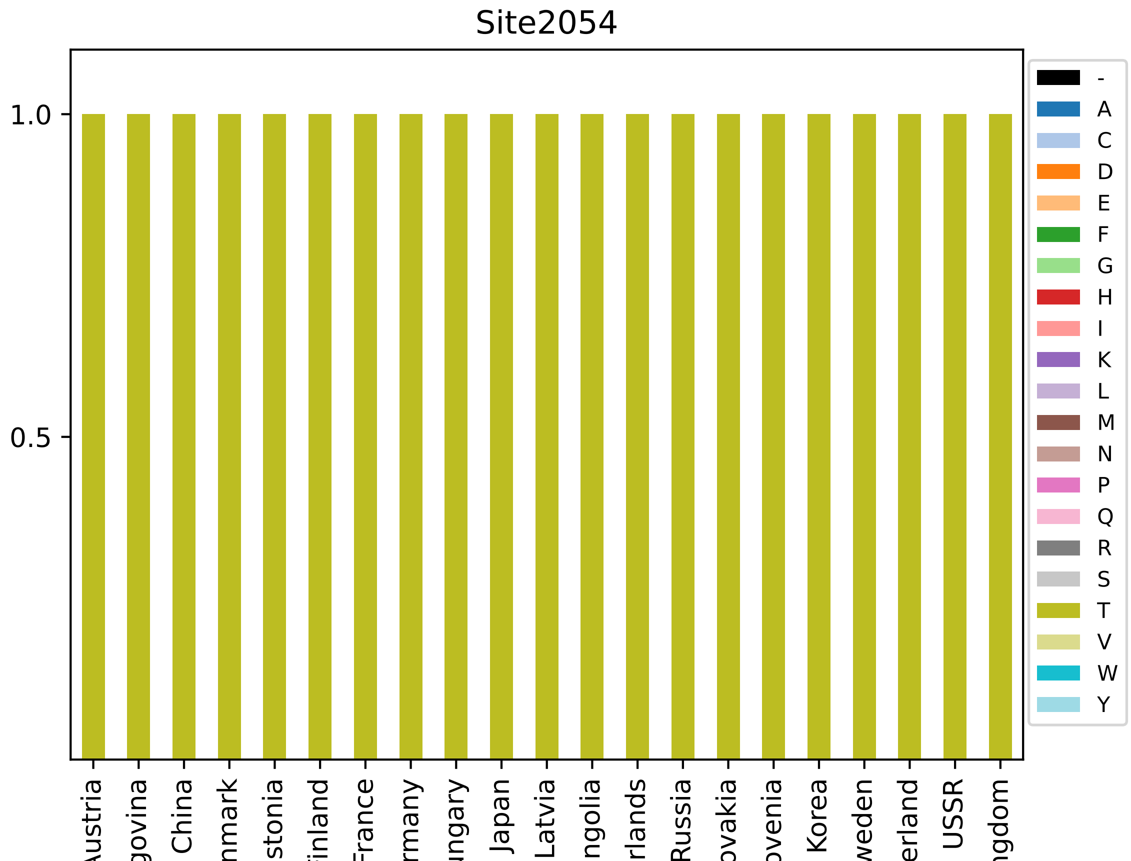

Site2054
1.0
0.5
-
A
C
D
E
F
G
H
I
K
L
M
N
P
Q
R
S
T
V
W
Y
Austria
govina
China
nmark
stonia
Finland
France
rmany
ungary
Japan
Latvia
ngolia
rlands
Russia
ovakia
ovenia
Korea
weden
erland
USSR
ngdom

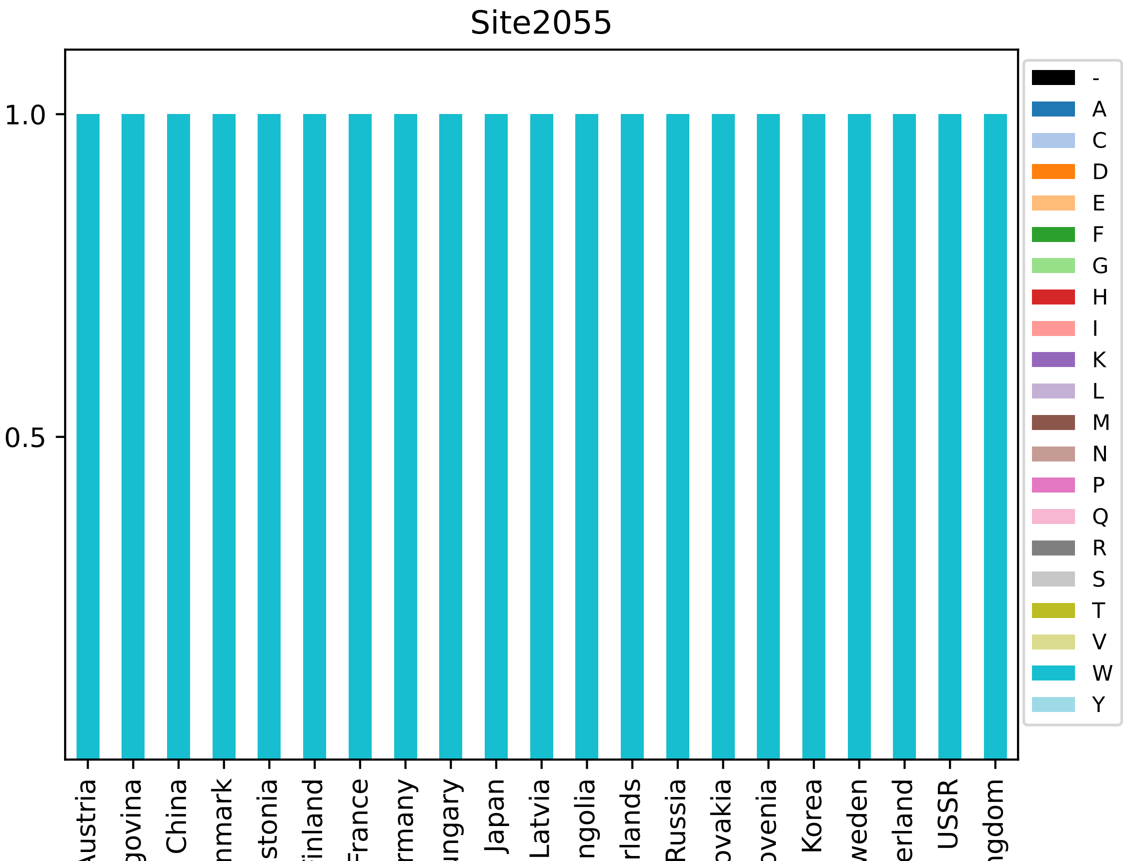
Site2055
1.0
0.5
-
A
C
D
E
F
G
H
I
K
L
M
N
P
Q
R
S
T
V
W
Y
China
Japan
Latvia
Russia
Korea
USSR

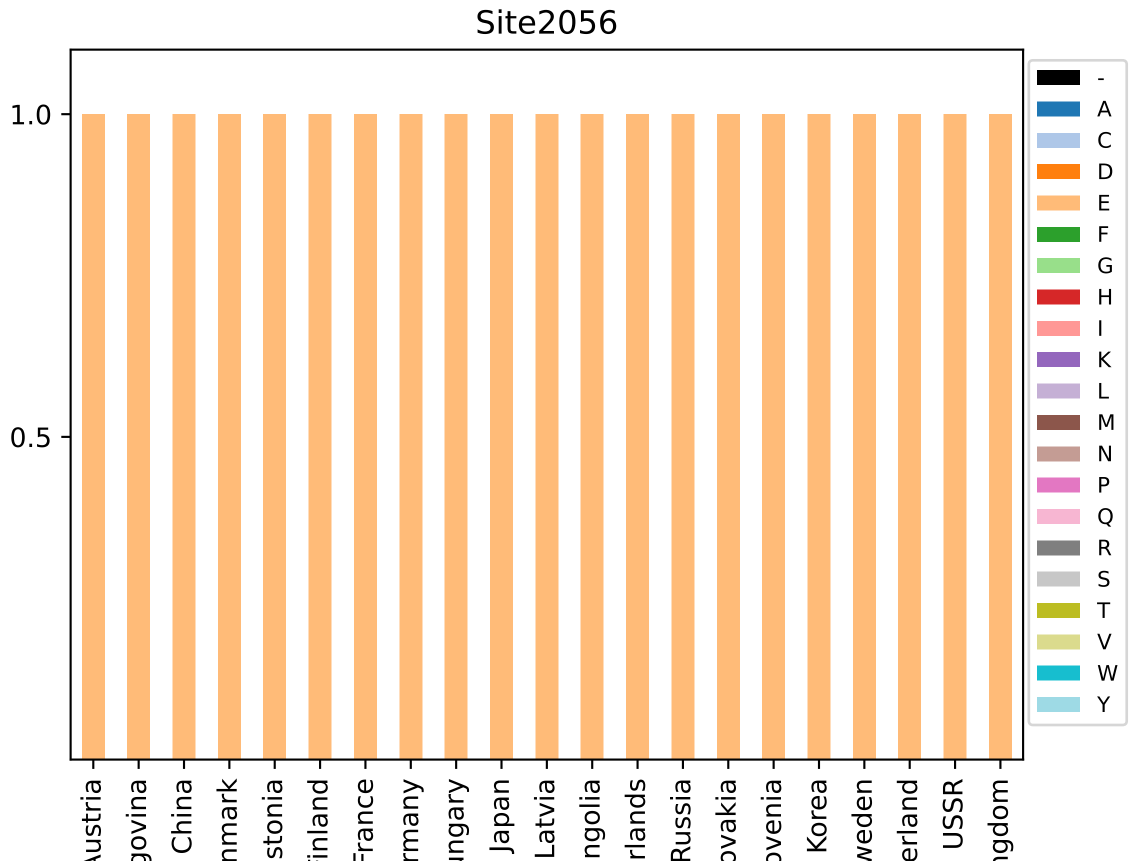

Site2056
1.0
0.5
-
A
C
D
E
F
G
H
I
K
L
M
N
P
Q
R
S
T
V
W
Y
China
Japan
Latvia
Russia
Korea
USSR

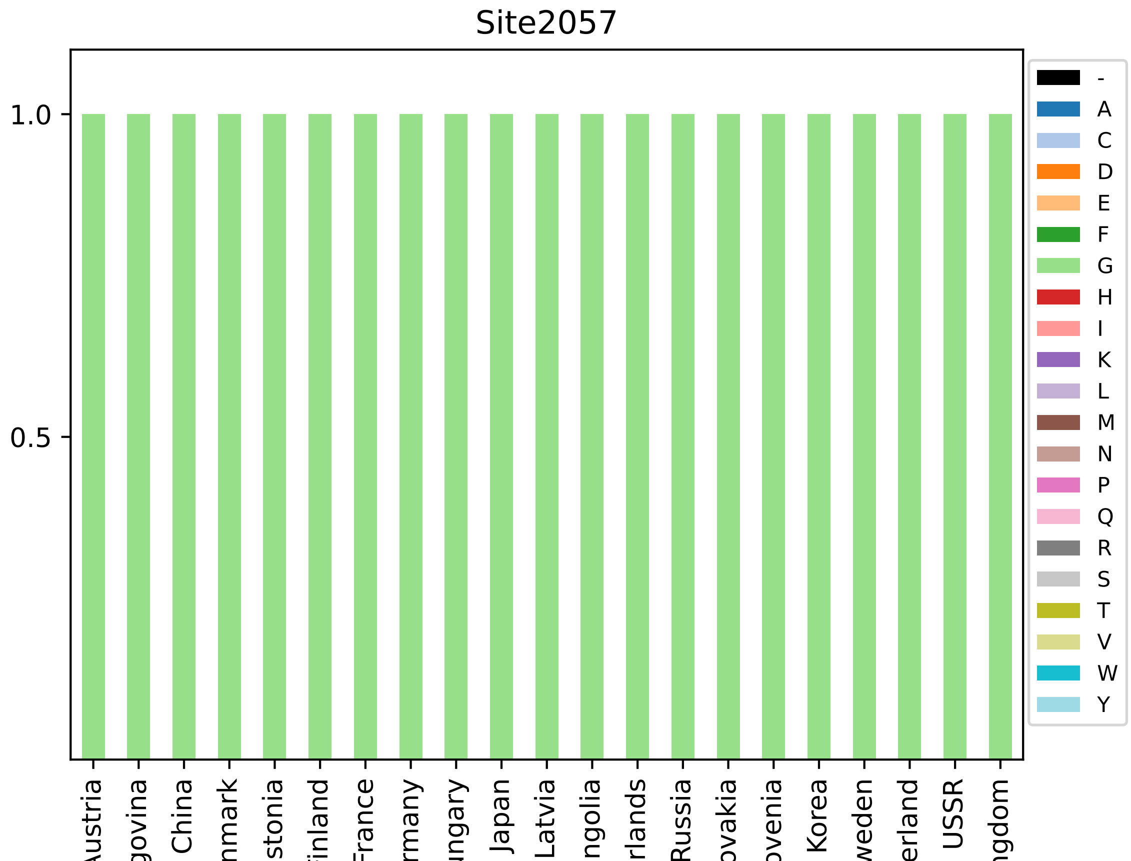
Site2057
1.0
0.5
-
A
C
D
E
F
G
H
I
K
L
M
N
P
Q
R
S
T
V
W
Y
China
Japan
Latvia
Russia
Korea
USSR

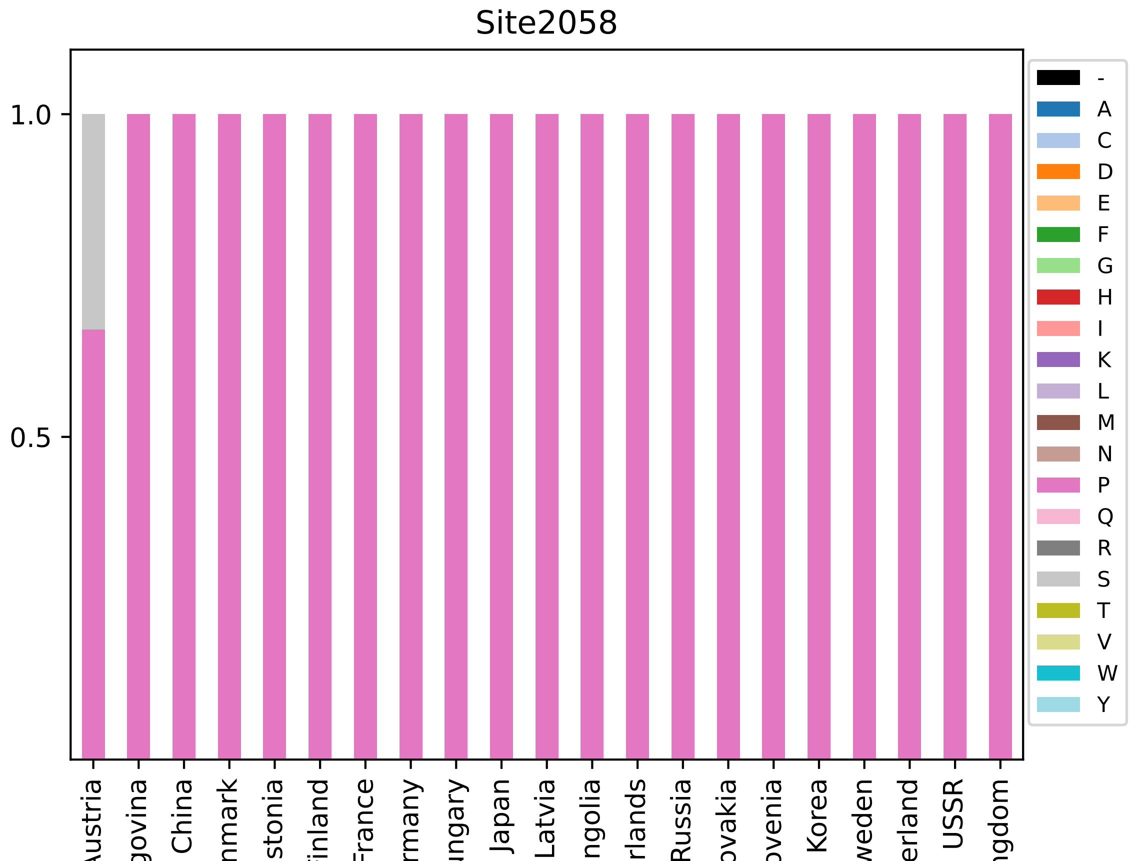

Site2058
1.0
0.5
-
A
C
D
E
F
G
H
I
K
L
M
N
P
Q
R
S
T
V
W
Y
Austria
govina
China
nmark
stonia
Finland
France
rmany
ungary
Japan
Latvia
ngolia
rlands
Russia
ovakia
ovenia
Korea
weden
erland
USSR
ngdom

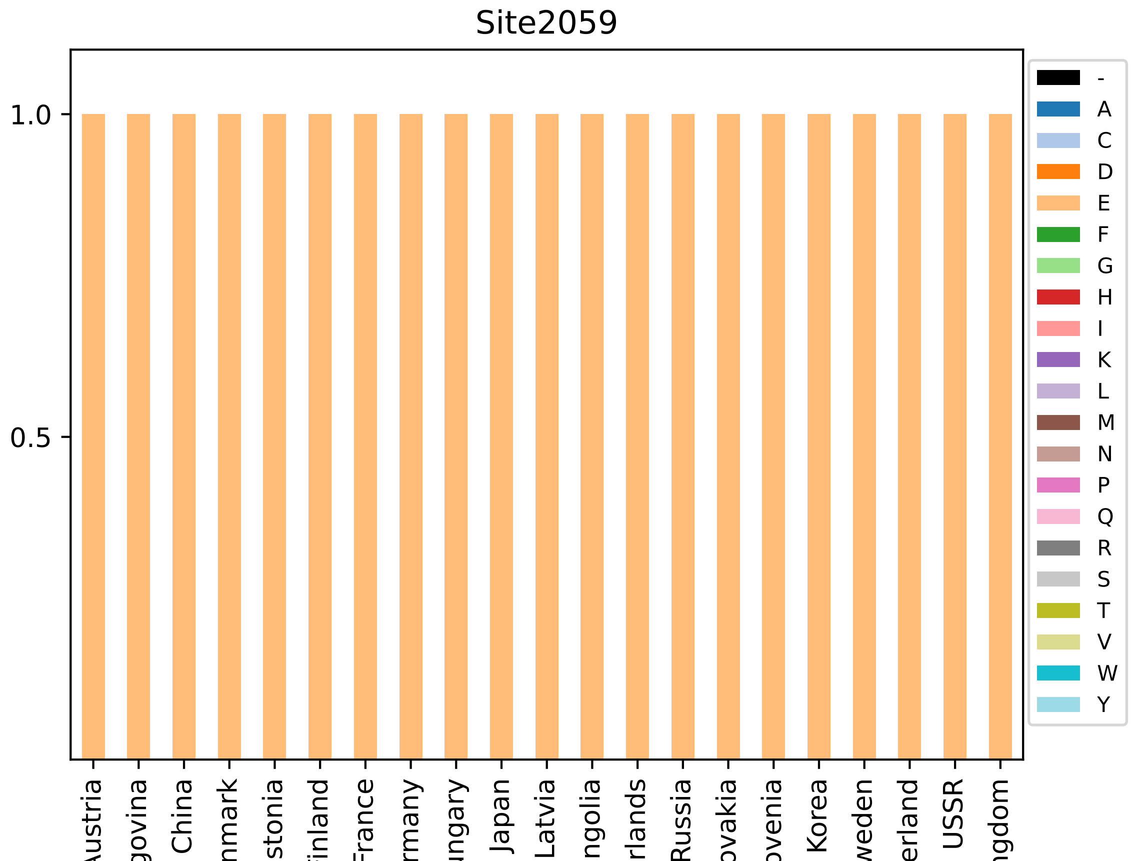

Site2059
1.0
0.5
-
A
C
D
E
F
G
H
I
K
L
M
N
P
Q
R
S
T
V
W
Y
China
Japan
Latvia
Russia
Korea
USSR

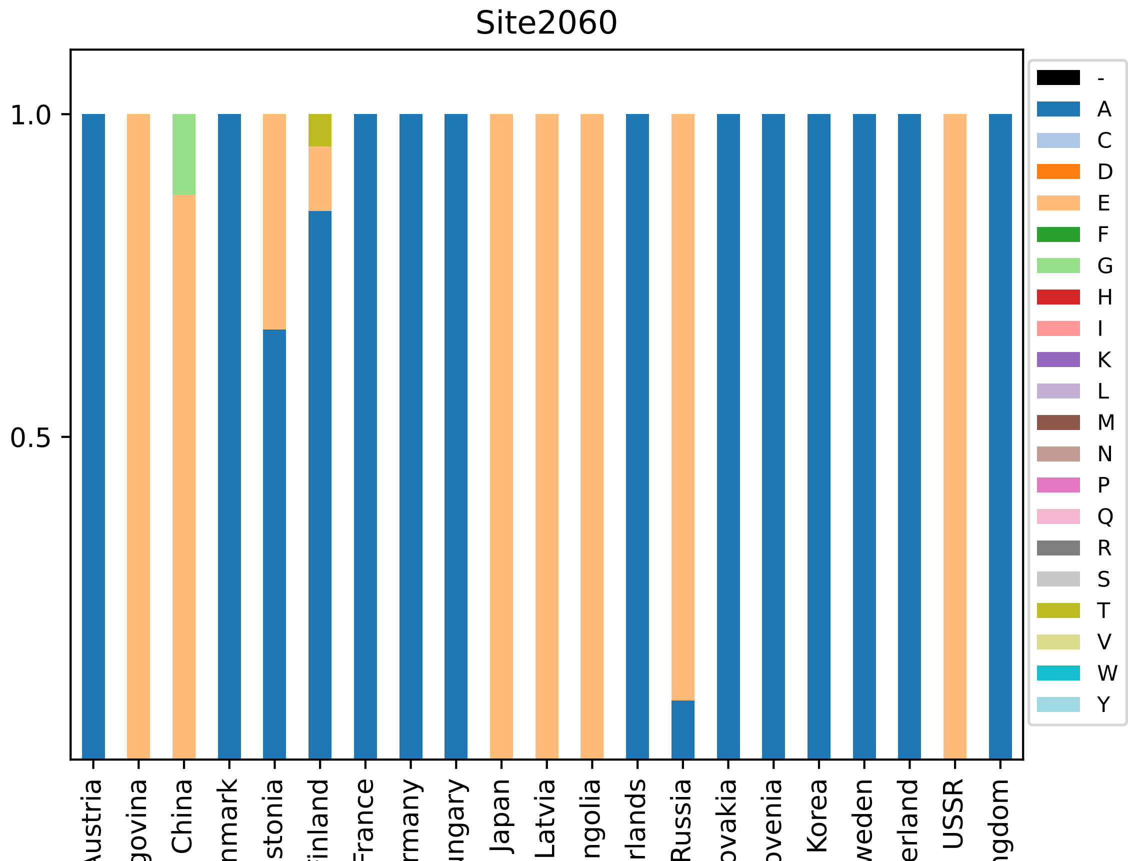

Site2060
1.0
0.5
-
A
C
D
E
F
G
H
I
K
L
M
N
P
Q
R
S
T
V
W
Y
China
Japan
Latvia
Russia
Korea
USSR

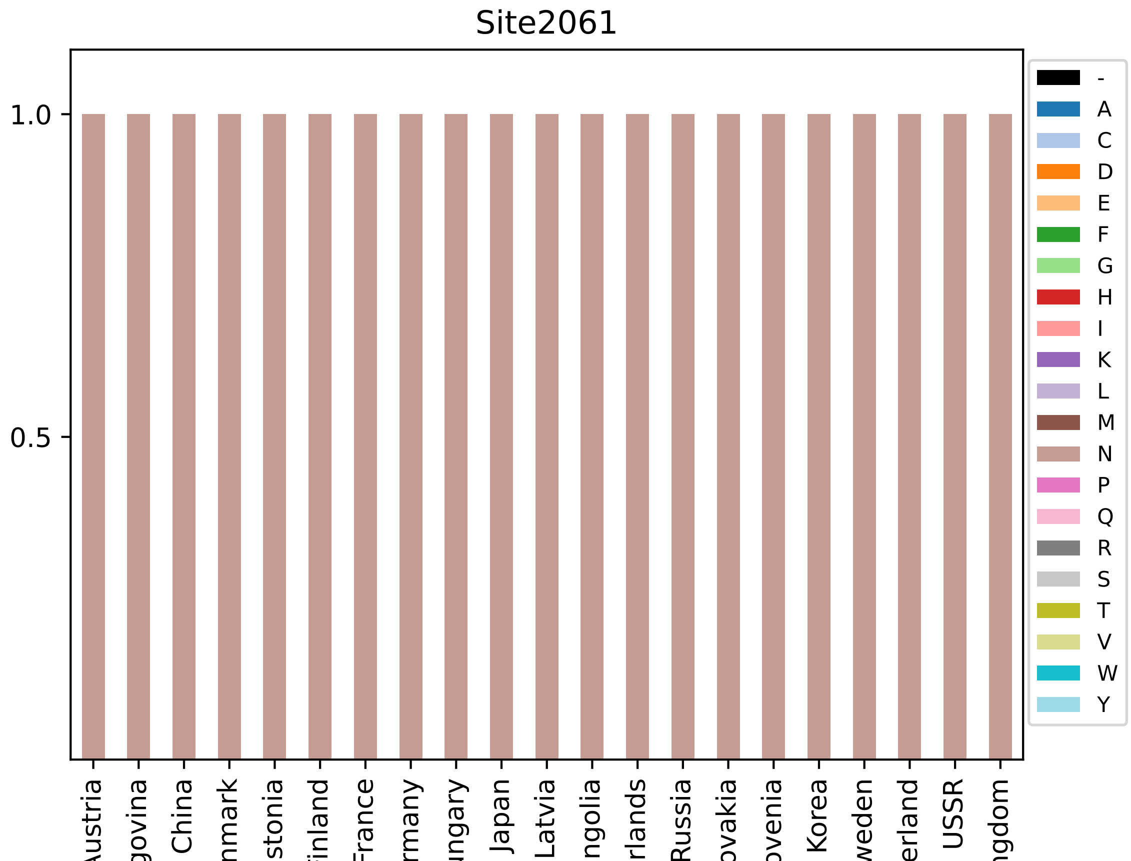
Site2061
1.0
0.5
Austria
govina
China
nmark
stonia
Finland
France
rmany
ungary
Japan
Latvia
ngolia
rlands
Russia
ovakia
ovenia
Korea
weden
erland
USSR
ngdom
-
A
C
D
E
F
G
H
I
K
L
M
N
P
Q
R
S
T
V
W
Y

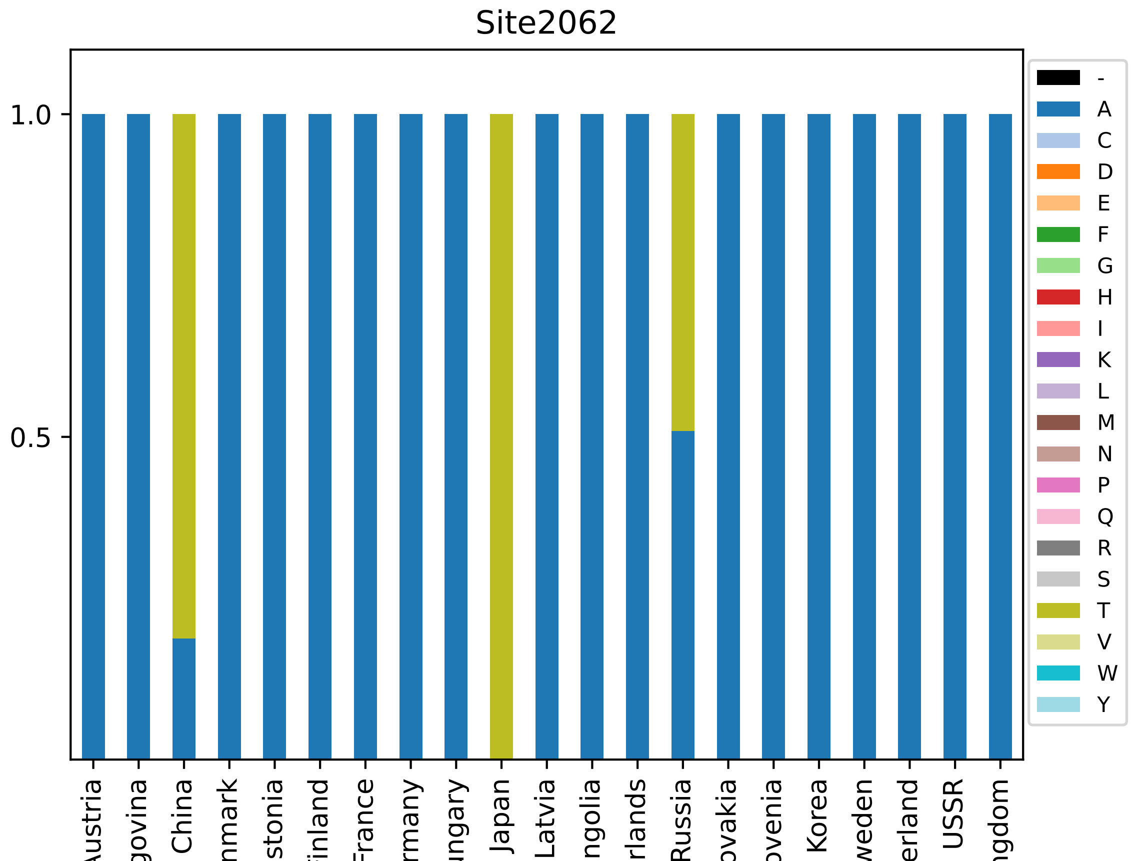
Site2062
1.0
0.5
-
A
C
D
E
F
G
H
I
K
L
M
N
P
Q
R
S
T
V
W
Y
Austria
govina
China
nmark
stonia
Finland
France
rmany
ungary
Japan
Latvia
ngolia
rlands
Russia
ovakia
ovenia
Korea
weden
erland
USSR
ngdom

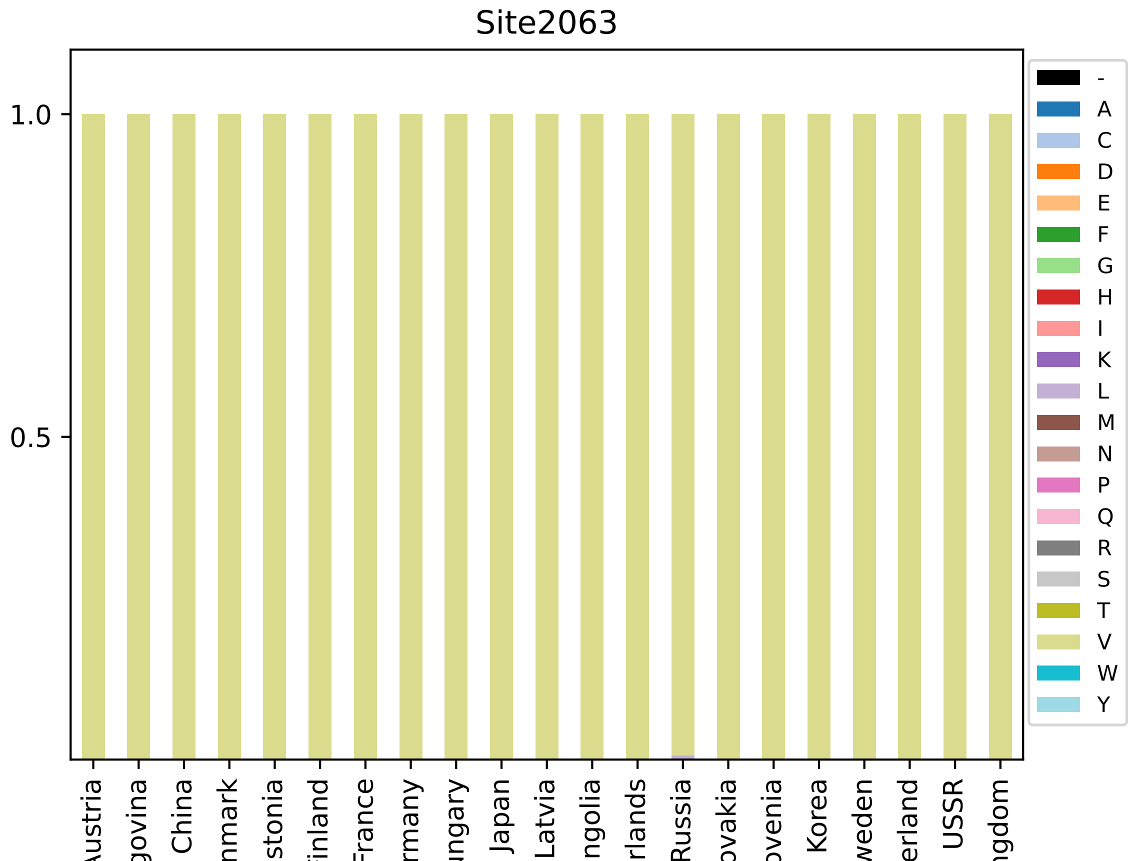

Site2063
1.0
0.5
-
A
C
D
E
F
G
H
I
K
L
M
N
P
Q
R
S
T
V
W
Y
Austria
China
Japan
Latvia
Russia
Korea
USSR

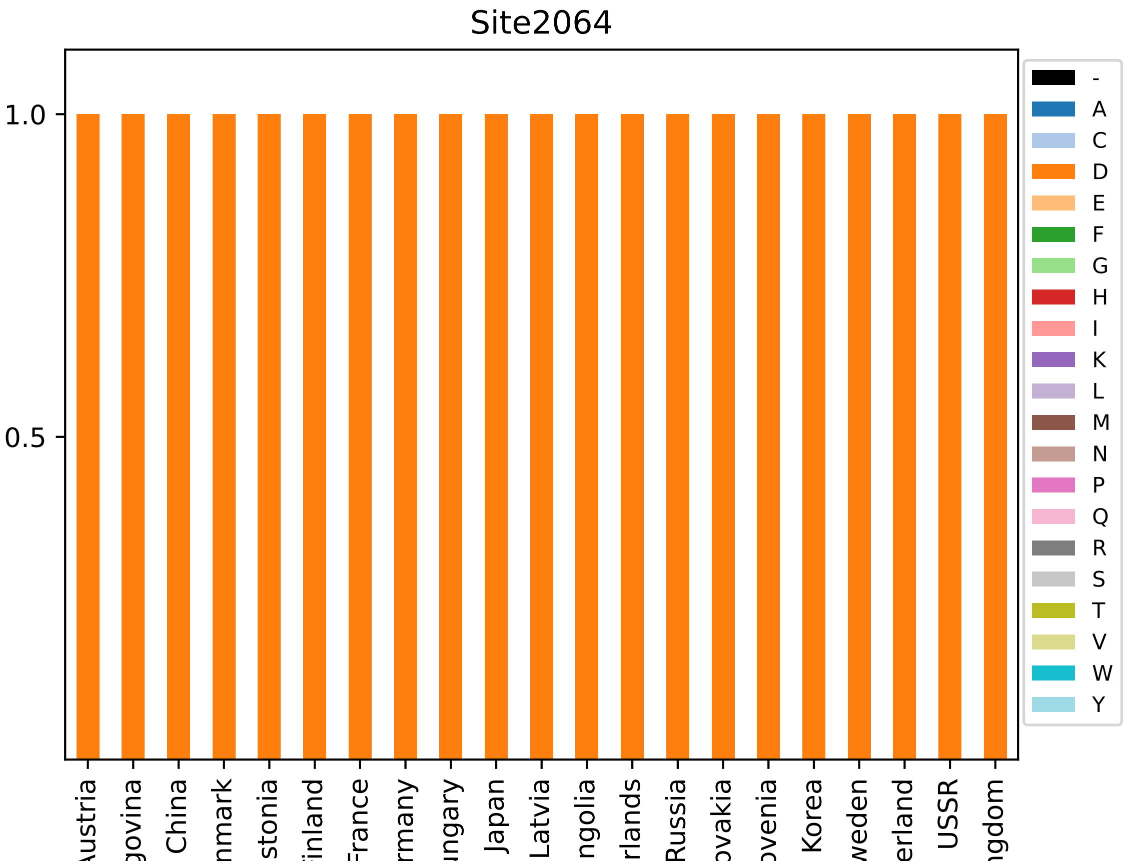

Site2064
1.0
0.5
-
A
C
D
E
F
G
H
I
K
L
M
N
P
Q
R
S
T
V
W
Y
Austria
govina
China
nmark
stonia
inland
France
rmany
ungary
Japan
Latvia
ngolia
rlands
Russia
ovakia
ovenia
Korea
weden
erland
USSR
ngdom

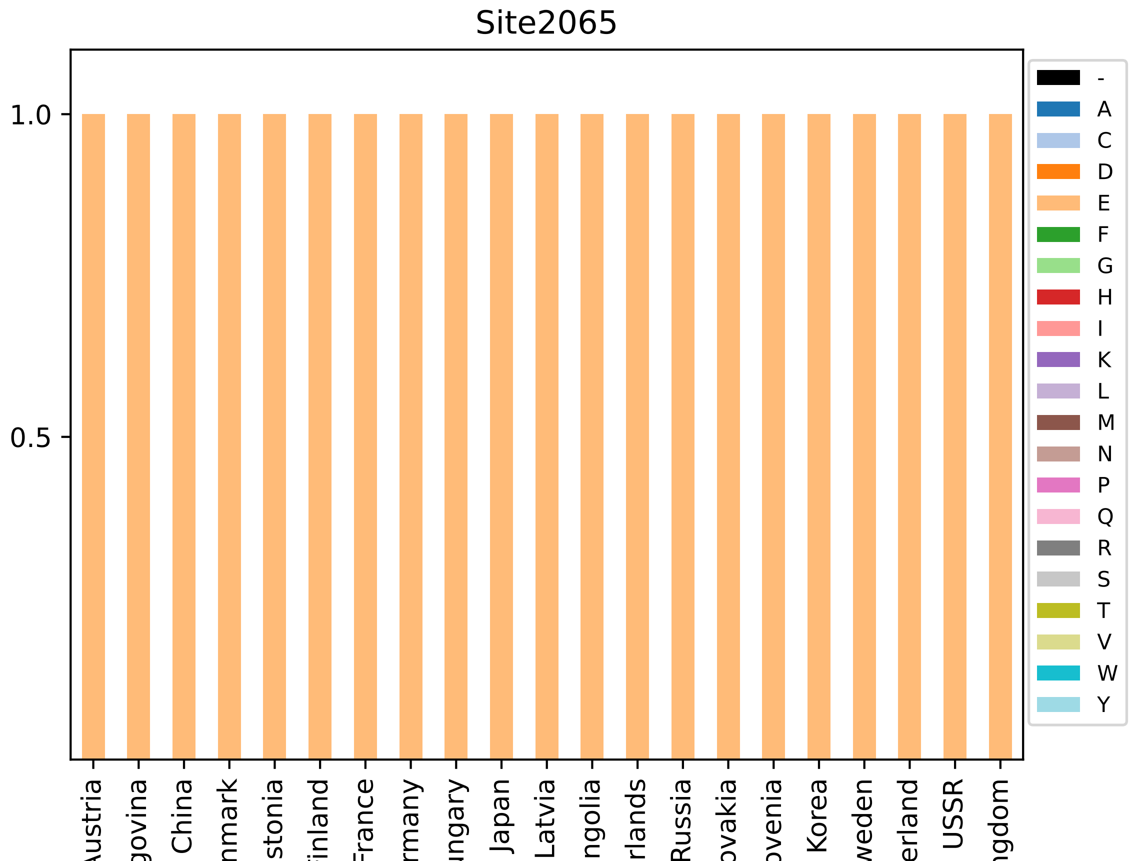
Site2065
1.0
0.5
-
A
C
D
E
F
G
H
I
K
L
M
N
P
Q
R
S
T
V
W
Y
Austria
govina
China
nmark
stonia
inland
France
rmany
ungary
Japan
Latvia
ngolia
rlands
Russia
ovakia
ovenia
Korea
weden
erland
USSR
ngdom

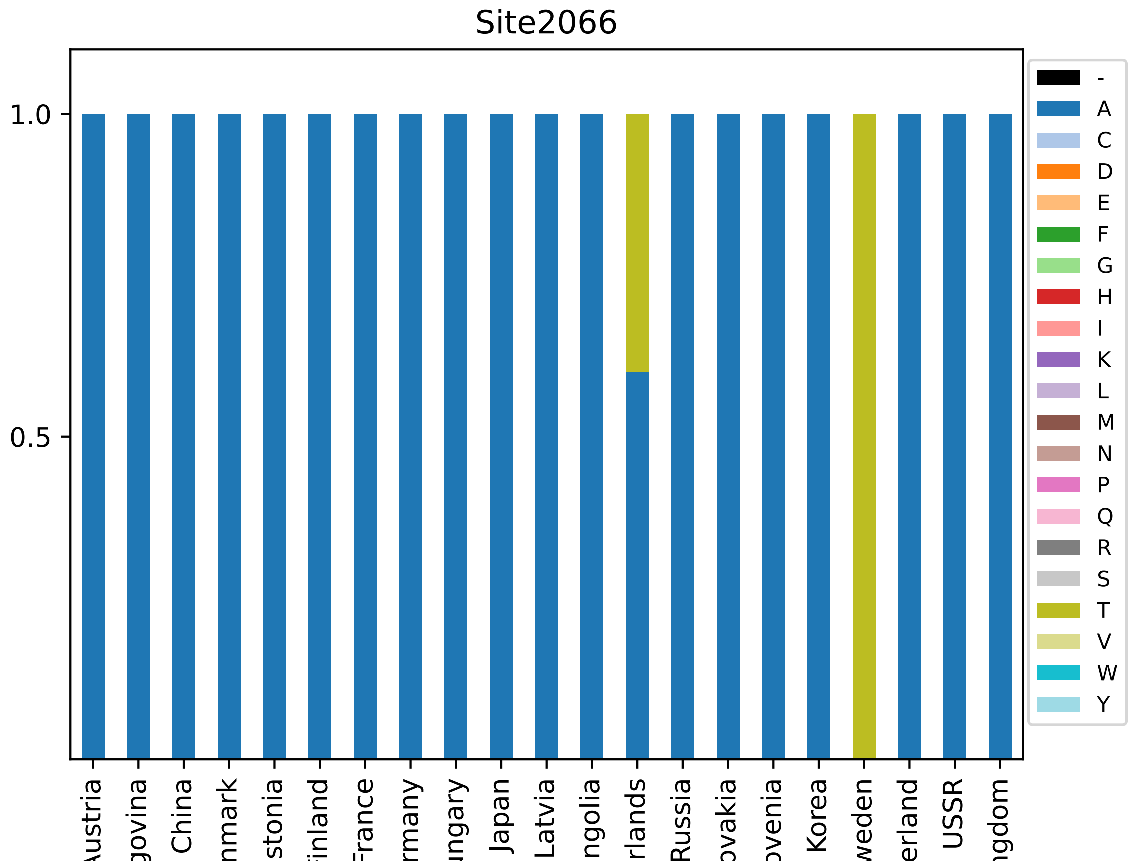
Site2066
1.0
0.5
-
A
C
D
E
F
G
H
I
K
L
M
N
P
Q
R
S
T
V
W
Y
Austria
govina
China
nmark
stonia
inland
France
rmany
ungary
Japan
Latvia
ngolia
rlands
Russia
ovakia
ovenia
Korea
weden
erland
USSR
ngdom

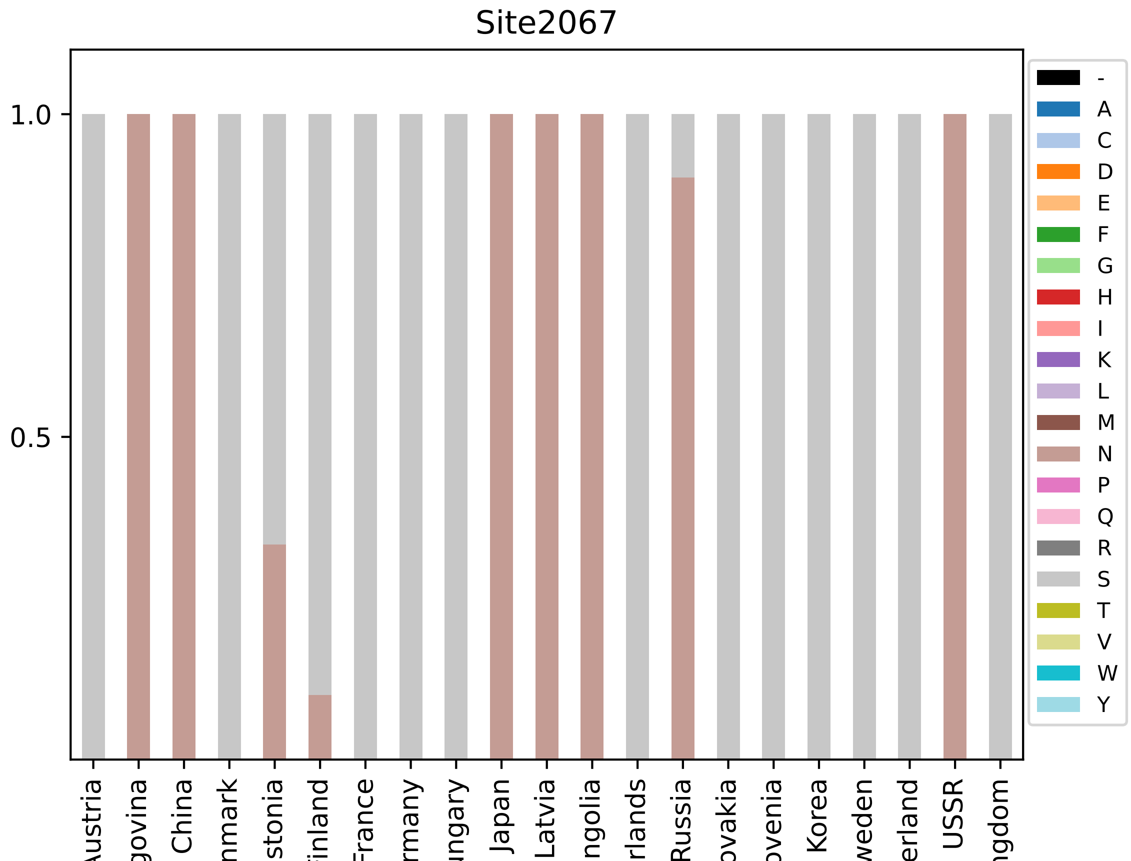
Site2067
1.0
0.5
-
A
C
D
E
F
G
H
I
K
L
M
N
P
Q
R
S
T
V
W
Y
China
Japan
Latvia
Russia
Korea
USSR

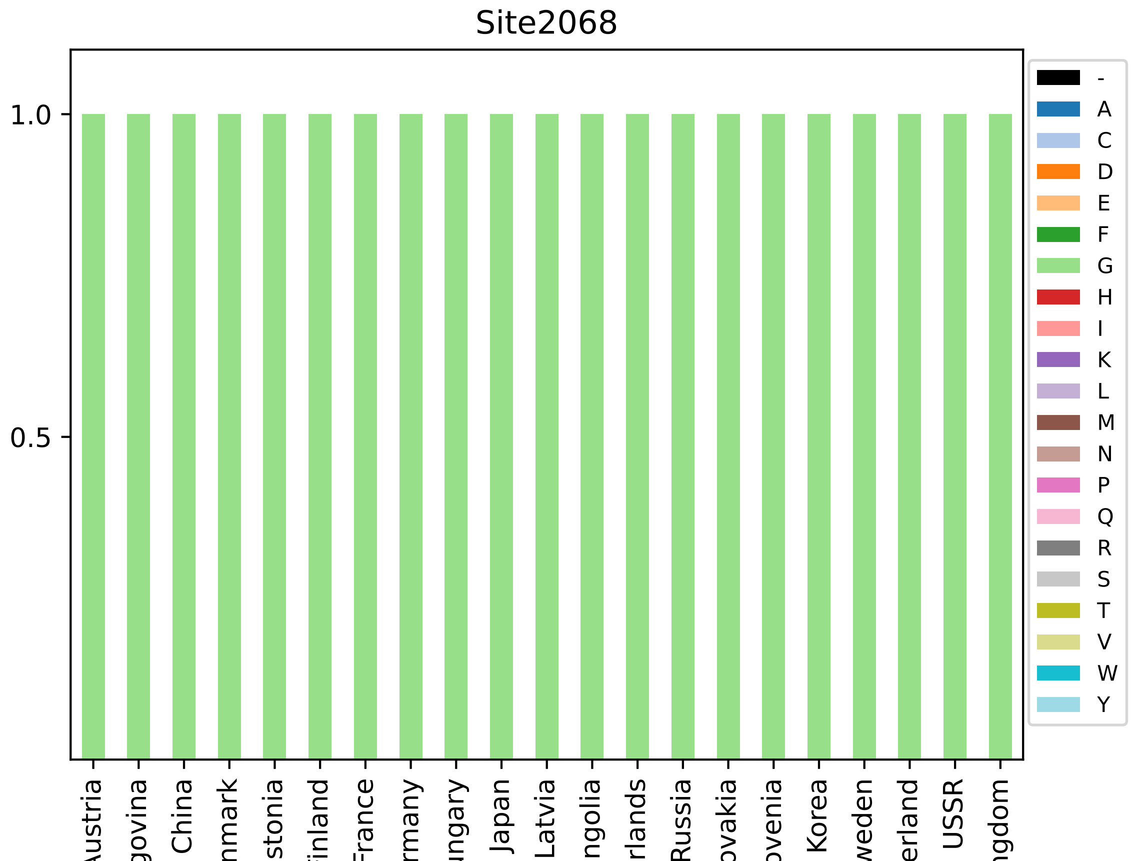
Site2068
1.0
0.5
-
A
C
D
E
F
G
H
I
K
L
M
N
P
Q
R
S
T
V
W
Y
China
Japan
Latvia
Russia
Korea
USSR

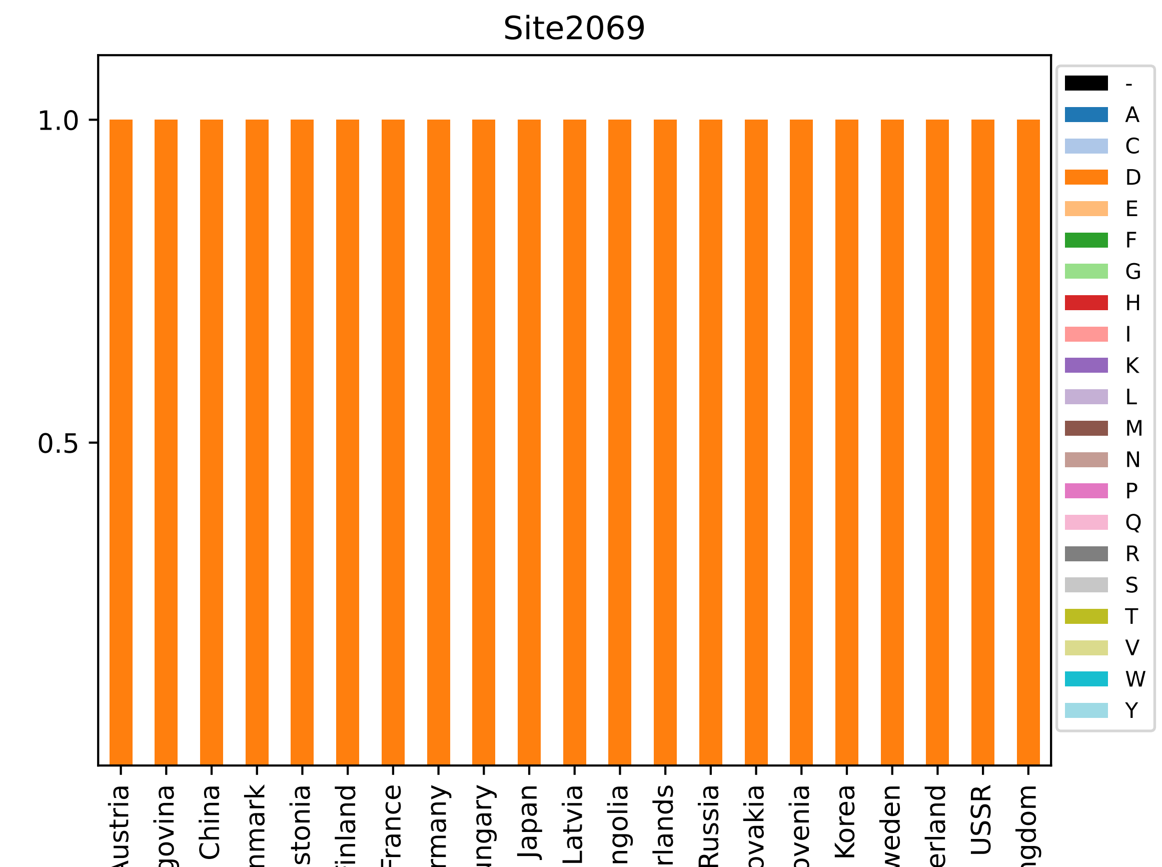

Site2069
1.0
0.5
-
A
C
D
E
F
G
H
I
K
L
M
N
P
Q
R
S
T
V
W
Y
China
Japan
Latvia
Russia
Korea
USSR

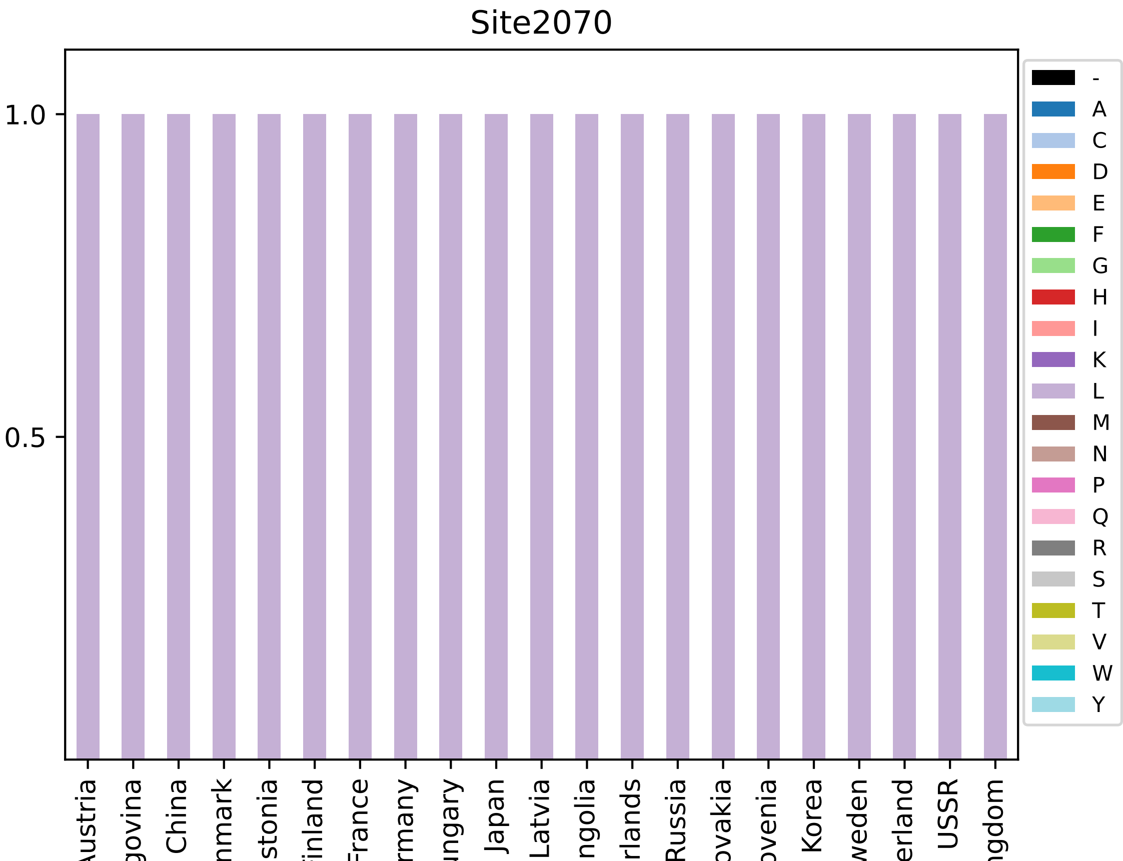

Site2070
1.0
0.5
-
A
C
D
E
F
G
H
I
K
L
M
N
P
Q
R
S
T
V
W
Y
China
Japan
Latvia
Russia
Korea
USSR

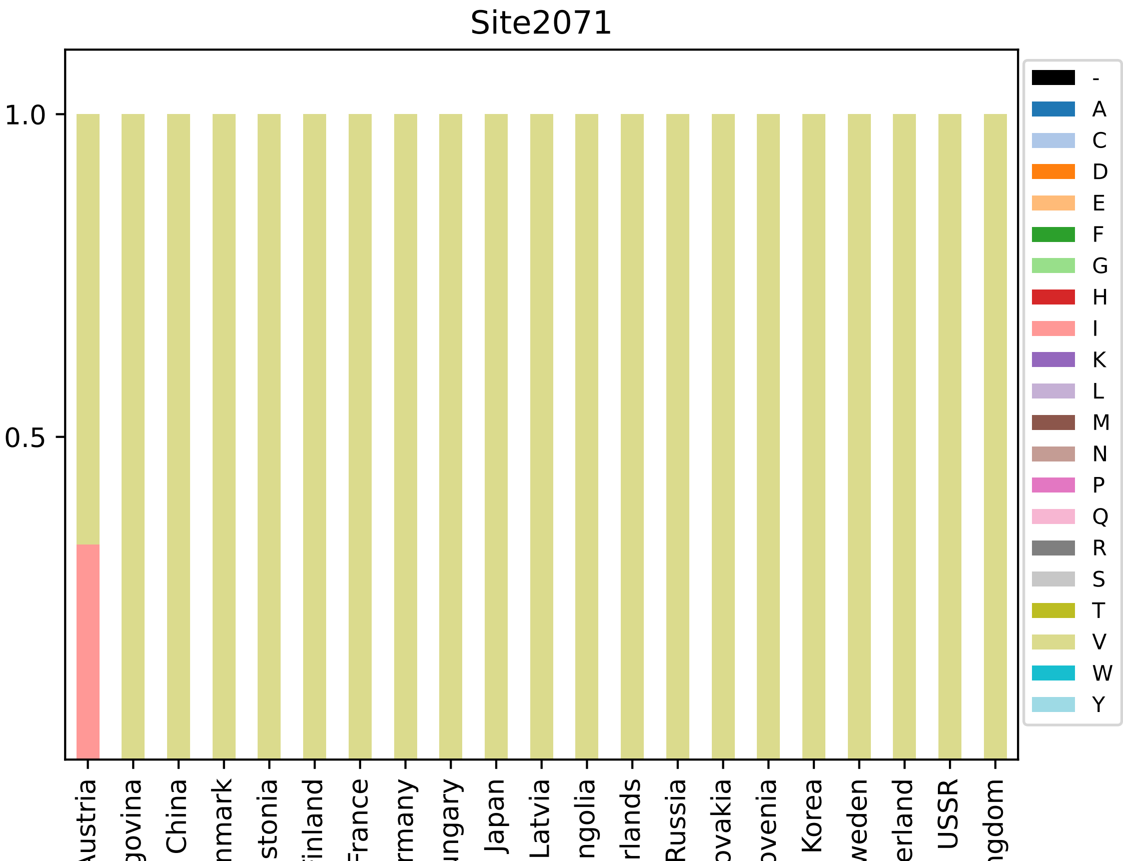

Site2071
1.0
0.5
-
A
C
D
E
F
G
H
I
K
L
M
N
P
Q
R
S
T
V
W
Y
Austria
China
Japan
Latvia
Russia
Korea
USSR

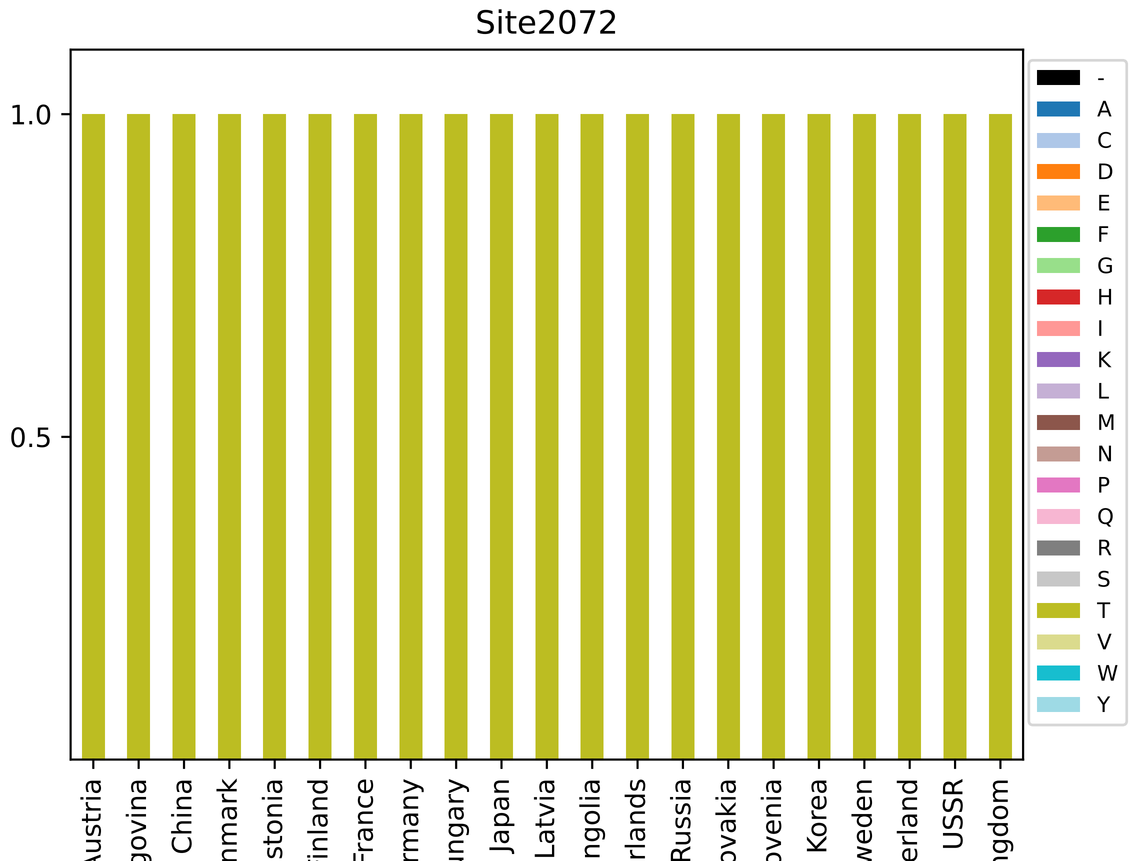

Site2072
1.0
0.5
-
A
C
D
E
F
G
H
I
K
L
M
N
P
Q
R
S
T
V
W
Y
Austria
govina
China
nmark
stonia
Finland
France
rmany
ungary
Japan
Latvia
ngolia
rlands
Russia
ovakia
ovenia
Korea
weden
erland
USSR
ngdom

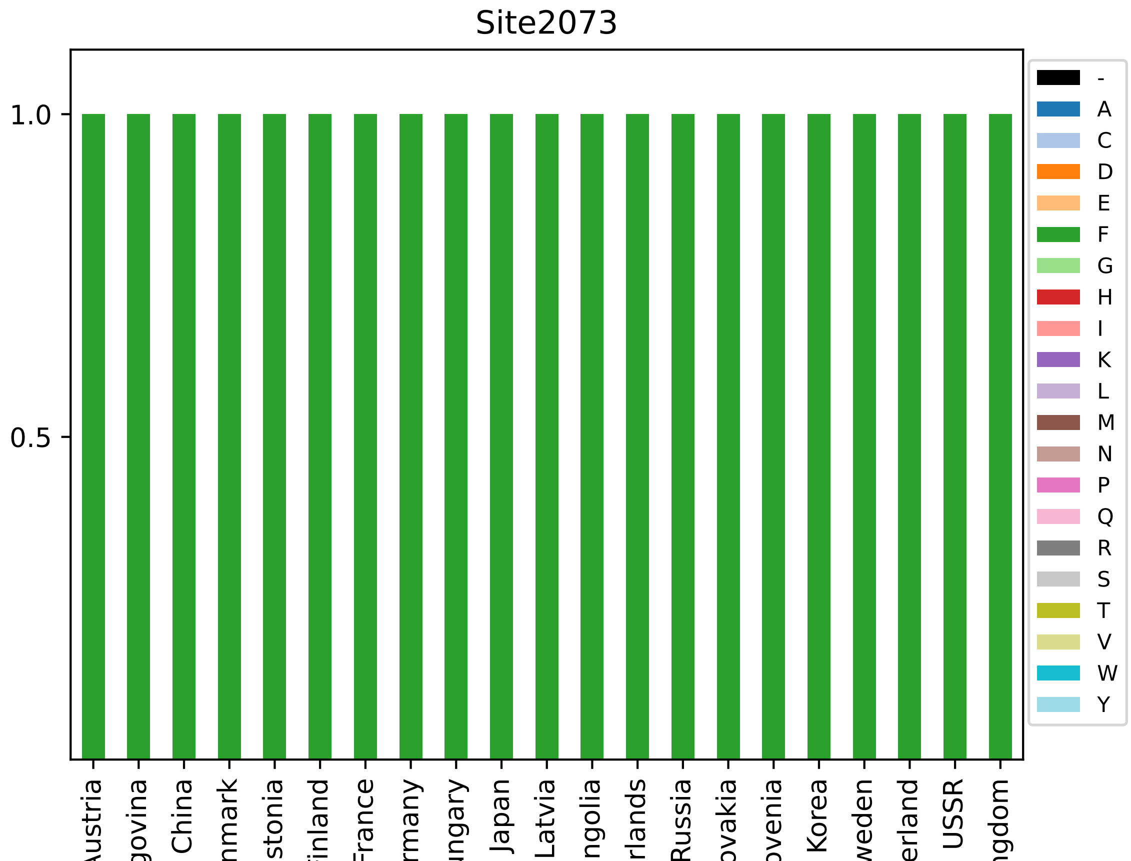
Site2073
1.0
0.5
-
A
C
D
E
F
G
H
I
K
L
M
N
P
Q
R
S
T
V
W
Y
Austria
govina
China
nmark
stonia
Finland
France
rmany
ungary
Japan
Latvia
ngolia
rlands
Russia
ovakia
ovenia
Korea
weden
erland
USSR
ngdom

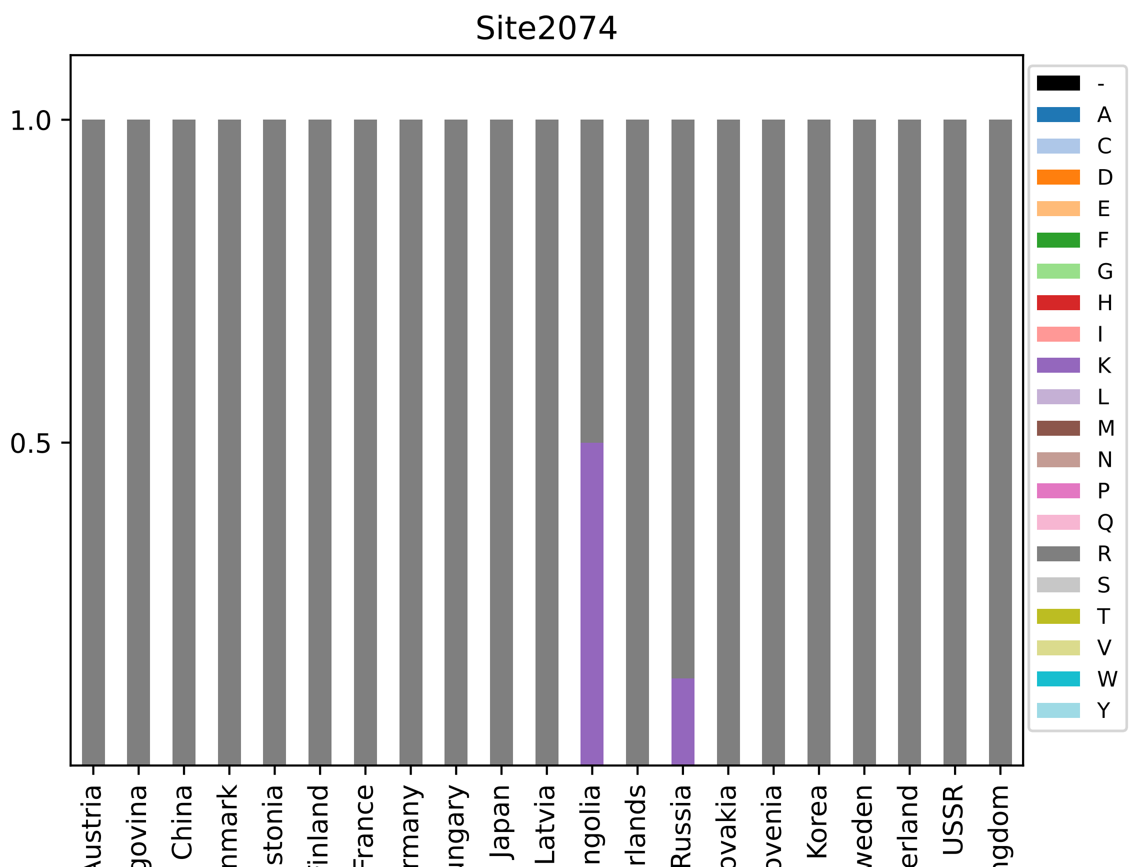
Site2074
1.0
0.5
Austria
govina
China
nmark
stonia
Finland
France
rmany
ungary
Japan
Latvia
ngolia
rlands
Russia
ovakia
ovenia
Korea
weden
erland
USSR
ngdom
-
A
C
D
E
F
G
H
I
K
L
M
N
P
Q
R
S
T
V
W
Y

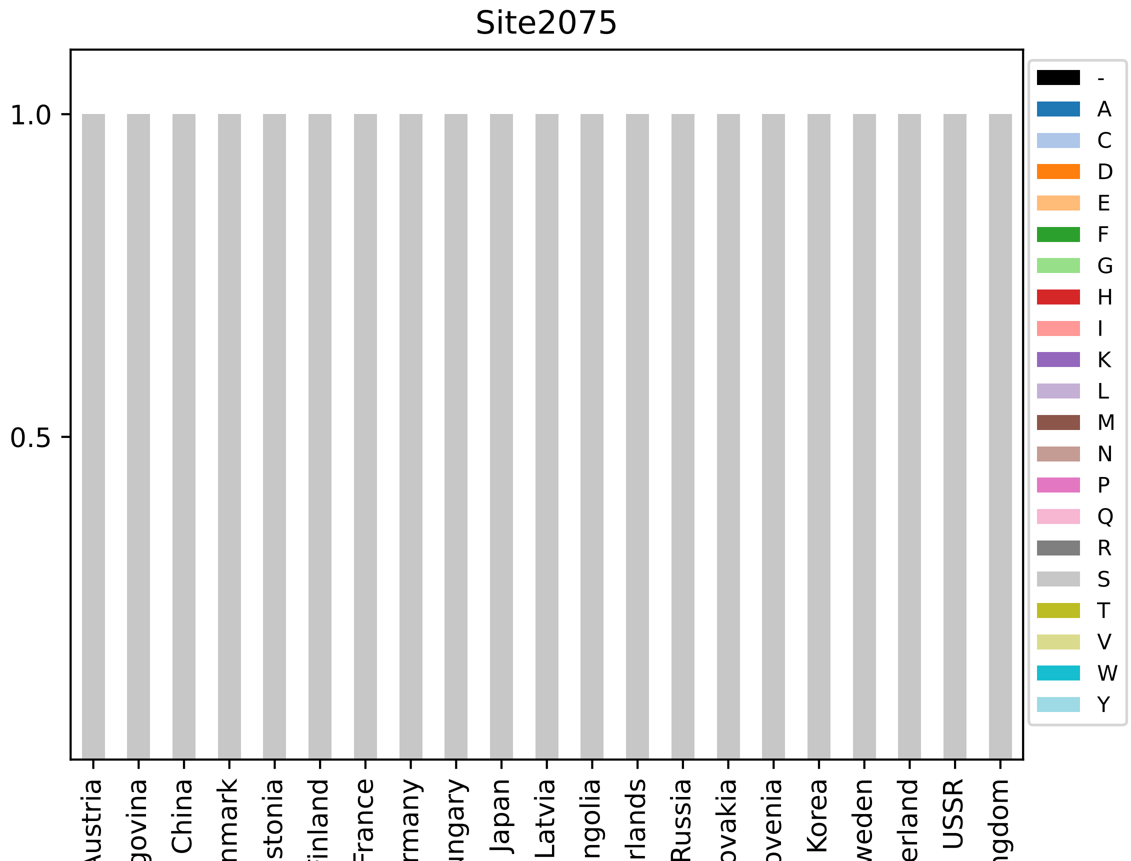
Site2075
1.0
0.5
-
A
C
D
E
F
G
H
I
K
L
M
N
P
Q
R
S
T
V
W
Y
China
Finland
Japan
Latvia
Russia
Korea
USSR

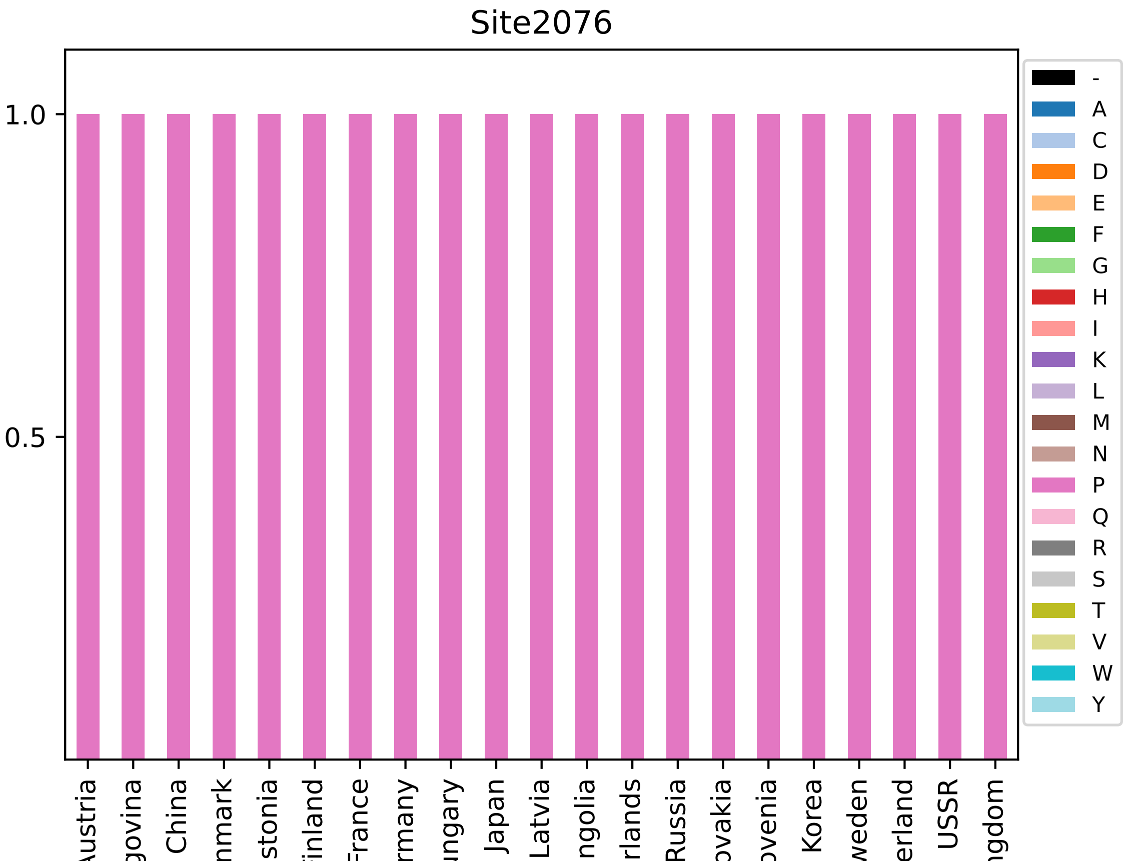
Site2076
1.0
0.5
-
A
C
D
E
F
G
H
I
K
L
M
N
P
Q
R
S
T
V
W
Y
Austria
China
Japan
Latvia
Russia
Korea
USSR

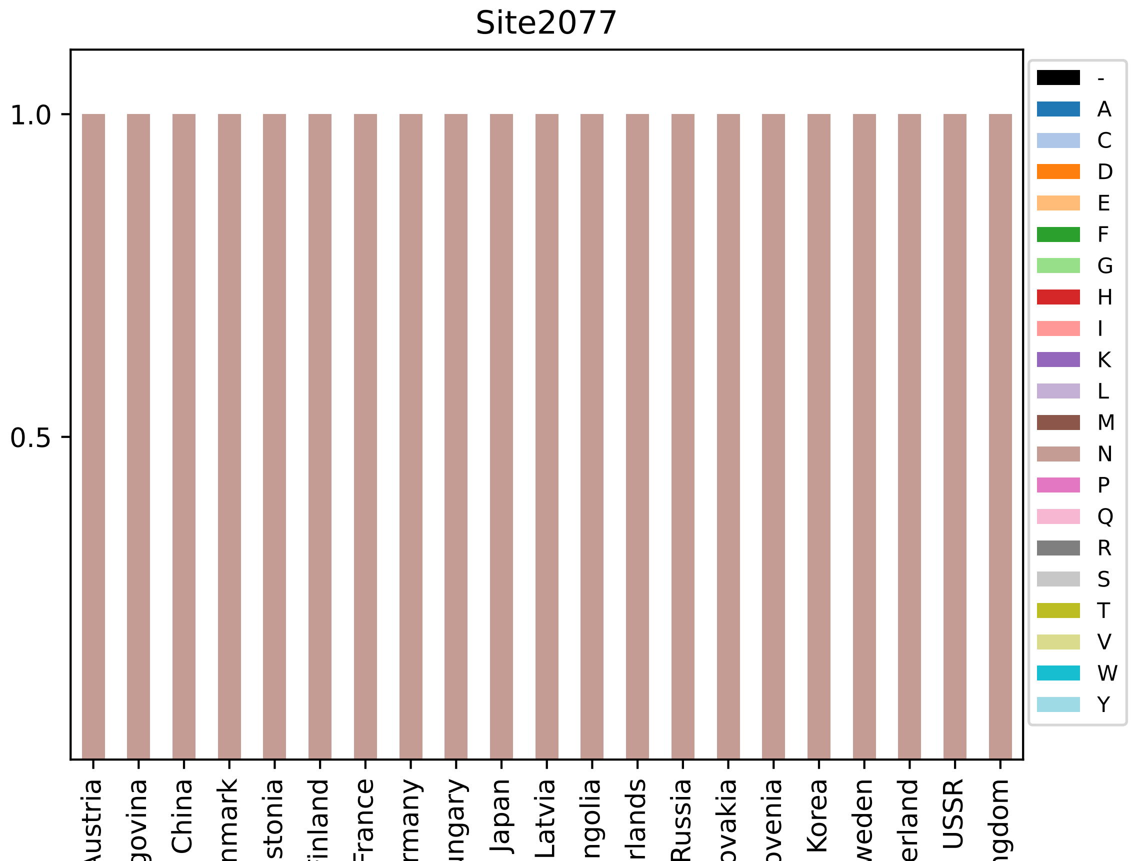

Site2077
1.0
0.5
-
A
C
D
E
F
G
H
I
K
L
M
N
P
Q
R
S
T
V
W
Y
Austria
China
Japan
Latvia
Russia
Korea
USSR

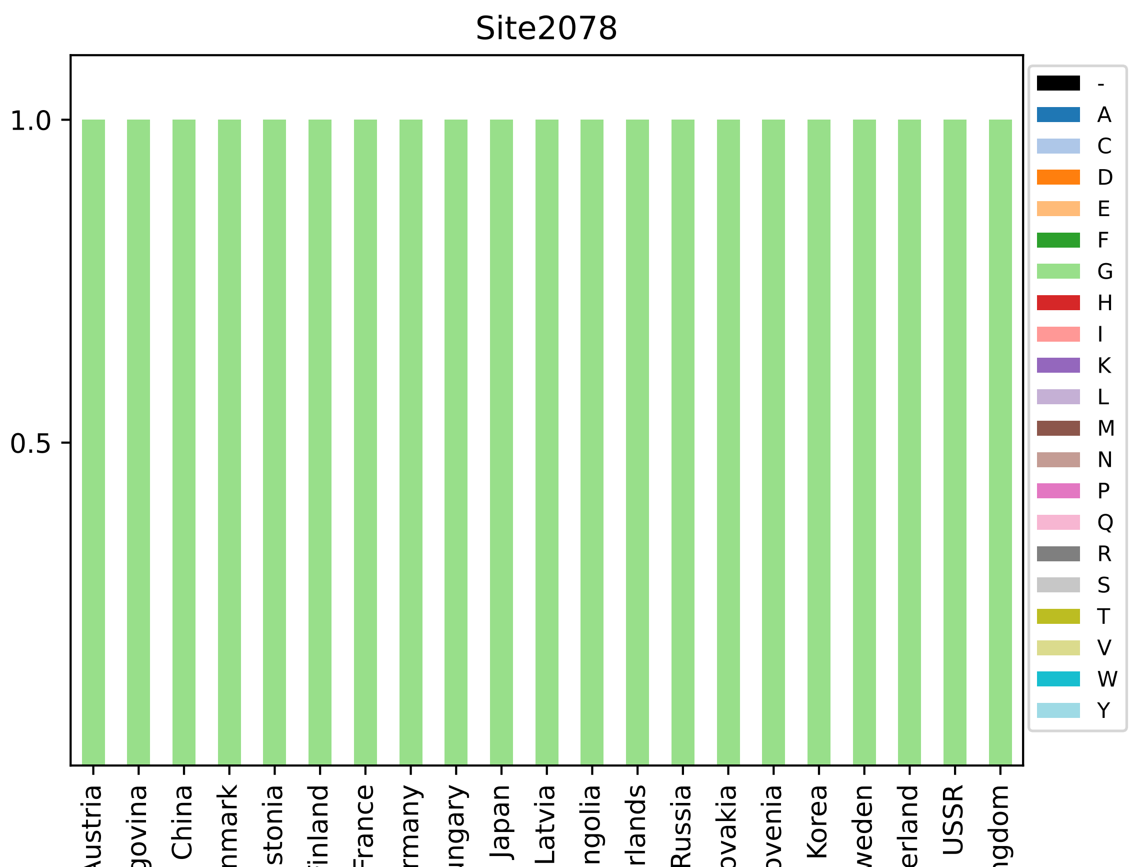
Site2078
1.0
0.5
-
A
C
D
E
F
G
H
I
K
L
M
N
P
Q
R
S
T
V
W
Y
Austria
govina
China
nmark
stonia
inland
France
rmany
ungary
Japan
Latvia
ngolia
rlands
Russia
ovakia
ovenia
Korea
weden
erland
USSR
ngdom

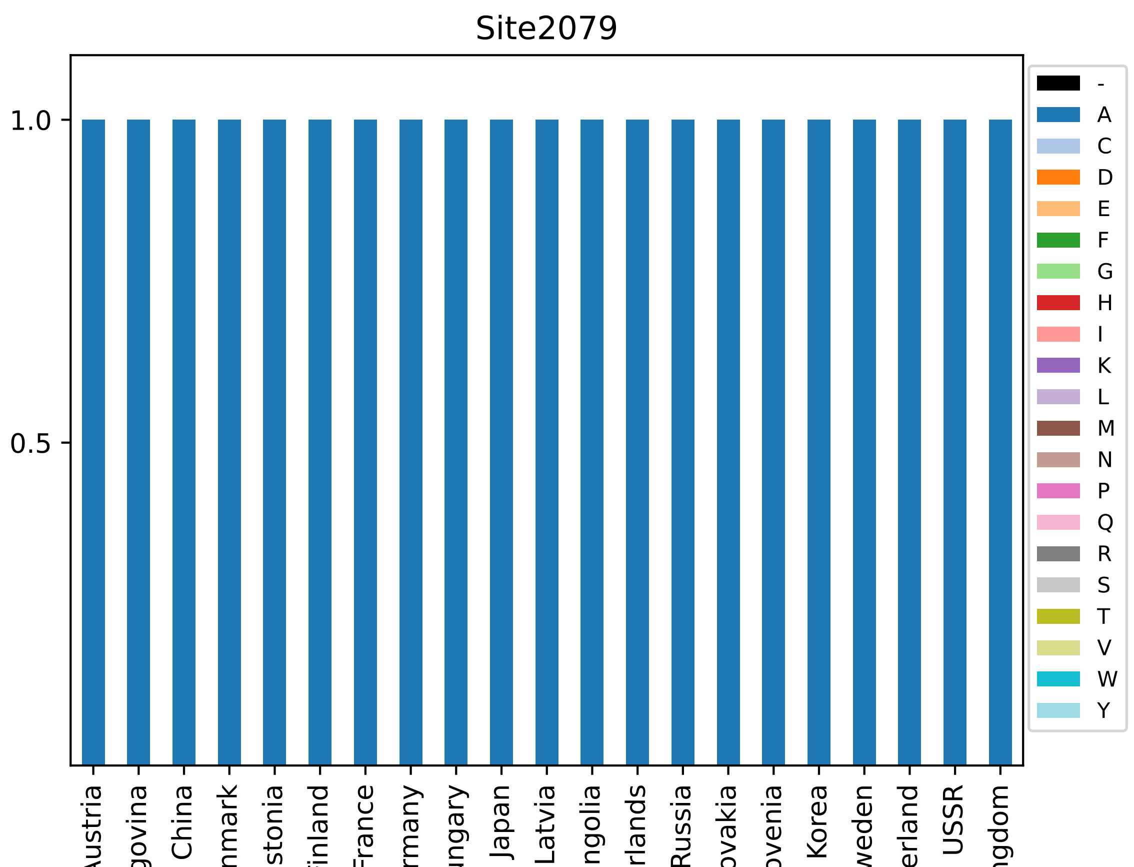

Site2079
1.0
0.5
-
A
C
D
E
F
G
H
I
K
L
M
N
P
Q
R
S
T
V
W
Y
Austria
govina
China
nmark
stonia
Finland
France
rmany
ungary
Japan
Latvia
ngolia
rlands
Russia
ovakia
ovenia
Korea
weden
erland
USSR
ngdom

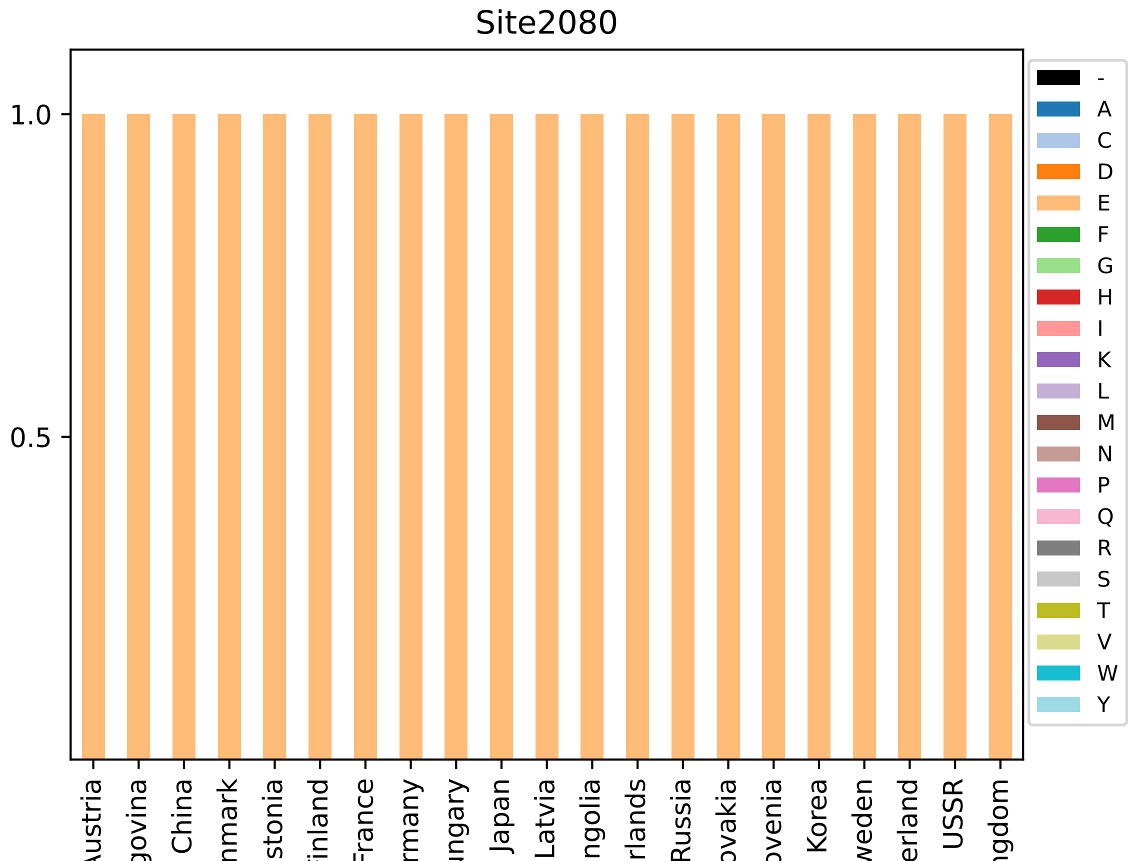
Site2080
1.0
0.5
Austria
govina
China
nmark
stonia
Finland
France
rmany
ungary
Japan
Latvia
ngolia
rlands
Russia
ovakia
ovenia
Korea
weden
erland
USSR
ngdom
-
A
C
D
E
F
G
H
I
K
L
M
N
P
Q
R
S
T
V
W
Y

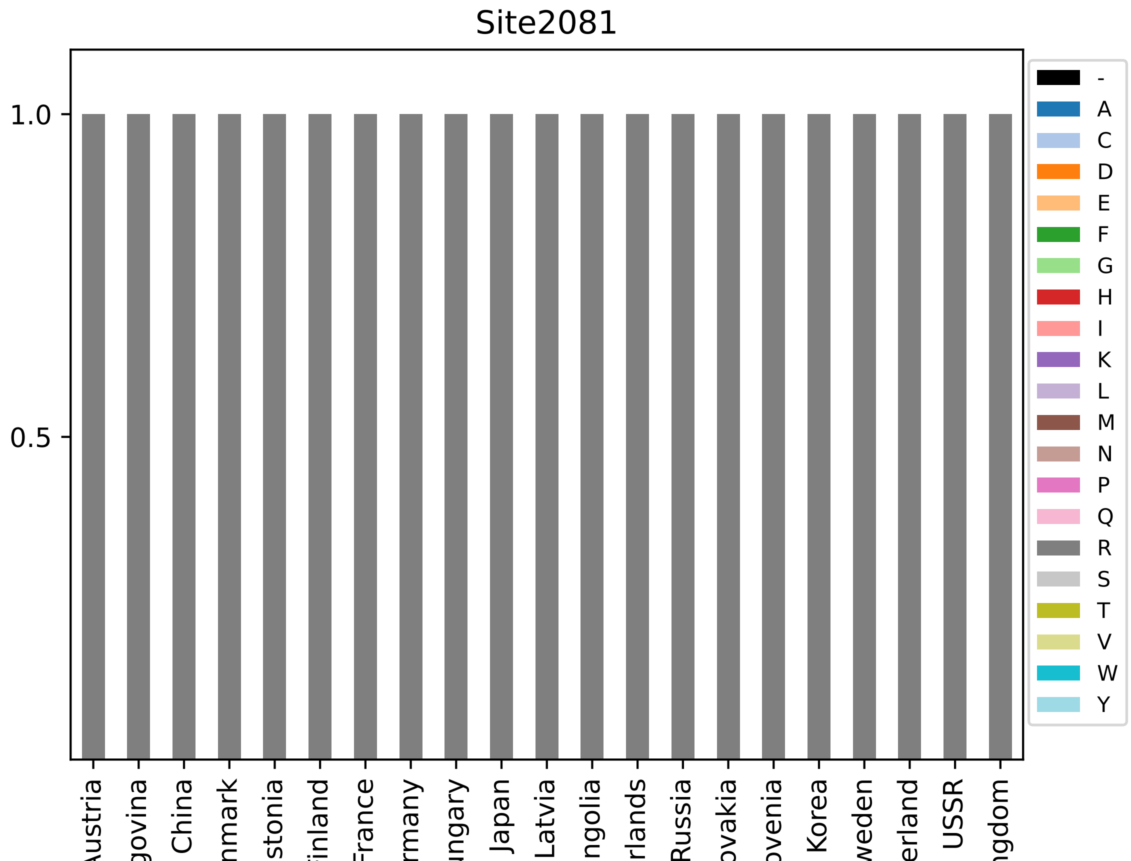

Site2081
1.0
0.5
-
A
C
D
E
F
G
H
I
K
L
M
N
P
Q
R
S
T
V
W
Y
Austria
China
Japan
Latvia
Russia
Korea
USSR

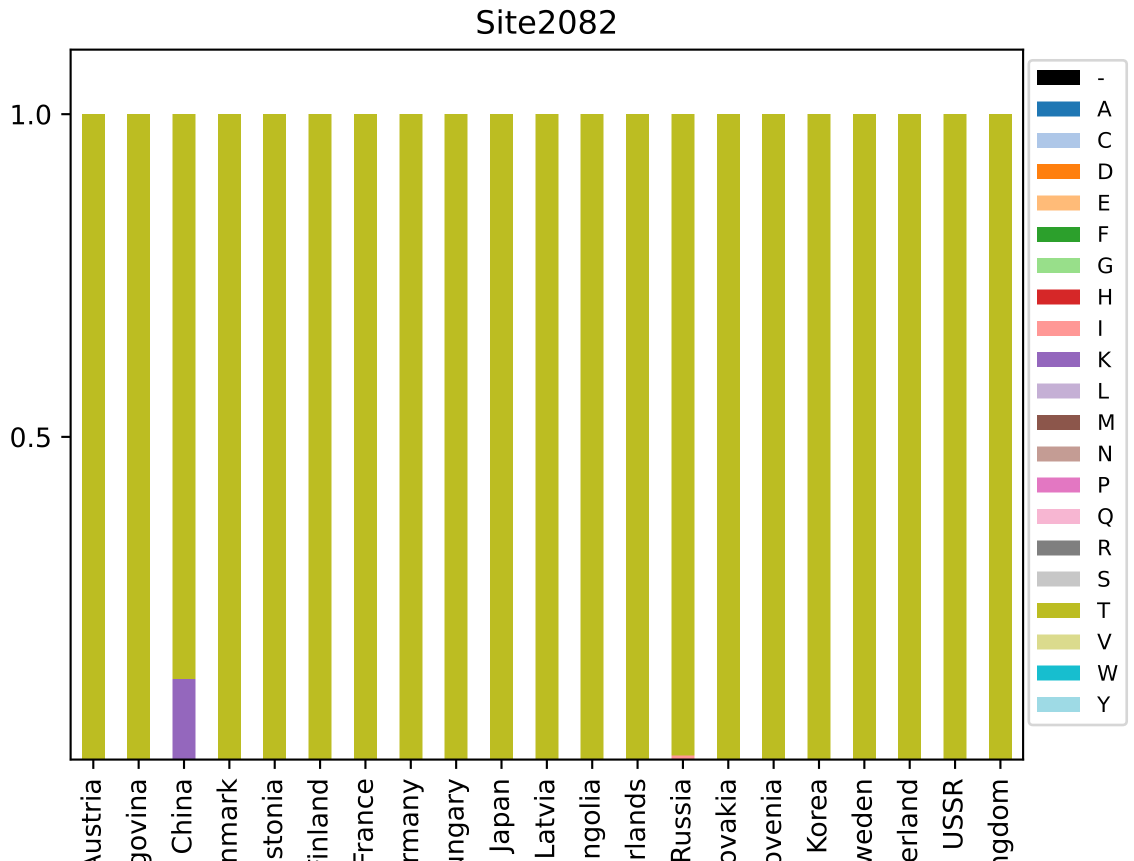
Site2082
1.0
0.5
-
A
C
D
E
F
G
H
I
K
L
M
N
P
Q
R
S
T
V
W
Y
Austria
China
Japan
Latvia
Russia
Korea
USSR

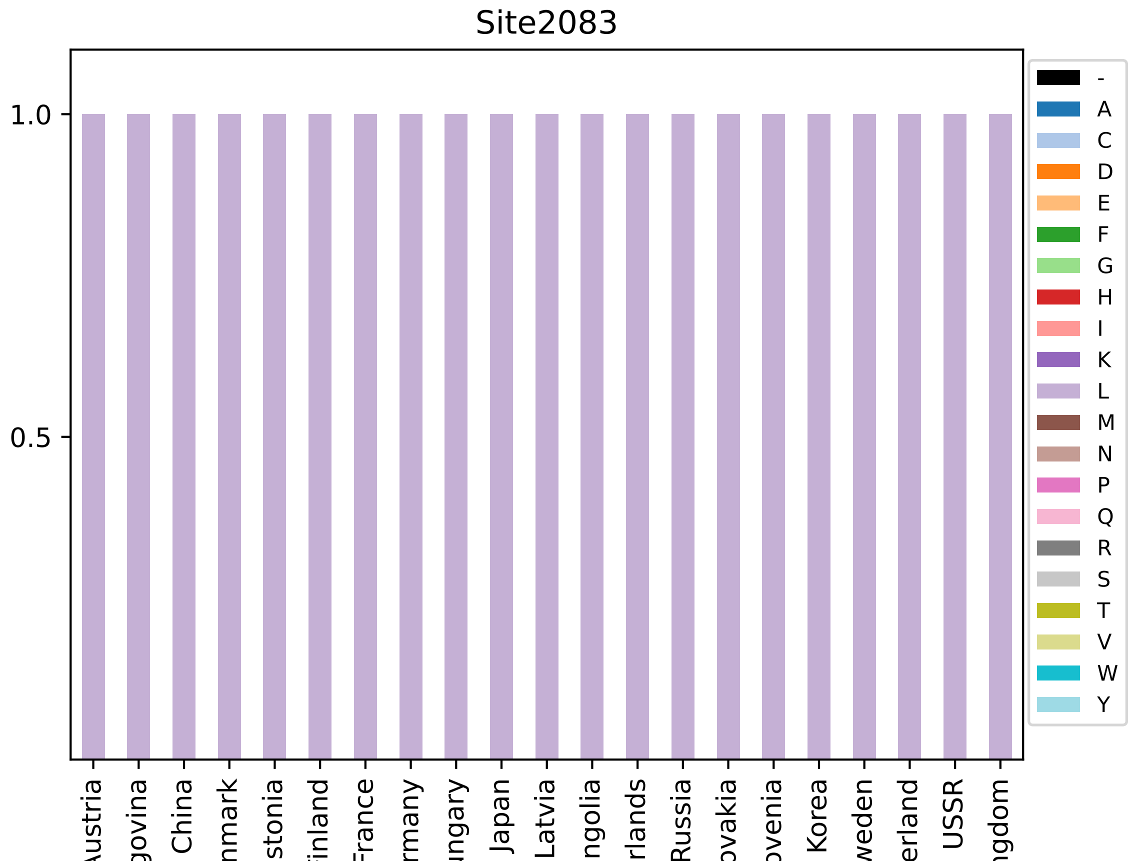

Site2083
1.0
0.5
-
A
C
D
E
F
G
H
I
K
L
M
N
P
Q
R
S
T
V
W
Y
China
Japan
Latvia
Russia
Korea
USSR

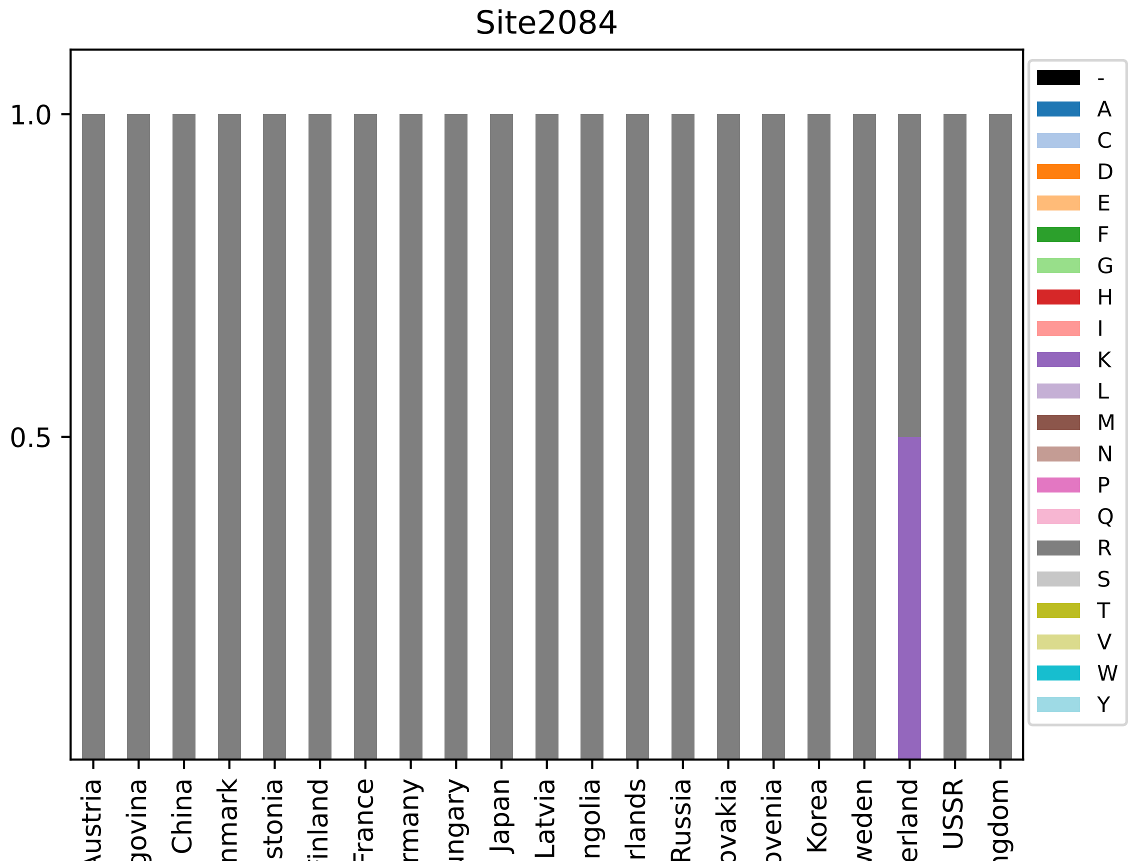
Site2084
1.0
0.5
-
A
C
D
E
F
G
H
I
K
L
M
N
P
Q
R
S
T
V
W
Y
China
Japan
Latvia
Russia
Korea
USSR

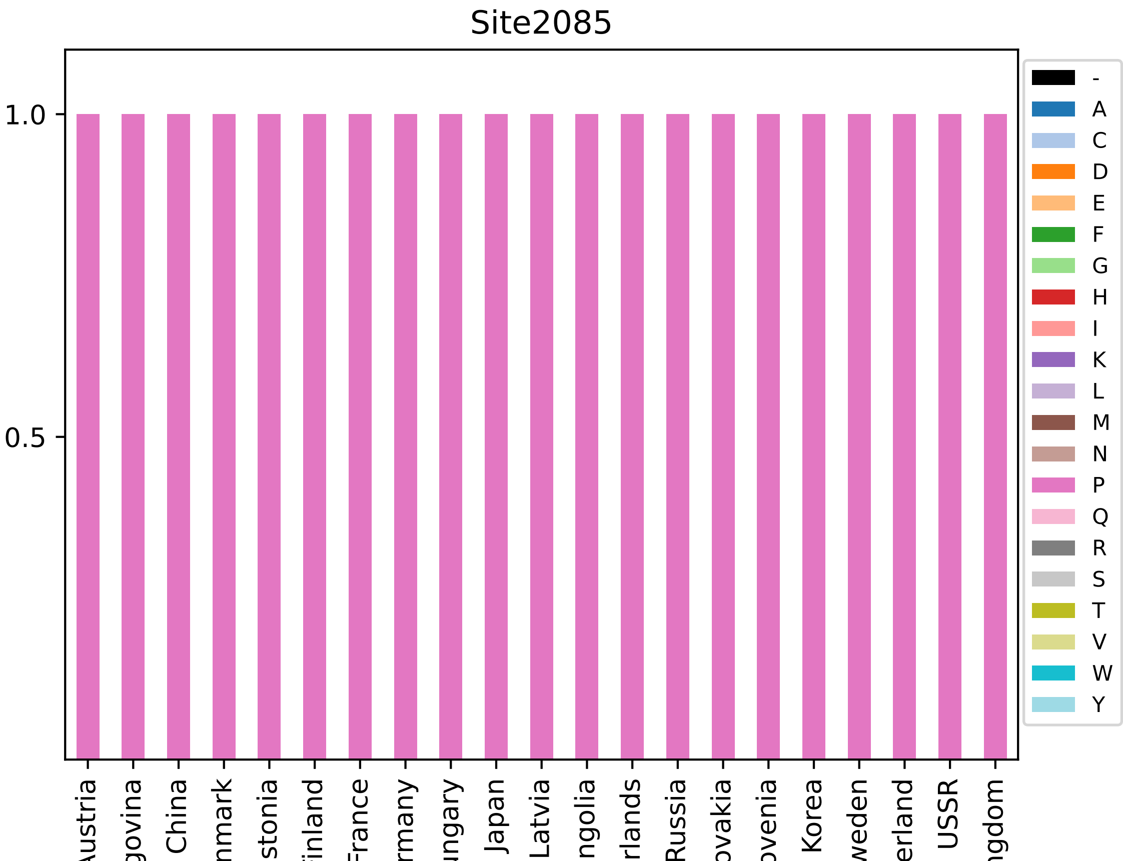

Site2085
1.0
0.5
-
A
C
D
E
F
G
H
I
K
L
M
N
P
Q
R
S
T
V
W
Y
Austria
China
Japan
Latvia
Russia
Korea
USSR

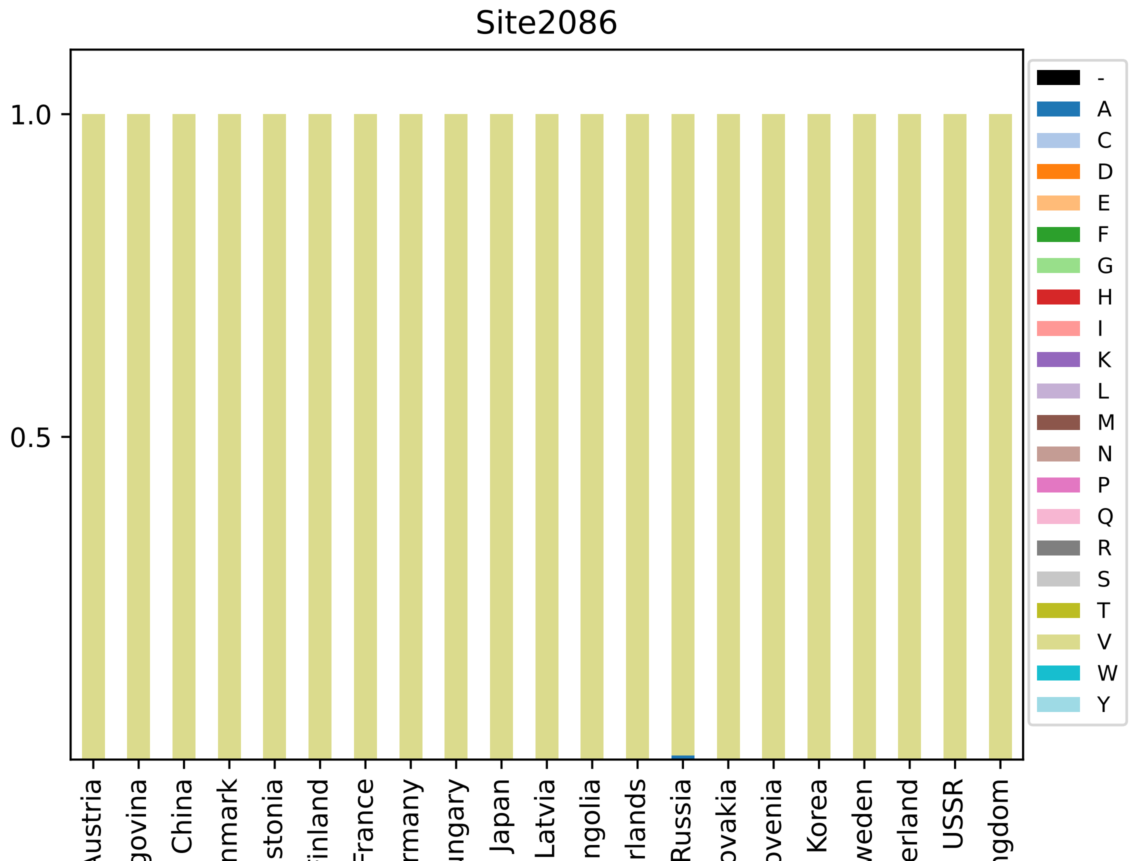
Site2086
1.0
0.5
-
A
C
D
E
F
G
H
I
K
L
M
N
P
Q
R
S
T
V
W
Y
Austria
China
Japan
Latvia
Russia
Korea
USSR

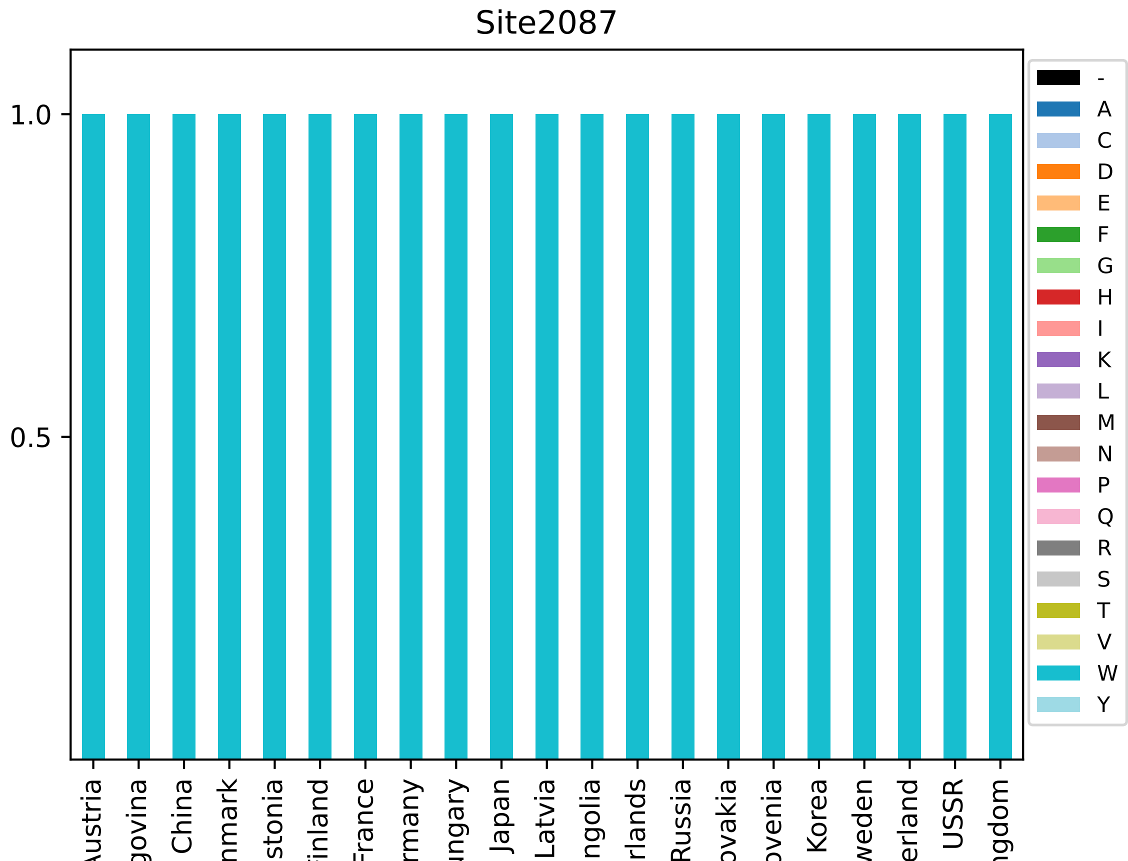
Site2087
1.0
0.5
-
A
C
D
E
F
G
H
I
K
L
M
N
P
Q
R
S
T
V
W
Y
China
Japan
Latvia
Russia
Korea
USSR

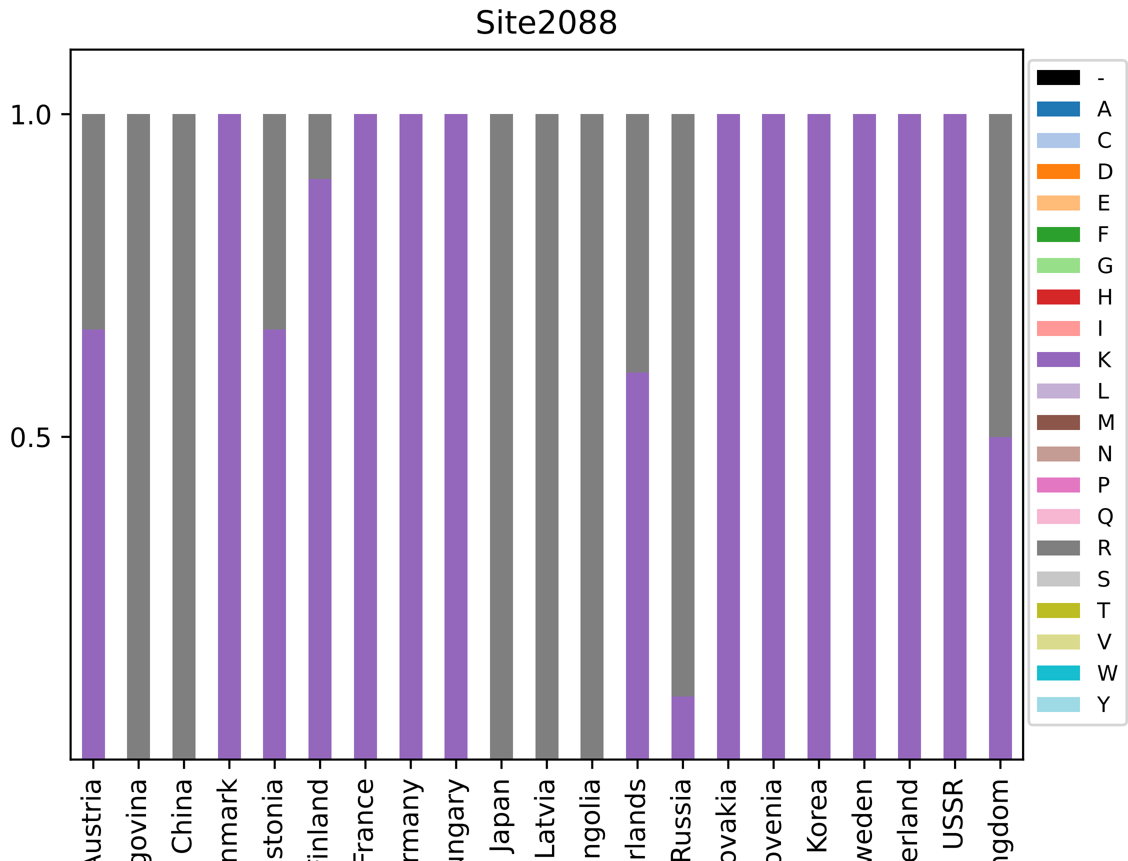

Site2088
1.0
0.5
-
A
C
D
E
F
G
H
I
K
L
M
N
P
Q
R
S
T
V
W
Y
Austria
govina
China
nmark
stonia
Finland
France
rmany
ungary
Japan
Latvia
ngolia
rlands
Russia
ovakia
ovenia
Korea
weden
erland
USSR
ngdom

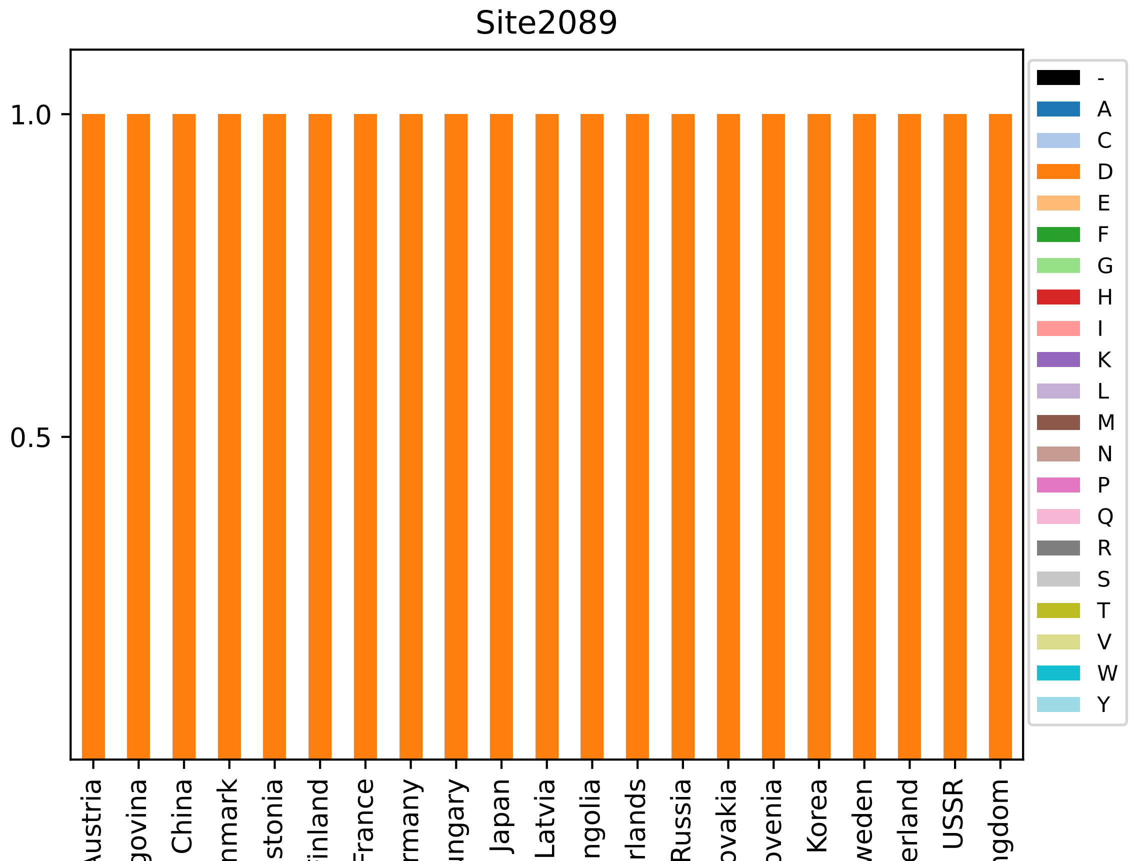

Site2089
1.0
0.5
-
A
C
D
E
F
G
H
I
K
L
M
N
P
Q
R
S
T
V
W
Y
Austria
govina
China
nmark
stonia
inland
France
rmany
ungary
Japan
Latvia
ngolia
rlands
Russia
ovakia
ovenia
Korea
weden
erland
USSR
ngdom

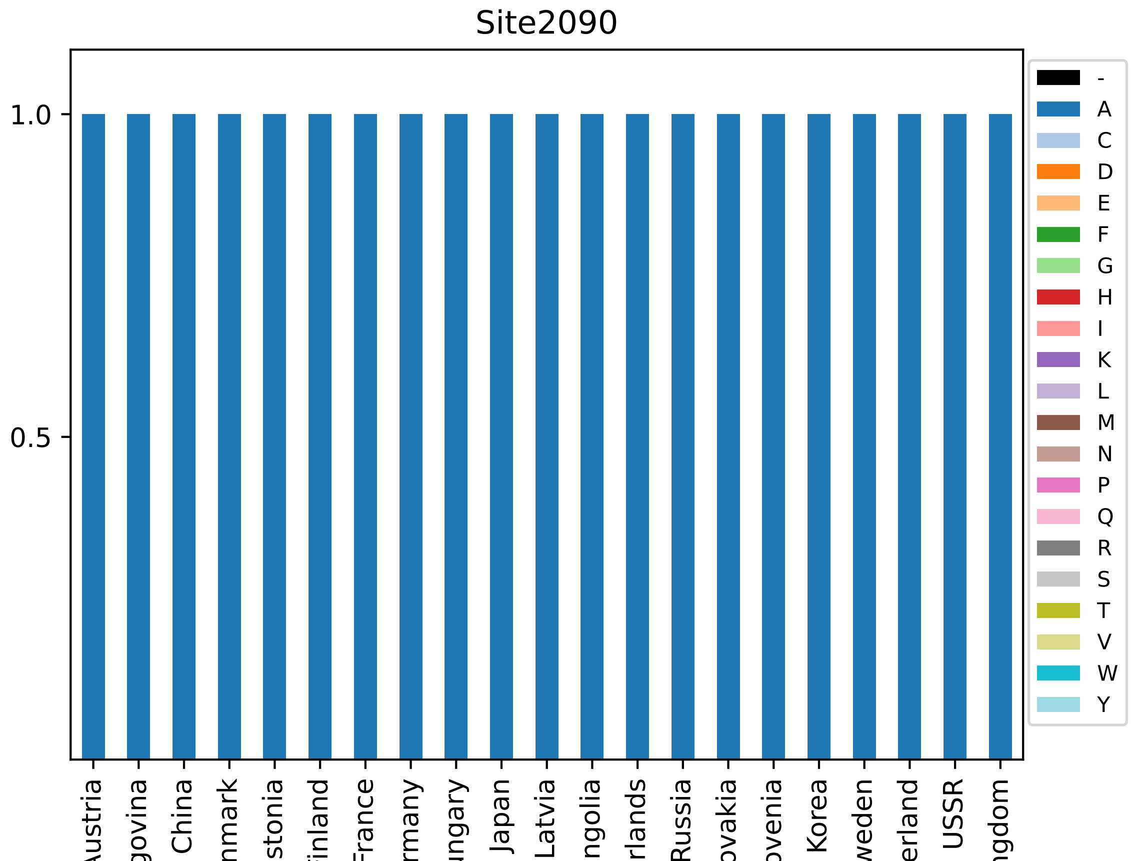
Site2090
1.0
0.5
-
A
C
D
E
F
G
H
I
K
L
M
N
P
Q
R
S
T
V
W
Y
China
Japan
Latvia
Russia
Korea
USSR

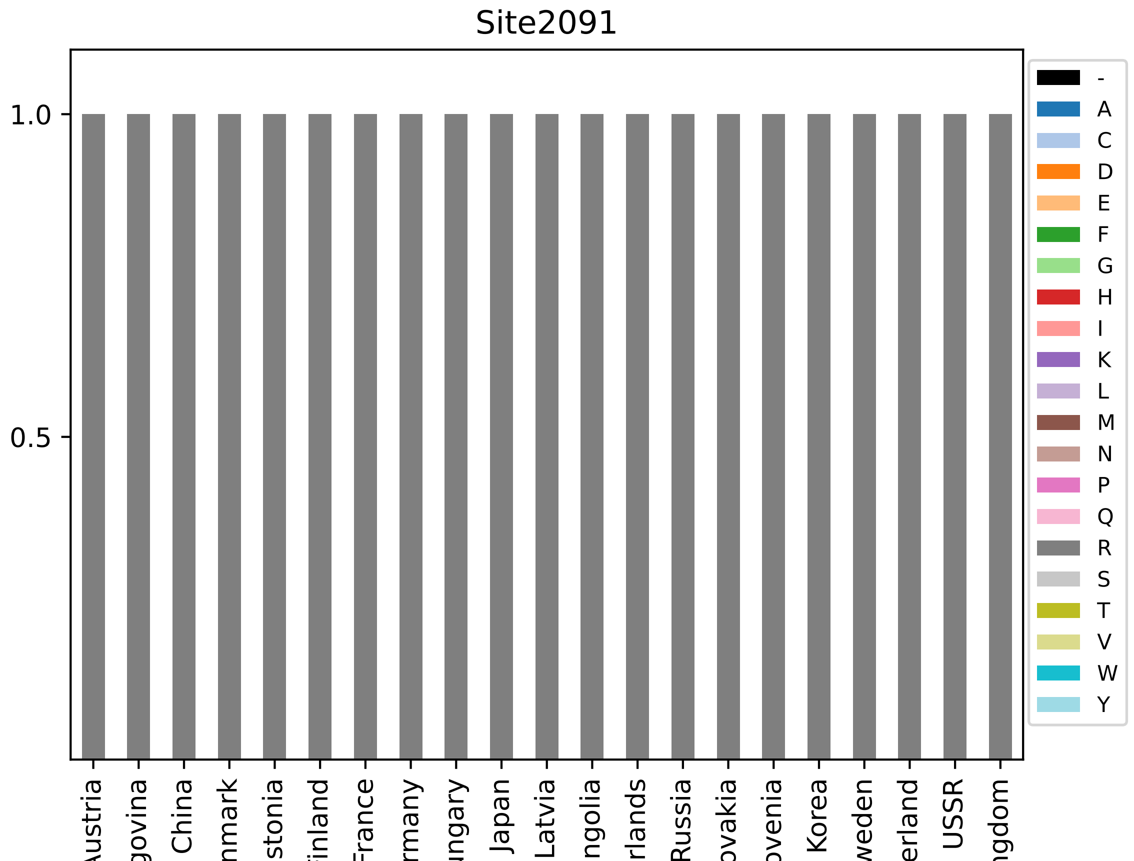
Site2091
1.0
0.5
-
A
C
D
E
F
G
H
I
K
L
M
N
P
Q
R
S
T
V
W
Y
Austria
China
Japan
Latvia
Russia
Korea
USSR

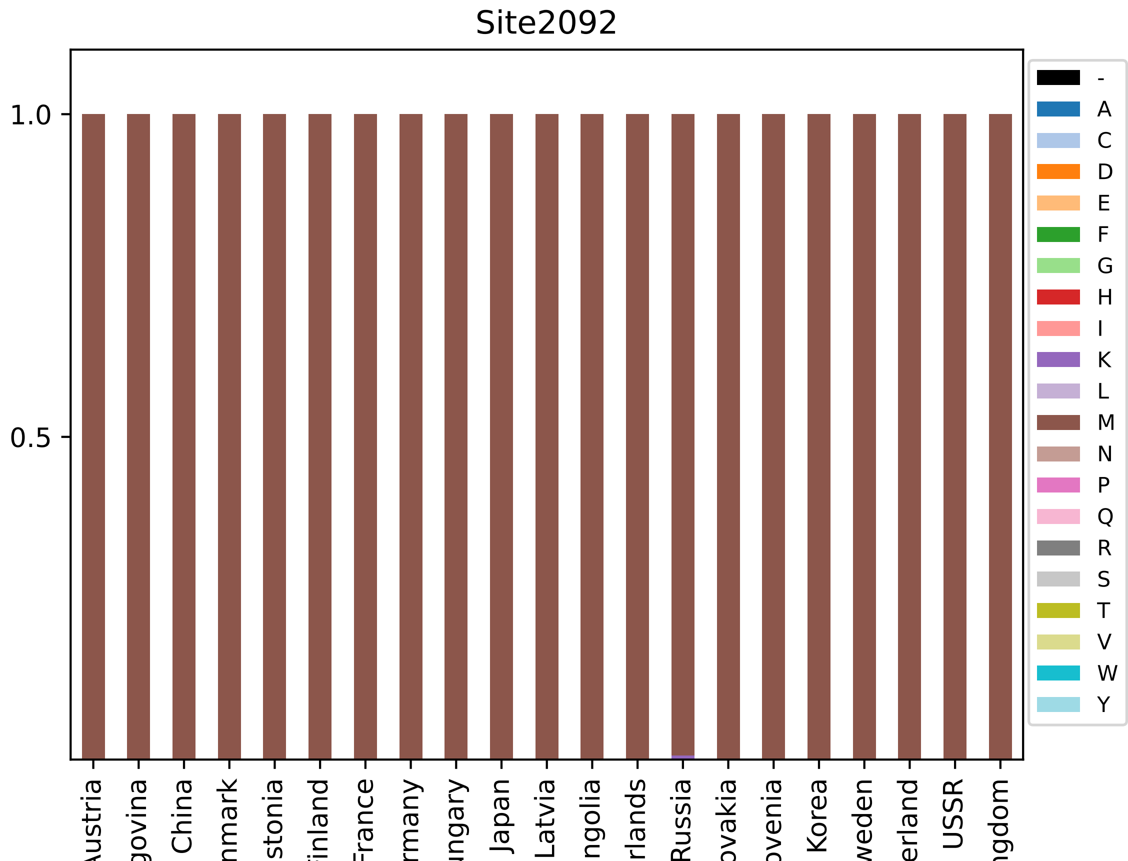

Site2092
1.0
0.5
-
A
C
D
E
F
G
H
I
K
L
M
N
P
Q
R
S
T
V
W
Y
Austria
govina
China
nmark
stonia
Finland
France
rmany
ungary
Japan
Latvia
ngolia
rlands
Russia
ovakia
ovenia
Korea
weden
erland
USSR
ngdom

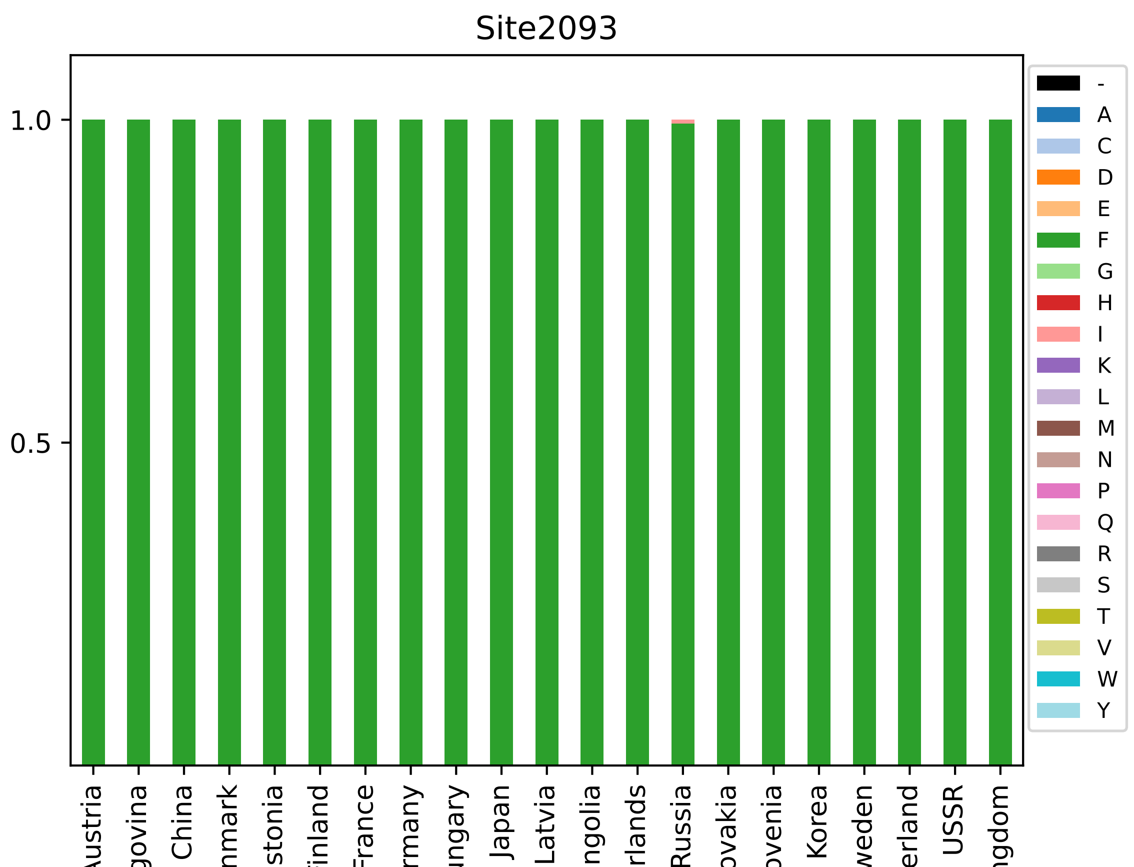

Site2093
1.0
0.5
-
A
C
D
E
F
G
H
I
K
L
M
N
P
Q
R
S
T
V
W
Y
Austria
govina
China
nmark
stonia
inland
France
rmany
ungary
Japan
Latvia
ngolia
rlands
Russia
ovakia
ovenia
Korea
weden
erland
USSR
ngdom

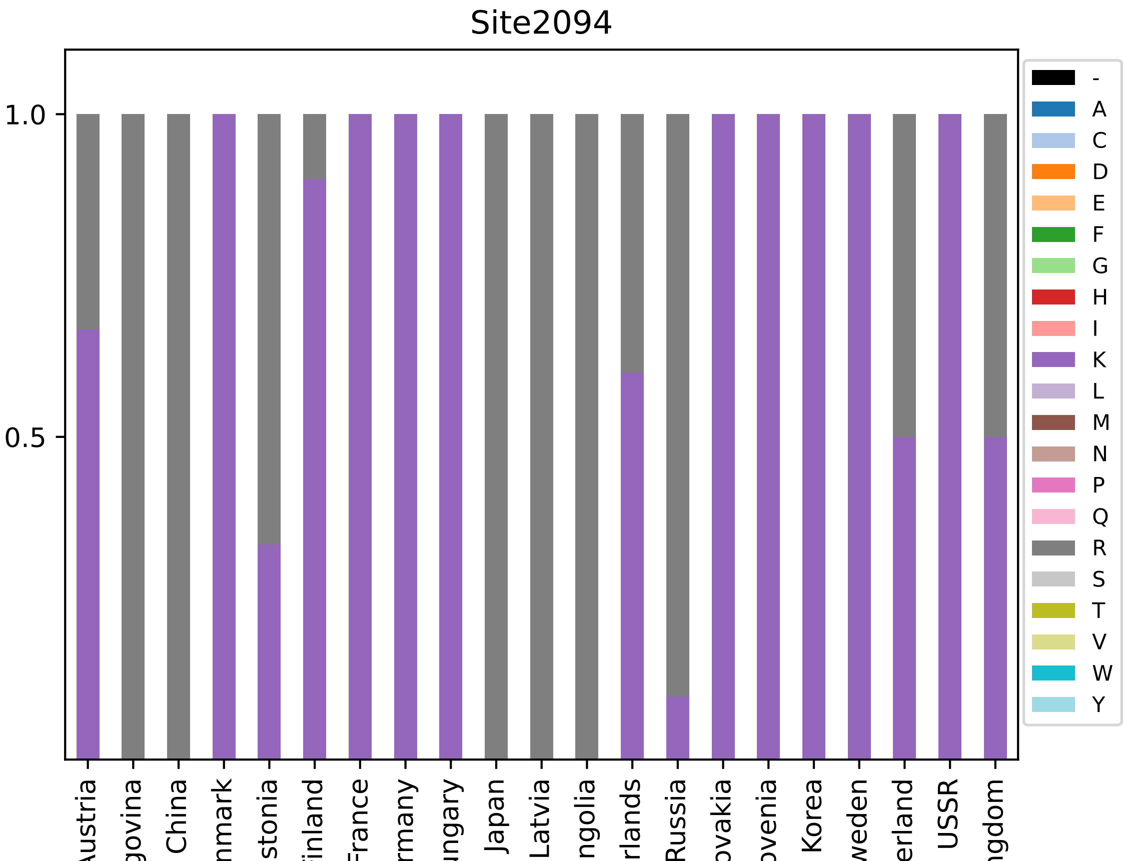
Site2094
1.0
0.5
-
A
C
D
E
F
G
H
I
K
L
M
N
P
Q
R
S
T
V
W
Y
Austria
China
Latvia
Japan
Russia
Korea
USSR

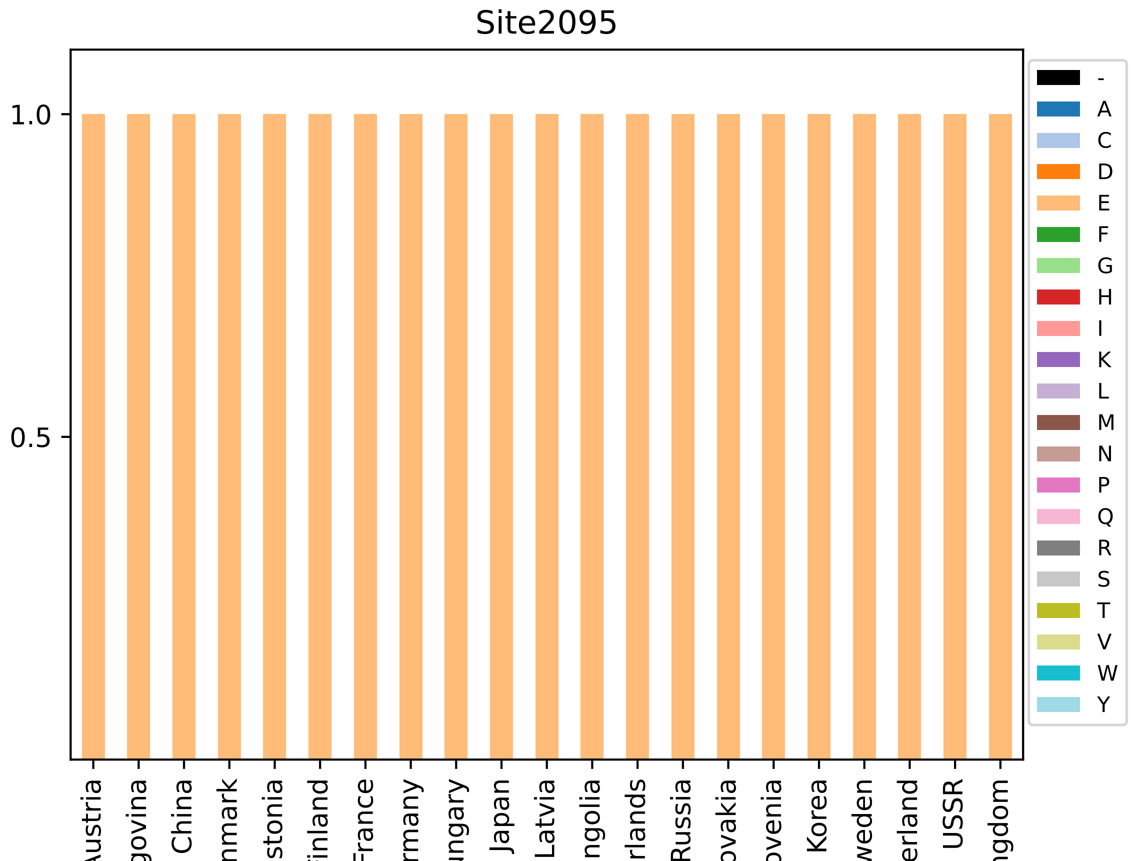

Site2095
1.0
0.5
-
A
C
D
E
F
G
H
I
K
L
M
N
P
Q
R
S
T
V
W
Y
Austria
govina
China
nmark
stonia
Finland
France
rmany
ungary
Japan
Latvia
ngolia
rlands
Russia
ovakia
ovenia
Korea
weden
erland
USSR
ngdom

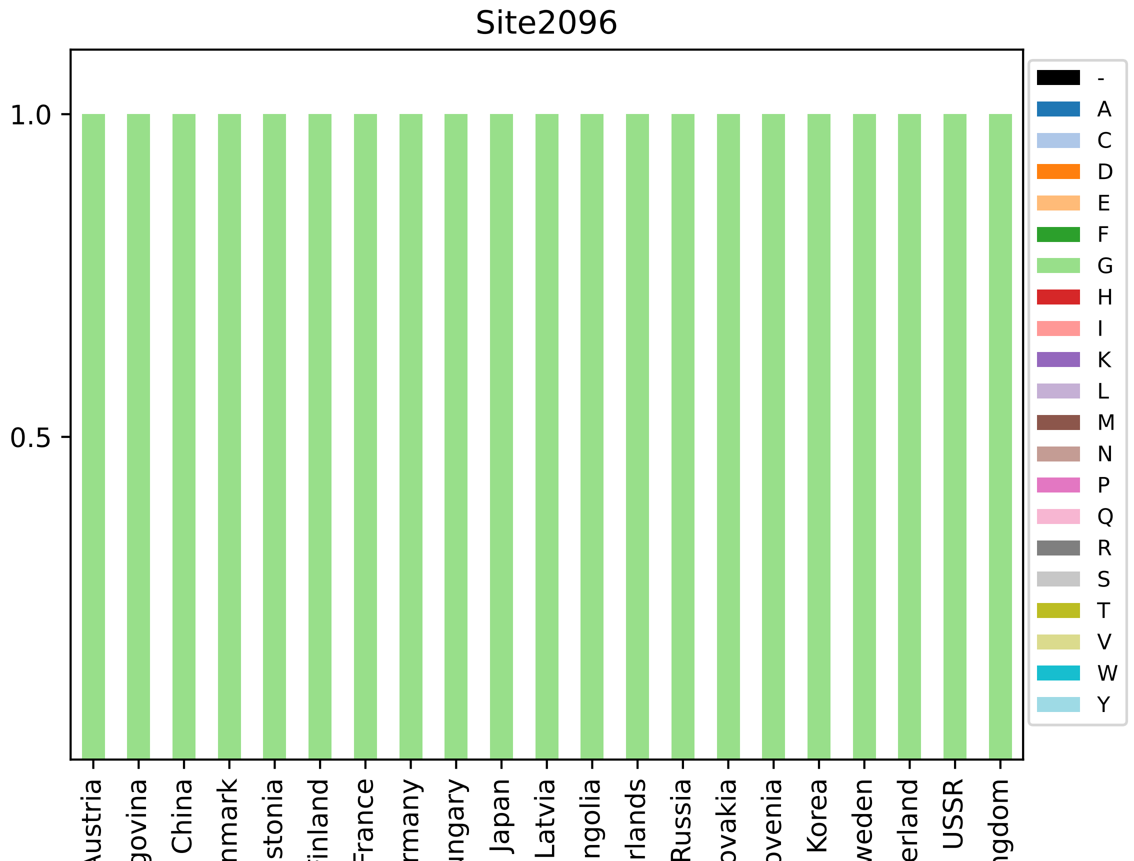
Site2096
1.0
0.5
-
A
C
D
E
F
G
H
I
K
L
M
N
P
Q
R
S
T
V
W
Y
Austria
China
Estonia
Finland
France
Japan
Latvia
Russia
Korea
USSR

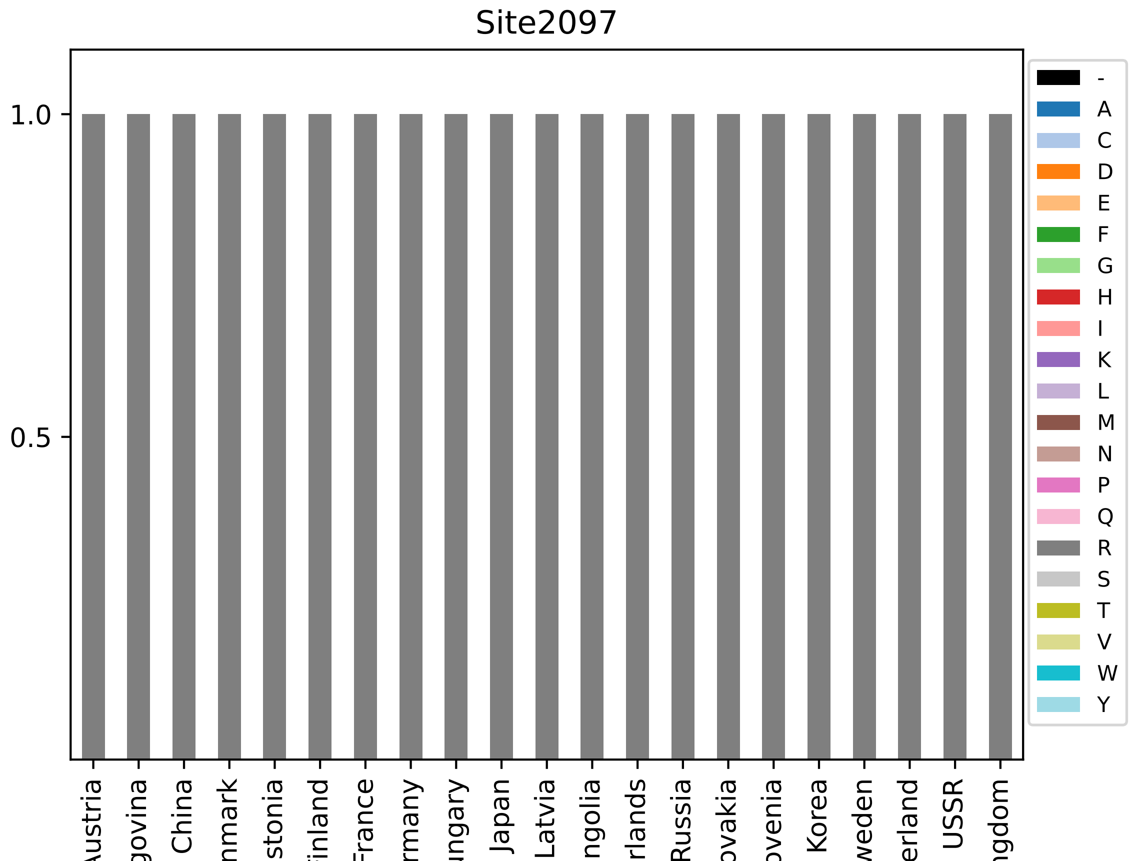

Site2097
1.0
0.5
-
A
C
D
E
F
G
H
I
K
L
M
N
P
Q
R
S
T
V
W
Y
Austria
govina
China
nmark
stonia
Finland
France
rmany
ungary
Japan
Latvia
ngolia
rlands
Russia
ovakia
ovenia
Korea
weden
erland
USSR
ngdom

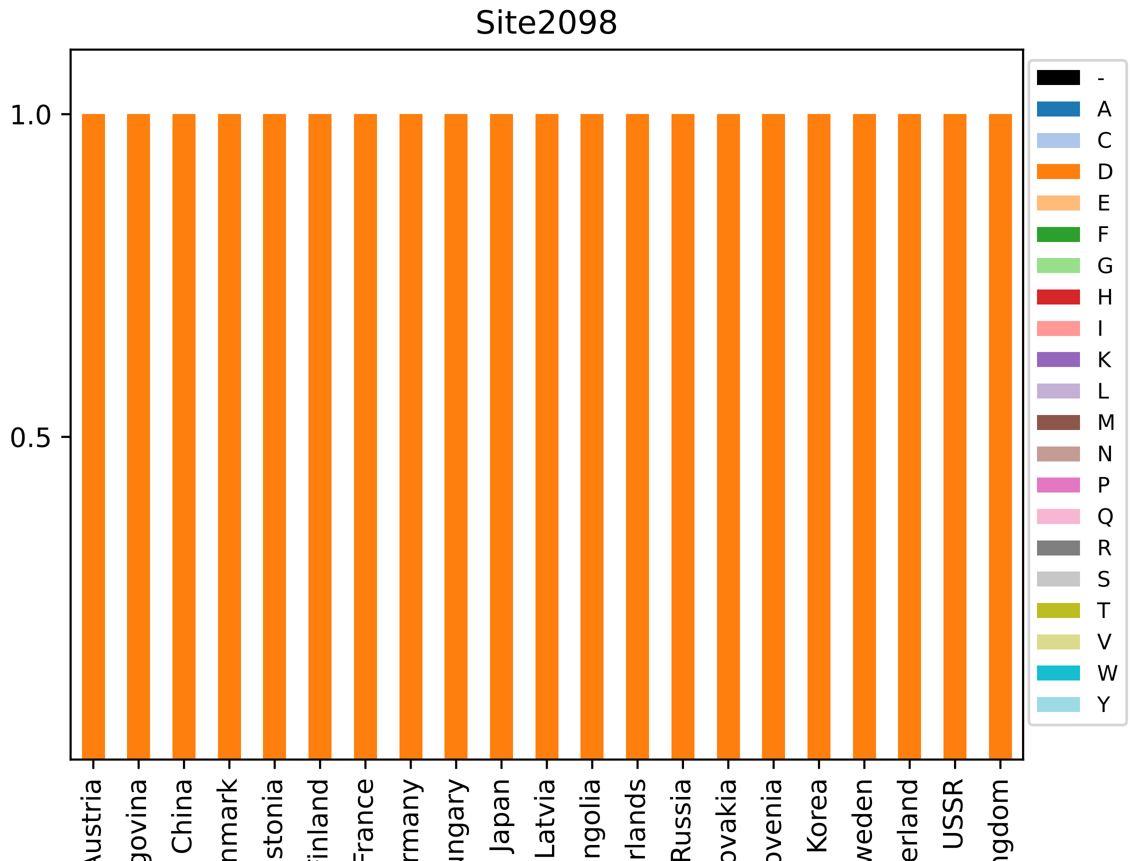

Site2098
1.0
0.5
-
A
C
D
E
F
G
H
I
K
L
M
N
P
Q
R
S
T
V
W
Y
Austria
China
Estonia
Finland
France
Hungary
Japan
Latvia
Russia
Korea
Sweden
USSR

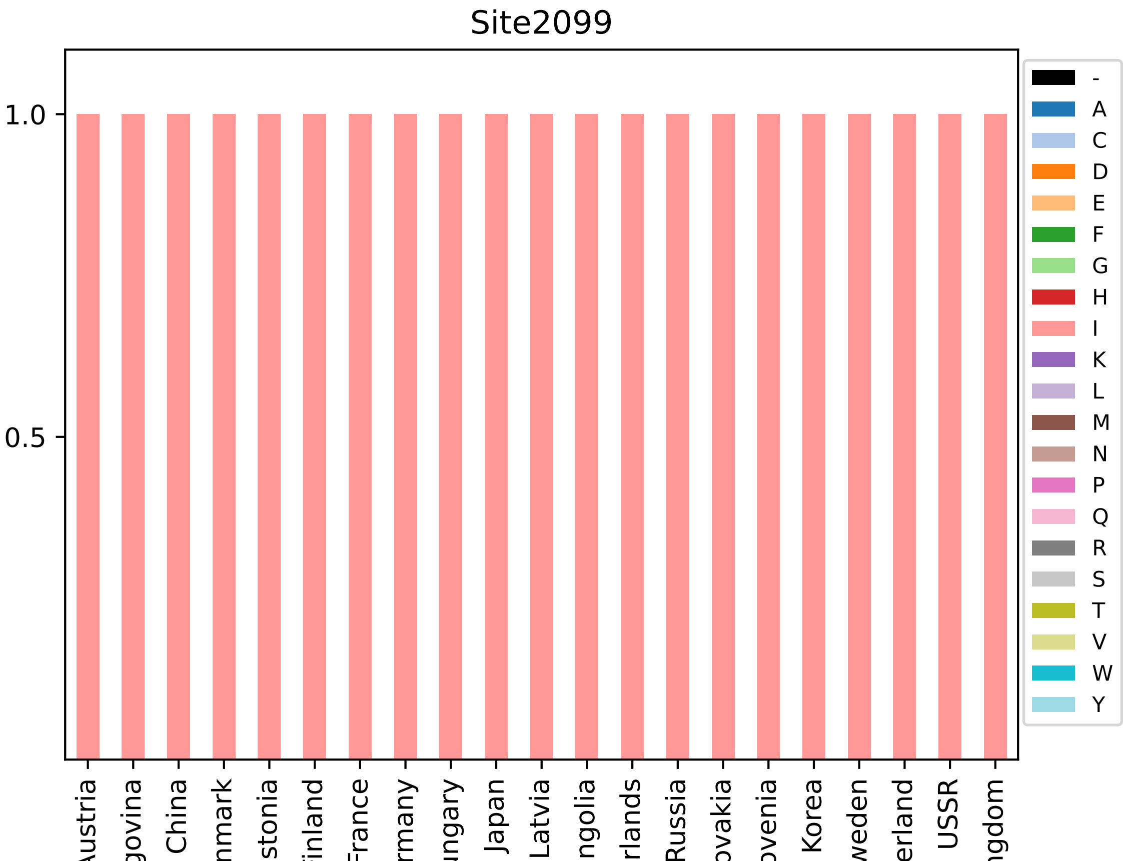
Site2099
1.0
0.5
-
A
C
D
E
F
G
H
I
K
L
M
N
P
Q
R
S
T
V
W
Y
China
Japan
Latvia
Russia
Korea
USSR

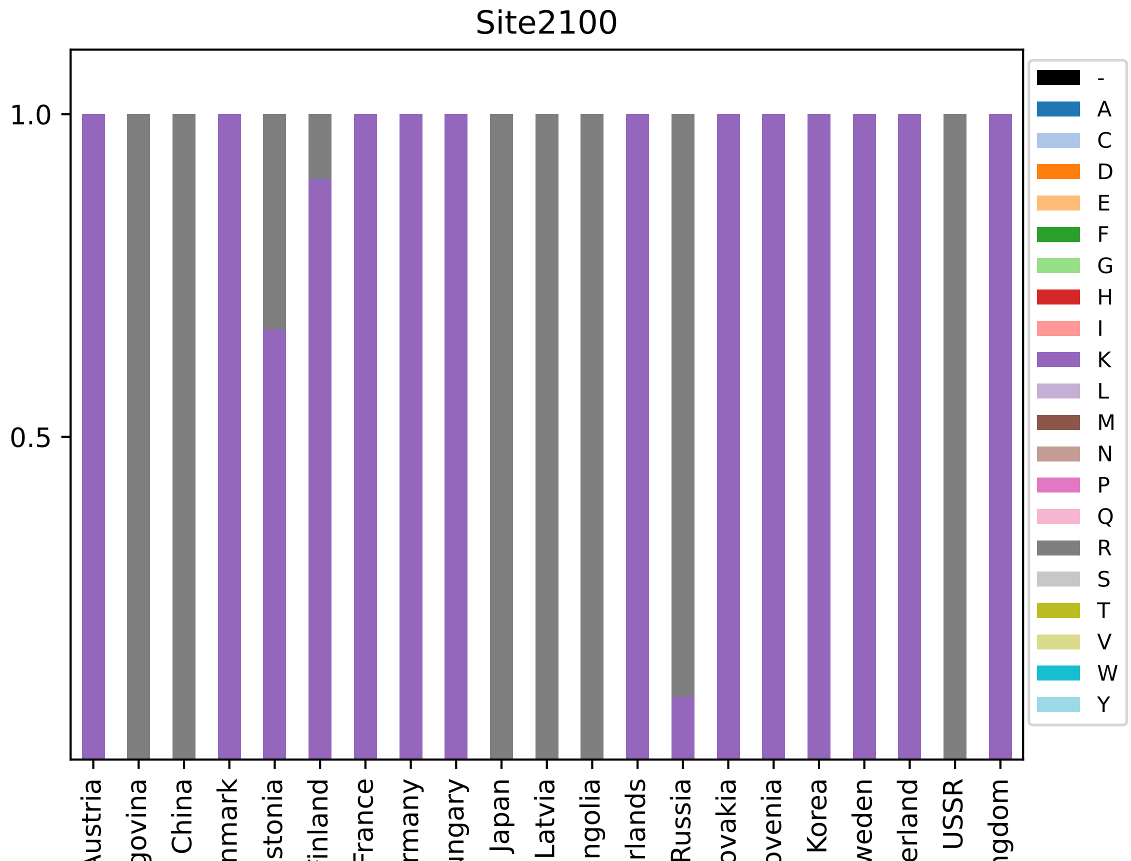

Site2100
1.0
0.5
-
A
C
D
E
F
G
H
I
K
L
M
N
P
Q
R
S
T
V
W
Y
China
Finland
France
Japan
Latvia
Russia
Korea
USSR

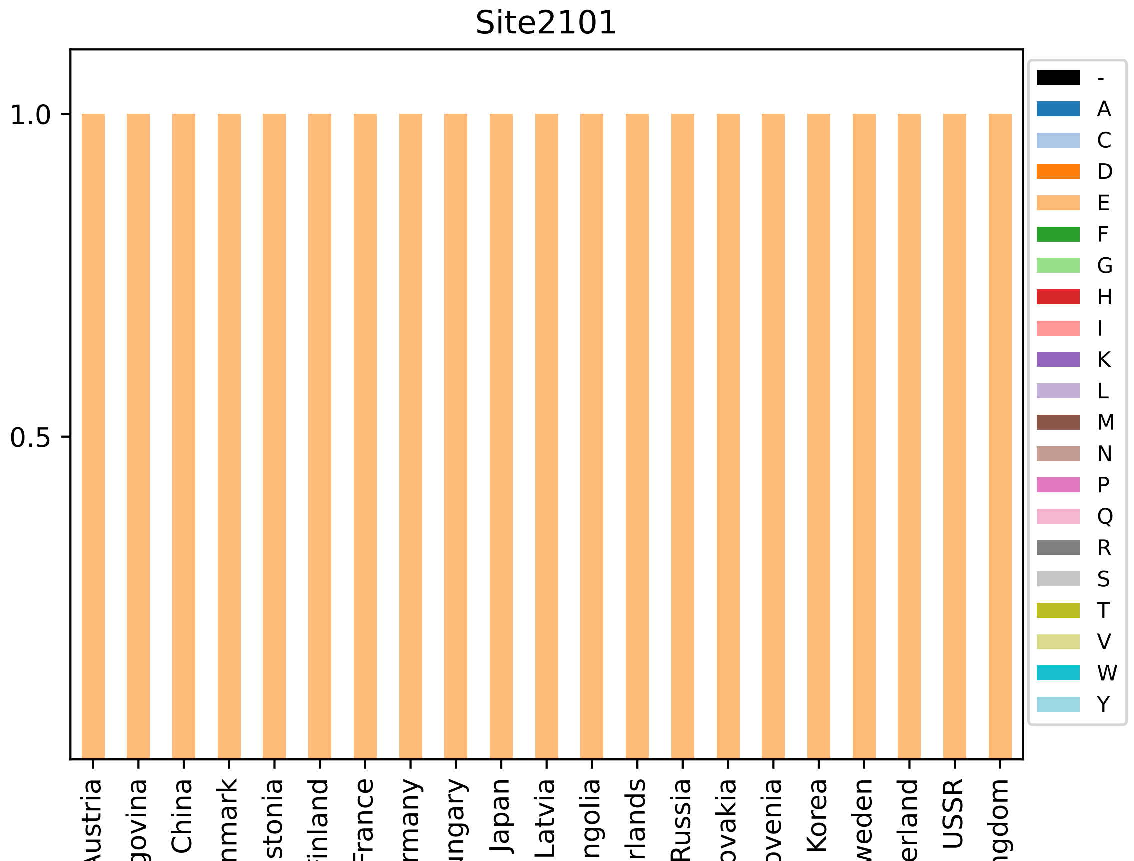

Site2101
1.0
0.5
-
A
C
D
E
F
G
H
I
K
L
M
N
P
Q
R
S
T
V
W
Y
Austria
govina
China
nmark
stonia
Finland
France
rmany
ungary
Japan
Latvia
ngolia
rlands
Russia
ovakia
ovenia
Korea
weden
erland
USSR
ngdom

Site2102

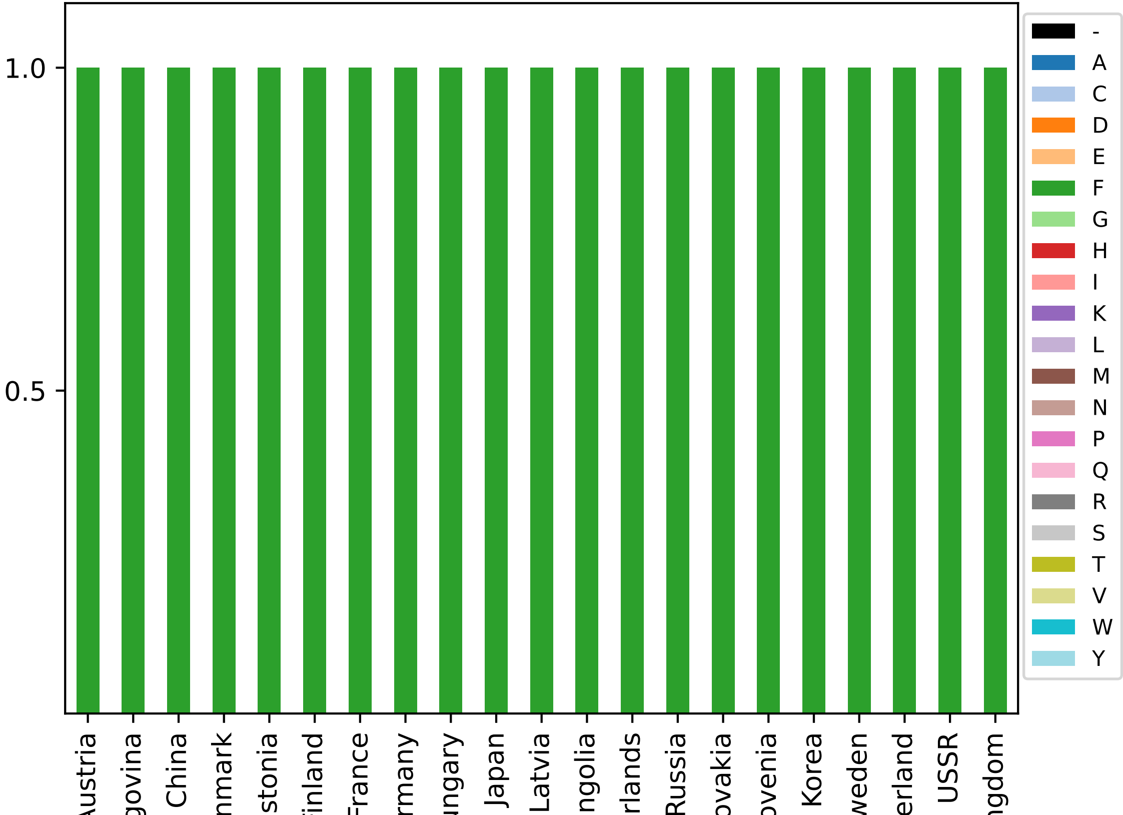

1.0
0.5
-
A
C
D
E
F
G
H
I
K
L
M
N
P
Q
R
S
T
V
W
Y
Austria
govina
China
nmark
stonia
Finland
France
rmany
ungary
Japan
Latvia
ngolia
rlands
Russia
ovakia
ovenia
Korea
weden
erland
USSR
ngdom

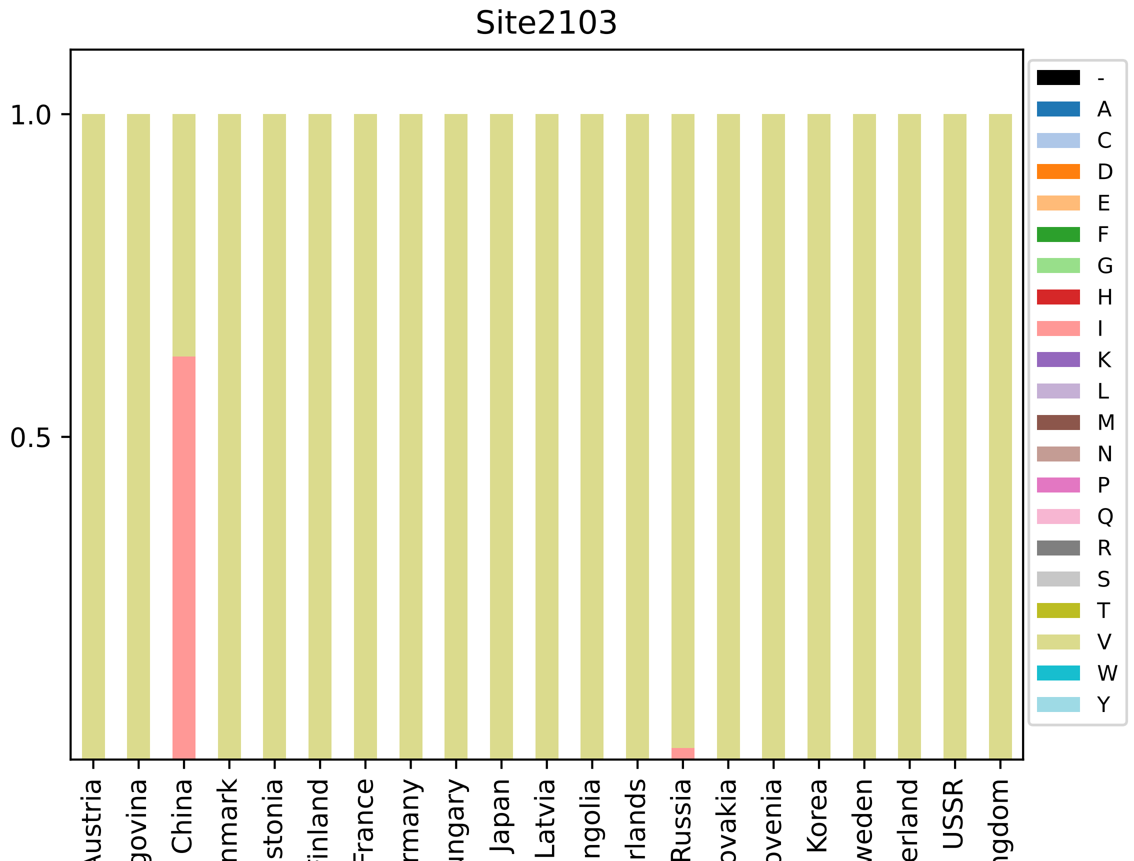
Site2103
1.0
0.5
-
A
C
D
E
F
G
H
I
K
L
M
N
P
Q
R
S
T
V
W
Y
China
Japan
Latvia
Russia
Korea
USSR

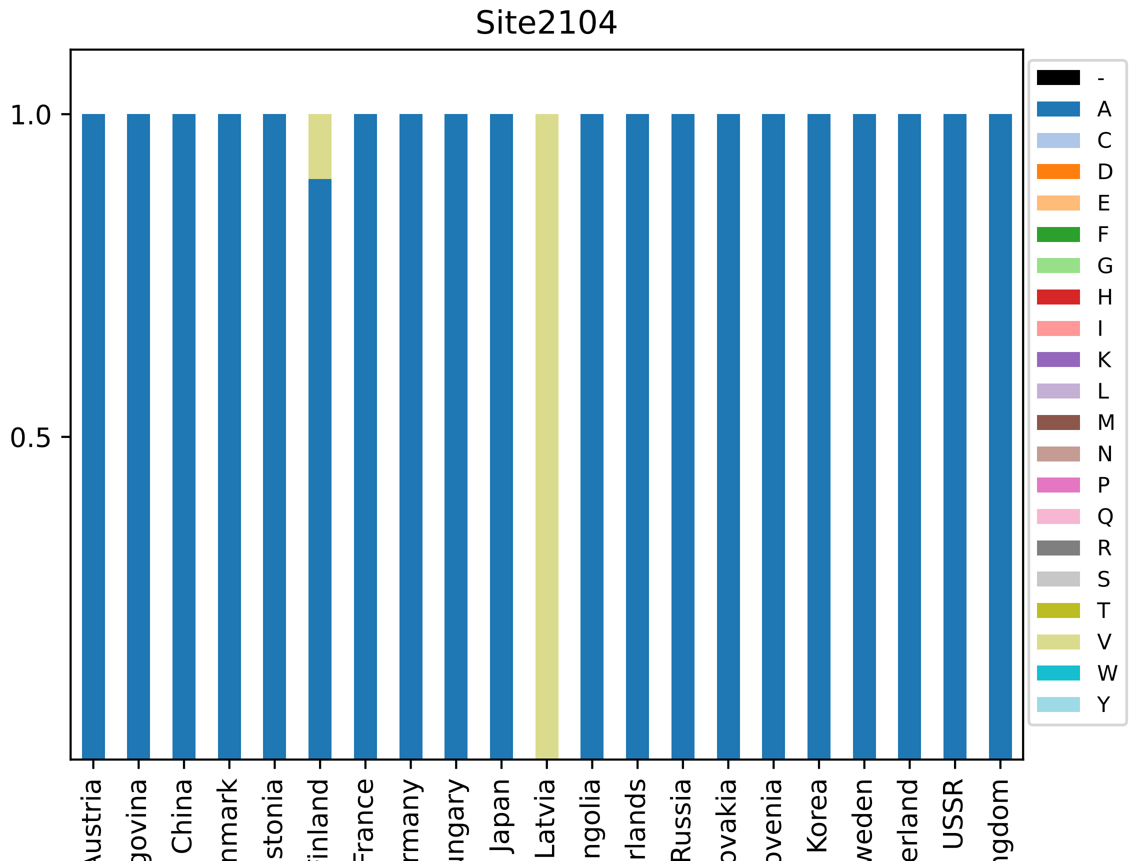

Site2104
1.0
0.5
-
A
C
D
E
F
G
H
I
K
L
M
N
P
Q
R
S
T
V
W
Y
Austria
govina
China
nmark
stonia
inland
France
rmany
ungary
Japan
Latvia
ngolia
rlands
Russia
ovakia
ovenia
Korea
weden
erland
USSR
ngdom

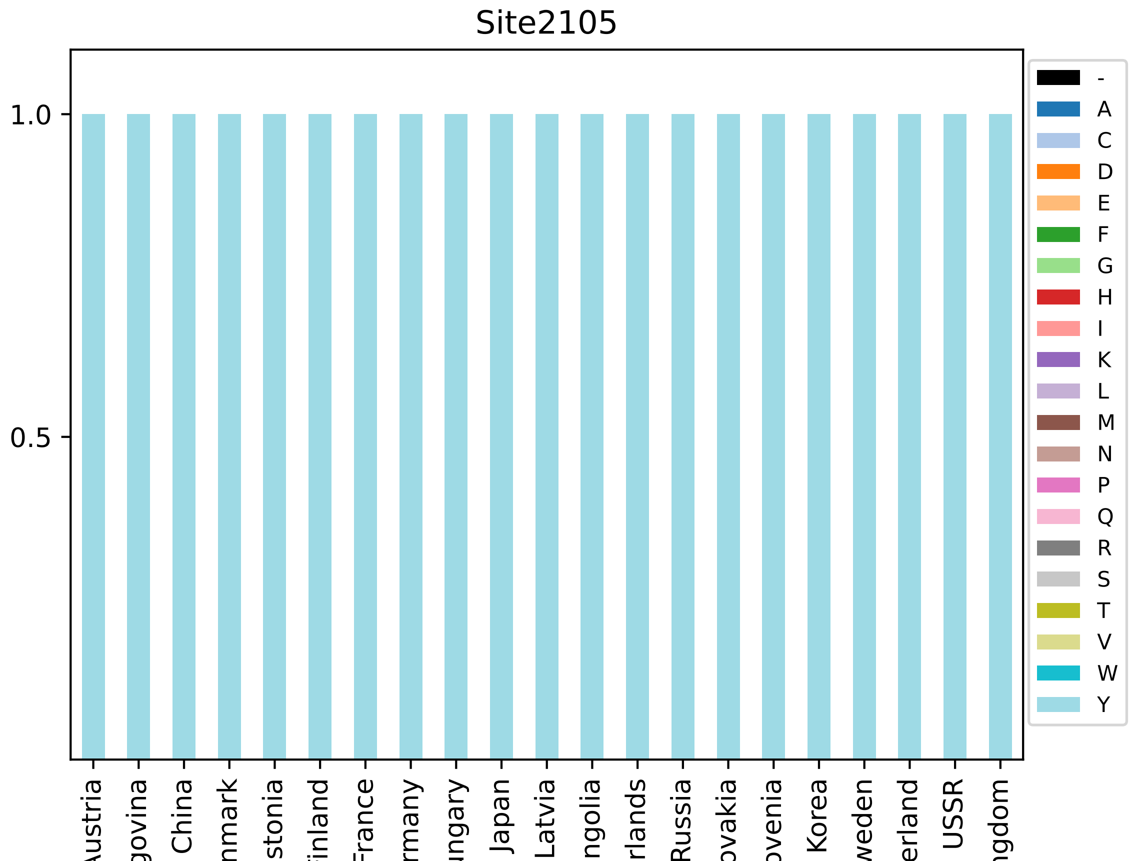
Site2105
1.0
0.5
-
A
C
D
E
F
G
H
I
K
L
M
N
P
Q
R
S
T
V
W
Y
Austria
govina
China
nmark
stonia
Finland
France
rmany
ungary
Japan
Latvia
ngolia
rlands
Russia
ovakia
ovenia
Korea
weden
erland
USSR
ngdom

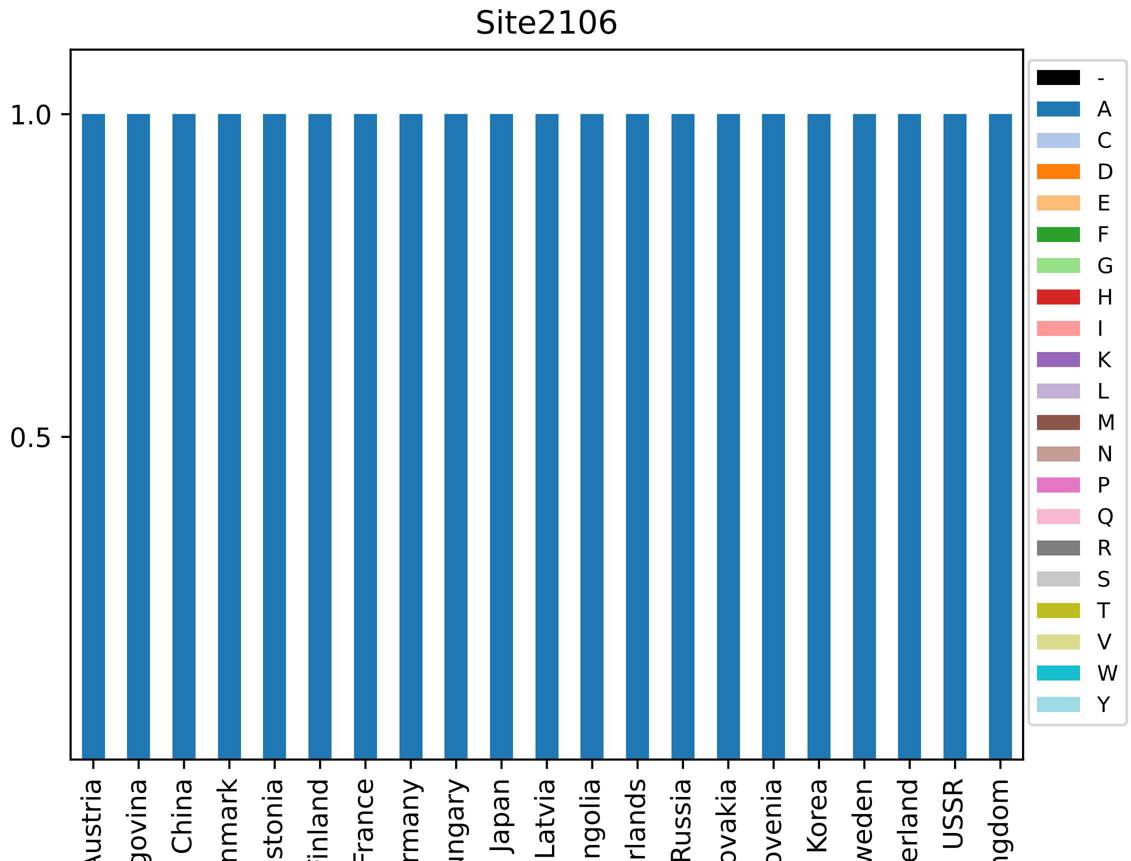
Site2106
1.0
0.5
Austria
govina
China
nmark
stonia
inland
France
rmany
ungary
Japan
Latvia
ngolia
rlands
Russia
ovakia
ovenia
Korea
weden
erland
USSR
ngdom
-
A
C
D
E
F
G
H
I
K
L
M
N
P
Q
R
S
T
V
W
Y

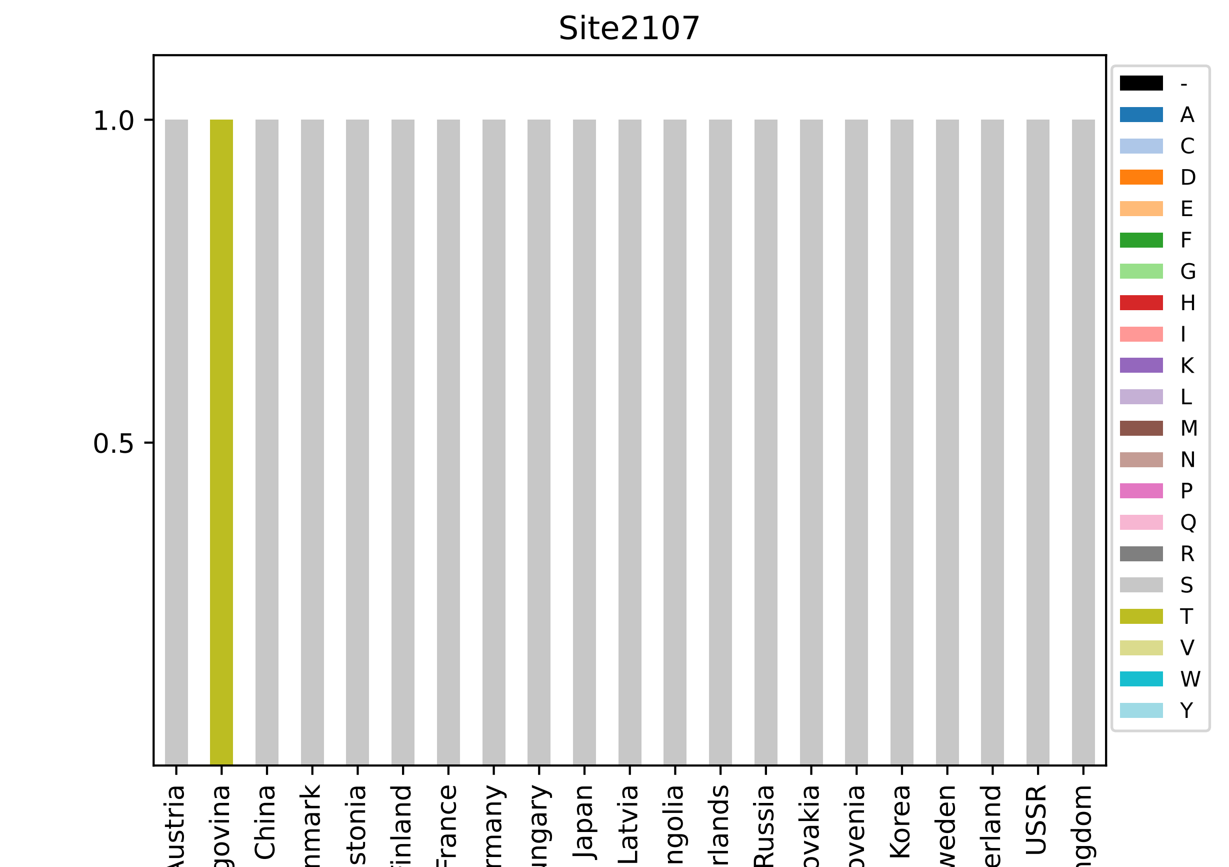

Site2107
1.0
0.5
-
A
C
D
E
F
G
H
I
K
L
M
N
P
Q
R
S
T
V
W
Y
Austria
govina
China
nmark
stonia
inland
France
rmany
ungary
Japan
Latvia
ngolia
rlands
Russia
ovakia
ovenia
Korea
weden
erland
USSR
ngdom

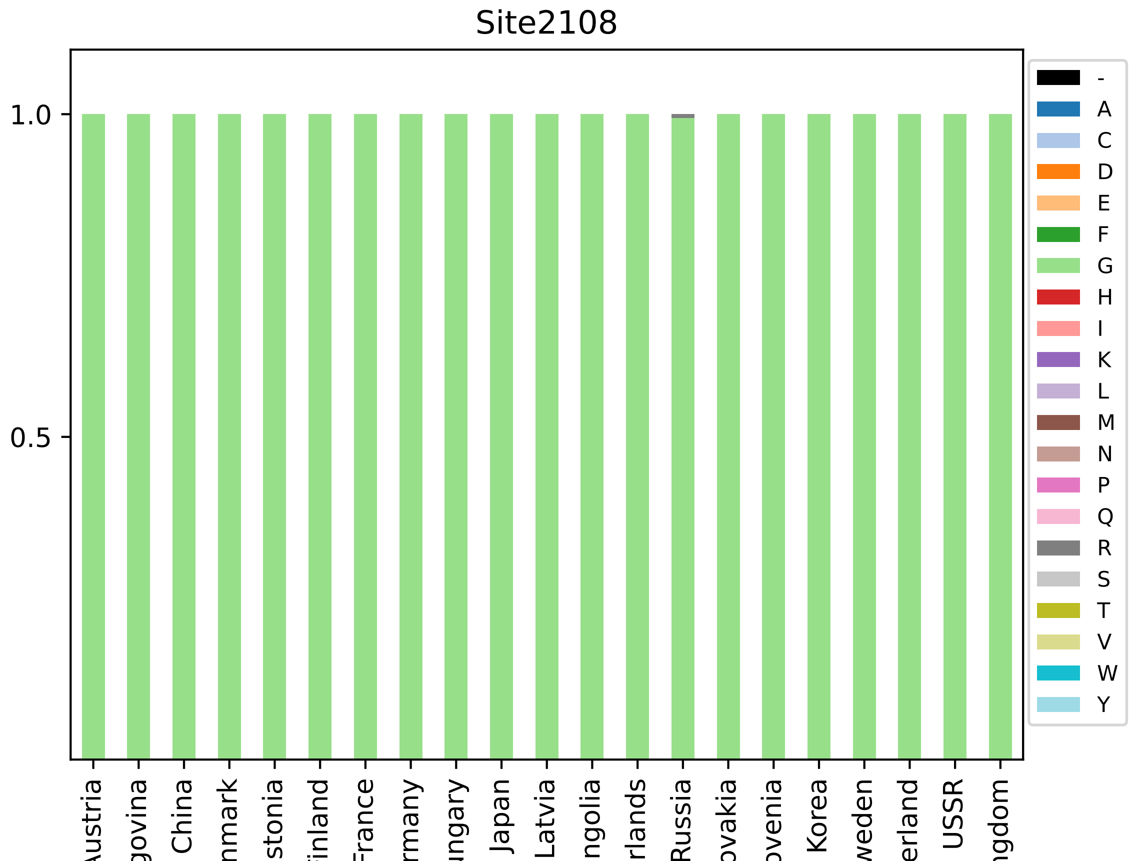
Site2108
1.0
0.5
-
A
C
D
E
F
G
H
I
K
L
M
N
P
Q
R
S
T
V
W
Y
Austria
China
Finland
France
Japan
Latvia
Russia
Korea
USSR

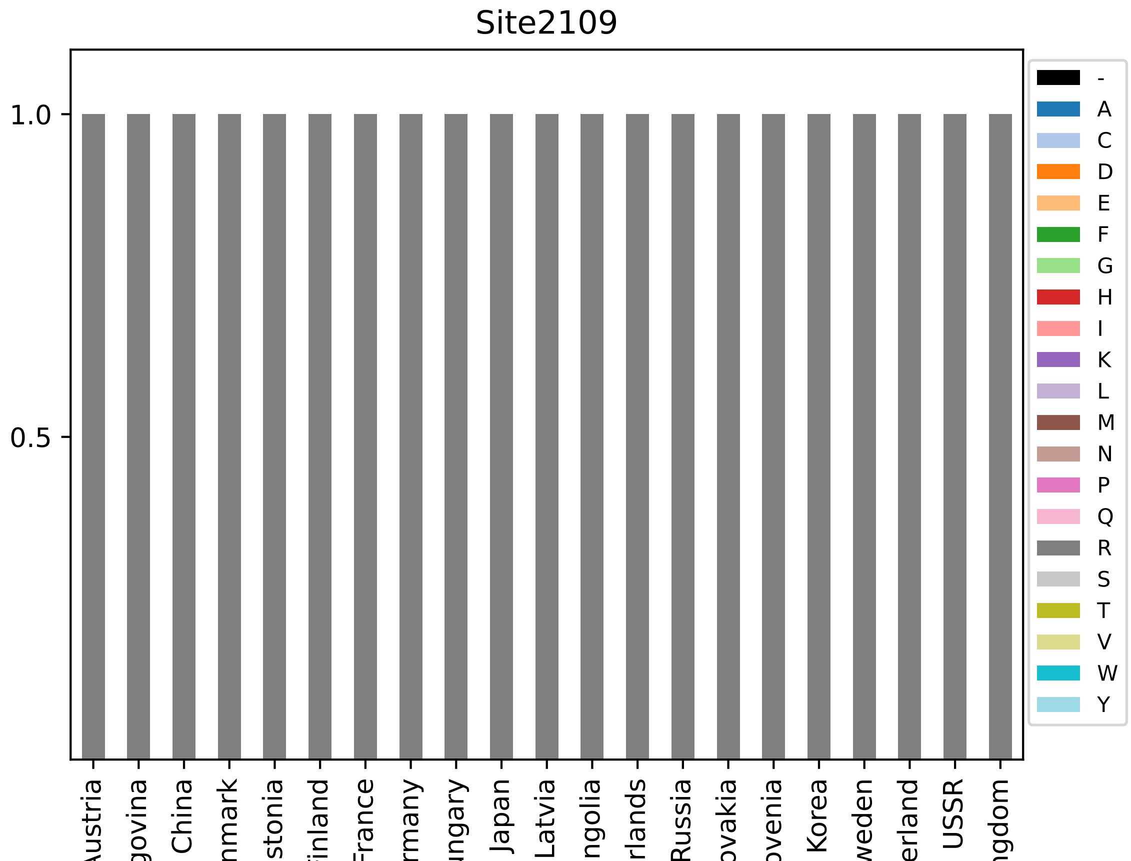

Site2109
1.0
0.5
-
A
C
D
E
F
G
H
I
K
L
M
N
P
Q
R
S
T
V
W
Y
Austria
China
Estonia
Finland
France
Germany
Hungary
Japan
Latvia
Russia
Korea
Sweden
USSR

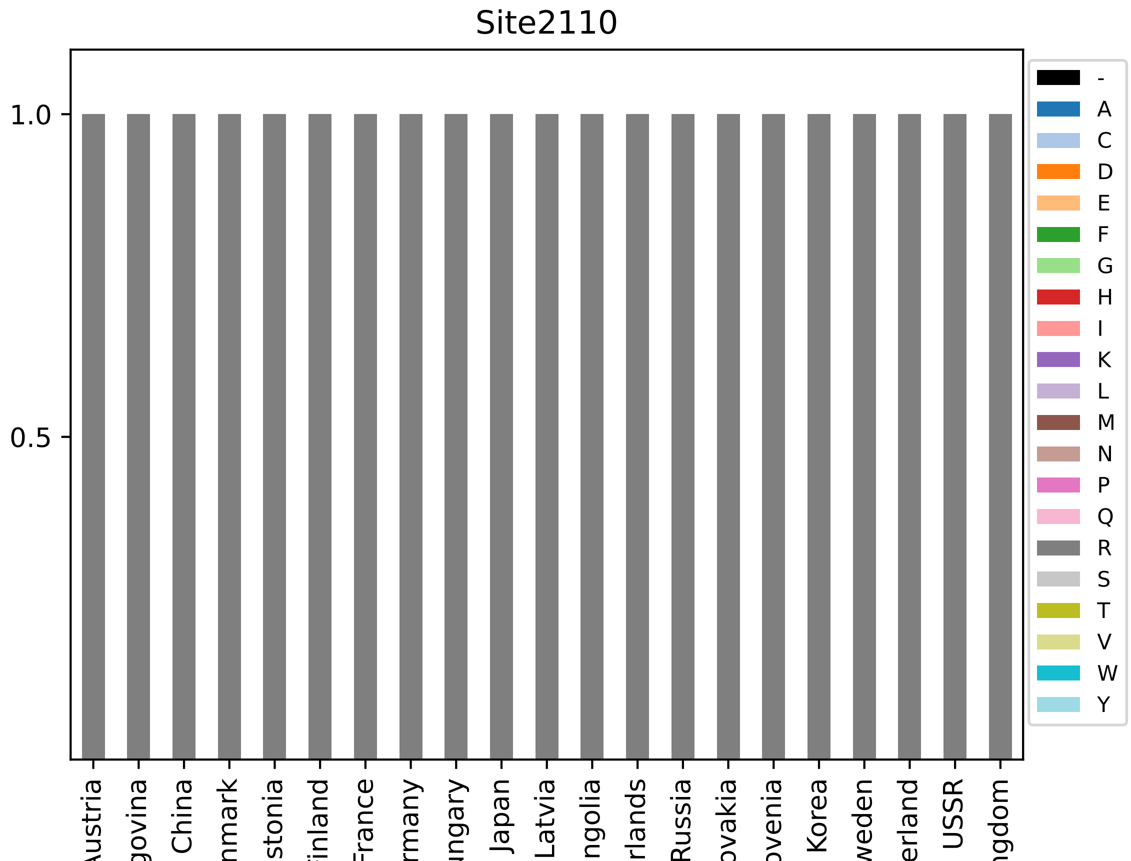

Site2110
1.0
0.5
-
A
C
D
E
F
G
H
I
K
L
M
N
P
Q
R
S
T
V
W
Y
Austria
China
Japan
Latvia
Russia
Korea
USSR

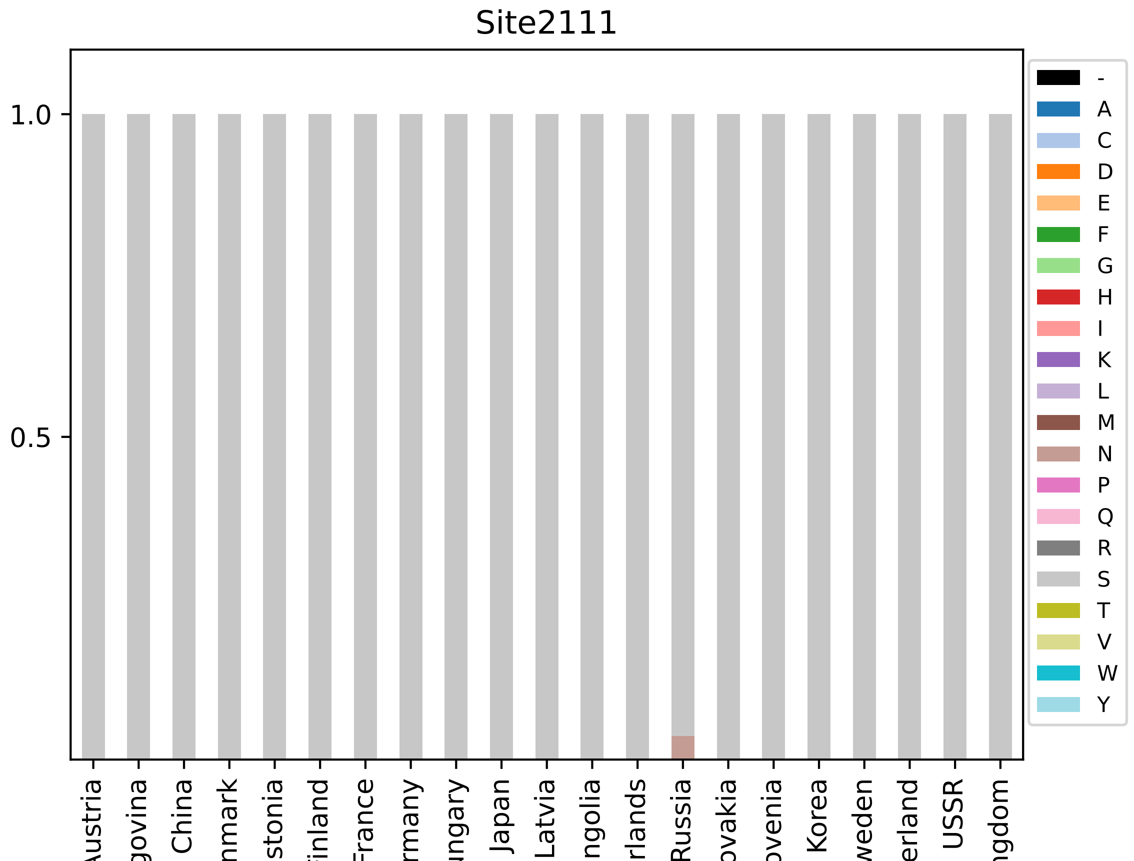
Site2111
1.0
0.5
-
A
C
D
E
F
G
H
I
K
L
M
N
P
Q
R
S
T
V
W
Y
Austria
China
Finland
France
Japan
Latvia
Russia
Korea
USSR

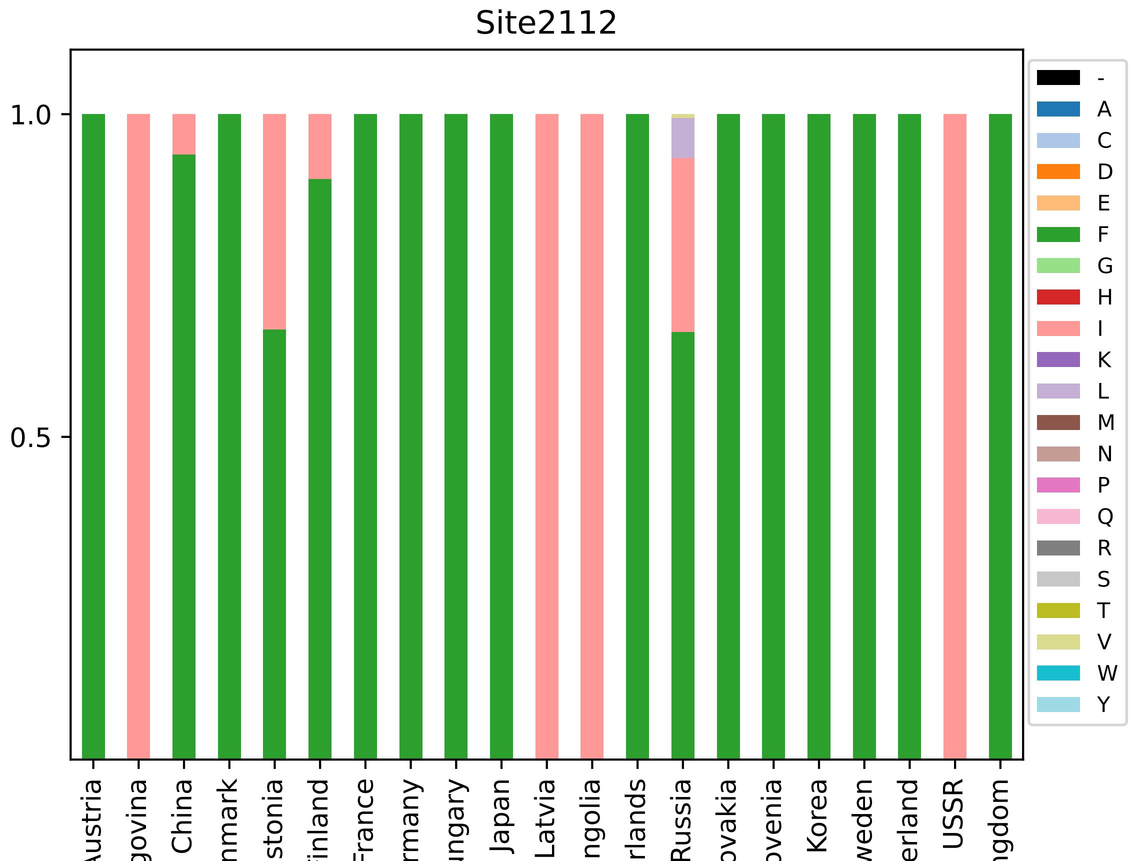
Site2112
1.0
0.5
-
A
C
D
E
F
G
H
I
K
L
M
N
P
Q
R
S
T
V
W
Y
China
Japan
Latvia
Russia
Korea
USSR

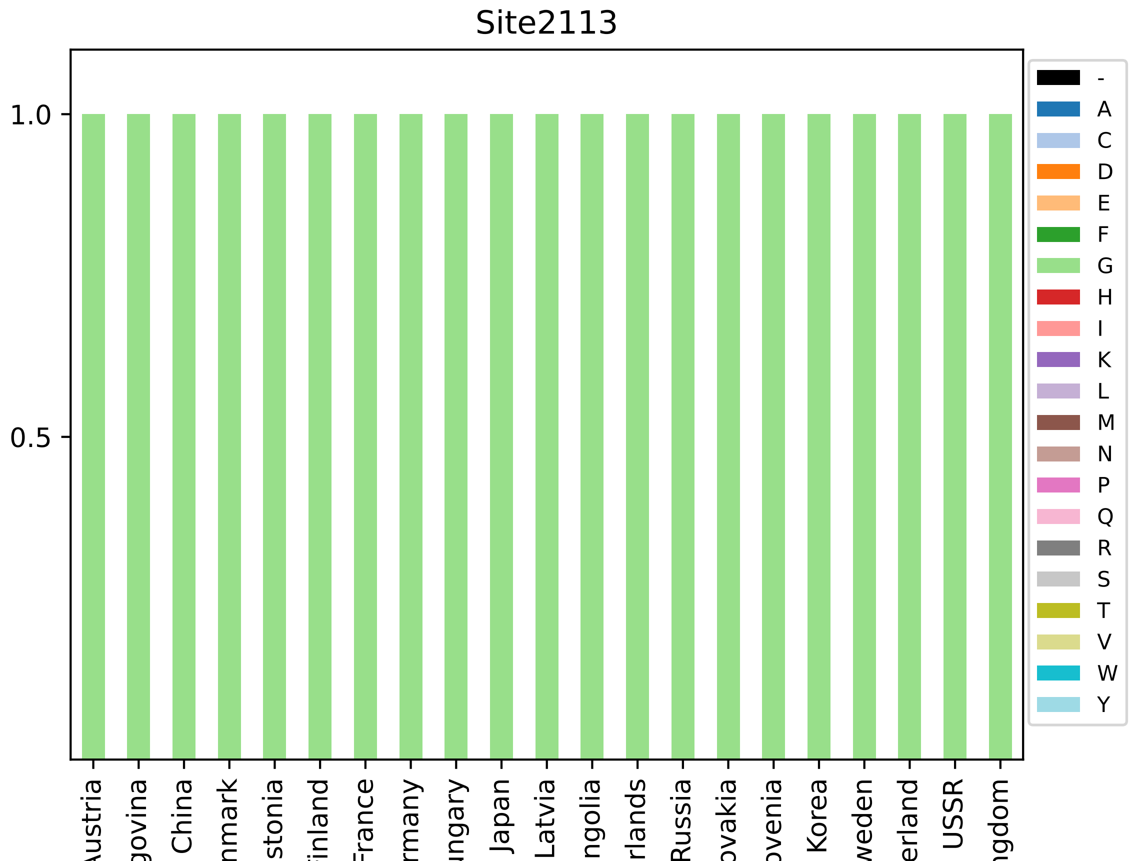

Site2113
1.0
0.5
-
A
C
D
E
F
G
H
I
K
L
M
N
P
Q
R
S
T
V
W
Y
China
Japan
Latvia
Russia
Korea
USSR

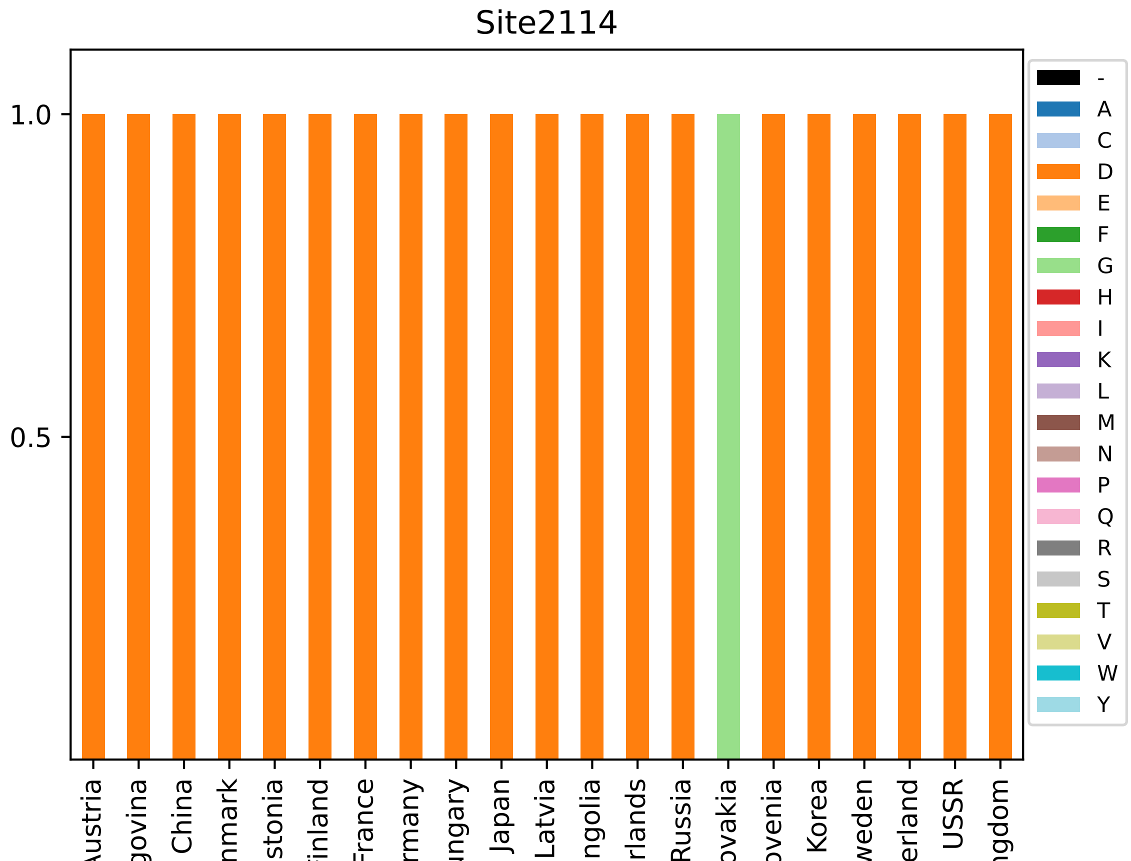

Site2114
1.0
0.5
-
A
C
D
E
F
G
H
I
K
L
M
N
P
Q
R
S
T
V
W
Y
China
Japan
Latvia
Russia
Korea
USSR

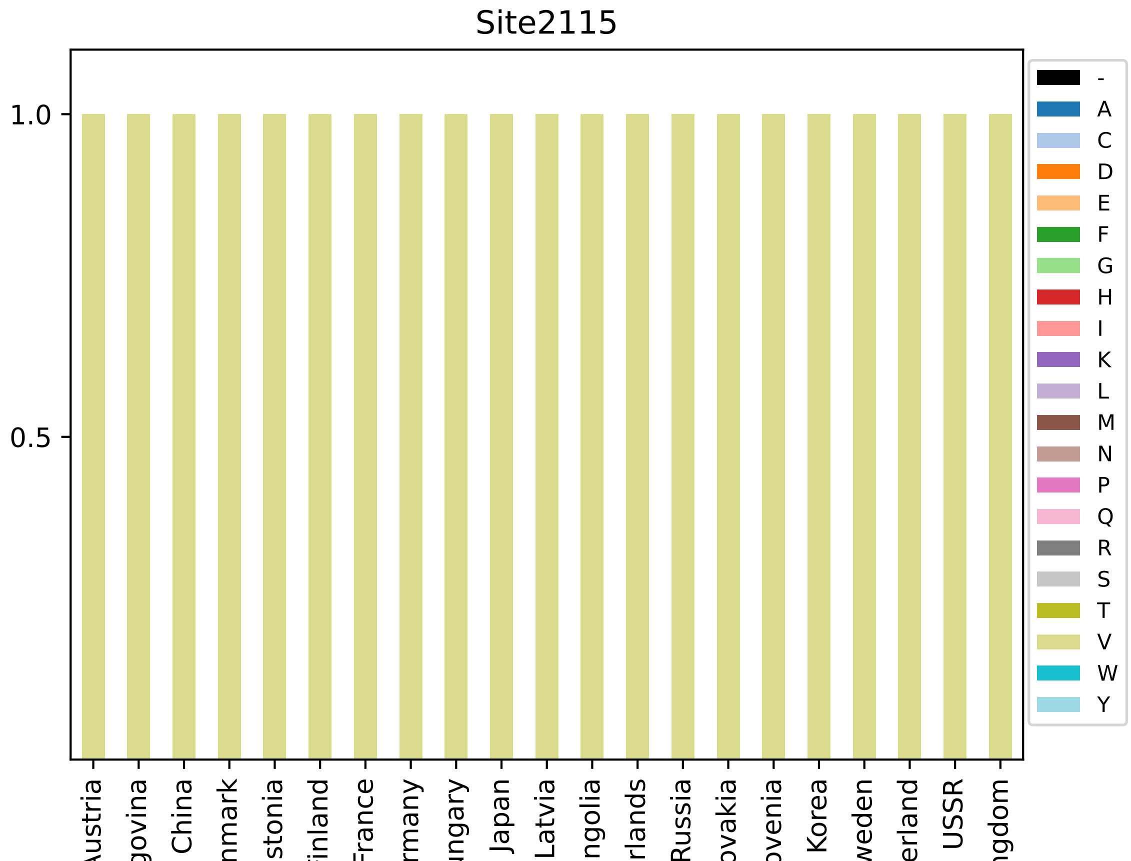

Site2115
1.0
0.5
-
A
C
D
E
F
G
H
I
K
L
M
N
P
Q
R
S
T
V
W
Y
China
Japan
Latvia
Russia
Korea
USSR

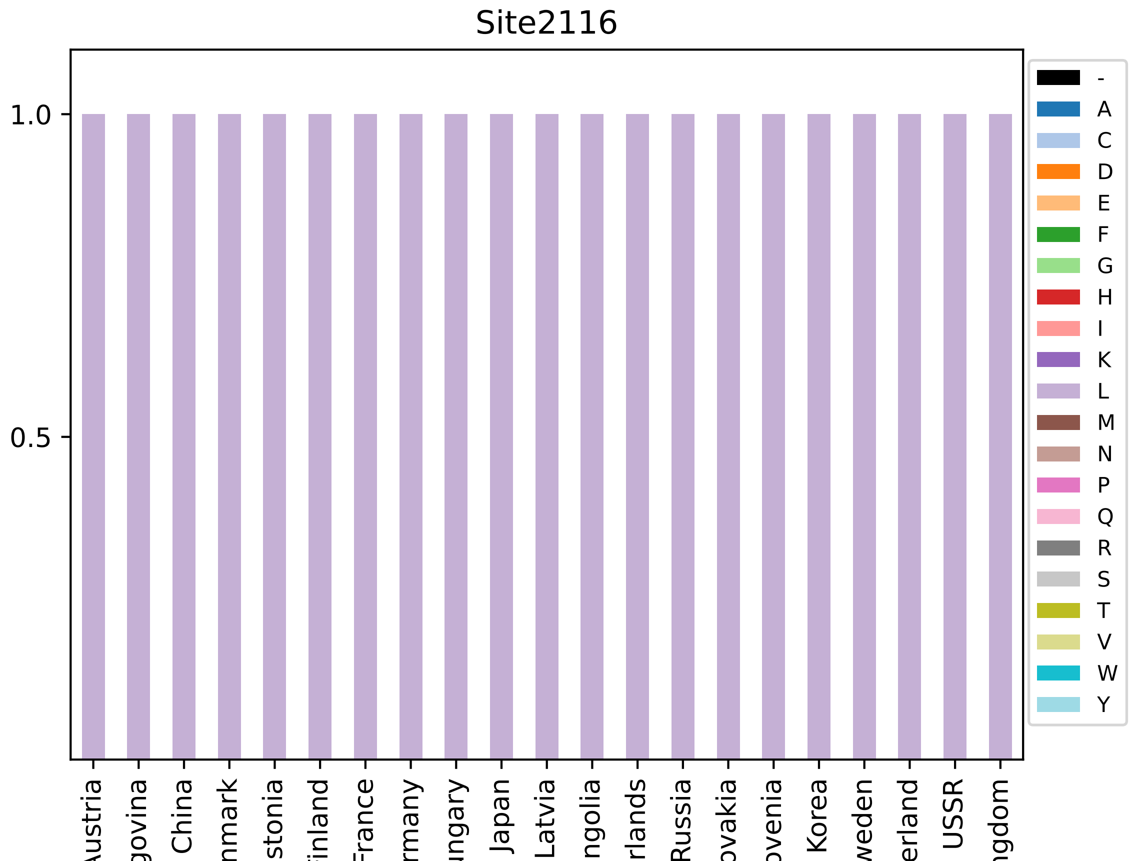

Site2116
1.0
0.5
-
A
C
D
E
F
G
H
I
K
L
M
N
P
Q
R
S
T
V
W
Y
China
Japan
Latvia
Russia
Korea
USSR

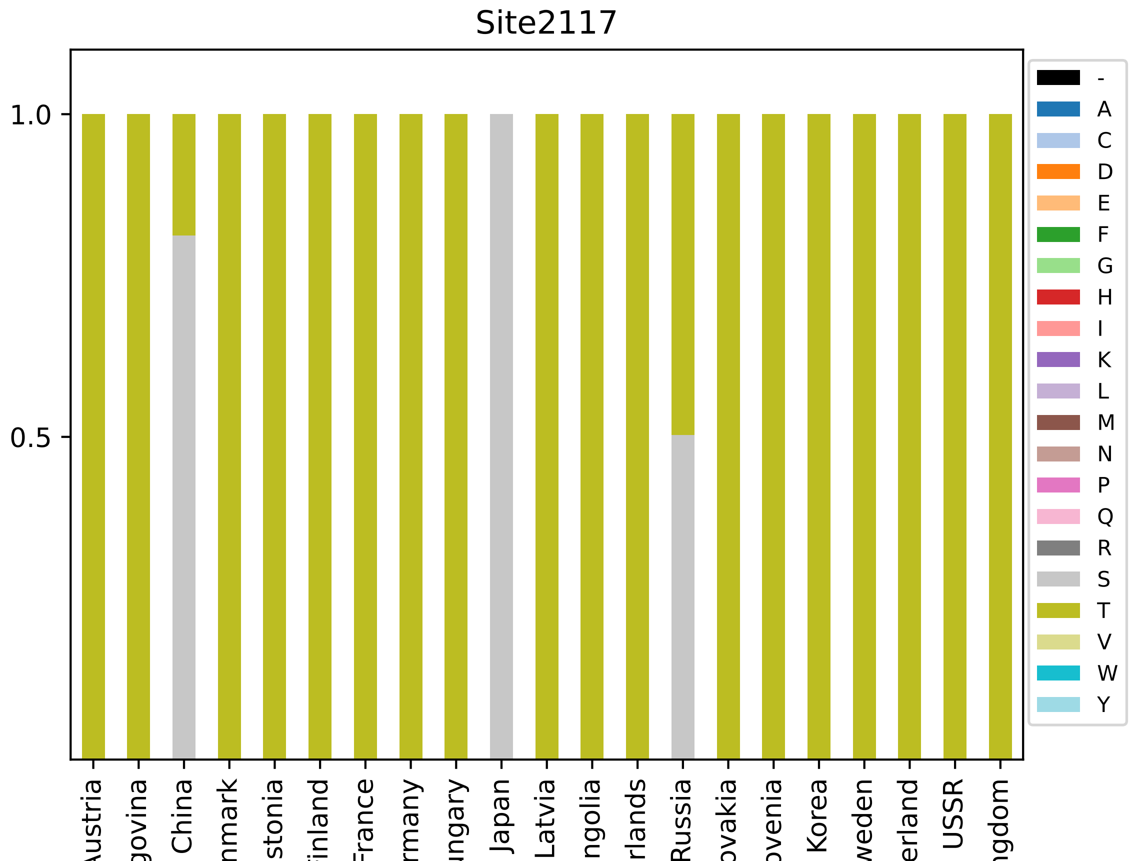

Site2117
1.0
0.5
-
A
C
D
E
F
G
H
I
K
L
M
N
P
Q
R
S
T
V
W
Y
Austria
govina
China
nmark
stonia
Finland
France
rmany
ungary
Japan
Latvia
ngolia
rlands
Russia
ovakia
ovenia
Korea
weden
erland
USSR
ngdom

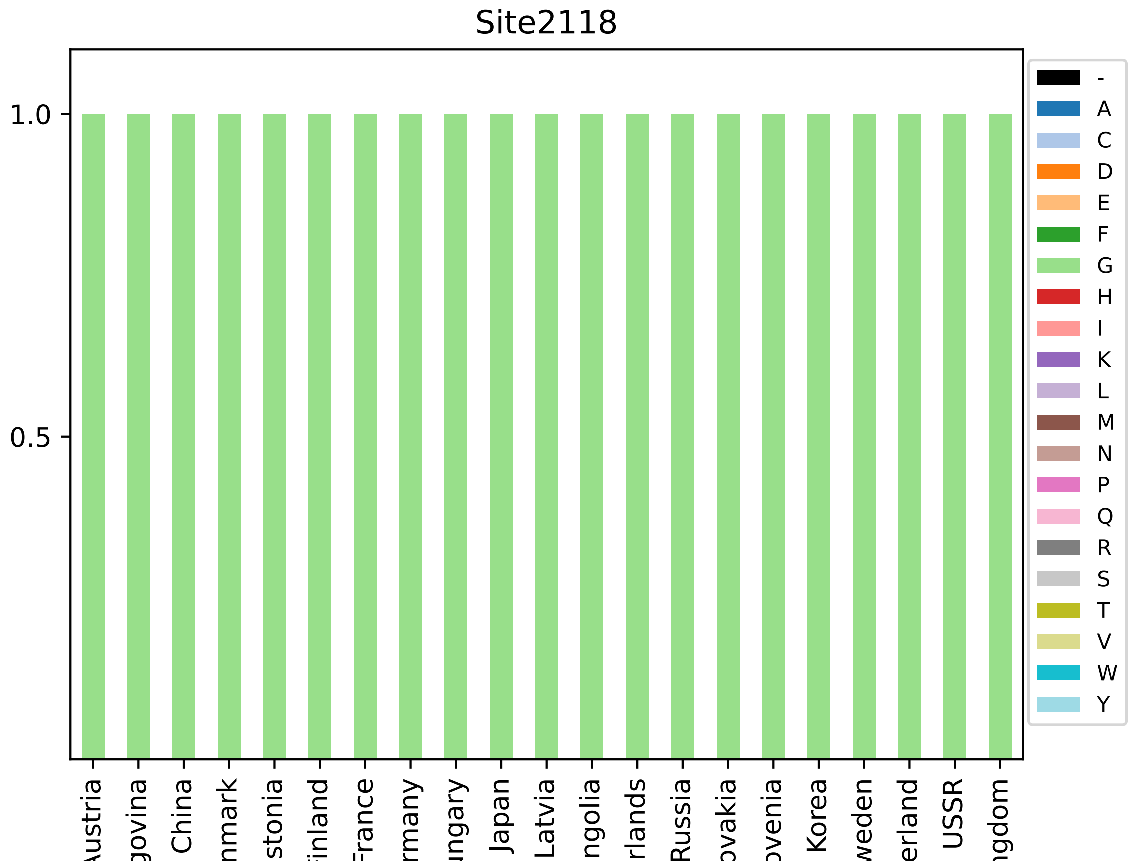

Site2118
1.0
0.5
-
A
C
D
E
F
G
H
I
K
L
M
N
P
Q
R
S
T
V
W
Y
China
Japan
Latvia
Russia
Korea
USSR

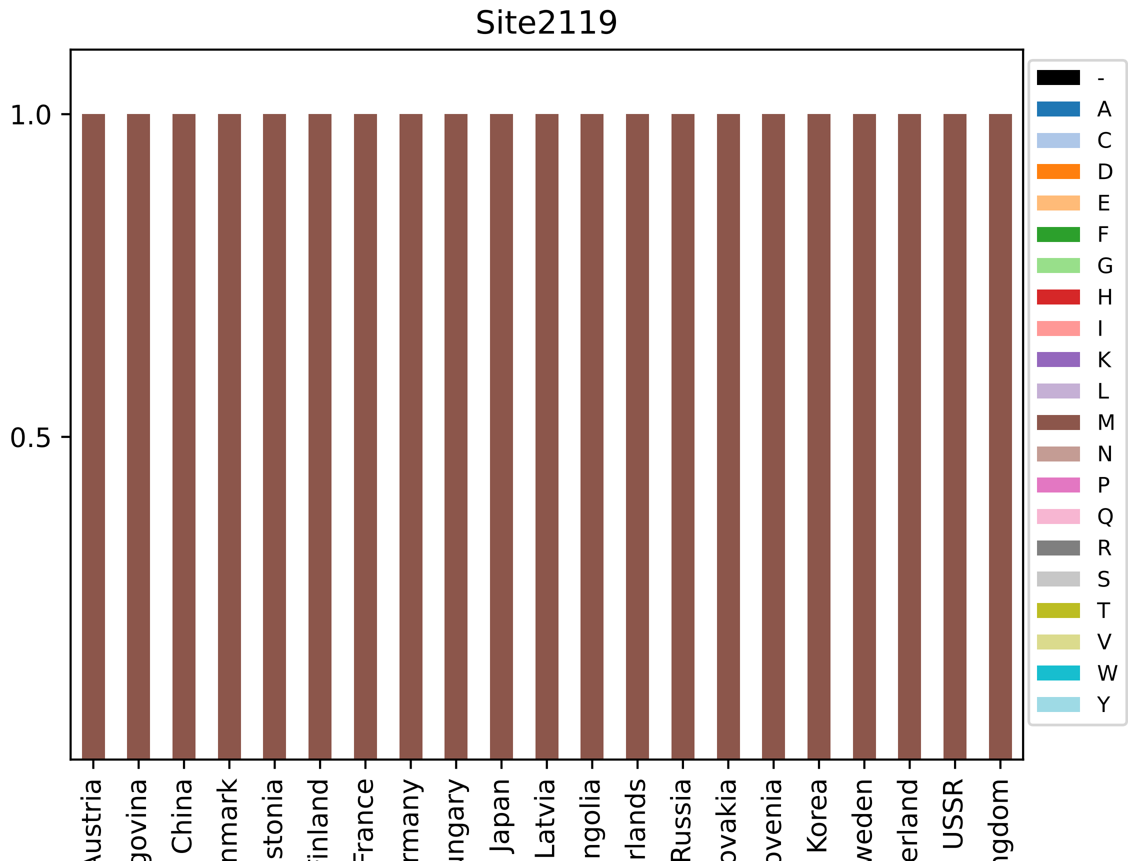
Site2119
1.0
0.5
-
A
C
D
E
F
G
H
I
K
L
M
N
P
Q
R
S
T
V
W
Y
China
Japan
Latvia
Russia
Korea
USSR

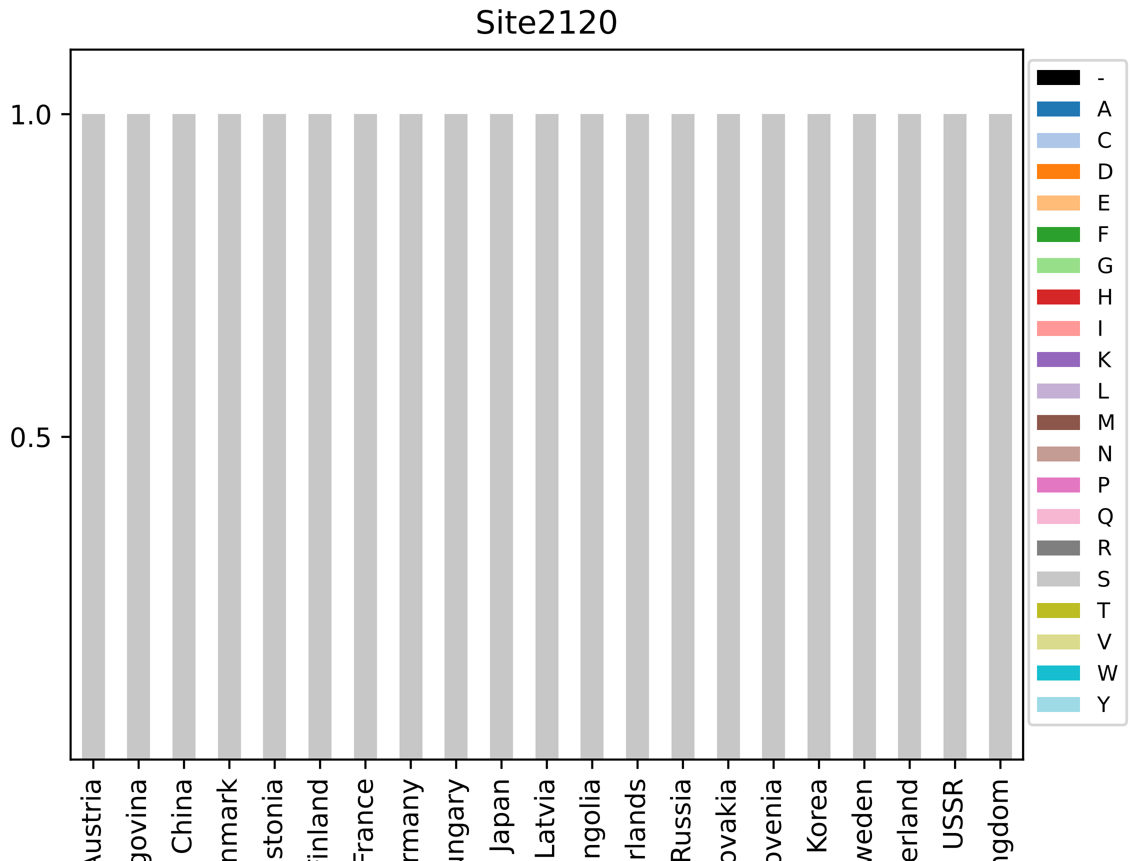
Site2120
1.0
0.5
Austria
govina
China
nmark
stonia
Finland
France
rmany
ungary
Japan
Latvia
ngolia
rlands
Russia
ovakia
ovenia
Korea
weden
erland
USSR
ngdom
-
A
C
D
E
F
G
H
I
K
L
M
N
P
Q
R
S
T
V
W
Y

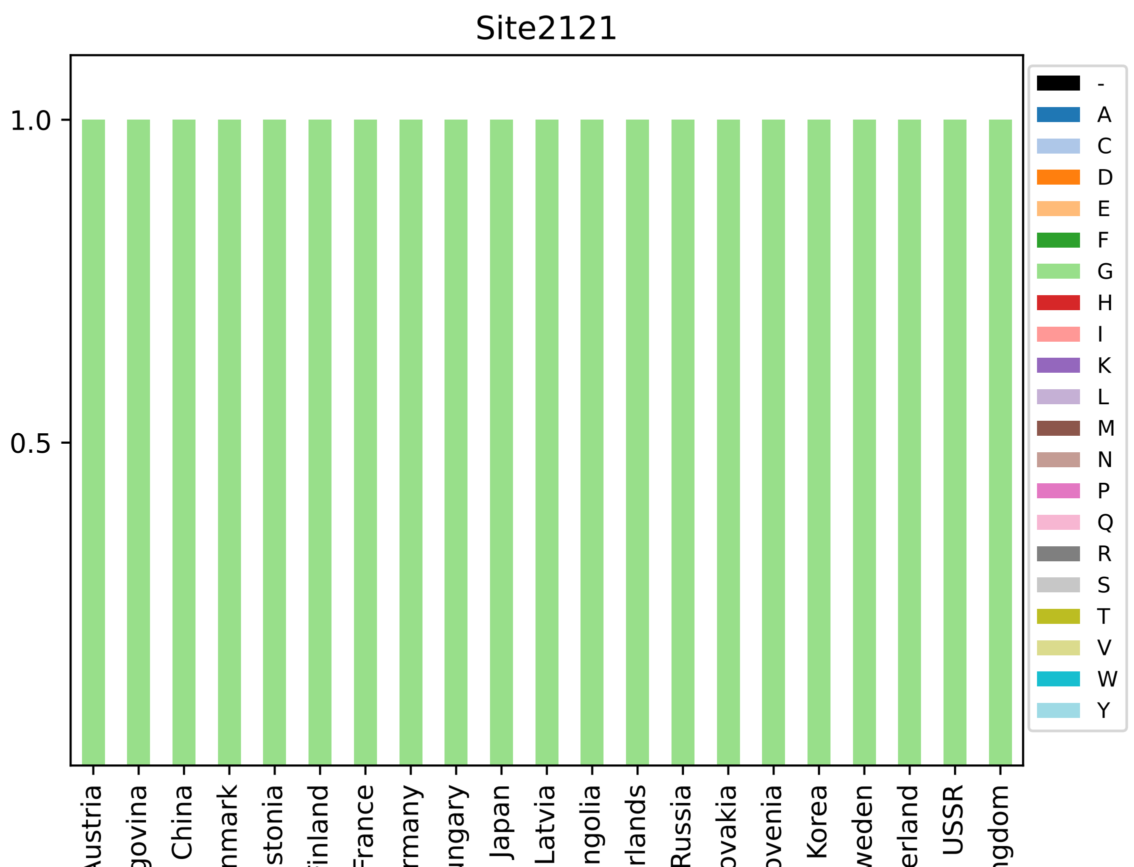
Site2121
1.0
0.5
-
A
C
D
E
F
G
H
I
K
L
M
N
P
Q
R
S
T
V
W
Y
China
Japan
Latvia
Russia
Korea
USSR

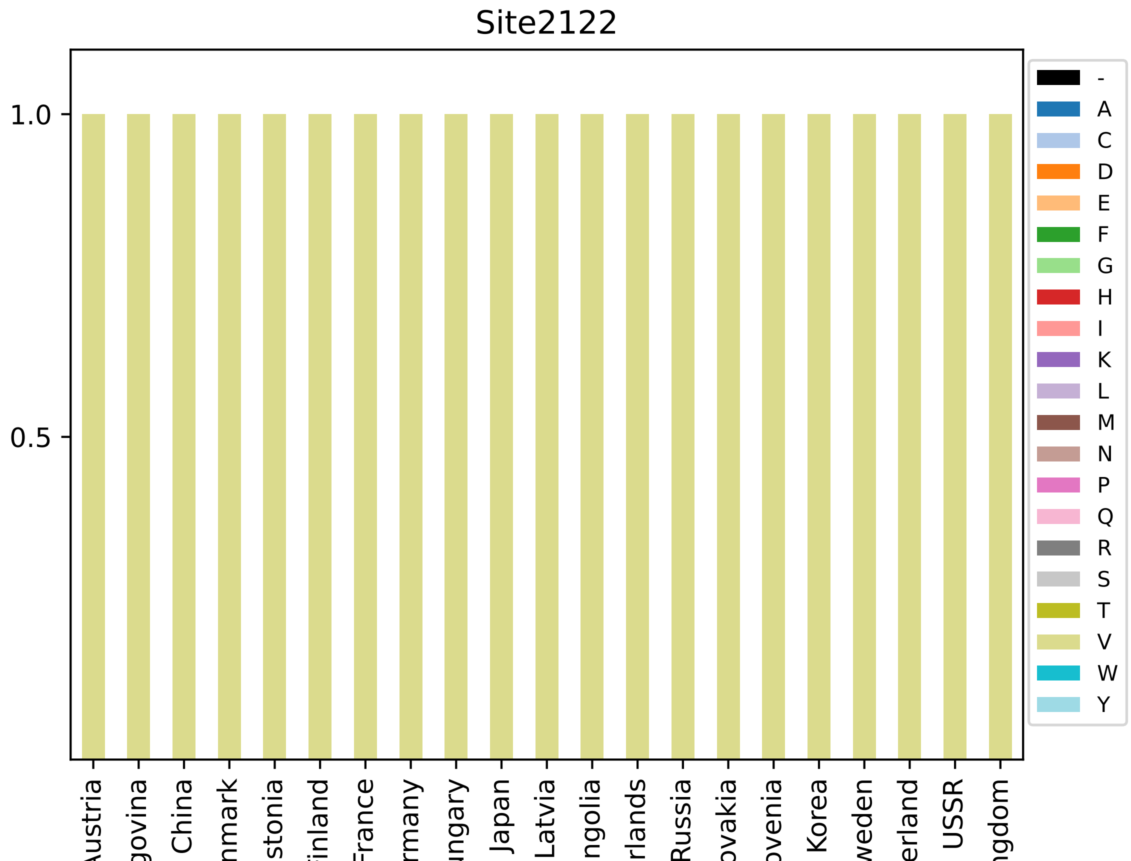

Site2122
1.0
0.5
-
A
C
D
E
F
G
H
I
K
L
M
N
P
Q
R
S
T
V
W
Y
Austria
China
Finland
France
Japan
Latvia
Russia
Korea
USSR

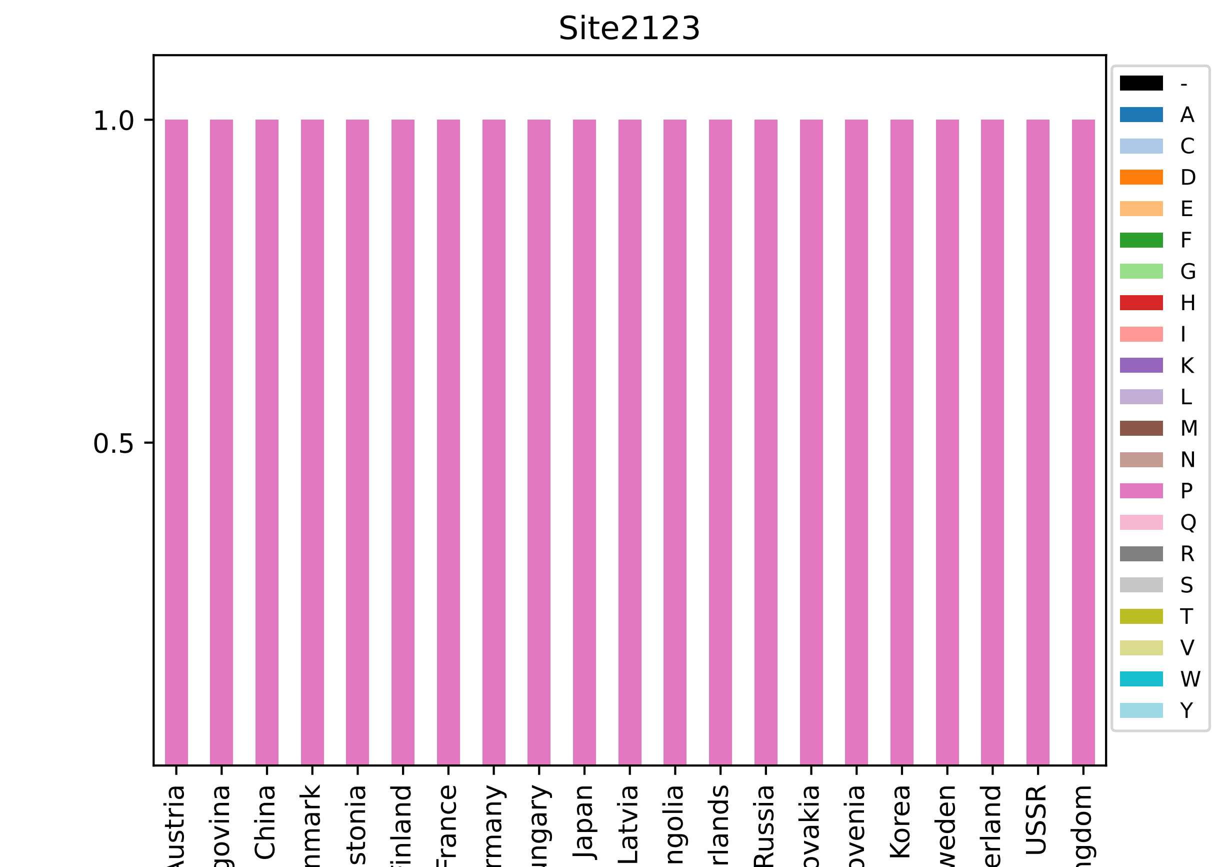

Site2123
1.0
0.5
-
A
C
D
E
F
G
H
I
K
L
M
N
P
Q
R
S
T
V
W
Y
China
Japan
Latvia
Russia
Korea
USSR

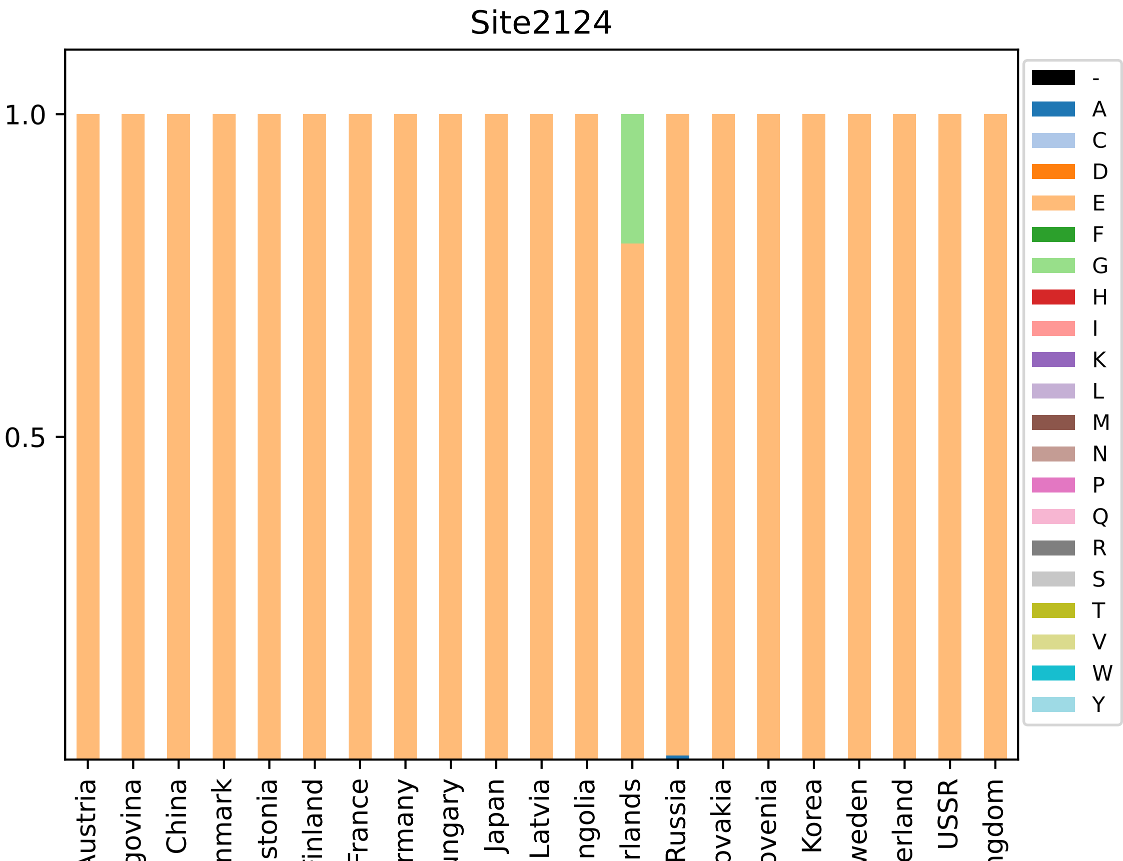
Site2124
1.0
0.5
-
A
C
D
E
F
G
H
I
K
L
M
N
P
Q
R
S
T
V
W
Y
China
Japan
Latvia
Russia
Korea
USSR

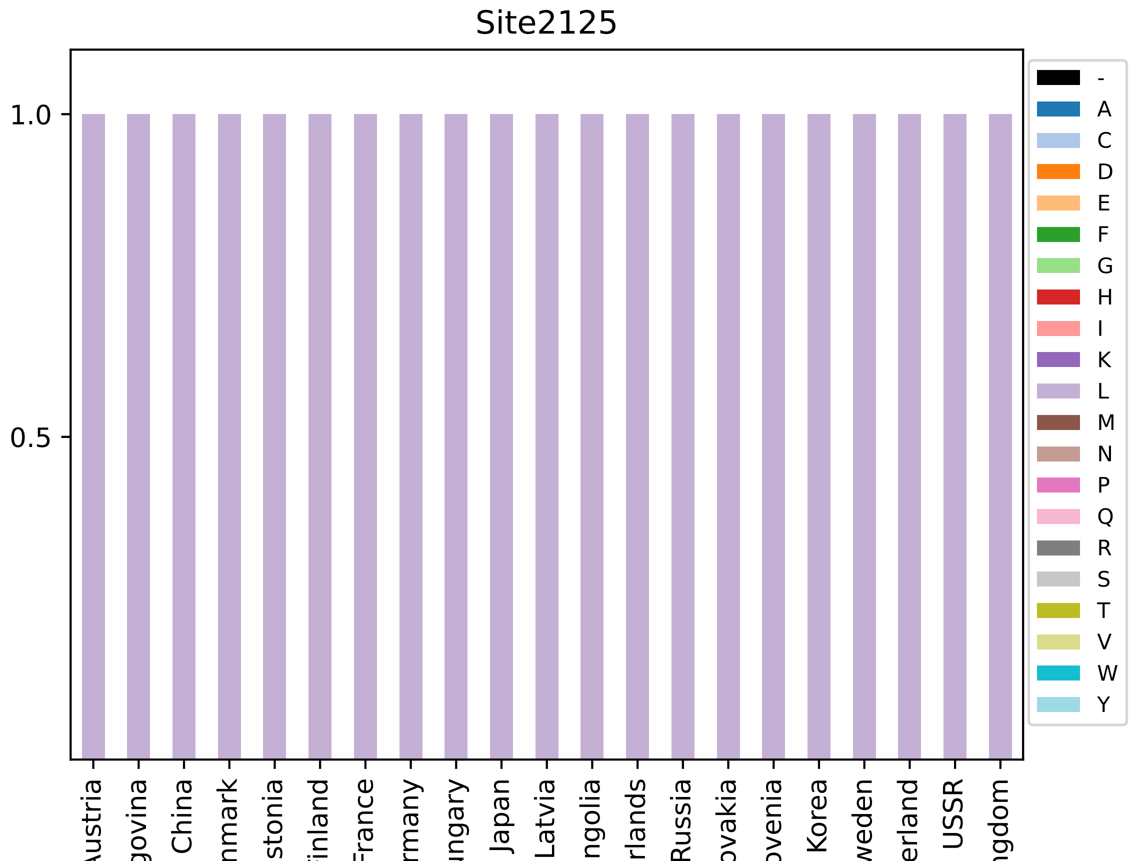
Site2125
1.0
0.5
-
A
C
D
E
F
G
H
I
K
L
M
N
P
Q
R
S
T
V
W
Y
Austria
govina
China
nmark
stonia
inland
France
rmany
ungary
Japan
Latvia
ngolia
rlands
Russia
ovakia
ovenia
Korea
weden
erland
USSR
ngdom

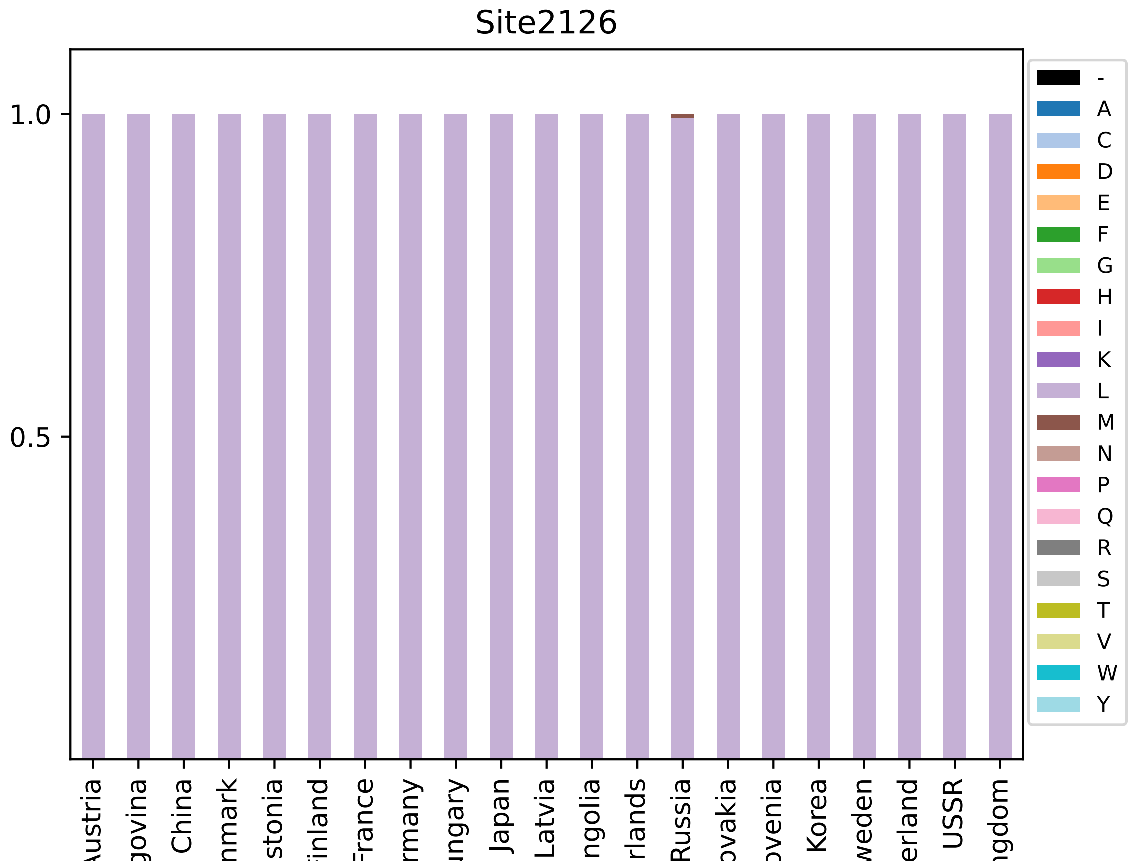

Site2126
1.0
0.5
-
A
C
D
E
F
G
H
I
K
L
M
N
P
Q
R
S
T
V
W
Y
China
Japan
Latvia
Russia
Korea
USSR

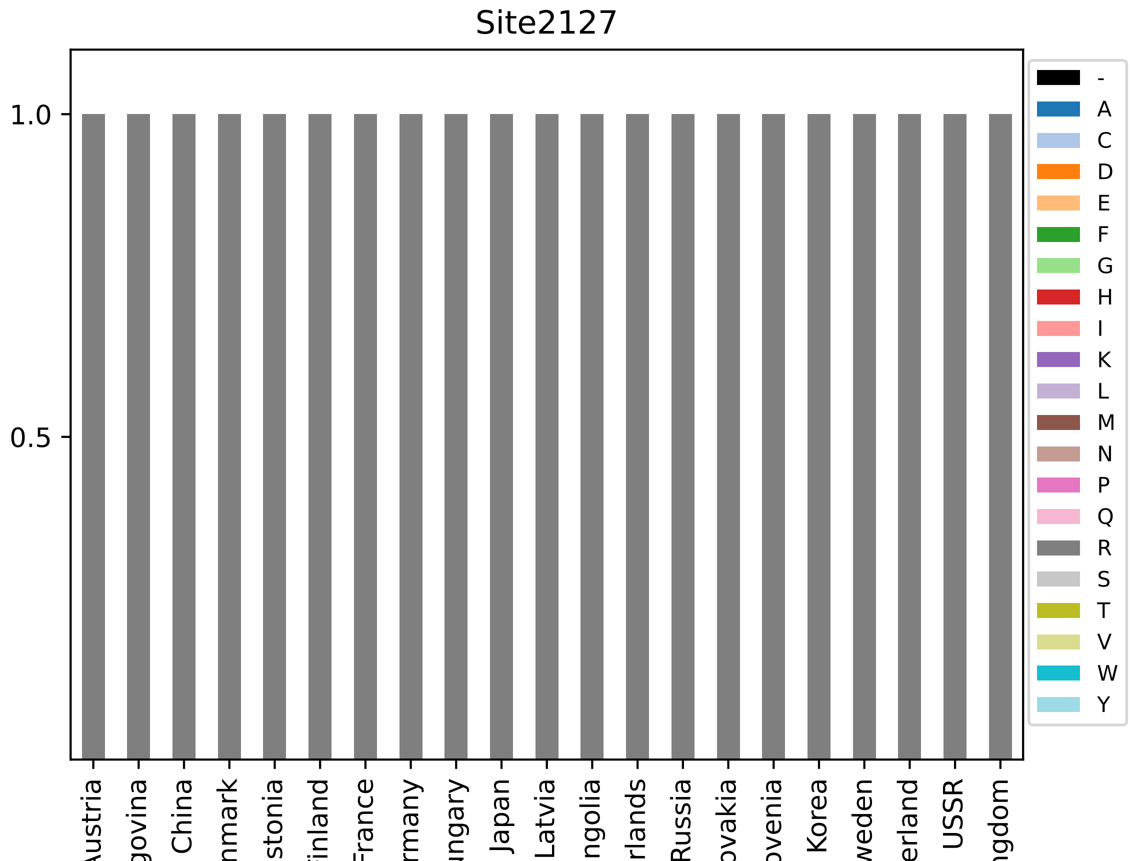

Site2127
1.0
0.5
-
A
C
D
E
F
G
H
I
K
L
M
N
P
Q
R
S
T
V
W
Y
Austria
govina
China
nmark
stonia
inland
France
rmany
ungary
Japan
Latvia
ngolia
rlands
Russia
ovakia
ovenia
Korea
weden
erland
USSR
ngdom

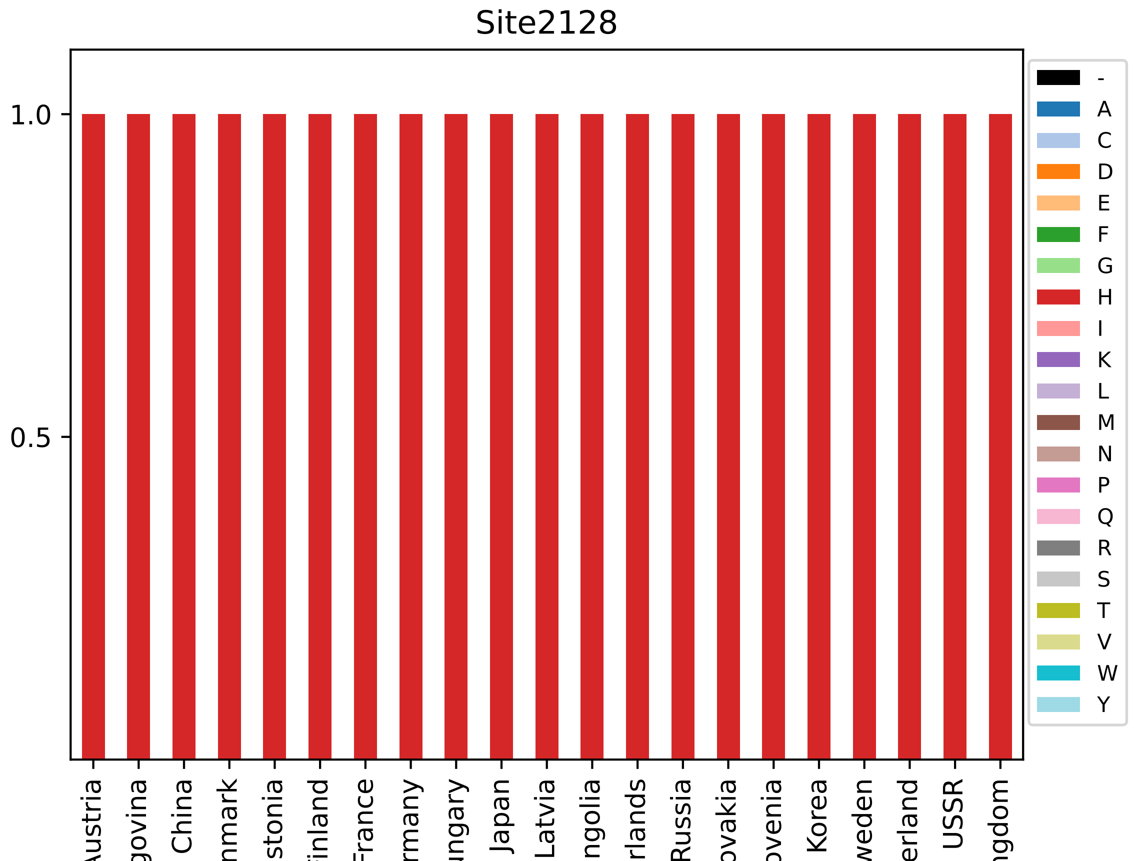

Site2128
1.0
0.5
-
A
C
D
E
F
G
H
I
K
L
M
N
P
Q
R
S
T
V
W
Y
Austria
govina
China
nmark
stonia
inland
France
rmany
ungary
Japan
Latvia
ngolia
rlands
Russia
ovakia
ovenia
Korea
weden
erland
USSR
ngdom

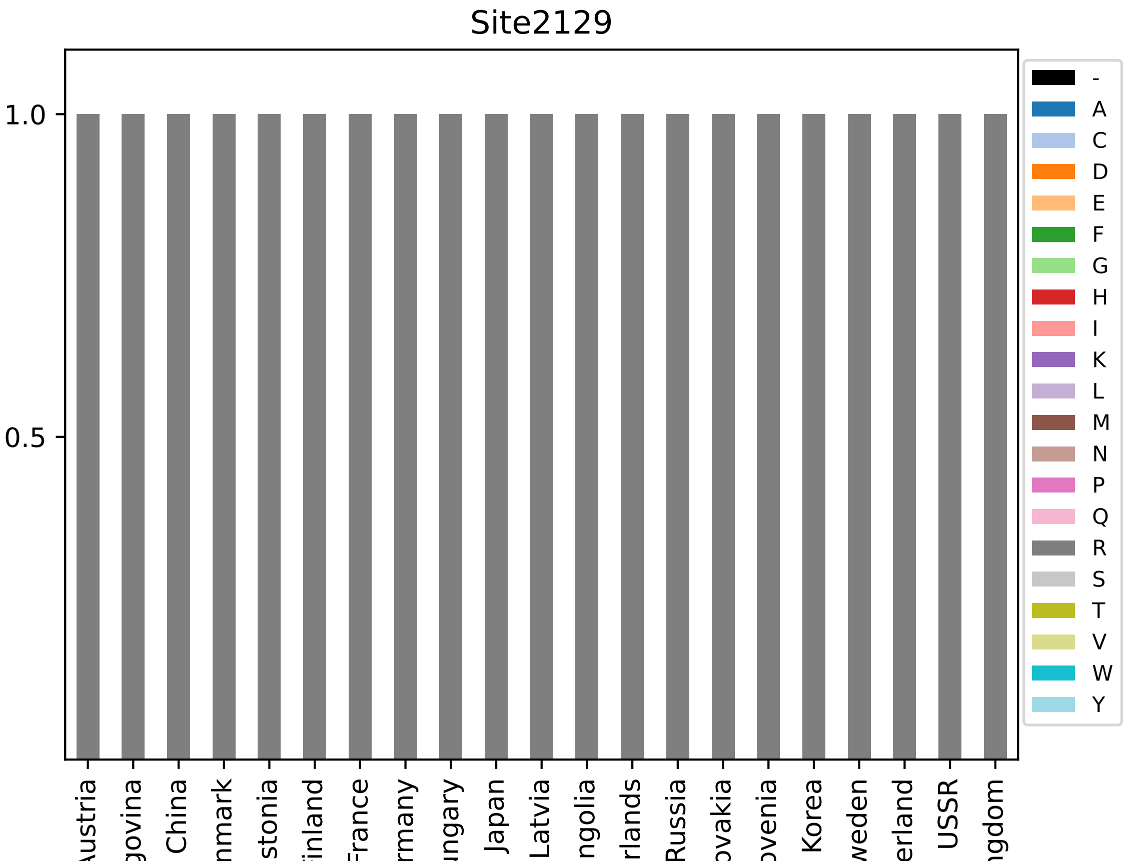

Site2129
1.0
0.5
-
A
C
D
E
F
G
H
I
K
L
M
N
P
Q
R
S
T
V
W
Y
Austria
govina
China
nmark
stonia
Finland
France
rmany
ungary
Japan
Latvia
ngolia
rlands
Russia
ovakia
ovenia
Korea
weden
erland
USSR
ngdom

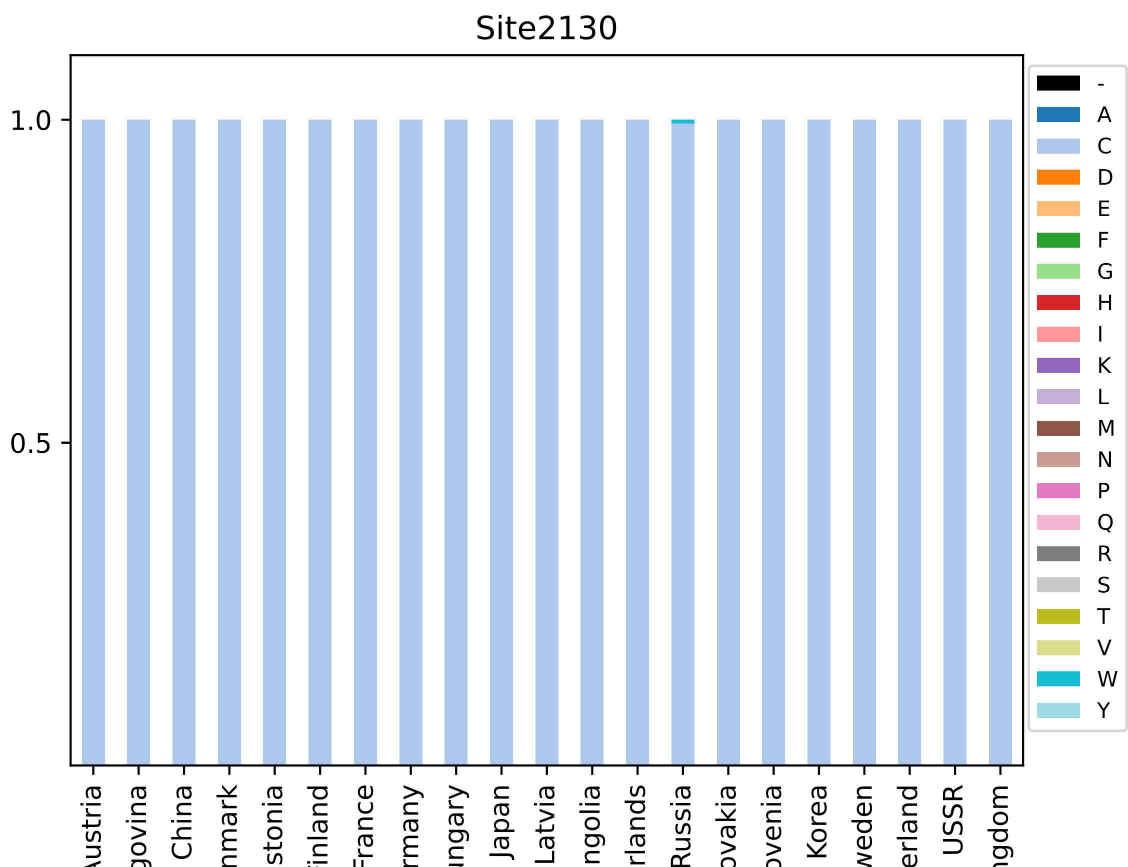

Site2130
1.0
0.5
-
A
C
D
E
F
G
H
I
K
L
M
N
P
Q
R
S
T
V
W
Y
Austria
govina
China
nmark
stonia
Finland
France
rmany
ungary
Japan
Latvia
ngolia
rlands
Russia
ovakia
ovenia
Korea
weden
erland
USSR
ngdom

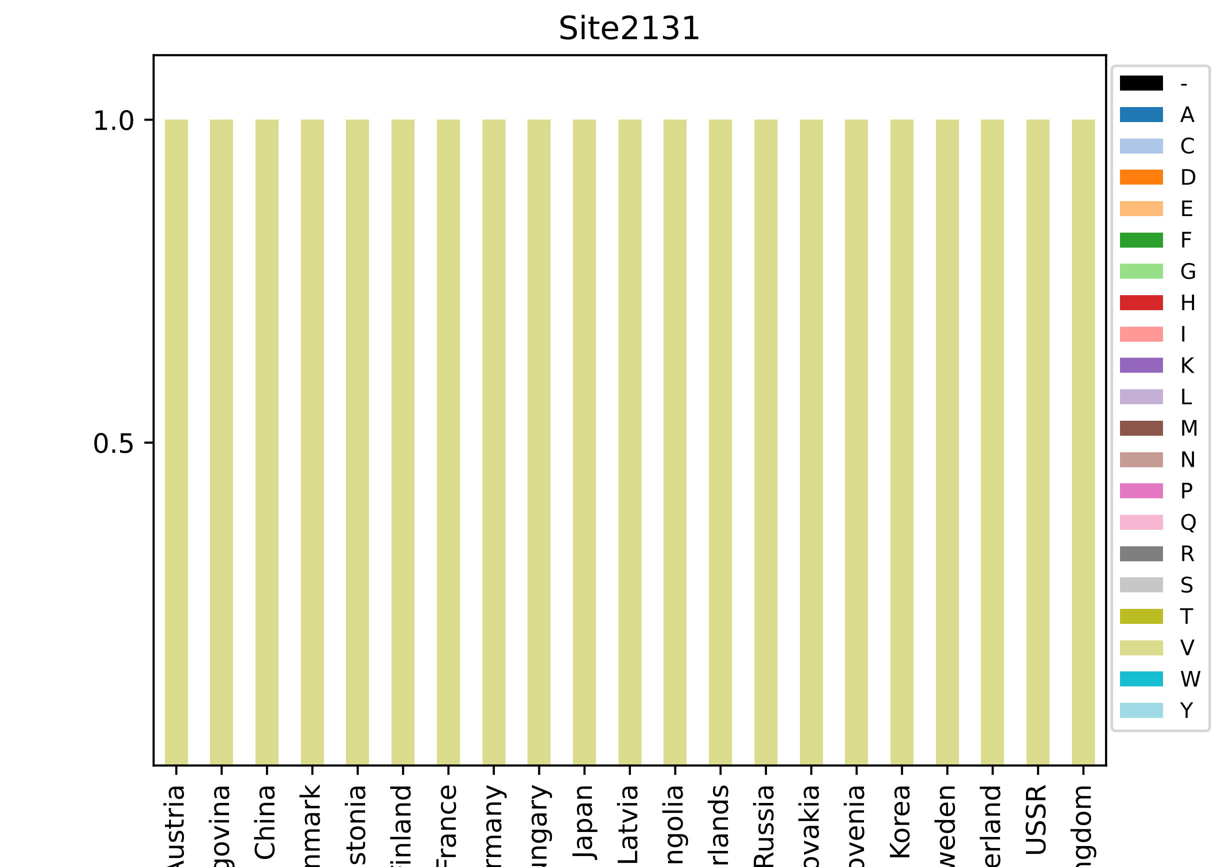

Site2131
1.0
0.5
Austria
govina
China
nmark
stonia
Finland
France
rmany
ungary
Japan
Latvia
ngolia
rlands
Russia
ovakia
ovenia
Korea
weden
erland
USSR
ngdom
-
A
C
D
E
F
G
H
I
K
L
M
N
P
Q
R
S
T
V
W
Y

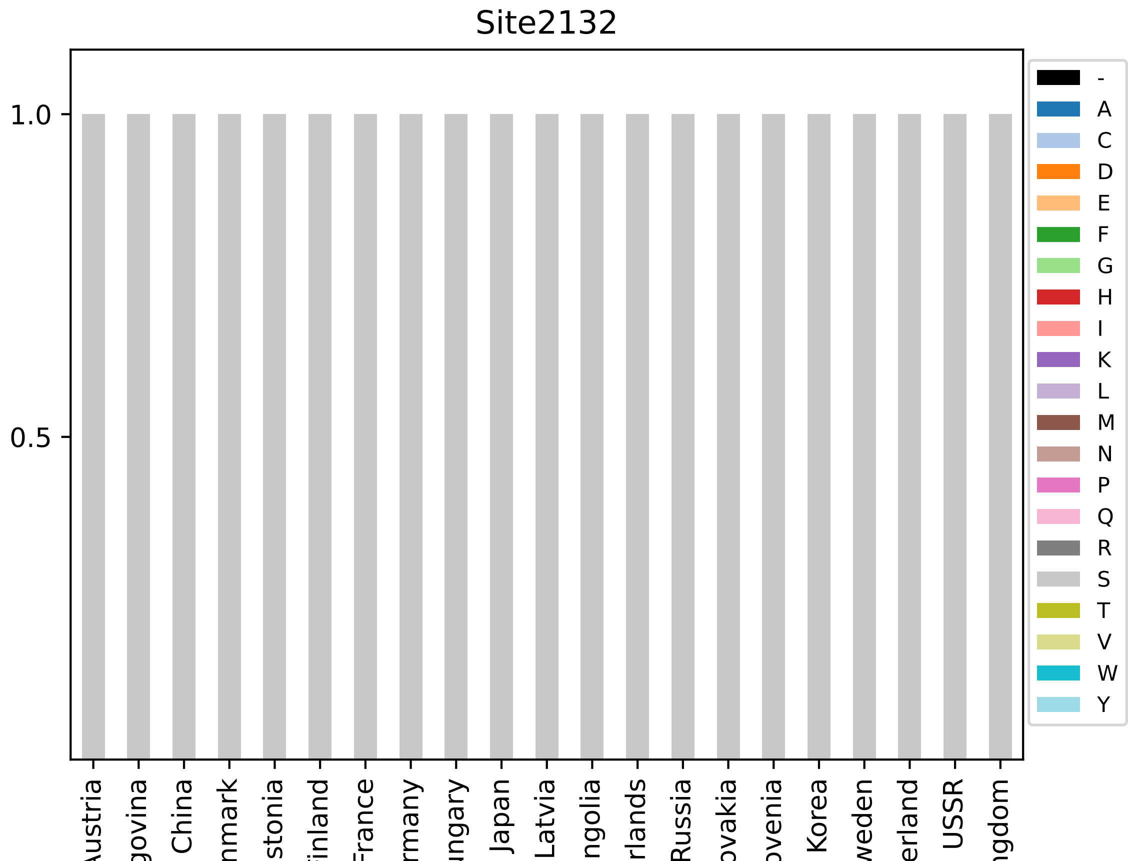

Site2132
1.0
0.5
-
A
C
D
E
F
G
H
I
K
L
M
N
P
Q
R
S
T
V
W
Y
Austria
govina
China
nmark
stonia
Finland
France
rmany
ungary
Japan
Latvia
ngolia
rlands
Russia
ovakia
ovenia
Korea
weden
erland
USSR
ngdom

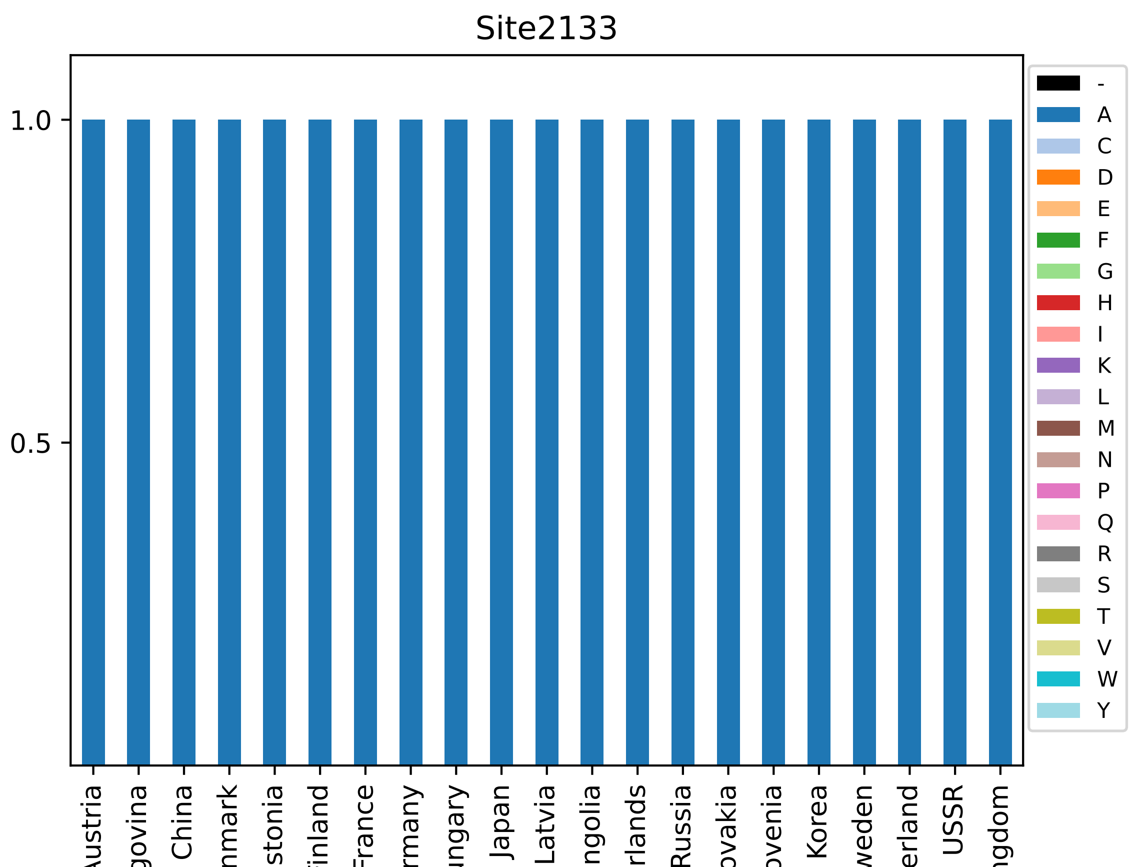

Site2133
1.0
0.5
-
A
C
D
E
F
G
H
I
K
L
M
N
P
Q
R
S
T
V
W
Y
Austria
China
Japan
Latvia
Russia
Korea
USSR

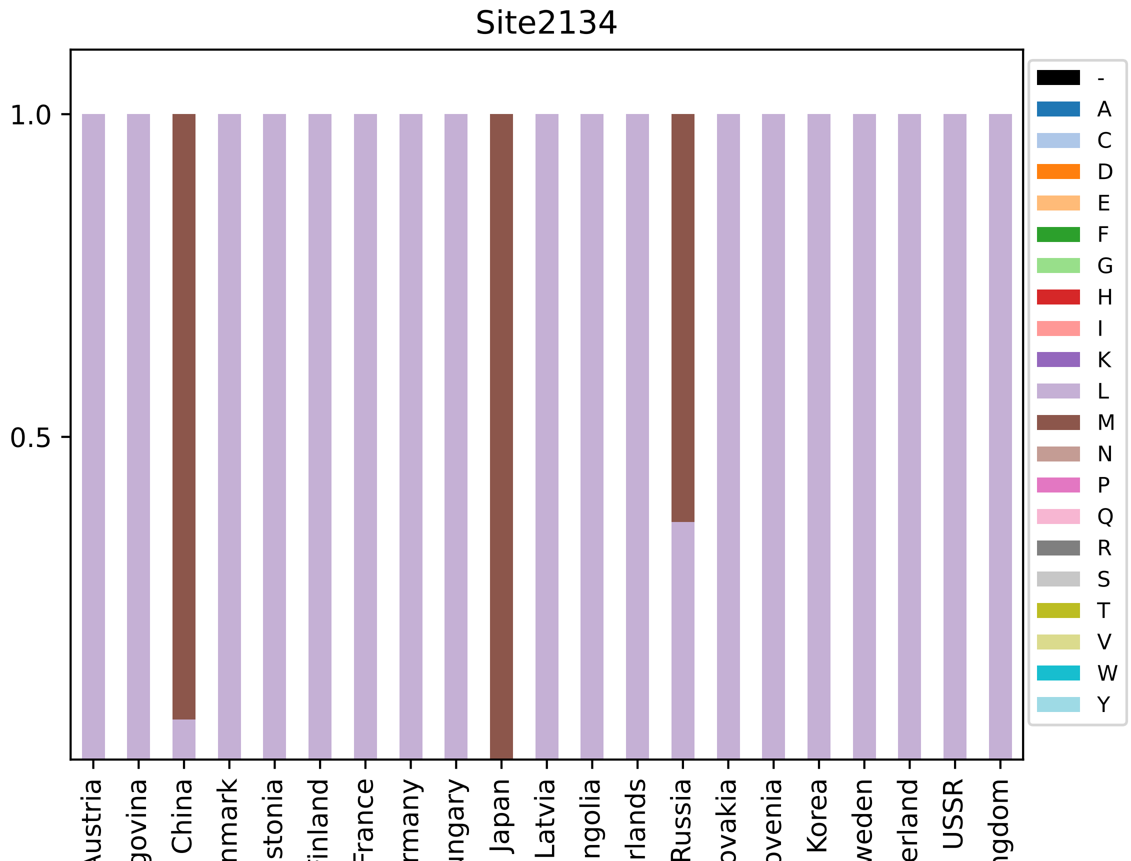

Site2134
1.0
0.5
-
A
C
D
E
F
G
H
I
K
L
M
N
P
Q
R
S
T
V
W
Y
China
Japan
Latvia
Russia
Korea
USSR

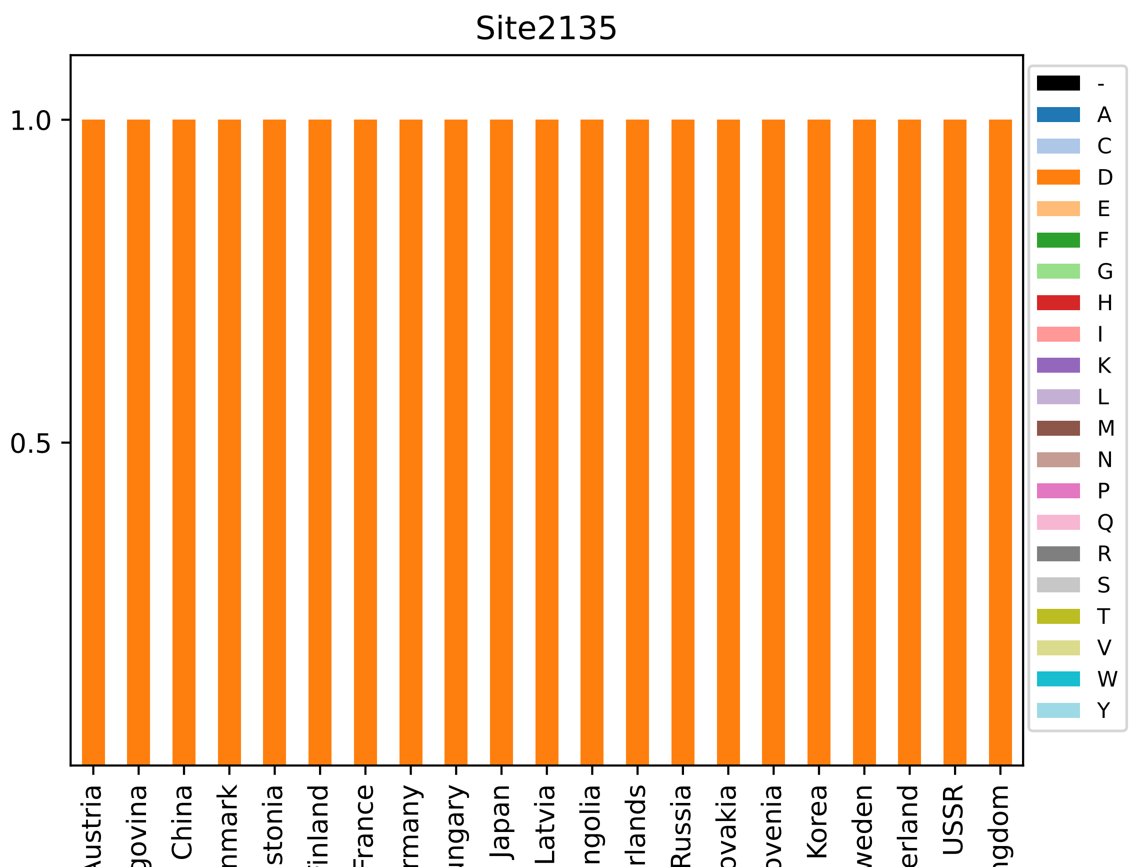

Site2135
1.0
0.5
-
A
C
D
E
F
G
H
I
K
L
M
N
P
Q
R
S
T
V
W
Y
China
Japan
Latvia
Russia
Korea
USSR

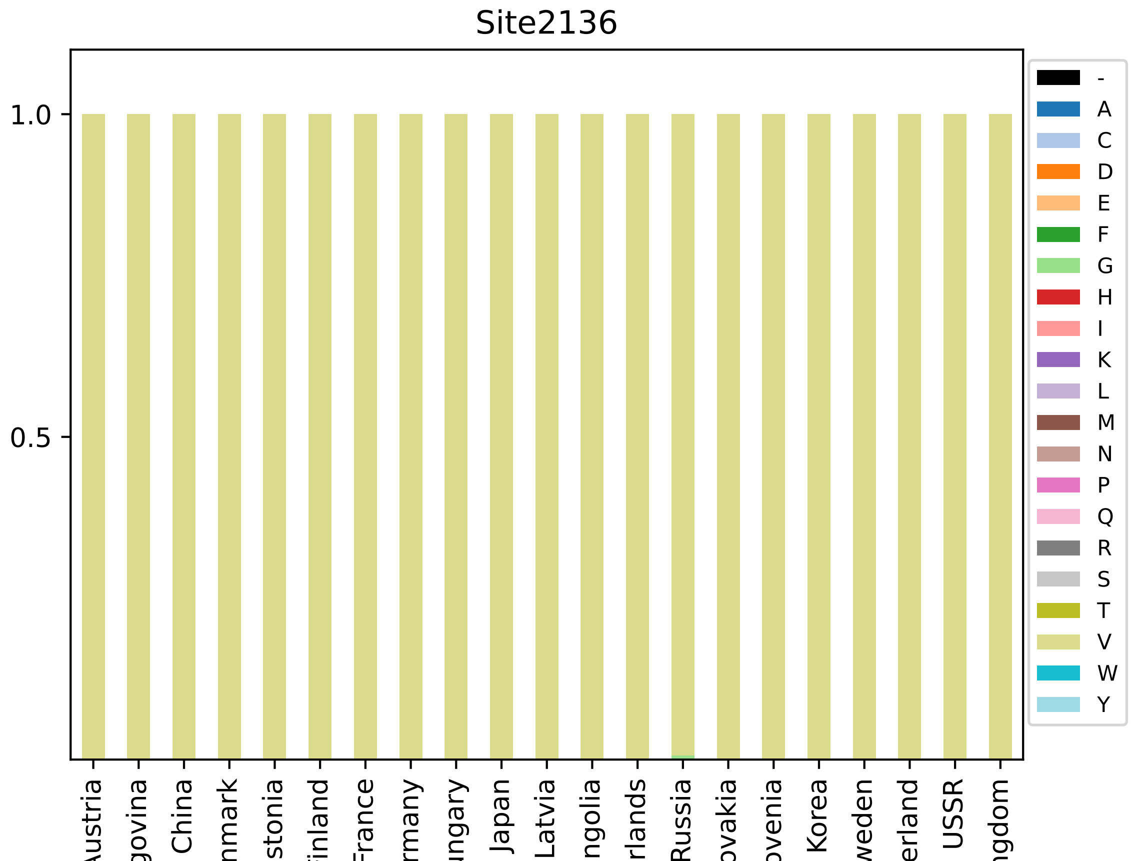
Site2136
1.0
0.5
-
A
C
D
E
F
G
H
I
K
L
M
N
P
Q
R
S
T
V
W
Y
China
Japan
Latvia
Russia
Korea
USSR

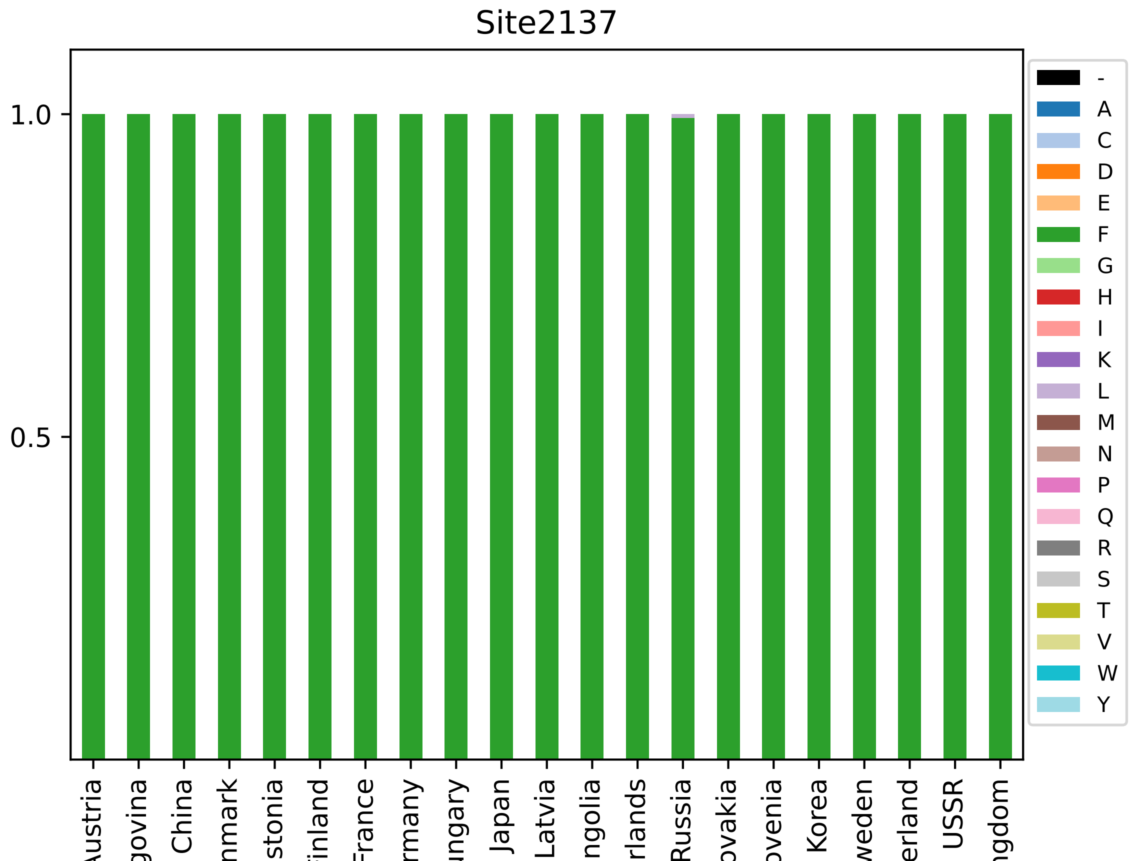
Site2137
1.0
0.5
-
A
C
D
E
F
G
H
I
K
L
M
N
P
Q
R
S
T
V
W
Y
Austria
govina
China
nmark
stonia
inland
France
rmany
ungary
Japan
Latvia
ngolia
rlands
Russia
ovakia
ovenia
Korea
weden
erland
USSR
ngdom

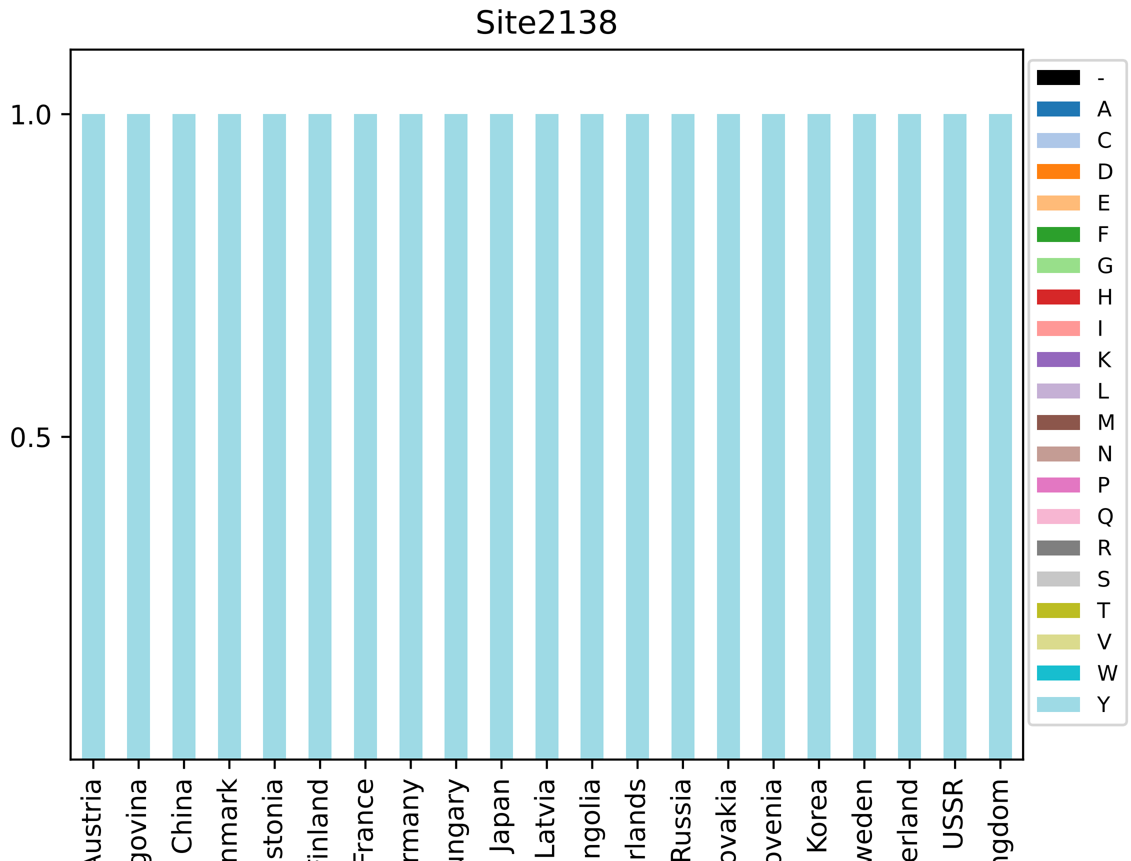
Site2138
1.0
0.5
-
A
C
D
E
F
G
H
I
K
L
M
N
P
Q
R
S
T
V
W
Y
China
Japan
Latvia
Russia
Korea
USSR

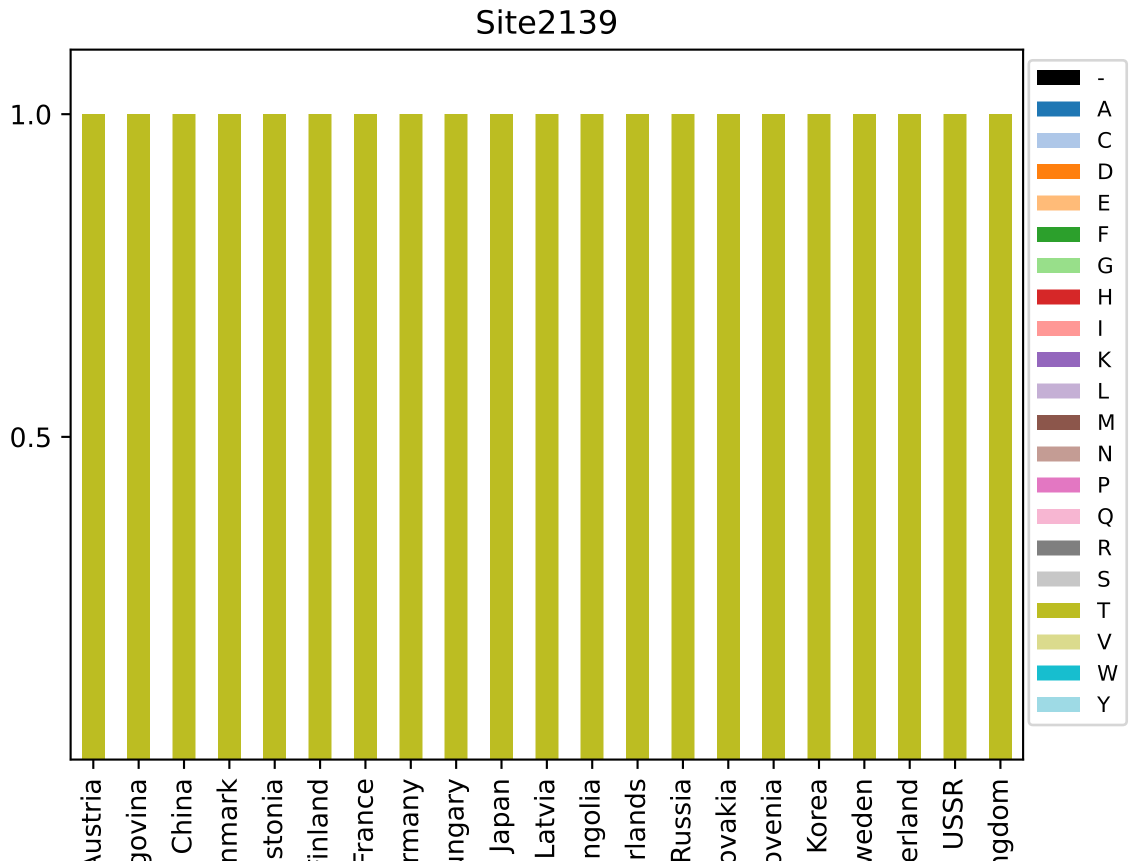
Site2139
1.0
0.5
-
A
C
D
E
F
G
H
I
K
L
M
N
P
Q
R
S
T
V
W
Y
Austria
China
Japan
Latvia
Russia
Korea
USSR

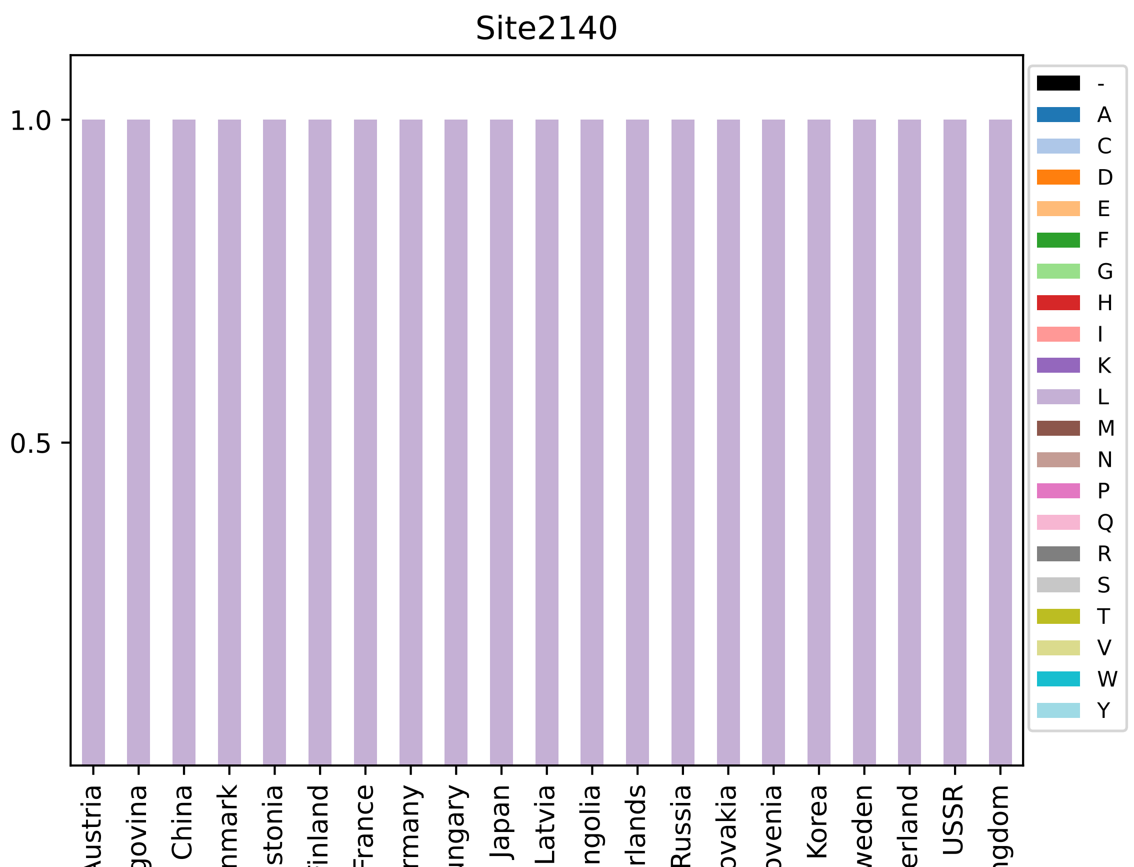

Site2140
1.0
0.5
-
A
C
D
E
F
G
H
I
K
L
M
N
P
Q
R
S
T
V
W
Y
China
Japan
Latvia
Russia
Korea
USSR

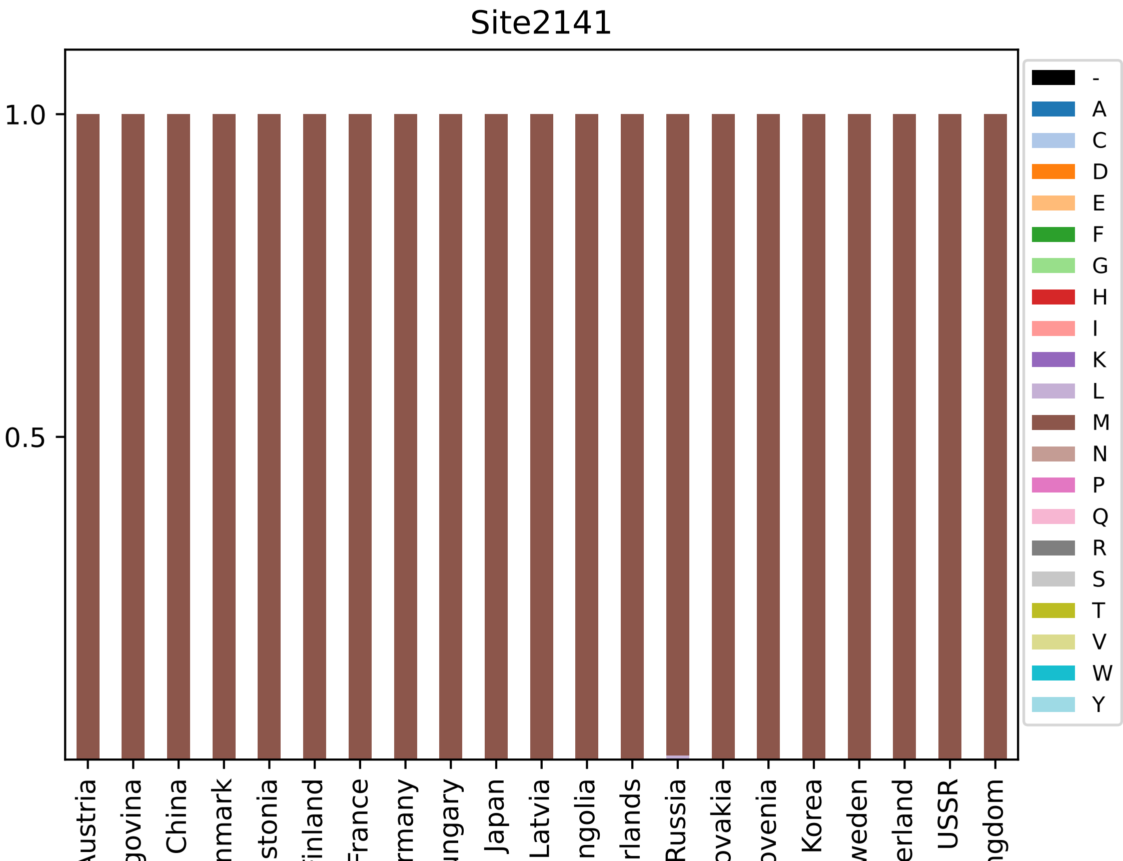
Site2141
1.0
0.5
-
A
C
D
E
F
G
H
I
K
L
M
N
P
Q
R
S
T
V
W
Y
Austria
govina
China
nmark
stonia
inland
France
rmany
ungary
Japan
Latvia
ngolia
rlands
Russia
ovakia
ovenia
Korea
weden
erland
USSR
ngdom

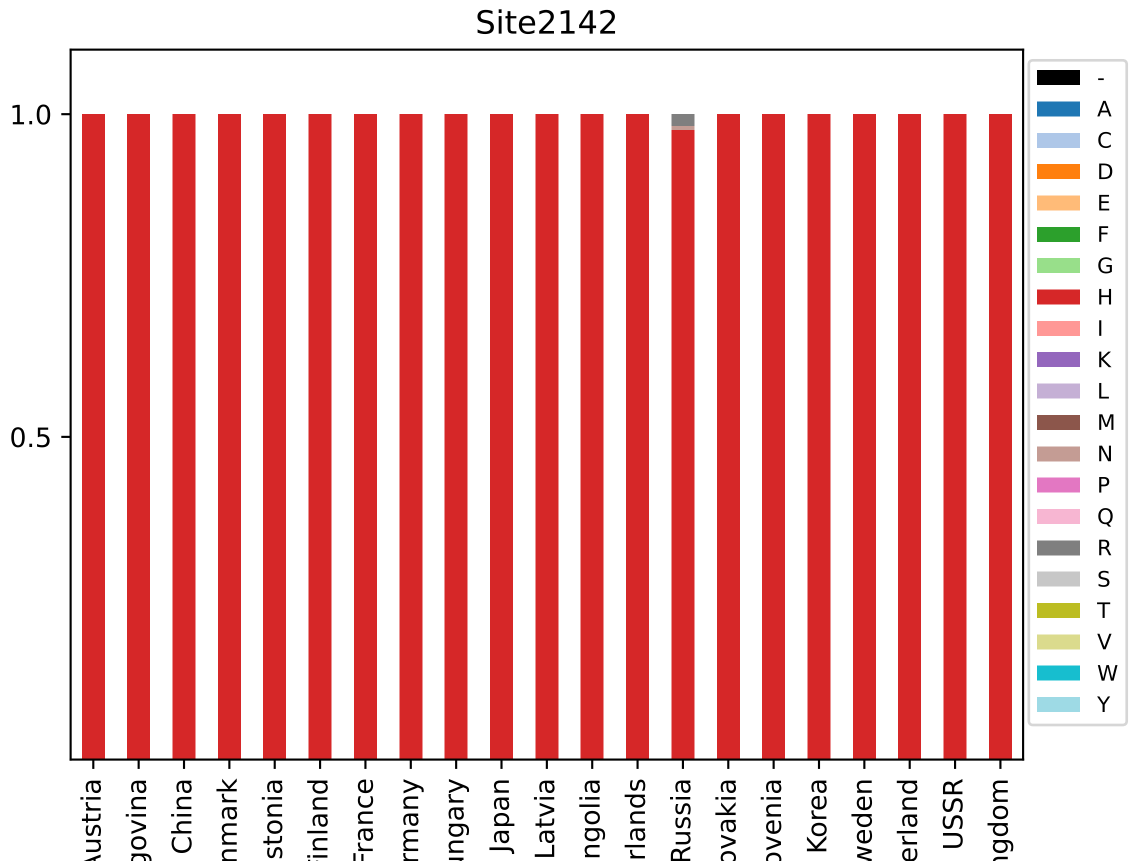

Site2142
1.0
0.5
-
A
C
D
E
F
G
H
I
K
L
M
N
P
Q
R
S
T
V
W
Y
Austria
govina
China
nmark
stonia
inland
France
rmany
ungary
Japan
Latvia
ngolia
rlands
Russia
ovakia
ovenia
Korea
weden
erland
USSR
ngdom

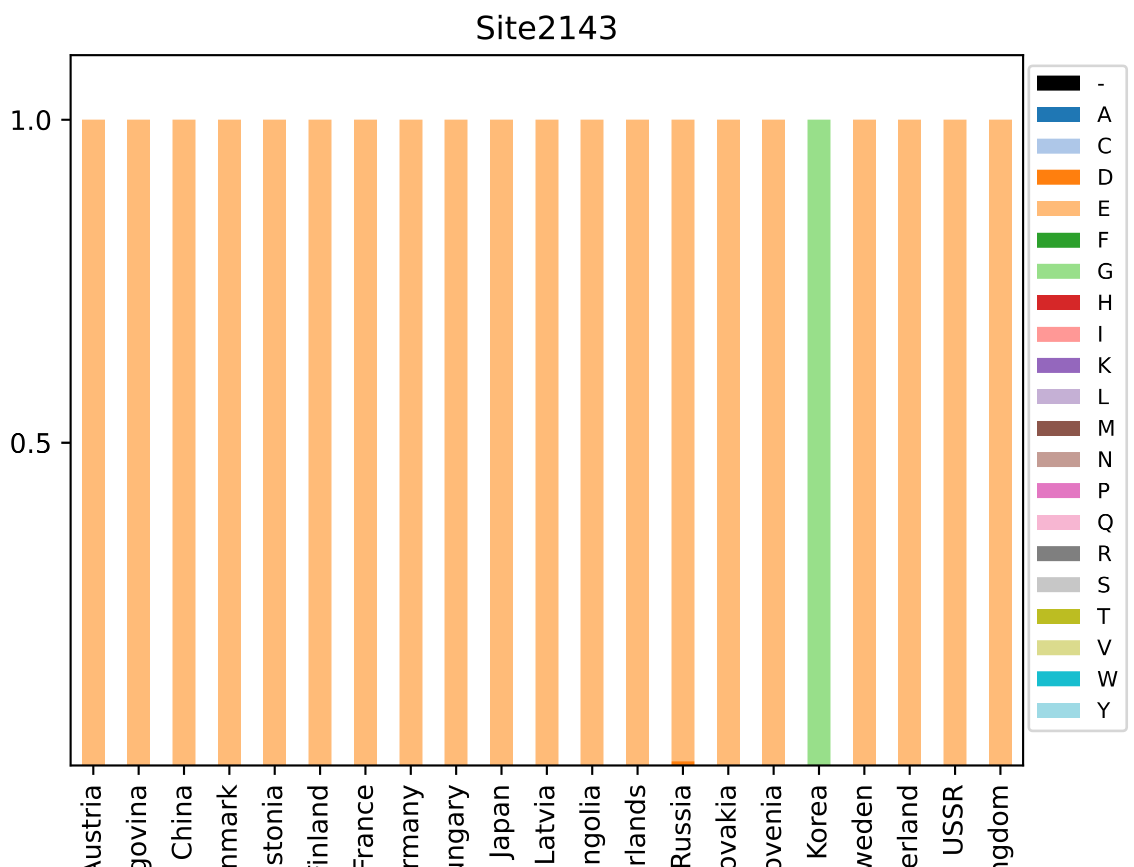

Site2143
1.0
0.5
-
A
C
D
E
F
G
H
I
K
L
M
N
P
Q
R
S
T
V
W
Y
Austria
govina
China
nmark
stonia
inland
France
rmany
ungary
Japan
Latvia
ngolia
rlands
Russia
ovakia
ovenia
Korea
weden
erland
USSR
ngdom

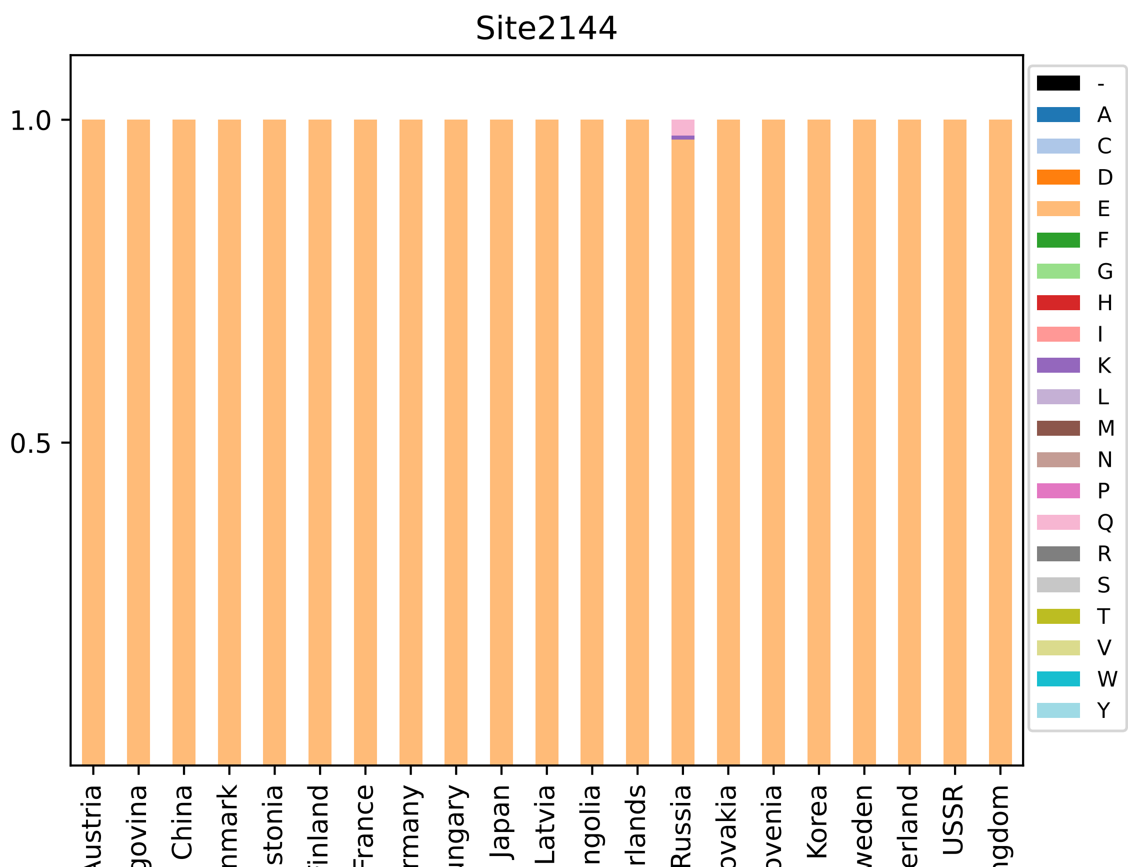

Site2144
1.0
0.5
-
A
C
D
E
F
G
H
I
K
L
M
N
P
Q
R
S
T
V
W
Y
Austria
China
Japan
Latvia
Russia
Korea
USSR

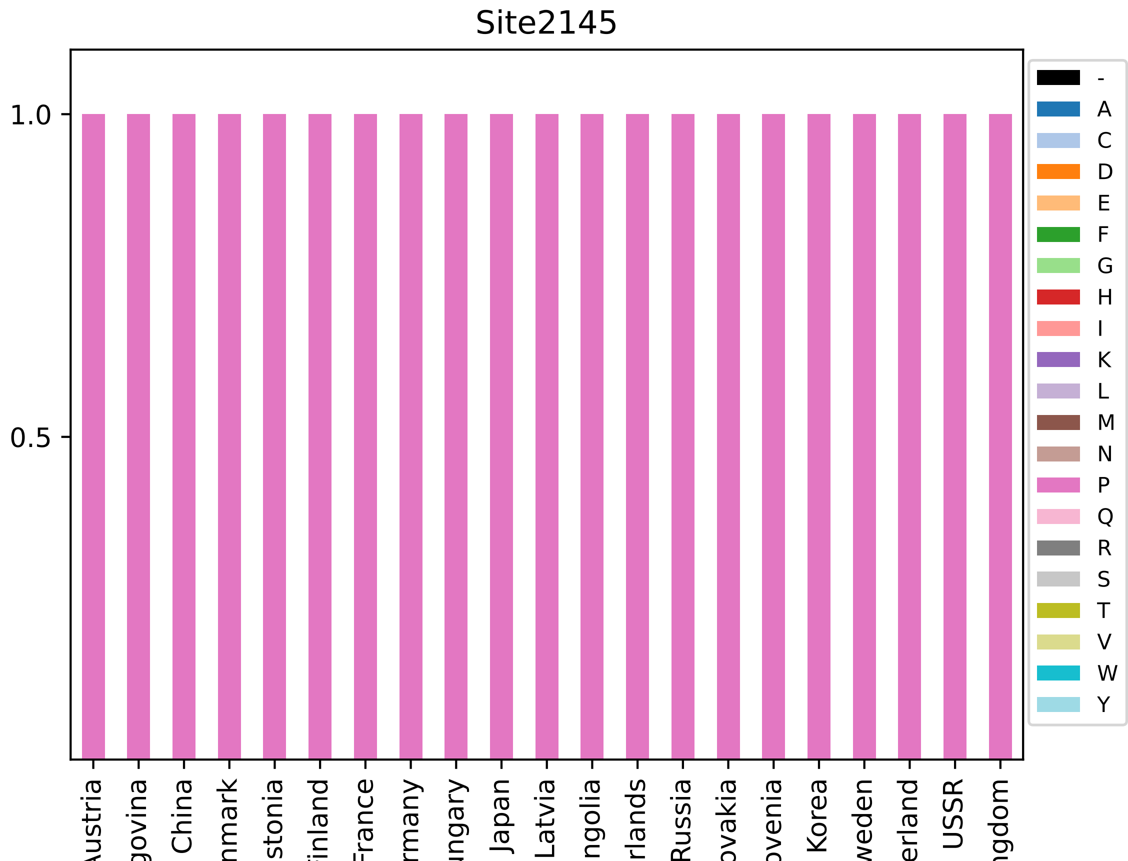
Site2145
1.0
0.5
-
A
C
D
E
F
G
H
I
K
L
M
N
P
Q
R
S
T
V
W
Y
China
Japan
Latvia
Russia
Korea
USSR

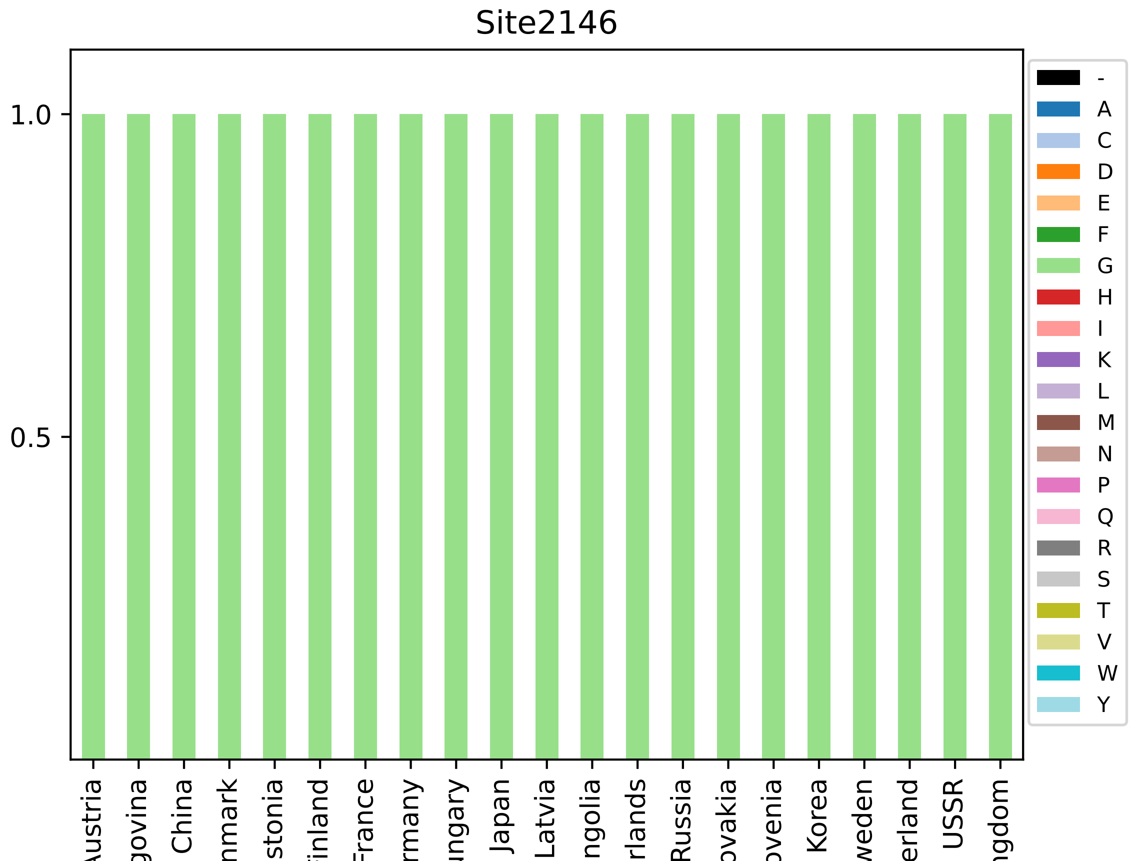

Site2146
1.0
0.5
-
A
C
D
E
F
G
H
I
K
L
M
N
P
Q
R
S
T
V
W
Y
China
Japan
Latvia
Russia
Korea
USSR

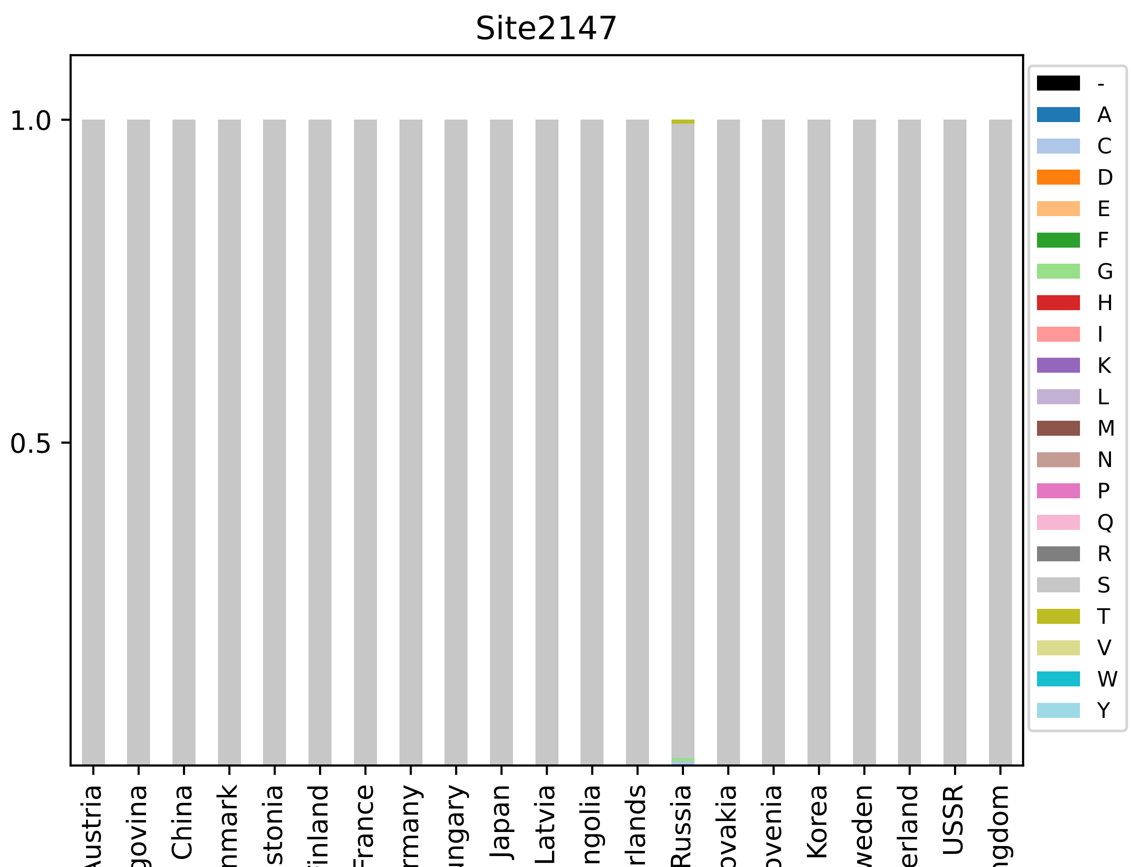
Site2147
1.0
0.5
-
A
C
D
E
F
G
H
I
K
L
M
N
P
Q
R
S
T
V
W
Y
Austria
China
Finland
France
Japan
Latvia
Russia
Korea
USSR

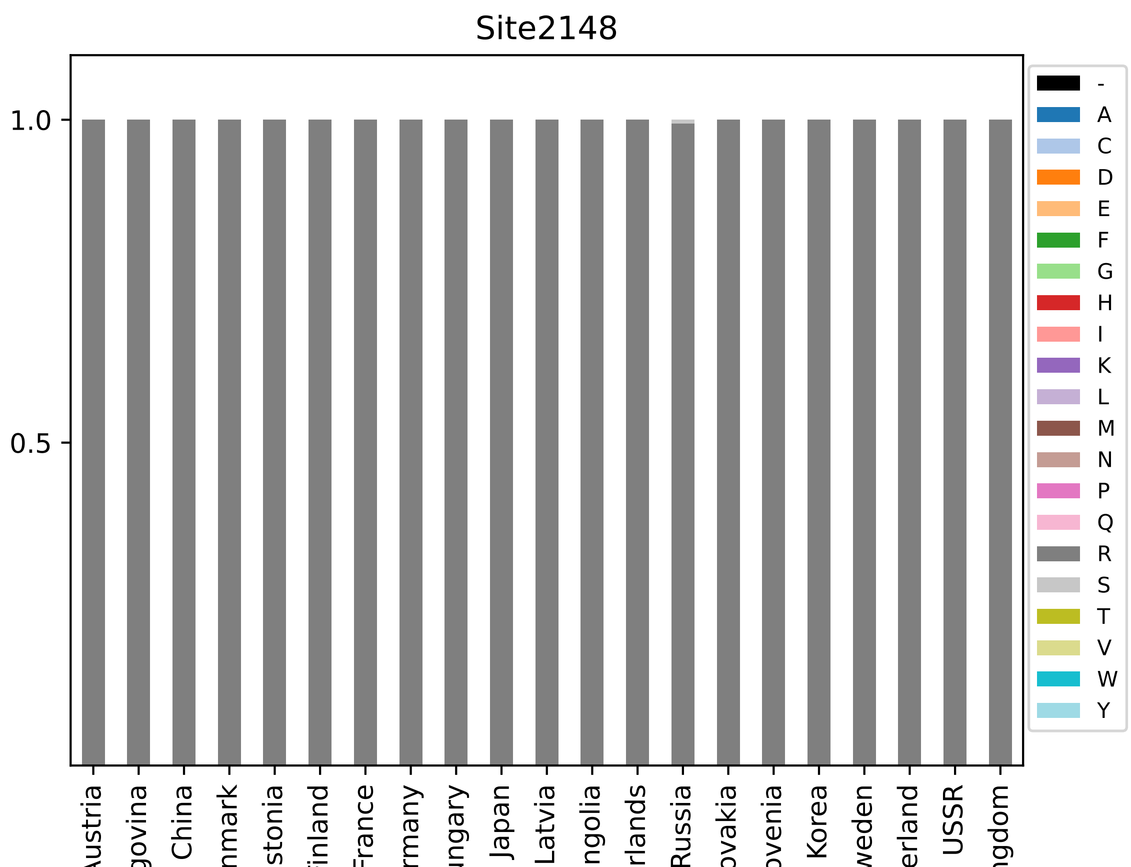
Site2148
1.0
0.5
-
A
C
D
E
F
G
H
I
K
L
M
N
P
Q
R
S
T
V
W
Y
China
Japan
Latvia
Russia
Korea
USSR

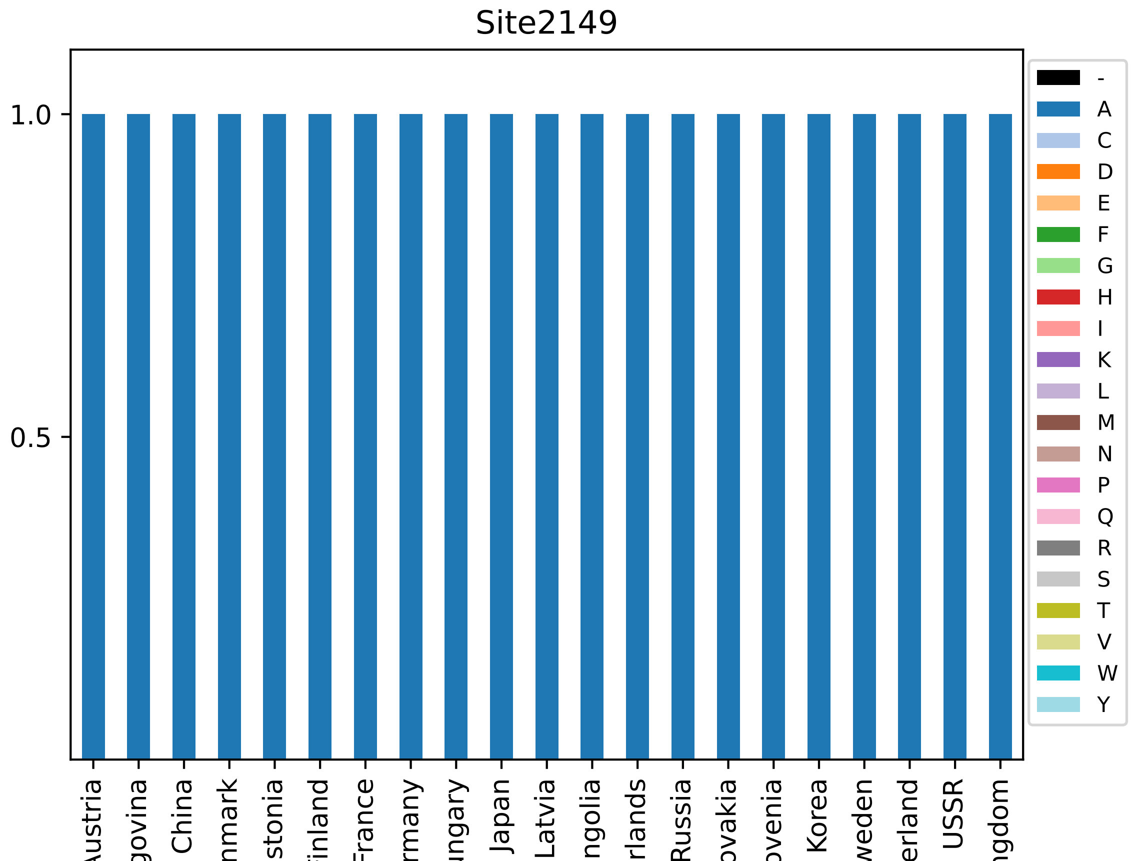
Site2149
1.0
0.5
Austria
govina
China
nmark
stonia
inland
France
rmany
ungary
Japan
Latvia
ngolia
rlands
Russia
ovakia
ovenia
Korea
weden
erland
USSR
ngdom
-
A
C
D
E
F
G
H
I
K
L
M
N
P
Q
R
S
T
V
W
Y

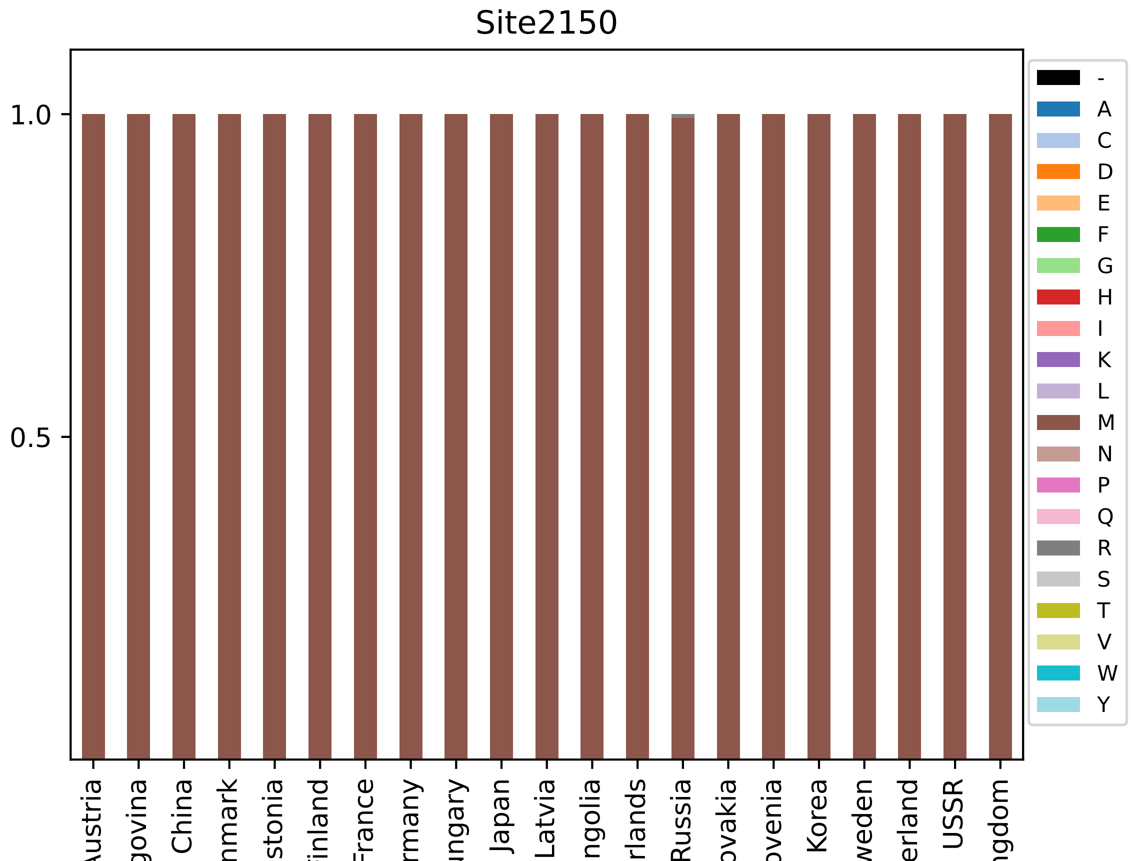

Site2150
1.0
0.5
-
A
C
D
E
F
G
H
I
K
L
M
N
P
Q
R
S
T
V
W
Y
China
Japan
Latvia
Russia
Korea
USSR

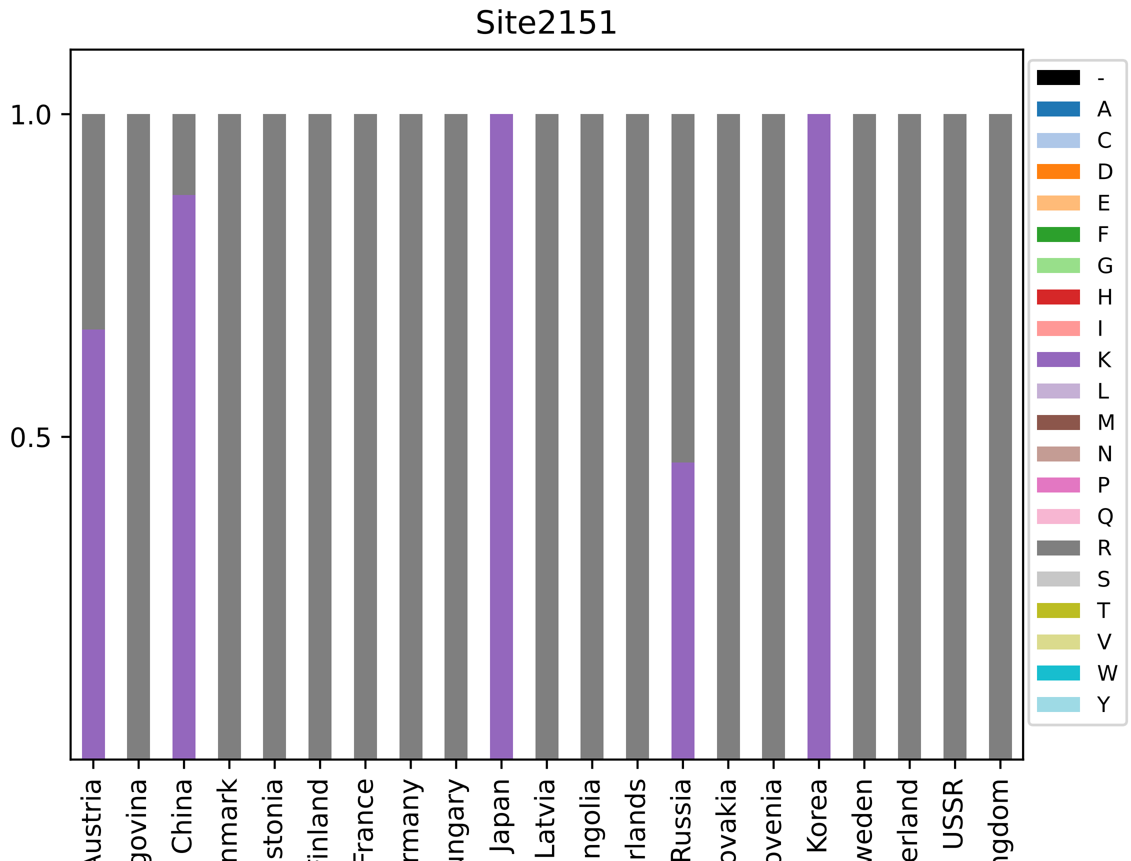

Site2151
1.0
0.5
-
A
C
D
E
F
G
H
I
K
L
M
N
P
Q
R
S
T
V
W
Y
Austria
govina
China
nmark
stonia
inland
France
rmany
ungary
Japan
Latvia
ngolia
rlands
Russia
ovakia
ovenia
Korea
weden
erland
USSR
ngdom

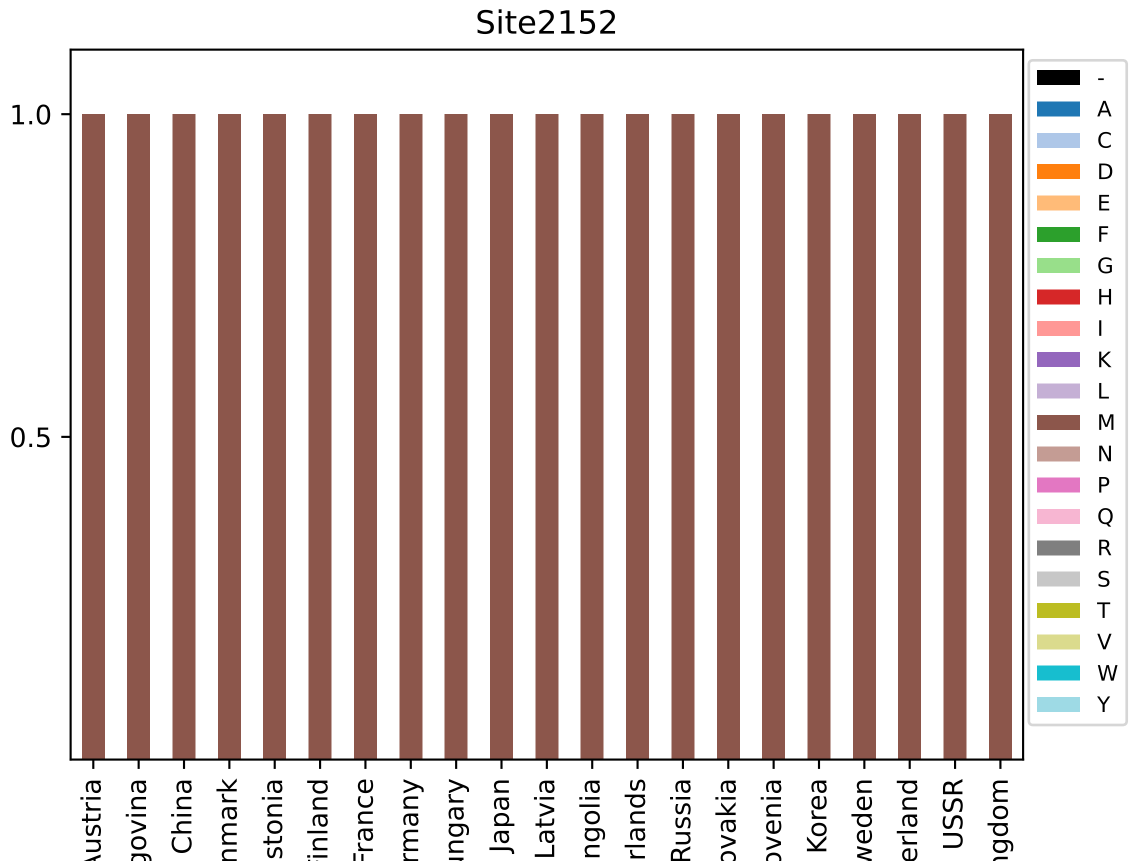
Site2152
1.0
0.5
-
A
C
D
E
F
G
H
I
K
L
M
N
P
Q
R
S
T
V
W
Y
Austria
govina
China
nmark
stonia
inland
France
rmany
ungary
Japan
Latvia
ngolia
rlands
Russia
ovakia
ovenia
Korea
weden
erland
USSR
ngdom

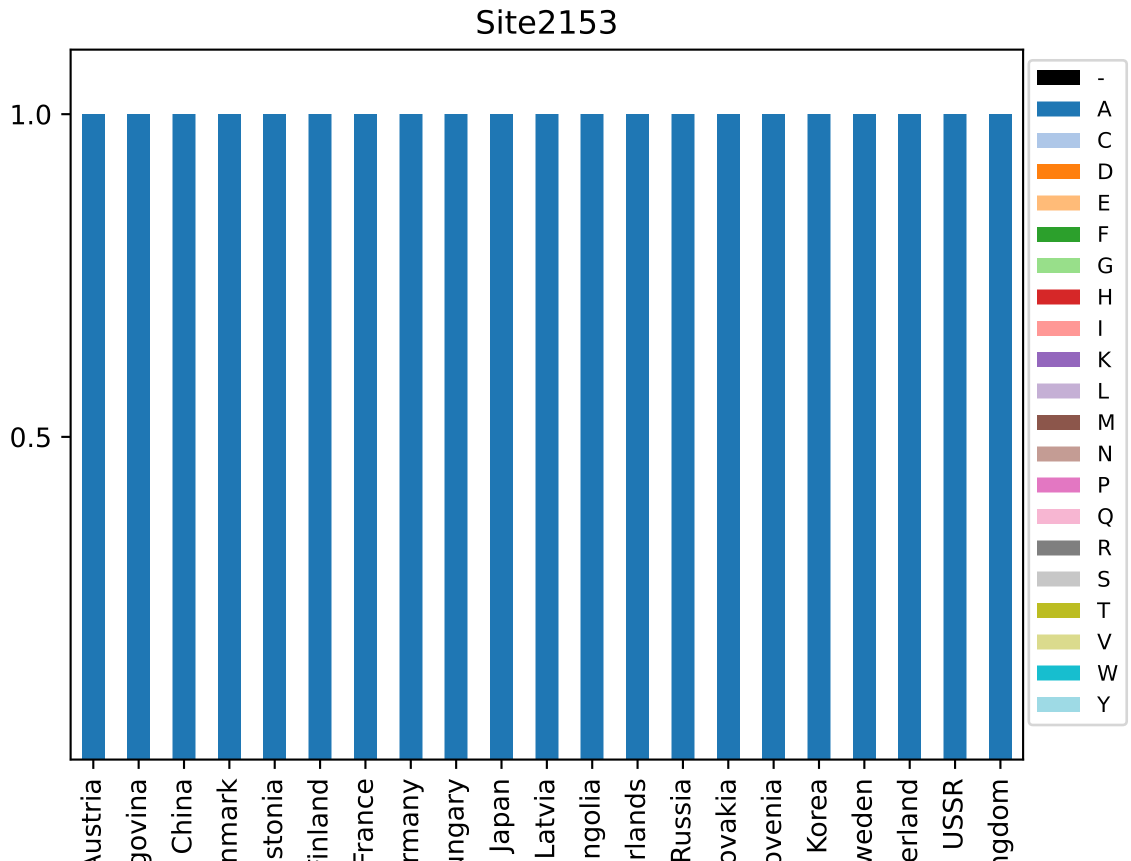

Site2153
1.0
0.5
Austria
govina
China
nmark
stonia
inland
France
rmany
ungary
Japan
Latvia
ngolia
rlands
Russia
ovakia
ovenia
Korea
weden
erland
USSR
ngdom
-
A
C
D
E
F
G
H
I
K
L
M
N
P
Q
R
S
T
V
W
Y

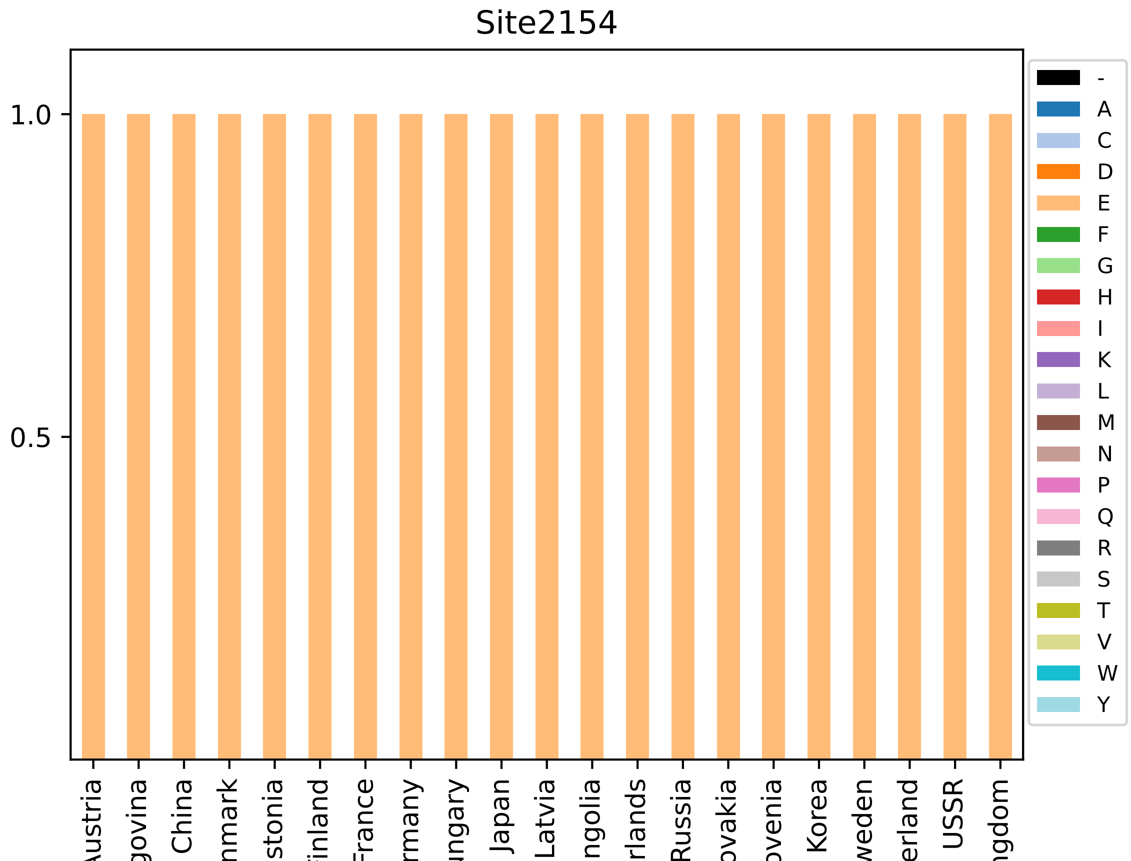
Site2154
1.0
0.5
-
A
C
D
E
F
G
H
I
K
L
M
N
P
Q
R
S
T
V
W
Y
China
Japan
Latvia
Russia
Korea
USSR

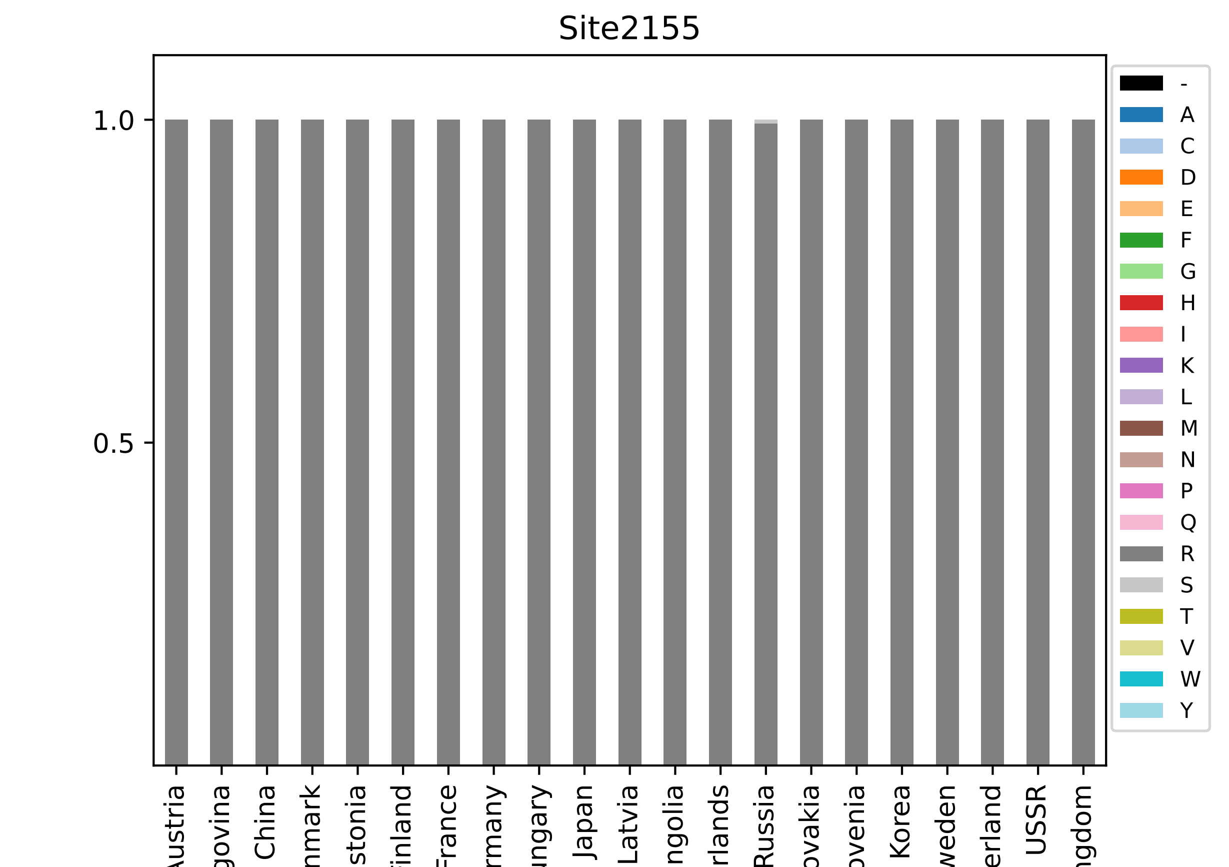

Site2155
1.0
0.5
-
A
C
D
E
F
G
H
I
K
L
M
N
P
Q
R
S
T
V
W
Y
Austria
govina
China
nmark
stonia
Finland
France
rmany
ungary
Japan
Latvia
ngolia
rlands
Russia
ovakia
ovenia
Korea
weden
erland
USSR
ngdom

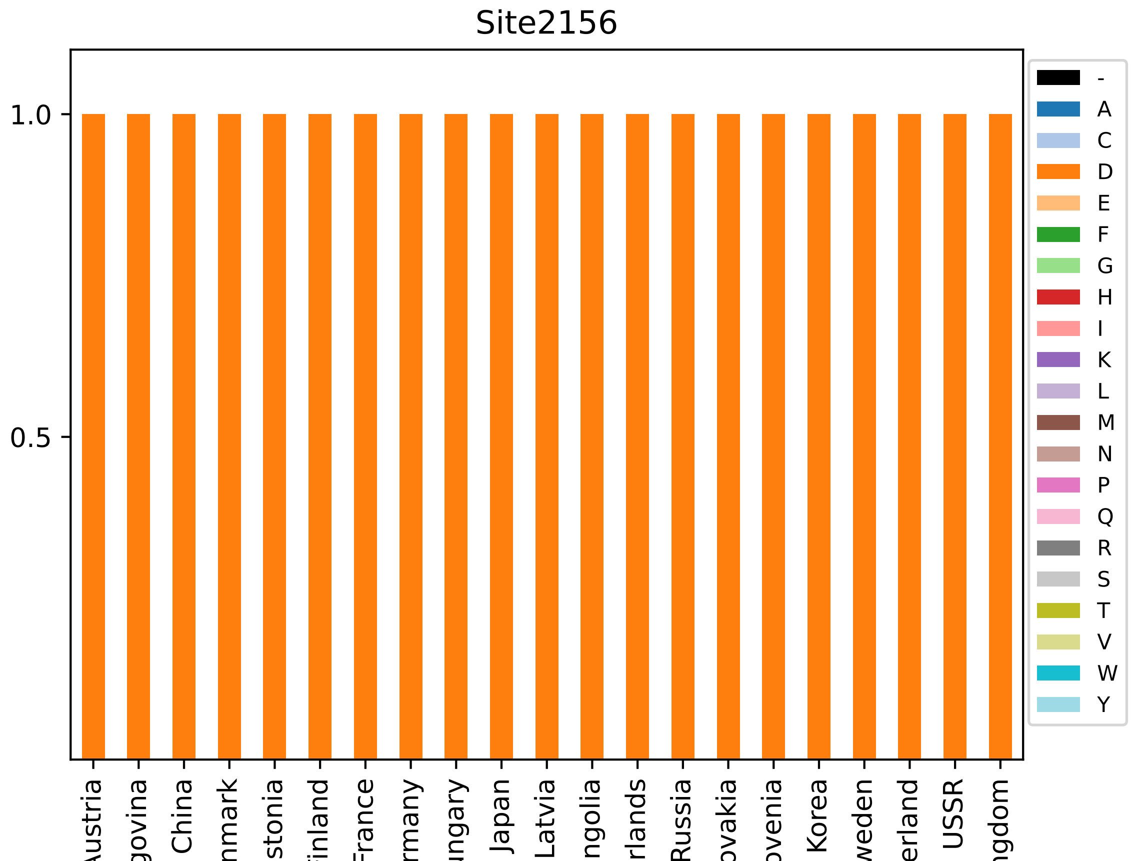
Site2156
1.0
0.5
-
A
C
D
E
F
G
H
I
K
L
M
N
P
Q
R
S
T
V
W
Y
China
Japan
Latvia
Russia
Korea
USSR

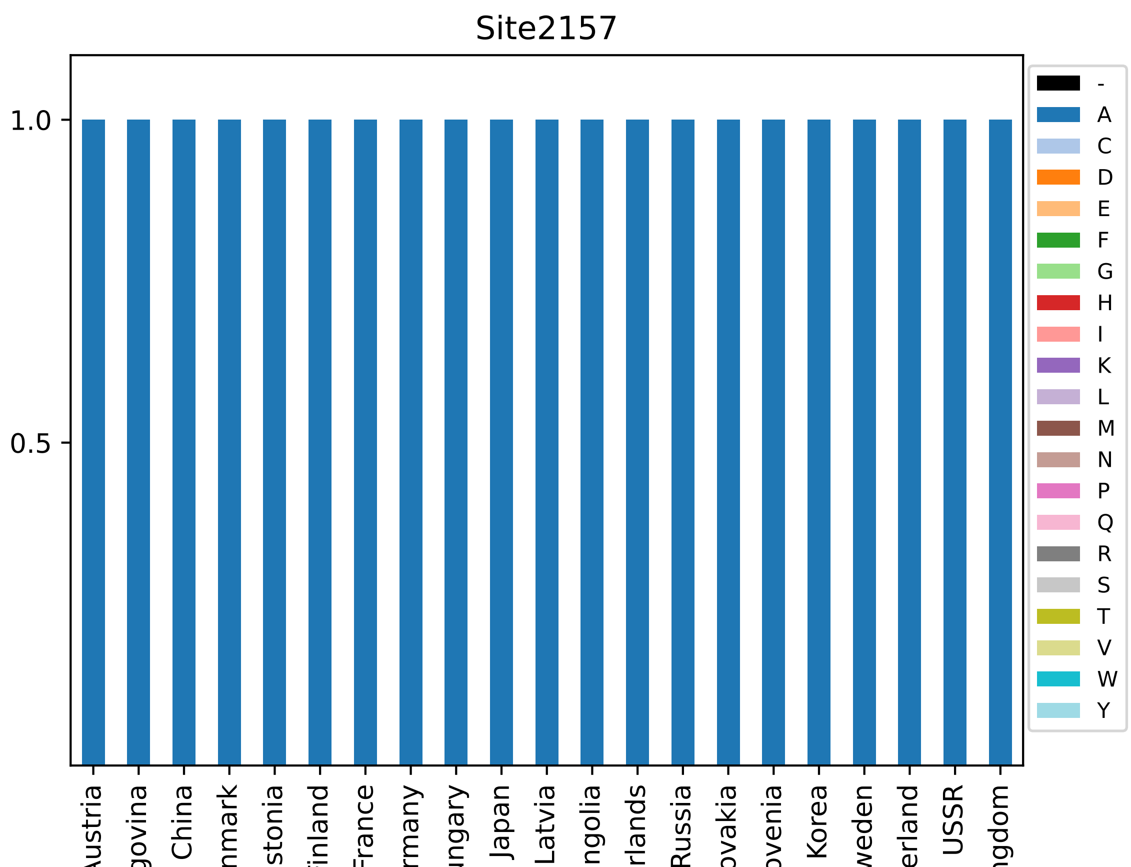

Site2157
1.0
0.5
-
A
C
D
E
F
G
H
I
K
L
M
N
P
Q
R
S
T
V
W
Y
Austria
govina
China
nmark
stonia
Finland
France
rmany
ungary
Japan
Latvia
ngolia
rlands
Russia
ovakia
ovenia
Korea
weden
erland
USSR
ngdom

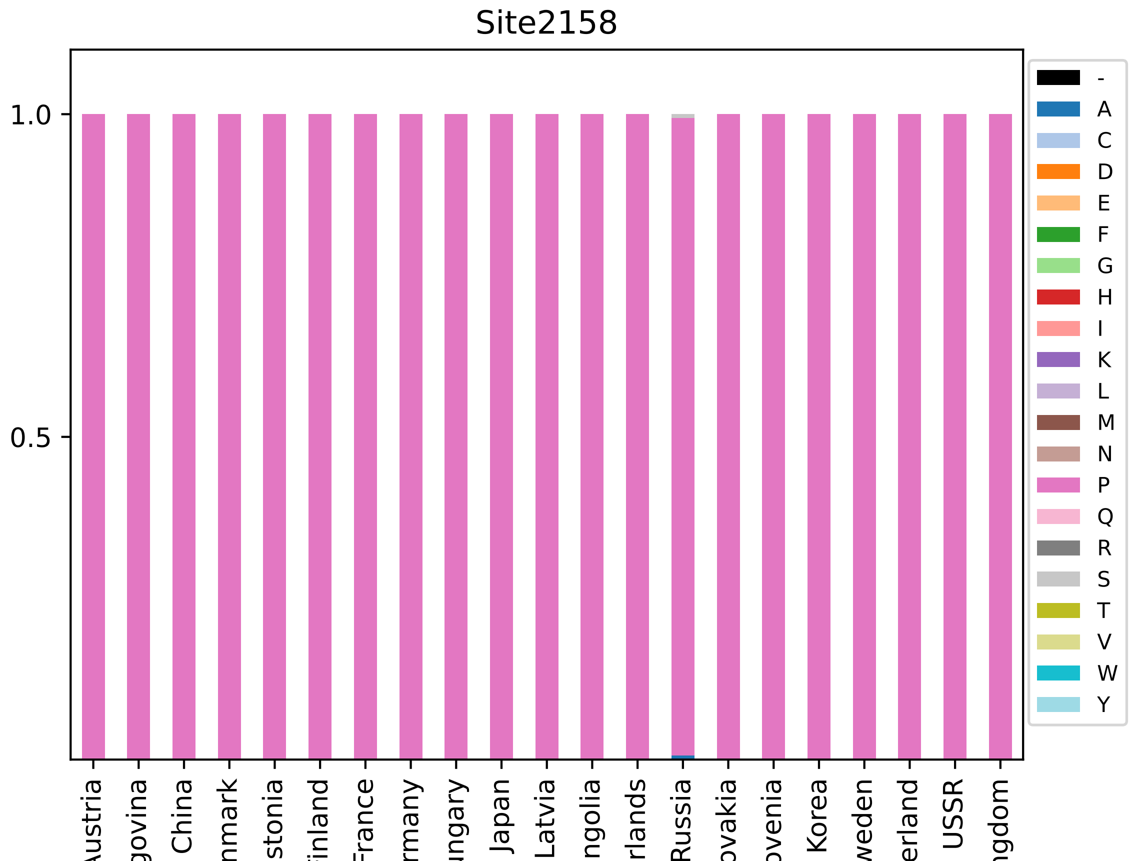
Site2158
1.0
0.5
-
A
C
D
E
F
G
H
I
K
L
M
N
P
Q
R
S
T
V
W
Y
China
Japan
Latvia
Russia
Korea
USSR

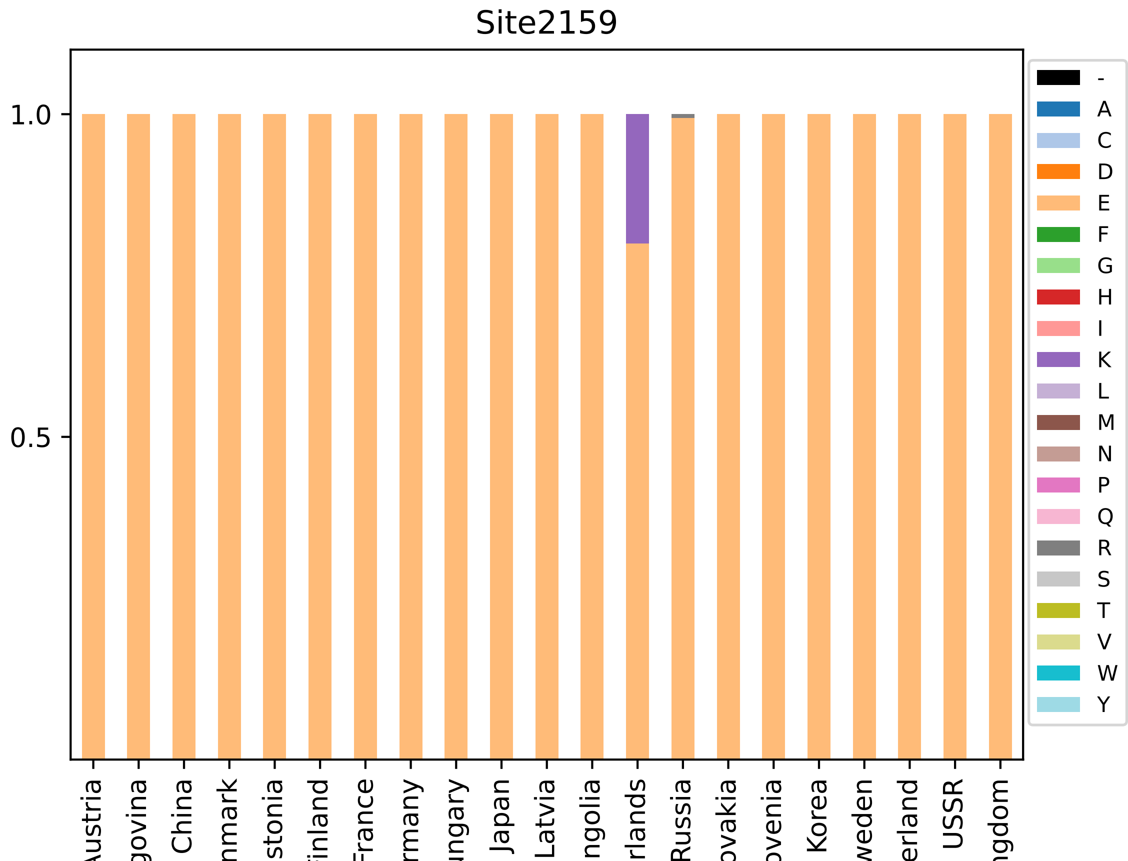
Site2159
1.0
0.5
-
A
C
D
E
F
G
H
I
K
L
M
N
P
Q
R
S
T
V
W
Y
Austria
govina
China
nmark
stonia
inland
France
rmany
ungary
Japan
Latvia
ngolia
rlands
Russia
ovakia
ovenia
Korea
weden
erland
USSR
ngdom

Site2160

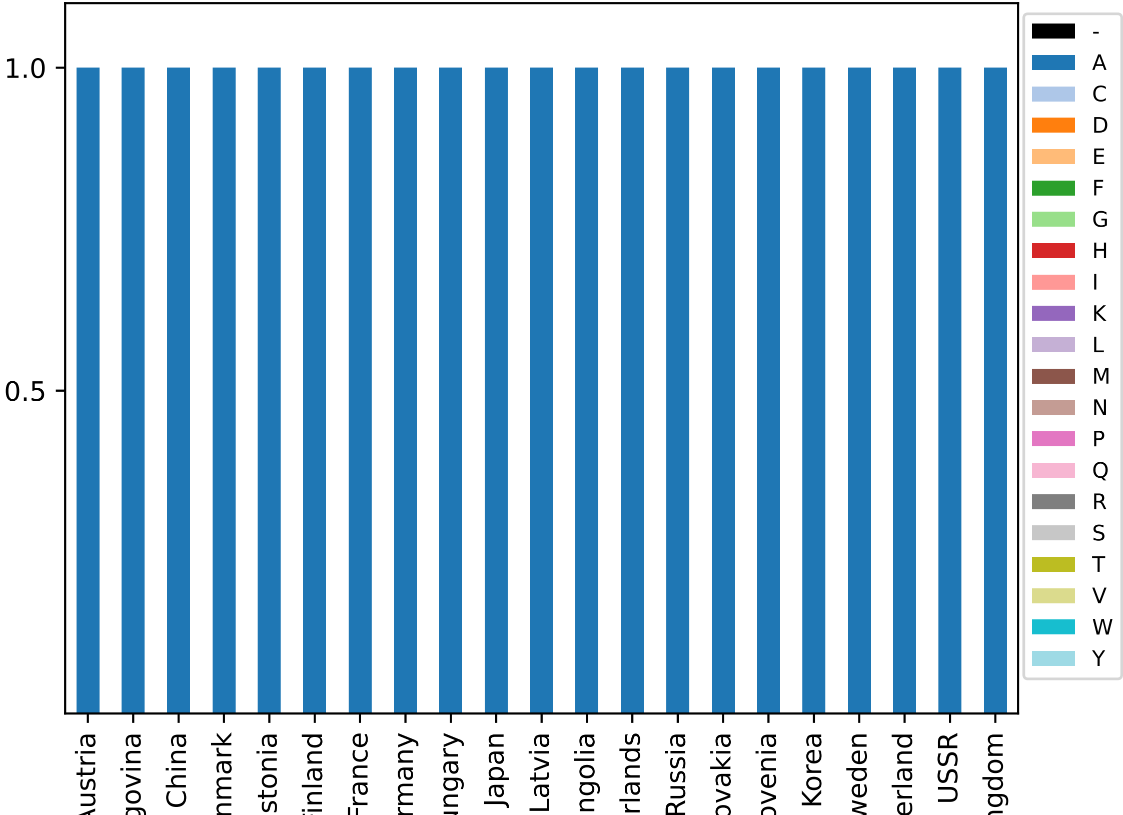
1.0
0.5
-
A
C
D
E
F
G
H
I
K
L
M
N
P
Q
R
S
T
V
W
Y
China
Japan
Latvia
Russia
Korea
USSR

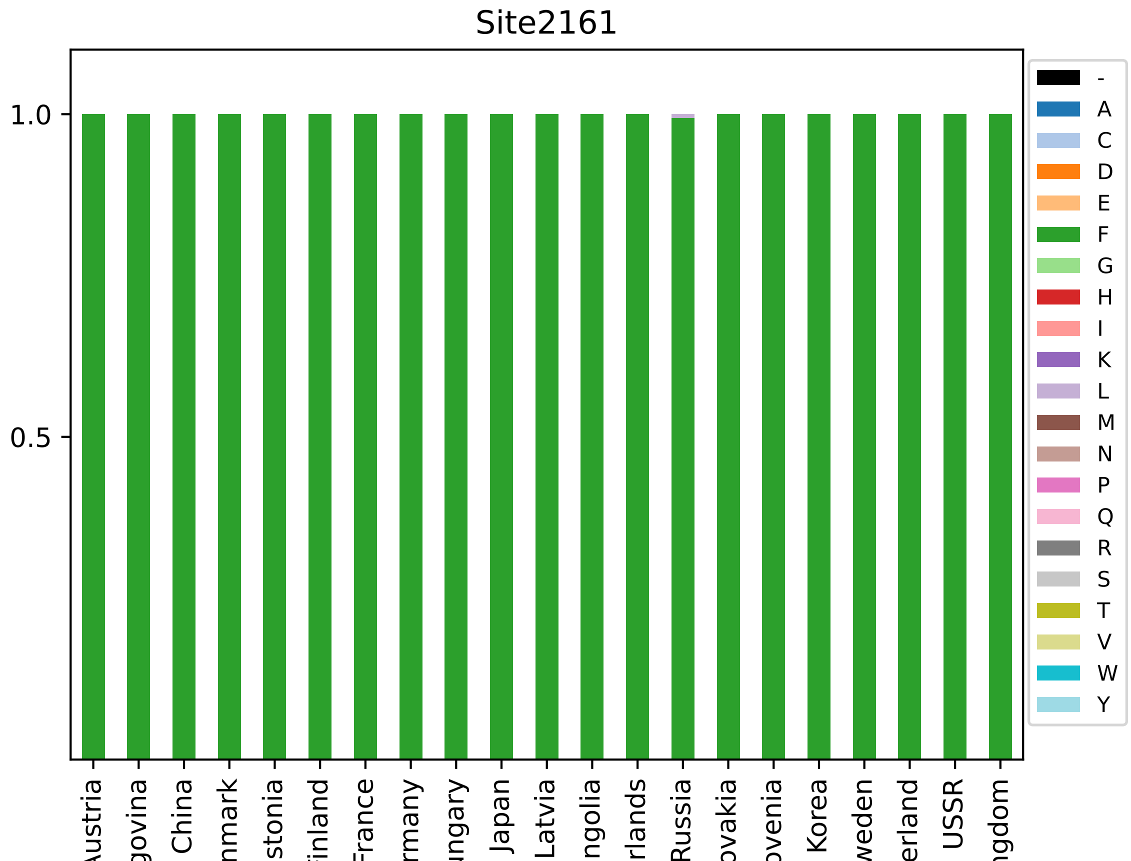

Site2161
1.0
0.5
-
A
C
D
E
F
G
H
I
K
L
M
N
P
Q
R
S
T
V
W
Y
Austria
China
Japan
Latvia
Russia
Korea
USSR

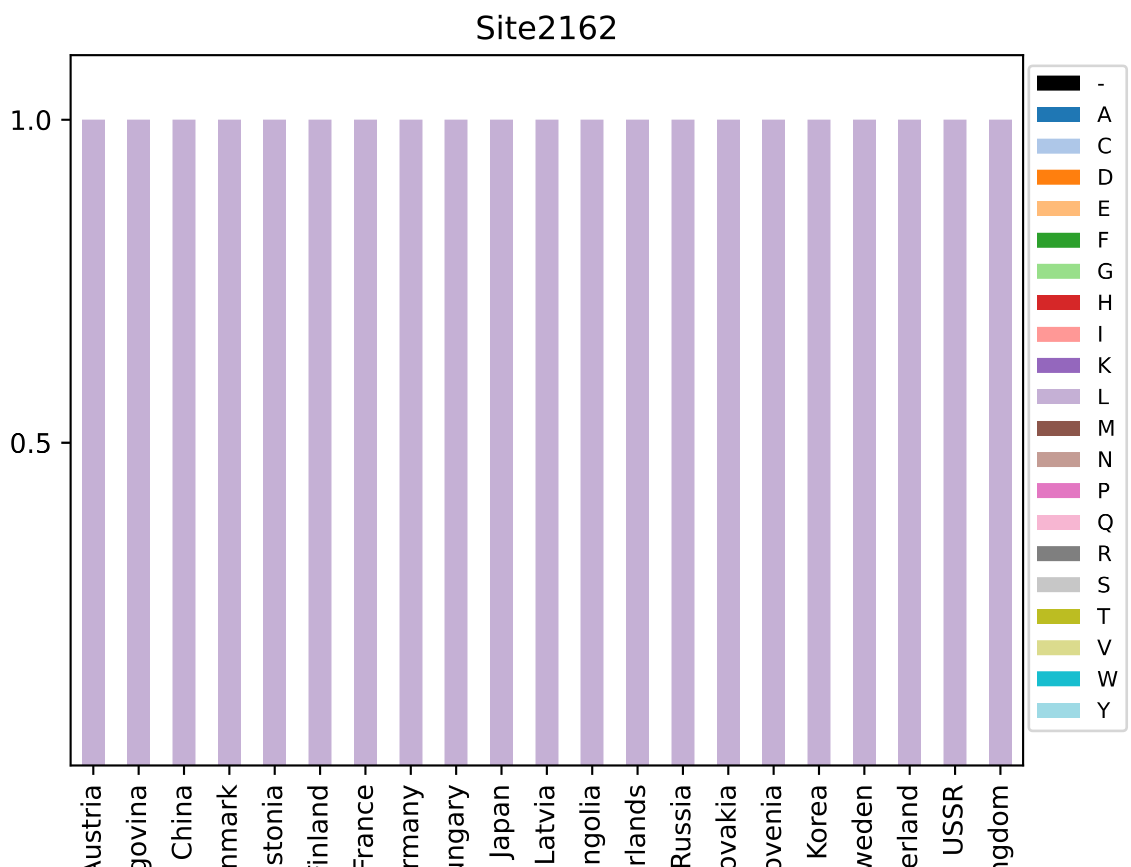
Site2162
1.0
0.5
-
A
C
D
E
F
G
H
I
K
L
M
N
P
Q
R
S
T
V
W
Y
Austria
China
Japan
Latvia
Russia
Korea
USSR

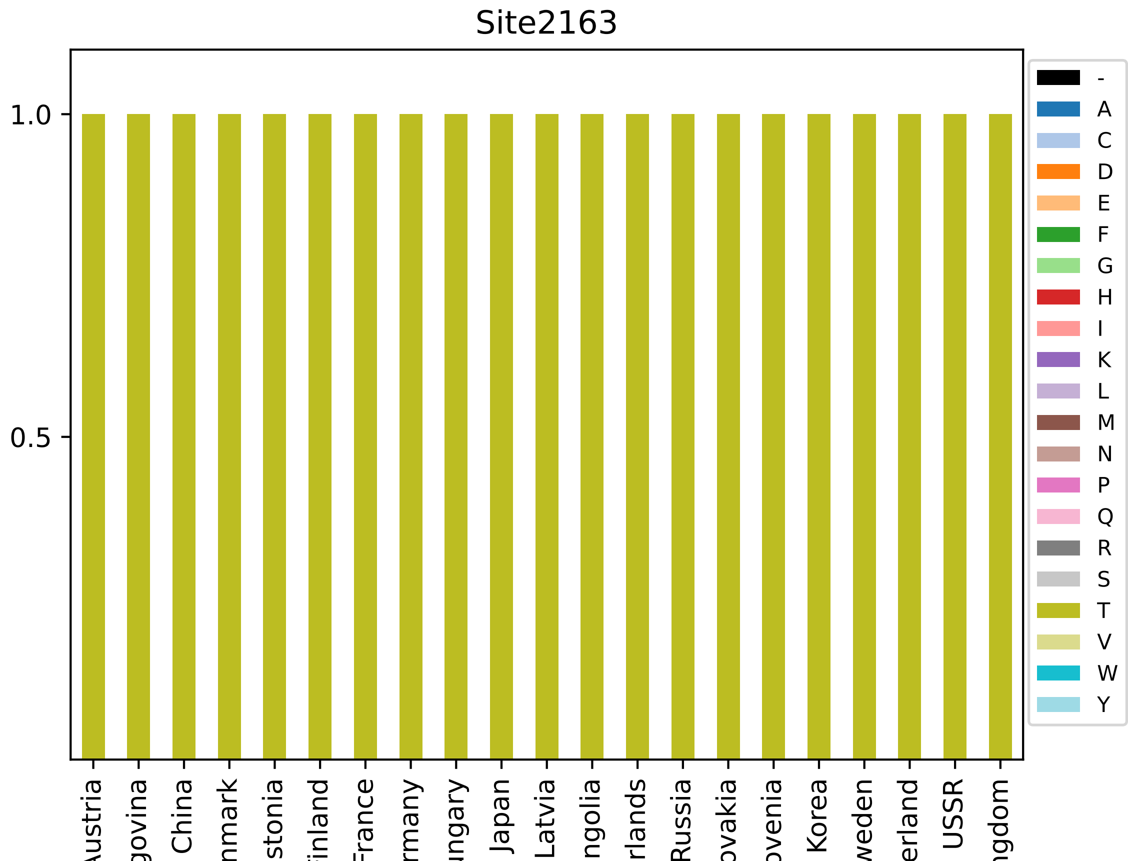
Site2163
1.0
0.5
-
A
C
D
E
F
G
H
I
K
L
M
N
P
Q
R
S
T
V
W
Y
Austria
govina
China
nmark
stonia
Finland
France
rmany
ungary
Japan
Latvia
ngolia
rlands
Russia
ovakia
ovenia
Korea
weden
erland
USSR
ngdom

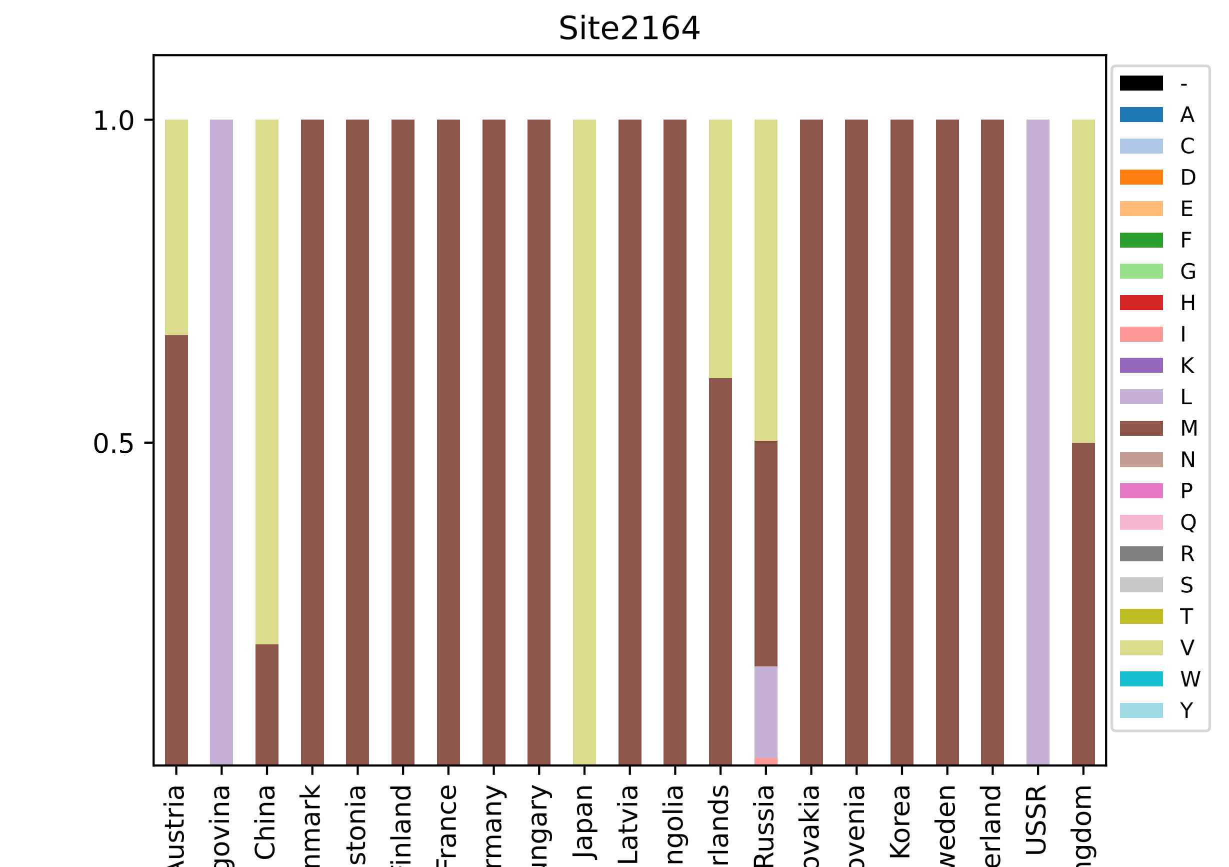
Site2164
1.0
0.5
-
A
C
D
E
F
G
H
I
K
L
M
N
P
Q
R
S
T
V
W
Y
Austria
govina
China
nmark
stonia
inland
France
rmany
ungary
Japan
Latvia
ngolia
rlands
Russia
ovakia
ovenia
Korea
weden
erland
USSR
ngdom

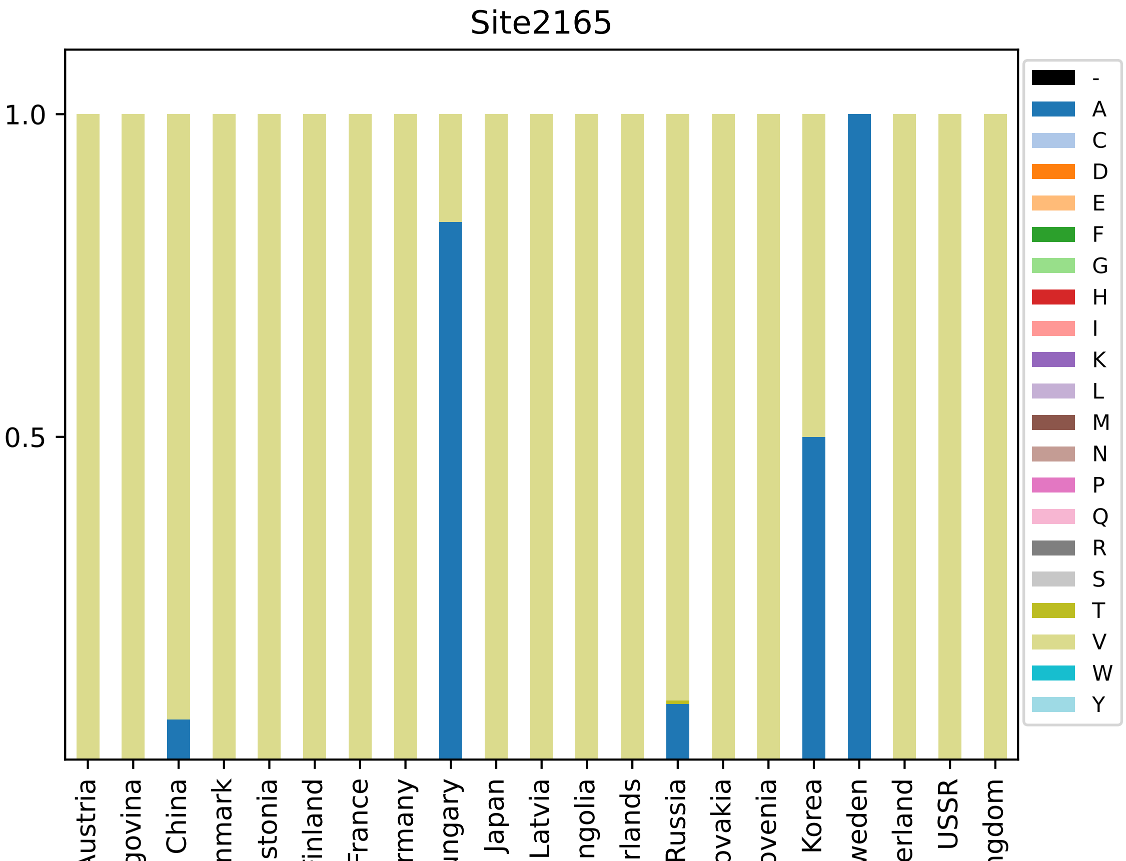

Site2165
1.0
0.5
-
A
C
D
E
F
G
H
I
K
L
M
N
P
Q
R
S
T
V
W
Y
China
Japan
Latvia
Russia
Korea
USSR

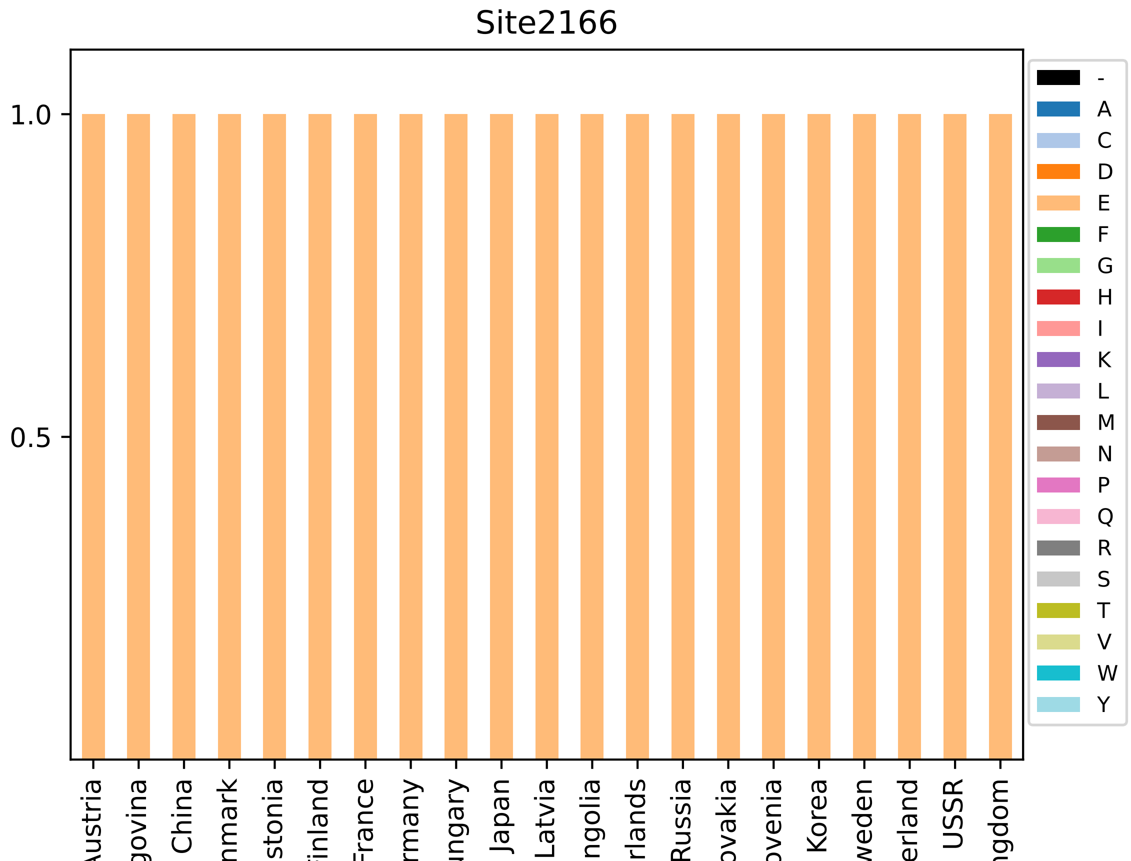
Site2166
1.0
0.5
-
A
C
D
E
F
G
H
I
K
L
M
N
P
Q
R
S
T
V
W
Y
China
Japan
Latvia
Russia
Korea
USSR

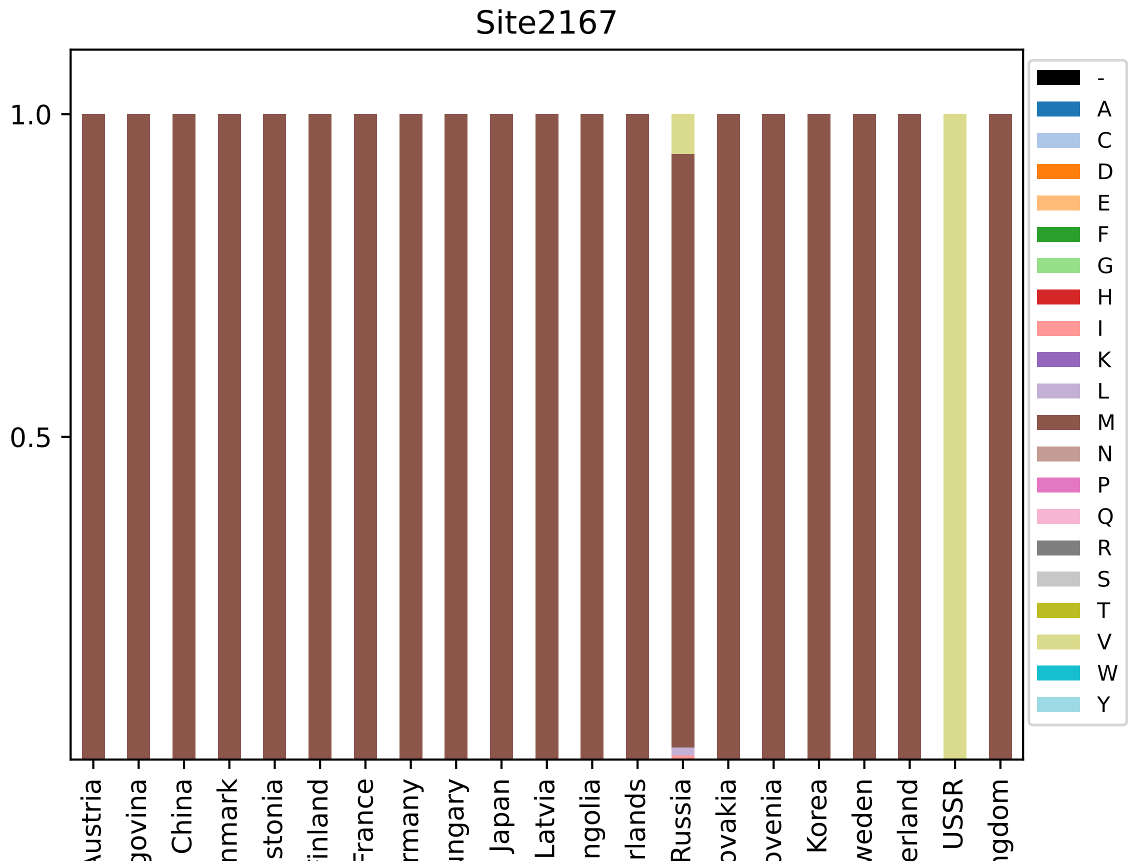

Site2167
1.0
0.5
-
A
C
D
E
F
G
H
I
K
L
M
N
P
Q
R
S
T
V
W
Y
China
Japan
Latvia
Russia
Korea
USSR

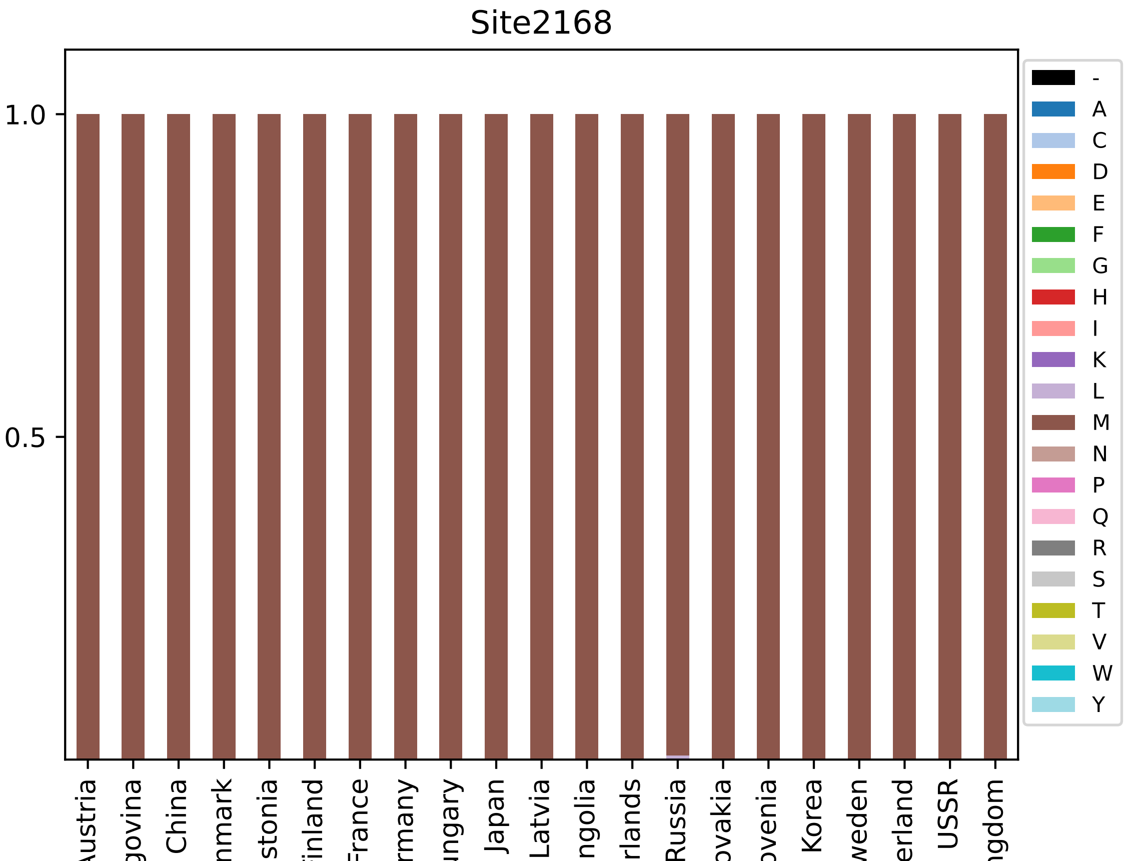
Site2168
1.0
0.5
-
A
C
D
E
F
G
H
I
K
L
M
N
P
Q
R
S
T
V
W
Y
Austria
govina
China
nmark
stonia
inland
France
rmany
ungary
Japan
Latvia
ngolia
rlands
Russia
ovakia
ovenia
Korea
weden
erland
USSR
ngdom

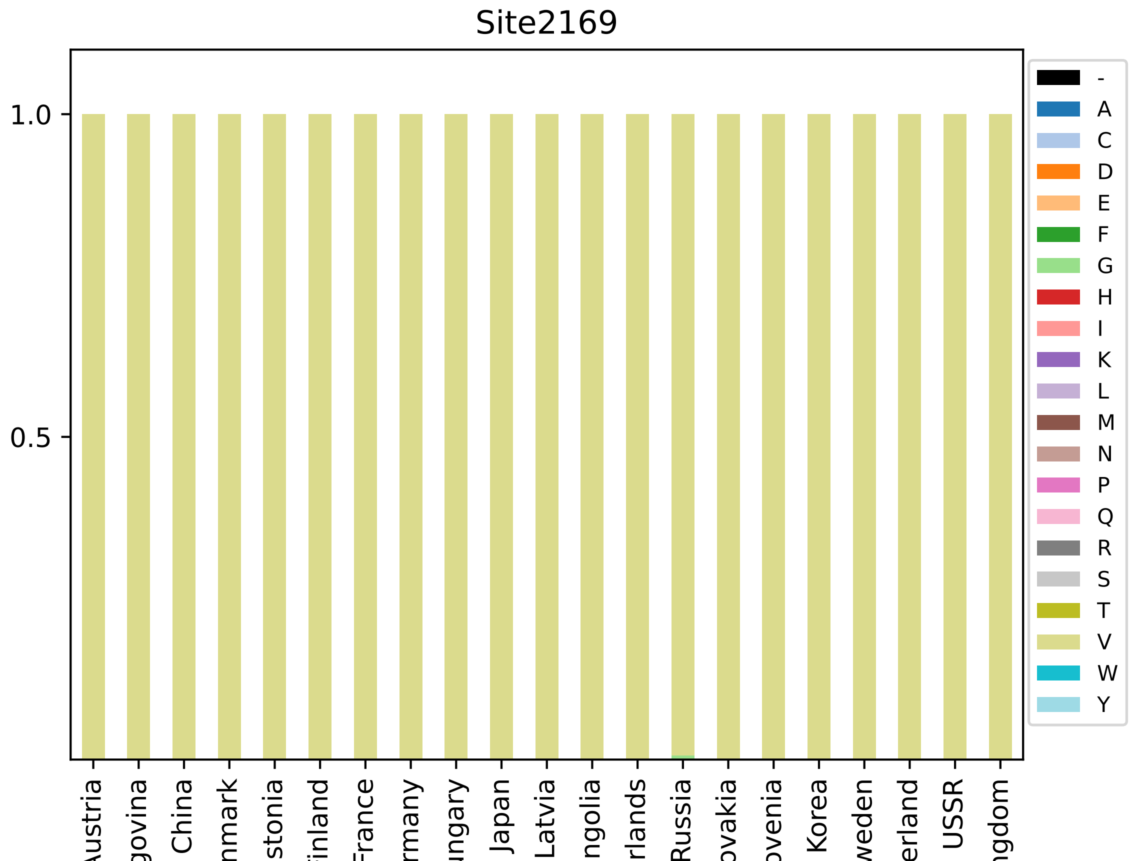
Site2169
1.0
0.5
-
A
C
D
E
F
G
H
I
K
L
M
N
P
Q
R
S
T
V
W
Y
China
Japan
Latvia
Russia
Korea
USSR

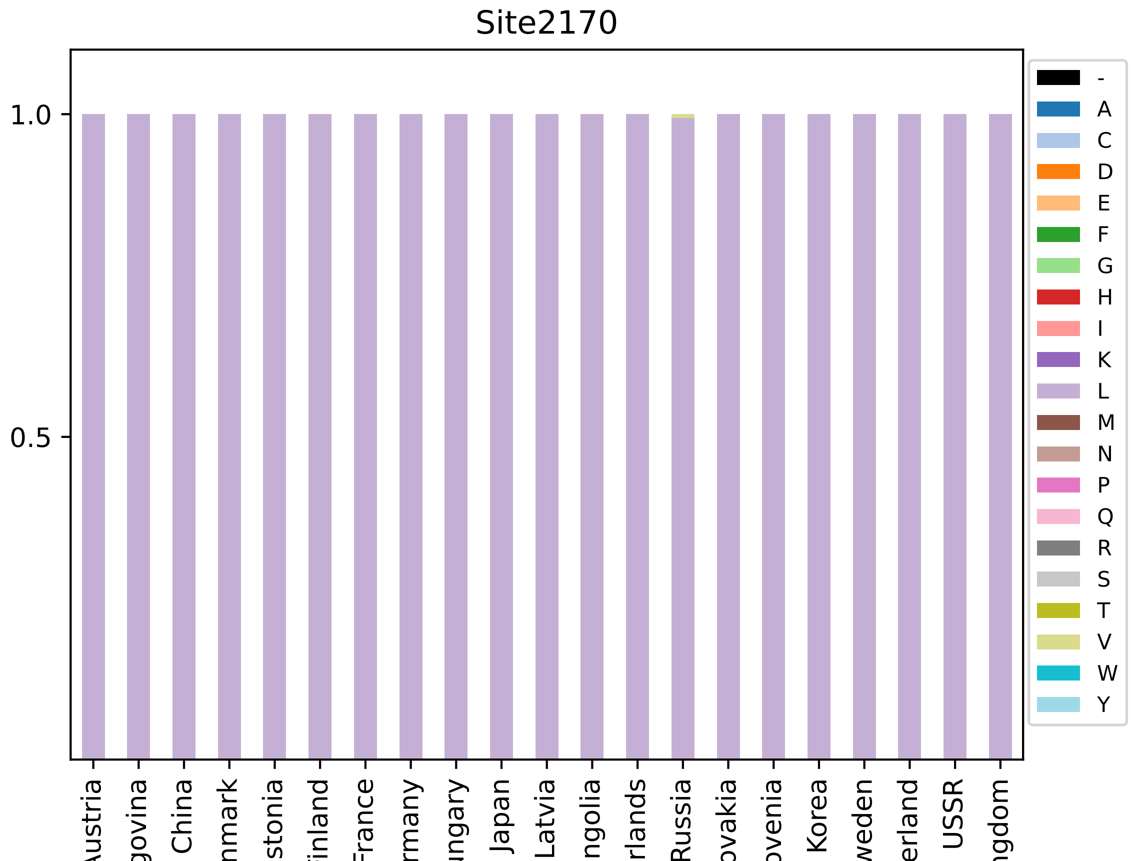
Site2170
1.0
0.5
-
A
C
D
E
F
G
H
I
K
L
M
N
P
Q
R
S
T
V
W
Y
China
Japan
Latvia
Russia
Korea
USSR

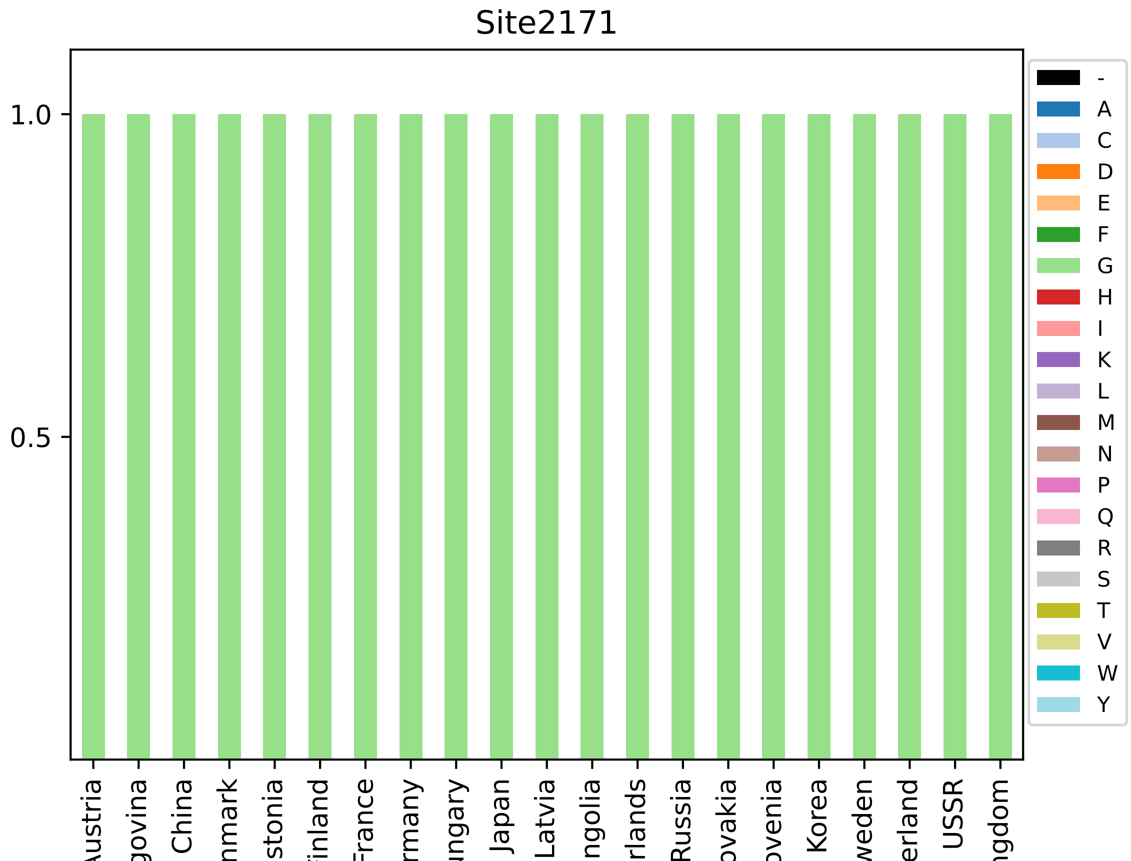
Site2171
1.0
0.5
-
A
C
D
E
F
G
H
I
K
L
M
N
P
Q
R
S
T
V
W
Y
China
Japan
Latvia
Russia
Korea
USSR

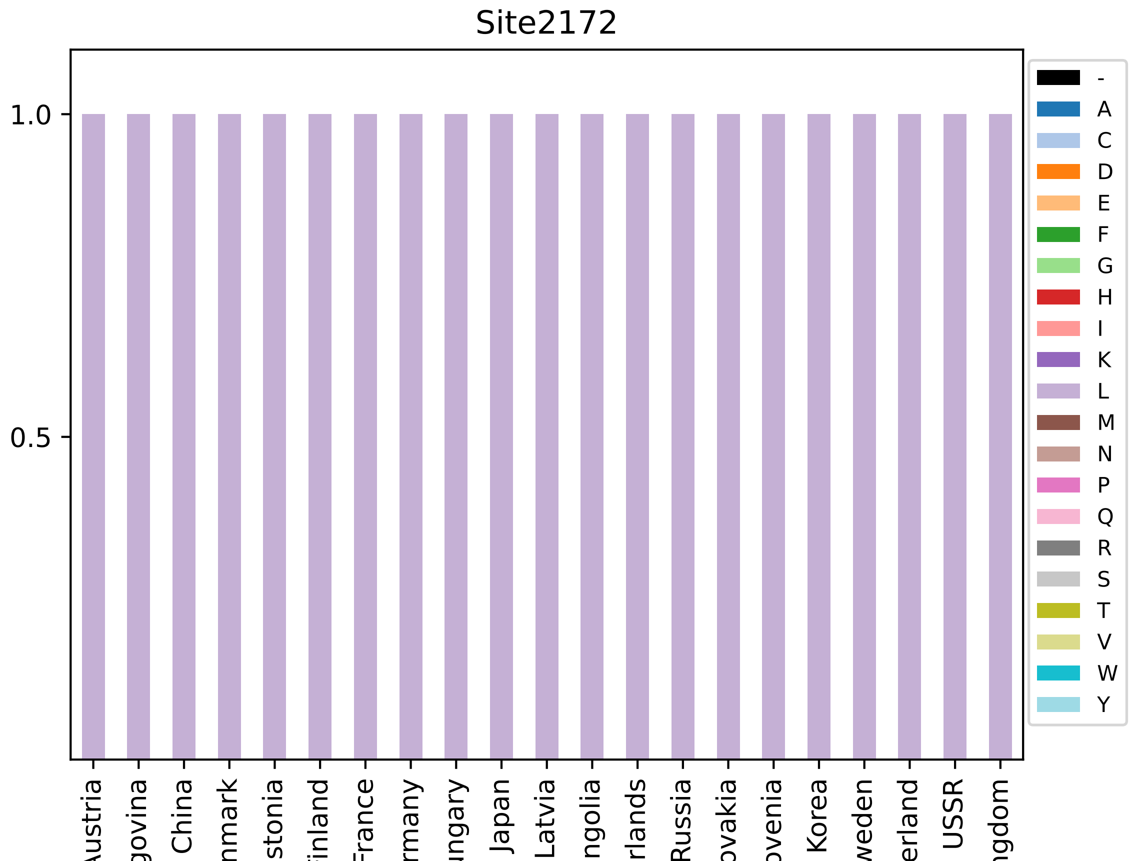

Site2172
1.0
0.5
-
A
C
D
E
F
G
H
I
K
L
M
N
P
Q
R
S
T
V
W
Y
China
Japan
Latvia
Russia
Korea
USSR

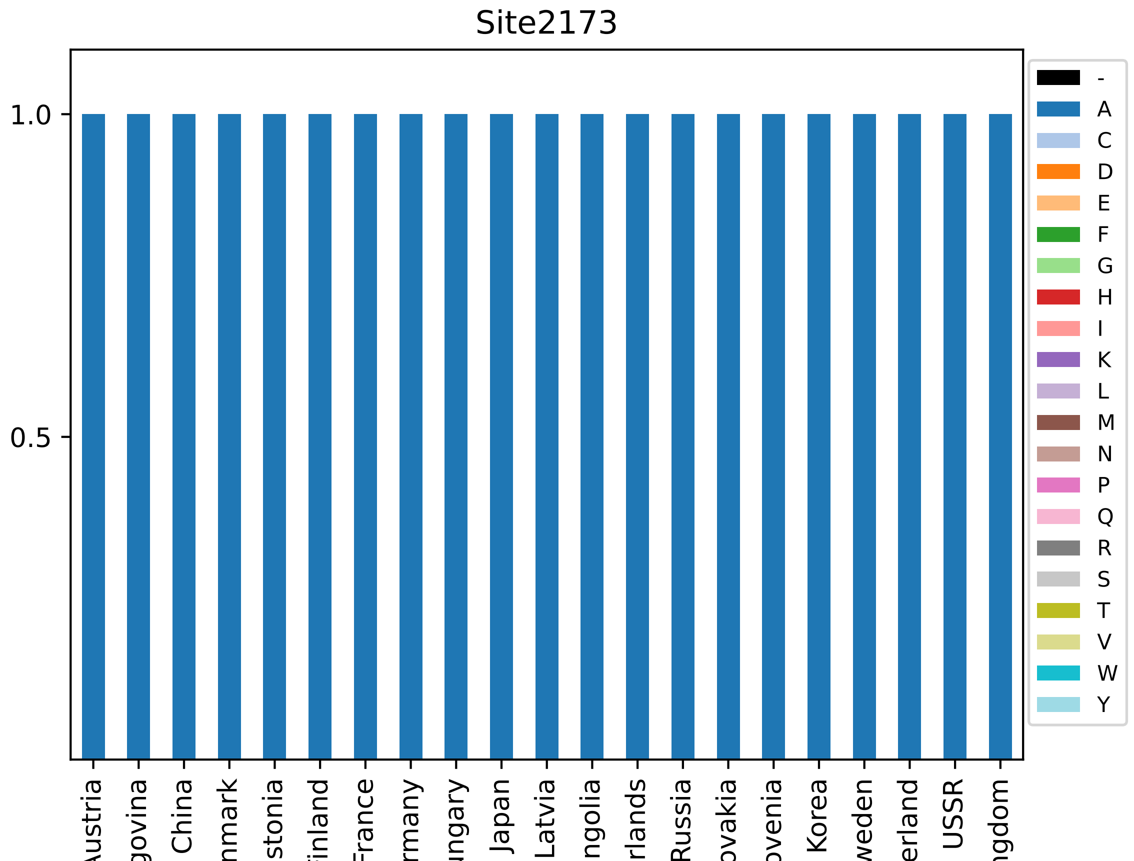
Site2173
1.0
0.5
-
A
C
D
E
F
G
H
I
K
L
M
N
P
Q
R
S
T
V
W
Y
Austria
China
Finland
France
Japan
Latvia
Russia
Korea
USSR

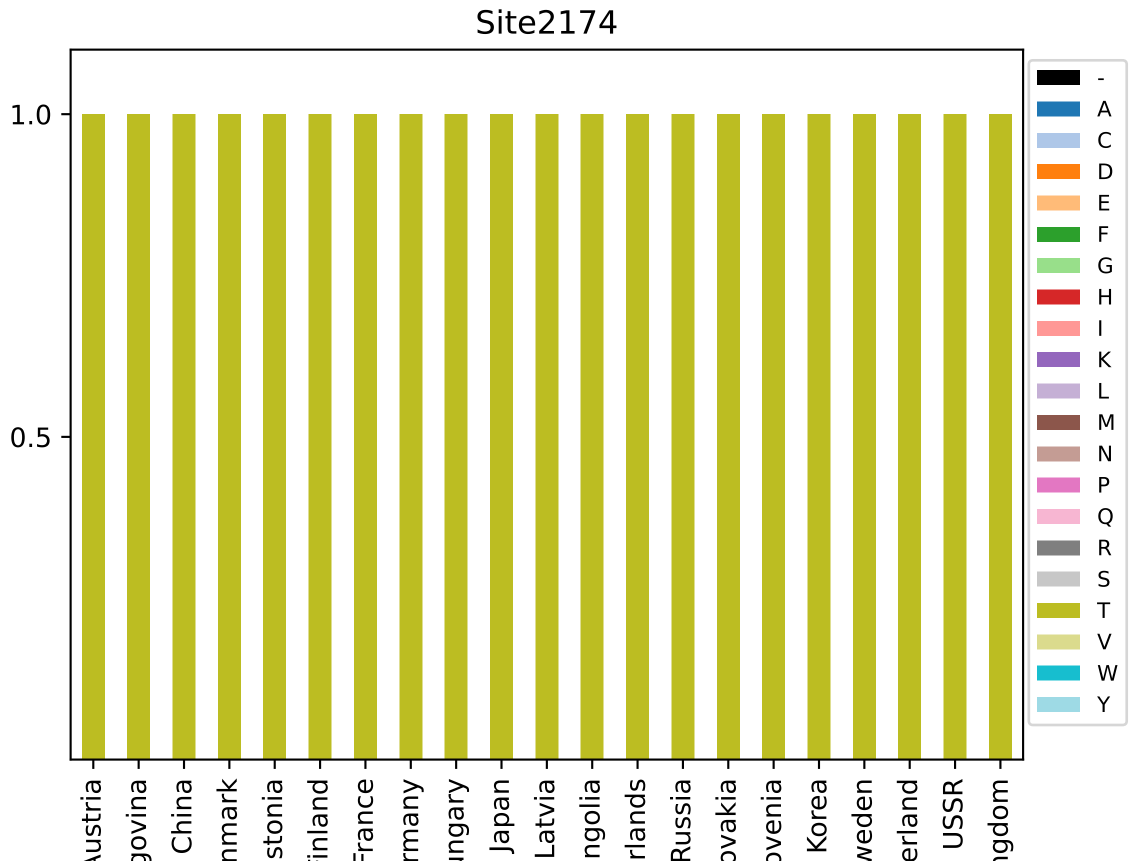
Site2174
1.0
0.5
-
A
C
D
E
F
G
H
I
K
L
M
N
P
Q
R
S
T
V
W
Y
China
Japan
Latvia
Russia
Korea
USSR

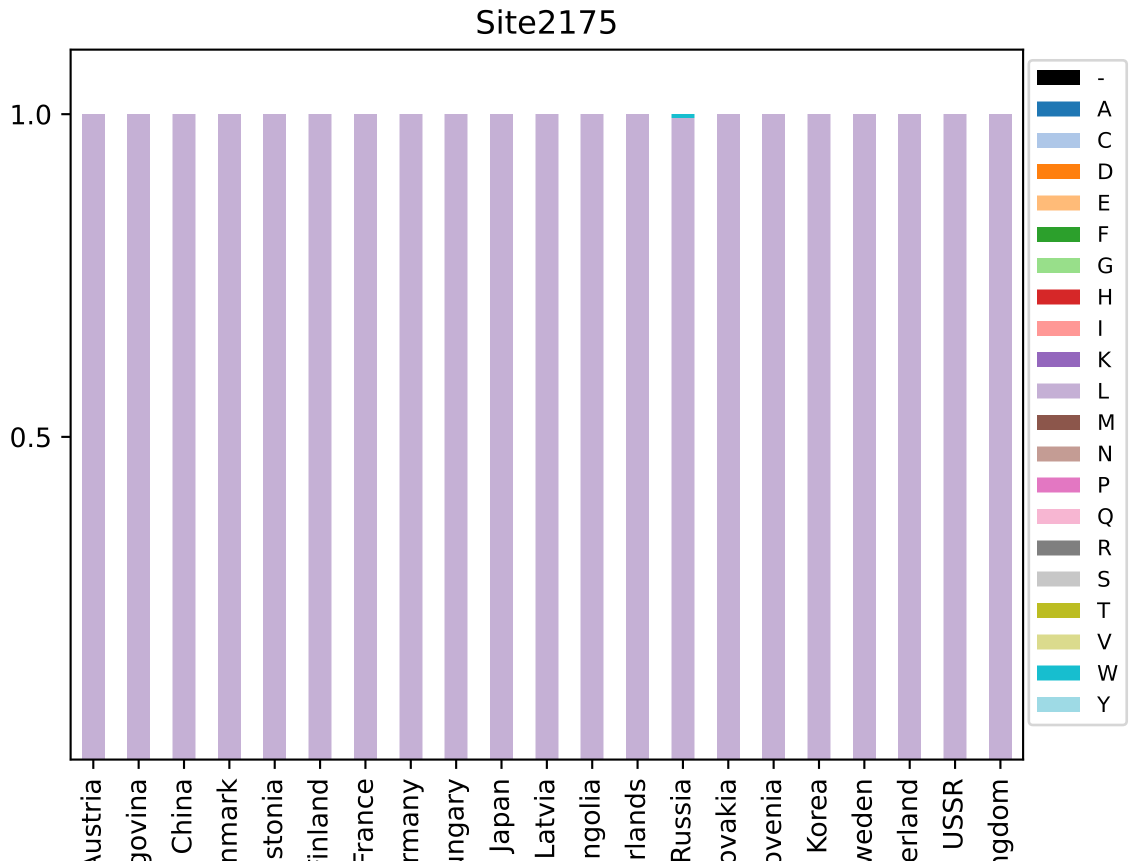
Site2175
1.0
0.5
-
A
C
D
E
F
G
H
I
K
L
M
N
P
Q
R
S
T
V
W
Y
China
Japan
Latvia
Russia
Korea
USSR

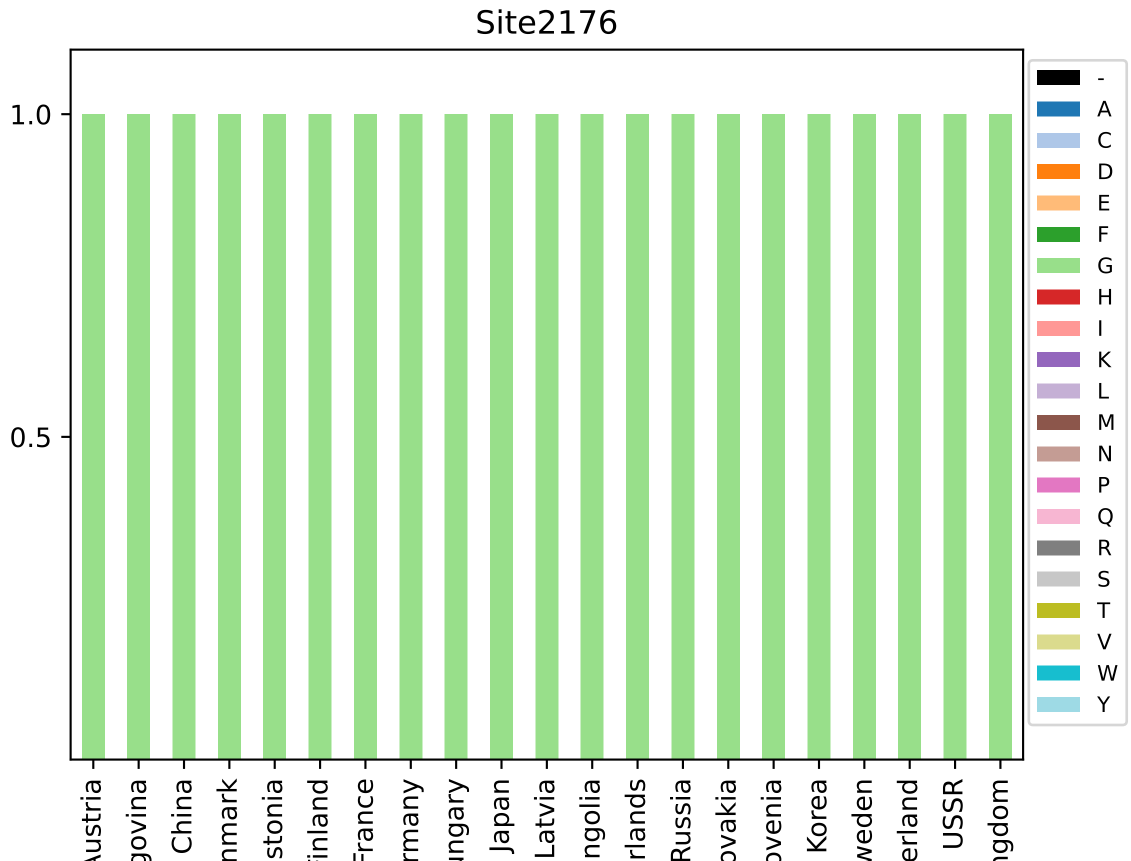

Site2176
1.0
0.5
-
A
C
D
E
F
G
H
I
K
L
M
N
P
Q
R
S
T
V
W
Y
China
Japan
Latvia
Russia
Korea
USSR

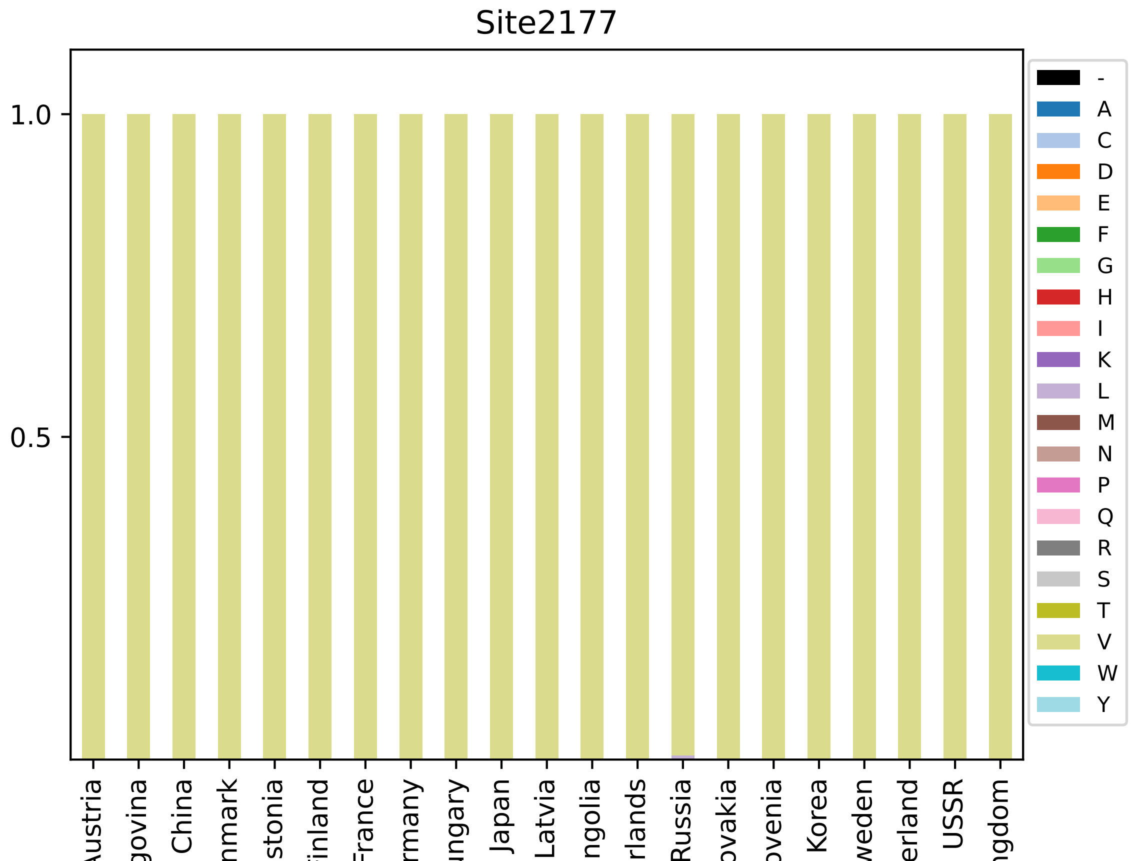
Site2177
1.0
0.5
-
A
C
D
E
F
G
H
I
K
L
M
N
P
Q
R
S
T
V
W
Y
China
Japan
Latvia
Russia
Korea
USSR

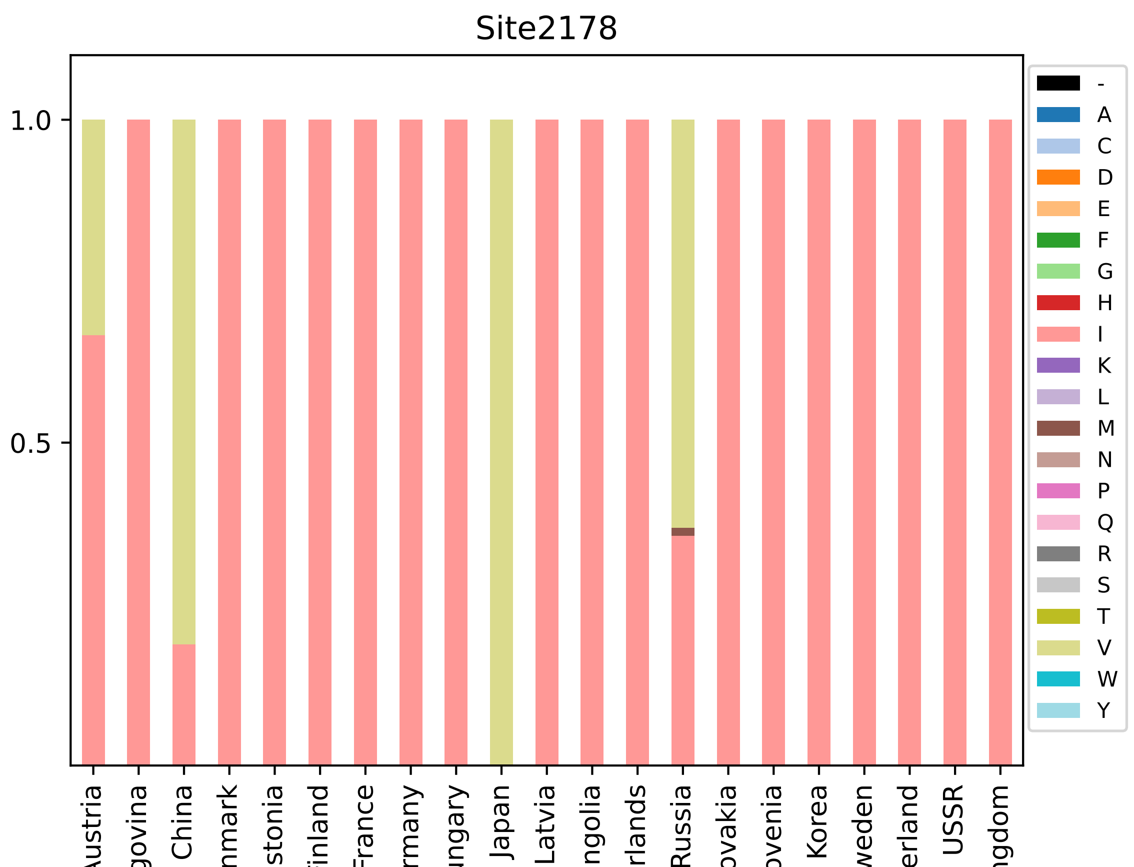
Site2178
1.0
0.5
-
A
C
D
E
F
G
H
I
K
L
M
N
P
Q
R
S
T
V
W
Y
Austria
China
Finland
France
Japan
Latvia
Russia
Korea
USSR

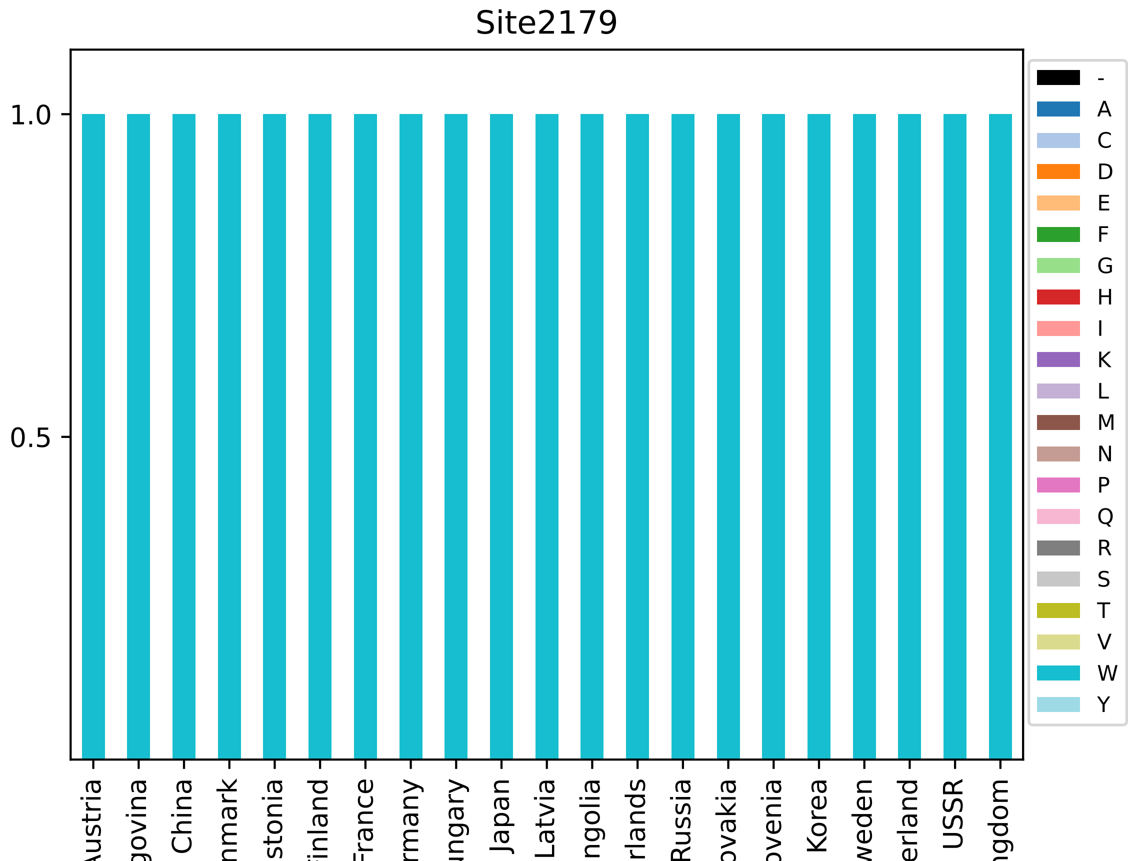
Site2179
1.0
0.5
-
A
C
D
E
F
G
H
I
K
L
M
N
P
Q
R
S
T
V
W
Y
China
Japan
Latvia
Russia
Korea
USSR

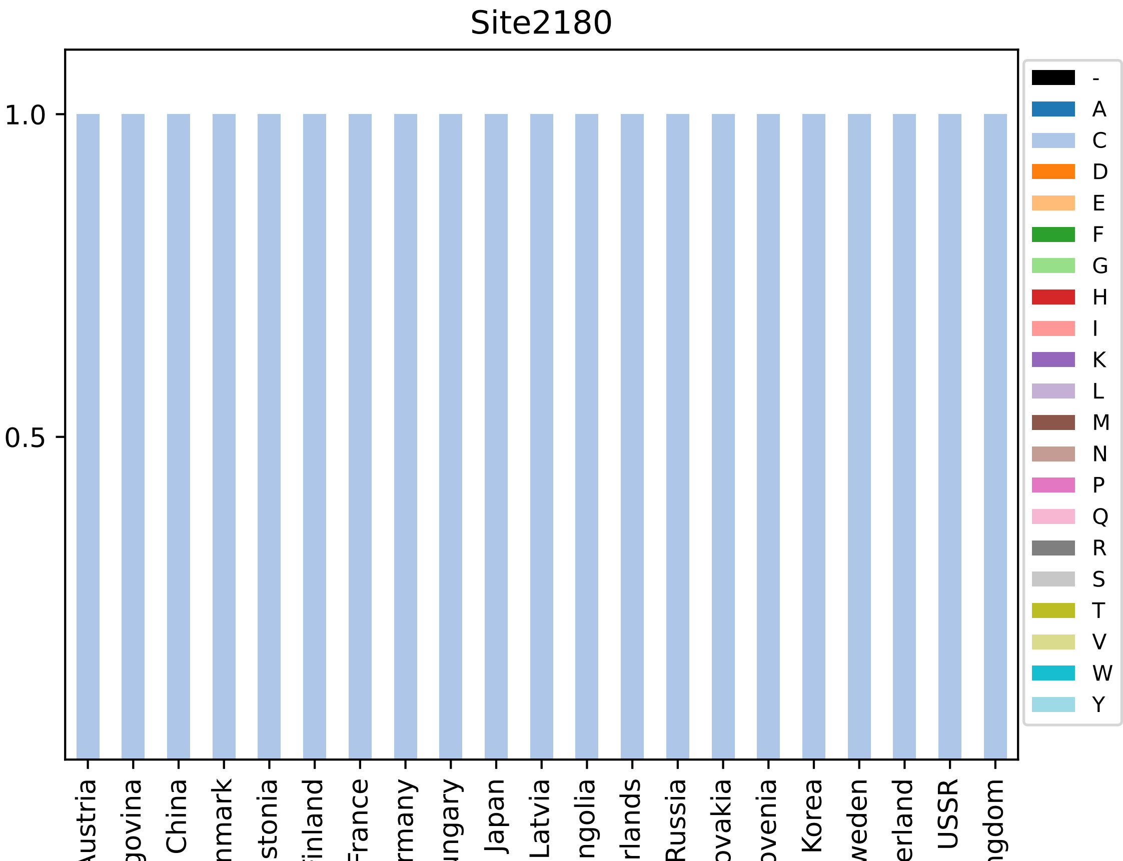
Site2180
1.0
0.5
Austria
China
Japan
Latvia
Russia
Korea
USSR
-
A
C
D
E
F
G
H
I
K
L
M
N
P
Q
R
S
T
V
W
Y

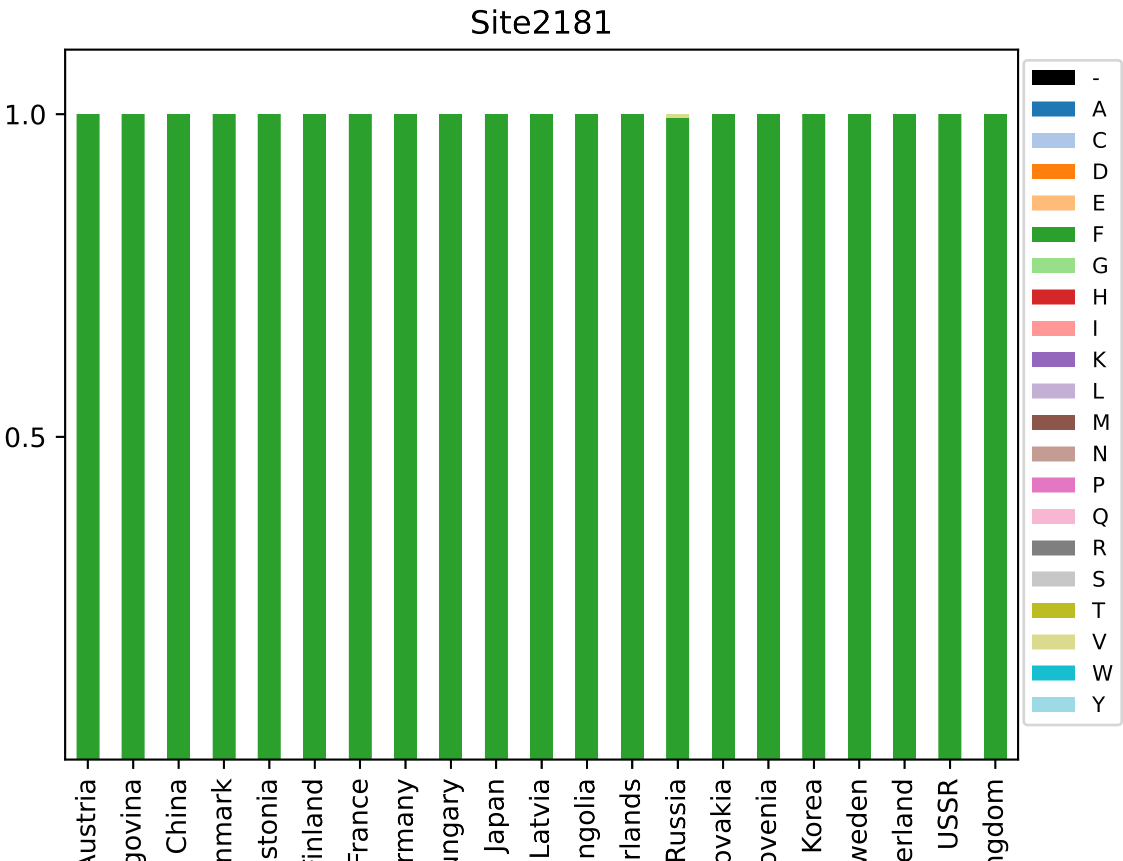

Site2181
1.0
0.5
-
A
C
D
E
F
G
H
I
K
L
M
N
P
Q
R
S
T
V
W
Y
Austria
govina
China
nmark
stonia
inland
France
rmany
ungary
Japan
Latvia
ngolia
rlands
Russia
ovakia
ovenia
Korea
weden
erland
USSR
ngdom

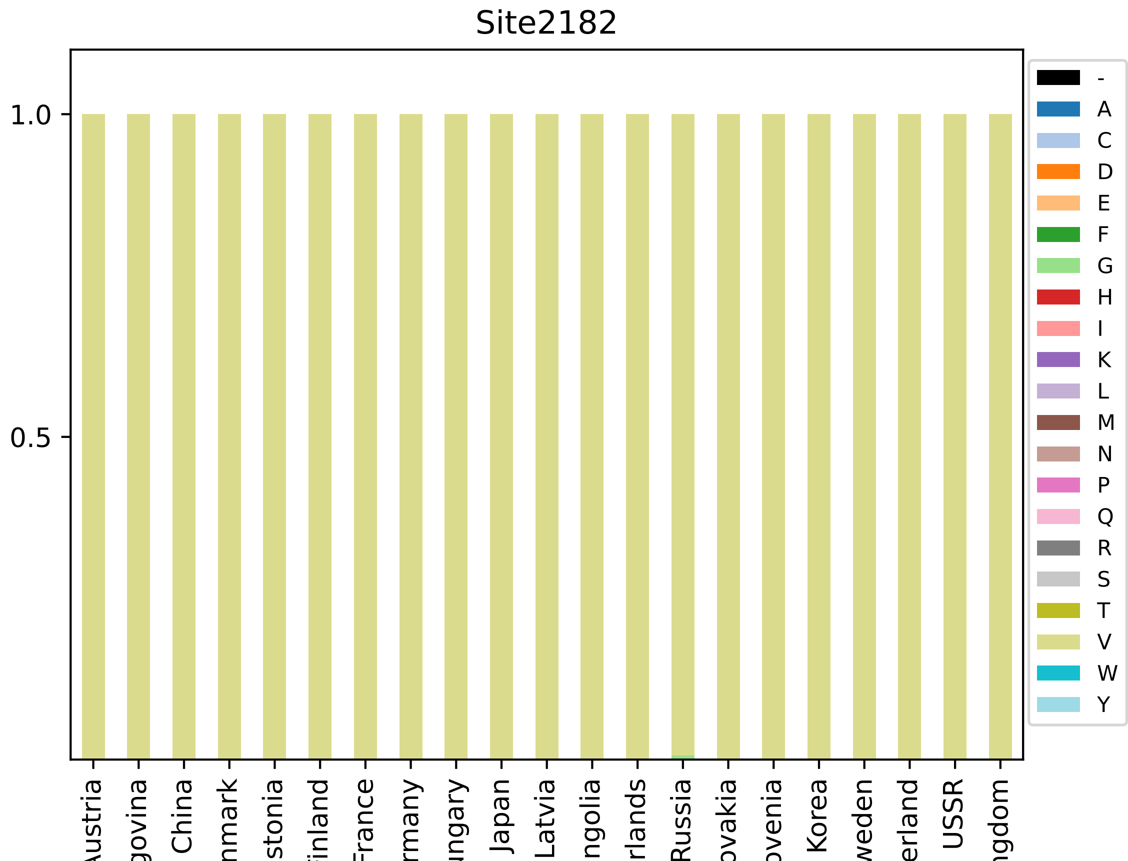
Site2182
1.0
0.5
-
A
C
D
E
F
G
H
I
K
L
M
N
P
Q
R
S
T
V
W
Y
Austria
govina
China
nmark
stonia
Finland
France
rmany
ungary
Japan
Latvia
ngolia
rlands
Russia
ovakia
ovenia
Korea
weden
erland
USSR
ngdom

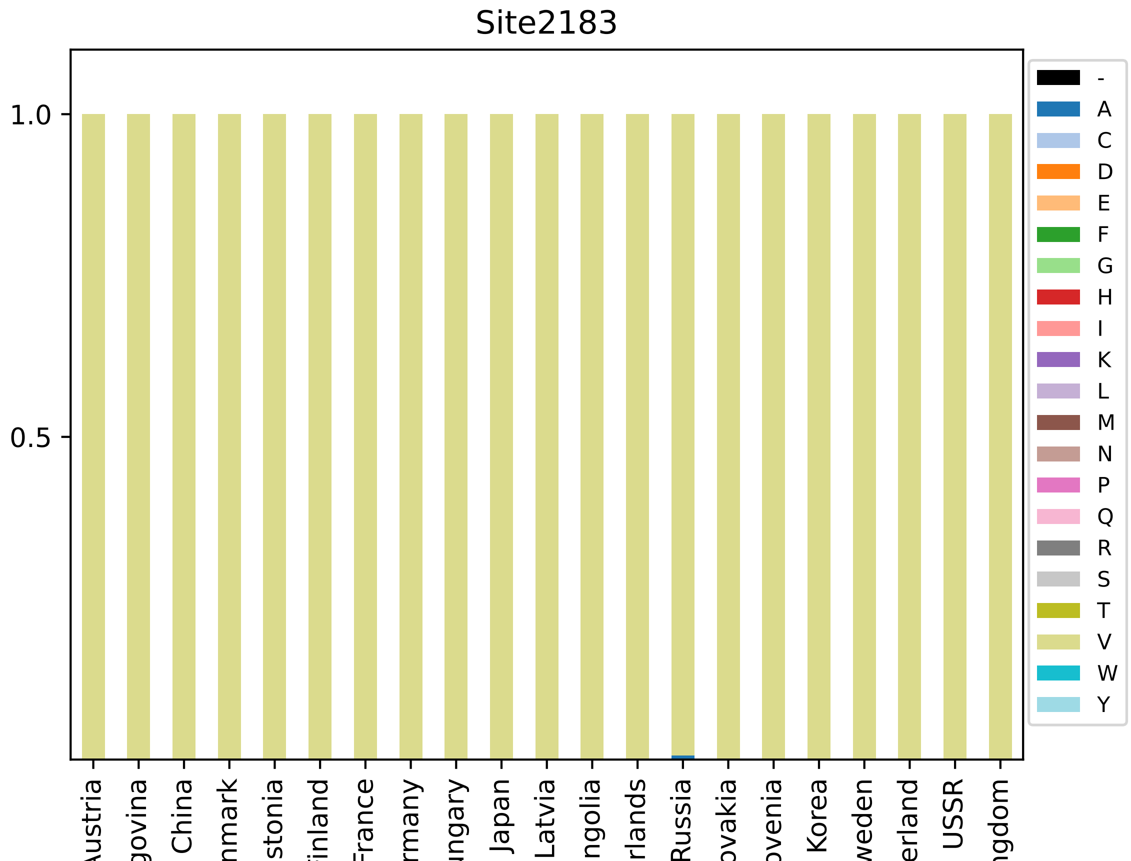
Site2183
1.0
0.5
-
A
C
D
E
F
G
H
I
K
L
M
N
P
Q
R
S
T
V
W
Y
Austria
govina
China
nmark
stonia
Finland
France
rmany
ungary
Japan
Latvia
ngolia
rlands
Russia
ovakia
ovenia
Korea
weden
erland
USSR
ngdom

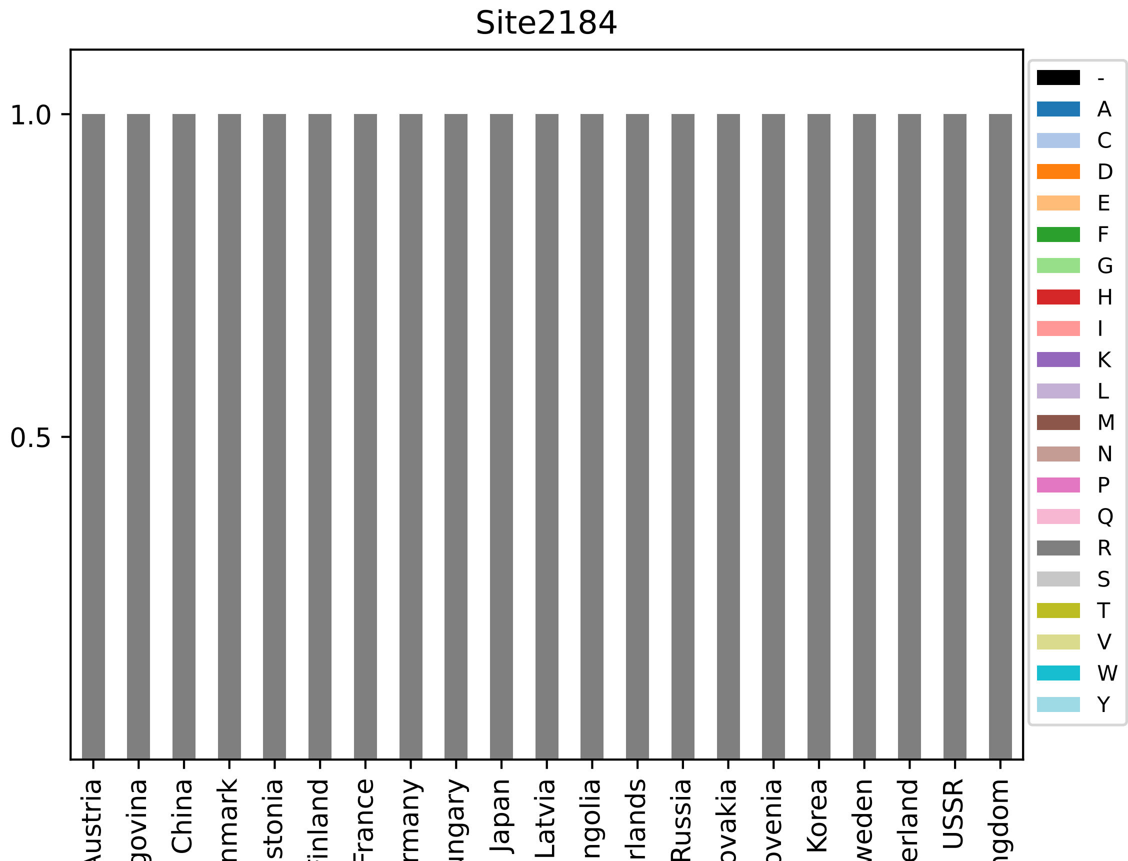
Site2184
1.0
0.5
Austria
govina
China
nmark
stonia
inland
rance
rmany
ungary
Japan
Latvia
ngolia
rlands
Russia
ovakia
ovenia
Korea
weden
erland
USSR
ngdom
-
A
C
D
E
F
G
H
I
K
L
M
N
P
Q
R
S
T
V
W
Y

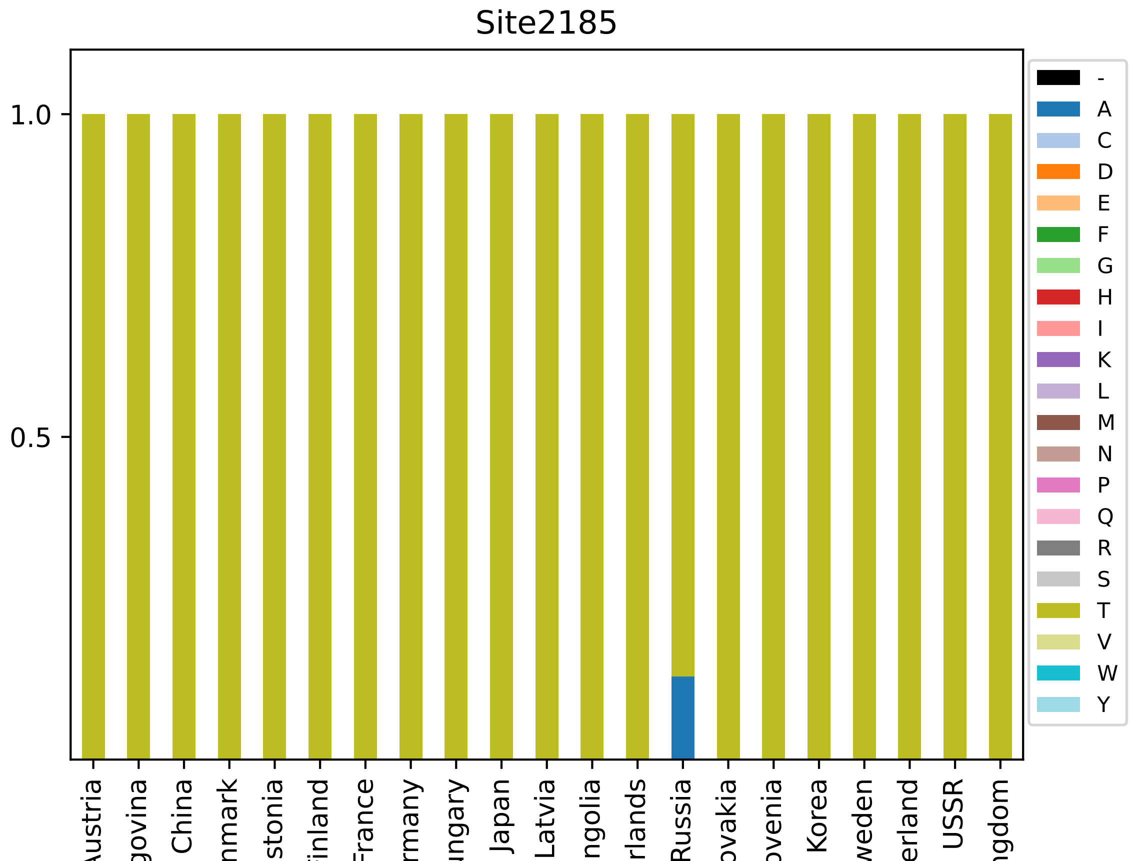

Site2185
1.0
0.5
-
A
C
D
E
F
G
H
I
K
L
M
N
P
Q
R
S
T
V
W
Y
Austria
govina
China
nmark
stonia
Finland
France
rmany
ungary
Japan
Latvia
ngolia
rlands
Russia
ovakia
ovenia
Korea
weden
erland
USSR
ngdom

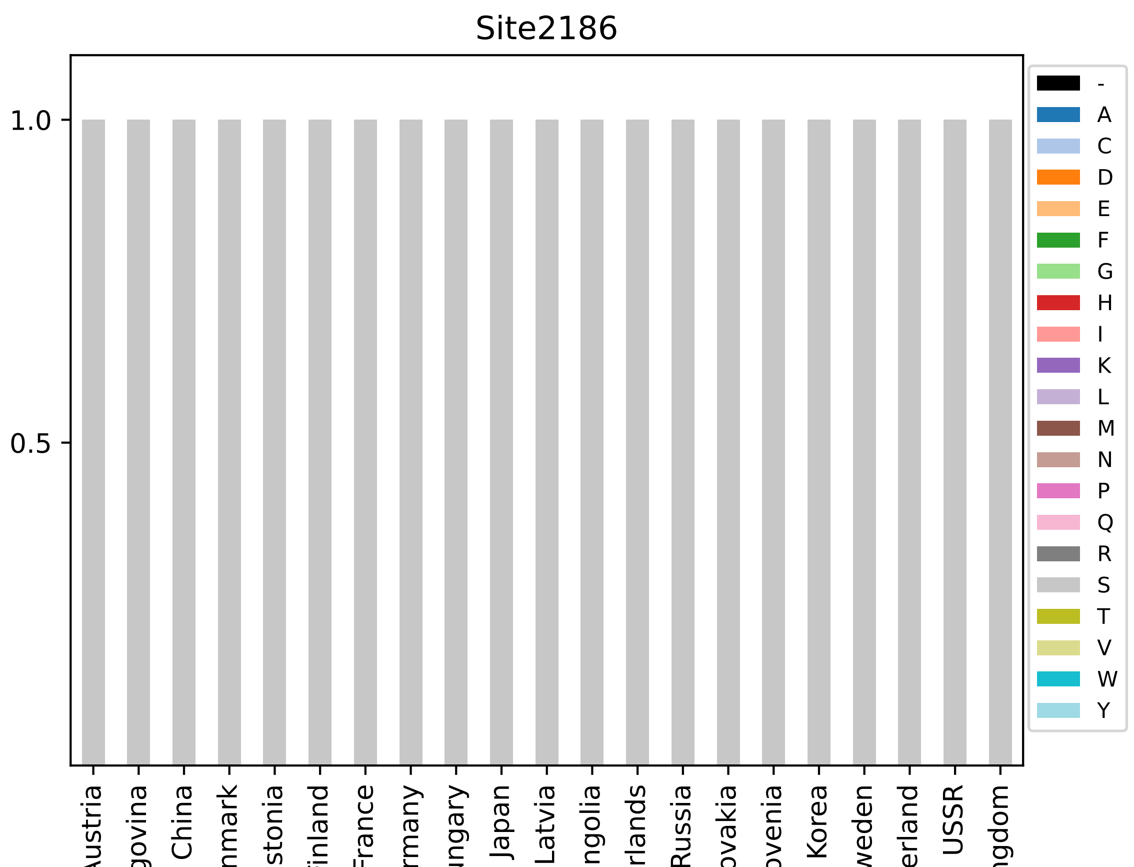
Site2186
1.0
0.5
-
A
C
D
E
F
G
H
I
K
L
M
N
P
Q
R
S
T
V
W
Y
China
Japan
Latvia
Russia
Korea
USSR

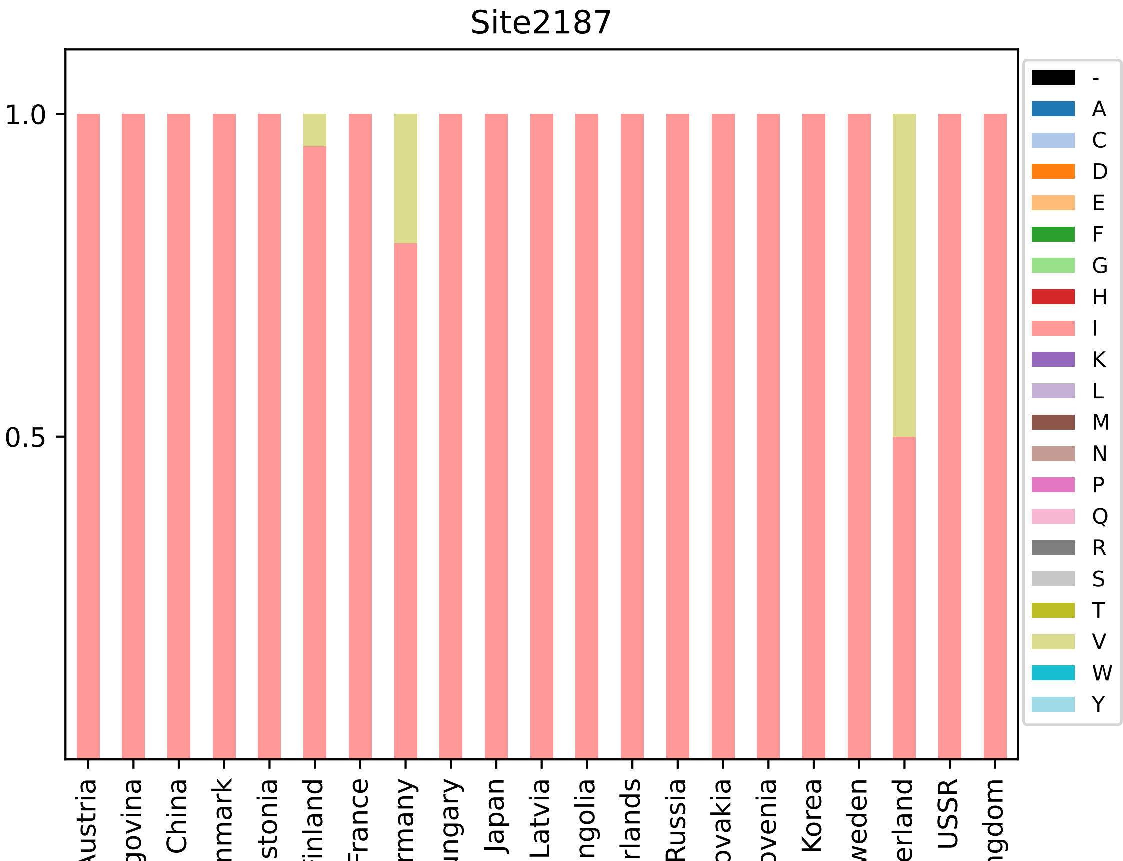

Site2187
1.0
0.5
-
A
C
D
E
F
G
H
I
K
L
M
N
P
Q
R
S
T
V
W
Y
China
Japan
Latvia
Russia
Korea
USSR

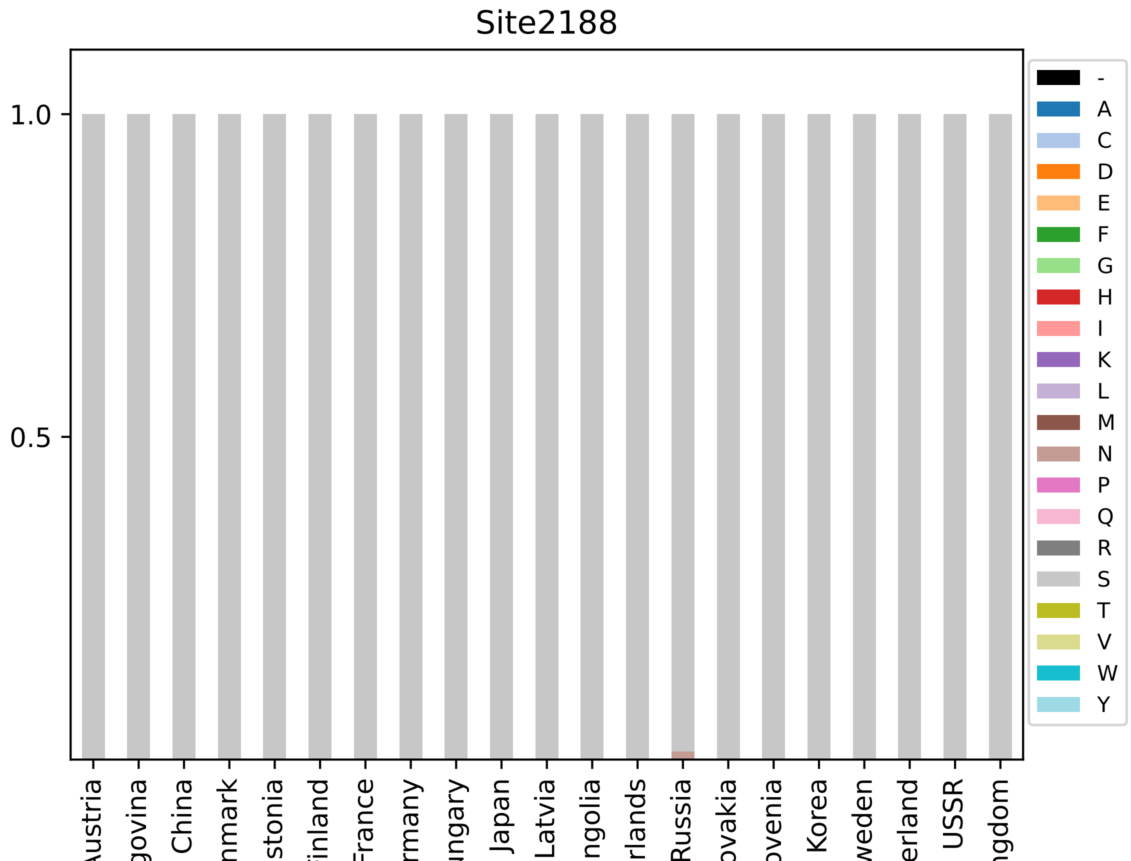

Site2188
1.0
0.5
-
A
C
D
E
F
G
H
I
K
L
M
N
P
Q
R
S
T
V
W
Y
Austria
govina
China
nmark
stonia
Finland
France
rmany
ungary
Japan
Latvia
ngolia
rlands
Russia
ovakia
ovenia
Korea
weden
erland
USSR
ngdom

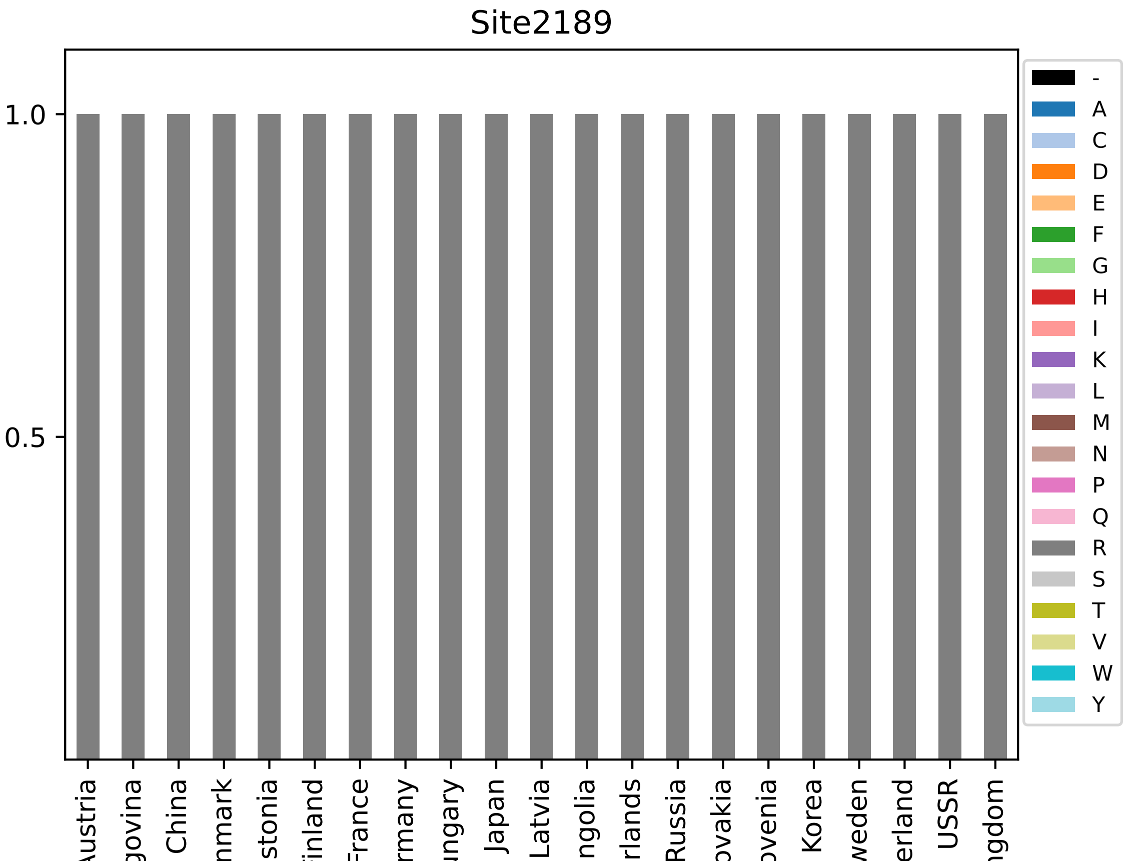
Site2189
1.0
0.5
-
A
C
D
E
F
G
H
I
K
L
M
N
P
Q
R
S
T
V
W
Y
China
Japan
Latvia
Russia
Korea
USSR

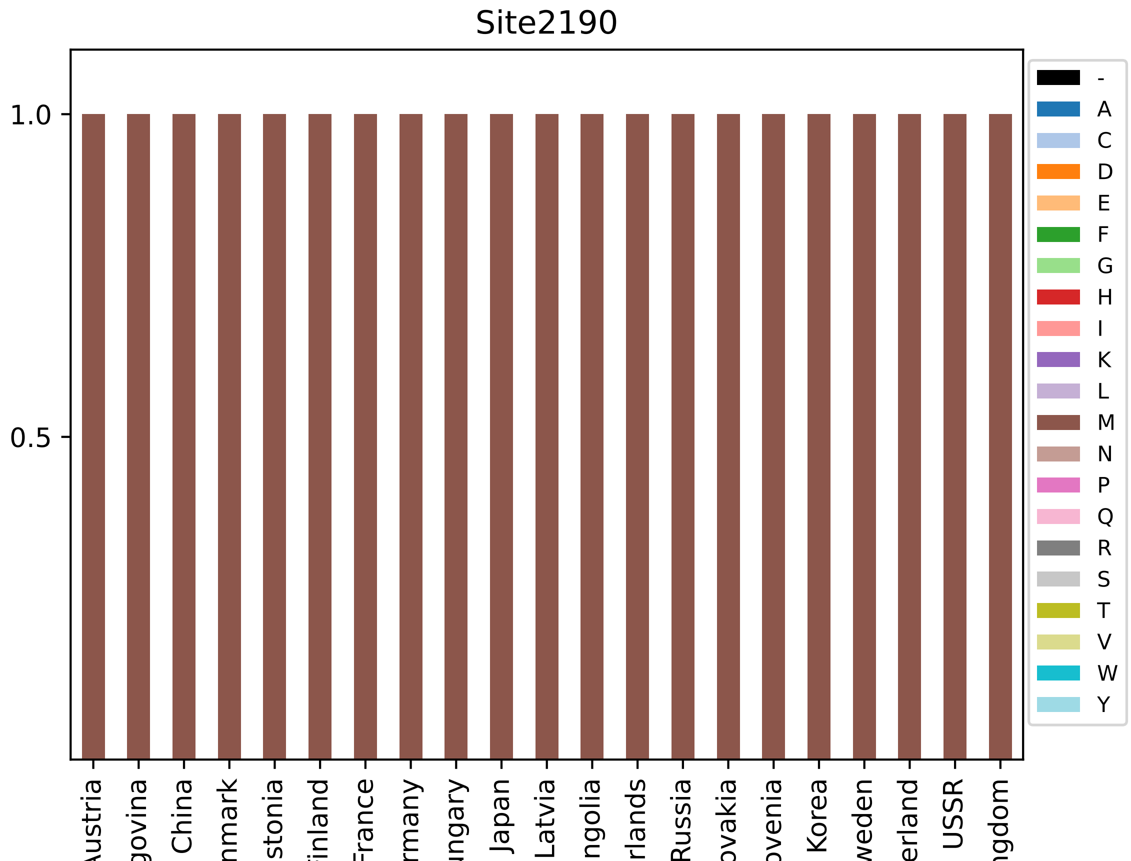

Site2190
1.0
0.5
-
A
C
D
E
F
G
H
I
K
L
M
N
P
Q
R
S
T
V
W
Y
Austria
China
Japan
Latvia
Russia
Korea
USSR

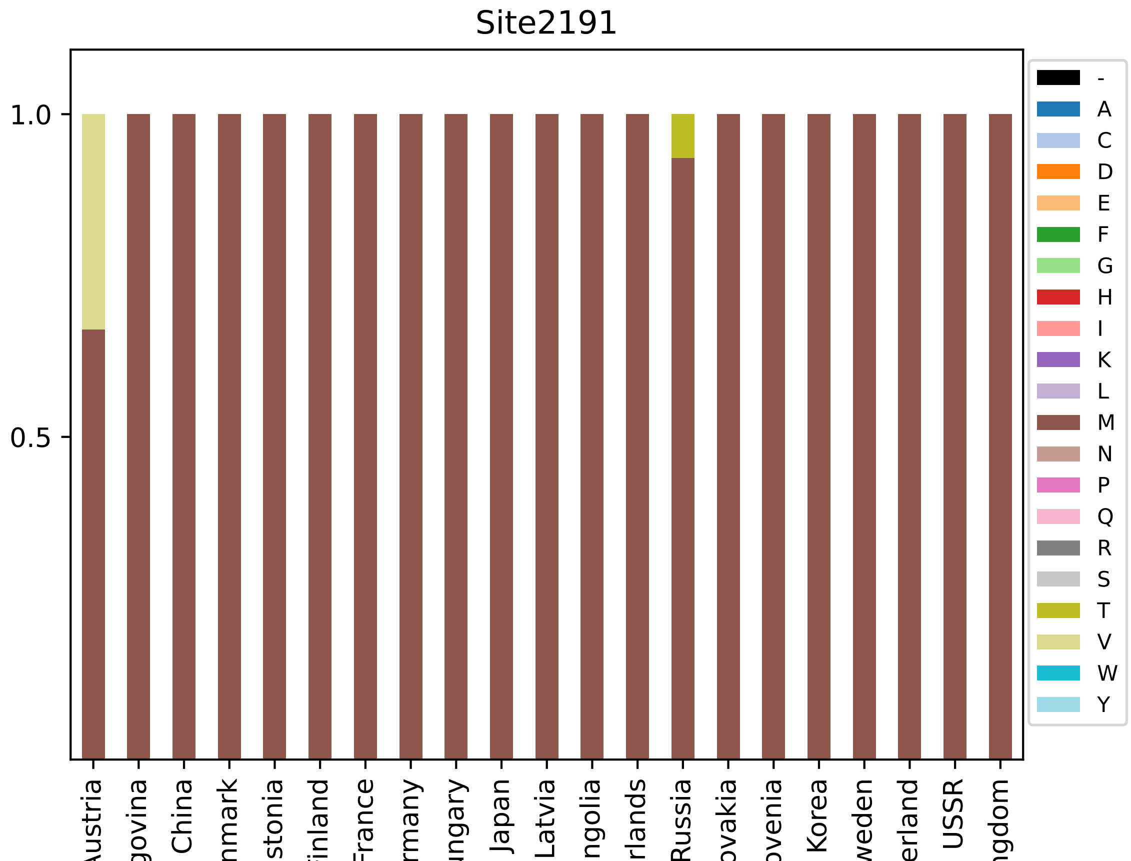

Site2191
1.0
0.5
-
A
C
D
E
F
G
H
I
K
L
M
N
P
Q
R
S
T
V
W
Y
Austria
govina
China
nmark
stonia
Finland
France
rmany
ungary
Japan
Latvia
ngolia
rlands
Russia
ovakia
ovenia
Korea
weden
erland
USSR
ngdom

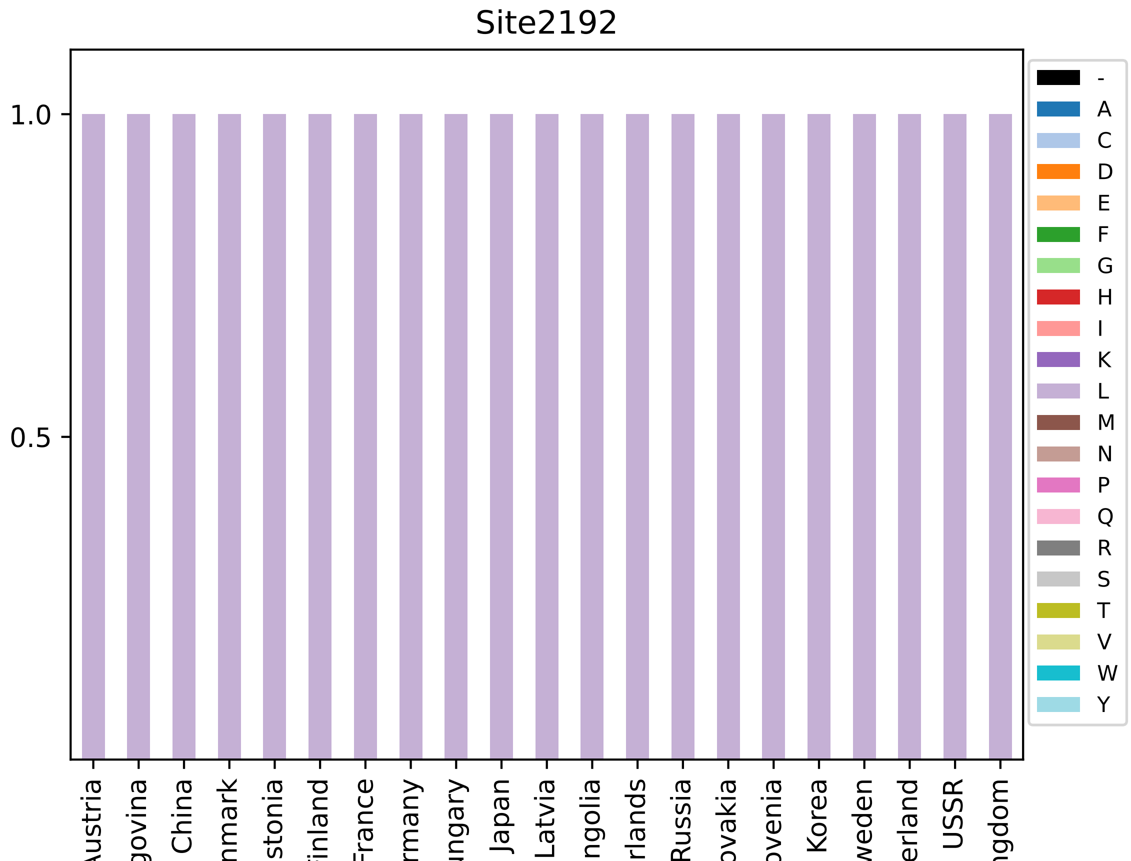

Site2192
1.0
0.5
China
Japan
Latvia
Russia
Korea
USSR
-
A
C
D
E
F
G
H
I
K
L
M
N
P
Q
R
S
T
V
W
Y

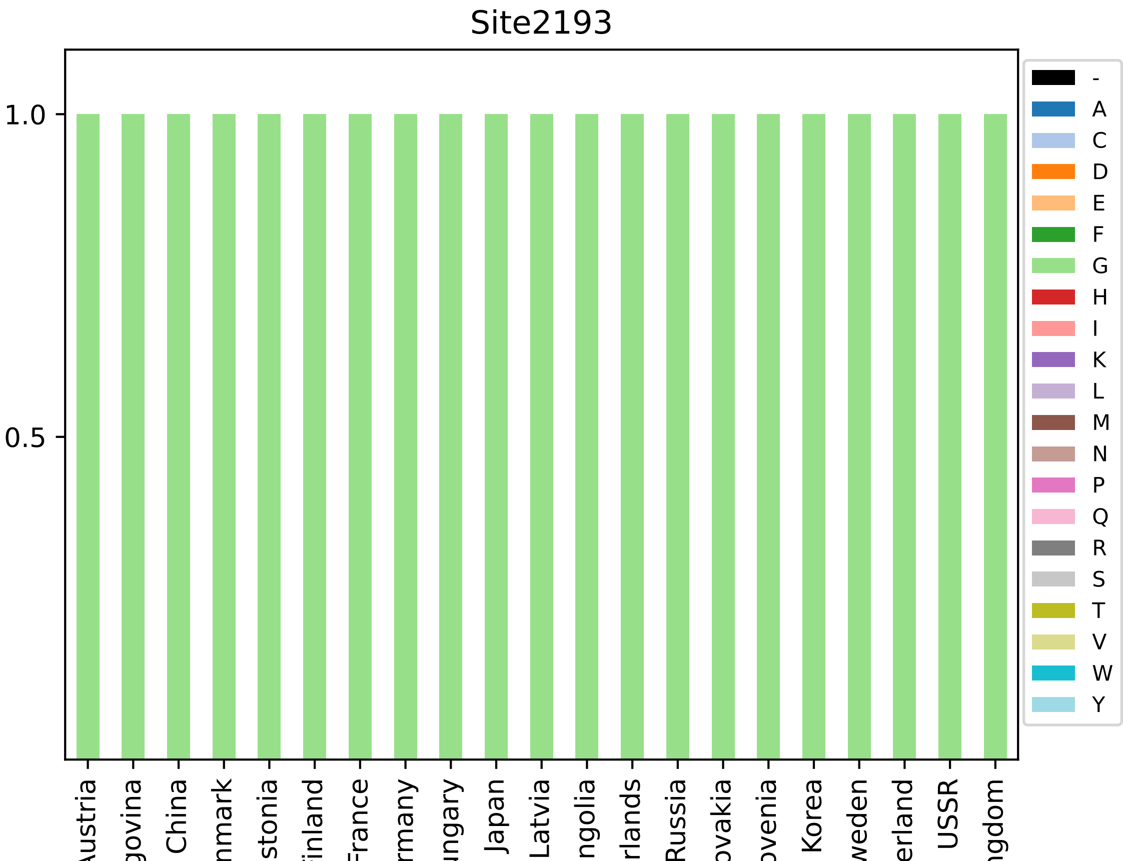

Site2193
1.0
0.5
-
A
C
D
E
F
G
H
I
K
L
M
N
P
Q
R
S
T
V
W
Y
China
Japan
Latvia
Russia
Korea
USSR

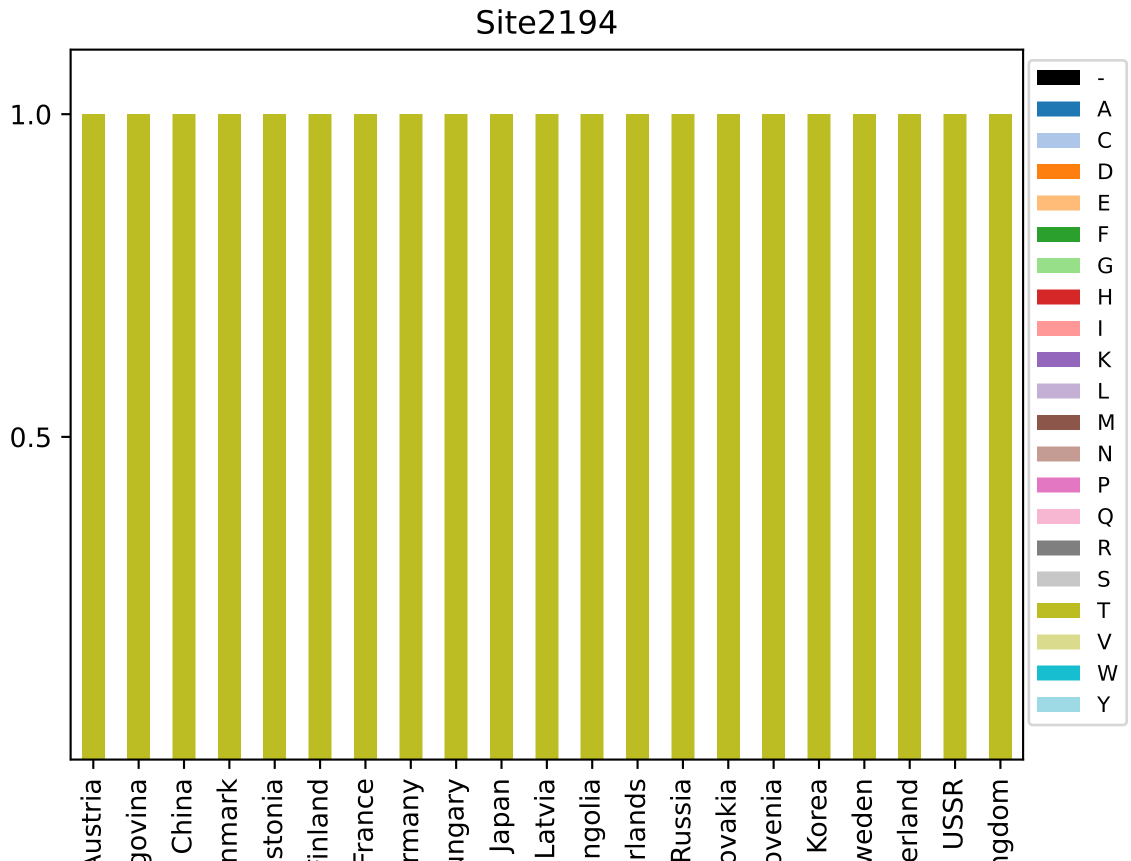
Site2194
1.0
0.5
Austria
China
Japan
Latvia
Russia
Korea
USSR
-
A
C
D
E
F
G
H
I
K
L
M
N
P
Q
R
S
T
V
W
Y

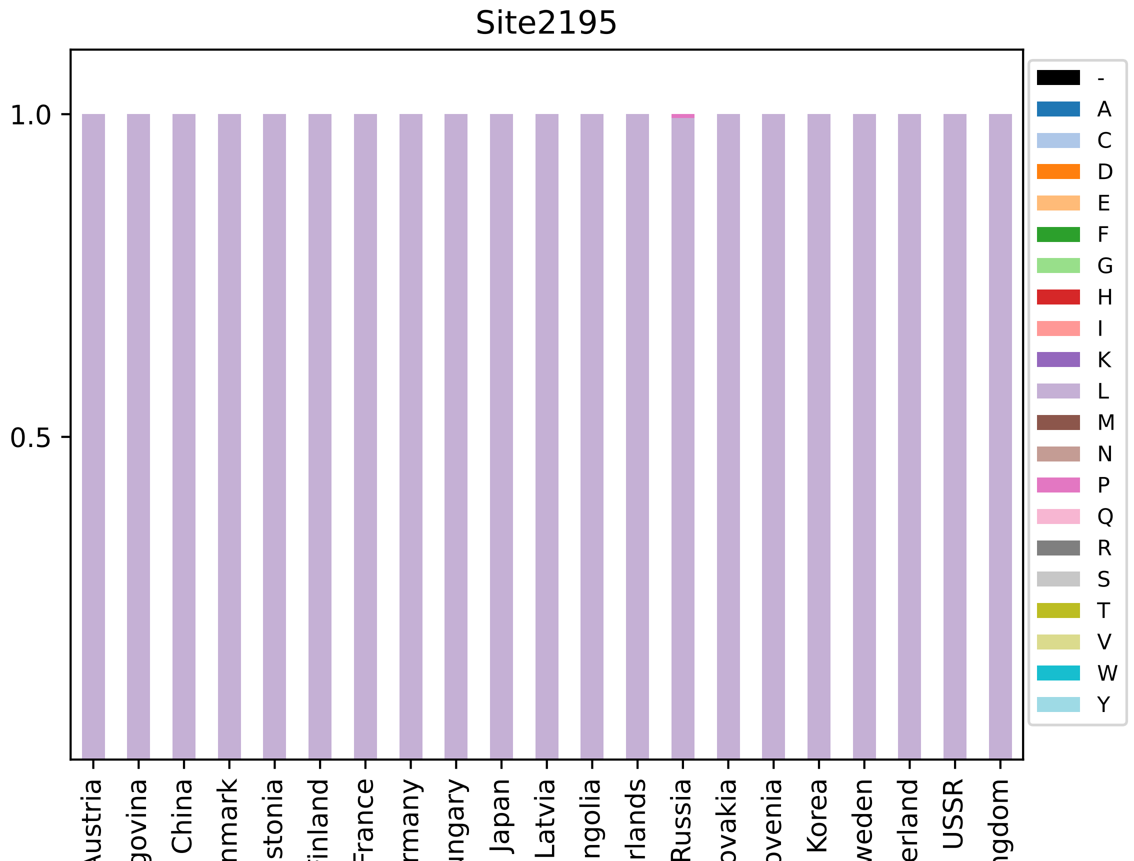
Site2195
1.0
0.5
-
A
C
D
E
F
G
H
I
K
L
M
N
P
Q
R
S
T
V
W
Y
China
Japan
Latvia
Russia
Korea
USSR

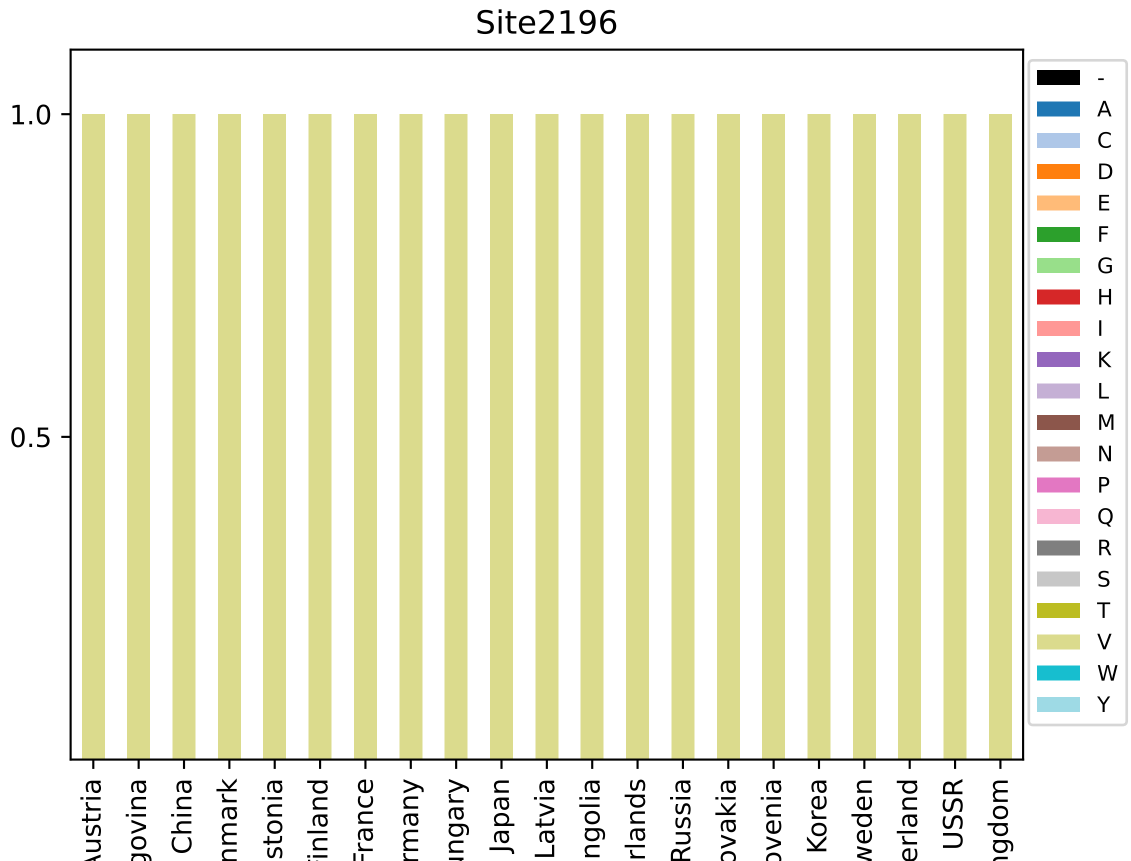

Site2196
1.0
0.5
-
A
C
D
E
F
G
H
I
K
L
M
N
P
Q
R
S
T
V
W
Y
Austria
China
Japan
Latvia
Russia
Korea
USSR

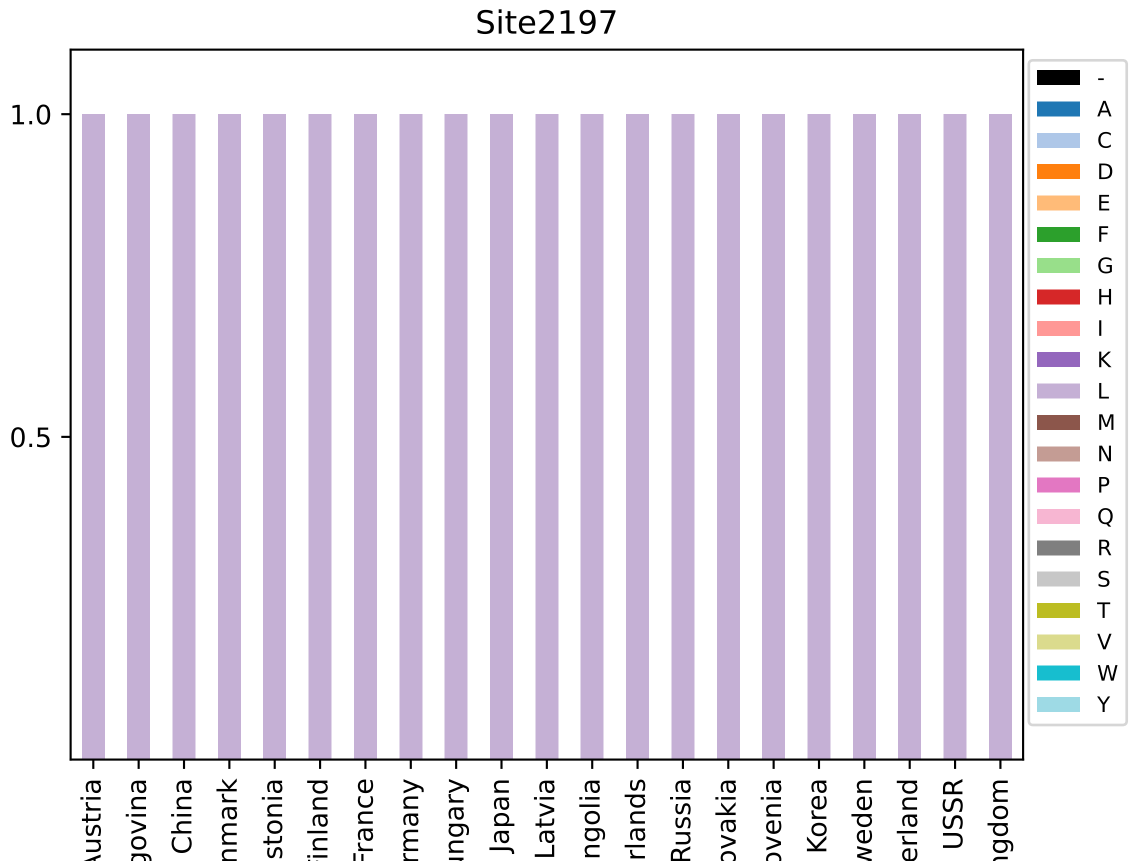

Site2197
1.0
0.5
-
A
C
D
E
F
G
H
I
K
L
M
N
P
Q
R
S
T
V
W
Y
China
Japan
Latvia
Russia
Korea
USSR

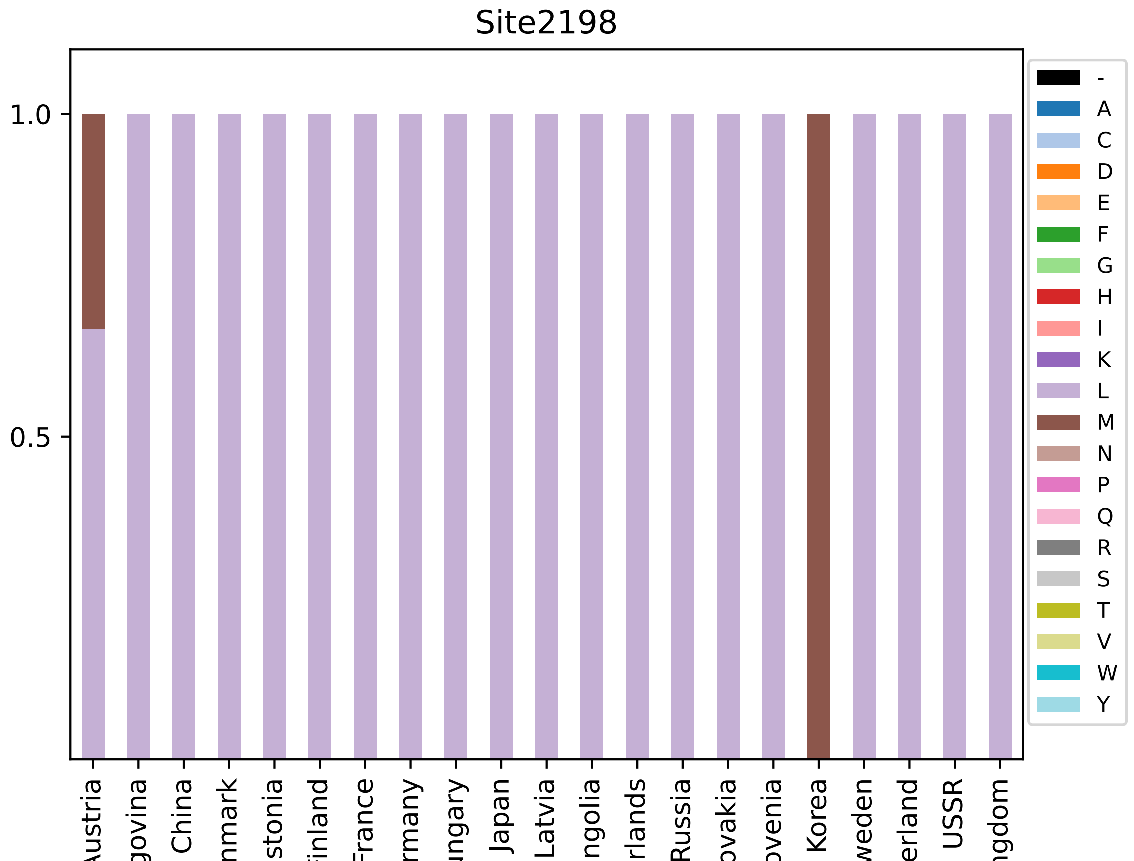
Site2198
1.0
0.5
China
Japan
Latvia
Russia
Korea
USSR
-
A
C
D
E
F
G
H
I
K
L
M
N
P
Q
R
S
T
V
W
Y

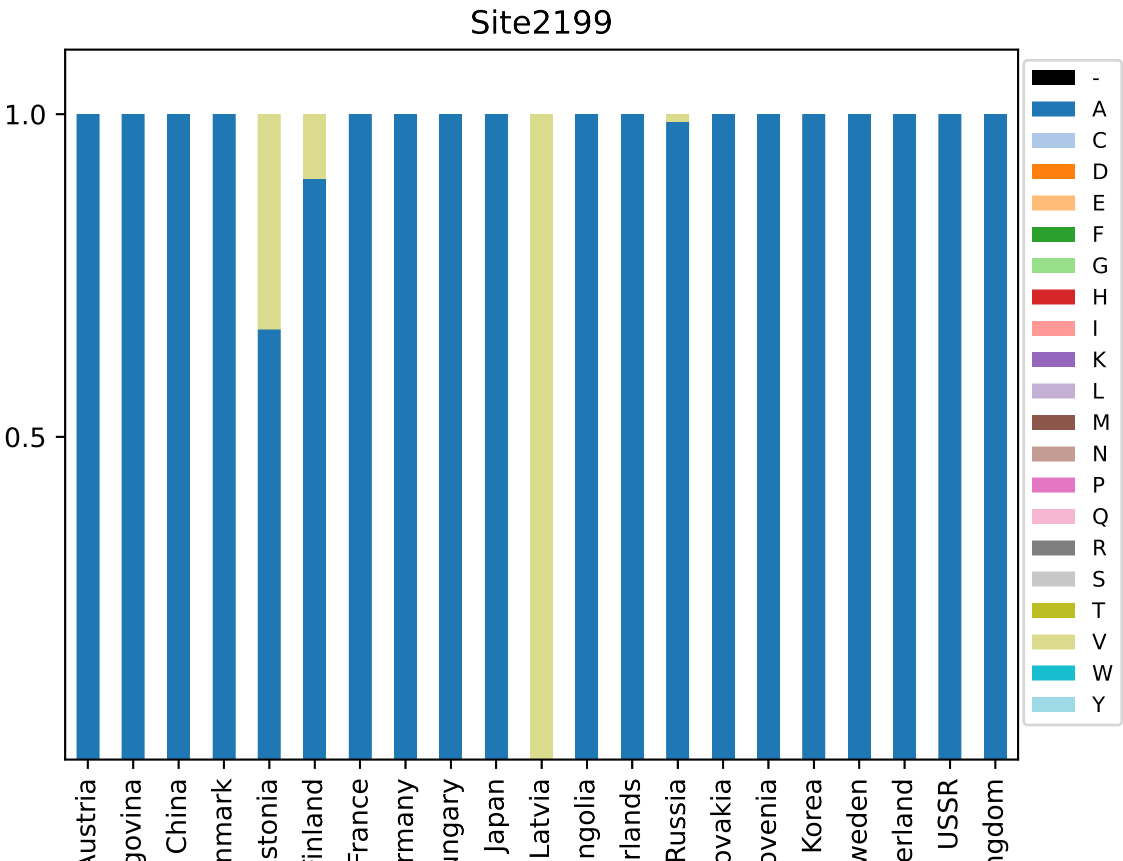

Site2199
1.0
0.5
-
A
C
D
E
F
G
H
I
K
L
M
N
P
Q
R
S
T
V
W
Y
Austria
govina
China
nmark
stonia
inland
France
rmany
ungary
Japan
Latvia
ngolia
rlands
Russia
ovakia
ovenia
Korea
weden
erland
USSR
ngdom

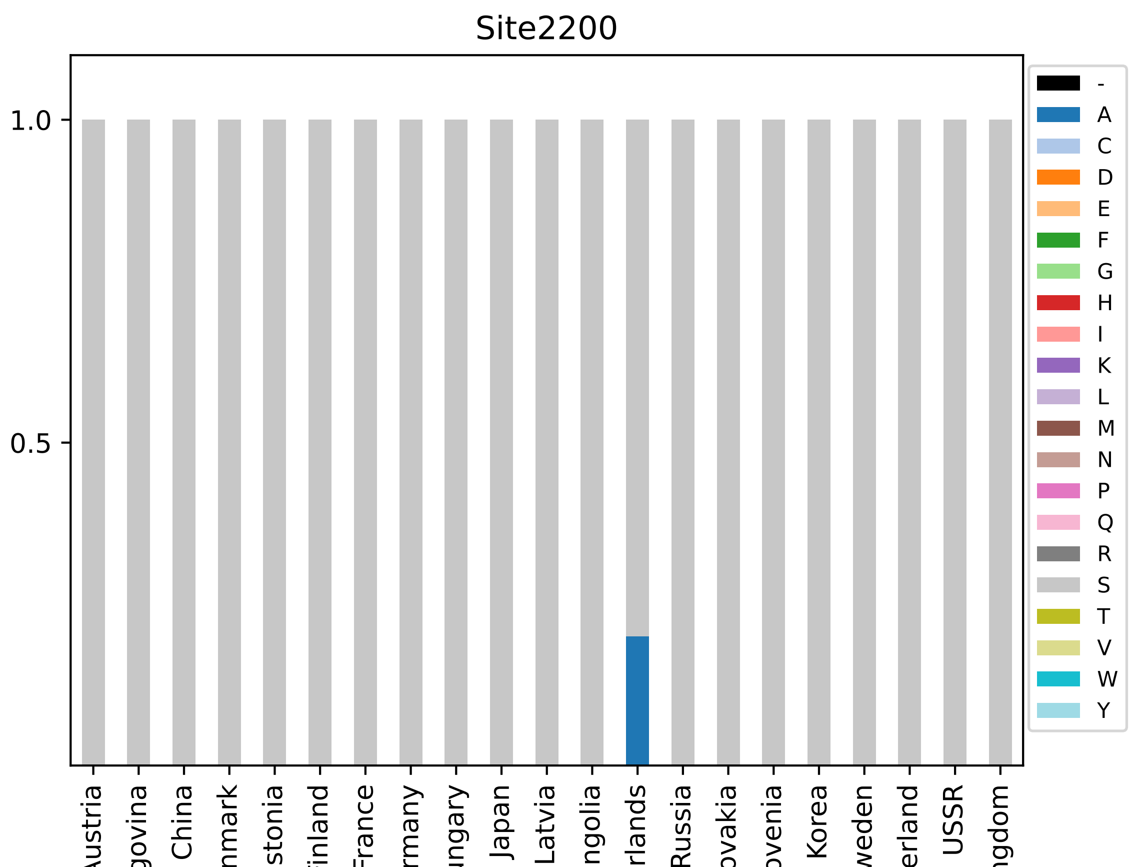

Site2200
1.0
0.5
-
A
C
D
E
F
G
H
I
K
L
M
N
P
Q
R
S
T
V
W
Y
Austria
govina
China
nmark
stonia
Finland
France
rmany
ungary
Japan
Latvia
ngolia
rlands
Russia
ovakia
ovenia
Korea
weden
erland
USSR
ngdom

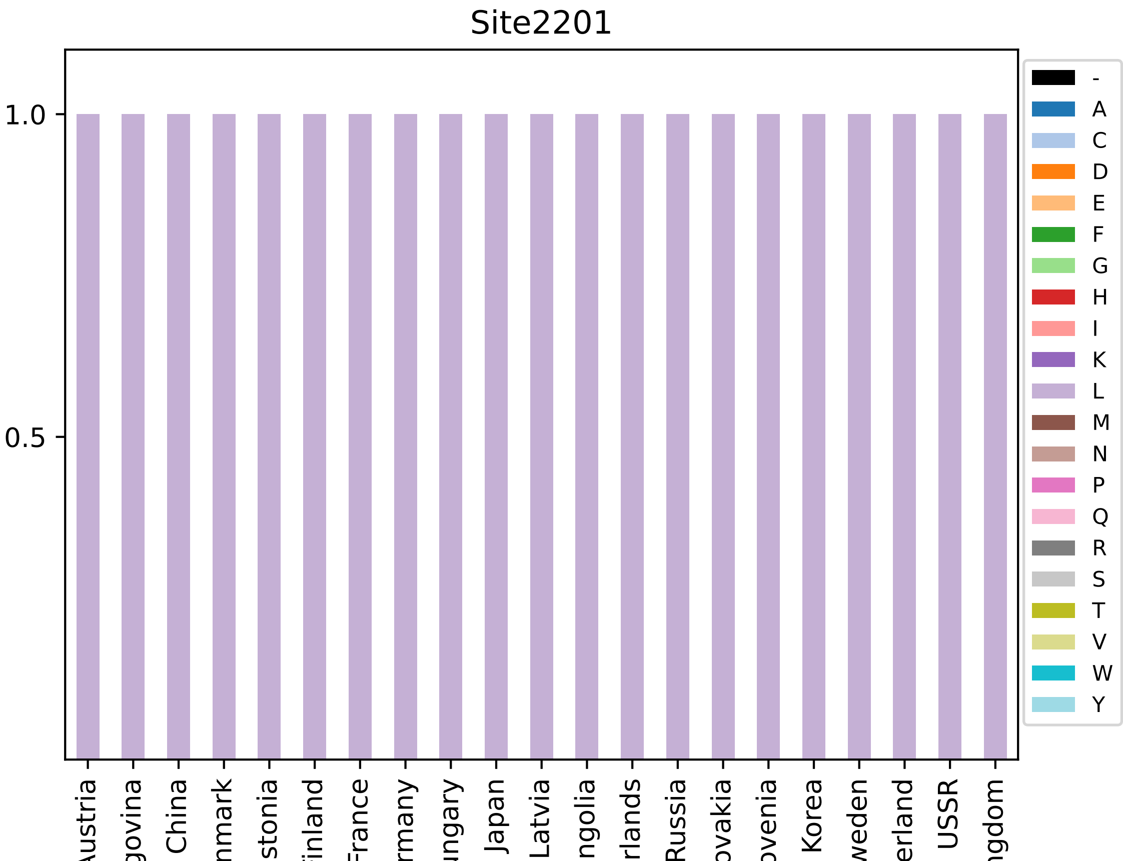

Site2201
1.0
0.5
-
A
C
D
E
F
G
H
I
K
L
M
N
P
Q
R
S
T
V
W
Y
Austria
govina
China
nmark
stonia
inland
France
rmany
ungary
Japan
Latvia
ngolia
rlands
Russia
ovakia
ovenia
Korea
weden
erland
USSR
ngdom

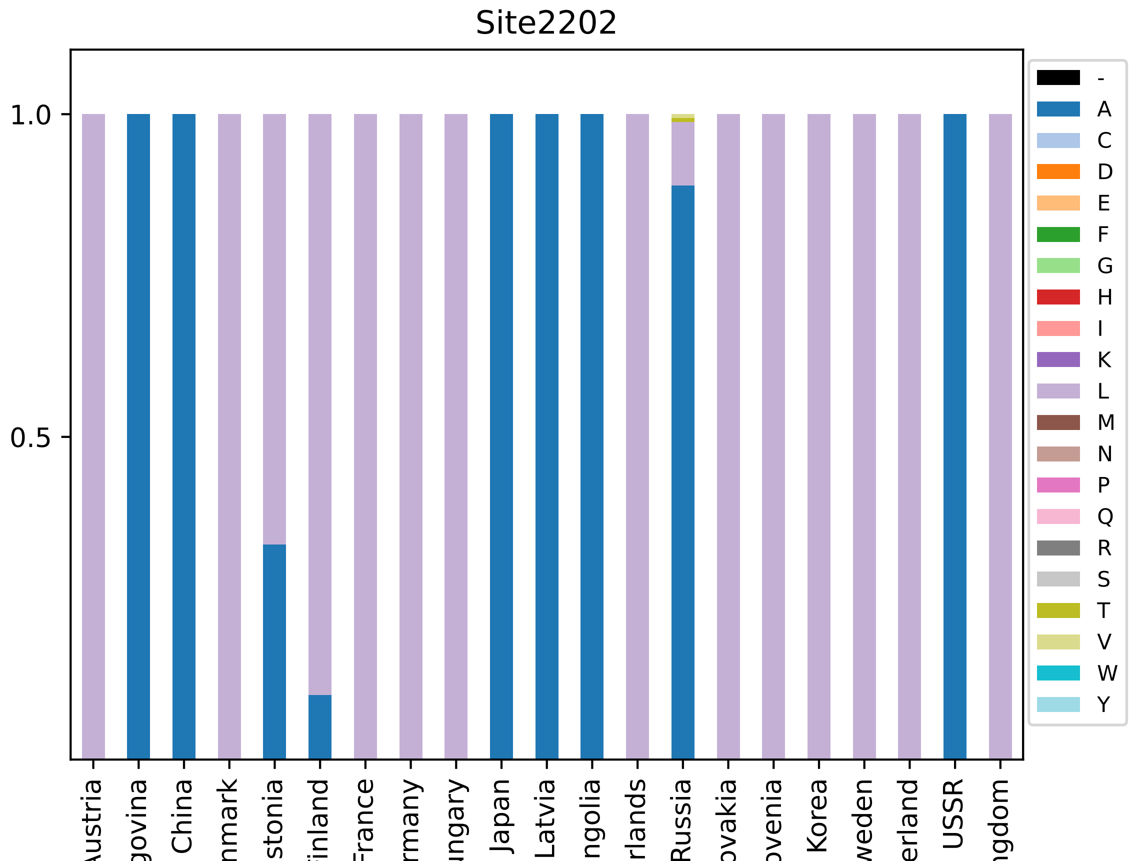
Site2202
1.0
0.5
-
A
C
D
E
F
G
H
I
K
L
M
N
P
Q
R
S
T
V
W
Y
Austria
govina
China
nmark
stonia
inland
France
rmany
ungary
Japan
Latvia
ngolia
rlands
Russia
ovakia
ovenia
Korea
weden
erland
USSR
ngdom

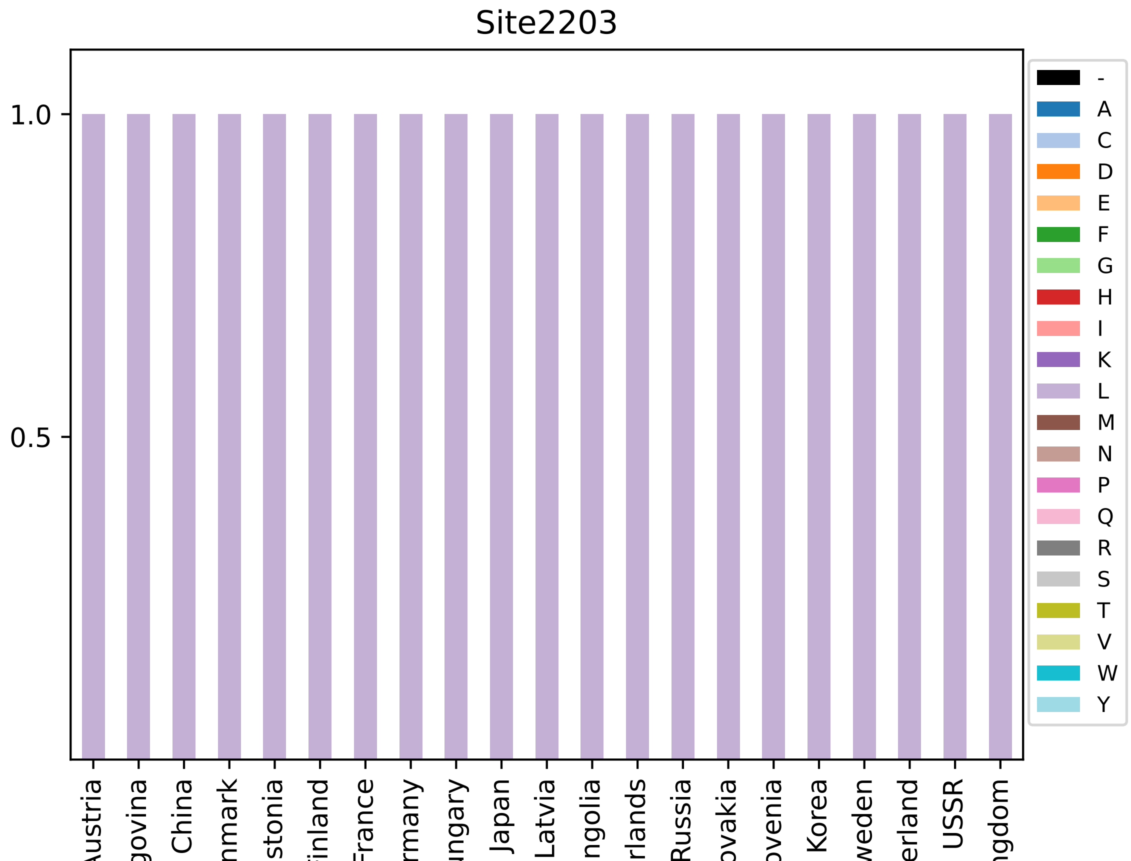

Site2203
1.0
0.5
-
A
C
D
E
F
G
H
I
K
L
M
N
P
Q
R
S
T
V
W
Y
China
Japan
Latvia
Russia
Korea
USSR

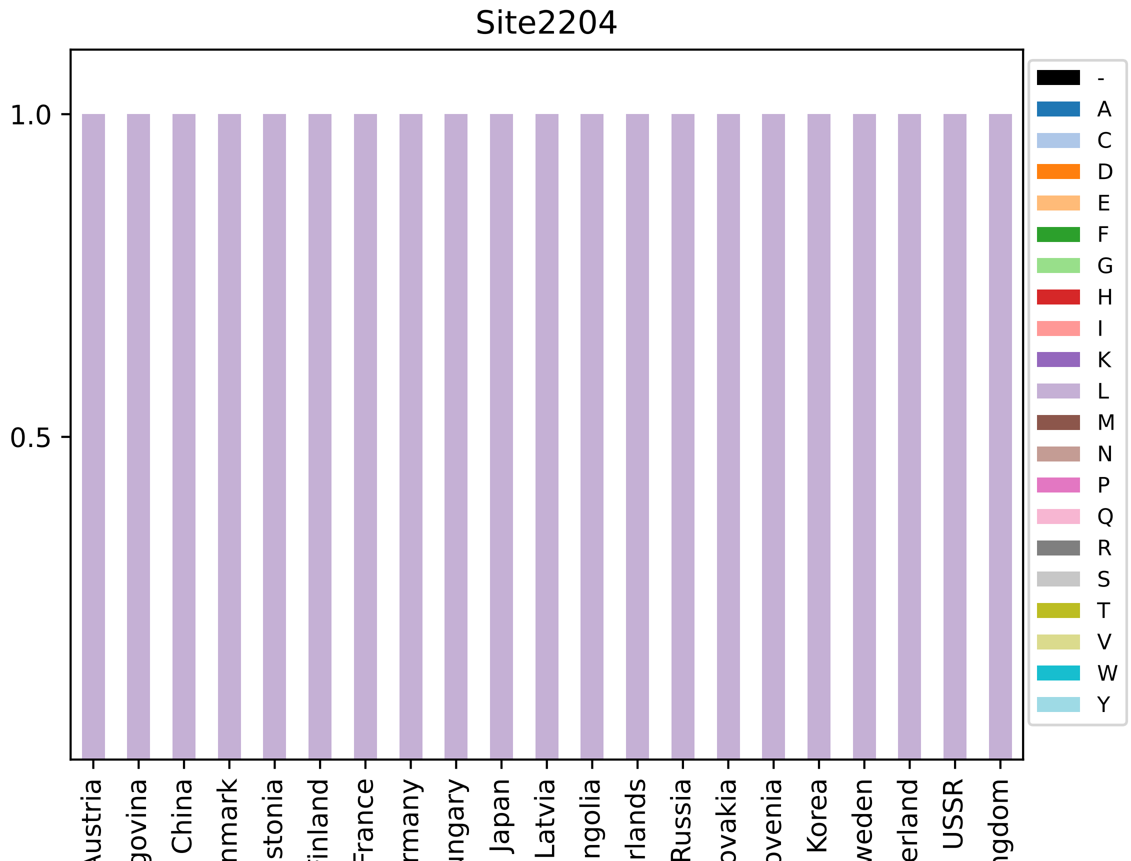

Site2204
1.0
0.5
-
A
C
D
E
F
G
H
I
K
L
M
N
P
Q
R
S
T
V
W
Y
China
Japan
Latvia
Russia
Korea
USSR

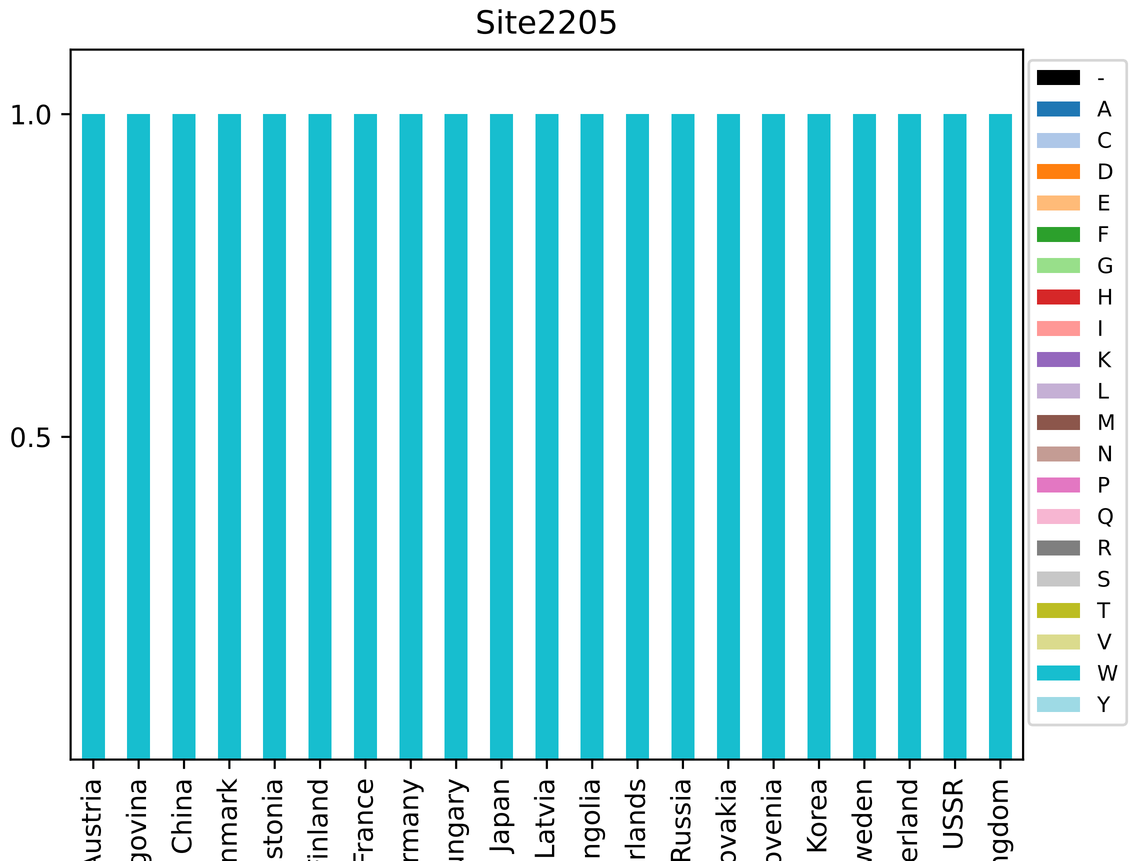

Site2205
1.0
0.5
-
A
C
D
E
F
G
H
I
K
L
M
N
P
Q
R
S
T
V
W
Y
China
Japan
Latvia
Russia
Korea
USSR

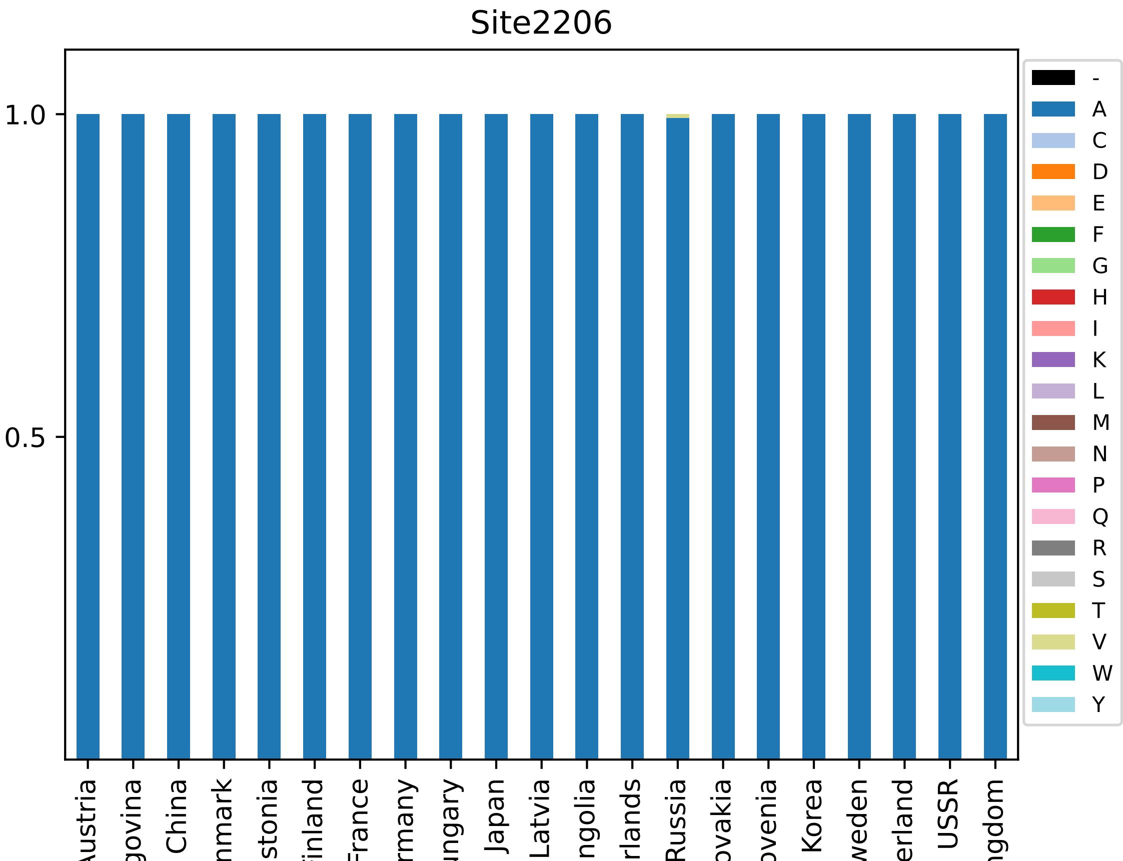

Site2206
1.0
0.5
-
A
C
D
E
F
G
H
I
K
L
M
N
P
Q
R
S
T
V
W
Y
China
Japan
Latvia
Russia
Korea
USSR

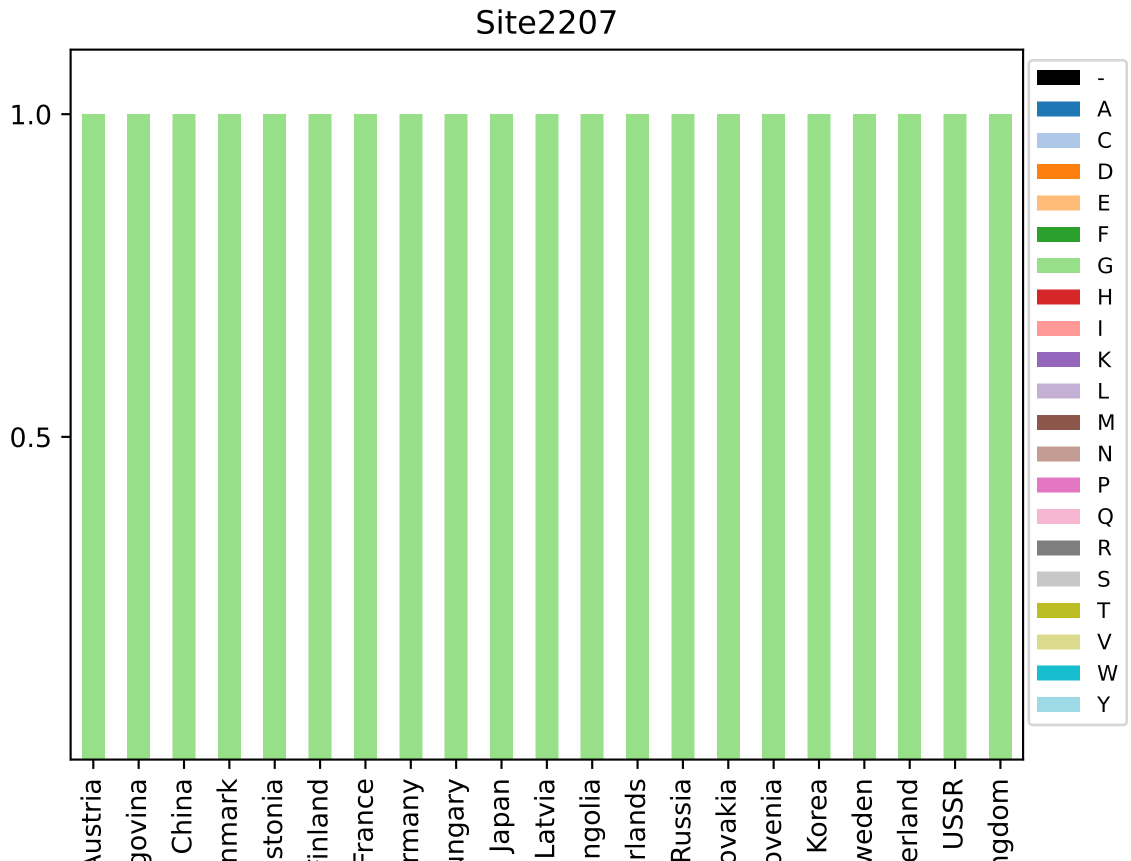
Site2207
1.0
0.5
China
Japan
Latvia
Russia
Korea
USSR
-
A
C
D
E
F
G
H
I
K
L
M
N
P
Q
R
S
T
V
W
Y

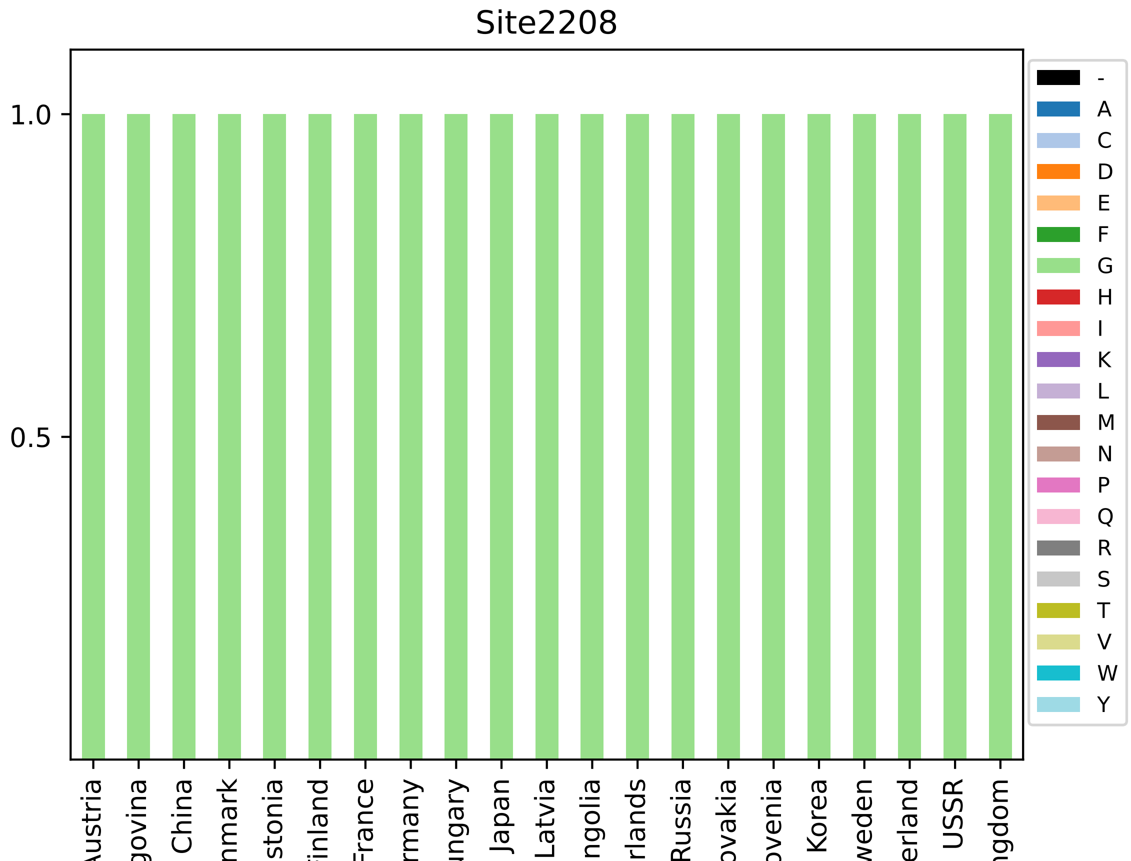
Site2208
1.0
0.5
-
A
C
D
E
F
G
H
I
K
L
M
N
P
Q
R
S
T
V
W
Y
Austria
govina
China
nmark
stonia
inland
France
rmany
ungary
Japan
Latvia
ngolia
rlands
Russia
ovakia
ovenia
Korea
weden
erland
USSR
ngdom

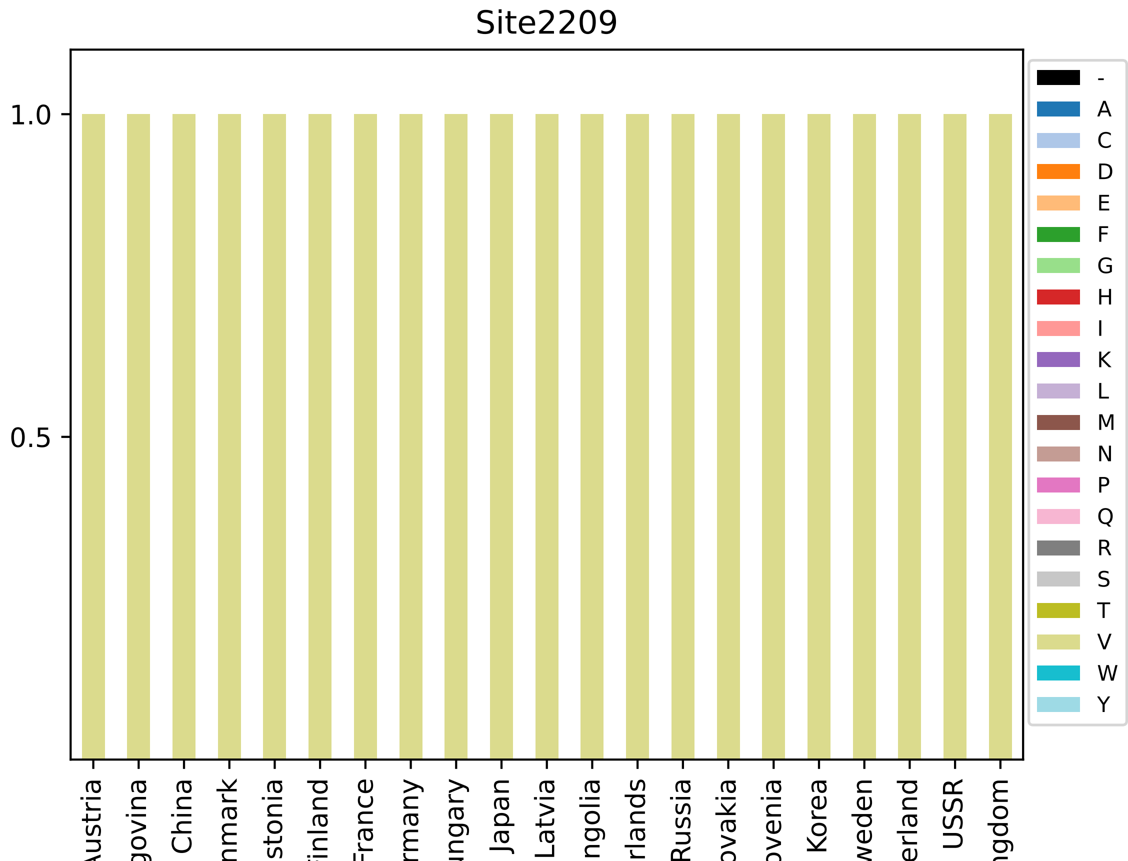

Site2209
1.0
0.5
-
A
C
D
E
F
G
H
I
K
L
M
N
P
Q
R
S
T
V
W
Y
Austria
China
Japan
Latvia
Russia
Korea
USSR

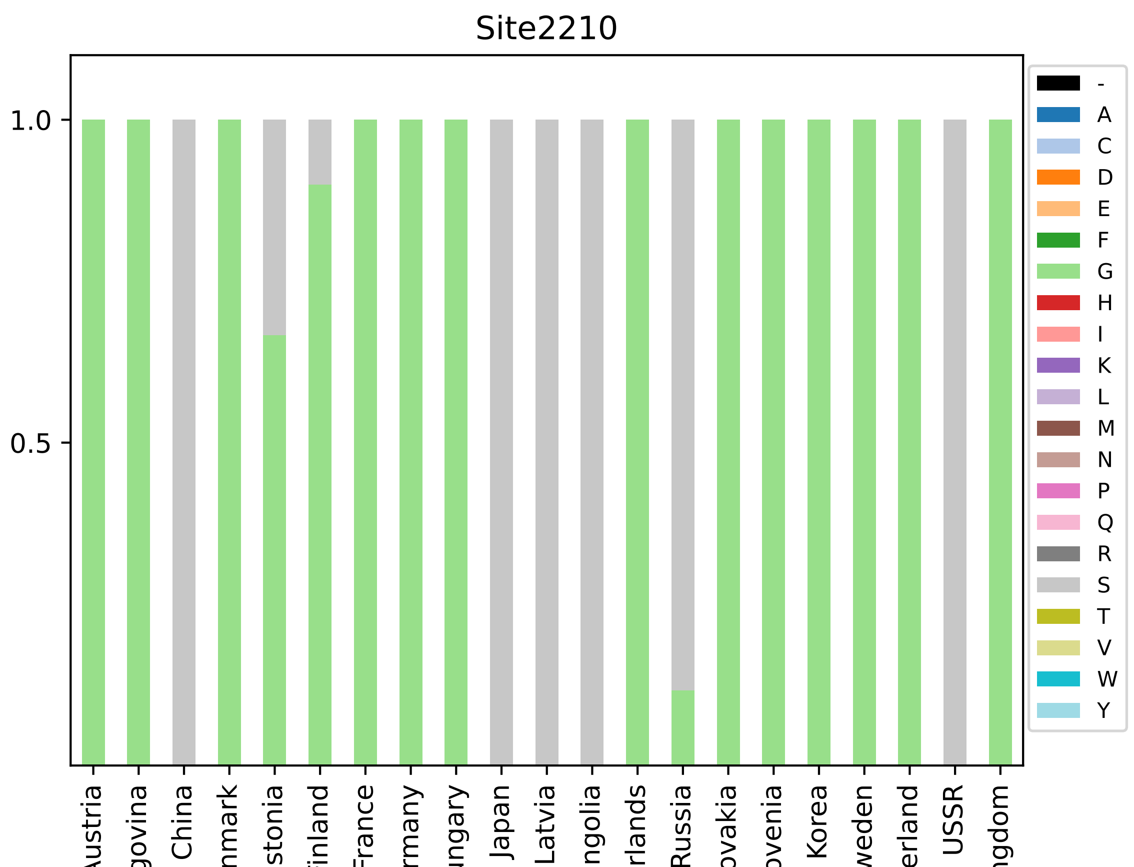

Site2210
1.0
0.5
Austria
govina
China
nmark
stonia
Finland
France
rmany
ungary
Japan
Latvia
ngolia
rlands
Russia
ovakia
ovenia
Korea
weden
erland
USSR
ngdom
-
A
C
D
E
F
G
H
I
K
L
M
N
P
Q
R
S
T
V
W
Y

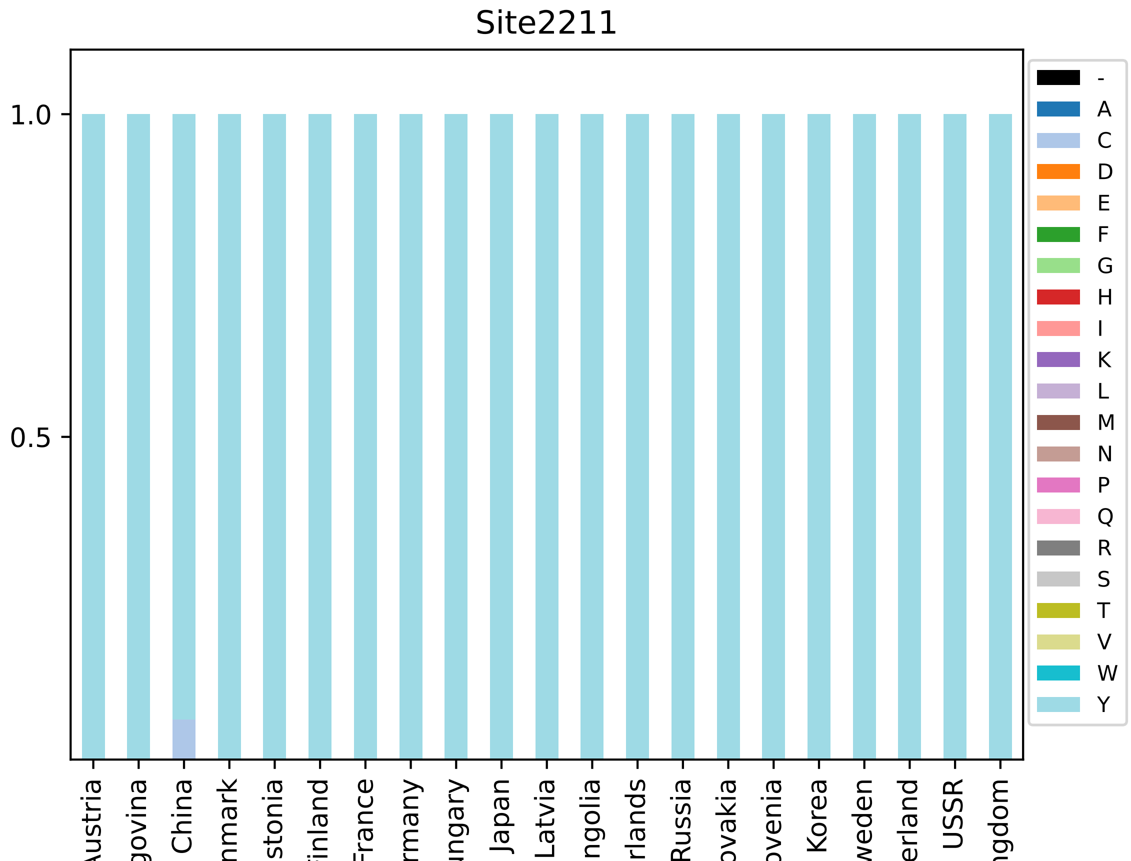

Site2211
1.0
0.5
-
A
C
D
E
F
G
H
I
K
L
M
N
P
Q
R
S
T
V
W
Y
Austria
govina
China
nmark
stonia
Finland
France
rmany
ungary
Japan
Latvia
ngolia
rlands
Russia
ovakia
ovenia
Korea
weden
erland
USSR
ngdom

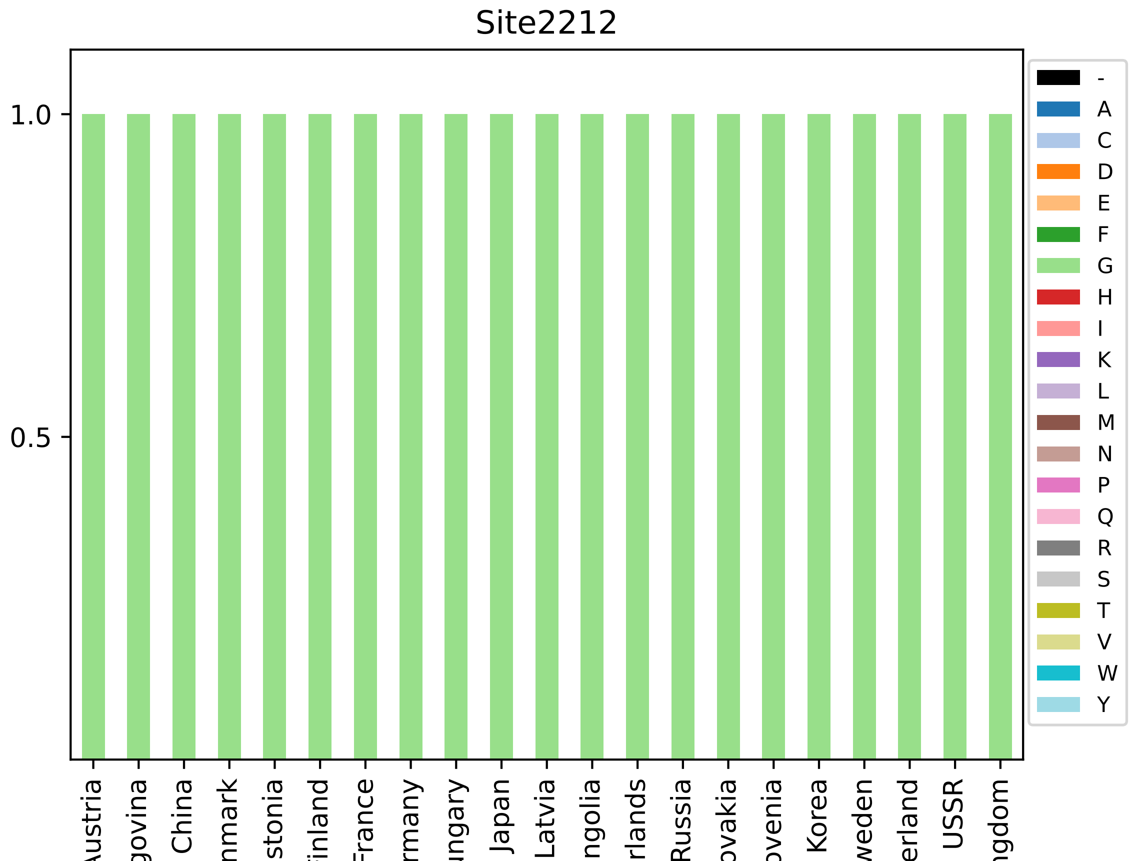

Site2212
1.0
0.5
-
A
C
D
E
F
G
H
I
K
L
M
N
P
Q
R
S
T
V
W
Y
China
Japan
Latvia
Russia
Korea
USSR

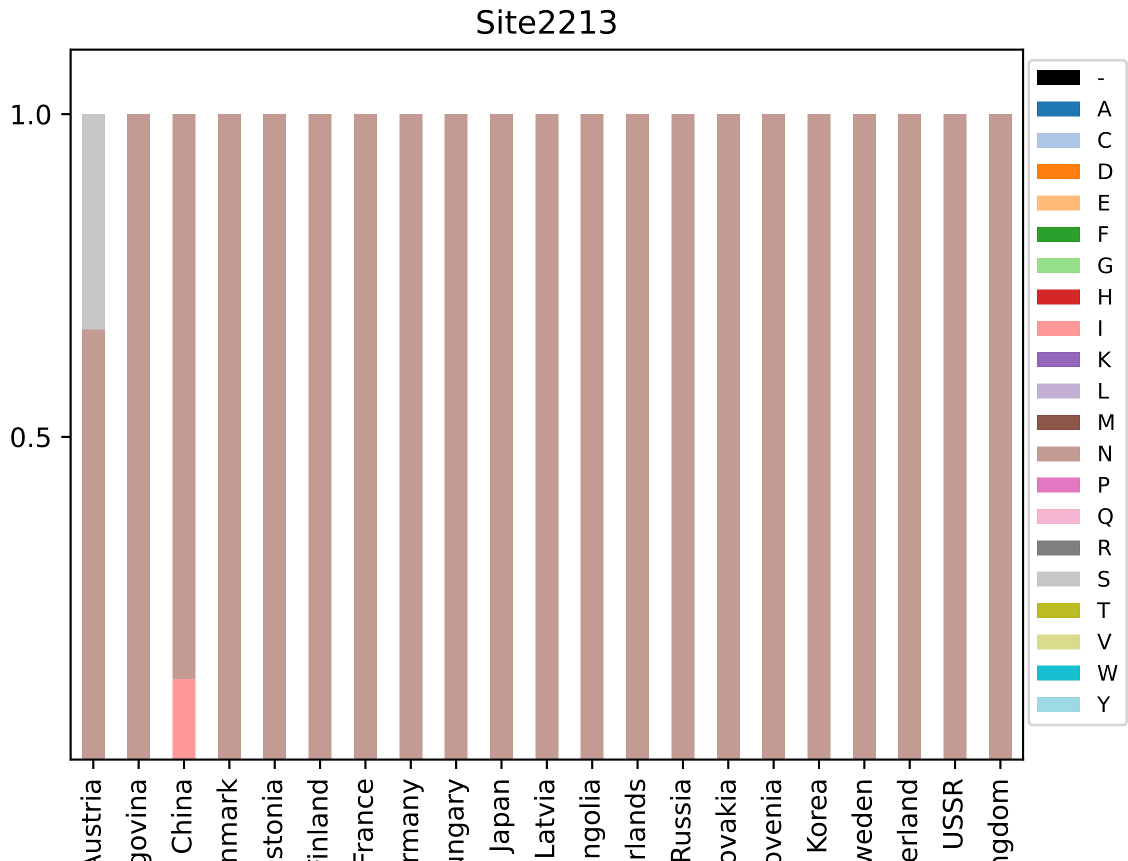
Site2213
1.0
0.5
-
A
C
D
E
F
G
H
I
K
L
M
N
P
Q
R
S
T
V
W
Y
Austria
govina
China
nmark
stonia
Finland
France
rmany
ungary
Japan
Latvia
ngolia
rlands
Russia
ovakia
ovenia
Korea
weden
erland
USSR
ngdom

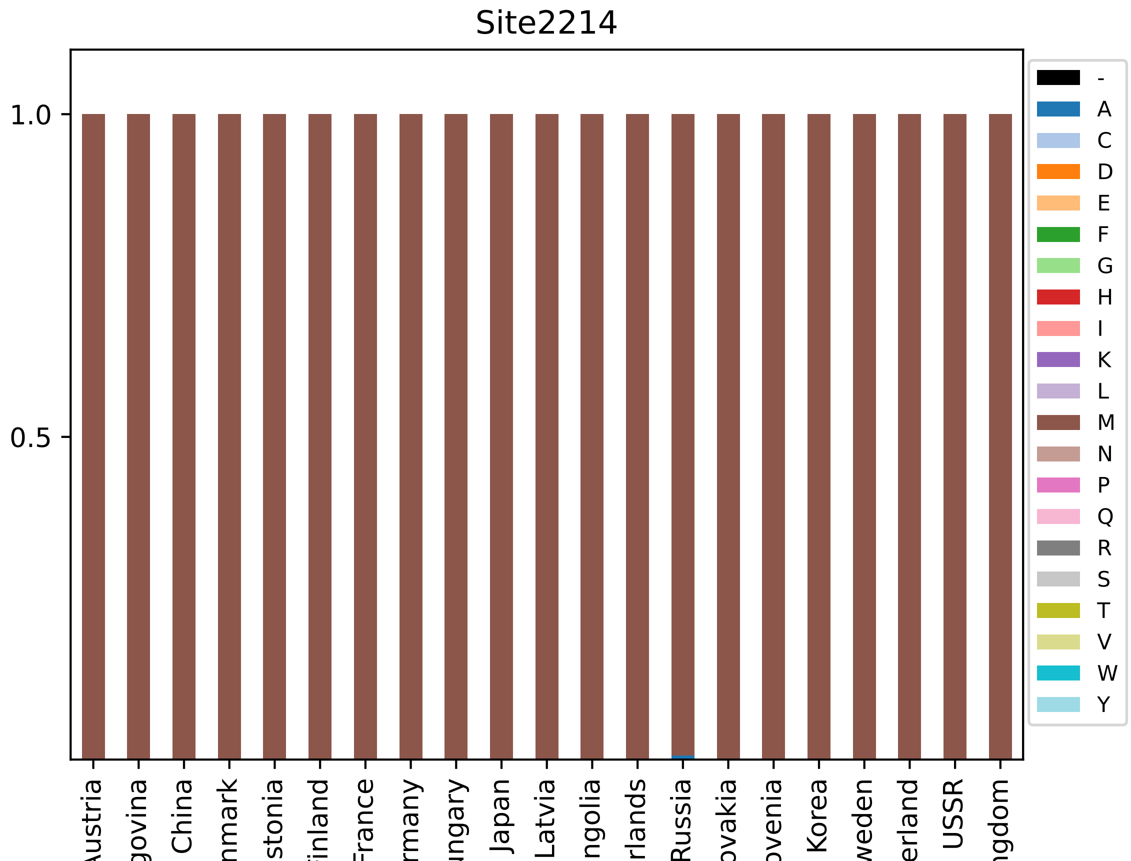
Site2214
1.0
0.5
-
A
C
D
E
F
G
H
I
K
L
M
N
P
Q
R
S
T
V
W
Y
China
Japan
Latvia
Russia
Korea
USSR

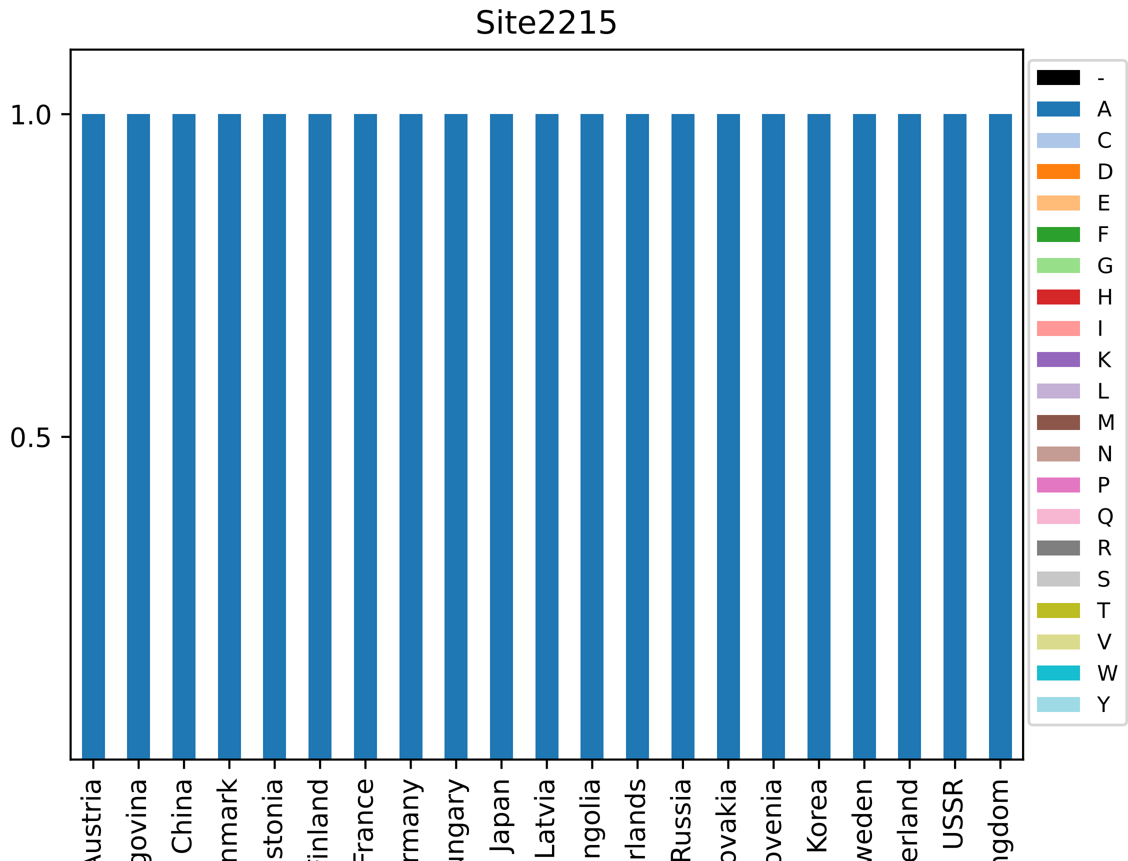
Site2215
1.0
0.5
-
A
C
D
E
F
G
H
I
K
L
M
N
P
Q
R
S
T
V
W
Y
Austria
govina
China
nmark
stonia
inland
France
rmany
ungary
Japan
Latvia
ngolia
rlands
Russia
ovakia
ovenia
Korea
weden
erland
USSR
ngdom

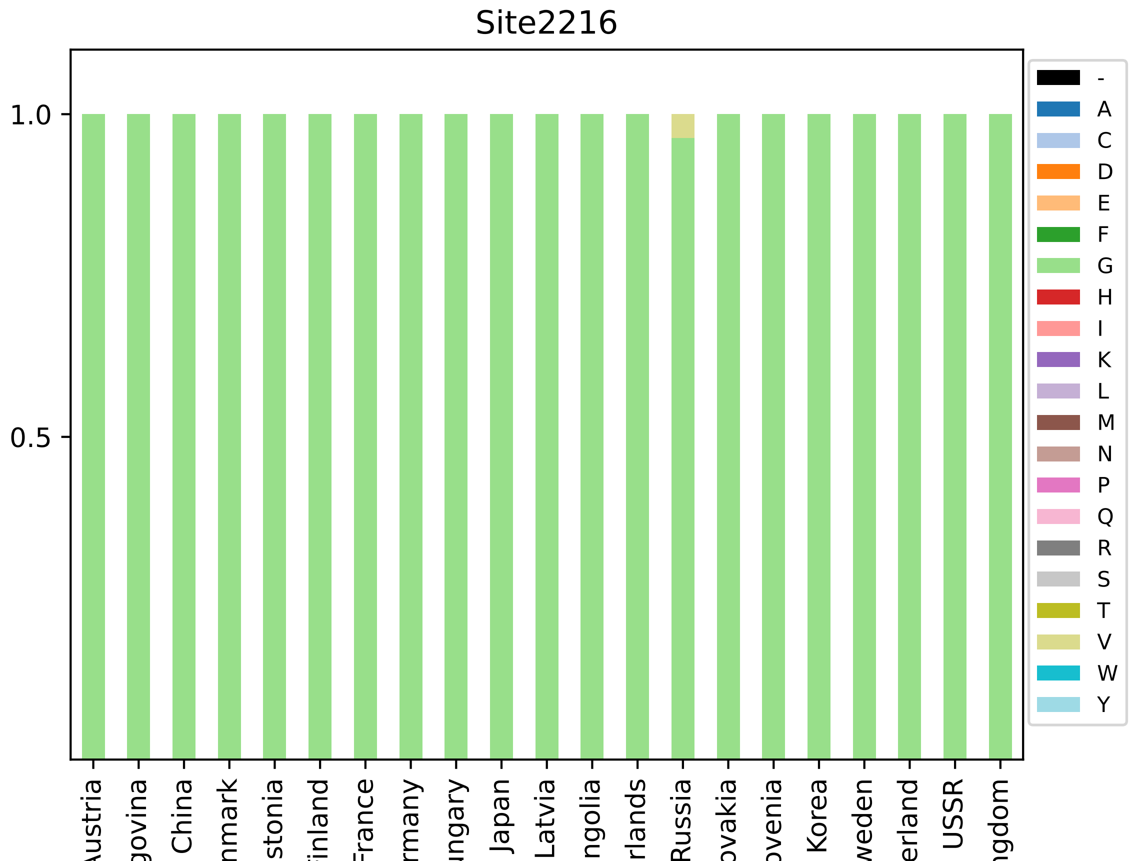
Site2216
1.0
0.5
-
A
C
D
E
F
G
H
I
K
L
M
N
P
Q
R
S
T
V
W
Y
China
Japan
Latvia
Russia
Korea
USSR

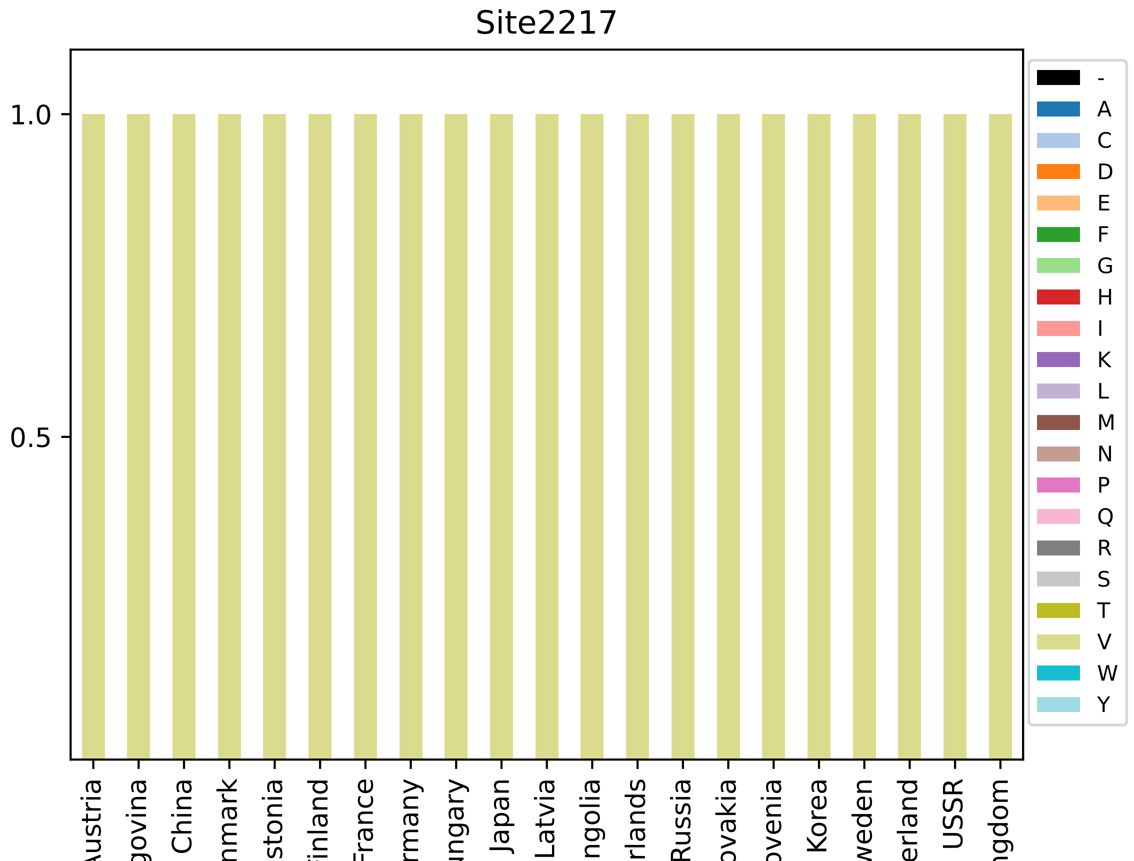
Site2217
1.0
0.5
-
A
C
D
E
F
G
H
I
K
L
M
N
P
Q
R
S
T
V
W
Y
Austria
govina
China
nmark
stonia
Finland
France
rmany
ungary
Japan
Latvia
ngolia
rlands
Russia
ovakia
ovenia
Korea
weden
erland
USSR
ngdom

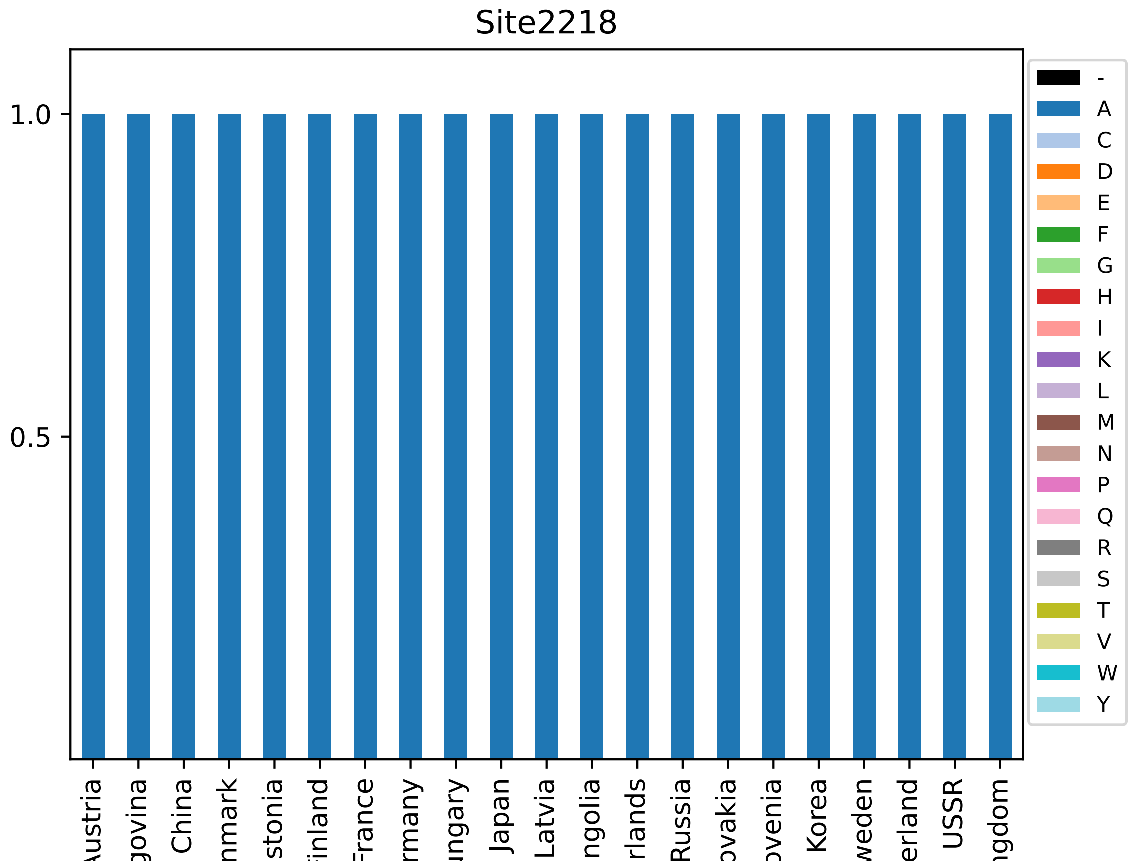

Site2218
1.0
0.5
-
A
C
D
E
F
G
H
I
K
L
M
N
P
Q
R
S
T
V
W
Y
China
Japan
Latvia
Russia
Korea
USSR

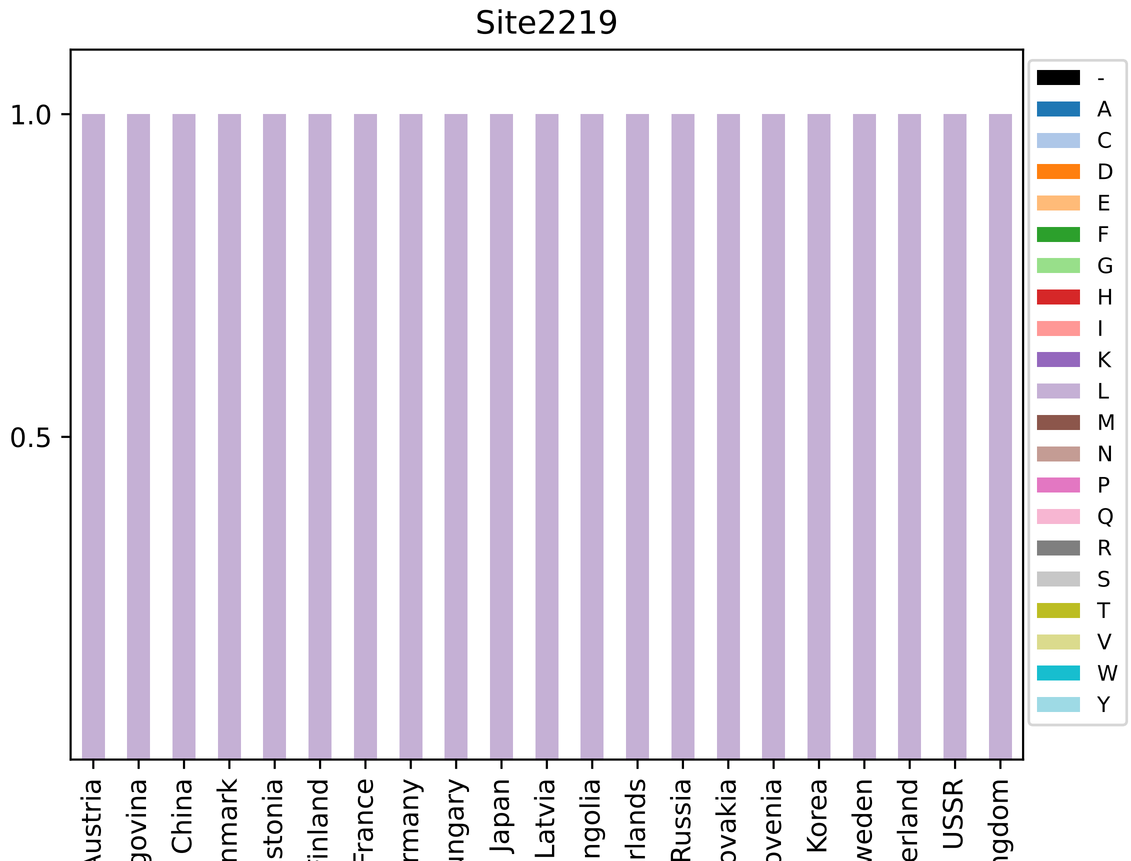
Site2219
1.0
0.5
-
A
C
D
E
F
G
H
I
K
L
M
N
P
Q
R
S
T
V
W
Y
China
Japan
Latvia
Russia
Korea
USSR

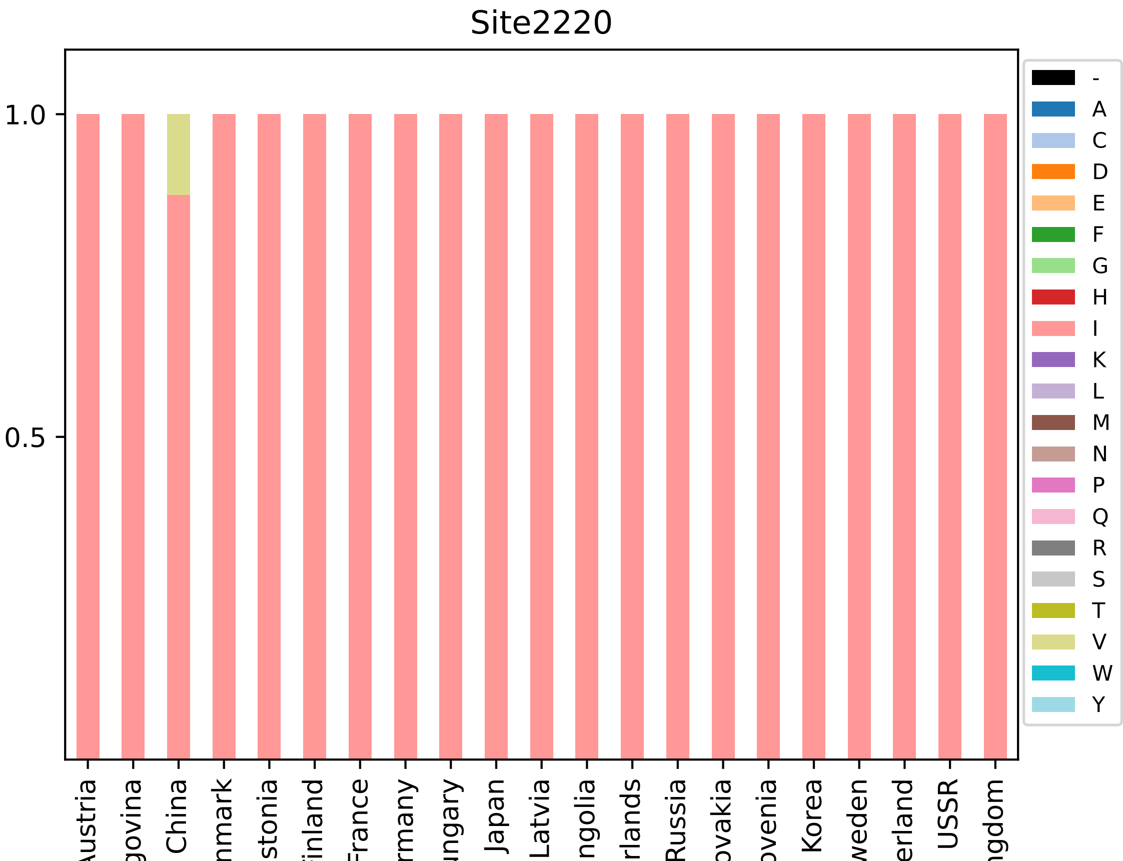
Site2220
1.0
0.5
-
A
C
D
E
F
G
H
I
K
L
M
N
P
Q
R
S
T
V
W
Y
China
Japan
Latvia
Russia
Korea
USSR

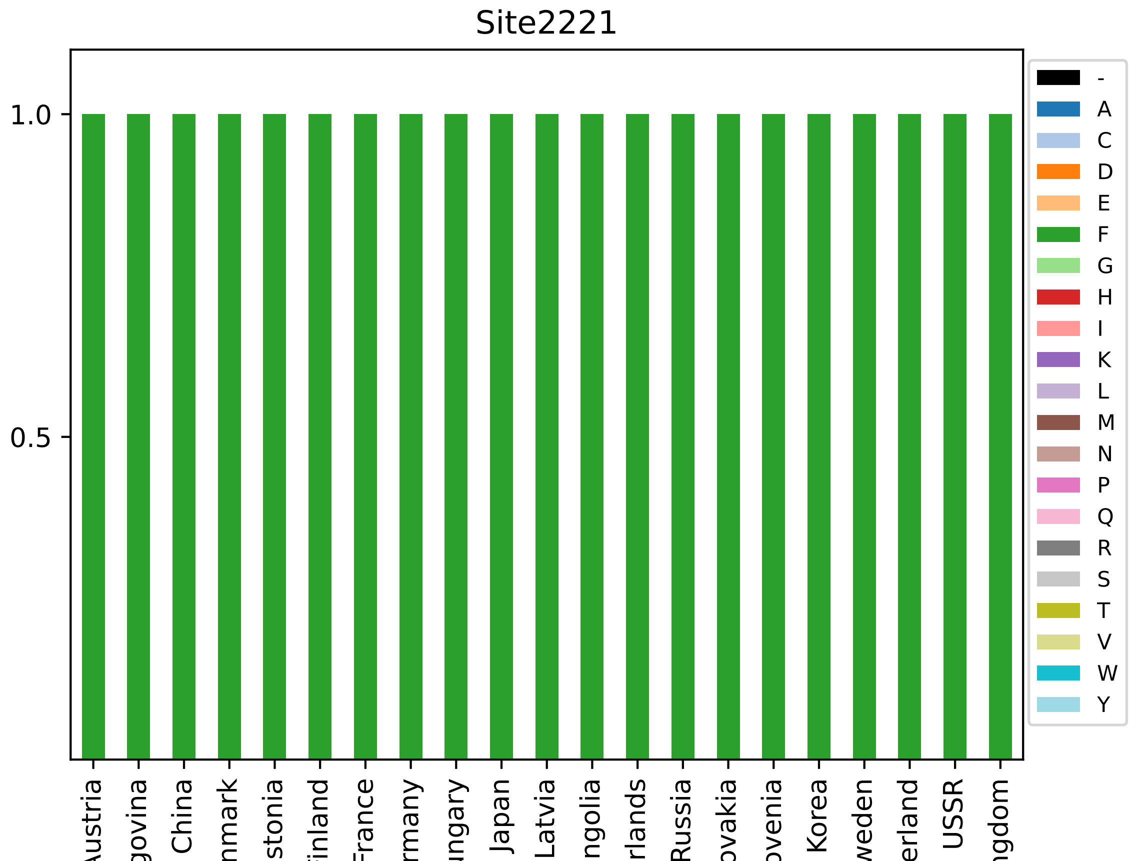
Site2221
1.0
0.5
-
A
C
D
E
F
G
H
I
K
L
M
N
P
Q
R
S
T
V
W
Y
Austria
China
Japan
Latvia
Russia
Korea
USSR

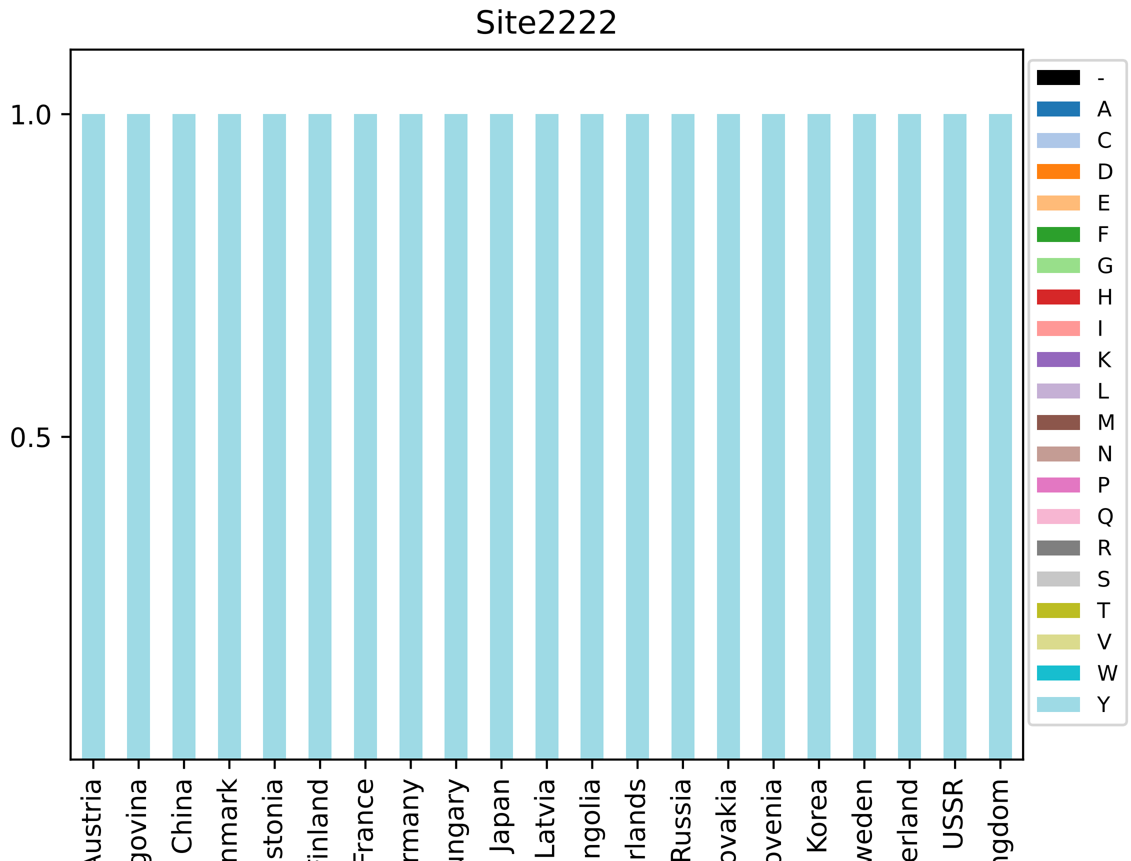
Site2222
1.0
0.5
Austria
govina
China
nmark
stonia
Finland
France
rmany
ungary
Japan
Latvia
ngolia
rlands
Russia
ovakia
ovenia
Korea
weden
erland
USSR
ngdom
-
A
C
D
E
F
G
H
I
K
L
M
N
P
Q
R
S
T
V
W
Y

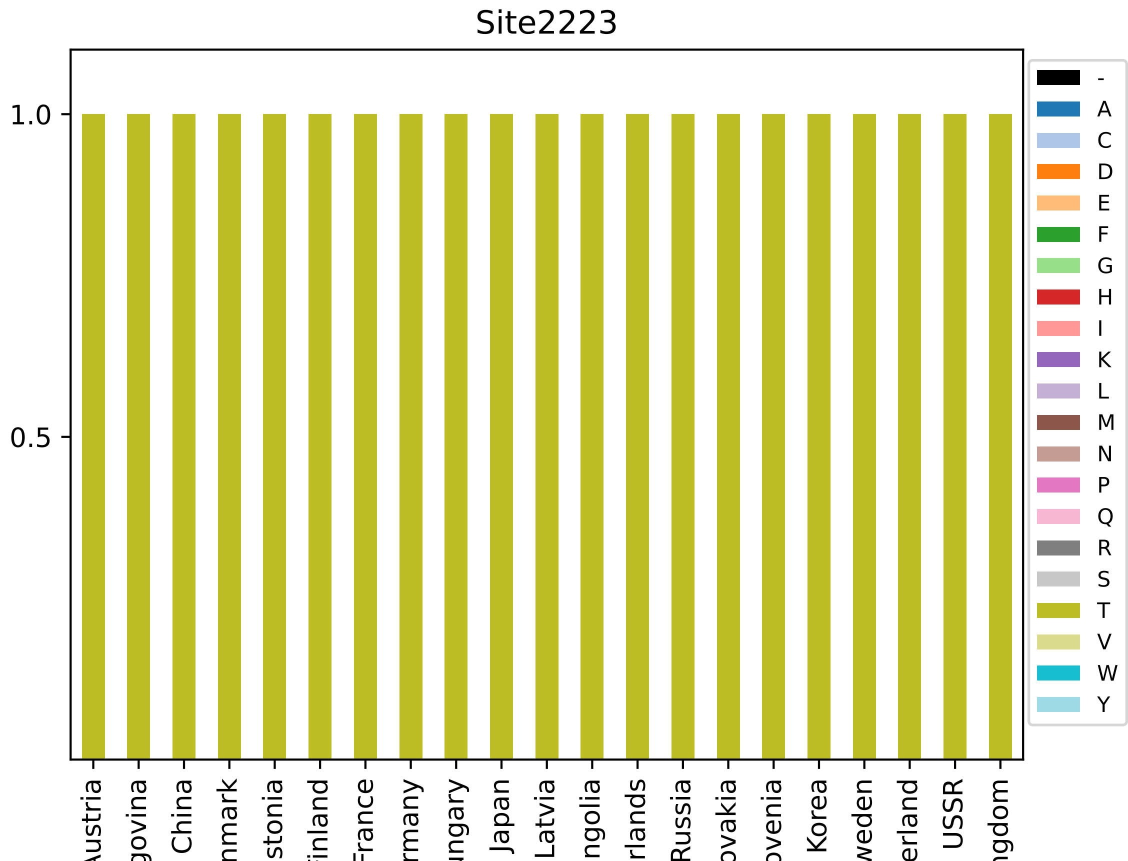

Site2223
1.0
0.5
-
A
C
D
E
F
G
H
I
K
L
M
N
P
Q
R
S
T
V
W
Y
Austria
China
Finland
France
Japan
Latvia
Russia
Korea
USSR

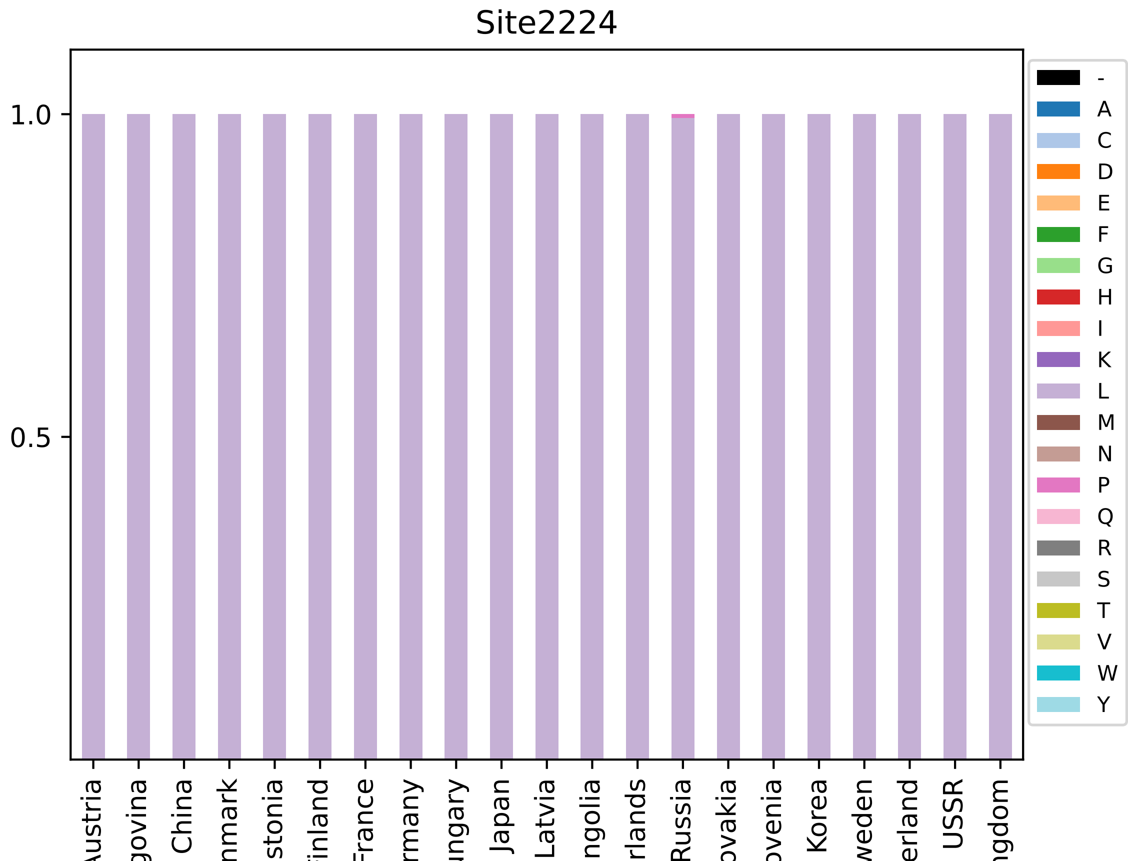
Site2224
1.0
0.5
-
A
C
D
E
F
G
H
I
K
L
M
N
P
Q
R
S
T
V
W
Y
Austria
govina
China
nmark
stonia
Finland
France
rmany
ungary
Japan
Latvia
ngolia
rlands
Russia
ovakia
ovenia
Korea
weden
erland
USSR
ngdom

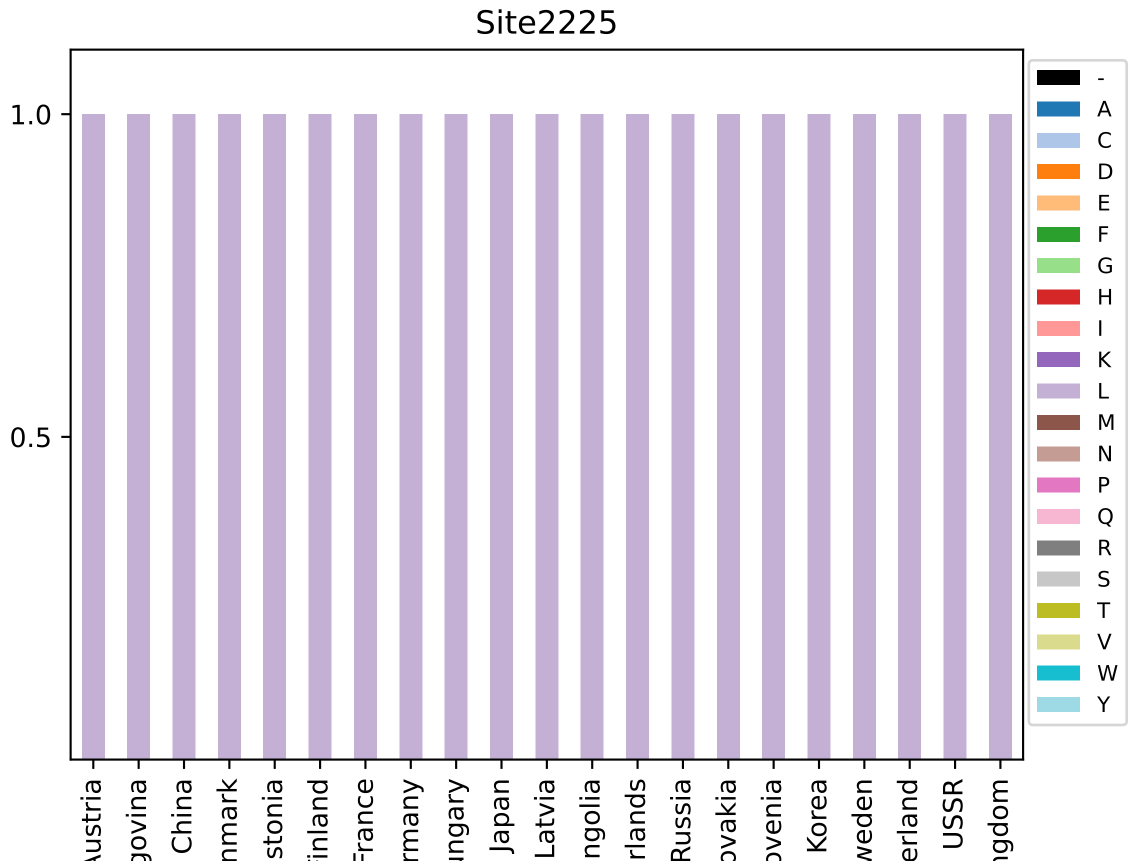

Site2225
1.0
0.5
-
A
C
D
E
F
G
H
I
K
L
M
N
P
Q
R
S
T
V
W
Y
China
Japan
Latvia
Russia
Korea
USSR

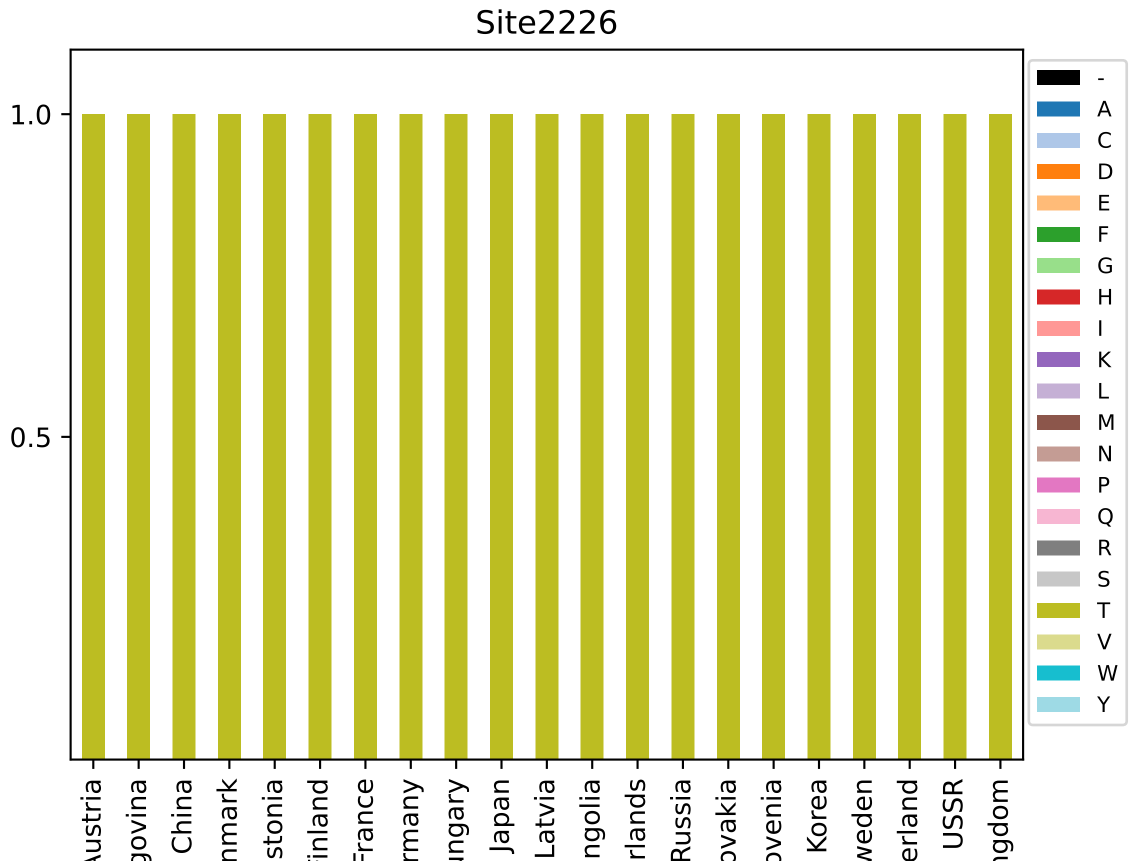

Site2226
1.0
0.5
-
A
C
D
E
F
G
H
I
K
L
M
N
P
Q
R
S
T
V
W
Y
Austria
govina
China
nmark
stonia
inland
France
rmany
ungary
Japan
Latvia
ngolia
rlands
Russia
ovakia
ovenia
Korea
weden
erland
USSR
ngdom

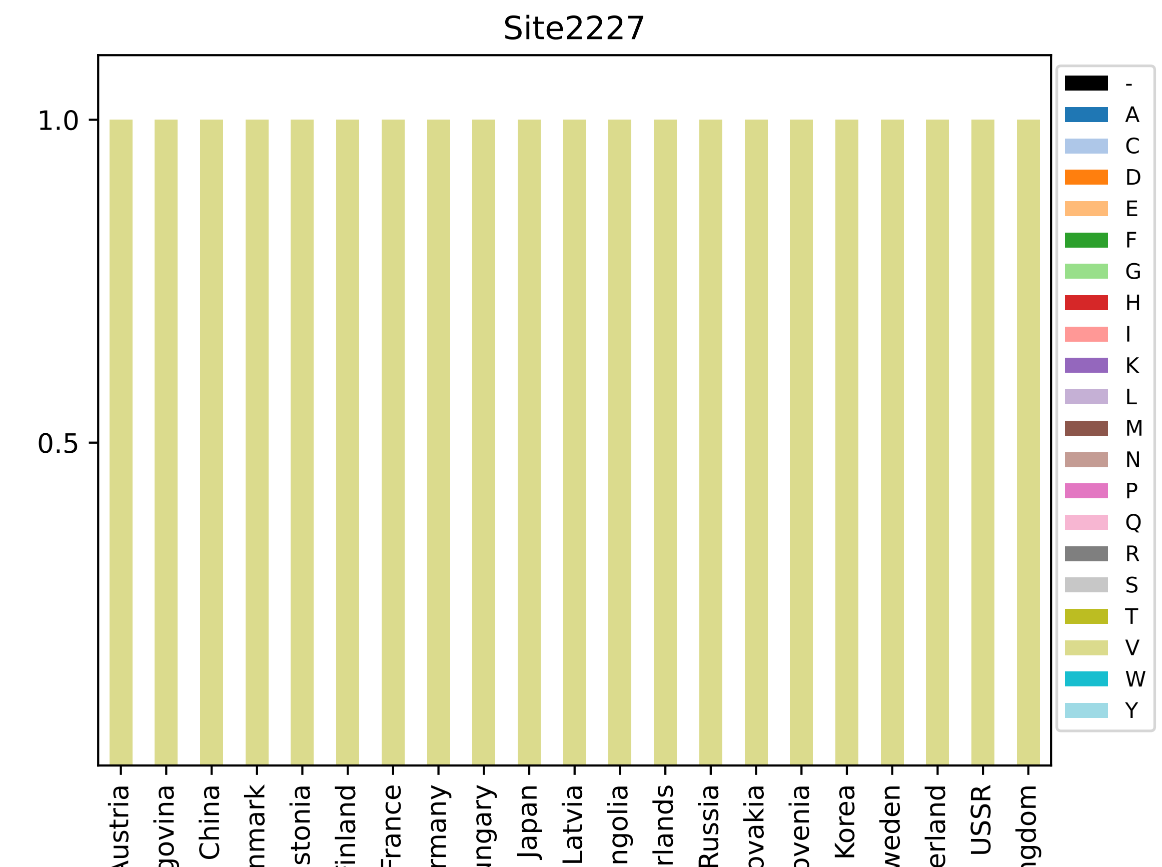

Site2227
1.0
0.5
-
A
C
D
E
F
G
H
I
K
L
M
N
P
Q
R
S
T
V
W
Y
Austria
China
Finland
France
Japan
Latvia
Russia
Korea
USSR

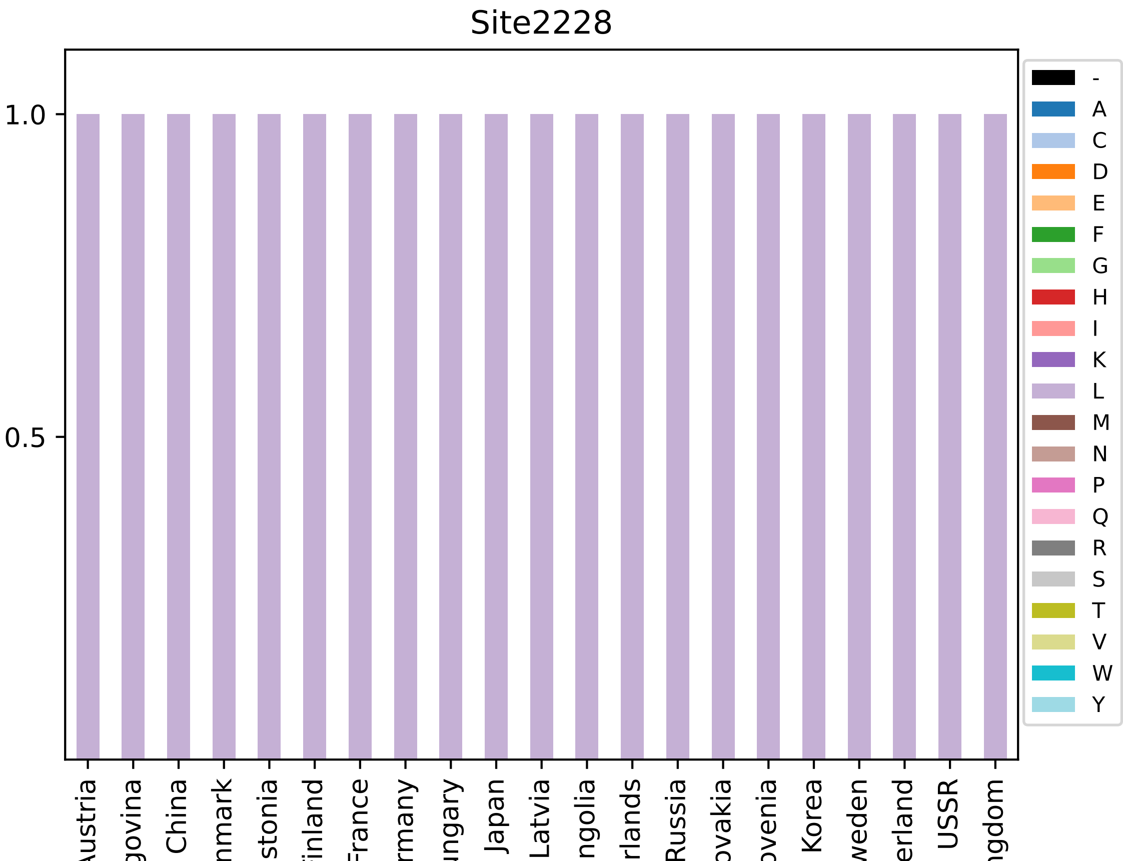

Site2228
1.0
0.5
-
A
C
D
E
F
G
H
I
K
L
M
N
P
Q
R
S
T
V
W
Y
Austria
govina
China
nmark
stonia
inland
France
rmany
ungary
Japan
Latvia
ngolia
rlands
Russia
ovakia
ovenia
Korea
weden
erland
USSR
ngdom

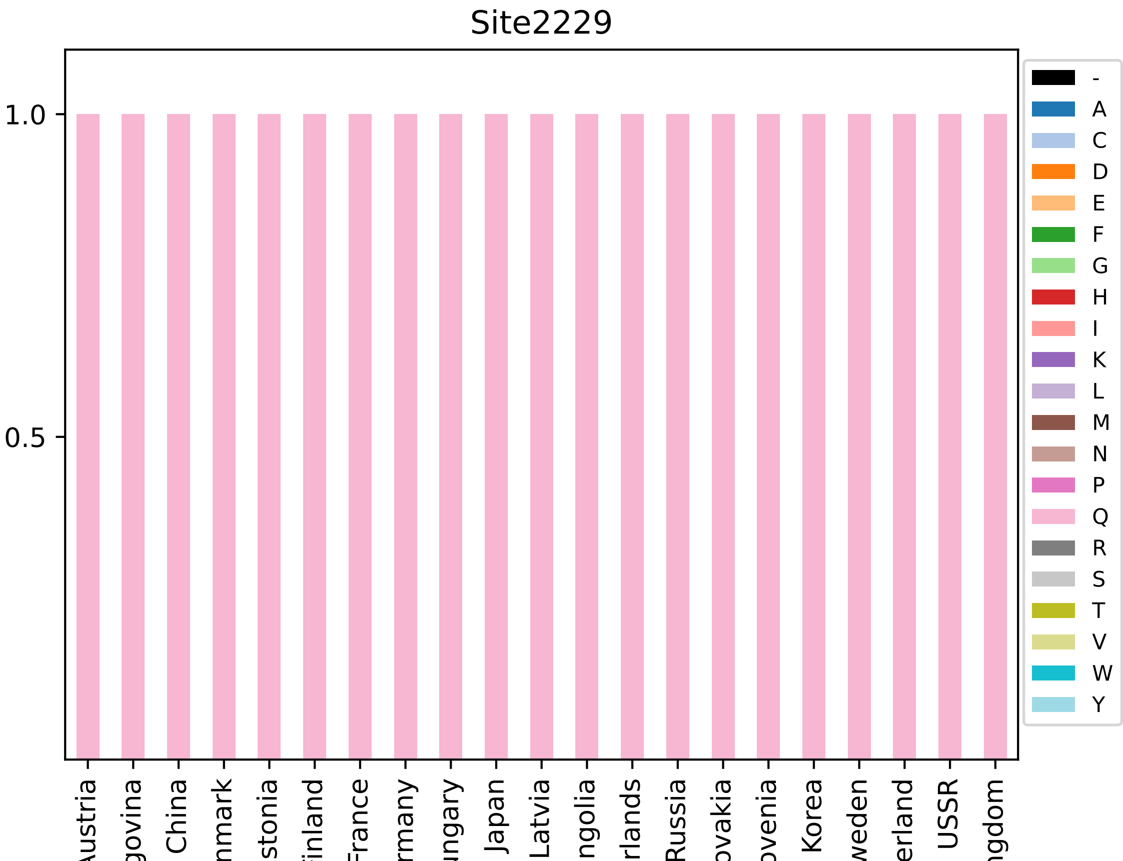

Site2229
1.0
0.5
-
A
C
D
E
F
G
H
I
K
L
M
N
P
Q
R
S
T
V
W
Y
China
Japan
Latvia
Russia
Korea
USSR

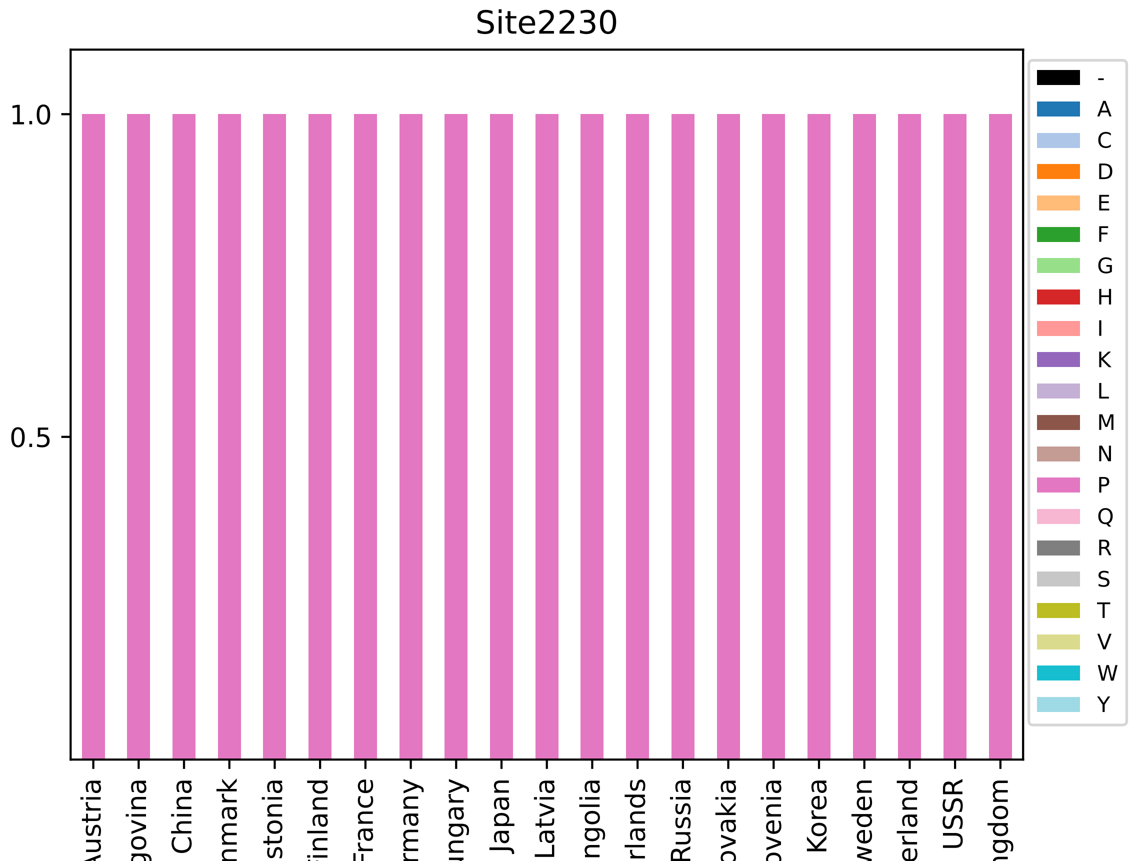
Site2230
1.0
0.5
-
A
C
D
E
F
G
H
I
K
L
M
N
P
Q
R
S
T
V
W
Y
China
Japan
Latvia
Russia
Korea
USSR

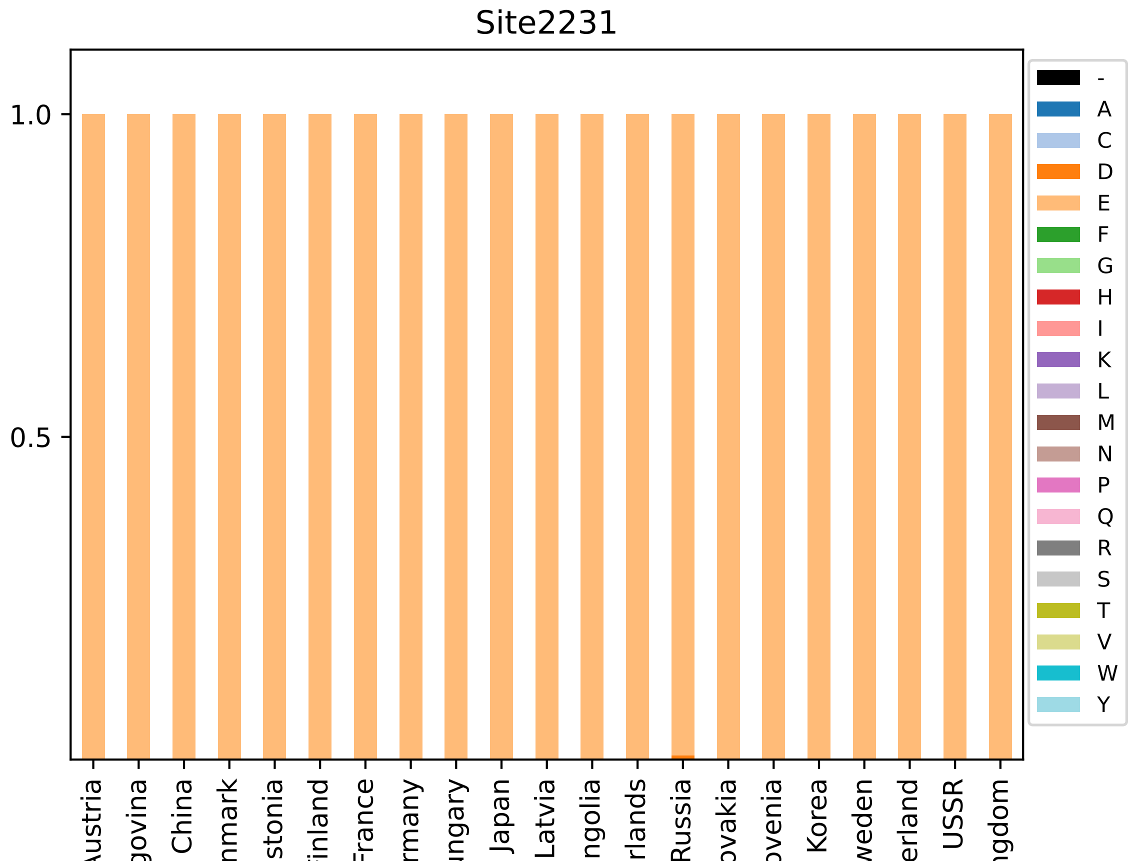
Site2231
1.0
0.5
-
A
C
D
E
F
G
H
I
K
L
M
N
P
Q
R
S
T
V
W
Y
Austria
govina
China
nmark
stonia
Finland
France
rmany
ungary
Japan
Latvia
ngolia
rlands
Russia
ovakia
ovenia
Korea
weden
erland
USSR
ngdom

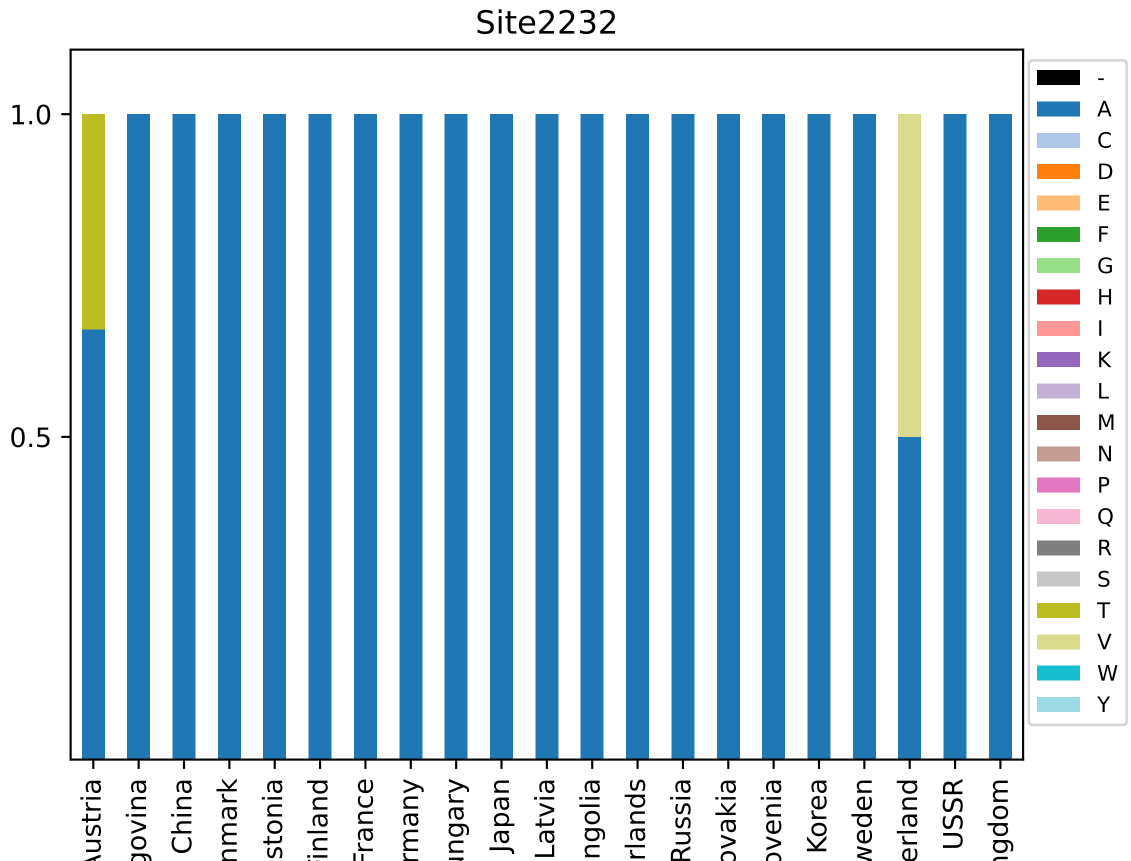
Site2232
1.0
0.5
-
A
C
D
E
F
G
H
I
K
L
M
N
P
Q
R
S
T
V
W
Y
China
Japan
Latvia
Russia
Korea
USSR

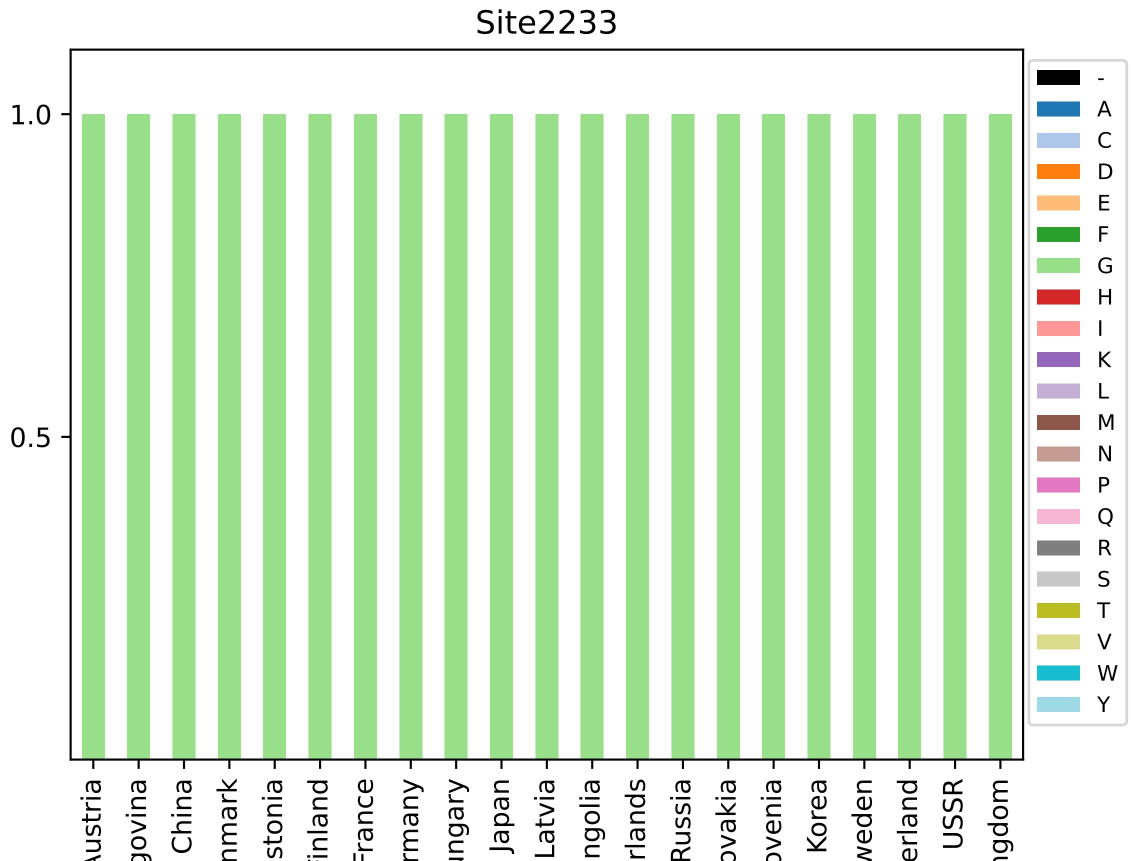
Site2233
1.0
0.5
-
A
C
D
E
F
G
H
I
K
L
M
N
P
Q
R
S
T
V
W
Y
China
Japan
Latvia
Russia
Korea
USSR

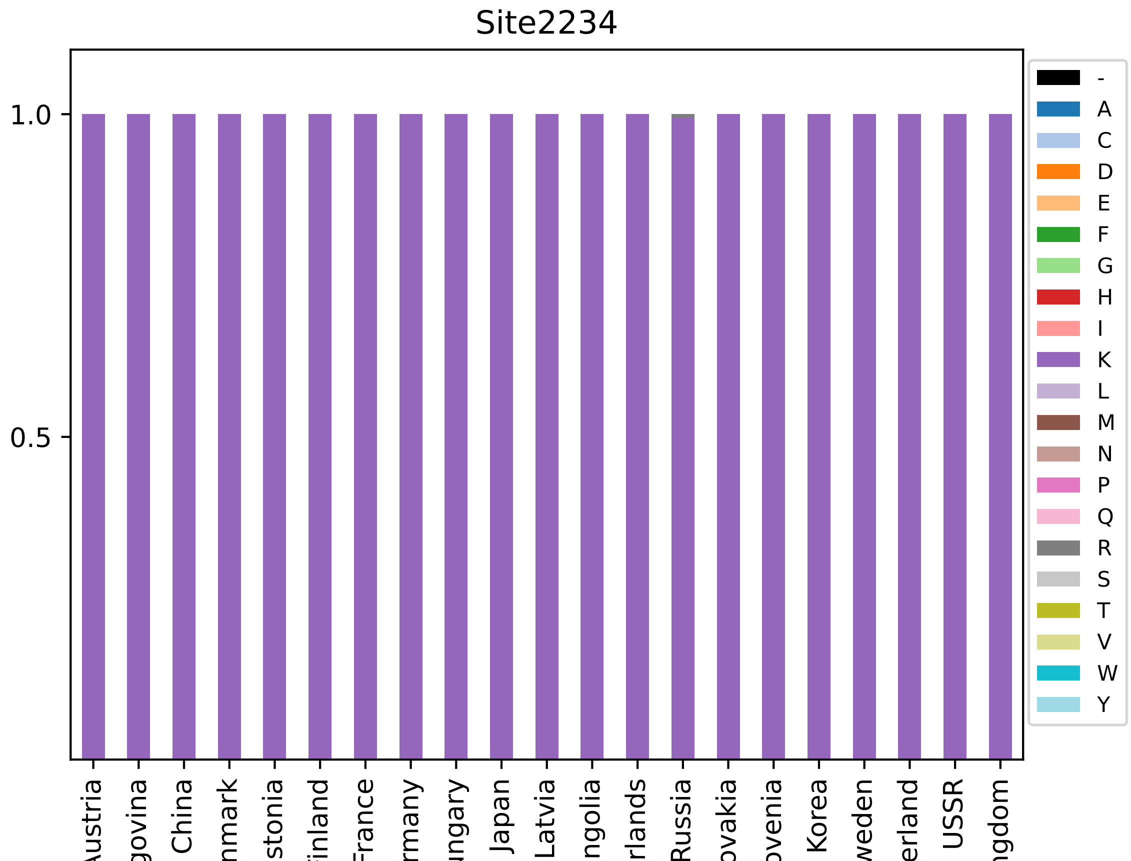

Site2234
1.0
0.5
-
A
C
D
E
F
G
H
I
K
L
M
N
P
Q
R
S
T
V
W
Y
China
Japan
Latvia
Russia
Korea
USSR

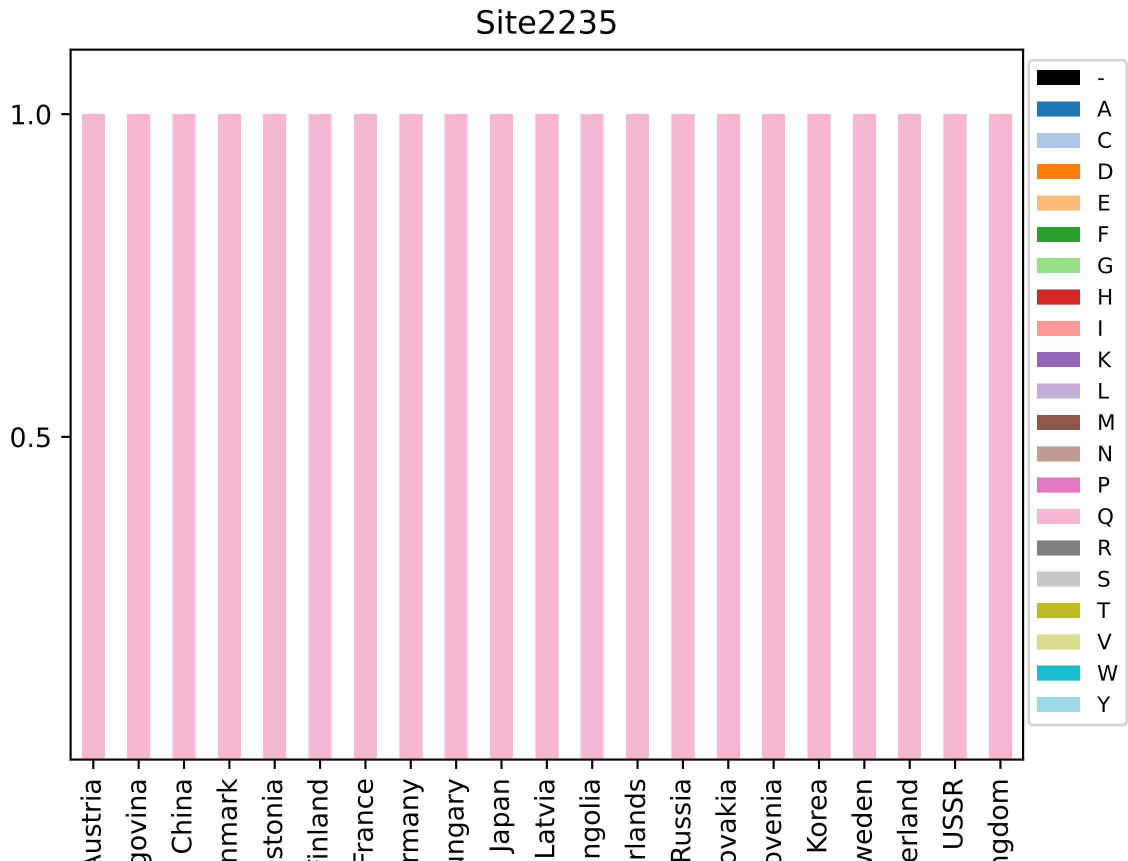

Site2235
1.0
0.5
Austria
govina
China
nmark
stonia
Finland
France
rmany
ungary
Japan
Latvia
ngolia
rlands
Russia
ovakia
ovenia
Korea
weden
erland
USSR
ngdom
-
A
C
D
E
F
G
H
I
K
L
M
N
P
Q
R
S
T
V
W
Y

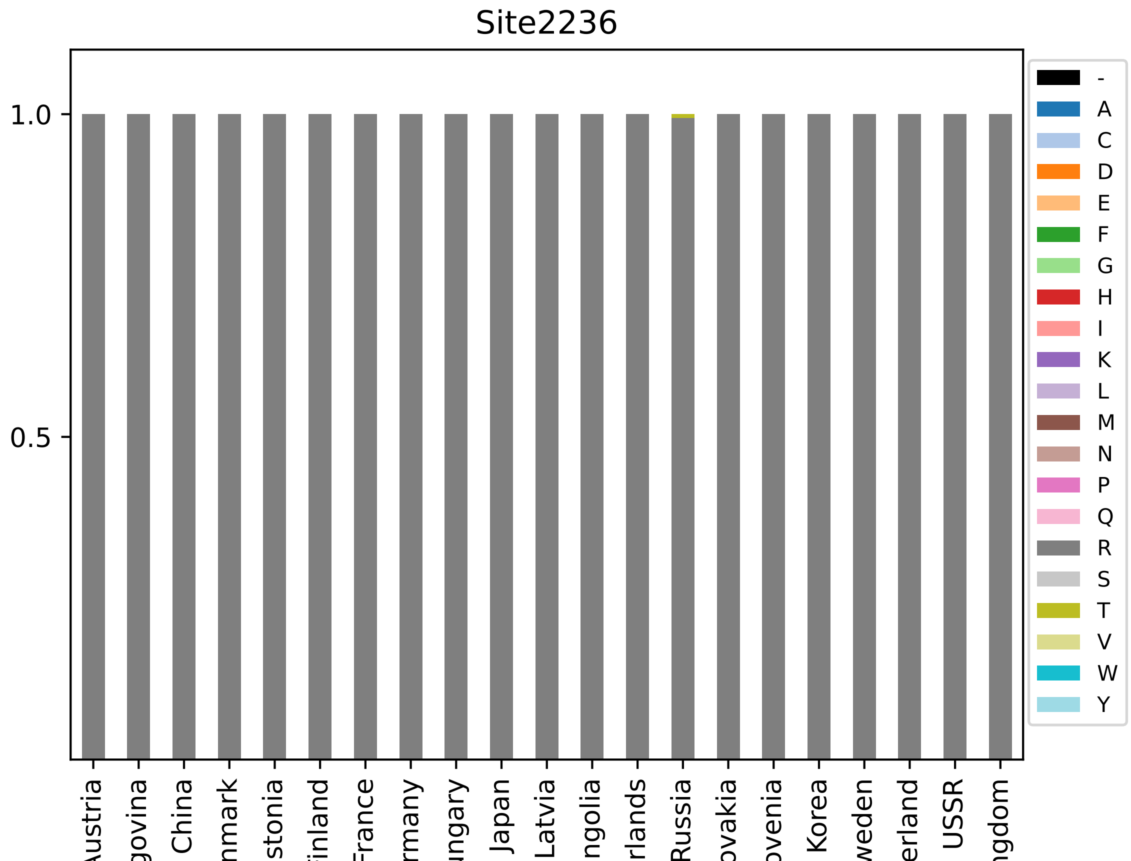

Site2236
1.0
0.5
-
A
C
D
E
F
G
H
I
K
L
M
N
P
Q
R
S
T
V
W
Y
Austria
govina
China
nmark
stonia
Finland
France
rmany
ungary
Japan
Latvia
ngolia
rlands
Russia
ovakia
ovenia
Korea
weden
erland
USSR
ngdom

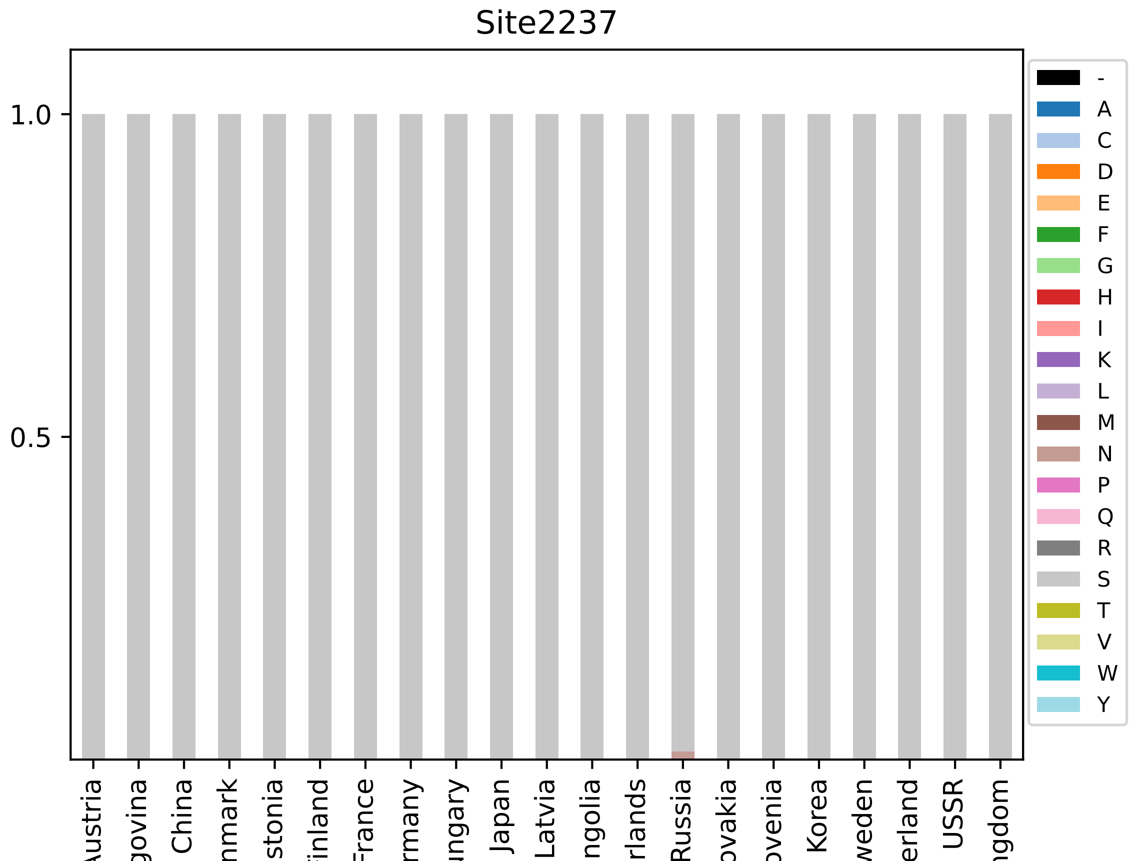

Site2237
1.0
0.5
-
A
C
D
E
F
G
H
I
K
L
M
N
P
Q
R
S
T
V
W
Y
Austria
govina
China
nmark
stonia
Finland
France
rmany
ungary
Japan
Latvia
ngolia
rlands
Russia
ovakia
ovenia
Korea
weden
erland
USSR
ngdom

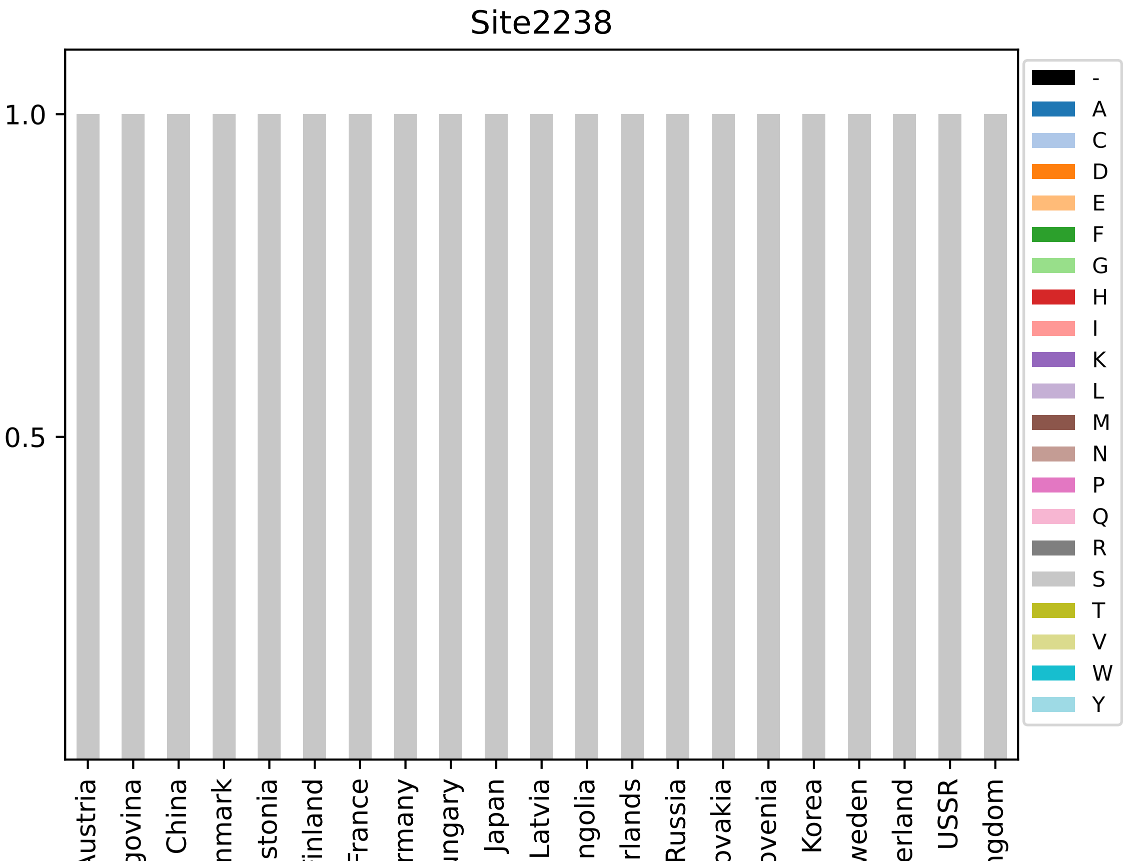
Site2238
1.0
0.5
Austria
govina
China
nmark
stonia
Finland
France
rmany
ungary
Japan
Latvia
ngolia
rlands
Russia
ovakia
ovenia
Korea
weden
erland
USSR
ngdom
-
A
C
D
E
F
G
H
I
K
L
M
N
P
Q
R
S
T
V
W
Y

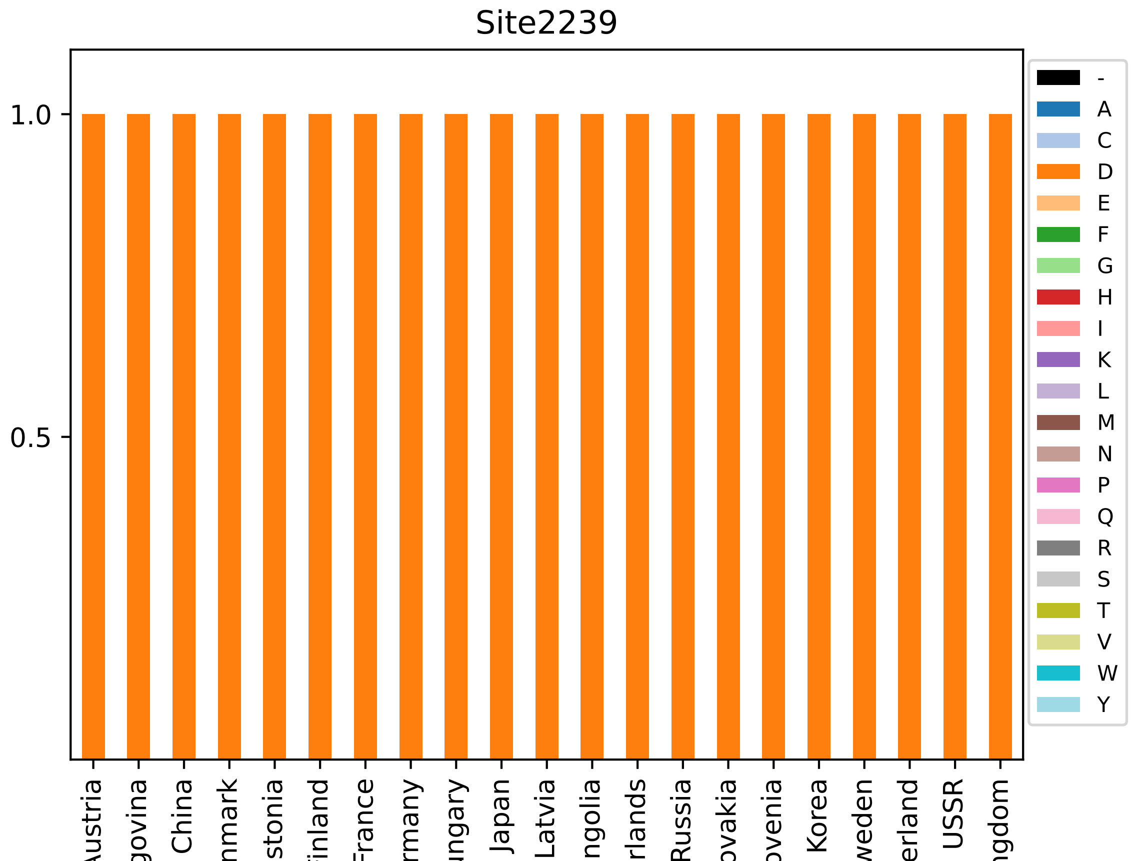
Site2239
1.0
0.5
-
A
C
D
E
F
G
H
I
K
L
M
N
P
Q
R
S
T
V
W
Y
Austria
China
Japan
Latvia
Russia
Korea
USSR

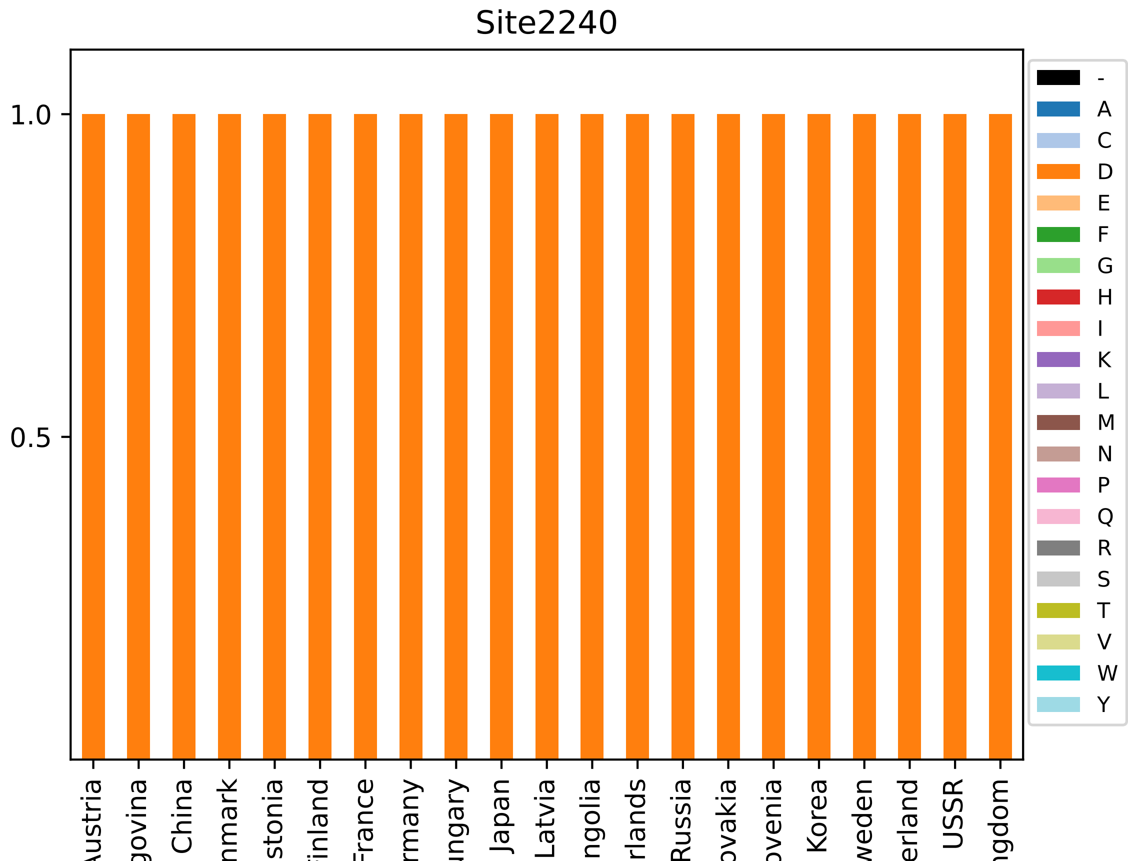
Site2240
1.0
0.5
-
A
C
D
E
F
G
H
I
K
L
M
N
P
Q
R
S
T
V
W
Y
Austria
govina
China
nmark
stonia
inland
France
rmany
ungary
Japan
Latvia
ngolia
rlands
Russia
ovakia
ovenia
Korea
weden
erland
USSR
ngdom

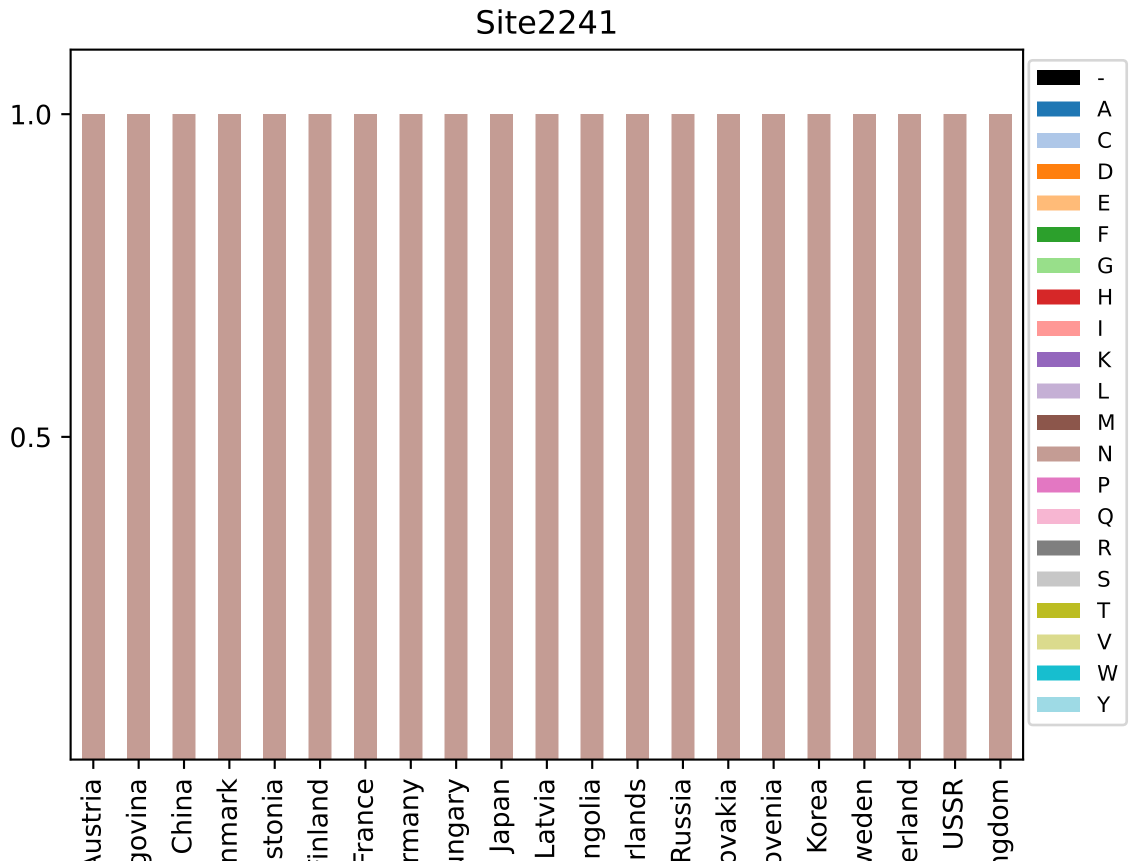
Site2241
1.0
0.5
-
A
C
D
E
F
G
H
I
K
L
M
N
P
Q
R
S
T
V
W
Y
Austria
China
Japan
Latvia
Russia
Korea
USSR

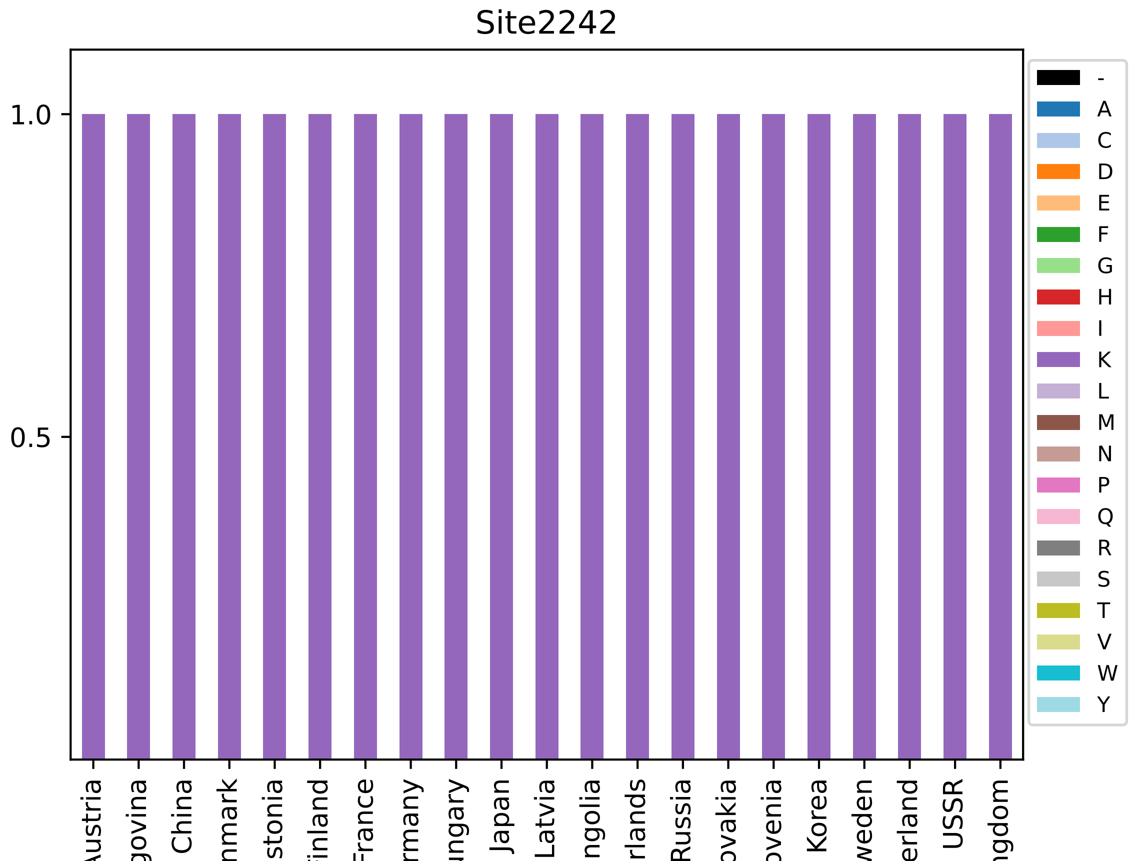

Site2242
1.0
0.5
-
A
C
D
E
F
G
H
I
K
L
M
N
P
Q
R
S
T
V
W
Y
Austria
govina
China
nmark
stonia
inland
France
rmany
ungary
Japan
Latvia
ngolia
rlands
Russia
ovakia
ovenia
Korea
weden
erland
USSR
ngdom

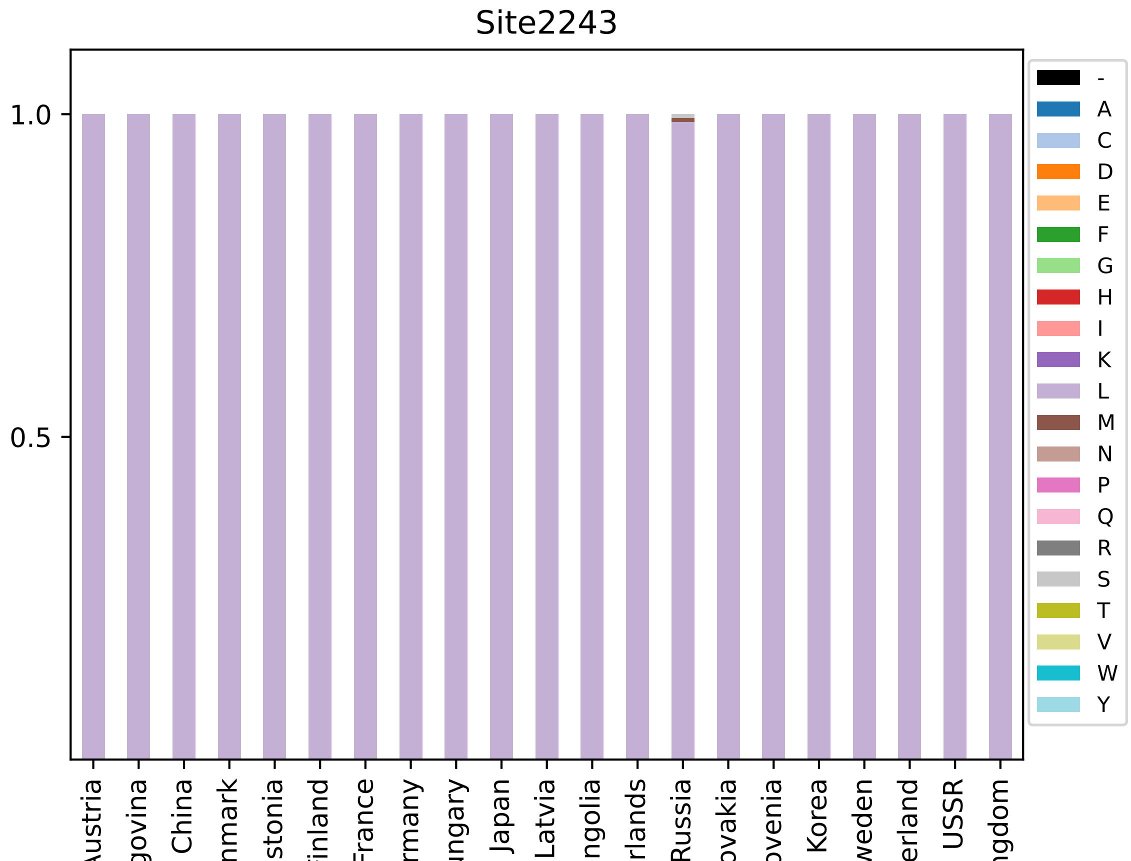
Site2243
1.0
0.5
China
Japan
Latvia
Russia
Korea
USSR
-
A
C
D
E
F
G
H
I
K
L
M
N
P
Q
R
S
T
V
W
Y

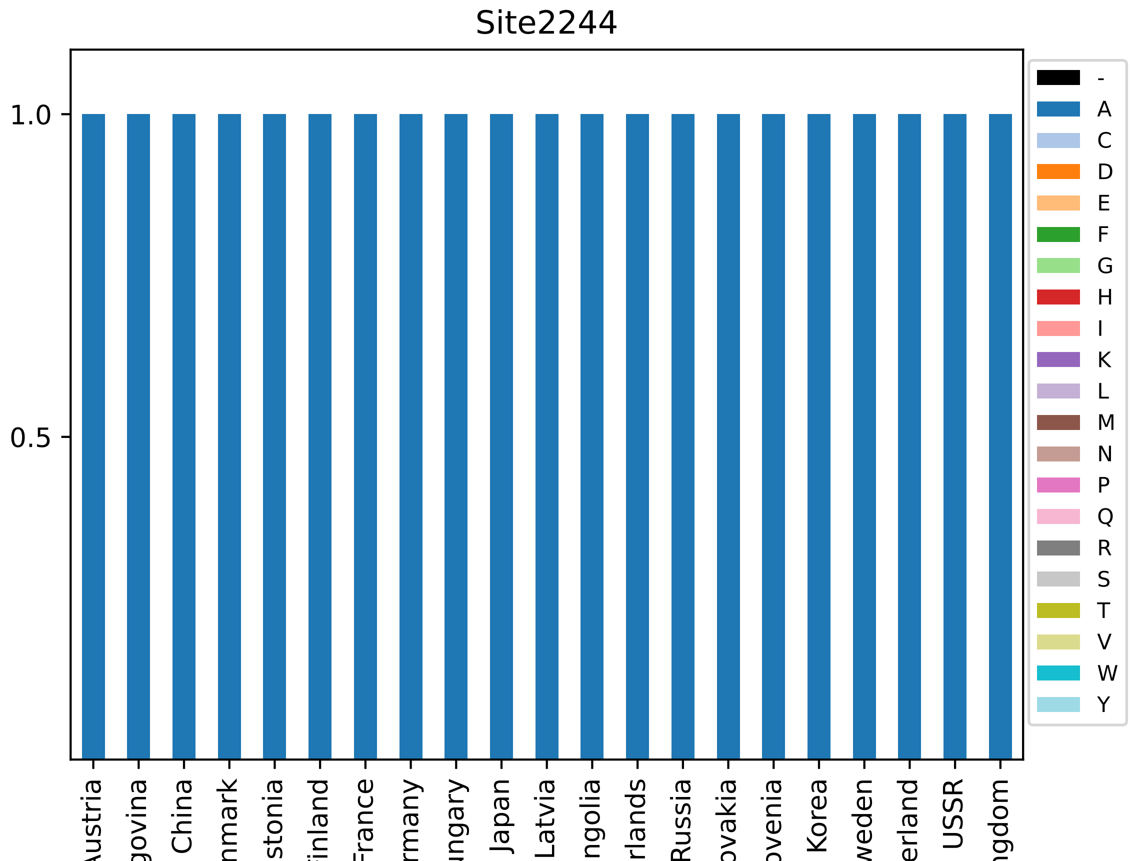

Site2244
1.0
0.5
-
A
C
D
E
F
G
H
I
K
L
M
N
P
Q
R
S
T
V
W
Y
Austria
China
Estonia
Finland
France
Hungary
Japan
Latvia
Russia
Korea
USSR

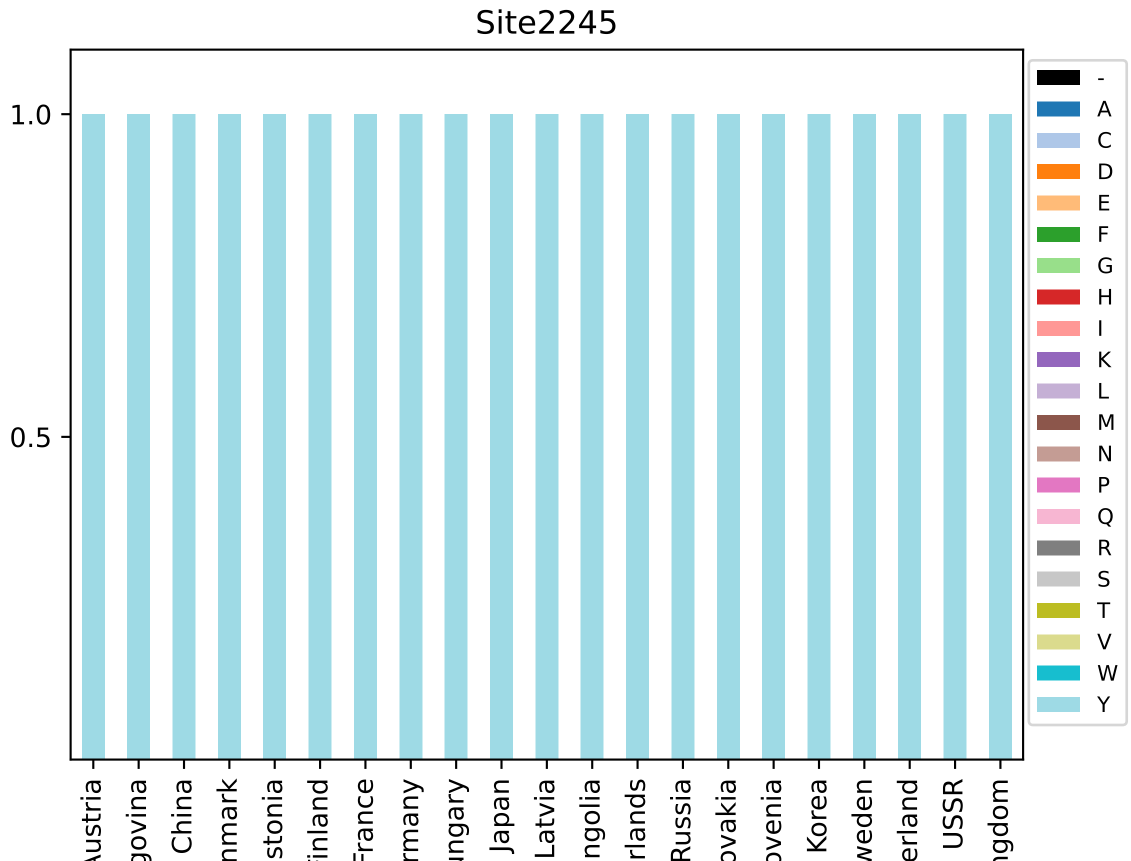
Site2245
1.0
0.5
China
Japan
Latvia
Korea
USSR
-
A
C
D
E
F
G
H
I
K
L
M
N
P
Q
R
S
T
V
W
Y

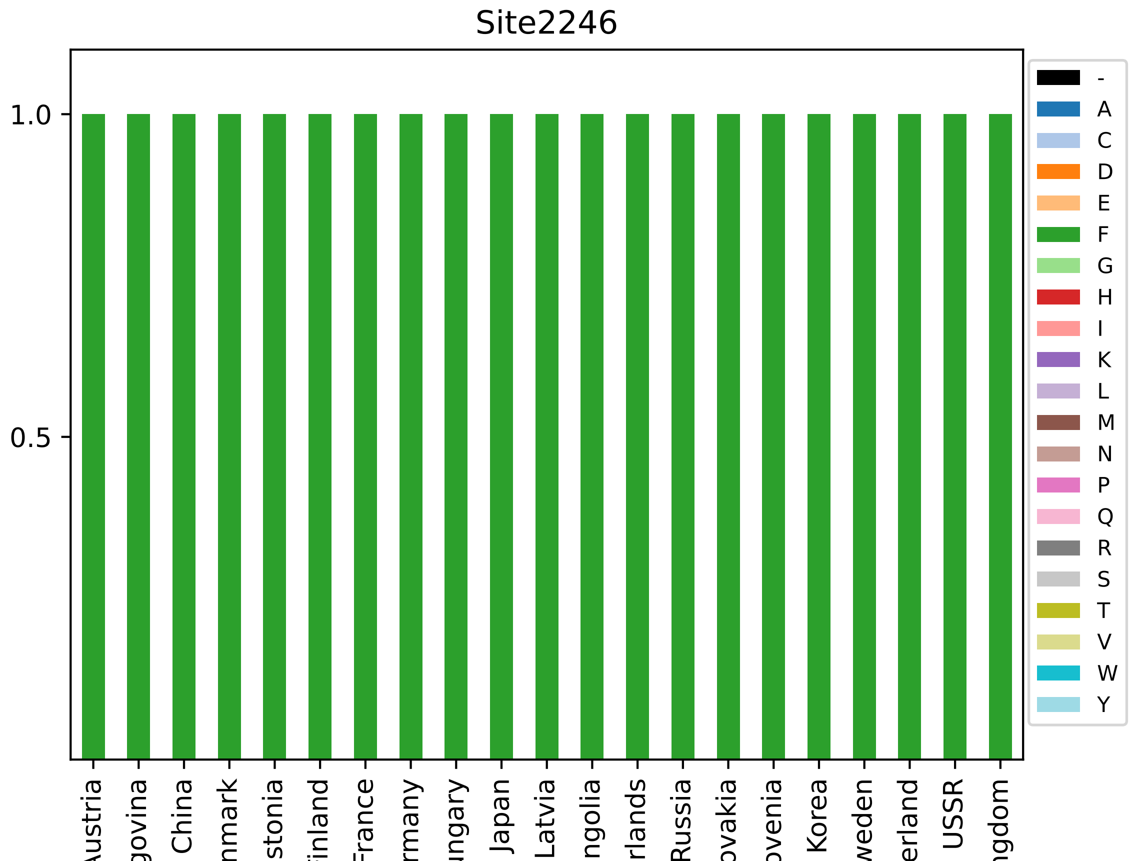

Site2246
1.0
0.5
-
A
C
D
E
F
G
H
I
K
L
M
N
P
Q
R
S
T
V
W
Y
Austria
China
Estonia
Finland
France
Hungary
Japan
Latvia
Mongolia
Russia
Slovenia
Korea
Sweden
USSR

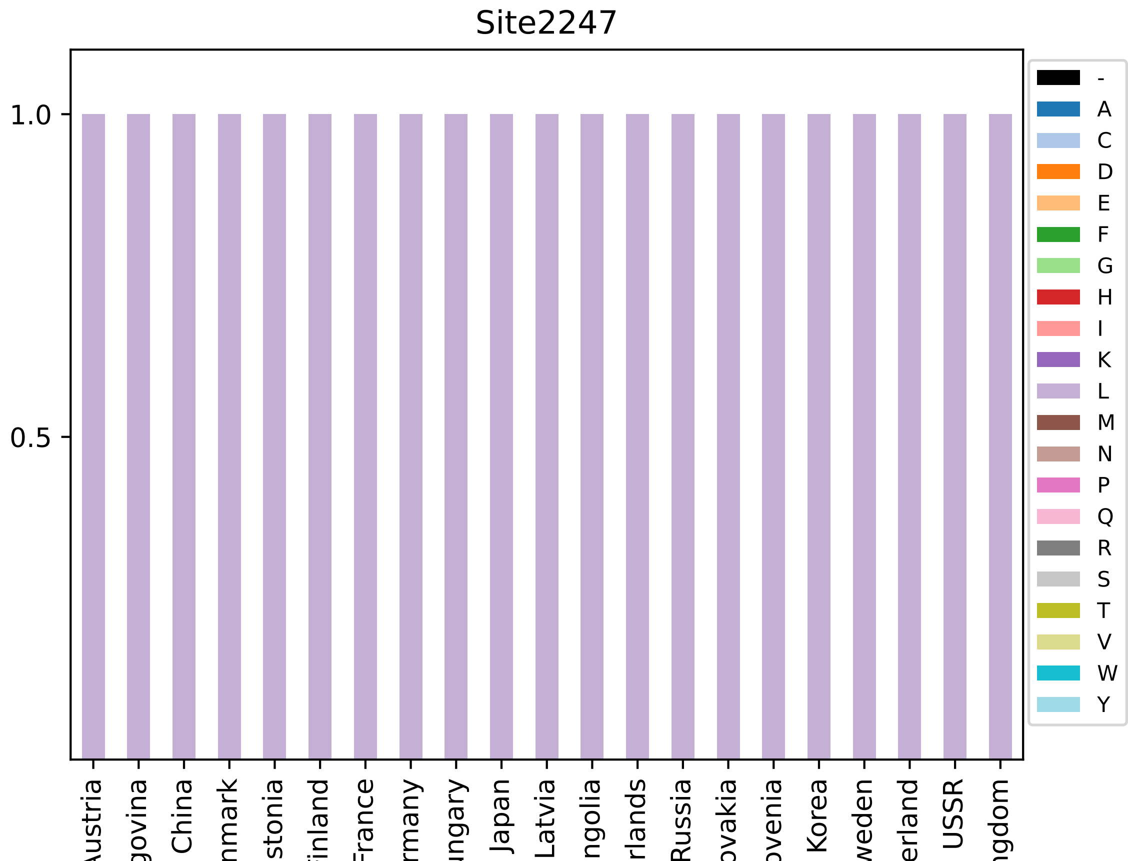

Site2247
1.0
0.5
-
A
C
D
E
F
G
H
I
K
L
M
N
P
Q
R
S
T
V
W
Y
China
Japan
Latvia
Russia
Korea
USSR

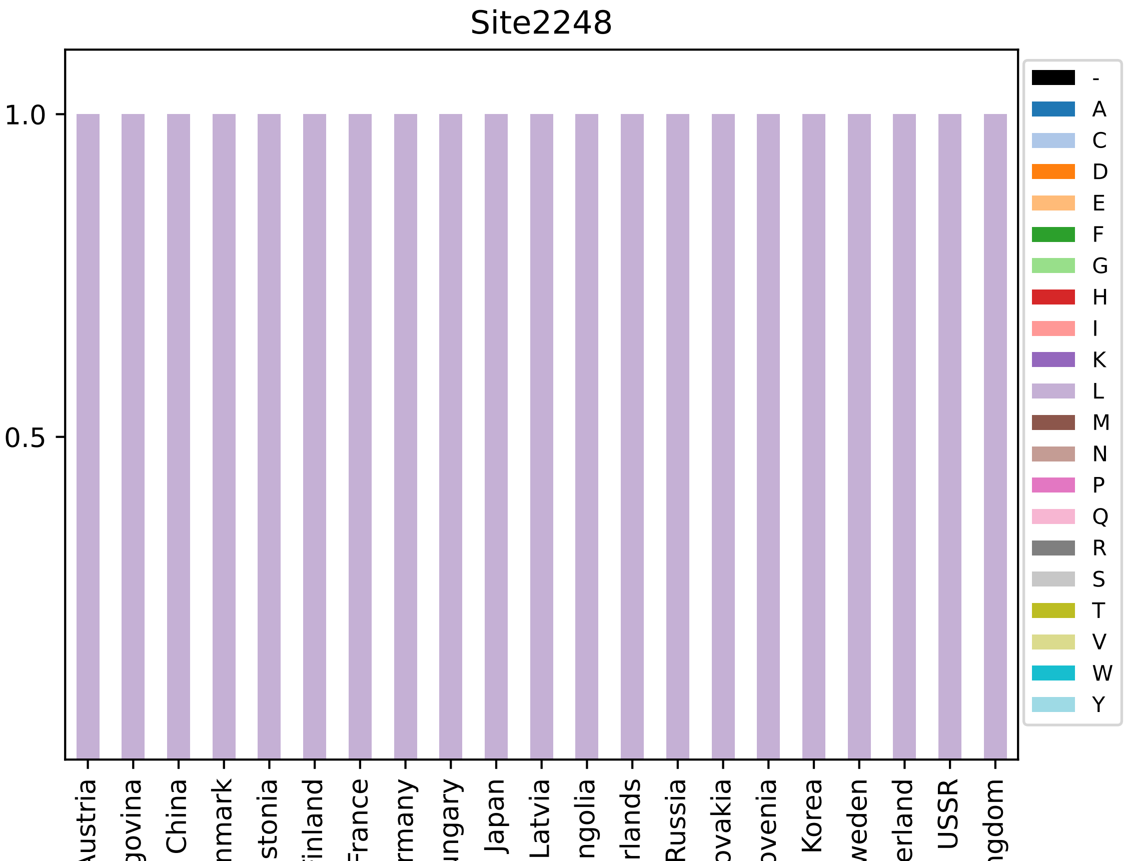
Site2248
1.0
0.5
-
A
C
D
E
F
G
H
I
K
L
M
N
P
Q
R
S
T
V
W
Y
China
Japan
Latvia
Russia
Korea
USSR

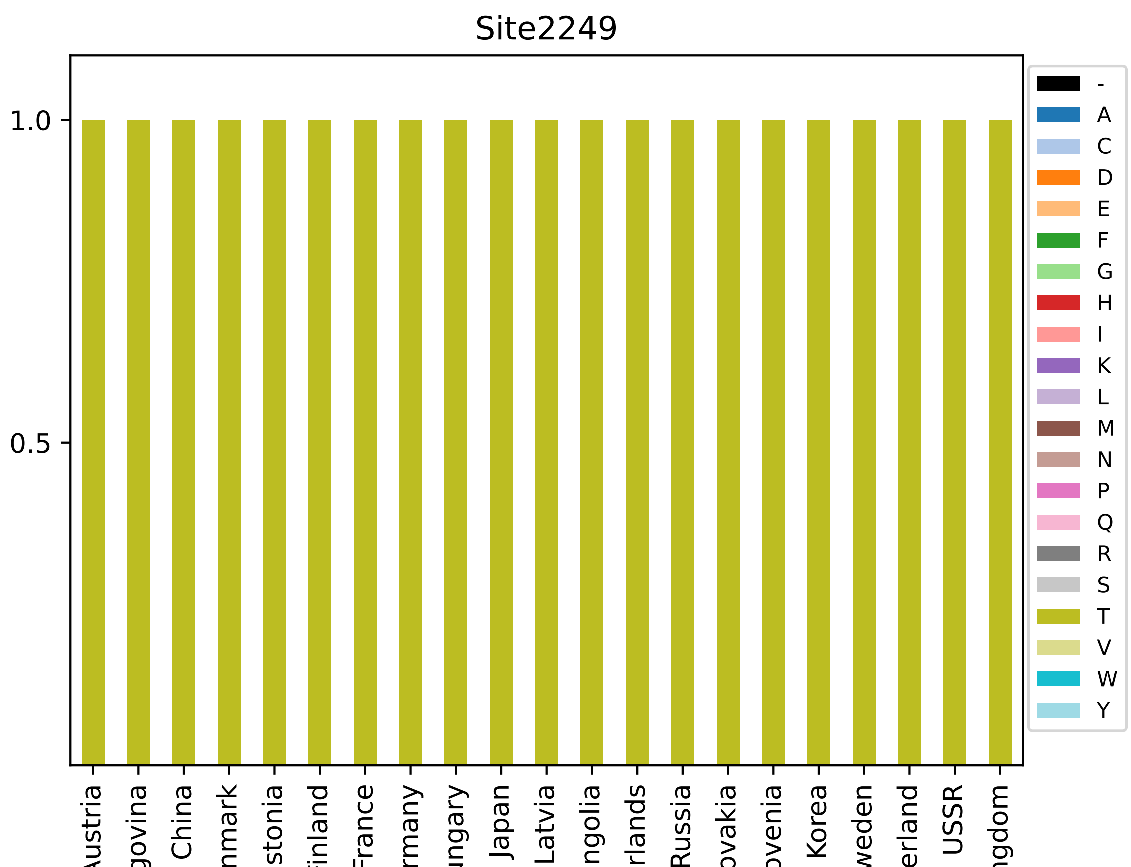

Site2249
1.0
0.5
-
A
C
D
E
F
G
H
I
K
L
M
N
P
Q
R
S
T
V
W
Y
Austria
govina
China
nmark
stonia
Finland
France
rmany
ungary
Japan
Latvia
ngolia
rlands
Russia
ovakia
ovenia
Korea
weden
erland
USSR
ngdom

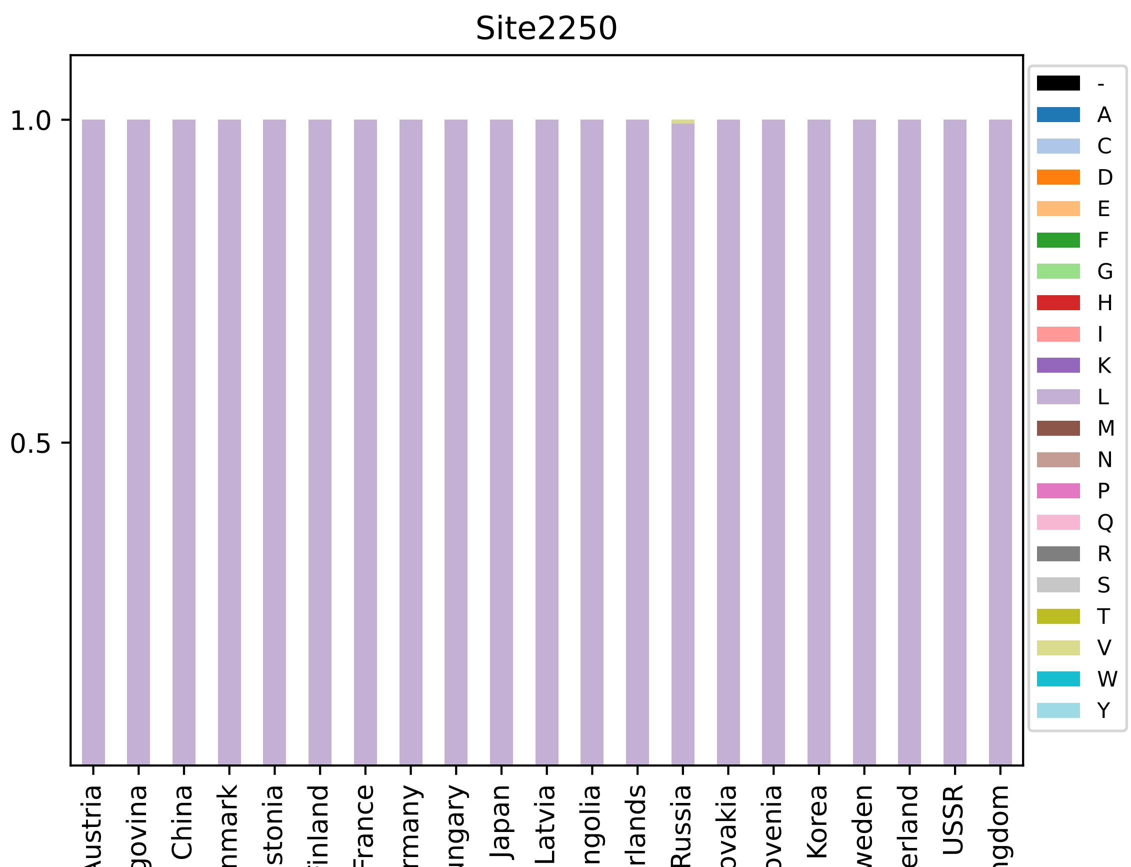
Site2250
1.0
0.5
-
A
C
D
E
F
G
H
I
K
L
M
N
P
Q
R
S
T
V
W
Y
China
Japan
Latvia
Russia
Korea
USSR

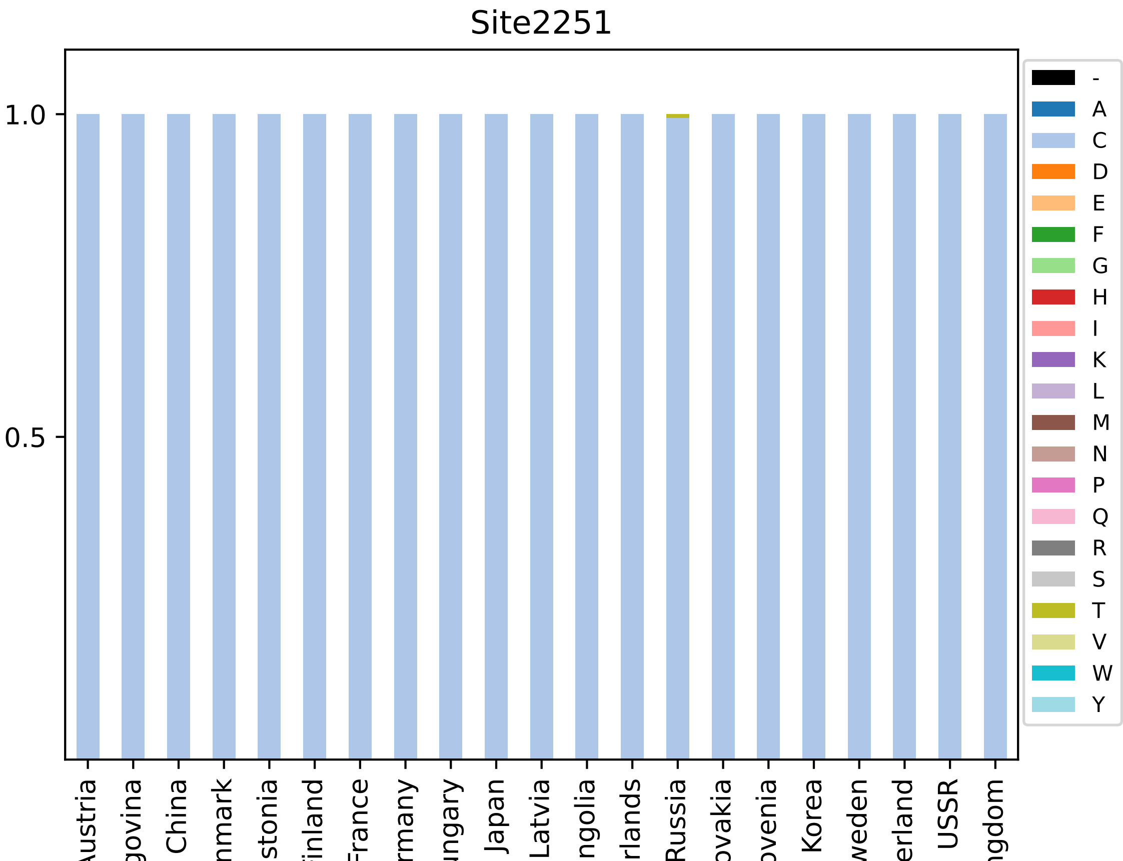

Site2251
1.0
0.5
ustria
govina
China
nmark
stonia
inland
rance
rmany
ungary
Japan
Latvia
ngolia
rlands
Russia
ovakia
ovenia
Korea
weden
erland
USSR
ngdom
-
A
C
D
E
F
G
H
I
K
L
M
N
P
Q
R
S
T
V
W
Y

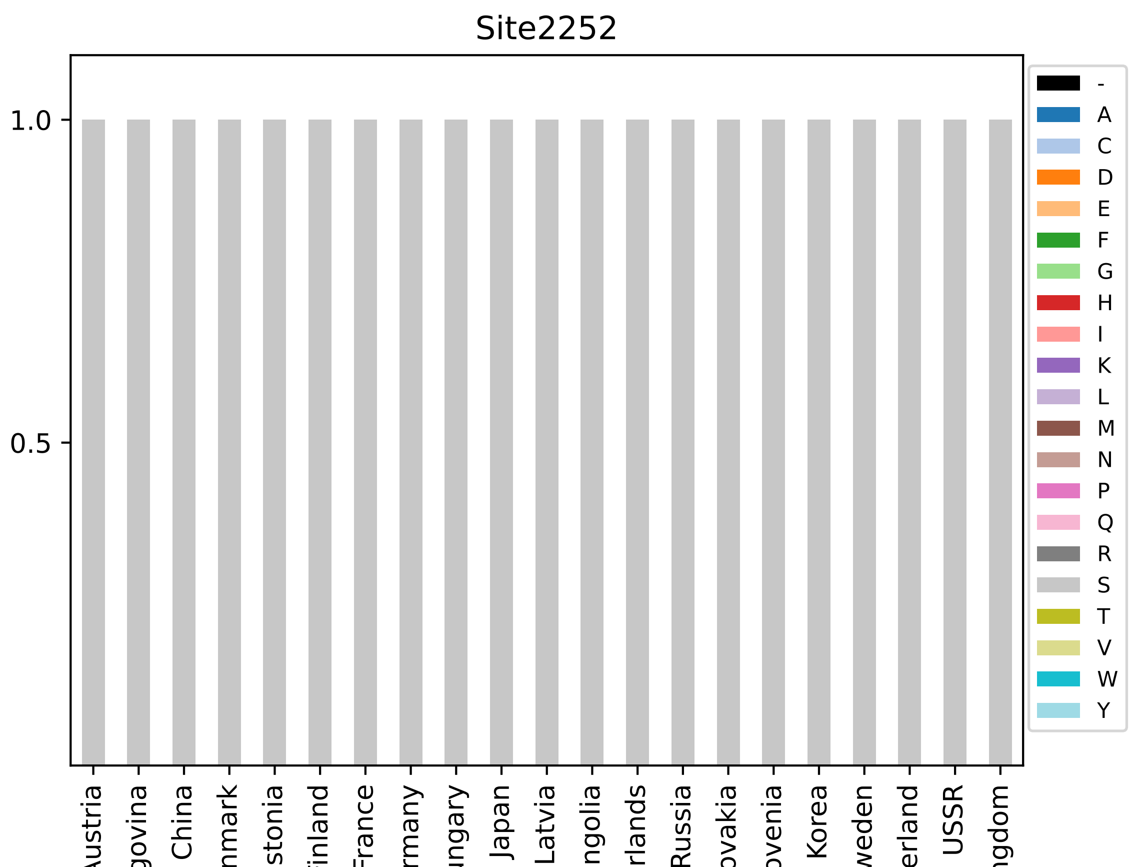
Site2252
1.0
0.5
-
A
C
D
E
F
G
H
I
K
L
M
N
P
Q
R
S
T
V
W
Y
China
Japan
Latvia
Korea
USSR

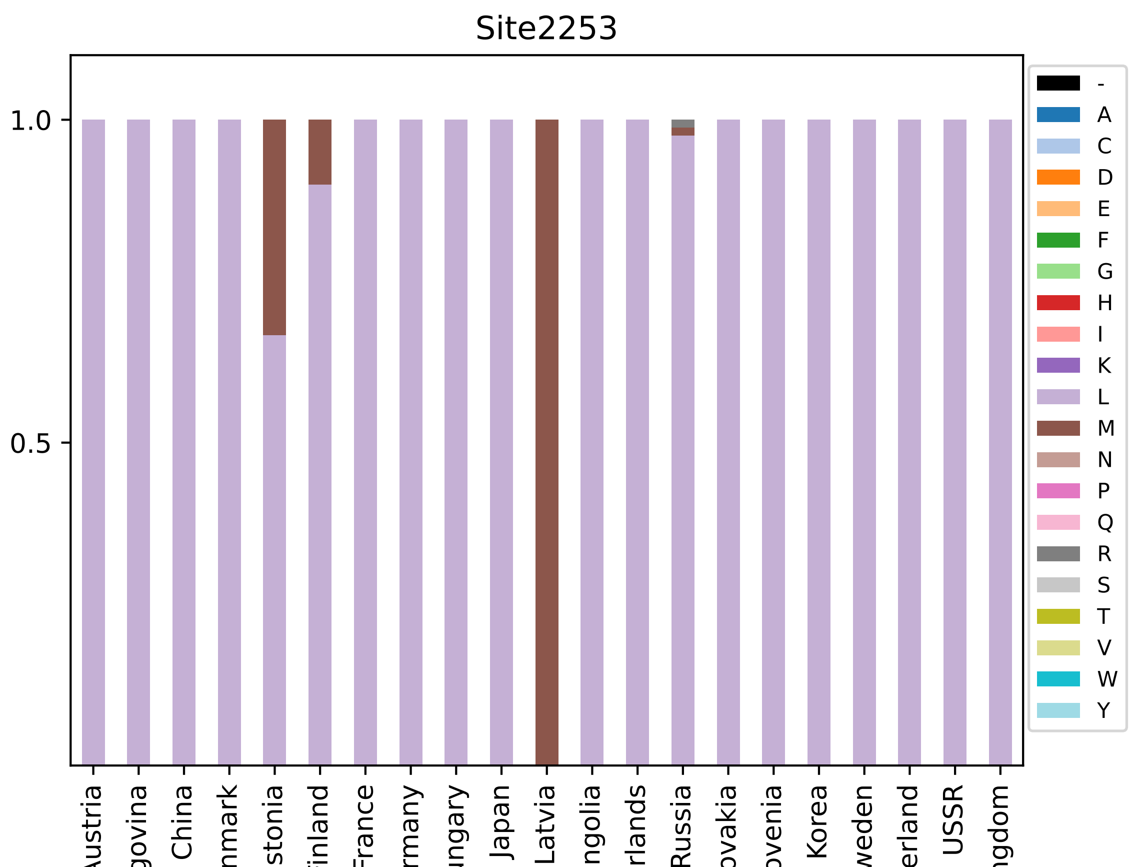
Site2253
1.0
0.5
-
A
C
D
E
F
G
H
I
K
L
M
N
P
Q
R
S
T
V
W
Y
China
Japan
Latvia
Russia
Korea
USSR

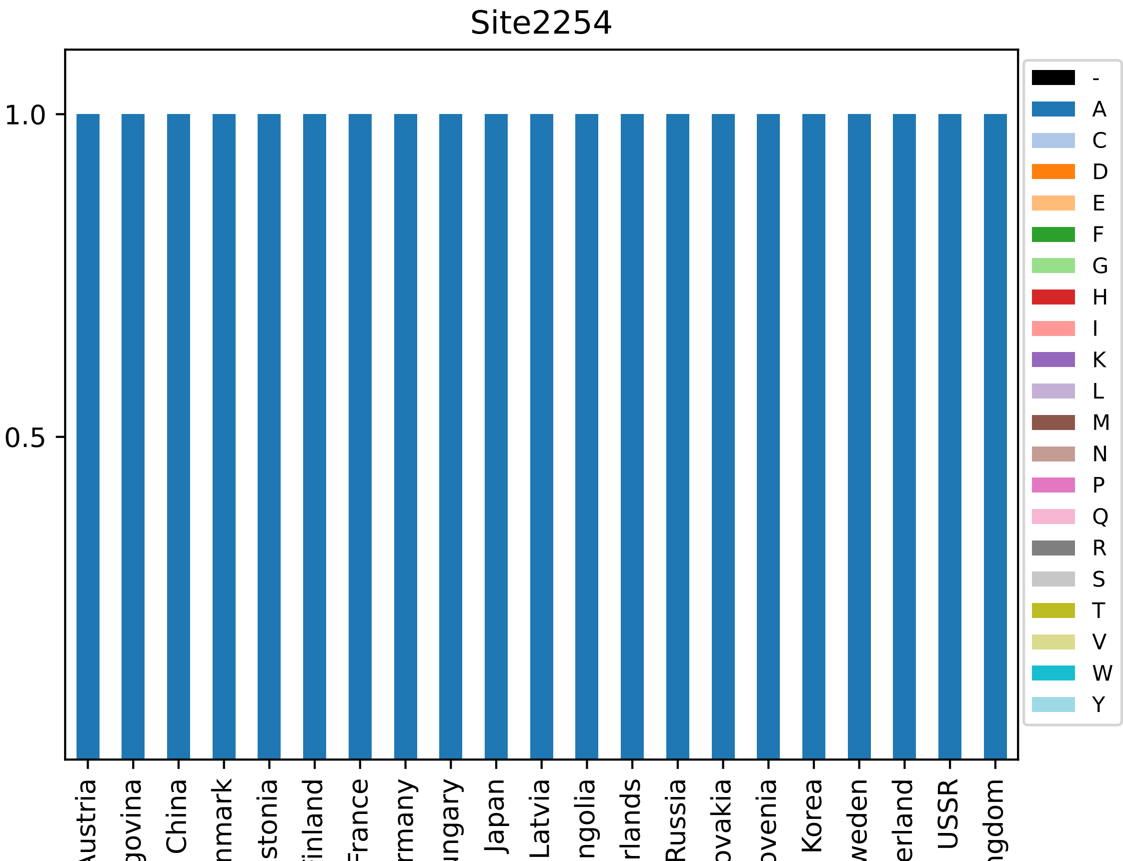

Site2254
1.0
0.5
-
A
C
D
E
F
G
H
I
K
L
M
N
P
Q
R
S
T
V
W
Y
China
Japan
Latvia
Russia
Korea
USSR

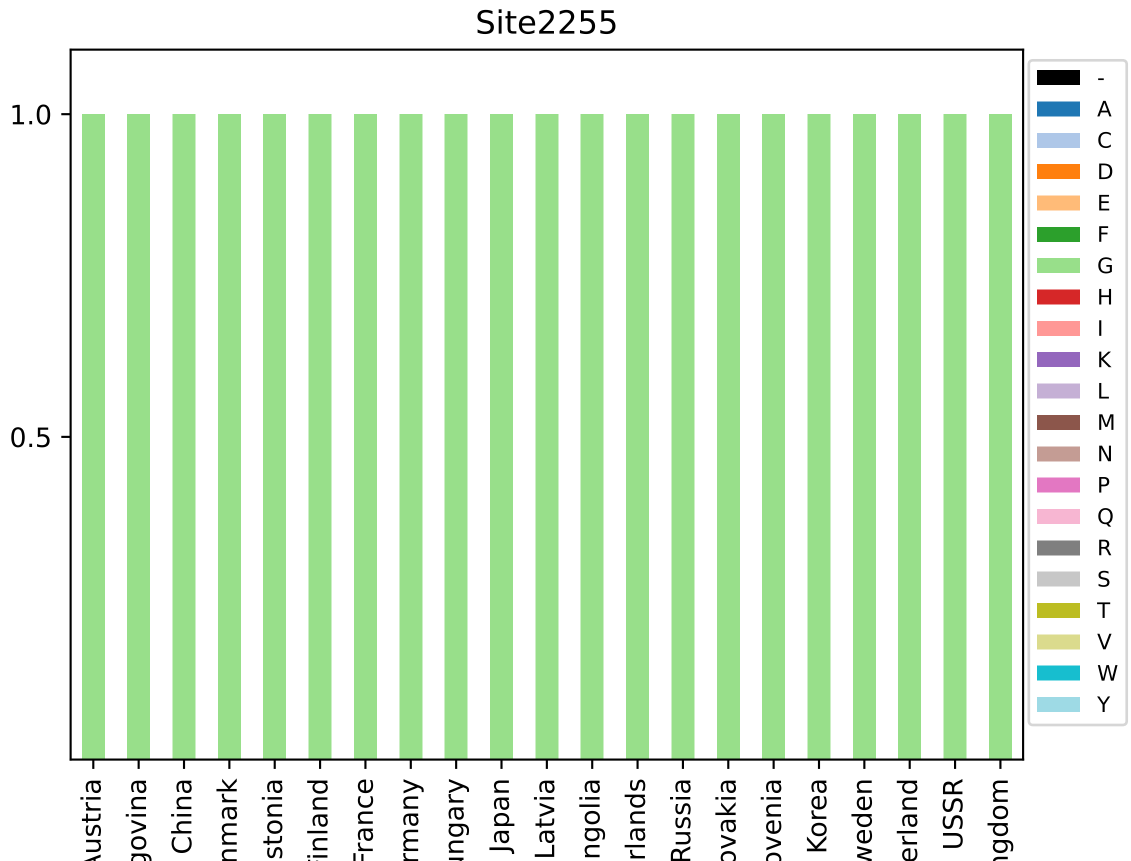
Site2255
1.0
0.5
-
A
C
D
E
F
G
H
I
K
L
M
N
P
Q
R
S
T
V
W
Y
Austria
govina
China
nmark
stonia
Finland
France
rmany
ungary
Japan
Latvia
ngolia
rlands
Russia
ovakia
ovenia
Korea
weden
erland
USSR
ngdom

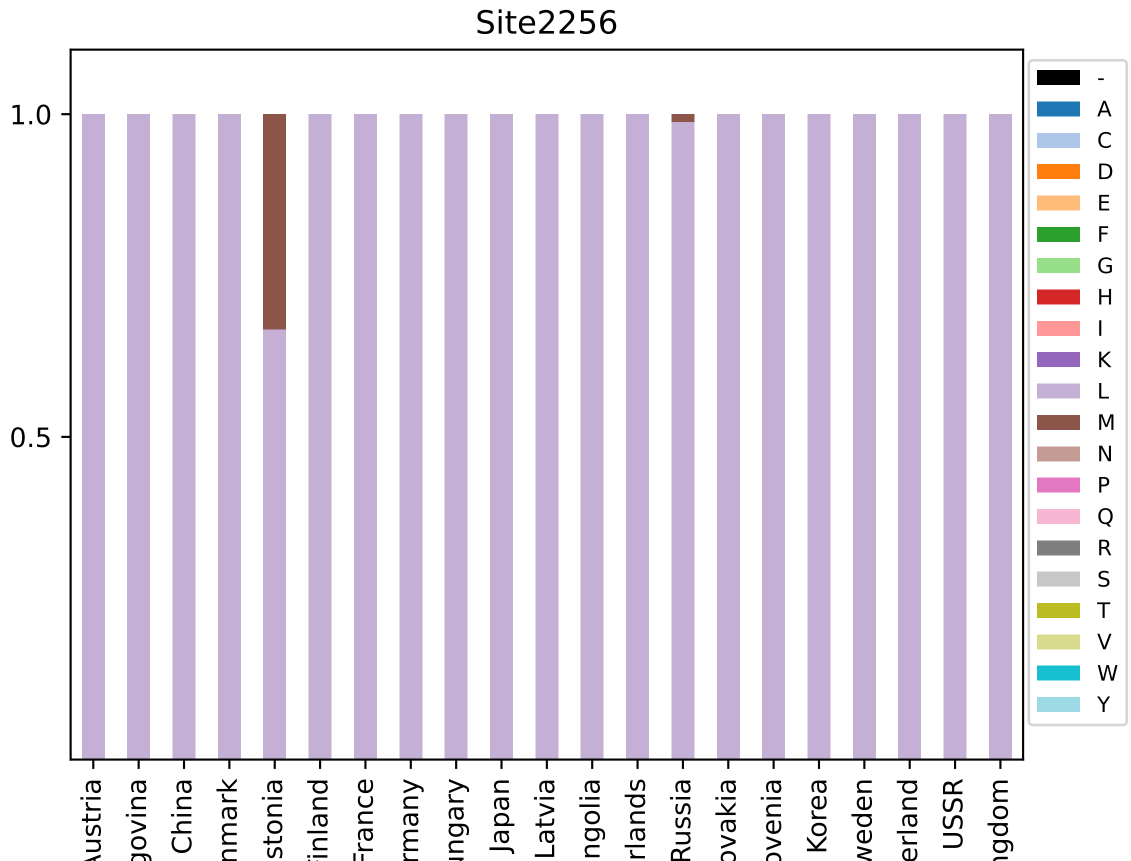
Site2256
1.0
0.5
-
A
C
D
E
F
G
H
I
K
L
M
N
P
Q
R
S
T
V
W
Y
Austria
China
Japan
Latvia
Russia
Korea
USSR

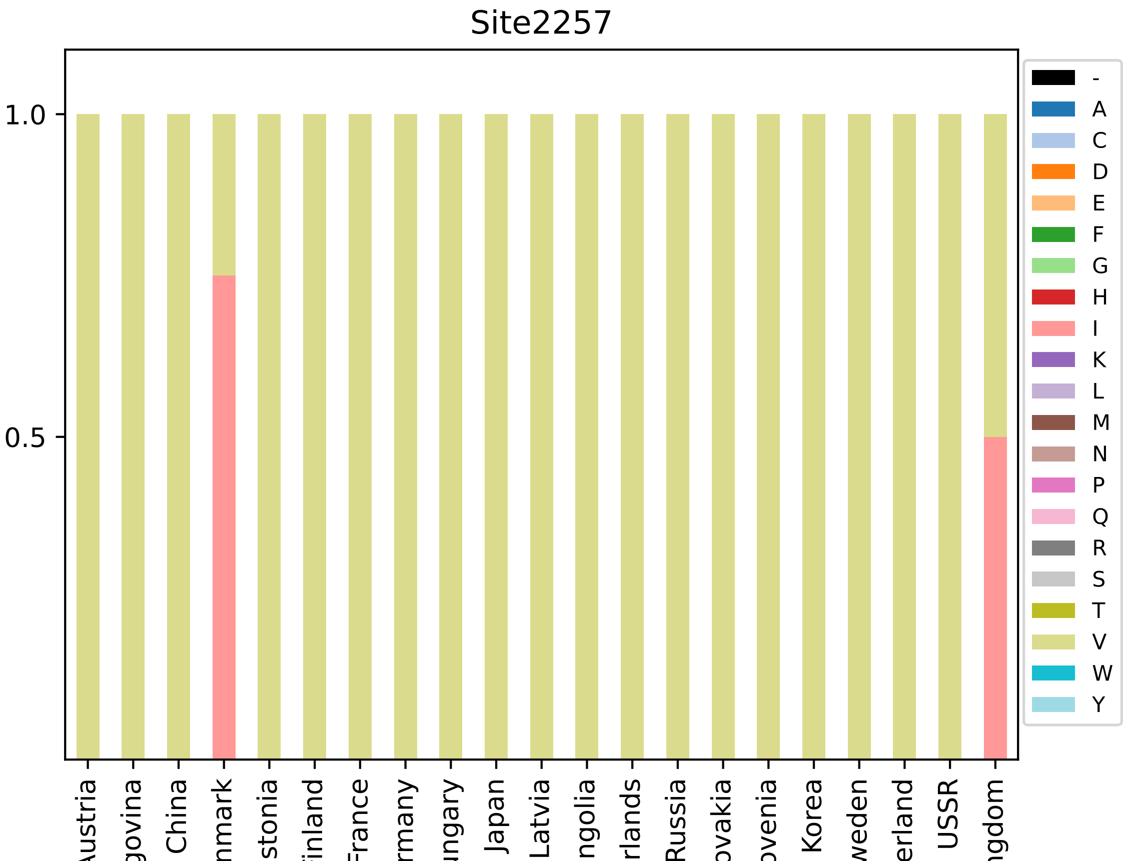
Site2257
1.0
0.5
-
A
C
D
E
F
G
H
I
K
L
M
N
P
Q
R
S
T
V
W
Y
Austria
China
Japan
Latvia
Russia
Korea
USSR

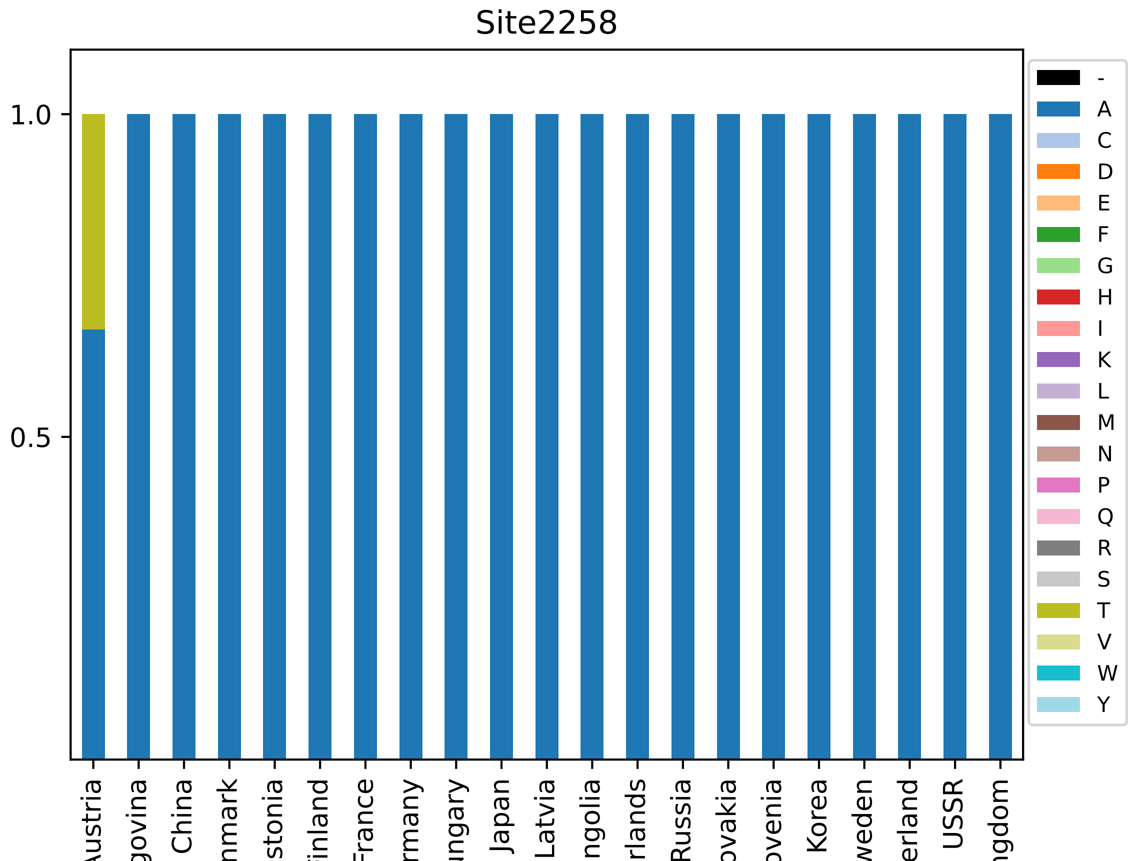

Site2258
1.0
0.5
-
A
C
D
E
F
G
H
I
K
L
M
N
P
Q
R
S
T
V
W
Y
Austria
govina
China
nmark
stonia
inland
France
rmany
ungary
Japan
Latvia
ngolia
rlands
Russia
ovakia
ovenia
Korea
weden
erland
USSR
ngdom

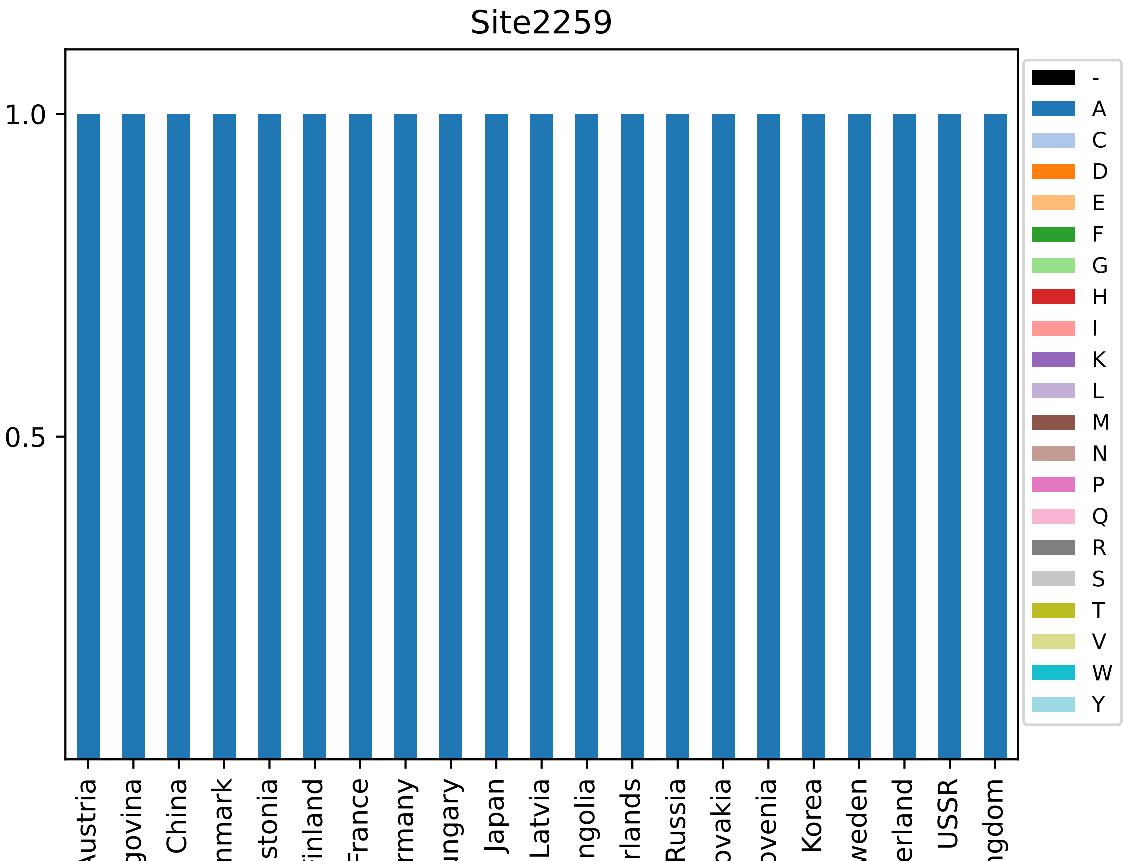

Site2259
1.0
0.5
-
A
C
D
E
F
G
H
I
K
L
M
N
P
Q
R
S
T
V
W
Y
China
Japan
Latvia
Russia
Korea
USSR

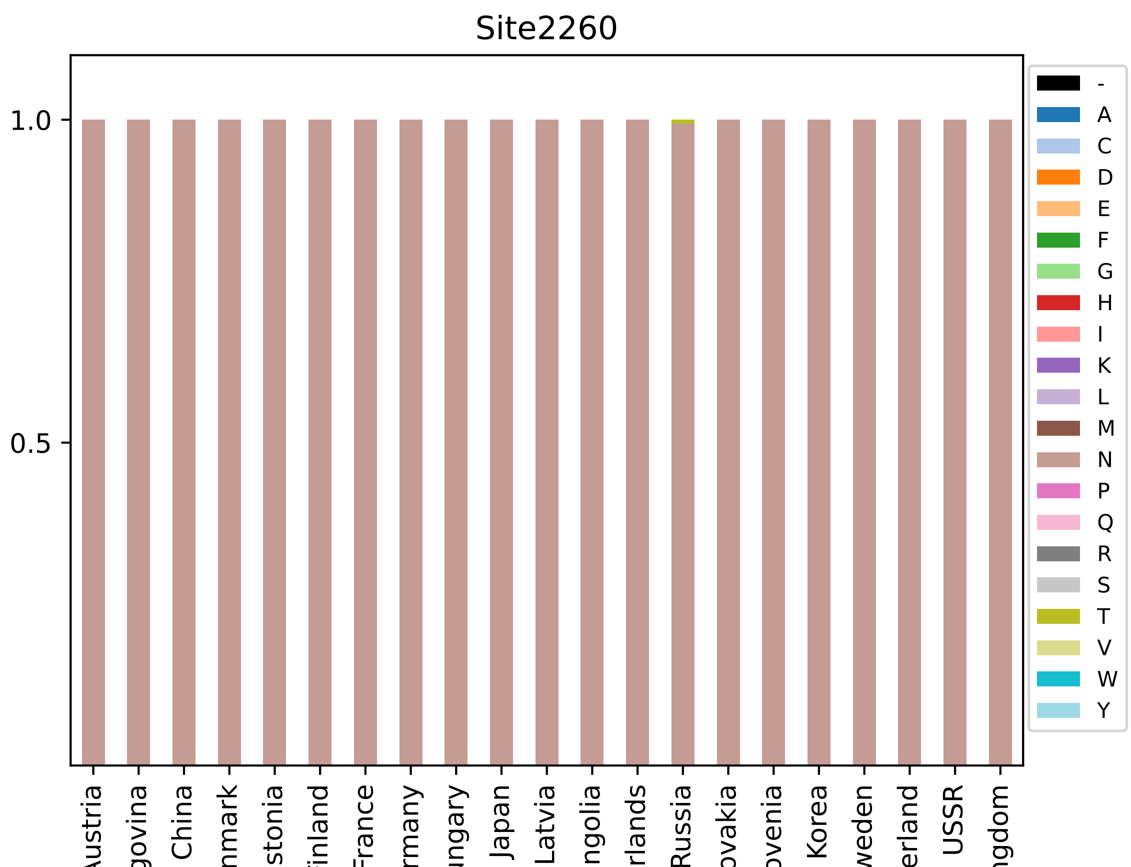
Site2260
1.0
0.5
Austria
govina
China
nmark
stonia
Finland
France
rmany
ungary
Japan
Latvia
ngolia
rlands
Russia
ovakia
ovenia
Korea
weden
erland
USSR
ngdom
-
A
C
D
E
F
G
H
I
K
L
M
N
P
Q
R
S
T
V
W
Y

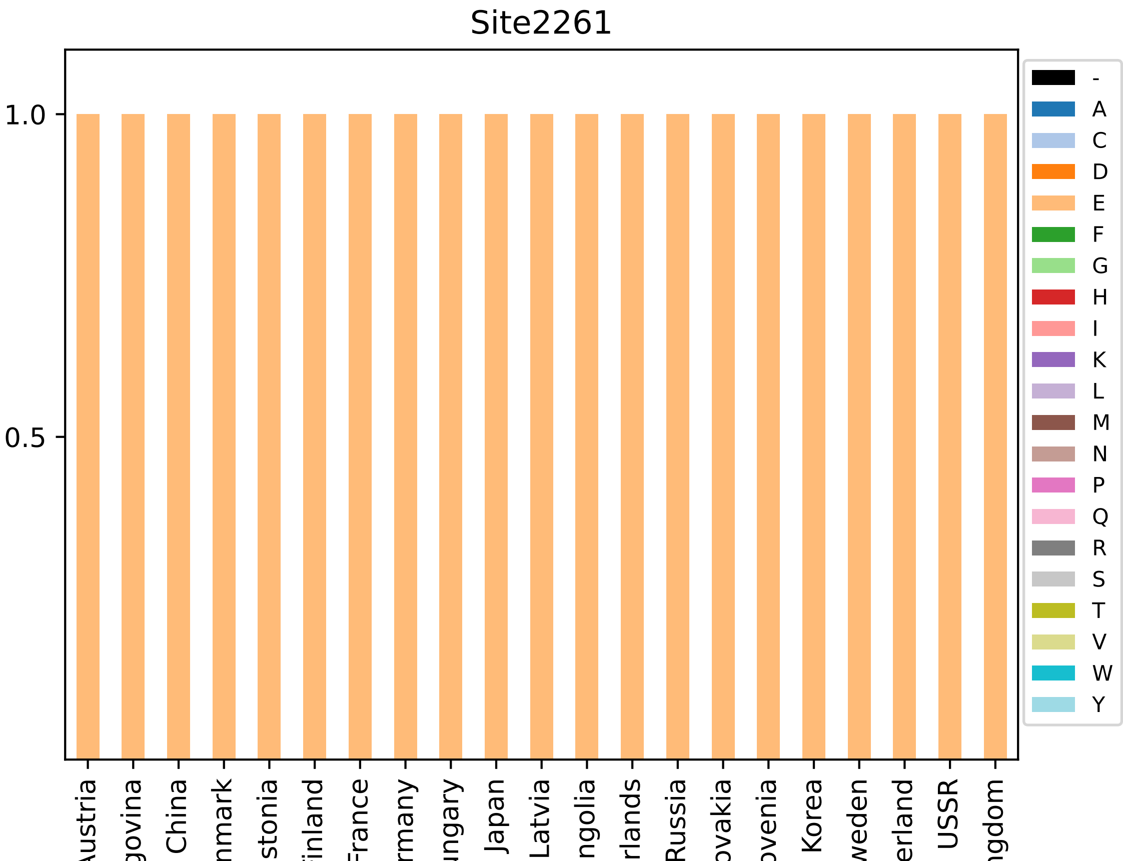
Site2261
1.0
0.5
-
A
C
D
E
F
G
H
I
K
L
M
N
P
Q
R
S
T
V
W
Y
China
Japan
Latvia
Russia
Korea
USSR

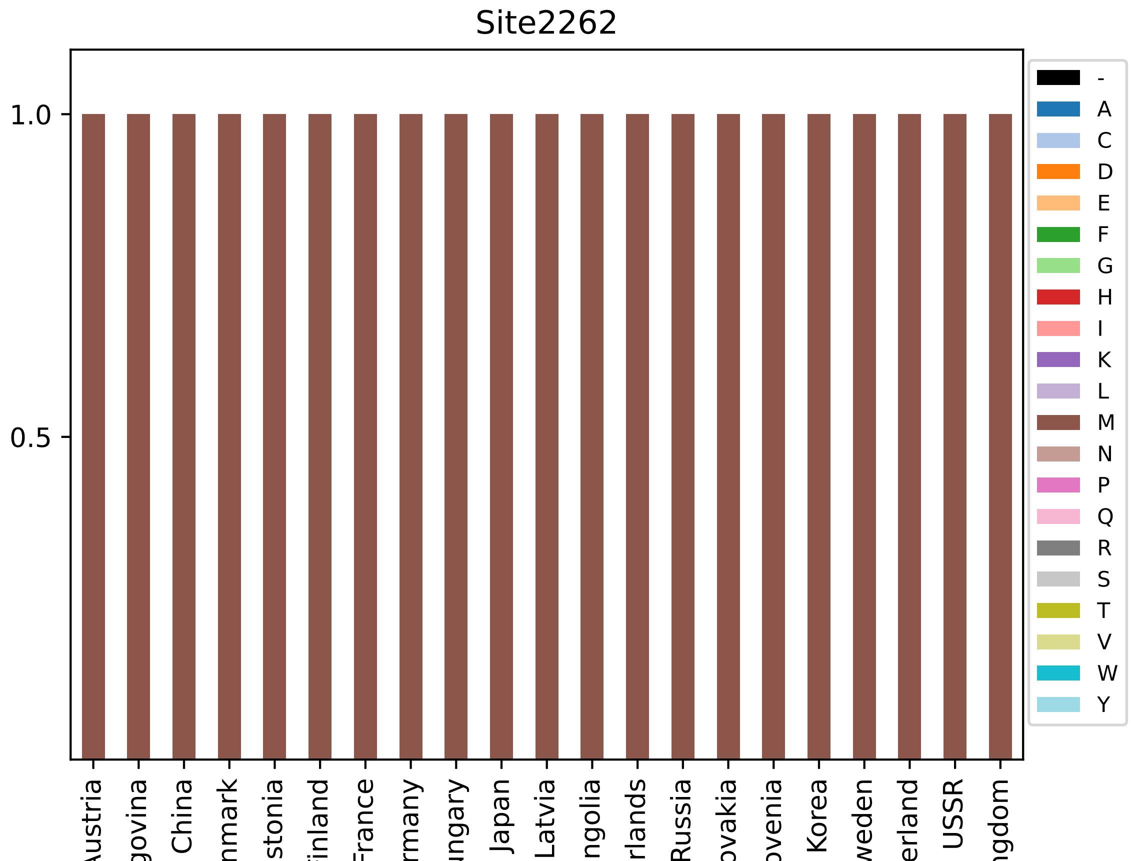
Site2262
1.0
0.5
-
A
C
D
E
F
G
H
I
K
L
M
N
P
Q
R
S
T
V
W
Y
Austria
China
Japan
Latvia
Russia
Korea
USSR

Site2263

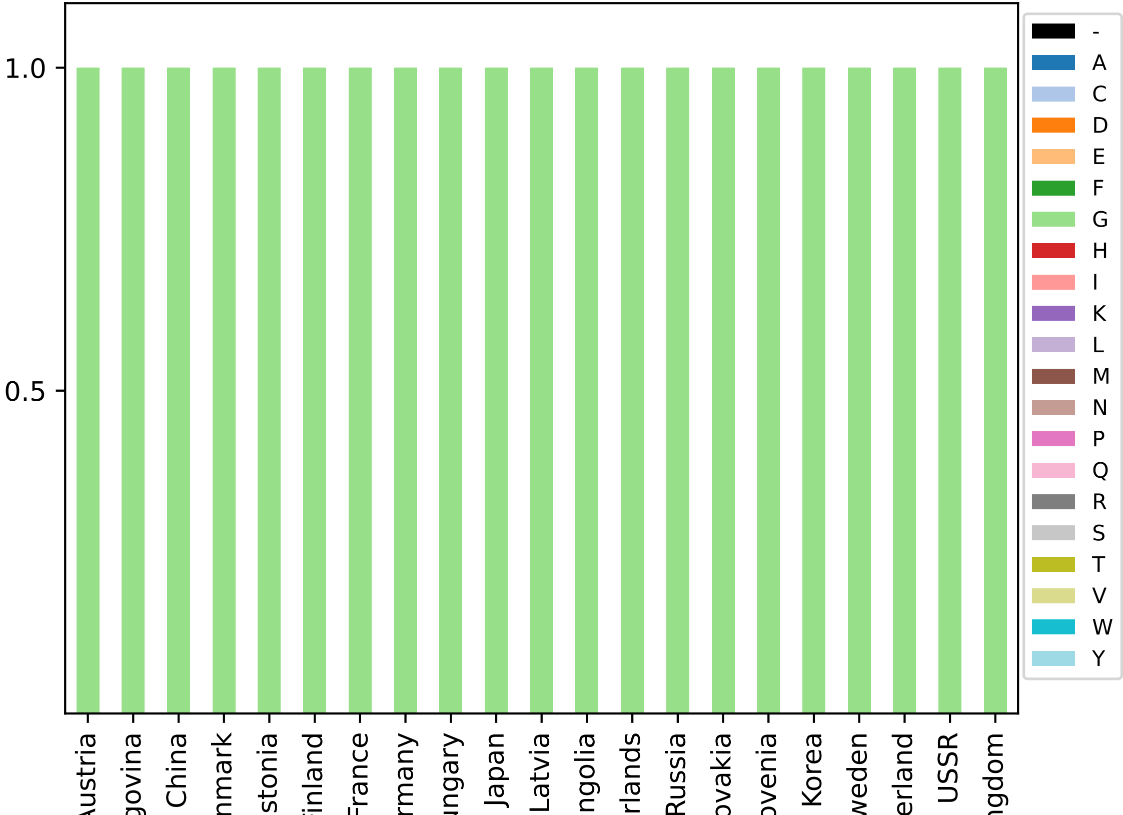
1.0
0.5
-
A
C
D
E
F
G
H
I
K
L
M
N
P
Q
R
S
T
V
W
Y
Austria
govina
China
nmark
stonia
Finland
France
rmany
ungary
Japan
Latvia
ngolia
rlands
Russia
ovakia
ovenia
Korea
weden
erland
USSR
ngdom

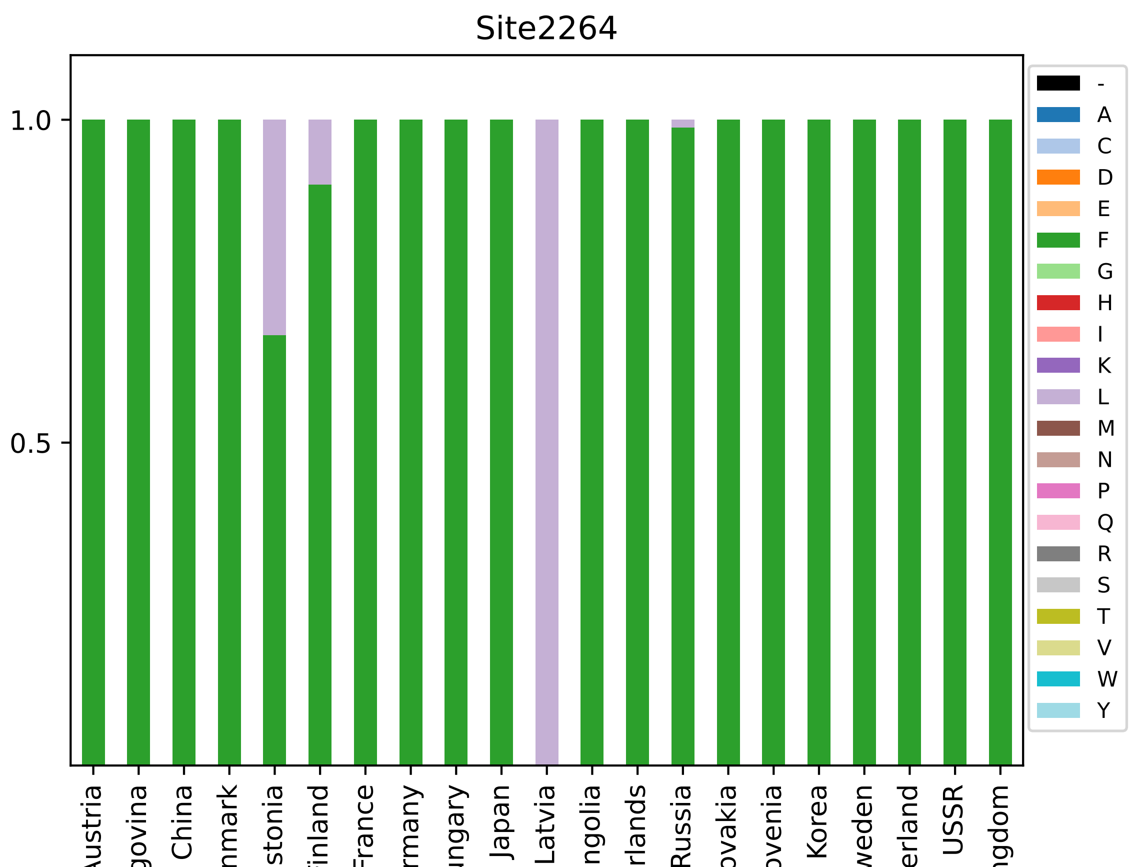
Site2264
1.0
0.5
-
A
C
D
E
F
G
H
I
K
L
M
N
P
Q
R
S
T
V
W
Y
Austria
China
Finland
France
Japan
Latvia
Russia
Korea
USSR

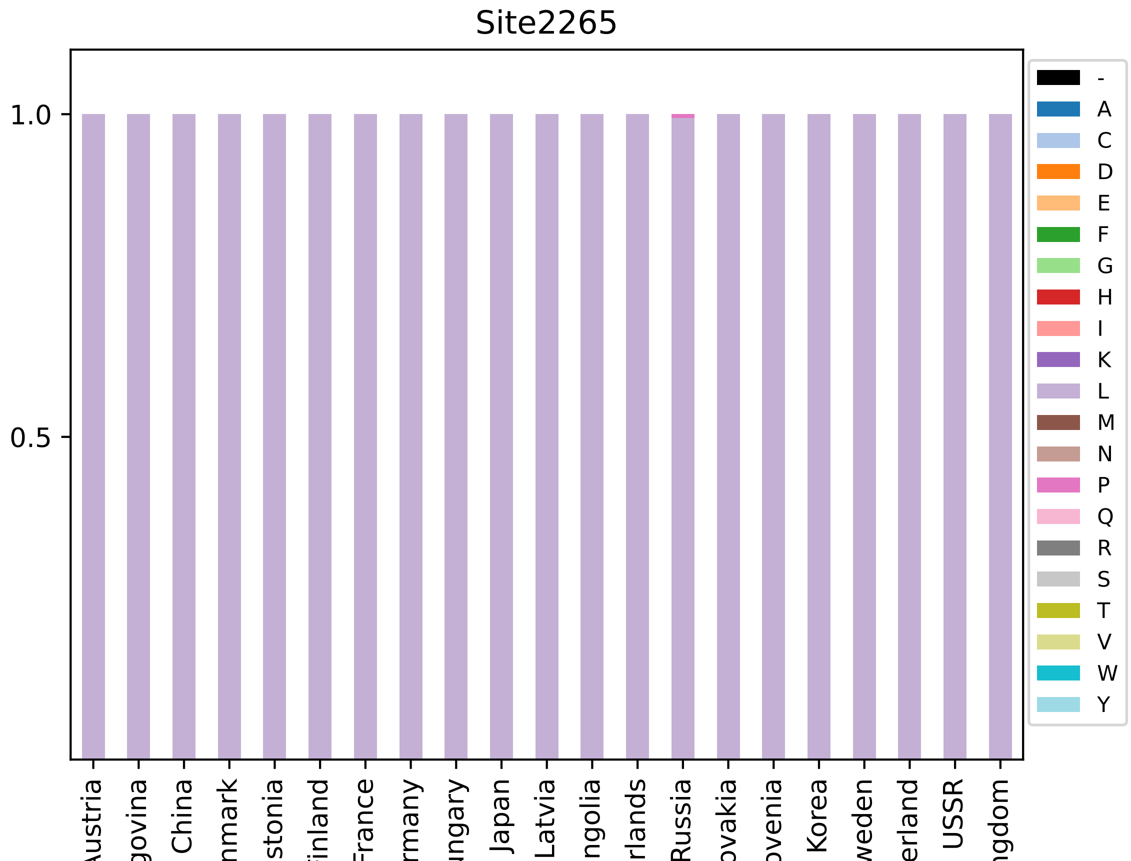

Site2265
1.0
0.5
Austria
govina
China
nmark
stonia
Finland
France
rmany
ungary
Japan
Latvia
ngolia
rlands
Russia
ovakia
ovenia
Korea
weden
erland
USSR
ngdom
-
A
C
D
E
F
G
H
I
K
L
M
N
P
Q
R
S
T
V
W
Y

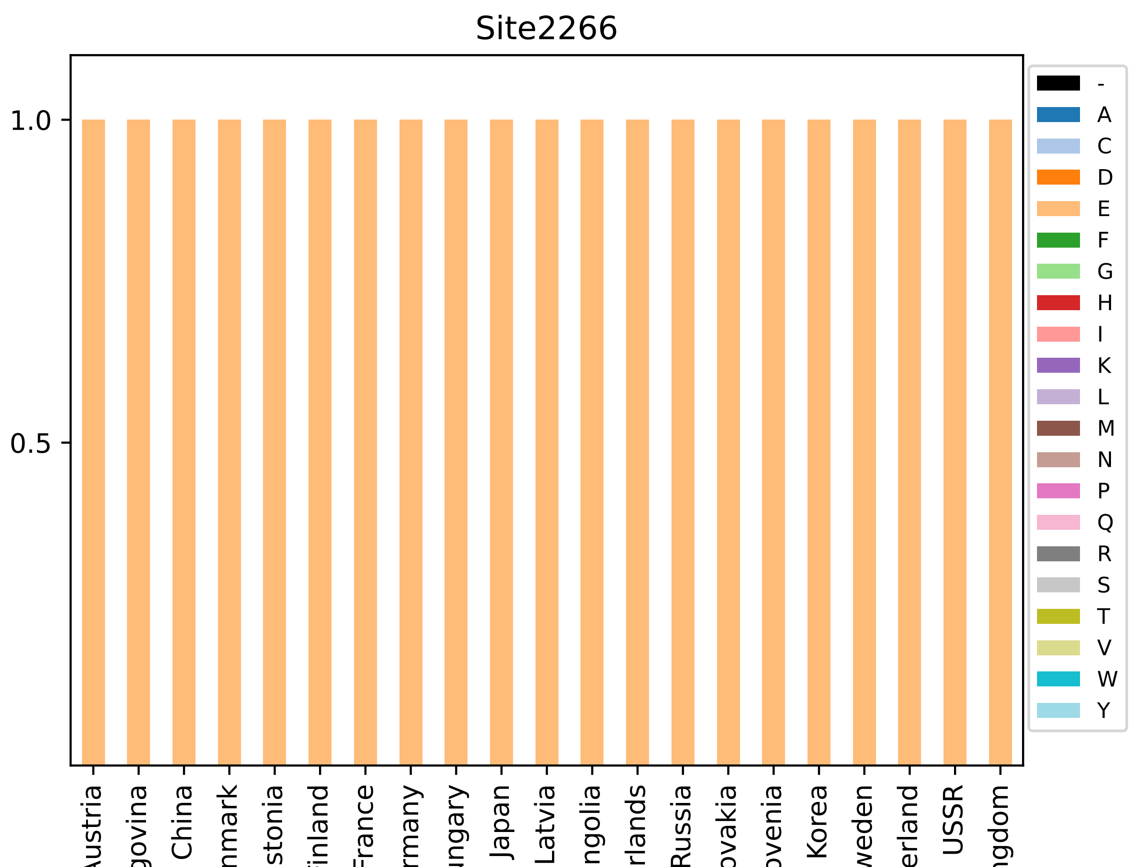
Site2266
1.0
0.5
-
A
C
D
E
F
G
H
I
K
L
M
N
P
Q
R
S
T
V
W
Y
China
Japan
Latvia
Russia
Korea
USSR

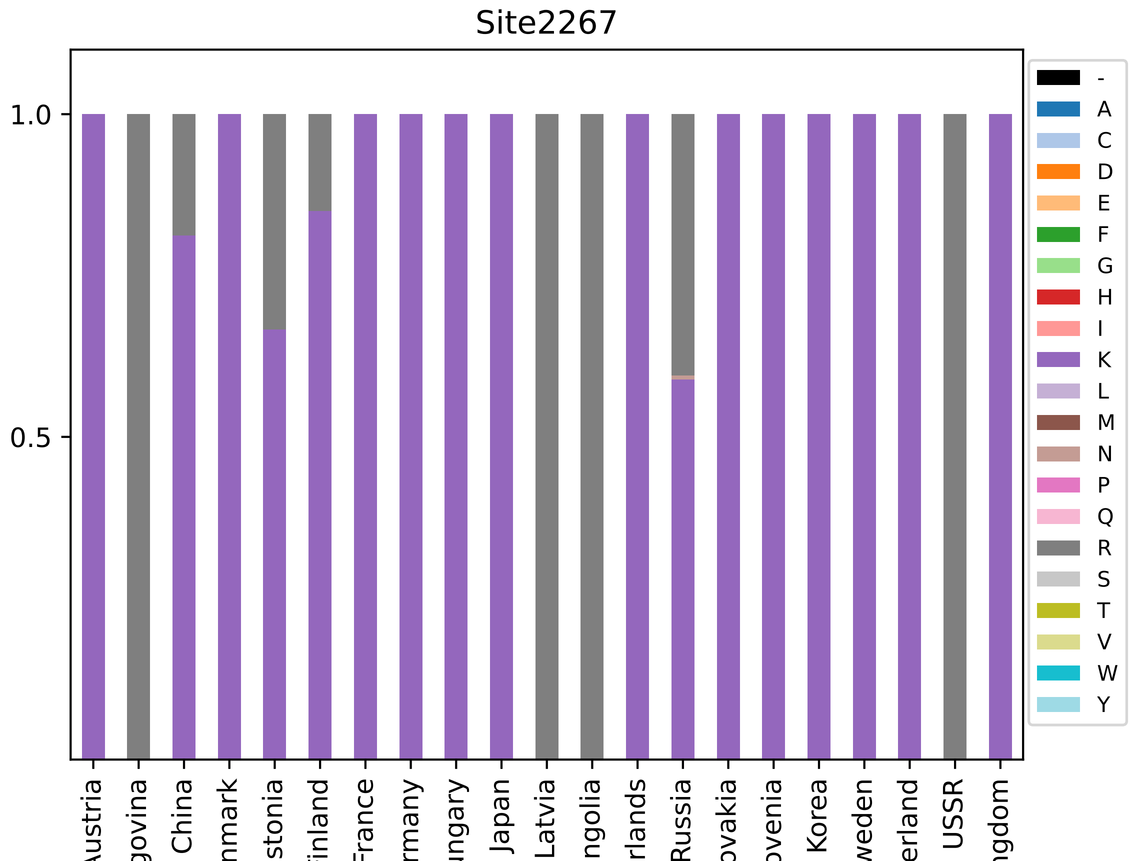
Site2267
1.0
0.5
-
A
C
D
E
F
G
H
I
K
L
M
N
P
Q
R
S
T
V
W
Y
Austria
govina
China
nmark
stonia
inland
France
rmany
ungary
Japan
Latvia
ngolia
rlands
Russia
ovakia
ovenia
Korea
weden
erland
USSR
ngdom

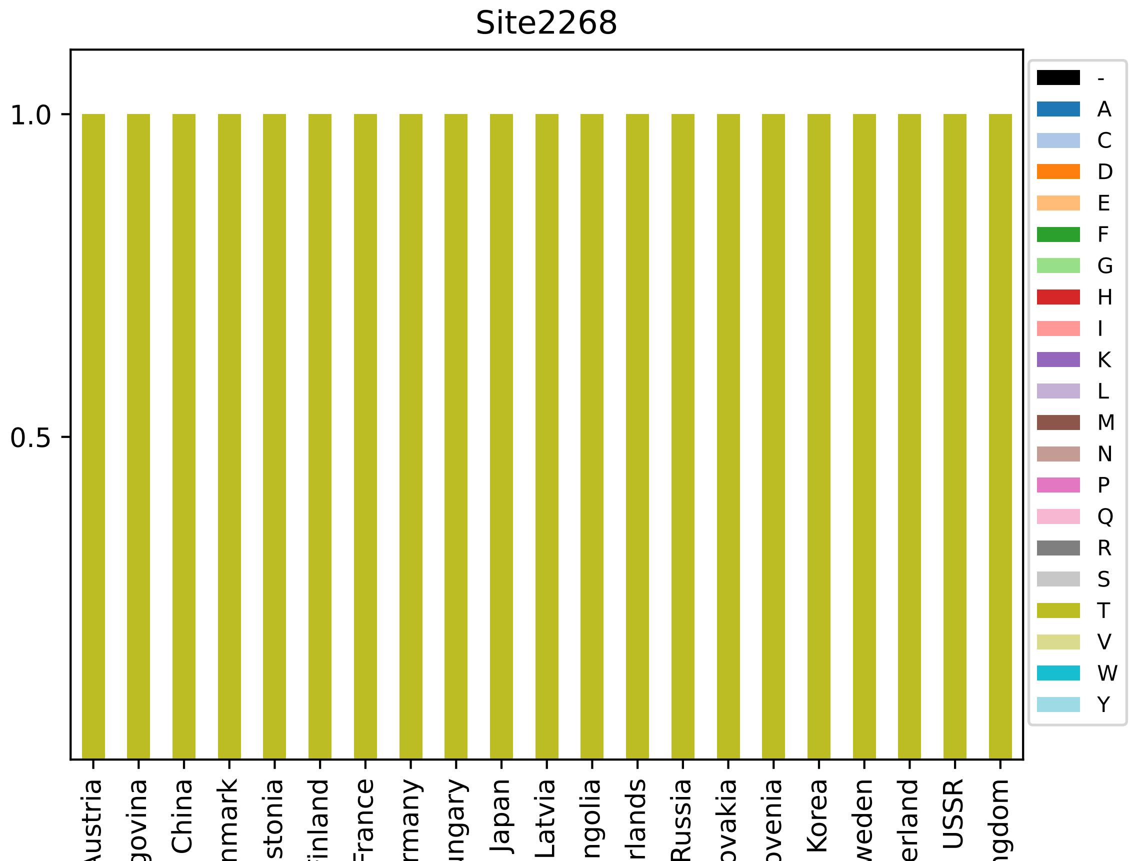

Site2268
1.0
0.5
-
A
C
D
E
F
G
H
I
K
L
M
N
P
Q
R
S
T
V
W
Y
China
Japan
Latvia
Russia
Korea
USSR

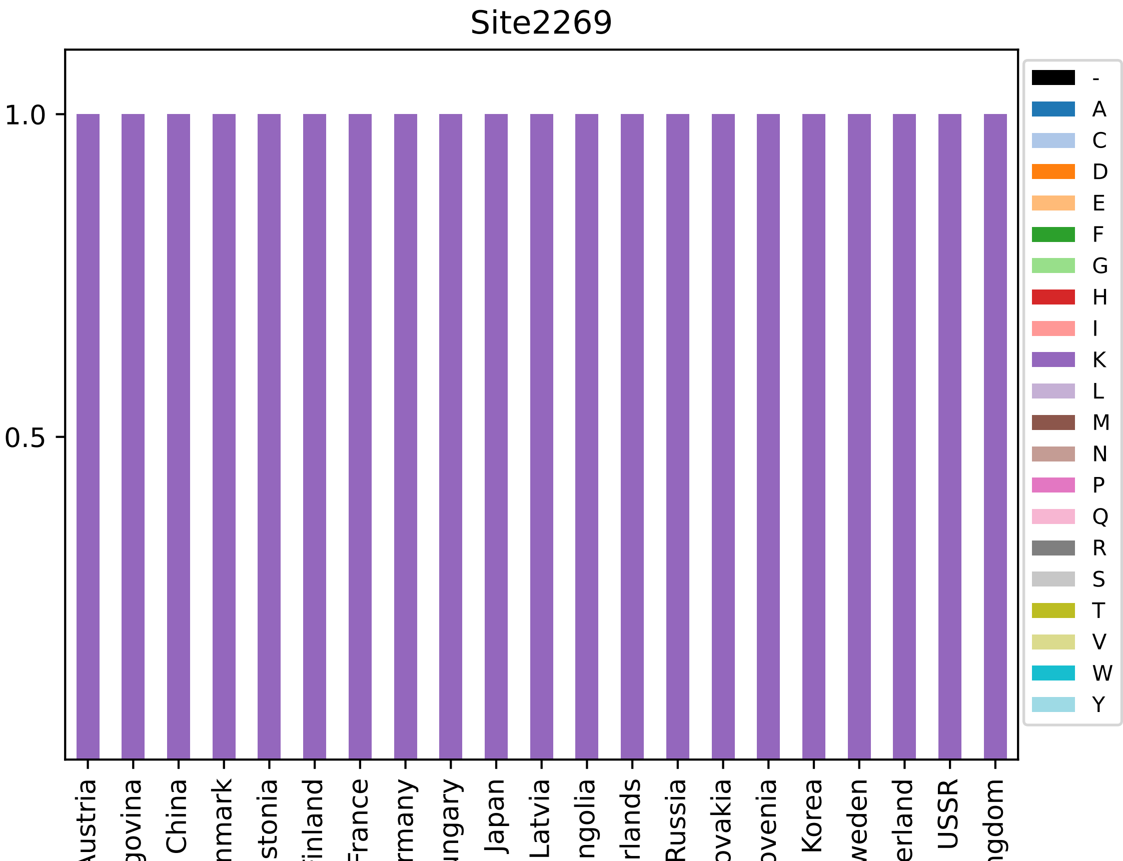
Site2269
1.0
0.5
-
A
C
D
E
F
G
H
I
K
L
M
N
P
Q
R
S
T
V
W
Y
Austria
govina
China
nmark
stonia
inland
France
rmany
ungary
Japan
Latvia
ngolia
rlands
Russia
ovakia
ovenia
Korea
weden
erland
USSR
ngdom

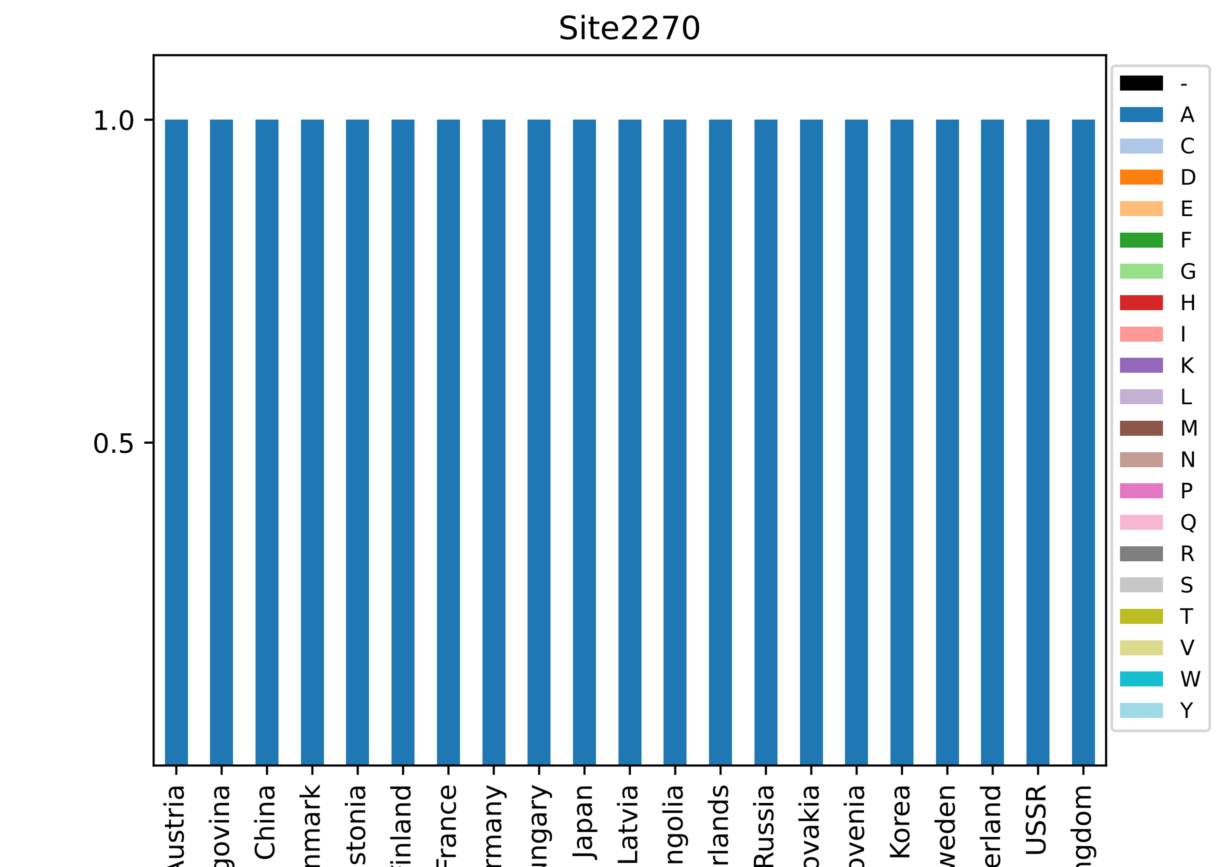

Site2270
1.0
0.5
-
A
C
D
E
F
G
H
I
K
L
M
N
P
Q
R
S
T
V
W
Y
China
Japan
Latvia
Russia
Korea
USSR

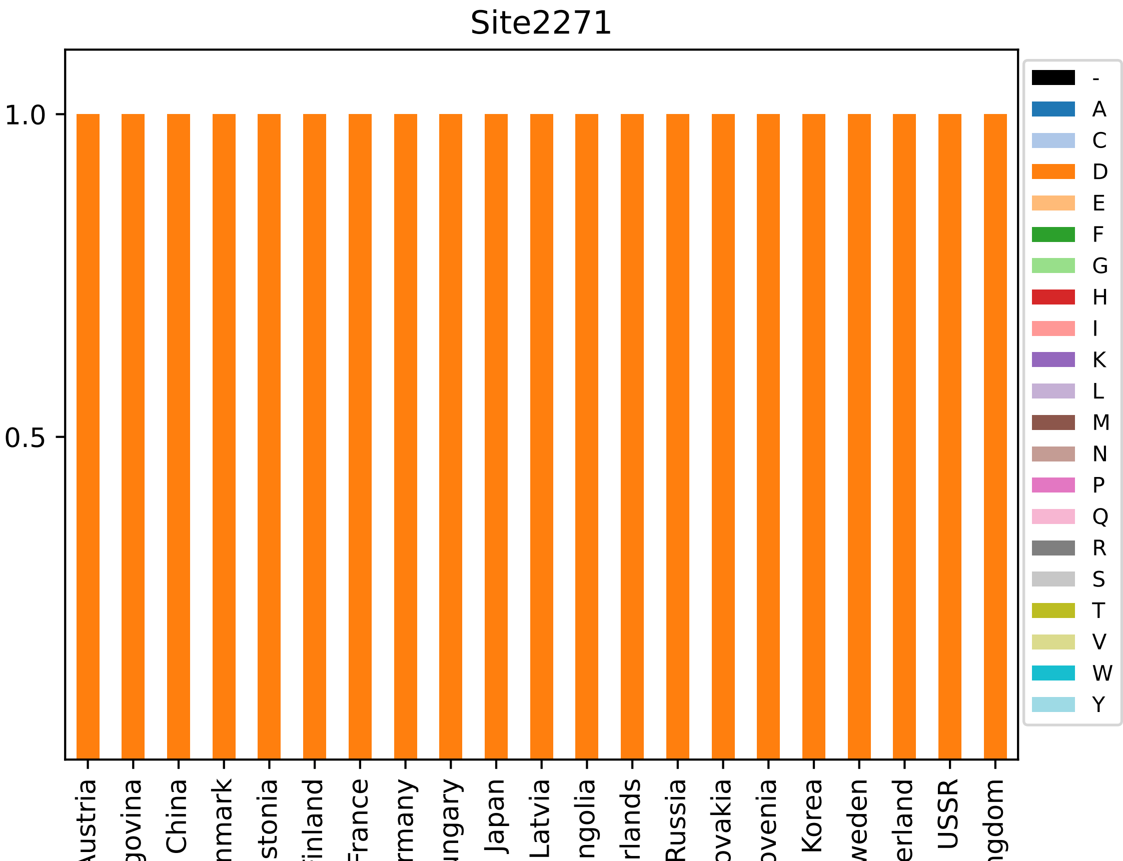
Site2271
1.0
0.5
-
A
C
D
E
F
G
H
I
K
L
M
N
P
Q
R
S
T
V
W
Y
China
Japan
Latvia
Russia
Korea
USSR

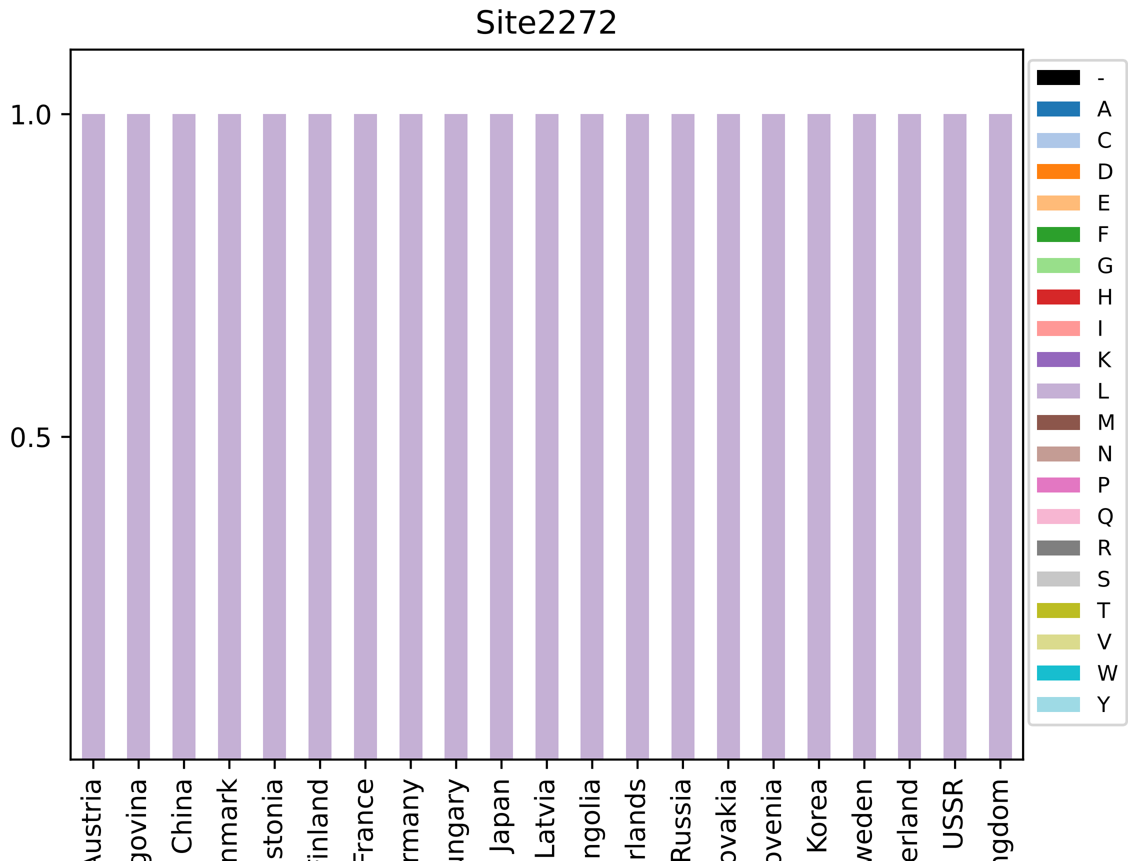
Site2272
1.0
0.5
China
Japan
Latvia
Russia
Korea
USSR
-
A
C
D
E
F
G
H
I
K
L
M
N
P
Q
R
S
T
V
W
Y

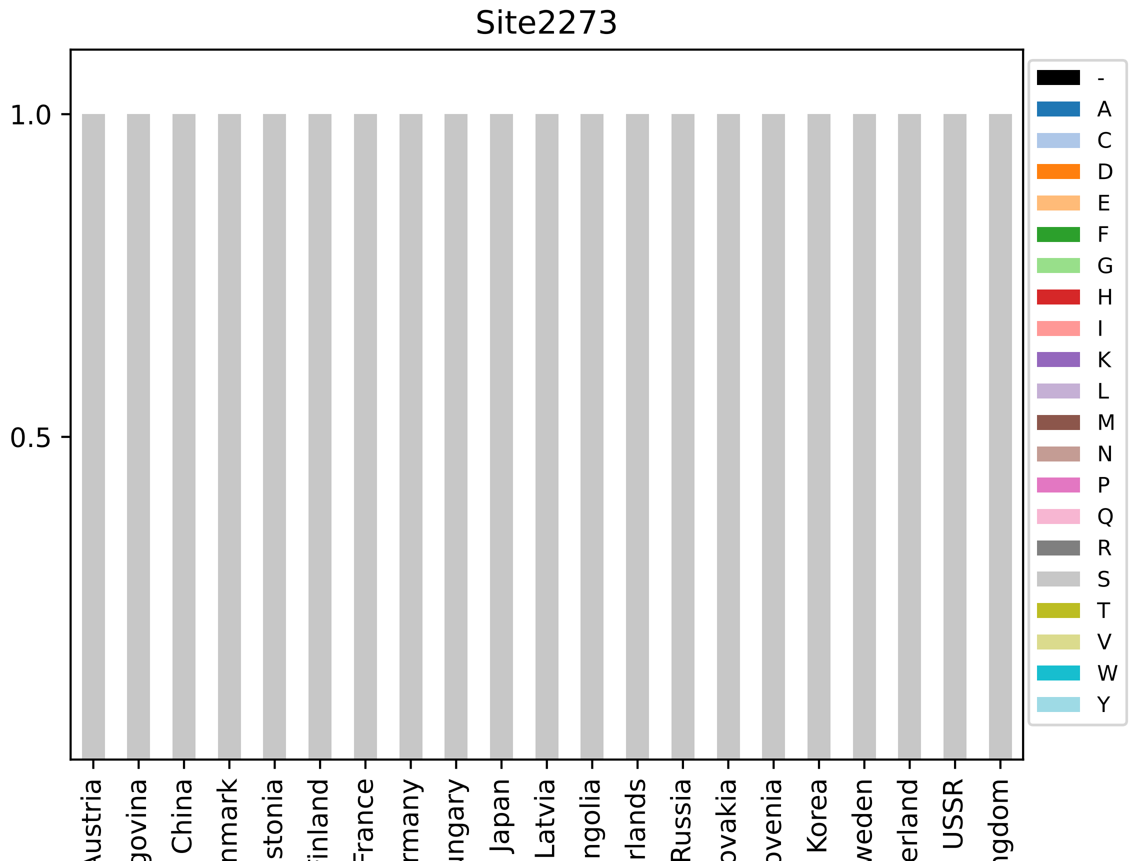
Site2273
1.0
0.5
-
A
C
D
E
F
G
H
I
K
L
M
N
P
Q
R
S
T
V
W
Y
Austria
China
Japan
Latvia
Russia
Korea
USSR

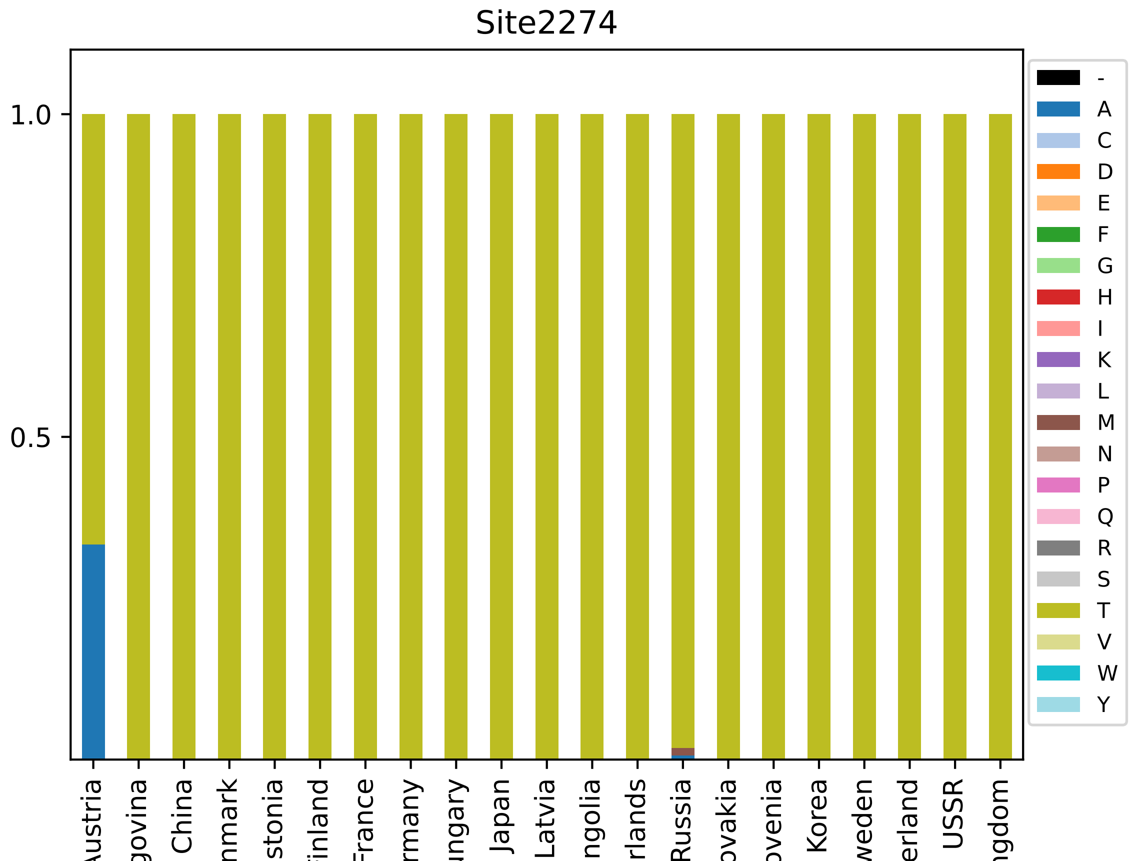
Site2274
1.0
0.5
-
A
C
D
E
F
G
H
I
K
L
M
N
P
Q
R
S
T
V
W
Y
China
Japan
Latvia
Russia
Korea
USSR

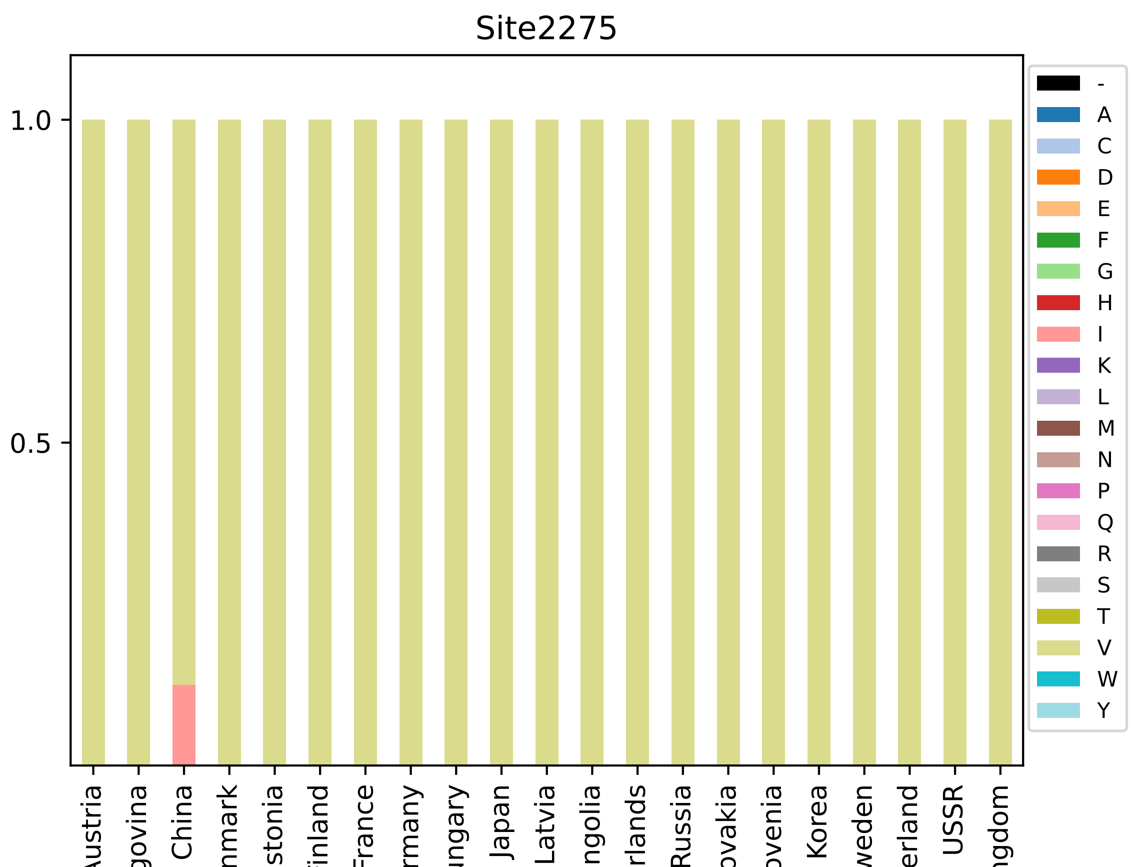

Site2275
1.0
0.5
-
A
C
D
E
F
G
H
I
K
L
M
N
P
Q
R
S
T
V
W
Y
Austria
govina
China
nmark
stonia
inland
France
rmany
ungary
Japan
Latvia
ngolia
rlands
Russia
ovakia
ovenia
Korea
weden
erland
USSR
ngdom

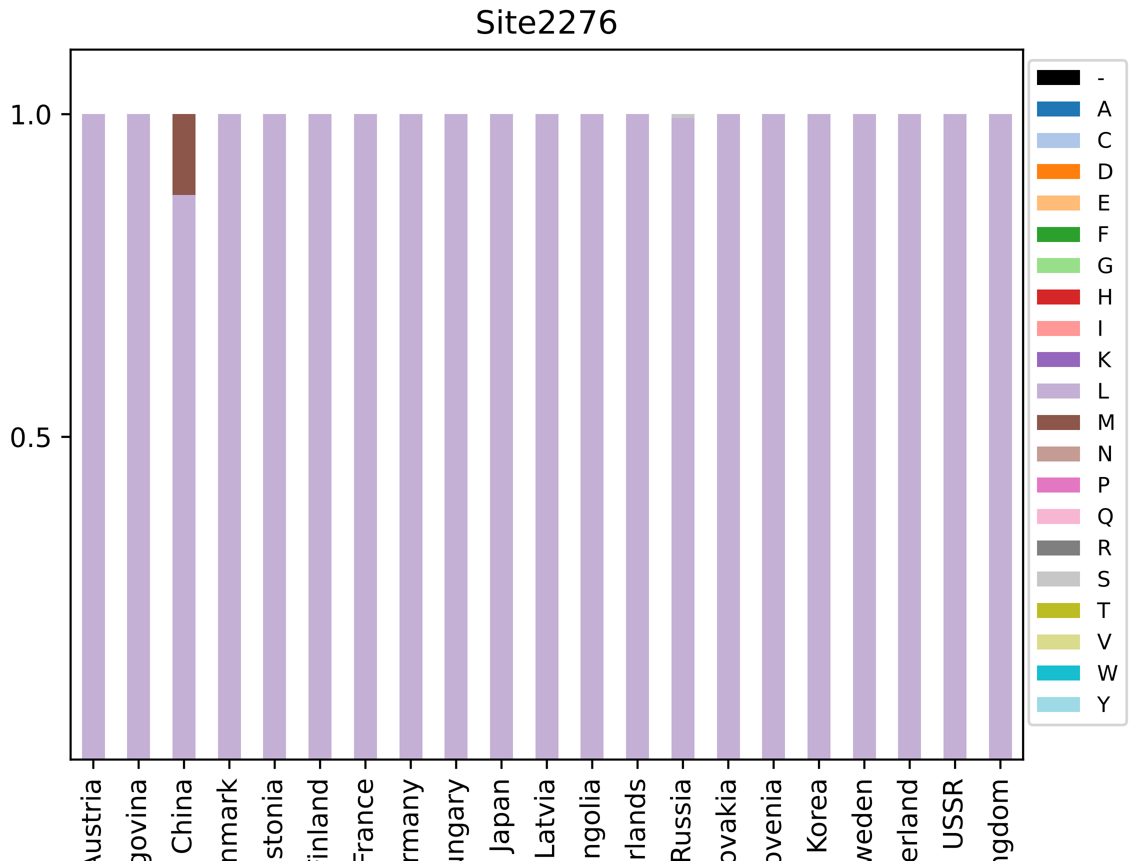
Site2276
1.0
0.5
-
A
C
D
E
F
G
H
I
K
L
M
N
P
Q
R
S
T
V
W
Y
China
Japan
Latvia
Russia
Korea
USSR

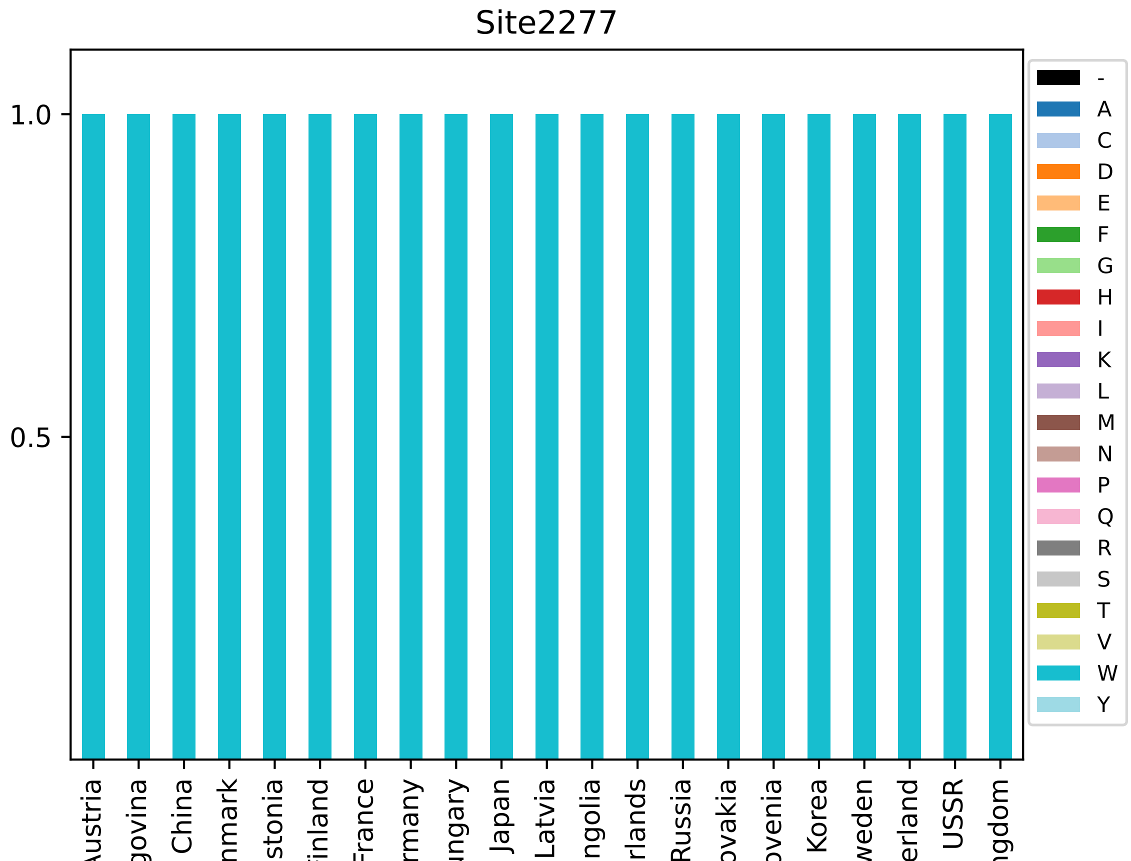
Site2277
1.0
0.5
Austria
govina
China
nmark
stonia
inland
rance
rmany
ungary
Japan
Latvia
ngolia
rlands
Russia
ovakia
ovenia
Korea
weden
erland
USSR
ngdom
-
A
C
D
E
F
G
H
I
K
L
M
N
P
Q
R
S
T
V
W
Y

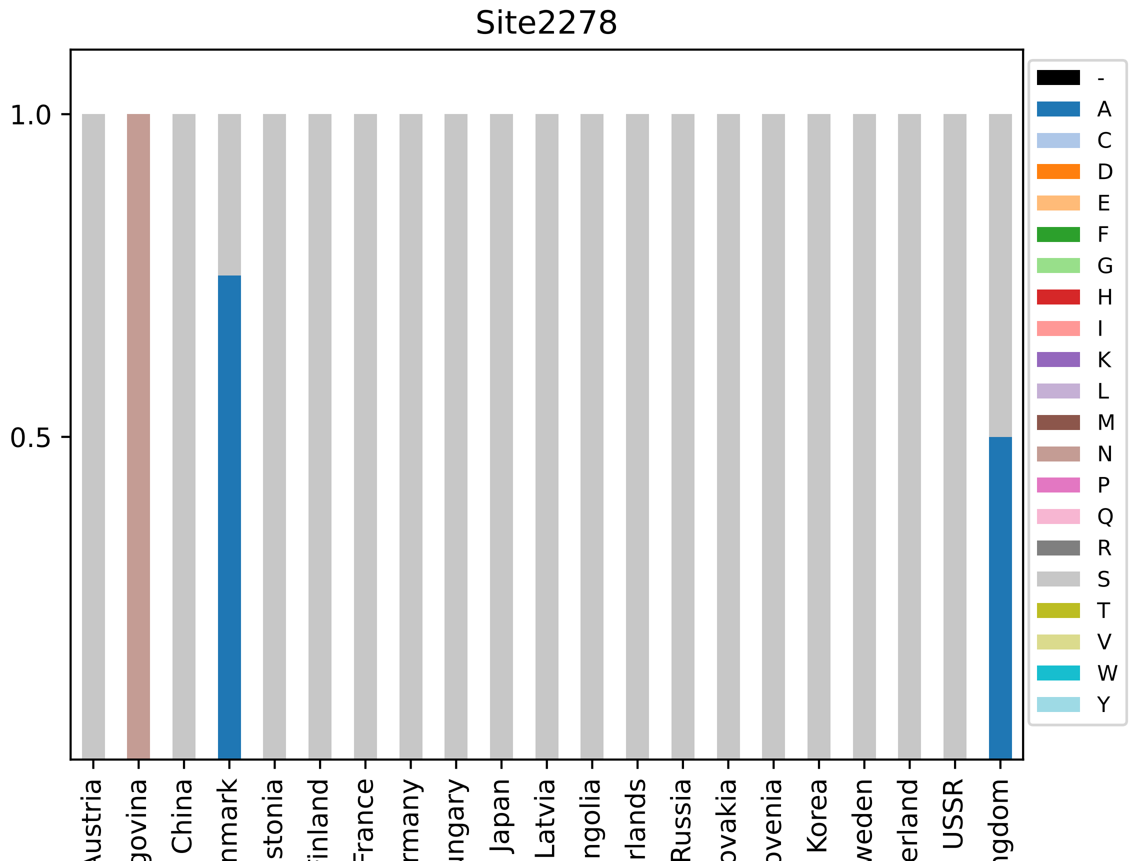

Site2278
1.0
0.5
-
A
C
D
E
F
G
H
I
K
L
M
N
P
Q
R
S
T
V
W
Y
Austria
China
Japan
Latvia
Russia
Korea
USSR

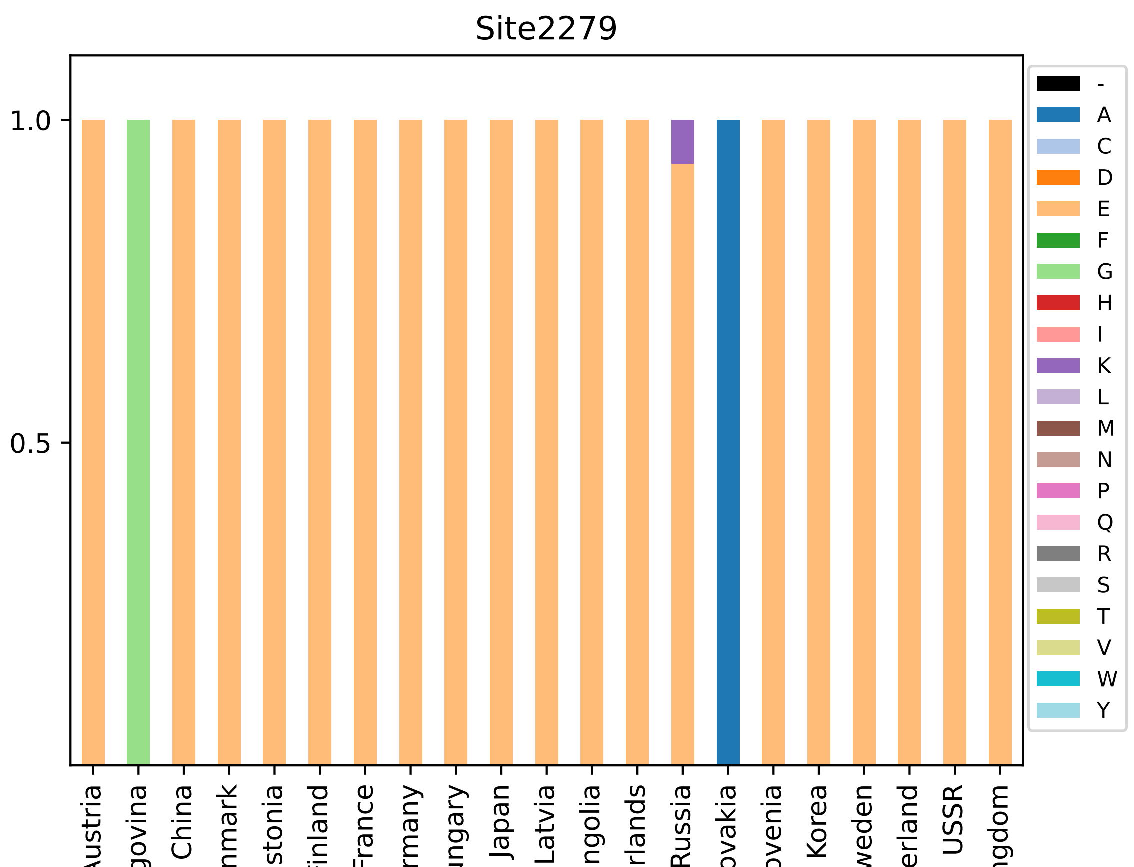
Site2279
1.0
0.5
-
A
C
D
E
F
G
H
I
K
L
M
N
P
Q
R
S
T
V
W
Y
Austria
China
Japan
Latvia
Russia
Korea
USSR

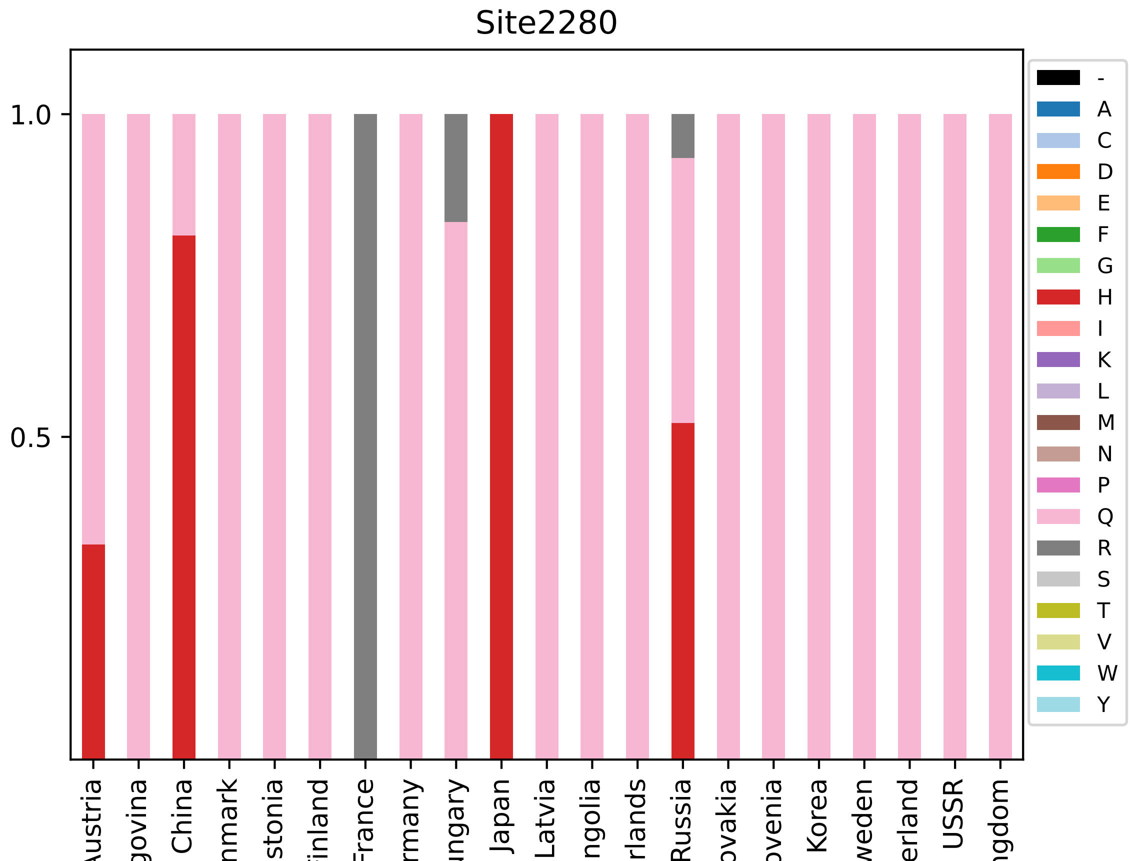
Site2280
1.0
0.5
-
A
C
D
E
F
G
H
I
K
L
M
N
P
Q
R
S
T
V
W
Y
Austria
govina
China
nmark
stonia
inland
France
rmany
ungary
Japan
Latvia
ngolia
rlands
Russia
ovakia
ovenia
Korea
weden
erland
USSR
ngdom

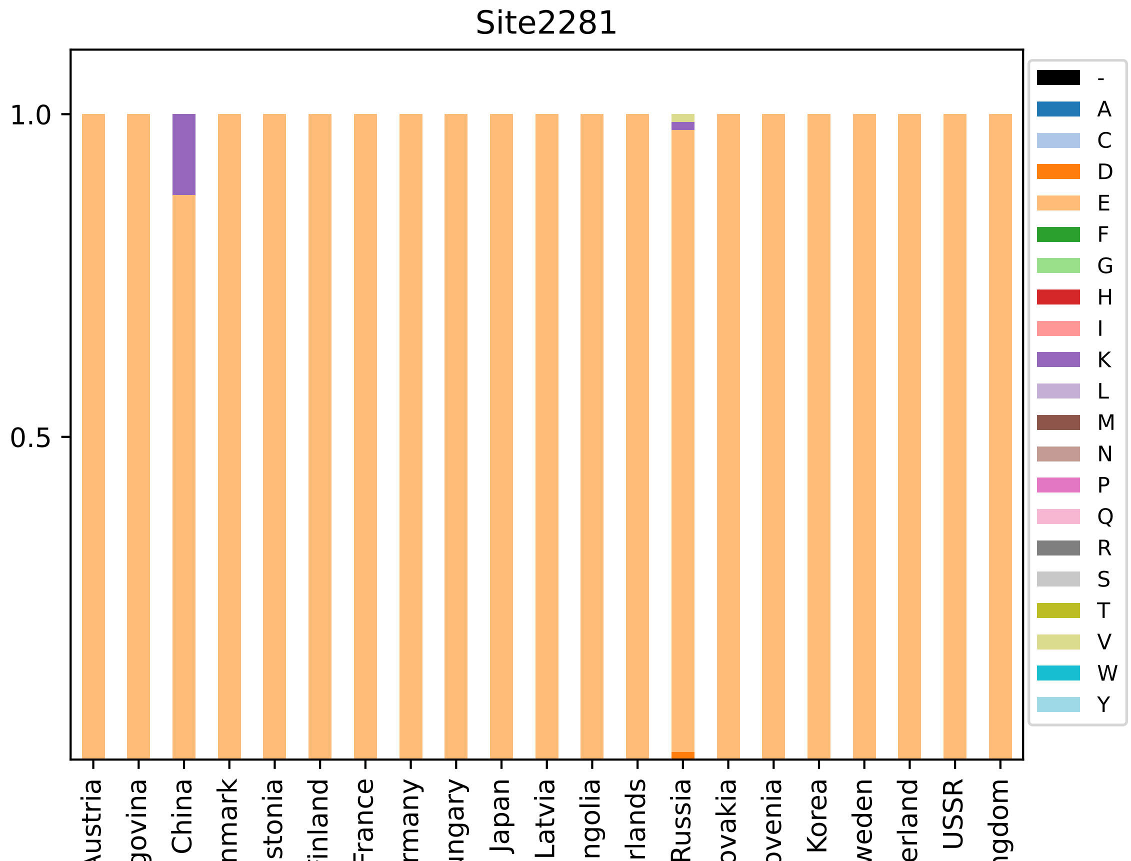
Site2281
1.0
0.5
-
A
C
D
E
F
G
H
I
K
L
M
N
P
Q
R
S
T
V
W
Y
China
Japan
Latvia
Russia
Korea
USSR

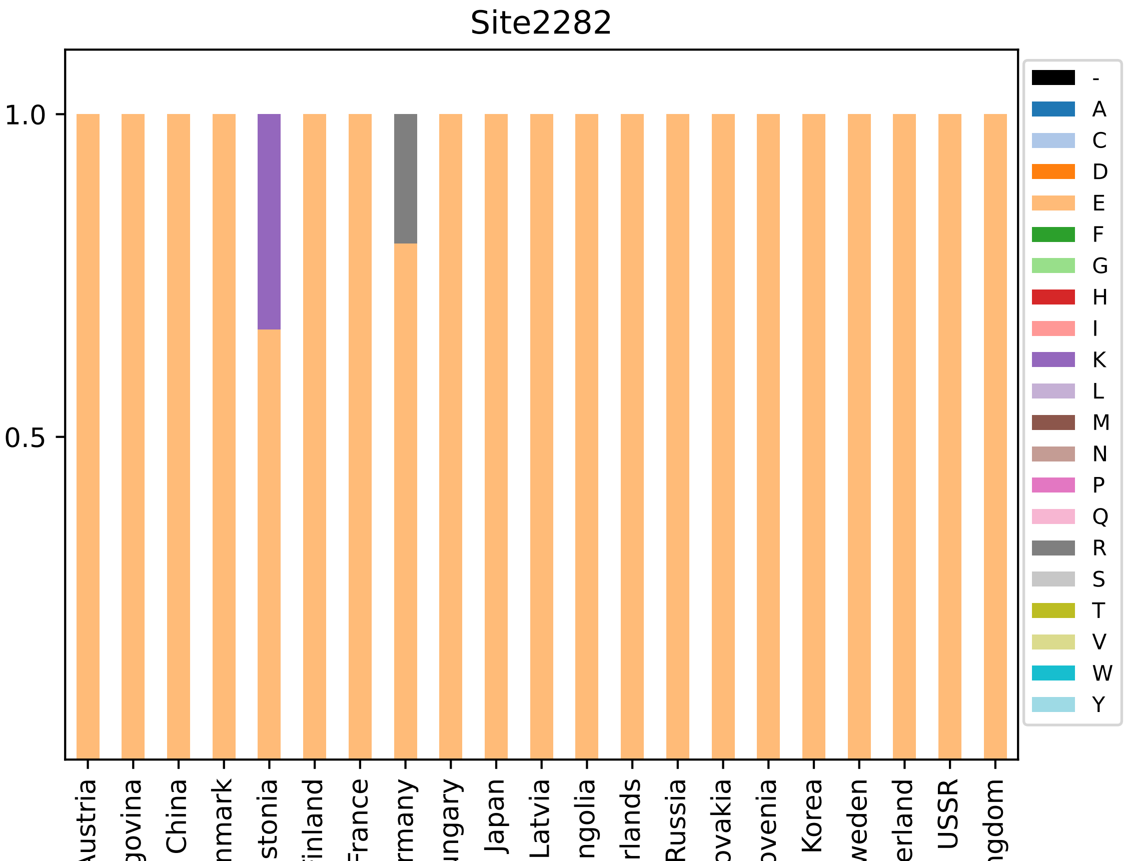

Site2282
1.0
0.5
-
A
C
D
E
F
G
H
I
K
L
M
N
P
Q
R
S
T
V
W
Y
China
Japan
Latvia
Russia
Korea
USSR

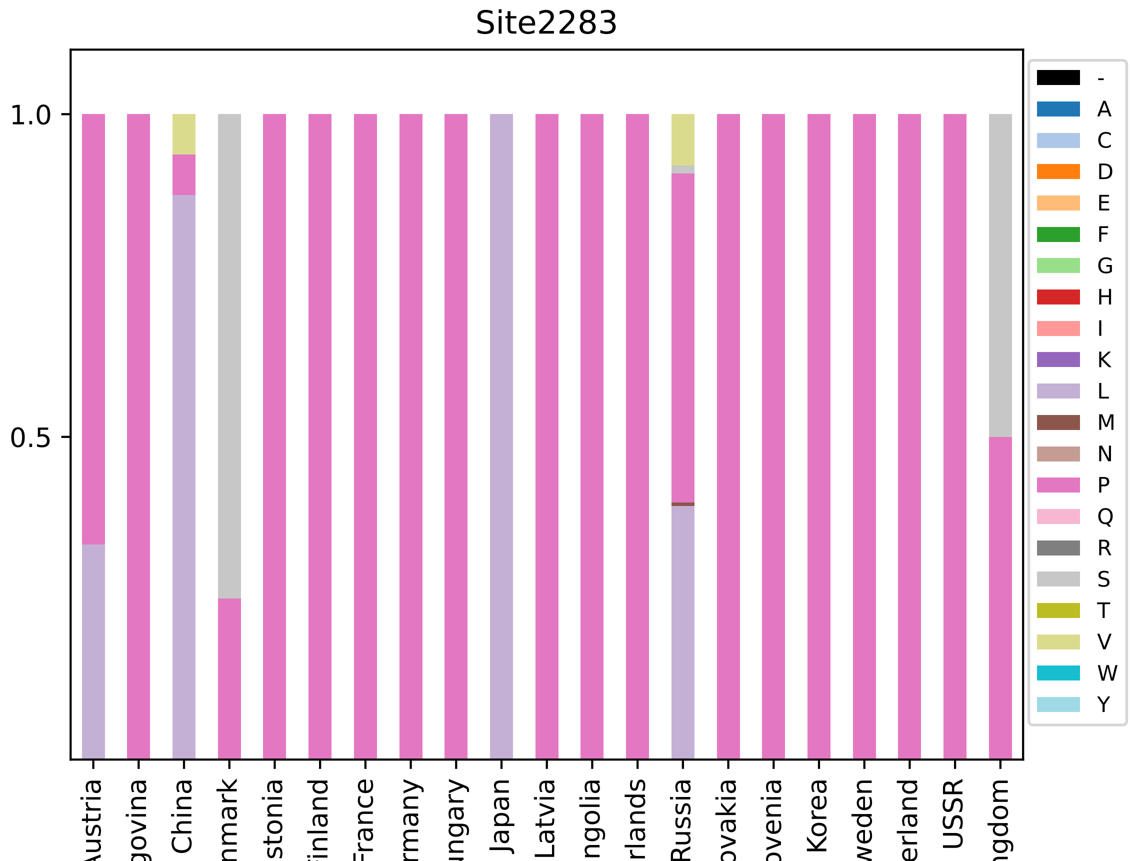
Site2283
1.0
0.5
-
A
C
D
E
F
G
H
I
K
L
M
N
P
Q
R
S
T
V
W
Y
China
Japan
Latvia
Russia
Korea
USSR

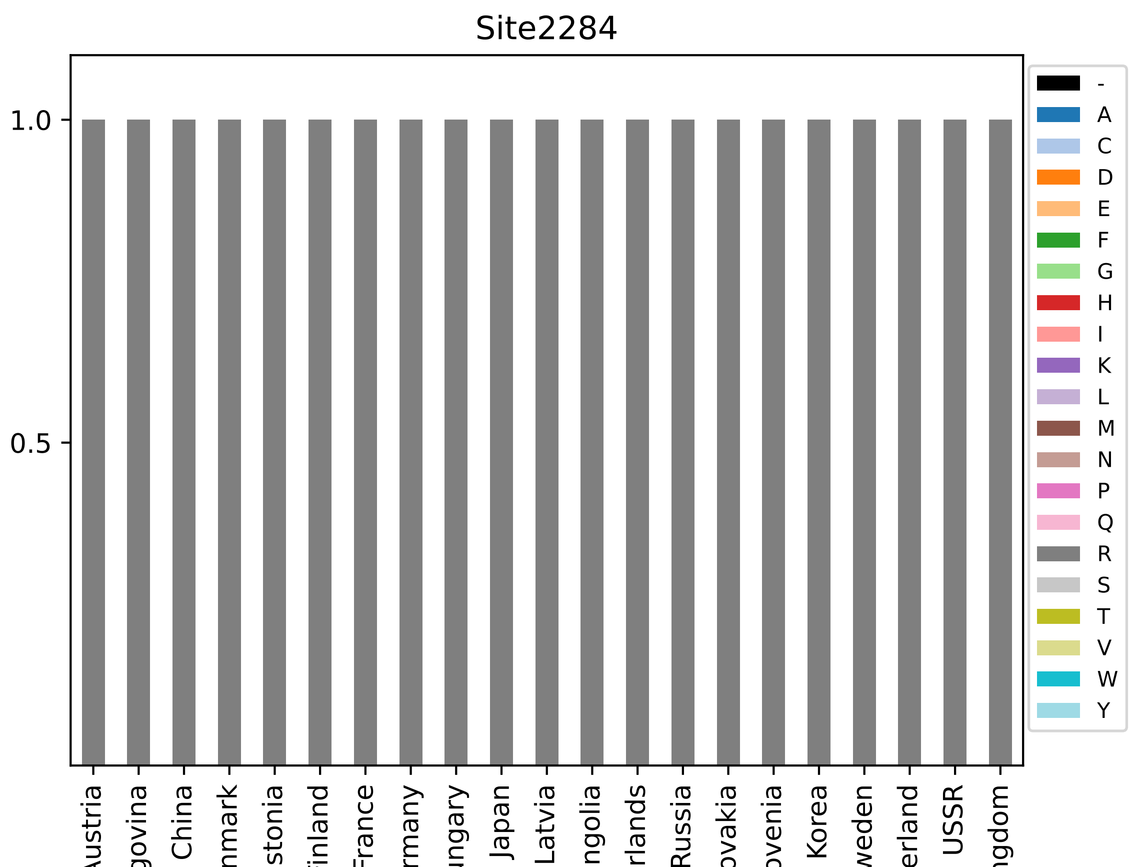
Site2284
1.0
0.5
China
Japan
Latvia
Korea
USSR
-
A
C
D
E
F
G
H
I
K
L
M
N
P
Q
R
S
T
V
W
Y

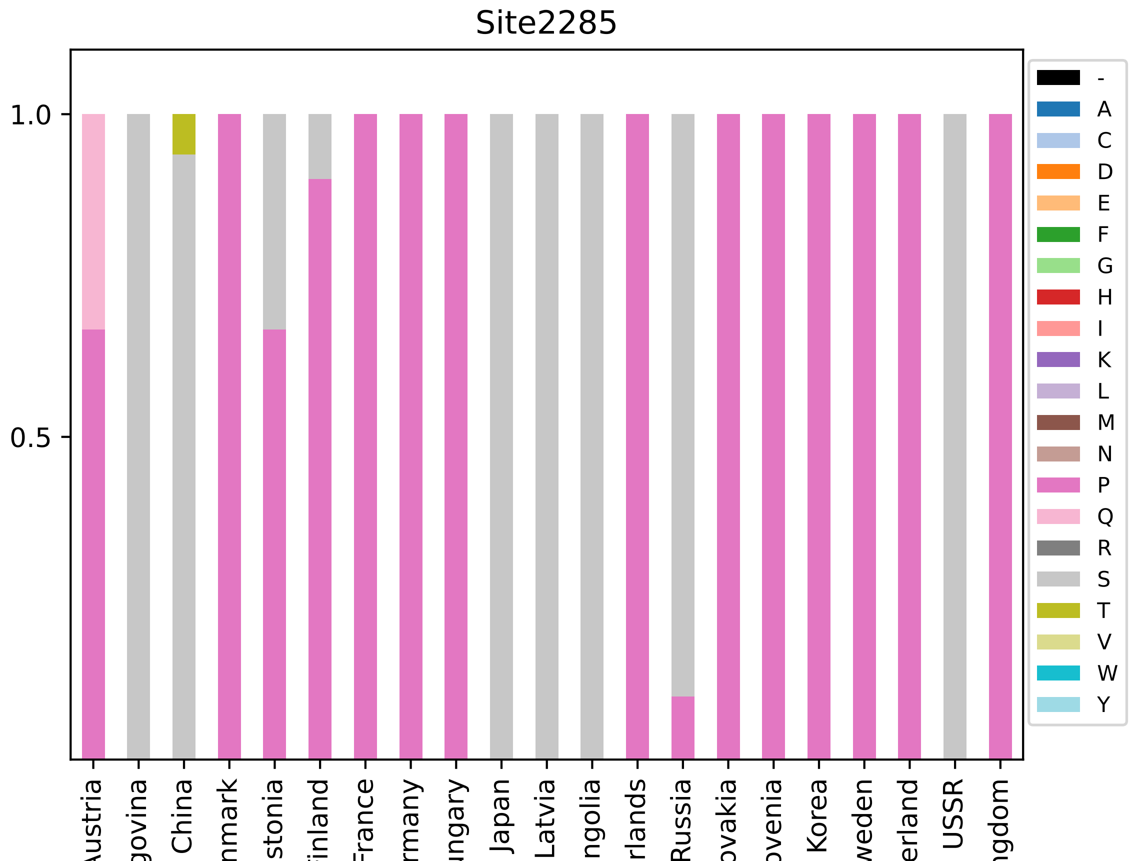
Site2285
1.0
0.5
-
A
C
D
E
F
G
H
I
K
L
M
N
P
Q
R
S
T
V
W
Y
Austria
govina
China
nmark
stonia
inland
France
rmany
ungary
Japan
Latvia
ngolia
rlands
Russia
ovakia
ovenia
Korea
weden
erland
USSR
ngdom

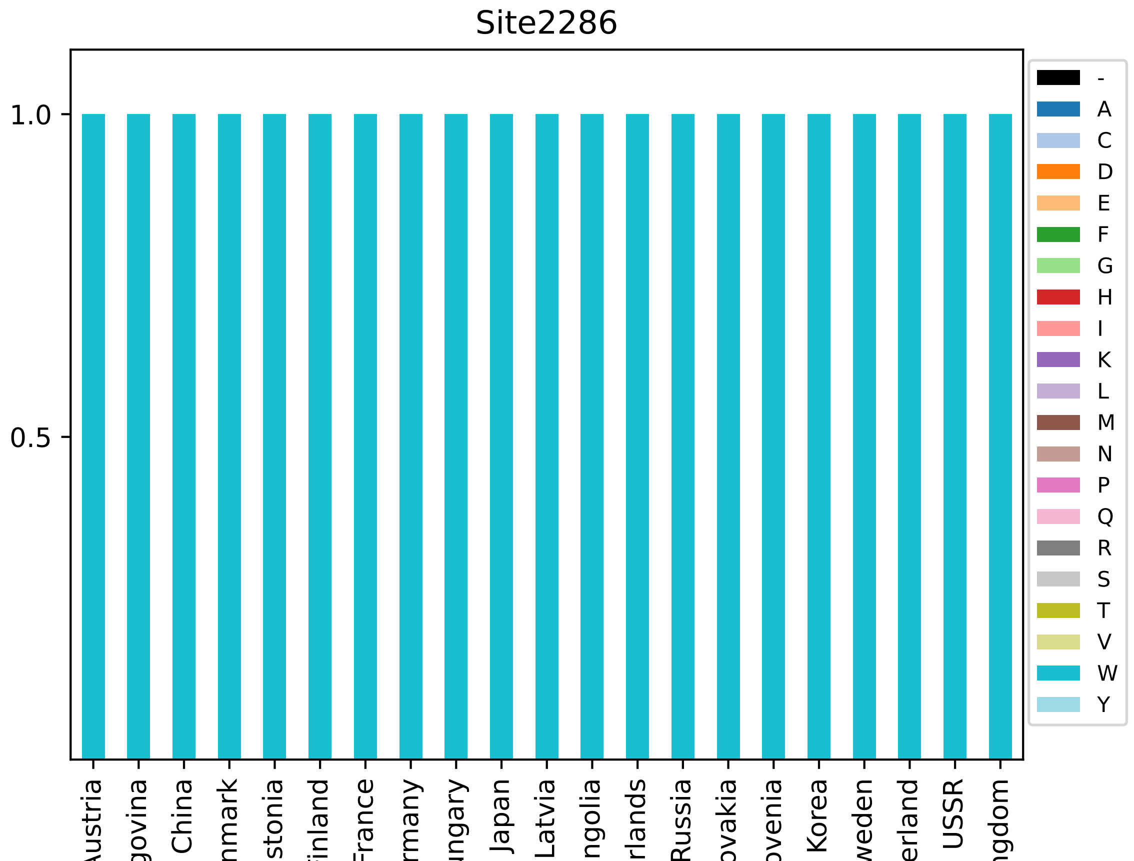

Site2286
1.0
0.5
-
A
C
D
E
F
G
H
I
K
L
M
N
P
Q
R
S
T
V
W
Y
China
Japan
Latvia
Russia
Korea
USSR

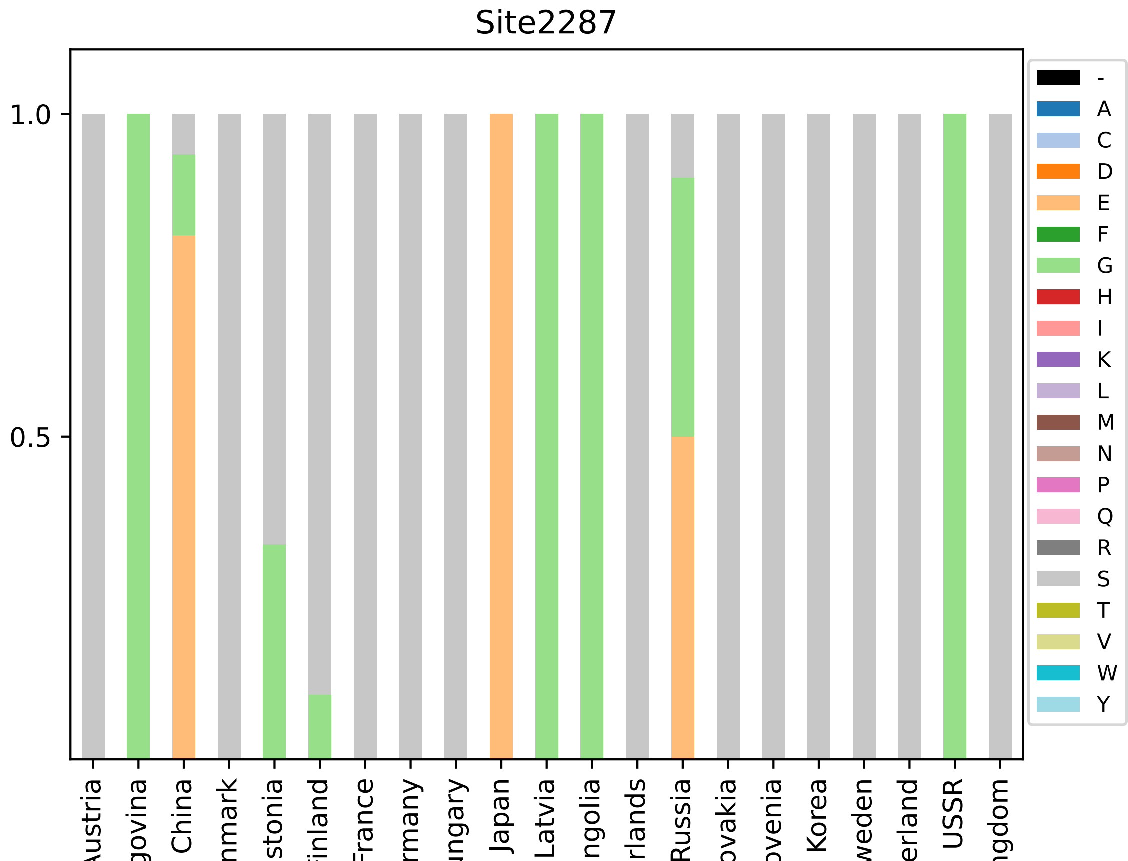

Site2287
1.0
0.5
-
A
C
D
E
F
G
H
I
K
L
M
N
P
Q
R
S
T
V
W
Y
China
Japan
Latvia
Russia
Korea
USSR

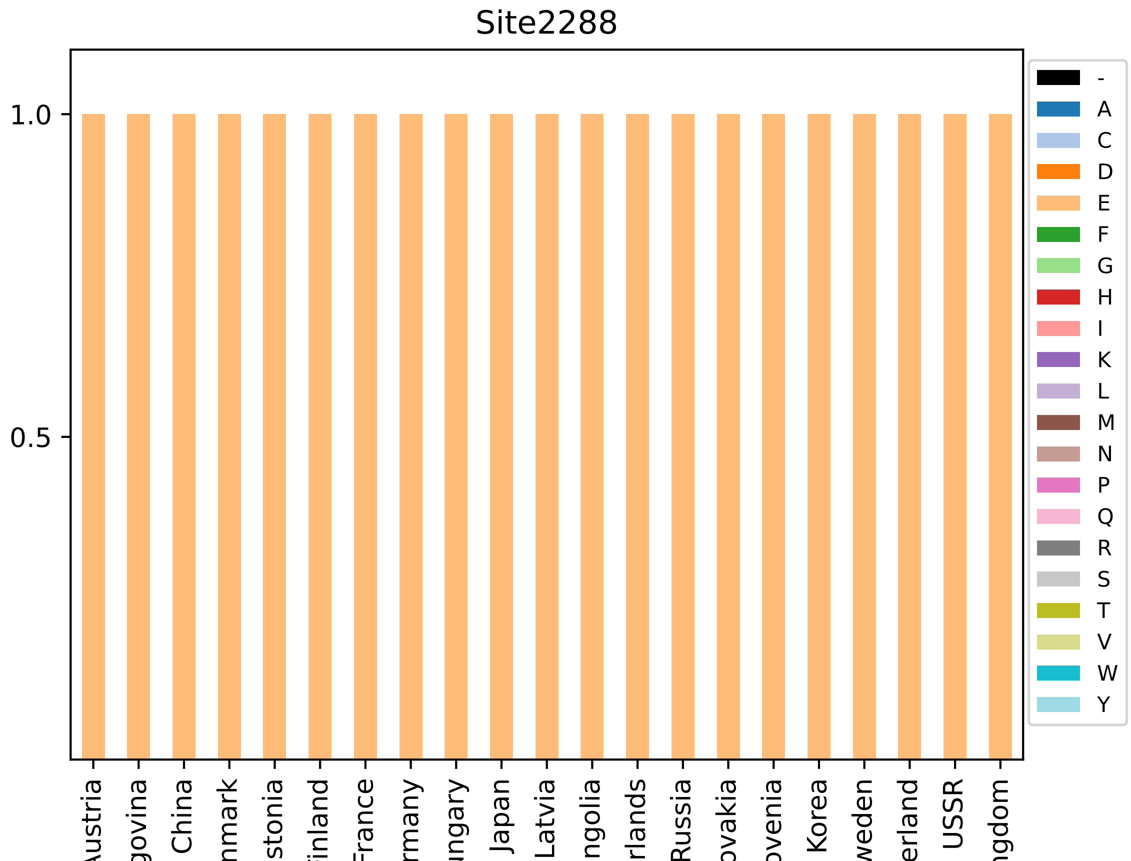
Site2288
1.0
0.5
-
A
C
D
E
F
G
H
I
K
L
M
N
P
Q
R
S
T
V
W
Y
China
Japan
Latvia
Russia
Korea
USSR

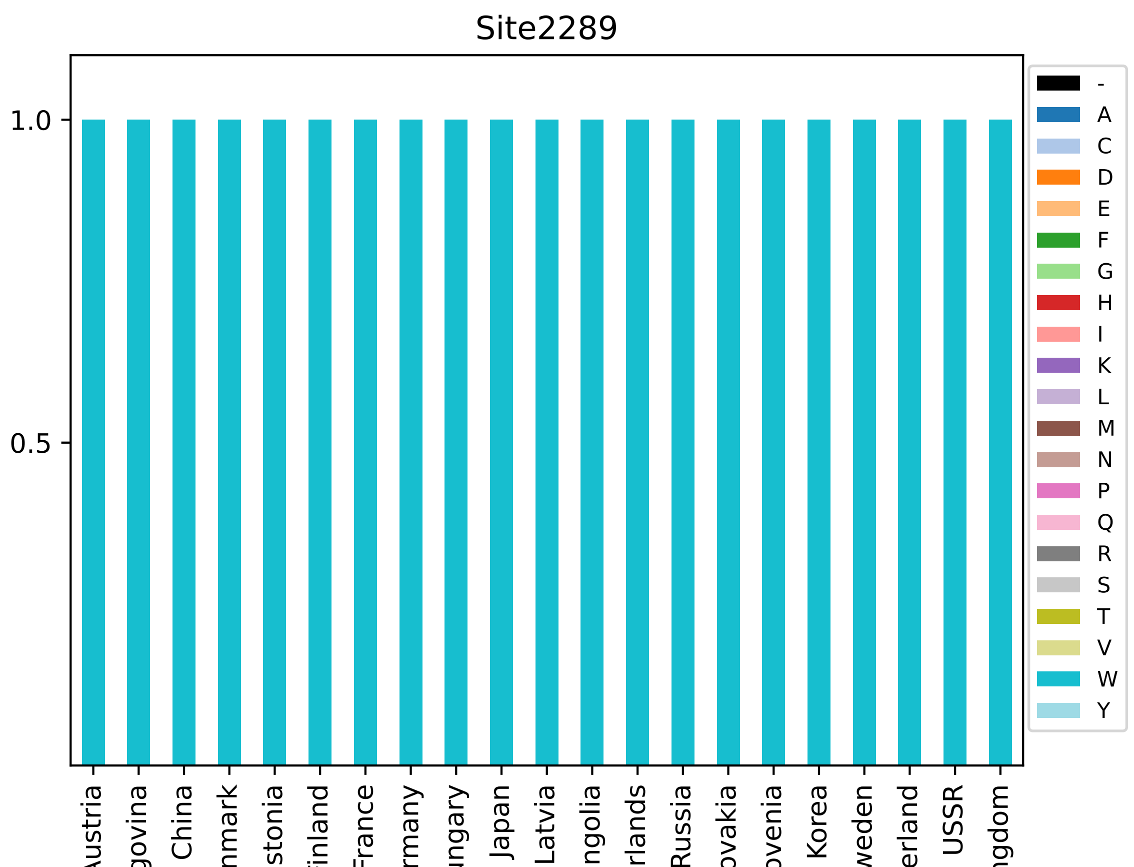
Site2289
1.0
0.5
Austria
govina
China
nmark
stonia
Finland
France
rmany
ungary
Japan
Latvia
ngolia
rlands
Russia
ovakia
ovenia
Korea
weden
erland
USSR
ngdom
-
A
C
D
E
F
G
H
I
K
L
M
N
P
Q
R
S
T
V
W
Y

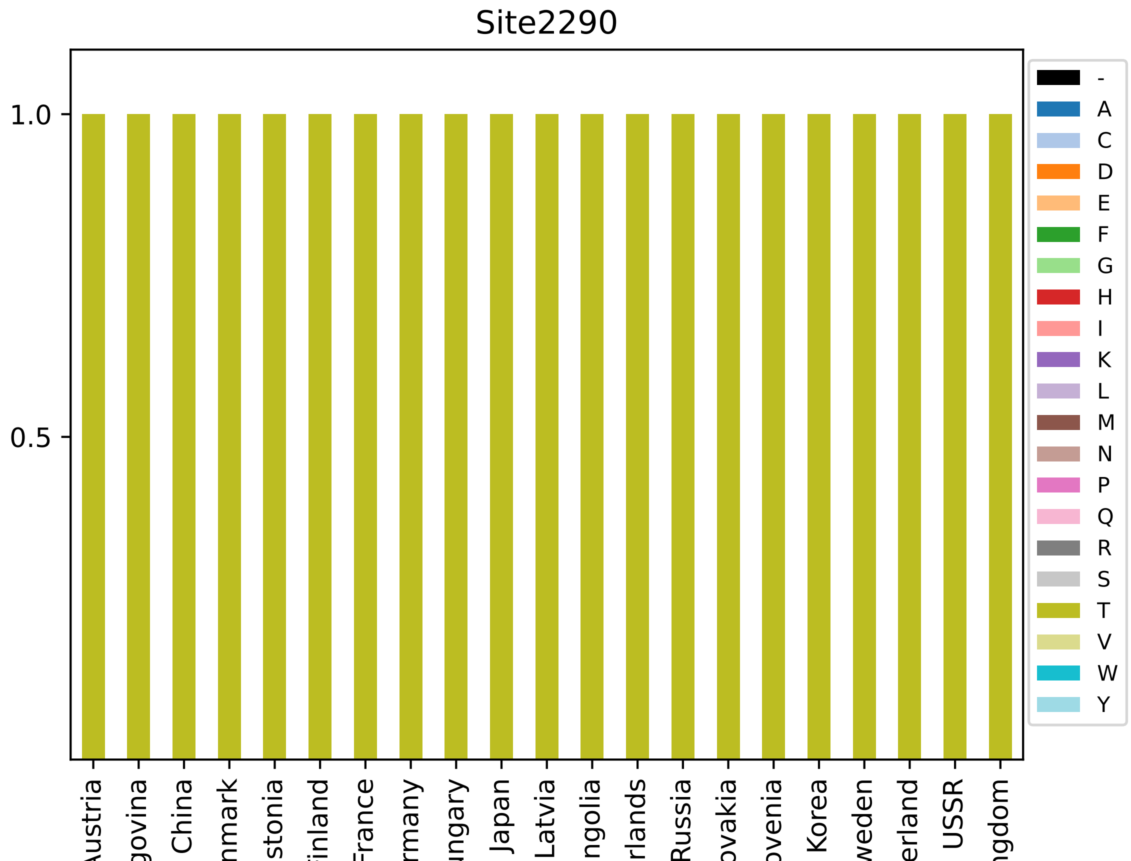
Site2290
1.0
0.5
-
A
C
D
E
F
G
H
I
K
L
M
N
P
Q
R
S
T
V
W
Y
China
Japan
Latvia
Russia
Korea
USSR

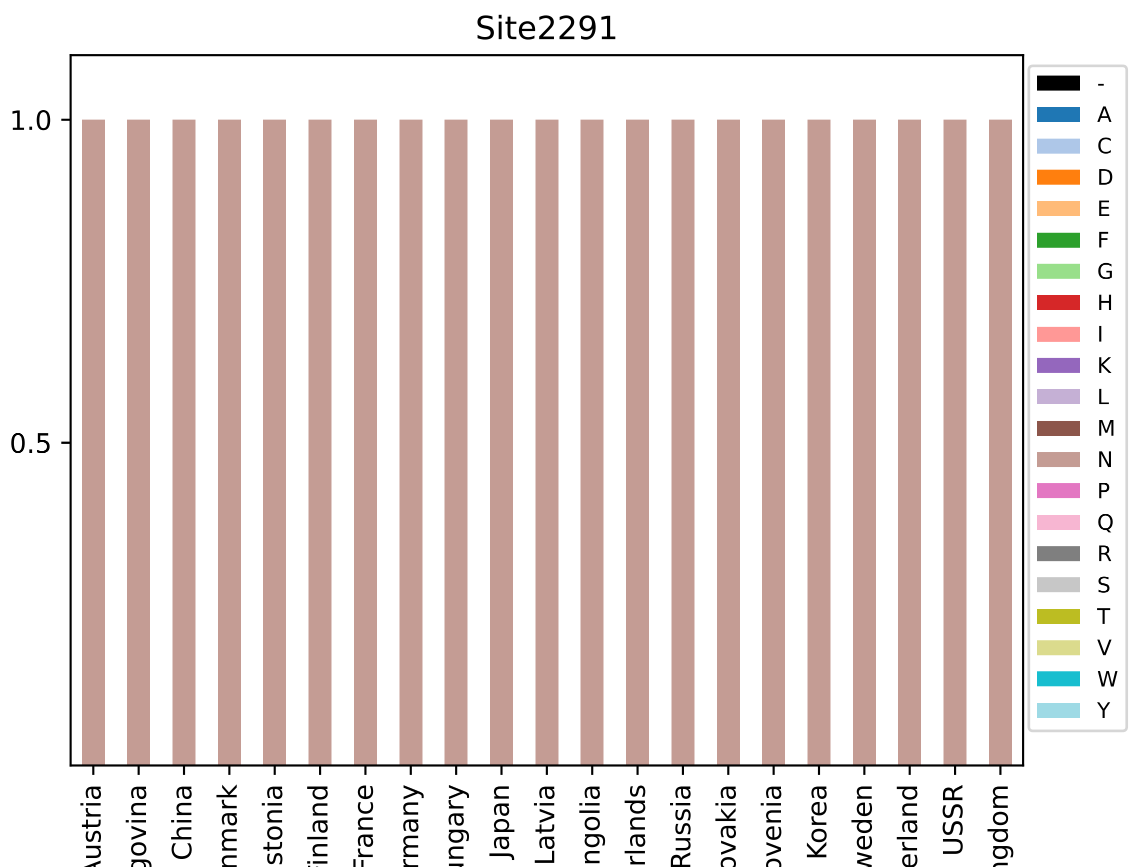
Site2291
1.0
0.5
-
A
C
D
E
F
G
H
I
K
L
M
N
P
Q
R
S
T
V
W
Y
China
Japan
Latvia
Russia
Korea
USSR

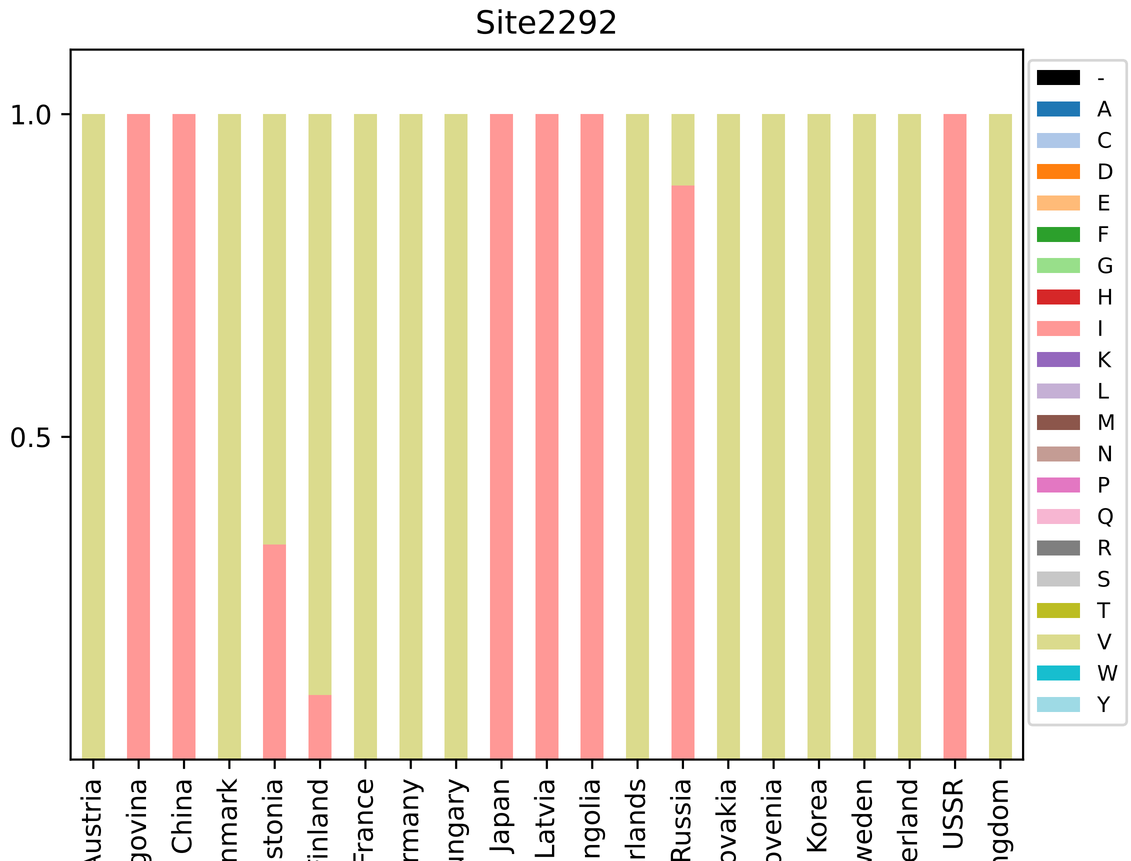

Site2292
1.0
0.5
-
A
C
D
E
F
G
H
I
K
L
M
N
P
Q
R
S
T
V
W
Y
China
Latvia
Russia
Korea
USSR
Japan

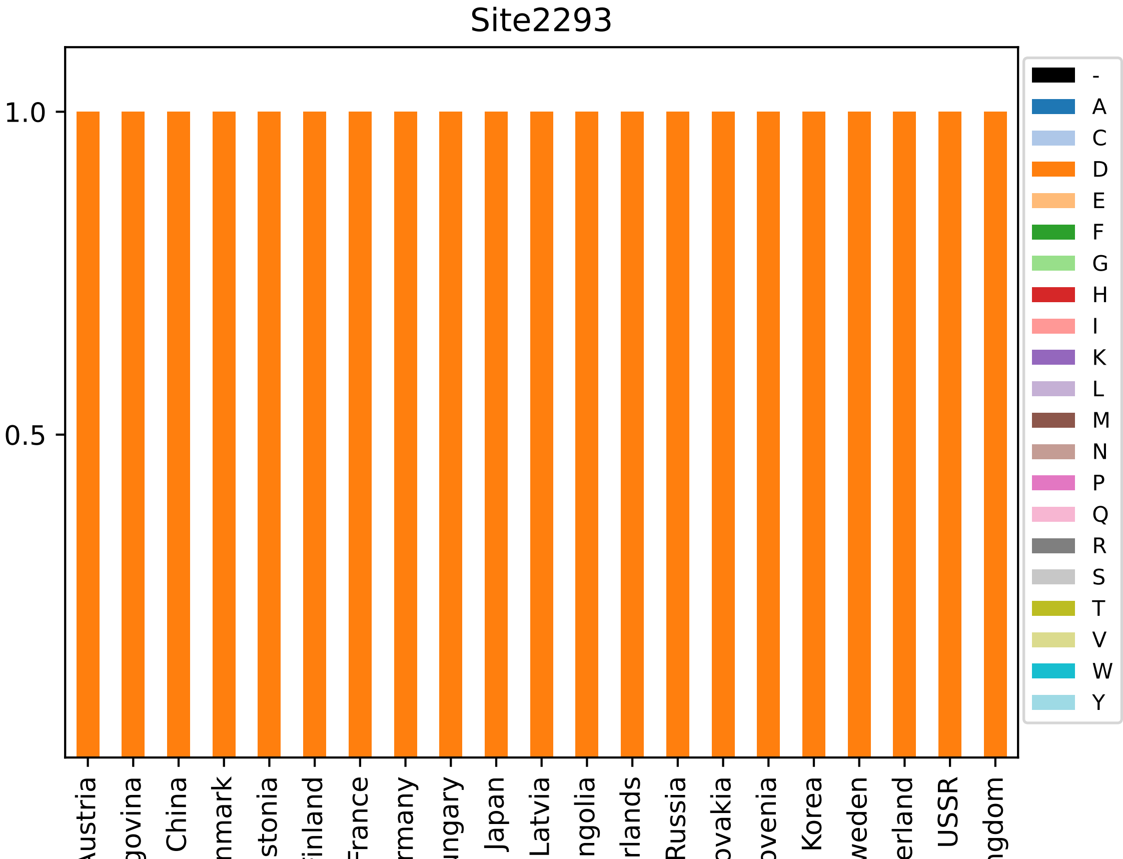

Site2293
1.0
0.5
-
A
C
D
E
F
G
H
I
K
L
M
N
P
Q
R
S
T
V
W
Y
Austria
govina
China
nmark
stonia
inland
France
rmany
ungary
Japan
Latvia
ngolia
rlands
Russia
ovakia
ovenia
Korea
weden
erland
USSR
ngdom

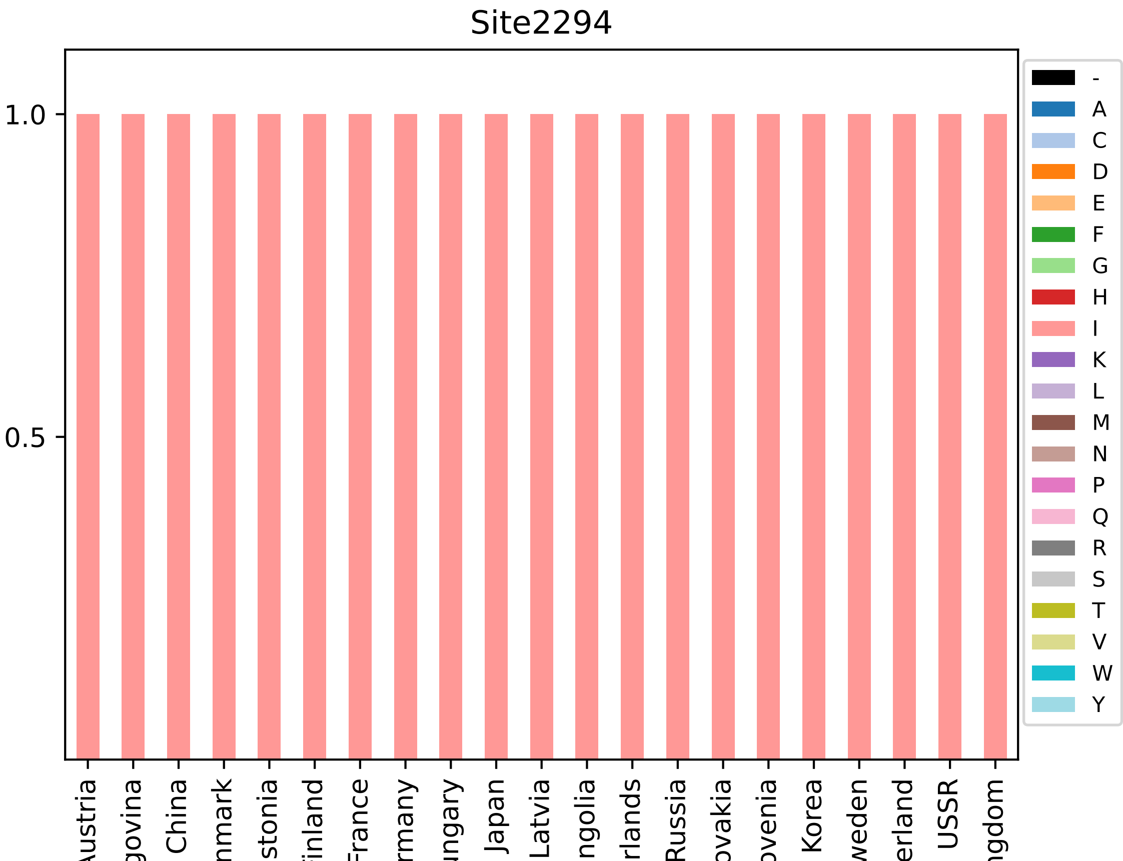

Site2294
1.0
0.5
-
A
C
D
E
F
G
H
I
K
L
M
N
P
Q
R
S
T
V
W
Y
China
Japan
Latvia
Russia
Korea
USSR

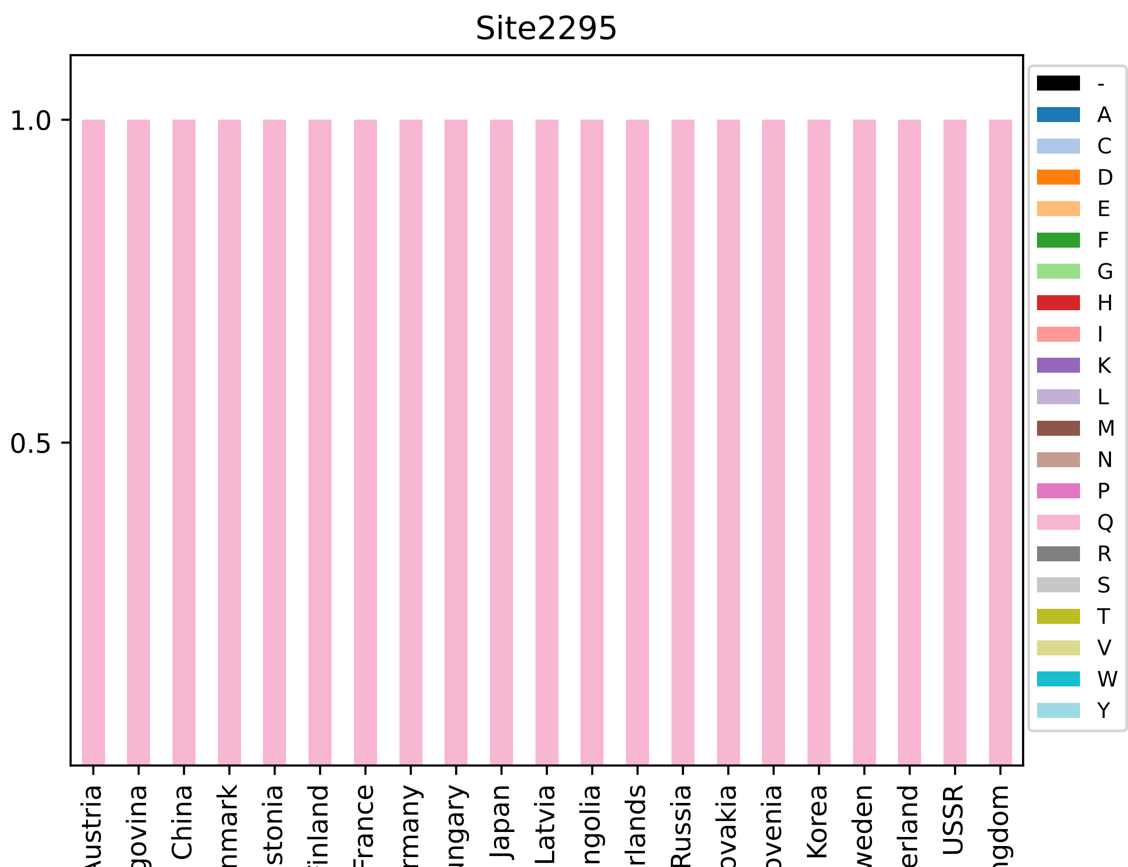

Site2295
1.0
0.5
-
A
C
D
E
F
G
H
I
K
L
M
N
P
Q
R
S
T
V
W
Y
Austria
govina
China
nmark
stonia
Finland
France
rmany
ungary
Japan
Latvia
ngolia
rlands
Russia
ovakia
ovenia
Korea
weden
erland
USSR
ngdom

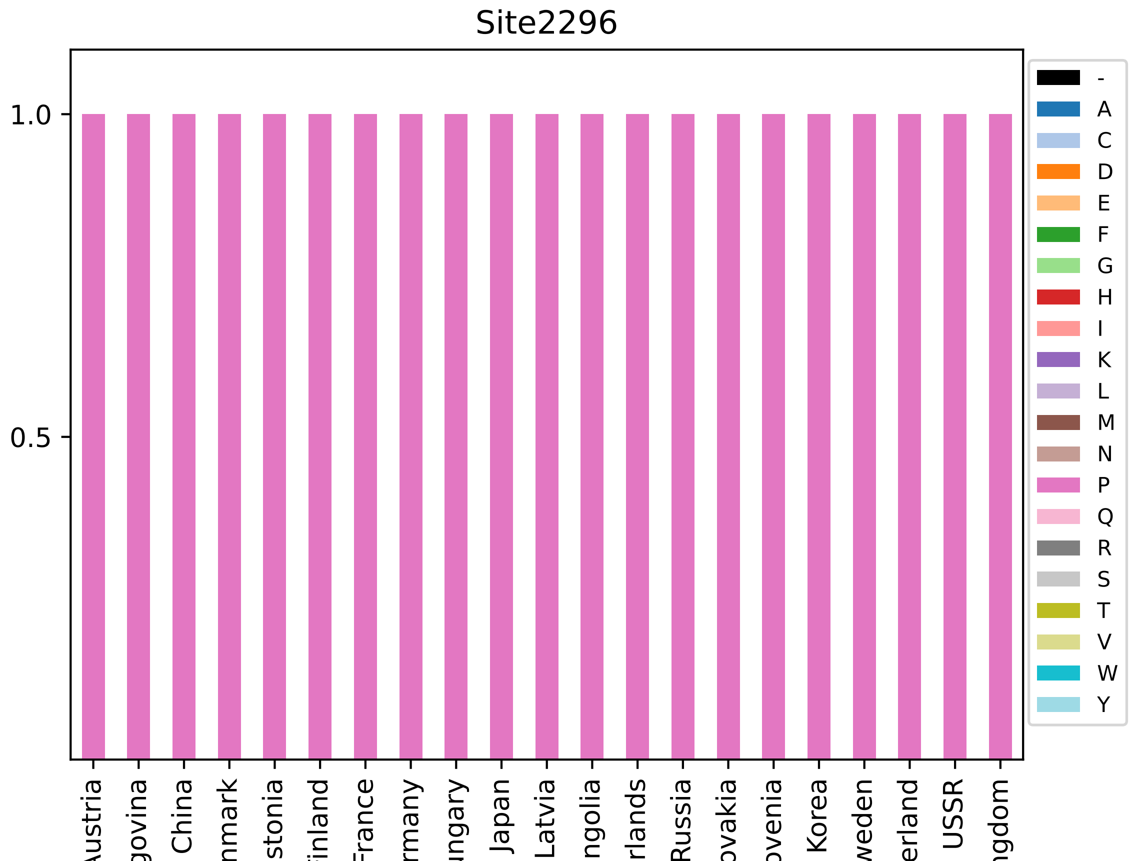

Site2296
1.0
0.5
-
A
C
D
E
F
G
H
I
K
L
M
N
P
Q
R
S
T
V
W
Y
China
Japan
Latvia
Russia
Korea
USSR

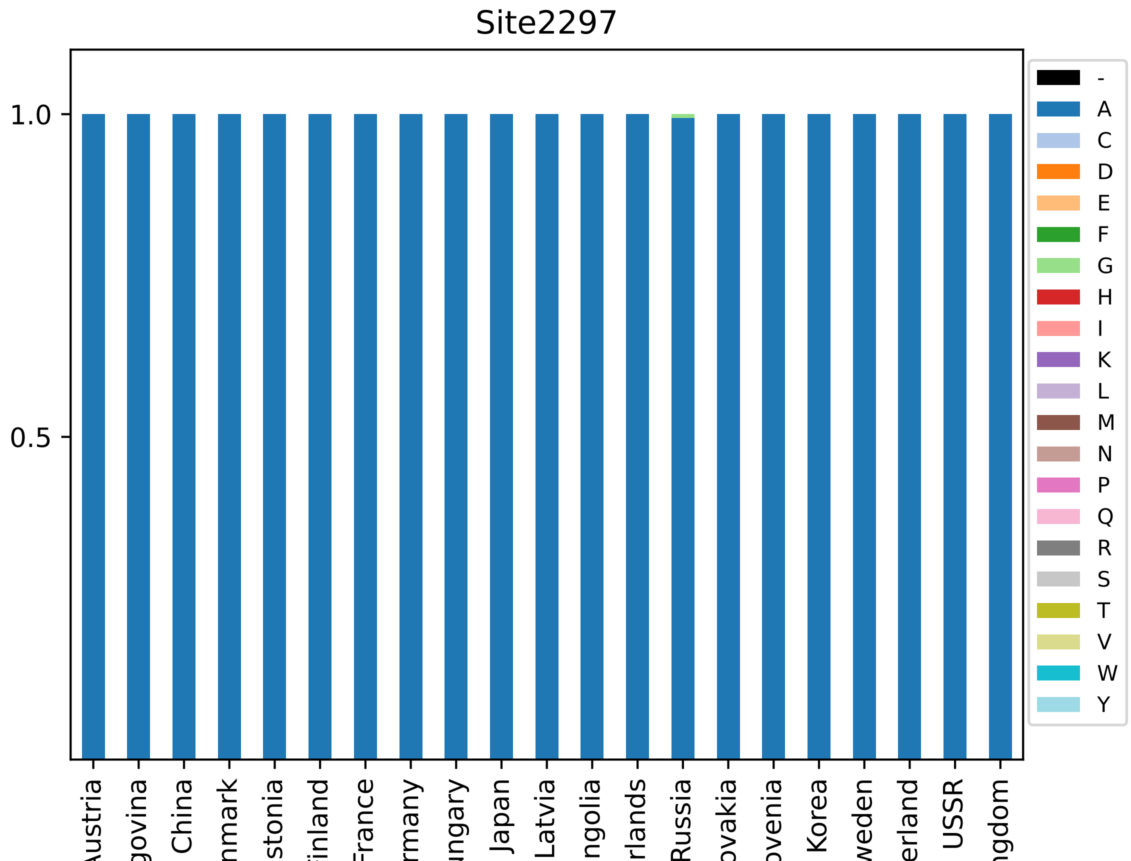

Site2297
1.0
0.5
-
A
C
D
E
F
G
H
I
K
L
M
N
P
Q
R
S
T
V
W
Y
Austria
govina
China
nmark
stonia
Finland
France
rmany
ungary
Japan
Latvia
ngolia
rlands
Russia
ovakia
ovenia
Korea
weden
erland
USSR
ngdom

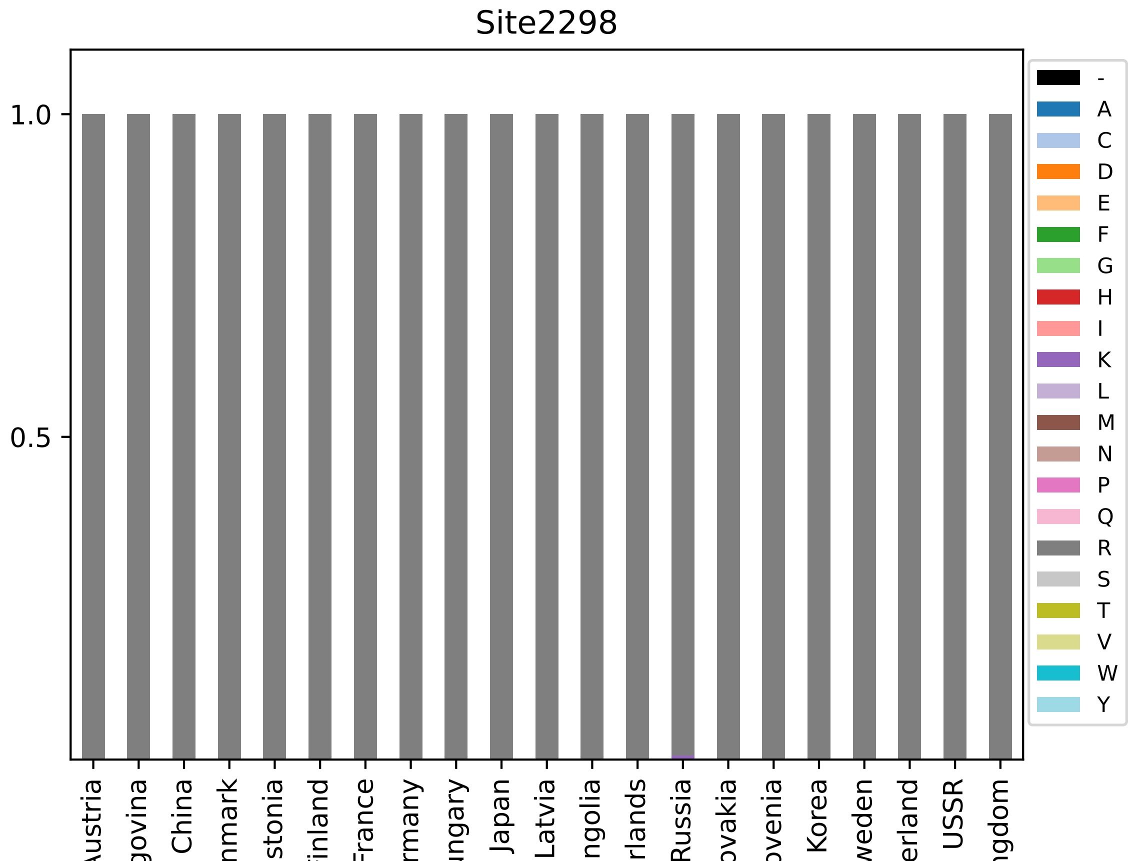
Site2298
1.0
0.5
-
A
C
D
E
F
G
H
I
K
L
M
N
P
Q
R
S
T
V
W
Y
Austria
govina
China
nmark
stonia
Finland
France
rmany
ungary
Japan
Latvia
ngolia
rlands
Russia
ovakia
ovenia
Korea
weden
erland
USSR
ngdom

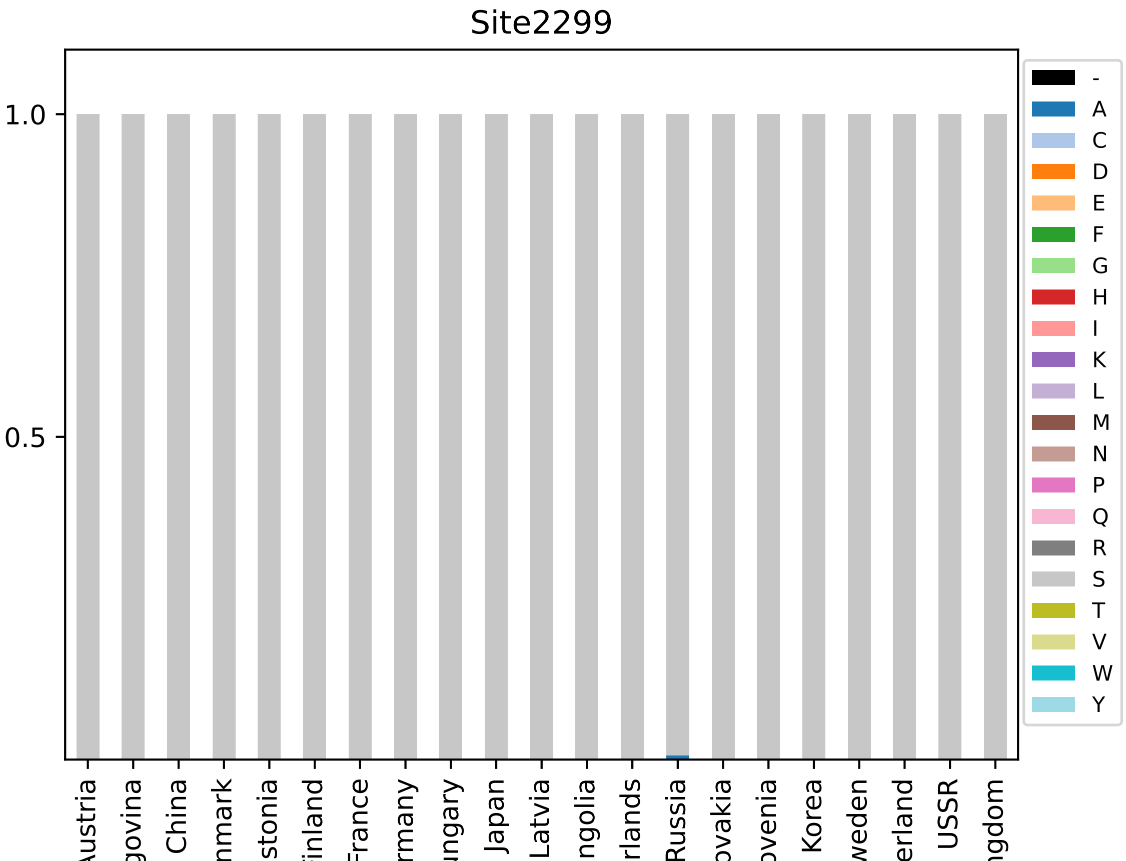

Site2299
1.0
0.5
-
A
C
D
E
F
G
H
I
K
L
M
N
P
Q
R
S
T
V
W
Y
Austria
govina
China
nmark
stonia
Finland
France
rmany
ungary
Japan
Latvia
ngolia
rlands
Russia
ovakia
ovenia
Korea
weden
erland
USSR
ngdom

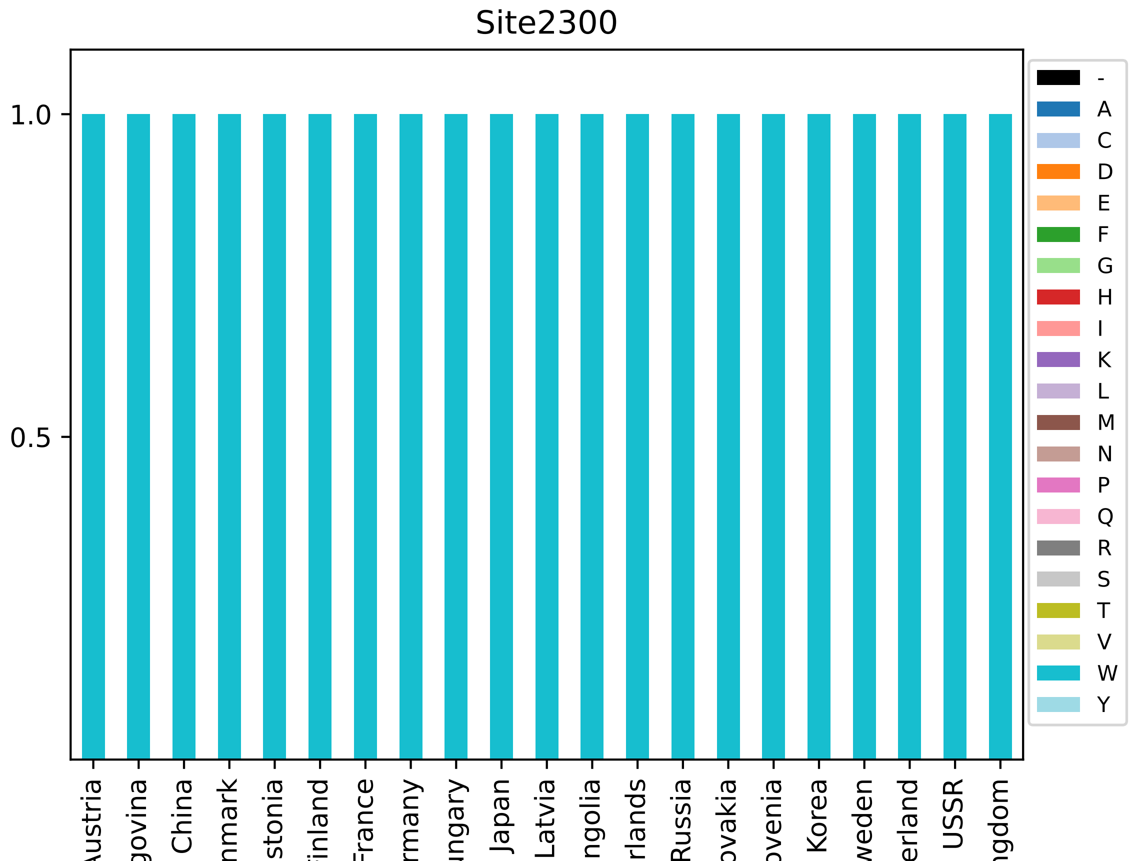
Site2300
1.0
0.5
-
A
C
D
E
F
G
H
I
K
L
M
N
P
Q
R
S
T
V
W
Y
Austria
govina
China
nmark
stonia
inland
France
rmany
ungary
Japan
Latvia
ngolia
rlands
Russia
ovakia
ovenia
Korea
weden
erland
USSR
ngdom

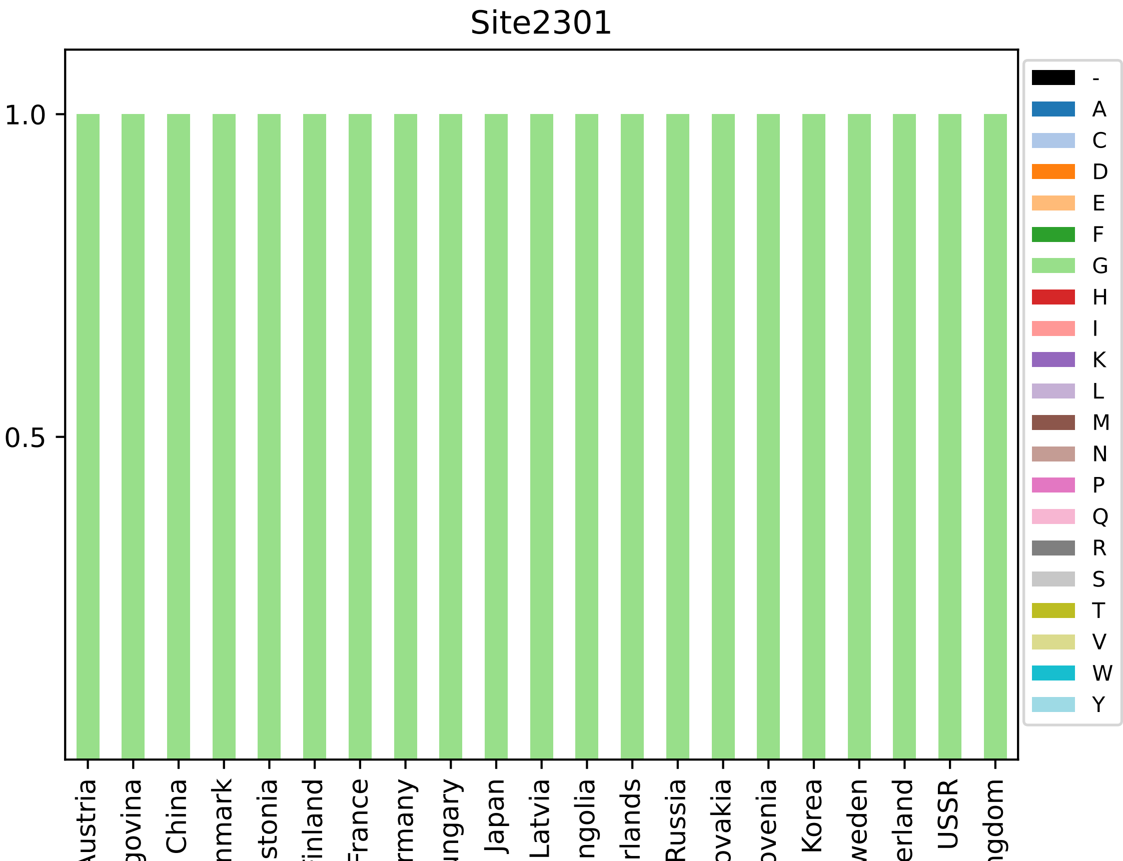

Site2301
1.0
0.5
-
A
C
D
E
F
G
H
I
K
L
M
N
P
Q
R
S
T
V
W
Y
Austria
govina
China
nmark
stonia
inland
France
rmany
ungary
Japan
Latvia
ngolia
rlands
Russia
ovakia
ovenia
Korea
weden
erland
USSR
ngdom

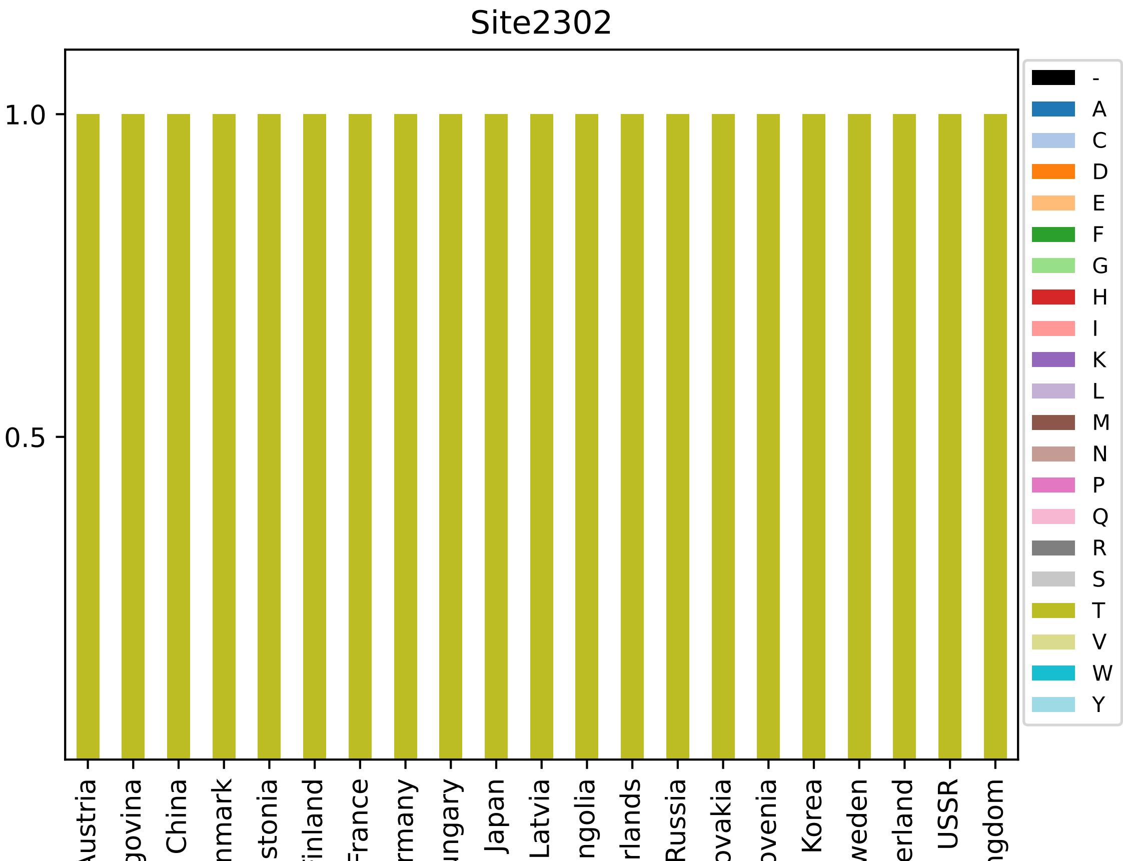
Site2302
1.0
0.5
-
A
C
D
E
F
G
H
I
K
L
M
N
P
Q
R
S
T
V
W
Y
China
Japan
Latvia
Russia
Korea
USSR

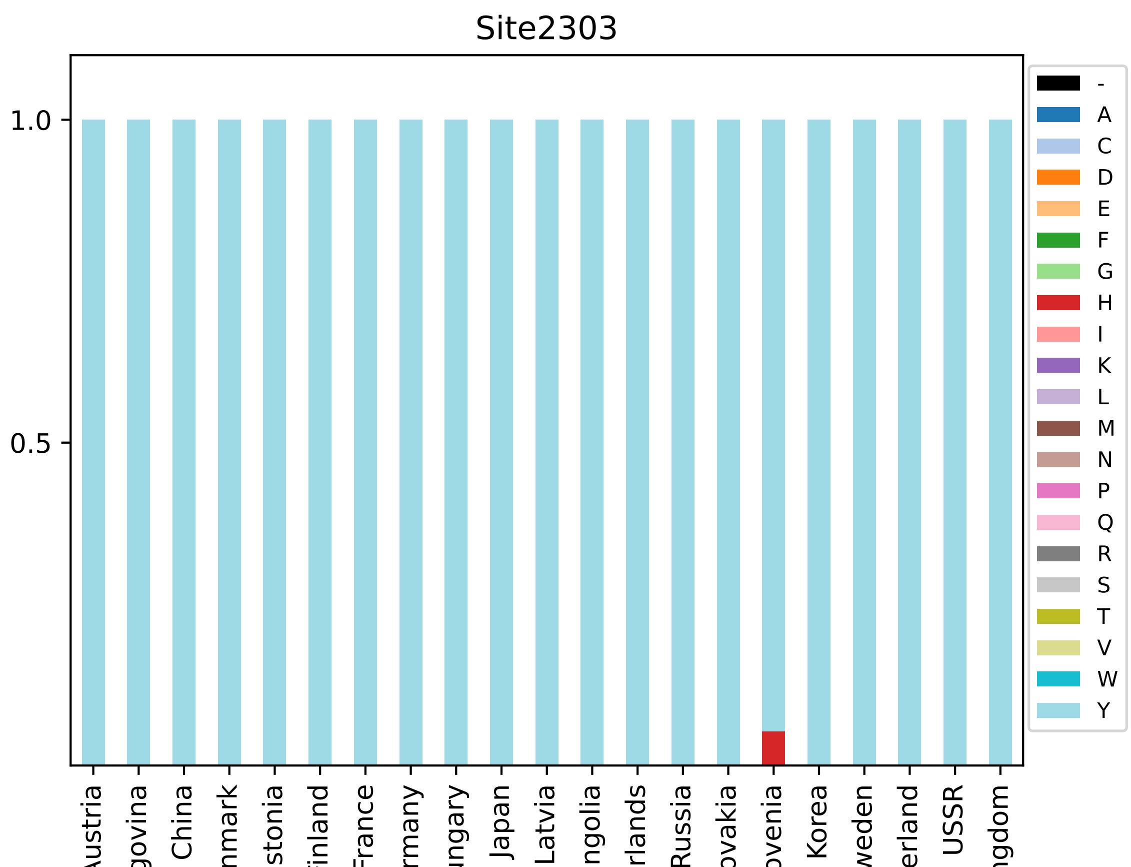

Site2303
1.0
0.5
-
A
C
D
E
F
G
H
I
K
L
M
N
P
Q
R
S
T
V
W
Y
China
Japan
Latvia
Russia
Korea
USSR

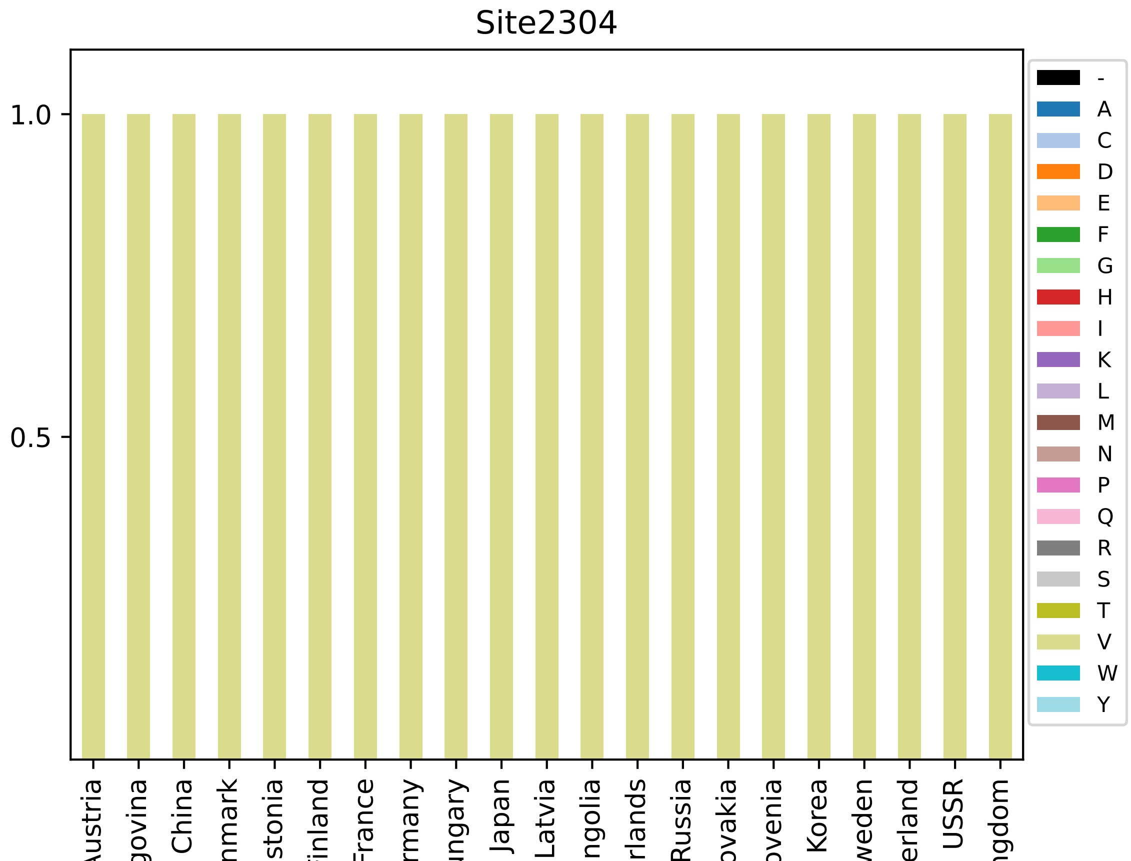

Site2304
1.0
0.5
-
A
C
D
E
F
G
H
I
K
L
M
N
P
Q
R
S
T
V
W
Y
Austria
China
Finland
France
Japan
Latvia
Russia
Korea
USSR

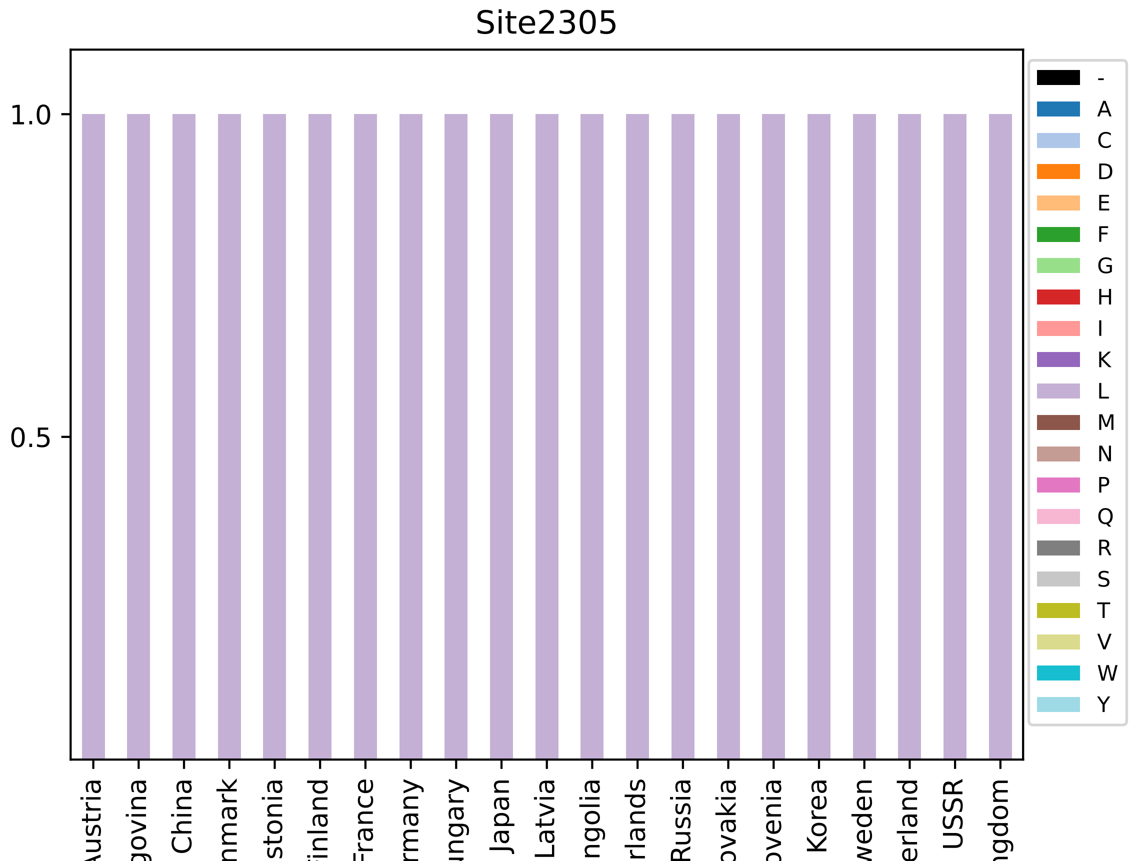

Site2305
1.0
0.5
-
A
C
D
E
F
G
H
I
K
L
M
N
P
Q
R
S
T
V
W
Y
China
Japan
Latvia
Russia
Korea
USSR

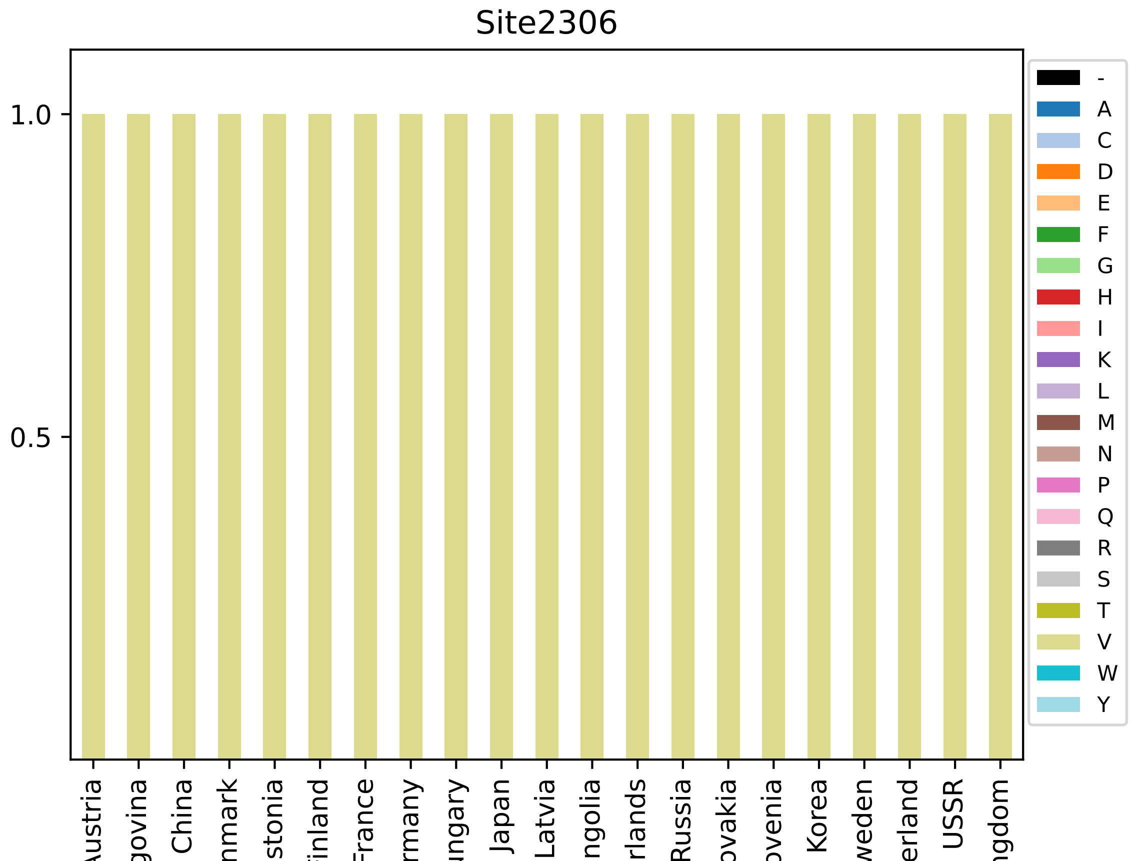

Site2306
1.0
0.5
-
A
C
D
E
F
G
H
I
K
L
M
N
P
Q
R
S
T
V
W
Y
China
Japan
Latvia
Russia
Korea
USSR

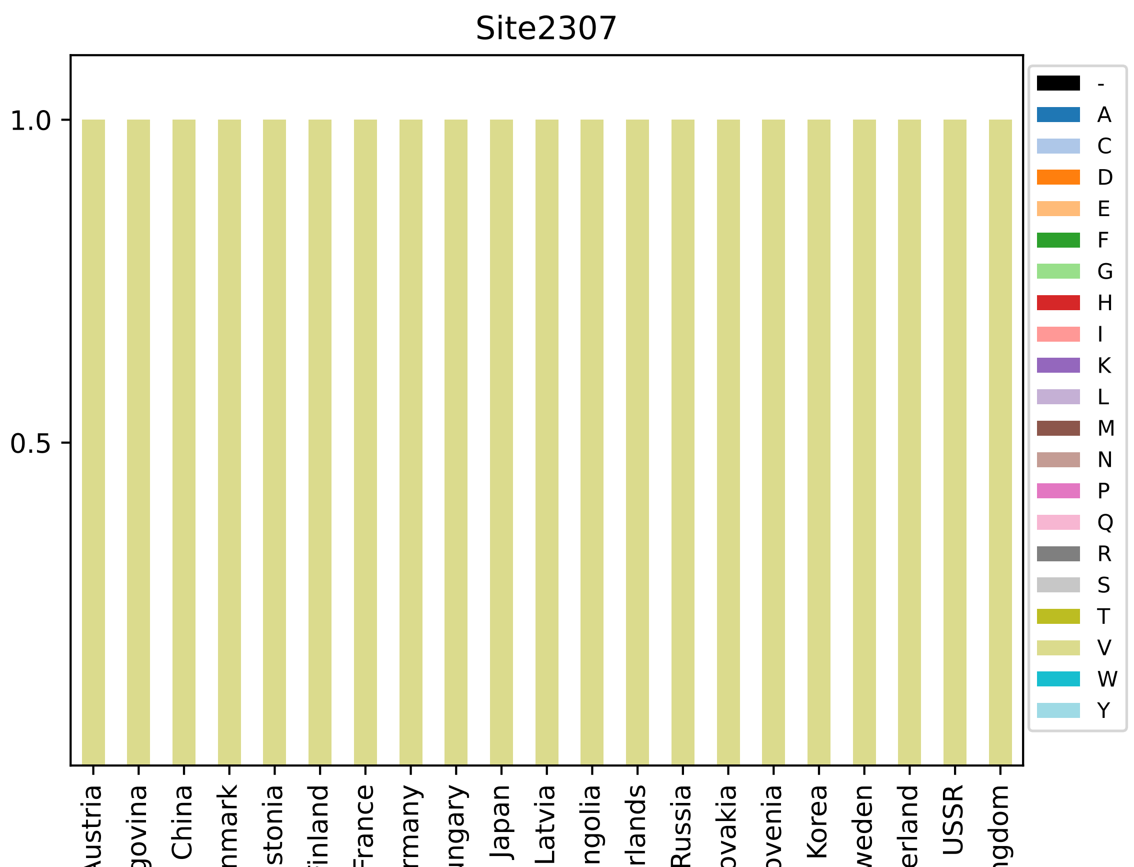
Site2307
1.0
0.5
-
A
C
D
E
F
G
H
I
K
L
M
N
P
Q
R
S
T
V
W
Y
Austria
govina
China
nmark
stonia
Finland
France
rmany
ungary
Japan
Latvia
ngolia
rlands
Russia
ovakia
ovenia
Korea
weden
erland
USSR
ngdom

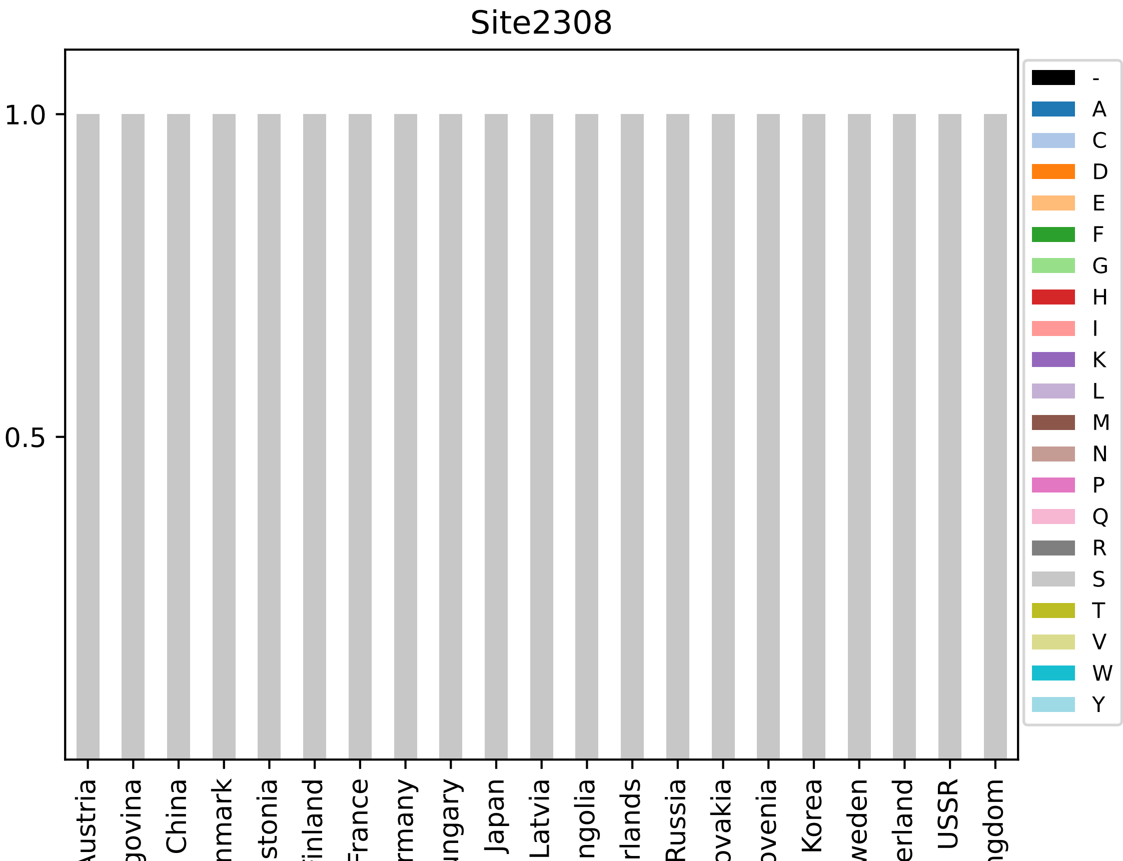
Site2308
1.0
0.5
Austria
govina
China
nmark
stonia
Finland
France
rmany
ungary
Japan
Latvia
ngolia
rlands
Russia
ovakia
ovenia
Korea
weden
erland
USSR
ngdom
-
A
C
D
E
F
G
H
I
K
L
M
N
P
Q
R
S
T
V
W
Y

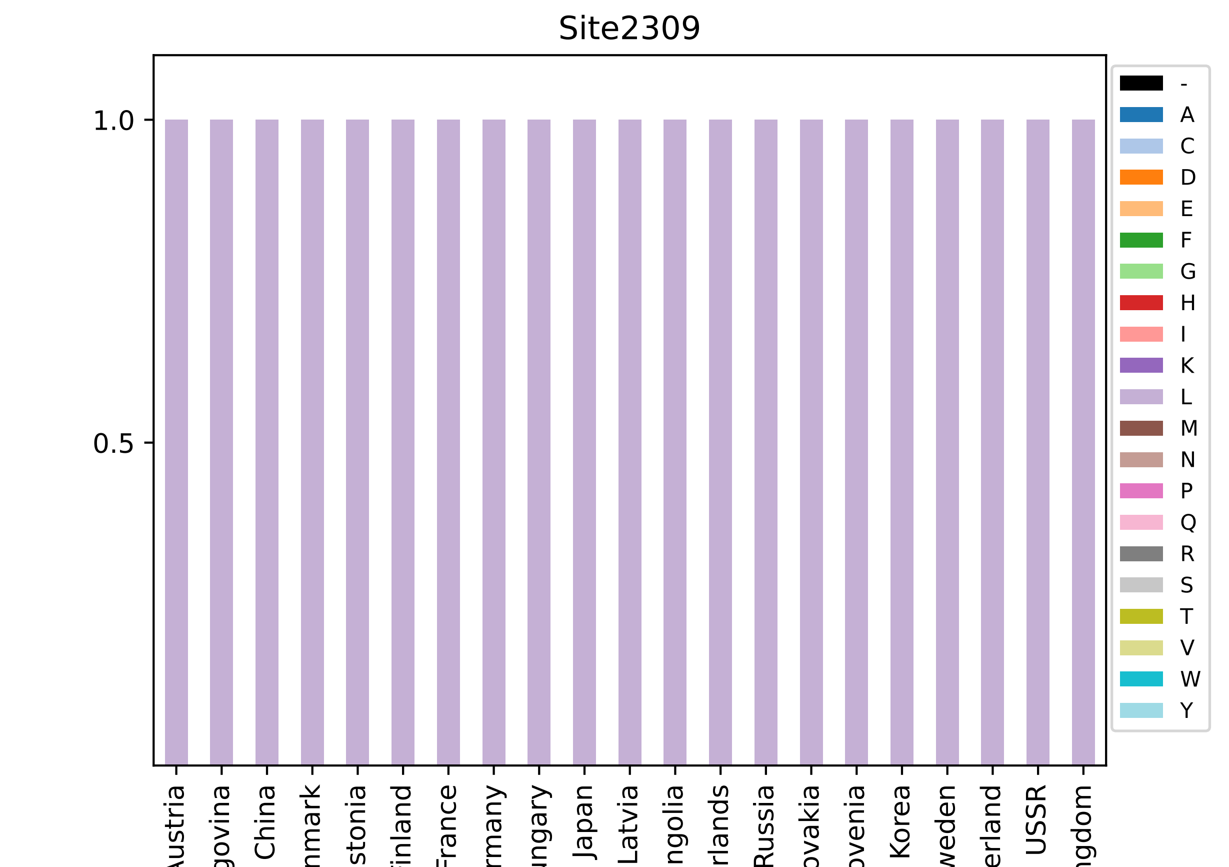
Site2309
1.0
0.5
-
A
C
D
E
F
G
H
I
K
L
M
N
P
Q
R
S
T
V
W
Y
Austria
govina
China
nmark
stonia
Finland
France
rmany
ungary
Japan
Latvia
ngolia
rlands
Russia
ovakia
ovenia
Korea
weden
erland
USSR
ngdom

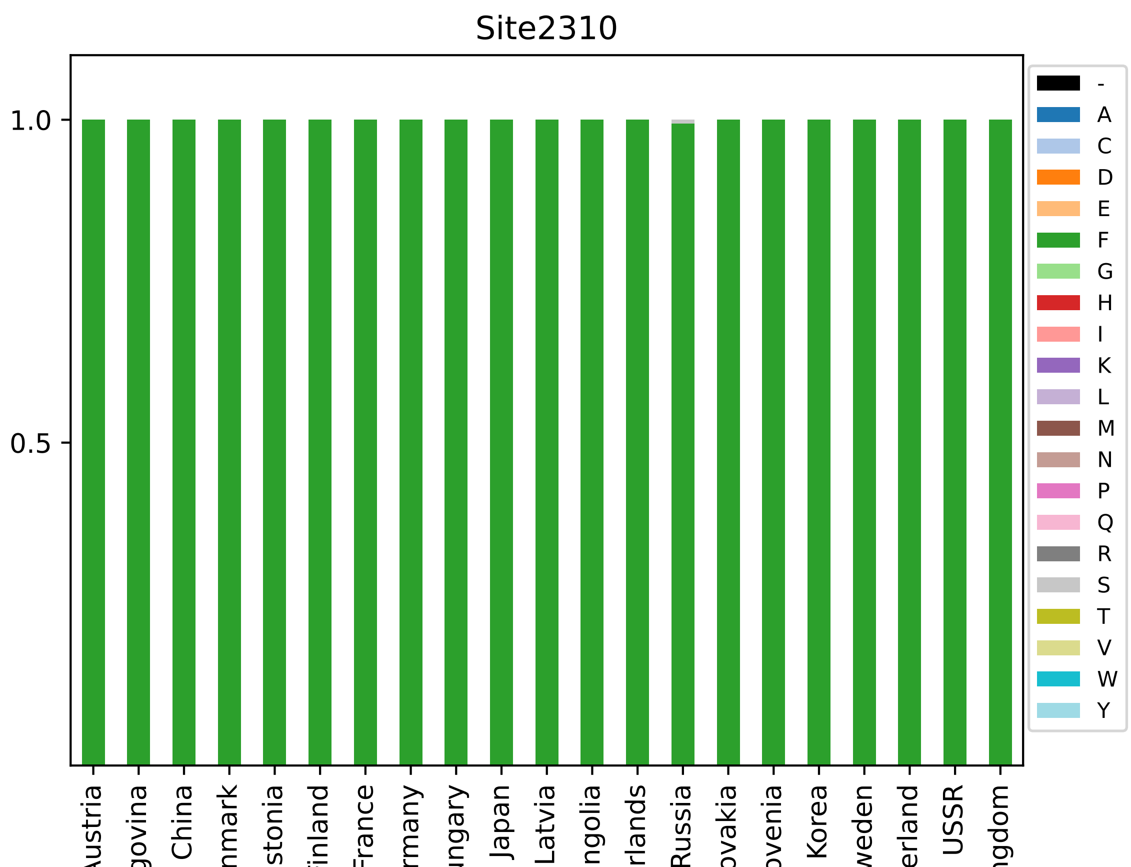

Site2310
1.0
0.5
-
A
C
D
E
F
G
H
I
K
L
M
N
P
Q
R
S
T
V
W
Y
Austria
govina
China
nmark
stonia
Finland
France
rmany
ungary
Japan
Latvia
ngolia
rlands
Russia
ovakia
ovenia
Korea
weden
erland
USSR
ngdom

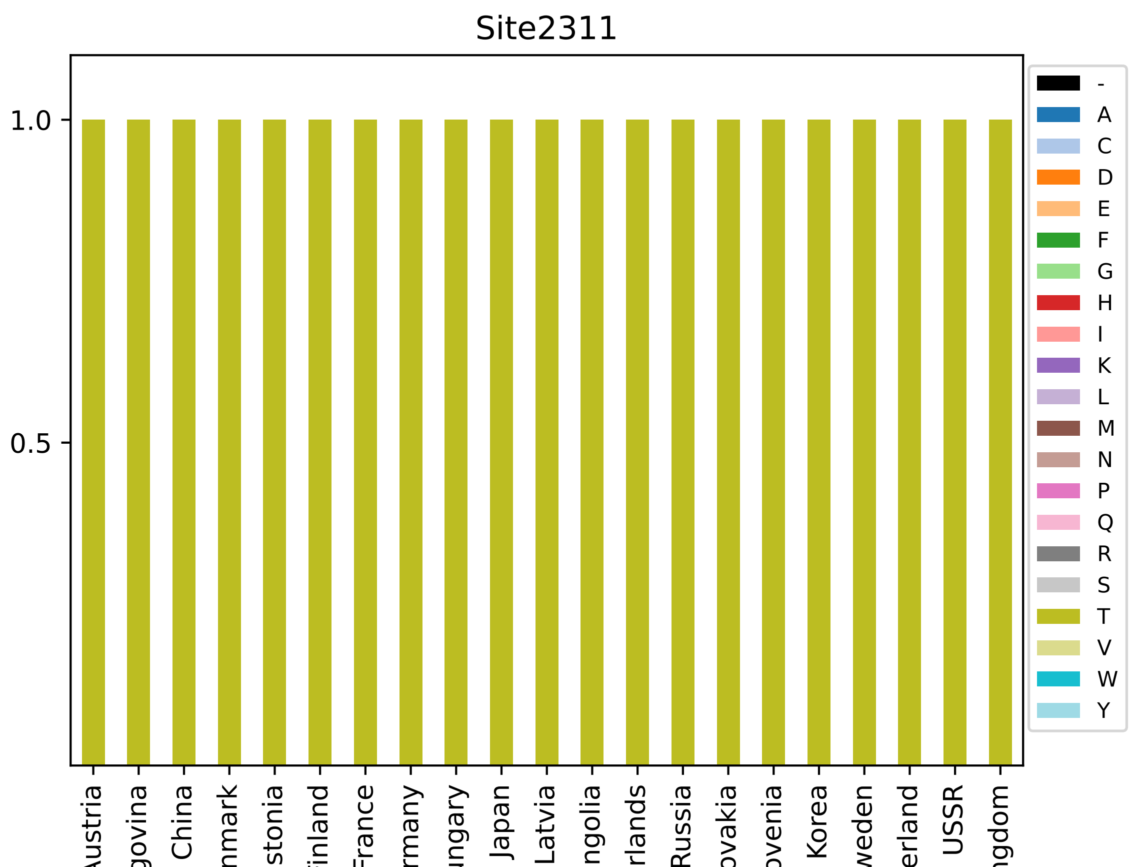
Site2311
1.0
0.5
-
A
C
D
E
F
G
H
I
K
L
M
N
P
Q
R
S
T
V
W
Y
Austria
govina
China
nmark
stonia
inland
France
rmany
ungary
Japan
Latvia
ngolia
rlands
Russia
ovakia
ovenia
Korea
weden
erland
USSR
ngdom

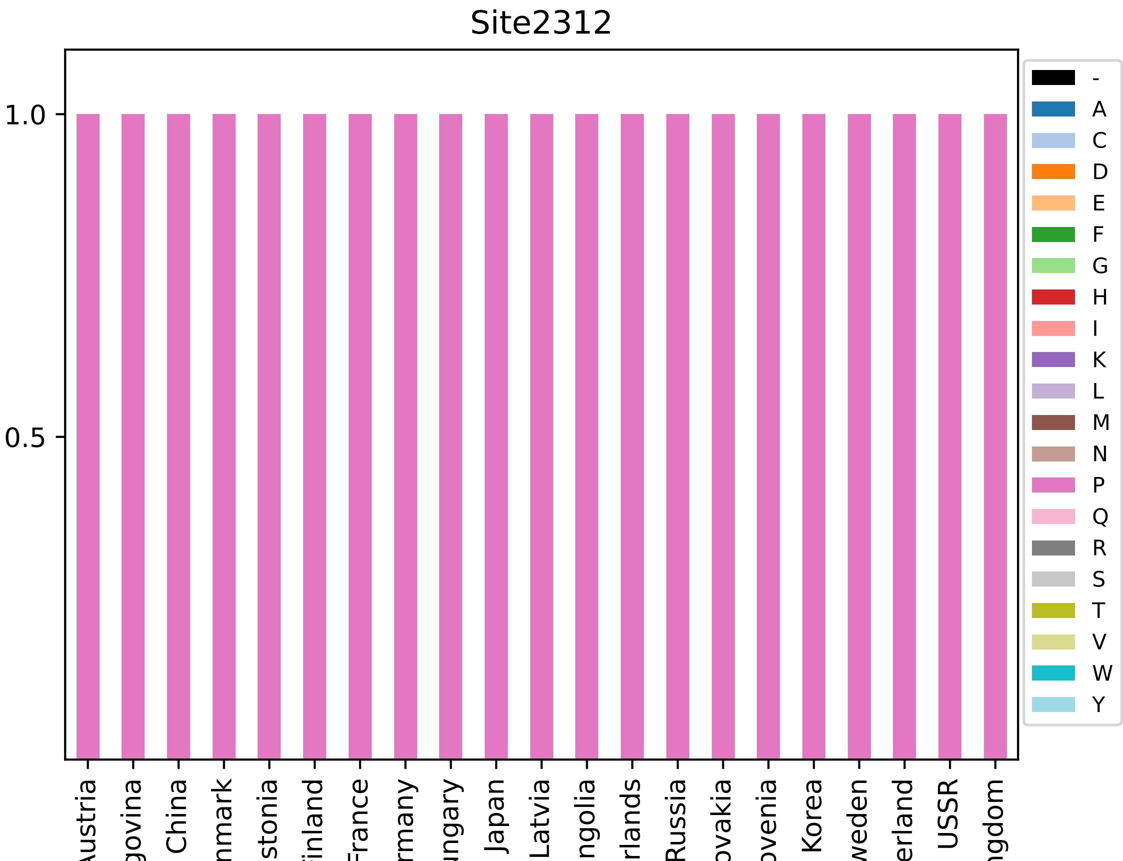
Site2312
1.0
0.5
-
A
C
D
E
F
G
H
I
K
L
M
N
P
Q
R
S
T
V
W
Y
China
Japan
Latvia
Russia
Korea
USSR

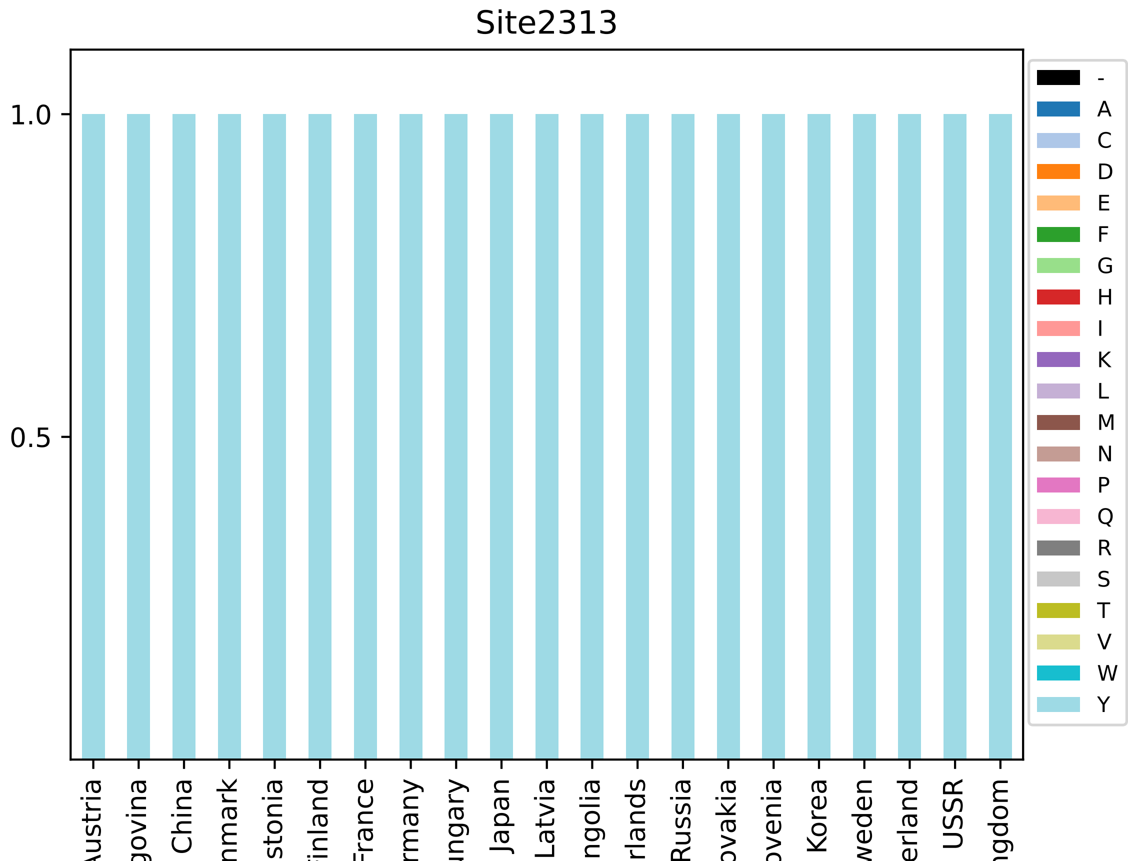
Site2313
1.0
0.5
-
A
C
D
E
F
G
H
I
K
L
M
N
P
Q
R
S
T
V
W
Y
China
Japan
Latvia
Russia
Korea
USSR

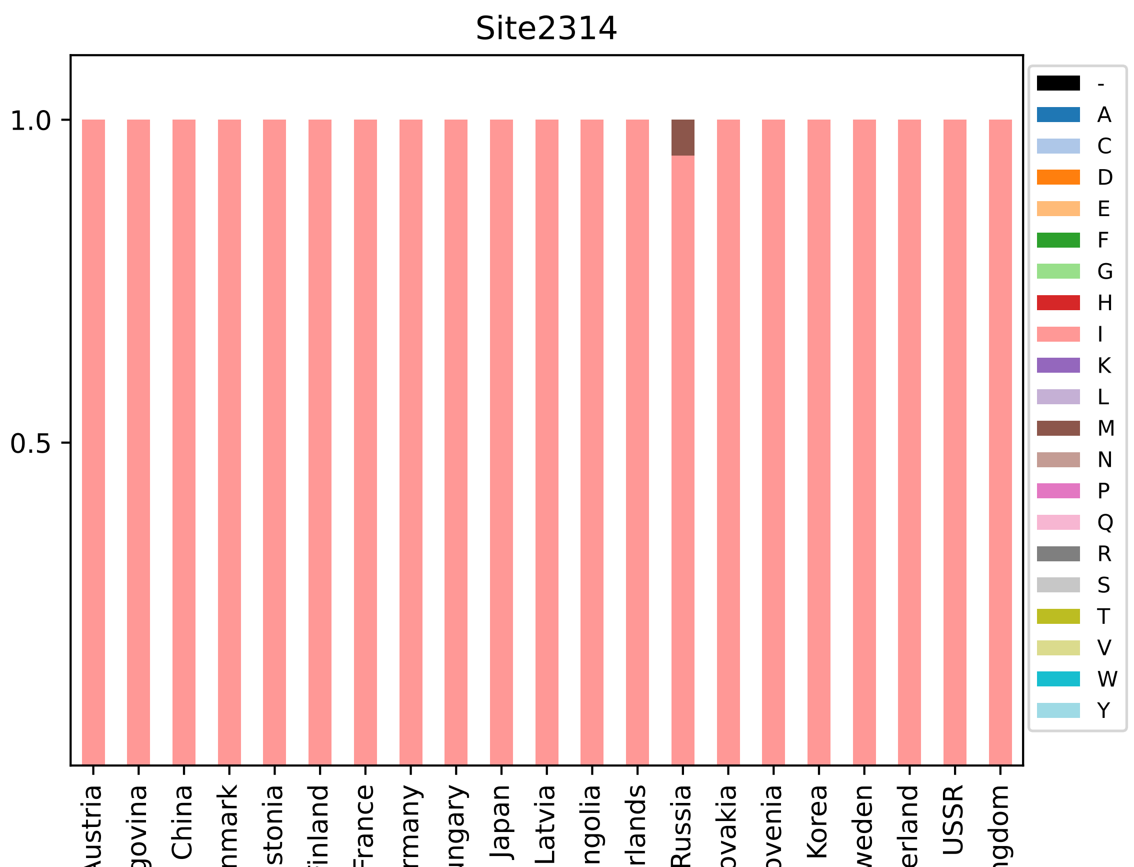

Site2314
1.0
0.5
-
A
C
D
E
F
G
H
I
K
L
M
N
P
Q
R
S
T
V
W
Y
China
Japan
Latvia
Russia
Korea
USSR

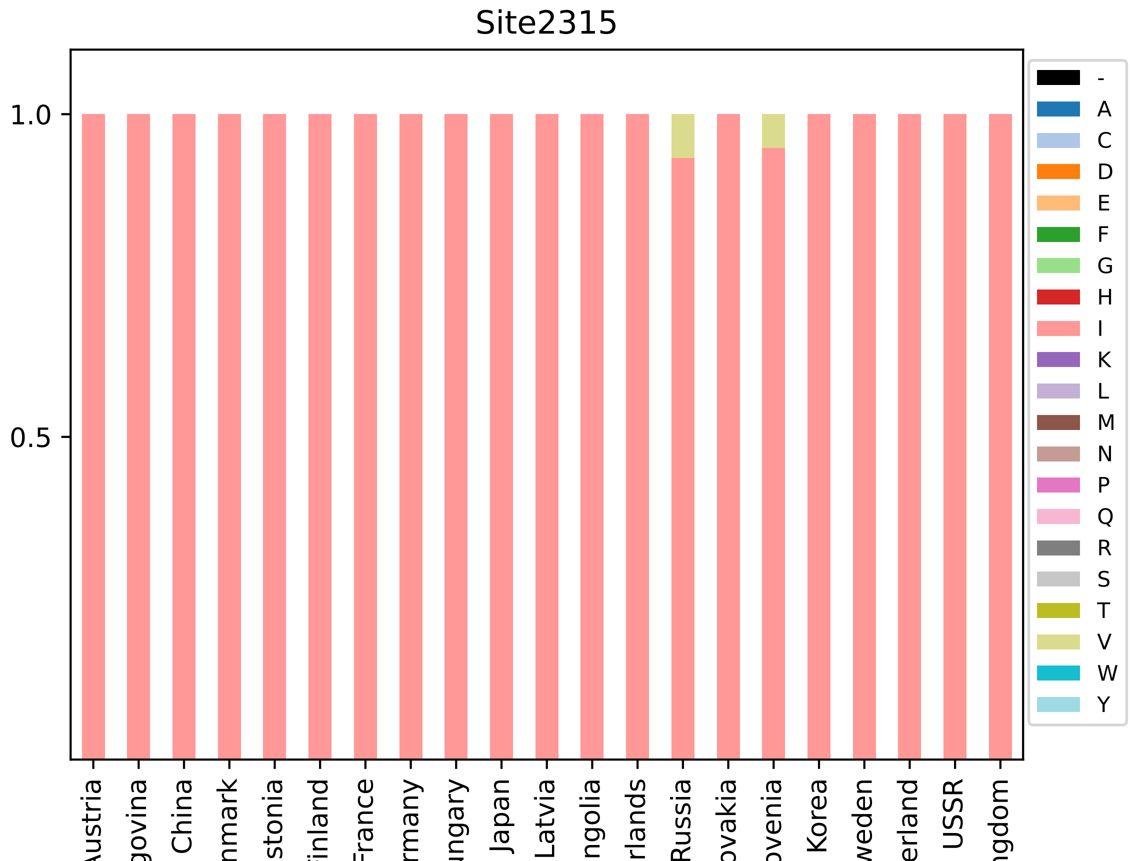

Site2315
1.0
0.5
-
A
C
D
E
F
G
H
I
K
L
M
N
P
Q
R
S
T
V
W
Y
Austria
govina
China
nmark
stonia
Finland
France
rmany
ungary
Japan
Latvia
ngolia
rlands
Russia
ovakia
ovenia
Korea
weden
erland
USSR
ngdom

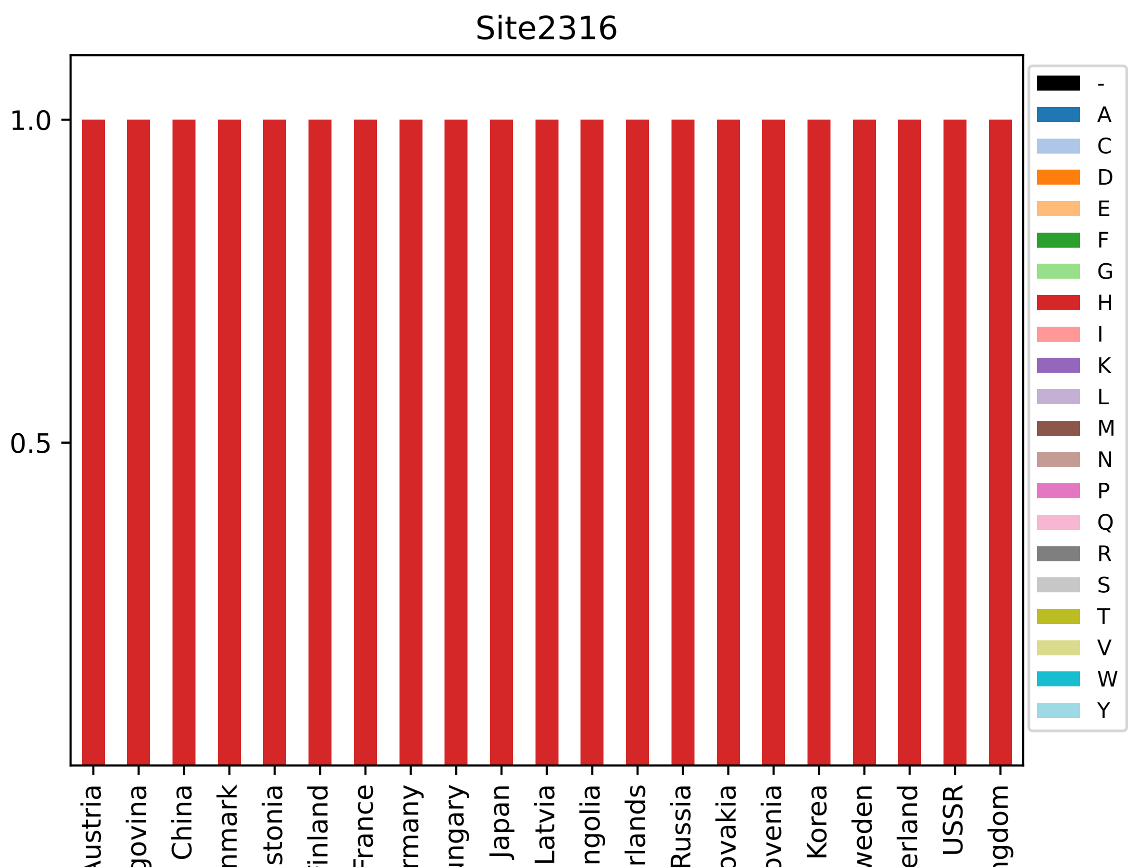

Site2316
1.0
0.5
-
A
C
D
E
F
G
H
I
K
L
M
N
P
Q
R
S
T
V
W
Y
China
Japan
Latvia
Russia
Korea
USSR

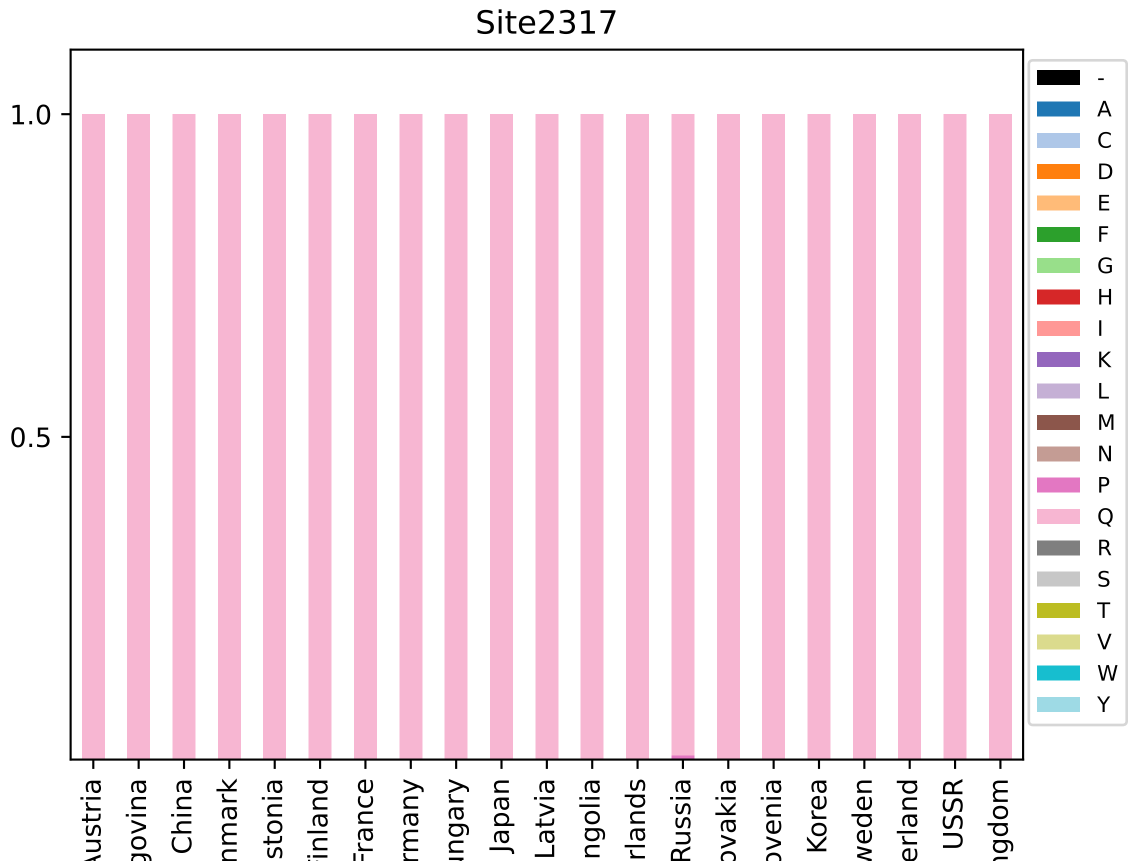

Site2317
1.0
0.5
-
A
C
D
E
F
G
H
I
K
L
M
N
P
Q
R
S
T
V
W
Y
Austria
govina
China
nmark
stonia
inland
France
rmany
ungary
Japan
Latvia
ngolia
rlands
Russia
ovakia
ovenia
Korea
weden
erland
USSR
ngdom

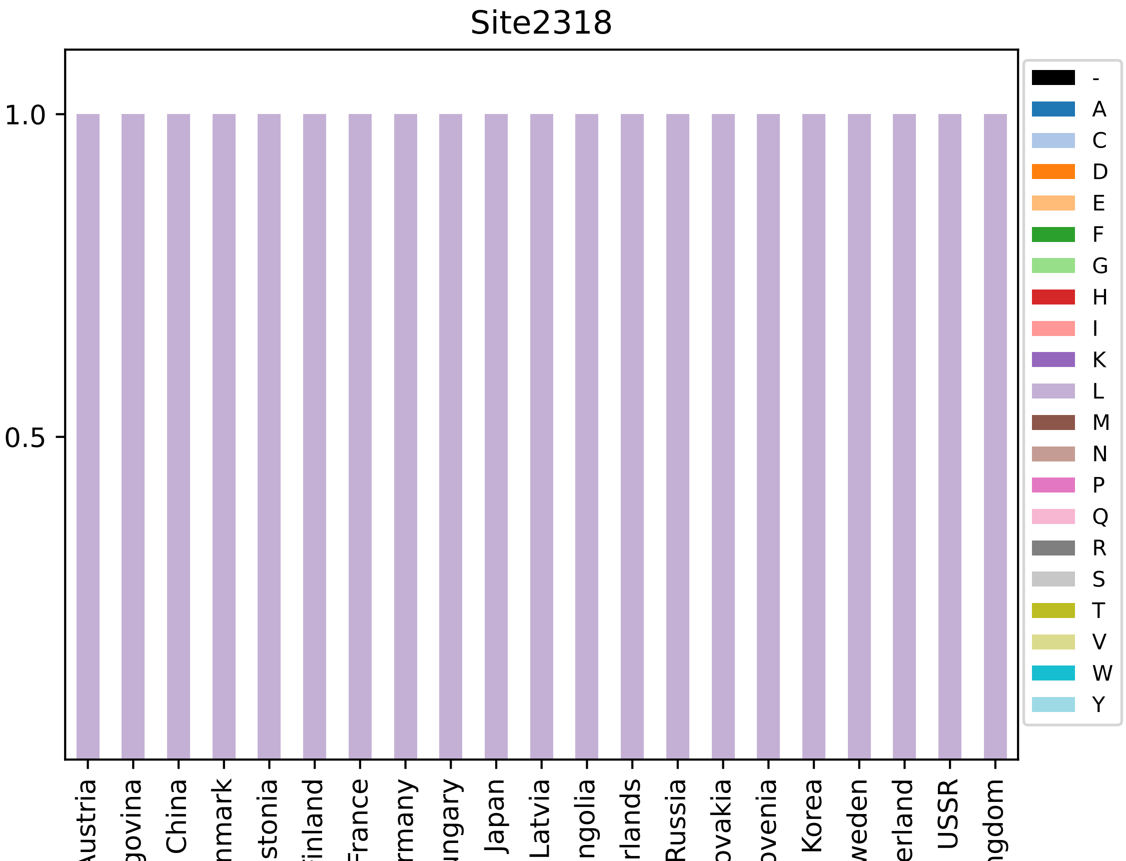

Site2318
1.0
0.5
-
A
C
D
E
F
G
H
I
K
L
M
N
P
Q
R
S
T
V
W
Y
Austria
China
Japan
Latvia
Russia
Korea
USSR

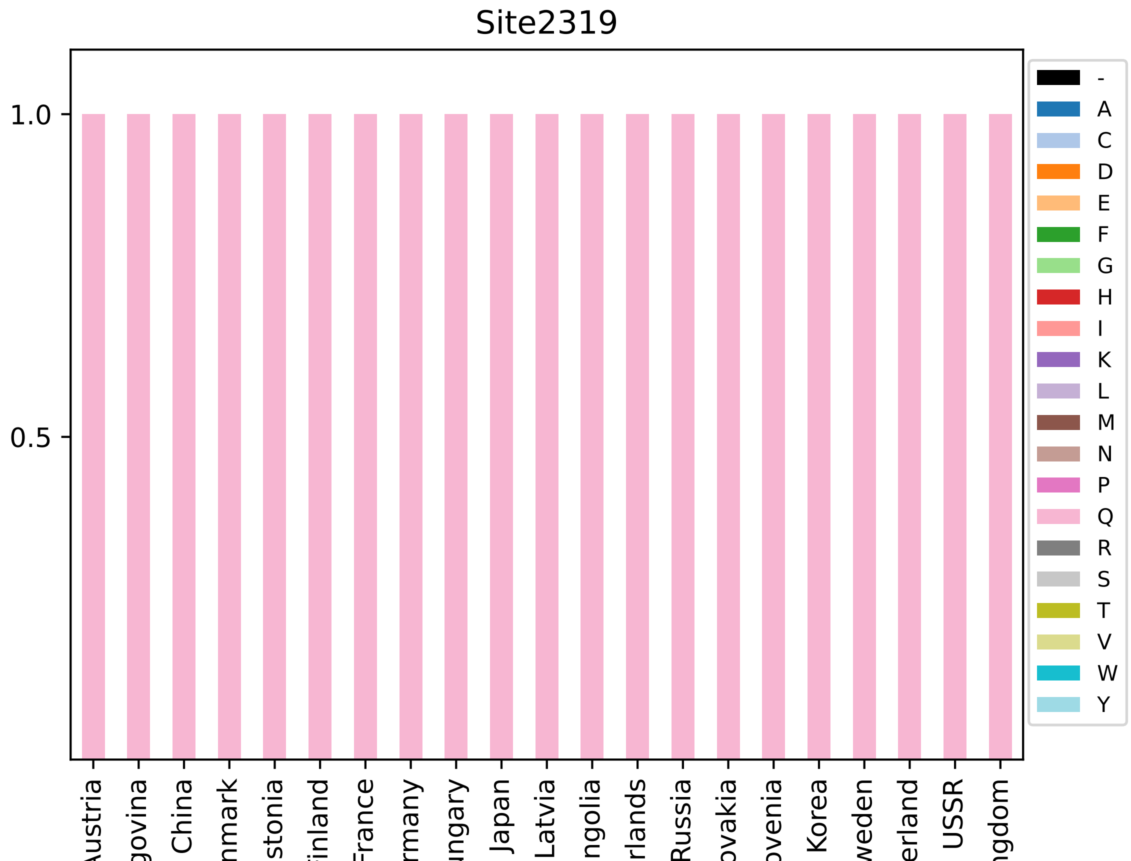
Site2319
1.0
0.5
Austria
govina
China
nmark
stonia
Finland
France
rmany
ungary
Japan
Latvia
ngolia
rlands
Russia
ovakia
ovenia
Korea
weden
erland
USSR
ngdom
-
A
C
D
E
F
G
H
I
K
L
M
N
P
Q
R
S
T
V
W
Y

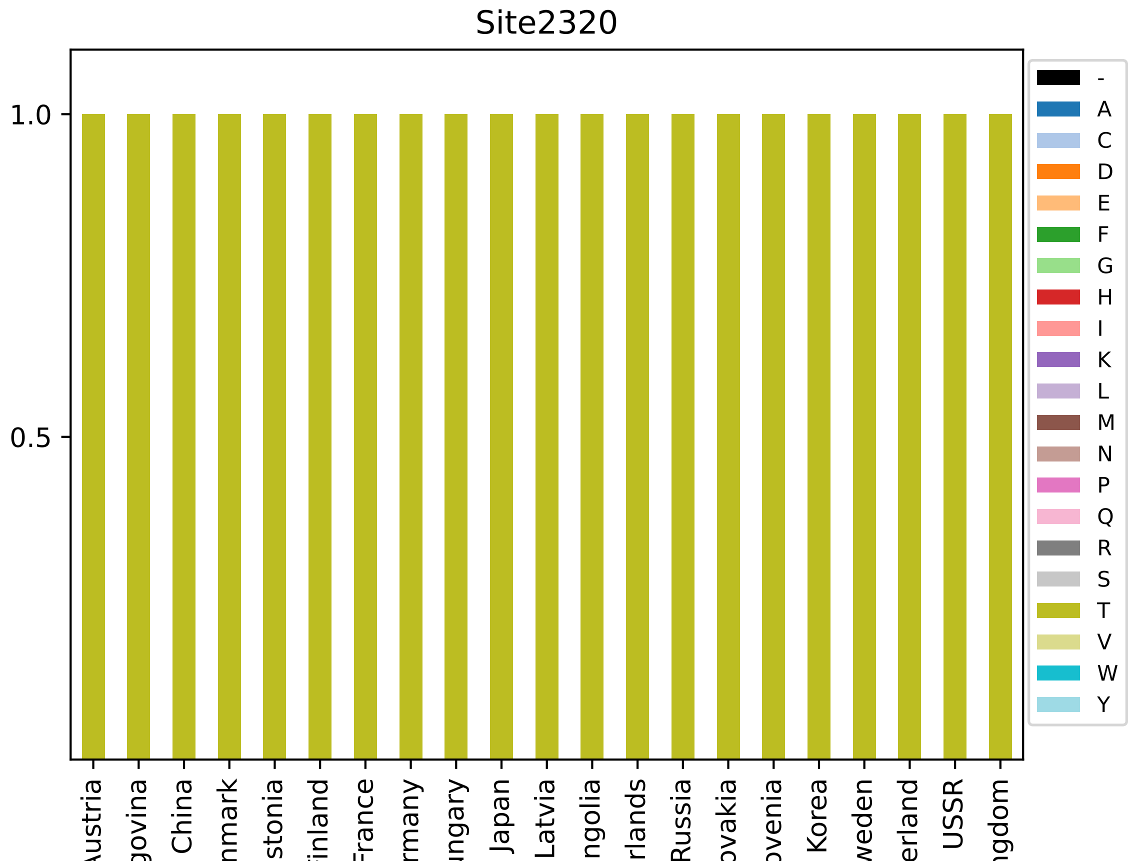

Site2320
1.0
0.5
-
A
C
D
E
F
G
H
I
K
L
M
N
P
Q
R
S
T
V
W
Y
Austria
govina
China
nmark
stonia
Finland
France
rmany
ungary
Japan
Latvia
ngolia
rlands
Russia
ovakia
ovenia
Korea
weden
erland
USSR
ngdom

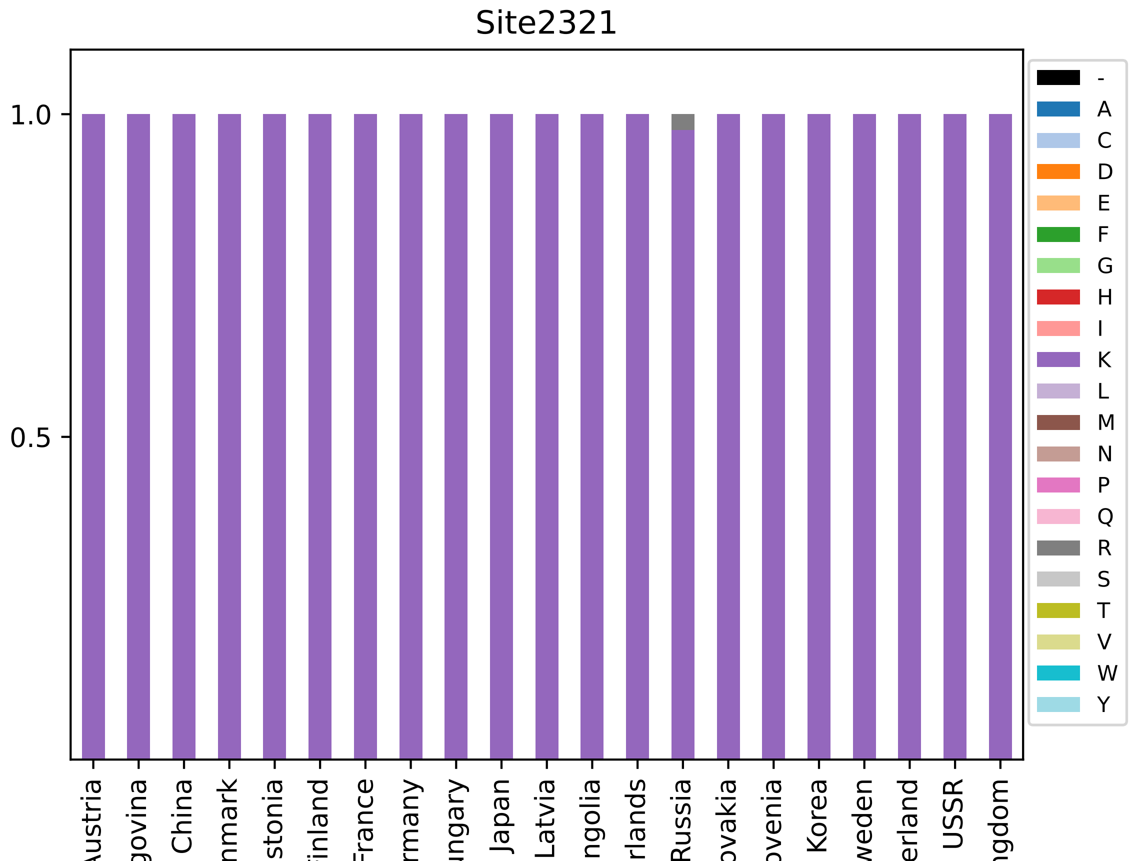
Site2321
1.0
0.5
-
A
C
D
E
F
G
H
I
K
L
M
N
P
Q
R
S
T
V
W
Y
China
Japan
Latvia
Russia
Korea
USSR

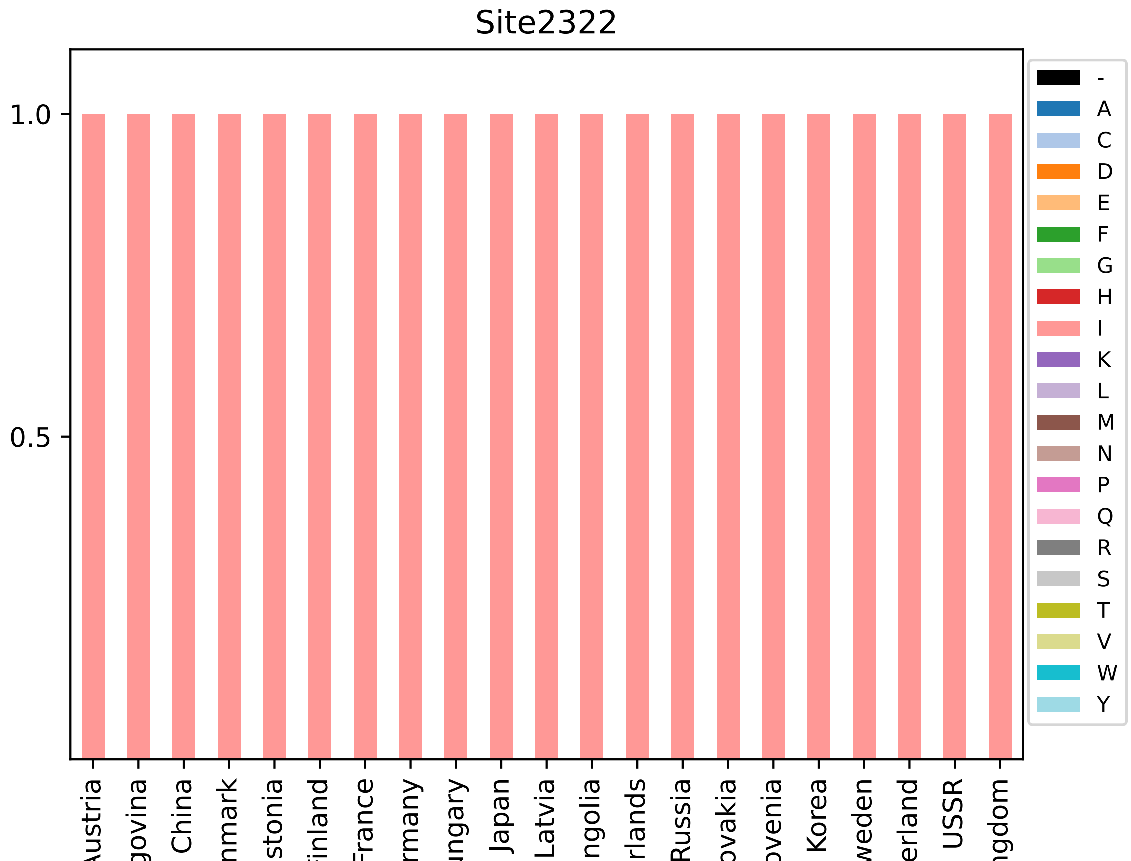
Site2322
1.0
0.5
China
Japan
Latvia
Russia
Korea
USSR
-
A
C
D
E
F
G
H
I
K
L
M
N
P
Q
R
S
T
V
W
Y

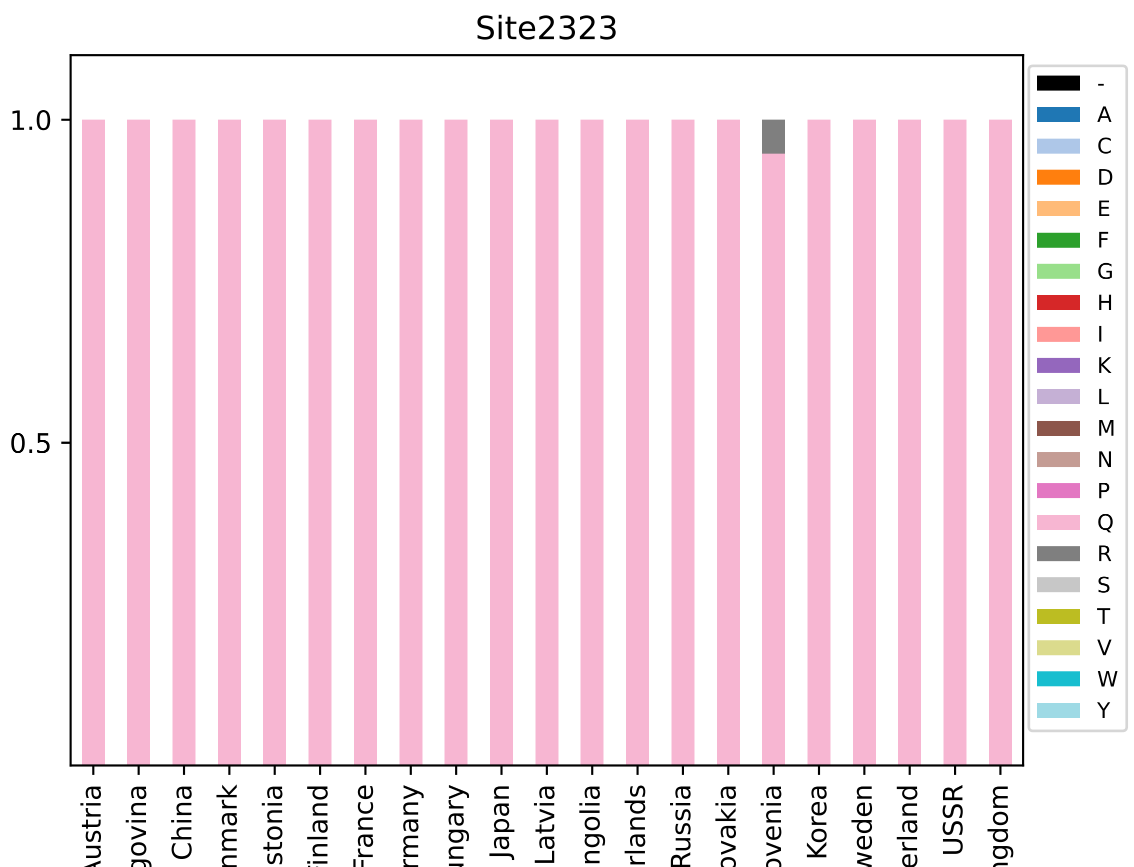

Site2323
1.0
0.5
-
A
C
D
E
F
G
H
I
K
L
M
N
P
Q
R
S
T
V
W
Y
China
Japan
Latvia
Russia
Korea
USSR

Site2324

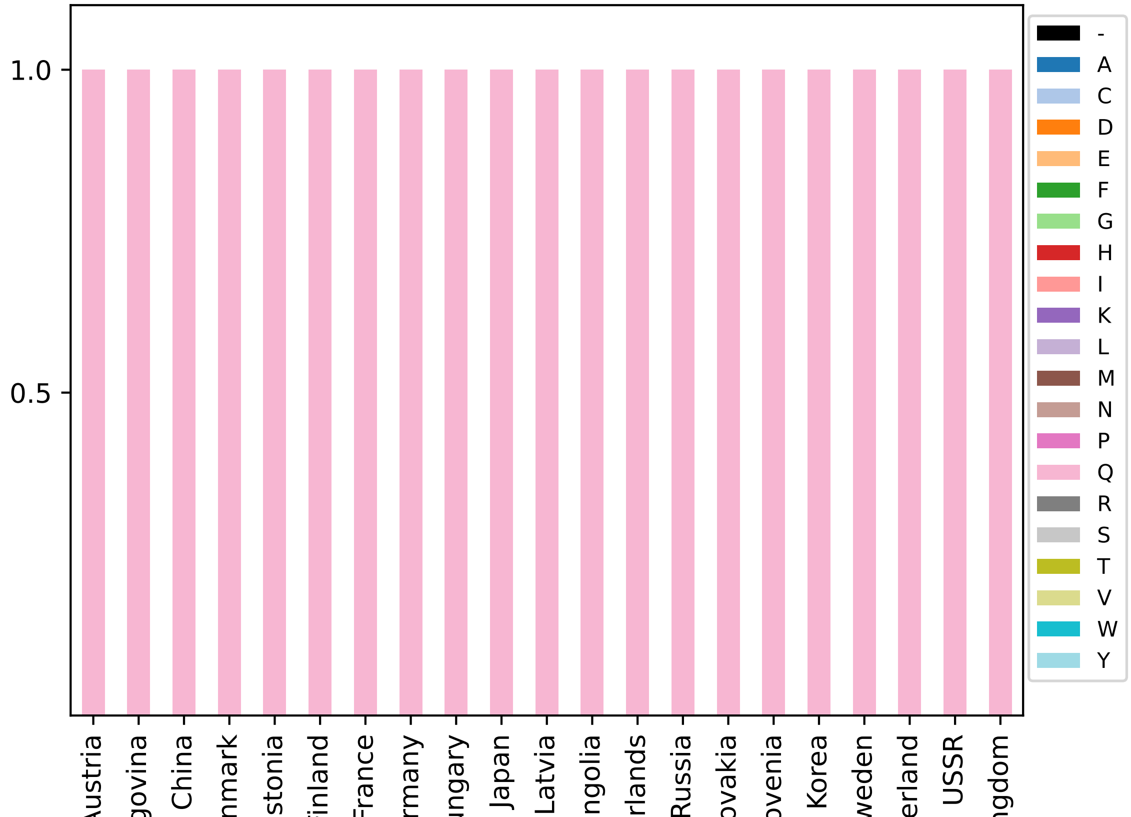

1.0
0.5
China
Japan
Latvia
Russia
Korea
USSR
-
A
C
D
E
F
G
H
I
K
L
M
N
P
Q
R
S
T
V
W
Y

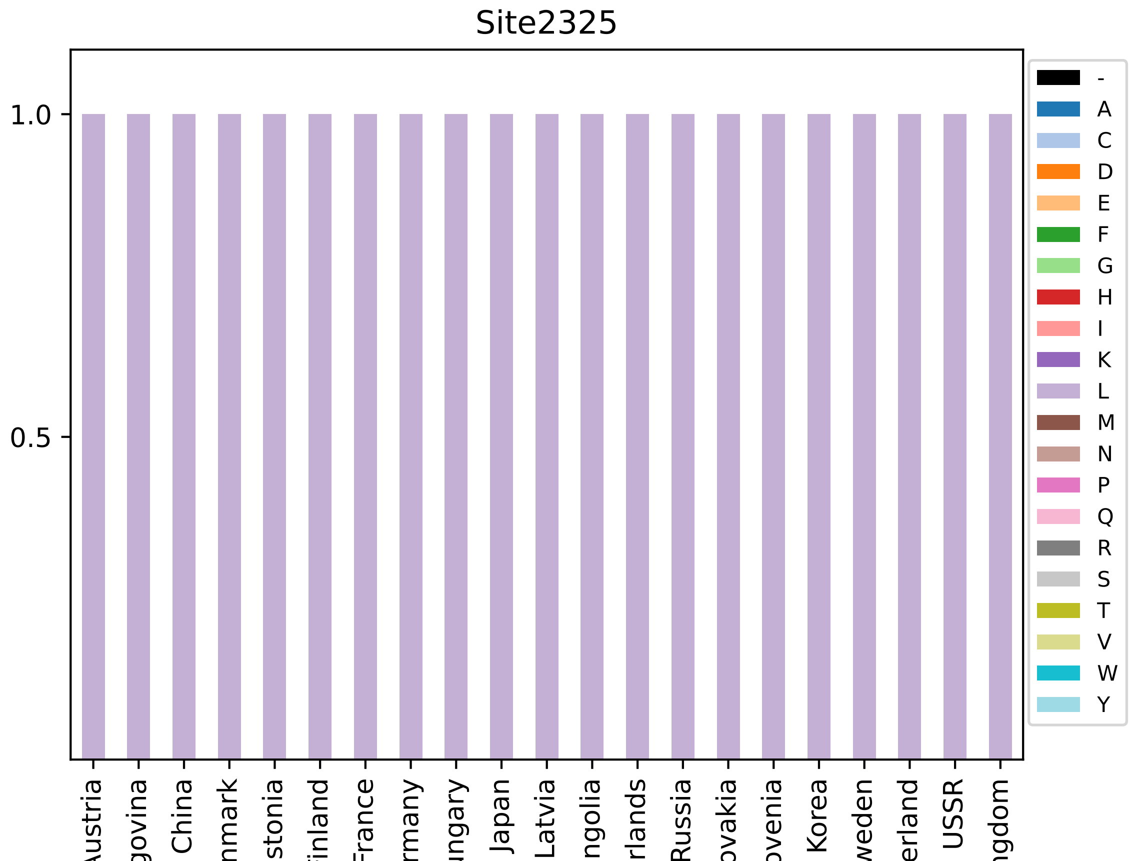
Site2325
1.0
0.5
-
A
C
D
E
F
G
H
I
K
L
M
N
P
Q
R
S
T
V
W
Y
China
Japan
Latvia
Russia
Korea
USSR

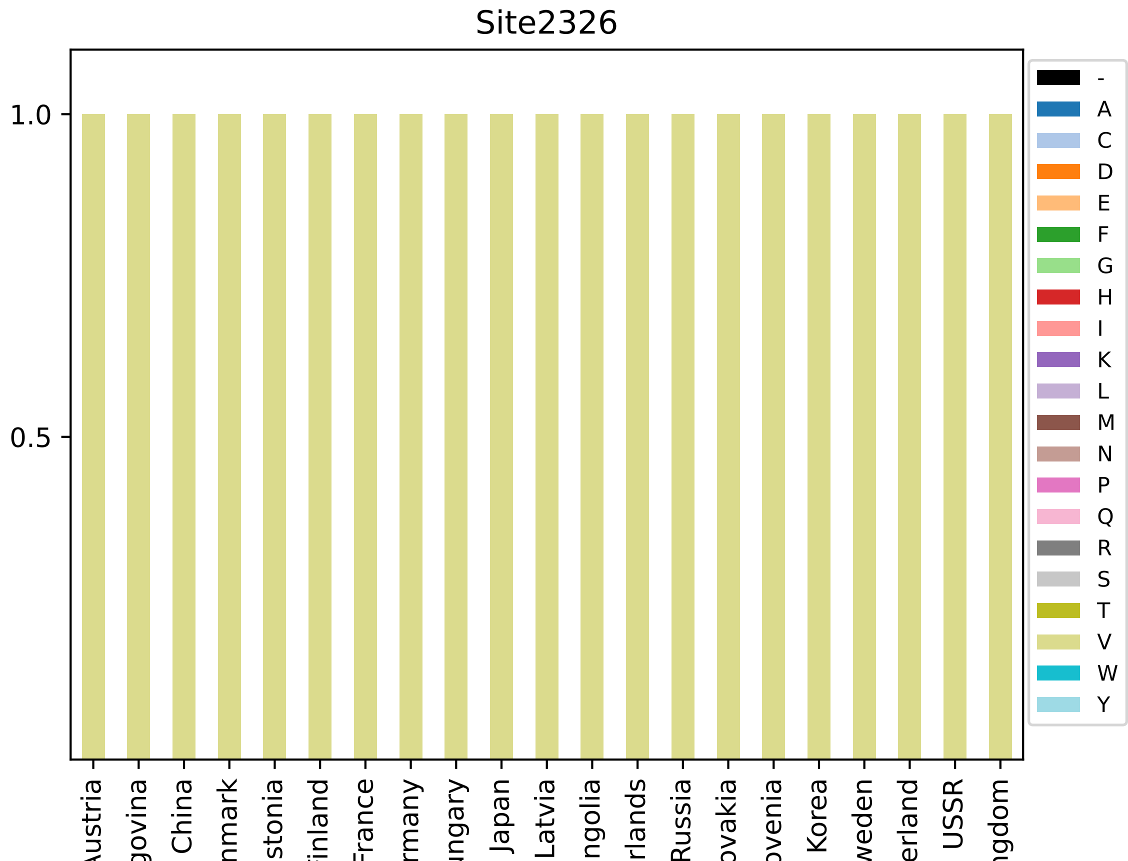
Site2326
1.0
0.5
-
A
C
D
E
F
G
H
I
K
L
M
N
P
Q
R
S
T
V
W
Y
China
Japan
Latvia
Russia
Korea
USSR

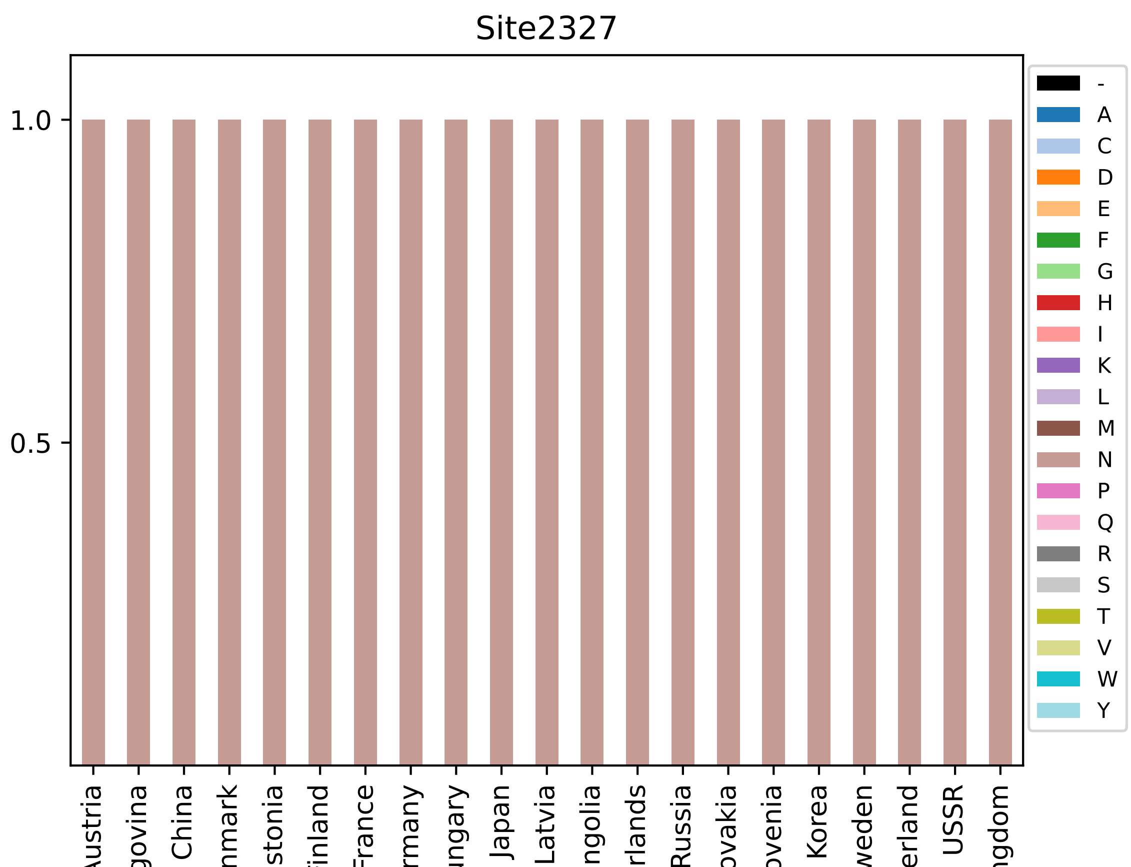
Site2327
1.0
0.5
-
A
C
D
E
F
G
H
I
K
L
M
N
P
Q
R
S
T
V
W
Y
China
Japan
Latvia
Russia
Korea
USSR

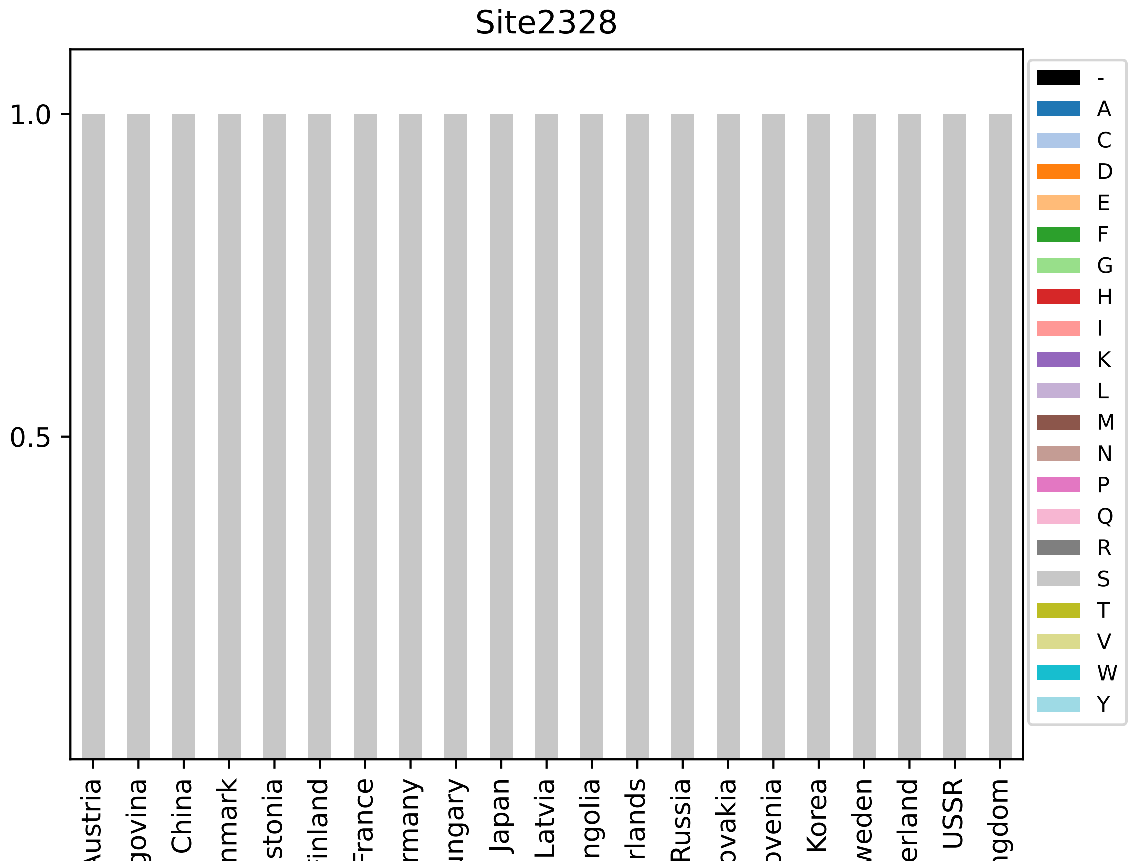
Site2328
1.0
0.5
Austria
govina
China
nmark
stonia
Finland
France
rmany
ungary
Japan
Latvia
ngolia
rlands
Russia
ovakia
ovenia
Korea
weden
erland
USSR
ngdom
-
A
C
D
E
F
G
H
I
K
L
M
N
P
Q
R
S
T
V
W
Y

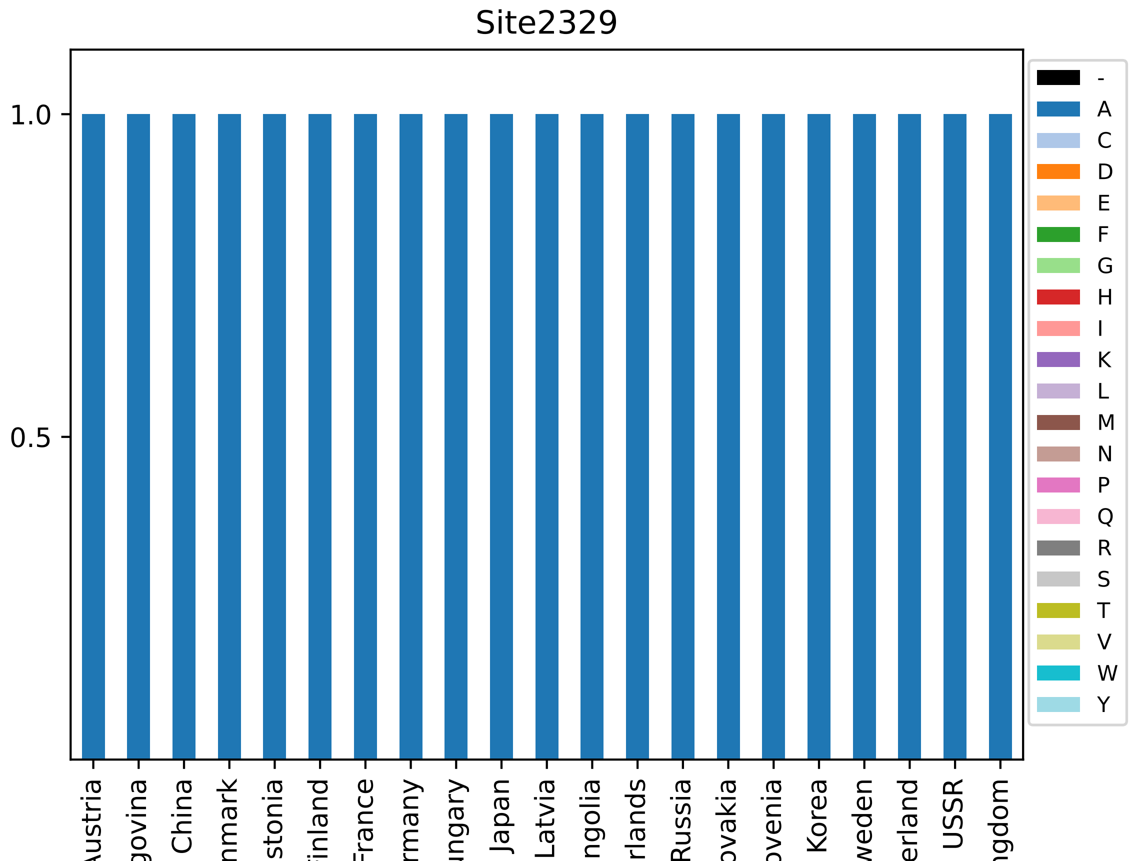

Site2329
1.0
0.5
-
A
C
D
E
F
G
H
I
K
L
M
N
P
Q
R
S
T
V
W
Y
Austria
govina
China
nmark
stonia
inland
France
rmany
ungary
Japan
Latvia
ngolia
rlands
Russia
ovakia
ovenia
Korea
weden
erland
USSR
ngdom

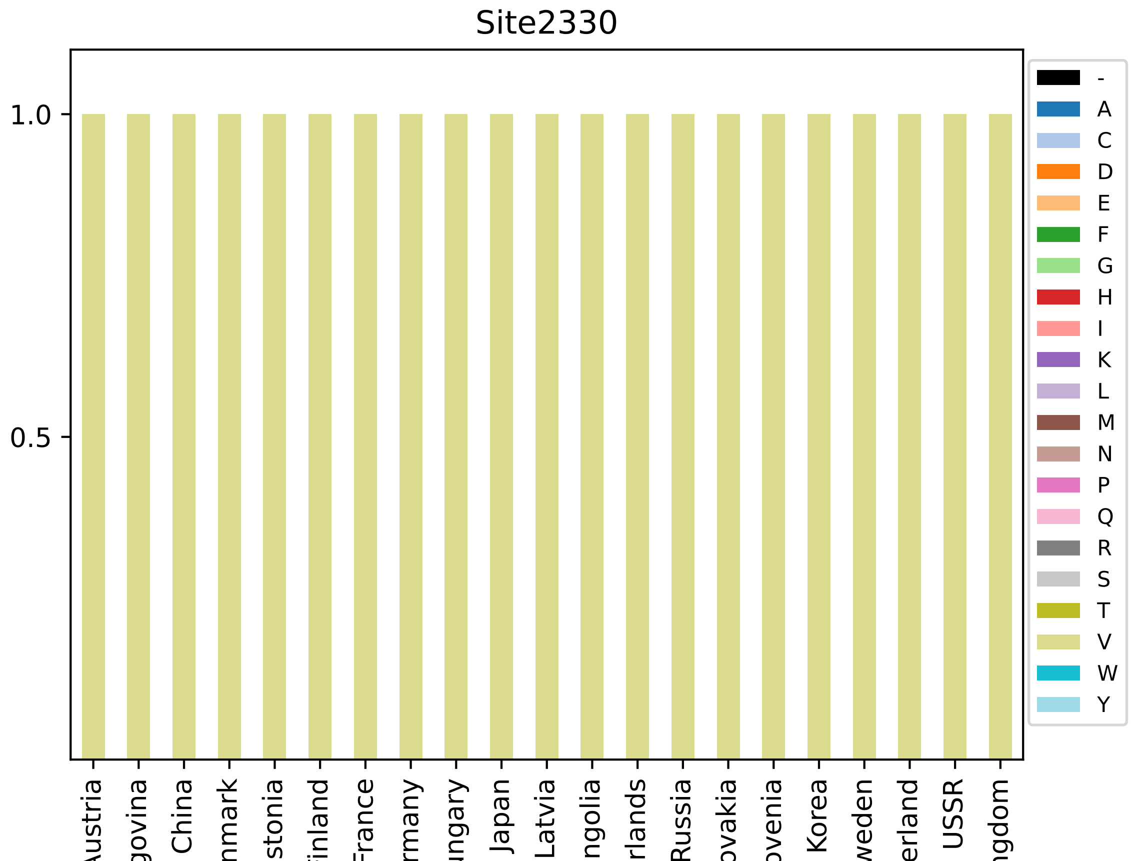

Site2330
1.0
0.5
-
A
C
D
E
F
G
H
I
K
L
M
N
P
Q
R
S
T
V
W
Y
Austria
China
Japan
Latvia
Russia
Korea
USSR

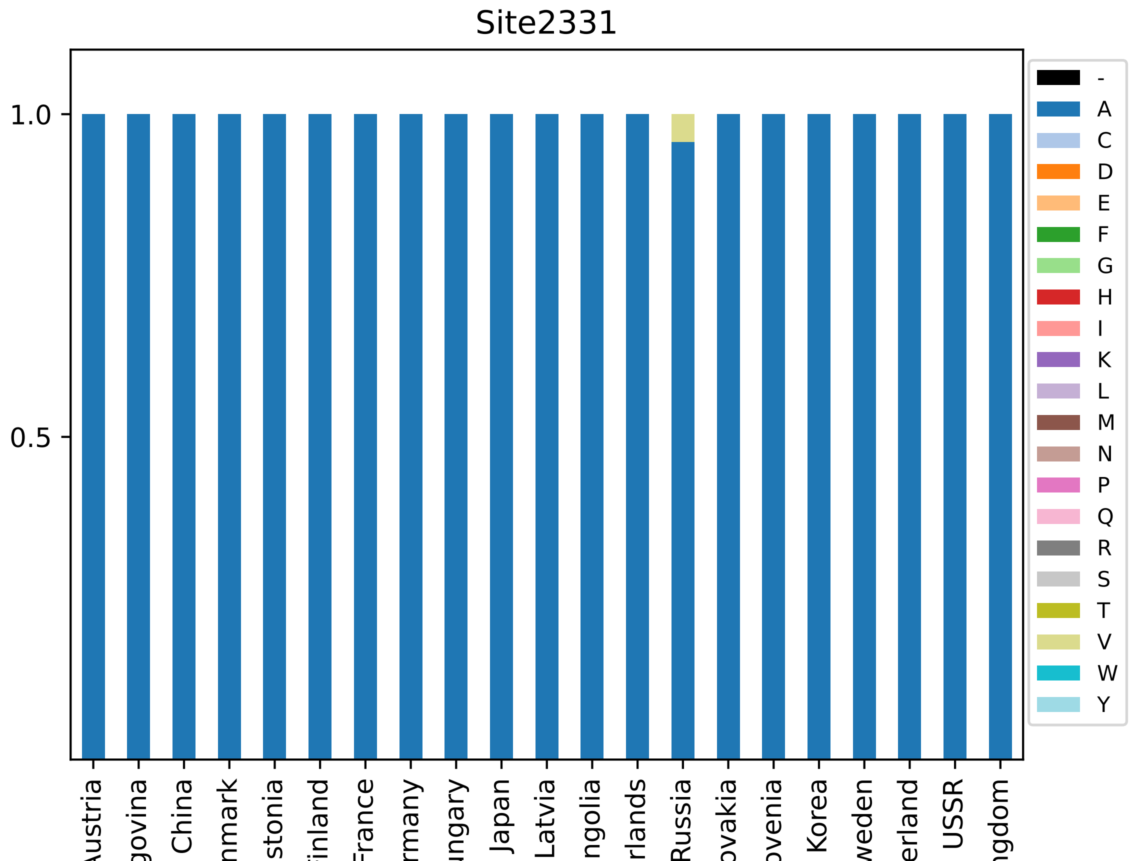
Site2331
1.0
0.5
-
A
C
D
E
F
G
H
I
K
L
M
N
P
Q
R
S
T
V
W
Y
Austria
govina
China
nmark
stonia
inland
France
rmany
ungary
Japan
Latvia
ngolia
rlands
Russia
ovakia
ovenia
Korea
weden
erland
USSR
ngdom

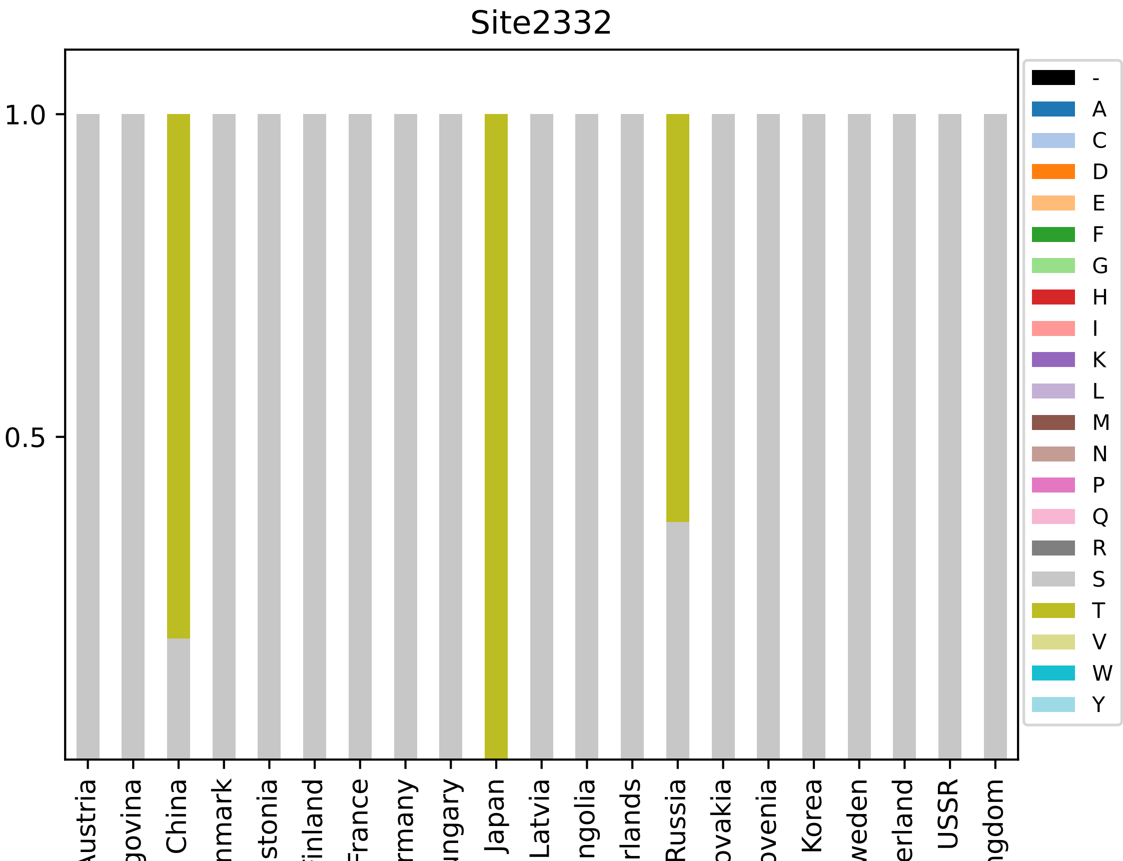

Site2332
1.0
0.5
-
A
C
D
E
F
G
H
I
K
L
M
N
P
Q
R
S
T
V
W
Y
Austria
govina
China
nmark
stonia
inland
France
rmany
ungary
Japan
Latvia
ngolia
rlands
Russia
ovakia
ovenia
Korea
weden
erland
USSR
ngdom

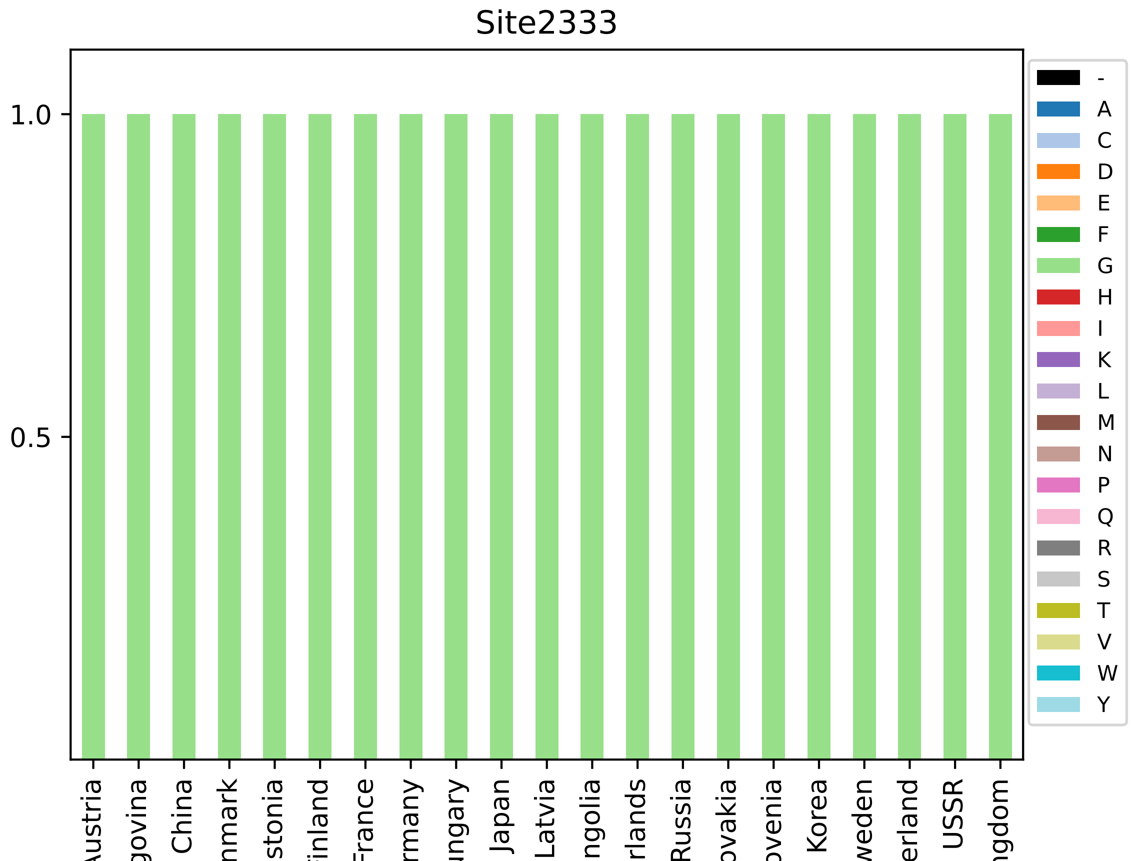
Site2333
1.0
0.5
-
A
C
D
E
F
G
H
I
K
L
M
N
P
Q
R
S
T
V
W
Y
Austria
China
Japan
Latvia
Russia
Korea
USSR

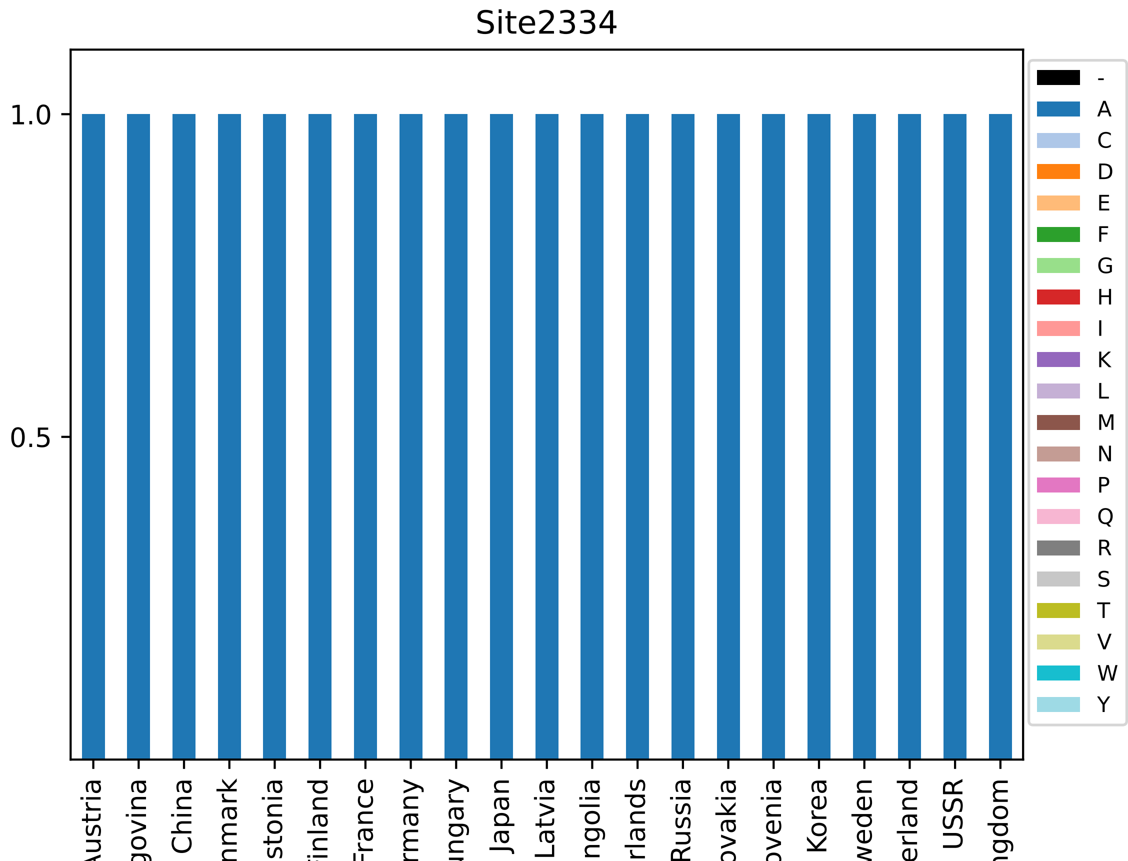

Site2334
1.0
0.5
-
A
C
D
E
F
G
H
I
K
L
M
N
P
Q
R
S
T
V
W
Y
Austria
govina
China
nmark
stonia
inland
France
rmany
ungary
Japan
Latvia
ngolia
rlands
Russia
ovakia
ovenia
Korea
weden
erland
USSR
ngdom

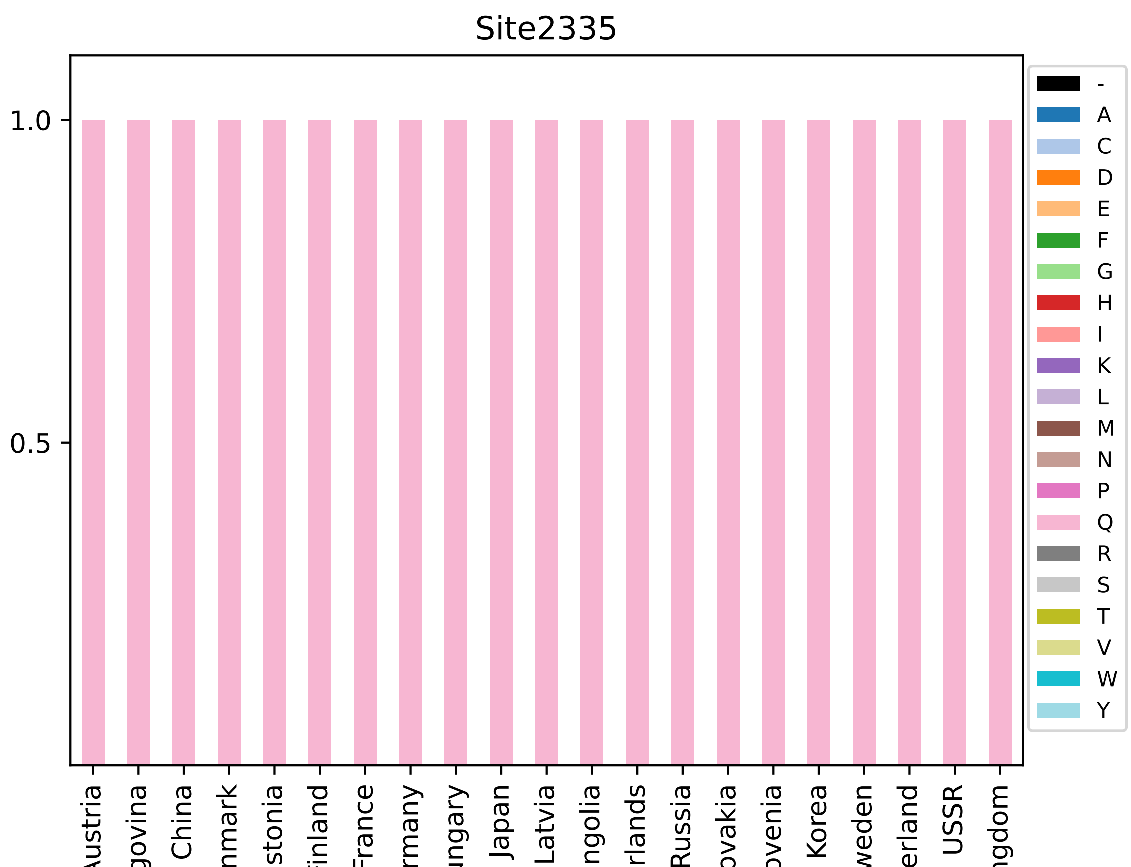

Site2335
1.0
0.5
-
A
C
D
E
F
G
H
I
K
L
M
N
P
Q
R
S
T
V
W
Y
Austria
govina
China
nmark
stonia
inland
France
rmany
ungary
Japan
Latvia
ngolia
rlands
Russia
ovakia
ovenia
Korea
weden
erland
USSR
ngdom

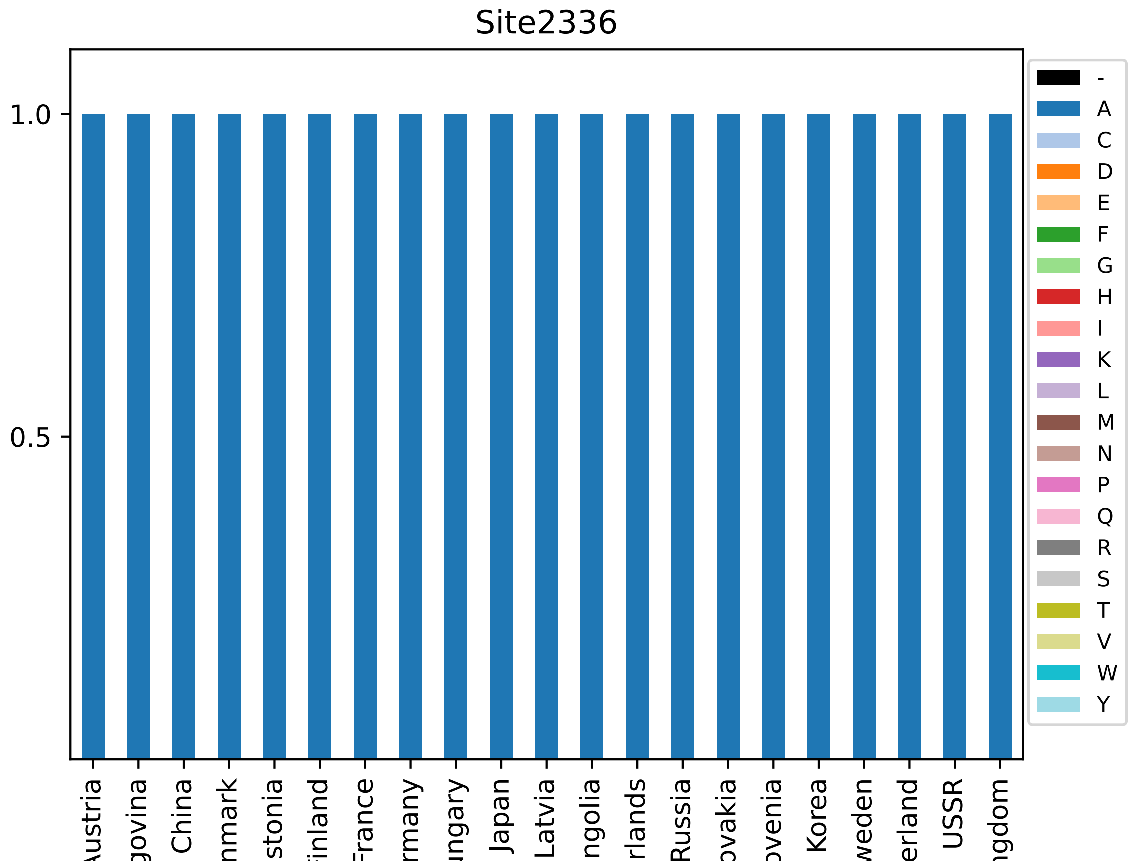

Site2336
1.0
0.5
-
A
C
D
E
F
G
H
I
K
L
M
N
P
Q
R
S
T
V
W
Y
Austria
China
Japan
Latvia
Russia
Korea
USSR

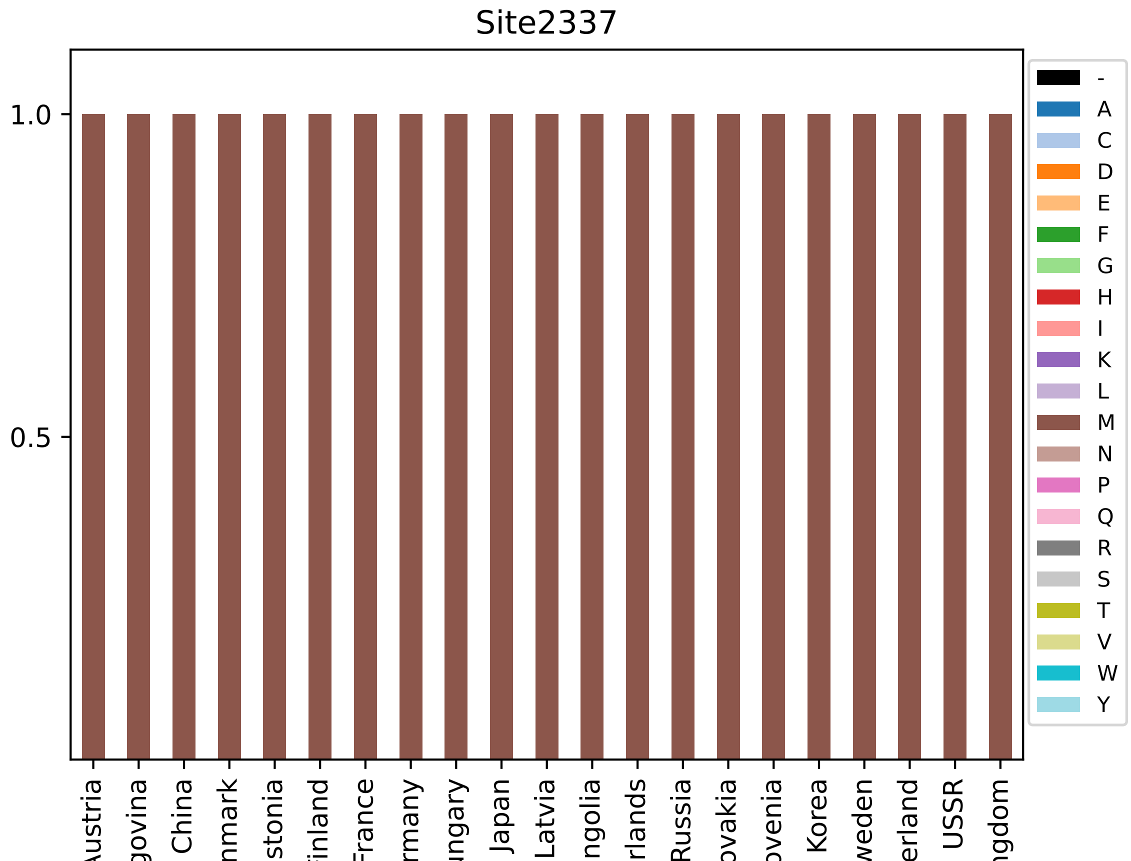
Site2337
1.0
0.5
-
A
C
D
E
F
G
H
I
K
L
M
N
P
Q
R
S
T
V
W
Y
China
Japan
Latvia
Russia
Korea
USSR

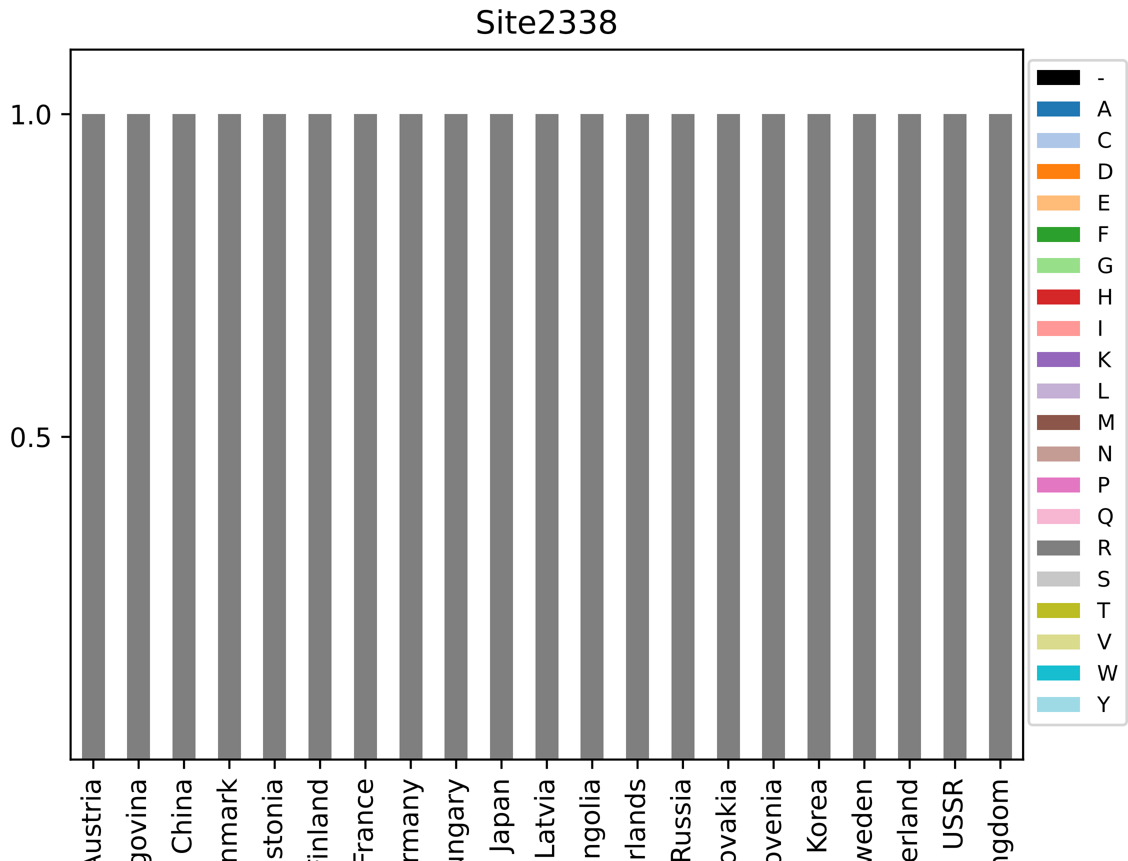
Site2338
1.0
0.5
China
Japan
Latvia
Russia
Korea
USSR
-
A
C
D
E
F
G
H
I
K
L
M
N
P
Q
R
S
T
V
W
Y

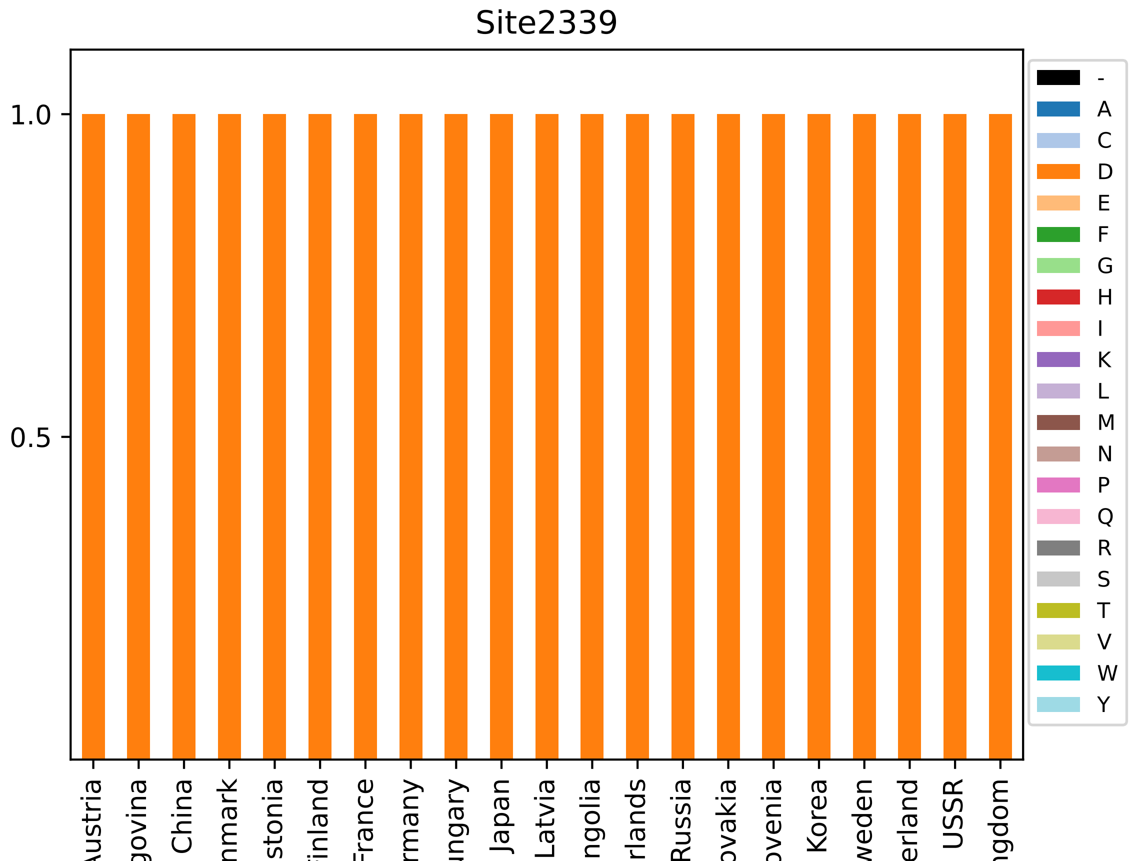
Site2339
1.0
0.5
-
A
C
D
E
F
G
H
I
K
L
M
N
P
Q
R
S
T
V
W
Y
China
Japan
Latvia
Russia
Korea
USSR

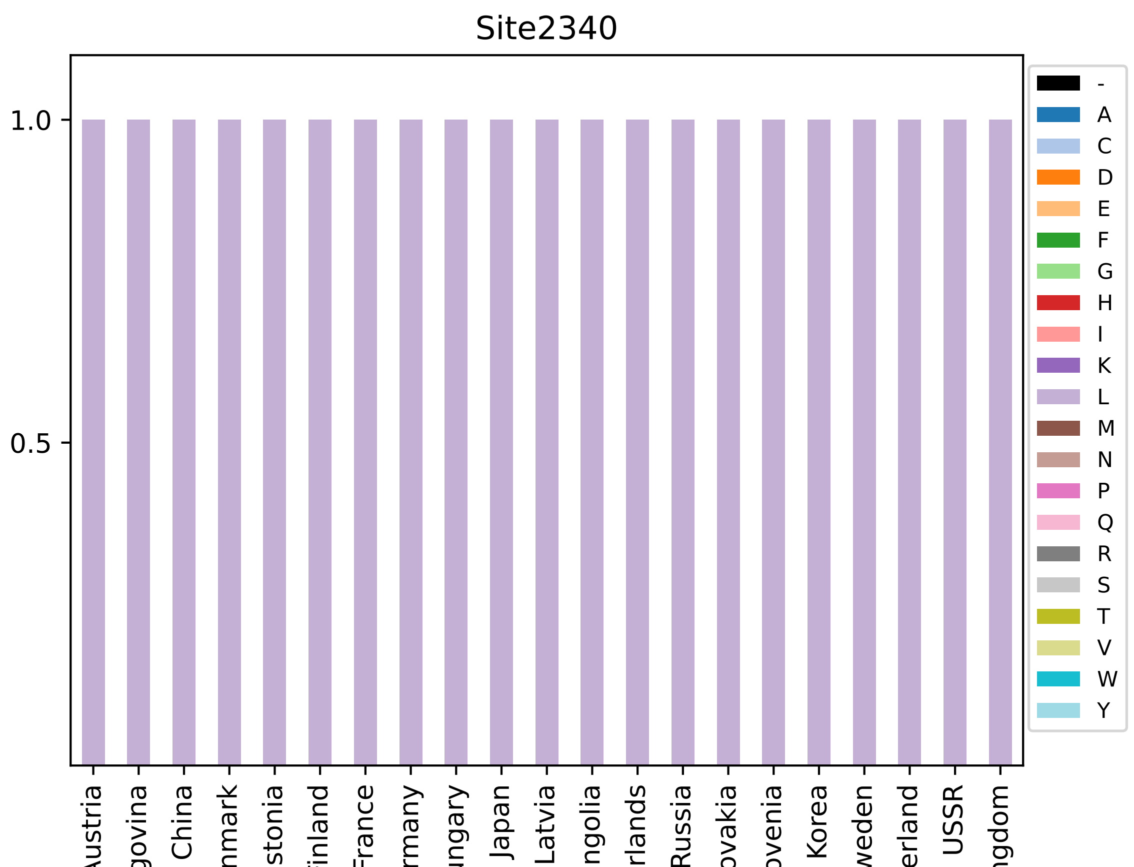

Site2340
1.0
0.5
-
A
C
D
E
F
G
H
I
K
L
M
N
P
Q
R
S
T
V
W
Y
Austria
China
Japan
Latvia
Russia
Korea
USSR

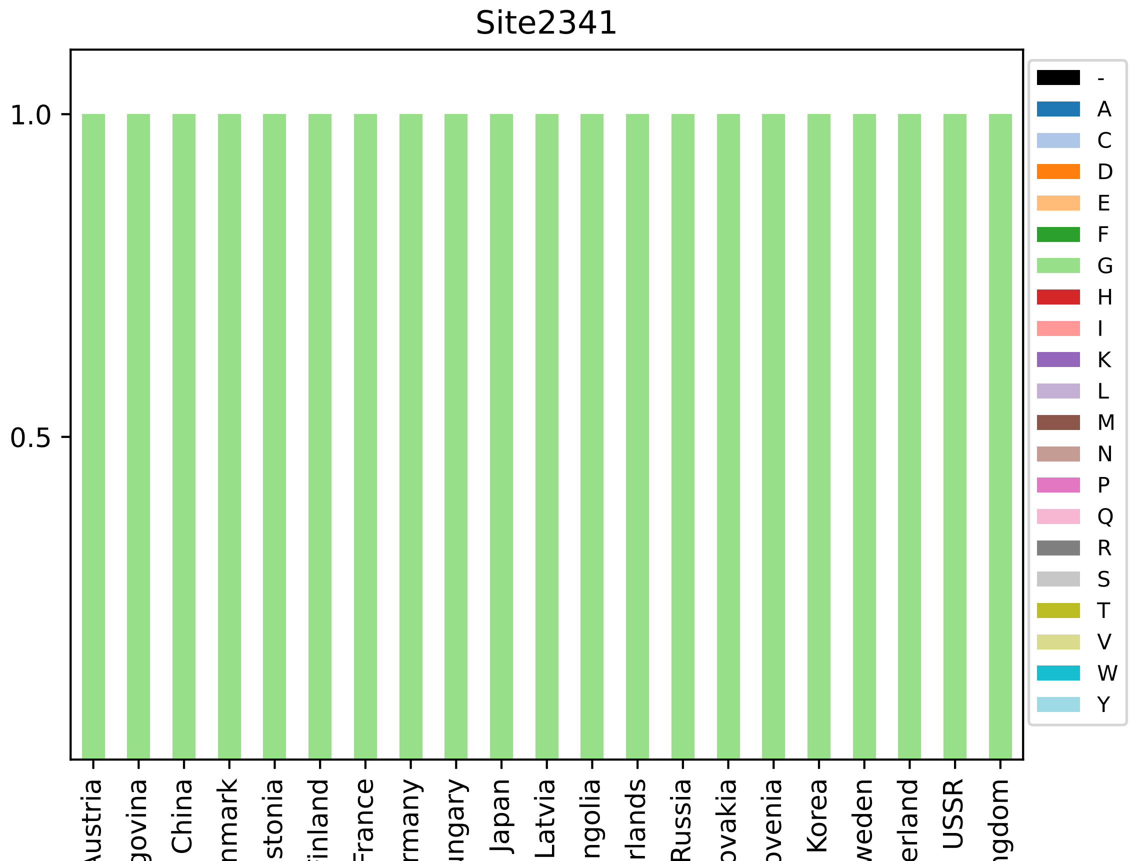

Site2341
1.0
0.5
-
A
C
D
E
F
G
H
I
K
L
M
N
P
Q
R
S
T
V
W
Y
Austria
govina
China
nmark
stonia
inland
France
rmany
ungary
Japan
Latvia
ngolia
rlands
Russia
ovakia
ovenia
Korea
weden
erland
USSR
ngdom

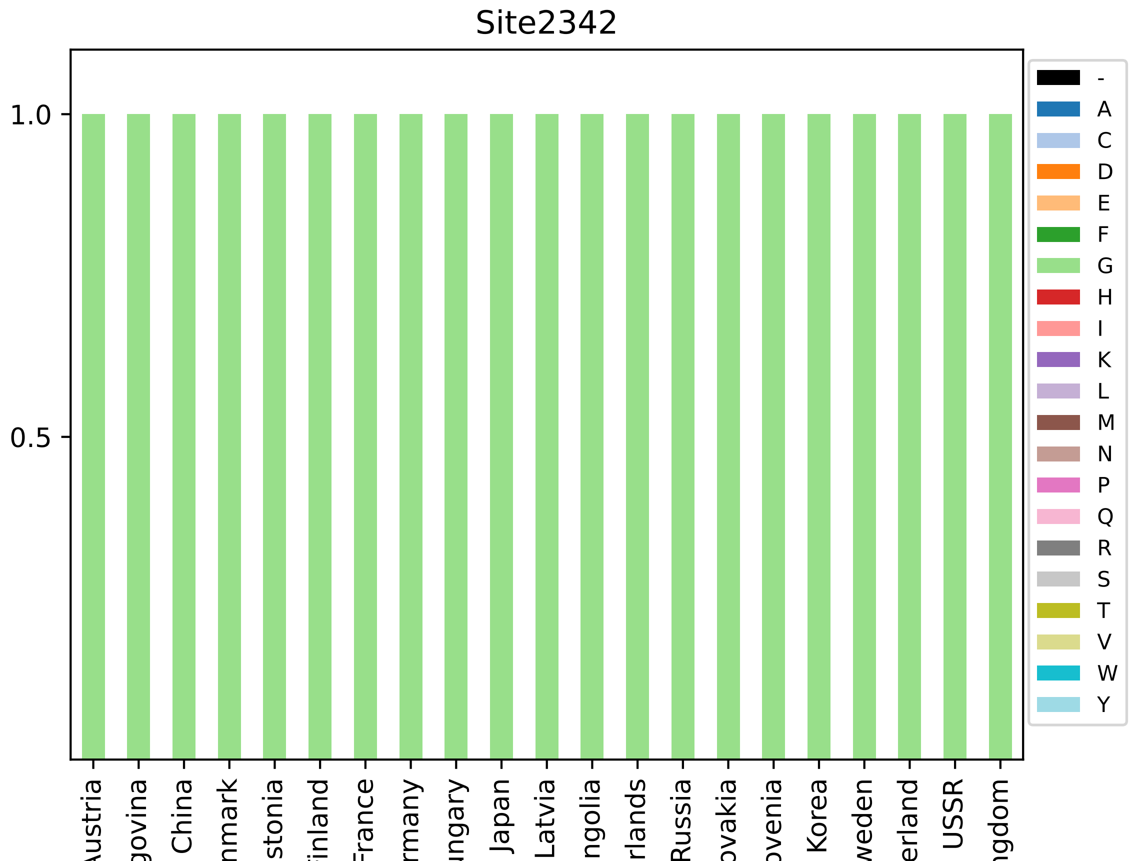
Site2342
1.0
0.5
-
A
C
D
E
F
G
H
I
K
L
M
N
P
Q
R
S
T
V
W
Y
China
Japan
Latvia
Russia
Korea
USSR

Site2343

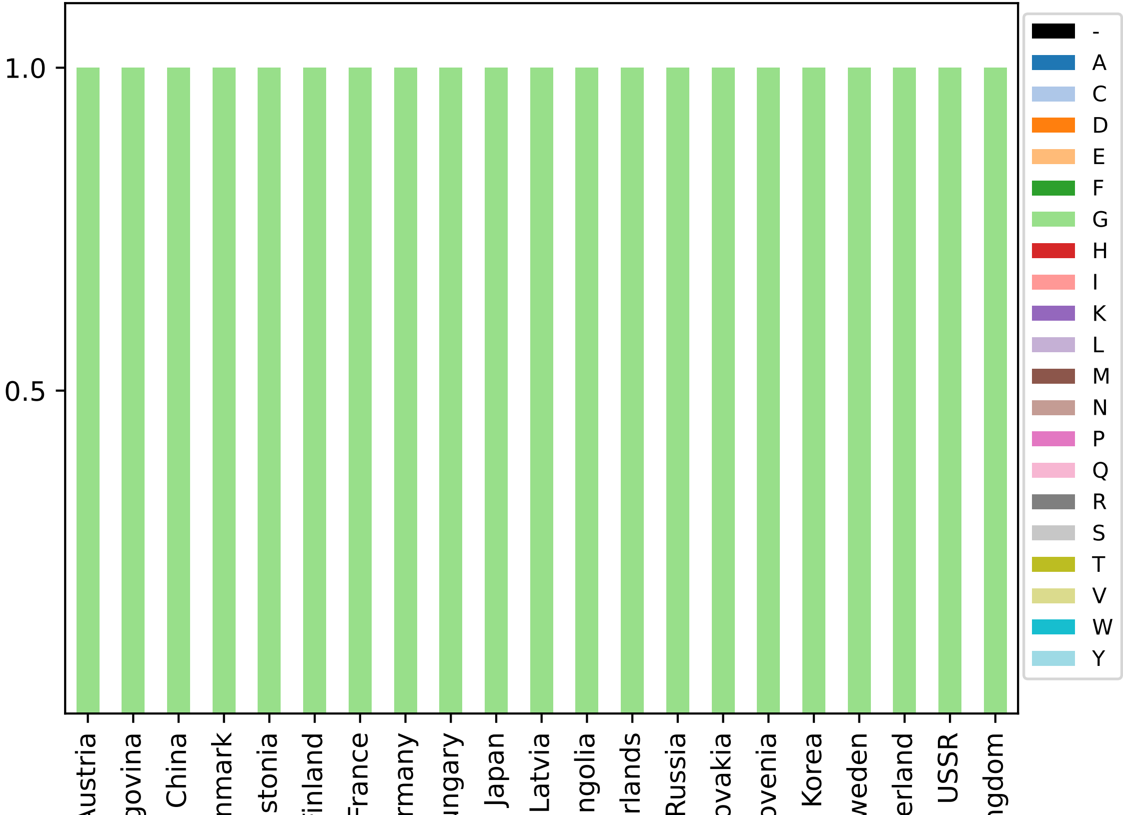

1.0
0.5
Austria
govina
China
nmark
stonia
inland
France
rmany
ungary
Japan
Latvia
ngolia
rlands
Russia
ovakia
ovenia
Korea
weden
erland
USSR
ngdom
-
A
C
D
E
F
G
H
I
K
L
M
N
P
Q
R
S
T
V
W
Y

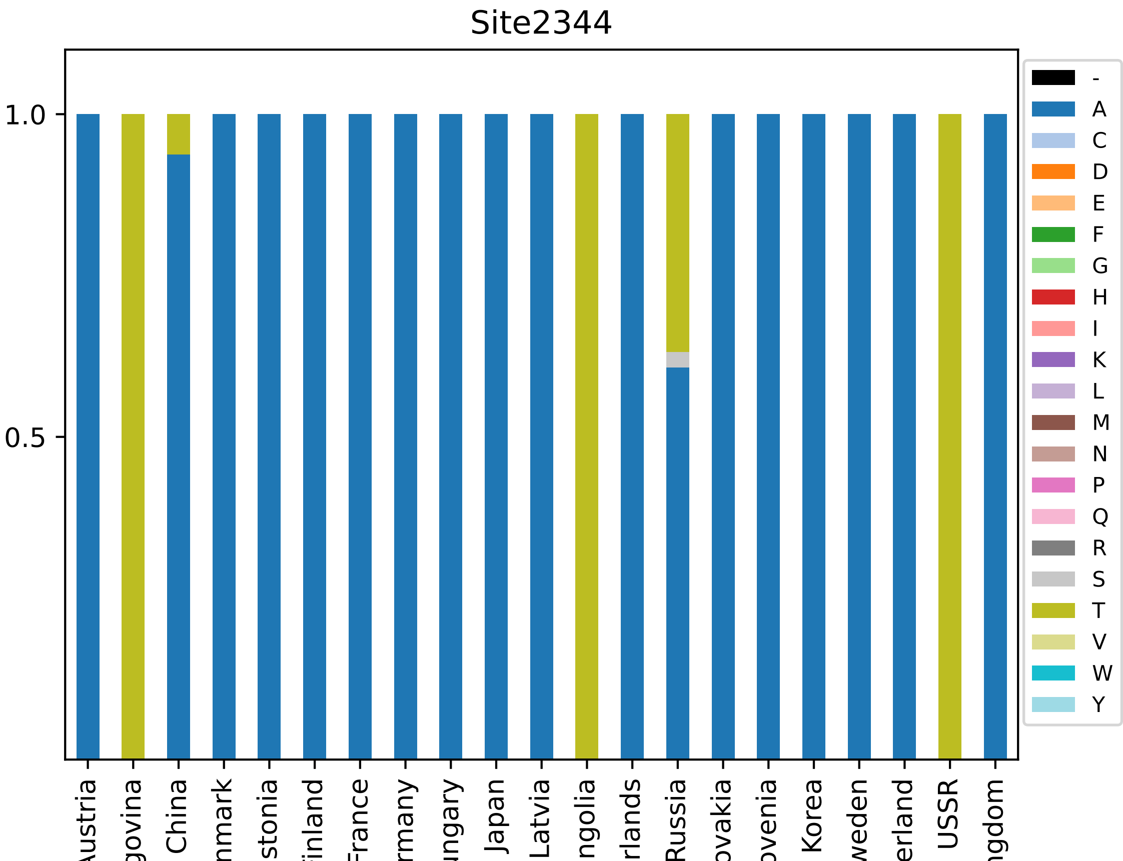
Site2344
1.0
0.5
-
A
C
D
E
F
G
H
I
K
L
M
N
P
Q
R
S
T
V
W
Y
China
Japan
Latvia
Russia
Korea
USSR

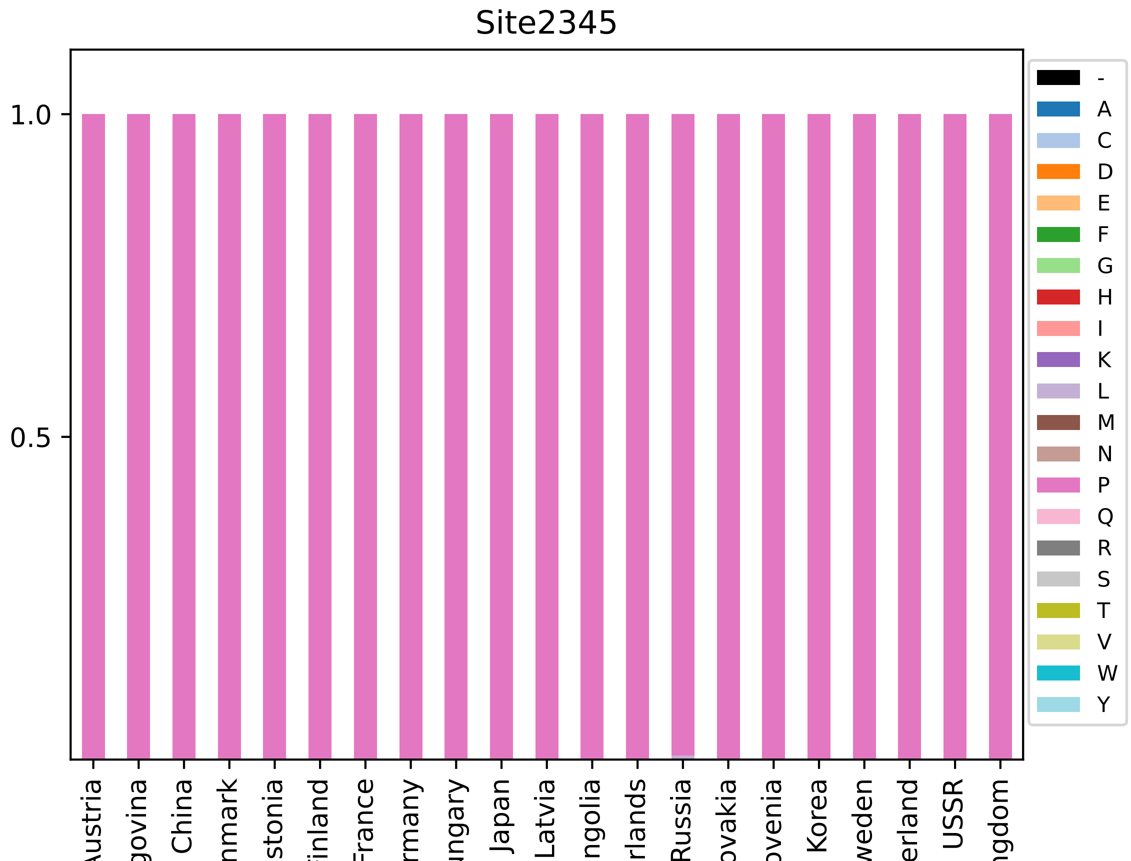

Site2345
1.0
0.5
-
A
C
D
E
F
G
H
I
K
L
M
N
P
Q
R
S
T
V
W
Y
China
Japan
Latvia
Russia
Korea
USSR

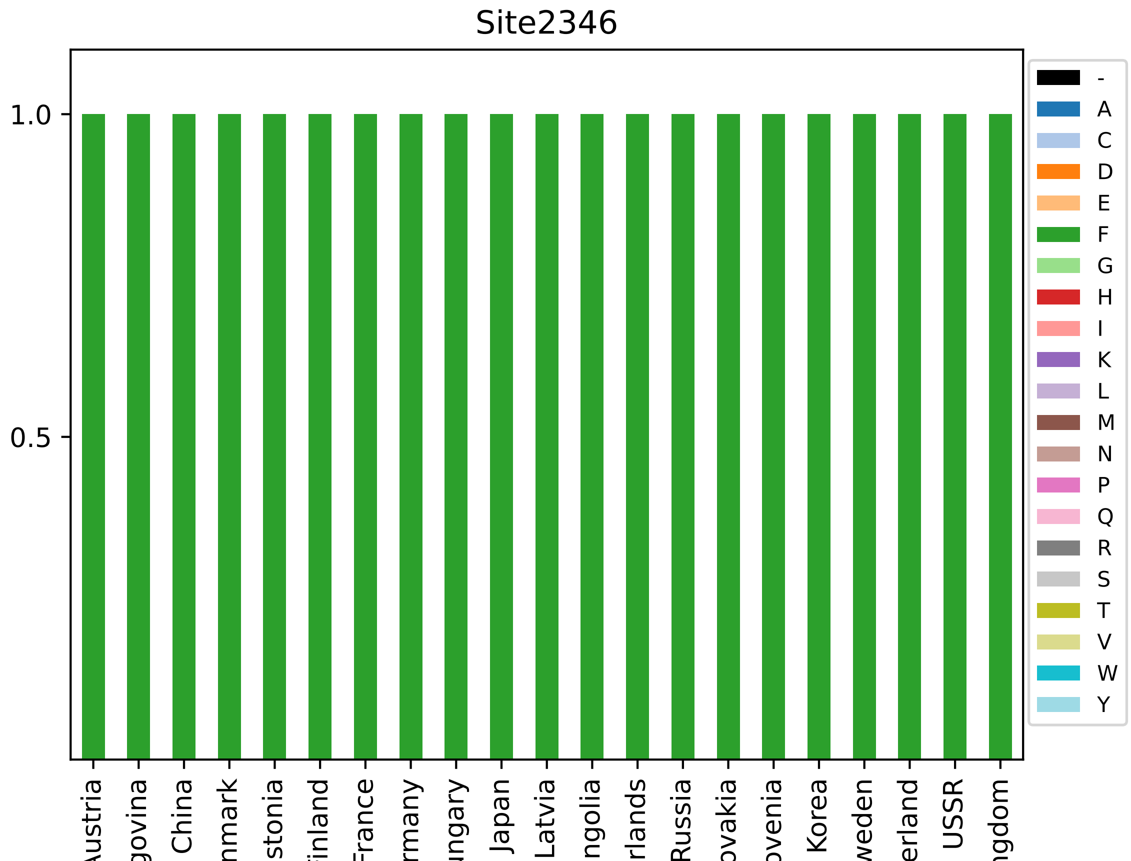

Site2346
1.0
0.5
-
A
C
D
E
F
G
H
I
K
L
M
N
P
Q
R
S
T
V
W
Y
Austria
govina
China
nmark
stonia
inland
France
rmany
ungary
Japan
Latvia
ngolia
rlands
Russia
ovakia
ovenia
Korea
weden
erland
USSR
ngdom

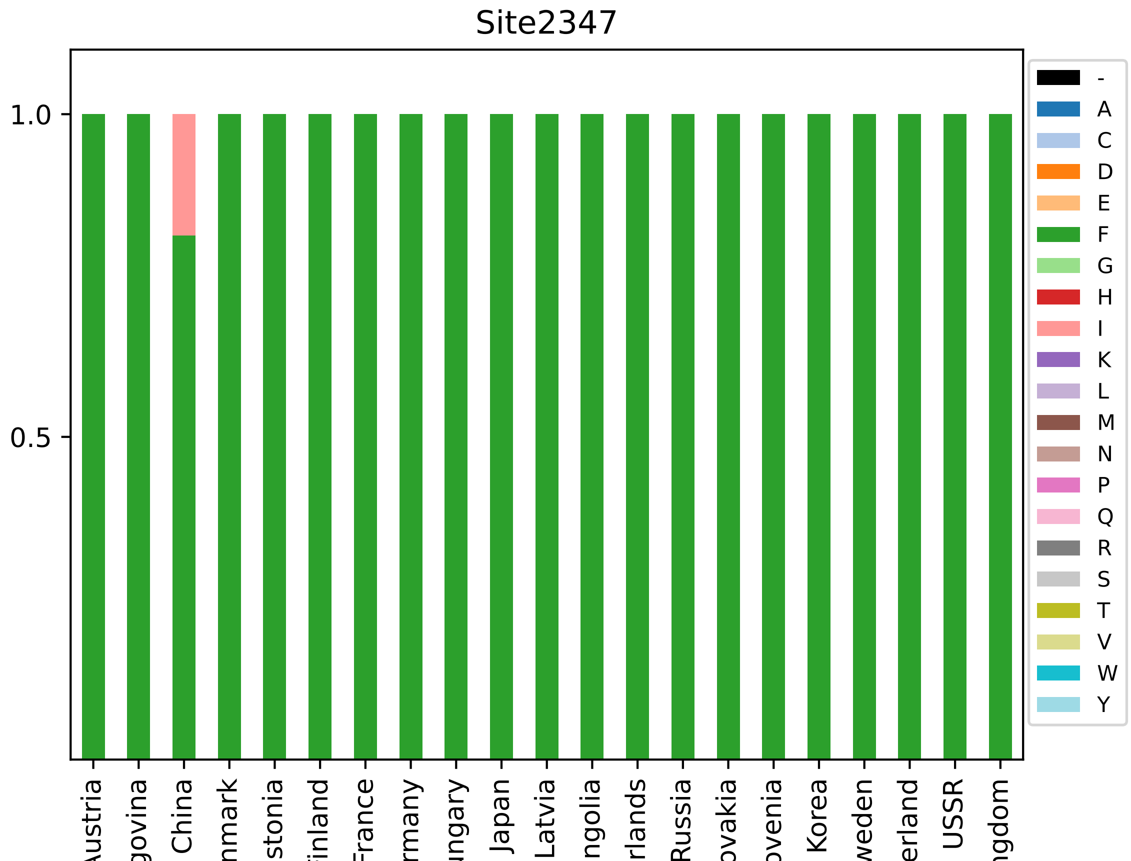

Site2347
1.0
0.5
-
A
C
D
E
F
G
H
I
K
L
M
N
P
Q
R
S
T
V
W
Y
Austria
China
Japan
Latvia
Russia
Korea
USSR

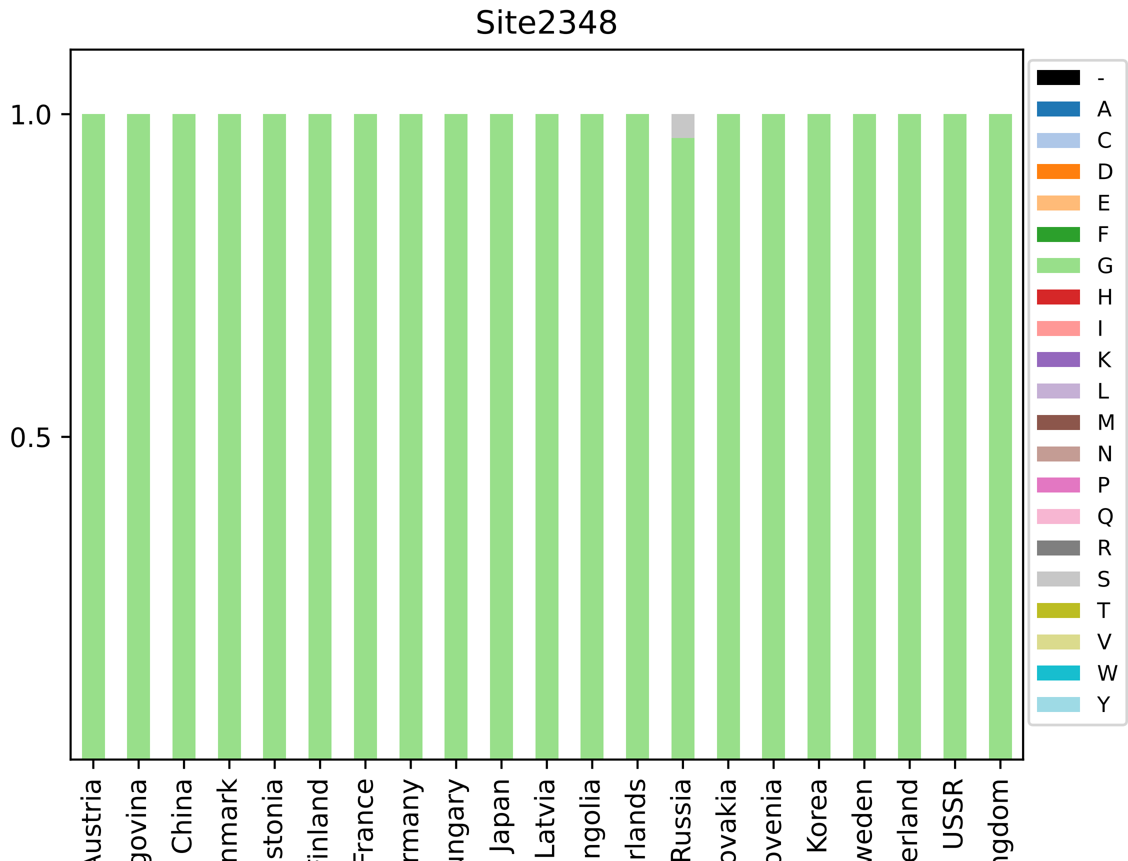
Site2348
1.0
0.5
-
A
C
D
E
F
G
H
I
K
L
M
N
P
Q
R
S
T
V
W
Y
China
Japan
Latvia
Russia
Korea
USSR

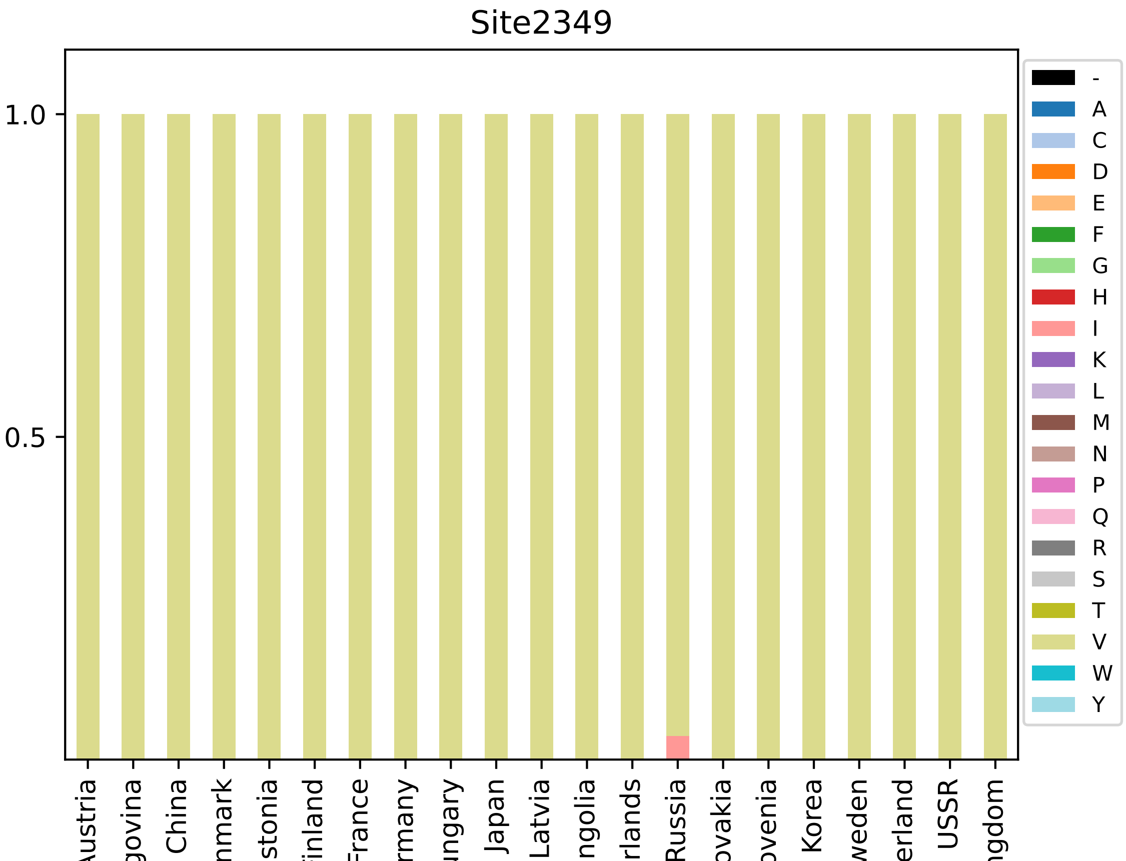
Site2349
1.0
0.5
-
A
C
D
E
F
G
H
I
K
L
M
N
P
Q
R
S
T
V
W
Y
China
Japan
Latvia
Russia
Korea
USSR

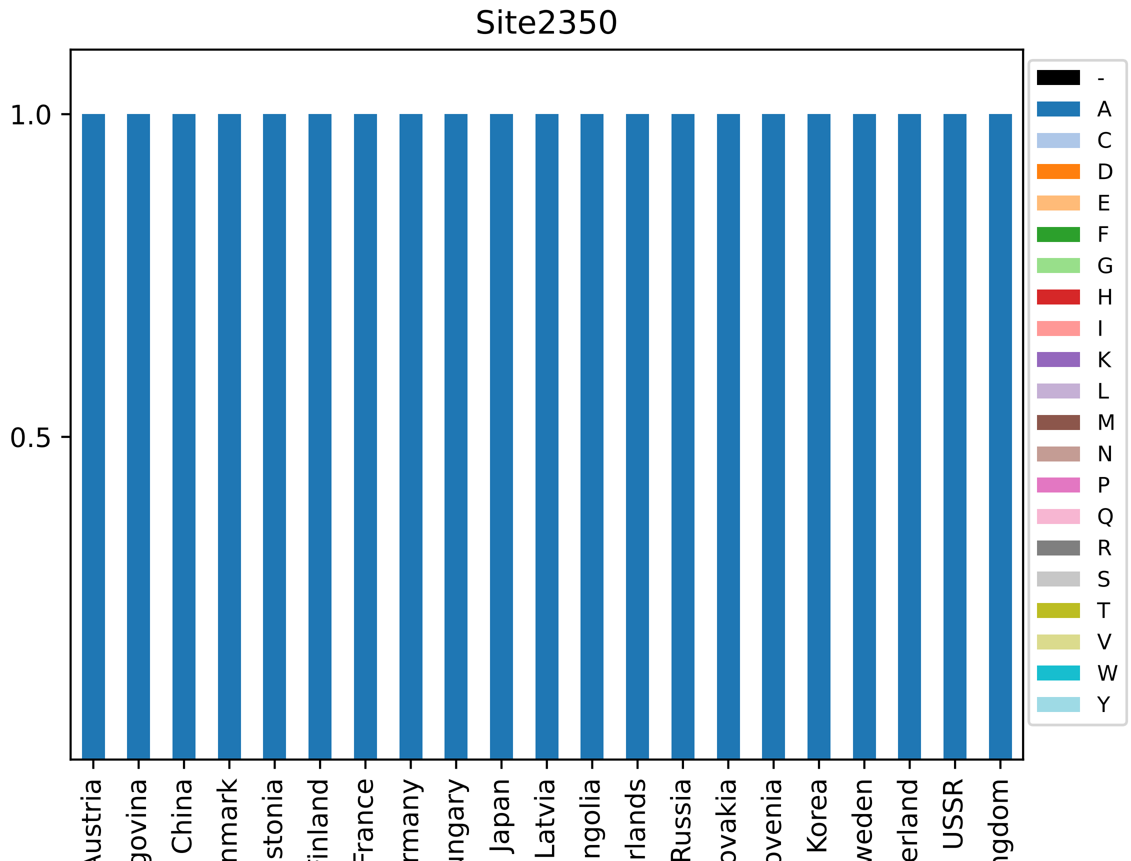
Site2350
1.0
0.5
-
A
C
D
E
F
G
H
I
K
L
M
N
P
Q
R
S
T
V
W
Y
China
Japan
Latvia
Russia
Korea
USSR

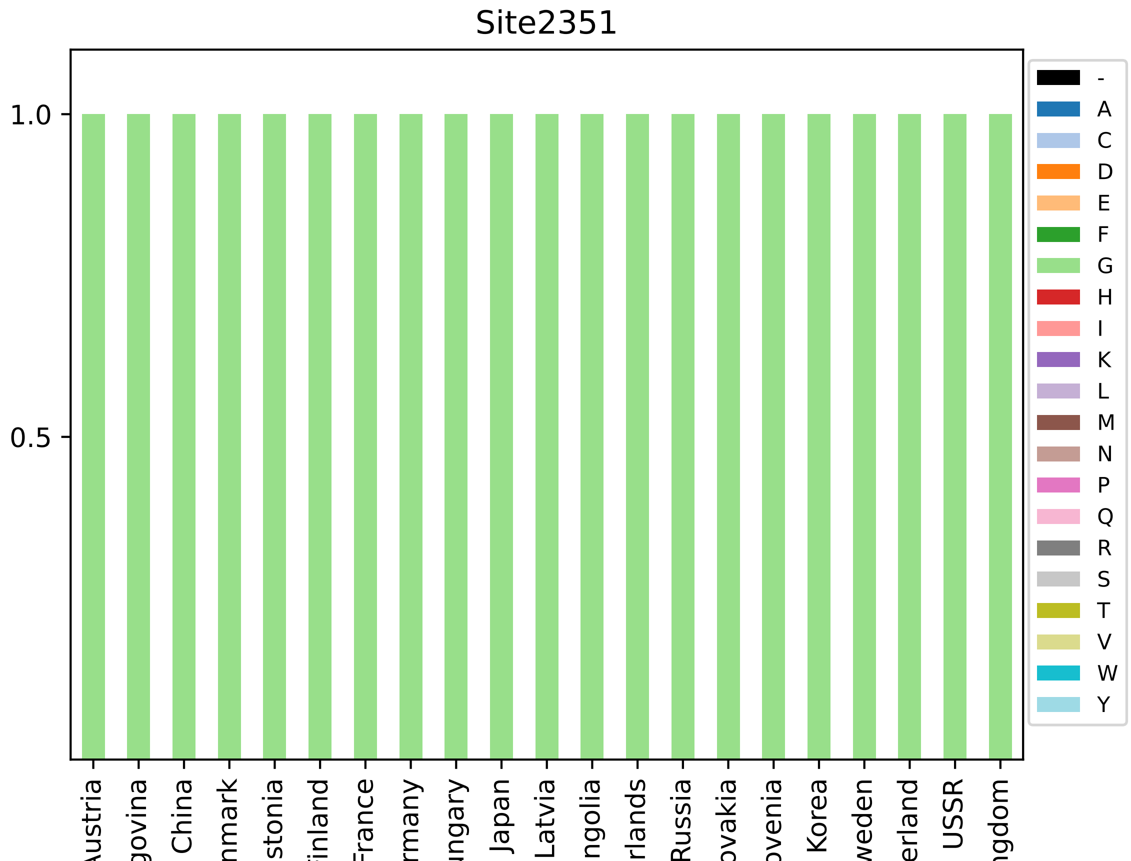
Site2351
1.0
0.5
-
A
C
D
E
F
G
H
I
K
L
M
N
P
Q
R
S
T
V
W
Y
China
Japan
Latvia
Russia
Korea
USSR

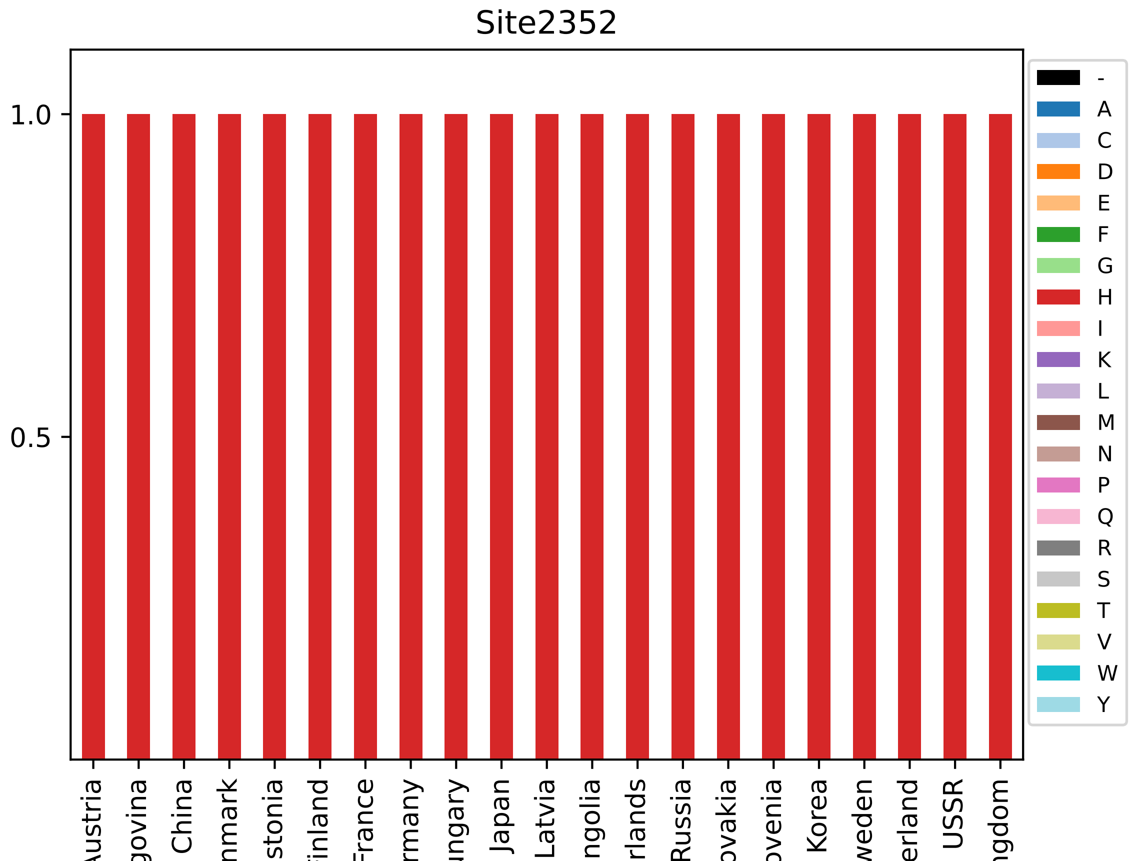

Site2352
1.0
0.5
-
A
C
D
E
F
G
H
I
K
L
M
N
P
Q
R
S
T
V
W
Y
Austria
China
Japan
Latvia
Russia
Korea
USSR

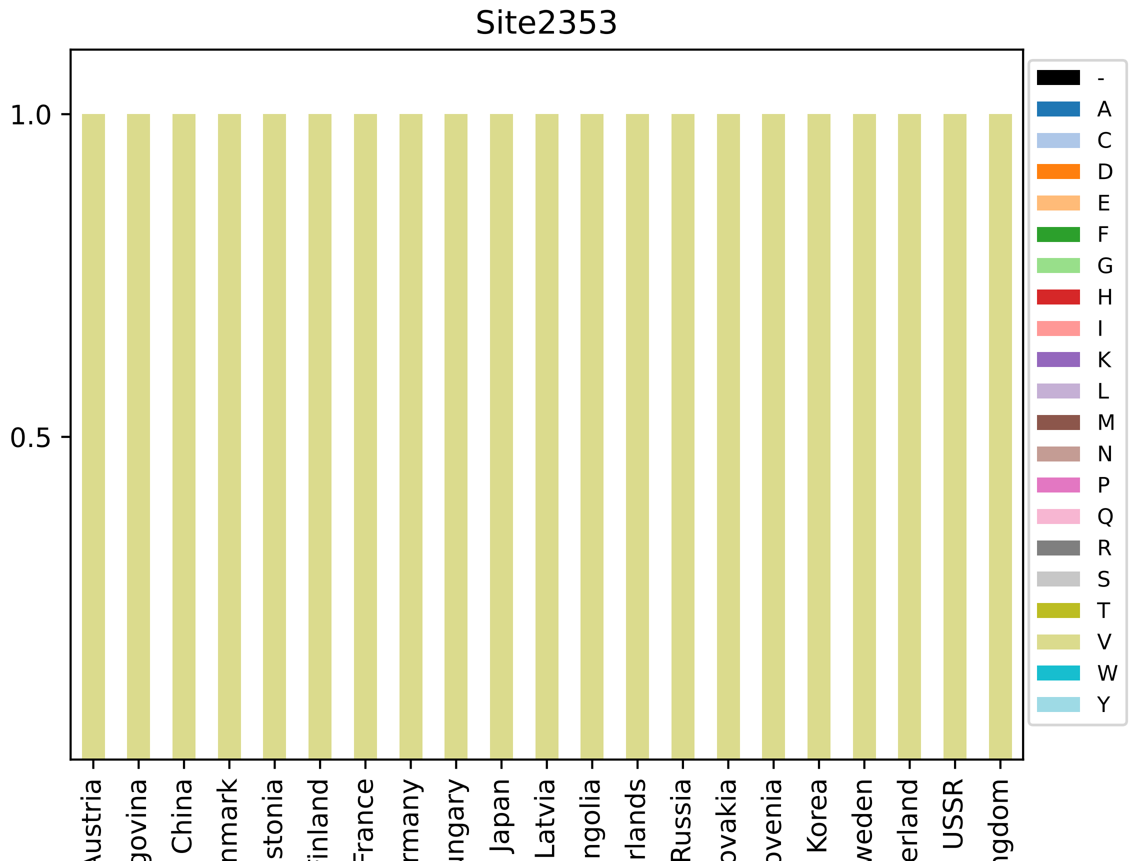
Site2353
1.0
0.5
-
A
C
D
E
F
G
H
I
K
L
M
N
P
Q
R
S
T
V
W
Y
China
Japan
Latvia
Russia
Korea
USSR

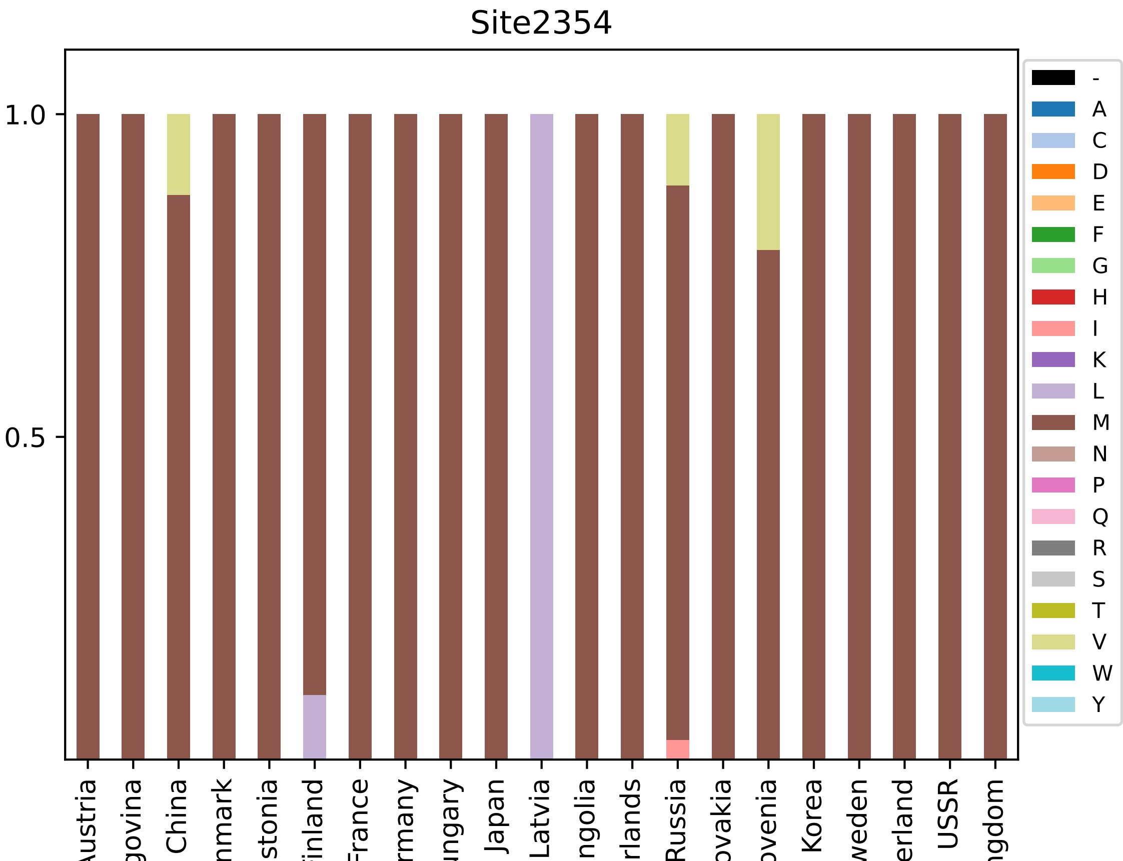

Site2354
1.0
0.5
-
A
C
D
E
F
G
H
I
K
L
M
N
P
Q
R
S
T
V
W
Y
Austria
govina
China
nmark
stonia
inland
France
rmany
ungary
Japan
Latvia
ngolia
rlands
Russia
ovakia
ovenia
Korea
weden
erland
USSR
ngdom

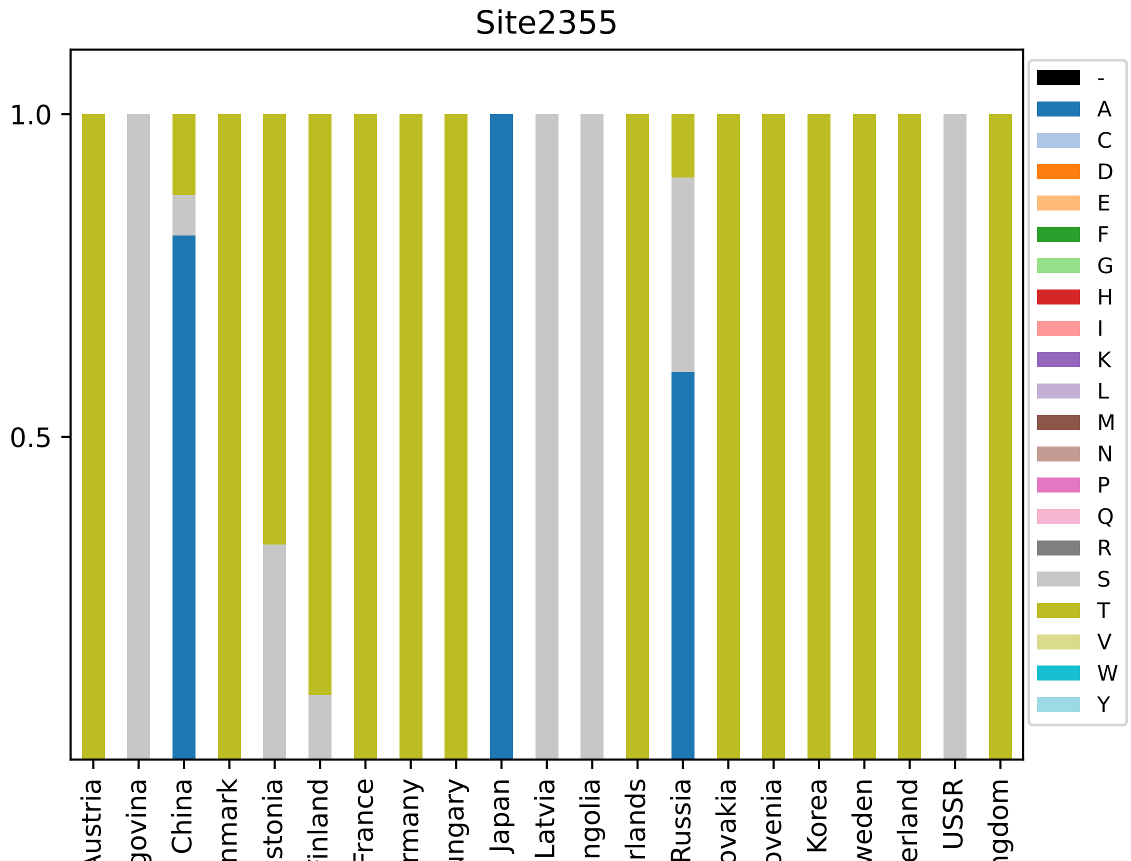

Site2355
1.0
0.5
-
A
C
D
E
F
G
H
I
K
L
M
N
P
Q
R
S
T
V
W
Y
Austria
govina
China
nmark
stonia
inland
France
rmany
ungary
Japan
Latvia
ngolia
rlands
Russia
ovakia
ovenia
Korea
weden
erland
USSR
ngdom

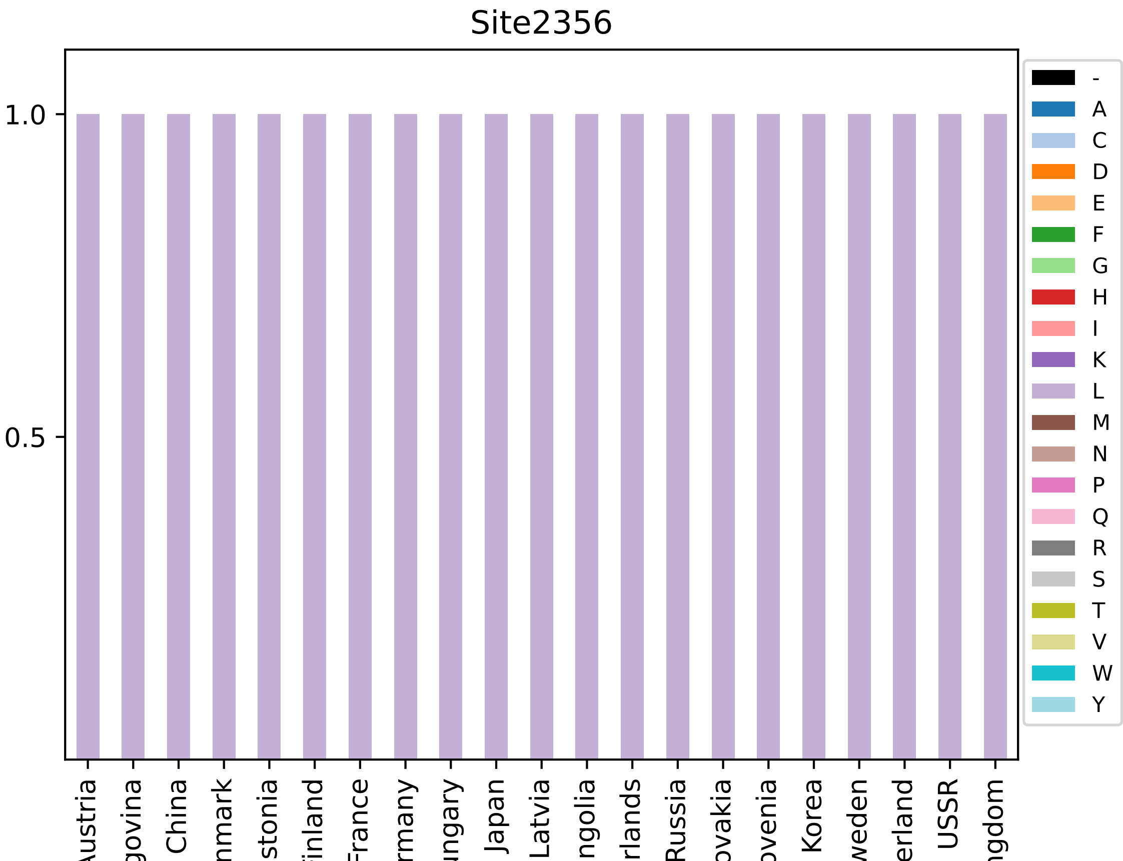
Site2356
1.0
0.5
-
A
C
D
E
F
G
H
I
K
L
M
N
P
Q
R
S
T
V
W
Y
China
Japan
Latvia
Russia
Korea
USSR

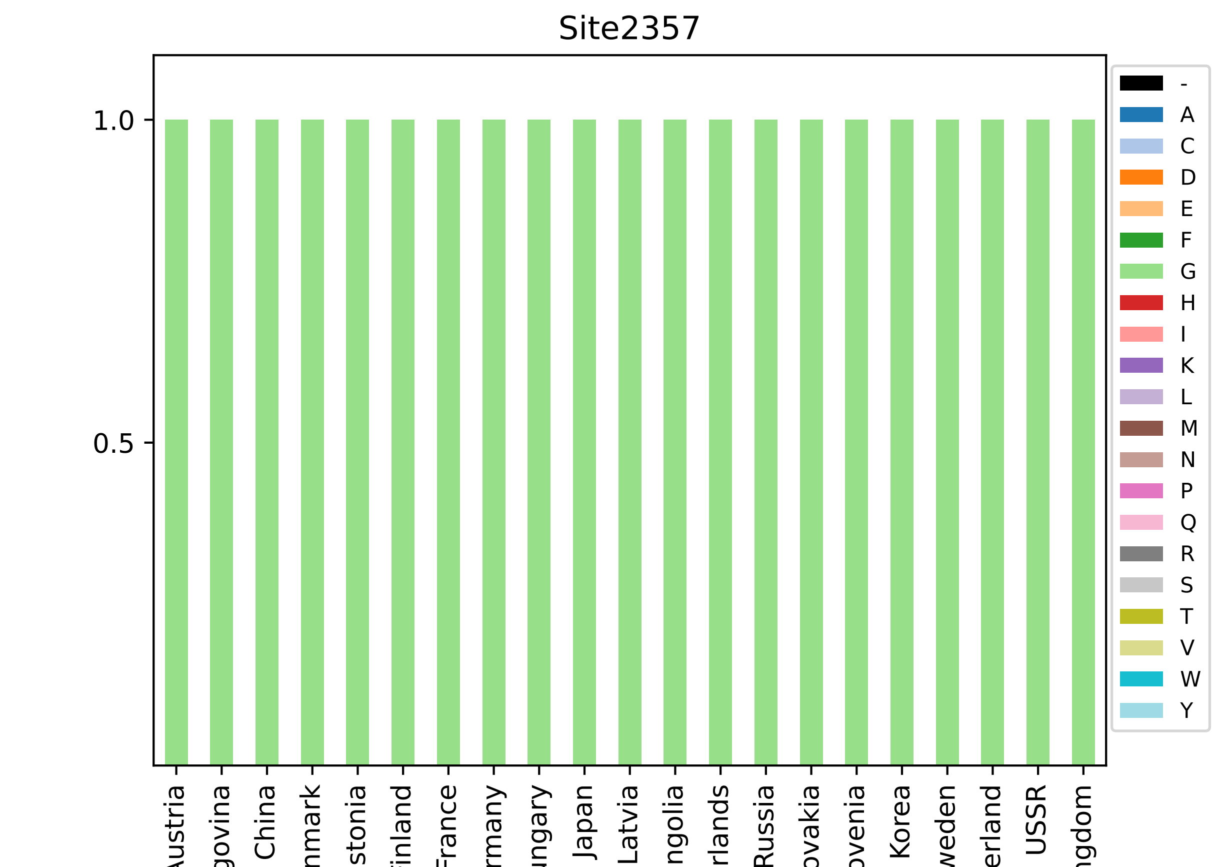
Site2357
1.0
0.5
-
A
C
D
E
F
G
H
I
K
L
M
N
P
Q
R
S
T
V
W
Y
Austria
govina
China
nmark
stonia
Finland
France
rmany
ungary
Japan
Latvia
ngolia
rlands
Russia
ovakia
ovenia
Korea
weden
erland
USSR
ngdom

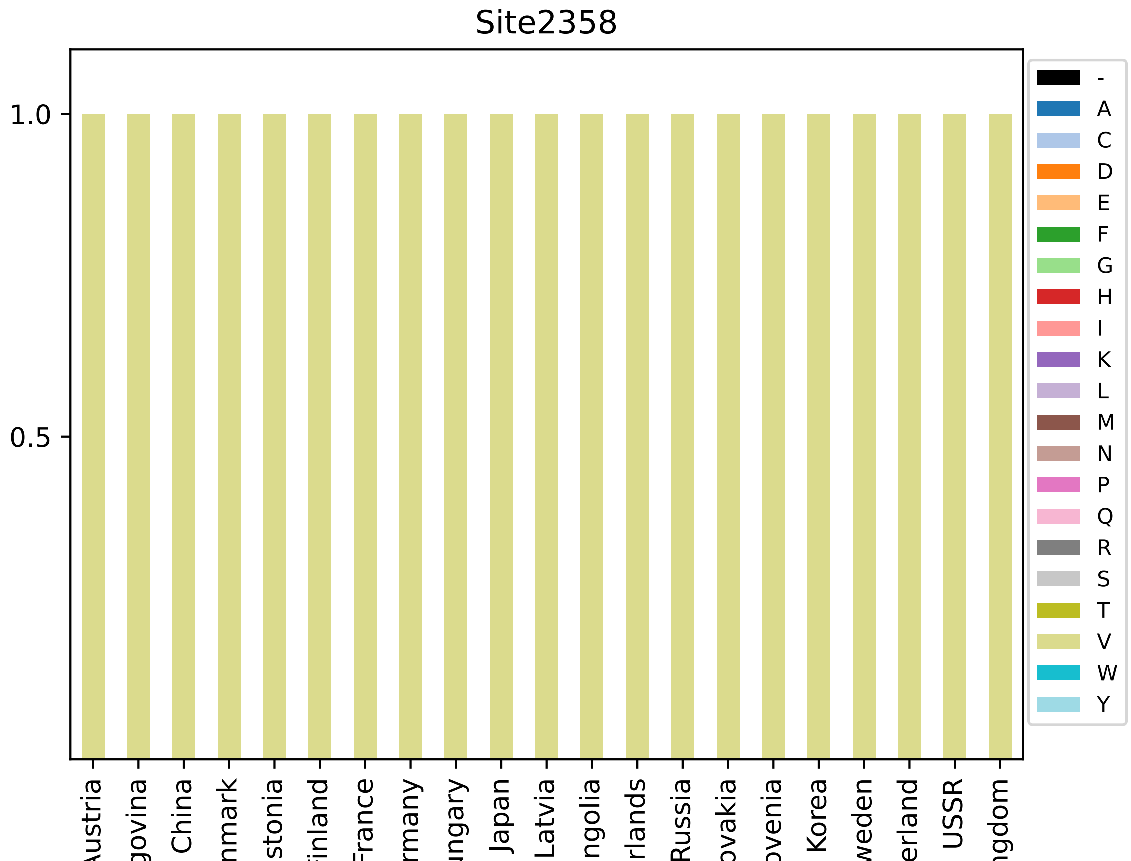
Site2358
1.0
0.5
-
A
C
D
E
F
G
H
I
K
L
M
N
P
Q
R
S
T
V
W
Y
Austria
China
Japan
Latvia
Russia
Korea
USSR

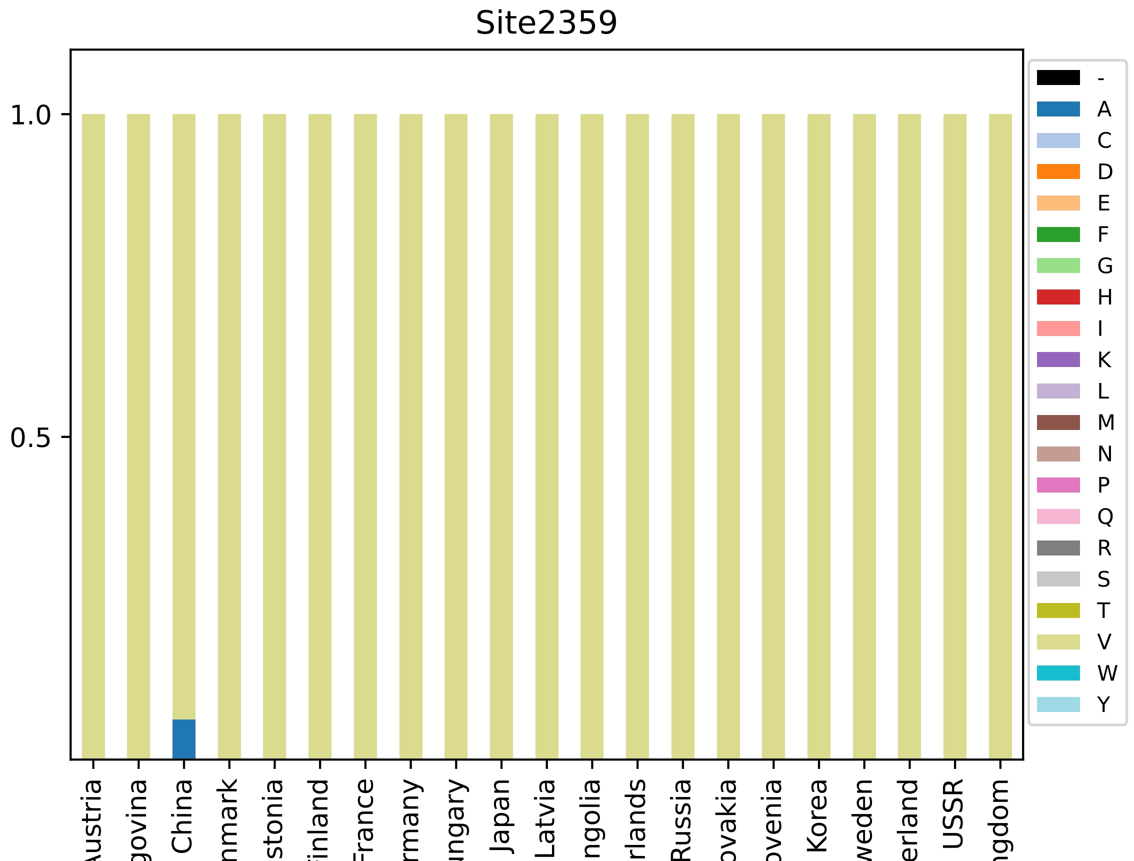
Site2359
1.0
0.5
Austria
govina
China
nmark
stonia
inland
France
rmany
ungary
Japan
Latvia
ngolia
rlands
Russia
ovakia
ovenia
Korea
weden
erland
USSR
ngdom
-
A
C
D
E
F
G
H
I
K
L
M
N
P
Q
R
S
T
V
W
Y

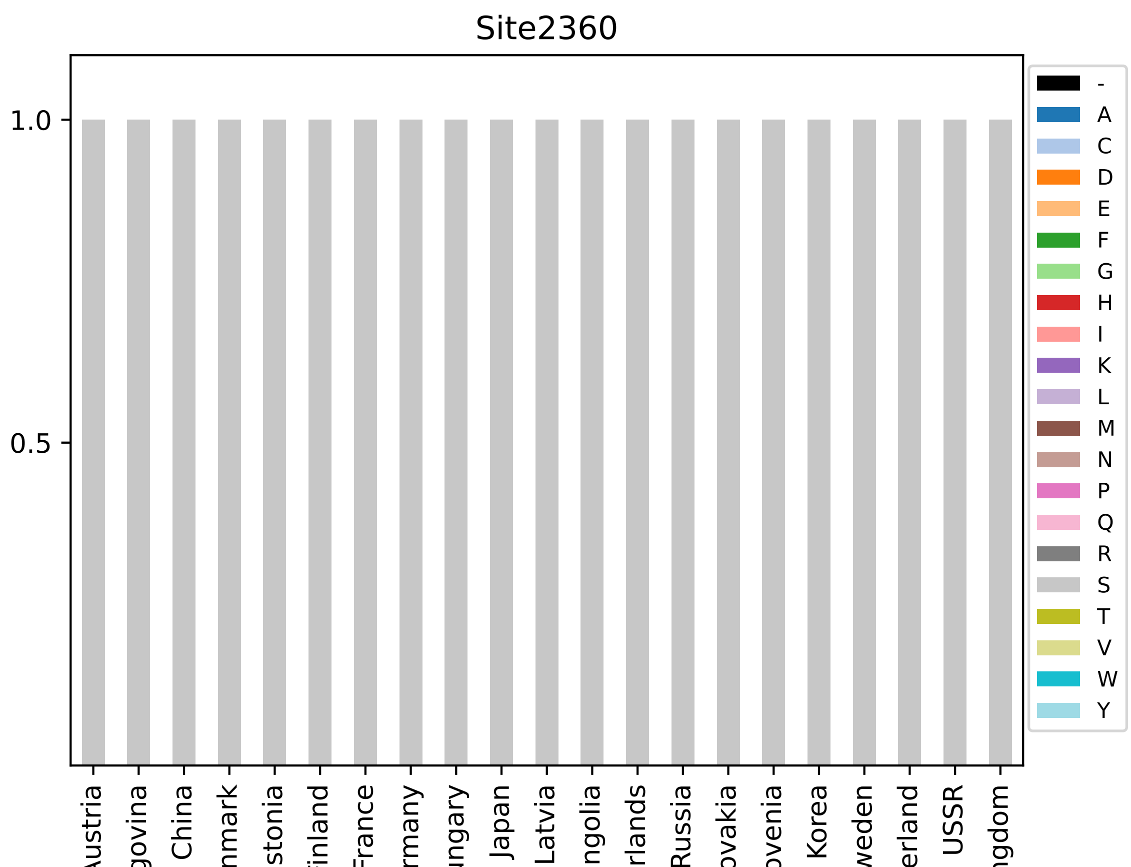
Site2360
1.0
0.5
-
A
C
D
E
F
G
H
I
K
L
M
N
P
Q
R
S
T
V
W
Y
ustria
govina
China
nmark
stonia
inland
rance
rmany
ungary
Japan
Latvia
ngolia
rlands
Russia
ovakia
ovenia
Korea
weden
erland
USSR
ngdom

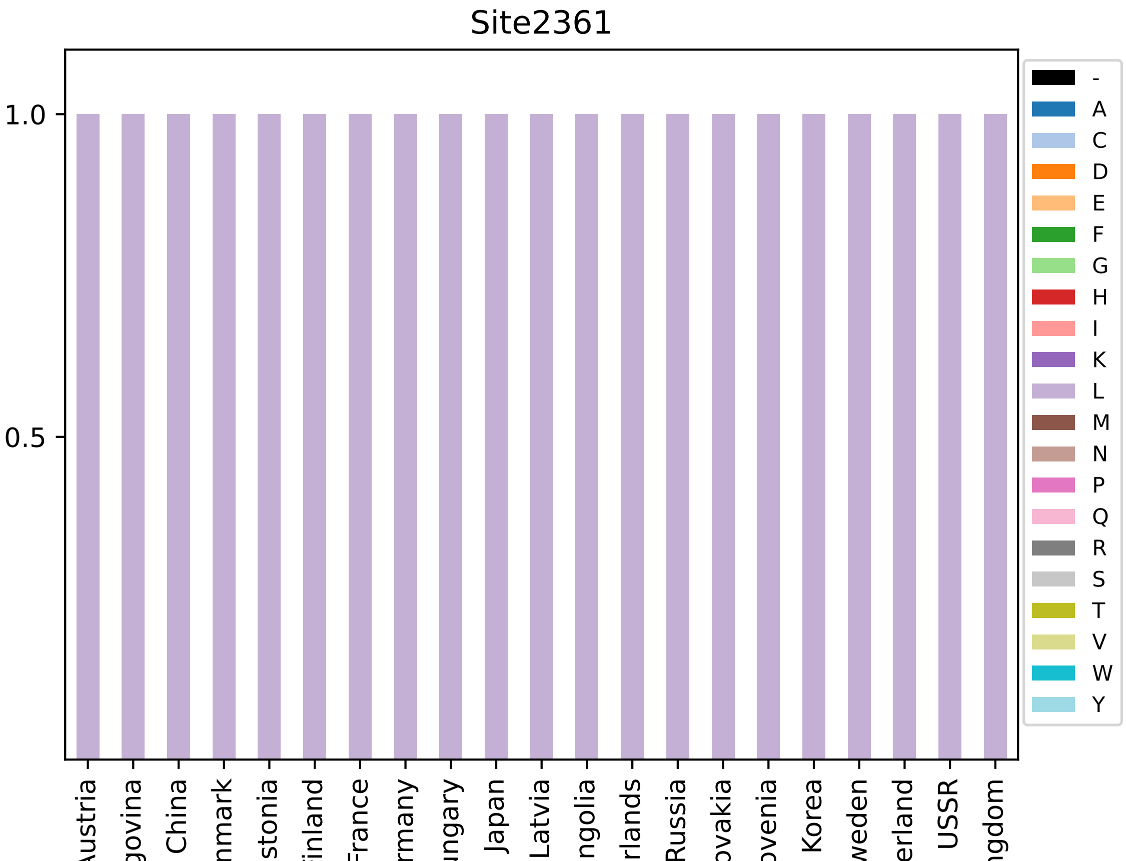
Site2361
1.0
0.5
China
Japan
Latvia
Russia
Korea
USSR
-
A
C
D
E
F
G
H
I
K
L
M
N
P
Q
R
S
T
V
W
Y

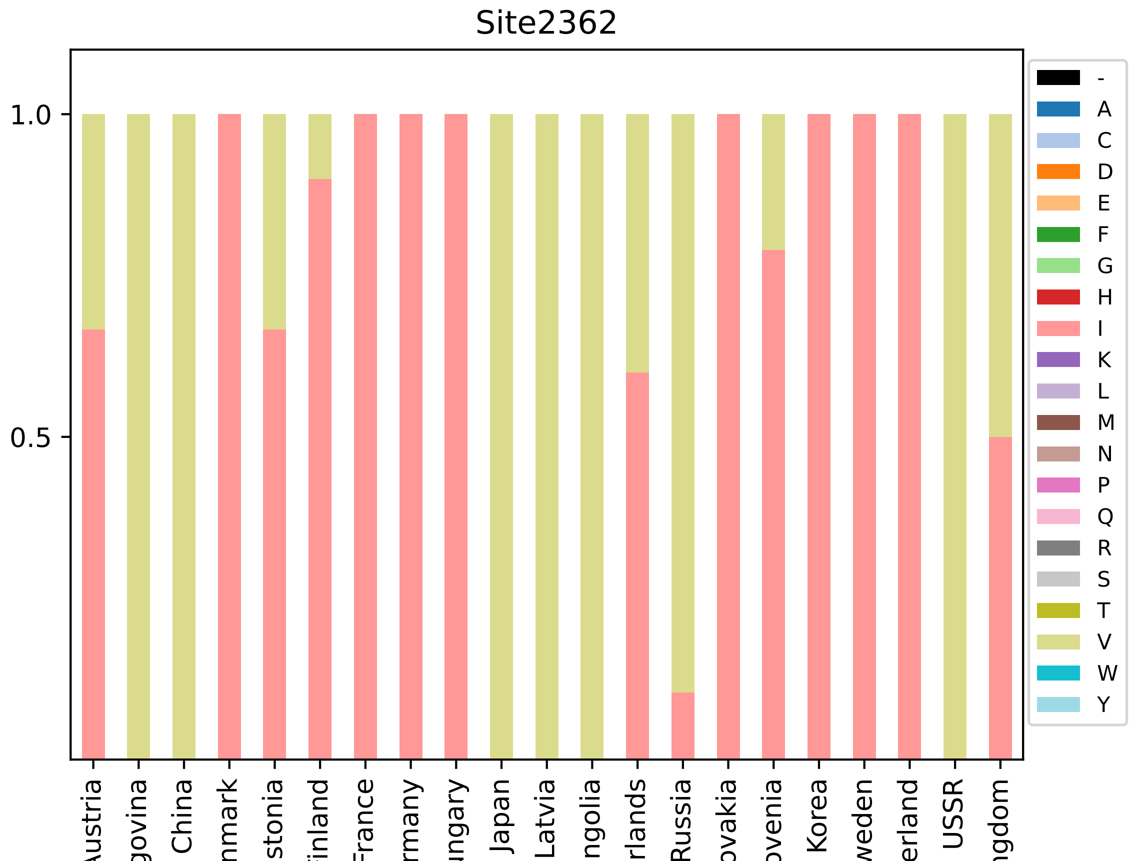

Site2362
1.0
0.5
-
A
C
D
E
F
G
H
I
K
L
M
N
P
Q
R
S
T
V
W
Y
Austria
govina
China
nmark
stonia
inland
France
rmany
ungary
Japan
Latvia
ngolia
rlands
Russia
ovakia
ovenia
Korea
weden
erland
USSR
ngdom

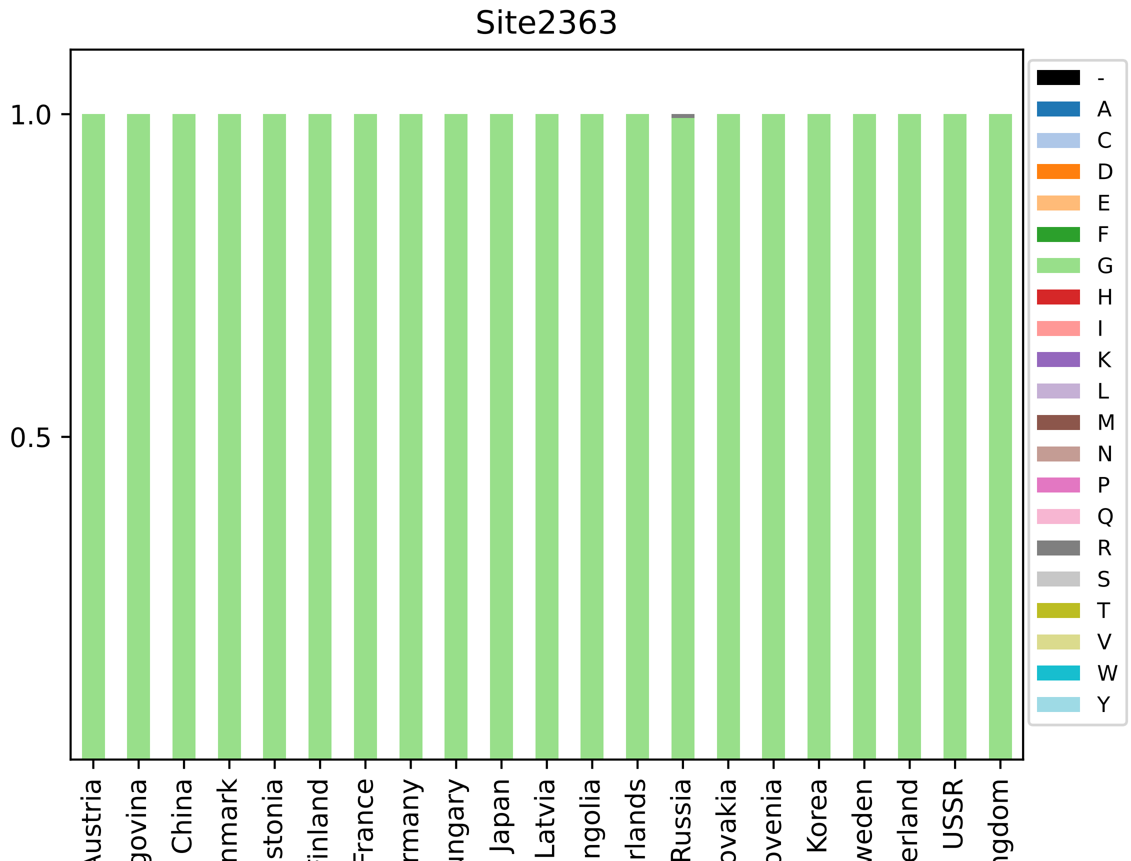

Site2363
1.0
0.5
-
A
C
D
E
F
G
H
I
K
L
M
N
P
Q
R
S
T
V
W
Y
China
Japan
Latvia
Russia
Korea
USSR

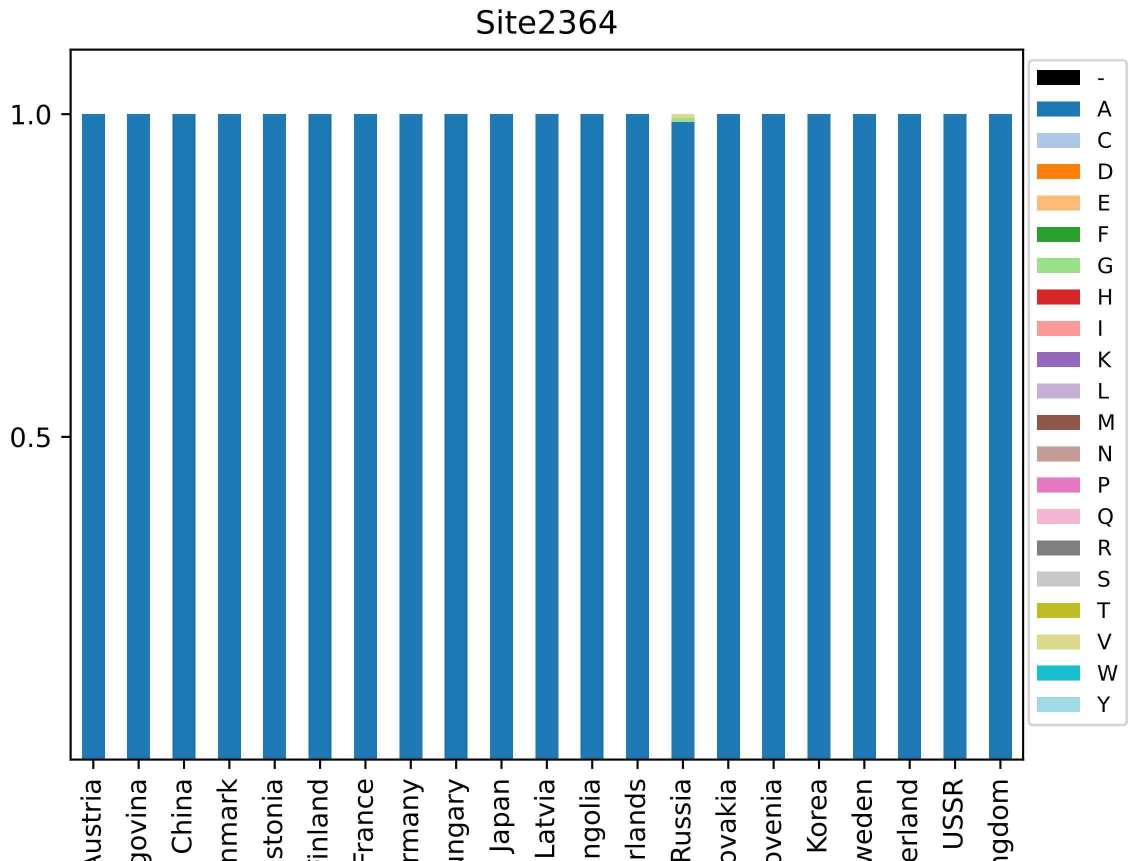

Site2364
1.0
0.5
-
A
C
D
E
F
G
H
I
K
L
M
N
P
Q
R
S
T
V
W
Y
Austria
govina
China
nmark
stonia
inland
France
rmany
ungary
Japan
Latvia
ngolia
rlands
Russia
ovakia
ovenia
Korea
weden
erland
USSR
ngdom

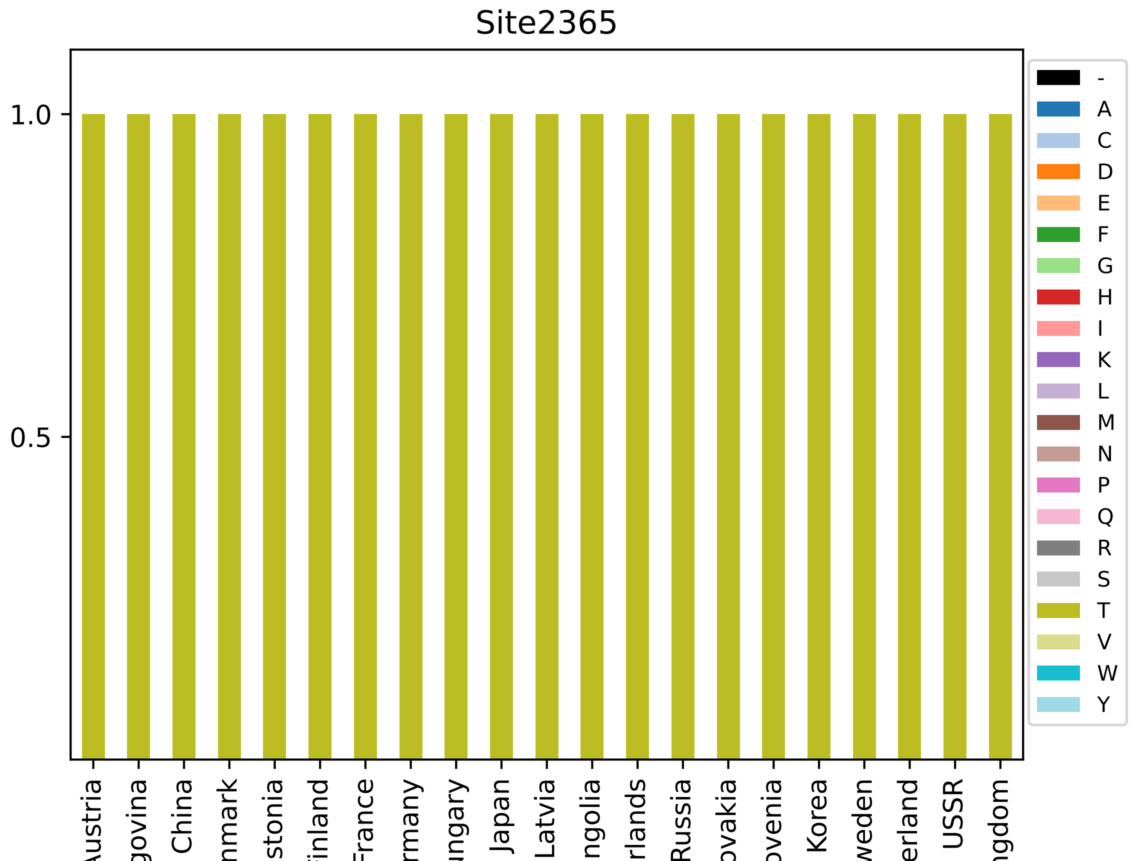

Site2365
1.0
0.5
-
A
C
D
E
F
G
H
I
K
L
M
N
P
Q
R
S
T
V
W
Y
Austria
govina
China
nmark
stonia
inland
France
rmany
ungary
Japan
Latvia
ngolia
rlands
Russia
ovakia
ovenia
Korea
weden
erland
USSR
ngdom

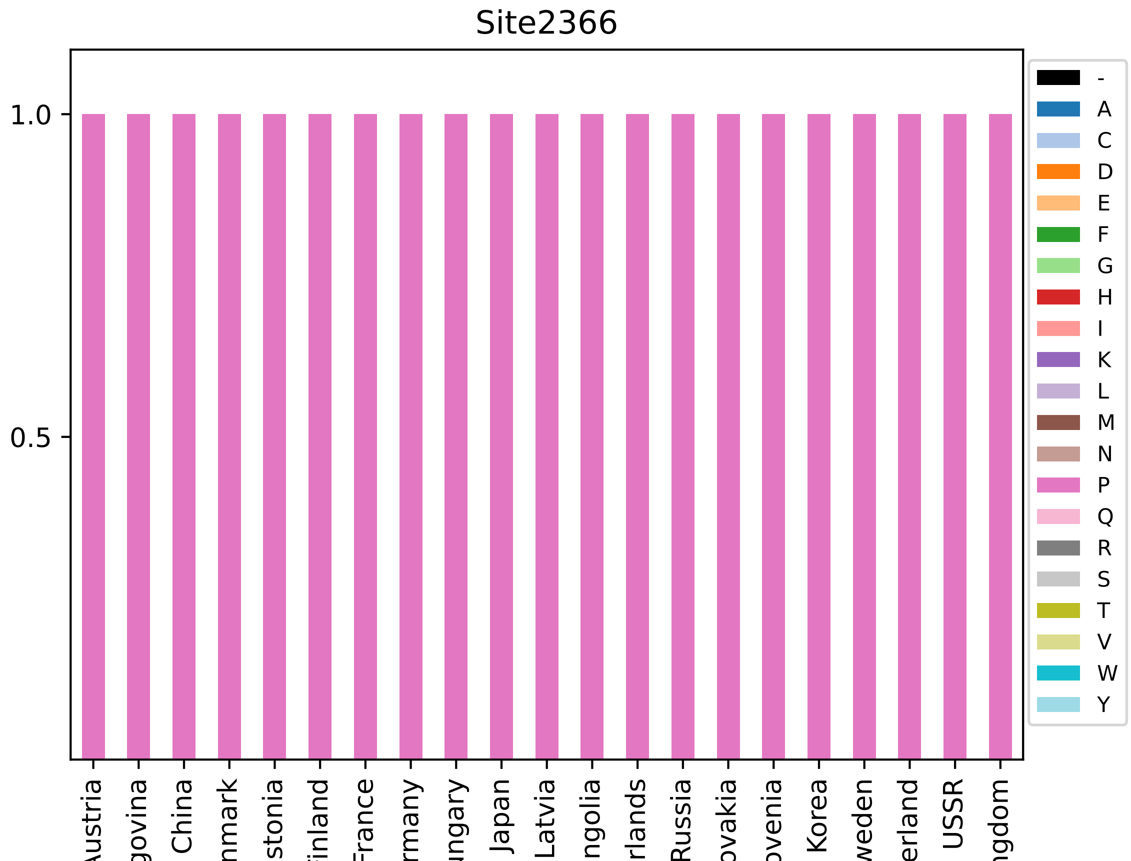
Site2366
1.0
0.5
-
A
C
D
E
F
G
H
I
K
L
M
N
P
Q
R
S
T
V
W
Y
China
Japan
Latvia
Russia
Korea
USSR

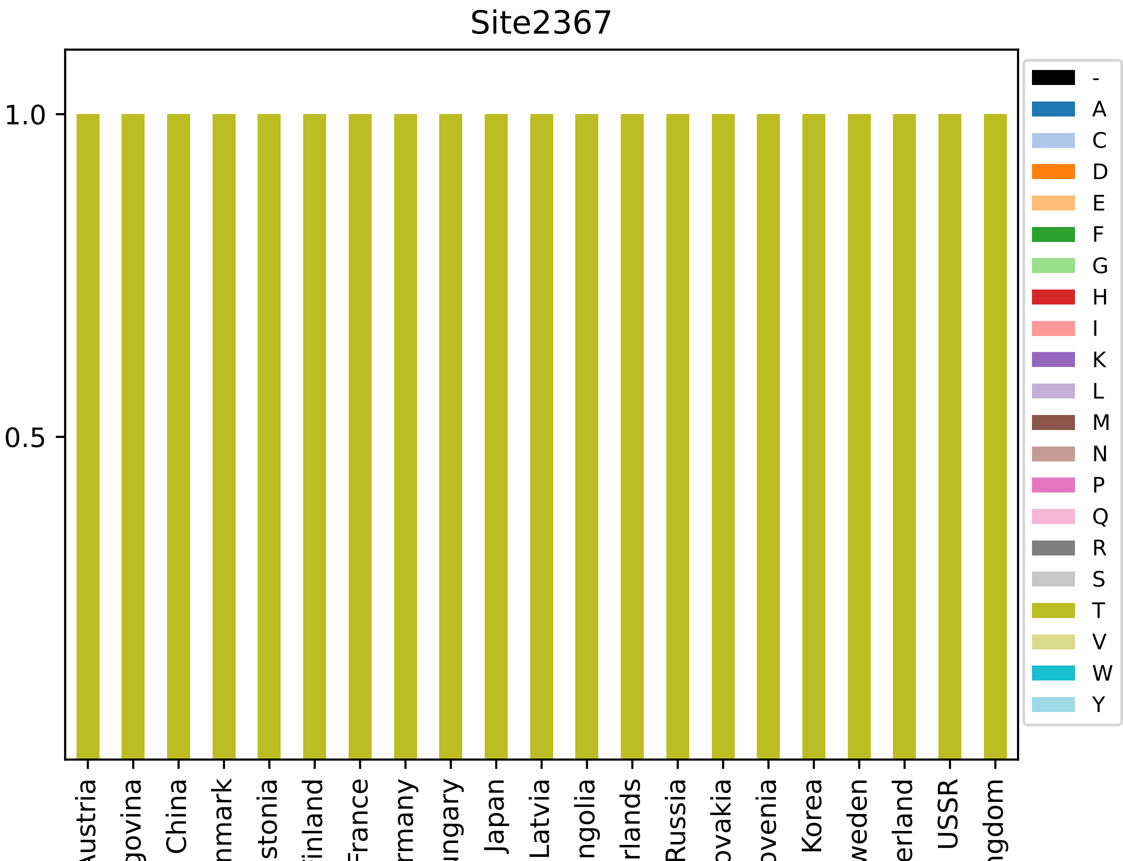
Site2367
1.0
0.5
-
A
C
D
E
F
G
H
I
K
L
M
N
P
Q
R
S
T
V
W
Y
China
Japan
Latvia
Russia
Korea
USSR

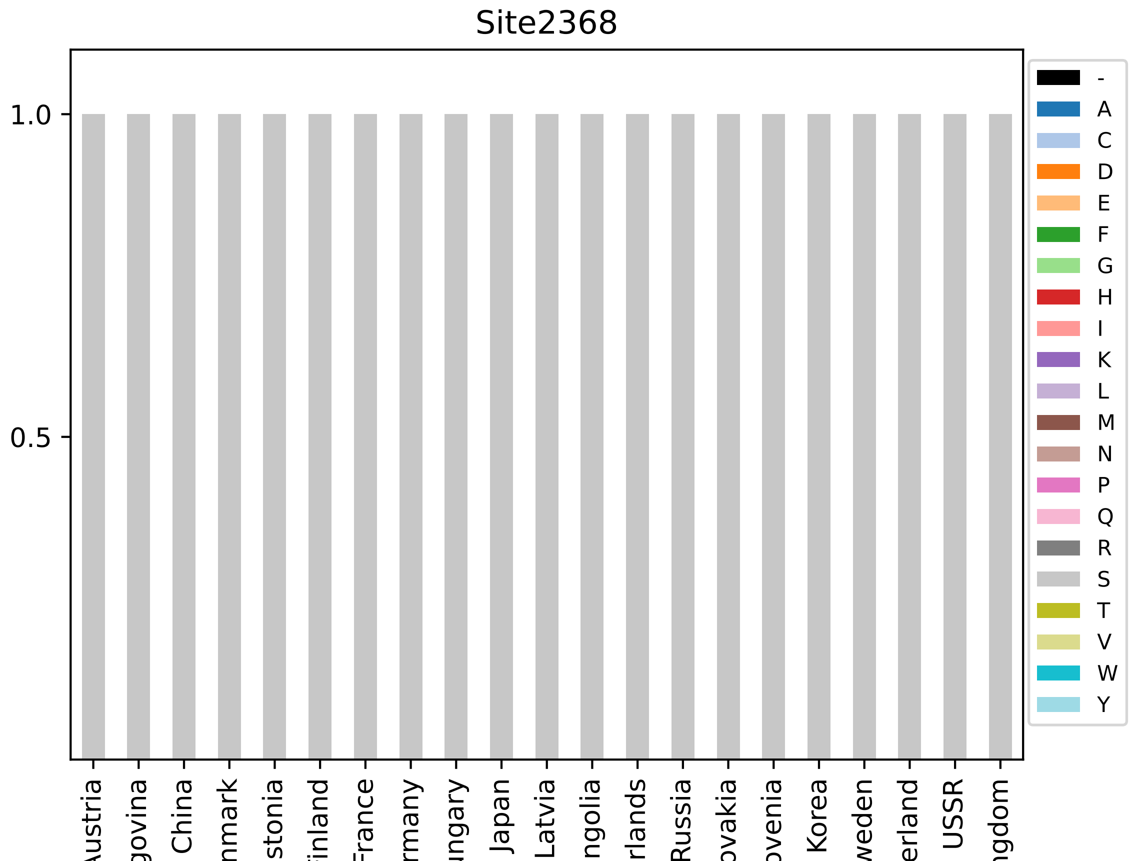
Site2368
1.0
0.5
Austria
govina
China
nmark
stonia
Finland
France
rmany
ungary
Japan
Latvia
ngolia
rlands
Russia
ovakia
ovenia
Korea
weden
erland
USSR
ngdom
-
A
C
D
E
F
G
H
I
K
L
M
N
P
Q
R
S
T
V
W
Y

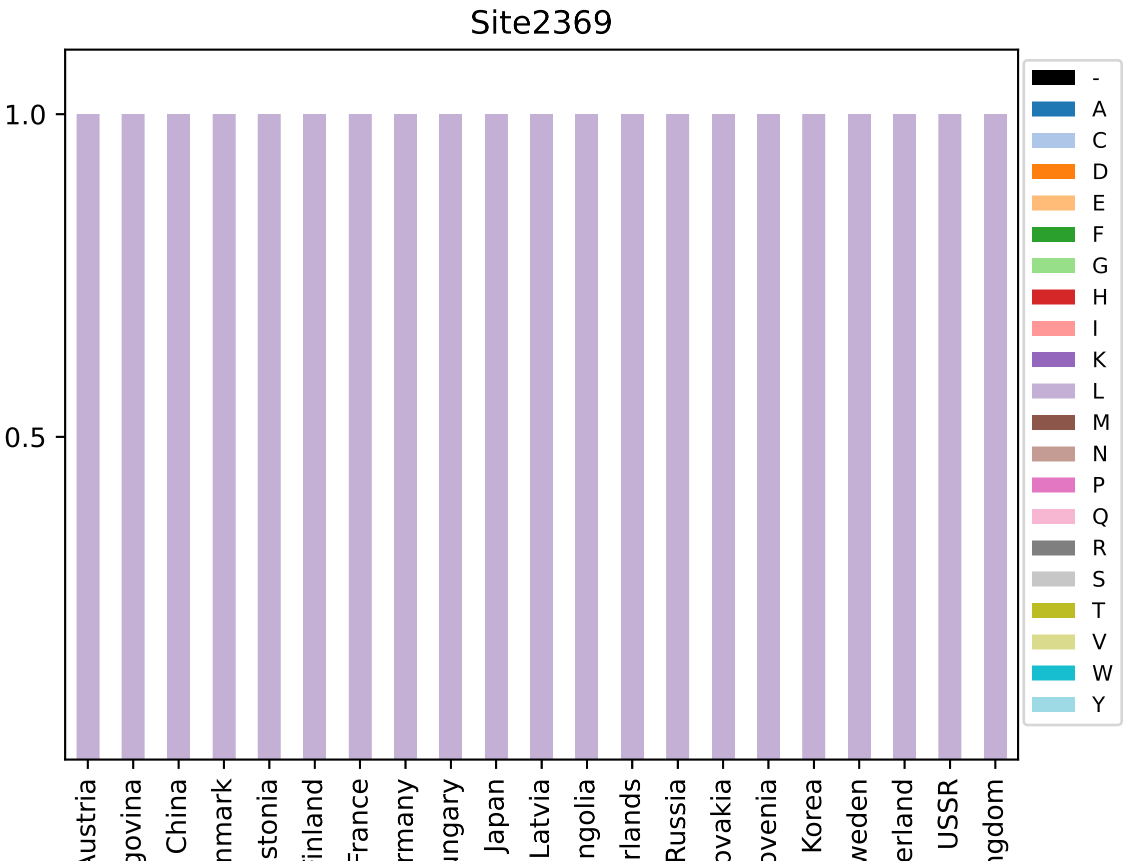

Site2369
1.0
0.5
-
A
C
D
E
F
G
H
I
K
L
M
N
P
Q
R
S
T
V
W
Y
Austria
China
Japan
Latvia
Russia
Korea
USSR

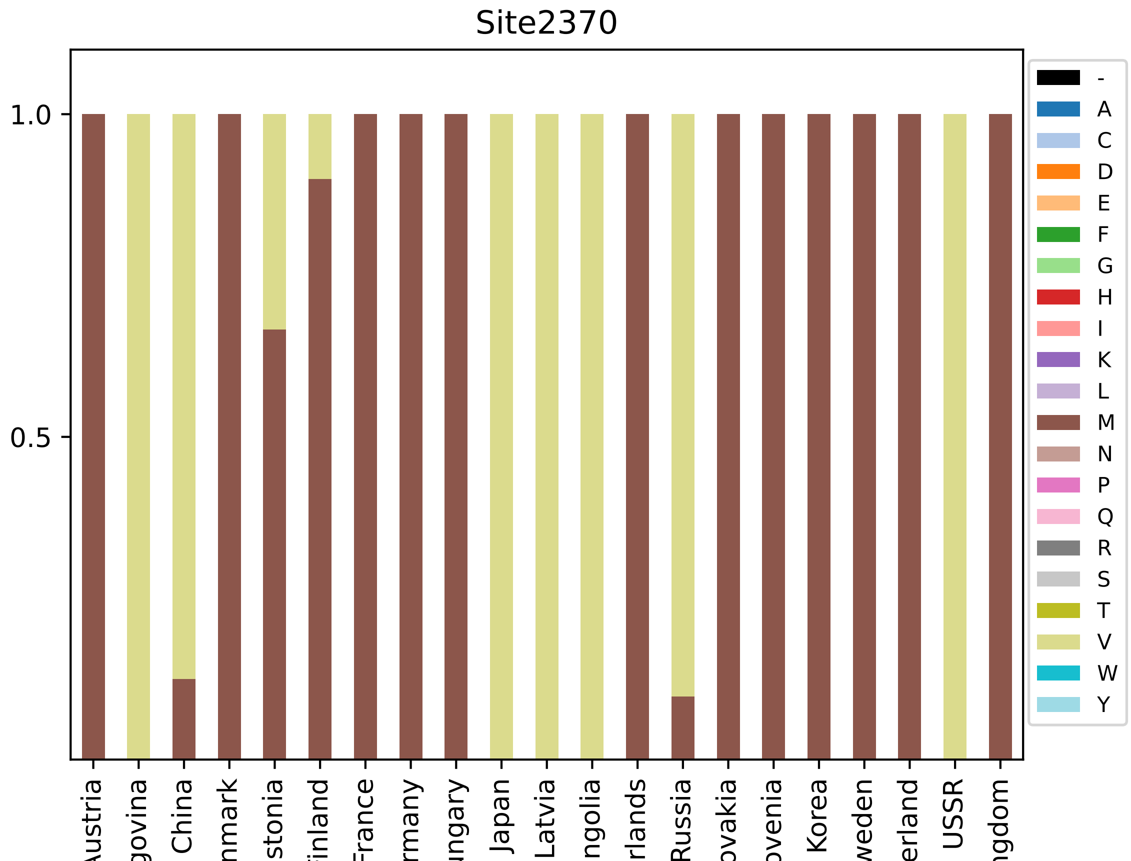

Site2370
1.0
0.5
-
A
C
D
E
F
G
H
I
K
L
M
N
P
Q
R
S
T
V
W
Y
Austria
govina
China
nmark
stonia
inland
France
rmany
ungary
Japan
Latvia
ngolia
rlands
Russia
ovakia
ovenia
Korea
weden
erland
USSR
ngdom

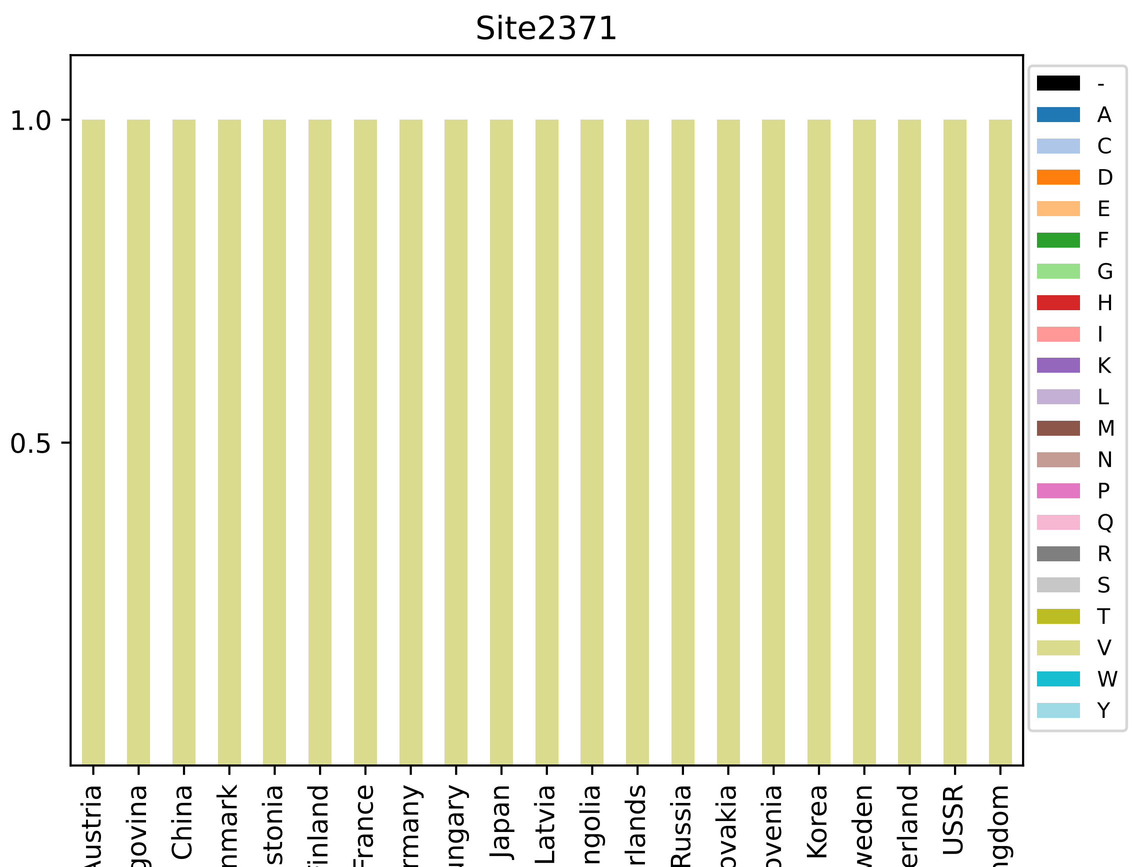

Site2371
1.0
0.5
-
A
C
D
E
F
G
H
I
K
L
M
N
P
Q
R
S
T
V
W
Y
Austria
China
Japan
Latvia
Russia
Korea
USSR

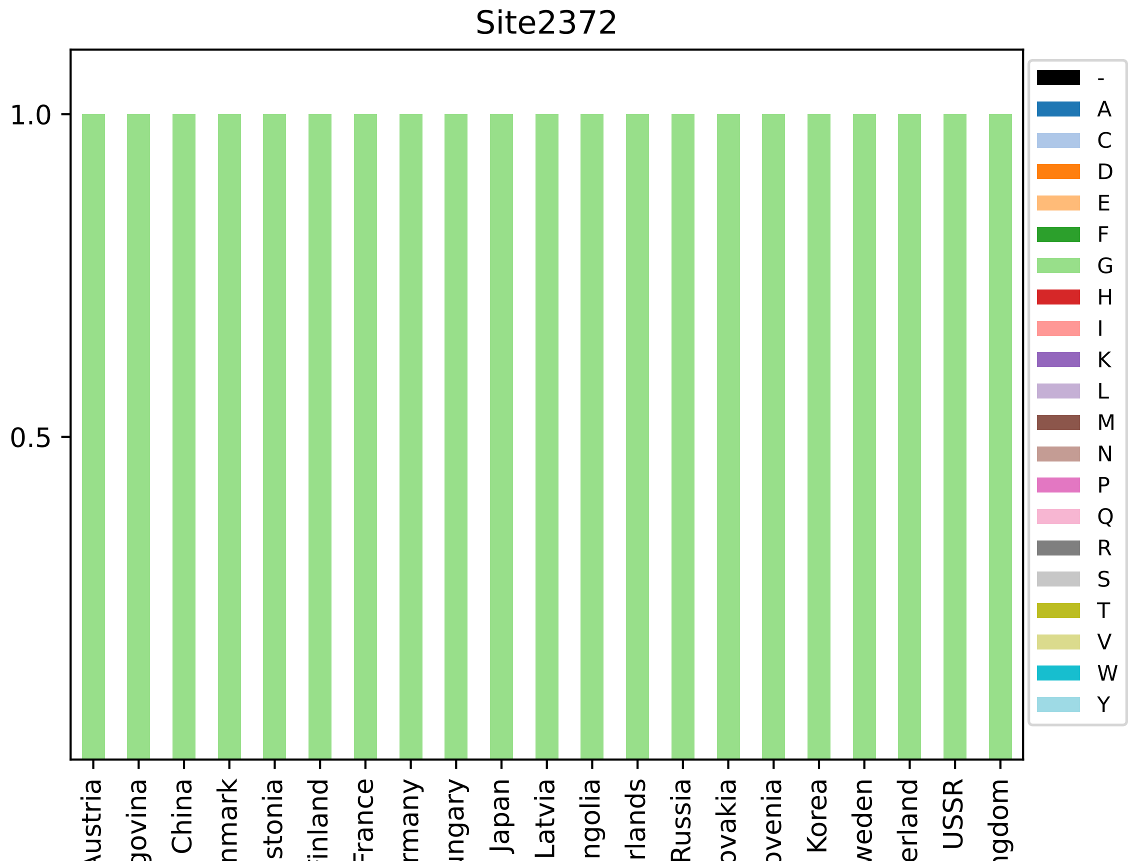

Site2372
1.0
0.5
-
A
C
D
E
F
G
H
I
K
L
M
N
P
Q
R
S
T
V
W
Y
Austria
govina
China
nmark
stonia
Finland
France
rmany
ungary
Japan
Latvia
ngolia
rlands
Russia
ovakia
ovenia
Korea
weden
erland
USSR
ngdom

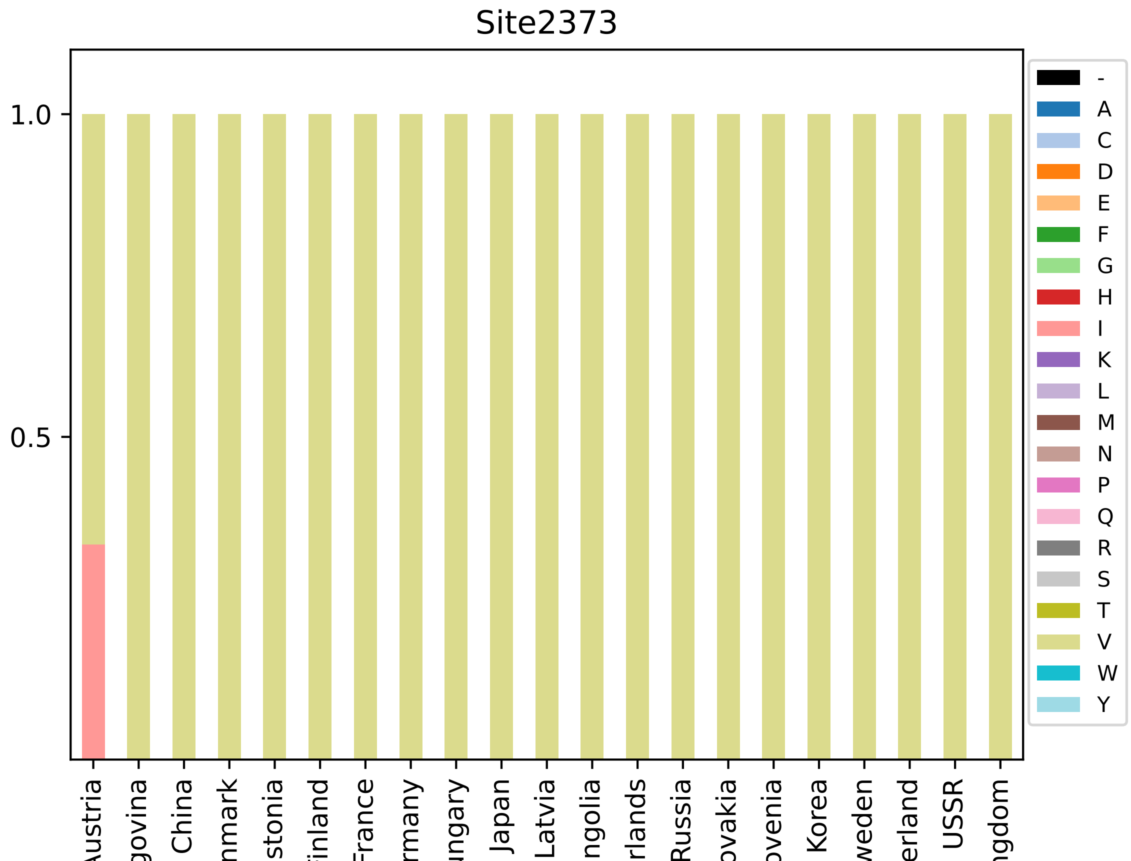
Site2373
1.0
0.5
-
A
C
D
E
F
G
H
I
K
L
M
N
P
Q
R
S
T
V
W
Y
Austria
govina
China
nmark
stonia
Finland
France
rmany
ungary
Japan
Latvia
ngolia
rlands
Russia
ovakia
ovenia
Korea
weden
erland
USSR
ngdom

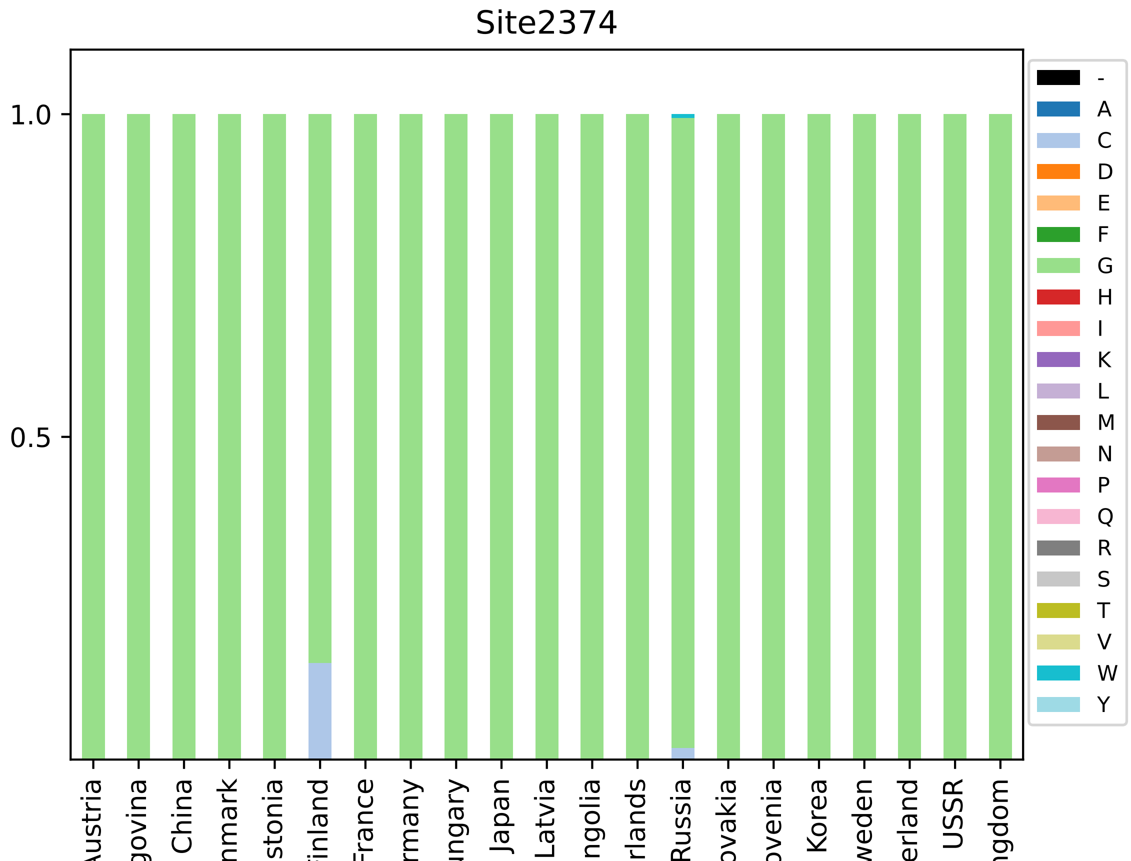

Site2374
1.0
0.5
-
A
C
D
E
F
G
H
I
K
L
M
N
P
Q
R
S
T
V
W
Y
China
Japan
Latvia
Russia
Korea
USSR

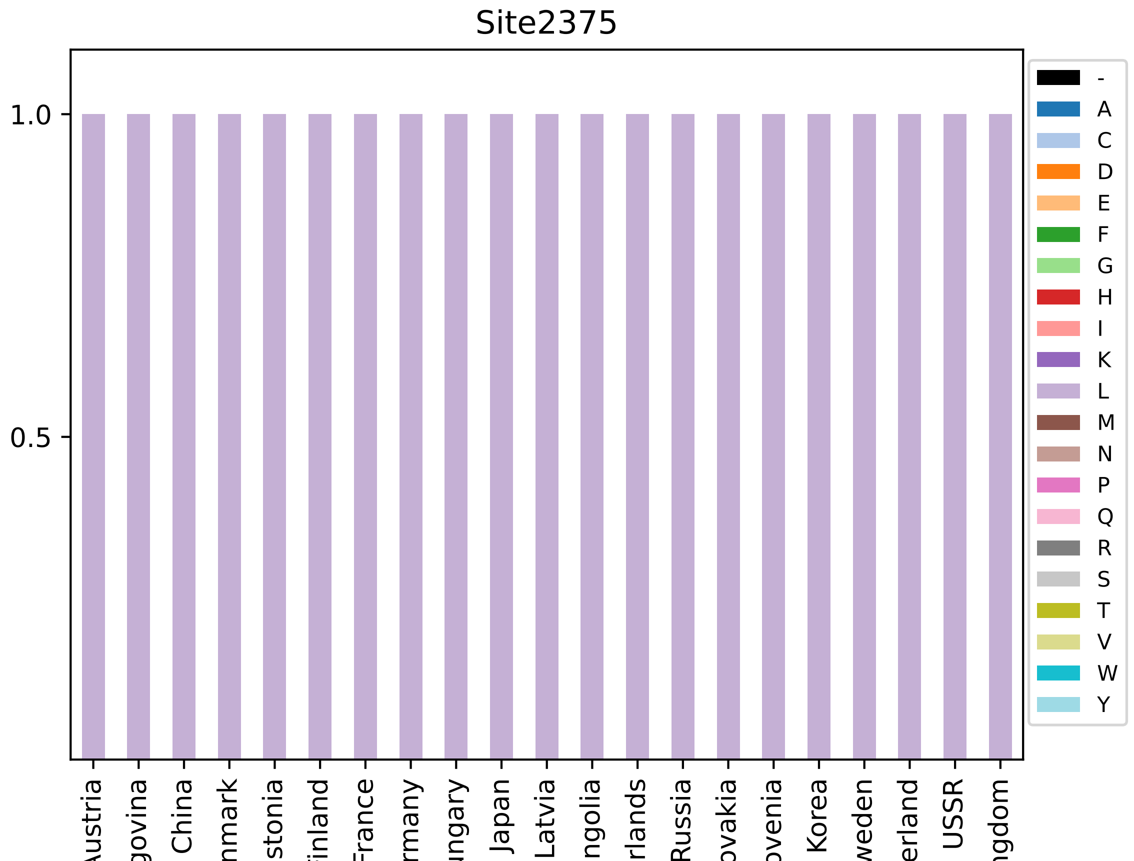
Site2375
1.0
0.5
-
A
C
D
E
F
G
H
I
K
L
M
N
P
Q
R
S
T
V
W
Y
China
Japan
Latvia
Russia
Korea
USSR

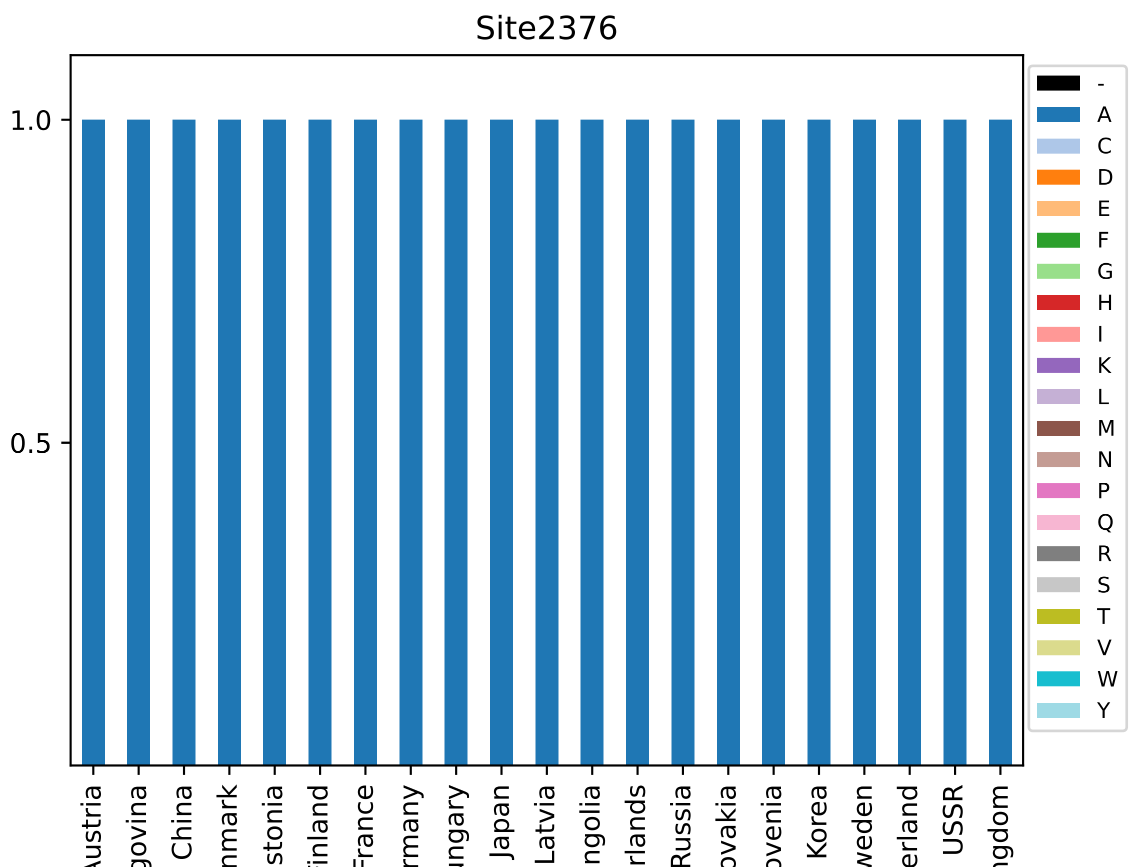
Site2376
1.0
0.5
-
A
C
D
E
F
G
H
I
K
L
M
N
P
Q
R
S
T
V
W
Y
China
Japan
Latvia
Russia
Korea
USSR

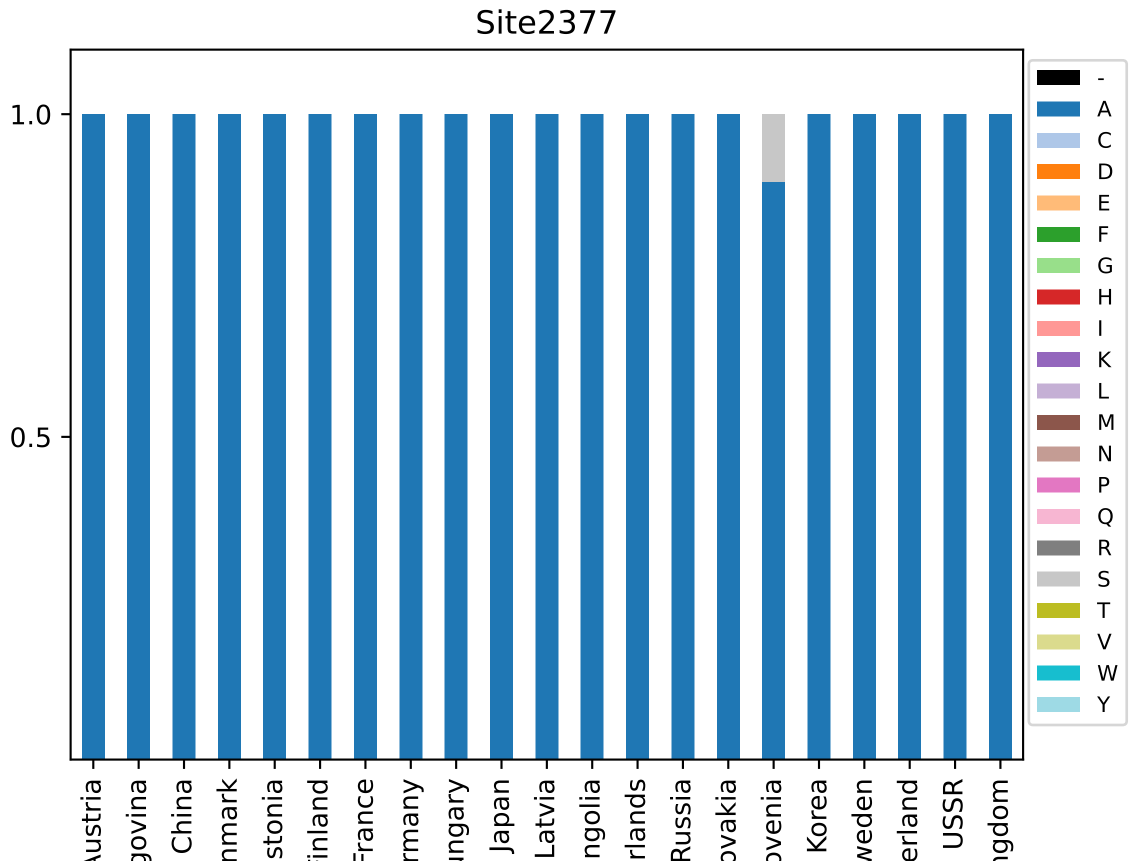
Site2377
1.0
0.5
-
A
C
D
E
F
G
H
I
K
L
M
N
P
Q
R
S
T
V
W
Y
Austria
govina
China
nmark
stonia
inland
France
rmany
ungary
Japan
Latvia
ngolia
rlands
Russia
ovakia
ovenia
Korea
weden
erland
USSR
ngdom

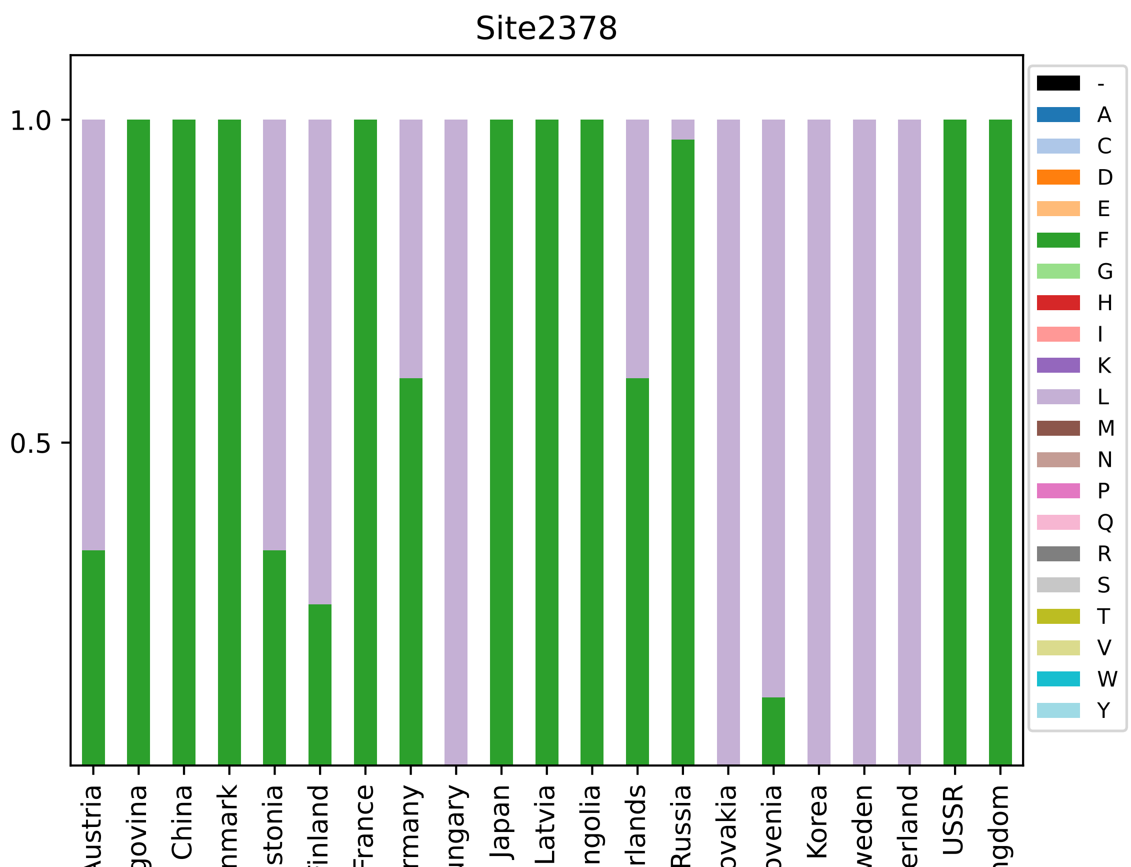

Site2378
1.0
0.5
-
A
C
D
E
F
G
H
I
K
L
M
N
P
Q
R
S
T
V
W
Y
Austria
govina
China
nmark
stonia
inland
France
rmany
ungary
Japan
Latvia
ngolia
rlands
Russia
ovakia
ovenia
Korea
weden
erland
USSR
ngdom

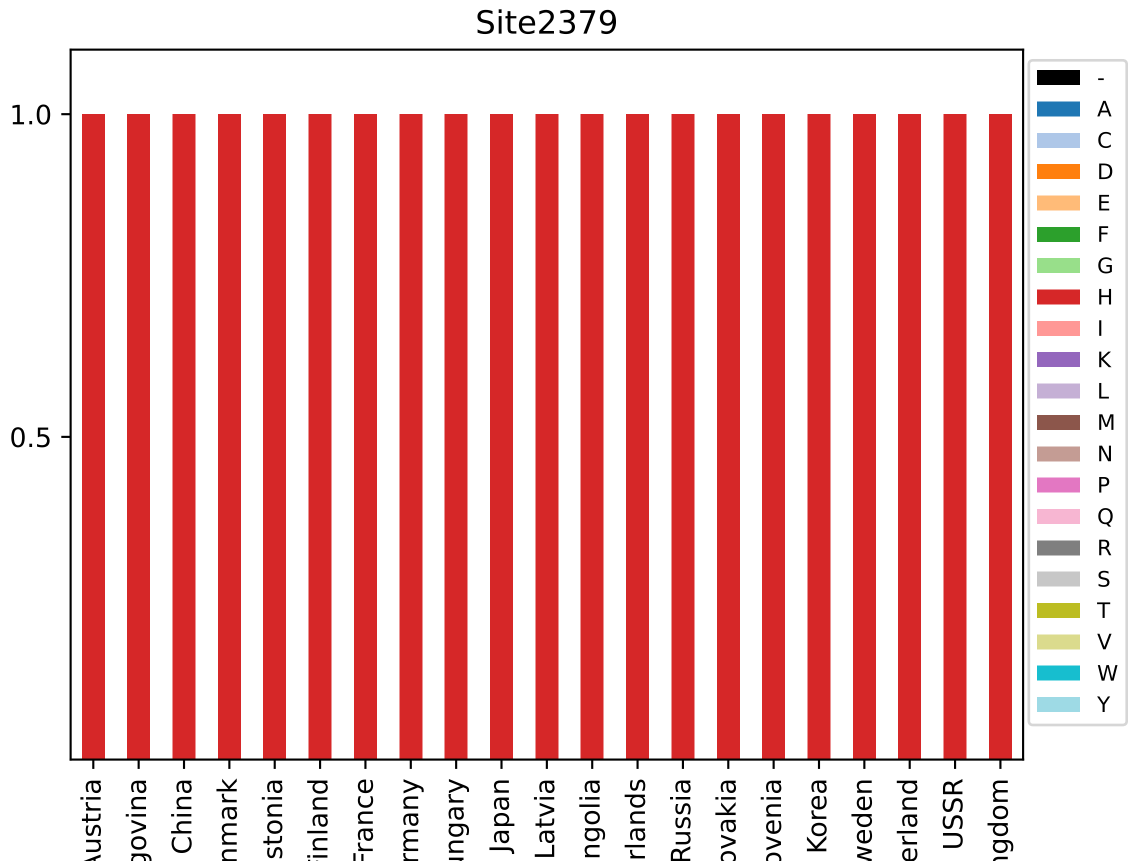
Site2379
1.0
0.5
-
A
C
D
E
F
G
H
I
K
L
M
N
P
Q
R
S
T
V
W
Y
China
Japan
Latvia
Korea
USSR

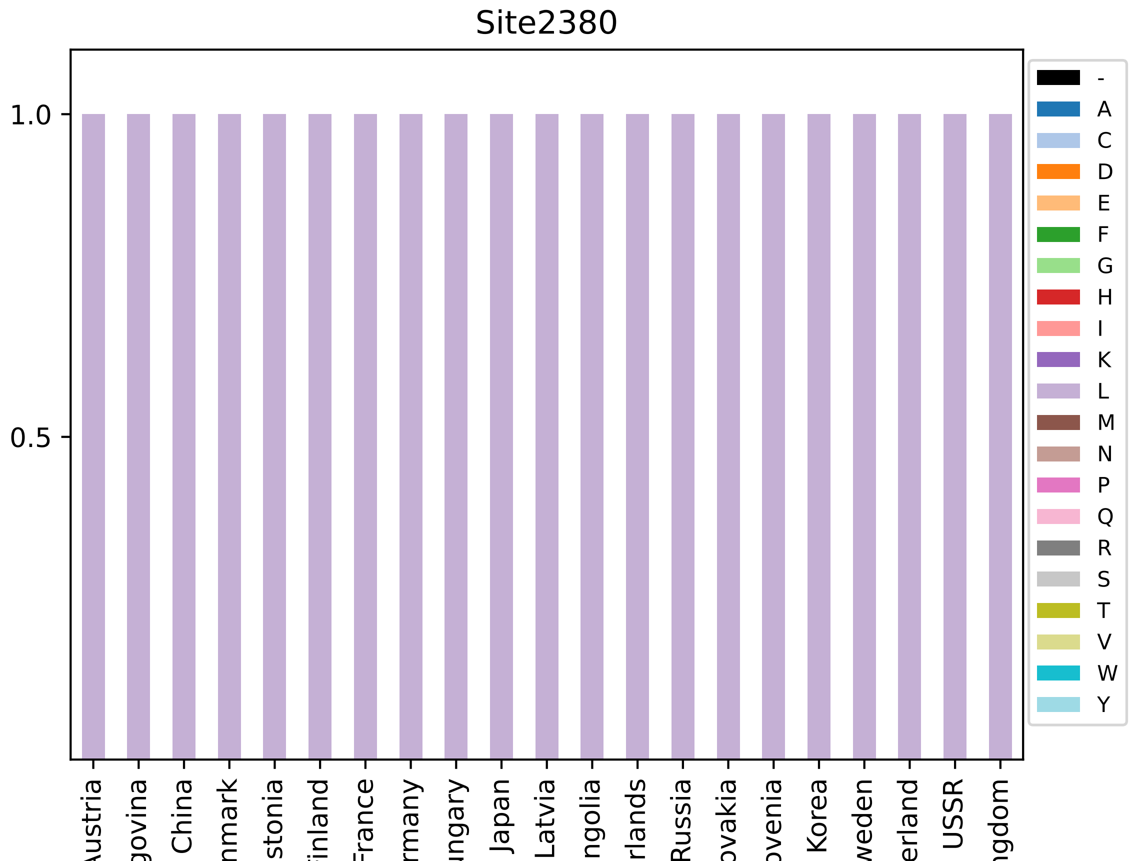
Site2380
1.0
0.5
-
A
C
D
E
F
G
H
I
K
L
M
N
P
Q
R
S
T
V
W
Y
China
Japan
Latvia
Russia
Korea
USSR

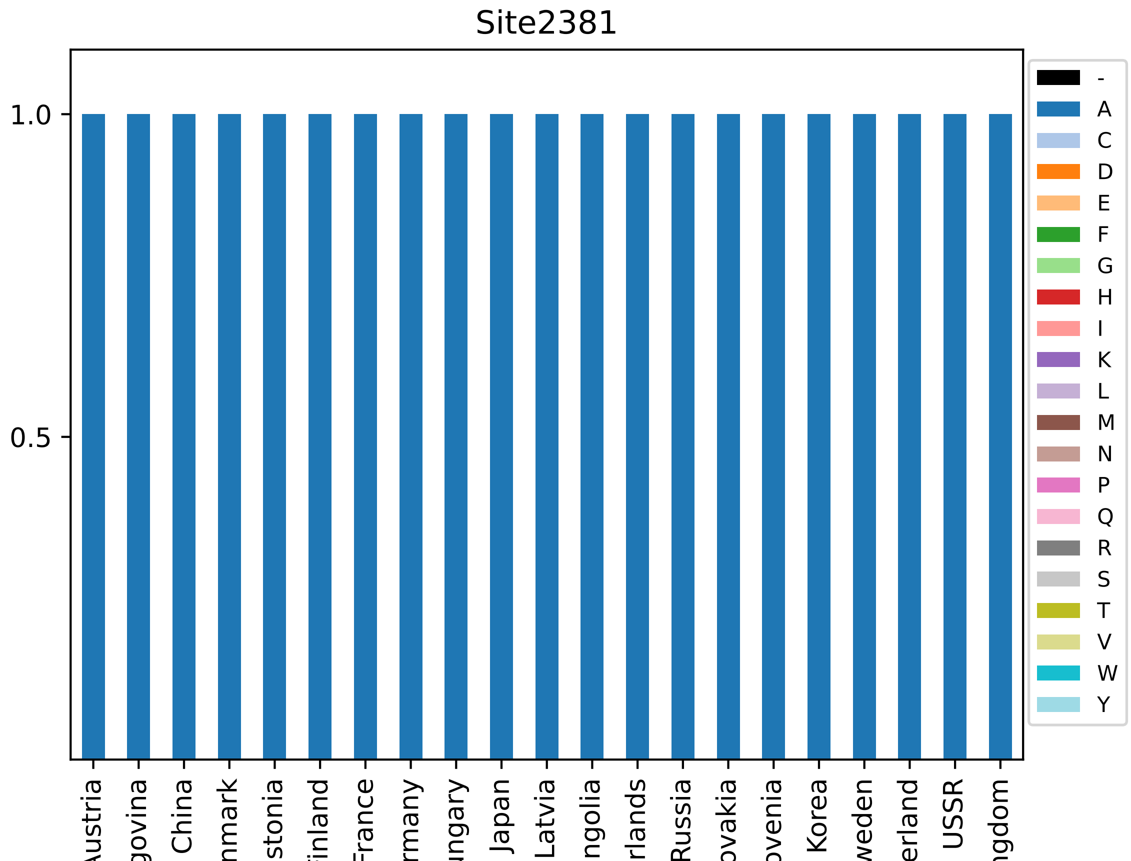
Site2381
1.0
0.5
-
A
C
D
E
F
G
H
I
K
L
M
N
P
Q
R
S
T
V
W
Y
China
Japan
Latvia
Russia
Korea
USSR

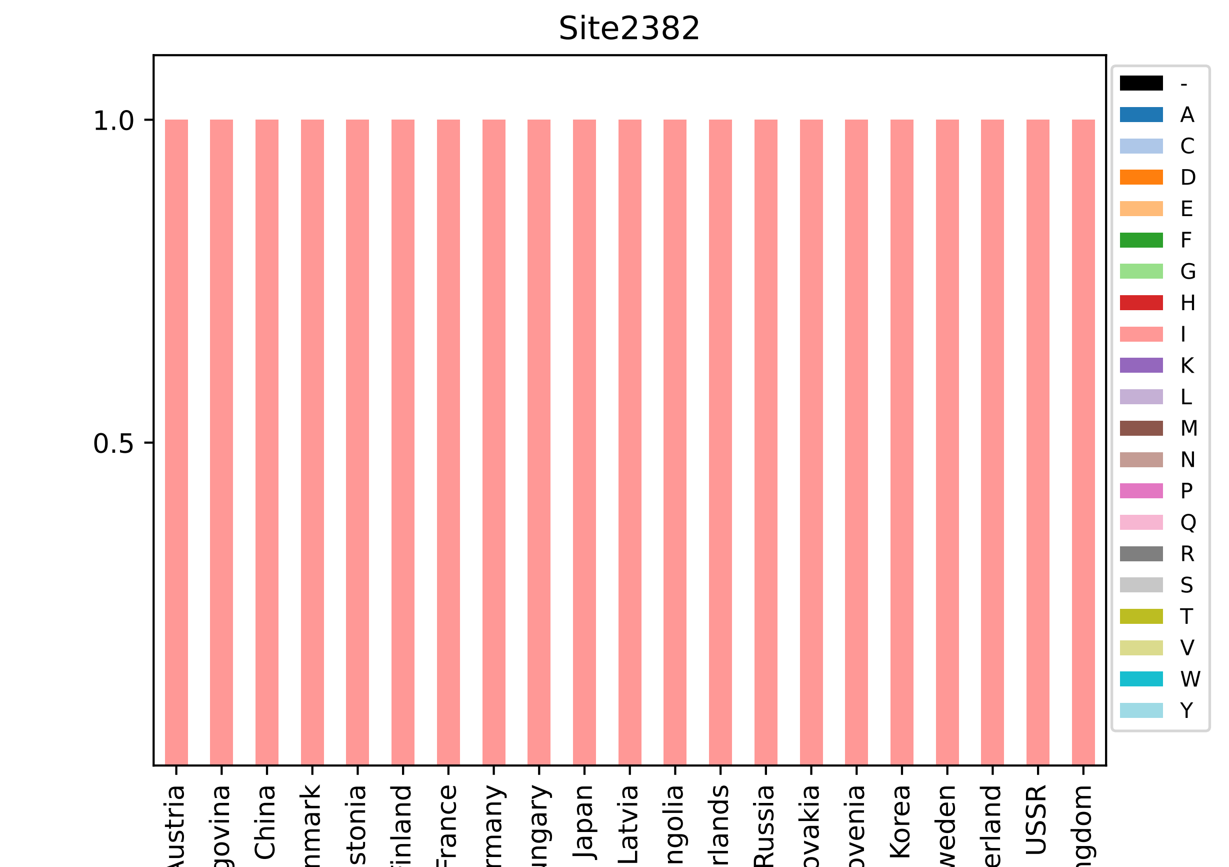
Site2382
1.0
0.5
-
A
C
D
E
F
G
H
I
K
L
M
N
P
Q
R
S
T
V
W
Y
Austria
govina
China
nmark
stonia
Finland
France
rmany
ungary
Japan
Latvia
ngolia
rlands
Russia
ovakia
ovenia
Korea
weden
erland
USSR
ngdom

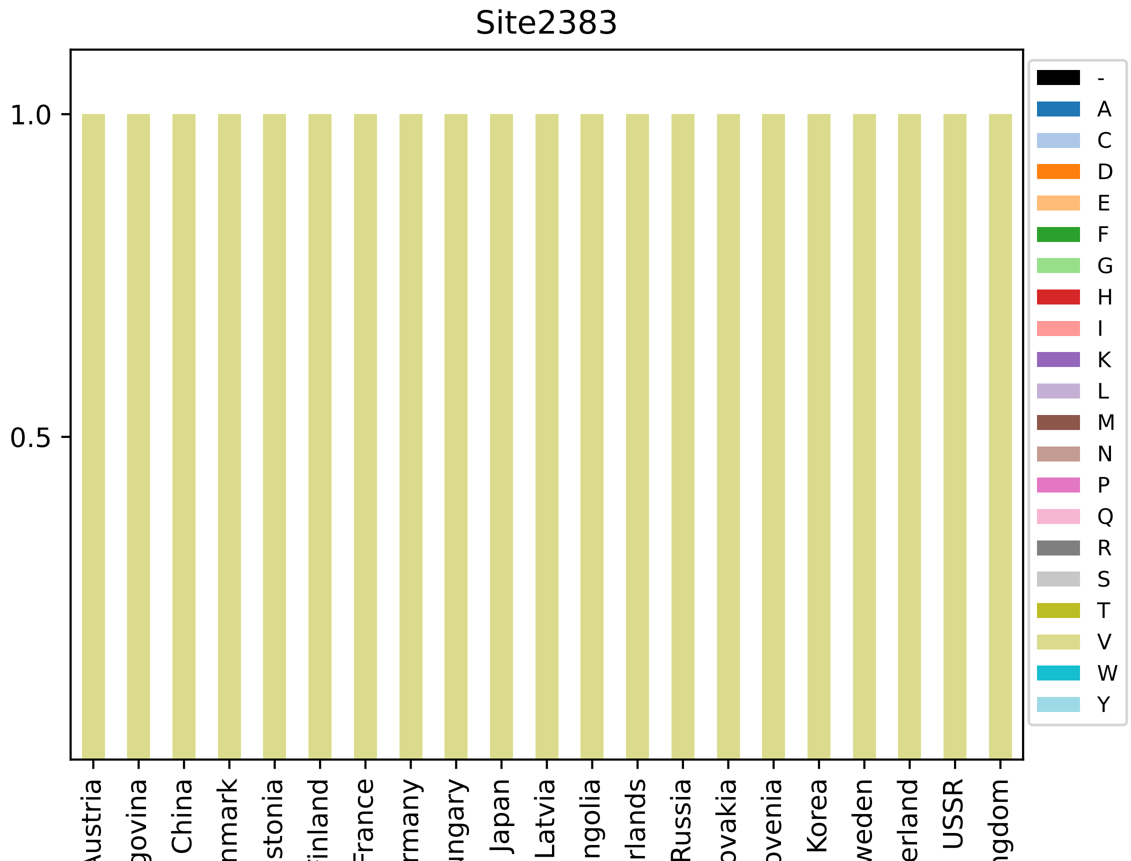

Site2383
1.0
0.5
-
A
C
D
E
F
G
H
I
K
L
M
N
P
Q
R
S
T
V
W
Y
Austria
China
Japan
Latvia
Russia
Korea
USSR

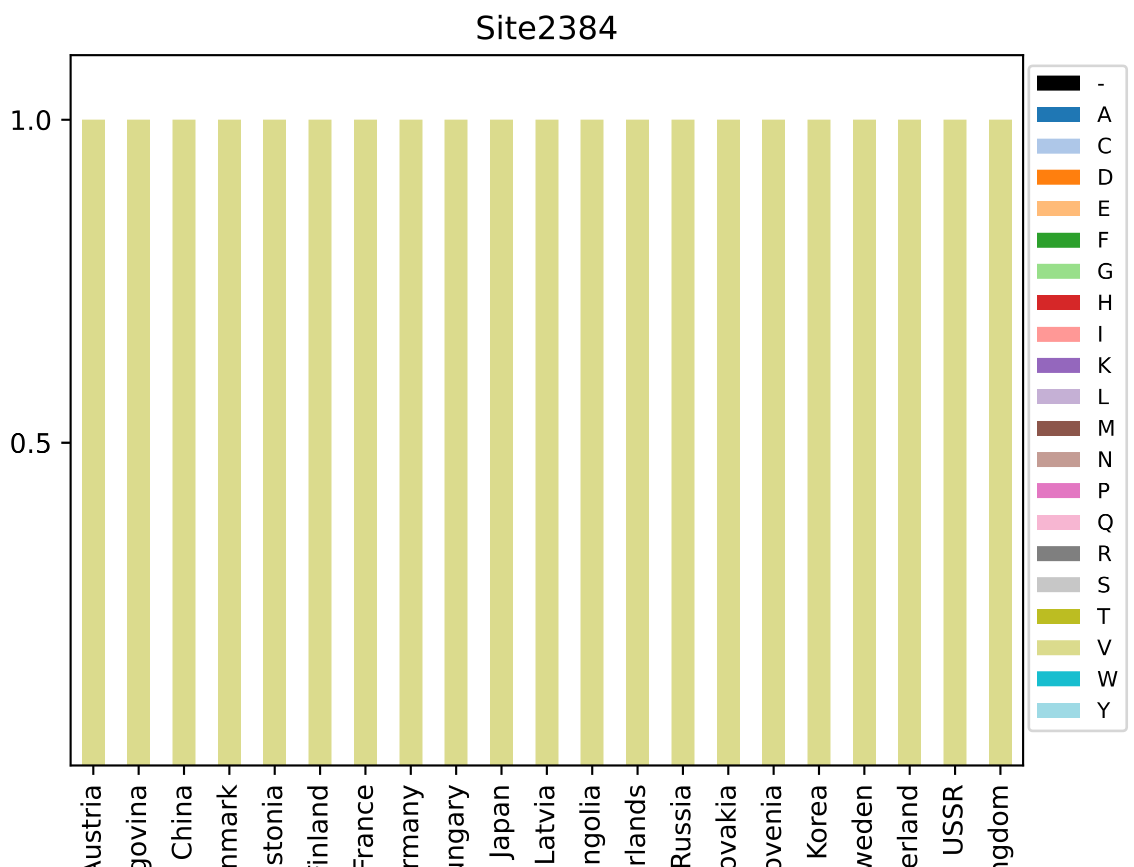
Site2384
1.0
0.5
-
A
C
D
E
F
G
H
I
K
L
M
N
P
Q
R
S
T
V
W
Y
Austria
govina
China
nmark
stonia
Finland
France
rmany
ungary
Japan
Latvia
ngolia
rlands
Russia
ovakia
ovenia
Korea
weden
erland
USSR
ngdom

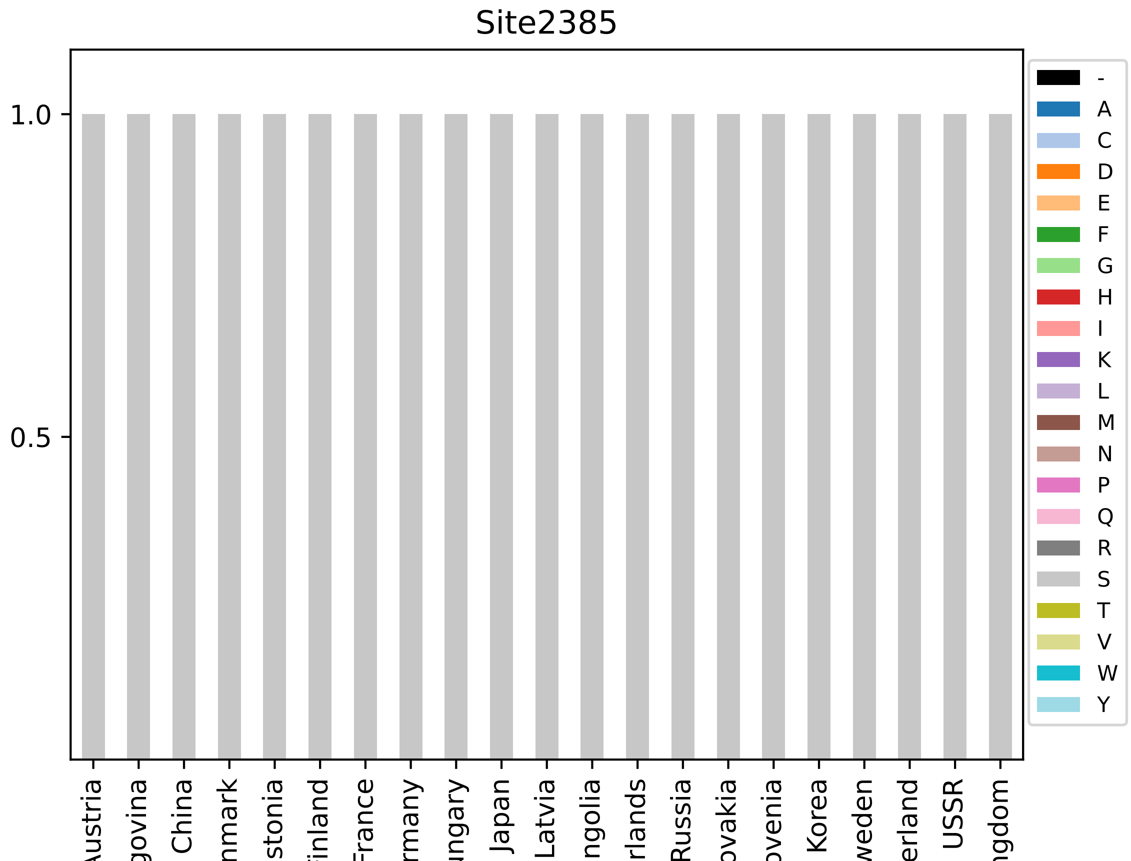
Site2385
1.0
0.5
-
A
C
D
E
F
G
H
I
K
L
M
N
P
Q
R
S
T
V
W
Y
Austria
govina
China
nmark
stonia
Finland
France
rmany
ungary
Japan
Latvia
ngolia
rlands
Russia
ovakia
ovenia
Korea
weden
erland
USSR
ngdom

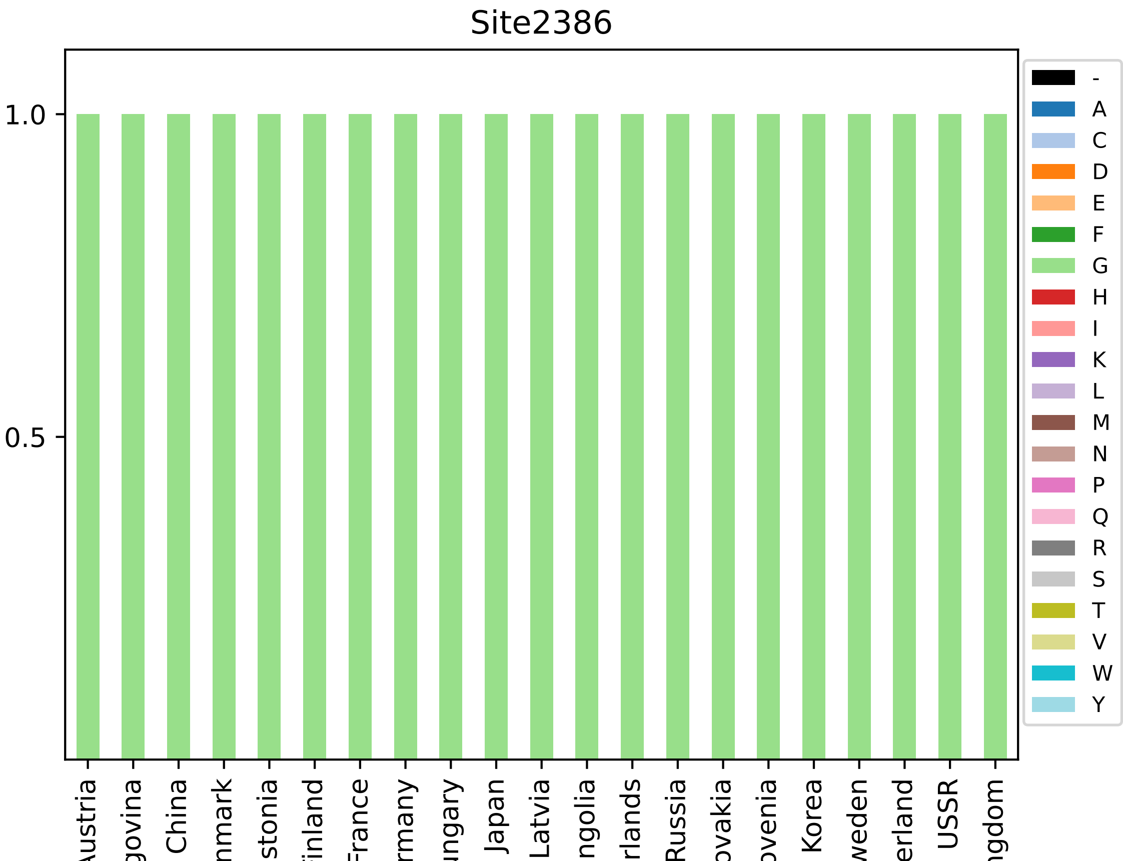
Site2386
1.0
0.5
-
A
C
D
E
F
G
H
I
K
L
M
N
P
Q
R
S
T
V
W
Y
China
Japan
Latvia
Russia
Korea
USSR

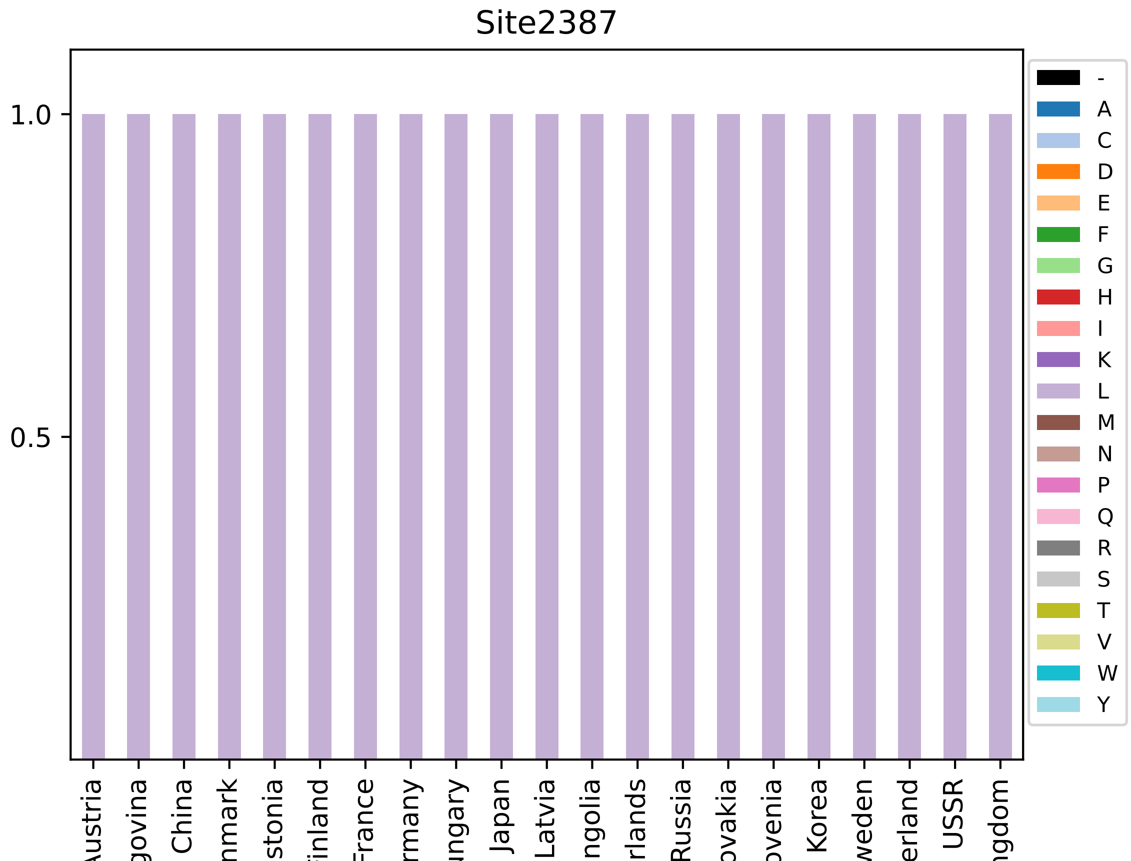
Site2387
1.0
0.5
-
A
C
D
E
F
G
H
I
K
L
M
N
P
Q
R
S
T
V
W
Y
Austria
govina
China
nmark
stonia
Finland
France
rmany
ungary
Japan
Latvia
ngolia
rlands
Russia
ovakia
ovenia
Korea
weden
erland
USSR
ngdom

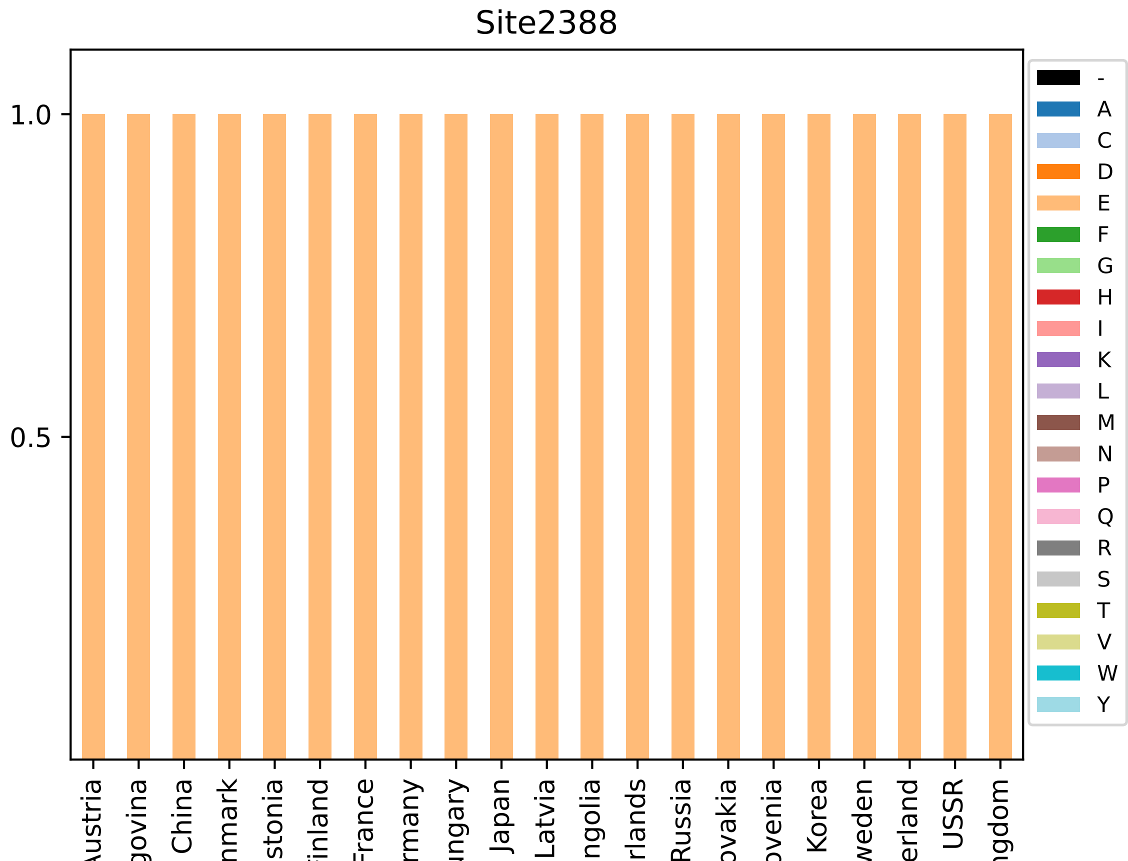

Site2388
1.0
0.5
-
A
C
D
E
F
G
H
I
K
L
M
N
P
Q
R
S
T
V
W
Y
China
Japan
Latvia
Russia
Korea
USSR

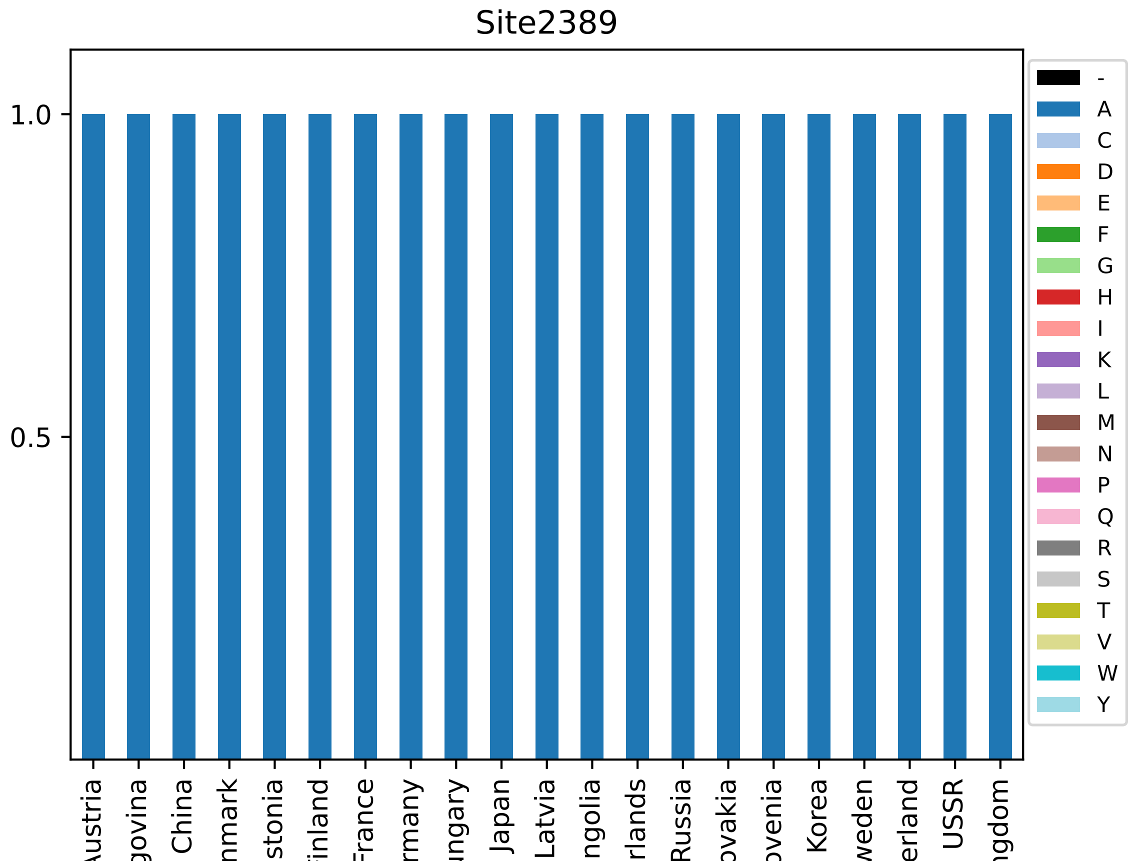

Site2389
1.0
0.5
-
A
C
D
E
F
G
H
I
K
L
M
N
P
Q
R
S
T
V
W
Y
China
Japan
Latvia
Russia
Korea
USSR

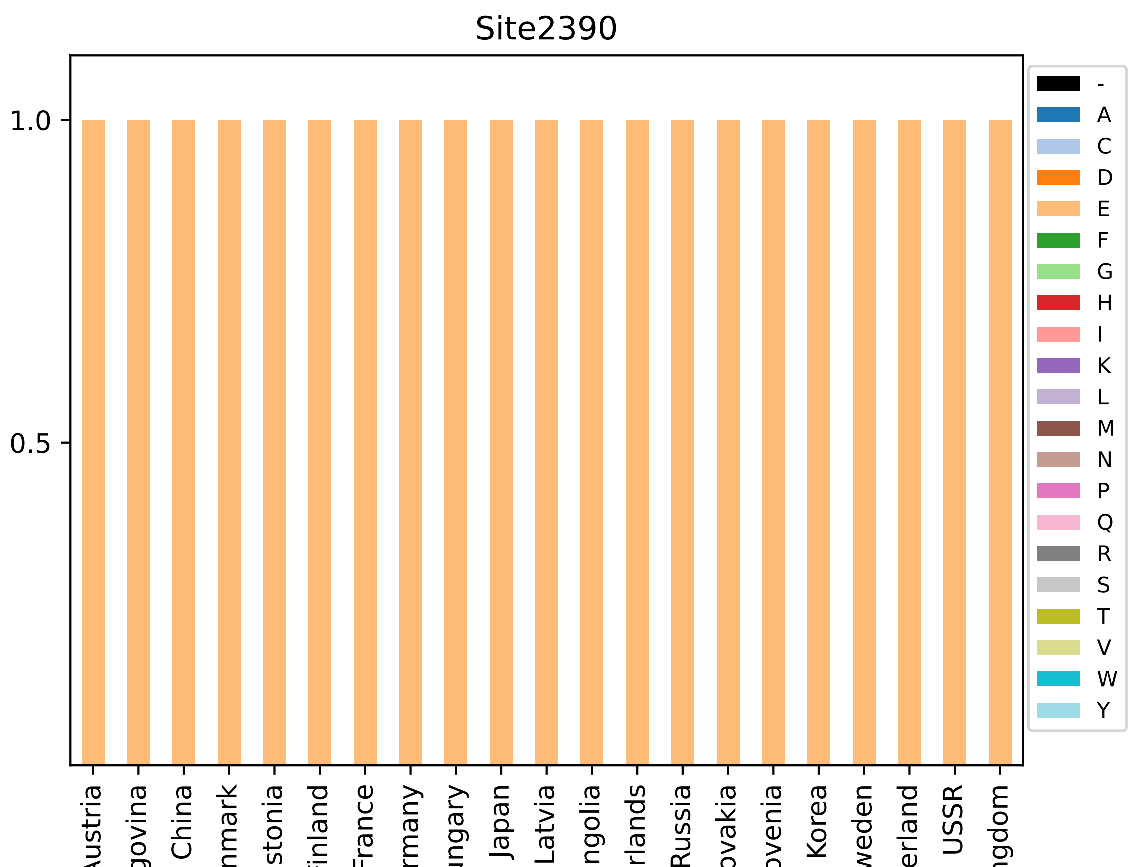
Site2390
1.0
0.5
-
A
C
D
E
F
G
H
I
K
L
M
N
P
Q
R
S
T
V
W
Y
Austria
China
Japan
Latvia
Russia
Korea
USSR

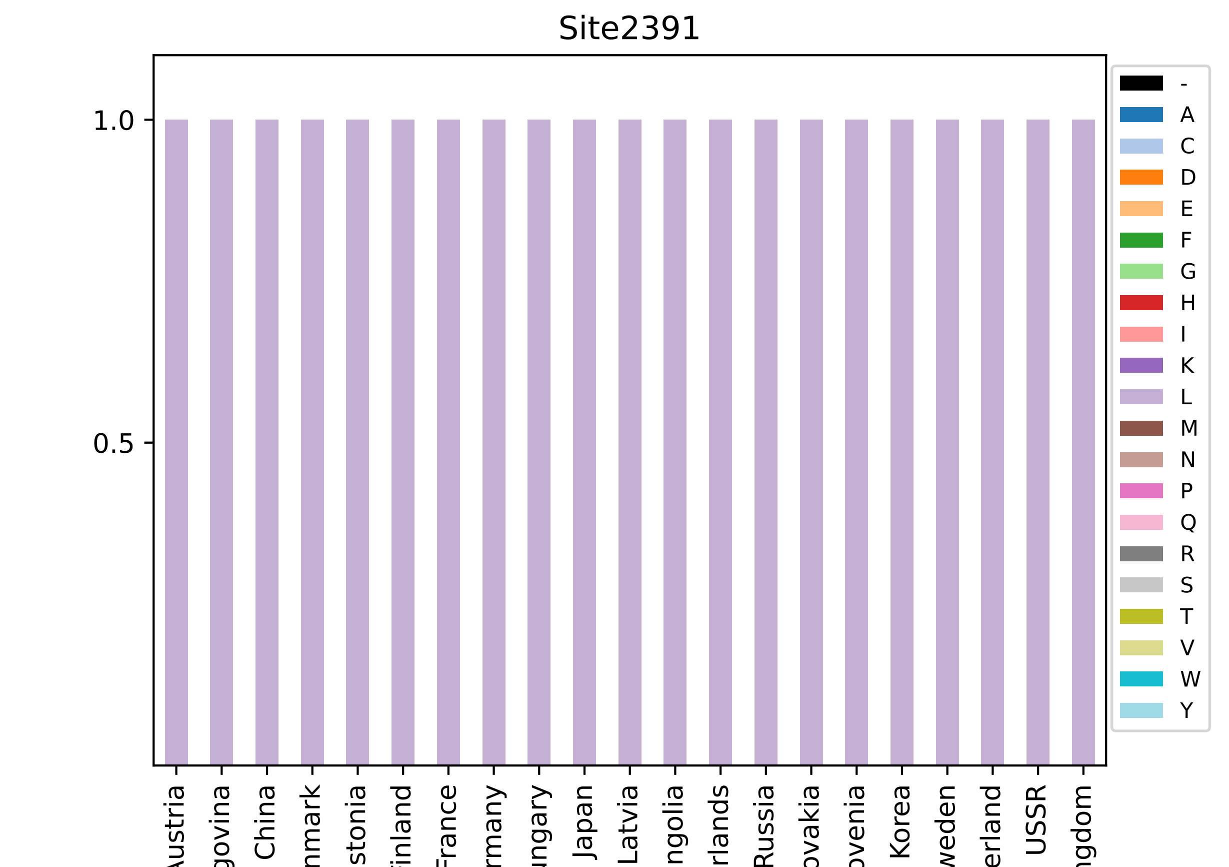

Site2391
1.0
0.5
-
A
C
D
E
F
G
H
I
K
L
M
N
P
Q
R
S
T
V
W
Y
China
Japan
Latvia
Russia
Korea
USSR

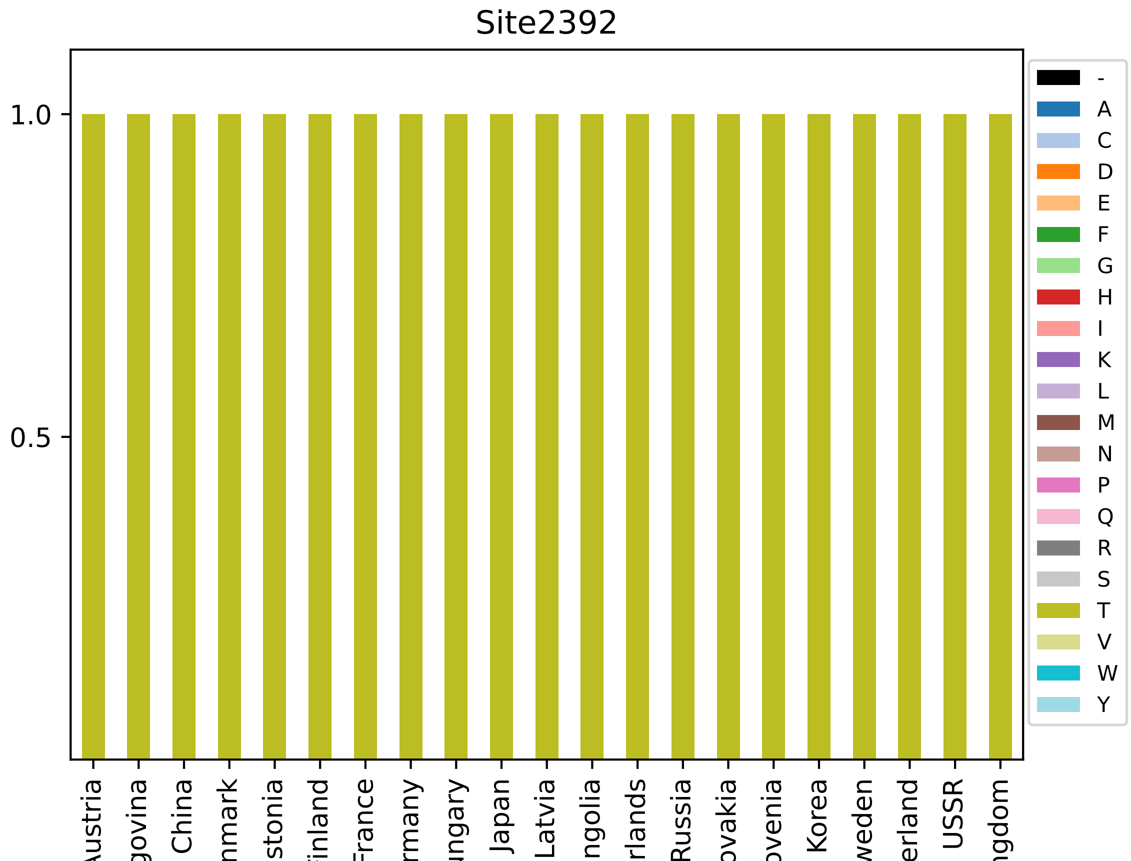

Site2392
1.0
0.5
-
A
C
D
E
F
G
H
I
K
L
M
N
P
Q
R
S
T
V
W
Y
China
Japan
Latvia
Russia
Korea
USSR

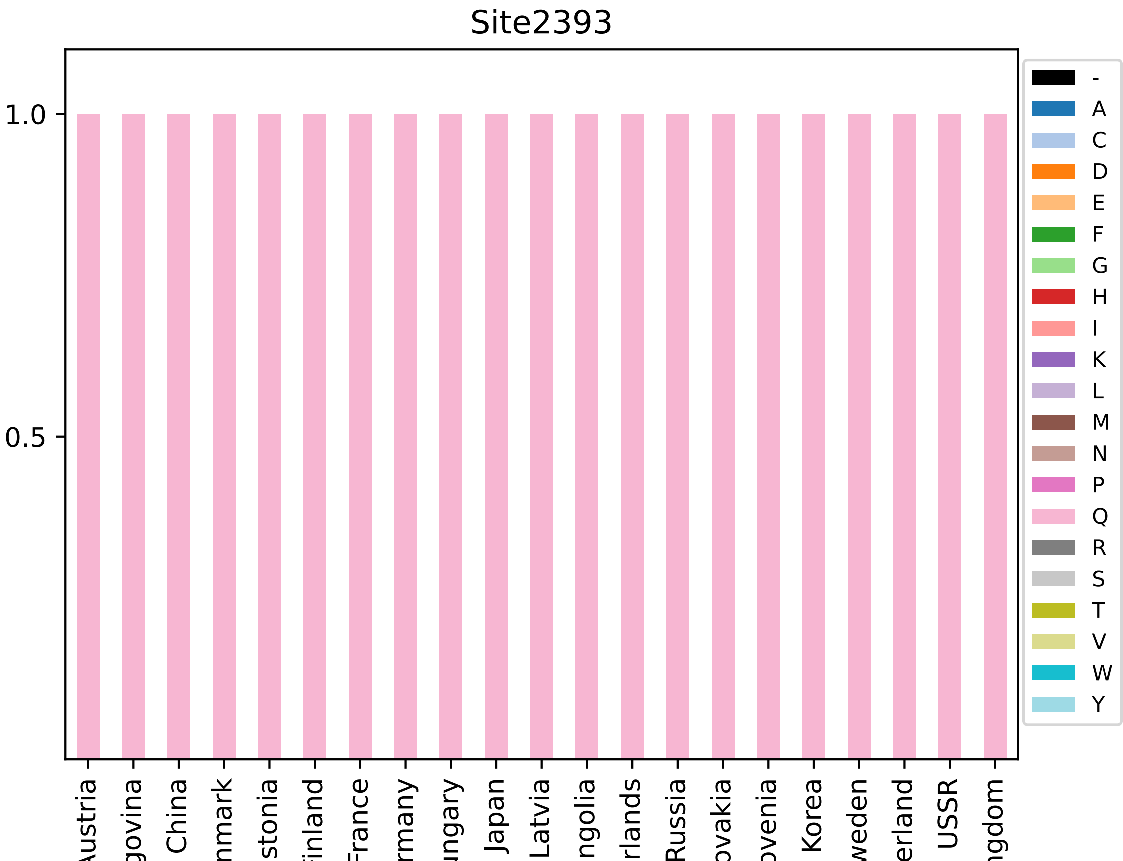
Site2393
1.0
0.5
-
A
C
D
E
F
G
H
I
K
L
M
N
P
Q
R
S
T
V
W
Y
ustria
govina
China
nmark
stonia
inland
rance
rmany
ungary
Japan
Latvia
ngolia
rlands
Russia
ovakia
ovenia
Korea
weden
erland
USSR
ngdom

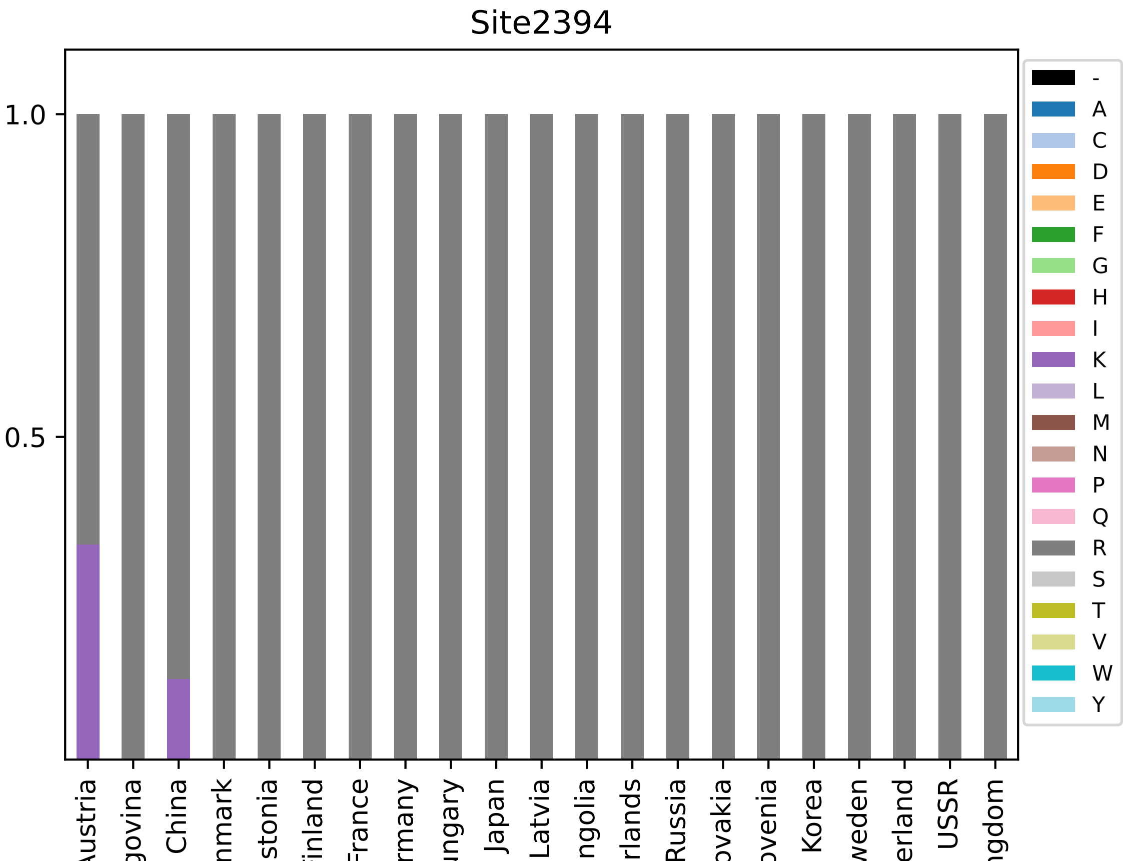

Site2394
1.0
0.5
-
A
C
D
E
F
G
H
I
K
L
M
N
P
Q
R
S
T
V
W
Y
Austria
govina
China
nmark
stonia
Finland
France
rmany
ungary
Japan
Latvia
ngolia
rlands
Russia
ovakia
ovenia
Korea
weden
erland
USSR
ngdom

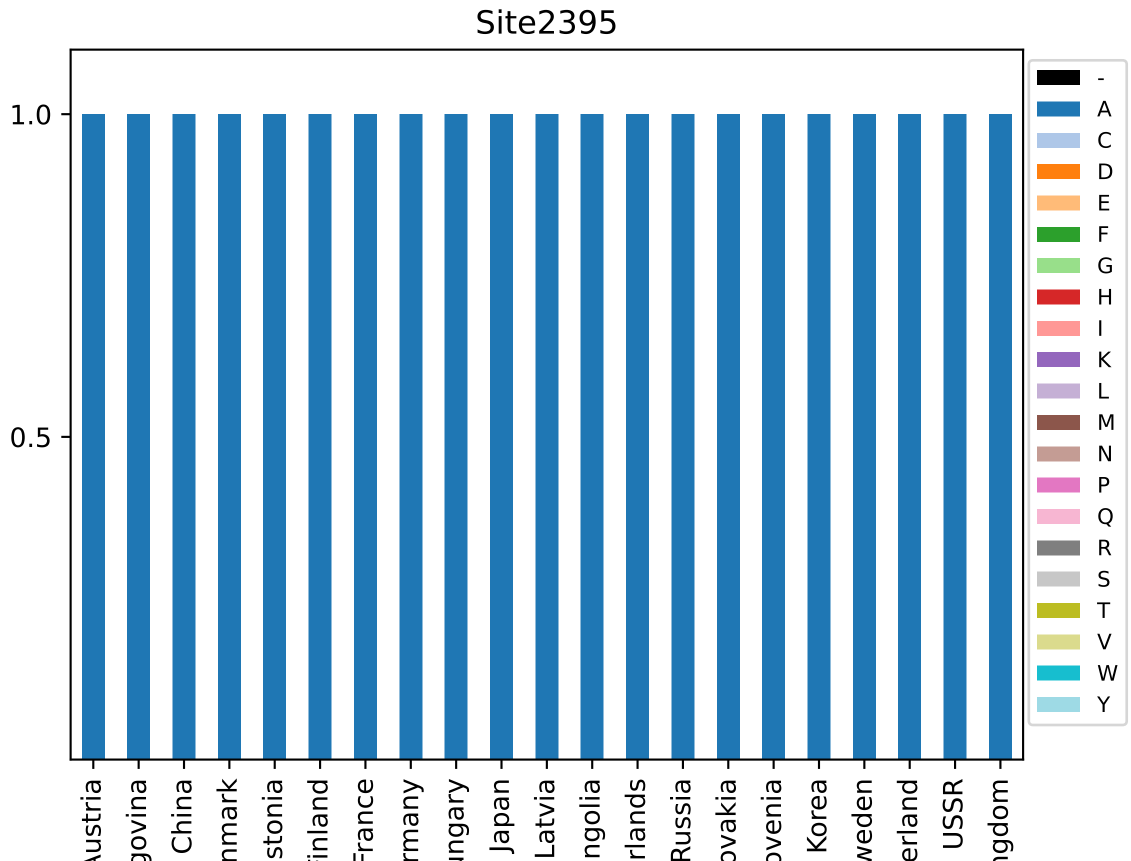
Site2395
1.0
0.5
China
Japan
Latvia
Russia
Korea
USSR
-
A
C
D
E
F
G
H
I
K
L
M
N
P
Q
R
S
T
V
W
Y

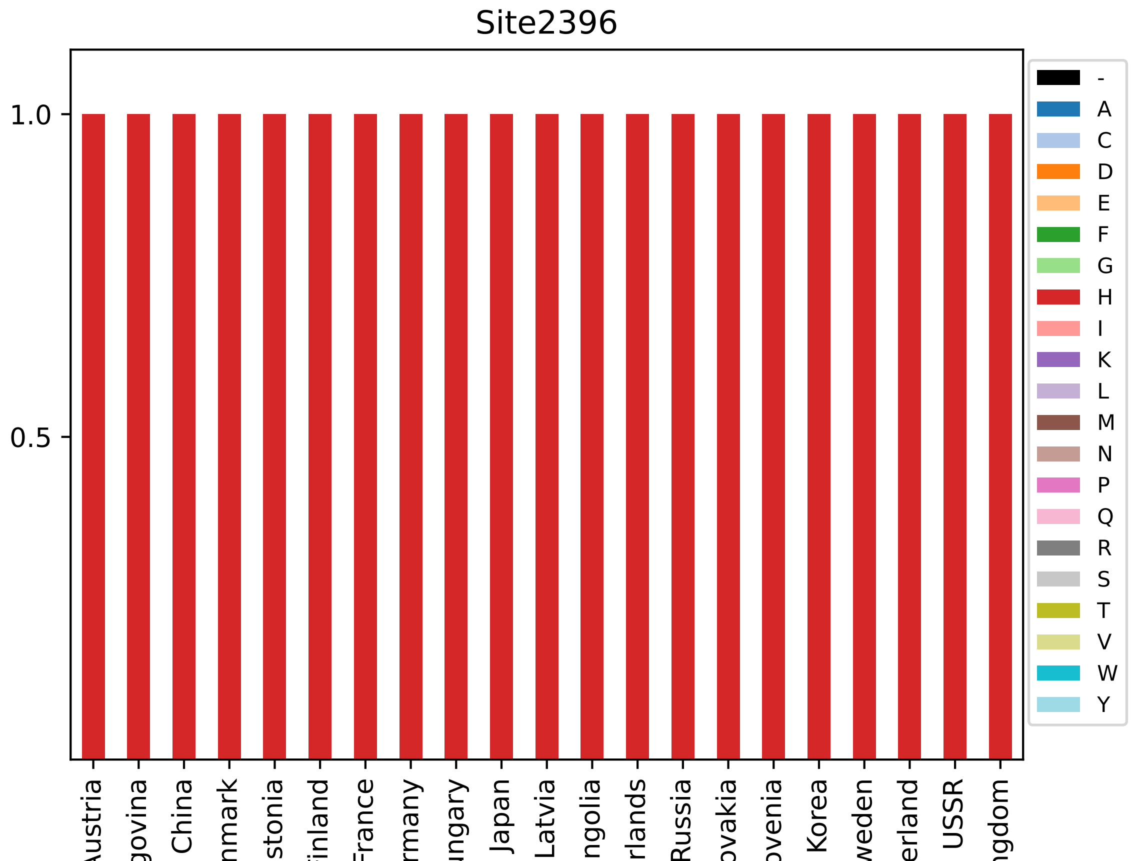

Site2396
1.0
0.5
-
A
C
D
E
F
G
H
I
K
L
M
N
P
Q
R
S
T
V
W
Y
China
Japan
Latvia
Russia
Korea
USSR

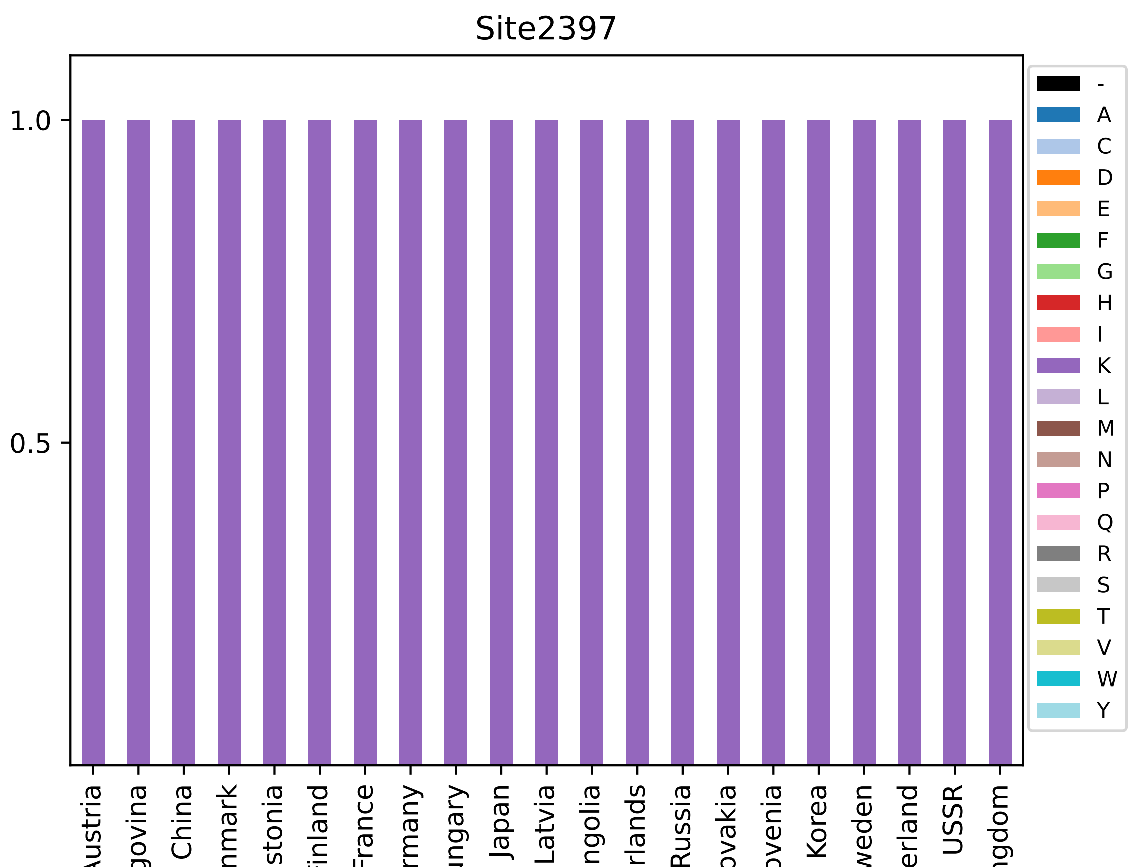
Site2397
1.0
0.5
Austria
govina
China
nmark
stonia
Finland
France
rmany
ungary
Japan
Latvia
ngolia
rlands
Russia
ovakia
ovenia
Korea
weden
erland
USSR
ngdom
-
A
C
D
E
F
G
H
I
K
L
M
N
P
Q
R
S
T
V
W
Y

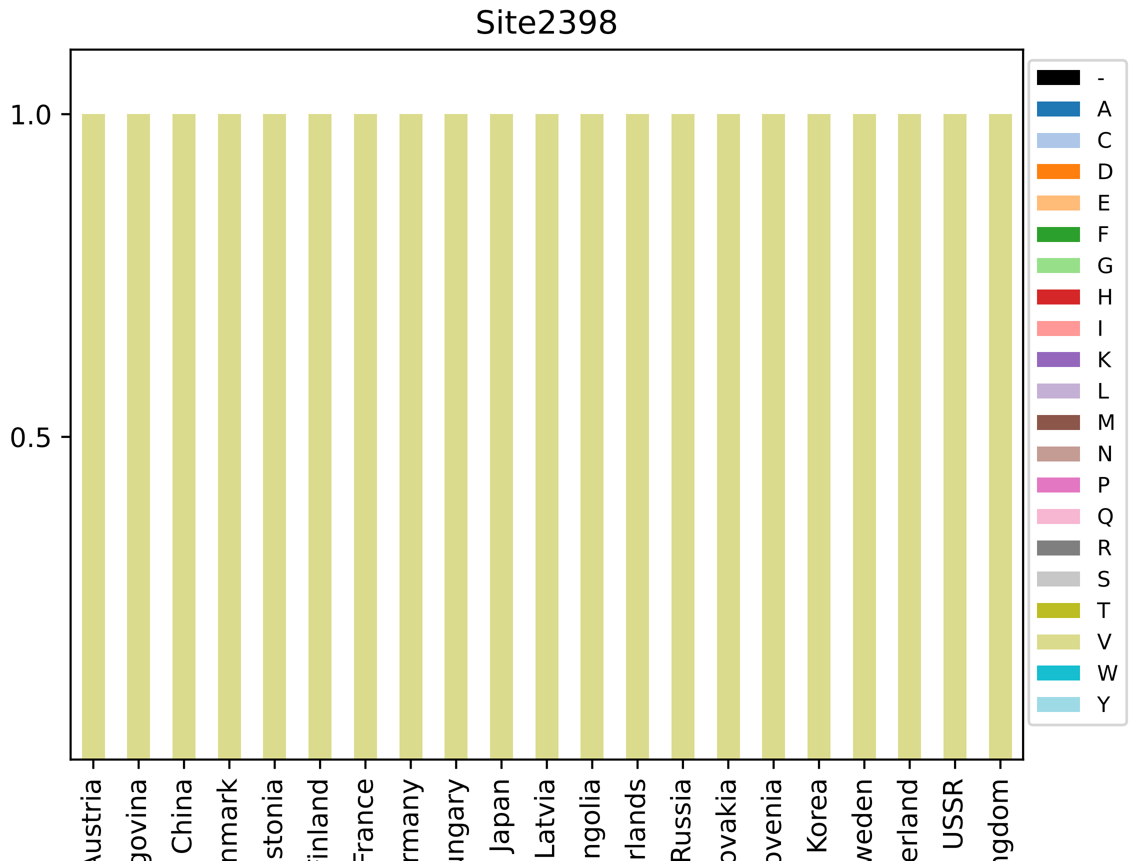

Site2398
1.0
0.5
-
A
C
D
E
F
G
H
I
K
L
M
N
P
Q
R
S
T
V
W
Y
China
Japan
Latvia
Russia
Korea
USSR

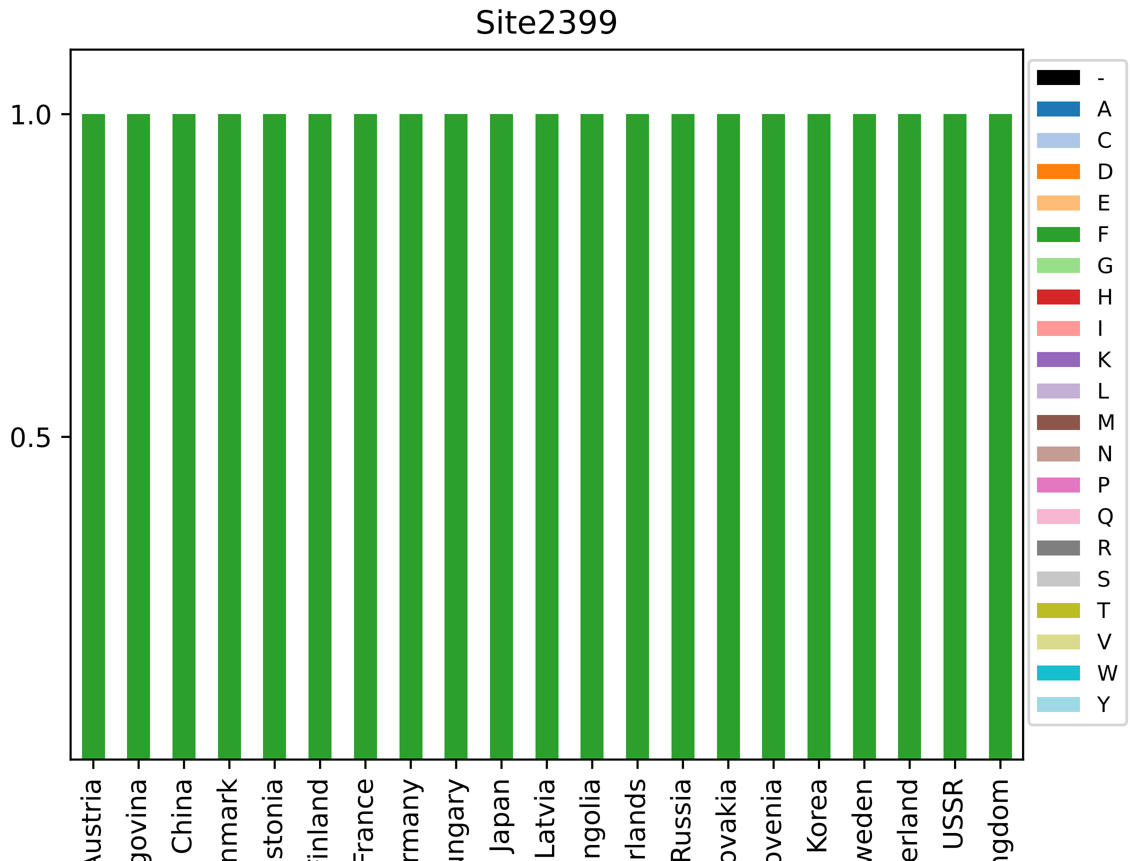

Site2399
1.0
0.5
-
A
C
D
E
F
G
H
I
K
L
M
N
P
Q
R
S
T
V
W
Y
Austria
govina
China
nmark
stonia
inland
France
rmany
ungary
Japan
Latvia
ngolia
rlands
Russia
ovakia
ovenia
Korea
weden
erland
USSR
ngdom

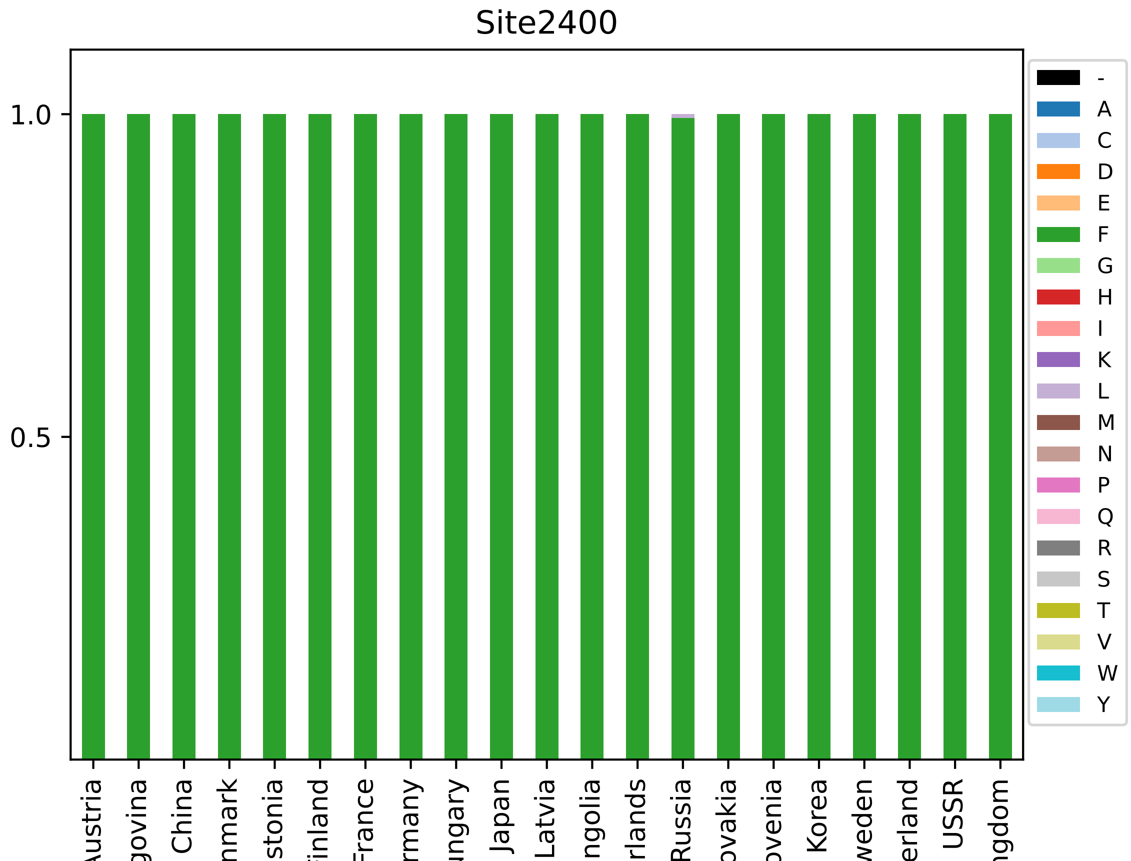
Site2400
1.0
0.5
-
A
C
D
E
F
G
H
I
K
L
M
N
P
Q
R
S
T
V
W
Y
China
Japan
Latvia
Russia
Korea
USSR

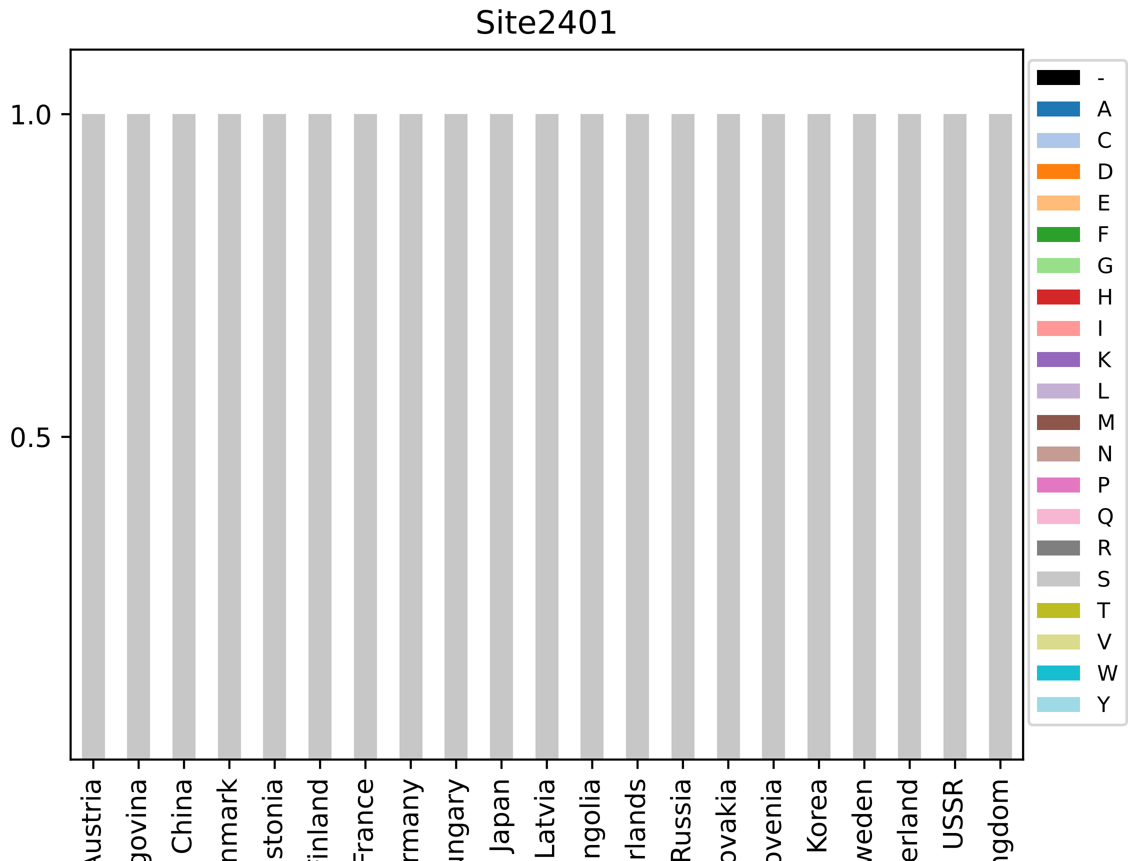
Site2401
1.0
0.5
-
A
C
D
E
F
G
H
I
K
L
M
N
P
Q
R
S
T
V
W
Y
China
Japan
Latvia
Russia
Korea
USSR

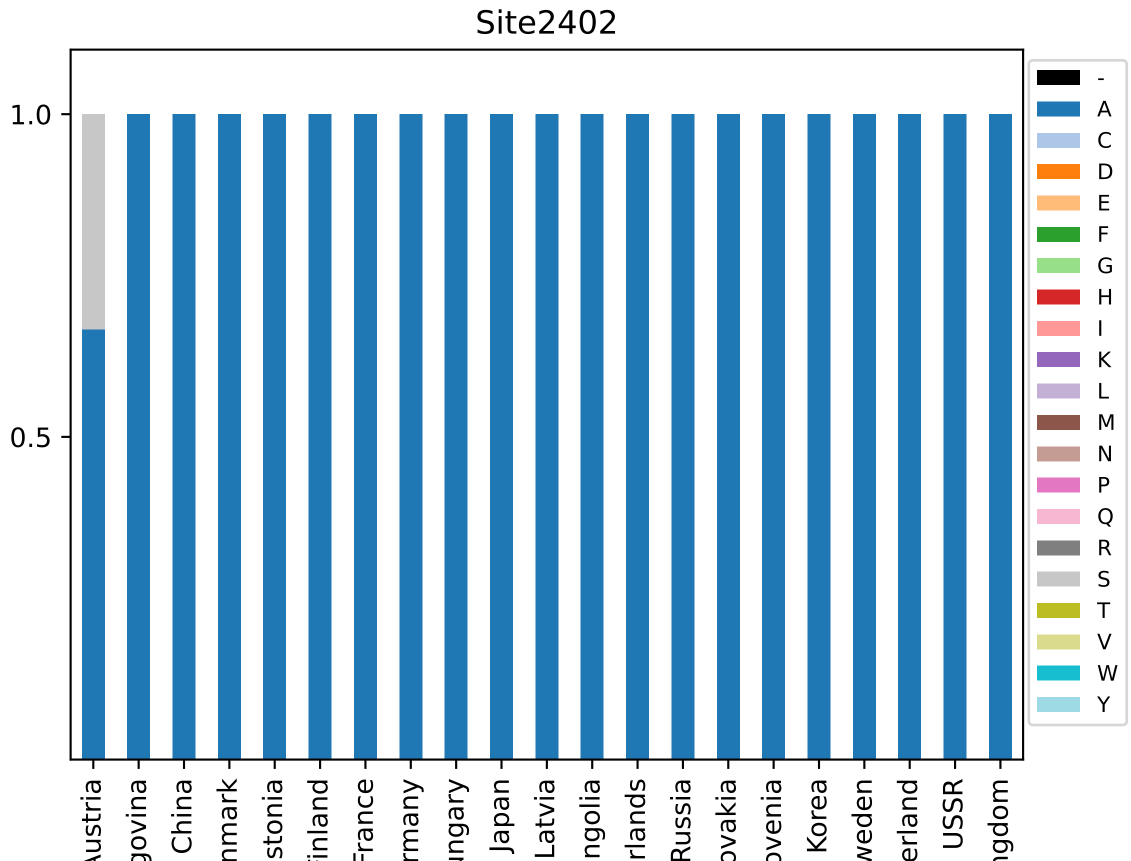

Site2402
1.0
0.5
-
A
C
D
E
F
G
H
I
K
L
M
N
P
Q
R
S
T
V
W
Y
China
Japan
Latvia
Russia
Korea
USSR

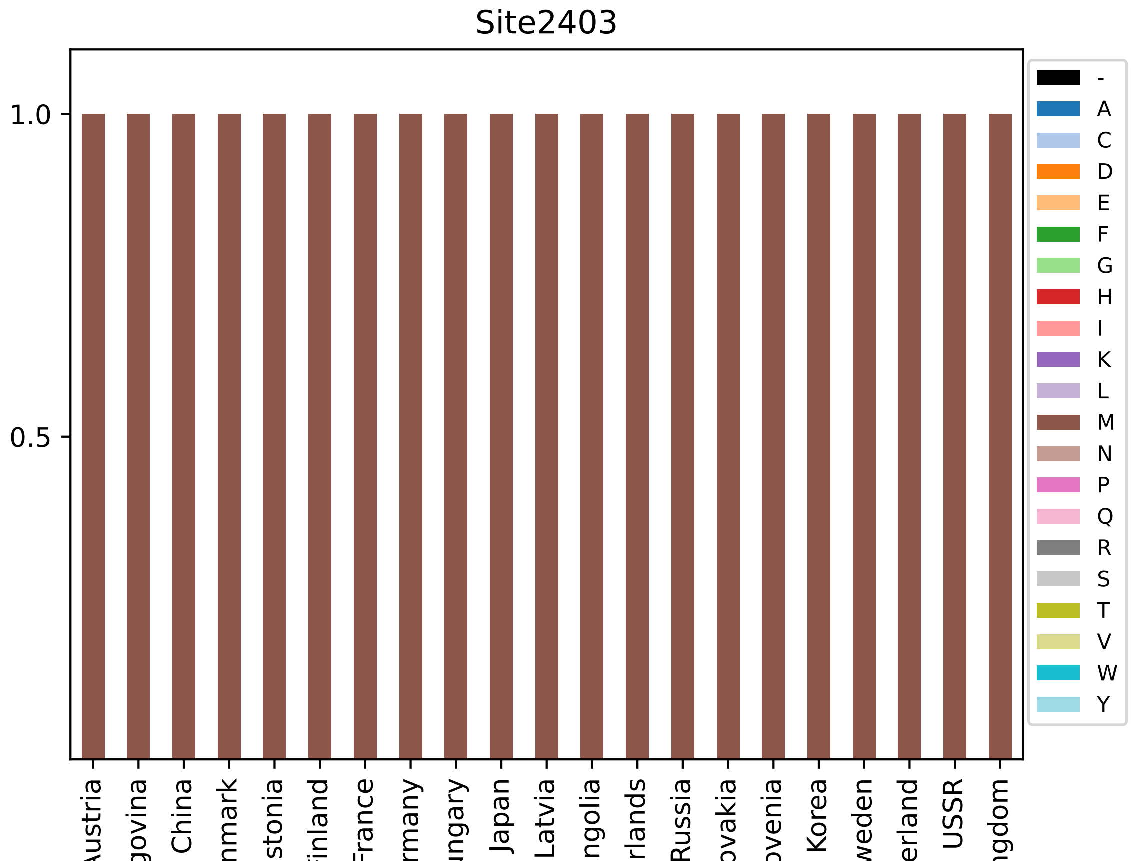

Site2403
1.0
0.5
-
A
C
D
E
F
G
H
I
K
L
M
N
P
Q
R
S
T
V
W
Y
China
Japan
Latvia
Russia
Korea
USSR

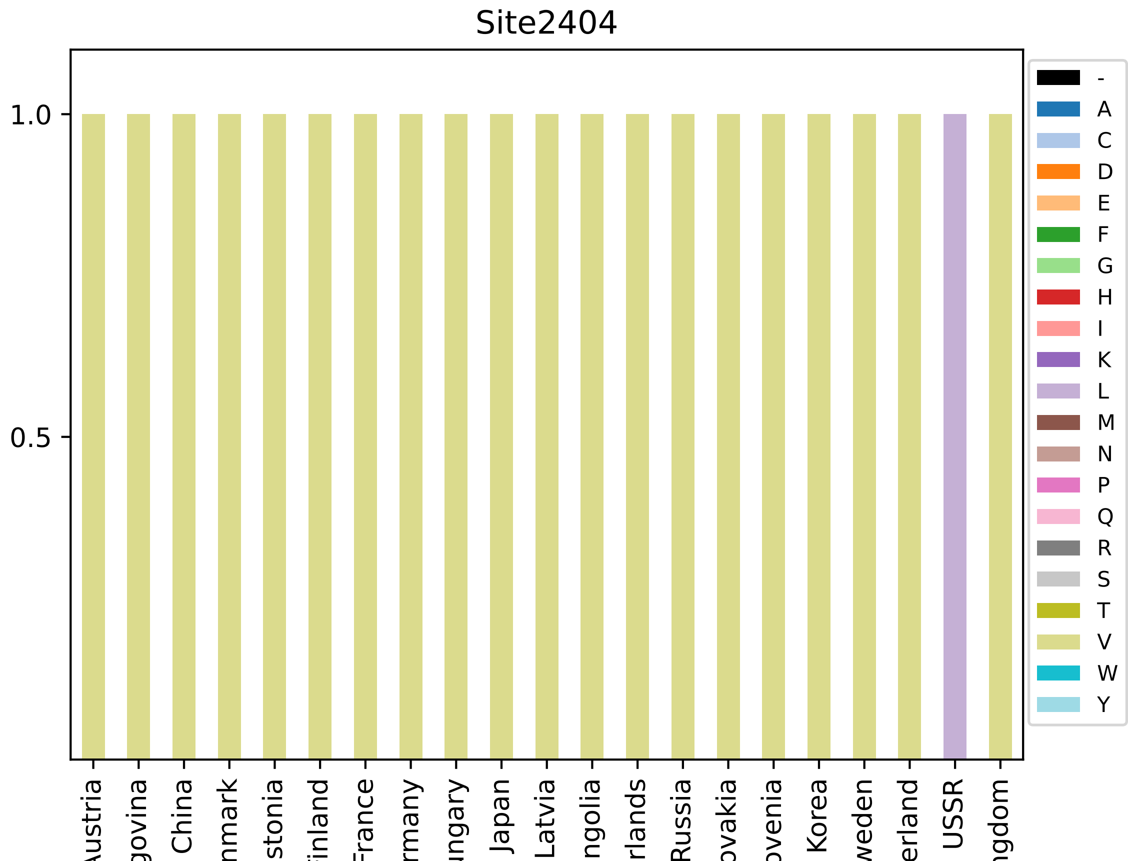

Site2404
1.0
0.5
-
A
C
D
E
F
G
H
I
K
L
M
N
P
Q
R
S
T
V
W
Y
Austria
China
Japan
Latvia
Russia
Korea
USSR

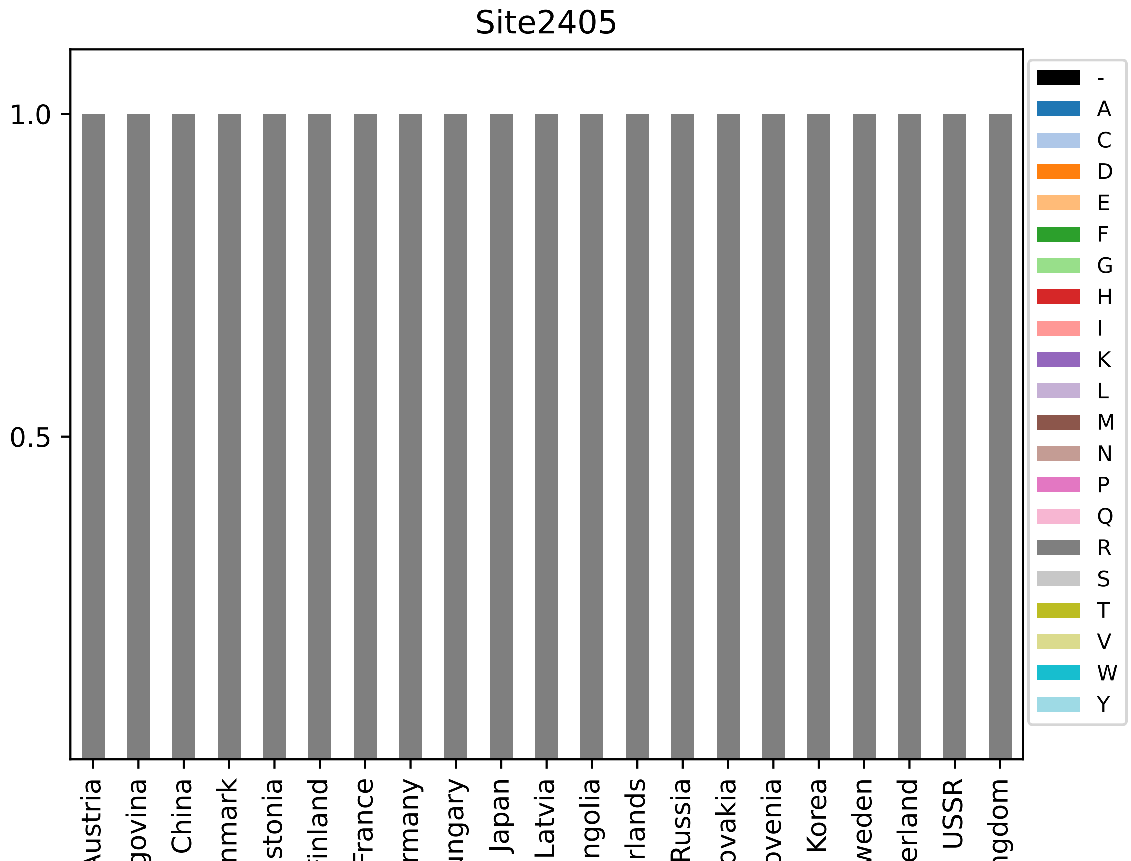
Site2405
1.0
0.5
-
A
C
D
E
F
G
H
I
K
L
M
N
P
Q
R
S
T
V
W
Y
China
Japan
Latvia
Russia
Korea
USSR

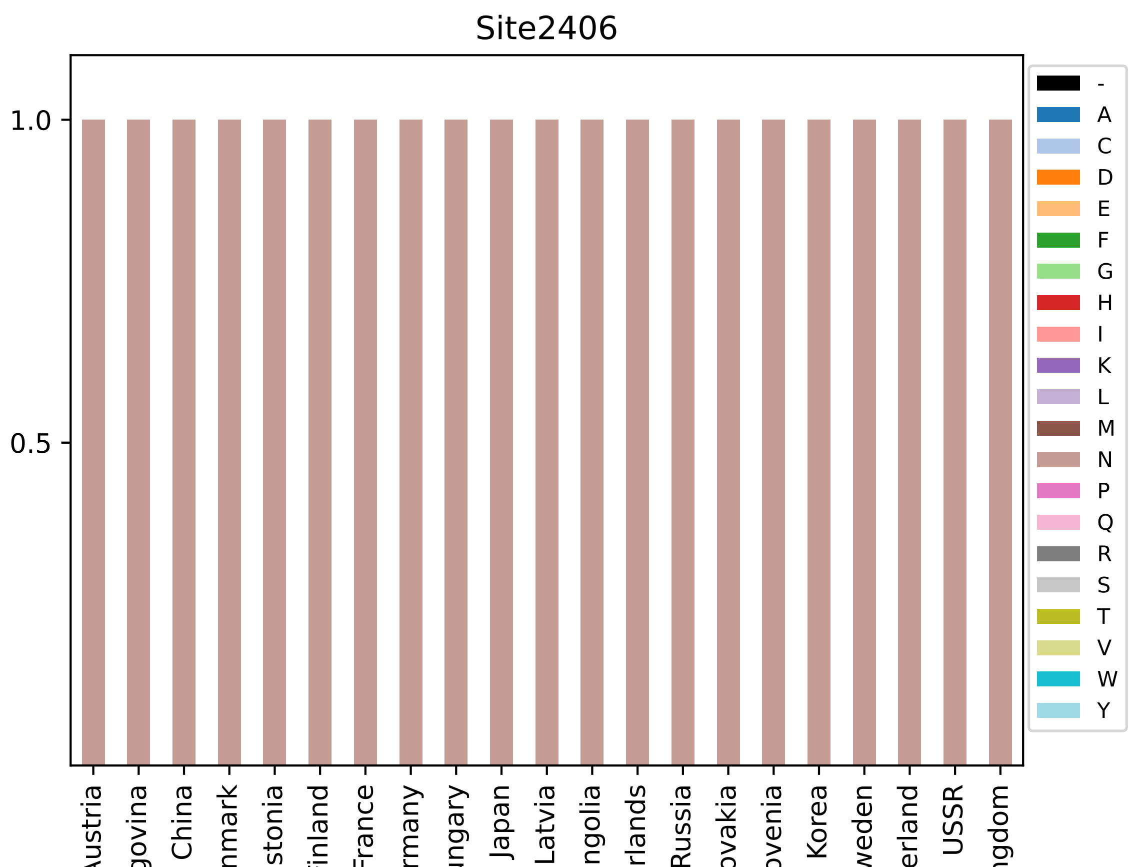
Site2406
1.0
0.5
-
A
C
D
E
F
G
H
I
K
L
M
N
P
Q
R
S
T
V
W
Y
ustria
govina
China
nmark
stonia
inland
rance
rmany
ungary
Japan
Latvia
ngolia
rlands
Russia
ovakia
ovenia
Korea
weden
erland
USSR
ngdom

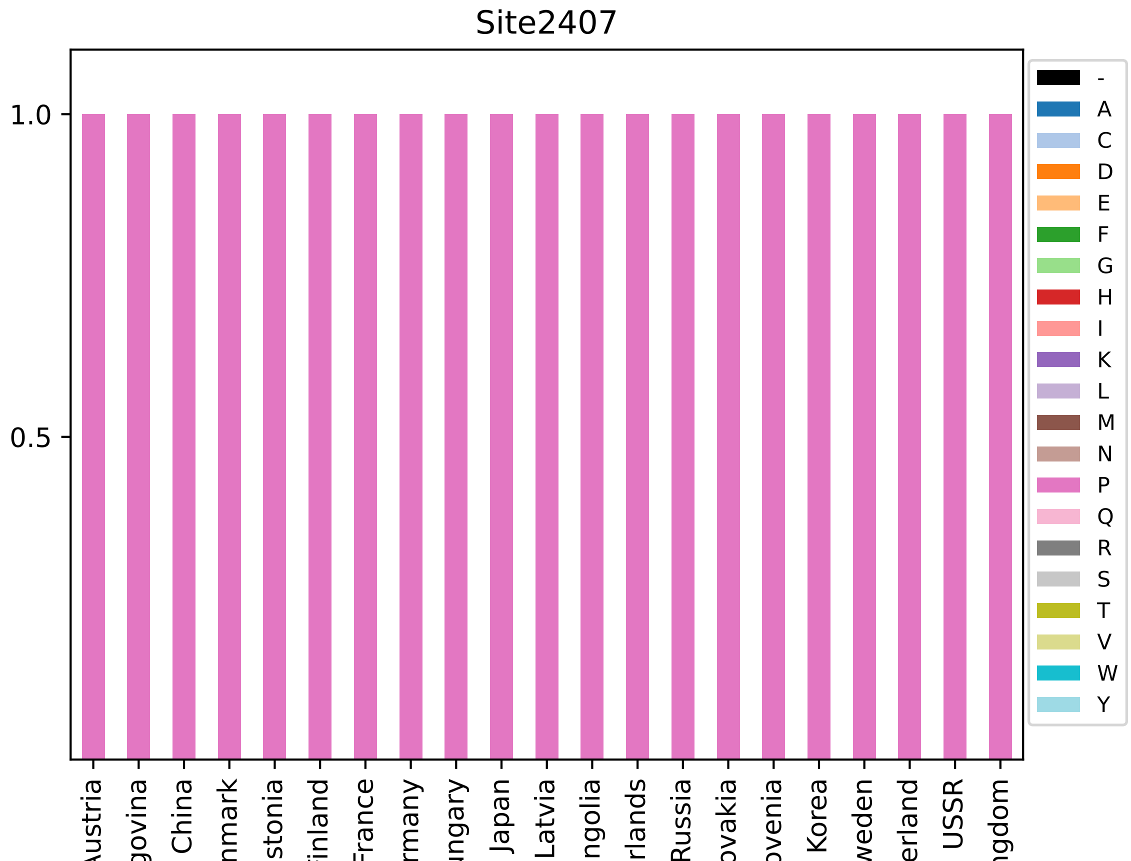

Site2407
1.0
0.5
-
A
C
D
E
F
G
H
I
K
L
M
N
P
Q
R
S
T
V
W
Y
China
Japan
Latvia
Russia
Korea
USSR

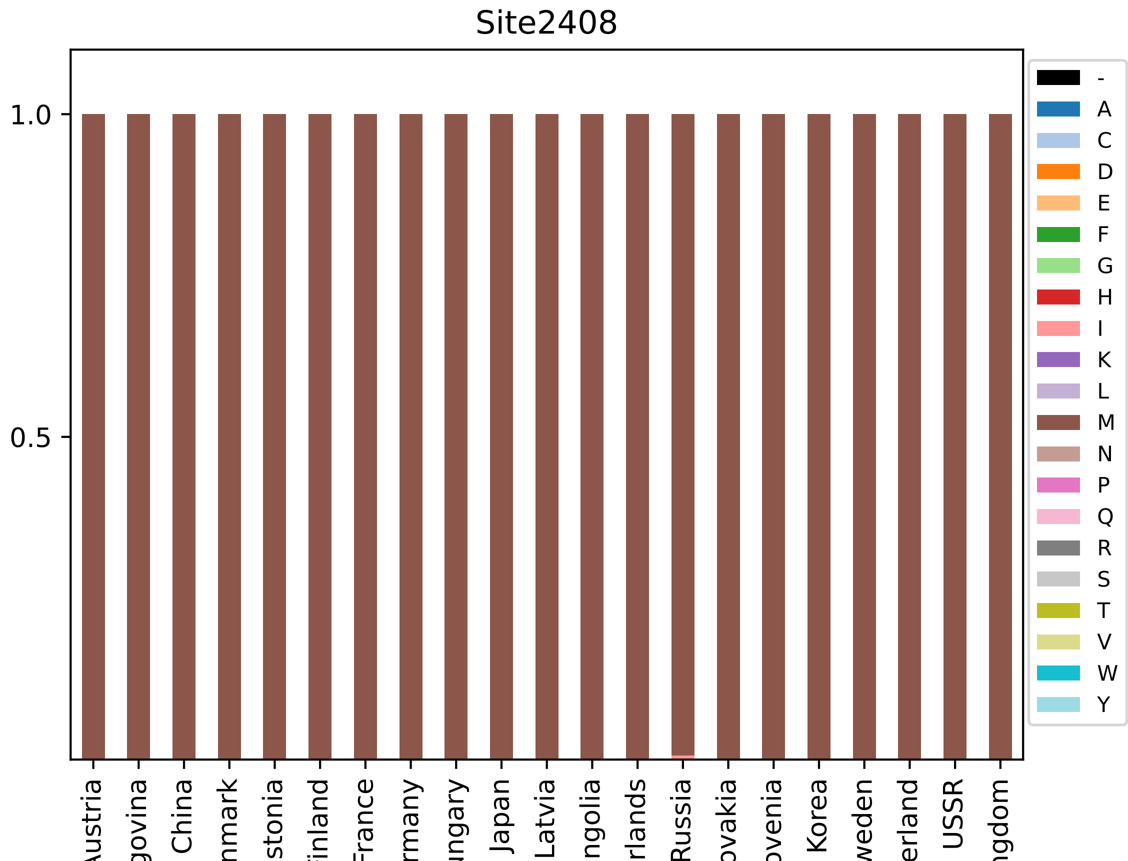
Site2408
1.0
0.5
-
A
C
D
E
F
G
H
I
K
L
M
N
P
Q
R
S
T
V
W
Y
China
Japan
Latvia
Russia
Korea
USSR

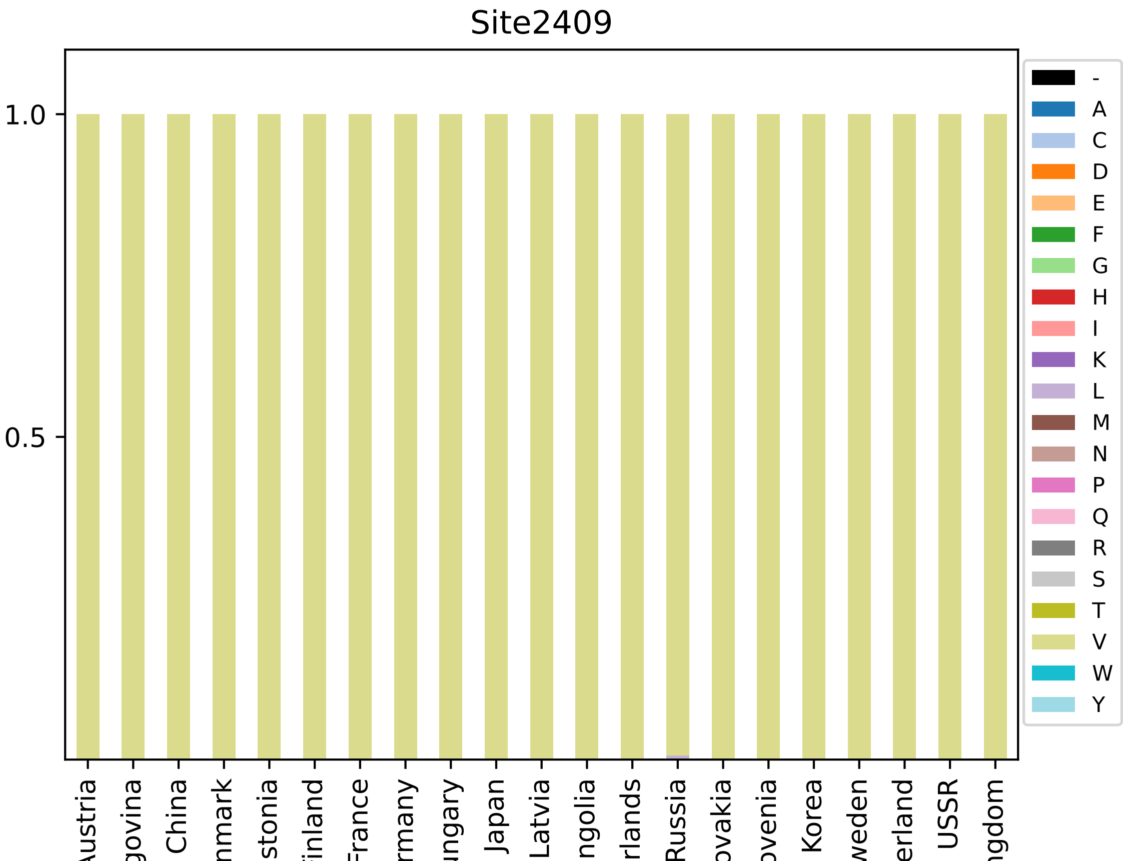
Site2409
1.0
0.5
-
A
C
D
E
F
G
H
I
K
L
M
N
P
Q
R
S
T
V
W
Y
China
Japan
Latvia
Russia
Korea
USSR

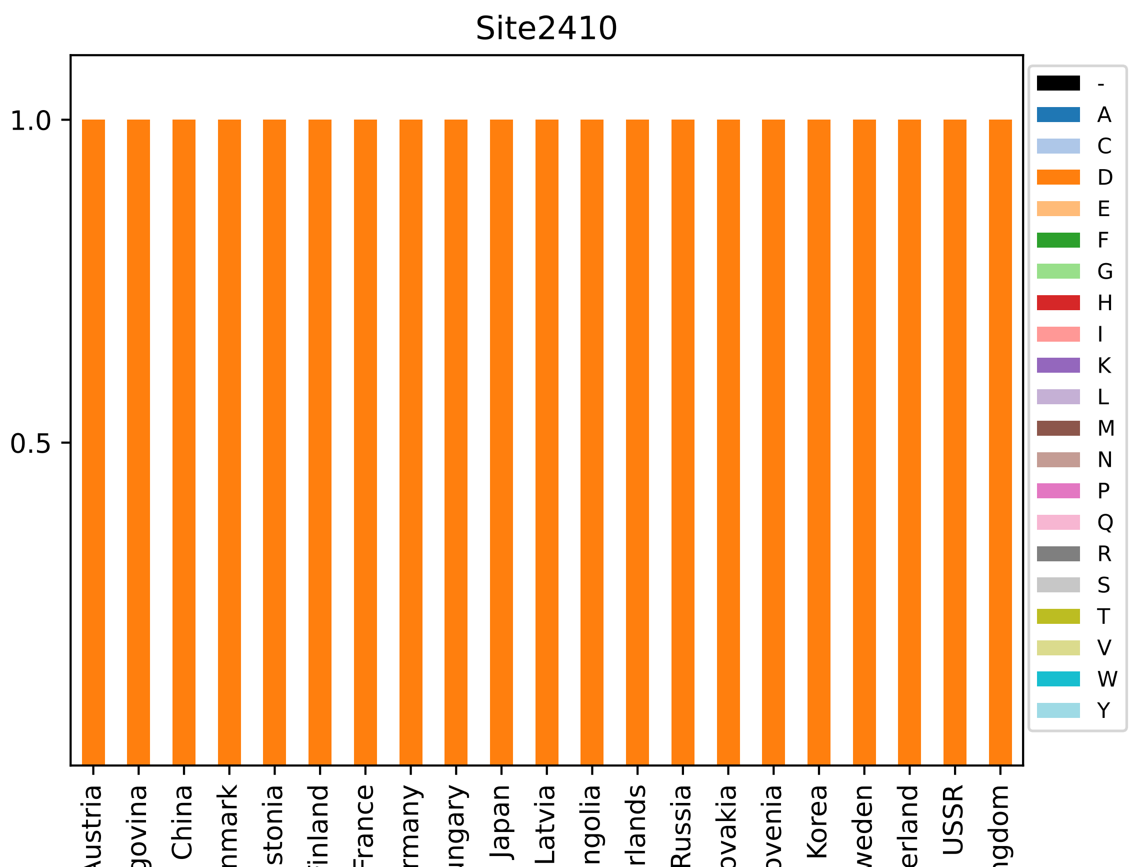

Site2410
1.0
0.5
-
A
C
D
E
F
G
H
I
K
L
M
N
P
Q
R
S
T
V
W
Y
Austria
govina
China
nmark
stonia
Finland
France
rmany
ungary
Japan
Latvia
ngolia
rlands
Russia
ovakia
ovenia
Korea
weden
erland
USSR
ngdom

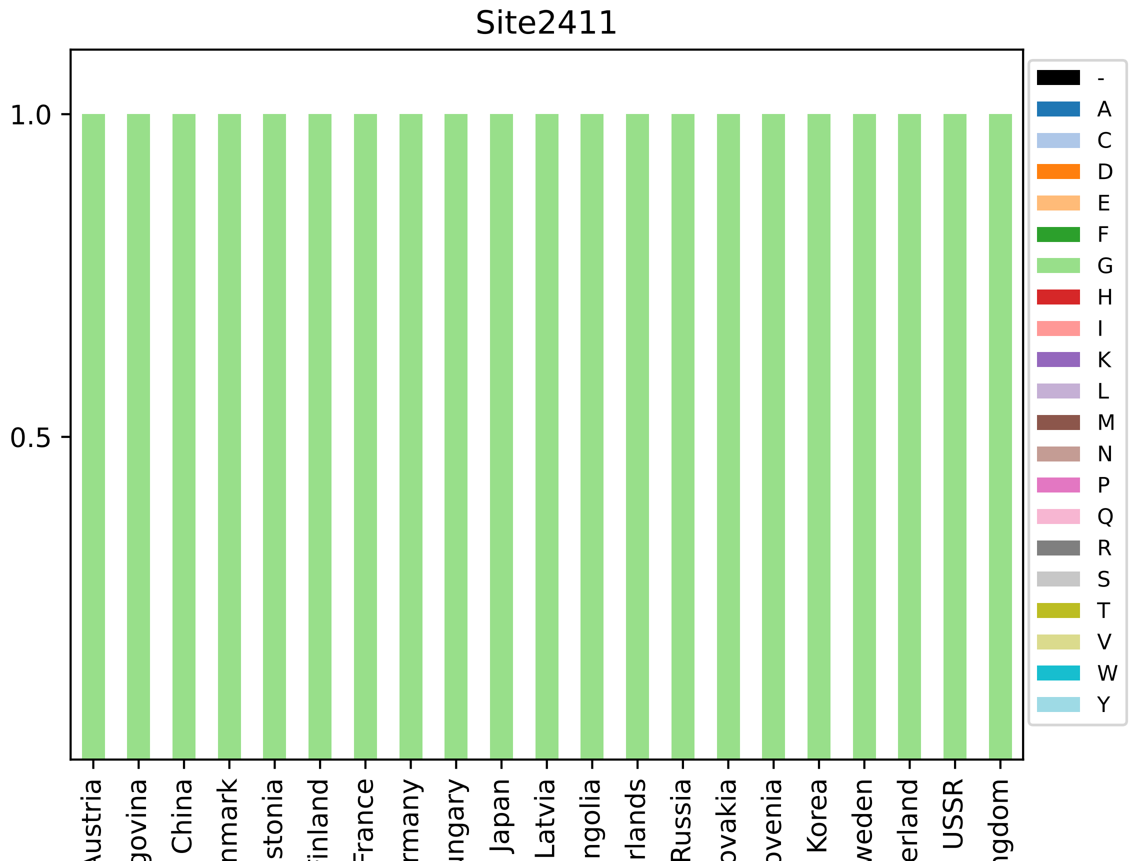

Site2411
1.0
0.5
-
A
C
D
E
F
G
H
I
K
L
M
N
P
Q
R
S
T
V
W
Y
Austria
govina
China
nmark
stonia
Finland
France
rmany
ungary
Japan
Latvia
ngolia
rlands
Russia
ovakia
ovenia
Korea
weden
erland
USSR
ngdom

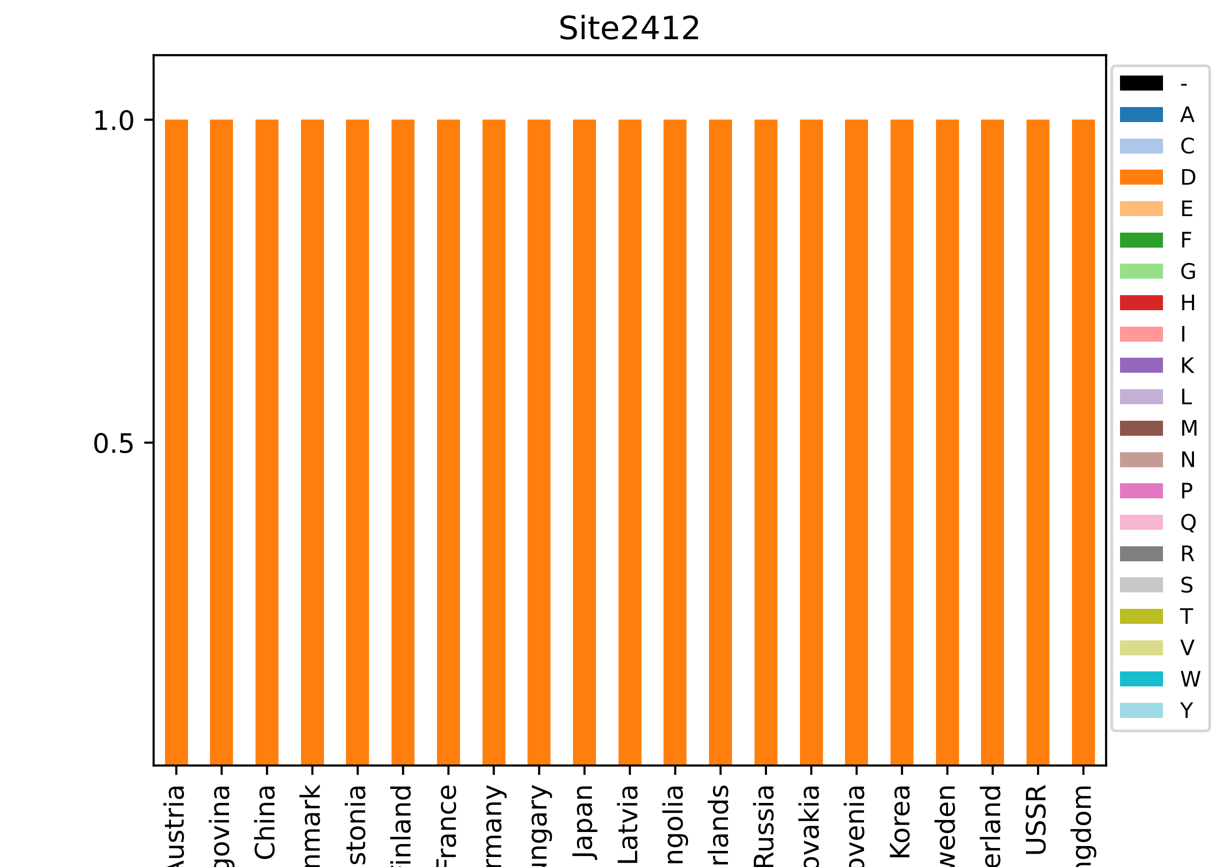
Site2412
1.0
0.5
-
A
C
D
E
F
G
H
I
K
L
M
N
P
Q
R
S
T
V
W
Y
Austria
govina
China
nmark
stonia
Finland
France
rmany
ungary
Japan
Latvia
ngolia
rlands
Russia
ovakia
ovenia
Korea
weden
erland
USSR
ngdom

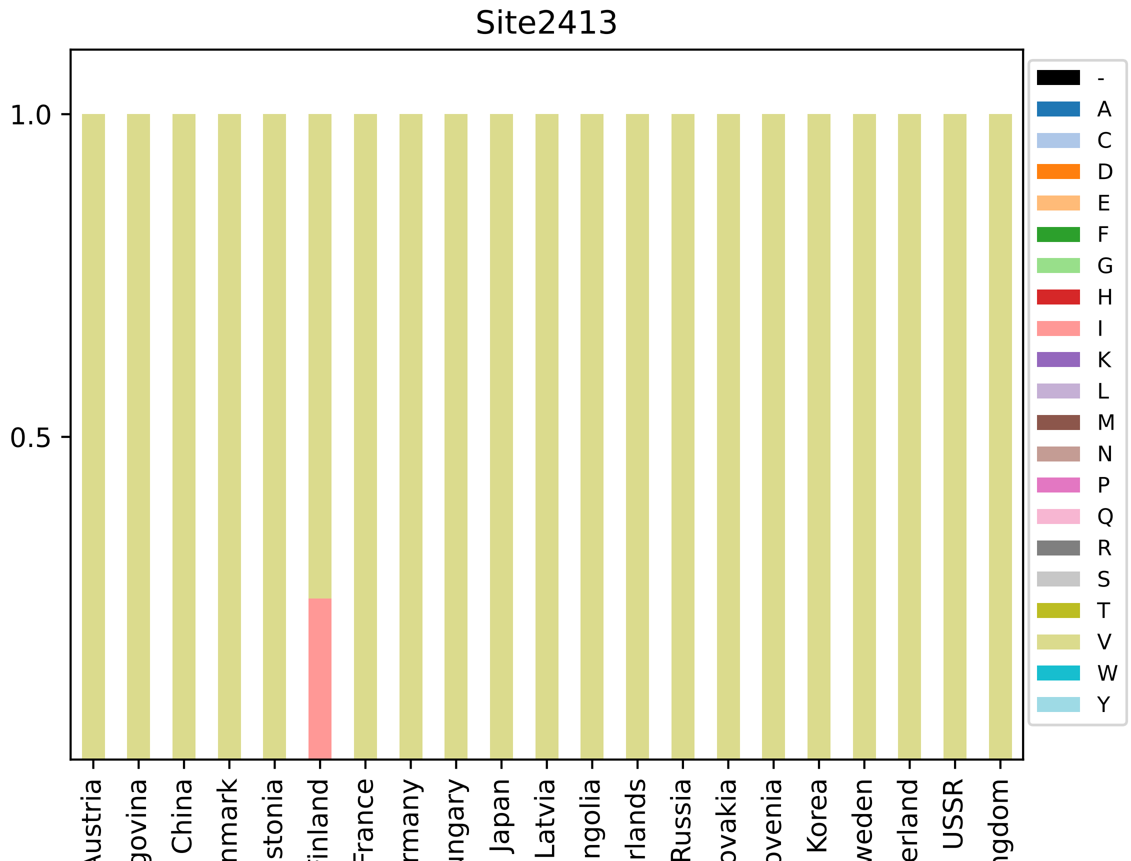
Site2413
1.0
0.5
-
A
C
D
E
F
G
H
I
K
L
M
N
P
Q
R
S
T
V
W
Y
China
Japan
Latvia
Russia
Korea
USSR

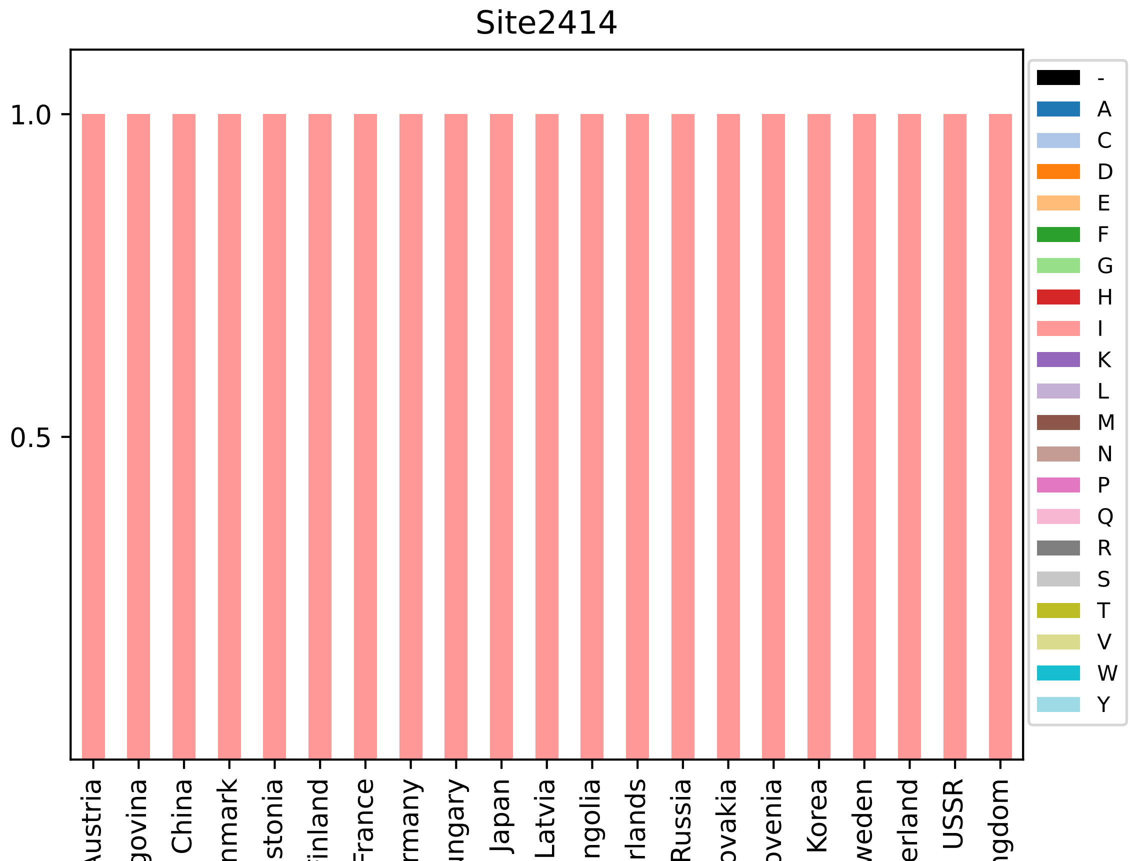
Site2414
1.0
0.5
China
Japan
Latvia
Russia
Korea
USSR
-
A
C
D
E
F
G
H
I
K
L
M
N
P
Q
R
S
T
V
W
Y

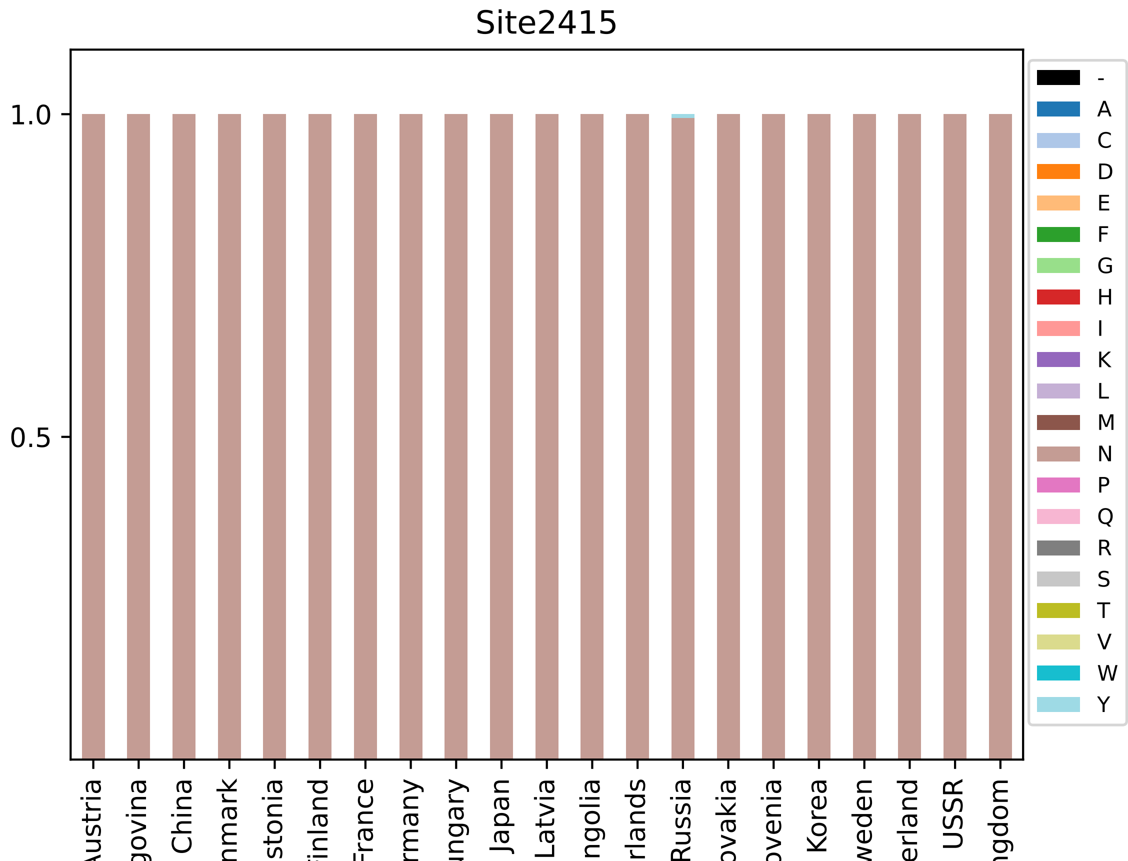

Site2415
1.0
0.5
-
A
C
D
E
F
G
H
I
K
L
M
N
P
Q
R
S
T
V
W
Y
Austria
govina
China
nmark
stonia
inland
France
rmany
ungary
Japan
Latvia
ngolia
rlands
Russia
ovakia
ovenia
Korea
weden
erland
USSR
ngdom

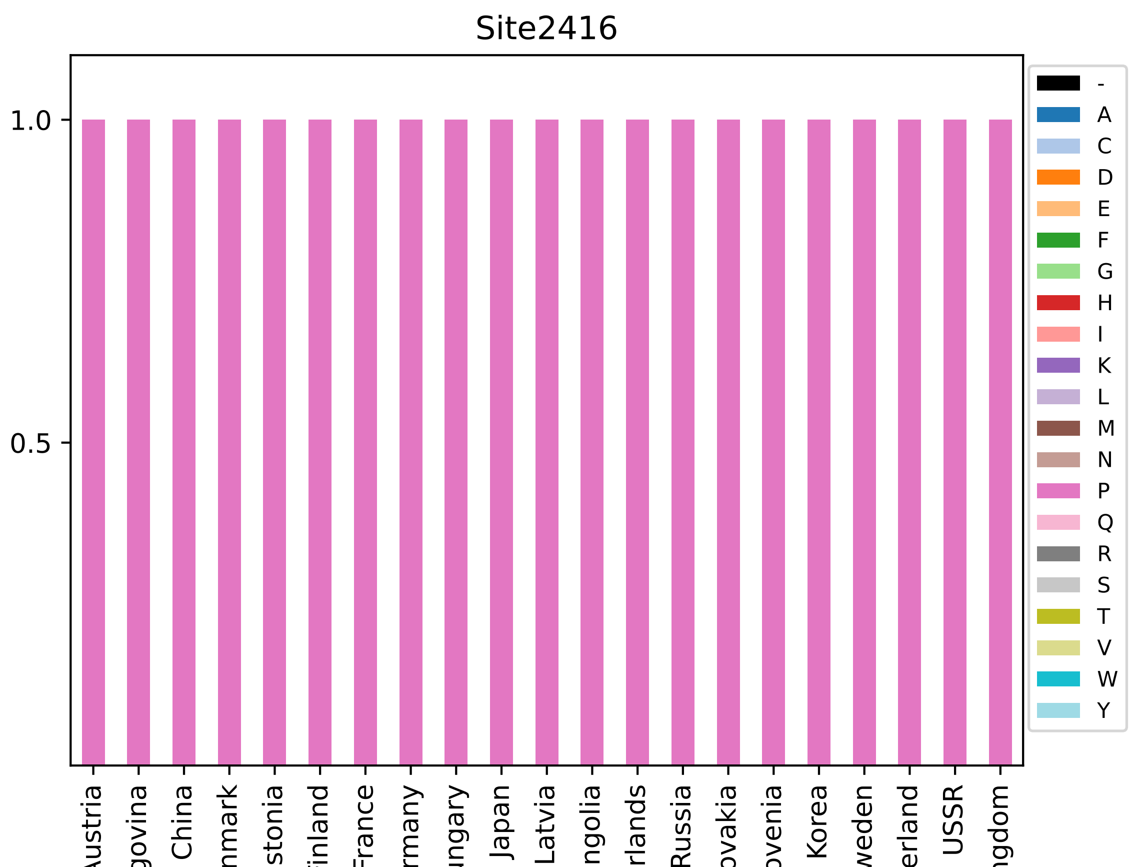

Site2416
1.0
0.5
-
A
C
D
E
F
G
H
I
K
L
M
N
P
Q
R
S
T
V
W
Y
Austria
China
Japan
Latvia
Russia
Korea
USSR

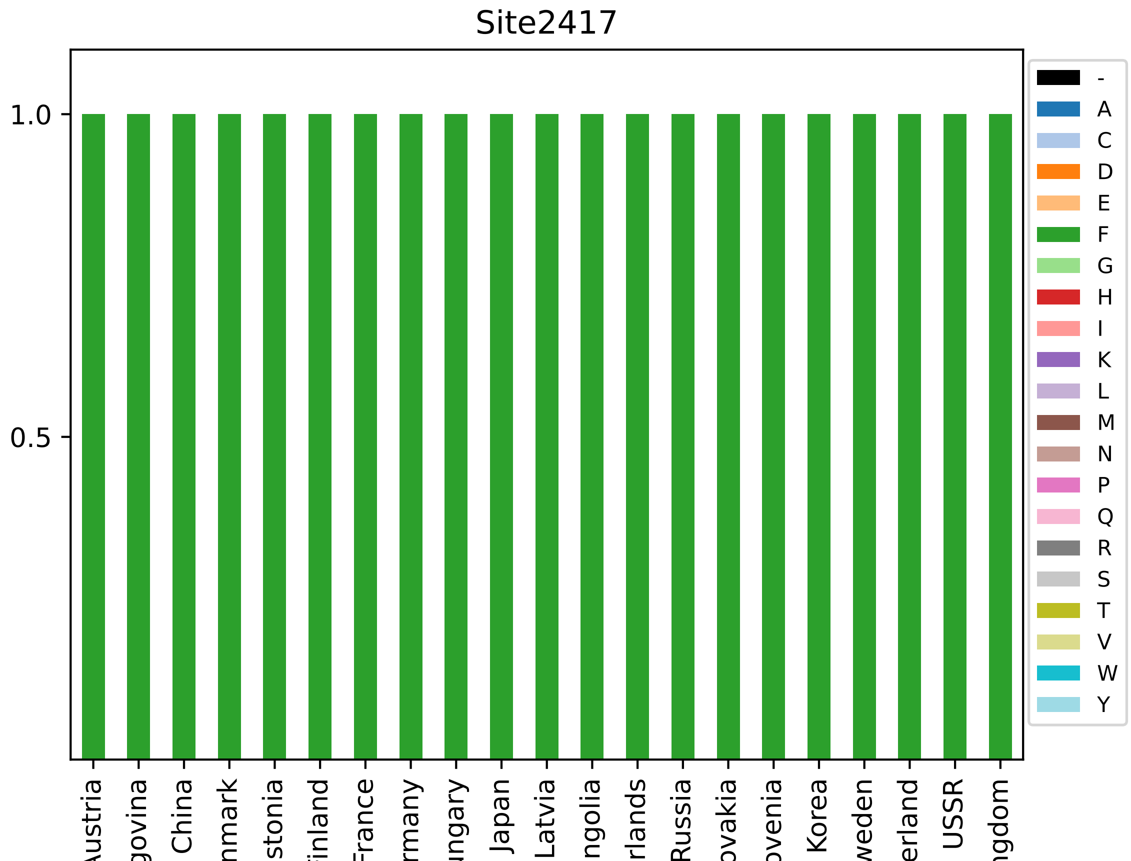
Site2417
1.0
0.5
-
A
C
D
E
F
G
H
I
K
L
M
N
P
Q
R
S
T
V
W
Y
China
Japan
Latvia
Russia
Korea
USSR

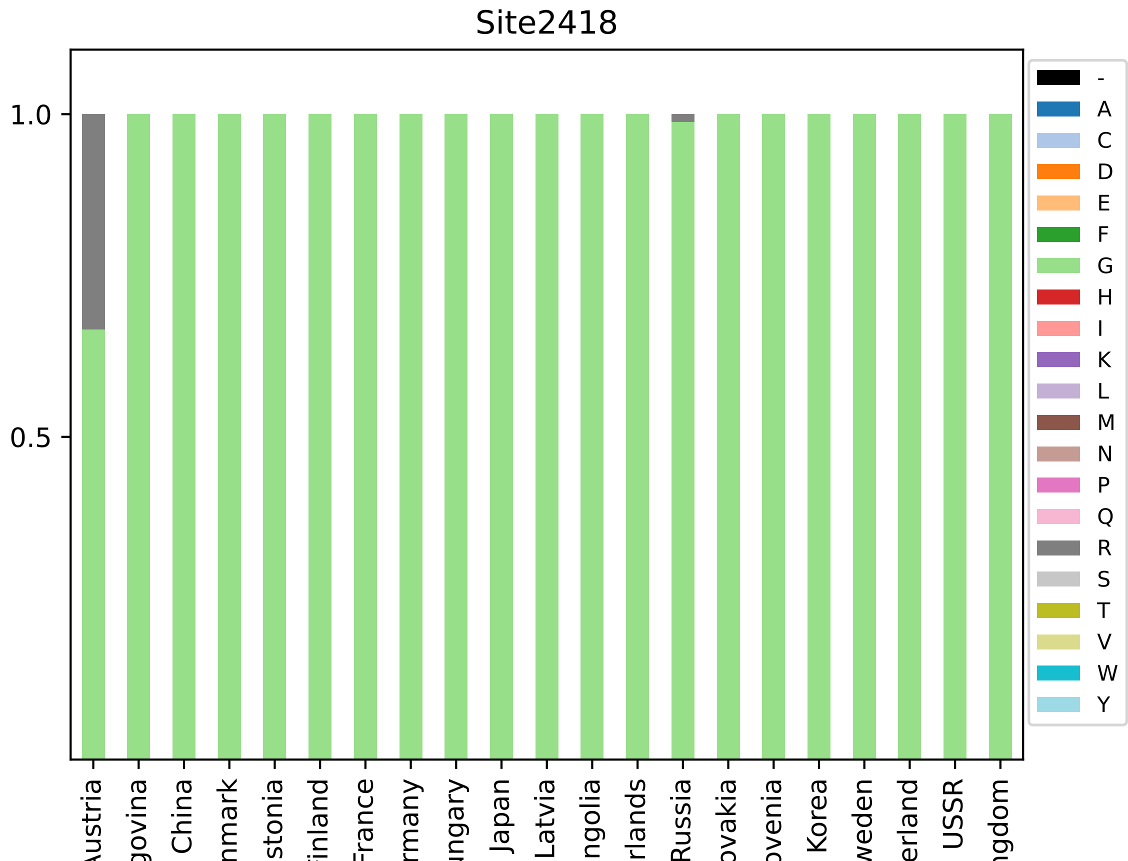

Site2418
1.0
0.5
-
A
C
D
E
F
G
H
I
K
L
M
N
P
Q
R
S
T
V
W
Y
Austria
govina
China
nmark
stonia
inland
France
rmany
ungary
Japan
Latvia
ngolia
rlands
Russia
ovakia
ovenia
Korea
weden
erland
USSR
ngdom

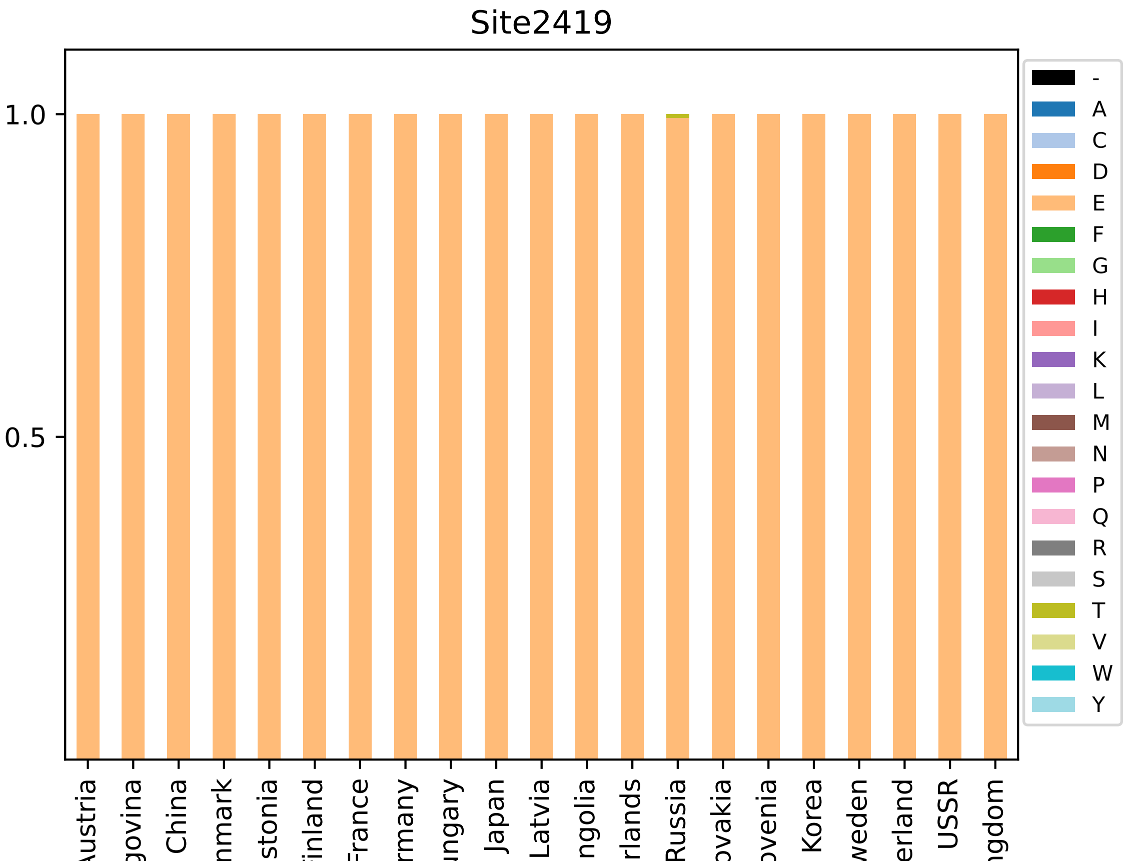
Site2419
1.0
0.5
-
A
C
D
E
F
G
H
I
K
L
M
N
P
Q
R
S
T
V
W
Y
China
Japan
Latvia
Russia
Korea
USSR

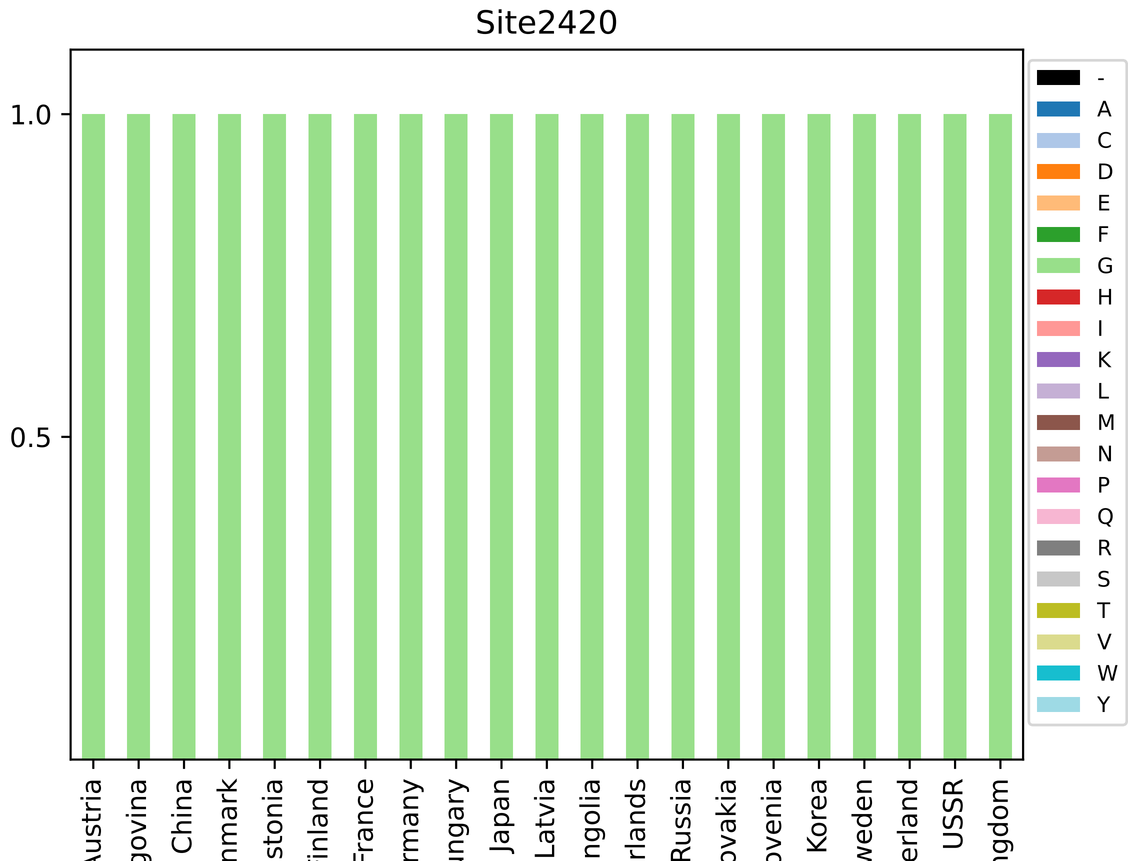
Site2420
1.0
0.5
-
A
C
D
E
F
G
H
I
K
L
M
N
P
Q
R
S
T
V
W
Y
Austria
China
Japan
Latvia
Russia
Korea
USSR

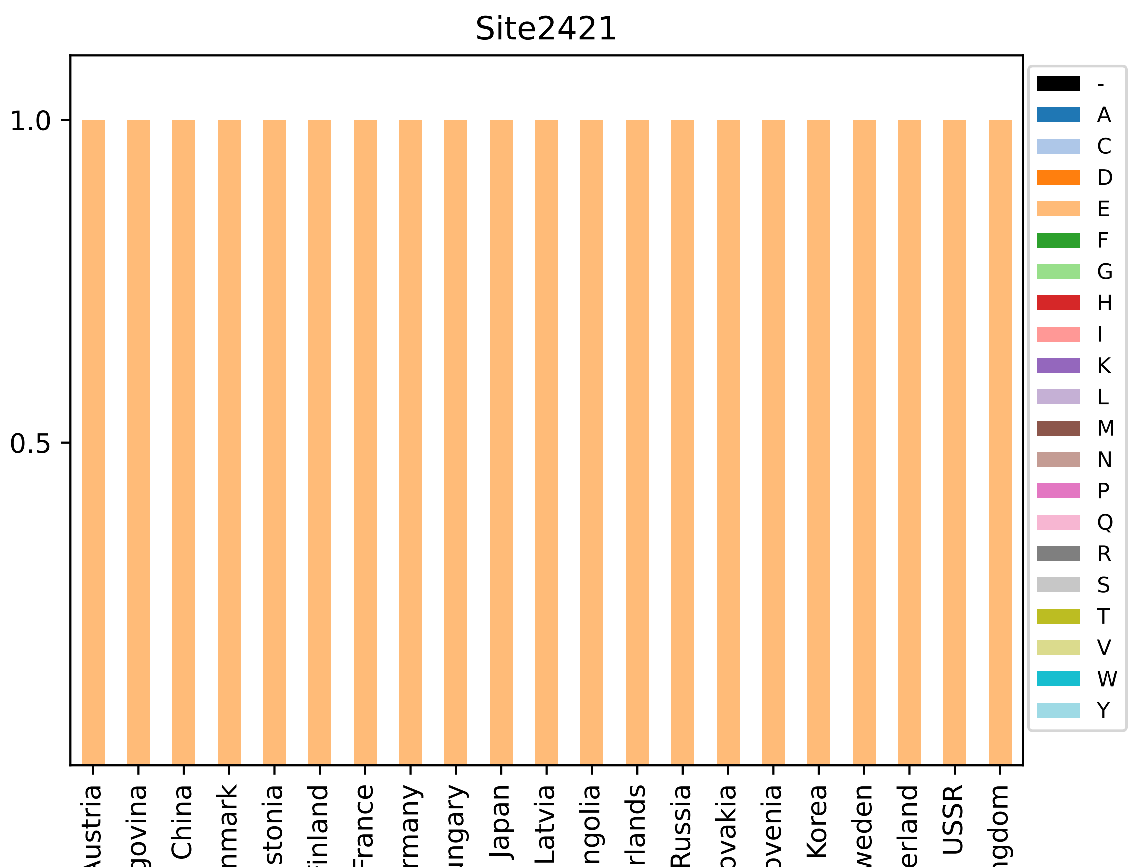

Site2421
1.0
0.5
-
A
C
D
E
F
G
H
I
K
L
M
N
P
Q
R
S
T
V
W
Y
China
Japan
Latvia
Russia
Korea
USSR

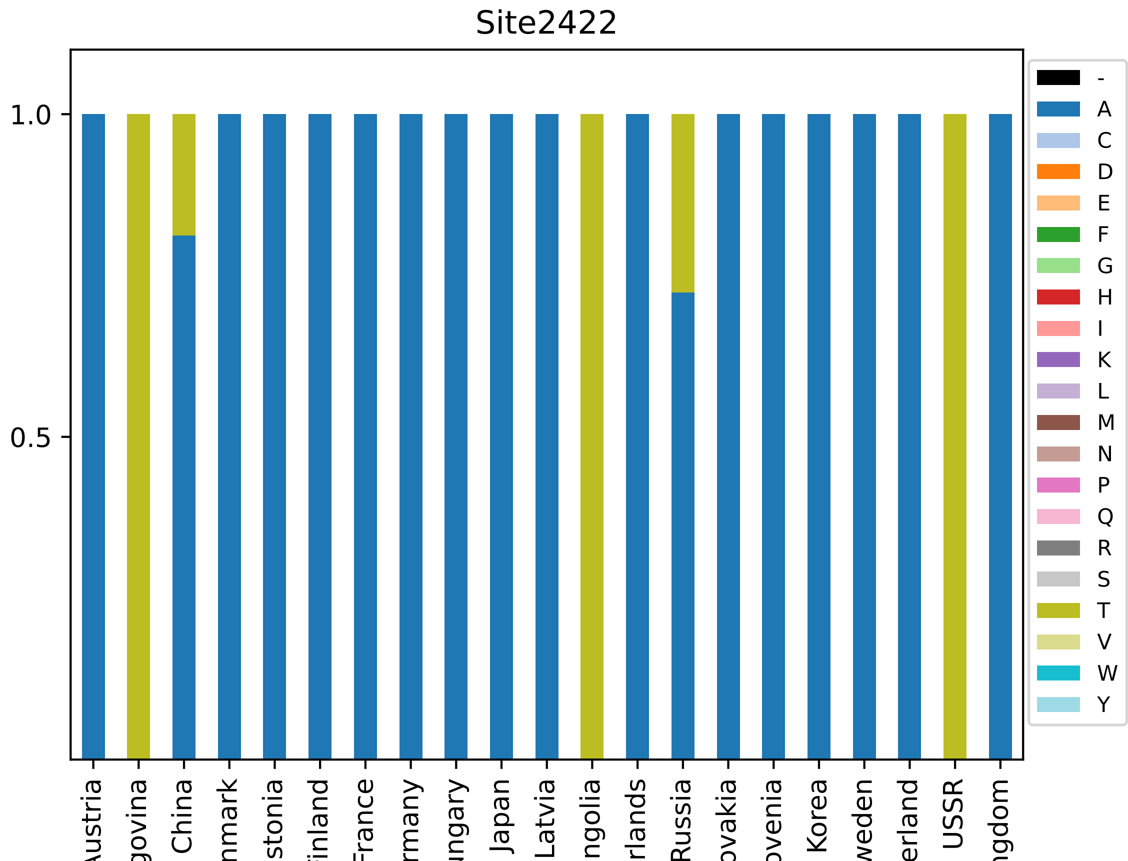

Site2422
1.0
0.5
Austria
China
Japan
Latvia
Russia
Korea
USSR
-
A
C
D
E
F
G
H
I
K
L
M
N
P
Q
R
S
T
V
W
Y

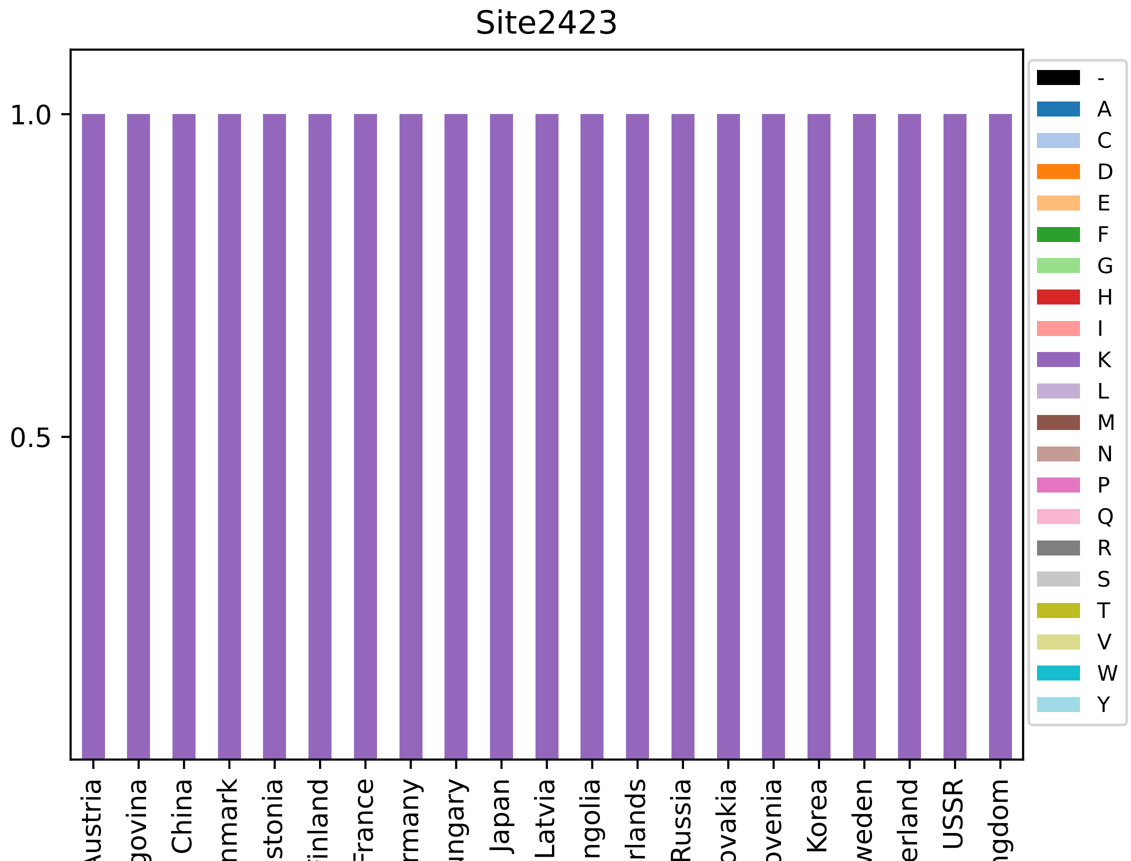

Site2423
1.0
0.5
-
A
C
D
E
F
G
H
I
K
L
M
N
P
Q
R
S
T
V
W
Y
China
Japan
Latvia
Russia
Korea
USSR

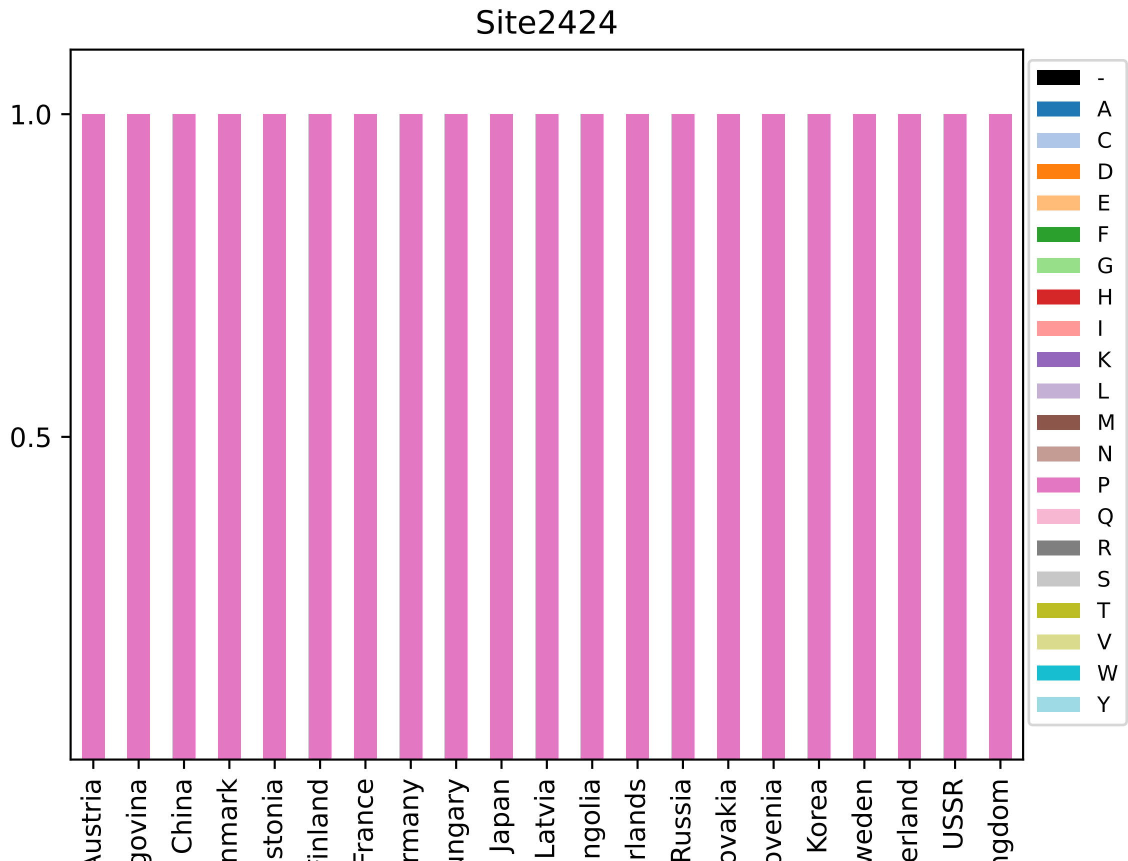

Site2424
1.0
0.5
-
A
C
D
E
F
G
H
I
K
L
M
N
P
Q
R
S
T
V
W
Y
Austria
govina
China
nmark
stonia
inland
France
rmany
ungary
Japan
Latvia
ngolia
rlands
Russia
ovakia
ovenia
Korea
weden
erland
USSR
ngdom

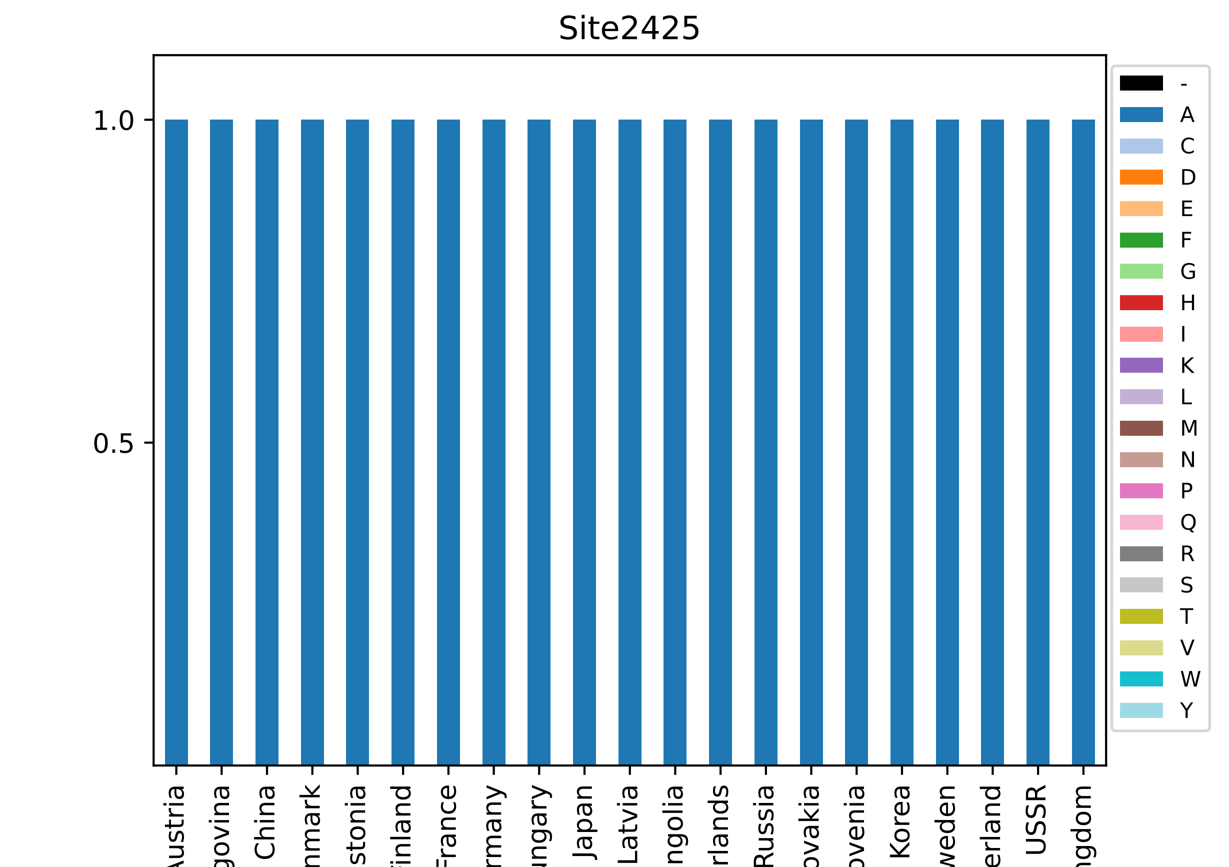
Site2425
1.0
0.5
-
A
C
D
E
F
G
H
I
K
L
M
N
P
Q
R
S
T
V
W
Y
China
Japan
Latvia
Russia
Korea
USSR

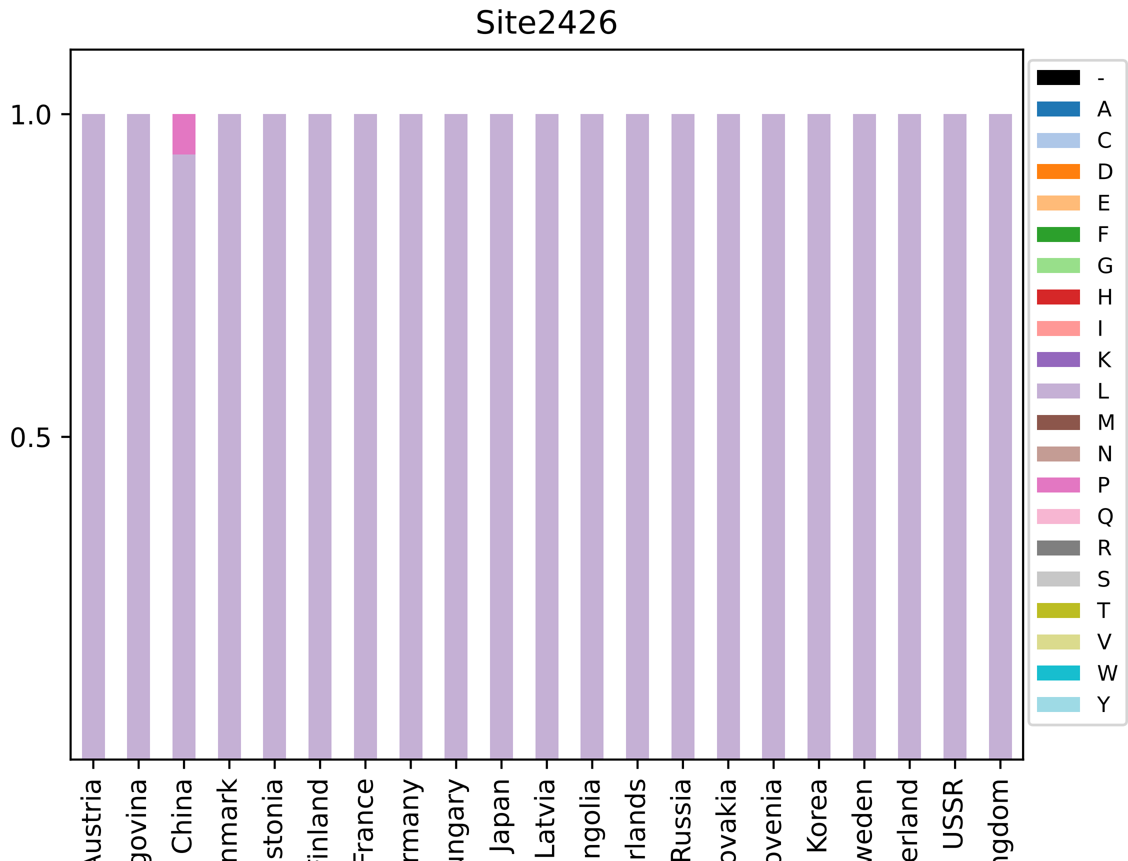
Site2426
1.0
0.5
-
A
C
D
E
F
G
H
I
K
L
M
N
P
Q
R
S
T
V
W
Y
China
Japan
Latvia
Russia
Korea
USSR

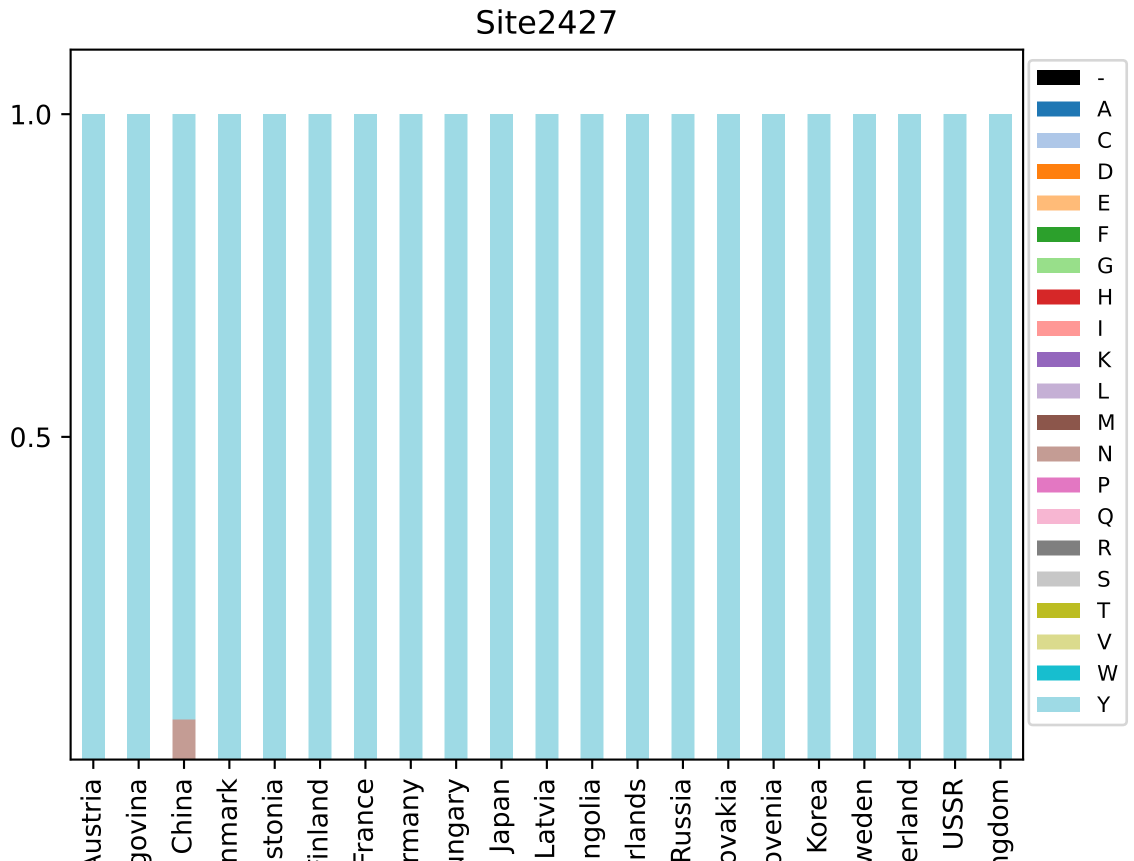
Site2427
1.0
0.5
Austria
govina
China
nmark
stonia
Finland
France
rmany
ungary
Japan
Latvia
ngolia
rlands
Russia
ovakia
ovenia
Korea
weden
erland
USSR
ngdom
-
A
C
D
E
F
G
H
I
K
L
M
N
P
Q
R
S
T
V
W
Y

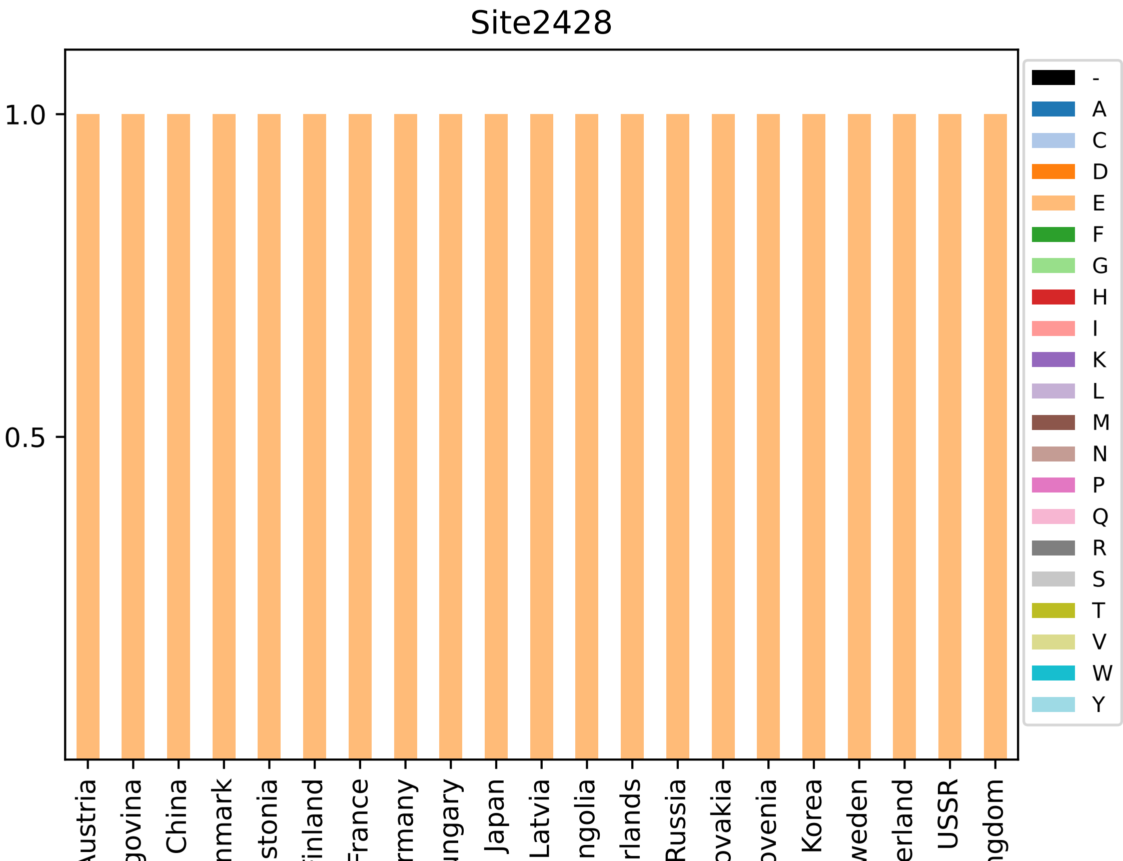
Site2428
1.0
0.5
-
A
C
D
E
F
G
H
I
K
L
M
N
P
Q
R
S
T
V
W
Y
China
Japan
Latvia
Russia
Korea
USSR

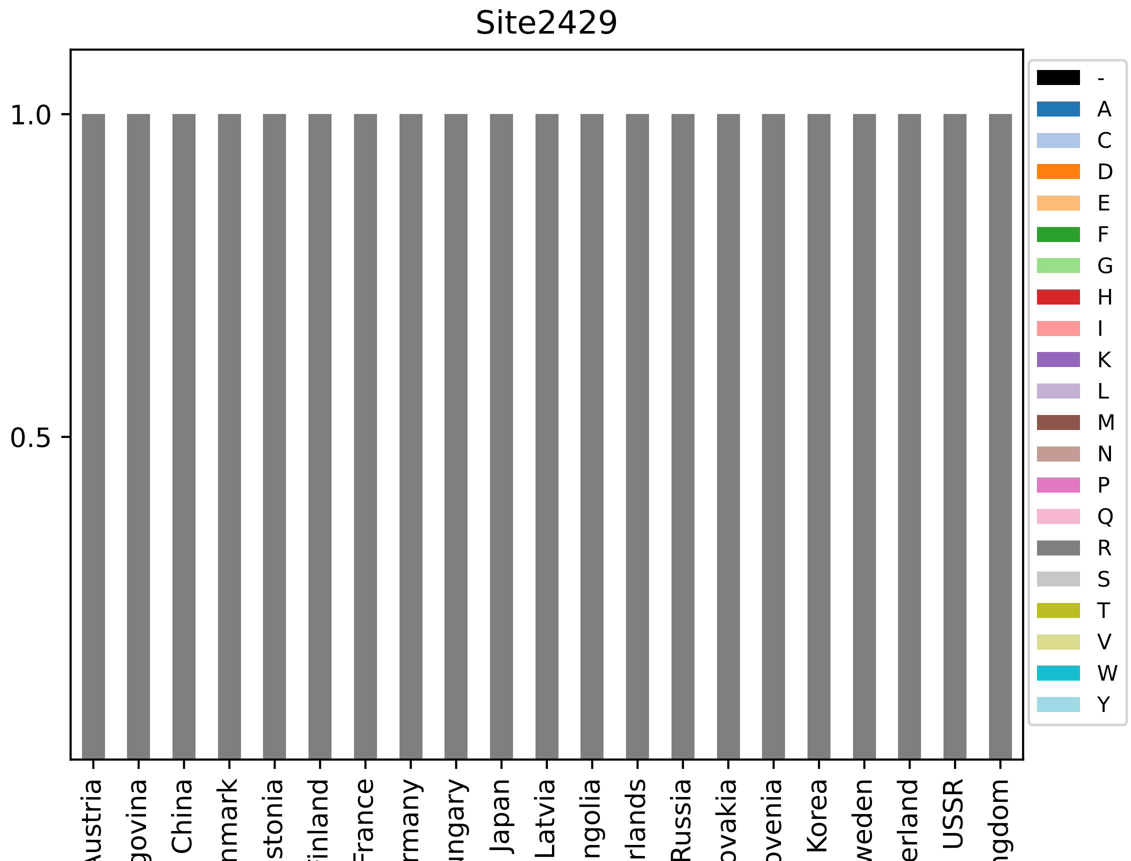

Site2429
1.0
0.5
-
A
C
D
E
F
G
H
I
K
L
M
N
P
Q
R
S
T
V
W
Y
China
Japan
Latvia
Russia
Korea
USSR

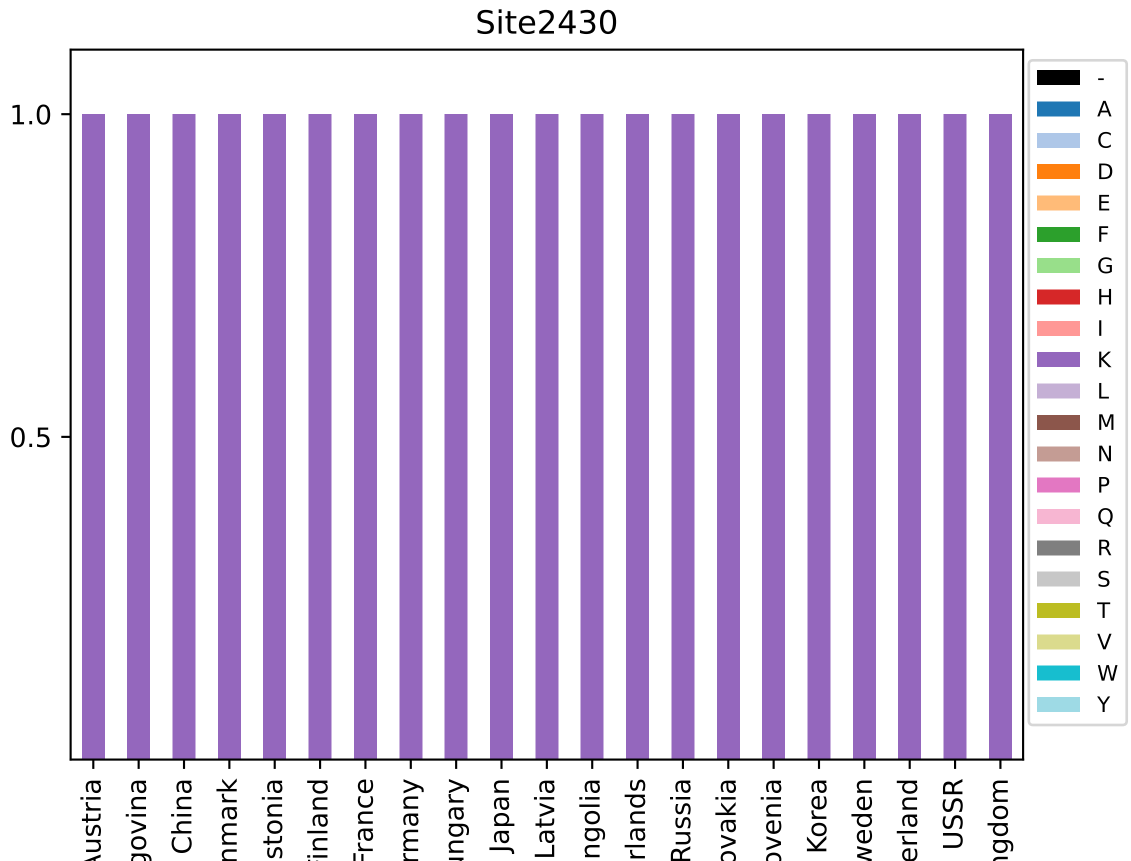
Site2430
1.0
0.5
-
A
C
D
E
F
G
H
I
K
L
M
N
P
Q
R
S
T
V
W
Y
China
Japan
Latvia
Russia
Korea
USSR

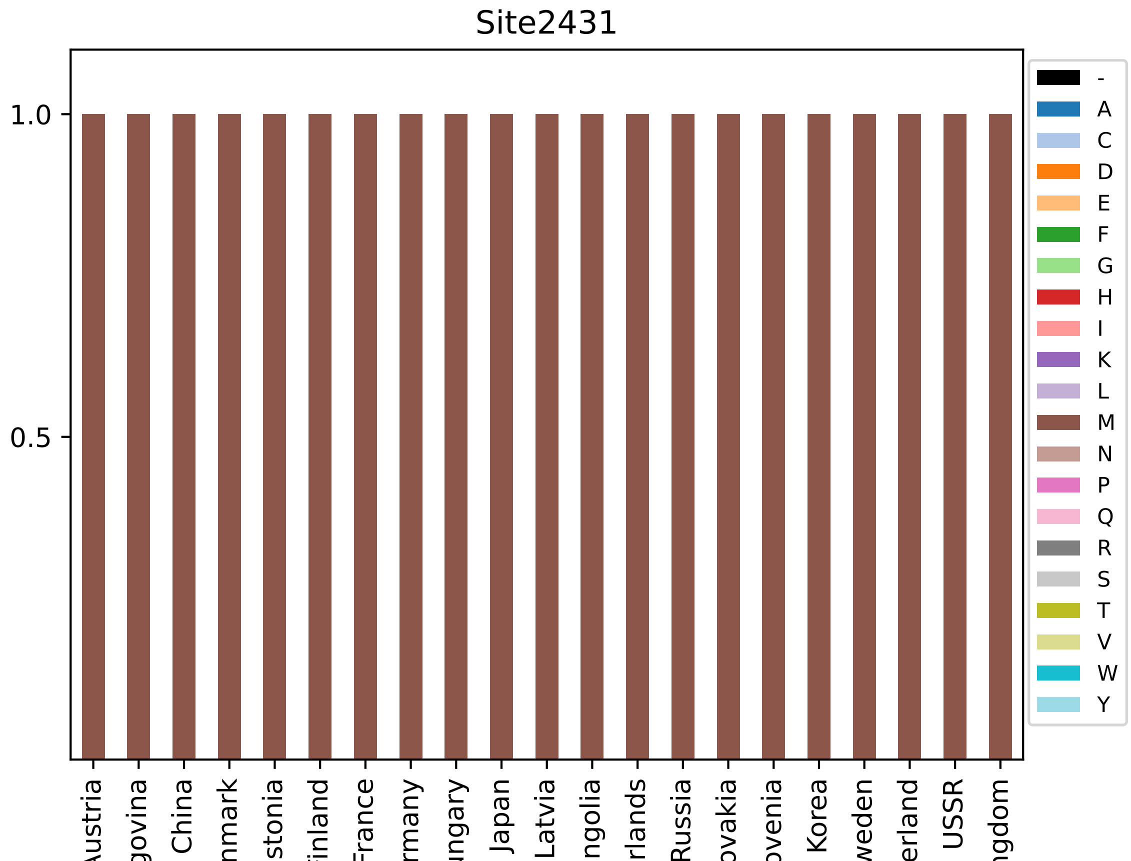
Site2431
1.0
0.5
-
A
C
D
E
F
G
H
I
K
L
M
N
P
Q
R
S
T
V
W
Y
China
Japan
Latvia
Russia
Korea
USSR

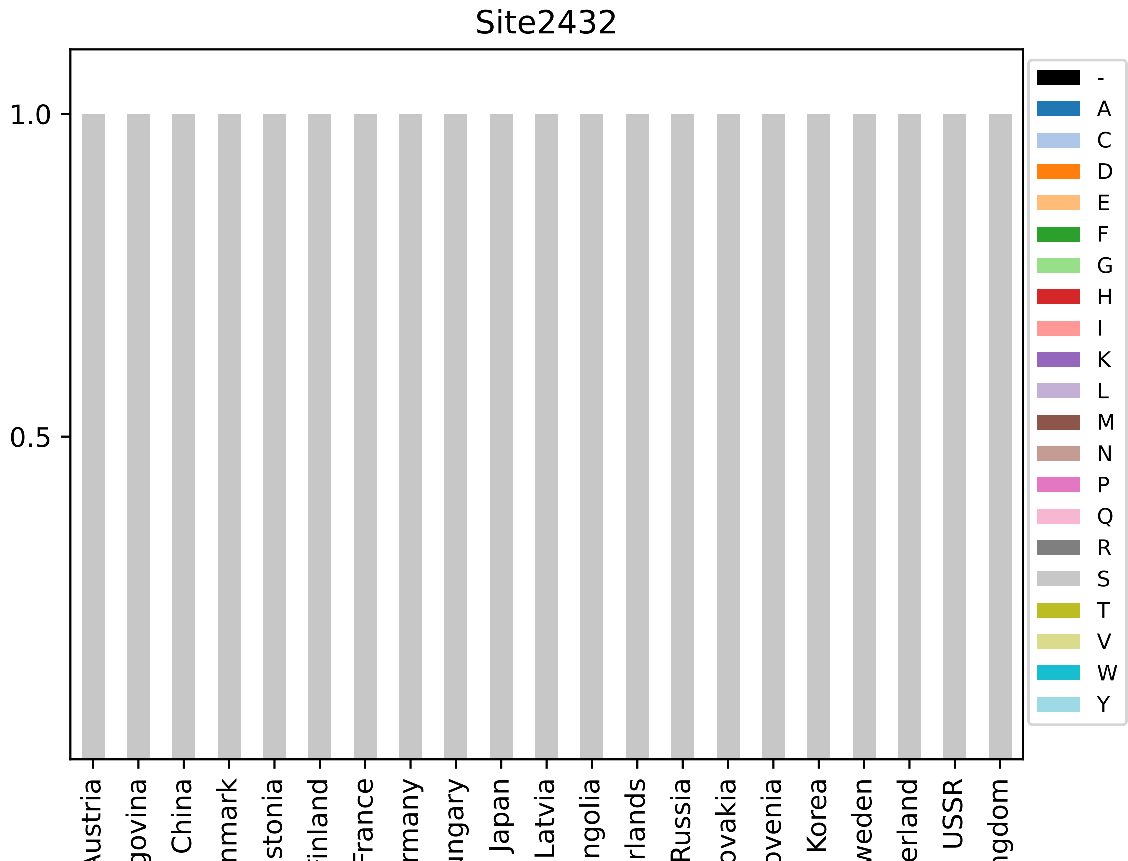

Site2432
1.0
0.5
Austria
govina
China
nmark
stonia
Finland
France
rmany
ungary
Japan
Latvia
ngolia
rlands
Russia
ovakia
ovenia
Korea
weden
erland
USSR
ngdom
-
A
C
D
E
F
G
H
I
K
L
M
N
P
Q
R
S
T
V
W
Y

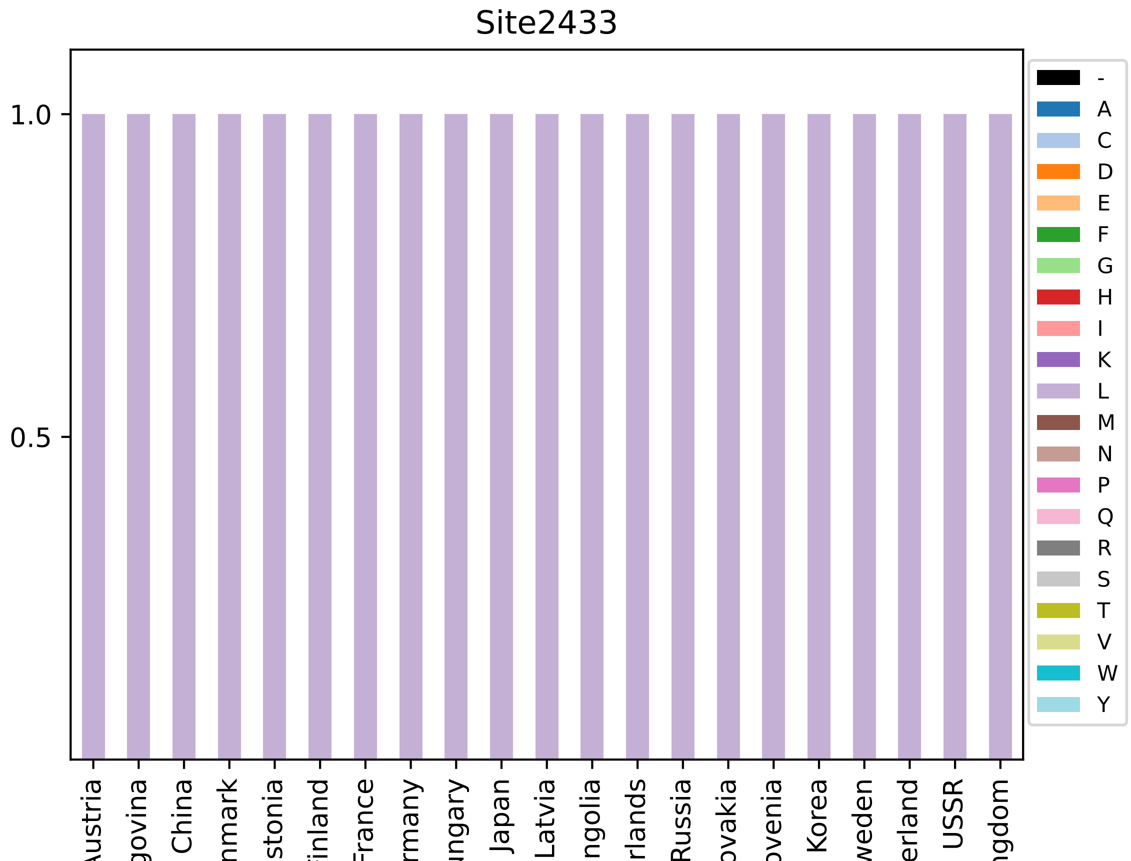
Site2433
1.0
0.5
-
A
C
D
E
F
G
H
I
K
L
M
N
P
Q
R
S
T
V
W
Y
China
Japan
Latvia
Russia
Korea
USSR

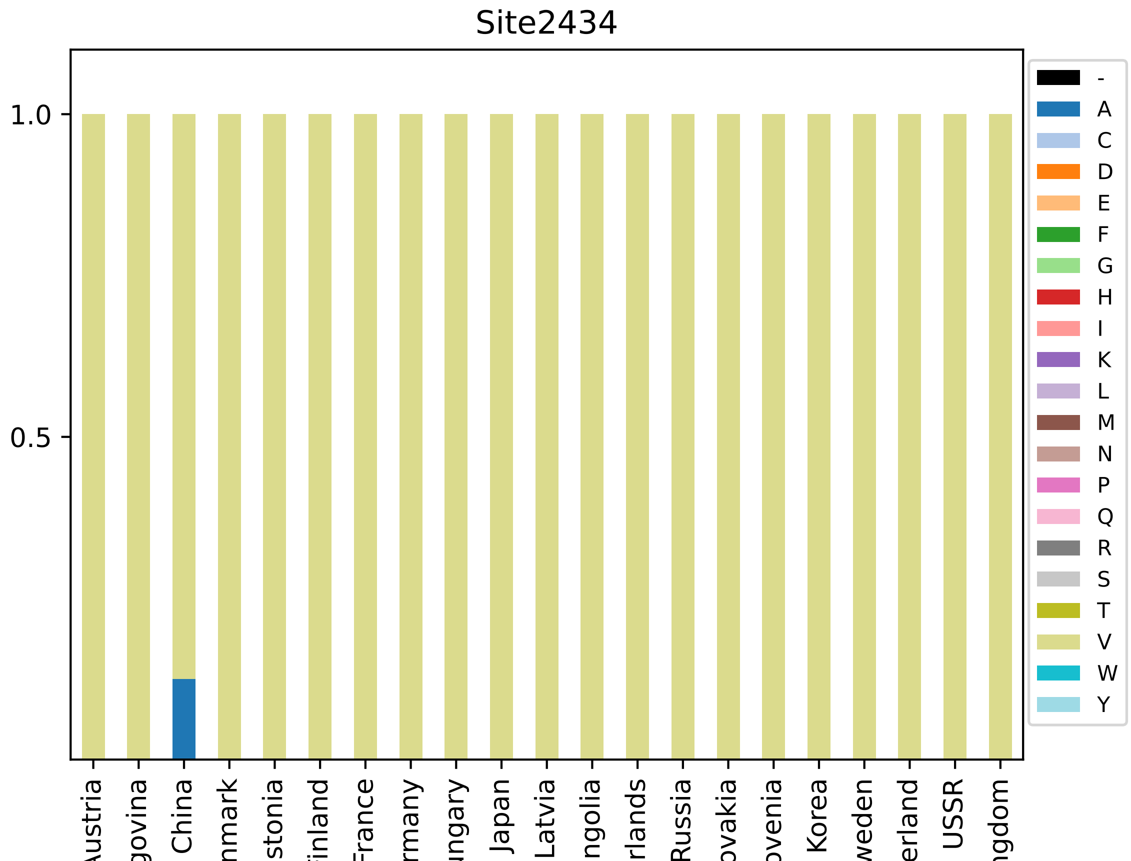

Site2434
1.0
0.5
-
A
C
D
E
F
G
H
I
K
L
M
N
P
Q
R
S
T
V
W
Y
China
Japan
Latvia
Russia
Korea
USSR

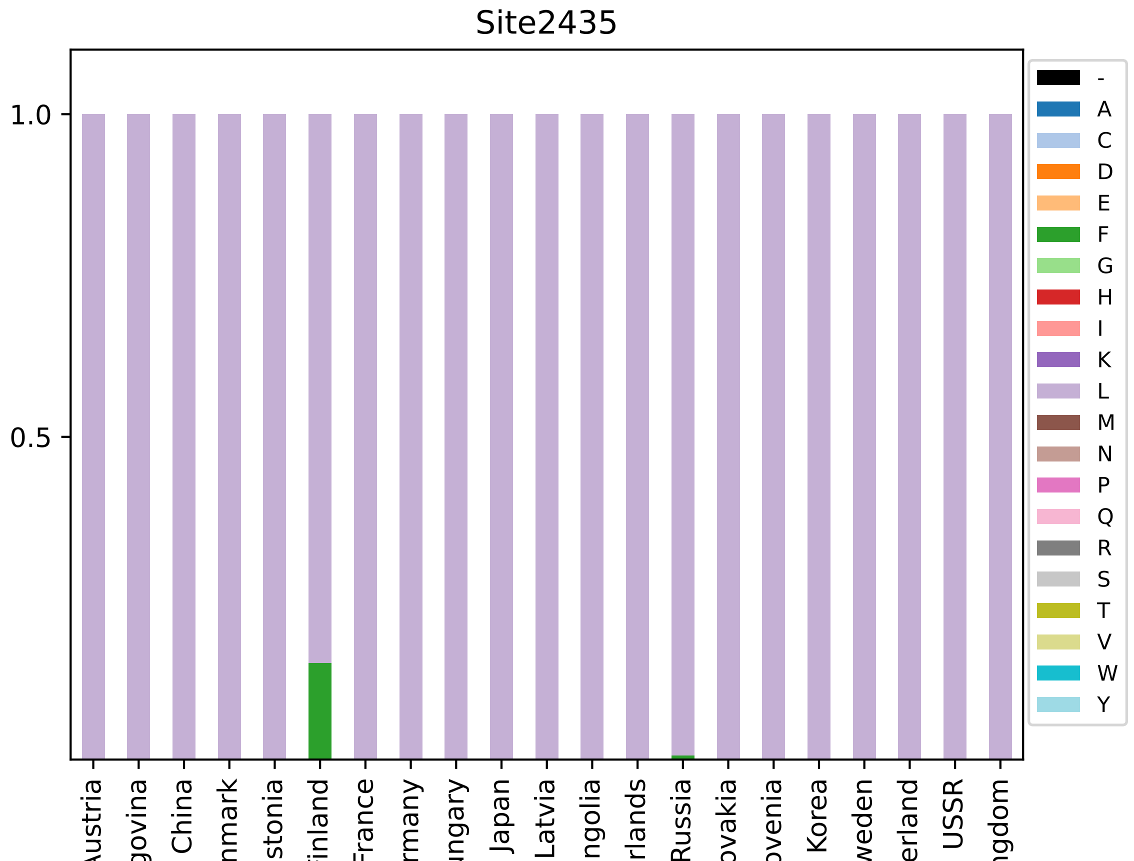
Site2435
1.0
0.5
-
A
C
D
E
F
G
H
I
K
L
M
N
P
Q
R
S
T
V
W
Y
China
Japan
Latvia
Russia
Korea
USSR

Site2436

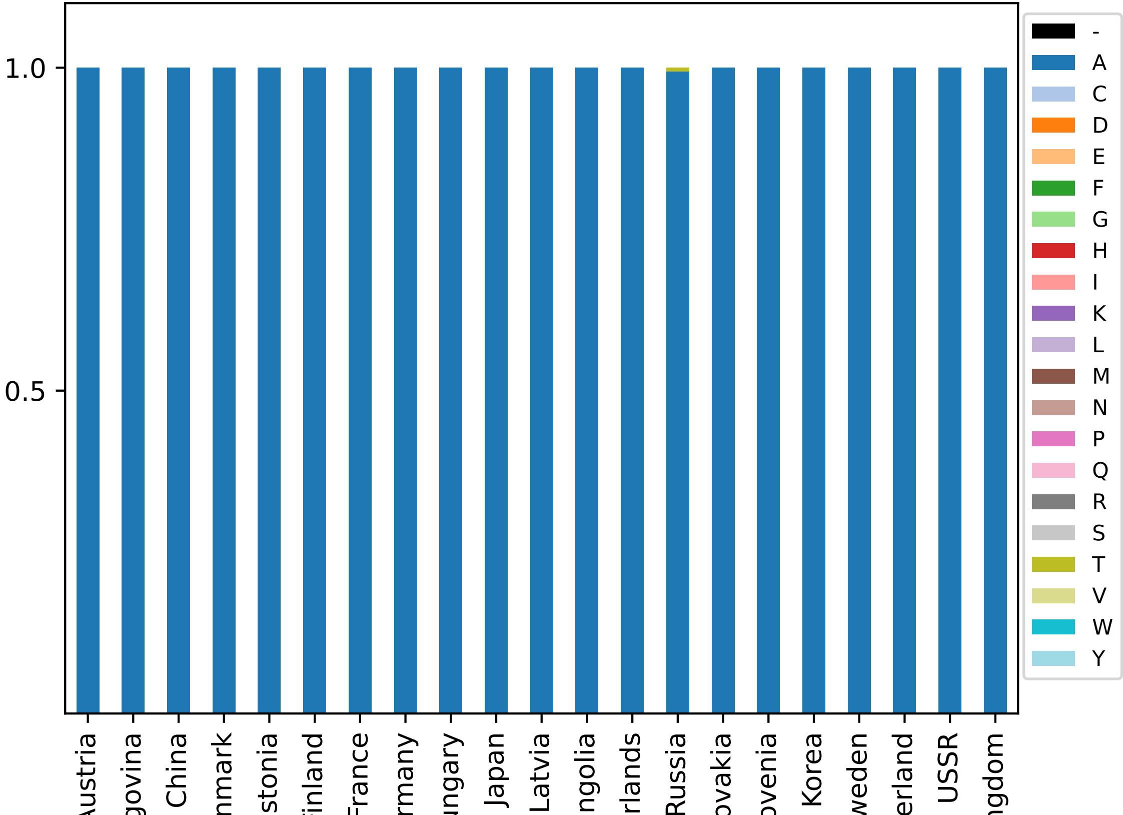
1.0
0.5
-
A
C
D
E
F
G
H
I
K
L
M
N
P
Q
R
S
T
V
W
Y
ustria
govina
China
nmark
stonia
inland
rance
rmany
ungary
Japan
Latvia
ngolia
rlands
Russia
ovakia
ovenia
Korea
weden
erland
USSR
ngdom

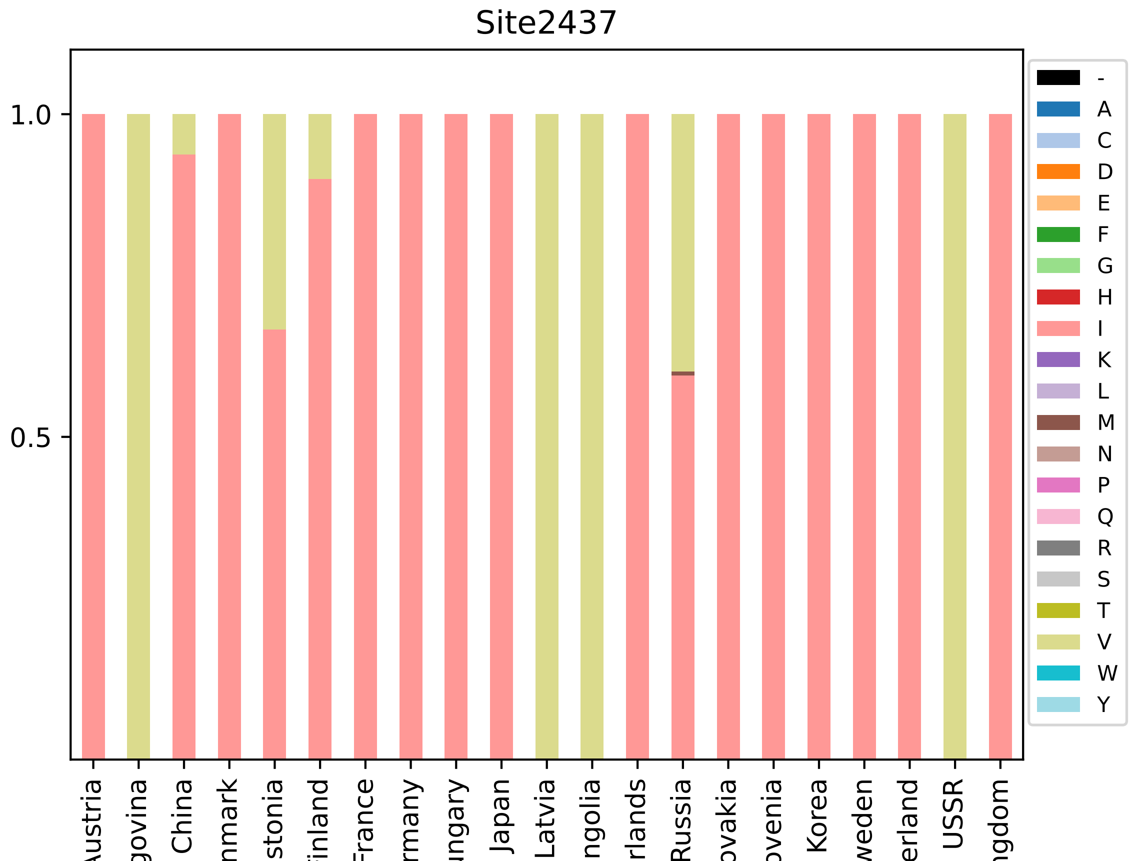

Site2437
1.0
0.5
-
A
C
D
E
F
G
H
I
K
L
M
N
P
Q
R
S
T
V
W
Y
China
Japan
Latvia
Russia
Korea
USSR

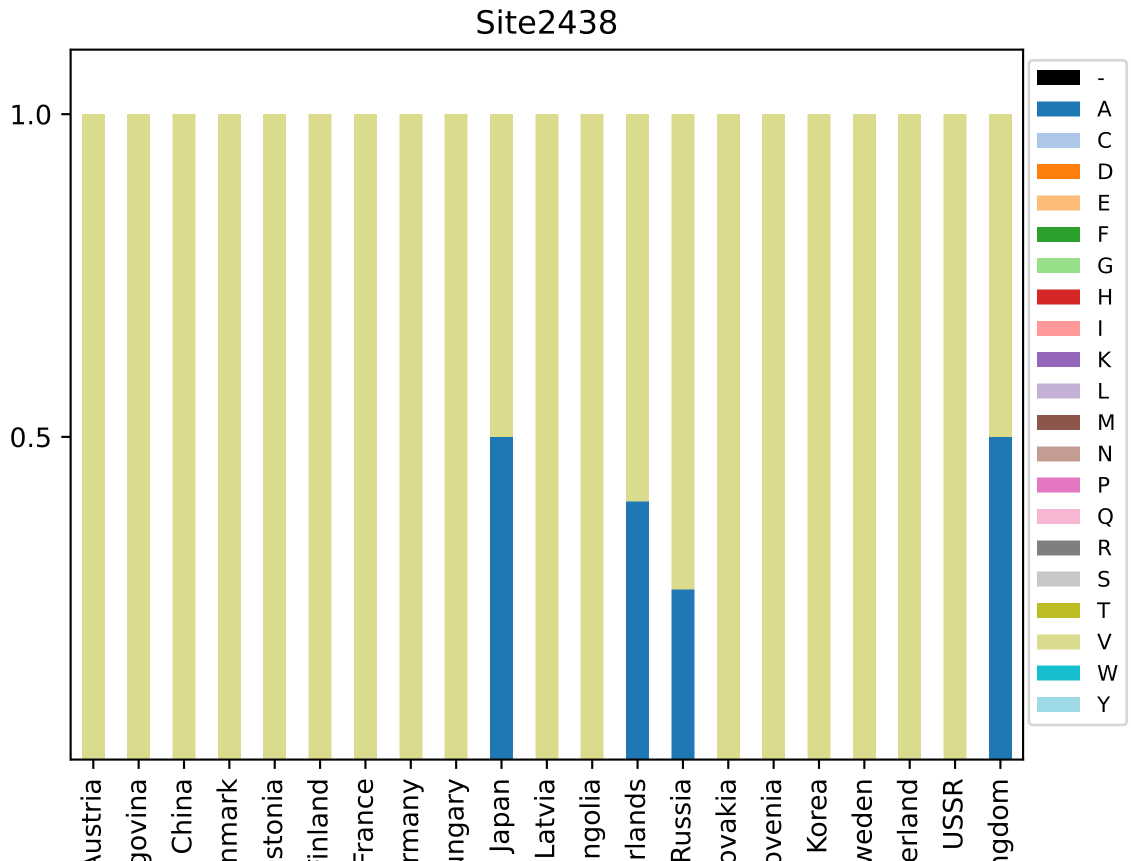

Site2438
1.0
0.5
-
A
C
D
E
F
G
H
I
K
L
M
N
P
Q
R
S
T
V
W
Y
Austria
govina
China
nmark
stonia
Finland
France
rmany
ungary
Japan
Latvia
ngolia
rlands
Russia
ovakia
ovenia
Korea
weden
erland
USSR
ngdom

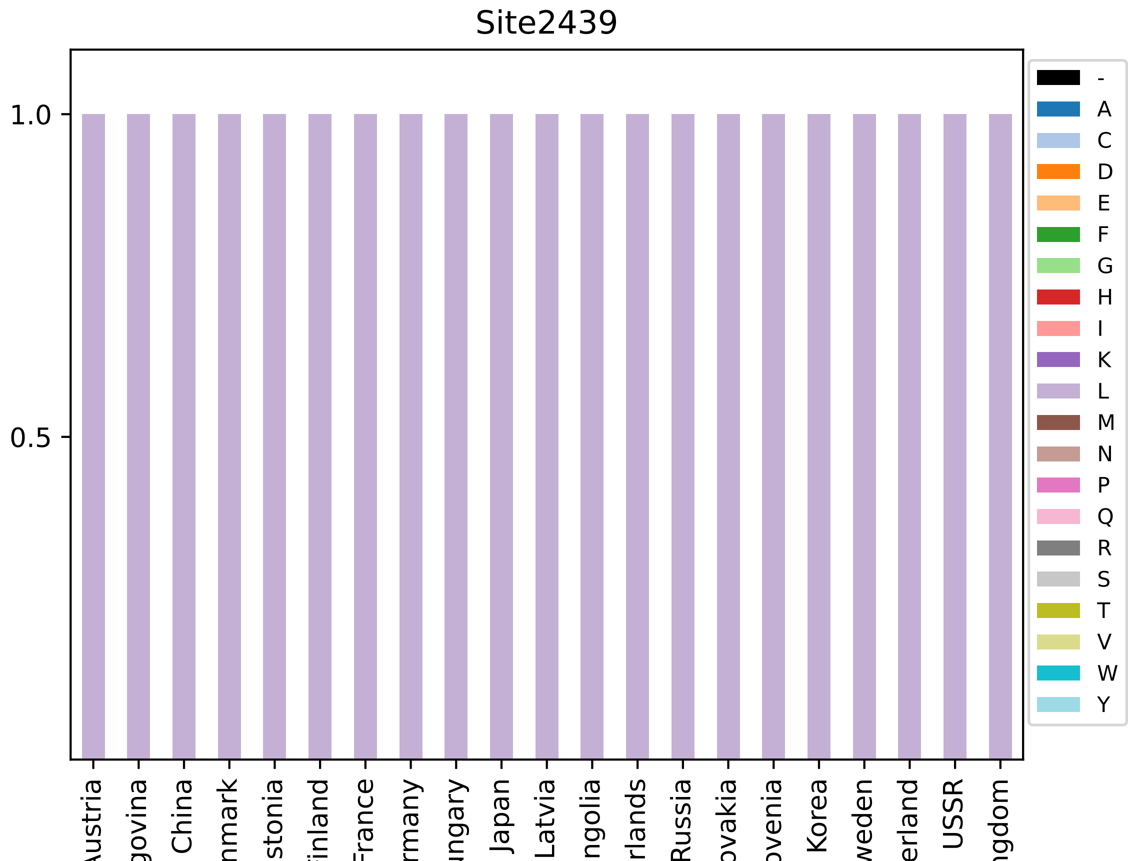
Site2439
1.0
0.5
-
A
C
D
E
F
G
H
I
K
L
M
N
P
Q
R
S
T
V
W
Y
China
Japan
Latvia
Russia
Korea
USSR

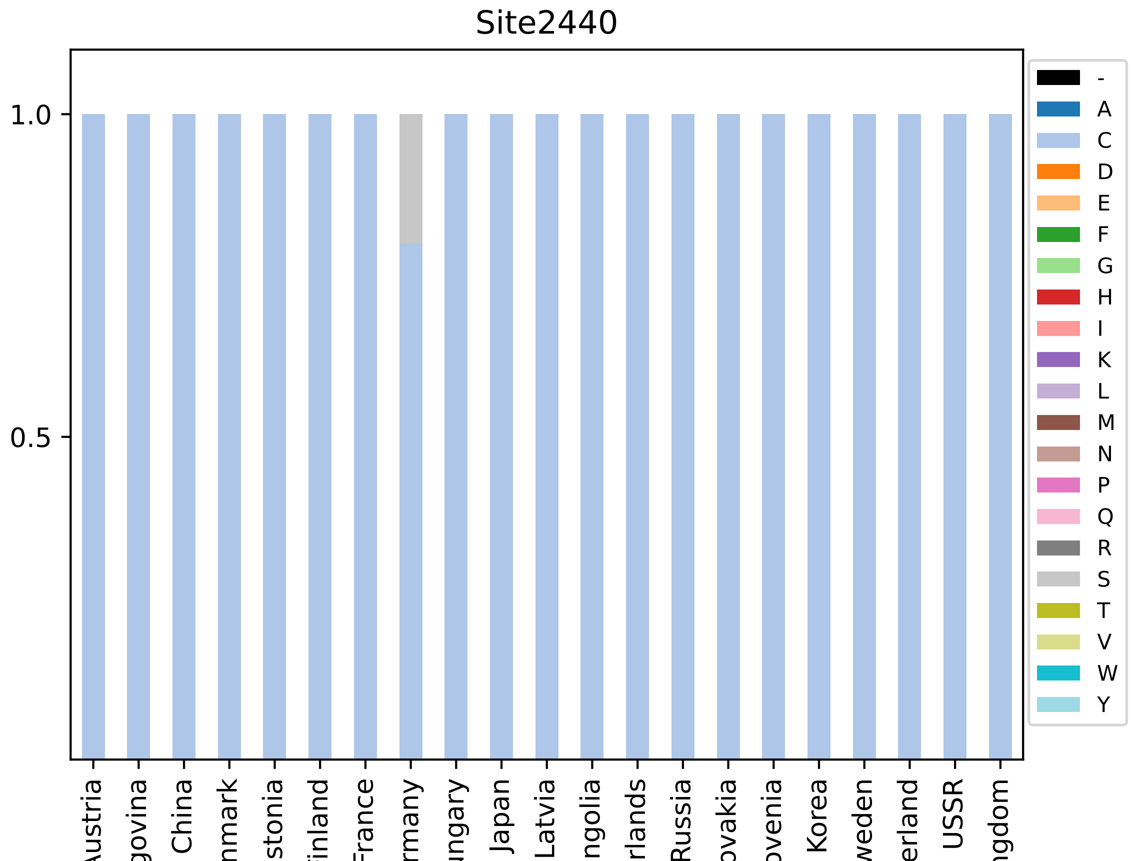

Site2440
1.0
0.5
-
A
C
D
E
F
G
H
I
K
L
M
N
P
Q
R
S
T
V
W
Y
China
Japan
Latvia
Russia
Korea
USSR

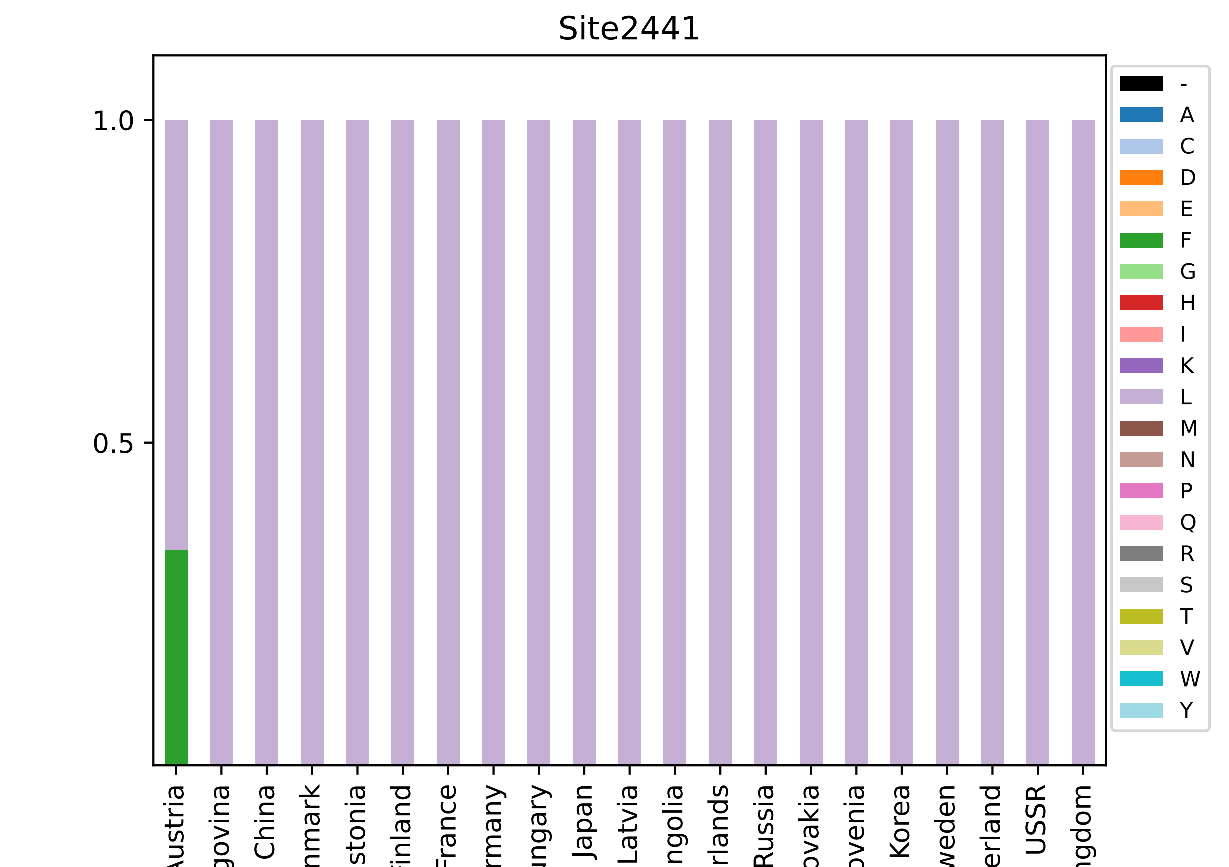

Site2441
1.0
0.5
-
A
C
D
E
F
G
H
I
K
L
M
N
P
Q
R
S
T
V
W
Y
China
Japan
Latvia
Russia
Korea
USSR

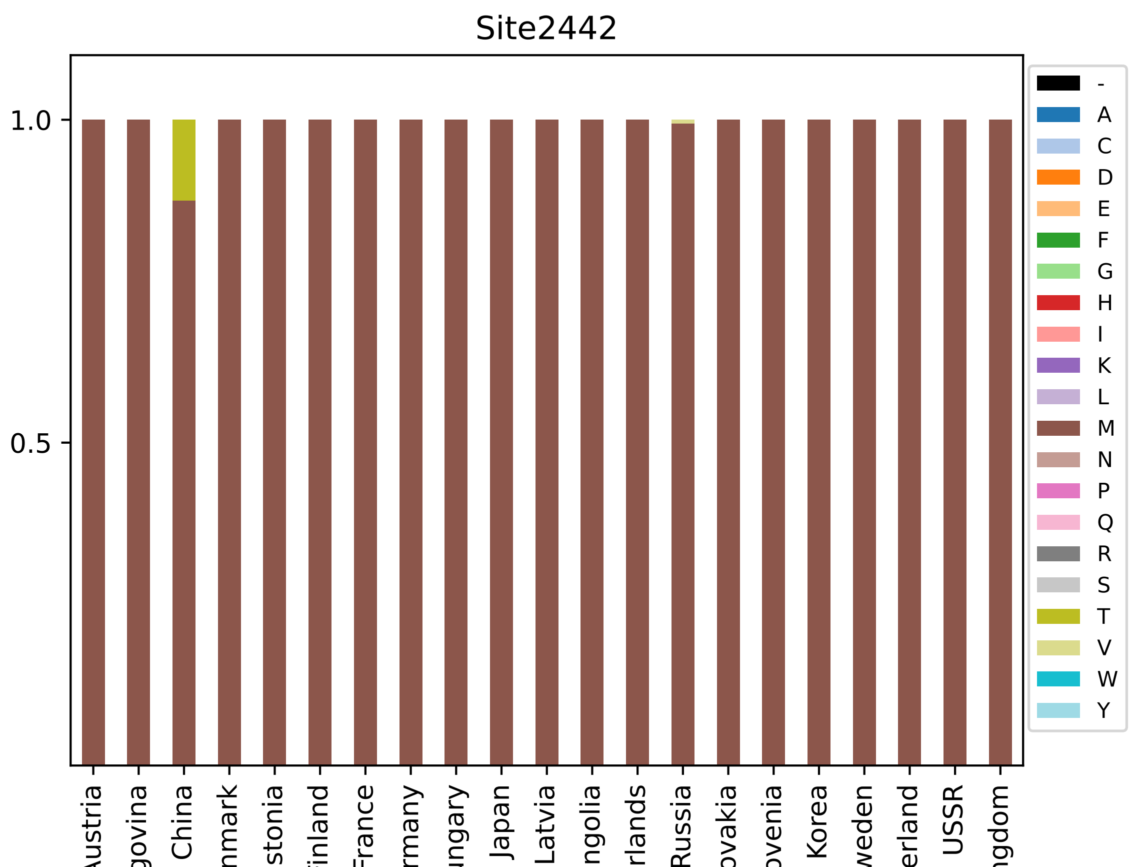
Site2442
1.0
0.5
-
A
C
D
E
F
G
H
I
K
L
M
N
P
Q
R
S
T
V
W
Y
China
Japan
Latvia
Russia
Korea
USSR

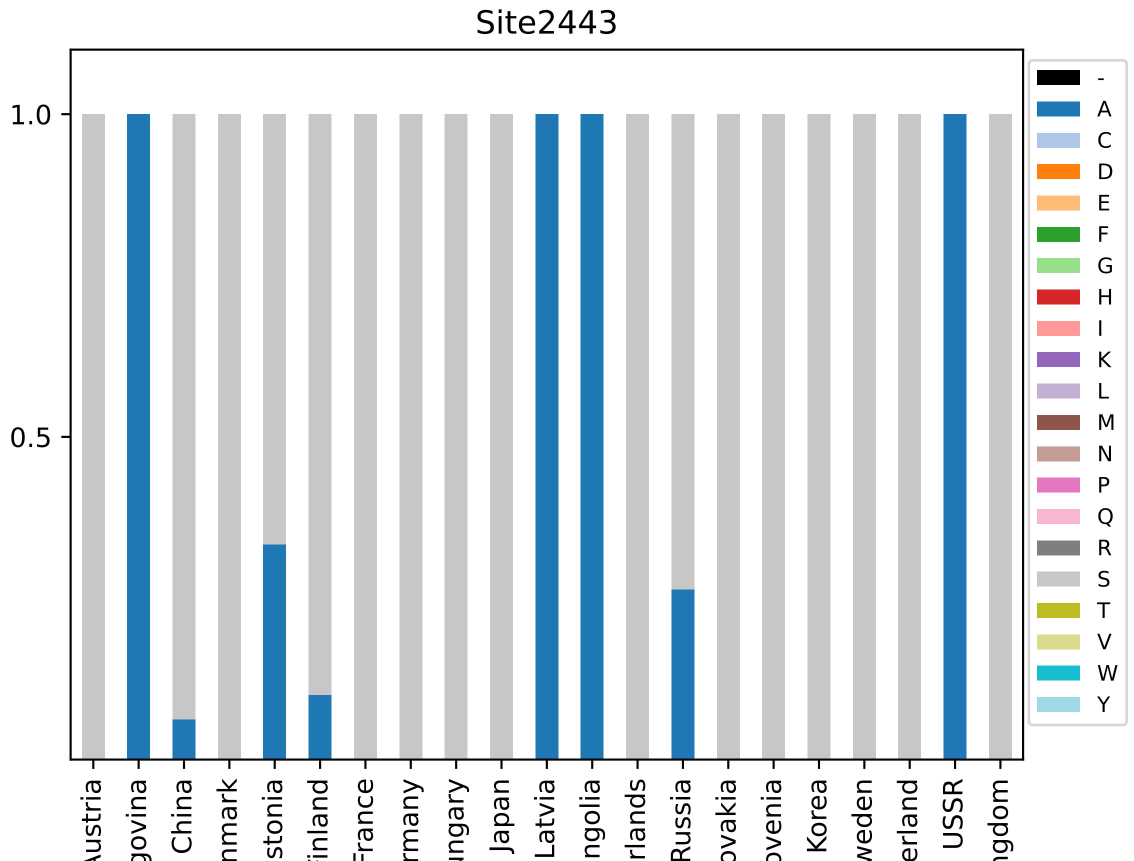

Site2443
1.0
0.5
-
A
C
D
E
F
G
H
I
K
L
M
N
P
Q
R
S
T
V
W
Y
China
Japan
Latvia
Russia
Korea
USSR

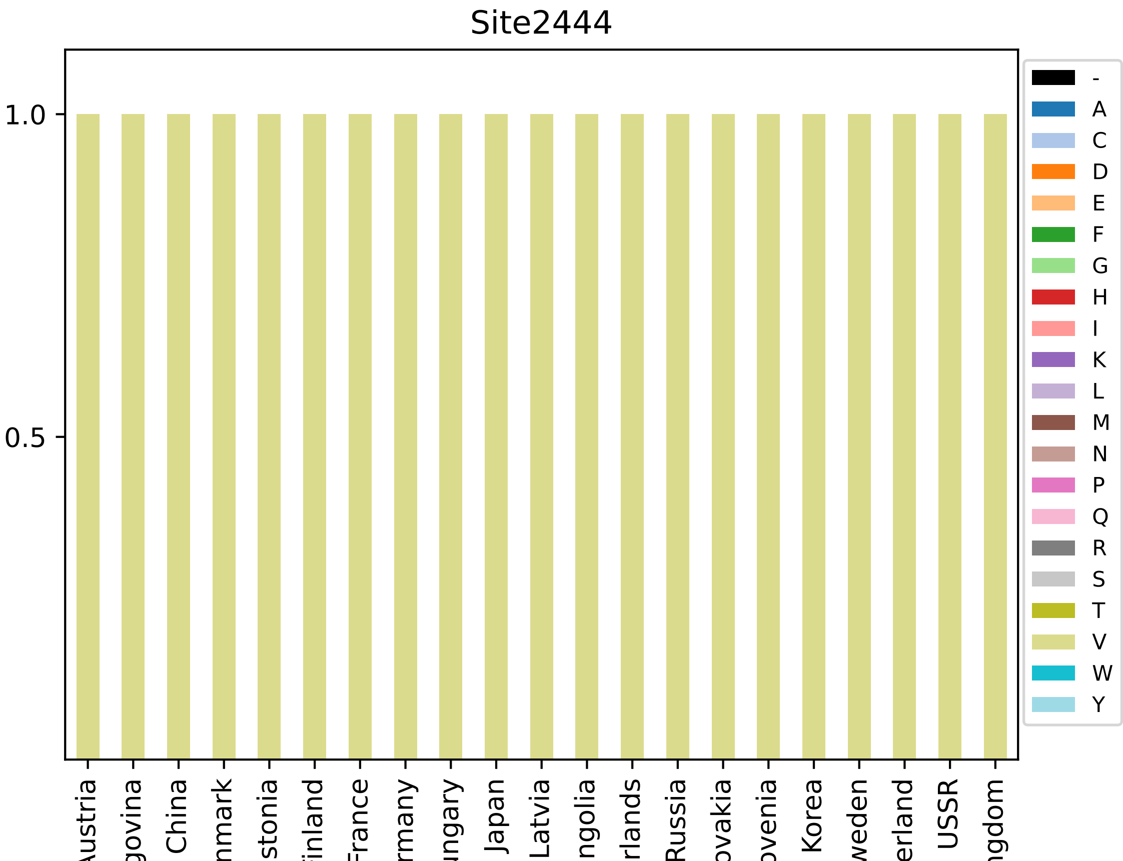
Site2444
1.0
0.5
-
A
C
D
E
F
G
H
I
K
L
M
N
P
Q
R
S
T
V
W
Y
China
Japan
Latvia
Russia
Korea
USSR

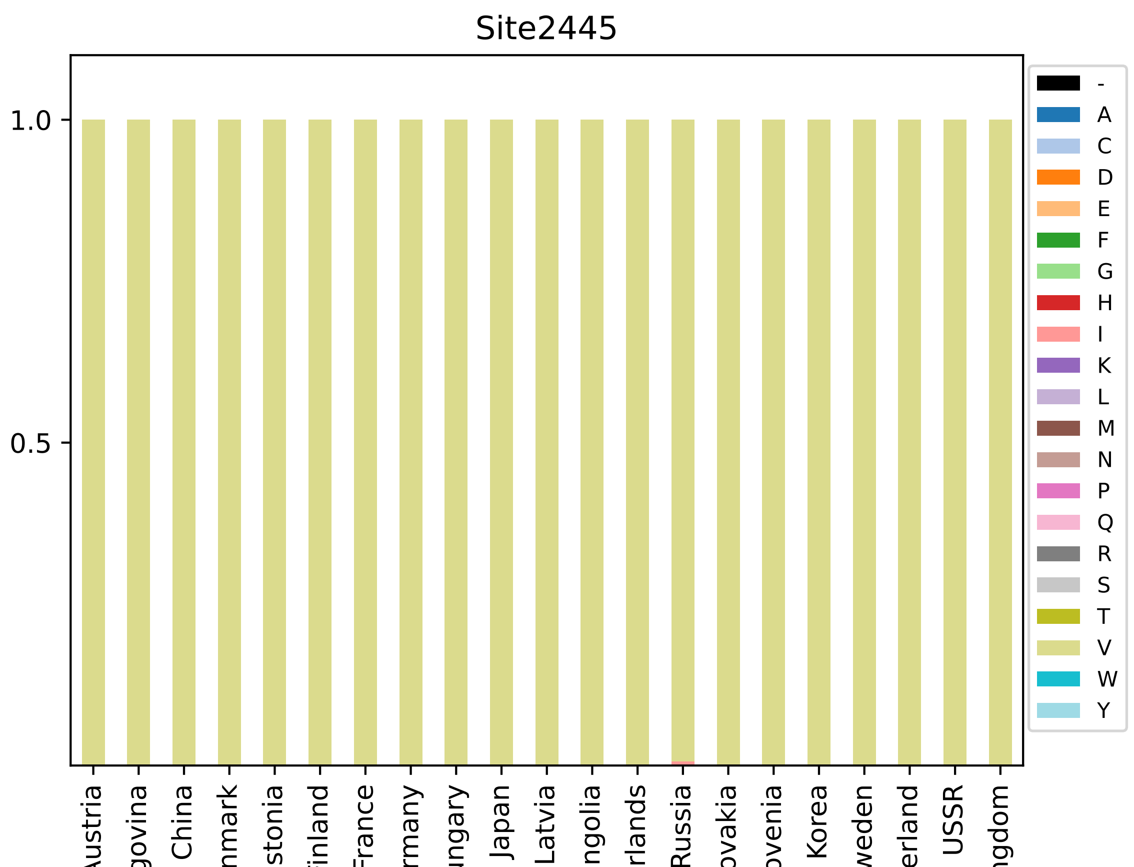

Site2445
1.0
0.5
-
A
C
D
E
F
G
H
I
K
L
M
N
P
Q
R
S
T
V
W
Y
Austria
govina
China
nmark
stonia
inland
France
rmany
ungary
Japan
Latvia
ngolia
rlands
Russia
ovakia
ovenia
Korea
weden
erland
USSR
ngdom

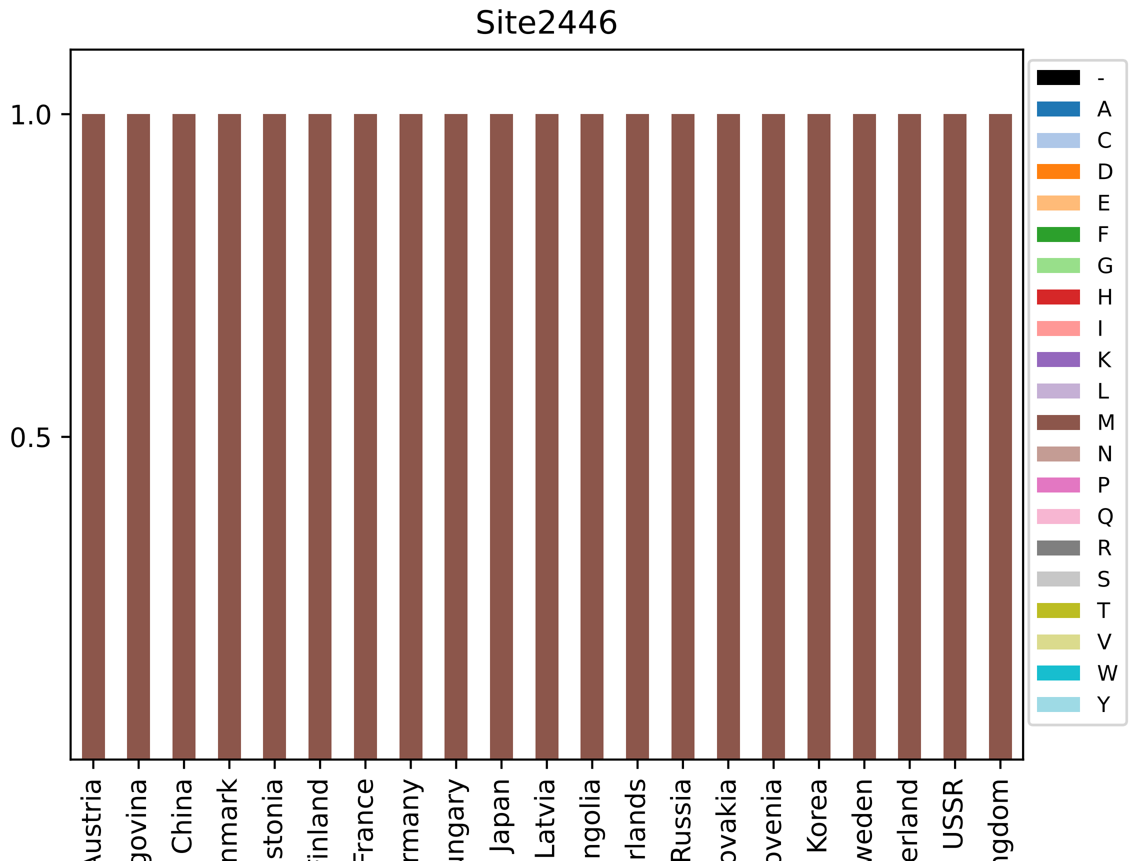
Site2446
1.0
0.5
China
Japan
Latvia
Russia
Korea
USSR
-
A
C
D
E
F
G
H
I
K
L
M
N
P
Q
R
S
T
V
W
Y

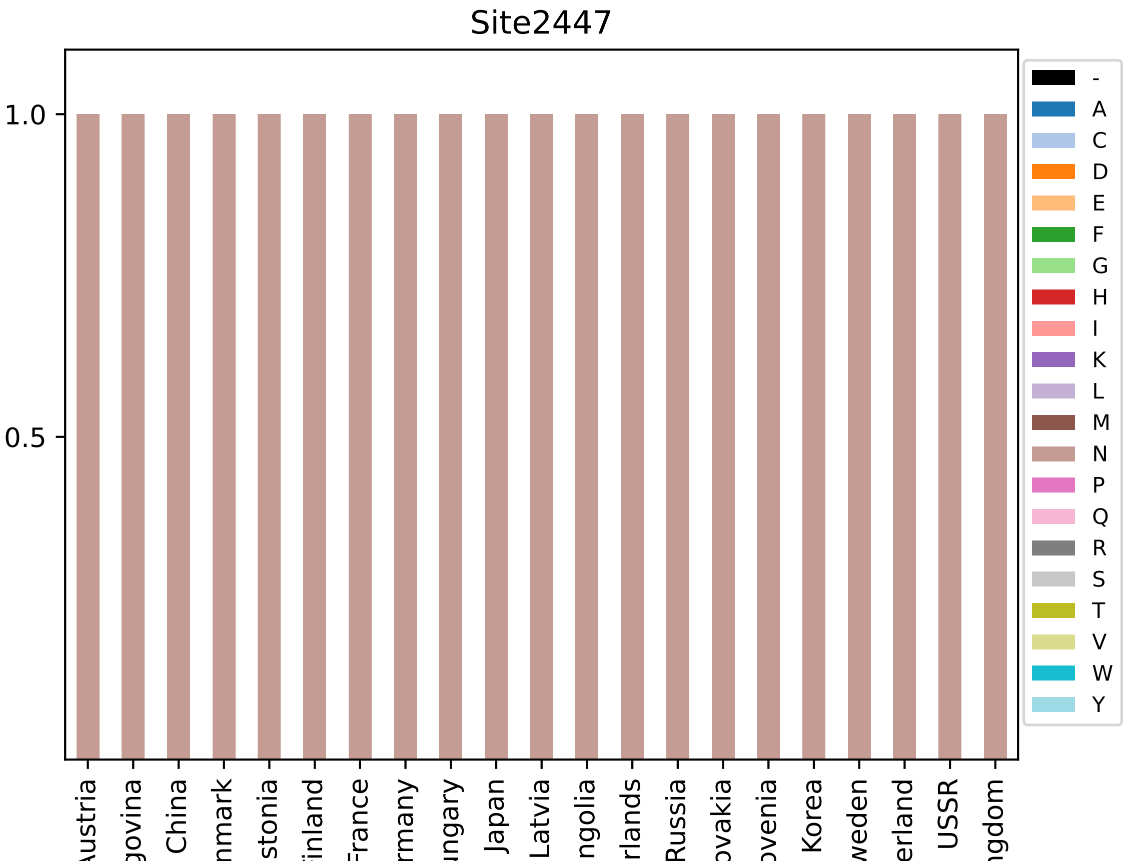
Site2447
1.0
0.5
China
Japan
Latvia
Russia
Korea
USSR
-
A
C
D
E
F
G
H
I
K
L
M
N
P
Q
R
S
T
V
W
Y

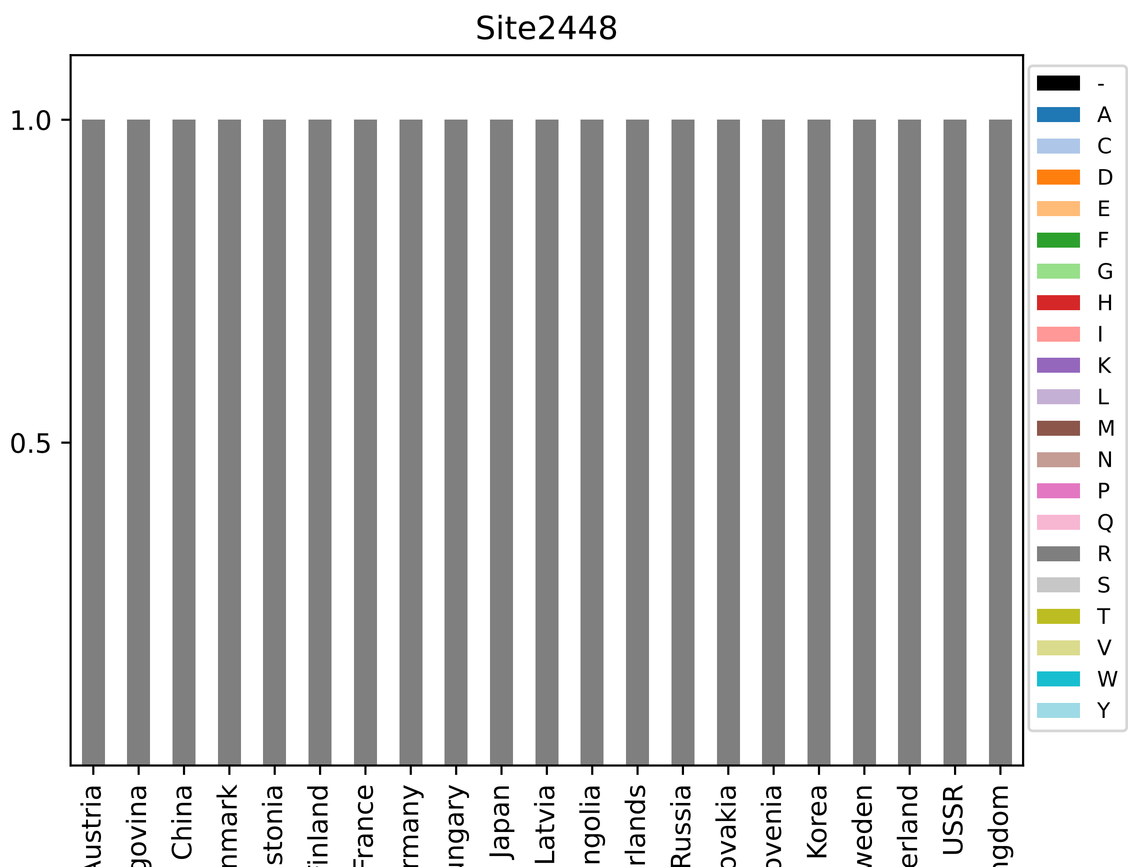

Site2448
1.0
0.5
-
A
C
D
E
F
G
H
I
K
L
M
N
P
Q
R
S
T
V
W
Y
Austria
China
Japan
Latvia
Russia
Korea
USSR

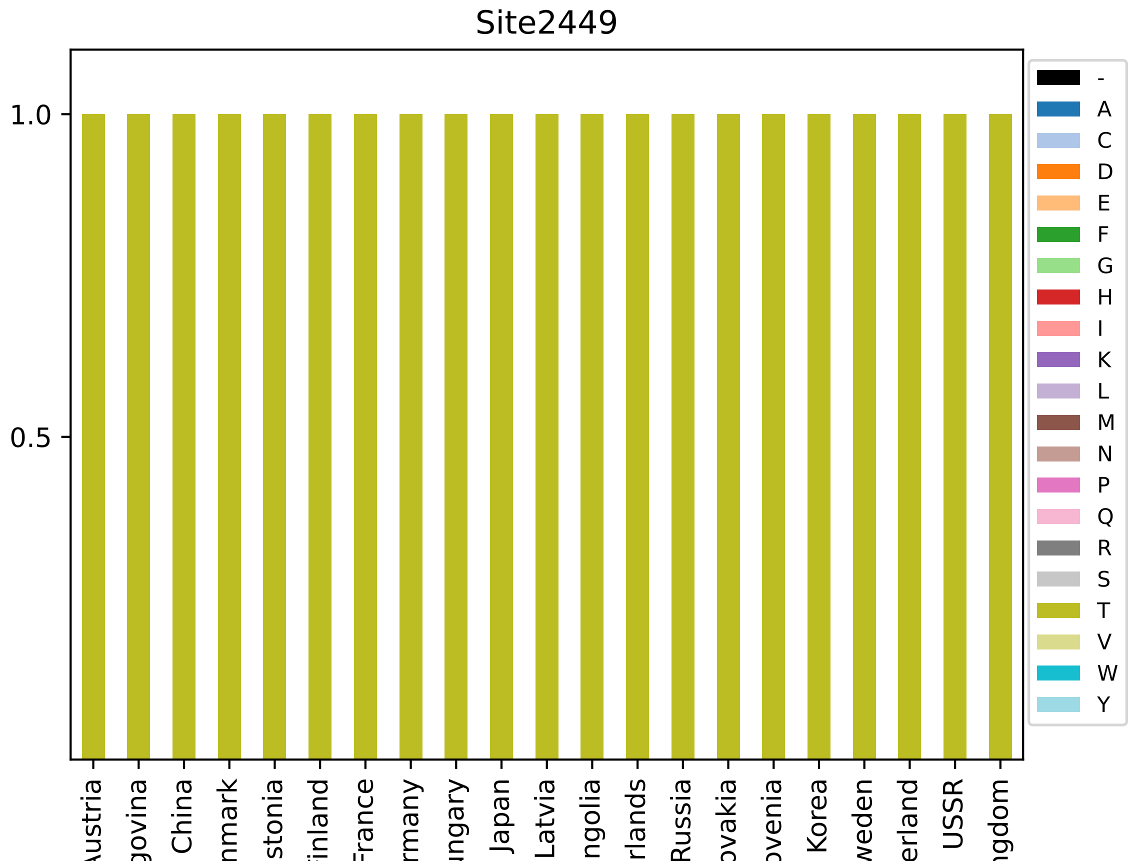
Site2449
1.0
0.5
-
A
C
D
E
F
G
H
I
K
L
M
N
P
Q
R
S
T
V
W
Y
China
Japan
Latvia
Russia
Korea
USSR

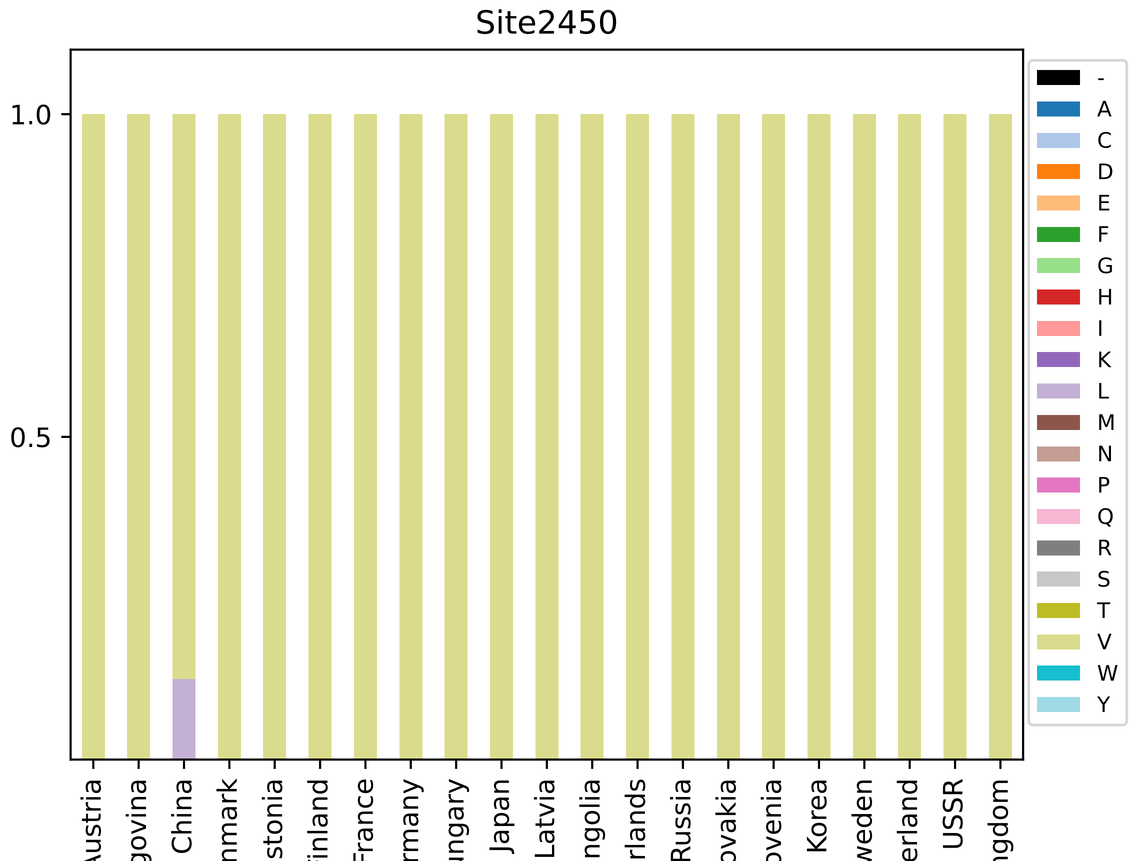

Site2450
1.0
0.5
Austria
govina
China
nmark
stonia
Finland
France
rmany
ungary
Japan
Latvia
ngolia
rlands
Russia
ovakia
ovenia
Korea
weden
erland
USSR
ngdom
-
A
C
D
E
F
G
H
I
K
L
M
N
P
Q
R
S
T
V
W
Y

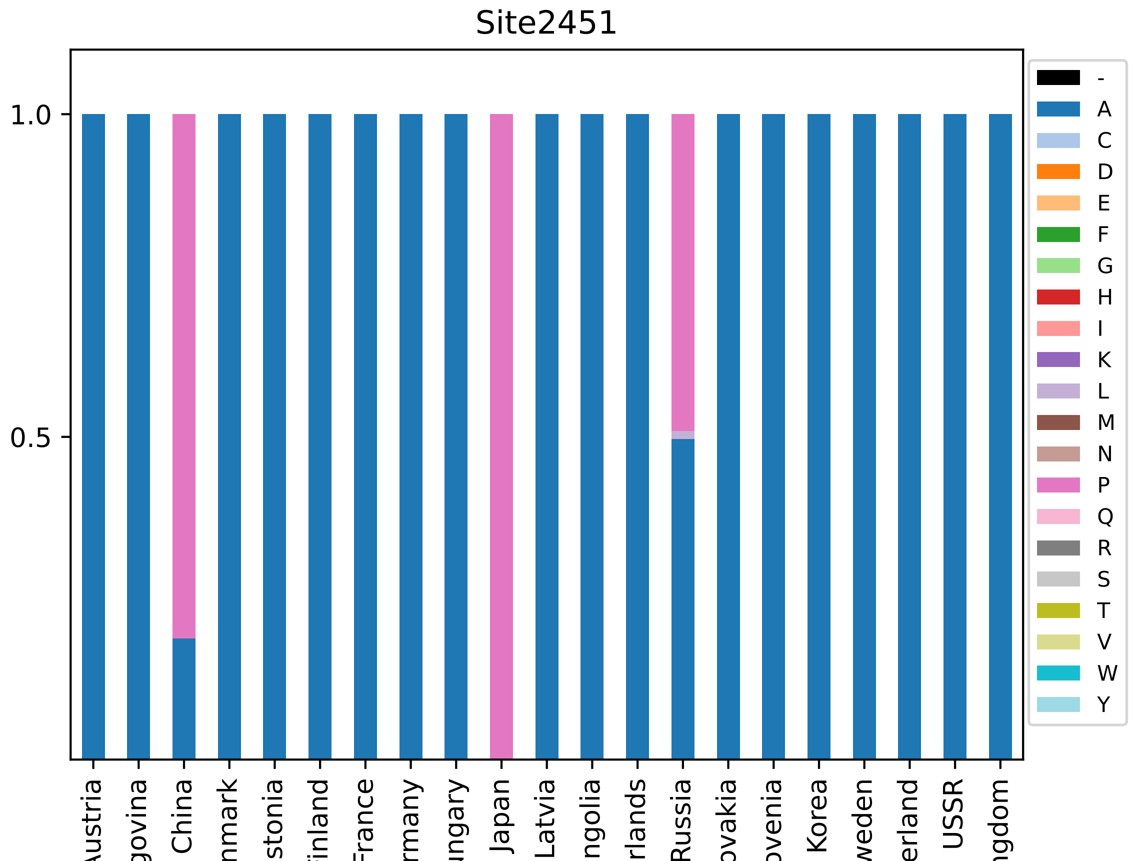
Site2451
1.0
0.5
-
A
C
D
E
F
G
H
I
K
L
M
N
P
Q
R
S
T
V
W
Y
Austria
govina
China
nmark
stonia
Finland
France
rmany
ungary
Japan
Latvia
ngolia
rlands
Russia
ovakia
ovenia
Korea
weden
erland
USSR
ngdom

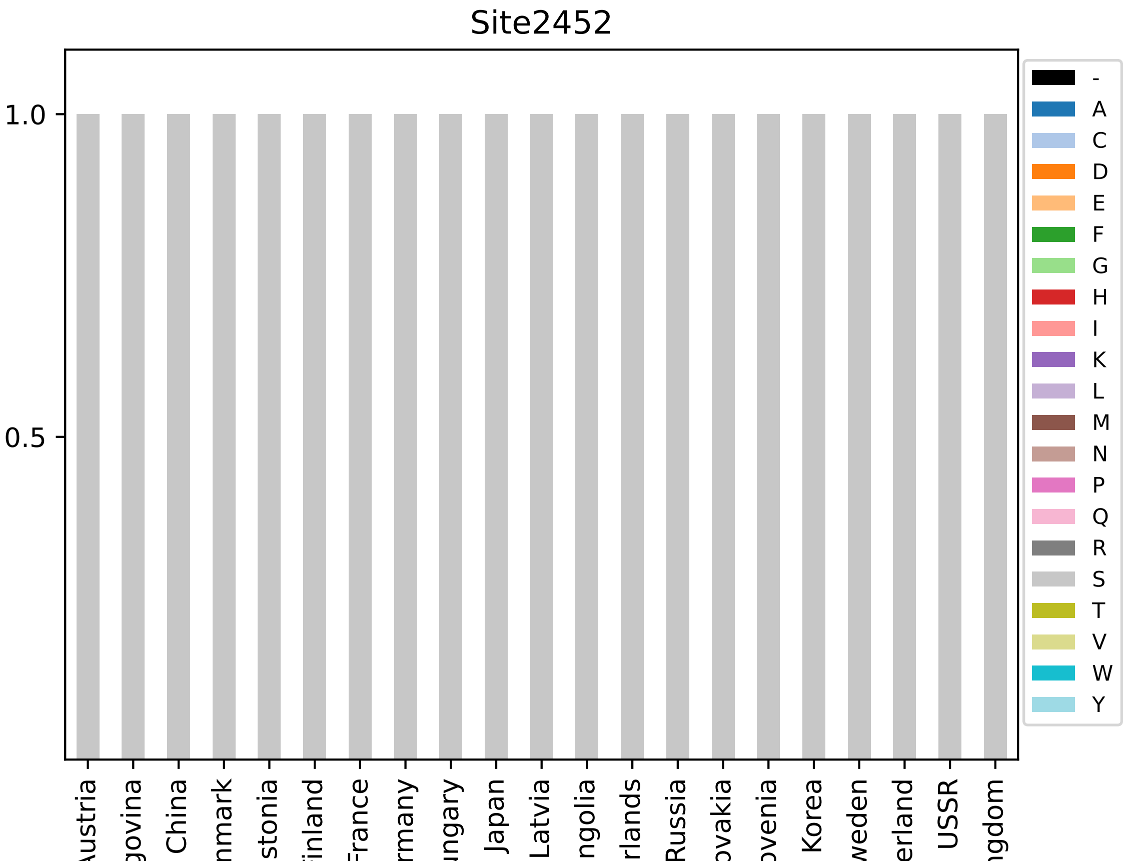

Site2452
1.0
0.5
-
A
C
D
E
F
G
H
I
K
L
M
N
P
Q
R
S
T
V
W
Y
China
Japan
Latvia
Russia
Korea
USSR

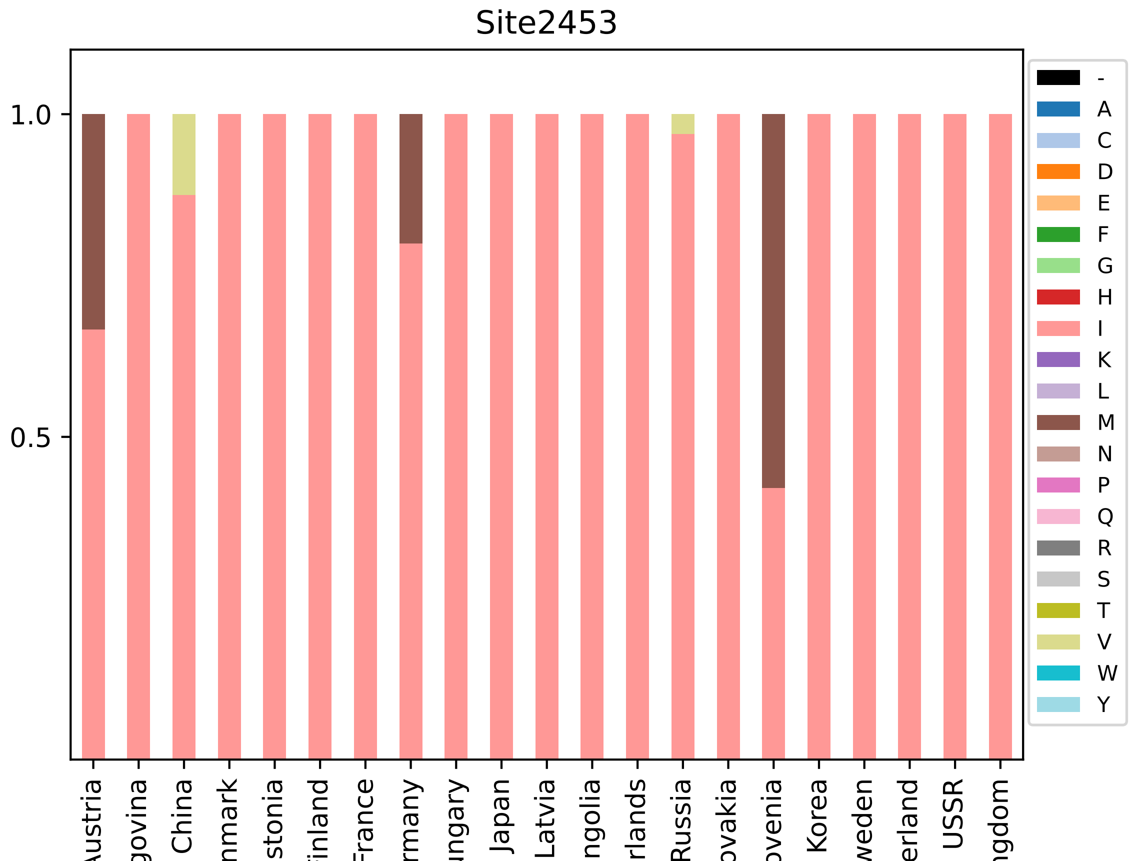
Site2453
1.0
0.5
-
A
C
D
E
F
G
H
I
K
L
M
N
P
Q
R
S
T
V
W
Y
Austria
China
Finland
France
Japan
Latvia
Russia
Korea
USSR

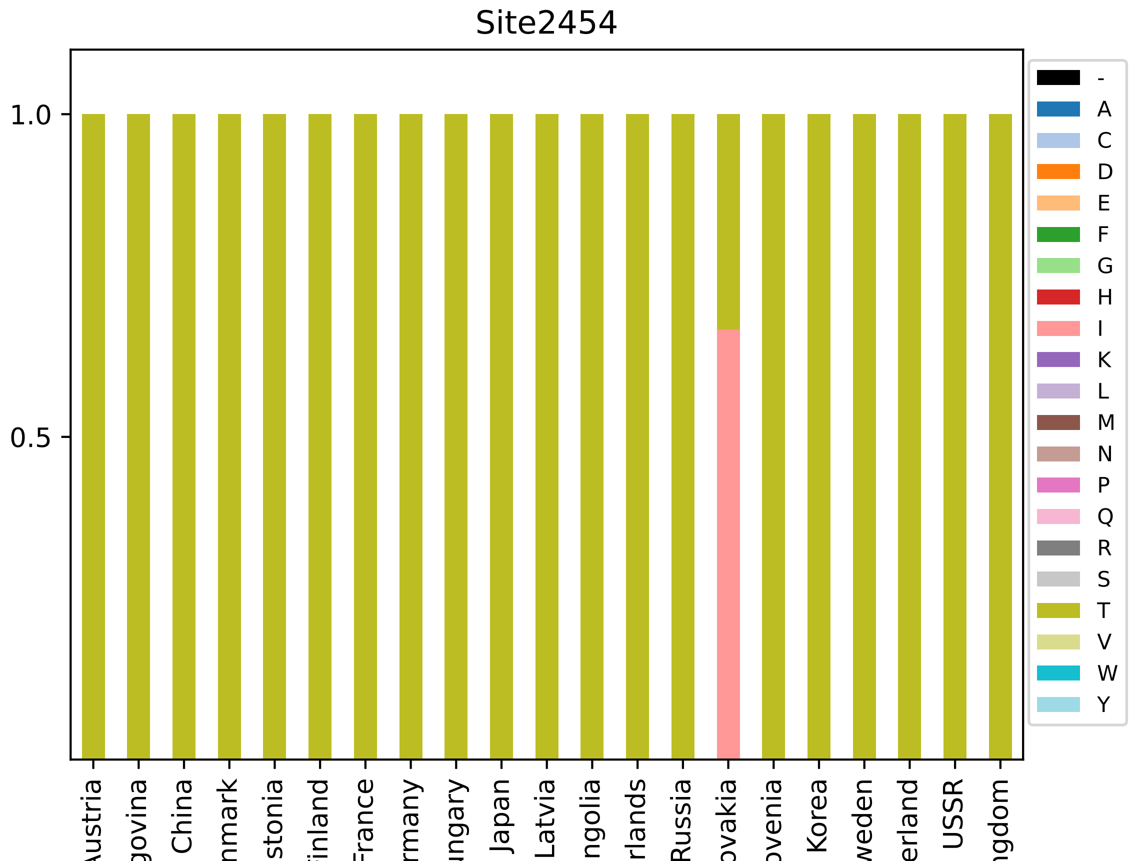

Site2454
1.0
0.5
-
A
C
D
E
F
G
H
I
K
L
M
N
P
Q
R
S
T
V
W
Y
Austria
China
Estonia
Finland
France
Germany
Hungary
Japan
Latvia
Russia
Korea
Sweden
USSR

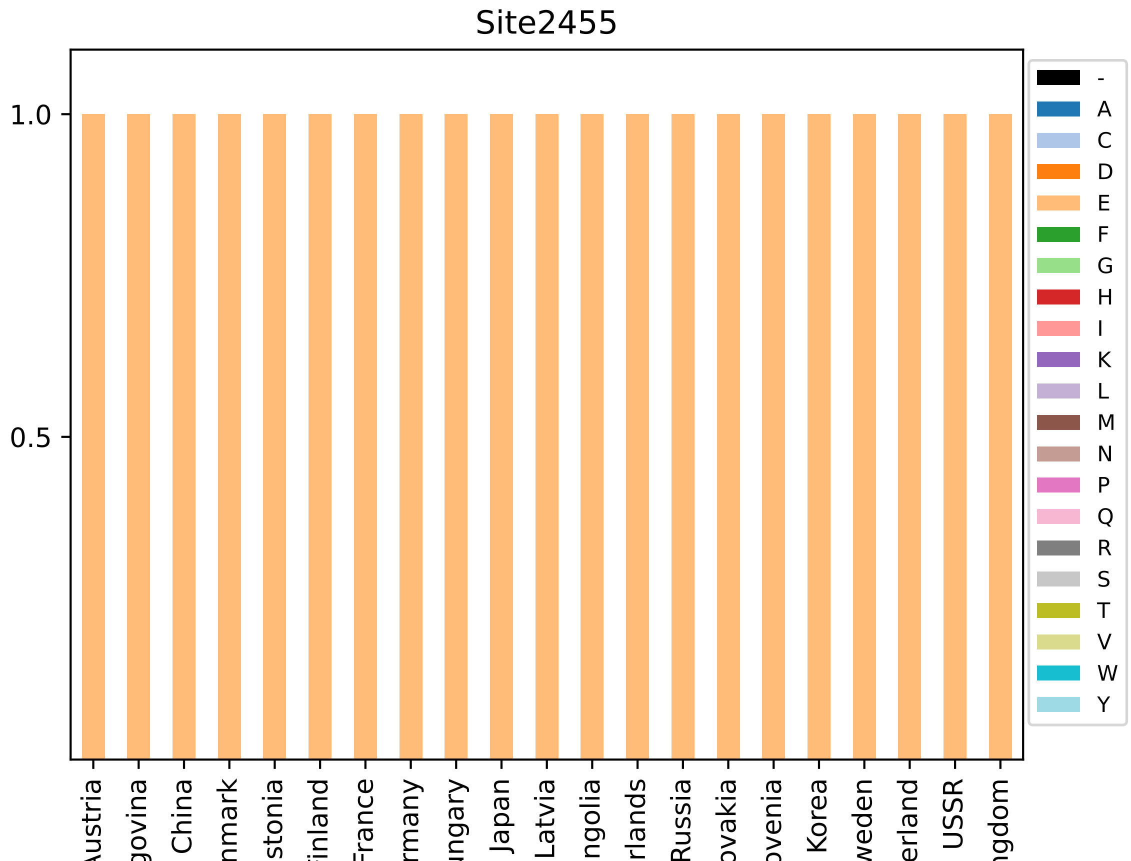
Site2455
1.0
0.5
China
Japan
Latvia
Russia
Korea
USSR
-
A
C
D
E
F
G
H
I
K
L
M
N
P
Q
R
S
T
V
W
Y

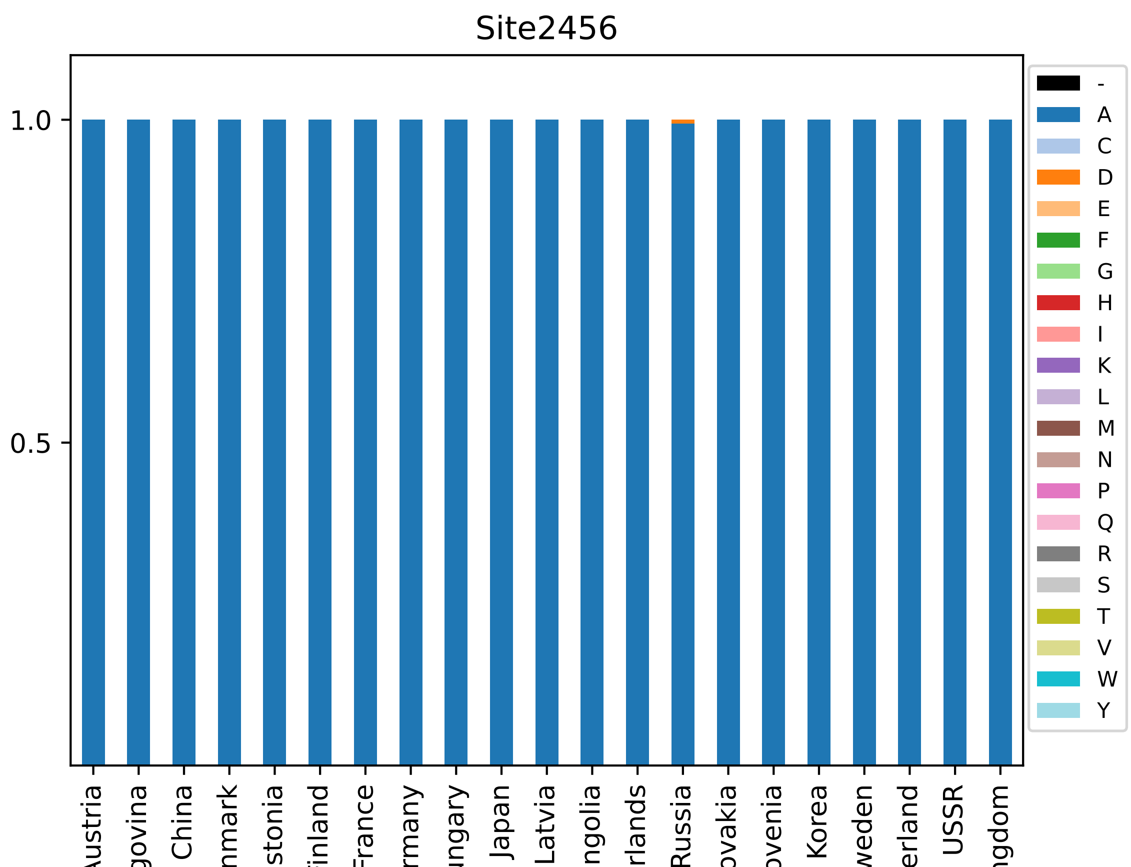

Site2456
1.0
0.5
Austria
govina
China
nmark
stonia
Finland
France
rmany
ungary
Japan
Latvia
ngolia
rlands
Russia
ovakia
ovenia
Korea
weden
erland
USSR
ngdom
-
A
C
D
E
F
G
H
I
K
L
M
N
P
Q
R
S
T
V
W
Y

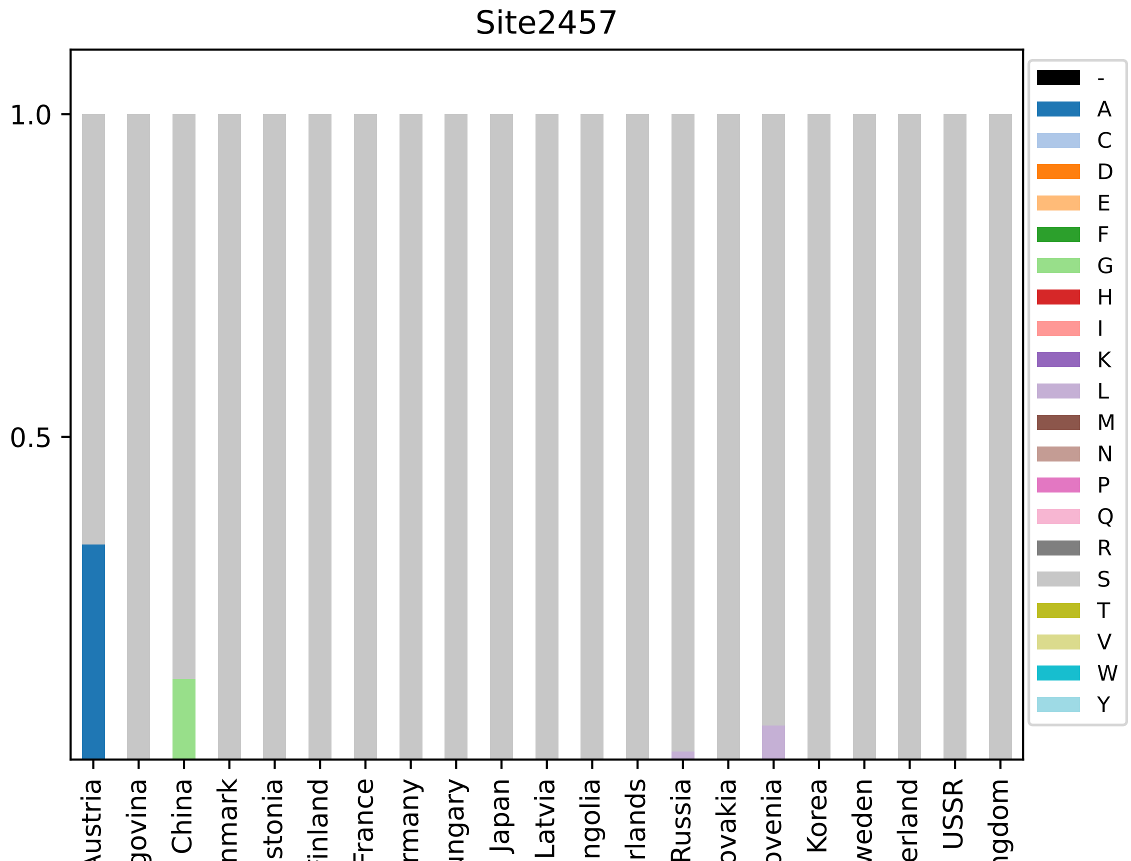

Site2457
1.0
0.5
-
A
C
D
E
F
G
H
I
K
L
M
N
P
Q
R
S
T
V
W
Y
Austria
govina
China
nmark
stonia
inland
France
rmany
ungary
Japan
Latvia
ngolia
rlands
Russia
ovakia
ovenia
Korea
weden
erland
USSR
ngdom

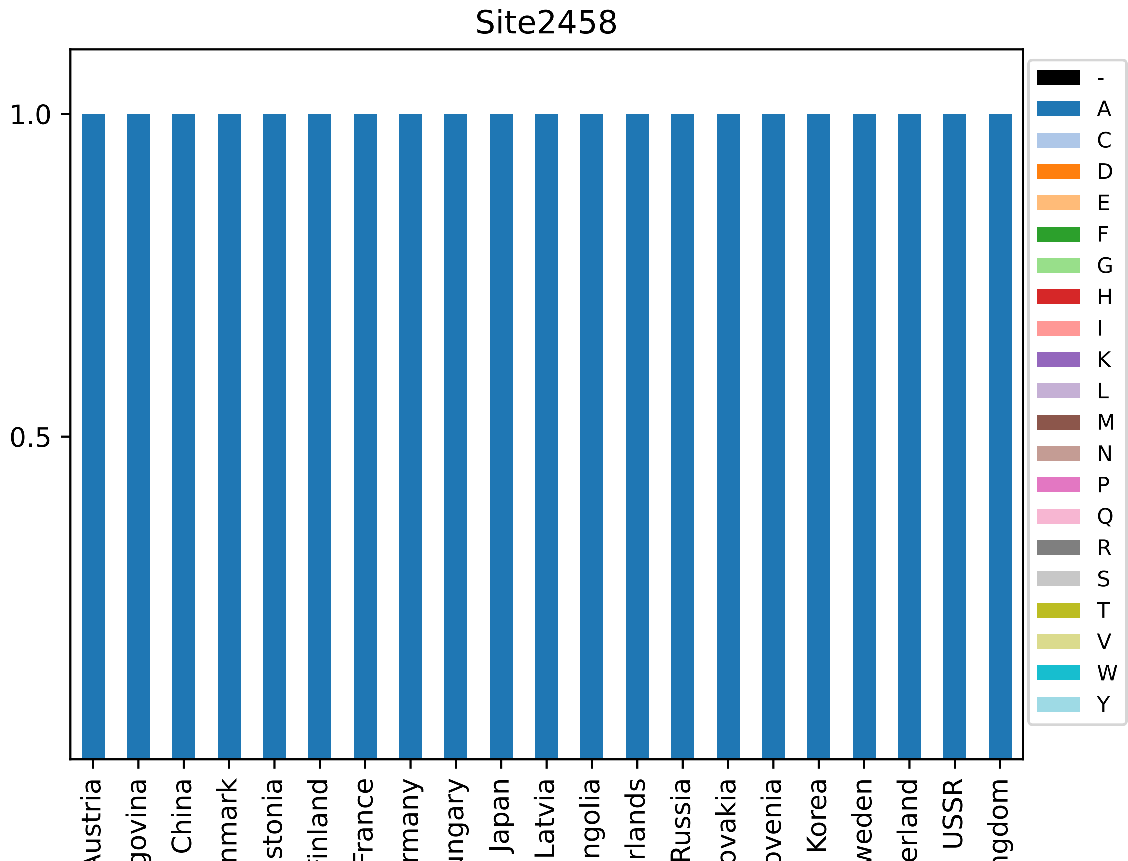
Site2458
1.0
0.5
-
A
C
D
E
F
G
H
I
K
L
M
N
P
Q
R
S
T
V
W
Y
Austria
China
Japan
Latvia
Russia
Korea
USSR

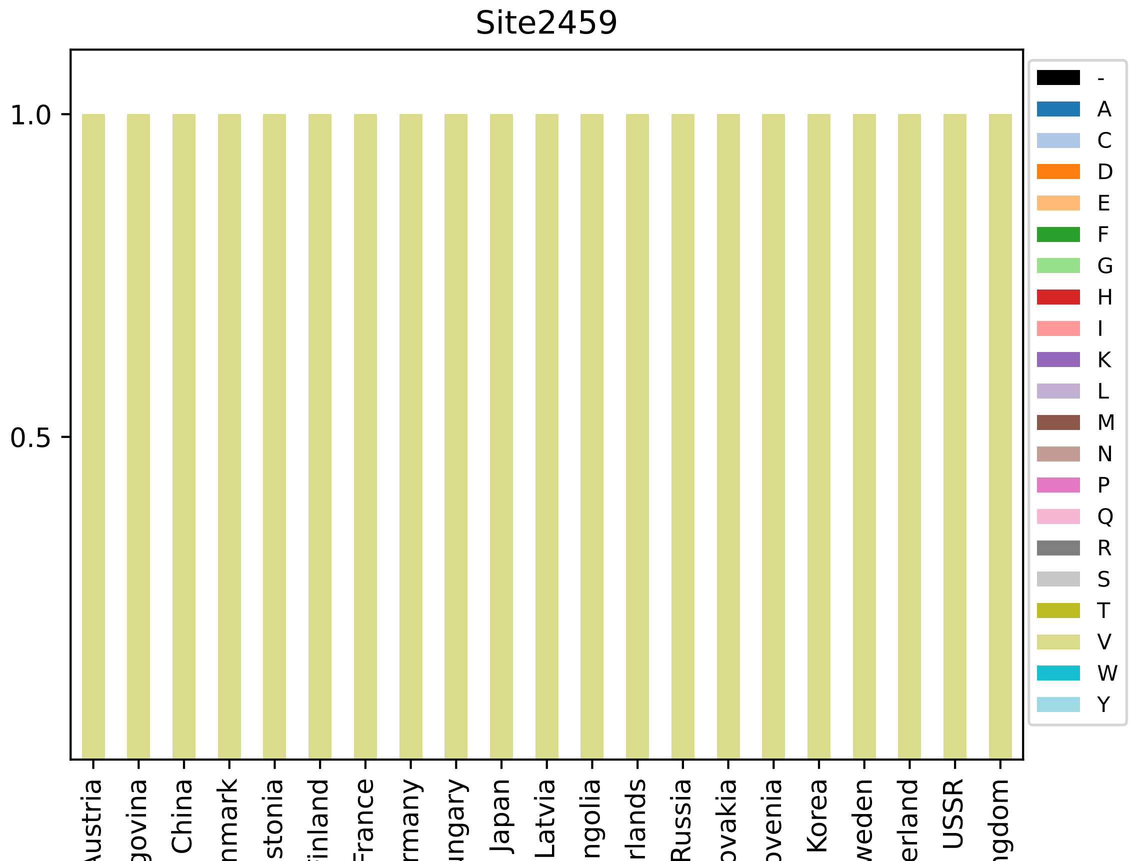
Site2459
1.0
0.5
-
A
C
D
E
F
G
H
I
K
L
M
N
P
Q
R
S
T
V
W
Y
Austria
govina
China
nmark
stonia
inland
France
rmany
ungary
Japan
Latvia
ngolia
rlands
Russia
ovakia
ovenia
Korea
weden
erland
USSR
ngdom

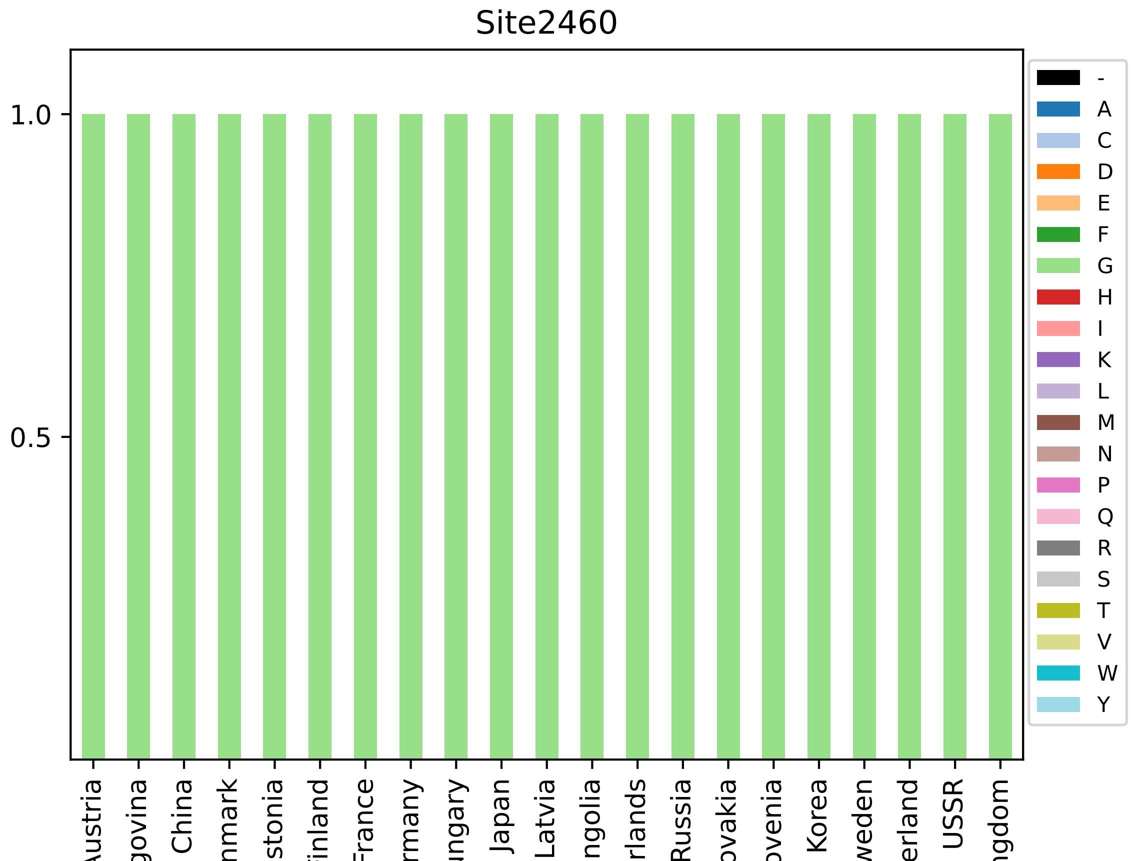

Site2460
1.0
0.5
-
A
C
D
E
F
G
H
I
K
L
M
N
P
Q
R
S
T
V
W
Y
Austria
govina
China
nmark
stonia
Finland
France
rmany
ungary
Japan
Latvia
ngolia
rlands
Russia
ovakia
ovenia
Korea
weden
erland
USSR
ngdom

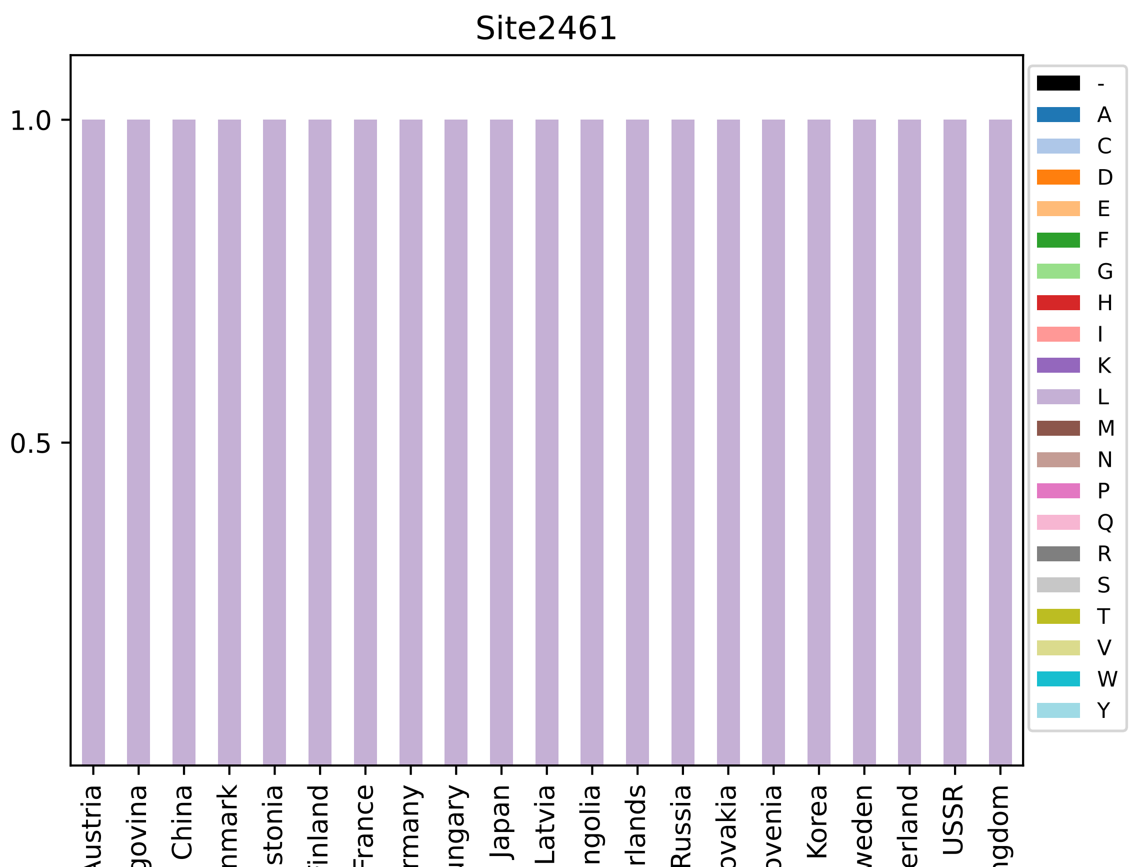
Site2461
1.0
0.5
-
A
C
D
E
F
G
H
I
K
L
M
N
P
Q
R
S
T
V
W
Y
Austria
govina
China
nmark
stonia
Finland
France
rmany
ungary
Japan
Latvia
ngolia
rlands
Russia
ovakia
ovenia
Korea
weden
erland
USSR
ngdom

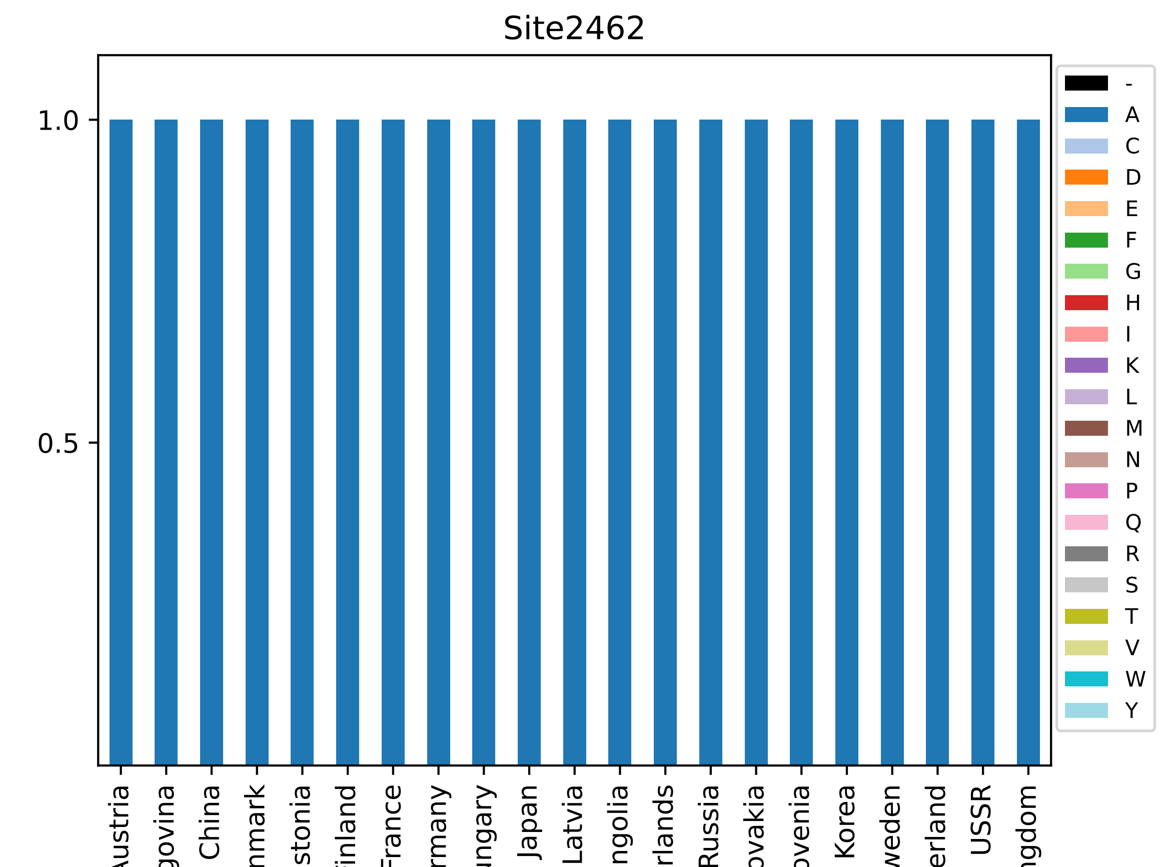

Site2462
1.0
0.5
-
A
C
D
E
F
G
H
I
K
L
M
N
P
Q
R
S
T
V
W
Y
Austria
China
Finland
France
Japan
Latvia
Russia
Korea
USSR

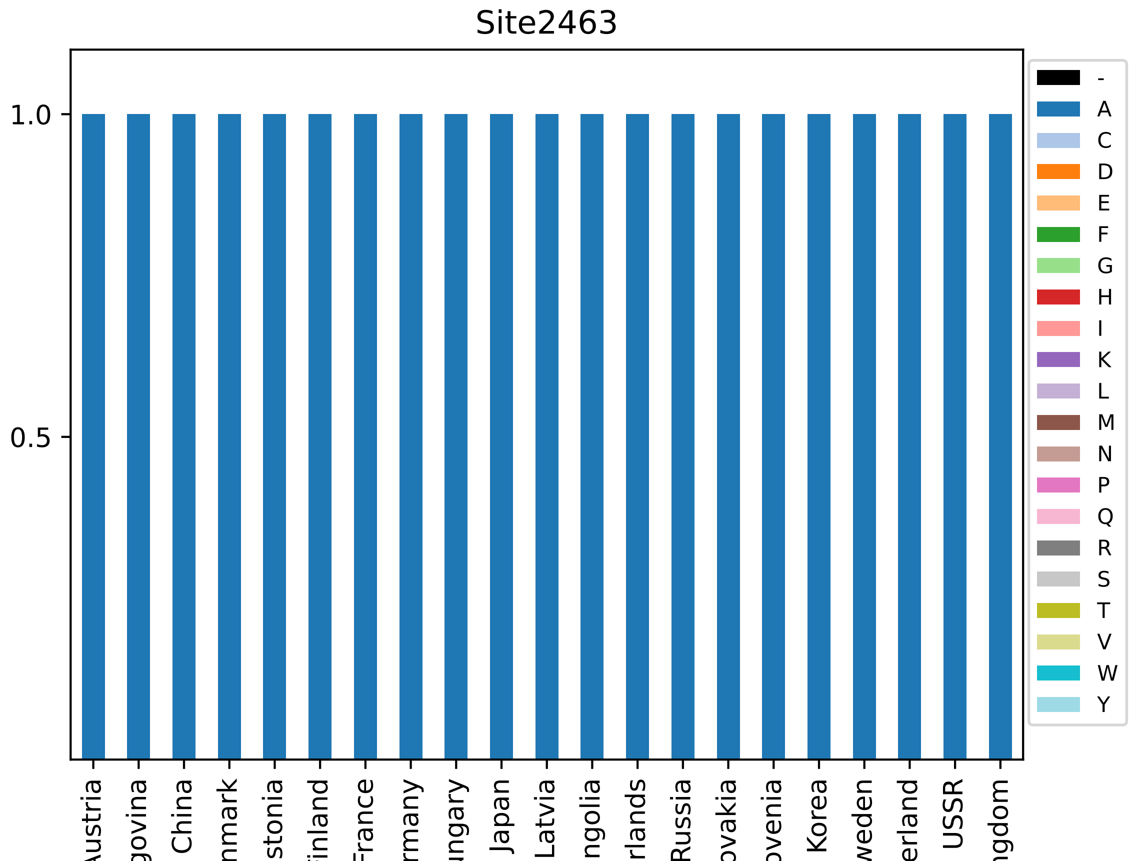

Site2463
1.0
0.5
-
A
C
D
E
F
G
H
I
K
L
M
N
P
Q
R
S
T
V
W
Y
China
Japan
Latvia
Russia
Korea
USSR

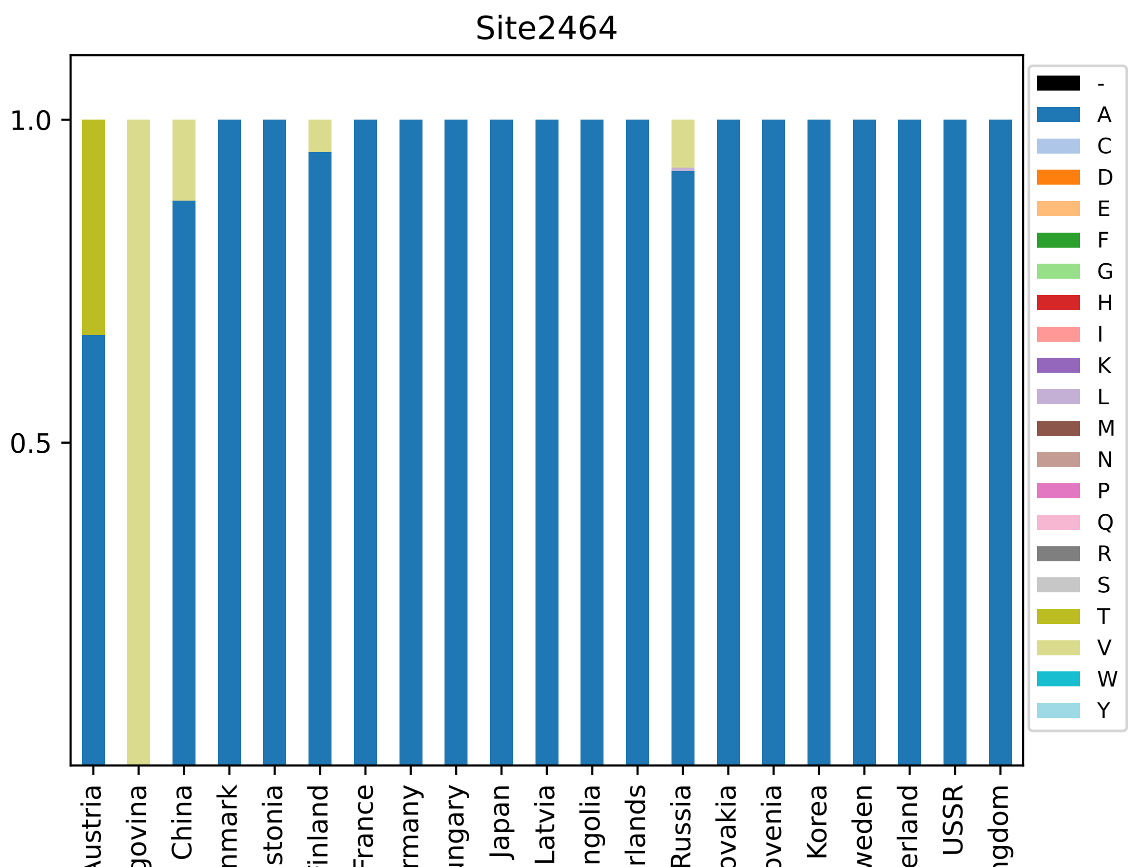

Site2464
1.0
0.5
-
A
C
D
E
F
G
H
I
K
L
M
N
P
Q
R
S
T
V
W
Y
China
Japan
Latvia
Russia
Korea
USSR

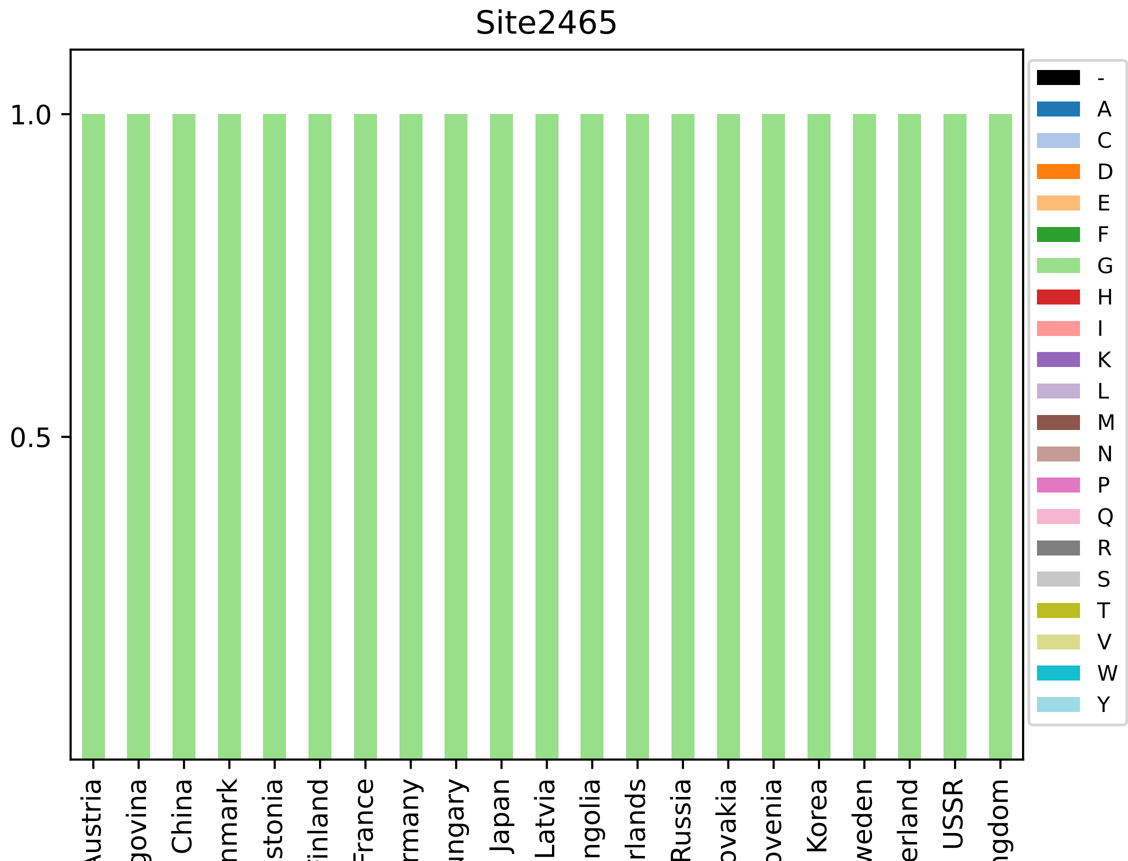
Site2465
1.0
0.5
-
A
C
D
E
F
G
H
I
K
L
M
N
P
Q
R
S
T
V
W
Y
China
Japan
Latvia
Russia
Korea
USSR

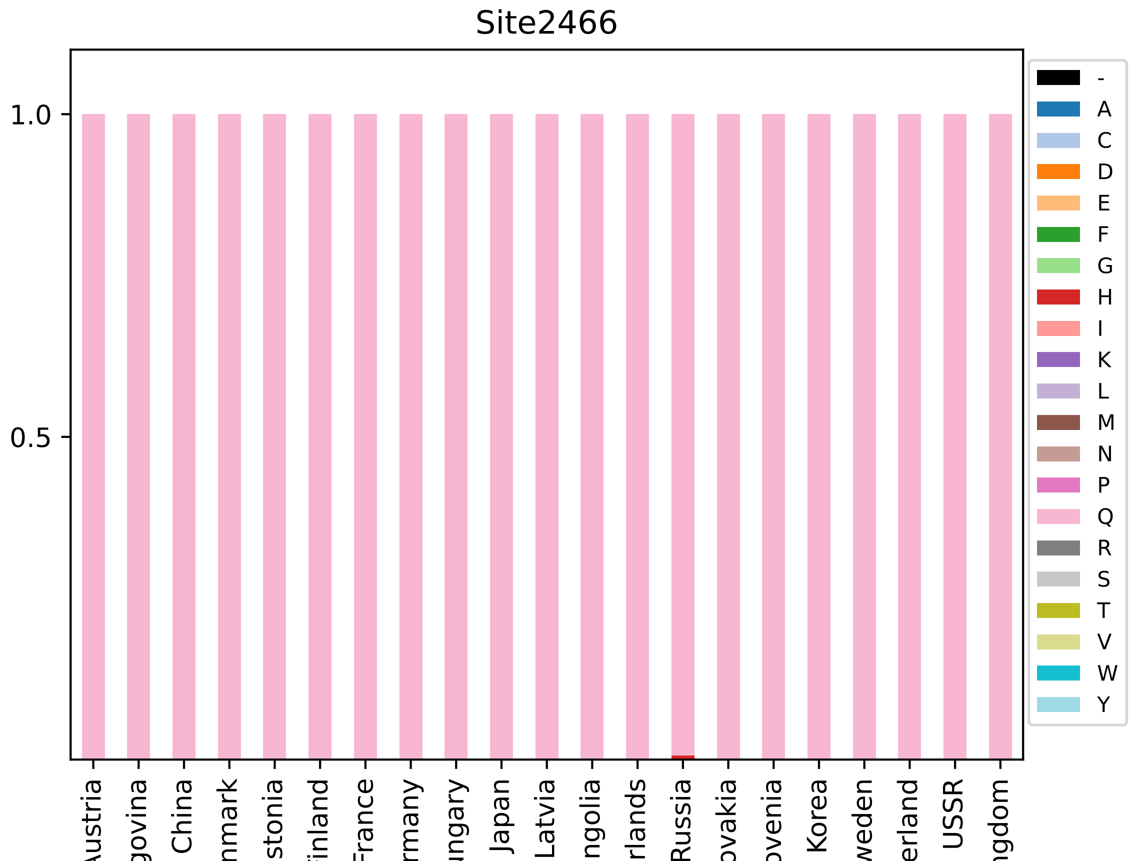

Site2466
1.0
0.5
-
A
C
D
E
F
G
H
I
K
L
M
N
P
Q
R
S
T
V
W
Y
Austria
China
Finland
France
Japan
Latvia
Russia
Korea
USSR

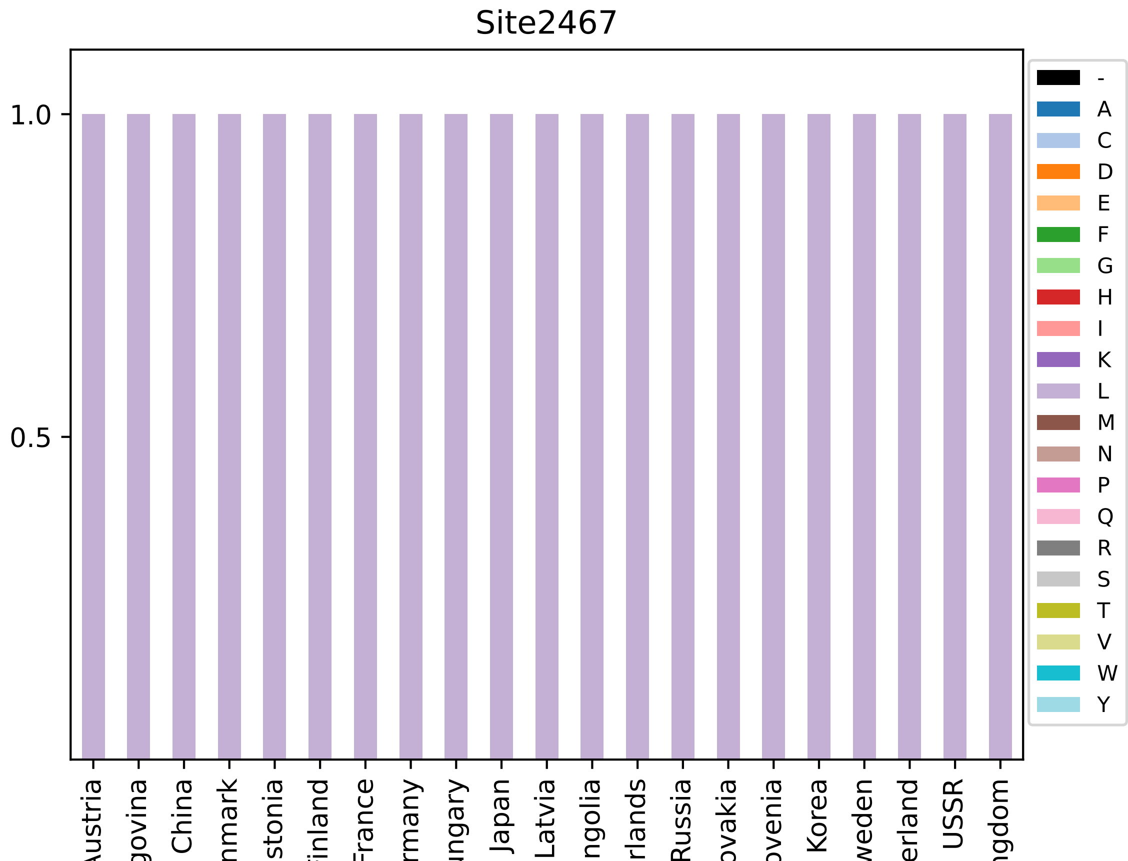

Site2467
1.0
0.5
-
A
C
D
E
F
G
H
I
K
L
M
N
P
Q
R
S
T
V
W
Y
Austria
govina
China
nmark
stonia
inland
France
rmany
ungary
Japan
Latvia
ngolia
rlands
Russia
ovakia
ovenia
Korea
weden
erland
USSR
ngdom

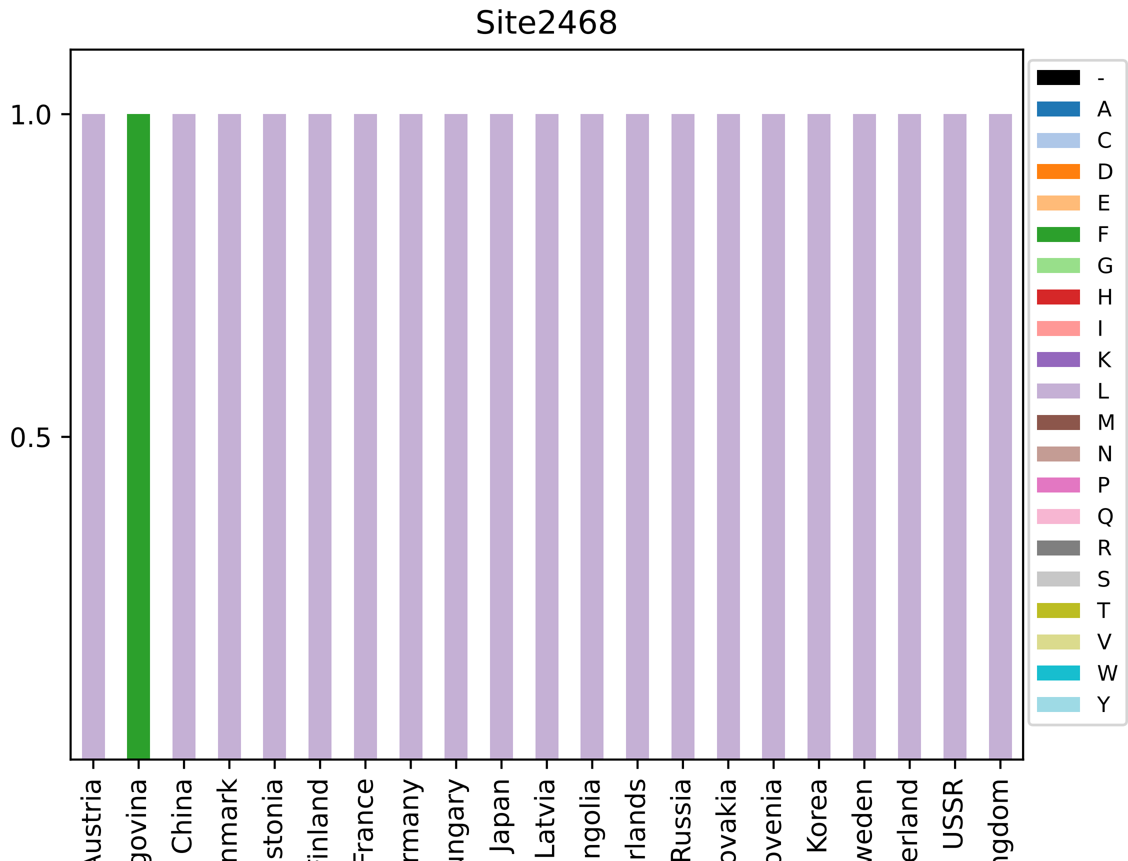
Site2468
1.0
0.5
-
A
C
D
E
F
G
H
I
K
L
M
N
P
Q
R
S
T
V
W
Y
China
Japan
Latvia
Russia
Korea
USSR

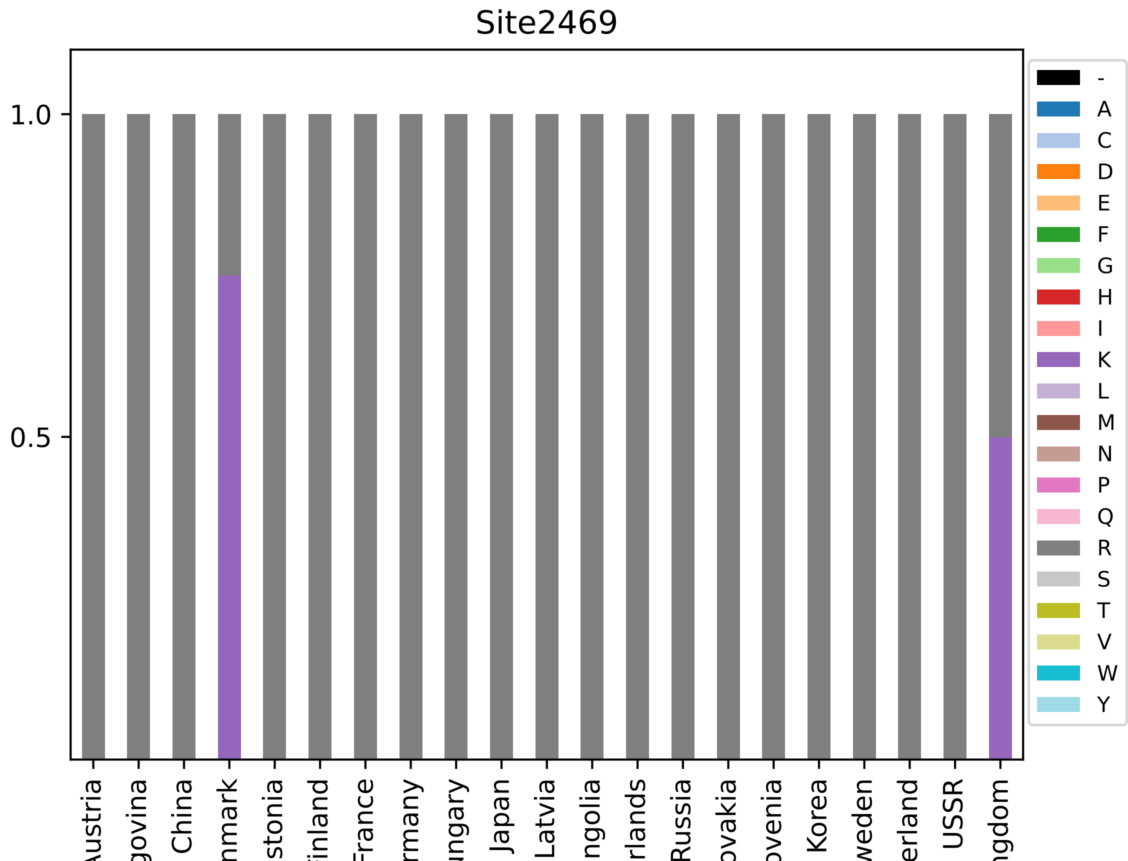

Site2469
1.0
0.5
-
A
C
D
E
F
G
H
I
K
L
M
N
P
Q
R
S
T
V
W
Y
Austria
govina
China
nmark
stonia
inland
France
rmany
ungary
Japan
Latvia
ngolia
rlands
Russia
ovakia
ovenia
Korea
weden
erland
USSR
ngdom

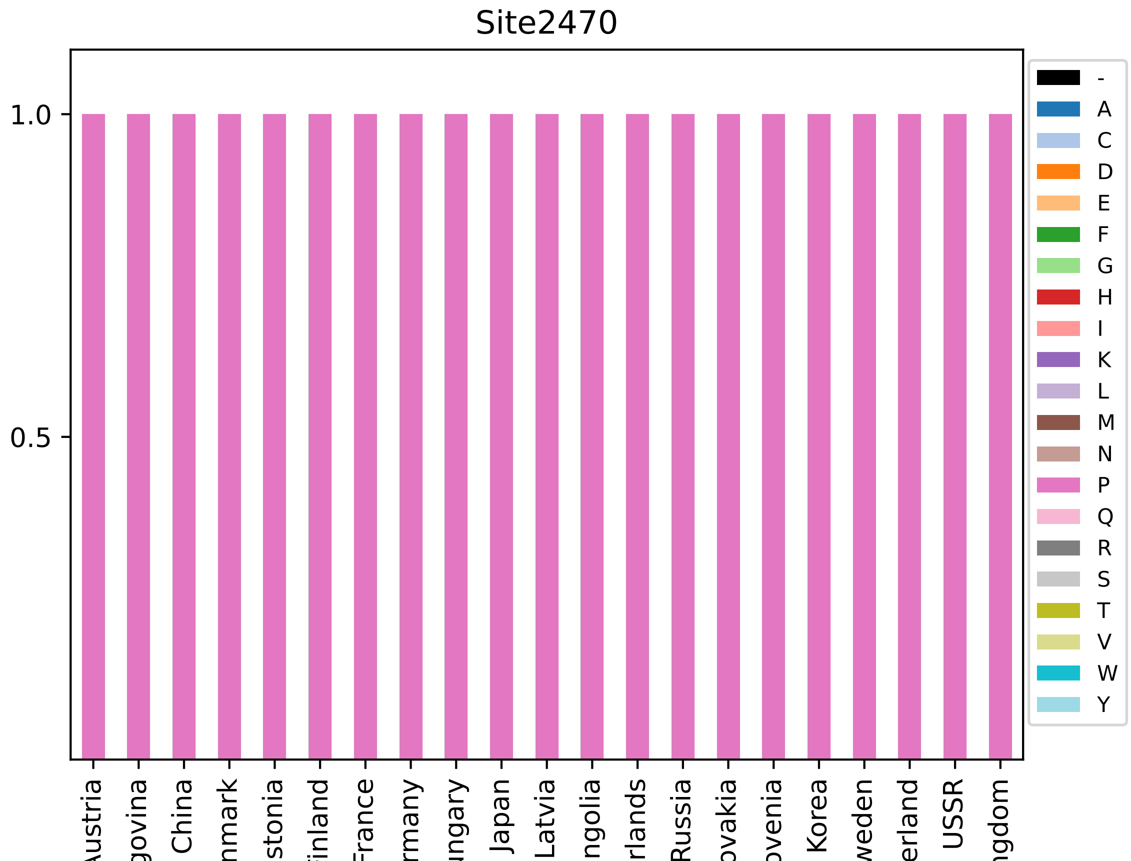

Site2470
1.0
0.5
-
A
C
D
E
F
G
H
I
K
L
M
N
P
Q
R
S
T
V
W
Y
Austria
China
Finland
Japan
Latvia
Russia
Korea
USSR

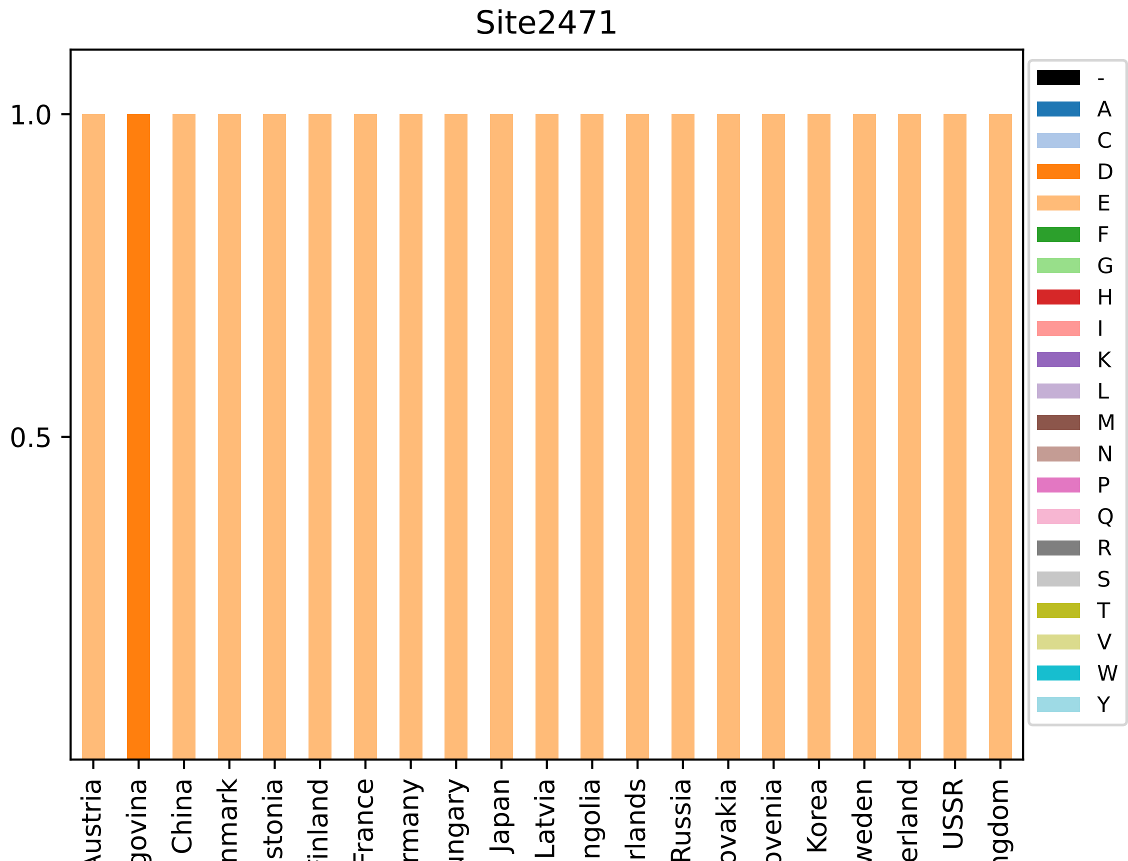
Site2471
1.0
0.5
-
A
C
D
E
F
G
H
I
K
L
M
N
P
Q
R
S
T
V
W
Y
China
Japan
Latvia
Russia
Korea
USSR

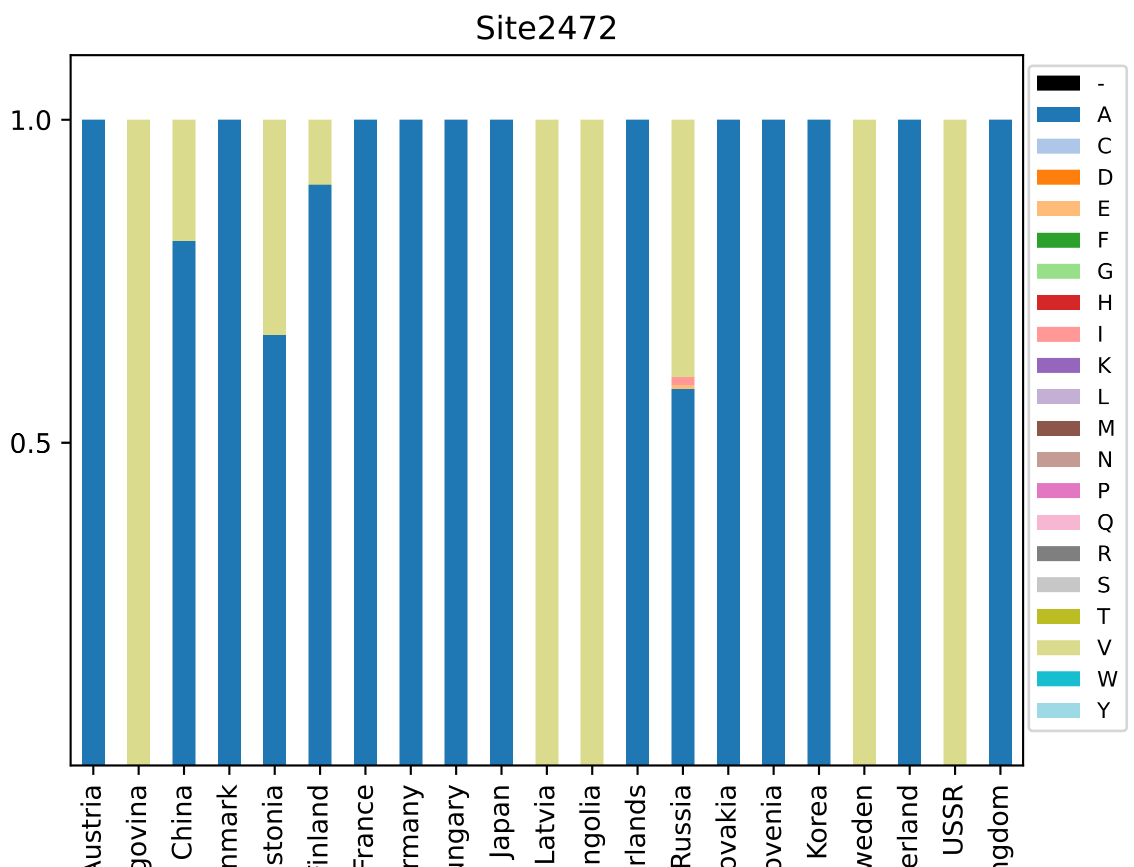

Site2472
1.0
0.5
-
A
C
D
E
F
G
H
I
K
L
M
N
P
Q
R
S
T
V
W
Y
Austria
China
Japan
Latvia
Russia
Korea
USSR

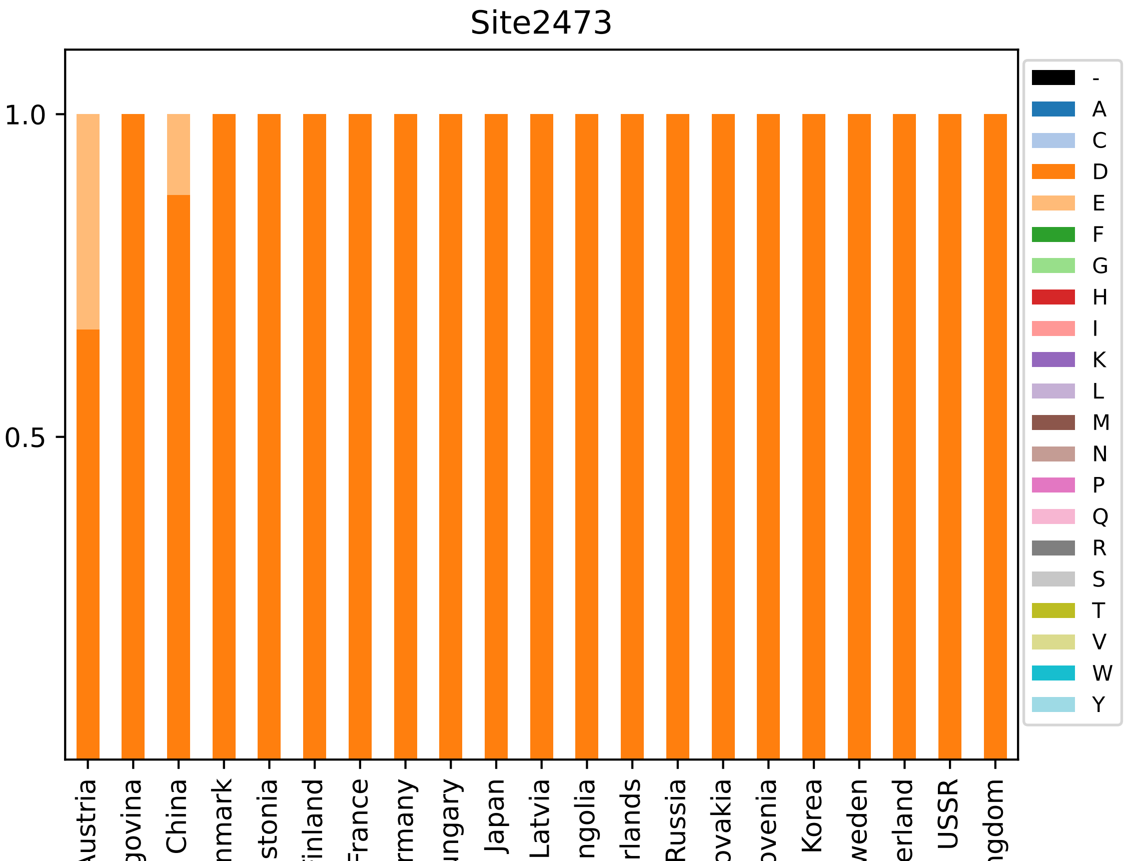

Site2473
1.0
0.5
-
A
C
D
E
F
G
H
I
K
L
M
N
P
Q
R
S
T
V
W
Y
China
Japan
Latvia
Russia
Korea
USSR

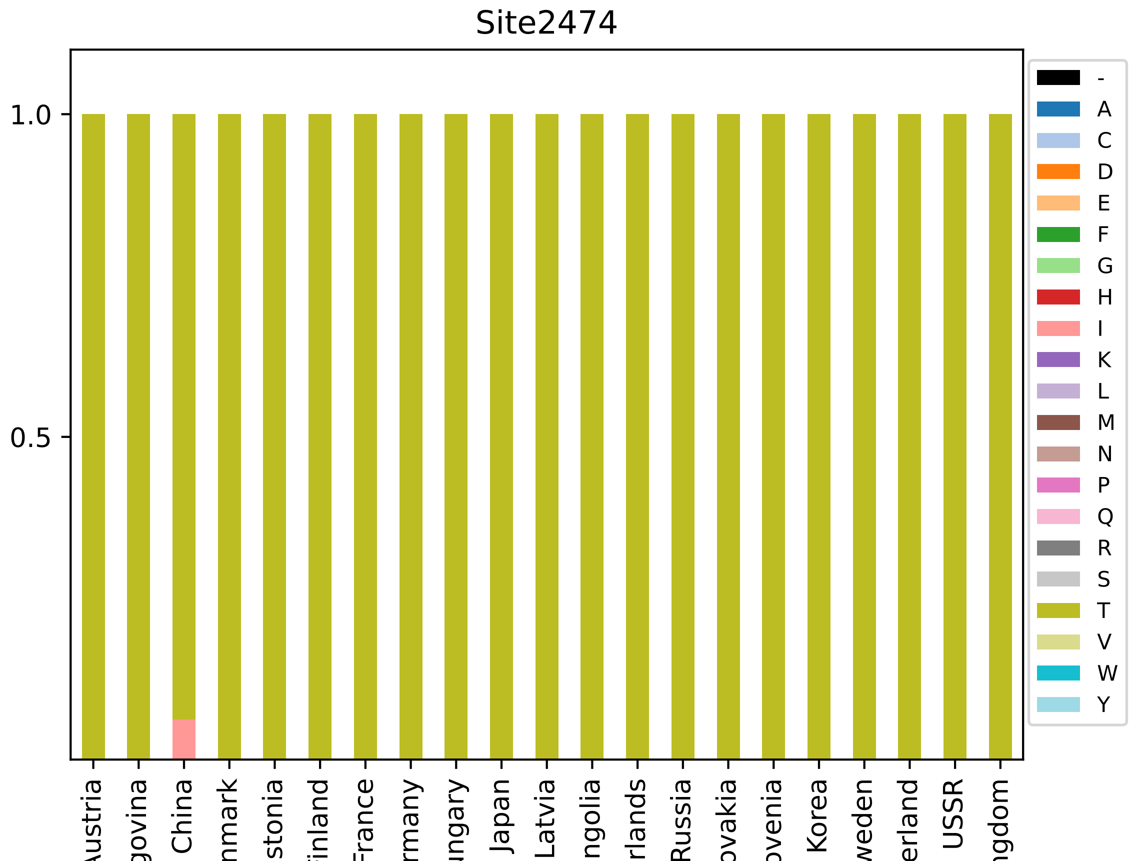
Site2474
1.0
0.5
-
A
C
D
E
F
G
H
I
K
L
M
N
P
Q
R
S
T
V
W
Y
China
Japan
Latvia
Russia
Korea
USSR

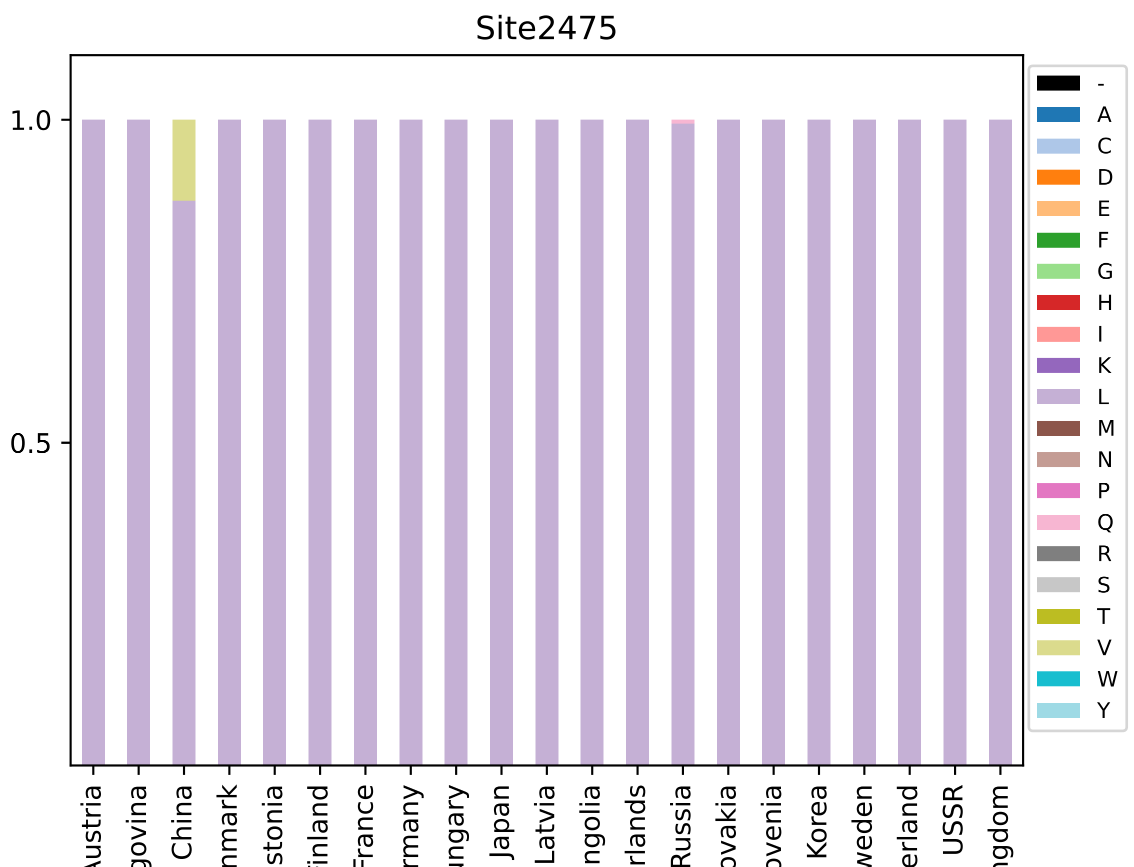
Site2475
1.0
0.5
-
A
C
D
E
F
G
H
I
K
L
M
N
P
Q
R
S
T
V
W
Y
China
Japan
Latvia
Russia
Korea
USSR

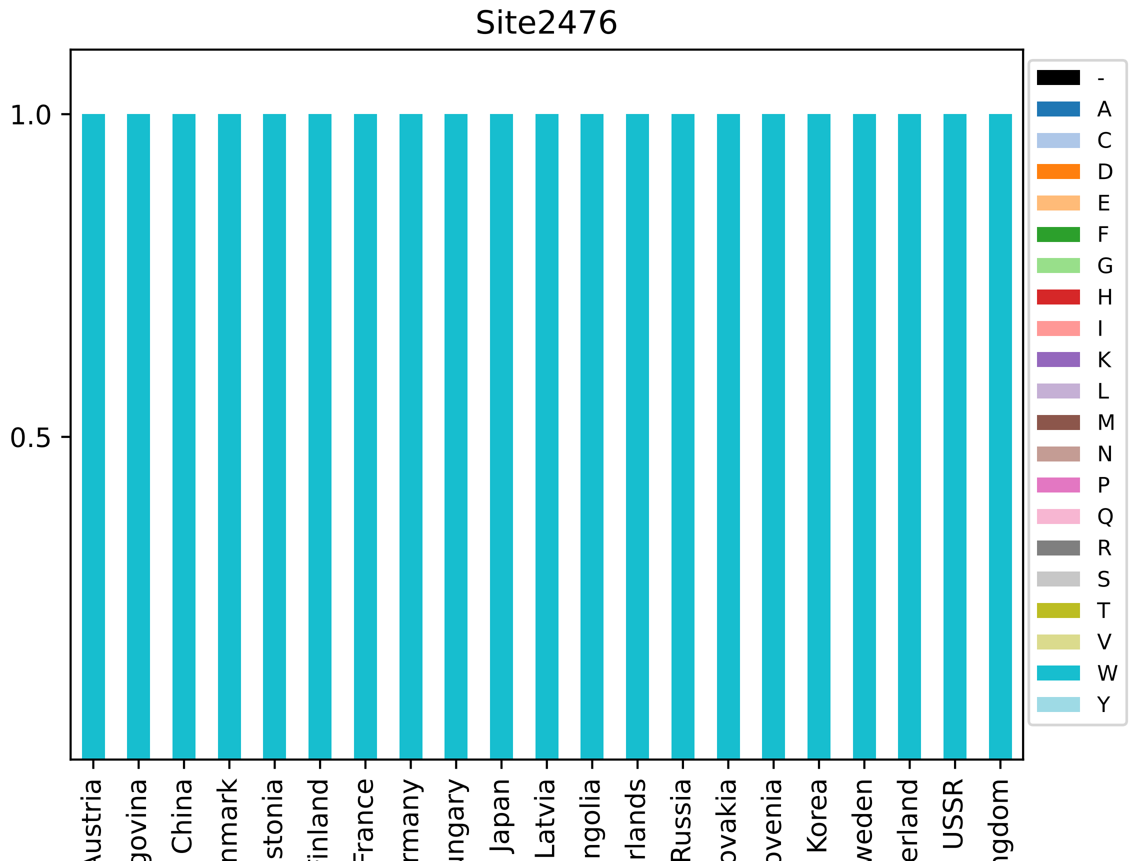
Site2476
1.0
0.5
-
A
C
D
E
F
G
H
I
K
L
M
N
P
Q
R
S
T
V
W
Y
Austria
govina
China
nmark
stonia
inland
France
rmany
ungary
Japan
Latvia
ngolia
rlands
Russia
ovakia
ovenia
Korea
weden
erland
USSR
ngdom

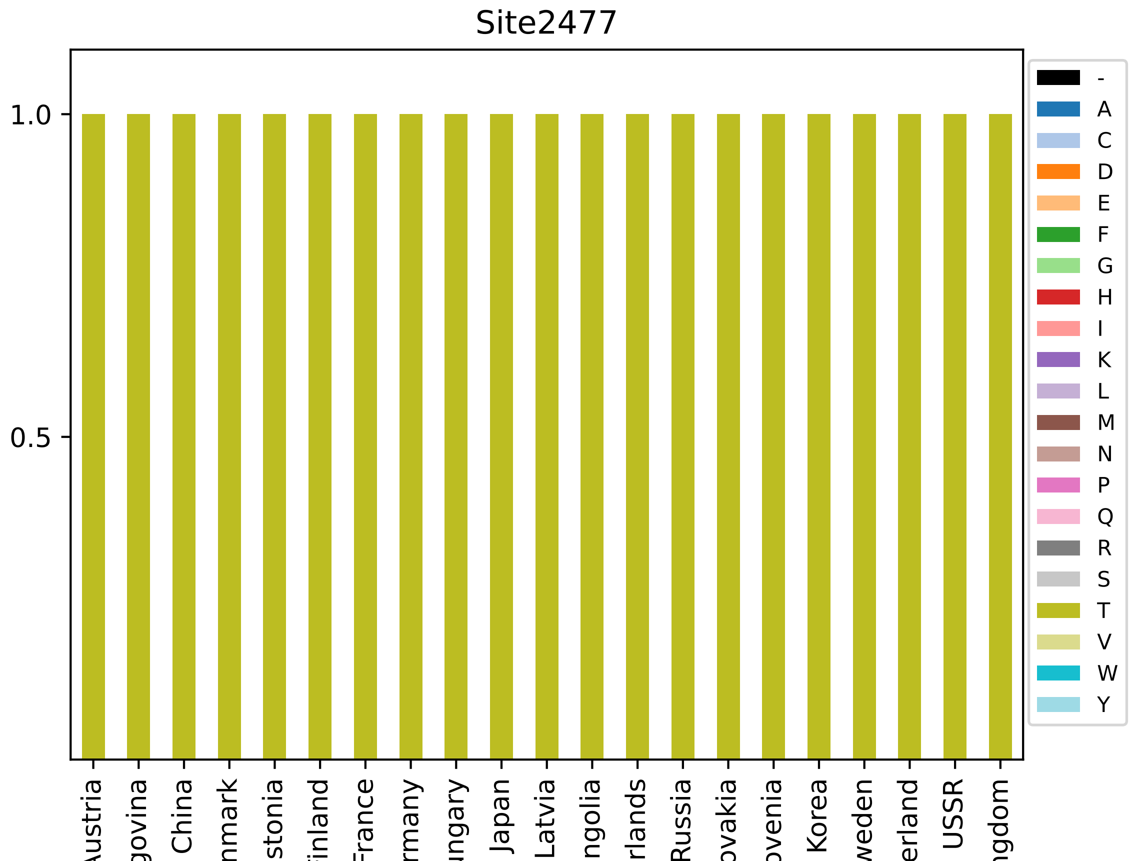
Site2477
1.0
0.5
-
A
C
D
E
F
G
H
I
K
L
M
N
P
Q
R
S
T
V
W
Y
China
Japan
Latvia
Russia
Korea
USSR

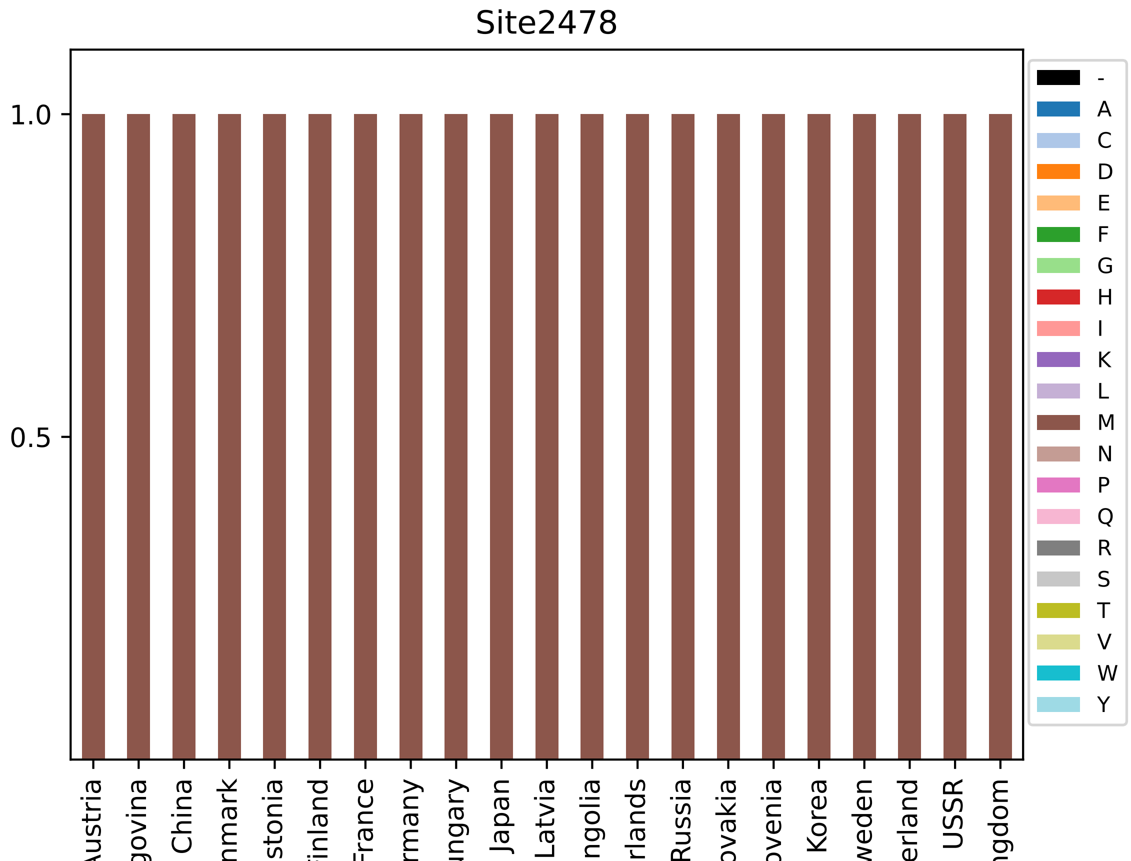

Site2478
1.0
0.5
-
A
C
D
E
F
G
H
I
K
L
M
N
P
Q
R
S
T
V
W
Y
ustria
govina
China
nmark
stonia
inland
rance
rmany
ungary
Japan
Latvia
ngolia
rlands
Russia
ovakia
ovenia
Korea
weden
erland
USSR
ngdom

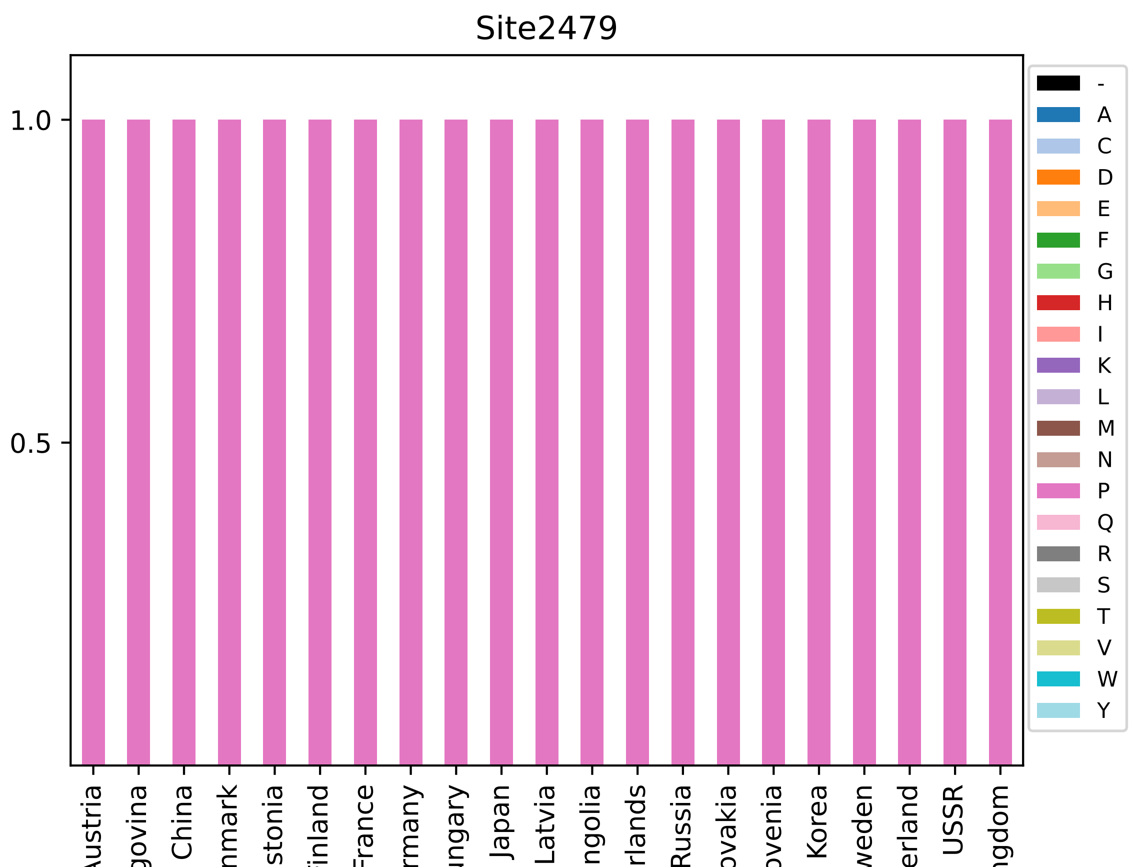

Site2479
1.0
0.5
-
A
C
D
E
F
G
H
I
K
L
M
N
P
Q
R
S
T
V
W
Y
Austria
govina
China
nmark
stonia
Finland
France
rmany
ungary
Japan
Latvia
ngolia
rlands
Russia
ovakia
ovenia
Korea
weden
erland
USSR
ngdom

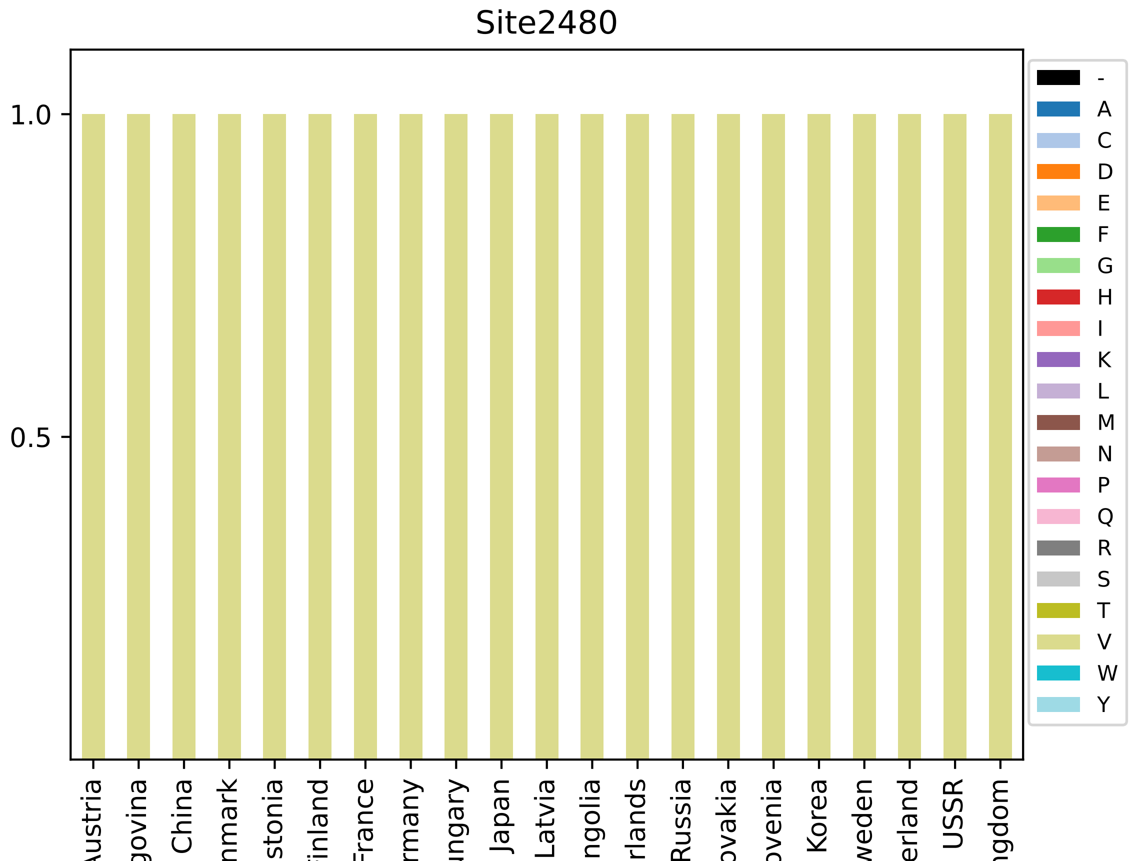

Site2480
1.0
0.5
-
A
C
D
E
F
G
H
I
K
L
M
N
P
Q
R
S
T
V
W
Y
Austria
govina
China
nmark
stonia
Finland
France
rmany
ungary
Japan
Latvia
ngolia
rlands
Russia
ovakia
ovenia
Korea
weden
erland
USSR
ngdom

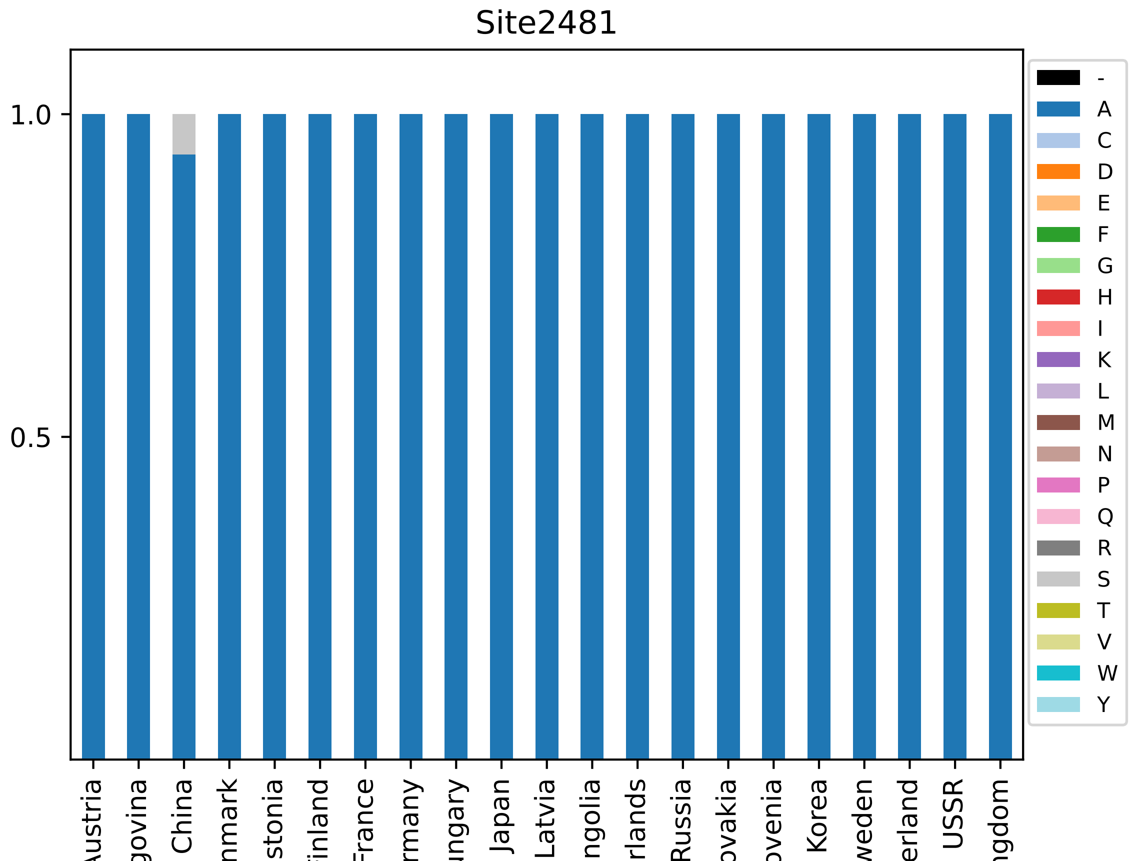

Site2481
1.0
0.5
-
A
C
D
E
F
G
H
I
K
L
M
N
P
Q
R
S
T
V
W
Y
Austria
govina
China
nmark
stonia
Finland
France
rmany
ungary
Japan
Latvia
ngolia
rlands
Russia
ovakia
ovenia
Korea
weden
erland
USSR
ngdom

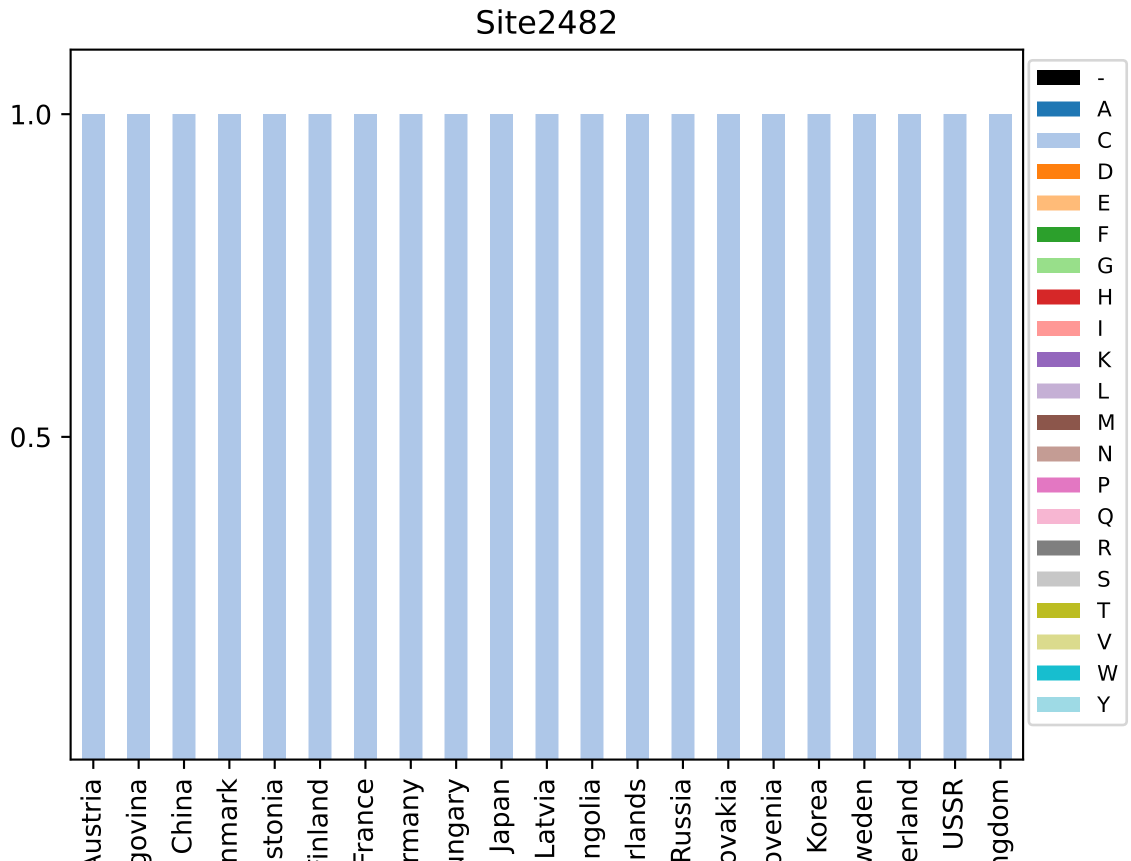
Site2482
1.0
0.5
-
A
C
D
E
F
G
H
I
K
L
M
N
P
Q
R
S
T
V
W
Y
China
Japan
Latvia
Russia
Korea
USSR

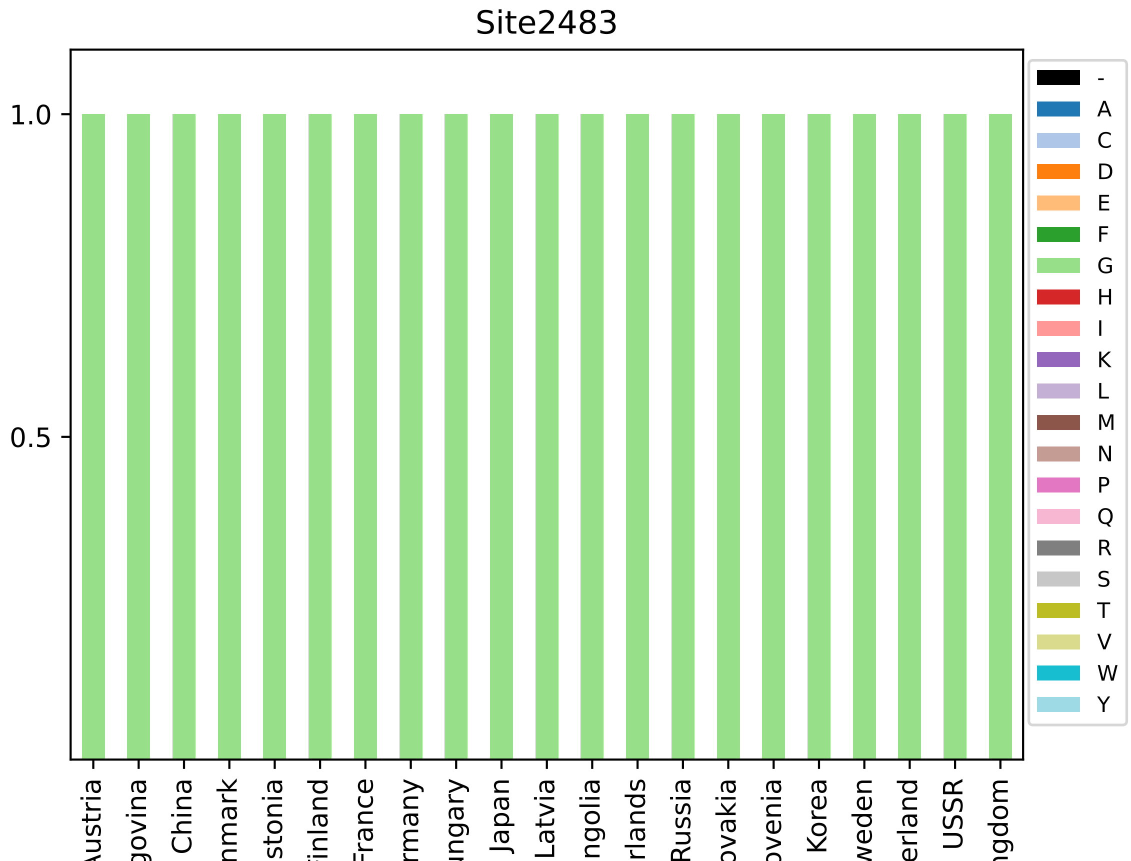

Site2483
1.0
0.5
-
A
C
D
E
F
G
H
I
K
L
M
N
P
Q
R
S
T
V
W
Y
China
Japan
Latvia
Russia
Korea
USSR

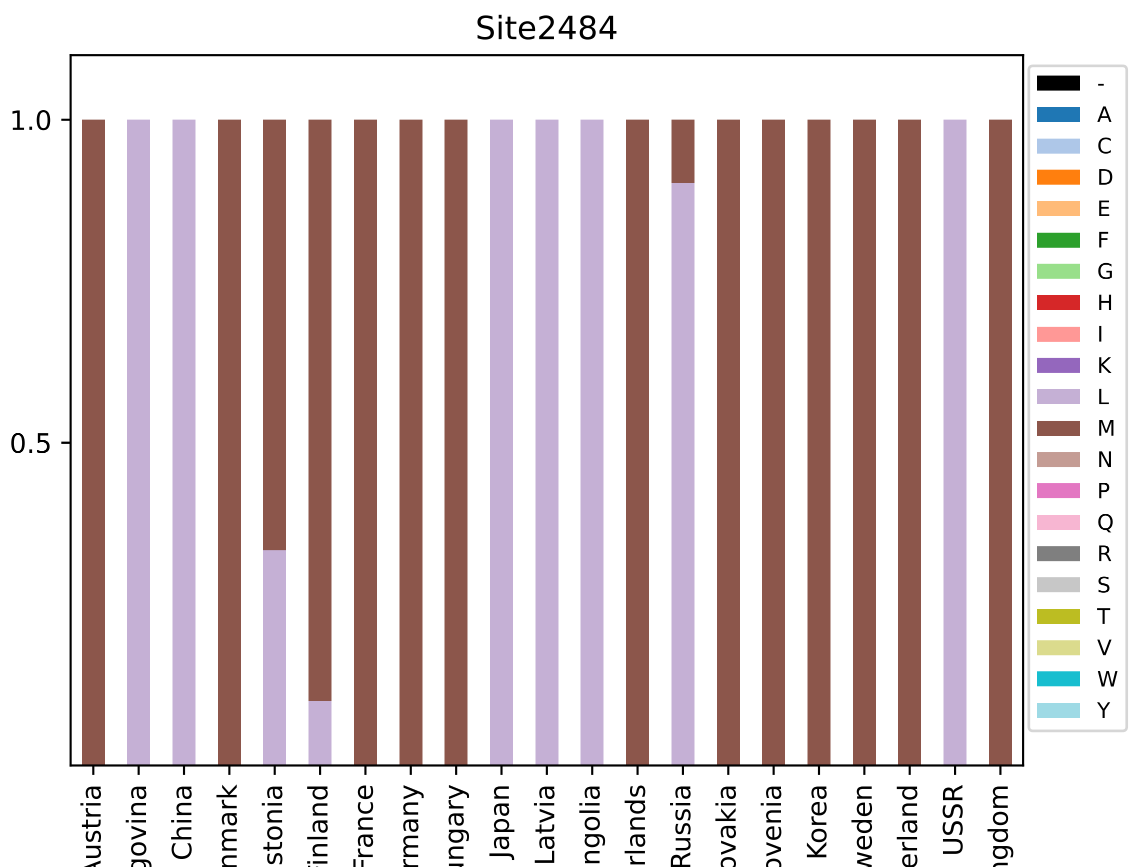
Site2484
1.0
0.5
Austria
govina
China
nmark
stonia
Finland
France
rmany
ungary
Japan
Latvia
ngolia
rlands
Russia
ovakia
ovenia
Korea
weden
erland
USSR
ngdom
-
A
C
D
E
F
G
H
I
K
L
M
N
P
Q
R
S
T
V
W
Y

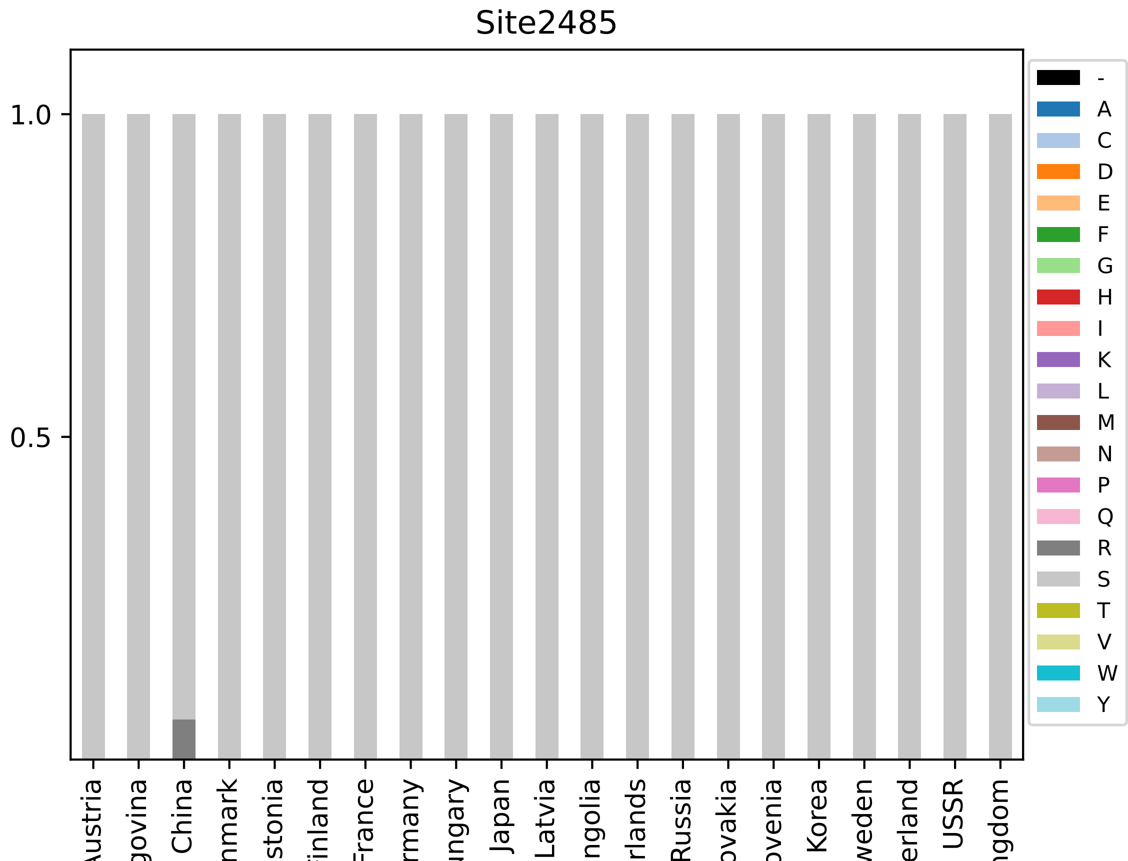
Site2485
1.0
0.5
-
A
C
D
E
F
G
H
I
K
L
M
N
P
Q
R
S
T
V
W
Y
China
Japan
Latvia
Russia
Korea
USSR

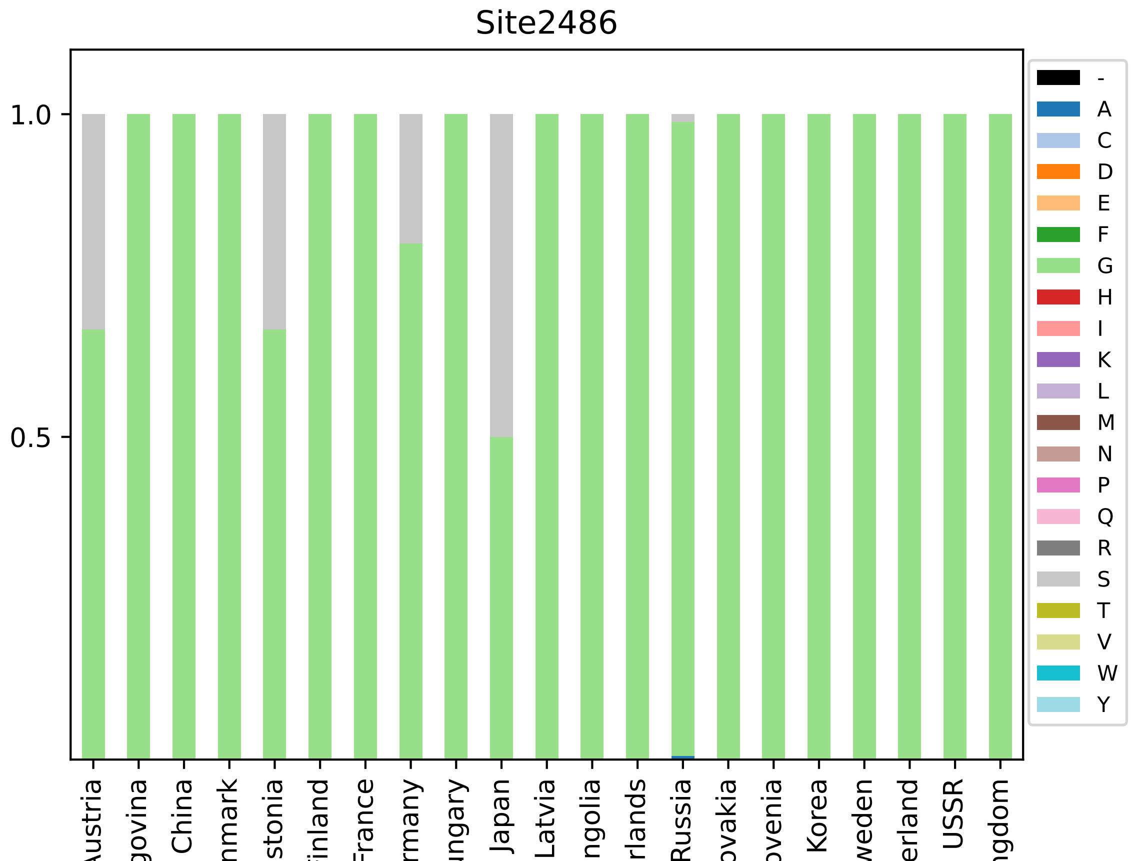
Site2486
1.0
0.5
-
A
C
D
E
F
G
H
I
K
L
M
N
P
Q
R
S
T
V
W
Y
Austria
govina
China
nmark
stonia
Finland
France
rmany
ungary
Japan
Latvia
ngolia
rlands
Russia
ovakia
ovenia
Korea
weden
erland
USSR
ngdom

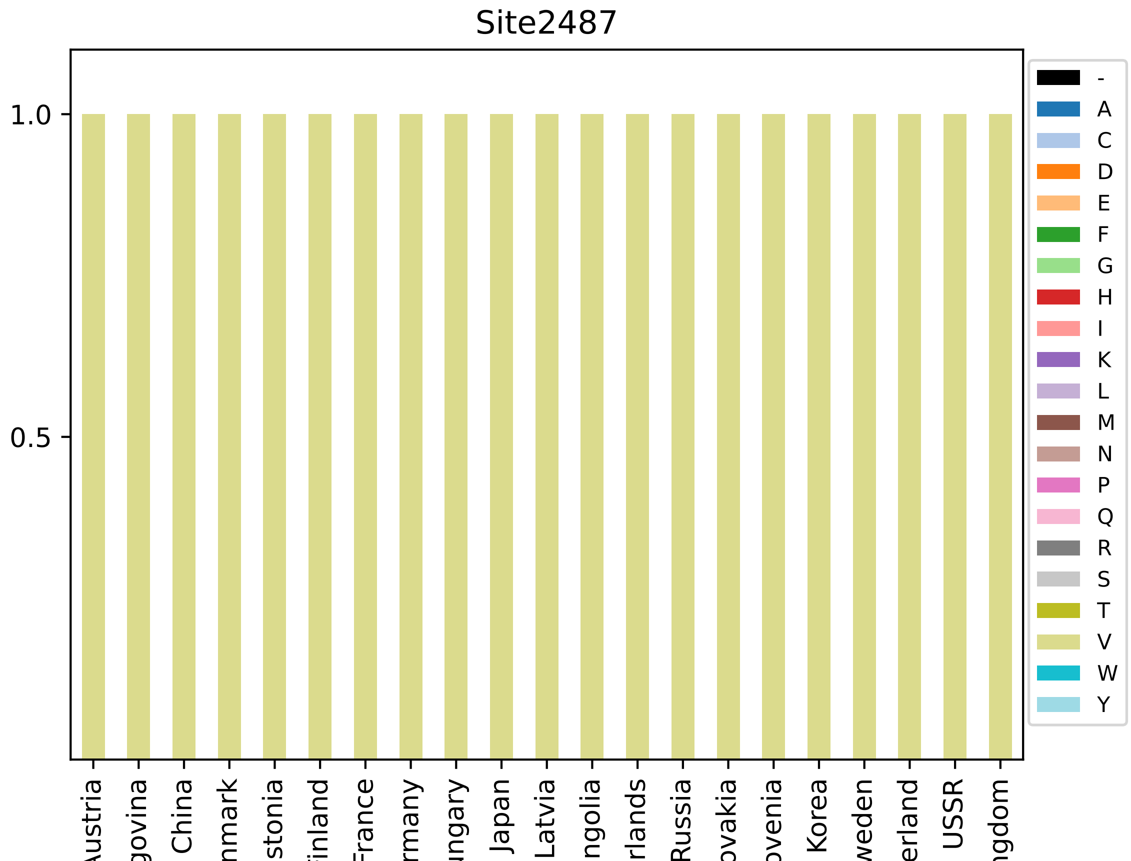

Site2487
1.0
0.5
-
A
C
D
E
F
G
H
I
K
L
M
N
P
Q
R
S
T
V
W
Y
China
Japan
Latvia
Russia
Korea
USSR

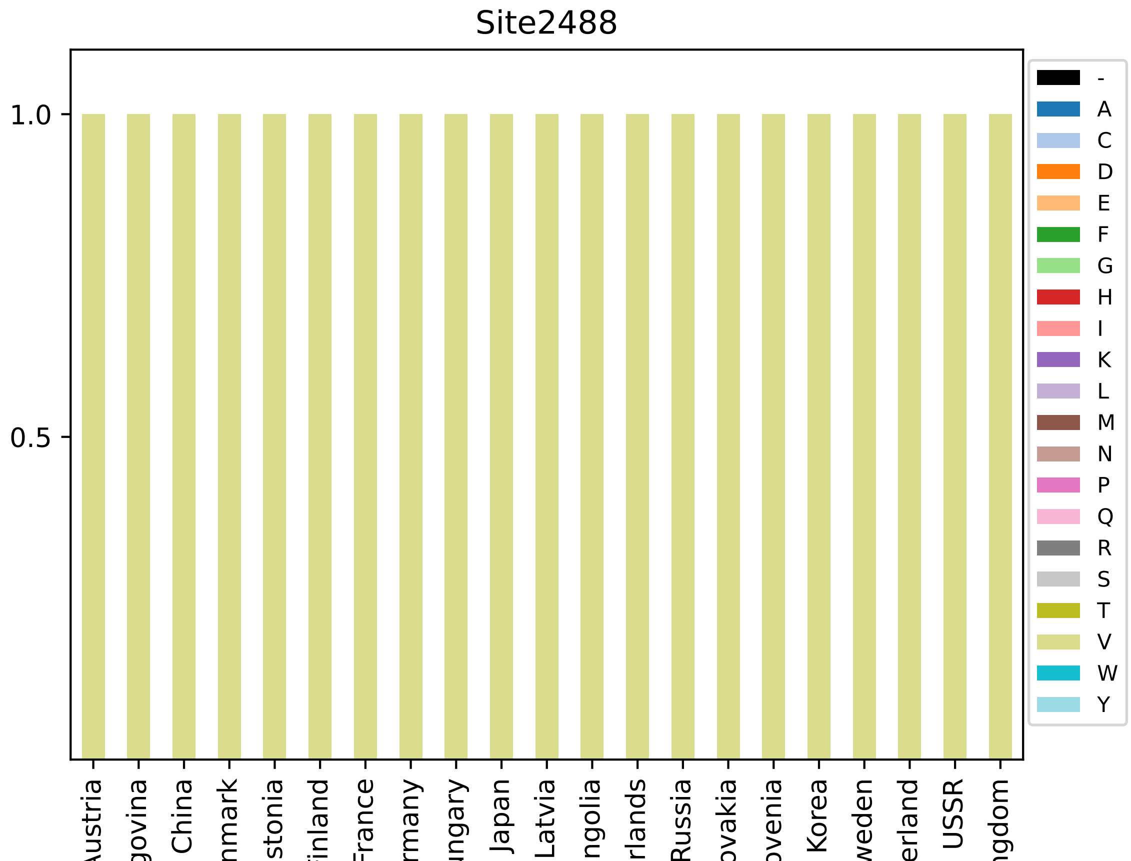
Site2488
1.0
0.5
Austria
govina
China
nmark
stonia
Finland
France
rmany
ungary
Japan
Latvia
ngolia
rlands
Russia
ovakia
ovenia
Korea
weden
erland
USSR
ngdom
-
A
C
D
E
F
G
H
I
K
L
M
N
P
Q
R
S
T
V
W
Y

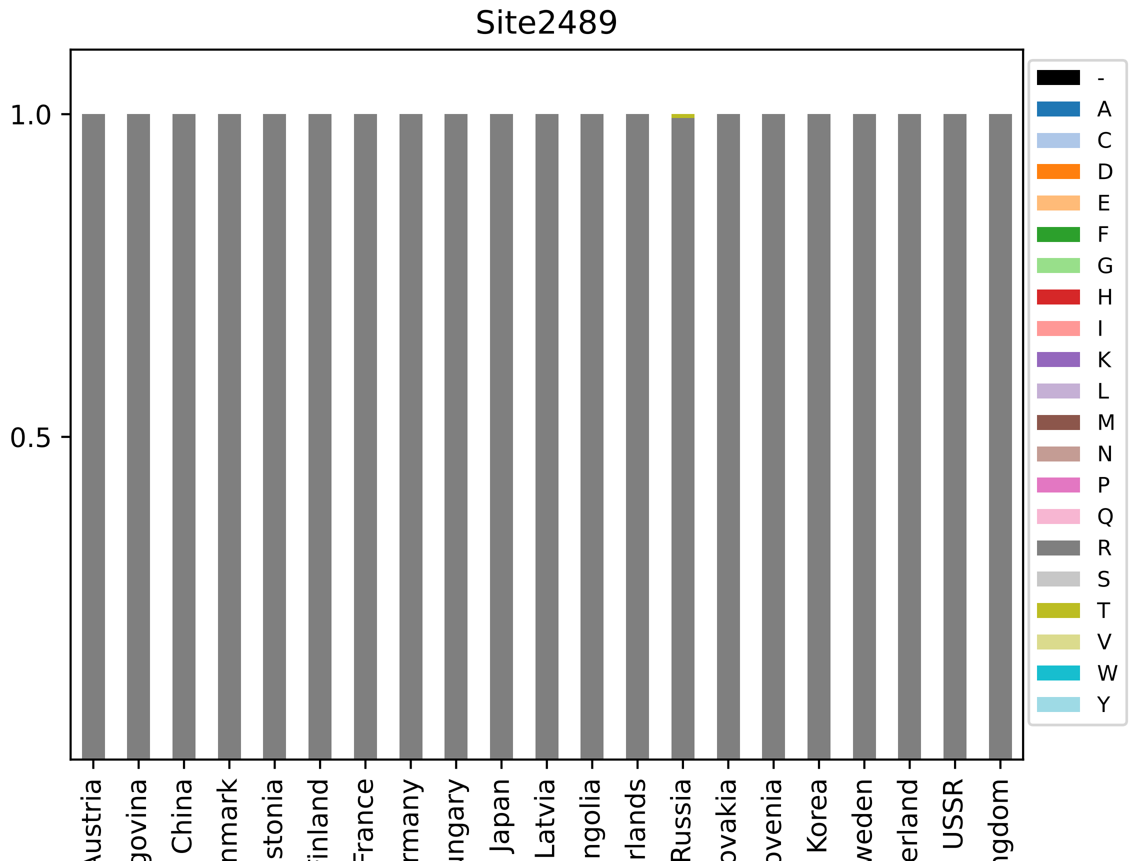

Site2489
1.0
0.5
-
A
C
D
E
F
G
H
I
K
L
M
N
P
Q
R
S
T
V
W
Y
Austria
govina
China
nmark
stonia
Finland
France
rmany
ungary
Japan
Latvia
ngolia
rlands
Russia
ovakia
ovenia
Korea
weden
erland
USSR
ngdom

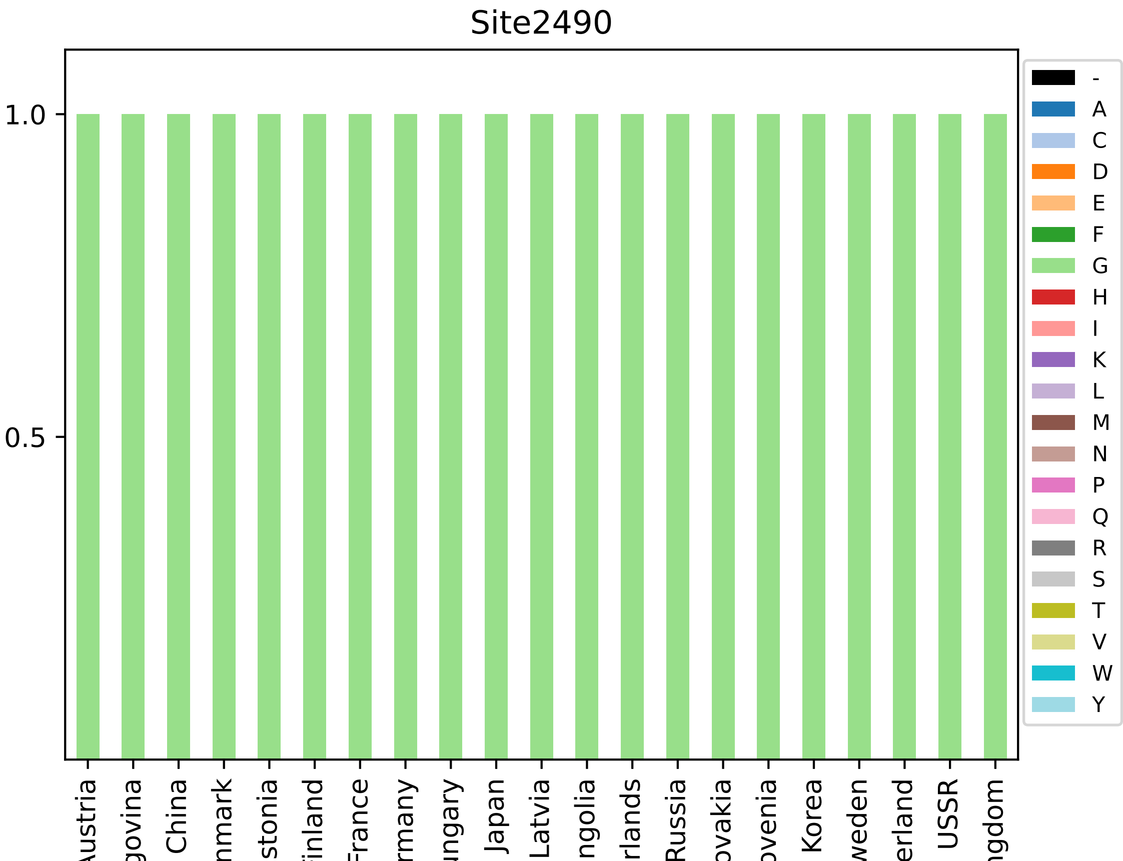
Site2490
1.0
0.5
-
A
C
D
E
F
G
H
I
K
L
M
N
P
Q
R
S
T
V
W
Y
China
Japan
Latvia
Russia
Korea
USSR

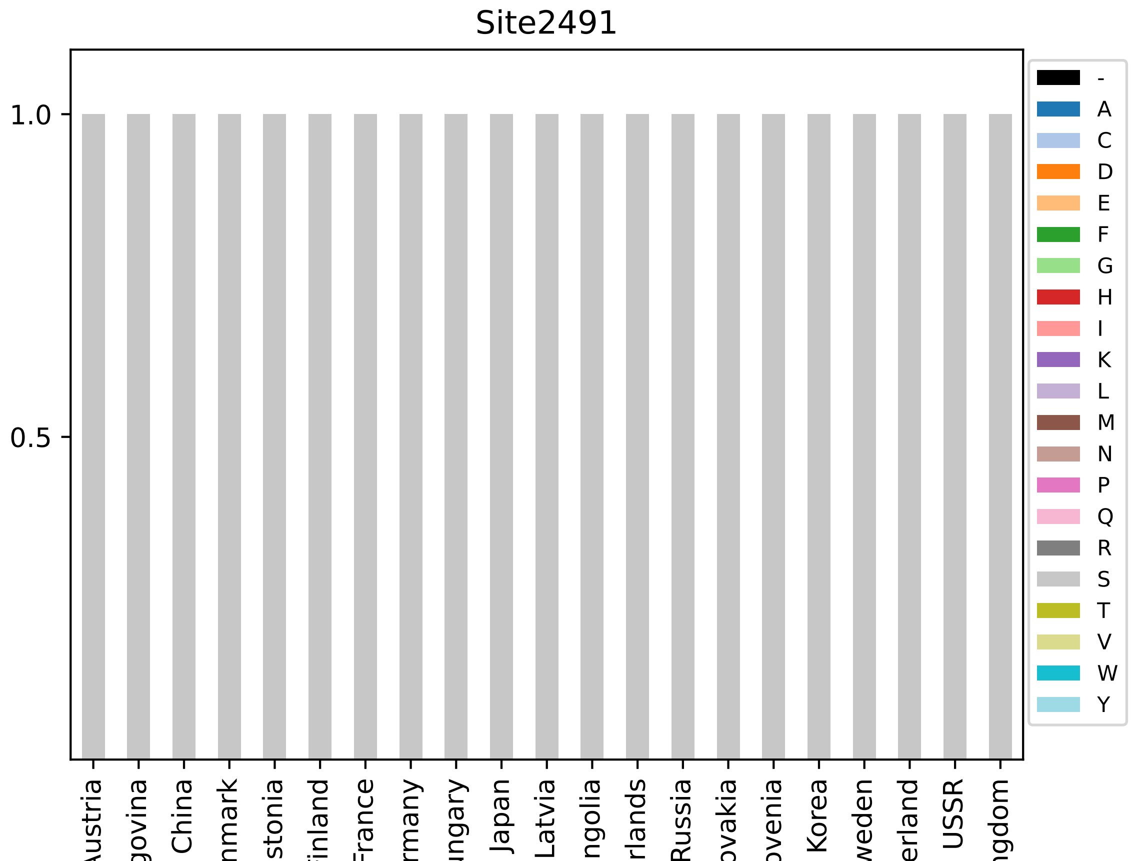

Site2491
1.0
0.5
-
A
C
D
E
F
G
H
I
K
L
M
N
P
Q
R
S
T
V
W
Y
Austria
China
Japan
Latvia
Russia
Korea
USSR

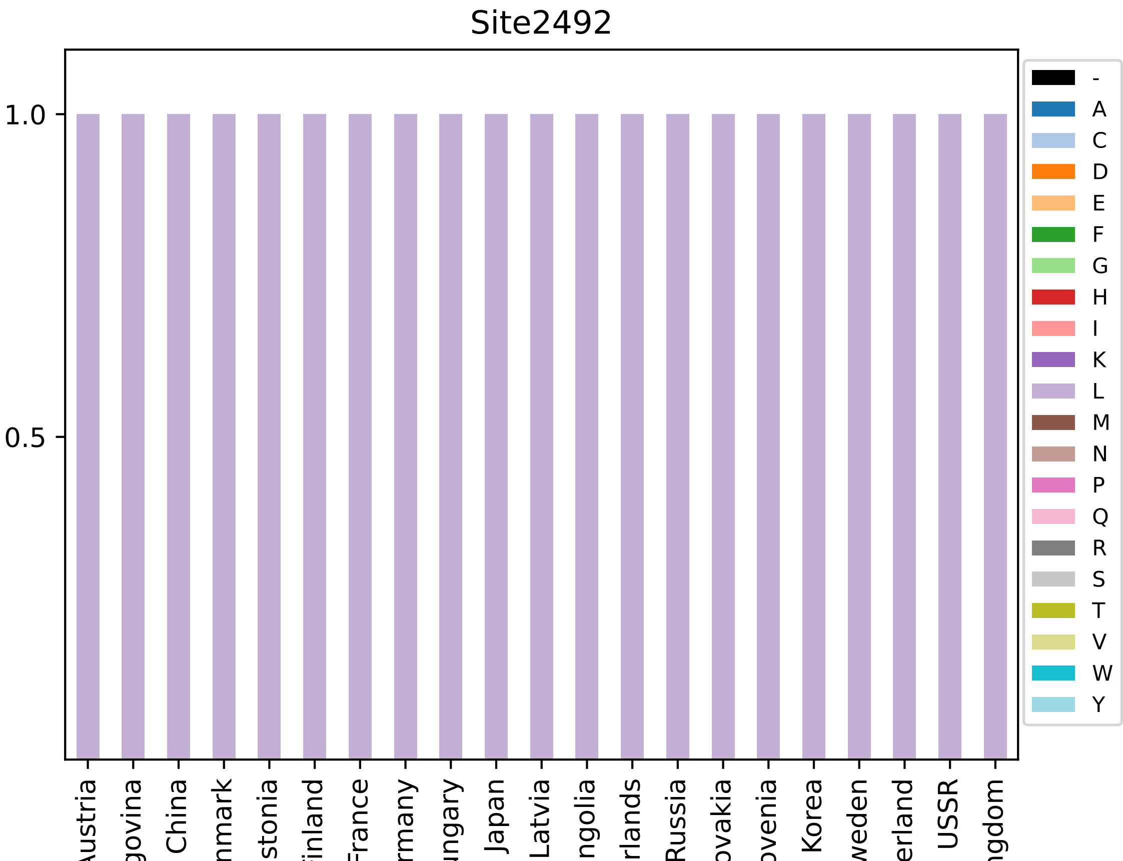

Site2492
1.0
0.5
-
A
C
D
E
F
G
H
I
K
L
M
N
P
Q
R
S
T
V
W
Y
China
Japan
Latvia
Russia
Korea
USSR

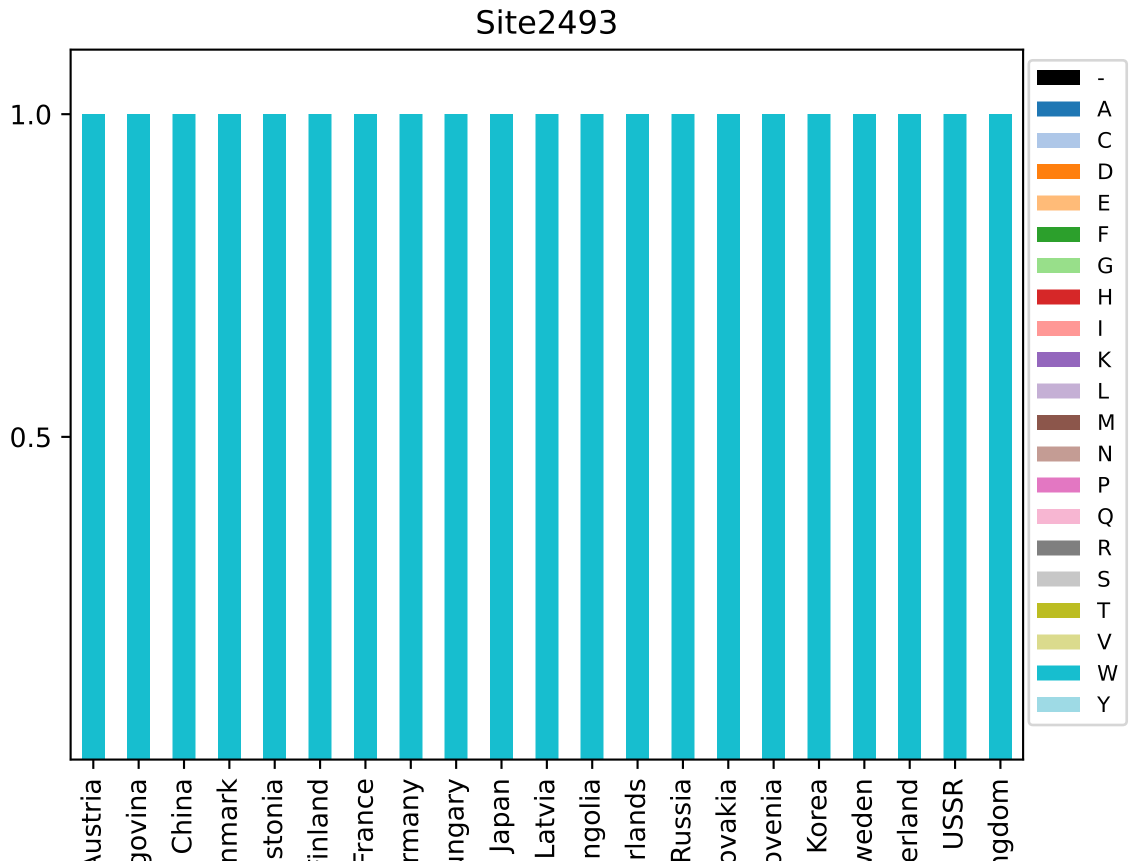
Site2493
1.0
0.5
-
A
C
D
E
F
G
H
I
K
L
M
N
P
Q
R
S
T
V
W
Y
China
Japan
Latvia
Russia
Korea
USSR

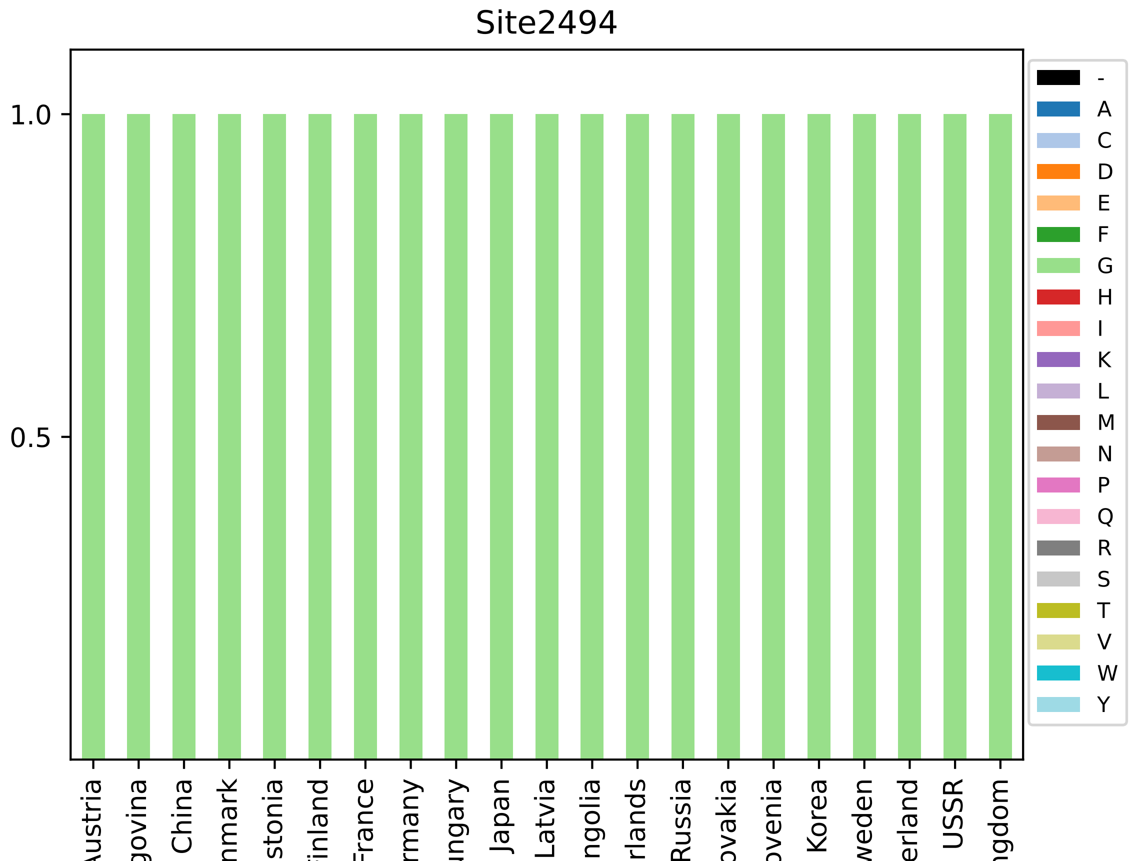

Site2494
1.0
0.5
-
A
C
D
E
F
G
H
I
K
L
M
N
P
Q
R
S
T
V
W
Y
Austria
China
Japan
Latvia
Russia
Korea
USSR

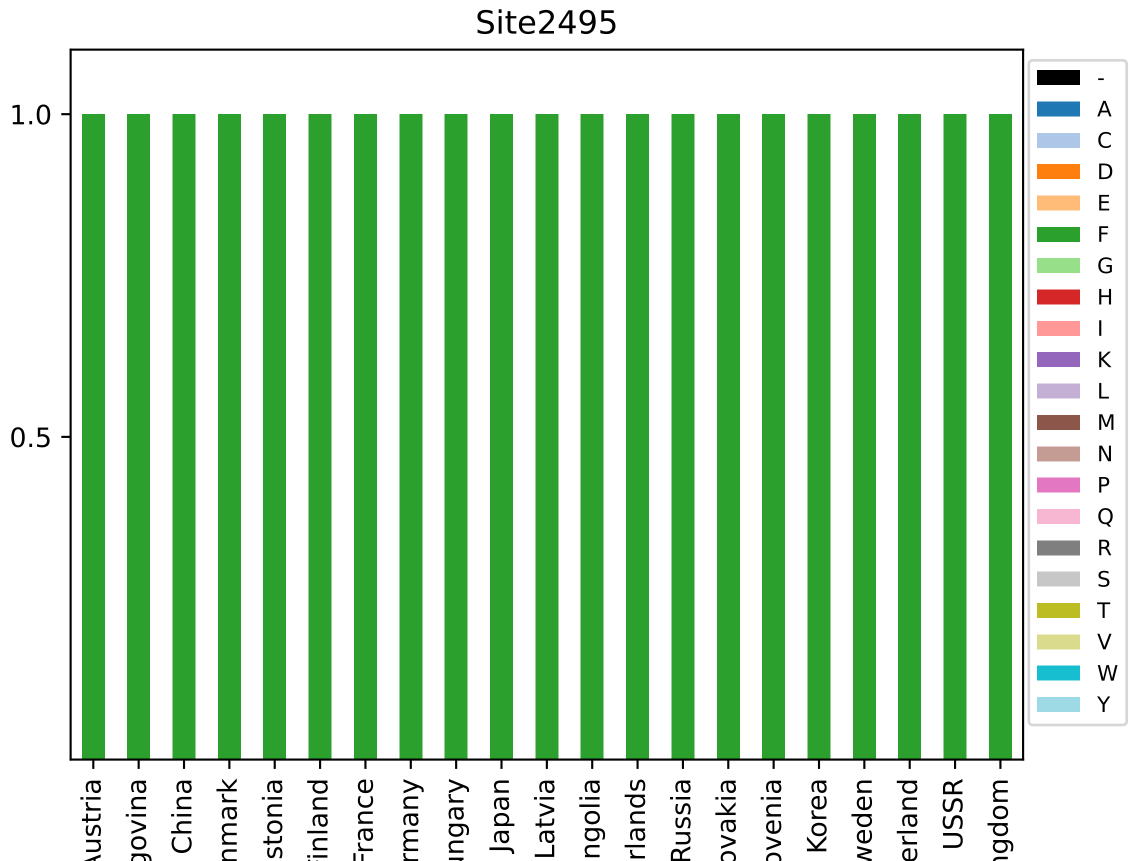
Site2495
1.0
0.5
-
A
C
D
E
F
G
H
I
K
L
M
N
P
Q
R
S
T
V
W
Y
China
Japan
Latvia
Russia
Korea
USSR

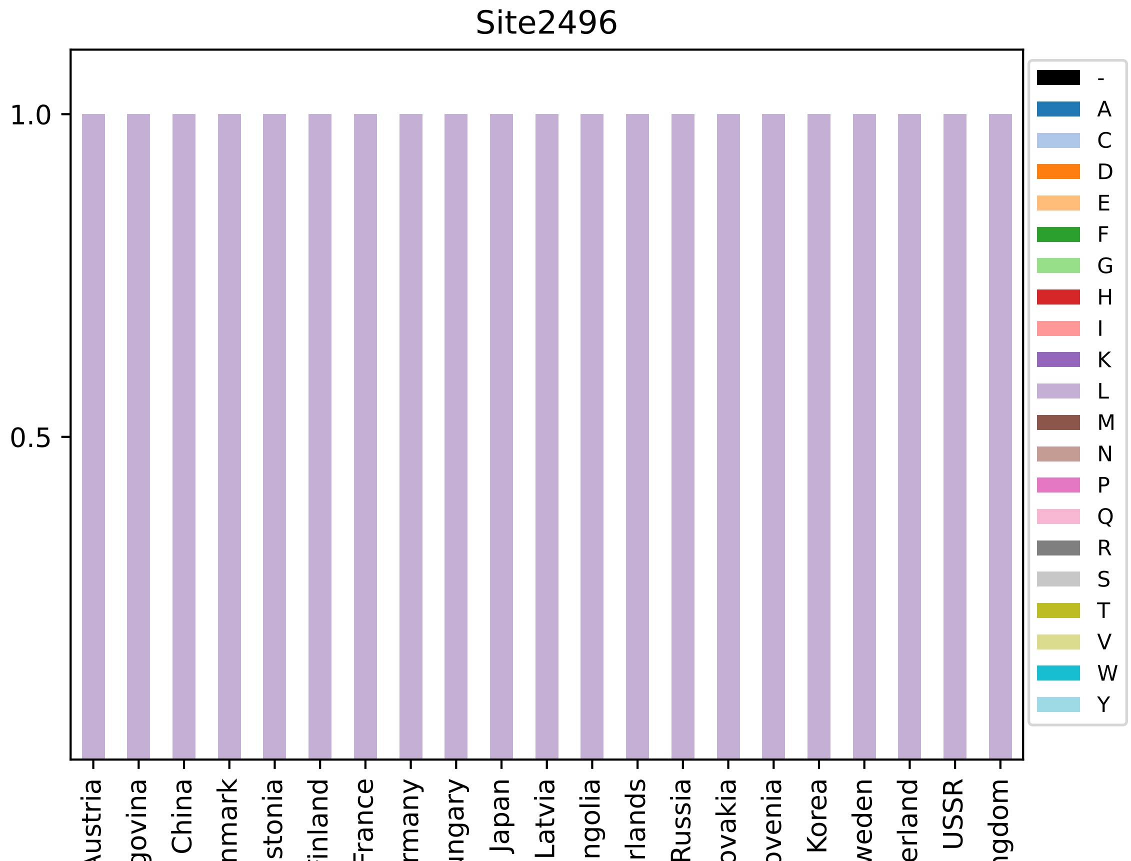

Site2496
1.0
0.5
-
A
C
D
E
F
G
H
I
K
L
M
N
P
Q
R
S
T
V
W
Y
China
Japan
Latvia
Russia
Korea
USSR

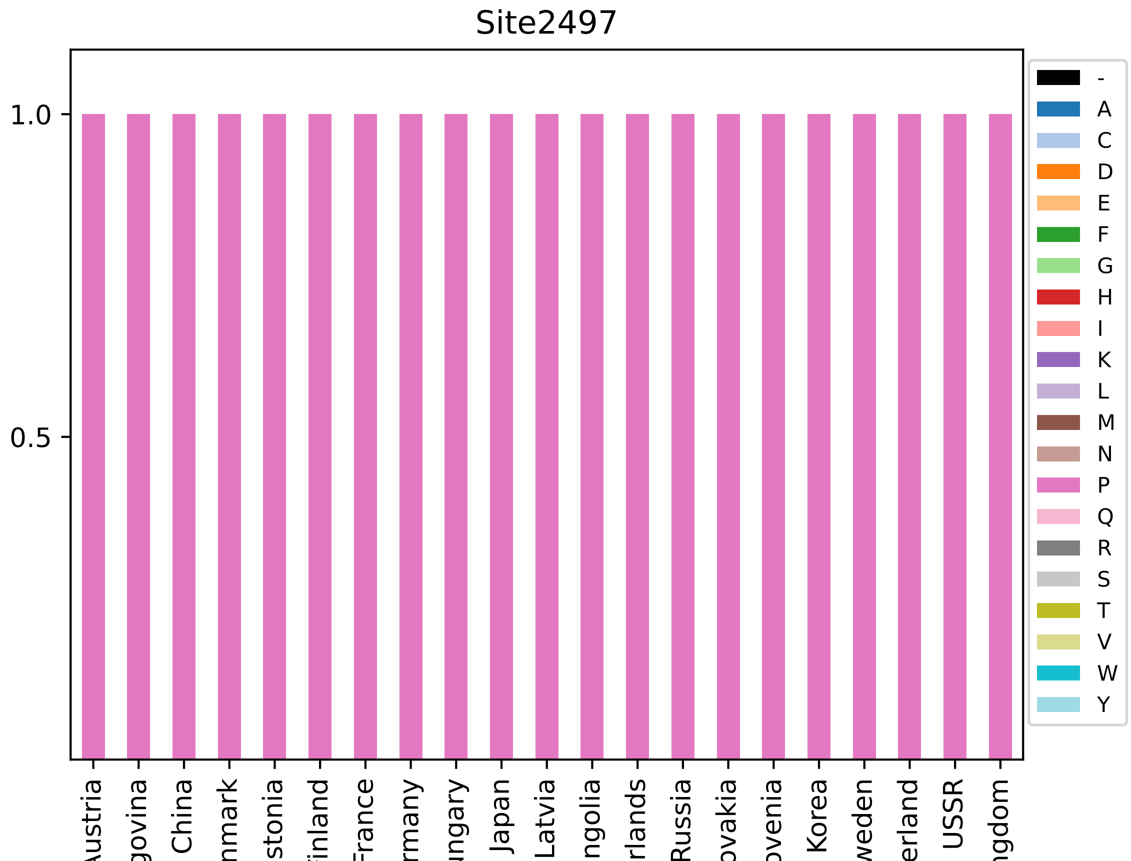
Site2497
1.0
0.5
-
A
C
D
E
F
G
H
I
K
L
M
N
P
Q
R
S
T
V
W
Y
Austria
China
Japan
Latvia
Russia
Korea
USSR

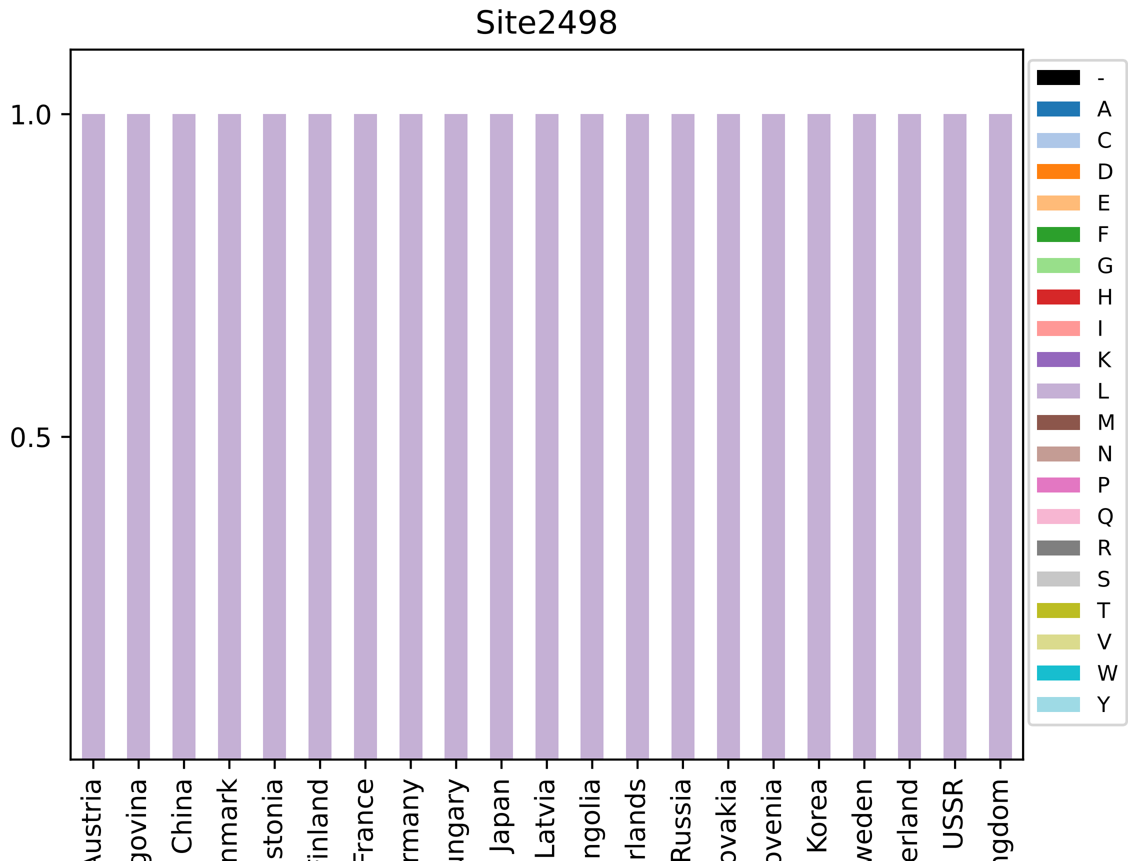
Site2498
1.0
0.5
-
A
C
D
E
F
G
H
I
K
L
M
N
P
Q
R
S
T
V
W
Y
Austria
govina
China
nmark
stonia
Finland
France
rmany
ungary
Japan
Latvia
ngolia
rlands
Russia
ovakia
ovenia
Korea
weden
erland
USSR
ngdom

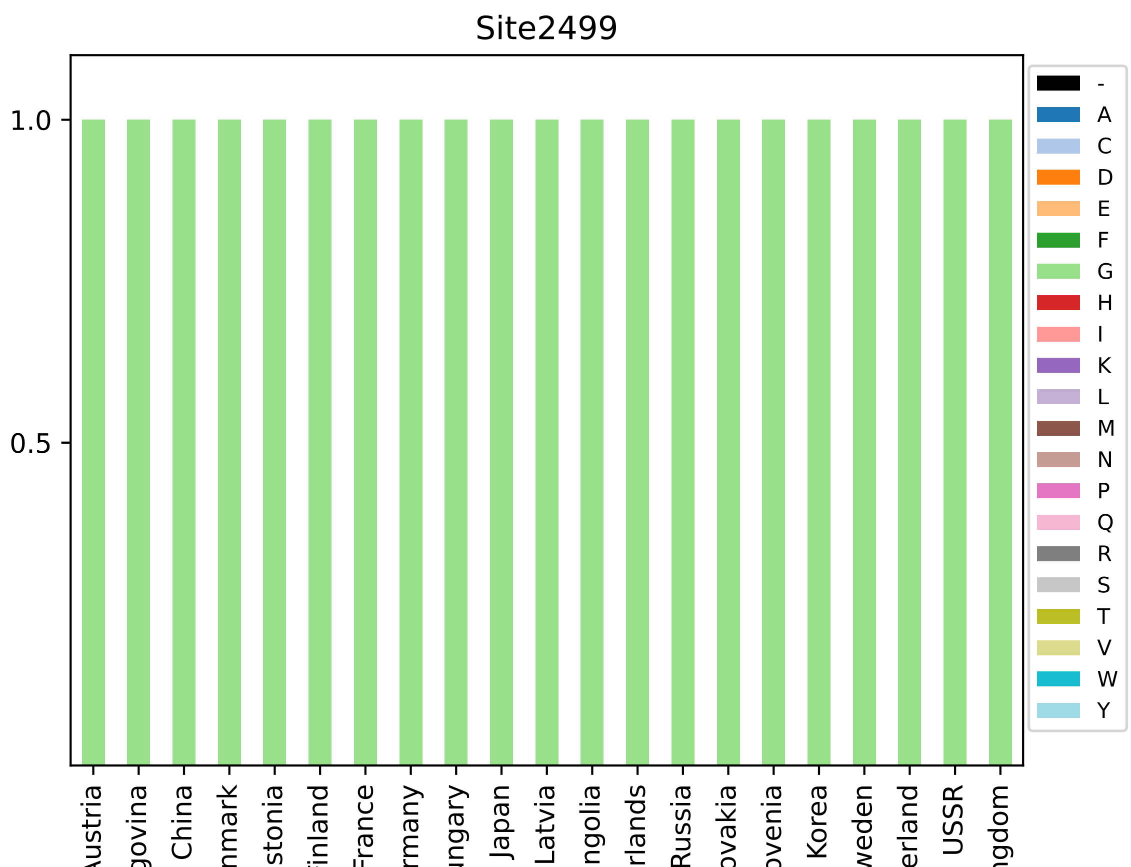

Site2499
1.0
0.5
-
A
C
D
E
F
G
H
I
K
L
M
N
P
Q
R
S
T
V
W
Y
China
Japan
Latvia
Russia
Korea
USSR

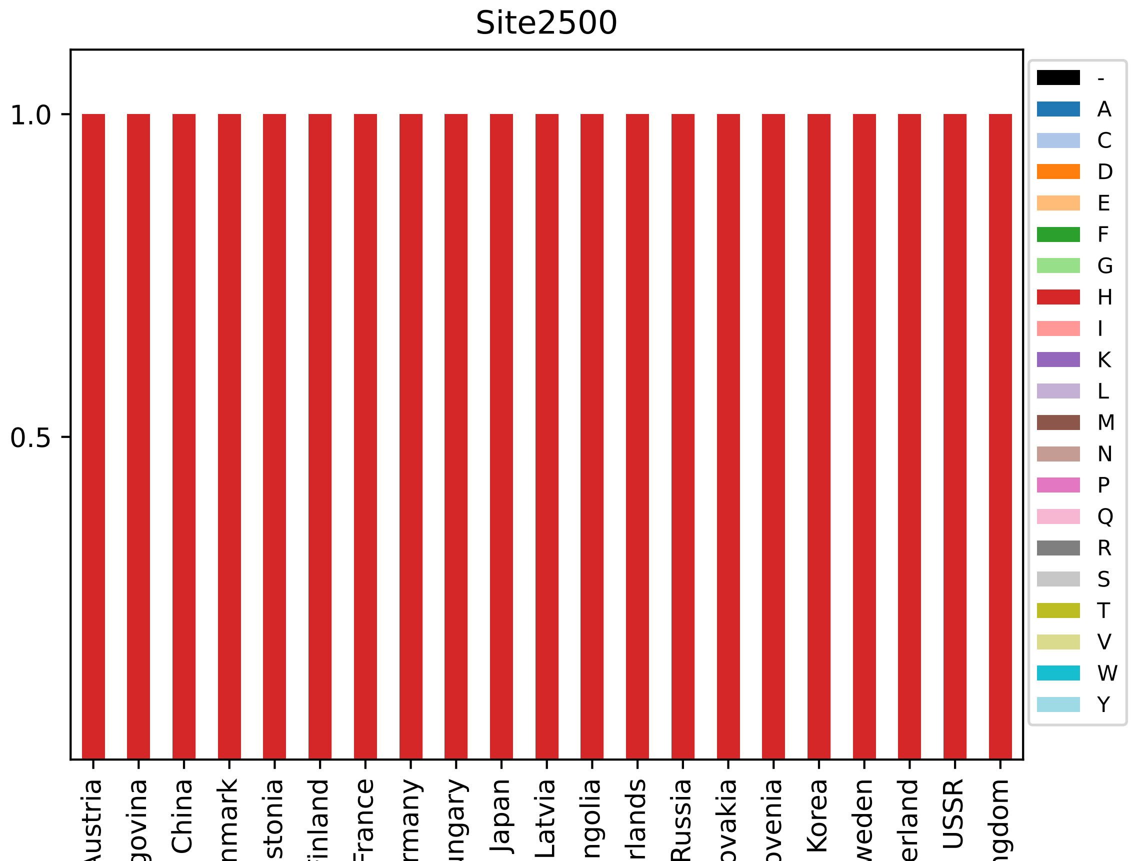

Site2500
1.0
0.5
-
A
C
D
E
F
G
H
I
K
L
M
N
P
Q
R
S
T
V
W
Y
China
Japan
Latvia
Russia
Korea
USSR

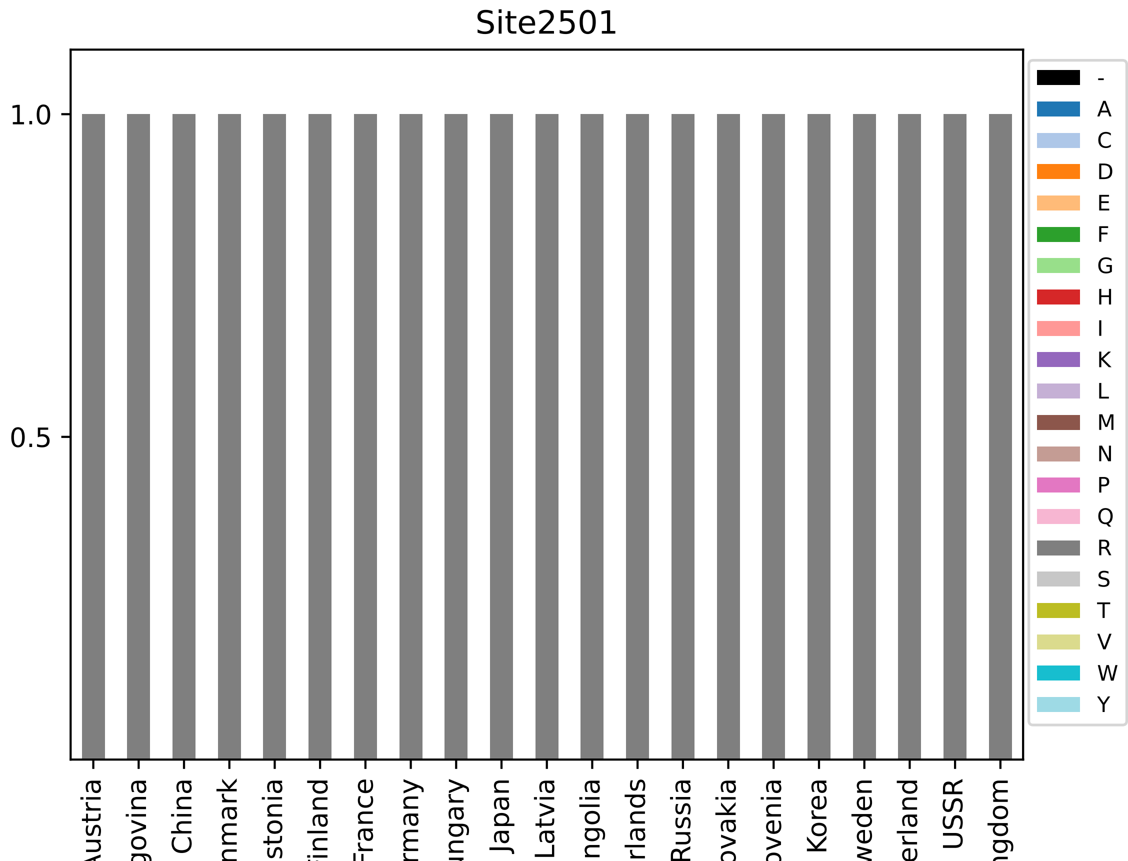

Site2501
1.0
0.5
-
A
C
D
E
F
G
H
I
K
L
M
N
P
Q
R
S
T
V
W
Y
China
Japan
Latvia
Russia
Korea
USSR

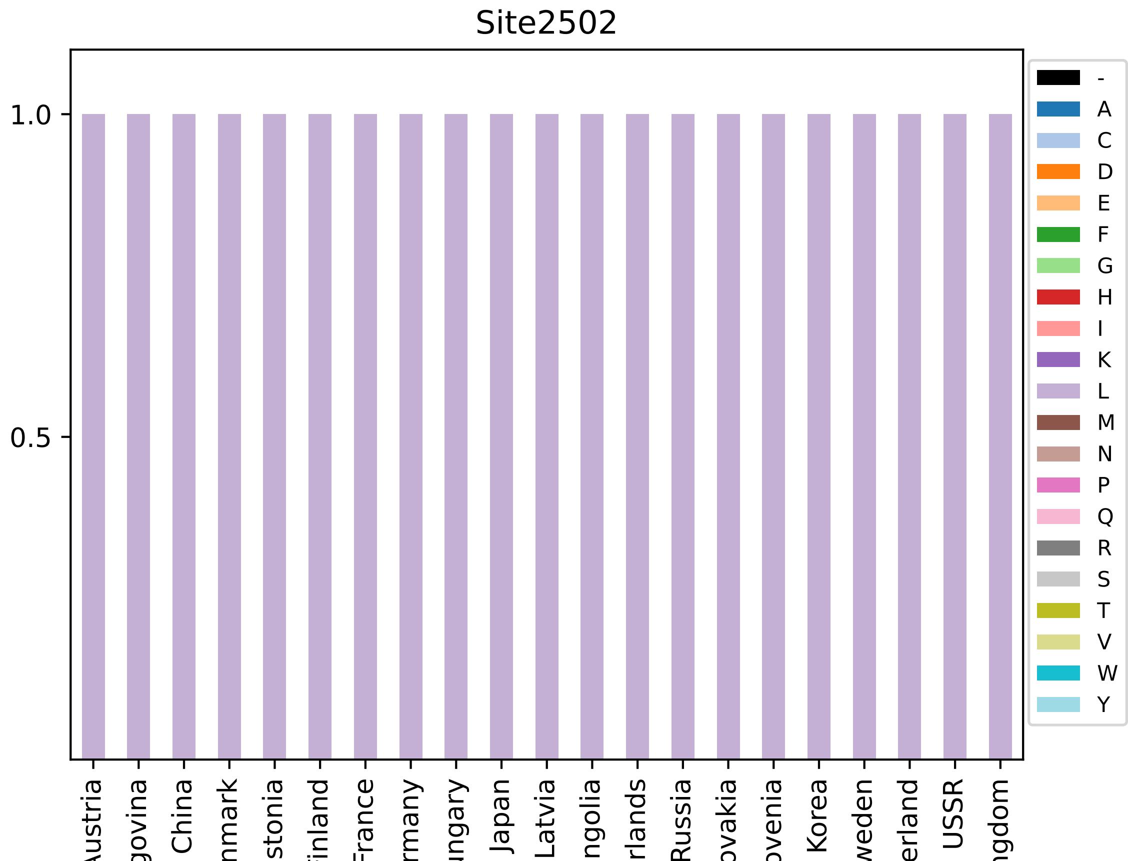
Site2502
1.0
0.5
-
A
C
D
E
F
G
H
I
K
L
M
N
P
Q
R
S
T
V
W
Y
China
Japan
Latvia
Russia
Korea
USSR

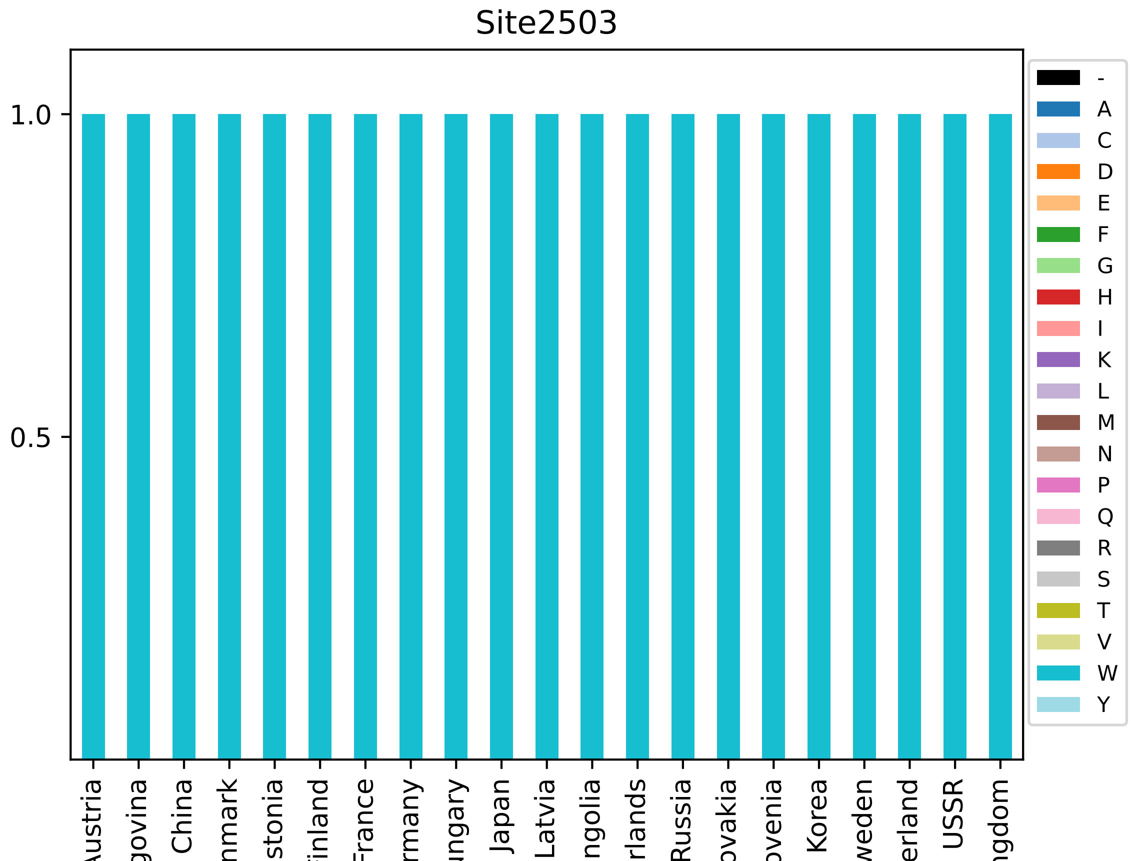

Site2503
1.0
0.5
-
A
C
D
E
F
G
H
I
K
L
M
N
P
Q
R
S
T
V
W
Y
China
Japan
Latvia
Russia
Korea
USSR

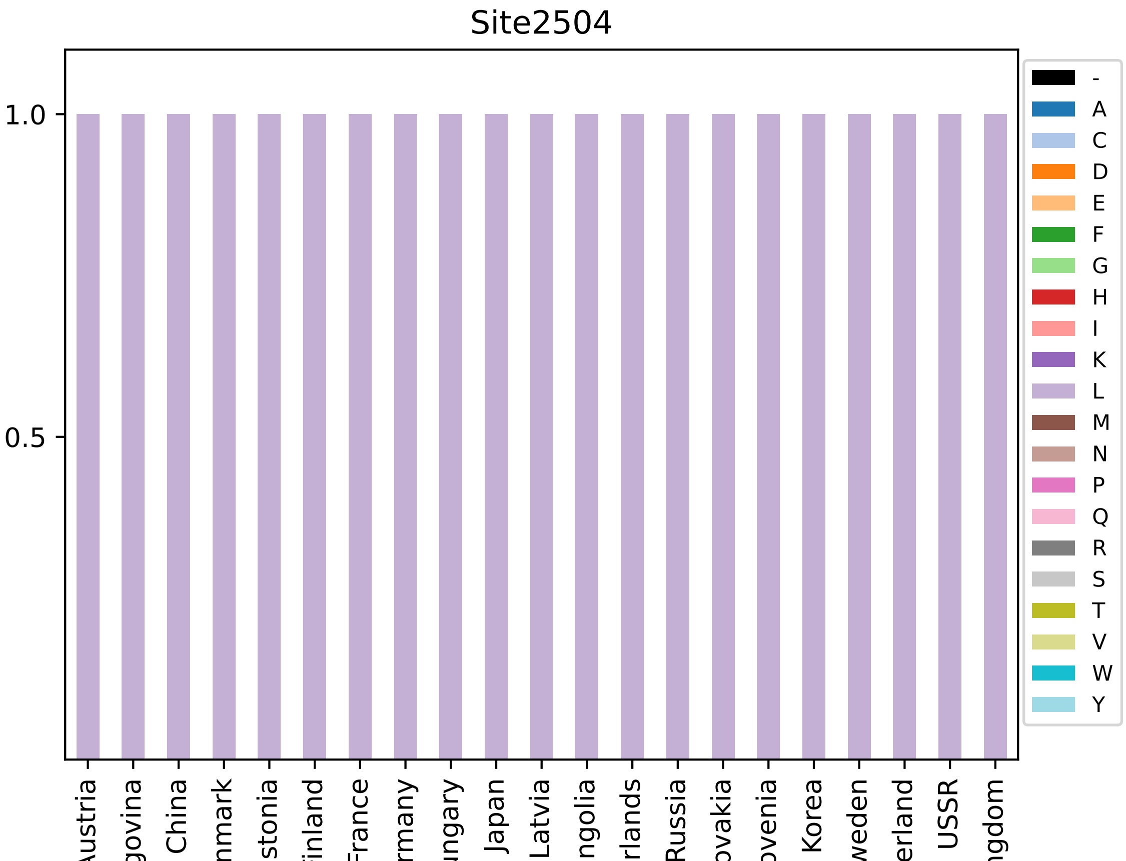
Site2504
1.0
0.5
-
A
C
D
E
F
G
H
I
K
L
M
N
P
Q
R
S
T
V
W
Y
Austria
China
Japan
Latvia
Russia
Korea
USSR

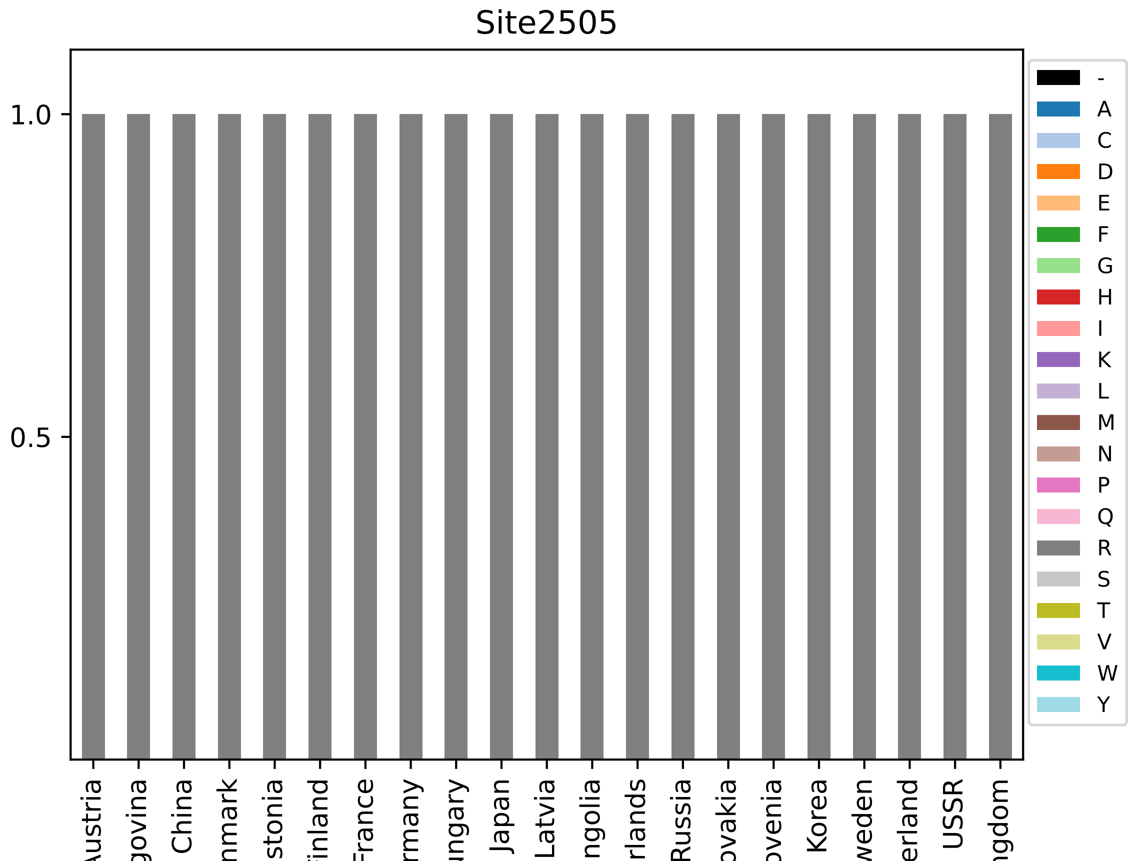
Site2505
1.0
0.5
-
A
C
D
E
F
G
H
I
K
L
M
N
P
Q
R
S
T
V
W
Y
Austria
China
Japan
Latvia
Russia
Korea
USSR

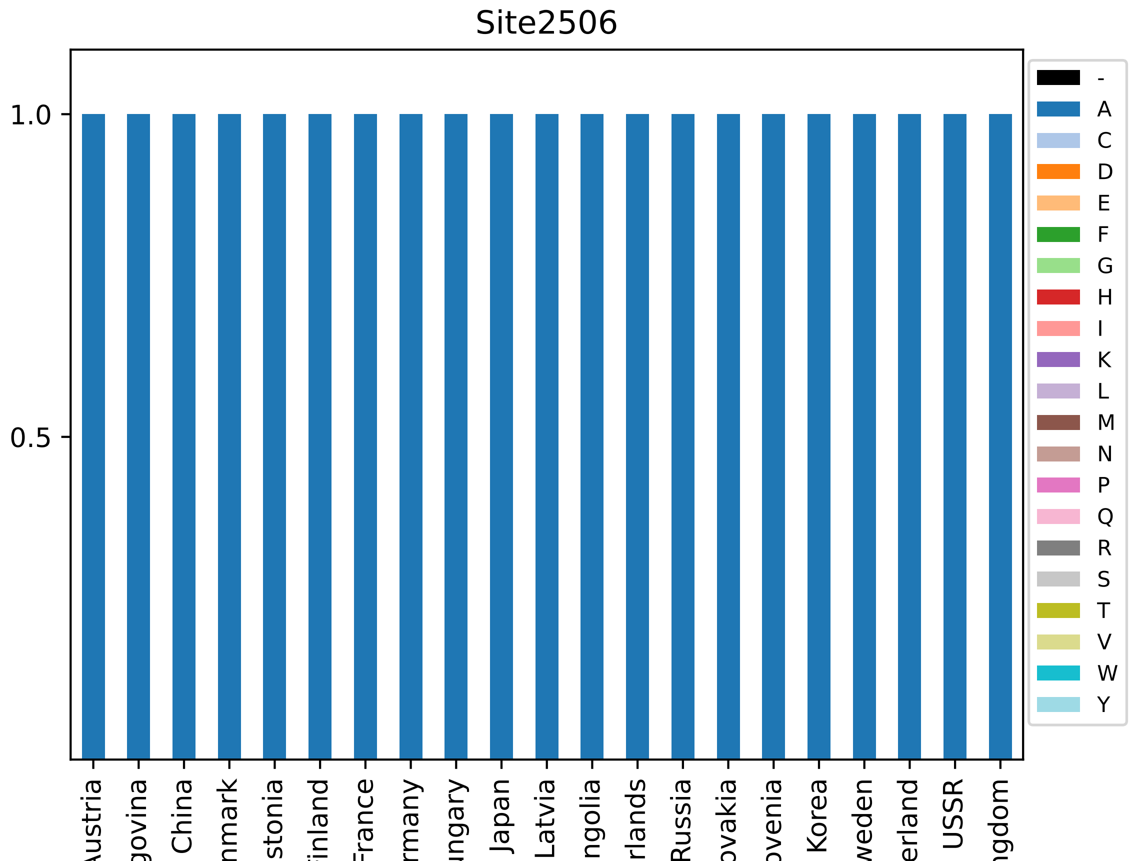
Site2506
1.0
0.5
-
A
C
D
E
F
G
H
I
K
L
M
N
P
Q
R
S
T
V
W
Y
China
Japan
Latvia
Russia
Korea
USSR

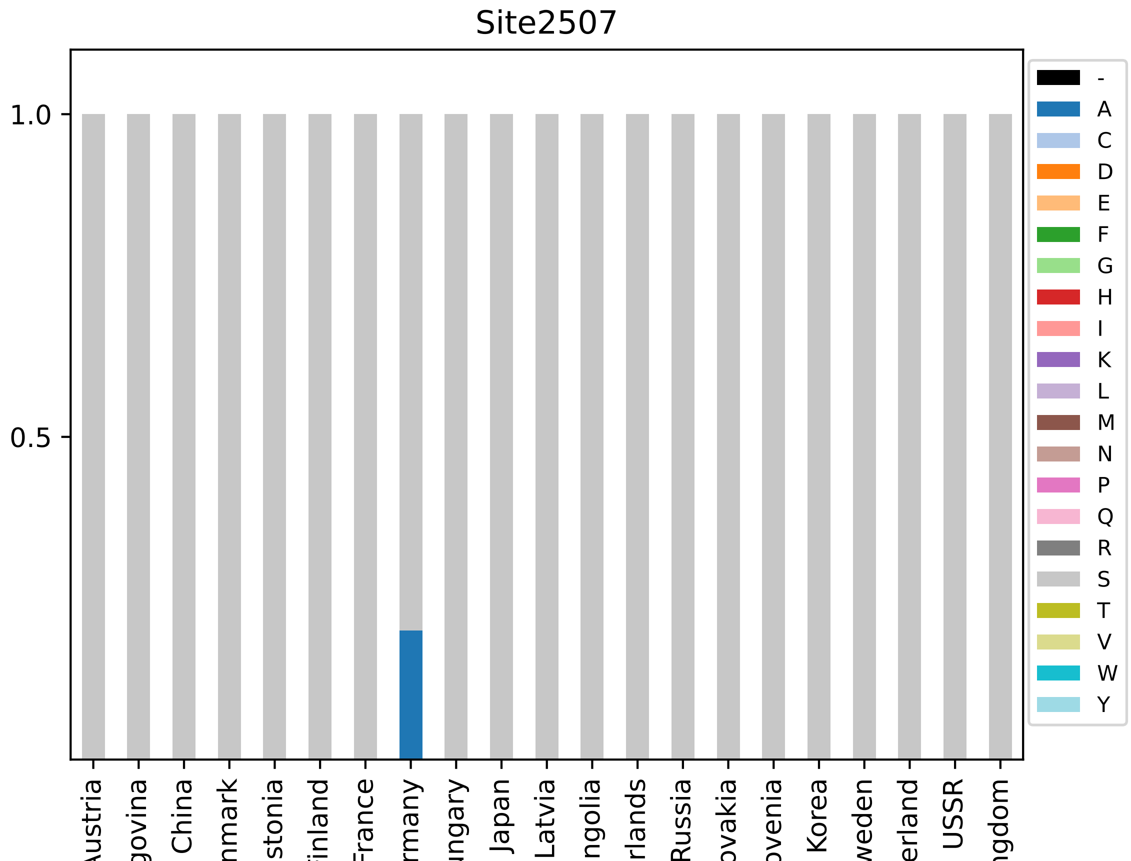

Site2507
1.0
0.5
-
A
C
D
E
F
G
H
I
K
L
M
N
P
Q
R
S
T
V
W
Y
Austria
govina
China
nmark
stonia
Finland
France
rmany
ungary
Japan
Latvia
ngolia
rlands
Russia
ovakia
ovenia
Korea
weden
erland
USSR
ngdom

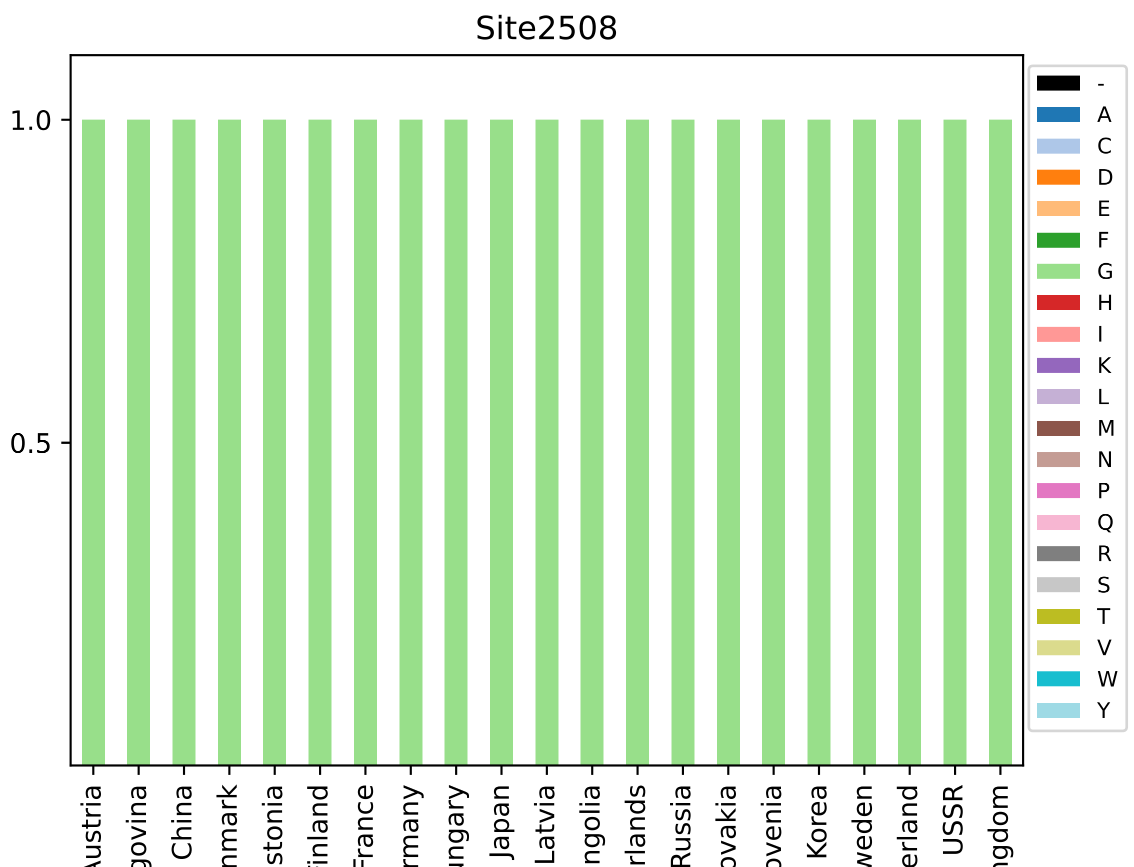
Site2508
1.0
0.5
-
A
C
D
E
F
G
H
I
K
L
M
N
P
Q
R
S
T
V
W
Y
Austria
China
Japan
Latvia
Russia
Korea
USSR

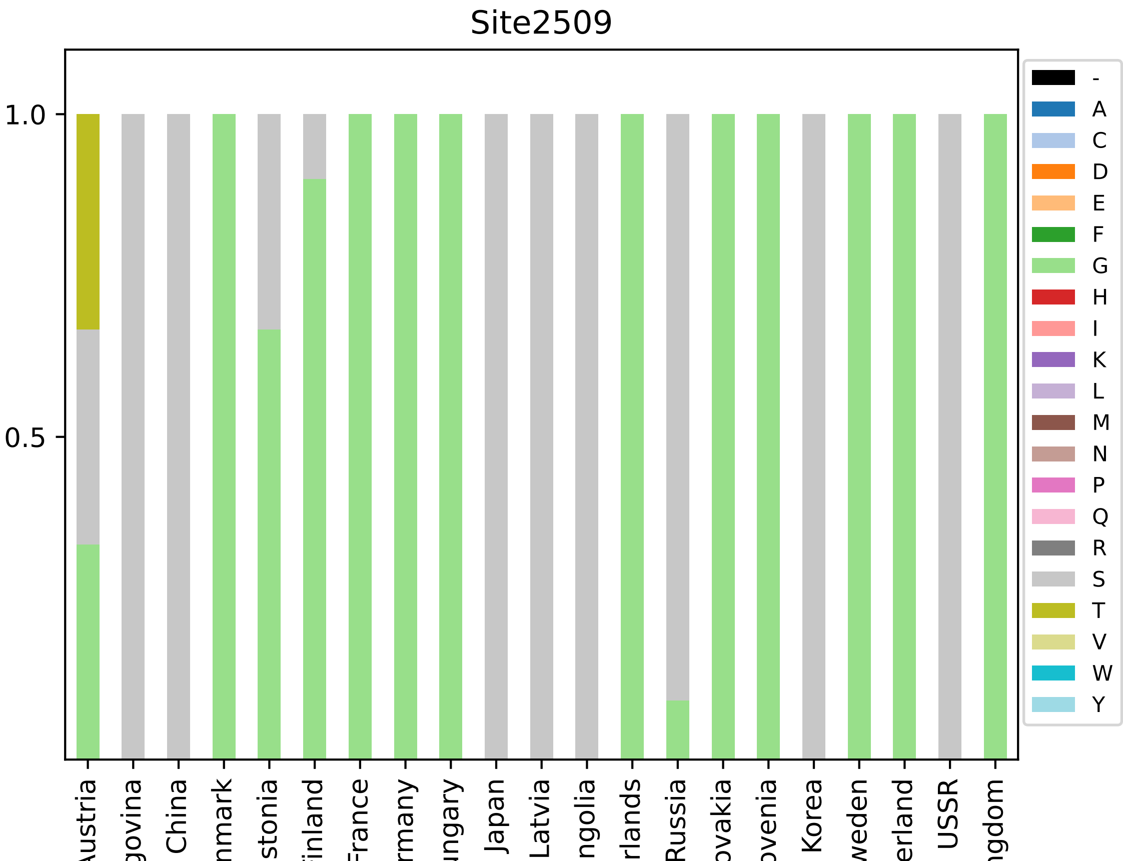
Site2509
1.0
0.5
-
A
C
D
E
F
G
H
I
K
L
M
N
P
Q
R
S
T
V
W
Y
Austria
China
Japan
Latvia
Russia
Korea
USSR

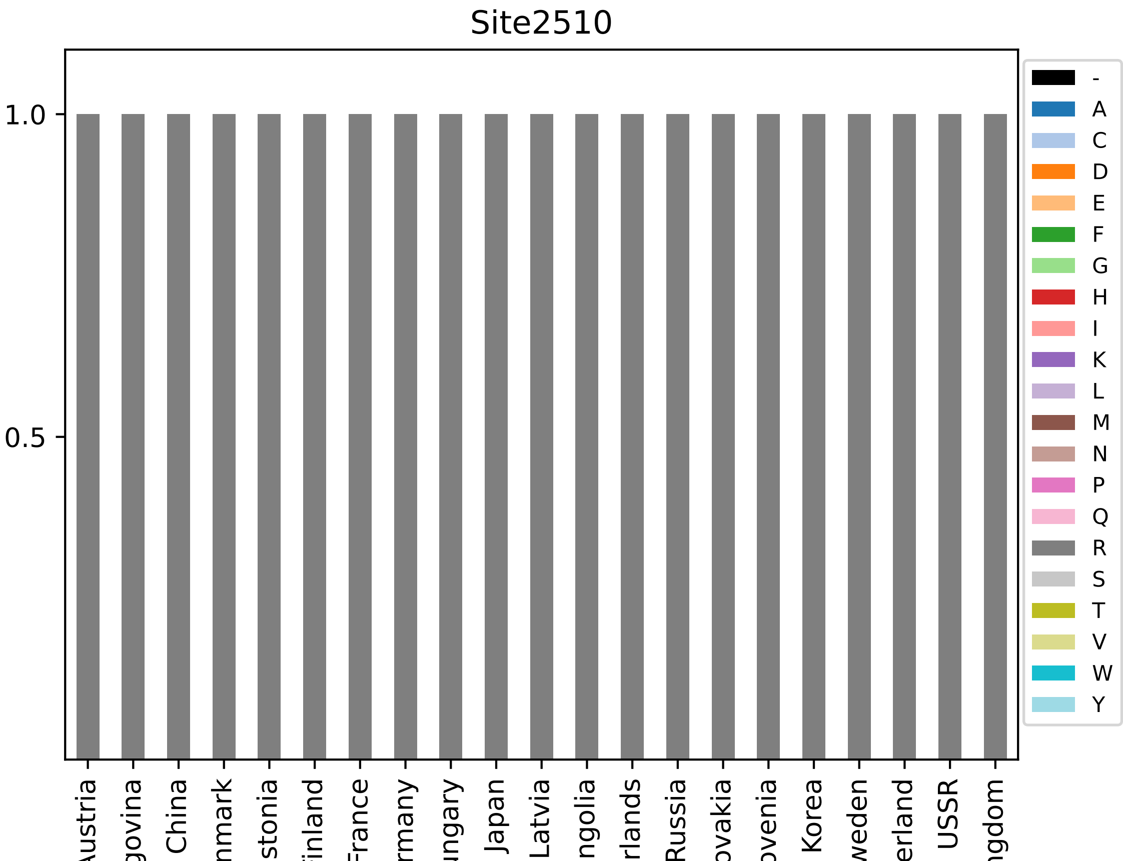
Site2510
1.0
0.5
-
A
C
D
E
F
G
H
I
K
L
M
N
P
Q
R
S
T
V
W
Y
China
Japan
Latvia
Russia
Korea
USSR

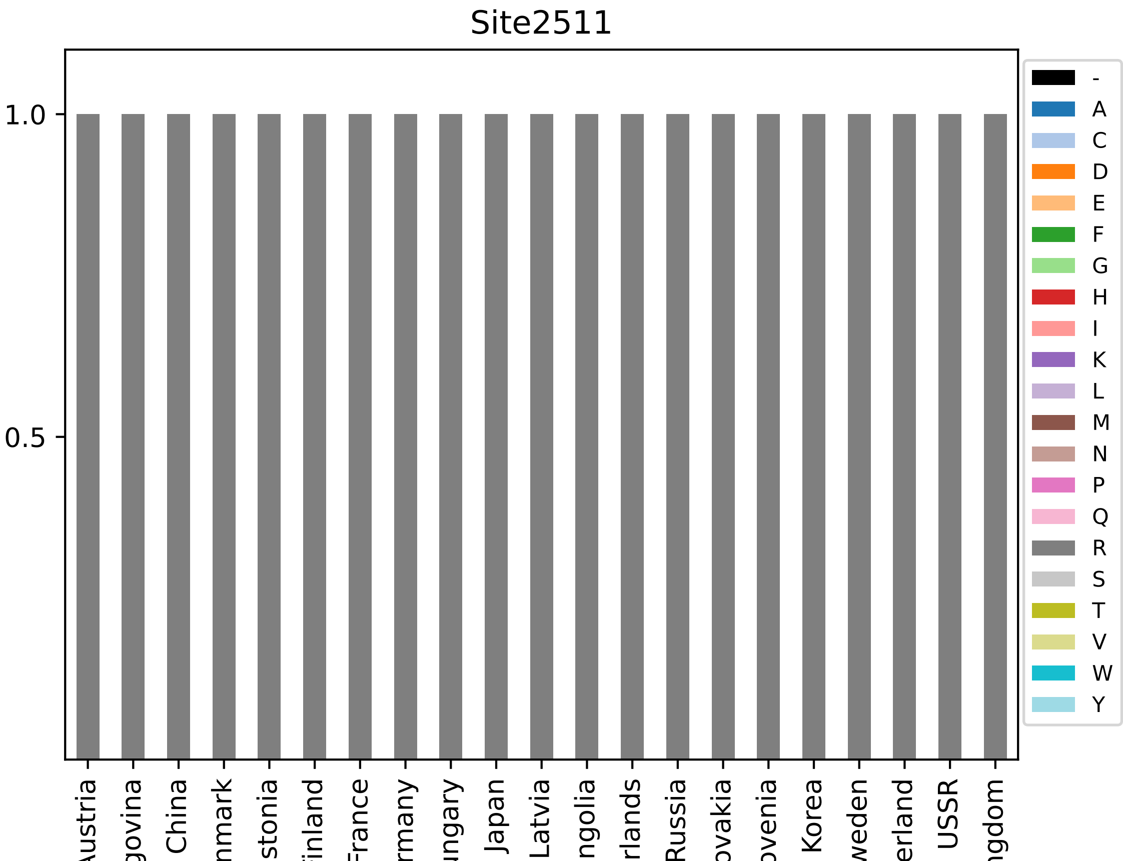
Site2511
1.0
0.5
-
A
C
D
E
F
G
H
I
K
L
M
N
P
Q
R
S
T
V
W
Y
China
Japan
Latvia
Russia
Korea
USSR

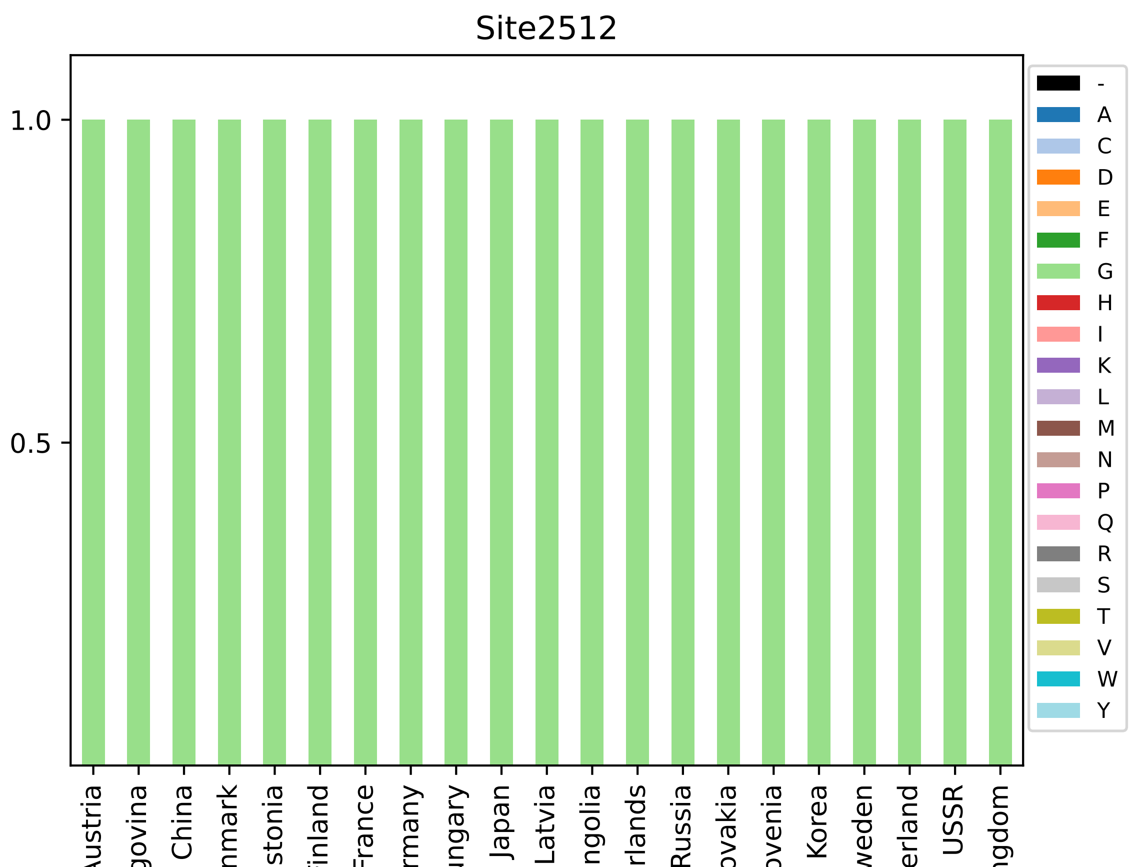
Site2512
1.0
0.5
-
A
C
D
E
F
G
H
I
K
L
M
N
P
Q
R
S
T
V
W
Y
Austria
govina
China
nmark
stonia
inland
France
rmany
ungary
Japan
Latvia
ngolia
rlands
Russia
ovakia
ovenia
Korea
weden
erland
USSR
ngdom

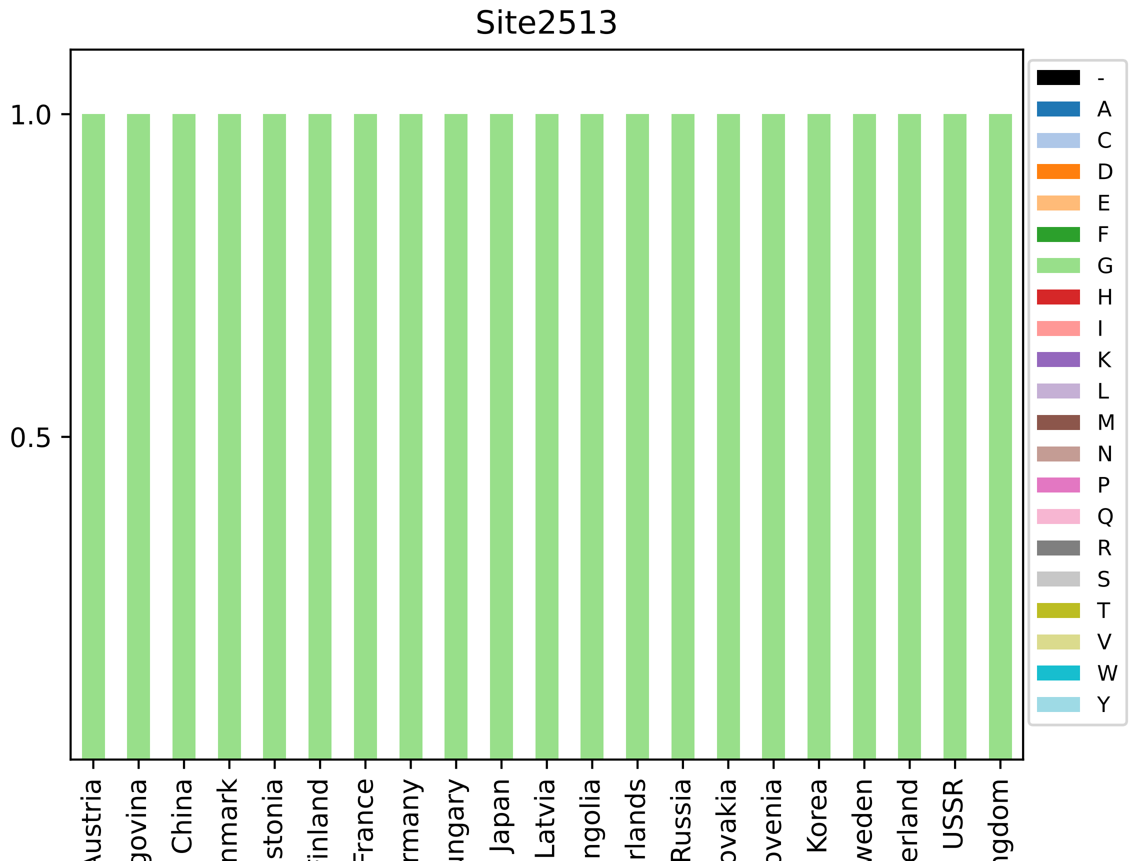
Site2513
1.0
0.5
-
A
C
D
E
F
G
H
I
K
L
M
N
P
Q
R
S
T
V
W
Y
Austria
China
Finland
France
Japan
Latvia
Russia
Korea
USSR

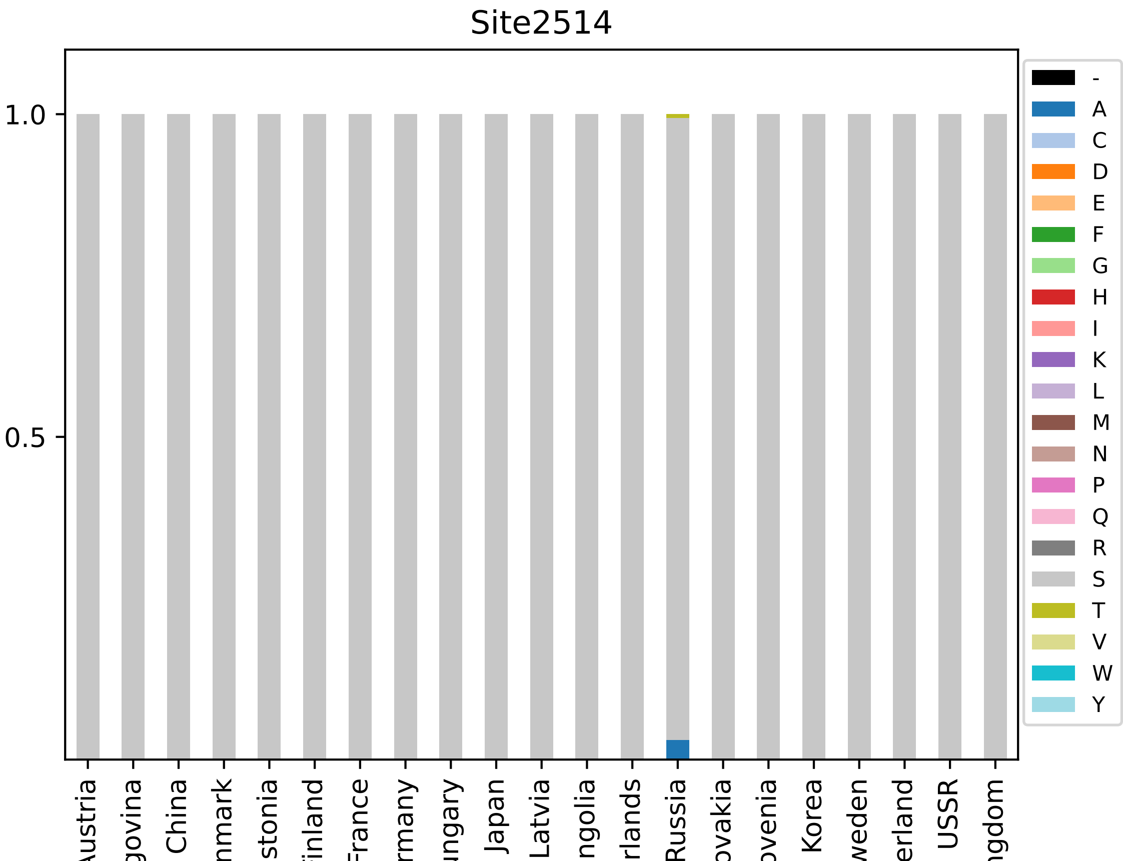
Site2514
1.0
0.5
-
A
C
D
E
F
G
H
I
K
L
M
N
P
Q
R
S
T
V
W
Y
Austria
govina
China
nmark
stonia
Finland
France
rmany
ungary
Japan
Latvia
ngolia
rlands
Russia
ovakia
ovenia
Korea
weden
erland
USSR
ngdom

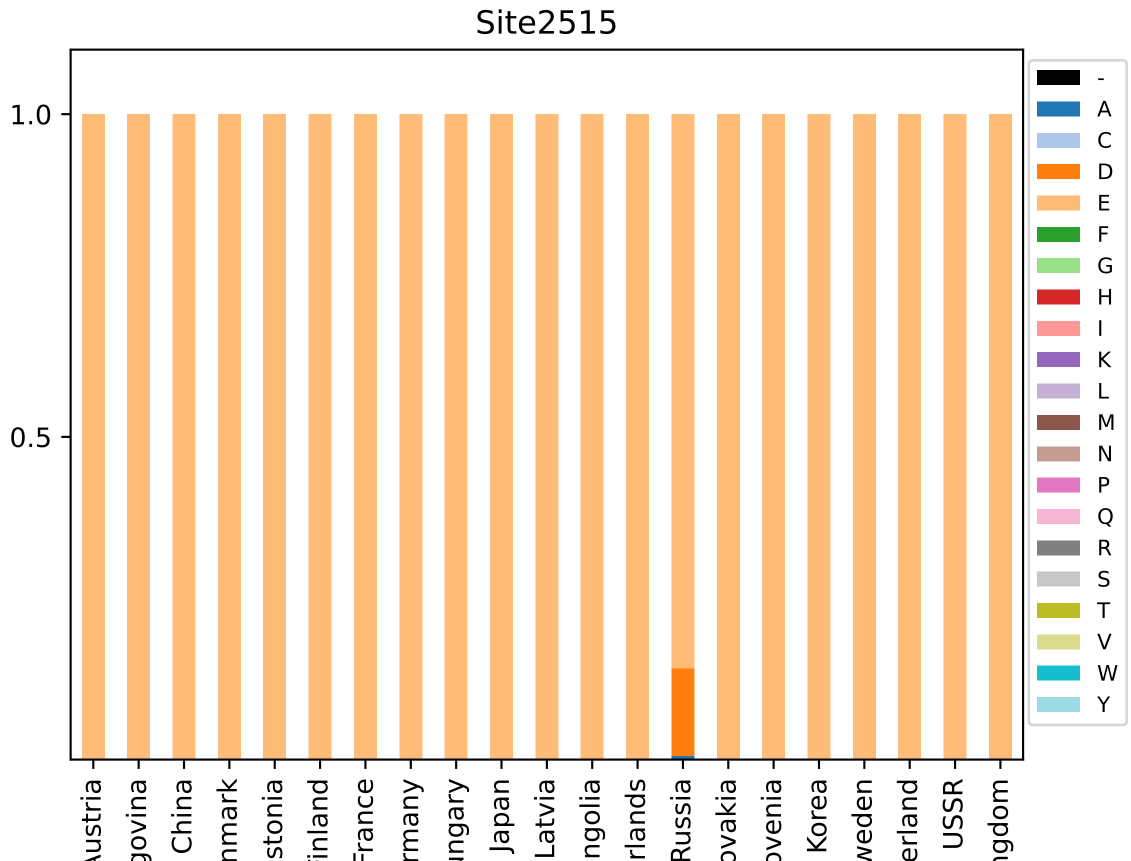

Site2515
1.0
0.5
-
A
C
D
E
F
G
H
I
K
L
M
N
P
Q
R
S
T
V
W
Y
China
Japan
Latvia
Russia
Korea
USSR

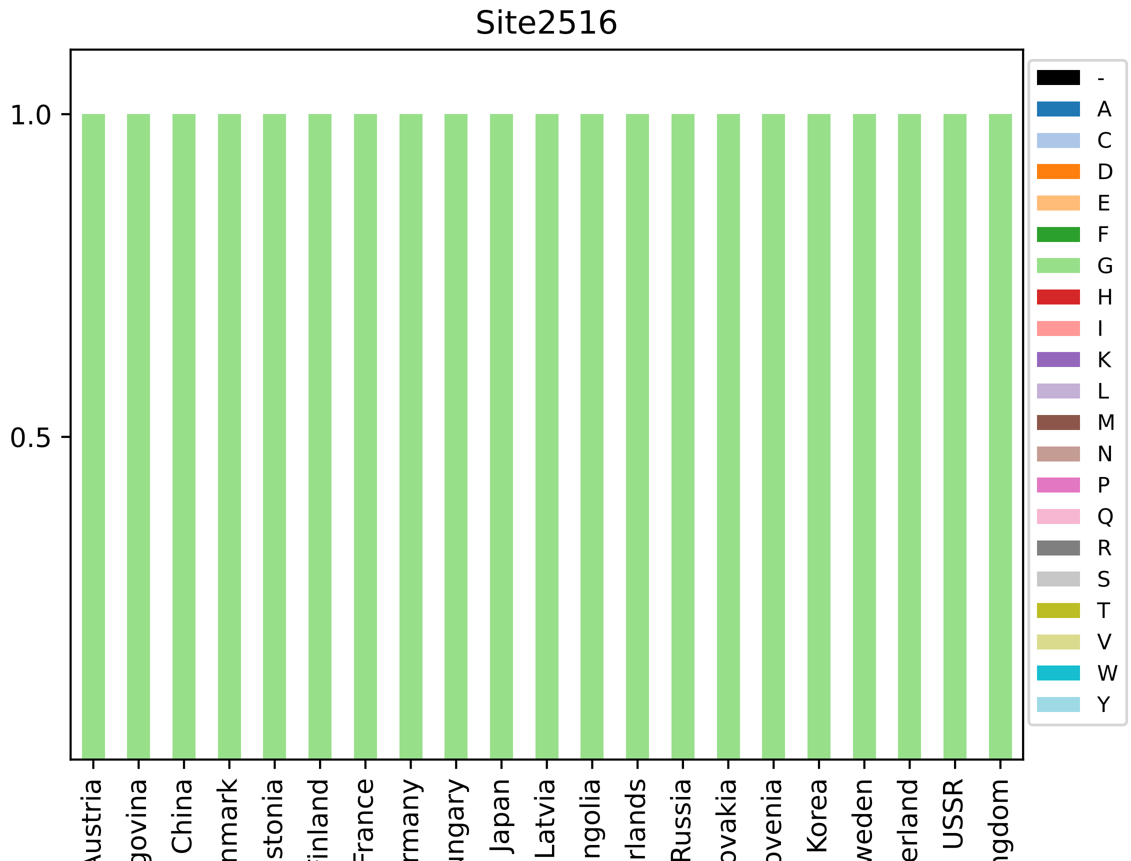
Site2516
1.0
0.5
-
A
C
D
E
F
G
H
I
K
L
M
N
P
Q
R
S
T
V
W
Y
China
Japan
Latvia
Russia
Korea
USSR

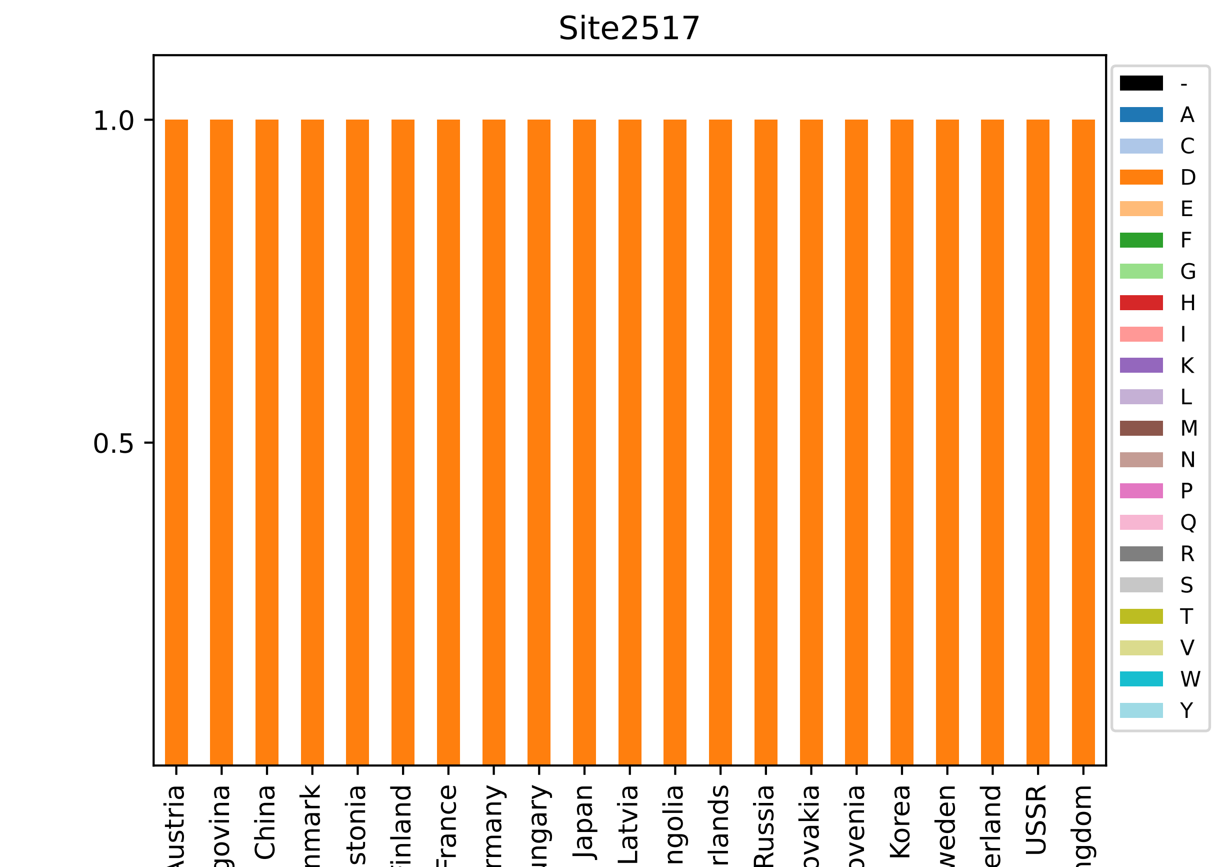
Site2517
1.0
0.5
-
A
C
D
E
F
G
H
I
K
L
M
N
P
Q
R
S
T
V
W
Y
Austria
govina
China
nmark
stonia
Finland
France
rmany
ungary
Japan
Latvia
ngolia
rlands
Russia
ovakia
ovenia
Korea
weden
erland
USSR
ngdom

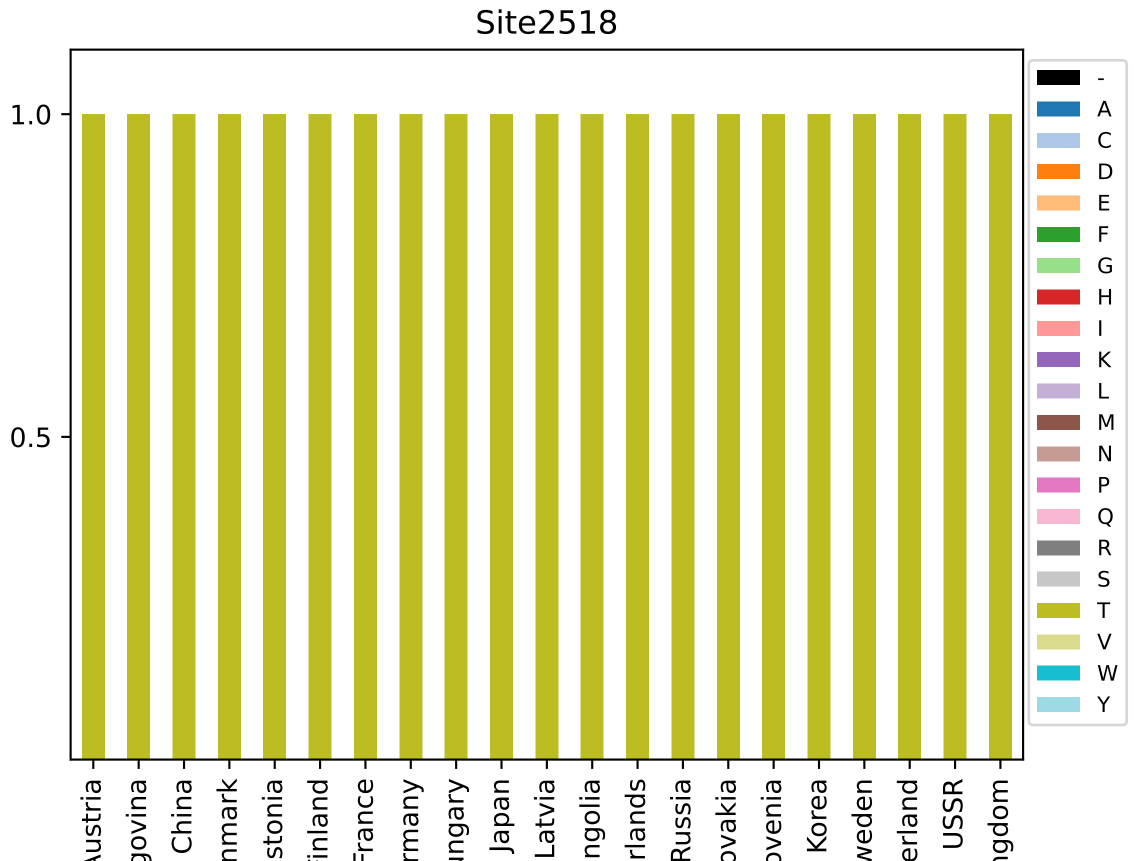
Site2518
1.0
0.5
China
Japan
Latvia
Russia
Korea
USSR
-
A
C
D
E
F
G
H
I
K
L
M
N
P
Q
R
S
T
V
W
Y

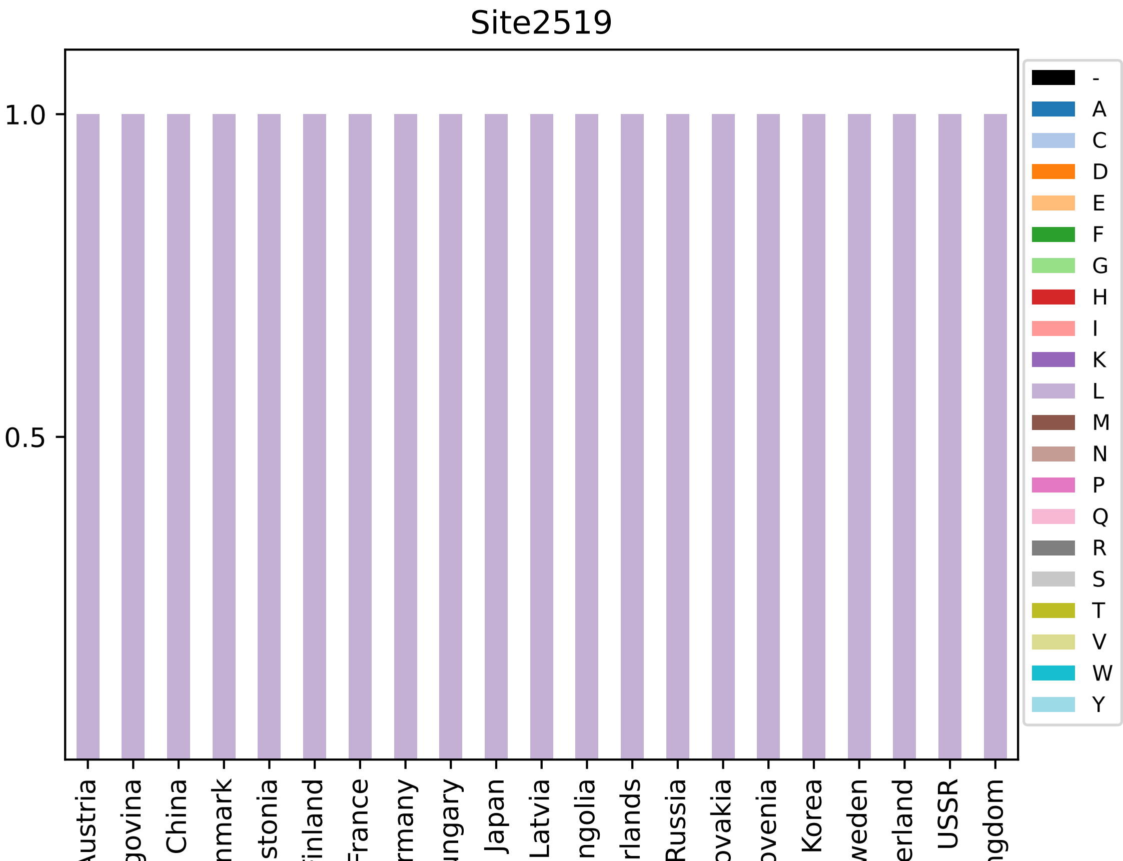
Site2519
1.0
0.5
-
A
C
D
E
F
G
H
I
K
L
M
N
P
Q
R
S
T
V
W
Y
China
Japan
Latvia
Russia
Korea
USSR

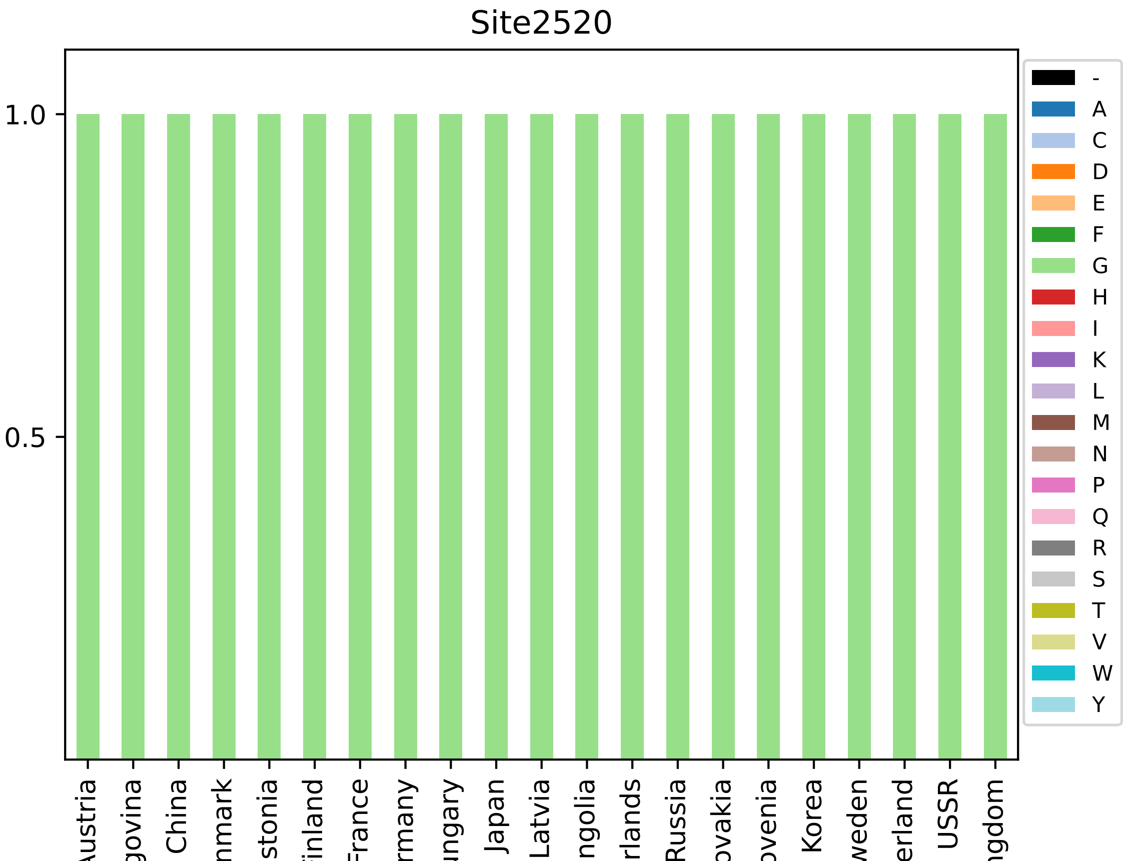

Site2520
1.0
0.5
-
A
C
D
E
F
G
H
I
K
L
M
N
P
Q
R
S
T
V
W
Y
Austria
govina
China
nmark
stonia
Finland
France
rmany
ungary
Japan
Latvia
ngolia
rlands
Russia
ovakia
ovenia
Korea
weden
erland
USSR
ngdom

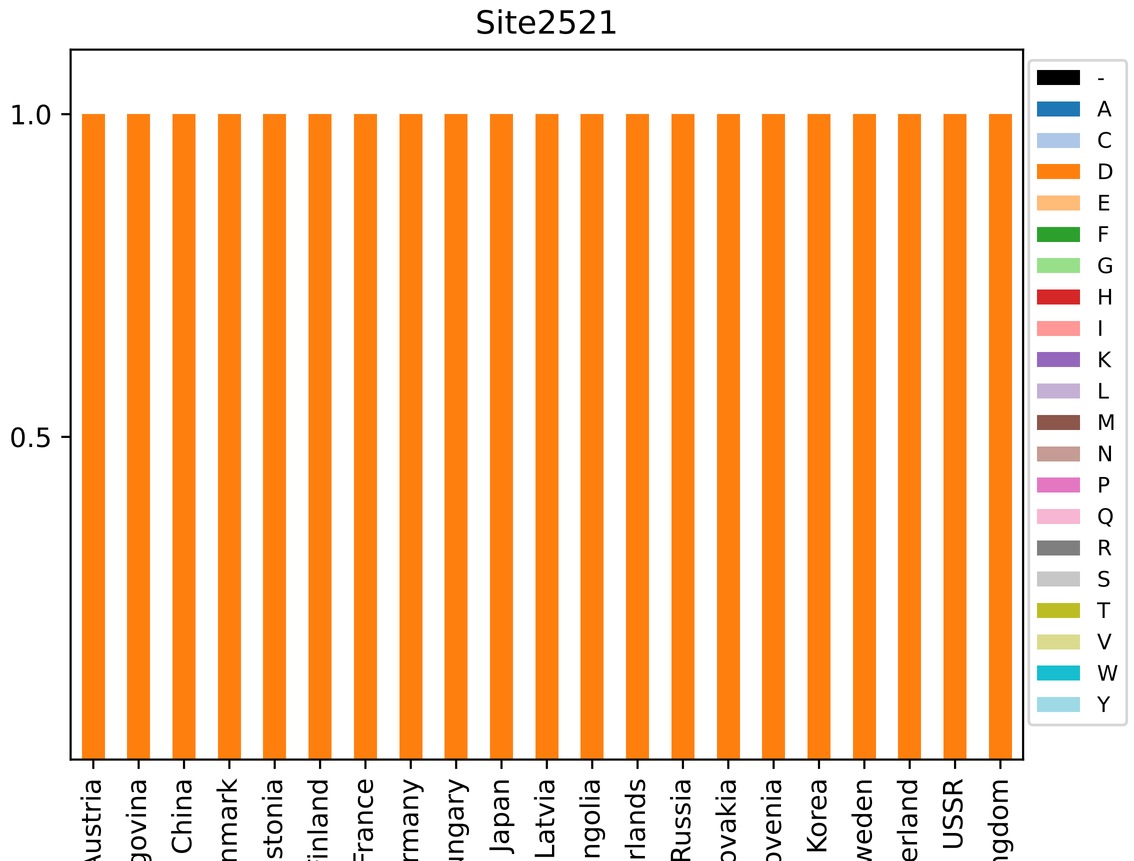
Site2521
1.0
0.5
-
A
C
D
E
F
G
H
I
K
L
M
N
P
Q
R
S
T
V
W
Y
Austria
govina
China
nmark
stonia
inland
France
rmany
ungary
Japan
Latvia
ngolia
rlands
Russia
ovakia
ovenia
Korea
weden
erland
USSR
ngdom

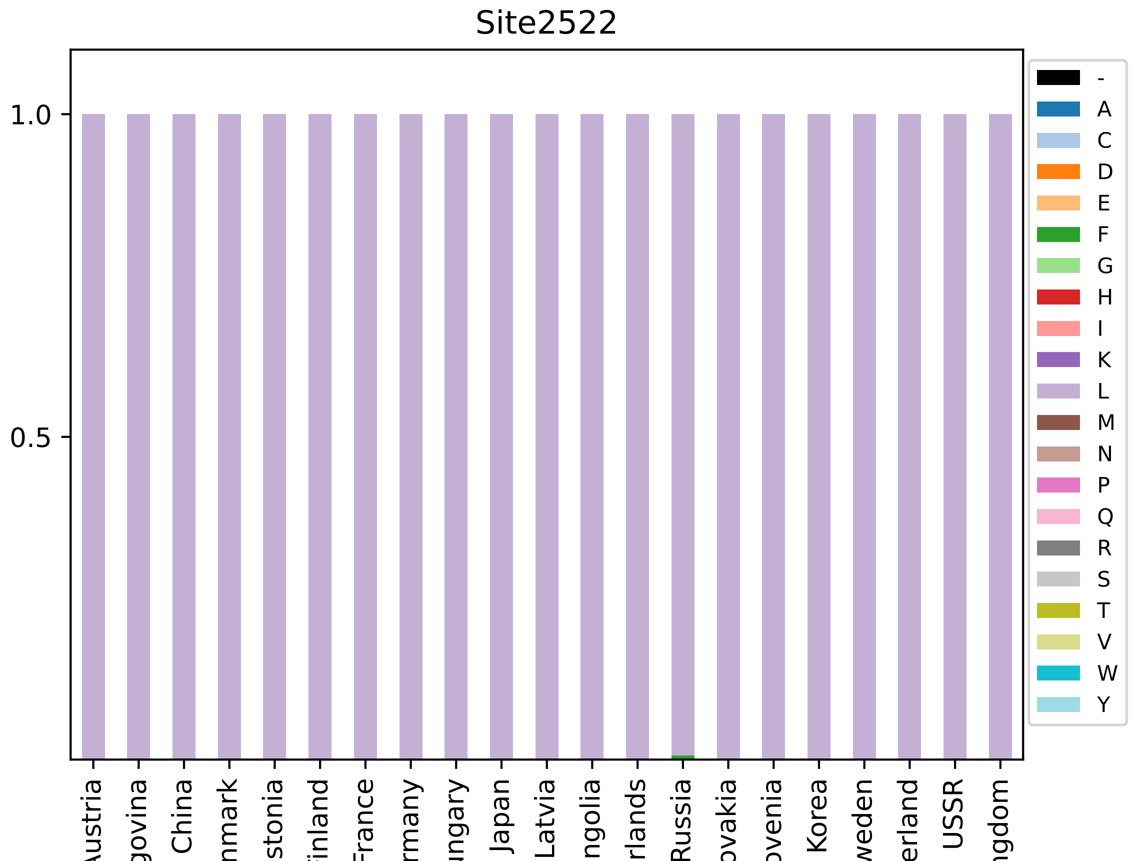

Site2522
1.0
0.5
Austria
govina
China
nmark
stonia
Finland
France
rmany
ungary
Japan
Latvia
ngolia
rlands
Russia
ovakia
ovenia
Korea
weden
erland
USSR
ngdom
-
A
C
D
E
F
G
H
I
K
L
M
N
P
Q
R
S
T
V
W
Y

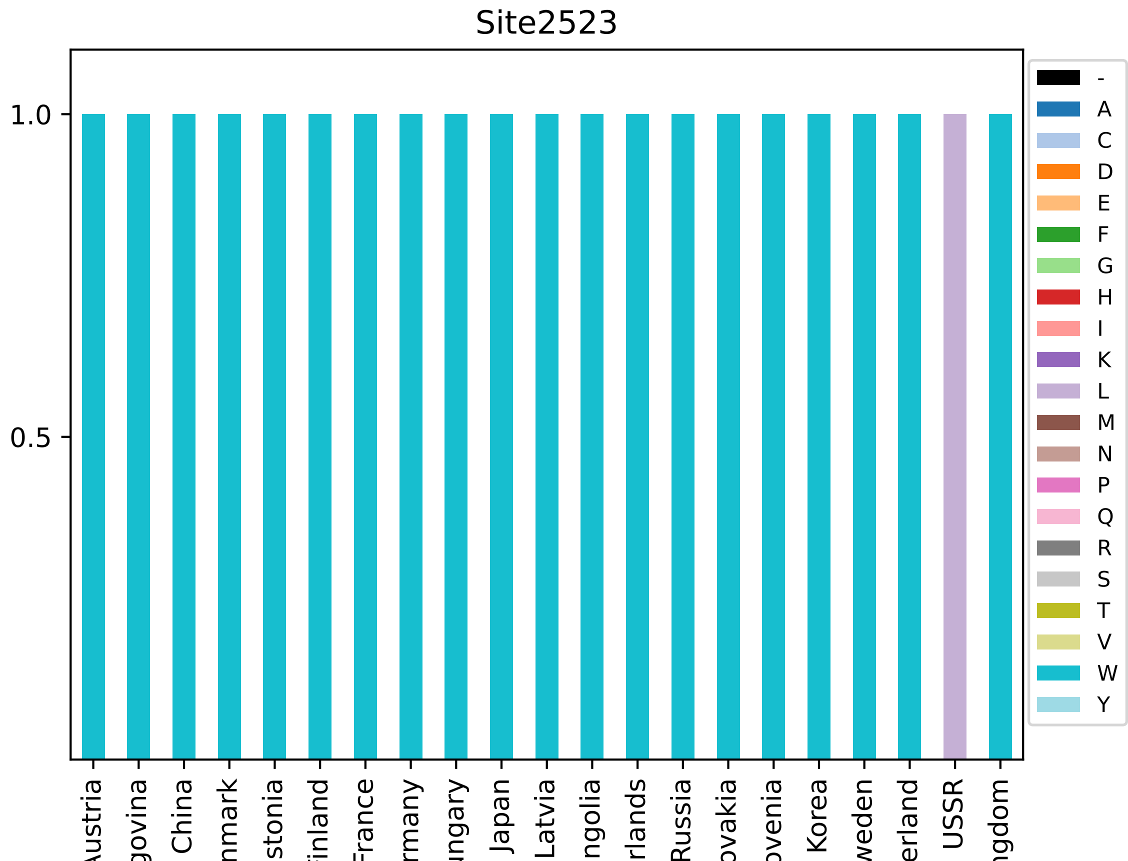
Site2523
1.0
0.5
-
A
C
D
E
F
G
H
I
K
L
M
N
P
Q
R
S
T
V
W
Y
China
Japan
Latvia
Russia
Korea
USSR

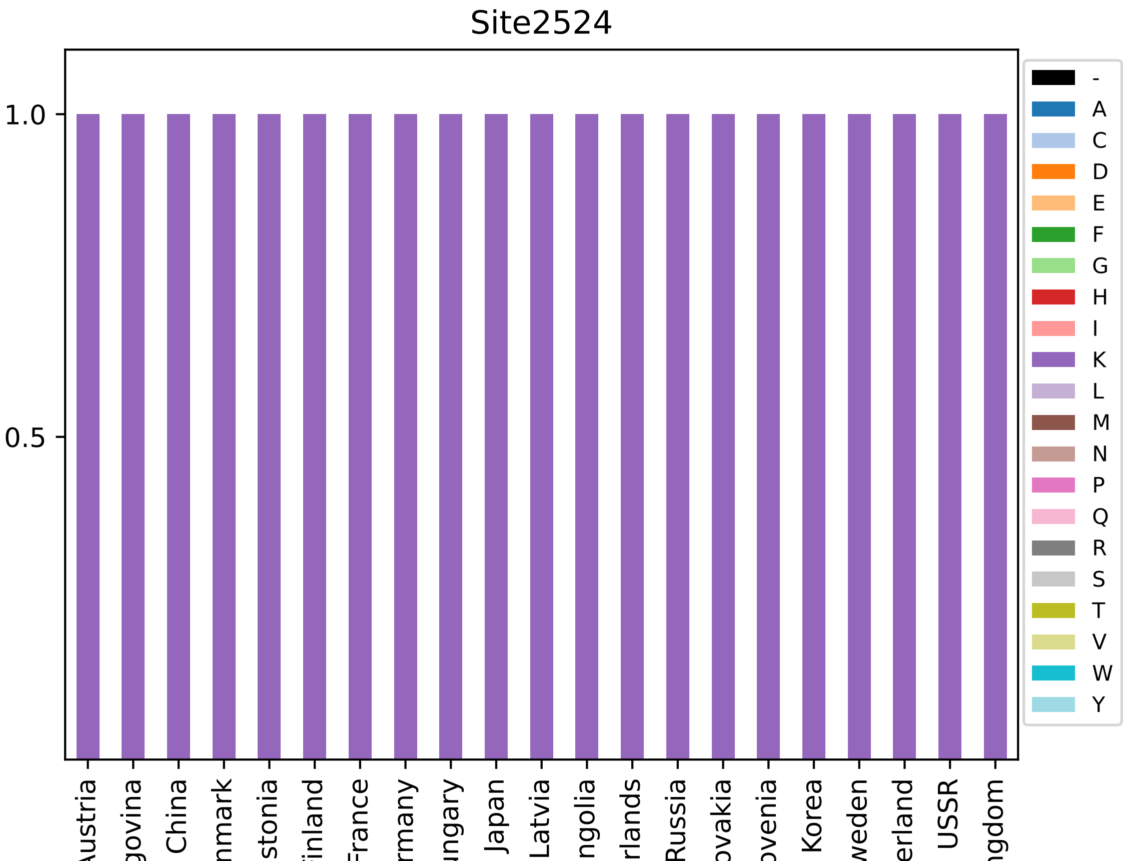

Site2524
1.0
0.5
-
A
C
D
E
F
G
H
I
K
L
M
N
P
Q
R
S
T
V
W
Y
China
Japan
Latvia
Russia
Korea
USSR

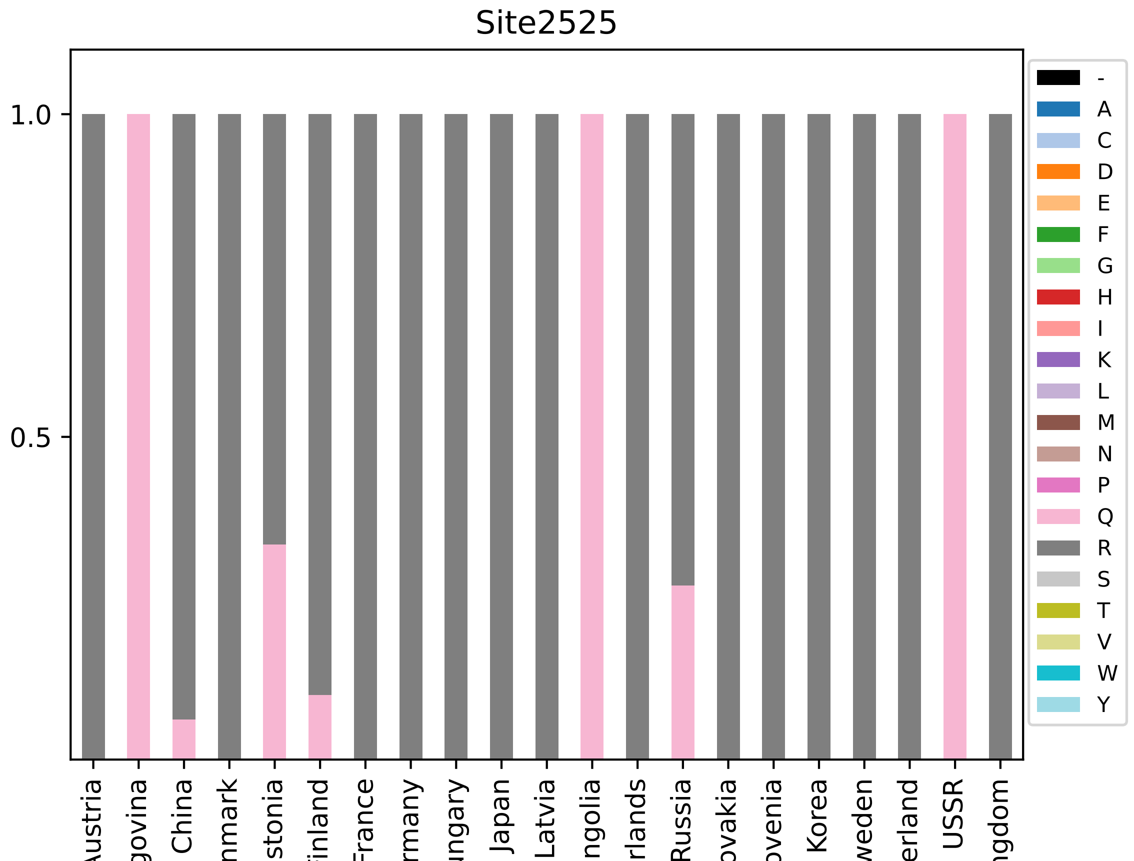

Site2525
1.0
0.5
Austria
govina
China
nmark
stonia
Finland
France
rmany
ungary
Japan
Latvia
ngolia
rlands
Russia
ovakia
ovenia
Korea
weden
erland
USSR
ngdom
-
A
C
D
E
F
G
H
I
K
L
M
N
P
Q
R
S
T
V
W
Y

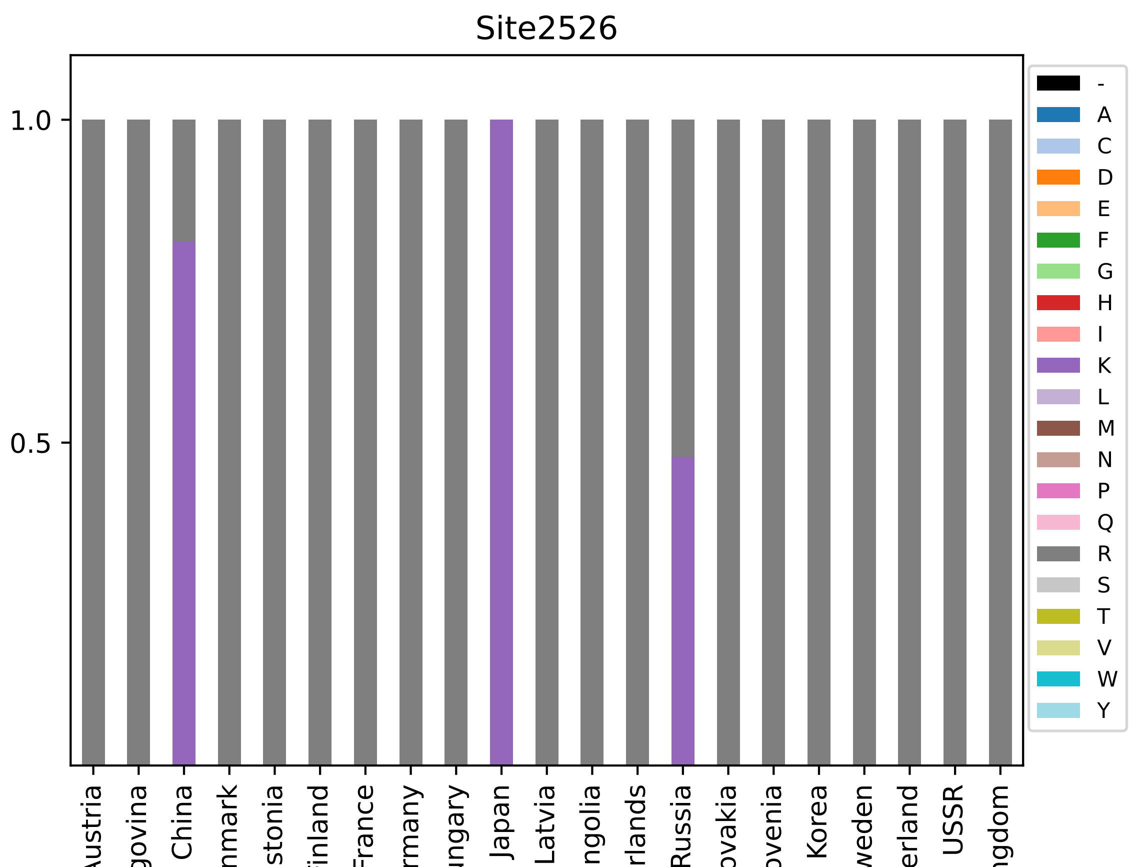

Site2526
1.0
0.5
-
A
C
D
E
F
G
H
I
K
L
M
N
P
Q
R
S
T
V
W
Y
Austria
govina
China
nmark
stonia
inland
France
rmany
ungary
Japan
Latvia
ngolia
rlands
Russia
ovakia
ovenia
Korea
weden
erland
USSR
ngdom

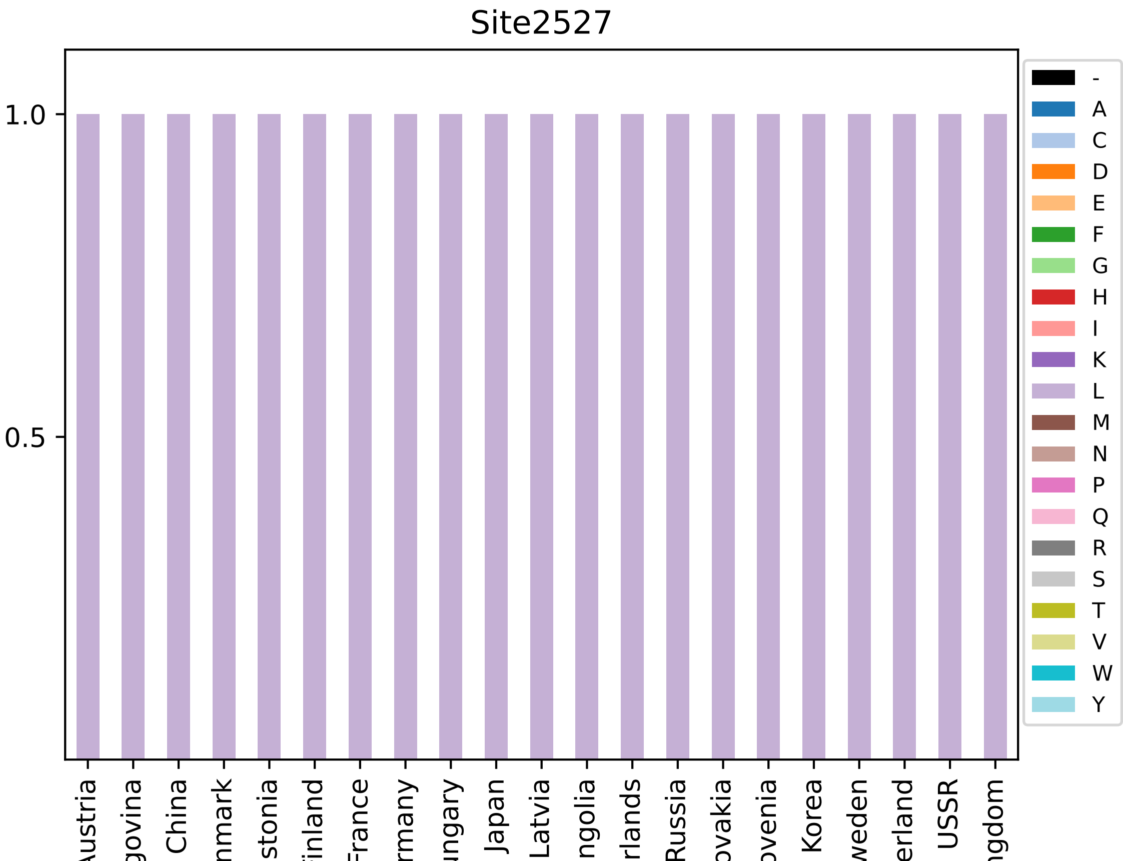
Site2527
1.0
0.5
-
A
C
D
E
F
G
H
I
K
L
M
N
P
Q
R
S
T
V
W
Y
China
Japan
Latvia
Russia
Korea
USSR

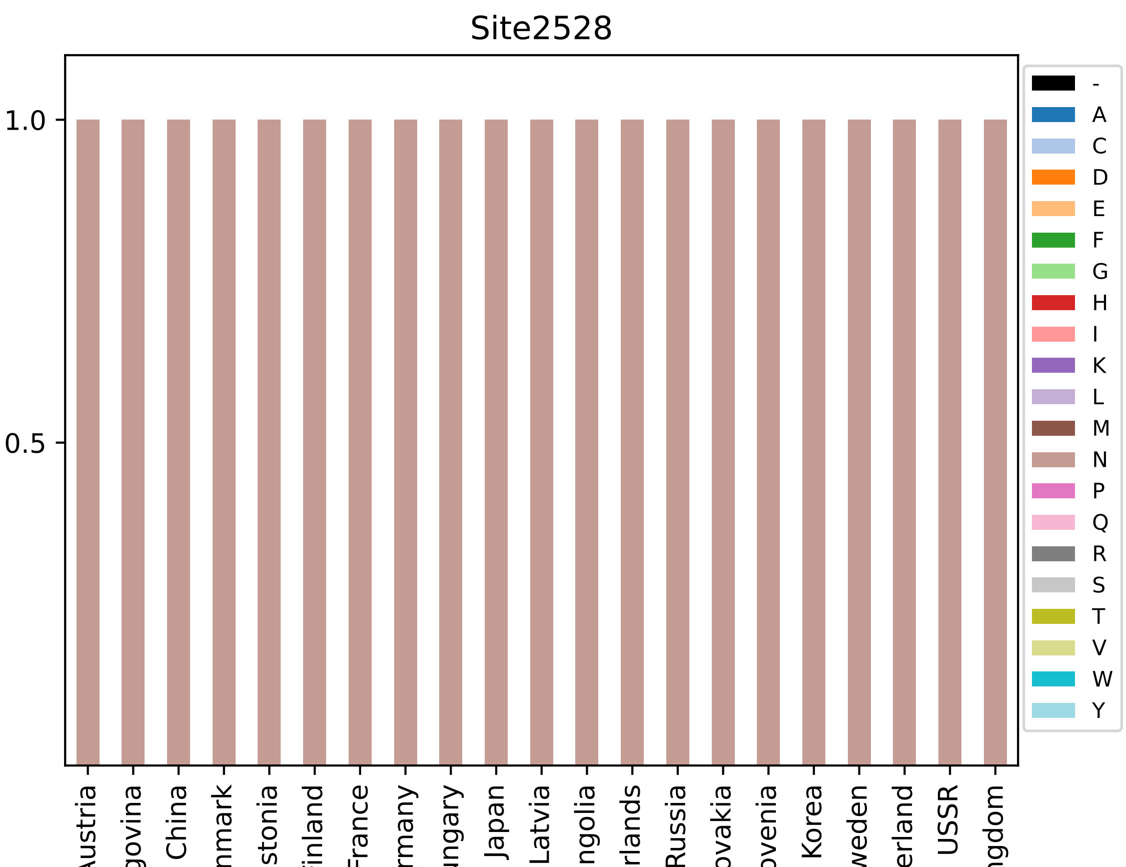

Site2528
1.0
0.5
-
A
C
D
E
F
G
H
I
K
L
M
N
P
Q
R
S
T
V
W
Y
ustria
govina
China
nmark
stonia
inland
rance
rmany
ungary
Japan
Latvia
ngolia
rlands
Russia
ovakia
ovenia
Korea
weden
erland
USSR
ngdom

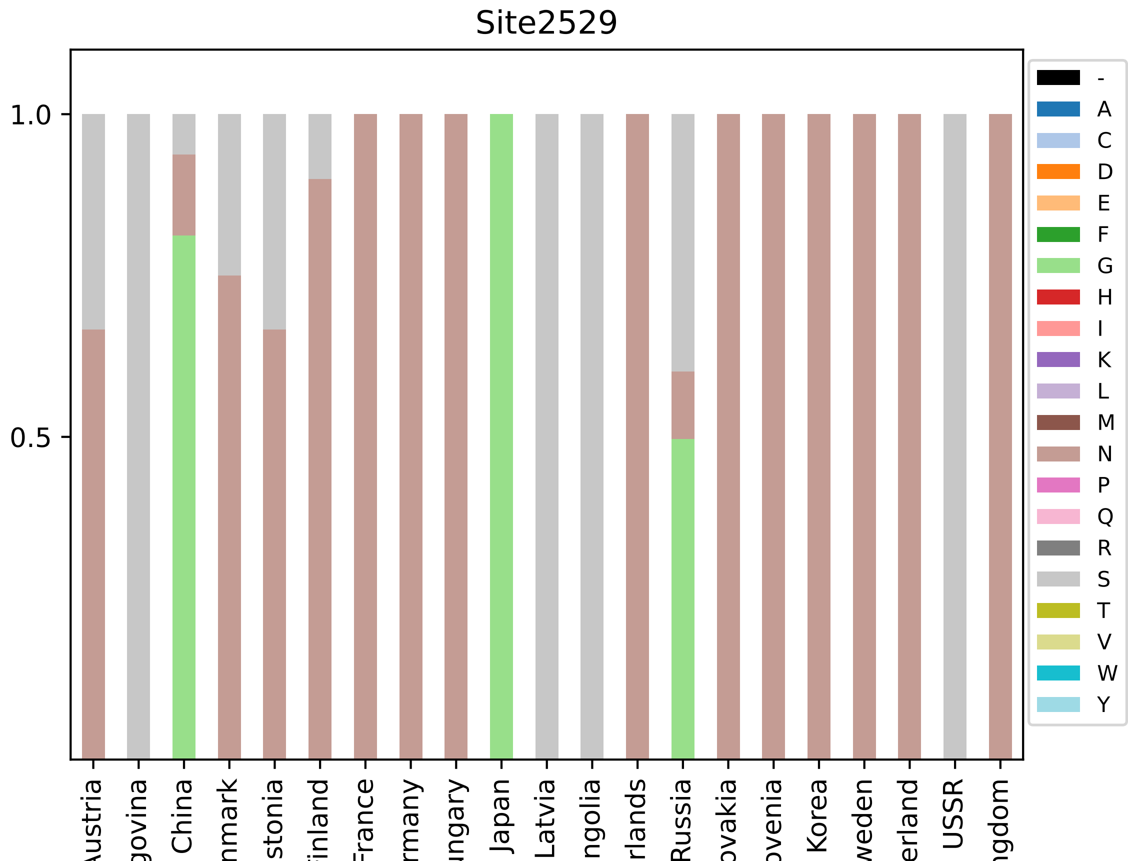

Site2529
1.0
0.5
-
A
C
D
E
F
G
H
I
K
L
M
N
P
Q
R
S
T
V
W
Y
Austria
China
Finland
France
Japan
Latvia
Russia
Korea
USSR

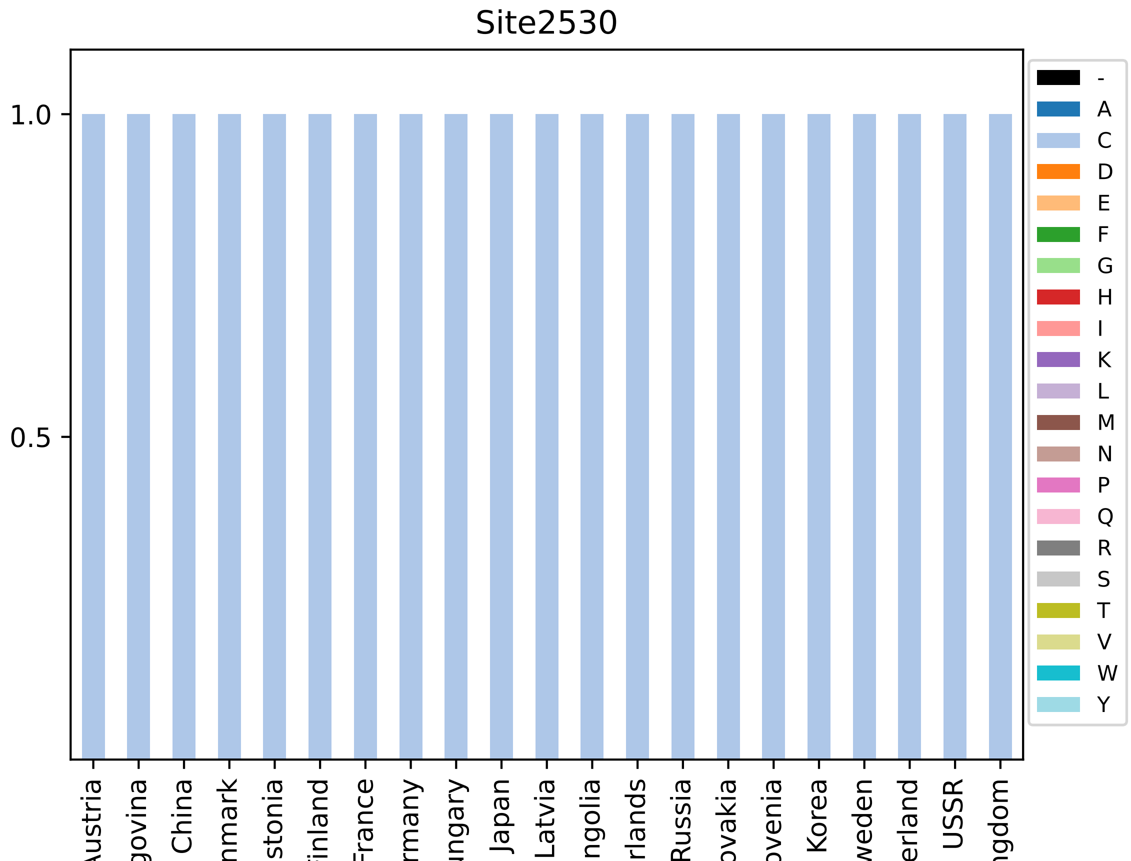
Site2530
1.0
0.5
-
A
C
D
E
F
G
H
I
K
L
M
N
P
Q
R
S
T
V
W
Y
China
Japan
Latvia
Russia
Korea
USSR

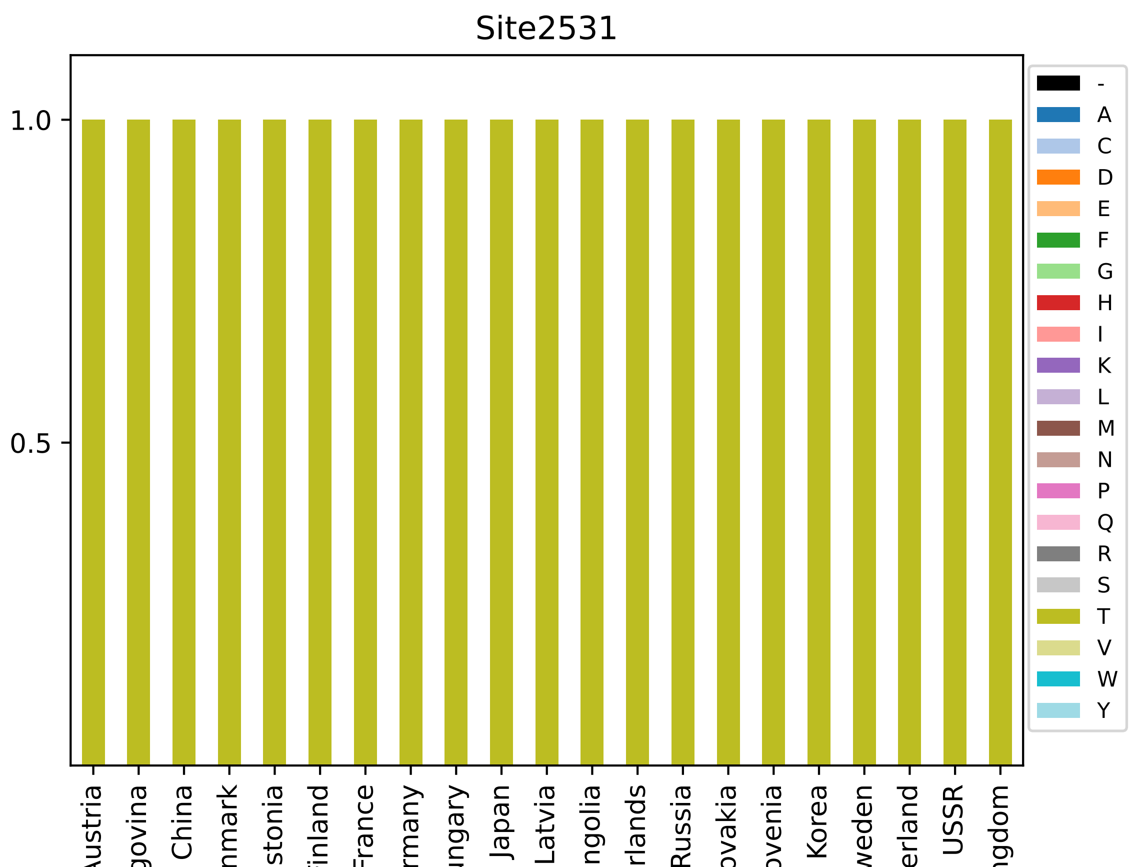

Site2531
1.0
0.5
Austria
govina
China
nmark
stonia
Finland
France
rmany
ungary
Japan
Latvia
ngolia
rlands
Russia
ovakia
ovenia
Korea
weden
erland
USSR
ngdom
-
A
C
D
E
F
G
H
I
K
L
M
N
P
Q
R
S
T
V
W
Y

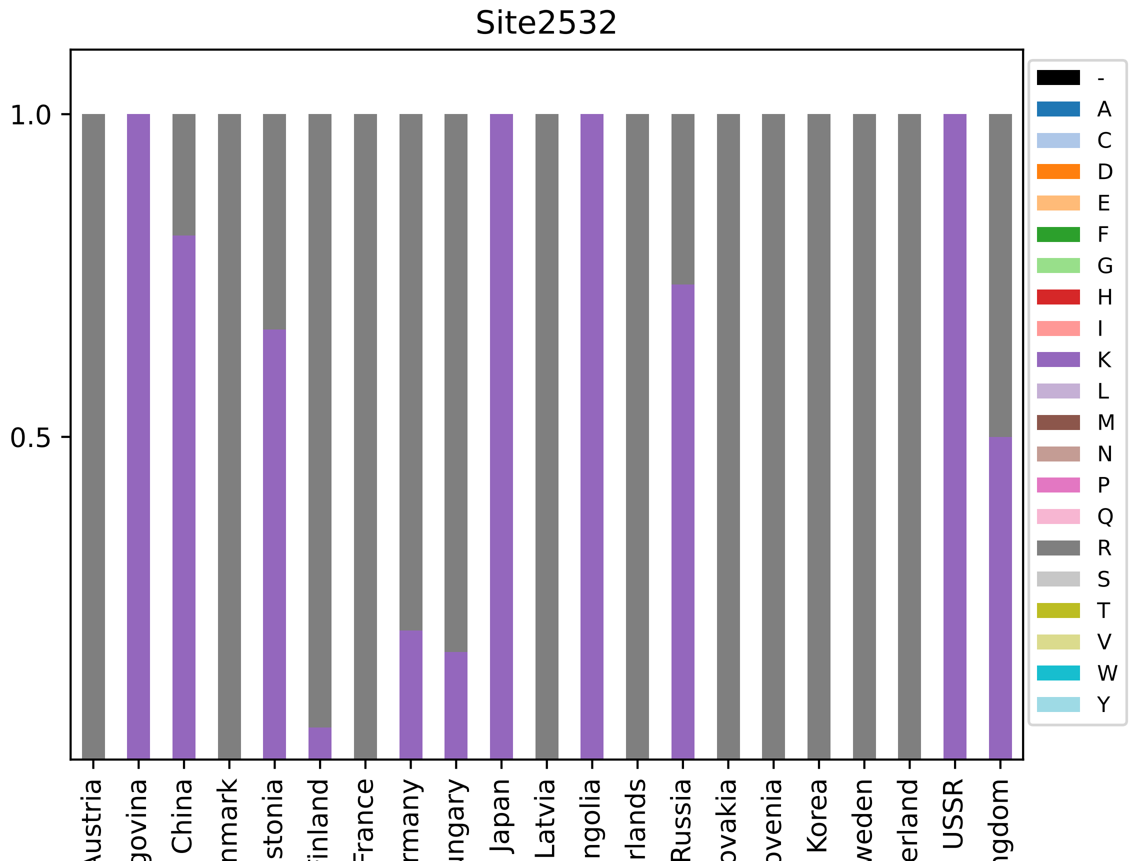

Site2532
1.0
0.5
Austria
govina
China
nmark
stonia
inland
France
rmany
ungary
Japan
Latvia
ngolia
rlands
Russia
ovakia
ovenia
Korea
weden
erland
USSR
ngdom
-
A
C
D
E
F
G
H
I
K
L
M
N
P
Q
R
S
T
V
W
Y

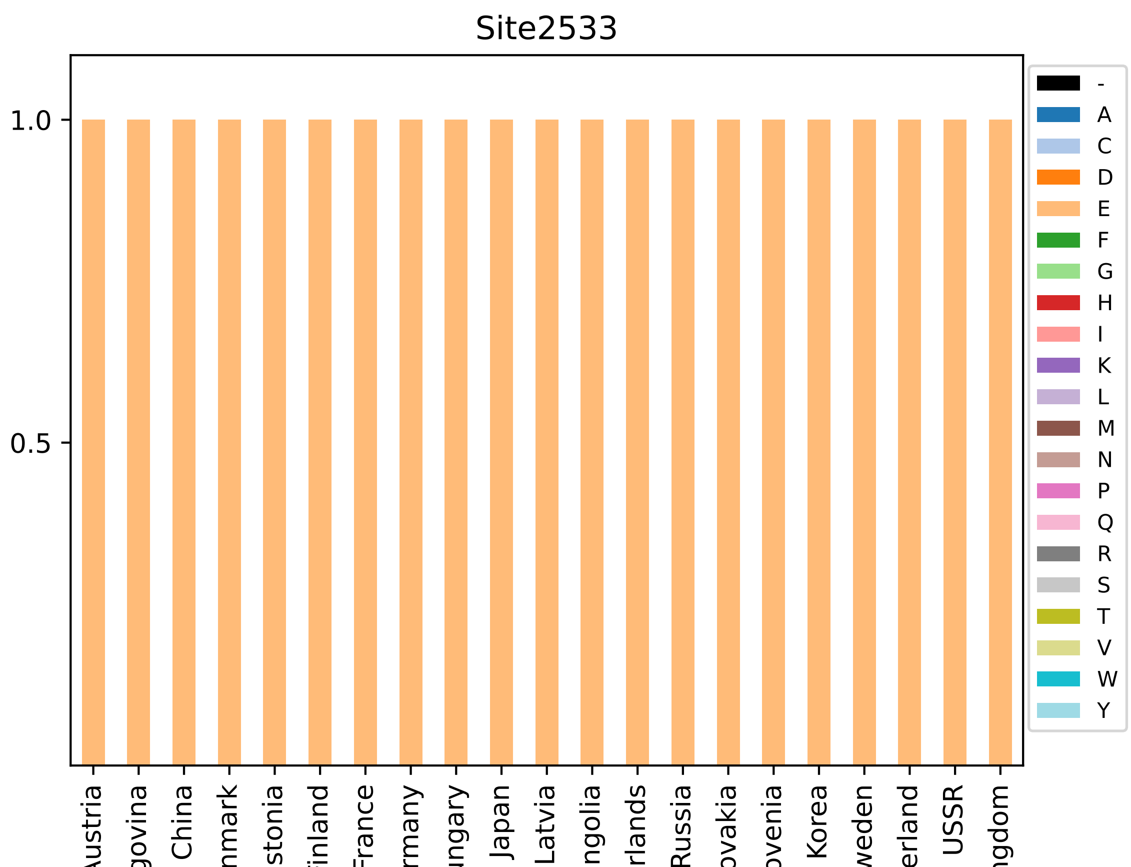
Site2533
1.0
0.5
-
A
C
D
E
F
G
H
I
K
L
M
N
P
Q
R
S
T
V
W
Y
China
Japan
Latvia
Russia
Korea
USSR

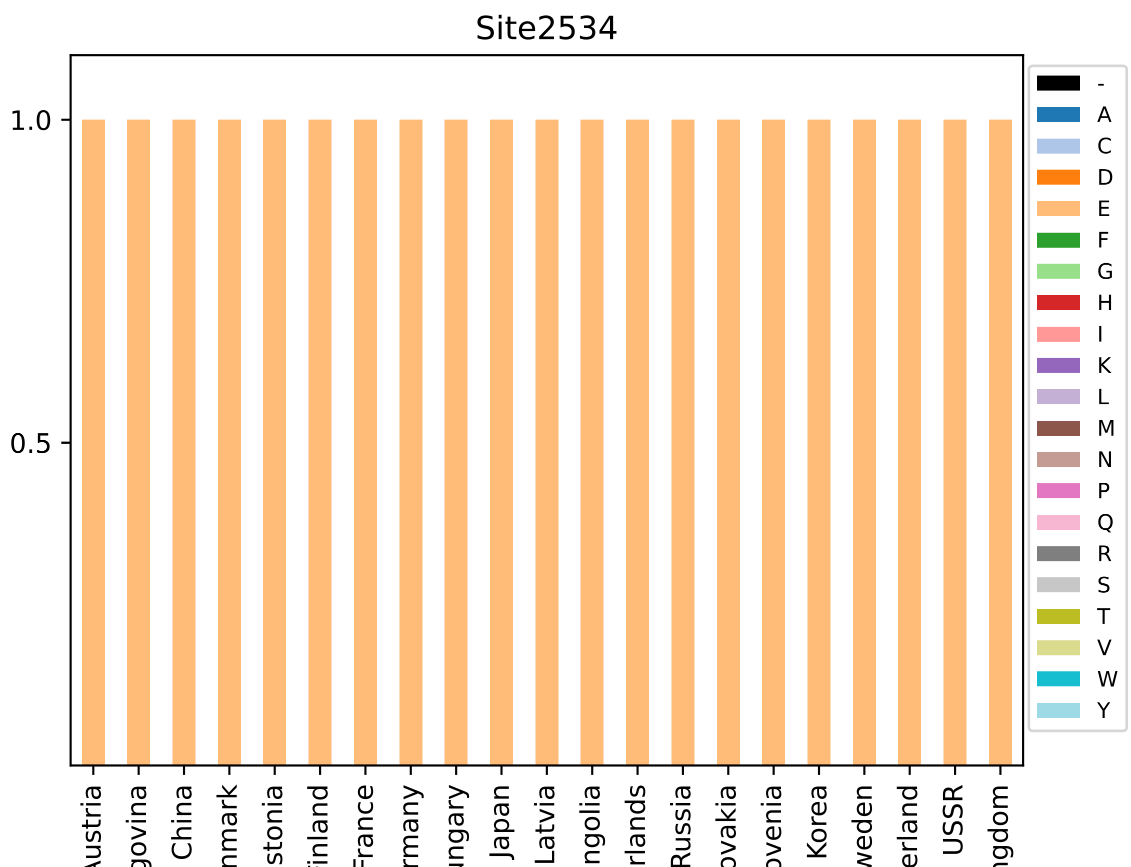
Site2534
1.0
0.5
-
A
C
D
E
F
G
H
I
K
L
M
N
P
Q
R
S
T
V
W
Y
China
Japan
Latvia
Russia
Korea
USSR

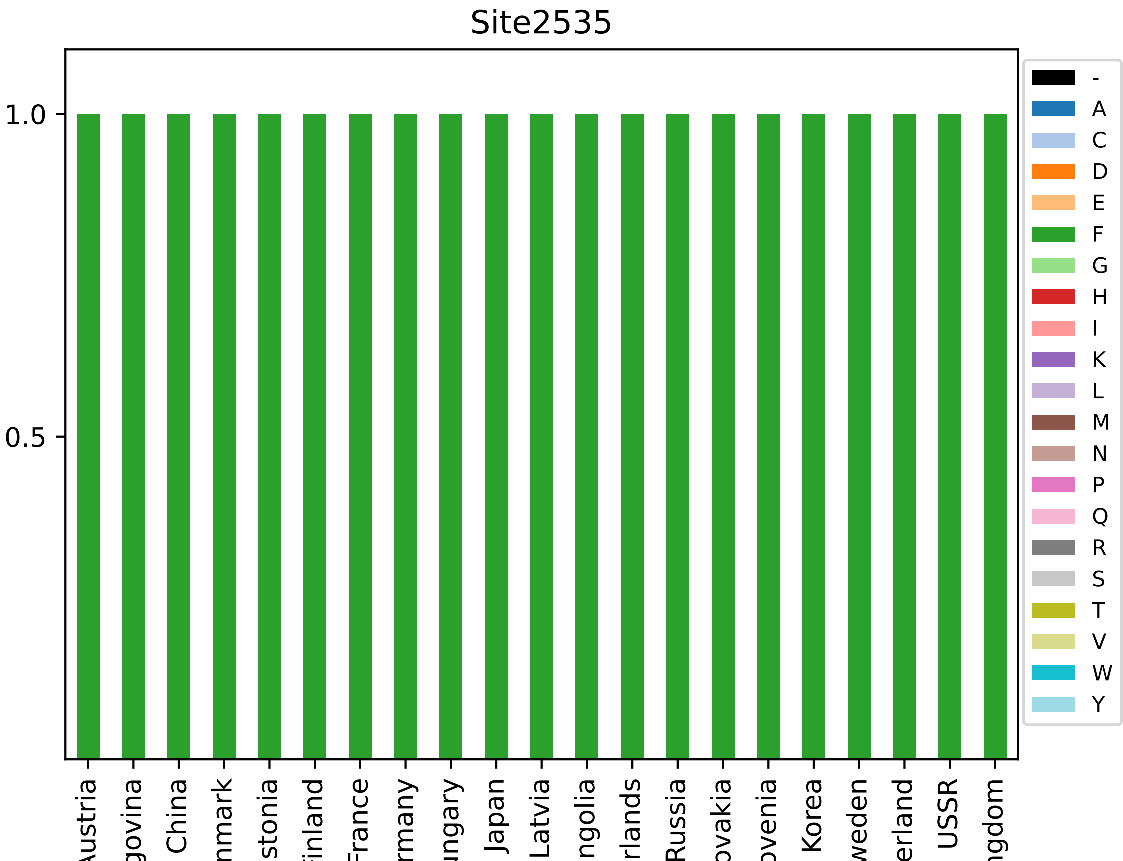
Site2535
1.0
0.5
-
A
C
D
E
F
G
H
I
K
L
M
N
P
Q
R
S
T
V
W
Y
China
Japan
Latvia
Russia
Korea
USSR

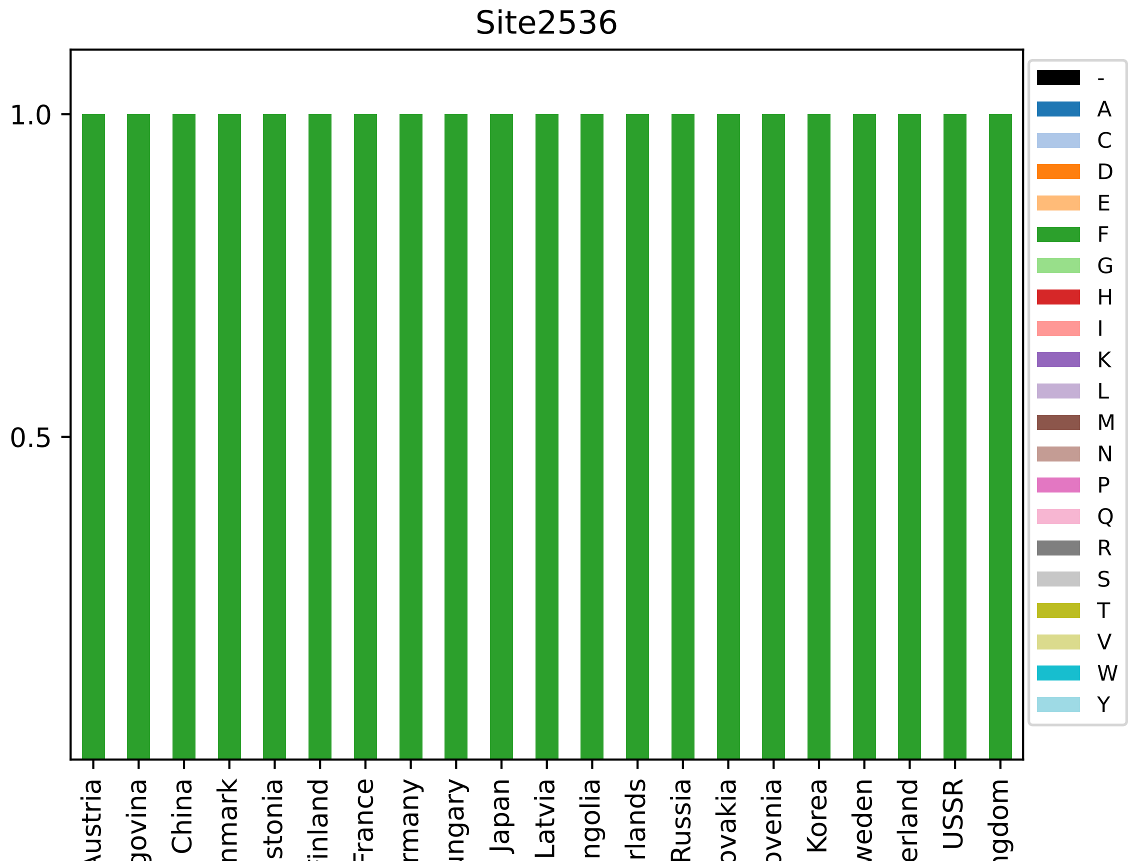

Site2536
1.0
0.5
-
A
C
D
E
F
G
H
I
K
L
M
N
P
Q
R
S
T
V
W
Y
China
Japan
Latvia
Russia
Korea
USSR

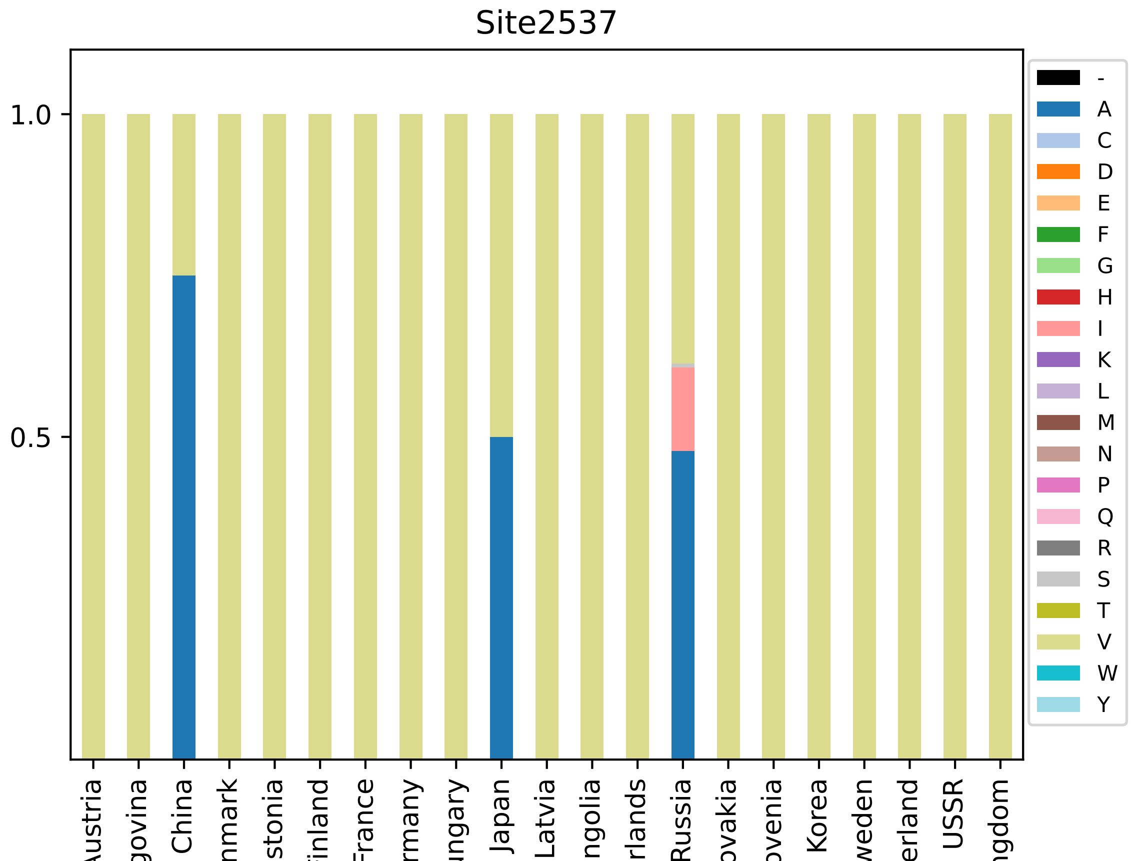

Site2537
1.0
0.5
-
A
C
D
E
F
G
H
I
K
L
M
N
P
Q
R
S
T
V
W
Y
China
Japan
Latvia
Russia
Korea
USSR

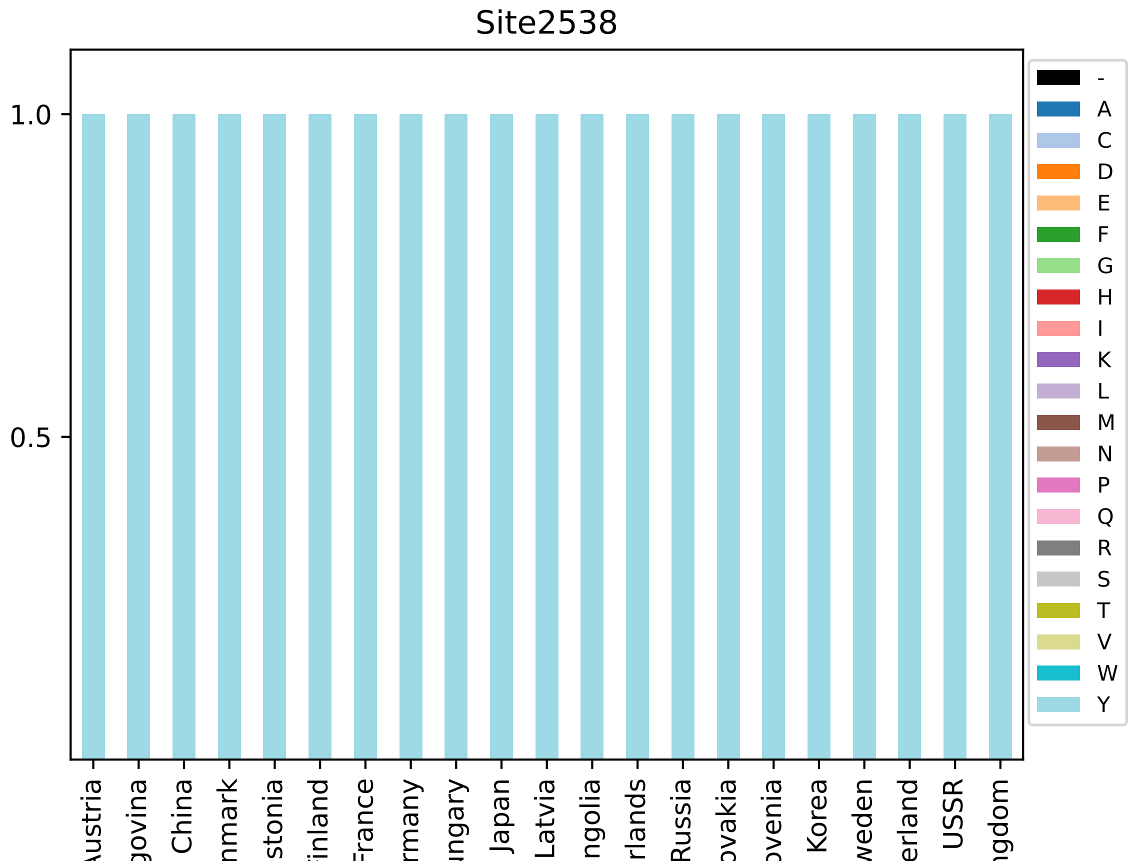
Site2538
1.0
0.5
-
A
C
D
E
F
G
H
I
K
L
M
N
P
Q
R
S
T
V
W
Y
China
Japan
Latvia
Russia
Korea
USSR

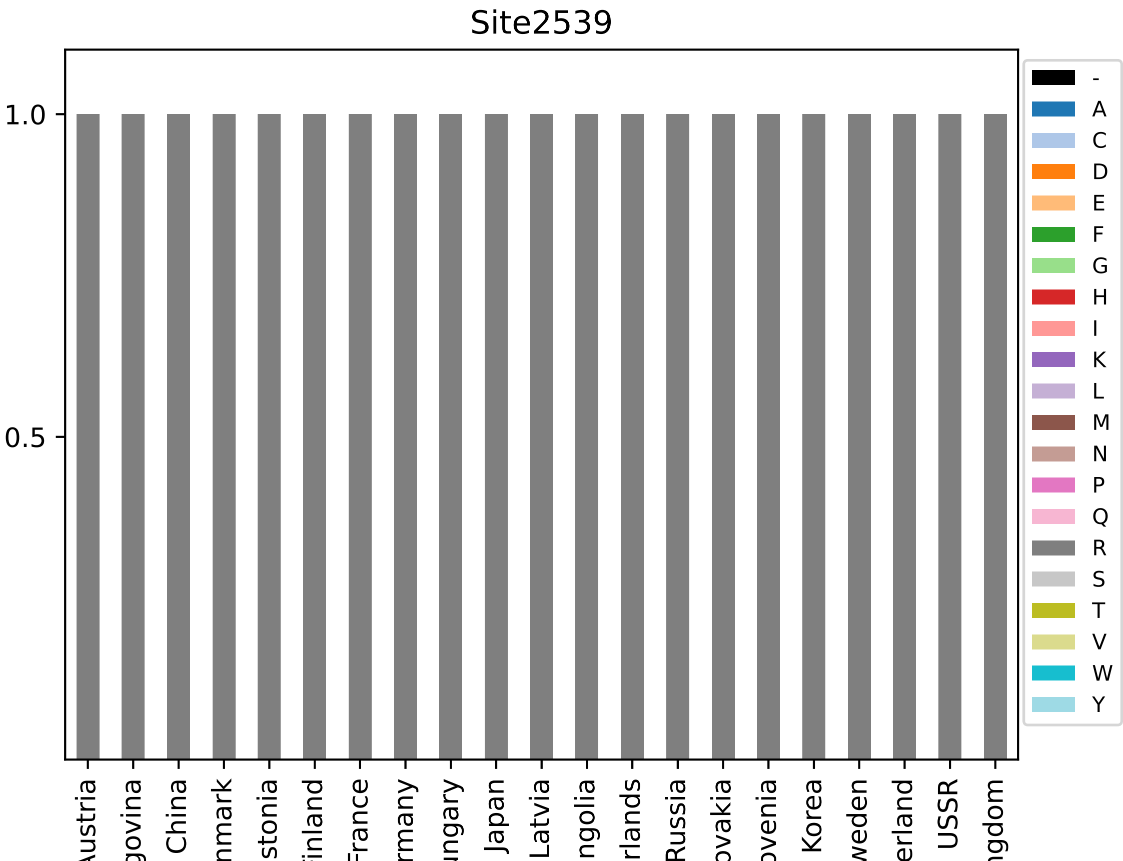
Site2539
1.0
0.5
-
A
C
D
E
F
G
H
I
K
L
M
N
P
Q
R
S
T
V
W
Y
China
Japan
Latvia
Russia
Korea
USSR

Site2540

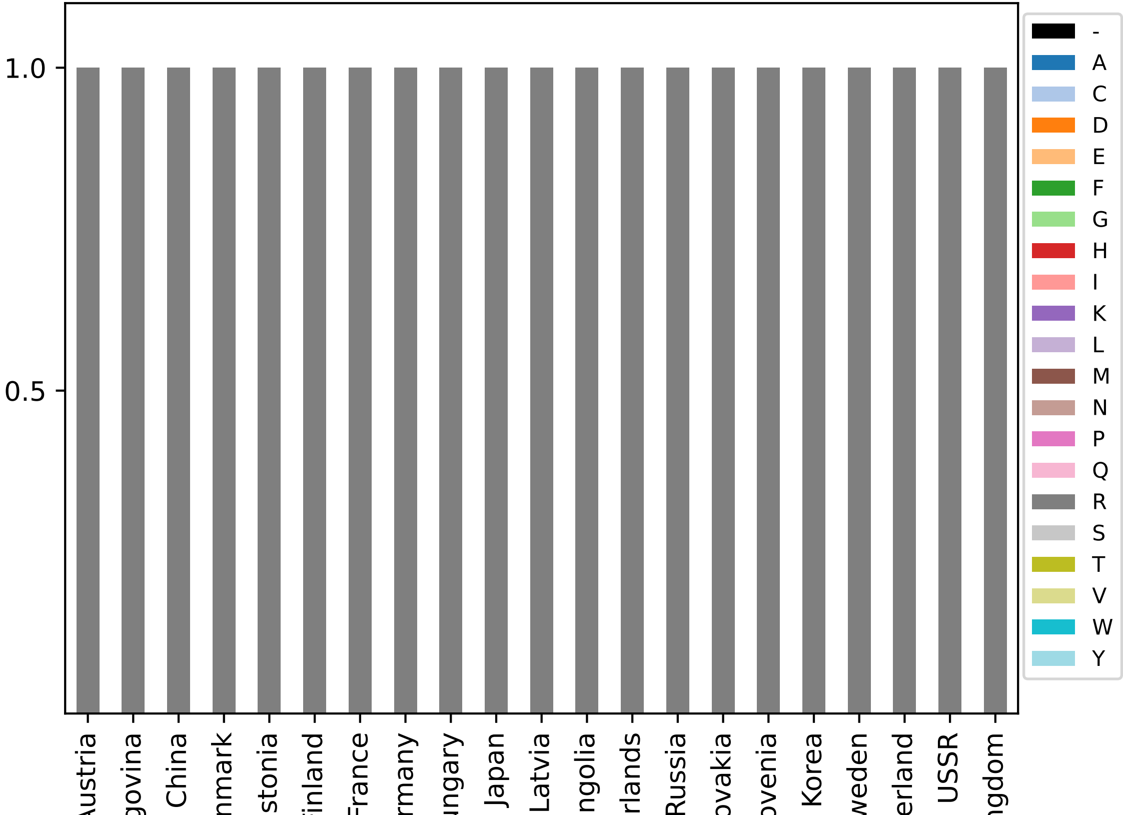
1.0
0.5
-
A
C
D
E
F
G
H
I
K
L
M
N
P
Q
R
S
T
V
W
Y
China
Japan
Latvia
Russia
Korea
USSR

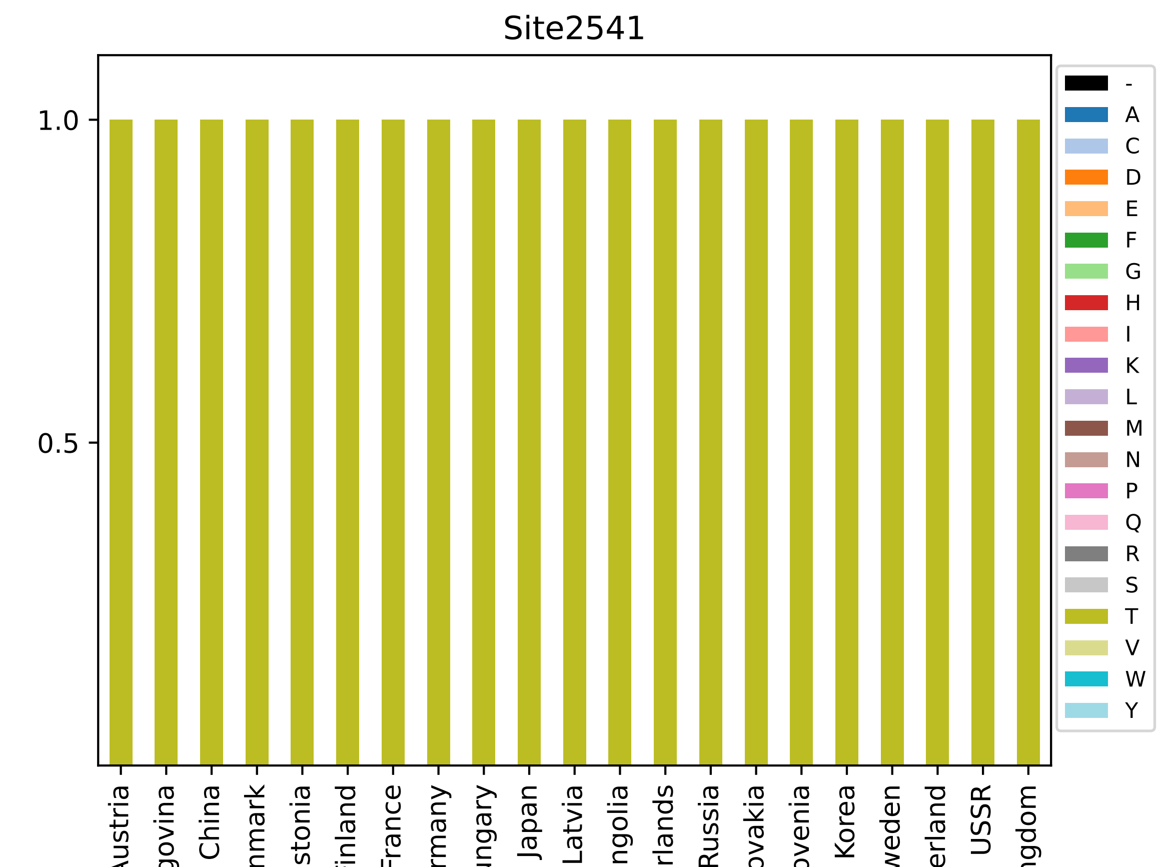

Site2541
1.0
0.5
-
A
C
D
E
F
G
H
I
K
L
M
N
P
Q
R
S
T
V
W
Y
Austria
China
Finland
France
Japan
Latvia
Russia
Korea
USSR

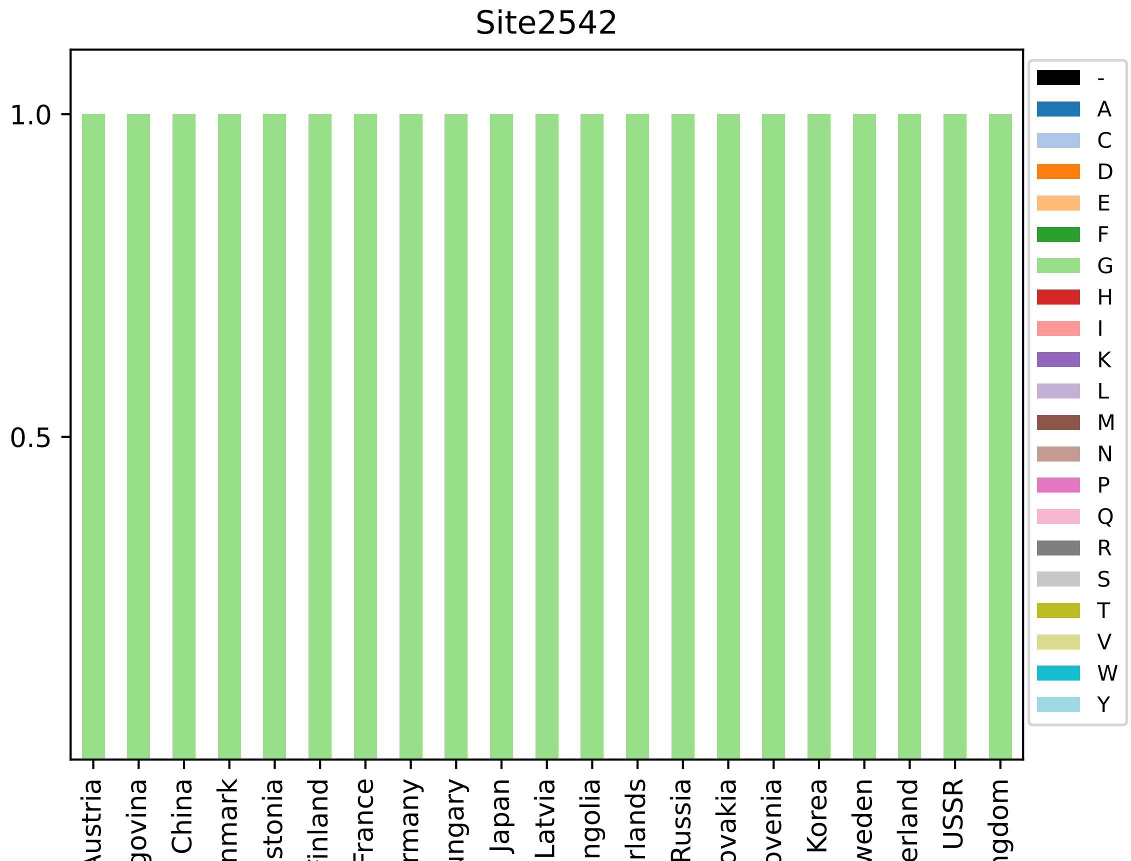
Site2542
1.0
0.5
-
A
C
D
E
F
G
H
I
K
L
M
N
P
Q
R
S
T
V
W
Y
China
France
Japan
Latvia
Russia
Korea
USSR

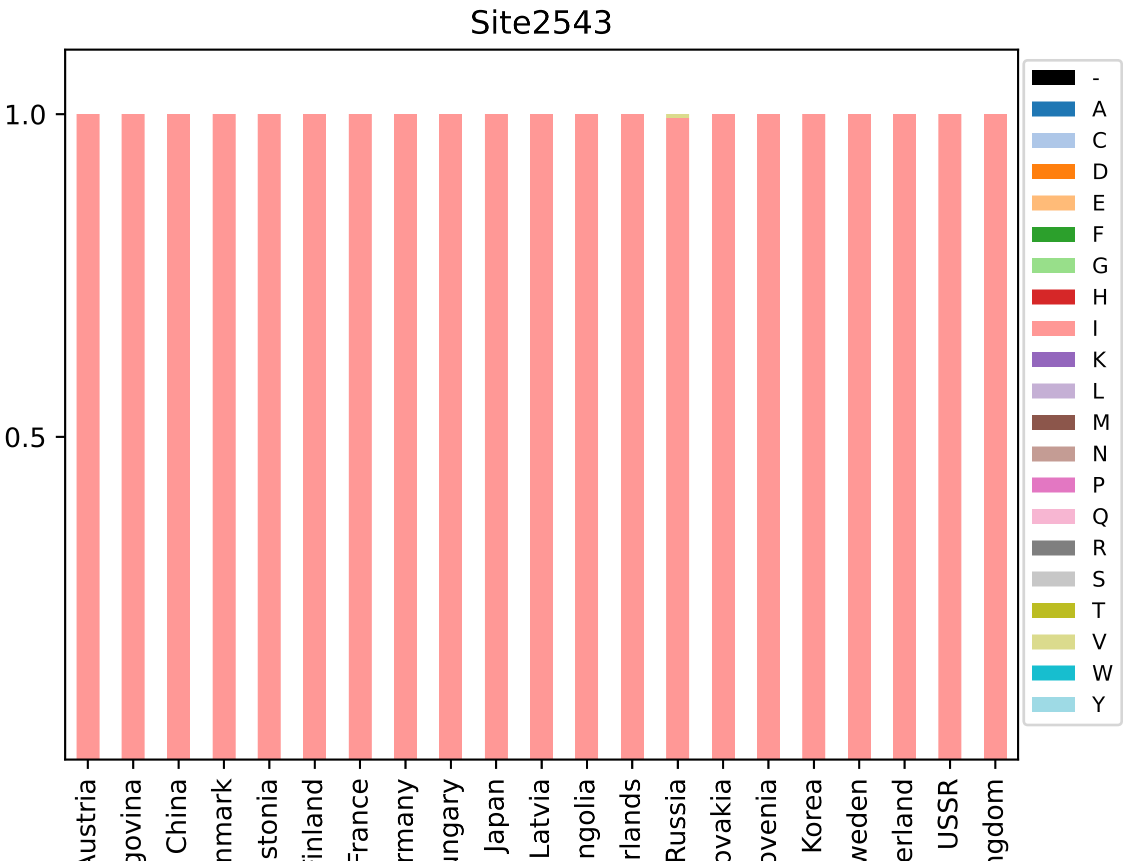

Site2543
1.0
0.5
-
A
C
D
E
F
G
H
I
K
L
M
N
P
Q
R
S
T
V
W
Y
China
Japan
Latvia
Russia
Korea
USSR

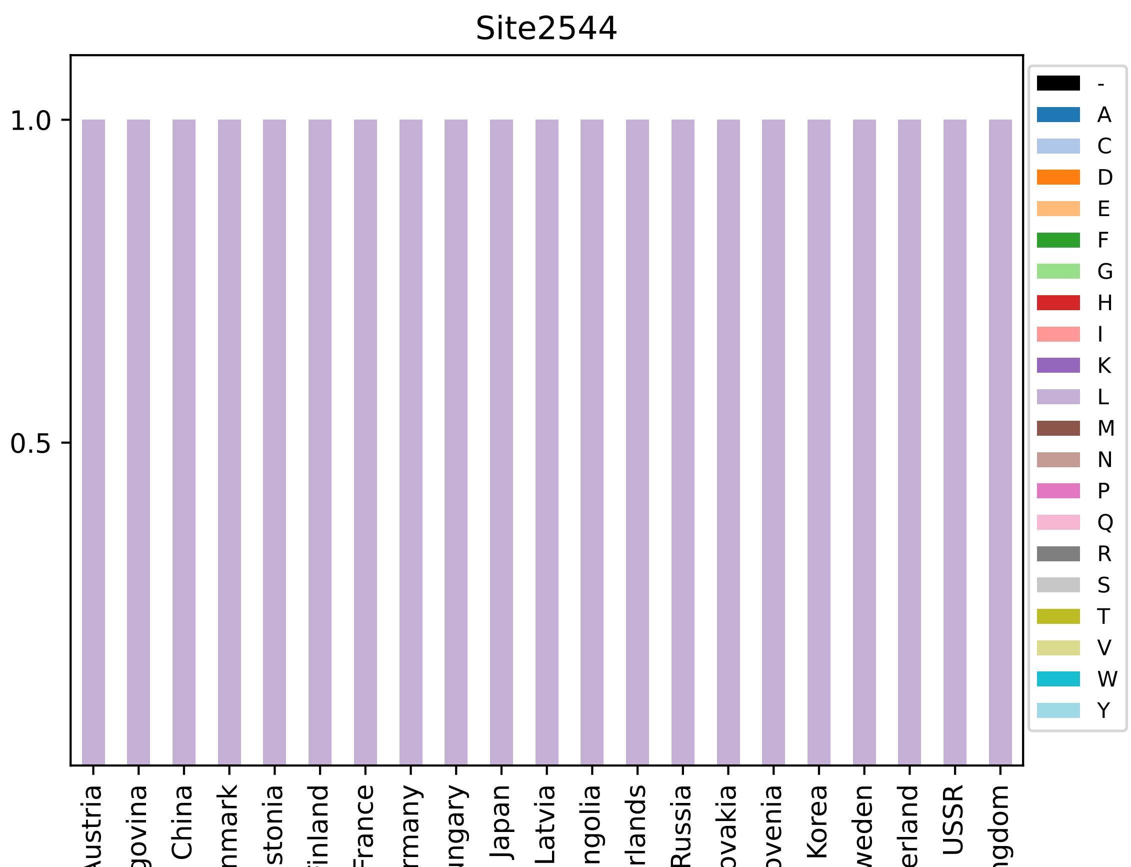

Site2544
1.0
0.5
-
A
C
D
E
F
G
H
I
K
L
M
N
P
Q
R
S
T
V
W
Y
ustria
govina
China
nmark
stonia
inland
rance
rmany
ungary
Japan
Latvia
ngolia
rlands
Russia
ovakia
ovenia
Korea
weden
erland
USSR
ngdom

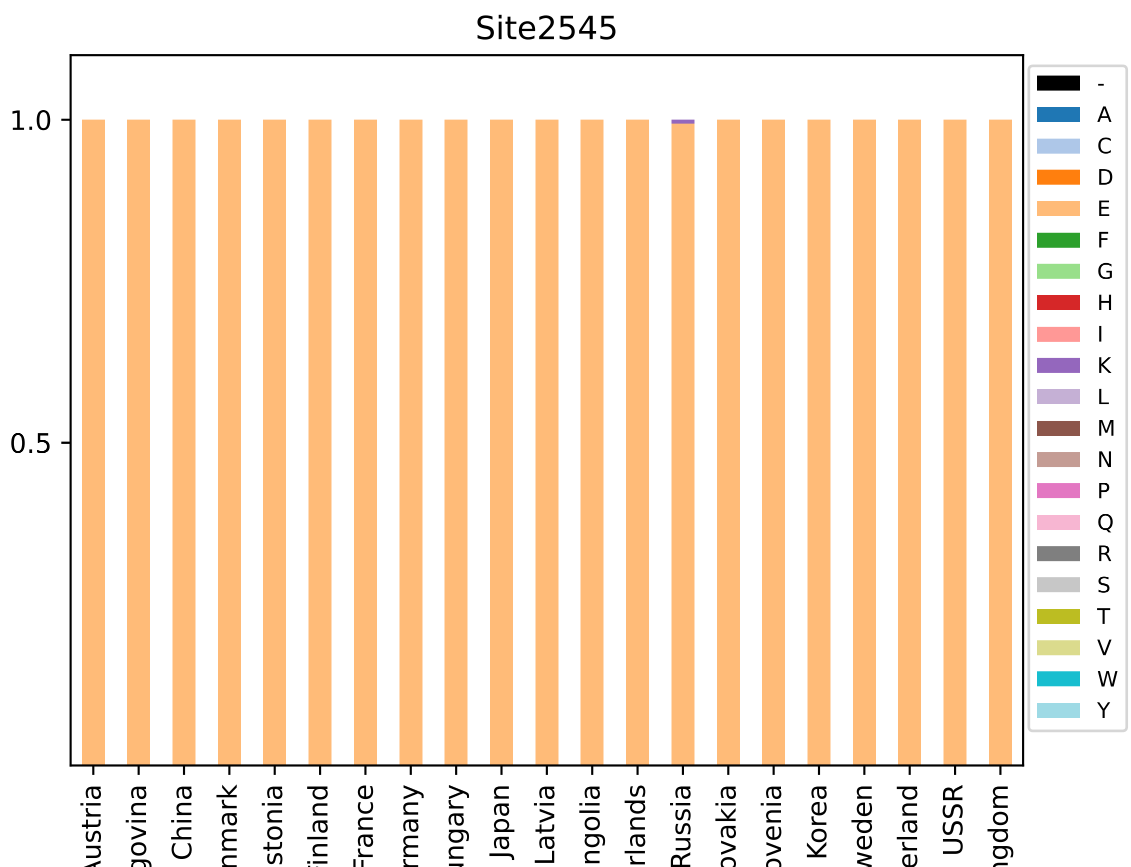

Site2545
1.0
0.5
-
A
C
D
E
F
G
H
I
K
L
M
N
P
Q
R
S
T
V
W
Y
China
Japan
Latvia
Russia
Korea
USSR

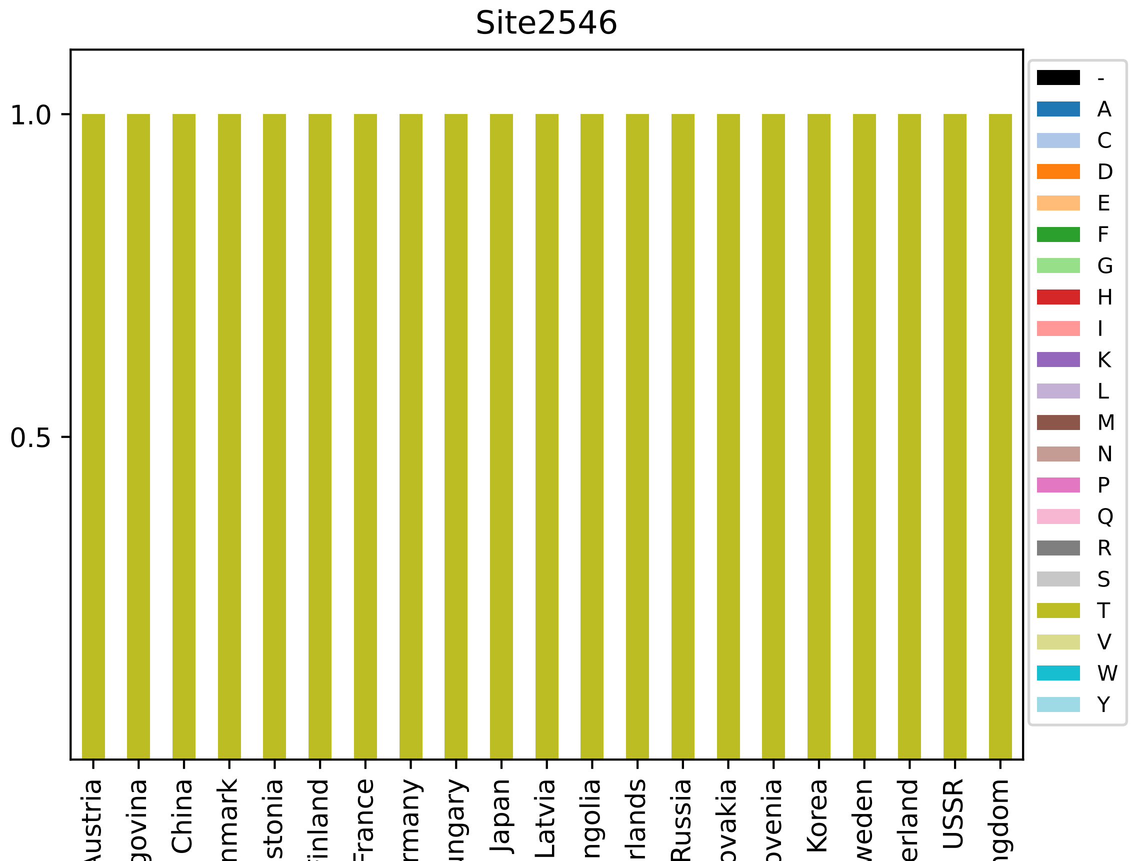
Site2546
1.0
0.5
-
A
C
D
E
F
G
H
I
K
L
M
N
P
Q
R
S
T
V
W
Y
China
Japan
Latvia
Russia
Korea
USSR

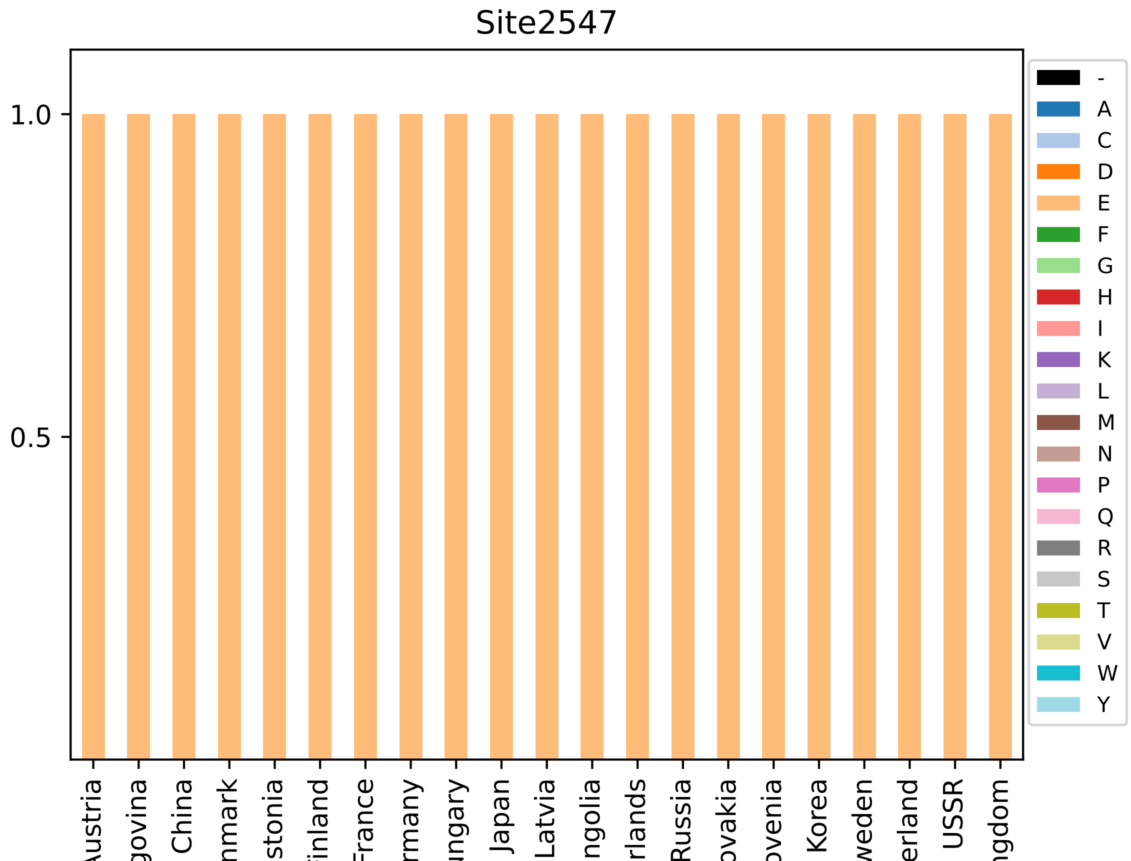
Site2547
1.0
0.5
-
A
C
D
E
F
G
H
I
K
L
M
N
P
Q
R
S
T
V
W
Y
China
Japan
Latvia
Russia
Korea
USSR

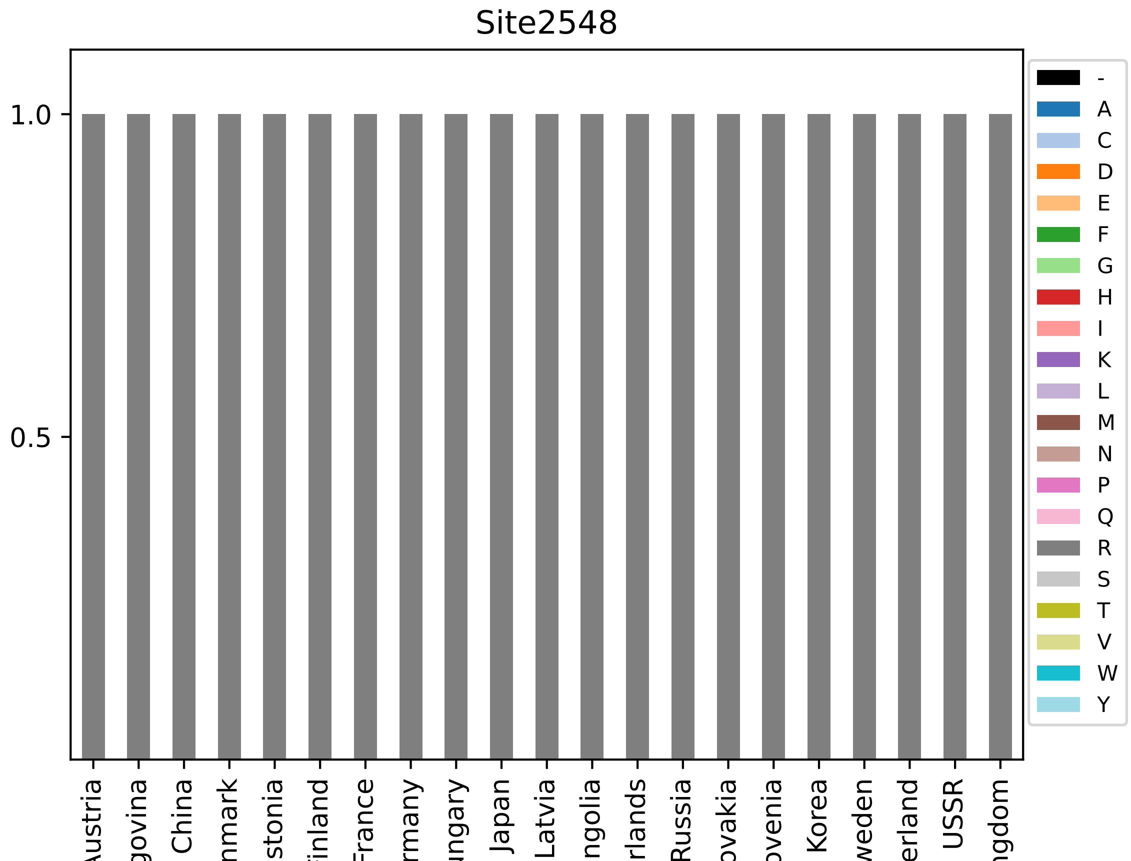

Site2548
1.0
0.5
-
A
C
D
E
F
G
H
I
K
L
M
N
P
Q
R
S
T
V
W
Y
Austria
China
Finland
France
Japan
Latvia
Russia
Korea
USSR

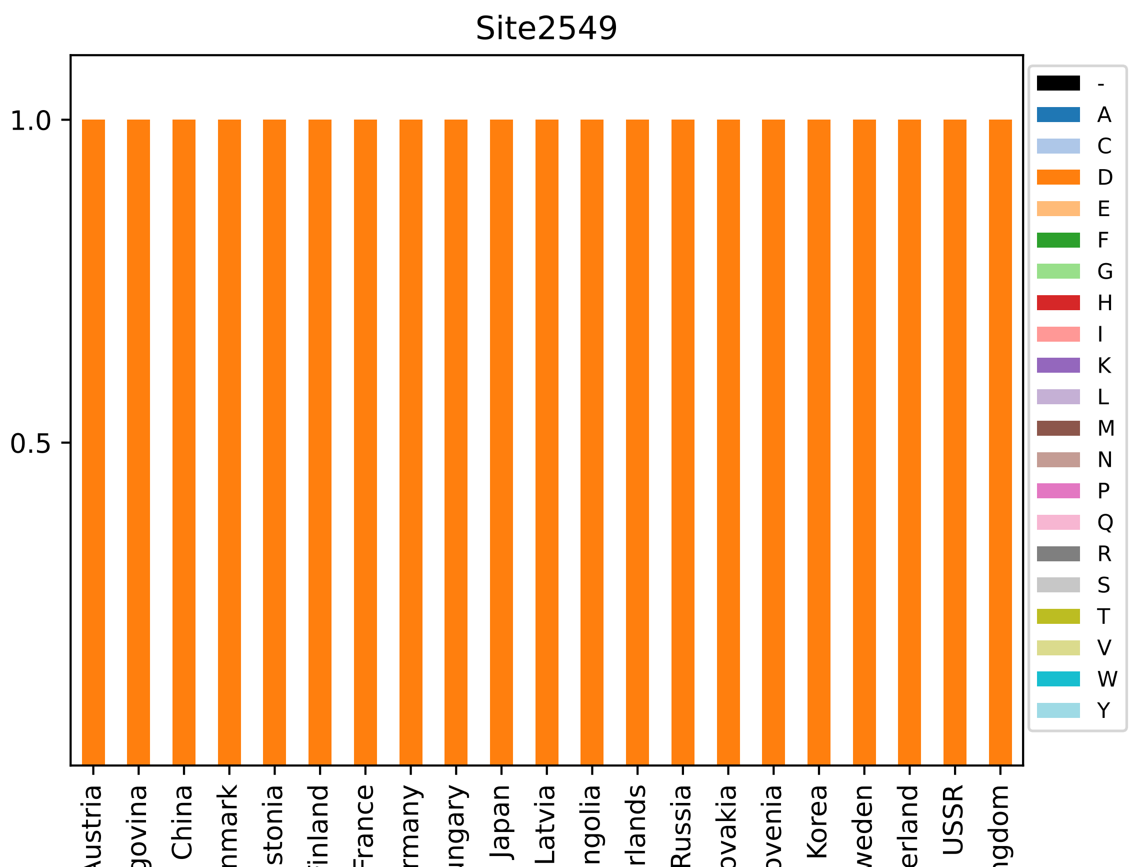

Site2549
1.0
0.5
-
A
C
D
E
F
G
H
I
K
L
M
N
P
Q
R
S
T
V
W
Y
China
Japan
Latvia
Russia
Korea
USSR

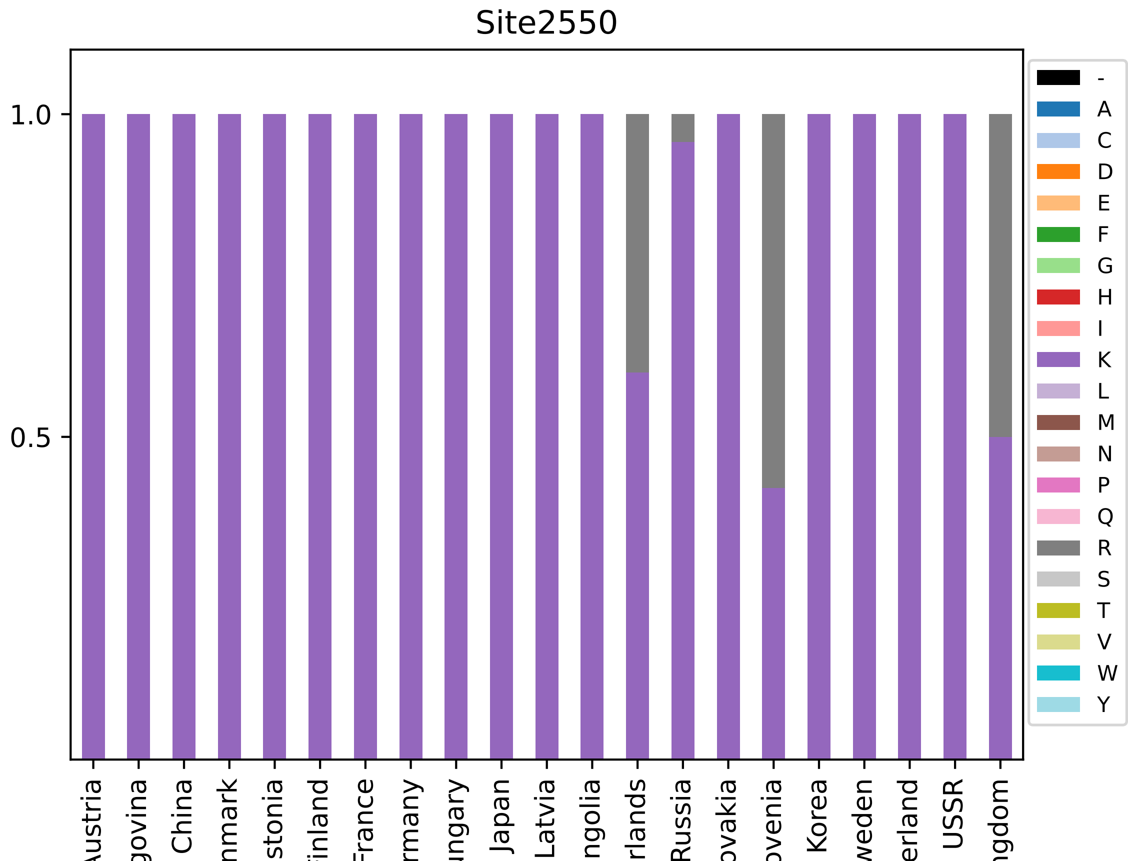
Site2550
1.0
0.5
Austria
govina
China
nmark
stonia
inland
France
rmany
ungary
Japan
Latvia
ngolia
rlands
Russia
ovakia
ovenia
Korea
weden
erland
USSR
ngdom
-
A
C
D
E
F
G
H
I
K
L
M
N
P
Q
R
S
T
V
W
Y

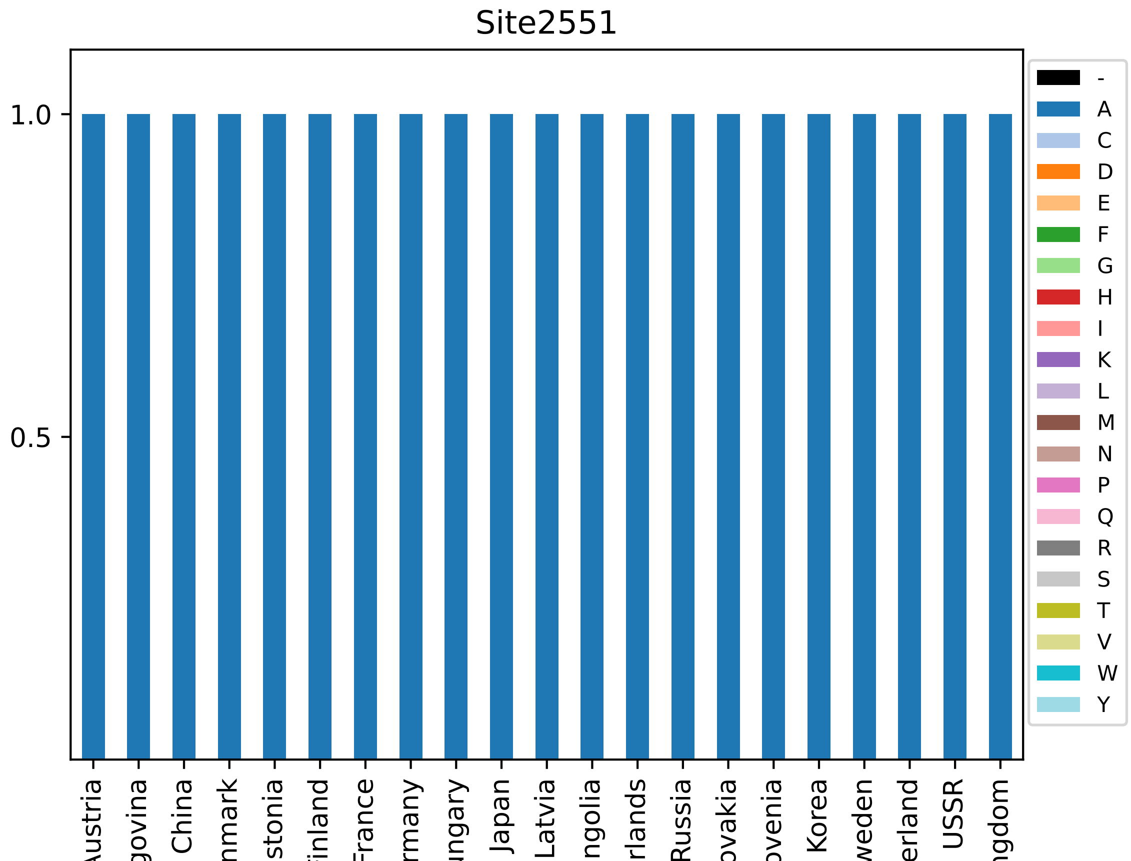
Site2551
1.0
0.5
-
A
C
D
E
F
G
H
I
K
L
M
N
P
Q
R
S
T
V
W
Y
Austria
govina
China
nmark
stonia
inland
France
rmany
ungary
Japan
Latvia
ngolia
rlands
Russia
ovakia
ovenia
Korea
weden
erland
USSR
ngdom

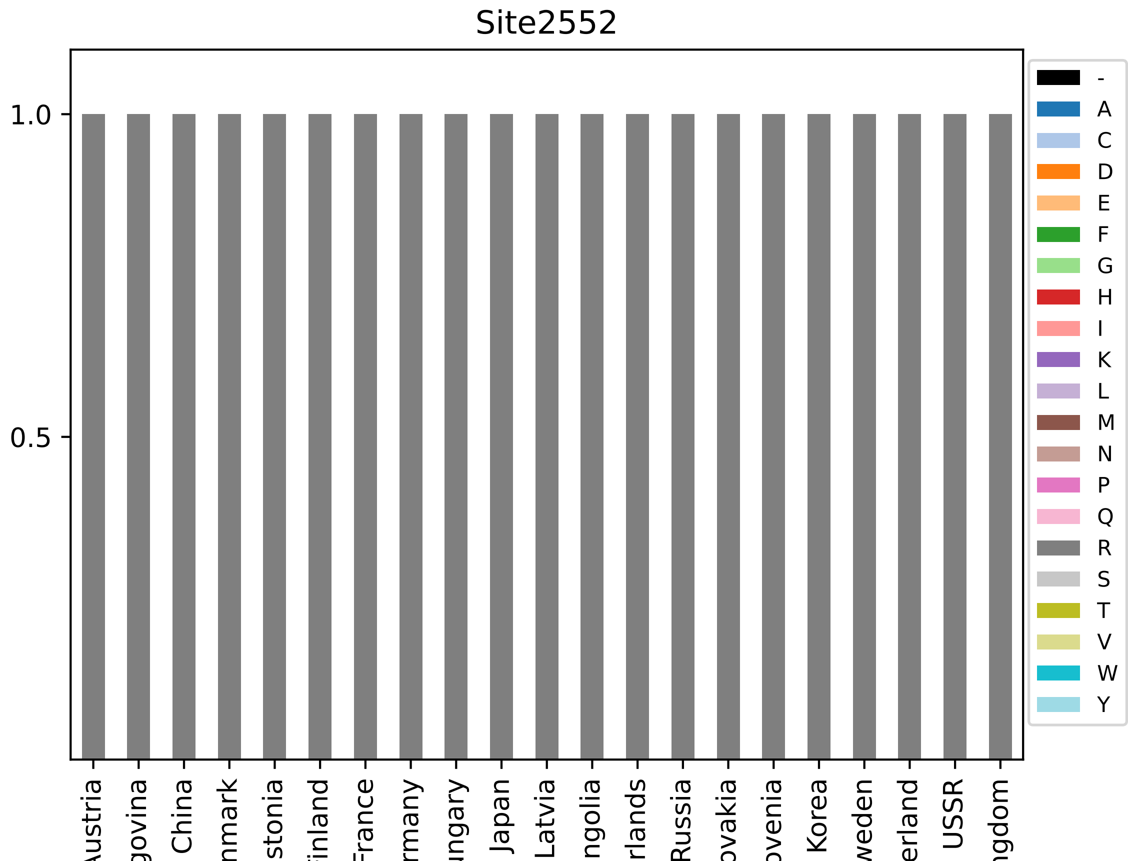

Site2552
1.0
0.5
-
A
C
D
E
F
G
H
I
K
L
M
N
P
Q
R
S
T
V
W
Y
Austria
China
Japan
Latvia
Russia
Korea
USSR

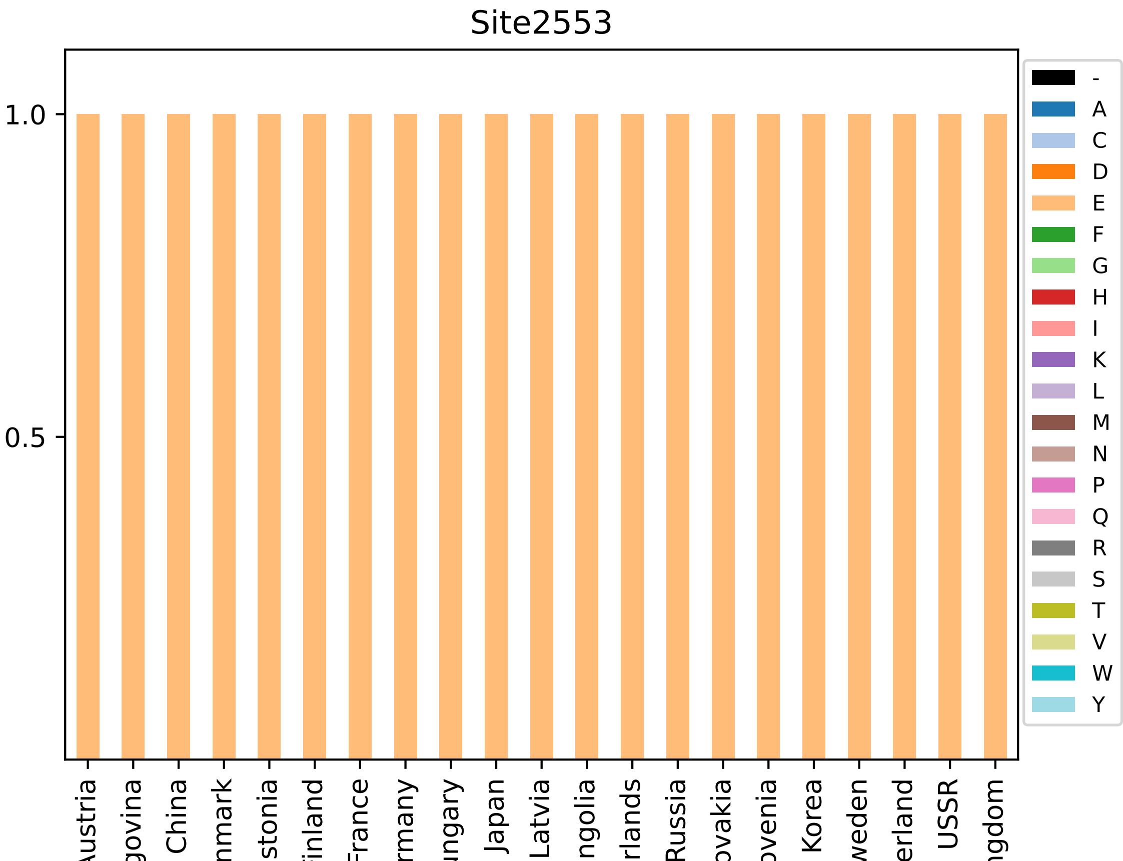
Site2553
1.0
0.5
-
A
C
D
E
F
G
H
I
K
L
M
N
P
Q
R
S
T
V
W
Y
China
Japan
Latvia
Russia
Korea
USSR

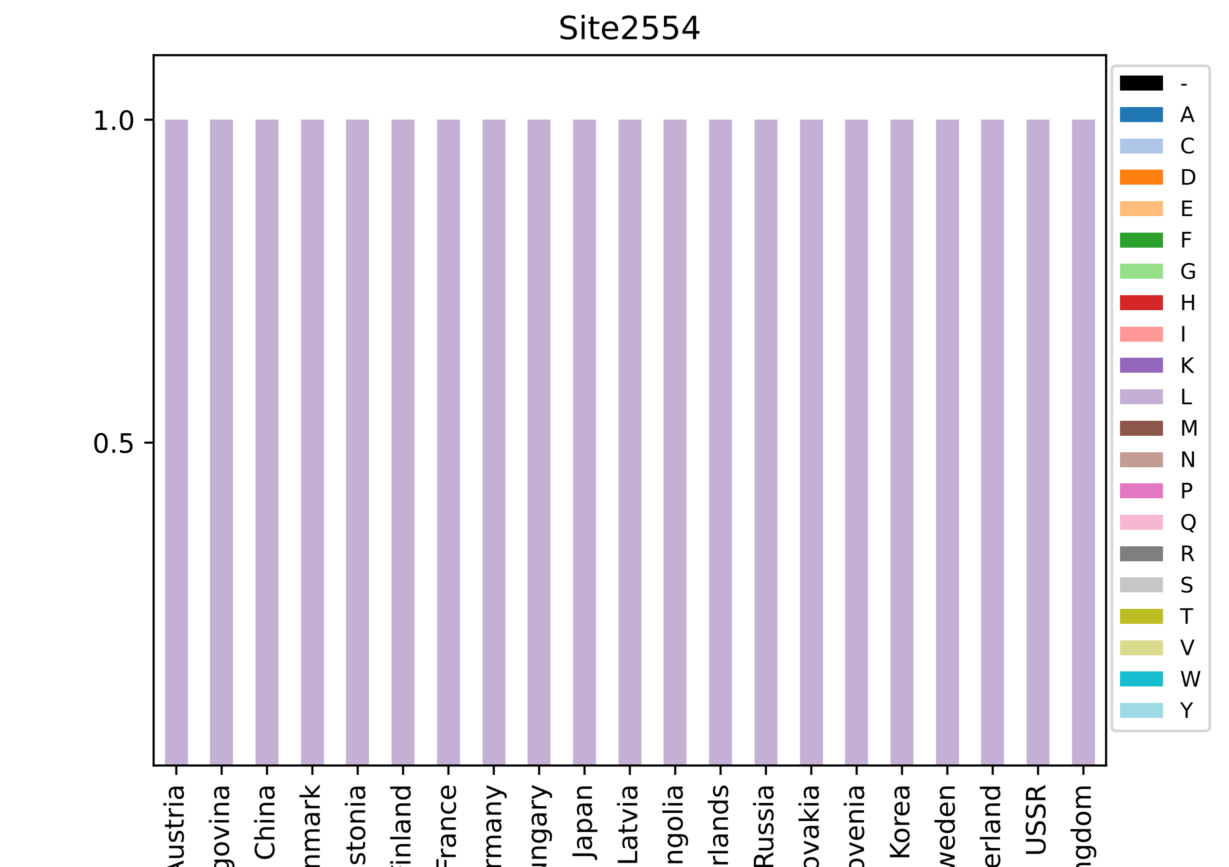

Site2554
1.0
0.5
-
A
C
D
E
F
G
H
I
K
L
M
N
P
Q
R
S
T
V
W
Y
Japan
Latvia
Russia
Korea
USSR

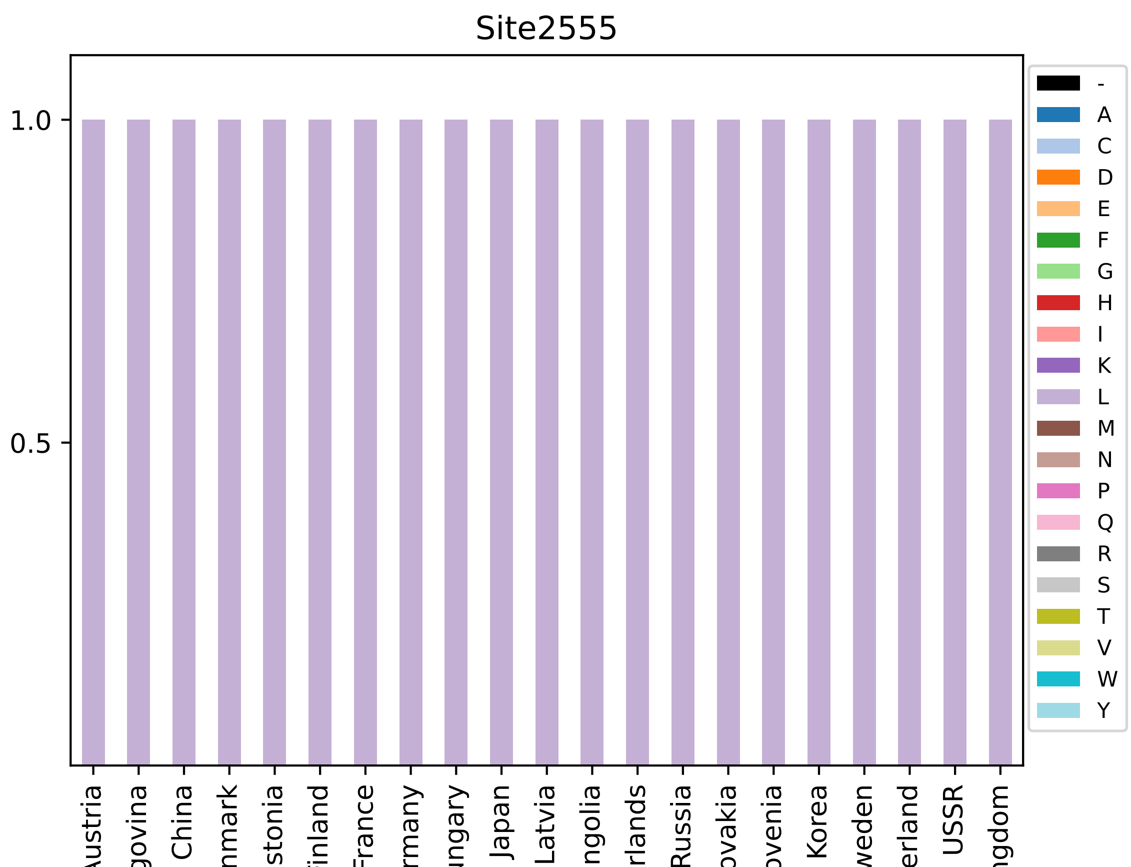
Site2555
1.0
0.5
-
A
C
D
E
F
G
H
I
K
L
M
N
P
Q
R
S
T
V
W
Y
China
Japan
Latvia
Russia
Korea
USSR

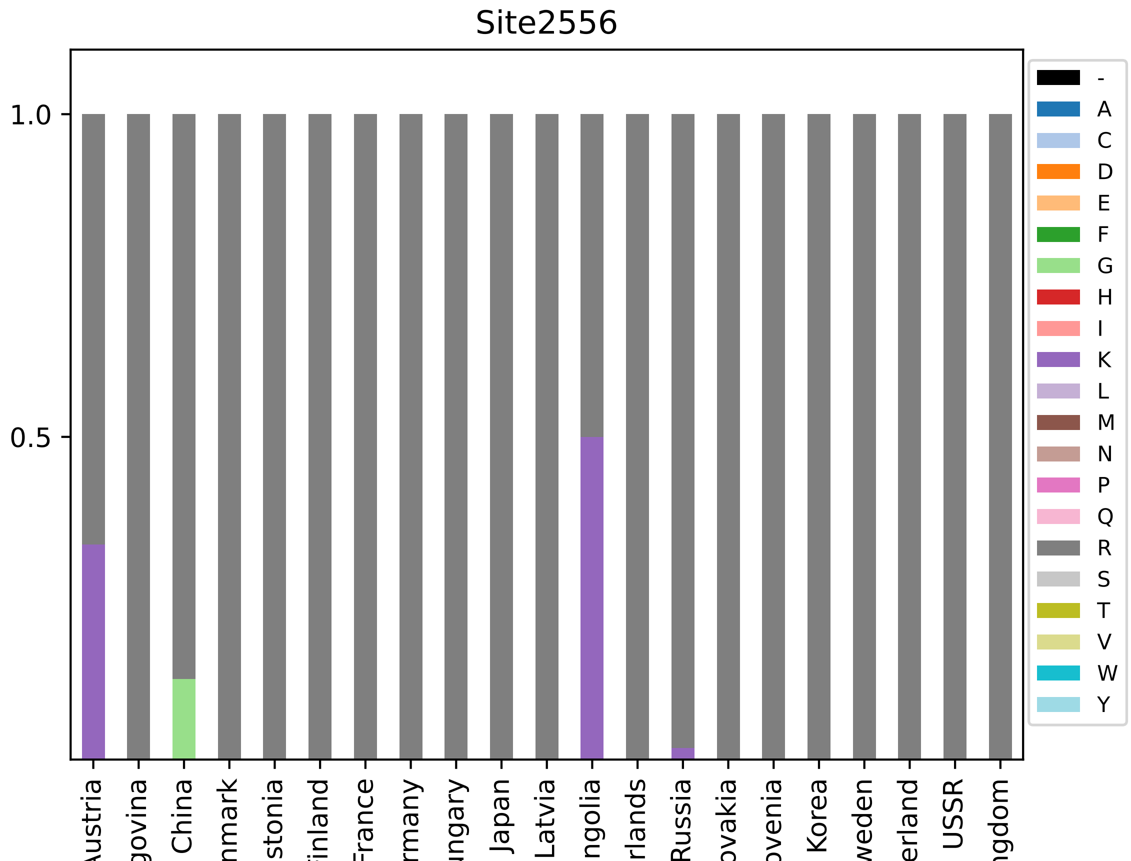
Site2556
1.0
0.5
-
A
C
D
E
F
G
H
I
K
L
M
N
P
Q
R
S
T
V
W
Y
China
Japan
Latvia
Russia
Korea
USSR

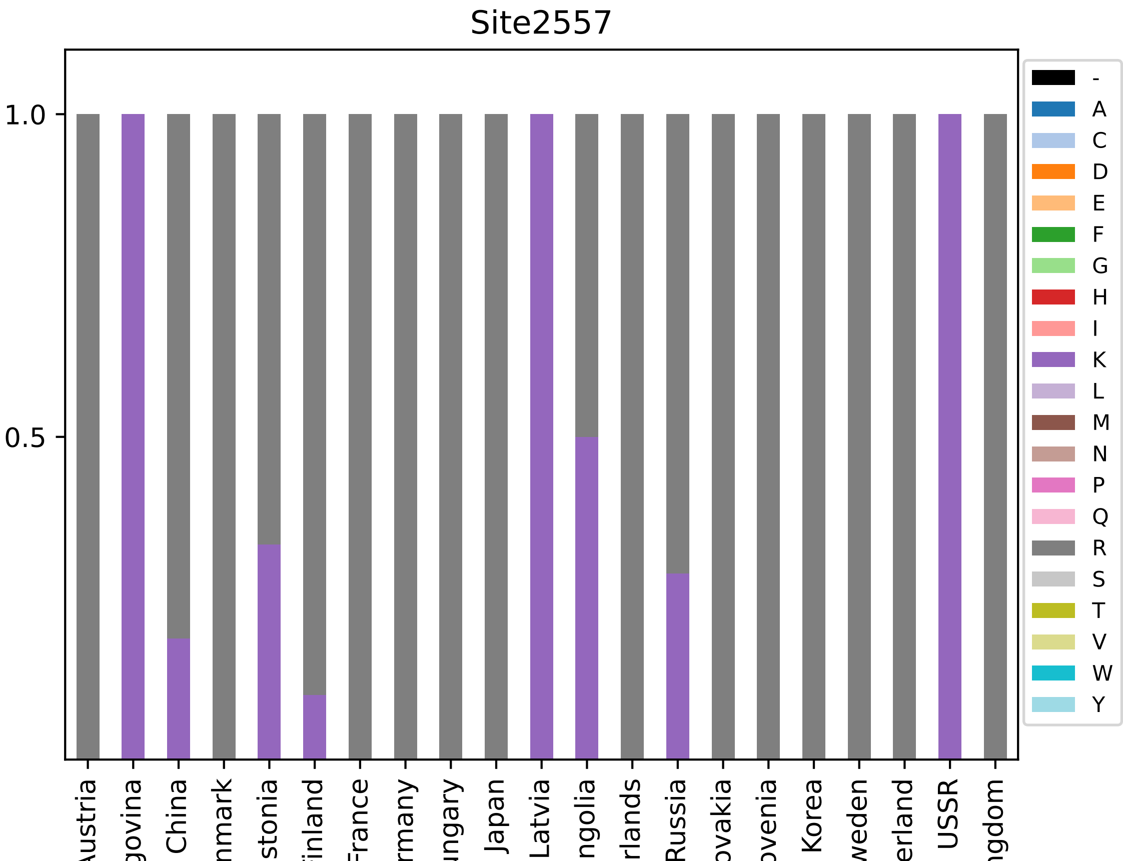
Site2557
1.0
0.5
-
A
C
D
E
F
G
H
I
K
L
M
N
P
Q
R
S
T
V
W
Y
China
Japan
Latvia
Russia
Korea
USSR

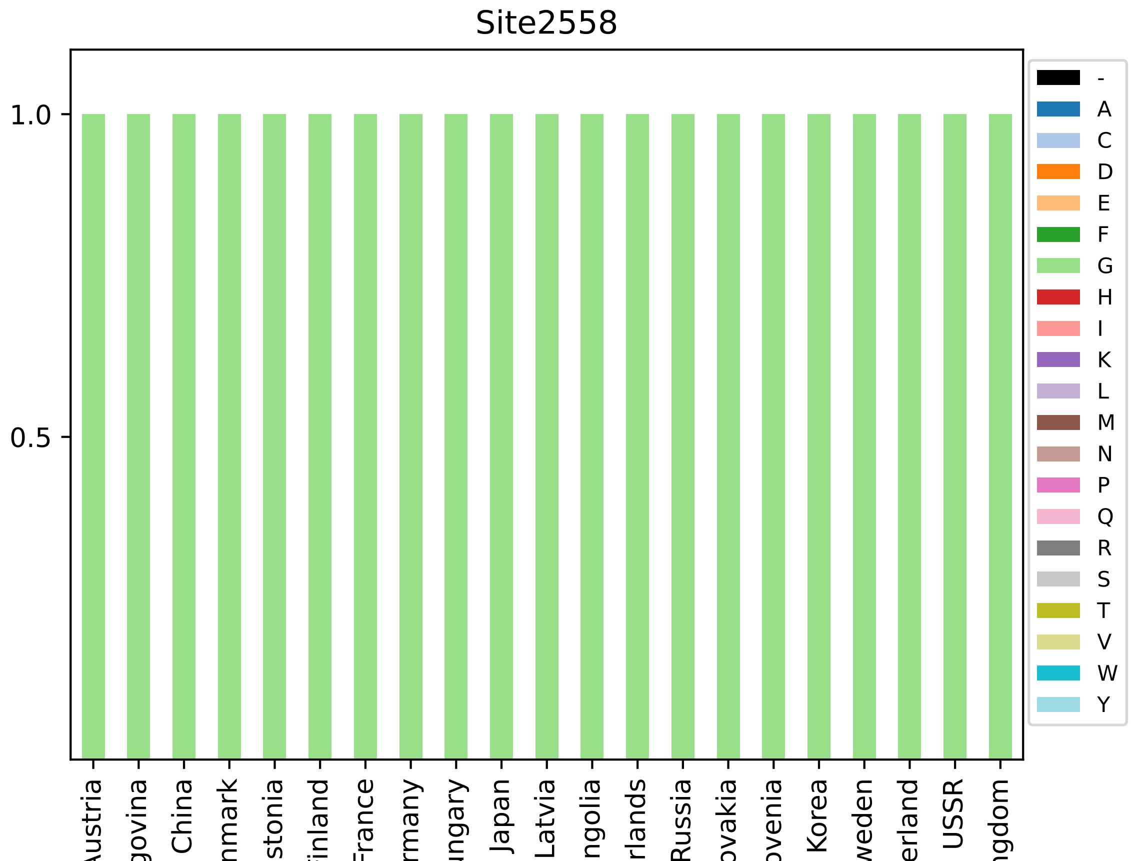
Site2558
1.0
0.5
-
A
C
D
E
F
G
H
I
K
L
M
N
P
Q
R
S
T
V
W
Y
China
Japan
Latvia
Russia
Korea
USSR

Site2559

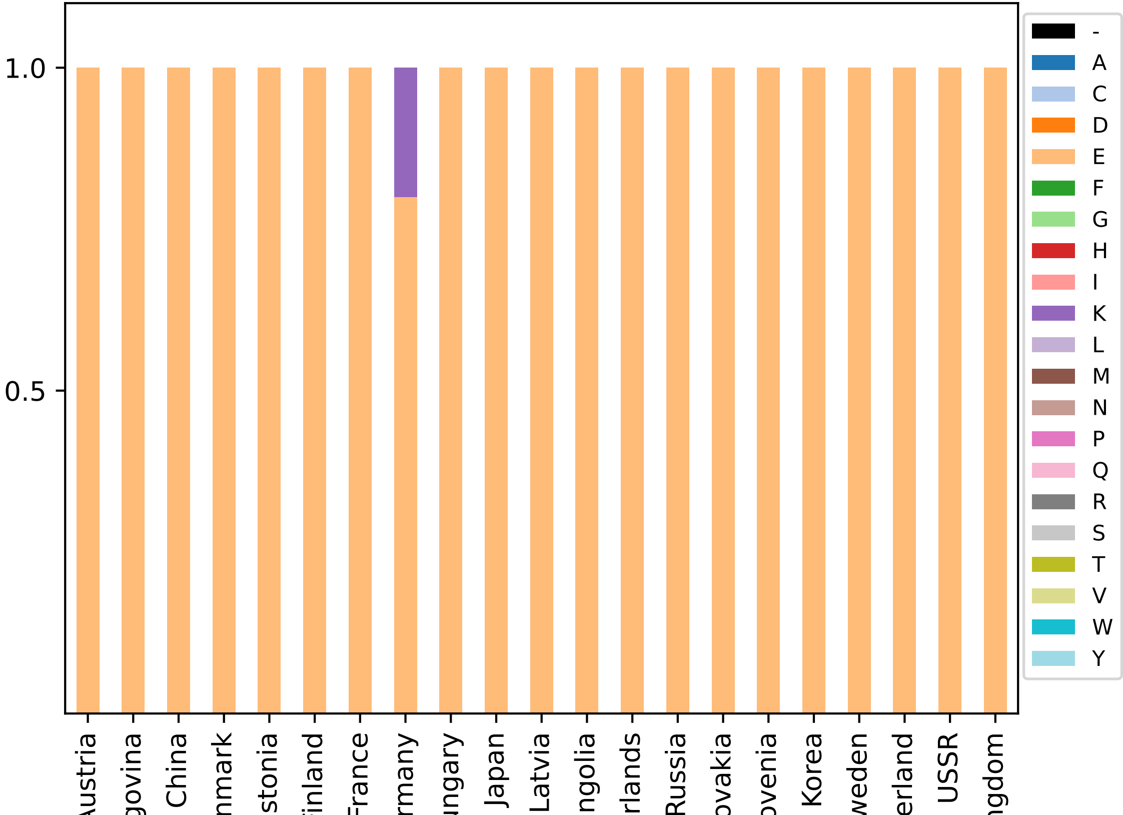
1.0
0.5
-
A
C
D
E
F
G
H
I
K
L
M
N
P
Q
R
S
T
V
W
Y
Austria
govina
China
nmark
stonia
Finland
France
rmany
ungary
Japan
Latvia
ngolia
rlands
Russia
ovakia
ovenia
Korea
weden
erland
USSR
ngdom

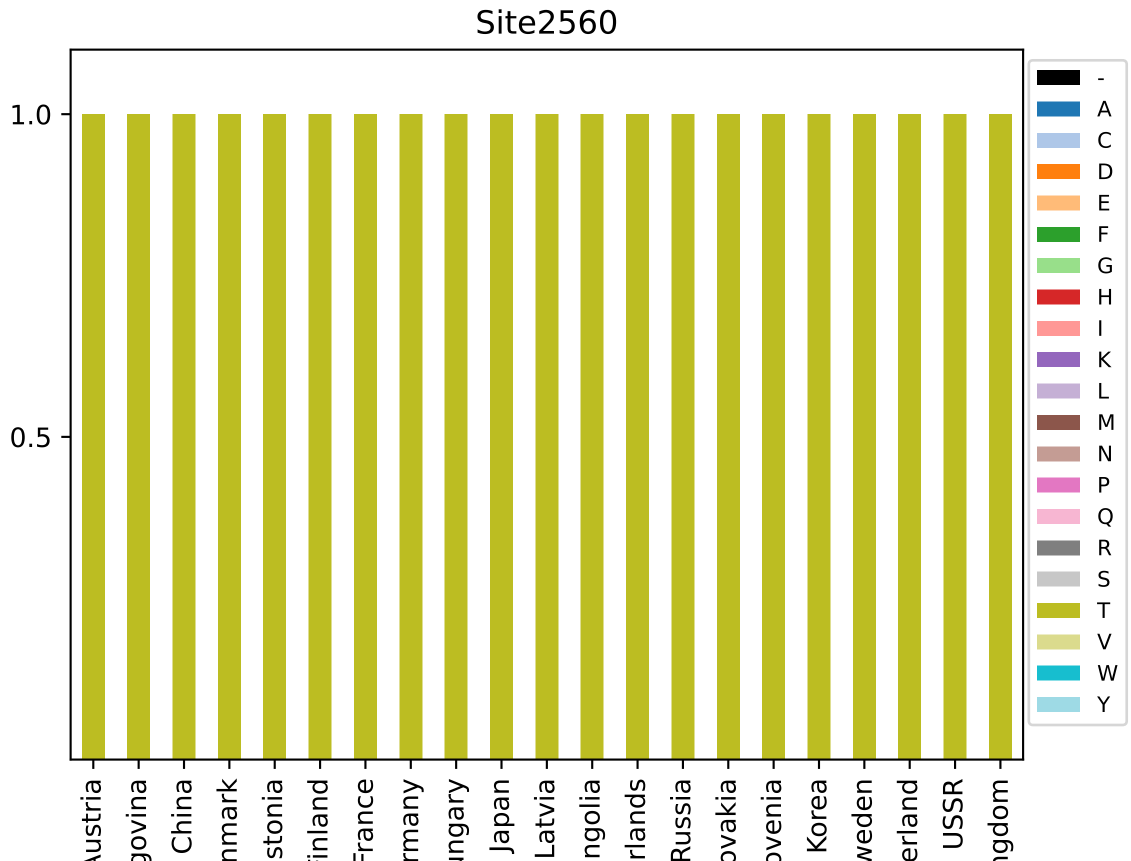

Site2560
1.0
0.5
-
A
C
D
E
F
G
H
I
K
L
M
N
P
Q
R
S
T
V
W
Y
govina
China
nmark
stonia
rmany
ungary
Japan
Latvia
ngolia
rlands
Russia
ovakia
ovenia
Korea
weden
erland
USSR
ngdom

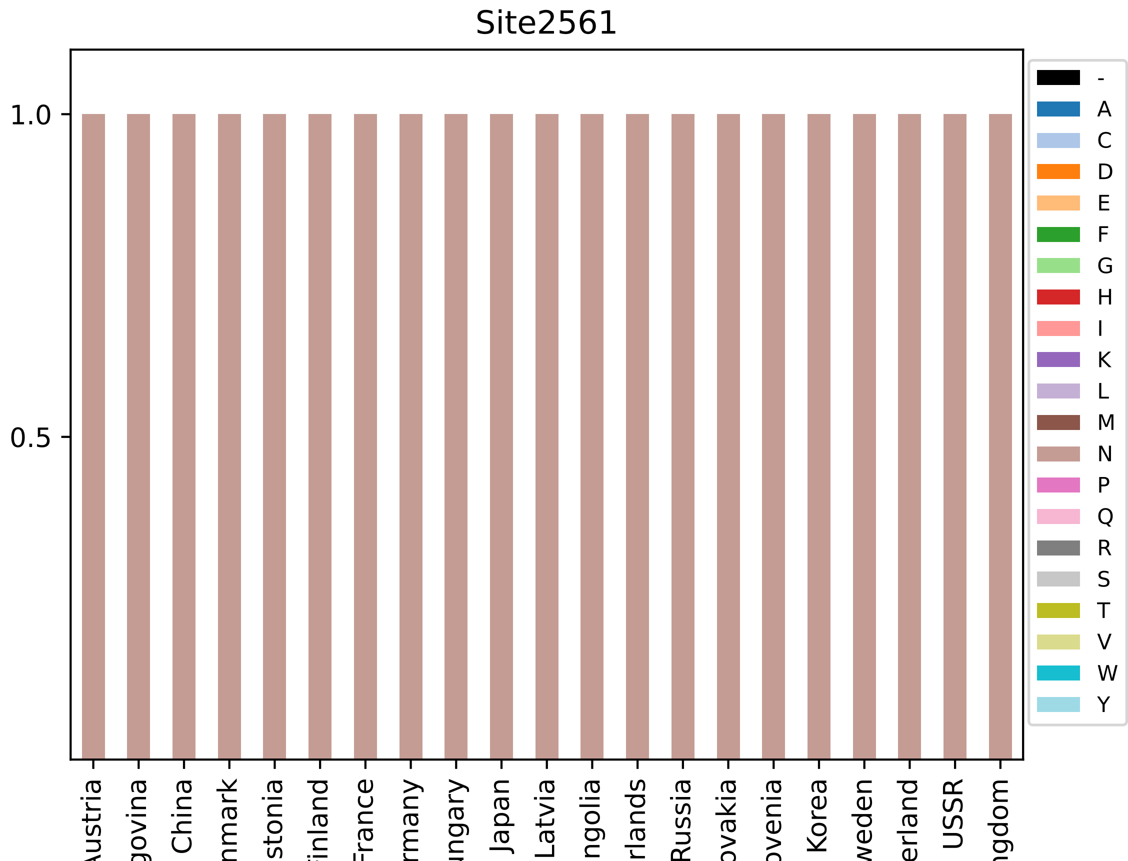

Site2561
1.0
0.5
Austria
China
Japan
Latvia
Russia
Korea
USSR
-
A
C
D
E
F
G
H
I
K
L
M
N
P
Q
R
S
T
V
W
Y

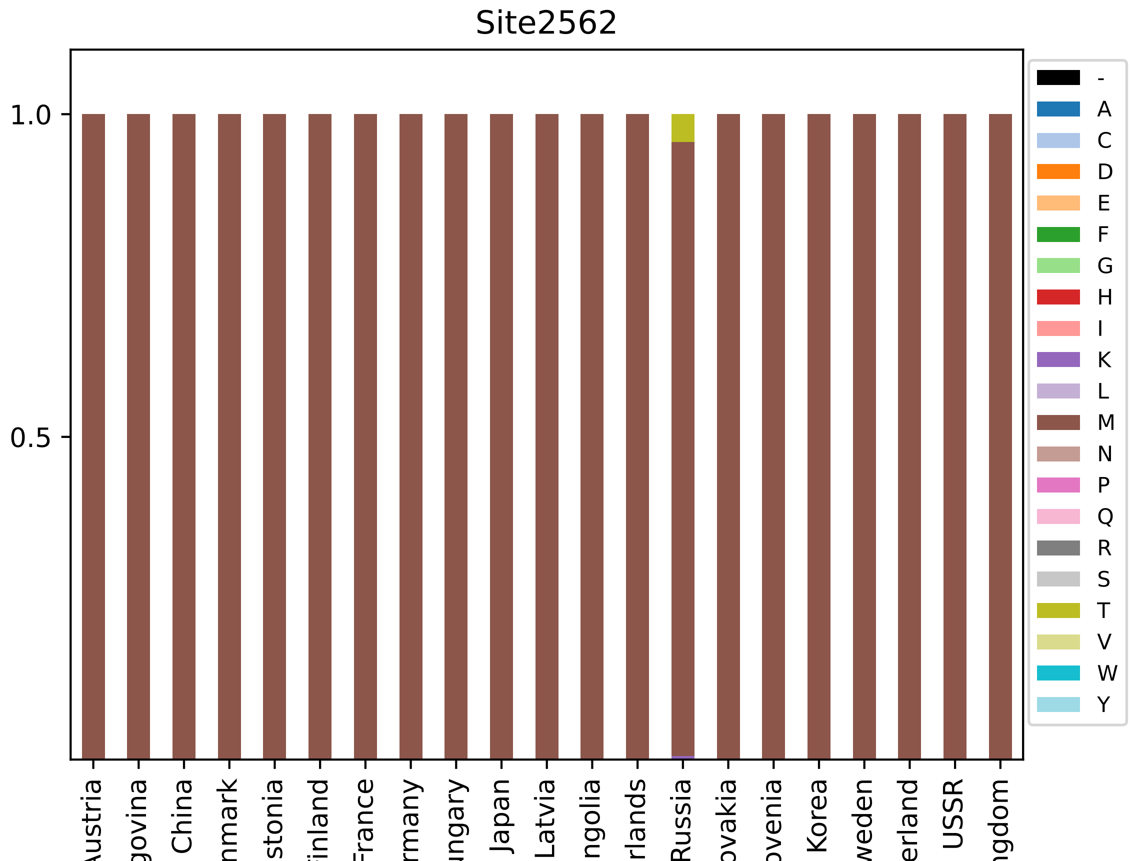

Site2562
1.0
0.5
-
A
C
D
E
F
G
H
I
K
L
M
N
P
Q
R
S
T
V
W
Y
Austria
China
Japan
Latvia
Russia
Korea
USSR

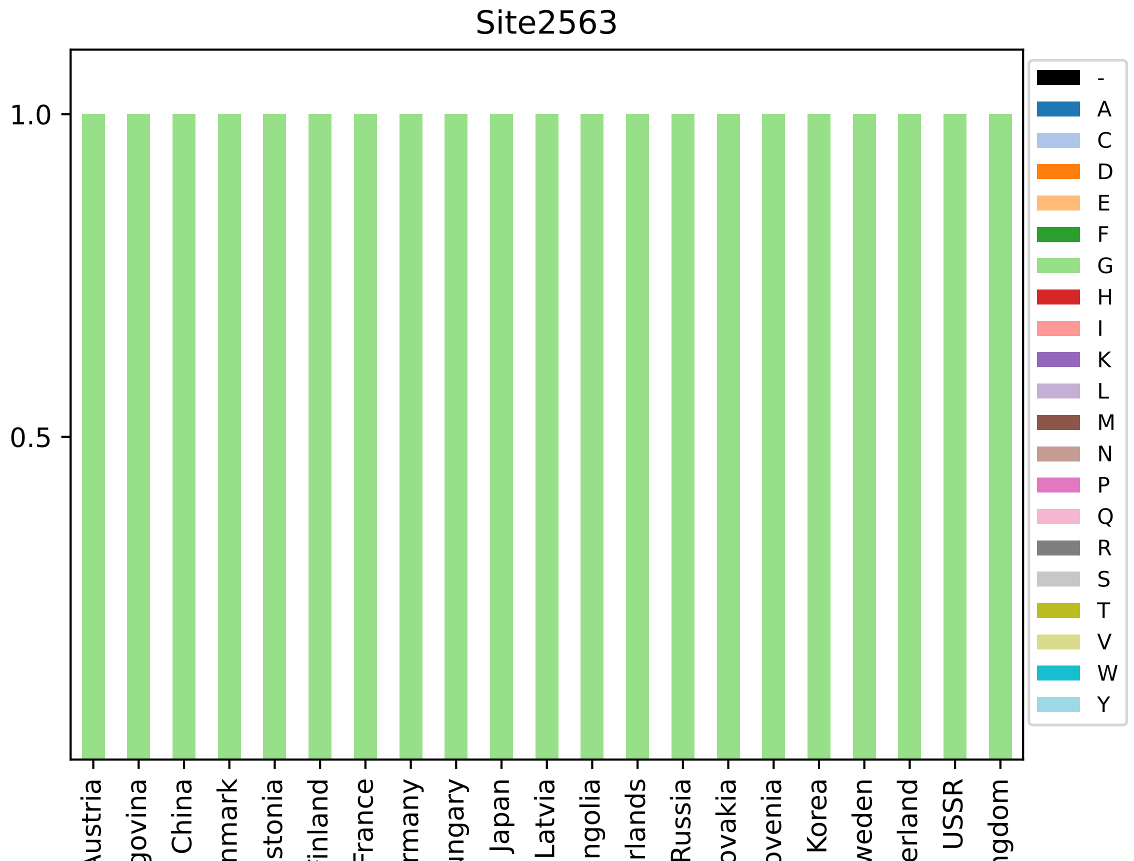

Site2563
1.0
0.5
-
A
C
D
E
F
G
H
I
K
L
M
N
P
Q
R
S
T
V
W
Y
Austria
govina
China
nmark
stonia
inland
France
rmany
ungary
Japan
Latvia
ngolia
rlands
Russia
ovakia
ovenia
Korea
weden
erland
USSR
ngdom

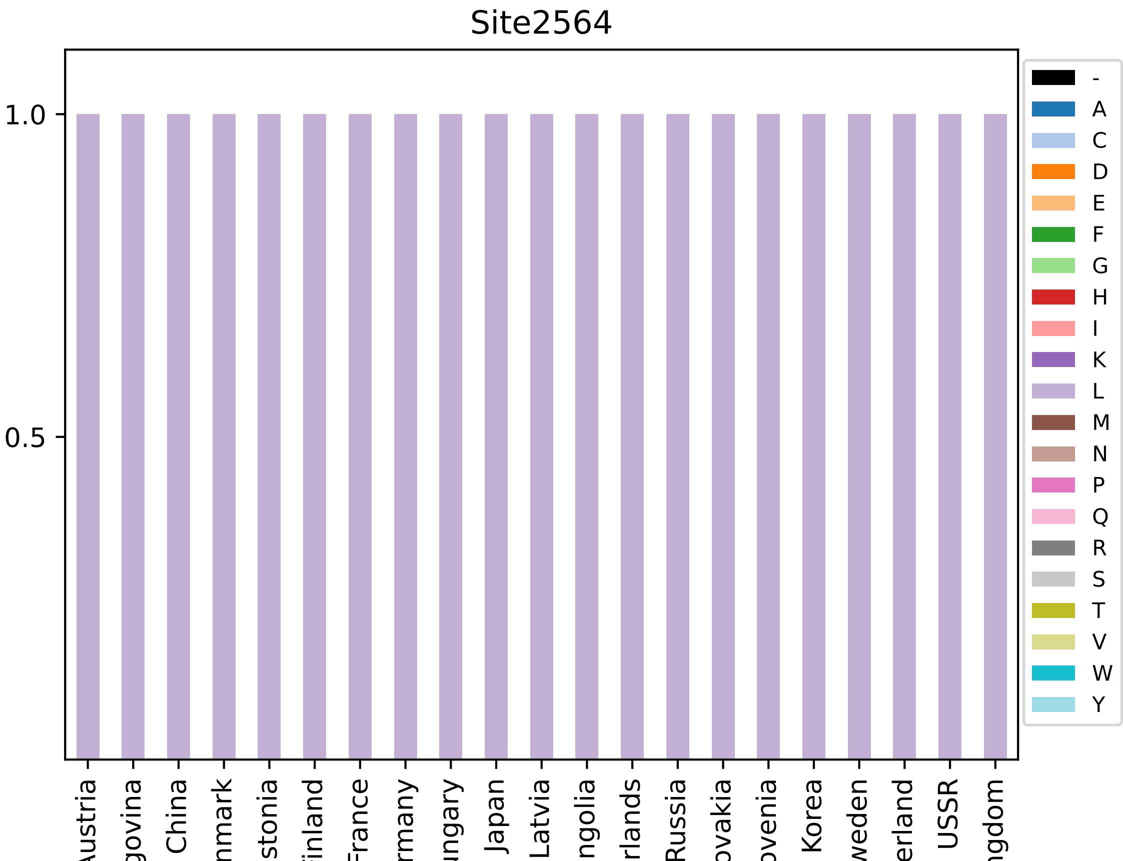
Site2564
1.0
0.5
China
Japan
Latvia
Russia
Korea
USSR
-
A
C
D
E
F
G
H
I
K
L
M
N
P
Q
R
S
T
V
W
Y

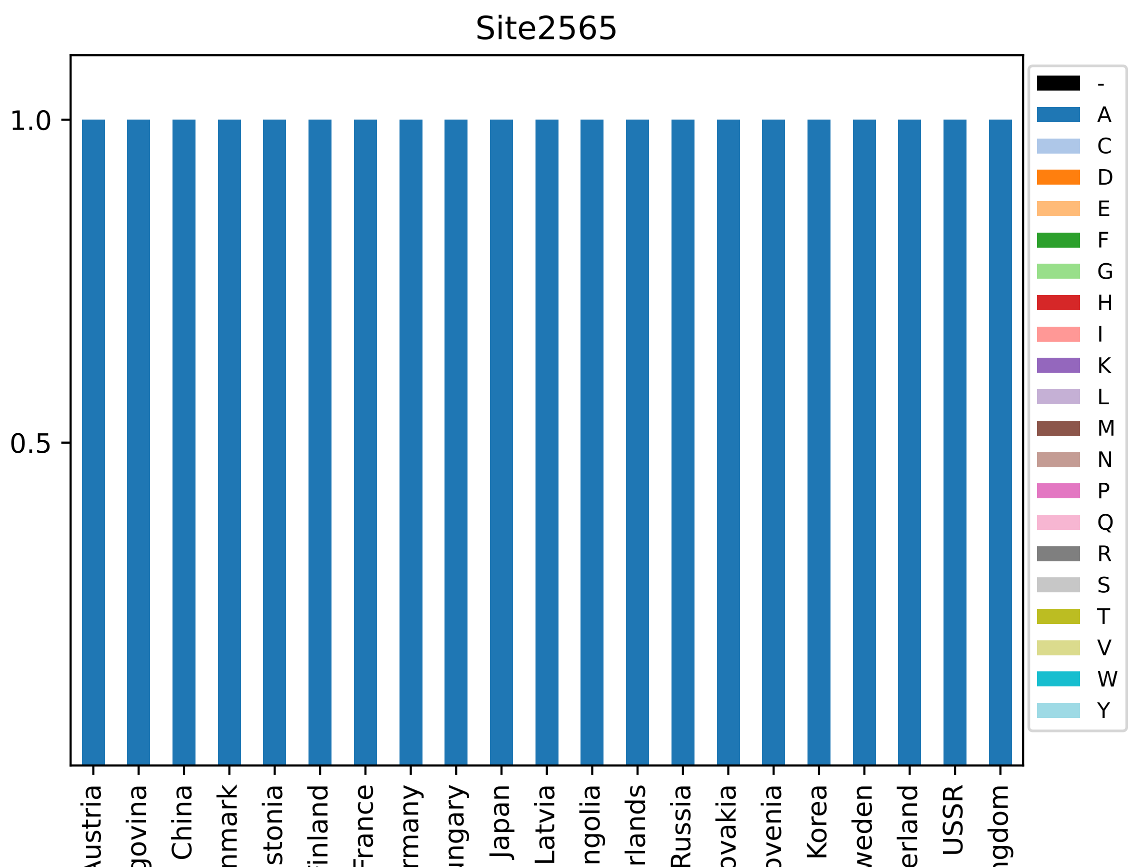

Site2565
1.0
0.5
-
A
C
D
E
F
G
H
I
K
L
M
N
P
Q
R
S
T
V
W
Y
China
Japan
Latvia
Russia
Korea
USSR

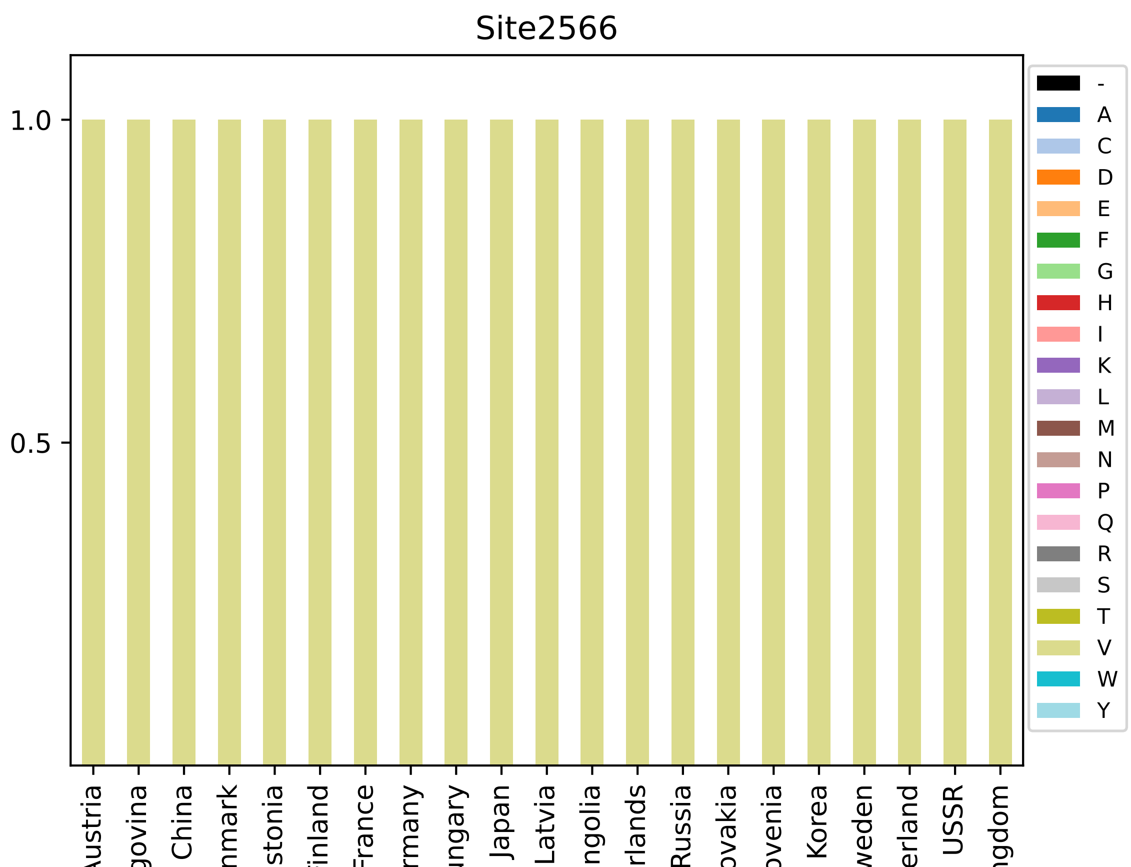

Site2566
1.0
0.5
-
A
C
D
E
F
G
H
I
K
L
M
N
P
Q
R
S
T
V
W
Y
China
Japan
Latvia
Russia
Korea
USSR

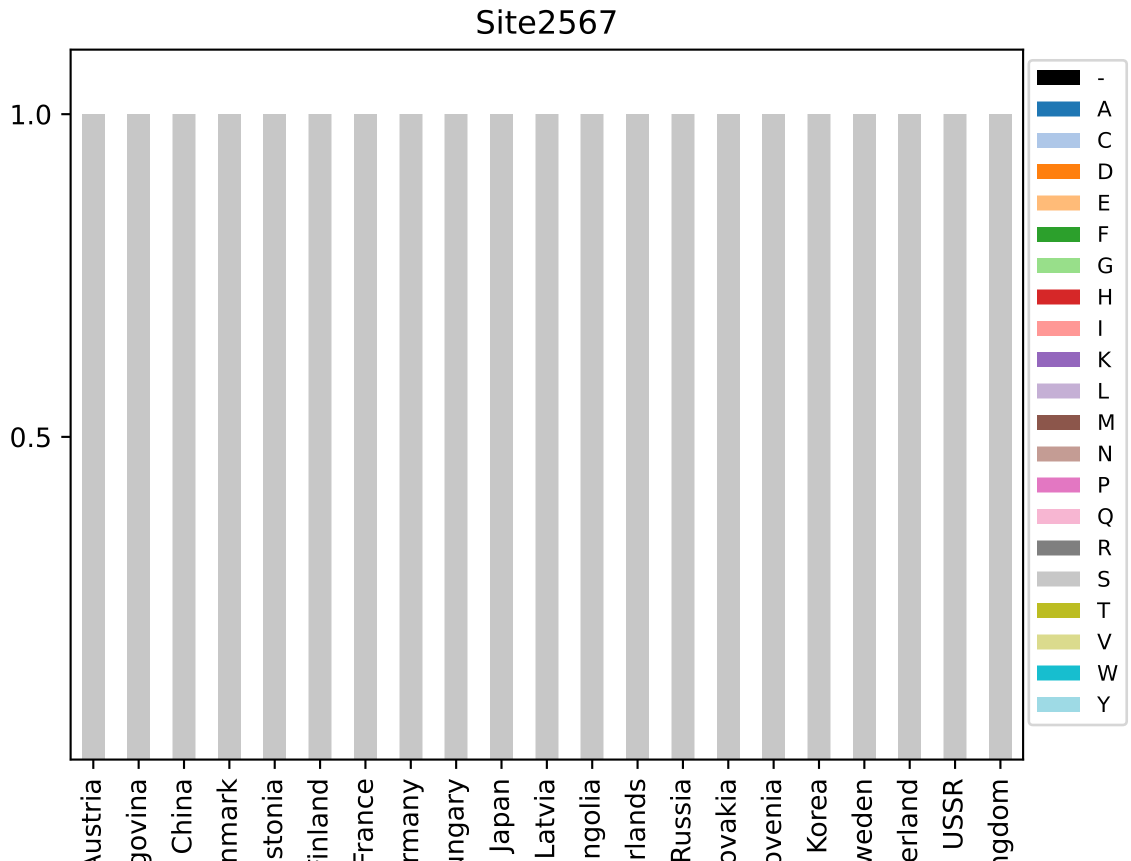
Site2567
1.0
0.5
Austria
govina
China
nmark
stonia
Finland
France
rmany
ungary
Japan
Latvia
ngolia
rlands
Russia
ovakia
ovenia
Korea
weden
erland
USSR
ngdom
-
A
C
D
E
F
G
H
I
K
L
M
N
P
Q
R
S
T
V
W
Y

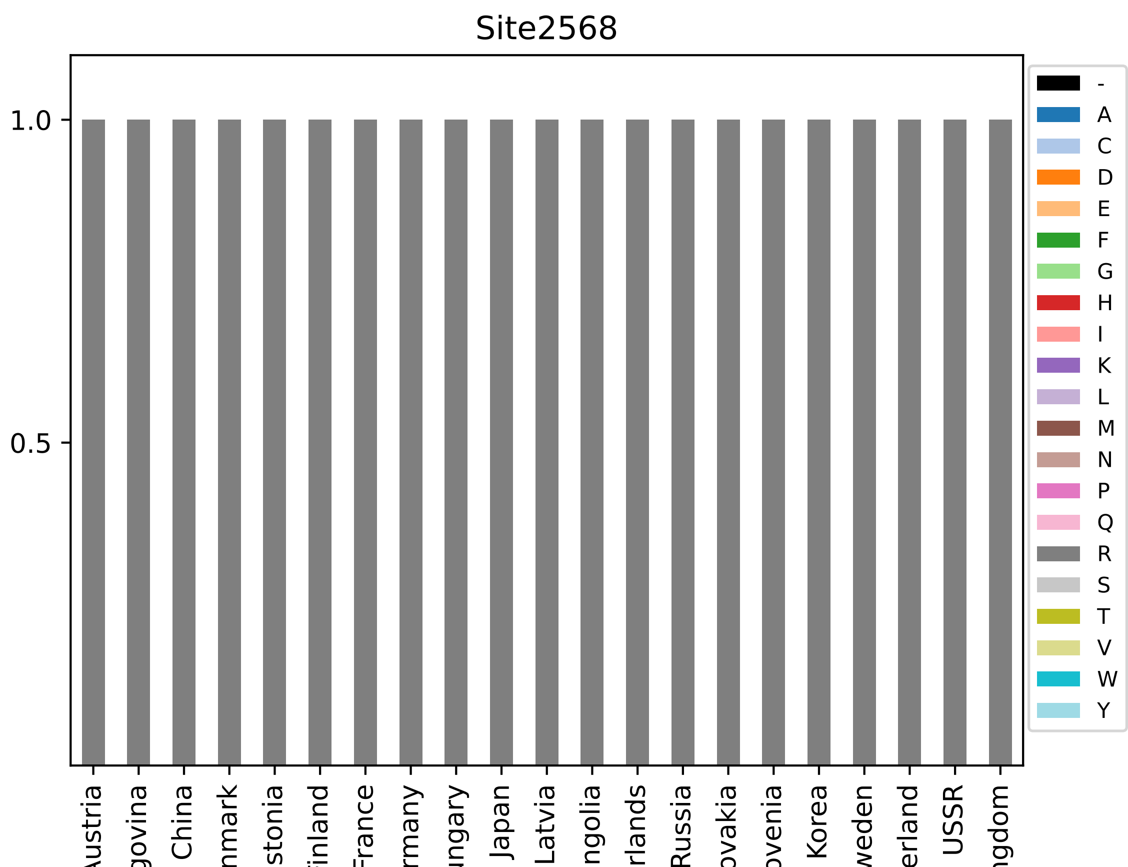
Site2568
1.0
0.5
-
A
C
D
E
F
G
H
I
K
L
M
N
P
Q
R
S
T
V
W
Y
China
Japan
Latvia
Russia
Korea
USSR

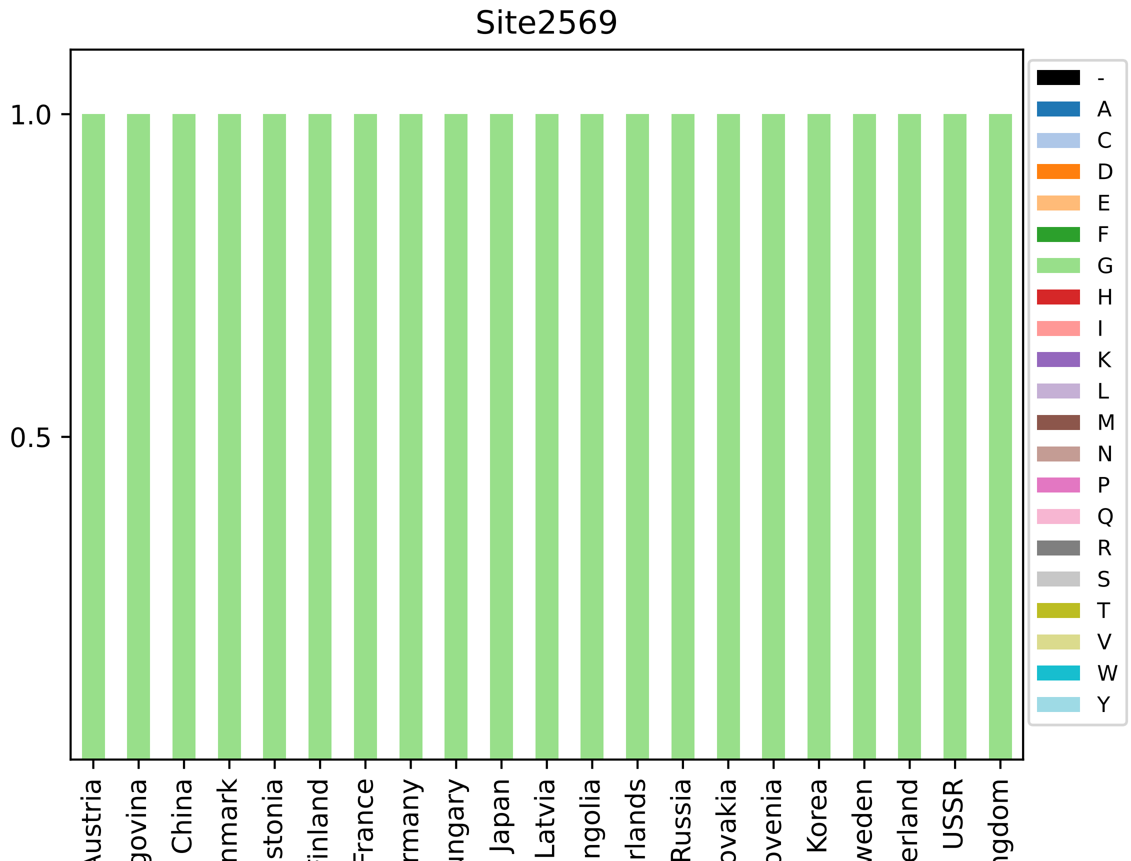

Site2569
1.0
0.5
-
A
C
D
E
F
G
H
I
K
L
M
N
P
Q
R
S
T
V
W
Y
Austria
govina
China
nmark
stonia
inland
France
rmany
ungary
Japan
Latvia
ngolia
rlands
Russia
ovakia
ovenia
Korea
weden
erland
USSR
ngdom

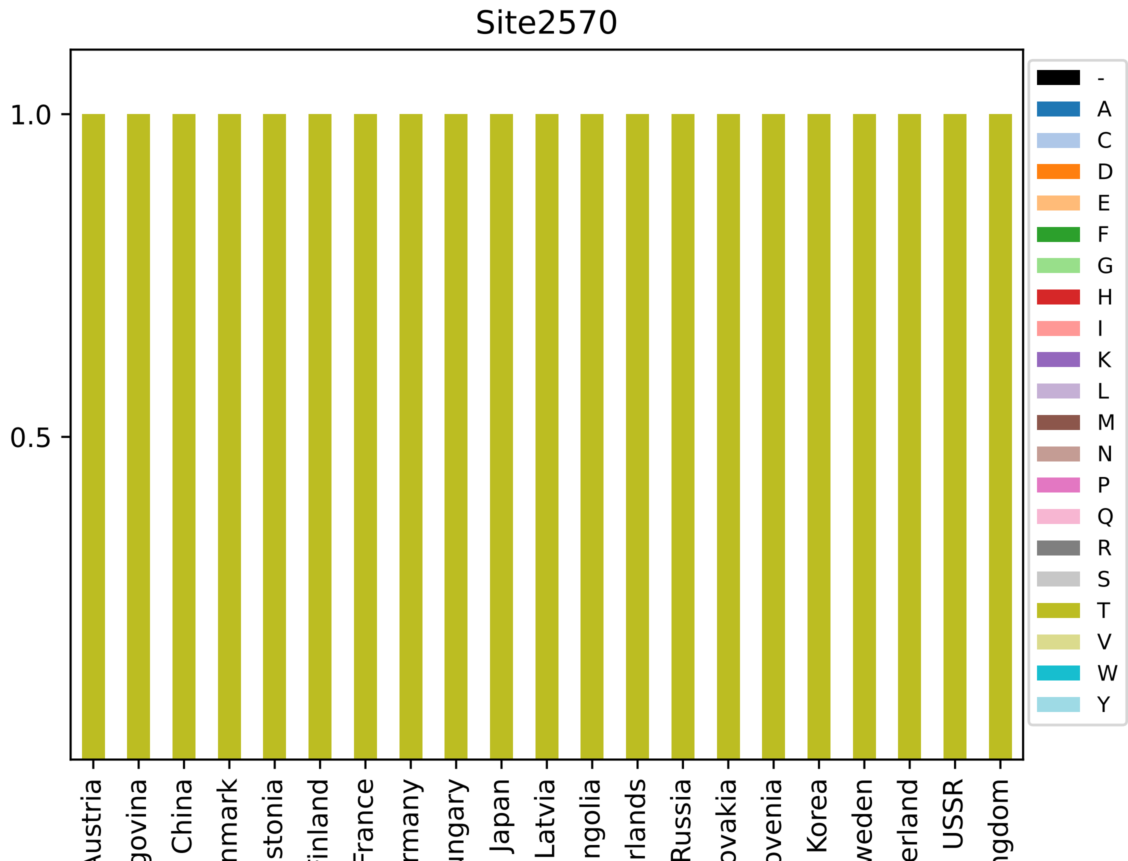
Site2570
1.0
0.5
-
A
C
D
E
F
G
H
I
K
L
M
N
P
Q
R
S
T
V
W
Y
China
Japan
Latvia
Russia
Korea
USSR

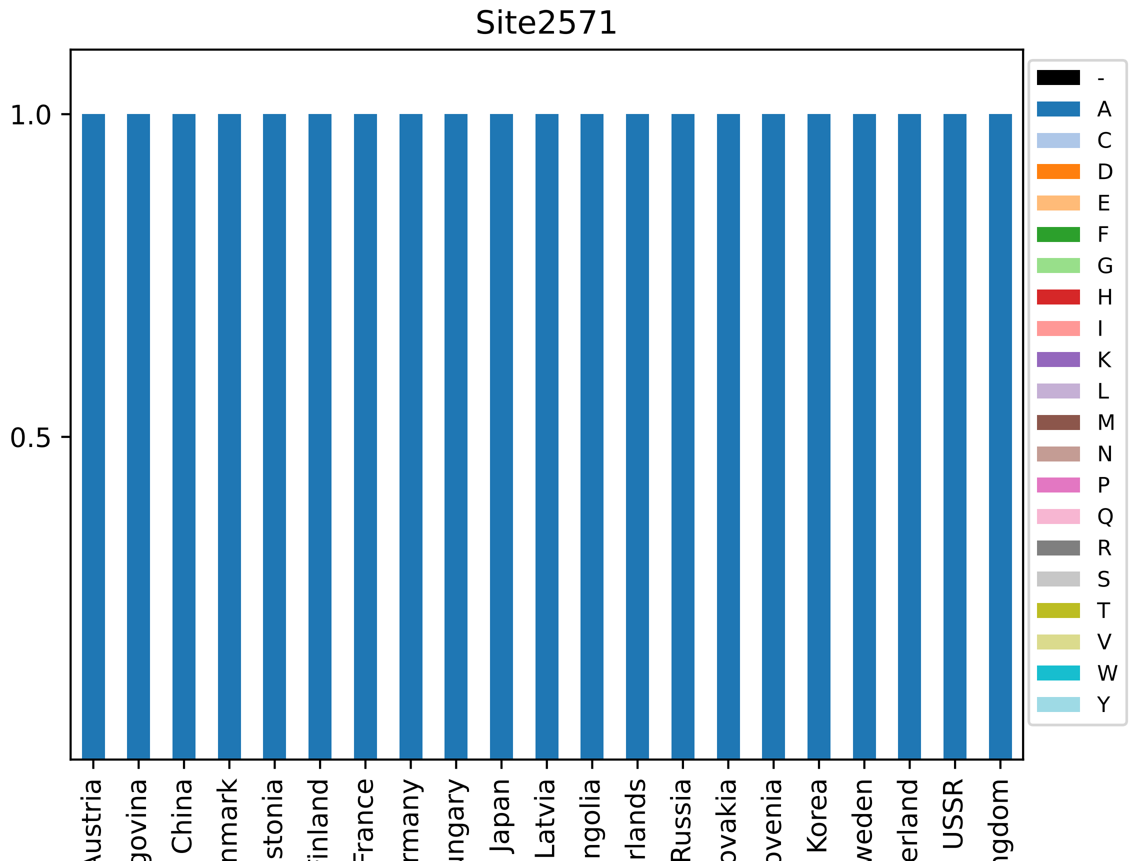
Site2571
1.0
0.5
-
A
C
D
E
F
G
H
I
K
L
M
N
P
Q
R
S
T
V
W
Y
Austria
China
Japan
Latvia
Russia
Korea
USSR

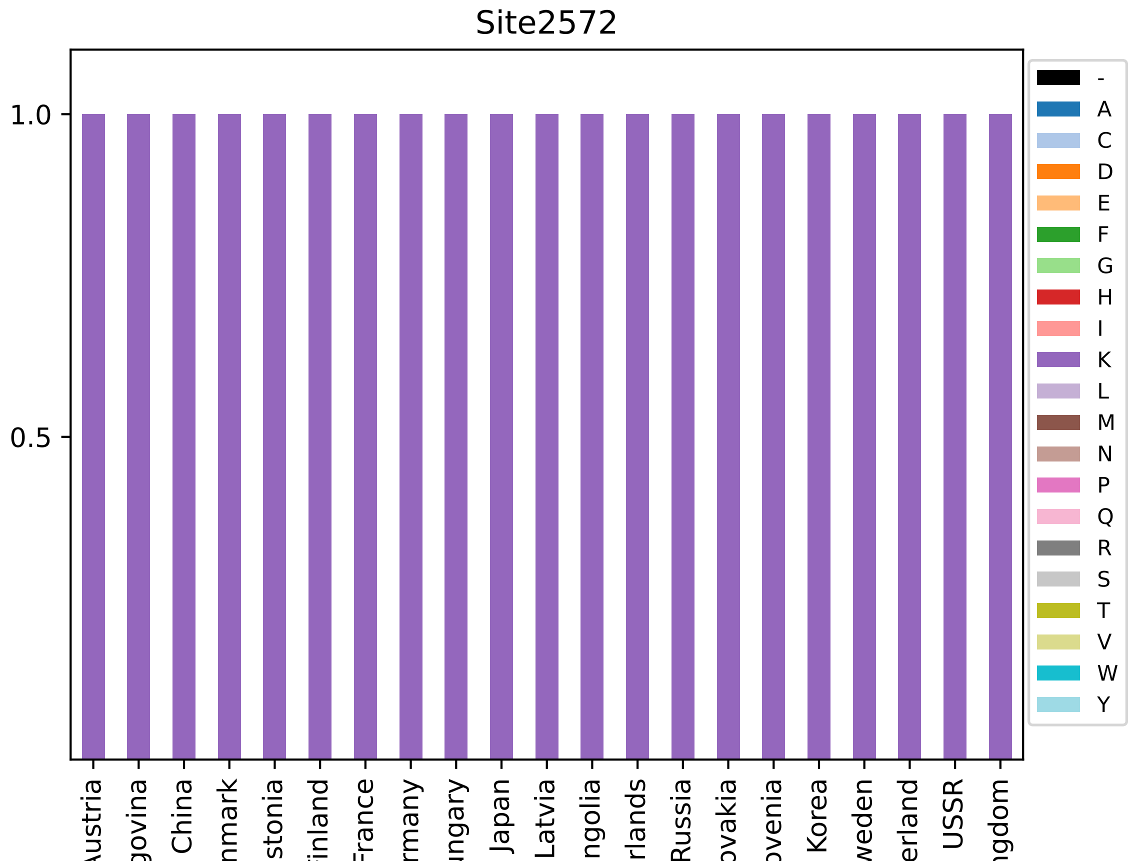

Site2572
1.0
0.5
-
A
C
D
E
F
G
H
I
K
L
M
N
P
Q
R
S
T
V
W
Y
China
France
Japan
Latvia
Russia
Korea
USSR

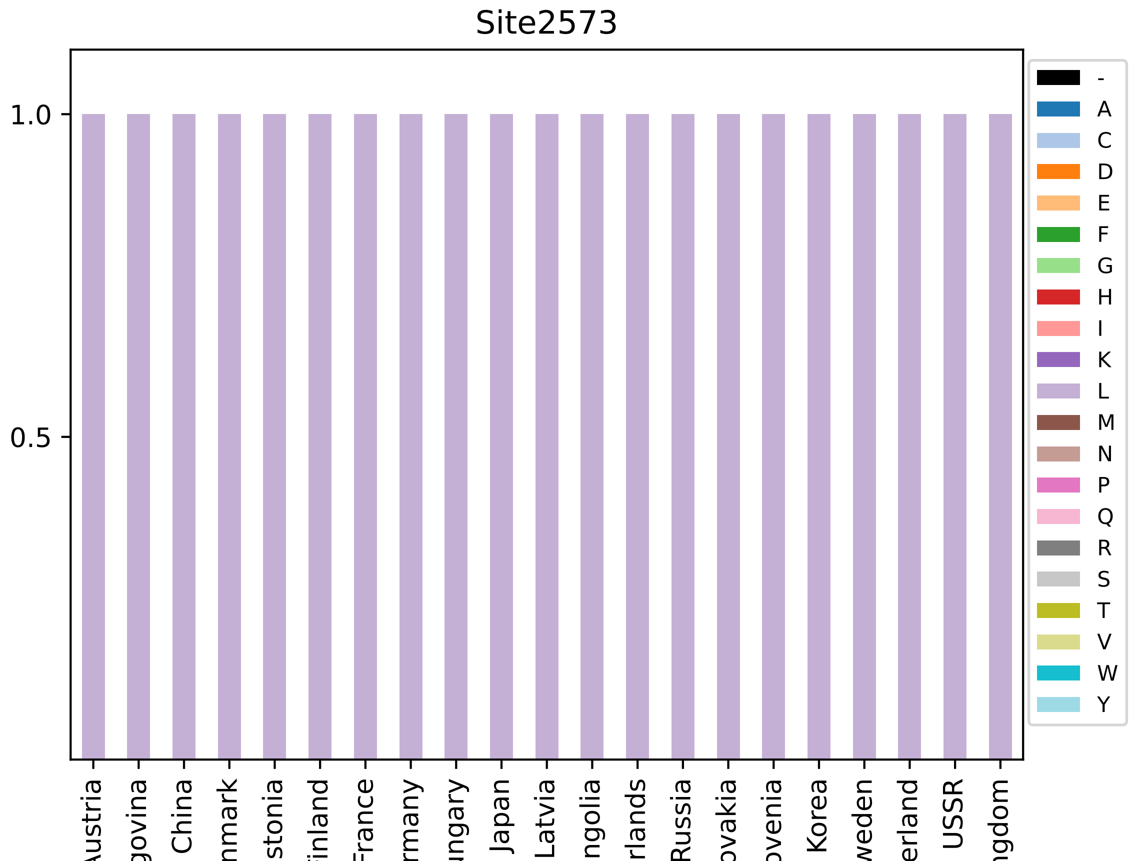
Site2573
1.0
0.5
-
A
C
D
E
F
G
H
I
K
L
M
N
P
Q
R
S
T
V
W
Y
China
Japan
Latvia
Russia
Korea
USSR

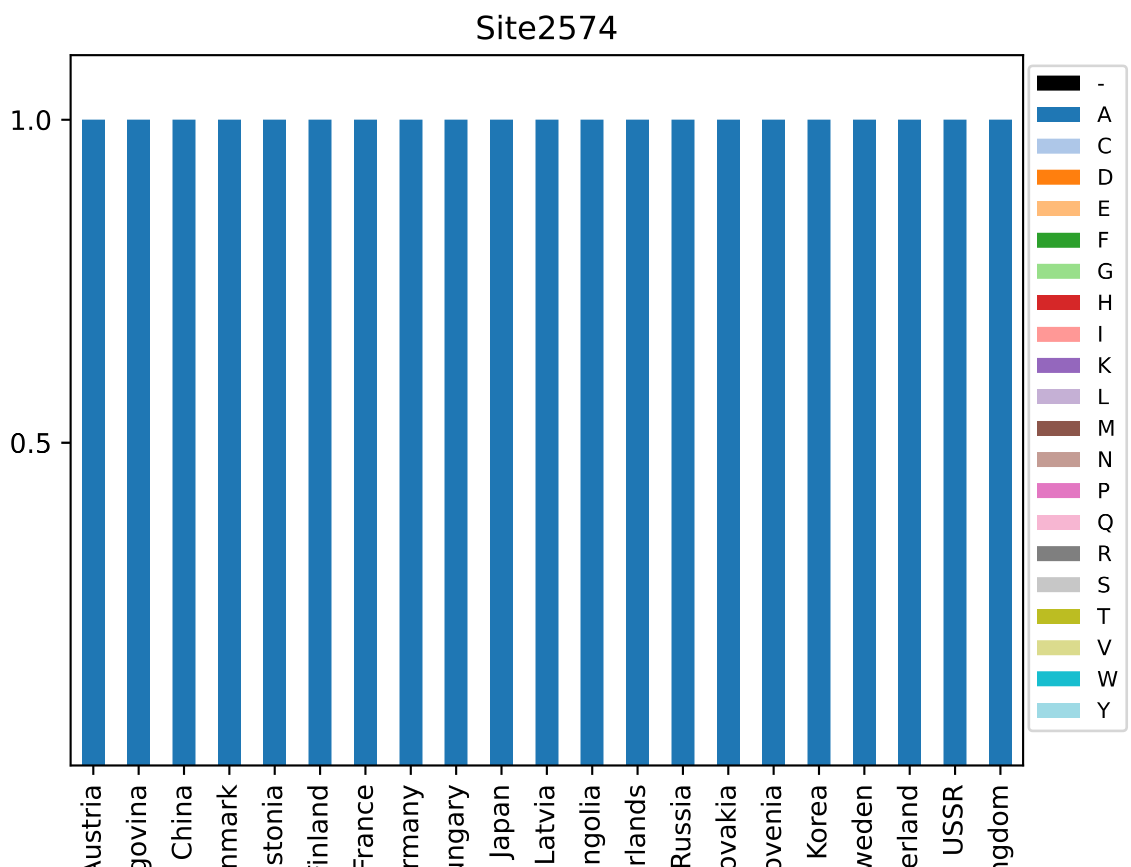
Site2574
1.0
0.5
-
A
C
D
E
F
G
H
I
K
L
M
N
P
Q
R
S
T
V
W
Y
China
Japan
Latvia
Russia
Korea
USSR

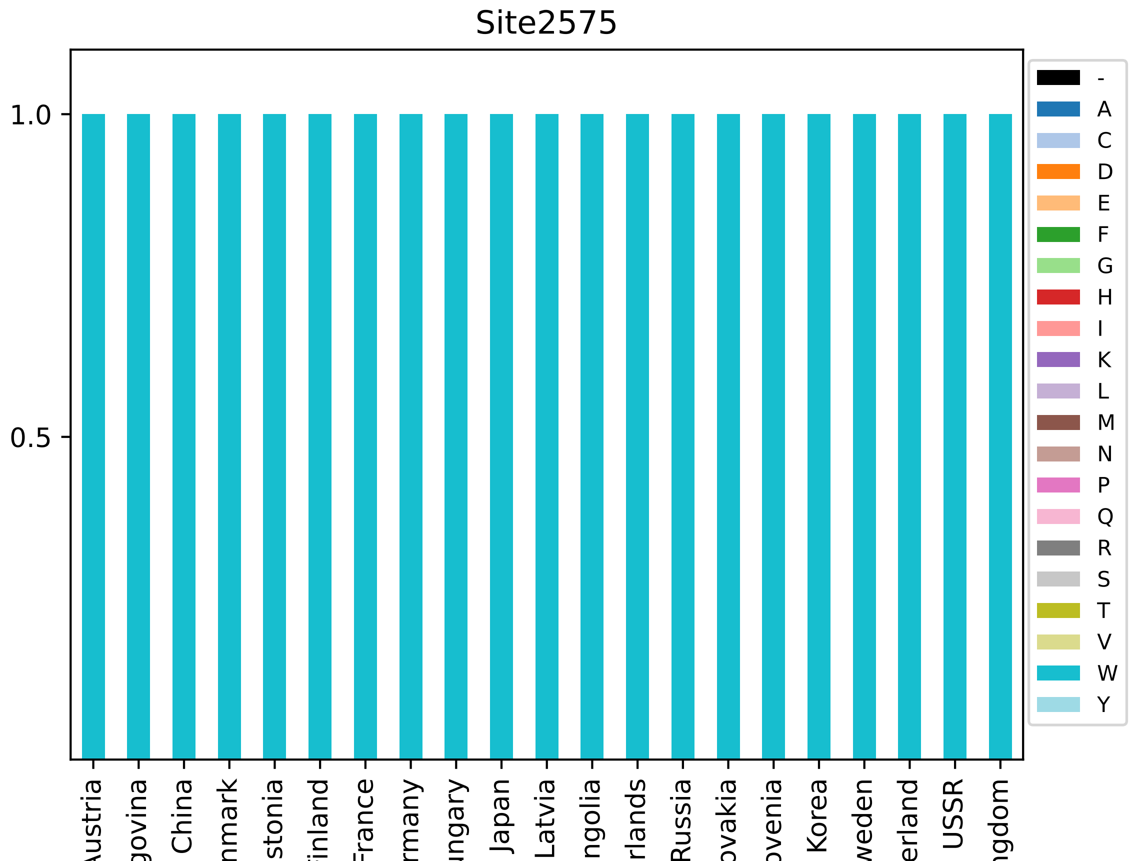

Site2575
1.0
0.5
-
A
C
D
E
F
G
H
I
K
L
M
N
P
Q
R
S
T
V
W
Y
China
Japan
Latvia
Russia
Korea
USSR

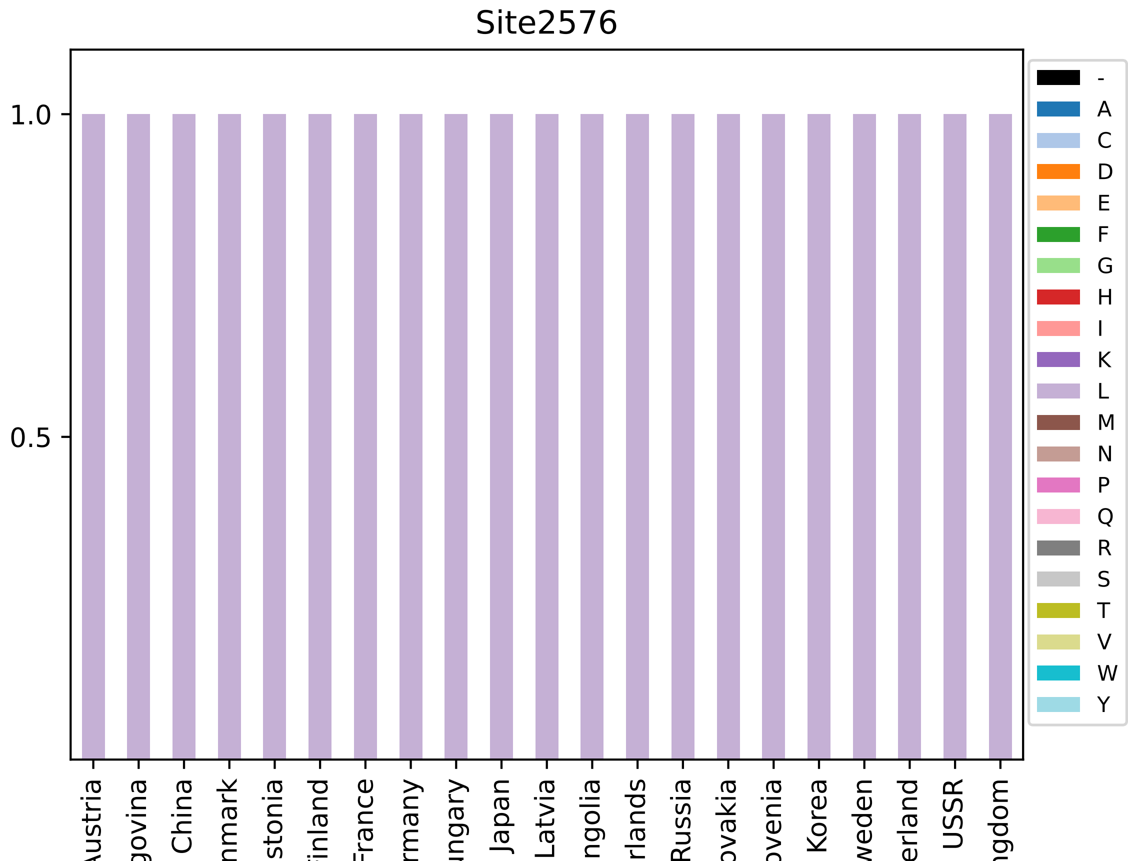
Site2576
1.0
0.5
-
A
C
D
E
F
G
H
I
K
L
M
N
P
Q
R
S
T
V
W
Y
China
Japan
Latvia
Russia
Korea
USSR

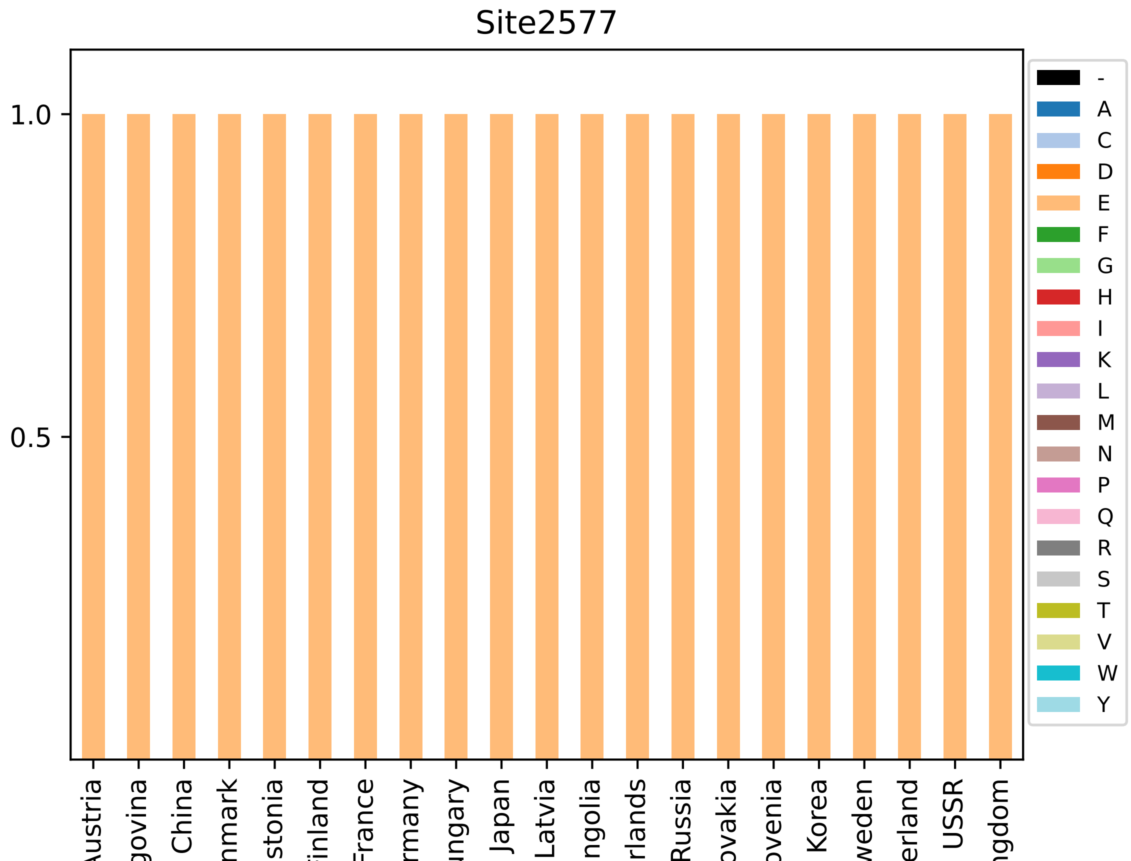

Site2577
1.0
0.5
-
A
C
D
E
F
G
H
I
K
L
M
N
P
Q
R
S
T
V
W
Y
China
Japan
Latvia
Korea
USSR

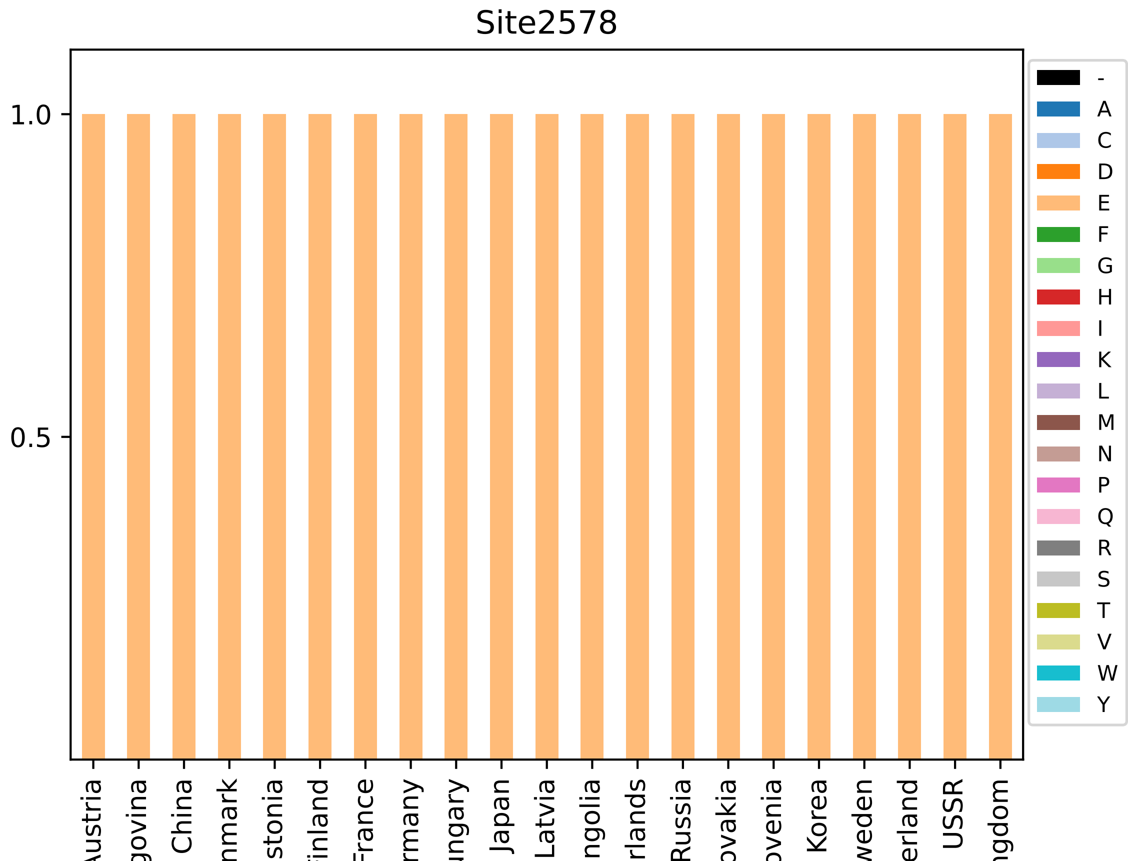
Site2578
1.0
0.5
China
Japan
Latvia
Russia
Korea
USSR
-
A
C
D
E
F
G
H
I
K
L
M
N
P
Q
R
S
T
V
W
Y

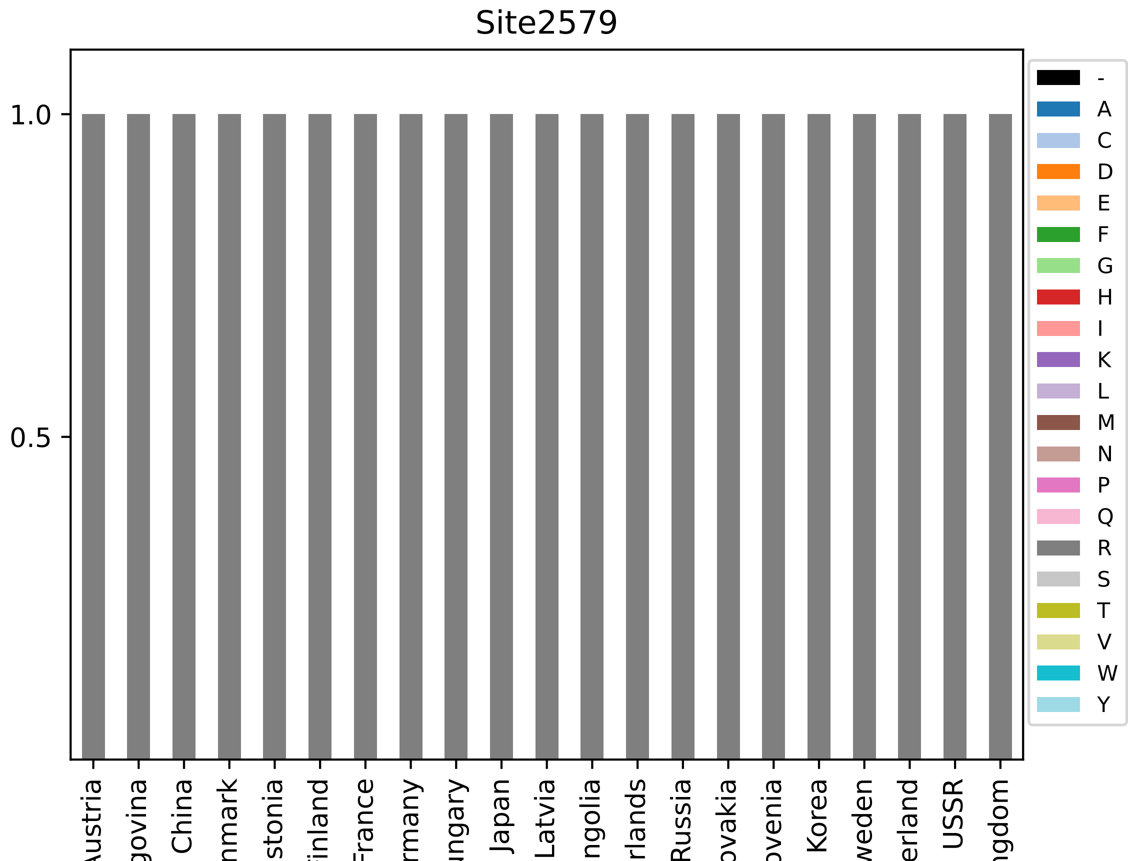
Site2579
1.0
0.5
Austria
govina
China
nmark
stonia
Finland
France
rmany
ungary
Japan
Latvia
ngolia
rlands
Russia
ovakia
ovenia
Korea
weden
erland
USSR
ngdom
-
A
C
D
E
F
G
H
I
K
L
M
N
P
Q
R
S
T
V
W
Y

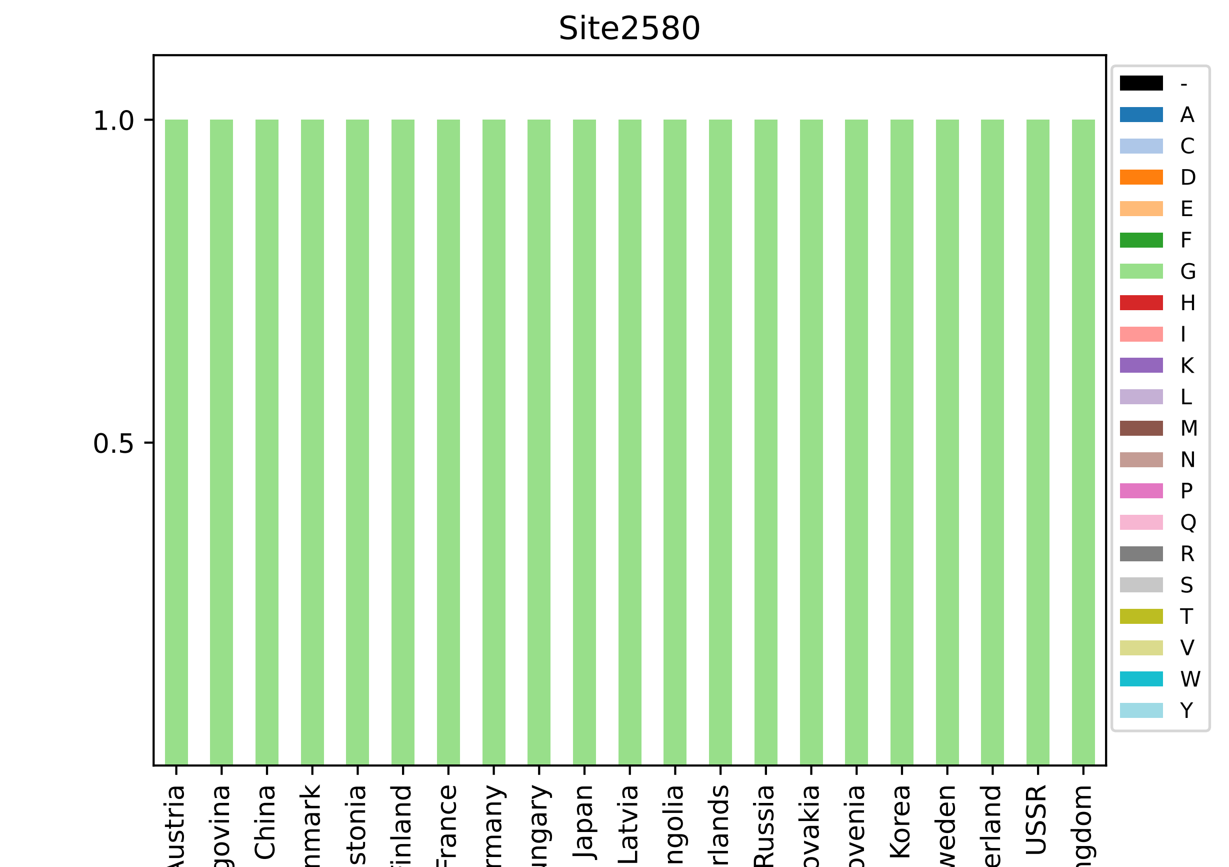
Site2580
1.0
0.5
-
A
C
D
E
F
G
H
I
K
L
M
N
P
Q
R
S
T
V
W
Y
Austria
govina
China
nmark
stonia
Finland
France
rmany
ungary
Japan
Latvia
ngolia
rlands
Russia
ovakia
ovenia
Korea
weden
erland
USSR
ngdom

Site2581

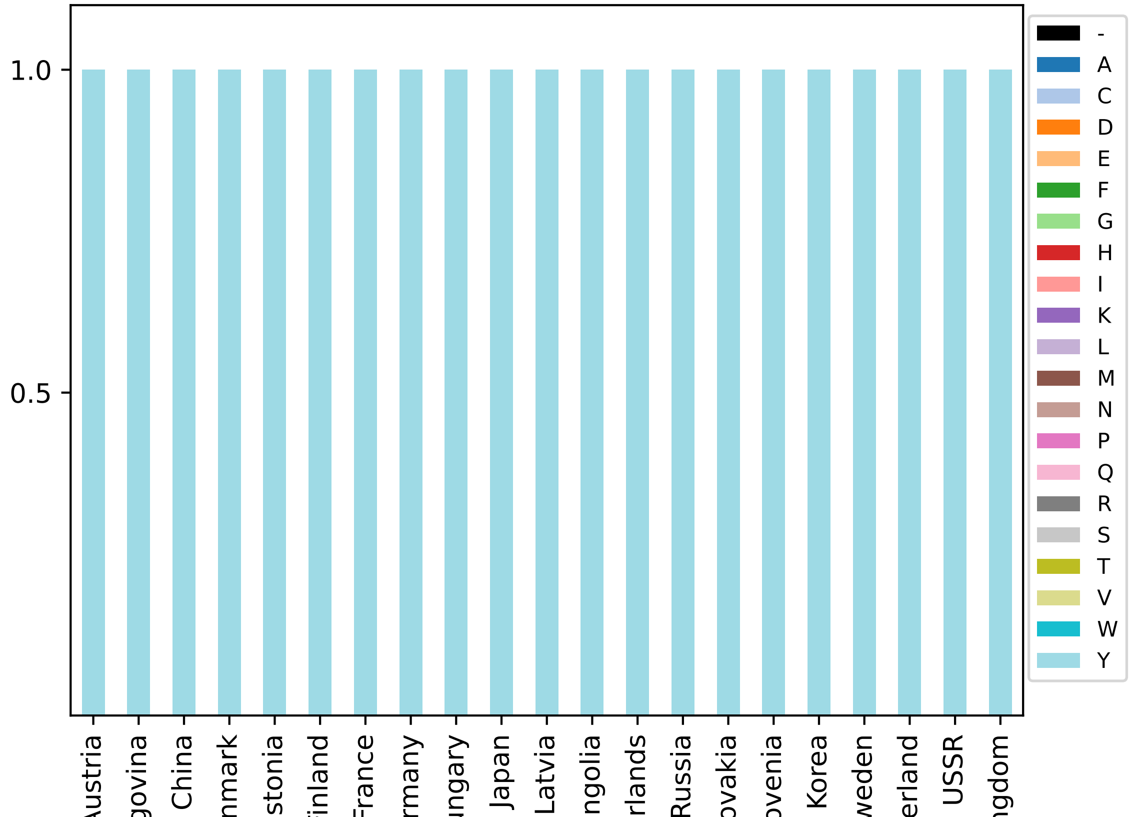

1.0
0.5
China
Japan
Latvia
Russia
Korea
USSR
-
A
C
D
E
F
G
H
I
K
L
M
N
P
Q
R
S
T
V
W
Y

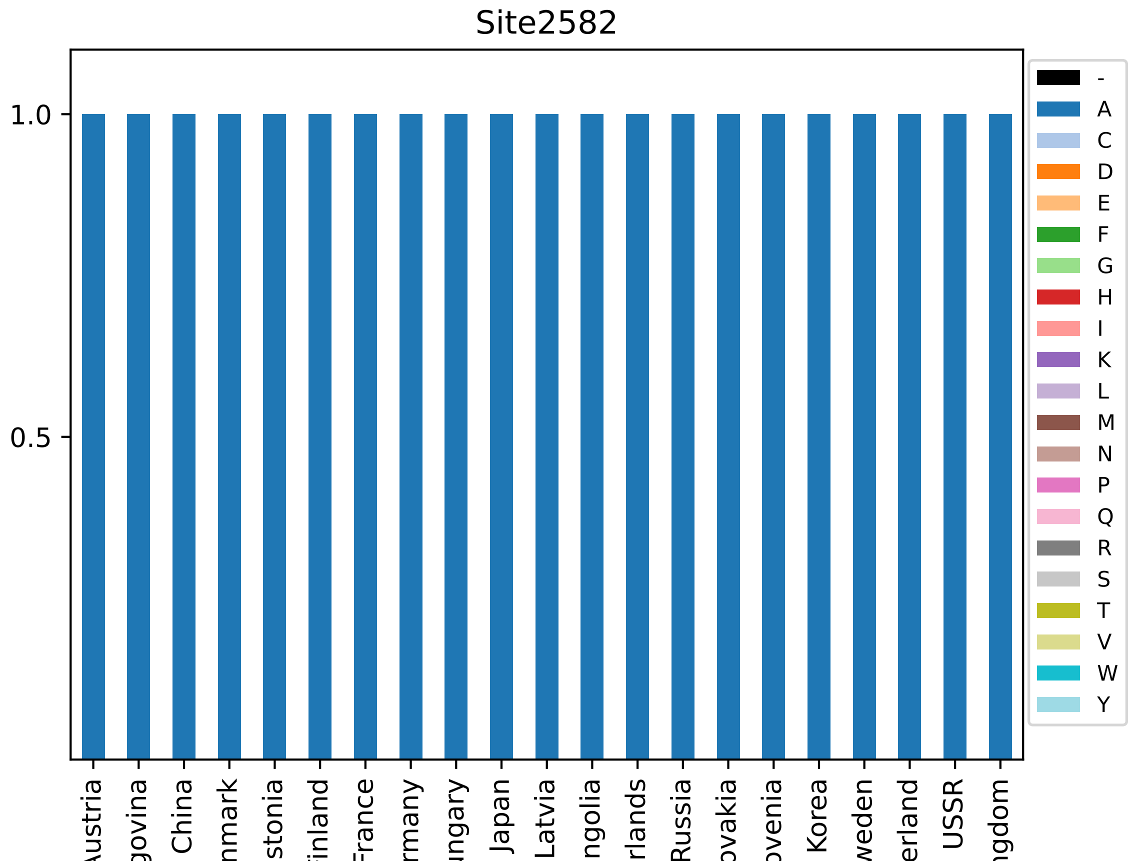

Site2582
1.0
0.5
-
A
C
D
E
F
G
H
I
K
L
M
N
P
Q
R
S
T
V
W
Y
China
Japan
Latvia
Russia
Korea
USSR

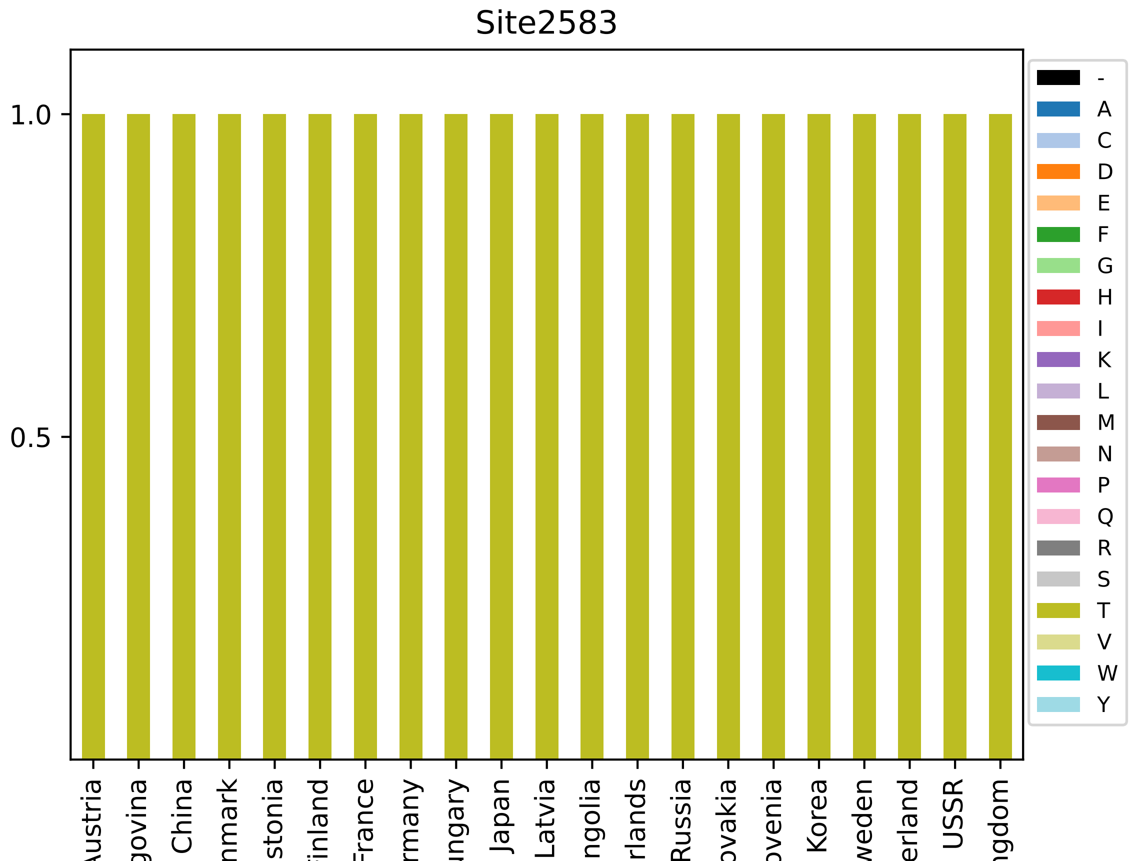
Site2583
1.0
0.5
Austria
govina
China
nmark
stonia
Finland
France
rmany
ungary
Japan
Latvia
ngolia
rlands
Russia
ovakia
ovenia
Korea
weden
erland
USSR
ngdom
-
A
C
D
E
F
G
H
I
K
L
M
N
P
Q
R
S
T
V
W
Y

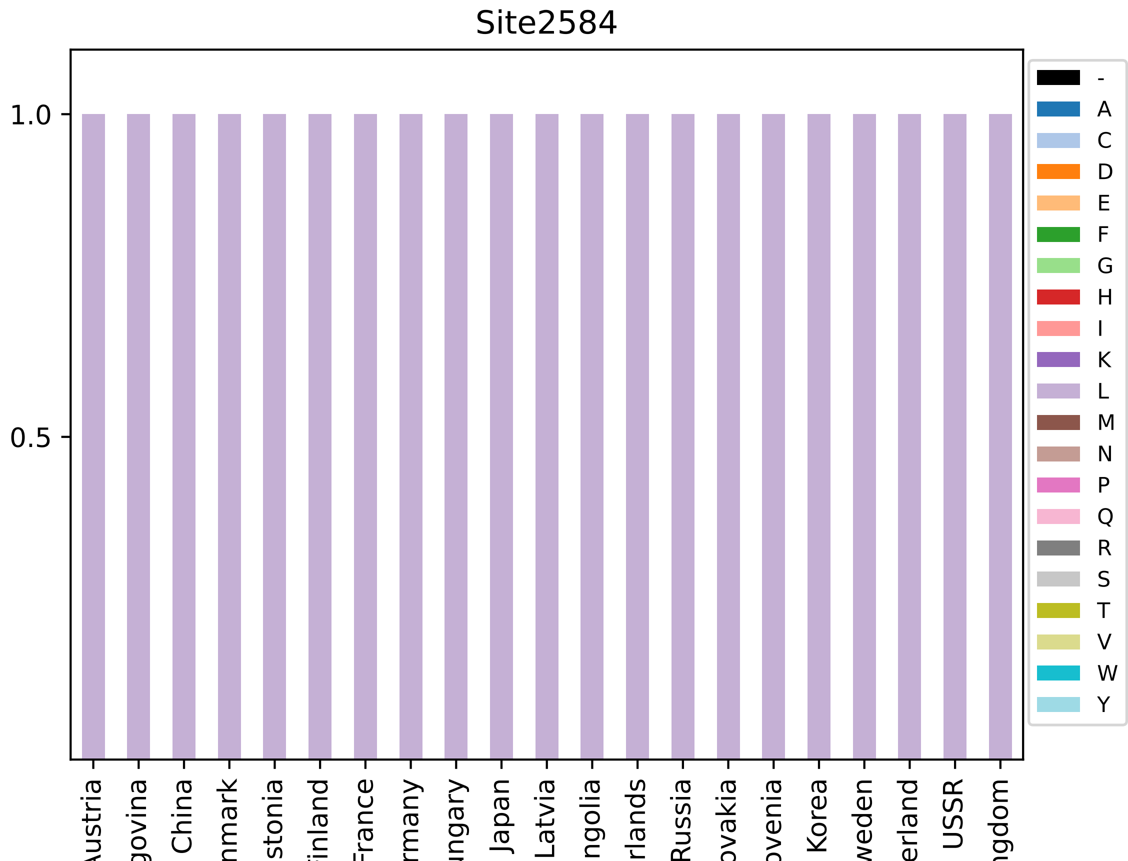
Site2584
1.0
0.5
-
A
C
D
E
F
G
H
I
K
L
M
N
P
Q
R
S
T
V
W
Y
China
Japan
Latvia
Russia
Korea
USSR

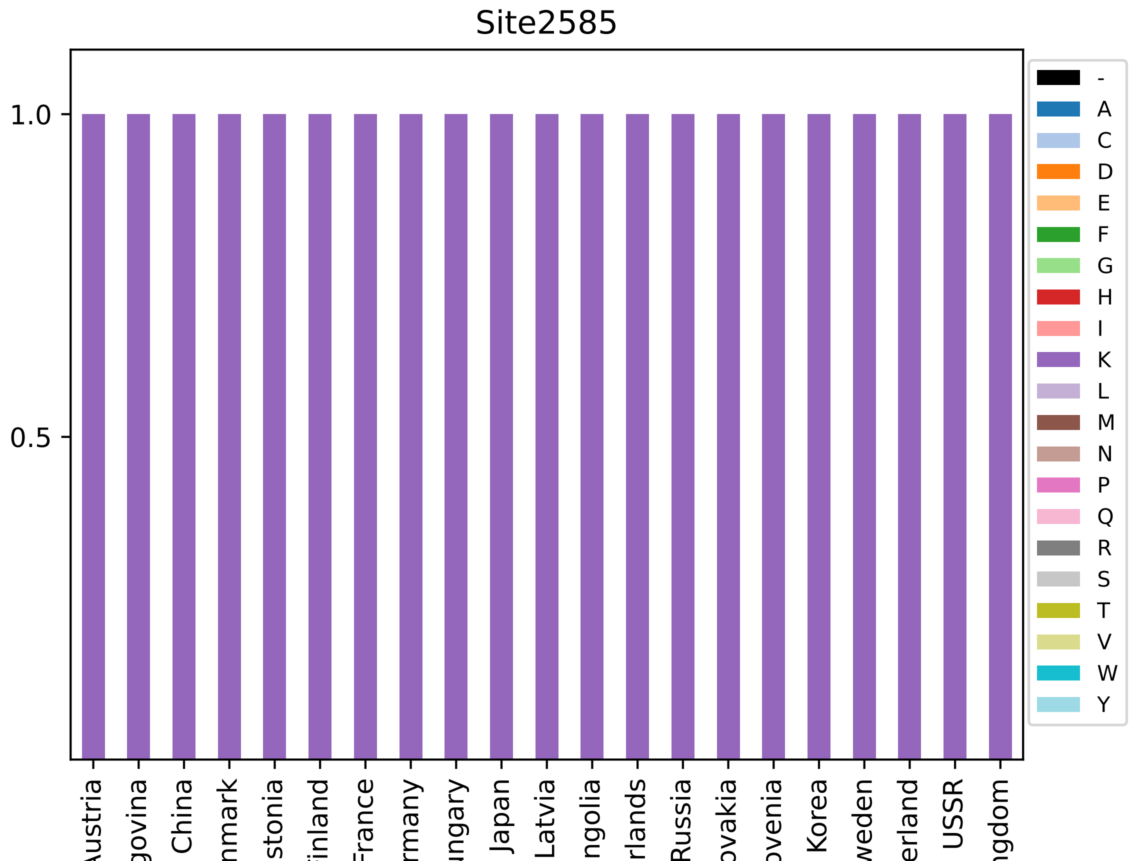
Site2585
1.0
0.5
-
A
C
D
E
F
G
H
I
K
L
M
N
P
Q
R
S
T
V
W
Y
Austria
govina
China
nmark
stonia
inland
France
rmany
ungary
Japan
Latvia
ngolia
rlands
Russia
ovakia
ovenia
Korea
weden
erland
USSR
ngdom

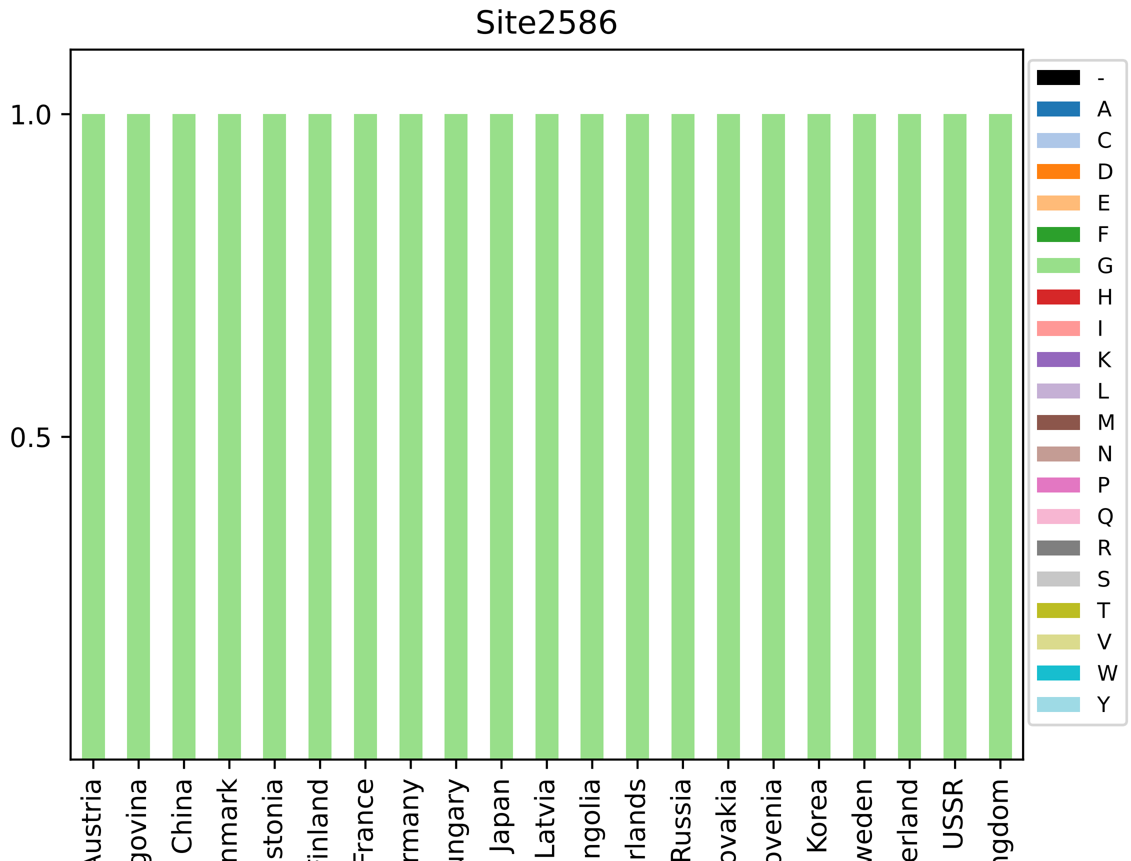
Site2586
1.0
0.5
-
A
C
D
E
F
G
H
I
K
L
M
N
P
Q
R
S
T
V
W
Y
China
Japan
Latvia
Russia
Korea
USSR

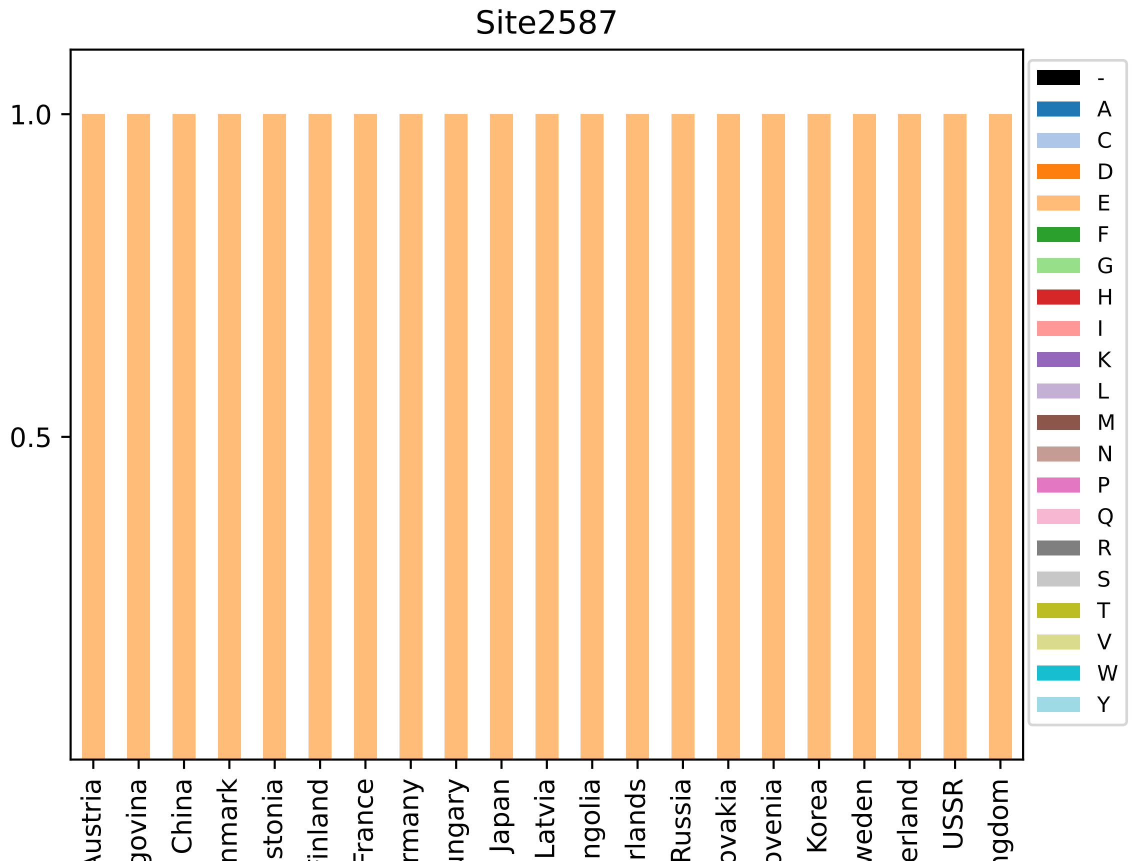
Site2587
1.0
0.5
-
A
C
D
E
F
G
H
I
K
L
M
N
P
Q
R
S
T
V
W
Y
Austria
govina
China
nmark
stonia
Finland
France
rmany
ungary
Japan
Latvia
ngolia
rlands
Russia
ovakia
ovenia
Korea
weden
erland
USSR
ngdom

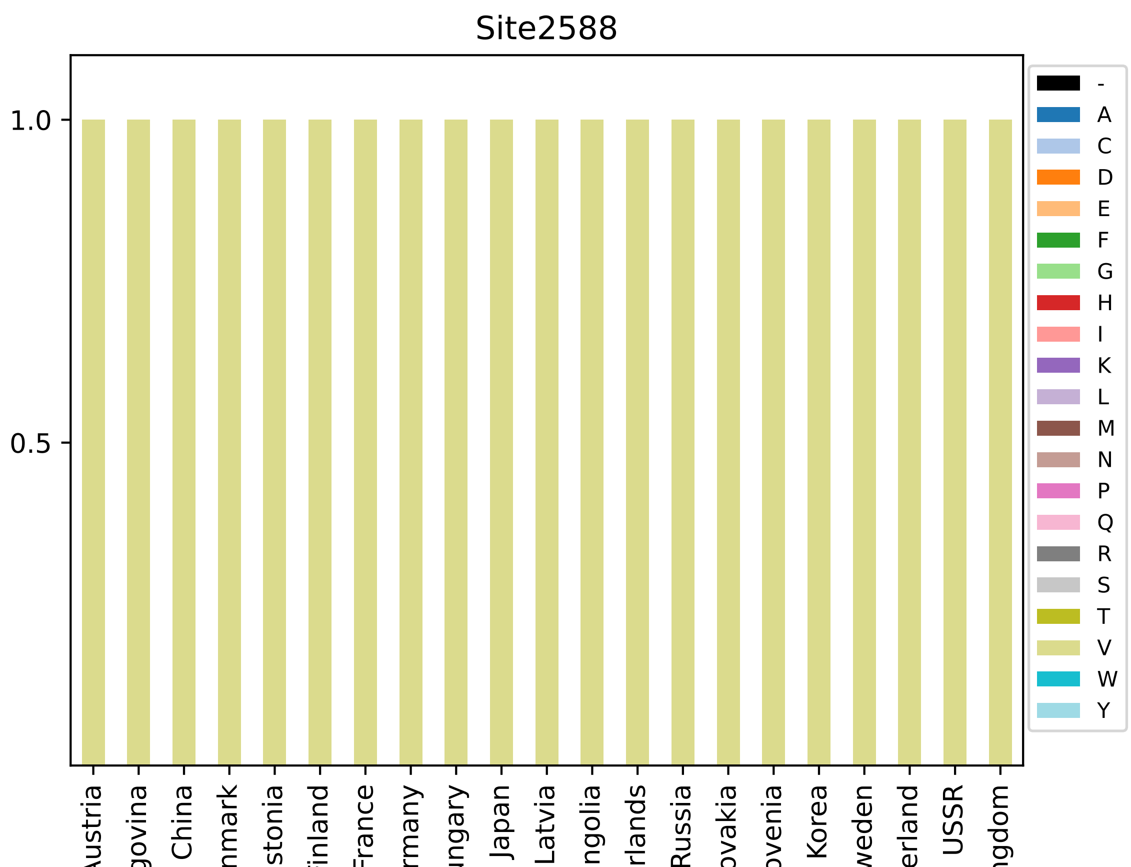
Site2588
1.0
0.5
-
A
C
D
E
F
G
H
I
K
L
M
N
P
Q
R
S
T
V
W
Y
China
Japan
Latvia
Russia
Korea
USSR

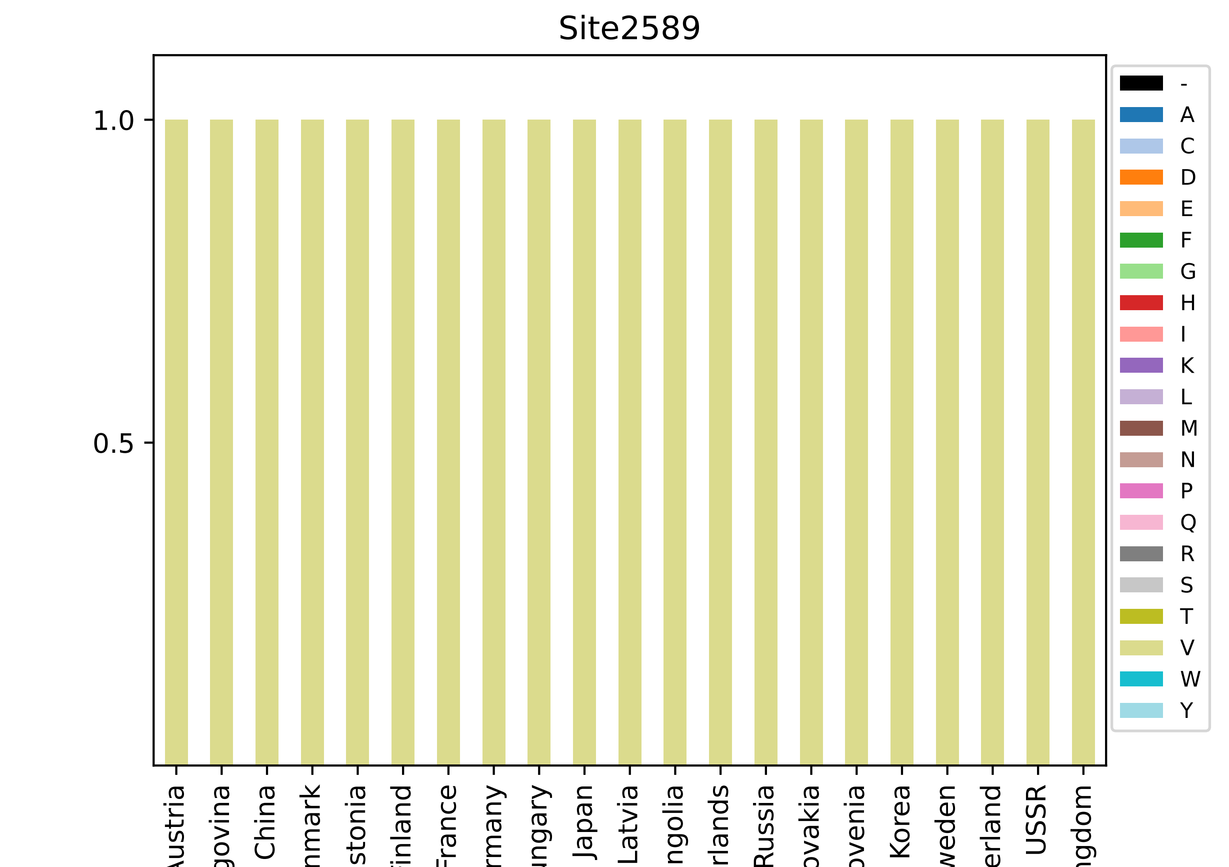

Site2589
1.0
0.5
-
A
C
D
E
F
G
H
I
K
L
M
N
P
Q
R
S
T
V
W
Y
Austria
China
Estonia
Finland
France
Japan
Latvia
Russia
Korea
USSR

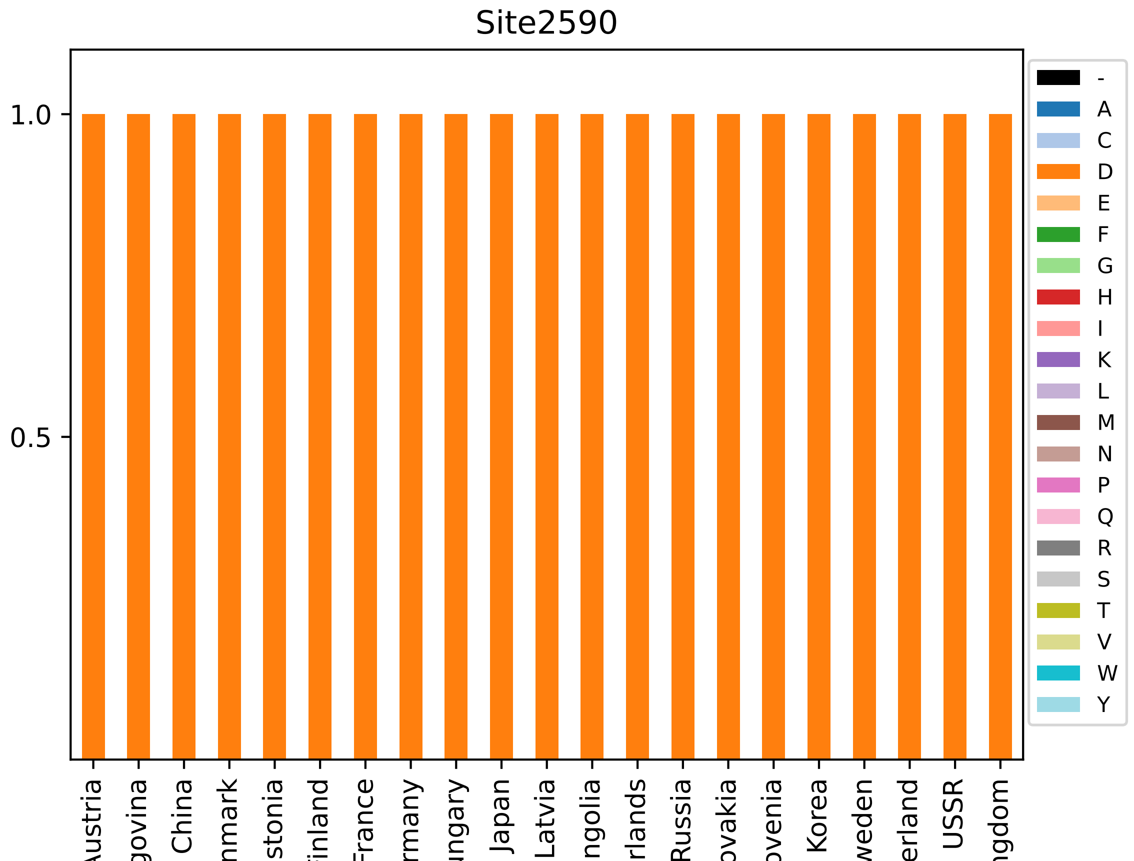
Site2590
1.0
0.5
-
A
C
D
E
F
G
H
I
K
L
M
N
P
Q
R
S
T
V
W
Y
Austria
China
Japan
Latvia
Russia
Korea
USSR

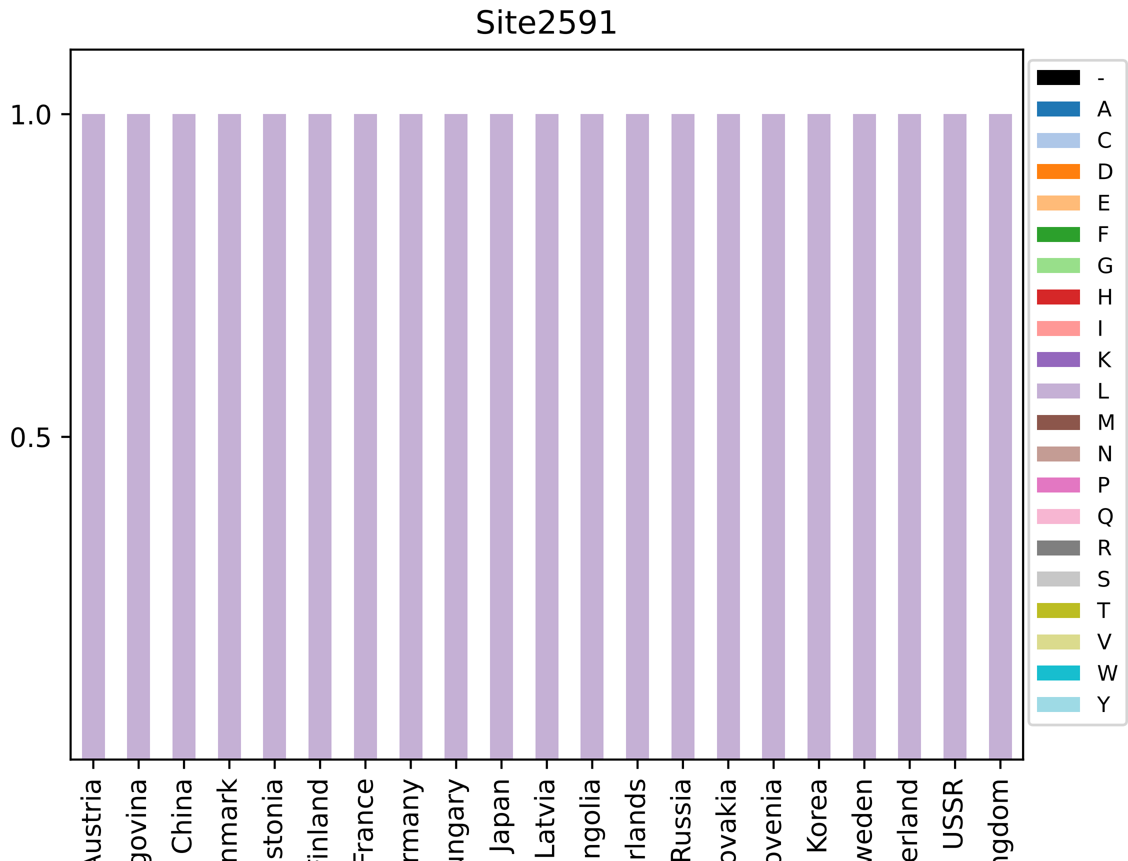
Site2591
1.0
0.5
-
A
C
D
E
F
G
H
I
K
L
M
N
P
Q
R
S
T
V
W
Y
China
Japan
Latvia
Russia
Korea
USSR

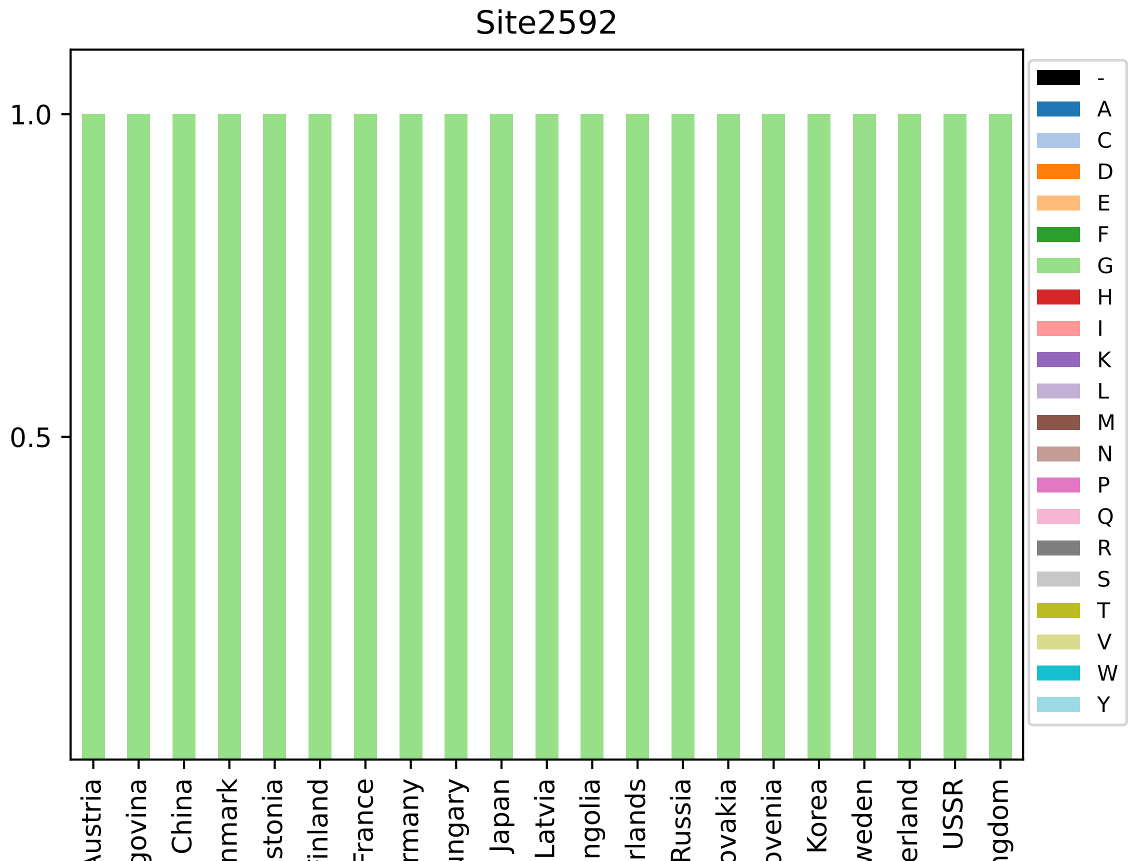
Site2592
1.0
0.5
-
A
C
D
E
F
G
H
I
K
L
M
N
P
Q
R
S
T
V
W
Y
China
Japan
Latvia
Russia
Korea
USSR

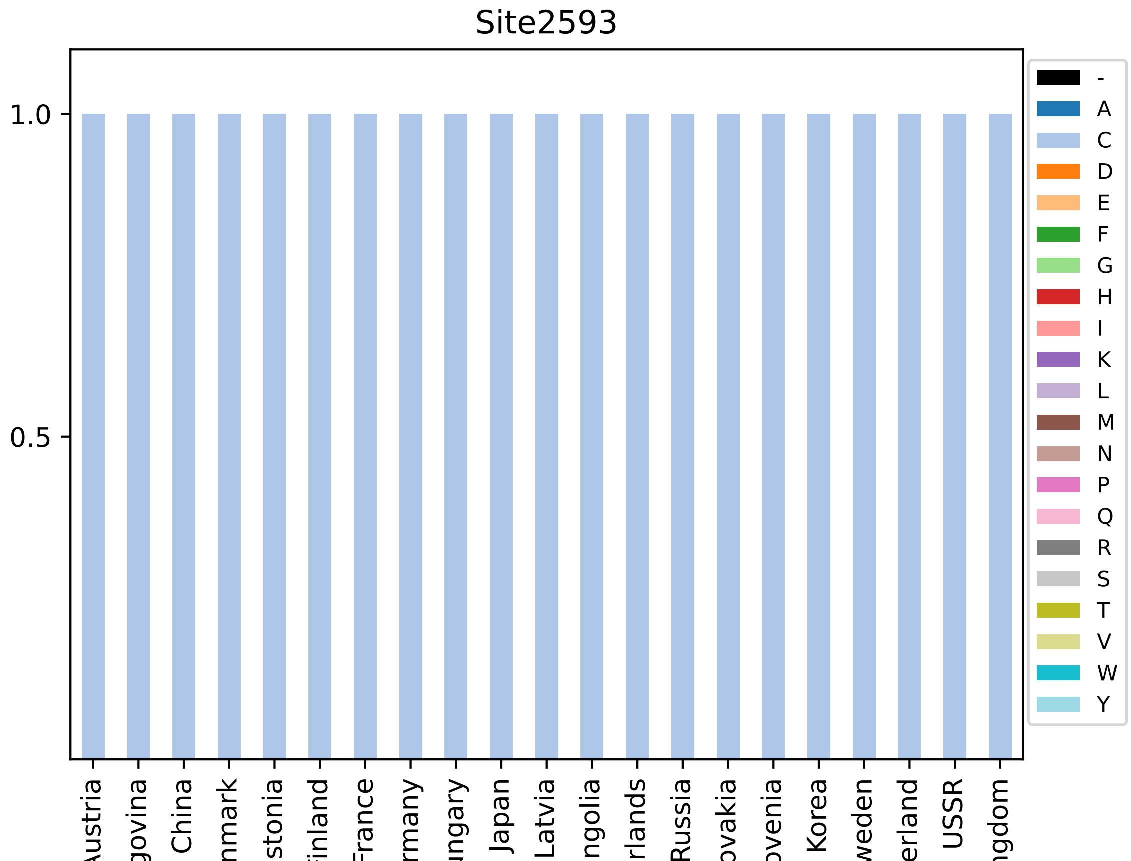
Site2593
1.0
0.5
China
Japan
Latvia
Russia
Korea
USSR
-
A
C
D
E
F
G
H
I
K
L
M
N
P
Q
R
S
T
V
W
Y

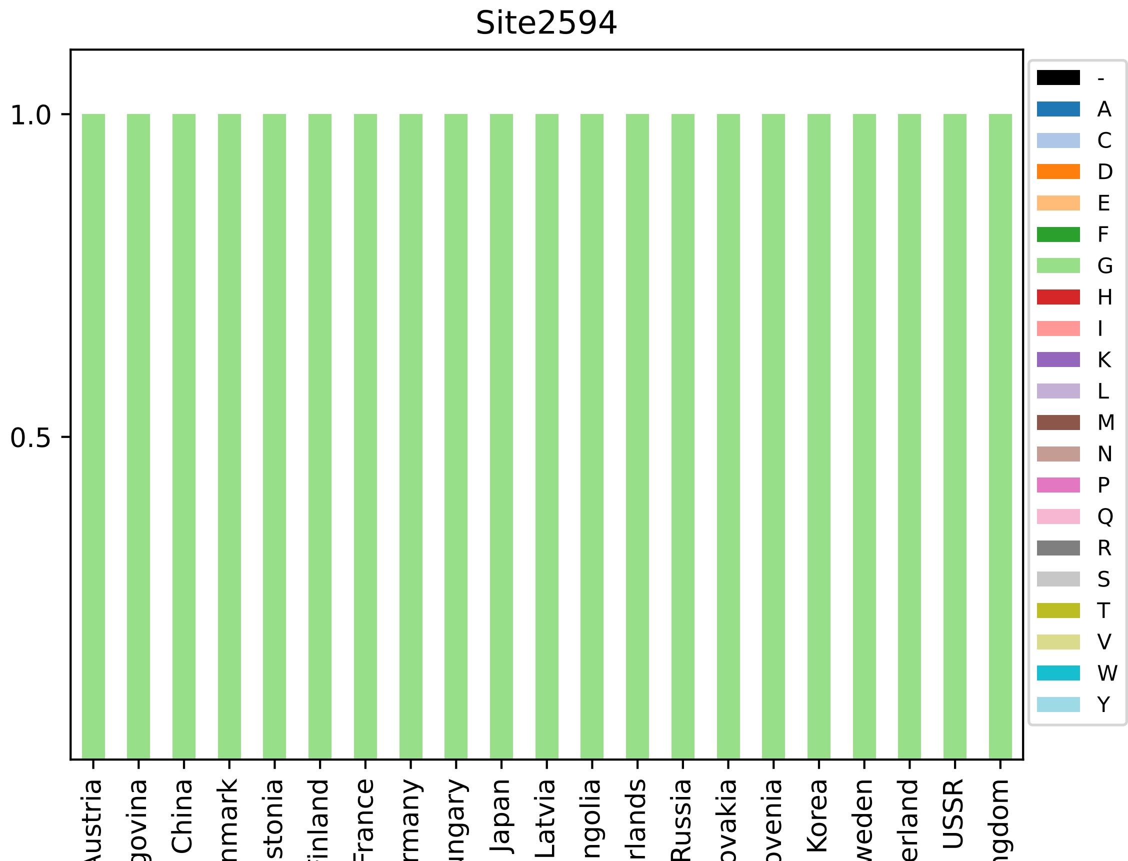
Site2594
1.0
0.5
-
A
C
D
E
F
G
H
I
K
L
M
N
P
Q
R
S
T
V
W
Y
China
Japan
Latvia
Russia
Korea
USSR

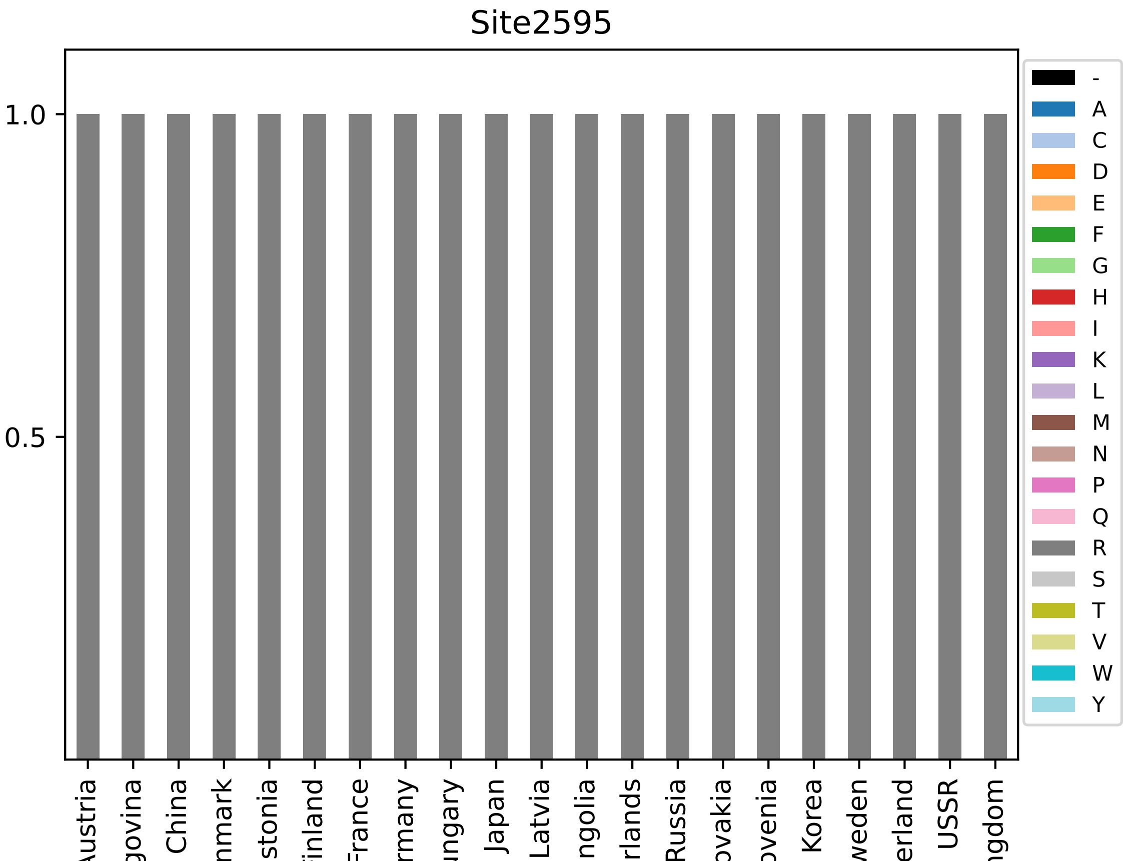
Site2595
1.0
0.5
-
A
C
D
E
F
G
H
I
K
L
M
N
P
Q
R
S
T
V
W
Y
Austria
govina
China
nmark
stonia
Finland
France
rmany
ungary
Japan
Latvia
ngolia
rlands
Russia
ovakia
ovenia
Korea
weden
erland
USSR
ngdom

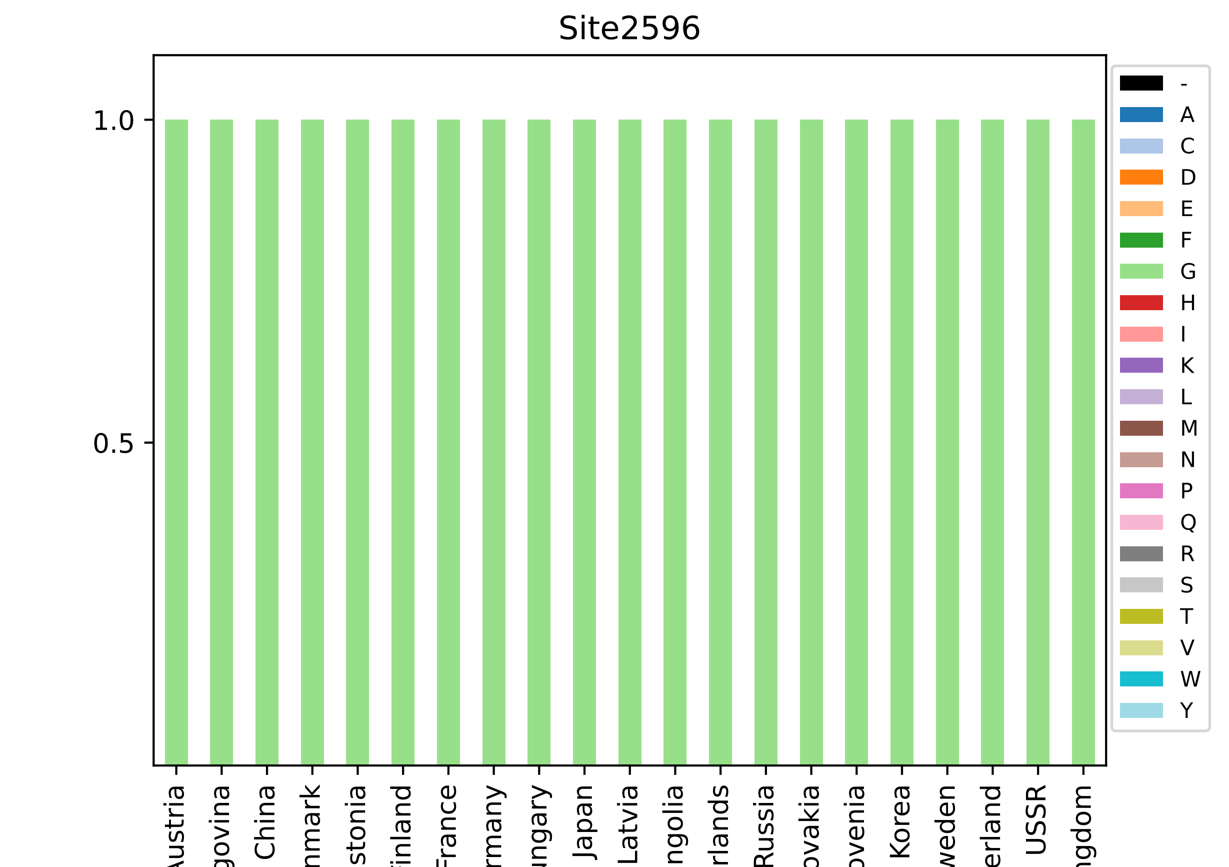
Site2596
1.0
0.5
-
A
C
D
E
F
G
H
I
K
L
M
N
P
Q
R
S
T
V
W
Y
China
Japan
Latvia
Russia
Korea
USSR

Site2597

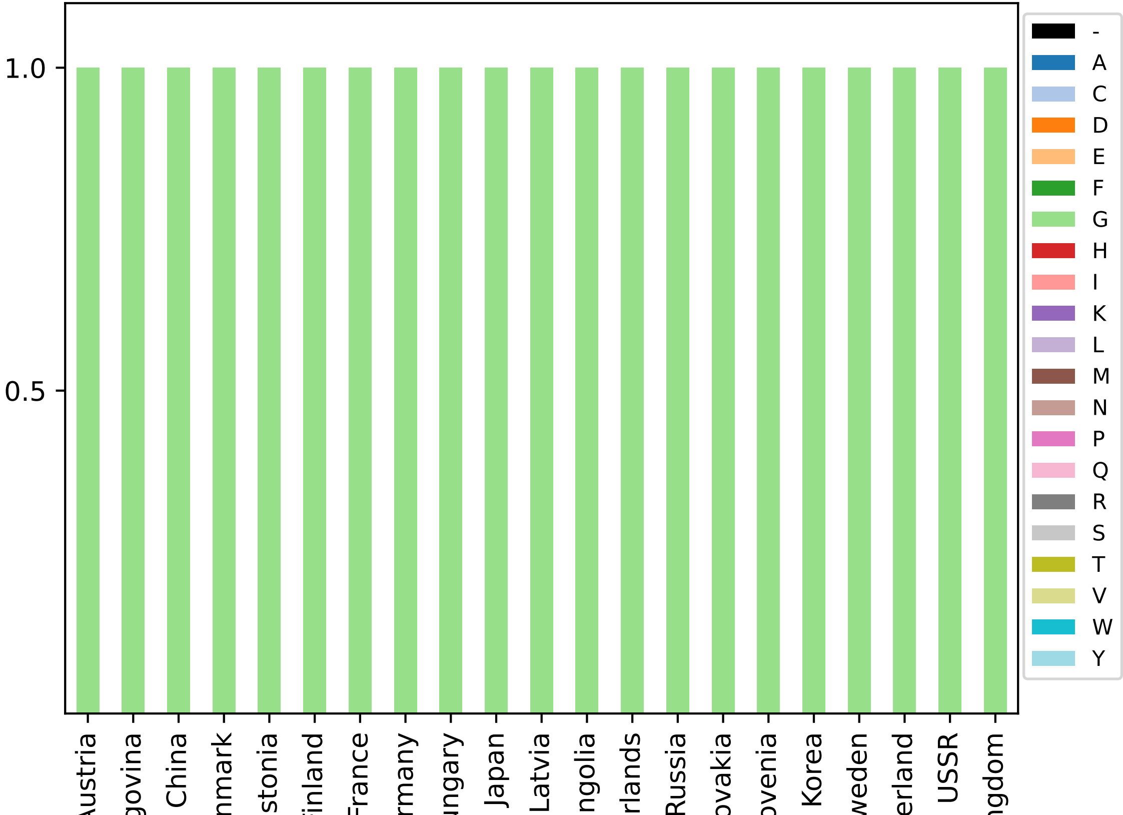
1.0
0.5
-
A
C
D
E
F
G
H
I
K
L
M
N
P
Q
R
S
T
V
W
Y
China
Japan
Latvia
Russia
Korea
USSR

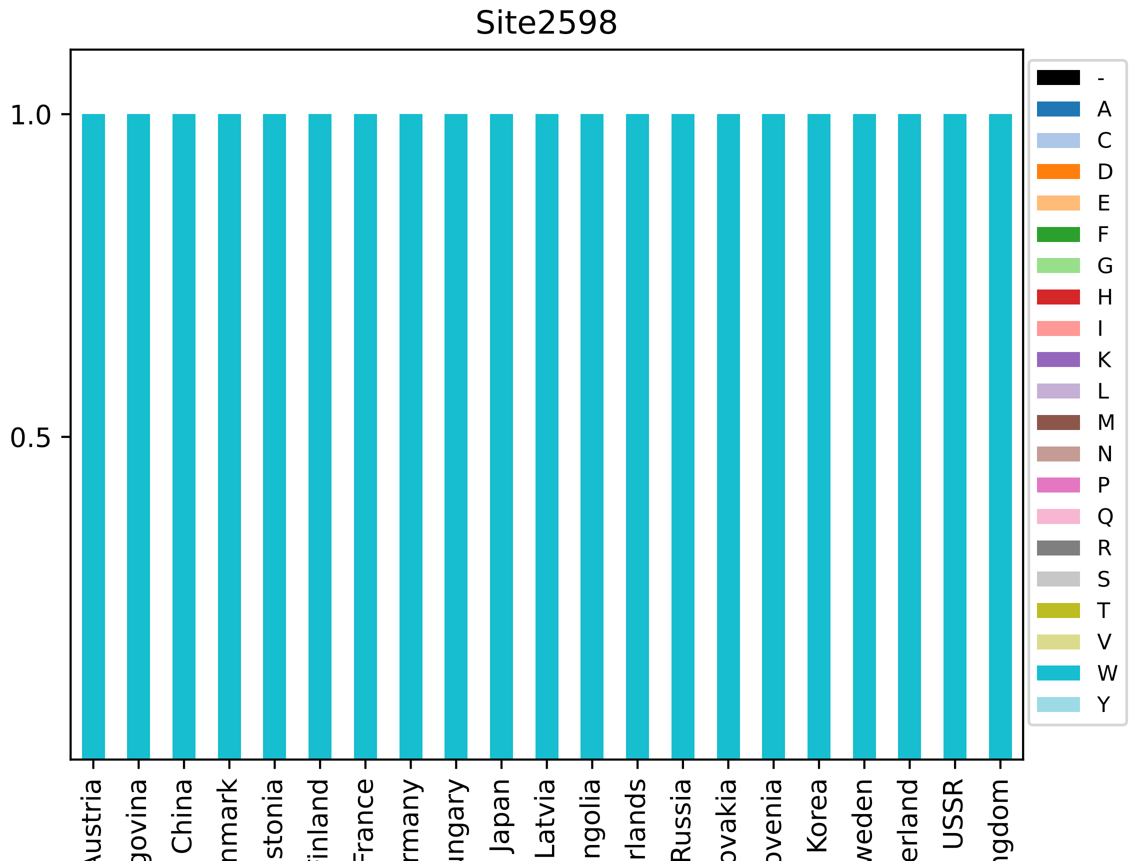
Site2598
1.0
0.5
-
A
C
D
E
F
G
H
I
K
L
M
N
P
Q
R
S
T
V
W
Y
China
Japan
Latvia
Russia
Korea
USSR

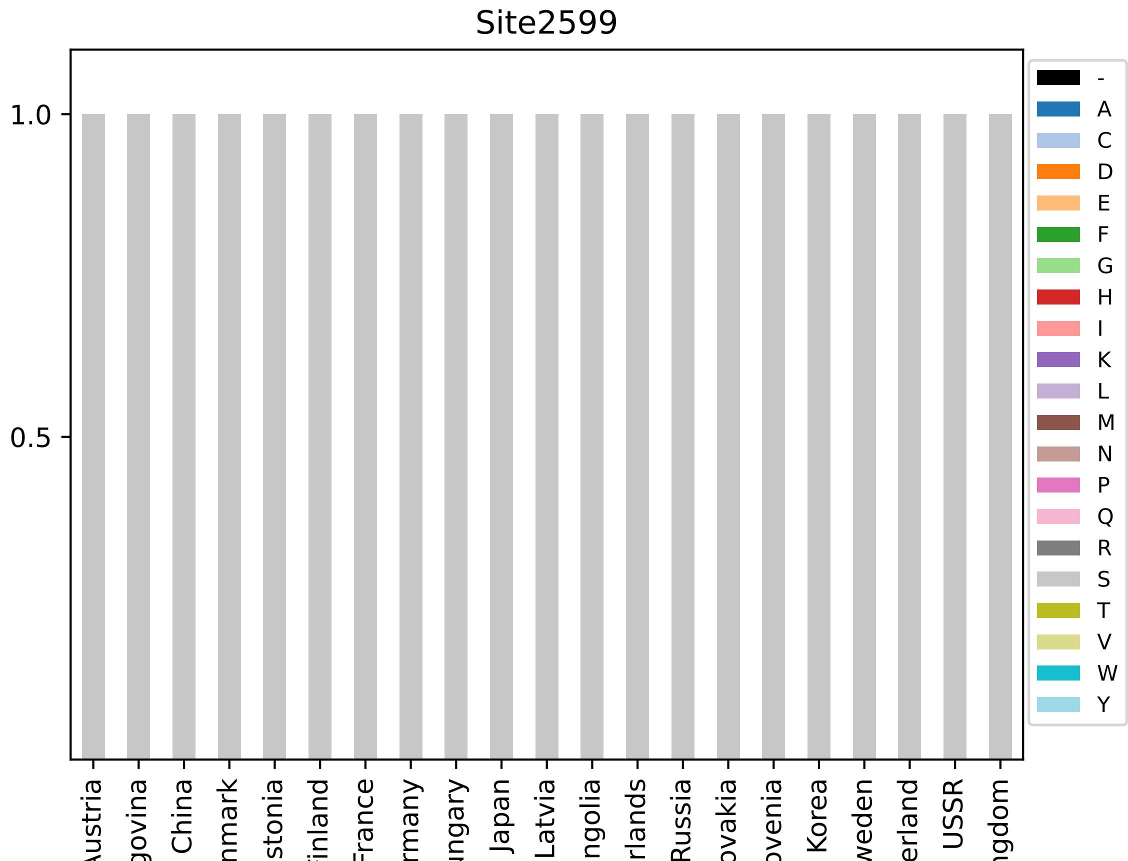
Site2599
1.0
0.5
-
A
C
D
E
F
G
H
I
K
L
M
N
P
Q
R
S
T
V
W
Y
Austria
govina
China
nmark
stonia
inland
France
rmany
ungary
Japan
Latvia
ngolia
rlands
Russia
ovakia
ovenia
Korea
weden
erland
USSR
ngdom

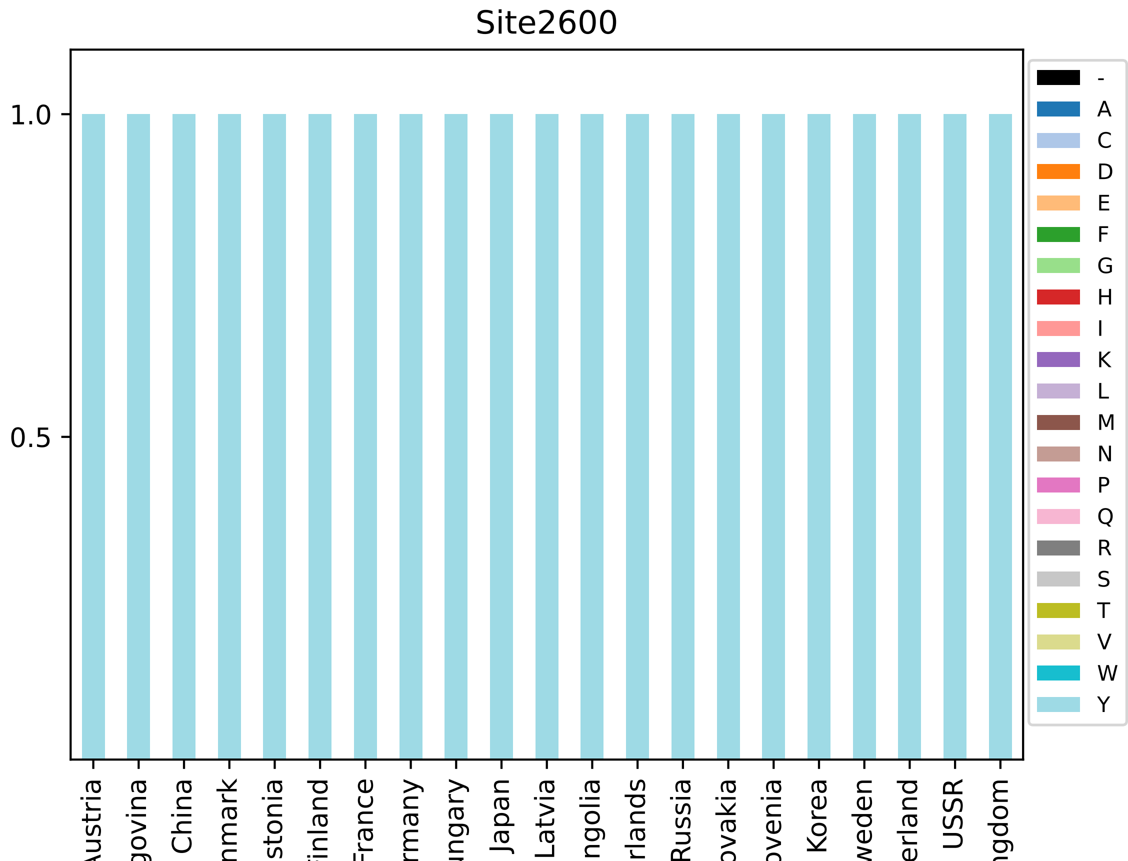
Site2600
1.0
0.5
-
A
C
D
E
F
G
H
I
K
L
M
N
P
Q
R
S
T
V
W
Y
Austria
govina
China
nmark
stonia
Finland
France
rmany
ungary
Japan
Latvia
ngolia
rlands
Russia
ovakia
ovenia
Korea
weden
erland
USSR
ngdom

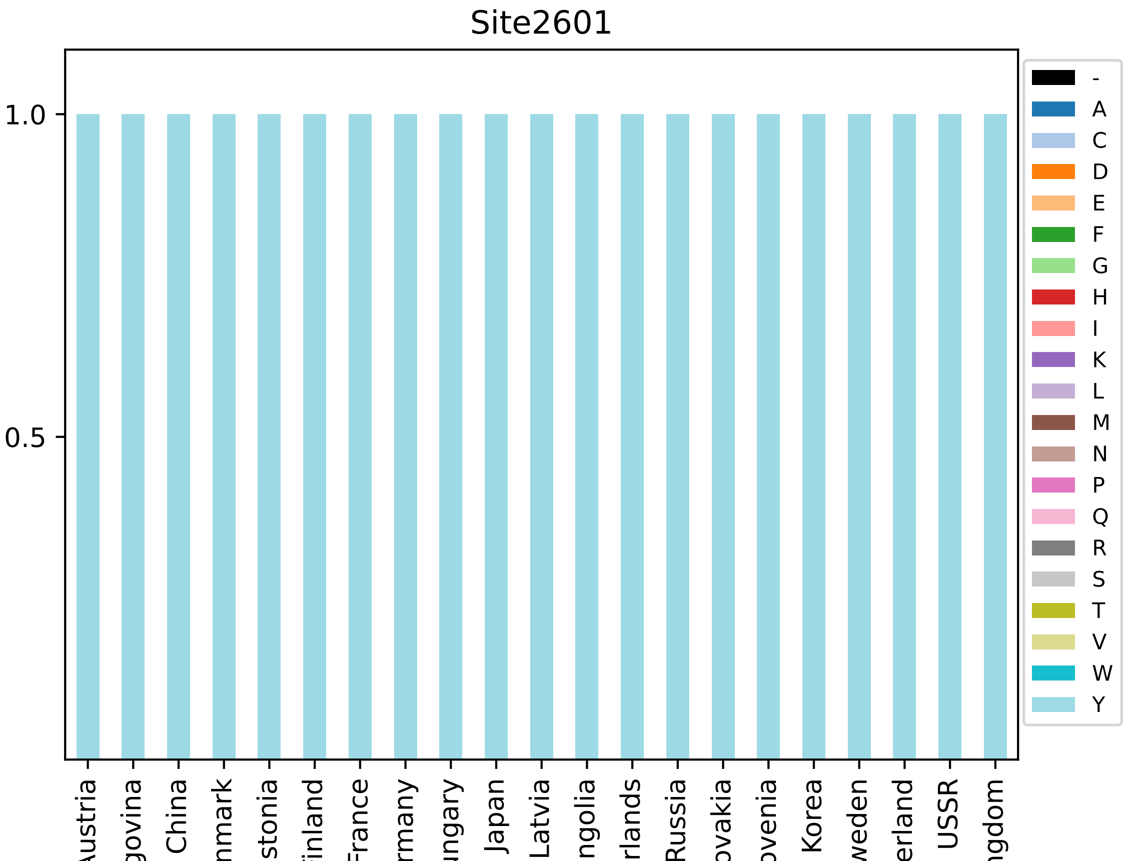

Site2601
1.0
0.5
-
A
C
D
E
F
G
H
I
K
L
M
N
P
Q
R
S
T
V
W
Y
Austria
China
Finland
France
Japan
Latvia
Russia
Korea
USSR

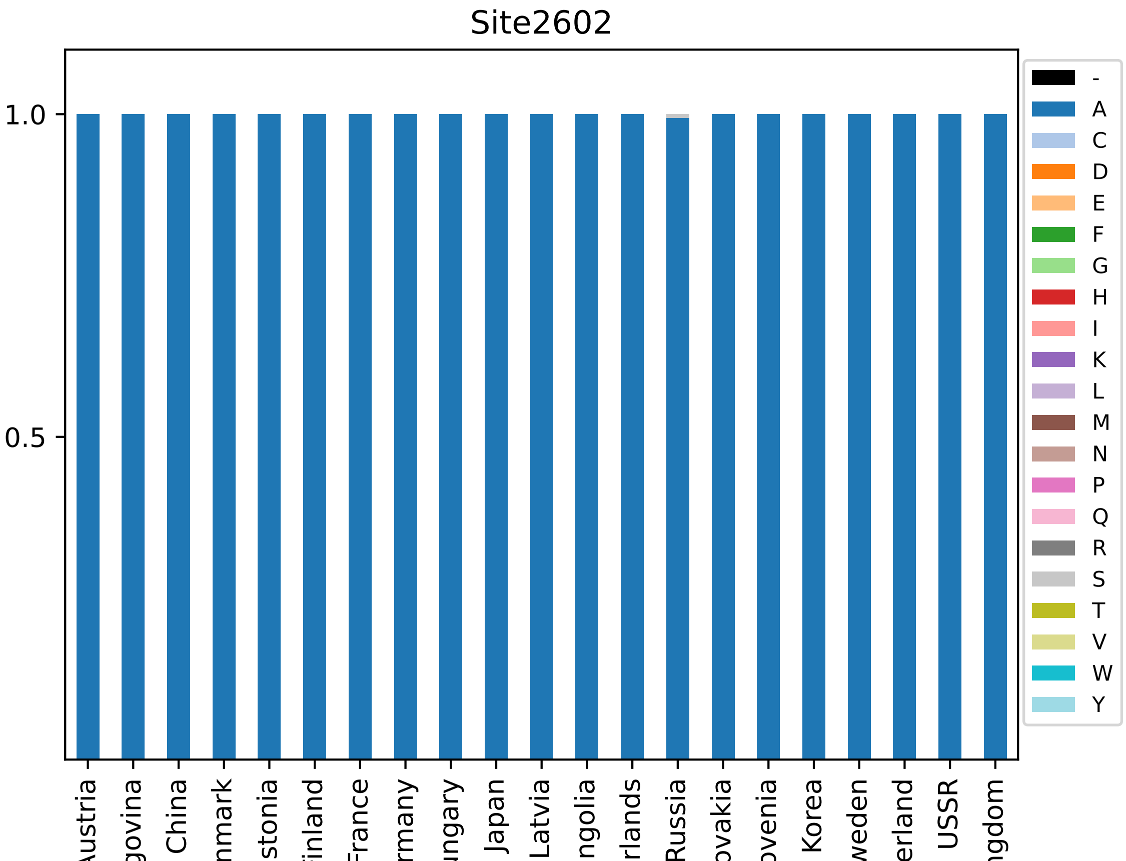
Site2602
1.0
0.5
-
A
C
D
E
F
G
H
I
K
L
M
N
P
Q
R
S
T
V
W
Y
China
Japan
Latvia
Russia
Korea
USSR

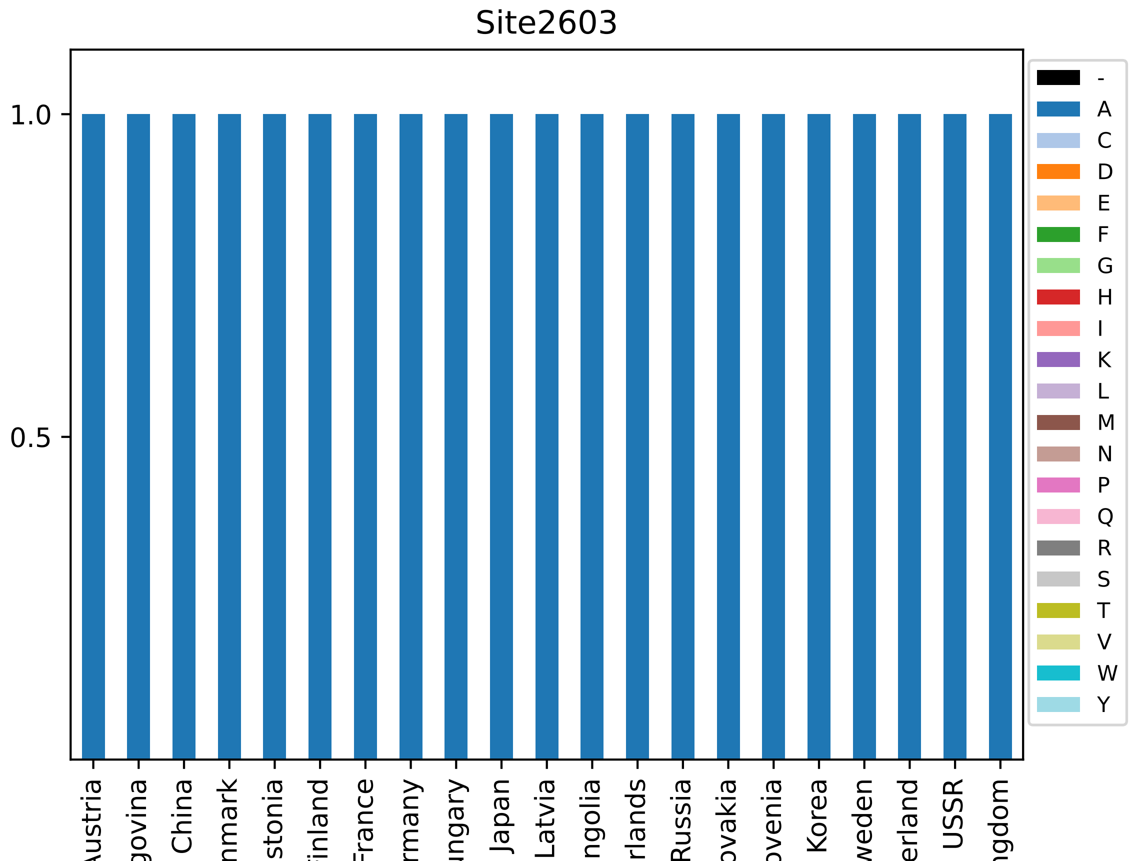

Site2603
1.0
0.5
-
A
C
D
E
F
G
H
I
K
L
M
N
P
Q
R
S
T
V
W
Y
Austria
govina
China
nmark
stonia
inland
France
rmany
ungary
Japan
Latvia
ngolia
rlands
Russia
ovakia
ovenia
Korea
weden
erland
USSR
ngdom

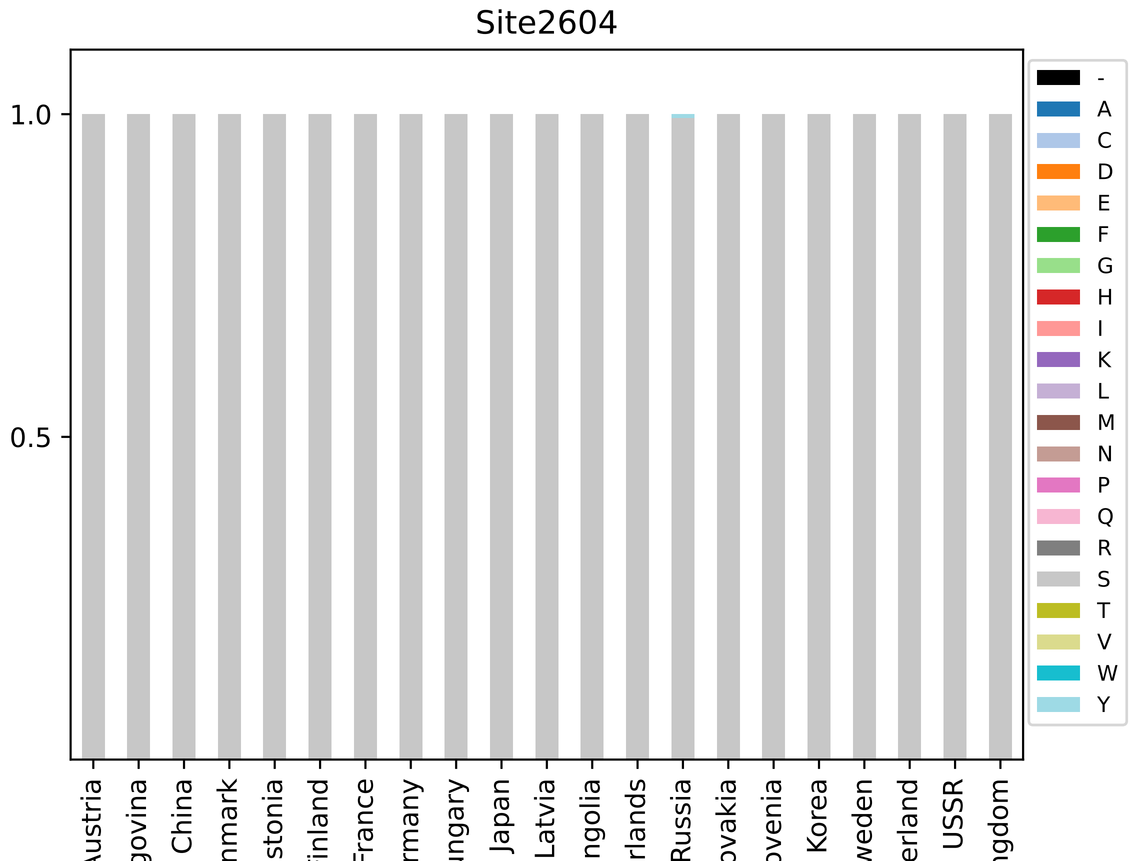

Site2604
1.0
0.5
-
A
C
D
E
F
G
H
I
K
L
M
N
P
Q
R
S
T
V
W
Y
Austria
govina
China
nmark
stonia
Finland
France
rmany
ungary
Japan
Latvia
ngolia
rlands
Russia
ovakia
ovenia
Korea
weden
erland
USSR
ngdom

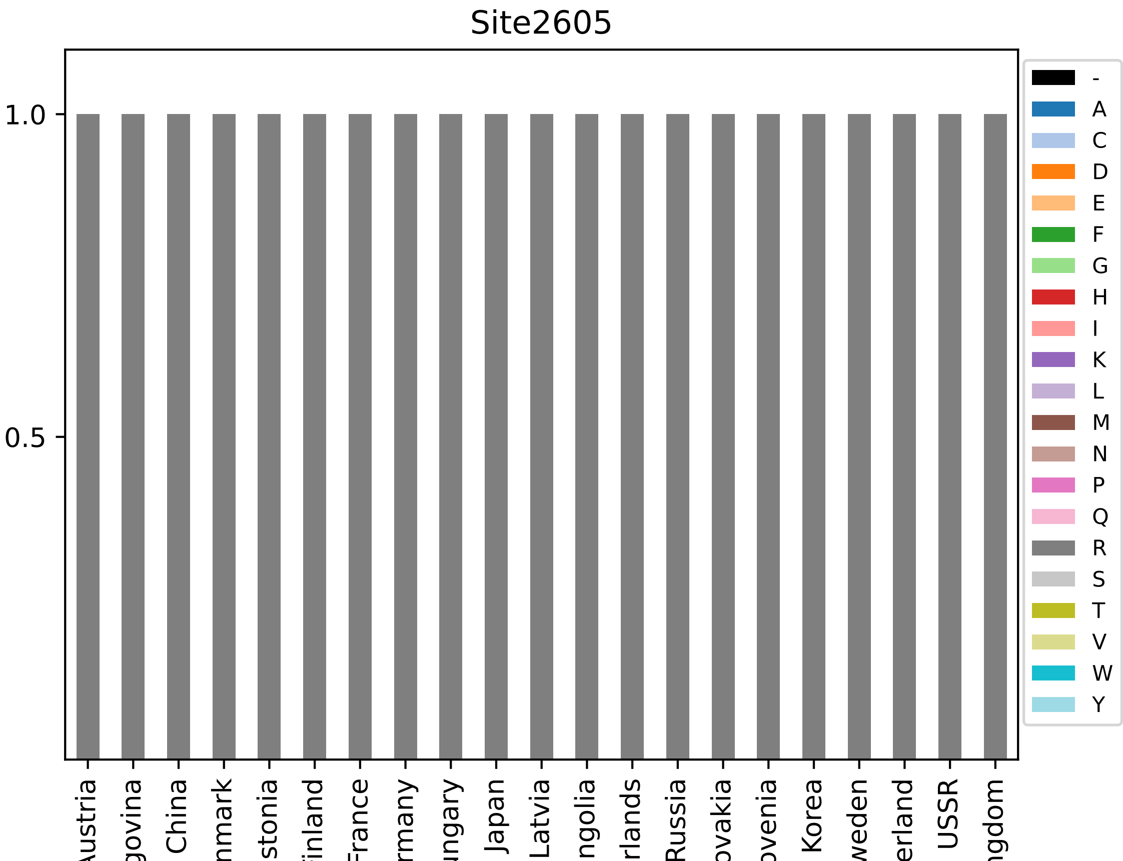

Site2605
1.0
0.5
-
A
C
D
E
F
G
H
I
K
L
M
N
P
Q
R
S
T
V
W
Y
Austria
govina
China
nmark
stonia
inland
France
rmany
ungary
Japan
Latvia
ngolia
rlands
Russia
ovakia
ovenia
Korea
weden
erland
USSR
ngdom

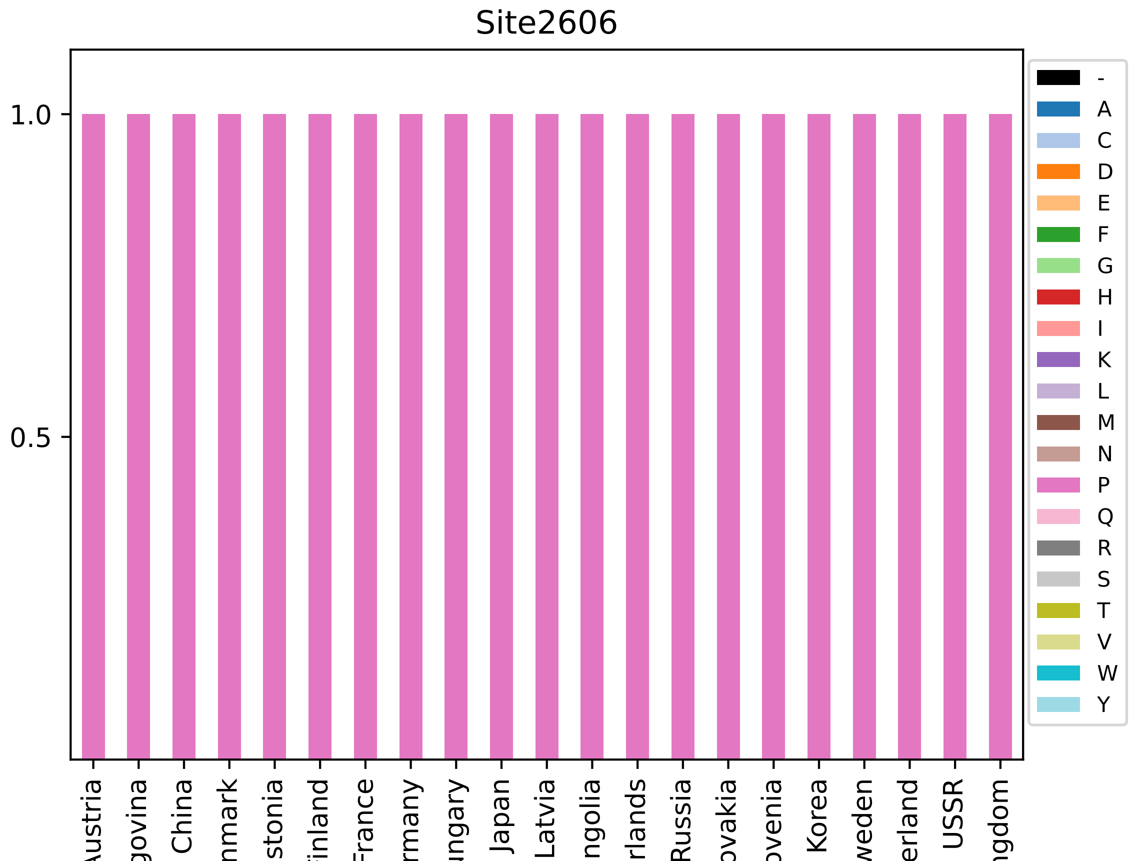
Site2606
1.0
0.5
-
A
C
D
E
F
G
H
I
K
L
M
N
P
Q
R
S
T
V
W
Y
Austria
govina
China
nmark
stonia
inland
France
rmany
ungary
Japan
Latvia
ngolia
rlands
Russia
ovakia
ovenia
Korea
weden
erland
USSR
ngdom

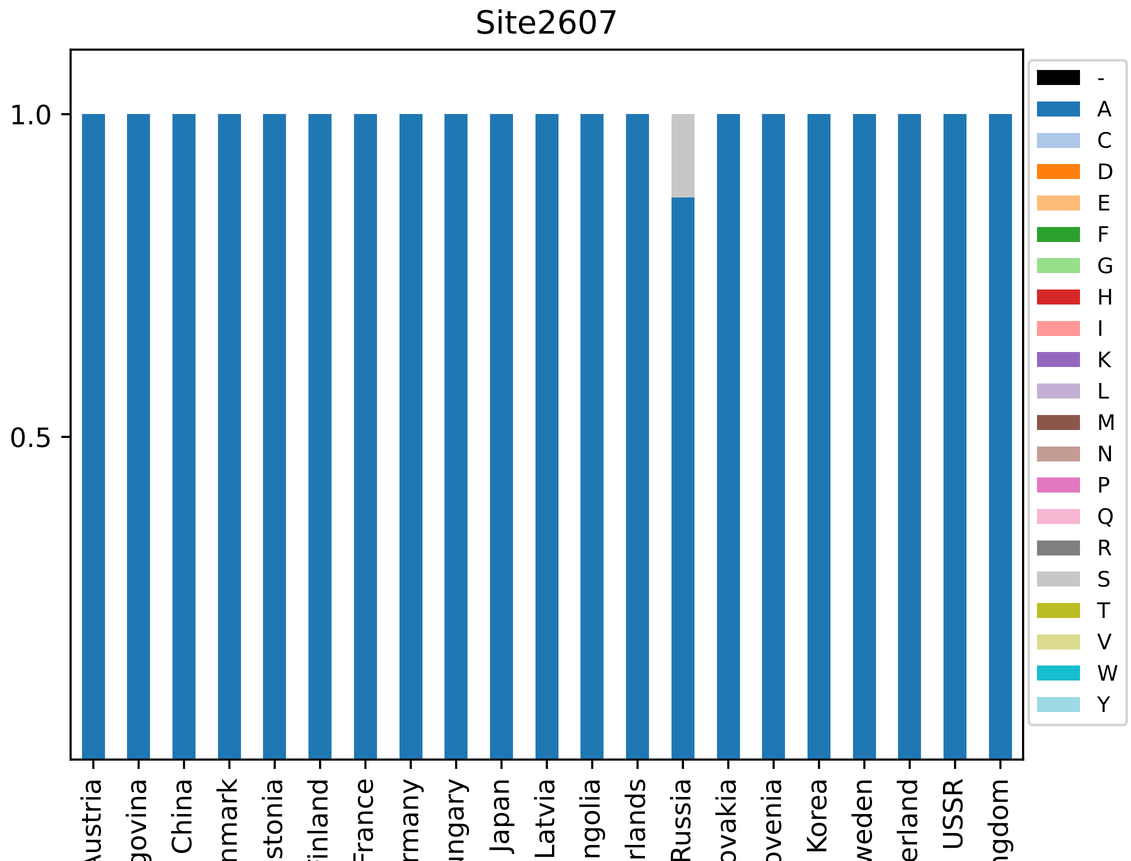
Site2607
1.0
0.5
-
A
C
D
E
F
G
H
I
K
L
M
N
P
Q
R
S
T
V
W
Y
Austria
China
Japan
Latvia
Russia
Korea
USSR

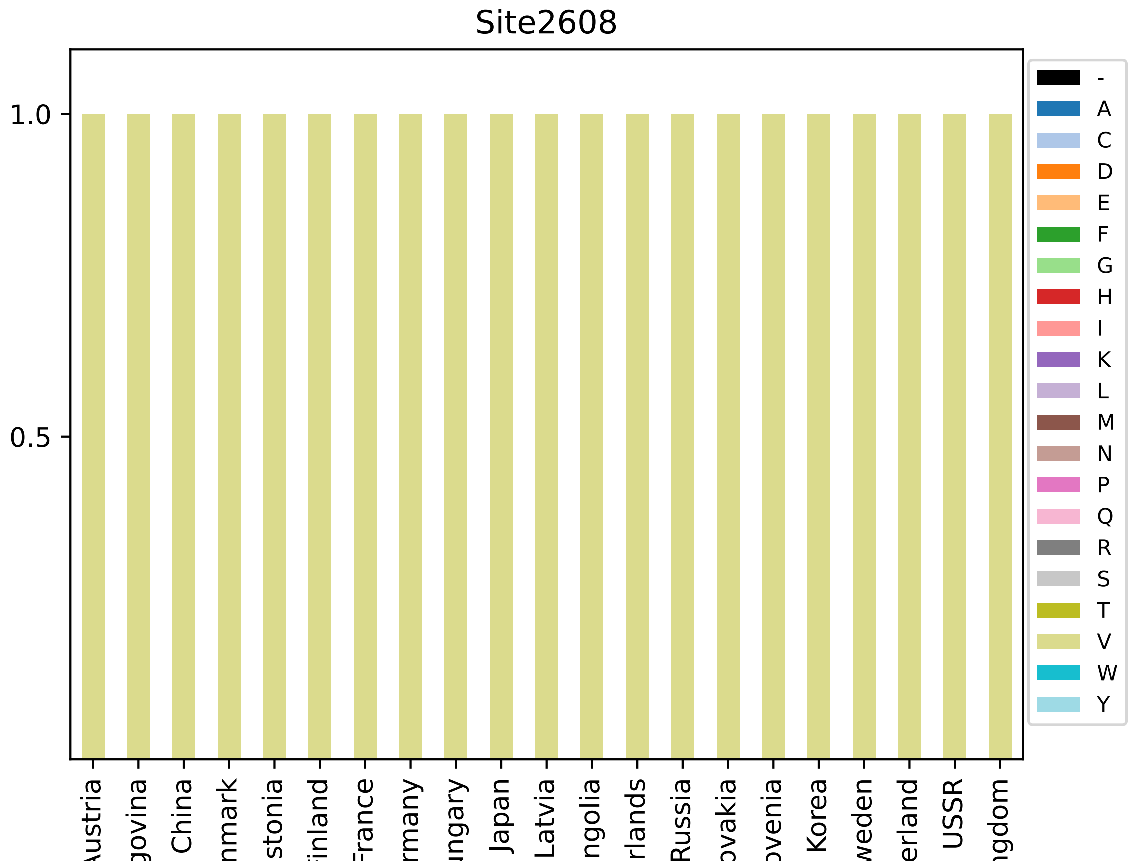
Site2608
1.0
0.5
China
Japan
Latvia
Russia
Korea
USSR
-
A
C
D
E
F
G
H
I
K
L
M
N
P
Q
R
S
T
V
W
Y

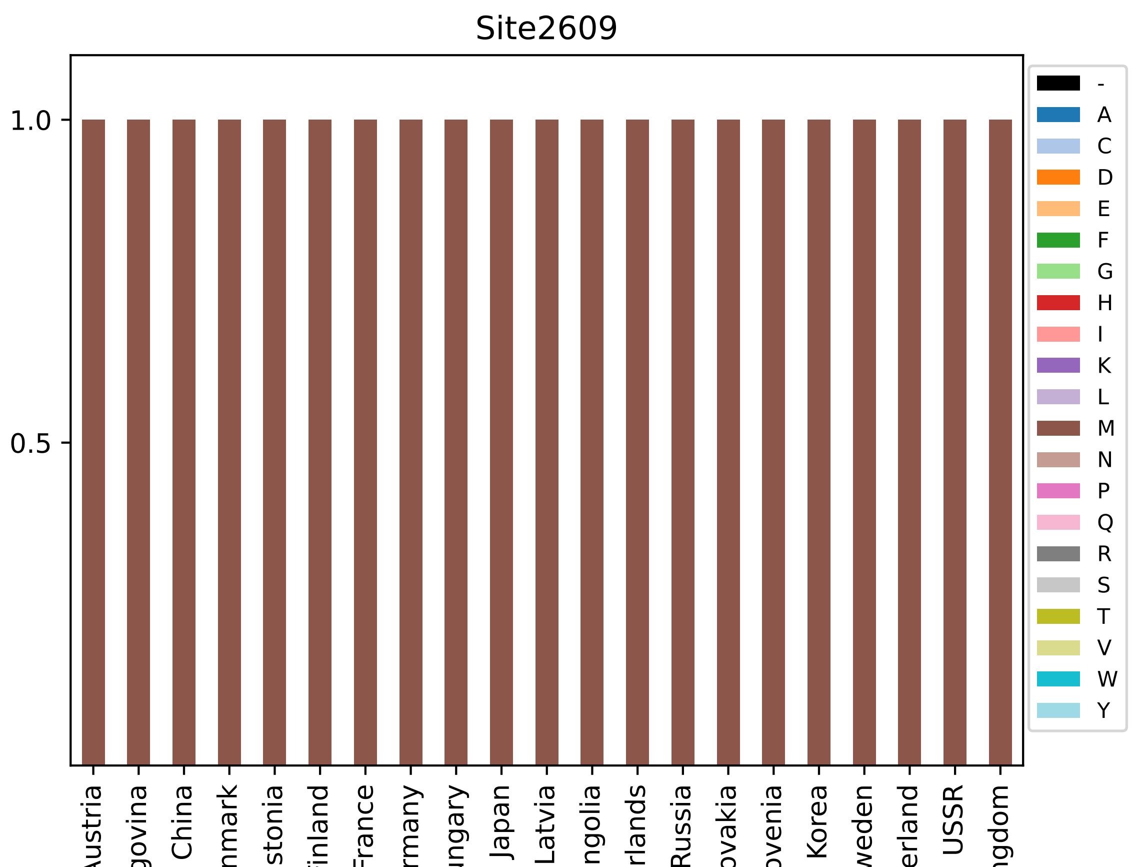
Site2609
1.0
0.5
-
A
C
D
E
F
G
H
I
K
L
M
N
P
Q
R
S
T
V
W
Y
Austria
China
Japan
Latvia
Russia
Korea
USSR

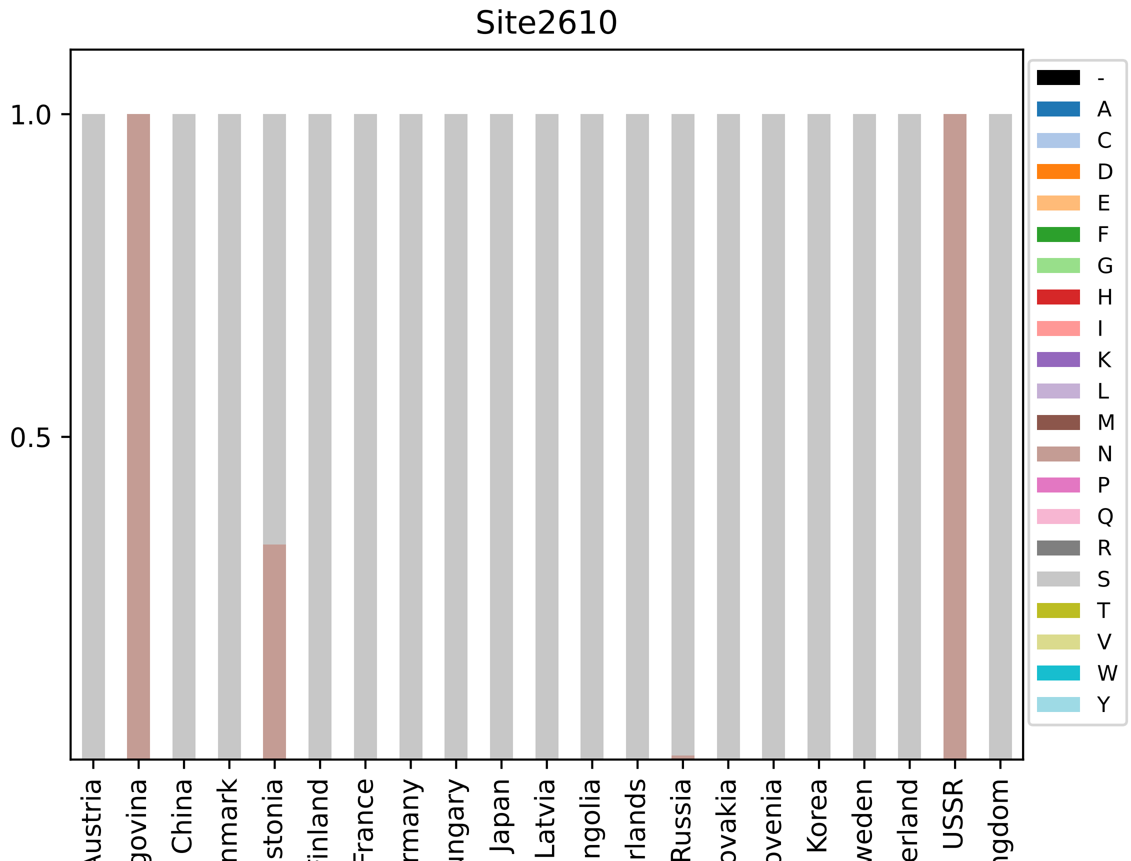
Site2610
1.0
0.5
-
A
C
D
E
F
G
H
I
K
L
M
N
P
Q
R
S
T
V
W
Y
Austria
govina
China
nmark
stonia
inland
France
rmany
ungary
Japan
Latvia
ngolia
rlands
Russia
ovakia
ovenia
Korea
weden
erland
USSR
ngdom

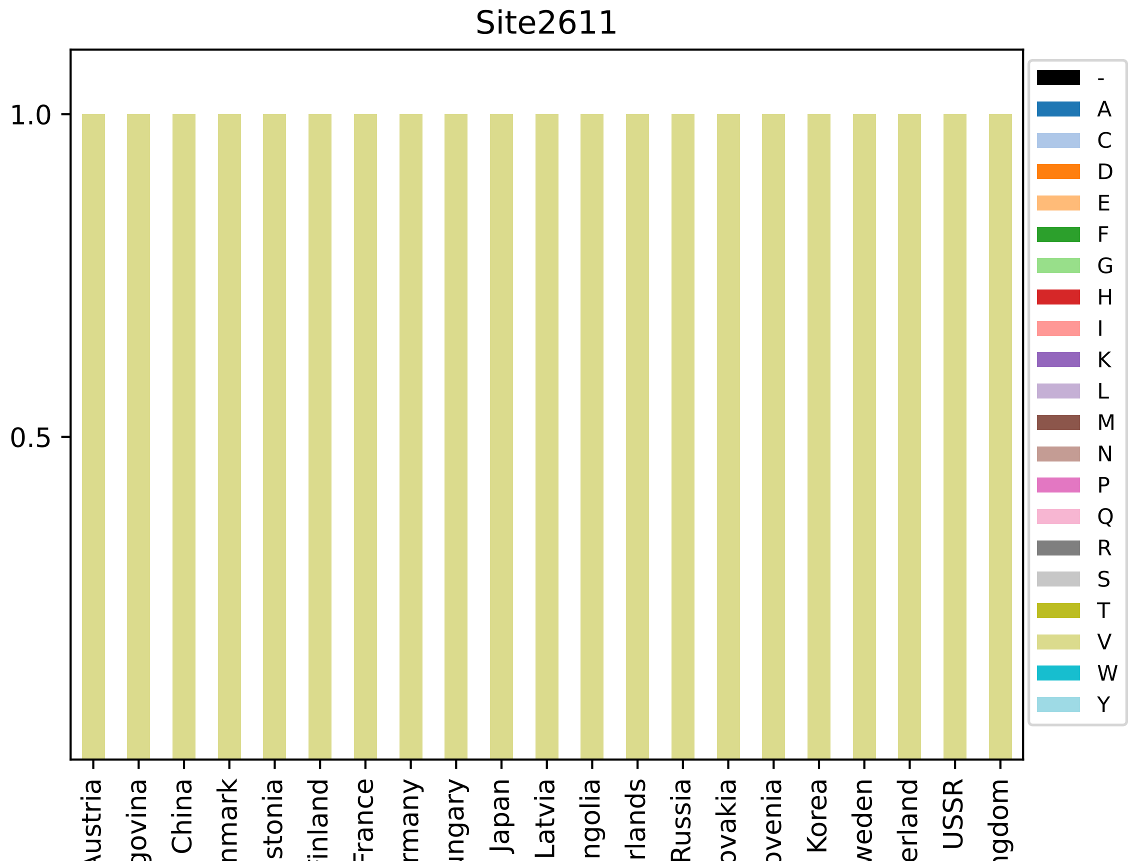
Site2611
1.0
0.5
-
A
C
D
E
F
G
H
I
K
L
M
N
P
Q
R
S
T
V
W
Y
China
Japan
Latvia
Russia
Korea
USSR

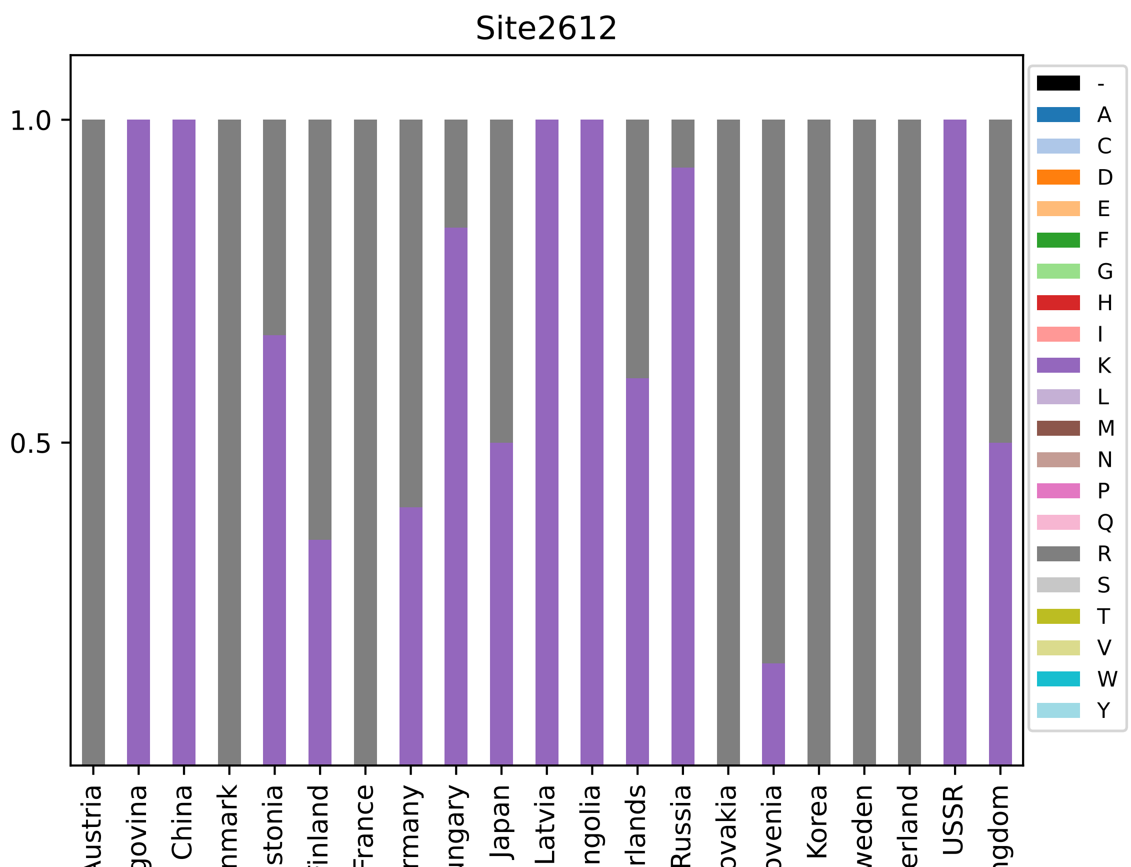

Site2612
1.0
0.5
-
A
C
D
E
F
G
H
I
K
L
M
N
P
Q
R
S
T
V
W
Y
Austria
China
Japan
Latvia
Russia
Korea
USSR

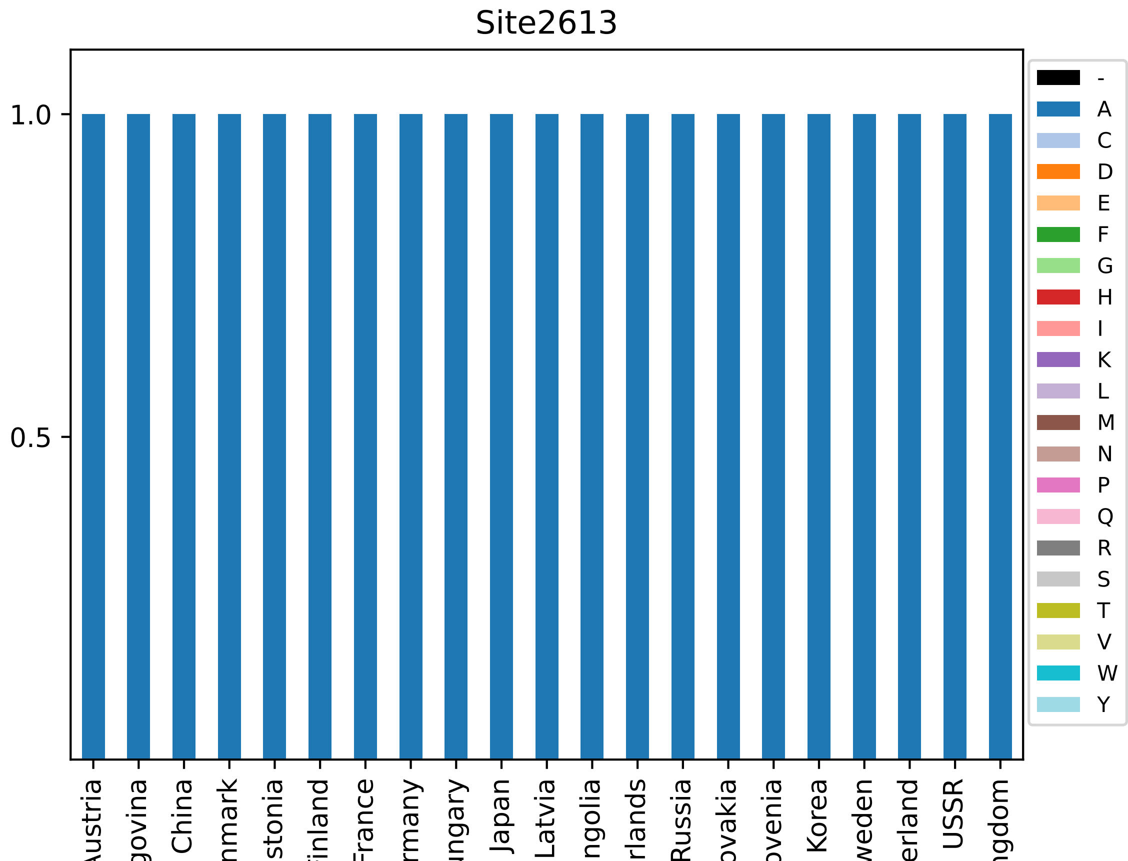
Site2613
1.0
0.5
-
A
C
D
E
F
G
H
I
K
L
M
N
P
Q
R
S
T
V
W
Y
China
Japan
Latvia
Russia
Korea
USSR

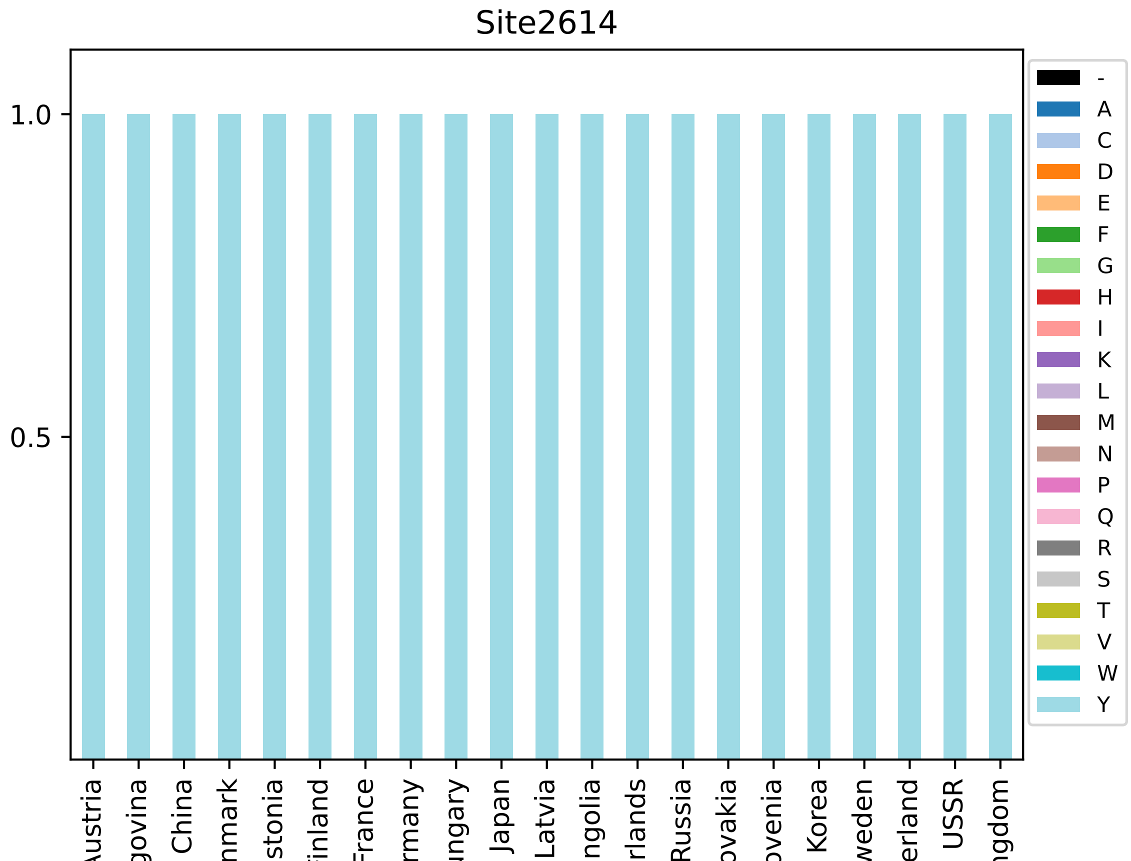
Site2614
1.0
0.5
-
A
C
D
E
F
G
H
I
K
L
M
N
P
Q
R
S
T
V
W
Y
China
Japan
Latvia
Russia
Korea
USSR

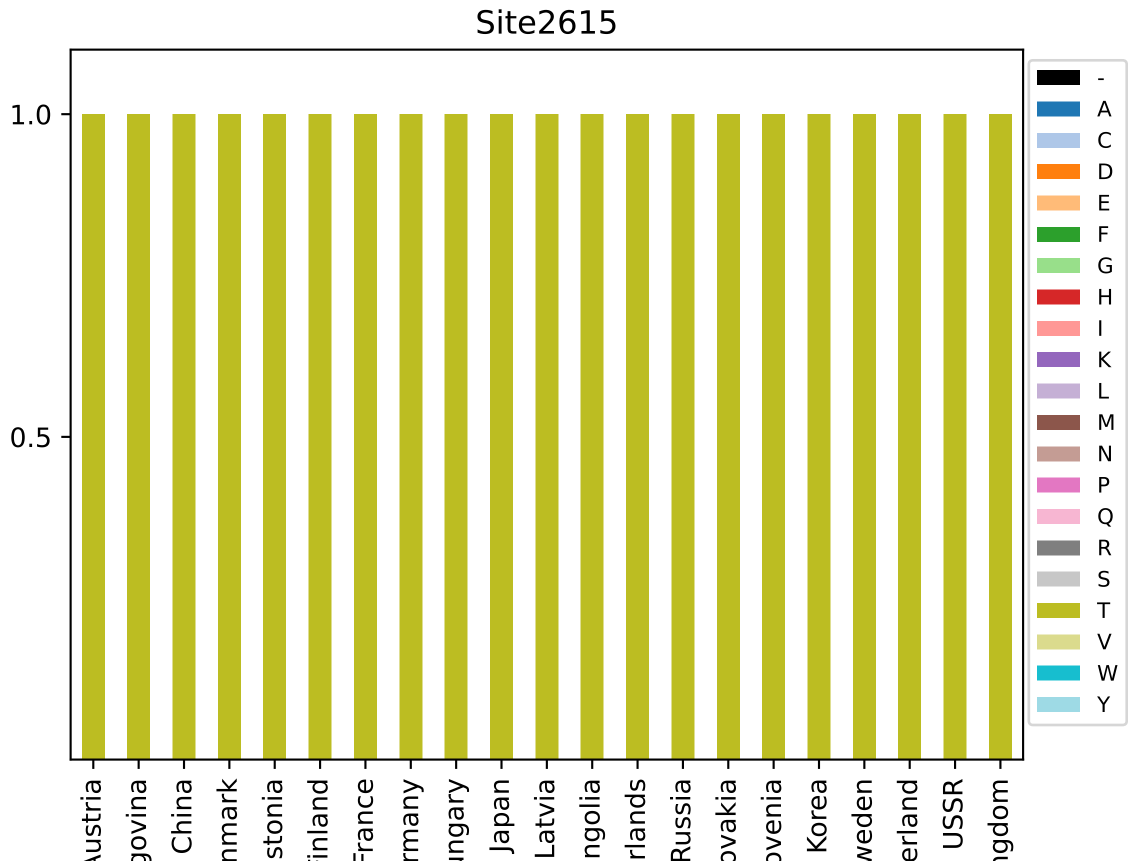

Site2615
1.0
0.5
-
A
C
D
E
F
G
H
I
K
L
M
N
P
Q
R
S
T
V
W
Y
Austria
govina
China
nmark
stonia
Finland
France
rmany
ungary
Japan
Latvia
ngolia
rlands
Russia
ovakia
ovenia
Korea
weden
erland
USSR
ngdom

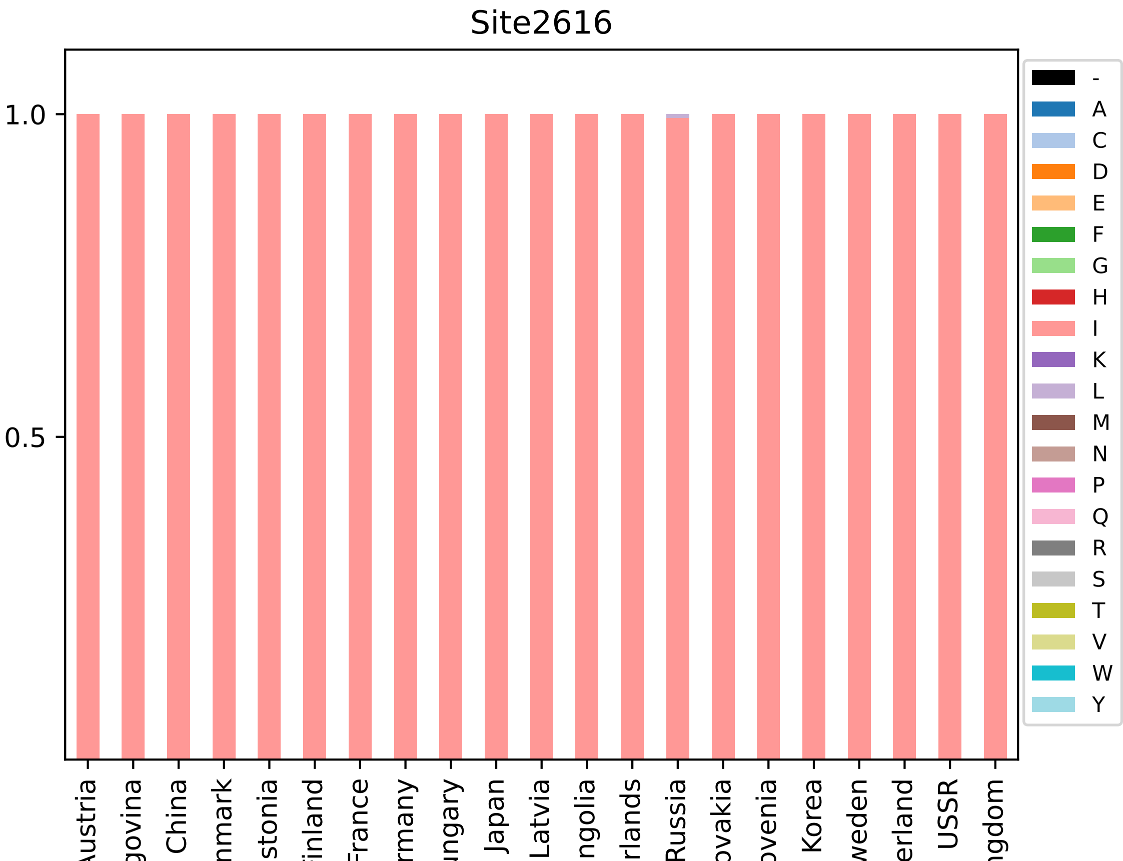

Site2616
1.0
0.5
-
A
C
D
E
F
G
H
I
K
L
M
N
P
Q
R
S
T
V
W
Y
Austria
govina
China
nmark
stonia
Finland
France
rmany
ungary
Japan
Latvia
ngolia
rlands
Russia
ovakia
ovenia
Korea
weden
erland
USSR
ngdom

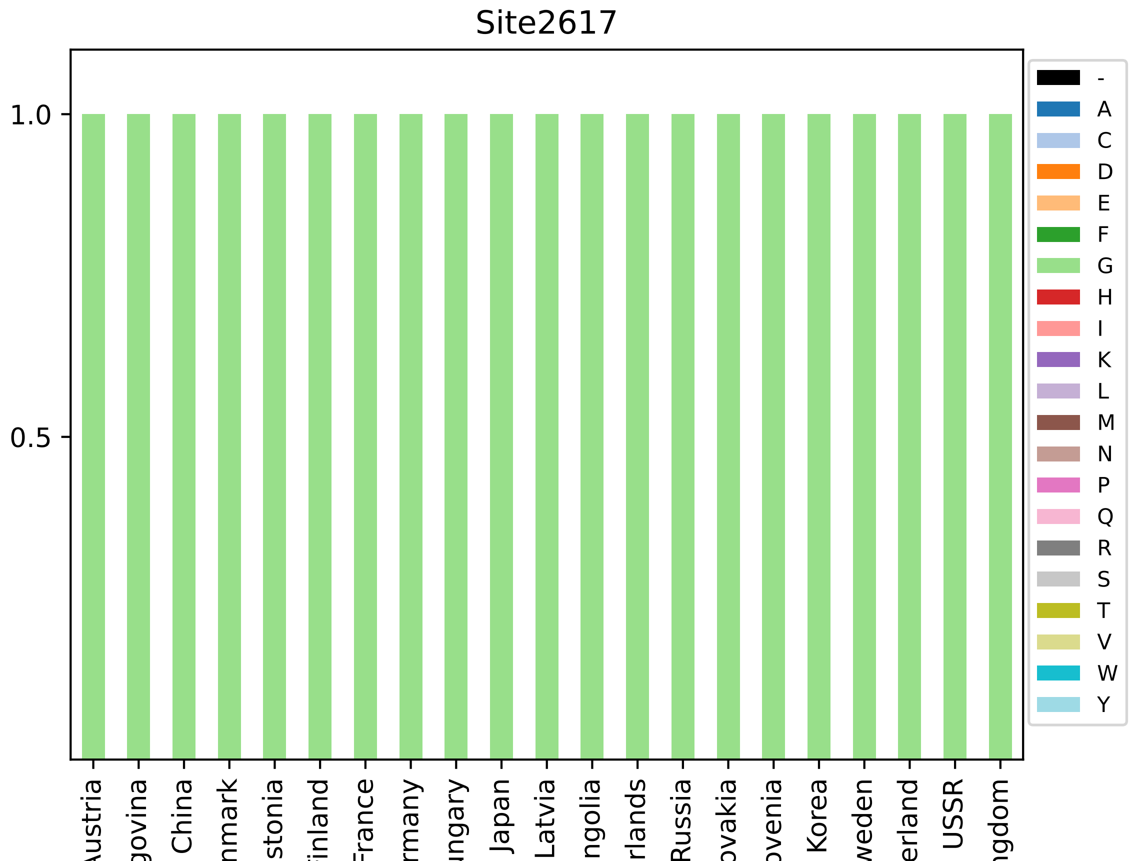
Site2617
1.0
0.5
-
A
C
D
E
F
G
H
I
K
L
M
N
P
Q
R
S
T
V
W
Y
Austria
govina
China
nmark
stonia
Finland
France
rmany
ungary
Japan
Latvia
ngolia
rlands
Russia
ovakia
ovenia
Korea
weden
erland
USSR
ngdom

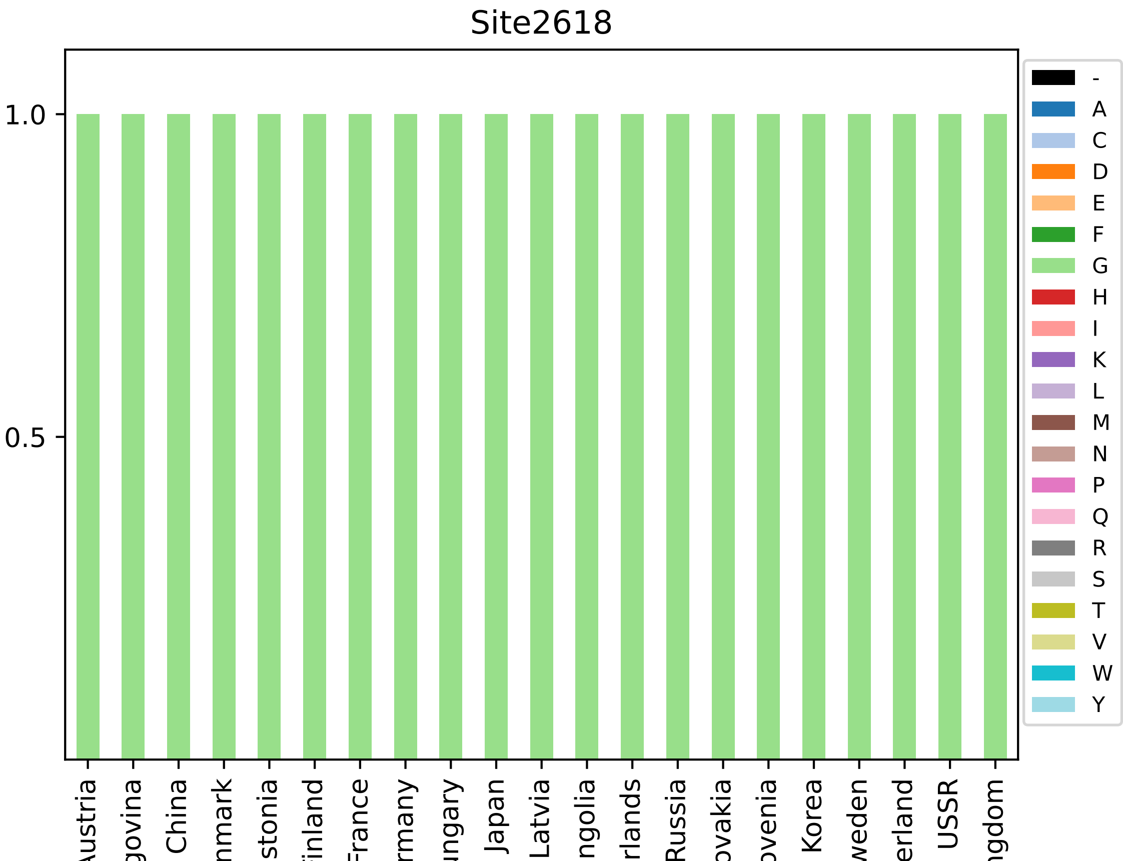
Site2618
1.0
0.5
-
A
C
D
E
F
G
H
I
K
L
M
N
P
Q
R
S
T
V
W
Y
China
Japan
Latvia
Russia
Korea
USSR

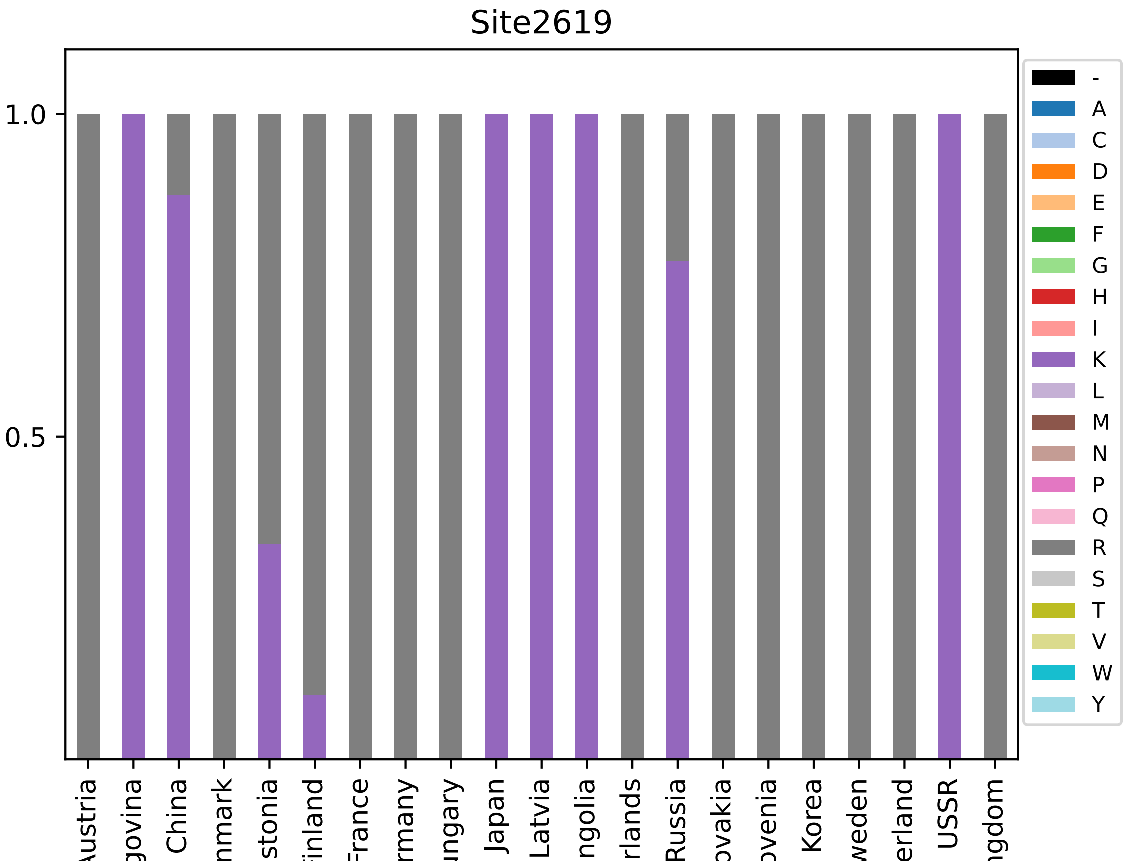

Site2619
1.0
0.5
-
A
C
D
E
F
G
H
I
K
L
M
N
P
Q
R
S
T
V
W
Y
Austria
China
Japan
Latvia
Russia
Korea
USSR

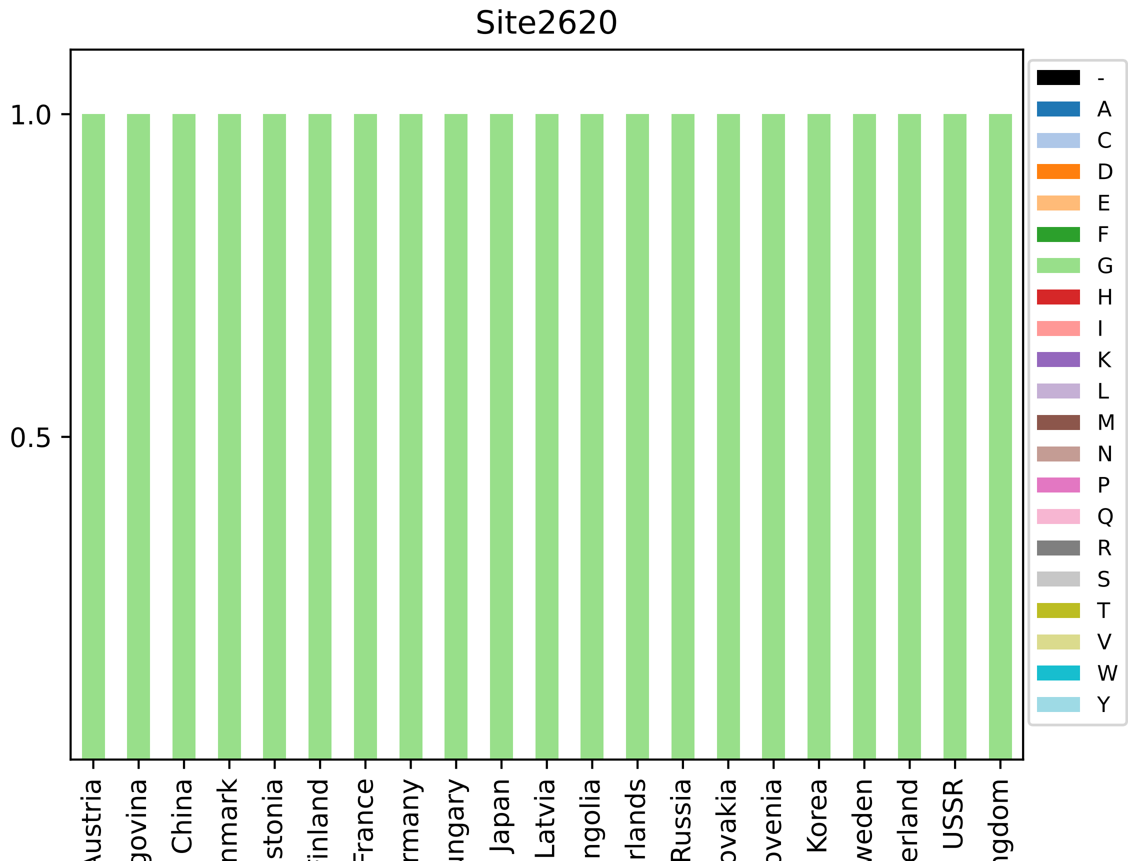
Site2620
1.0
0.5
-
A
C
D
E
F
G
H
I
K
L
M
N
P
Q
R
S
T
V
W
Y
China
Japan
Latvia
Russia
Korea
USSR

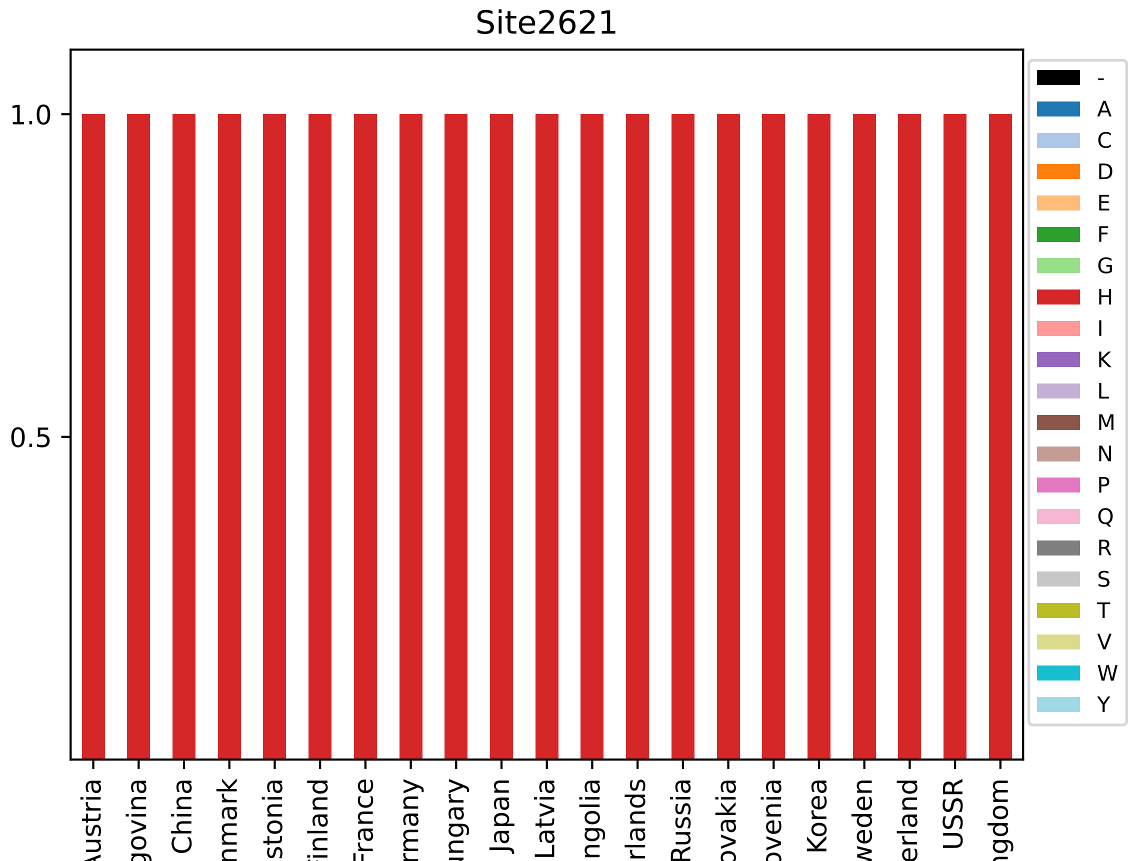

Site2621
1.0
0.5
-
A
C
D
E
F
G
H
I
K
L
M
N
P
Q
R
S
T
V
W
Y
Austria
China
Japan
Latvia
Russia
Korea
USSR

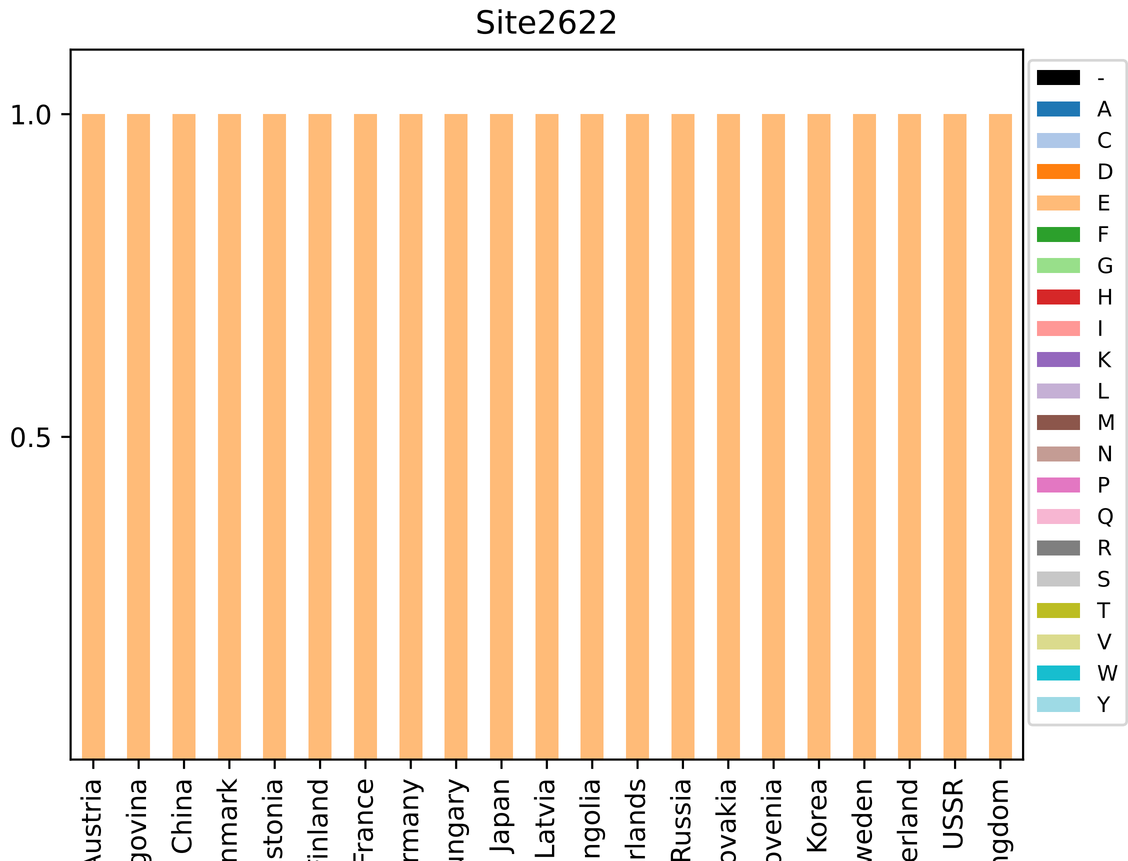

Site2622
1.0
0.5
Austria
govina
China
nmark
stonia
Finland
France
rmany
ungary
Japan
Latvia
ngolia
rlands
Russia
ovakia
ovenia
Korea
weden
erland
USSR
ngdom
-
A
C
D
E
F
G
H
I
K
L
M
N
P
Q
R
S
T
V
W
Y

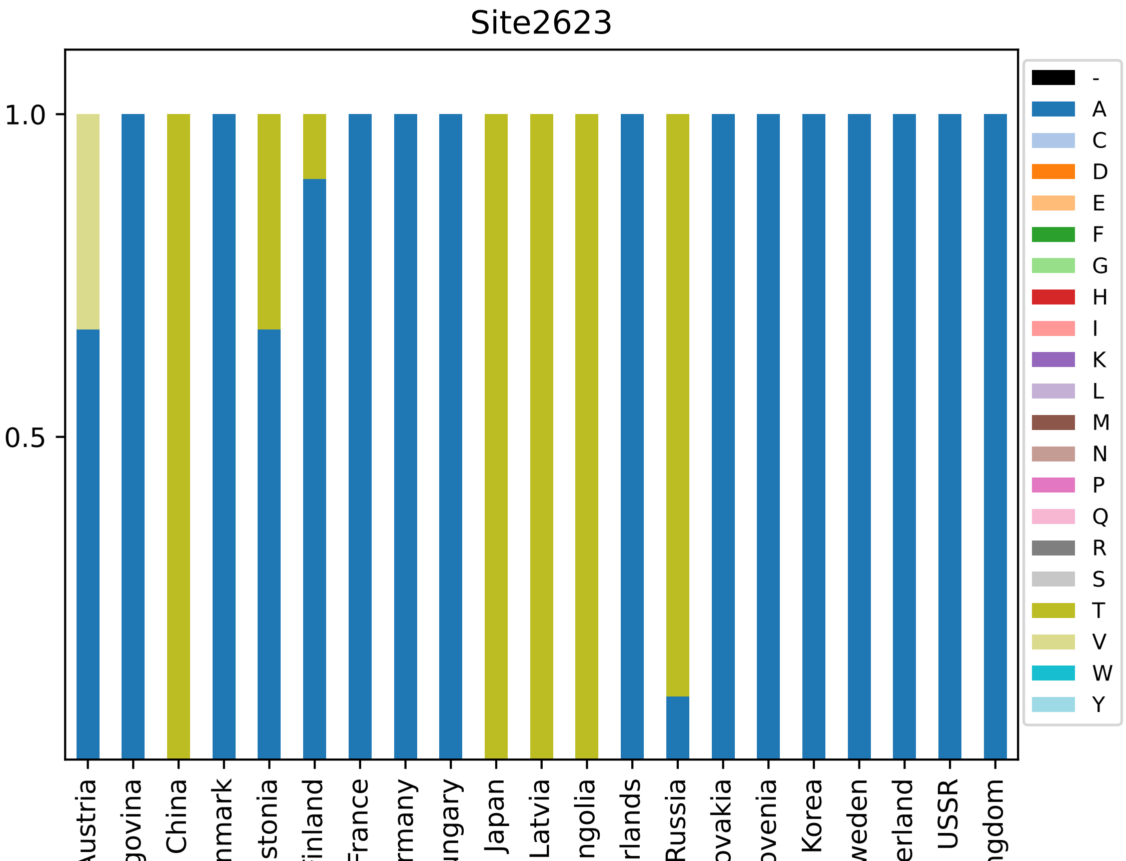

Site2623
1.0
0.5
-
A
C
D
E
F
G
H
I
K
L
M
N
P
Q
R
S
T
V
W
Y
China
Latvia
Japan
Russia
Korea
USSR

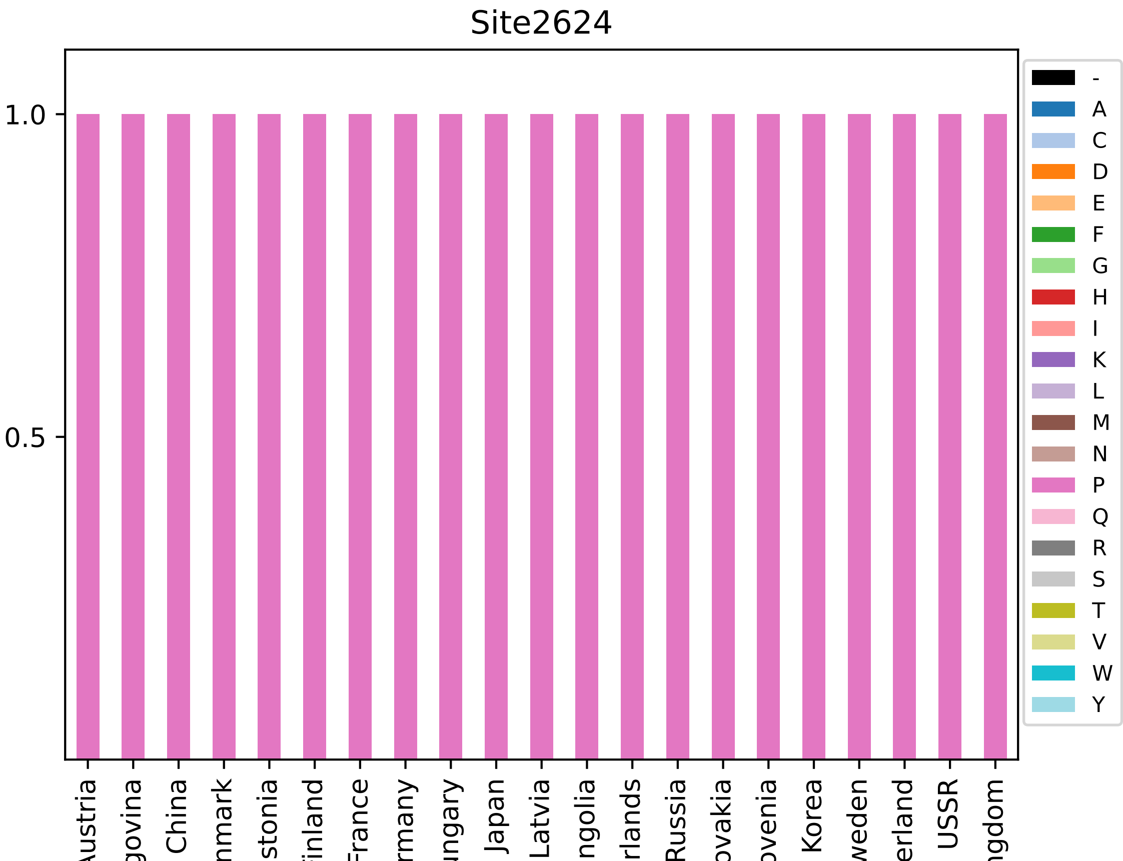

Site2624
1.0
0.5
-
A
C
D
E
F
G
H
I
K
L
M
N
P
Q
R
S
T
V
W
Y
China
Japan
Latvia
Russia
Korea
USSR

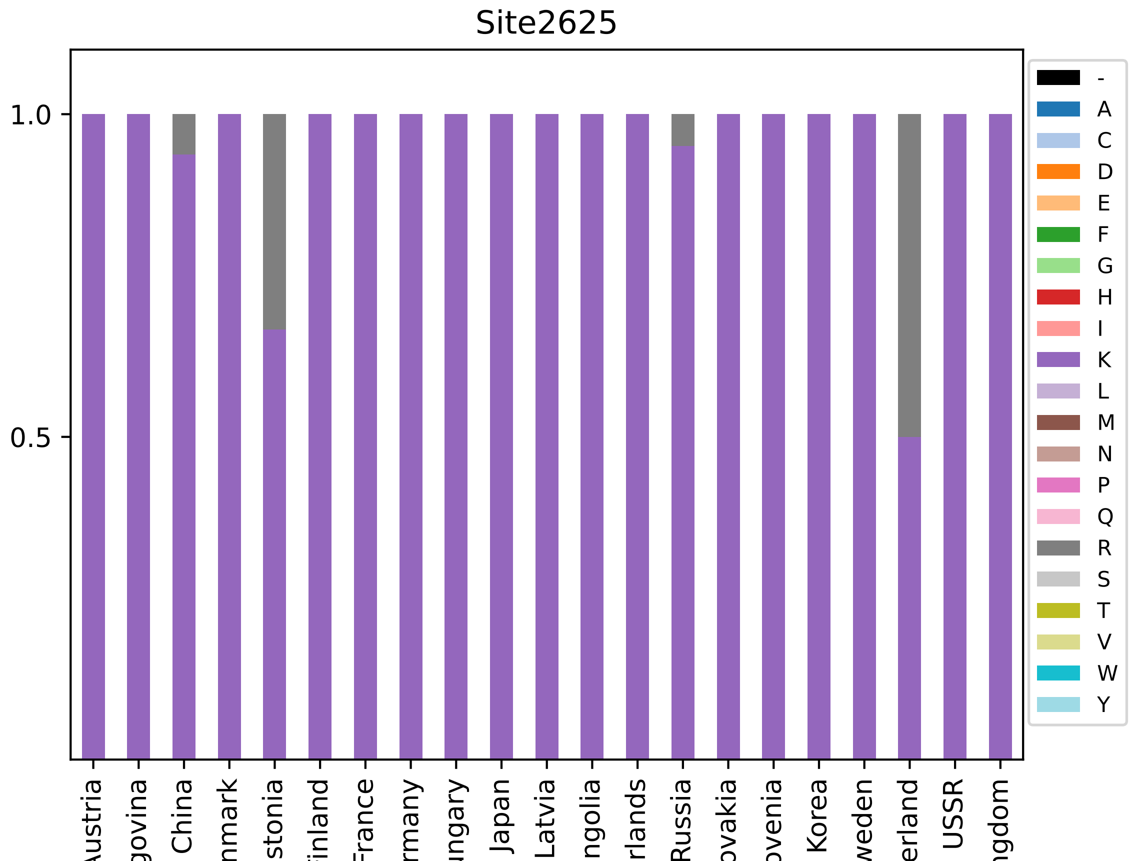
Site2625
1.0
0.5
-
A
C
D
E
F
G
H
I
K
L
M
N
P
Q
R
S
T
V
W
Y
China
Japan
Latvia
Russia
Korea
USSR

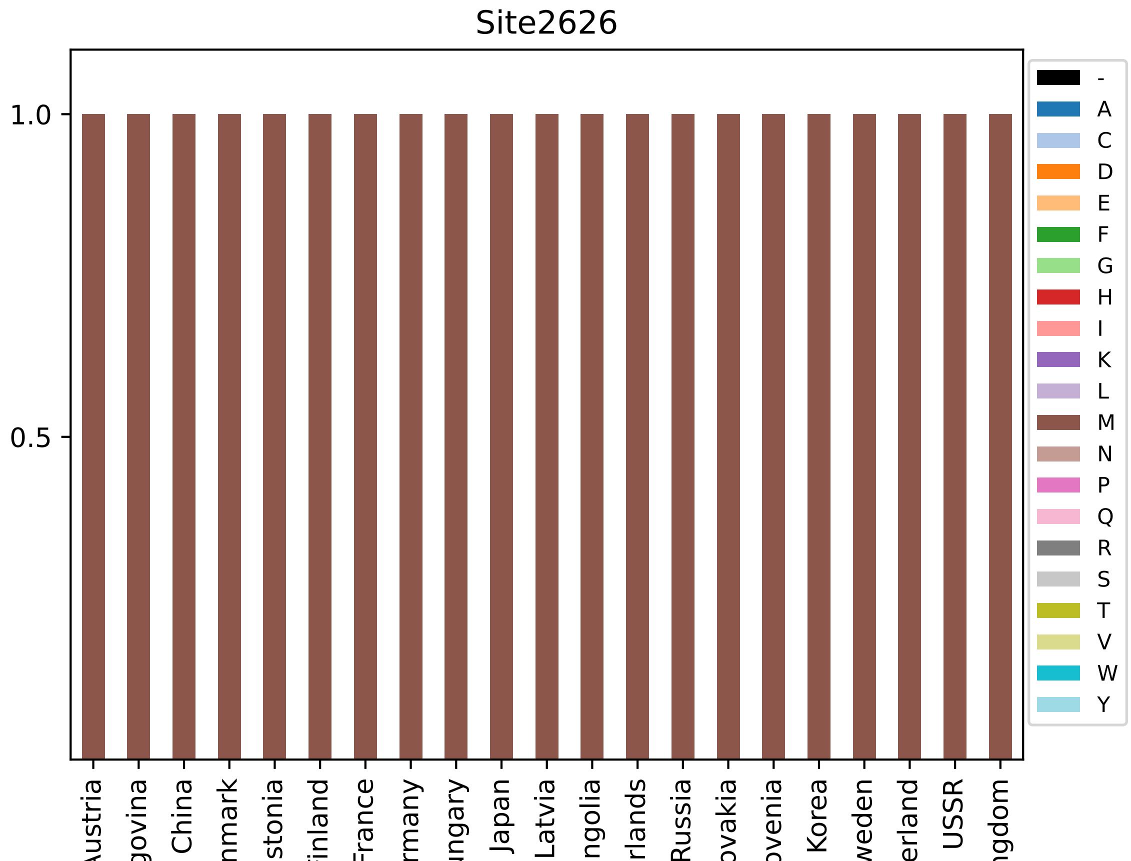
Site2626
1.0
0.5
-
A
C
D
E
F
G
H
I
K
L
M
N
P
Q
R
S
T
V
W
Y
Austria
China
Japan
Latvia
Russia
Korea
USSR

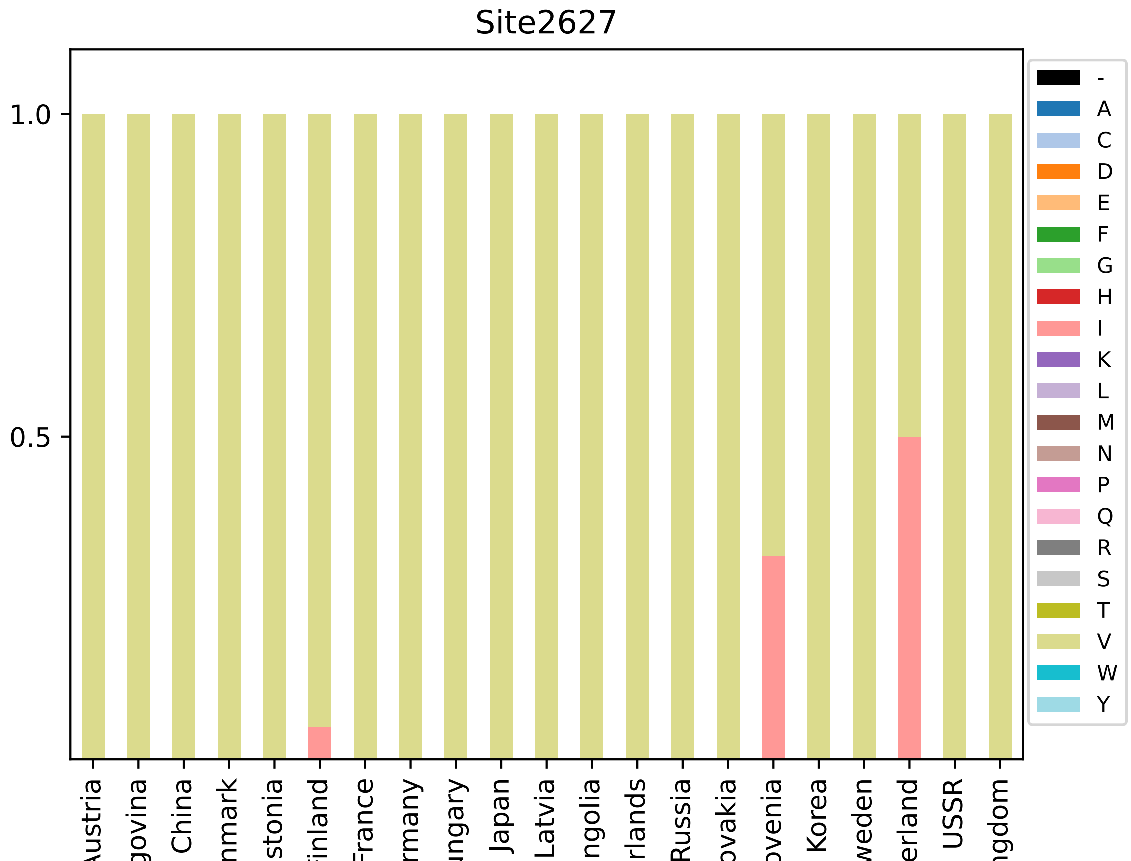
Site2627
1.0
0.5
-
A
C
D
E
F
G
H
I
K
L
M
N
P
Q
R
S
T
V
W
Y
Austria
govina
China
nmark
stonia
Finland
France
rmany
ungary
Japan
Latvia
ngolia
rlands
Russia
ovakia
ovenia
Korea
weden
erland
USSR
ngdom

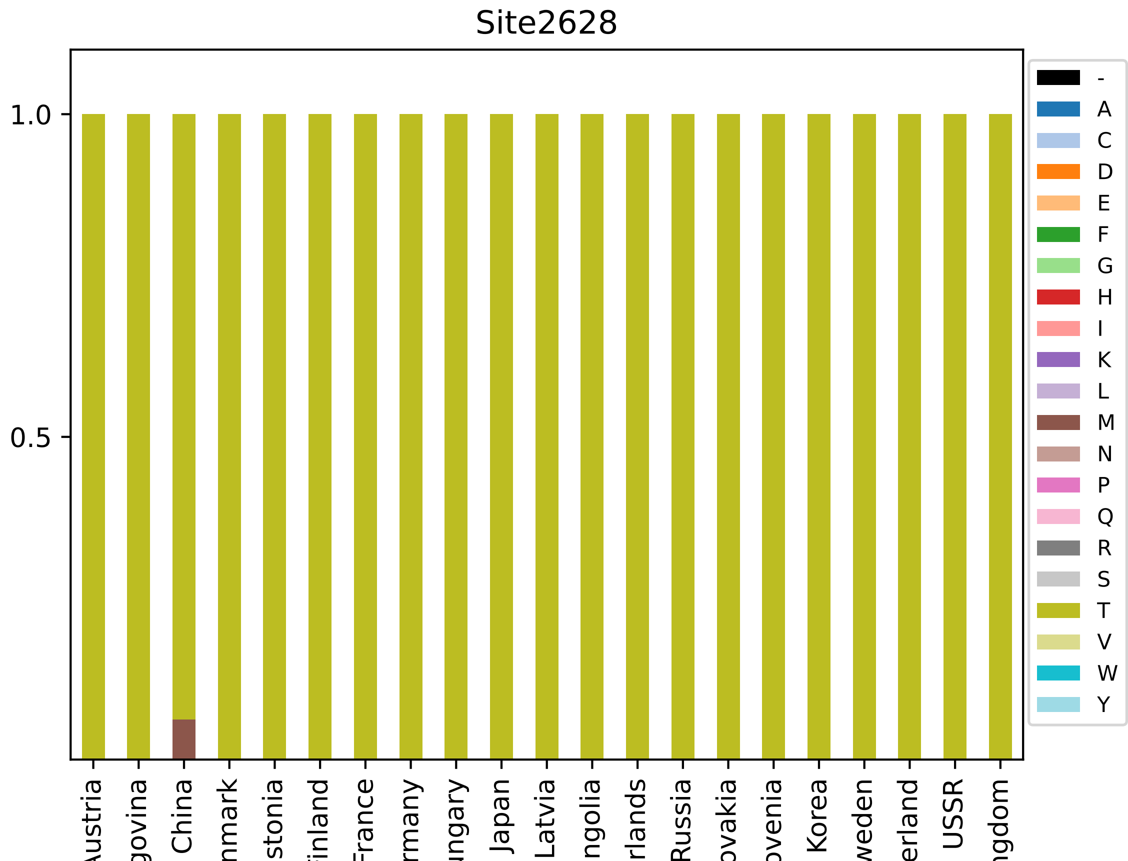

Site2628
1.0
0.5
-
A
C
D
E
F
G
H
I
K
L
M
N
P
Q
R
S
T
V
W
Y
China
Japan
Latvia
Russia
Korea
USSR

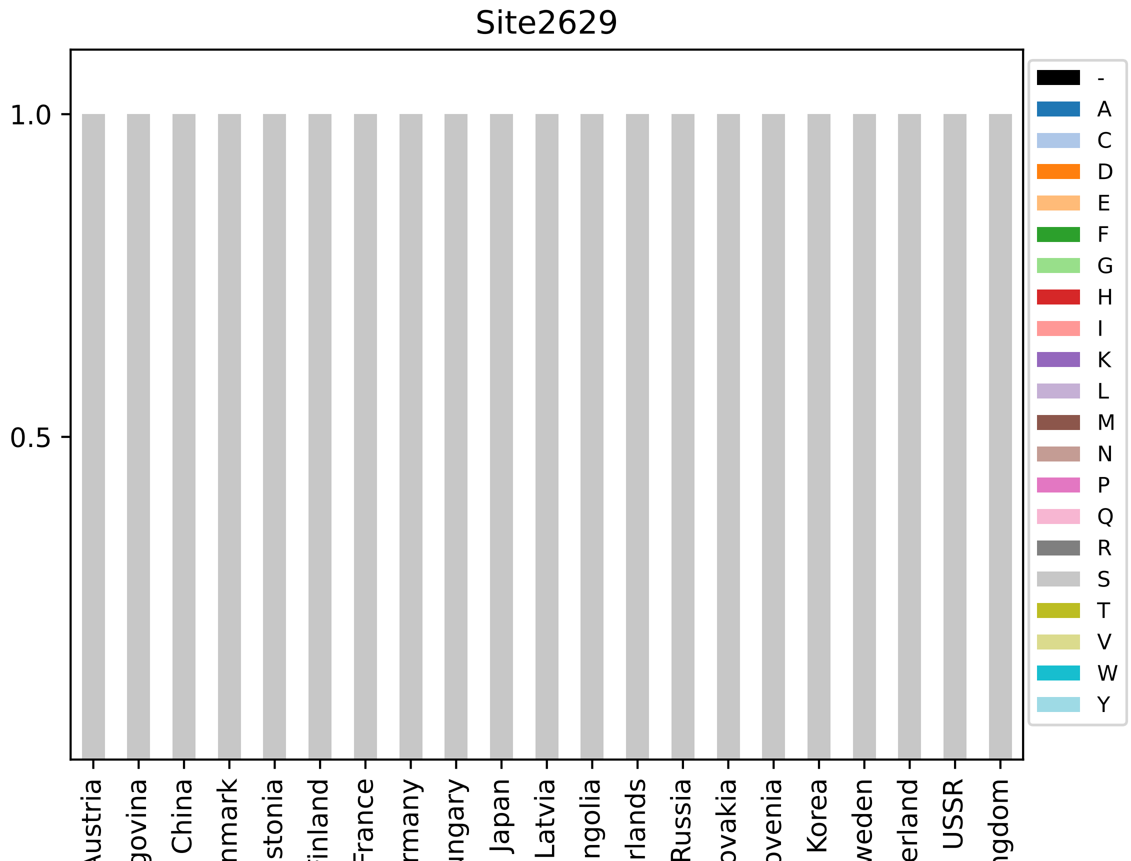
Site2629
1.0
0.5
-
A
C
D
E
F
G
H
I
K
L
M
N
P
Q
R
S
T
V
W
Y
Austria
China
Finland
France
Japan
Latvia
Russia
Korea
USSR

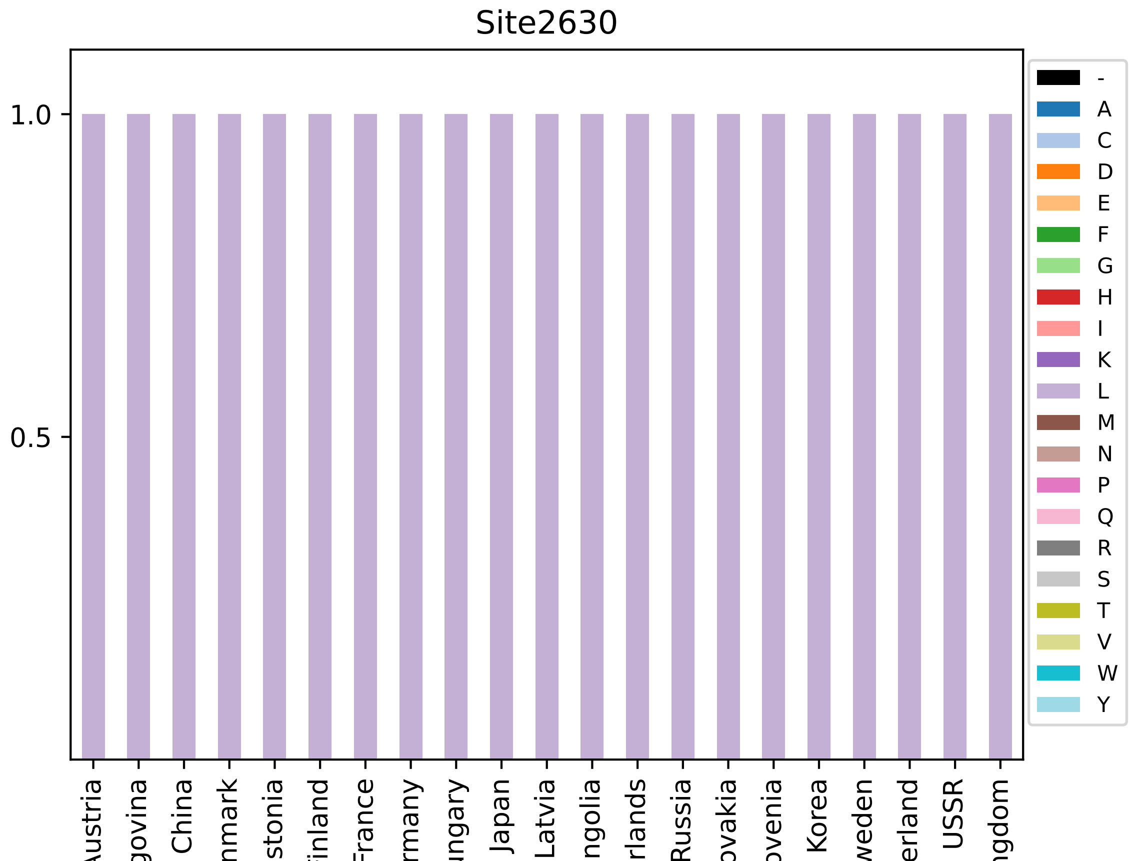
Site2630
1.0
0.5
-
A
C
D
E
F
G
H
I
K
L
M
N
P
Q
R
S
T
V
W
Y
Austria
China
Japan
Latvia
Russia
Korea
USSR

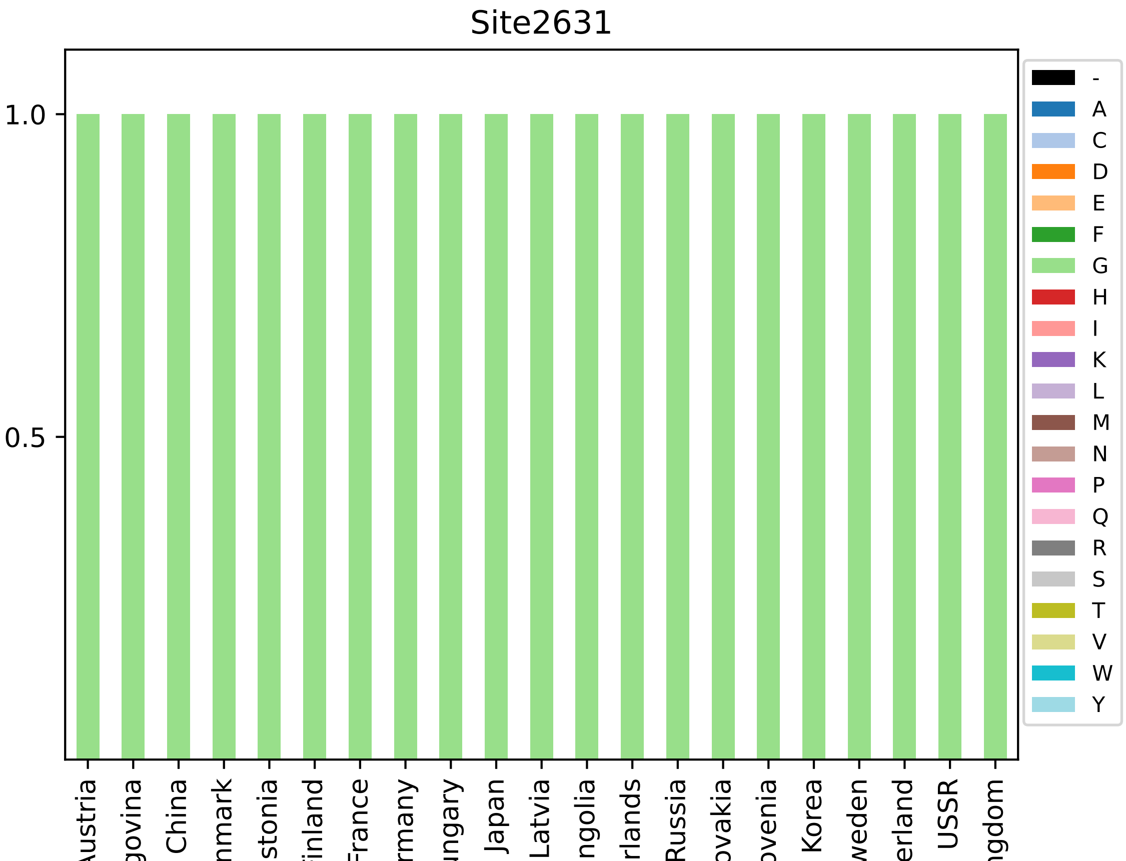

Site2631
1.0
0.5
-
A
C
D
E
F
G
H
I
K
L
M
N
P
Q
R
S
T
V
W
Y
Austria
China
Japan
Latvia
Russia
Korea
USSR

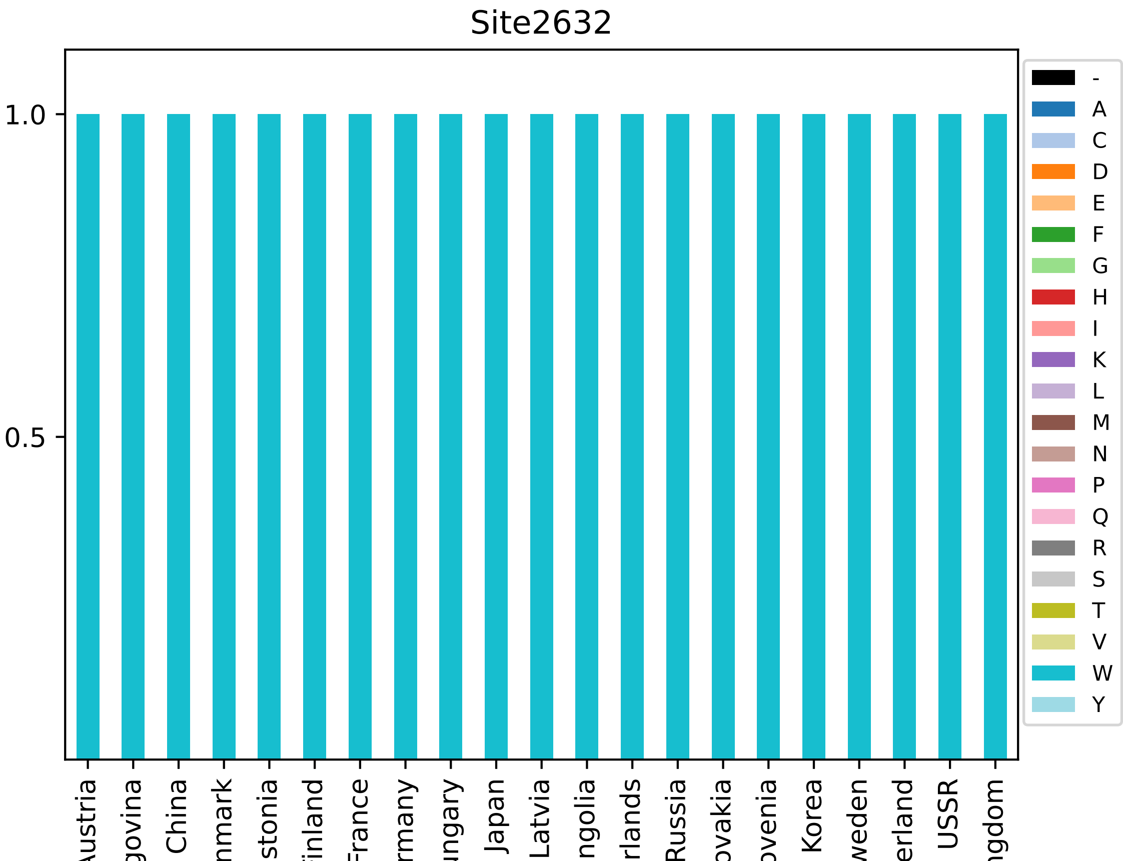

Site2632
1.0
0.5
-
A
C
D
E
F
G
H
I
K
L
M
N
P
Q
R
S
T
V
W
Y
Austria
govina
China
nmark
stonia
inland
France
rmany
ungary
Japan
Latvia
ngolia
rlands
Russia
ovakia
ovenia
Korea
weden
erland
USSR
ngdom

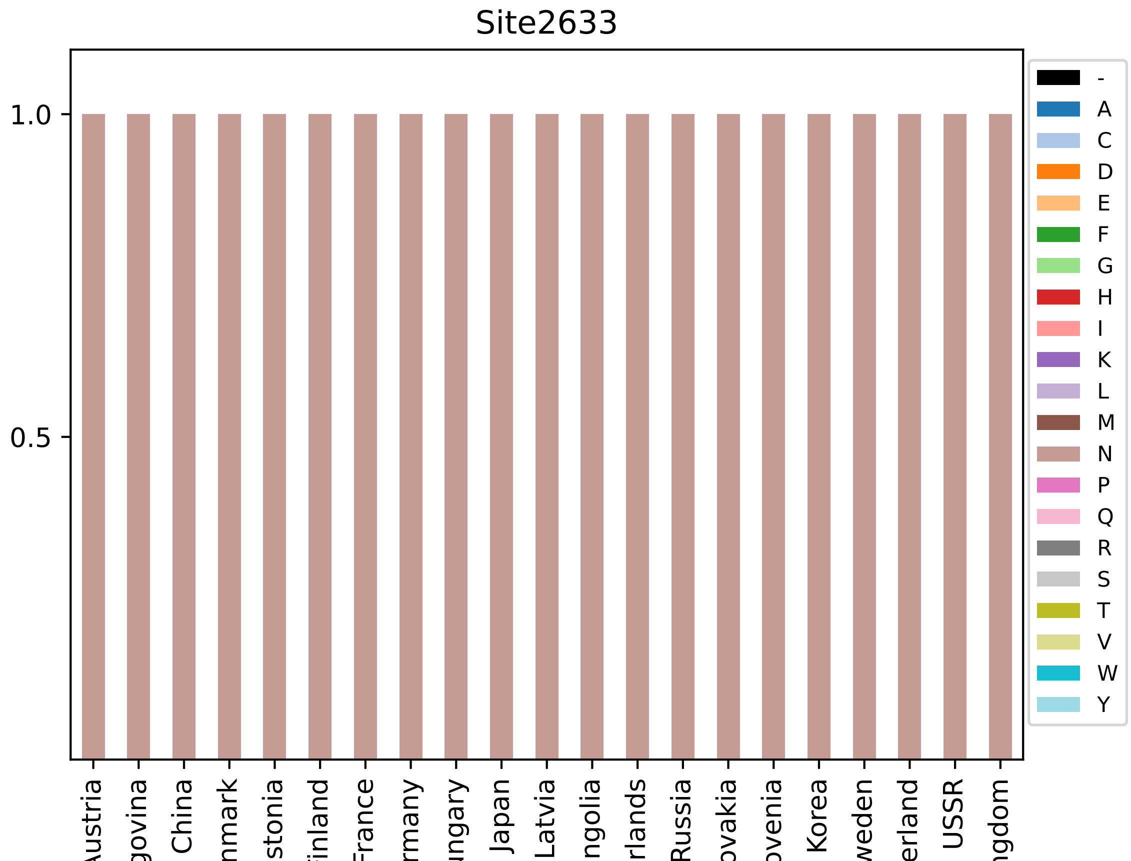

Site2633
1.0
0.5
-
A
C
D
E
F
G
H
I
K
L
M
N
P
Q
R
S
T
V
W
Y
Austria
China
Japan
Latvia
Russia
Korea
USSR

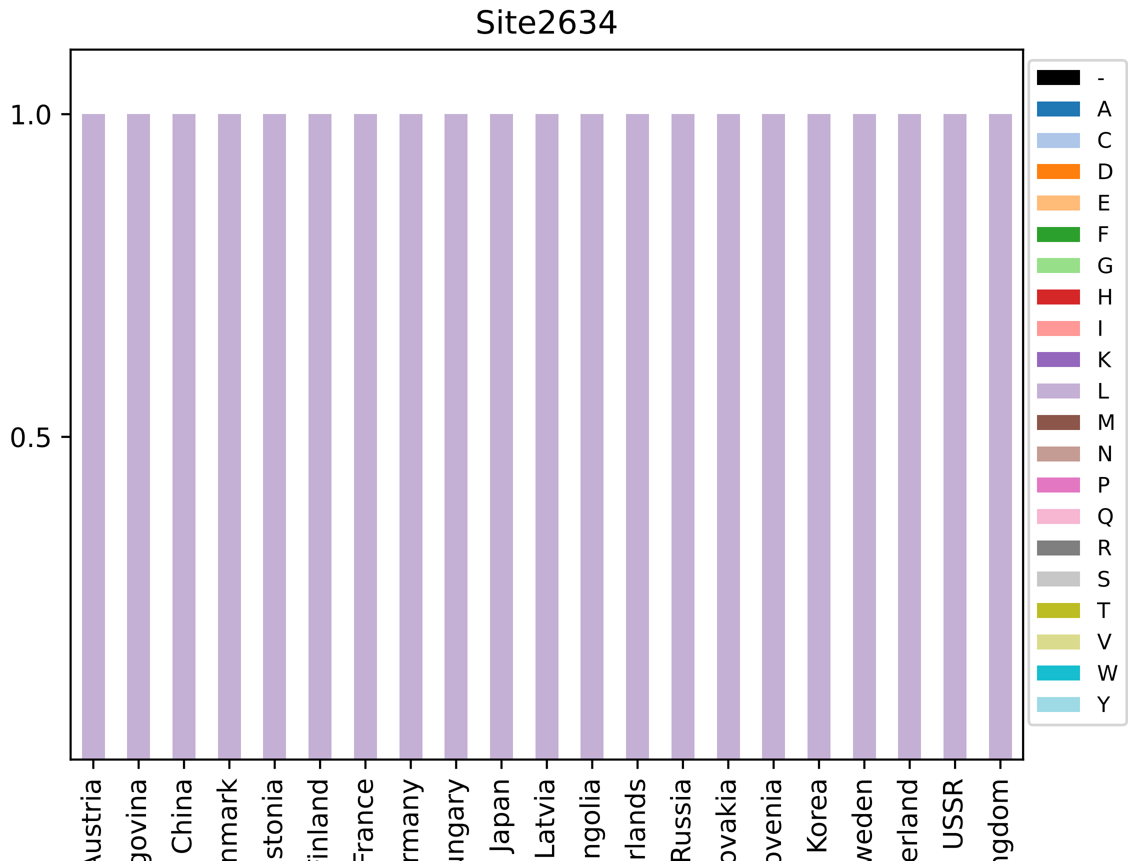

Site2634
1.0
0.5
China
Japan
Latvia
Russia
Korea
USSR
-
A
C
D
E
F
G
H
I
K
L
M
N
P
Q
R
S
T
V
W
Y

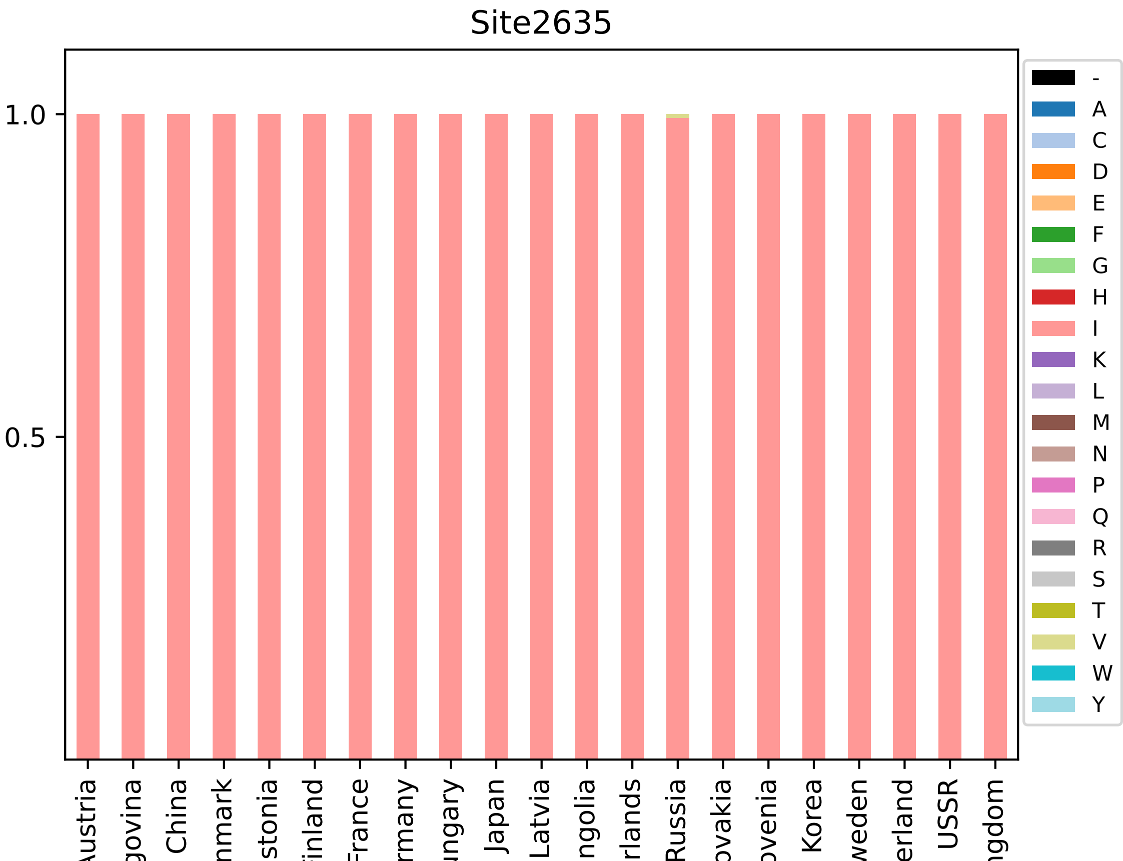

Site2635
1.0
0.5
-
A
C
D
E
F
G
H
I
K
L
M
N
P
Q
R
S
T
V
W
Y
Austria
China
Japan
Latvia
Russia
Korea
USSR

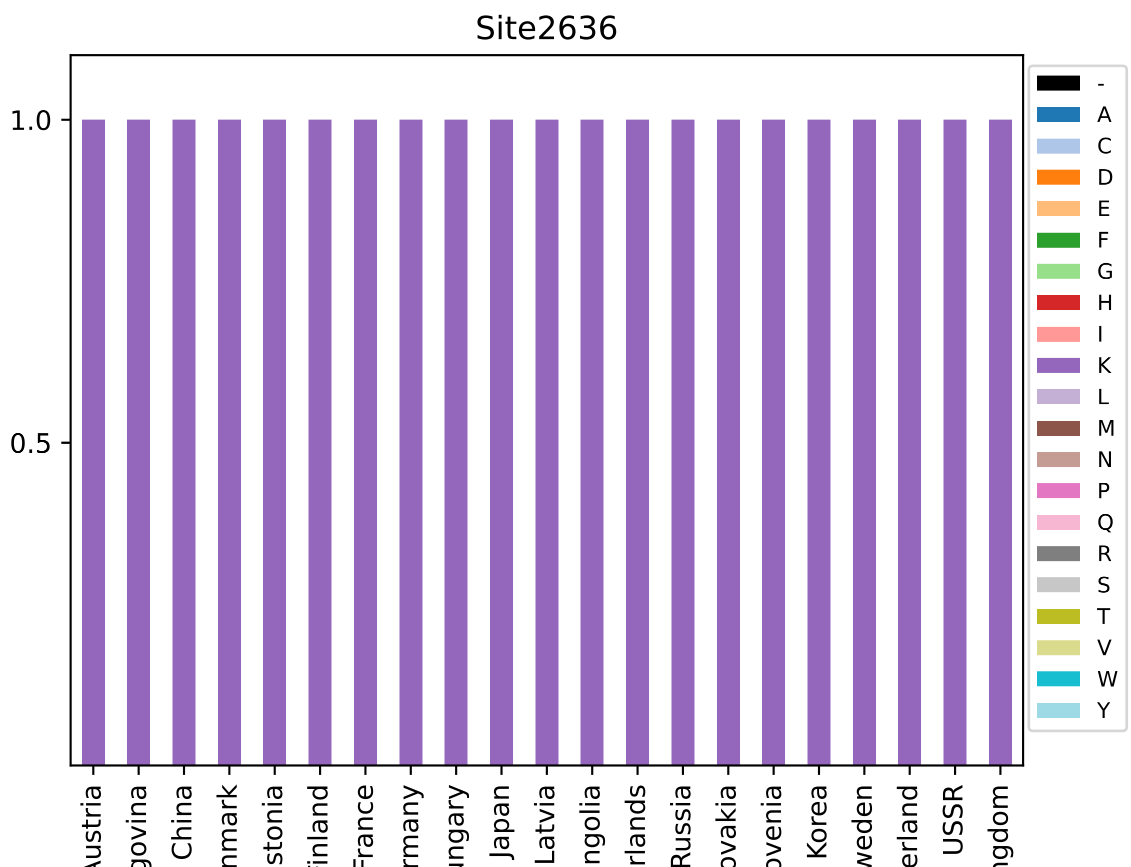

Site2636
1.0
0.5
-
A
C
D
E
F
G
H
I
K
L
M
N
P
Q
R
S
T
V
W
Y
China
Japan
Latvia
Russia
Korea
USSR

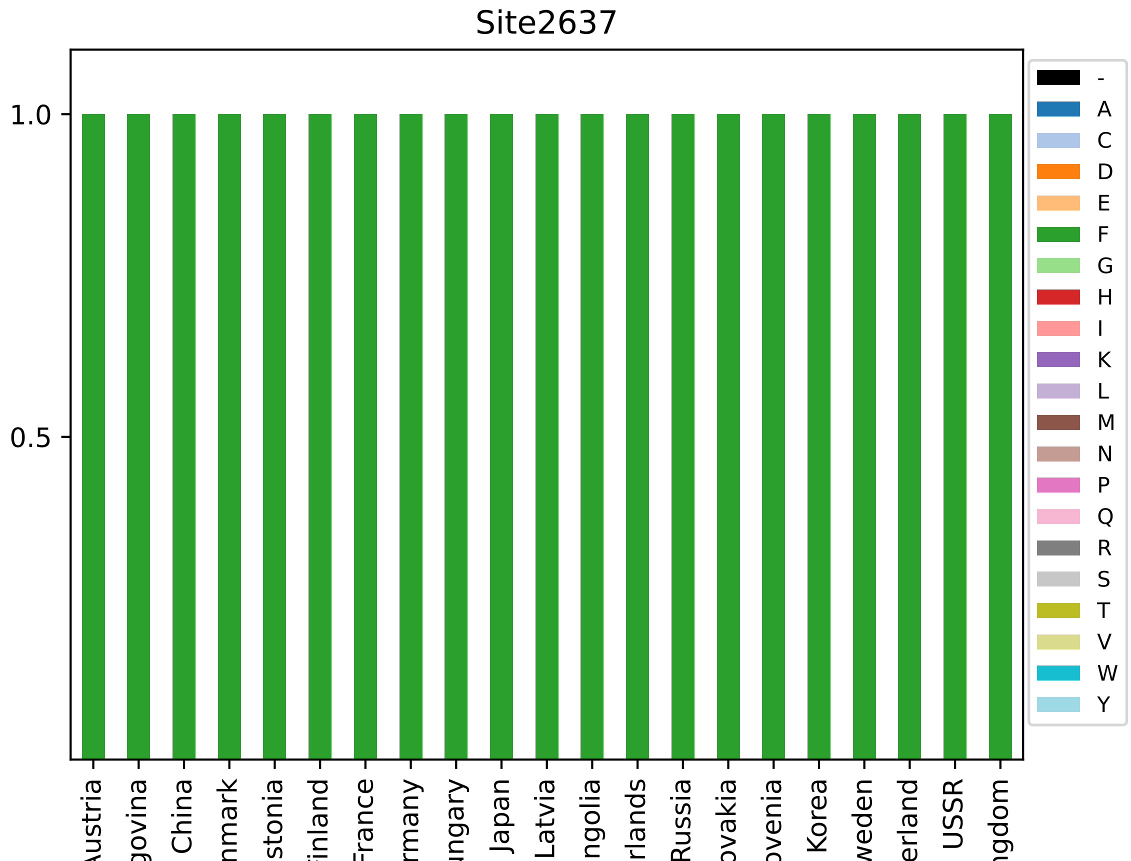

Site2637
1.0
0.5
-
A
C
D
E
F
G
H
I
K
L
M
N
P
Q
R
S
T
V
W
Y
Austria
China
Japan
Latvia
Russia
Korea
USSR

Site2638

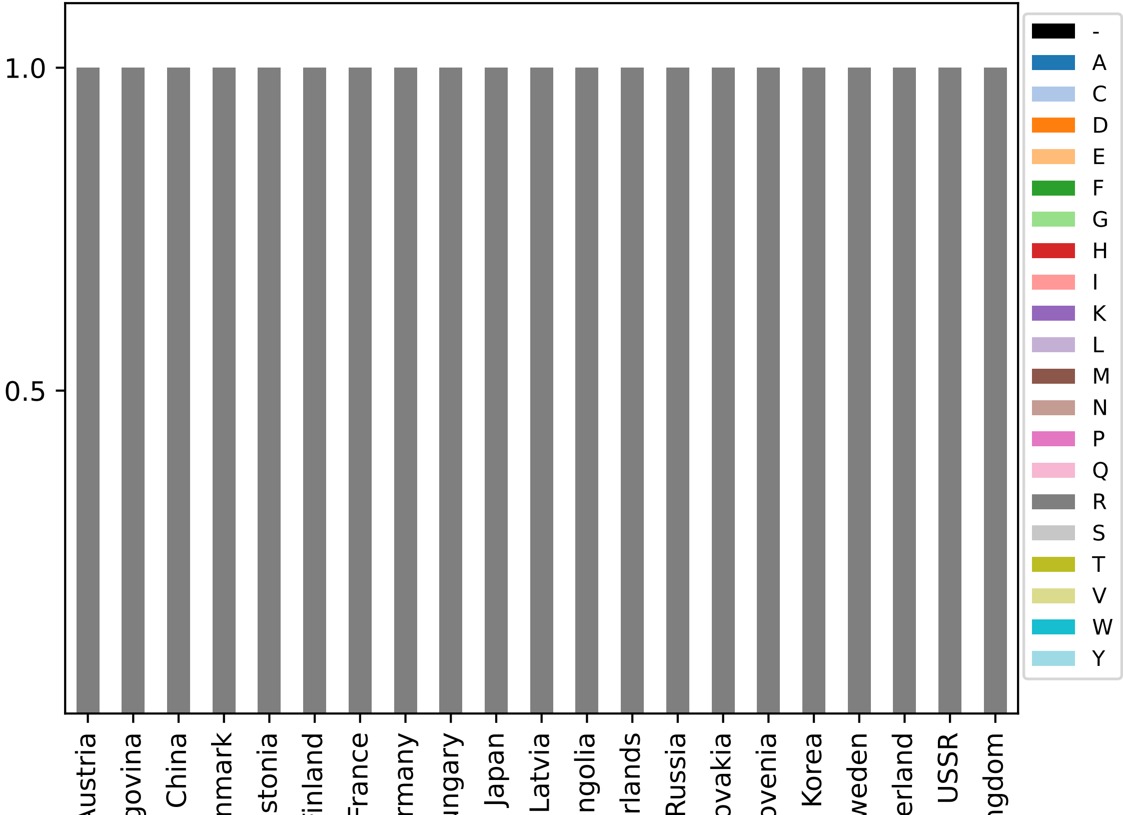
1.0
0.5
-
A
C
D
E
F
G
H
I
K
L
M
N
P
Q
R
S
T
V
W
Y
China
Japan
Latvia
Russia
Korea
USSR

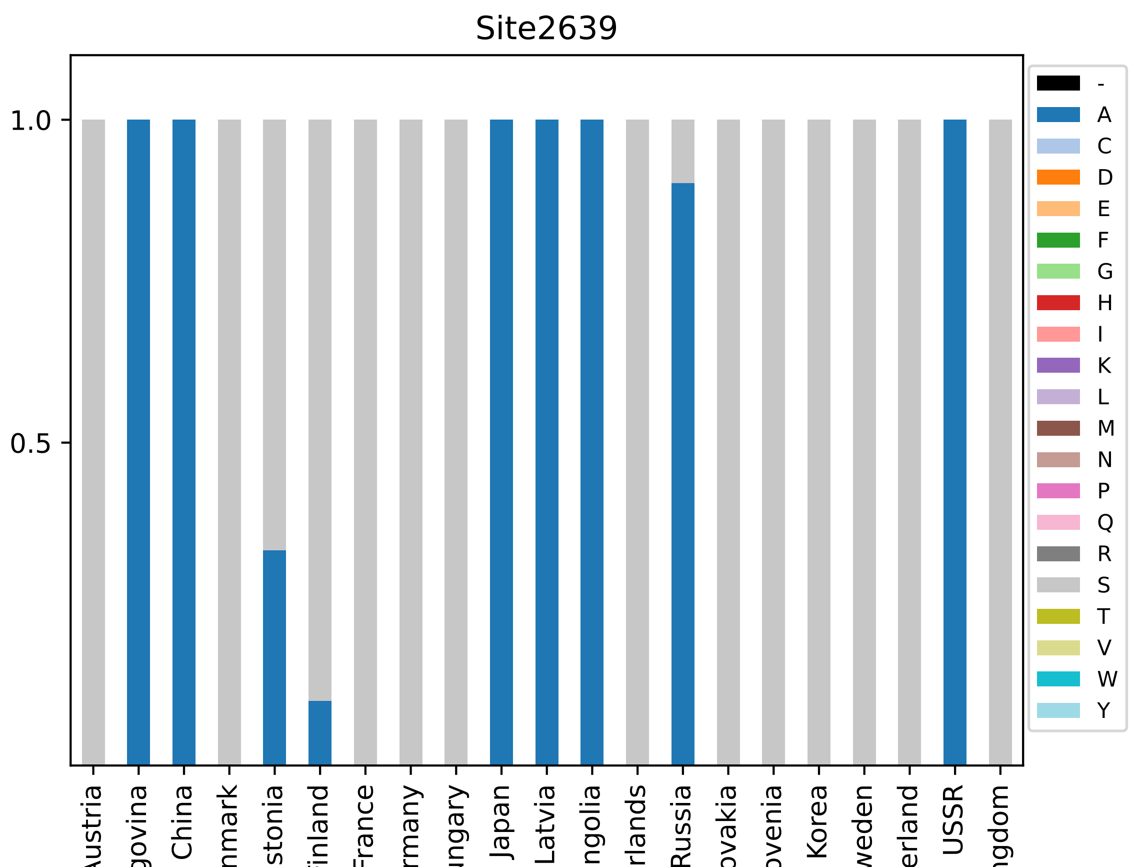

Site2639
1.0
0.5
-
A
C
D
E
F
G
H
I
K
L
M
N
P
Q
R
S
T
V
W
Y
China
Japan
Latvia
Russia
Korea
USSR

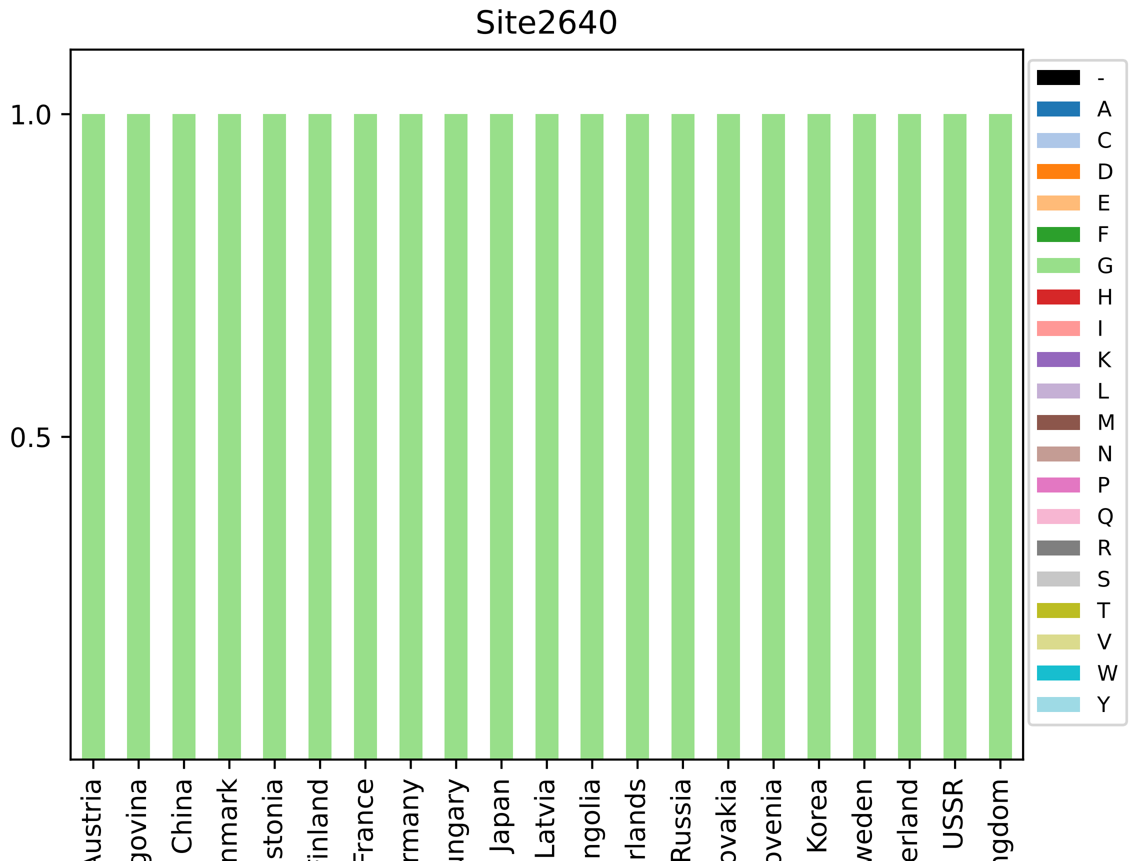
Site2640
1.0
0.5
-
A
C
D
E
F
G
H
I
K
L
M
N
P
Q
R
S
T
V
W
Y
Austria
govina
China
nmark
stonia
Finland
France
rmany
ungary
Japan
Latvia
ngolia
rlands
Russia
ovakia
ovenia
Korea
weden
erland
USSR
ngdom

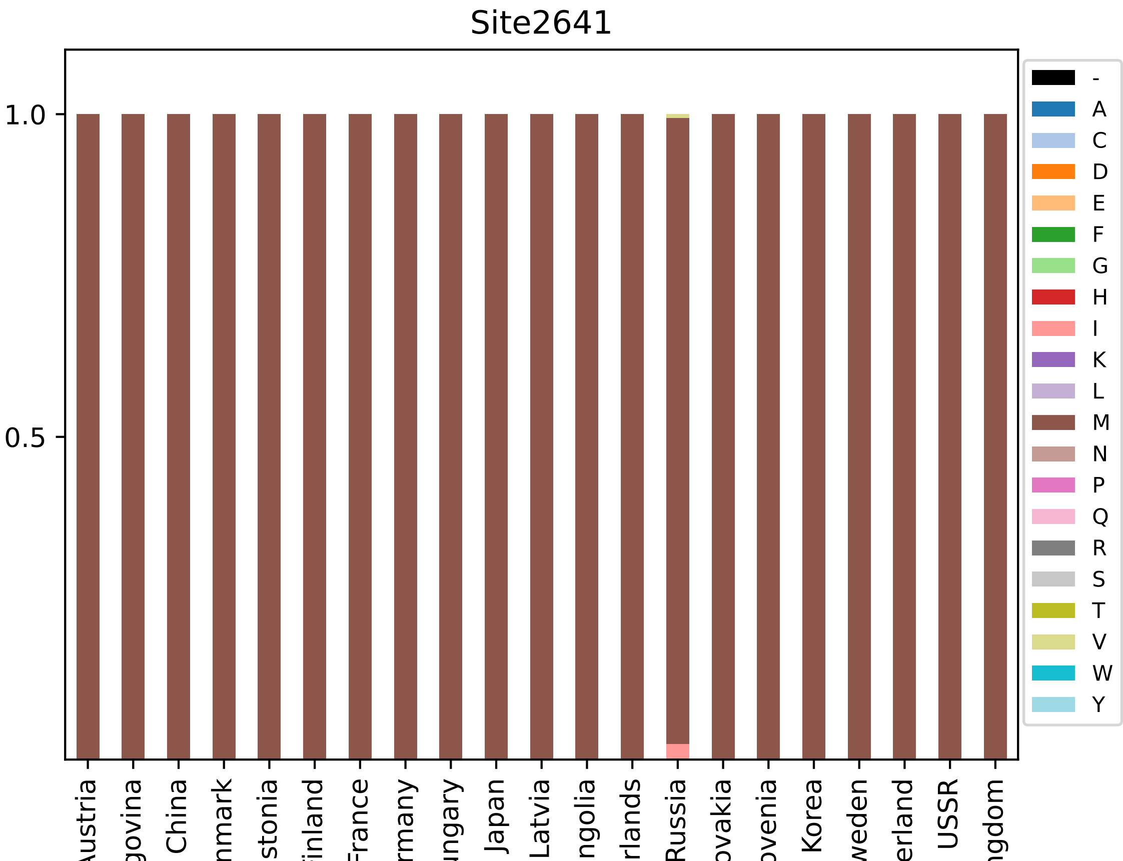
Site2641
1.0
0.5
-
A
C
D
E
F
G
H
I
K
L
M
N
P
Q
R
S
T
V
W
Y
China
Japan
Latvia
Russia
Korea
USSR

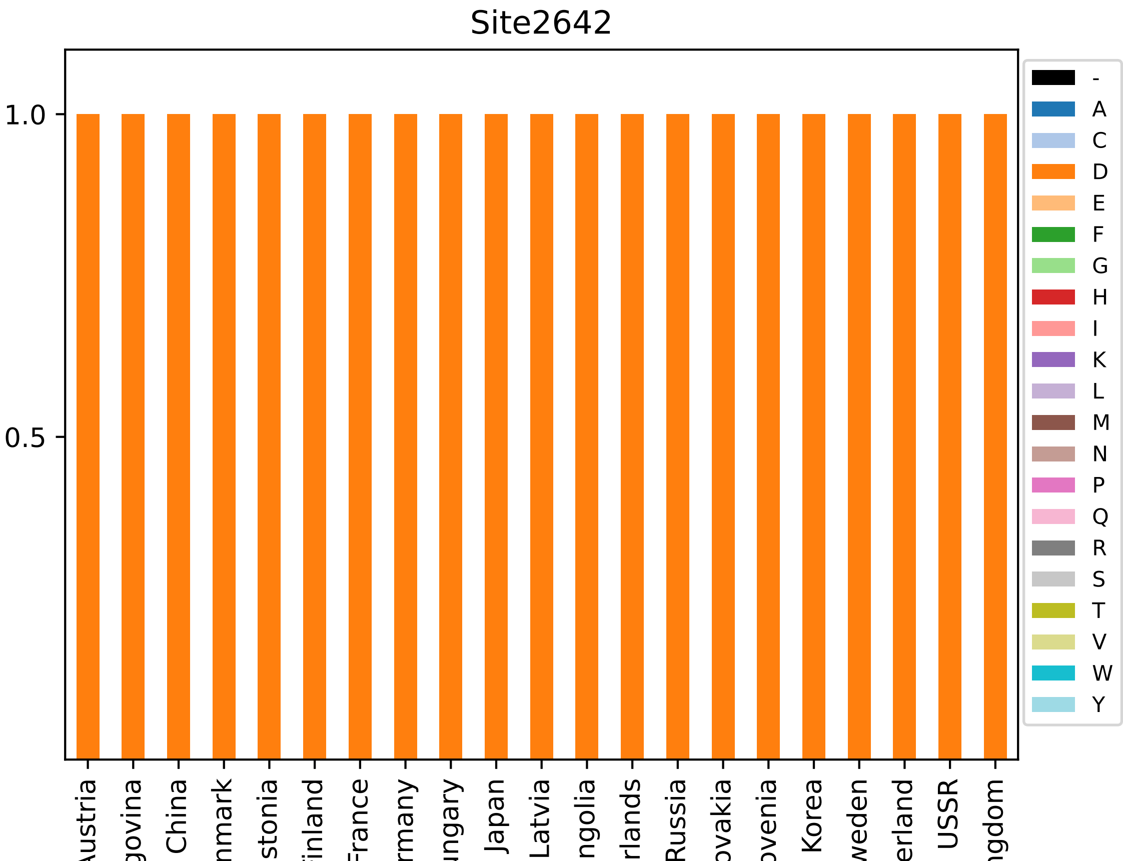
Site2642
1.0
0.5
-
A
C
D
E
F
G
H
I
K
L
M
N
P
Q
R
S
T
V
W
Y
China
Japan
Latvia
Russia
Korea
USSR

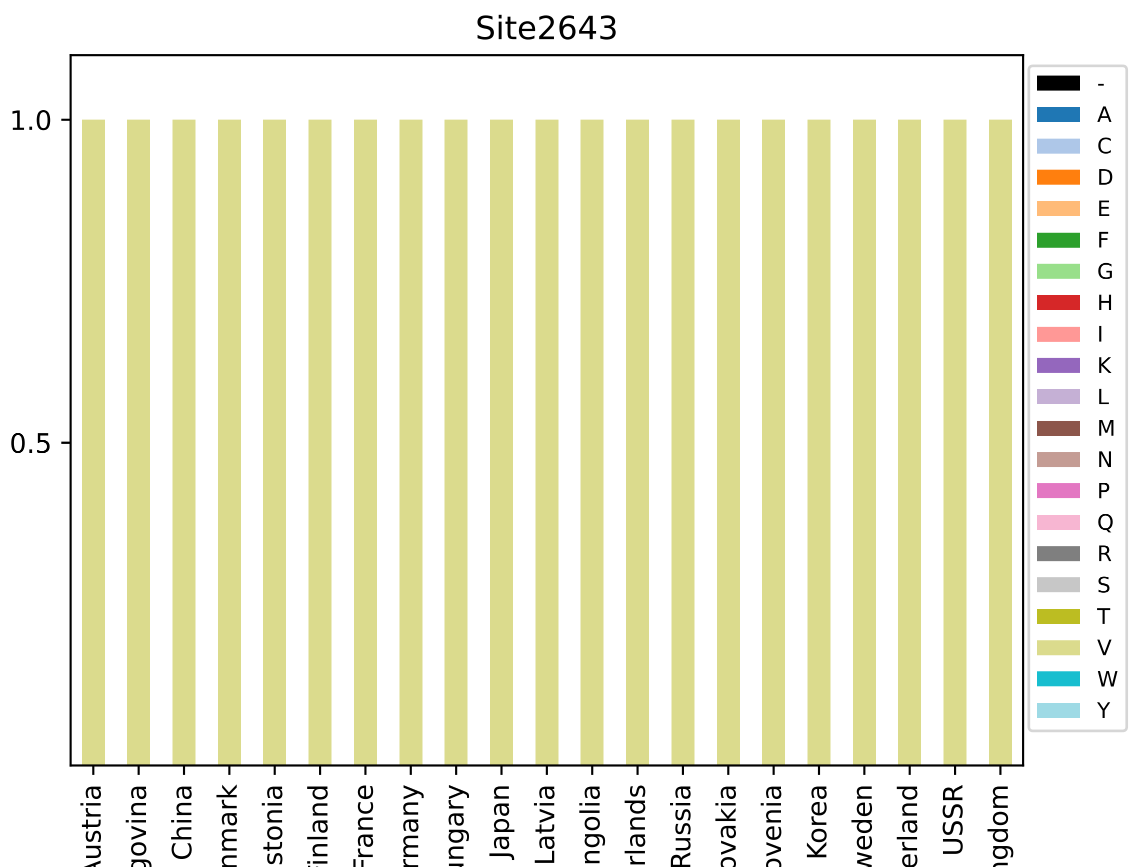

Site2643
1.0
0.5
-
A
C
D
E
F
G
H
I
K
L
M
N
P
Q
R
S
T
V
W
Y
China
Japan
Latvia
Russia
Korea
USSR

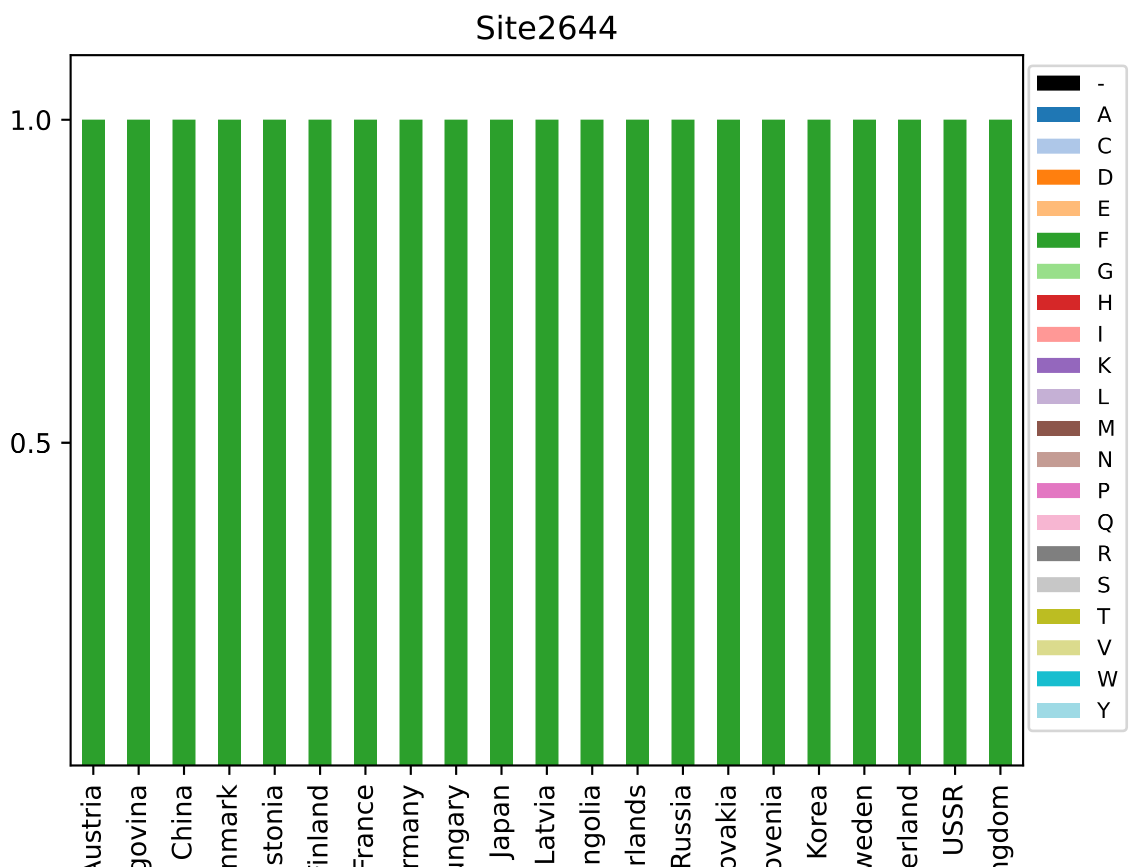

Site2644
1.0
0.5
-
A
C
D
E
F
G
H
I
K
L
M
N
P
Q
R
S
T
V
W
Y
China
Japan
Latvia
Russia
Korea
USSR

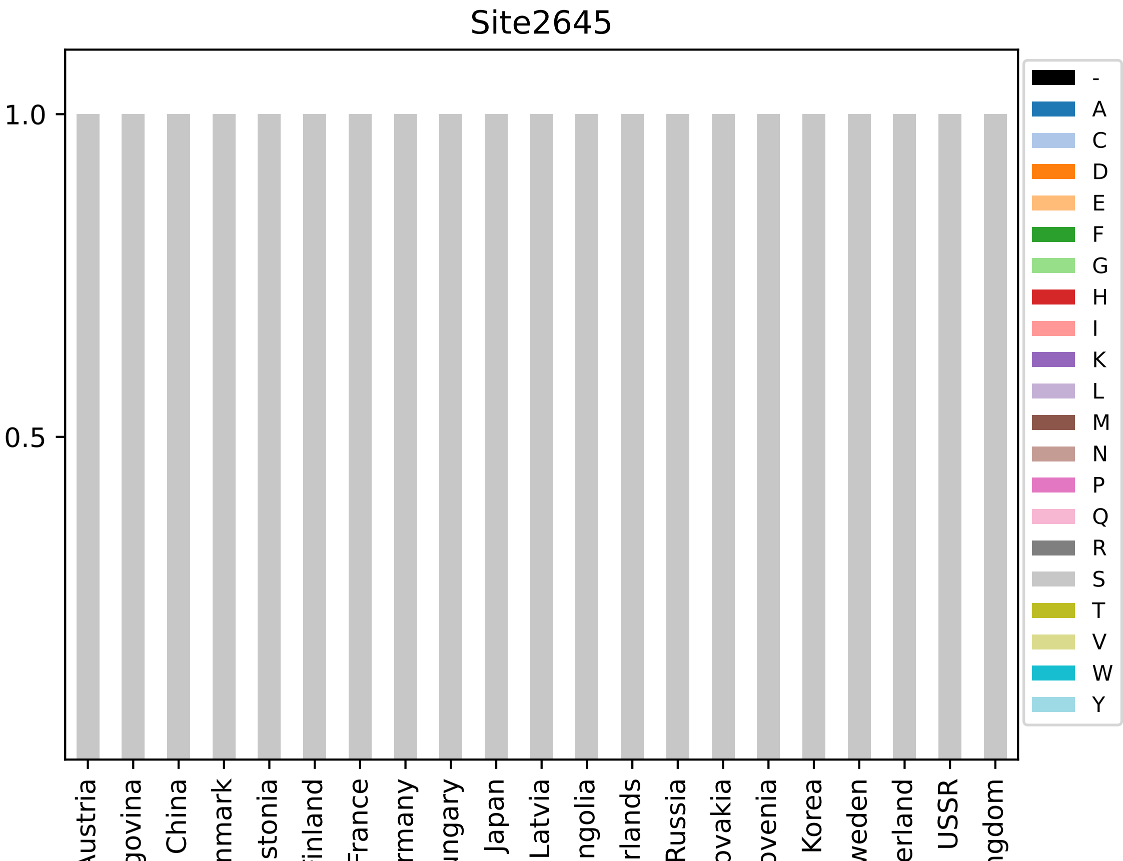
Site2645
1.0
0.5
-
A
C
D
E
F
G
H
I
K
L
M
N
P
Q
R
S
T
V
W
Y
China
Japan
Latvia
Russia
Korea
USSR

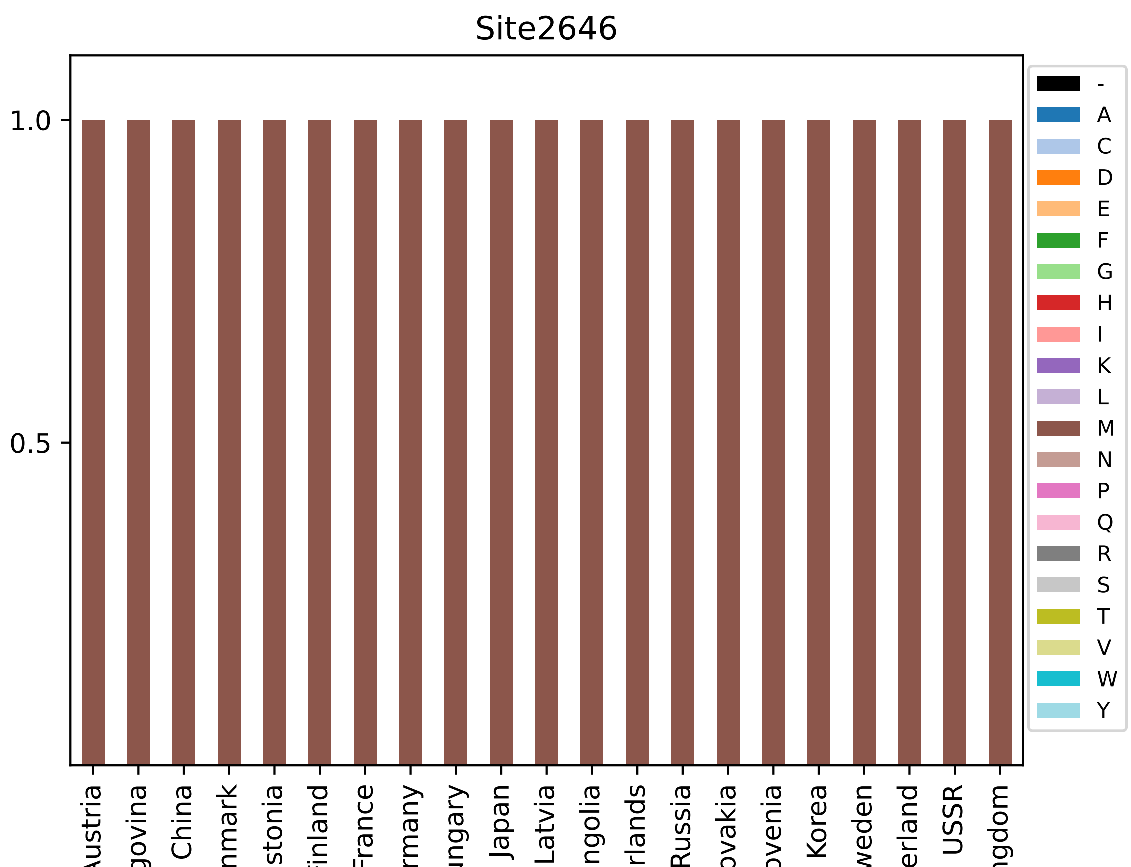

Site2646
1.0
0.5
-
A
C
D
E
F
G
H
I
K
L
M
N
P
Q
R
S
T
V
W
Y
China
Japan
Latvia
Russia
Korea
USSR

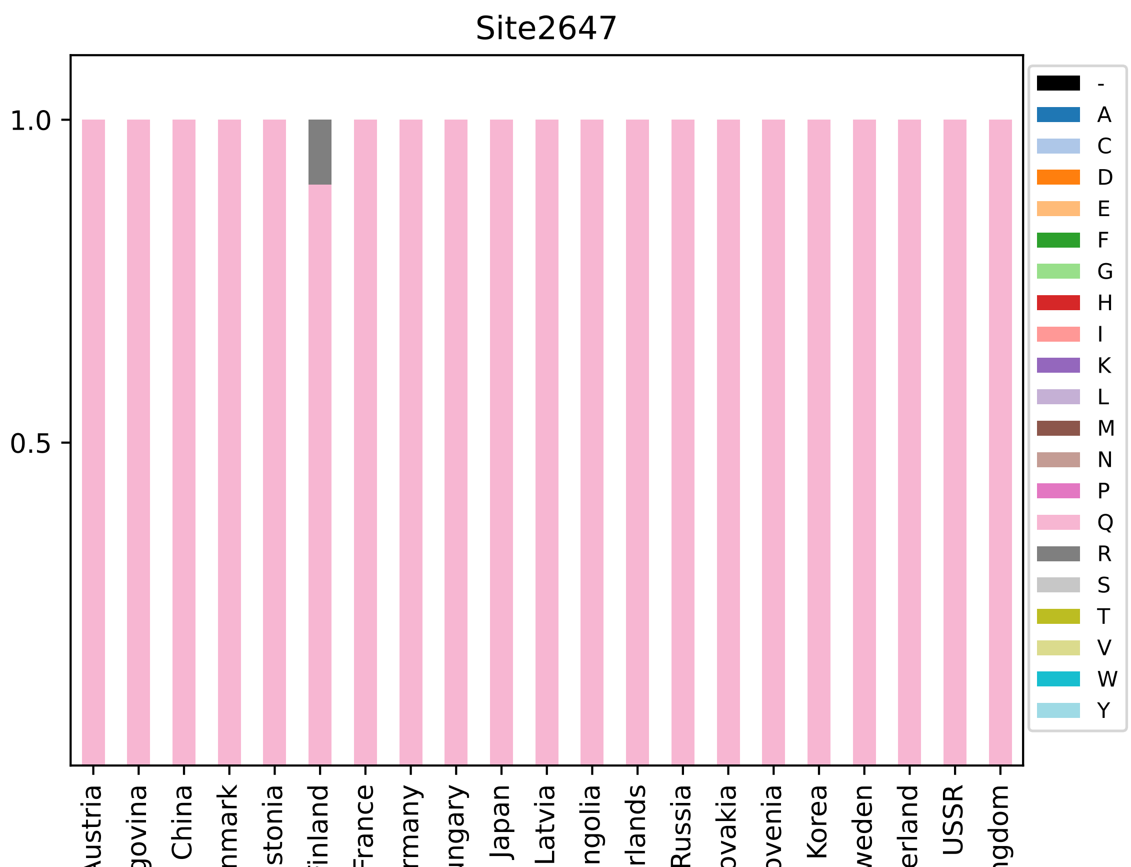
Site2647
1.0
0.5
-
A
C
D
E
F
G
H
I
K
L
M
N
P
Q
R
S
T
V
W
Y
China
Japan
Latvia
Russia
Korea
USSR

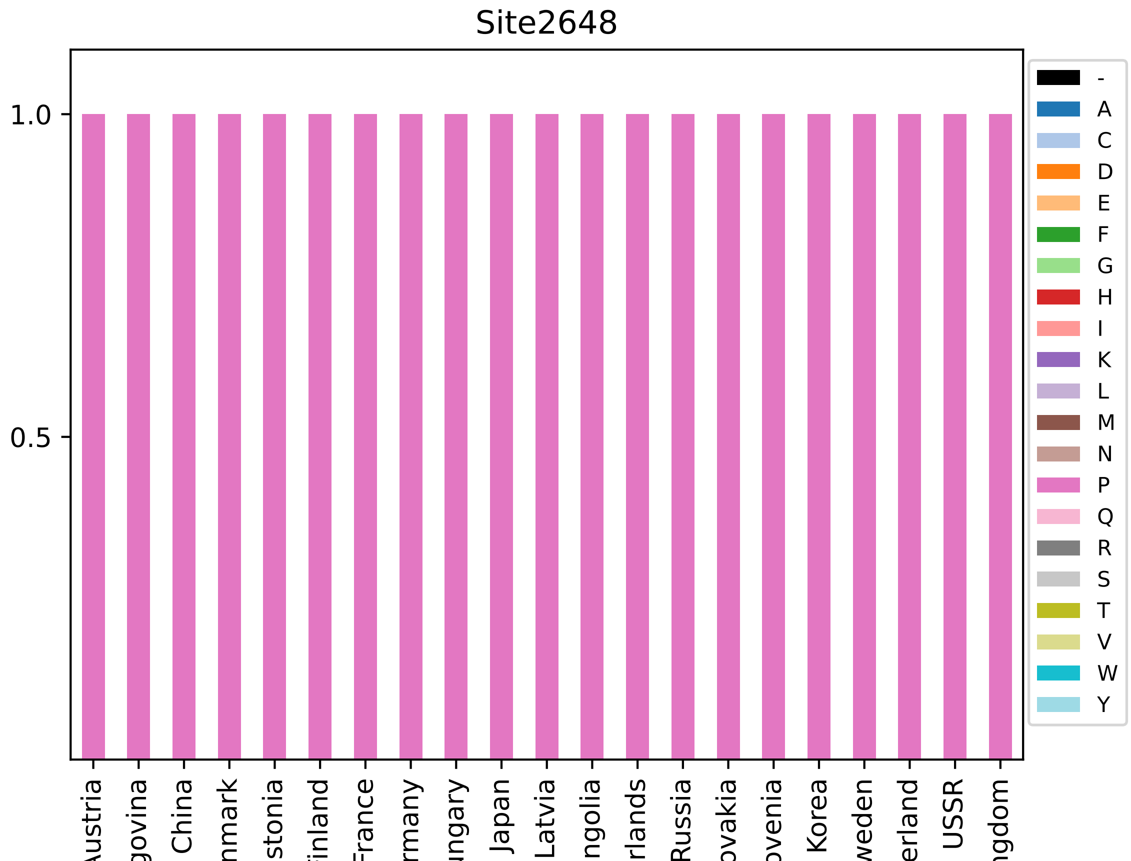
Site2648
1.0
0.5
-
A
C
D
E
F
G
H
I
K
L
M
N
P
Q
R
S
T
V
W
Y
China
Japan
Latvia
Russia
Korea
USSR

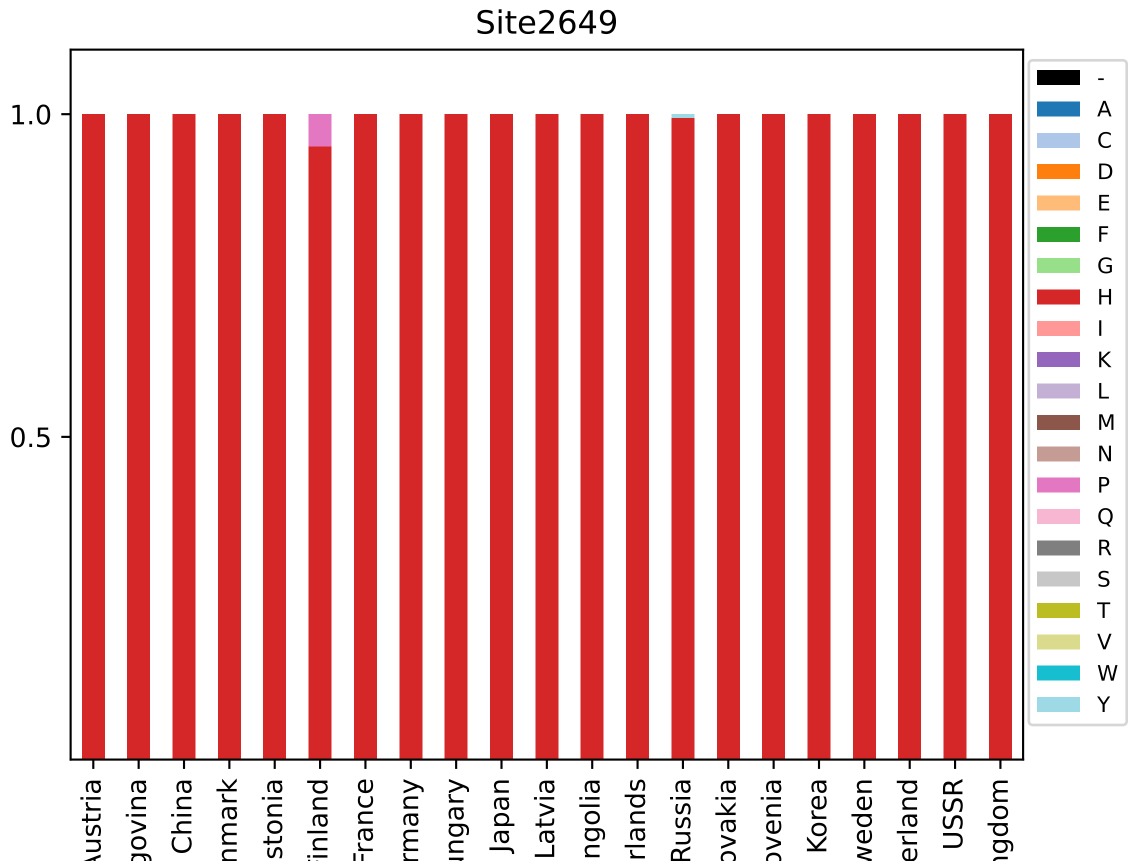

Site2649
1.0
0.5
-
A
C
D
E
F
G
H
I
K
L
M
N
P
Q
R
S
T
V
W
Y
China
Japan
Latvia
Russia
Korea
USSR

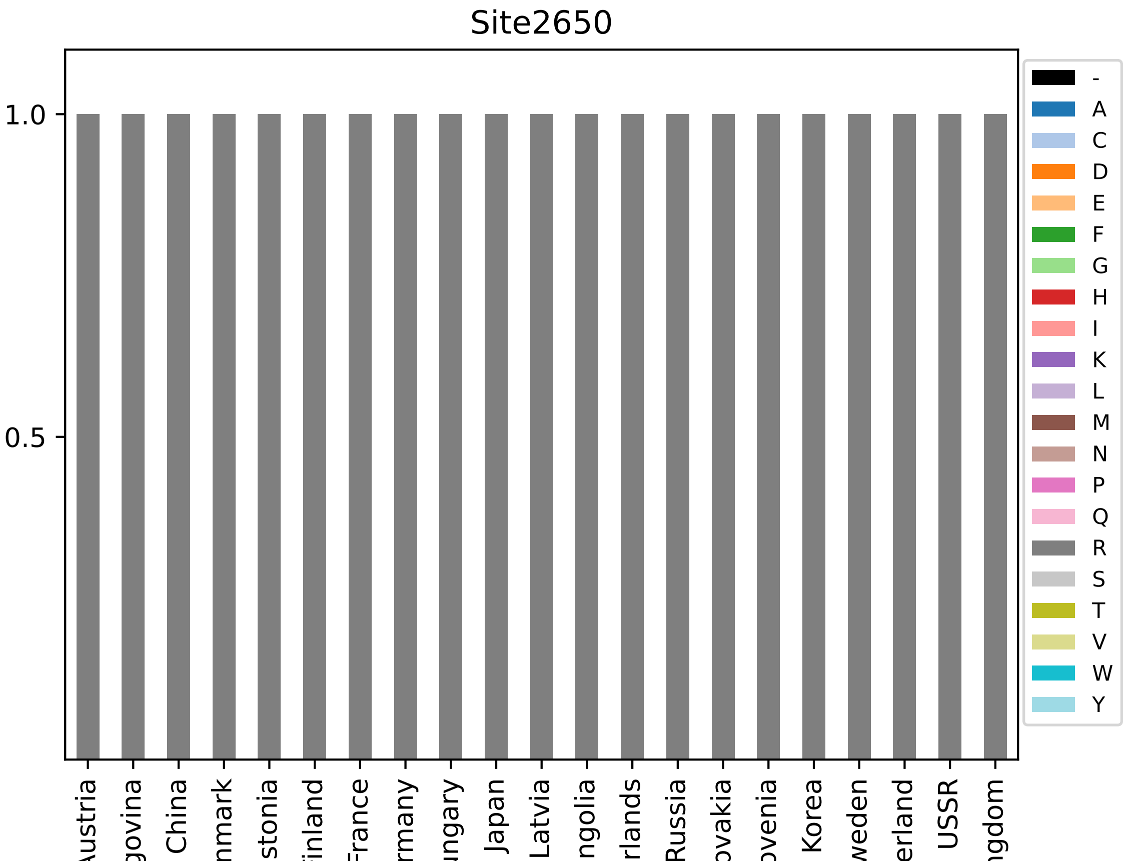

Site2650
1.0
0.5
-
A
C
D
E
F
G
H
I
K
L
M
N
P
Q
R
S
T
V
W
Y
China
Japan
Latvia
Russia
Korea
USSR

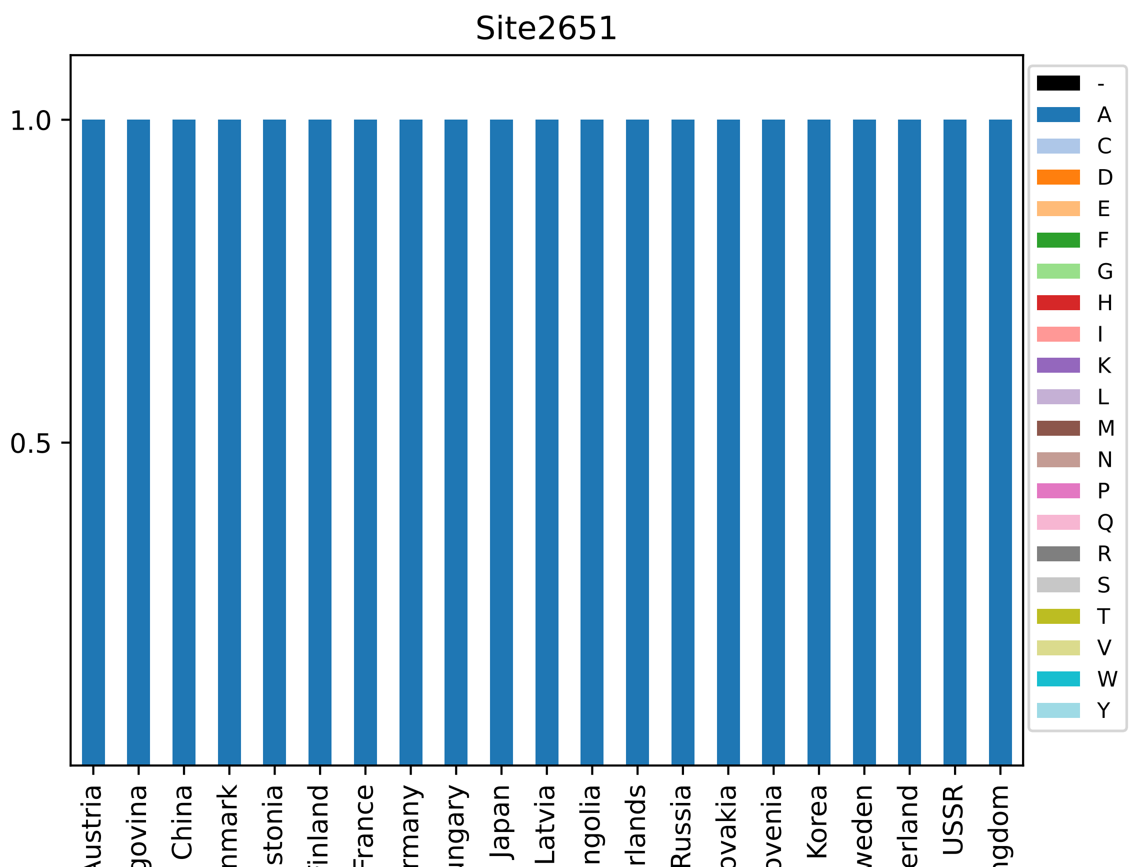
Site2651
1.0
0.5
-
A
C
D
E
F
G
H
I
K
L
M
N
P
Q
R
S
T
V
W
Y
Austria
China
Japan
Latvia
Russia
Korea
USSR

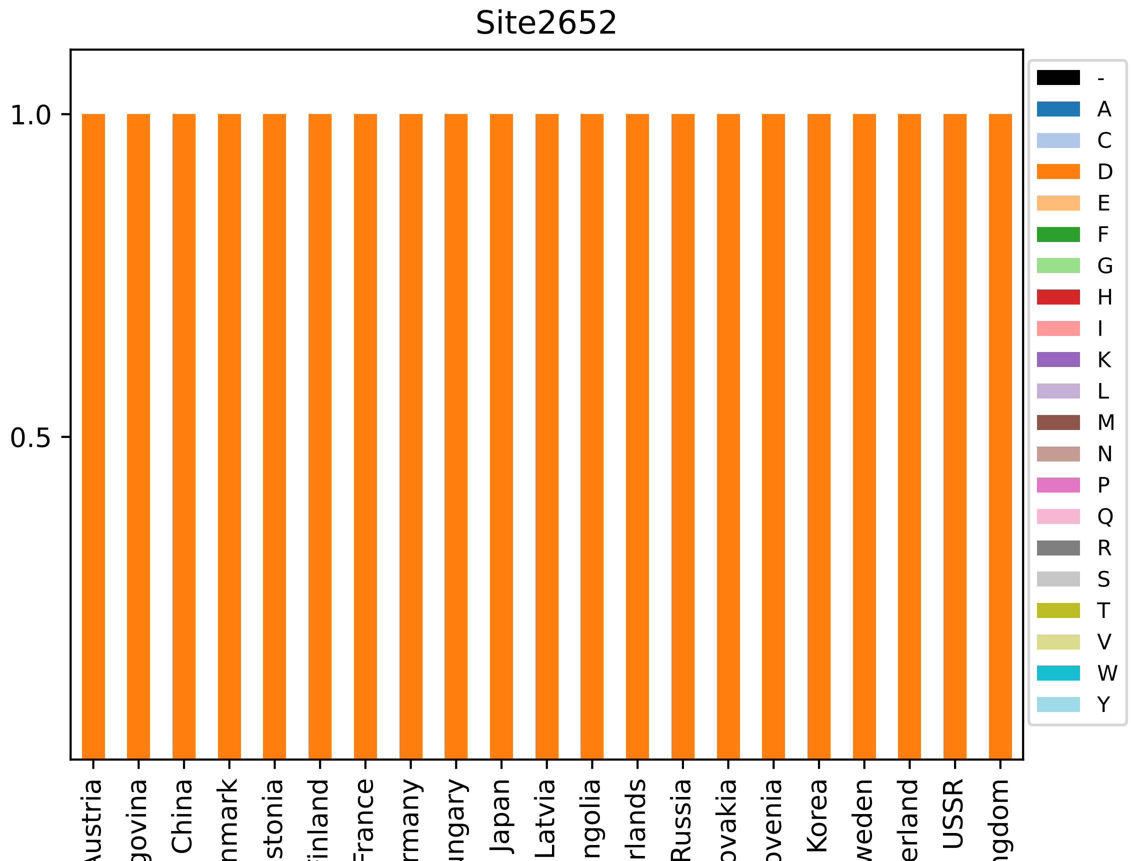

Site2652
1.0
0.5
-
A
C
D
E
F
G
H
I
K
L
M
N
P
Q
R
S
T
V
W
Y
Austria
govina
China
nmark
stonia
inland
France
rmany
ungary
Japan
Latvia
ngolia
rlands
Russia
ovakia
ovenia
Korea
weden
erland
USSR
ngdom

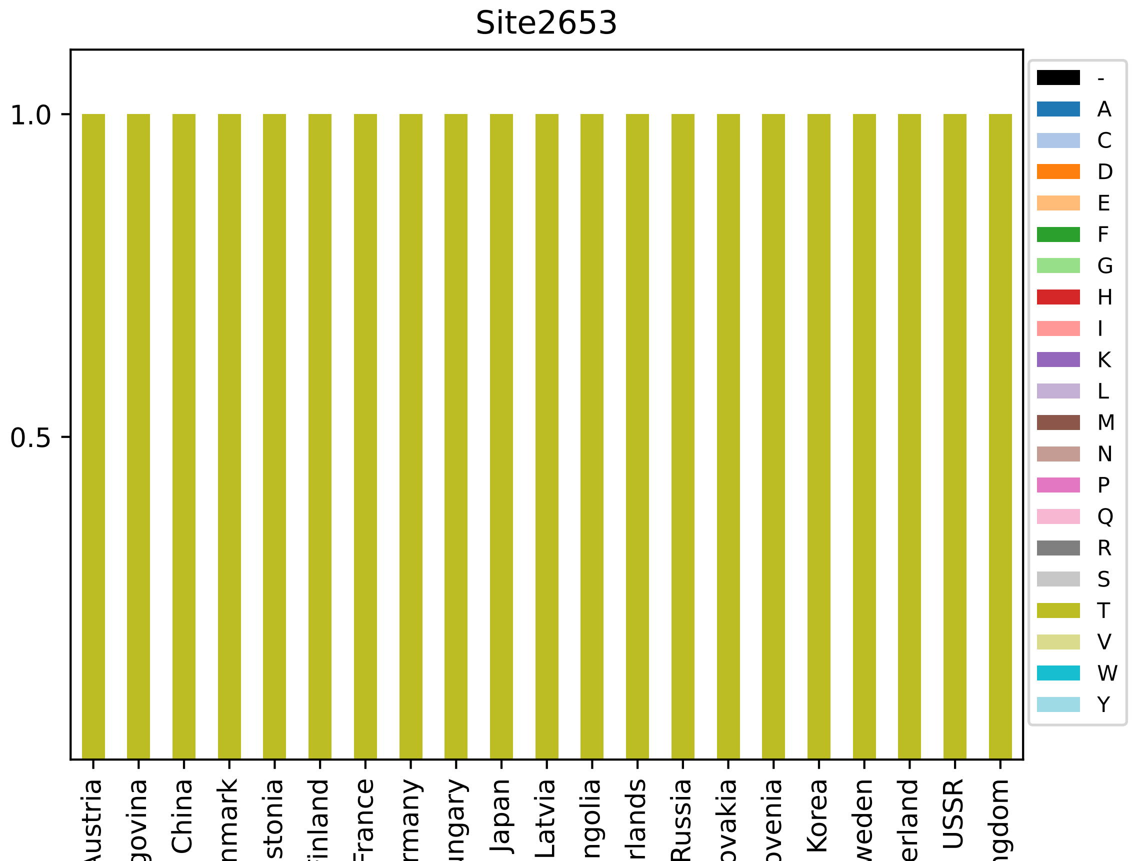
Site2653
1.0
0.5
-
A
C
D
E
F
G
H
I
K
L
M
N
P
Q
R
S
T
V
W
Y
Austria
China
Japan
Latvia
Russia
Korea
USSR

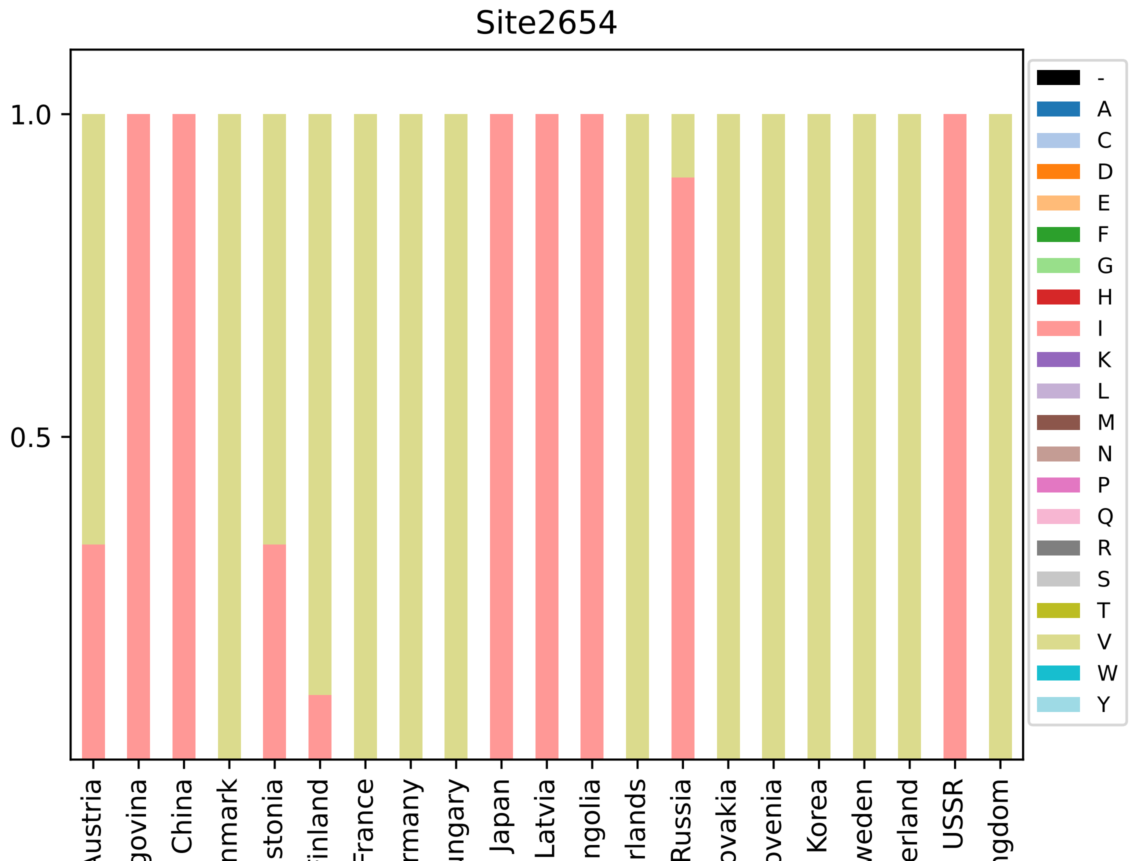

Site2654
1.0
0.5
-
A
C
D
E
F
G
H
I
K
L
M
N
P
Q
R
S
T
V
W
Y
Austria
China
Japan
Latvia
Russia
Korea
USSR

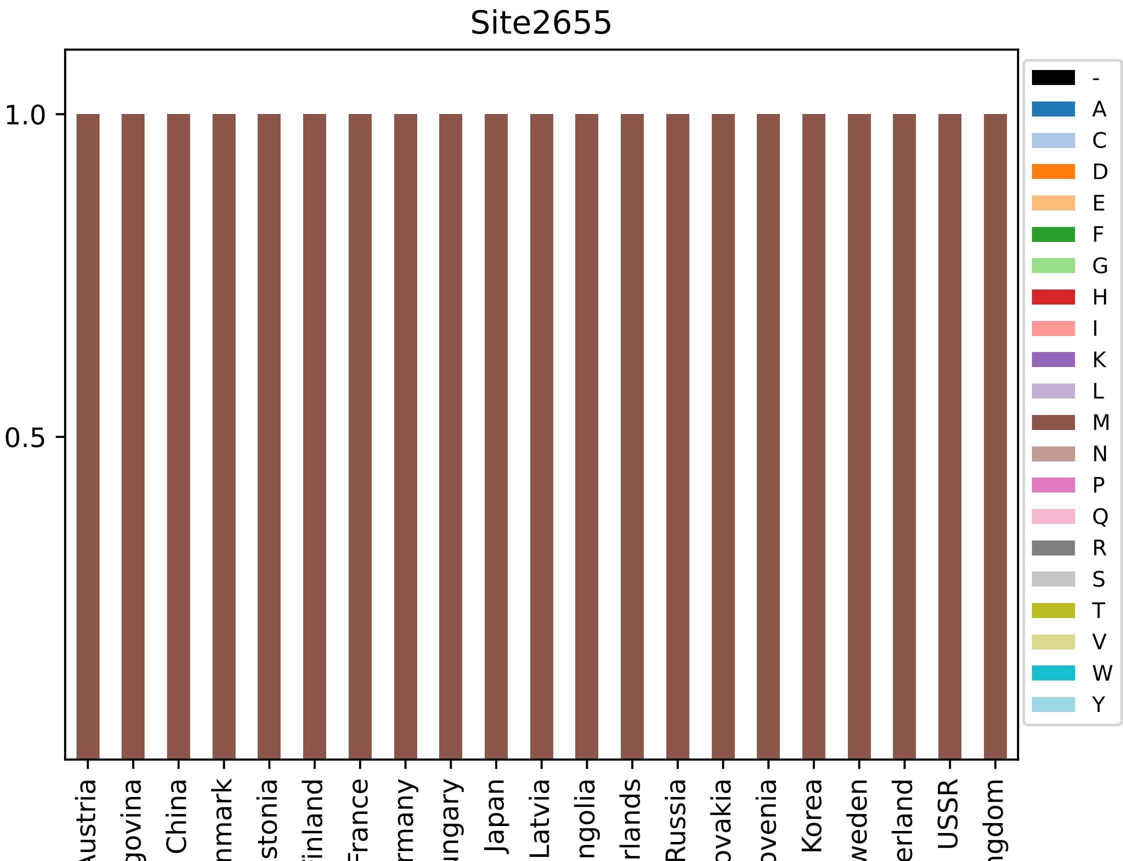

Site2655
1.0
0.5
-
A
C
D
E
F
G
H
I
K
L
M
N
P
Q
R
S
T
V
W
Y
Austria
China
Japan
Latvia
Russia
Korea
USSR

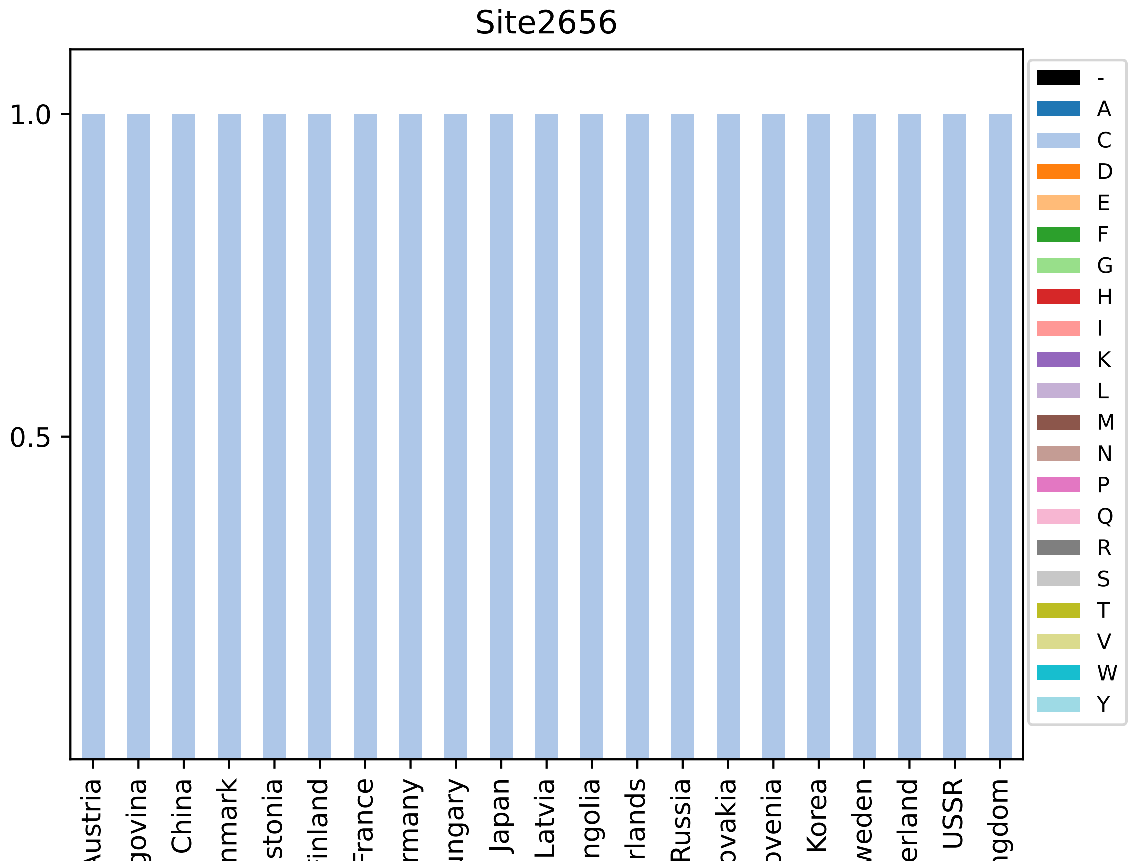
Site2656
1.0
0.5
China
Japan
Latvia
Russia
Korea
USSR
-
A
C
D
E
F
G
H
I
K
L
M
N
P
Q
R
S
T
V
W
Y

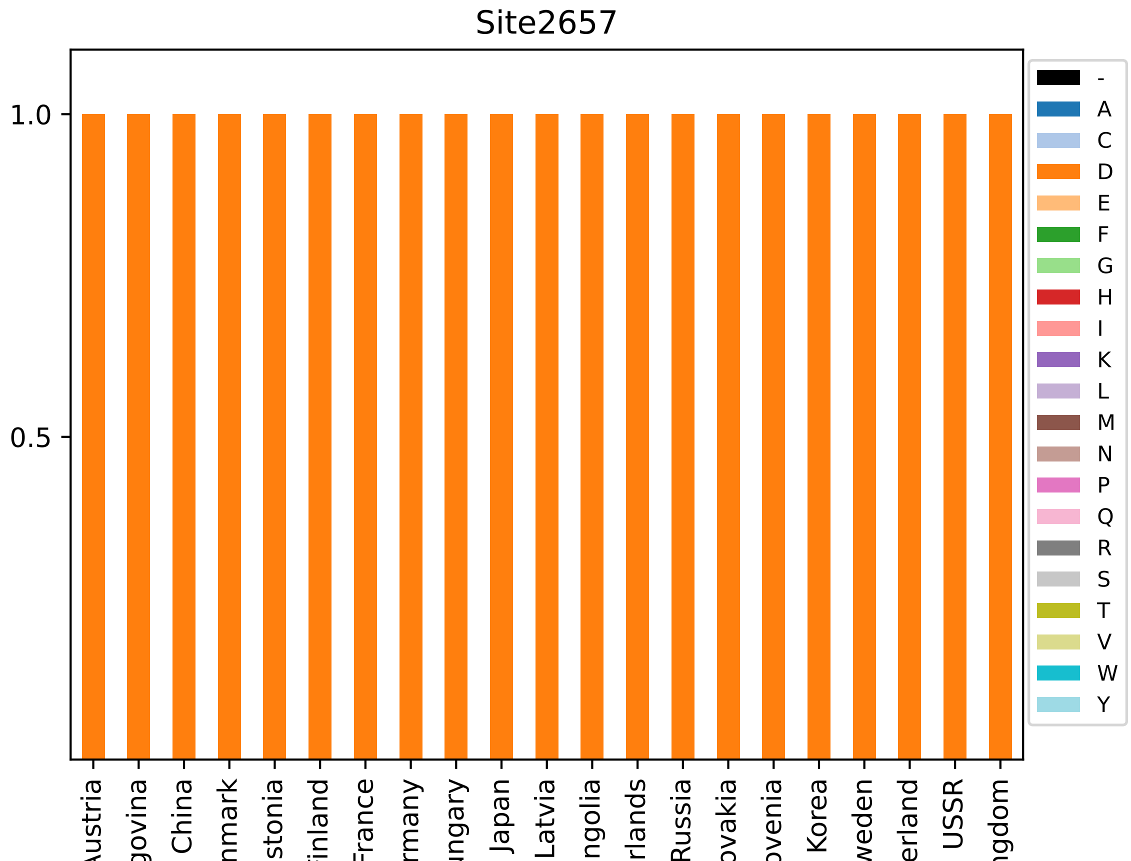
Site2657
1.0
0.5
China
Japan
Latvia
Russia
Korea
USSR
-
A
C
D
E
F
G
H
I
K
L
M
N
P
Q
R
S
T
V
W
Y

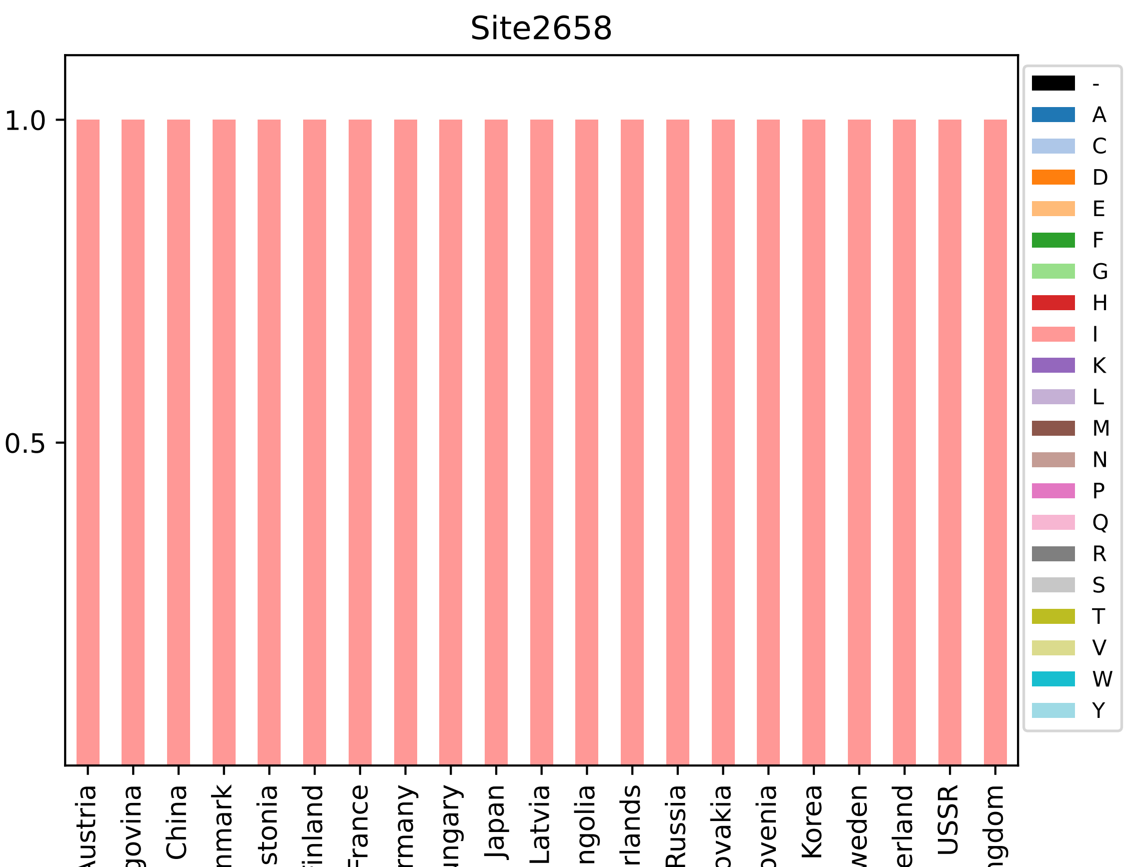

Site2658
1.0
0.5
-
A
C
D
E
F
G
H
I
K
L
M
N
P
Q
R
S
T
V
W
Y
Austria
govina
China
nmark
stonia
inland
France
rmany
ungary
Japan
Latvia
ngolia
rlands
Russia
ovakia
ovenia
Korea
weden
erland
USSR
ngdom

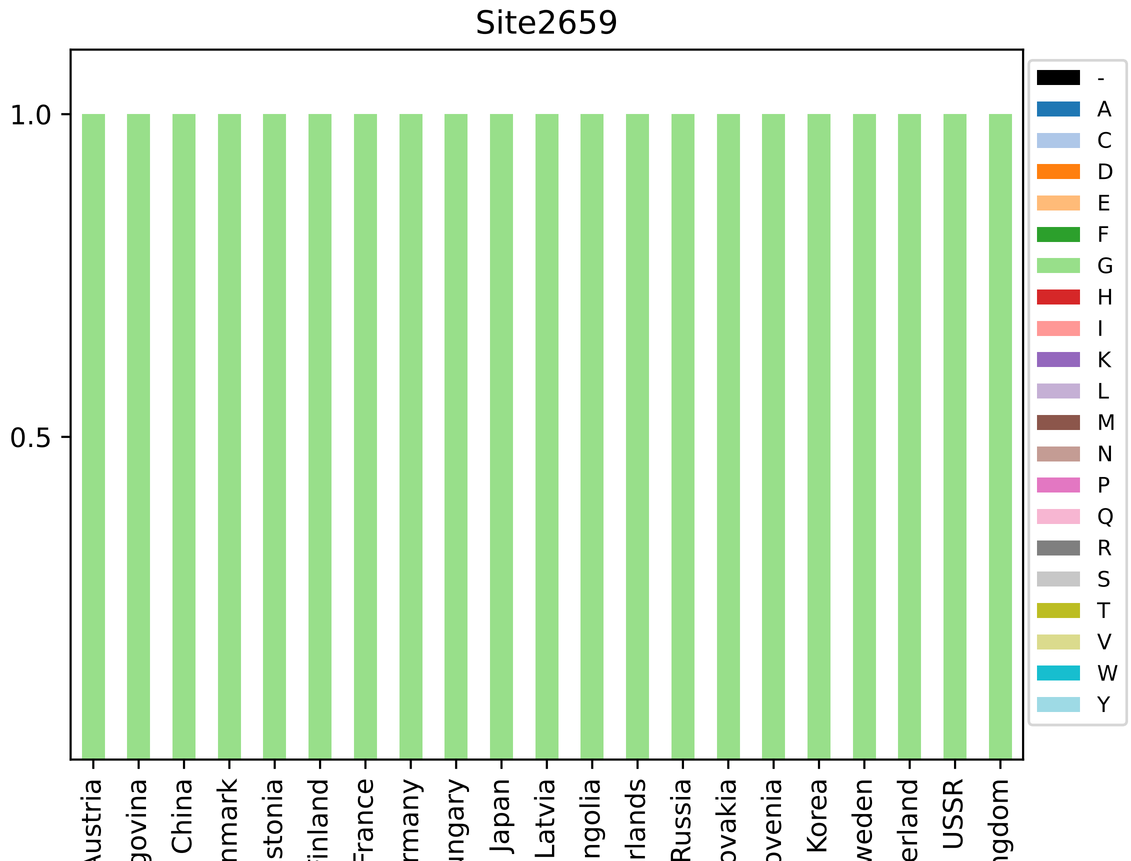
Site2659
1.0
0.5
-
A
C
D
E
F
G
H
I
K
L
M
N
P
Q
R
S
T
V
W
Y
China
Japan
Latvia
Russia
Korea
USSR

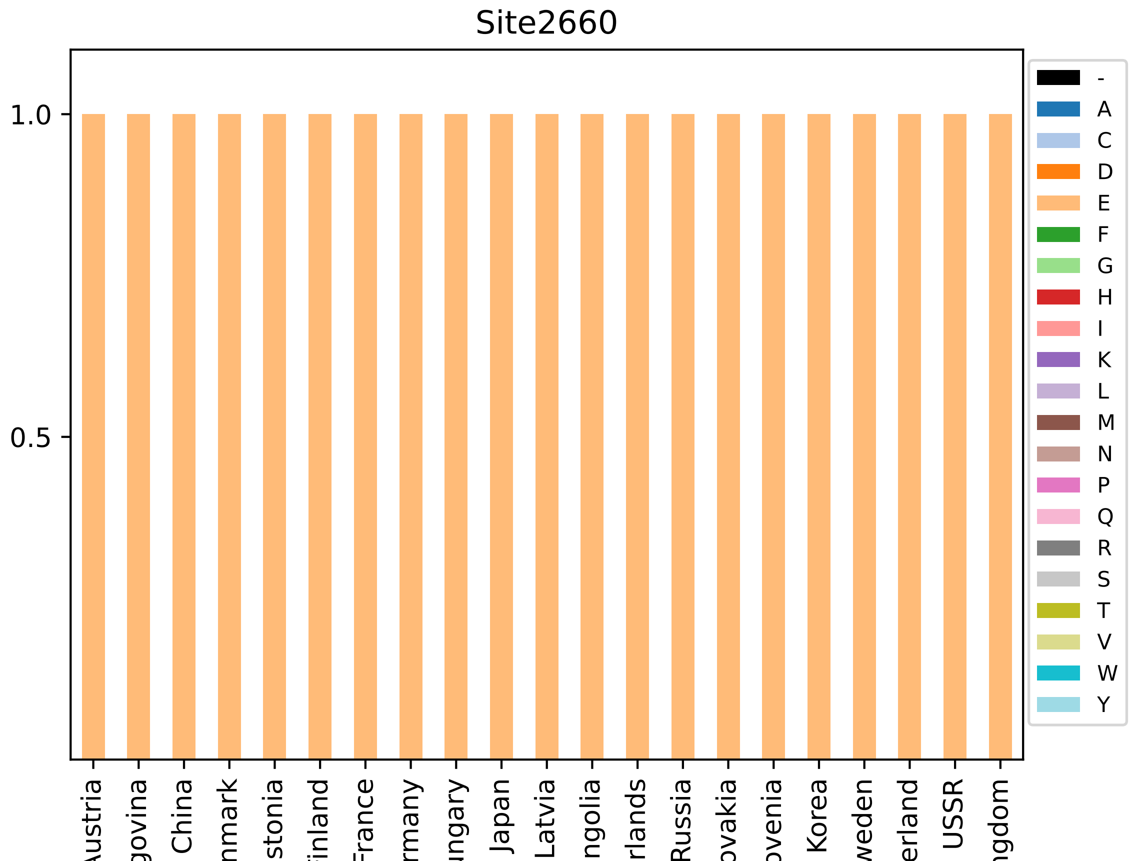
Site2660
1.0
0.5
-
A
C
D
E
F
G
H
I
K
L
M
N
P
Q
R
S
T
V
W
Y
China
Japan
Latvia
Russia
Korea
USSR

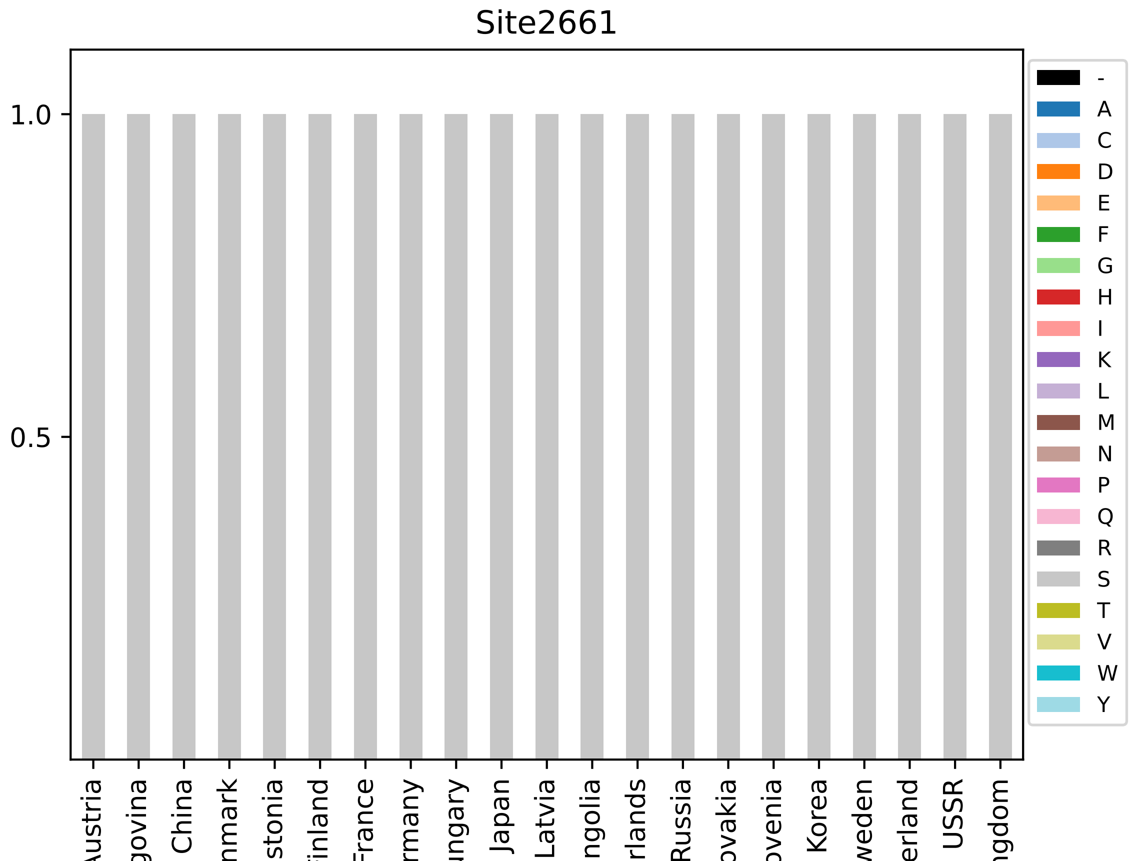

Site2661
1.0
0.5
-
A
C
D
E
F
G
H
I
K
L
M
N
P
Q
R
S
T
V
W
Y
China
Japan
Latvia
Russia
Korea
USSR

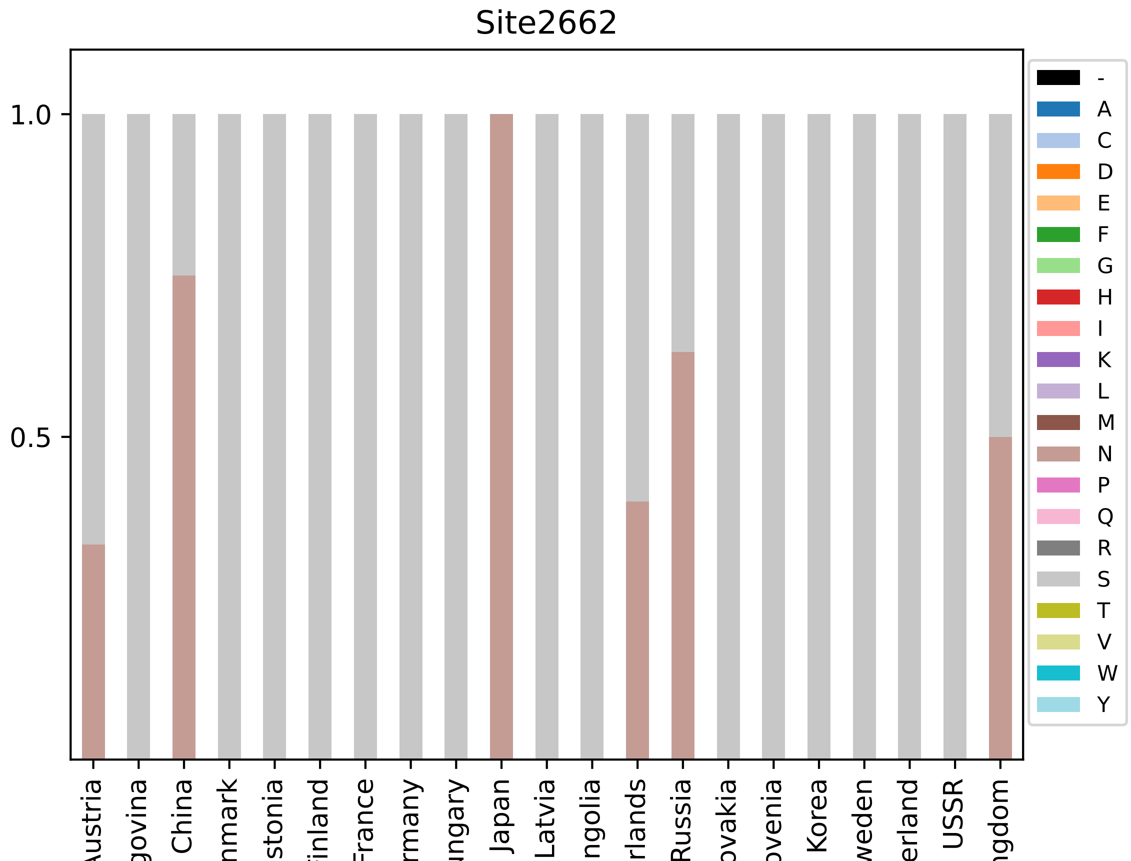
Site2662
1.0
0.5
-
A
C
D
E
F
G
H
I
K
L
M
N
P
Q
R
S
T
V
W
Y
China
Japan
Latvia
Russia
Korea
USSR

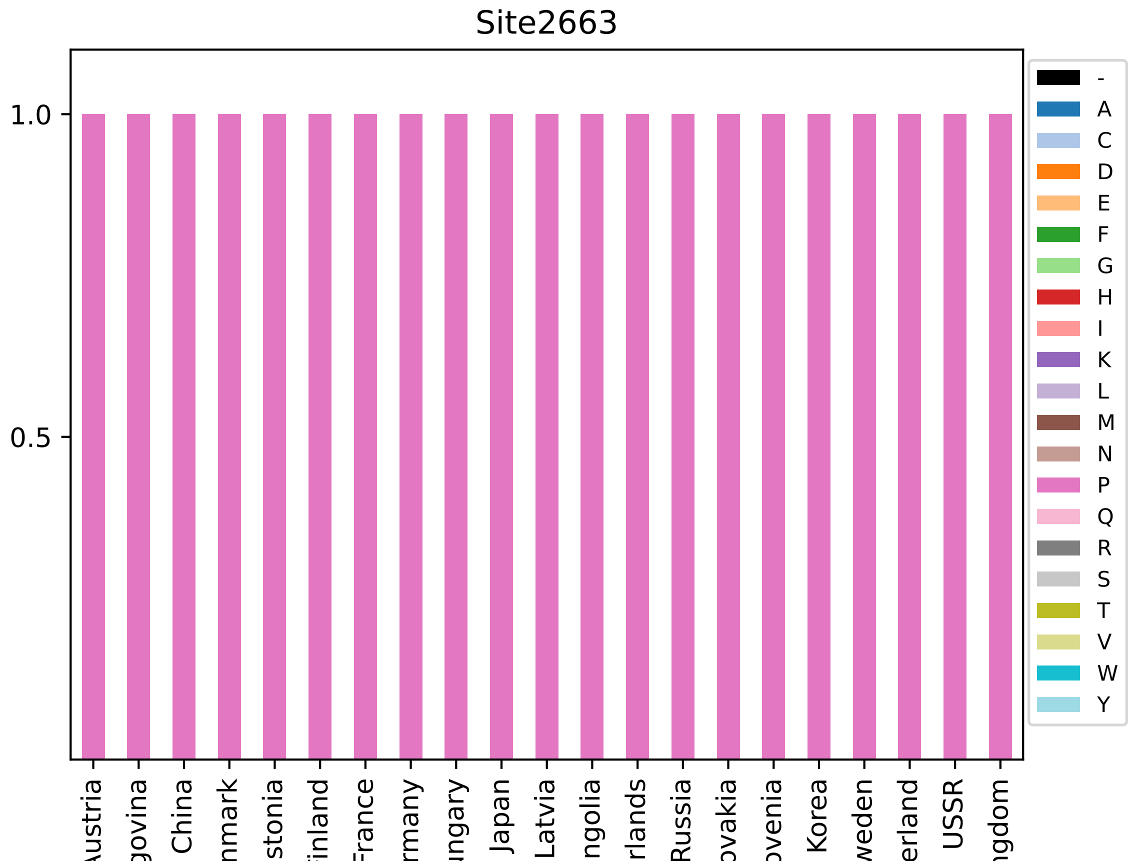
Site2663
1.0
0.5
-
A
C
D
E
F
G
H
I
K
L
M
N
P
Q
R
S
T
V
W
Y
China
Japan
Latvia
Russia
Korea
USSR

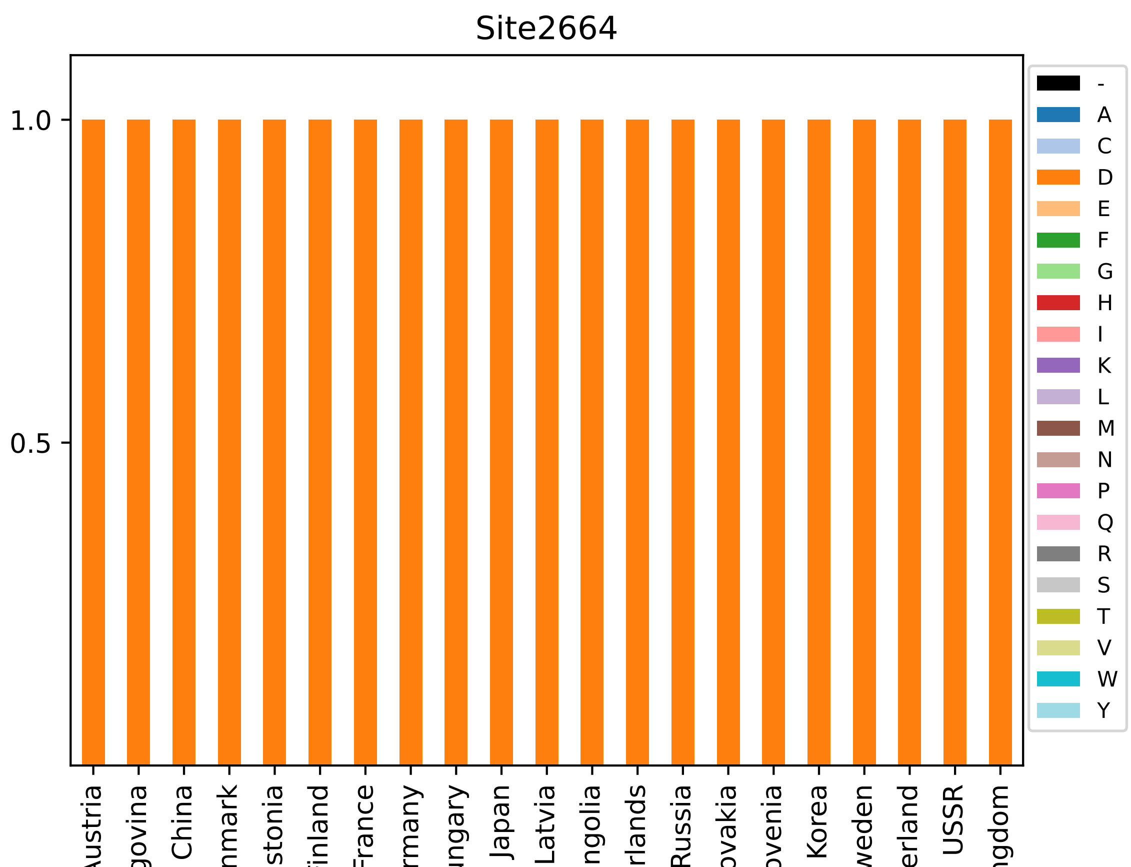

Site2664
1.0
0.5
-
A
C
D
E
F
G
H
I
K
L
M
N
P
Q
R
S
T
V
W
Y
China
Japan
Latvia
Russia
Korea
USSR

Site2665

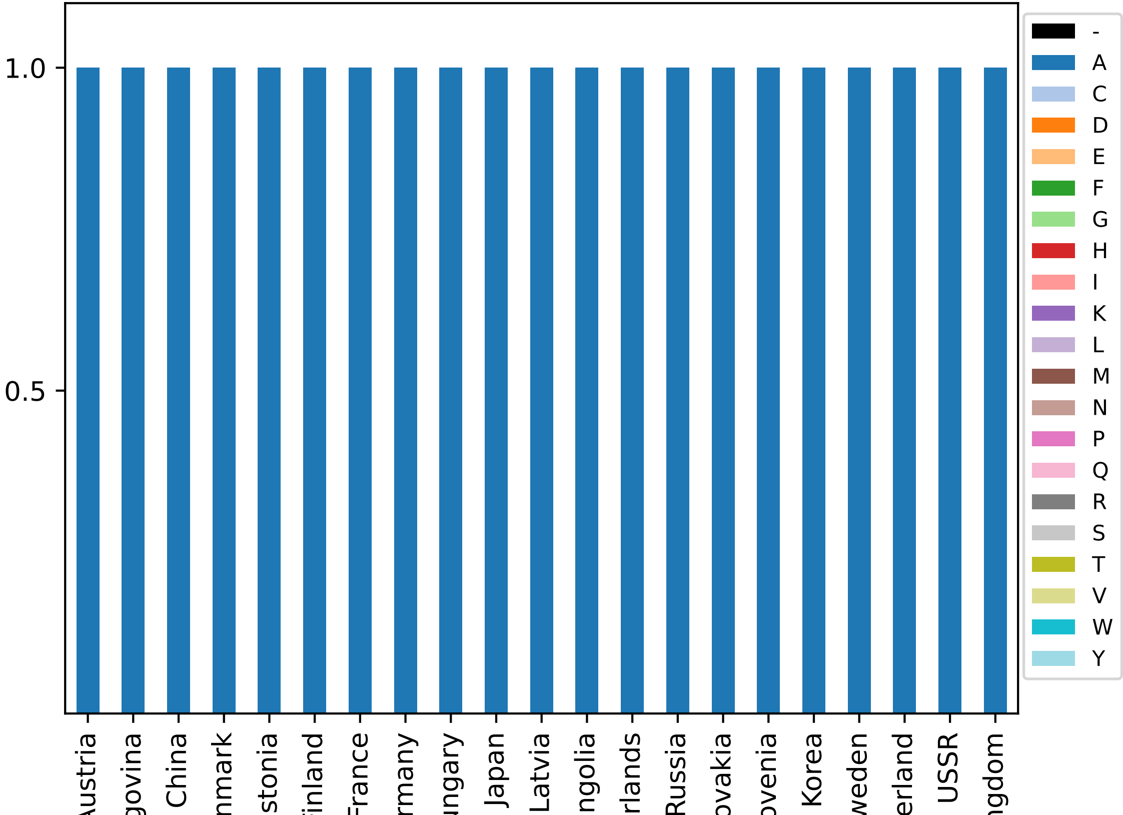
1.0
0.5
China
Japan
Latvia
Russia
Korea
USSR
-
A
C
D
E
F
G
H
I
K
L
M
N
P
Q
R
S
T
V
W
Y

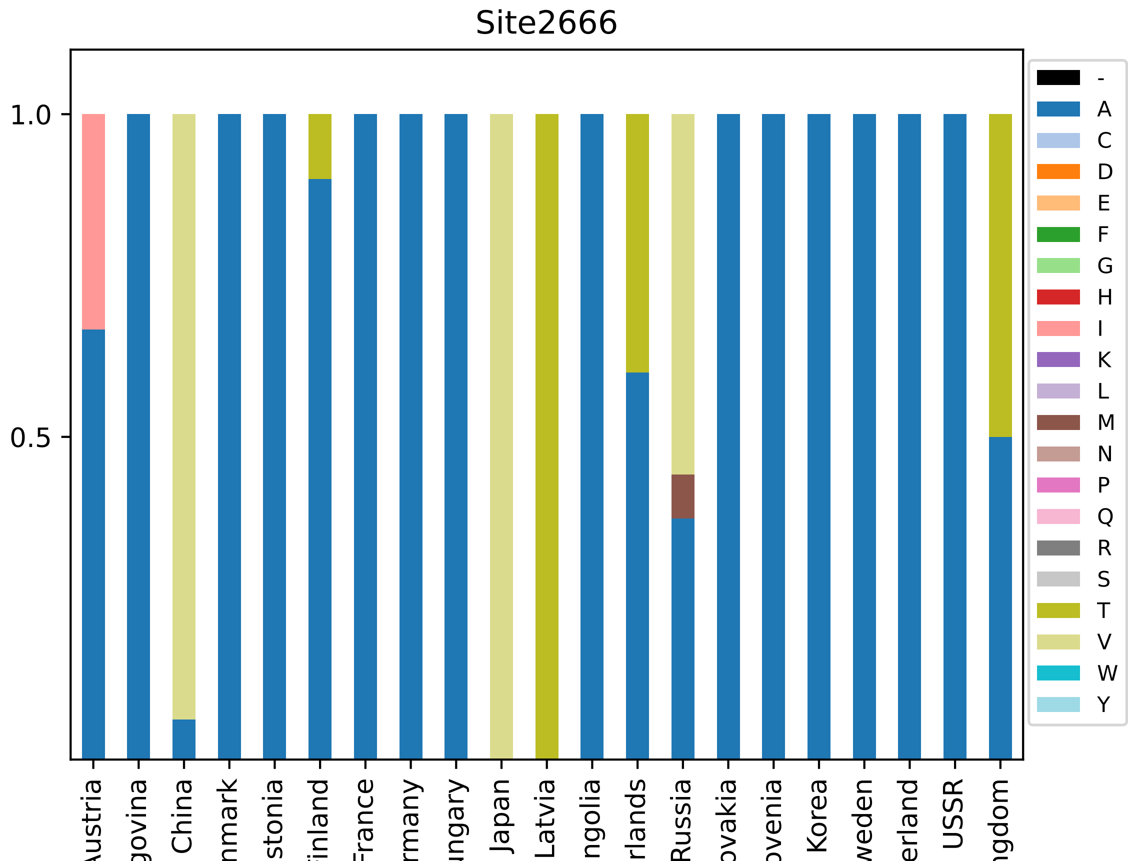

Site2666
1.0
0.5
Austria
govina
China
nmark
stonia
Finland
France
rmany
ungary
Japan
Latvia
ngolia
rlands
Russia
ovakia
ovenia
Korea
weden
erland
USSR
ngdom
-
A
C
D
E
F
G
H
I
K
L
M
N
P
Q
R
S
T
V
W
Y

Site2667

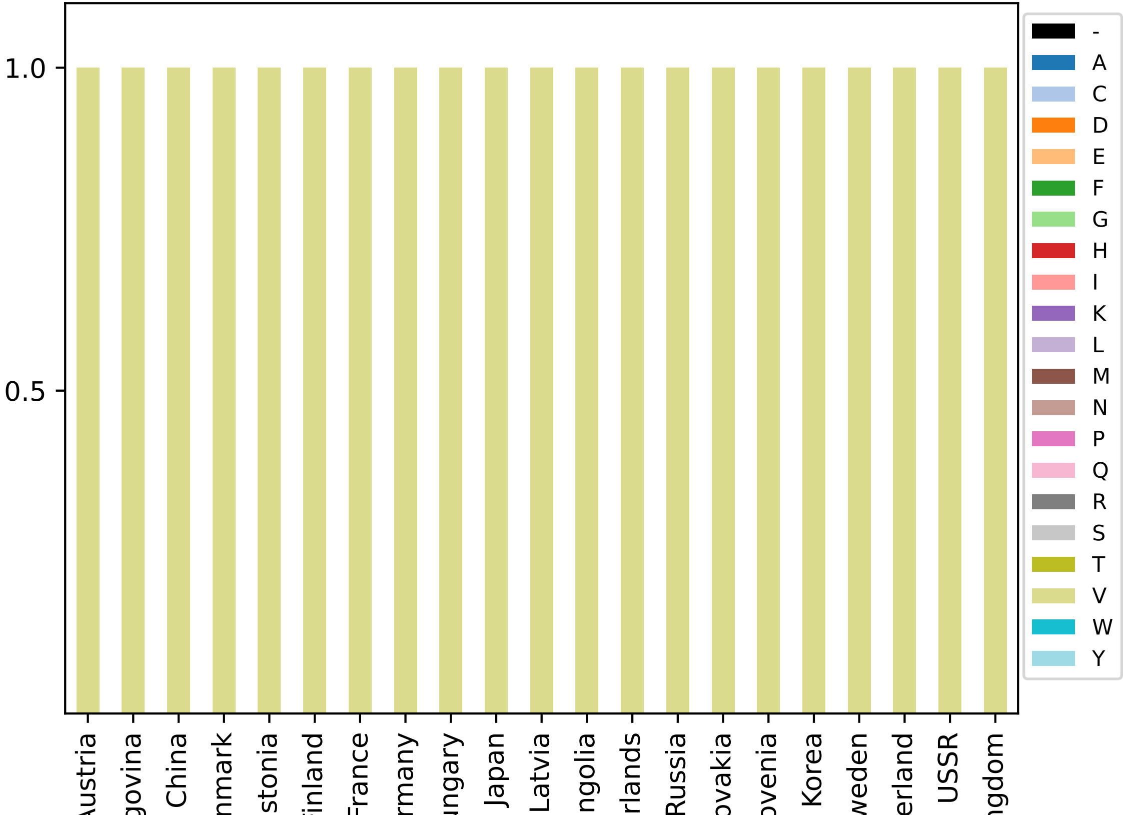
1.0
0.5
-
A
C
D
E
F
G
H
I
K
L
M
N
P
Q
R
S
T
V
W
Y
Austria
govina
China
nmark
stonia
inland
France
rmany
ungary
Japan
Latvia
ngolia
rlands
Russia
ovakia
ovenia
Korea
weden
erland
USSR
ngdom

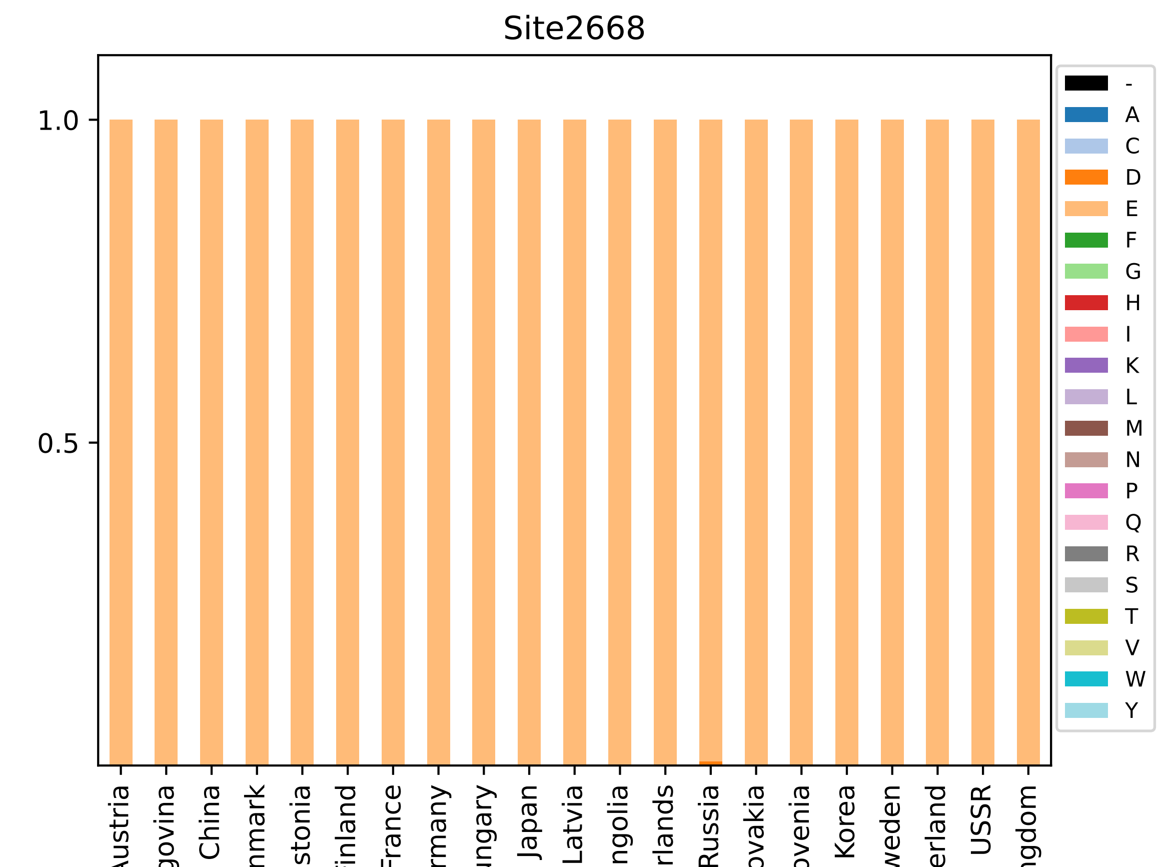
Site2668
1.0
0.5
-
A
C
D
E
F
G
H
I
K
L
M
N
P
Q
R
S
T
V
W
Y
Austria
govina
China
nmark
stonia
inland
France
rmany
ungary
Japan
Latvia
ngolia
rlands
Russia
ovakia
ovenia
Korea
weden
erland
USSR
ngdom

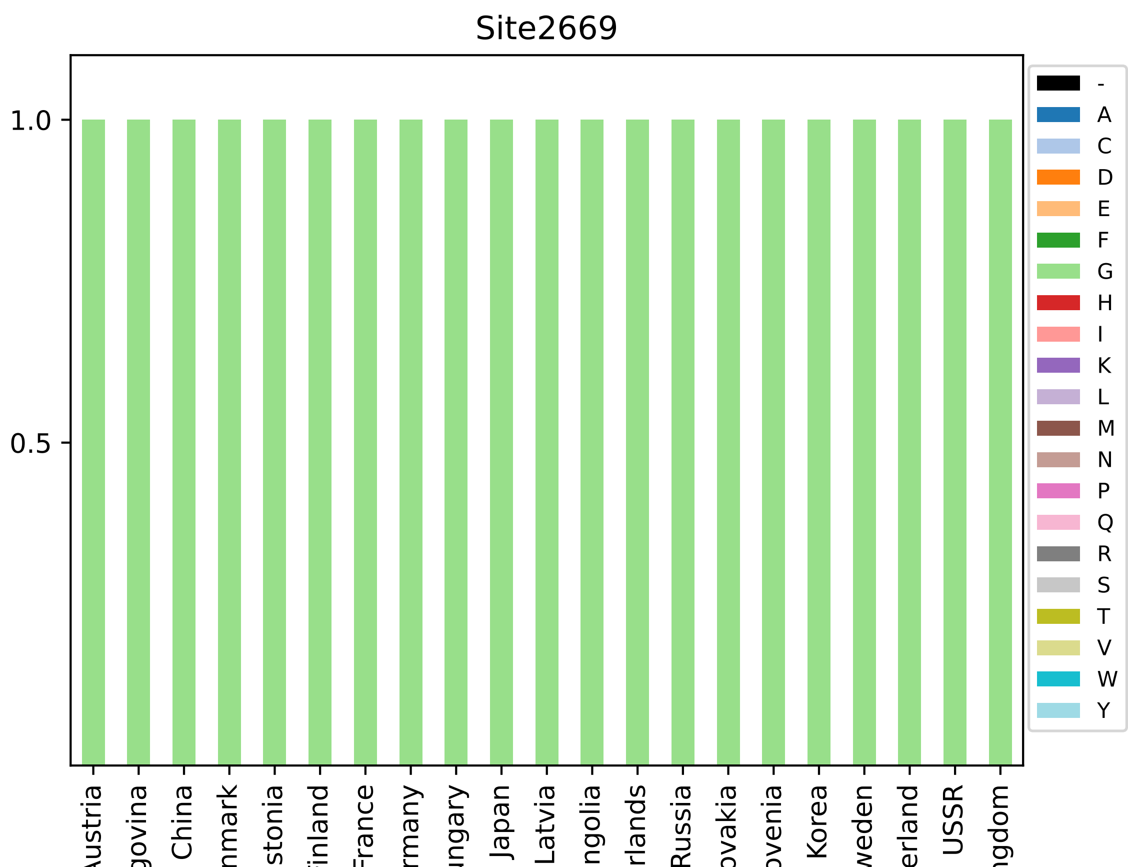
Site2669
1.0
0.5
-
A
C
D
E
F
G
H
I
K
L
M
N
P
Q
R
S
T
V
W
Y
Austria
govina
China
nmark
stonia
Finland
France
rmany
ungary
Japan
Latvia
ngolia
rlands
Russia
ovakia
ovenia
Korea
weden
erland
USSR
ngdom

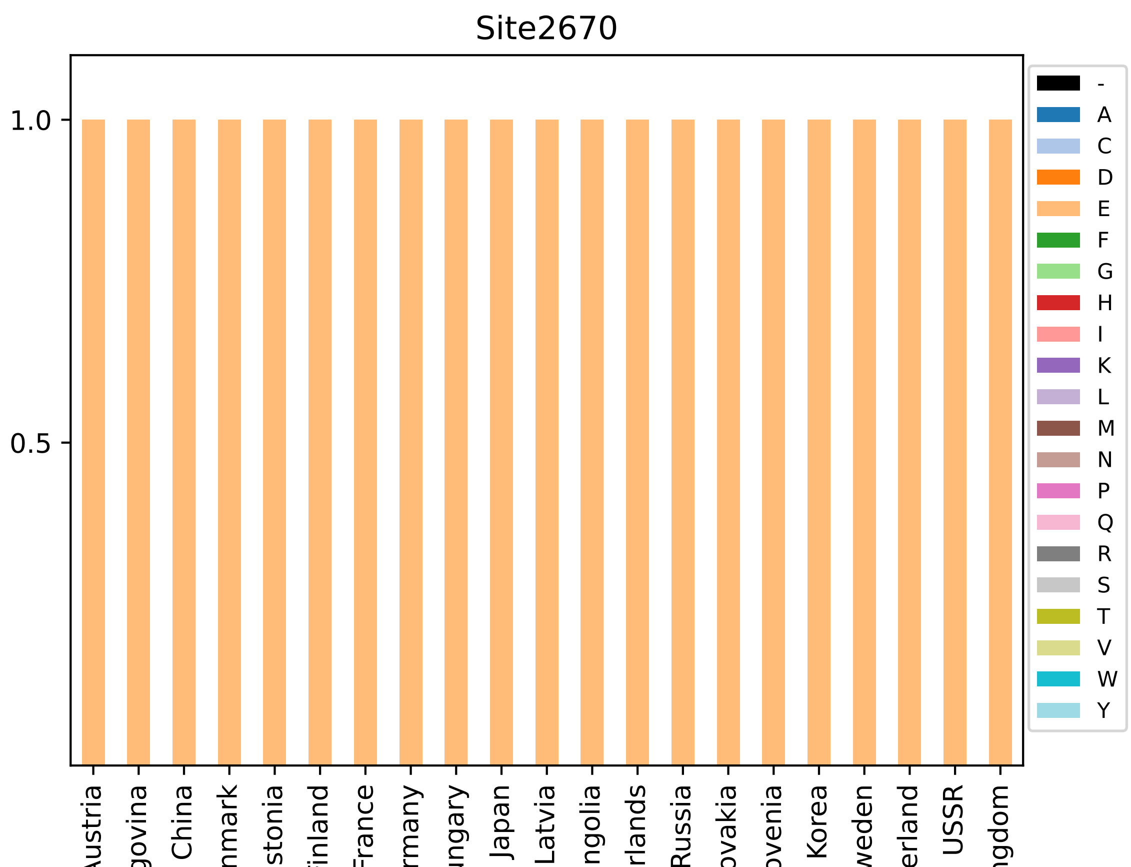
Site2670
1.0
0.5
-
A
C
D
E
F
G
H
I
K
L
M
N
P
Q
R
S
T
V
W
Y
China
Japan
Latvia
Russia
Korea
USSR

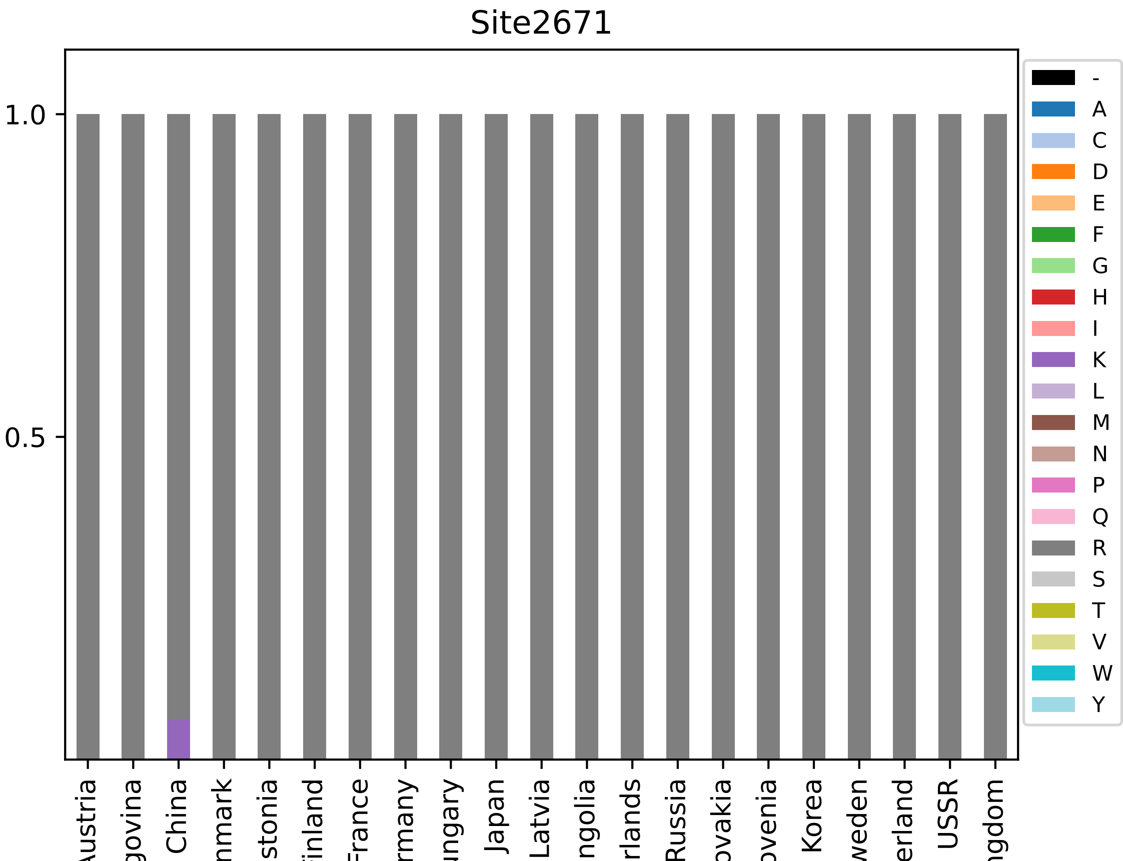

Site2671
1.0
0.5
-
A
C
D
E
F
G
H
I
K
L
M
N
P
Q
R
S
T
V
W
Y
Austria
China
Japan
Latvia
Russia
Korea
USSR

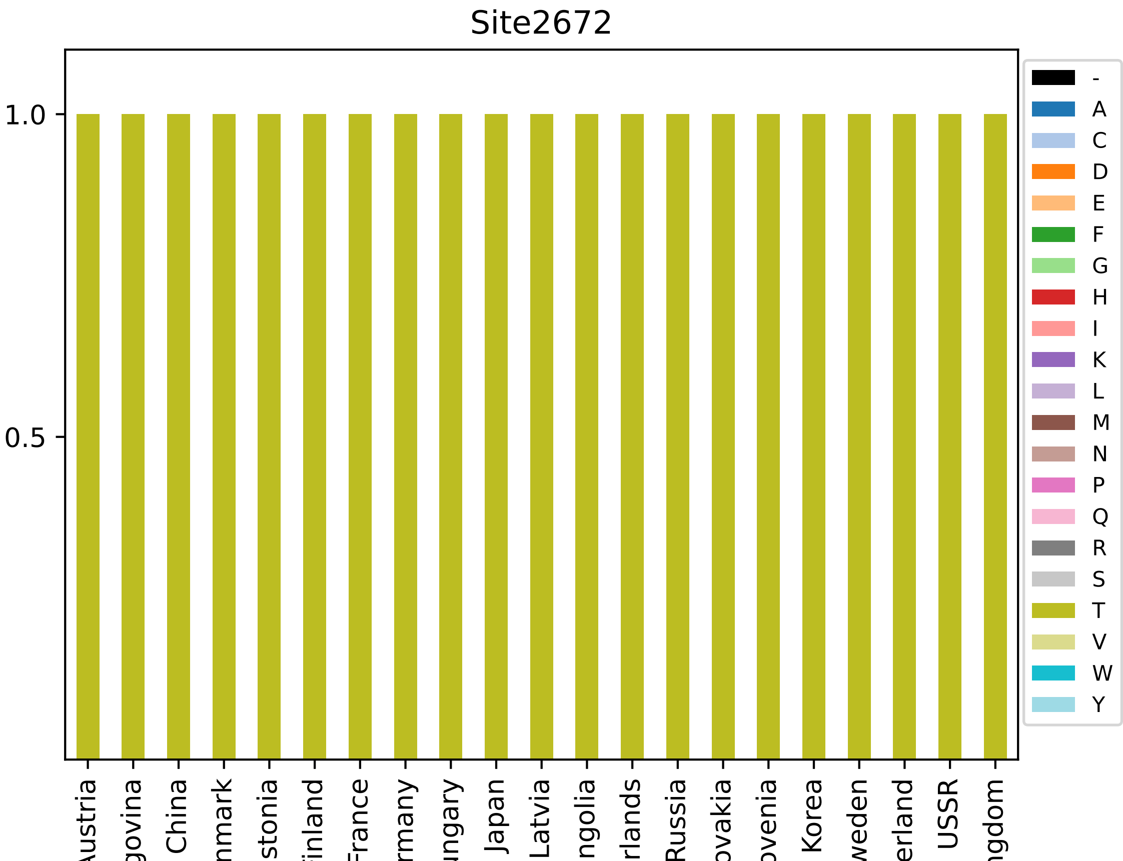

Site2672
1.0
0.5
-
A
C
D
E
F
G
H
I
K
L
M
N
P
Q
R
S
T
V
W
Y
China
Japan
Latvia
Russia
Korea
USSR

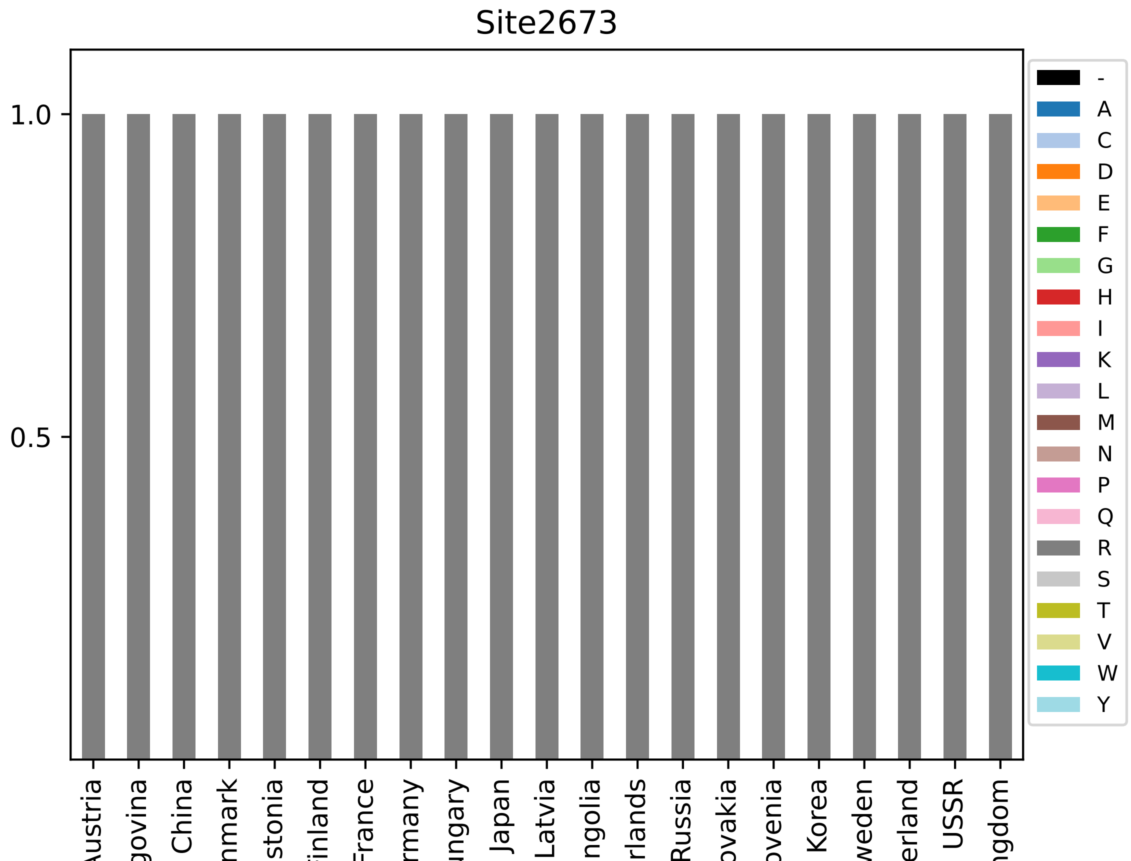

Site2673
1.0
0.5
-
A
C
D
E
F
G
H
I
K
L
M
N
P
Q
R
S
T
V
W
Y
Austria
govina
China
nmark
stonia
inland
France
rmany
ungary
Japan
Latvia
ngolia
rlands
Russia
ovakia
ovenia
Korea
weden
erland
USSR
ngdom

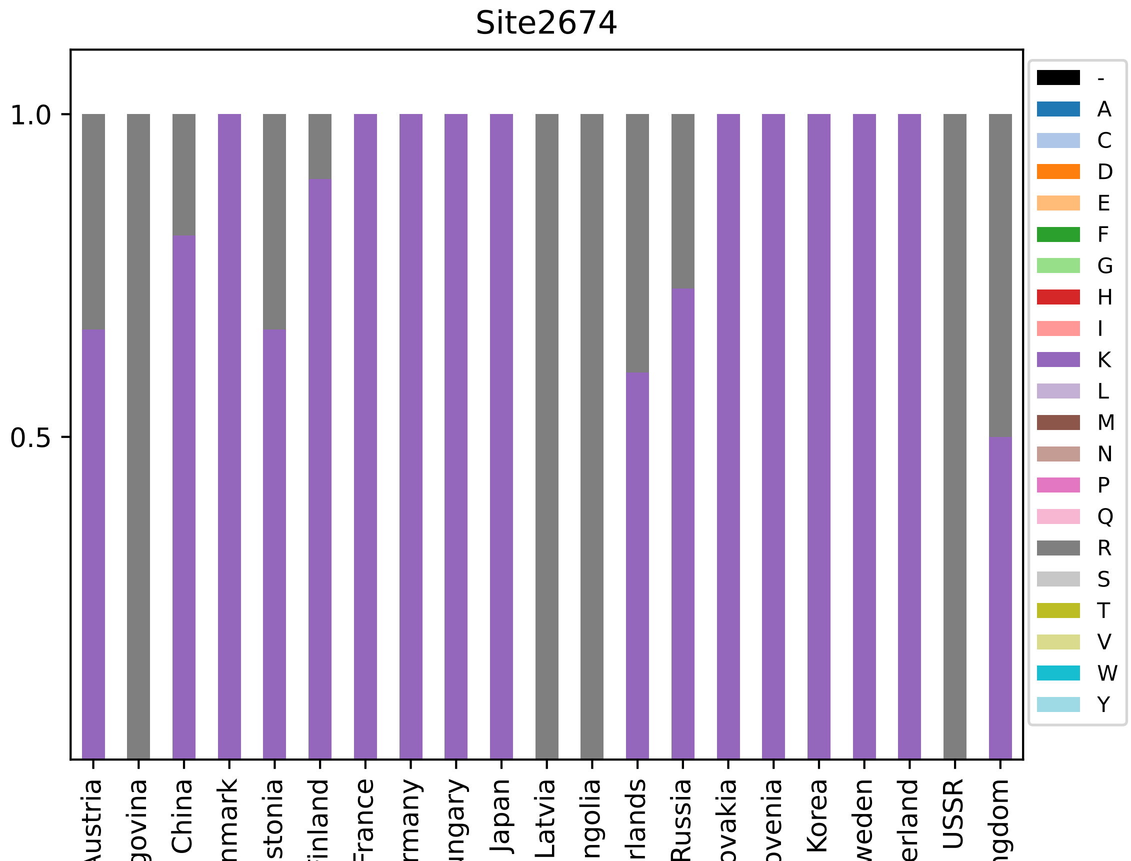

Site2674
1.0
0.5
-
A
C
D
E
F
G
H
I
K
L
M
N
P
Q
R
S
T
V
W
Y
Austria
govina
China
nmark
stonia
inland
France
rmany
ungary
Japan
Latvia
ngolia
rlands
Russia
ovakia
ovenia
Korea
weden
erland
USSR
ngdom

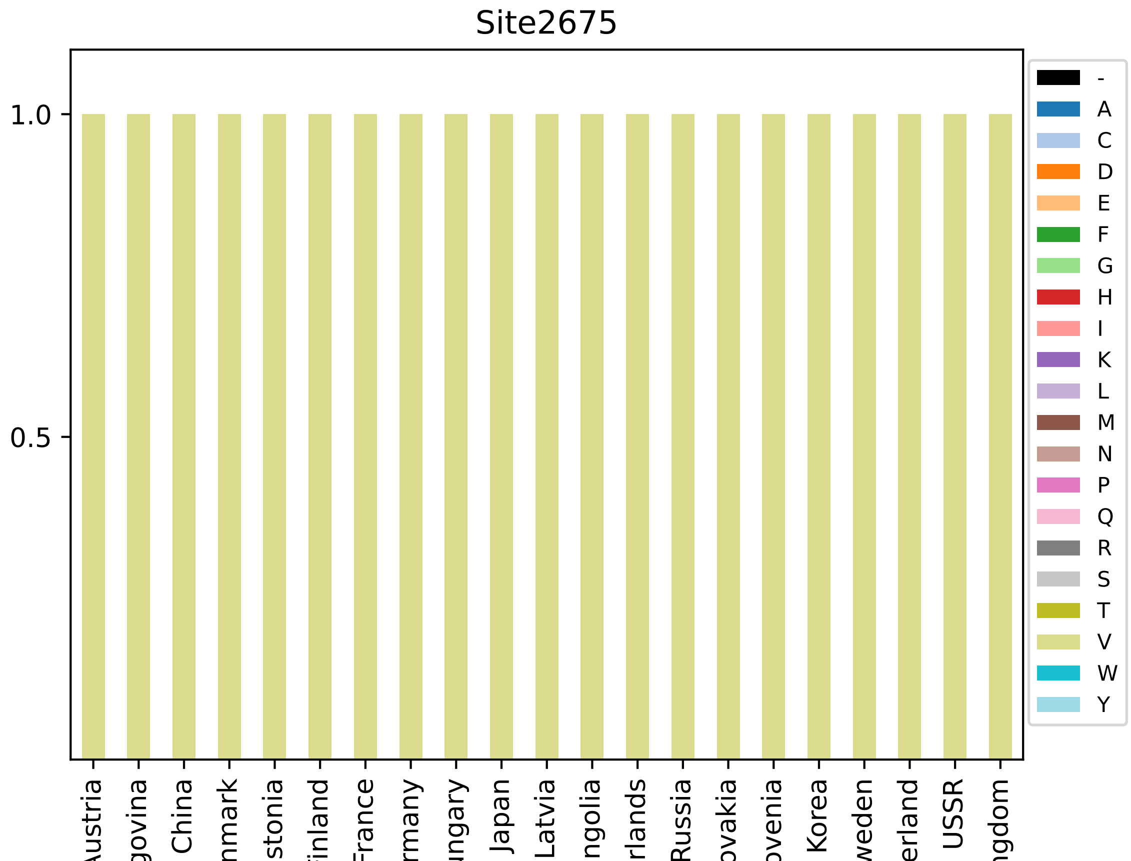
Site2675
1.0
0.5
-
A
C
D
E
F
G
H
I
K
L
M
N
P
Q
R
S
T
V
W
Y
China
Japan
Latvia
Russia
Korea
USSR

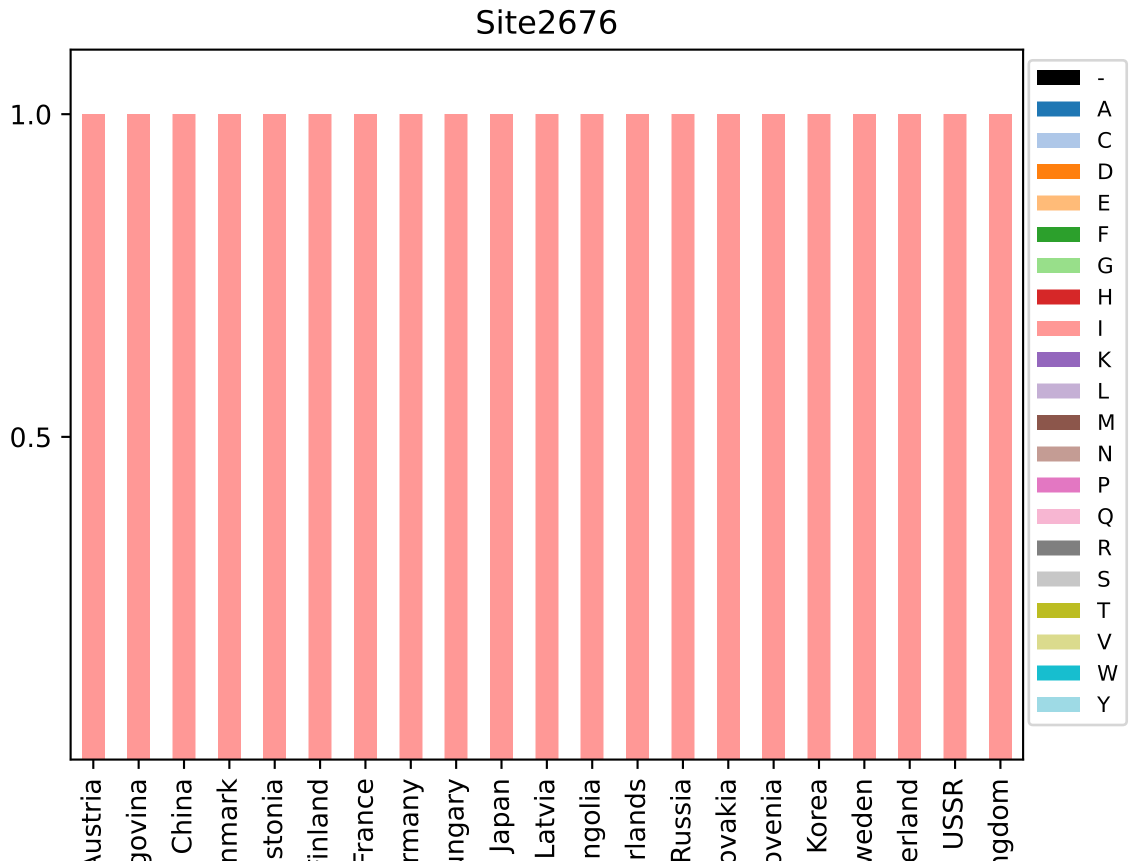

Site2676
1.0
0.5
Austria
govina
China
nmark
stonia
Finland
France
rmany
ungary
Japan
Latvia
ngolia
rlands
Russia
ovakia
ovenia
Korea
weden
erland
USSR
ngdom
-
A
C
D
E
F
G
H
I
K
L
M
N
P
Q
R
S
T
V
W
Y

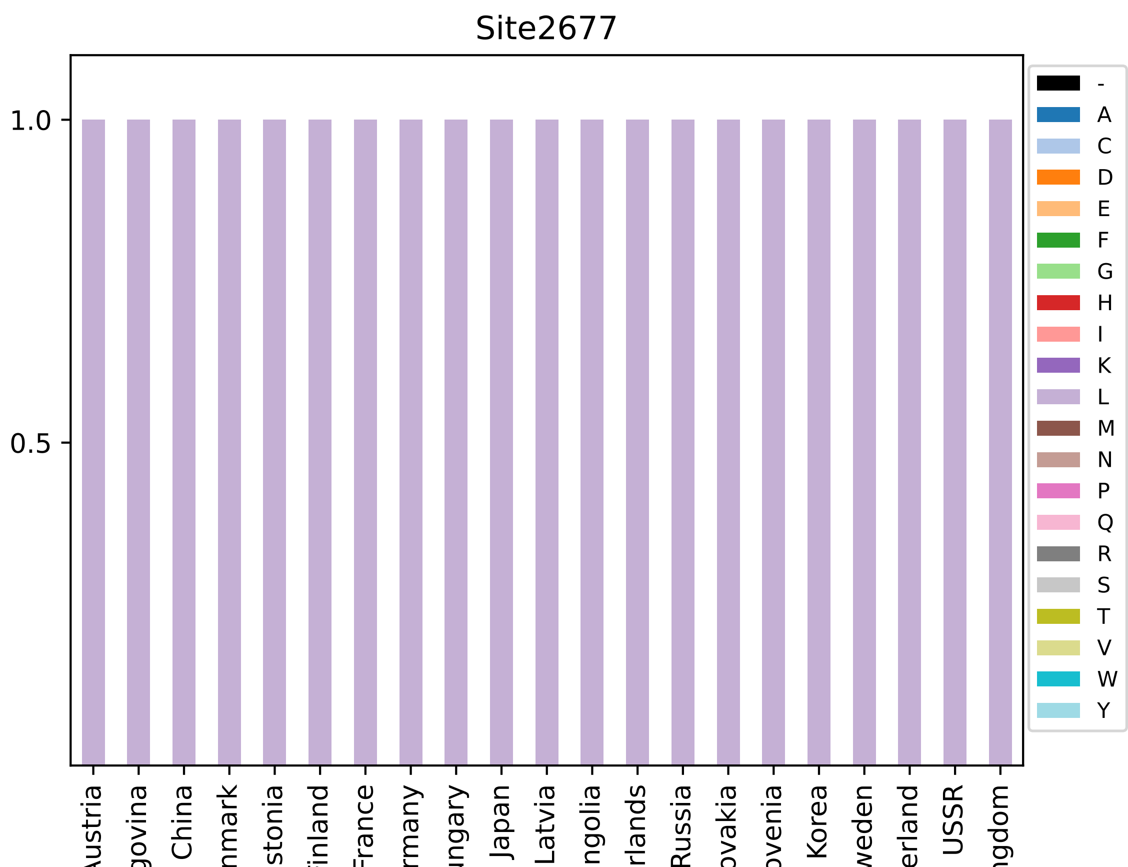

Site2677
1.0
0.5
-
A
C
D
E
F
G
H
I
K
L
M
N
P
Q
R
S
T
V
W
Y
China
Japan
Latvia
Russia
Korea
USSR

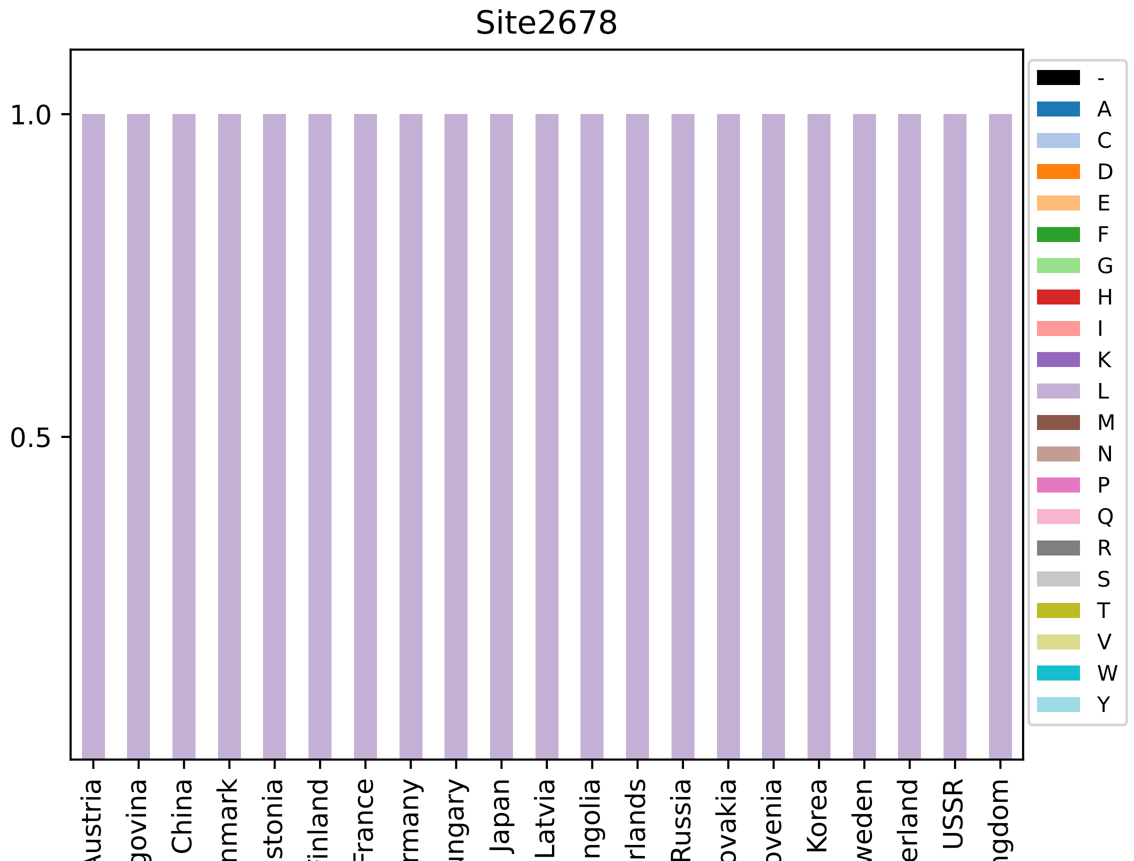

Site2678
1.0
0.5
-
A
C
D
E
F
G
H
I
K
L
M
N
P
Q
R
S
T
V
W
Y
China
Japan
Latvia
Russia
Korea
USSR

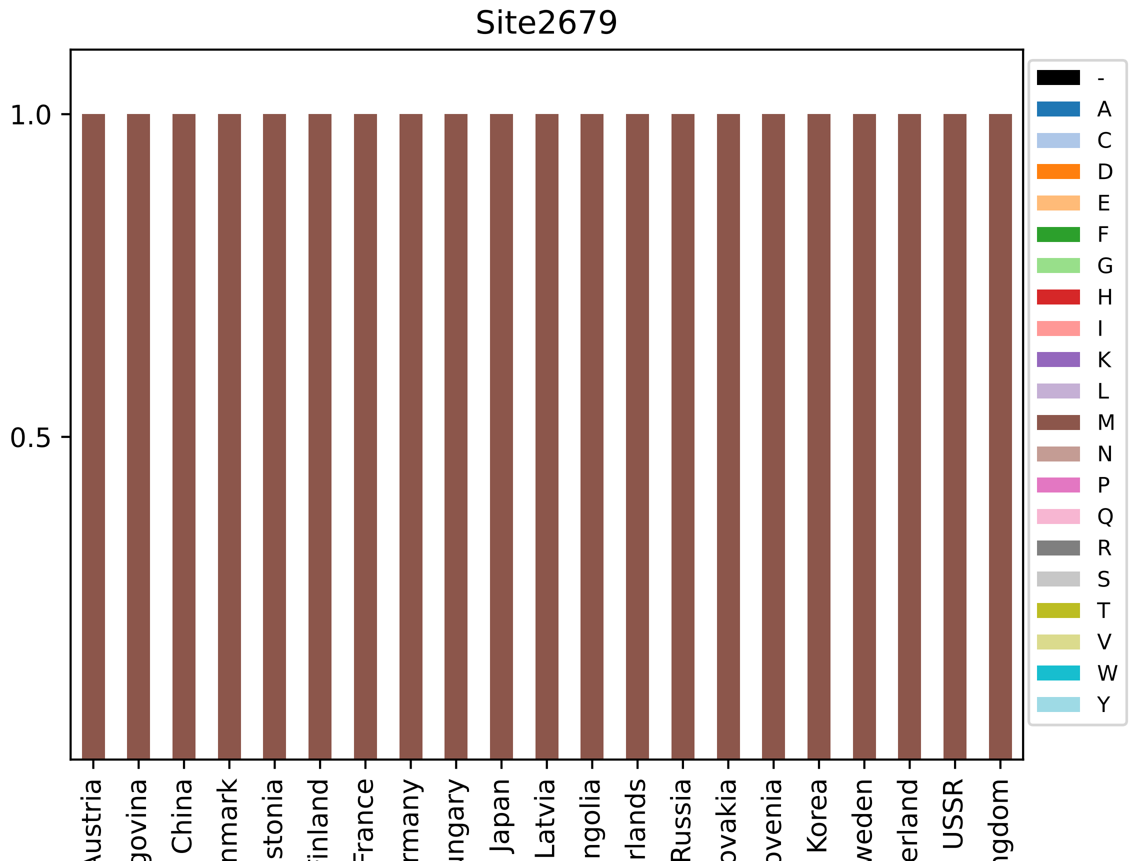
Site2679
1.0
0.5
-
A
C
D
E
F
G
H
I
K
L
M
N
P
Q
R
S
T
V
W
Y
China
Japan
Latvia
Russia
Korea
USSR

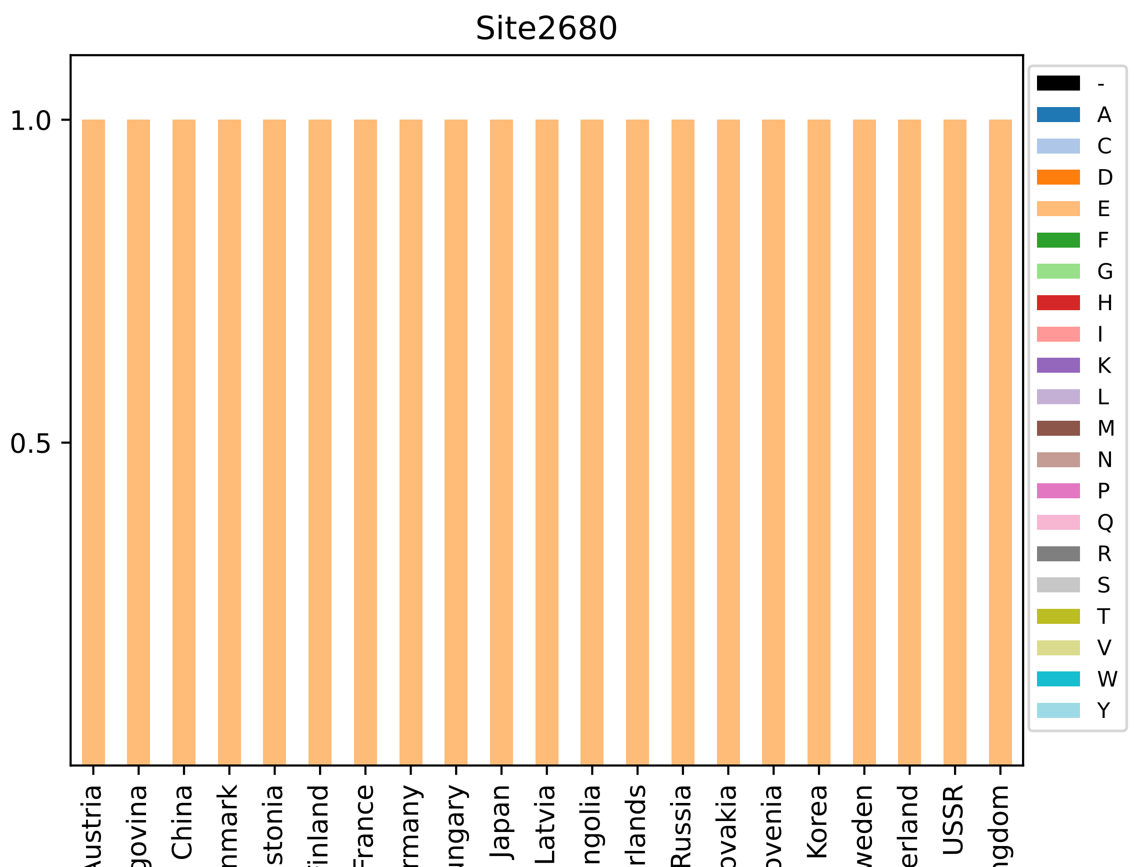

Site2680
1.0
0.5
Austria
govina
China
nmark
stonia
Finland
France
rmany
ungary
Japan
Latvia
ngolia
rlands
Russia
ovakia
ovenia
Korea
weden
erland
USSR
ngdom
-
A
C
D
E
F
G
H
I
K
L
M
N
P
Q
R
S
T
V
W
Y

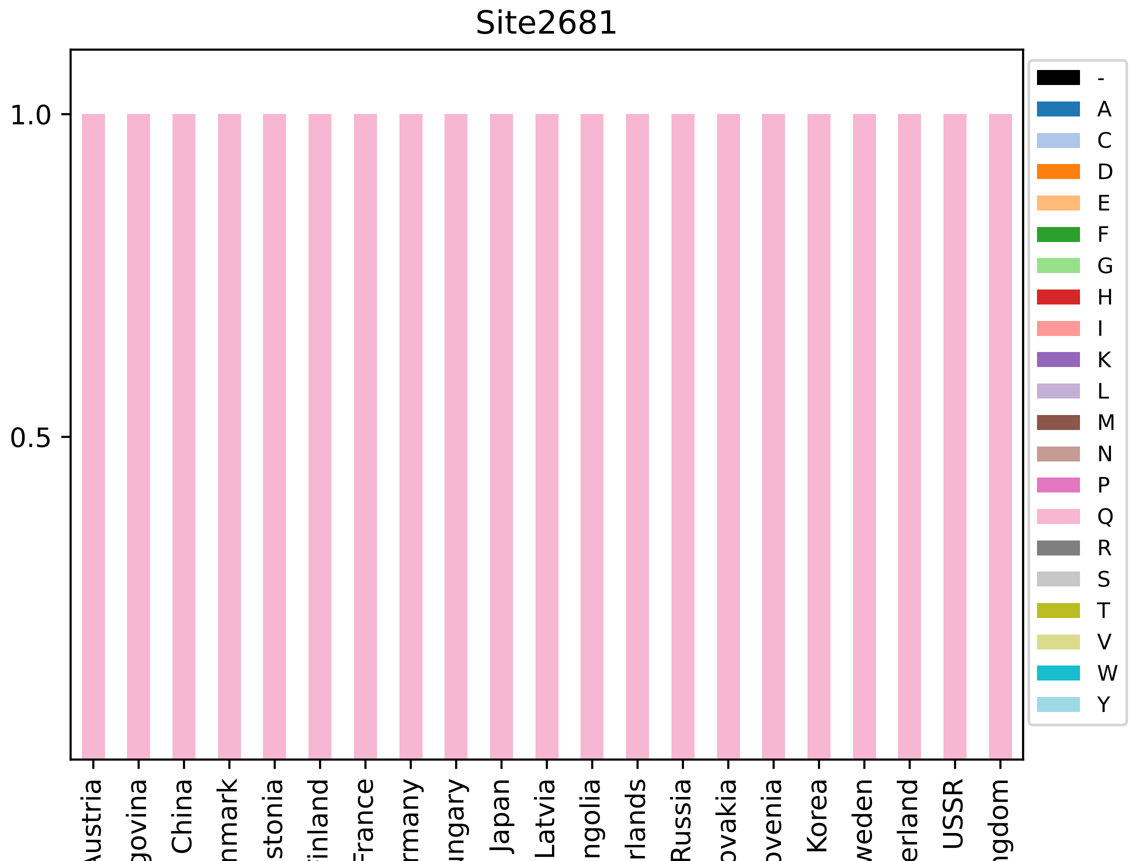

Site2681
1.0
0.5
-
A
C
D
E
F
G
H
I
K
L
M
N
P
Q
R
S
T
V
W
Y
China
Japan
Latvia
Russia
Korea
USSR

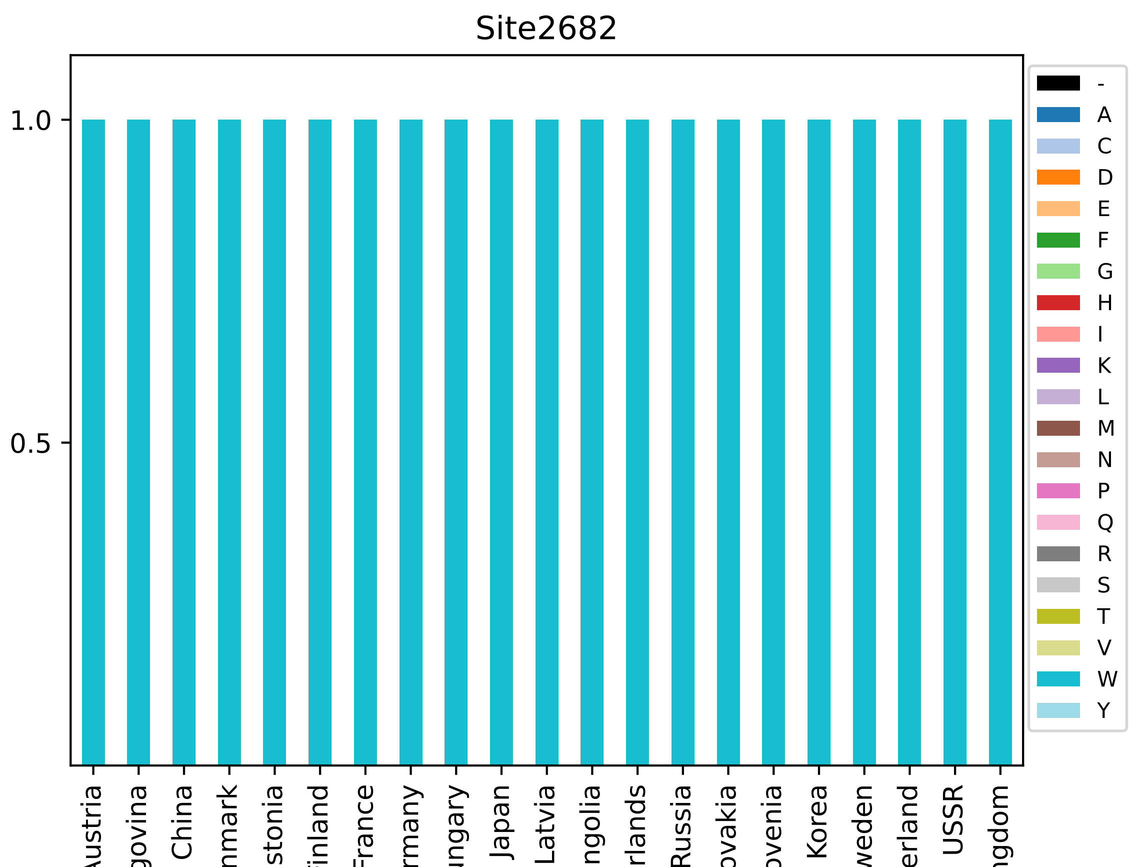
Site2682
1.0
0.5
-
A
C
D
E
F
G
H
I
K
L
M
N
P
Q
R
S
T
V
W
Y
China
Japan
Latvia
Russia
Korea
USSR

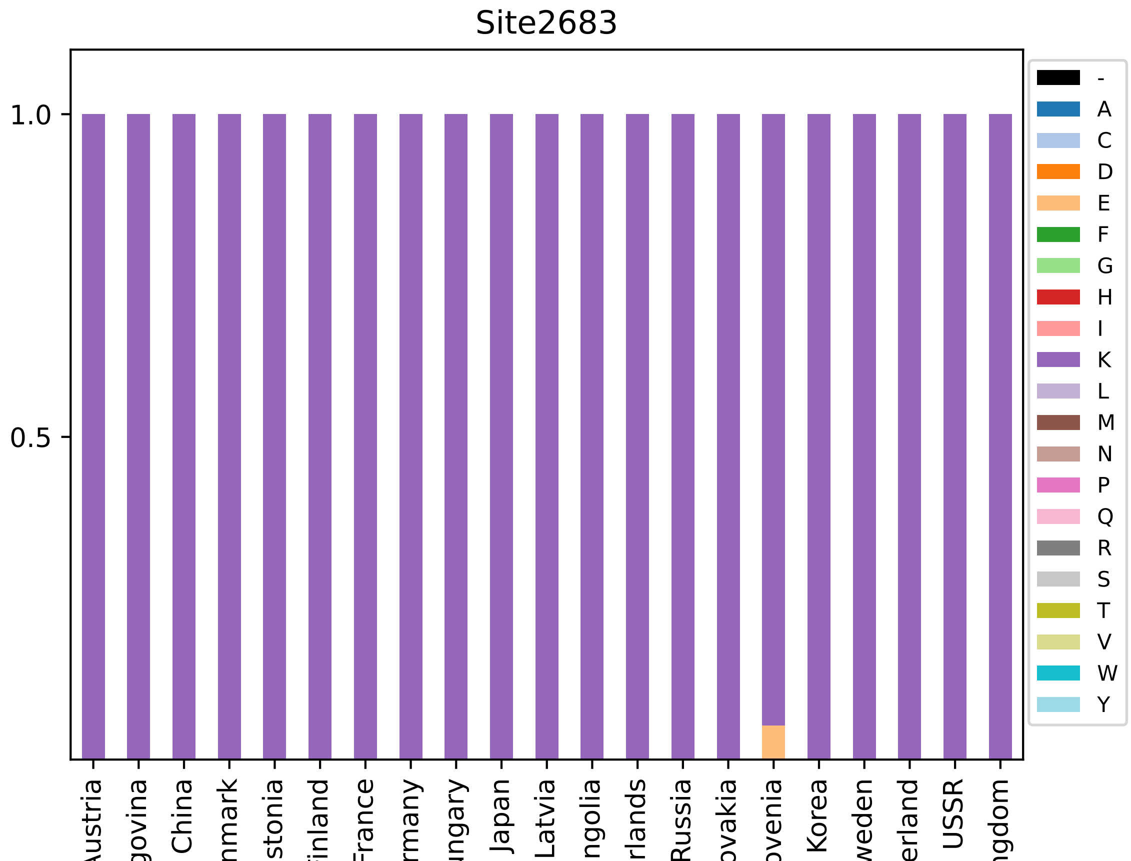
Site2683
1.0
0.5
-
A
C
D
E
F
G
H
I
K
L
M
N
P
Q
R
S
T
V
W
Y
China
Japan
Latvia
Russia
Korea
USSR

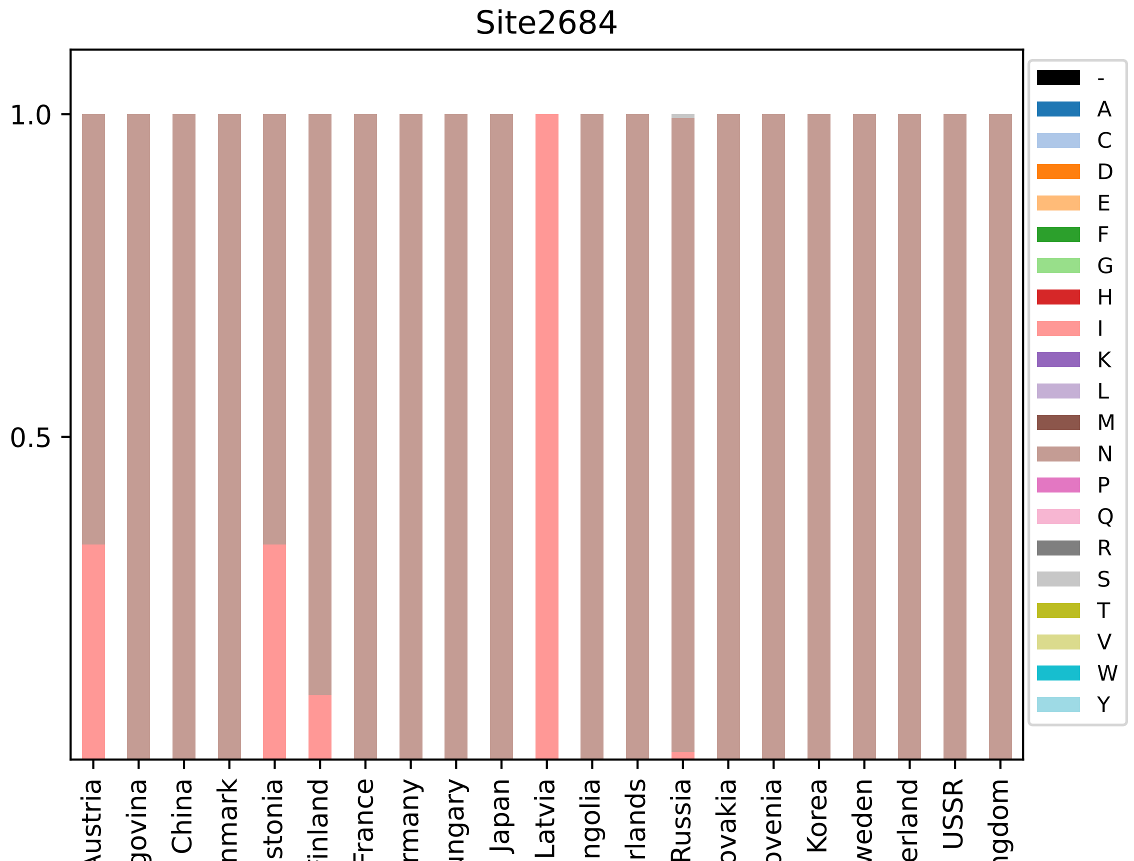

Site2684
1.0
0.5
-
A
C
D
E
F
G
H
I
K
L
M
N
P
Q
R
S
T
V
W
Y
Austria
govina
China
nmark
stonia
inland
France
rmany
ungary
Japan
Latvia
ngolia
rlands
Russia
ovakia
ovenia
Korea
weden
erland
USSR
ngdom

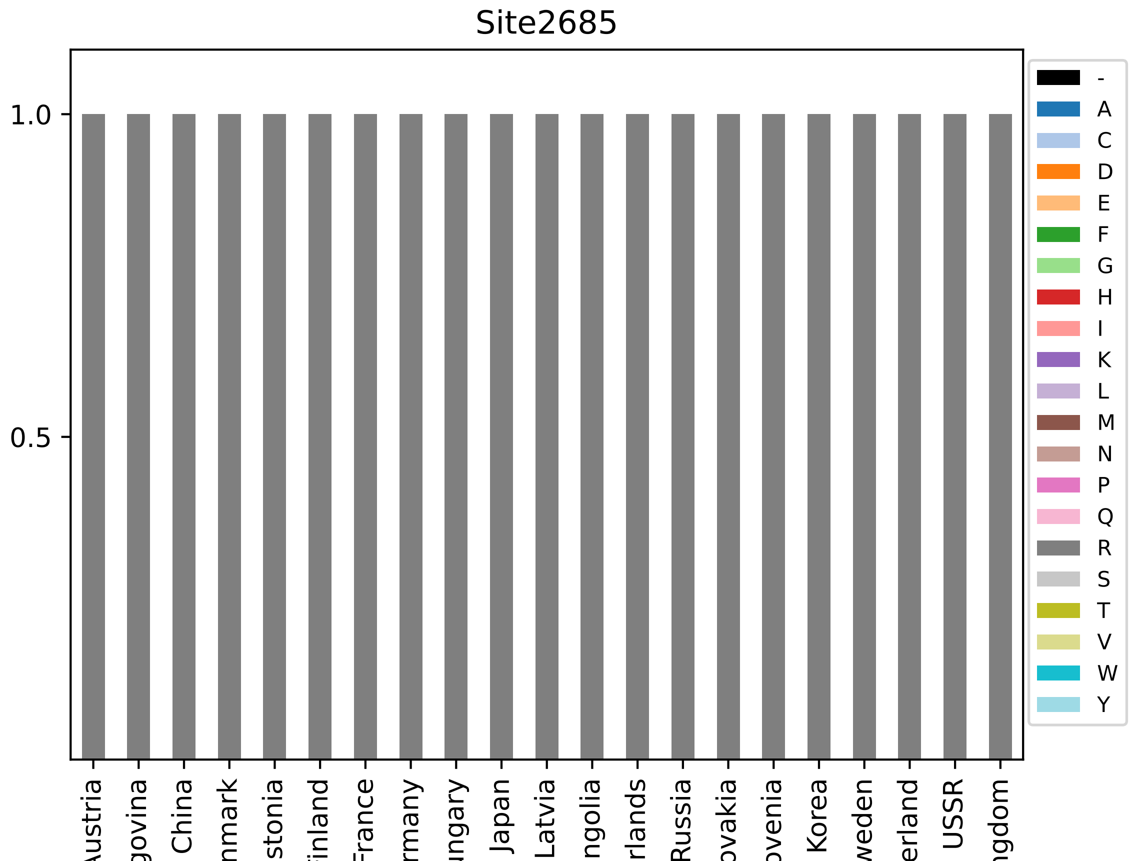

Site2685
1.0
0.5
China
Japan
Latvia
Russia
Korea
USSR
-
A
C
D
E
F
G
H
I
K
L
M
N
P
Q
R
S
T
V
W
Y

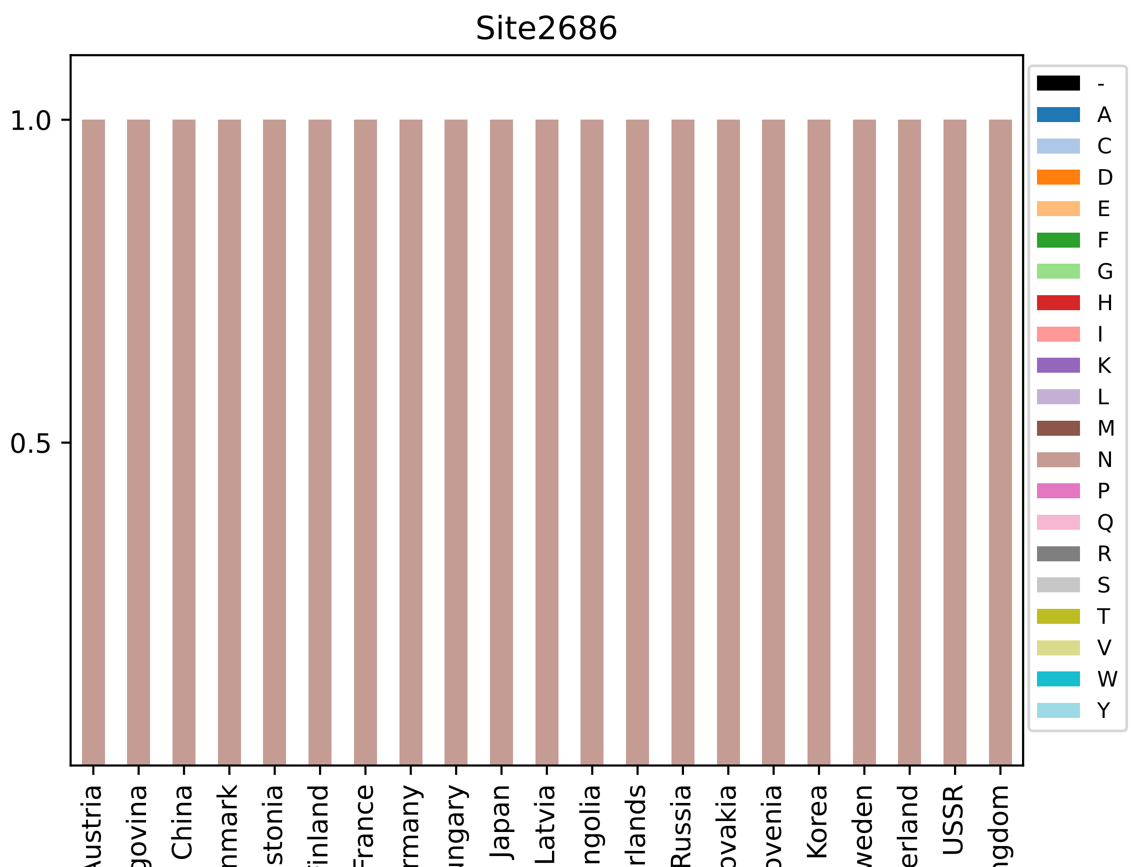

Site2686
1.0
0.5
-
A
C
D
E
F
G
H
I
K
L
M
N
P
Q
R
S
T
V
W
Y
China
Latvia
Russia
Korea
USSR

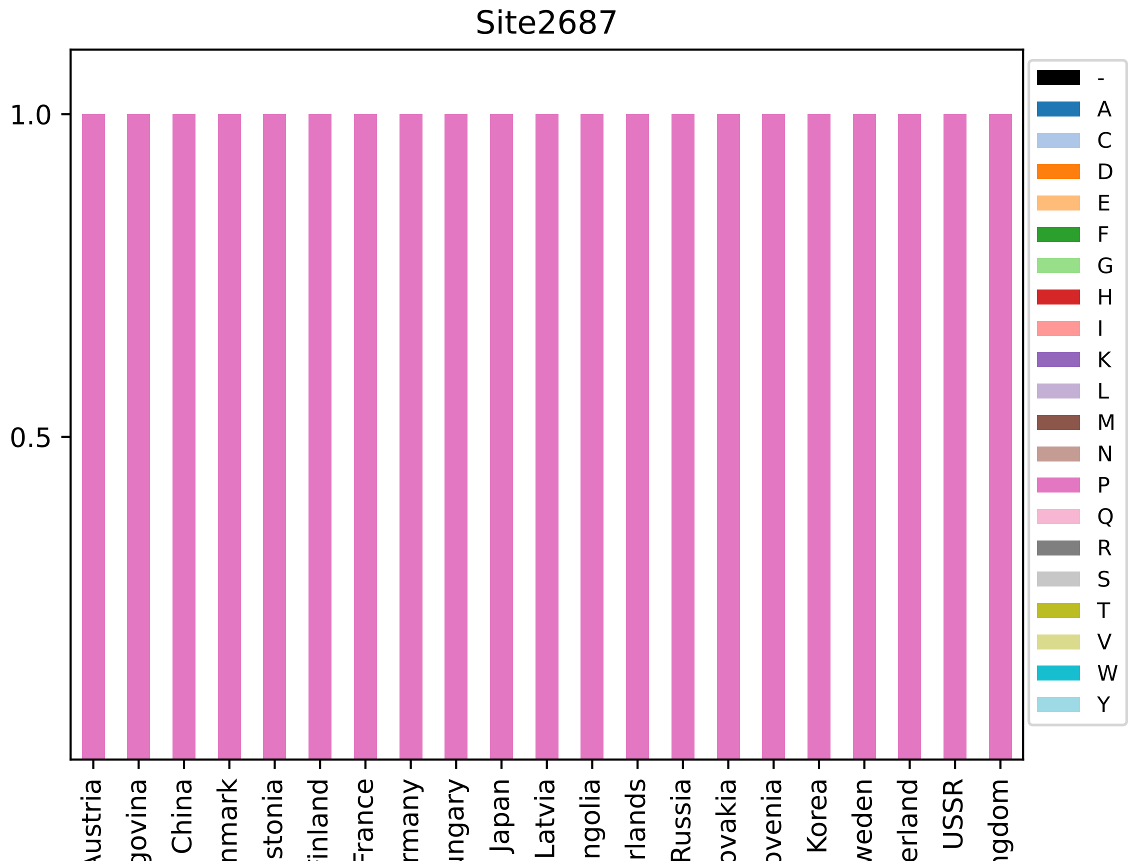

Site2687
1.0
0.5
-
A
C
D
E
F
G
H
I
K
L
M
N
P
Q
R
S
T
V
W
Y
Austria
govina
China
nmark
stonia
inland
France
rmany
ungary
Japan
Latvia
ngolia
rlands
Russia
ovakia
ovenia
Korea
weden
erland
USSR
ngdom

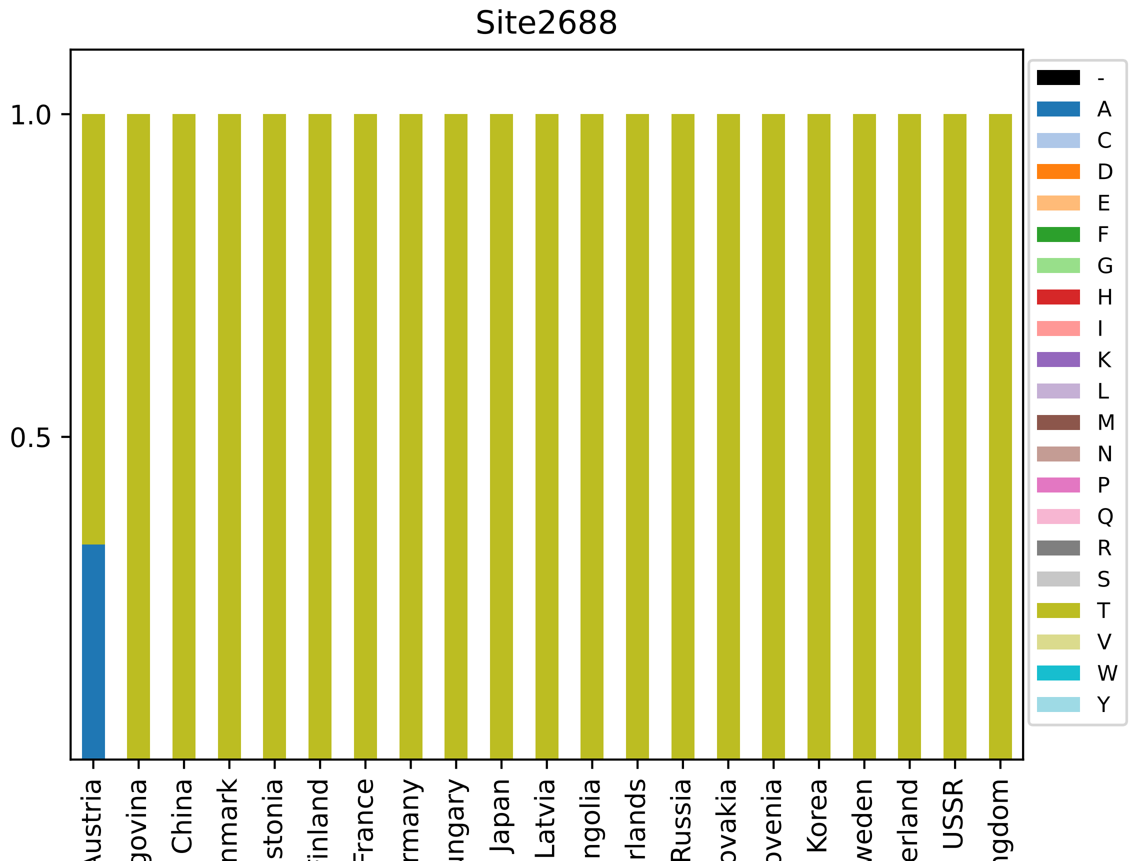
Site2688
1.0
0.5
-
A
C
D
E
F
G
H
I
K
L
M
N
P
Q
R
S
T
V
W
Y
Austria
govina
China
nmark
stonia
Finland
France
rmany
ungary
Japan
Latvia
ngolia
rlands
Russia
ovakia
ovenia
Korea
weden
erland
USSR
ngdom

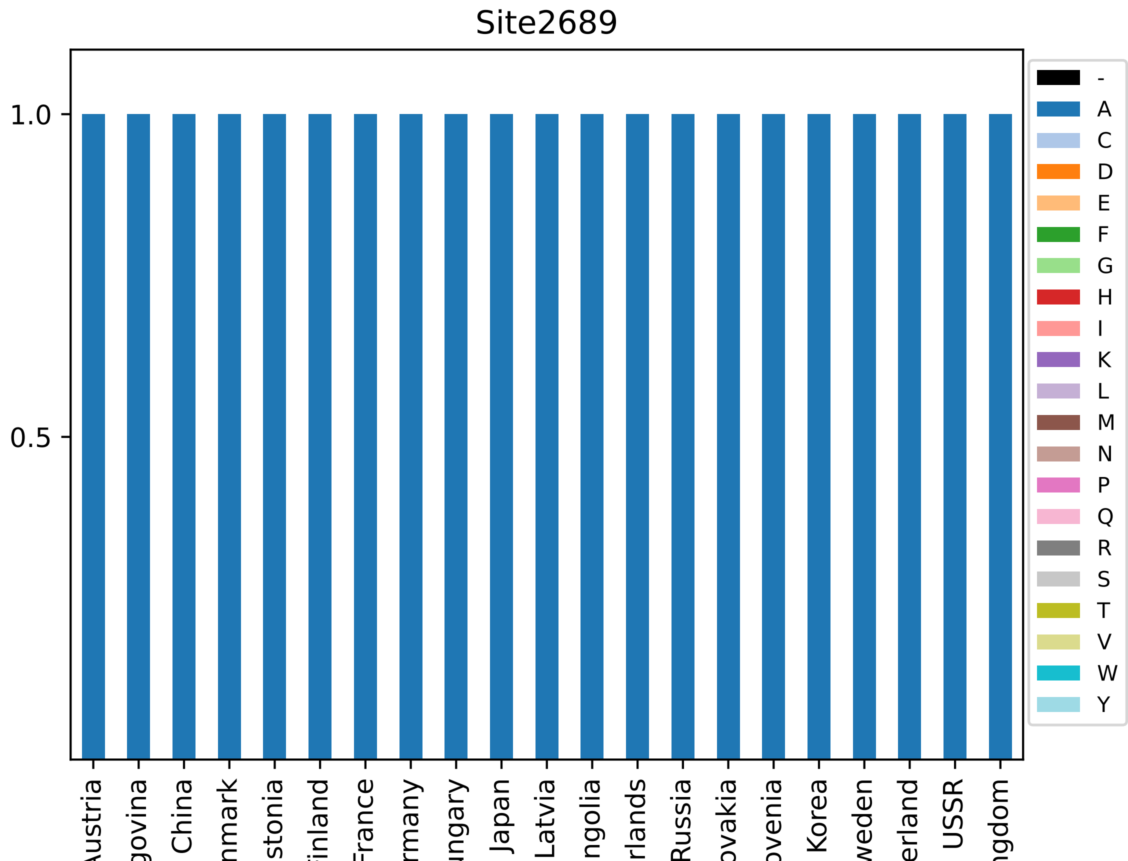
Site2689
1.0
0.5
-
A
C
D
E
F
G
H
I
K
L
M
N
P
Q
R
S
T
V
W
Y
Austria
govina
China
nmark
stonia
inland
France
rmany
ungary
Japan
Latvia
ngolia
rlands
Russia
ovakia
ovenia
Korea
weden
erland
USSR
ngdom

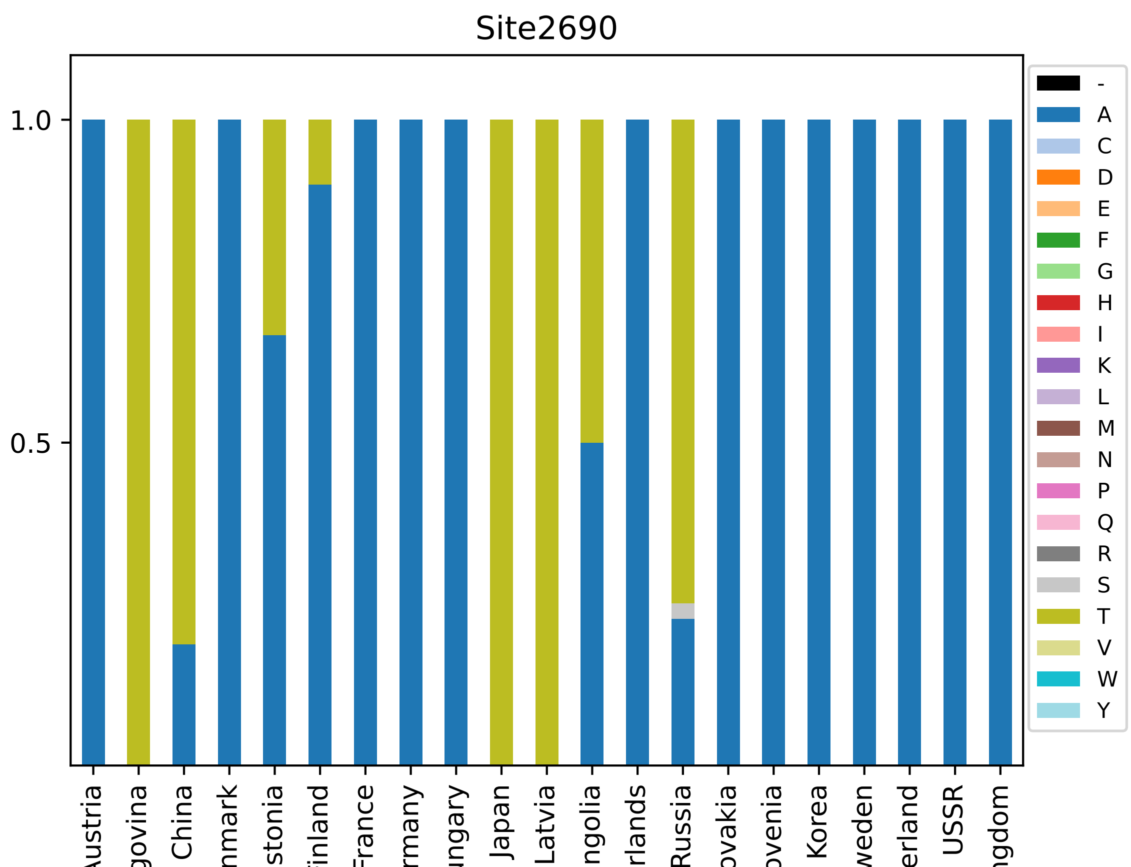
Site2690
1.0
0.5
-
A
C
D
E
F
G
H
I
K
L
M
N
P
Q
R
S
T
V
W
Y
China
Finland
France
Japan
Latvia
Russia
Korea
USSR

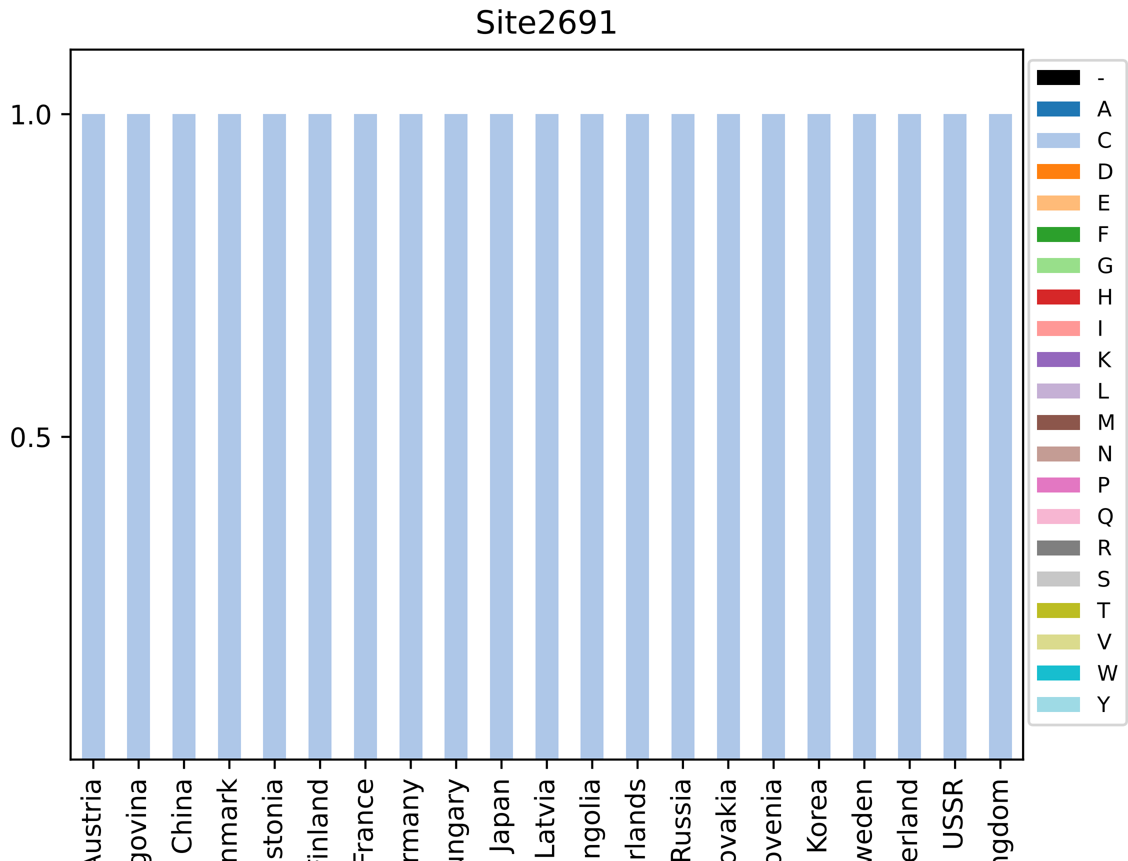

Site2691
1.0
0.5
-
A
C
D
E
F
G
H
I
K
L
M
N
P
Q
R
S
T
V
W
Y
China
Japan
Latvia
Russia
Korea
USSR

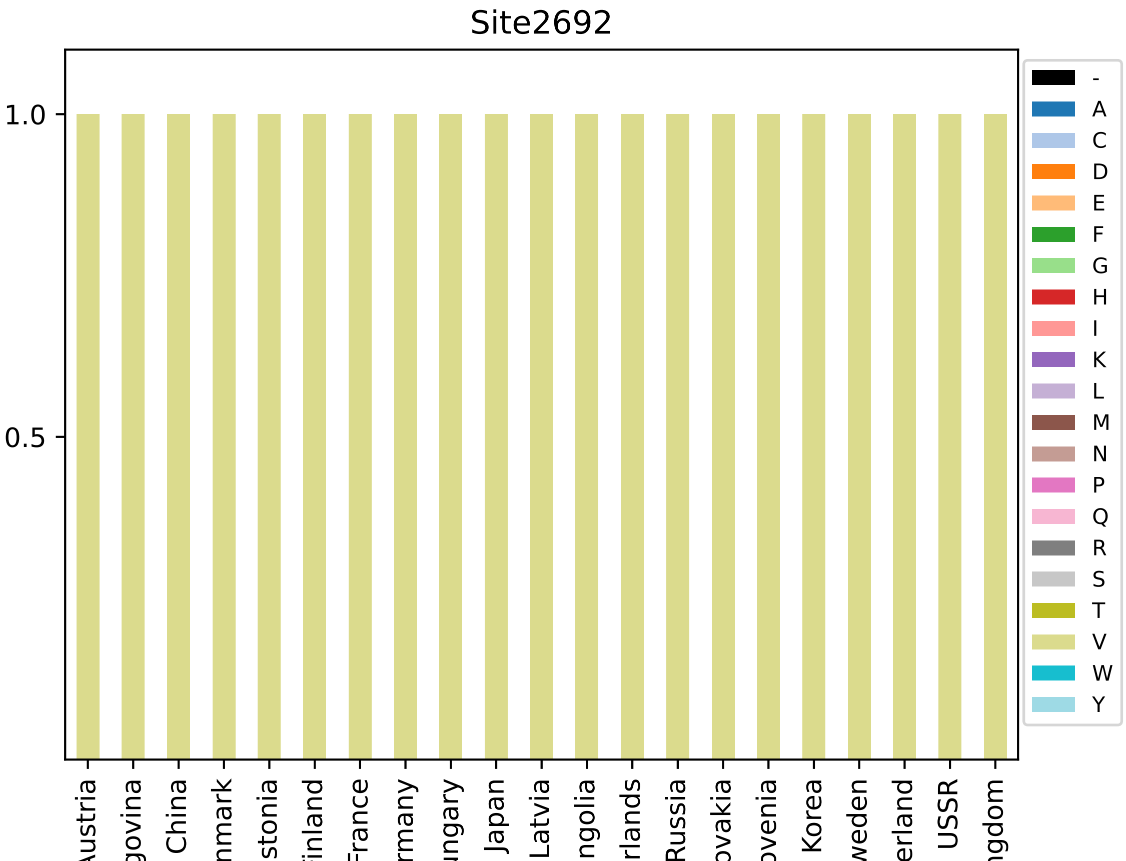
Site2692
1.0
0.5
-
A
C
D
E
F
G
H
I
K
L
M
N
P
Q
R
S
T
V
W
Y
Austria
China
Japan
Latvia
Russia
Korea
USSR

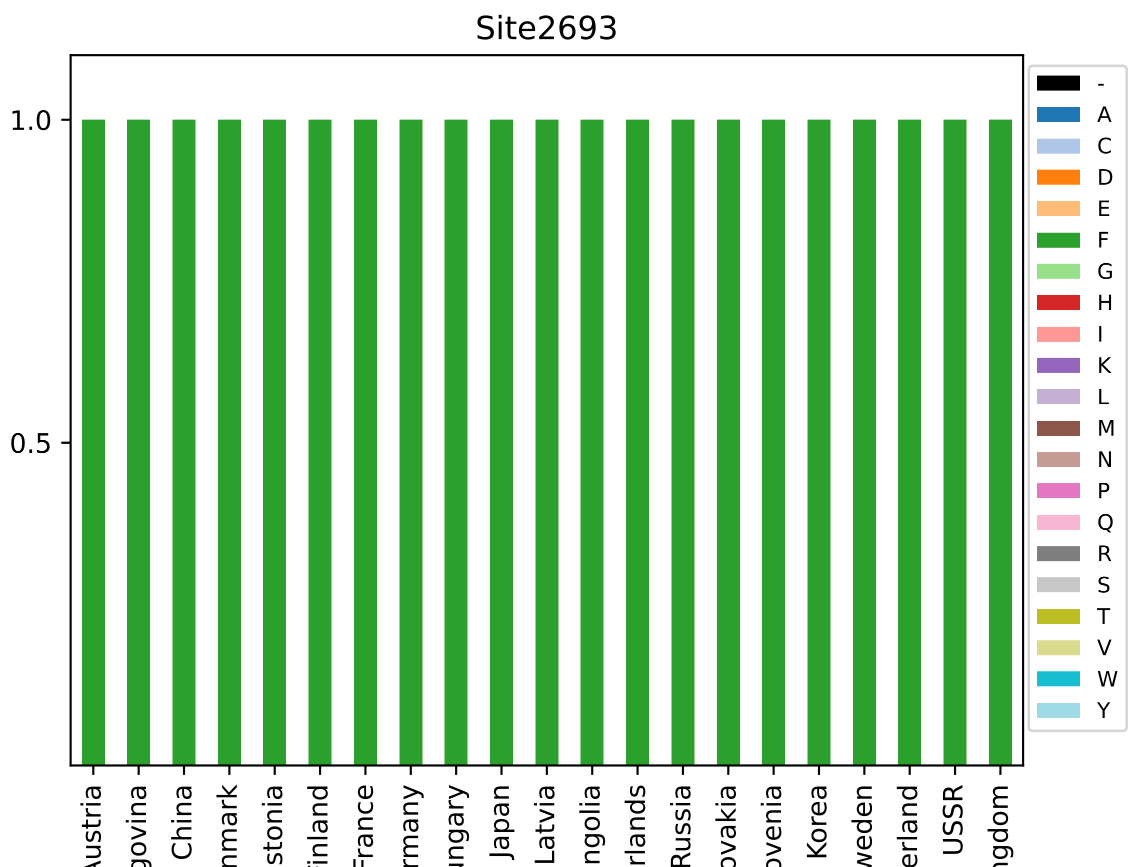
Site2693
1.0
0.5
-
A
C
D
E
F
G
H
I
K
L
M
N
P
Q
R
S
T
V
W
Y
Austria
China
Japan
Latvia
Russia
Korea
USSR

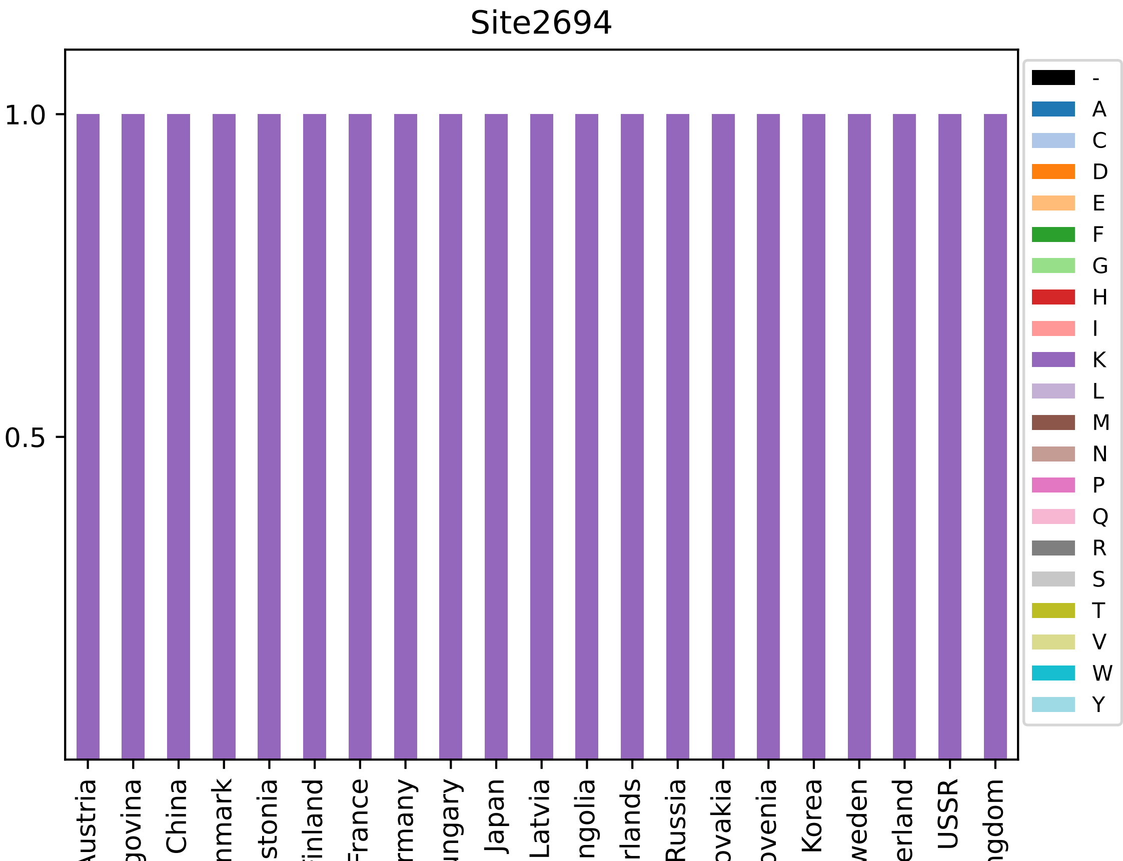
Site2694
1.0
0.5
-
A
C
D
E
F
G
H
I
K
L
M
N
P
Q
R
S
T
V
W
Y
Austria
China
Japan
Latvia
Russia
Korea
USSR

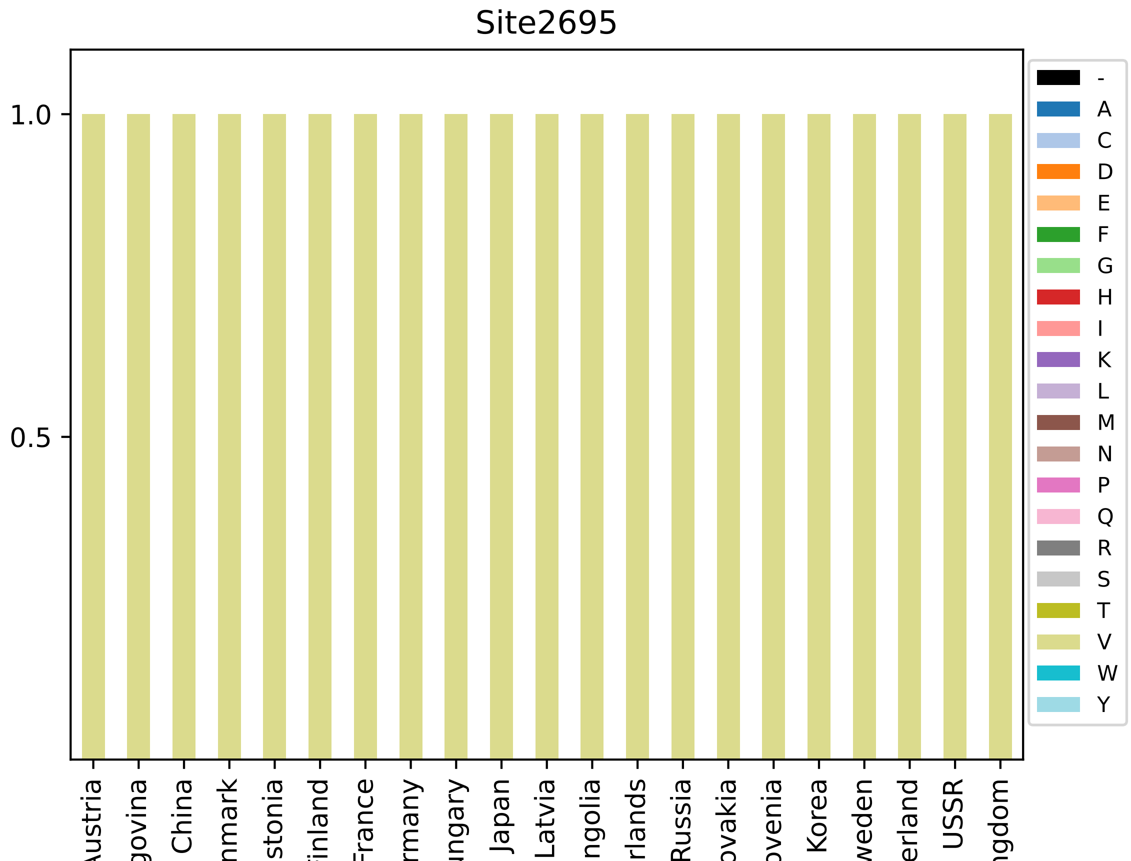
Site2695
1.0
0.5
Austria
govina
China
nmark
stonia
Finland
France
rmany
ungary
Japan
Latvia
ngolia
rlands
Russia
ovakia
ovenia
Korea
weden
erland
USSR
ngdom
-
A
C
D
E
F
G
H
I
K
L
M
N
P
Q
R
S
T
V
W
Y

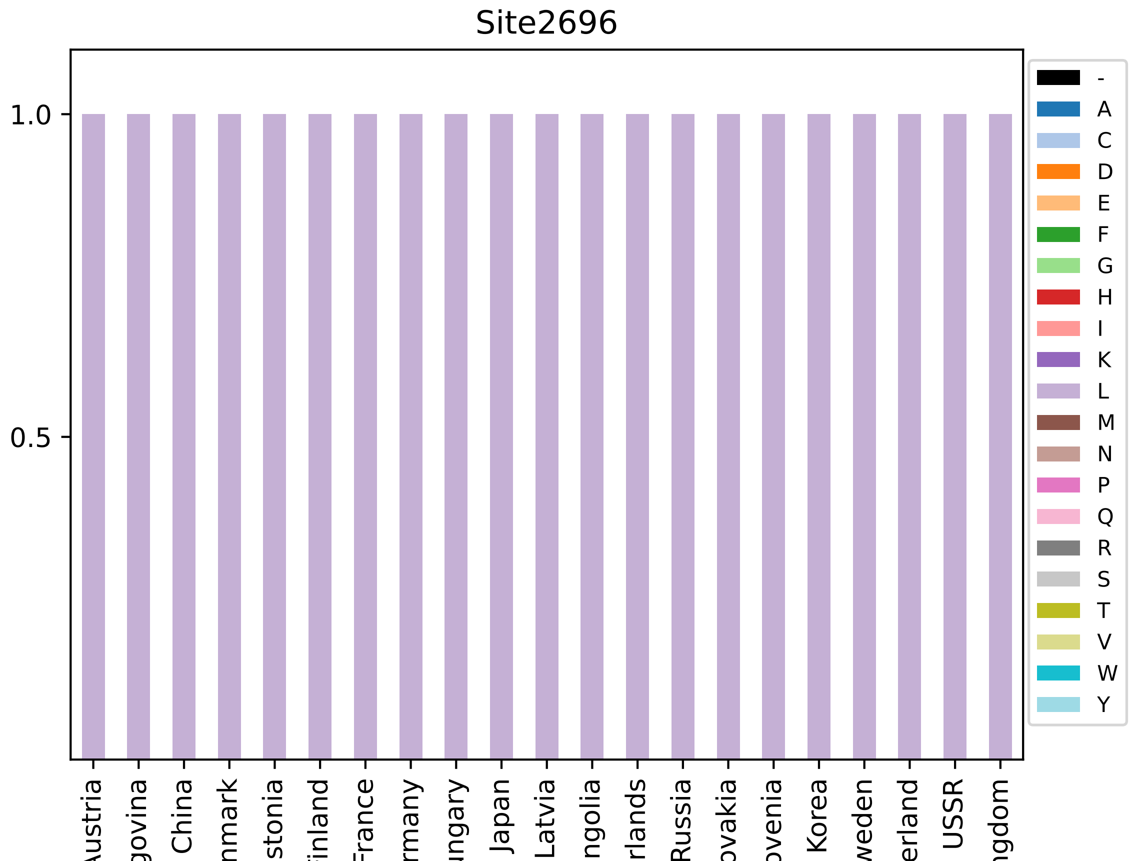
Site2696
1.0
0.5
China
Japan
Latvia
Russia
Korea
USSR
-
A
C
D
E
F
G
H
I
K
L
M
N
P
Q
R
S
T
V
W
Y

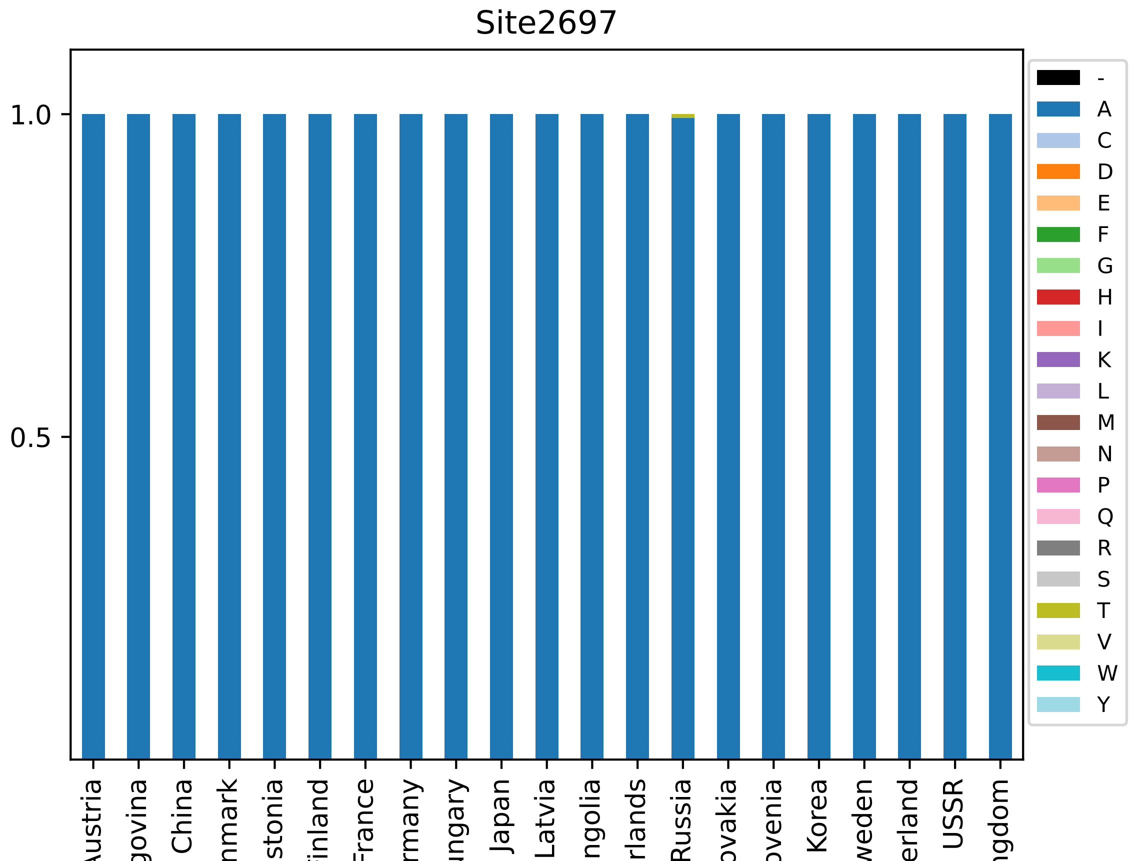

Site2697
1.0
0.5
-
A
C
D
E
F
G
H
I
K
L
M
N
P
Q
R
S
T
V
W
Y
Austria
govina
China
nmark
stonia
Finland
France
rmany
ungary
Japan
Latvia
ngolia
rlands
Russia
ovakia
ovenia
Korea
weden
erland
USSR
ngdom

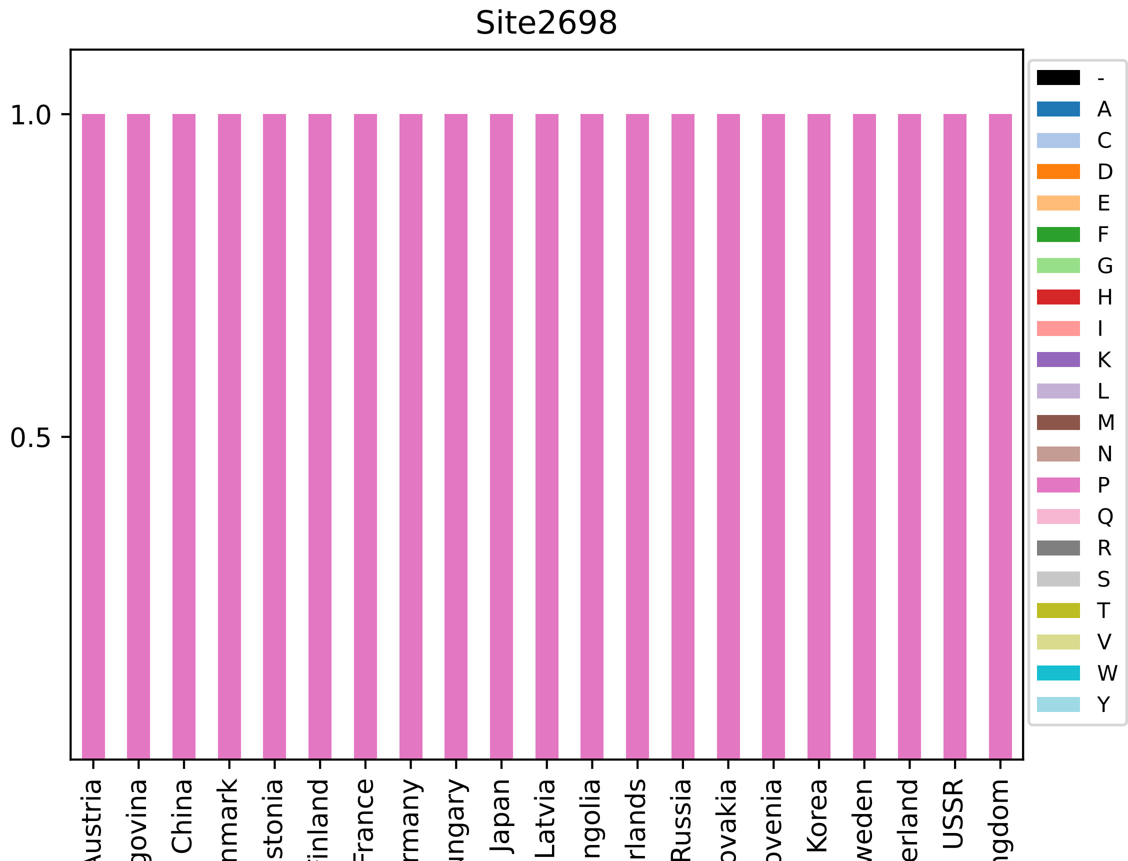

Site2698
1.0
0.5
-
A
C
D
E
F
G
H
I
K
L
M
N
P
Q
R
S
T
V
W
Y
Austria
govina
China
nmark
stonia
inland
France
rmany
ungary
Japan
Latvia
ngolia
rlands
Russia
ovakia
ovenia
Korea
weden
erland
USSR
ngdom

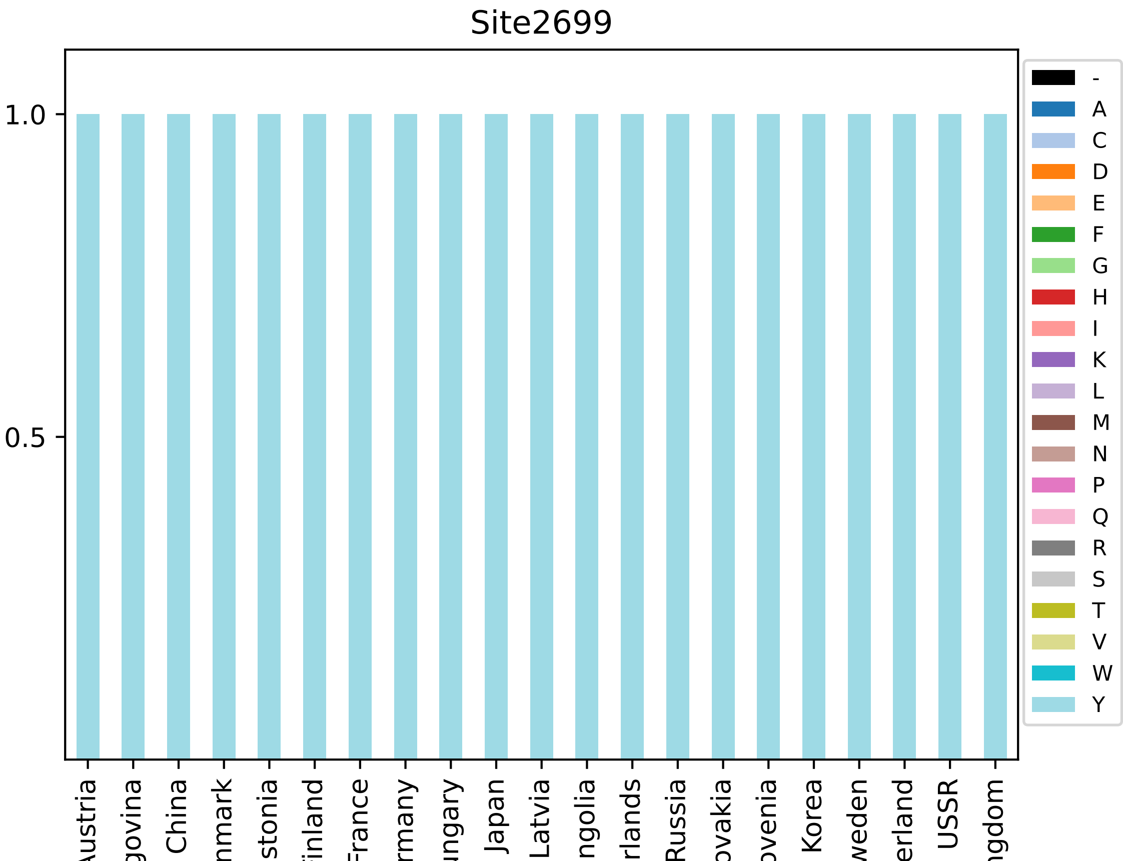
Site2699
1.0
0.5
-
A
C
D
E
F
G
H
I
K
L
M
N
P
Q
R
S
T
V
W
Y
ustria
govina
China
nmark
stonia
inland
rance
rmany
ungary
Japan
Latvia
ngolia
rlands
Russia
ovakia
ovenia
Korea
weden
erland
USSR
ngdom

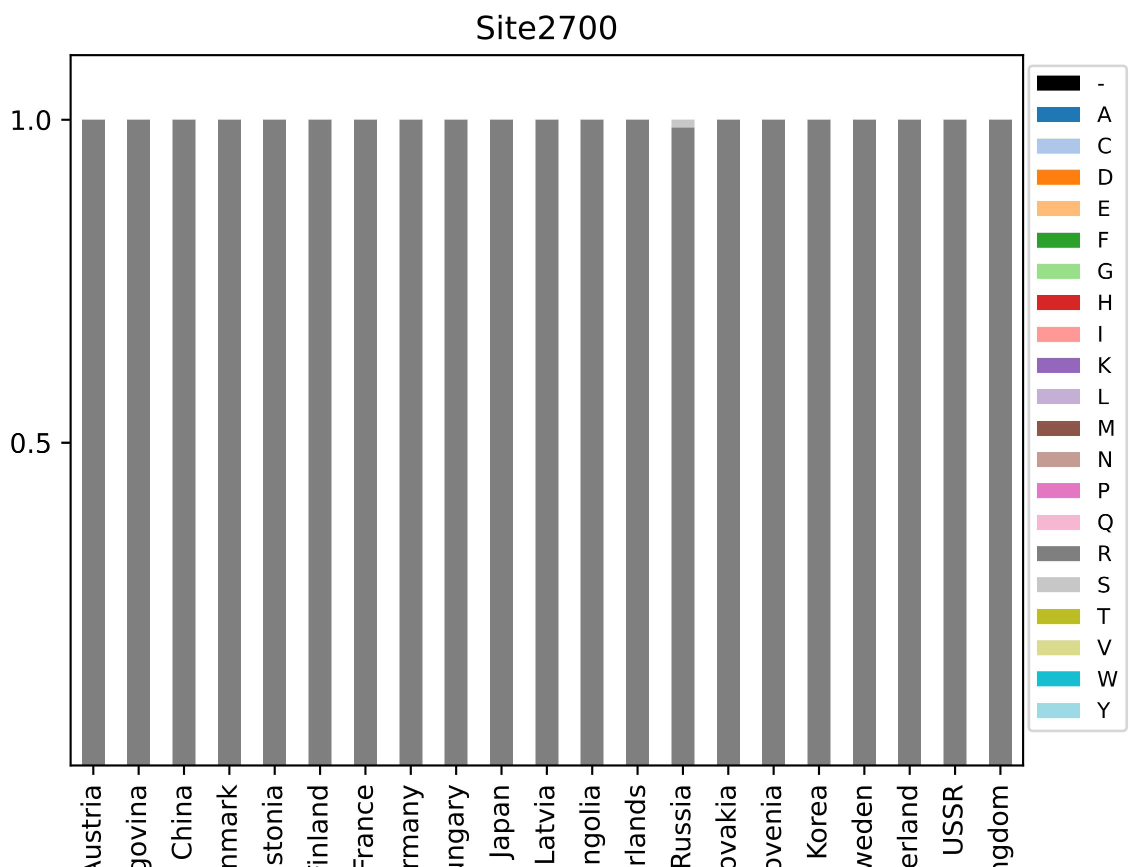
Site2700
1.0
0.5
-
A
C
D
E
F
G
H
I
K
L
M
N
P
Q
R
S
T
V
W
Y
Austria
govina
China
nmark
stonia
Finland
France
rmany
ungary
Japan
Latvia
ngolia
rlands
Russia
ovakia
ovenia
Korea
weden
erland
USSR
ngdom

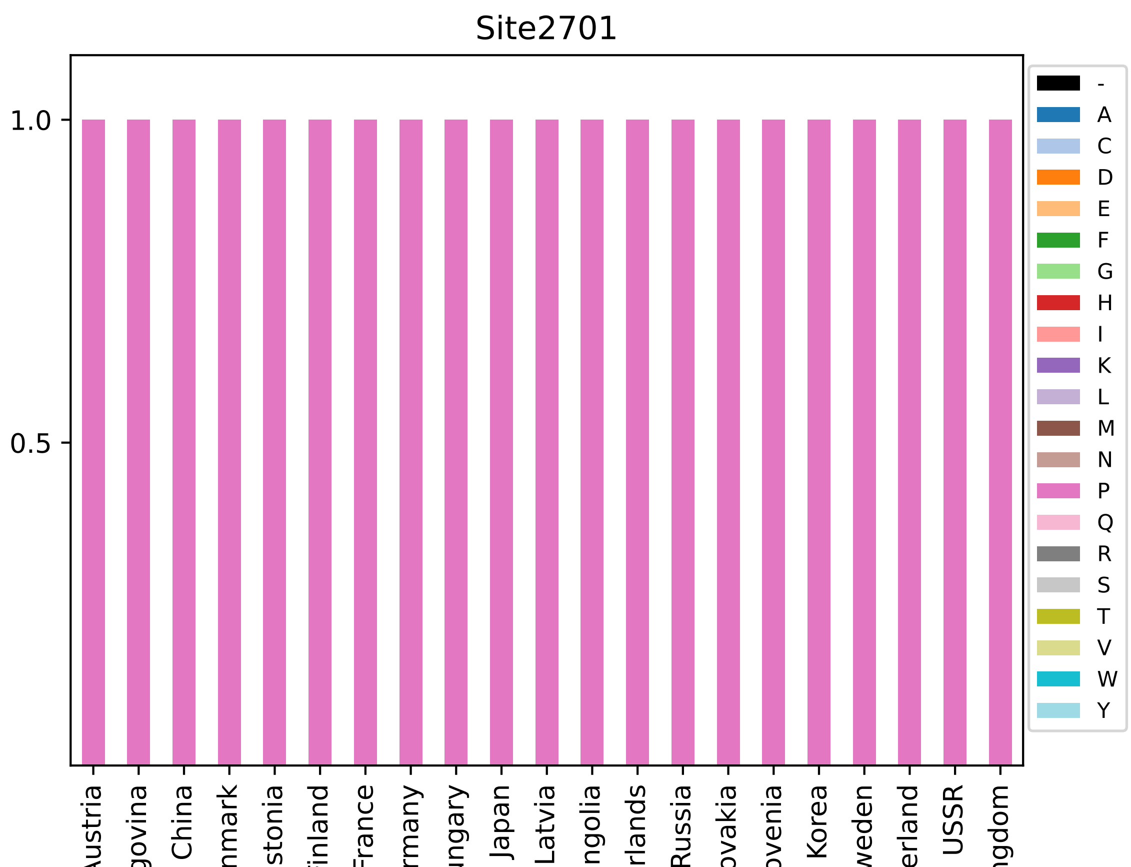
Site2701
1.0
0.5
-
A
C
D
E
F
G
H
I
K
L
M
N
P
Q
R
S
T
V
W
Y
China
Japan
Latvia
Russia
Korea
USSR

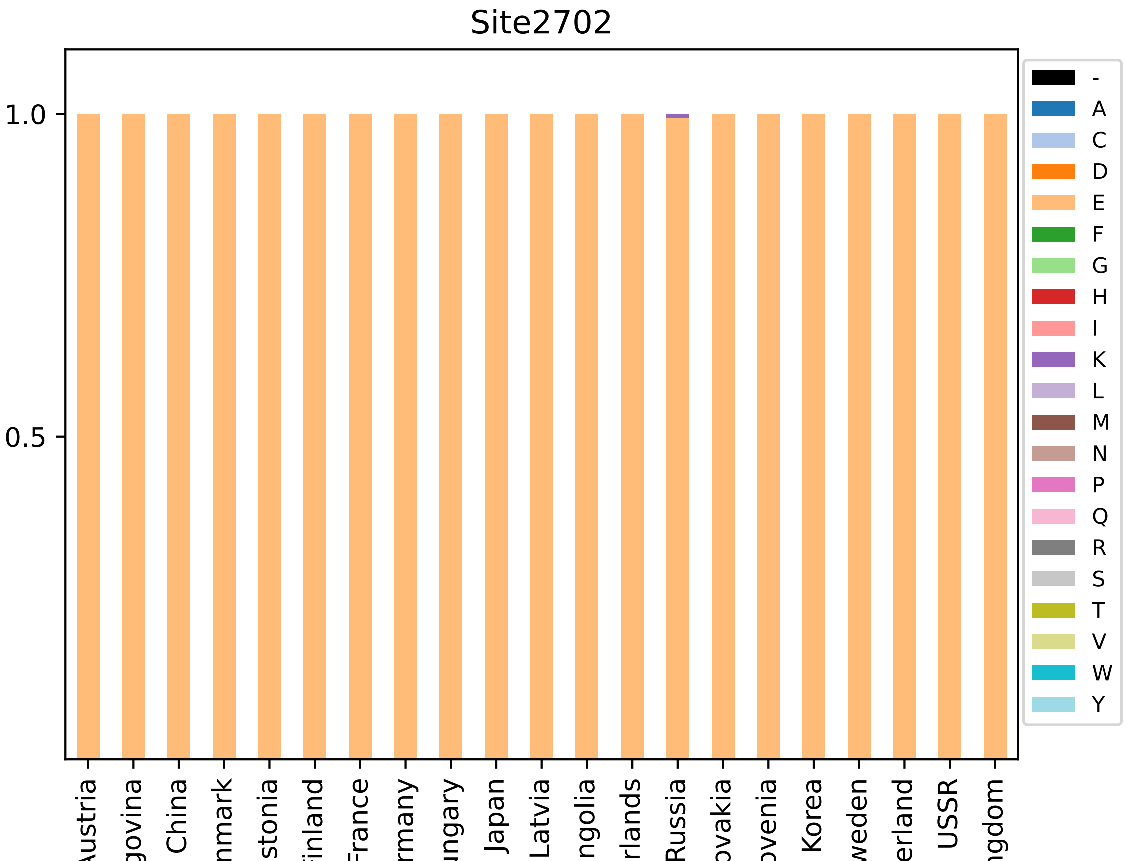

Site2702
1.0
0.5
-
A
C
D
E
F
G
H
I
K
L
M
N
P
Q
R
S
T
V
W
Y
Austria
govina
China
nmark
stonia
Finland
France
rmany
ungary
Japan
Latvia
ngolia
rlands
Russia
ovakia
ovenia
Korea
weden
erland
USSR
ngdom

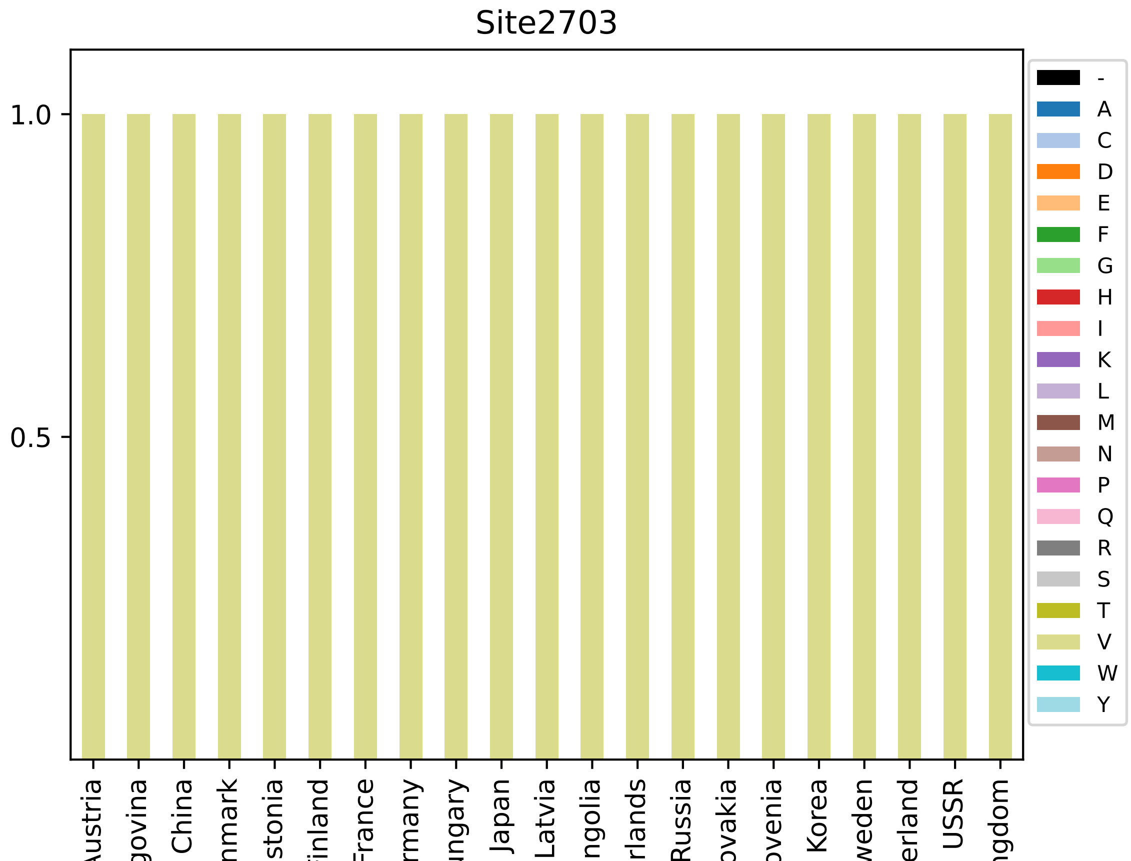
Site2703
1.0
0.5
-
A
C
D
E
F
G
H
I
K
L
M
N
P
Q
R
S
T
V
W
Y
China
Japan
Latvia
Korea
USSR

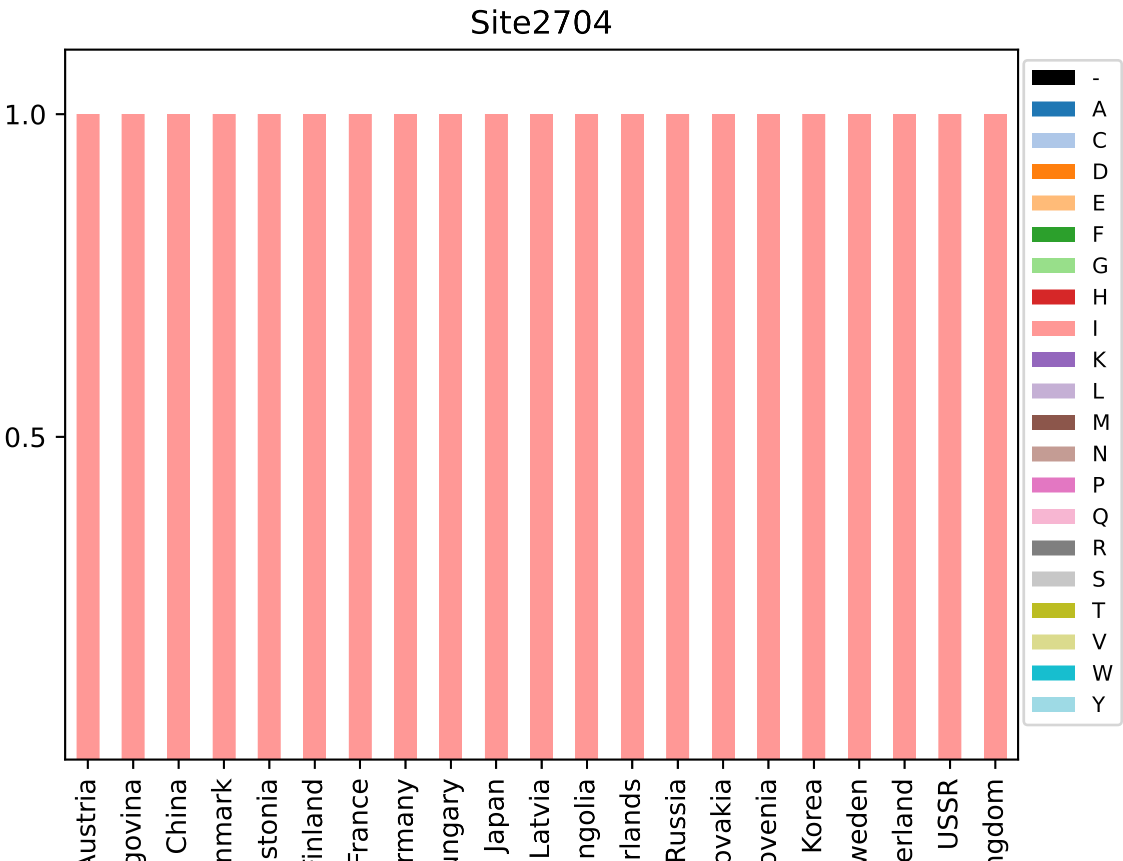

Site2704
1.0
0.5
-
A
C
D
E
F
G
H
I
K
L
M
N
P
Q
R
S
T
V
W
Y
China
Japan
Latvia
Russia
Korea
USSR

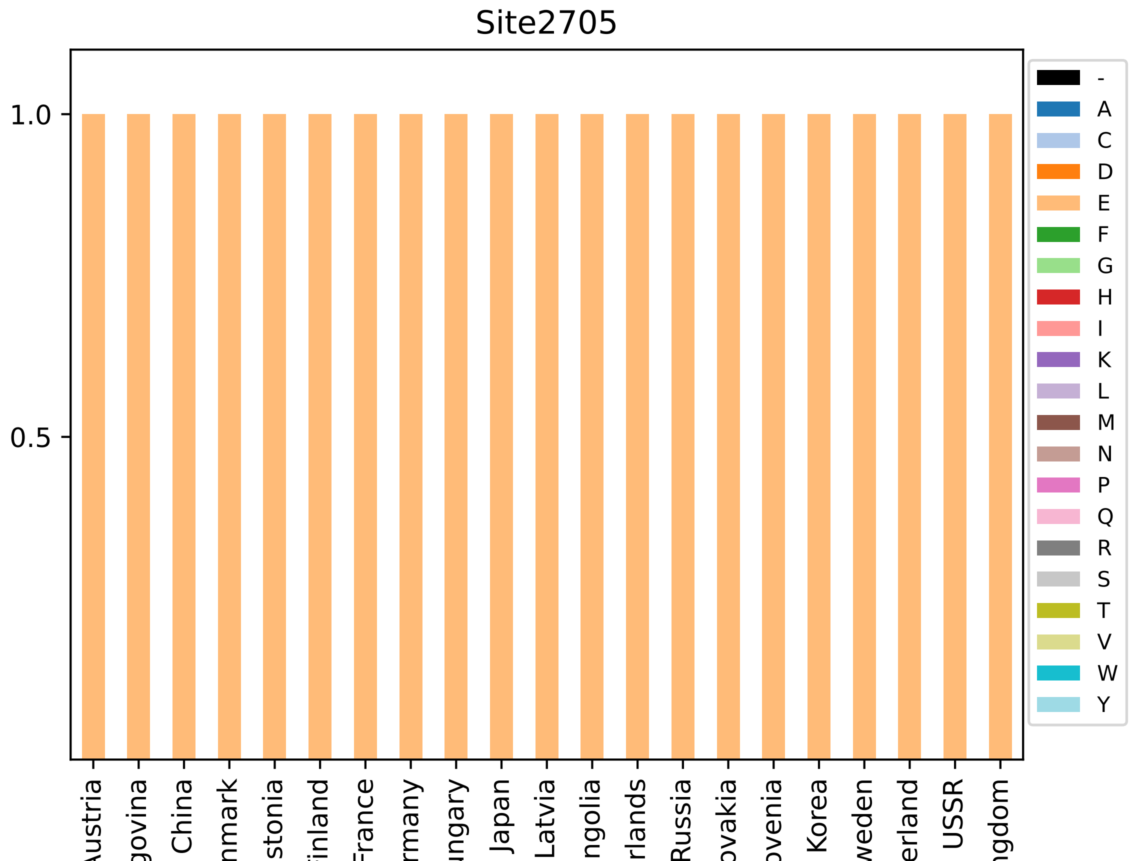
Site2705
1.0
0.5
-
A
C
D
E
F
G
H
I
K
L
M
N
P
Q
R
S
T
V
W
Y
China
Japan
Latvia
Russia
Korea
USSR

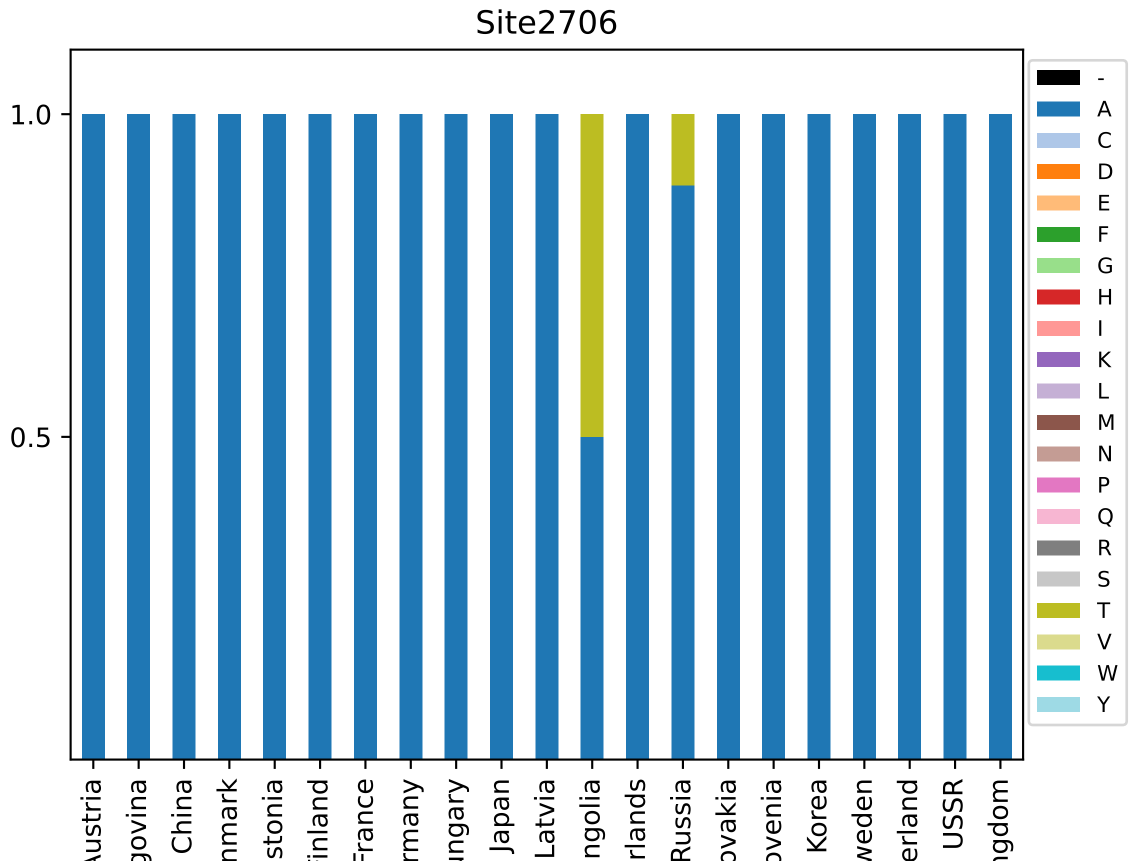

Site2706
1.0
0.5
-
A
C
D
E
F
G
H
I
K
L
M
N
P
Q
R
S
T
V
W
Y
Austria
govina
China
nmark
stonia
inland
France
rmany
ungary
Japan
Latvia
ngolia
rlands
Russia
ovakia
ovenia
Korea
weden
erland
USSR
ngdom

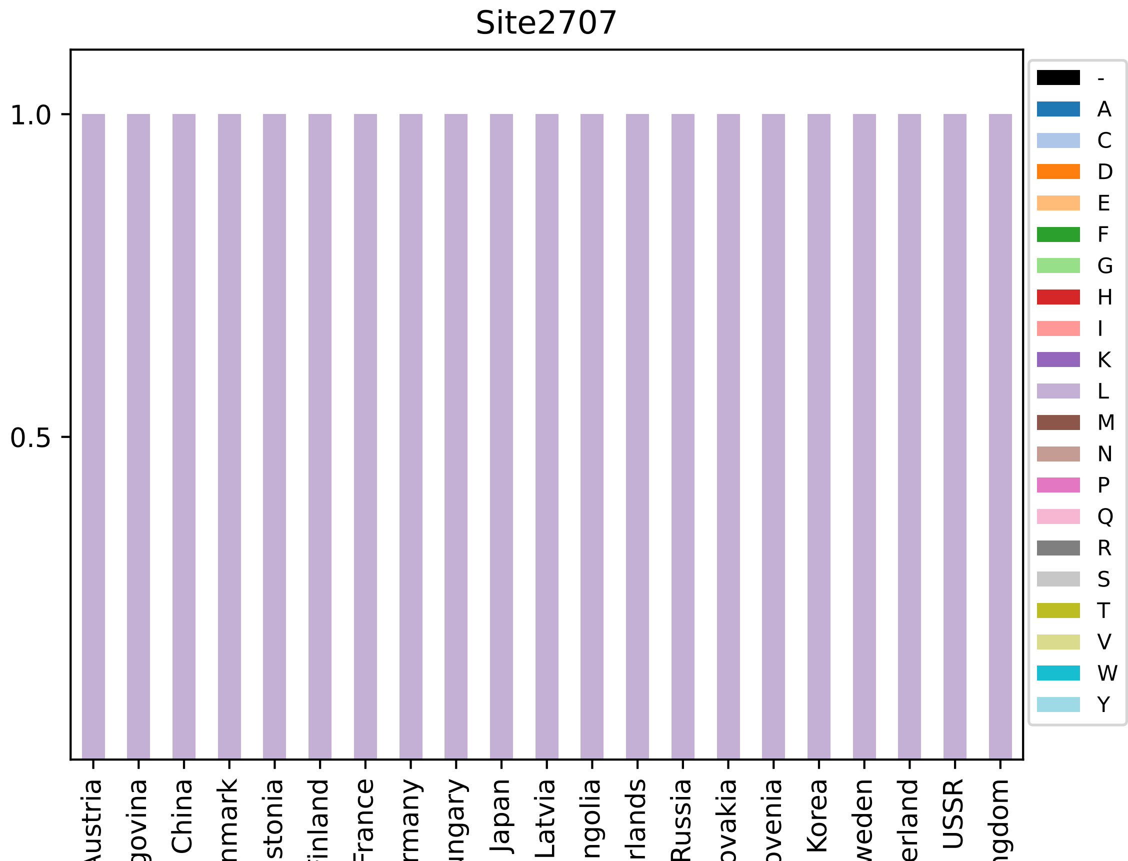
Site2707
1.0
0.5
-
A
C
D
E
F
G
H
I
K
L
M
N
P
Q
R
S
T
V
W
Y
Austria
govina
China
nmark
stonia
Finland
France
rmany
ungary
Japan
Latvia
ngolia
rlands
Russia
ovakia
ovenia
Korea
weden
erland
USSR
ngdom

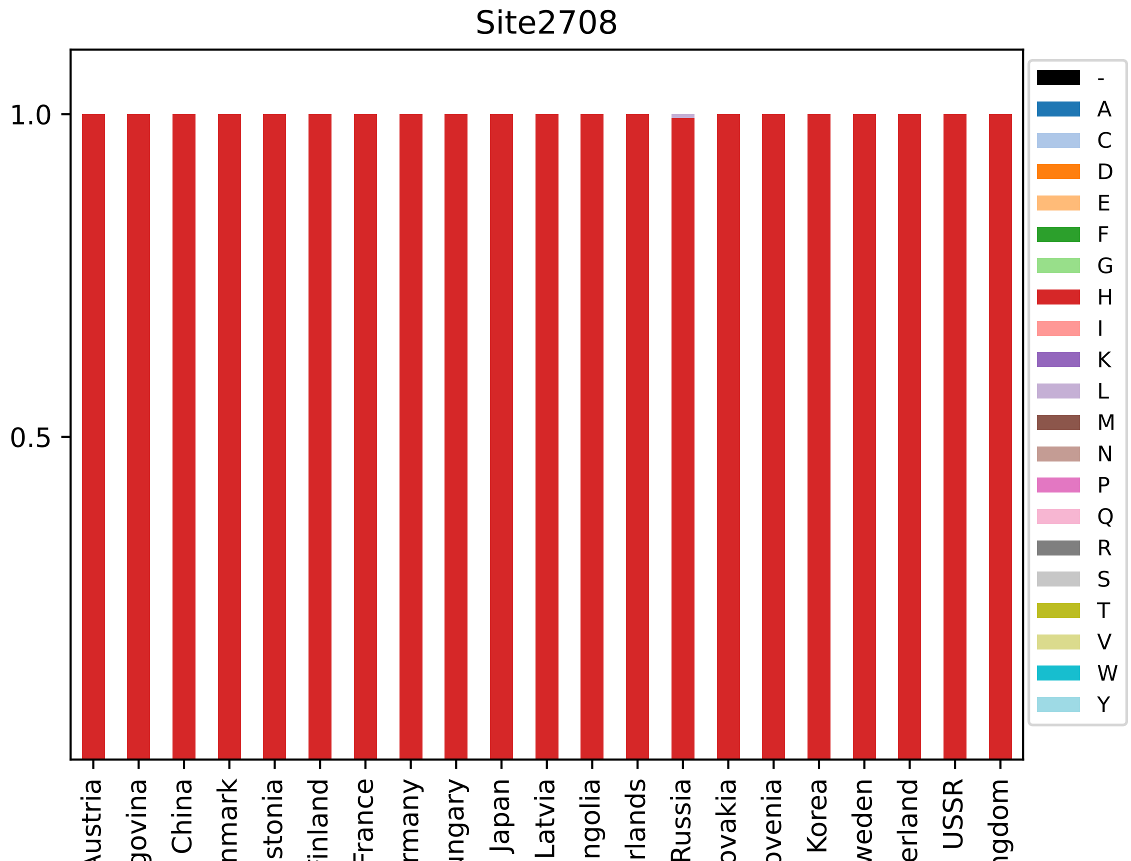
Site2708
1.0
0.5
-
A
C
D
E
F
G
H
I
K
L
M
N
P
Q
R
S
T
V
W
Y
China
Japan
Latvia
Russia
Korea
USSR

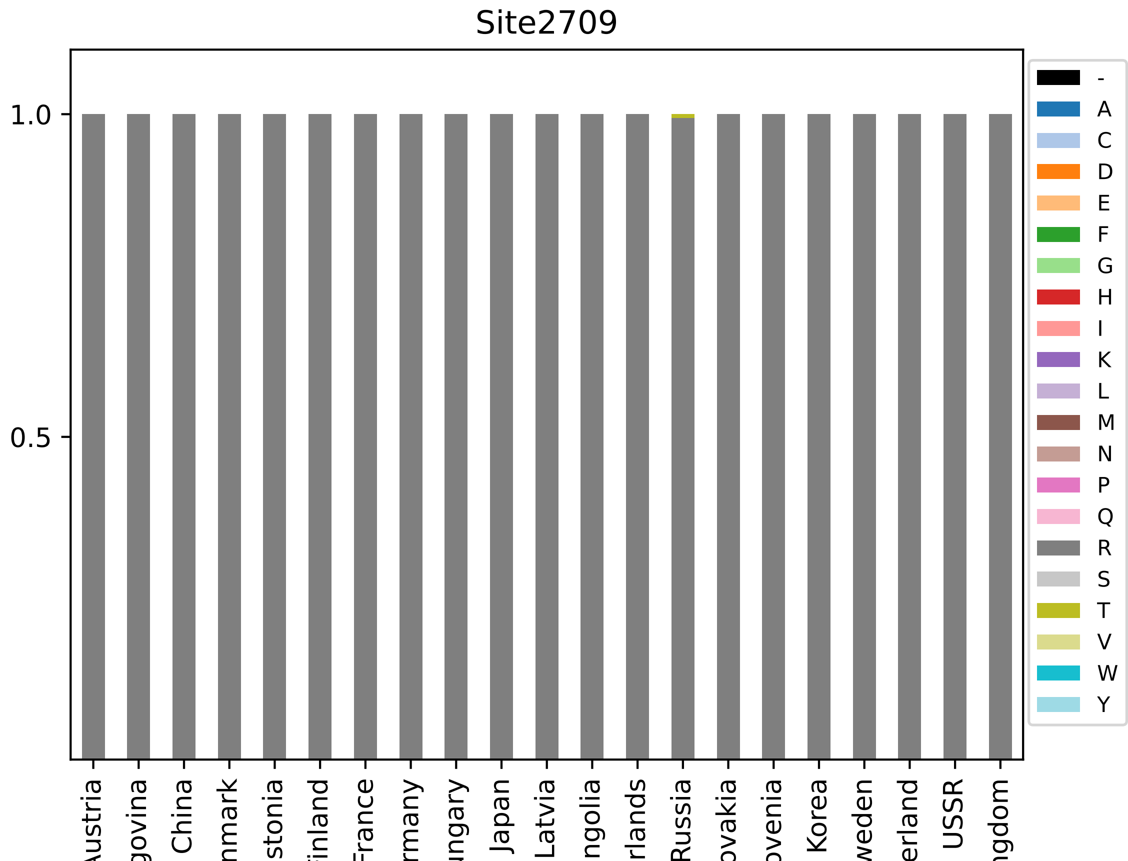

Site2709
1.0
0.5
-
A
C
D
E
F
G
H
I
K
L
M
N
P
Q
R
S
T
V
W
Y
Austria
govina
China
nmark
stonia
Finland
France
rmany
ungary
Japan
Latvia
ngolia
rlands
Russia
ovakia
ovenia
Korea
weden
erland
USSR
ngdom

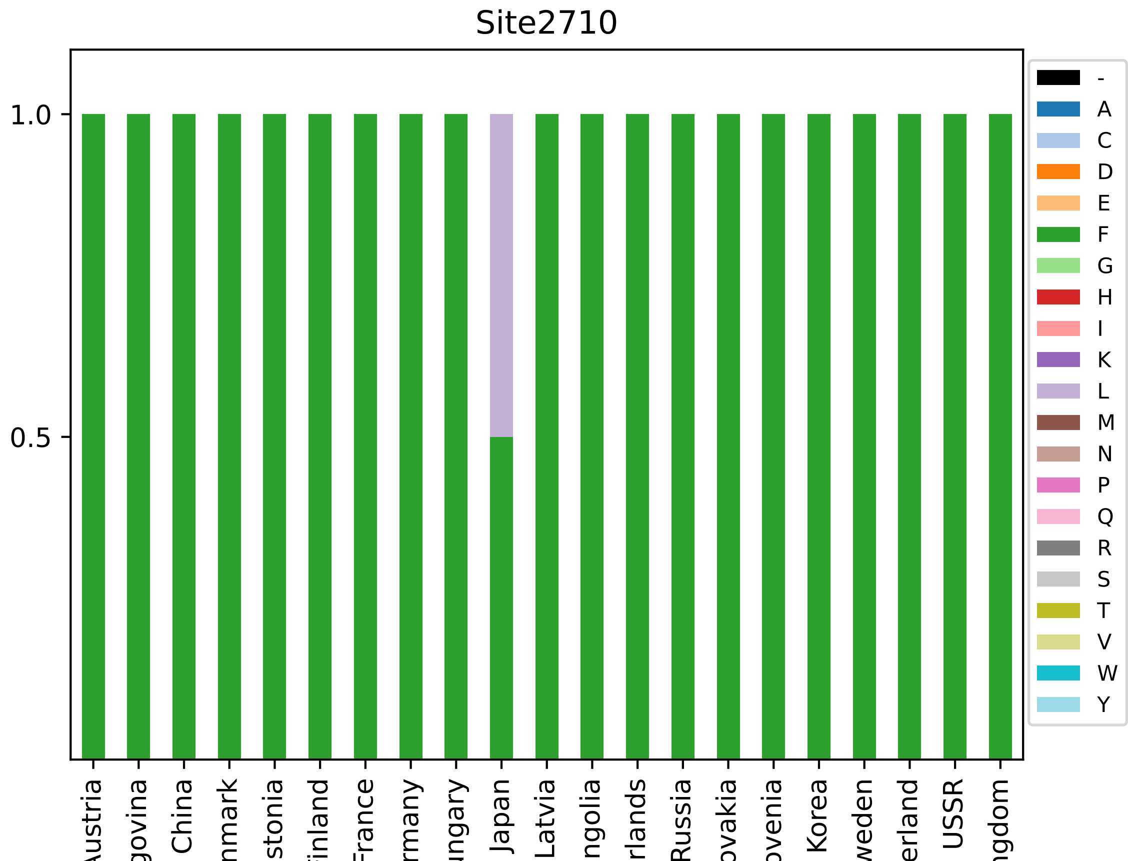

Site2710
1.0
0.5
-
A
C
D
E
F
G
H
I
K
L
M
N
P
Q
R
S
T
V
W
Y
Austria
govina
China
nmark
stonia
inland
France
rmany
ungary
Japan
Latvia
ngolia
rlands
Russia
ovakia
ovenia
Korea
weden
erland
USSR
ngdom

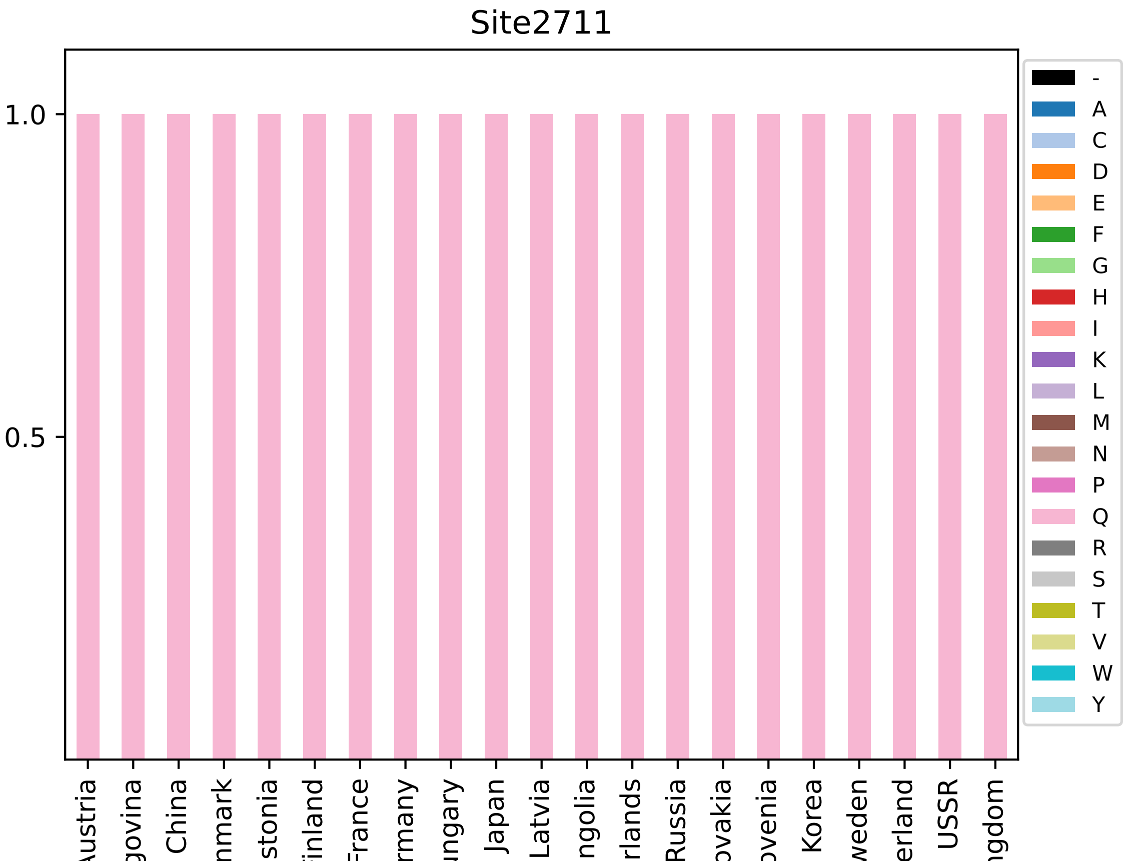

Site2711
1.0
0.5
-
A
C
D
E
F
G
H
I
K
L
M
N
P
Q
R
S
T
V
W
Y
China
Japan
Latvia
Russia
Korea
USSR

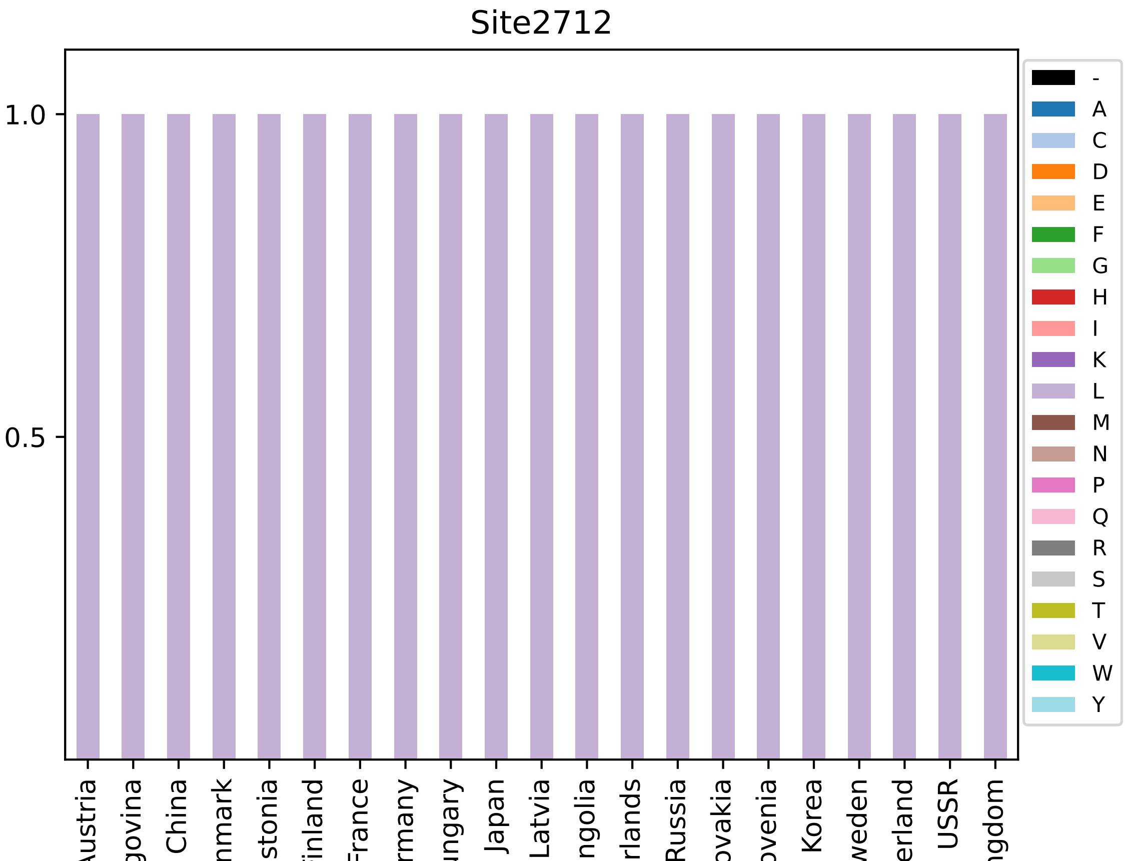

Site2712
1.0
0.5
-
A
C
D
E
F
G
H
I
K
L
M
N
P
Q
R
S
T
V
W
Y
China
Japan
Latvia
Russia
Korea
USSR

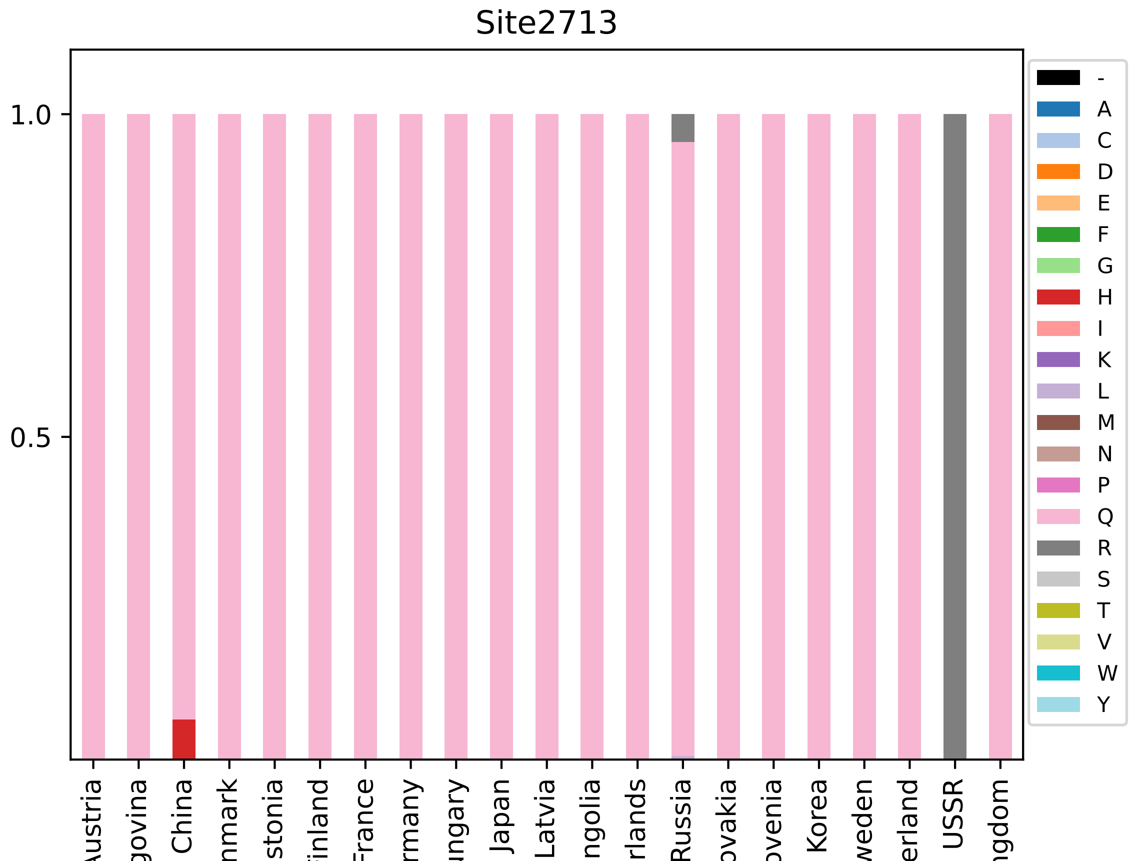
Site2713
1.0
0.5
-
A
C
D
E
F
G
H
I
K
L
M
N
P
Q
R
S
T
V
W
Y
Austria
China
Estonia
Finland
France
Japan
Latvia
Russia
Korea
USSR

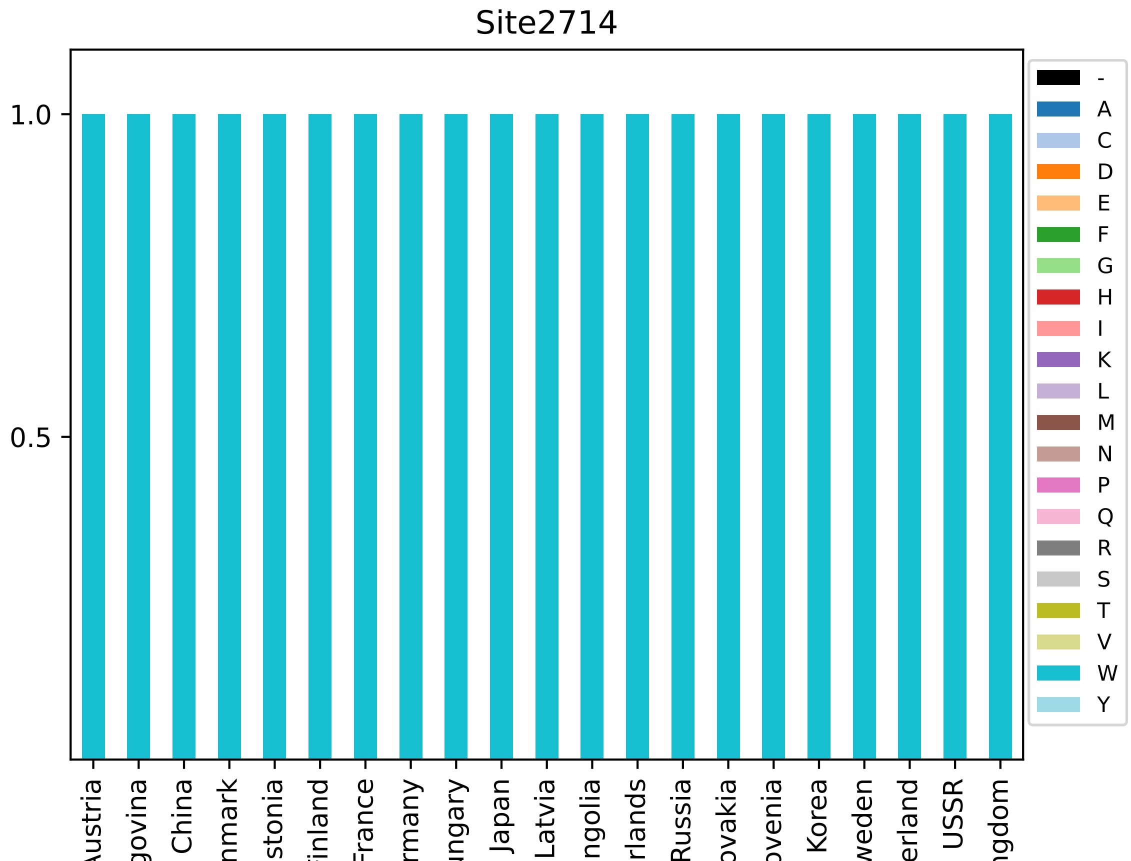
Site2714
1.0
0.5
-
A
C
D
E
F
G
H
I
K
L
M
N
P
Q
R
S
T
V
W
Y
China
Japan
Latvia
Russia
Korea
USSR

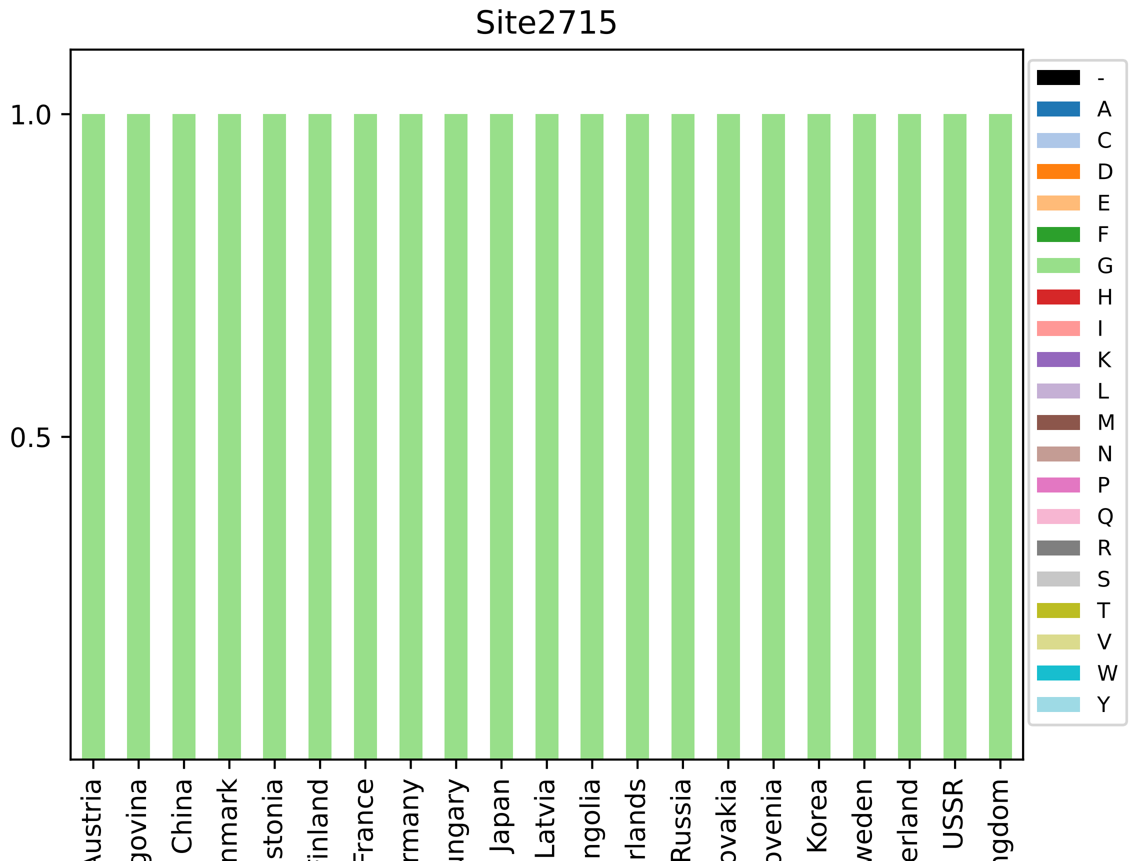
Site2715
1.0
0.5
-
A
C
D
E
F
G
H
I
K
L
M
N
P
Q
R
S
T
V
W
Y
China
Japan
Latvia
Russia
Korea
USSR

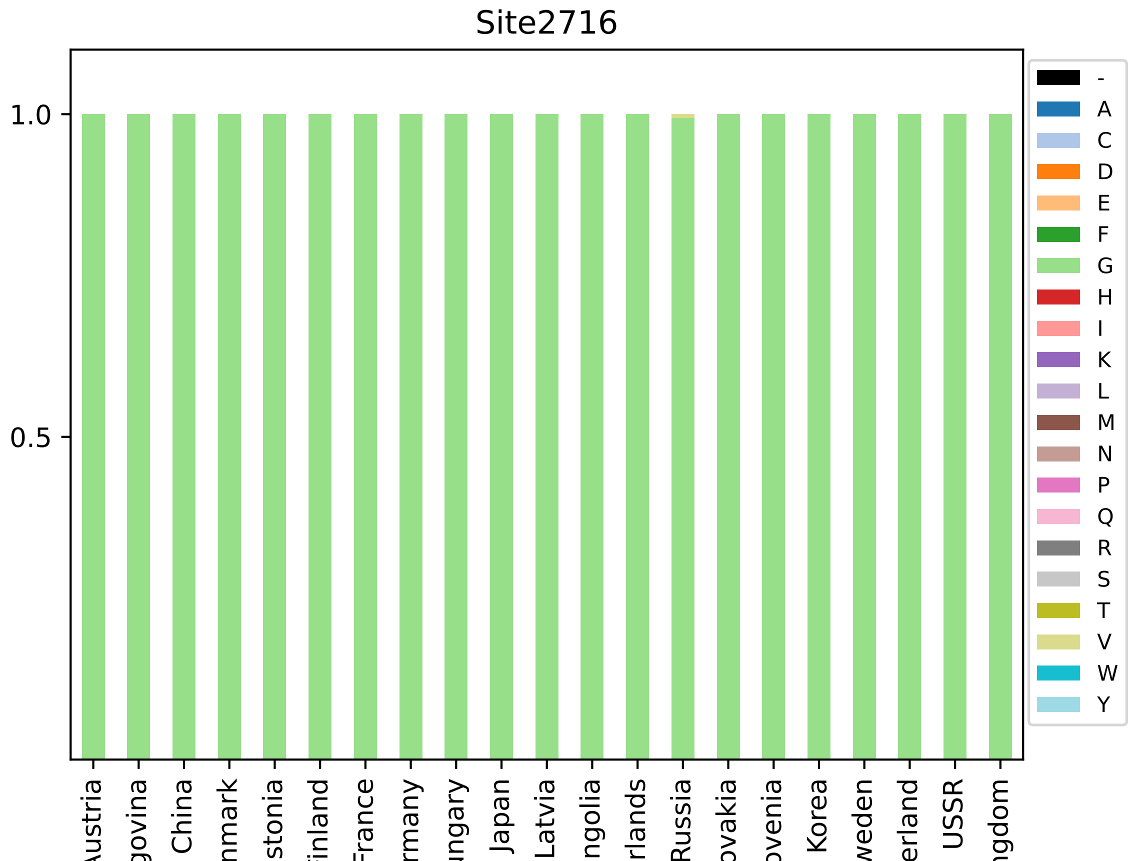
Site2716
1.0
0.5
-
A
C
D
E
F
G
H
I
K
L
M
N
P
Q
R
S
T
V
W
Y
China
Japan
Latvia
Russia
Korea
USSR

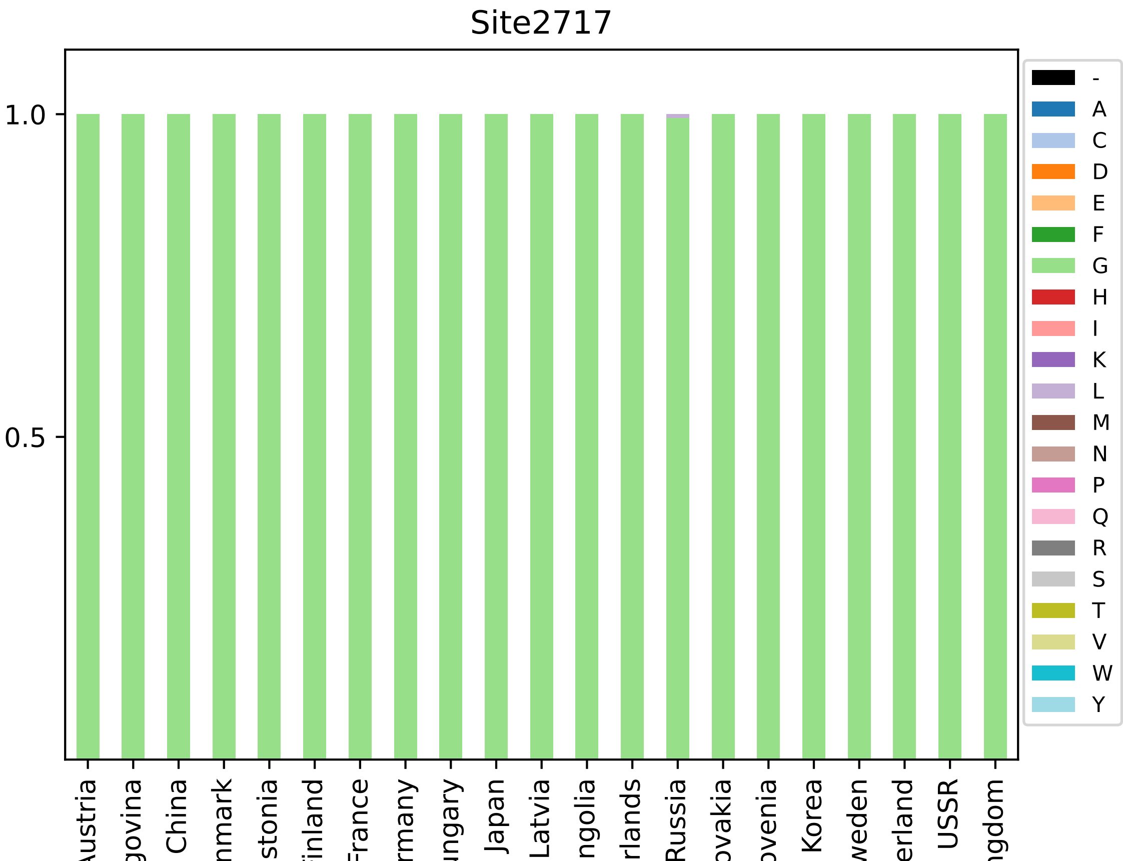

Site2717
1.0
0.5
-
A
C
D
E
F
G
H
I
K
L
M
N
P
Q
R
S
T
V
W
Y
Austria
China
Japan
Latvia
Russia
Korea
USSR

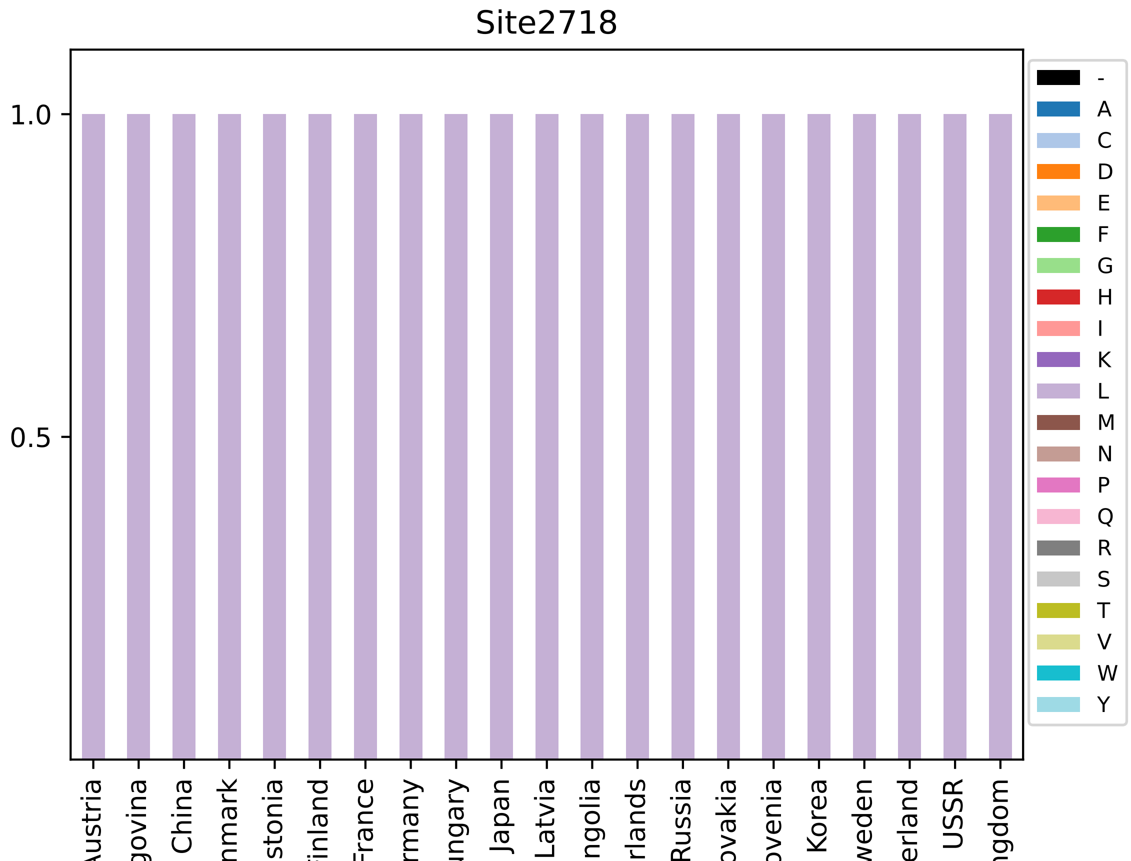
Site2718
1.0
0.5
-
A
C
D
E
F
G
H
I
K
L
M
N
P
Q
R
S
T
V
W
Y
China
Japan
Latvia
Russia
Korea
USSR

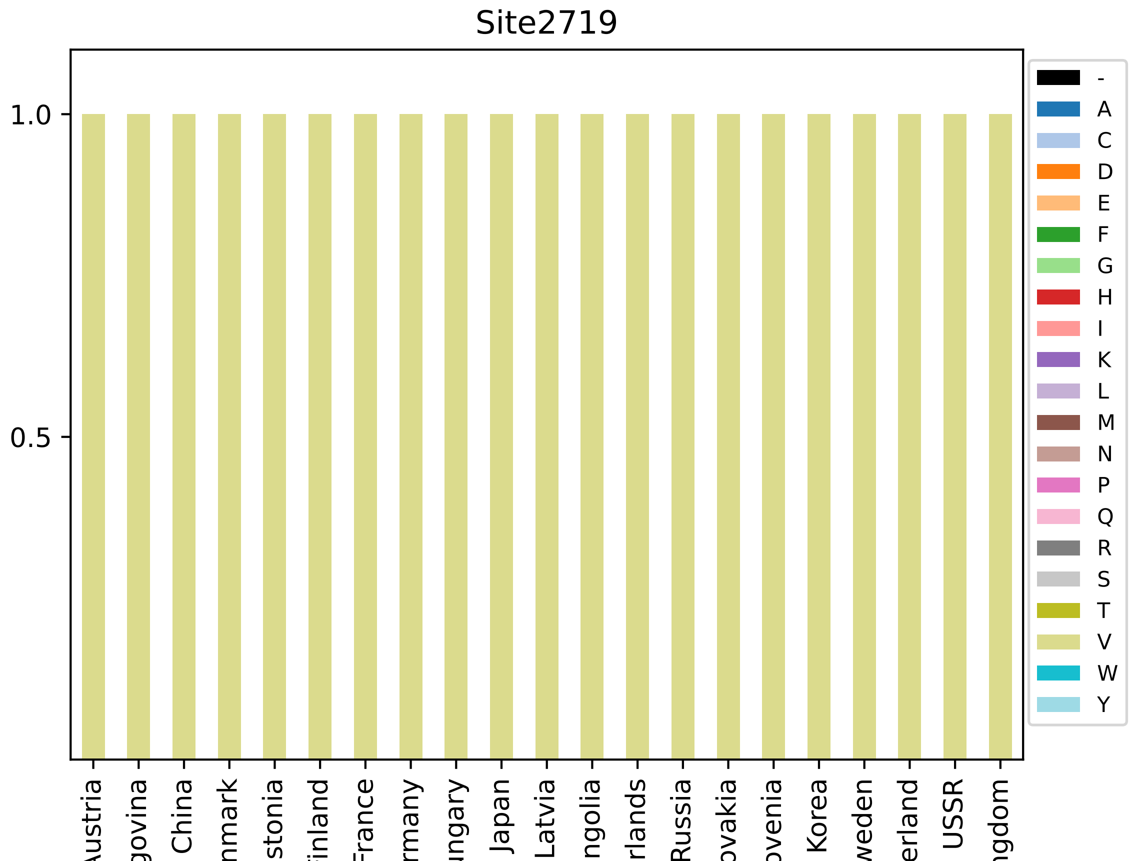
Site2719
1.0
0.5
-
A
C
D
E
F
G
H
I
K
L
M
N
P
Q
R
S
T
V
W
Y
China
Japan
Latvia
Russia
Korea
USSR

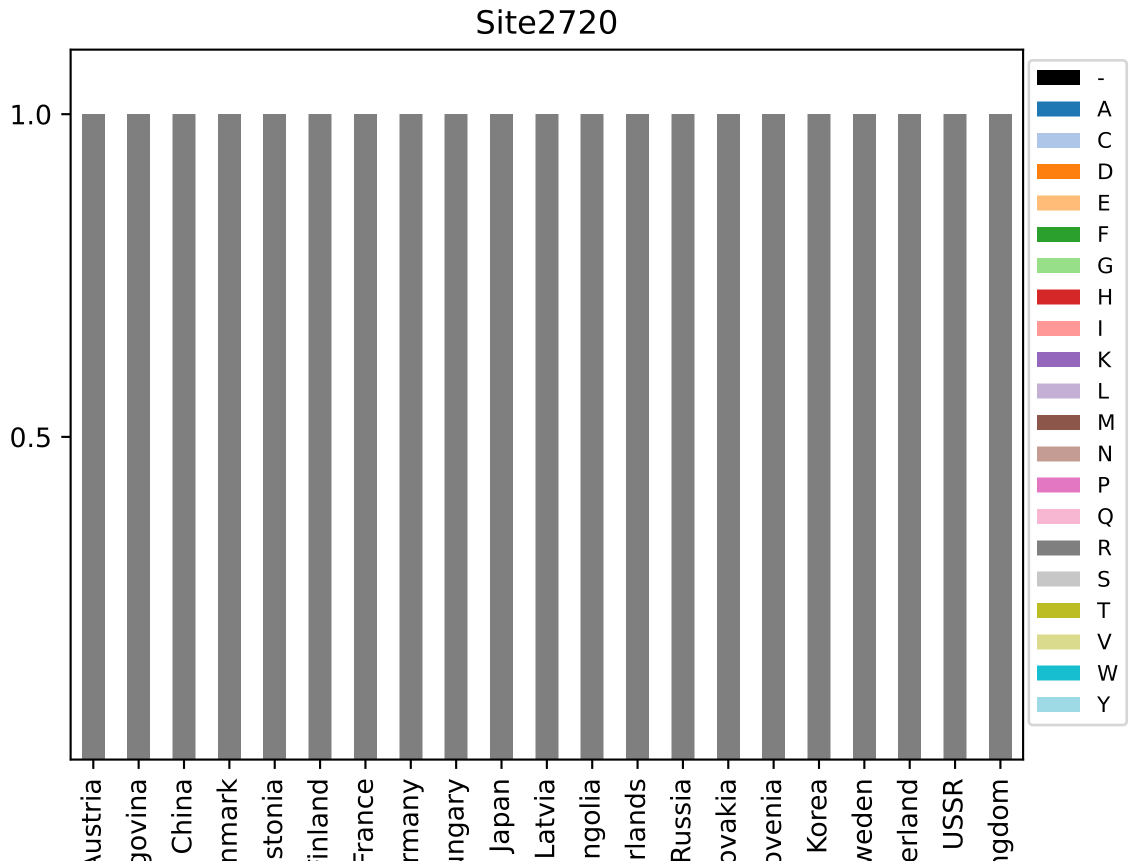
Site2720
1.0
0.5
-
A
C
D
E
F
G
H
I
K
L
M
N
P
Q
R
S
T
V
W
Y
Austria
China
Finland
France
Japan
Latvia
Russia
Korea
USSR

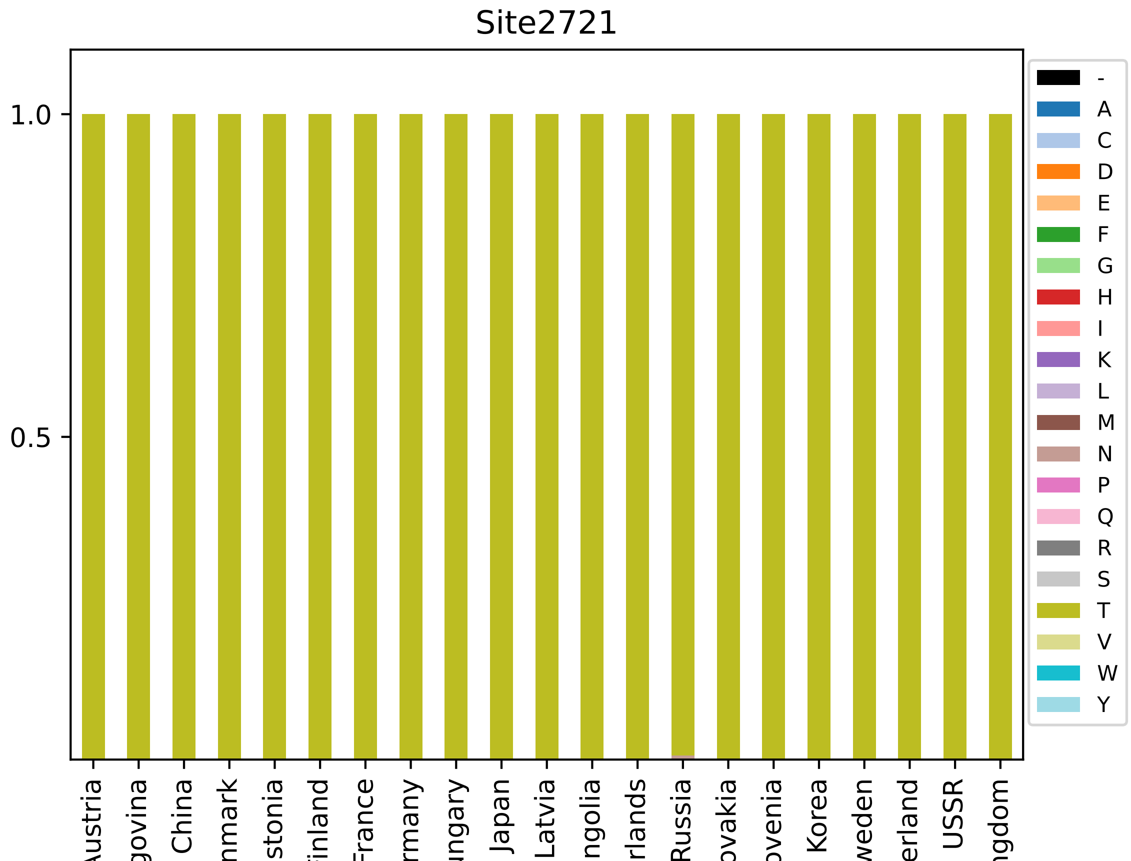

Site2721
1.0
0.5
-
A
C
D
E
F
G
H
I
K
L
M
N
P
Q
R
S
T
V
W
Y
Austria
govina
China
nmark
stonia
inland
France
rmany
ungary
Japan
Latvia
ngolia
rlands
Russia
ovakia
ovenia
Korea
weden
erland
USSR
ngdom

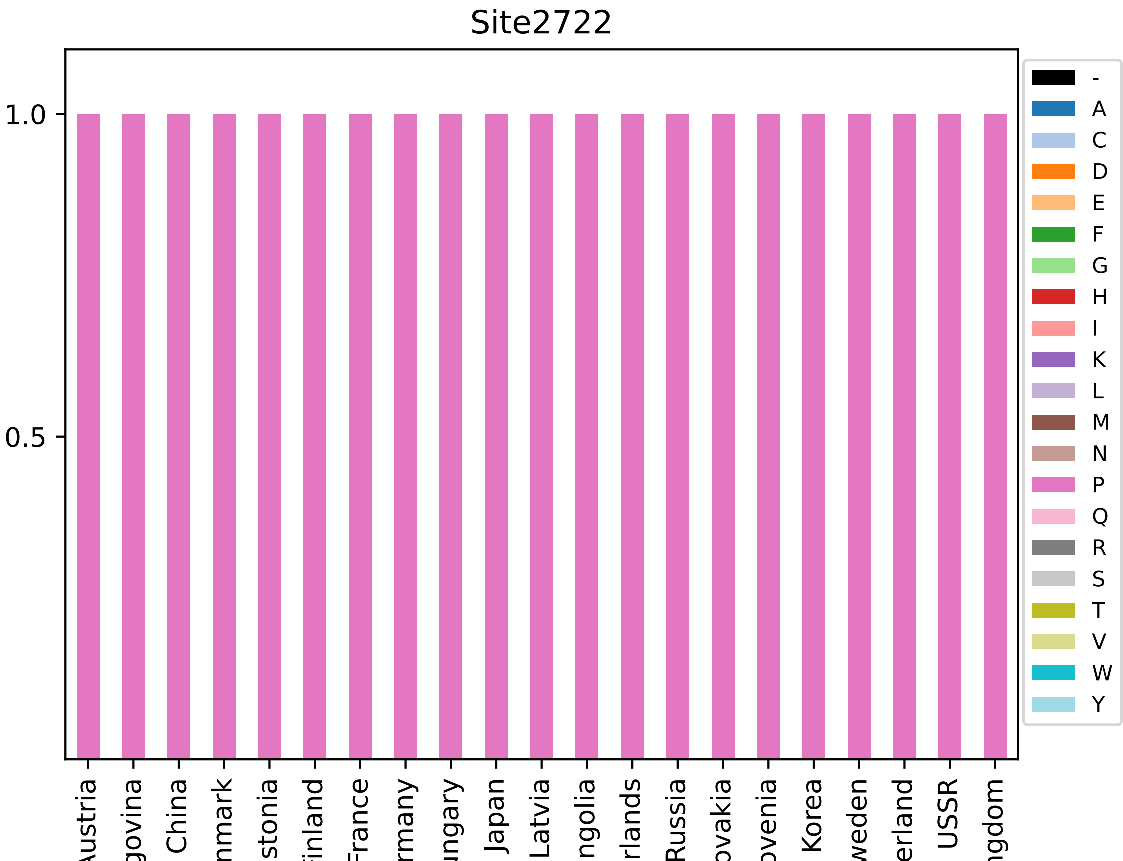
Site2722
1.0
0.5
-
A
C
D
E
F
G
H
I
K
L
M
N
P
Q
R
S
T
V
W
Y
China
Japan
Latvia
Russia
Korea
USSR

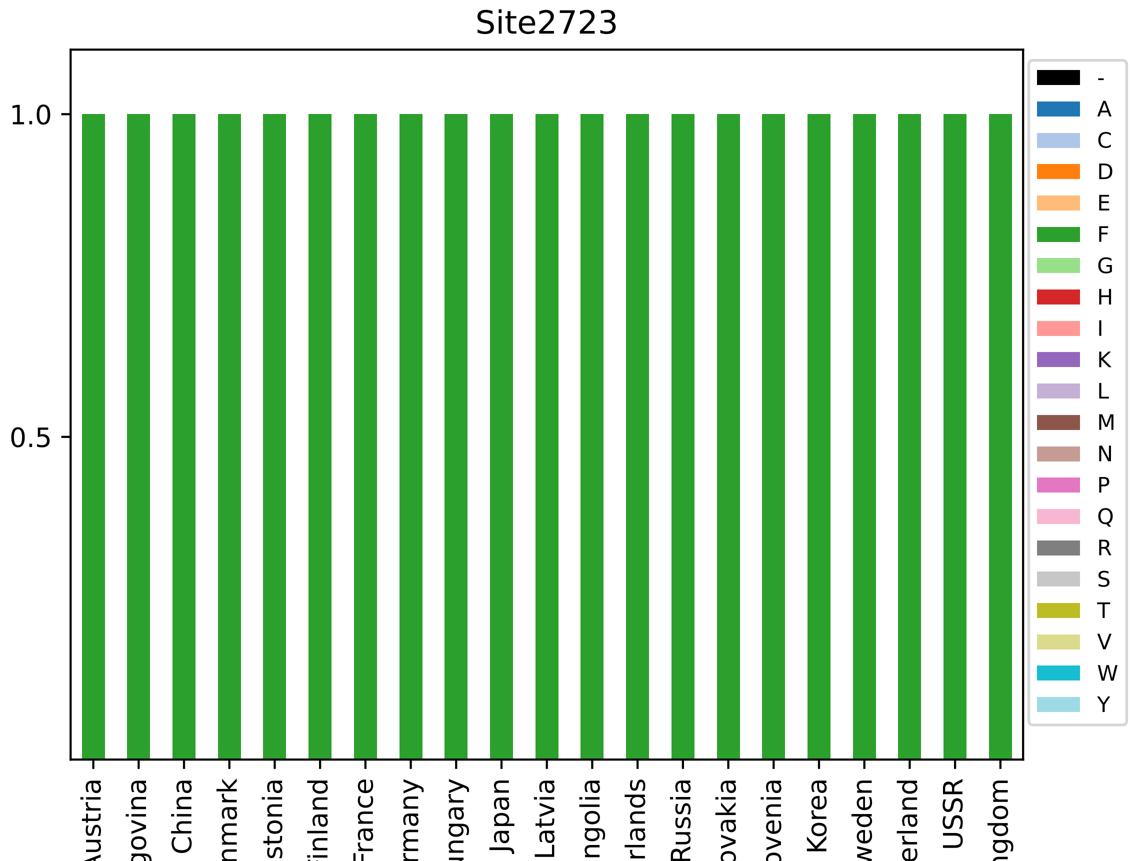
Site2723
1.0
0.5
-
A
C
D
E
F
G
H
I
K
L
M
N
P
Q
R
S
T
V
W
Y
Austria
govina
China
nmark
stonia
inland
France
rmany
ungary
Japan
Latvia
ngolia
rlands
Russia
ovakia
ovenia
Korea
weden
erland
USSR
ngdom

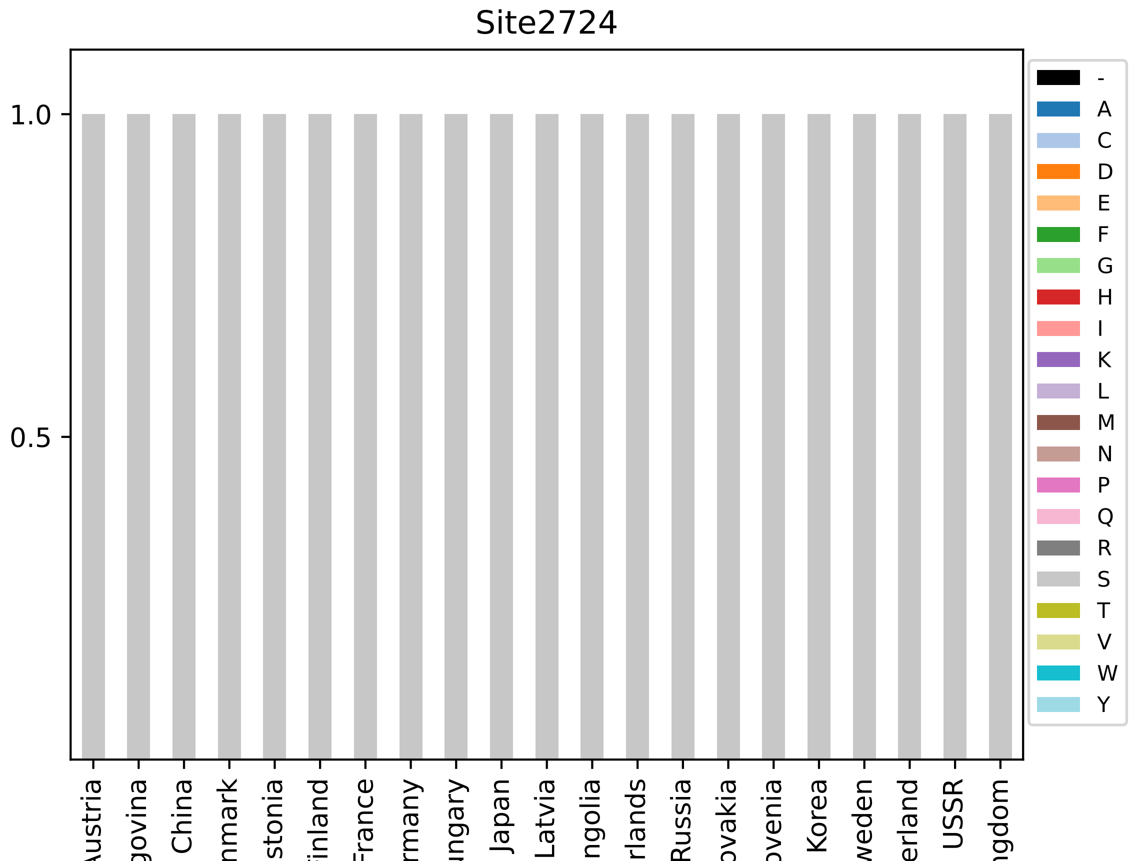
Site2724
1.0
0.5
-
A
C
D
E
F
G
H
I
K
L
M
N
P
Q
R
S
T
V
W
Y
China
Japan
Latvia
Russia
Korea
USSR

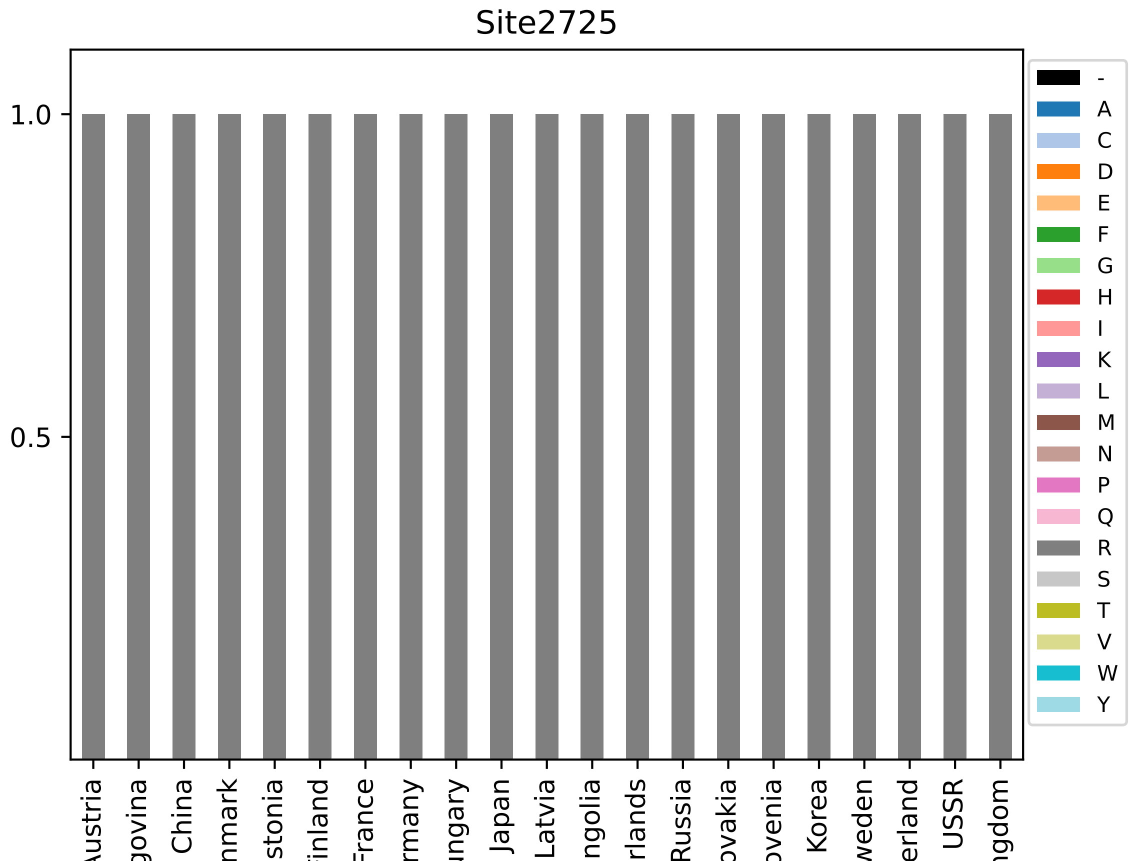

Site2725
1.0
0.5
-
A
C
D
E
F
G
H
I
K
L
M
N
P
Q
R
S
T
V
W
Y
China
Japan
Latvia
Russia
Korea
USSR

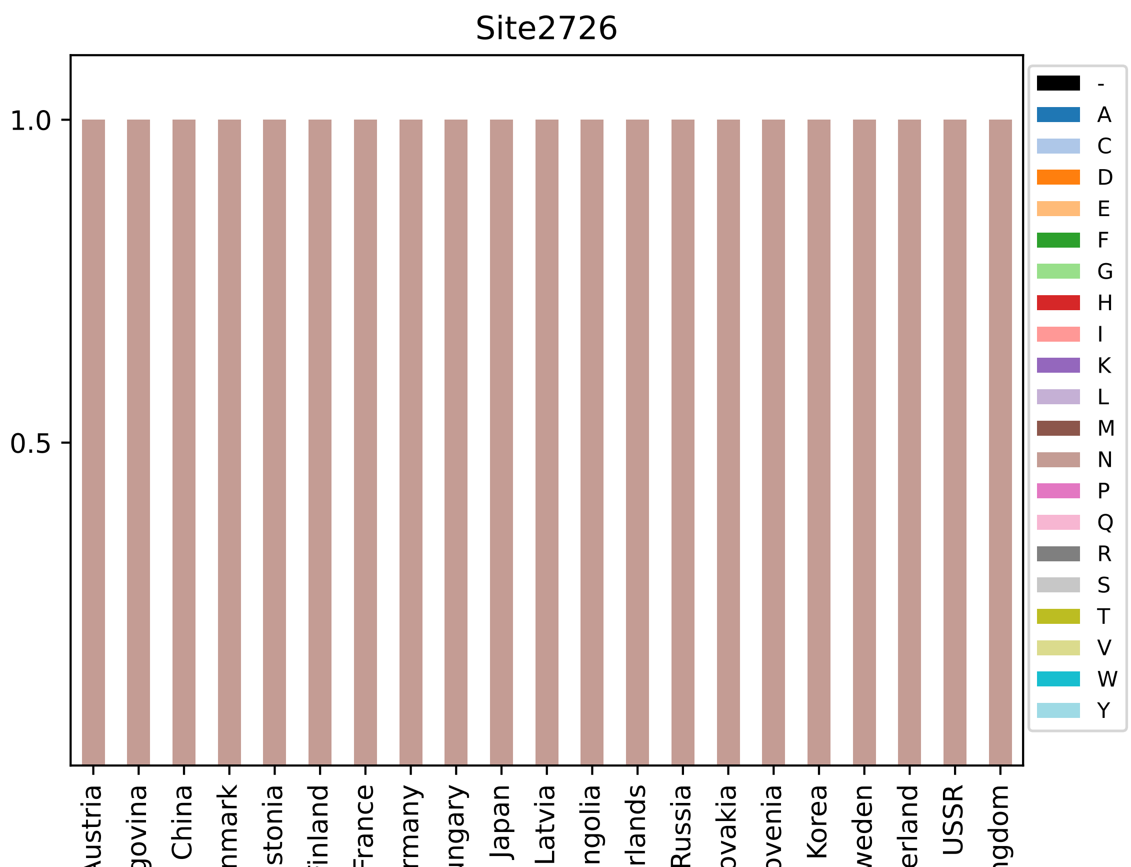

Site2726
1.0
0.5
-
A
C
D
E
F
G
H
I
K
L
M
N
P
Q
R
S
T
V
W
Y
Austria
China
Japan
Latvia
Russia
Korea
USSR

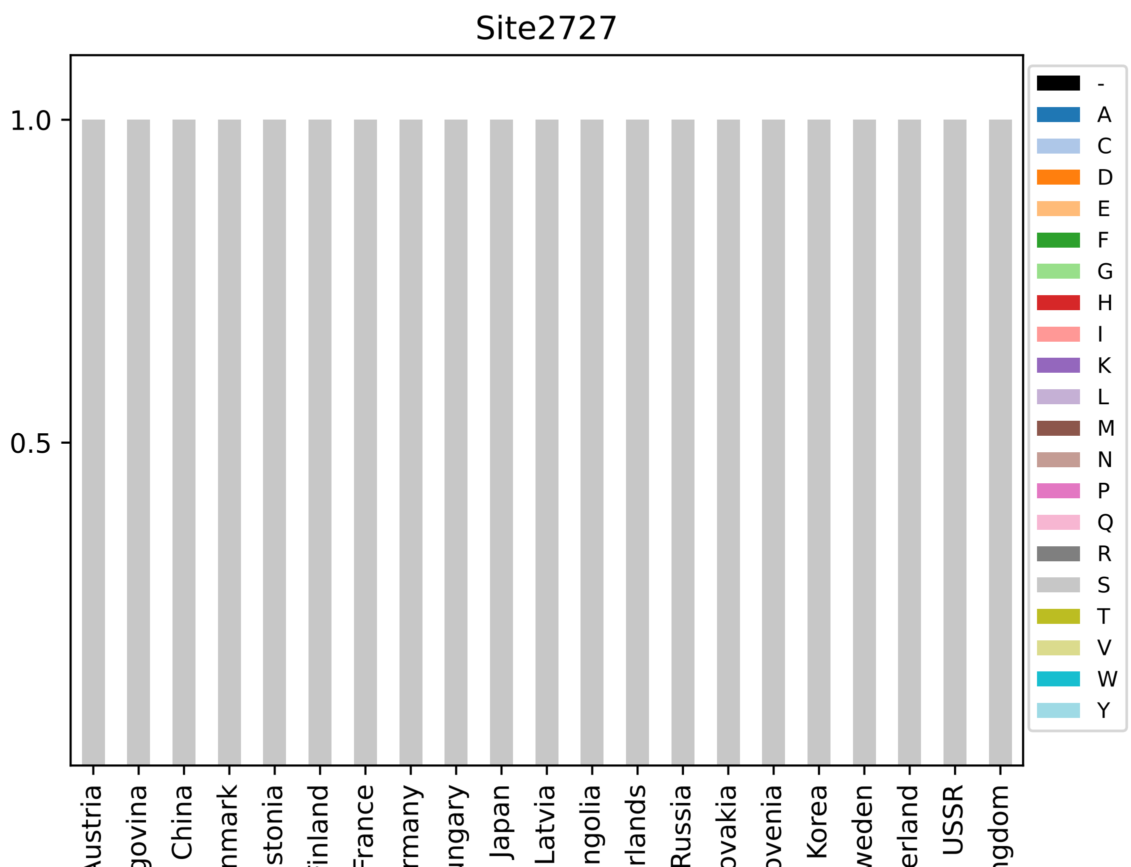

Site2727
1.0
0.5
Austria
govina
China
nmark
stonia
Finland
France
rmany
ungary
Japan
Latvia
ngolia
rlands
Russia
ovakia
ovenia
Korea
weden
erland
USSR
ngdom
-
A
C
D
E
F
G
H
I
K
L
M
N
P
Q
R
S
T
V
W
Y

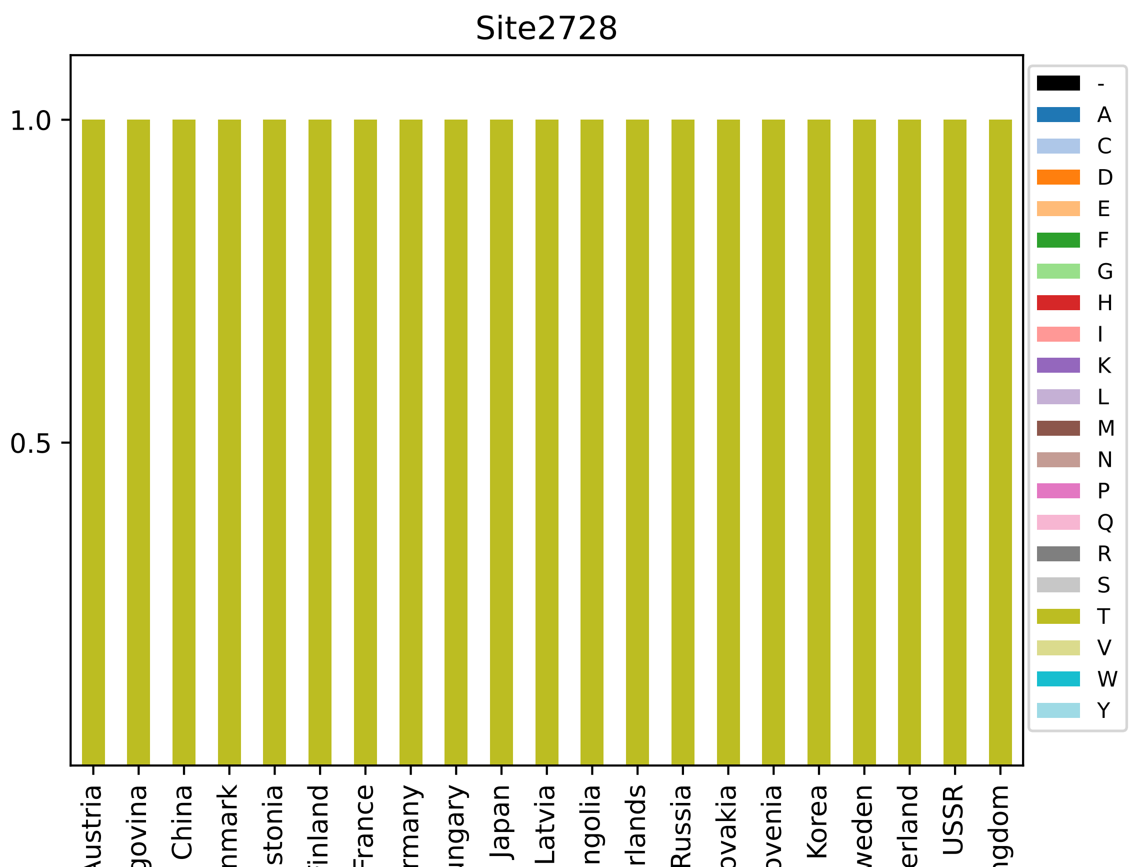

Site2728
1.0
0.5
-
A
C
D
E
F
G
H
I
K
L
M
N
P
Q
R
S
T
V
W
Y
Austria
govina
China
nmark
stonia
Finland
France
rmany
ungary
Japan
Latvia
ngolia
rlands
Russia
ovakia
ovenia
Korea
weden
erland
USSR
ngdom

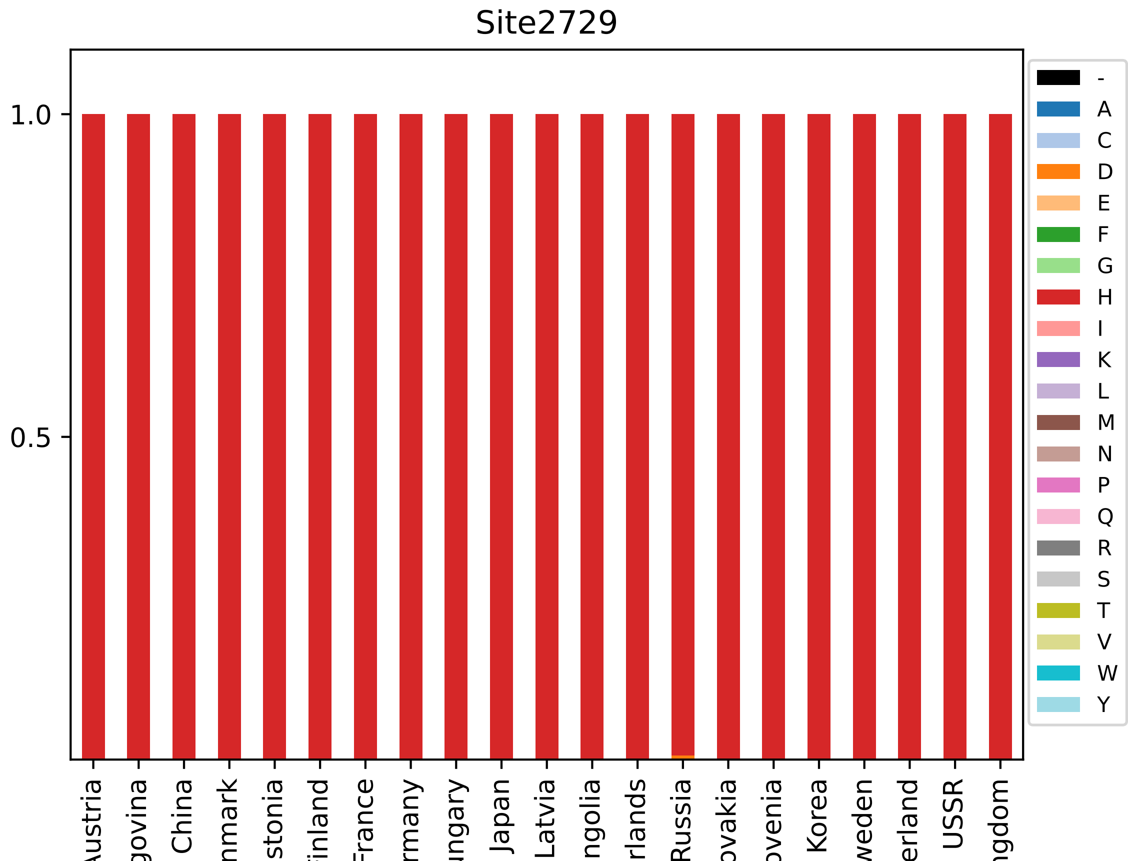

Site2729
1.0
0.5
China
Japan
Latvia
Russia
Korea
USSR
-
A
C
D
E
F
G
H
I
K
L
M
N
P
Q
R
S
T
V
W
Y

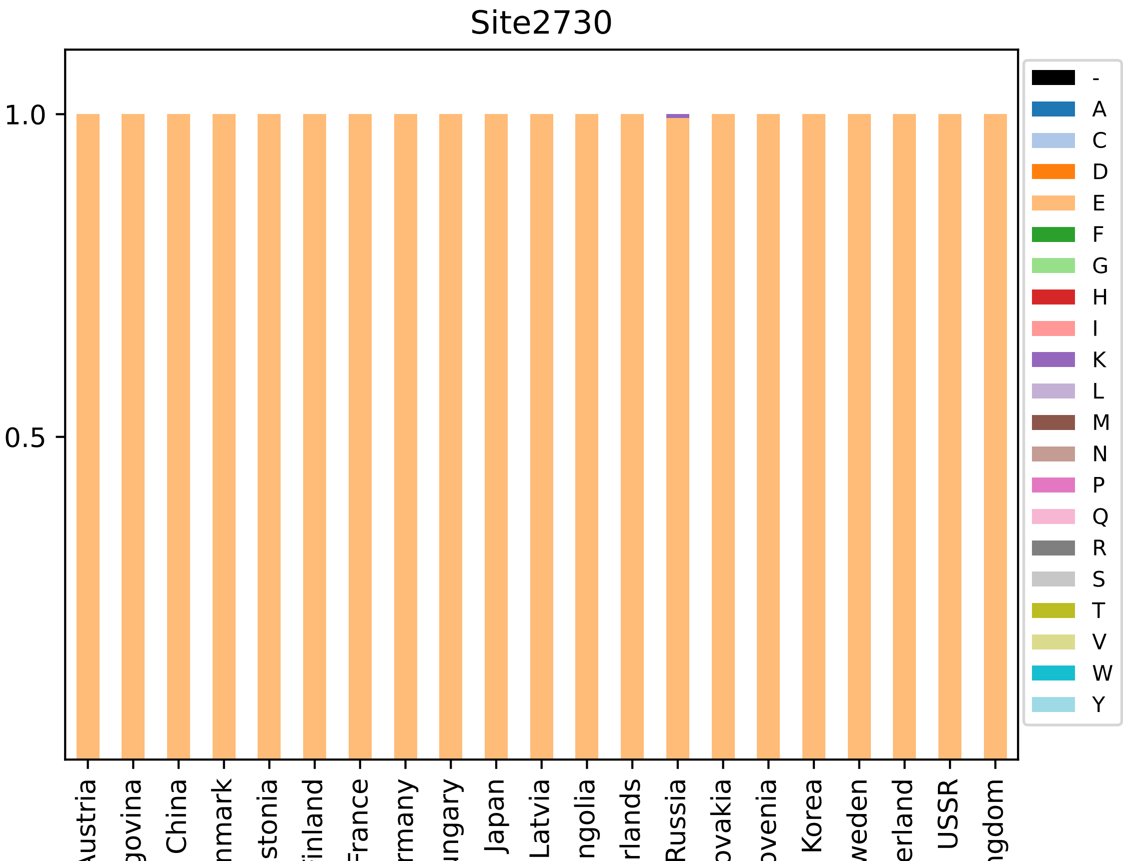

Site2730
1.0
0.5
-
A
C
D
E
F
G
H
I
K
L
M
N
P
Q
R
S
T
V
W
Y
Austria
China
Japan
Latvia
Russia
Korea
USSR

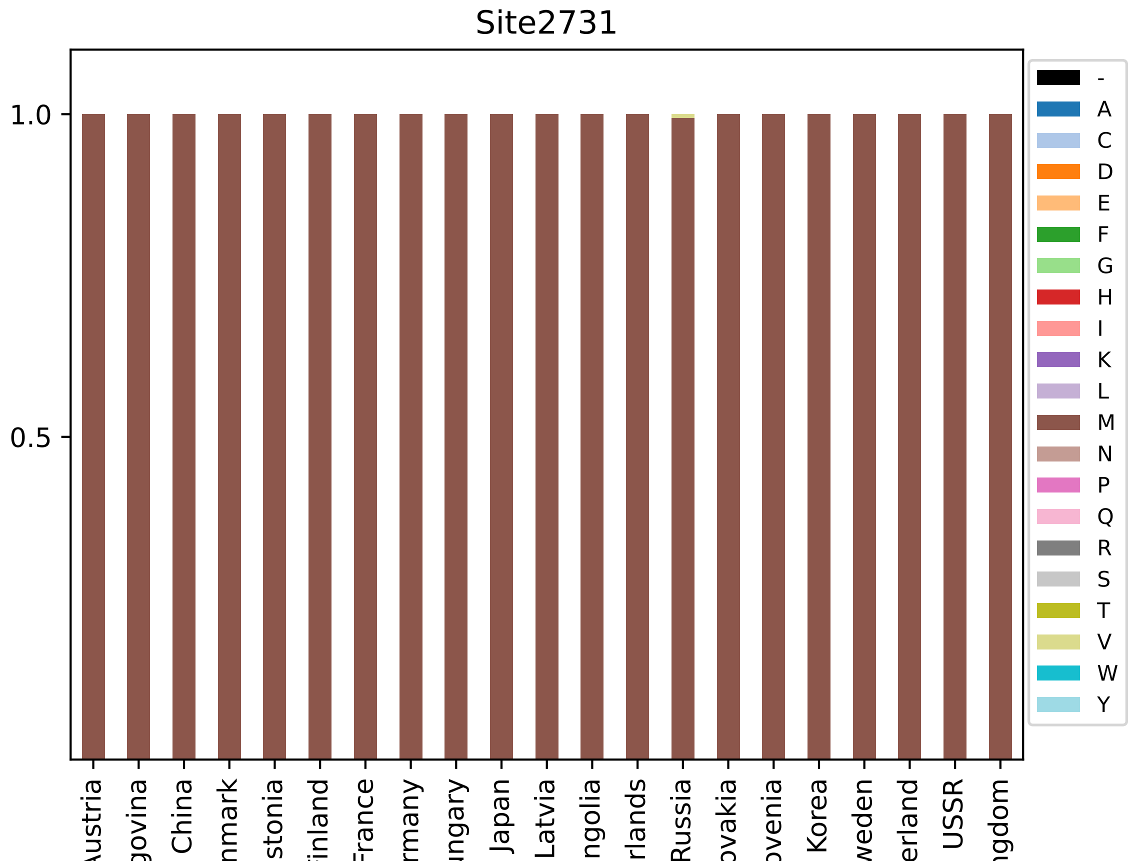

Site2731
1.0
0.5
-
A
C
D
E
F
G
H
I
K
L
M
N
P
Q
R
S
T
V
W
Y
Austria
govina
China
nmark
stonia
inland
France
rmany
ungary
Japan
Latvia
ngolia
rlands
Russia
ovakia
ovenia
Korea
weden
erland
USSR
ngdom

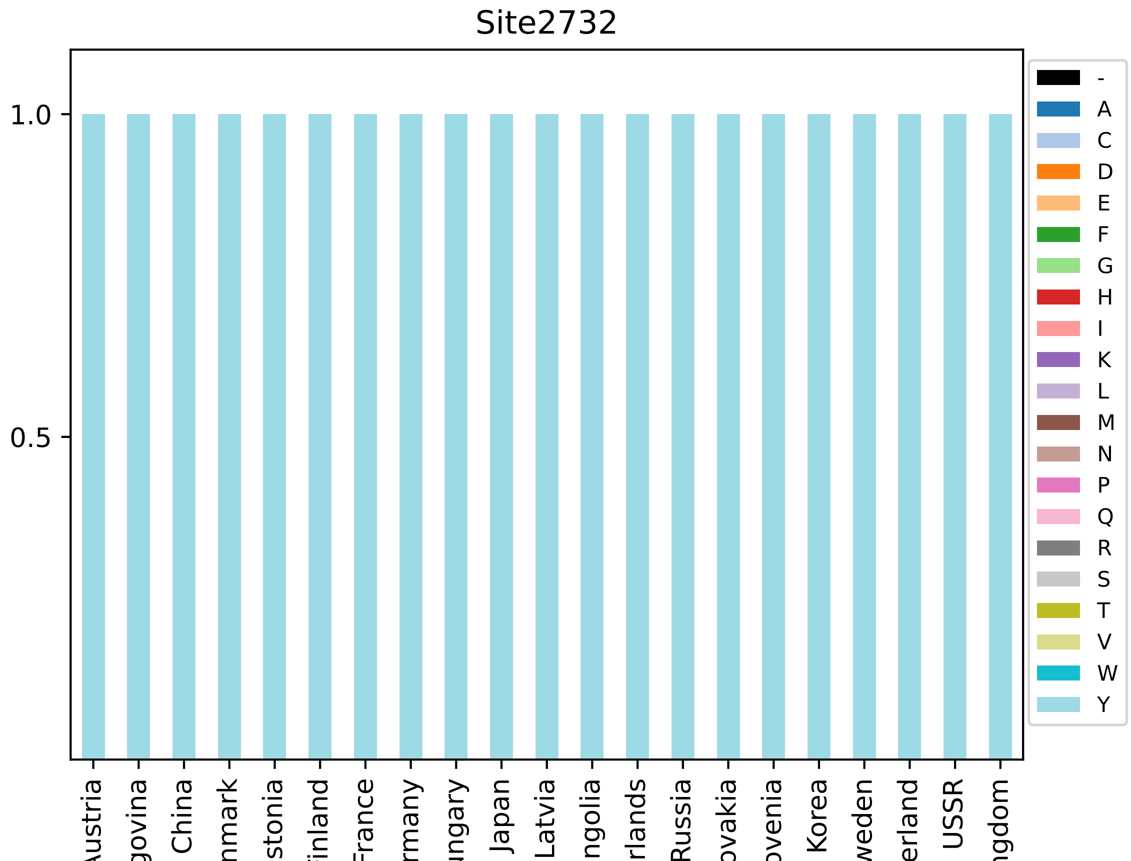

Site2732
1.0
0.5
-
A
C
D
E
F
G
H
I
K
L
M
N
P
Q
R
S
T
V
W
Y
China
Japan
Latvia
Russia
Korea
USSR

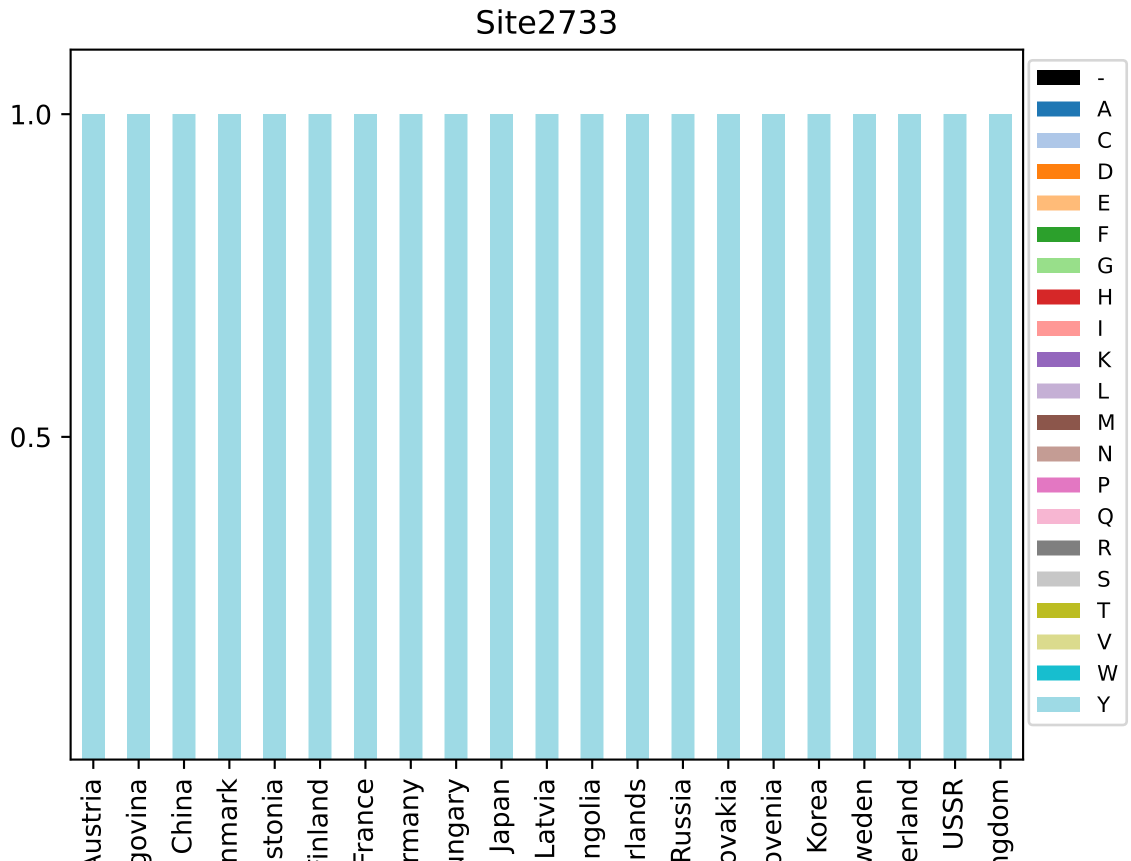

Site2733
1.0
0.5
Austria
govina
China
nmark
stonia
inland
France
rmany
ungary
Japan
Latvia
ngolia
rlands
Russia
ovakia
ovenia
Korea
weden
erland
USSR
ngdom
-
A
C
D
E
F
G
H
I
K
L
M
N
P
Q
R
S
T
V
W
Y

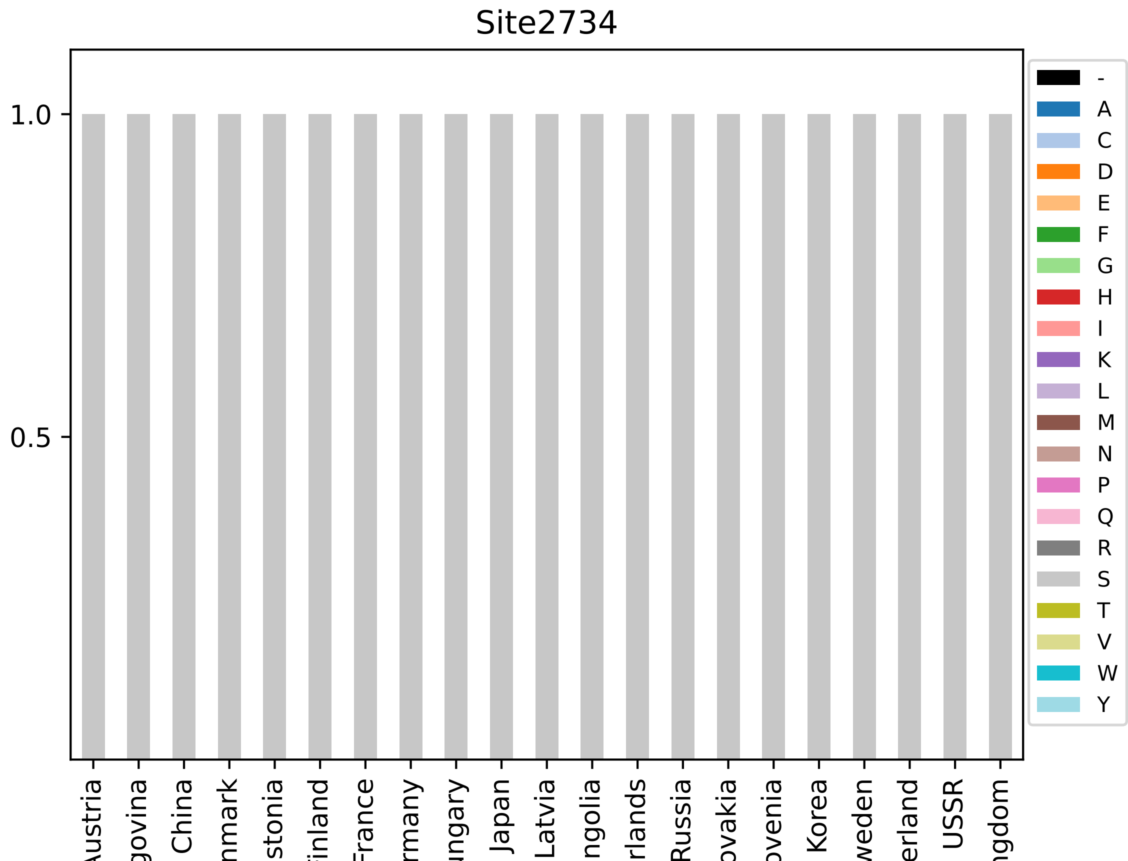
Site2734
1.0
0.5
-
A
C
D
E
F
G
H
I
K
L
M
N
P
Q
R
S
T
V
W
Y
China
Japan
Latvia
Russia
Korea
USSR

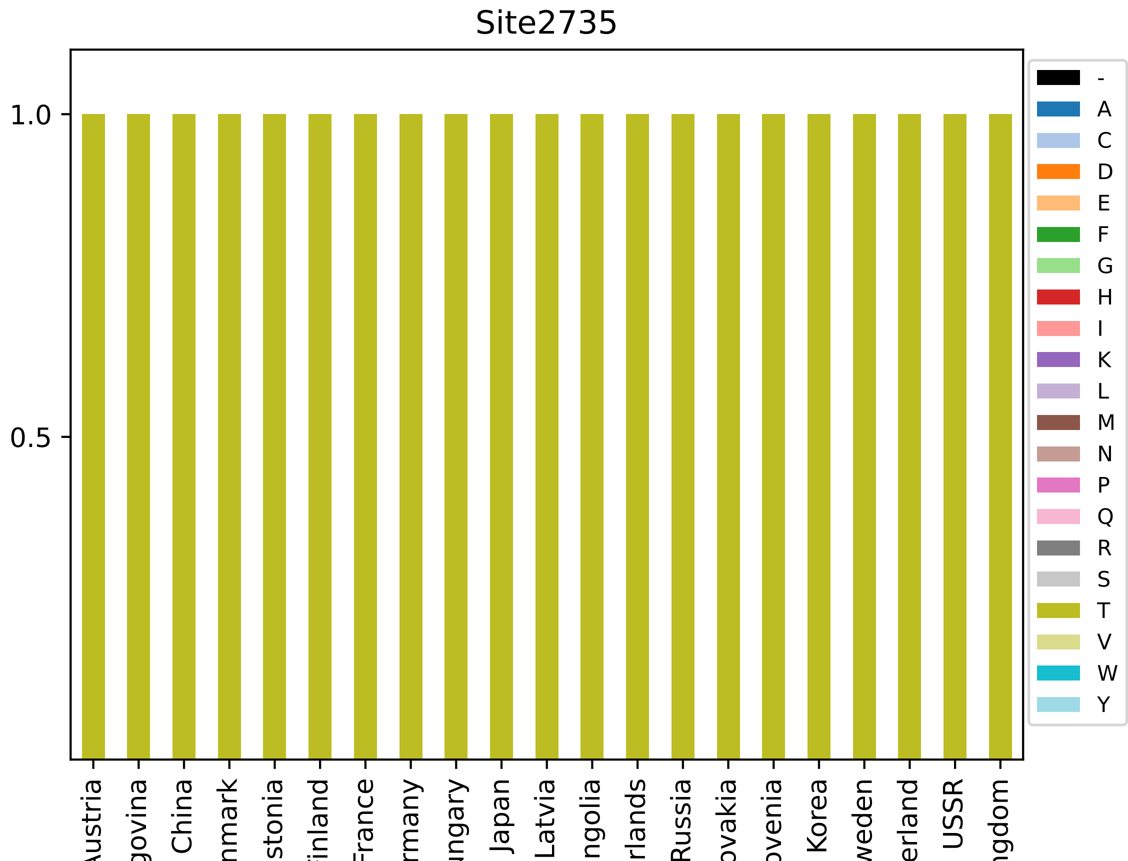
Site2735
1.0
0.5
-
A
C
D
E
F
G
H
I
K
L
M
N
P
Q
R
S
T
V
W
Y
China
Japan
Latvia
Russia
Korea
USSR

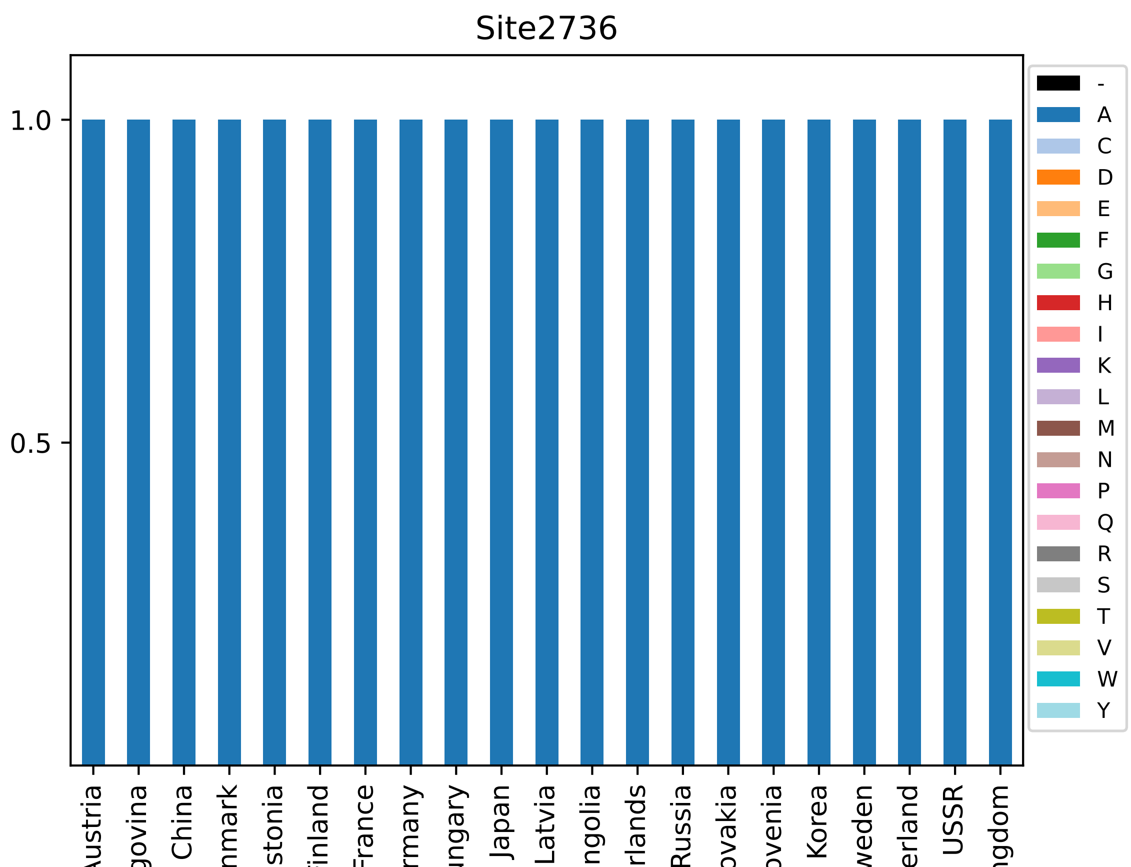
Site2736
1.0
0.5
-
A
C
D
E
F
G
H
I
K
L
M
N
P
Q
R
S
T
V
W
Y
Austria
govina
China
nmark
stonia
inland
France
rmany
ungary
Japan
Latvia
ngolia
rlands
Russia
ovakia
ovenia
Korea
weden
erland
USSR
ngdom

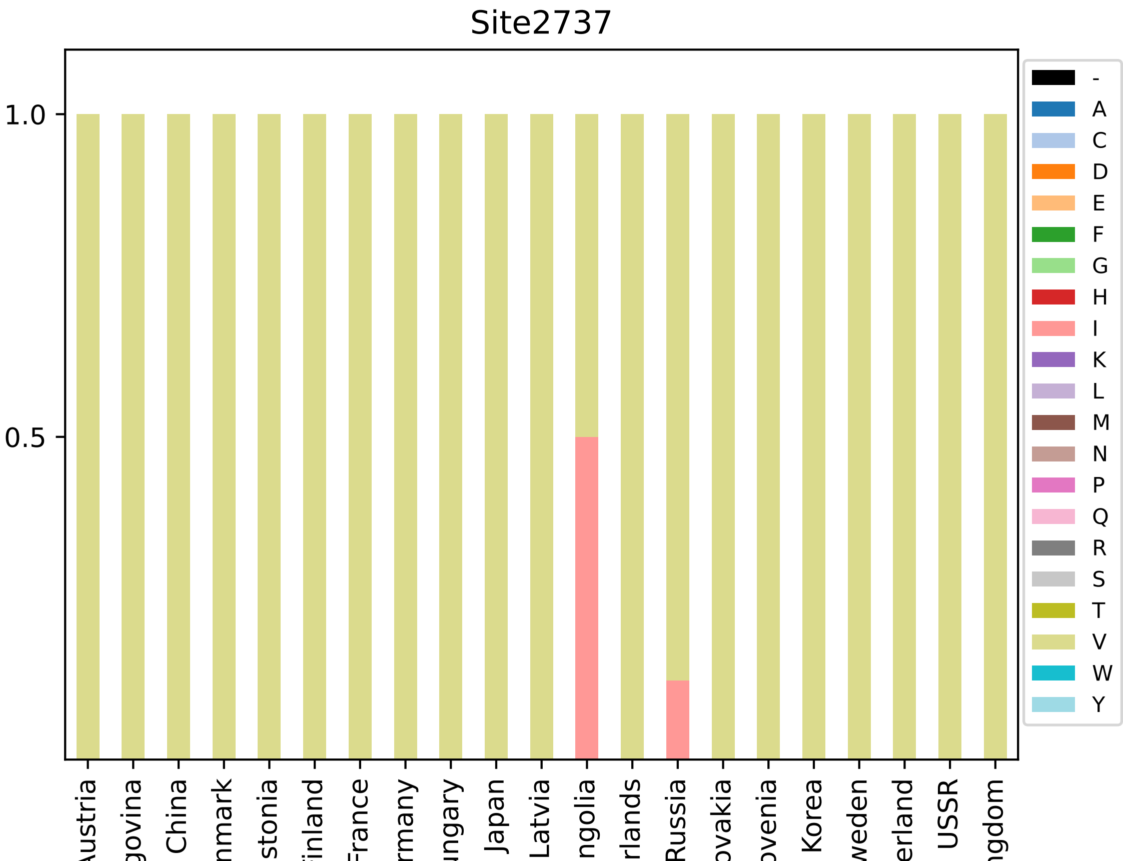

Site2737
1.0
0.5
-
A
C
D
E
F
G
H
I
K
L
M
N
P
Q
R
S
T
V
W
Y
Austria
govina
China
nmark
stonia
Finland
France
rmany
ungary
Japan
Latvia
ngolia
rlands
Russia
ovakia
ovenia
Korea
weden
erland
USSR
ngdom

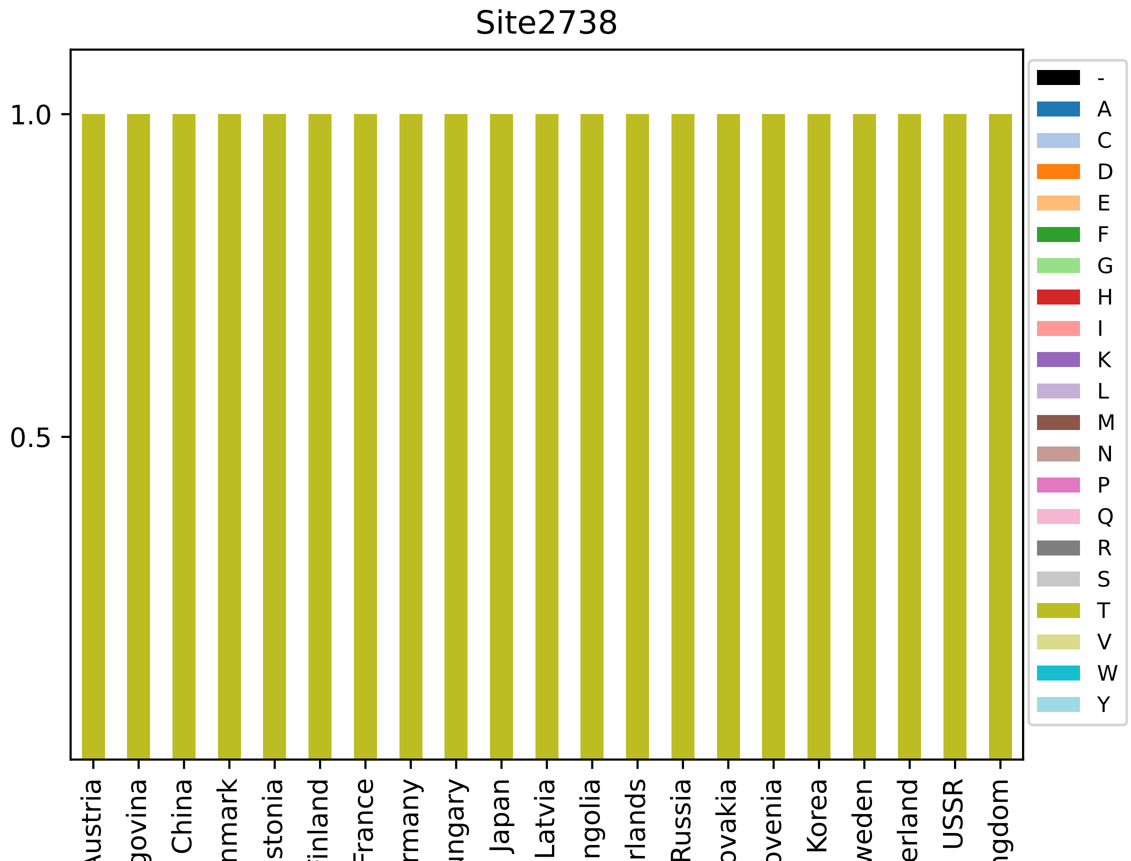
Site2738
1.0
0.5
-
A
C
D
E
F
G
H
I
K
L
M
N
P
Q
R
S
T
V
W
Y
China
Japan
Latvia
Russia
Korea
USSR

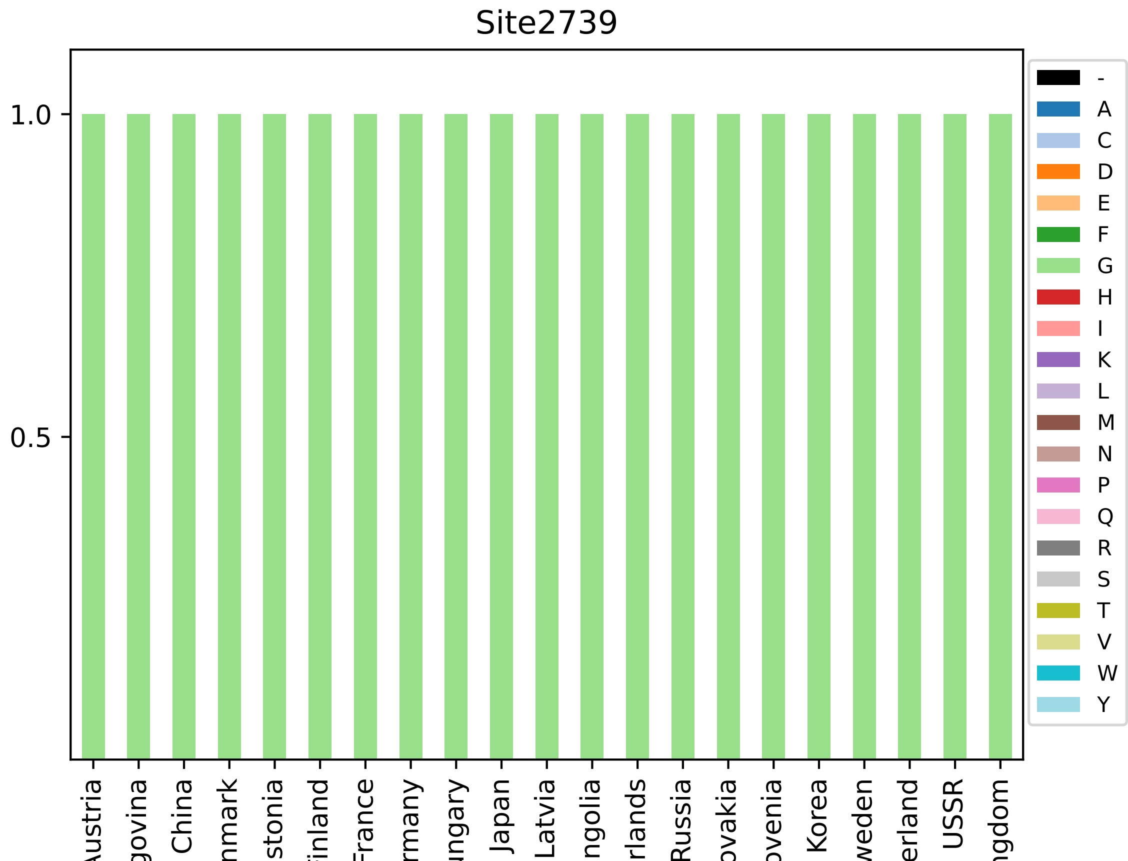
Site2739
1.0
0.5
-
A
C
D
E
F
G
H
I
K
L
M
N
P
Q
R
S
T
V
W
Y
China
Japan
Latvia
Russia
Korea
USSR

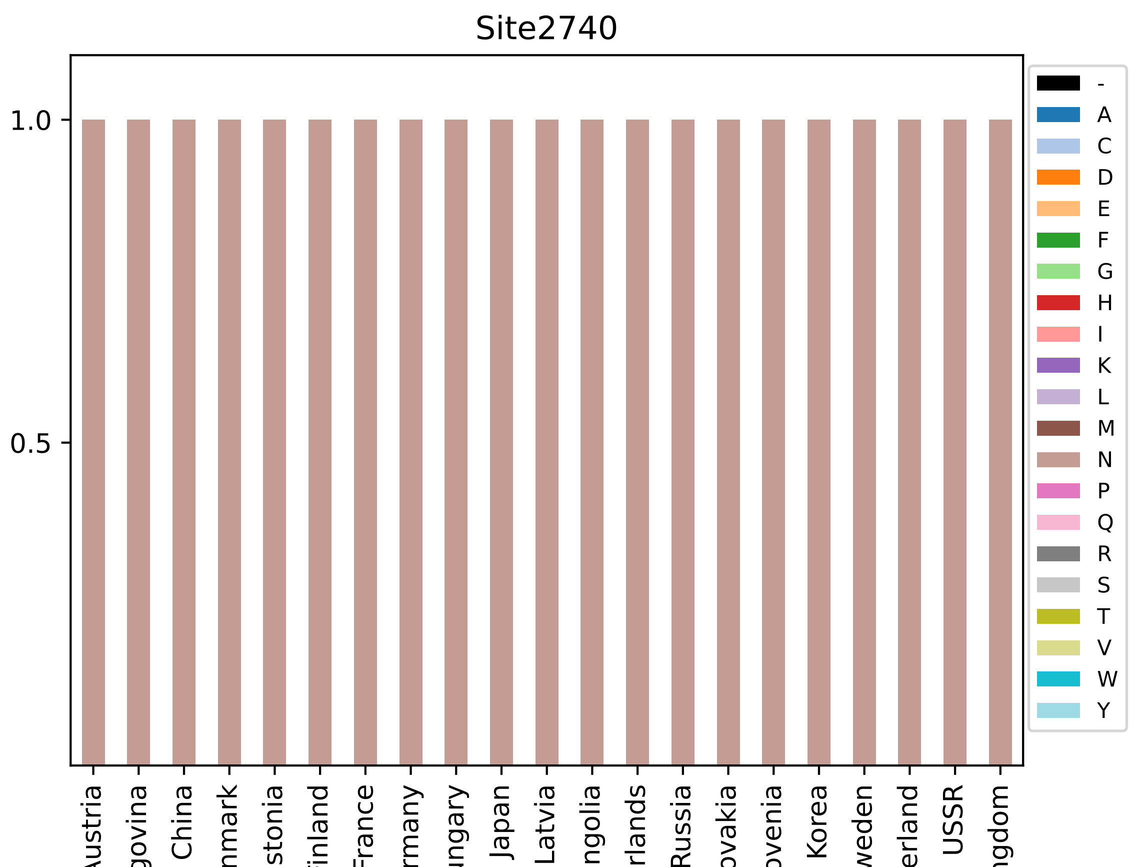

Site2740
1.0
0.5
-
A
C
D
E
F
G
H
I
K
L
M
N
P
Q
R
S
T
V
W
Y
Austria
govina
China
nmark
stonia
Finland
France
rmany
ungary
Japan
Latvia
ngolia
rlands
Russia
ovakia
ovenia
Korea
weden
erland
USSR
ngdom

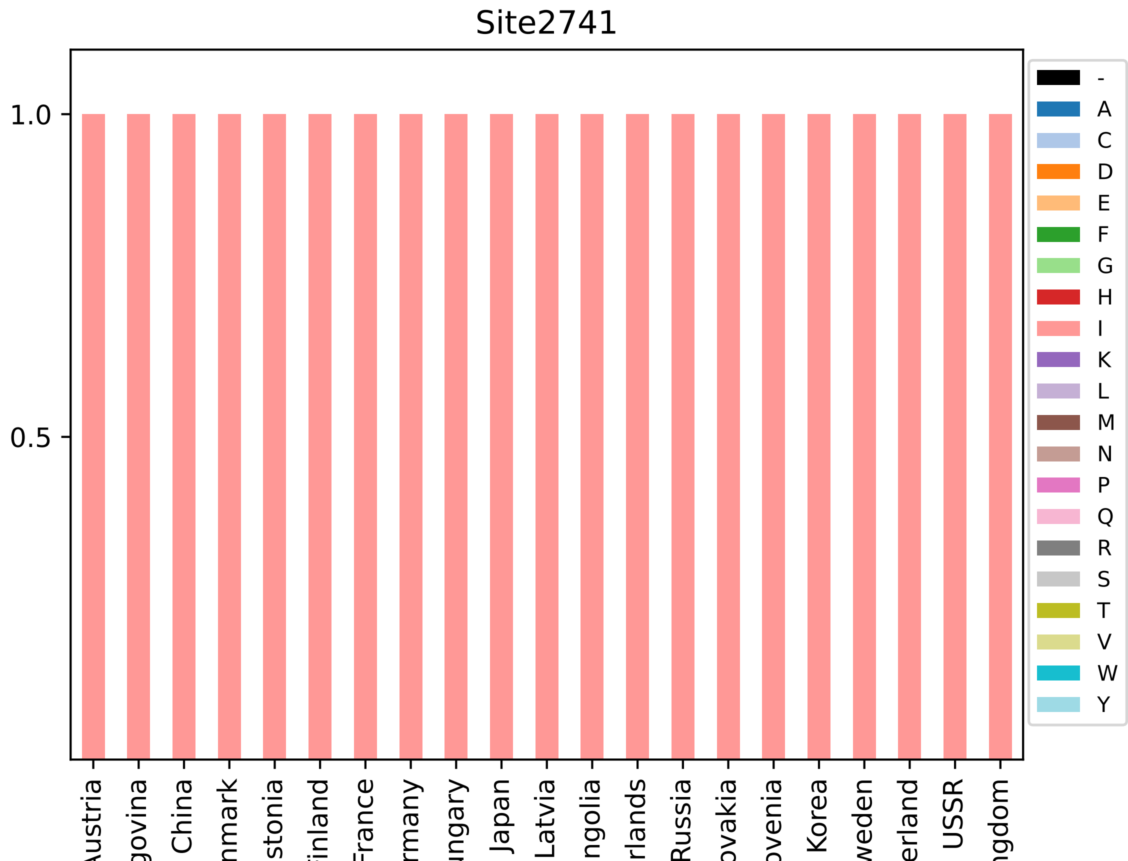

Site2741
1.0
0.5
-
A
C
D
E
F
G
H
I
K
L
M
N
P
Q
R
S
T
V
W
Y
Austria
China
Finland
France
Japan
Latvia
Russia
Korea
USSR

Site2742

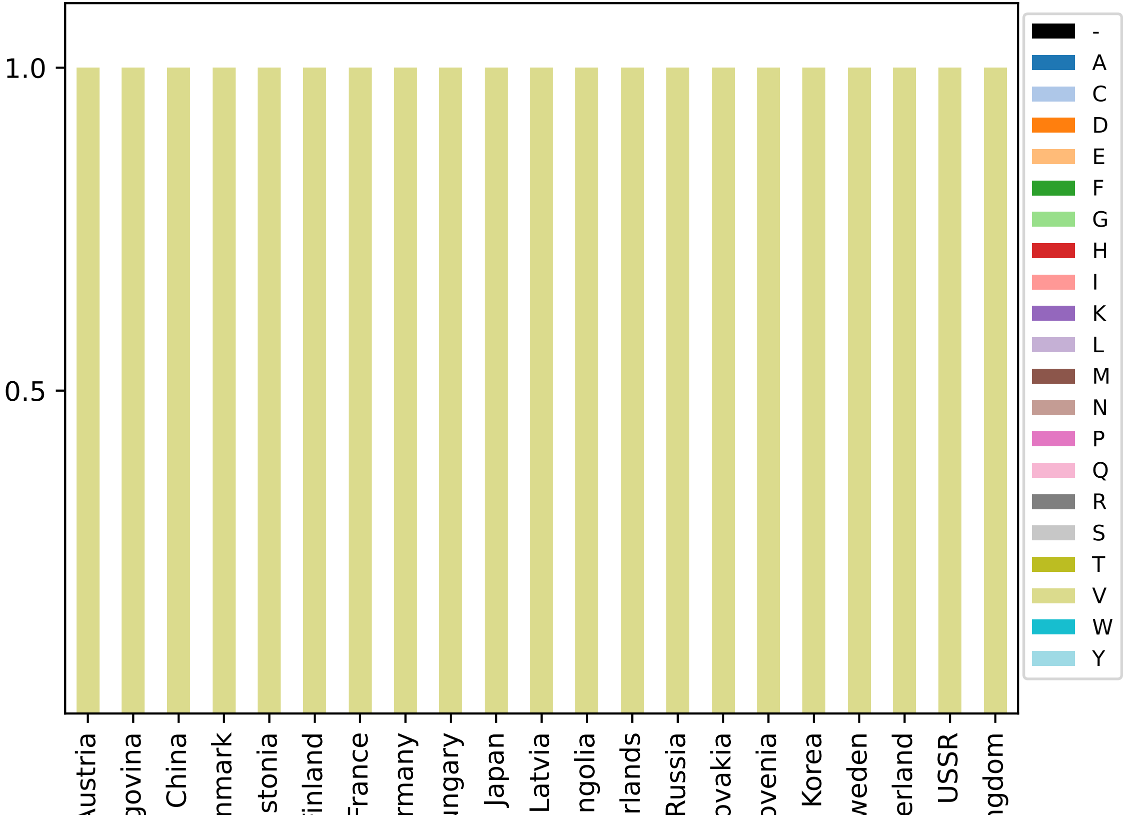

1.0
0.5
China
Japan
Latvia
Russia
Korea
USSR
-
A
C
D
E
F
G
H
I
K
L
M
N
P
Q
R
S
T
V
W
Y

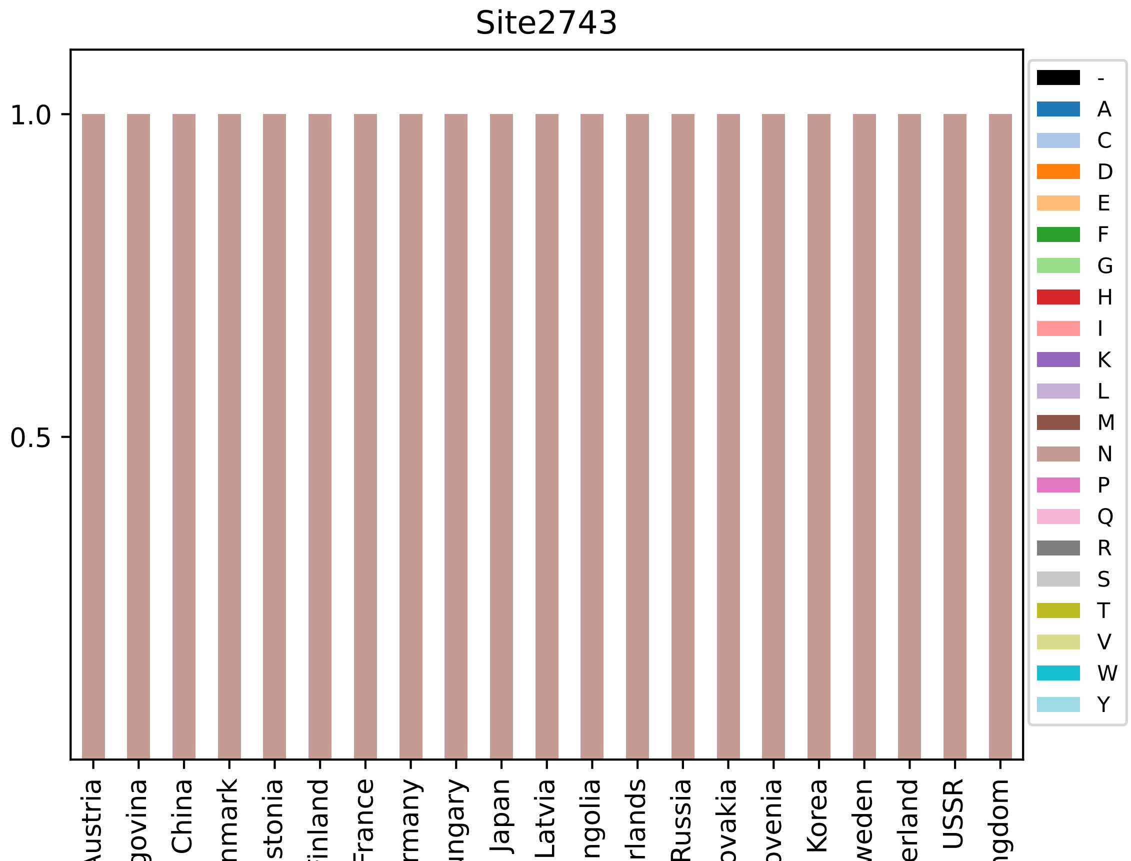

Site2743
1.0
0.5
-
A
C
D
E
F
G
H
I
K
L
M
N
P
Q
R
S
T
V
W
Y
China
Japan
Latvia
Russia
Korea
USSR

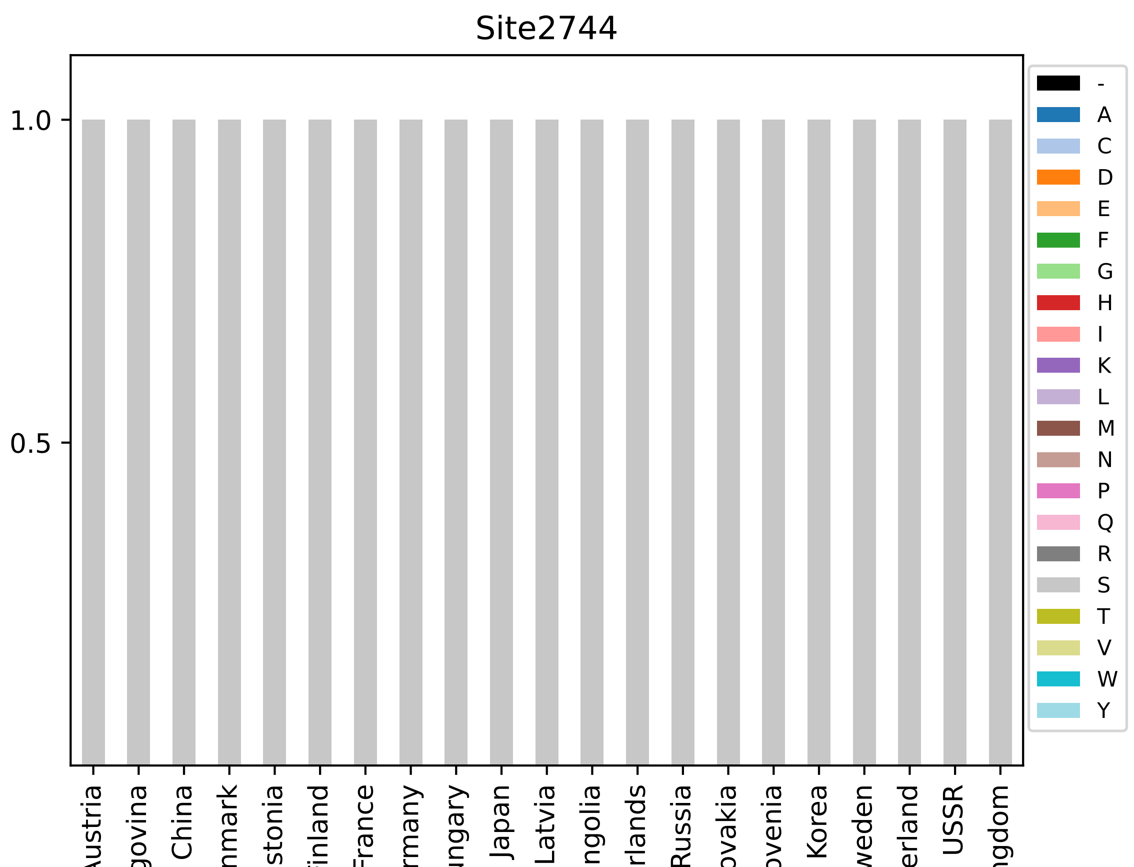
Site2744
1.0
0.5
-
A
C
D
E
F
G
H
I
K
L
M
N
P
Q
R
S
T
V
W
Y
Austria
govina
China
nmark
stonia
inland
France
rmany
ungary
Japan
Latvia
ngolia
rlands
Russia
ovakia
ovenia
Korea
weden
erland
USSR
ngdom

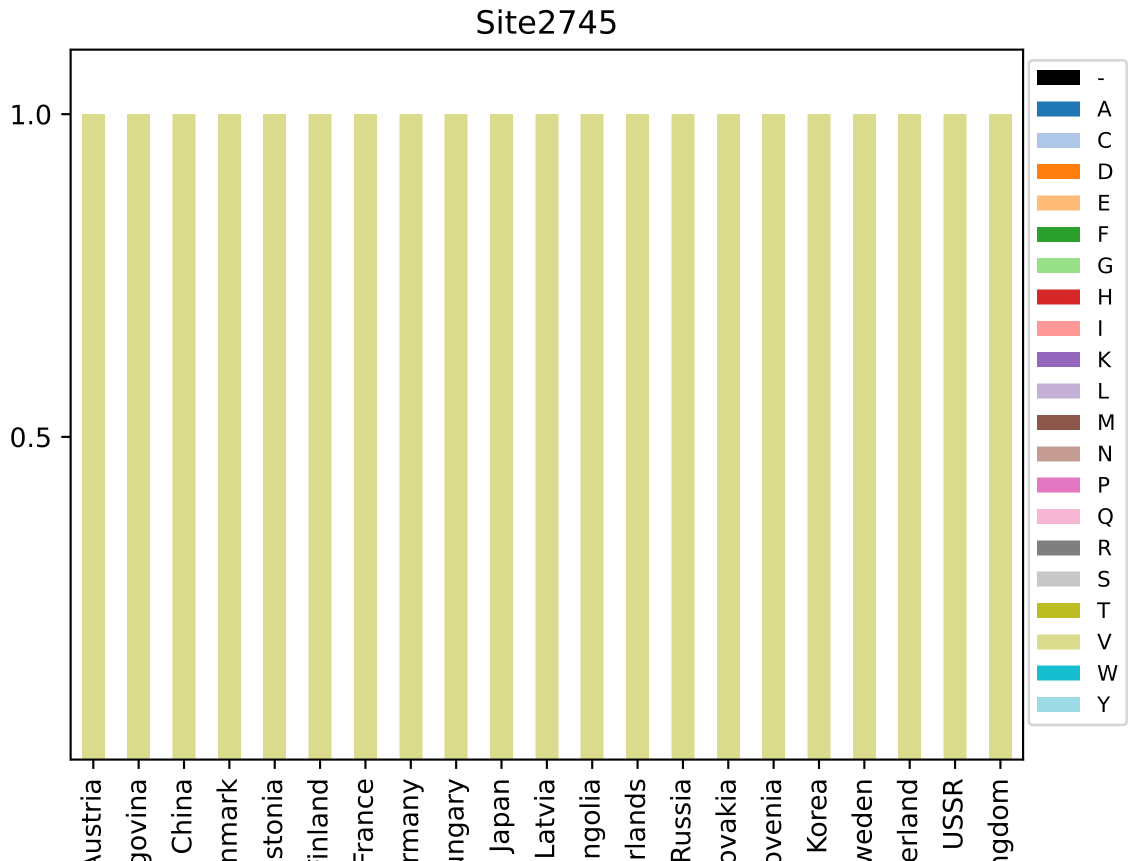
Site2745
1.0
0.5
China
Japan
Latvia
Russia
Korea
USSR
-
A
C
D
E
F
G
H
I
K
L
M
N
P
Q
R
S
T
V
W
Y

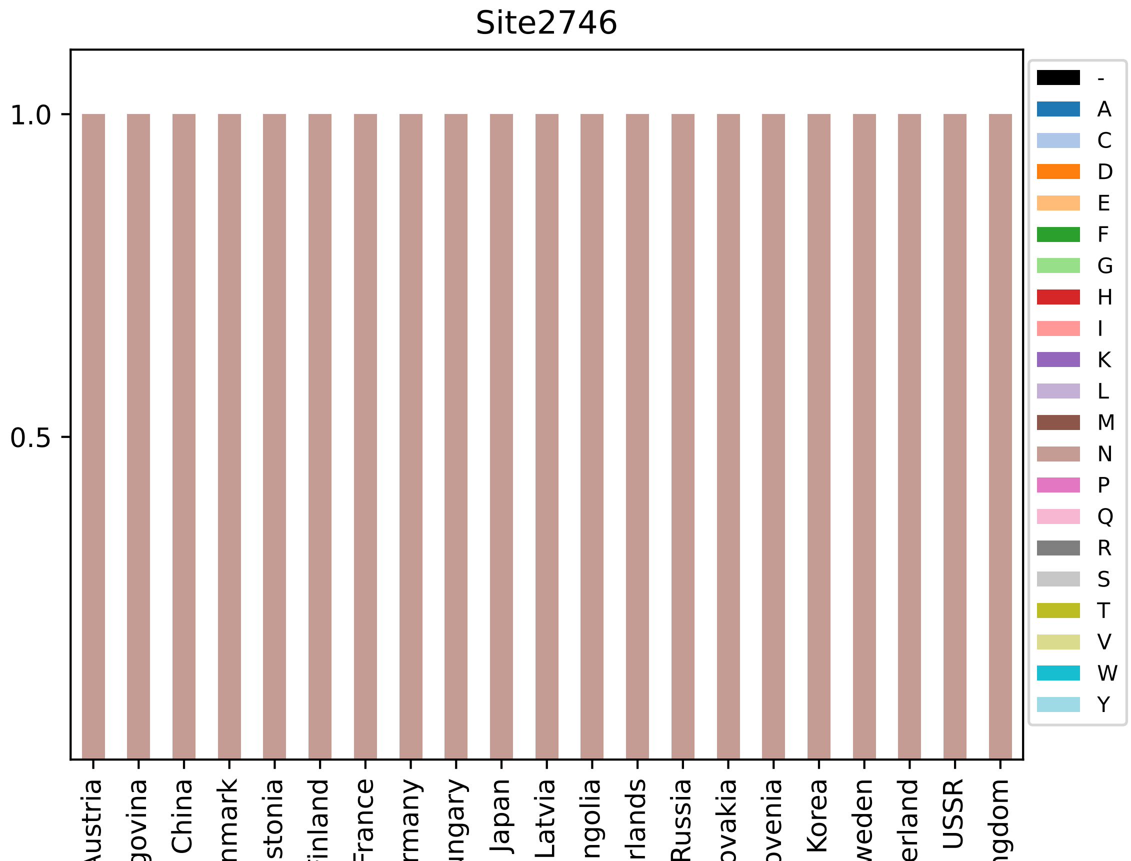

Site2746
1.0
0.5
-
A
C
D
E
F
G
H
I
K
L
M
N
P
Q
R
S
T
V
W
Y
China
Japan
Latvia
Russia
Korea
USSR

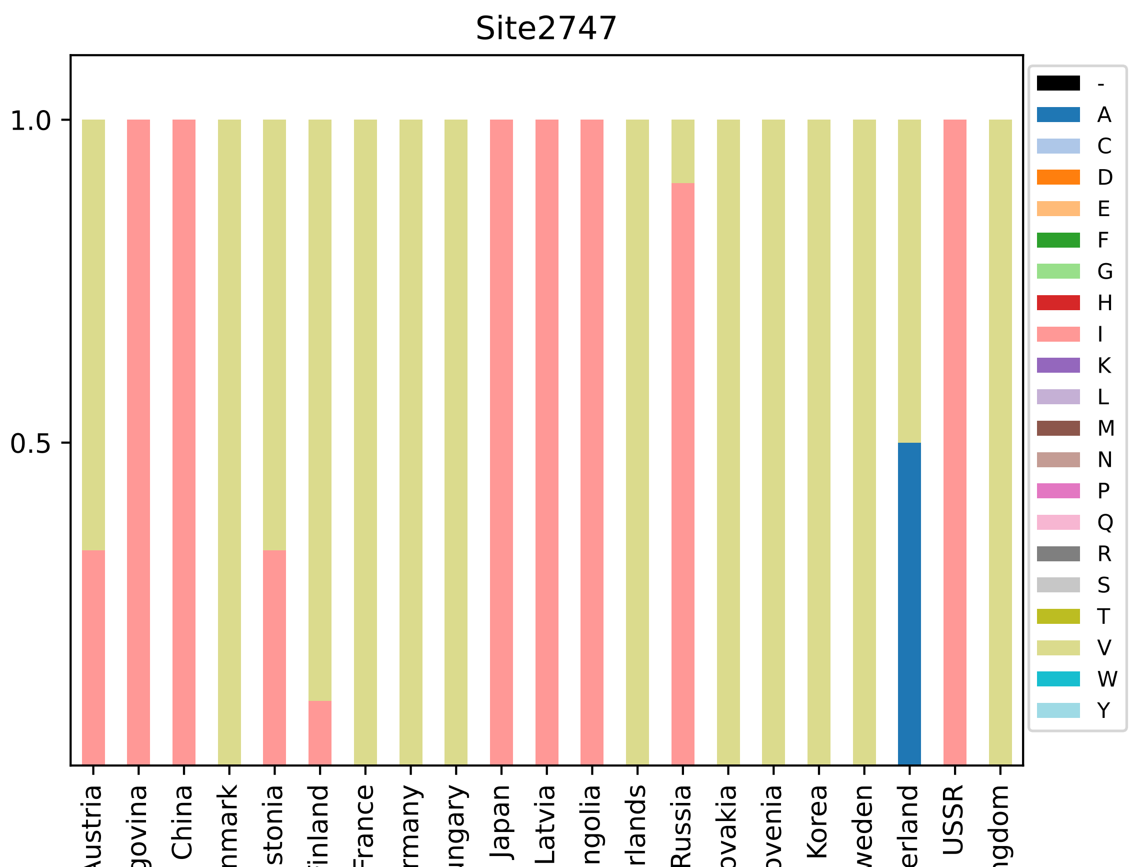

Site2747
1.0
0.5
-
A
C
D
E
F
G
H
I
K
L
M
N
P
Q
R
S
T
V
W
Y
Austria
govina
China
nmark
stonia
inland
France
rmany
ungary
Japan
Latvia
ngolia
rlands
Russia
ovakia
ovenia
Korea
weden
erland
USSR
ngdom

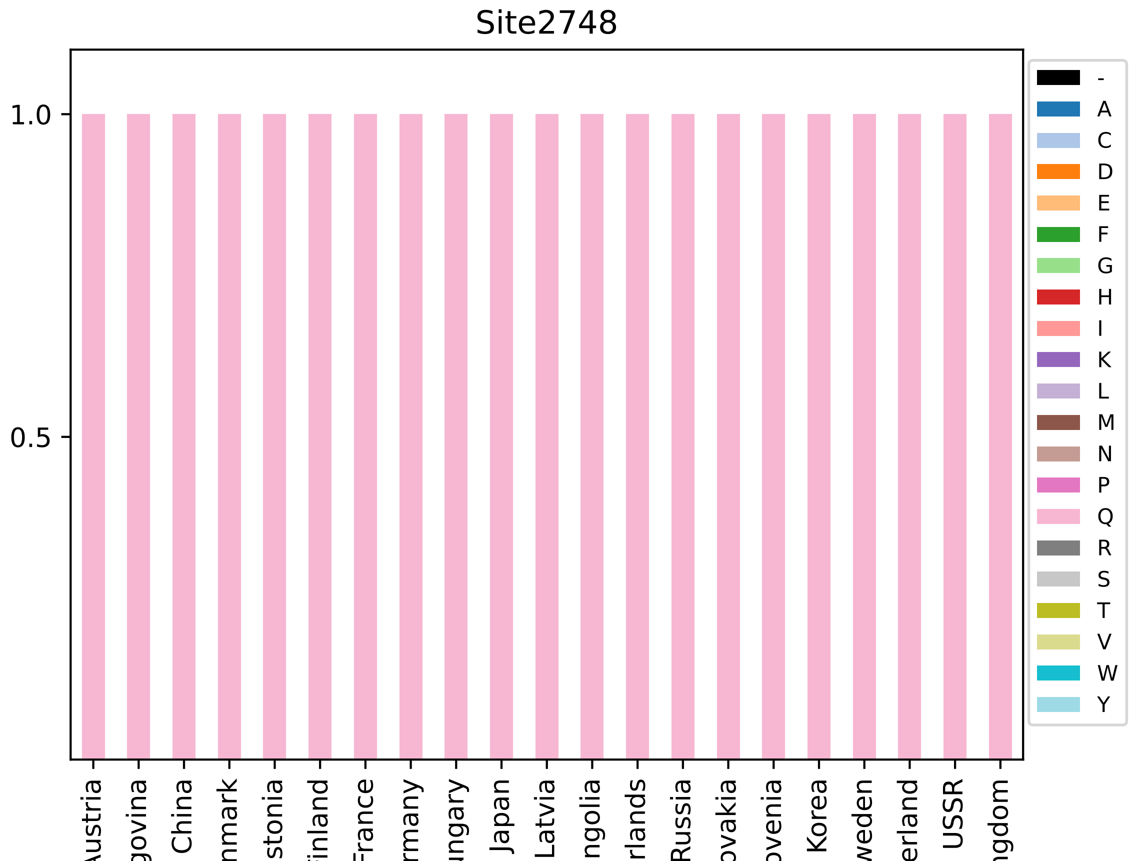

Site2748
1.0
0.5
Austria
govina
China
nmark
stonia
Finland
France
rmany
ungary
Japan
Latvia
ngolia
rlands
Russia
ovakia
ovenia
Korea
weden
erland
USSR
ngdom
-
A
C
D
E
F
G
H
I
K
L
M
N
P
Q
R
S
T
V
W
Y

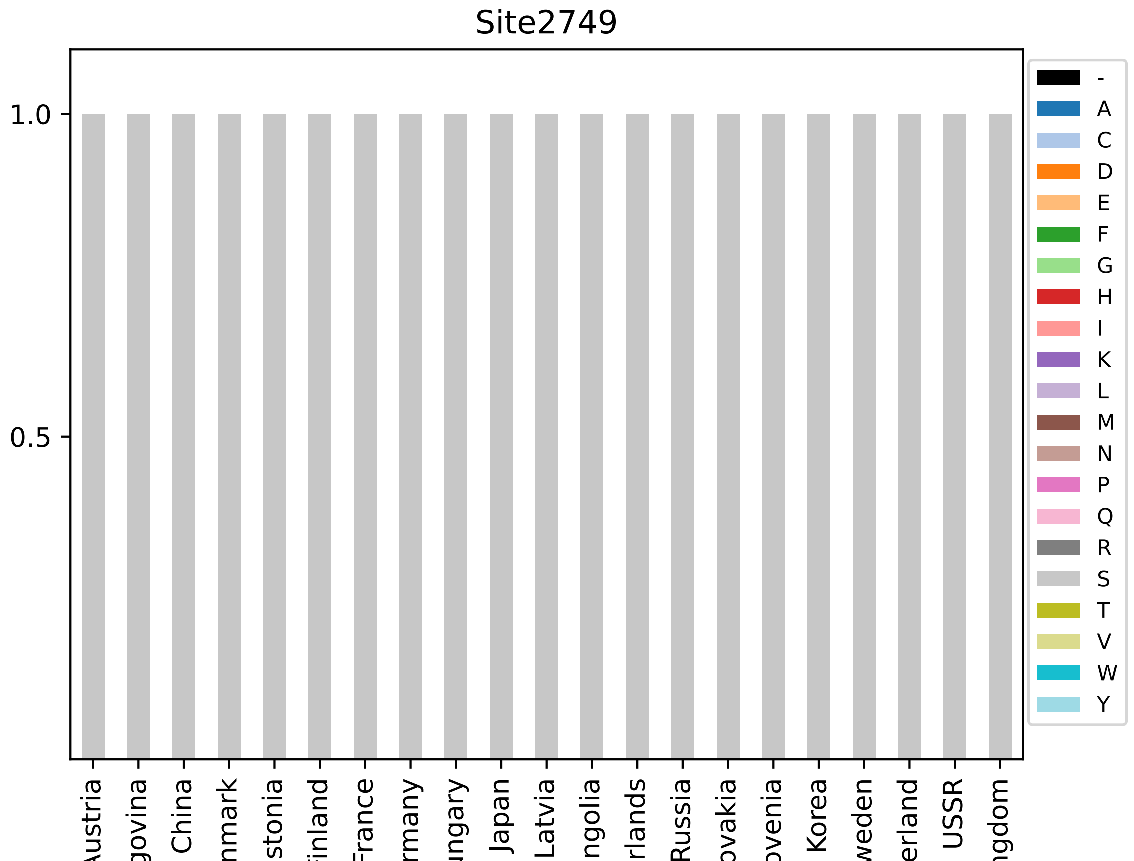
Site2749
1.0
0.5
-
A
C
D
E
F
G
H
I
K
L
M
N
P
Q
R
S
T
V
W
Y
China
Japan
Latvia
Russia
Korea
USSR

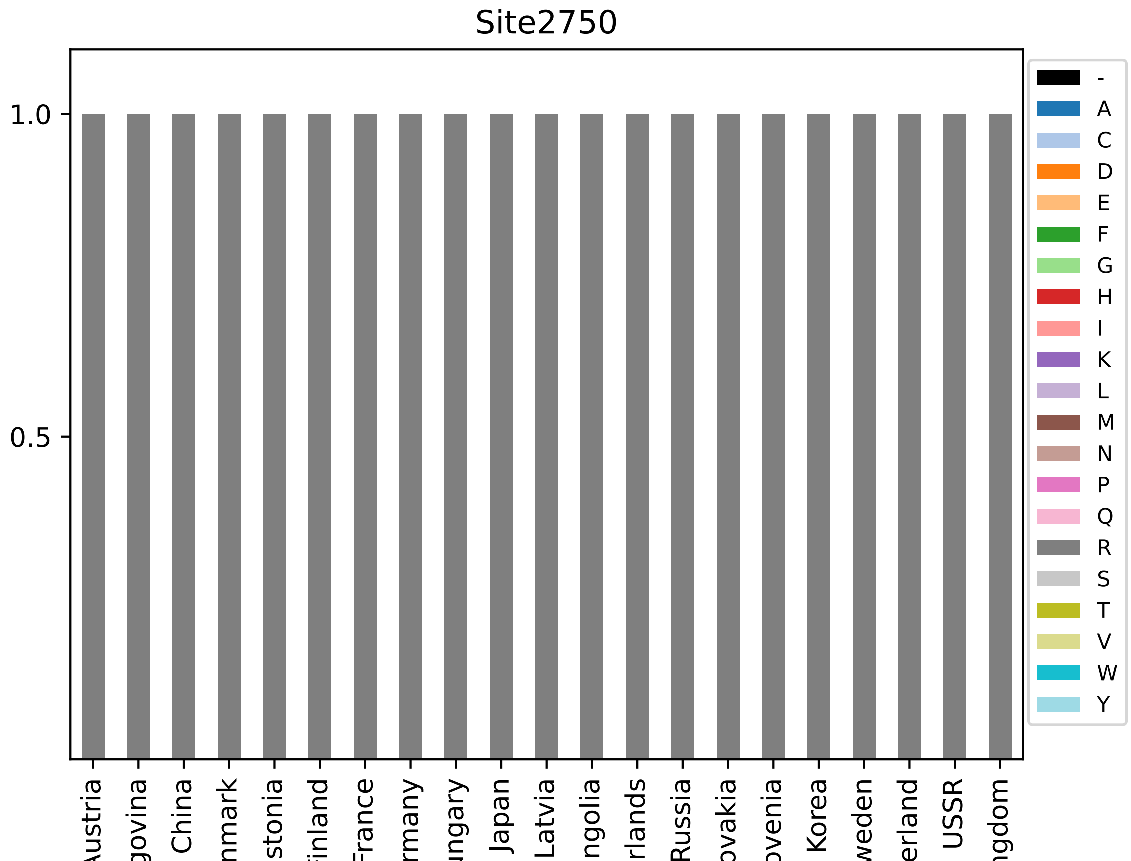

Site2750
1.0
0.5
-
A
C
D
E
F
G
H
I
K
L
M
N
P
Q
R
S
T
V
W
Y
Austria
govina
China
nmark
stonia
Finland
France
rmany
ungary
Japan
Latvia
ngolia
rlands
Russia
ovakia
ovenia
Korea
weden
erland
USSR
ngdom

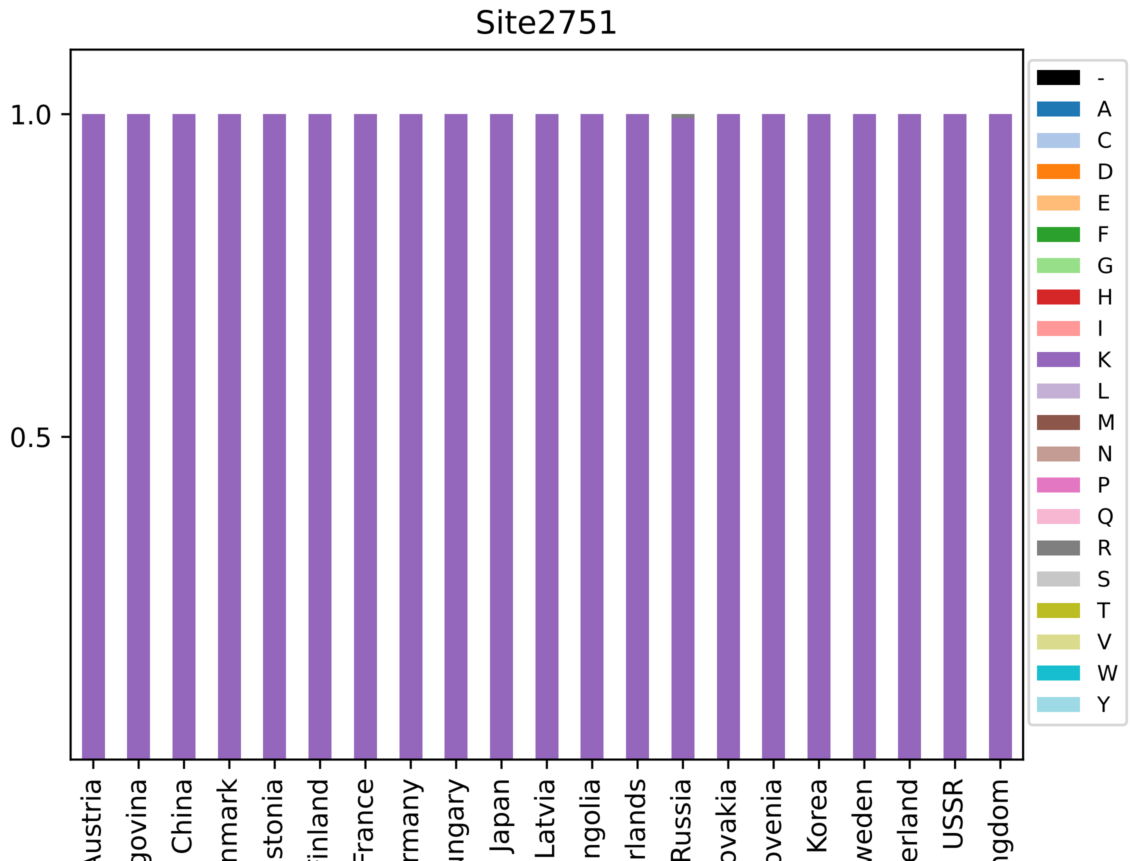
Site2751
1.0
0.5
-
A
C
D
E
F
G
H
I
K
L
M
N
P
Q
R
S
T
V
W
Y
Austria
govina
China
nmark
stonia
Finland
France
rmany
ungary
Japan
Latvia
ngolia
rlands
Russia
ovakia
ovenia
Korea
weden
erland
USSR
ngdom

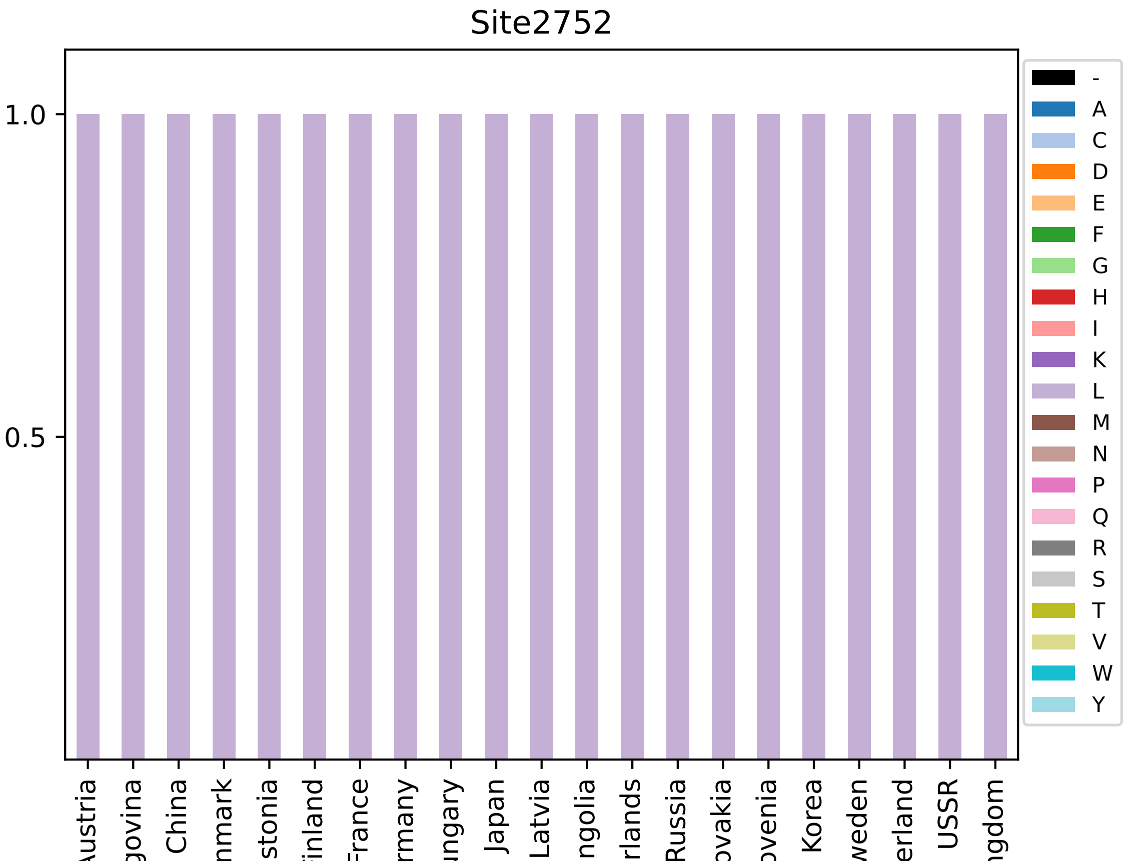
Site2752
1.0
0.5
-
A
C
D
E
F
G
H
I
K
L
M
N
P
Q
R
S
T
V
W
Y
China
Japan
Latvia
Russia
Korea
USSR

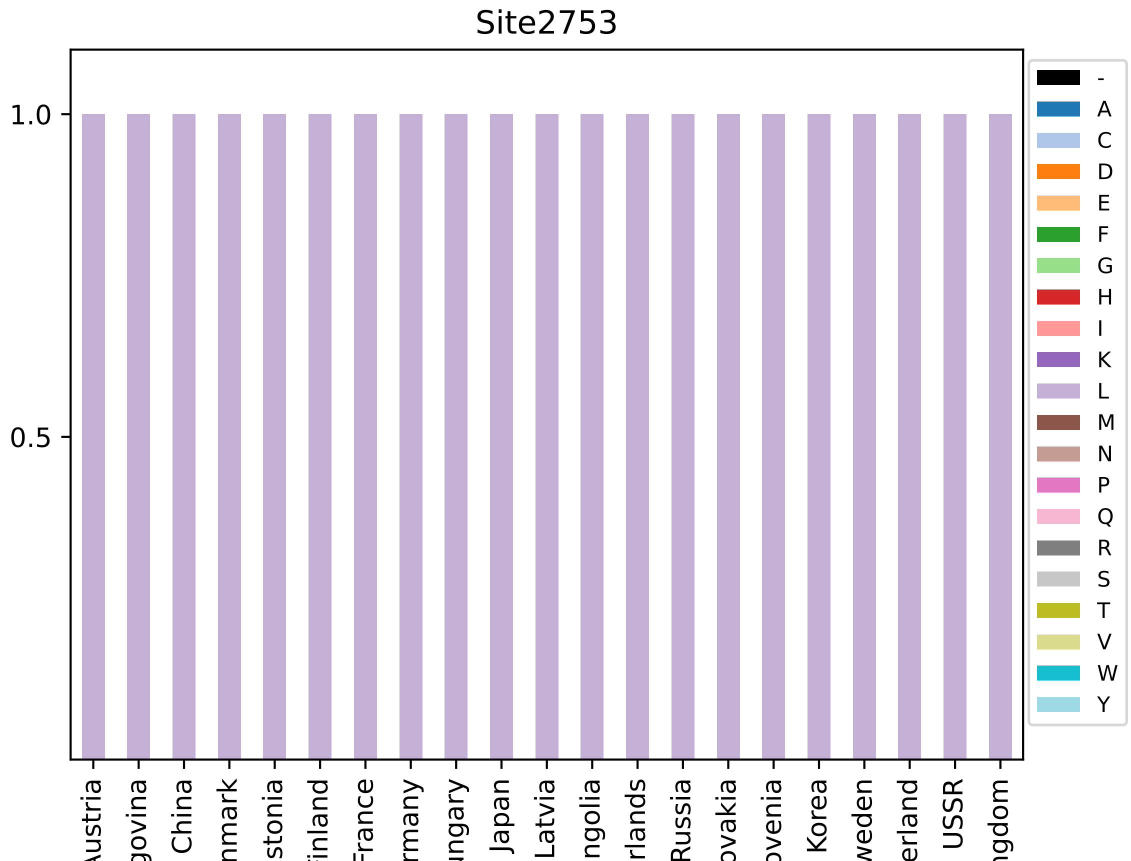
Site2753
1.0
0.5
-
A
C
D
E
F
G
H
I
K
L
M
N
P
Q
R
S
T
V
W
Y
Austria
govina
China
nmark
stonia
Finland
France
rmany
ungary
Japan
Latvia
ngolia
rlands
Russia
ovakia
ovenia
Korea
weden
erland
USSR
ngdom

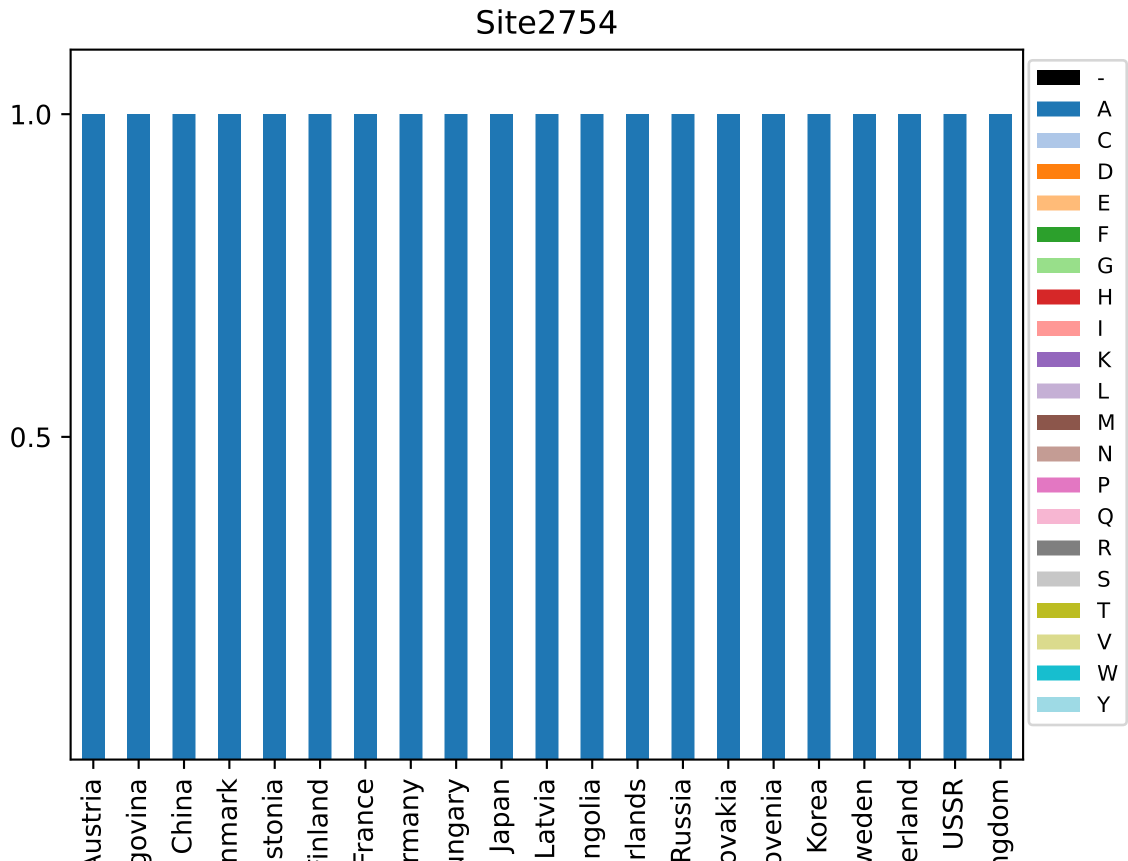
Site2754
1.0
0.5
-
A
C
D
E
F
G
H
I
K
L
M
N
P
Q
R
S
T
V
W
Y
China
Japan
Latvia
Russia
Korea
USSR

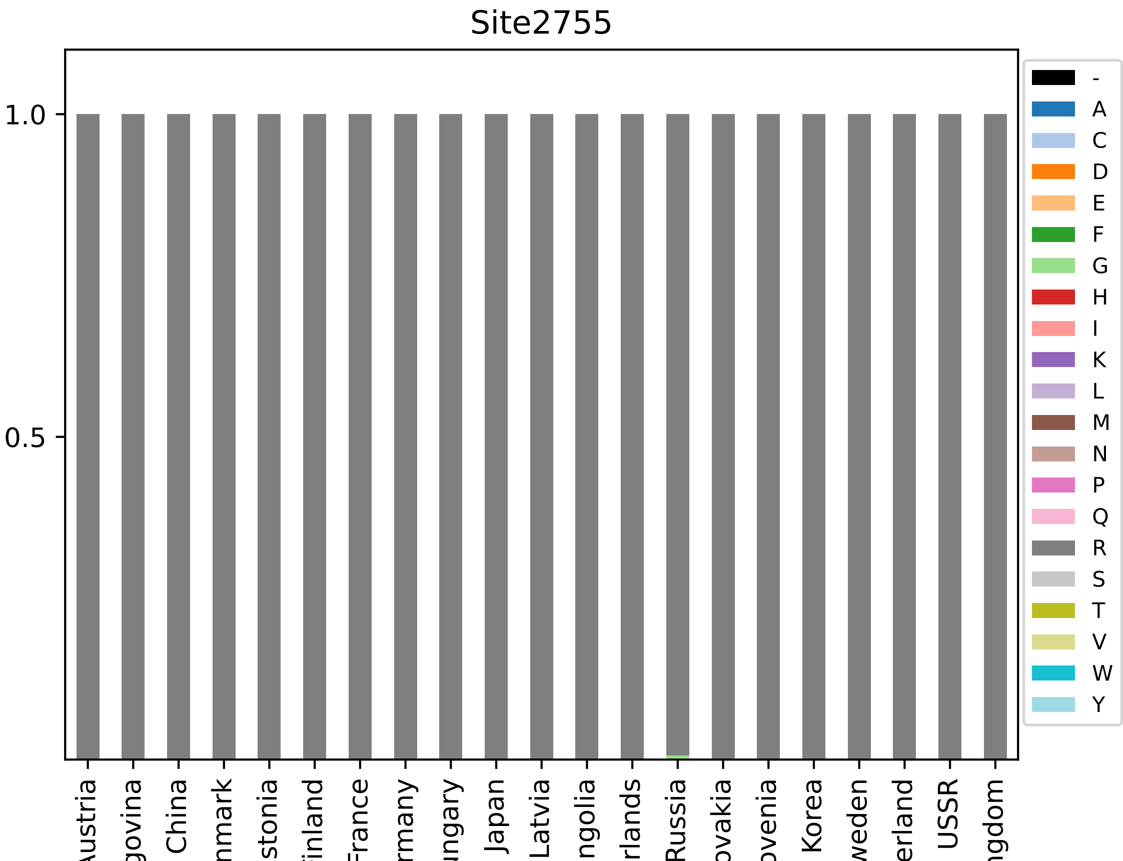
Site2755
1.0
0.5
-
A
C
D
E
F
G
H
I
K
L
M
N
P
Q
R
S
T
V
W
Y
China
Japan
Latvia
Russia
Korea
USSR

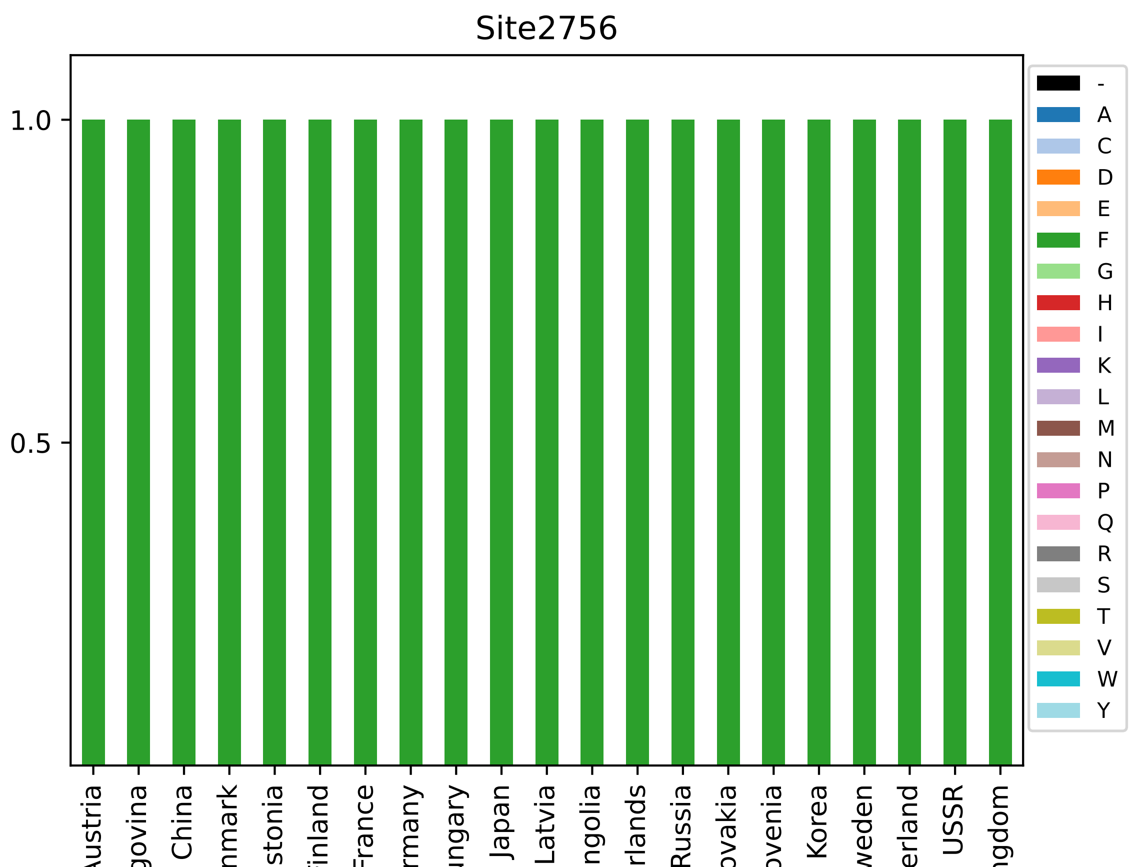

Site2756
1.0
0.5
-
A
C
D
E
F
G
H
I
K
L
M
N
P
Q
R
S
T
V
W
Y
Austria
govina
China
nmark
stonia
Finland
France
rmany
ungary
Japan
Latvia
ngolia
rlands
Russia
ovakia
ovenia
Korea
weden
erland
USSR
ngdom

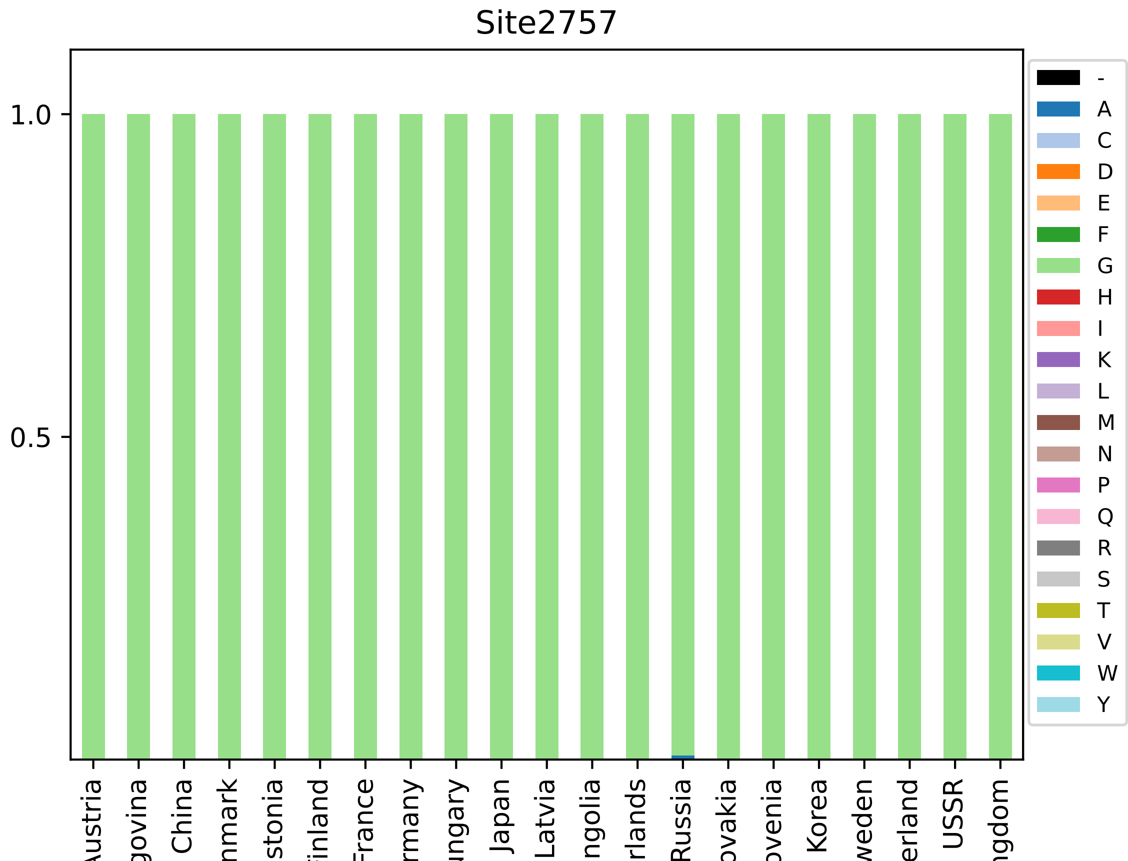
Site2757
1.0
0.5
-
A
C
D
E
F
G
H
I
K
L
M
N
P
Q
R
S
T
V
W
Y
Austria
govina
China
nmark
stonia
inland
France
rmany
ungary
Japan
Latvia
ngolia
rlands
Russia
ovakia
ovenia
Korea
weden
erland
USSR
ngdom

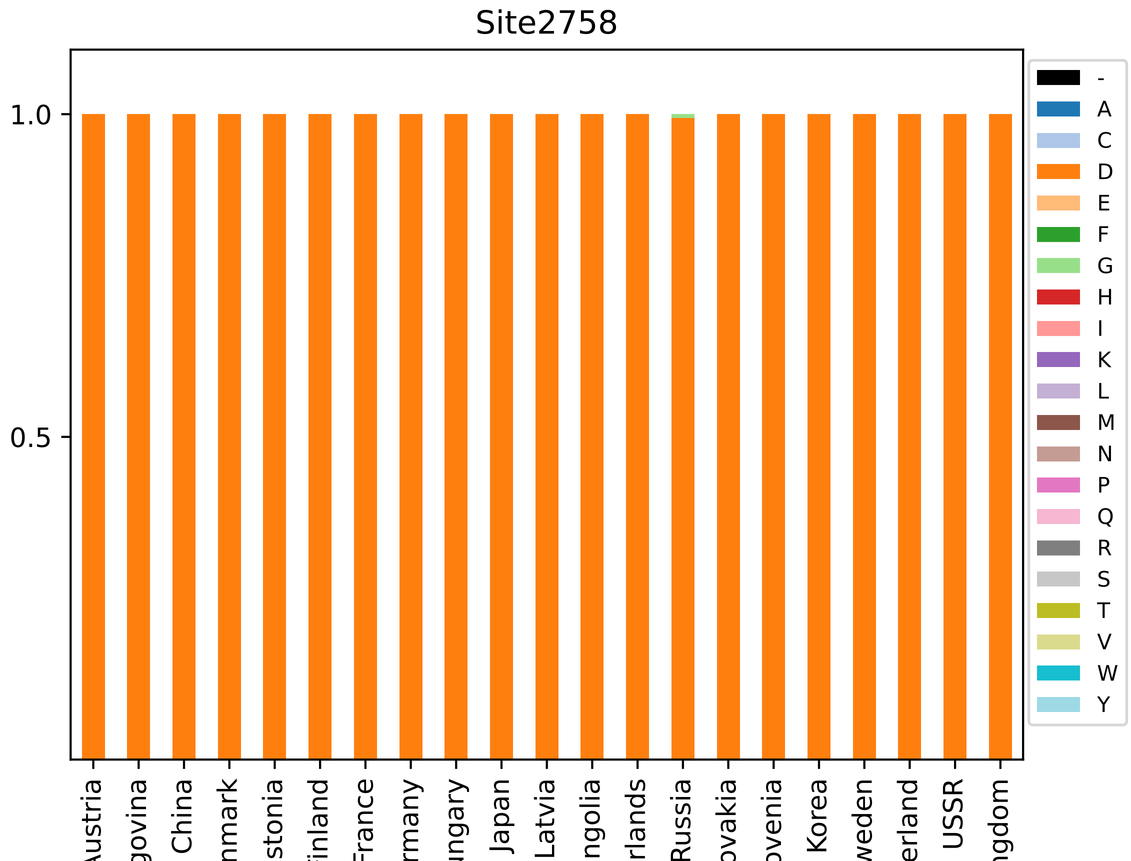

Site2758
1.0
0.5
-
A
C
D
E
F
G
H
I
K
L
M
N
P
Q
R
S
T
V
W
Y
China
Japan
Latvia
Russia
Korea
USSR

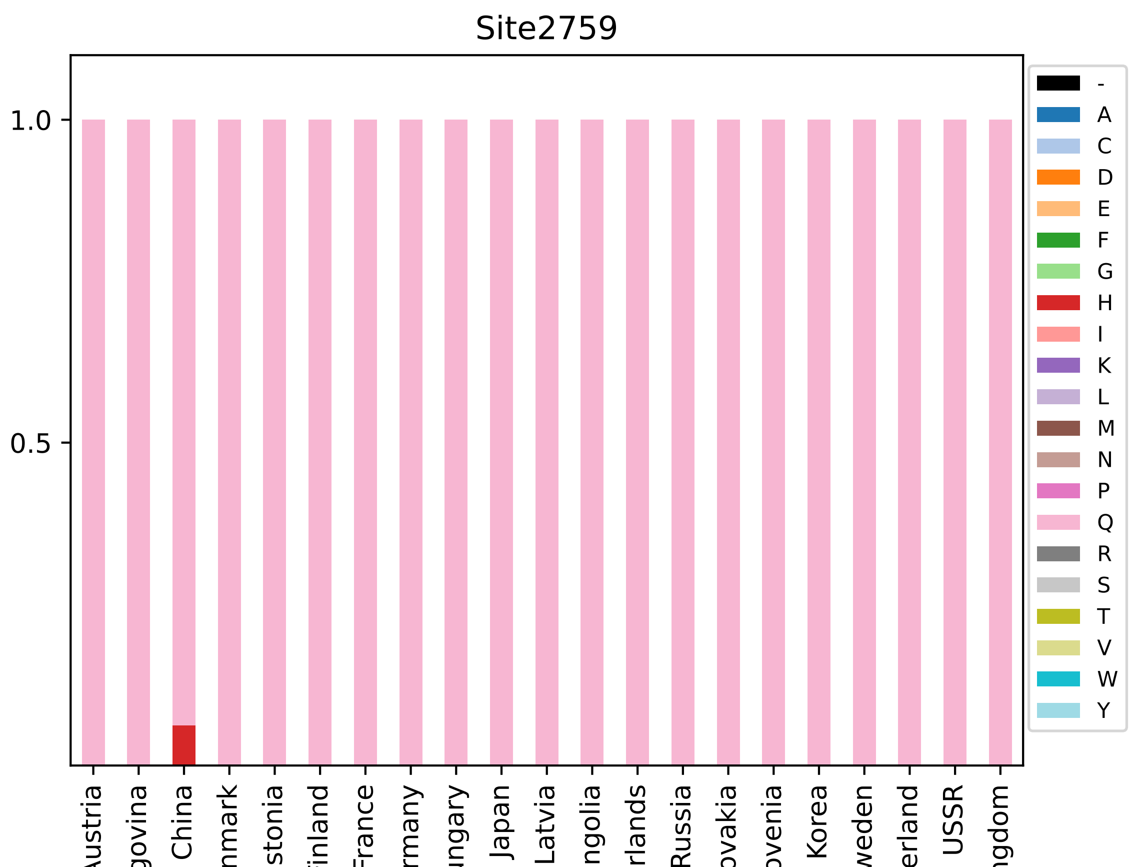
Site2759
1.0
0.5
-
A
C
D
E
F
G
H
I
K
L
M
N
P
Q
R
S
T
V
W
Y
China
Japan
Latvia
Russia
Korea
USSR

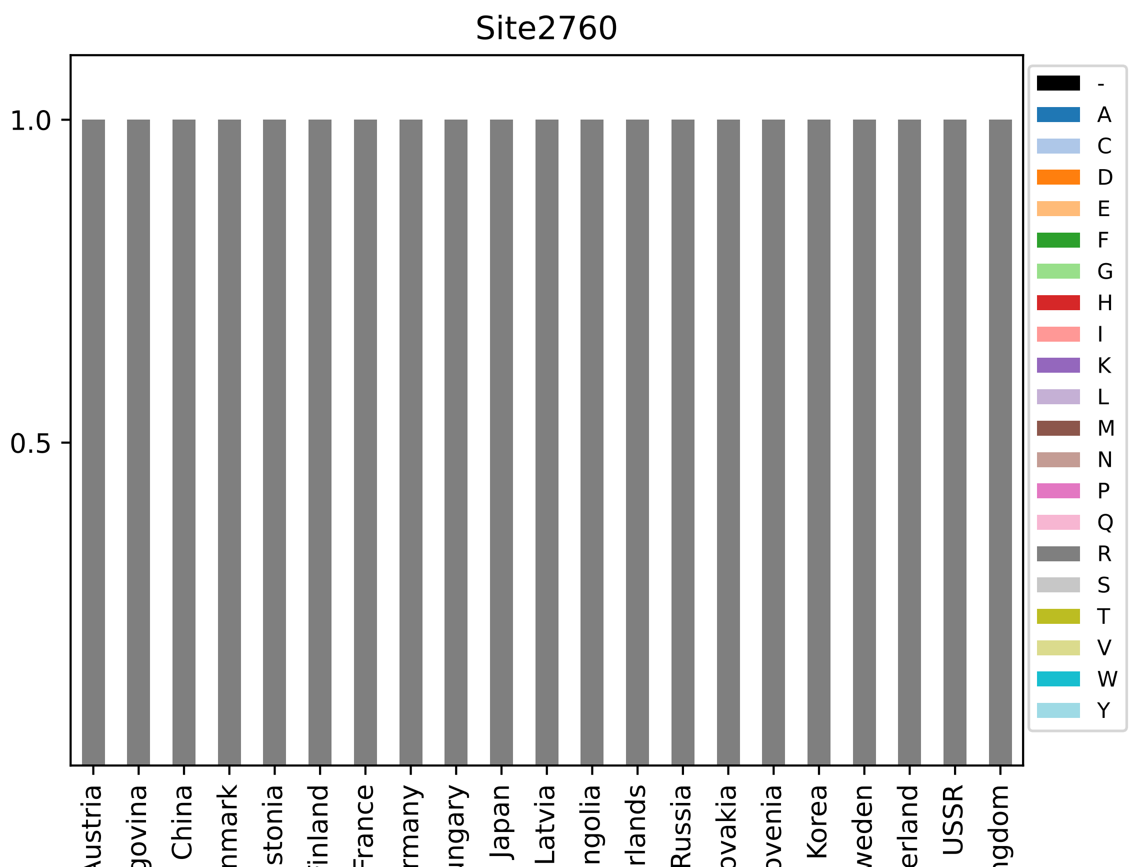
Site2760
1.0
0.5
-
A
C
D
E
F
G
H
I
K
L
M
N
P
Q
R
S
T
V
W
Y
China
Japan
Latvia
Russia
Korea
USSR

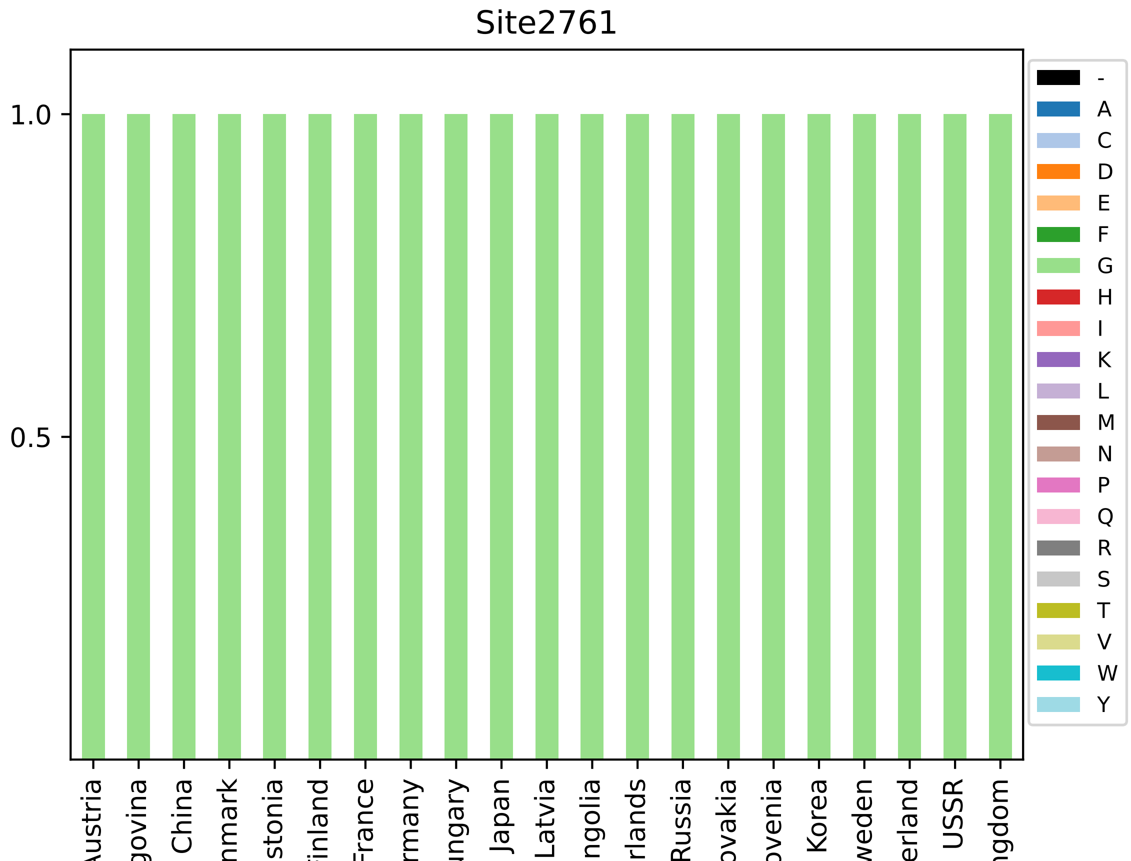
Site2761
1.0
0.5
-
A
C
D
E
F
G
H
I
K
L
M
N
P
Q
R
S
T
V
W
Y
China
Japan
Latvia
Russia
Korea
USSR

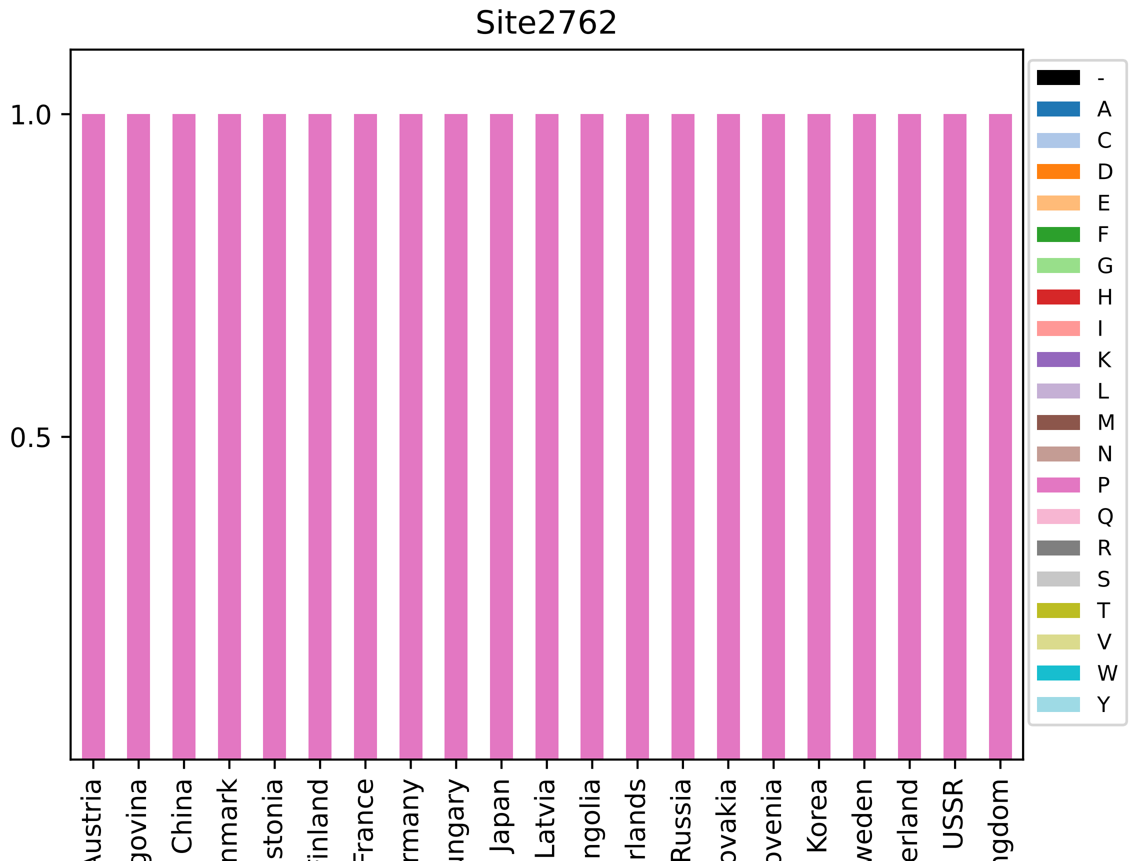

Site2762
1.0
0.5
ustria
govina
China
nmark
stonia
inland
rance
rmany
ungary
Japan
Latvia
ngolia
rlands
Russia
ovakia
ovenia
Korea
weden
erland
USSR
ngdom
-
A
C
D
E
F
G
H
I
K
L
M
N
P
Q
R
S
T
V
W
Y

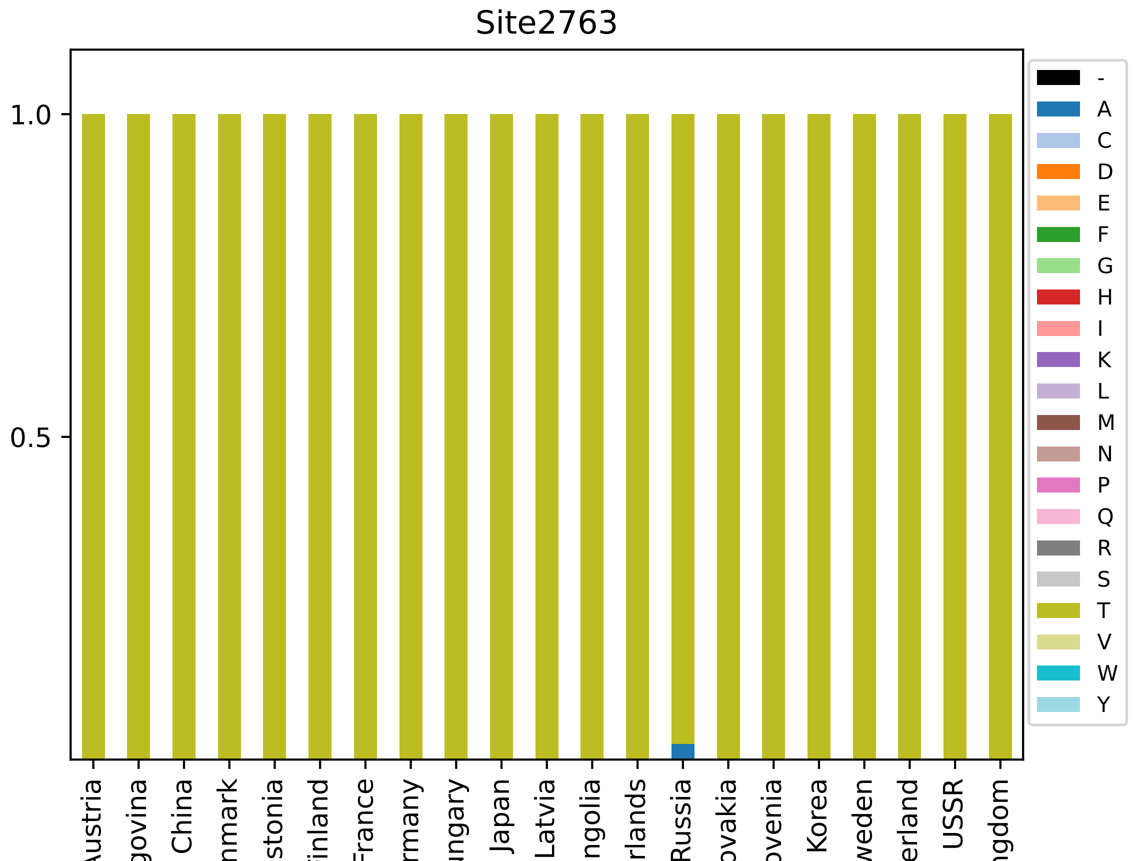

Site2763
1.0
0.5
-
A
C
D
E
F
G
H
I
K
L
M
N
P
Q
R
S
T
V
W
Y
Austria
govina
China
nmark
stonia
inland
France
rmany
ungary
Japan
Latvia
ngolia
rlands
Russia
ovakia
ovenia
Korea
weden
erland
USSR
ngdom

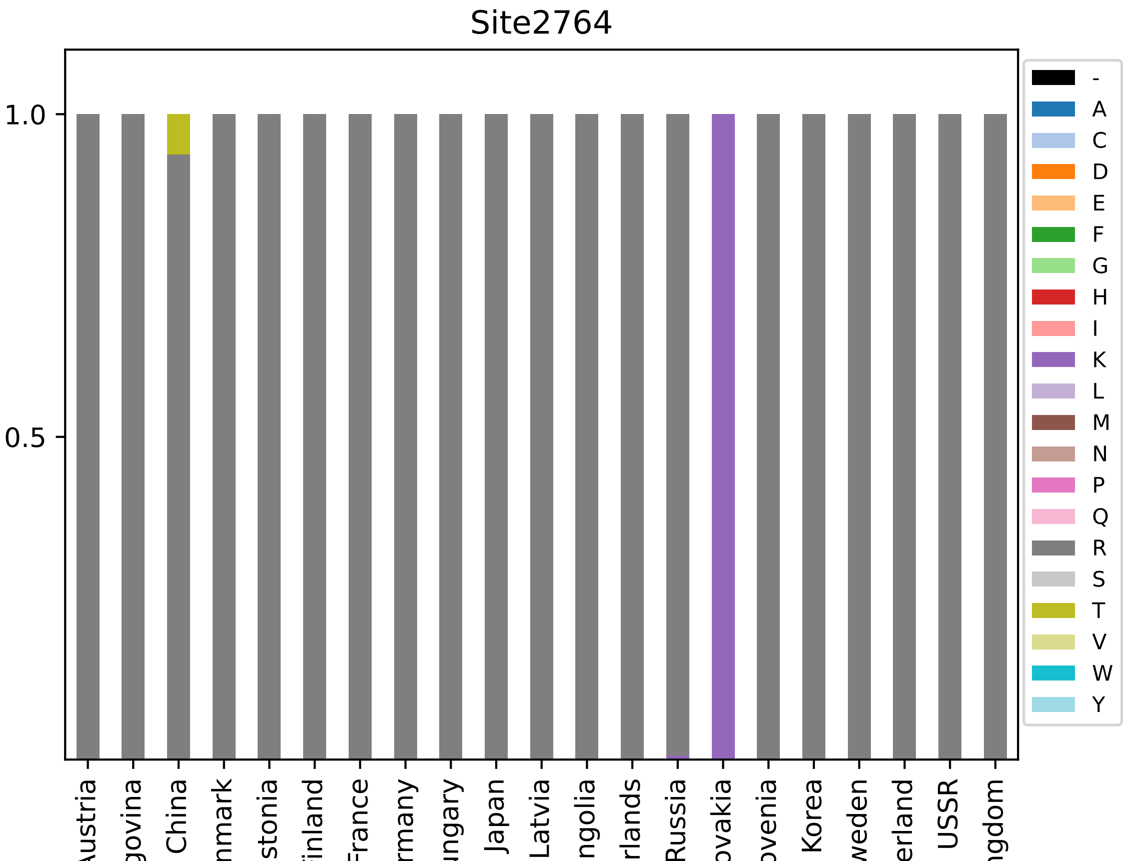

Site2764
1.0
0.5
-
A
C
D
E
F
G
H
I
K
L
M
N
P
Q
R
S
T
V
W
Y
China
Japan
Latvia
Russia
Korea
USSR

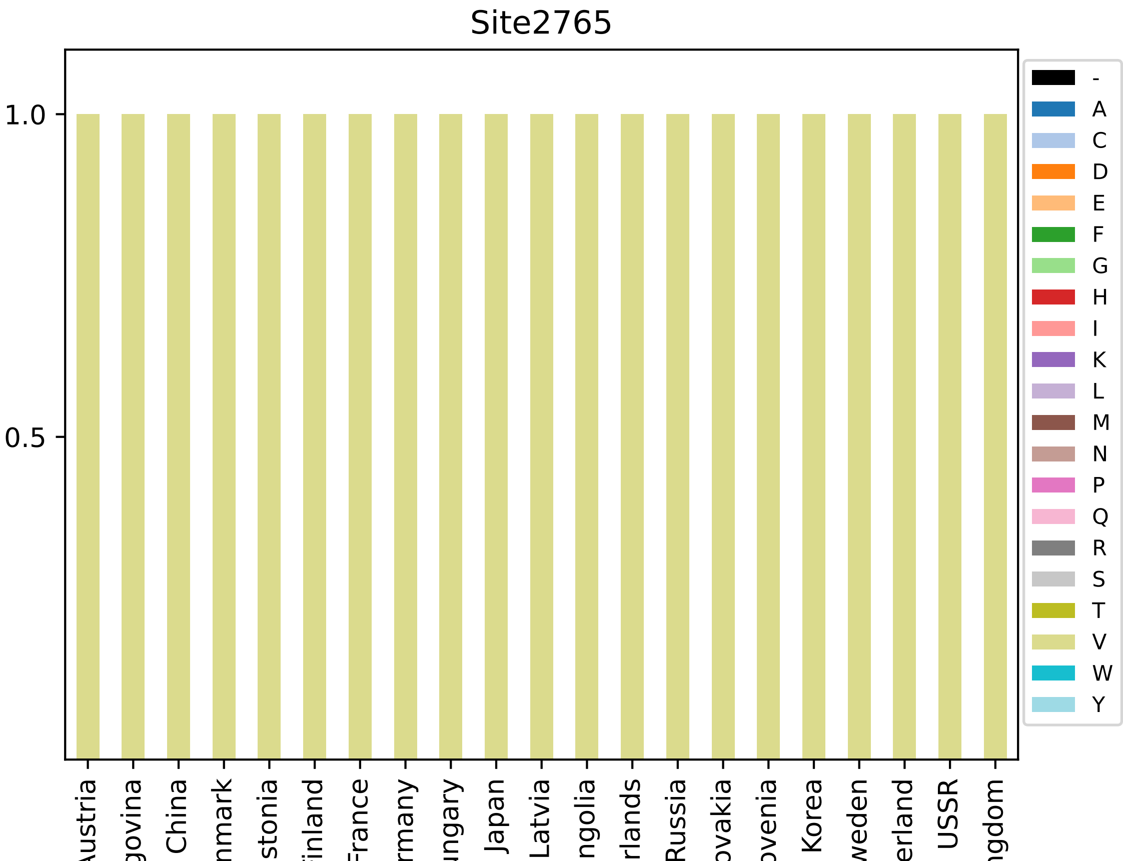
Site2765
1.0
0.5
-
A
C
D
E
F
G
H
I
K
L
M
N
P
Q
R
S
T
V
W
Y
China
Japan
Latvia
Russia
Korea
USSR

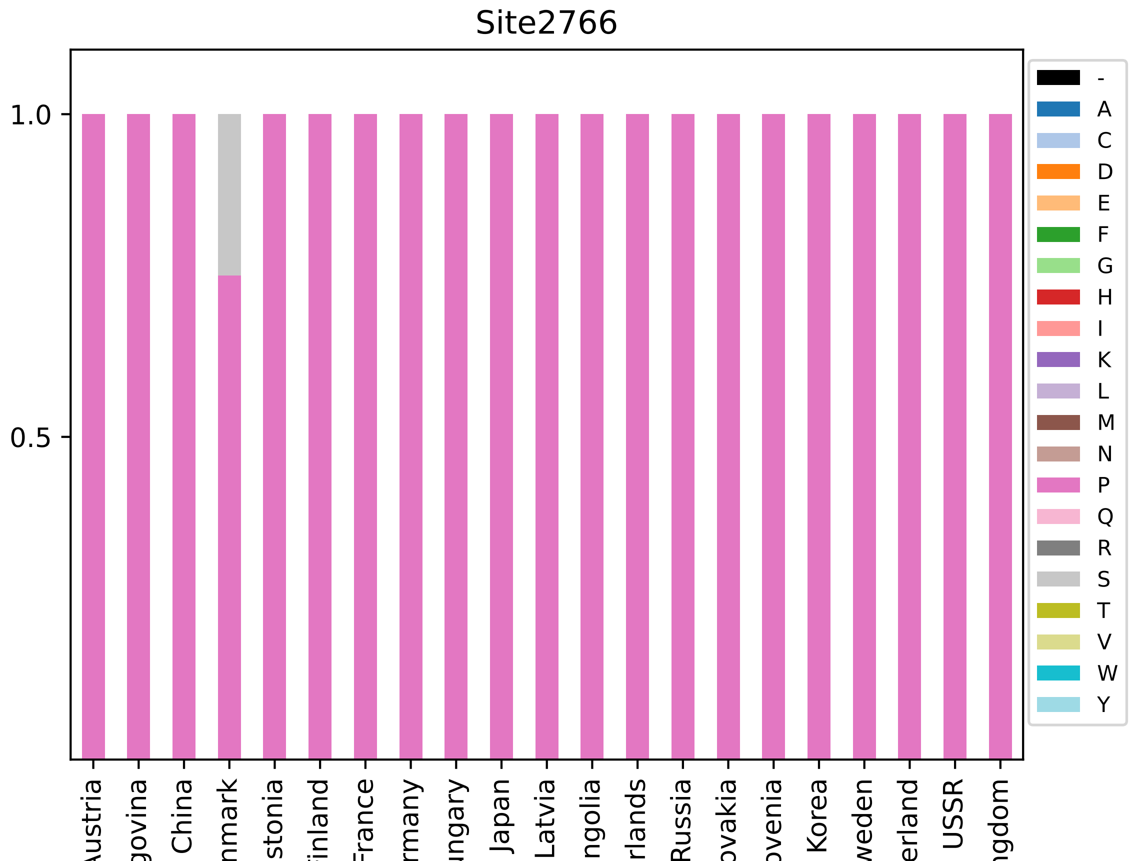
Site2766
1.0
0.5
-
A
C
D
E
F
G
H
I
K
L
M
N
P
Q
R
S
T
V
W
Y
Austria
govina
China
nmark
stonia
Finland
France
rmany
ungary
Japan
Latvia
ngolia
rlands
Russia
ovakia
ovenia
Korea
weden
erland
USSR
ngdom

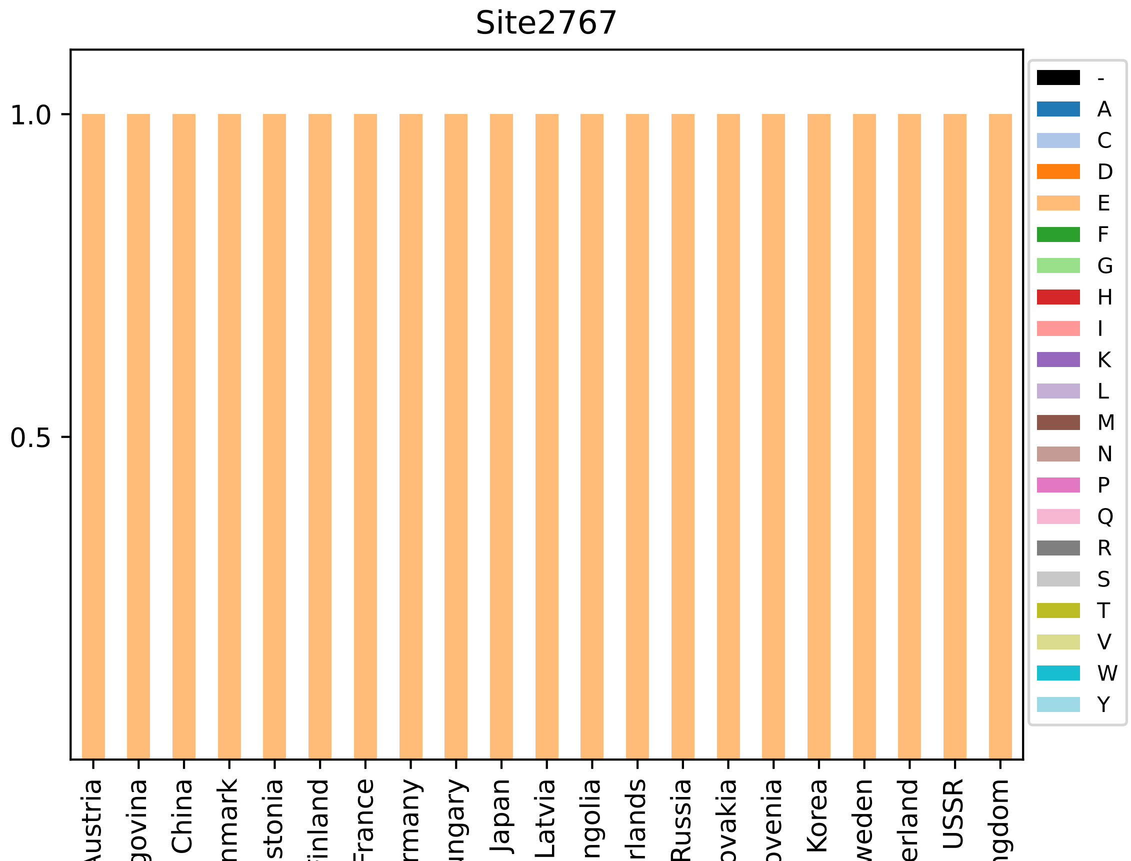

Site2767
1.0
0.5
-
A
C
D
E
F
G
H
I
K
L
M
N
P
Q
R
S
T
V
W
Y
Austria
govina
China
nmark
stonia
Finland
France
rmany
ungary
Japan
Latvia
ngolia
rlands
Russia
ovakia
ovenia
Korea
weden
erland
USSR
ngdom

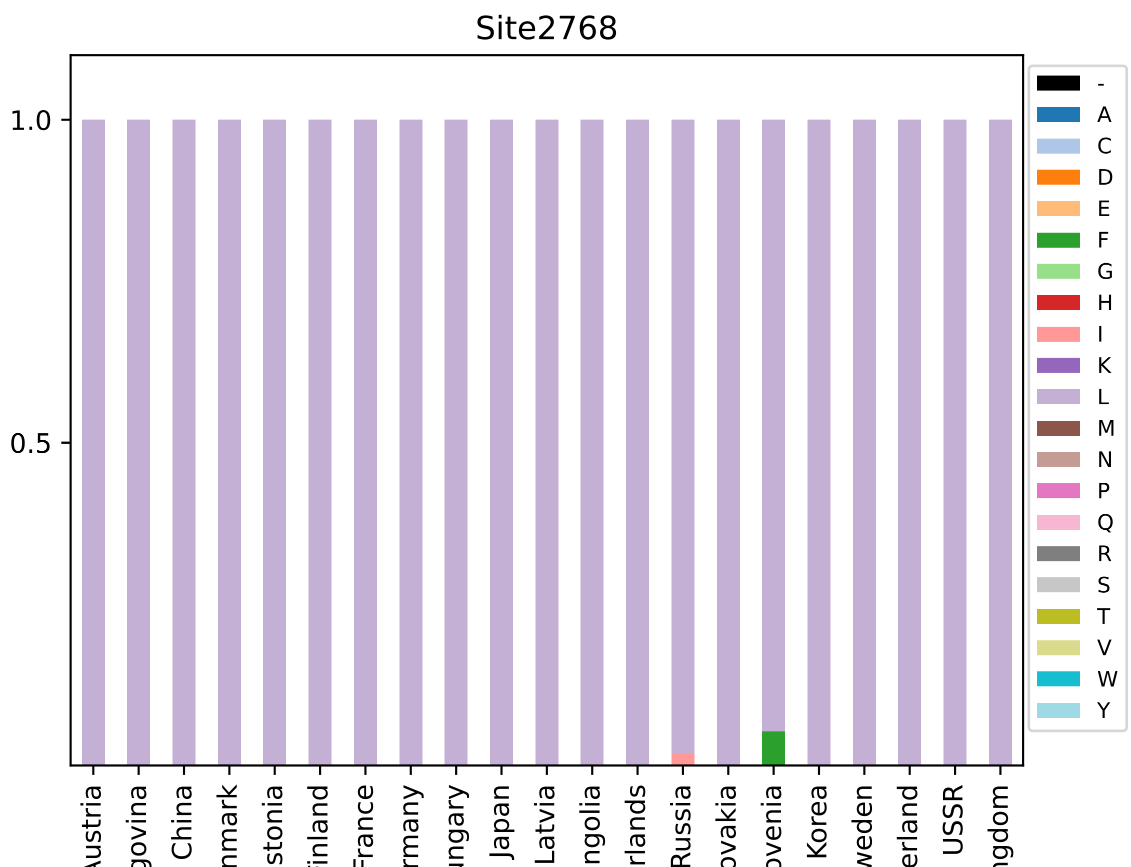
Site2768
1.0
0.5
-
A
C
D
E
F
G
H
I
K
L
M
N
P
Q
R
S
T
V
W
Y
Austria
China
Finland
France
Japan
Latvia
Russia
Korea
USSR

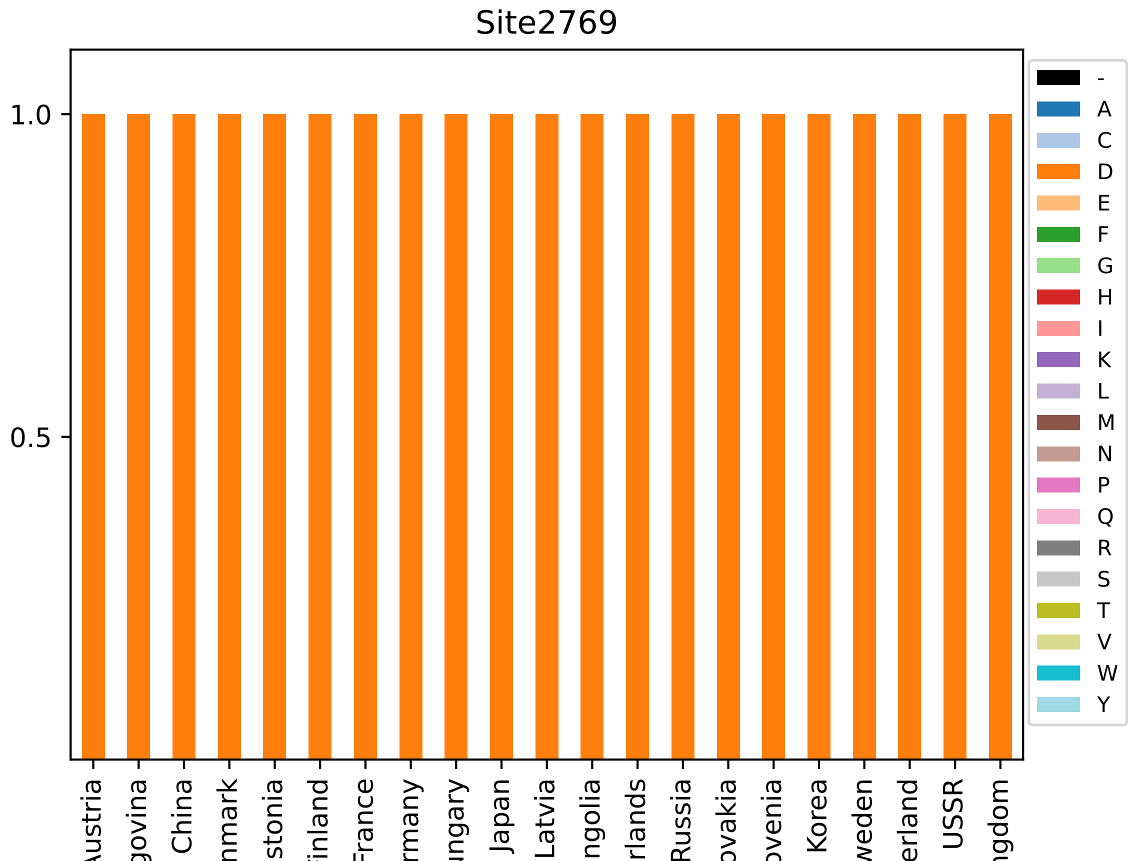

Site2769
1.0
0.5
-
A
C
D
E
F
G
H
I
K
L
M
N
P
Q
R
S
T
V
W
Y
Austria
China
Estonia
Finland
France
Japan
Latvia
Russia
Korea
USSR

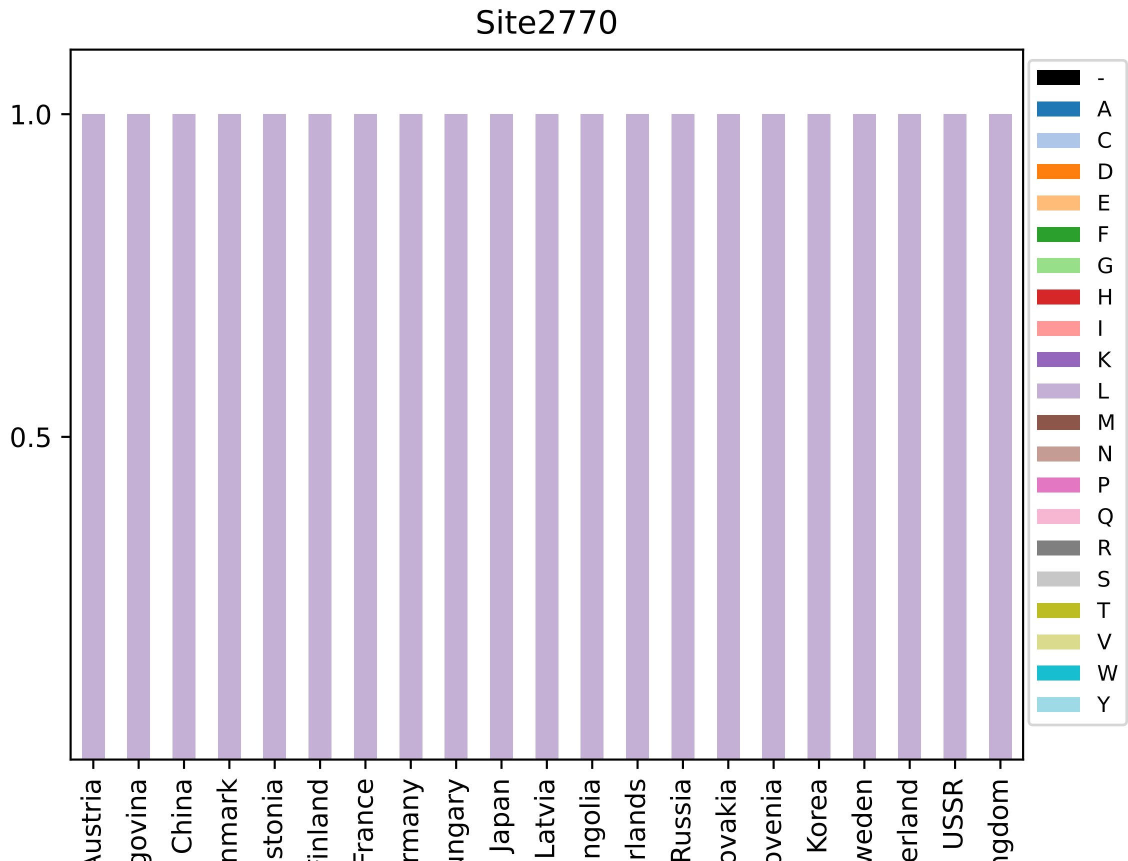

Site2770
1.0
0.5
-
A
C
D
E
F
G
H
I
K
L
M
N
P
Q
R
S
T
V
W
Y
China
Japan
Latvia
Russia
Korea
USSR

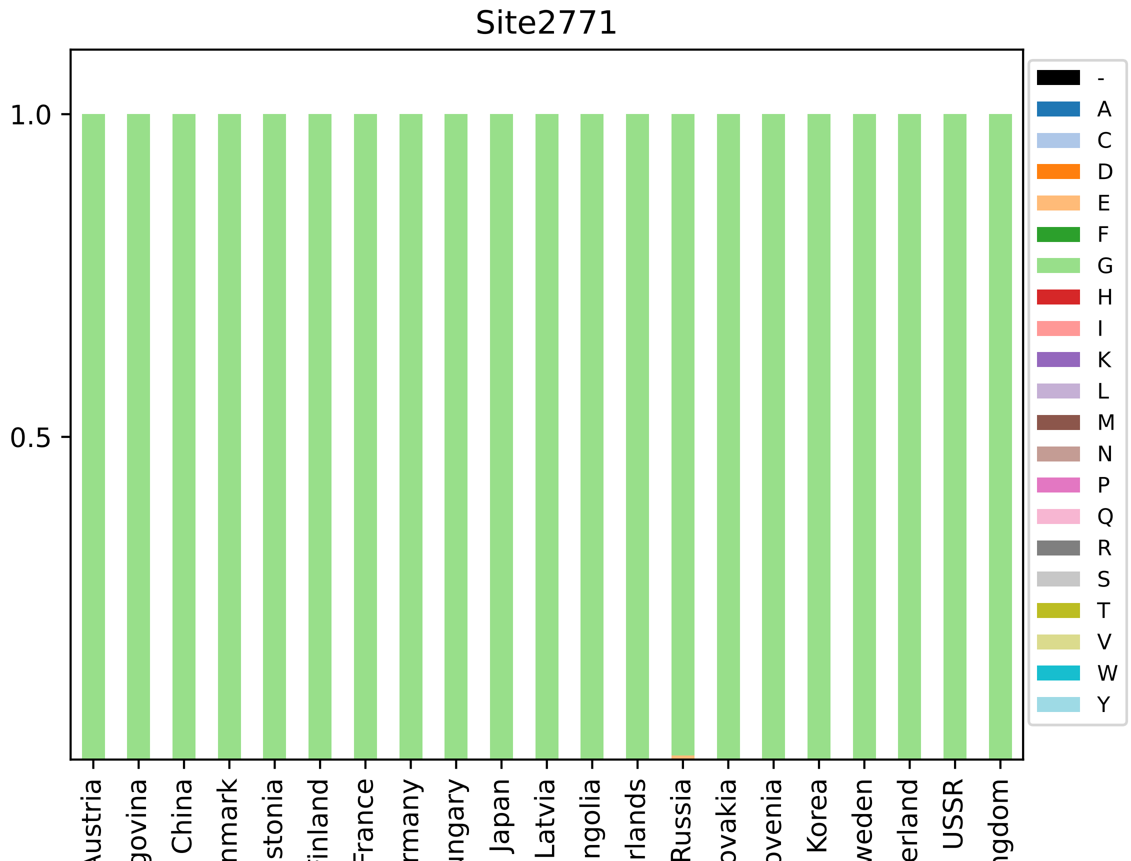

Site2771
1.0
0.5
-
A
C
D
E
F
G
H
I
K
L
M
N
P
Q
R
S
T
V
W
Y
China
Japan
Latvia
Russia
Korea
USSR

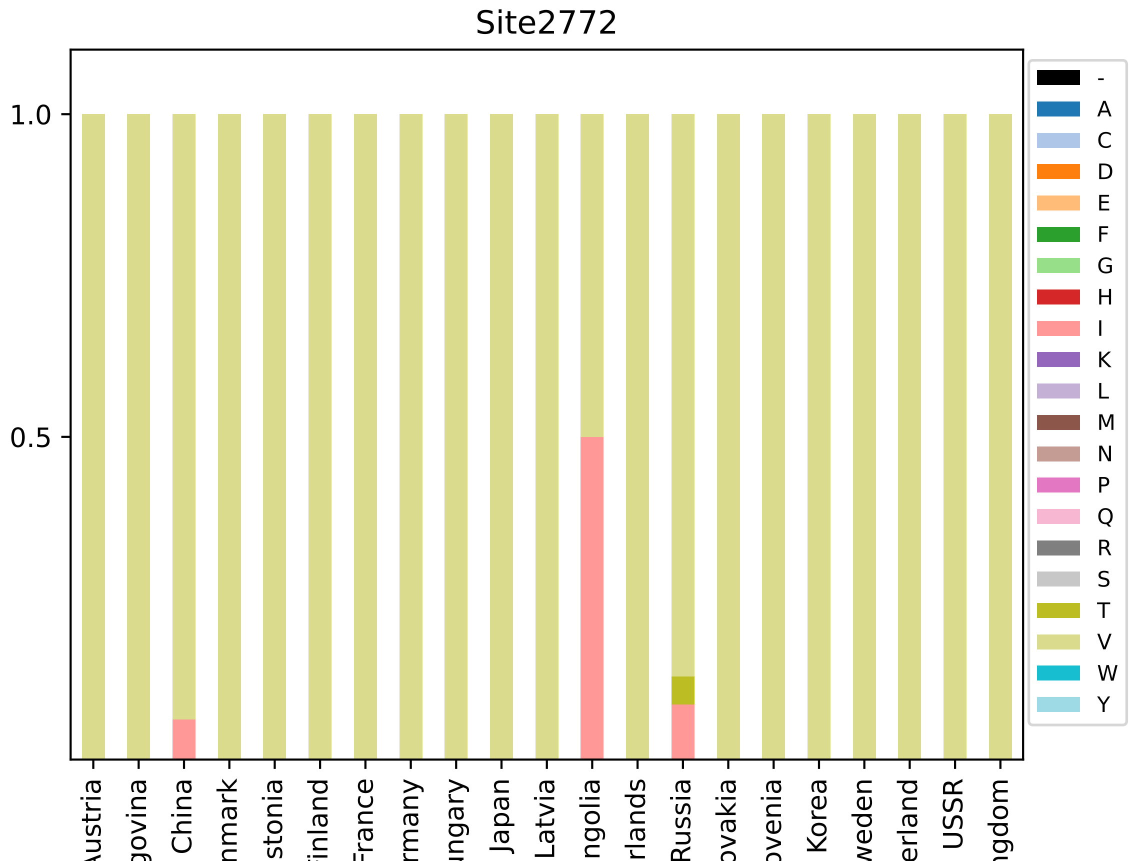
Site2772
1.0
0.5
-
A
C
D
E
F
G
H
I
K
L
M
N
P
Q
R
S
T
V
W
Y
Austria
govina
China
nmark
stonia
Finland
France
rmany
ungary
Japan
Latvia
ngolia
rlands
Russia
ovakia
ovenia
Korea
weden
erland
USSR
ngdom

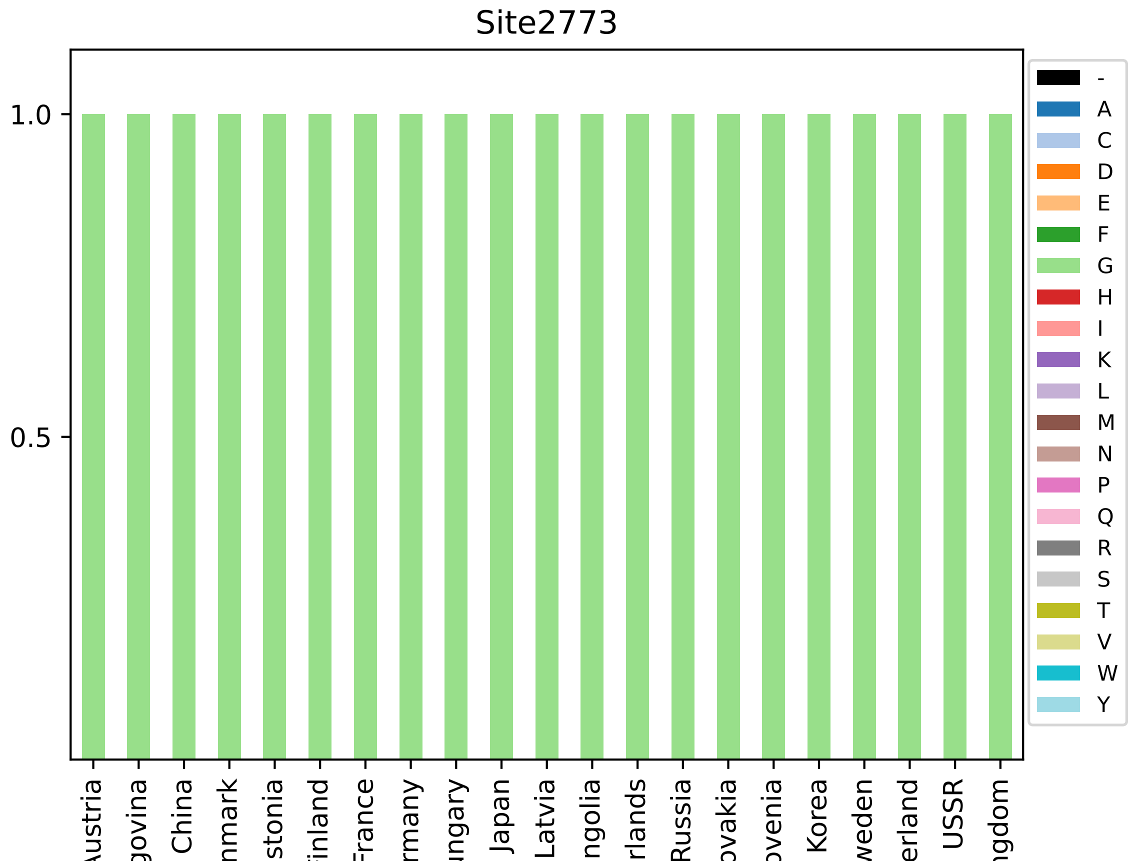
Site2773
1.0
0.5
-
A
C
D
E
F
G
H
I
K
L
M
N
P
Q
R
S
T
V
W
Y
China
Japan
Latvia
Russia
Korea
USSR

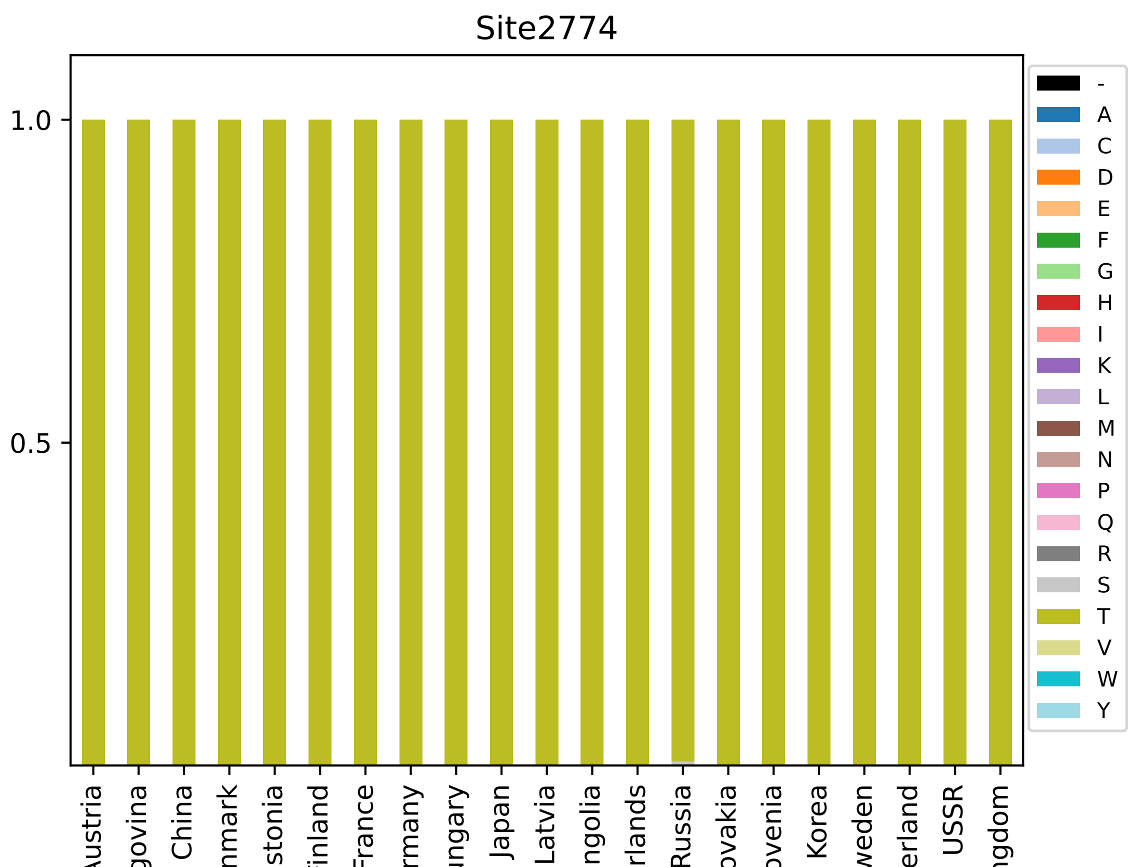
Site2774
1.0
0.5
-
A
C
D
E
F
G
H
I
K
L
M
N
P
Q
R
S
T
V
W
Y
Austria
China
Japan
Latvia
Russia
Korea
USSR

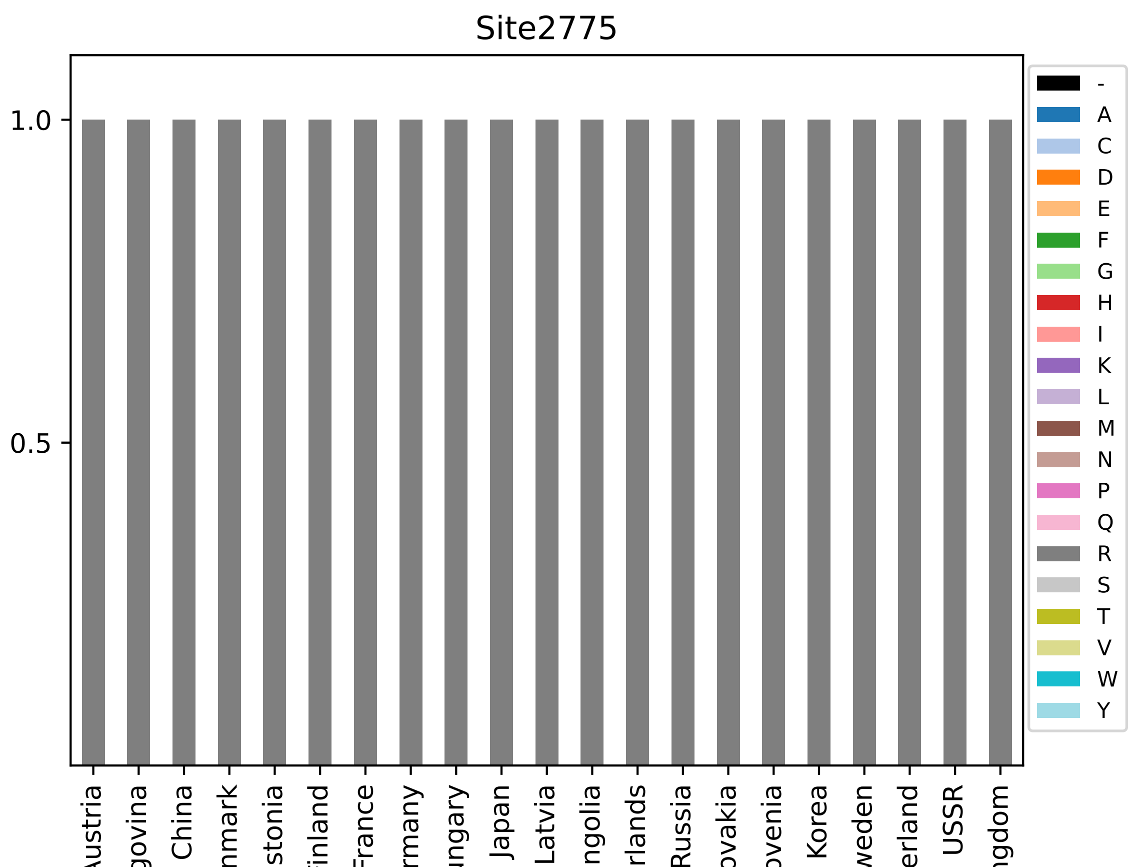
Site2775
1.0
0.5
-
A
C
D
E
F
G
H
I
K
L
M
N
P
Q
R
S
T
V
W
Y
China
Japan
Latvia
Russia
Korea
USSR

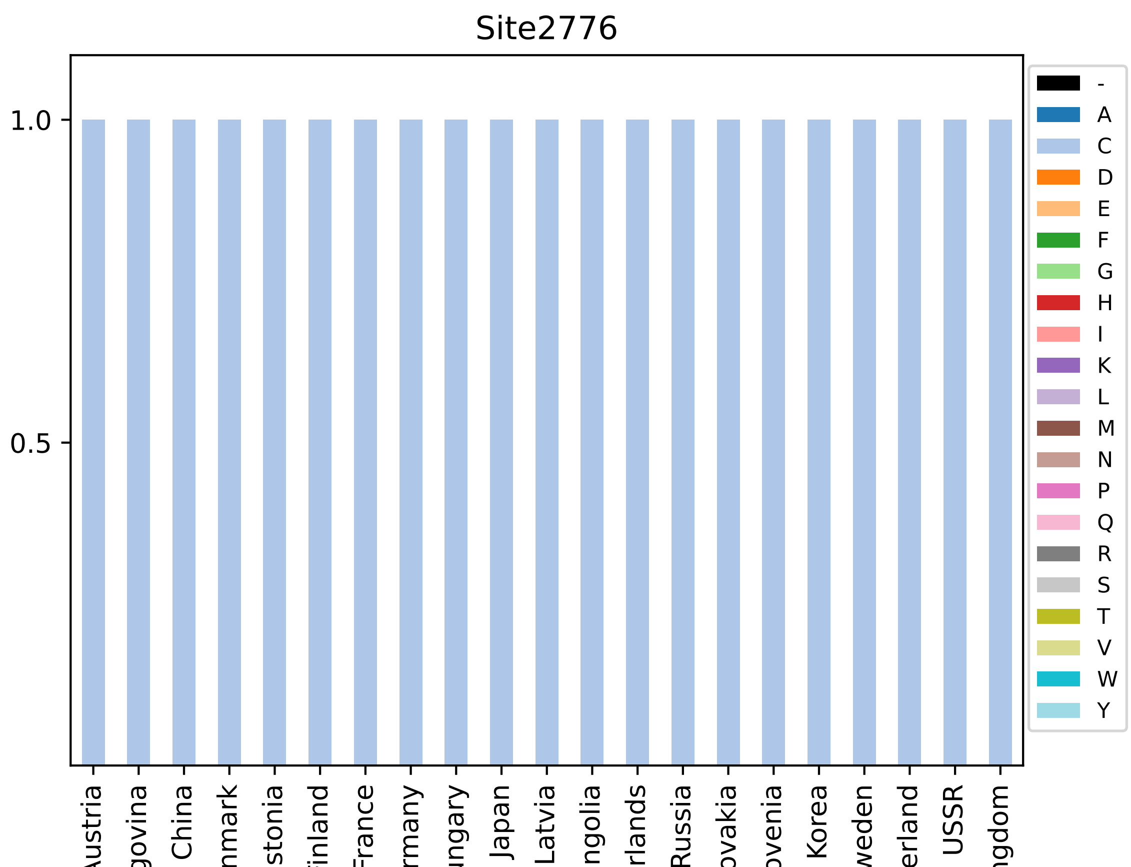
Site2776
1.0
0.5
-
A
C
D
E
F
G
H
I
K
L
M
N
P
Q
R
S
T
V
W
Y
China
Japan
Latvia
Russia
Korea
USSR

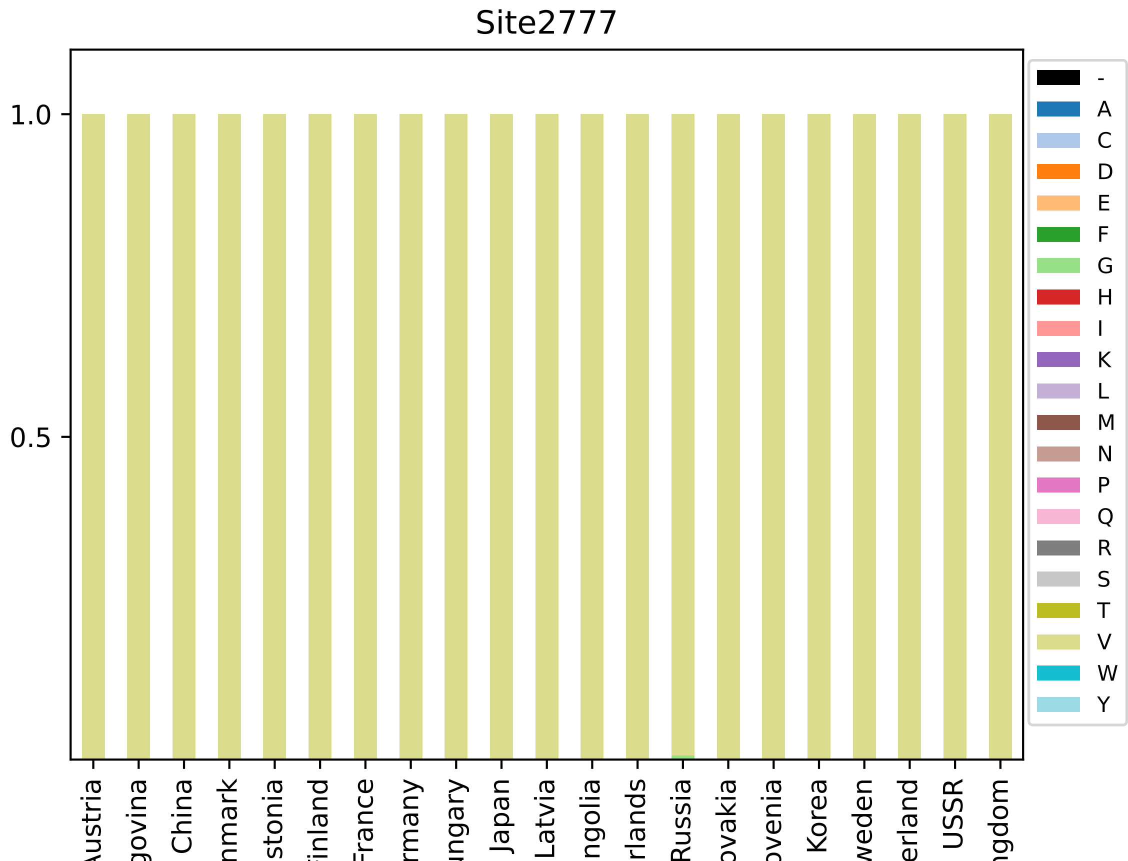

Site2777
1.0
0.5
-
A
C
D
E
F
G
H
I
K
L
M
N
P
Q
R
S
T
V
W
Y
Austria
govina
China
nmark
stonia
Finland
France
rmany
ungary
Japan
Latvia
ngolia
rlands
Russia
ovakia
ovenia
Korea
weden
erland
USSR
ngdom

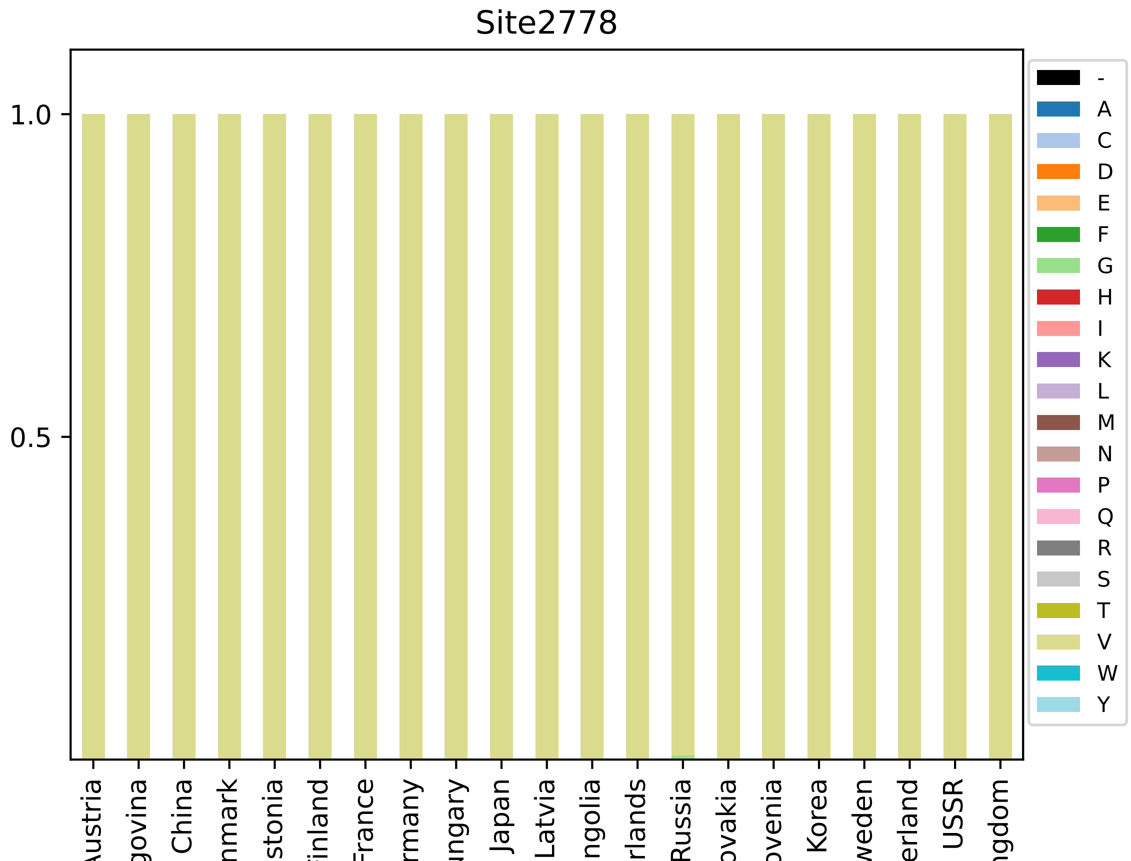
Site2778
1.0
0.5
Austria
govina
China
nmark
stonia
Finland
France
rmany
ungary
Japan
Latvia
ngolia
rlands
Russia
ovakia
ovenia
Korea
weden
erland
USSR
ngdom
-
A
C
D
E
F
G
H
I
K
L
M
N
P
Q
R
S
T
V
W
Y

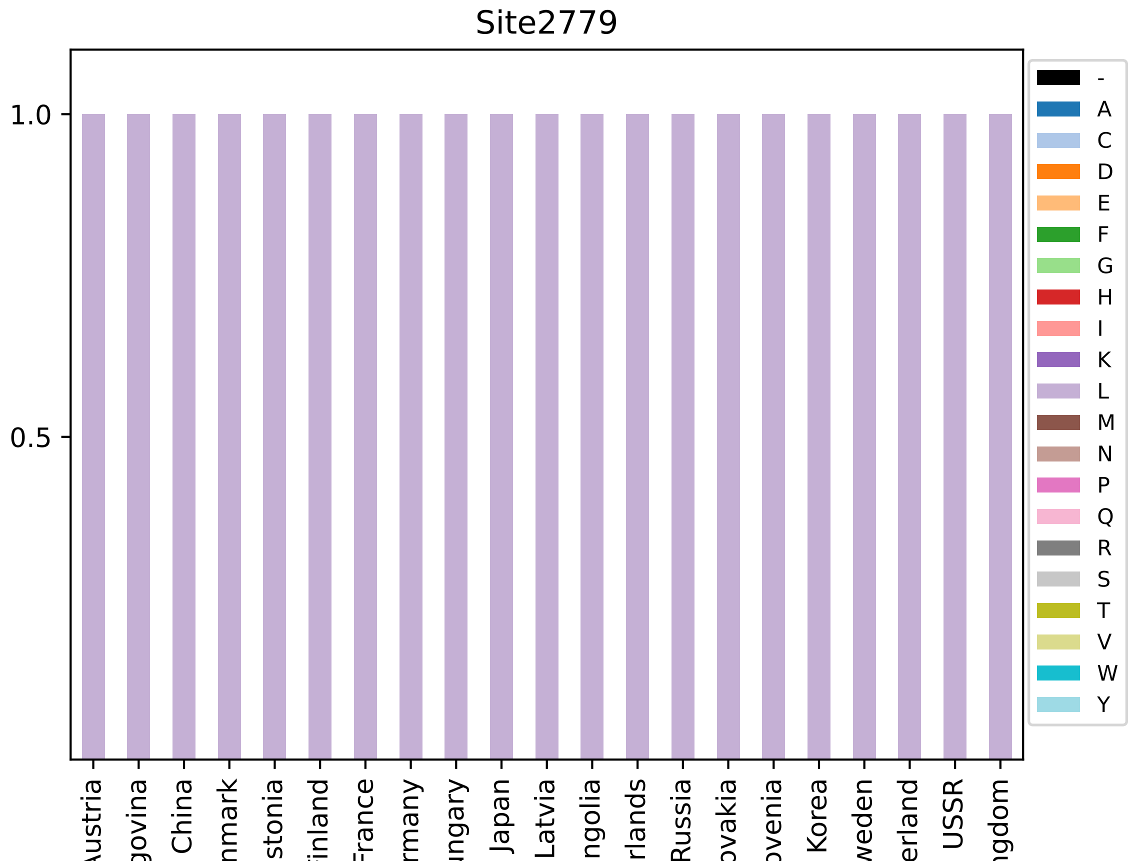
Site2779
1.0
0.5
-
A
C
D
E
F
G
H
I
K
L
M
N
P
Q
R
S
T
V
W
Y
China
Japan
Latvia
Russia
Korea
USSR

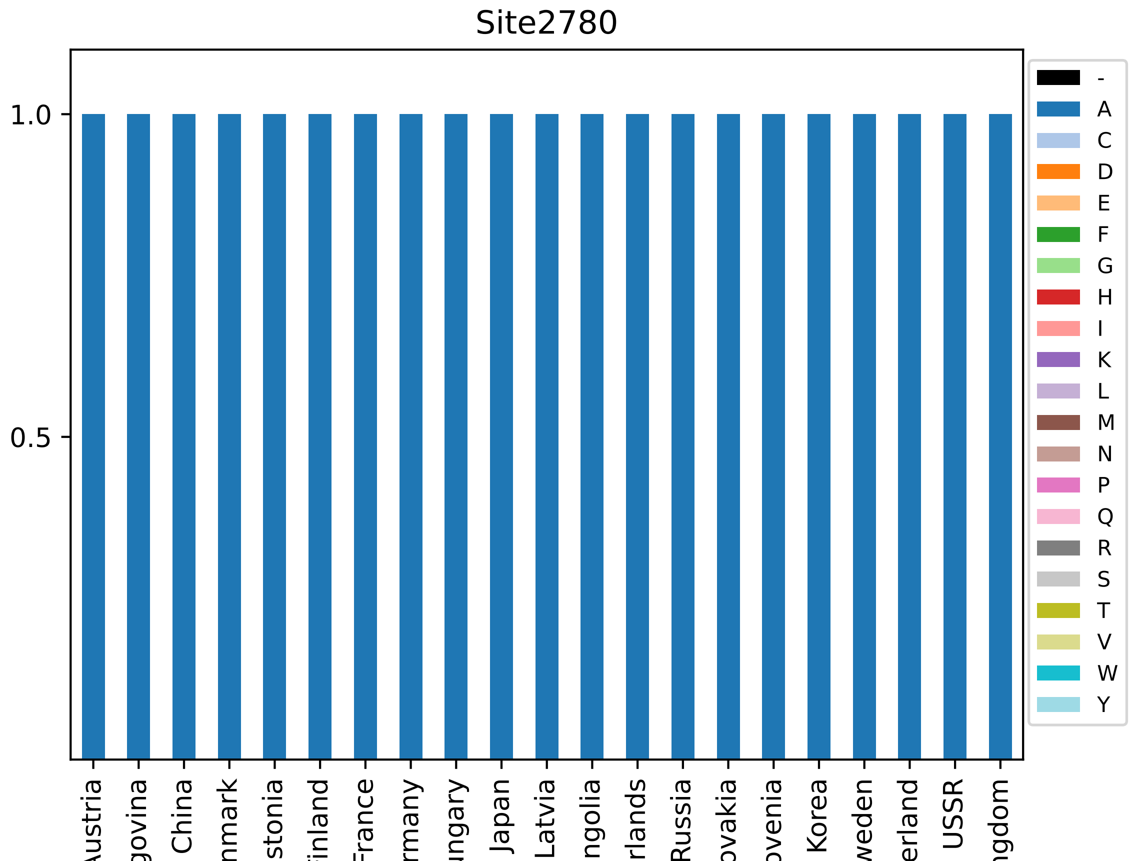

Site2780
1.0
0.5
-
A
C
D
E
F
G
H
I
K
L
M
N
P
Q
R
S
T
V
W
Y
Austria
govina
China
nmark
stonia
inland
France
rmany
ungary
Japan
Latvia
ngolia
rlands
Russia
ovakia
ovenia
Korea
weden
erland
USSR
ngdom

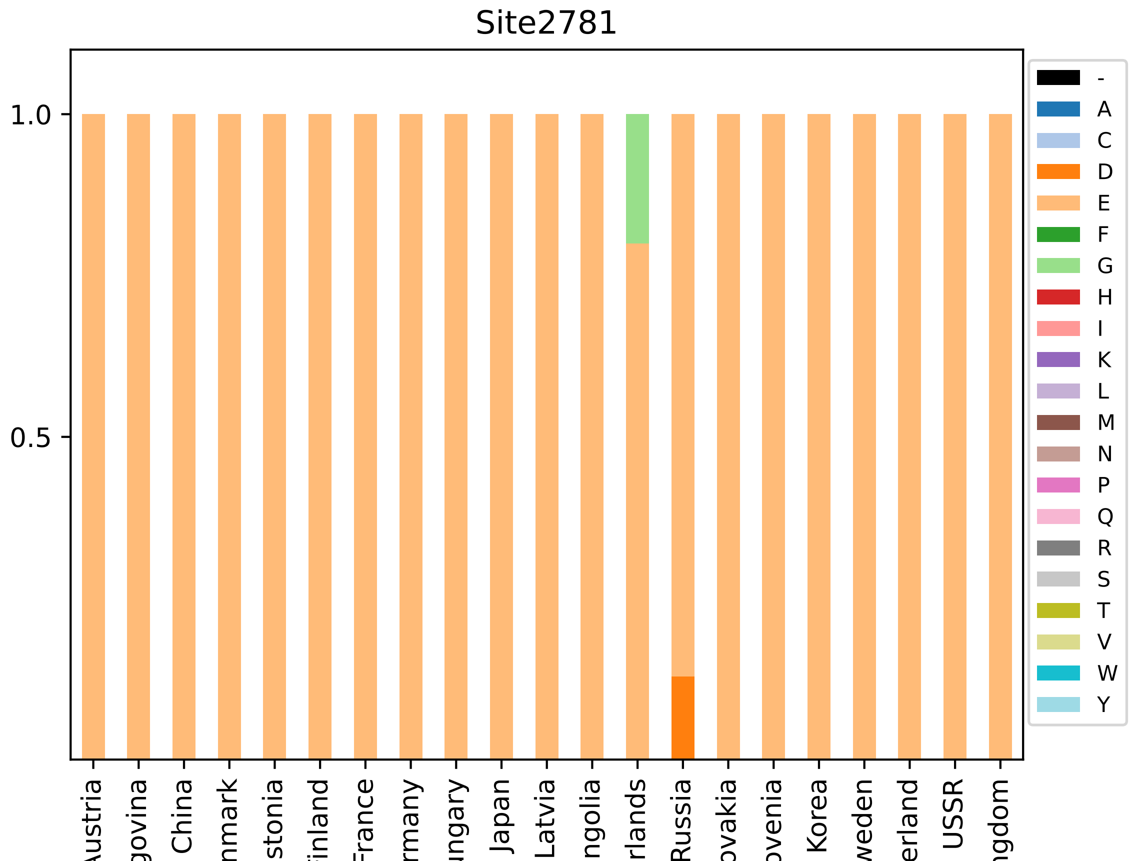

Site2781
1.0
0.5
-
A
C
D
E
F
G
H
I
K
L
M
N
P
Q
R
S
T
V
W
Y
China
Japan
Latvia
Russia
Korea
USSR

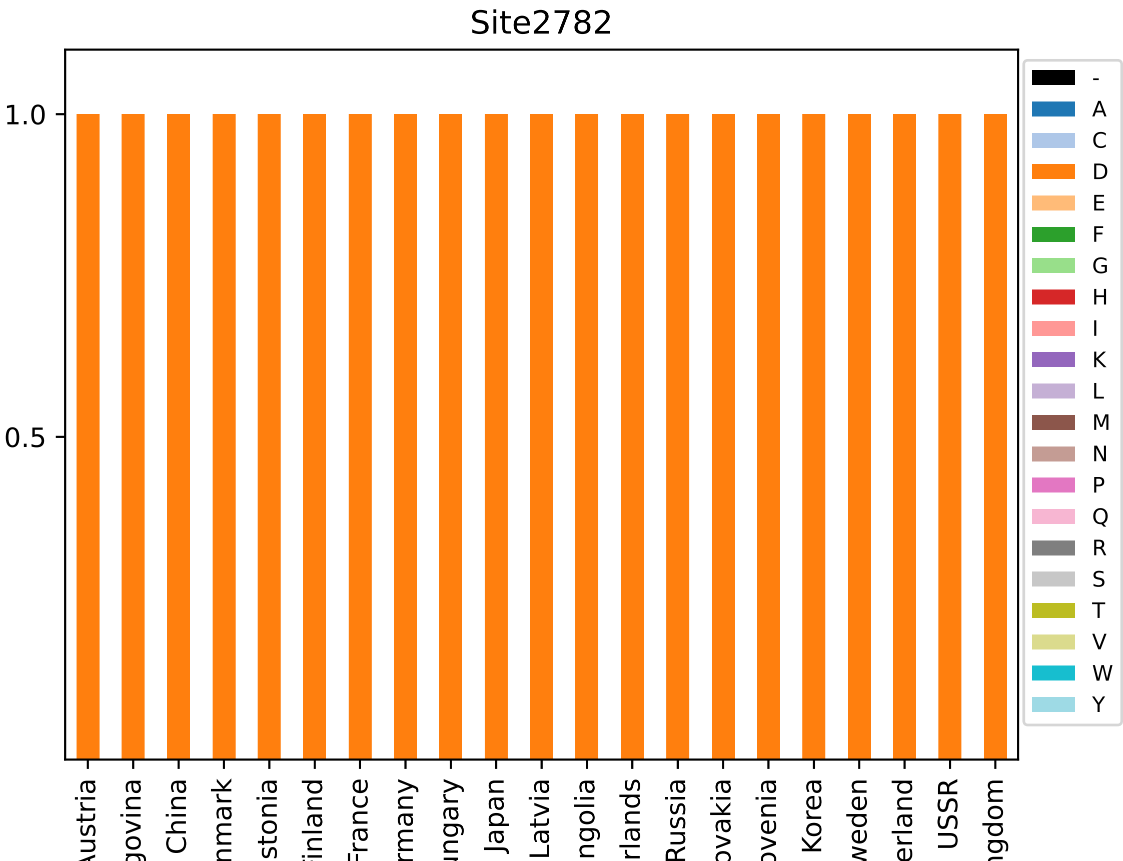

Site2782
1.0
0.5
-
A
C
D
E
F
G
H
I
K
L
M
N
P
Q
R
S
T
V
W
Y
China
Japan
Latvia
Russia
Korea
USSR

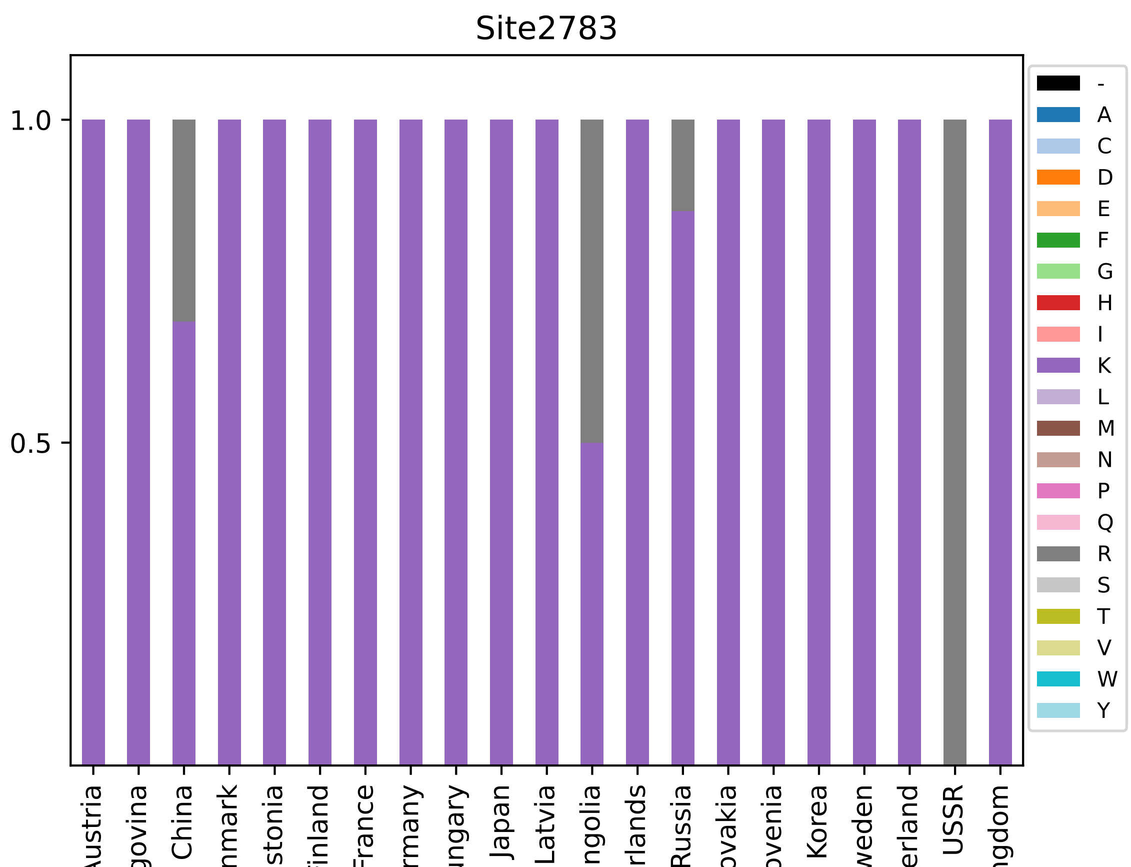
Site2783
1.0
0.5
-
A
C
D
E
F
G
H
I
K
L
M
N
P
Q
R
S
T
V
W
Y
China
Japan
Latvia
Russia
Korea
USSR

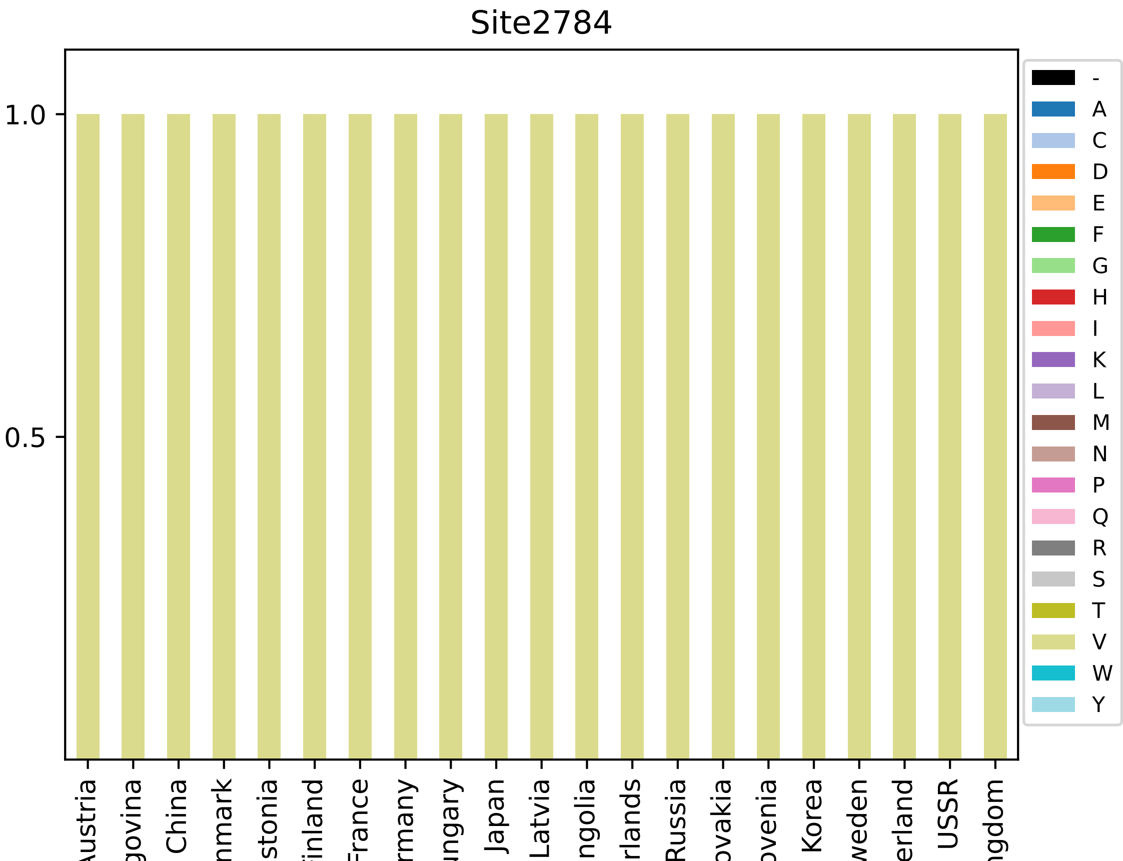
Site2784
1.0
0.5
-
A
C
D
E
F
G
H
I
K
L
M
N
P
Q
R
S
T
V
W
Y
China
Japan
Latvia
Russia
Korea
USSR

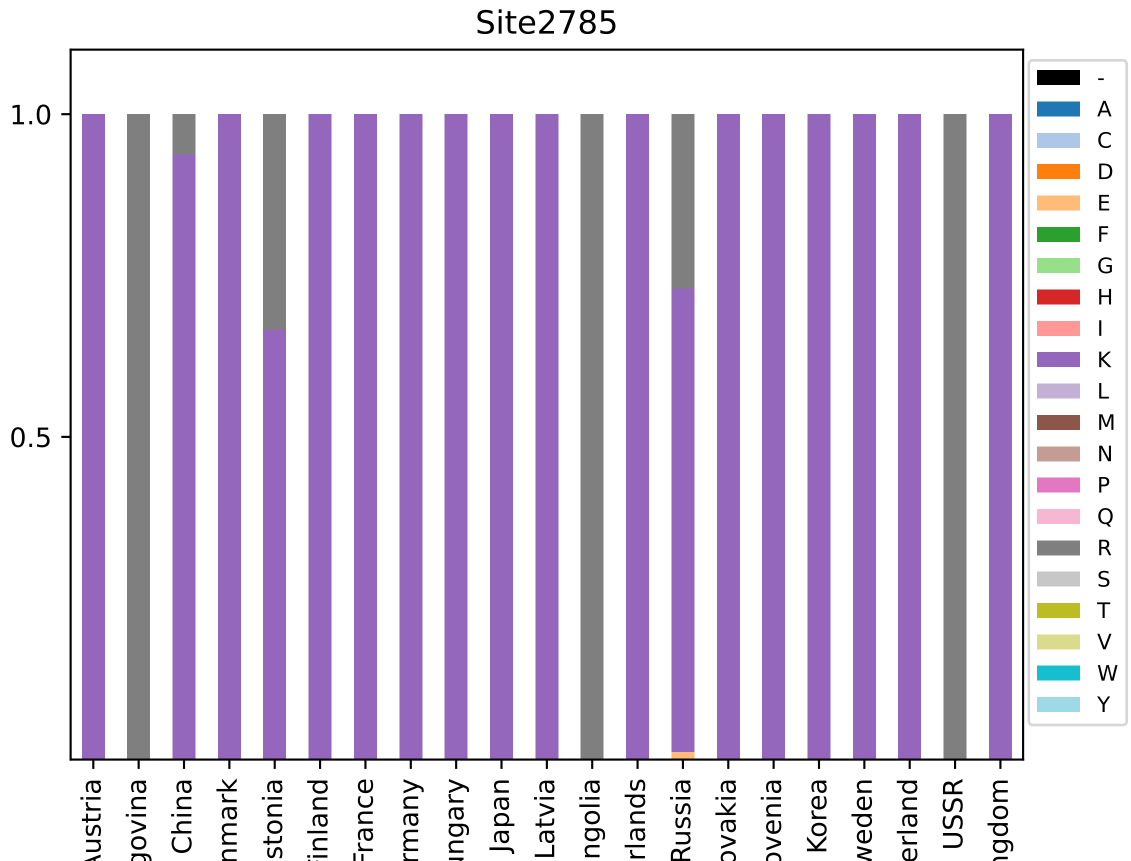
Site2785
1.0
0.5
-
A
C
D
E
F
G
H
I
K
L
M
N
P
Q
R
S
T
V
W
Y
Austria
govina
China
nmark
stonia
Finland
France
rmany
ungary
Japan
Latvia
ngolia
rlands
Russia
ovakia
ovenia
Korea
weden
erland
USSR
ngdom

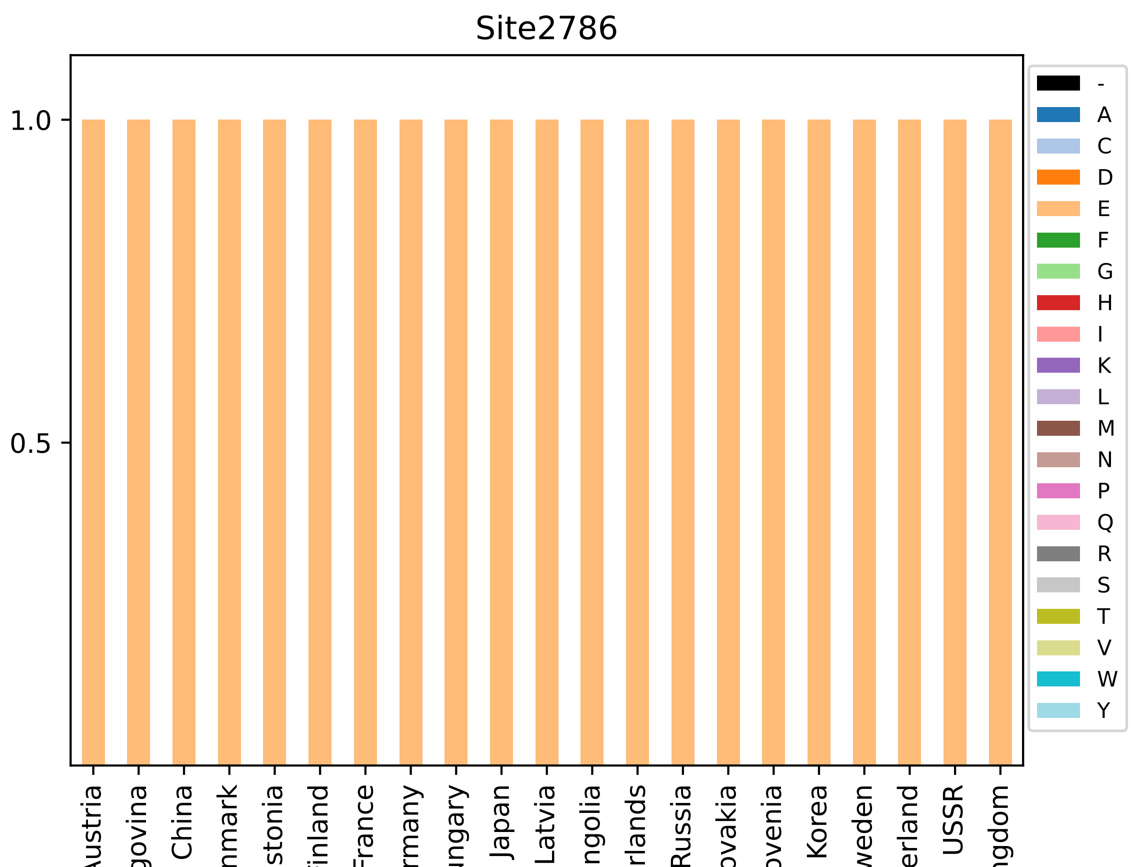
Site2786
1.0
0.5
-
A
C
D
E
F
G
H
I
K
L
M
N
P
Q
R
S
T
V
W
Y
Austria
China
Japan
Latvia
Russia
Korea
USSR

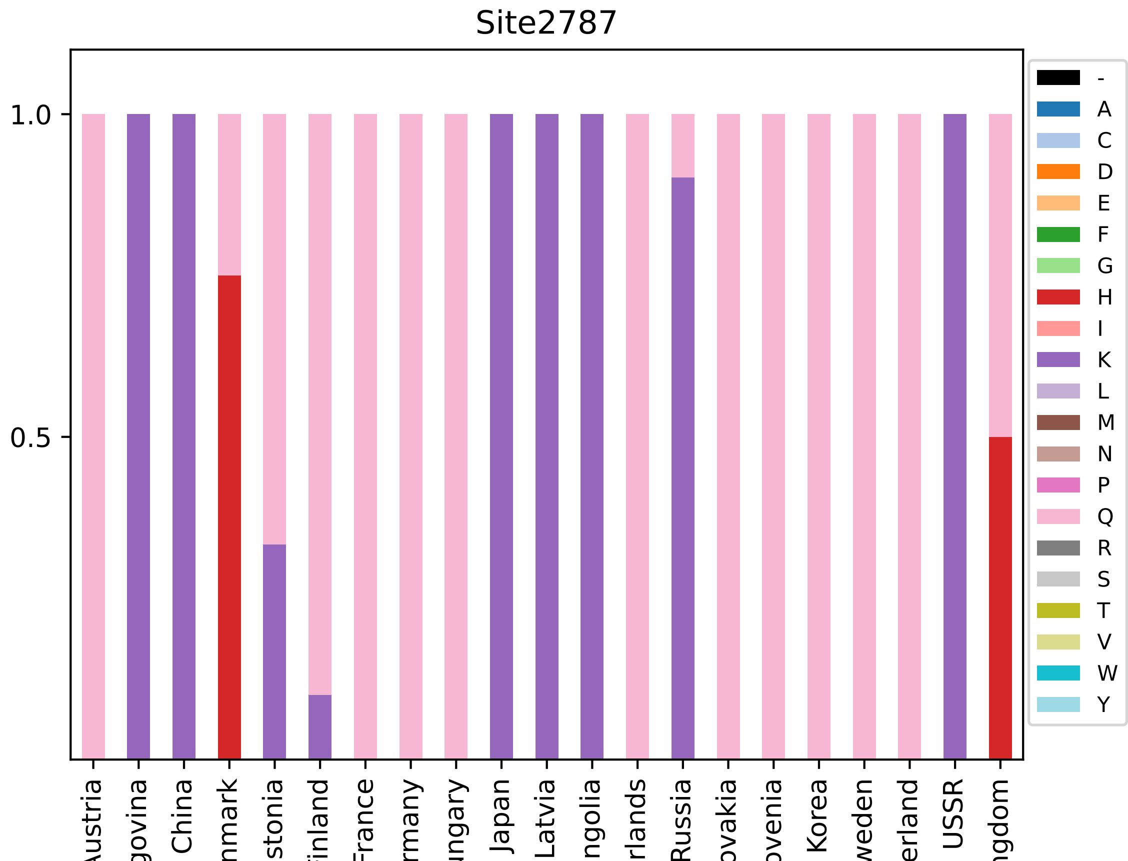
Site2787
1.0
0.5
-
A
C
D
E
F
G
H
I
K
L
M
N
P
Q
R
S
T
V
W
Y
Austria
govina
China
nmark
stonia
inland
France
rmany
ungary
Japan
Latvia
ngolia
rlands
Russia
ovakia
ovenia
Korea
weden
erland
USSR
ngdom

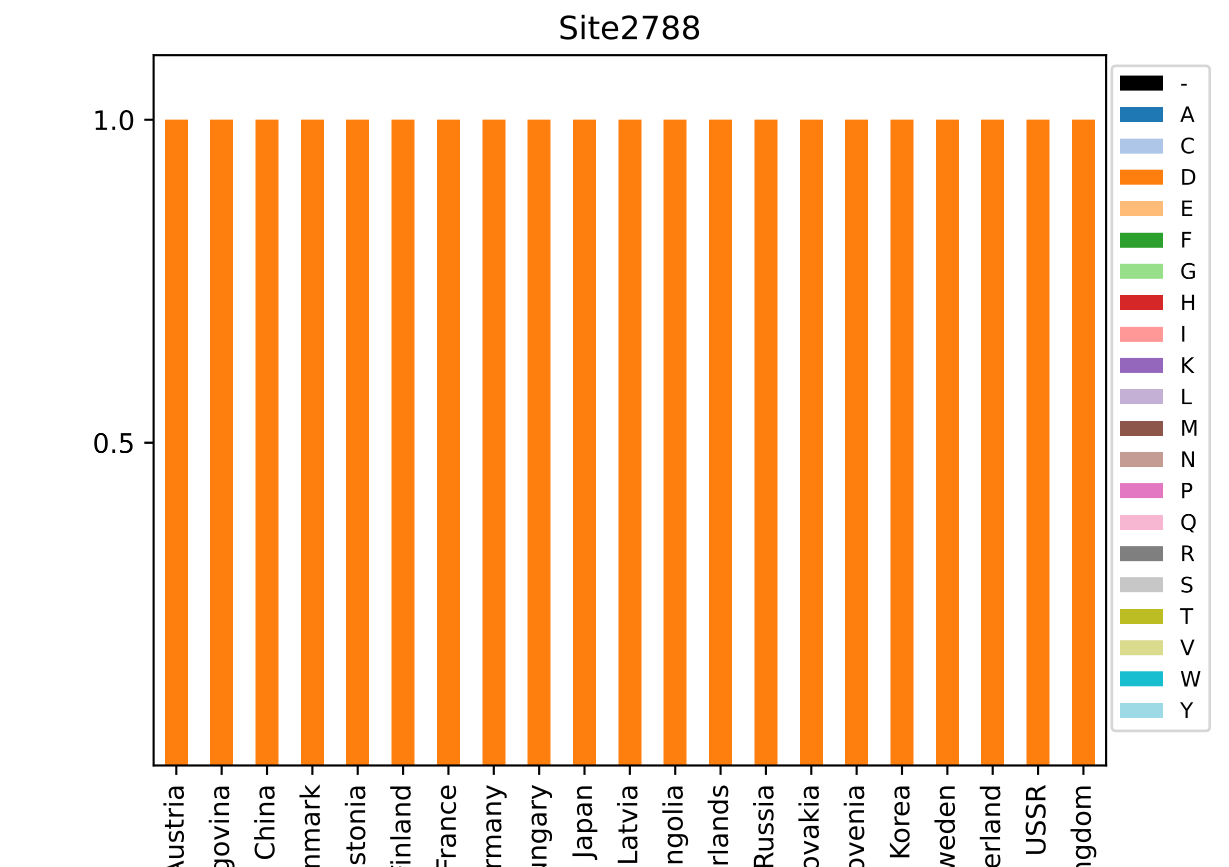

Site2788
1.0
0.5
-
A
C
D
E
F
G
H
I
K
L
M
N
P
Q
R
S
T
V
W
Y
Austria
govina
China
nmark
stonia
inland
France
rmany
ungary
Japan
Latvia
ngolia
rlands
Russia
ovakia
ovenia
Korea
weden
erland
USSR
ngdom

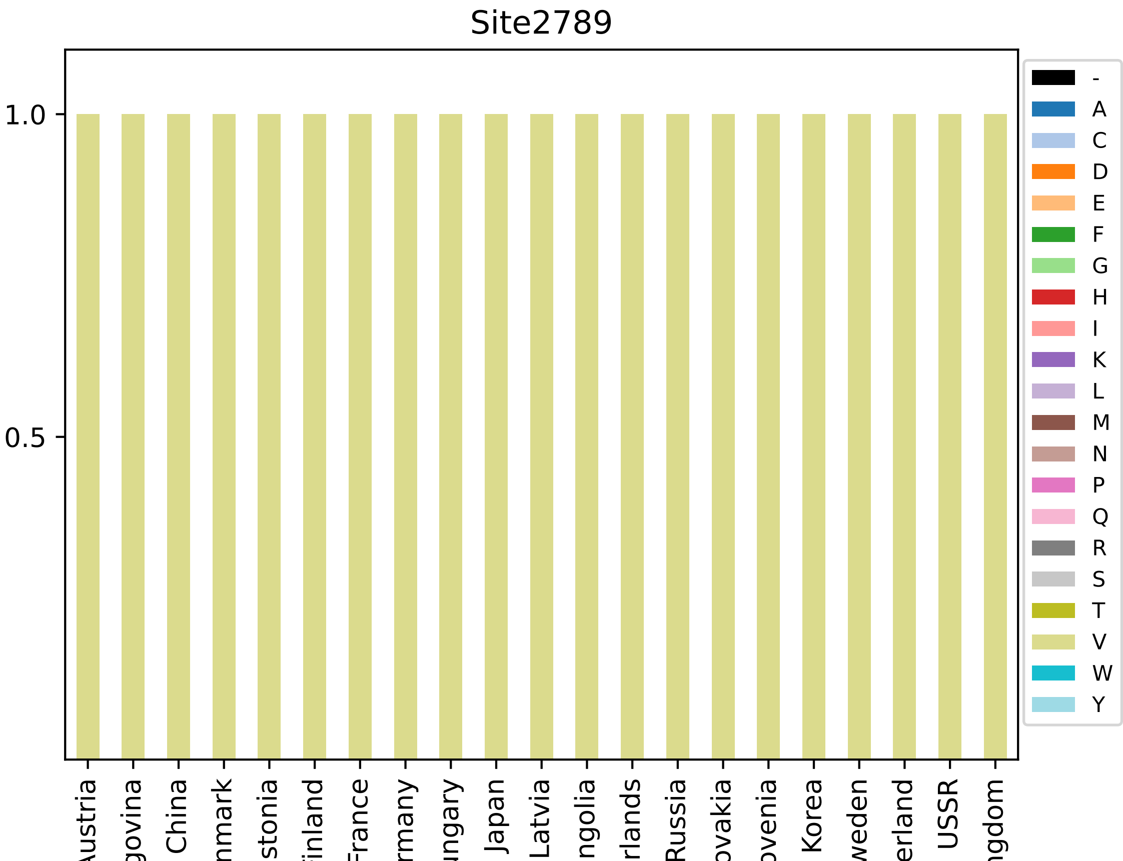

Site2789
1.0
0.5
-
A
C
D
E
F
G
H
I
K
L
M
N
P
Q
R
S
T
V
W
Y
Austria
China
Finland
France
Japan
Latvia
Russia
Korea
USSR

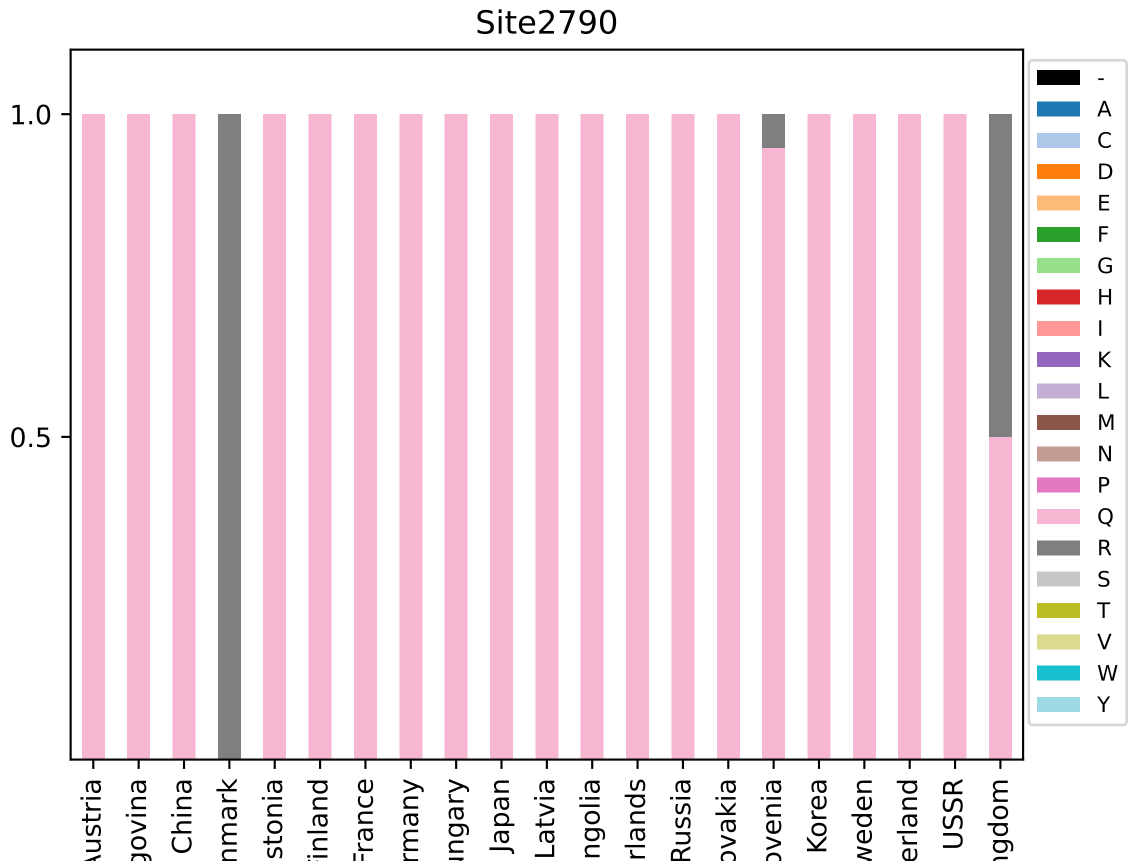
Site2790
1.0
0.5
-
A
C
D
E
F
G
H
I
K
L
M
N
P
Q
R
S
T
V
W
Y
China
Estonia
Finland
France
Japan
Latvia
Russia
Korea
USSR

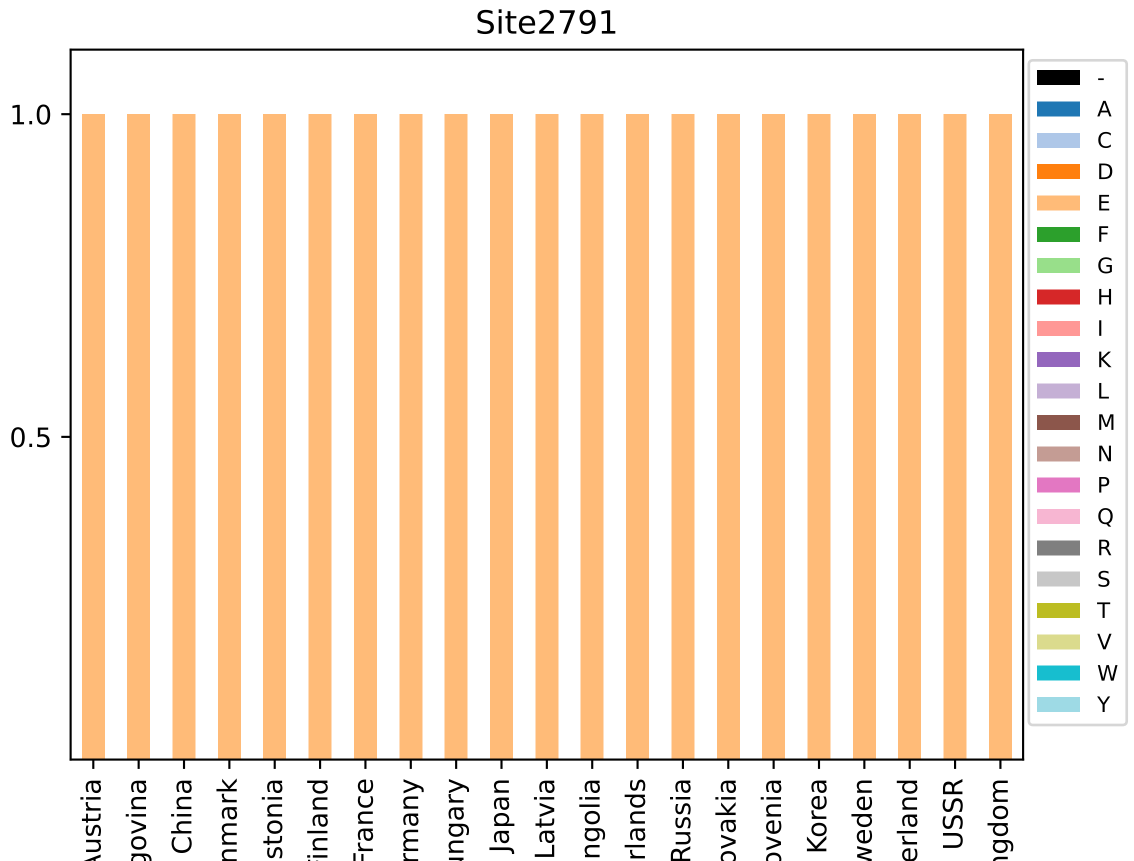

Site2791
1.0
0.5
-
A
C
D
E
F
G
H
I
K
L
M
N
P
Q
R
S
T
V
W
Y
China
Japan
Latvia
Russia
Korea
USSR

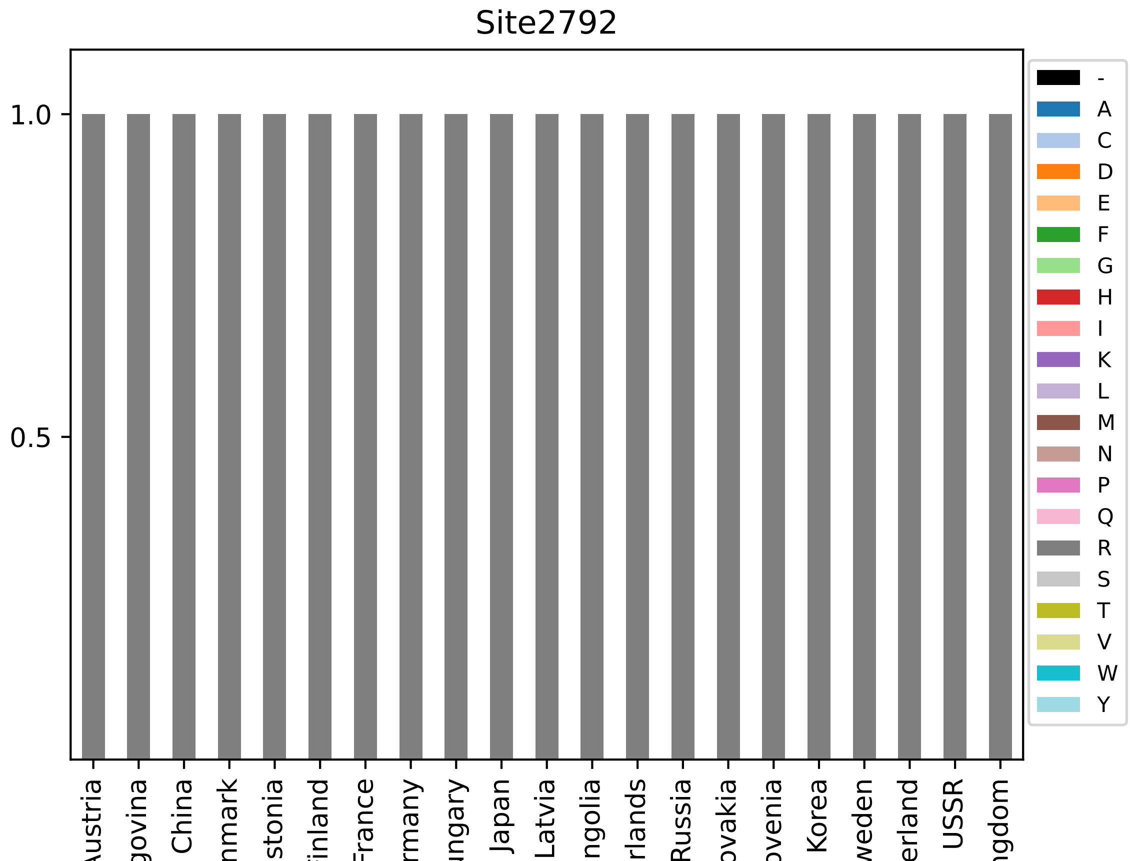
Site2792
1.0
0.5
-
A
C
D
E
F
G
H
I
K
L
M
N
P
Q
R
S
T
V
W
Y
Austria
China
Japan
Latvia
Russia
Korea
USSR

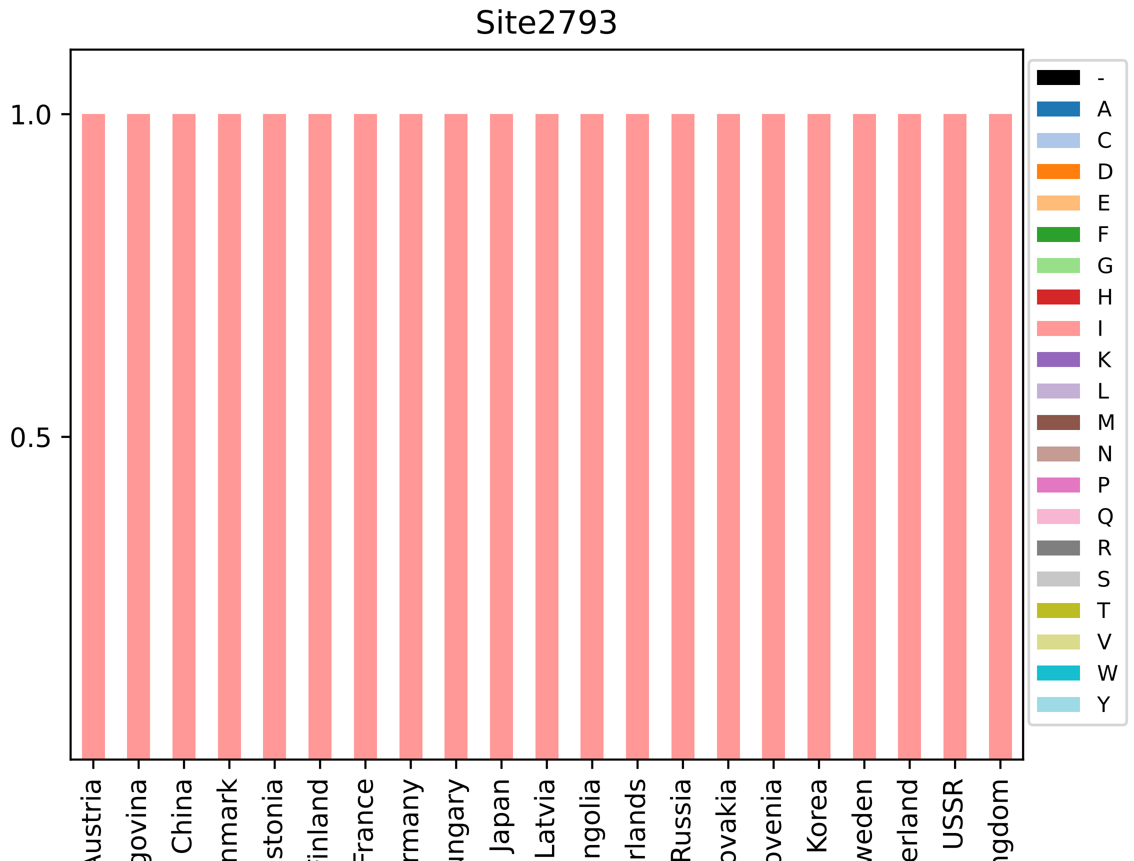

Site2793
1.0
0.5
-
A
C
D
E
F
G
H
I
K
L
M
N
P
Q
R
S
T
V
W
Y
Austria
govina
China
nmark
stonia
Finland
France
rmany
ungary
Japan
Latvia
ngolia
rlands
Russia
ovakia
ovenia
Korea
weden
erland
USSR
ngdom

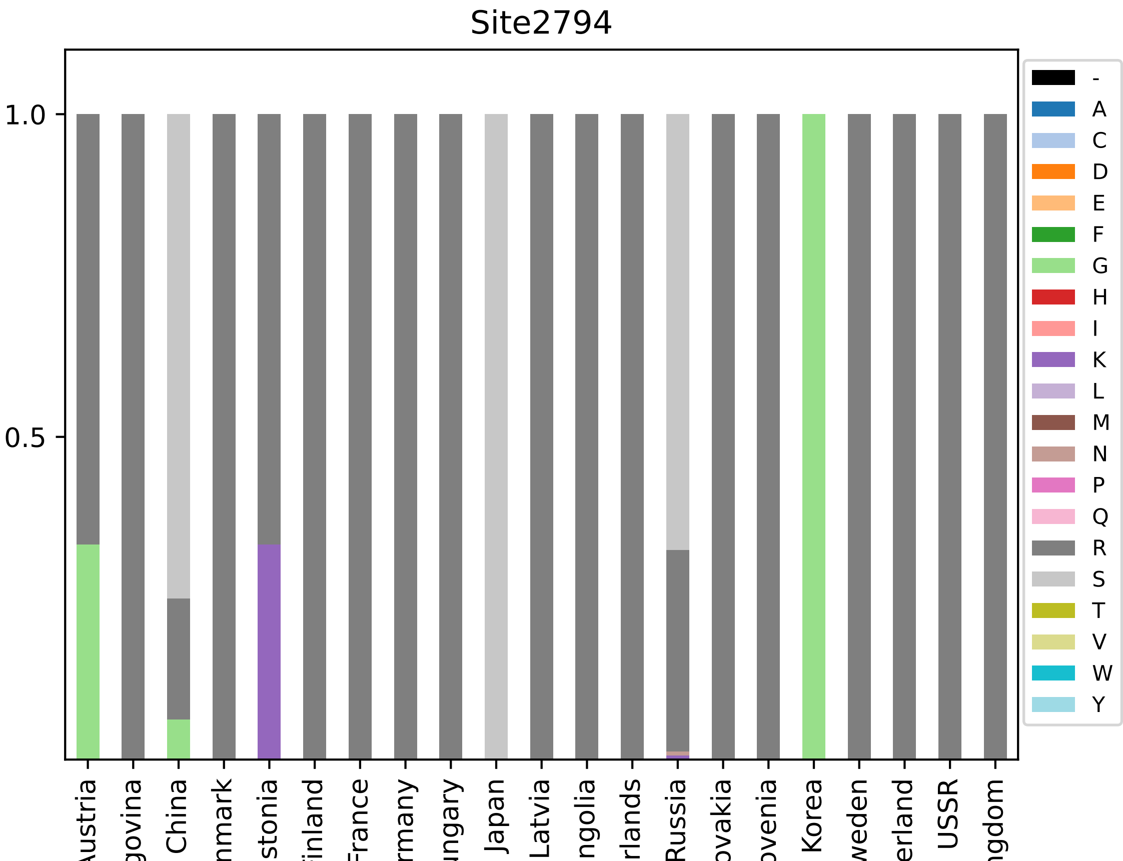
Site2794
1.0
0.5
-
A
C
D
E
F
G
H
I
K
L
M
N
P
Q
R
S
T
V
W
Y
China
Japan
Latvia
Russia
Korea
USSR

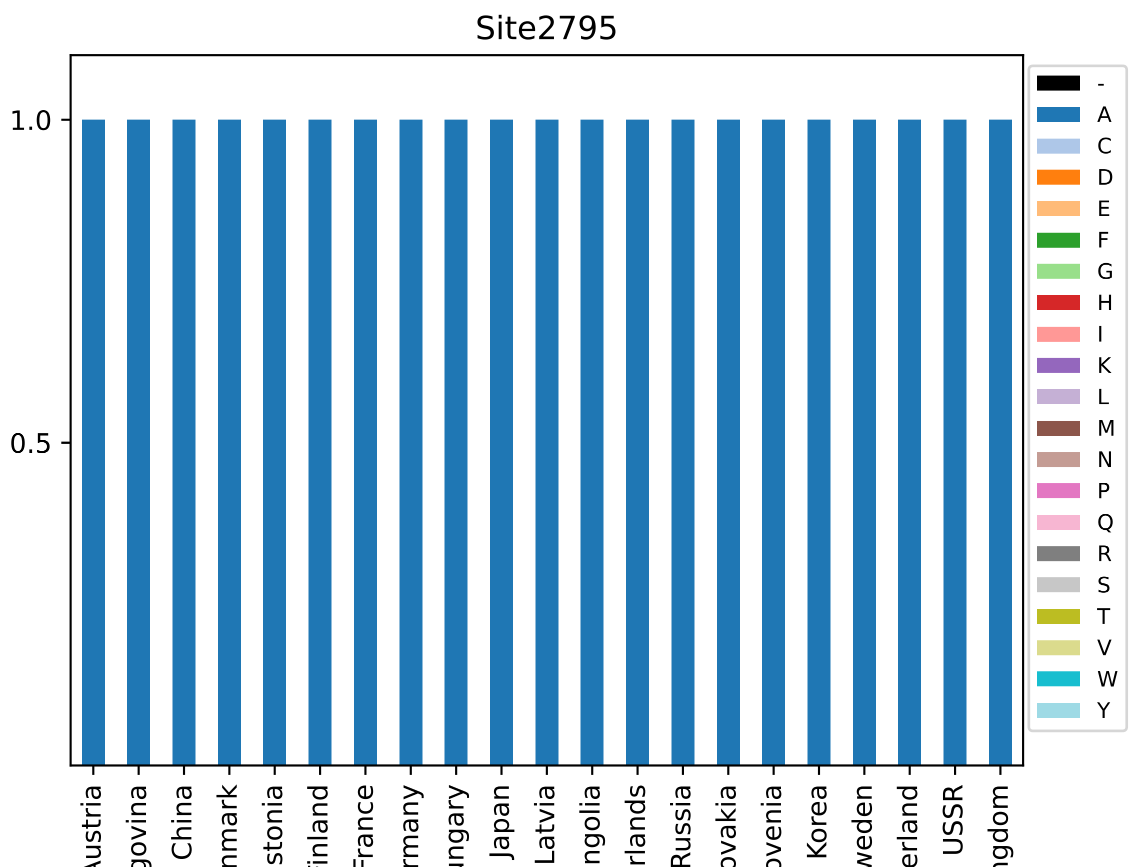

Site2795
1.0
0.5
-
A
C
D
E
F
G
H
I
K
L
M
N
P
Q
R
S
T
V
W
Y
China
Japan
Latvia
Russia
Korea
USSR

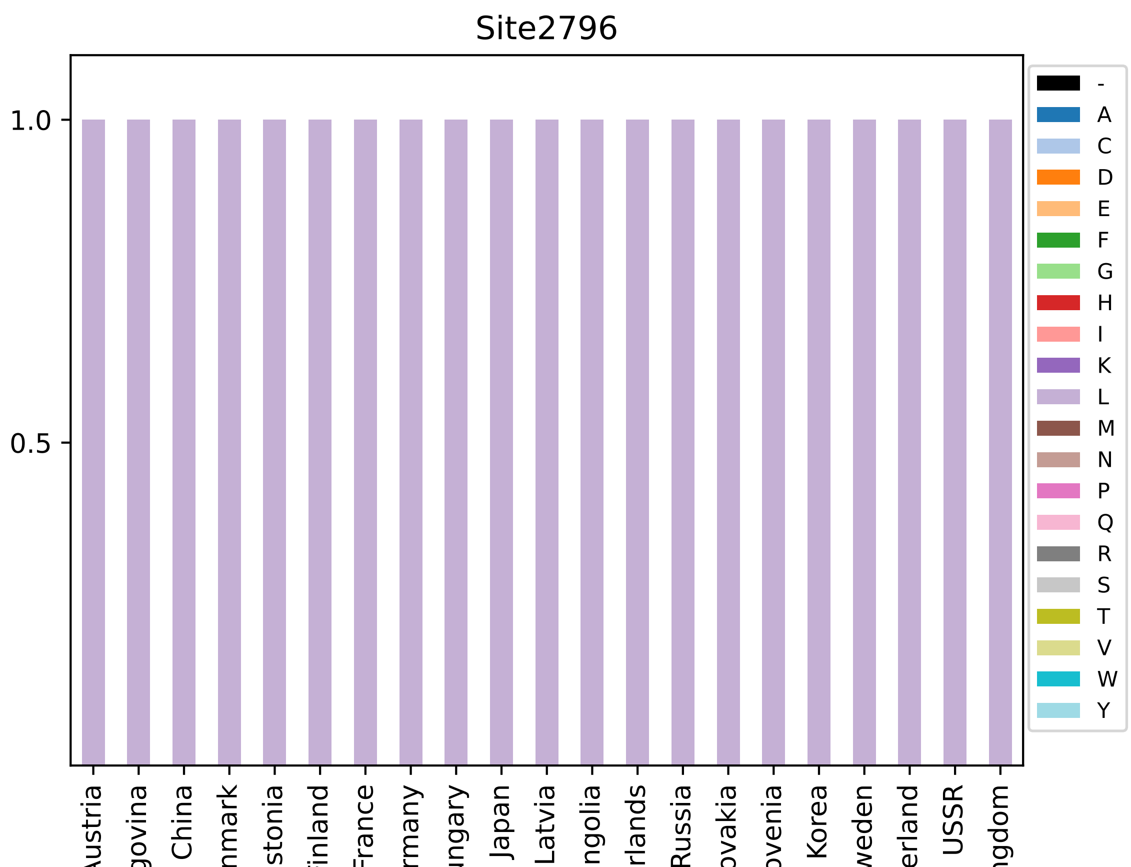
Site2796
1.0
0.5
-
A
C
D
E
F
G
H
I
K
L
M
N
P
Q
R
S
T
V
W
Y
China
Japan
Latvia
Russia
Korea
USSR

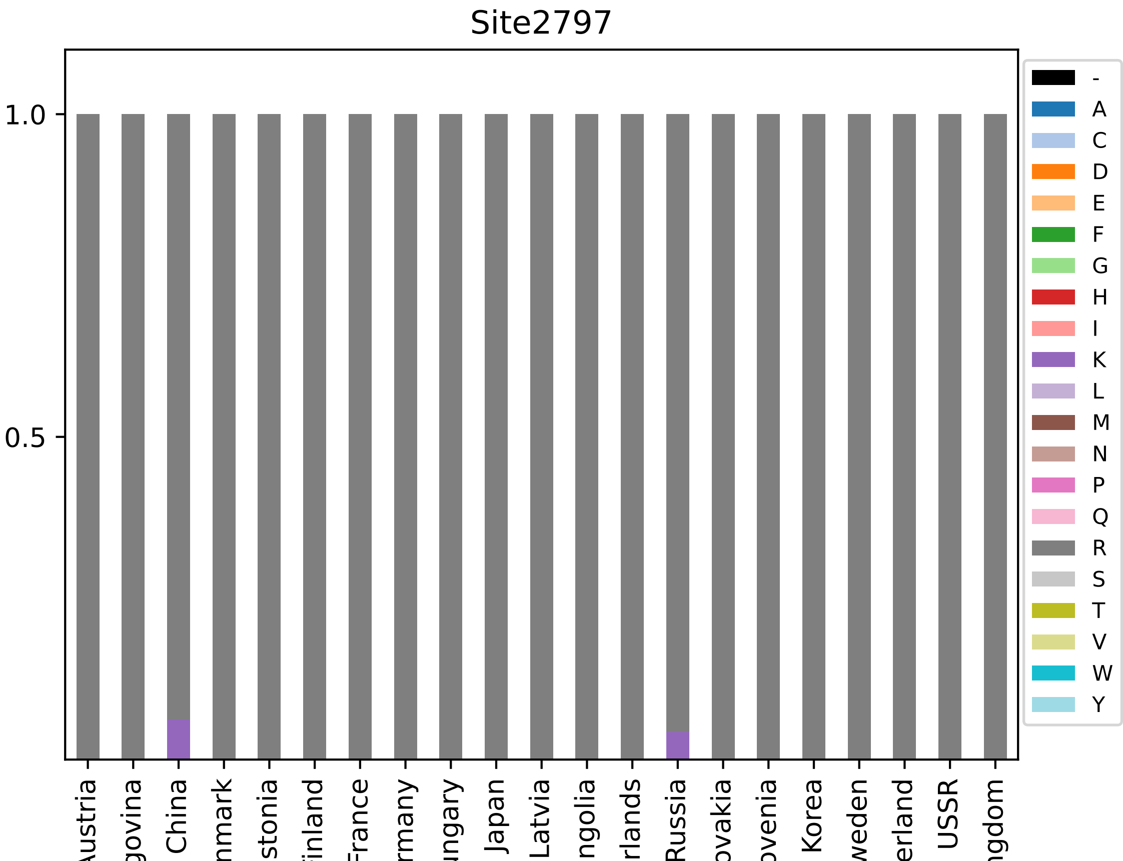
Site2797
1.0
0.5
-
A
C
D
E
F
G
H
I
K
L
M
N
P
Q
R
S
T
V
W
Y
Austria
govina
China
nmark
stonia
Finland
France
rmany
ungary
Japan
Latvia
ngolia
rlands
Russia
ovakia
ovenia
Korea
weden
erland
USSR
ngdom

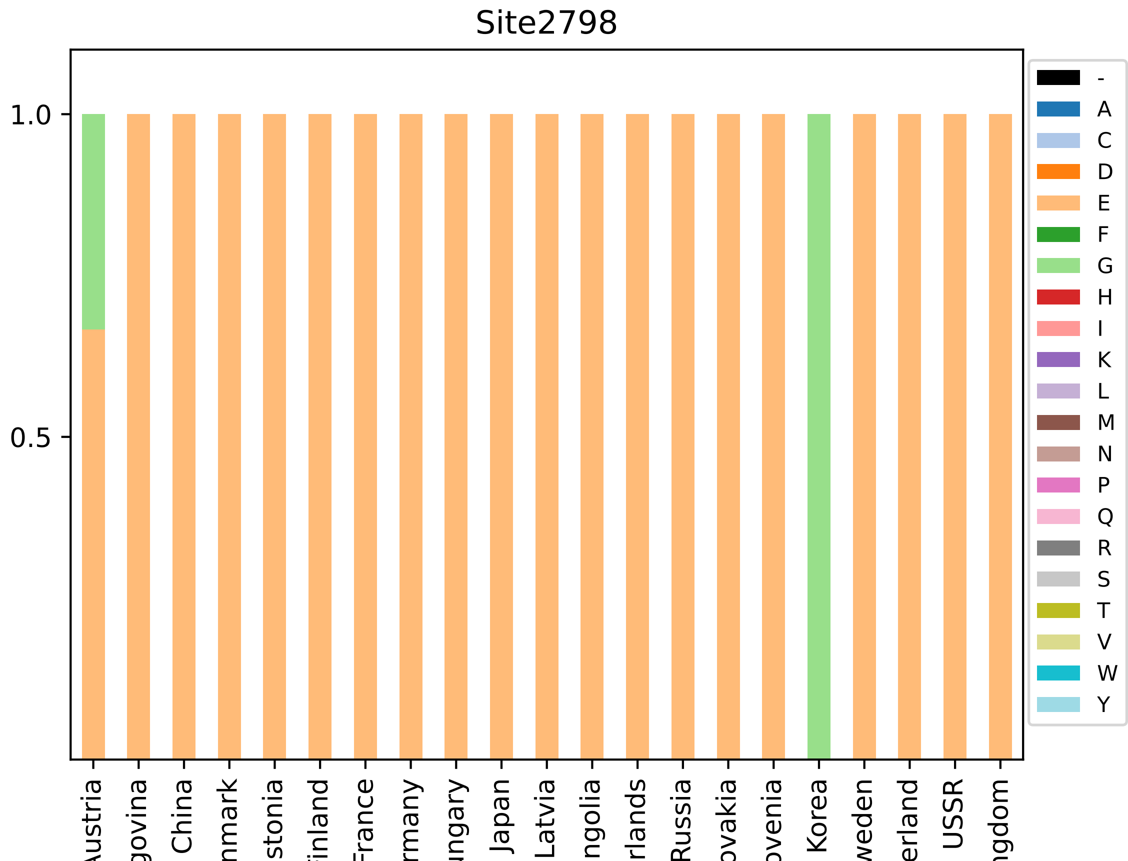

Site2798
1.0
0.5
-
A
C
D
E
F
G
H
I
K
L
M
N
P
Q
R
S
T
V
W
Y
Austria
govina
China
nmark
stonia
inland
France
rmany
ungary
Japan
Latvia
ngolia
rlands
Russia
ovakia
ovenia
Korea
weden
erland
USSR
ngdom

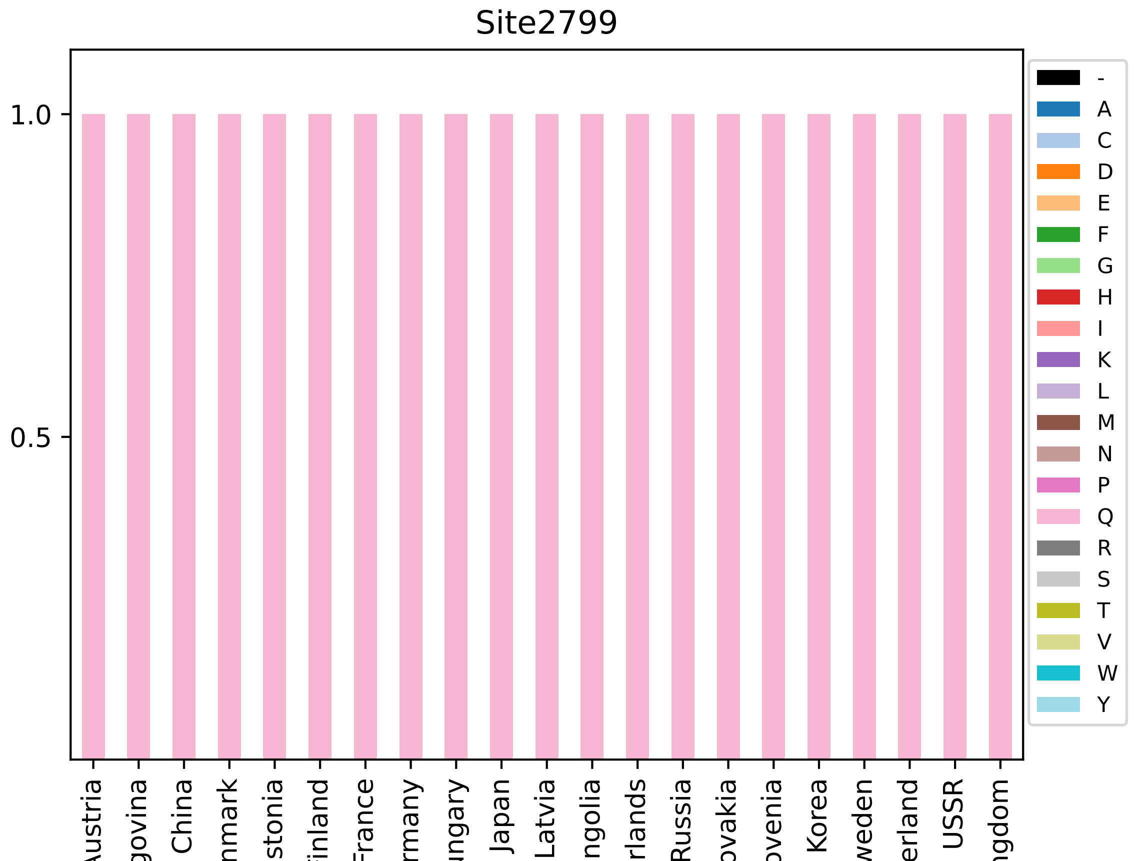

Site2799
1.0
0.5
-
A
C
D
E
F
G
H
I
K
L
M
N
P
Q
R
S
T
V
W
Y
Austria
China
Japan
Latvia
Russia
Korea
USSR

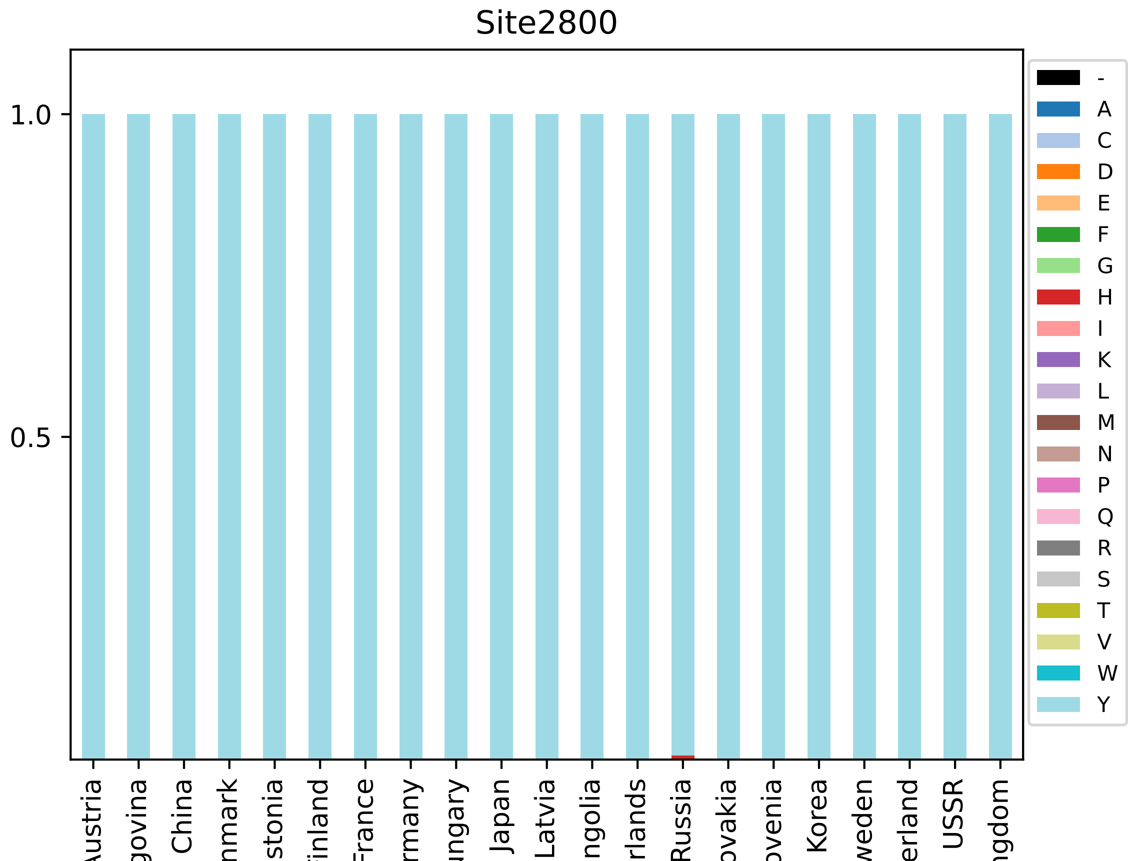

Site2800
1.0
0.5
-
A
C
D
E
F
G
H
I
K
L
M
N
P
Q
R
S
T
V
W
Y
China
Japan
Latvia
Russia
Korea
USSR

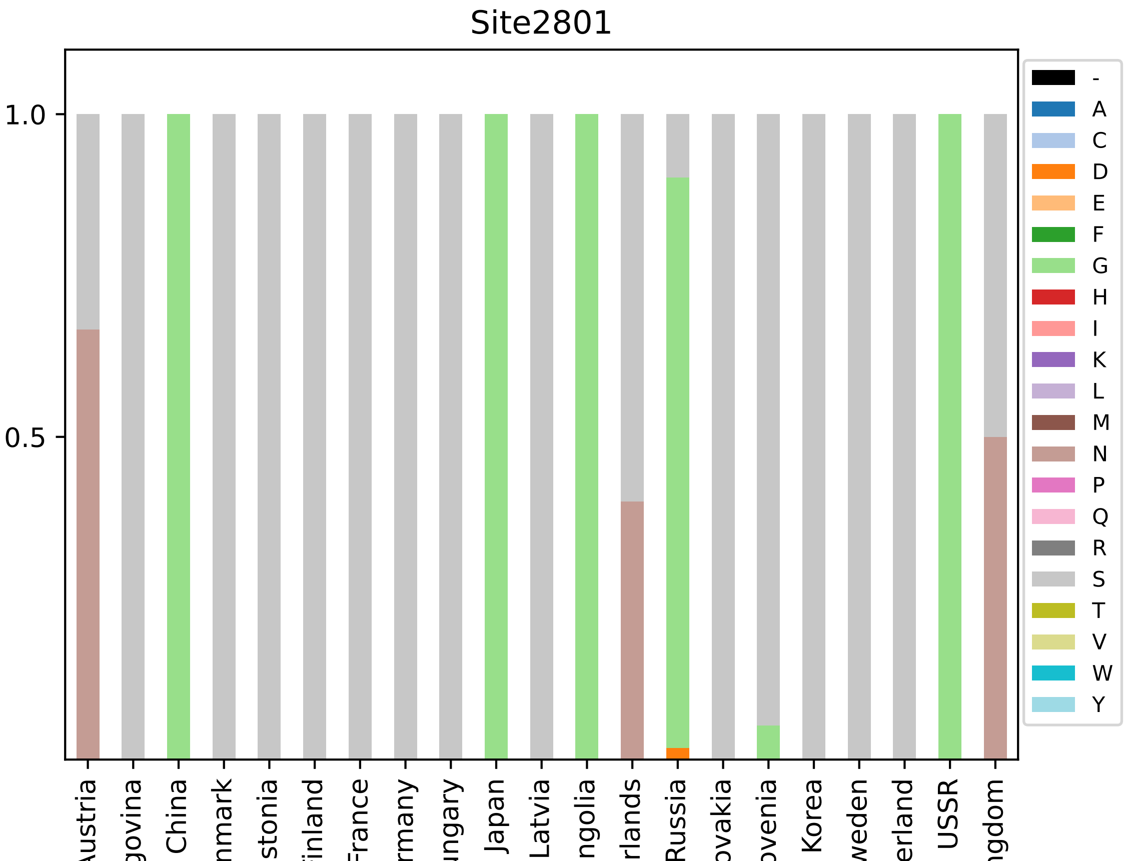

Site2801
1.0
0.5
-
A
C
D
E
F
G
H
I
K
L
M
N
P
Q
R
S
T
V
W
Y
China
Japan
Latvia
Russia
Korea
USSR

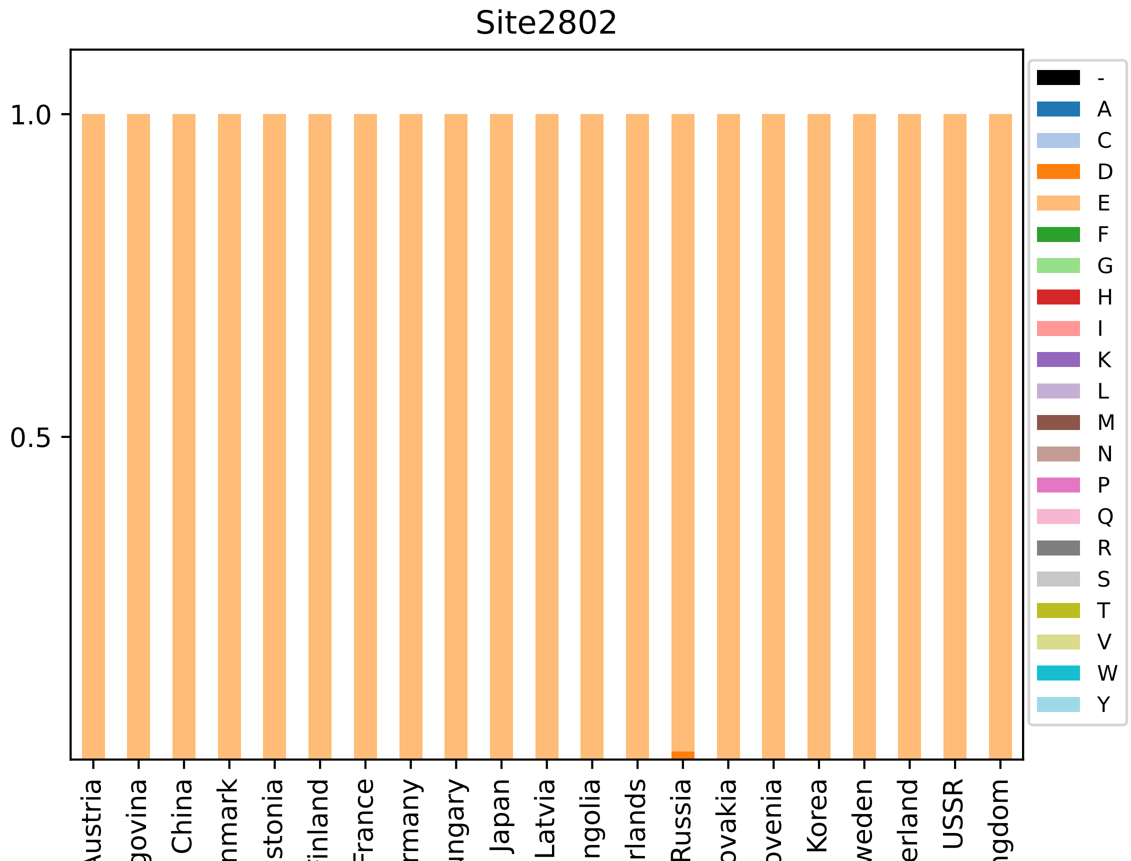
Site2802
1.0
0.5
-
A
C
D
E
F
G
H
I
K
L
M
N
P
Q
R
S
T
V
W
Y
China
Japan
Latvia
Russia
Korea
USSR

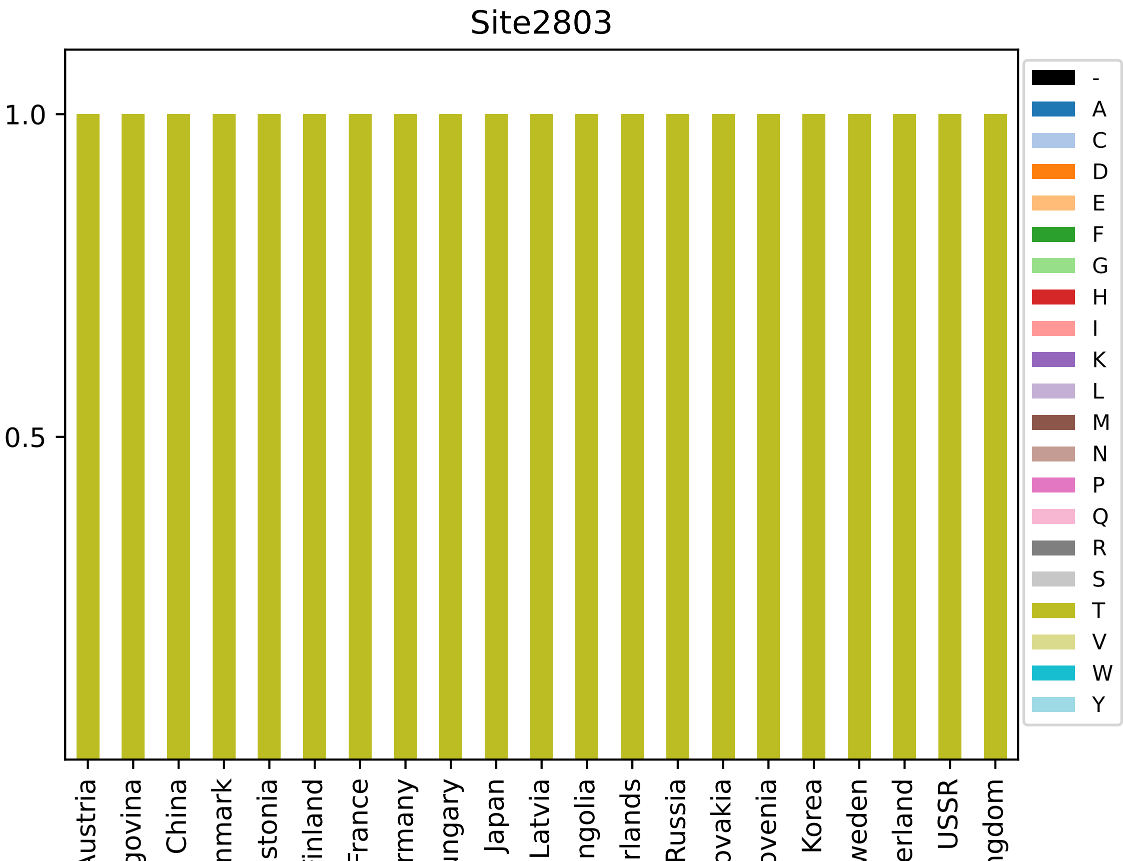
Site2803
1.0
0.5
-
A
C
D
E
F
G
H
I
K
L
M
N
P
Q
R
S
T
V
W
Y
China
Japan
Latvia
Russia
Korea
USSR

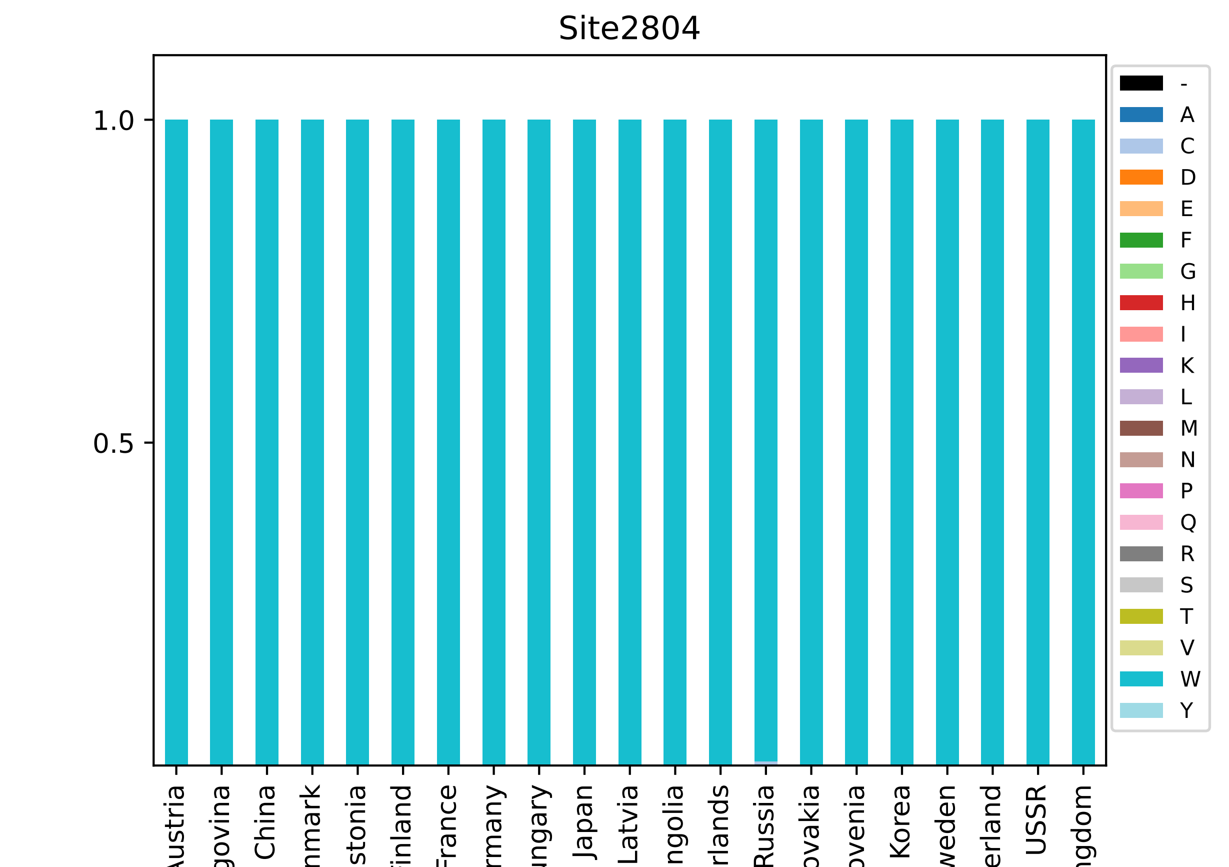

Site2804
1.0
0.5
-
A
C
D
E
F
G
H
I
K
L
M
N
P
Q
R
S
T
V
W
Y
Austria
govina
China
nmark
stonia
inland
France
rmany
ungary
Japan
Latvia
ngolia
rlands
Russia
ovakia
ovenia
Korea
weden
erland
USSR
ngdom

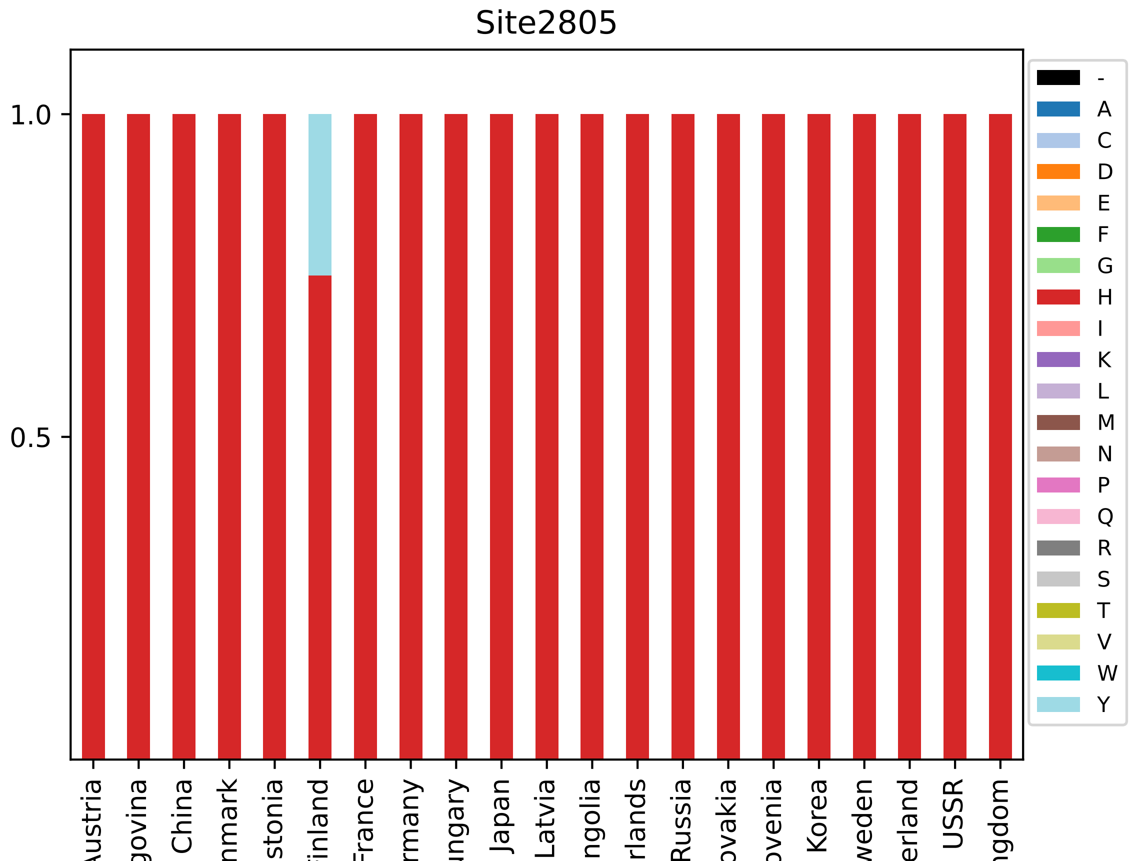

Site2805
1.0
0.5
-
A
C
D
E
F
G
H
I
K
L
M
N
P
Q
R
S
T
V
W
Y
China
Japan
Latvia
Russia
Korea
USSR

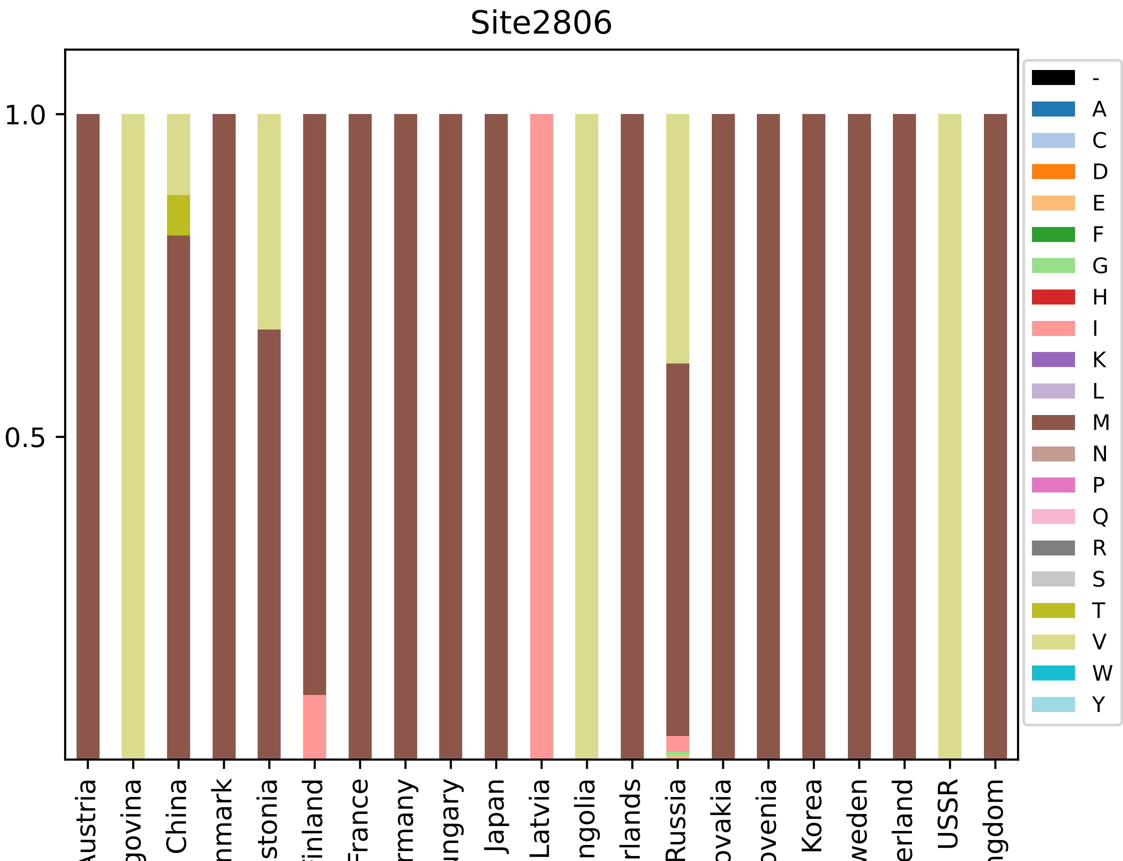

Site2806
1.0
0.5
-
A
C
D
E
F
G
H
I
K
L
M
N
P
Q
R
S
T
V
W
Y
Austria
China
Japan
Latvia
Russia
Korea
USSR

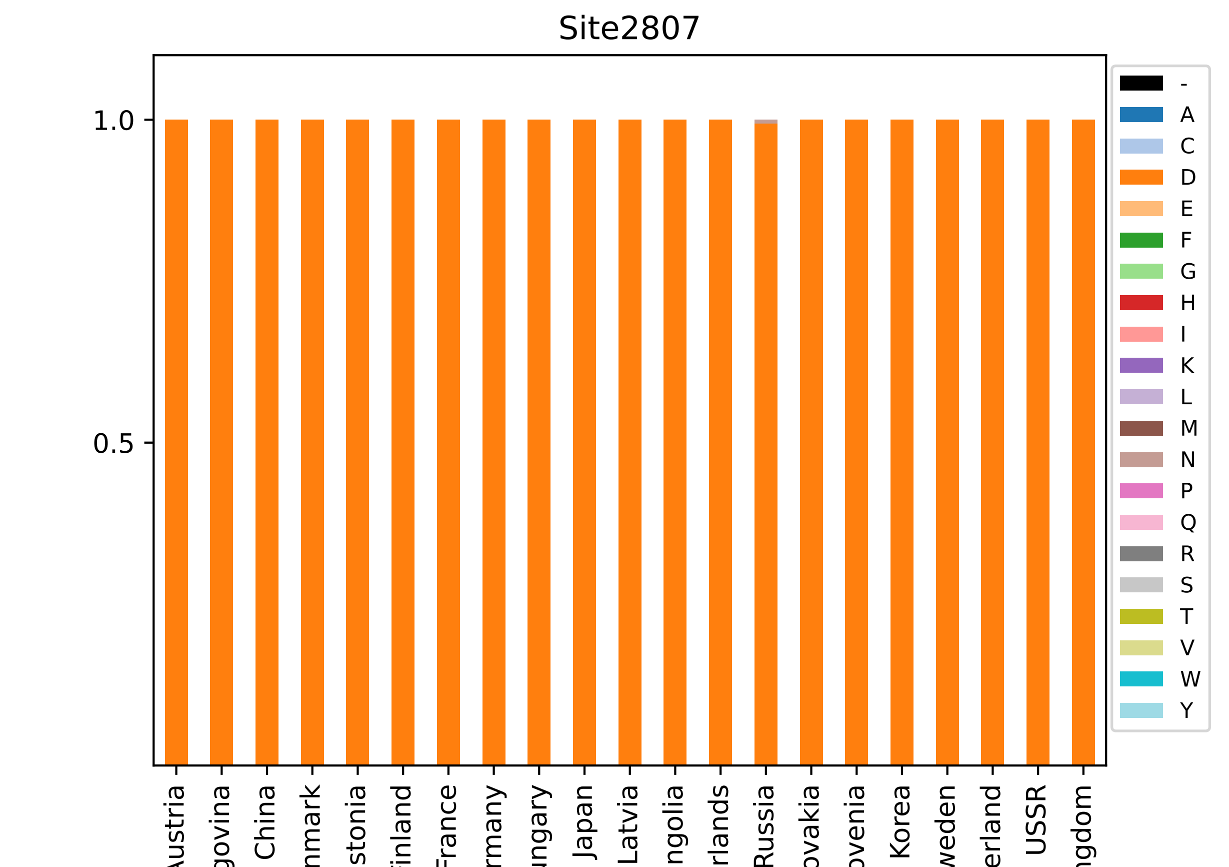

Site2807
1.0
0.5
-
A
C
D
E
F
G
H
I
K
L
M
N
P
Q
R
S
T
V
W
Y
Austria
govina
China
nmark
stonia
Finland
France
rmany
ungary
Japan
Latvia
ngolia
rlands
Russia
ovakia
ovenia
Korea
weden
erland
USSR
ngdom

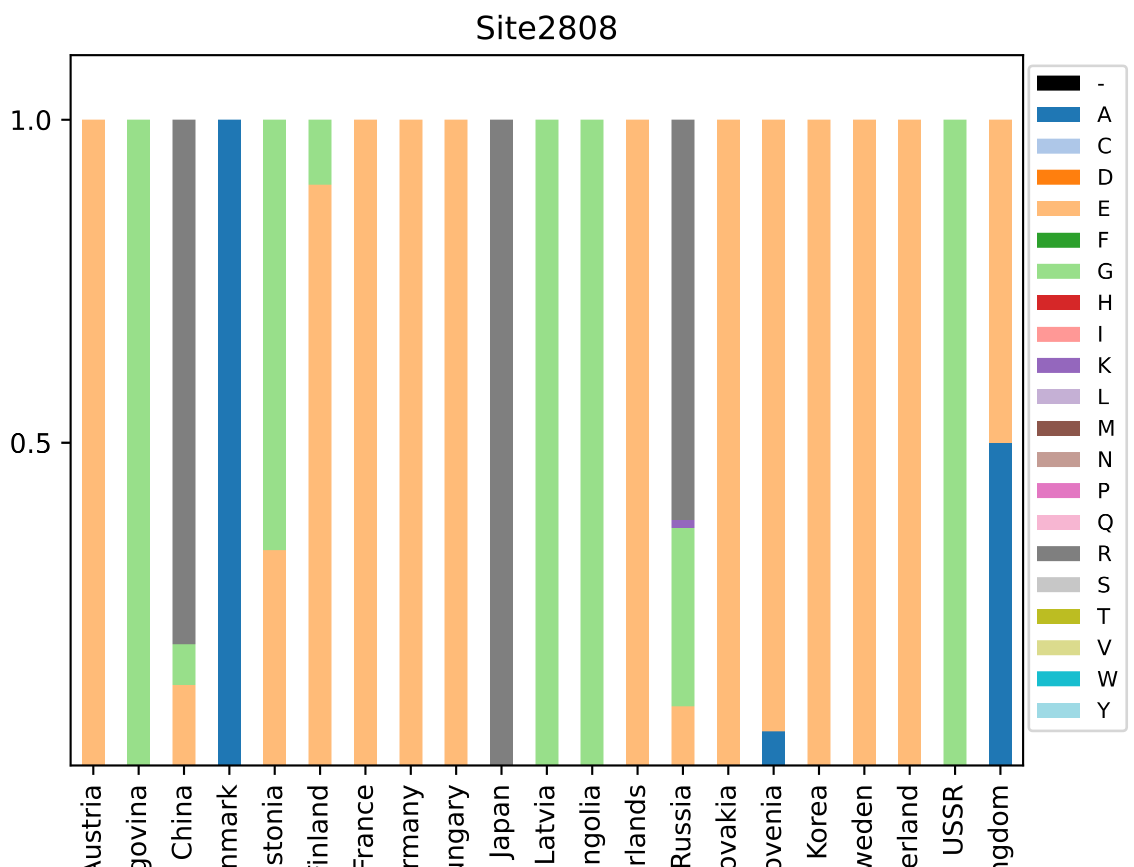
Site2808
1.0
0.5
Austria
govina
China
nmark
stonia
inland
France
rmany
ungary
Japan
Latvia
ngolia
rlands
Russia
ovakia
ovenia
Korea
weden
erland
USSR
ngdom
-
A
C
D
E
F
G
H
I
K
L
M
N
P
Q
R
S
T
V
W
Y

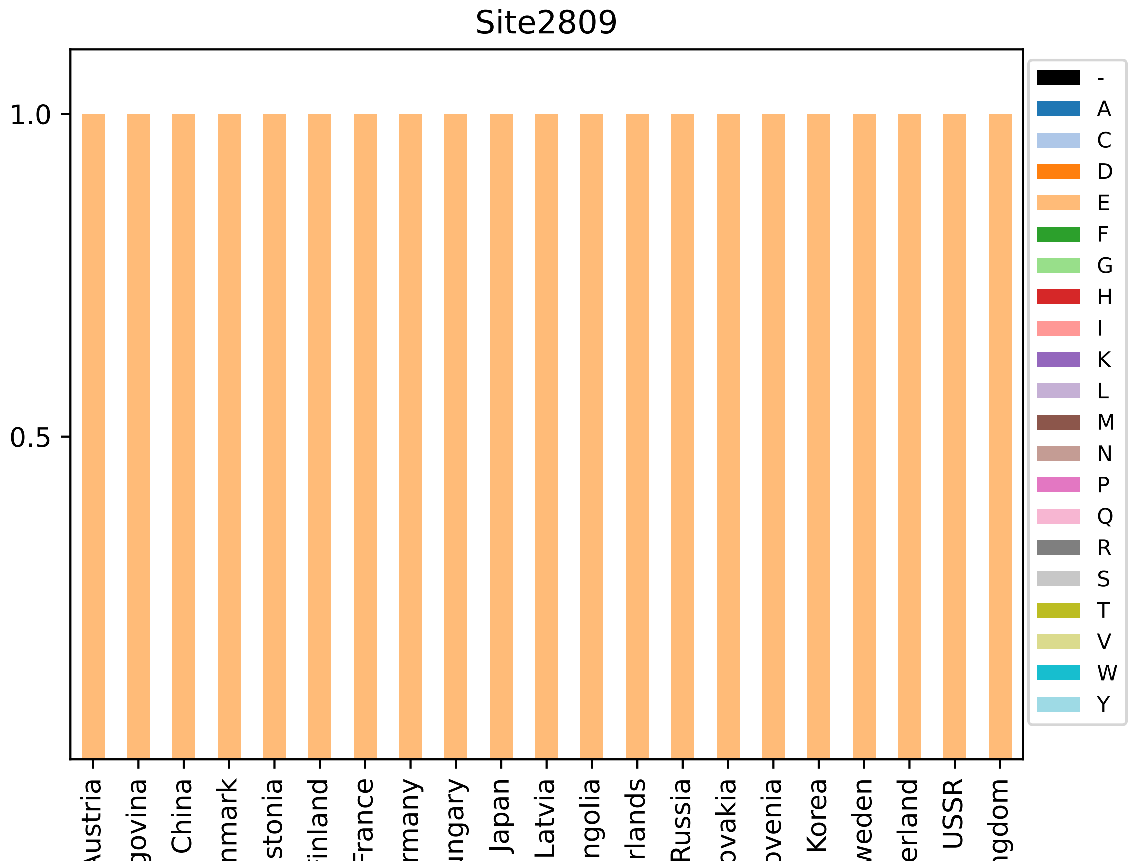
Site2809
1.0
0.5
-
A
C
D
E
F
G
H
I
K
L
M
N
P
Q
R
S
T
V
W
Y
China
Japan
Latvia
Russia
Korea
USSR

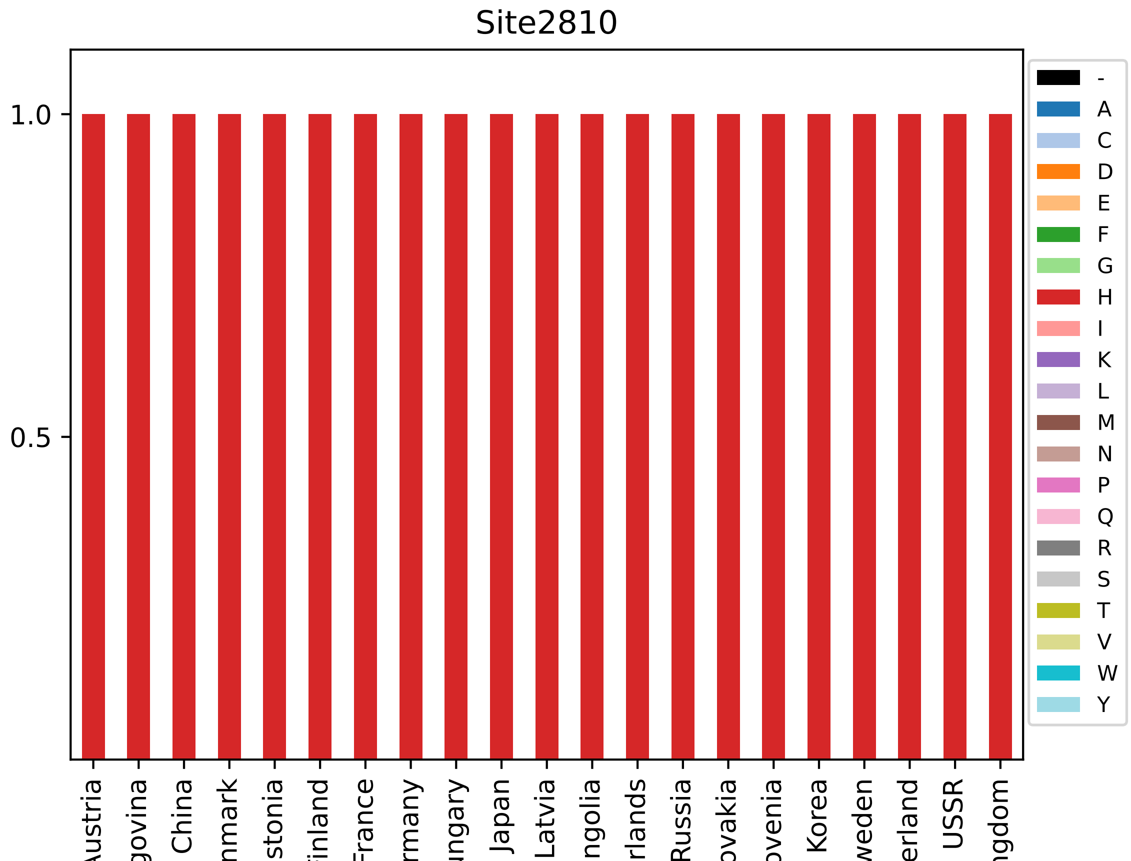
Site2810
1.0
0.5
-
A
C
D
E
F
G
H
I
K
L
M
N
P
Q
R
S
T
V
W
Y
China
Japan
Latvia
Russia
Korea
USSR

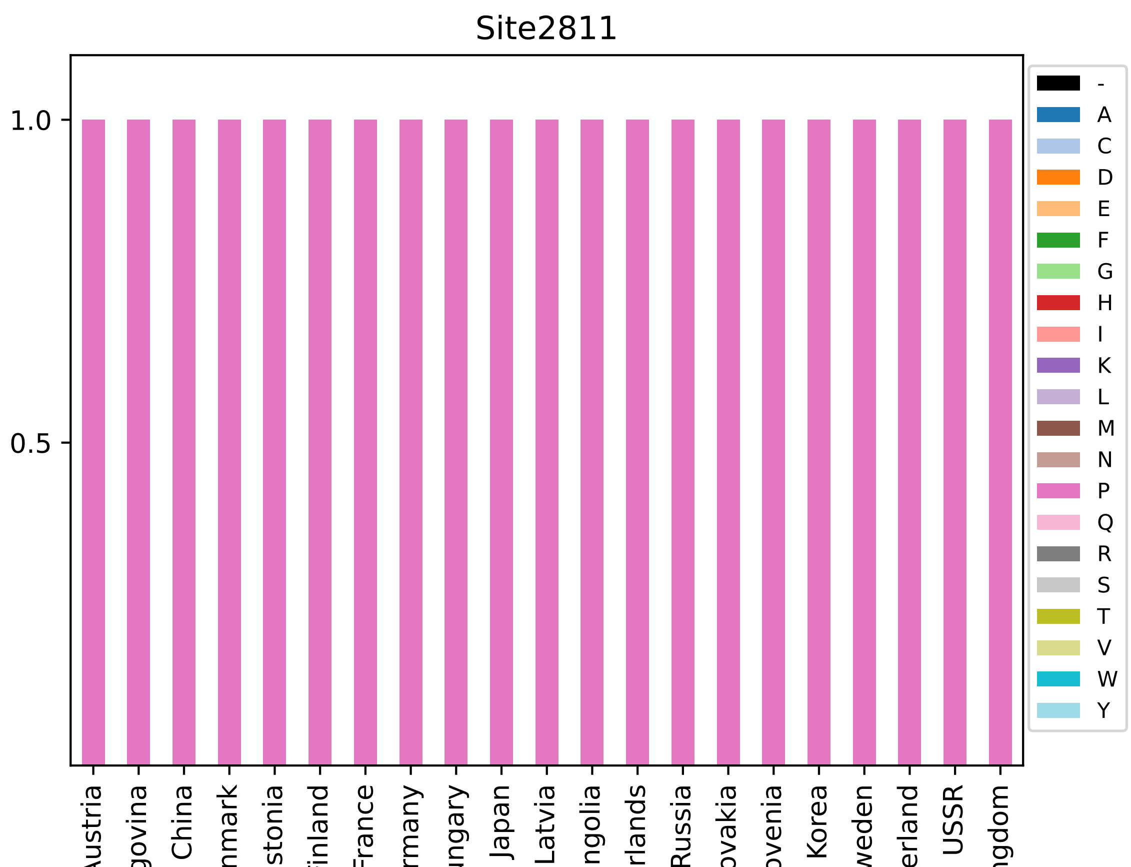
Site2811
1.0
0.5
-
A
C
D
E
F
G
H
I
K
L
M
N
P
Q
R
S
T
V
W
Y
China
Japan
Latvia
Russia
Korea
USSR

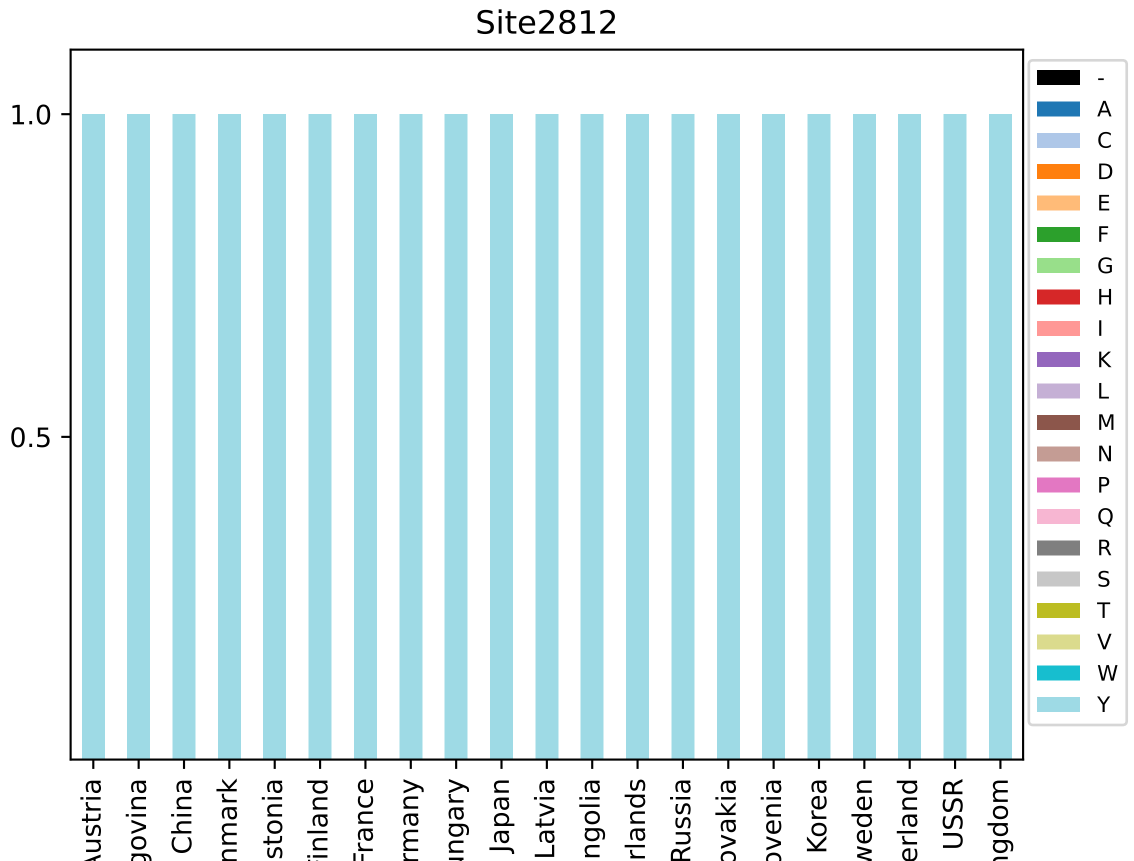

Site2812
1.0
0.5
-
A
C
D
E
F
G
H
I
K
L
M
N
P
Q
R
S
T
V
W
Y
Austria
govina
China
nmark
stonia
inland
France
rmany
ungary
Japan
Latvia
ngolia
rlands
Russia
ovakia
ovenia
Korea
weden
erland
USSR
ngdom

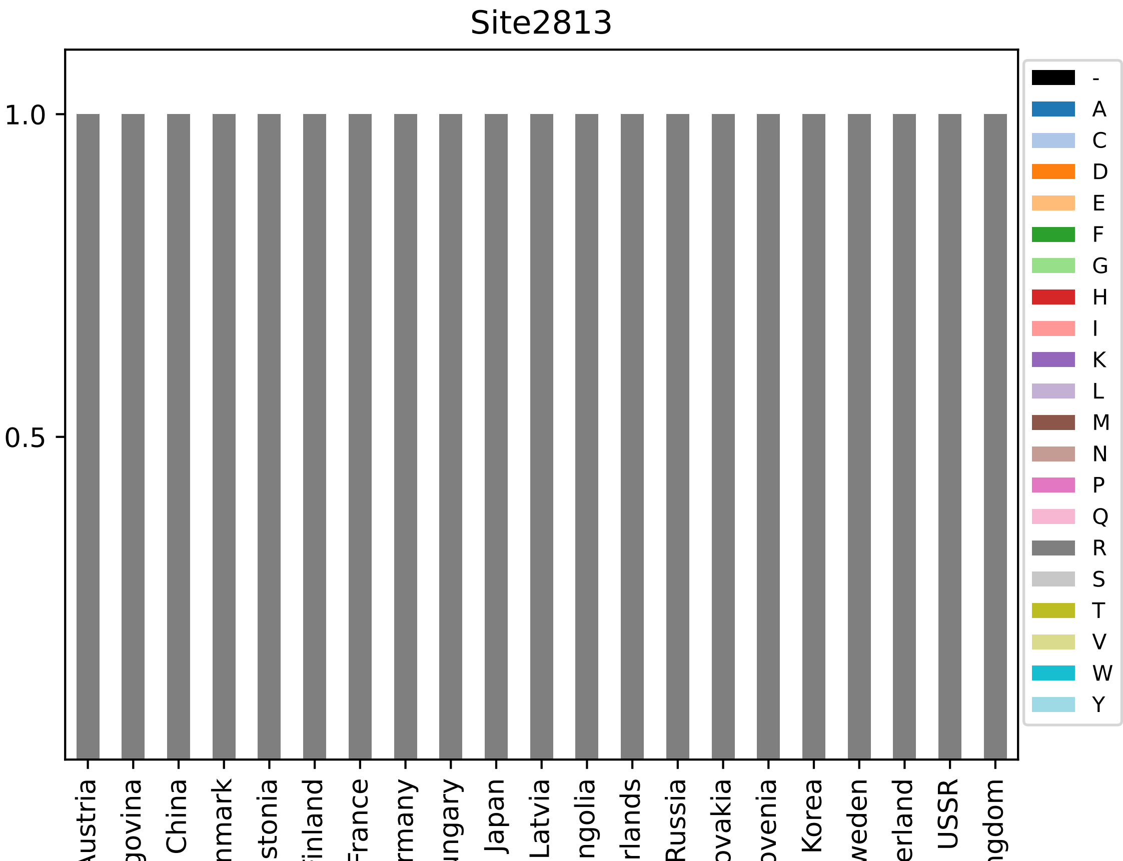

Site2813
1.0
0.5
-
A
C
D
E
F
G
H
I
K
L
M
N
P
Q
R
S
T
V
W
Y
China
Japan
Latvia
Russia
Korea
USSR

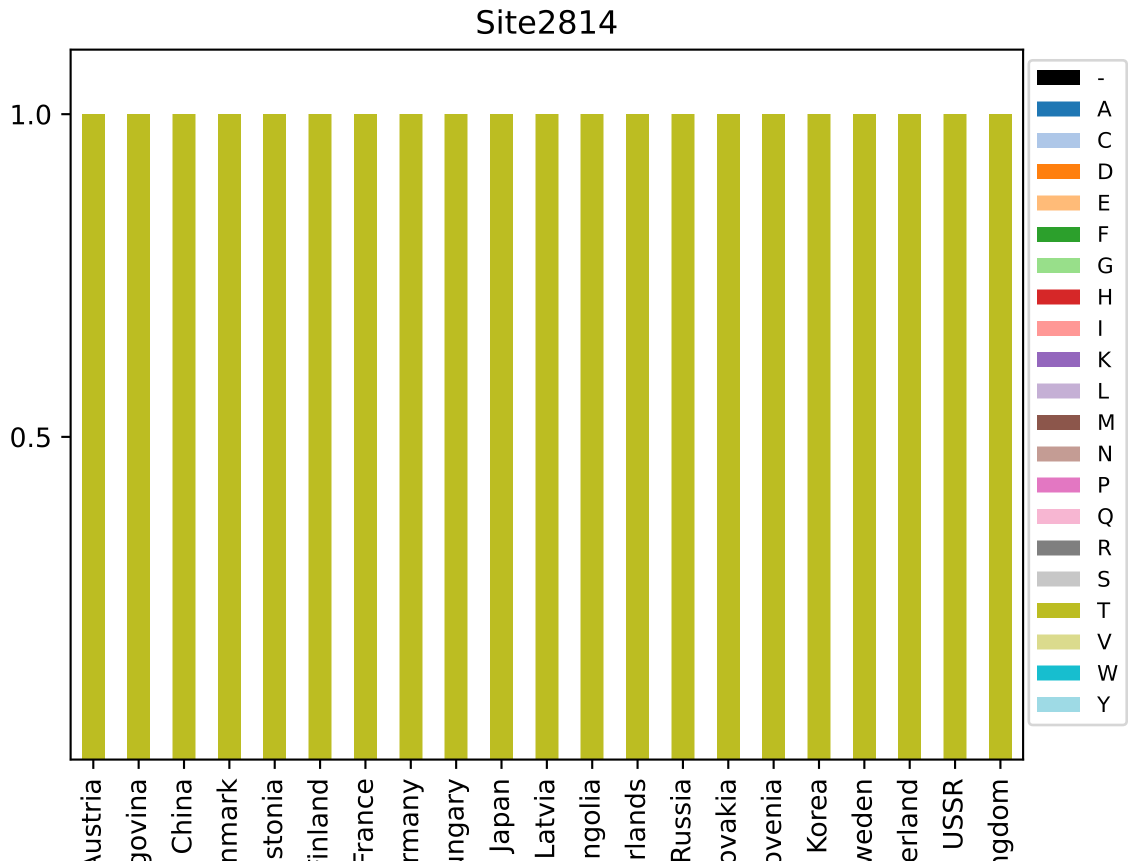
Site2814
1.0
0.5
-
A
C
D
E
F
G
H
I
K
L
M
N
P
Q
R
S
T
V
W
Y
China
Japan
Latvia
Russia
Korea
USSR

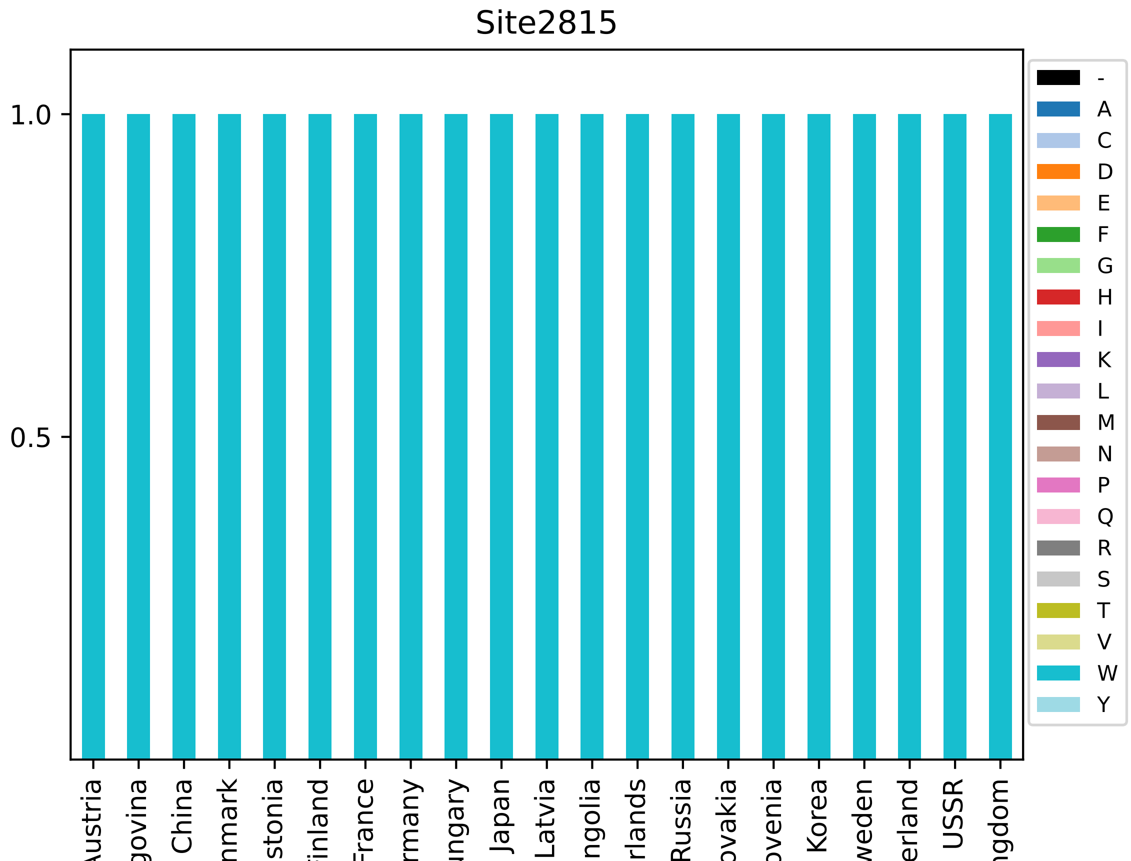

Site2815
1.0
0.5
-
A
C
D
E
F
G
H
I
K
L
M
N
P
Q
R
S
T
V
W
Y
China
Japan
Latvia
Russia
Korea
USSR

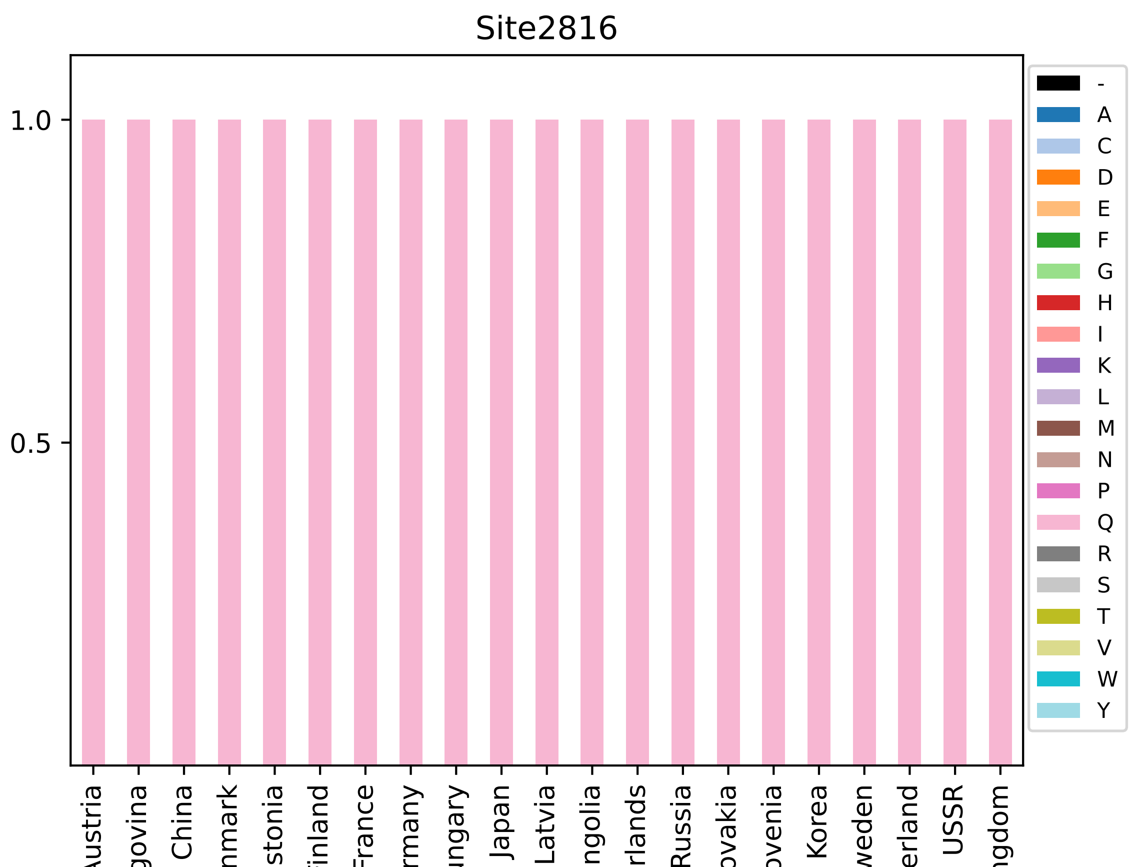

Site2816
1.0
0.5
-
A
C
D
E
F
G
H
I
K
L
M
N
P
Q
R
S
T
V
W
Y
Austria
China
Japan
Latvia
Russia
Korea
USSR

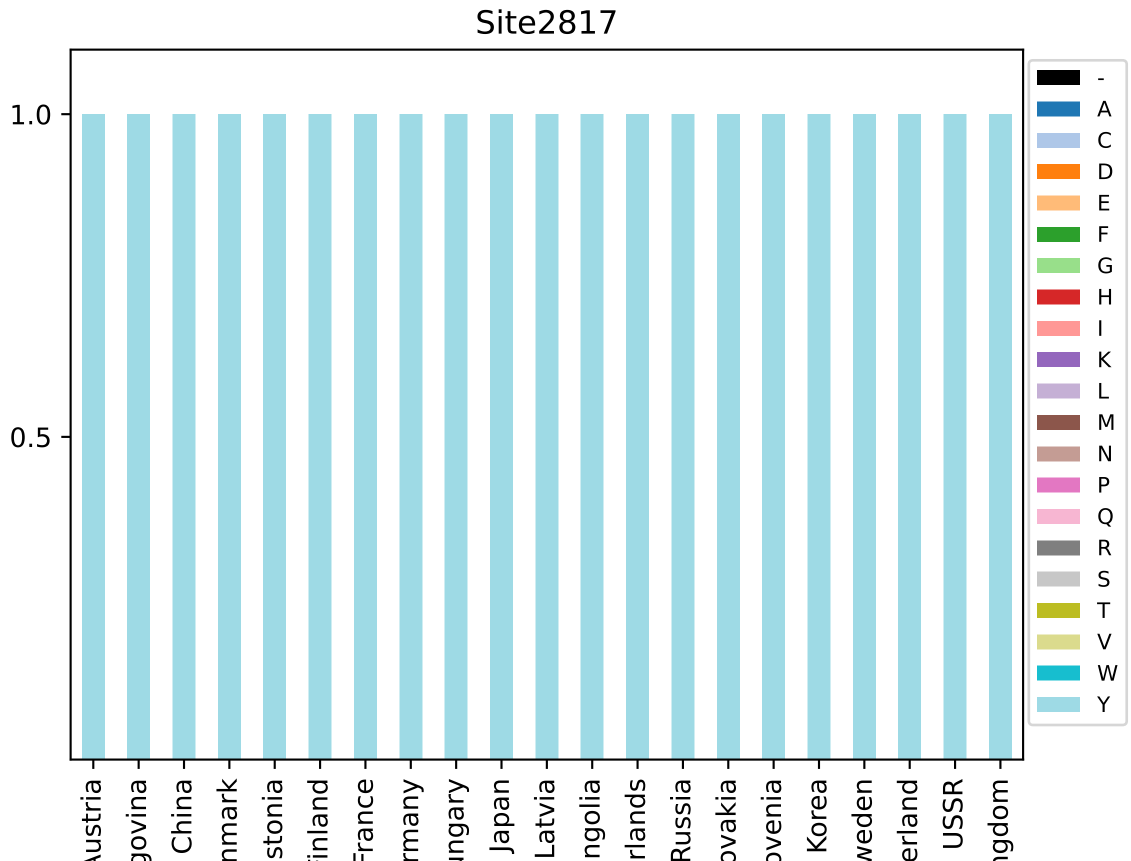
Site2817
1.0
0.5
-
A
C
D
E
F
G
H
I
K
L
M
N
P
Q
R
S
T
V
W
Y
China
Japan
Latvia
Russia
Korea
USSR

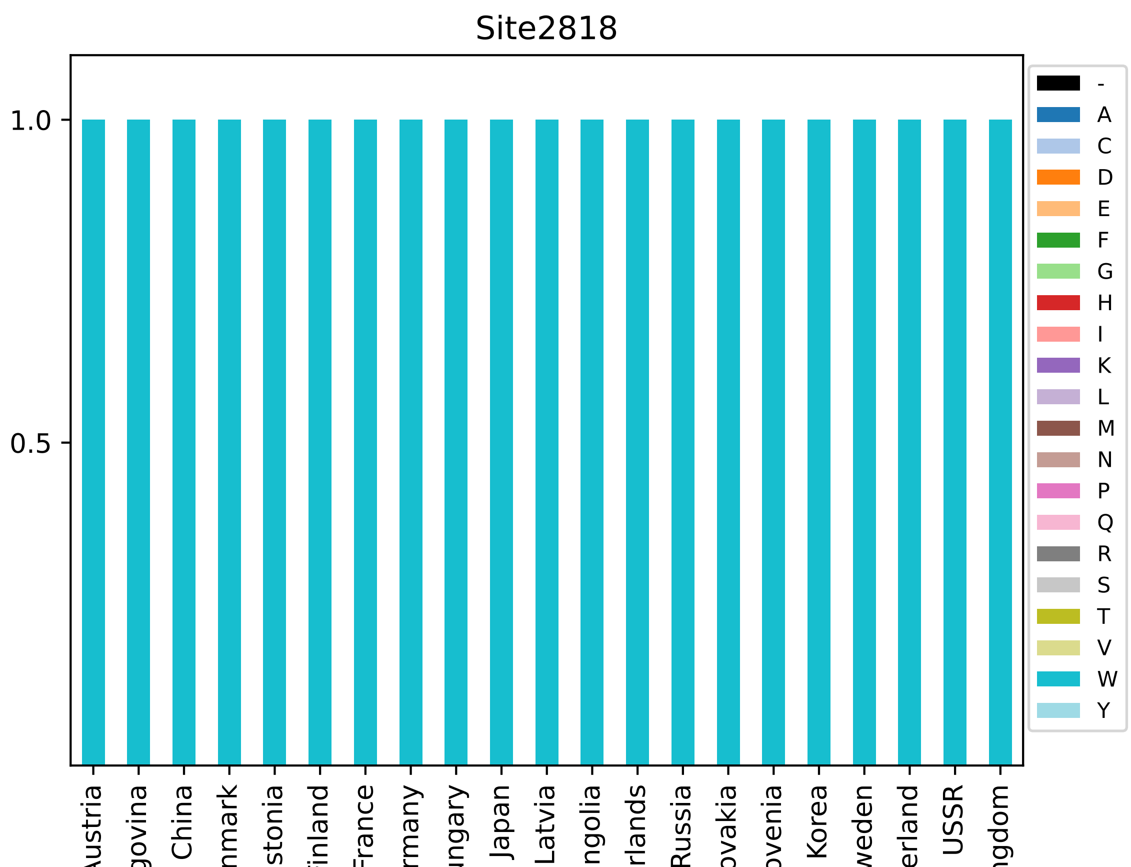
Site2818
1.0
0.5
-
A
C
D
E
F
G
H
I
K
L
M
N
P
Q
R
S
T
V
W
Y
China
Japan
Latvia
Russia
Korea
USSR

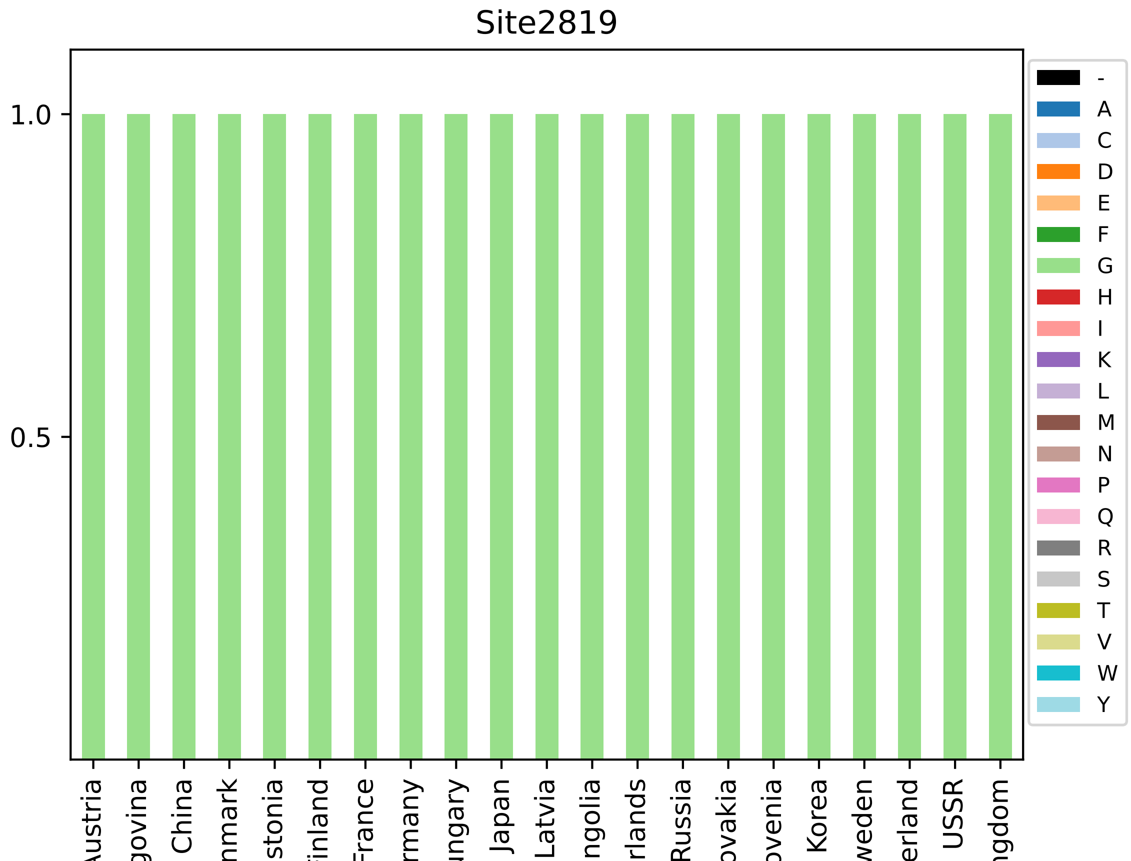
Site2819
1.0
0.5
China
Japan
Latvia
Russia
Korea
USSR
-
A
C
D
E
F
G
H
I
K
L
M
N
P
Q
R
S
T
V
W
Y

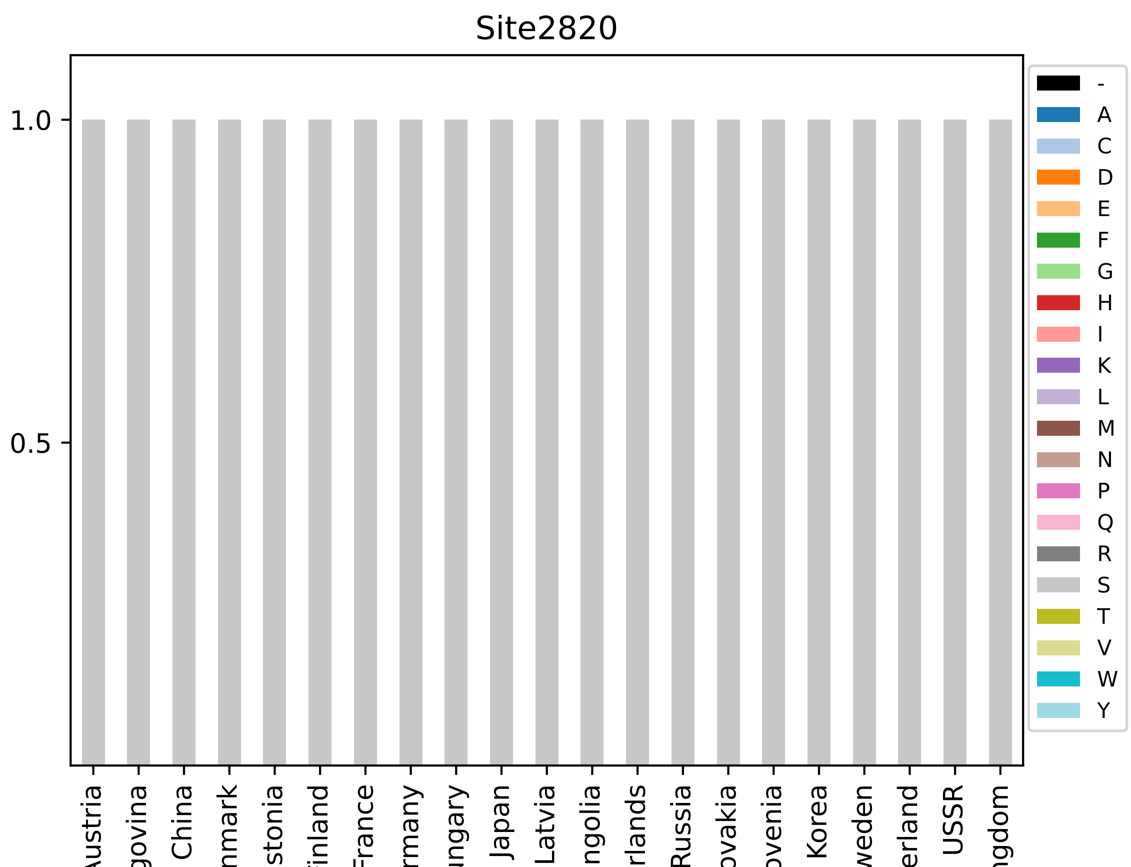
Site2820
1.0
0.5
-
A
C
D
E
F
G
H
I
K
L
M
N
P
Q
R
S
T
V
W
Y
China
Japan
Latvia
Russia
Korea
USSR

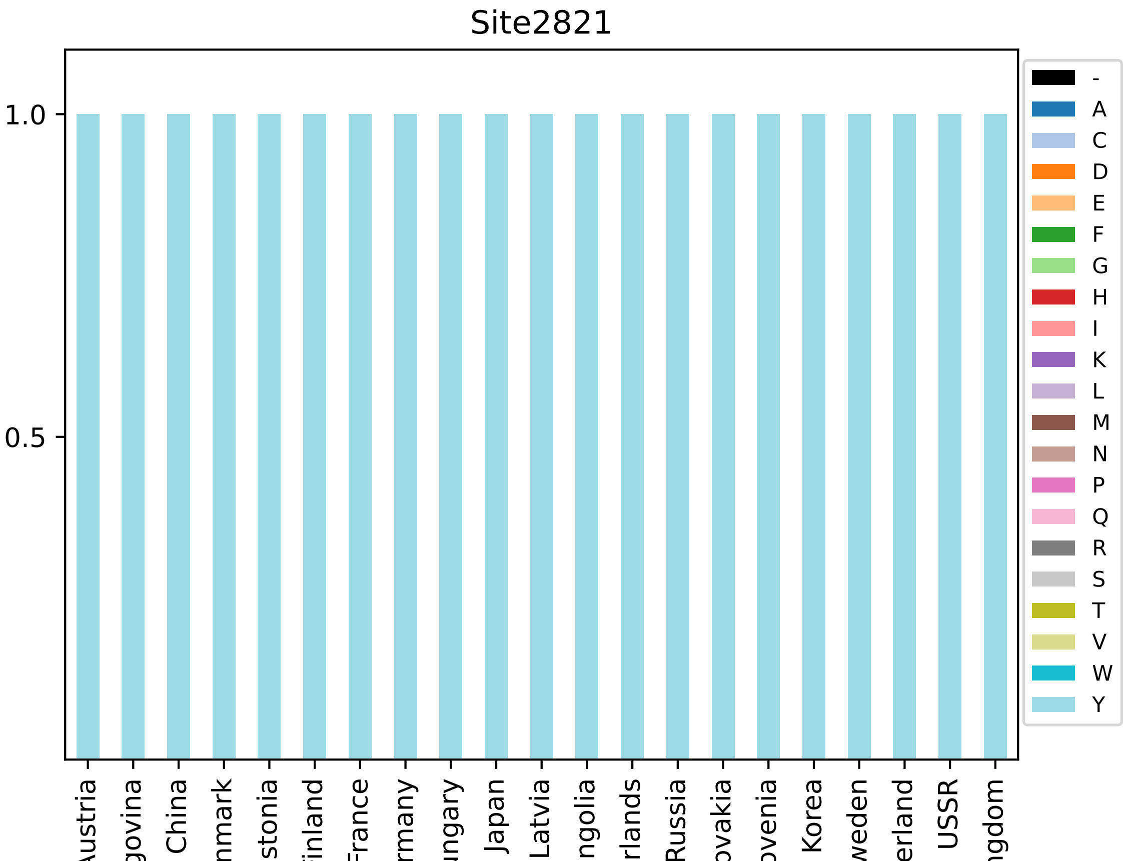

Site2821
1.0
0.5
-
A
C
D
E
F
G
H
I
K
L
M
N
P
Q
R
S
T
V
W
Y
Austria
China
Japan
Latvia
Russia
Korea
USSR

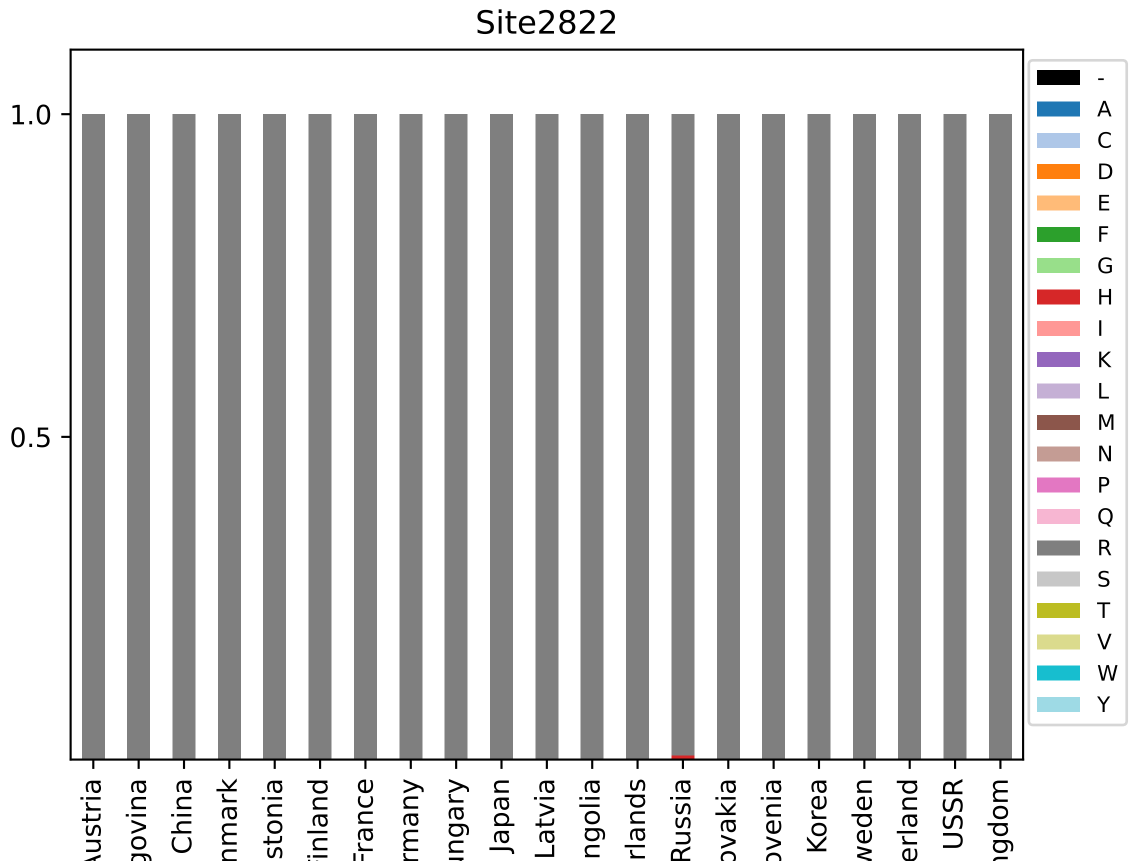

Site2822
1.0
0.5
-
A
C
D
E
F
G
H
I
K
L
M
N
P
Q
R
S
T
V
W
Y
Austria
govina
China
nmark
stonia
Finland
France
rmany
ungary
Japan
Latvia
ngolia
rlands
Russia
ovakia
ovenia
Korea
weden
erland
USSR
ngdom

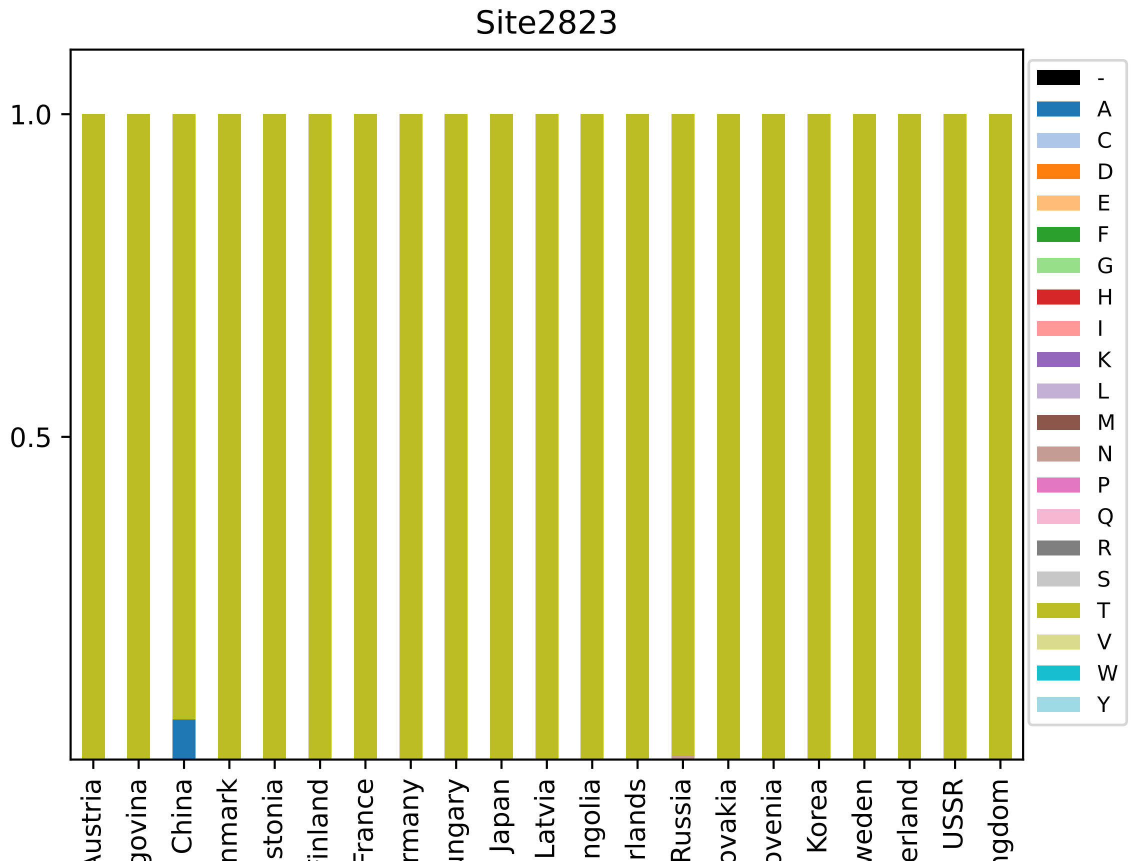

Site2823
1.0
0.5
-
A
C
D
E
F
G
H
I
K
L
M
N
P
Q
R
S
T
V
W
Y
Austria
govina
China
nmark
stonia
Finland
France
rmany
ungary
Japan
Latvia
ngolia
rlands
Russia
ovakia
ovenia
Korea
weden
erland
USSR
ngdom

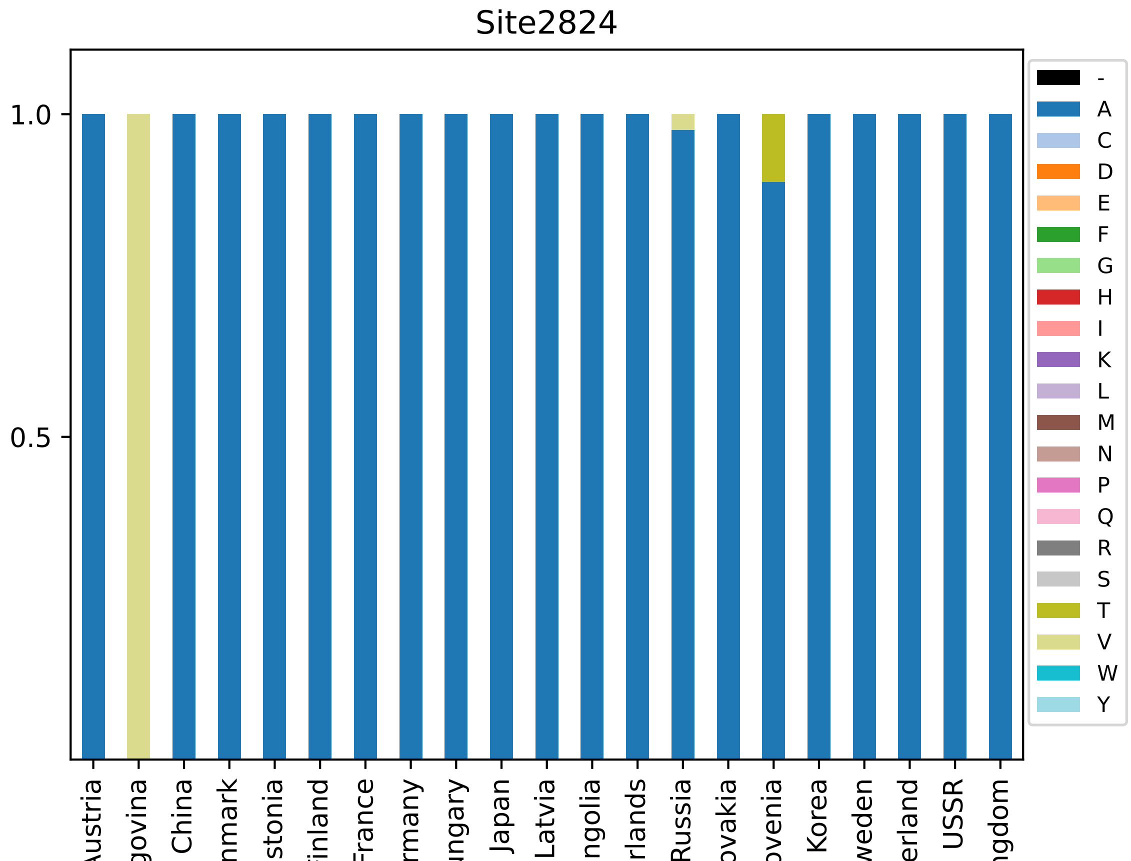

Site2824
1.0
0.5
-
A
C
D
E
F
G
H
I
K
L
M
N
P
Q
R
S
T
V
W
Y
Austria
govina
China
nmark
stonia
inland
France
rmany
ungary
Japan
Latvia
ngolia
rlands
Russia
ovakia
ovenia
Korea
weden
erland
USSR
ngdom

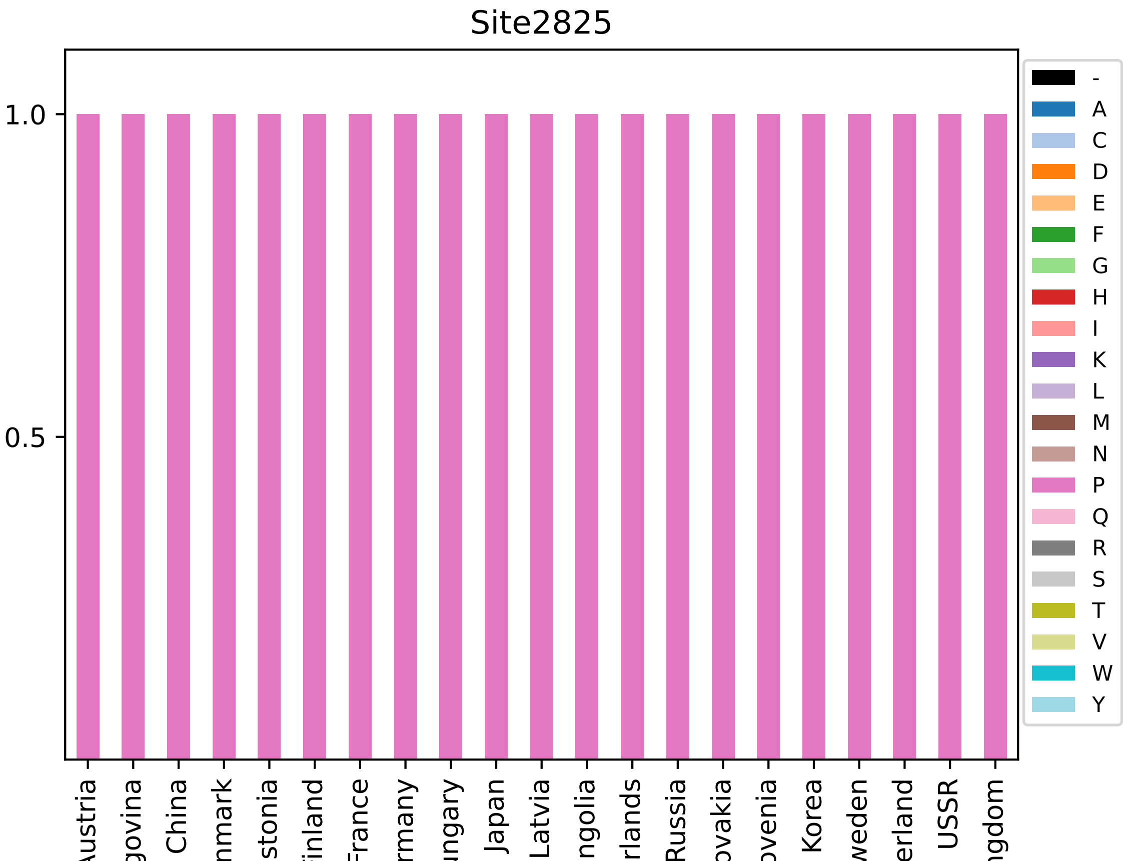

Site2825
1.0
0.5
Austria
govina
China
nmark
stonia
Finland
France
rmany
ungary
Japan
Latvia
ngolia
rlands
Russia
ovakia
ovenia
Korea
weden
erland
USSR
ngdom
-
A
C
D
E
F
G
H
I
K
L
M
N
P
Q
R
S
T
V
W
Y

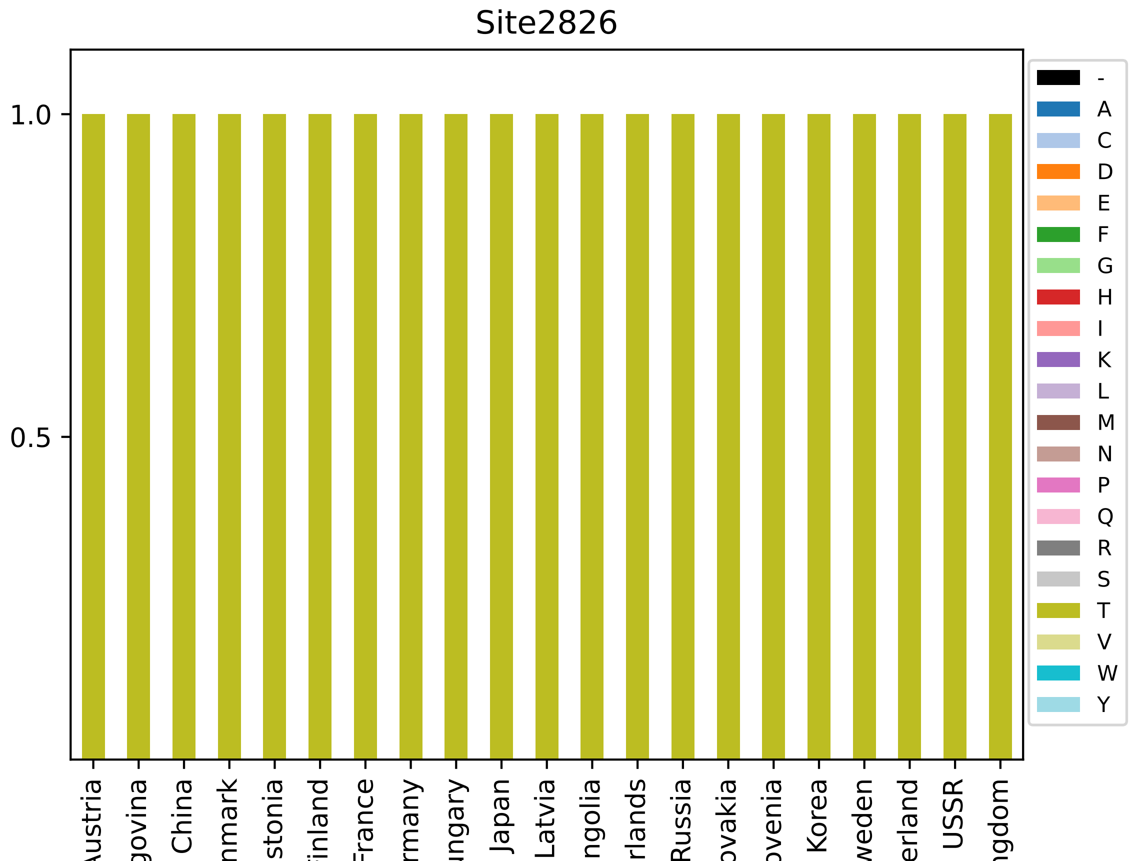
Site2826
1.0
0.5
-
A
C
D
E
F
G
H
I
K
L
M
N
P
Q
R
S
T
V
W
Y
Austria
govina
China
nmark
stonia
inland
France
rmany
ungary
Japan
Latvia
ngolia
rlands
Russia
ovakia
ovenia
Korea
weden
erland
USSR
ngdom

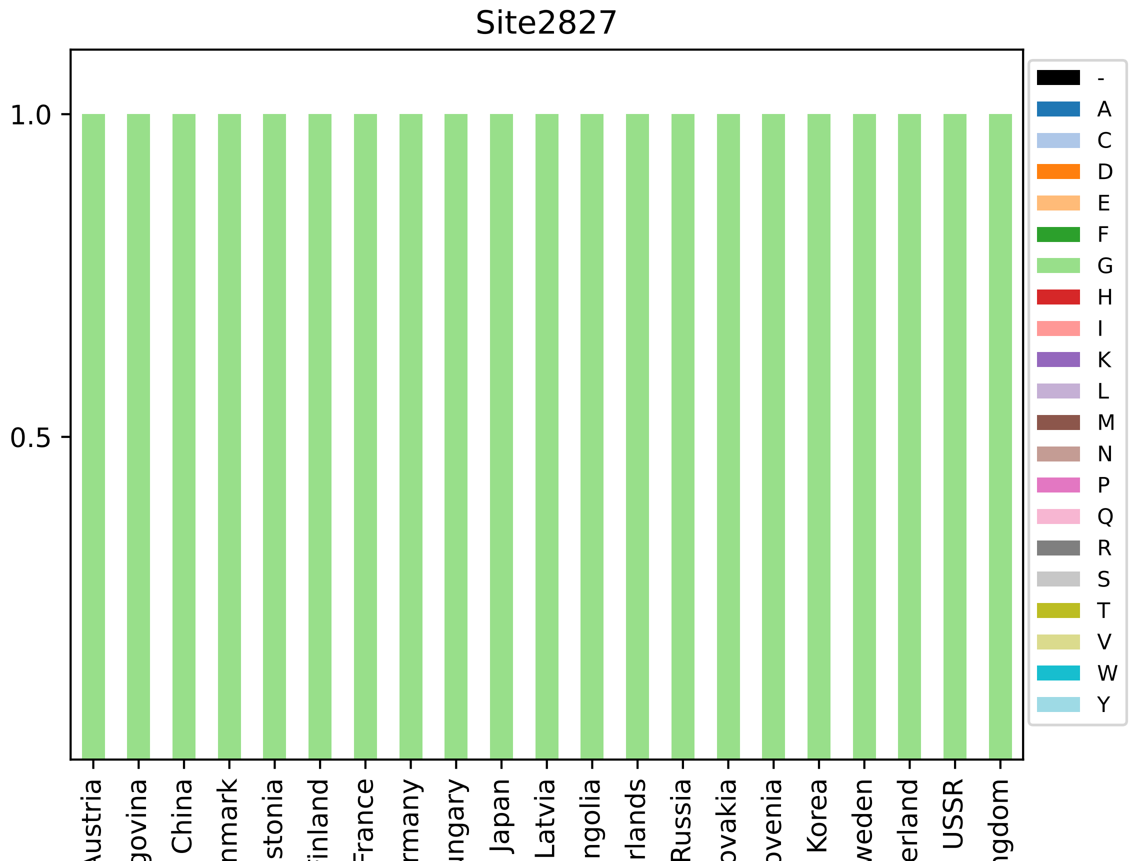

Site2827
1.0
0.5
-
A
C
D
E
F
G
H
I
K
L
M
N
P
Q
R
S
T
V
W
Y
China
Japan
Latvia
Russia
Korea
USSR

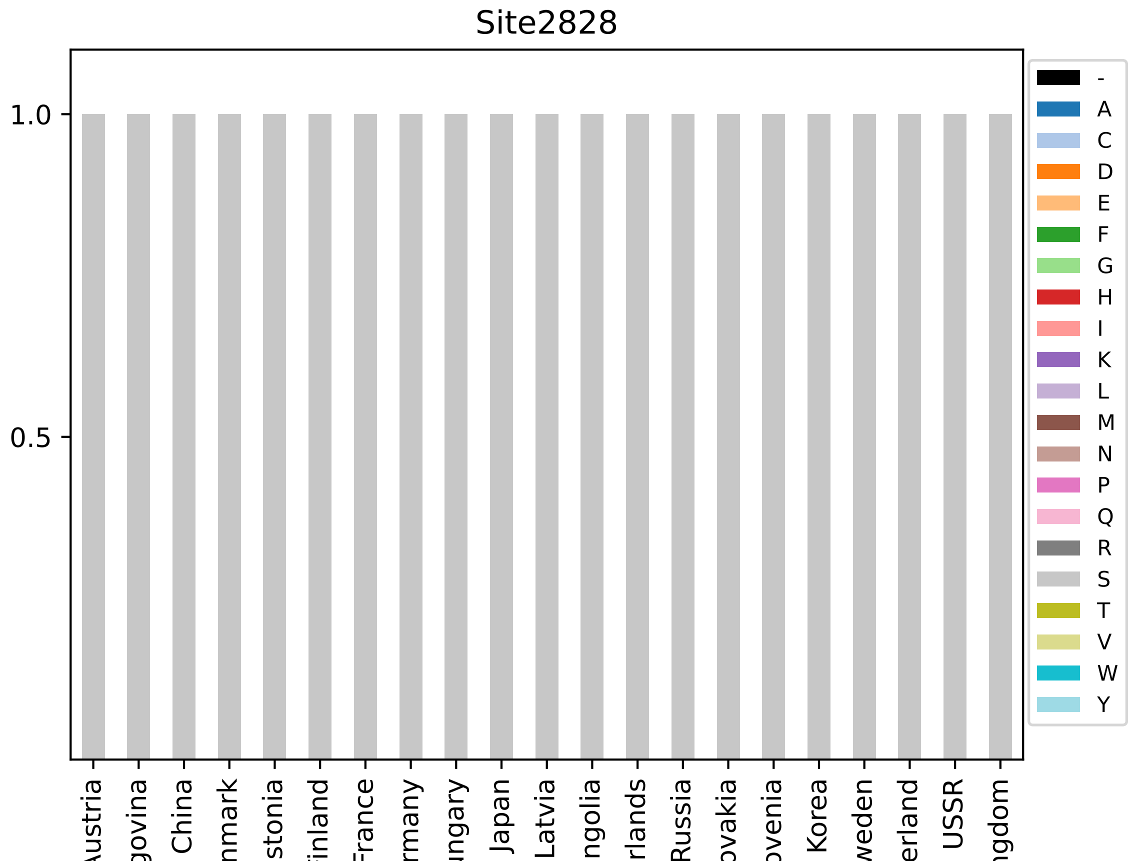
Site2828
1.0
0.5
Austria
govina
China
nmark
stonia
Finland
France
rmany
ungary
Japan
Latvia
ngolia
rlands
Russia
ovakia
ovenia
Korea
weden
erland
USSR
ngdom
-
A
C
D
E
F
G
H
I
K
L
M
N
P
Q
R
S
T
V
W
Y

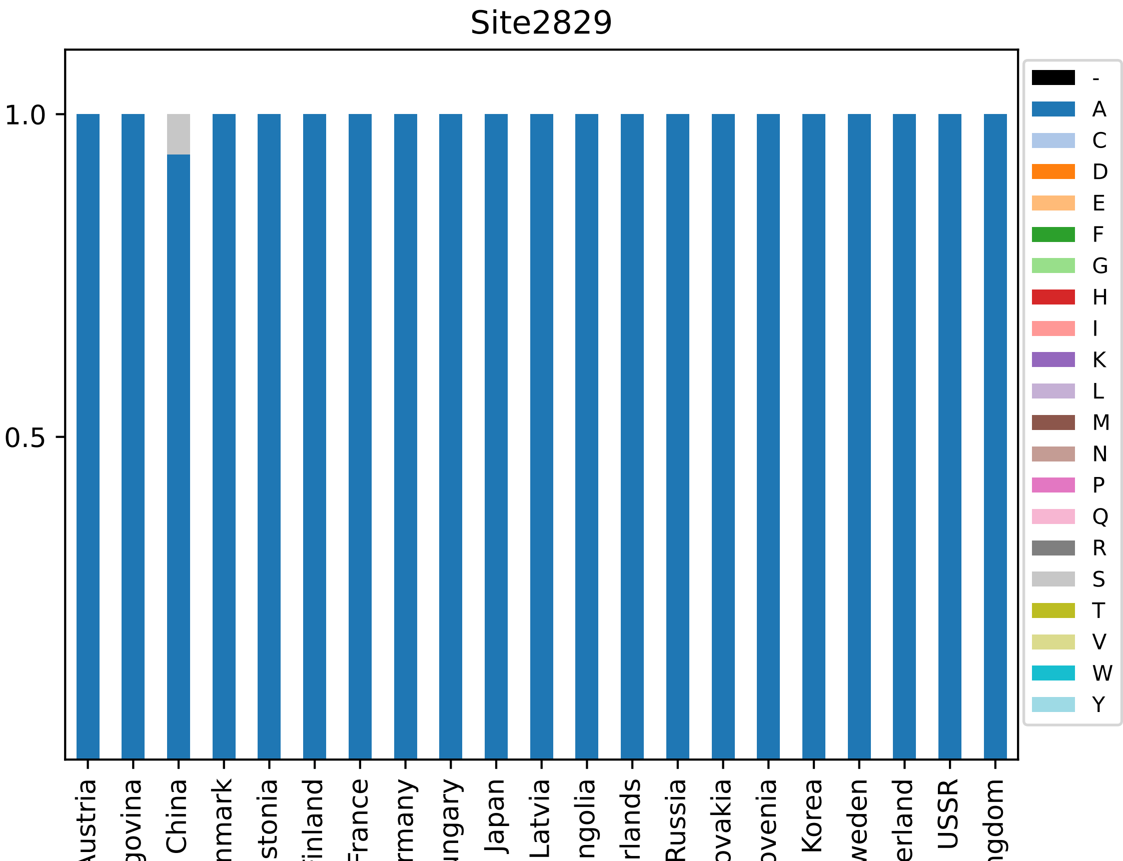
Site2829
1.0
0.5
-
A
C
D
E
F
G
H
I
K
L
M
N
P
Q
R
S
T
V
W
Y
China
Japan
Latvia
Russia
Korea
USSR

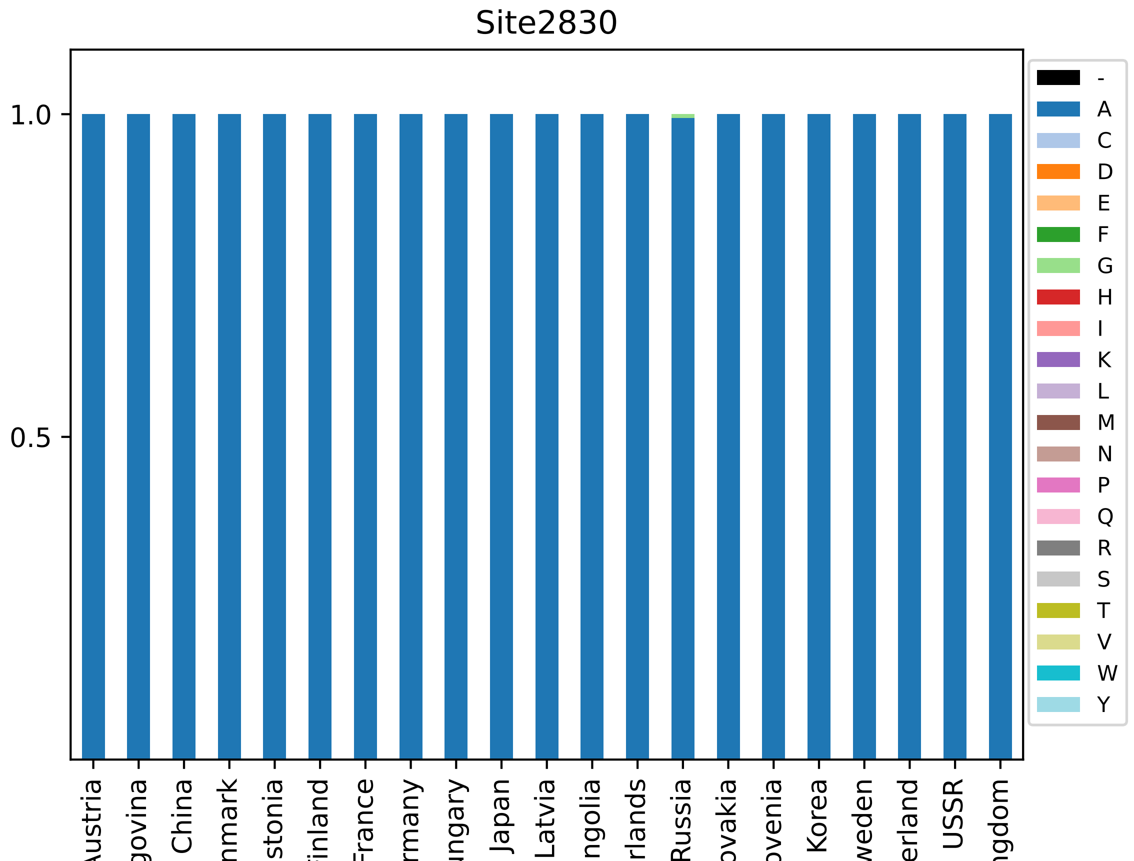
Site2830
1.0
0.5
-
A
C
D
E
F
G
H
I
K
L
M
N
P
Q
R
S
T
V
W
Y
Austria
govina
China
nmark
stonia
inland
France
rmany
ungary
Japan
Latvia
ngolia
rlands
Russia
ovakia
ovenia
Korea
weden
erland
USSR
ngdom

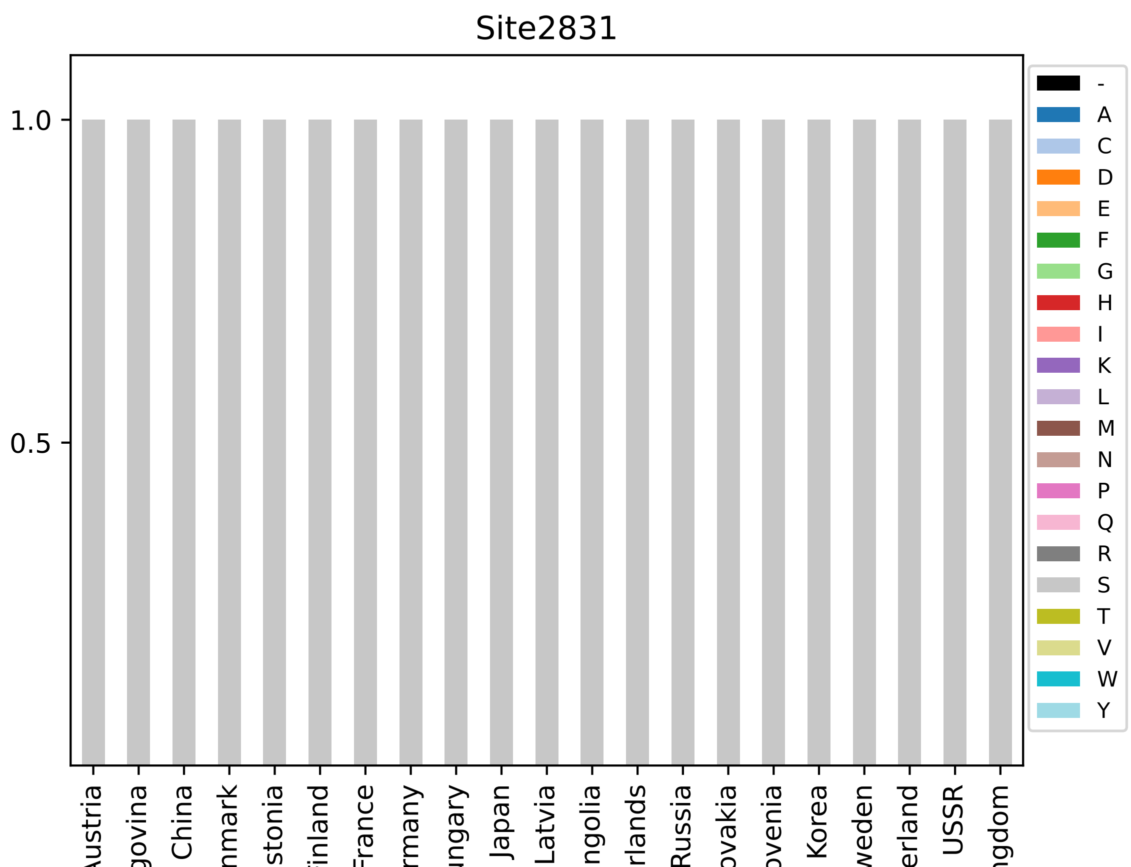

Site2831
1.0
0.5
-
A
C
D
E
F
G
H
I
K
L
M
N
P
Q
R
S
T
V
W
Y
Austria
China
Japan
Latvia
Russia
Korea
USSR

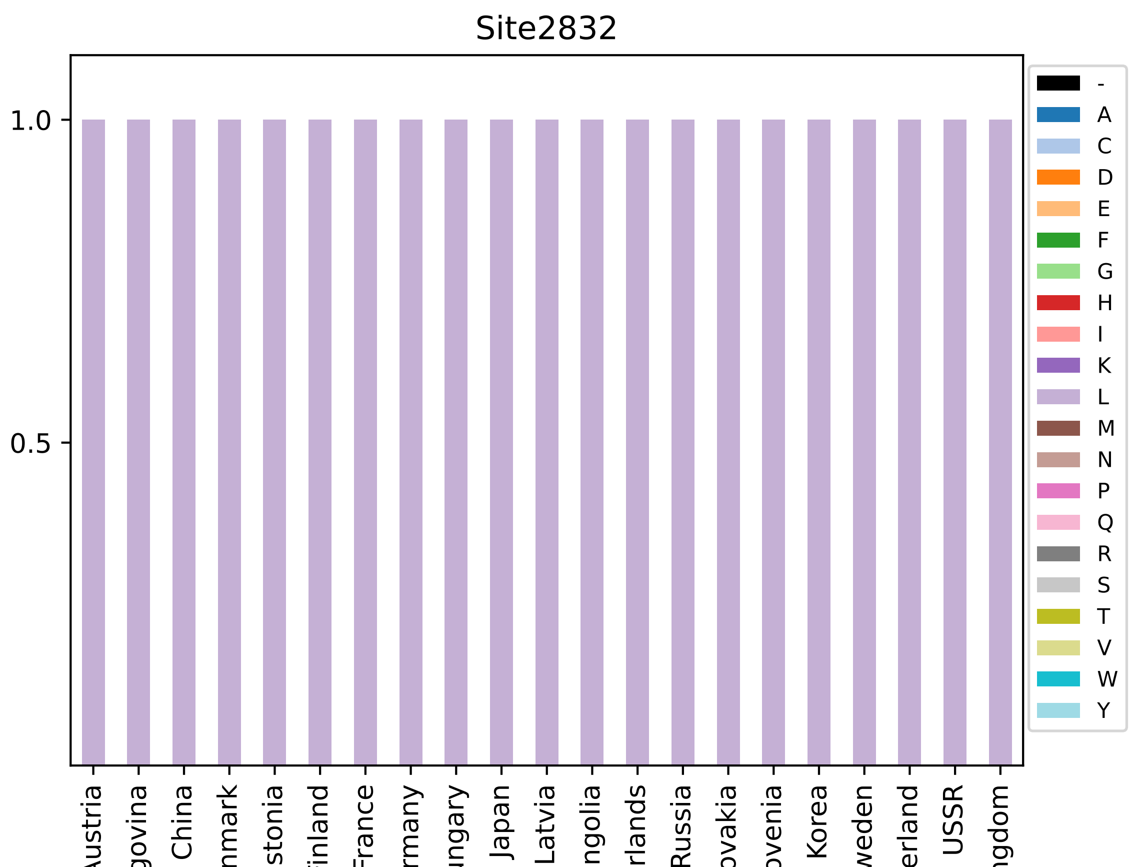
Site2832
1.0
0.5
-
A
C
D
E
F
G
H
I
K
L
M
N
P
Q
R
S
T
V
W
Y
Austria
govina
China
nmark
stonia
inland
France
rmany
ungary
Japan
Latvia
ngolia
rlands
Russia
ovakia
ovenia
Korea
weden
erland
USSR
ngdom

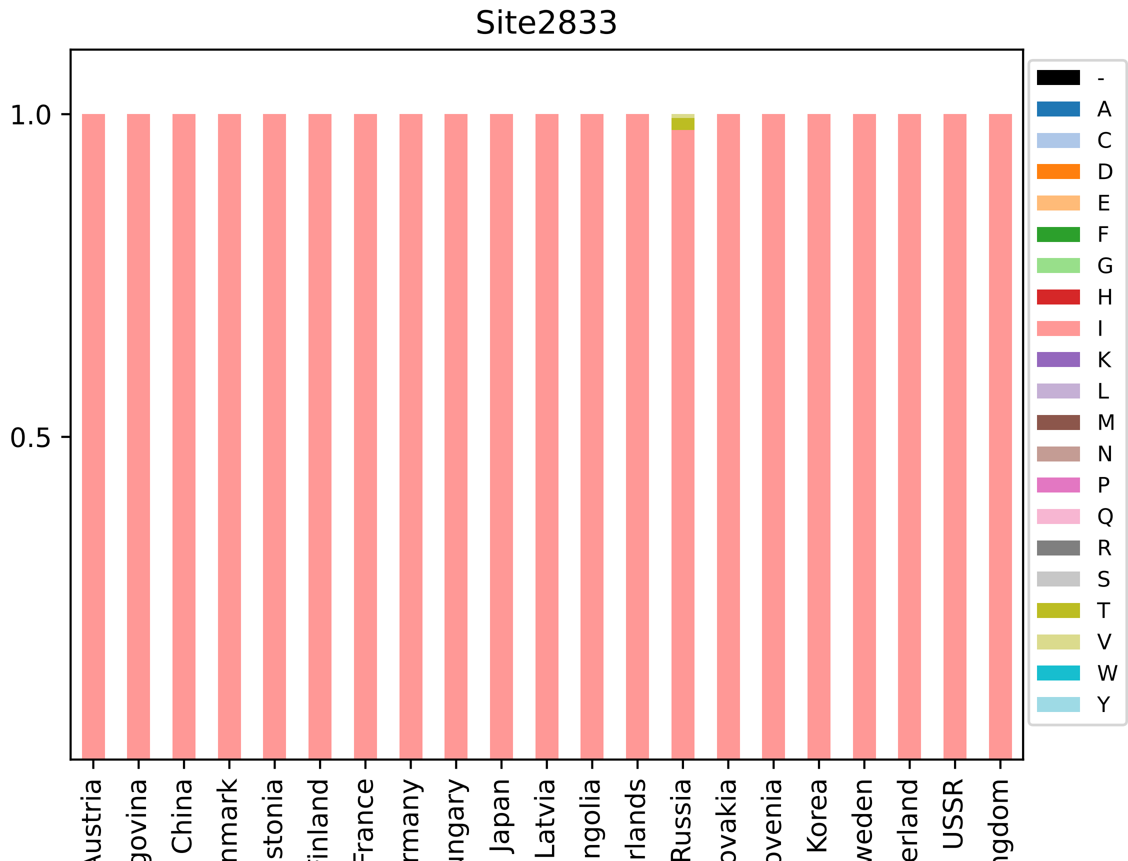

Site2833
1.0
0.5
-
A
C
D
E
F
G
H
I
K
L
M
N
P
Q
R
S
T
V
W
Y
Austria
govina
China
nmark
stonia
inland
France
rmany
ungary
Japan
Latvia
ngolia
rlands
Russia
ovakia
ovenia
Korea
weden
erland
USSR
ngdom

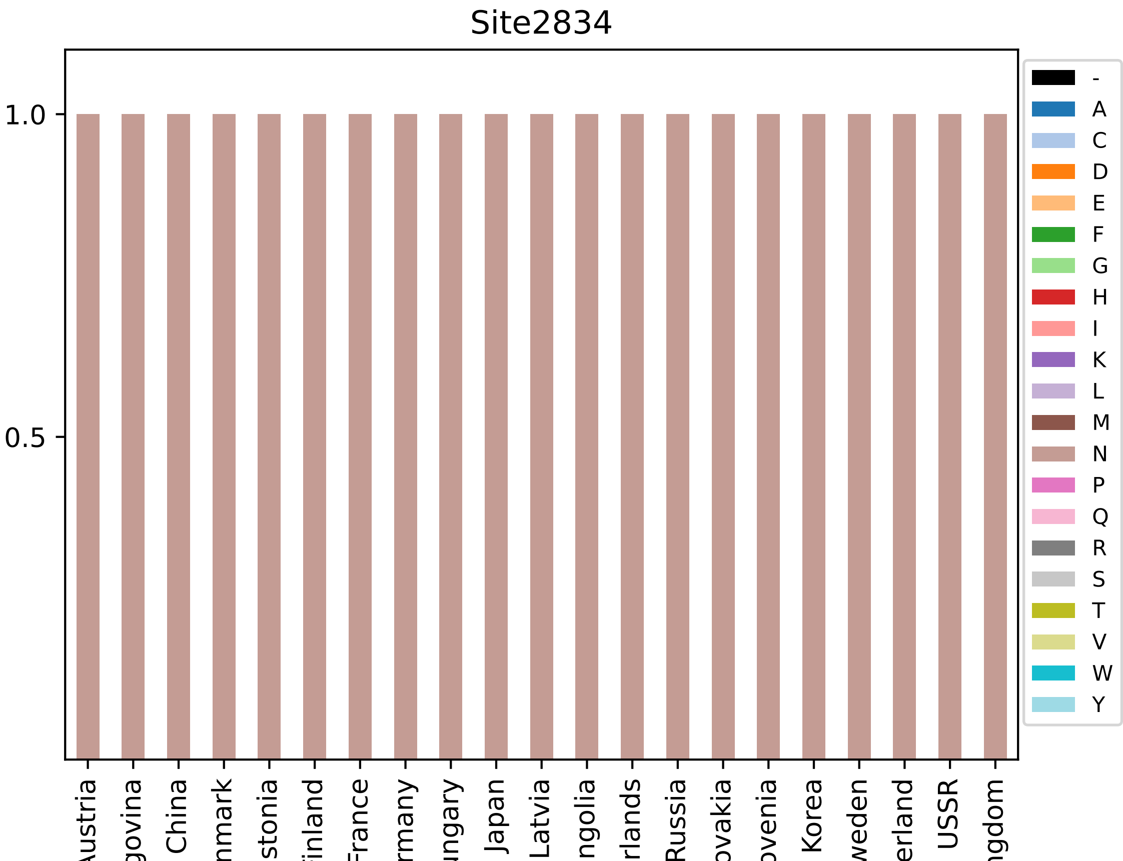

Site2834
1.0
0.5
-
A
C
D
E
F
G
H
I
K
L
M
N
P
Q
R
S
T
V
W
Y
Japan
Latvia
Russia
Korea
USSR

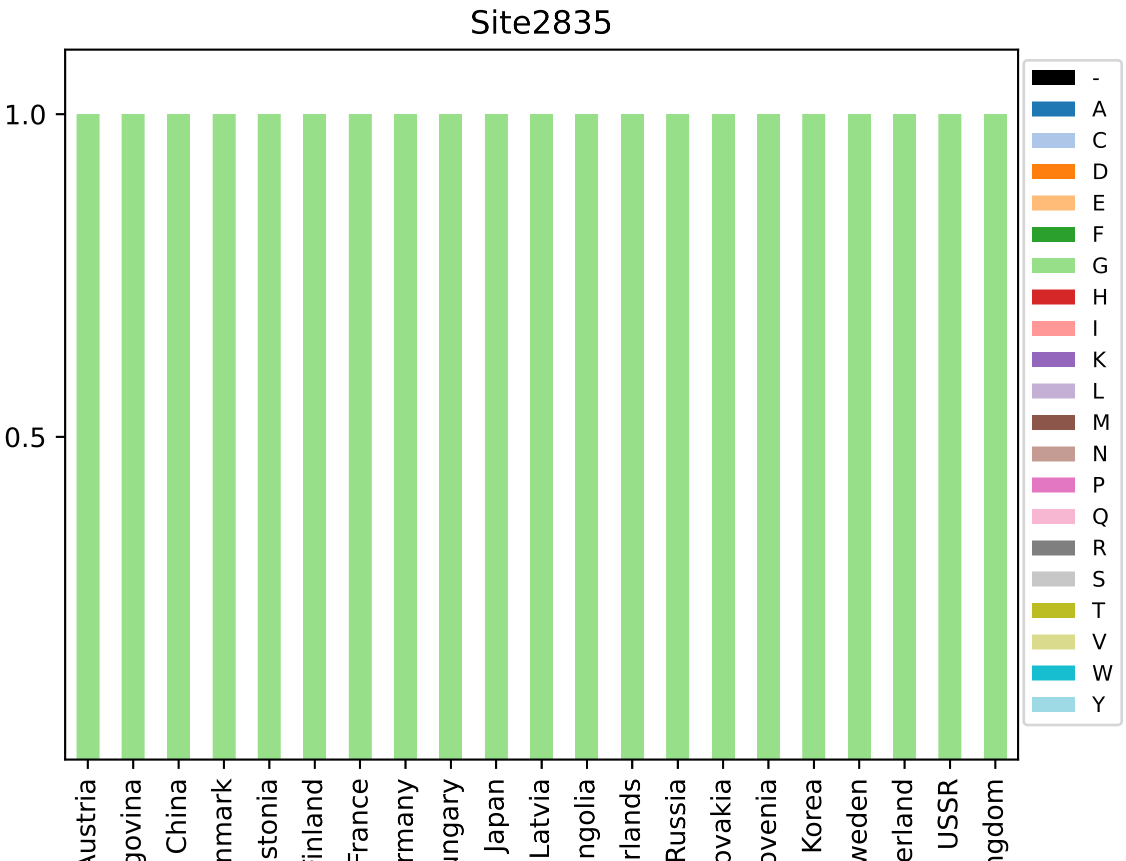

Site2835
1.0
0.5
-
A
C
D
E
F
G
H
I
K
L
M
N
P
Q
R
S
T
V
W
Y
China
Japan
Latvia
Russia
Korea
USSR

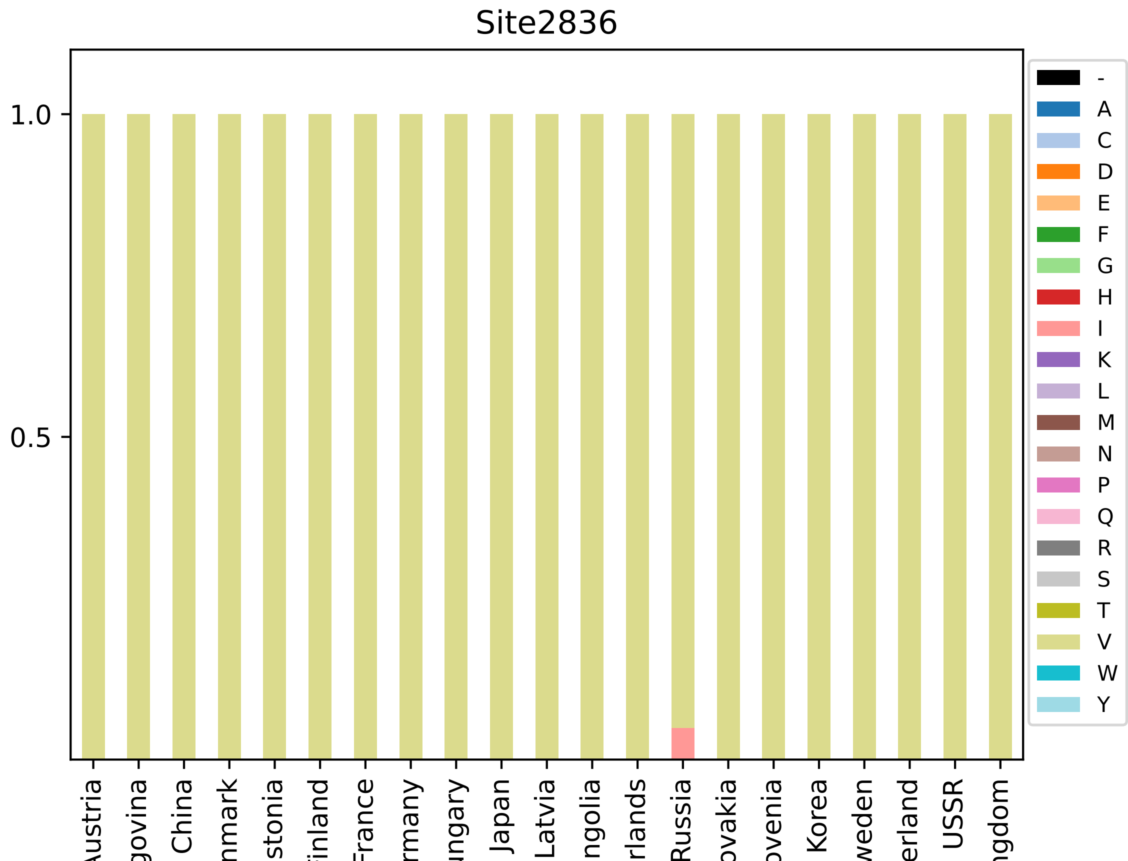
Site2836
1.0
0.5
-
A
C
D
E
F
G
H
I
K
L
M
N
P
Q
R
S
T
V
W
Y
Austria
govina
China
nmark
stonia
inland
France
rmany
ungary
Japan
Latvia
ngolia
rlands
Russia
ovakia
ovenia
Korea
weden
erland
USSR
ngdom

Site2837

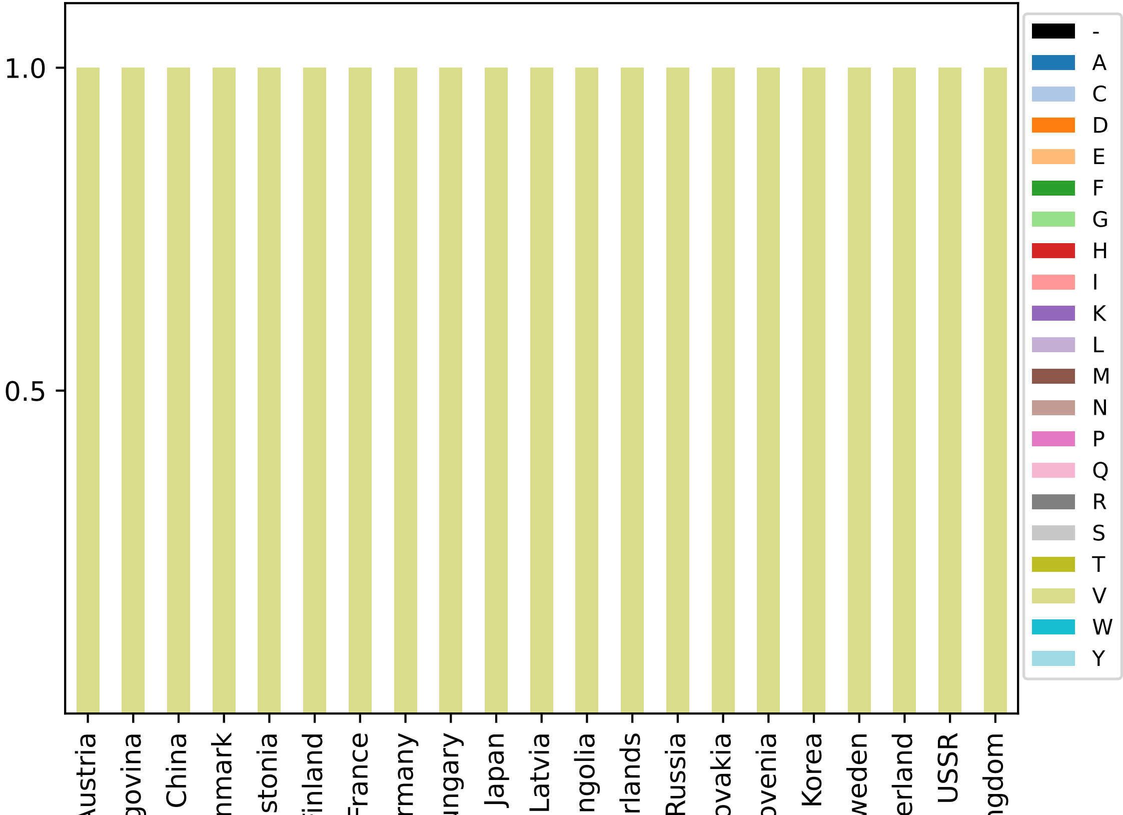
1.0
0.5
-
A
C
D
E
F
G
H
I
K
L
M
N
P
Q
R
S
T
V
W
Y
China
Japan
Latvia
Russia
Korea
USSR

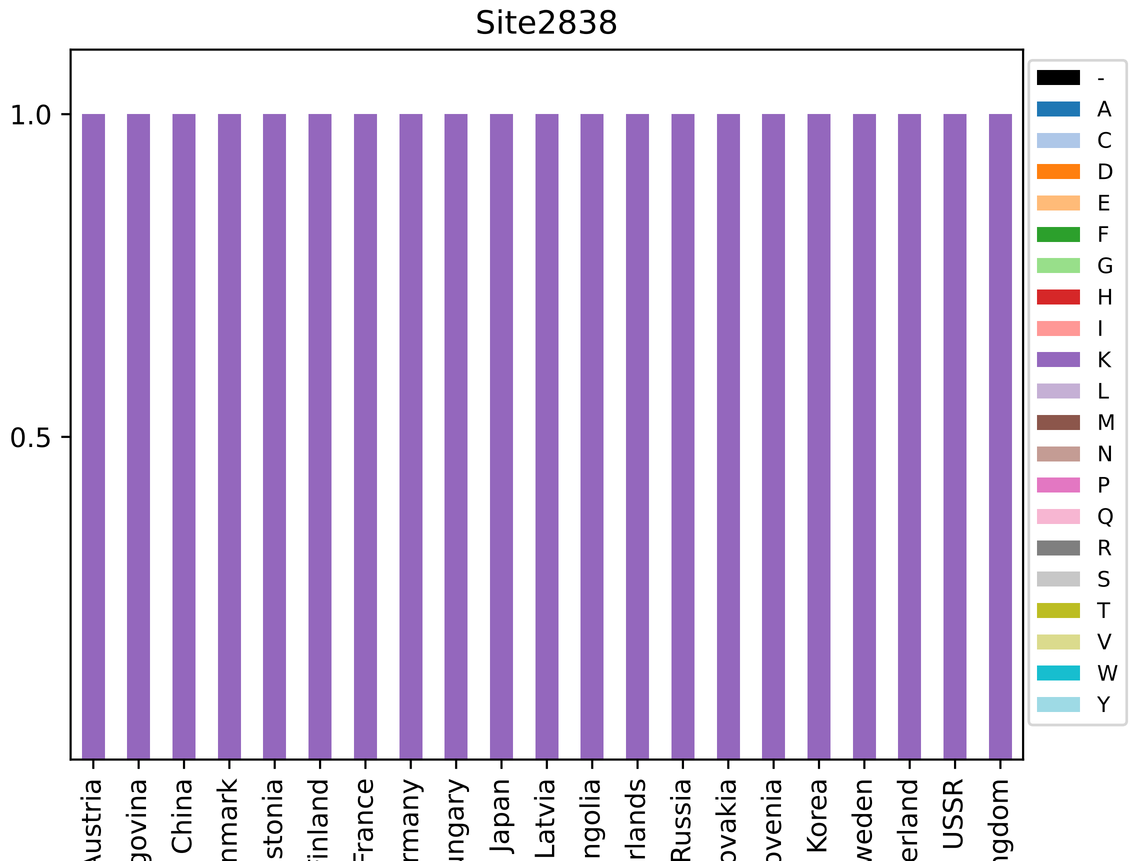

Site2838
1.0
0.5
-
A
C
D
E
F
G
H
I
K
L
M
N
P
Q
R
S
T
V
W
Y
Austria
govina
China
nmark
stonia
inland
France
rmany
ungary
Japan
Latvia
ngolia
rlands
Russia
ovakia
ovenia
Korea
weden
erland
USSR
ngdom

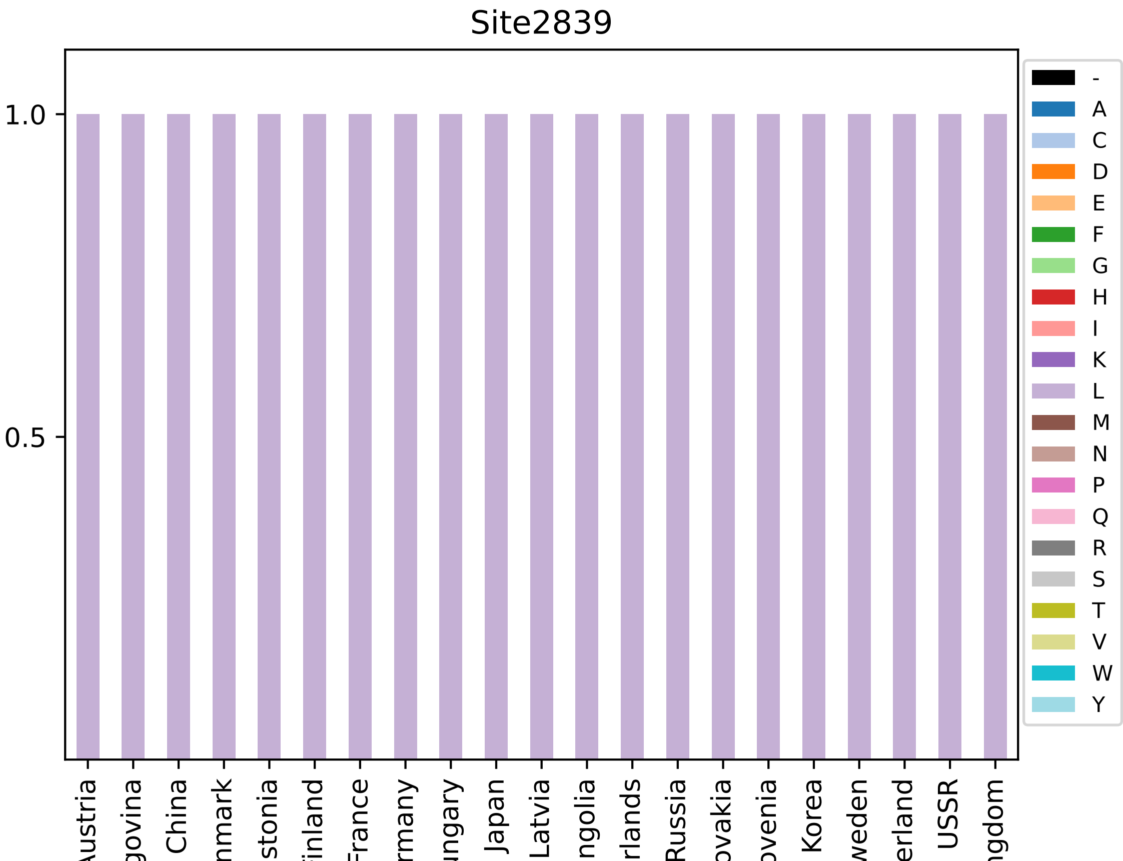

Site2839
1.0
0.5
-
A
C
D
E
F
G
H
I
K
L
M
N
P
Q
R
S
T
V
W
Y
China
Japan
Latvia
Russia
Korea
USSR

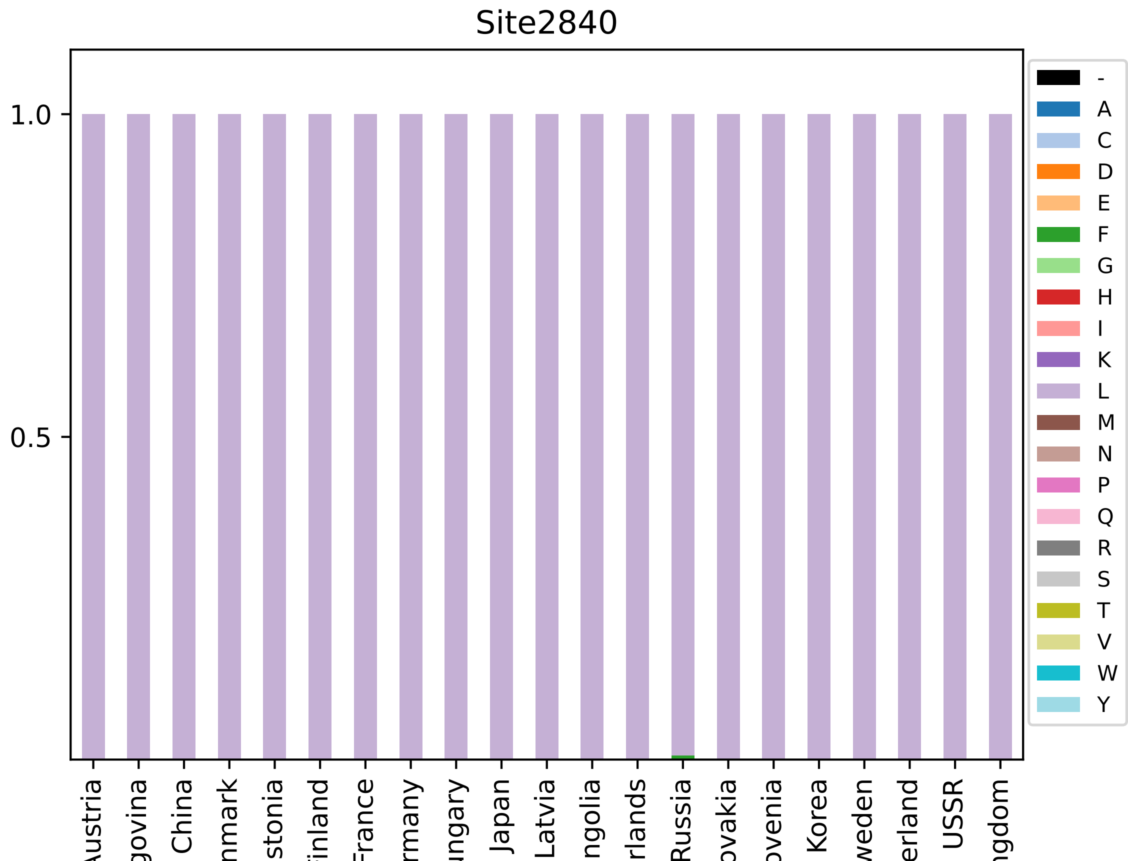

Site2840
1.0
0.5
-
A
C
D
E
F
G
H
I
K
L
M
N
P
Q
R
S
T
V
W
Y
Austria
govina
China
nmark
stonia
Finland
France
rmany
ungary
Japan
Latvia
ngolia
rlands
Russia
ovakia
ovenia
Korea
weden
erland
USSR
ngdom

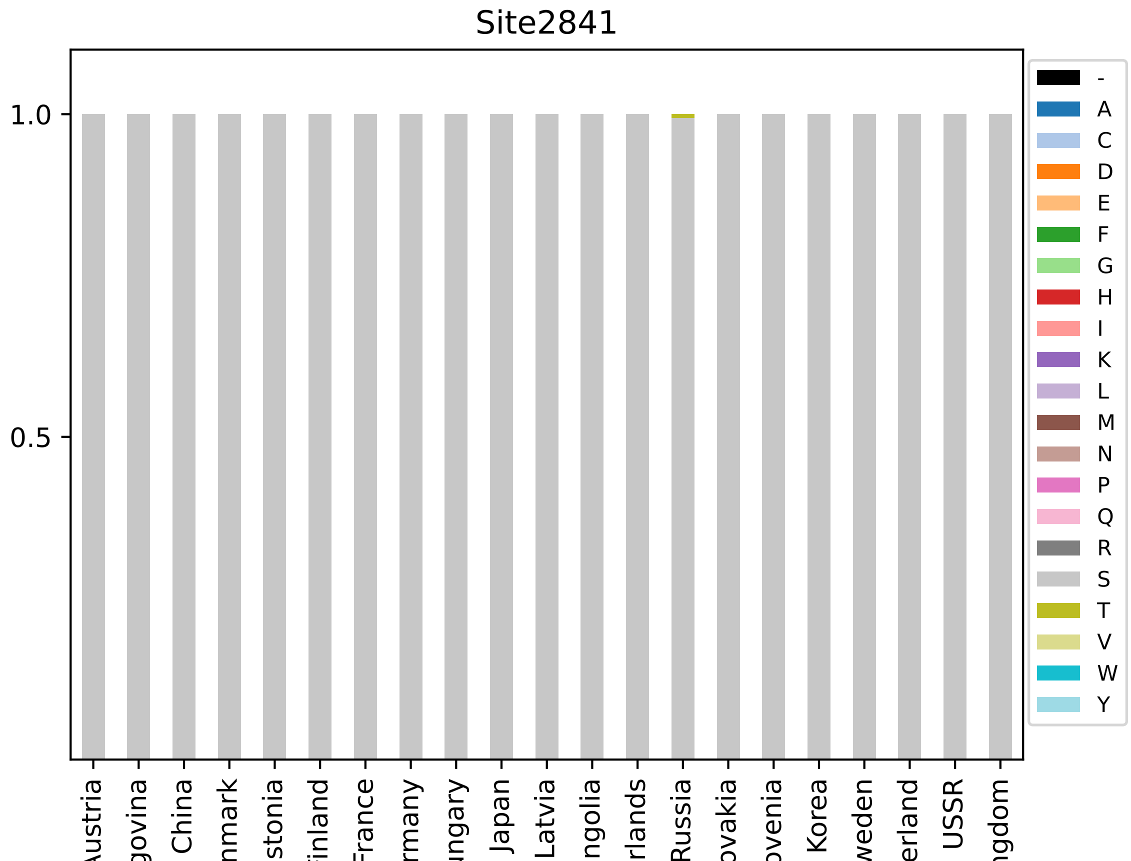
Site2841
1.0
0.5
-
A
C
D
E
F
G
H
I
K
L
M
N
P
Q
R
S
T
V
W
Y
Austria
govina
China
nmark
stonia
Finland
France
rmany
ungary
Japan
Latvia
ngolia
rlands
Russia
ovakia
ovenia
Korea
weden
erland
USSR
ngdom

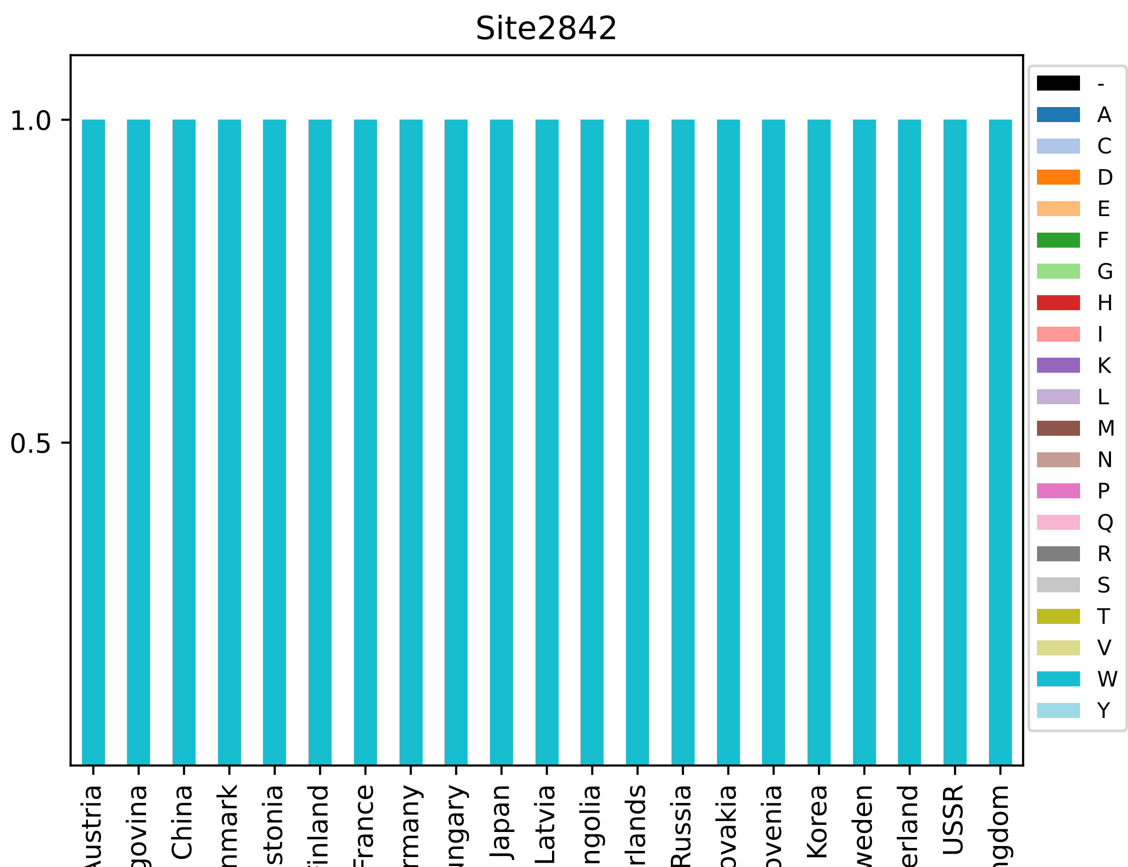

Site2842
1.0
0.5
Austria
govina
China
nmark
stonia
Finland
France
rmany
ungary
Japan
Latvia
ngolia
rlands
Russia
ovakia
ovenia
Korea
weden
erland
USSR
ngdom
-
A
C
D
E
F
G
H
I
K
L
M
N
P
Q
R
S
T
V
W
Y

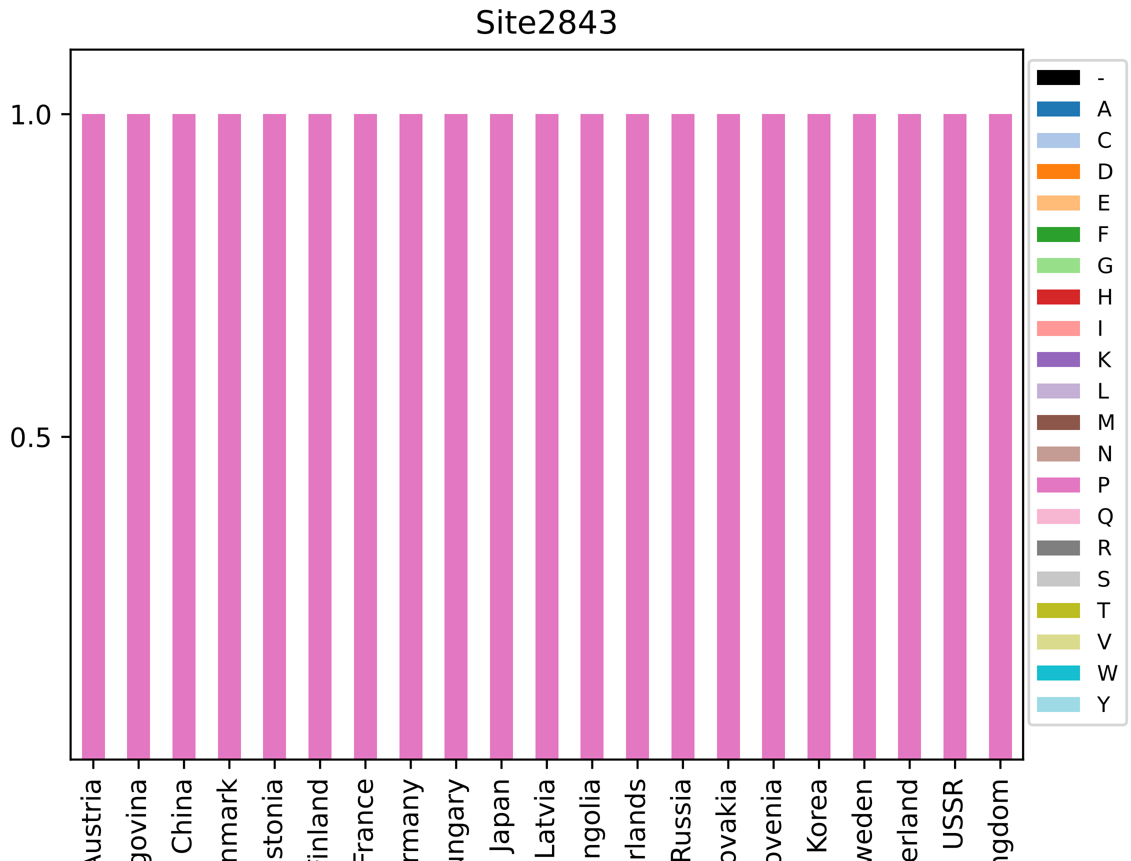
Site2843
1.0
0.5
China
Japan
Latvia
Russia
Korea
USSR
-
A
C
D
E
F
G
H
I
K
L
M
N
P
Q
R
S
T
V
W
Y

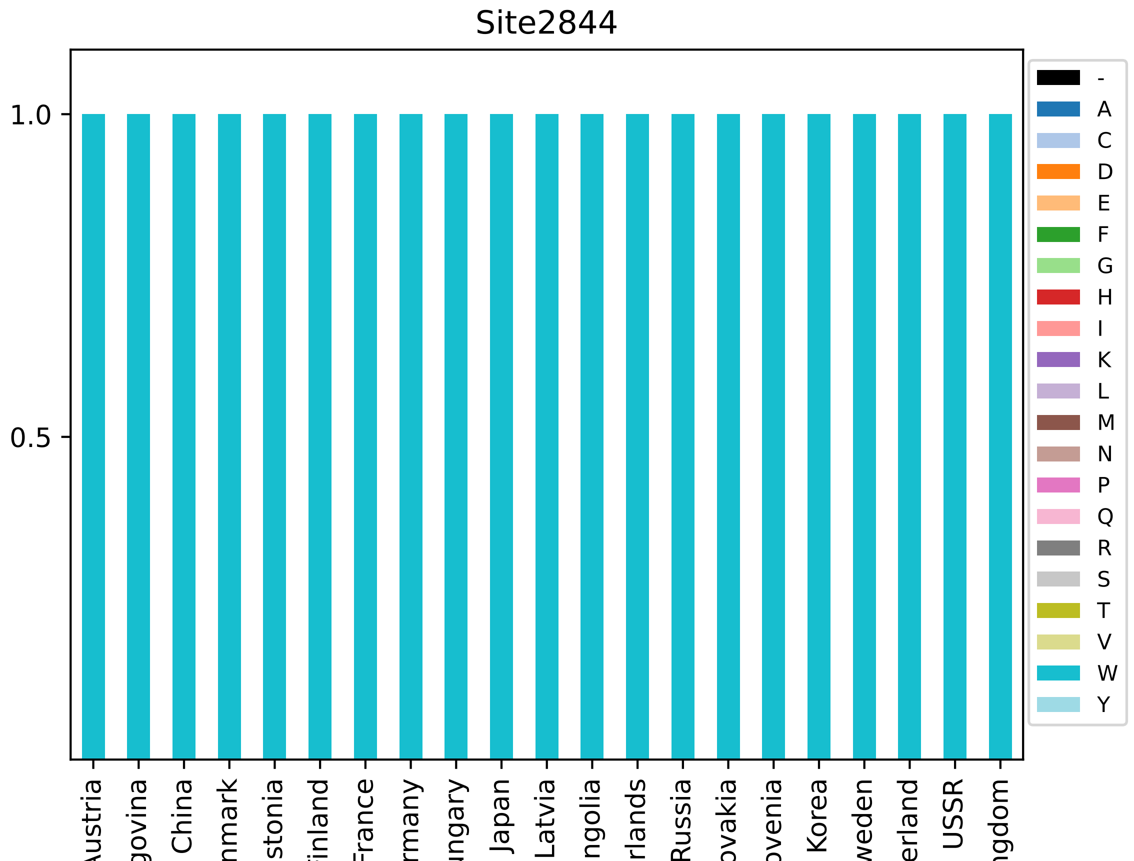
Site2844
1.0
0.5
-
A
C
D
E
F
G
H
I
K
L
M
N
P
Q
R
S
T
V
W
Y
China
Japan
Latvia
Russia
Korea
USSR

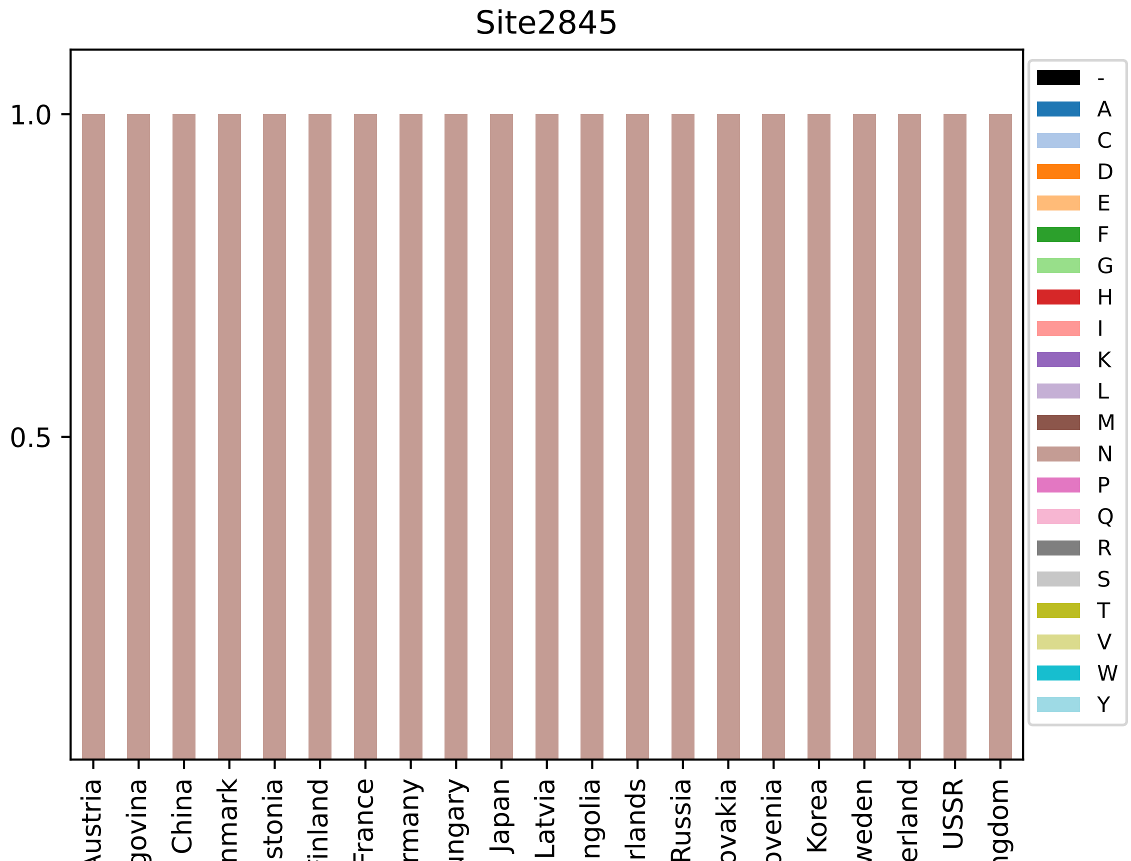
Site2845
1.0
0.5
-
A
C
D
E
F
G
H
I
K
L
M
N
P
Q
R
S
T
V
W
Y
Austria
govina
China
nmark
stonia
Finland
France
rmany
ungary
Japan
Latvia
ngolia
rlands
Russia
ovakia
ovenia
Korea
weden
erland
USSR
ngdom

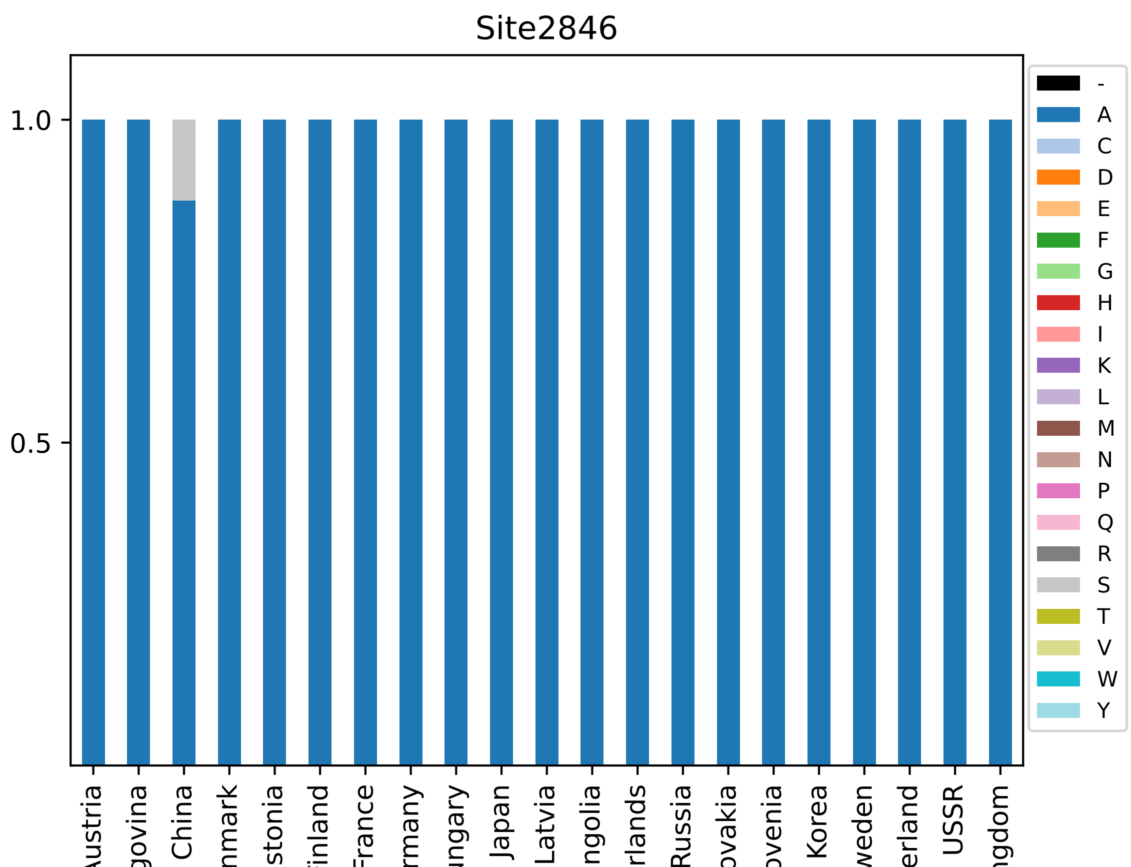

Site2846
1.0
0.5
Austria
govina
China
nmark
stonia
Finland
France
rmany
ungary
Japan
Latvia
ngolia
rlands
Russia
ovakia
ovenia
Korea
weden
erland
USSR
ngdom
-
A
C
D
E
F
G
H
I
K
L
M
N
P
Q
R
S
T
V
W
Y

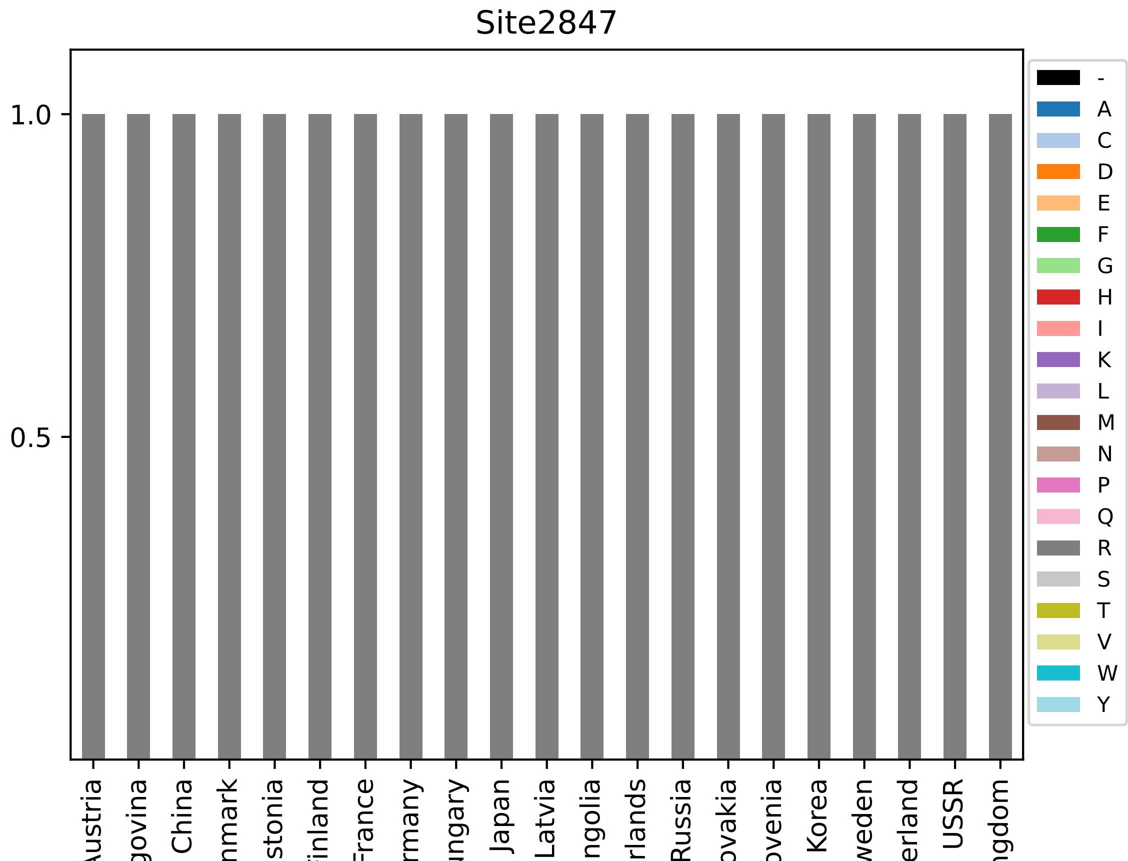

Site2847
1.0
0.5
-
A
C
D
E
F
G
H
I
K
L
M
N
P
Q
R
S
T
V
W
Y
China
Japan
Latvia
Russia
Korea
USSR

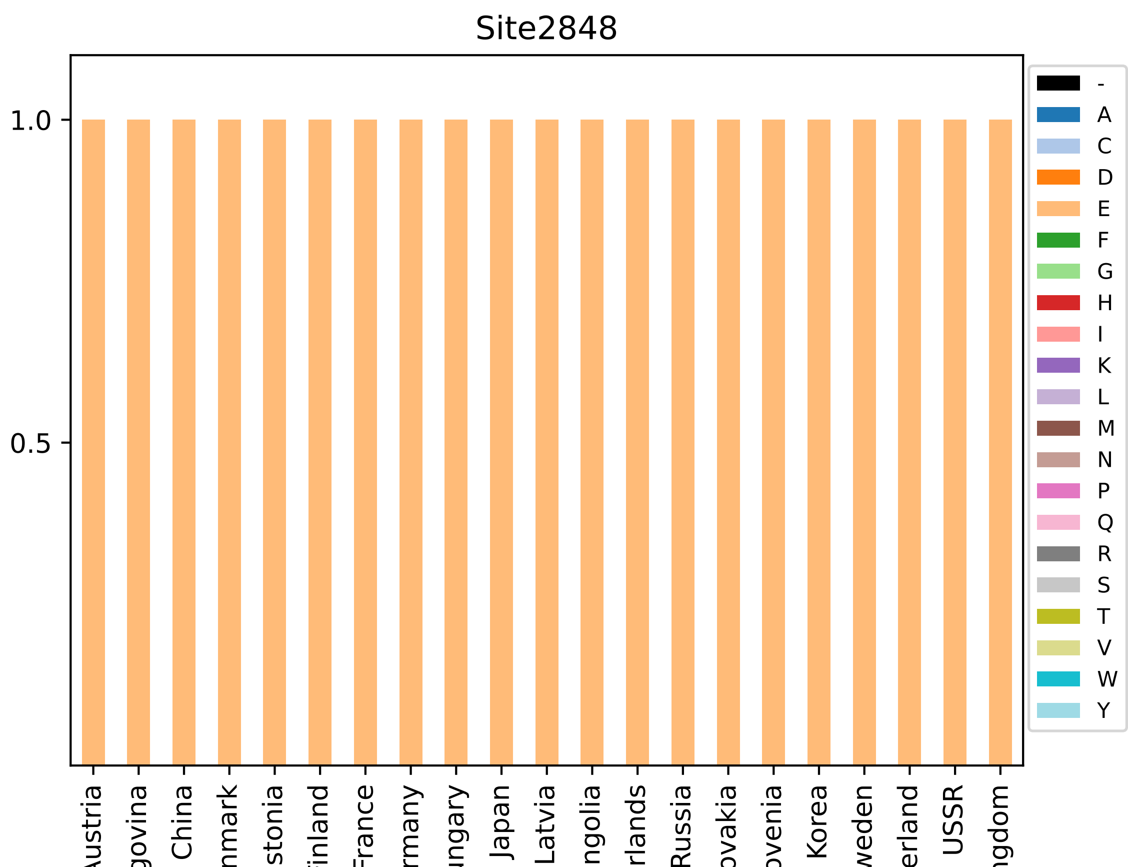

Site2848
1.0
0.5
-
A
C
D
E
F
G
H
I
K
L
M
N
P
Q
R
S
T
V
W
Y
China
Japan
Latvia
Russia
Korea
USSR

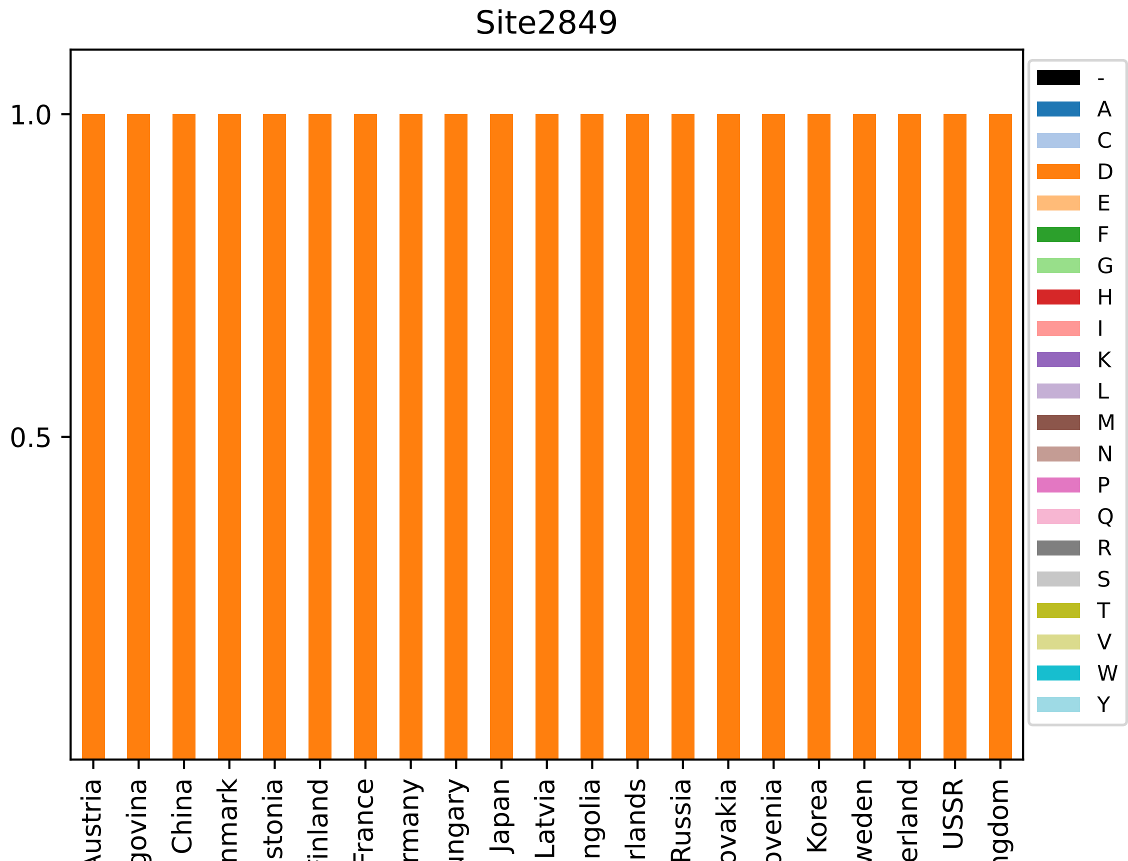

Site2849
1.0
0.5
-
A
C
D
E
F
G
H
I
K
L
M
N
P
Q
R
S
T
V
W
Y
China
Japan
Latvia
Russia
Korea
USSR

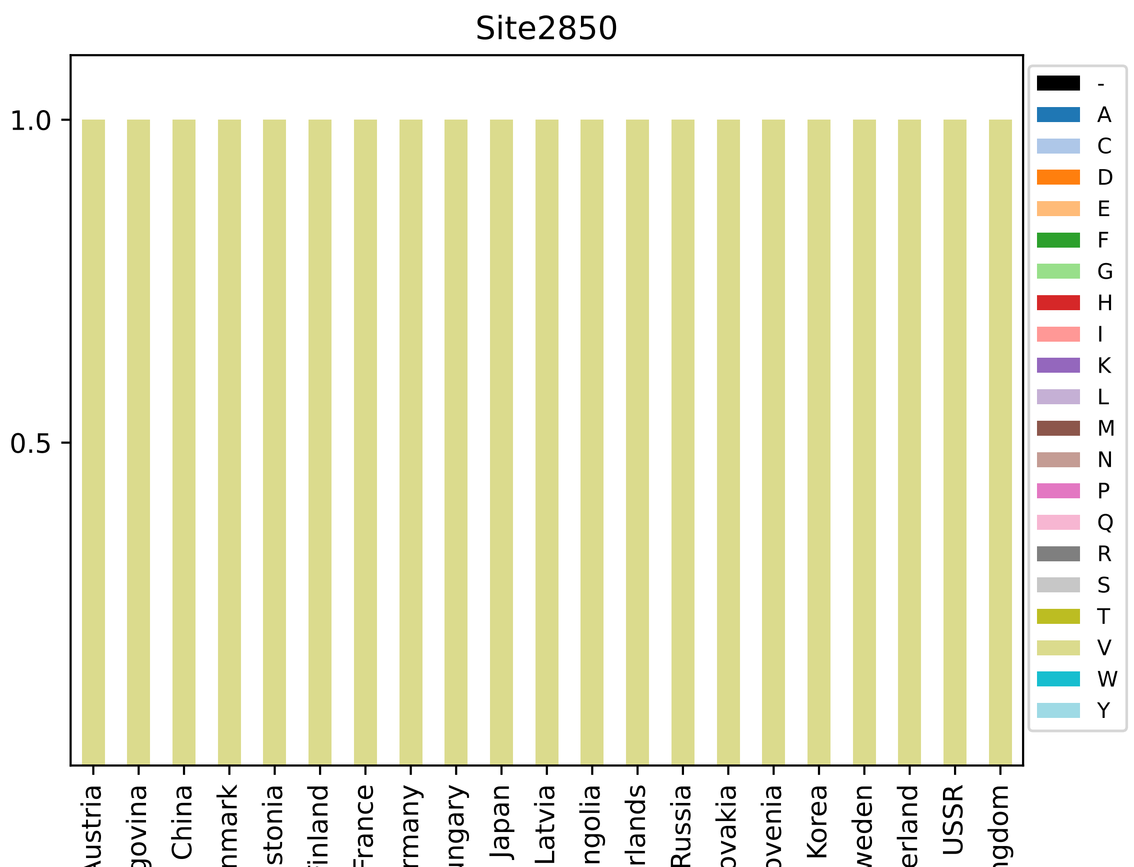

Site2850
1.0
0.5
-
A
C
D
E
F
G
H
I
K
L
M
N
P
Q
R
S
T
V
W
Y
Austria
China
Japan
Latvia
Russia
Korea
USSR

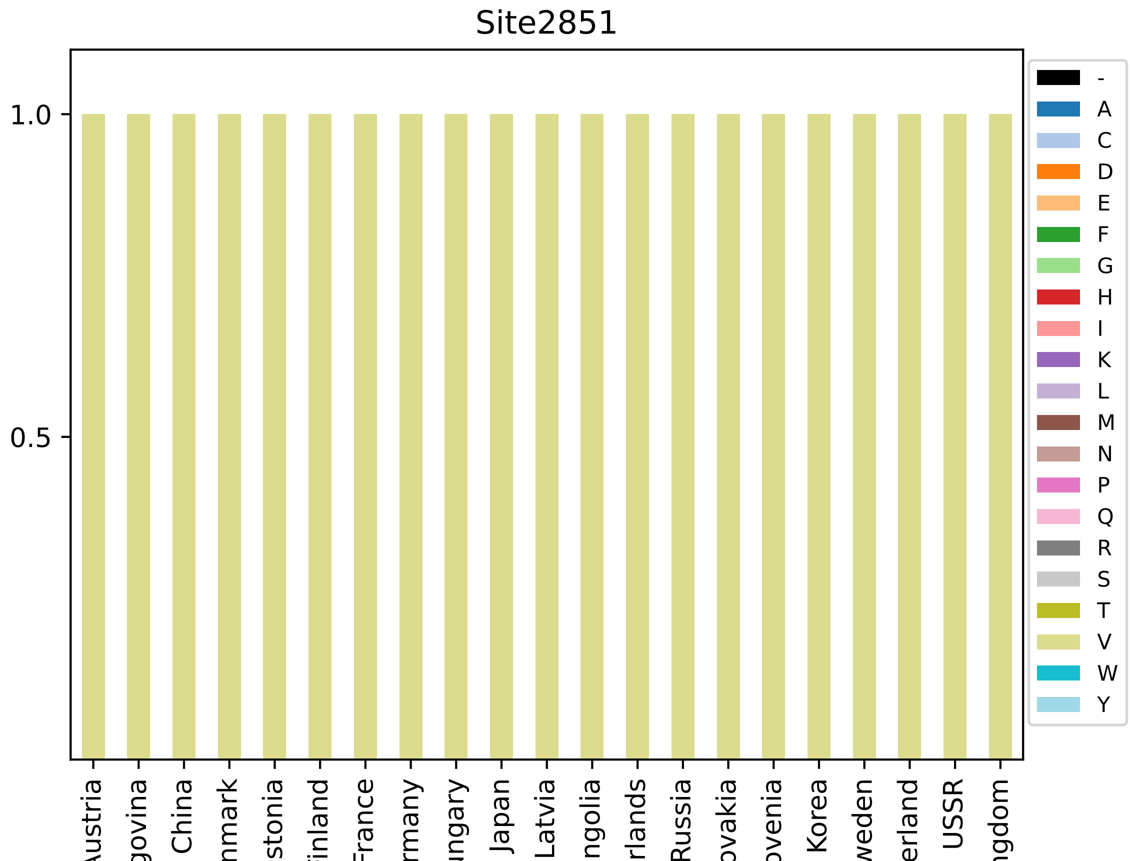
Site2851
1.0
0.5
-
A
C
D
E
F
G
H
I
K
L
M
N
P
Q
R
S
T
V
W
Y
Austria
China
Japan
Latvia
Russia
Korea
USSR

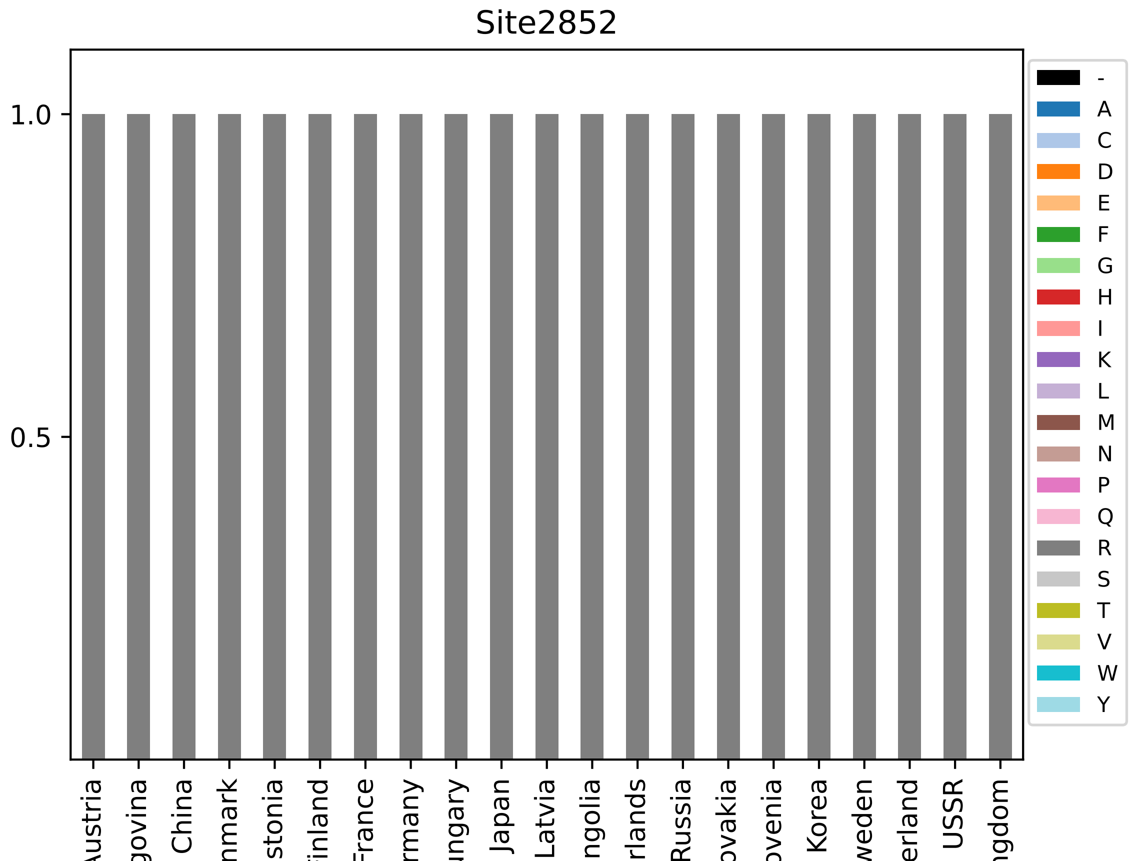

Site2852
1.0
0.5
-
A
C
D
E
F
G
H
I
K
L
M
N
P
Q
R
S
T
V
W
Y
China
Japan
Latvia
Russia
Korea
USSR

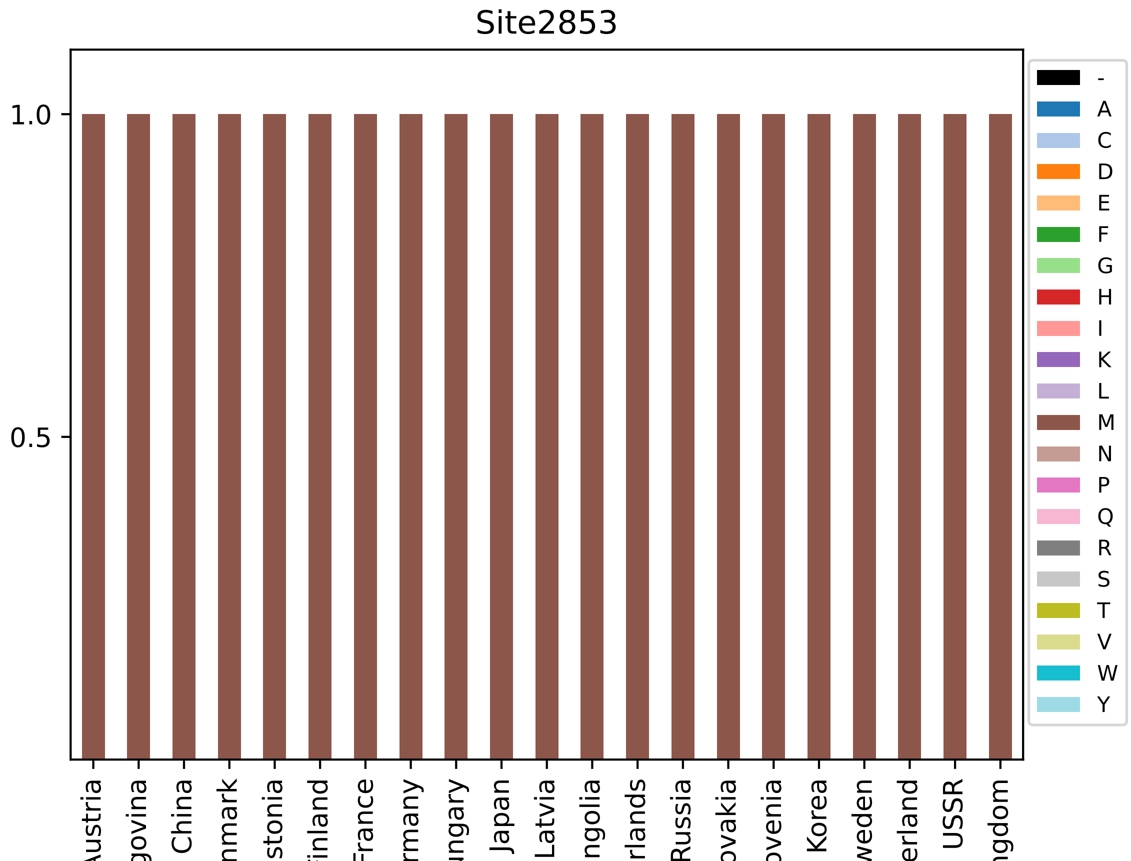
Site2853
1.0
0.5
-
A
C
D
E
F
G
H
I
K
L
M
N
P
Q
R
S
T
V
W
Y
Austria
China
Japan
Latvia
Russia
Korea
USSR

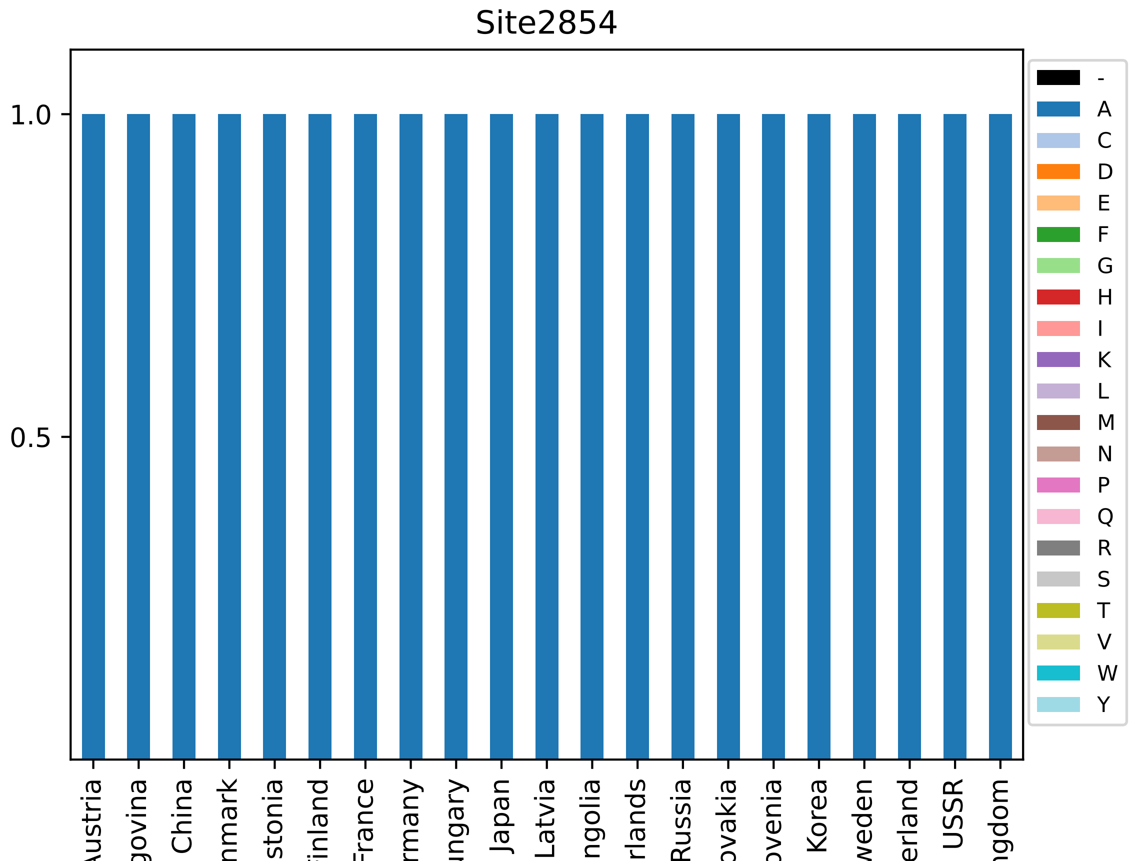

Site2854
1.0
0.5
Austria
China
Japan
Latvia
Russia
Korea
USSR
-
A
C
D
E
F
G
H
I
K
L
M
N
P
Q
R
S
T
V
W
Y

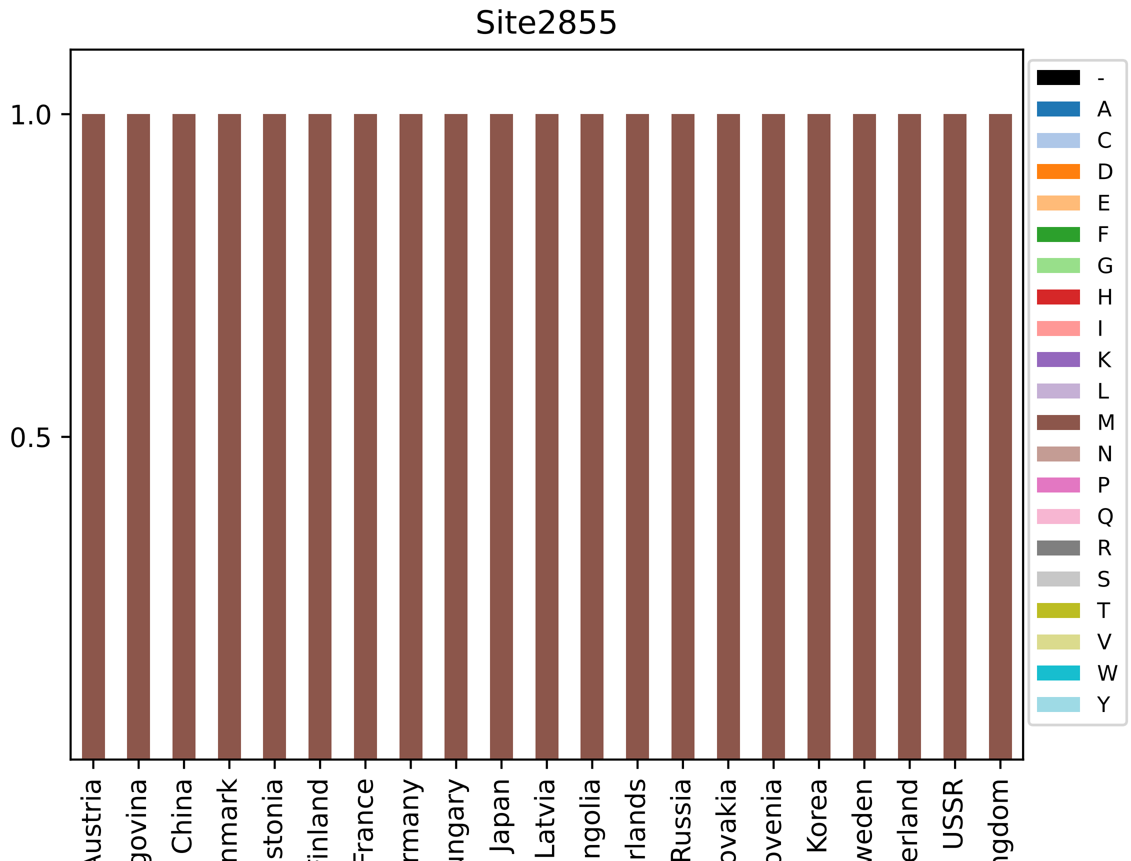
Site2855
1.0
0.5
-
A
C
D
E
F
G
H
I
K
L
M
N
P
Q
R
S
T
V
W
Y
Austria
govina
China
nmark
stonia
inland
France
rmany
ungary
Japan
Latvia
ngolia
rlands
Russia
ovakia
ovenia
Korea
weden
erland
USSR
ngdom

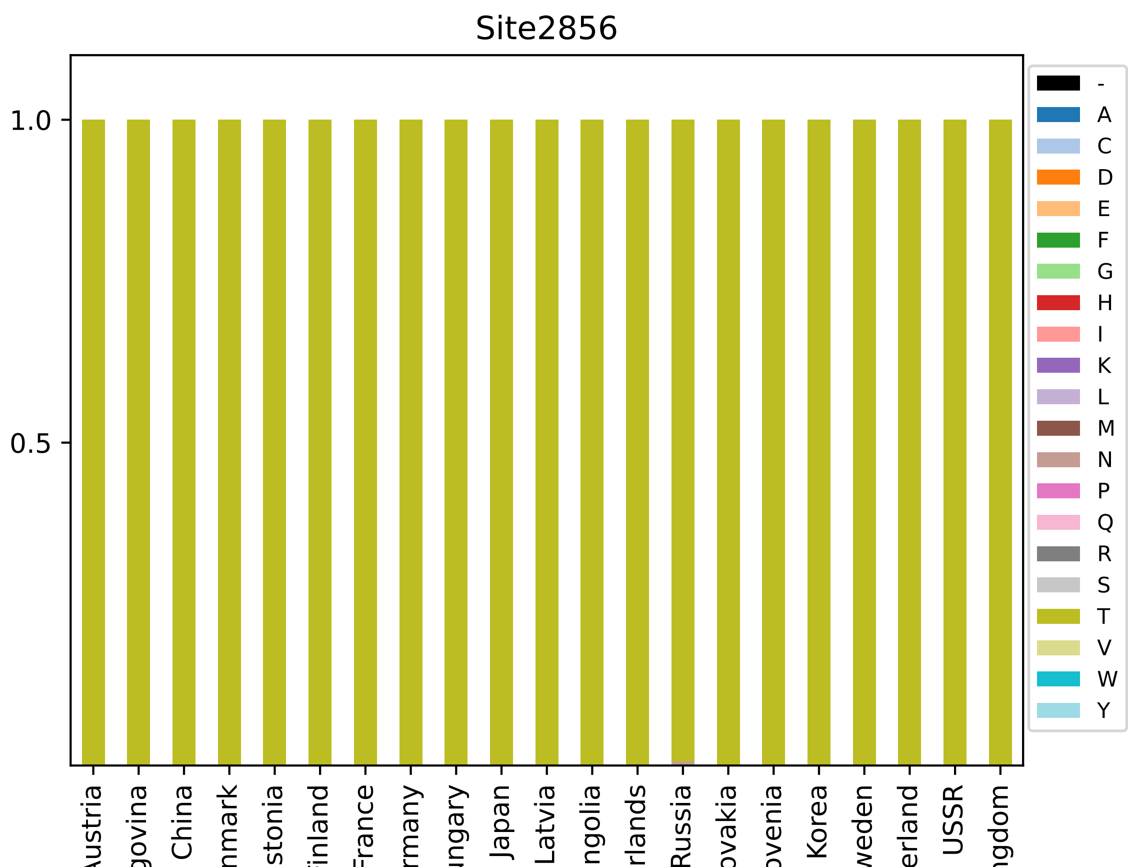

Site2856
1.0
0.5
-
A
C
D
E
F
G
H
I
K
L
M
N
P
Q
R
S
T
V
W
Y
Austria
China
Japan
Latvia
Russia
Korea
USSR

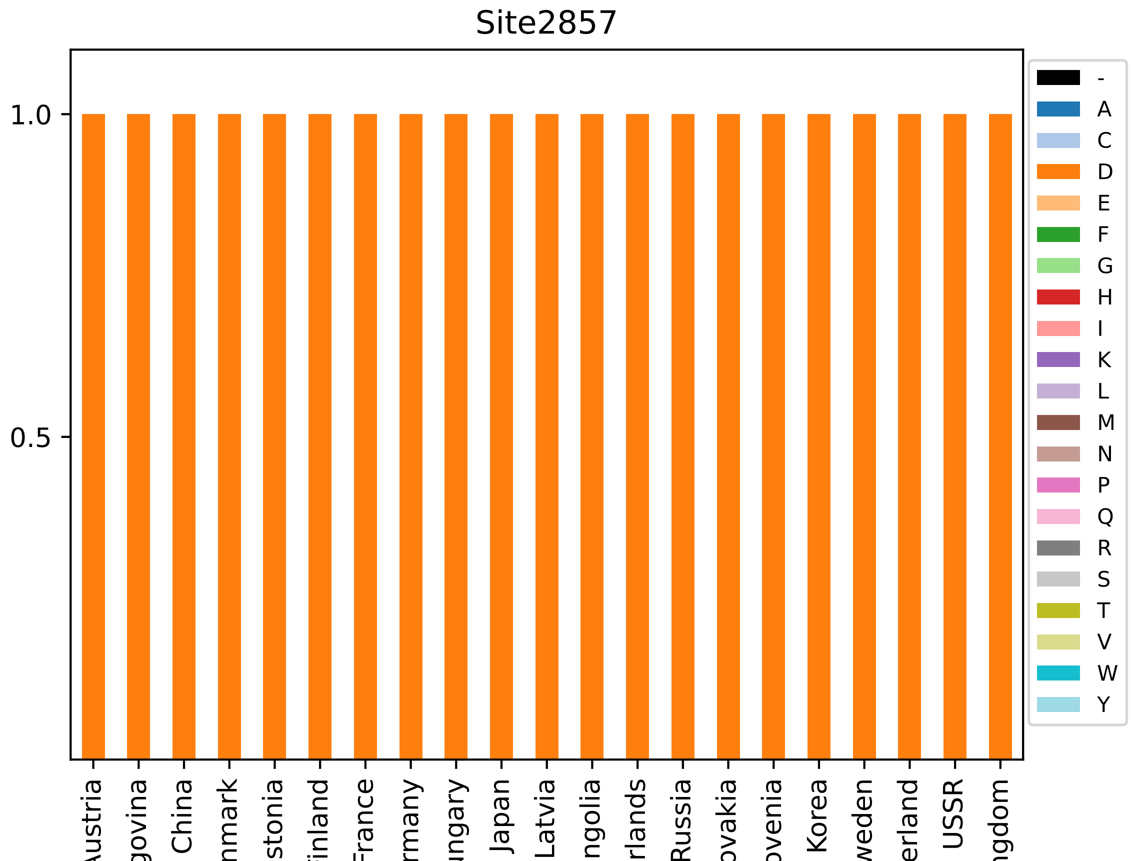

Site2857
1.0
0.5
-
A
C
D
E
F
G
H
I
K
L
M
N
P
Q
R
S
T
V
W
Y
China
Japan
Latvia
Russia
Korea
USSR

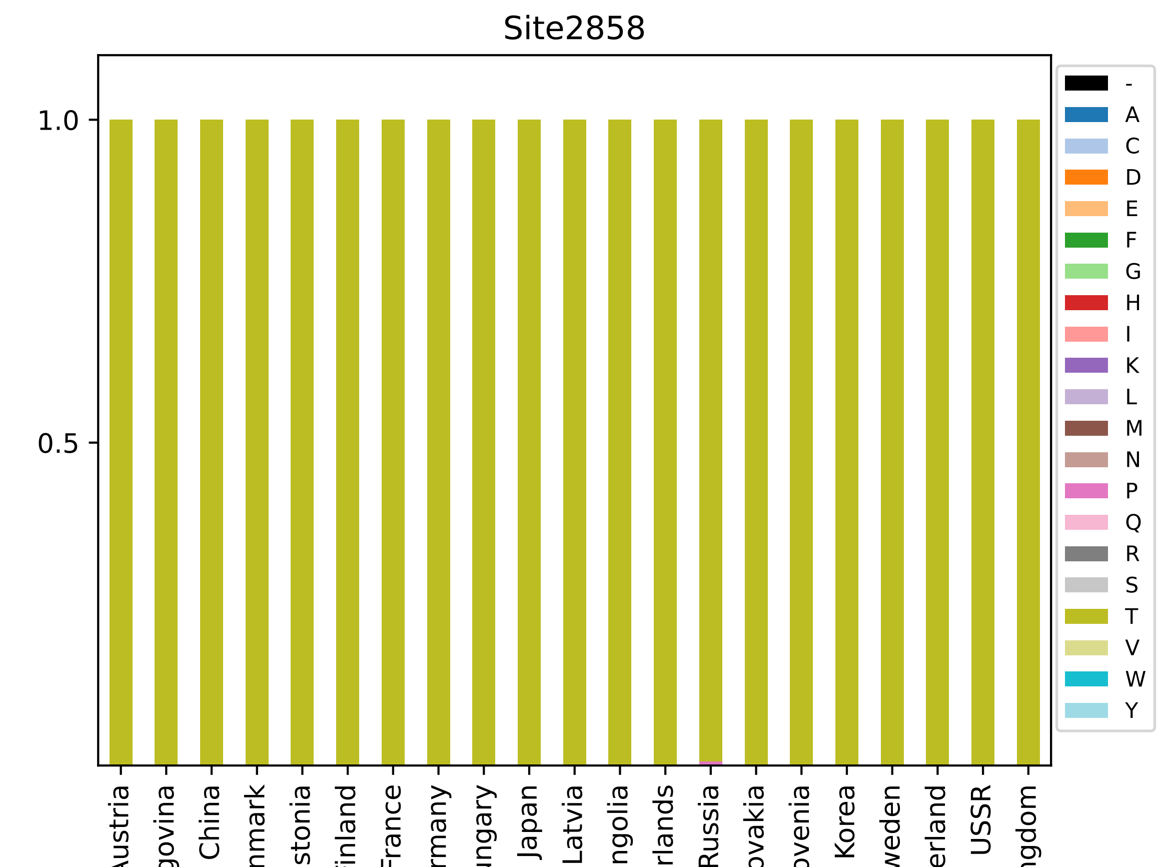

Site2858
1.0
0.5
-
A
C
D
E
F
G
H
I
K
L
M
N
P
Q
R
S
T
V
W
Y
Austria
govina
China
nmark
stonia
Finland
France
rmany
ungary
Japan
Latvia
ngolia
rlands
Russia
ovakia
ovenia
Korea
weden
erland
USSR
ngdom

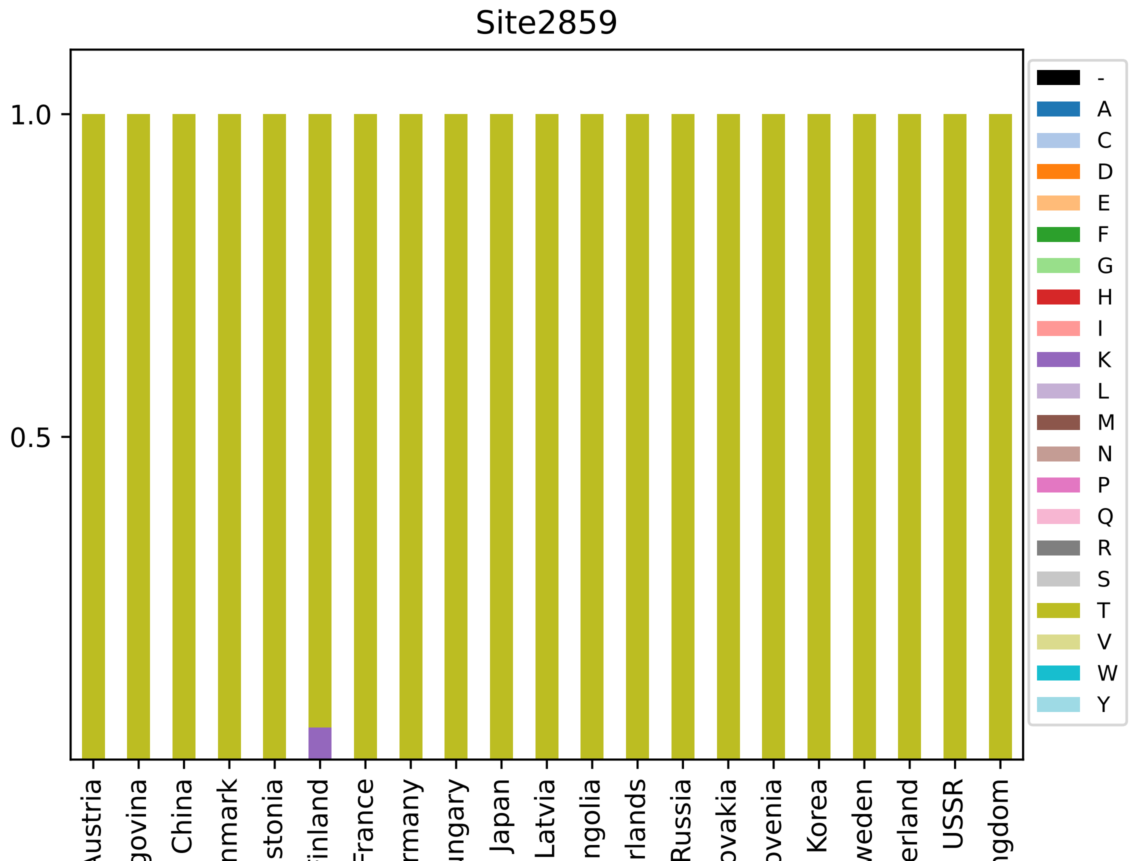
Site2859
1.0
0.5
-
A
C
D
E
F
G
H
I
K
L
M
N
P
Q
R
S
T
V
W
Y
Austria
govina
China
nmark
stonia
inland
France
rmany
ungary
Japan
Latvia
ngolia
rlands
Russia
ovakia
ovenia
Korea
weden
erland
USSR
ngdom

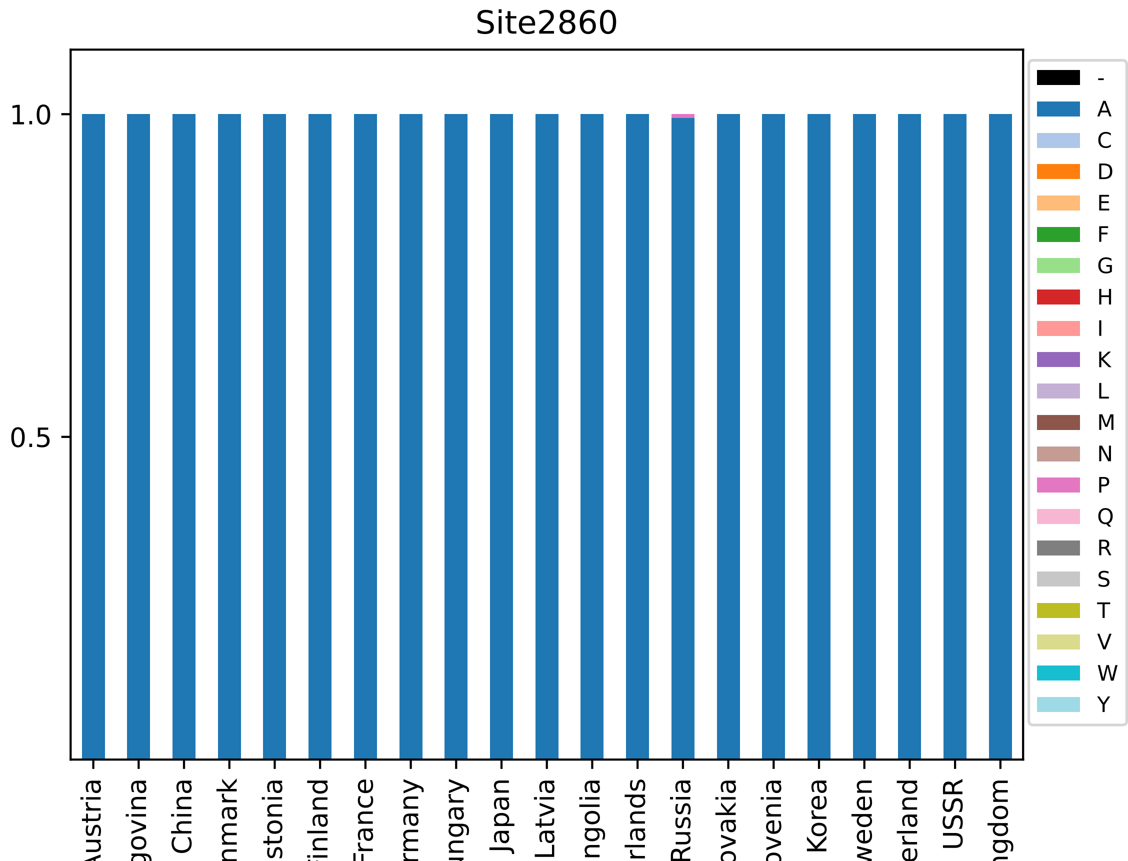

Site2860
1.0
0.5
-
A
C
D
E
F
G
H
I
K
L
M
N
P
Q
R
S
T
V
W
Y
Austria
govina
China
nmark
stonia
inland
France
rmany
ungary
Japan
Latvia
ngolia
rlands
Russia
ovakia
ovenia
Korea
weden
erland
USSR
ngdom

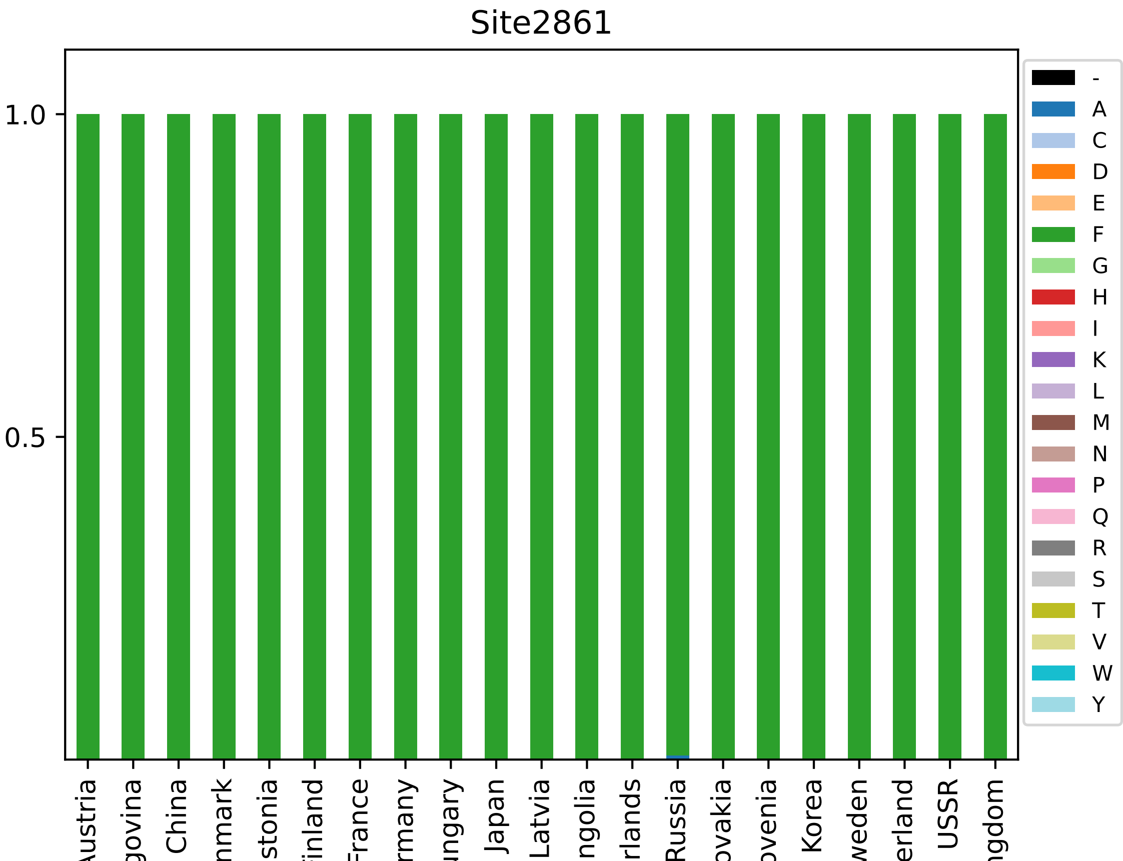

Site2861
1.0
0.5
-
A
C
D
E
F
G
H
I
K
L
M
N
P
Q
R
S
T
V
W
Y
Austria
govina
China
nmark
stonia
inland
France
rmany
ungary
Japan
Latvia
ngolia
rlands
Russia
ovakia
ovenia
Korea
weden
erland
USSR
ngdom

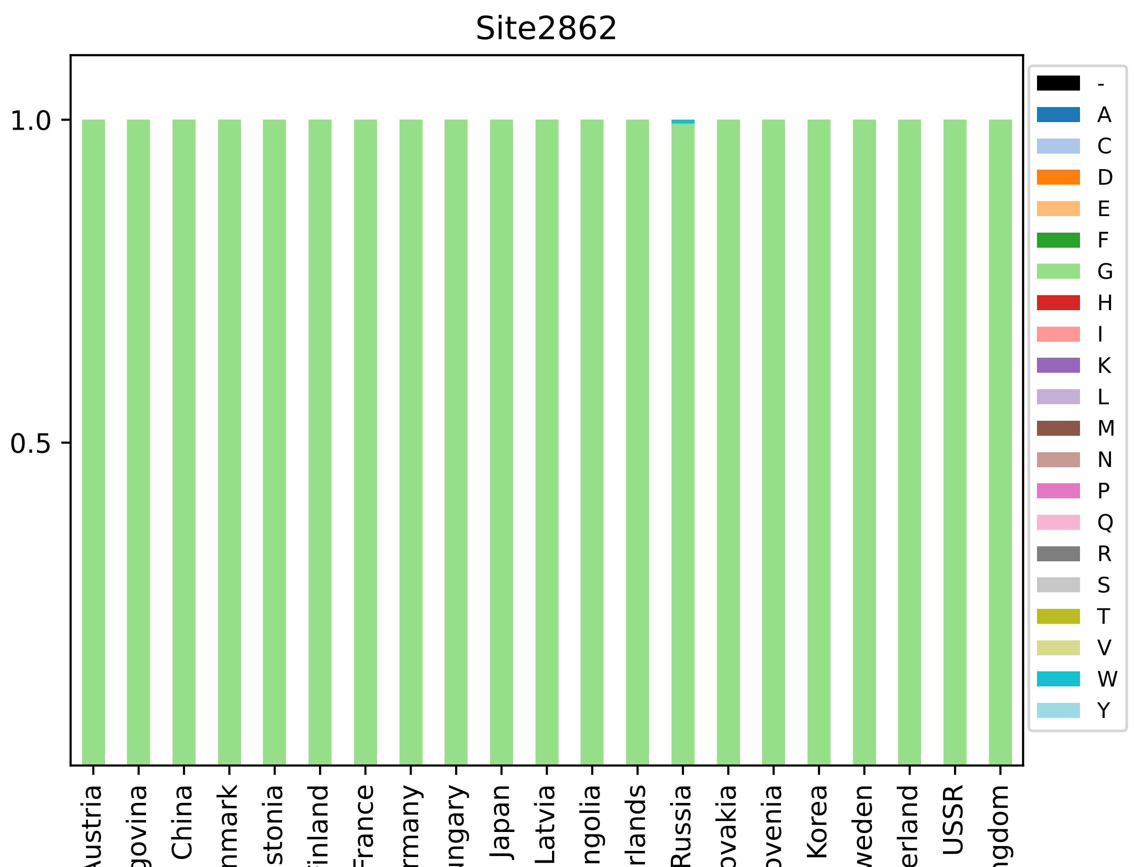

Site2862
1.0
0.5
-
A
C
D
E
F
G
H
I
K
L
M
N
P
Q
R
S
T
V
W
Y
Austria
China
Japan
Latvia
Russia
Korea
USSR

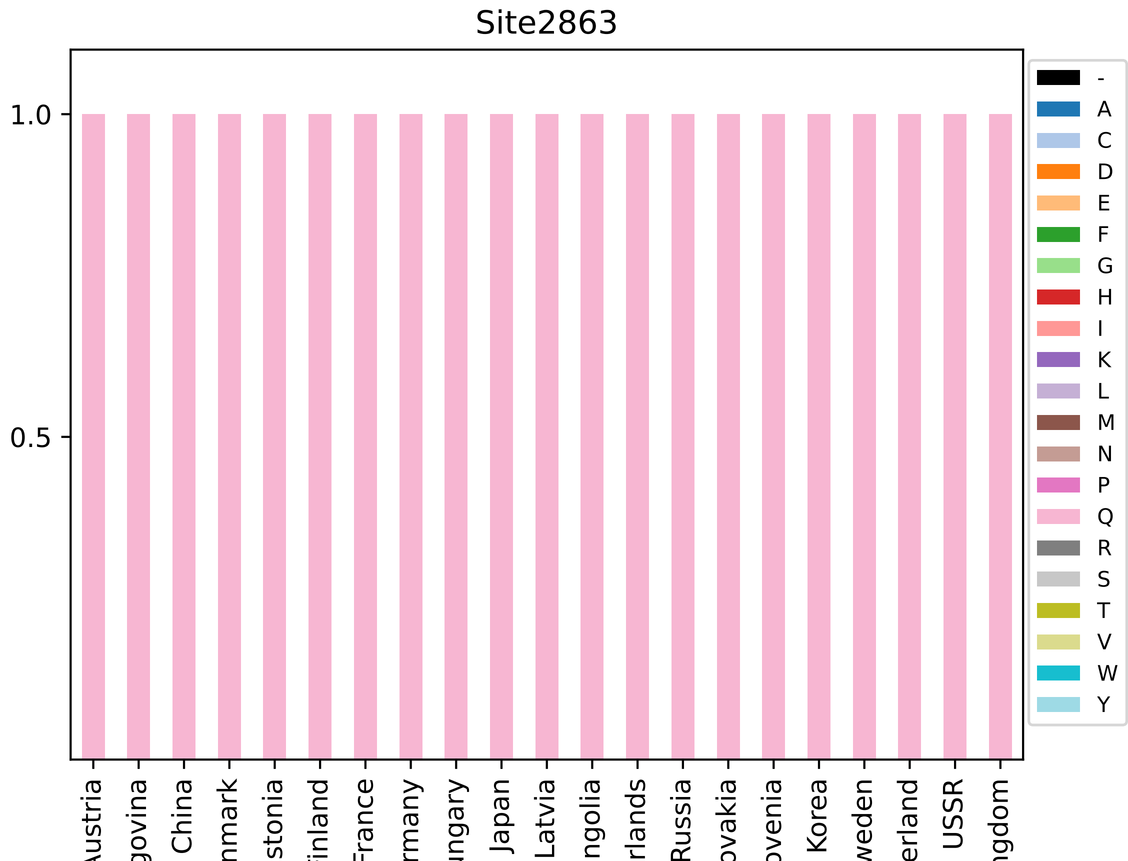

Site2863
1.0
0.5
-
A
C
D
E
F
G
H
I
K
L
M
N
P
Q
R
S
T
V
W
Y
China
Japan
Latvia
Russia
Korea
USSR

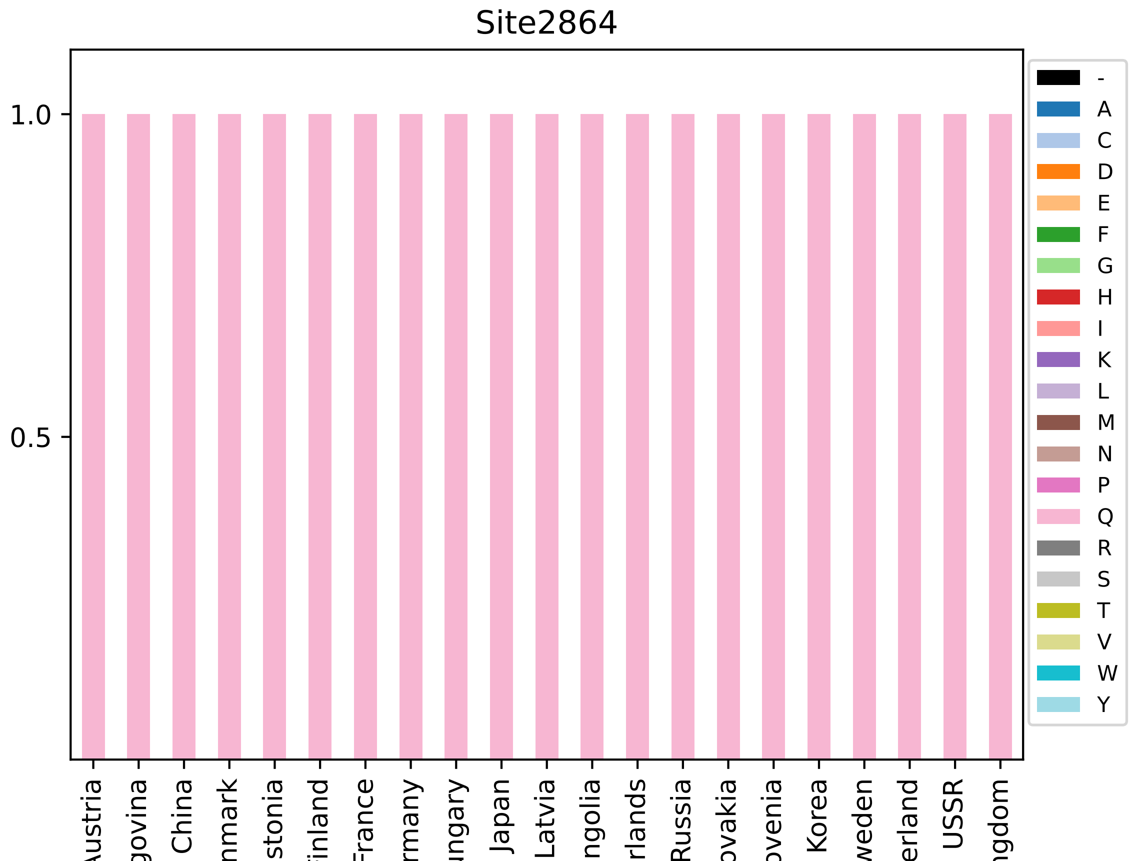

Site2864
1.0
0.5
-
A
C
D
E
F
G
H
I
K
L
M
N
P
Q
R
S
T
V
W
Y
Austria
China
Estonia
Finland
France
Japan
Latvia
Russia
Korea
USSR

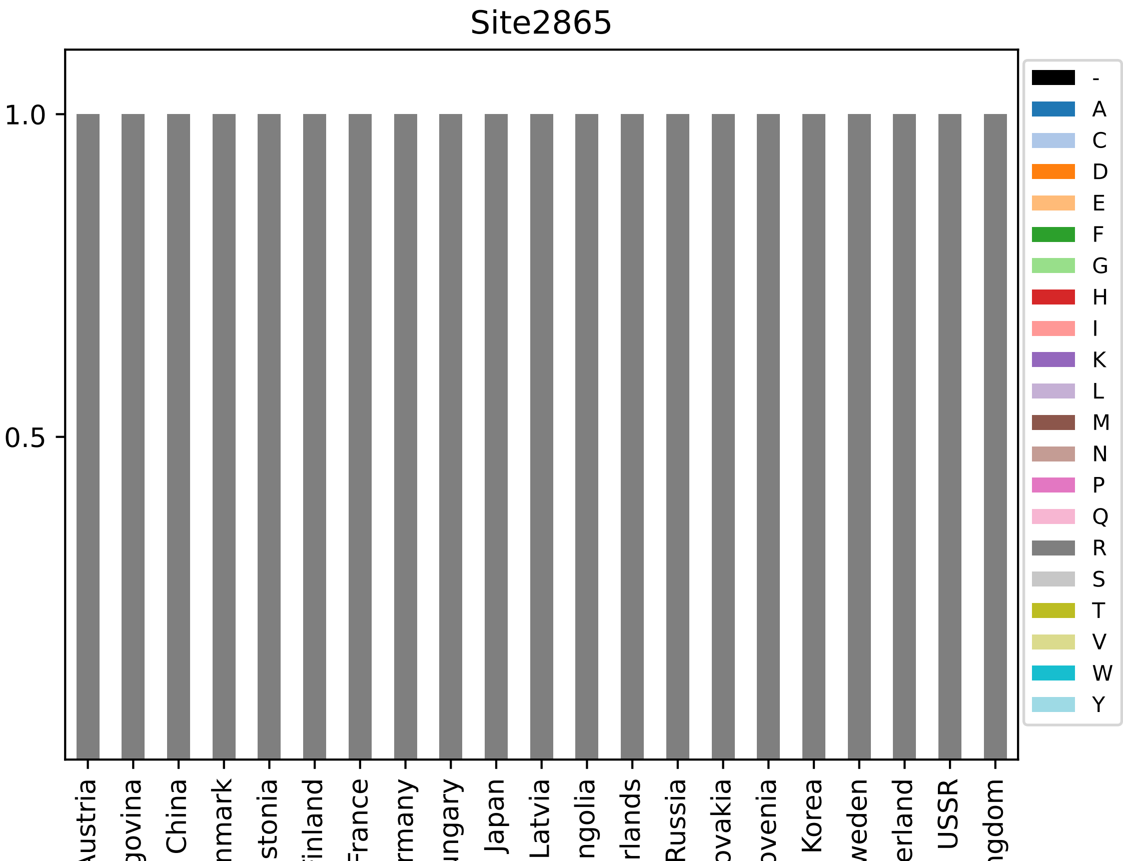
Site2865
1.0
0.5
-
A
C
D
E
F
G
H
I
K
L
M
N
P
Q
R
S
T
V
W
Y
China
Japan
Latvia
Russia
Korea
USSR

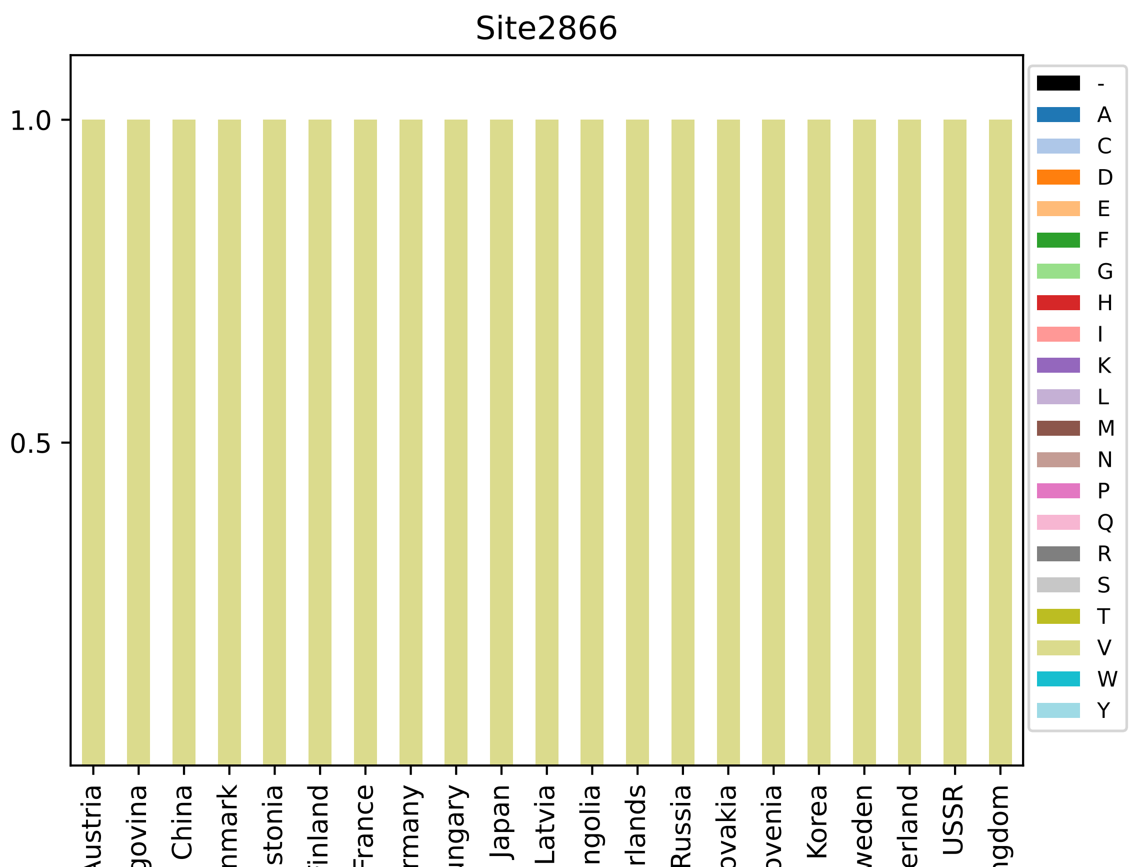
Site2866
1.0
0.5
-
A
C
D
E
F
G
H
I
K
L
M
N
P
Q
R
S
T
V
W
Y
China
Japan
Latvia
Russia
Korea
USSR

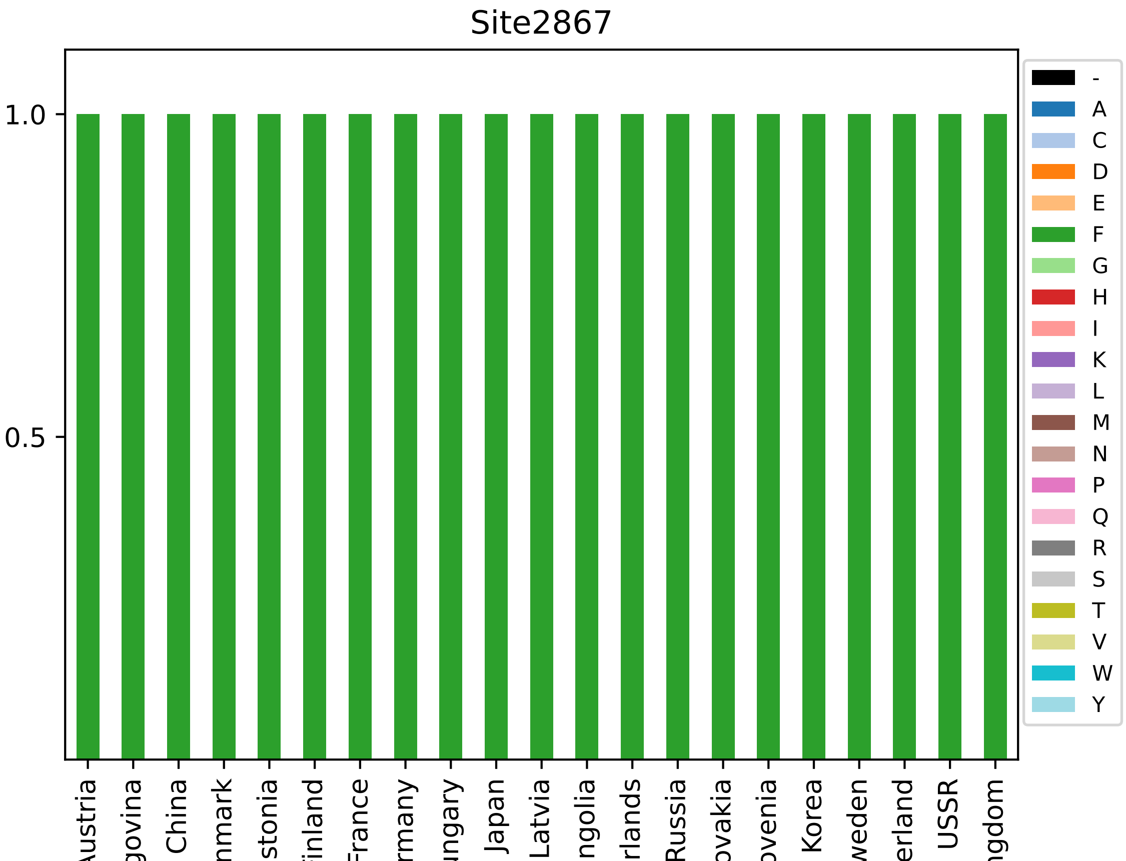
Site2867
1.0
0.5
-
A
C
D
E
F
G
H
I
K
L
M
N
P
Q
R
S
T
V
W
Y
China
Japan
Latvia
Russia
Korea
USSR

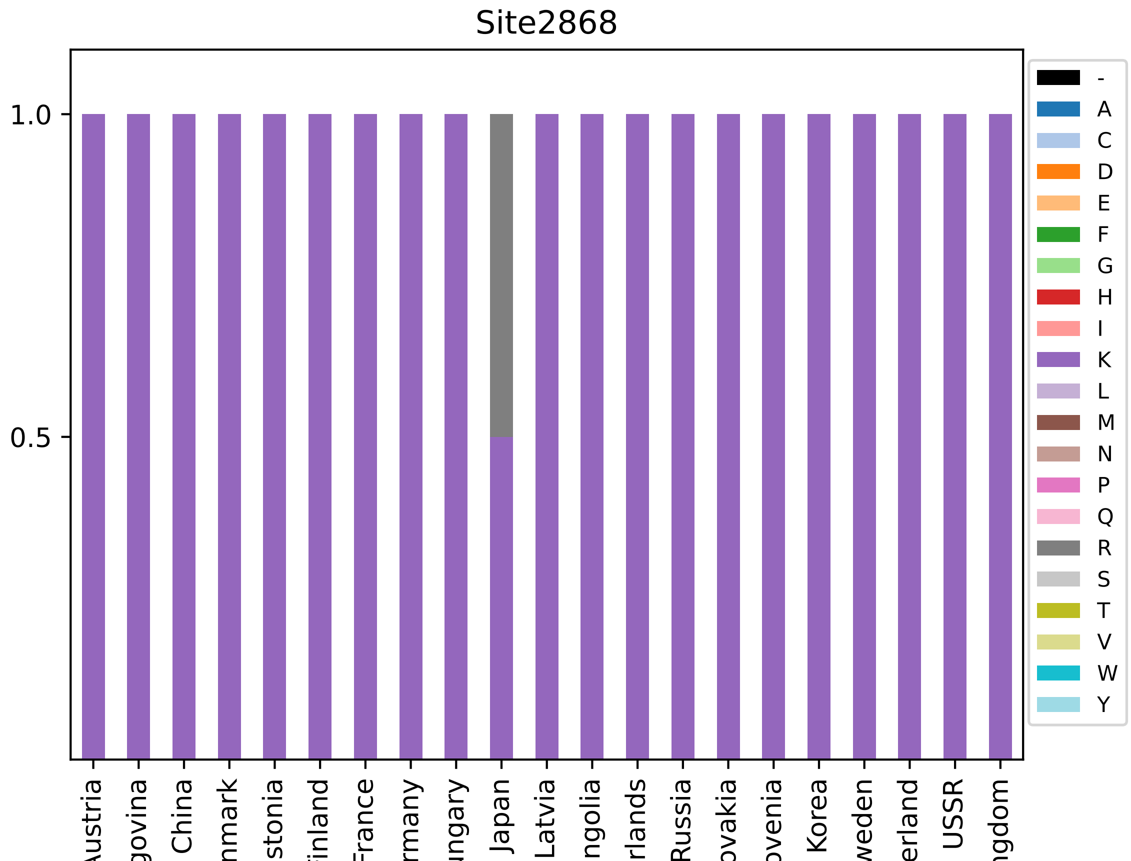
Site2868
1.0
0.5
-
A
C
D
E
F
G
H
I
K
L
M
N
P
Q
R
S
T
V
W
Y
China
Japan
Latvia
Russia
Korea
USSR

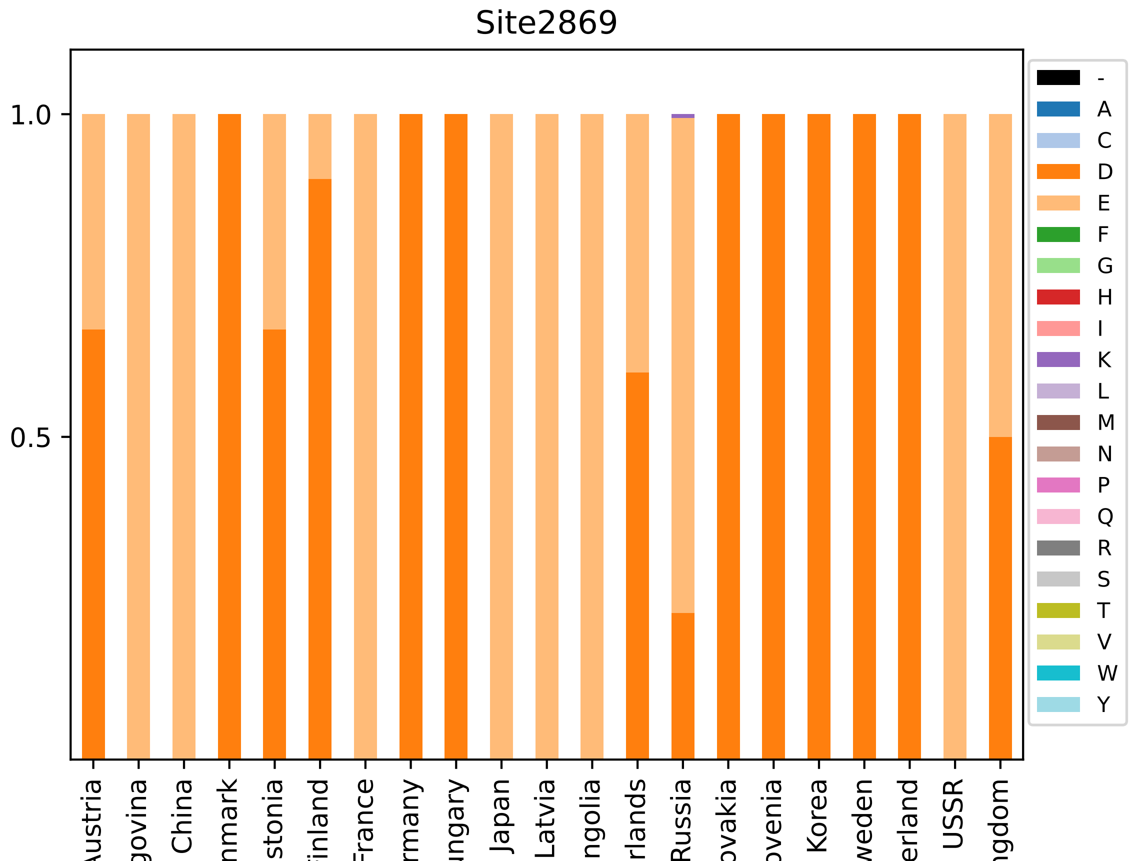

Site2869
1.0
0.5
-
A
C
D
E
F
G
H
I
K
L
M
N
P
Q
R
S
T
V
W
Y
Austria
govina
China
nmark
stonia
Finland
France
rmany
ungary
Japan
Latvia
ngolia
rlands
Russia
ovakia
ovenia
Korea
weden
erland
USSR
ngdom

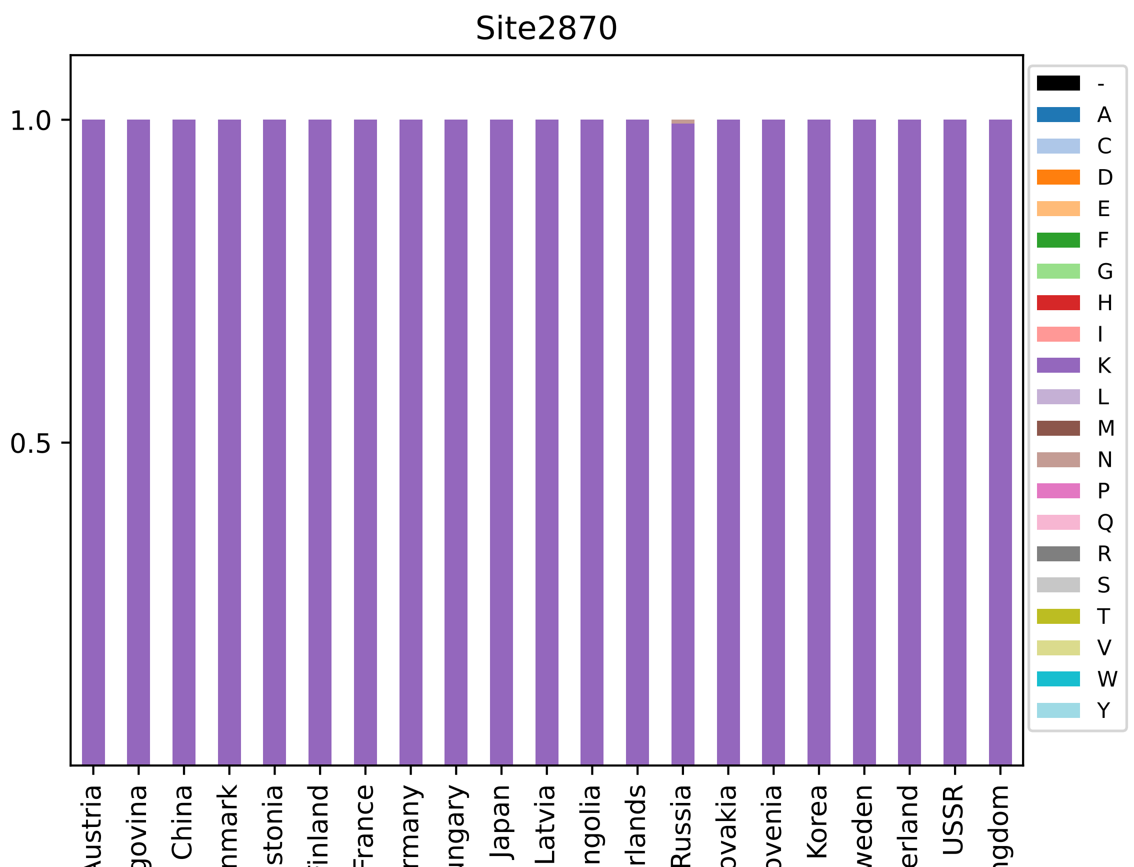

Site2870
1.0
0.5
-
A
C
D
E
F
G
H
I
K
L
M
N
P
Q
R
S
T
V
W
Y
China
Japan
Latvia
Russia
Korea
USSR

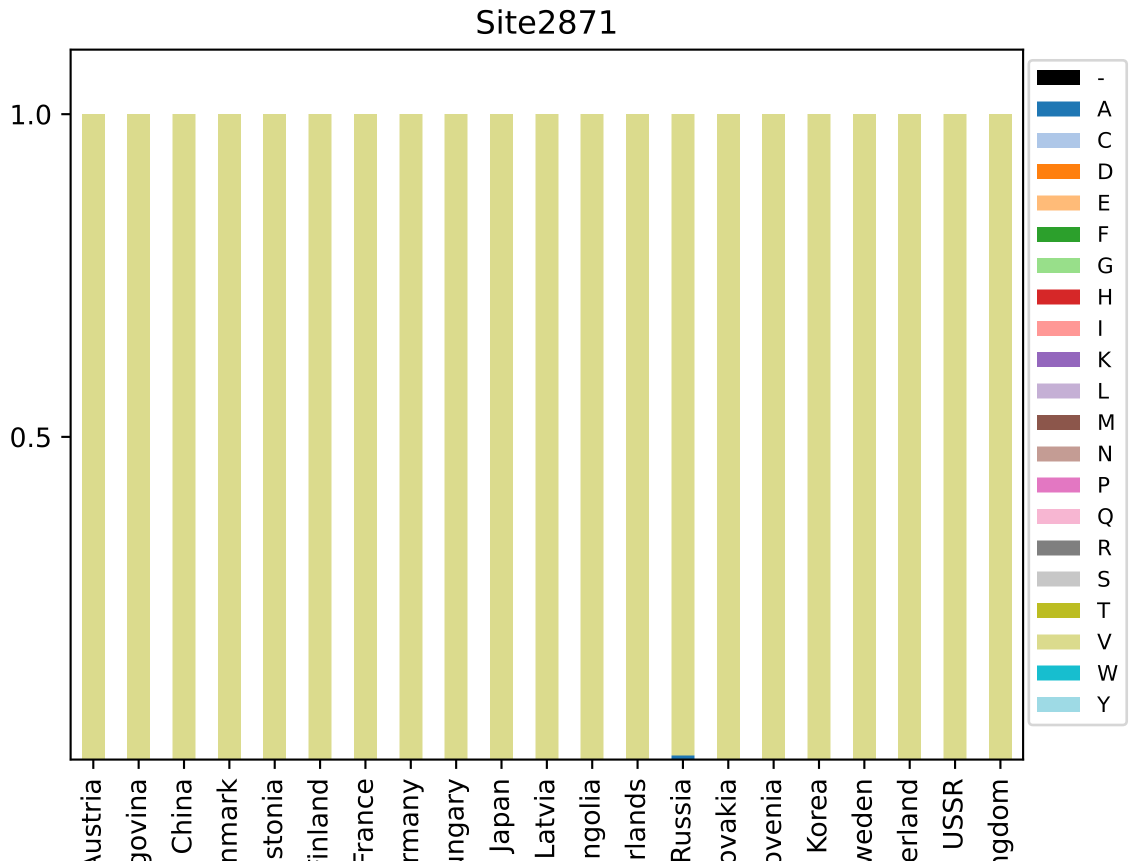

Site2871
1.0
0.5
-
A
C
D
E
F
G
H
I
K
L
M
N
P
Q
R
S
T
V
W
Y
China
Japan
Latvia
Russia
Korea
USSR

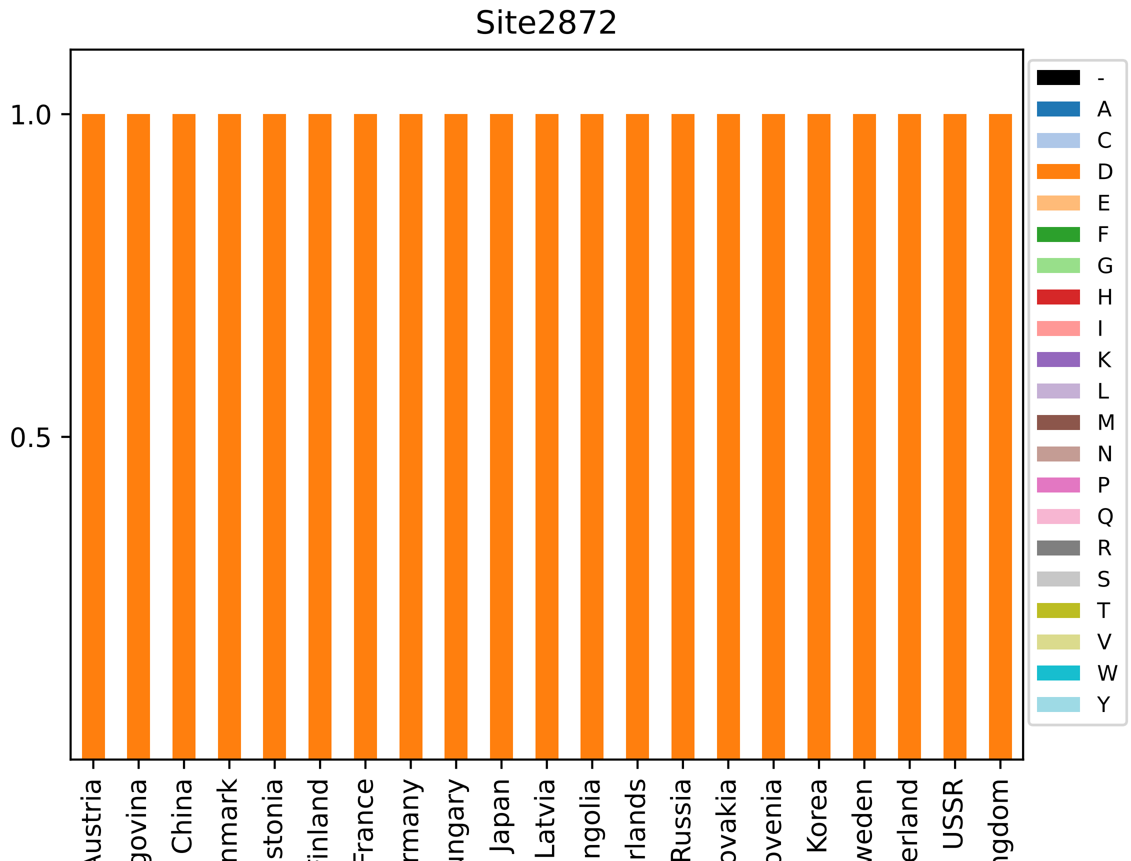

Site2872
1.0
0.5
-
A
C
D
E
F
G
H
I
K
L
M
N
P
Q
R
S
T
V
W
Y
Austria
China
Japan
Latvia
Russia
Korea
USSR

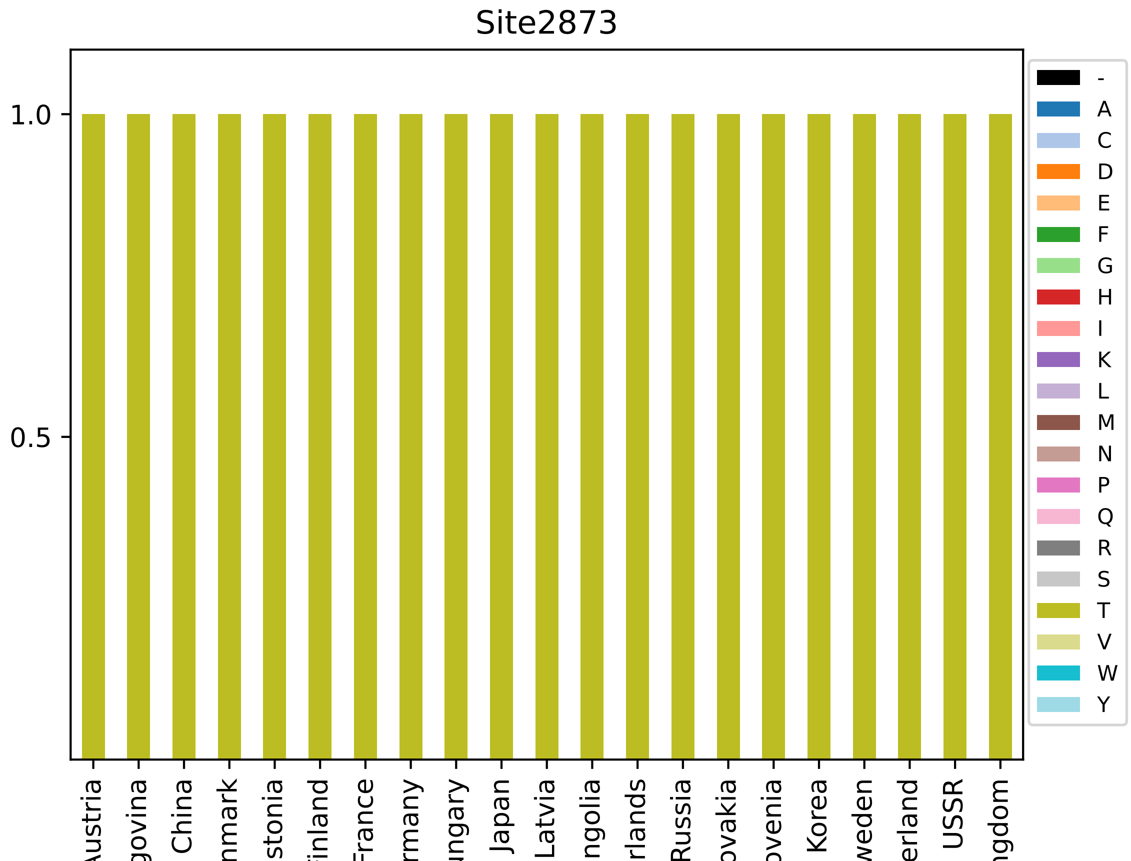
Site2873
1.0
0.5
-
A
C
D
E
F
G
H
I
K
L
M
N
P
Q
R
S
T
V
W
Y
China
Japan
Latvia
Russia
Korea
USSR

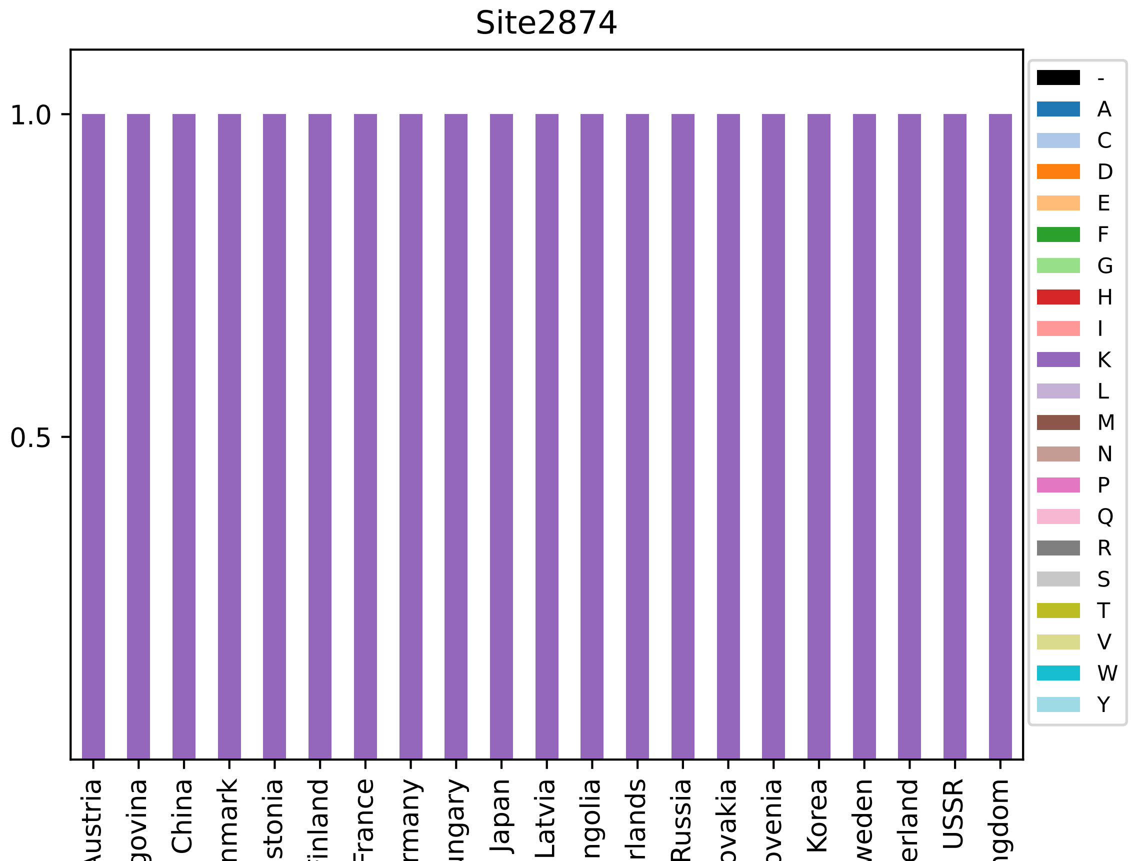
Site2874
1.0
0.5
China
Japan
Latvia
Russia
Korea
USSR
-
A
C
D
E
F
G
H
I
K
L
M
N
P
Q
R
S
T
V
W
Y

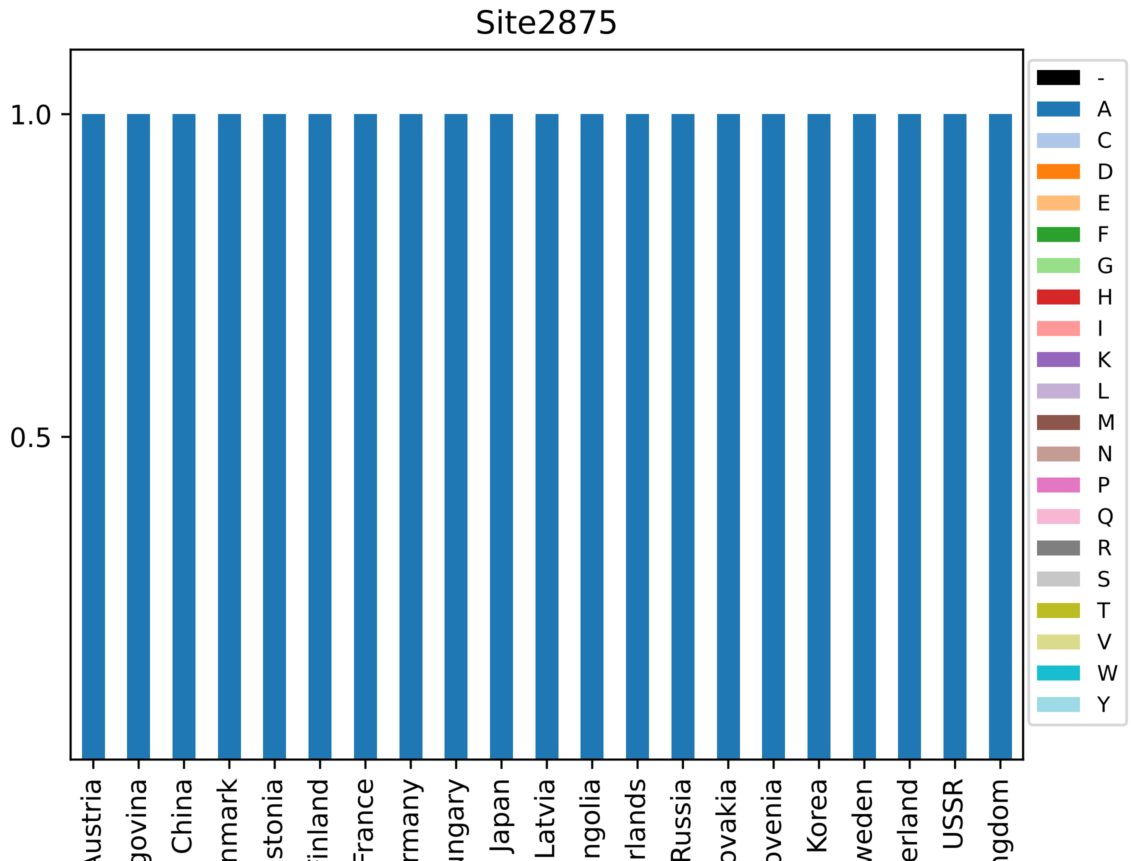
Site2875
1.0
0.5
Austria
govina
China
nmark
stonia
Finland
France
rmany
ungary
Japan
Latvia
ngolia
rlands
Russia
ovakia
ovenia
Korea
weden
erland
USSR
ngdom
-
A
C
D
E
F
G
H
I
K
L
M
N
P
Q
R
S
T
V
W
Y

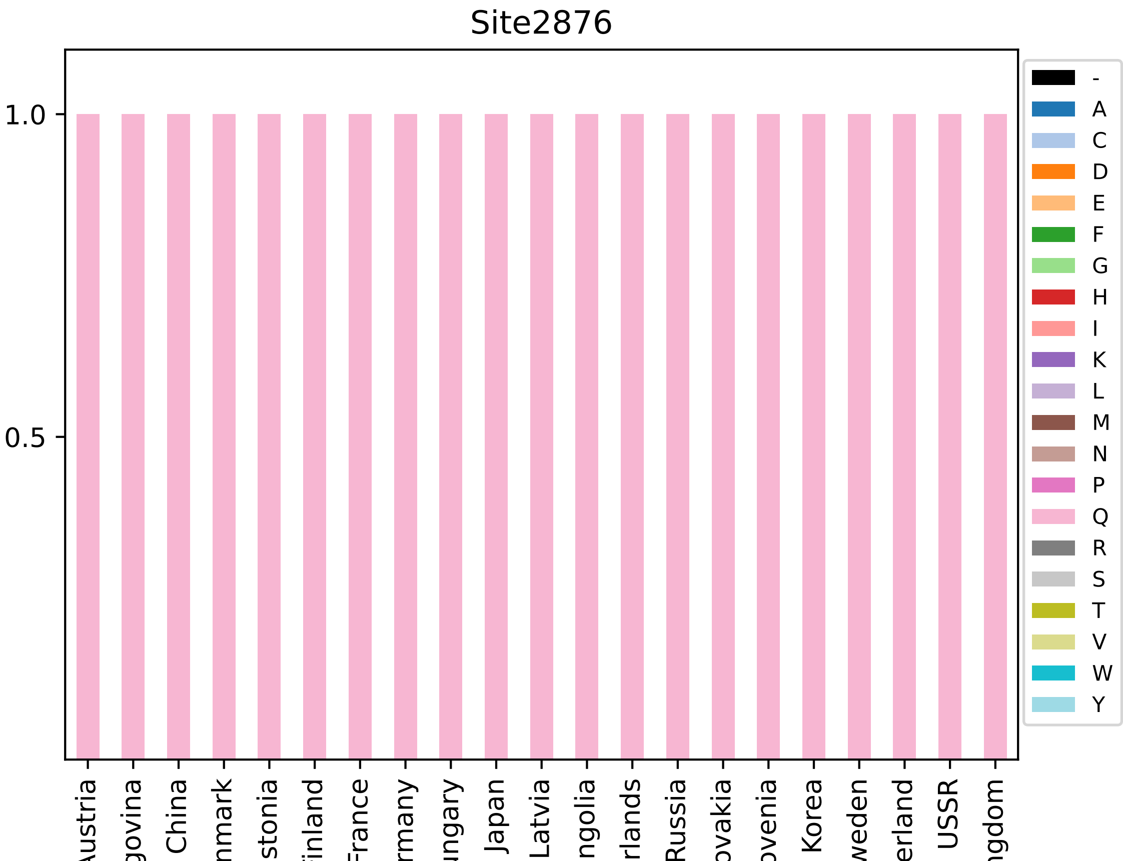
Site2876
1.0
0.5
-
A
C
D
E
F
G
H
I
K
L
M
N
P
Q
R
S
T
V
W
Y
China
Japan
Latvia
Russia
Korea
USSR

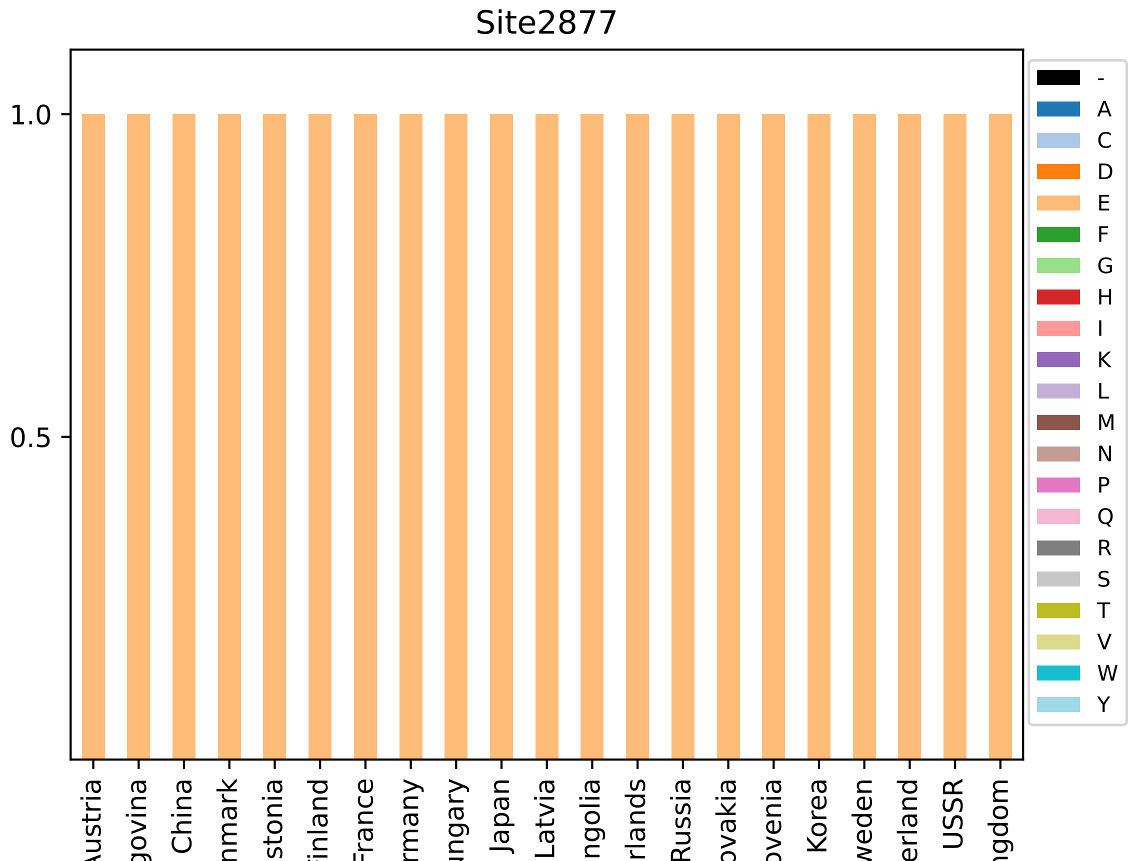

Site2877
1.0
0.5
-
A
C
D
E
F
G
H
I
K
L
M
N
P
Q
R
S
T
V
W
Y
Austria
govina
China
nmark
stonia
Finland
France
rmany
ungary
Japan
Latvia
ngolia
rlands
Russia
ovakia
ovenia
Korea
weden
erland
USSR
ngdom

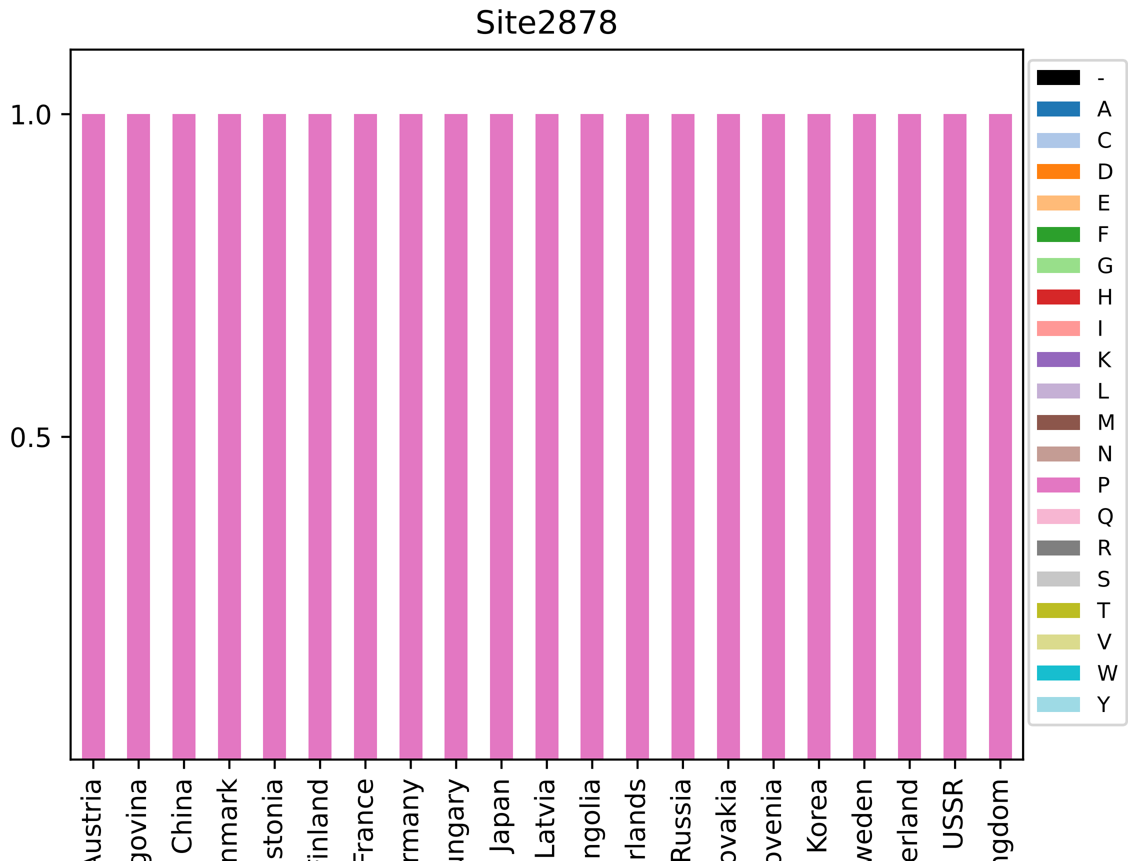

Site2878
1.0
0.5
-
A
C
D
E
F
G
H
I
K
L
M
N
P
Q
R
S
T
V
W
Y
China
Japan
Latvia
Russia
Korea
USSR

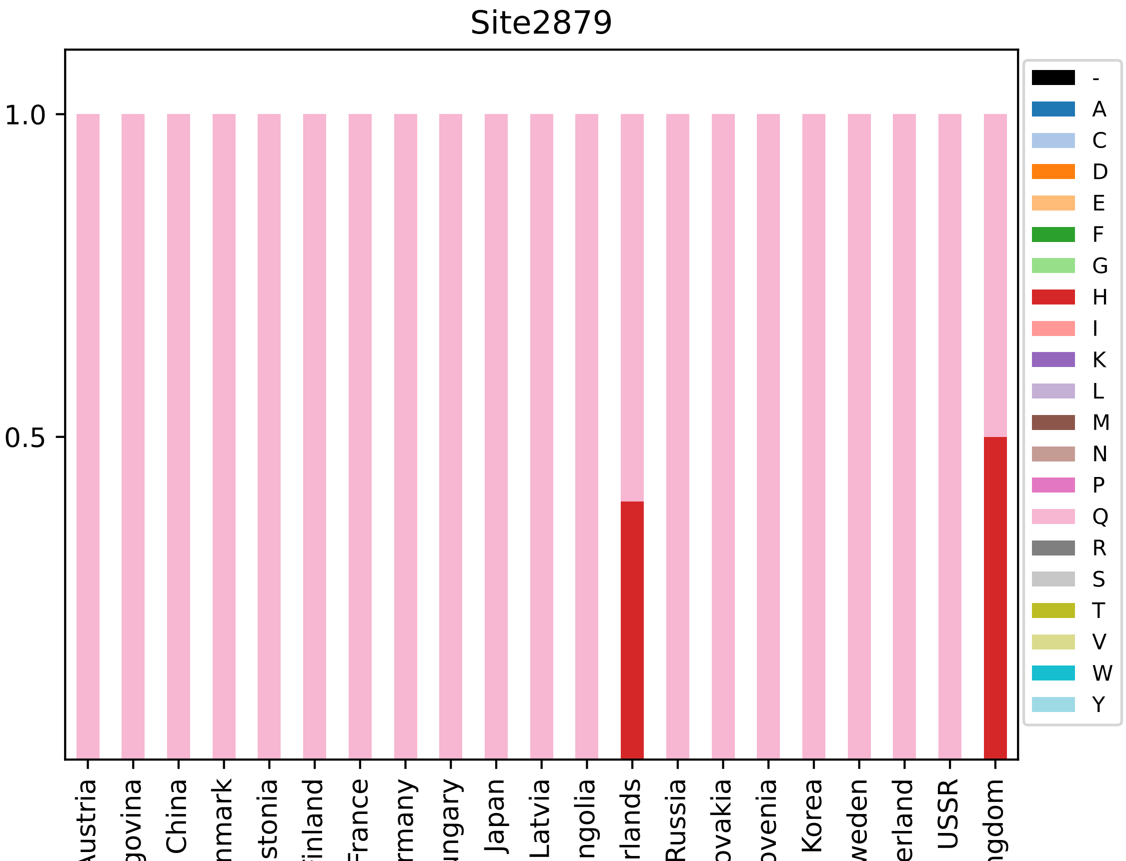

Site2879
1.0
0.5
-
A
C
D
E
F
G
H
I
K
L
M
N
P
Q
R
S
T
V
W
Y
Austria
govina
China
nmark
stonia
Finland
France
rmany
ungary
Japan
Latvia
ngolia
rlands
Russia
ovakia
ovenia
Korea
weden
erland
USSR
ngdom

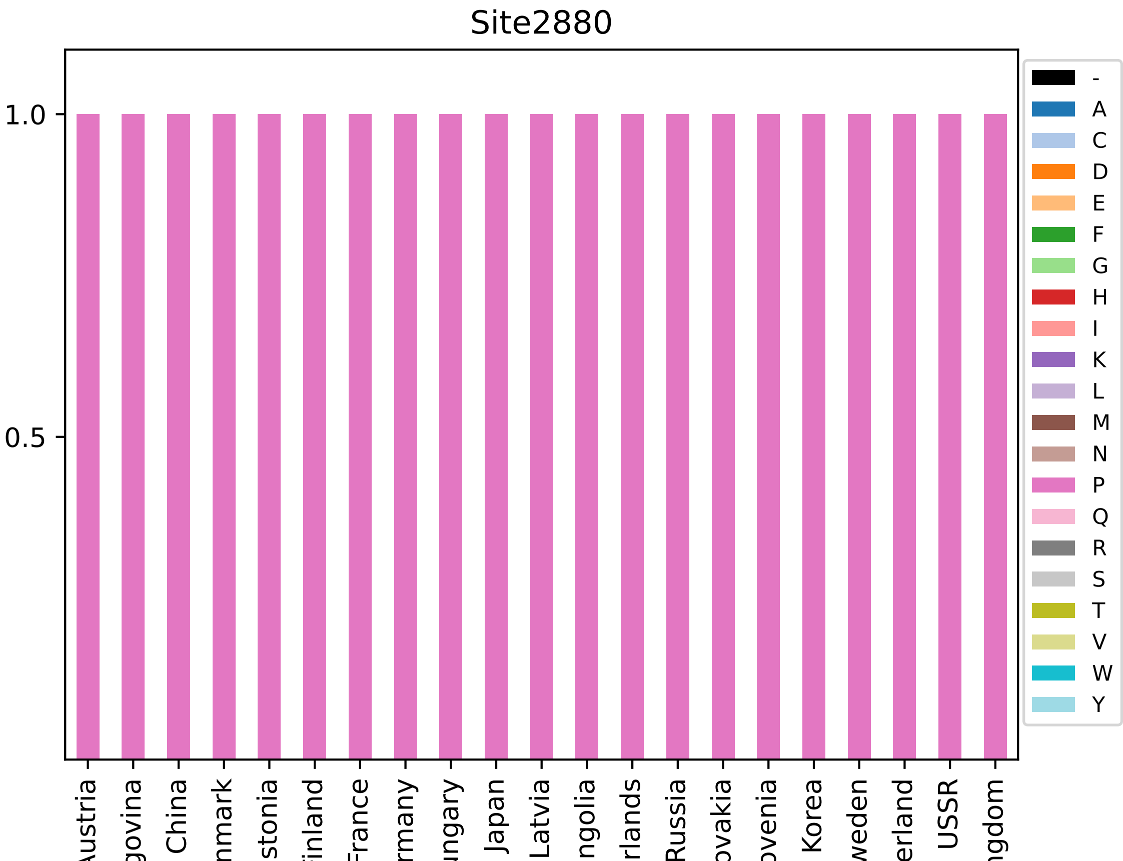
Site2880
1.0
0.5
-
A
C
D
E
F
G
H
I
K
L
M
N
P
Q
R
S
T
V
W
Y
China
Japan
Latvia
Russia
Korea
USSR

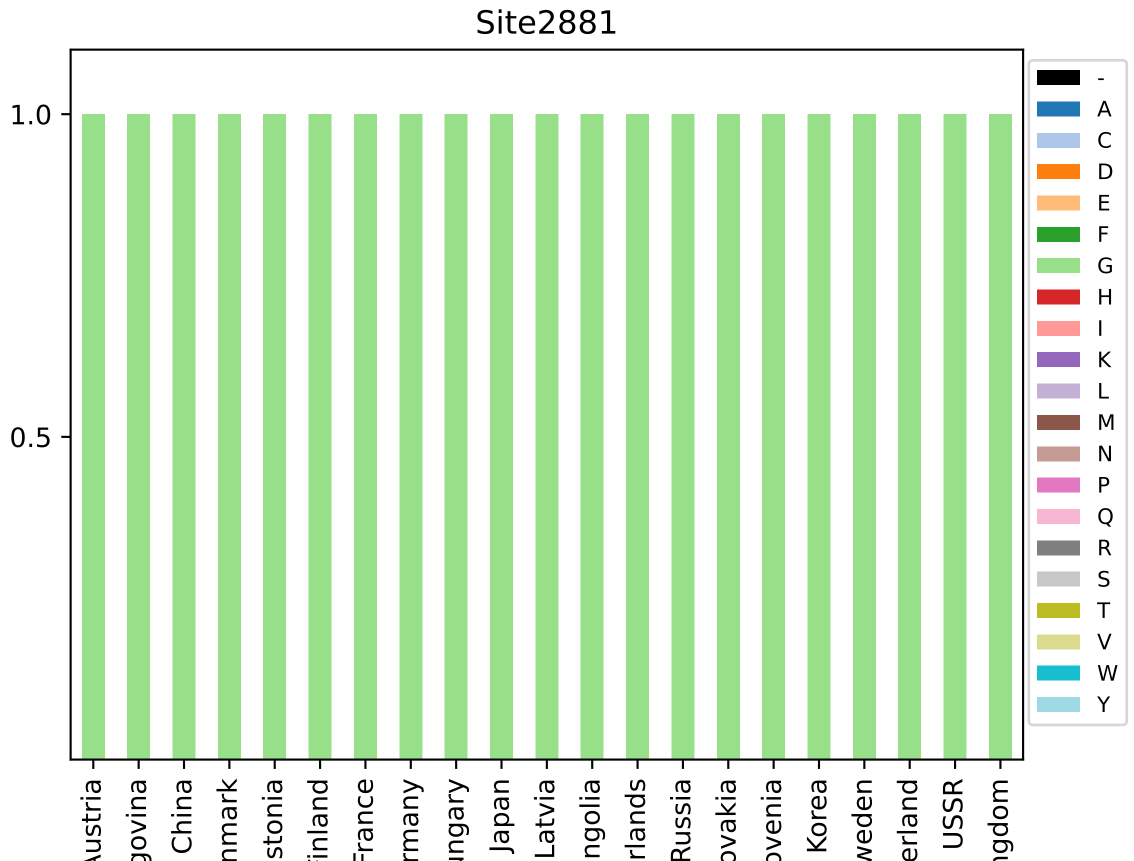

Site2881
1.0
0.5
-
A
C
D
E
F
G
H
I
K
L
M
N
P
Q
R
S
T
V
W
Y
China
Japan
Latvia
Russia
Korea
USSR

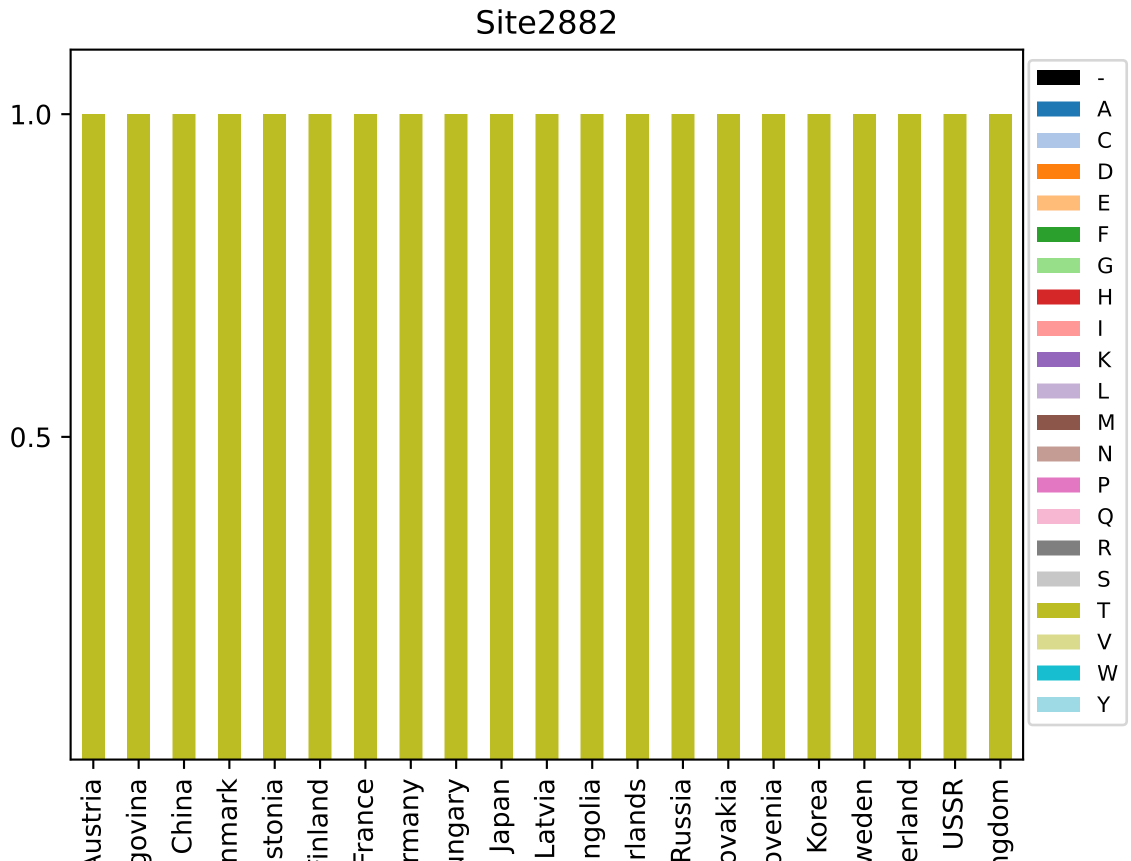
Site2882
1.0
0.5
-
A
C
D
E
F
G
H
I
K
L
M
N
P
Q
R
S
T
V
W
Y
Austria
govina
China
nmark
stonia
inland
rance
rmany
ungary
Japan
Latvia
ngolia
rlands
Russia
ovakia
ovenia
Korea
weden
erland
USSR
ngdom

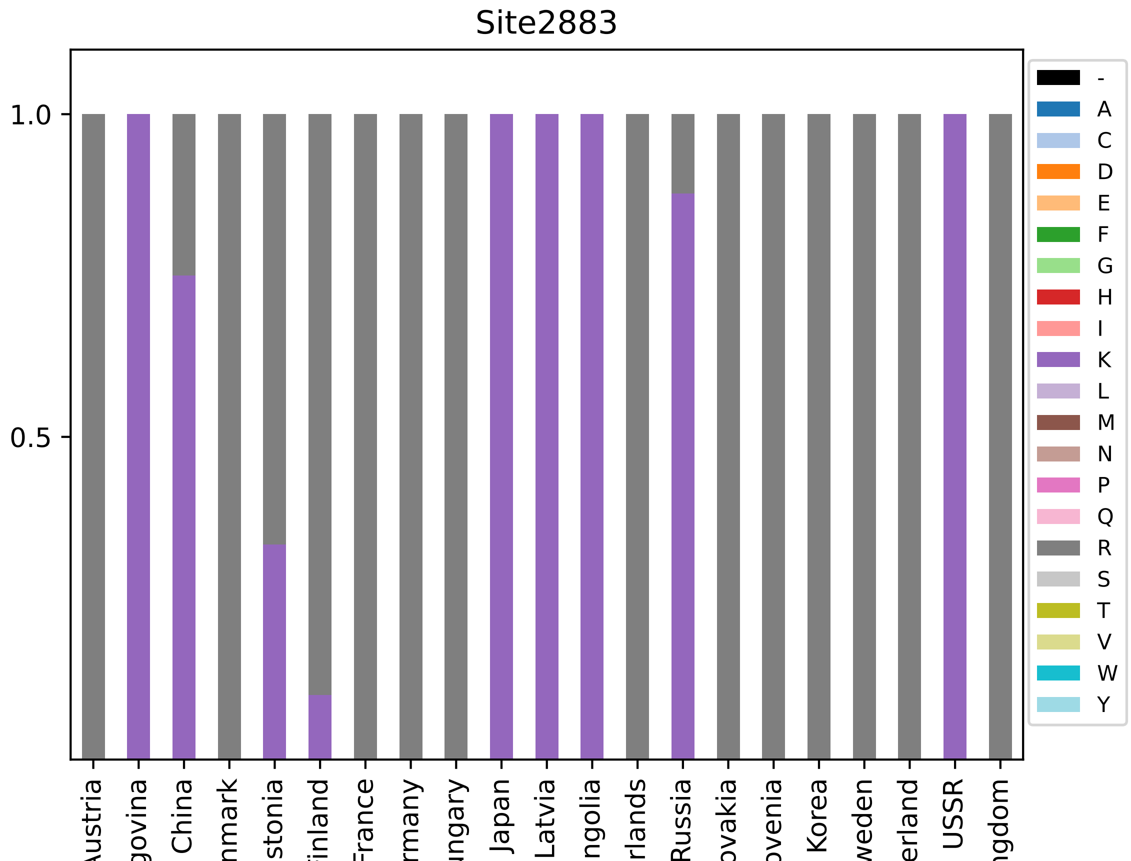

Site2883
1.0
0.5
-
A
C
D
E
F
G
H
I
K
L
M
N
P
Q
R
S
T
V
W
Y
Austria
China
Finland
France
Japan
Latvia
Russia
Korea
USSR

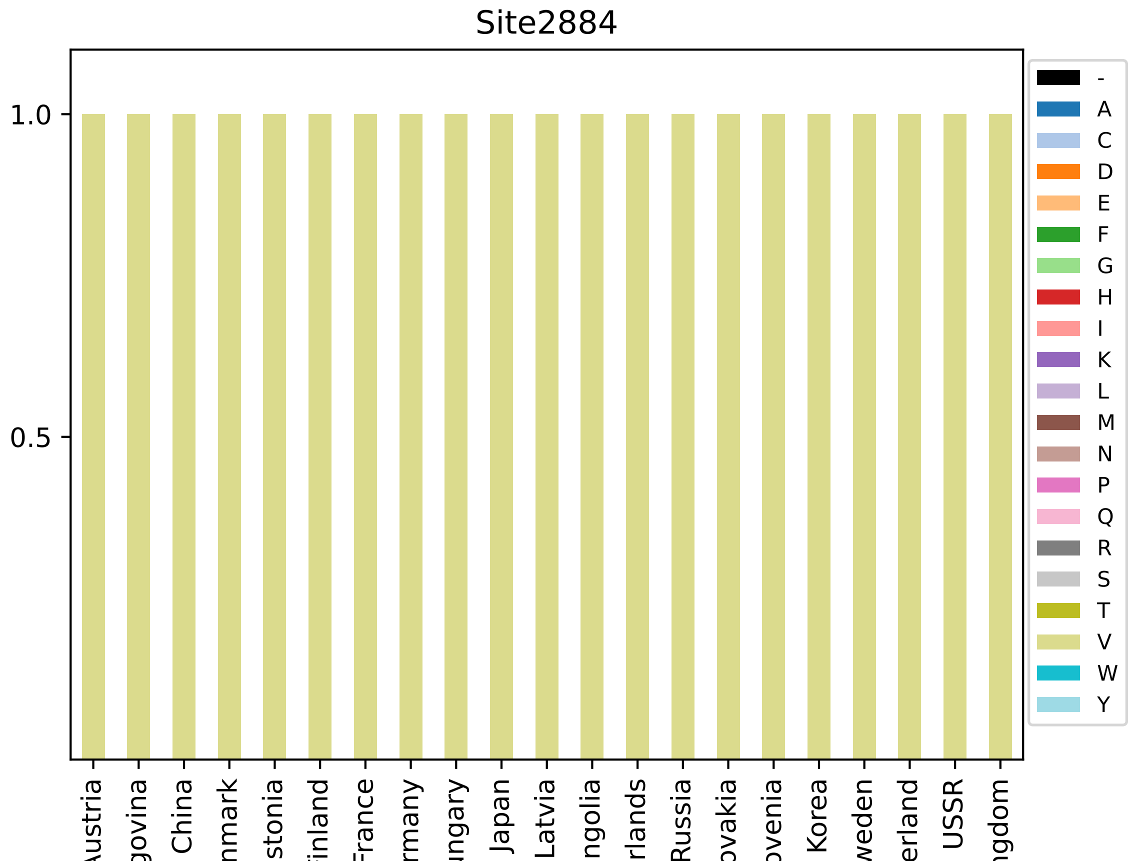
Site2884
1.0
0.5
-
A
C
D
E
F
G
H
I
K
L
M
N
P
Q
R
S
T
V
W
Y
China
Japan
Latvia
Russia
Korea
USSR

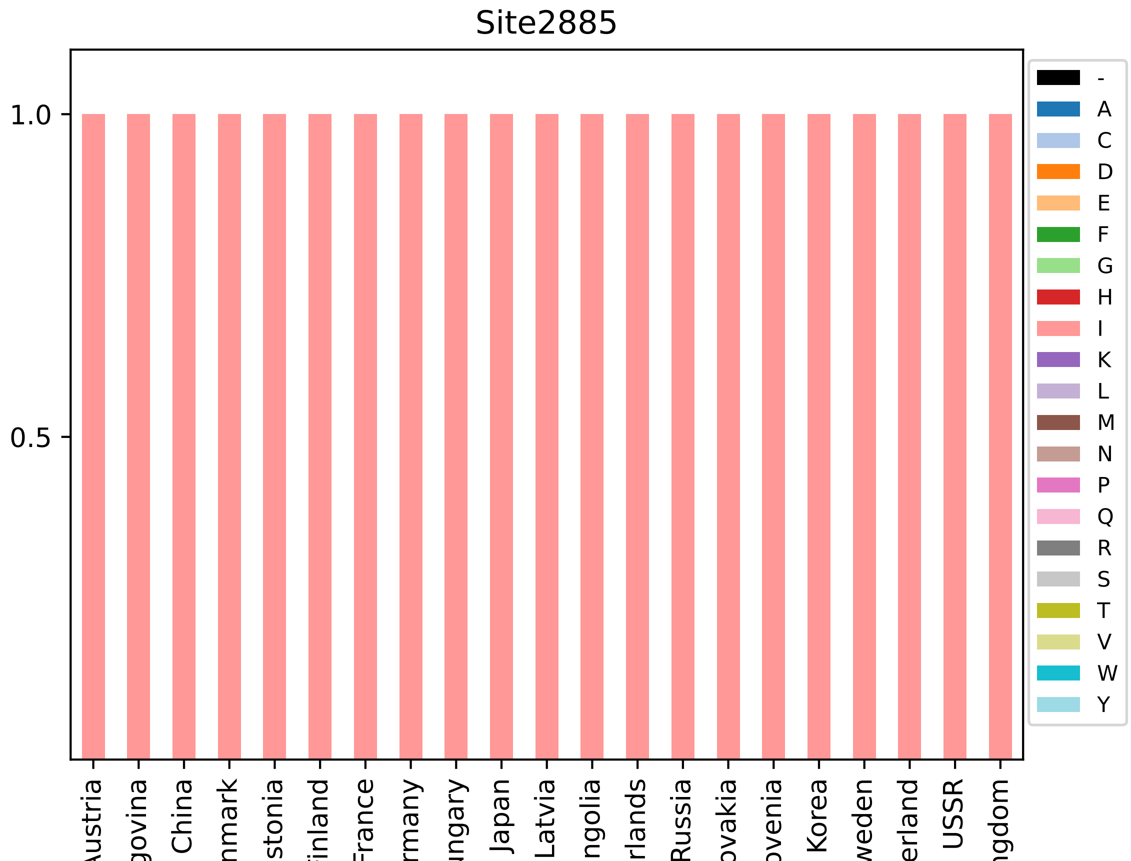

Site2885
1.0
0.5
-
A
C
D
E
F
G
H
I
K
L
M
N
P
Q
R
S
T
V
W
Y
China
Japan
Latvia
Russia
Korea
USSR

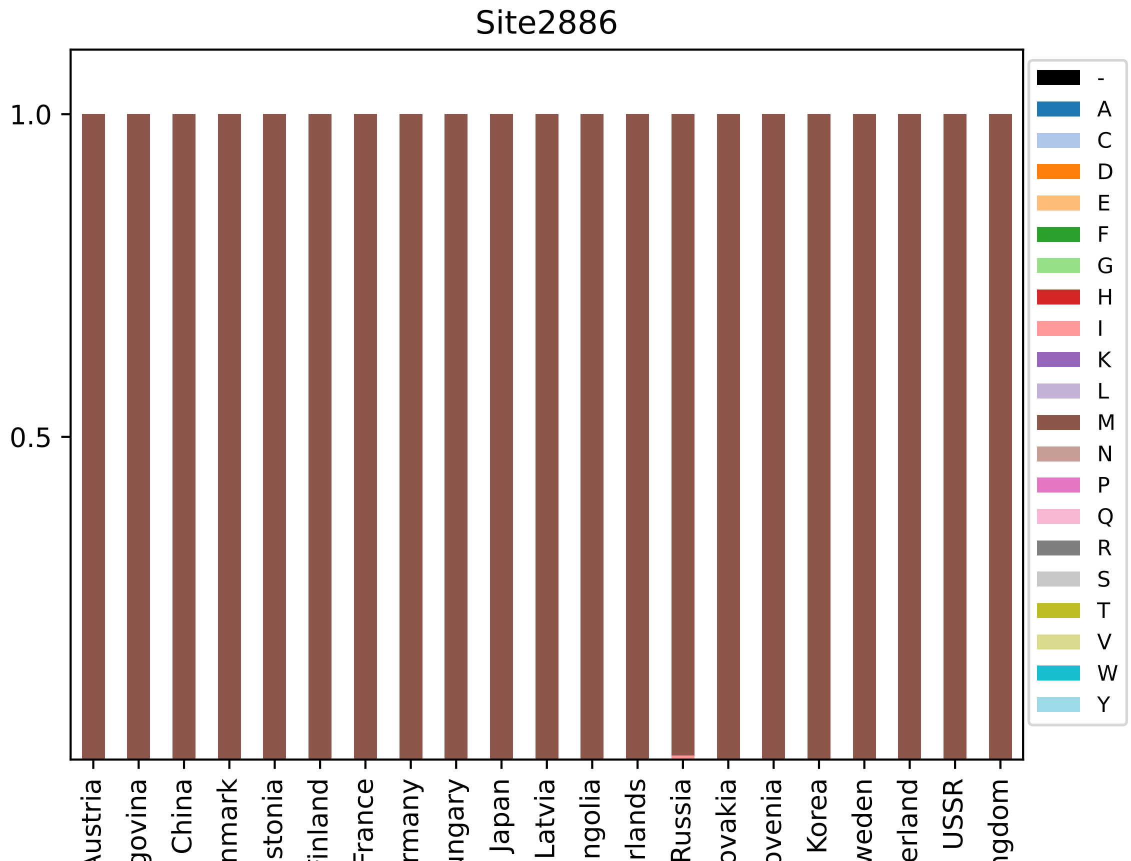
Site2886
1.0
0.5
-
A
C
D
E
F
G
H
I
K
L
M
N
P
Q
R
S
T
V
W
Y
Austria
govina
China
nmark
stonia
Finland
France
rmany
ungary
Japan
Latvia
ngolia
rlands
Russia
ovakia
ovenia
Korea
weden
erland
USSR
ngdom

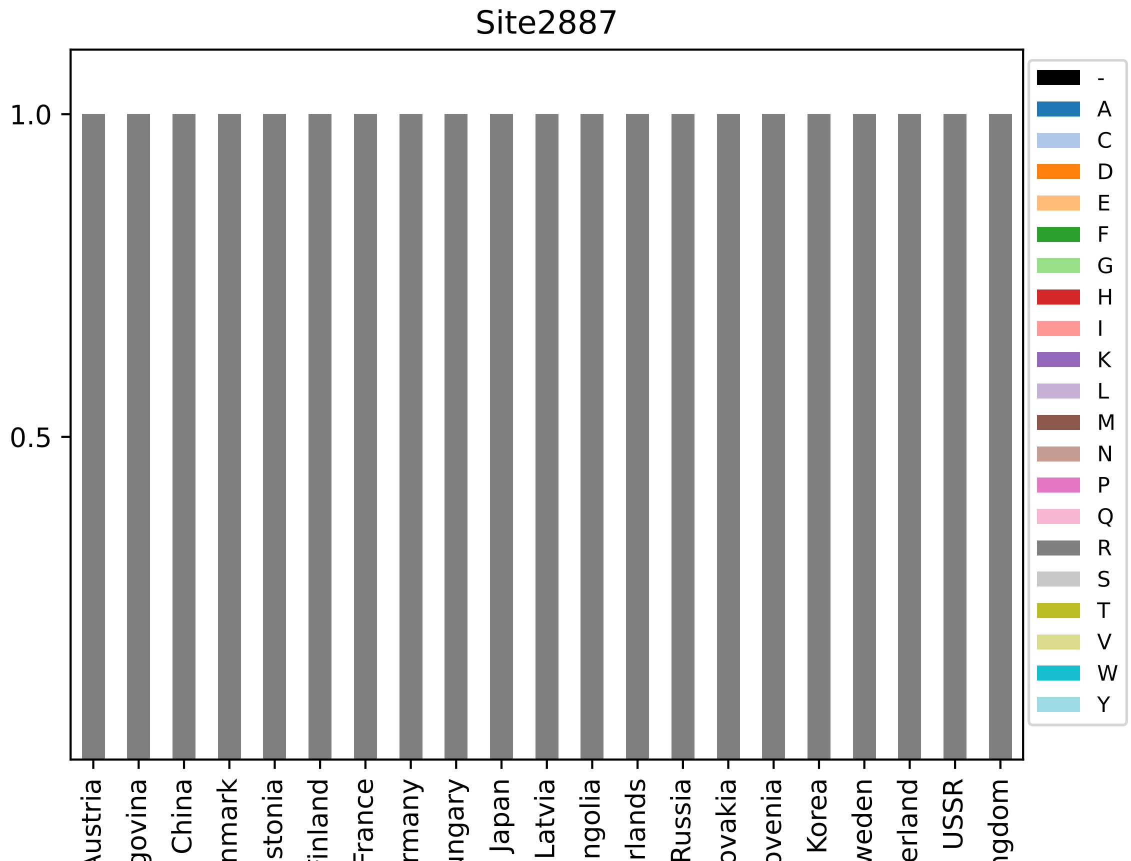

Site2887
1.0
0.5
-
A
C
D
E
F
G
H
I
K
L
M
N
P
Q
R
S
T
V
W
Y
Austria
China
Japan
Latvia
Russia
Korea
USSR

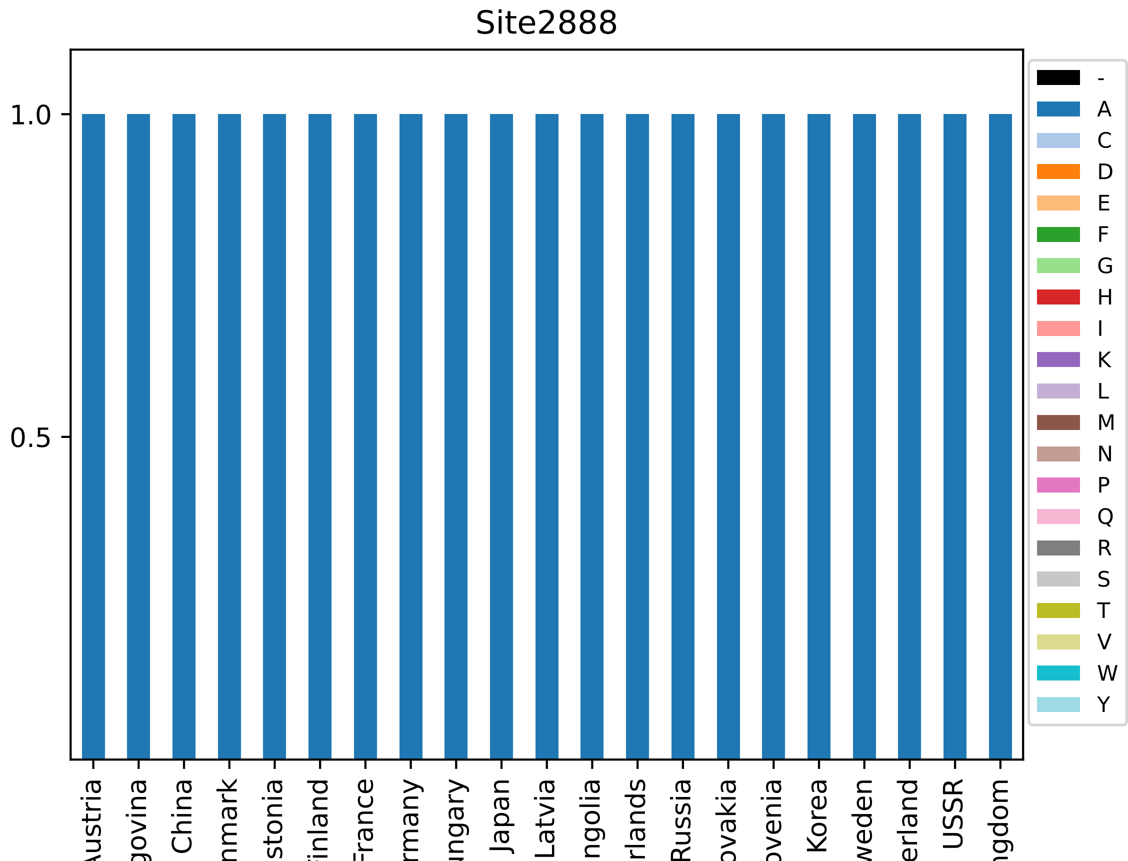

Site2888
1.0
0.5
-
A
C
D
E
F
G
H
I
K
L
M
N
P
Q
R
S
T
V
W
Y
Austria
govina
China
nmark
stonia
Finland
France
rmany
ungary
Japan
Latvia
ngolia
rlands
Russia
ovakia
ovenia
Korea
weden
erland
USSR
ngdom

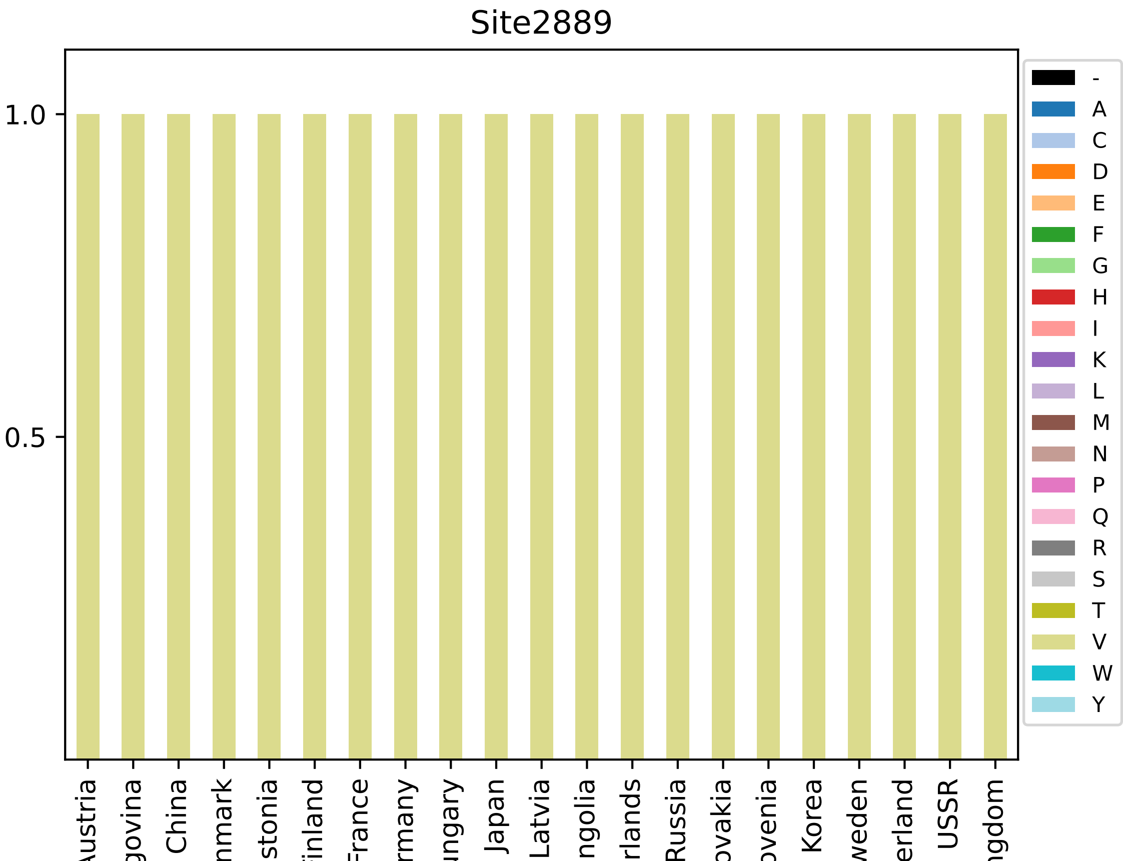
Site2889
1.0
0.5
-
A
C
D
E
F
G
H
I
K
L
M
N
P
Q
R
S
T
V
W
Y
Austria
govina
China
nmark
stonia
Finland
France
rmany
ungary
Japan
Latvia
ngolia
rlands
Russia
ovakia
ovenia
Korea
weden
erland
USSR
ngdom

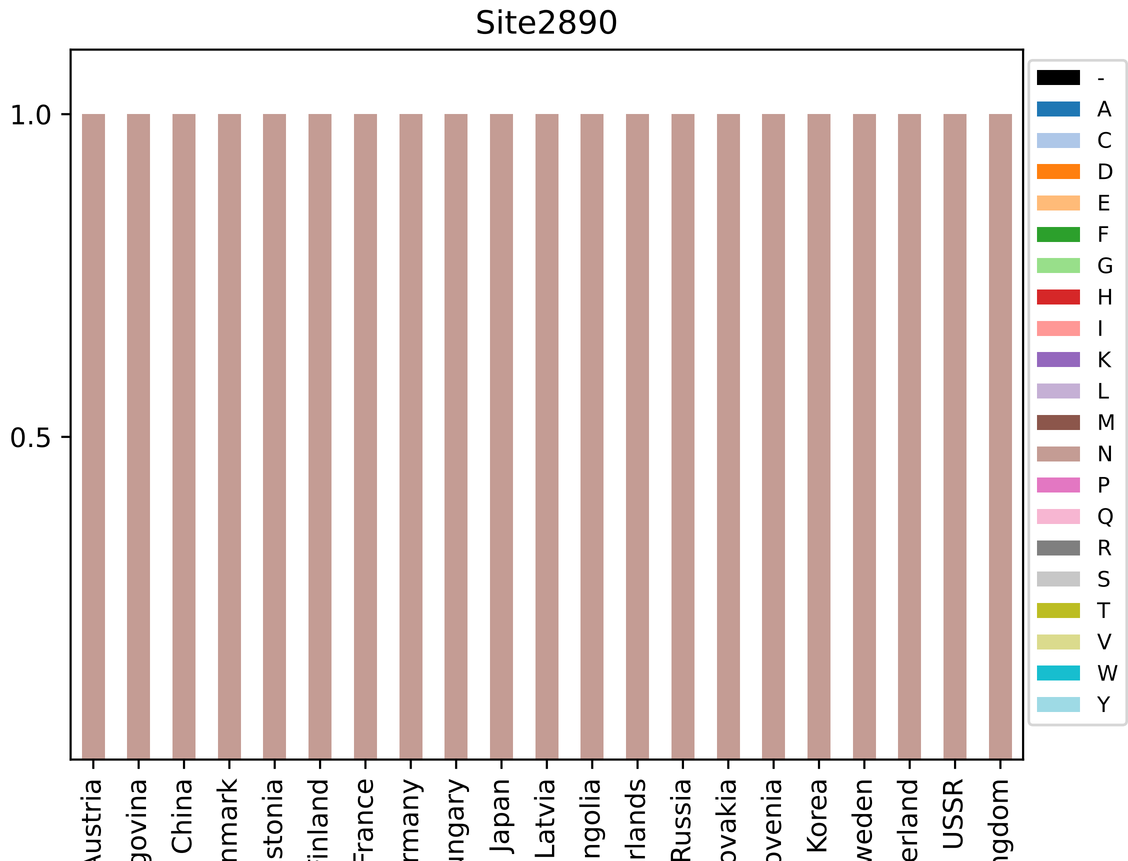
Site2890
1.0
0.5
-
A
C
D
E
F
G
H
I
K
L
M
N
P
Q
R
S
T
V
W
Y
China
Japan
Latvia
Russia
Korea
USSR

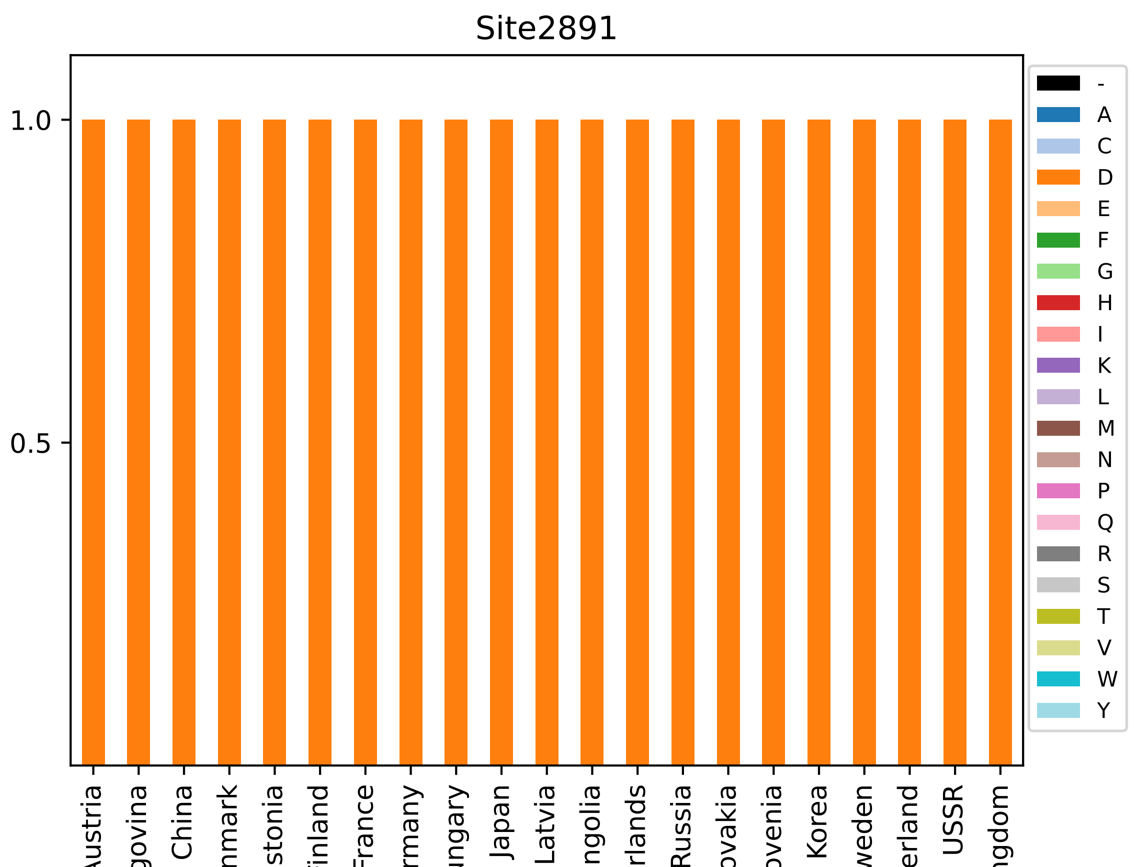

Site2891
1.0
0.5
-
A
C
D
E
F
G
H
I
K
L
M
N
P
Q
R
S
T
V
W
Y
Austria
govina
China
nmark
stonia
inland
France
rmany
ungary
Japan
Latvia
ngolia
rlands
Russia
ovakia
ovenia
Korea
weden
erland
USSR
ngdom

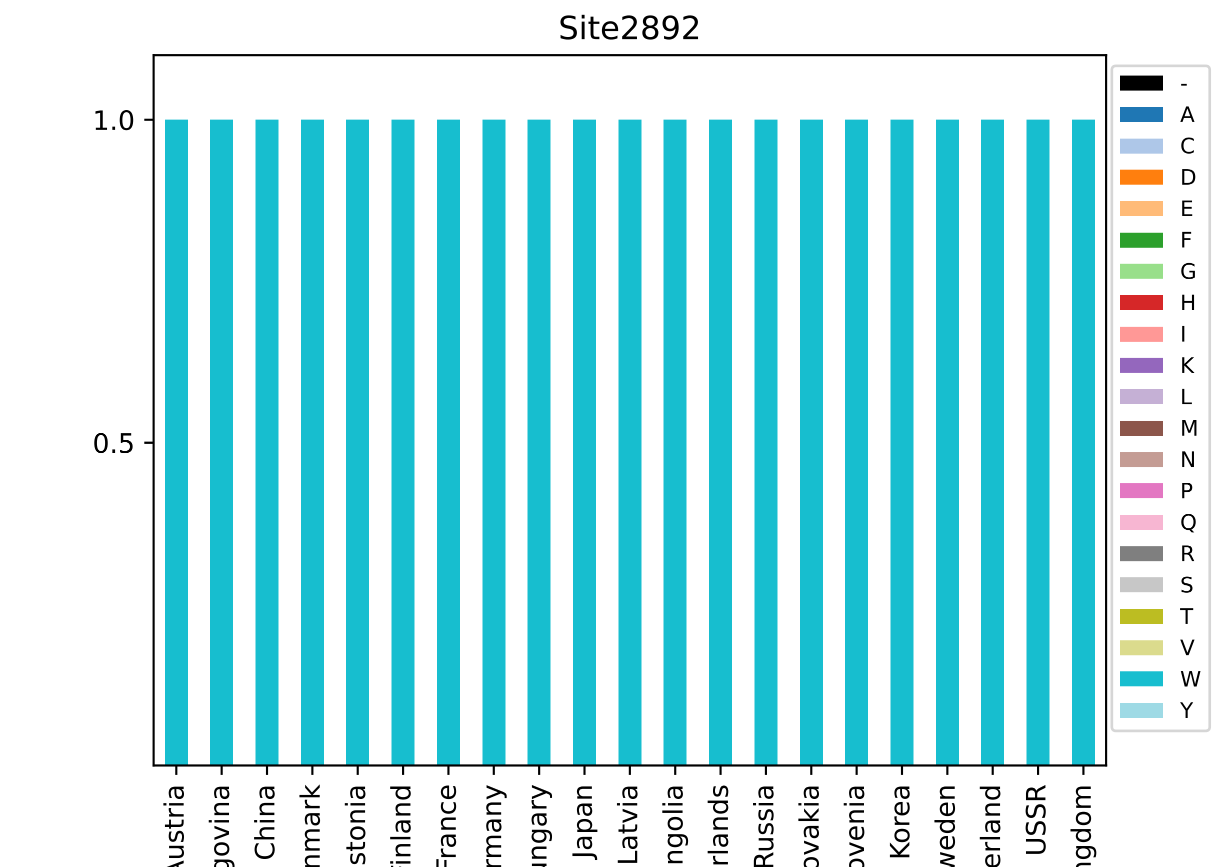
Site2892
1.0
0.5
-
A
C
D
E
F
G
H
I
K
L
M
N
P
Q
R
S
T
V
W
Y
Austria
govina
China
nmark
stonia
inland
France
rmany
ungary
Japan
Latvia
ngolia
rlands
Russia
ovakia
ovenia
Korea
weden
erland
USSR
ngdom

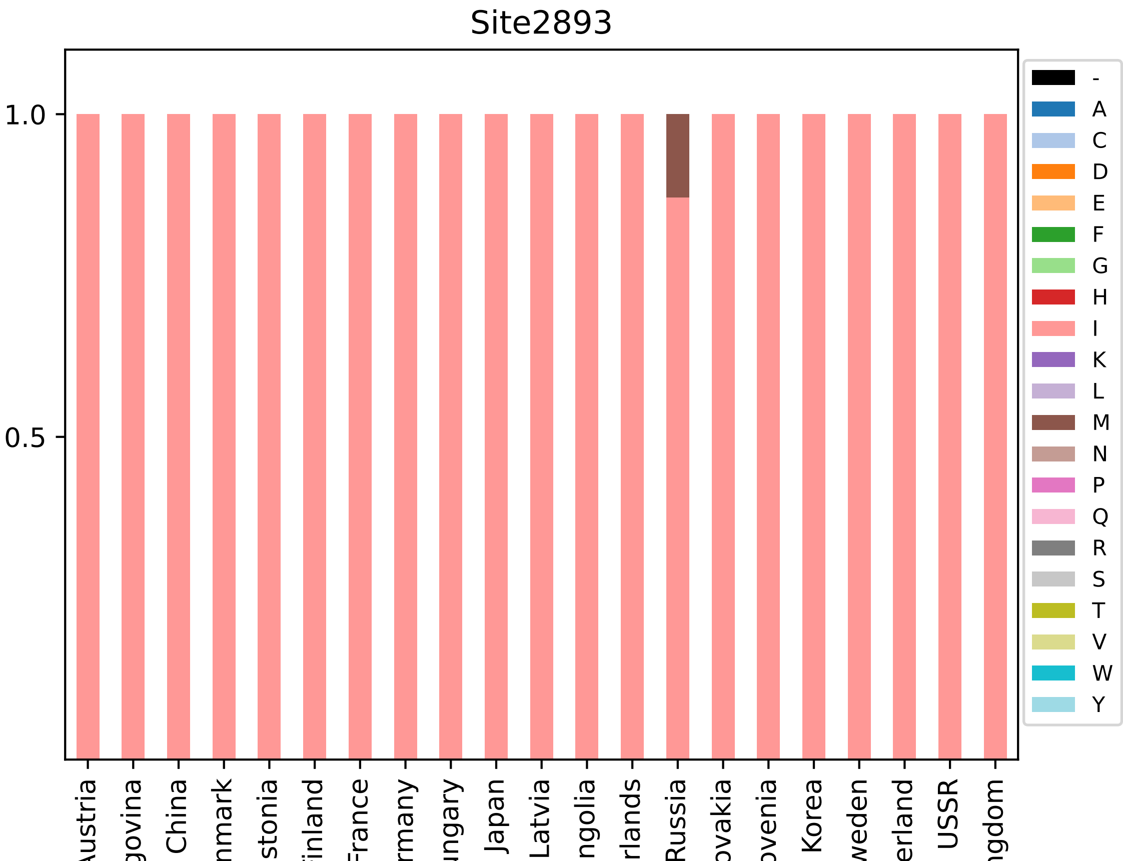

Site2893
1.0
0.5
-
A
C
D
E
F
G
H
I
K
L
M
N
P
Q
R
S
T
V
W
Y
Austria
govina
China
nmark
stonia
inland
rance
rmany
ungary
Japan
Latvia
ngolia
rlands
Russia
ovakia
ovenia
Korea
weden
erland
USSR
ngdom

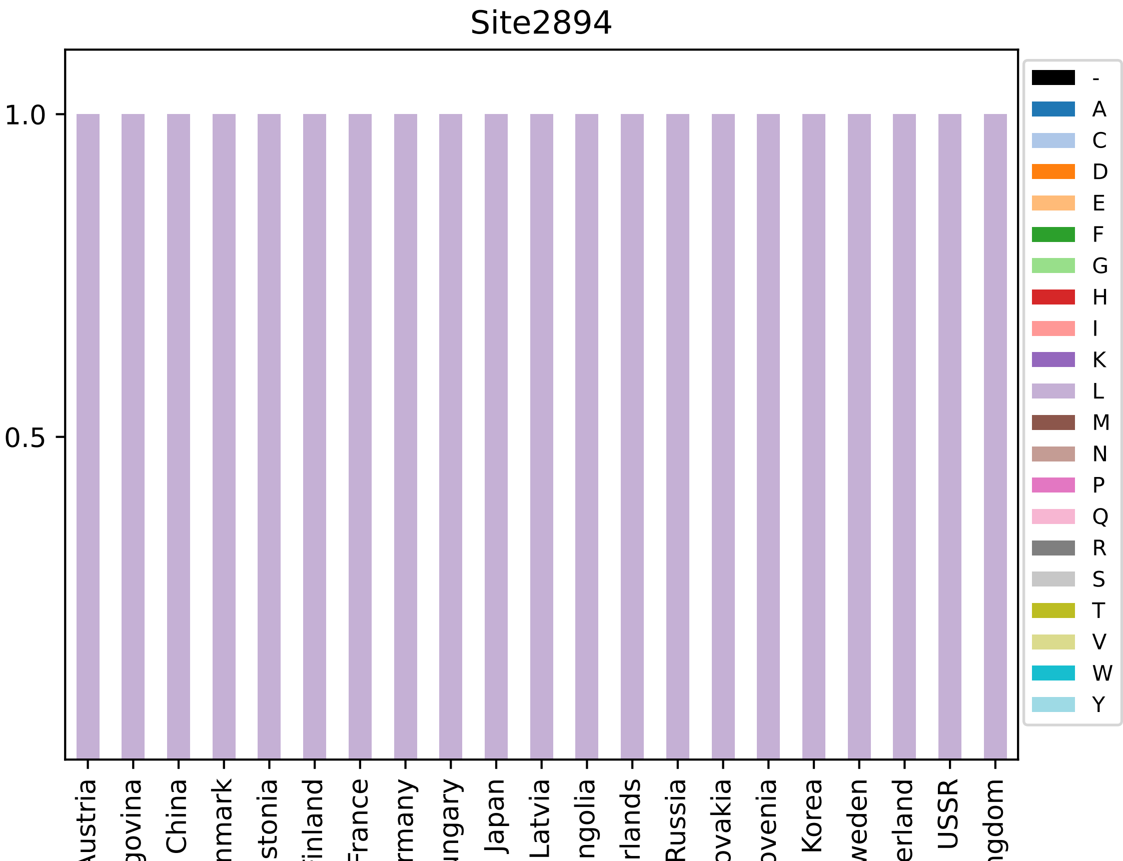

Site2894
1.0
0.5
-
A
C
D
E
F
G
H
I
K
L
M
N
P
Q
R
S
T
V
W
Y
China
Japan
Latvia
Russia
Korea
USSR

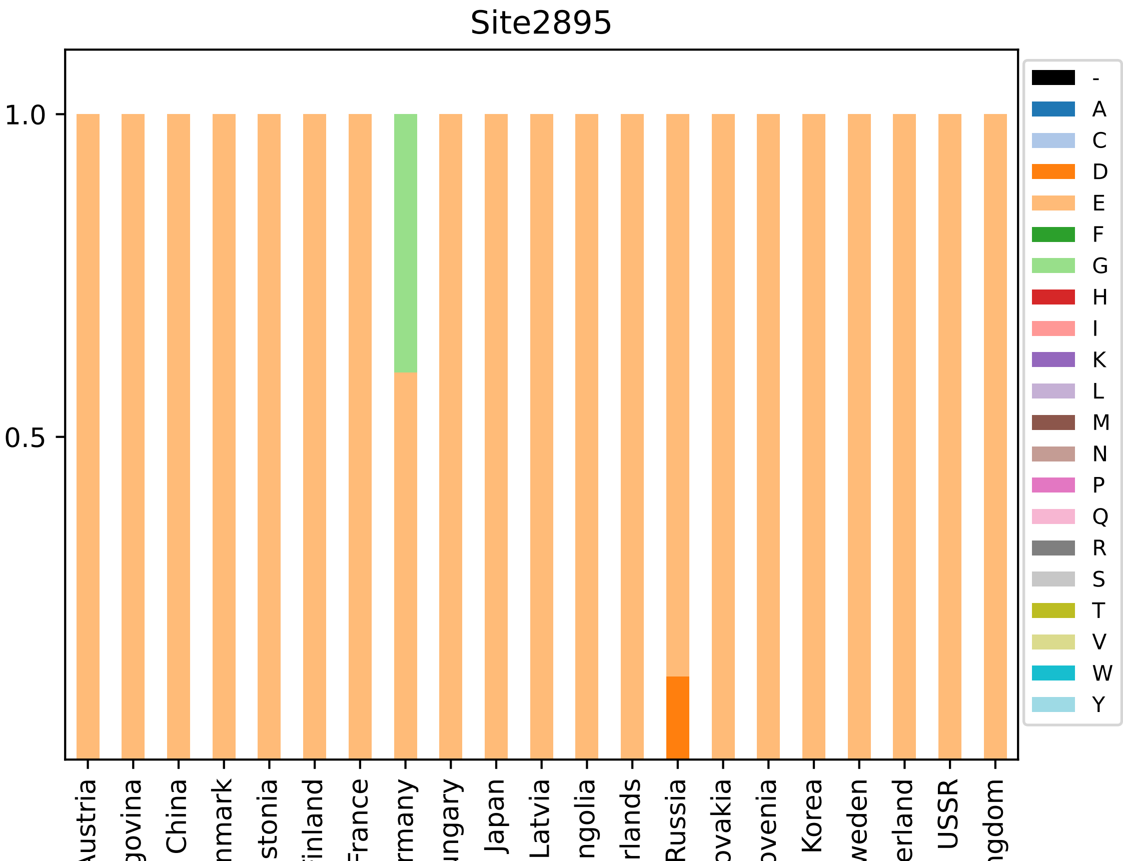

Site2895
1.0
0.5
-
A
C
D
E
F
G
H
I
K
L
M
N
P
Q
R
S
T
V
W
Y
China
Japan
Latvia
Russia
Korea
USSR

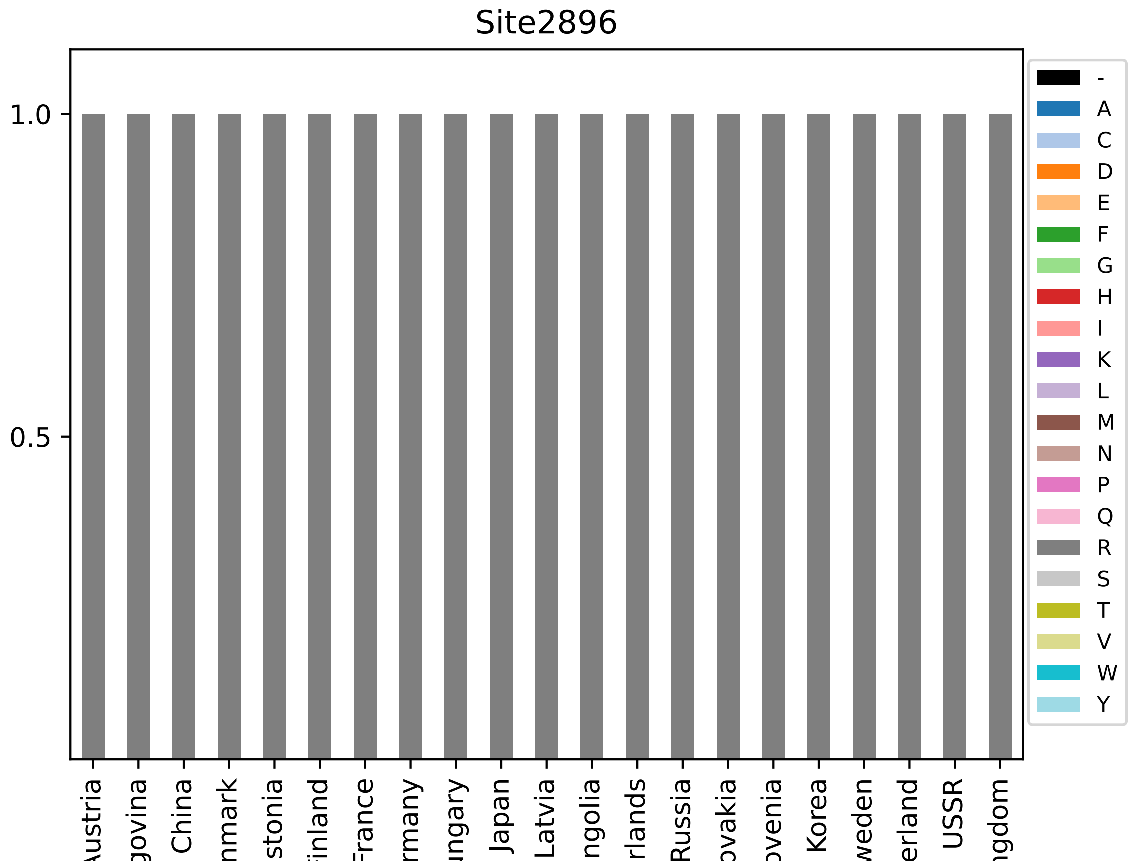

Site2896
1.0
0.5
-
A
C
D
E
F
G
H
I
K
L
M
N
P
Q
R
S
T
V
W
Y
Austria
China
Japan
Latvia
Russia
Korea
USSR

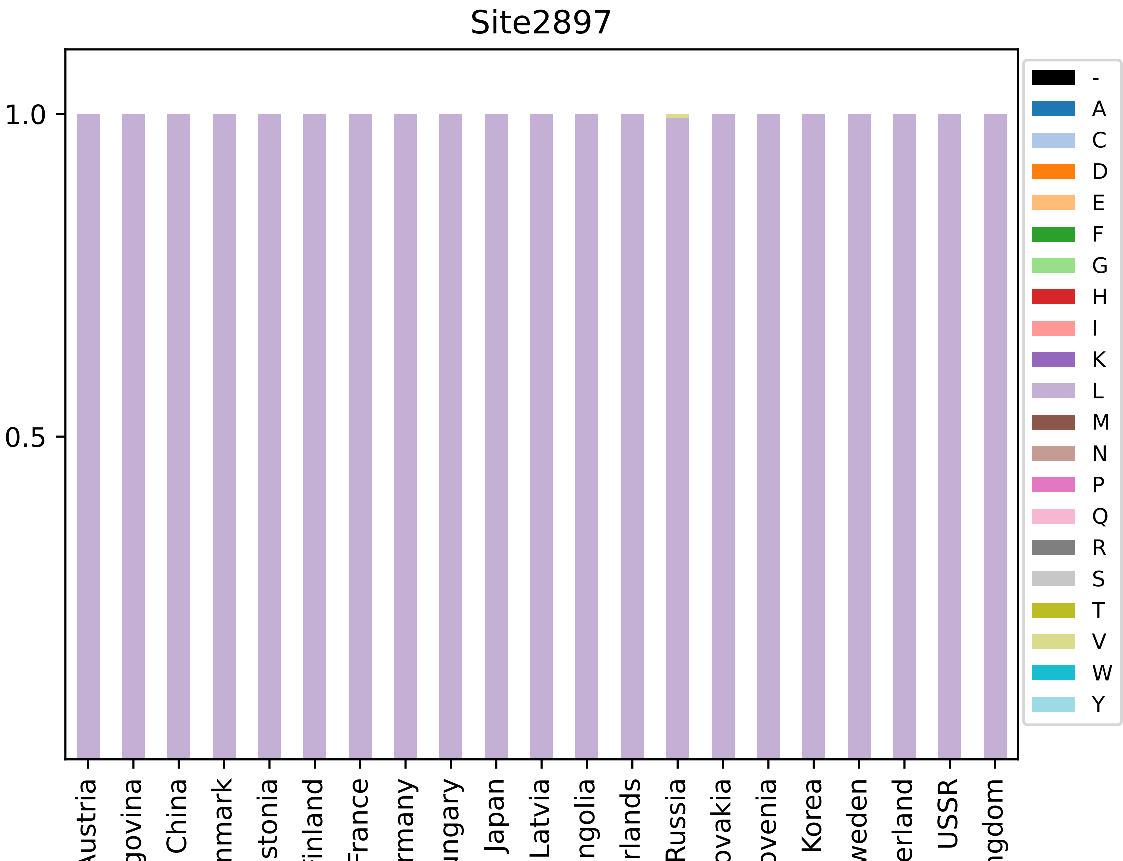

Site2897
1.0
0.5
-
A
C
D
E
F
G
H
I
K
L
M
N
P
Q
R
S
T
V
W
Y
China
Japan
Latvia
Russia
Korea
USSR

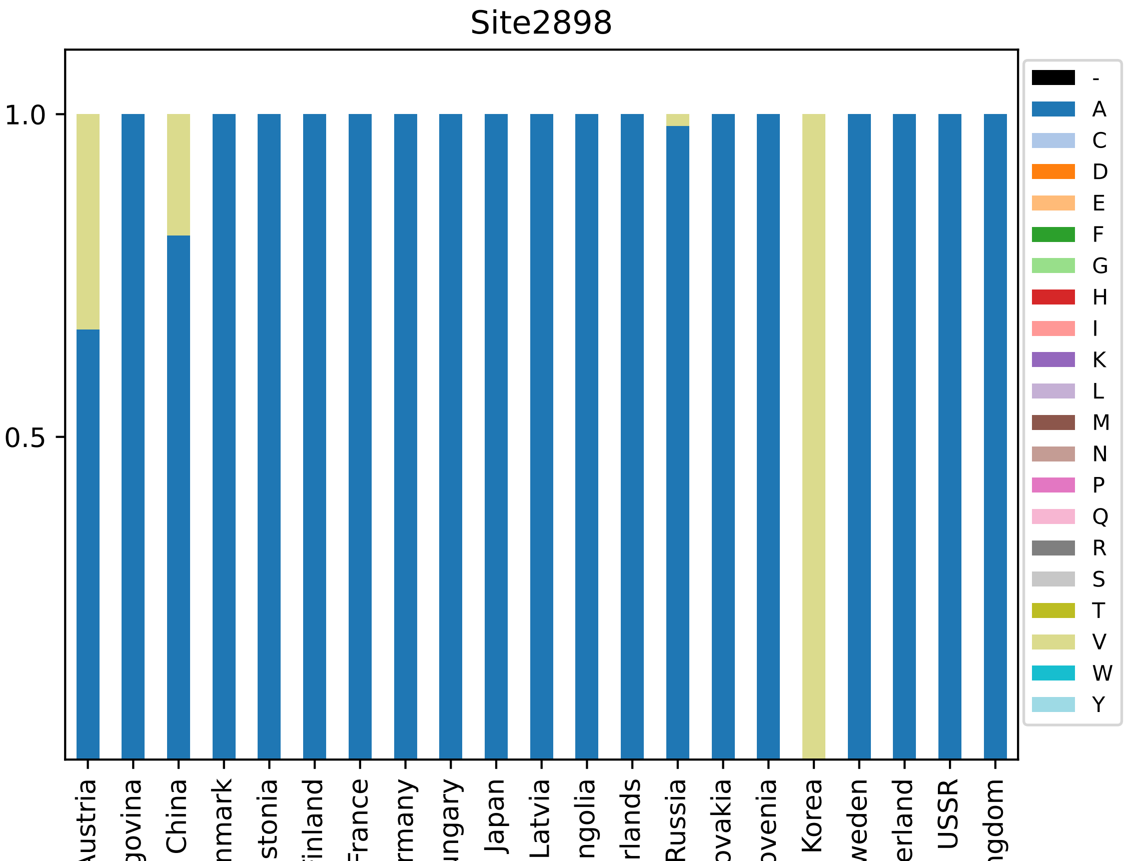

Site2898
1.0
0.5
-
A
C
D
E
F
G
H
I
K
L
M
N
P
Q
R
S
T
V
W
Y
China
Japan
Latvia
Russia
Korea
USSR

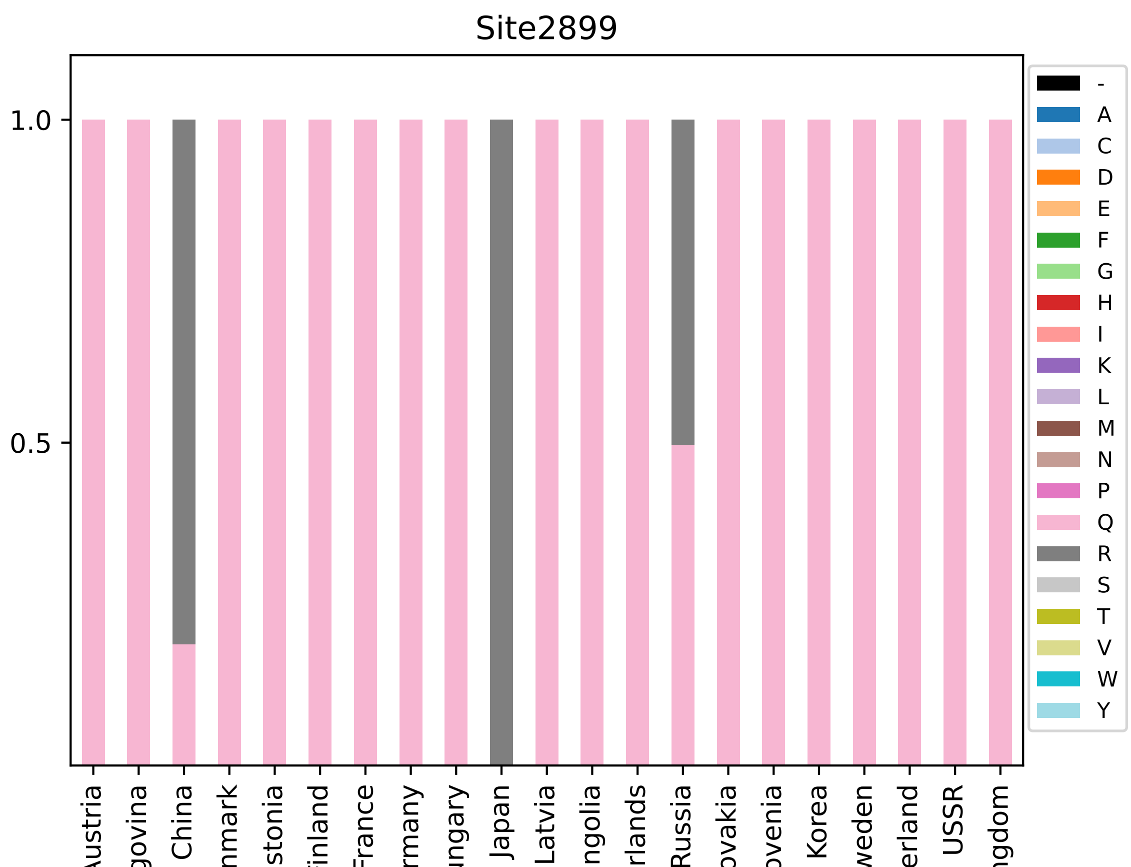

Site2899
1.0
0.5
-
A
C
D
E
F
G
H
I
K
L
M
N
P
Q
R
S
T
V
W
Y
China
Japan
Latvia
Russia
Korea
USSR

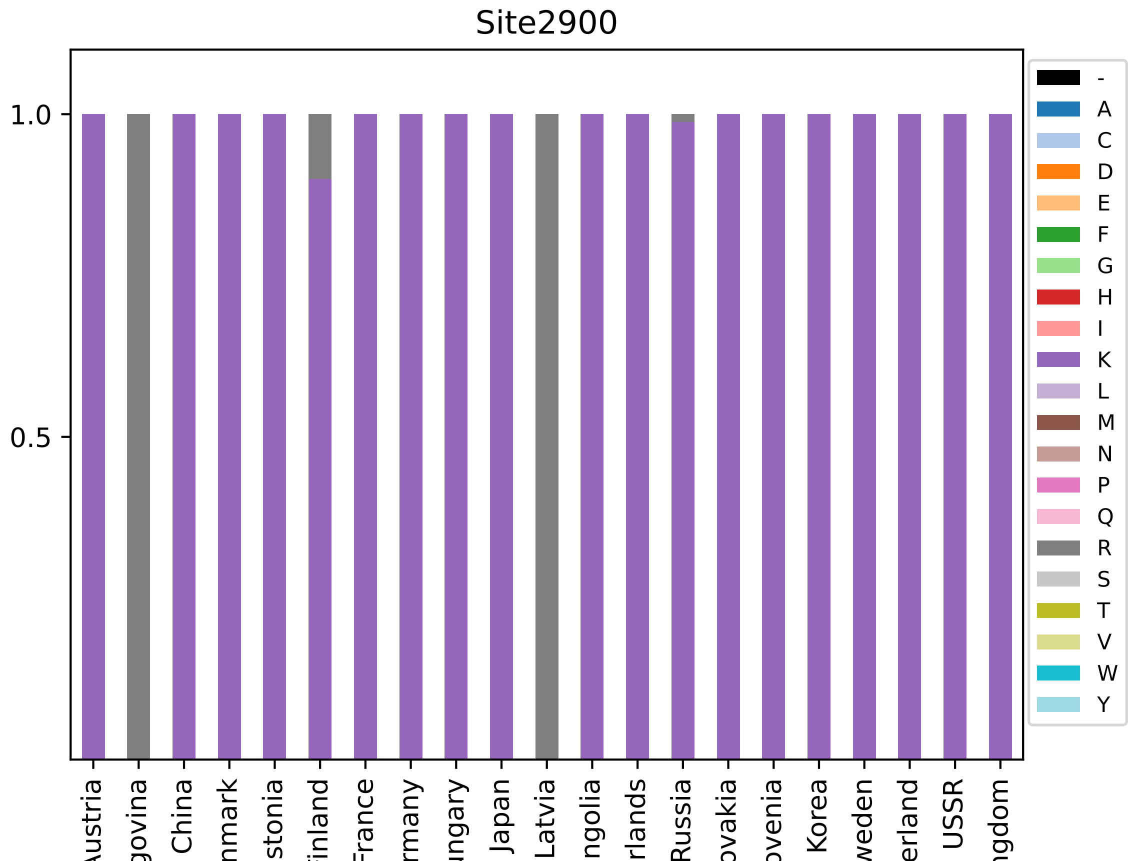

Site2900
1.0
0.5
-
A
C
D
E
F
G
H
I
K
L
M
N
P
Q
R
S
T
V
W
Y
Austria
govina
China
nmark
stonia
Finland
France
rmany
ungary
Japan
Latvia
ngolia
rlands
Russia
ovakia
ovenia
Korea
weden
erland
USSR
ngdom

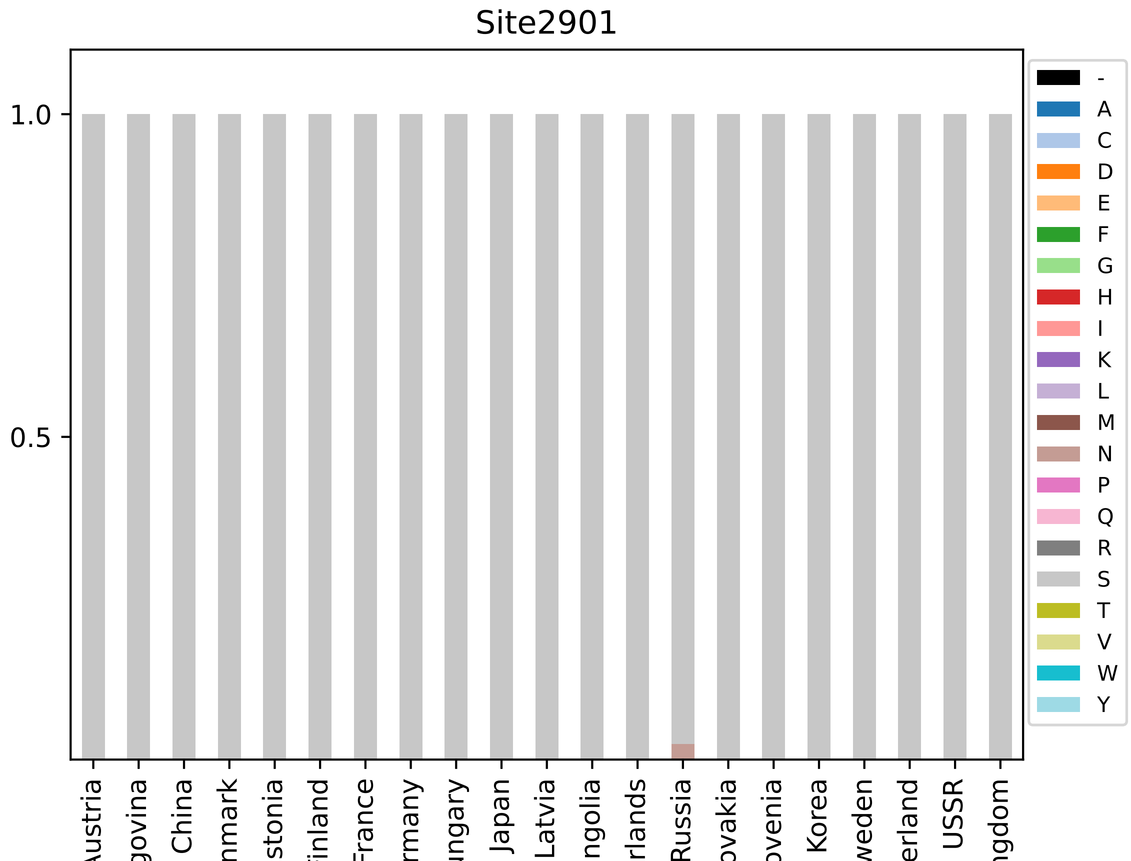

Site2901
1.0
0.5
-
A
C
D
E
F
G
H
I
K
L
M
N
P
Q
R
S
T
V
W
Y
Austria
China
Japan
Latvia
Russia
Korea
USSR

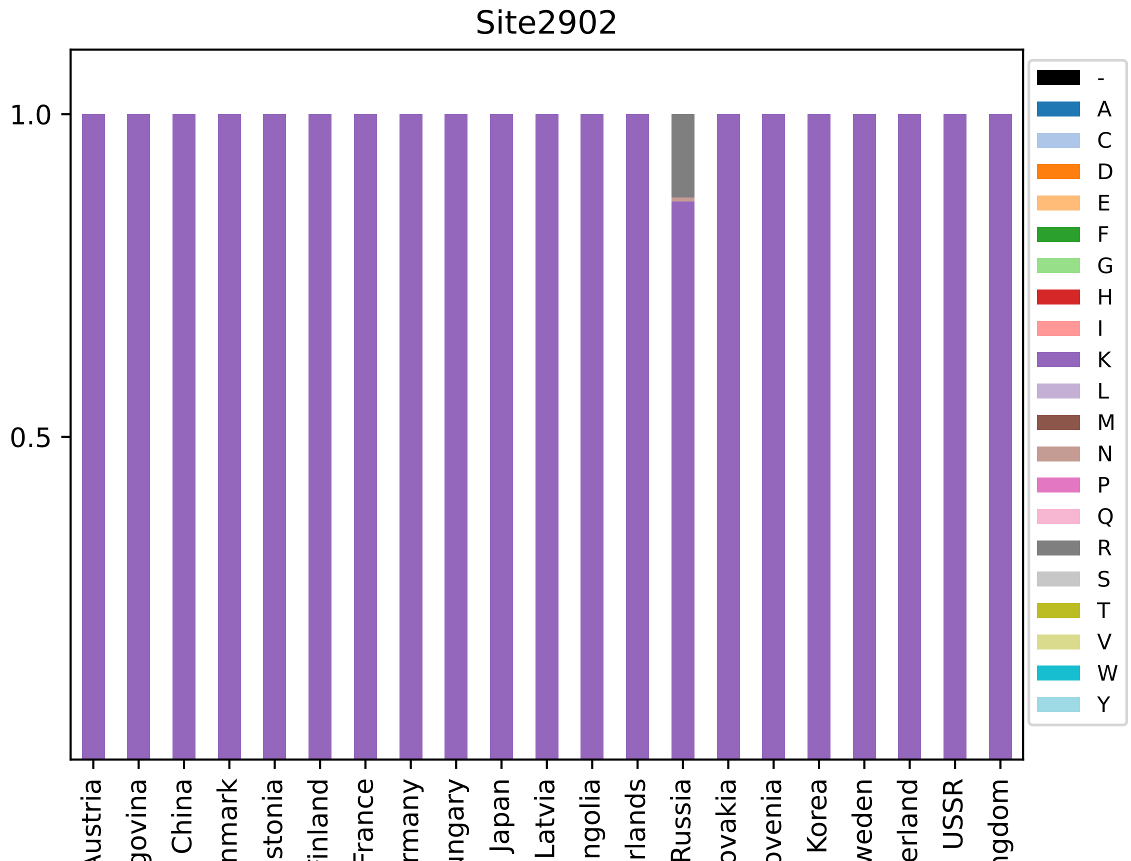

Site2902
1.0
0.5
Austria
govina
China
nmark
stonia
Finland
France
rmany
ungary
Japan
Latvia
ngolia
rlands
Russia
ovakia
ovenia
Korea
weden
erland
USSR
ngdom
-
A
C
D
E
F
G
H
I
K
L
M
N
P
Q
R
S
T
V
W
Y

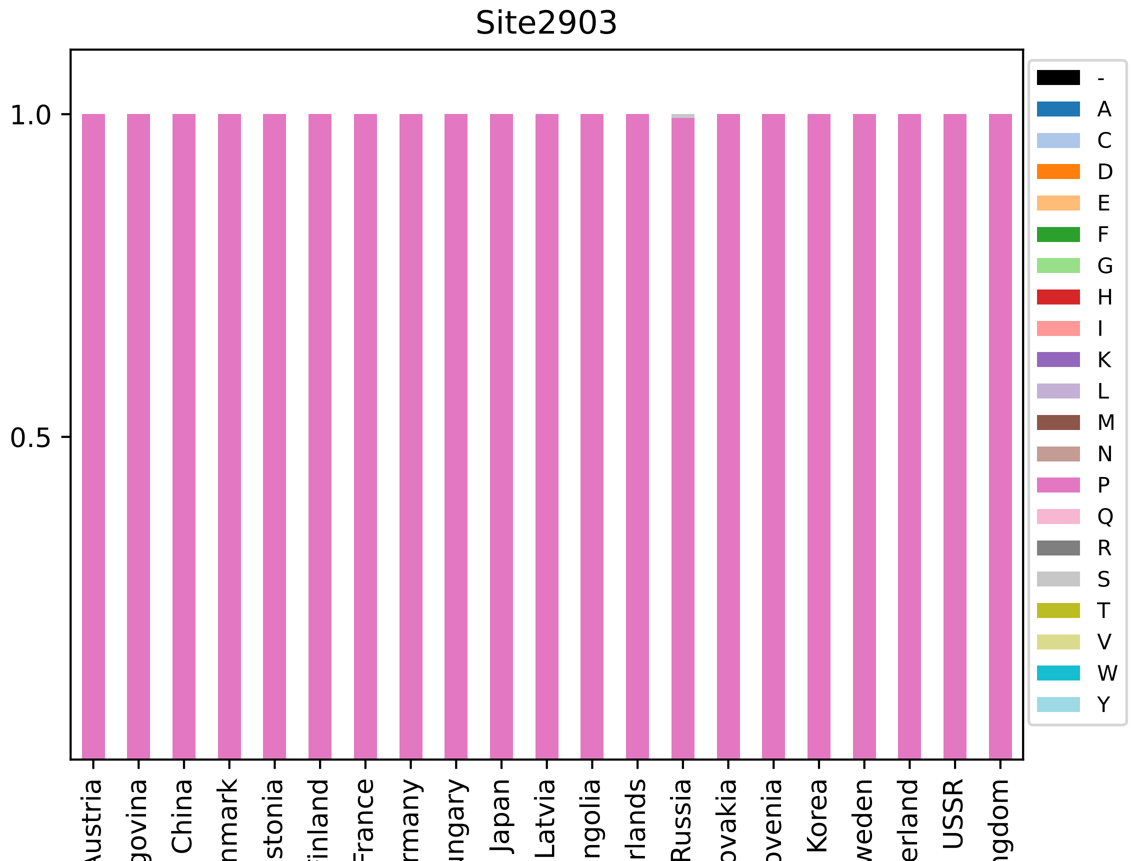

Site2903
1.0
0.5
-
A
C
D
E
F
G
H
I
K
L
M
N
P
Q
R
S
T
V
W
Y
Austria
China
Japan
Latvia
Russia
Korea
USSR

Site2904

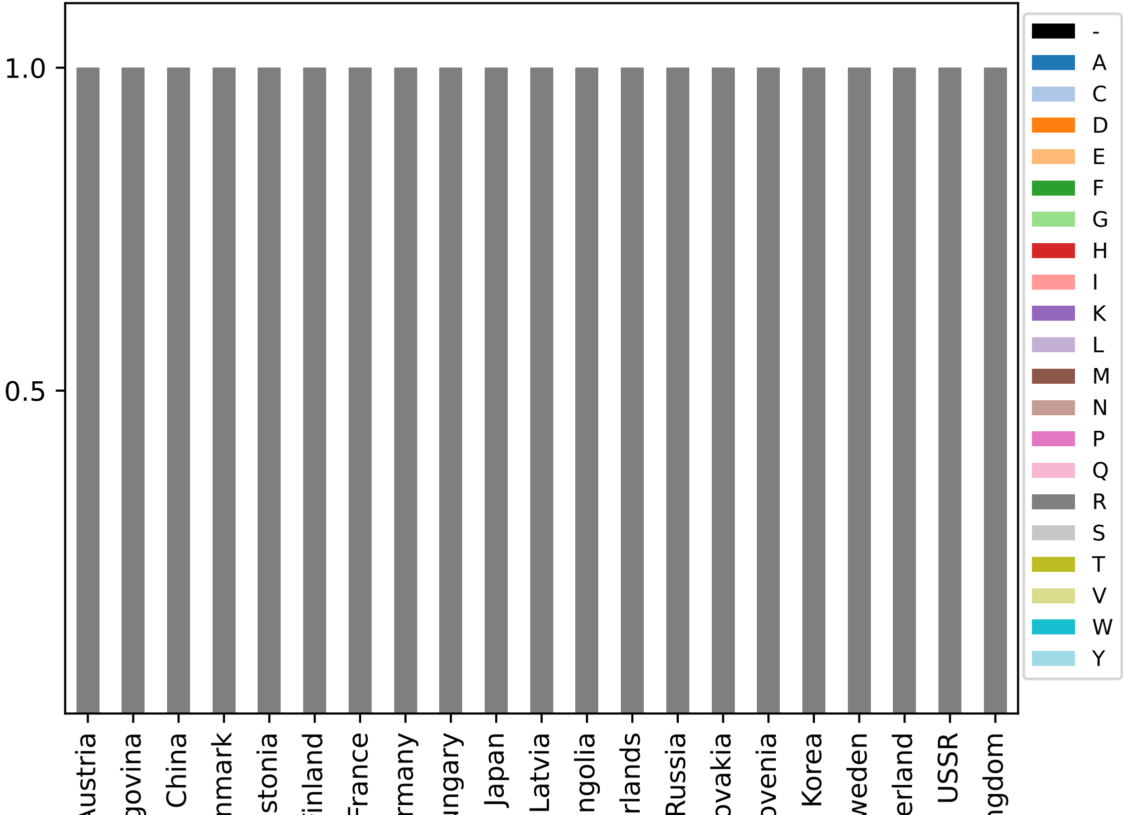

1.0
0.5
-
A
C
D
E
F
G
H
I
K
L
M
N
P
Q
R
S
T
V
W
Y
China
Japan
Latvia
Russia
Korea
USSR

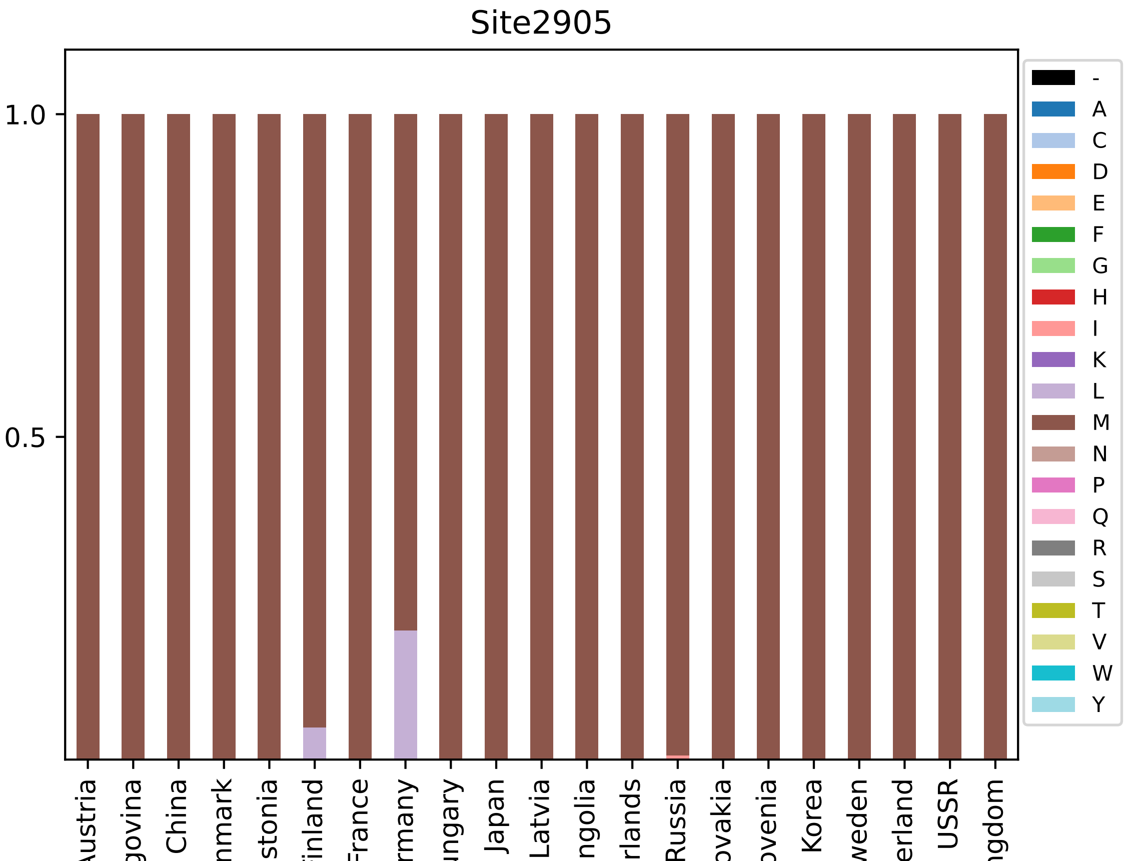
Site2905
1.0
0.5
-
A
C
D
E
F
G
H
I
K
L
M
N
P
Q
R
S
T
V
W
Y
Austria
China
Finland
France
Japan
Latvia
Russia
Korea
USSR

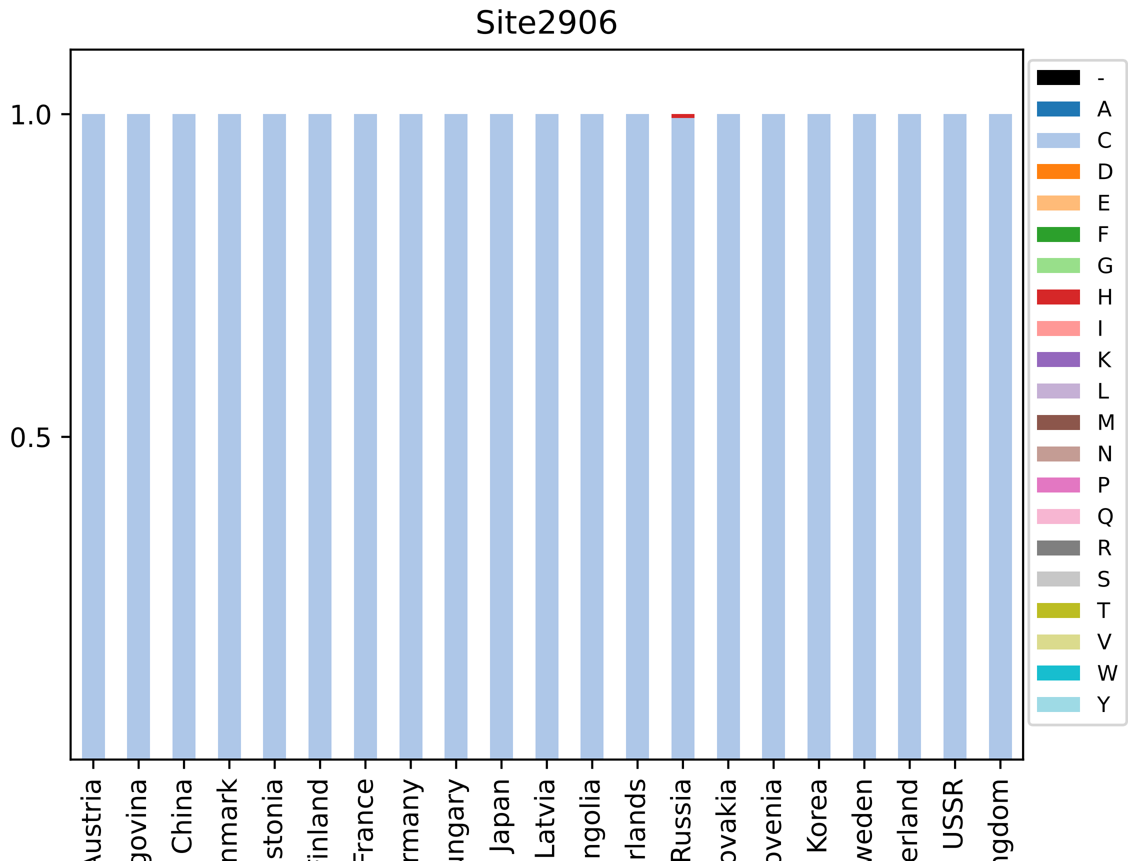

Site2906
1.0
0.5
-
A
C
D
E
F
G
H
I
K
L
M
N
P
Q
R
S
T
V
W
Y
Austria
govina
China
nmark
stonia
Finland
France
rmany
ungary
Japan
Latvia
ngolia
rlands
Russia
ovakia
ovenia
Korea
weden
erland
USSR
ngdom

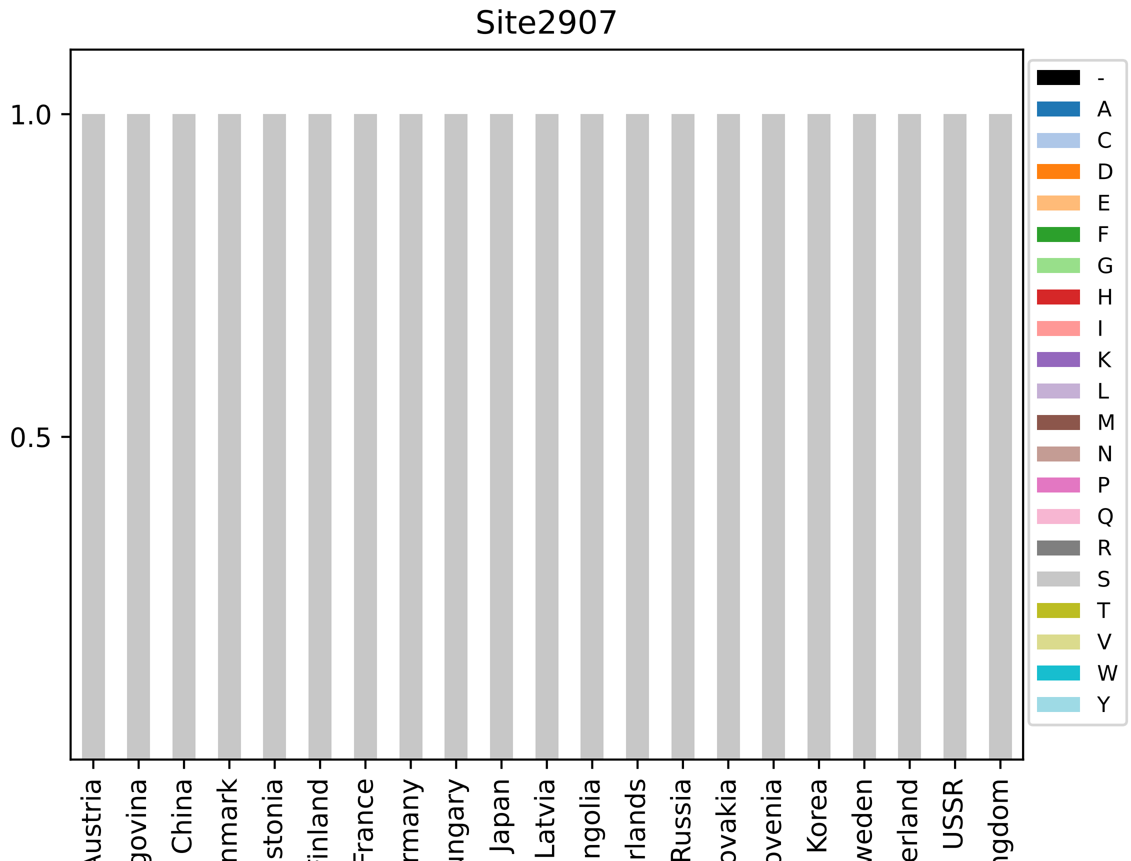
Site2907
1.0
0.5
-
A
C
D
E
F
G
H
I
K
L
M
N
P
Q
R
S
T
V
W
Y
Austria
govina
China
nmark
stonia
inland
France
rmany
ungary
Japan
Latvia
ngolia
rlands
Russia
ovakia
ovenia
Korea
weden
erland
USSR
ngdom

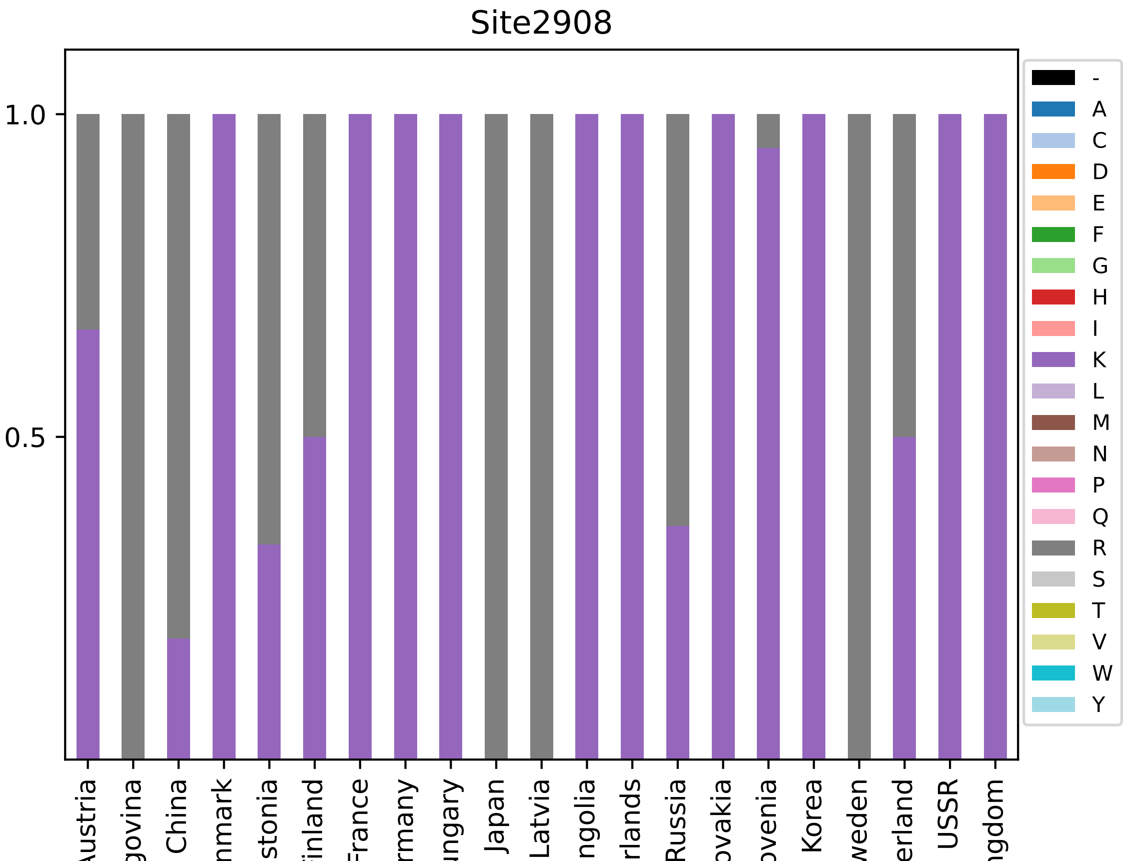
Site2908
1.0
0.5
-
A
C
D
E
F
G
H
I
K
L
M
N
P
Q
R
S
T
V
W
Y
China
Japan
Latvia
Russia
Korea
USSR

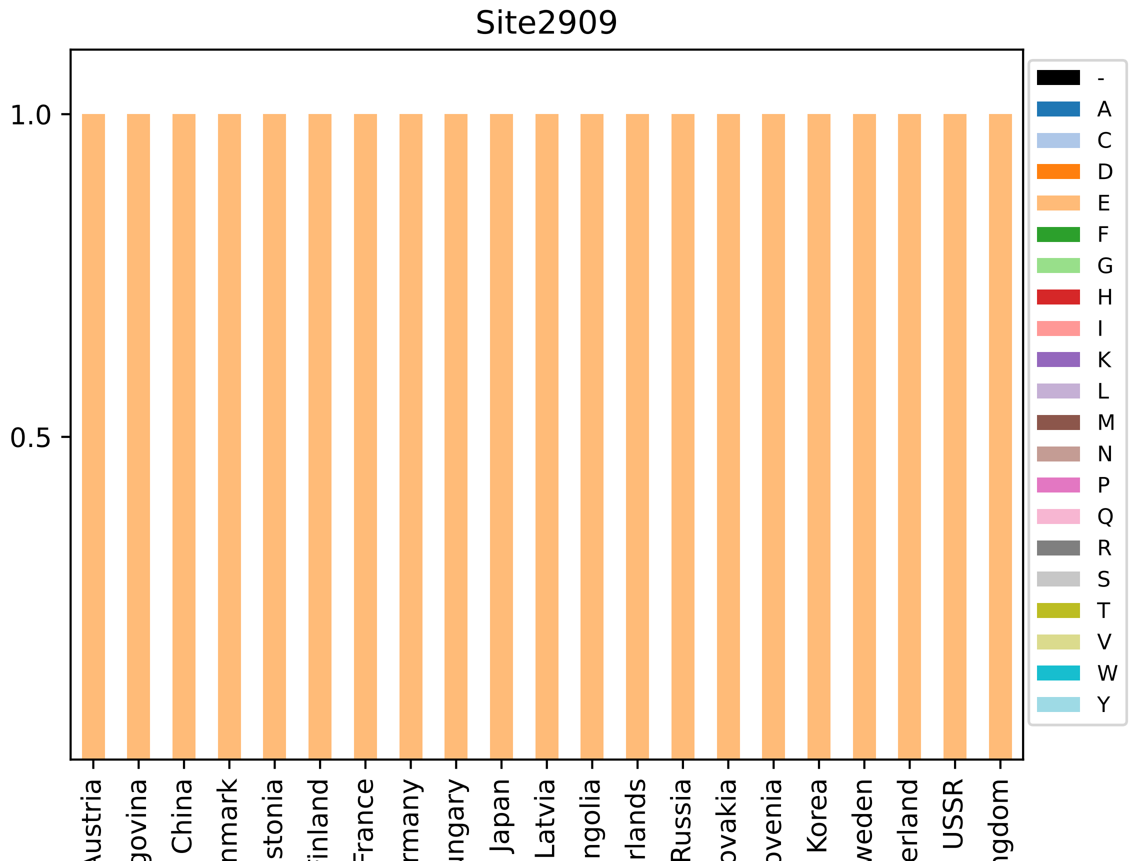

Site2909
1.0
0.5
-
A
C
D
E
F
G
H
I
K
L
M
N
P
Q
R
S
T
V
W
Y
China
Japan
Latvia
Russia
Korea
USSR

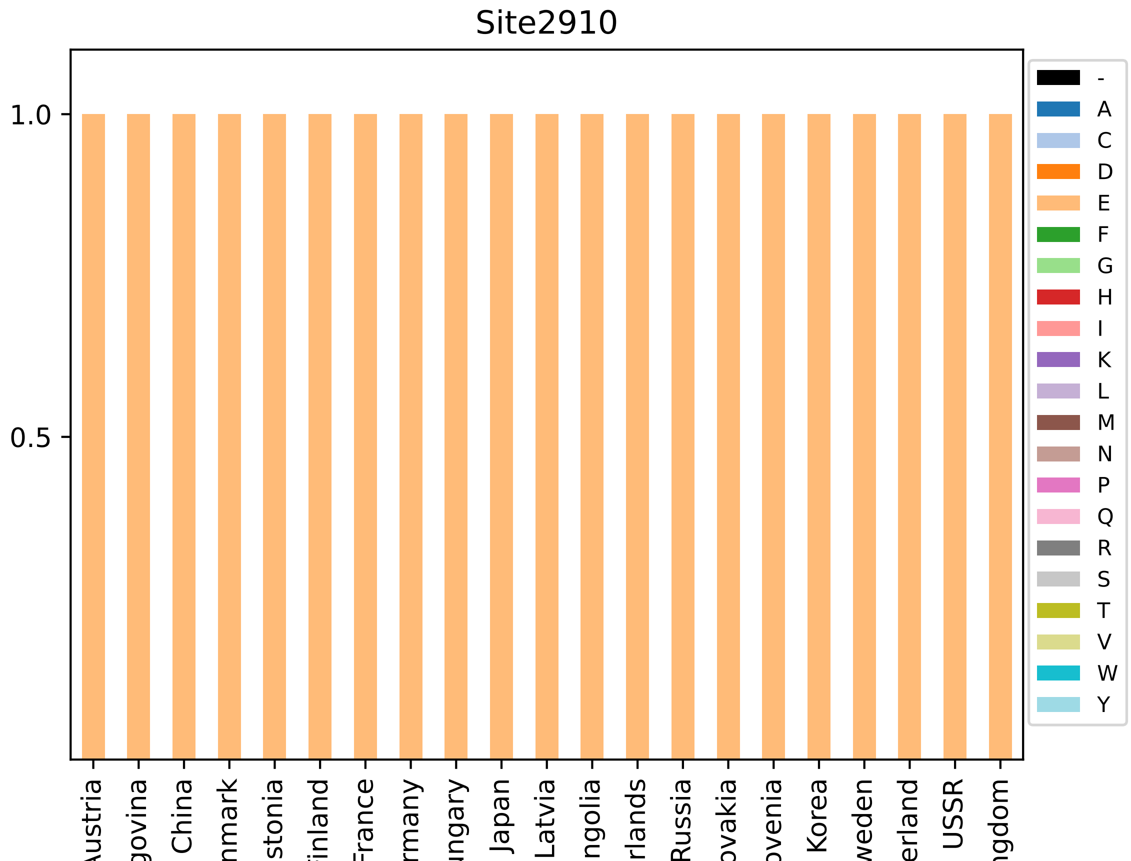

Site2910
1.0
0.5
-
A
C
D
E
F
G
H
I
K
L
M
N
P
Q
R
S
T
V
W
Y
China
Japan
Latvia
Russia
Korea
USSR

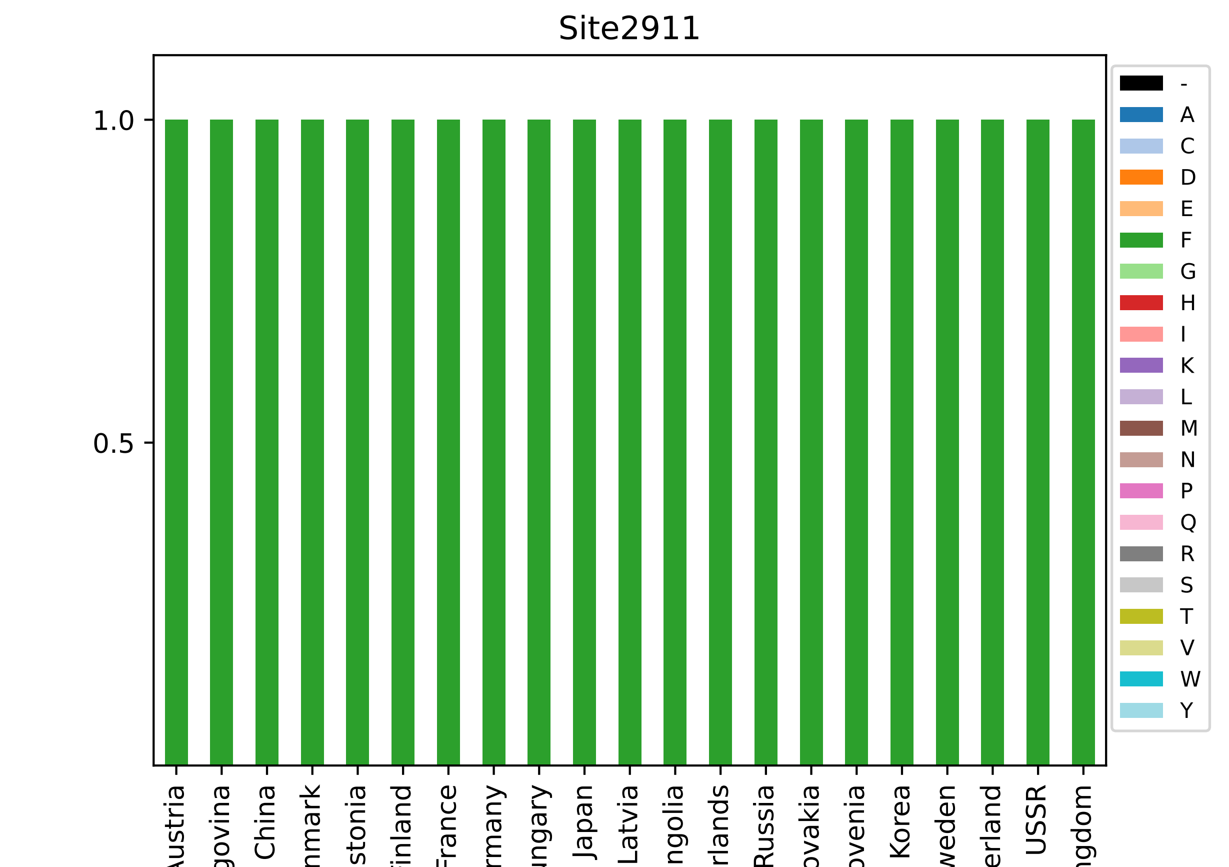
Site2911
1.0
0.5
-
A
C
D
E
F
G
H
I
K
L
M
N
P
Q
R
S
T
V
W
Y
Austria
govina
China
nmark
stonia
inland
France
rmany
ungary
Japan
Latvia
ngolia
rlands
Russia
ovakia
ovenia
Korea
weden
erland
USSR
ngdom

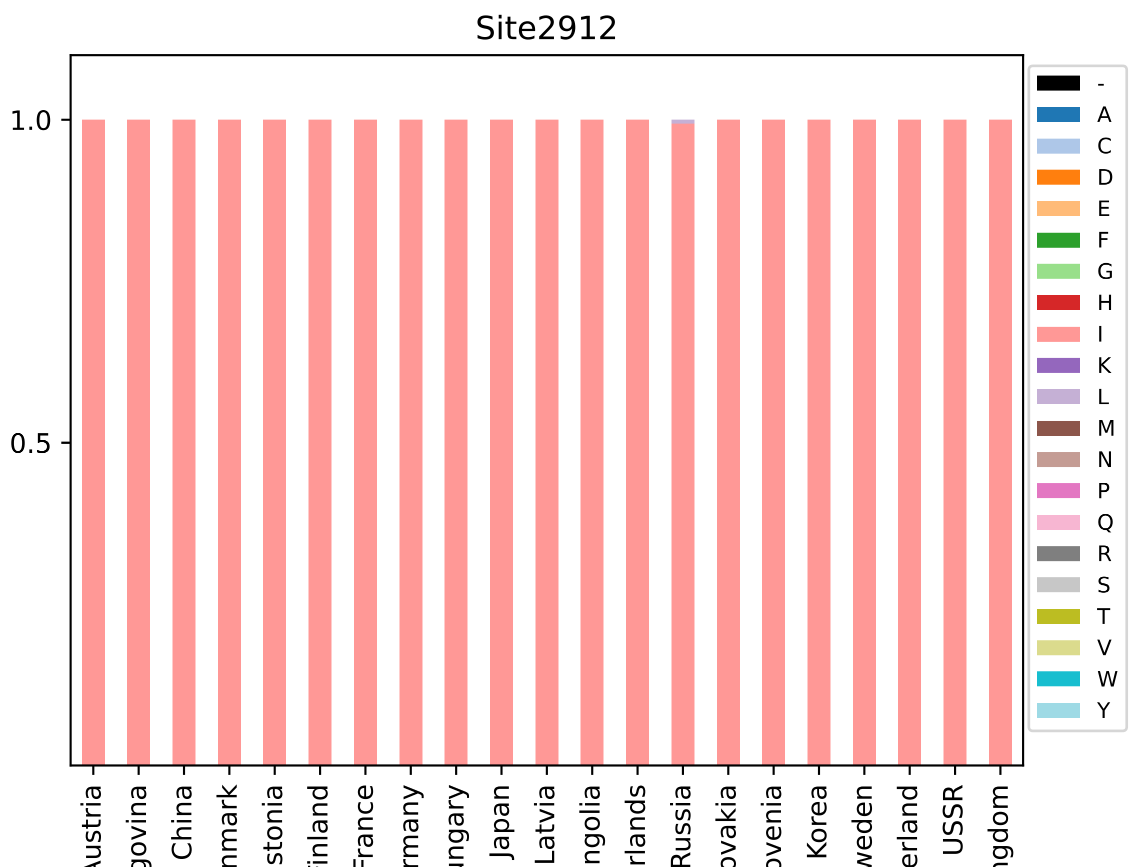
Site2912
1.0
0.5
-
A
C
D
E
F
G
H
I
K
L
M
N
P
Q
R
S
T
V
W
Y
China
Japan
Latvia
Russia
Korea
USSR

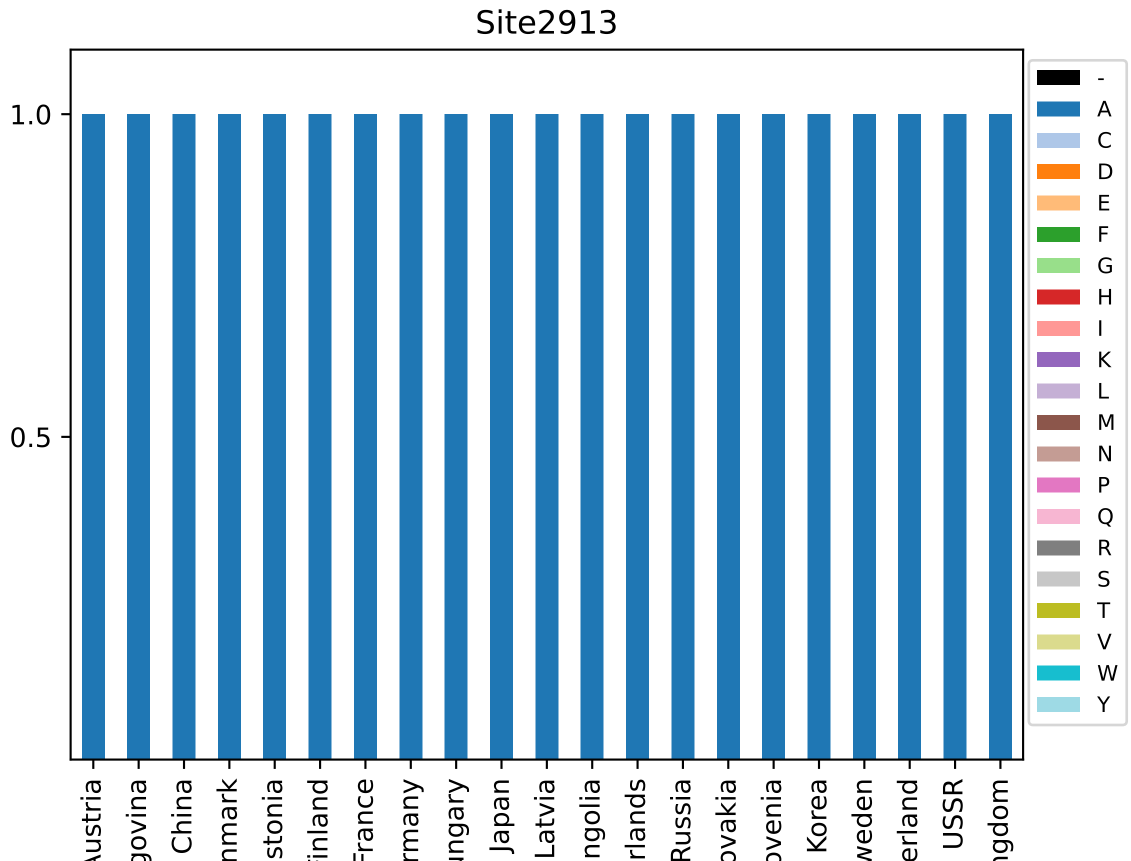
Site2913
1.0
0.5
China
Japan
Latvia
Russia
Korea
USSR
-
A
C
D
E
F
G
H
I
K
L
M
N
P
Q
R
S
T
V
W
Y

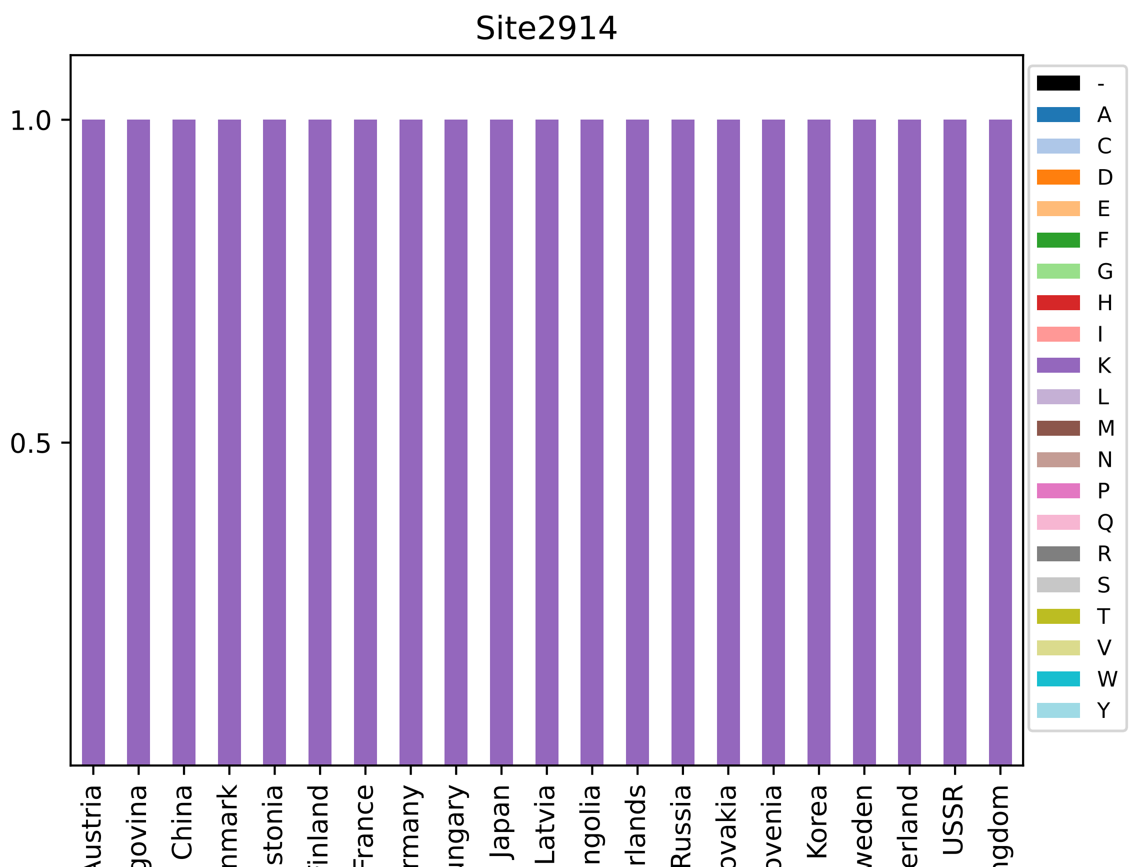

Site2914
1.0
0.5
-
A
C
D
E
F
G
H
I
K
L
M
N
P
Q
R
S
T
V
W
Y
Austria
China
Japan
Latvia
Russia
Korea
USSR

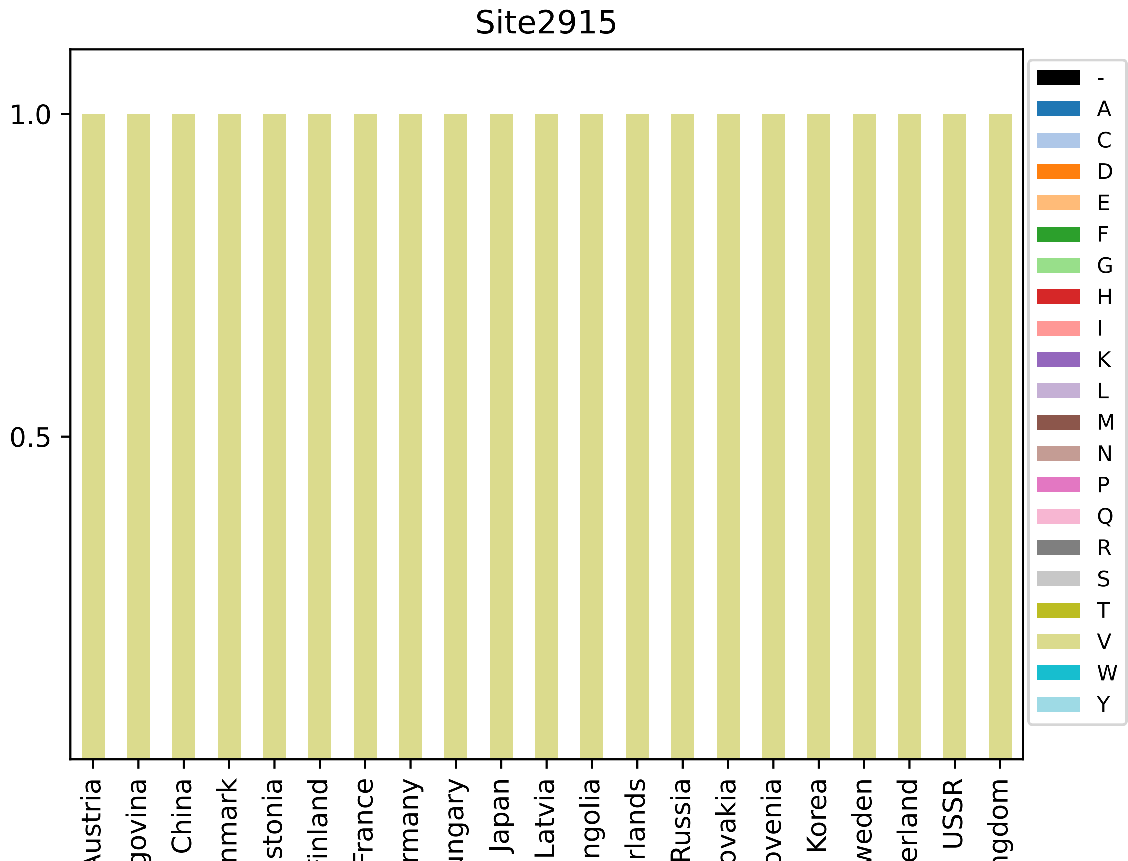
Site2915
1.0
0.5
-
A
C
D
E
F
G
H
I
K
L
M
N
P
Q
R
S
T
V
W
Y
China
Japan
Latvia
Russia
Korea
USSR

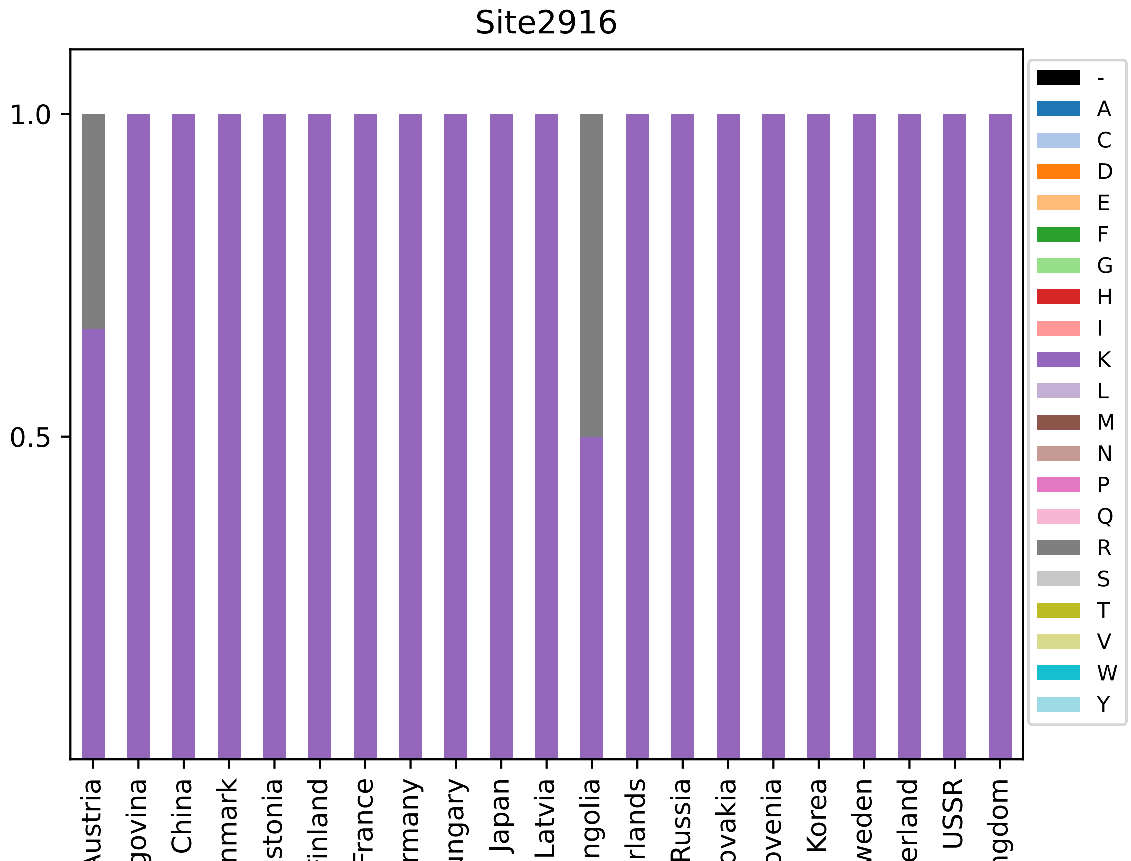

Site2916
1.0
0.5
-
A
C
D
E
F
G
H
I
K
L
M
N
P
Q
R
S
T
V
W
Y
China
Japan
Latvia
Russia
Korea
USSR

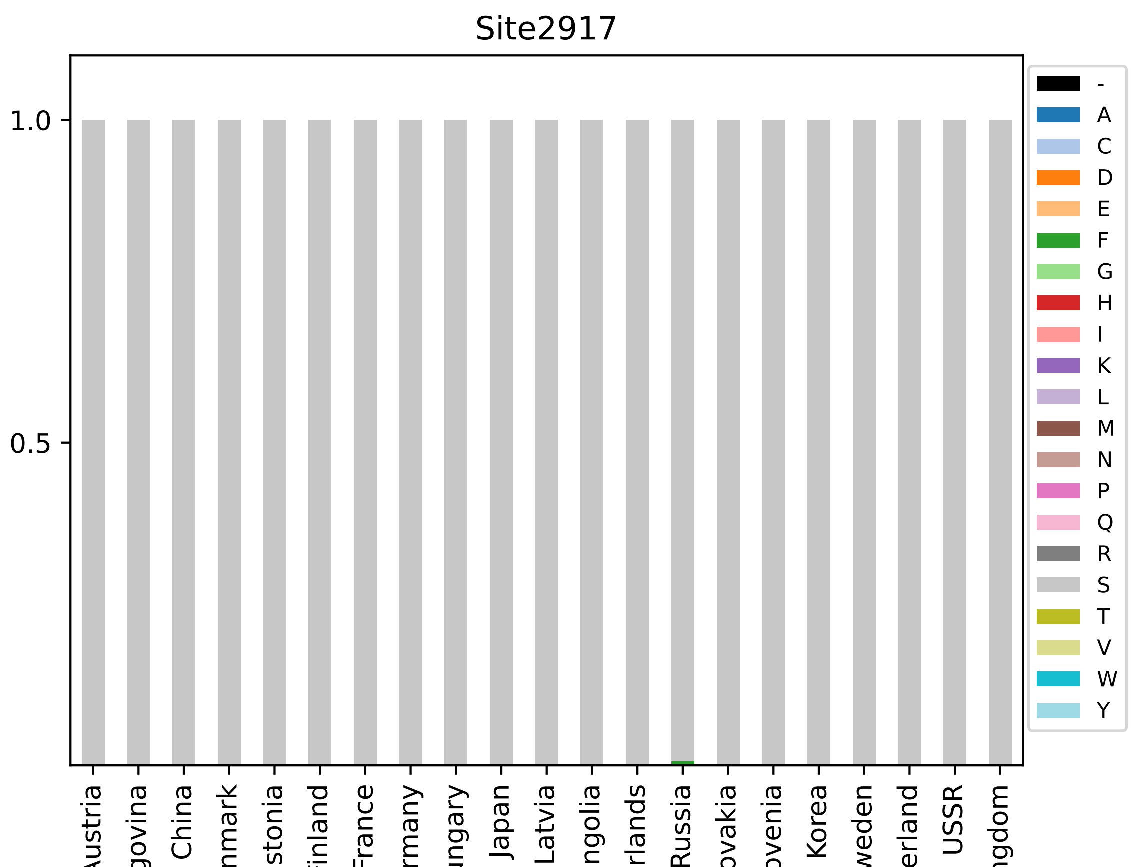

Site2917
1.0
0.5
Austria
govina
China
nmark
stonia
Finland
France
rmany
ungary
Japan
Latvia
ngolia
rlands
Russia
ovakia
ovenia
Korea
weden
erland
USSR
ngdom
-
A
C
D
E
F
G
H
I
K
L
M
N
P
Q
R
S
T
V
W
Y

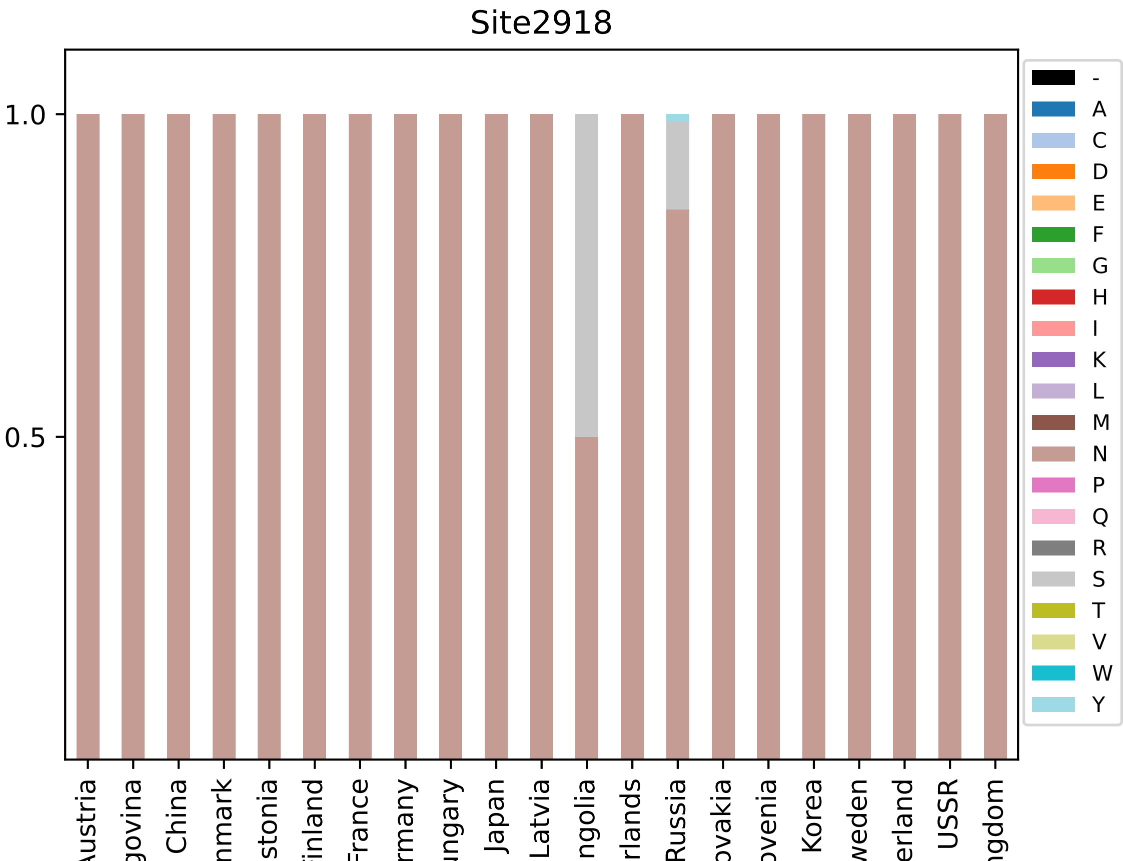

Site2918
1.0
0.5
-
A
C
D
E
F
G
H
I
K
L
M
N
P
Q
R
S
T
V
W
Y
China
Japan
Latvia
Russia
Korea
USSR

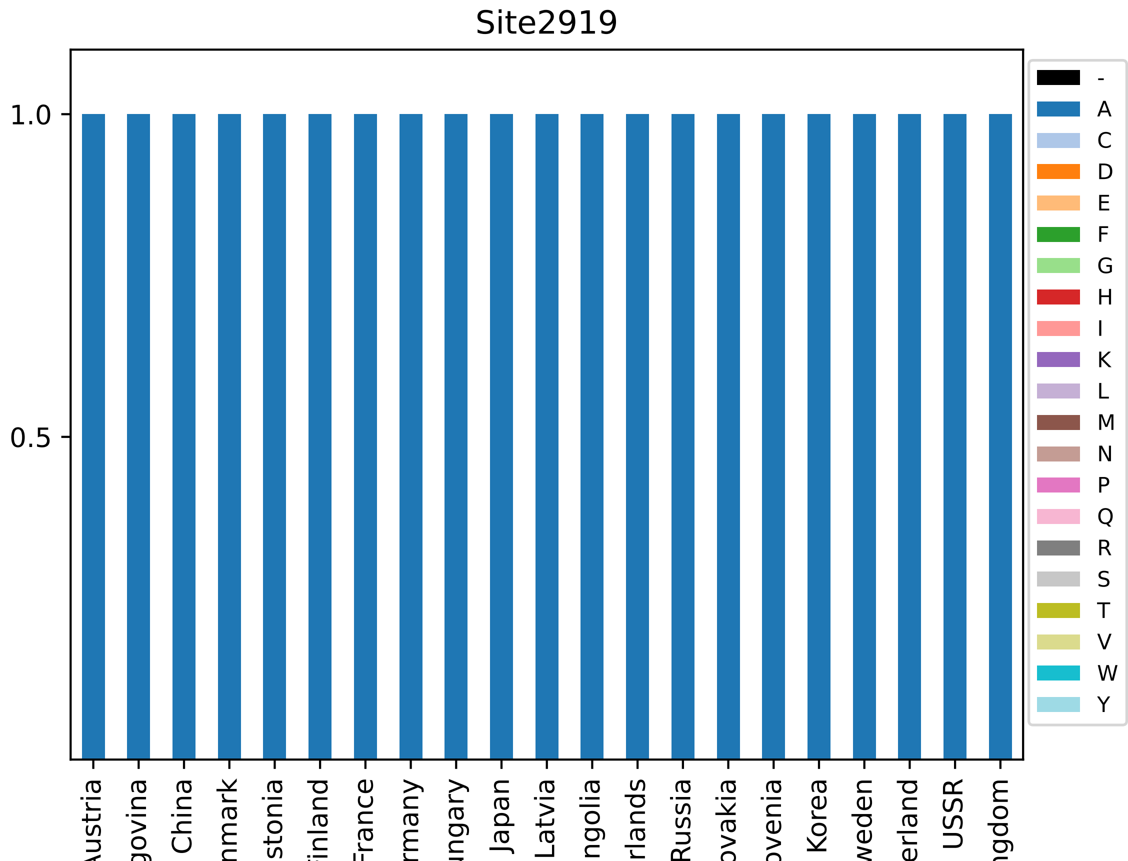

Site2919
1.0
0.5
-
A
C
D
E
F
G
H
I
K
L
M
N
P
Q
R
S
T
V
W
Y
Austria
govina
China
nmark
stonia
Finland
France
rmany
ungary
Japan
Latvia
ngolia
rlands
Russia
ovakia
ovenia
Korea
weden
erland
USSR
ngdom

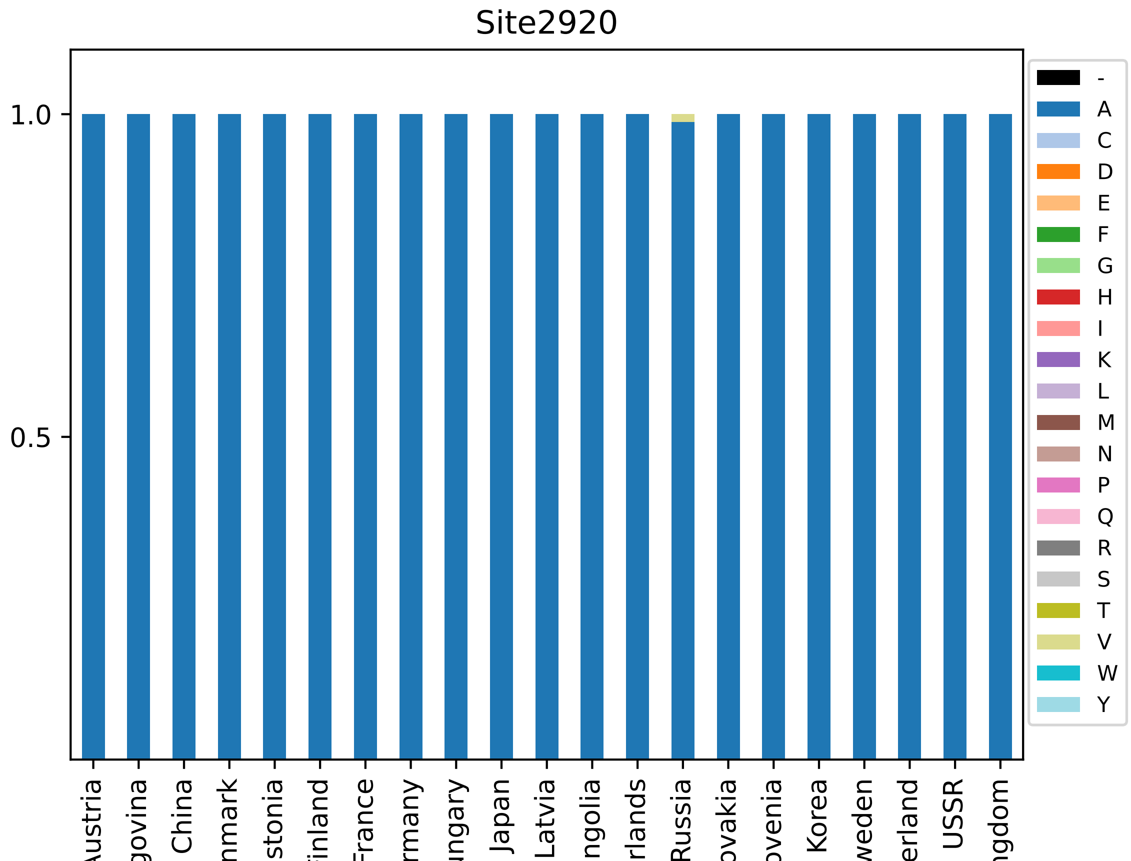
Site2920
1.0
0.5
-
A
C
D
E
F
G
H
I
K
L
M
N
P
Q
R
S
T
V
W
Y
China
Japan
Latvia
Russia
Korea
USSR

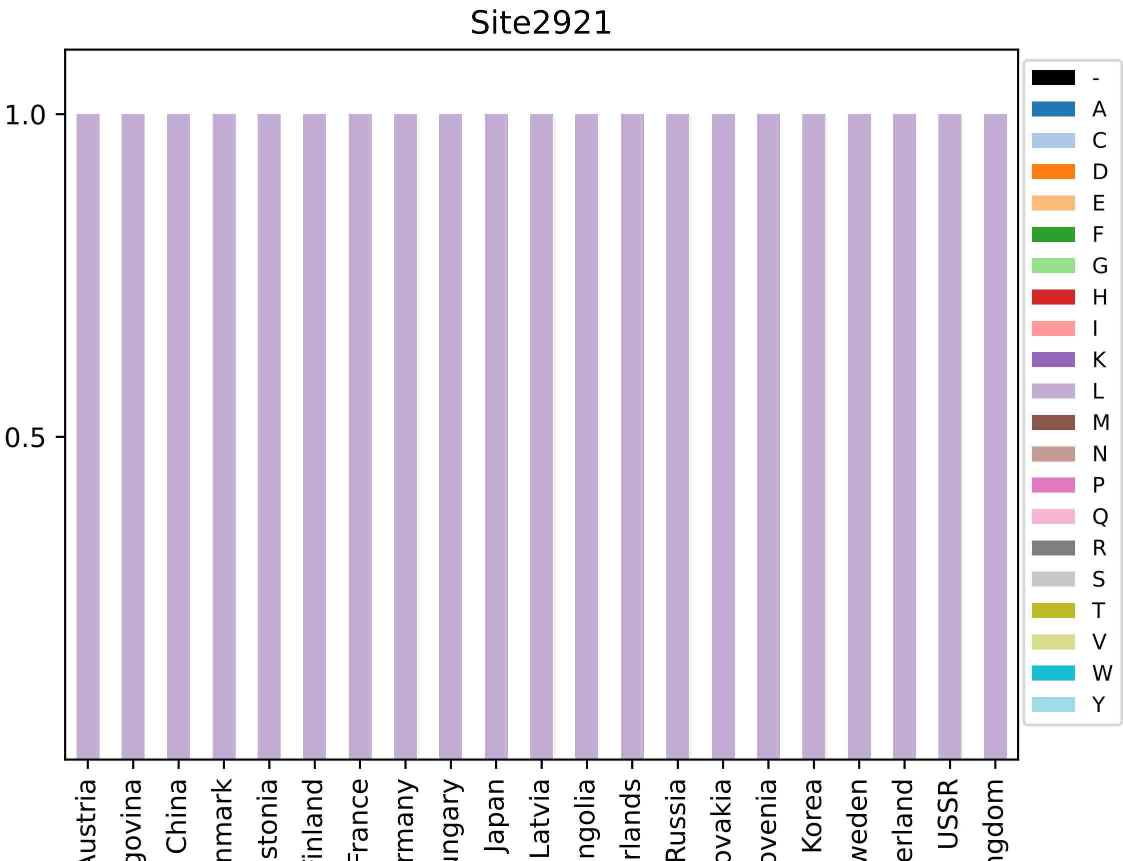

Site2921
1.0
0.5
Austria
govina
China
nmark
stonia
Finland
France
rmany
ungary
Japan
Latvia
ngolia
rlands
Russia
ovakia
ovenia
Korea
weden
erland
USSR
ngdom
-
A
C
D
E
F
G
H
I
K
L
M
N
P
Q
R
S
T
V
W
Y

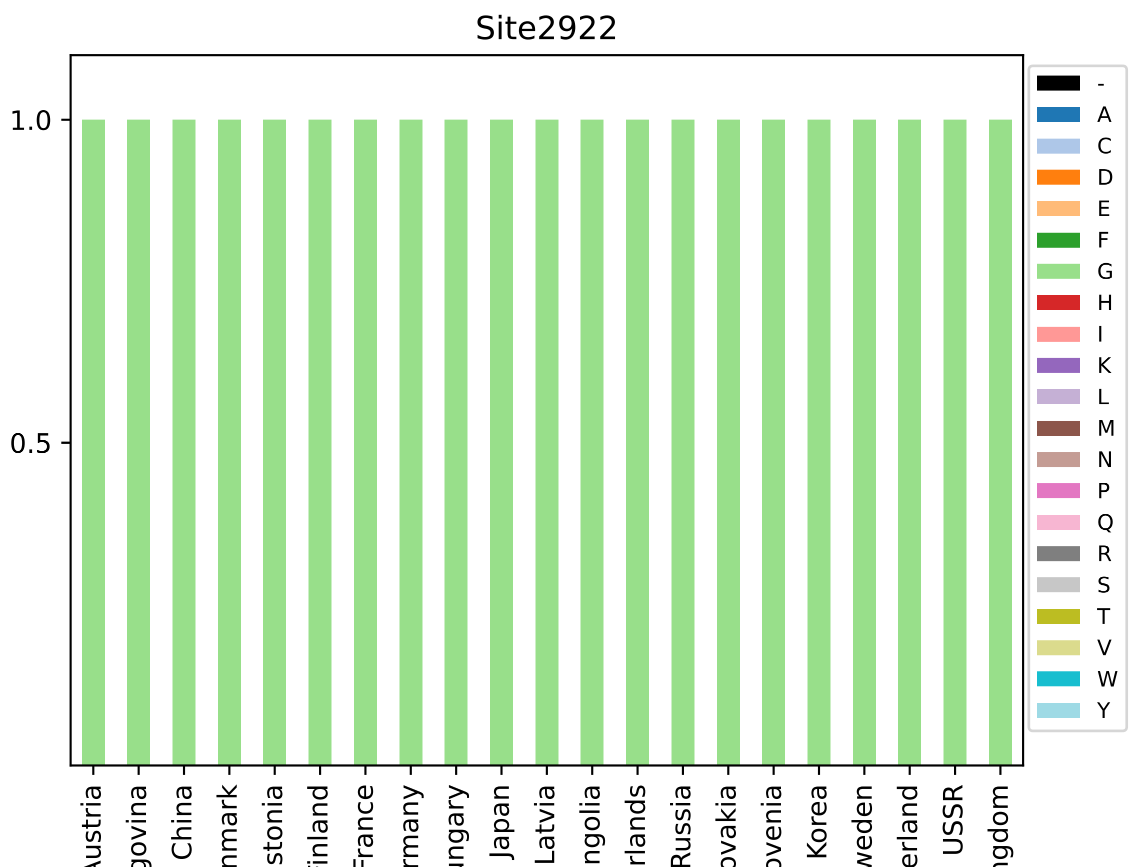

Site2922
1.0
0.5
-
A
C
D
E
F
G
H
I
K
L
M
N
P
Q
R
S
T
V
W
Y
Austria
China
Japan
Latvia
Russia
Korea
USSR

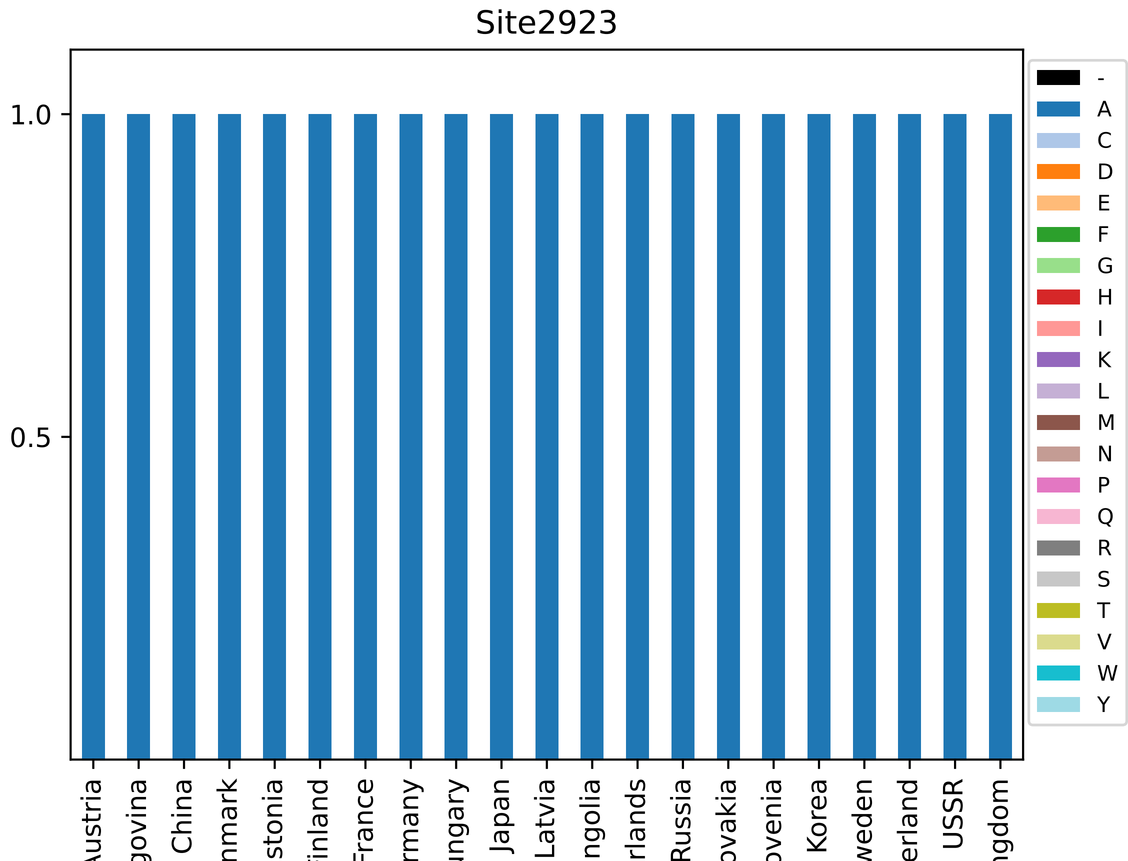
Site2923
1.0
0.5
-
A
C
D
E
F
G
H
I
K
L
M
N
P
Q
R
S
T
V
W
Y
China
Japan
Latvia
Russia
Korea
USSR

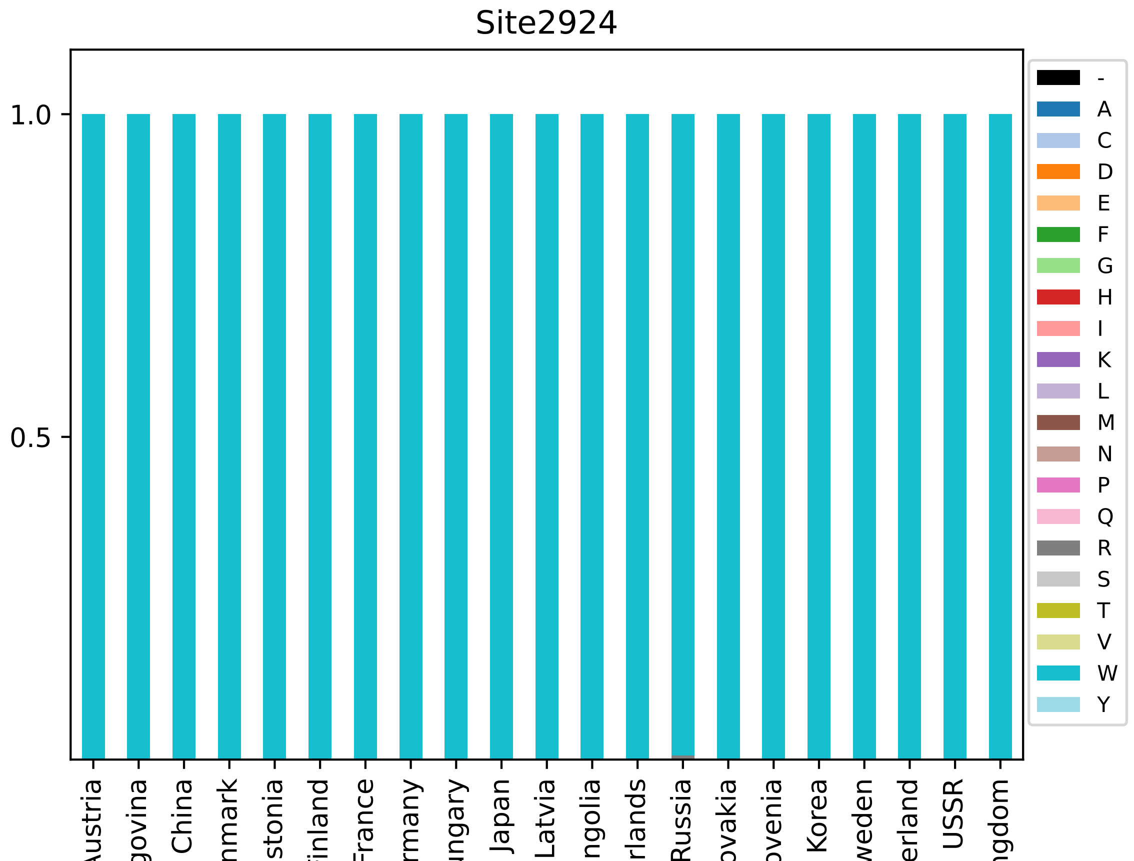
Site2924
1.0
0.5
-
A
C
D
E
F
G
H
I
K
L
M
N
P
Q
R
S
T
V
W
Y
China
Japan
Latvia
Russia
Korea
USSR

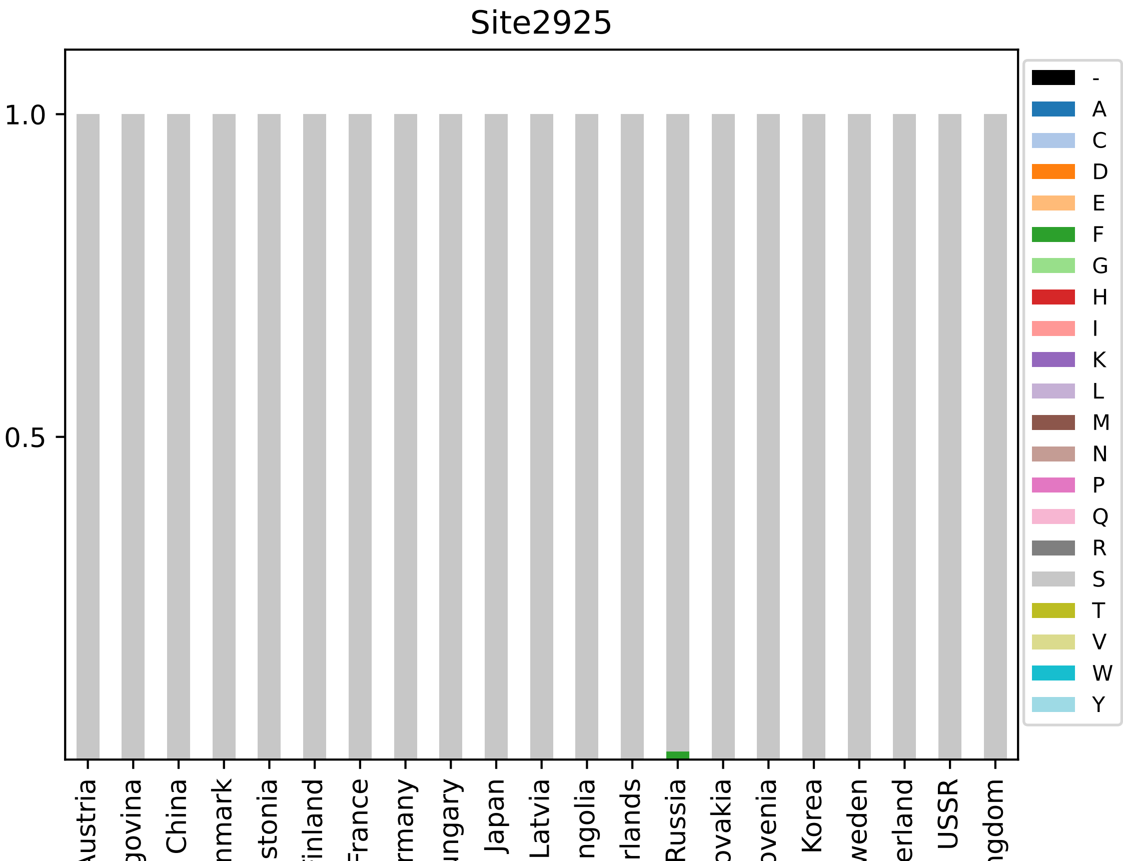

Site2925
1.0
0.5
-
A
C
D
E
F
G
H
I
K
L
M
N
P
Q
R
S
T
V
W
Y
Austria
govina
China
nmark
stonia
inland
France
rmany
ungary
Japan
Latvia
ngolia
rlands
Russia
ovakia
ovenia
Korea
weden
erland
USSR
ngdom

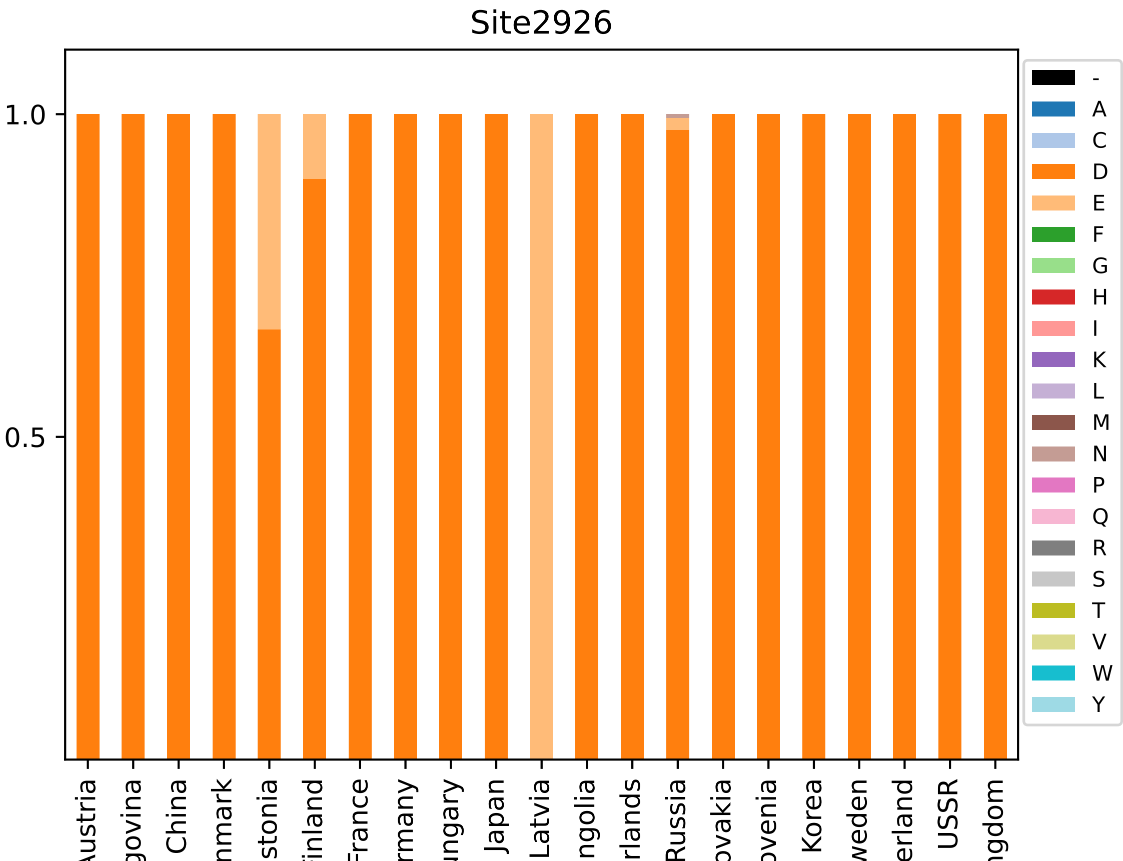

Site2926
1.0
0.5
-
A
C
D
E
F
G
H
I
K
L
M
N
P
Q
R
S
T
V
W
Y
Austria
China
Japan
Latvia
Russia
Korea
USSR

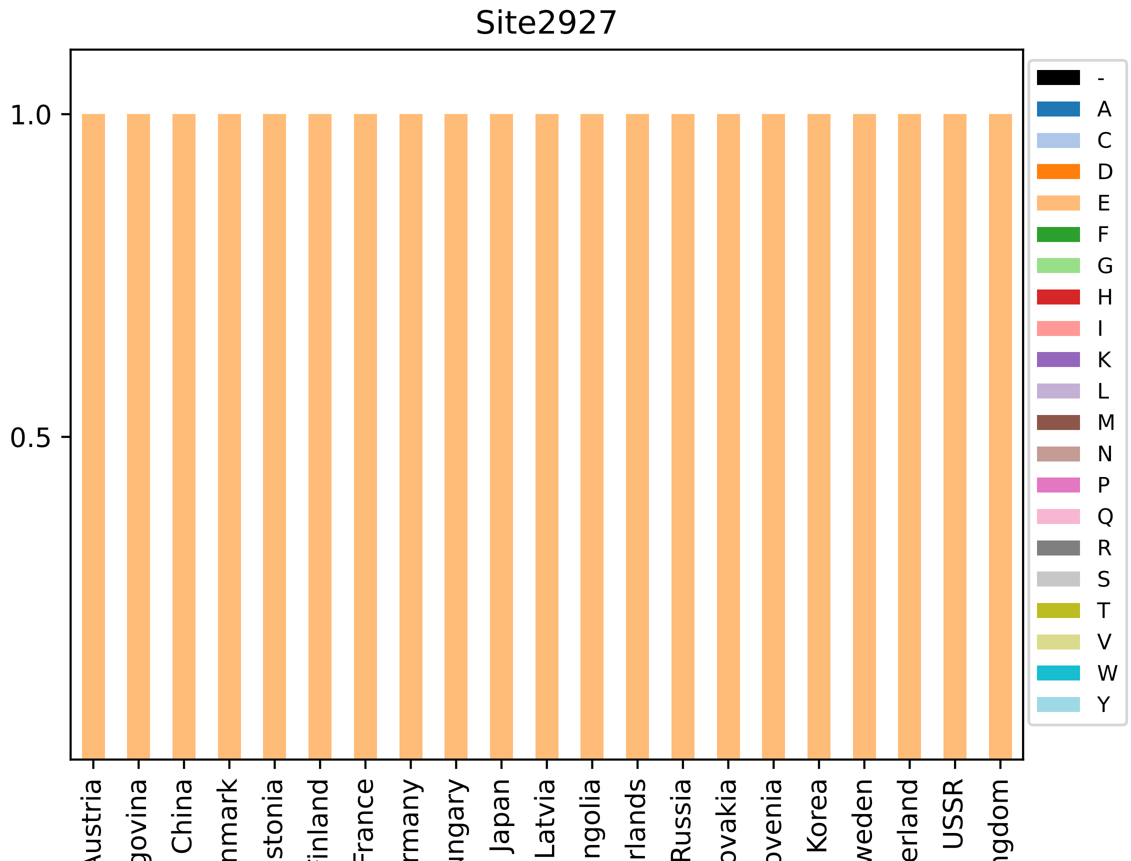

Site2927
1.0
0.5
-
A
C
D
E
F
G
H
I
K
L
M
N
P
Q
R
S
T
V
W
Y
ustria
govina
China
nmark
stonia
inland
rance
rmany
ungary
Japan
Latvia
ngolia
rlands
Russia
ovakia
ovenia
Korea
weden
erland
USSR
ngdom

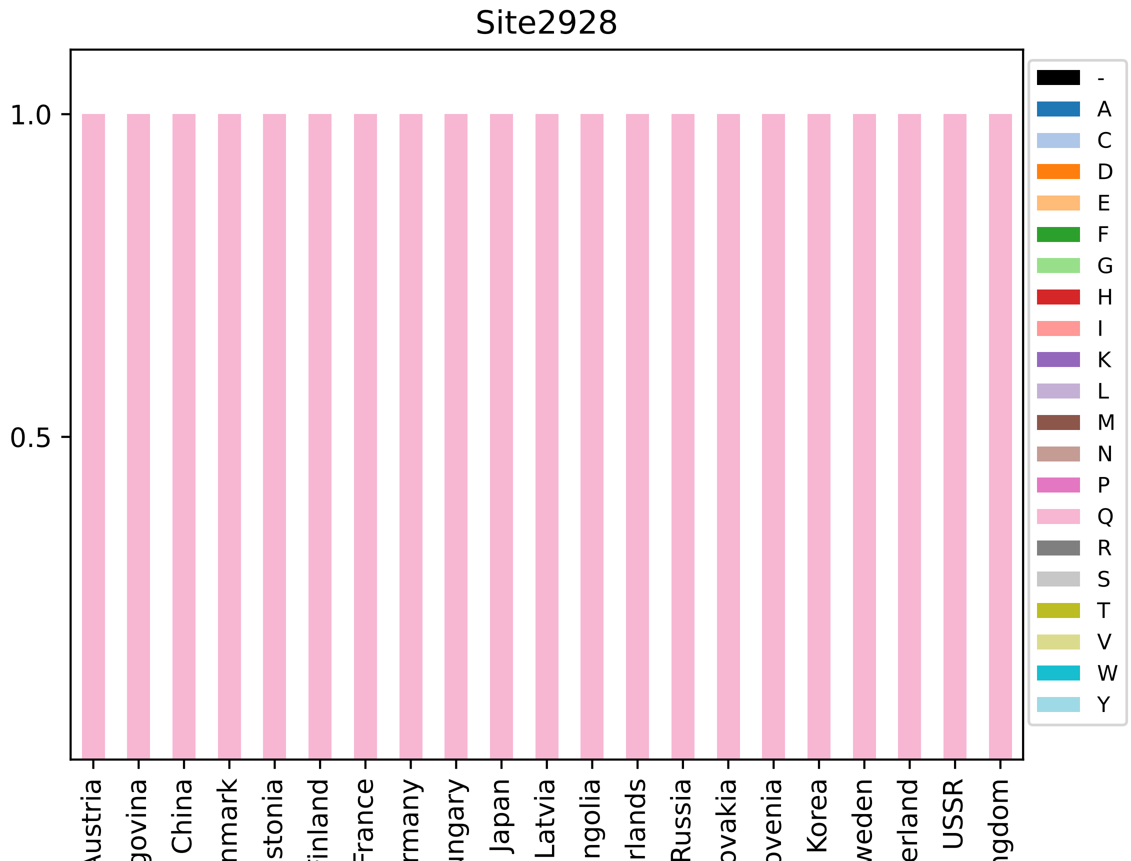
Site2928
1.0
0.5
-
A
C
D
E
F
G
H
I
K
L
M
N
P
Q
R
S
T
V
W
Y
China
Japan
Latvia
Russia
Korea
USSR

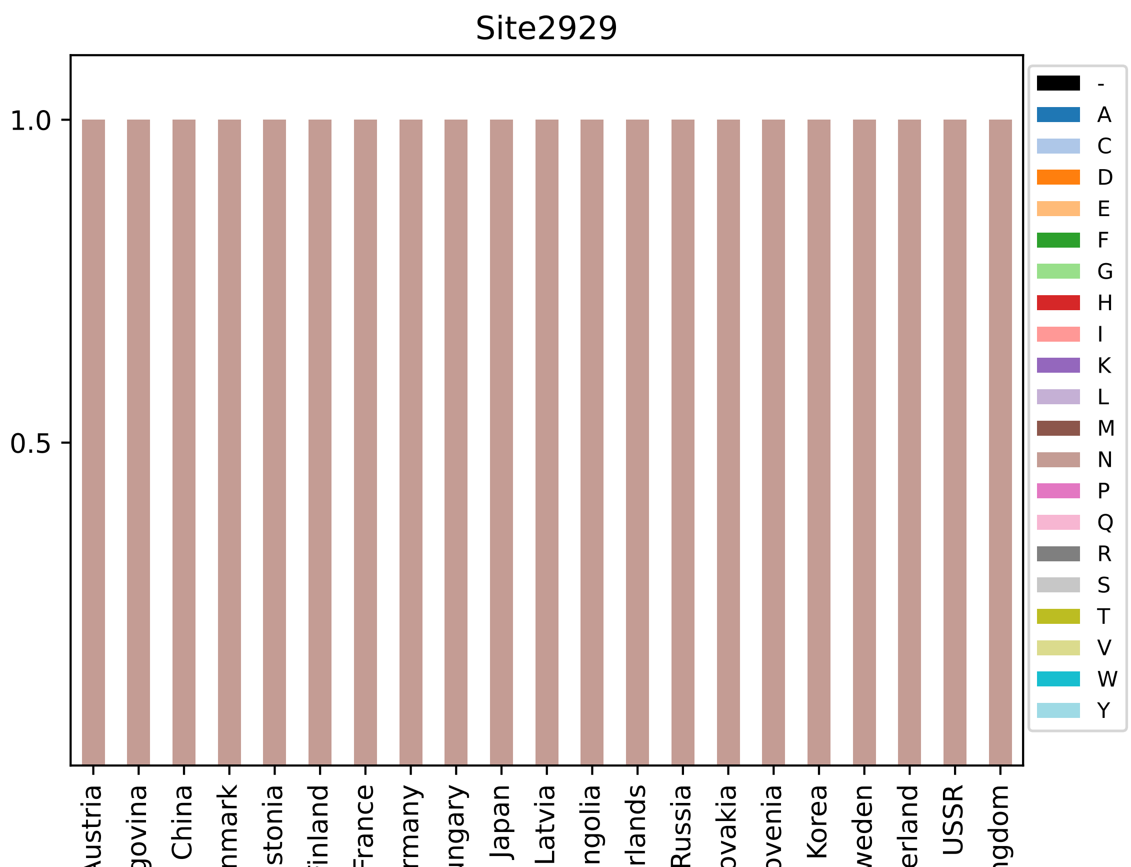

Site2929
1.0
0.5
-
A
C
D
E
F
G
H
I
K
L
M
N
P
Q
R
S
T
V
W
Y
China
Japan
Latvia
Russia
Korea
USSR

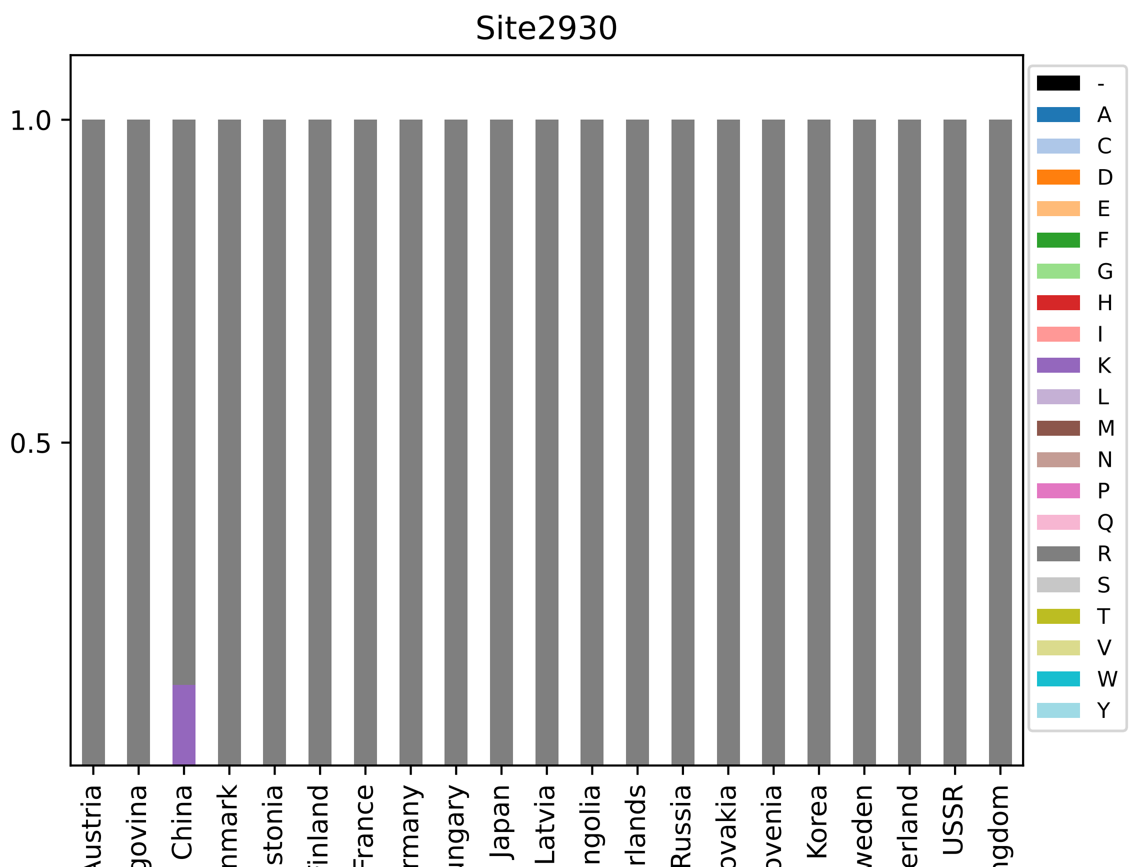
Site2930
1.0
0.5
-
A
C
D
E
F
G
H
I
K
L
M
N
P
Q
R
S
T
V
W
Y
Austria
govina
China
nmark
stonia
Finland
France
rmany
ungary
Japan
Latvia
ngolia
rlands
Russia
ovakia
ovenia
Korea
weden
erland
USSR
ngdom

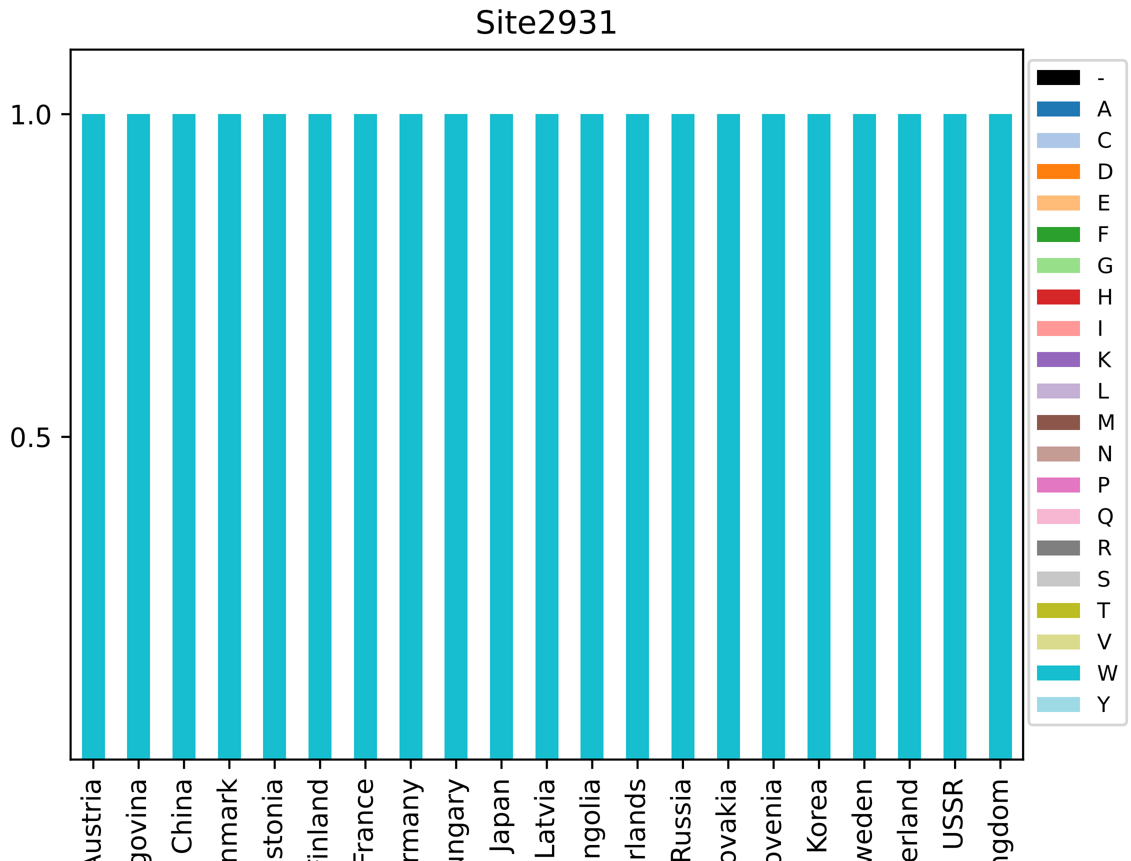

Site2931
1.0
0.5
-
A
C
D
E
F
G
H
I
K
L
M
N
P
Q
R
S
T
V
W
Y
Austria
China
Japan
Latvia
Russia
Korea
USSR

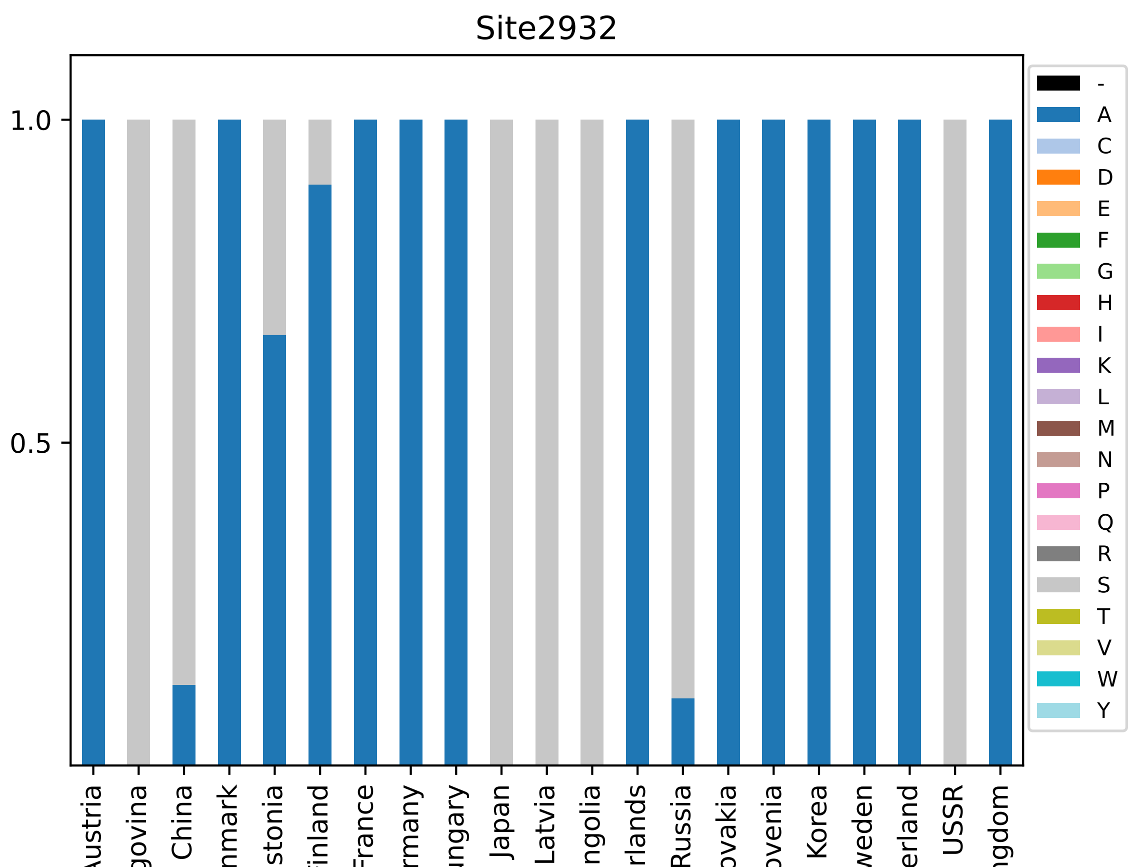

Site2932
1.0
0.5
-
A
C
D
E
F
G
H
I
K
L
M
N
P
Q
R
S
T
V
W
Y
Austria
govina
China
nmark
stonia
inland
France
rmany
ungary
Japan
Latvia
ngolia
rlands
Russia
ovakia
ovenia
Korea
weden
erland
USSR
ngdom

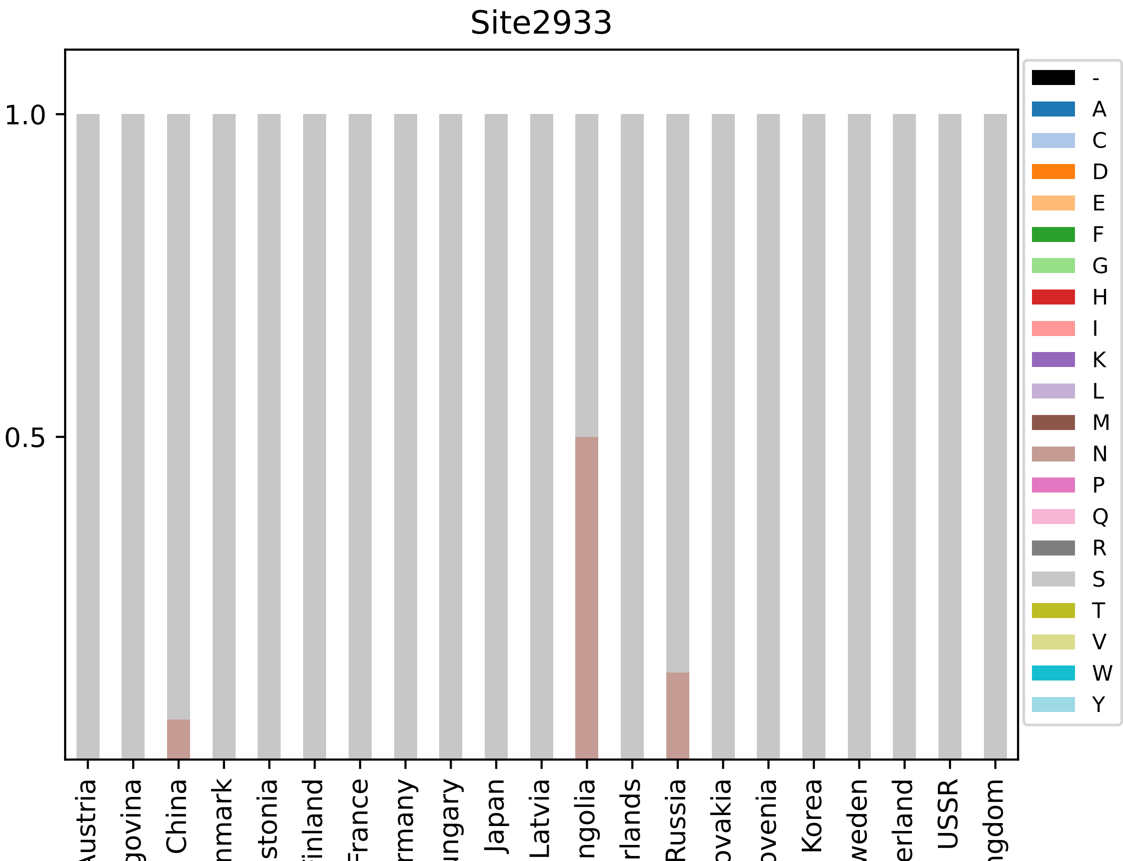

Site2933
1.0
0.5
-
A
C
D
E
F
G
H
I
K
L
M
N
P
Q
R
S
T
V
W
Y
Austria
govina
China
nmark
stonia
Finland
France
rmany
ungary
Japan
Latvia
ngolia
rlands
Russia
ovakia
ovenia
Korea
weden
erland
USSR
ngdom

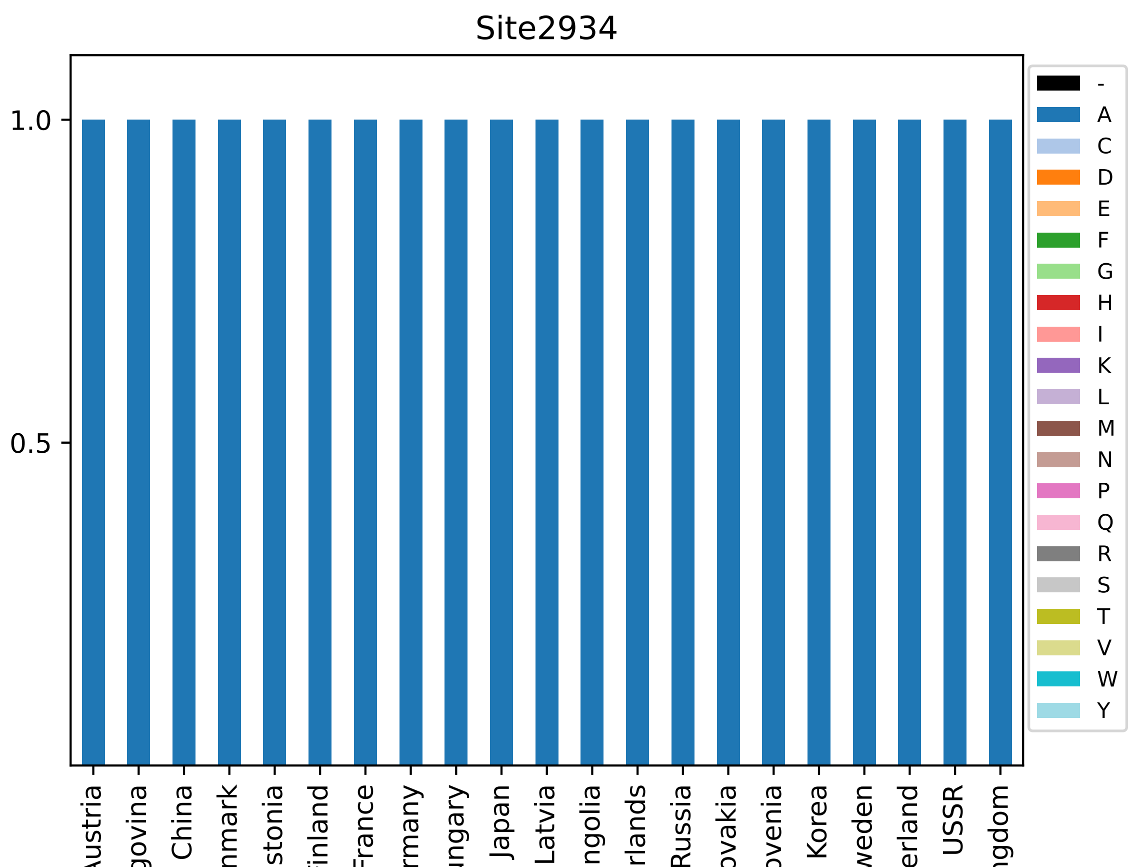
Site2934
1.0
0.5
-
A
C
D
E
F
G
H
I
K
L
M
N
P
Q
R
S
T
V
W
Y
China
Japan
Latvia
Russia
Korea
USSR

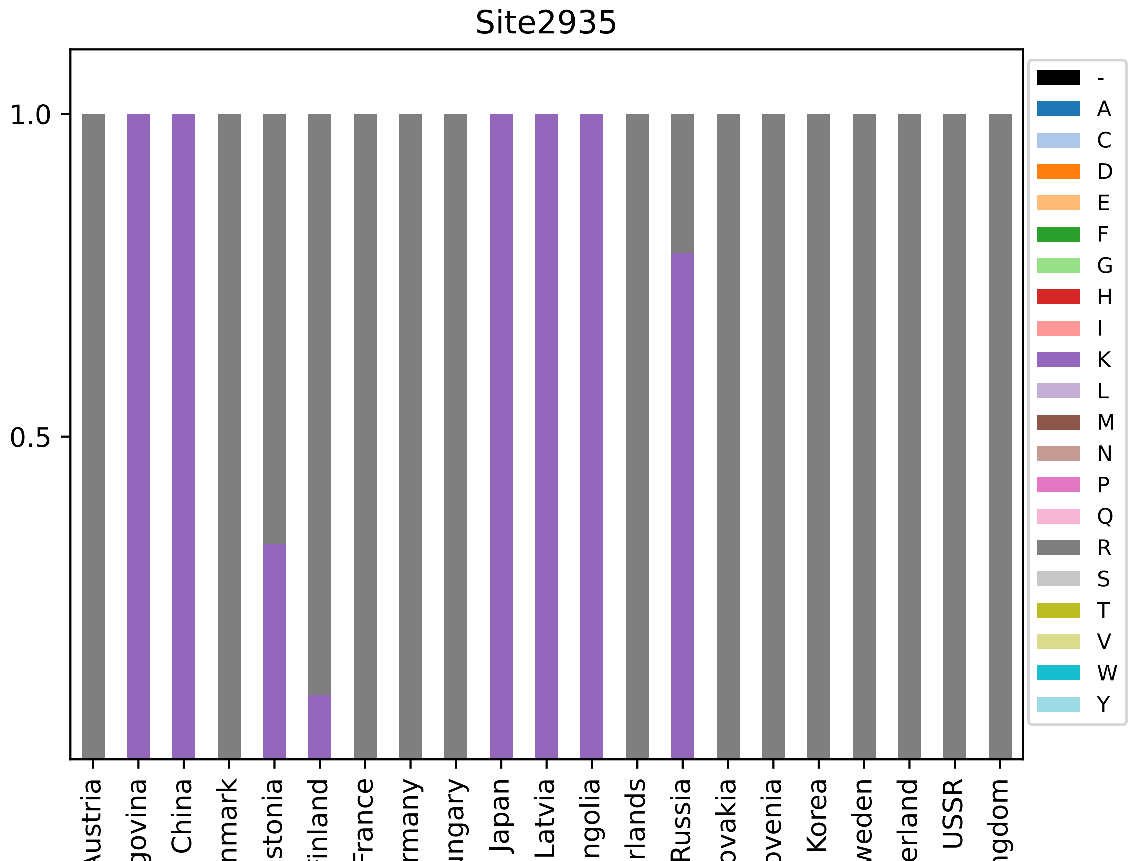

Site2935
1.0
0.5
-
A
C
D
E
F
G
H
I
K
L
M
N
P
Q
R
S
T
V
W
Y
Austria
govina
China
nmark
stonia
inland
France
rmany
ungary
Japan
Latvia
ngolia
rlands
Russia
ovakia
ovenia
Korea
weden
erland
USSR
ngdom

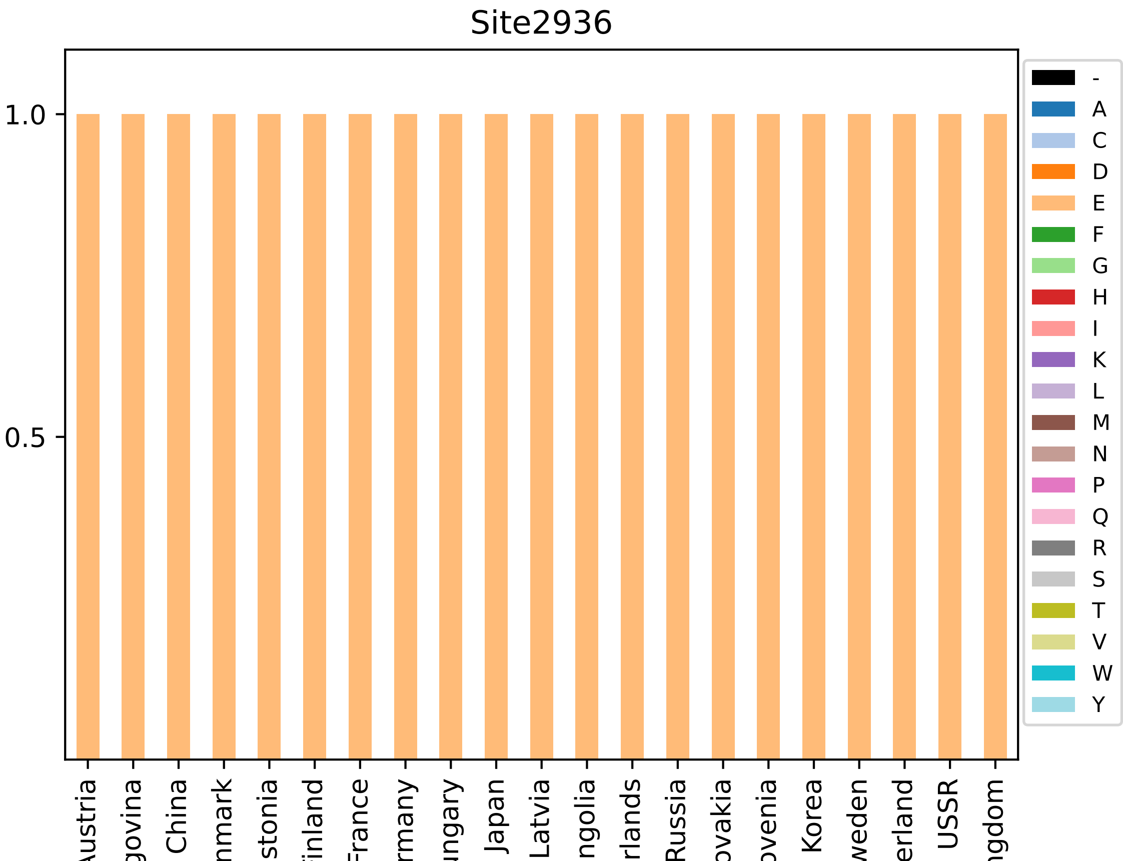
Site2936
1.0
0.5
-
A
C
D
E
F
G
H
I
K
L
M
N
P
Q
R
S
T
V
W
Y
Austria
govina
China
nmark
stonia
Finland
France
rmany
ungary
Japan
Latvia
ngolia
rlands
Russia
ovakia
ovenia
Korea
weden
erland
USSR
ngdom

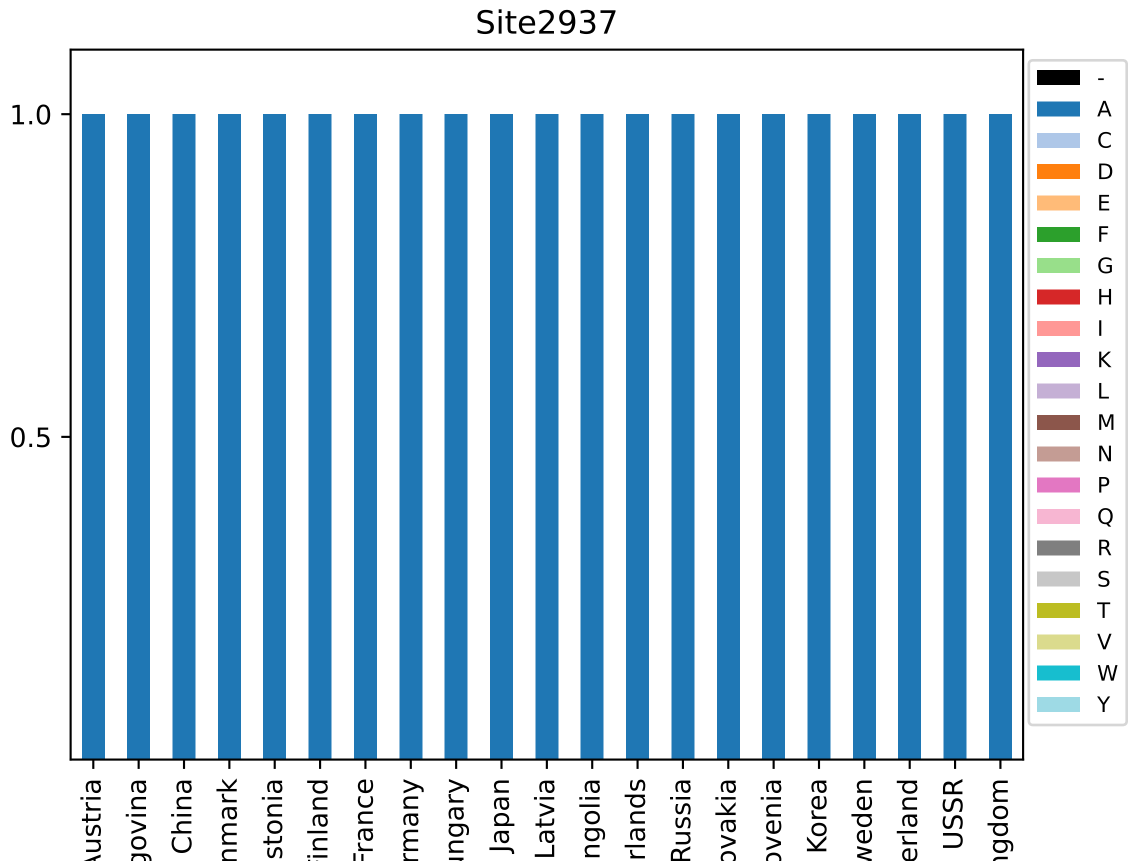

Site2937
1.0
0.5
-
A
C
D
E
F
G
H
I
K
L
M
N
P
Q
R
S
T
V
W
Y
Austria
China
Japan
Latvia
Russia
Korea
USSR

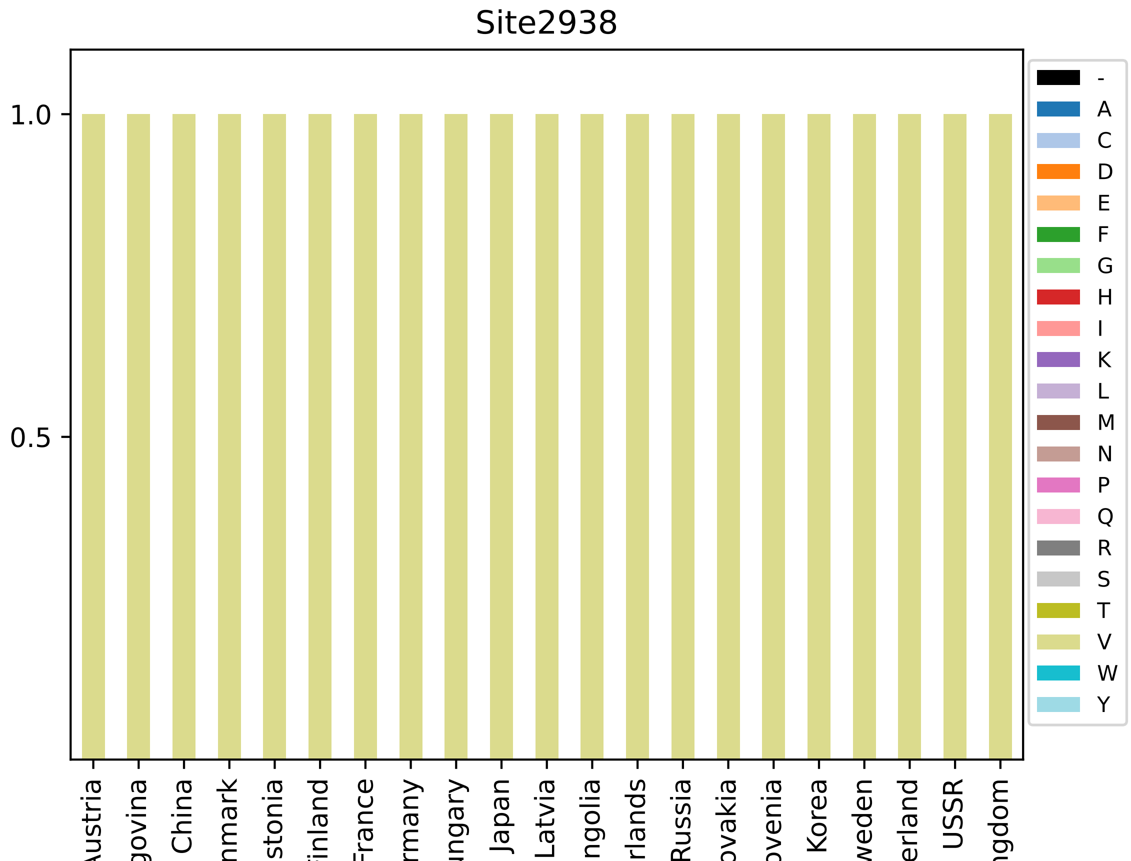
Site2938
1.0
0.5
-
A
C
D
E
F
G
H
I
K
L
M
N
P
Q
R
S
T
V
W
Y
Austria
China
Finland
France
Japan
Latvia
Russia
Korea
USSR

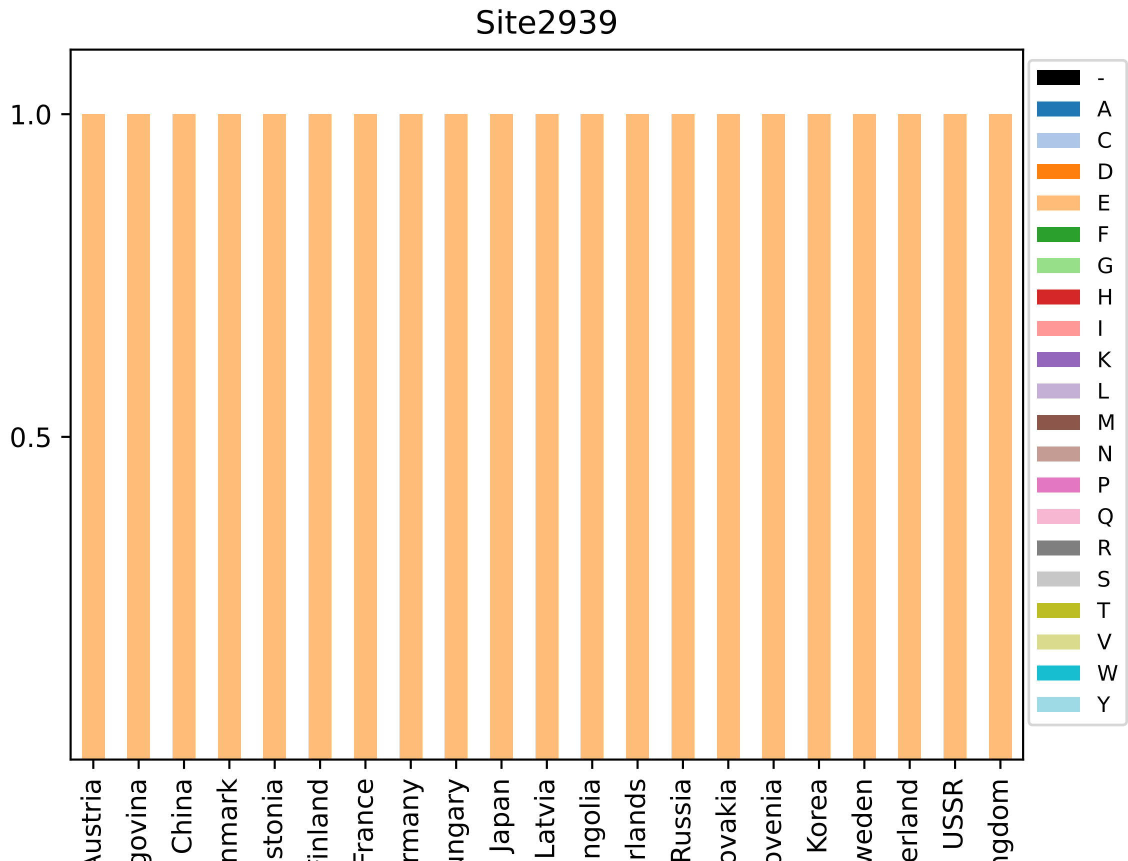
Site2939
1.0
0.5
-
A
C
D
E
F
G
H
I
K
L
M
N
P
Q
R
S
T
V
W
Y
Austria
China
Japan
Latvia
Russia
Korea
USSR

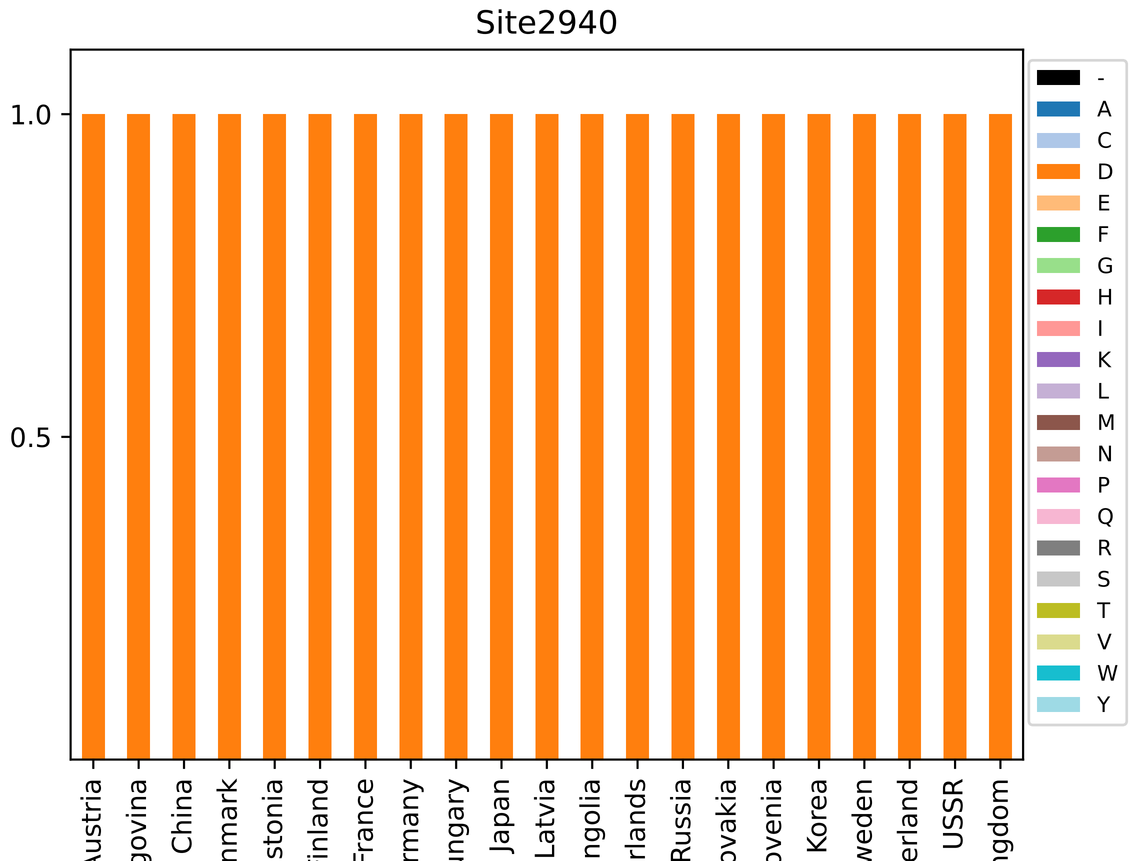
Site2940
1.0
0.5
-
A
C
D
E
F
G
H
I
K
L
M
N
P
Q
R
S
T
V
W
Y
China
Japan
Latvia
Russia
Korea
USSR

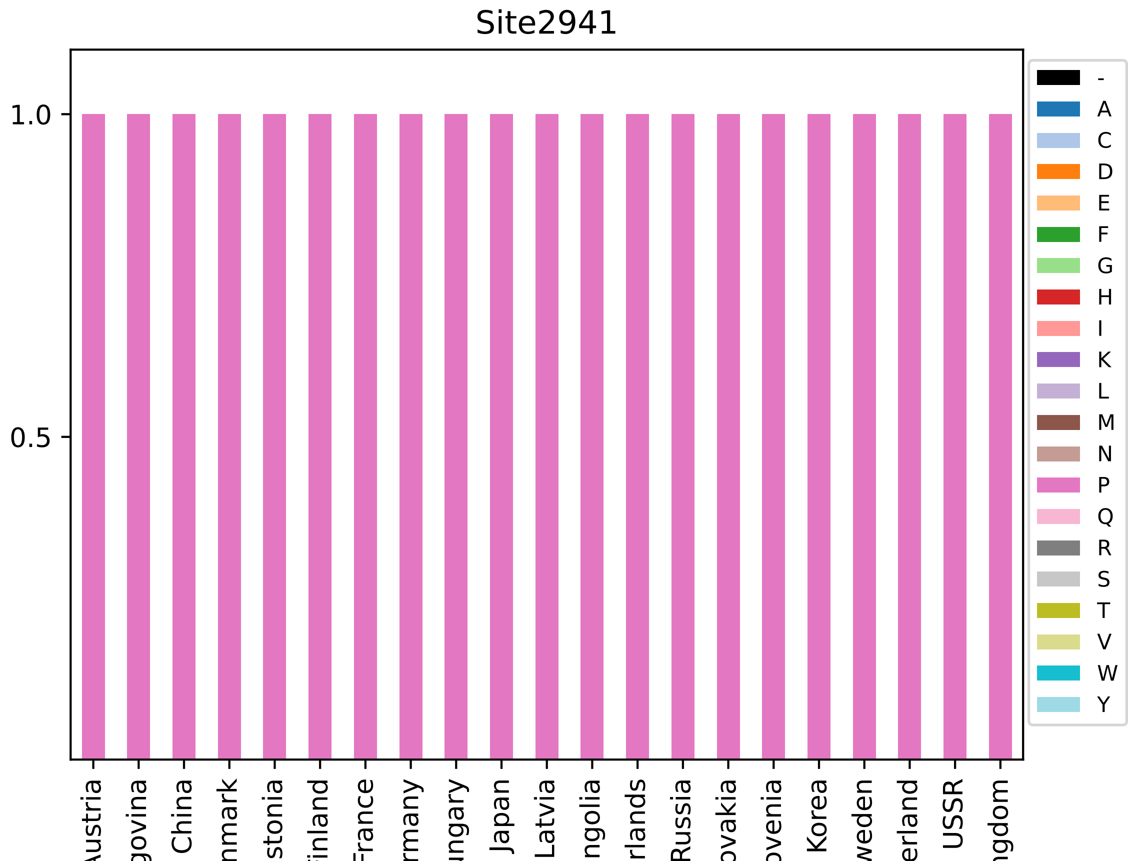
Site2941
1.0
0.5
-
A
C
D
E
F
G
H
I
K
L
M
N
P
Q
R
S
T
V
W
Y
Austria
China
Japan
Latvia
Russia
Korea
USSR

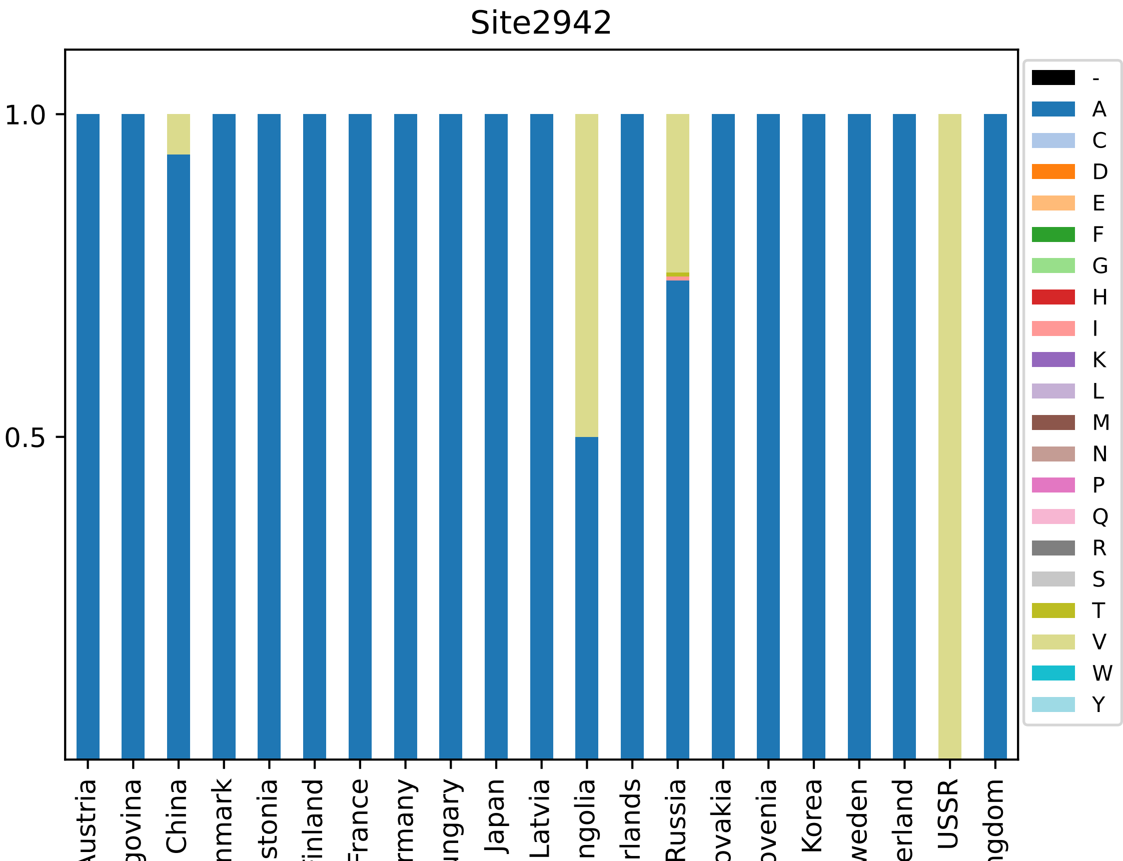

Site2942
1.0
0.5
-
A
C
D
E
F
G
H
I
K
L
M
N
P
Q
R
S
T
V
W
Y
Austria
govina
China
nmark
stonia
Finland
France
rmany
ungary
Japan
Latvia
ngolia
rlands
Russia
ovakia
ovenia
Korea
weden
erland
USSR
ngdom

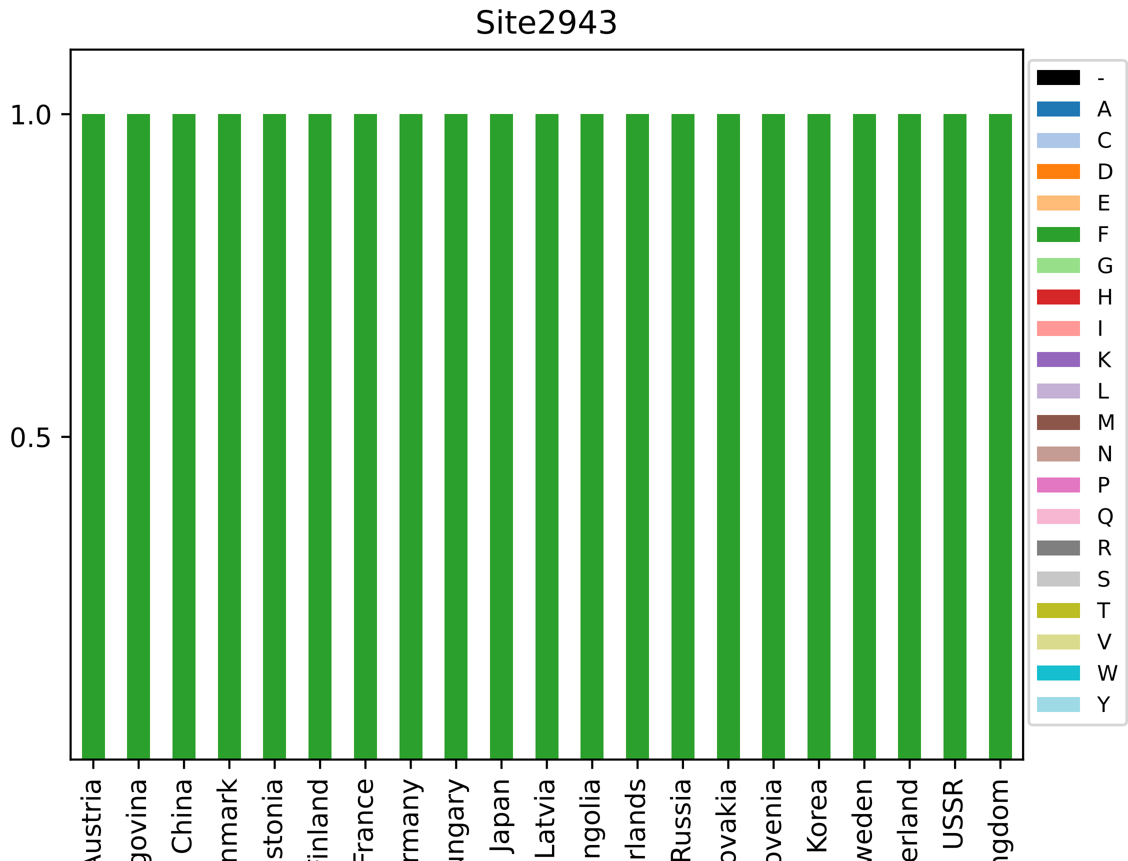

Site2943
1.0
0.5
-
A
C
D
E
F
G
H
I
K
L
M
N
P
Q
R
S
T
V
W
Y
China
Japan
Latvia
Russia
Korea
USSR

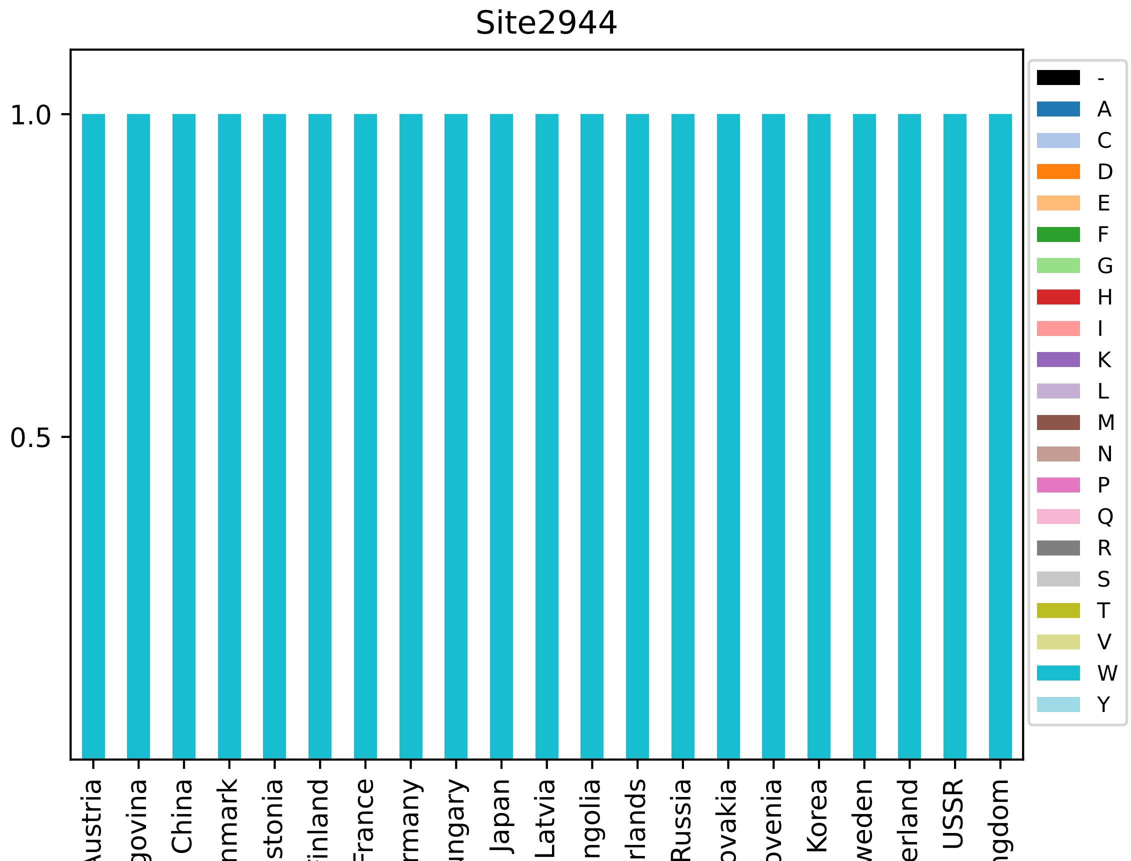

Site2944
1.0
0.5
-
A
C
D
E
F
G
H
I
K
L
M
N
P
Q
R
S
T
V
W
Y
China
Japan
Latvia
Russia
Korea
USSR

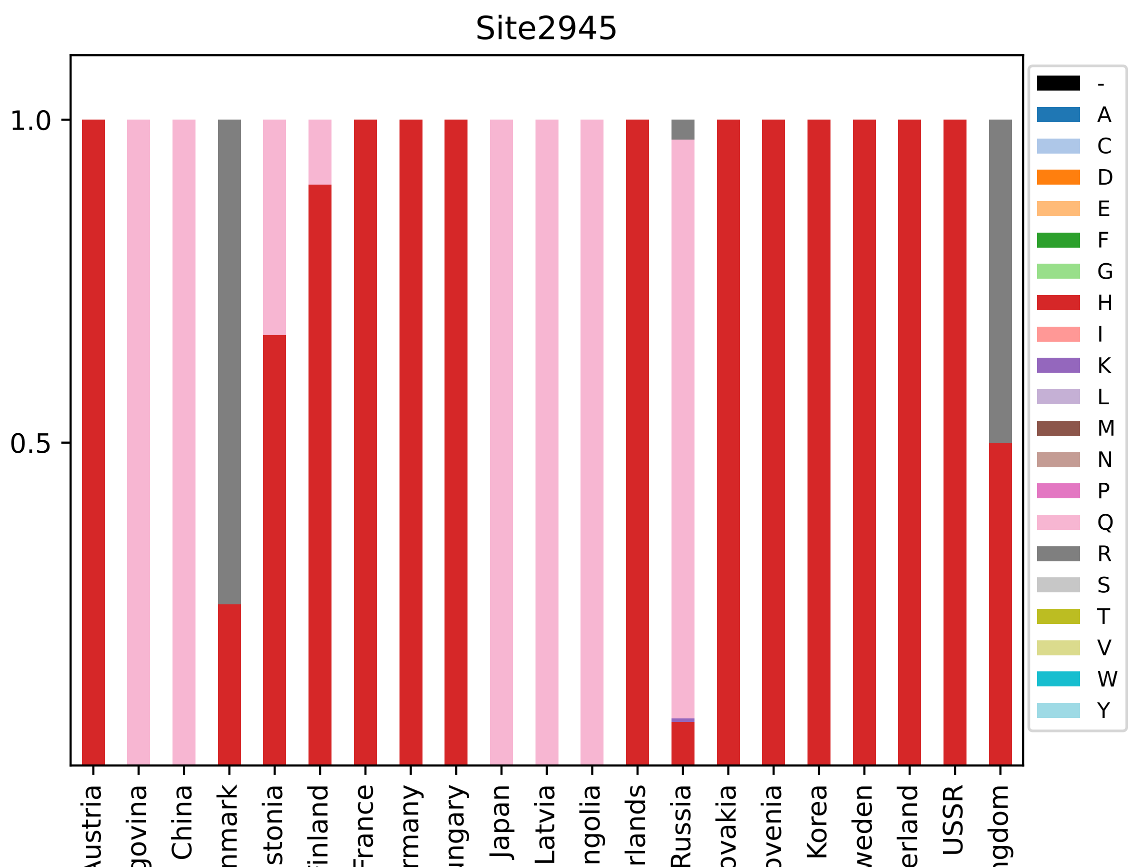

Site2945
1.0
0.5
-
A
C
D
E
F
G
H
I
K
L
M
N
P
Q
R
S
T
V
W
Y
China
Japan
Latvia
Russia
Korea
USSR

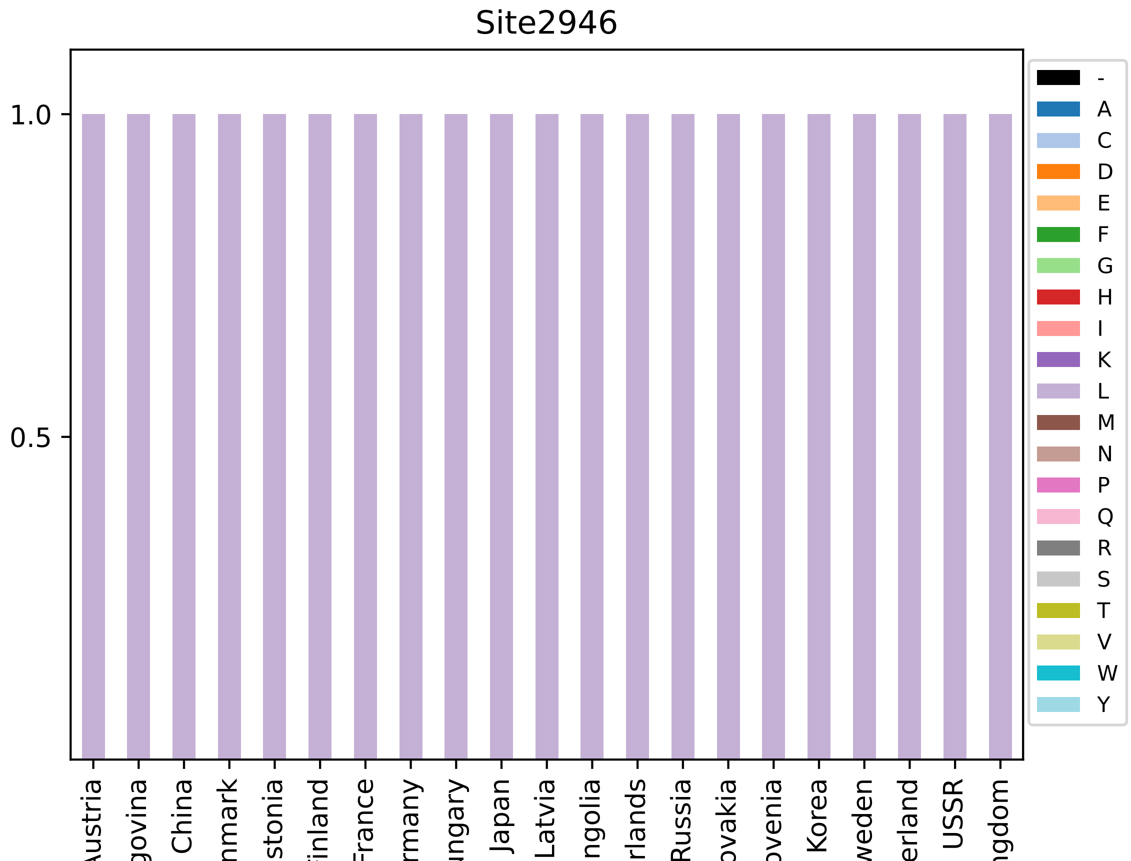
Site2946
1.0
0.5
-
A
C
D
E
F
G
H
I
K
L
M
N
P
Q
R
S
T
V
W
Y
China
Japan
Latvia
Russia
Korea
USSR

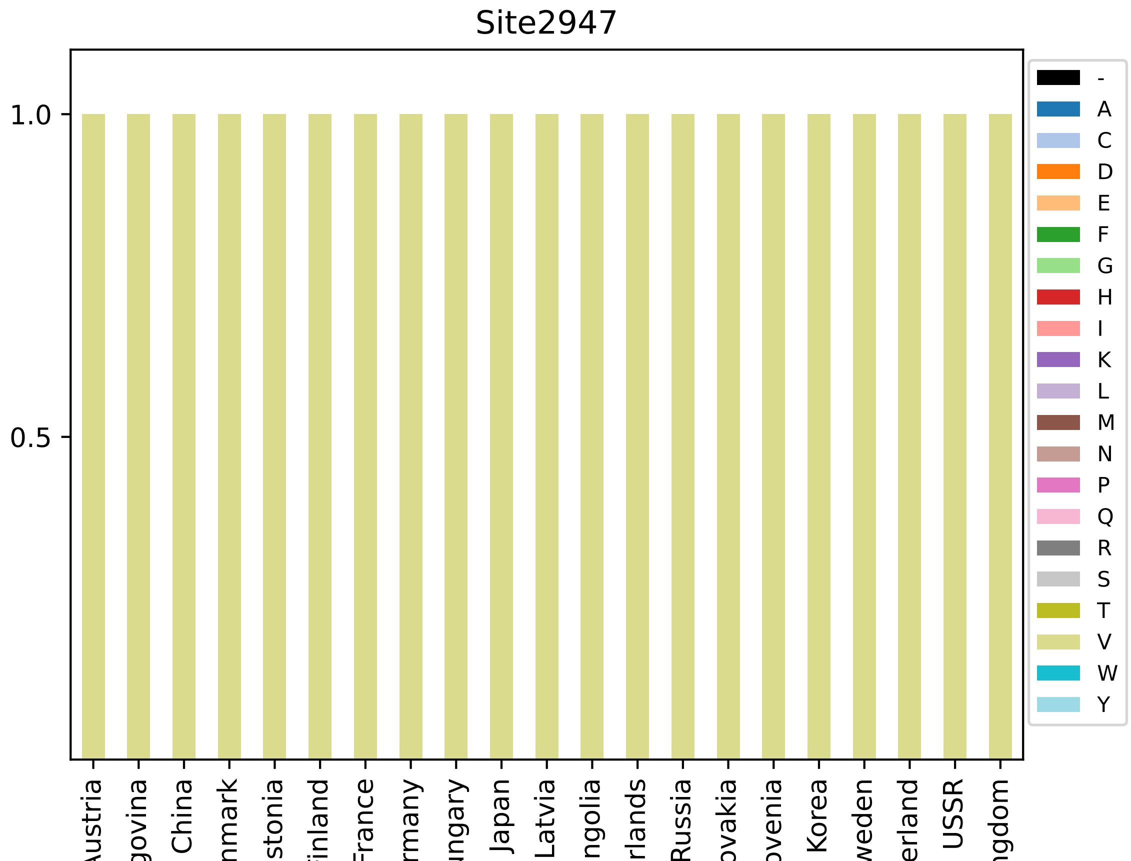
Site2947
1.0
0.5
-
A
C
D
E
F
G
H
I
K
L
M
N
P
Q
R
S
T
V
W
Y
Austria
China
Japan
Latvia
Russia
Korea
USSR

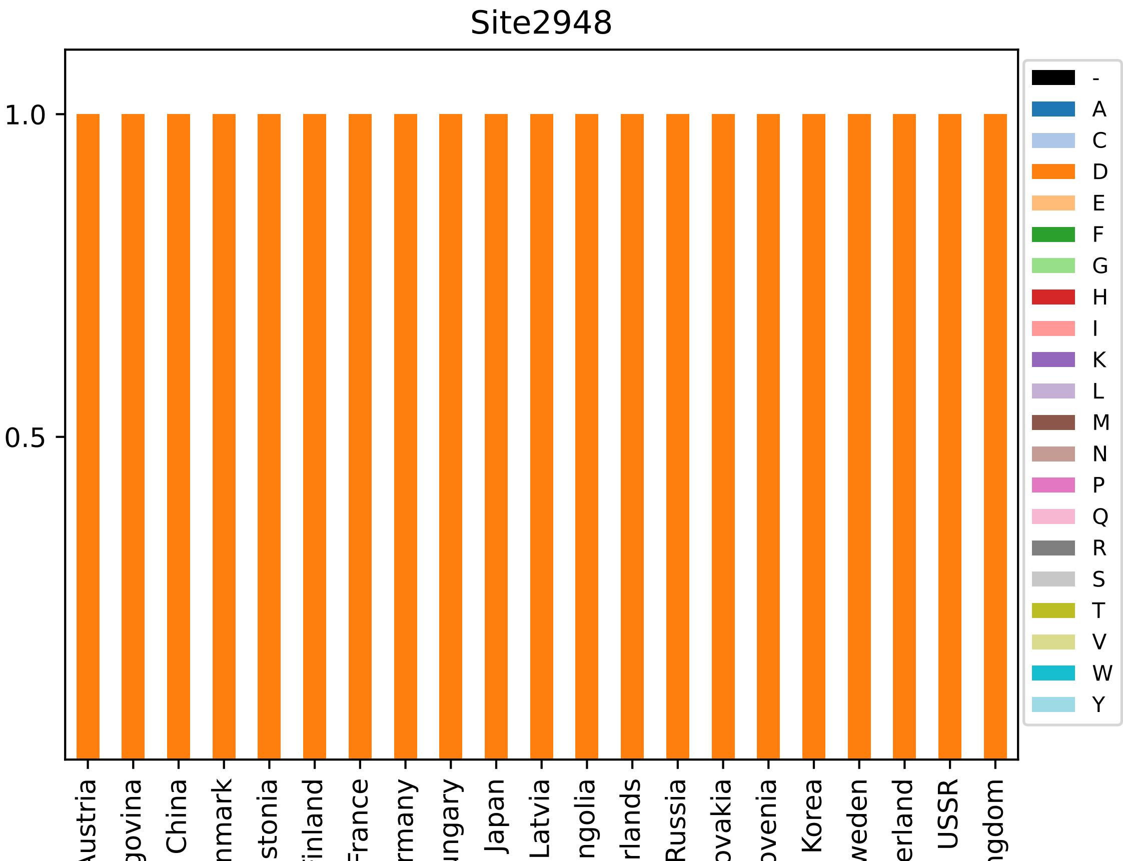

Site2948
1.0
0.5
-
A
C
D
E
F
G
H
I
K
L
M
N
P
Q
R
S
T
V
W
Y
China
Japan
Latvia
Russia
Korea
USSR

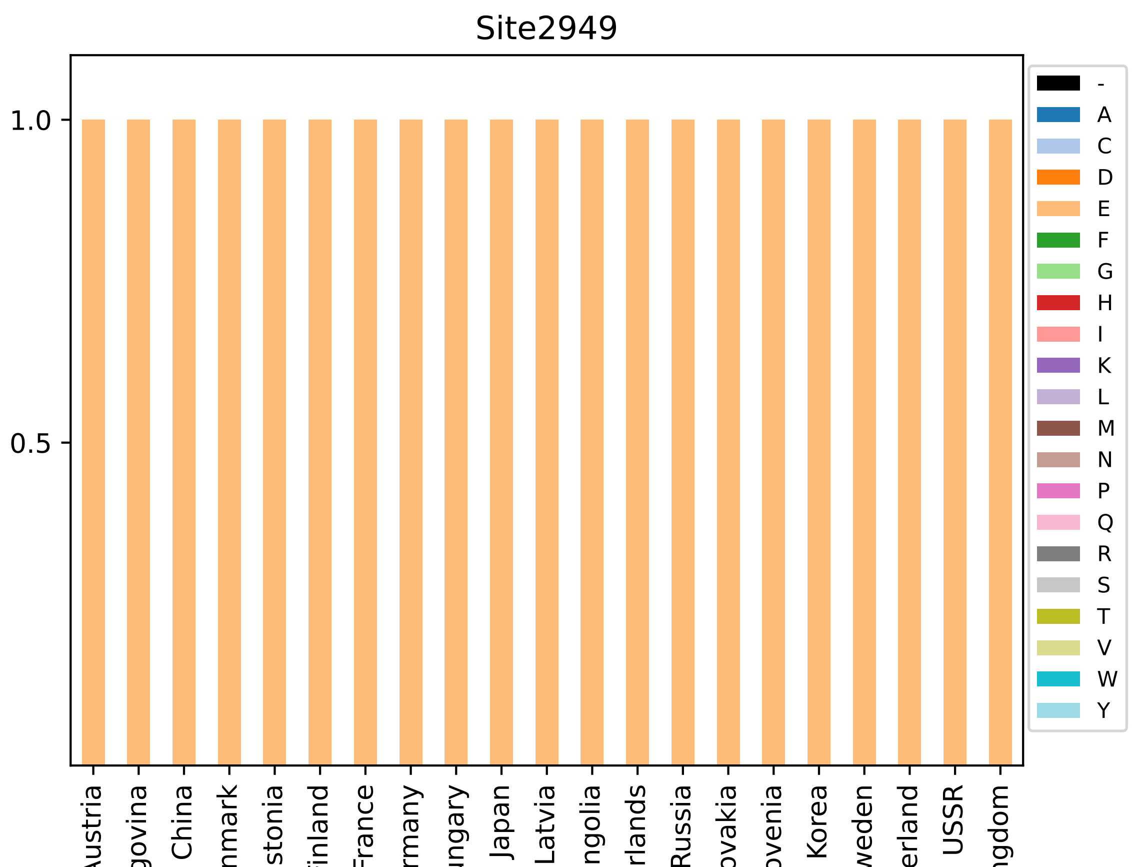

Site2949
1.0
0.5
-
A
C
D
E
F
G
H
I
K
L
M
N
P
Q
R
S
T
V
W
Y
Austria
China
Japan
Latvia
Russia
Korea
USSR

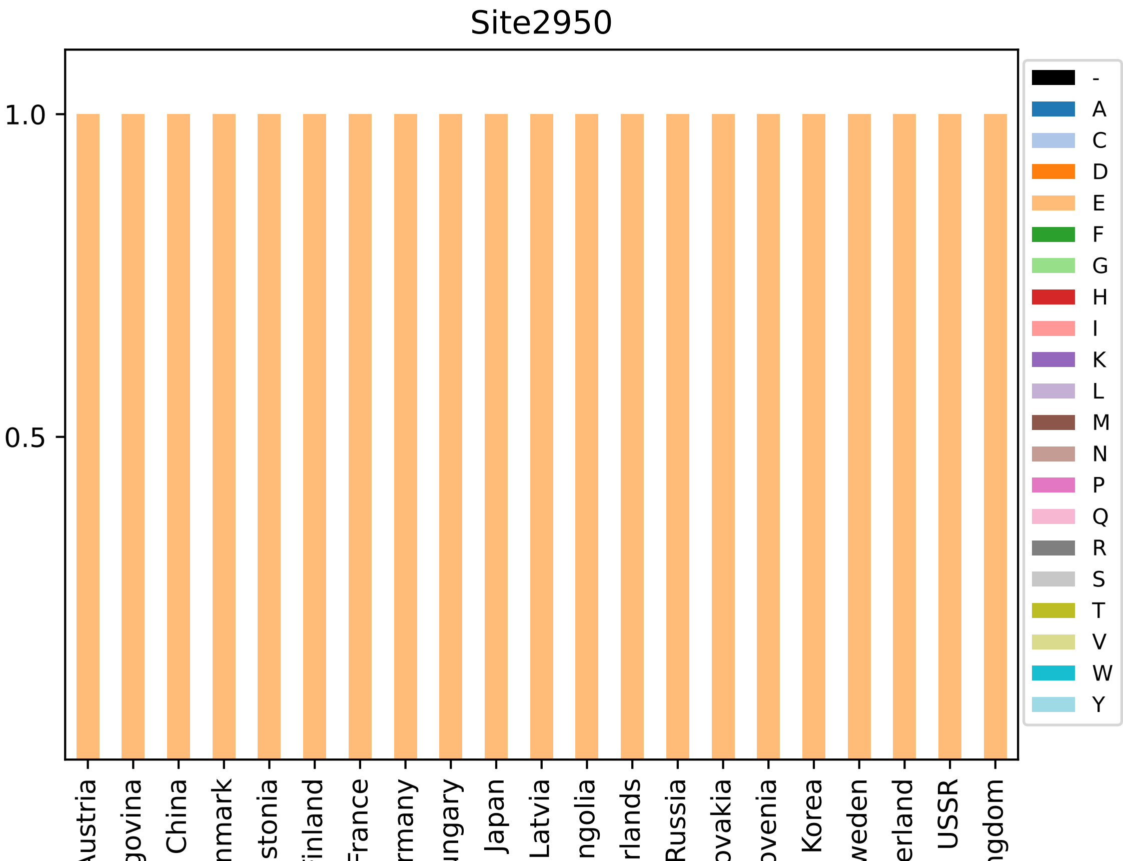

Site2950
1.0
0.5
-
A
C
D
E
F
G
H
I
K
L
M
N
P
Q
R
S
T
V
W
Y
China
Japan
Latvia
Russia
Korea
USSR

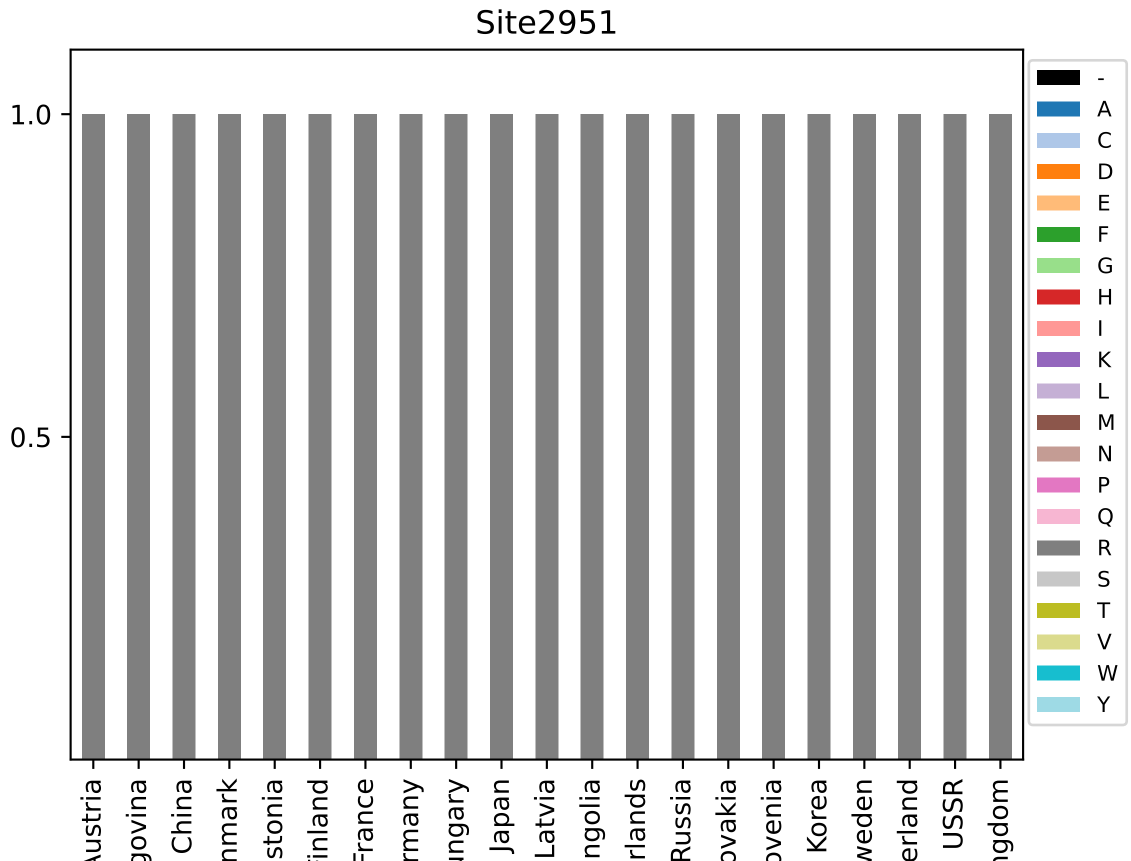
Site2951
1.0
0.5
-
A
C
D
E
F
G
H
I
K
L
M
N
P
Q
R
S
T
V
W
Y
Austria
govina
China
nmark
stonia
inland
France
rmany
ungary
Japan
Latvia
ngolia
rlands
Russia
ovakia
ovenia
Korea
weden
erland
USSR
ngdom

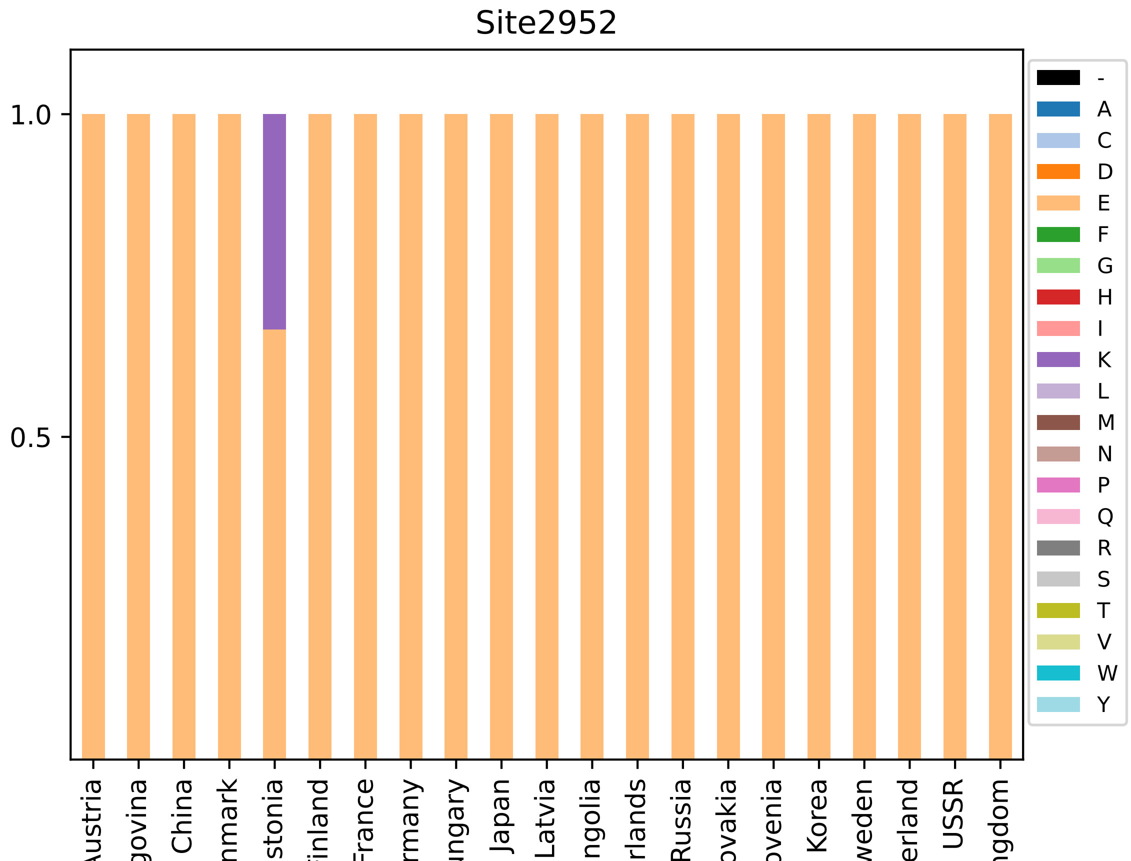

Site2952
1.0
0.5
-
A
C
D
E
F
G
H
I
K
L
M
N
P
Q
R
S
T
V
W
Y
China
Japan
Latvia
Russia
Korea
USSR

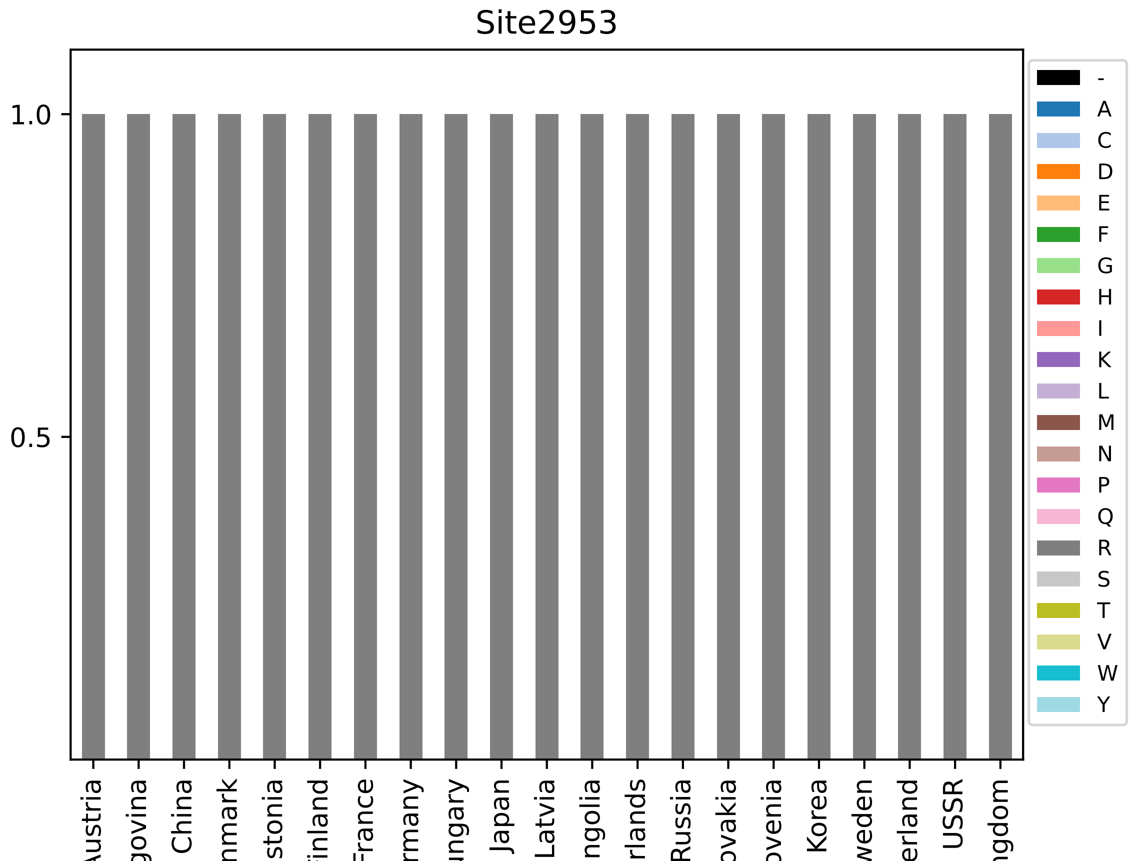
Site2953
1.0
0.5
-
A
C
D
E
F
G
H
I
K
L
M
N
P
Q
R
S
T
V
W
Y
Austria
China
Finland
France
Japan
Latvia
Russia
Korea
USSR

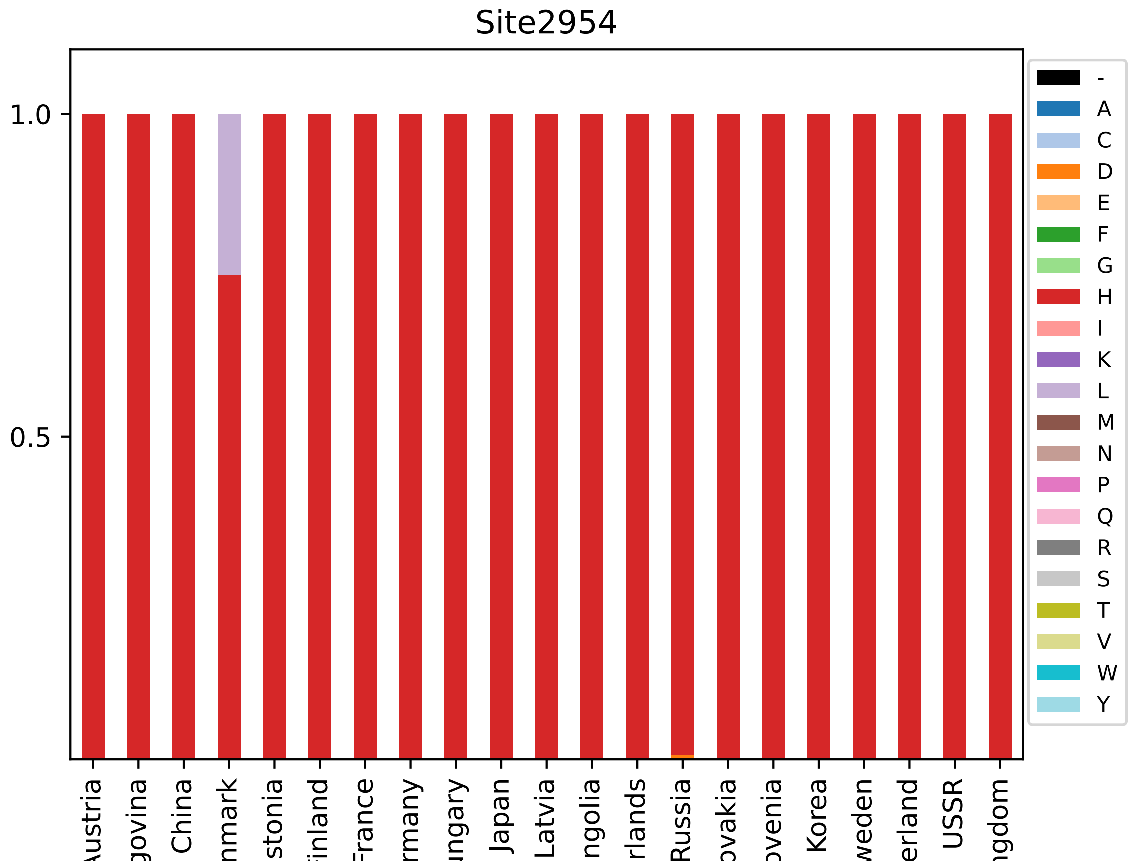
Site2954
1.0
0.5
-
A
C
D
E
F
G
H
I
K
L
M
N
P
Q
R
S
T
V
W
Y
Austria
govina
China
nmark
stonia
inland
France
rmany
ungary
Japan
Latvia
ngolia
rlands
Russia
ovakia
ovenia
Korea
weden
erland
USSR
ngdom

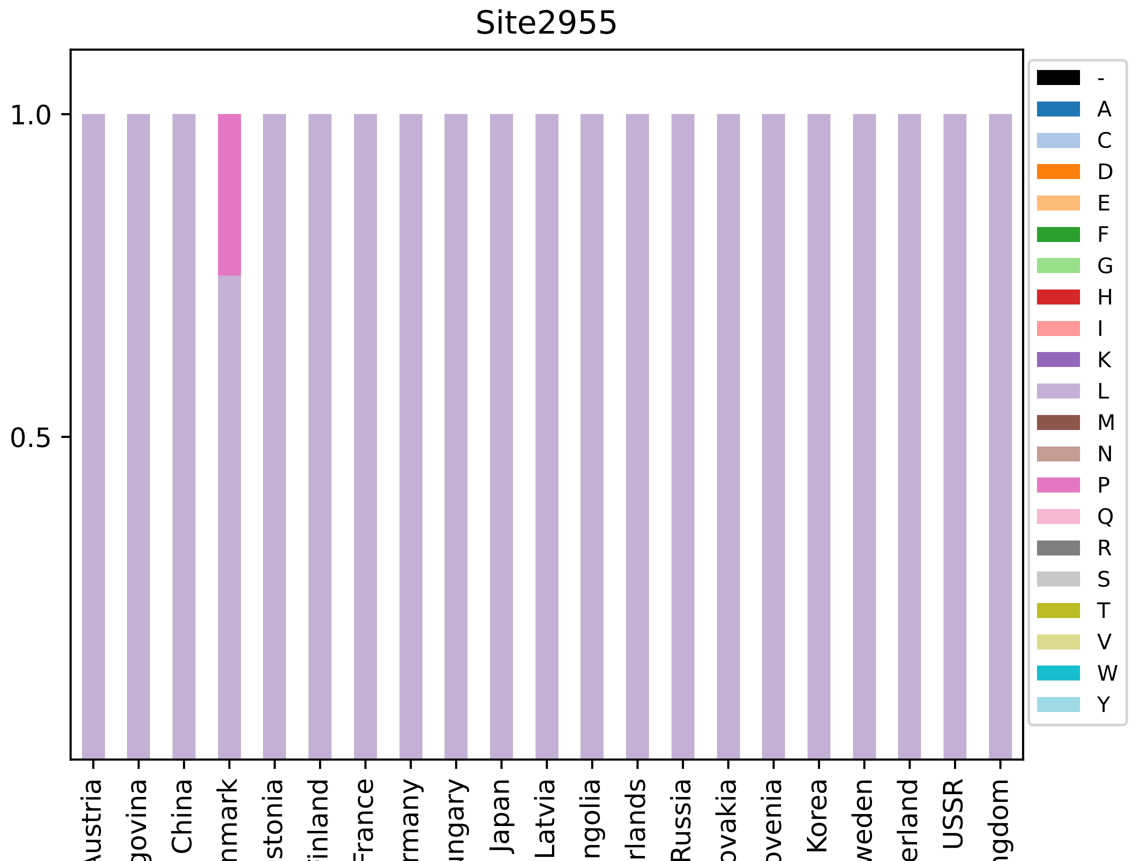

Site2955
1.0
0.5
Austria
govina
China
nmark
stonia
Finland
France
rmany
ungary
Japan
Latvia
ngolia
rlands
Russia
ovakia
ovenia
Korea
weden
erland
USSR
ngdom
-
A
C
D
E
F
G
H
I
K
L
M
N
P
Q
R
S
T
V
W
Y

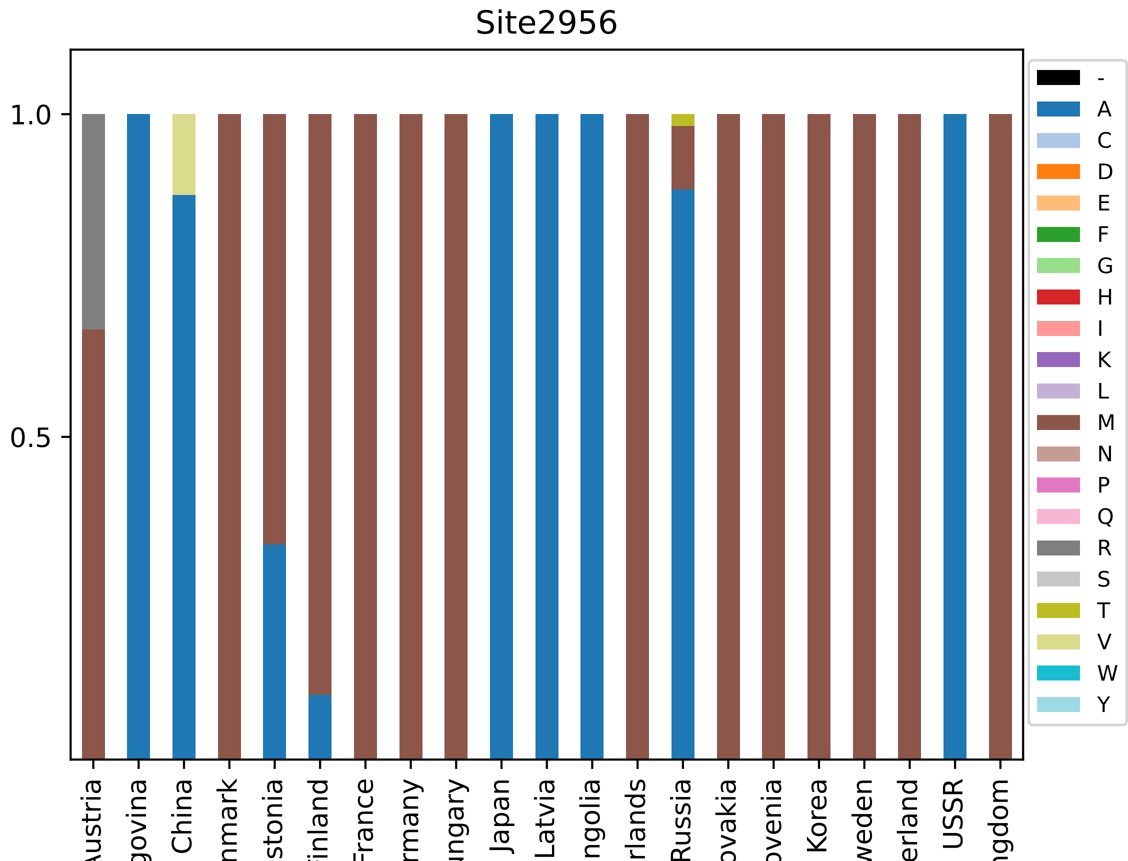

Site2956
1.0
0.5
-
A
C
D
E
F
G
H
I
K
L
M
N
P
Q
R
S
T
V
W
Y
Austria
China
Japan
Latvia
Russia
Korea
USSR

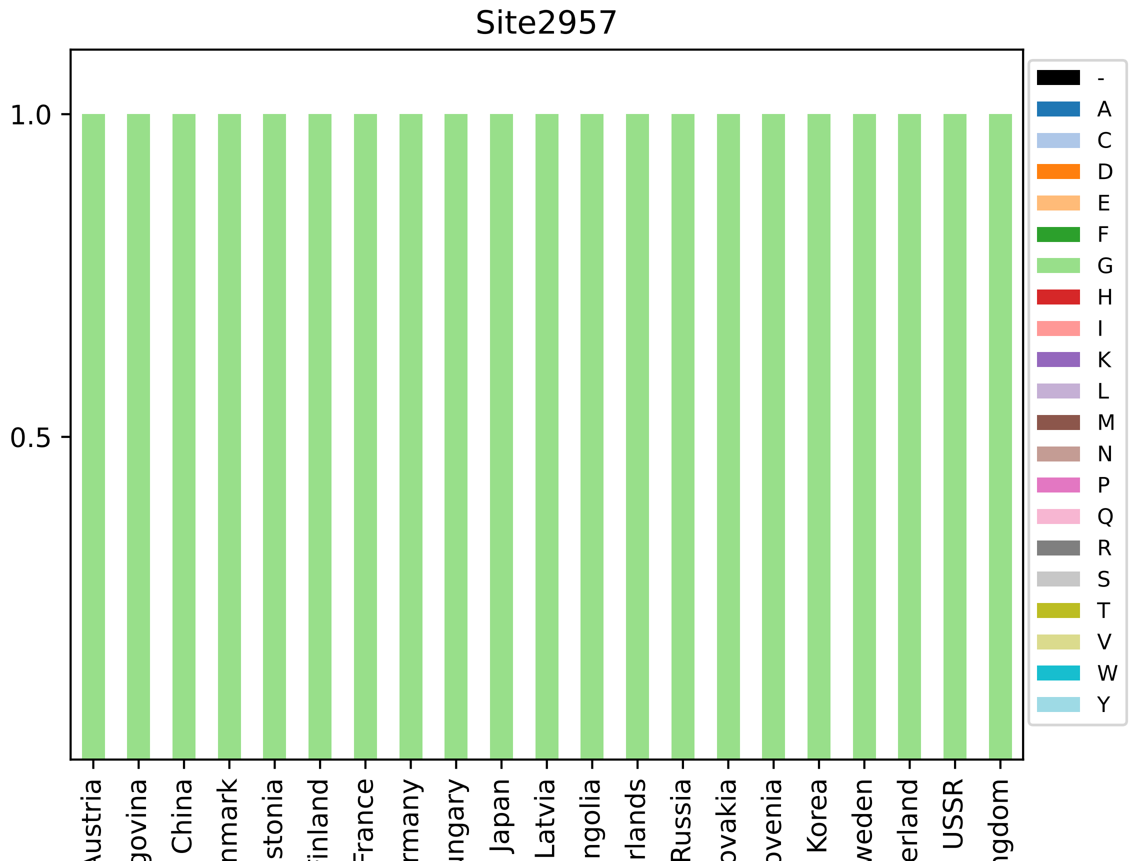

Site2957
1.0
0.5
-
A
C
D
E
F
G
H
I
K
L
M
N
P
Q
R
S
T
V
W
Y
China
Latvia
Japan
Korea
USSR

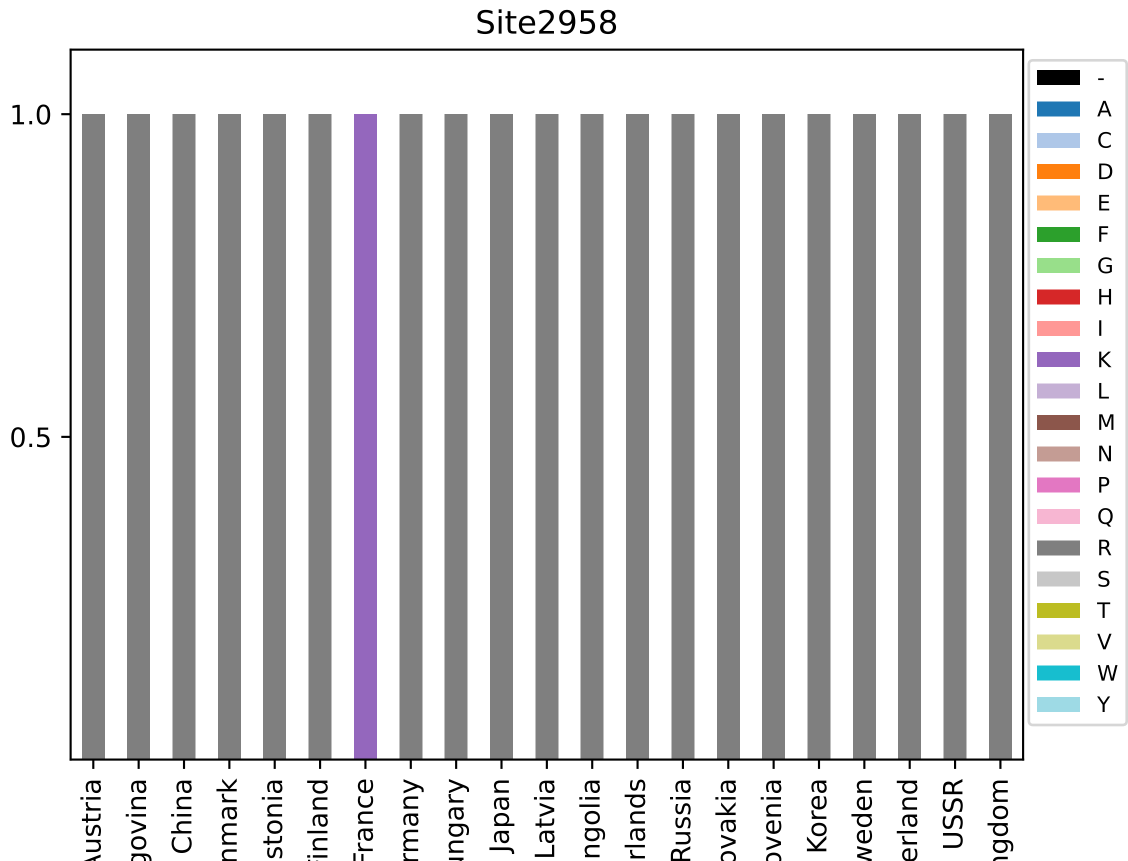

Site2958
1.0
0.5
-
A
C
D
E
F
G
H
I
K
L
M
N
P
Q
R
S
T
V
W
Y
Austria
China
Japan
Latvia
Russia
Korea
USSR

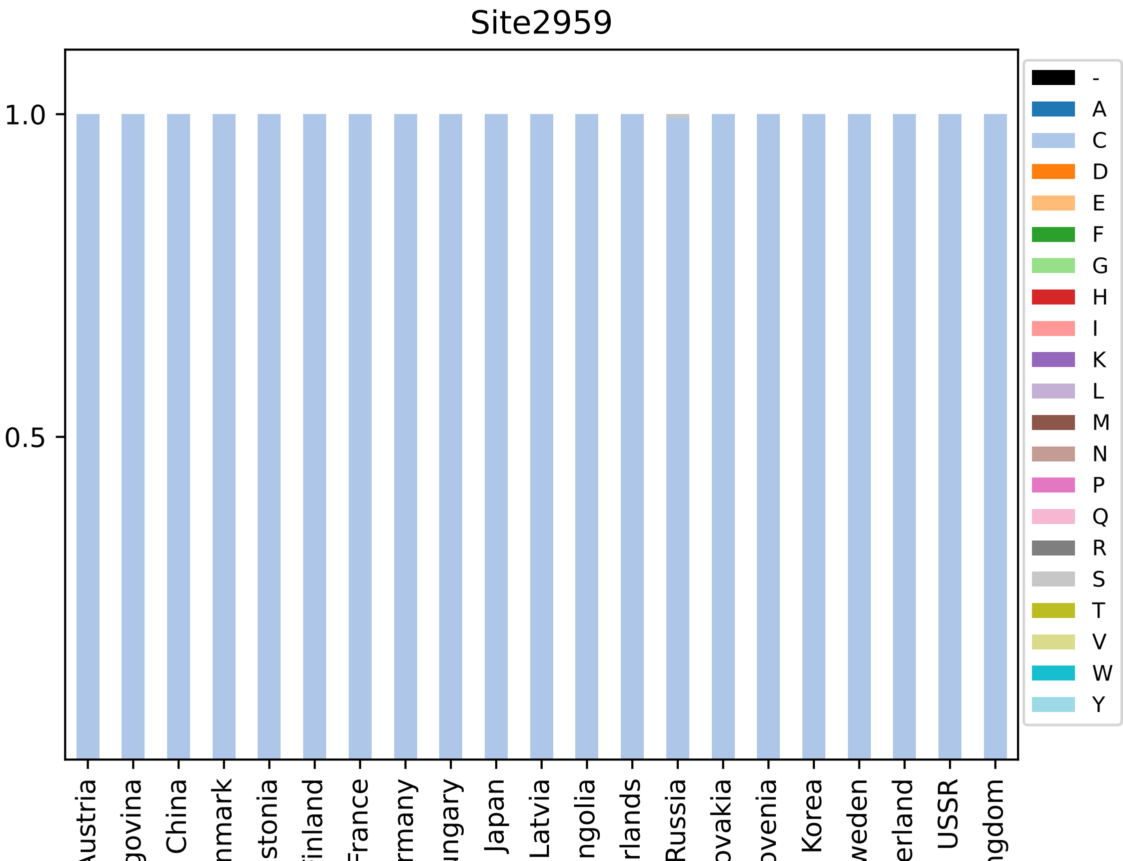
Site2959
1.0
0.5
-
A
C
D
E
F
G
H
I
K
L
M
N
P
Q
R
S
T
V
W
Y
Austria
govina
China
nmark
stonia
Finland
France
rmany
ungary
Japan
Latvia
ngolia
rlands
Russia
ovakia
ovenia
Korea
weden
erland
USSR
ngdom

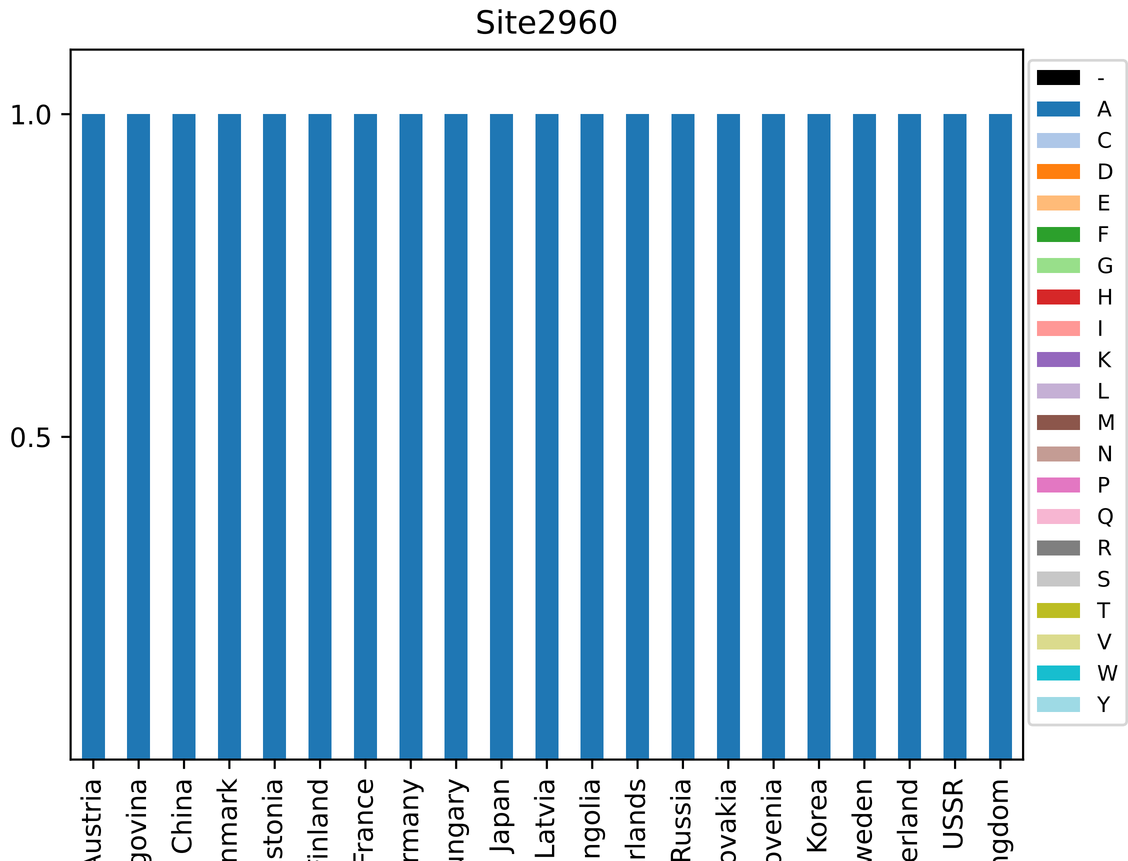
Site2960
1.0
0.5
-
A
C
D
E
F
G
H
I
K
L
M
N
P
Q
R
S
T
V
W
Y
China
Japan
Latvia
Russia
Korea
USSR

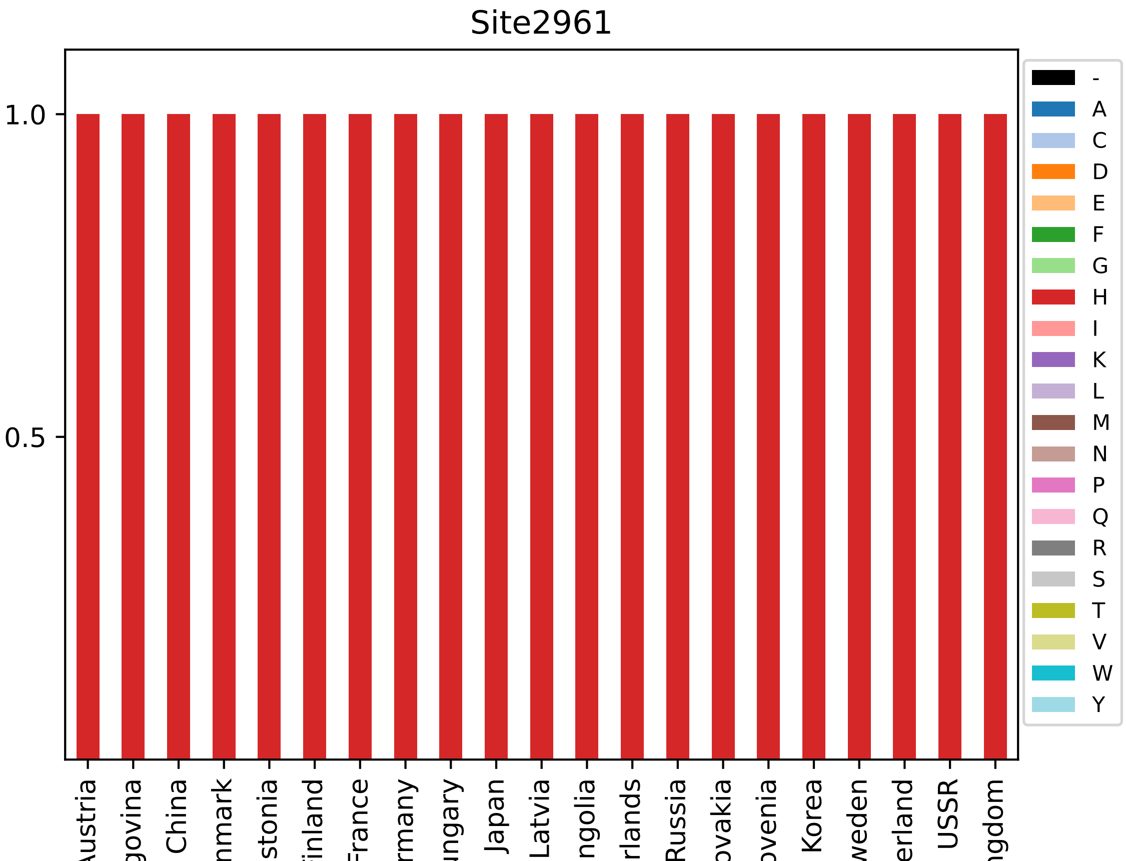

Site2961
1.0
0.5
-
A
C
D
E
F
G
H
I
K
L
M
N
P
Q
R
S
T
V
W
Y
China
Japan
Latvia
Russia
Korea
USSR

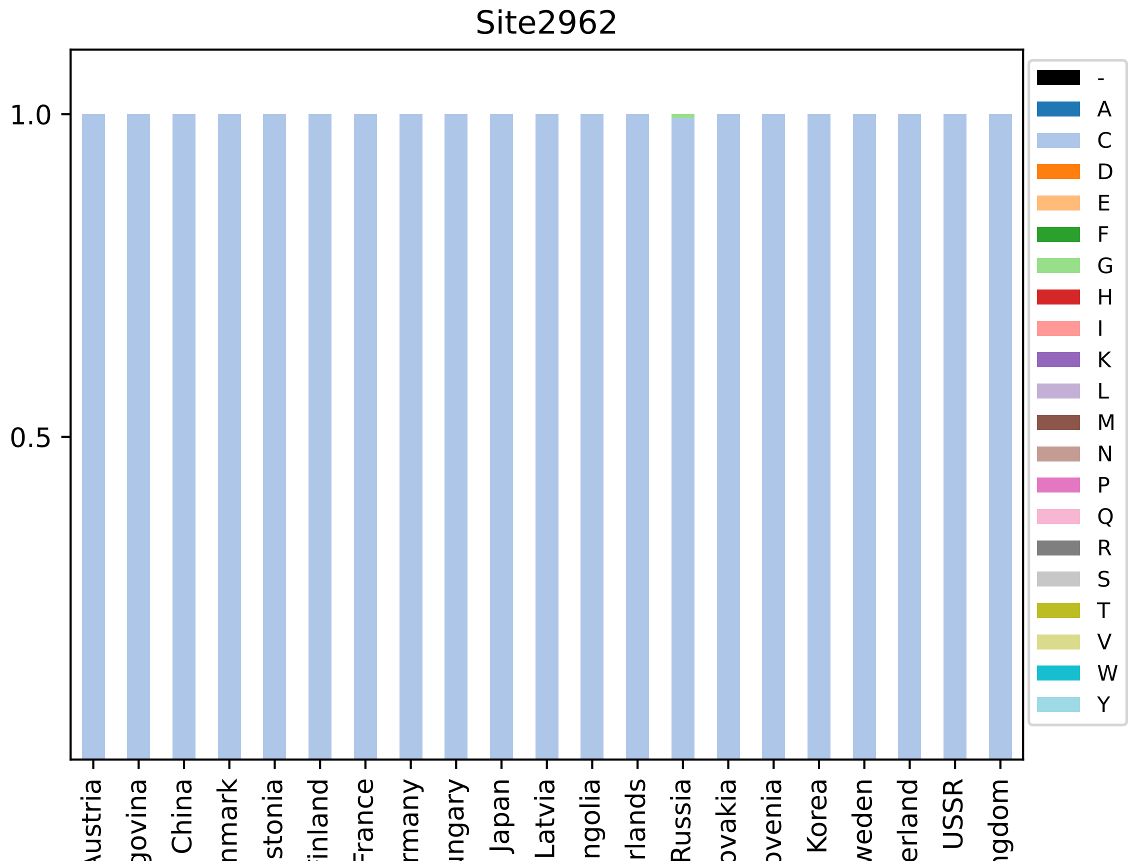
Site2962
1.0
0.5
-
A
C
D
E
F
G
H
I
K
L
M
N
P
Q
R
S
T
V
W
Y
China
Japan
Latvia
Russia
Korea
USSR

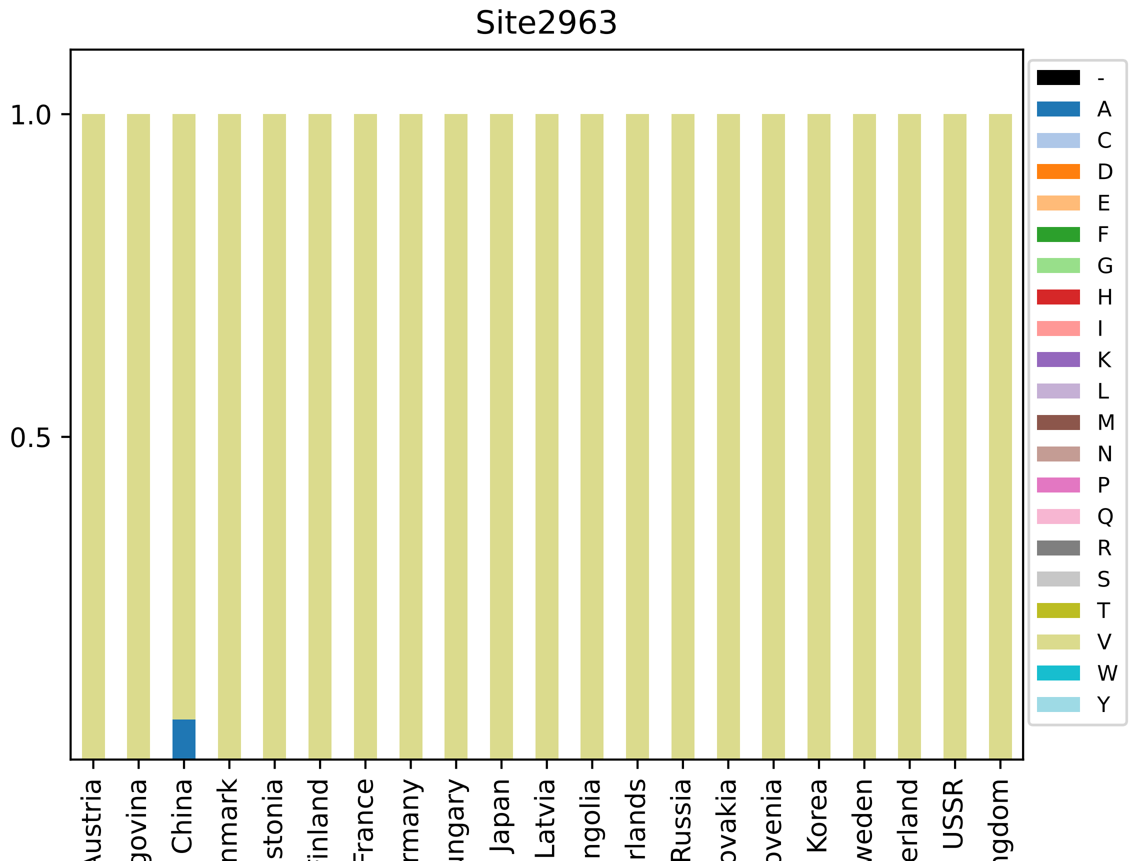

Site2963
1.0
0.5
-
A
C
D
E
F
G
H
I
K
L
M
N
P
Q
R
S
T
V
W
Y
Austria
govina
China
nmark
stonia
inland
France
rmany
ungary
Japan
Latvia
ngolia
rlands
Russia
ovakia
ovenia
Korea
weden
erland
USSR
ngdom

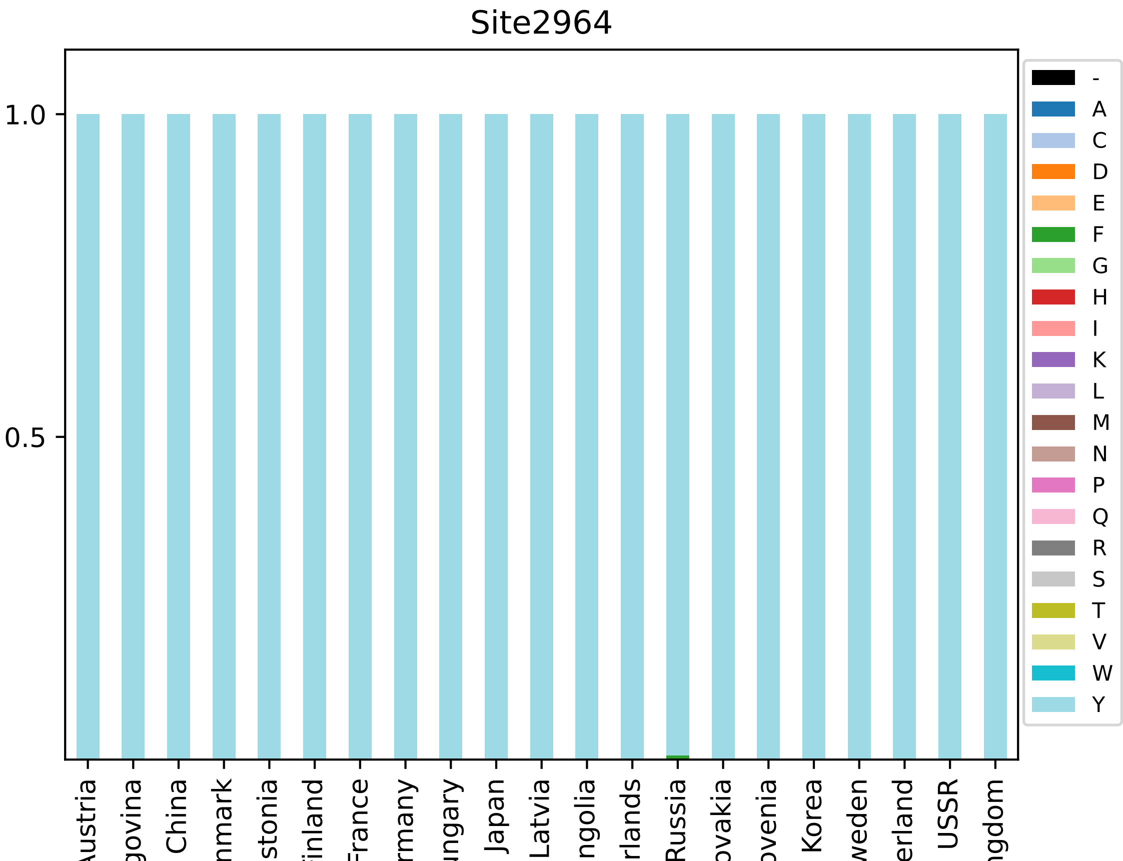
Site2964
1.0
0.5
-
A
C
D
E
F
G
H
I
K
L
M
N
P
Q
R
S
T
V
W
Y
Austria
govina
China
nmark
stonia
Finland
France
rmany
ungary
Japan
Latvia
ngolia
rlands
Russia
ovakia
ovenia
Korea
weden
erland
USSR
ngdom

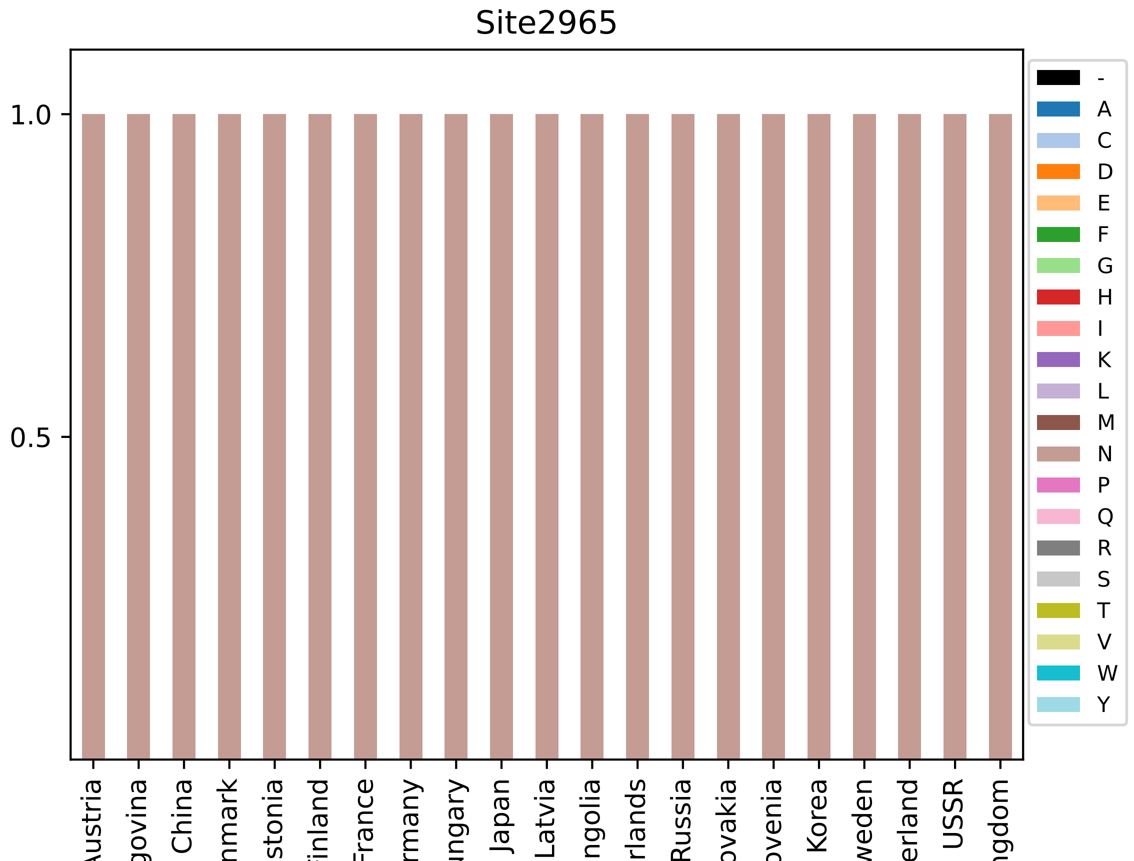

Site2965
1.0
0.5
-
A
C
D
E
F
G
H
I
K
L
M
N
P
Q
R
S
T
V
W
Y
Austria
govina
China
nmark
stonia
Finland
France
rmany
ungary
Japan
Latvia
ngolia
rlands
Russia
ovakia
ovenia
Korea
weden
erland
USSR
ngdom

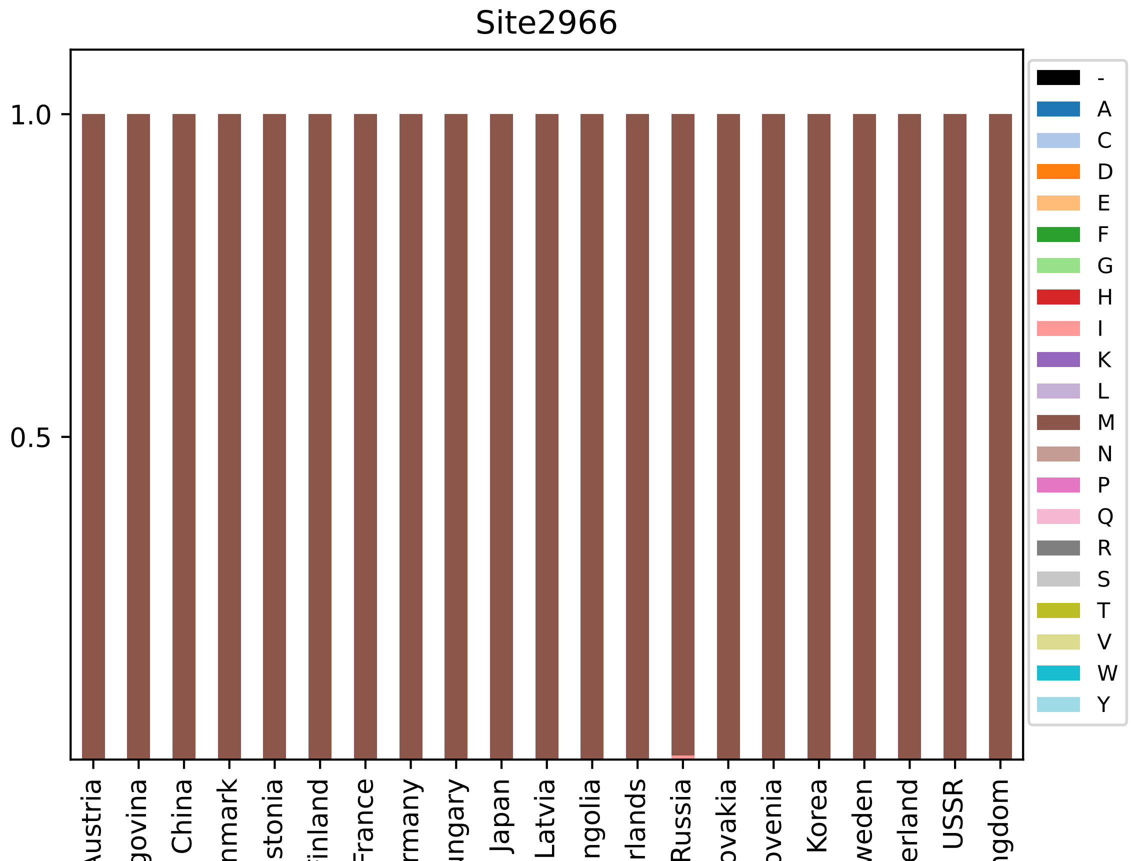

Site2966
1.0
0.5
-
A
C
D
E
F
G
H
I
K
L
M
N
P
Q
R
S
T
V
W
Y
Austria
govina
China
nmark
stonia
Finland
France
rmany
ungary
Japan
Latvia
ngolia
rlands
Russia
ovakia
ovenia
Korea
weden
erland
USSR
ngdom

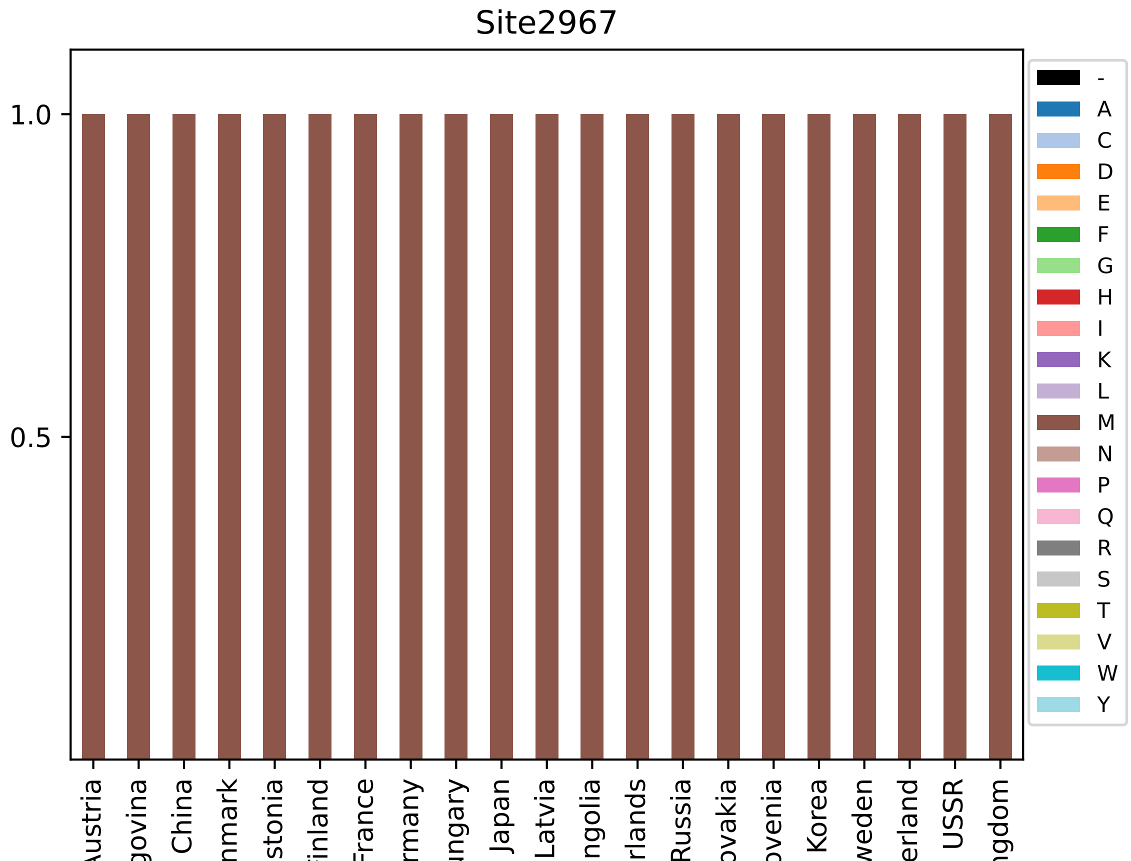
Site2967
1.0
0.5
-
A
C
D
E
F
G
H
I
K
L
M
N
P
Q
R
S
T
V
W
Y
Austria
govina
China
nmark
stonia
Finland
France
rmany
ungary
Japan
Latvia
ngolia
rlands
Russia
ovakia
ovenia
Korea
weden
erland
USSR
ngdom

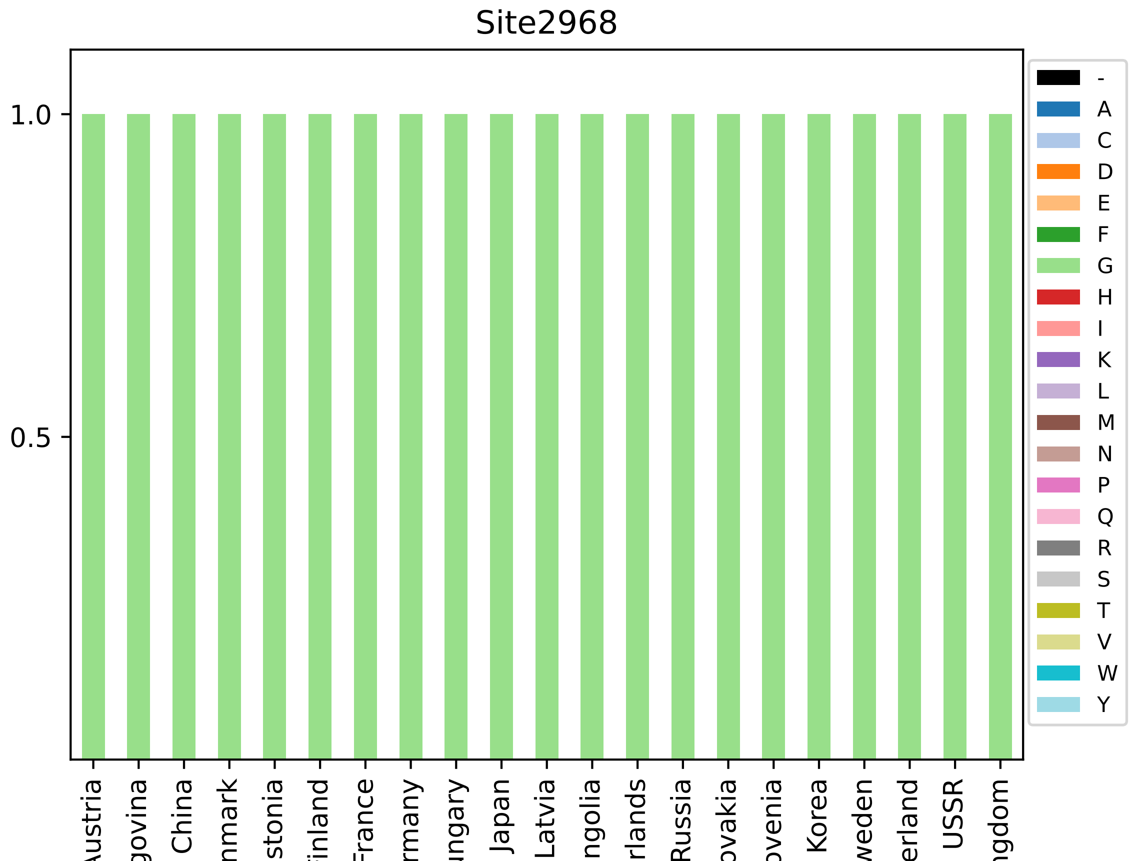
Site2968
1.0
0.5
Austria
govina
China
nmark
stonia
inland
France
rmany
ungary
Japan
Latvia
ngolia
rlands
Russia
ovakia
ovenia
Korea
weden
erland
USSR
ngdom
-
A
C
D
E
F
G
H
I
K
L
M
N
P
Q
R
S
T
V
W
Y

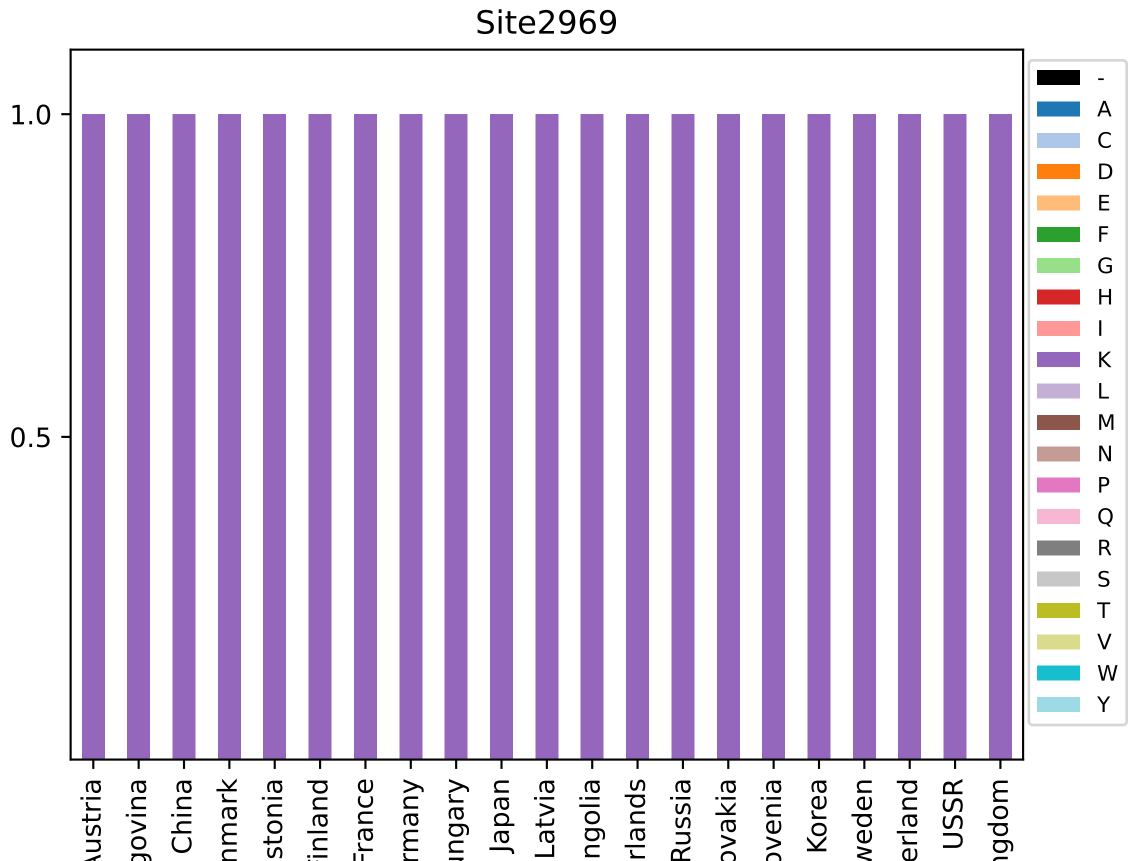

Site2969
1.0
0.5
-
A
C
D
E
F
G
H
I
K
L
M
N
P
Q
R
S
T
V
W
Y
China
Japan
Latvia
Russia
Korea
USSR

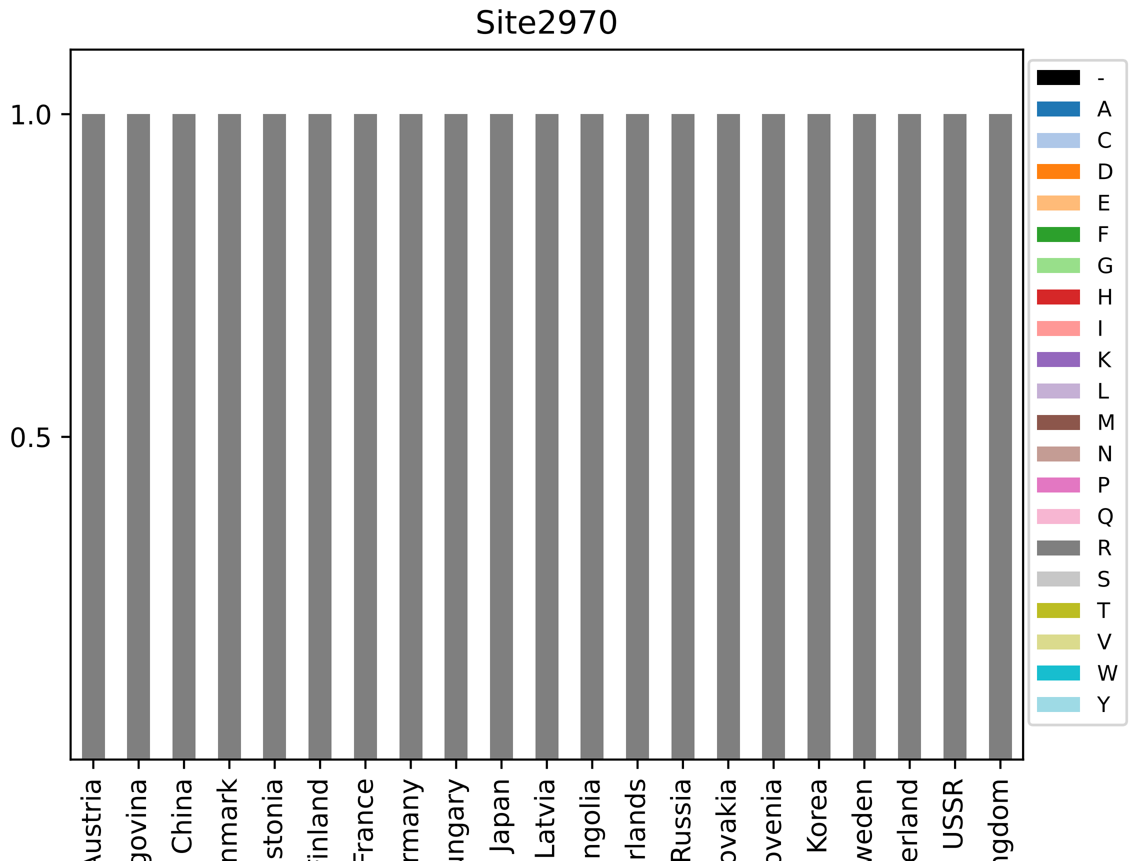

Site2970
1.0
0.5
-
A
C
D
E
F
G
H
I
K
L
M
N
P
Q
R
S
T
V
W
Y
China
Japan
Latvia
Russia
Korea
USSR

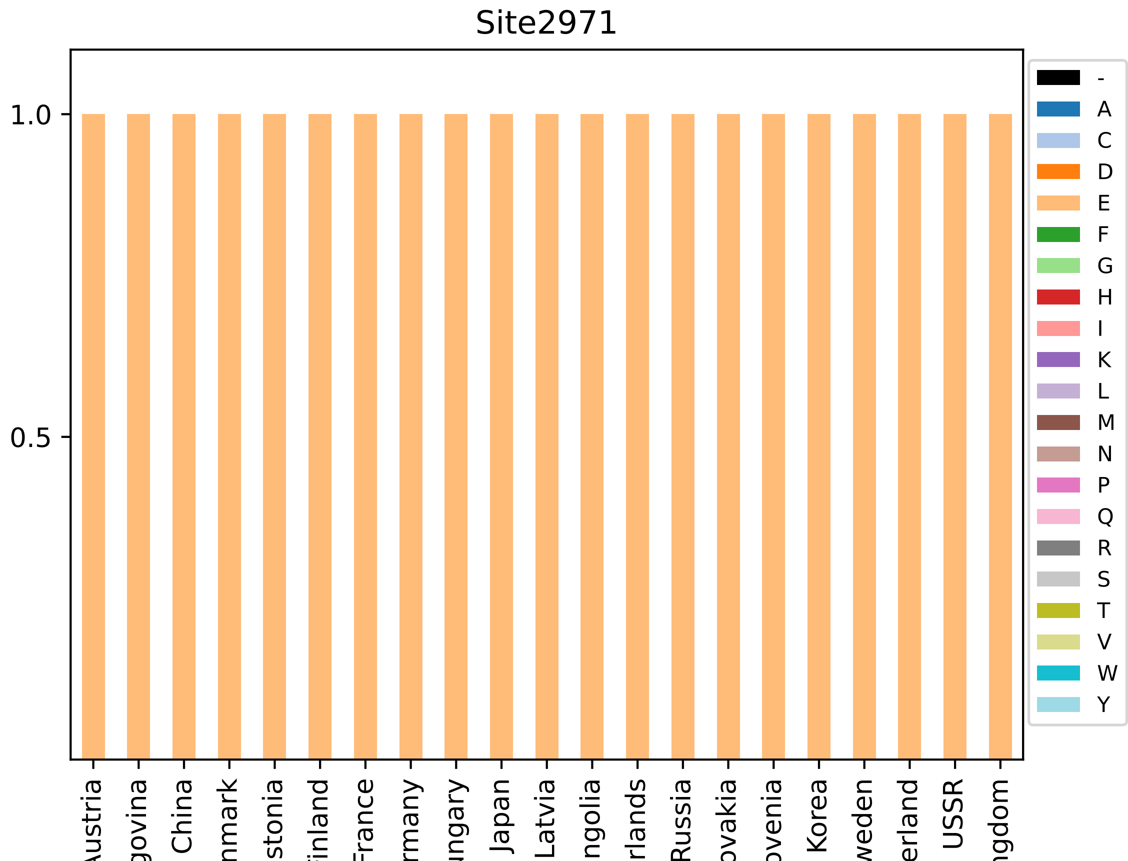

Site2971
1.0
0.5
-
A
C
D
E
F
G
H
I
K
L
M
N
P
Q
R
S
T
V
W
Y
China
Japan
Latvia
Russia
Korea
USSR

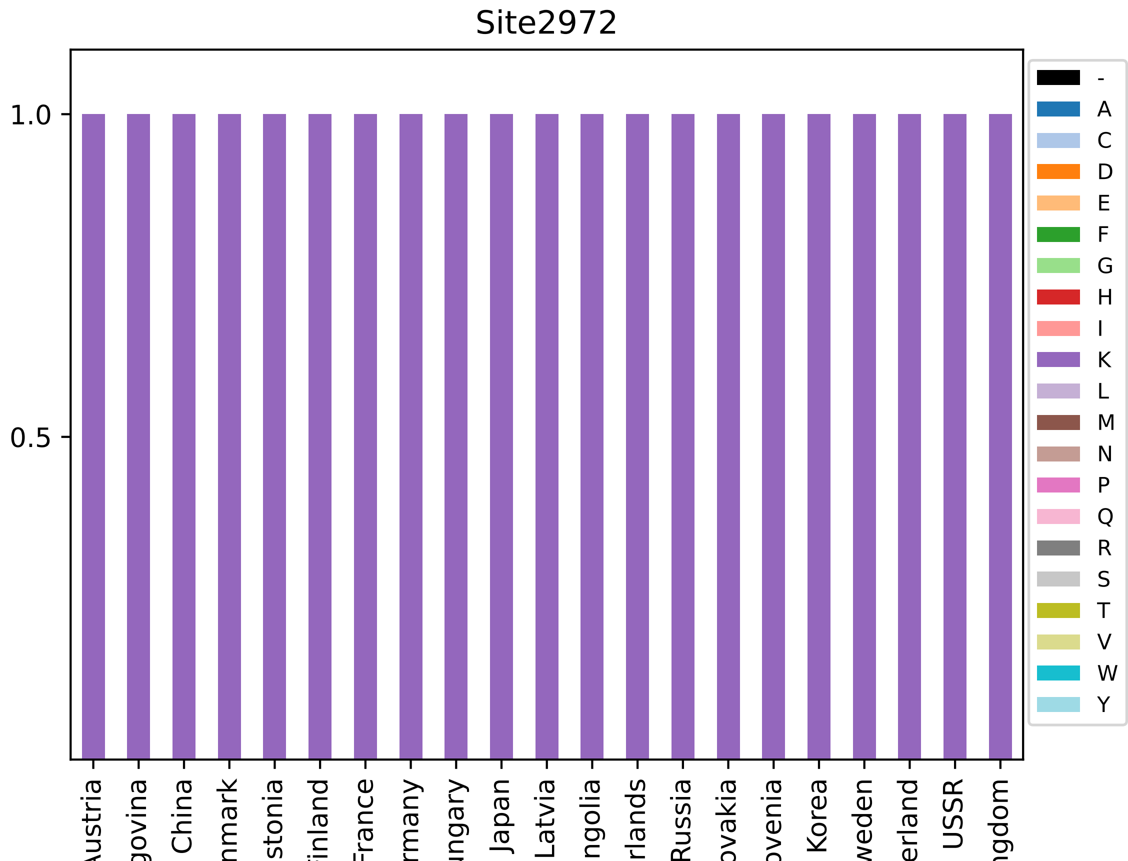

Site2972
1.0
0.5
-
A
C
D
E
F
G
H
I
K
L
M
N
P
Q
R
S
T
V
W
Y
Austria
govina
China
nmark
stonia
Finland
France
rmany
ungary
Japan
Latvia
ngolia
rlands
Russia
ovakia
ovenia
Korea
weden
erland
USSR
ngdom

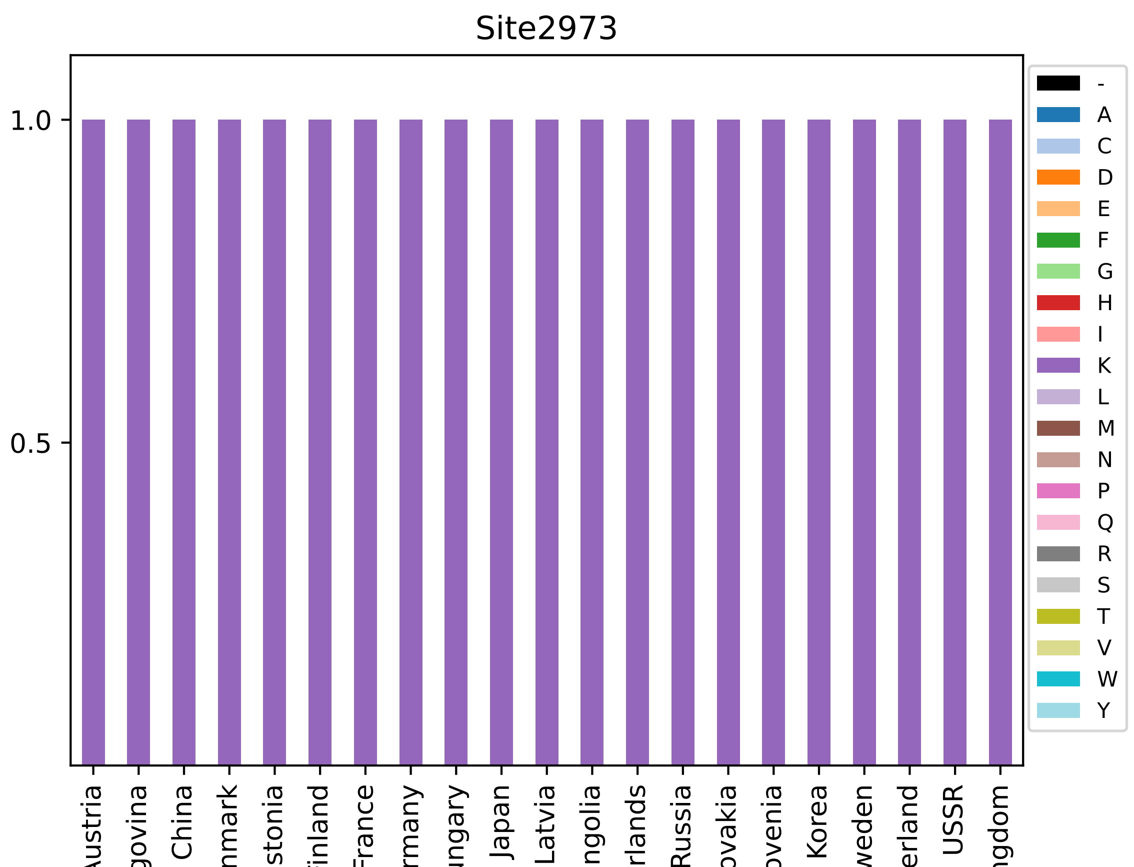

Site2973
1.0
0.5
-
A
C
D
E
F
G
H
I
K
L
M
N
P
Q
R
S
T
V
W
Y
China
Japan
Latvia
Russia
Korea
USSR

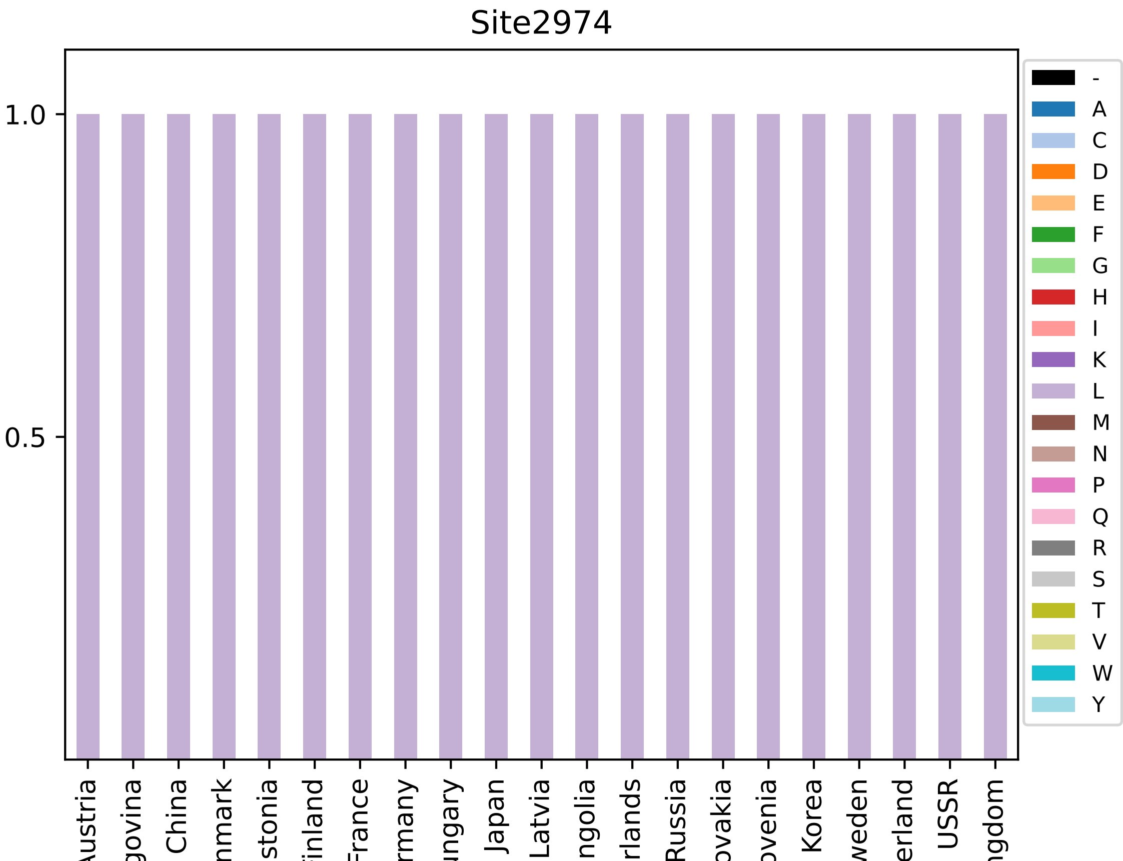
Site2974
1.0
0.5
-
A
C
D
E
F
G
H
I
K
L
M
N
P
Q
R
S
T
V
W
Y
China
Japan
Latvia
Russia
Korea
USSR

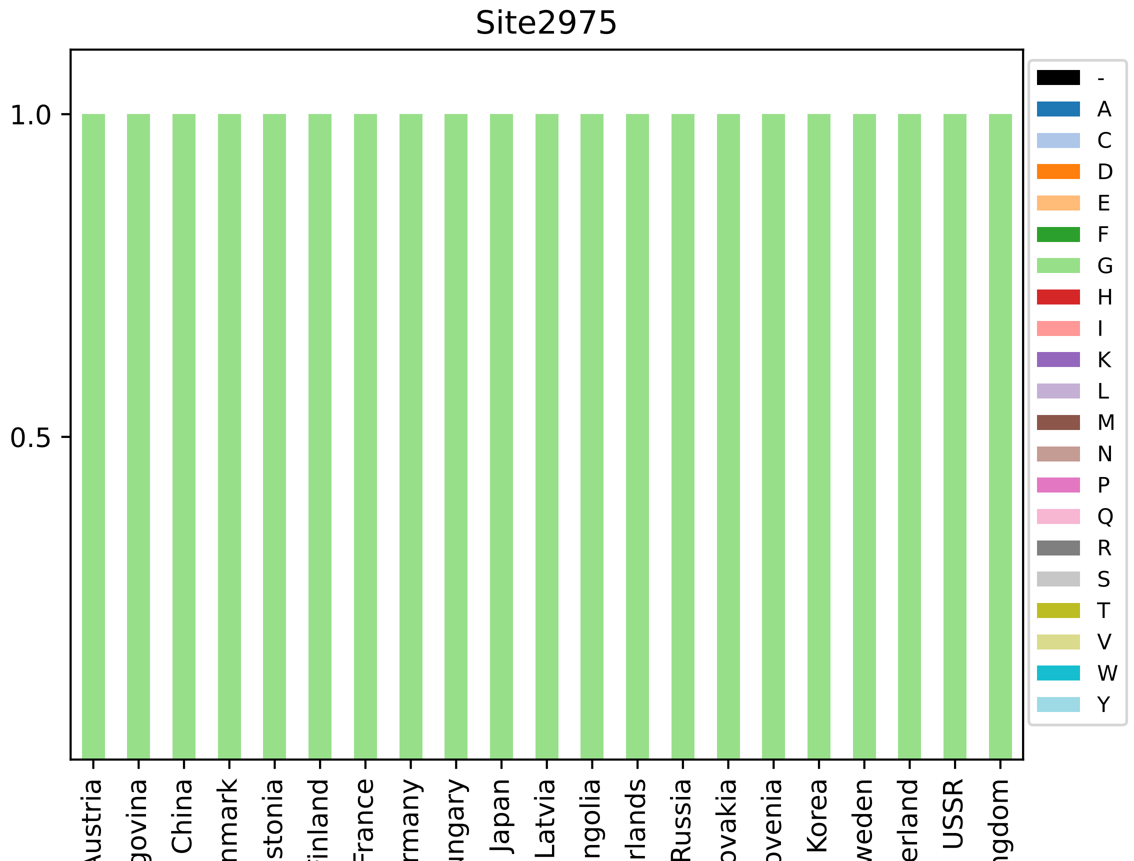

Site2975
1.0
0.5
-
A
C
D
E
F
G
H
I
K
L
M
N
P
Q
R
S
T
V
W
Y
China
Japan
Latvia
Russia
Korea
USSR

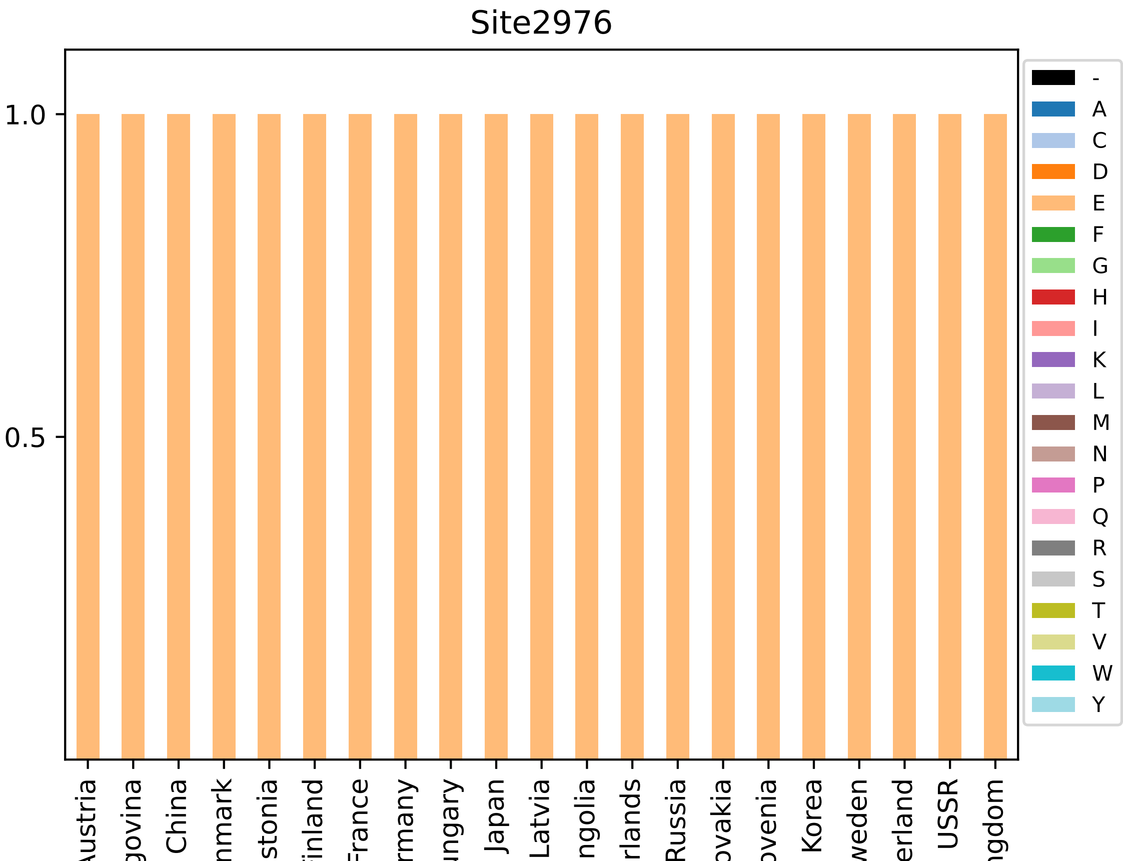

Site2976
1.0
0.5
-
A
C
D
E
F
G
H
I
K
L
M
N
P
Q
R
S
T
V
W
Y
China
France
Japan
Latvia
Russia
Korea
USSR

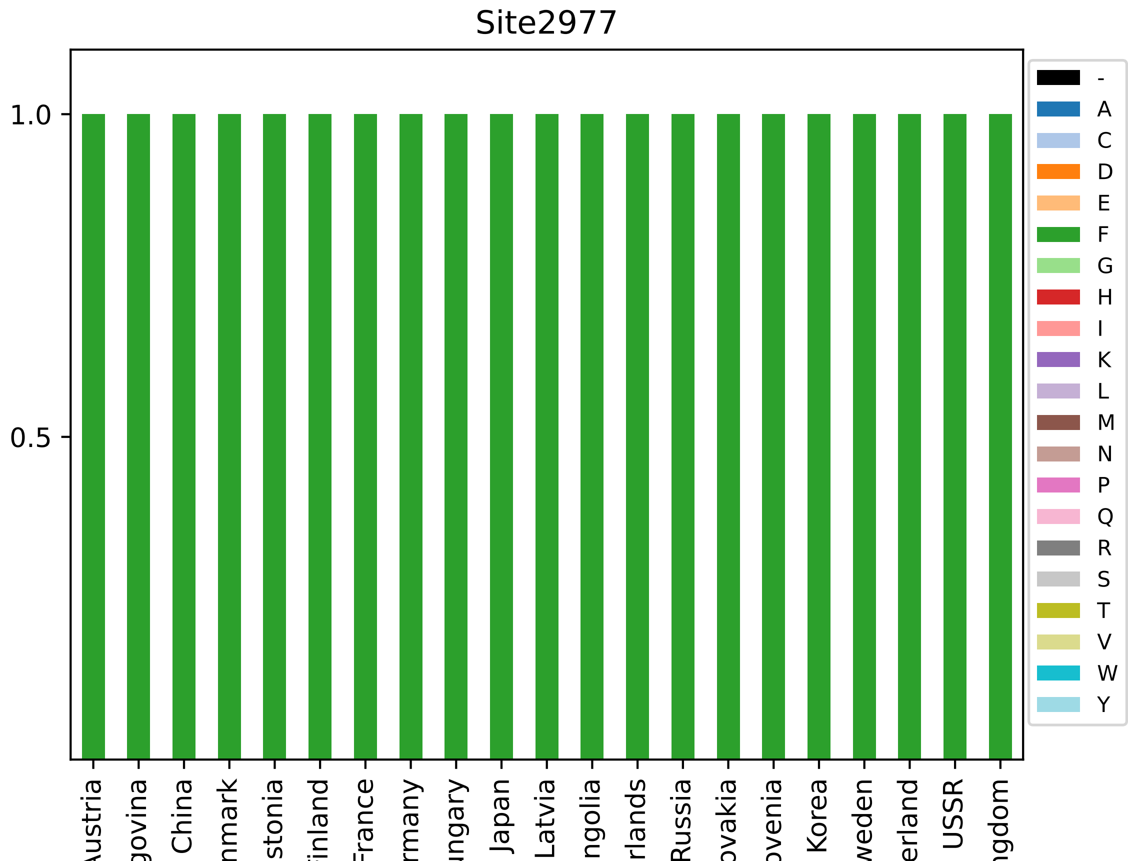

Site2977
1.0
0.5
-
A
C
D
E
F
G
H
I
K
L
M
N
P
Q
R
S
T
V
W
Y
China
Japan
Latvia
Russia
Korea
USSR

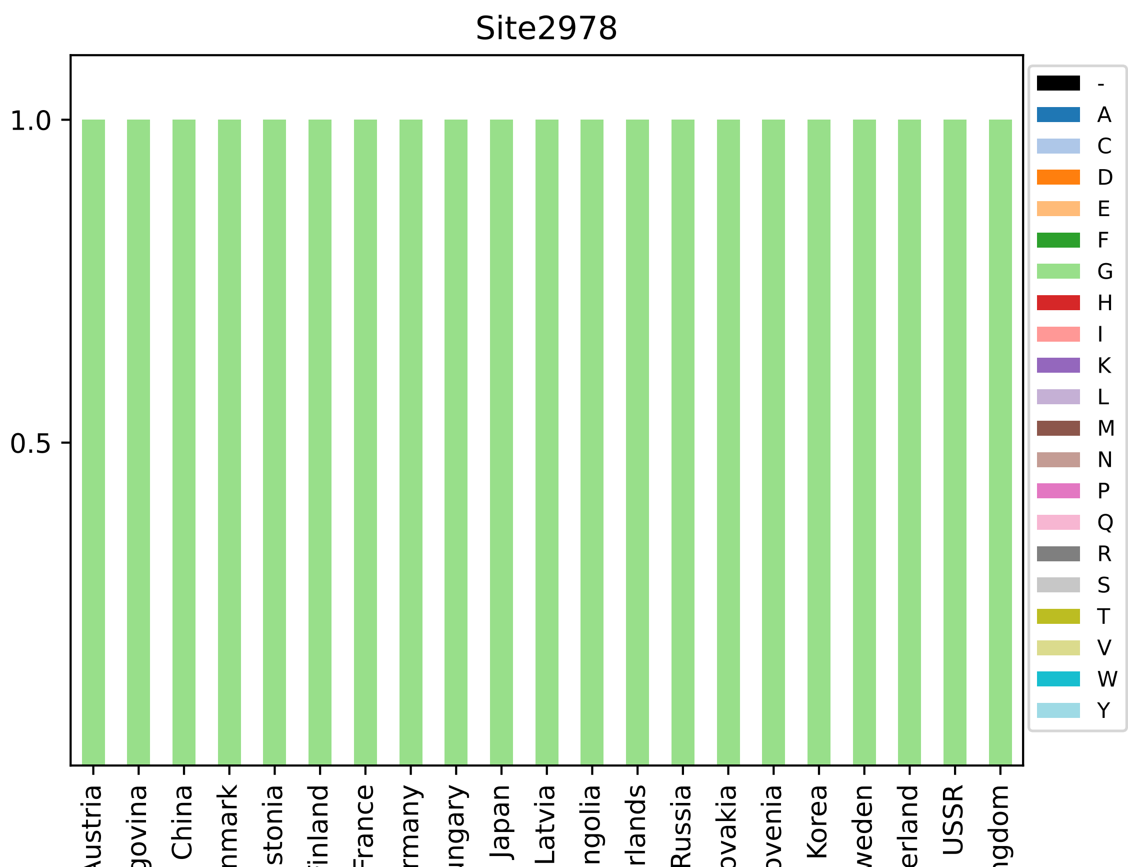

Site2978
1.0
0.5
-
A
C
D
E
F
G
H
I
K
L
M
N
P
Q
R
S
T
V
W
Y
Austria
China
Finland
France
Japan
Latvia
Russia
Korea
USSR

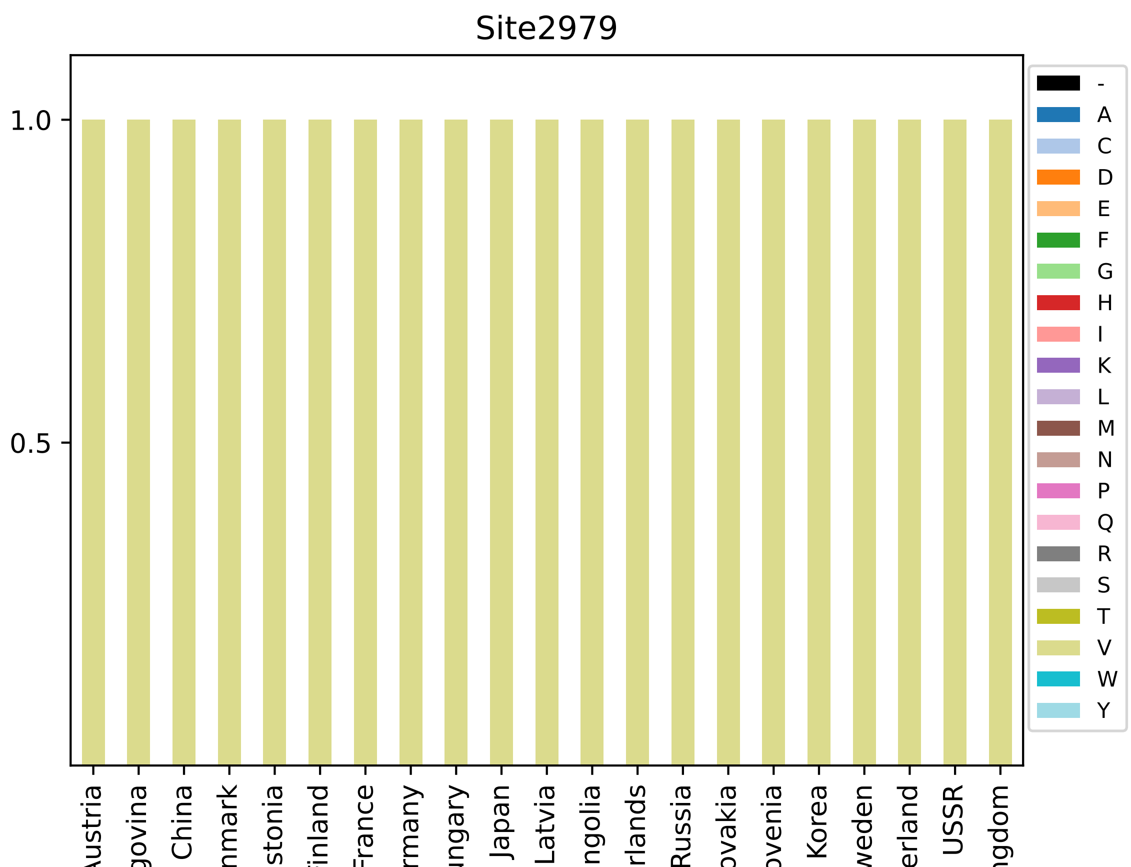
Site2979
1.0
0.5
-
A
C
D
E
F
G
H
I
K
L
M
N
P
Q
R
S
T
V
W
Y
Austria
China
Japan
Latvia
Russia
Korea
USSR

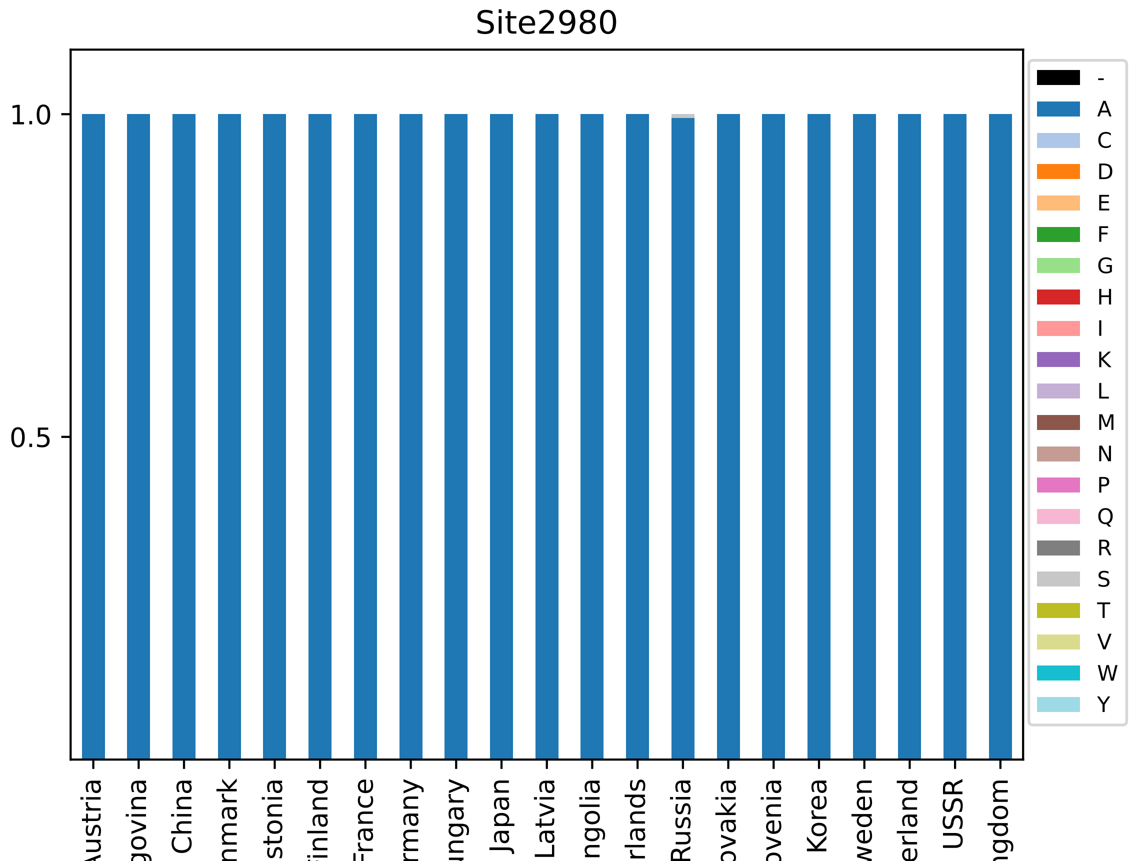

Site2980
1.0
0.5
-
A
C
D
E
F
G
H
I
K
L
M
N
P
Q
R
S
T
V
W
Y
China
Japan
Latvia
Russia
Korea
USSR

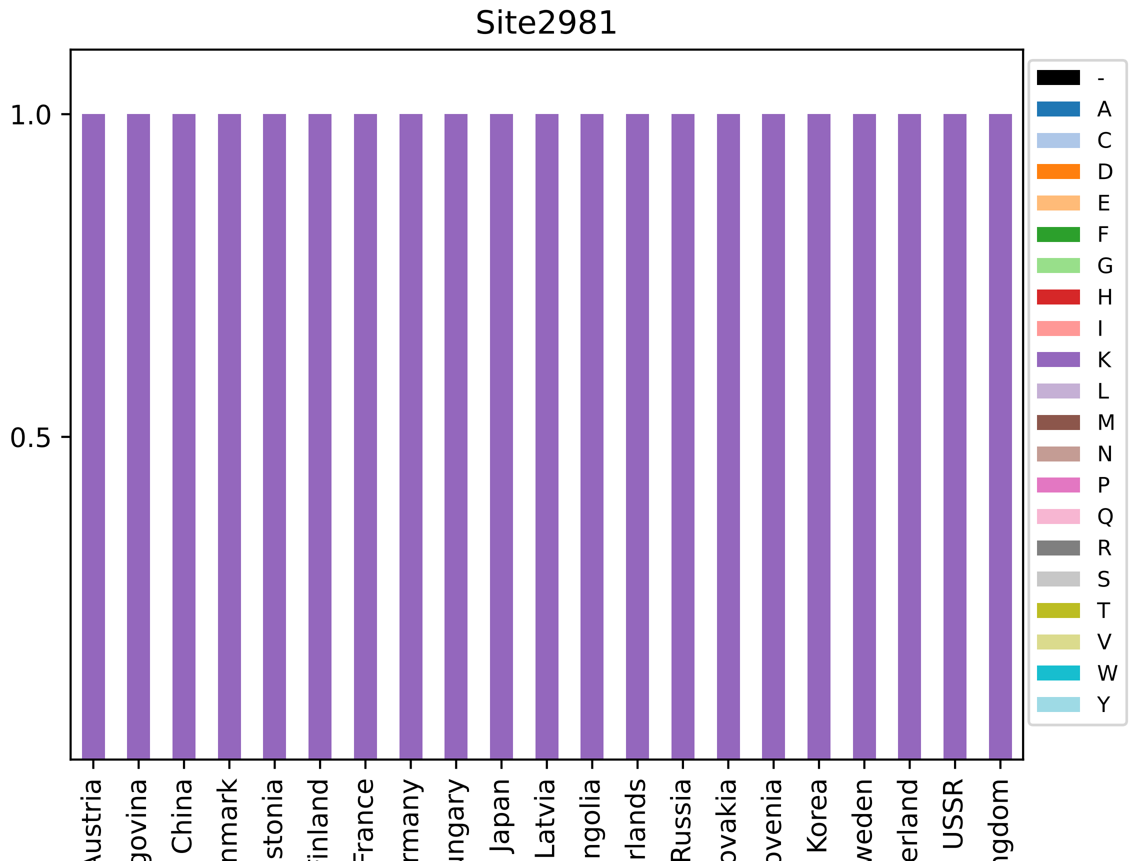

Site2981
1.0
0.5
-
A
C
D
E
F
G
H
I
K
L
M
N
P
Q
R
S
T
V
W
Y
China
Japan
Latvia
Russia
Korea
USSR

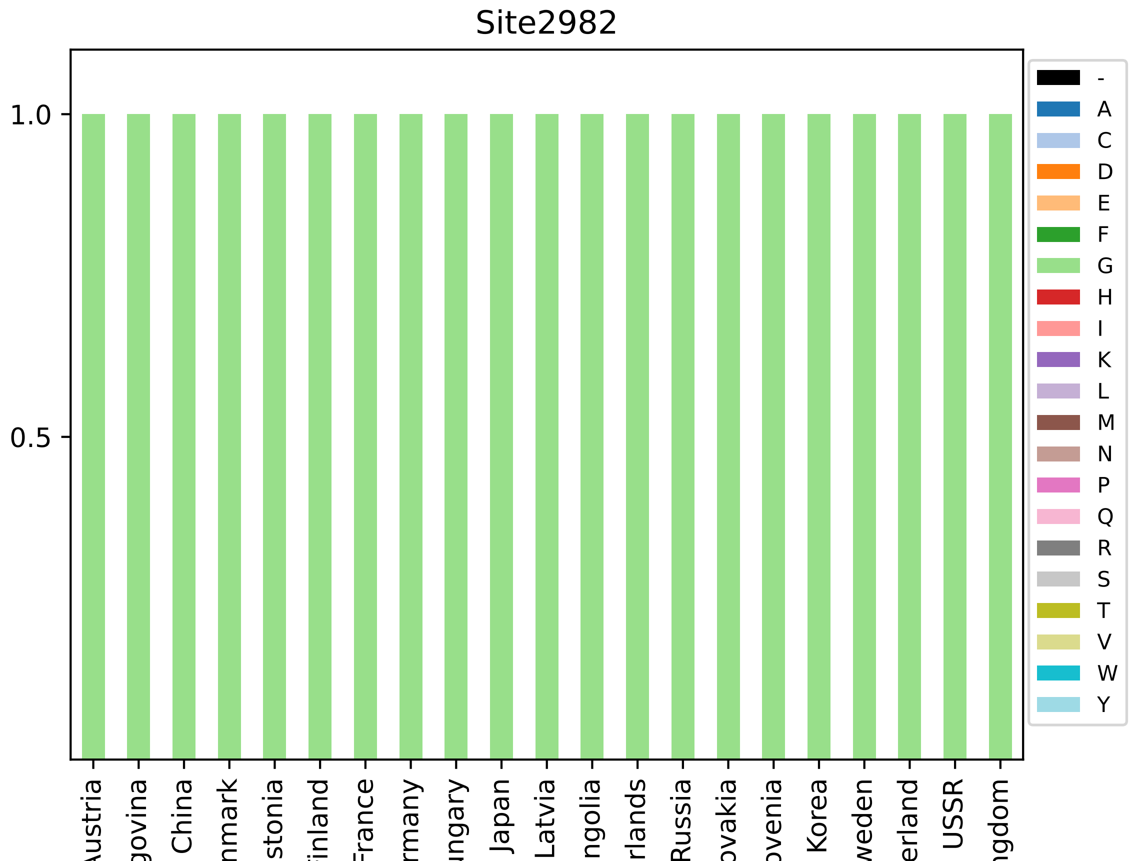
Site2982
1.0
0.5
-
A
C
D
E
F
G
H
I
K
L
M
N
P
Q
R
S
T
V
W
Y
Austria
govina
China
nmark
stonia
inland
France
rmany
ungary
Japan
Latvia
ngolia
rlands
Russia
ovakia
ovenia
Korea
weden
erland
USSR
ngdom

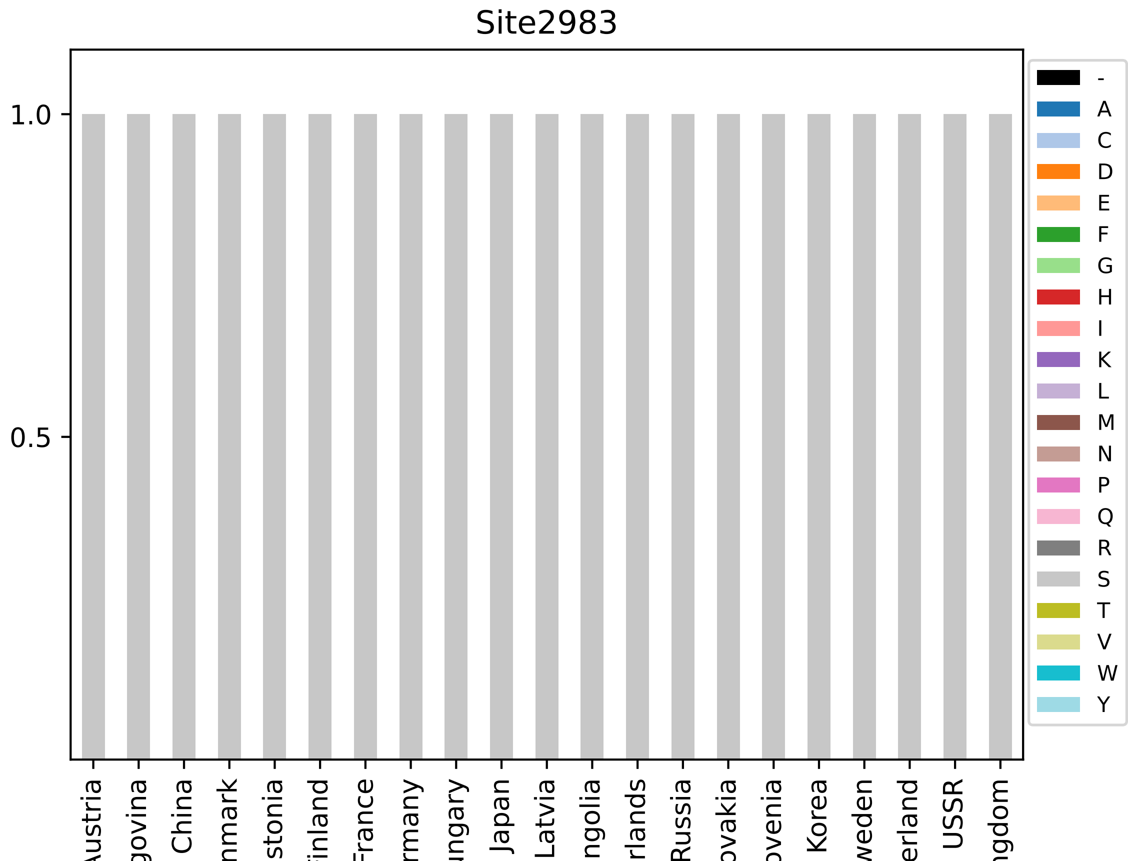
Site2983
1.0
0.5
-
A
C
D
E
F
G
H
I
K
L
M
N
P
Q
R
S
T
V
W
Y
China
Japan
Latvia
Russia
Korea
USSR

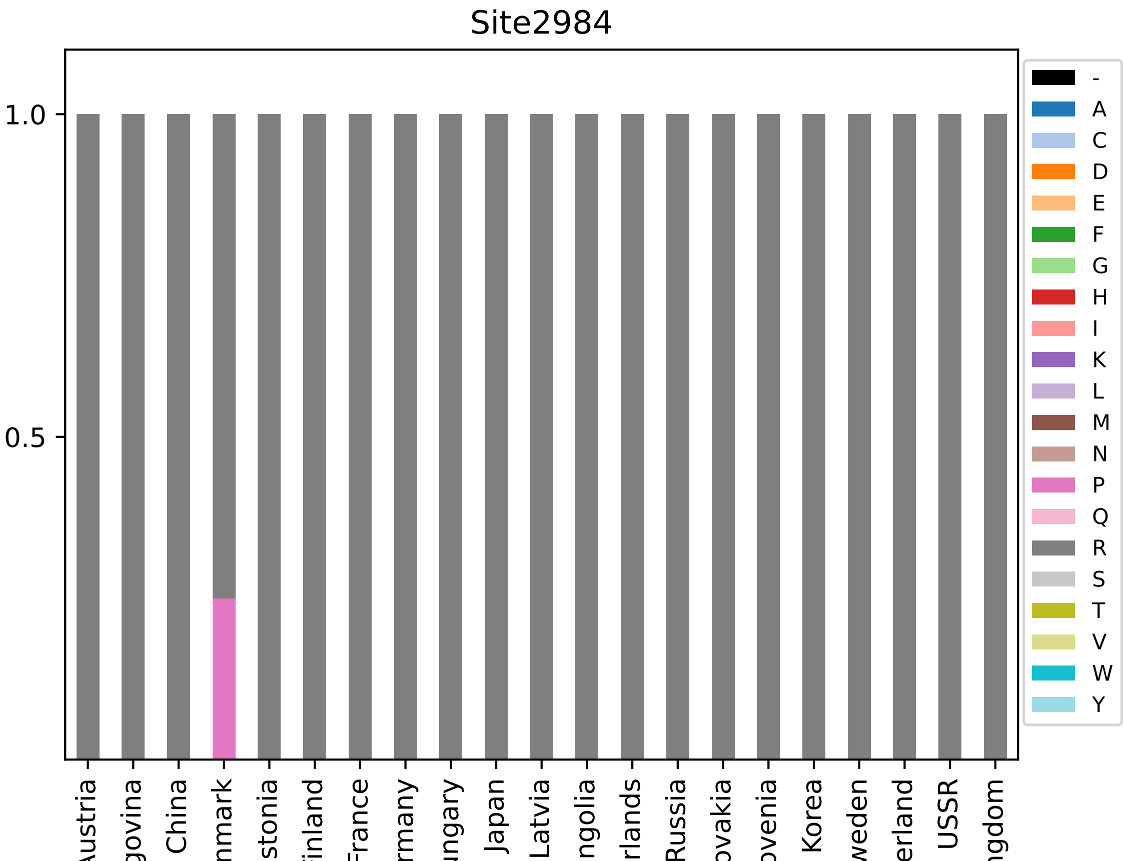
Site2984
1.0
0.5
-
A
C
D
E
F
G
H
I
K
L
M
N
P
Q
R
S
T
V
W
Y
China
Japan
Latvia
Russia
Korea
USSR

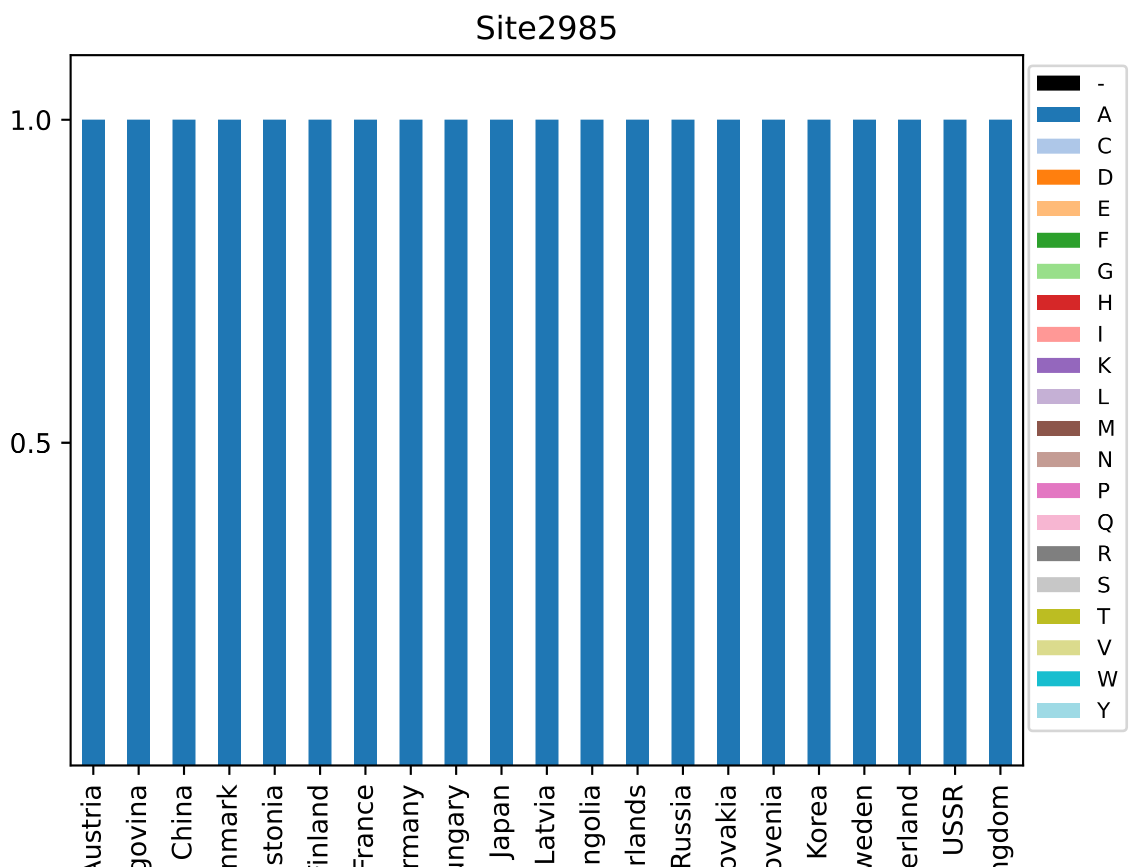
Site2985
1.0
0.5
-
A
C
D
E
F
G
H
I
K
L
M
N
P
Q
R
S
T
V
W
Y
Austria
China
Finland
Japan
Latvia
Russia
Korea
USSR

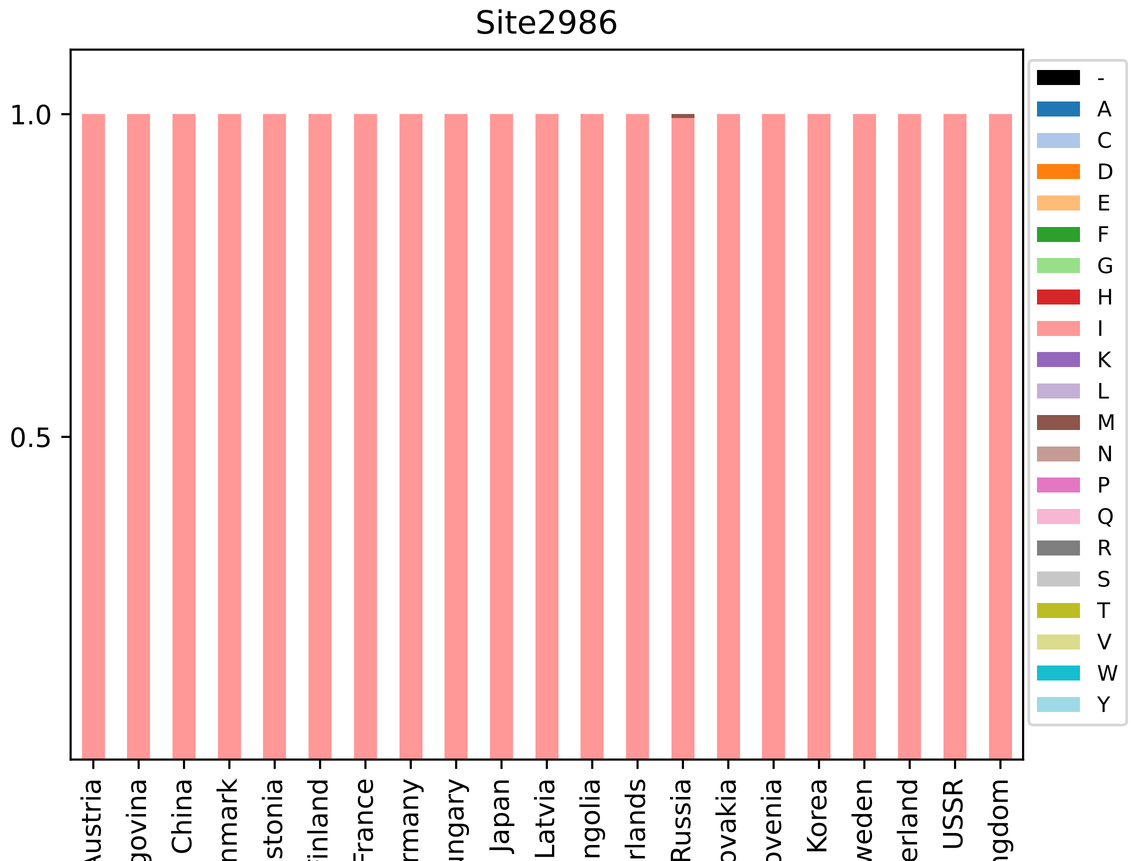
Site2986
1.0
0.5
-
A
C
D
E
F
G
H
I
K
L
M
N
P
Q
R
S
T
V
W
Y
China
Japan
Latvia
Russia
Korea
USSR

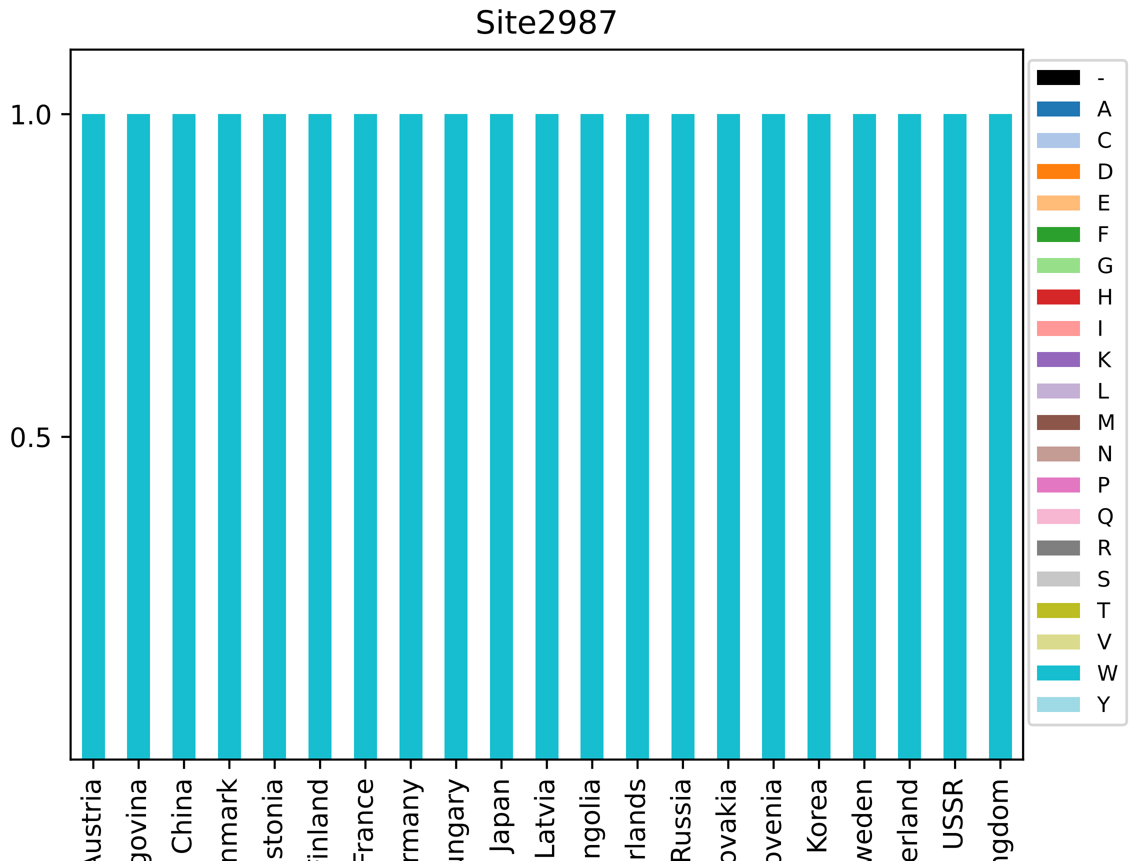

Site2987
1.0
0.5
-
A
C
D
E
F
G
H
I
K
L
M
N
P
Q
R
S
T
V
W
Y
China
Japan
Latvia
Russia
Korea
USSR

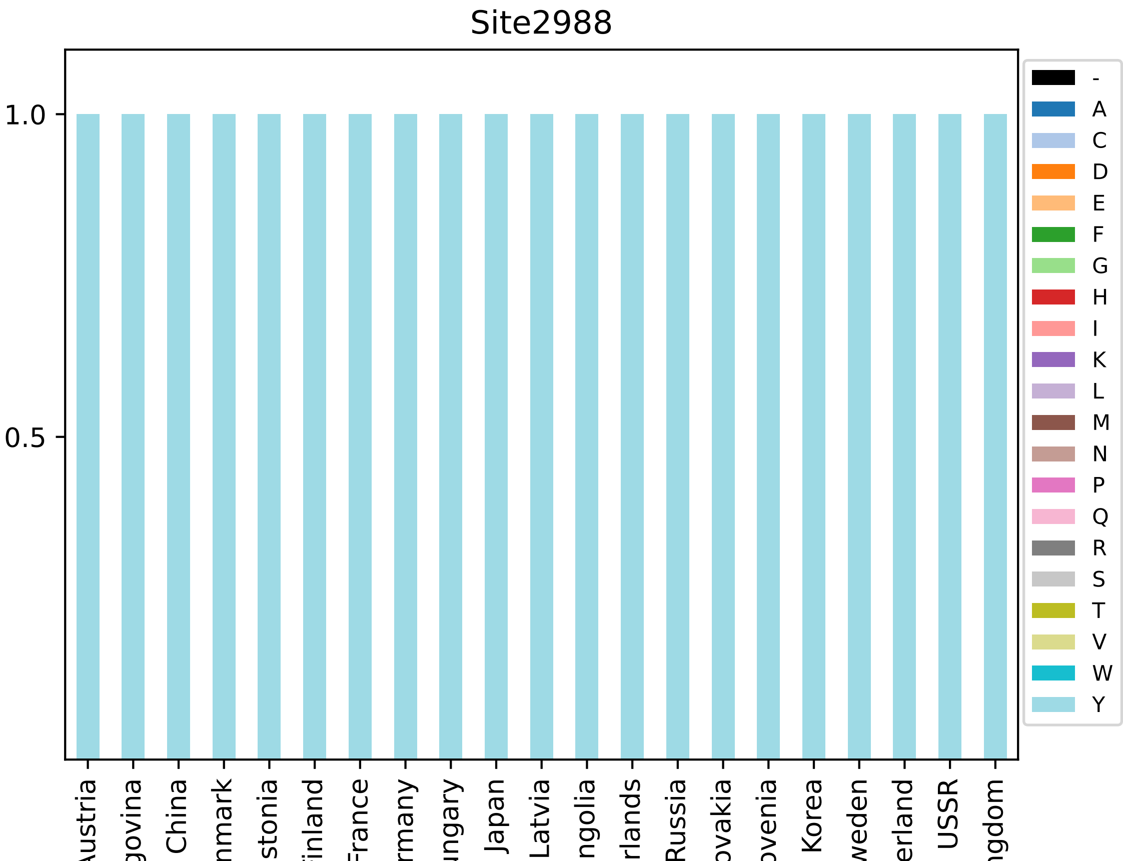
Site2988
1.0
0.5
-
A
C
D
E
F
G
H
I
K
L
M
N
P
Q
R
S
T
V
W
Y
Austria
China
Latvia
Russia
Korea
USSR

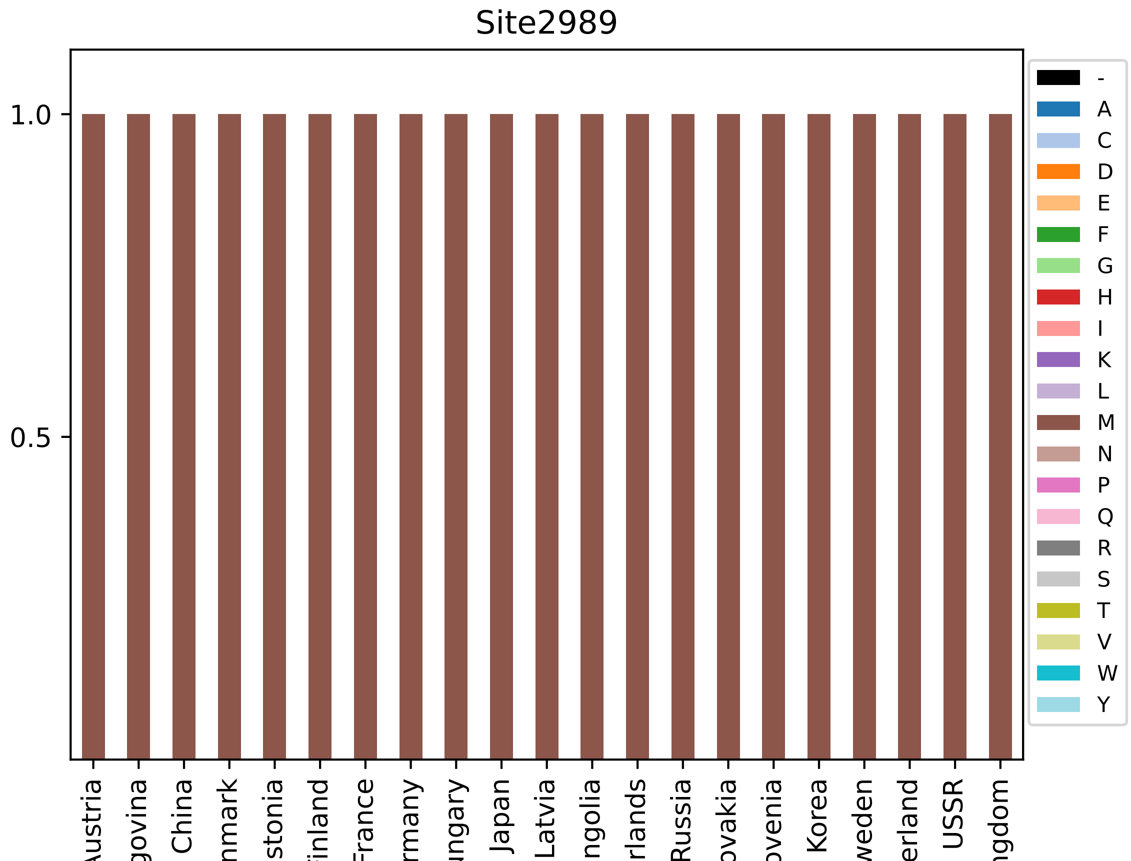

Site2989
1.0
0.5
Austria
govina
China
nmark
stonia
Finland
France
rmany
ungary
Japan
Latvia
ngolia
rlands
Russia
ovakia
ovenia
Korea
weden
erland
USSR
ngdom
-
A
C
D
E
F
G
H
I
K
L
M
N
P
Q
R
S
T
V
W
Y

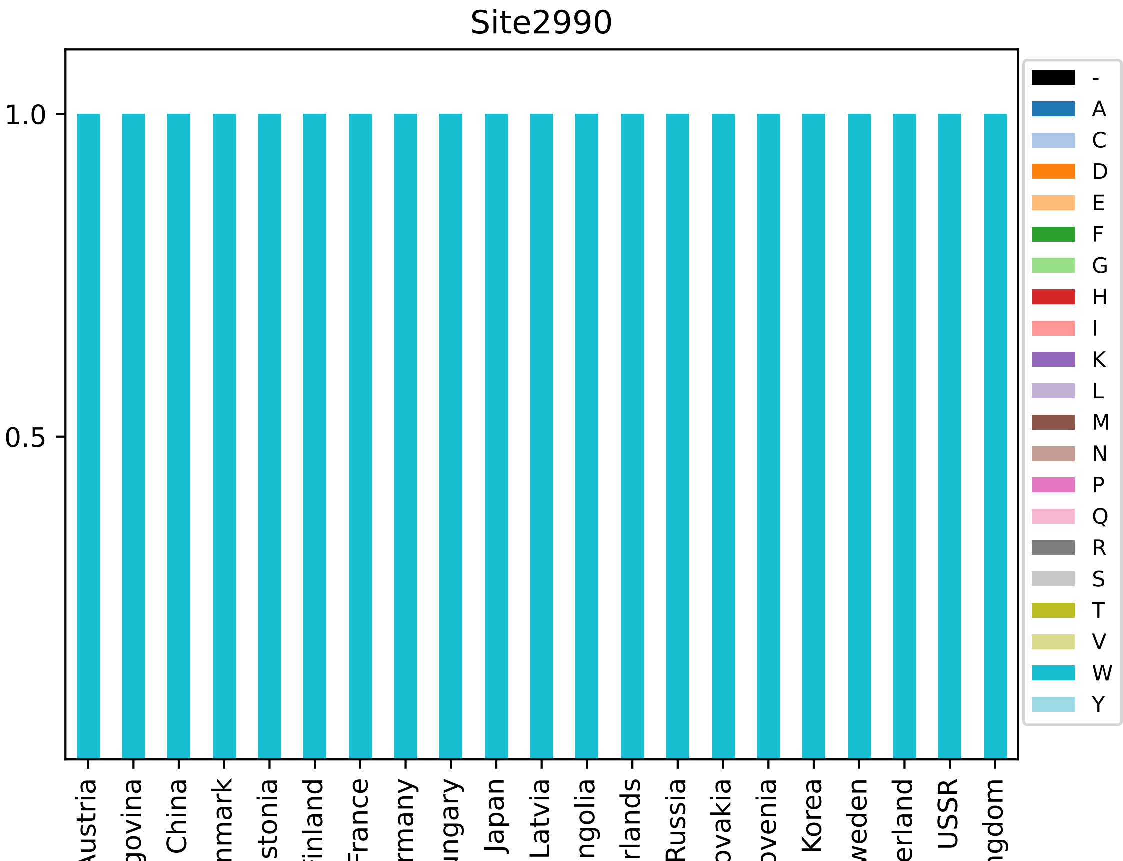
Site2990
1.0
0.5
-
A
C
D
E
F
G
H
I
K
L
M
N
P
Q
R
S
T
V
W
Y
China
Japan
Latvia
Russia
Korea
USSR

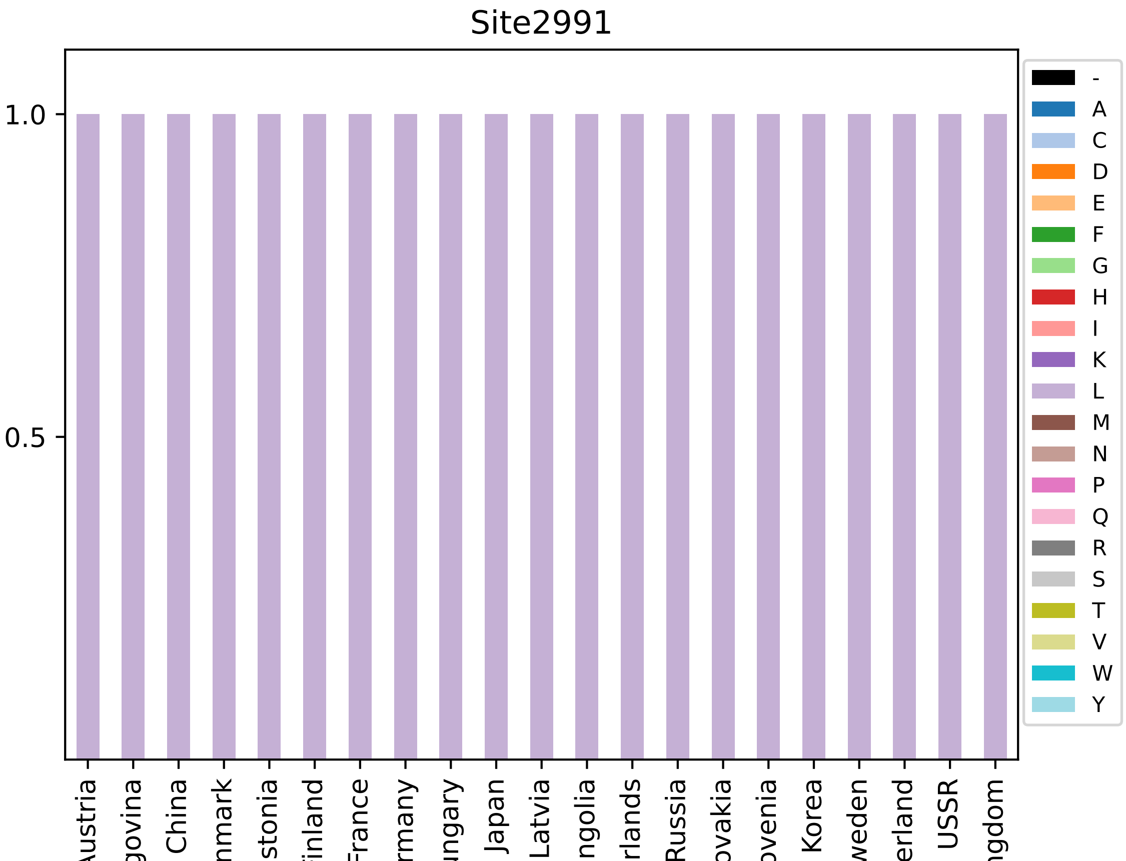
Site2991
1.0
0.5
-
A
C
D
E
F
G
H
I
K
L
M
N
P
Q
R
S
T
V
W
Y
China
Latvia
Russia
Korea
USSR
Japan

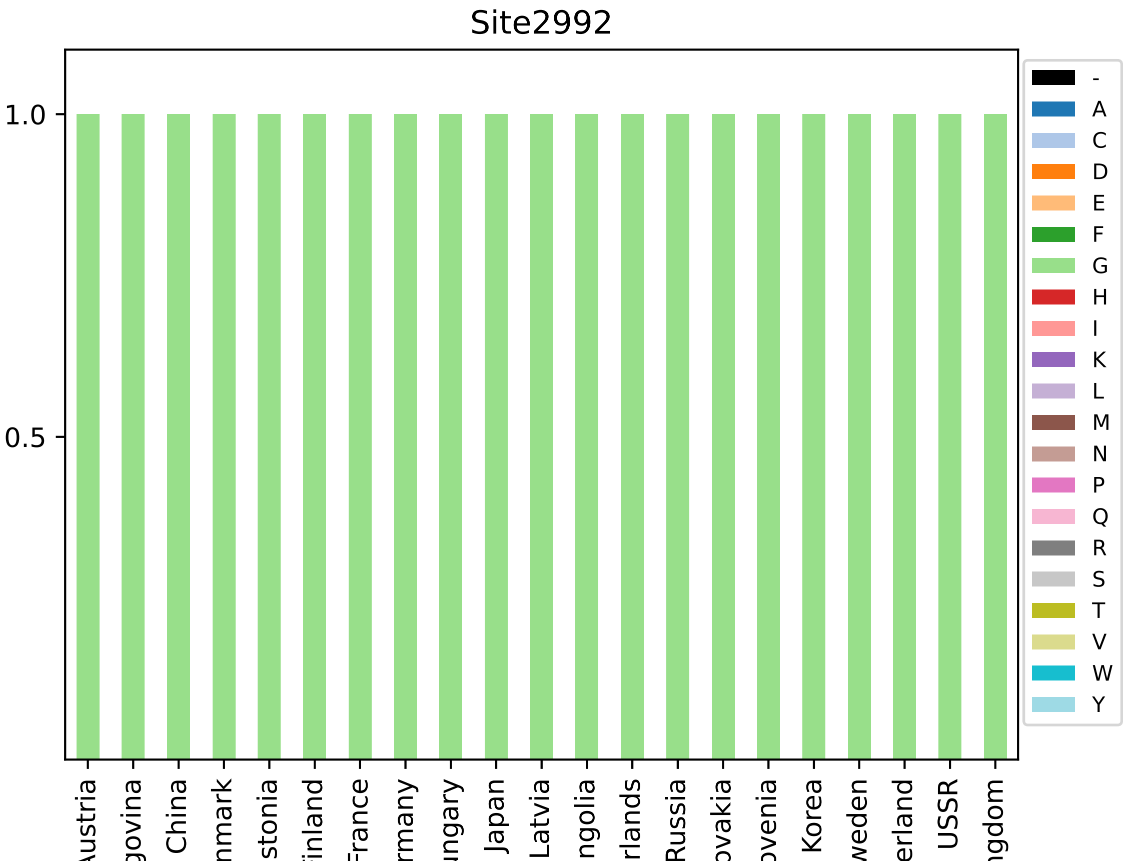
Site2992
1.0
0.5
-
A
C
D
E
F
G
H
I
K
L
M
N
P
Q
R
S
T
V
W
Y
Austria
govina
China
nmark
stonia
inland
France
rmany
ungary
Japan
Latvia
ngolia
rlands
Russia
ovakia
ovenia
Korea
weden
erland
USSR
ngdom

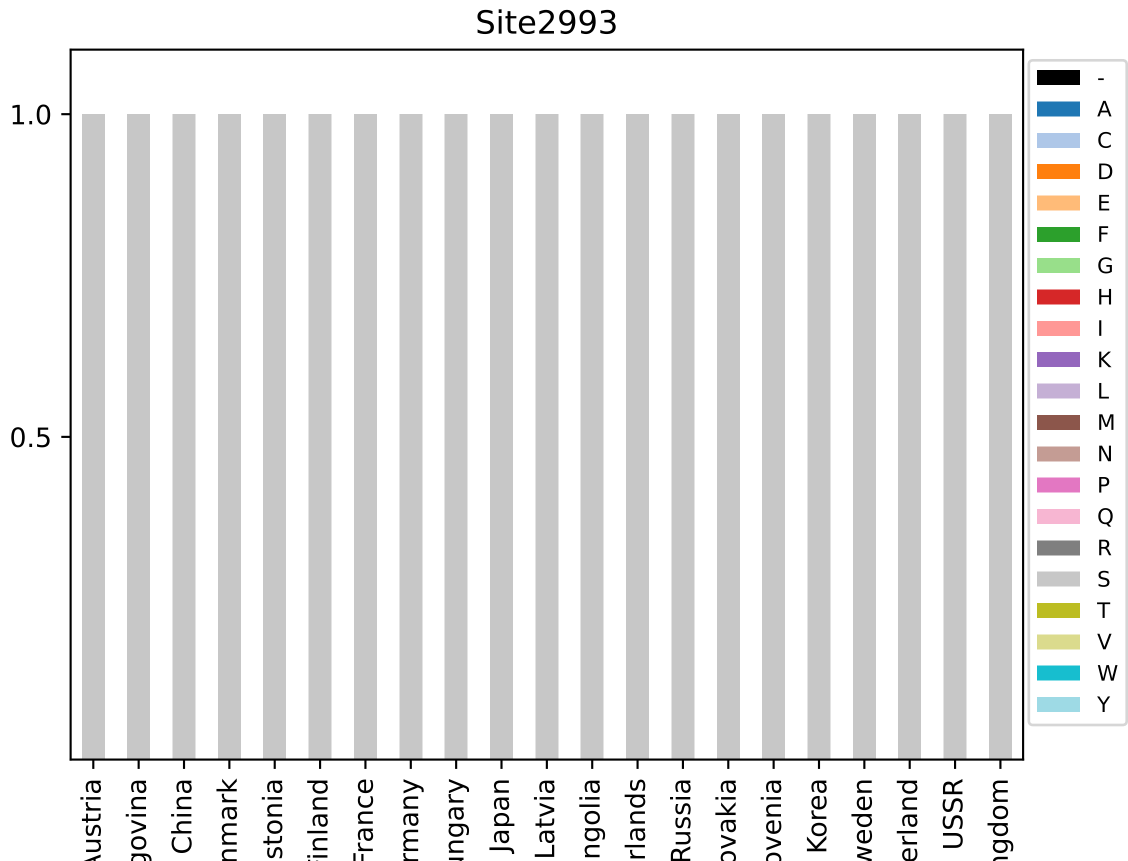

Site2993
1.0
0.5
-
A
C
D
E
F
G
H
I
K
L
M
N
P
Q
R
S
T
V
W
Y
Austria
China
Finland
France
Japan
Latvia
Russia
Korea
USSR

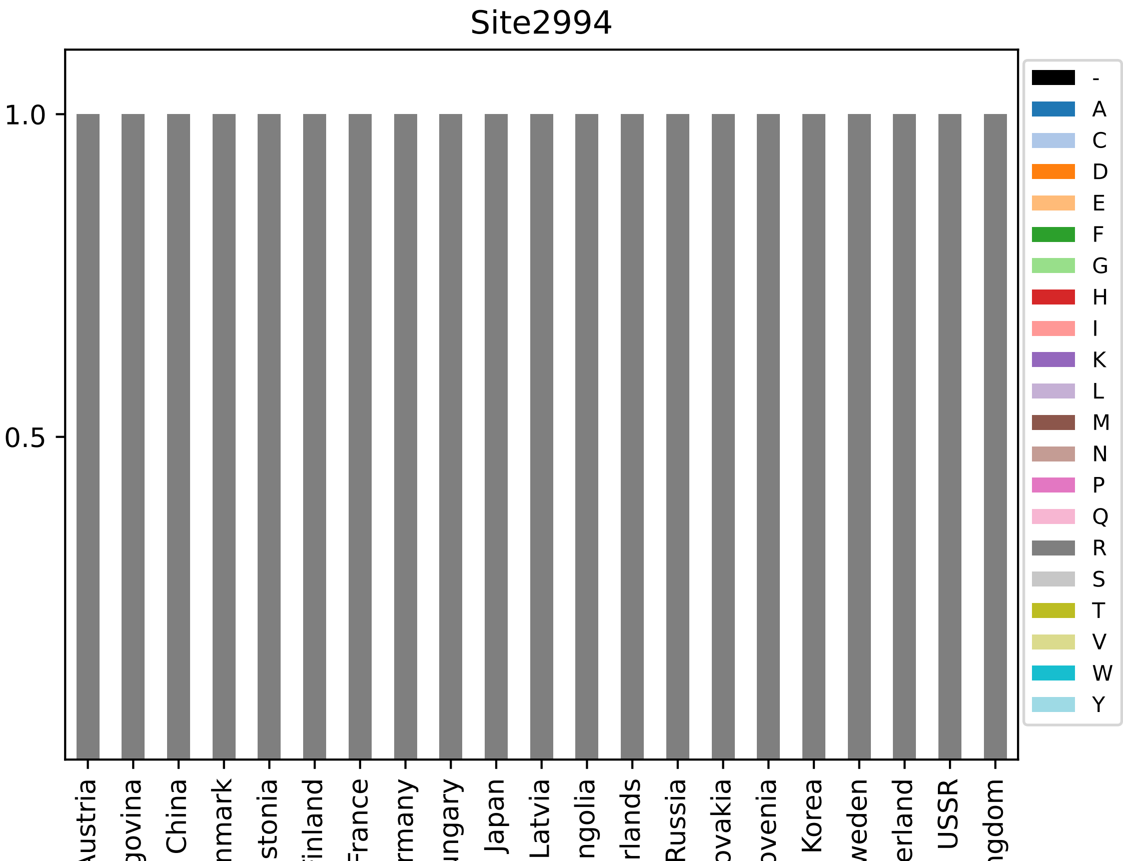

Site2994
1.0
0.5
-
A
C
D
E
F
G
H
I
K
L
M
N
P
Q
R
S
T
V
W
Y
China
Japan
Latvia
Russia
Korea
USSR

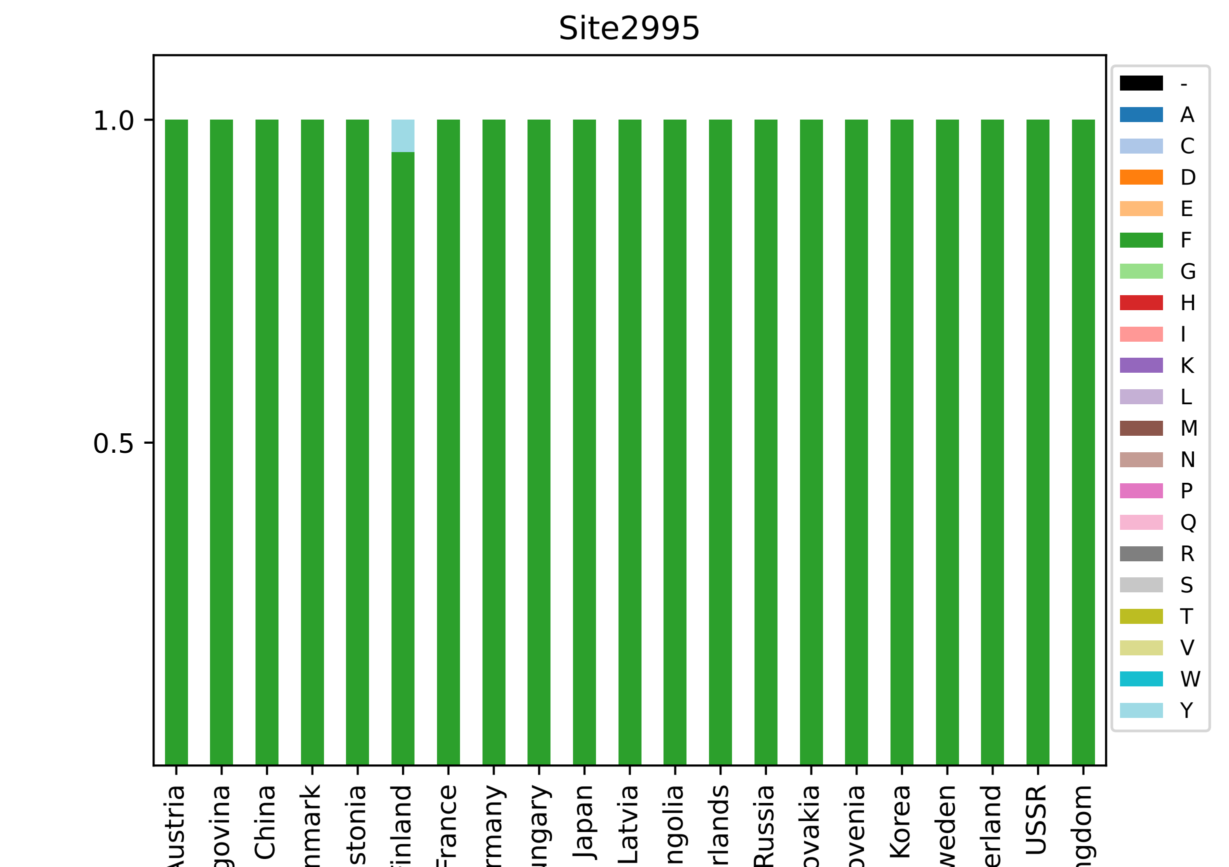

Site2995
1.0
0.5
-
A
C
D
E
F
G
H
I
K
L
M
N
P
Q
R
S
T
V
W
Y
Austria
govina
China
nmark
stonia
inland
France
rmany
ungary
Japan
Latvia
ngolia
rlands
Russia
ovakia
ovenia
Korea
weden
erland
USSR
ngdom

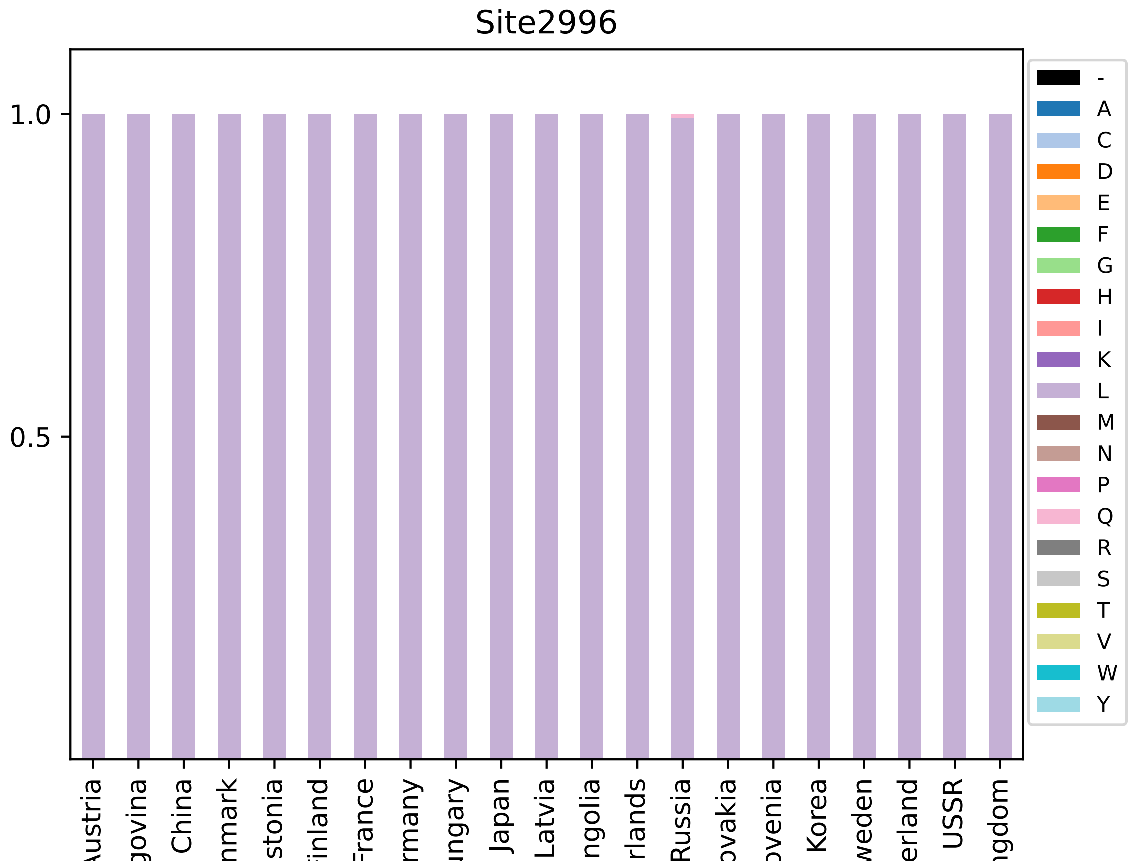

Site2996
1.0
0.5
-
A
C
D
E
F
G
H
I
K
L
M
N
P
Q
R
S
T
V
W
Y
Austria
China
Finland
France
Japan
Latvia
Russia
Korea
USSR

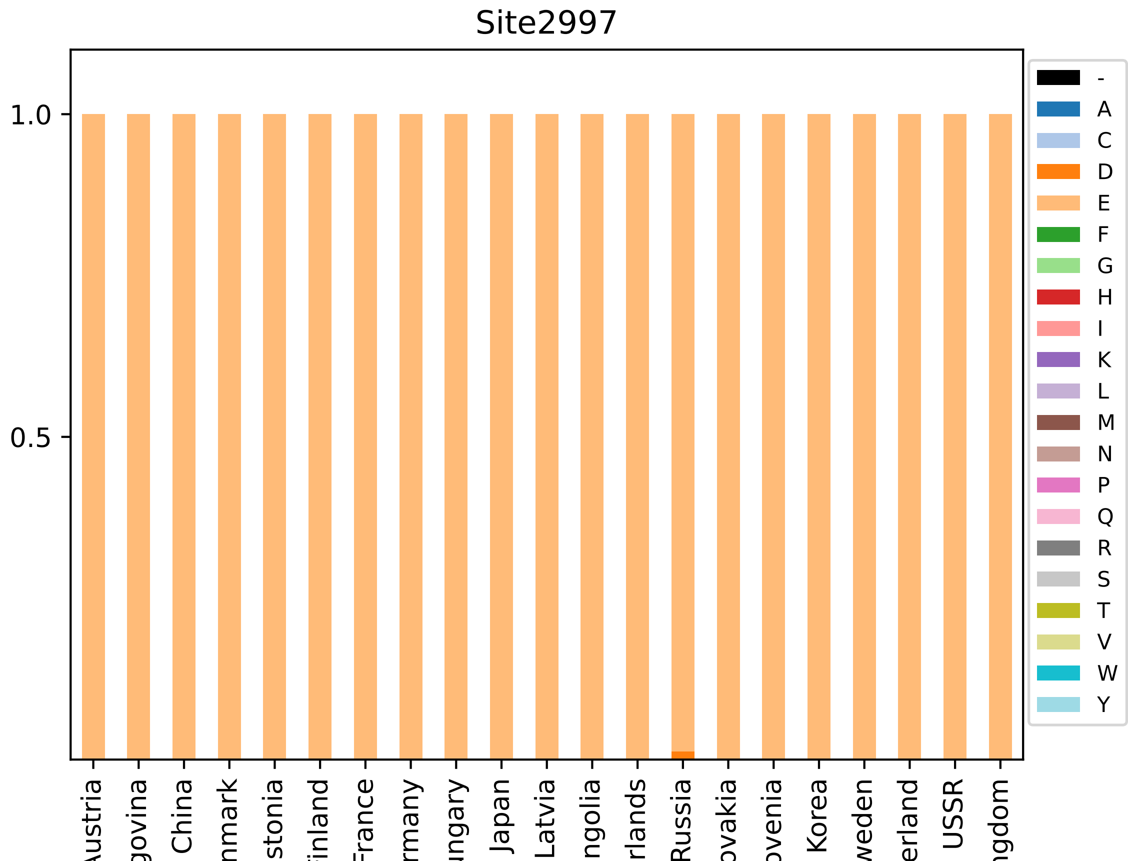
Site2997
1.0
0.5
China
France
Japan
Latvia
Russia
Korea
USSR
-
A
C
D
E
F
G
H
I
K
L
M
N
P
Q
R
S
T
V
W
Y

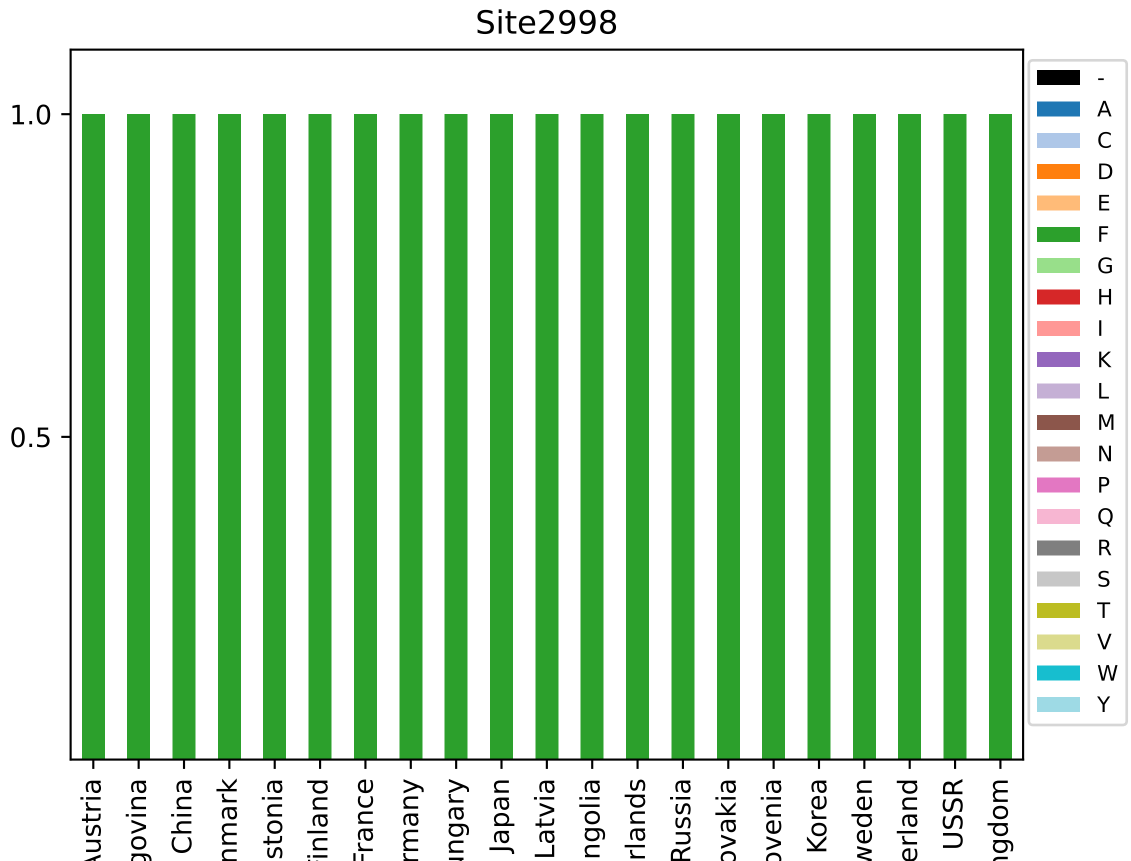
Site2998
1.0
0.5
-
A
C
D
E
F
G
H
I
K
L
M
N
P
Q
R
S
T
V
W
Y
China
Japan
Latvia
Russia
Korea
USSR

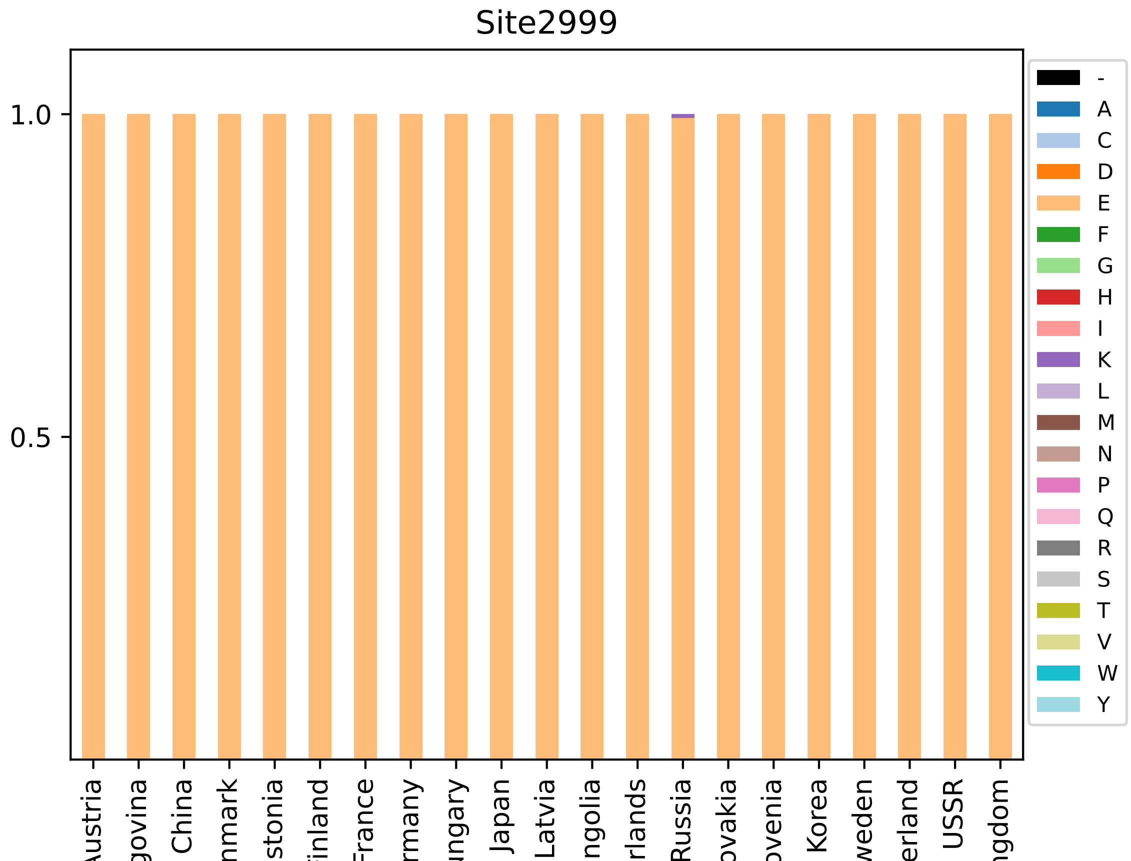
Site2999
1.0
0.5
China
Japan
Latvia
Korea
USSR
-
A
C
D
E
F
G
H
I
K
L
M
N
P
Q
R
S
T
V
W
Y

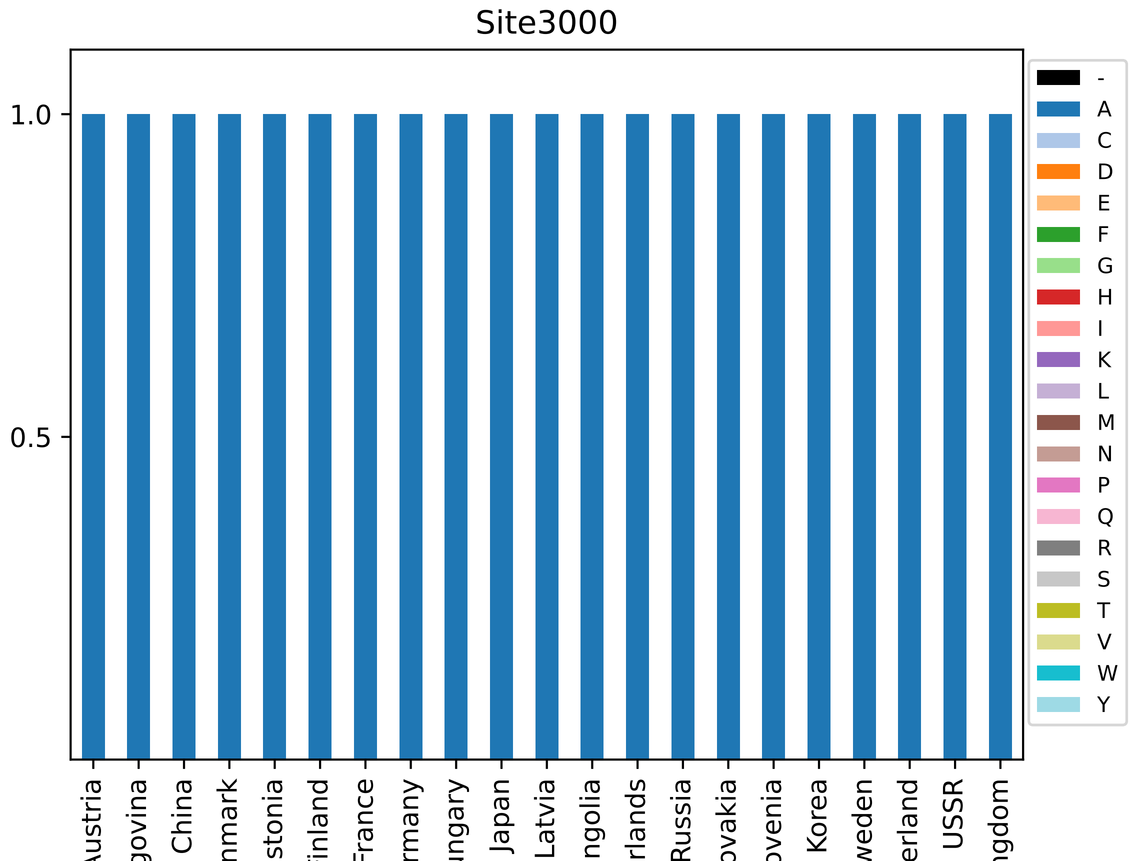
Site3000
1.0
0.5
-
A
C
D
E
F
G
H
I
K
L
M
N
P
Q
R
S
T
V
W
Y
China
Japan
Latvia
Russia
Korea
USSR

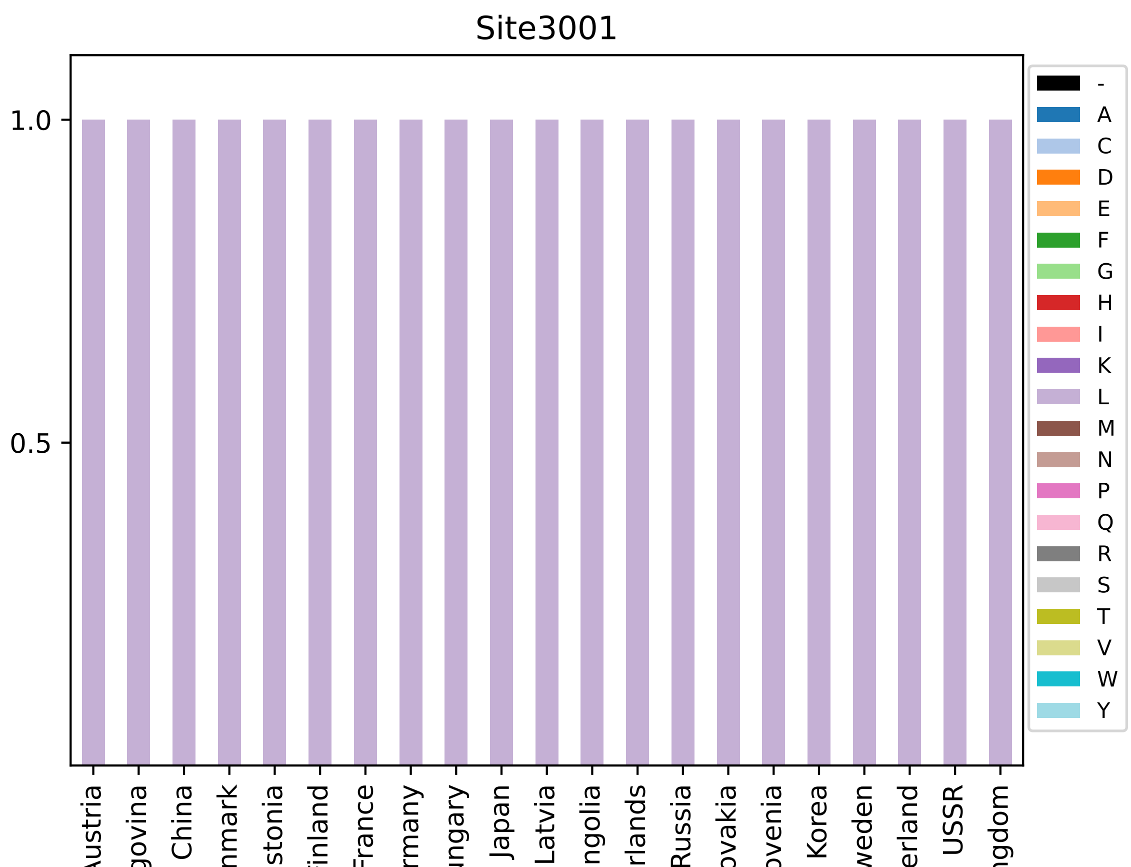

Site3001
1.0
0.5
-
A
C
D
E
F
G
H
I
K
L
M
N
P
Q
R
S
T
V
W
Y
Austria
China
Japan
Latvia
Russia
Korea
USSR

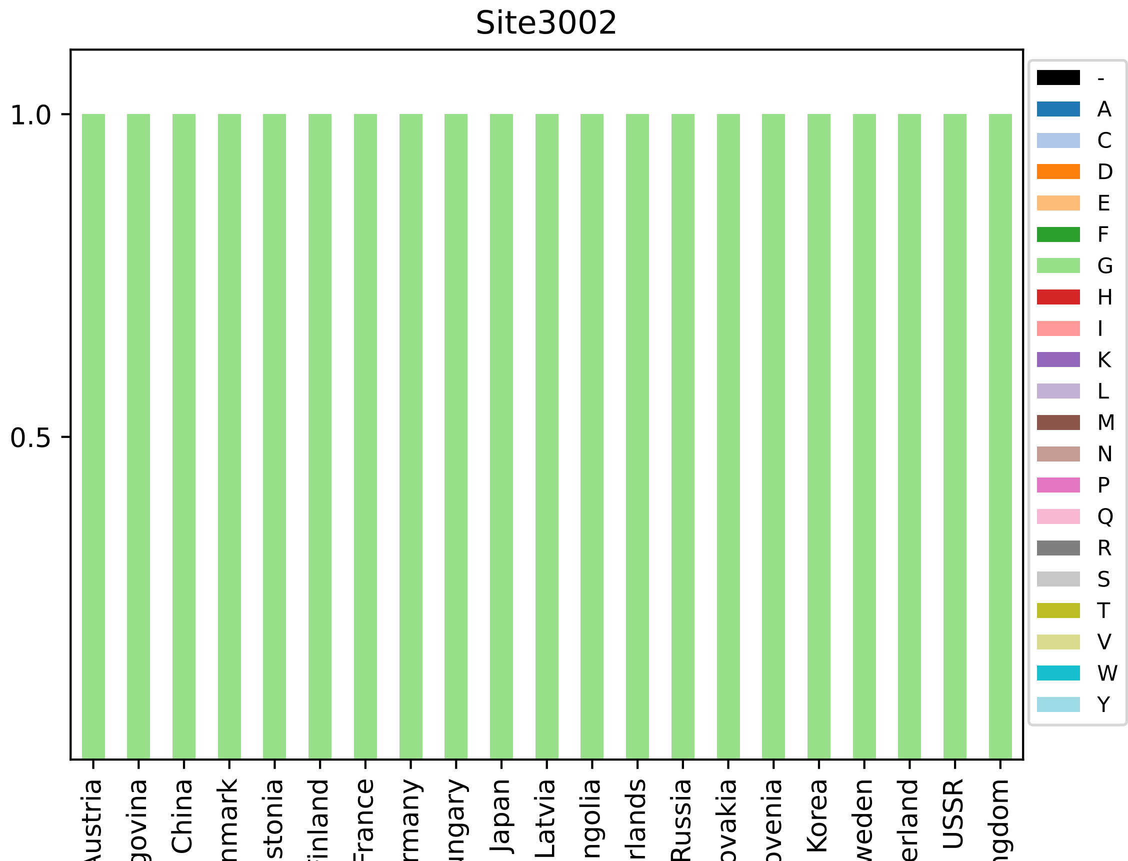
Site3002
1.0
0.5
-
A
C
D
E
F
G
H
I
K
L
M
N
P
Q
R
S
T
V
W
Y
Austria
govina
China
nmark
stonia
inland
France
rmany
ungary
Japan
Latvia
ngolia
rlands
Russia
ovakia
ovenia
Korea
weden
erland
USSR
ngdom

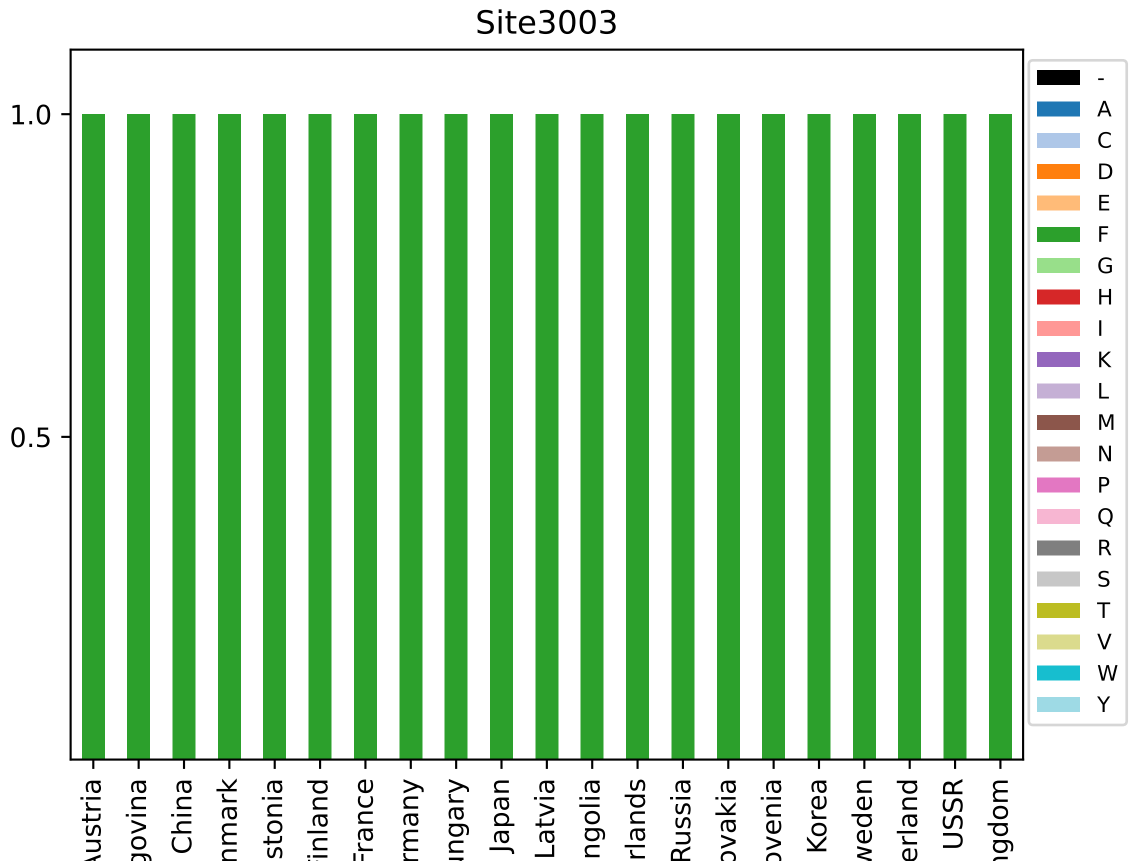
Site3003
1.0
0.5
-
A
C
D
E
F
G
H
I
K
L
M
N
P
Q
R
S
T
V
W
Y
Austria
govina
China
nmark
stonia
inland
France
rmany
ungary
Japan
Latvia
ngolia
rlands
Russia
ovakia
ovenia
Korea
weden
erland
USSR
ngdom

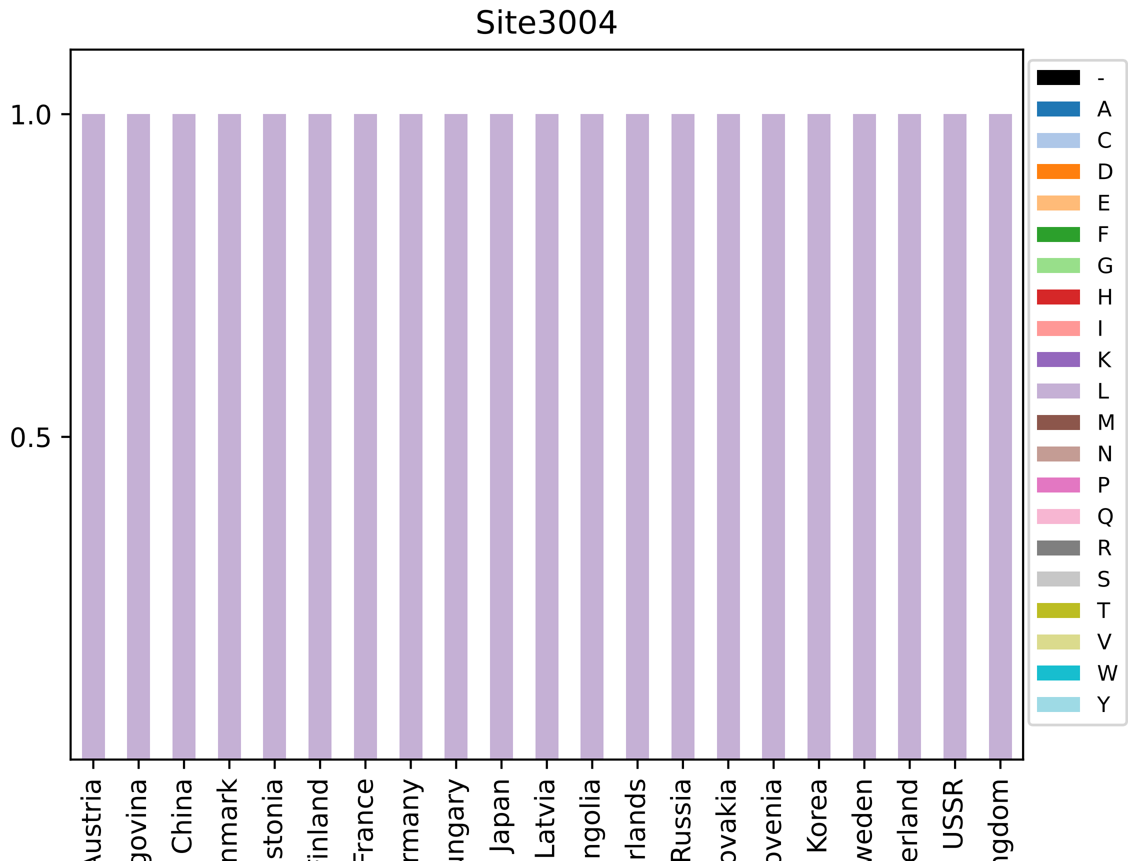
Site3004
1.0
0.5
-
A
C
D
E
F
G
H
I
K
L
M
N
P
Q
R
S
T
V
W
Y
China
Japan
Latvia
Russia
Korea
USSR

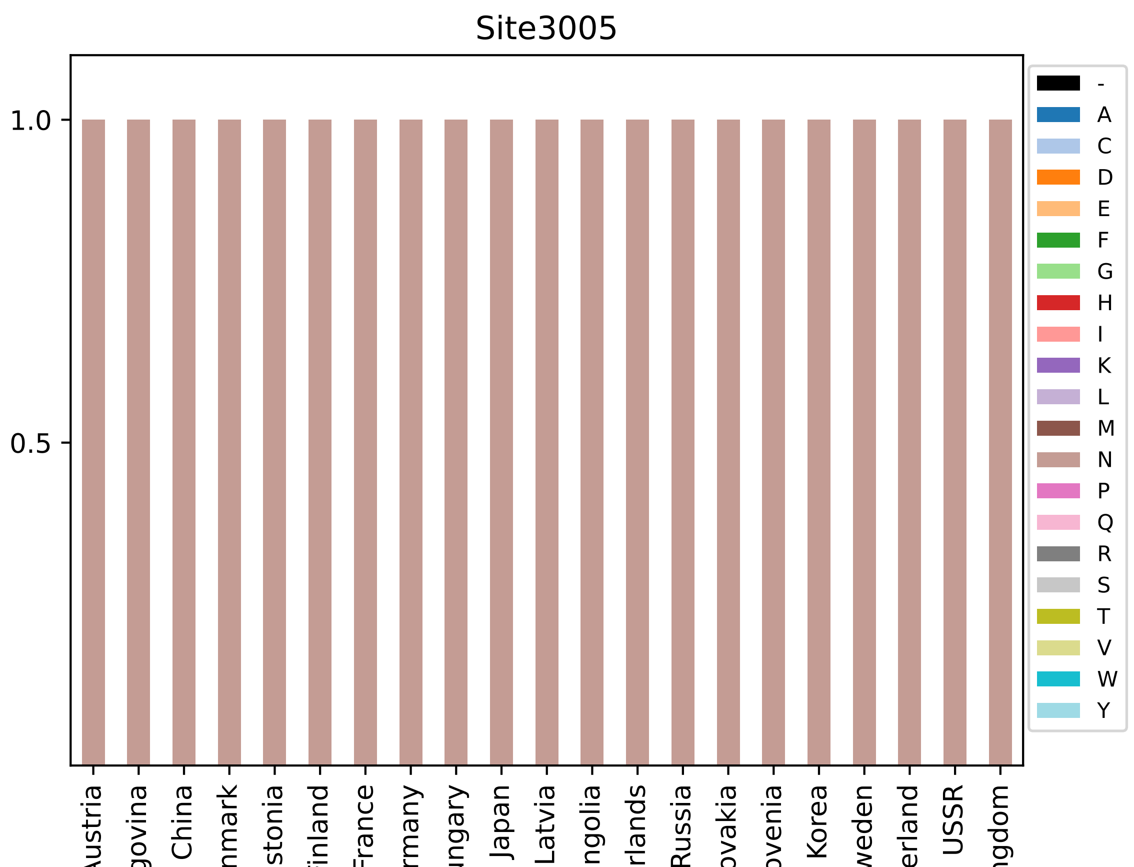
Site3005
1.0
0.5
-
A
C
D
E
F
G
H
I
K
L
M
N
P
Q
R
S
T
V
W
Y
Austria
govina
China
nmark
stonia
inland
France
rmany
ungary
Japan
Latvia
ngolia
rlands
Russia
ovakia
ovenia
Korea
weden
erland
USSR
ngdom

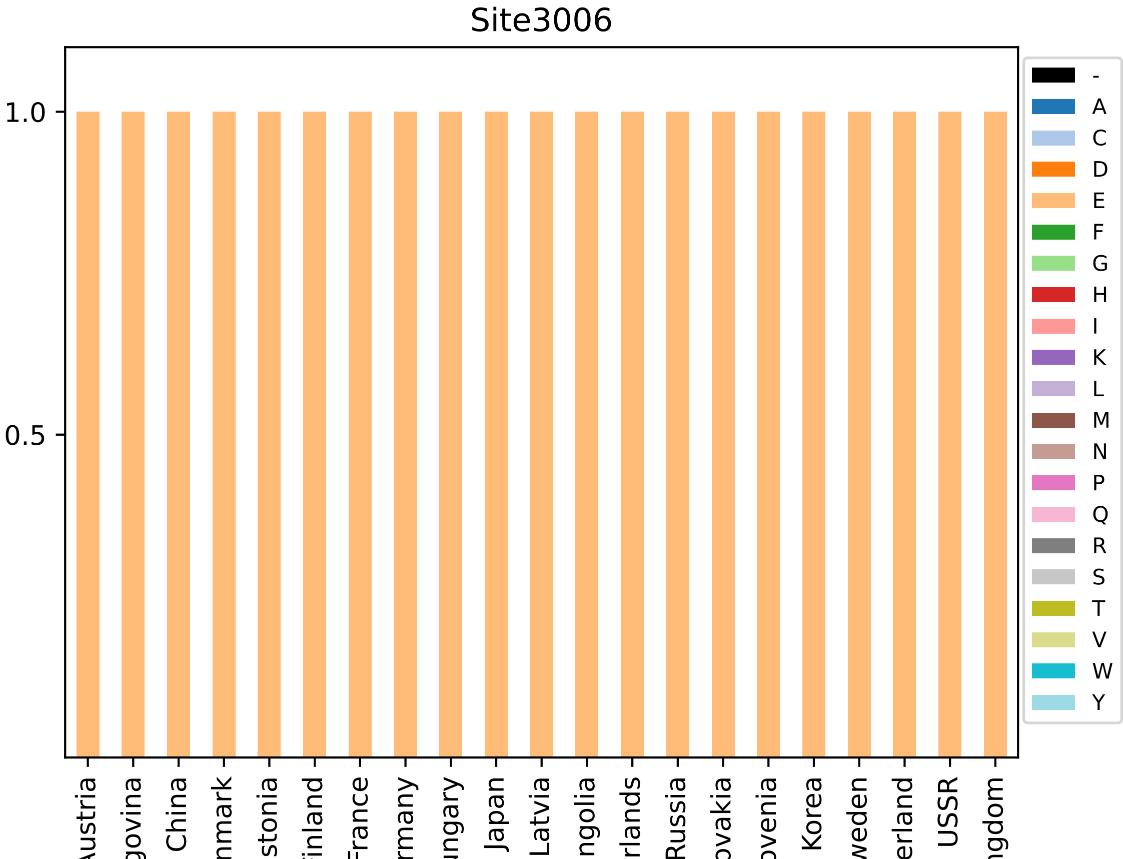
Site3006
1.0
0.5
-
A
C
D
E
F
G
H
I
K
L
M
N
P
Q
R
S
T
V
W
Y
China
Japan
Latvia
Russia
Korea
USSR

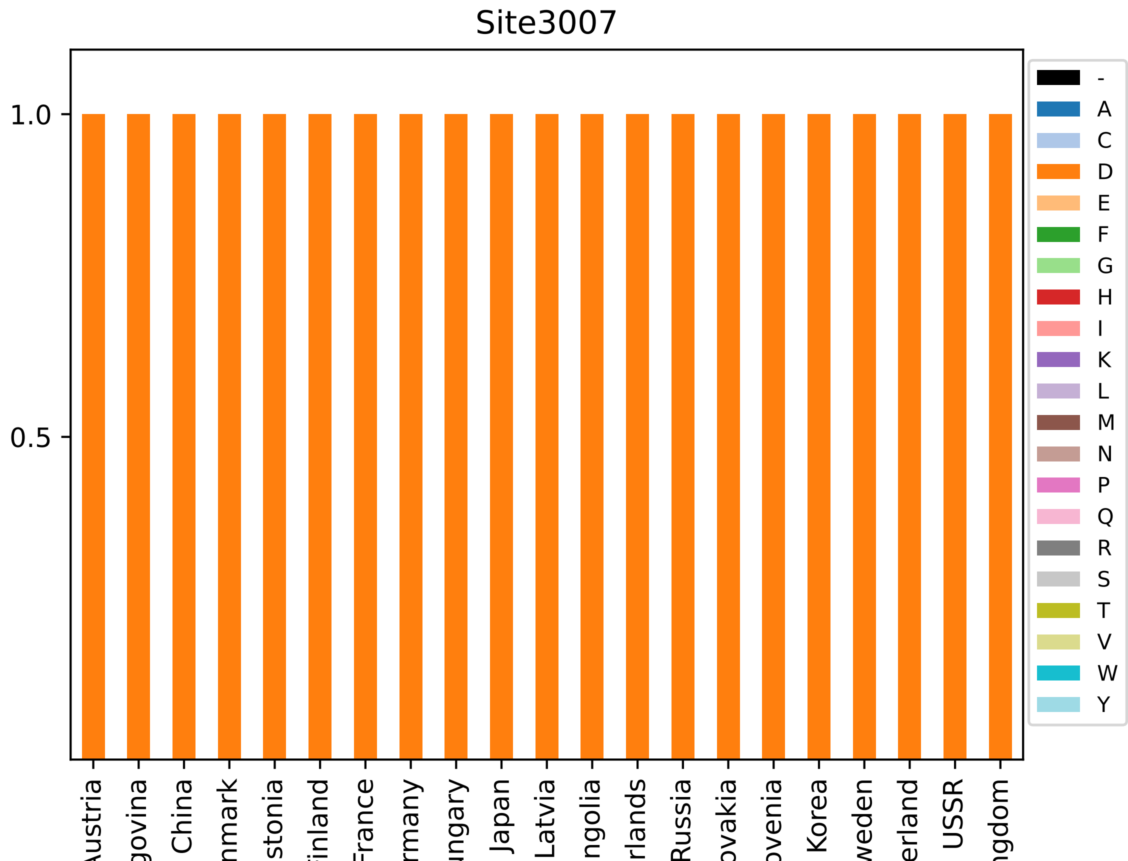
Site3007
1.0
0.5
-
A
C
D
E
F
G
H
I
K
L
M
N
P
Q
R
S
T
V
W
Y
China
Japan
Latvia
Russia
Korea
USSR

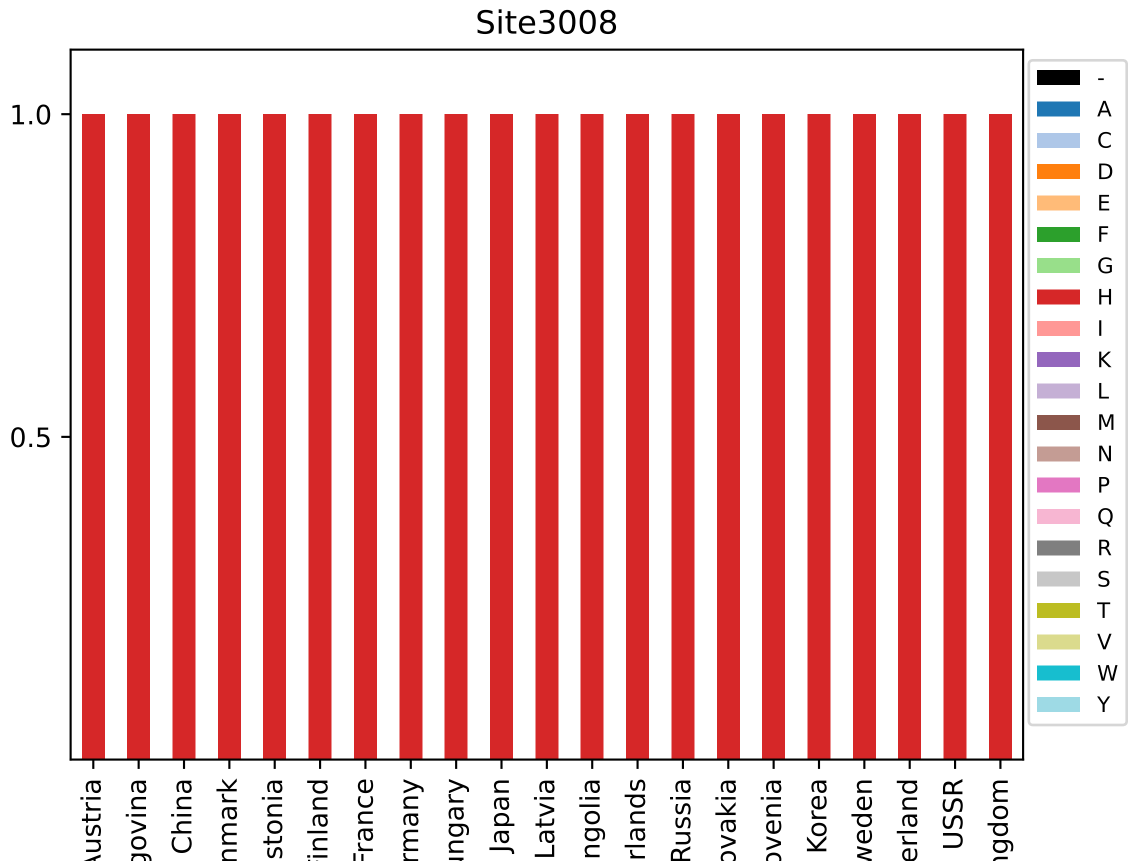
Site3008
1.0
0.5
-
A
C
D
E
F
G
H
I
K
L
M
N
P
Q
R
S
T
V
W
Y
China
Japan
Latvia
Russia
Korea
USSR

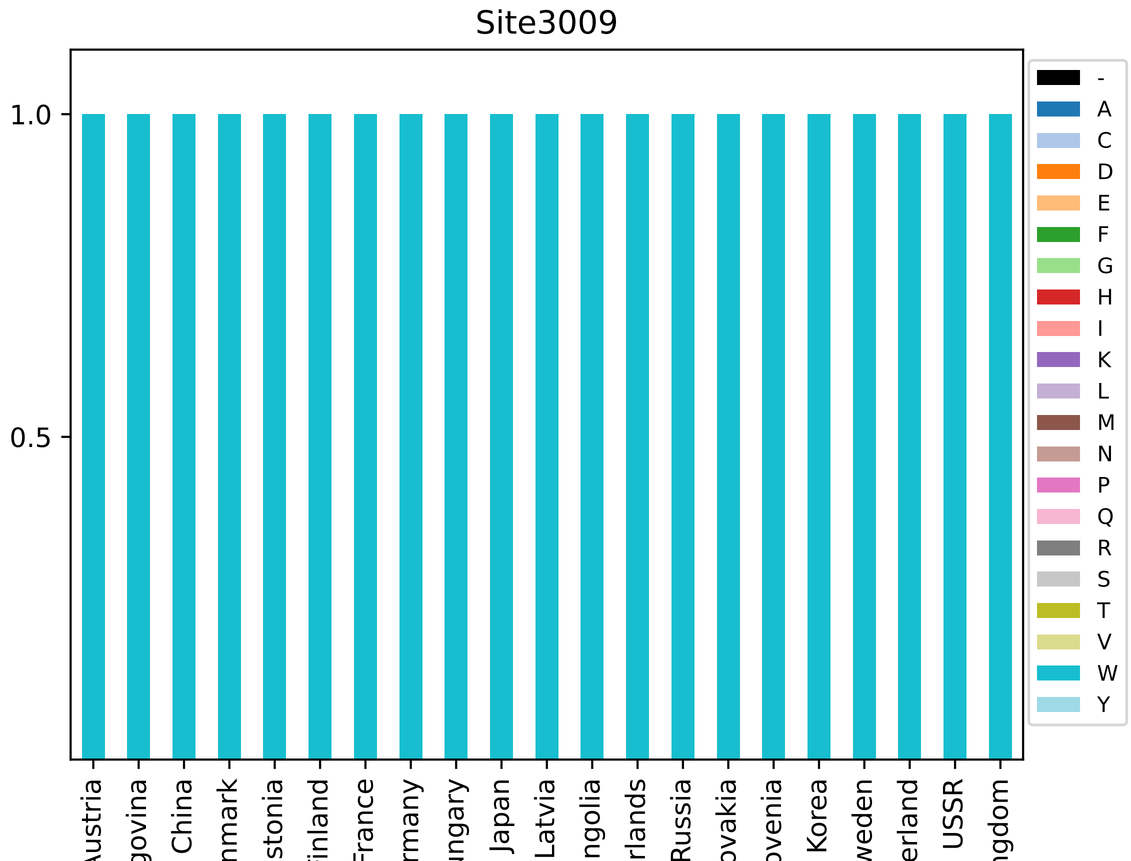

Site3009
1.0
0.5
-
A
C
D
E
F
G
H
I
K
L
M
N
P
Q
R
S
T
V
W
Y
Austria
govina
China
nmark
stonia
Finland
France
rmany
ungary
Japan
Latvia
ngolia
rlands
Russia
ovakia
ovenia
Korea
weden
erland
USSR
ngdom

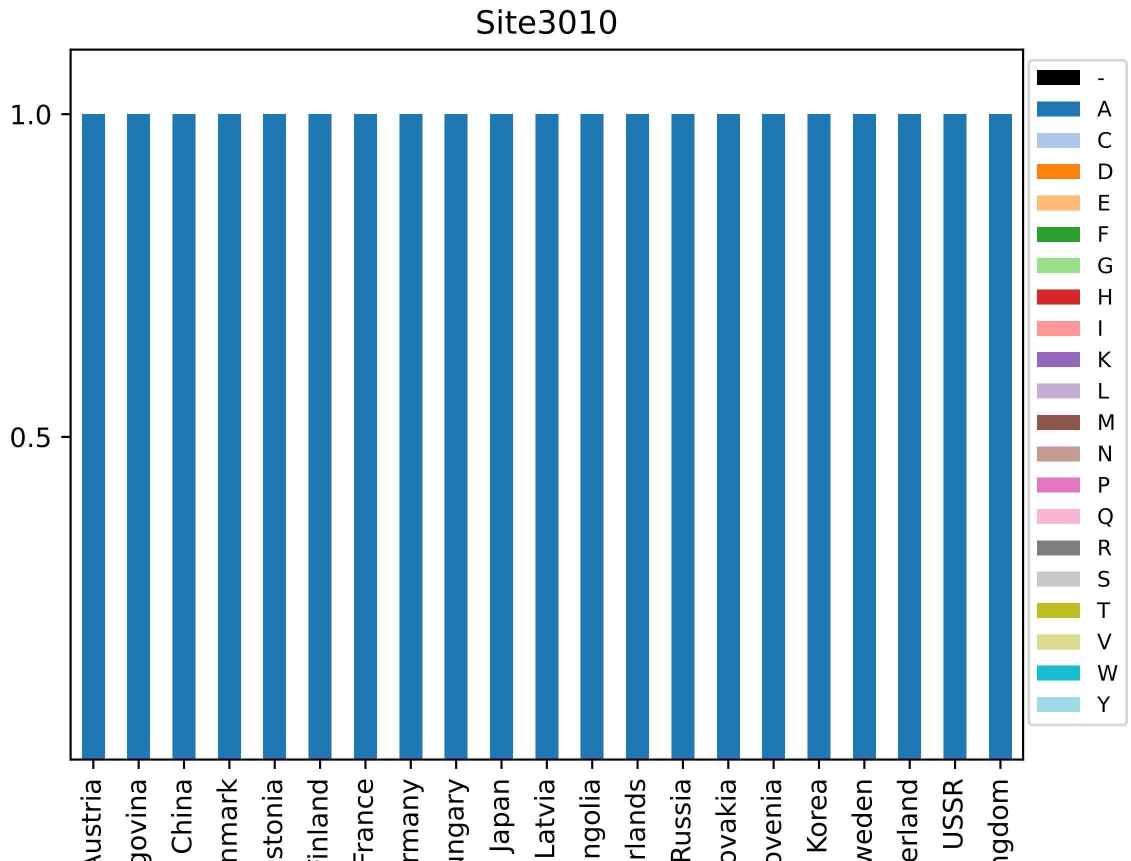

Site3010
1.0
0.5
-
A
C
D
E
F
G
H
I
K
L
M
N
P
Q
R
S
T
V
W
Y
China
Japan
Latvia
Russia
Korea
USSR

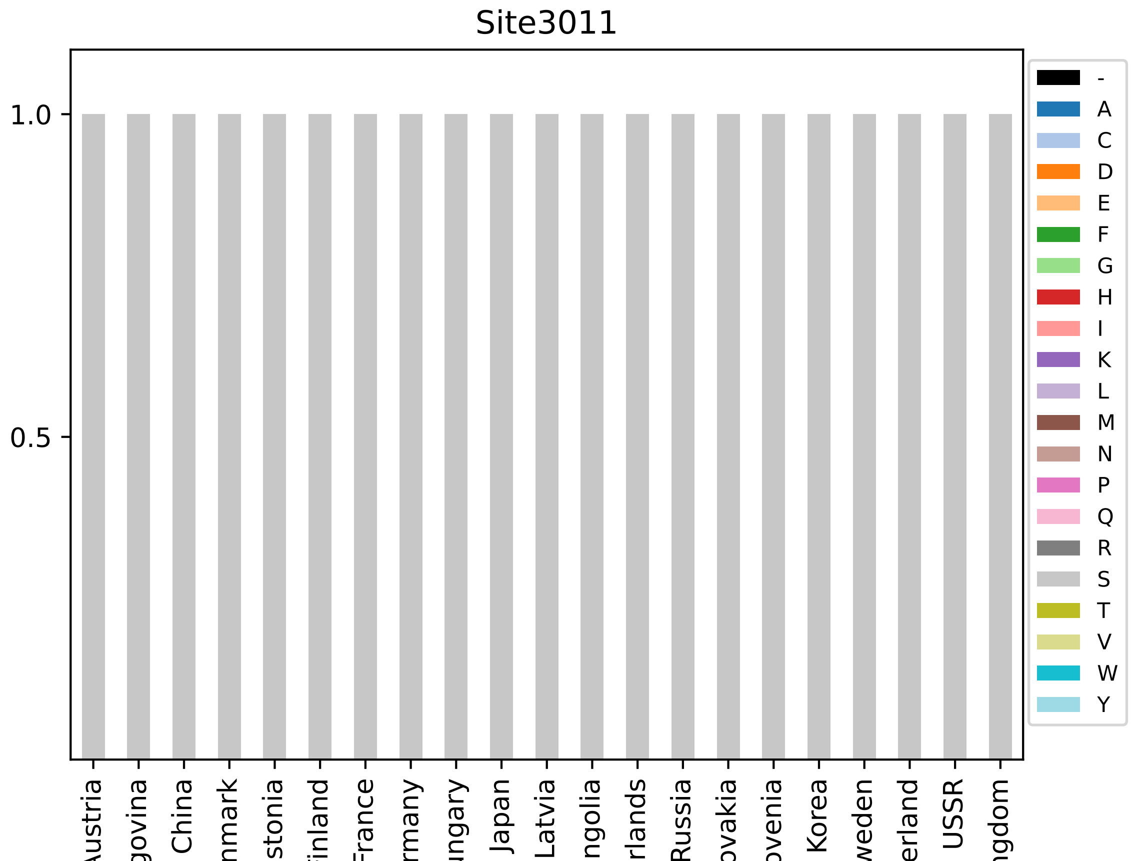
Site3011
1.0
0.5
-
A
C
D
E
F
G
H
I
K
L
M
N
P
Q
R
S
T
V
W
Y
China
Japan
Latvia
Russia
Korea
USSR

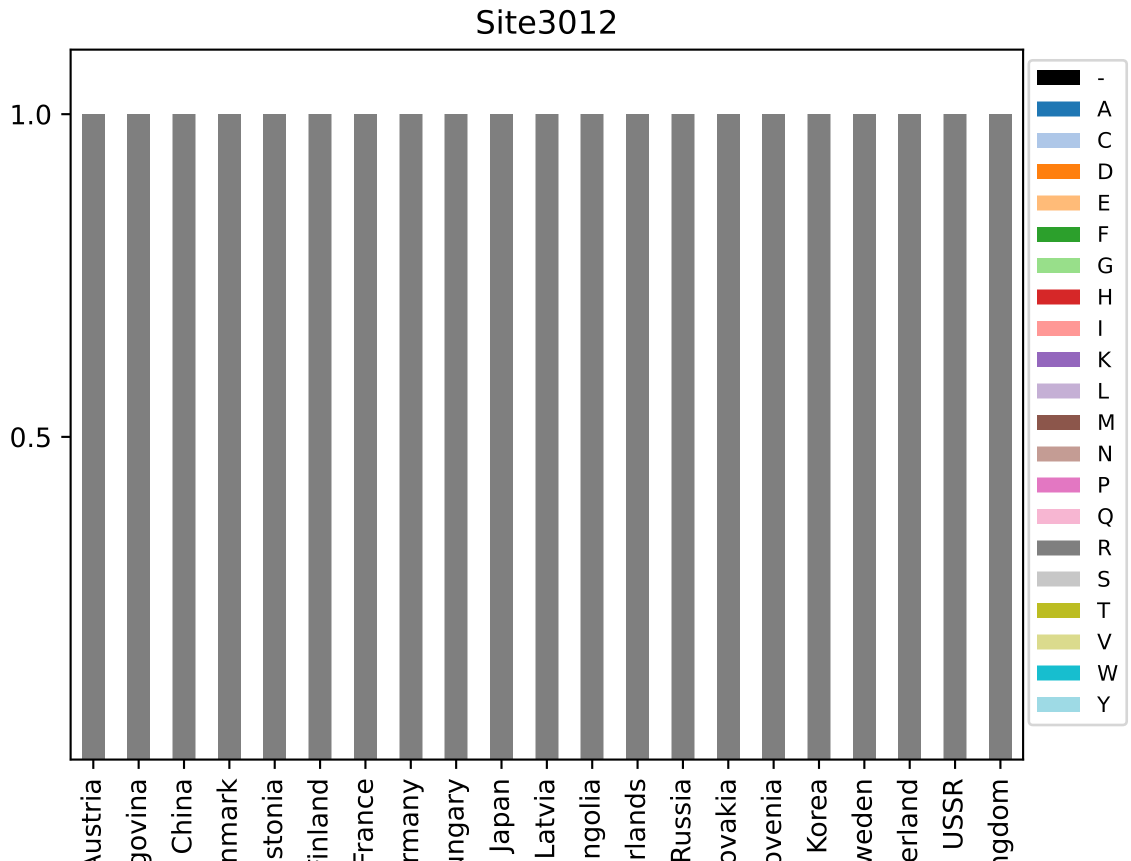

Site3012
1.0
0.5
-
A
C
D
E
F
G
H
I
K
L
M
N
P
Q
R
S
T
V
W
Y
China
Japan
Latvia
Russia
Korea
USSR

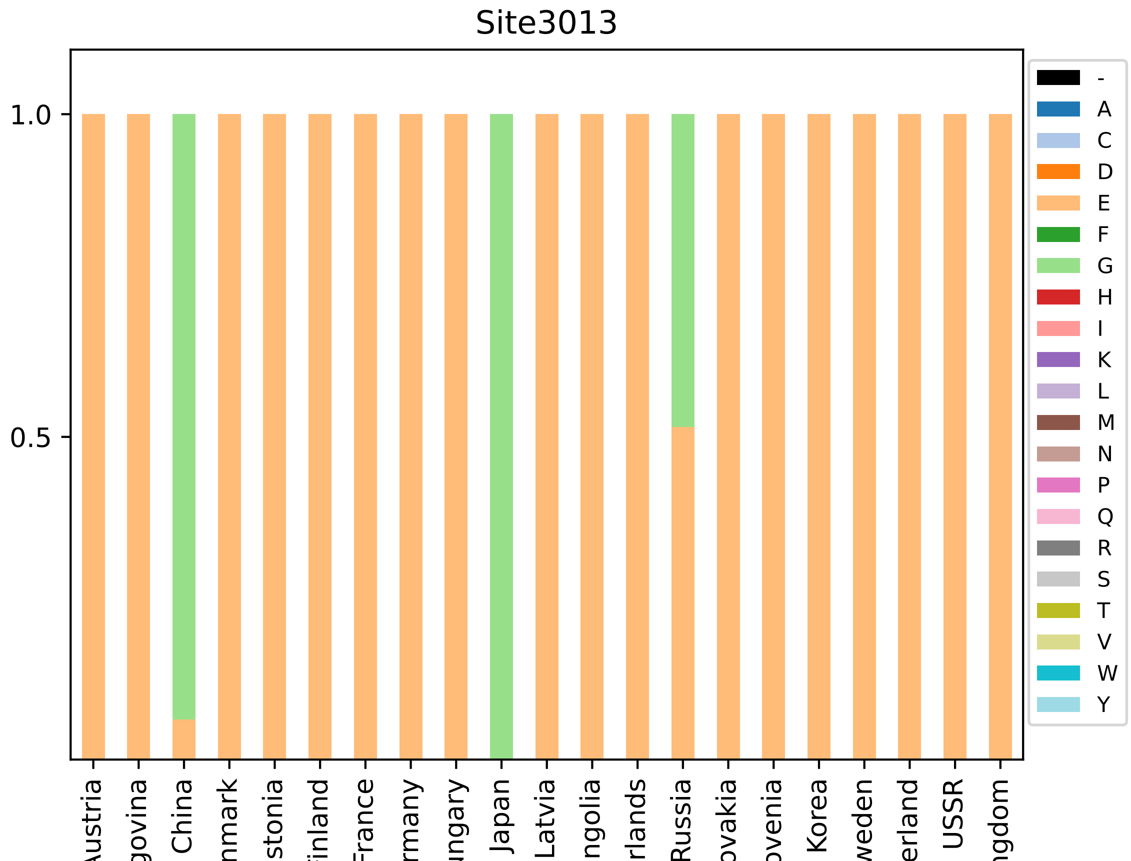
Site3013
1.0
0.5
-
A
C
D
E
F
G
H
I
K
L
M
N
P
Q
R
S
T
V
W
Y
China
Japan
Latvia
Russia
Korea
USSR

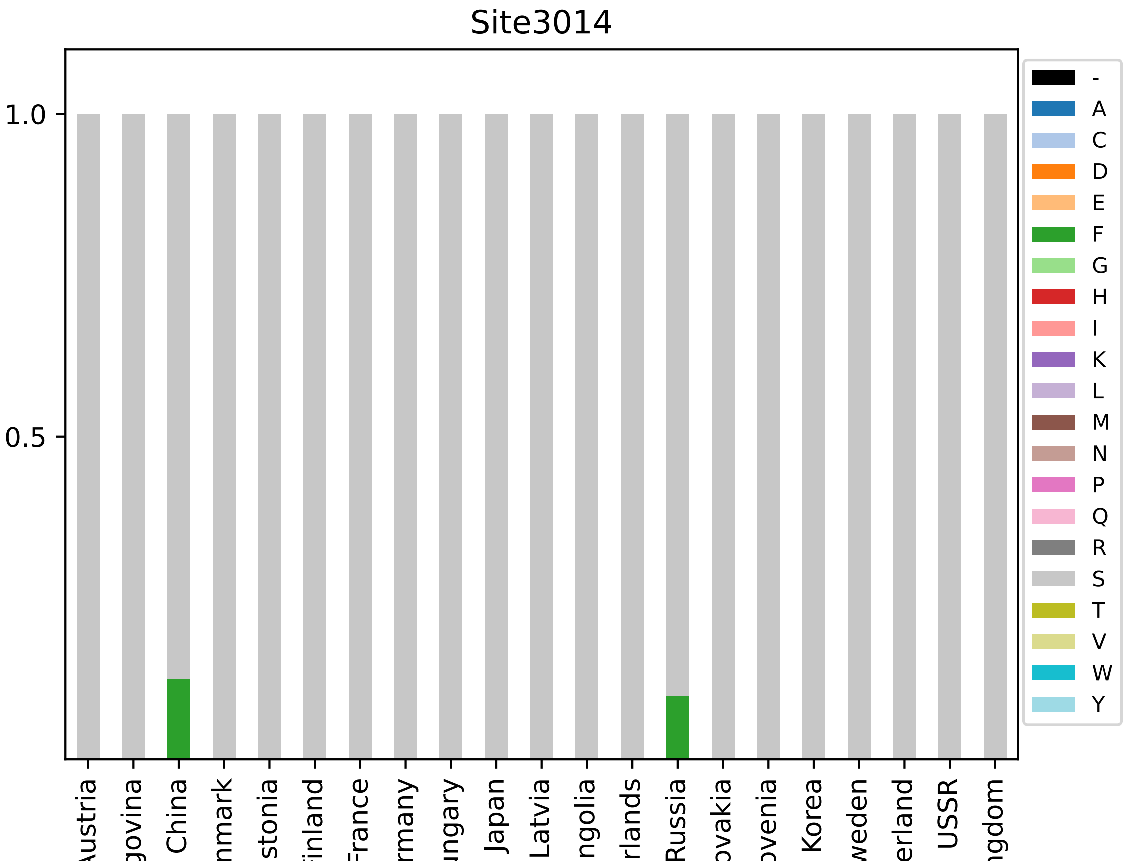

Site3014
1.0
0.5
-
A
C
D
E
F
G
H
I
K
L
M
N
P
Q
R
S
T
V
W
Y
China
Japan
Latvia
Russia
Korea
USSR

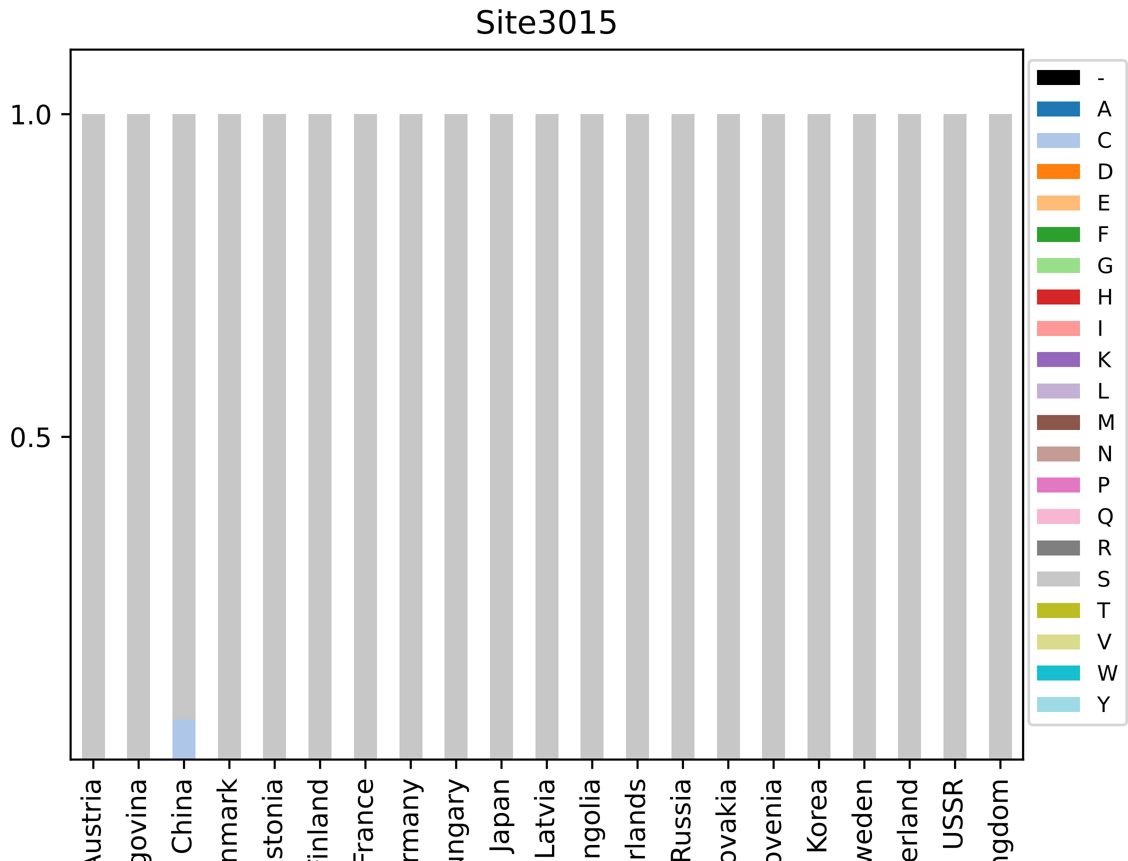
Site3015
1.0
0.5
-
A
C
D
E
F
G
H
I
K
L
M
N
P
Q
R
S
T
V
W
Y
Austria
China
Japan
Latvia
Russia
Korea
USSR

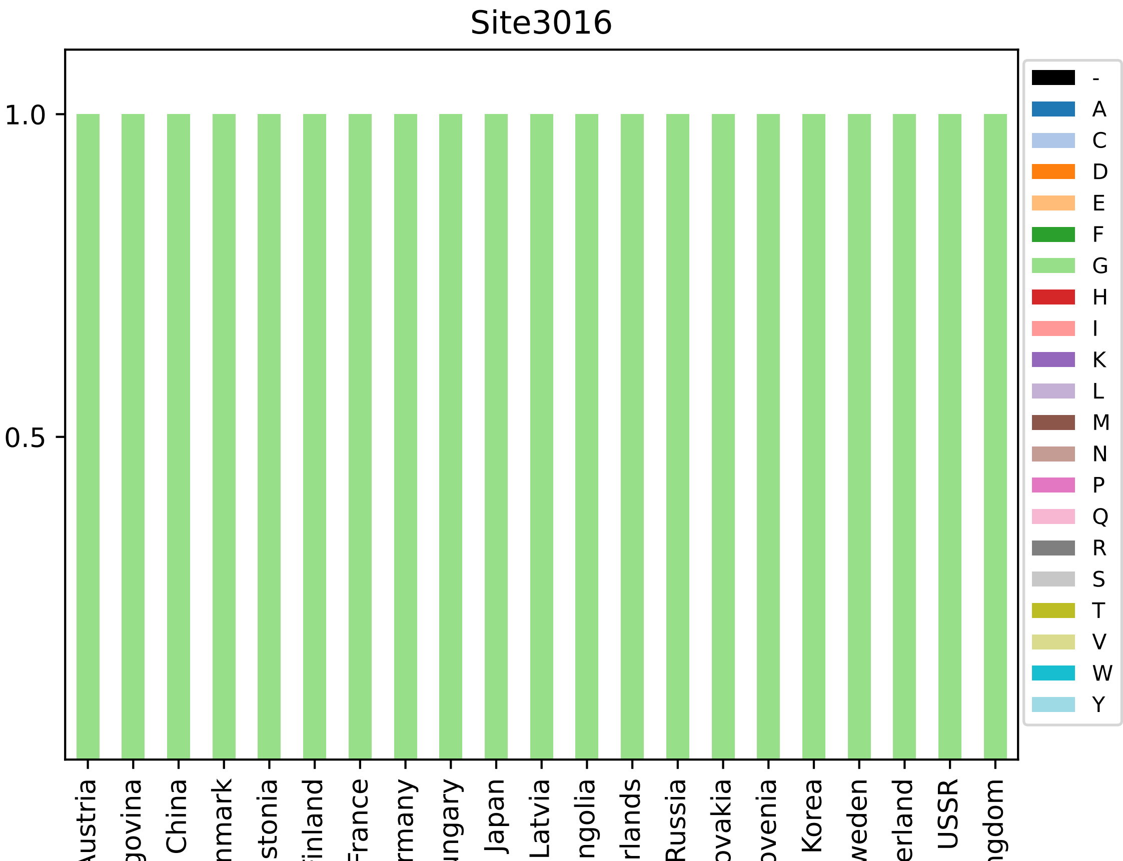

Site3016
1.0
0.5
-
A
C
D
E
F
G
H
I
K
L
M
N
P
Q
R
S
T
V
W
Y
China
Japan
Latvia
Russia
Korea
USSR

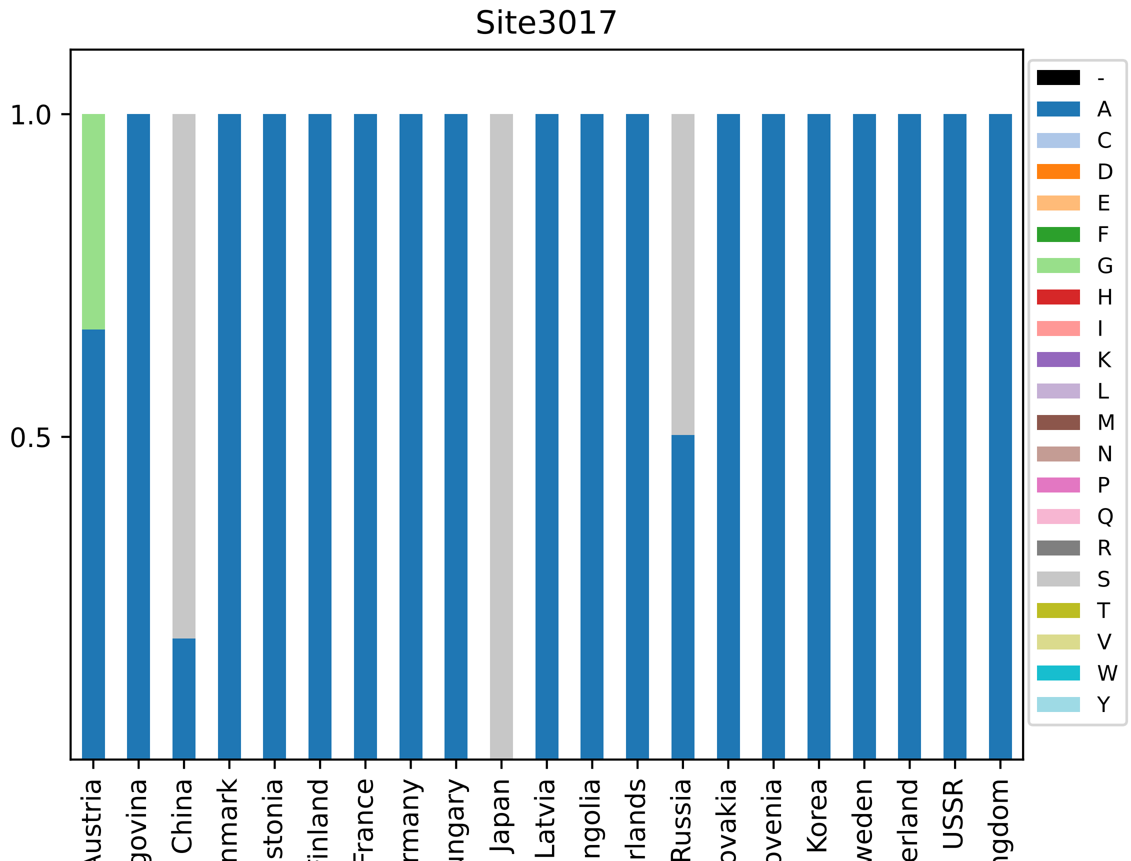
Site3017
1.0
0.5
-
A
C
D
E
F
G
H
I
K
L
M
N
P
Q
R
S
T
V
W
Y
Austria
govina
China
nmark
stonia
inland
France
rmany
ungary
Japan
Latvia
ngolia
rlands
Russia
ovakia
ovenia
Korea
weden
erland
USSR
ngdom

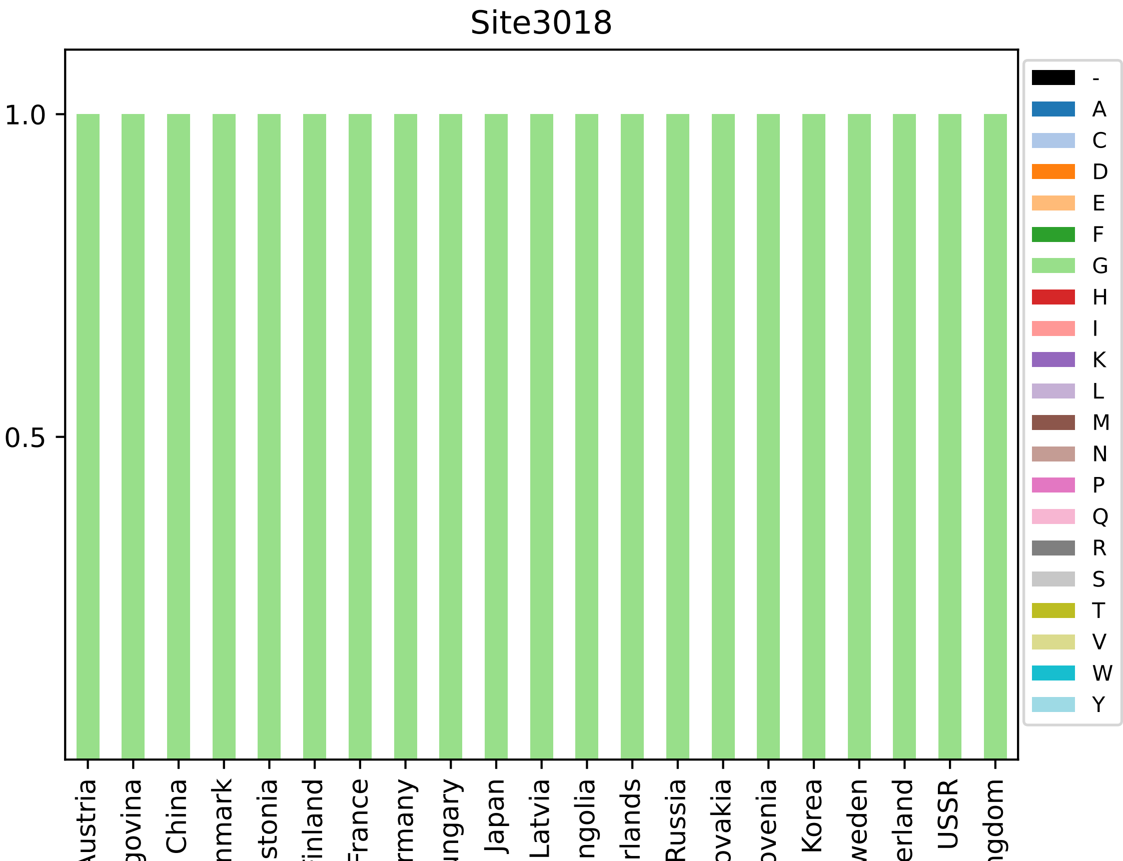
Site3018
1.0
0.5
-
A
C
D
E
F
G
H
I
K
L
M
N
P
Q
R
S
T
V
W
Y
Austria
China
Japan
Latvia
Russia
Korea
USSR

Site3019

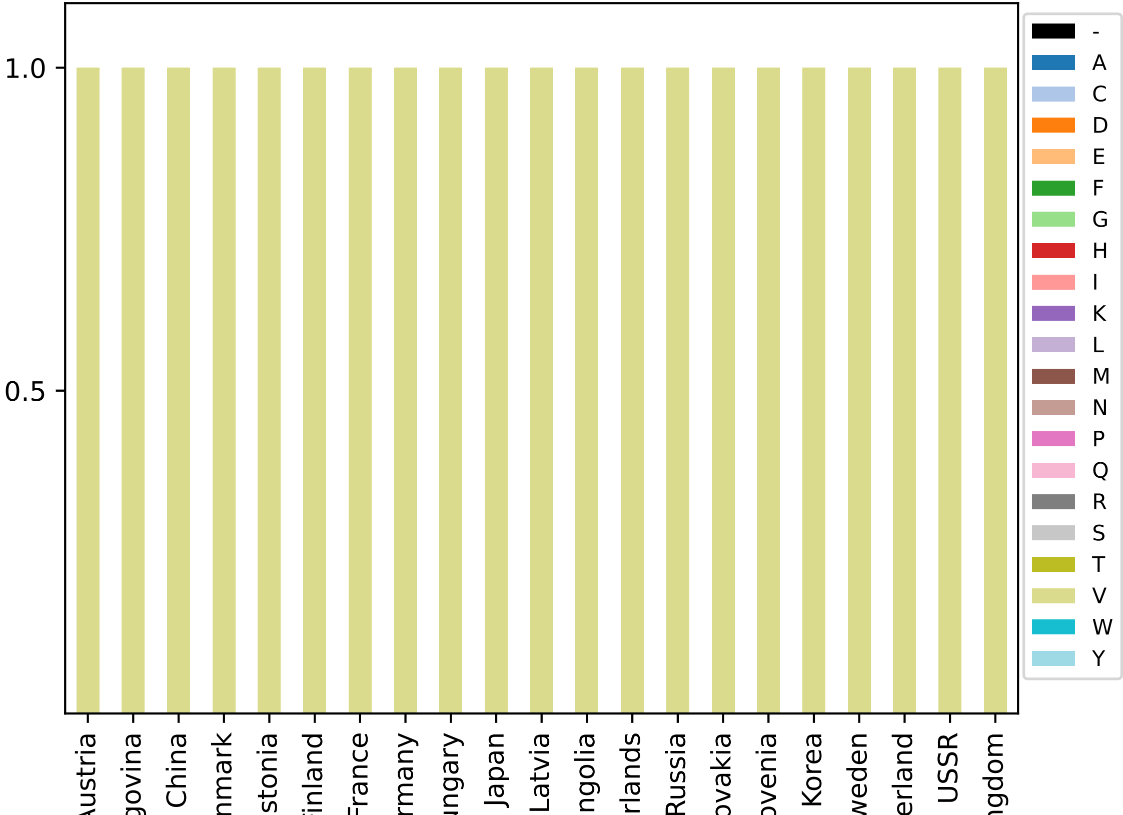
1.0
0.5
-
A
C
D
E
F
G
H
I
K
L
M
N
P
Q
R
S
T
V
W
Y
China
Japan
Latvia
Russia
Korea
USSR

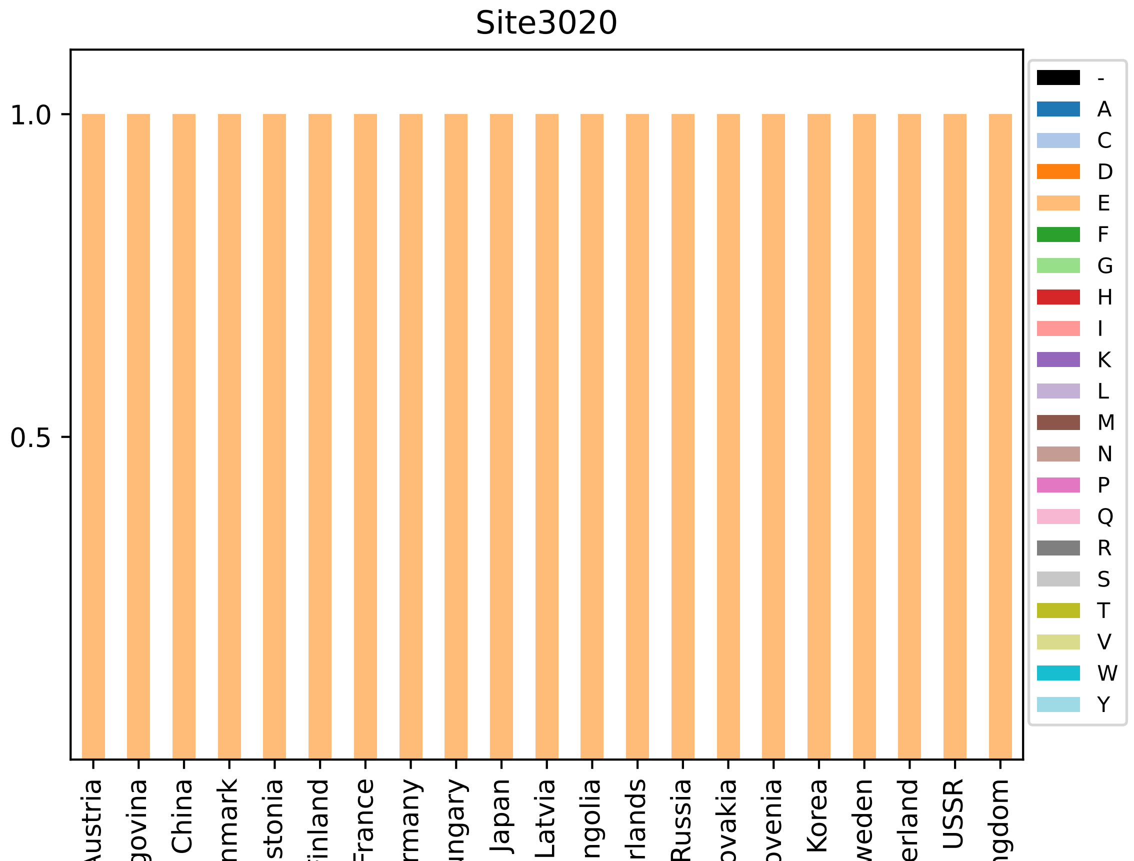

Site3020
1.0
0.5
-
A
C
D
E
F
G
H
I
K
L
M
N
P
Q
R
S
T
V
W
Y
China
Japan
Latvia
Russia
Korea
USSR

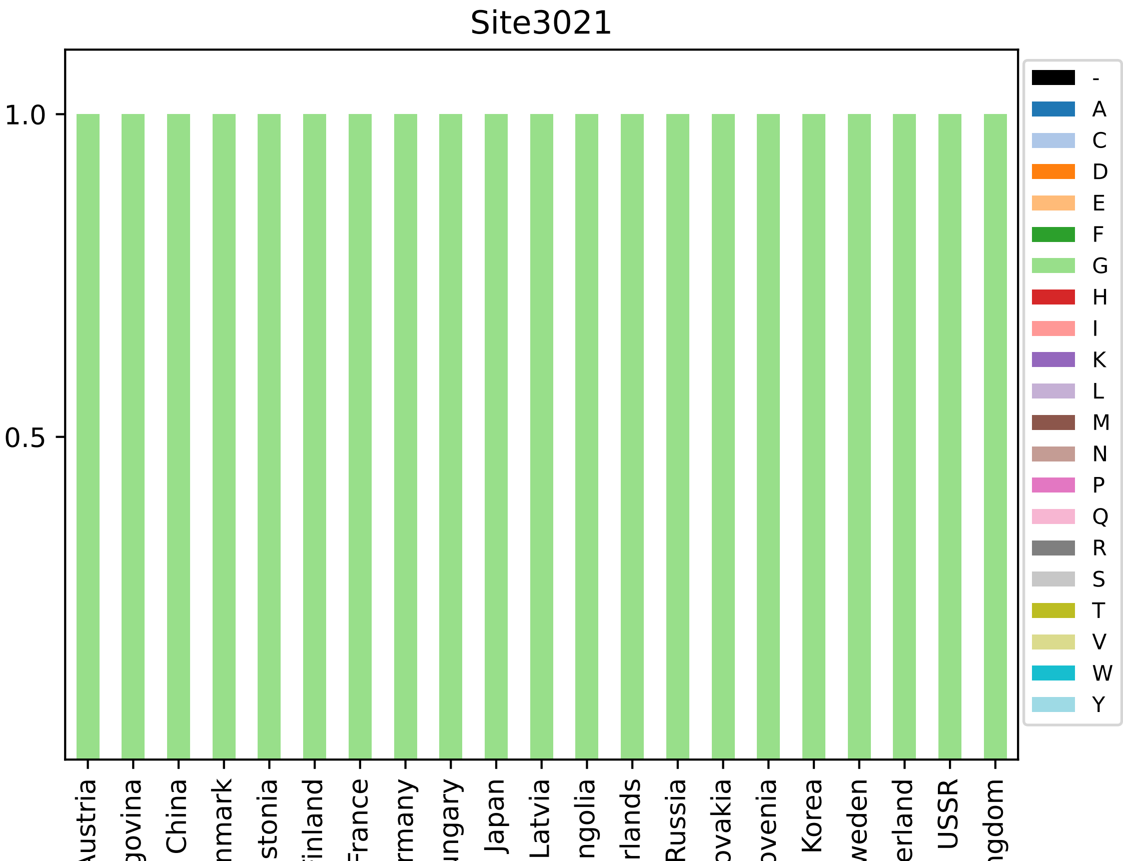

Site3021
1.0
0.5
-
A
C
D
E
F
G
H
I
K
L
M
N
P
Q
R
S
T
V
W
Y
Austria
China
Japan
Latvia
Russia
Korea
USSR

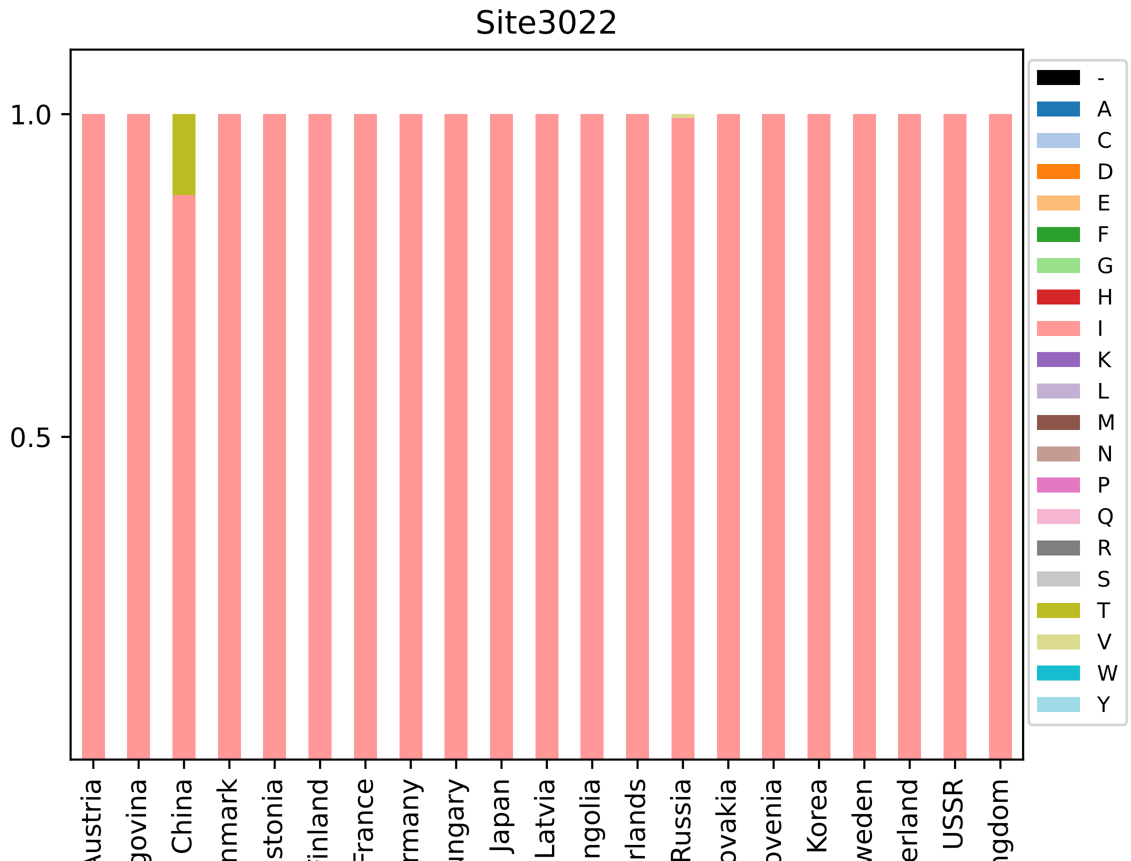

Site3022
1.0
0.5
-
A
C
D
E
F
G
H
I
K
L
M
N
P
Q
R
S
T
V
W
Y
Austria
govina
China
nmark
stonia
inland
France
rmany
ungary
Japan
Latvia
ngolia
rlands
Russia
ovakia
ovenia
Korea
weden
erland
USSR
ngdom

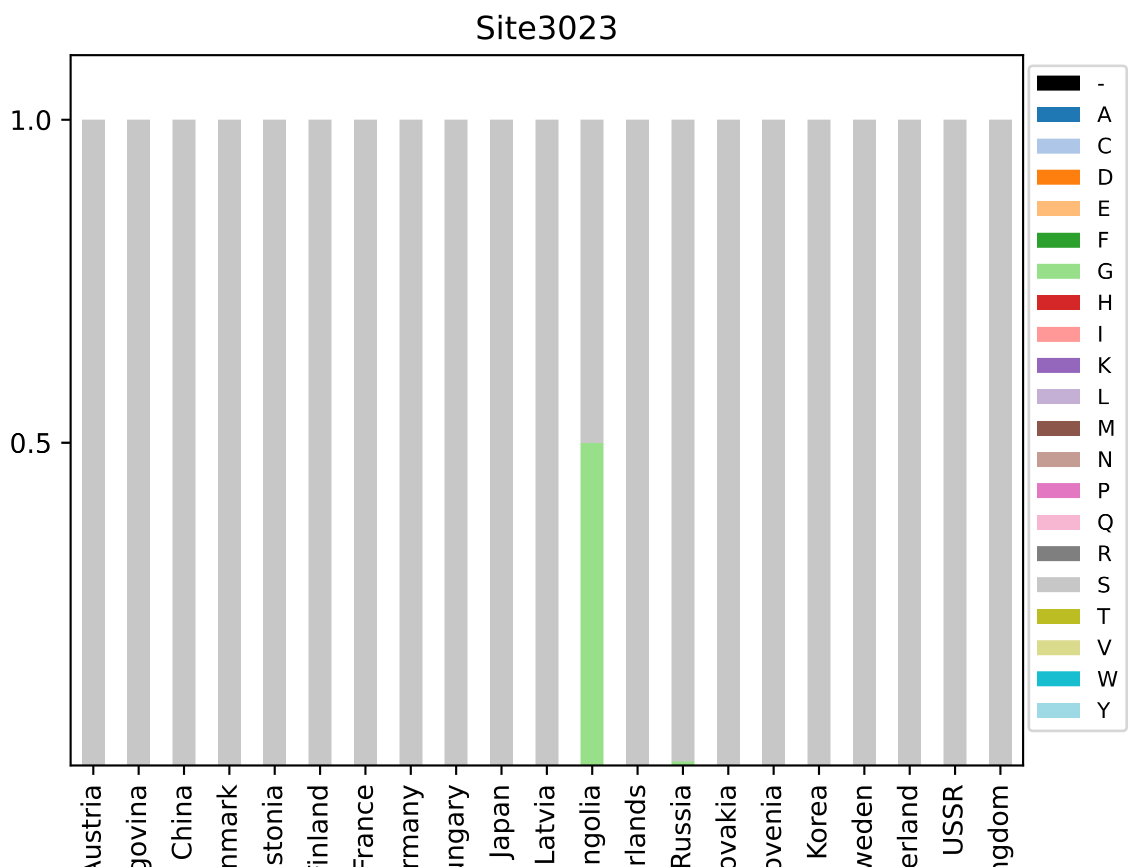

Site3023
1.0
0.5
-
A
C
D
E
F
G
H
I
K
L
M
N
P
Q
R
S
T
V
W
Y
Austria
govina
China
nmark
stonia
inland
France
rmany
ungary
Japan
Latvia
ngolia
rlands
Russia
ovakia
ovenia
Korea
weden
erland
USSR
ngdom

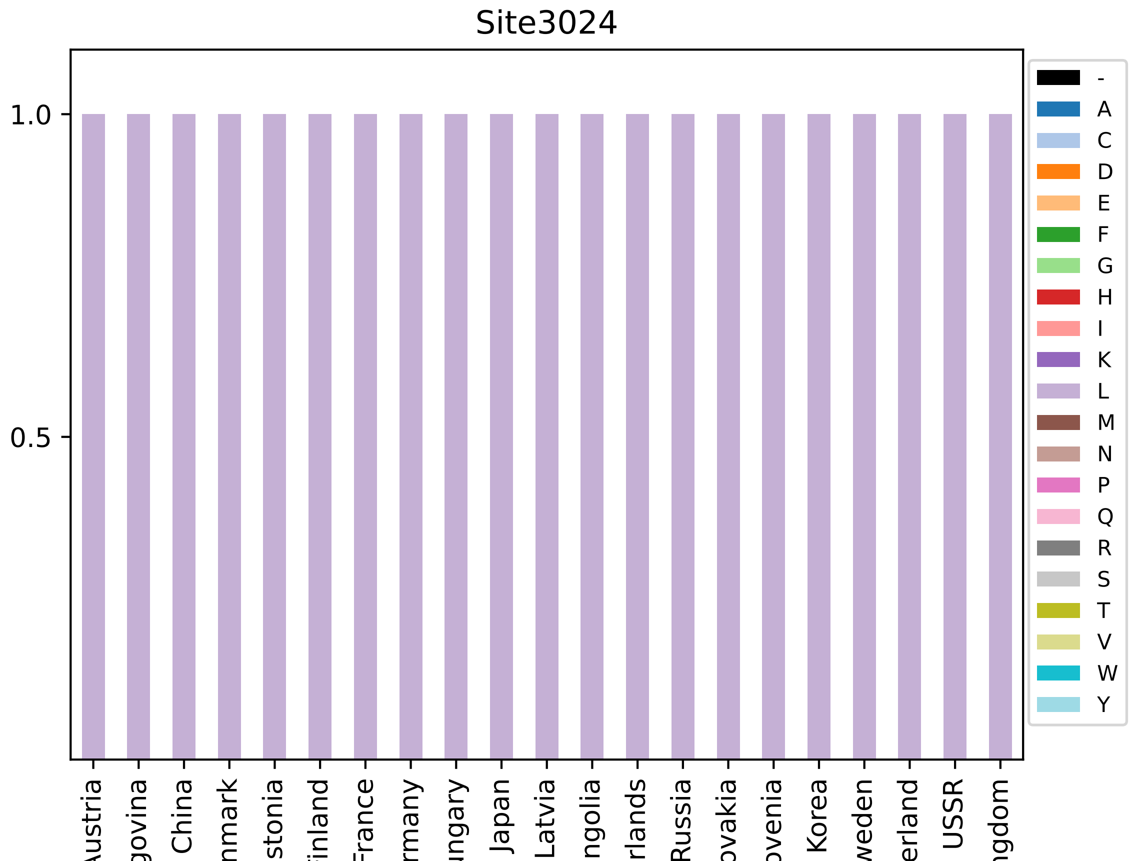

Site3024
1.0
0.5
-
A
C
D
E
F
G
H
I
K
L
M
N
P
Q
R
S
T
V
W
Y
China
Japan
Latvia
Russia
Korea
USSR

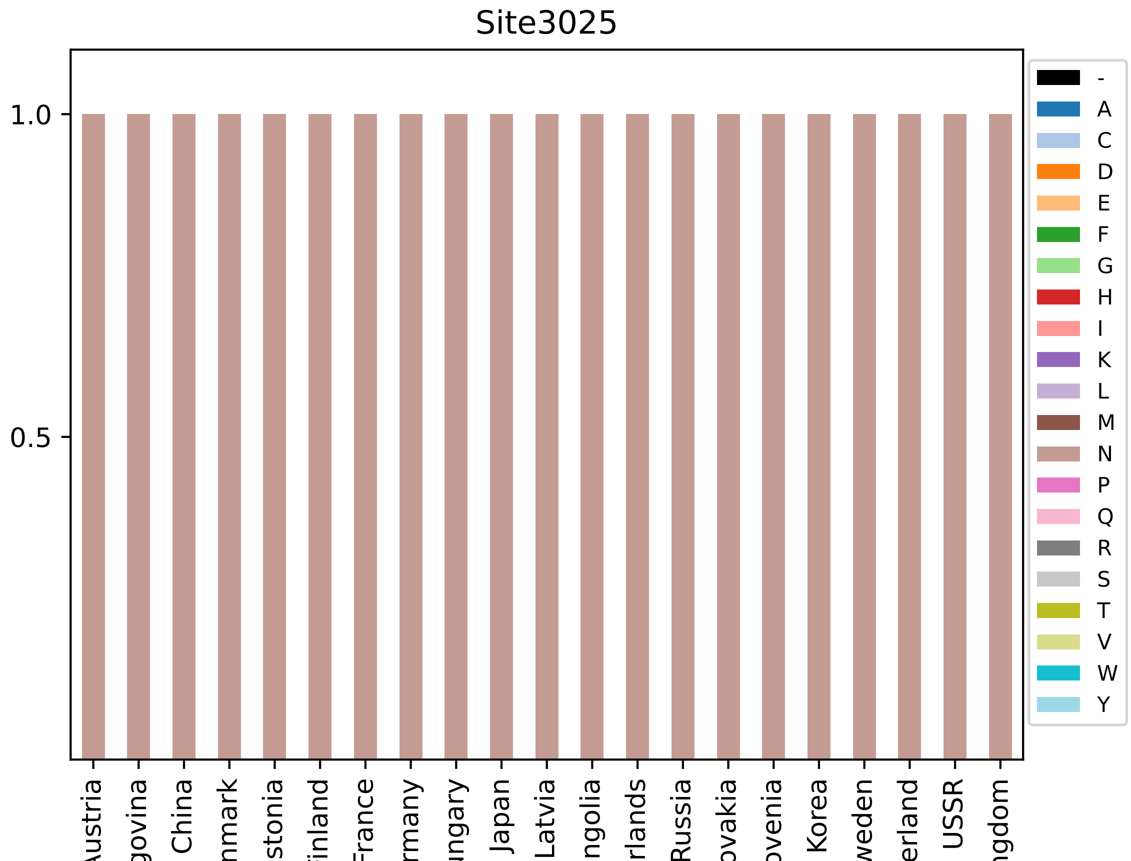

Site3025
1.0
0.5
-
A
C
D
E
F
G
H
I
K
L
M
N
P
Q
R
S
T
V
W
Y
Austria
govina
China
nmark
stonia
Finland
France
rmany
ungary
Japan
Latvia
ngolia
rlands
Russia
ovakia
ovenia
Korea
weden
erland
USSR
ngdom

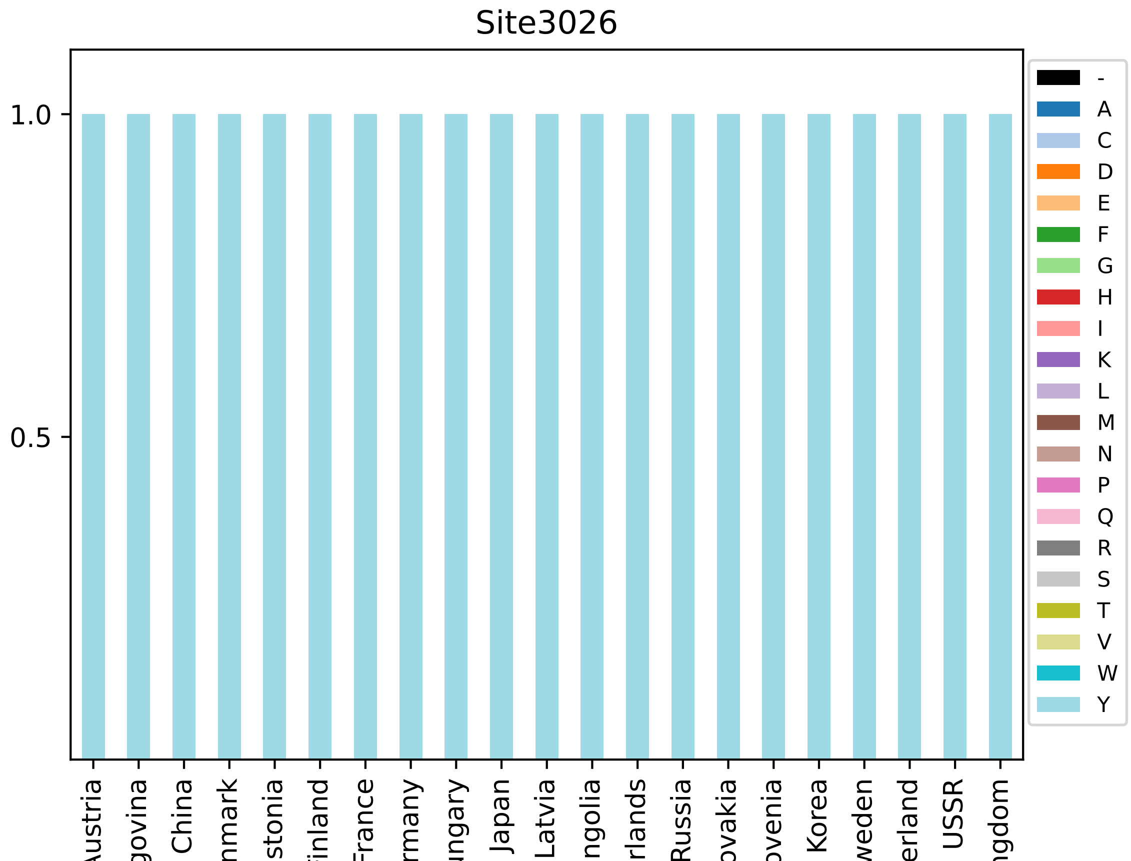

Site3026
1.0
0.5
-
A
C
D
E
F
G
H
I
K
L
M
N
P
Q
R
S
T
V
W
Y
Austria
govina
China
nmark
stonia
Finland
France
rmany
ungary
Japan
Latvia
ngolia
rlands
Russia
ovakia
ovenia
Korea
weden
erland
USSR
ngdom

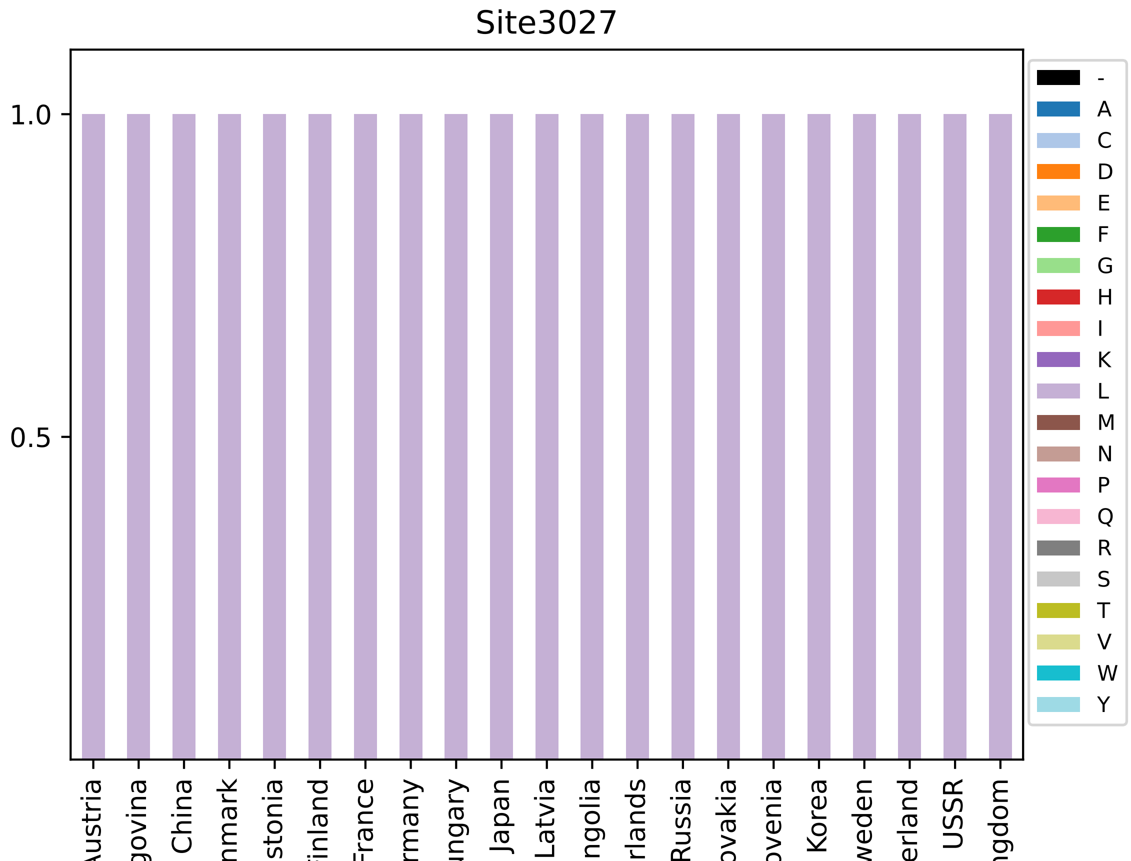
Site3027
1.0
0.5
-
A
C
D
E
F
G
H
I
K
L
M
N
P
Q
R
S
T
V
W
Y
China
Japan
Latvia
Russia
Korea
USSR

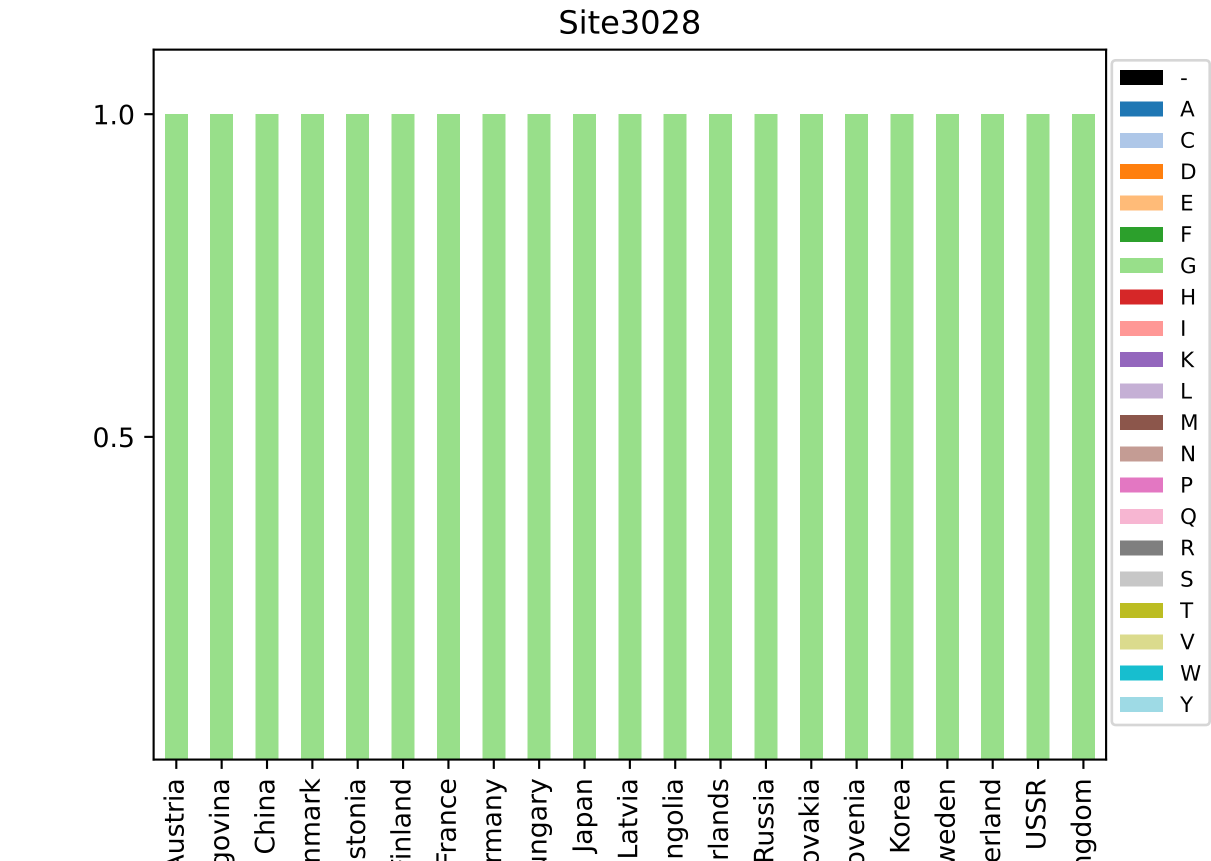
Site3028
1.0
0.5
-
A
C
D
E
F
G
H
I
K
L
M
N
P
Q
R
S
T
V
W
Y
China
Japan
Latvia
Russia
Korea
USSR

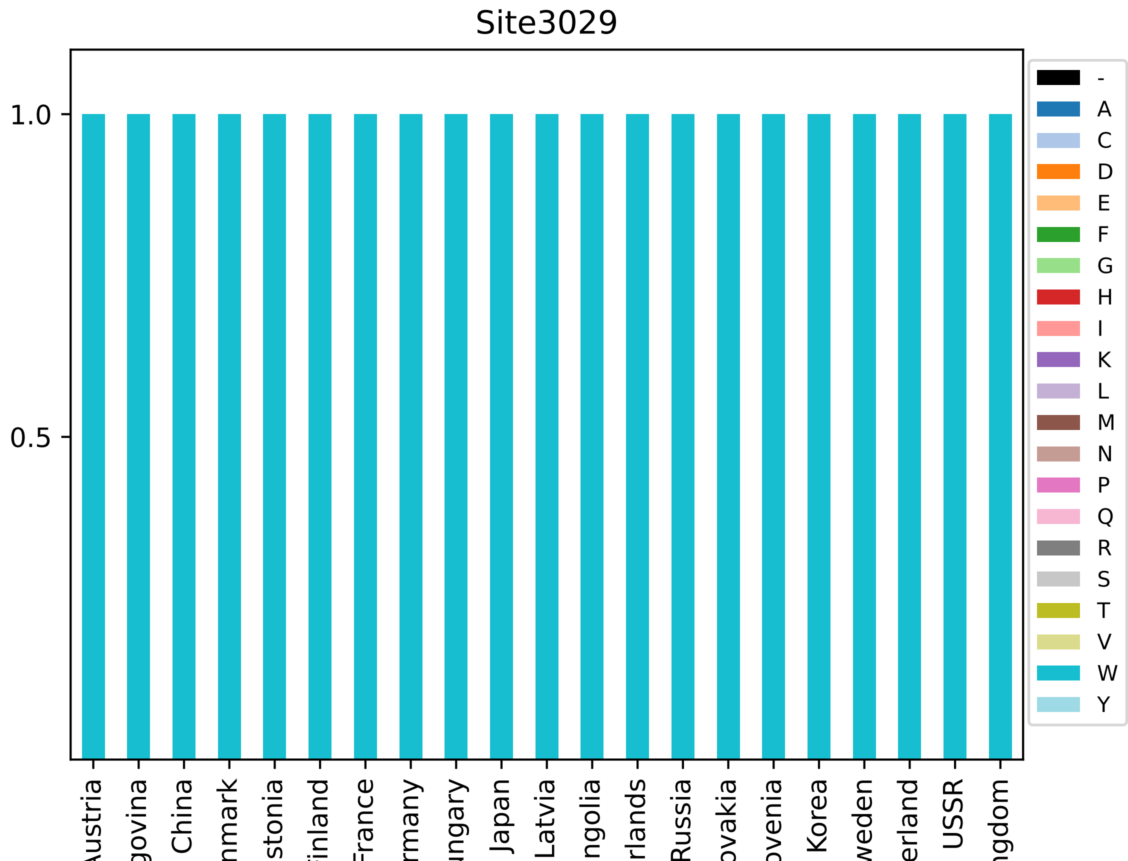
Site3029
1.0
0.5
-
A
C
D
E
F
G
H
I
K
L
M
N
P
Q
R
S
T
V
W
Y
China
Japan
Latvia
Russia
Korea
USSR

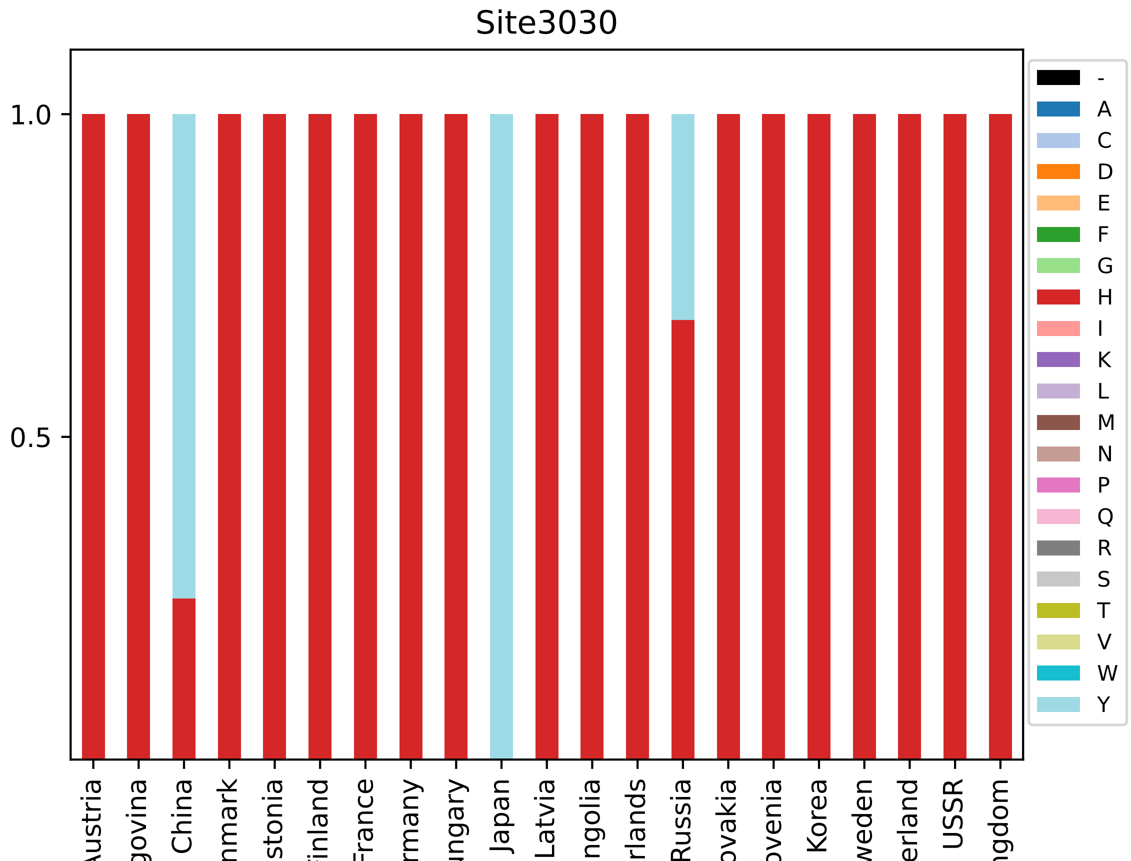

Site3030
1.0
0.5
-
A
C
D
E
F
G
H
I
K
L
M
N
P
Q
R
S
T
V
W
Y
China
Japan
Latvia
Russia
Korea
USSR

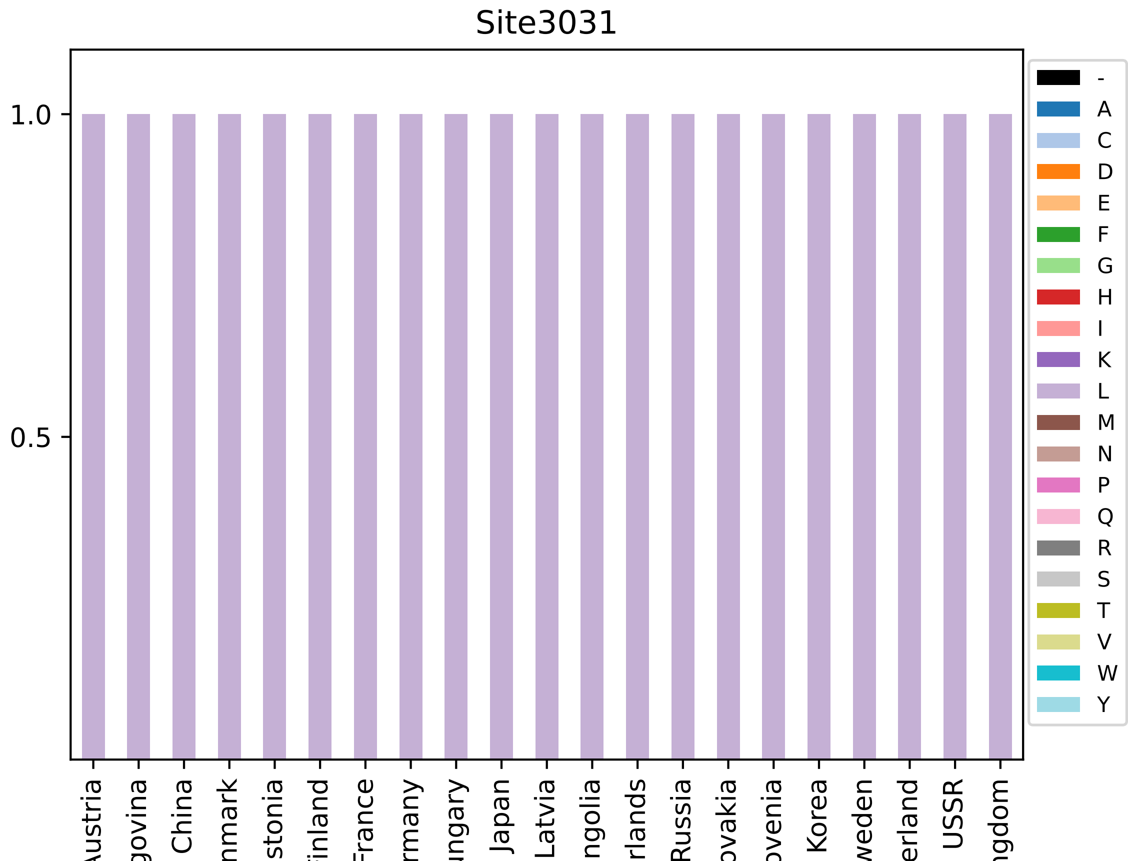

Site3031
1.0
0.5
-
A
C
D
E
F
G
H
I
K
L
M
N
P
Q
R
S
T
V
W
Y
China
Japan
Latvia
Russia
Korea
USSR

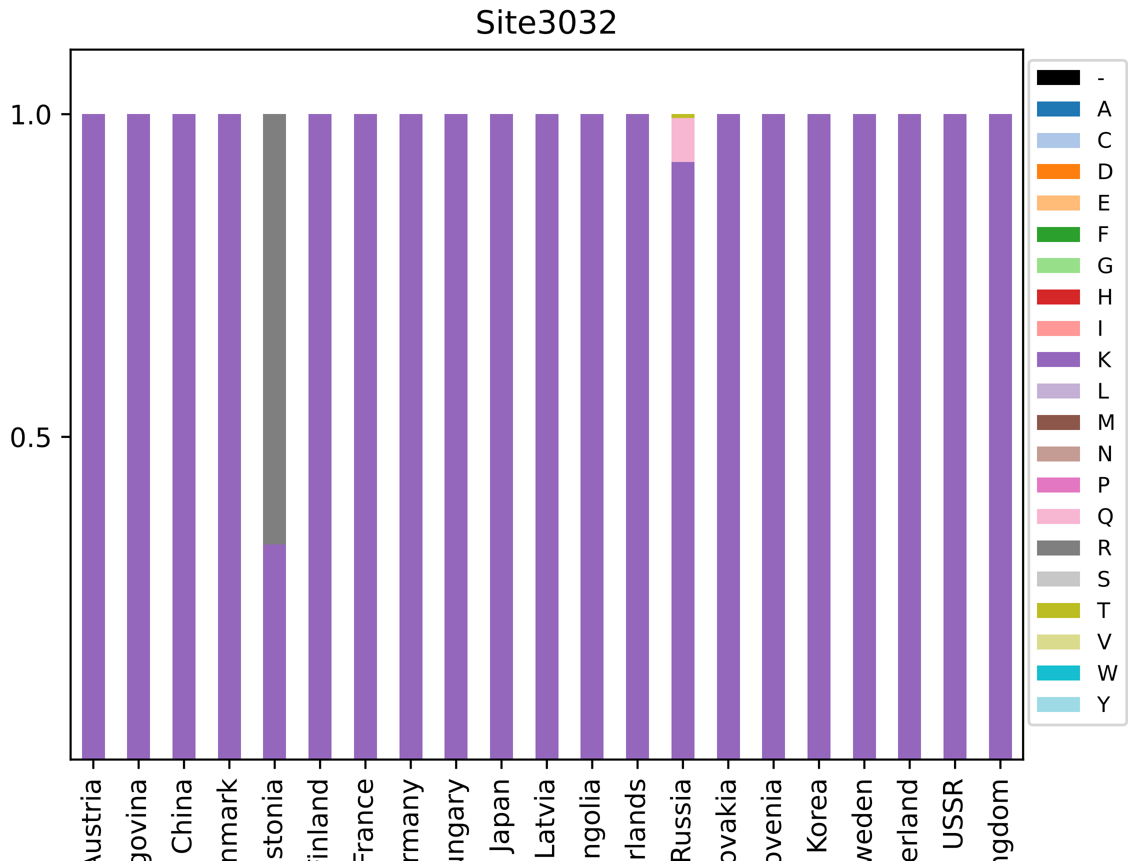
Site3032
1.0
0.5
-
A
C
D
E
F
G
H
I
K
L
M
N
P
Q
R
S
T
V
W
Y
Austria
govina
China
nmark
stonia
inland
France
rmany
ungary
Japan
Latvia
ngolia
rlands
Russia
ovakia
ovenia
Korea
weden
erland
USSR
ngdom

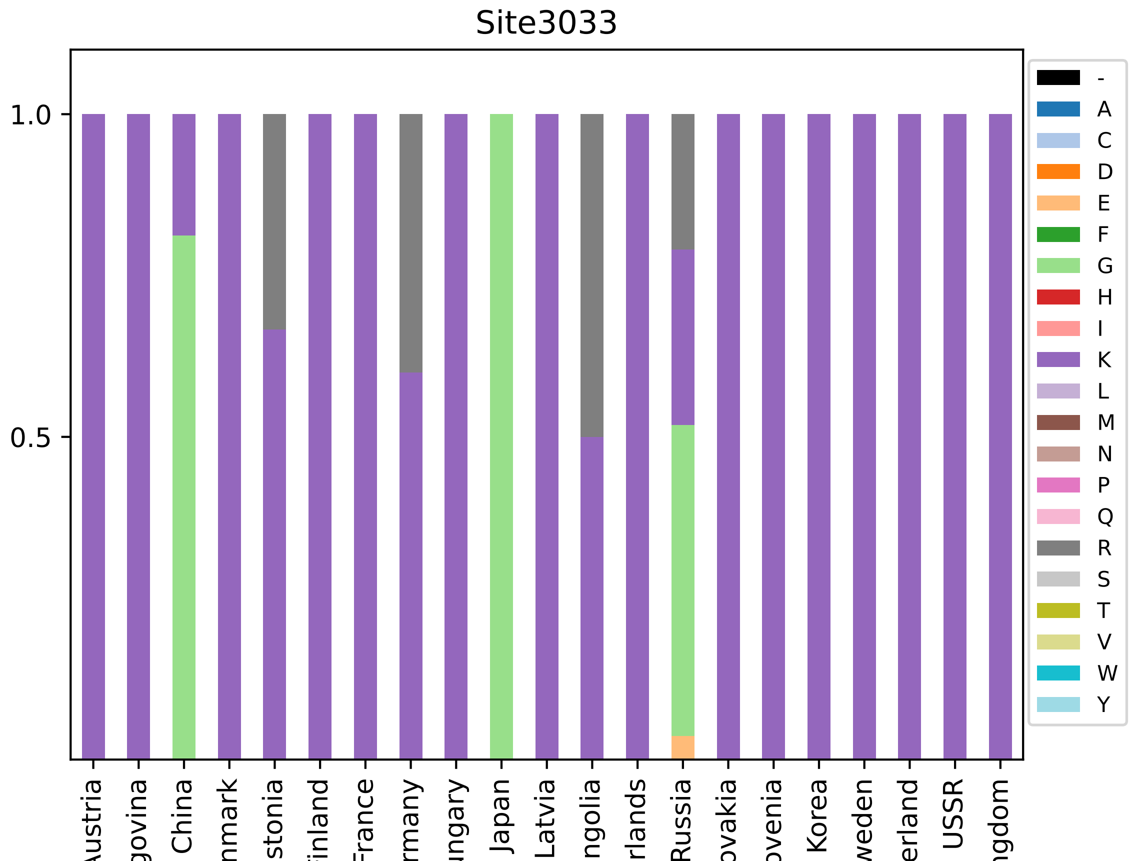
Site3033
1.0
0.5
-
A
C
D
E
F
G
H
I
K
L
M
N
P
Q
R
S
T
V
W
Y
China
Japan
Latvia
Russia
Korea
USSR

Site3034

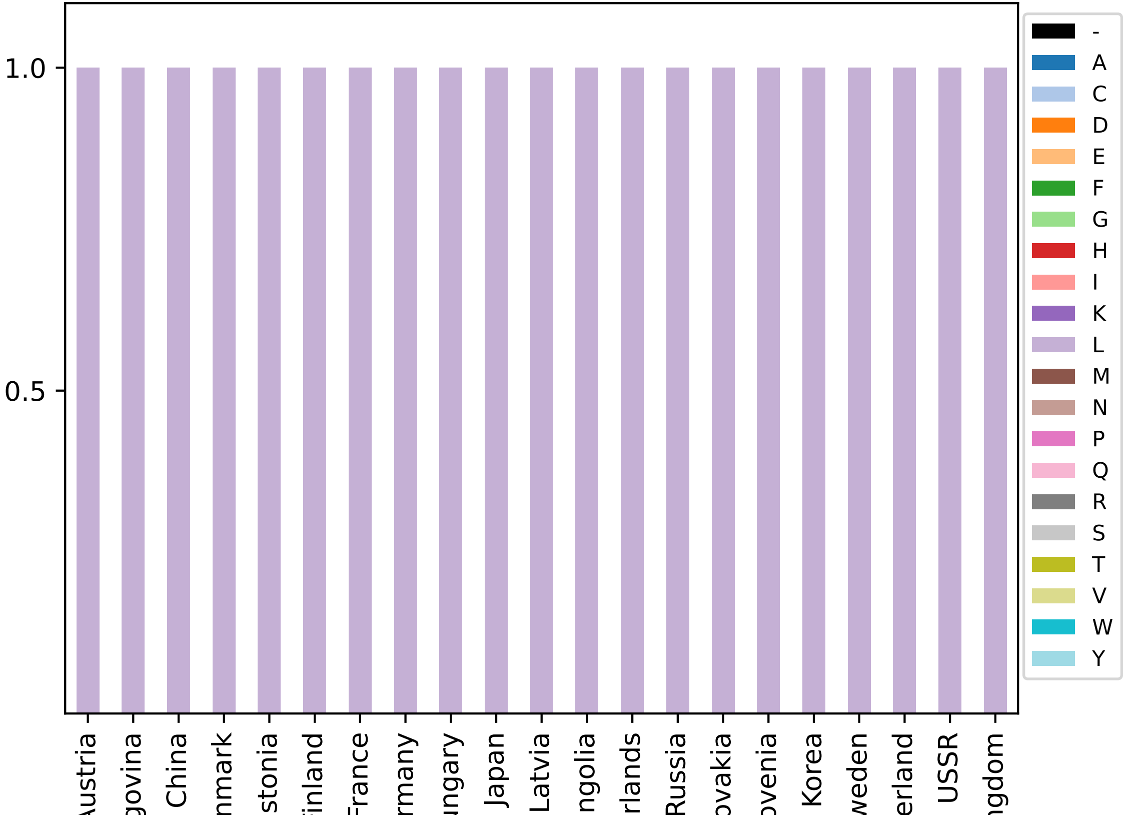

1.0
0.5
China
Japan
Latvia
Russia
Korea
USSR
-
A
C
D
E
F
G
H
I
K
L
M
N
P
Q
R
S
T
V
W
Y

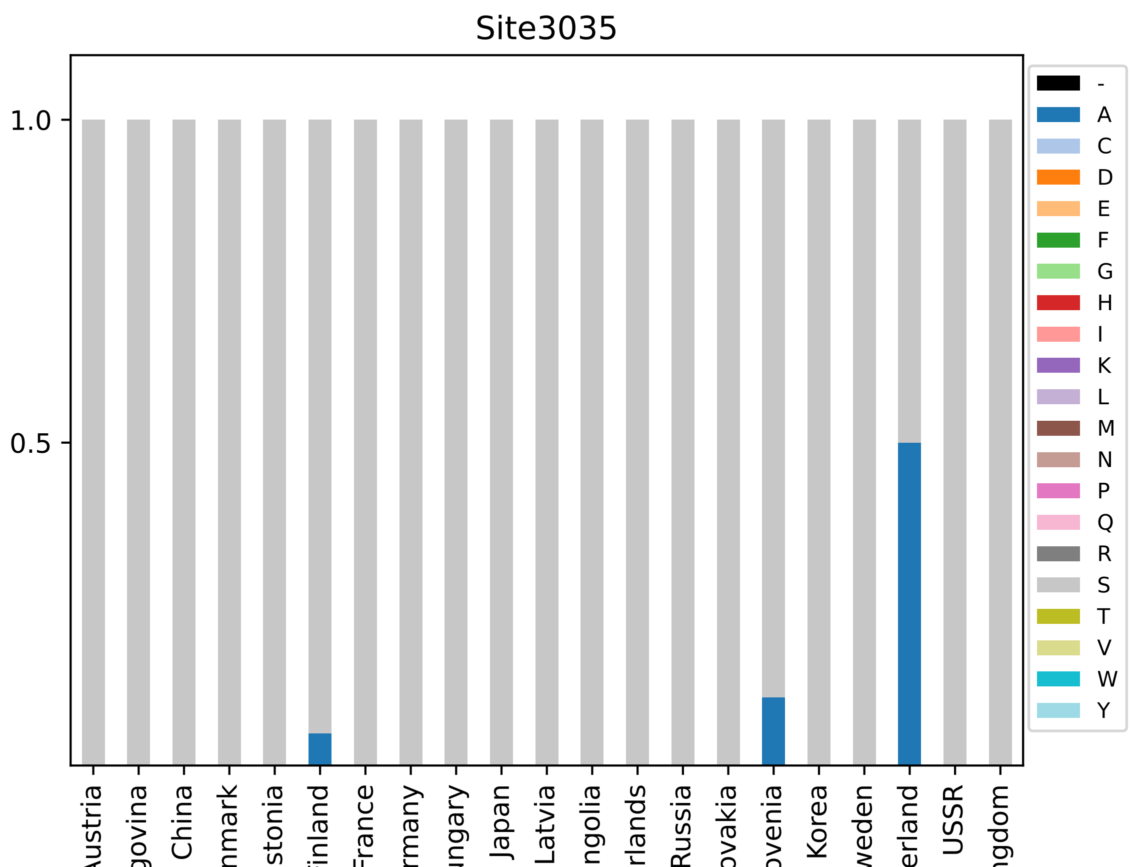

Site3035
1.0
0.5
-
A
C
D
E
F
G
H
I
K
L
M
N
P
Q
R
S
T
V
W
Y
Austria
China
Finland
France
Japan
Latvia
Russia
Korea
USSR

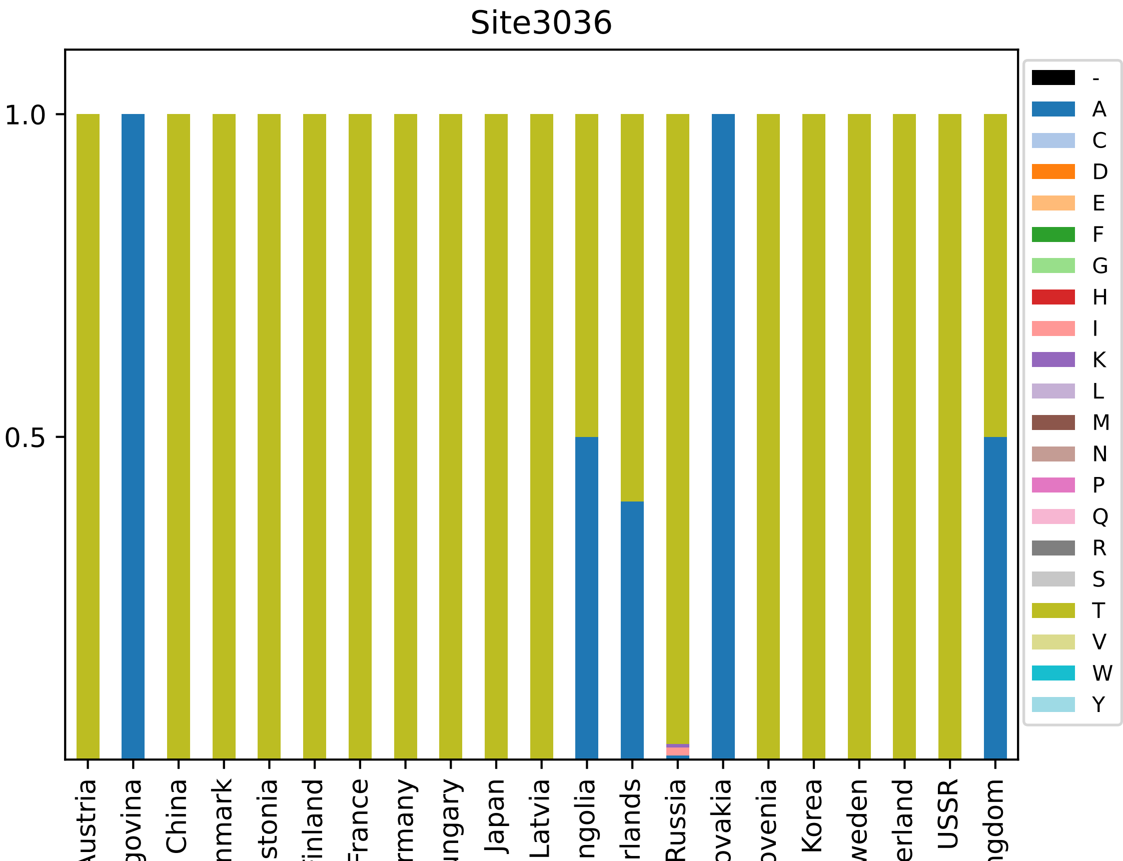
Site3036
1.0
0.5
-
A
C
D
E
F
G
H
I
K
L
M
N
P
Q
R
S
T
V
W
Y
Austria
govina
China
nmark
stonia
inland
France
rmany
ungary
Japan
Latvia
ngolia
rlands
Russia
ovakia
ovenia
Korea
weden
erland
USSR
ngdom

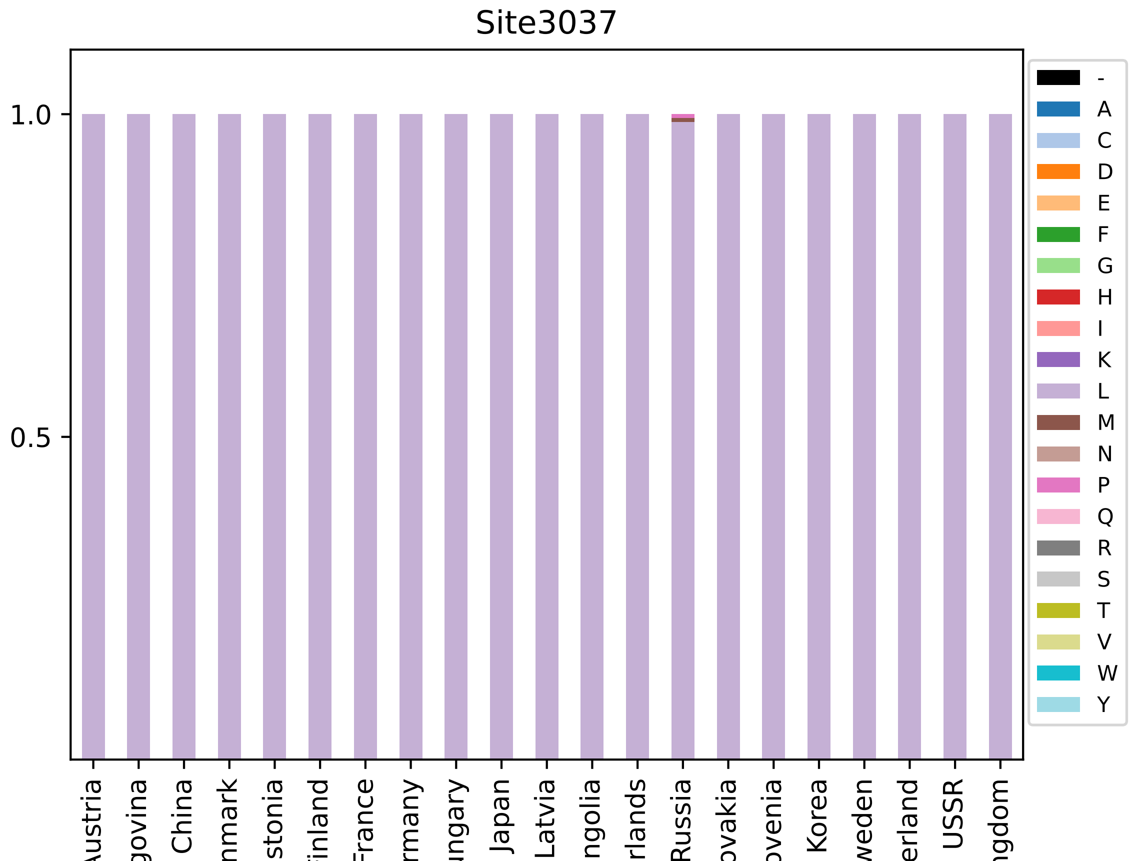
Site3037
1.0
0.5
-
A
C
D
E
F
G
H
I
K
L
M
N
P
Q
R
S
T
V
W
Y
China
Japan
Latvia
Russia
Korea
USSR

Site3038

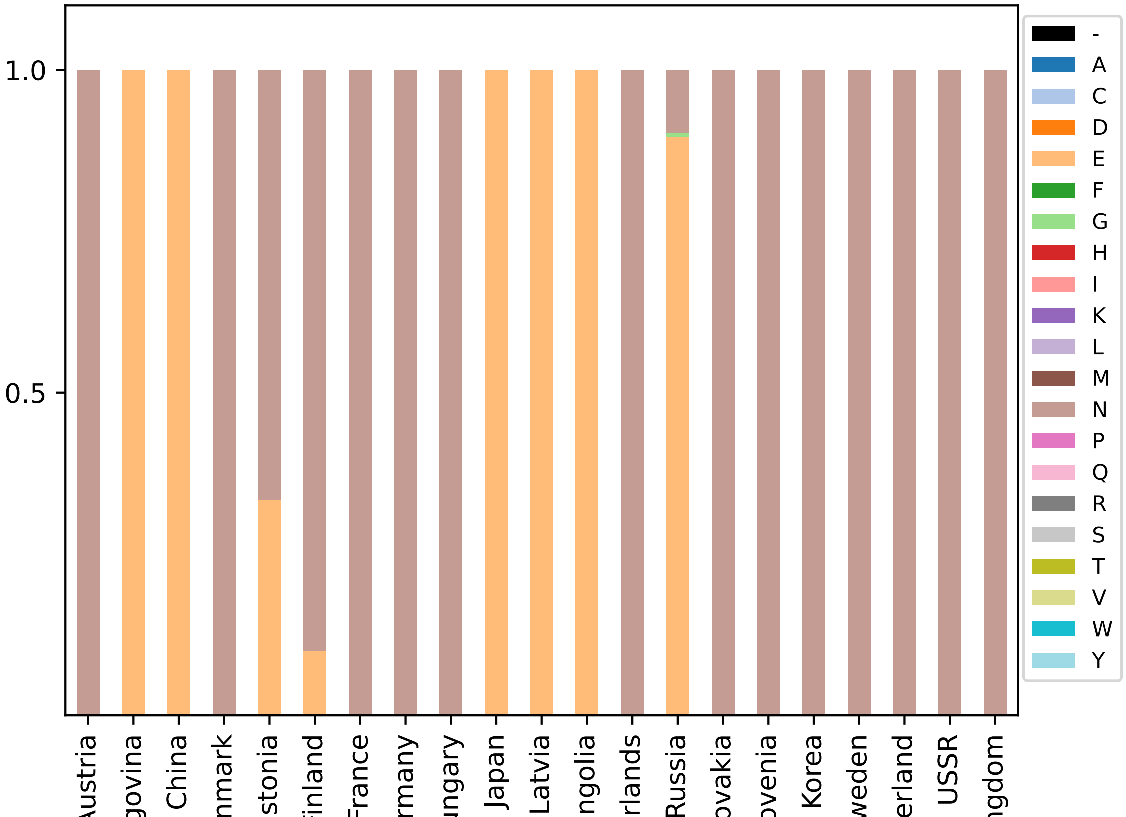

1.0
0.5
-
A
C
D
E
F
G
H
I
K
L
M
N
P
Q
R
S
T
V
W
Y
China
Latvia
Russia
Japan
Korea
USSR

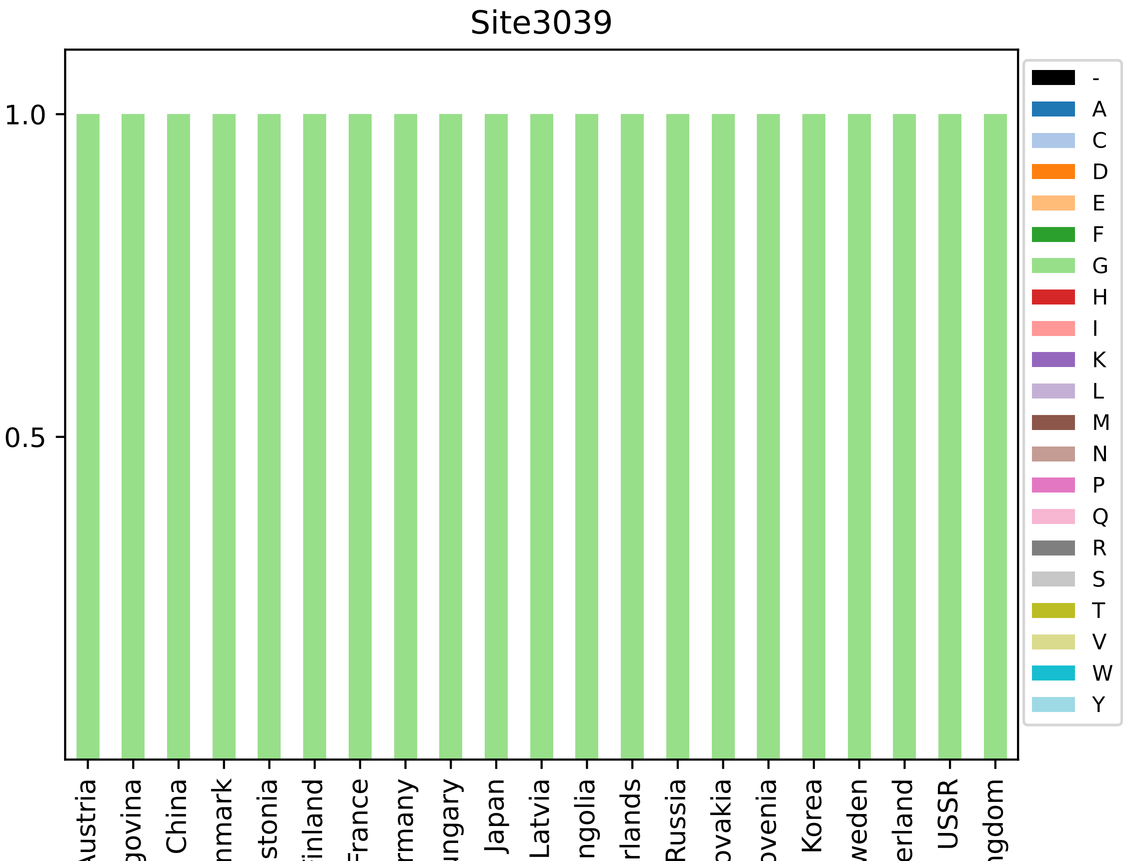
Site3039
1.0
0.5
-
A
C
D
E
F
G
H
I
K
L
M
N
P
Q
R
S
T
V
W
Y
China
Japan
Latvia
Russia
Korea
USSR

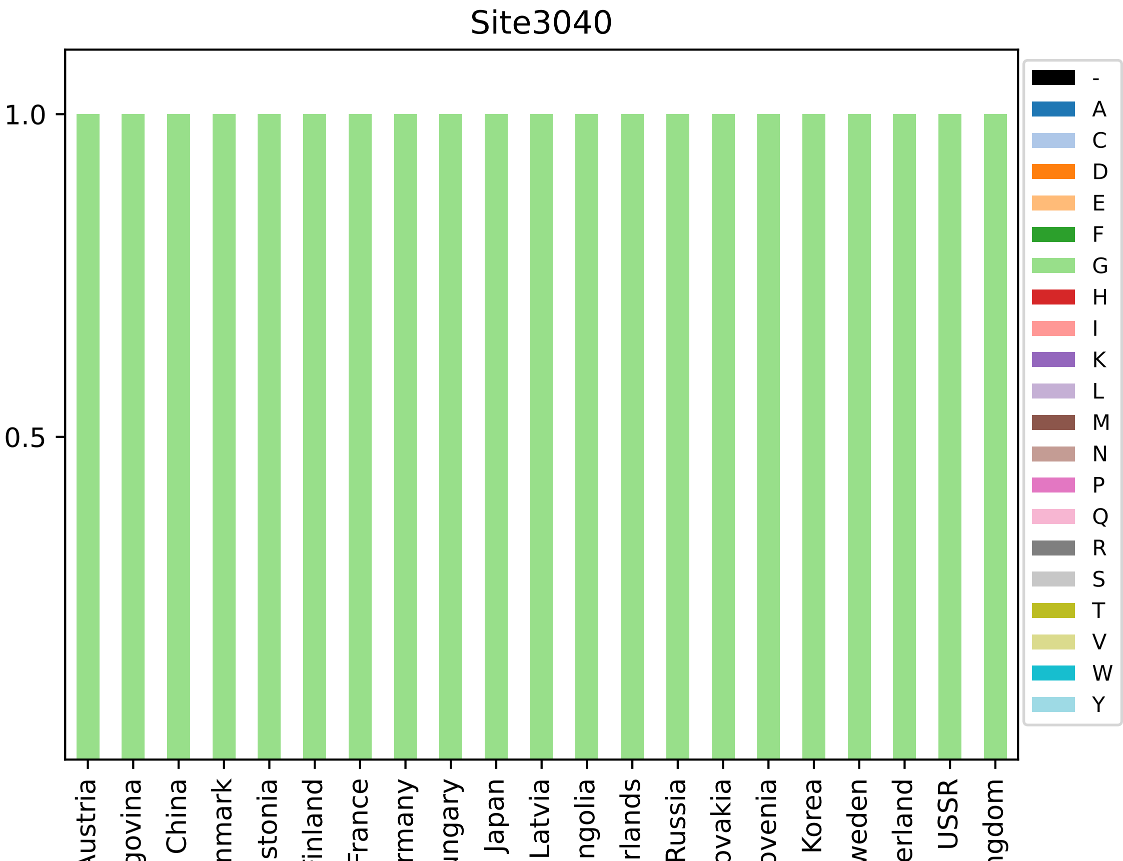
Site3040
1.0
0.5
-
A
C
D
E
F
G
H
I
K
L
M
N
P
Q
R
S
T
V
W
Y
Austria
govina
China
nmark
stonia
inland
France
rmany
ungary
Japan
Latvia
ngolia
rlands
Russia
ovakia
ovenia
Korea
weden
erland
USSR
ngdom

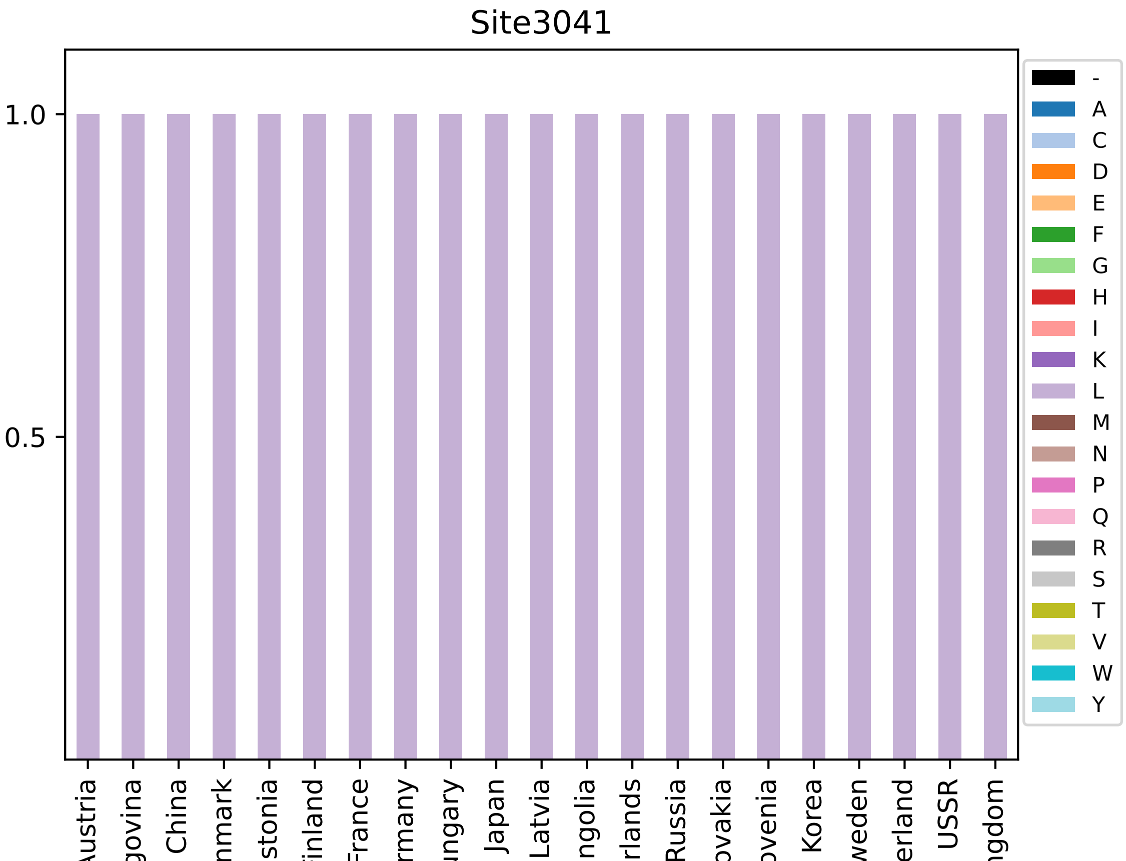

Site3041
1.0
0.5
China
Japan
Latvia
Russia
Korea
USSR
-
A
C
D
E
F
G
H
I
K
L
M
N
P
Q
R
S
T
V
W
Y

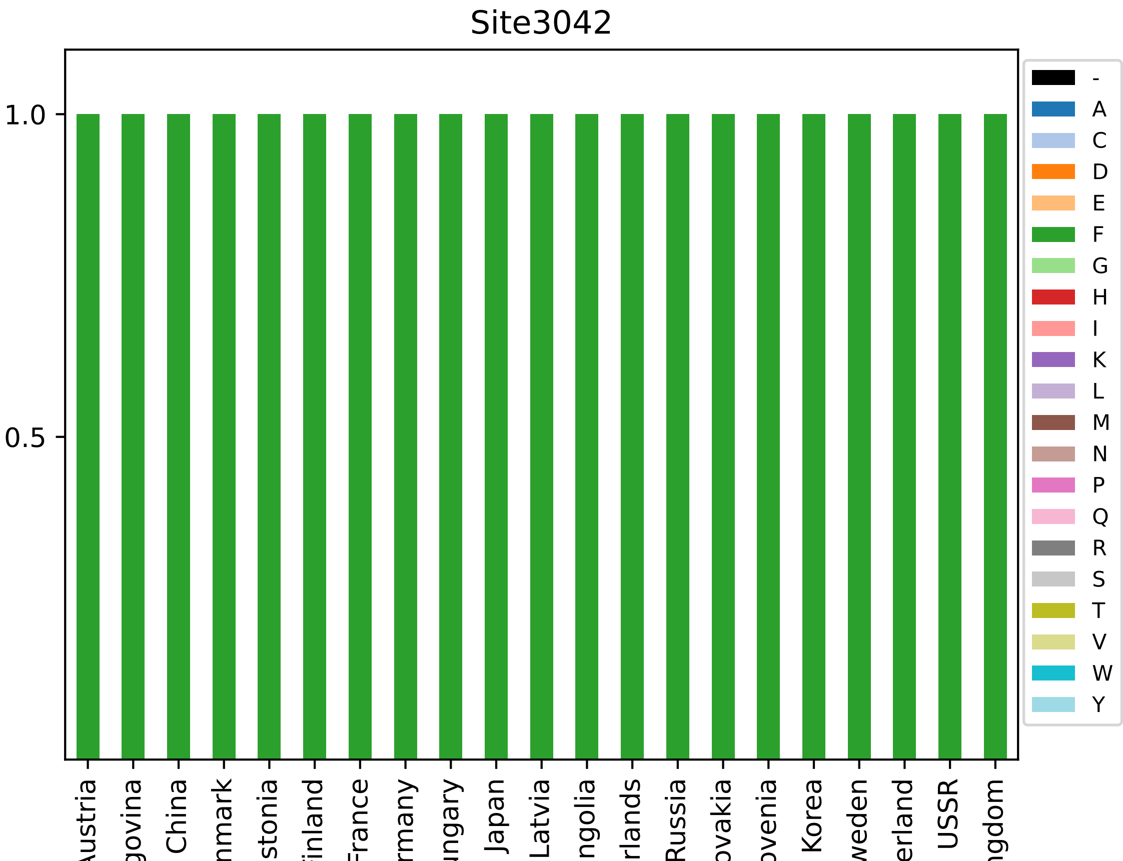
Site3042
1.0
0.5
-
A
C
D
E
F
G
H
I
K
L
M
N
P
Q
R
S
T
V
W
Y
China
Japan
Latvia
Russia
Korea
USSR

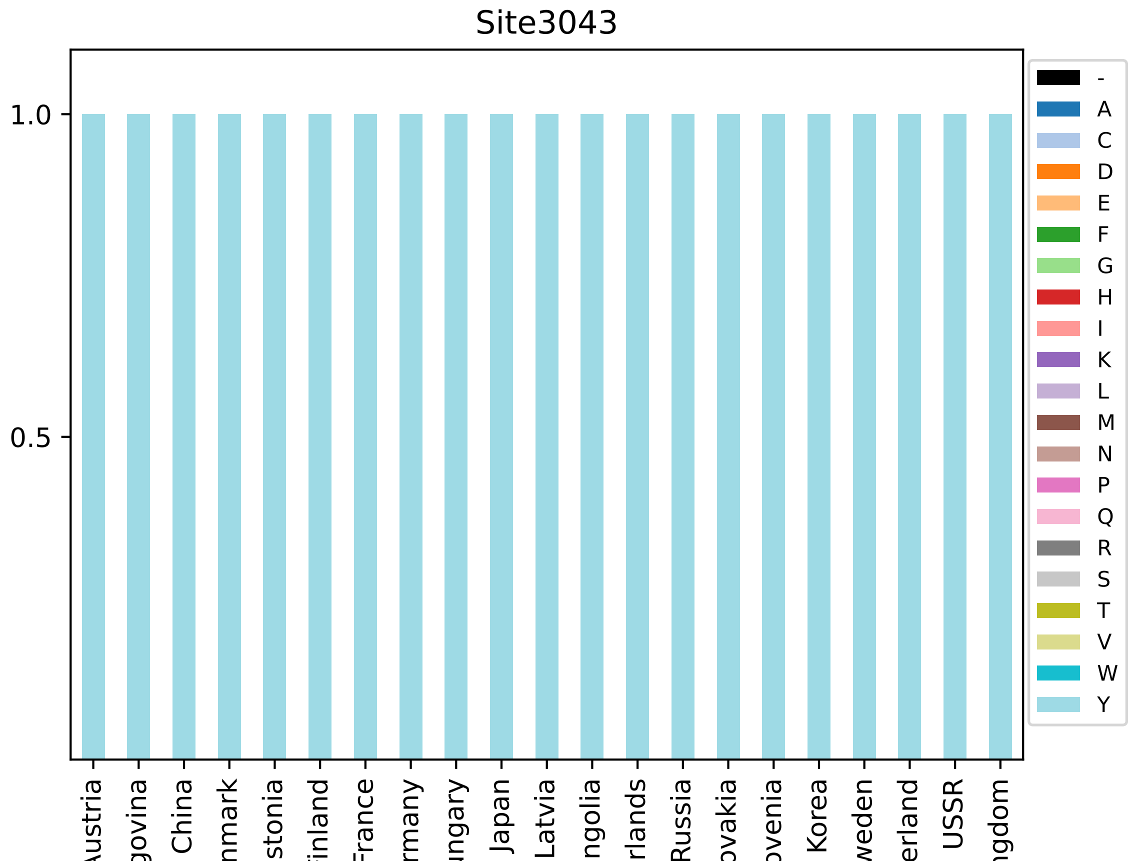

Site3043
1.0
0.5
-
A
C
D
E
F
G
H
I
K
L
M
N
P
Q
R
S
T
V
W
Y
Austria
govina
China
nmark
stonia
Finland
France
rmany
ungary
Japan
Latvia
ngolia
rlands
Russia
ovakia
ovenia
Korea
weden
erland
USSR
ngdom

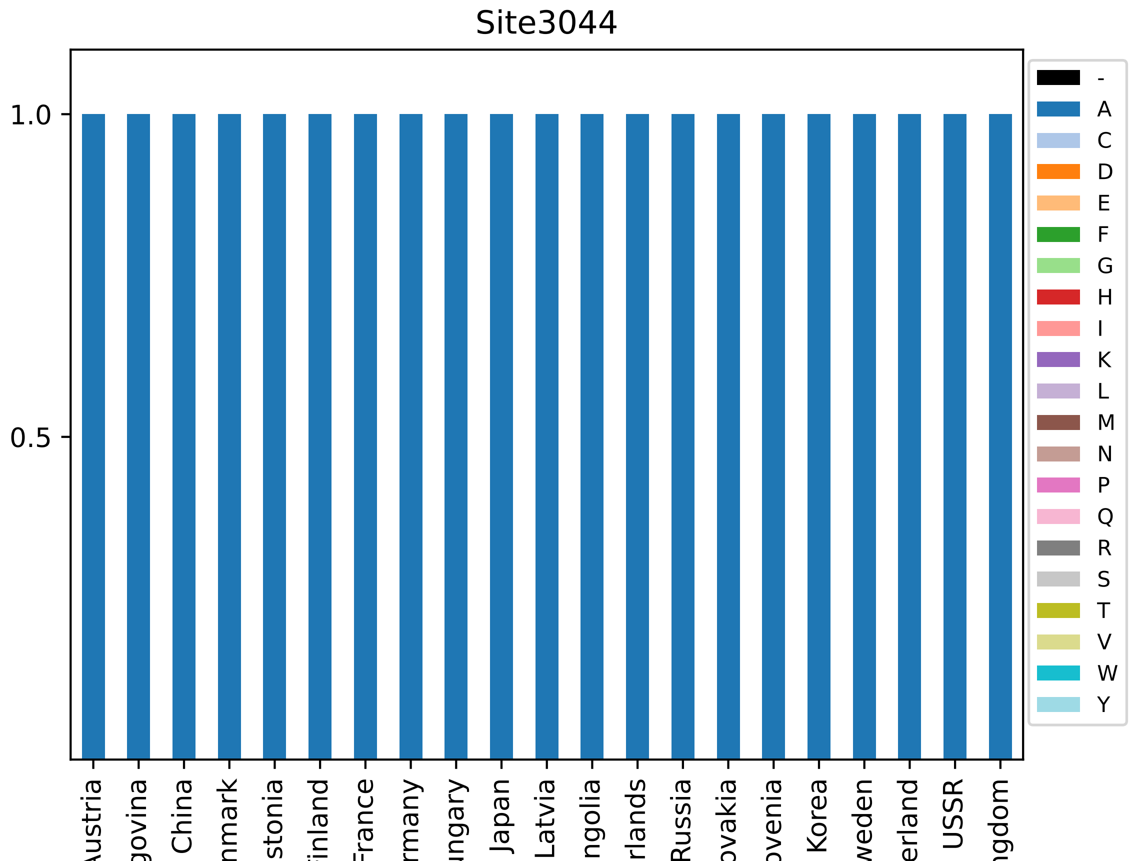
Site3044
1.0
0.5
-
A
C
D
E
F
G
H
I
K
L
M
N
P
Q
R
S
T
V
W
Y
China
Japan
Latvia
Russia
Korea
USSR

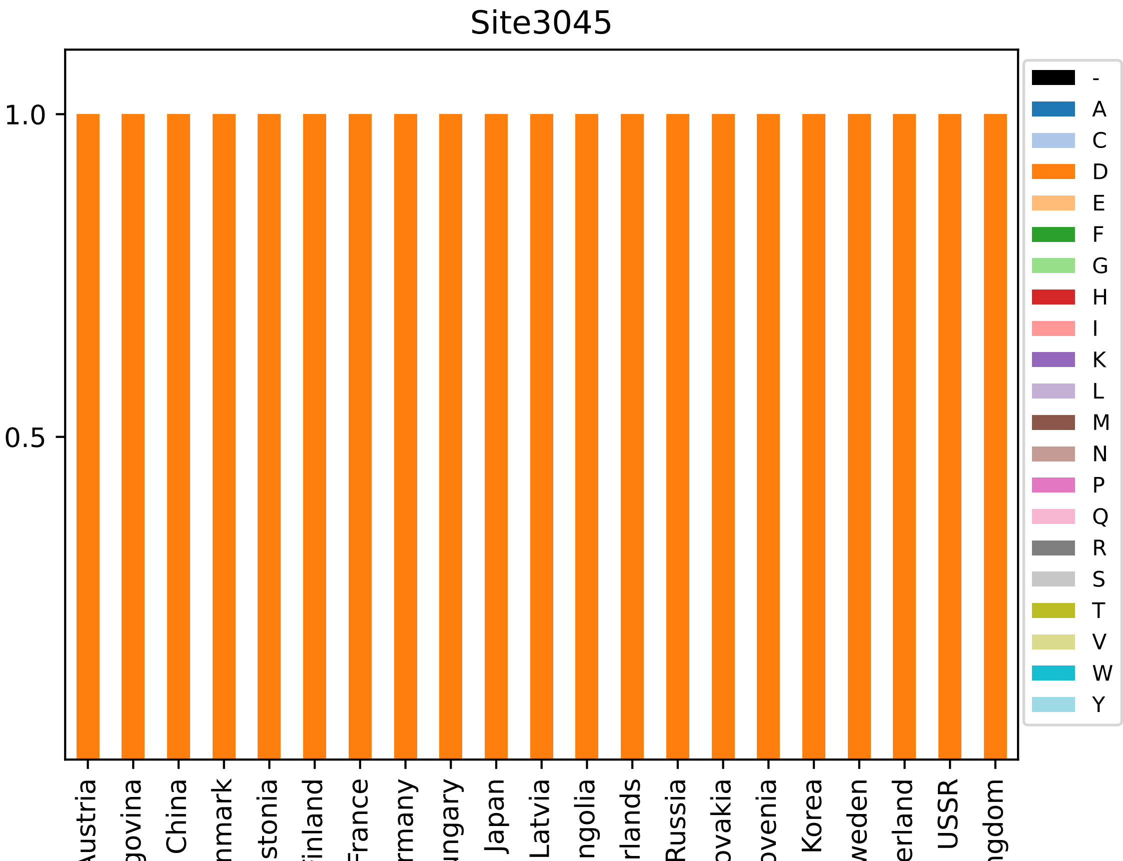
Site3045
1.0
0.5
-
A
C
D
E
F
G
H
I
K
L
M
N
P
Q
R
S
T
V
W
Y
Austria
govina
China
nmark
stonia
inland
France
rmany
ungary
Japan
Latvia
ngolia
rlands
Russia
ovakia
ovenia
Korea
weden
erland
USSR
ngdom

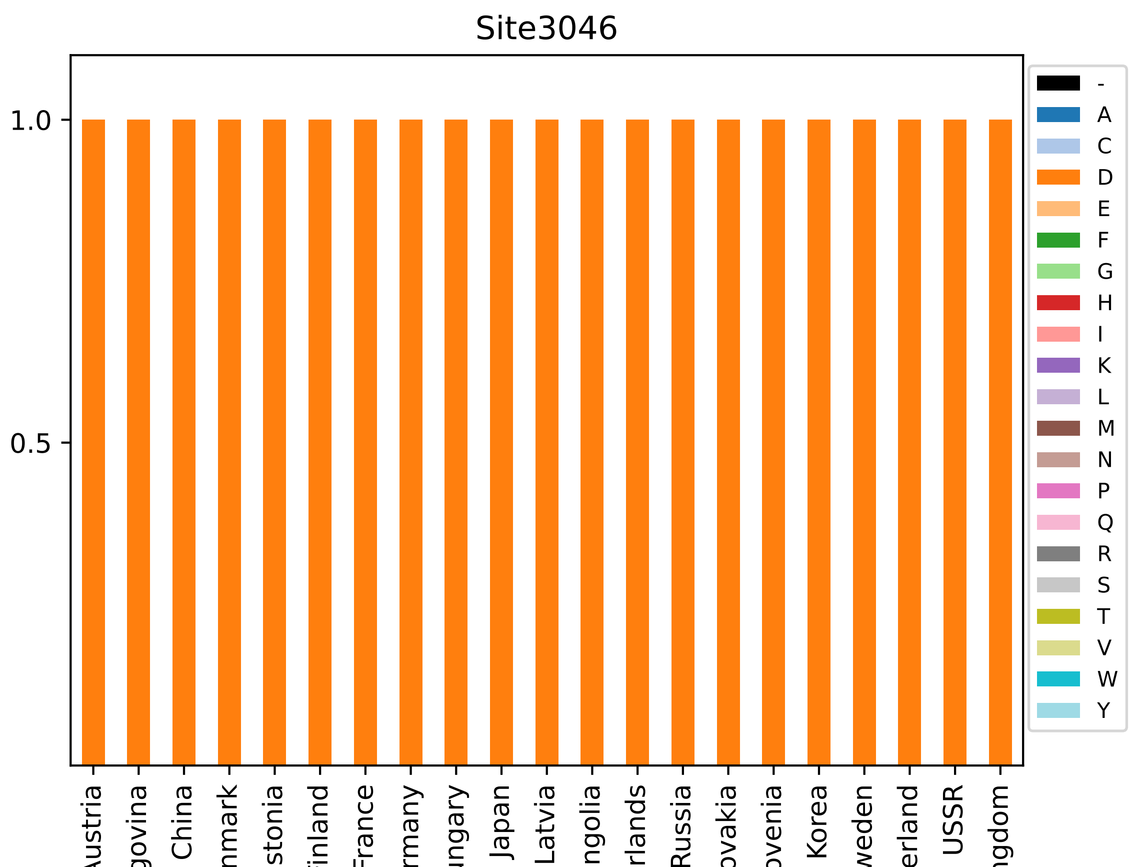
Site3046
1.0
0.5
-
A
C
D
E
F
G
H
I
K
L
M
N
P
Q
R
S
T
V
W
Y
China
Japan
Latvia
Russia
Korea
USSR

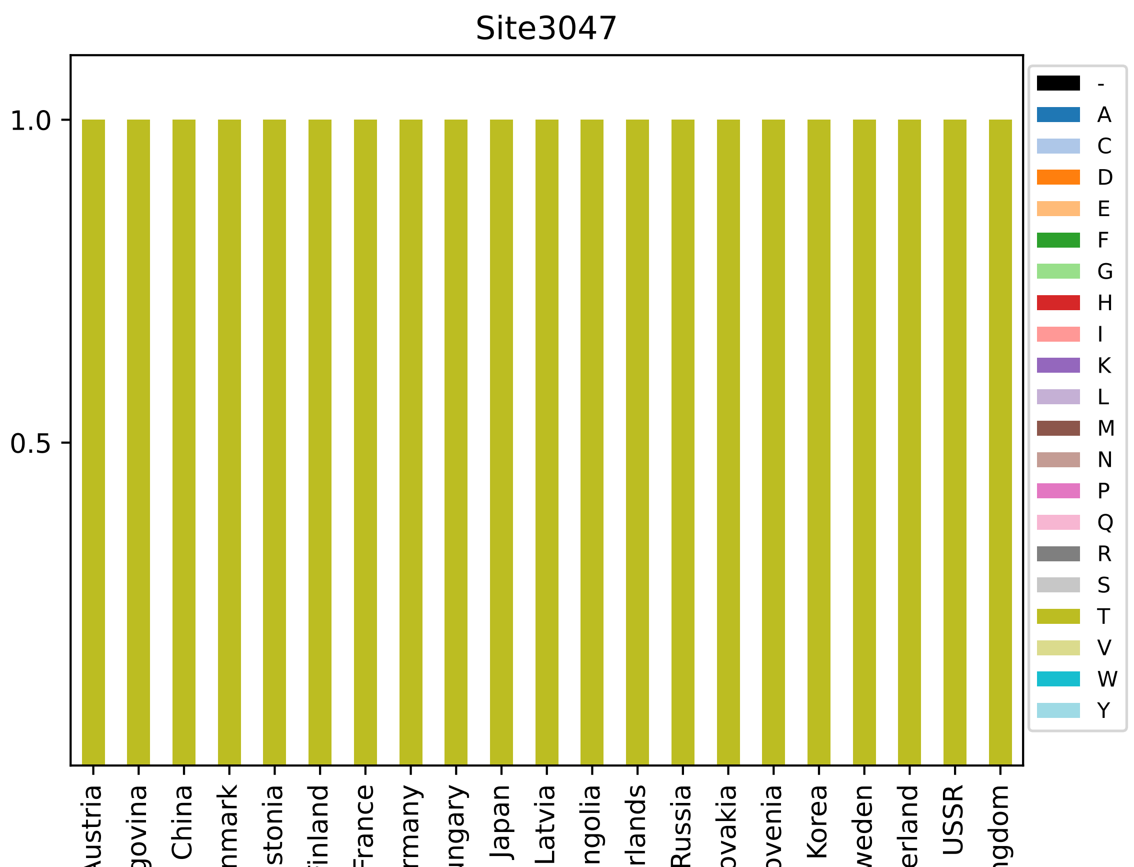

Site3047
1.0
0.5
-
A
C
D
E
F
G
H
I
K
L
M
N
P
Q
R
S
T
V
W
Y
China
Japan
Latvia
Russia
Korea
USSR

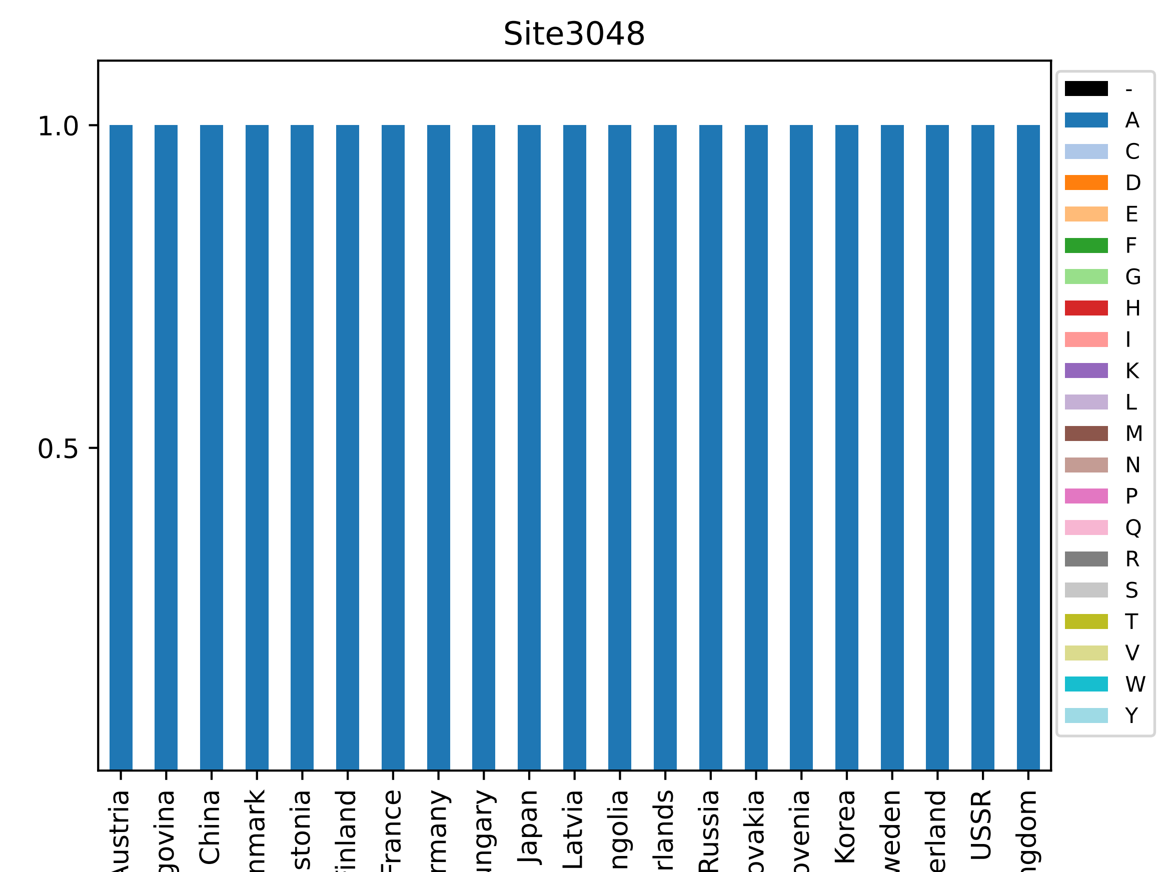
Site3048
1.0
0.5
-
A
C
D
E
F
G
H
I
K
L
M
N
P
Q
R
S
T
V
W
Y
Austria
govina
China
nmark
stonia
inland
France
rmany
ungary
Japan
Latvia
ngolia
rlands
Russia
ovakia
ovenia
Korea
weden
erland
USSR
ngdom

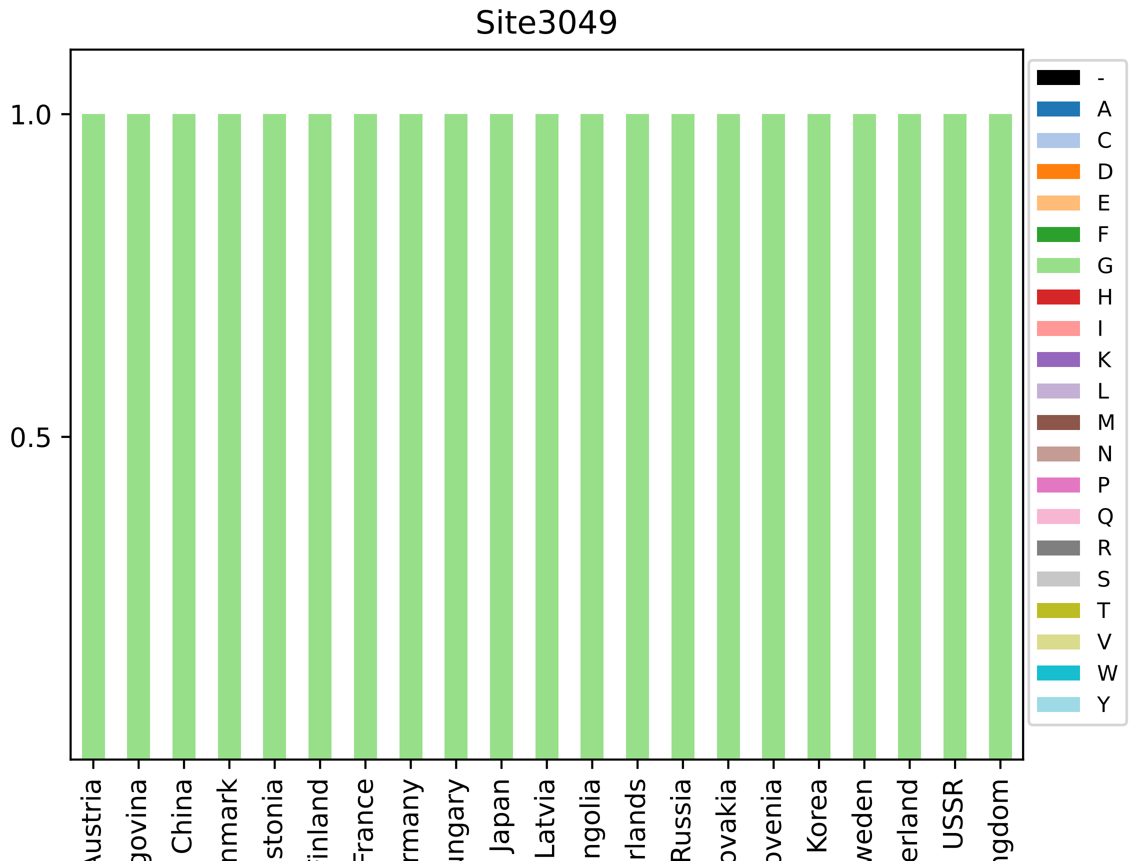

Site3049
1.0
0.5
-
A
C
D
E
F
G
H
I
K
L
M
N
P
Q
R
S
T
V
W
Y
Austria
govina
China
nmark
stonia
inland
France
rmany
ungary
Japan
Latvia
ngolia
rlands
Russia
ovakia
ovenia
Korea
weden
erland
USSR
ngdom

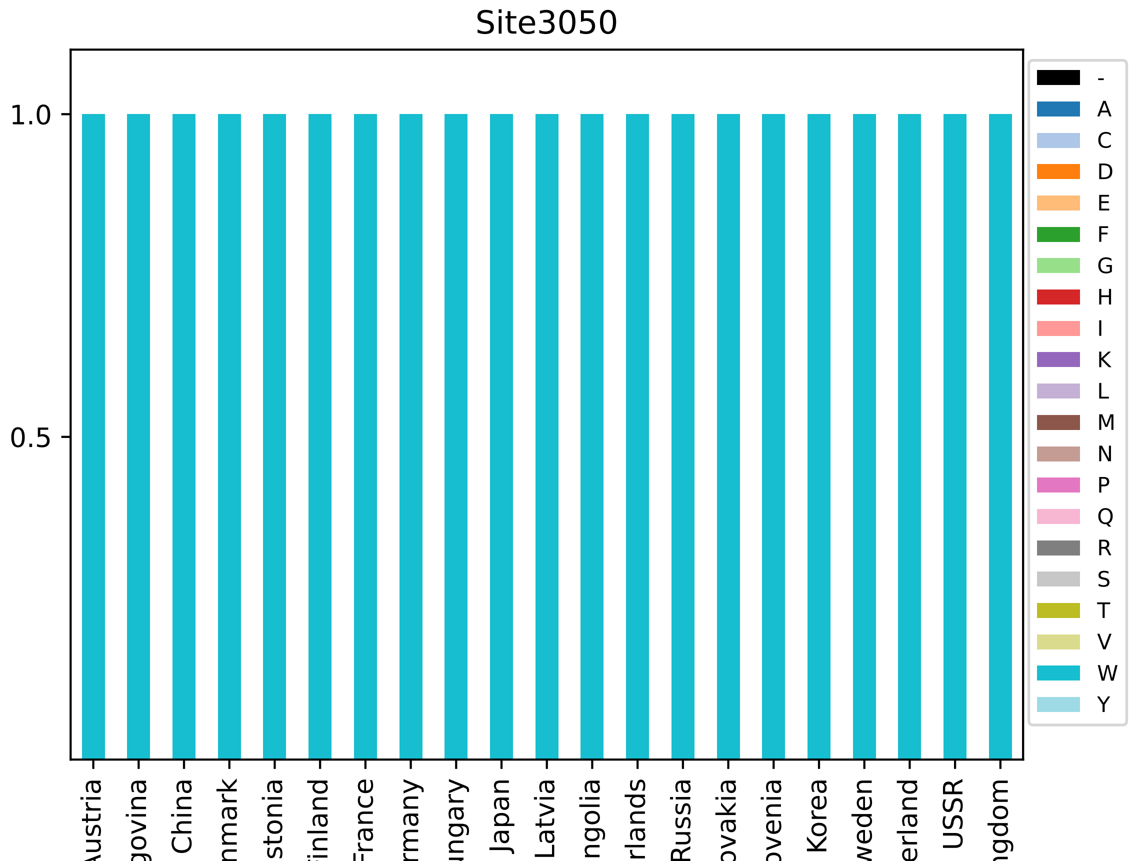

Site3050
1.0
0.5
-
A
C
D
E
F
G
H
I
K
L
M
N
P
Q
R
S
T
V
W
Y
ustria
govina
China
nmark
stonia
inland
rance
rmany
ungary
Japan
Latvia
ngolia
rlands
Russia
ovakia
ovenia
Korea
weden
erland
USSR
ngdom

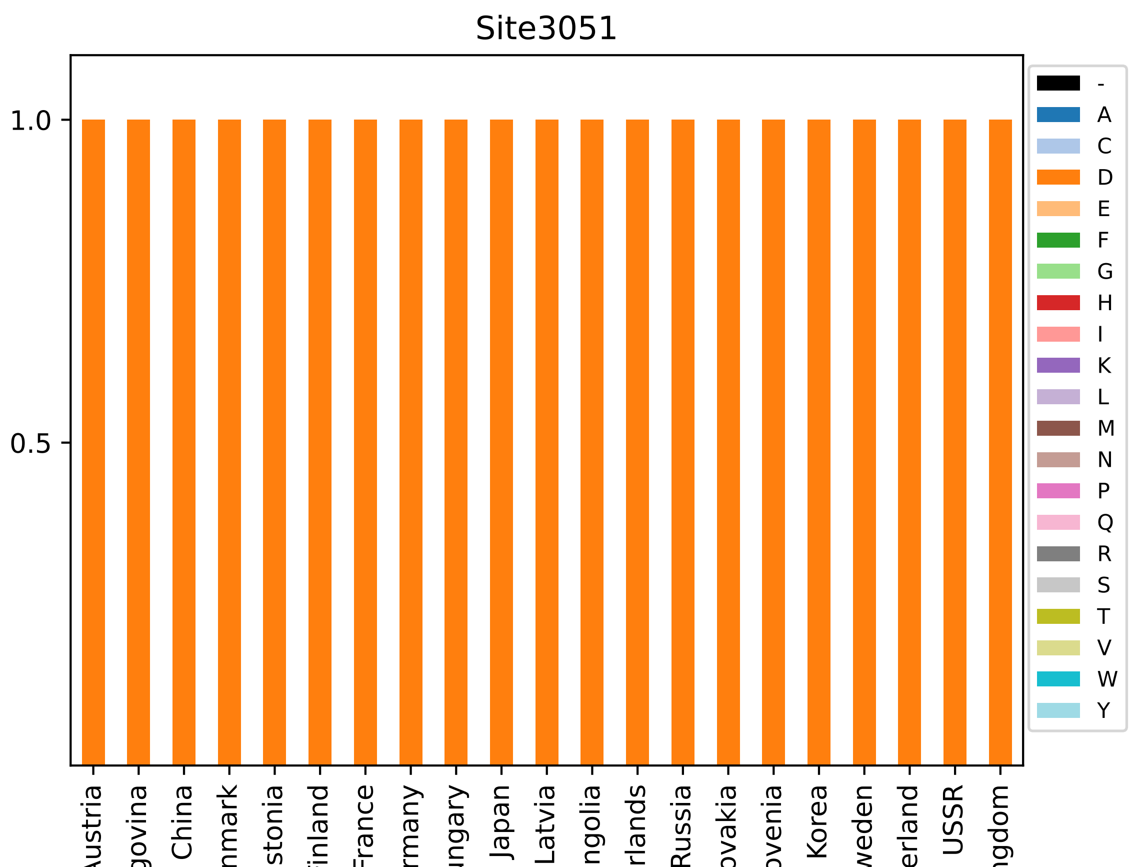

Site3051
1.0
0.5
-
A
C
D
E
F
G
H
I
K
L
M
N
P
Q
R
S
T
V
W
Y
Austria
govina
China
nmark
stonia
inland
France
rmany
ungary
Japan
Latvia
ngolia
rlands
Russia
ovakia
ovenia
Korea
weden
erland
USSR
ngdom

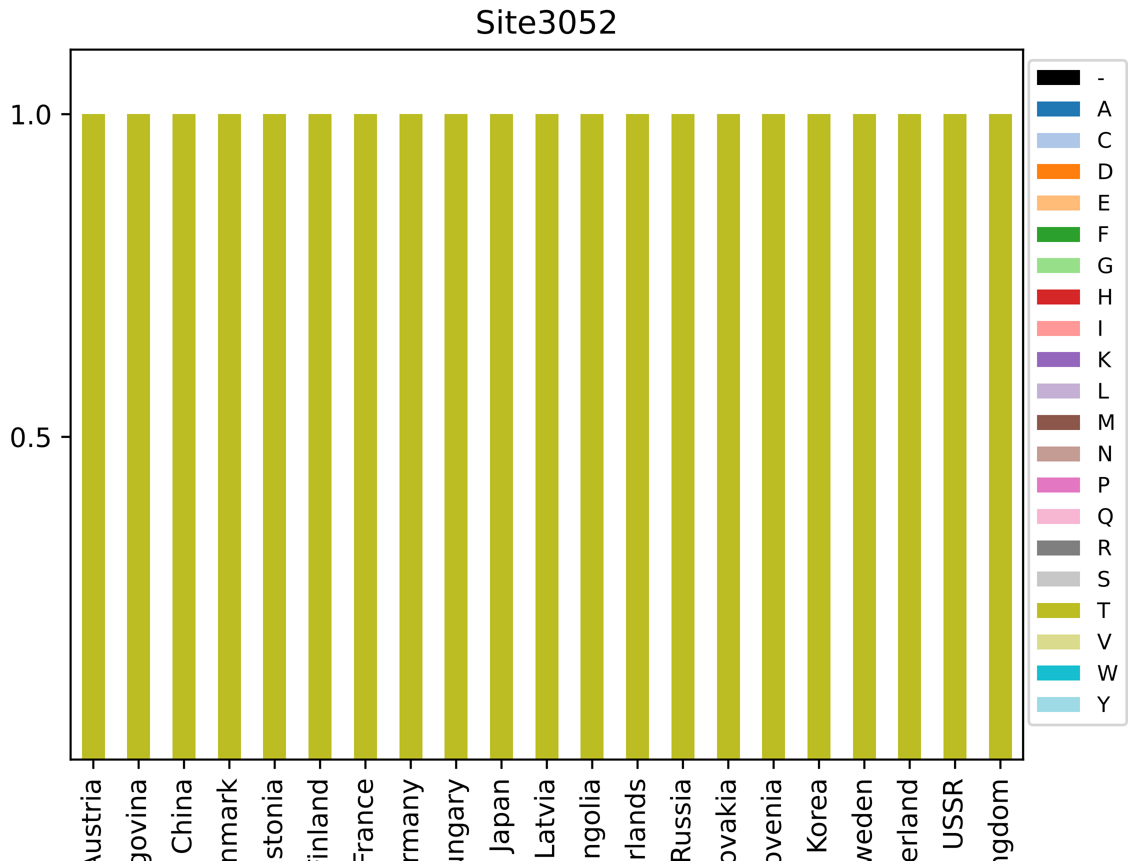
Site3052
1.0
0.5
-
A
C
D
E
F
G
H
I
K
L
M
N
P
Q
R
S
T
V
W
Y
Austria
govina
China
nmark
stonia
inland
France
rmany
ungary
Japan
Latvia
ngolia
rlands
Russia
ovakia
ovenia
Korea
weden
erland
USSR
ngdom

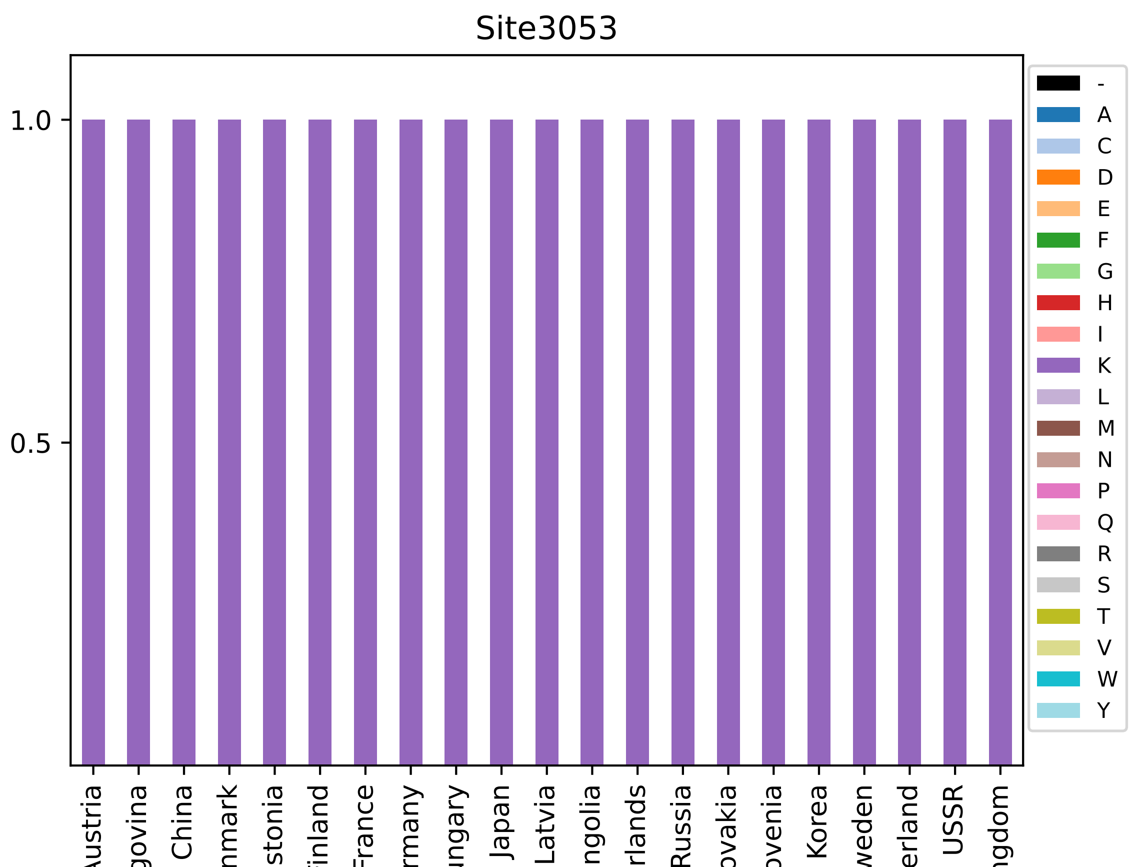
Site3053
1.0
0.5
-
A
C
D
E
F
G
H
I
K
L
M
N
P
Q
R
S
T
V
W
Y
China
Japan
Latvia
Russia
Korea
USSR

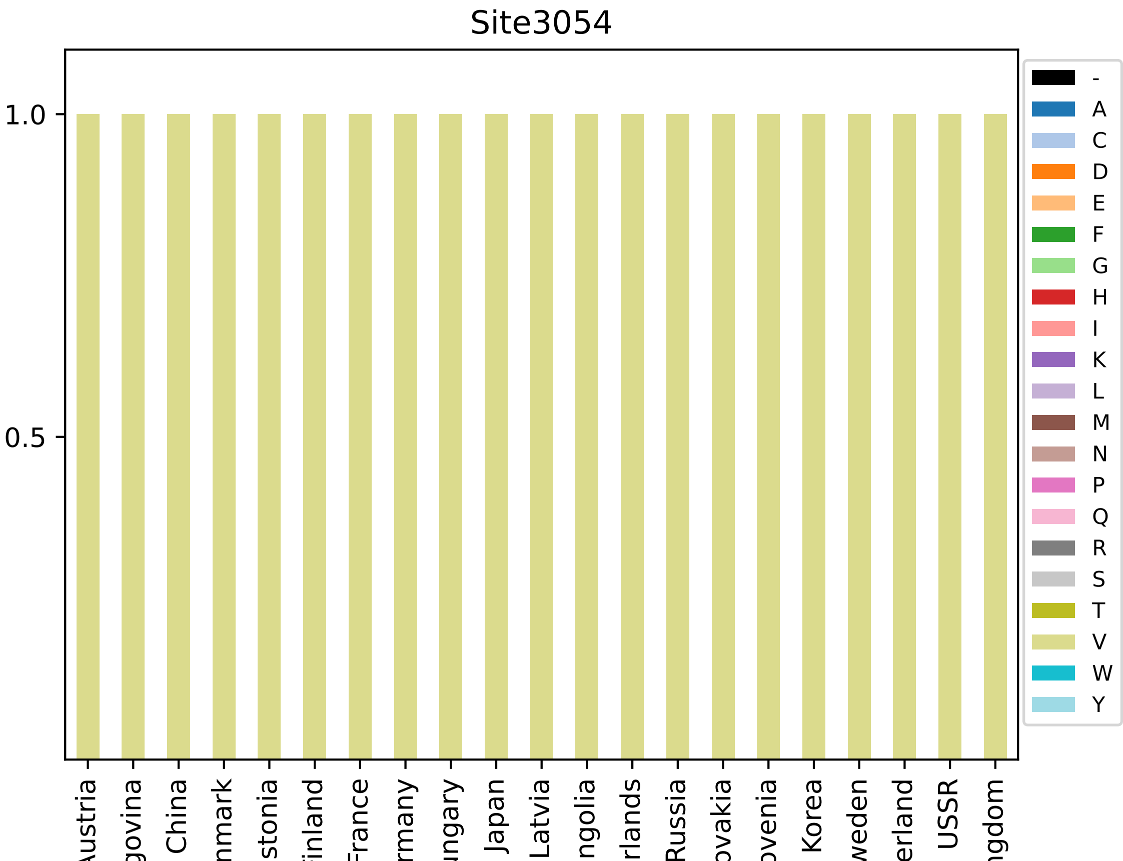
Site3054
1.0
0.5
-
A
C
D
E
F
G
H
I
K
L
M
N
P
Q
R
S
T
V
W
Y
China
Japan
Latvia
Russia
Korea
USSR

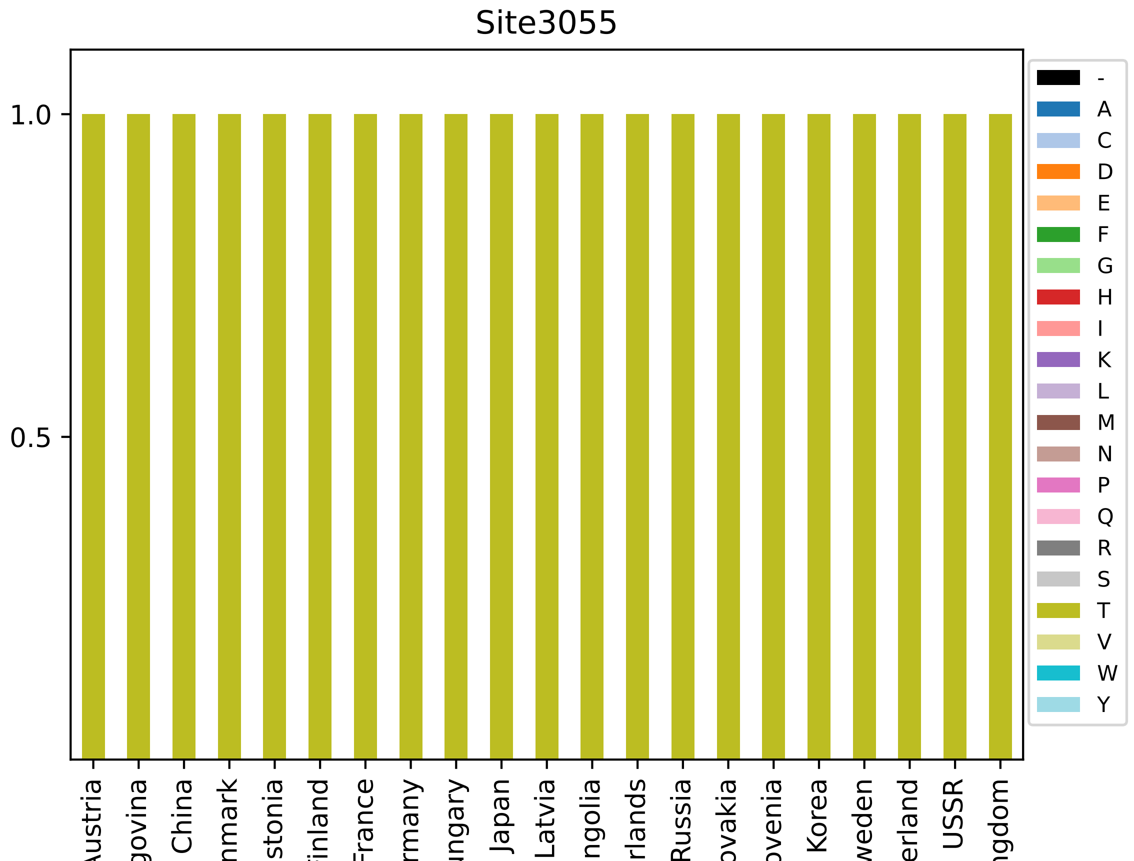

Site3055
1.0
0.5
-
A
C
D
E
F
G
H
I
K
L
M
N
P
Q
R
S
T
V
W
Y
Austria
govina
China
nmark
stonia
inland
France
rmany
ungary
Japan
Latvia
ngolia
rlands
Russia
ovakia
ovenia
Korea
weden
erland
USSR
ngdom

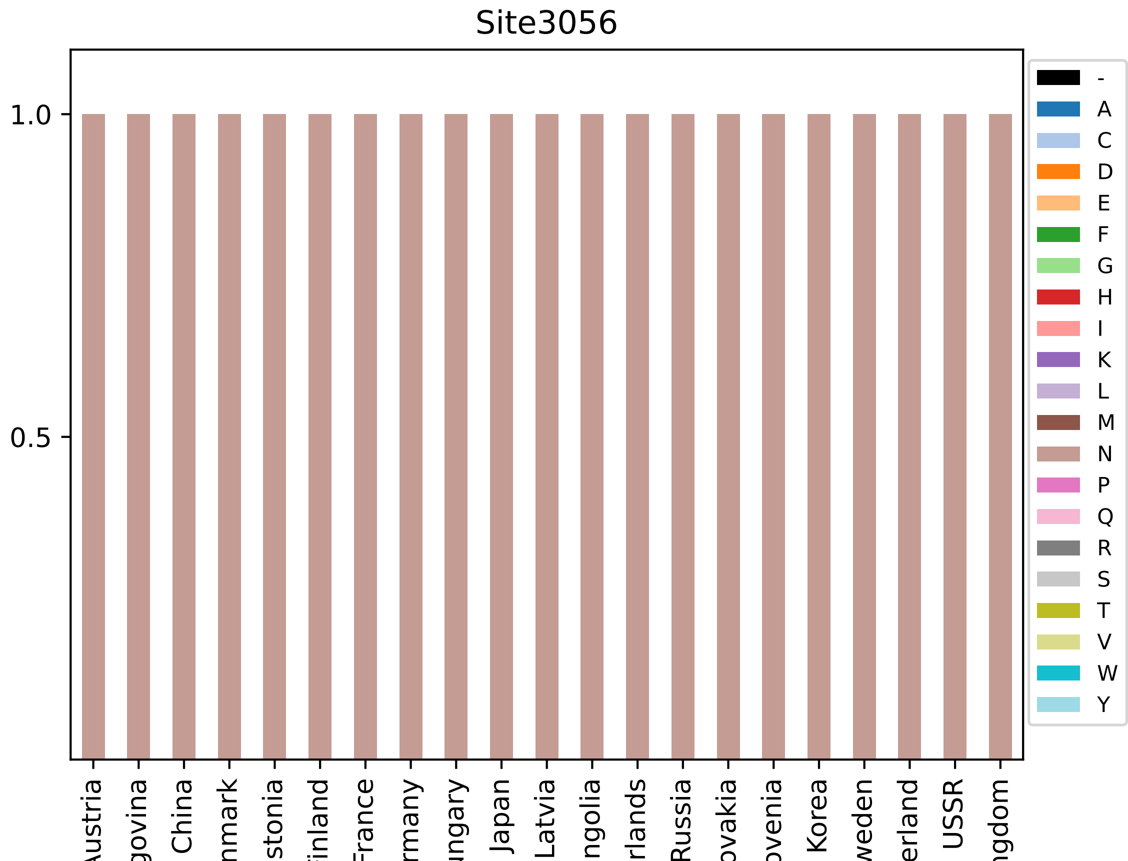
Site3056
1.0
0.5
-
A
C
D
E
F
G
H
I
K
L
M
N
P
Q
R
S
T
V
W
Y
Austria
China
Japan
Latvia
Russia
Korea
USSR

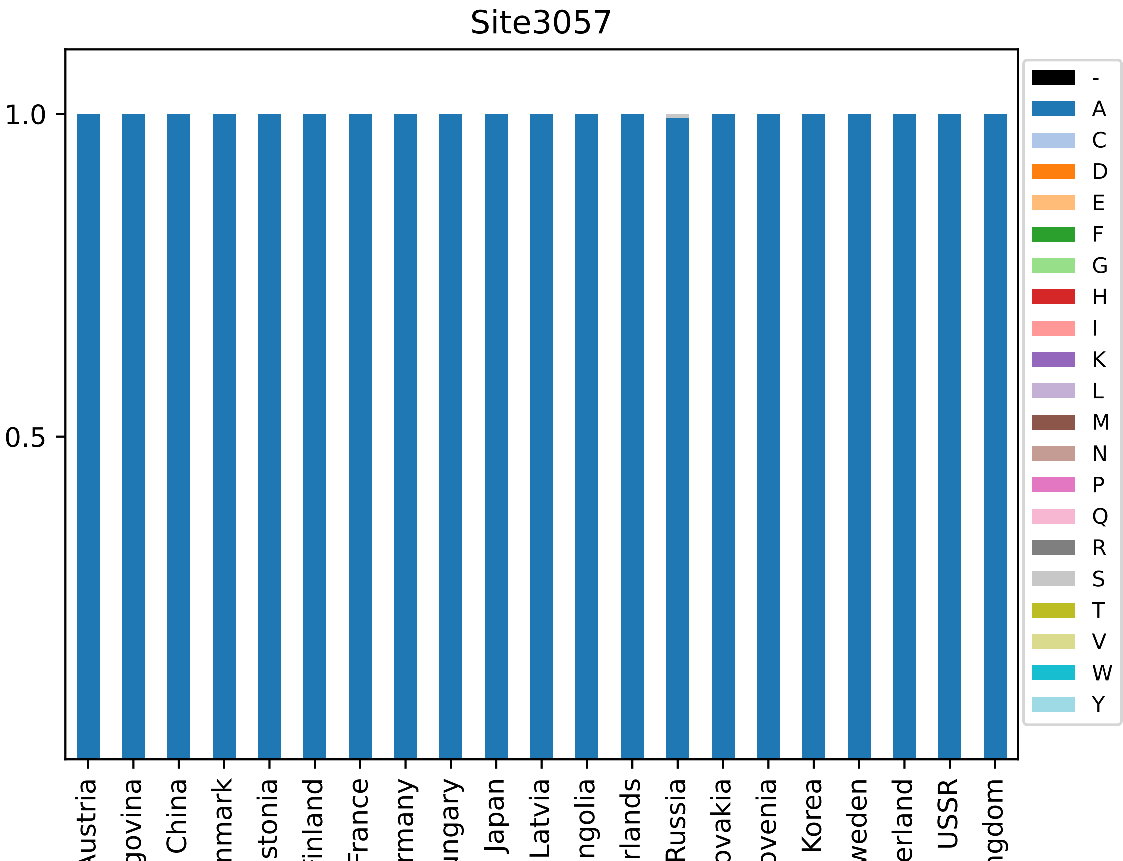
Site3057
1.0
0.5
-
A
C
D
E
F
G
H
I
K
L
M
N
P
Q
R
S
T
V
W
Y
Austria
govina
China
nmark
stonia
Finland
France
rmany
ungary
Japan
Latvia
ngolia
rlands
Russia
ovakia
ovenia
Korea
weden
erland
USSR
ngdom

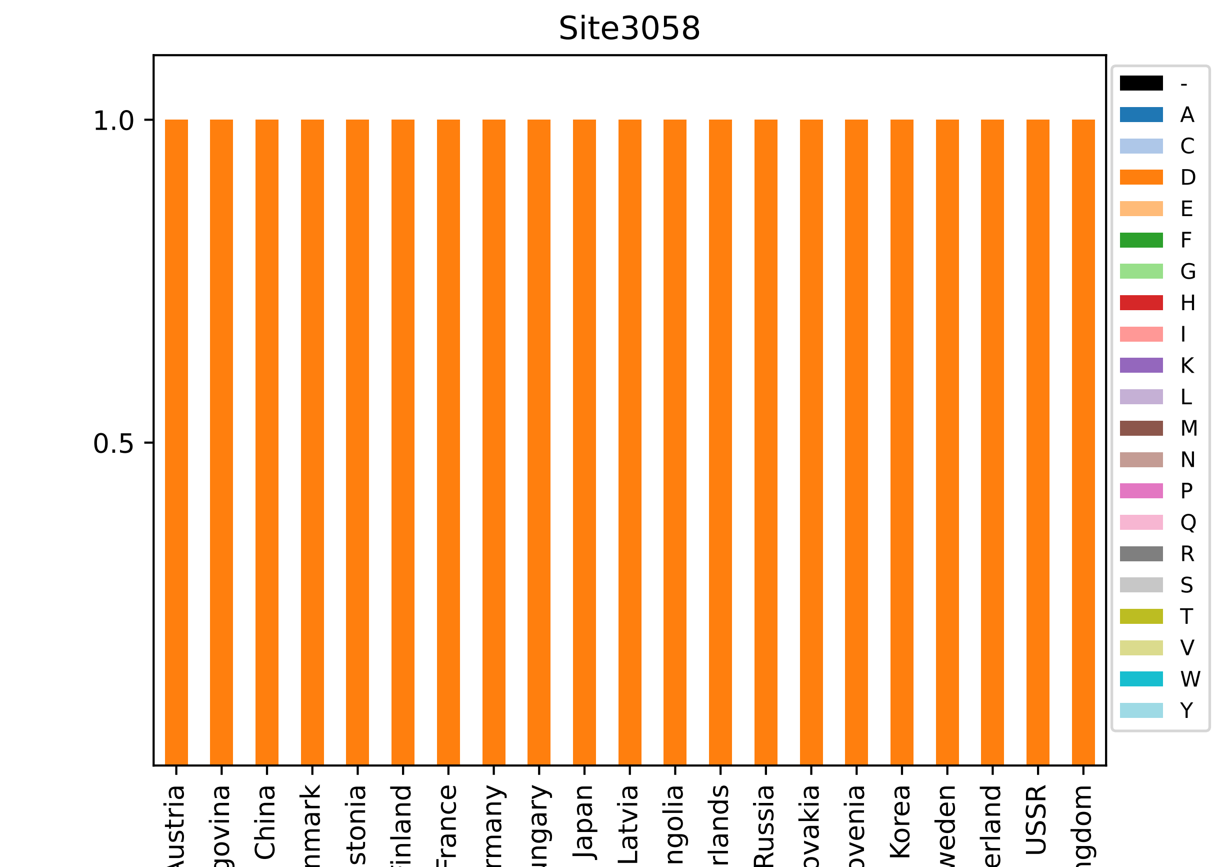

Site3058
1.0
0.5
-
A
C
D
E
F
G
H
I
K
L
M
N
P
Q
R
S
T
V
W
Y
Austria
govina
China
nmark
stonia
Finland
France
rmany
ungary
Japan
Latvia
ngolia
rlands
Russia
ovakia
ovenia
Korea
weden
erland
USSR
ngdom

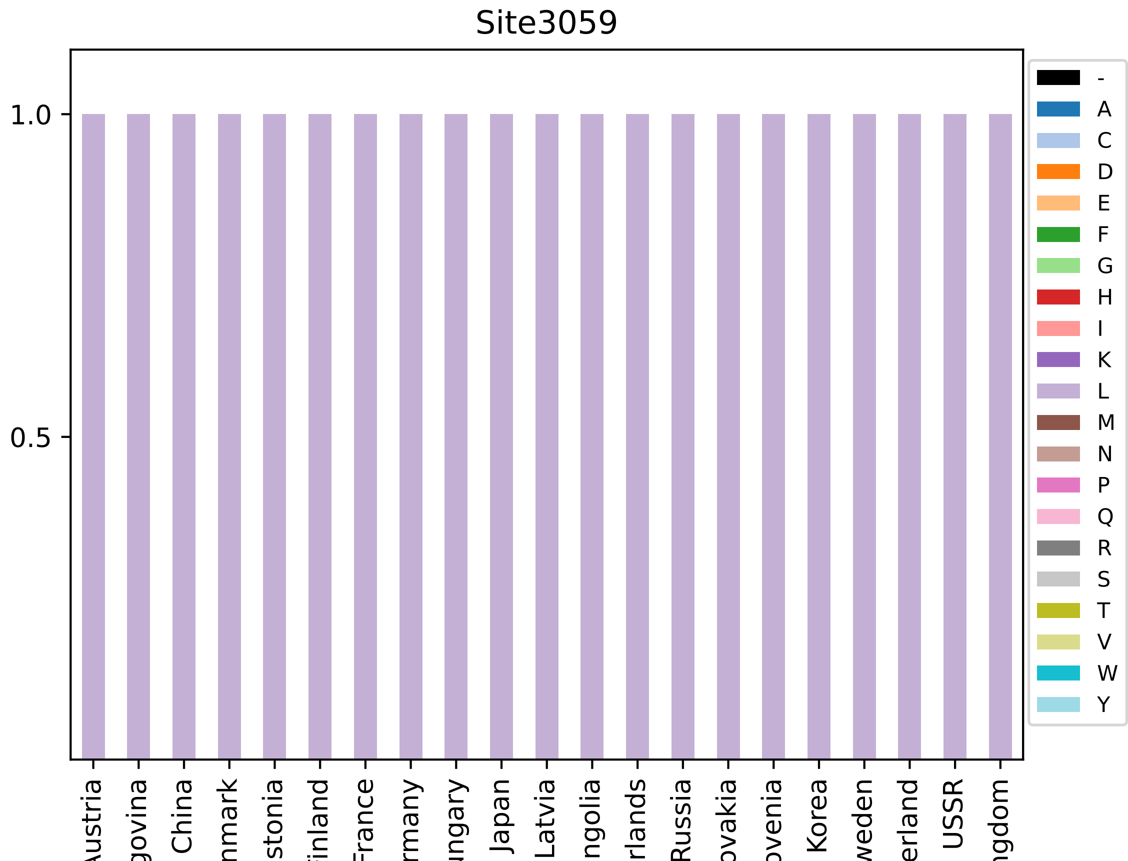

Site3059
1.0
0.5
-
A
C
D
E
F
G
H
I
K
L
M
N
P
Q
R
S
T
V
W
Y
Austria
China
Japan
Latvia
Russia
Korea
USSR

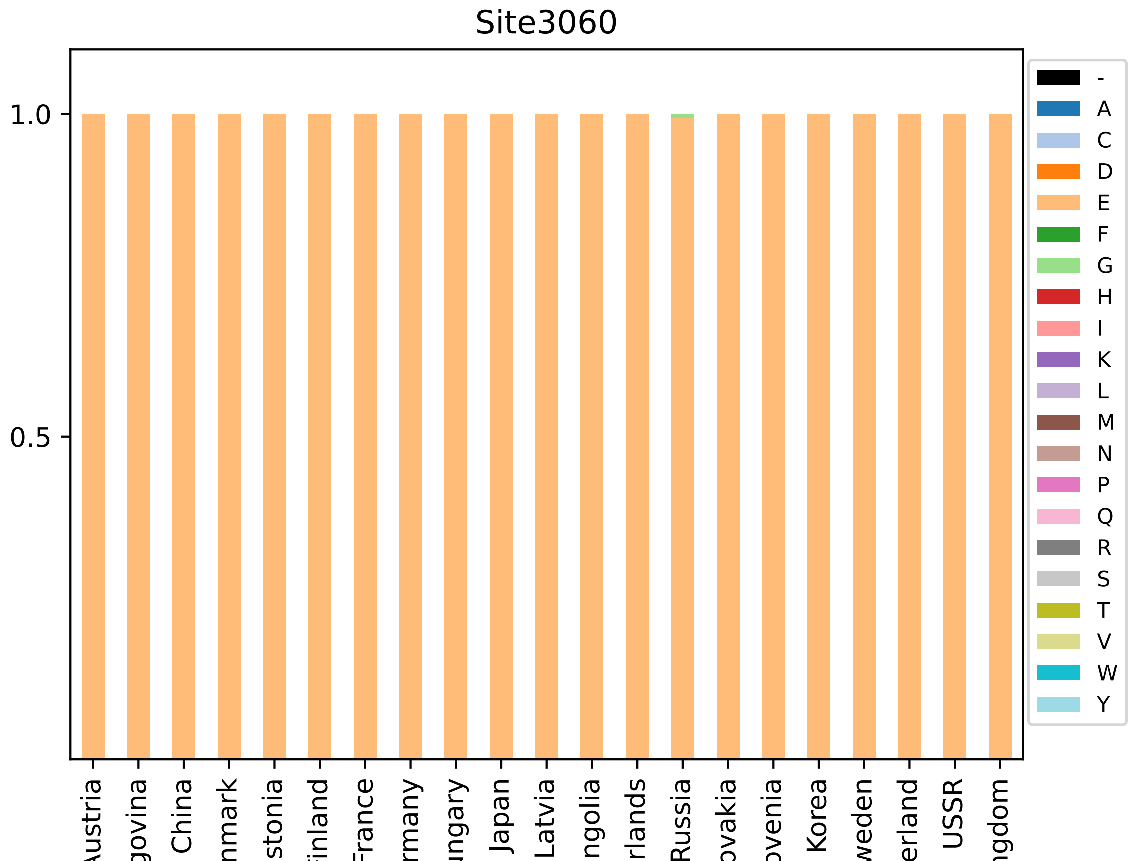

Site3060
1.0
0.5
-
A
C
D
E
F
G
H
I
K
L
M
N
P
Q
R
S
T
V
W
Y
Austria
govina
China
nmark
stonia
Finland
France
rmany
ungary
Japan
Latvia
ngolia
rlands
Russia
ovakia
ovenia
Korea
weden
erland
USSR
ngdom

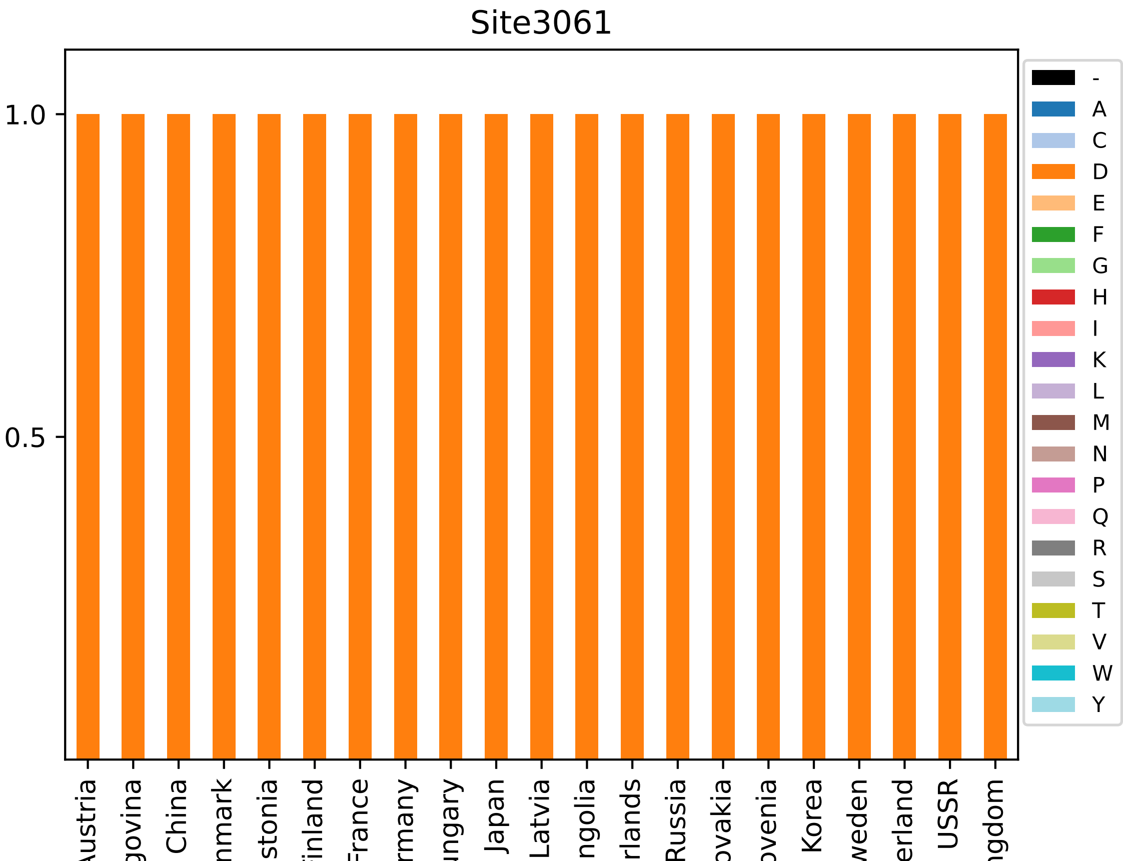
Site3061
1.0
0.5
-
A
C
D
E
F
G
H
I
K
L
M
N
P
Q
R
S
T
V
W
Y
China
Japan
Latvia
Russia
Korea
USSR

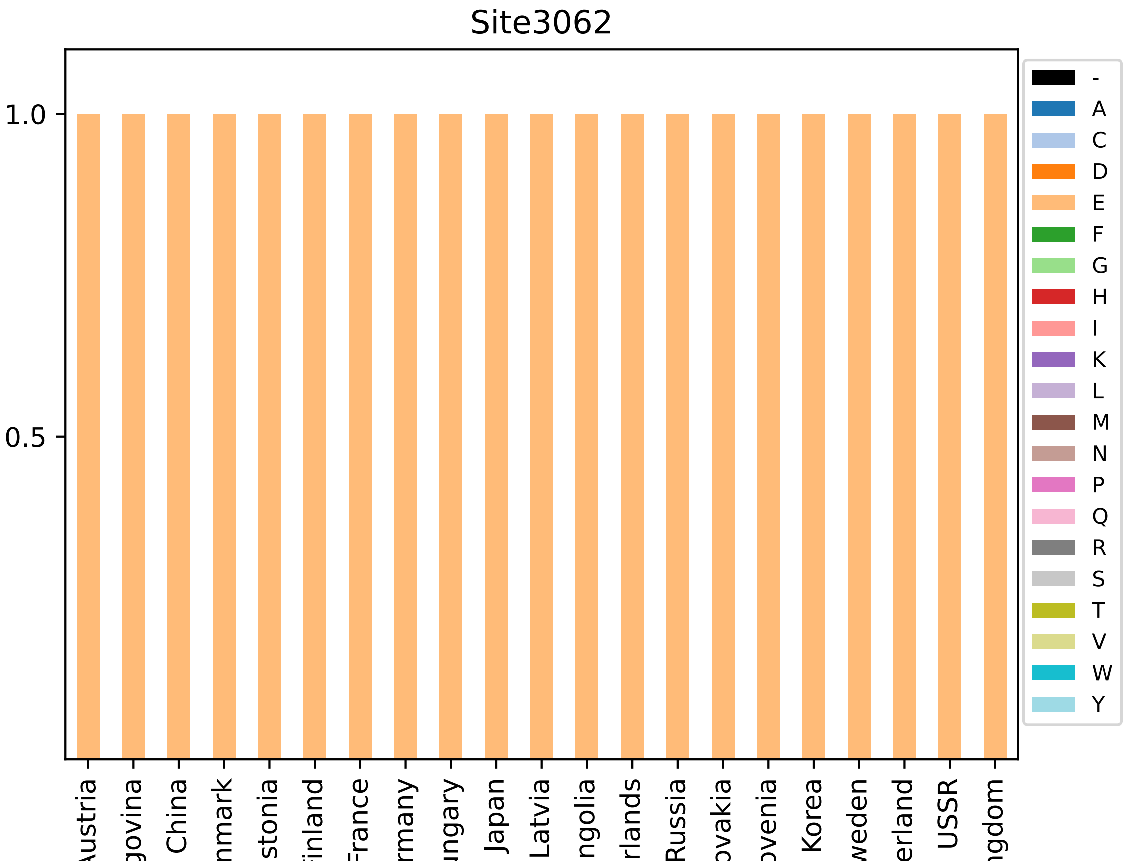
Site3062
1.0
0.5
-
A
C
D
E
F
G
H
I
K
L
M
N
P
Q
R
S
T
V
W
Y
China
Japan
Latvia
Russia
Korea
USSR

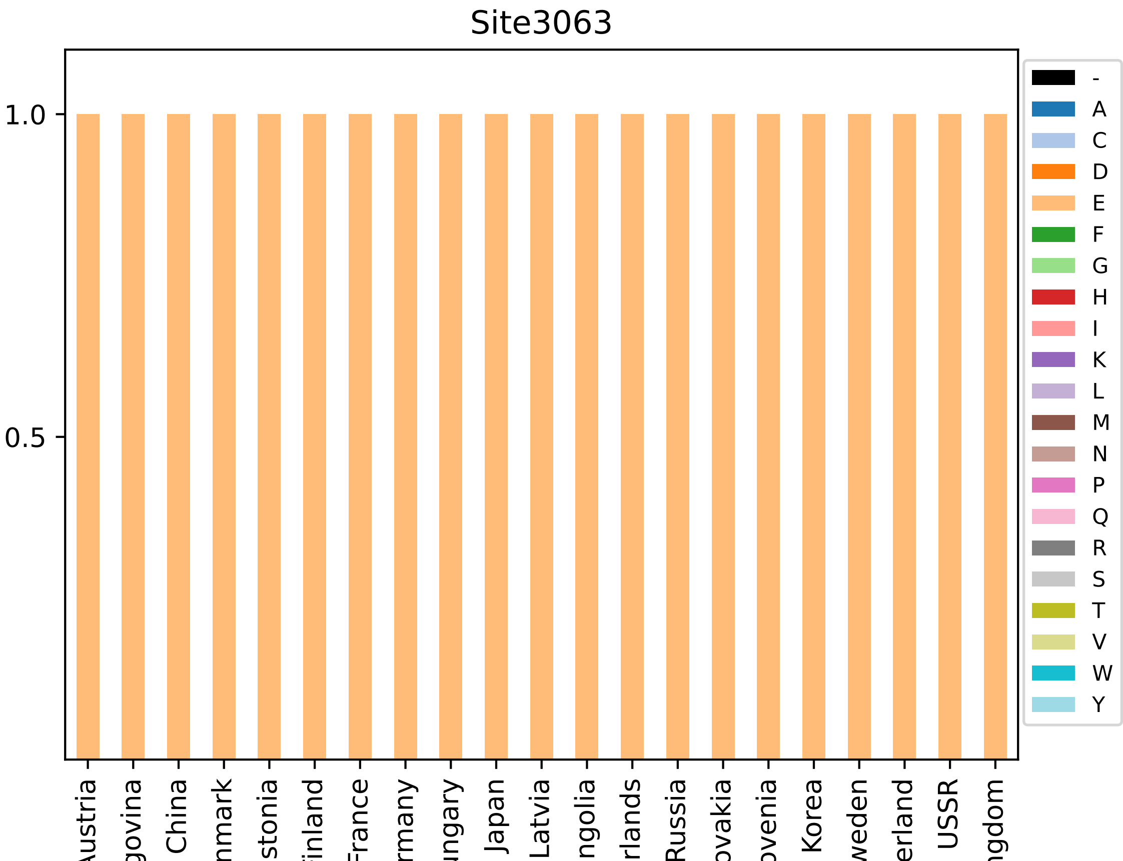

Site3063
1.0
0.5
-
A
C
D
E
F
G
H
I
K
L
M
N
P
Q
R
S
T
V
W
Y
Austria
govina
China
nmark
stonia
Finland
France
rmany
ungary
Japan
Latvia
ngolia
rlands
Russia
ovakia
ovenia
Korea
weden
erland
USSR
ngdom

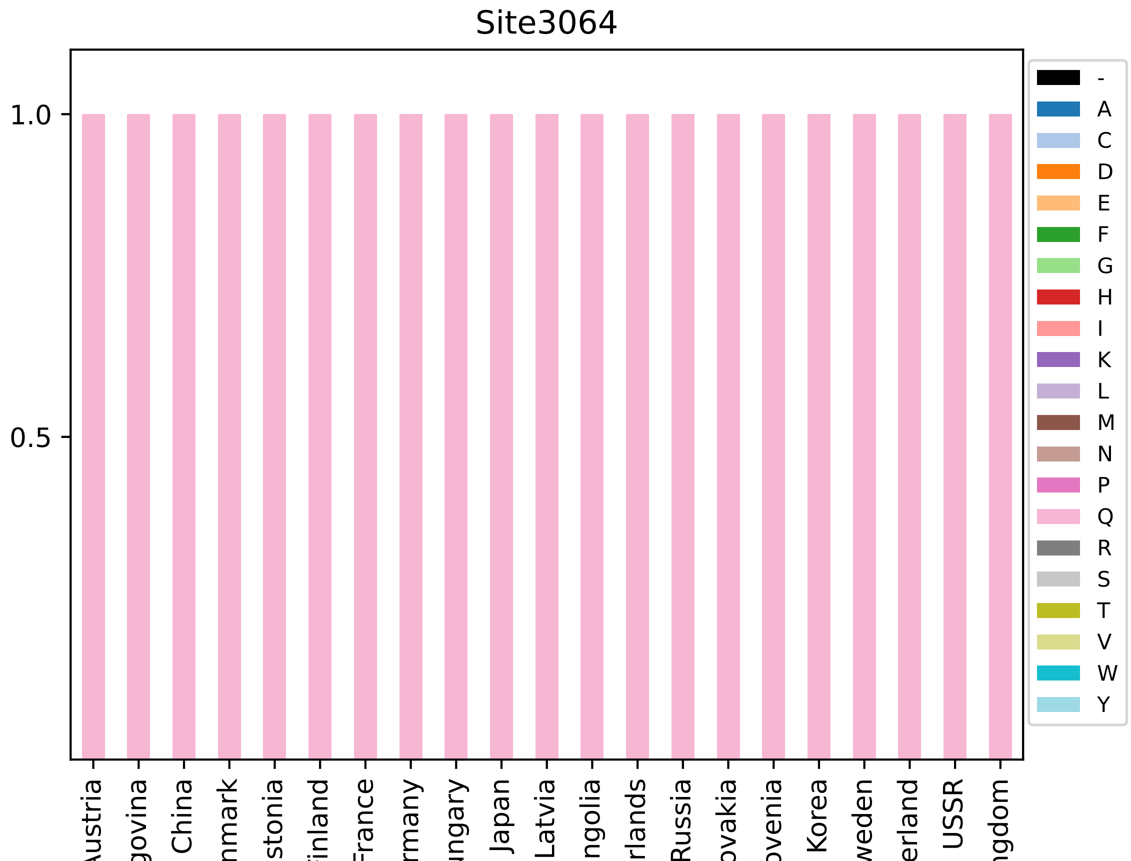
Site3064
1.0
0.5
-
A
C
D
E
F
G
H
I
K
L
M
N
P
Q
R
S
T
V
W
Y
Austria
govina
China
nmark
stonia
inland
France
rmany
ungary
Japan
Latvia
ngolia
rlands
Russia
ovakia
ovenia
Korea
weden
erland
USSR
ngdom

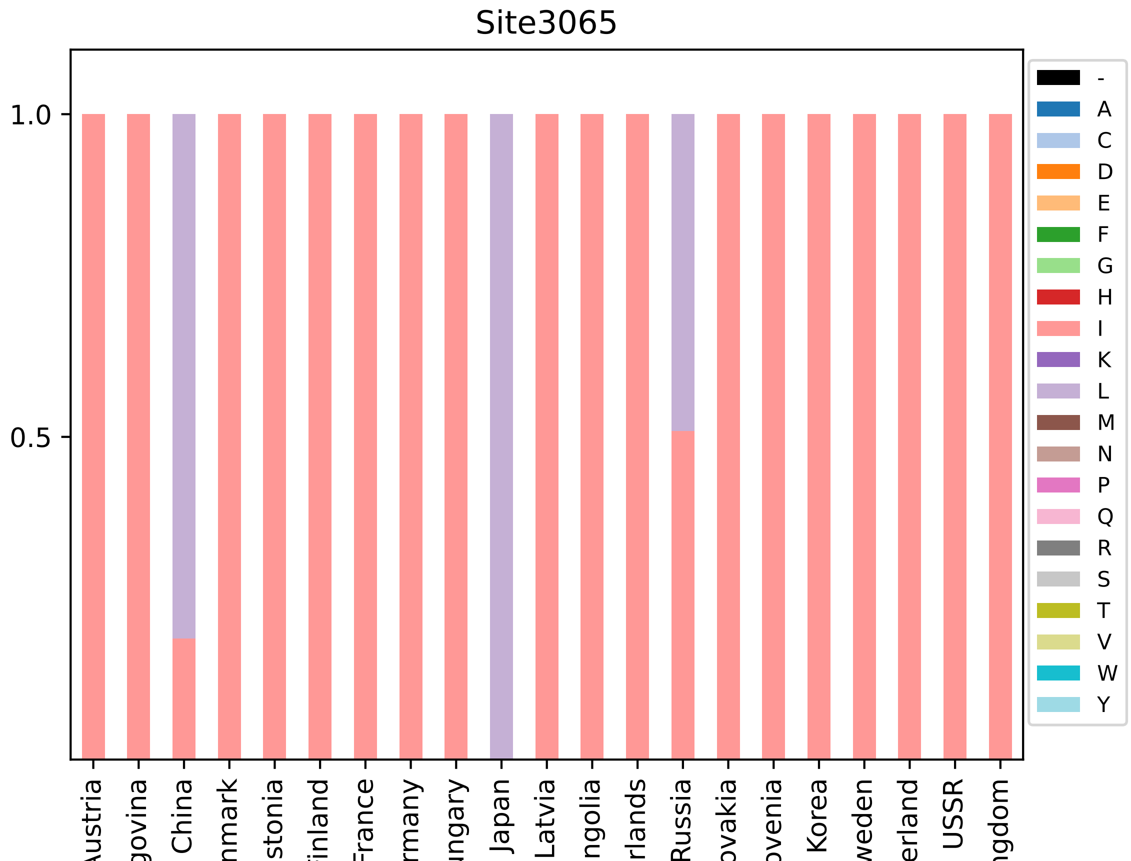
Site3065
1.0
0.5
-
A
C
D
E
F
G
H
I
K
L
M
N
P
Q
R
S
T
V
W
Y
Austria
govina
China
nmark
stonia
Finland
France
rmany
ungary
Japan
Latvia
ngolia
rlands
Russia
ovakia
ovenia
Korea
weden
erland
USSR
ngdom

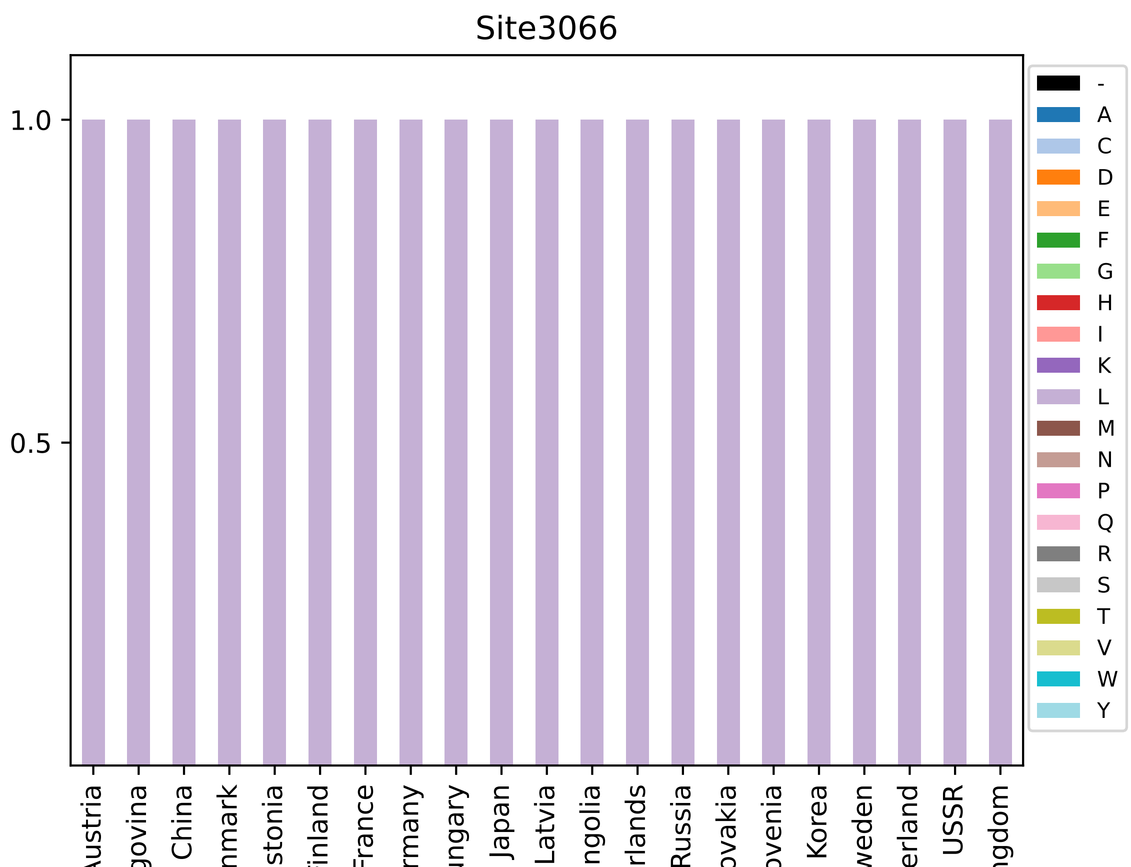

Site3066
1.0
0.5
-
A
C
D
E
F
G
H
I
K
L
M
N
P
Q
R
S
T
V
W
Y
China
Japan
Latvia
Russia
Korea
USSR

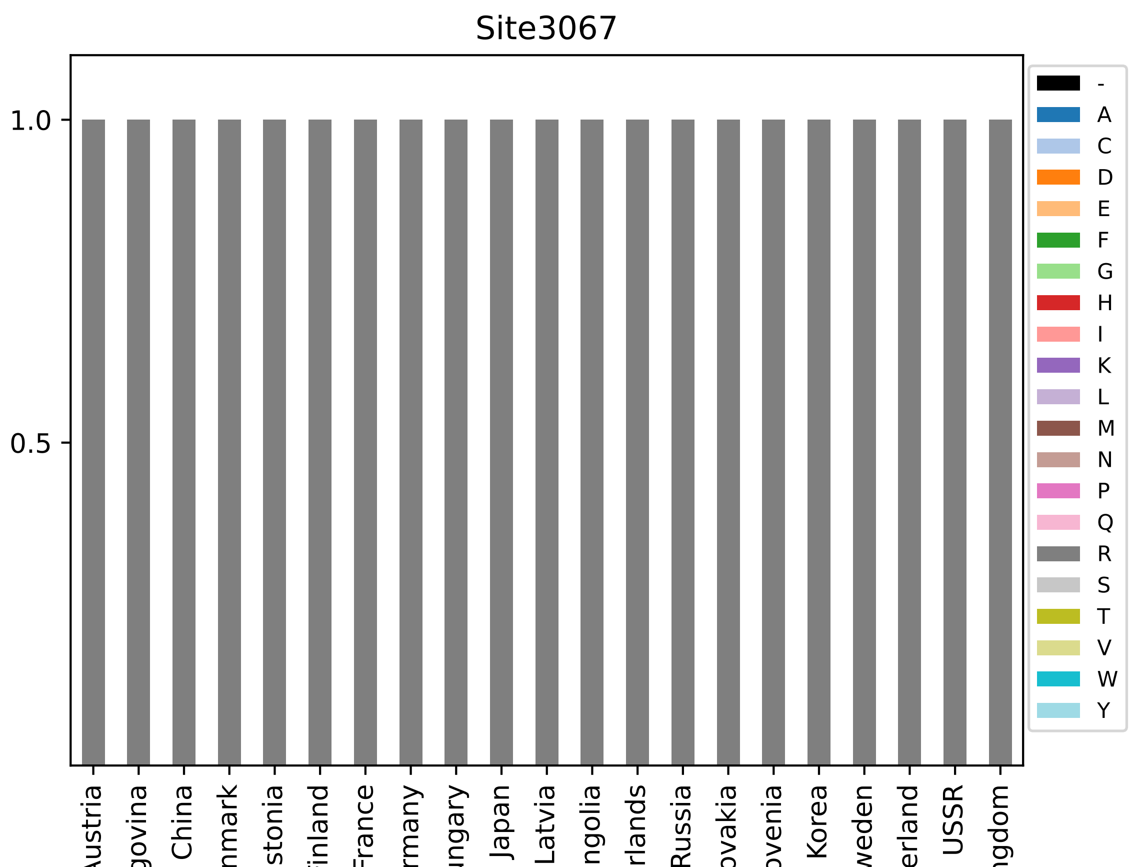
Site3067
1.0
0.5
Austria
govina
China
nmark
stonia
Finland
France
rmany
ungary
Japan
Latvia
ngolia
rlands
Russia
ovakia
ovenia
Korea
weden
erland
USSR
ngdom
-
A
C
D
E
F
G
H
I
K
L
M
N
P
Q
R
S
T
V
W
Y

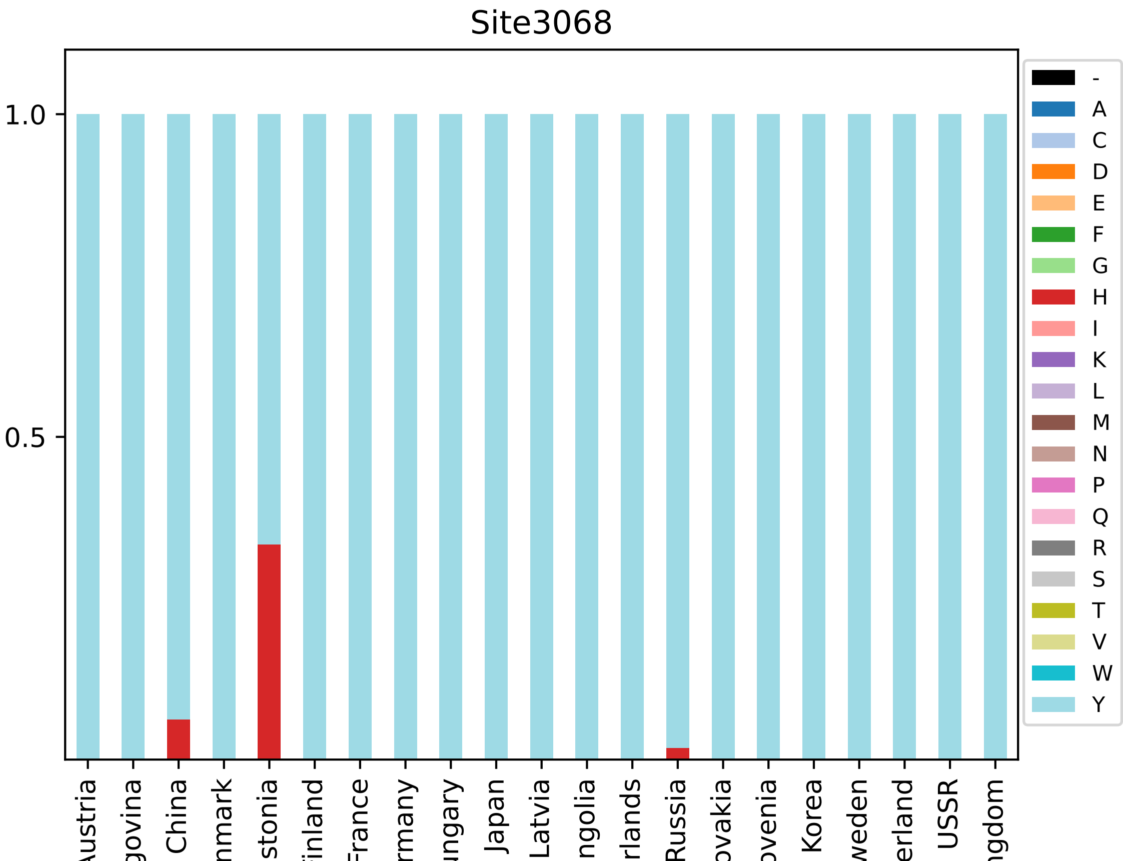

Site3068
1.0
0.5
-
A
C
D
E
F
G
H
I
K
L
M
N
P
Q
R
S
T
V
W
Y
Austria
govina
China
nmark
stonia
inland
France
rmany
ungary
Japan
Latvia
ngolia
rlands
Russia
ovakia
ovenia
Korea
weden
erland
USSR
ngdom

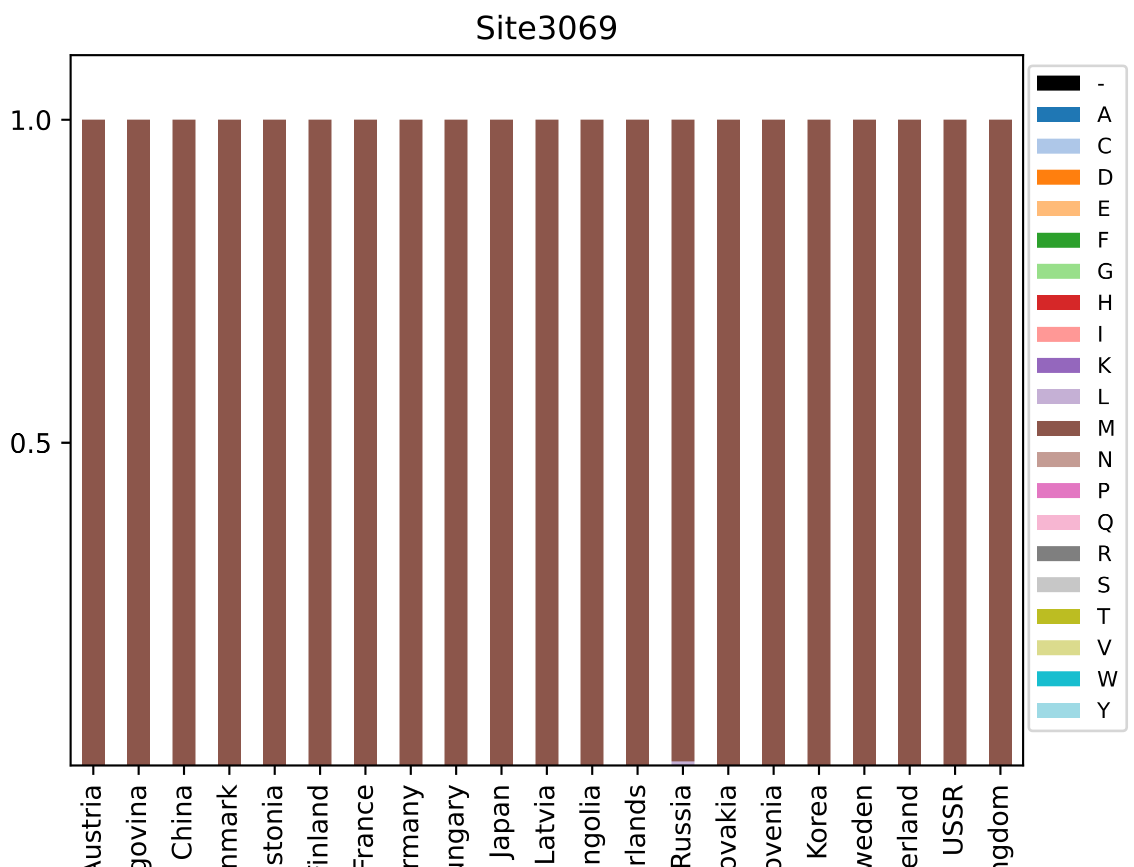
Site3069
1.0
0.5
-
A
C
D
E
F
G
H
I
K
L
M
N
P
Q
R
S
T
V
W
Y
Austria
govina
China
nmark
stonia
Finland
France
rmany
ungary
Japan
Latvia
ngolia
rlands
Russia
ovakia
ovenia
Korea
weden
erland
USSR
ngdom

Site3070

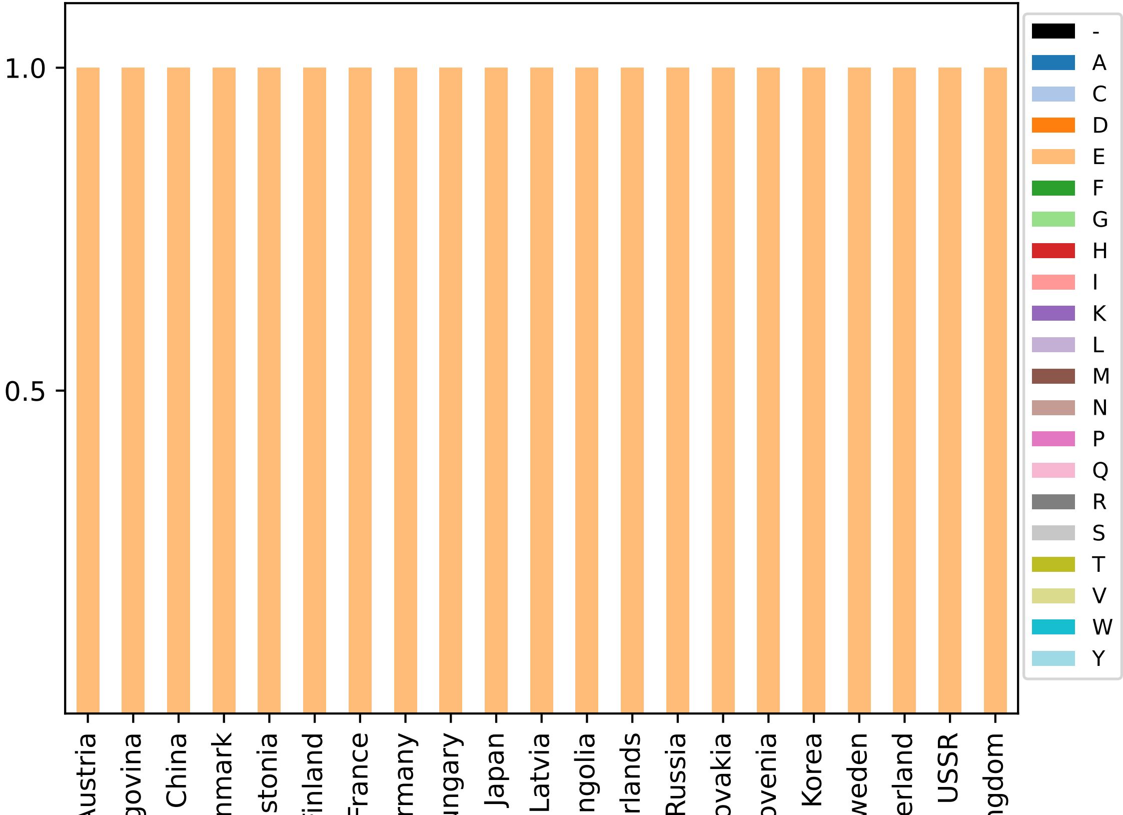

1.0
0.5
-
A
C
D
E
F
G
H
I
K
L
M
N
P
Q
R
S
T
V
W
Y
China
Japan
Latvia
Russia
Korea
USSR

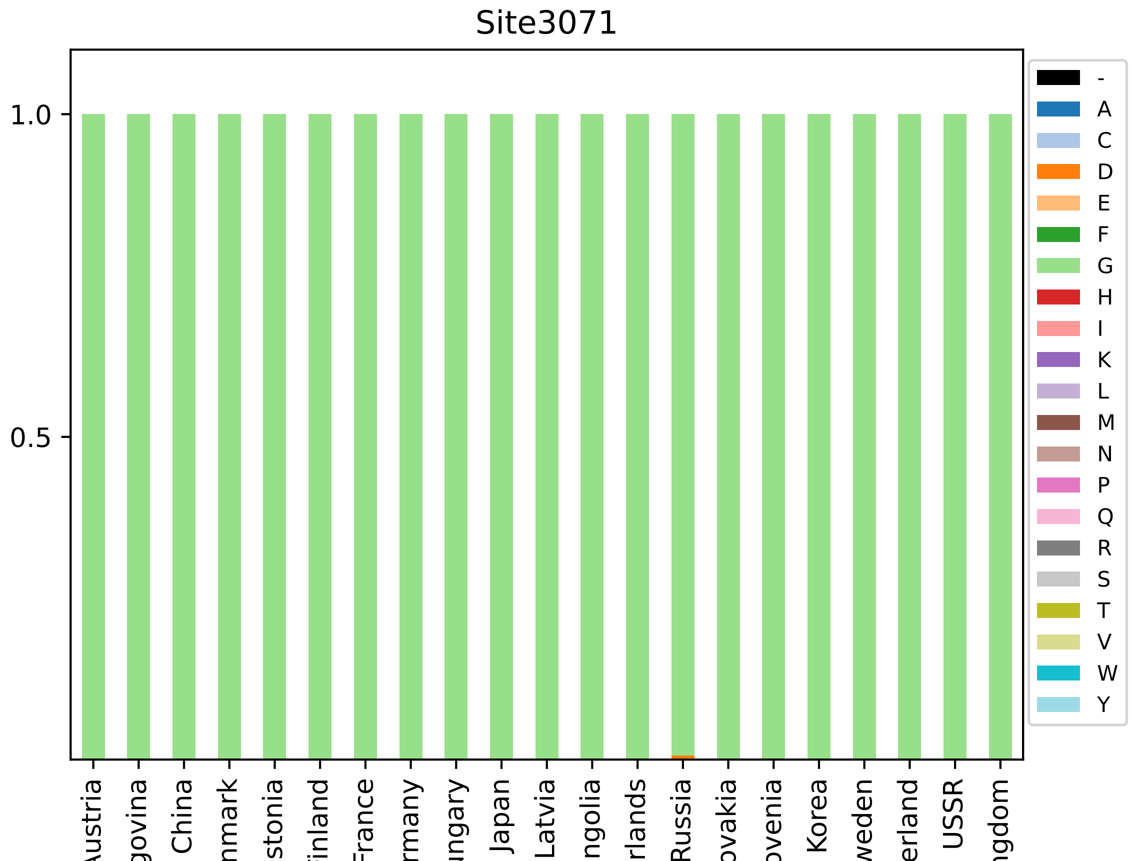

Site3071
1.0
0.5
-
A
C
D
E
F
G
H
I
K
L
M
N
P
Q
R
S
T
V
W
Y
Austria
China
Japan
Latvia
Russia
Korea
USSR

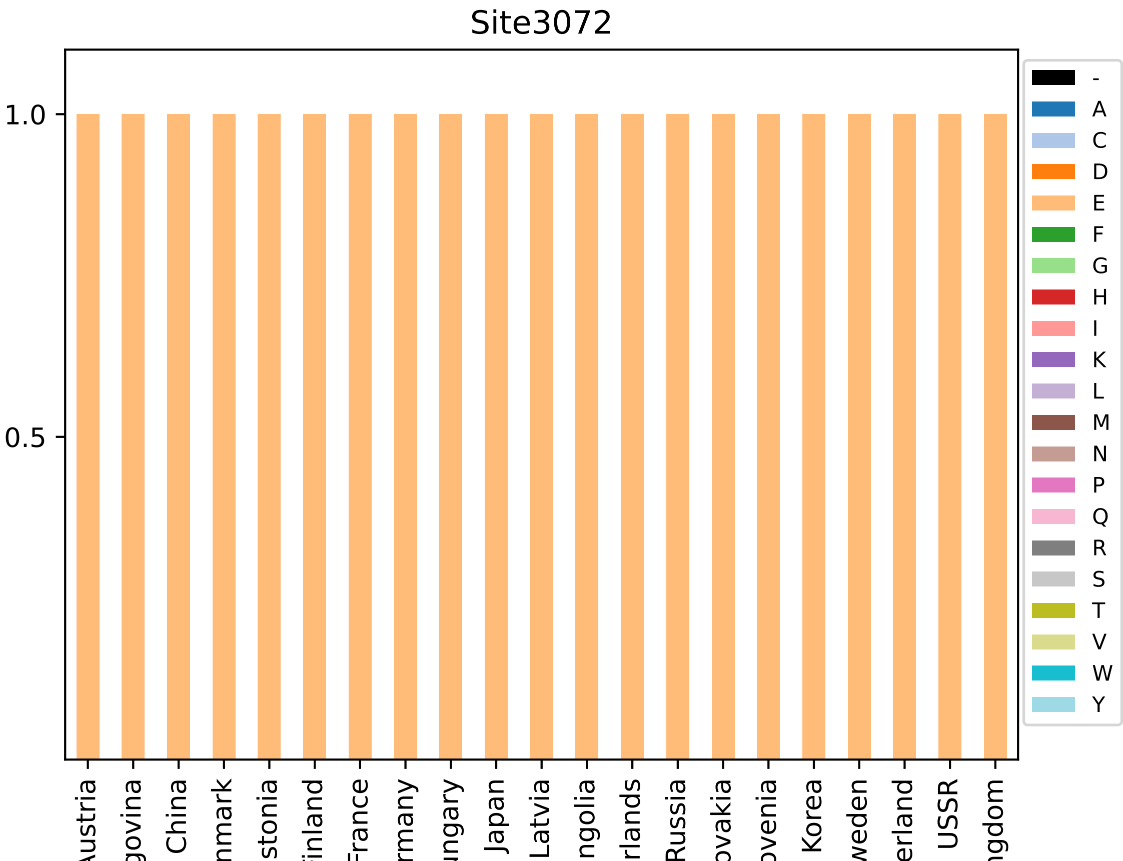

Site3072
1.0
0.5
-
A
C
D
E
F
G
H
I
K
L
M
N
P
Q
R
S
T
V
W
Y
China
Japan
Latvia
Russia
Korea
USSR

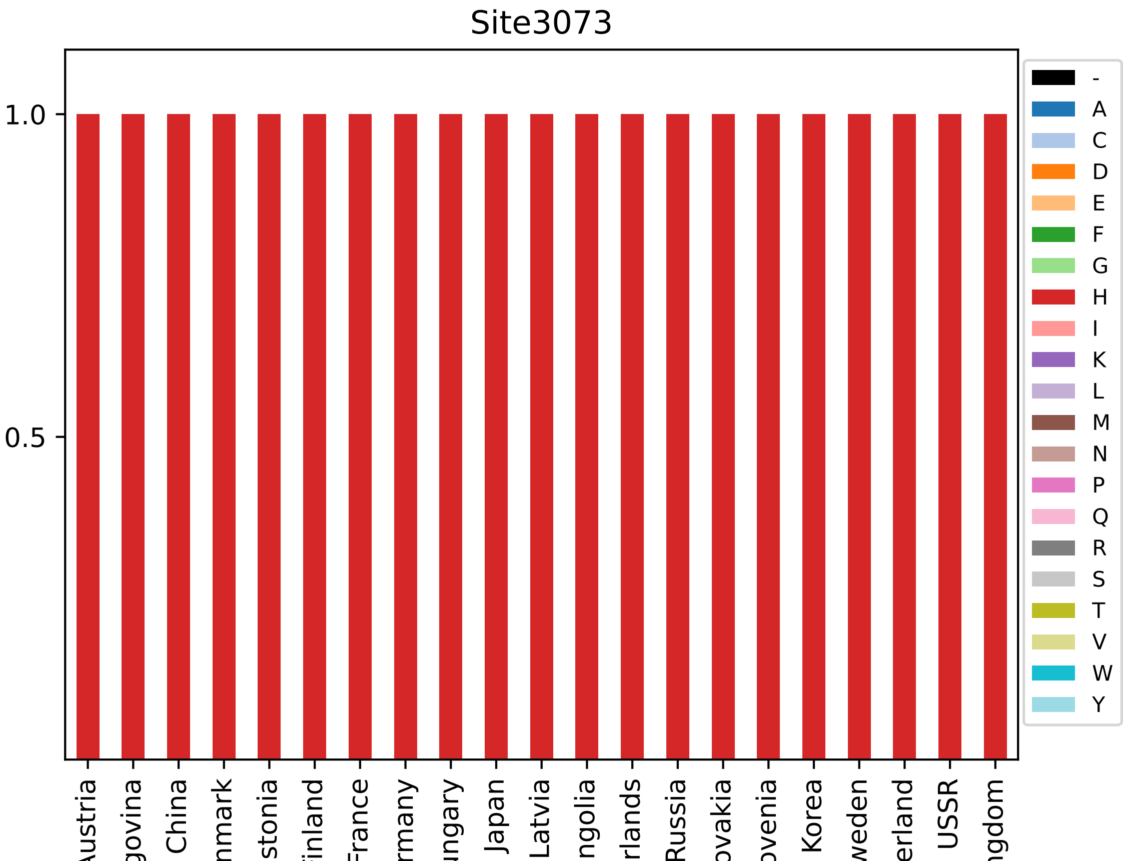

Site3073
1.0
0.5
-
A
C
D
E
F
G
H
I
K
L
M
N
P
Q
R
S
T
V
W
Y
China
Japan
Latvia
Russia
Korea
USSR

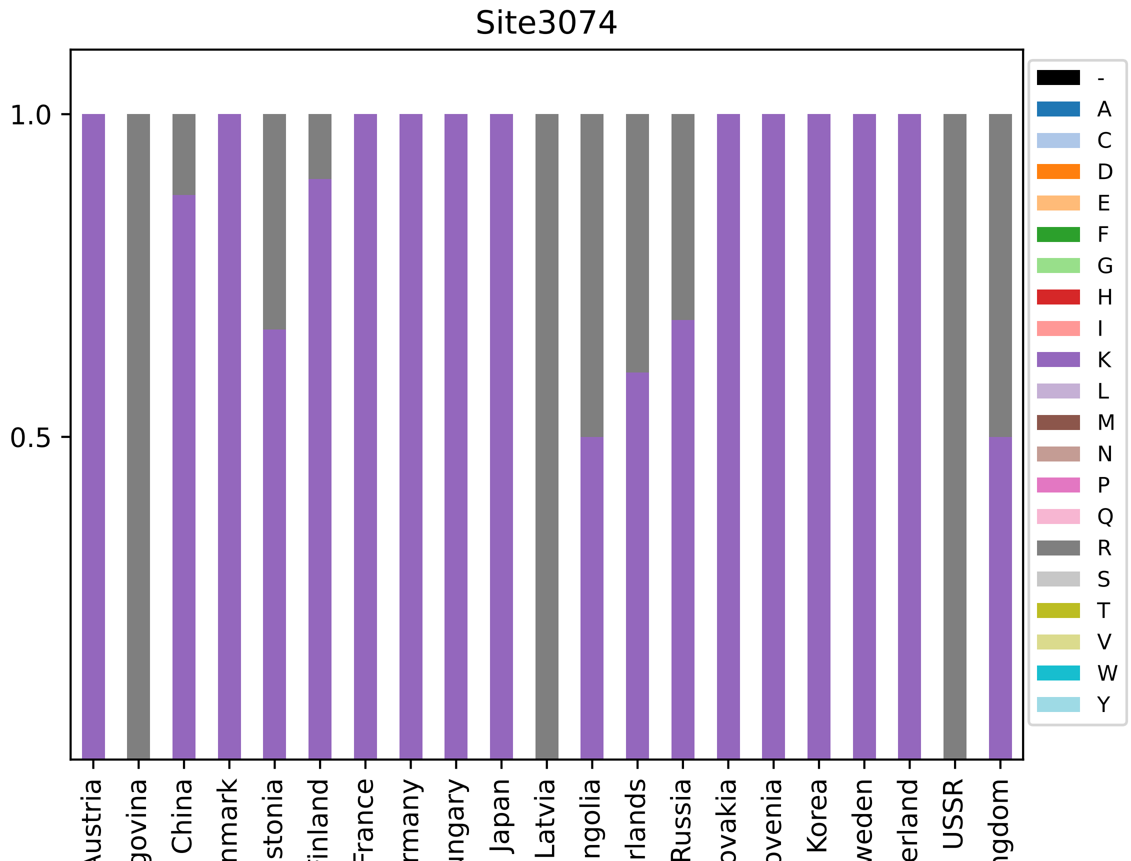
Site3074
1.0
0.5
-
A
C
D
E
F
G
H
I
K
L
M
N
P
Q
R
S
T
V
W
Y
Austria
China
Finland
France
Japan
Latvia
Russia
Korea
USSR

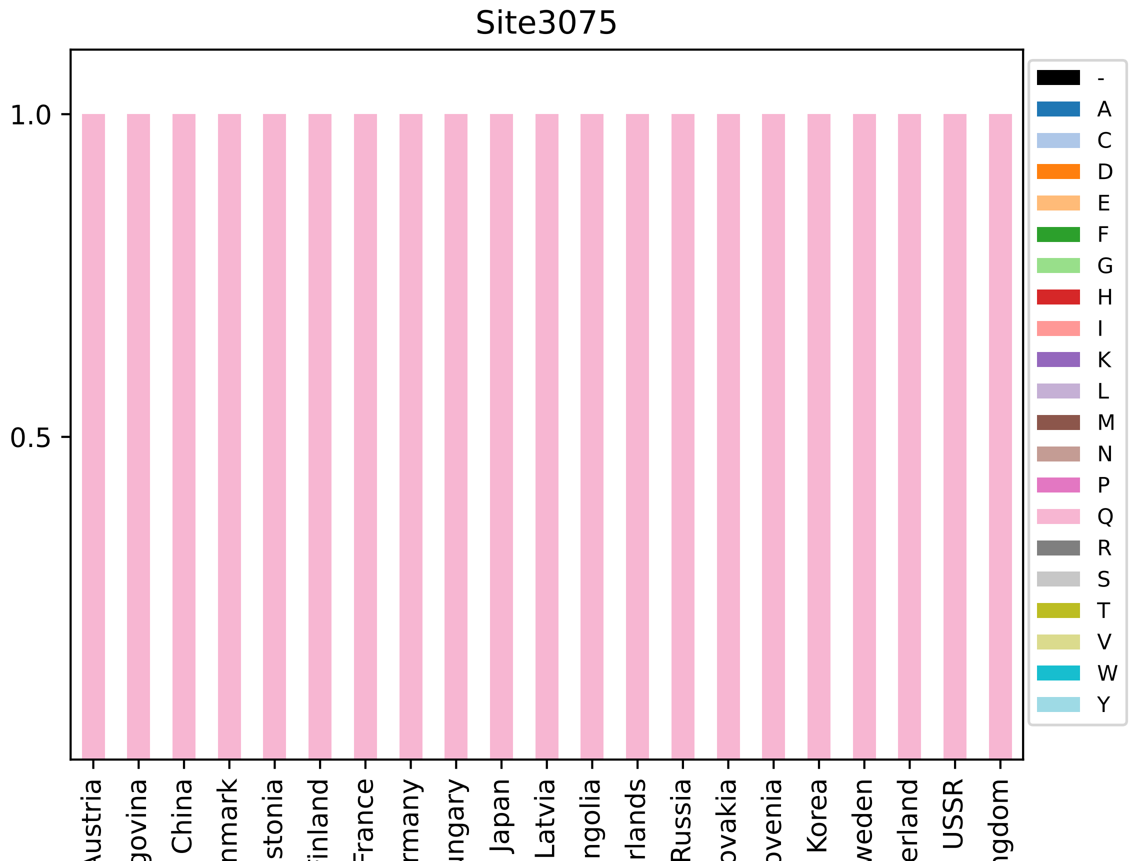

Site3075
1.0
0.5
-
A
C
D
E
F
G
H
I
K
L
M
N
P
Q
R
S
T
V
W
Y
Austria
govina
China
nmark
stonia
inland
France
rmany
ungary
Japan
Latvia
ngolia
rlands
Russia
ovakia
ovenia
Korea
weden
erland
USSR
ngdom

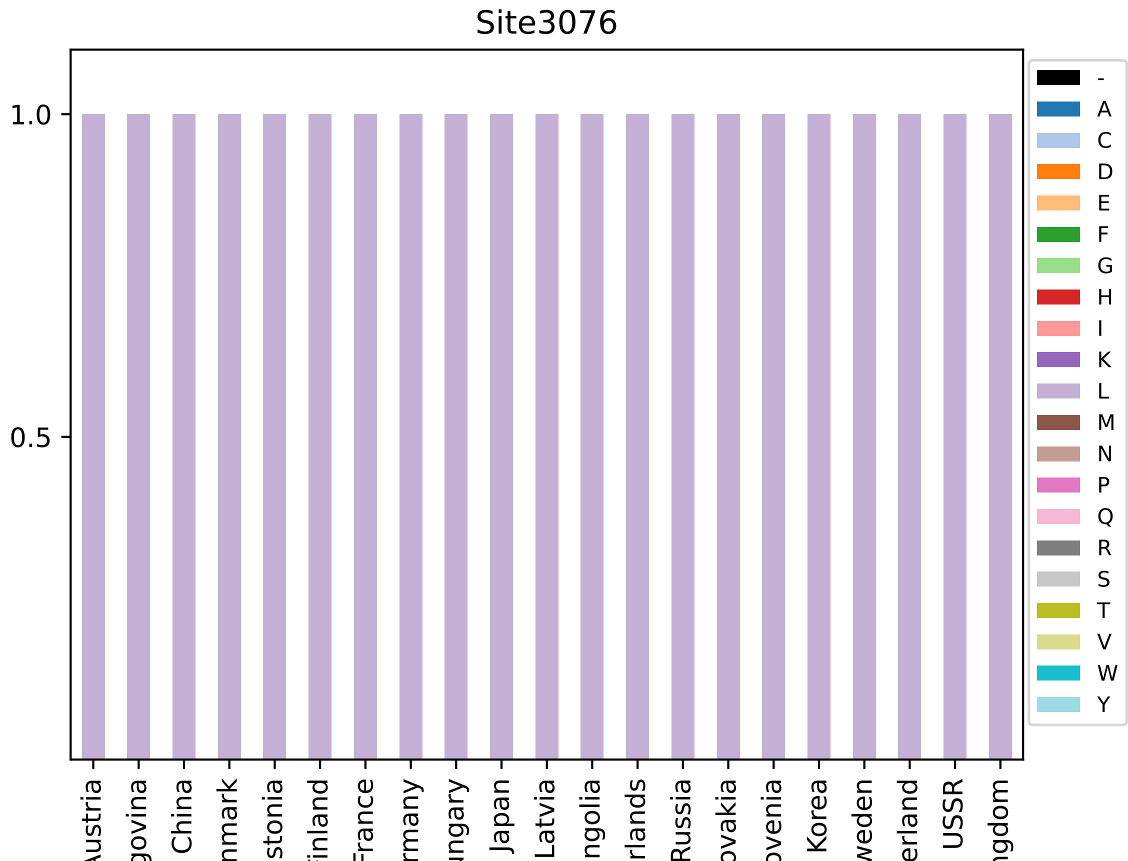
Site3076
1.0
0.5
-
A
C
D
E
F
G
H
I
K
L
M
N
P
Q
R
S
T
V
W
Y
China
Japan
Latvia
Russia
Korea
USSR

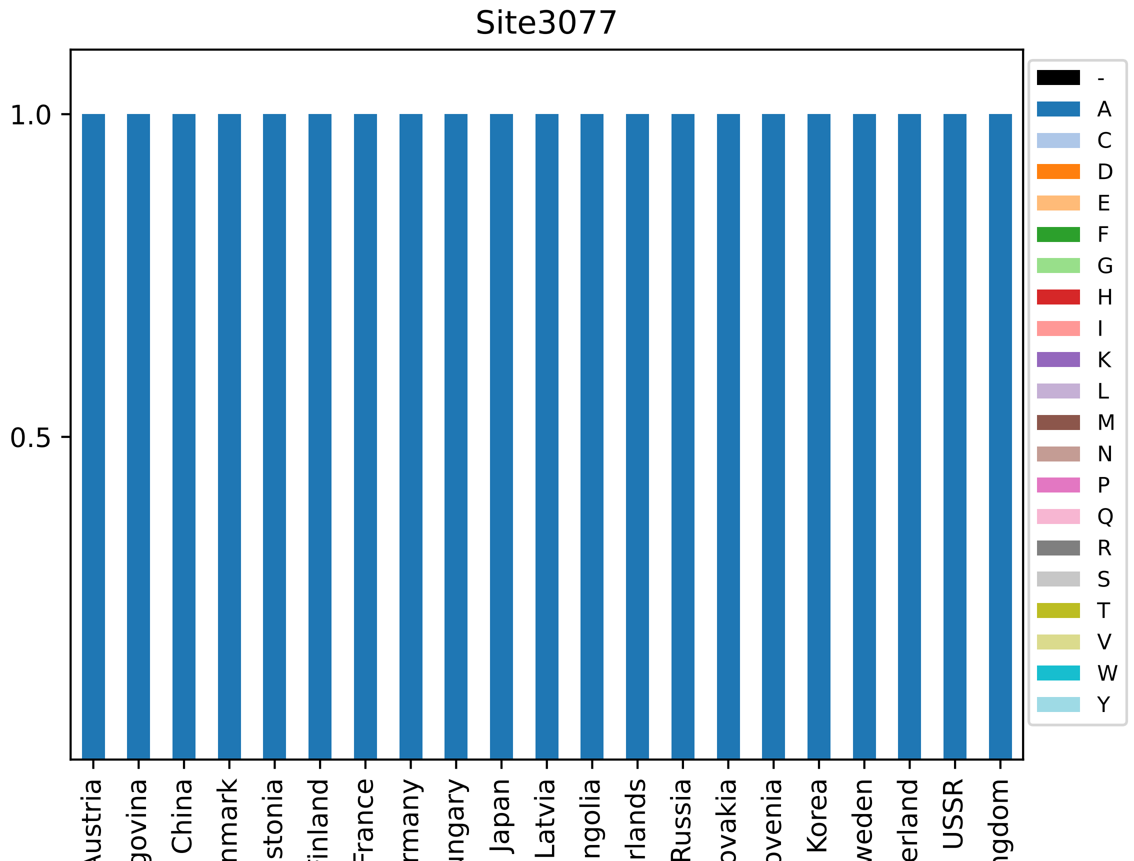

Site3077
1.0
0.5
-
A
C
D
E
F
G
H
I
K
L
M
N
P
Q
R
S
T
V
W
Y
China
Japan
Latvia
Korea
USSR

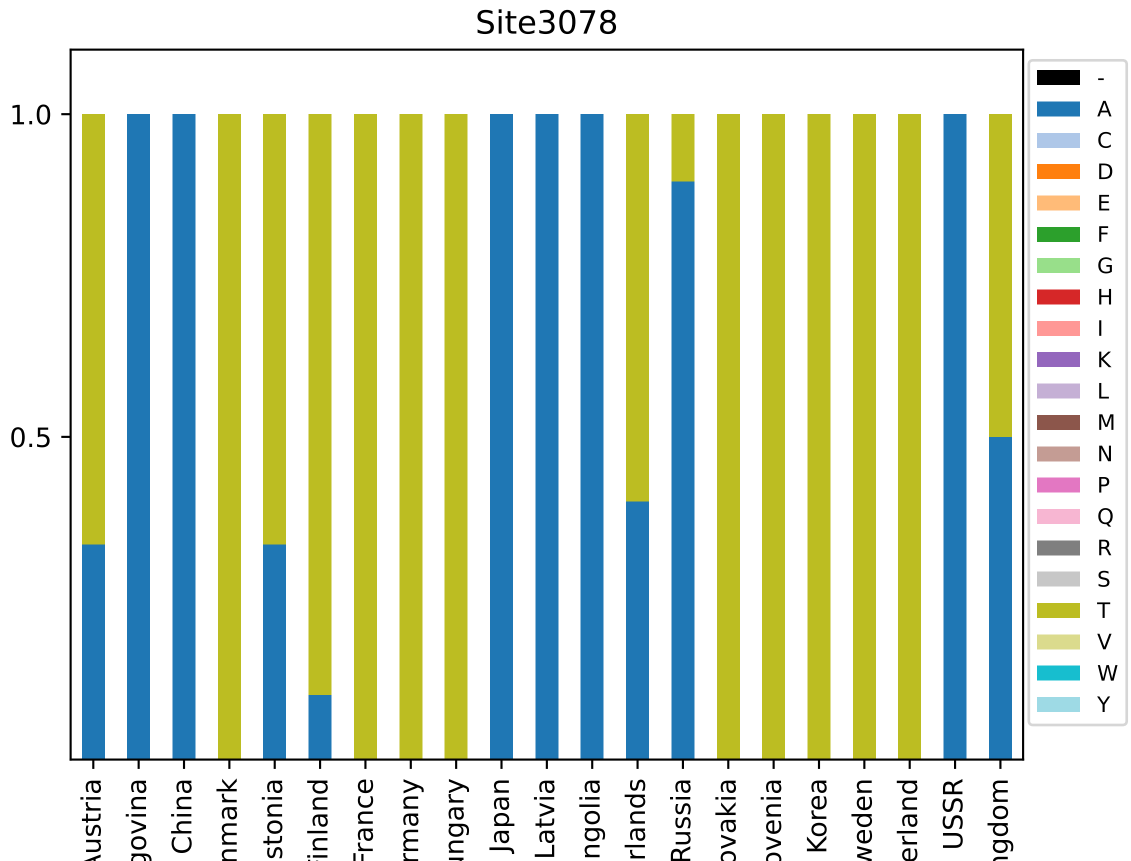
Site3078
1.0
0.5
-
A
C
D
E
F
G
H
I
K
L
M
N
P
Q
R
S
T
V
W
Y
China
stonia
France
Japan
Latvia
ngolia
rlands
Russia
ovakia
ovenia
Korea
weden
erland
USSR
ngdom

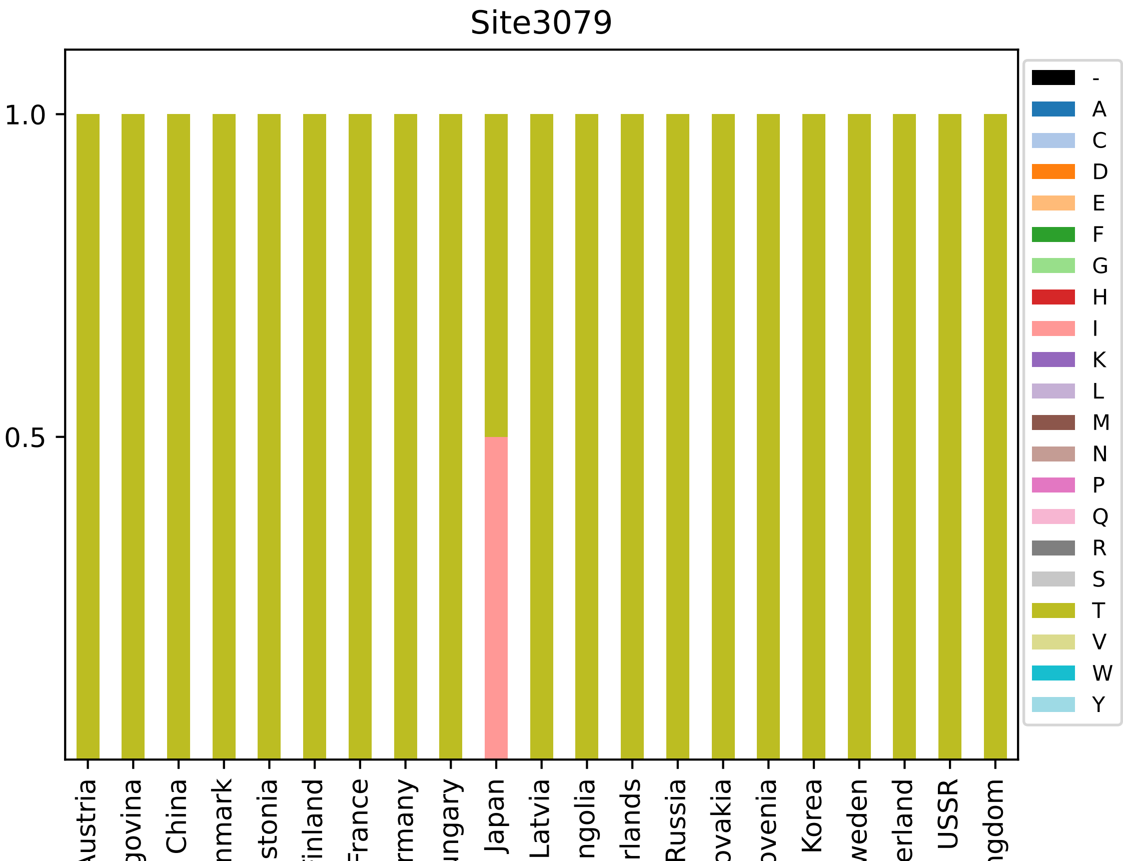

Site3079
1.0
0.5
-
A
C
D
E
F
G
H
I
K
L
M
N
P
Q
R
S
T
V
W
Y
Austria
China
Japan
Latvia
Russia
Korea
USSR

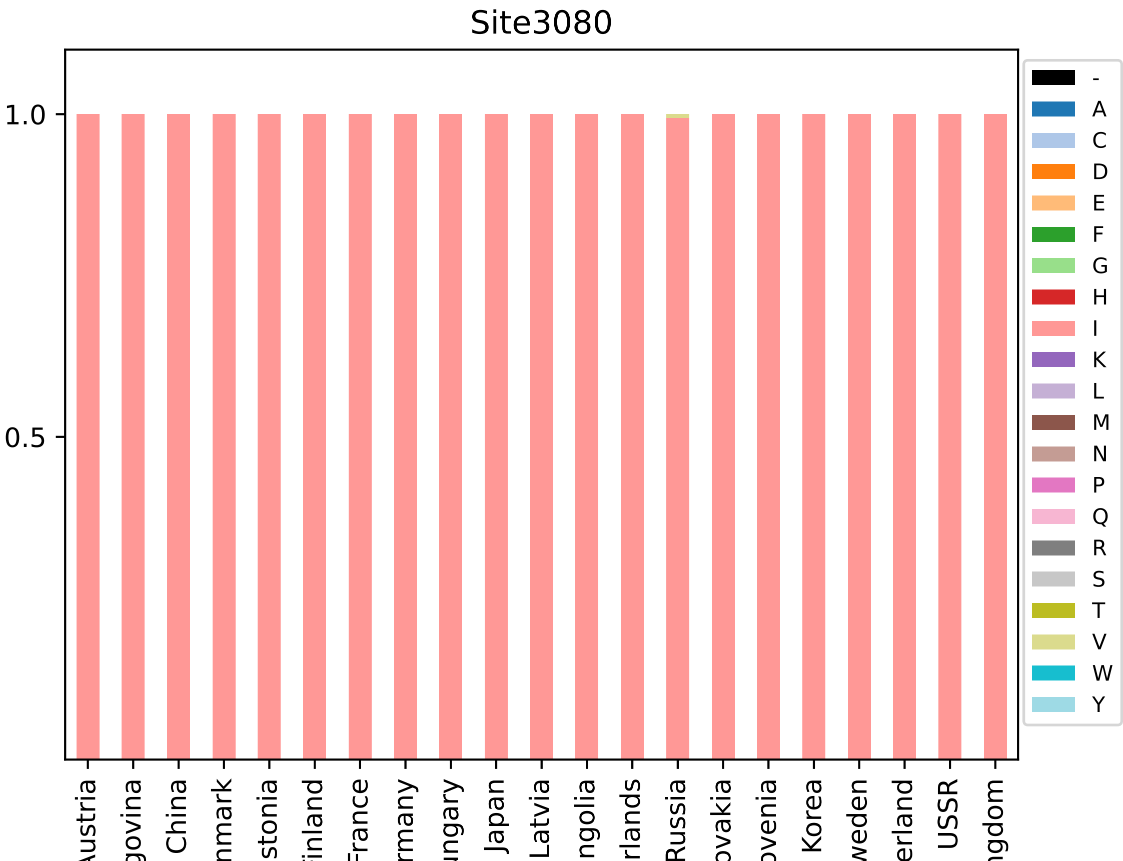
Site3080
1.0
0.5
-
A
C
D
E
F
G
H
I
K
L
M
N
P
Q
R
S
T
V
W
Y
Austria
govina
China
nmark
stonia
Finland
France
rmany
ungary
Japan
Latvia
ngolia
rlands
Russia
ovakia
ovenia
Korea
weden
erland
USSR
ngdom

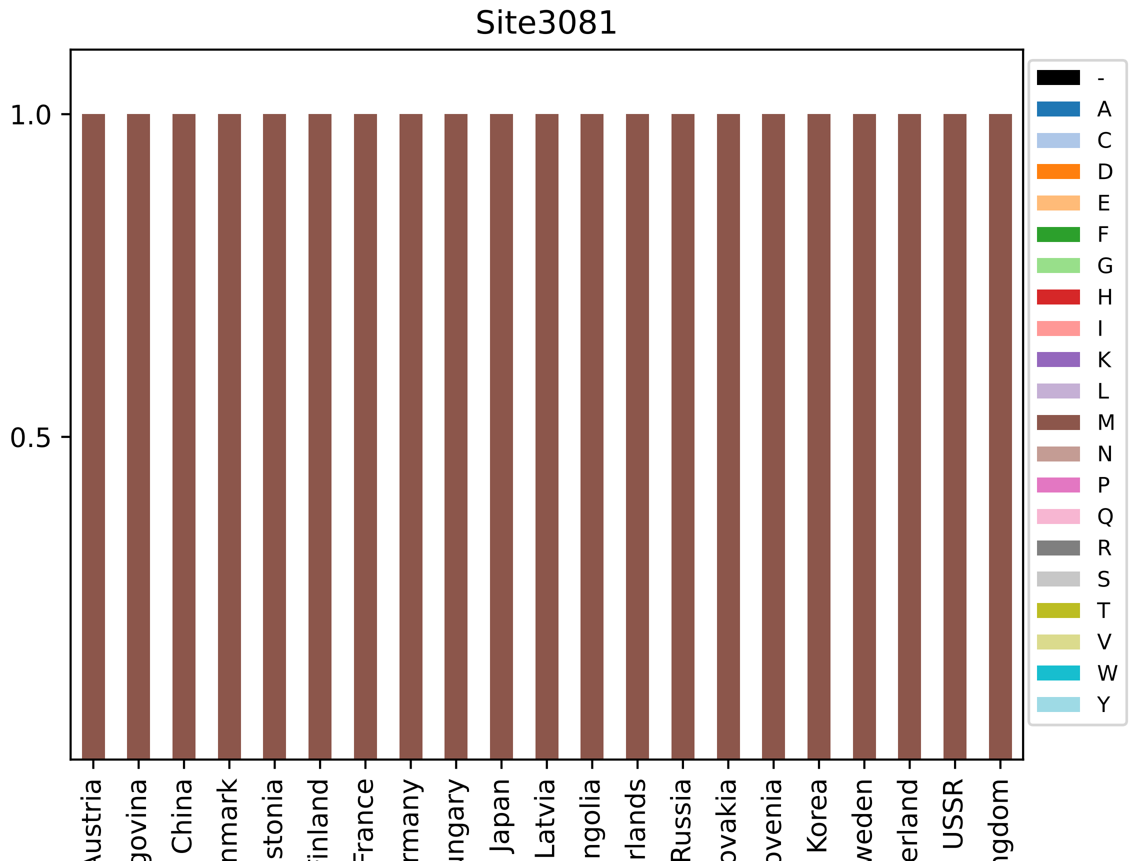
Site3081
1.0
0.5
-
A
C
D
E
F
G
H
I
K
L
M
N
P
Q
R
S
T
V
W
Y
Austria
China
Japan
Latvia
Russia
Korea
USSR

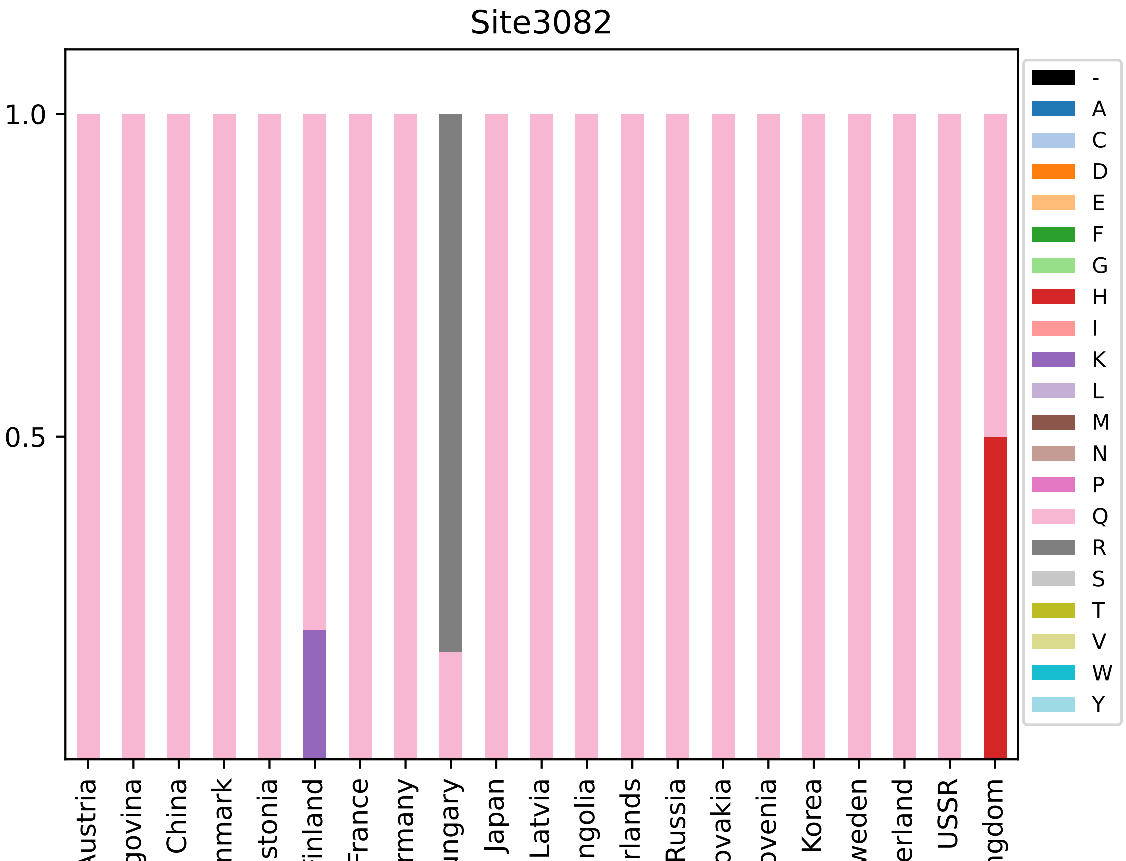

Site3082
1.0
0.5
-
A
C
D
E
F
G
H
I
K
L
M
N
P
Q
R
S
T
V
W
Y
Austria
govina
China
nmark
stonia
Finland
France
rmany
ungary
Japan
Latvia
ngolia
rlands
Russia
ovakia
ovenia
Korea
weden
erland
USSR
ngdom

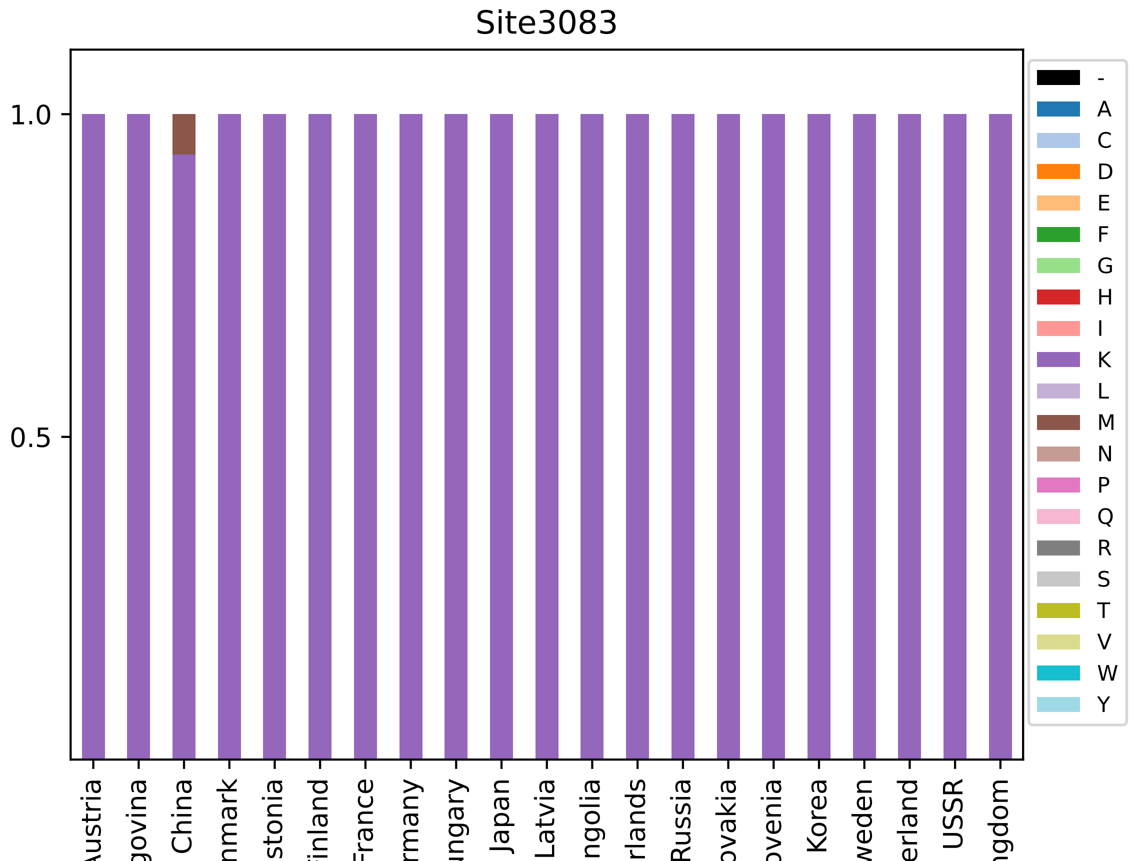

Site3083
1.0
0.5
-
A
C
D
E
F
G
H
I
K
L
M
N
P
Q
R
S
T
V
W
Y
China
Japan
Latvia
Russia
Korea
USSR

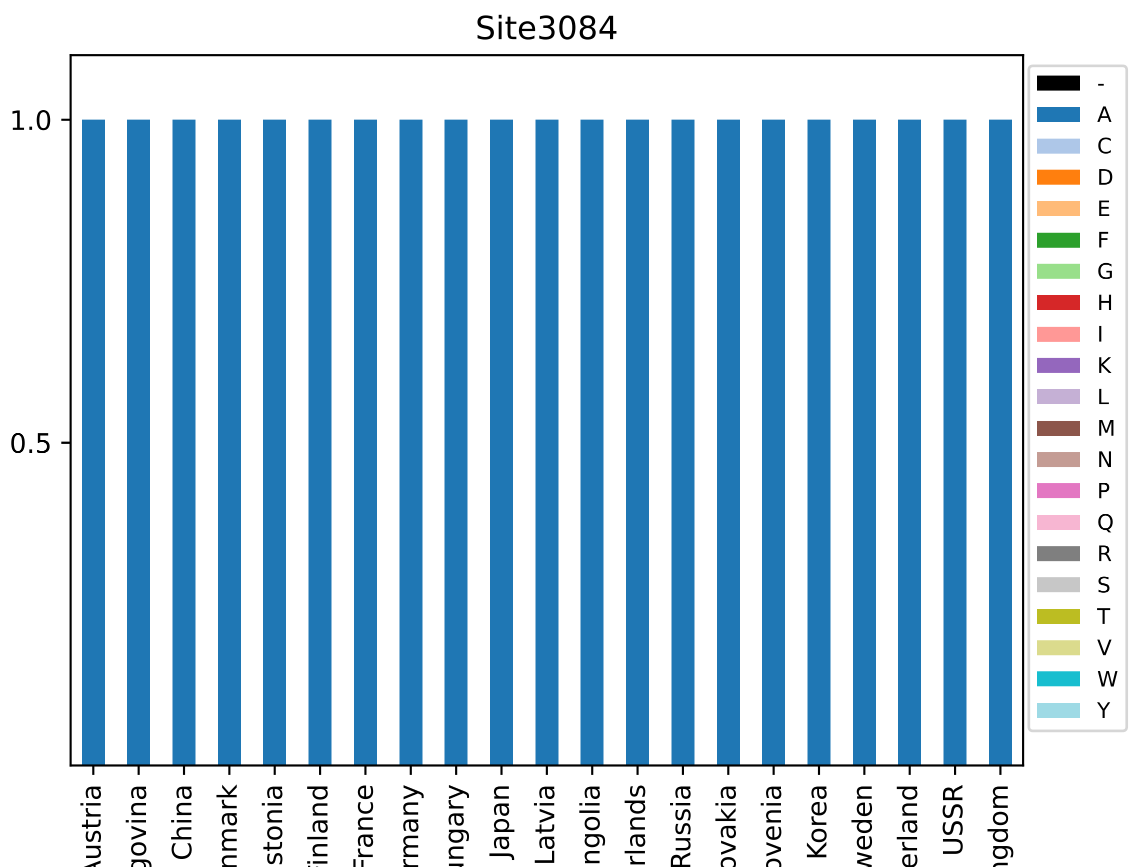

Site3084
1.0
0.5
-
A
C
D
E
F
G
H
I
K
L
M
N
P
Q
R
S
T
V
W
Y
China
Japan
Latvia
Russia
Korea
USSR

Site3085

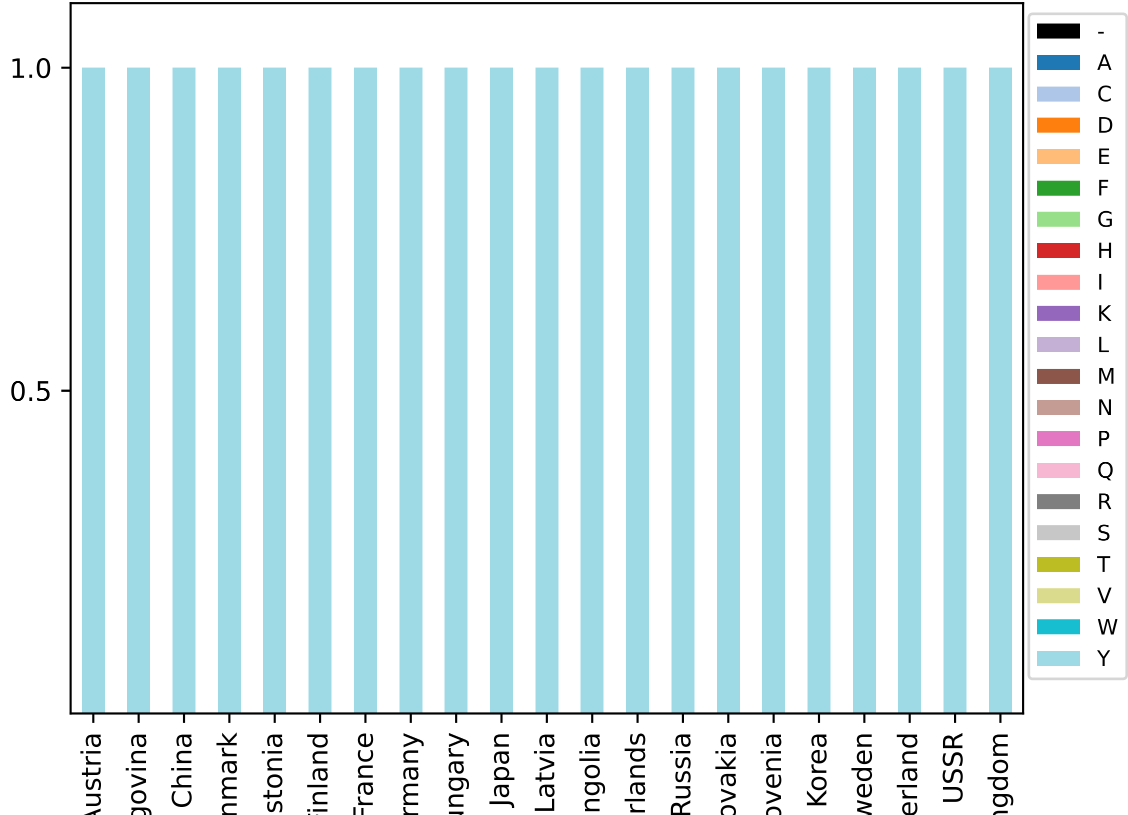

1.0
0.5
-
A
C
D
E
F
G
H
I
K
L
M
N
P
Q
R
S
T
V
W
Y
China
Japan
Latvia
Russia
Korea
USSR

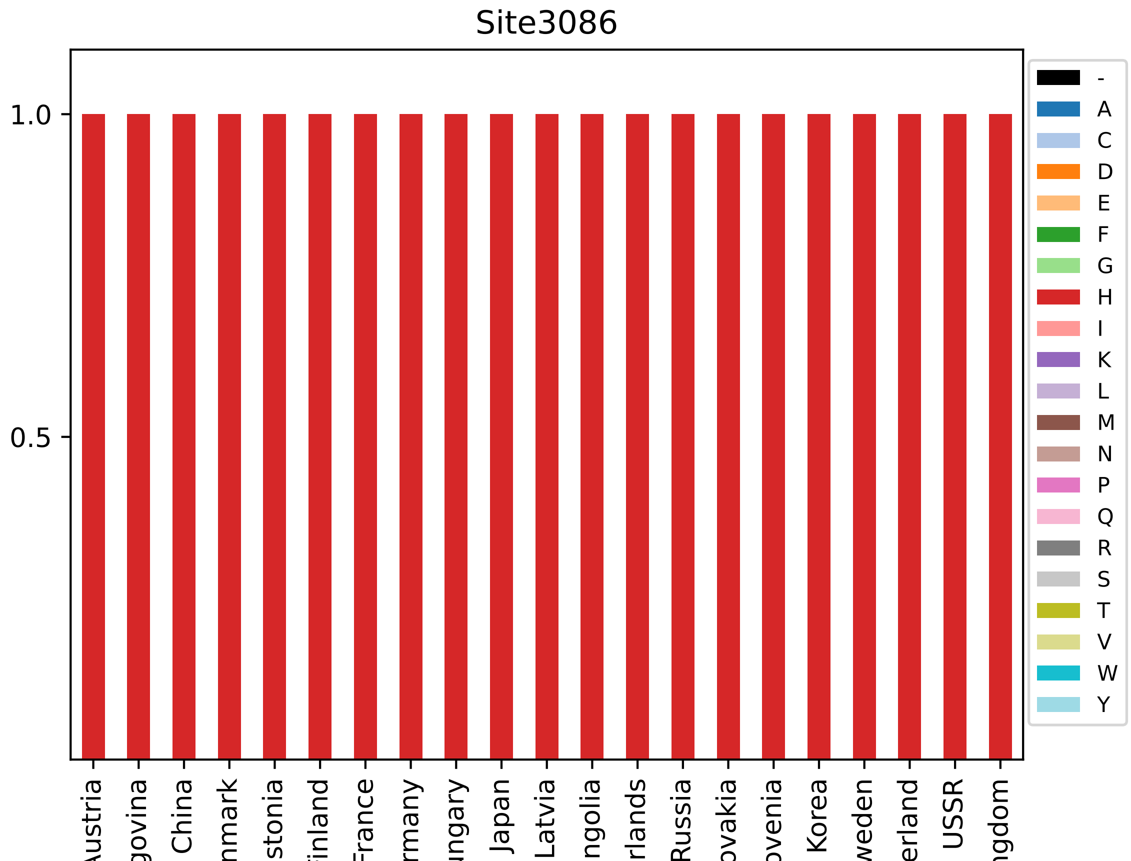

Site3086
1.0
0.5
Austria
govina
China
nmark
stonia
Finland
France
rmany
ungary
Japan
Latvia
ngolia
rlands
Russia
ovakia
ovenia
Korea
weden
erland
USSR
ngdom
-
A
C
D
E
F
G
H
I
K
L
M
N
P
Q
R
S
T
V
W
Y

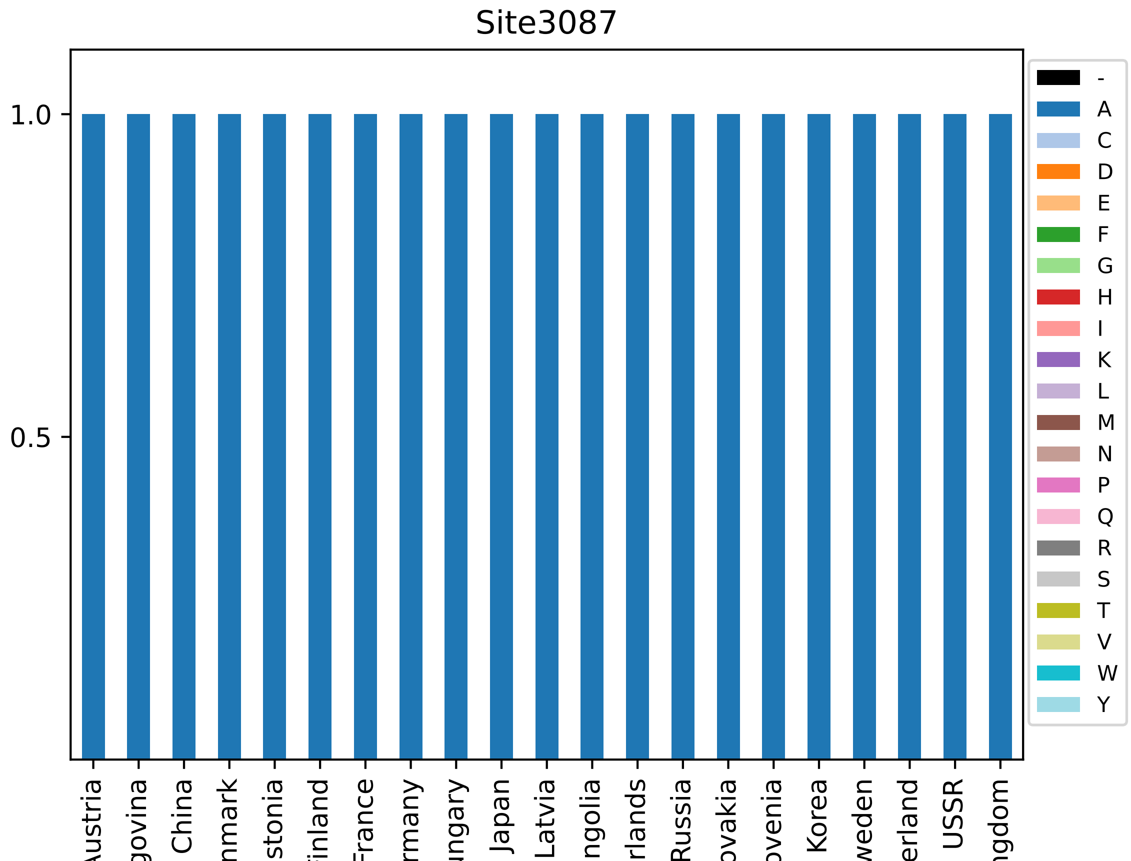
Site3087
1.0
0.5
-
A
C
D
E
F
G
H
I
K
L
M
N
P
Q
R
S
T
V
W
Y
China
Japan
Latvia
Russia
Korea
USSR

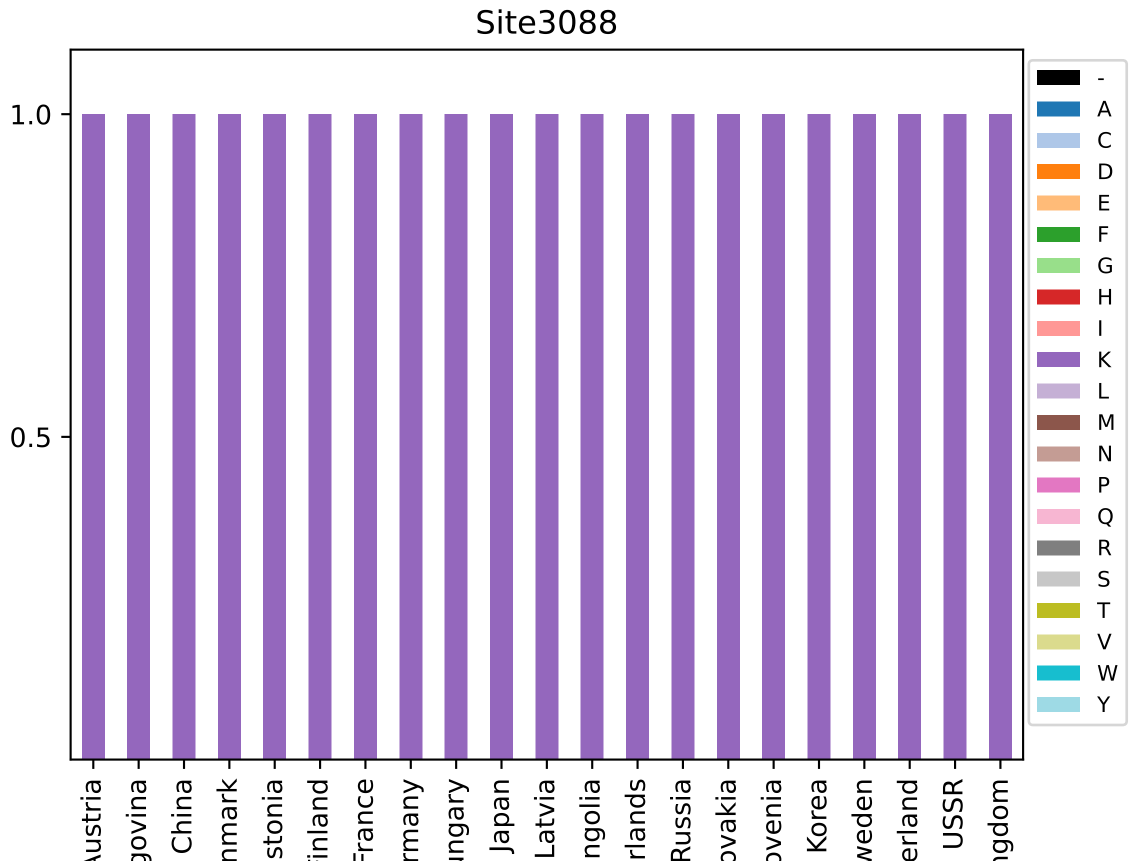
Site3088
1.0
0.5
-
A
C
D
E
F
G
H
I
K
L
M
N
P
Q
R
S
T
V
W
Y
China
Japan
Latvia
Russia
Korea
USSR

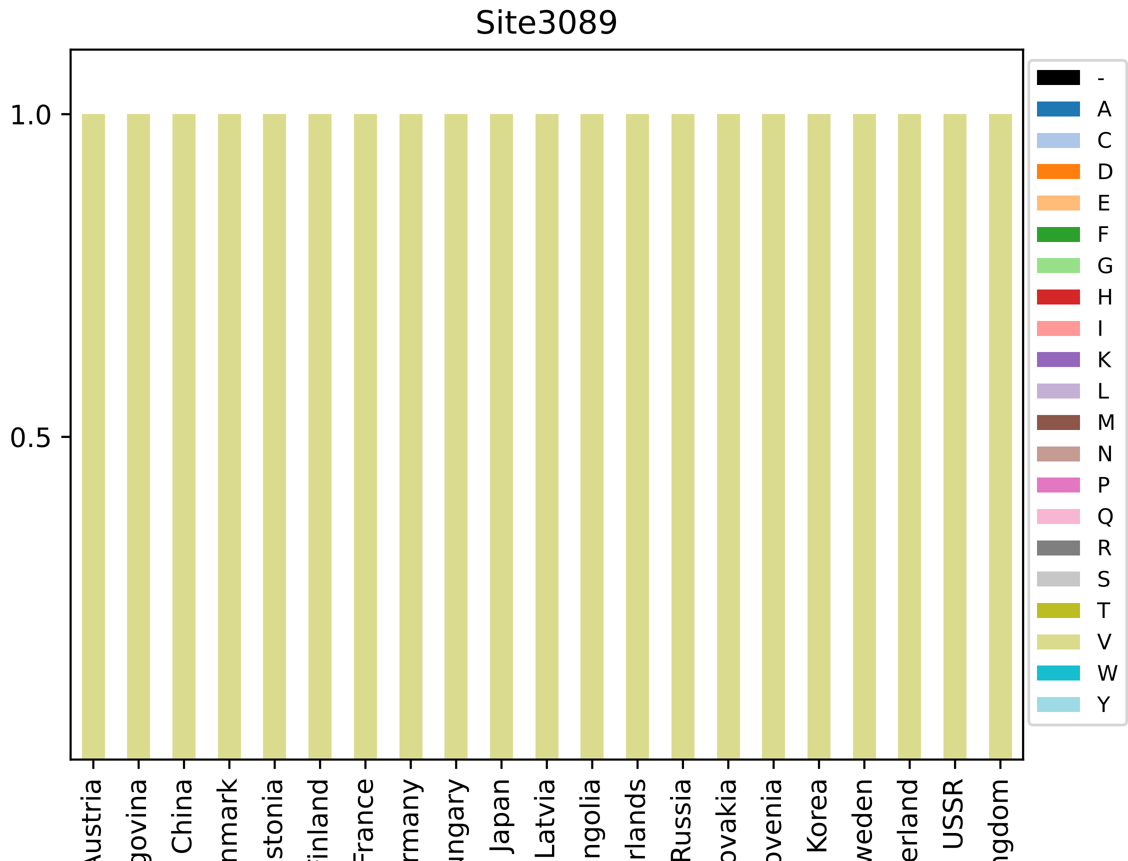

Site3089
1.0
0.5
-
A
C
D
E
F
G
H
I
K
L
M
N
P
Q
R
S
T
V
W
Y
China
Japan
Latvia
Russia
Korea
USSR

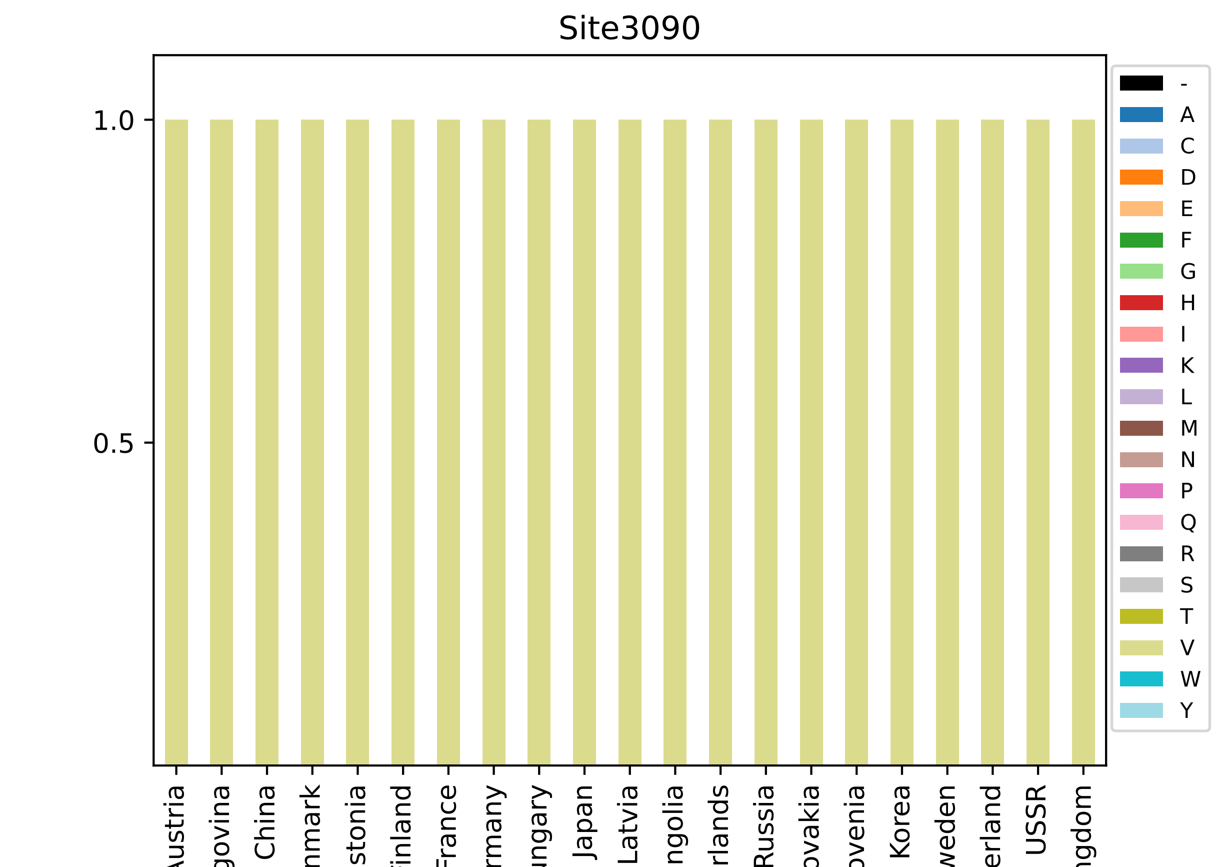

Site3090
1.0
0.5
-
A
C
D
E
F
G
H
I
K
L
M
N
P
Q
R
S
T
V
W
Y
Austria
govina
China
nmark
stonia
Finland
France
rmany
ungary
Japan
Latvia
ngolia
rlands
Russia
ovakia
ovenia
Korea
weden
erland
USSR
ngdom

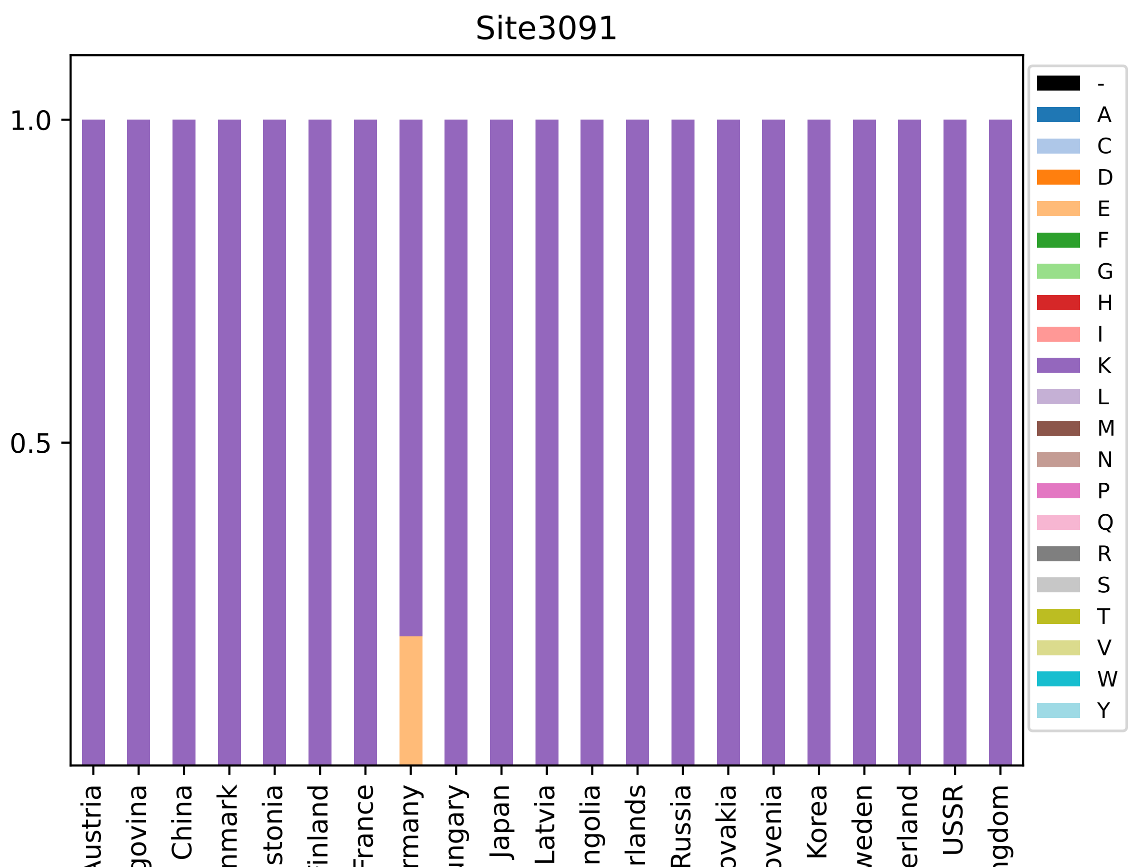
Site3091
1.0
0.5
-
A
C
D
E
F
G
H
I
K
L
M
N
P
Q
R
S
T
V
W
Y
Austria
govina
China
nmark
stonia
inland
France
rmany
ungary
Japan
Latvia
ngolia
rlands
Russia
ovakia
ovenia
Korea
weden
erland
USSR
ngdom

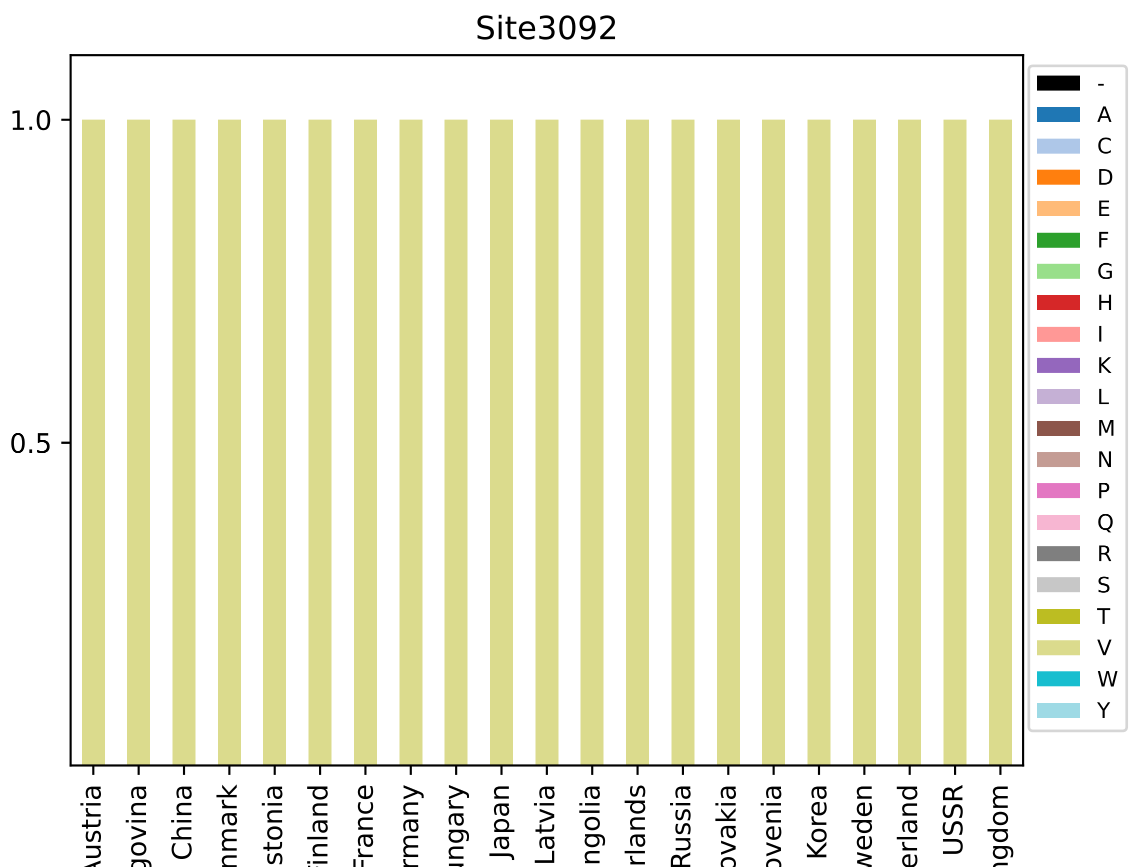
Site3092
1.0
0.5
-
A
C
D
E
F
G
H
I
K
L
M
N
P
Q
R
S
T
V
W
Y
Austria
China
Japan
Latvia
Russia
Korea
USSR

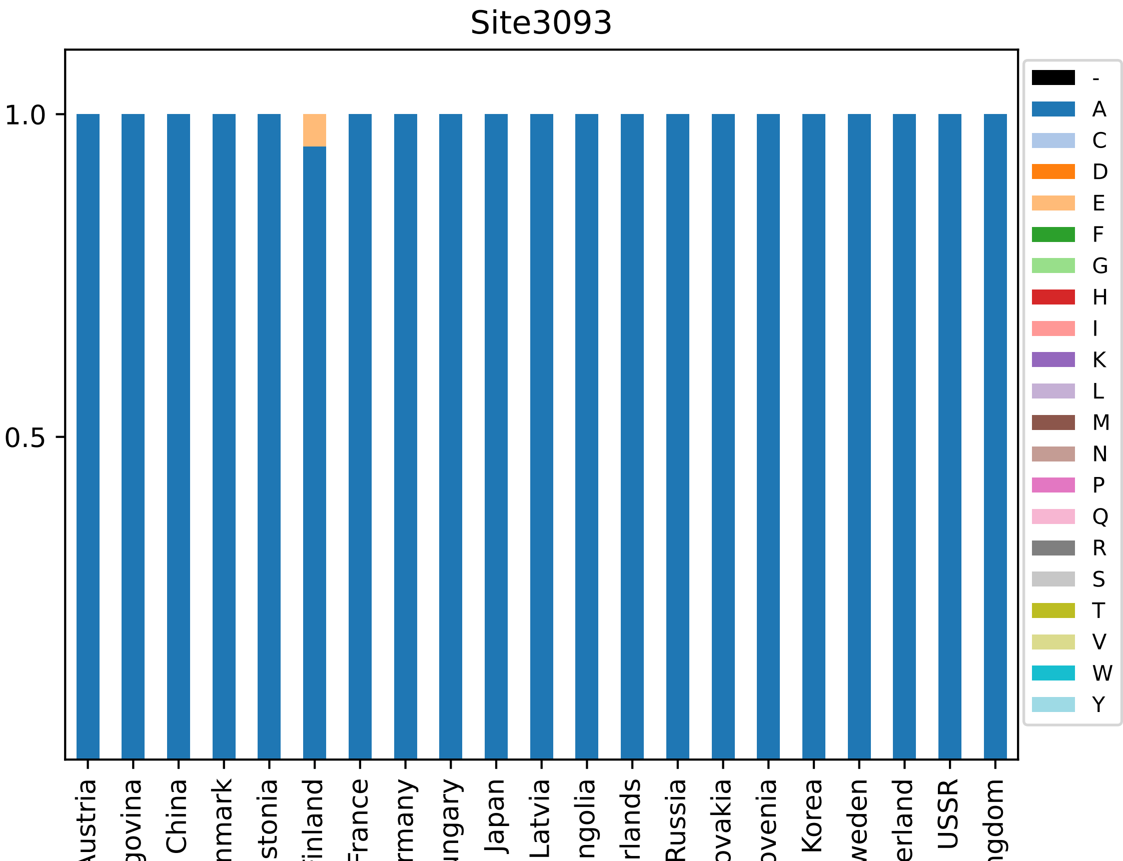

Site3093
1.0
0.5
-
A
C
D
E
F
G
H
I
K
L
M
N
P
Q
R
S
T
V
W
Y
Austria
govina
China
nmark
stonia
inland
France
rmany
ungary
Japan
Latvia
ngolia
rlands
Russia
ovakia
ovenia
Korea
weden
erland
USSR
ngdom

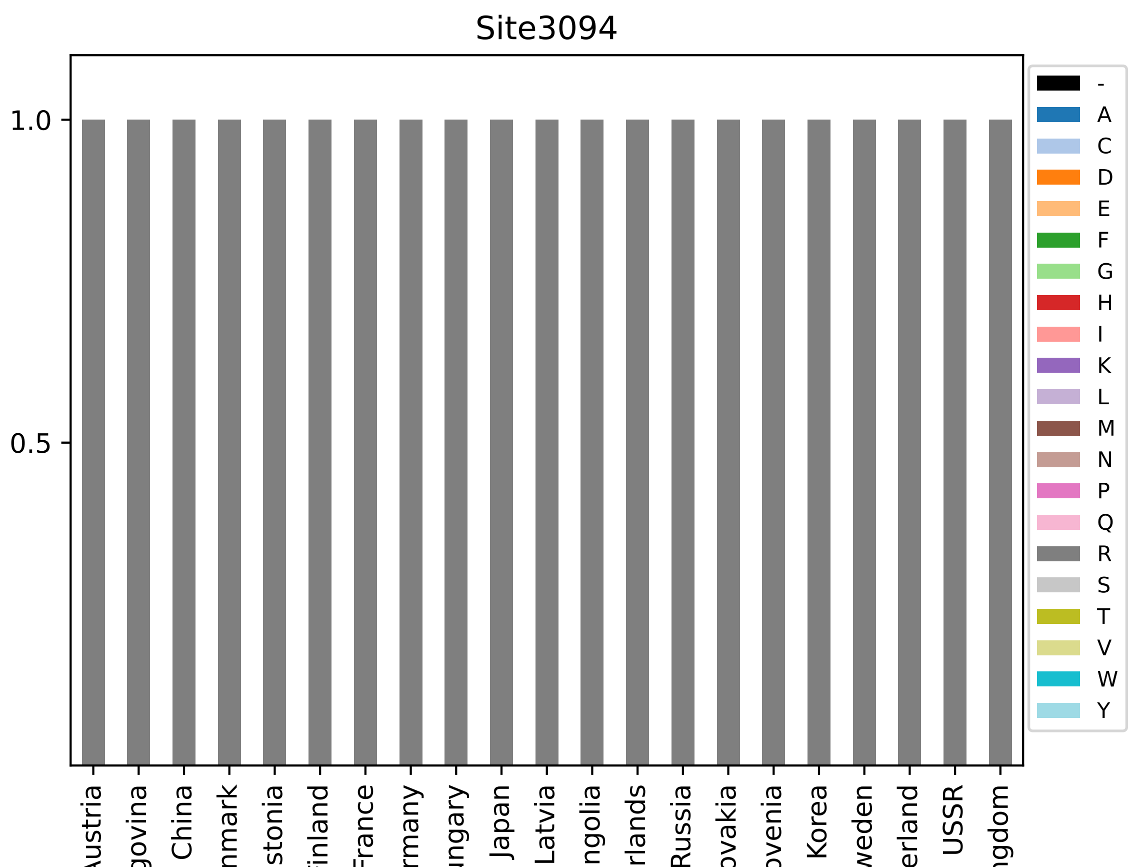

Site3094
1.0
0.5
-
A
C
D
E
F
G
H
I
K
L
M
N
P
Q
R
S
T
V
W
Y
China
Japan
Latvia
Russia
Korea
USSR

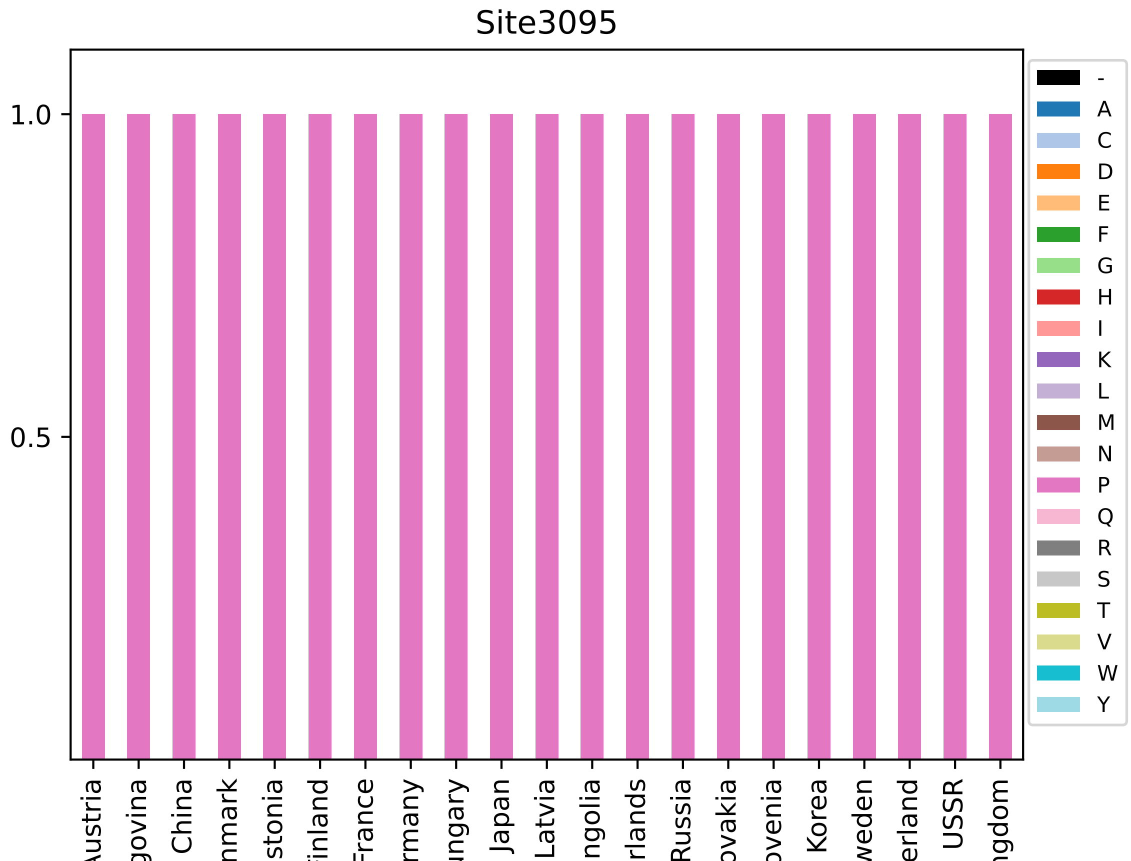

Site3095
1.0
0.5
-
A
C
D
E
F
G
H
I
K
L
M
N
P
Q
R
S
T
V
W
Y
China
Japan
Latvia
Russia
Korea
USSR

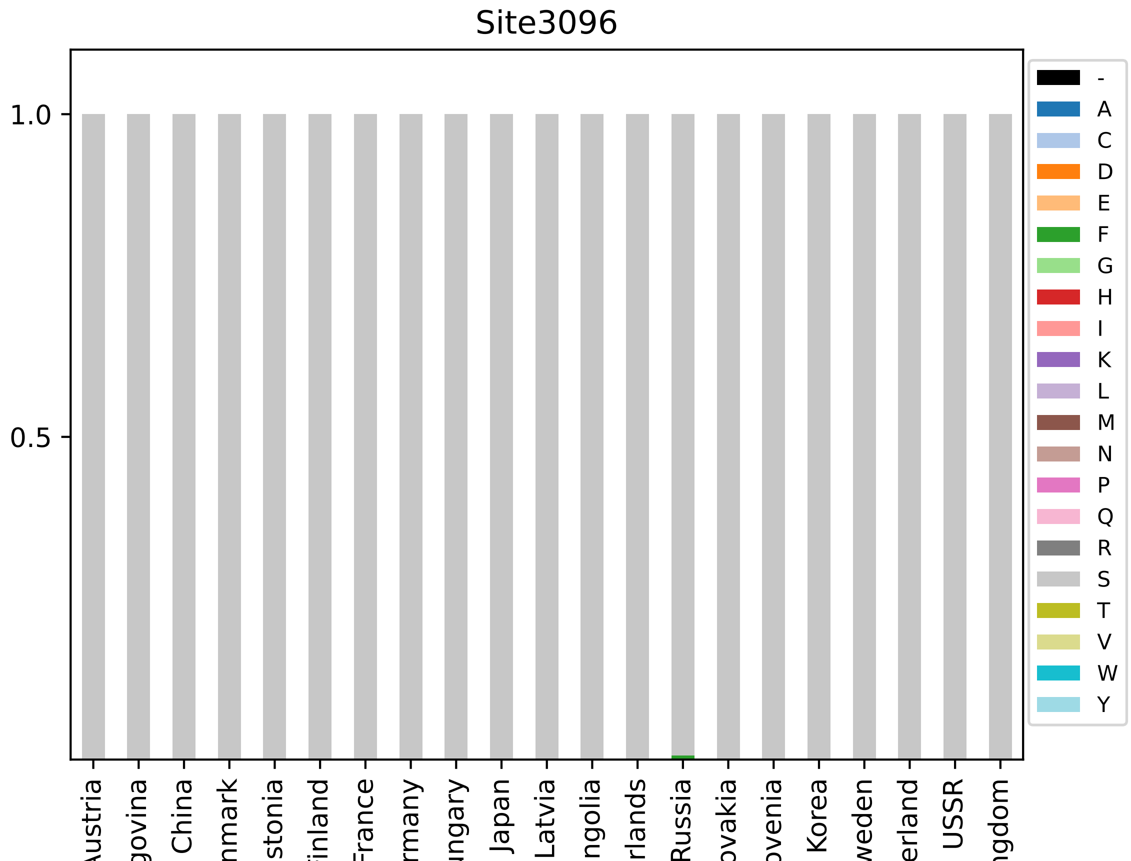

Site3096
1.0
0.5
-
A
C
D
E
F
G
H
I
K
L
M
N
P
Q
R
S
T
V
W
Y
Austria
China
Finland
France
Japan
Latvia
Russia
Korea
USSR

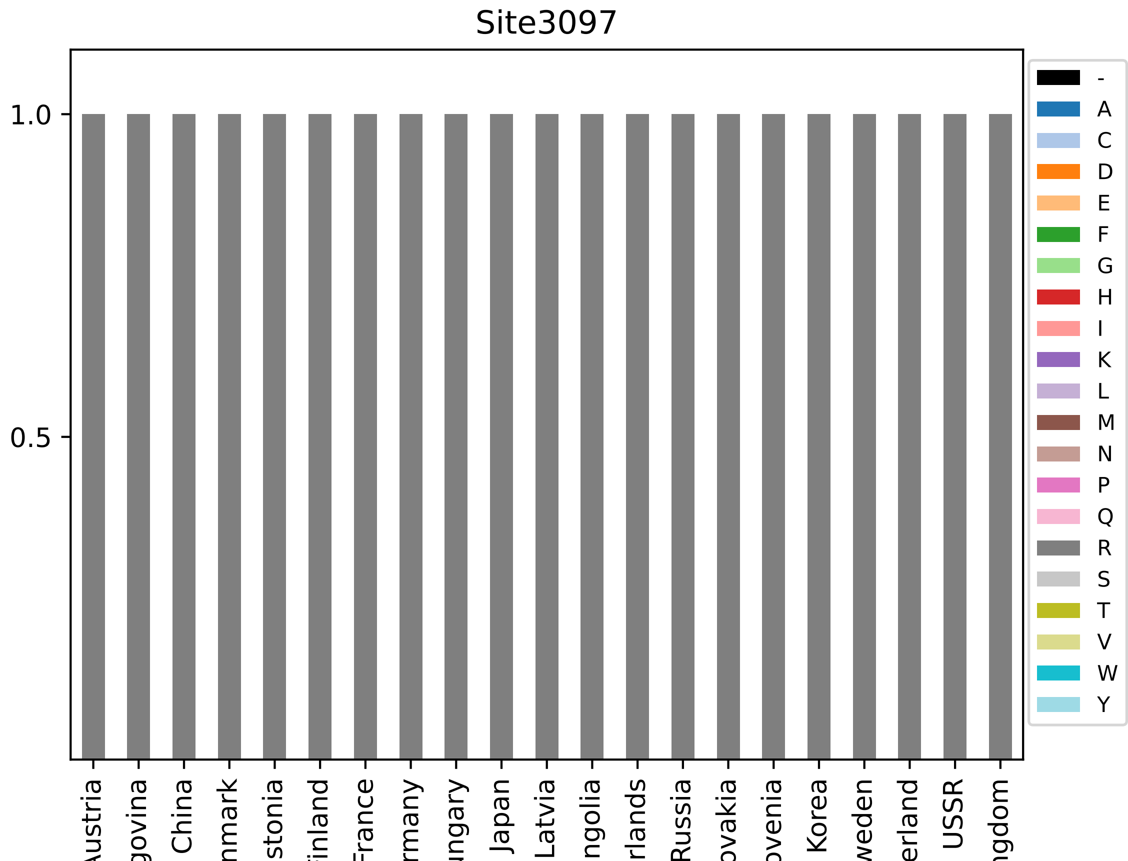
Site3097
1.0
0.5
-
A
C
D
E
F
G
H
I
K
L
M
N
P
Q
R
S
T
V
W
Y
Austria
China
Japan
Latvia
Russia
Korea
USSR

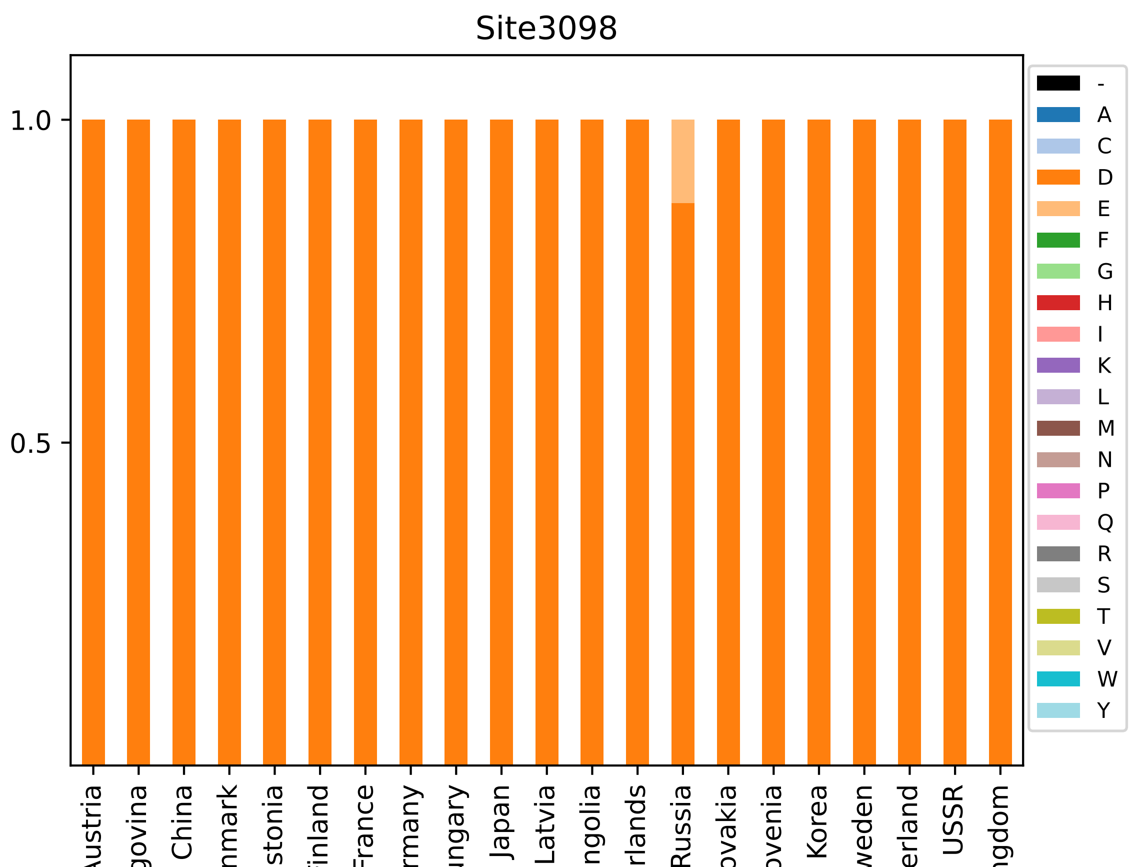

Site3098
1.0
0.5
-
A
C
D
E
F
G
H
I
K
L
M
N
P
Q
R
S
T
V
W
Y
China
Japan
Latvia
Russia
Korea
USSR

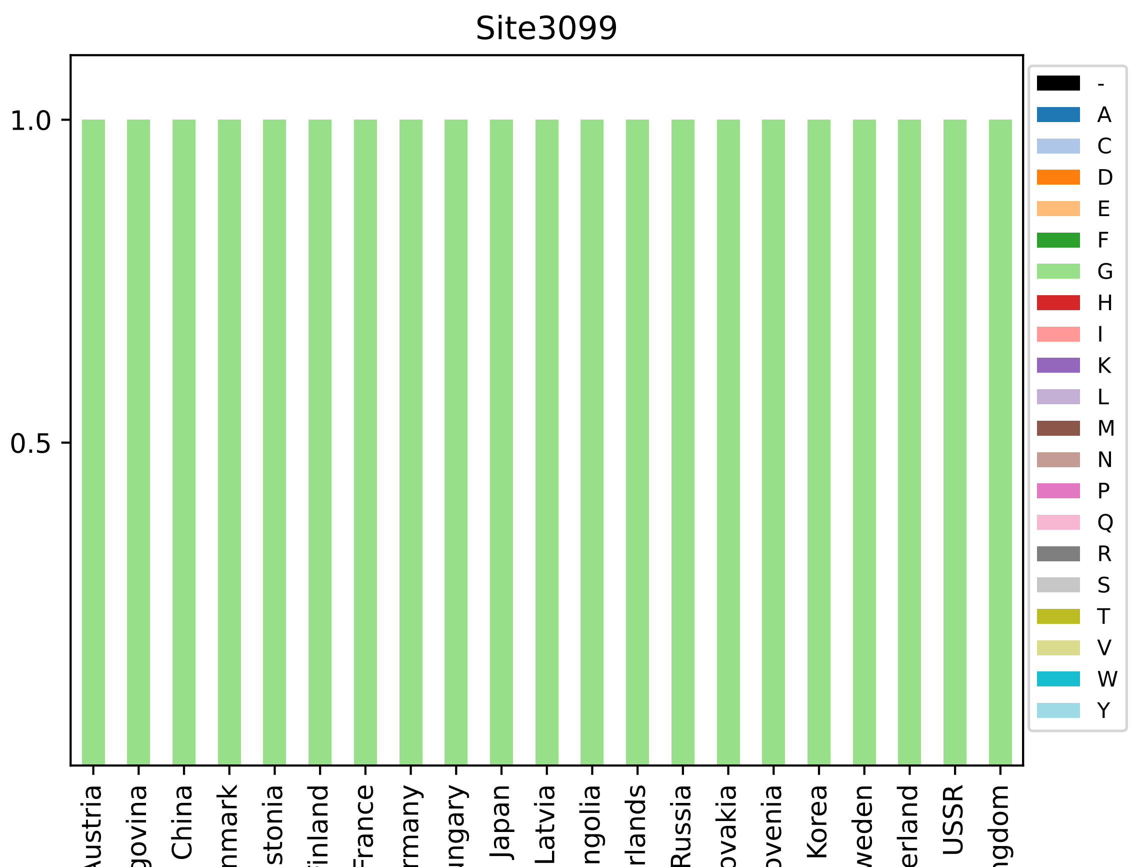
Site3099
1.0
0.5
-
A
C
D
E
F
G
H
I
K
L
M
N
P
Q
R
S
T
V
W
Y
Austria
govina
China
nmark
stonia
Finland
France
rmany
ungary
Japan
Latvia
ngolia
rlands
Russia
ovakia
ovenia
Korea
weden
erland
USSR
ngdom

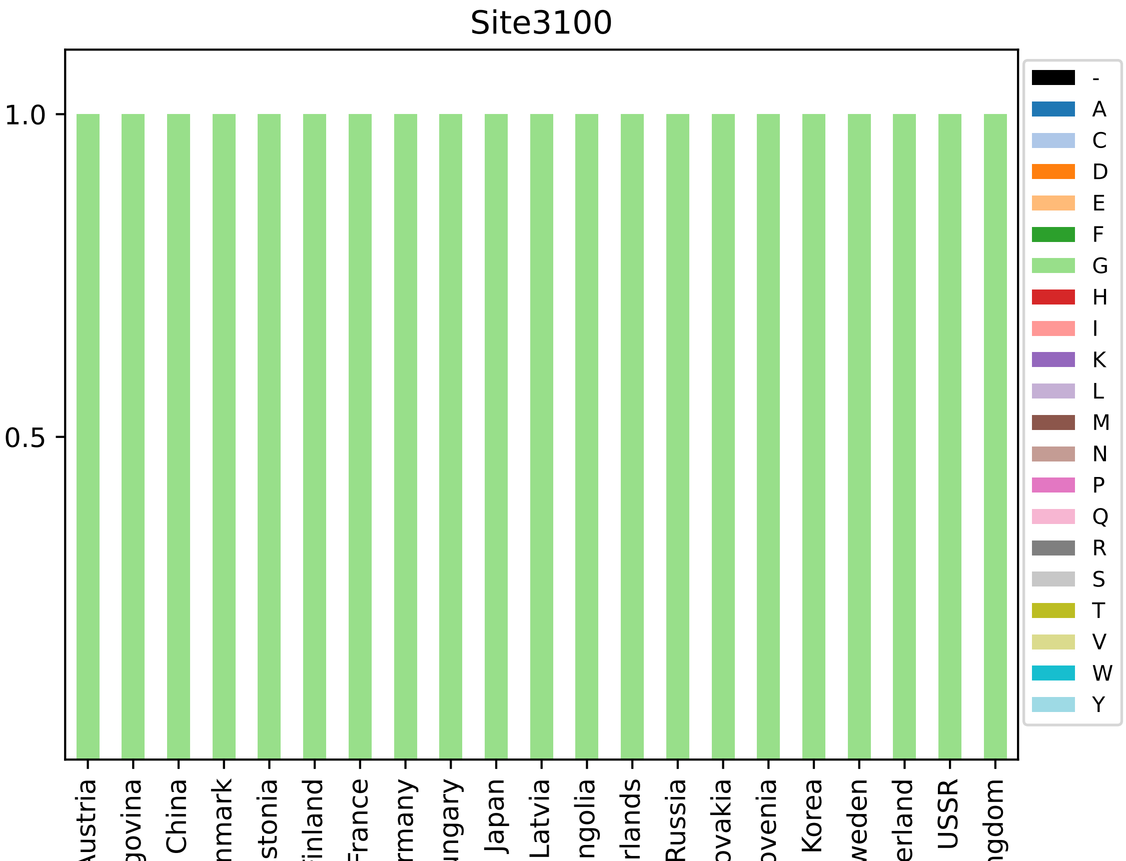
Site3100
1.0
0.5
-
A
C
D
E
F
G
H
I
K
L
M
N
P
Q
R
S
T
V
W
Y
China
Japan
Latvia
Russia
Korea
USSR

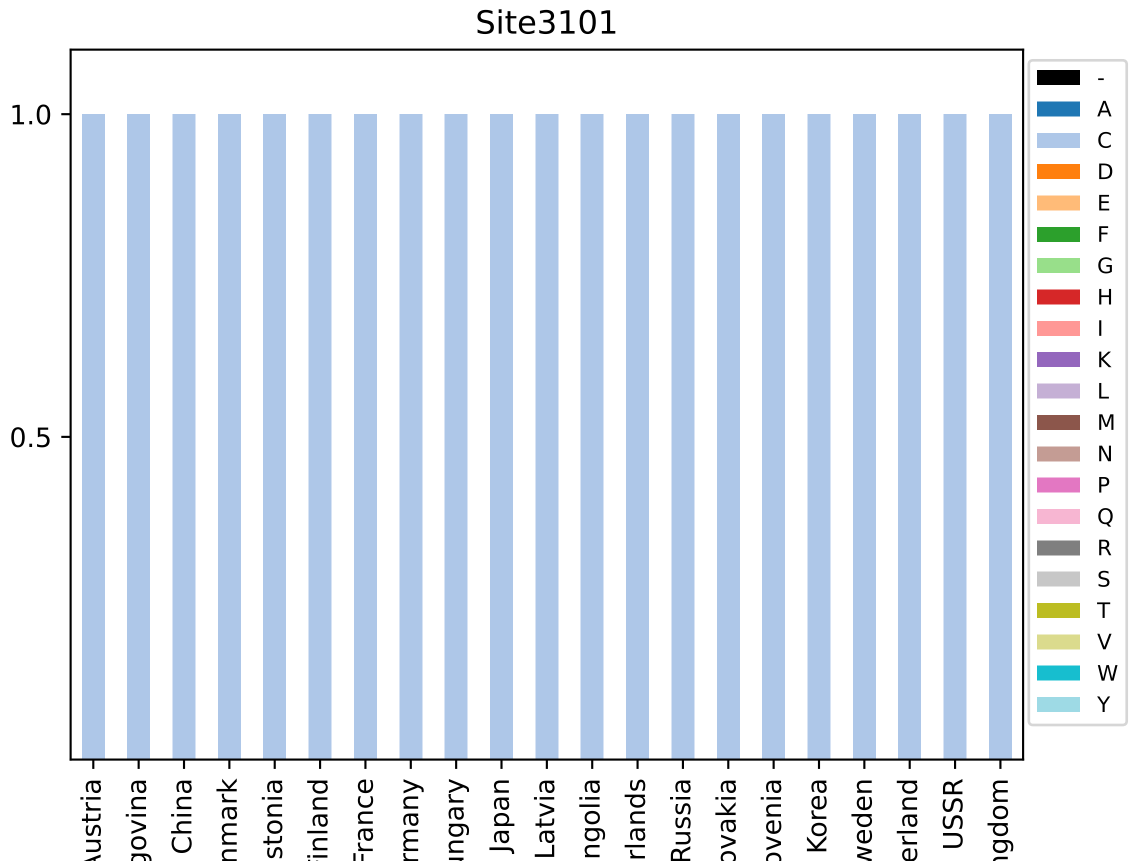

Site3101
1.0
0.5
-
A
C
D
E
F
G
H
I
K
L
M
N
P
Q
R
S
T
V
W
Y
Austria
govina
China
nmark
stonia
Finland
France
rmany
ungary
Japan
Latvia
ngolia
rlands
Russia
ovakia
ovenia
Korea
weden
erland
USSR
ngdom

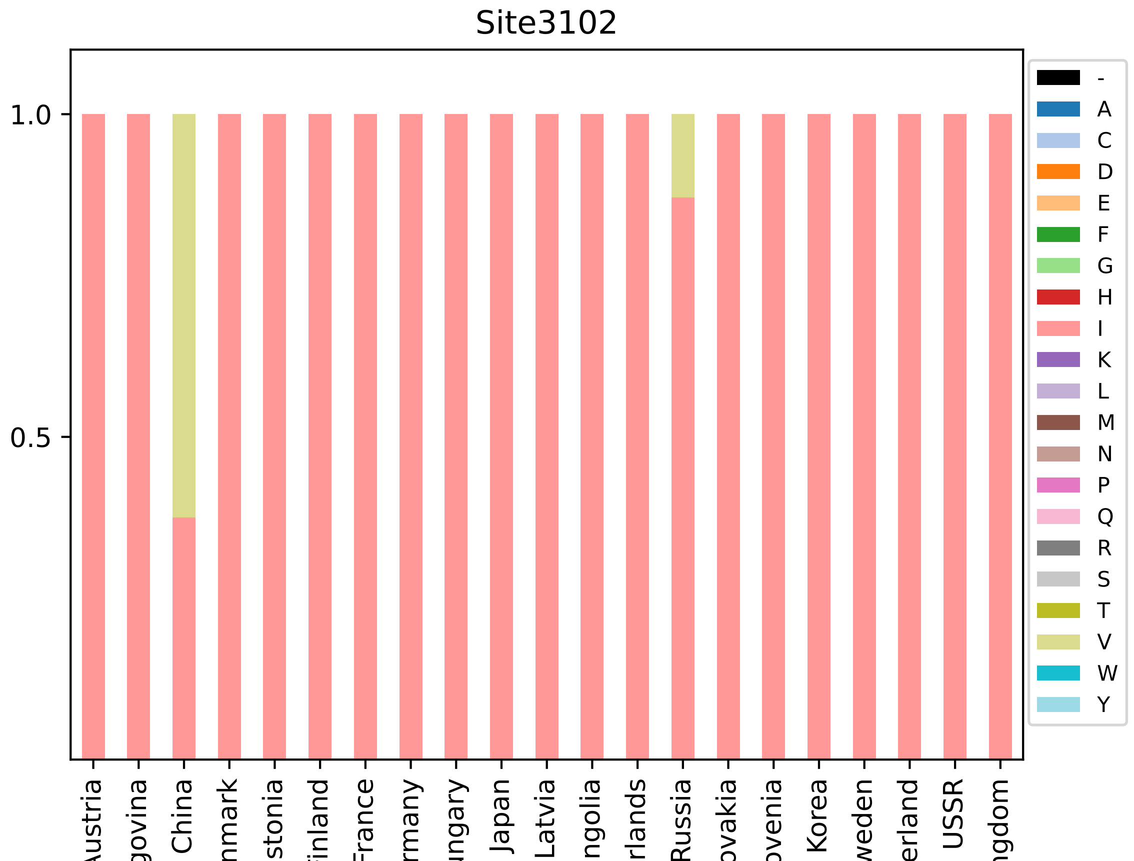

Site3102
1.0
0.5
-
A
C
D
E
F
G
H
I
K
L
M
N
P
Q
R
S
T
V
W
Y
China
Japan
Latvia
Russia
Korea
USSR

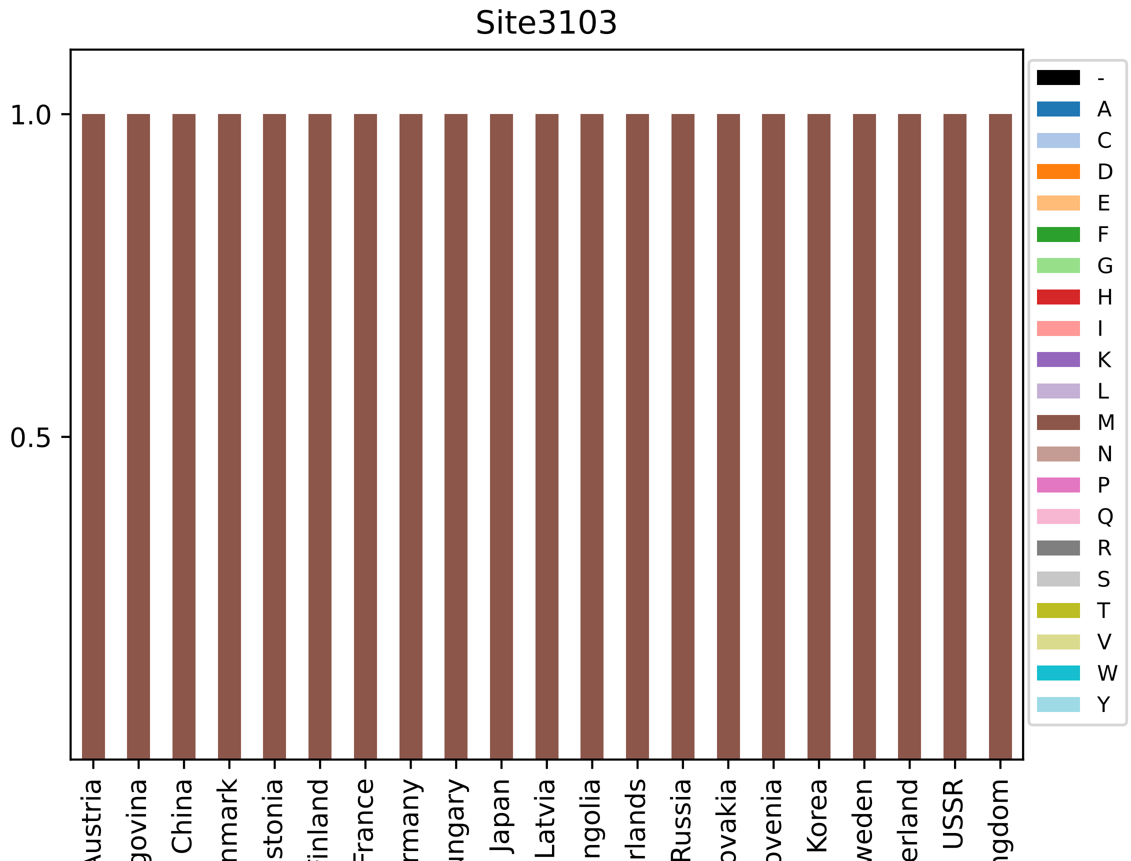
Site3103
1.0
0.5
-
A
C
D
E
F
G
H
I
K
L
M
N
P
Q
R
S
T
V
W
Y
Austria
China
Japan
Latvia
Russia
Korea
USSR

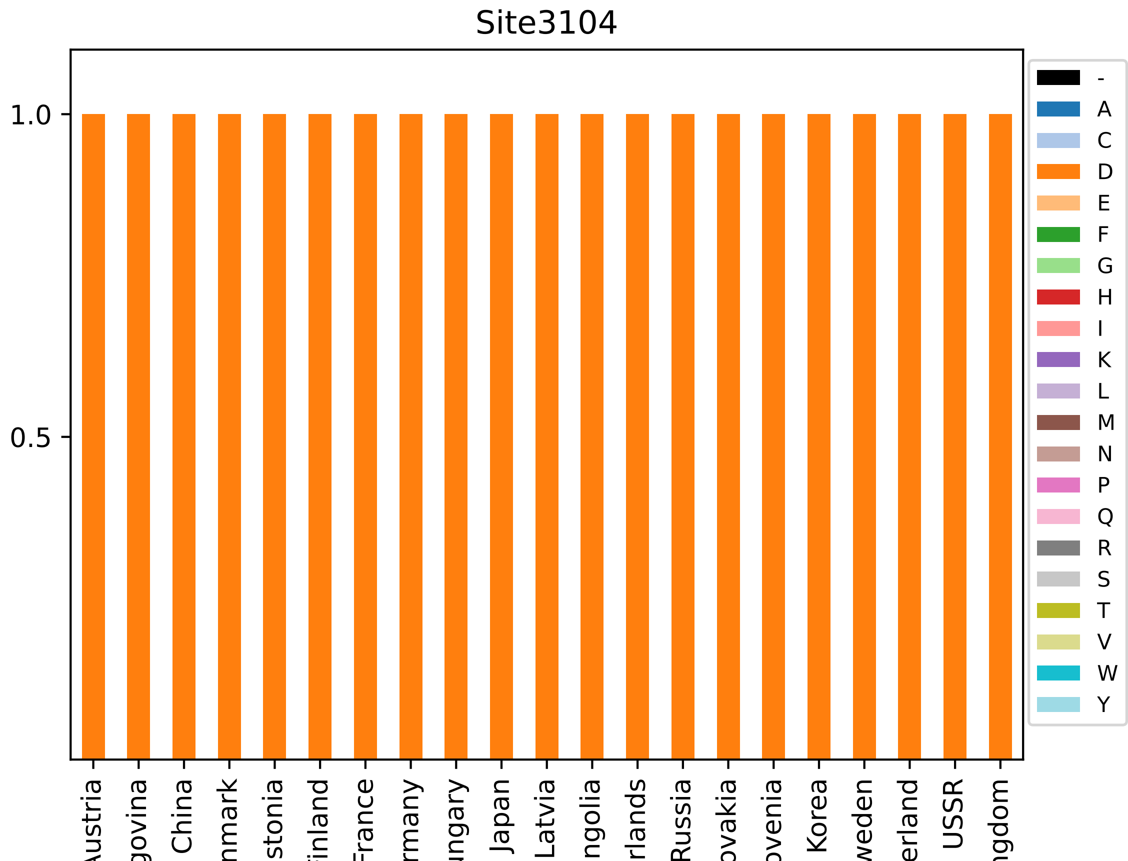
Site3104
1.0
0.5
-
A
C
D
E
F
G
H
I
K
L
M
N
P
Q
R
S
T
V
W
Y
China
Japan
Latvia
Russia
Korea
USSR

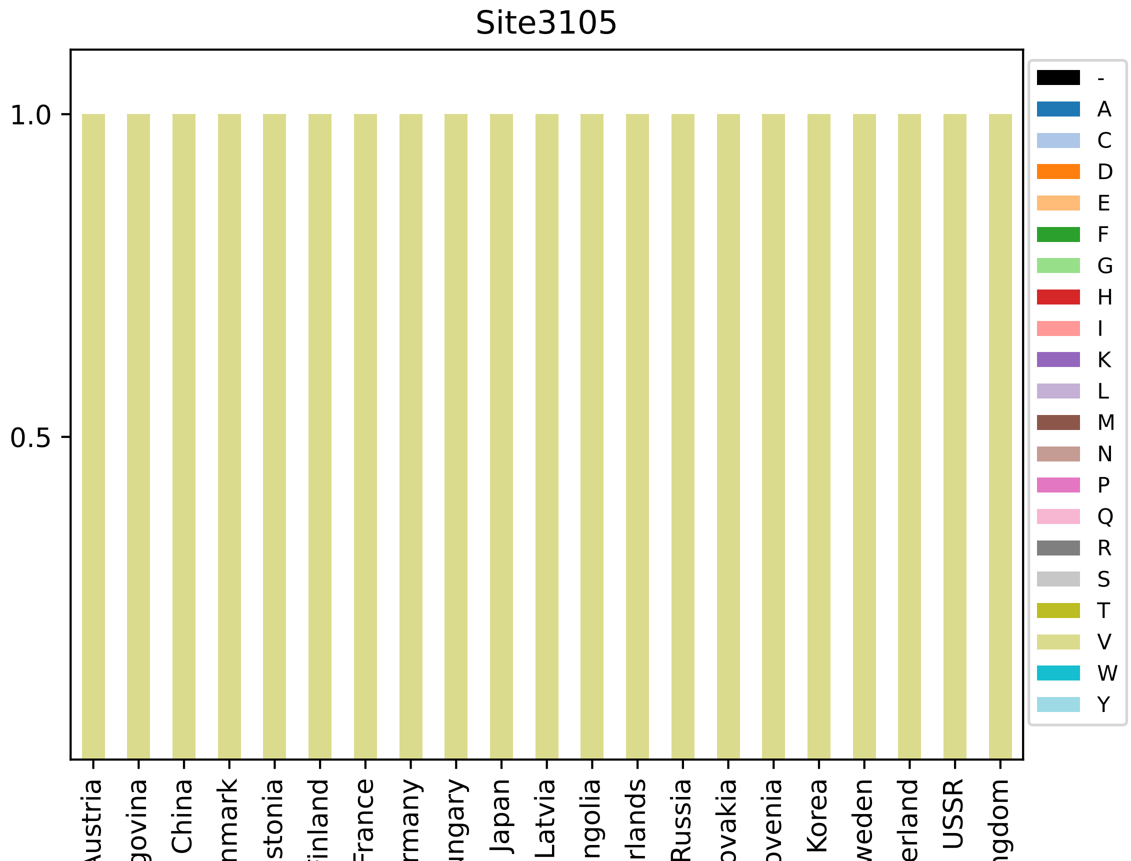
Site3105
1.0
0.5
-
A
C
D
E
F
G
H
I
K
L
M
N
P
Q
R
S
T
V
W
Y
China
Japan
Latvia
Russia
Korea
USSR

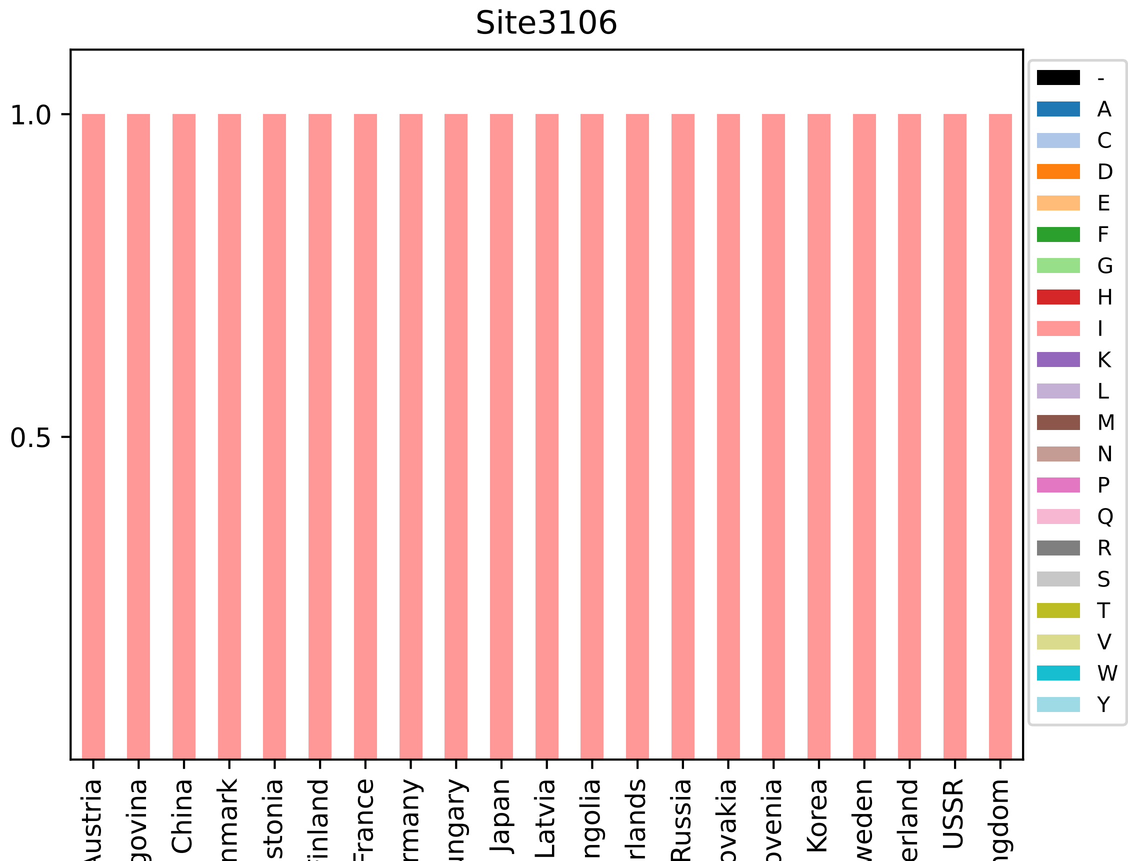
Site3106
1.0
0.5
-
A
C
D
E
F
G
H
I
K
L
M
N
P
Q
R
S
T
V
W
Y
China
Japan
Latvia
Russia
Korea
USSR

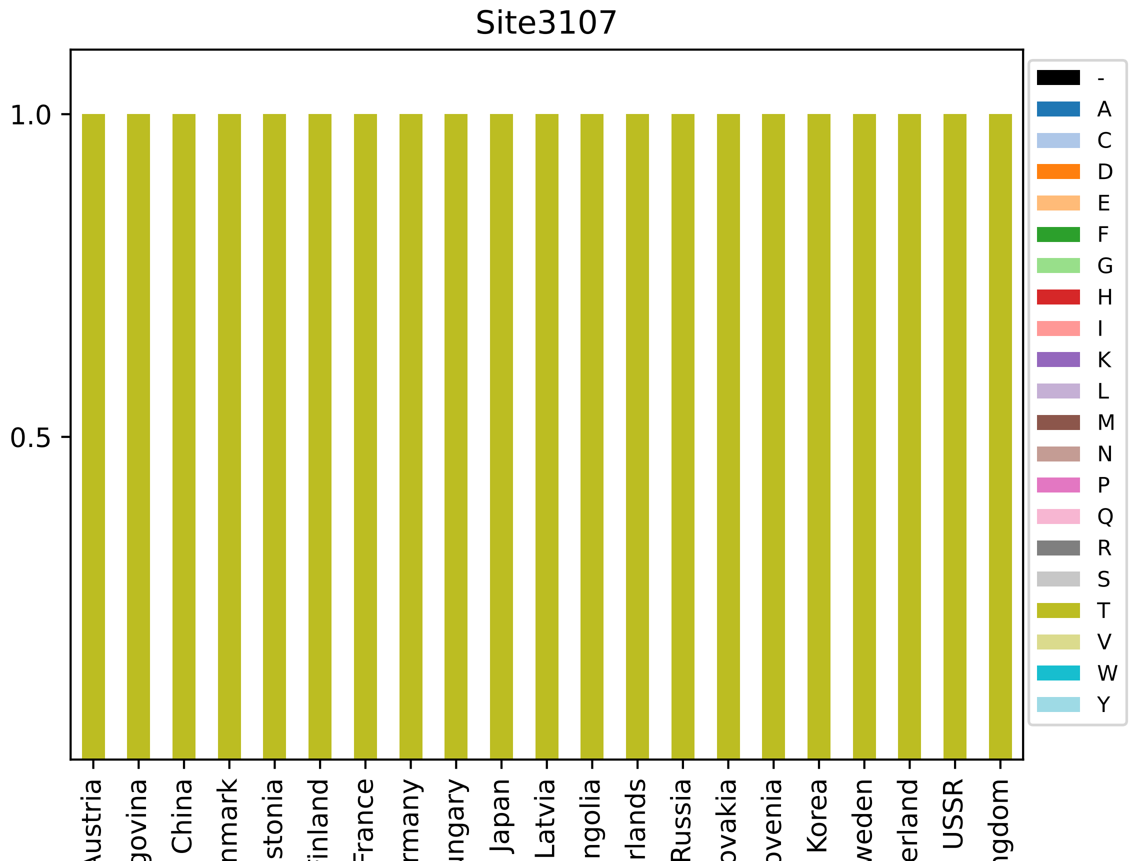

Site3107
1.0
0.5
-
A
C
D
E
F
G
H
I
K
L
M
N
P
Q
R
S
T
V
W
Y
Austria
govina
China
nmark
stonia
inland
France
rmany
ungary
Japan
Latvia
ngolia
rlands
Russia
ovakia
ovenia
Korea
weden
erland
USSR
ngdom

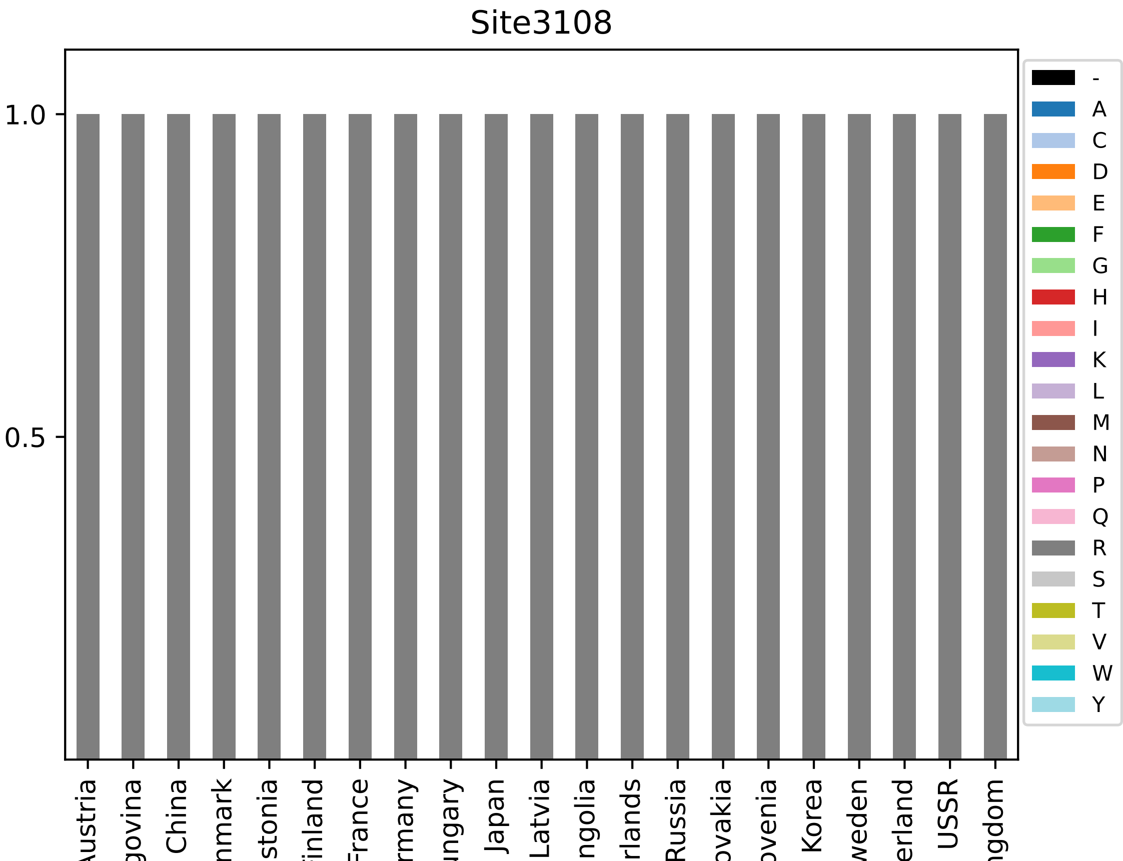

Site3108
1.0
0.5
-
A
C
D
E
F
G
H
I
K
L
M
N
P
Q
R
S
T
V
W
Y
Austria
govina
China
nmark
stonia
inland
France
rmany
ungary
Japan
Latvia
ngolia
rlands
Russia
ovakia
ovenia
Korea
weden
erland
USSR
ngdom

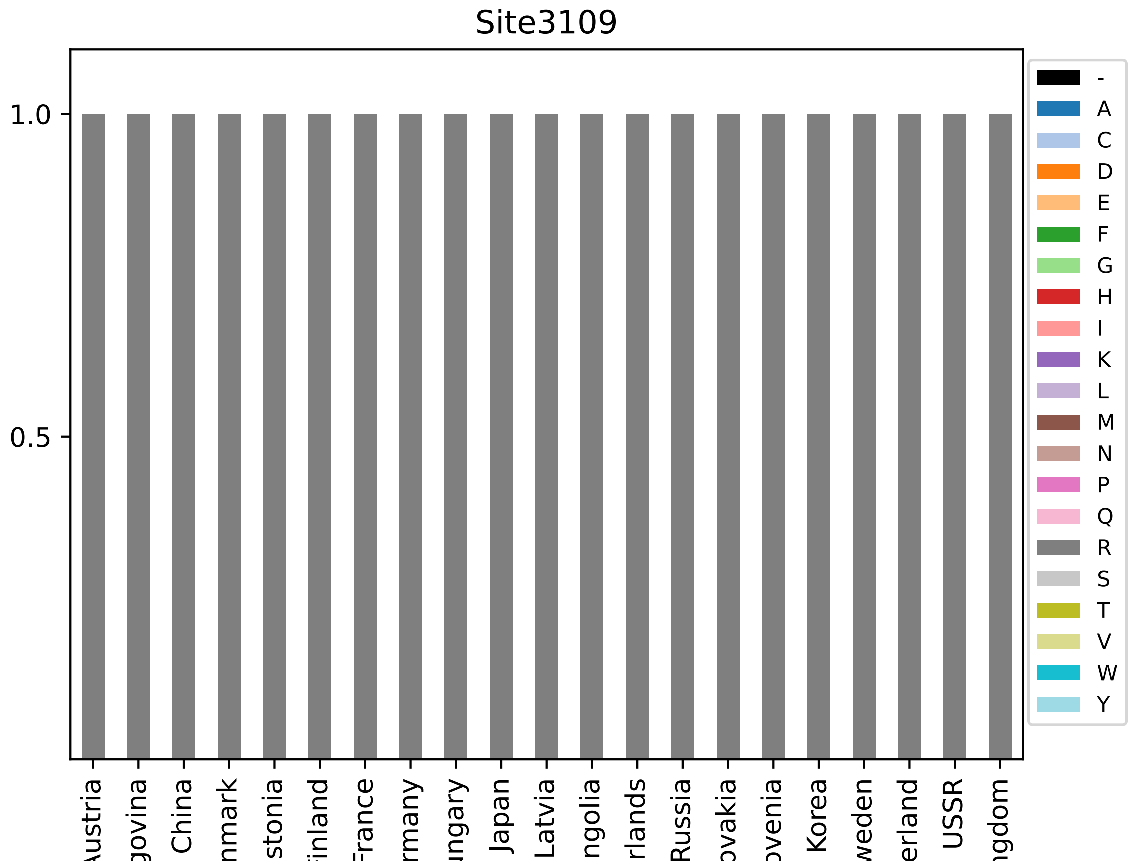
Site3109
1.0
0.5
Austria
govina
China
nmark
stonia
Finland
France
rmany
ungary
Japan
Latvia
ngolia
rlands
Russia
ovakia
ovenia
Korea
weden
erland
USSR
ngdom
-
A
C
D
E
F
G
H
I
K
L
M
N
P
Q
R
S
T
V
W
Y

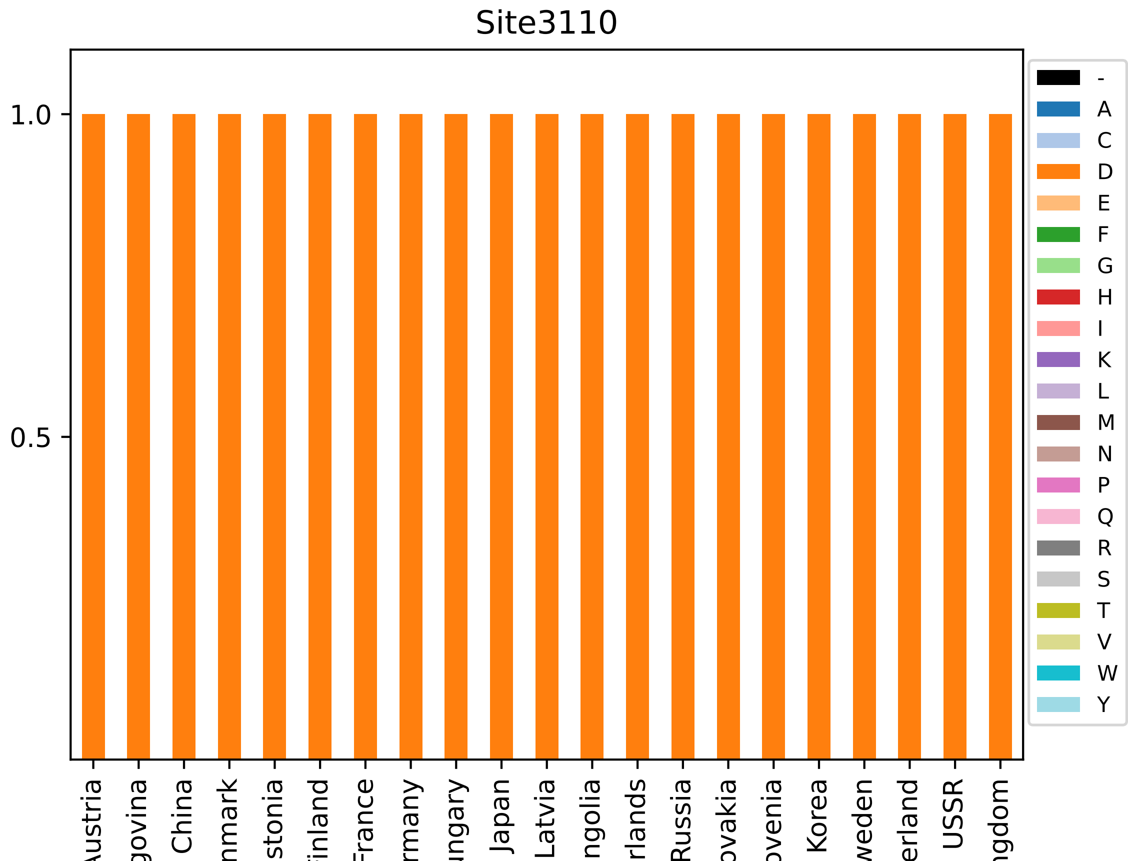
Site3110
1.0
0.5
-
A
C
D
E
F
G
H
I
K
L
M
N
P
Q
R
S
T
V
W
Y
China
Japan
Latvia
Russia
Korea
USSR

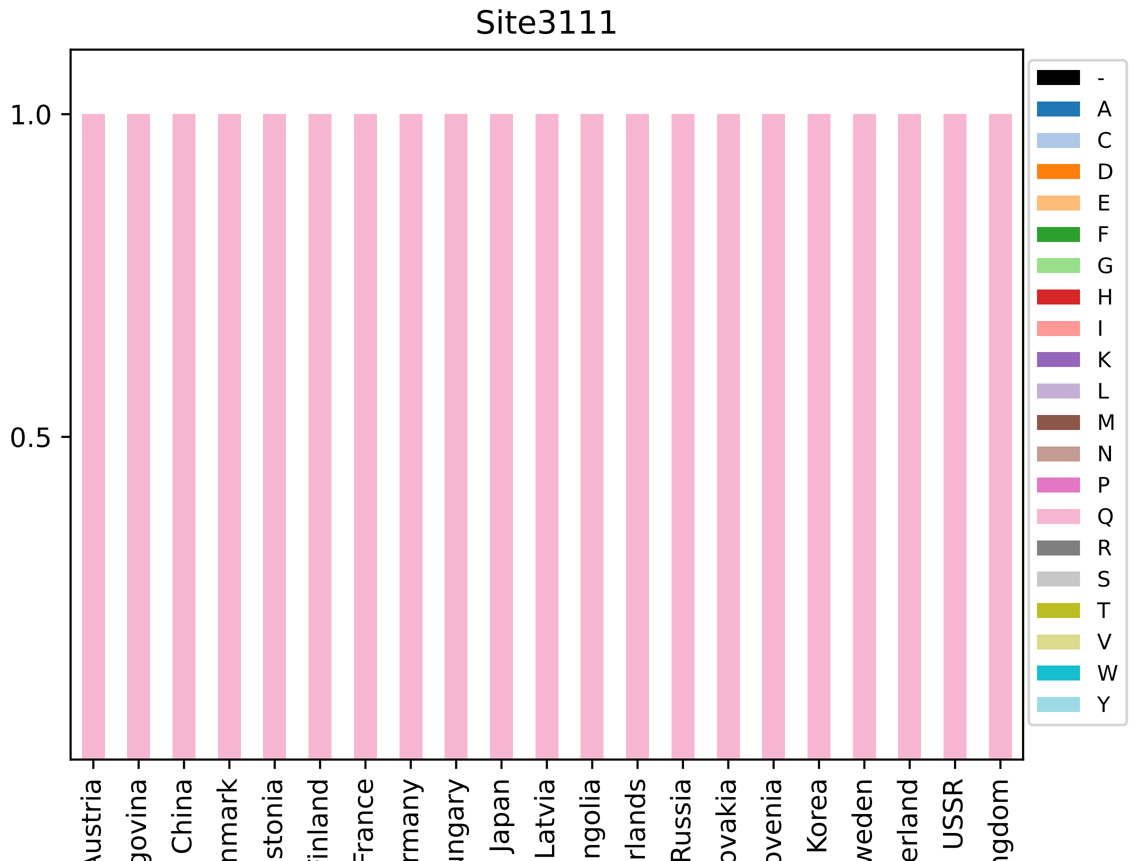
Site3111
1.0
0.5
-
A
C
D
E
F
G
H
I
K
L
M
N
P
Q
R
S
T
V
W
Y
Austria
China
Japan
Latvia
Russia
Korea
USSR

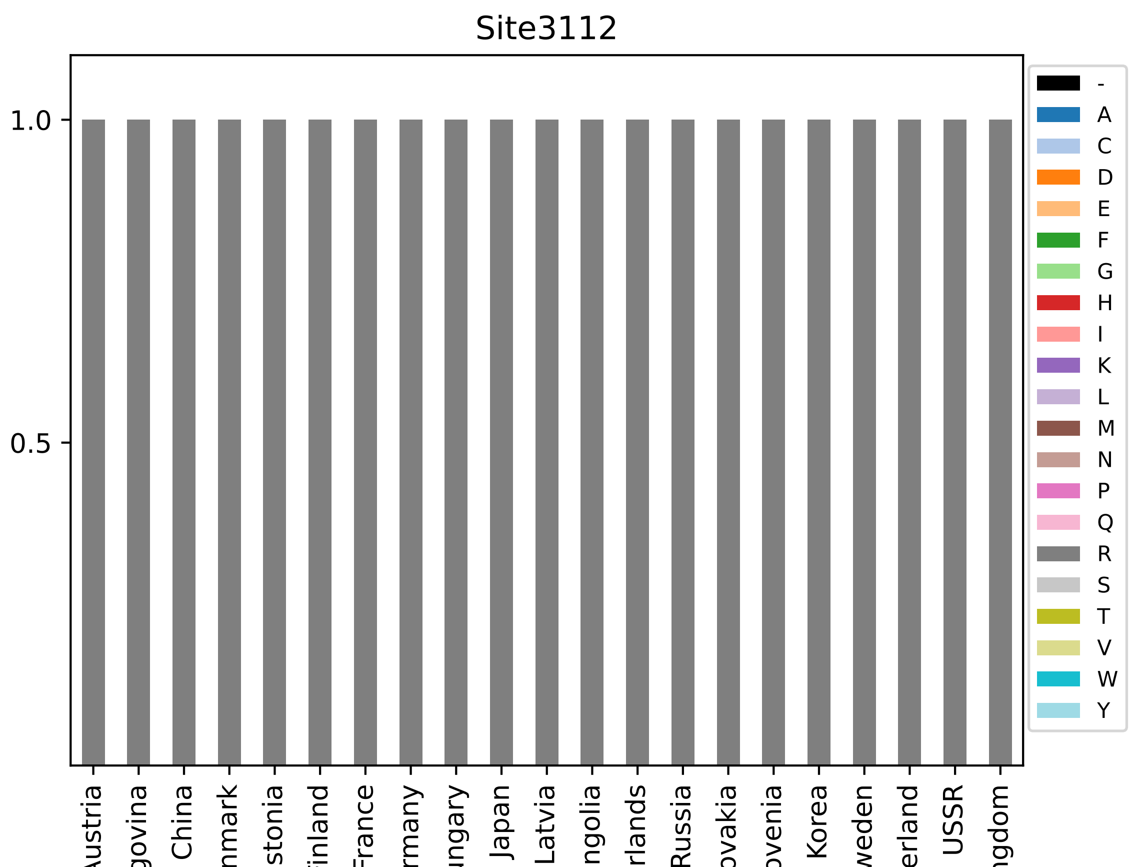
Site3112
1.0
0.5
-
A
C
D
E
F
G
H
I
K
L
M
N
P
Q
R
S
T
V
W
Y
China
Japan
Latvia
Russia
Korea
USSR

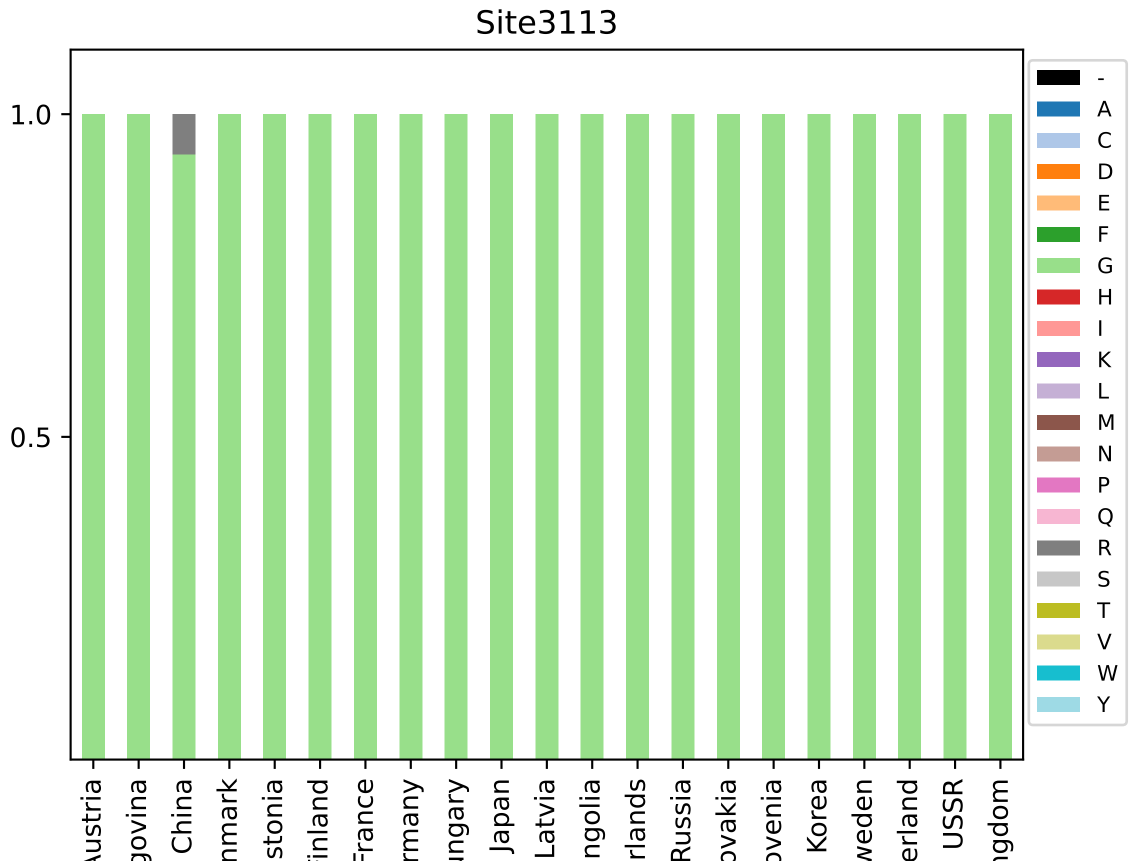
Site3113
1.0
0.5
-
A
C
D
E
F
G
H
I
K
L
M
N
P
Q
R
S
T
V
W
Y
China
Japan
Latvia
Russia
Korea
USSR

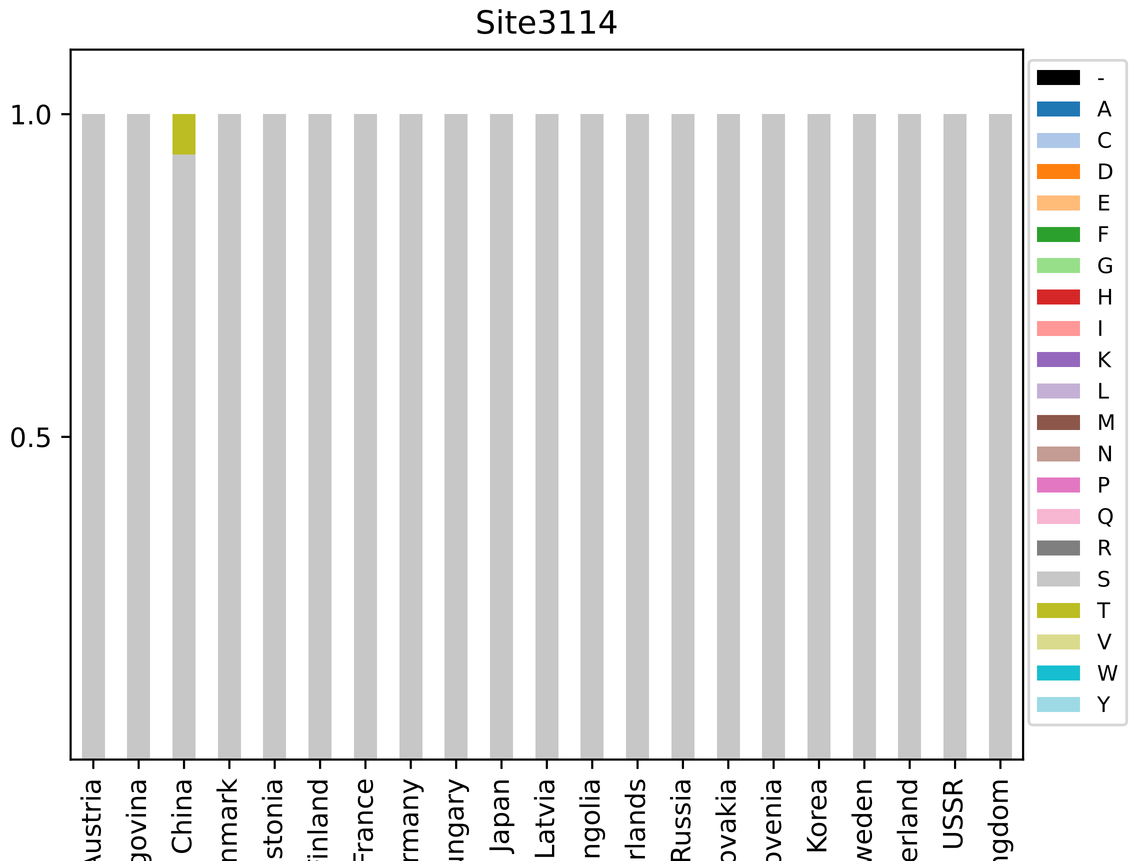
Site3114
1.0
0.5
-
A
C
D
E
F
G
H
I
K
L
M
N
P
Q
R
S
T
V
W
Y
China
Japan
Latvia
Russia
Korea
USSR

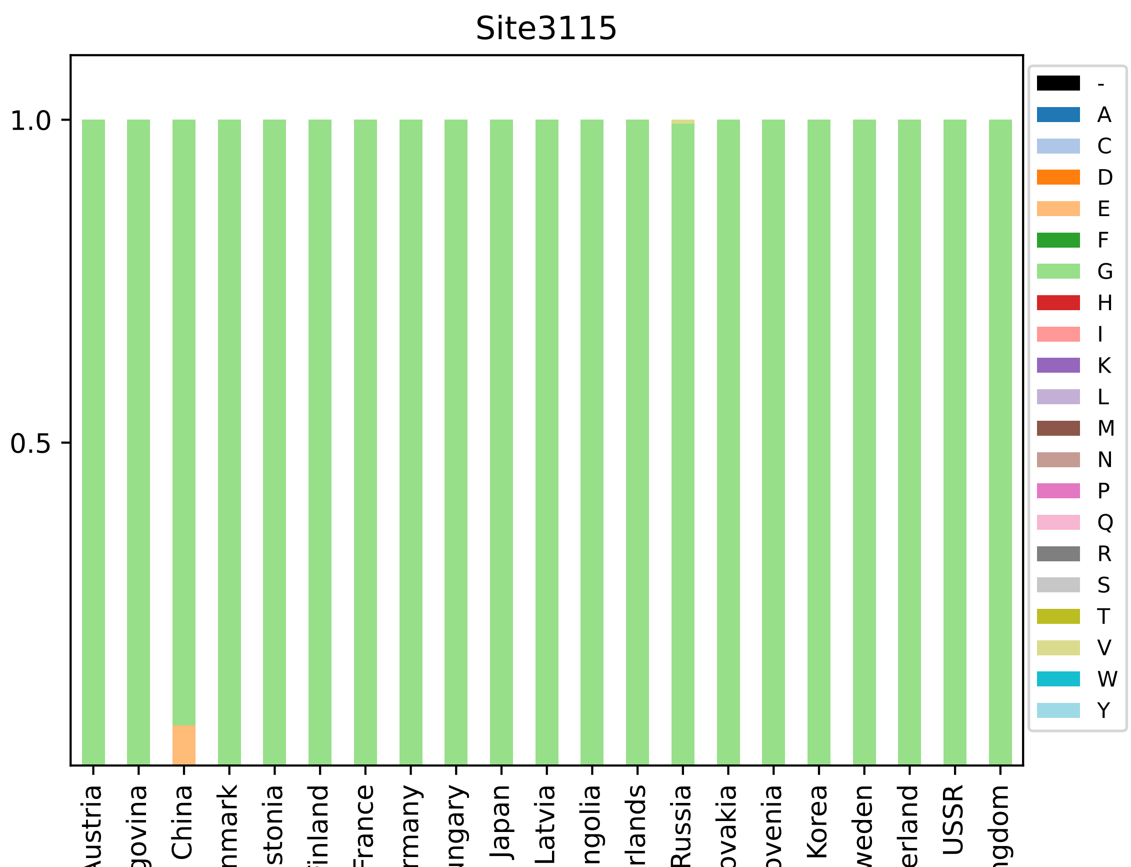

Site3115
1.0
0.5
-
A
C
D
E
F
G
H
I
K
L
M
N
P
Q
R
S
T
V
W
Y
China
Japan
Latvia
Russia
Korea
USSR

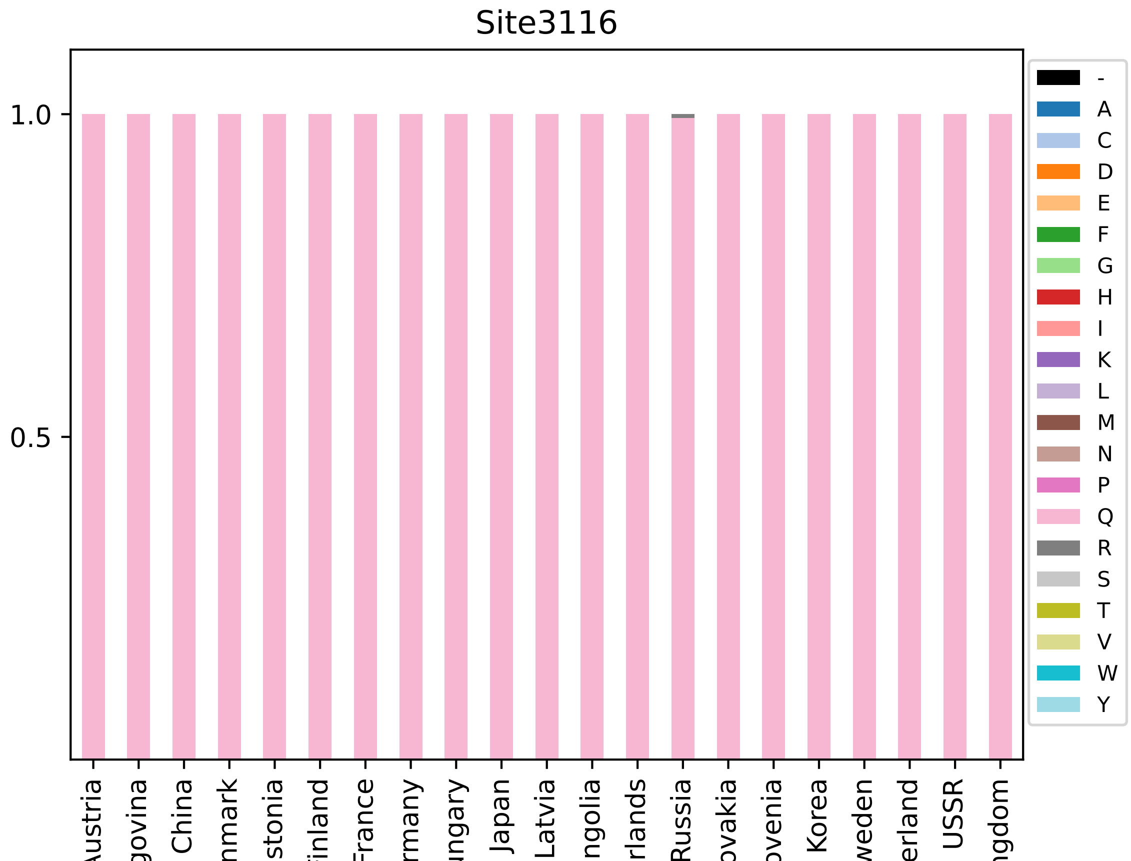

Site3116
1.0
0.5
-
A
C
D
E
F
G
H
I
K
L
M
N
P
Q
R
S
T
V
W
Y
China
Japan
Latvia
Russia
Korea
USSR

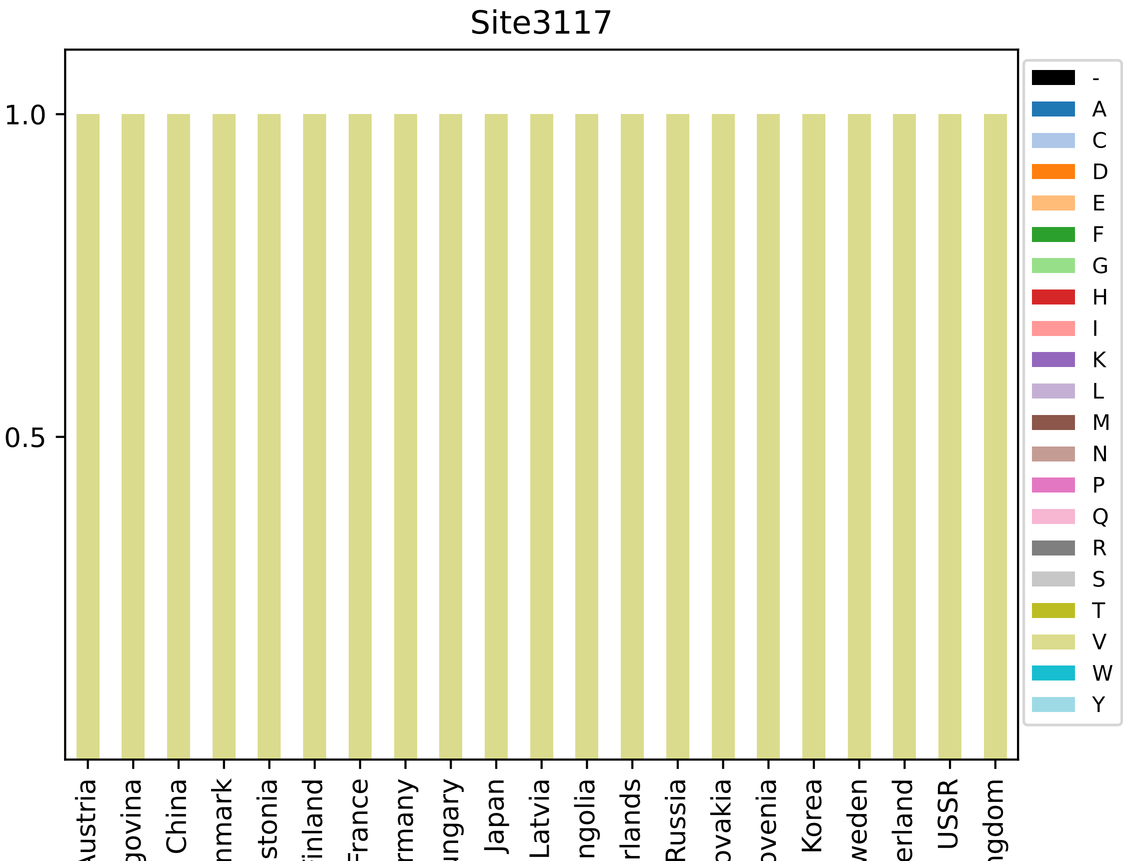
Site3117
1.0
0.5
-
A
C
D
E
F
G
H
I
K
L
M
N
P
Q
R
S
T
V
W
Y
China
Japan
Latvia
Russia
Korea
USSR

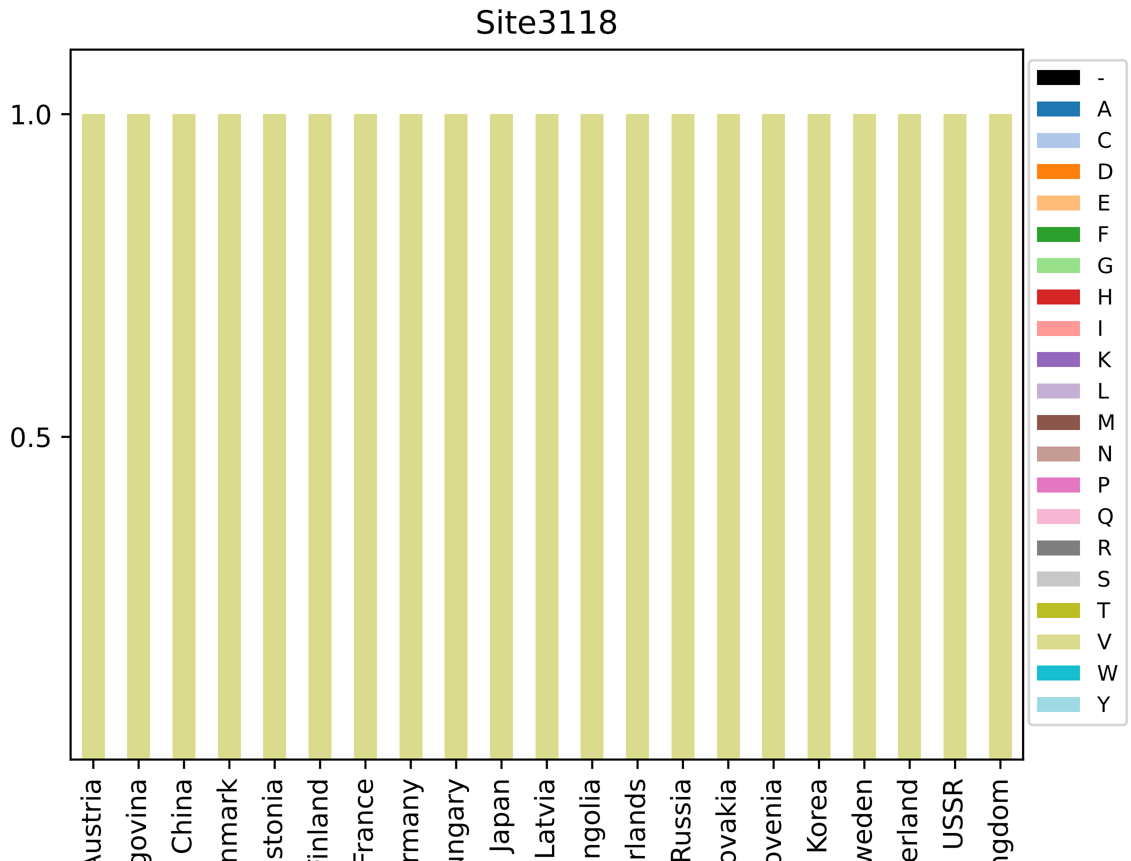
Site3118
1.0
0.5
-
A
C
D
E
F
G
H
I
K
L
M
N
P
Q
R
S
T
V
W
Y
China
Japan
Latvia
Russia
Korea
USSR

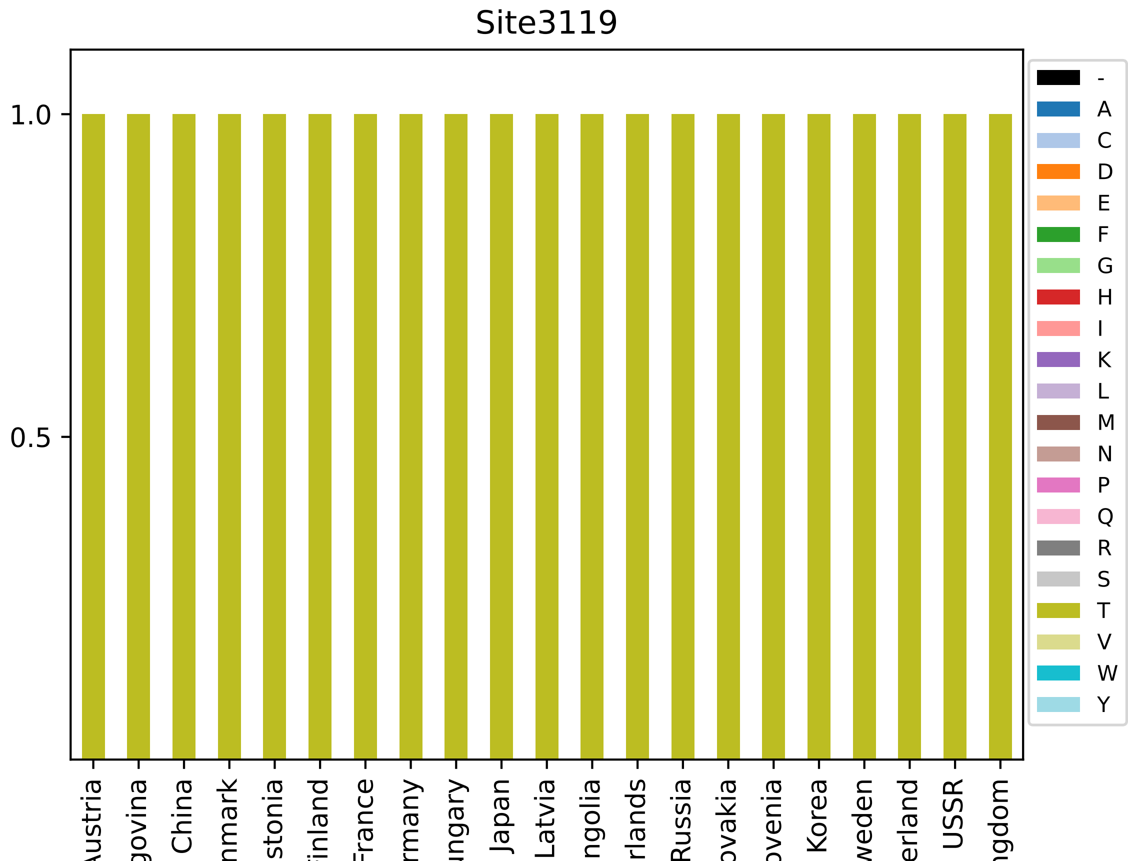

Site3119
1.0
0.5
-
A
C
D
E
F
G
H
I
K
L
M
N
P
Q
R
S
T
V
W
Y
Austria
govina
China
nmark
stonia
inland
France
rmany
ungary
Japan
Latvia
ngolia
rlands
Russia
ovakia
ovenia
Korea
weden
erland
USSR
ngdom

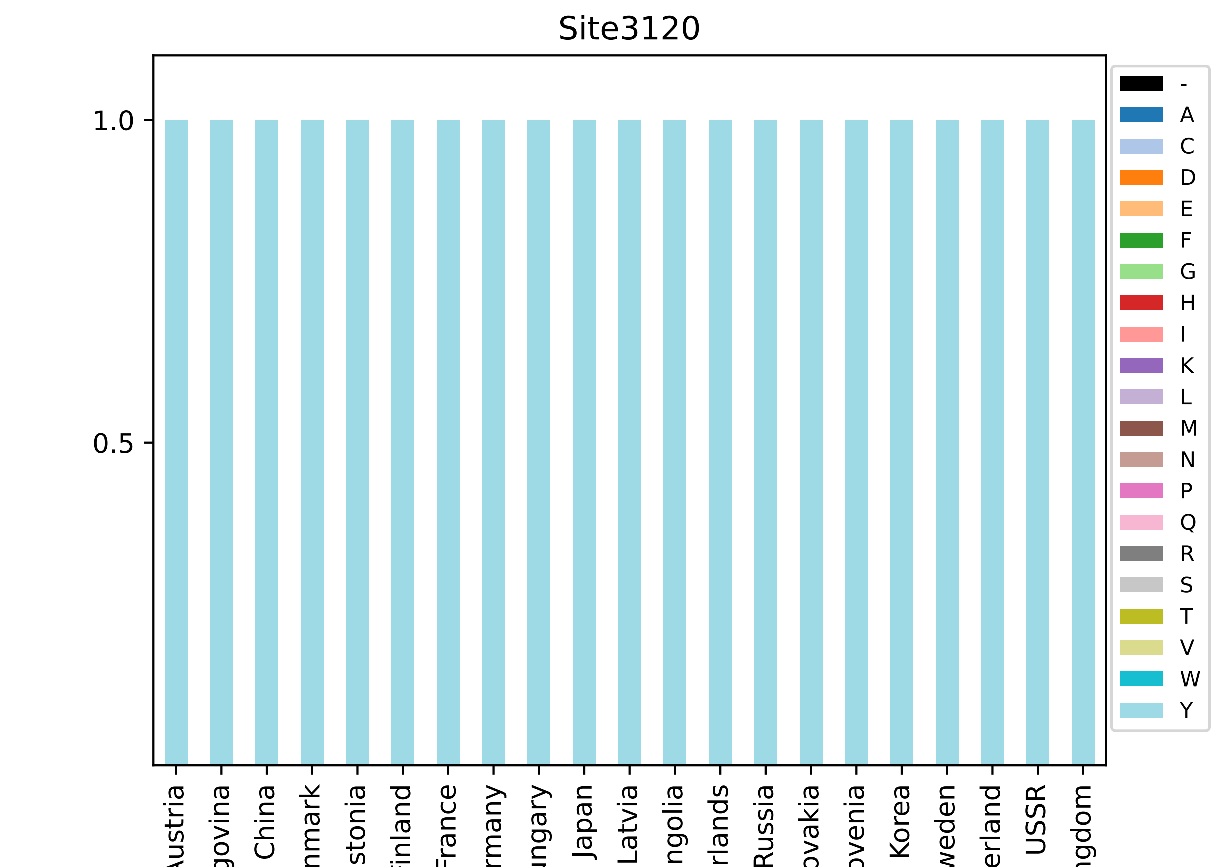

Site3120
1.0
0.5
Austria
govina
China
nmark
stonia
inland
France
rmany
ungary
Japan
Latvia
ngolia
rlands
Russia
ovakia
ovenia
Korea
weden
erland
USSR
ngdom
-
A
C
D
E
F
G
H
I
K
L
M
N
P
Q
R
S
T
V
W
Y

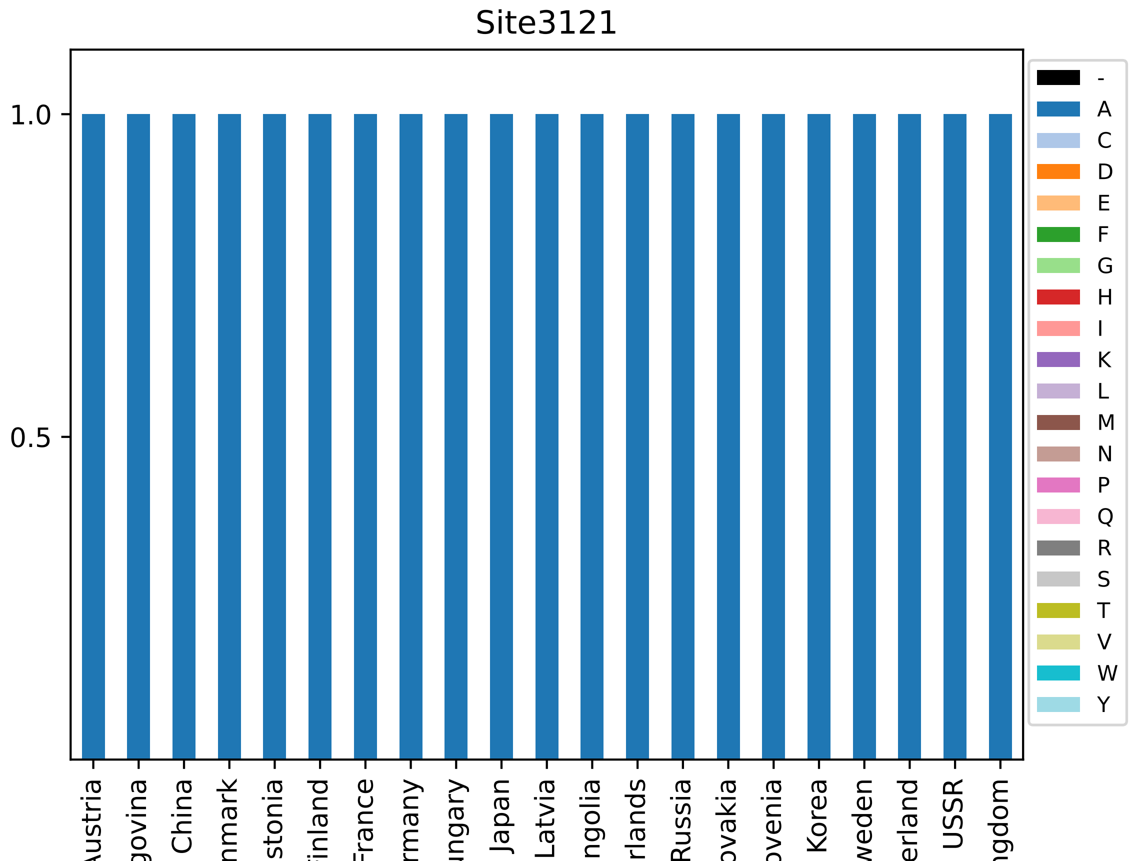
Site3121
1.0
0.5
-
A
C
D
E
F
G
H
I
K
L
M
N
P
Q
R
S
T
V
W
Y
Austria
govina
China
nmark
stonia
inland
France
rmany
ungary
Japan
Latvia
ngolia
rlands
Russia
ovakia
ovenia
Korea
weden
erland
USSR
ngdom

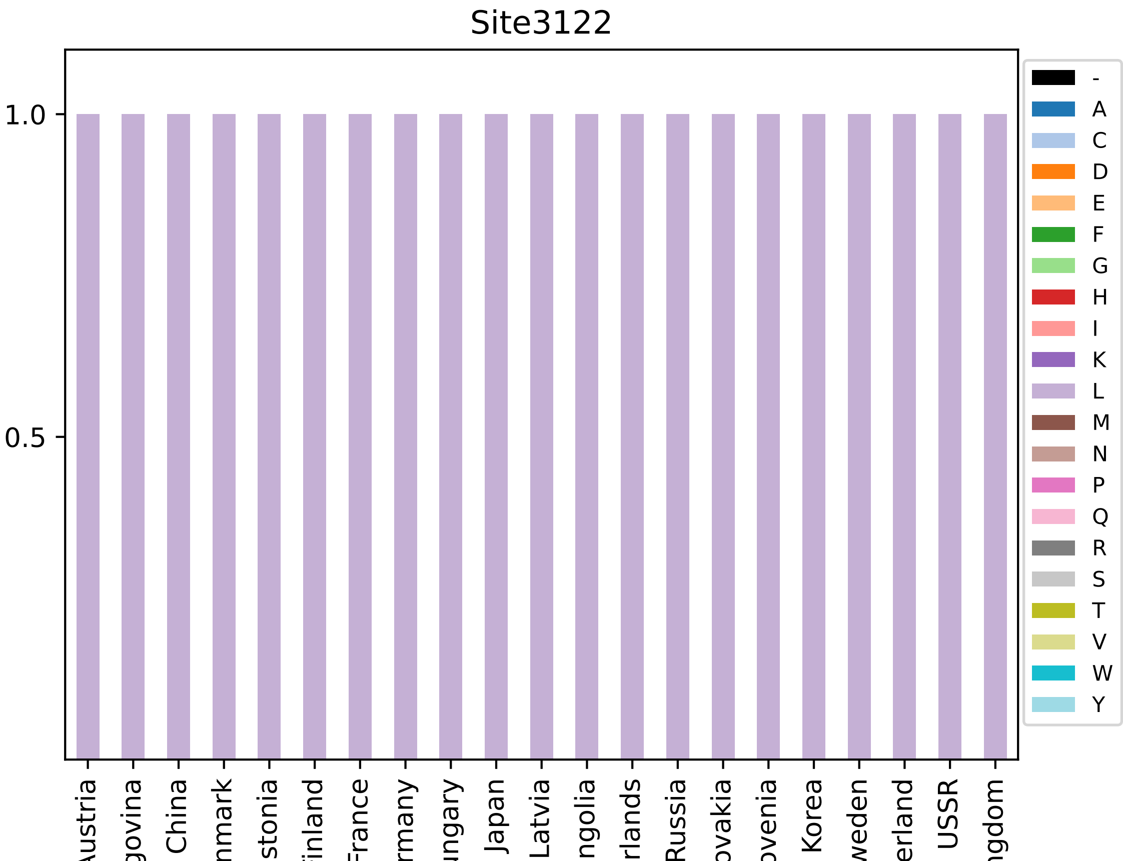

Site3122
1.0
0.5
-
A
C
D
E
F
G
H
I
K
L
M
N
P
Q
R
S
T
V
W
Y
China
Japan
Latvia
Russia
Korea
USSR

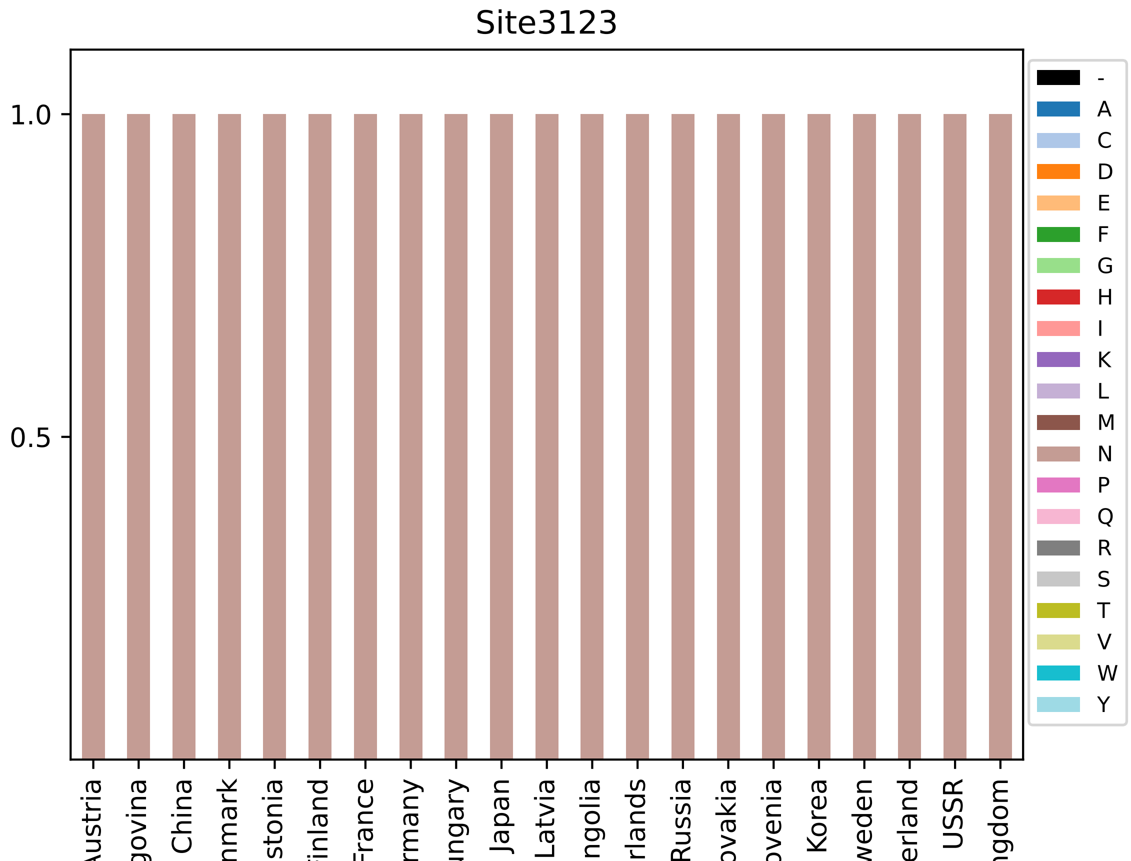

Site3123
1.0
0.5
-
A
C
D
E
F
G
H
I
K
L
M
N
P
Q
R
S
T
V
W
Y
China
Japan
Latvia
Russia
Korea
USSR

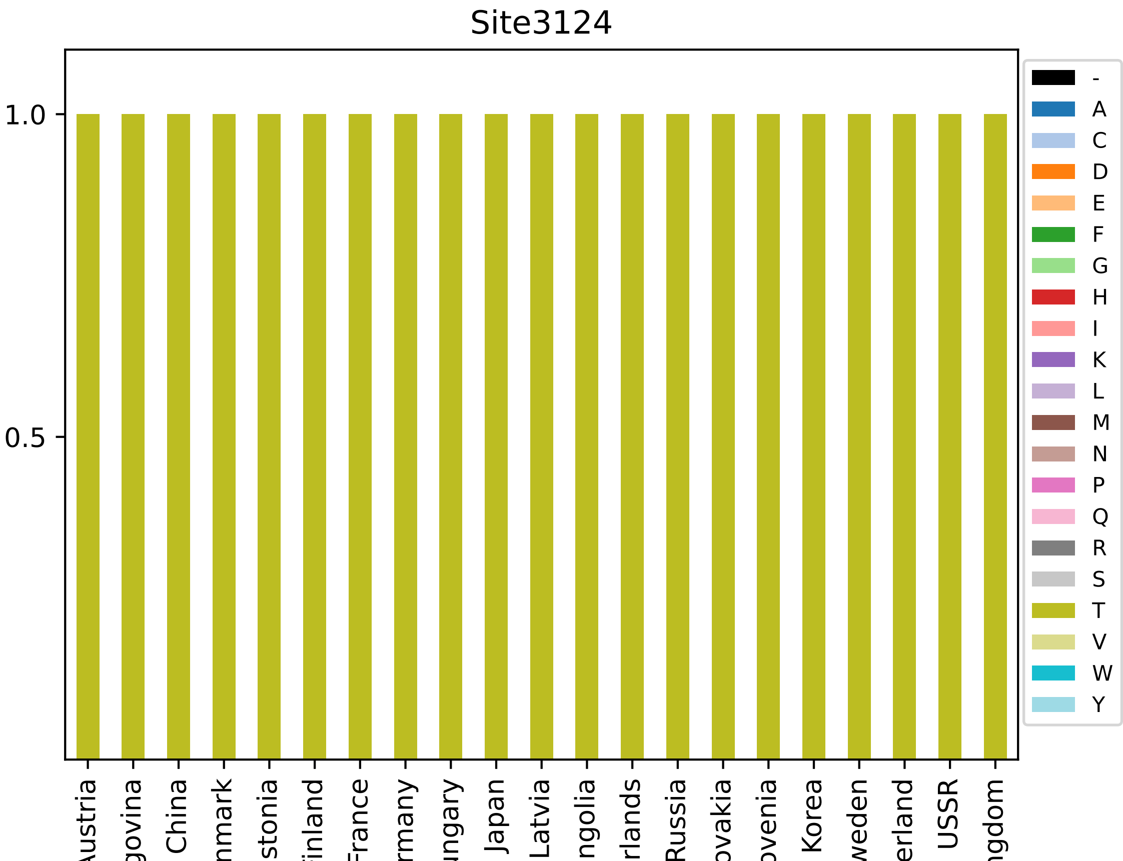
Site3124
1.0
0.5
China
Japan
Latvia
Russia
Korea
USSR
-
A
C
D
E
F
G
H
I
K
L
M
N
P
Q
R
S
T
V
W
Y

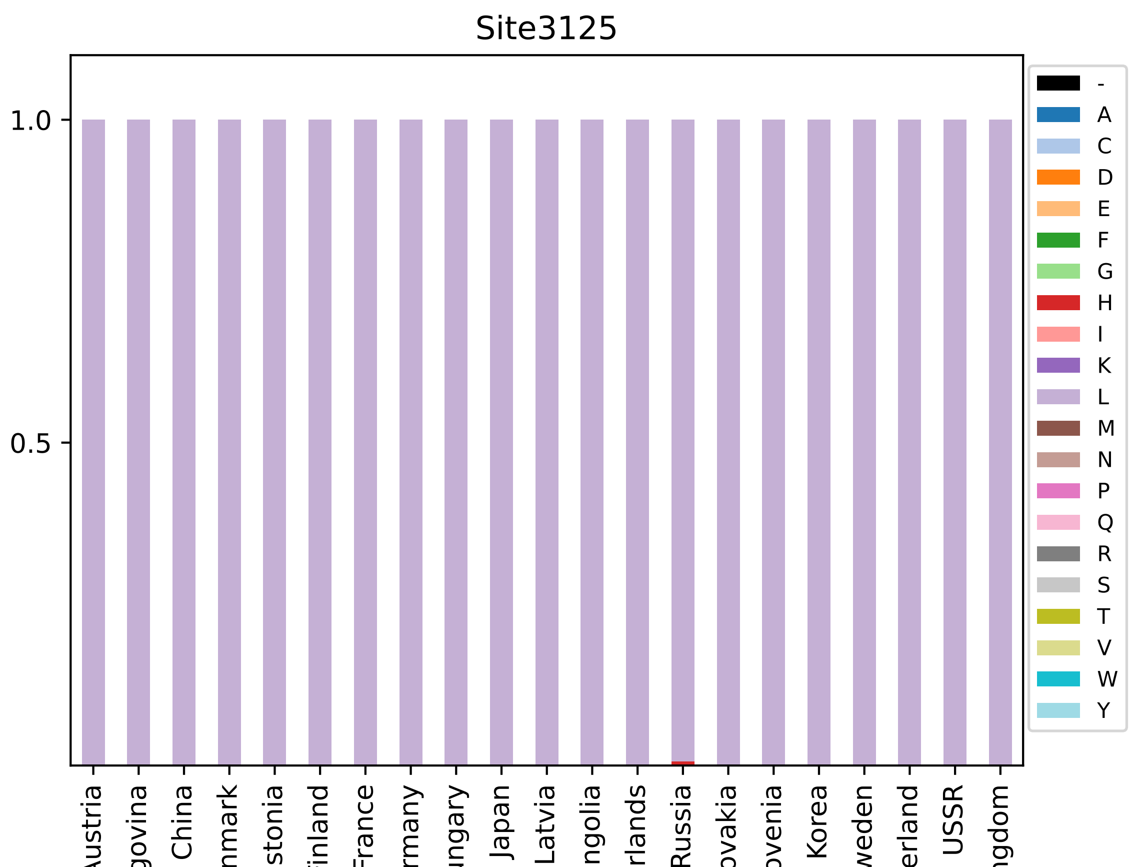

Site3125
1.0
0.5
-
A
C
D
E
F
G
H
I
K
L
M
N
P
Q
R
S
T
V
W
Y
Austria
govina
China
nmark
stonia
inland
France
rmany
ungary
Japan
Latvia
ngolia
rlands
Russia
ovakia
ovenia
Korea
weden
erland
USSR
ngdom

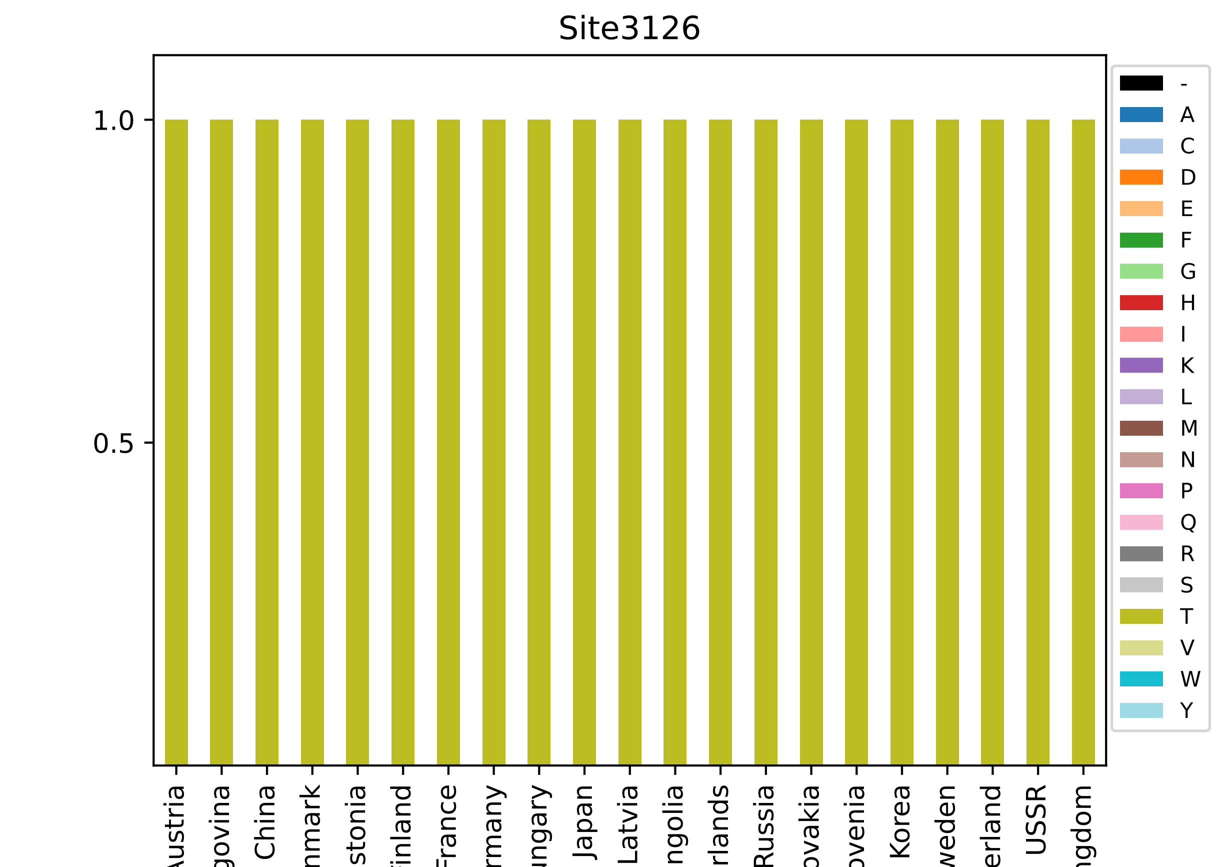

Site3126
1.0
0.5
-
A
C
D
E
F
G
H
I
K
L
M
N
P
Q
R
S
T
V
W
Y
Austria
govina
China
nmark
stonia
Finland
France
rmany
ungary
Japan
Latvia
ngolia
rlands
Russia
ovakia
ovenia
Korea
weden
erland
USSR
ngdom

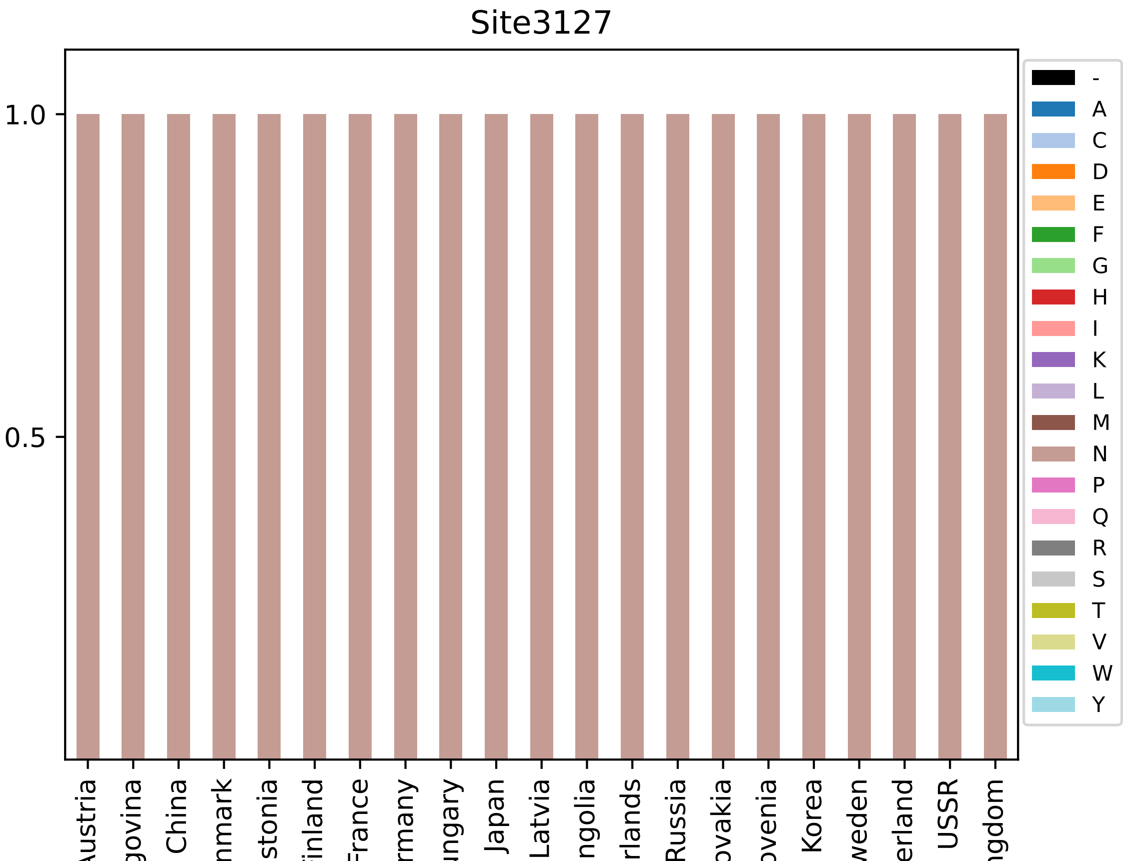
Site3127
1.0
0.5
-
A
C
D
E
F
G
H
I
K
L
M
N
P
Q
R
S
T
V
W
Y
Austria
China
Japan
Latvia
Russia
Korea
USSR

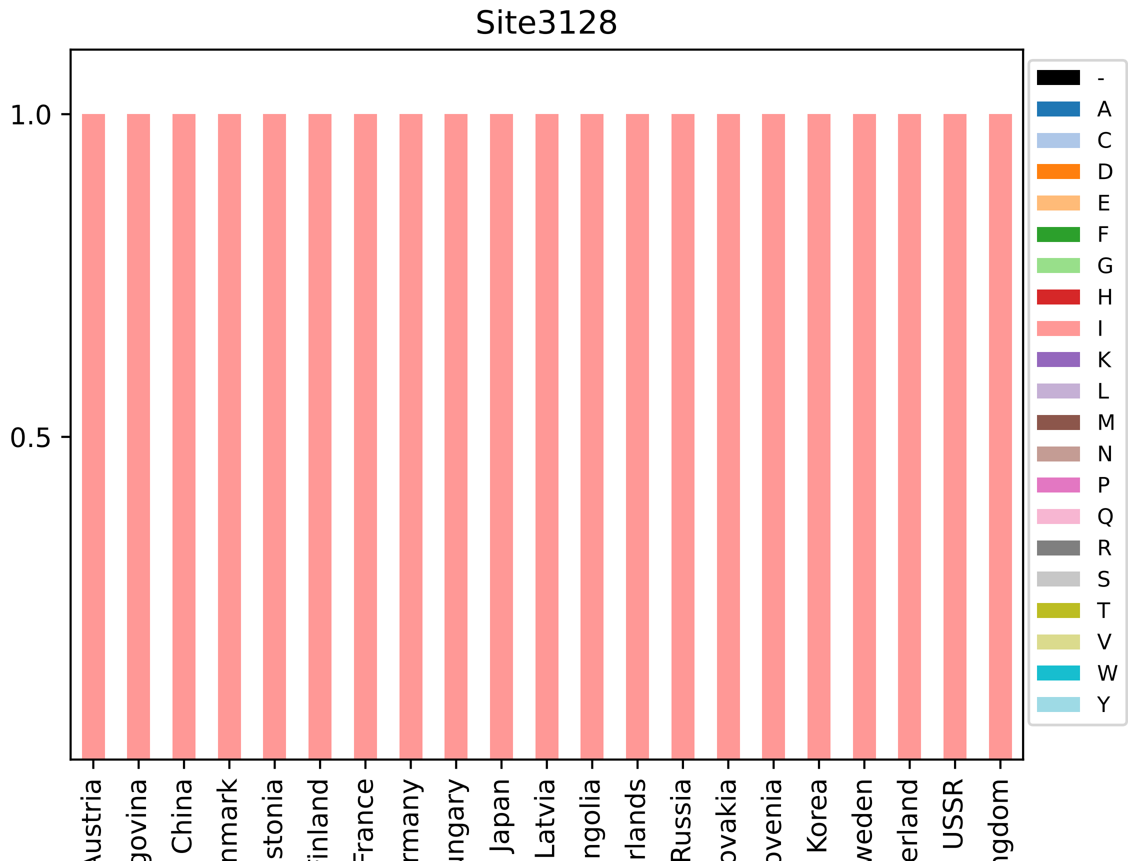

Site3128
1.0
0.5
-
A
C
D
E
F
G
H
I
K
L
M
N
P
Q
R
S
T
V
W
Y
Austria
govina
China
nmark
stonia
Finland
France
rmany
ungary
Japan
Latvia
ngolia
rlands
Russia
ovakia
ovenia
Korea
weden
erland
USSR
ngdom

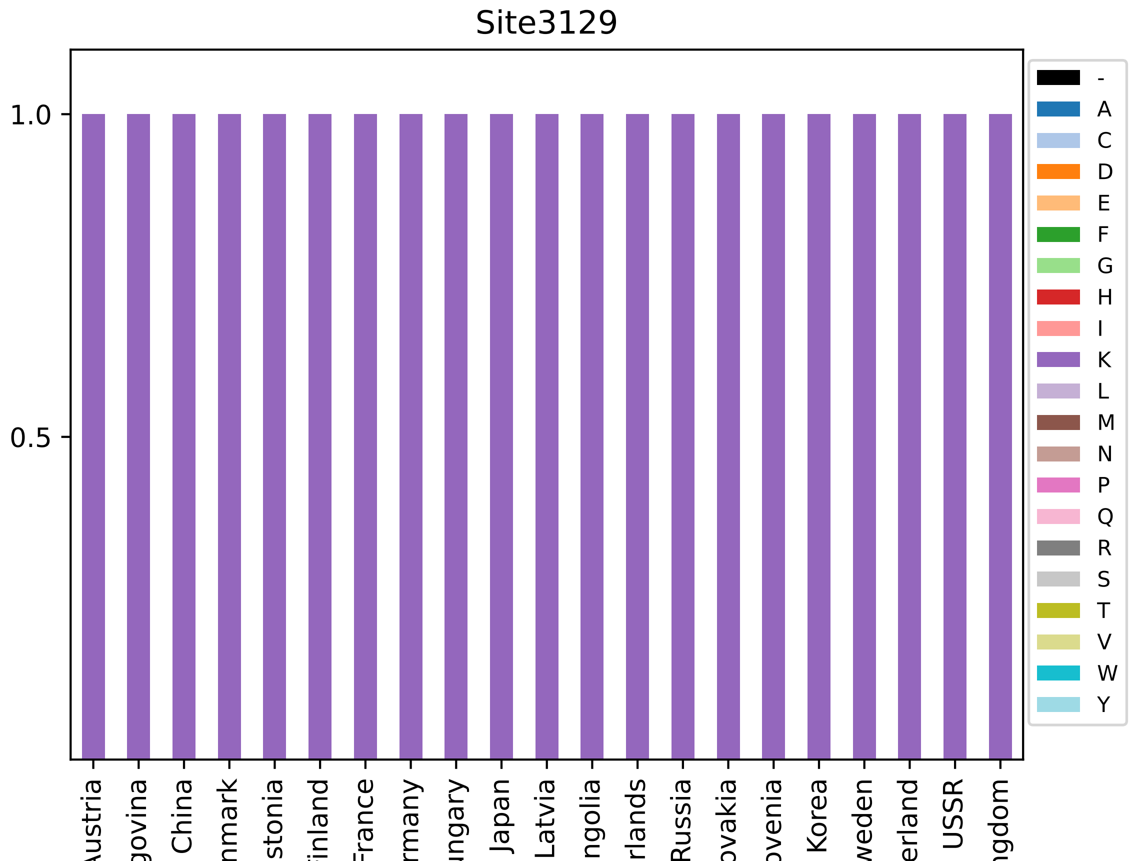
Site3129
1.0
0.5
-
A
C
D
E
F
G
H
I
K
L
M
N
P
Q
R
S
T
V
W
Y
Austria
China
Japan
Latvia
Russia
Korea
USSR

Site3130

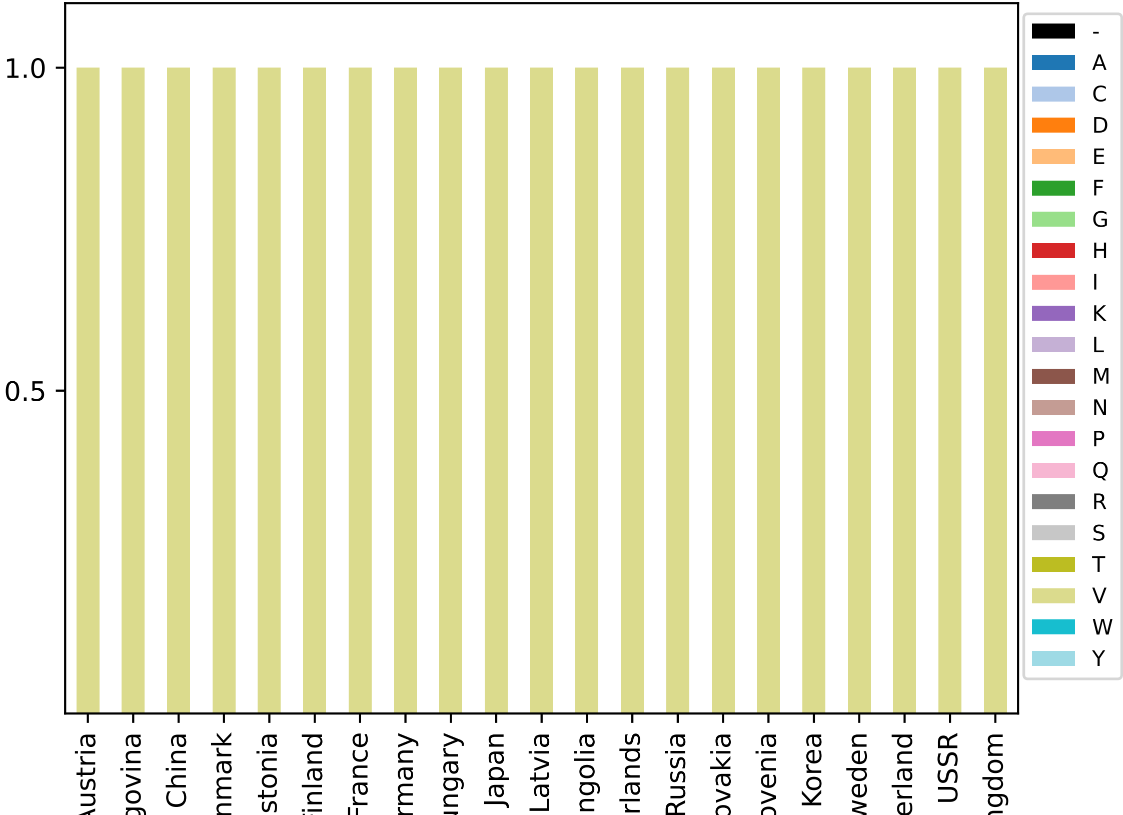

1.0
0.5
-
A
C
D
E
F
G
H
I
K
L
M
N
P
Q
R
S
T
V
W
Y
China
Japan
Latvia
Russia
Korea
USSR

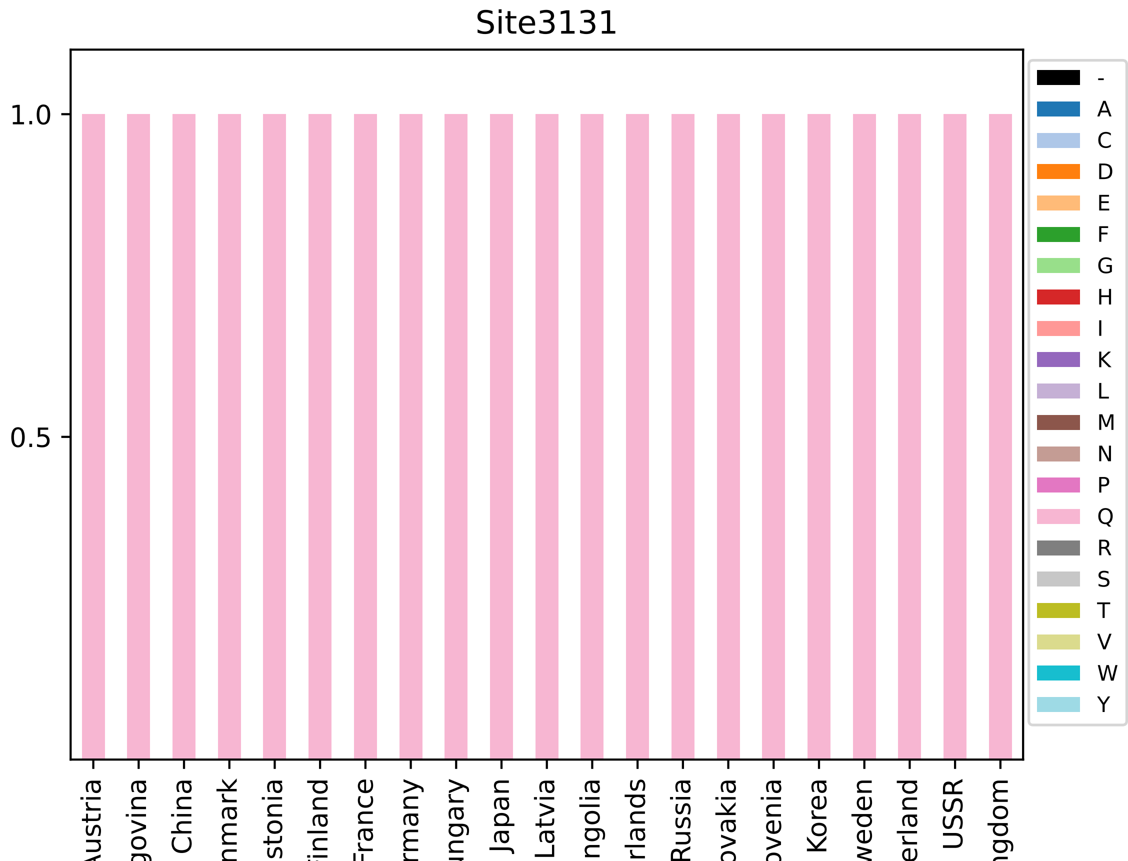

Site3131
1.0
0.5
-
A
C
D
E
F
G
H
I
K
L
M
N
P
Q
R
S
T
V
W
Y
Austria
govina
China
nmark
stonia
Finland
France
rmany
ungary
Japan
Latvia
ngolia
rlands
Russia
ovakia
ovenia
Korea
weden
erland
USSR
ngdom

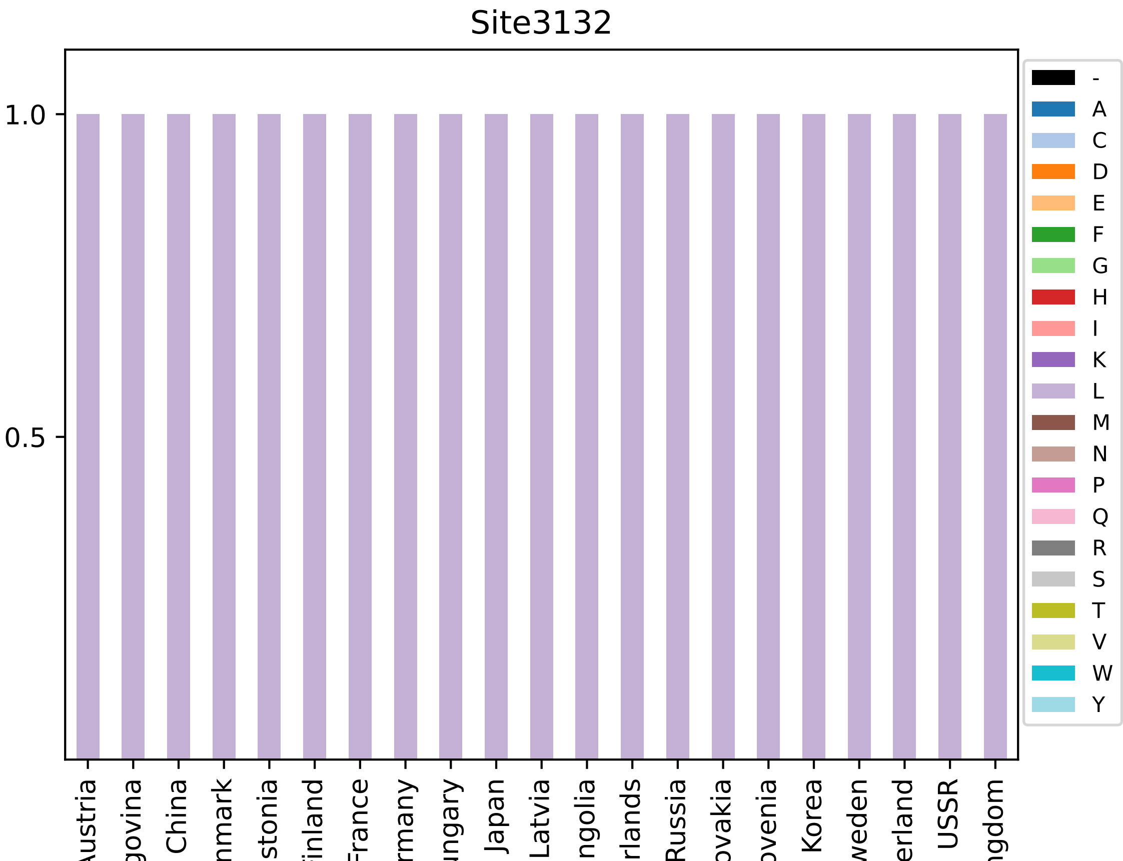
Site3132
1.0
0.5
-
A
C
D
E
F
G
H
I
K
L
M
N
P
Q
R
S
T
V
W
Y
China
Japan
Latvia
Russia
Korea
USSR

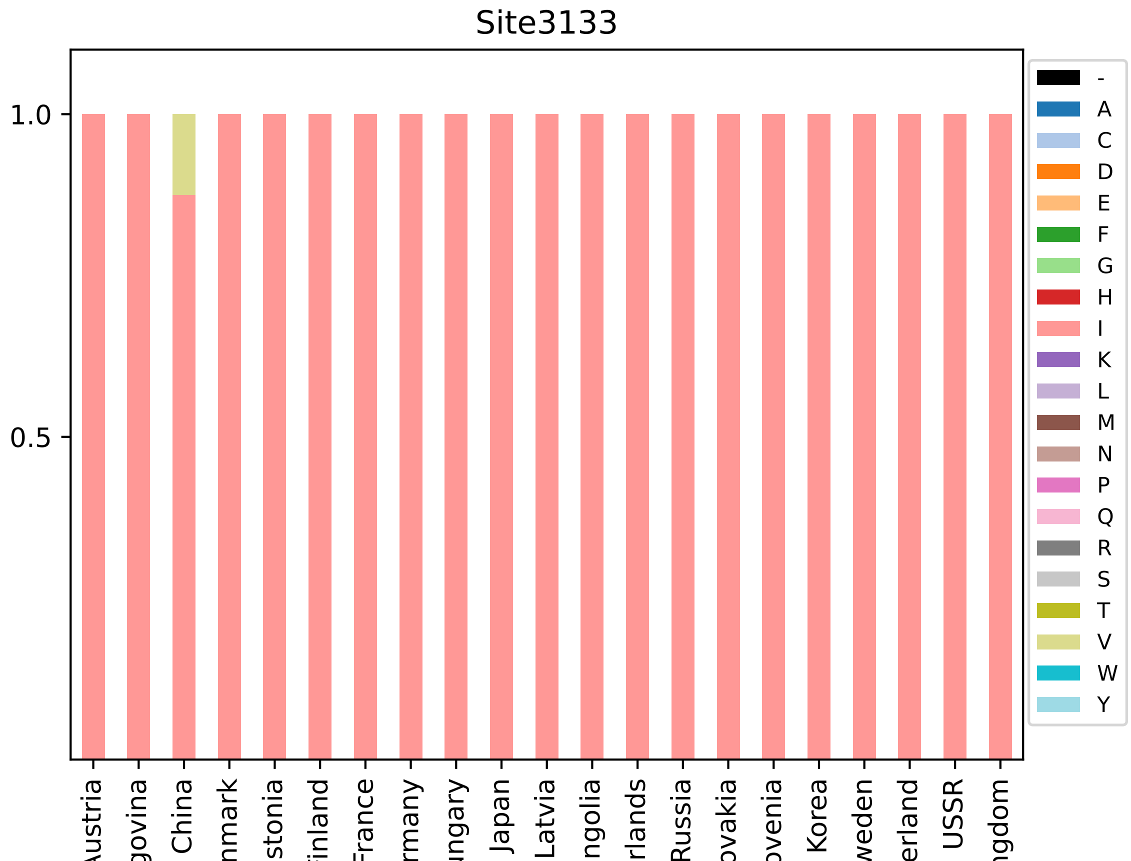
Site3133
1.0
0.5
-
A
C
D
E
F
G
H
I
K
L
M
N
P
Q
R
S
T
V
W
Y
China
Japan
Latvia
Russia
Korea
USSR

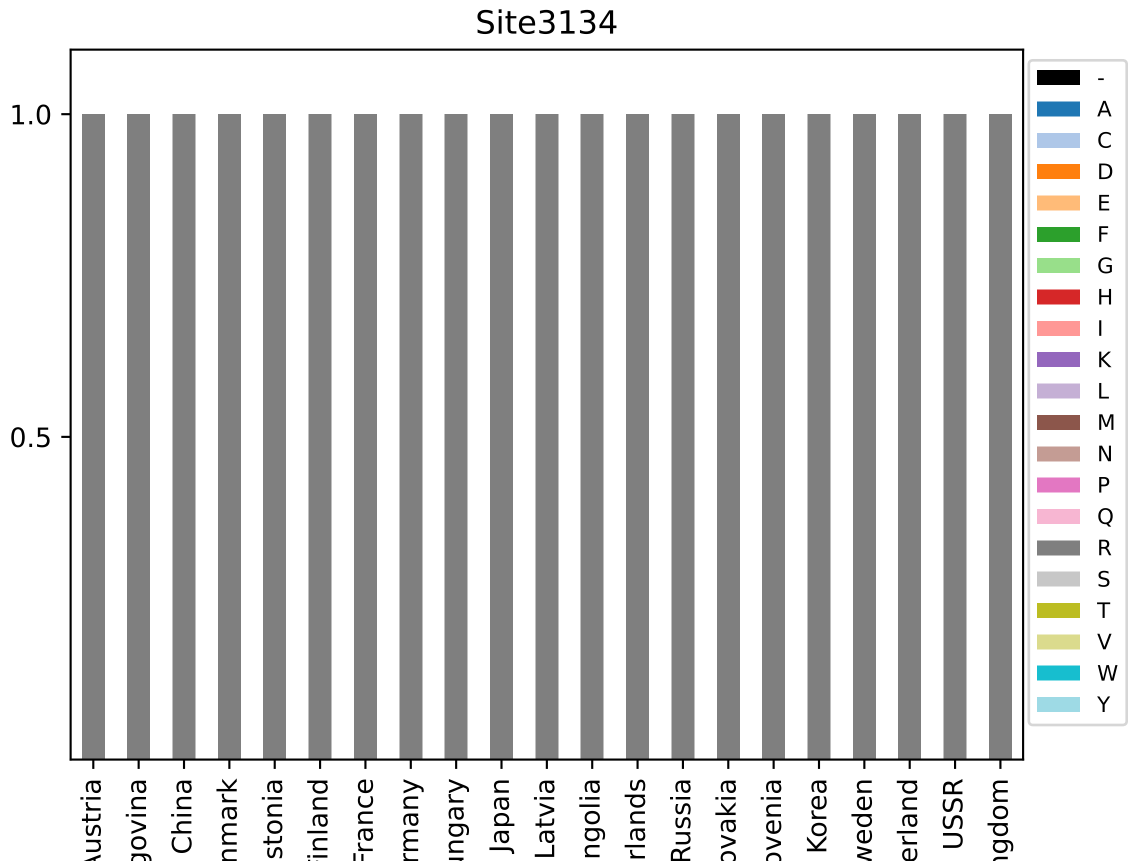
Site3134
1.0
0.5
-
A
C
D
E
F
G
H
I
K
L
M
N
P
Q
R
S
T
V
W
Y
Austria
govina
China
nmark
stonia
Finland
France
rmany
ungary
Japan
Latvia
ngolia
rlands
Russia
ovakia
ovenia
Korea
weden
erland
USSR
ngdom

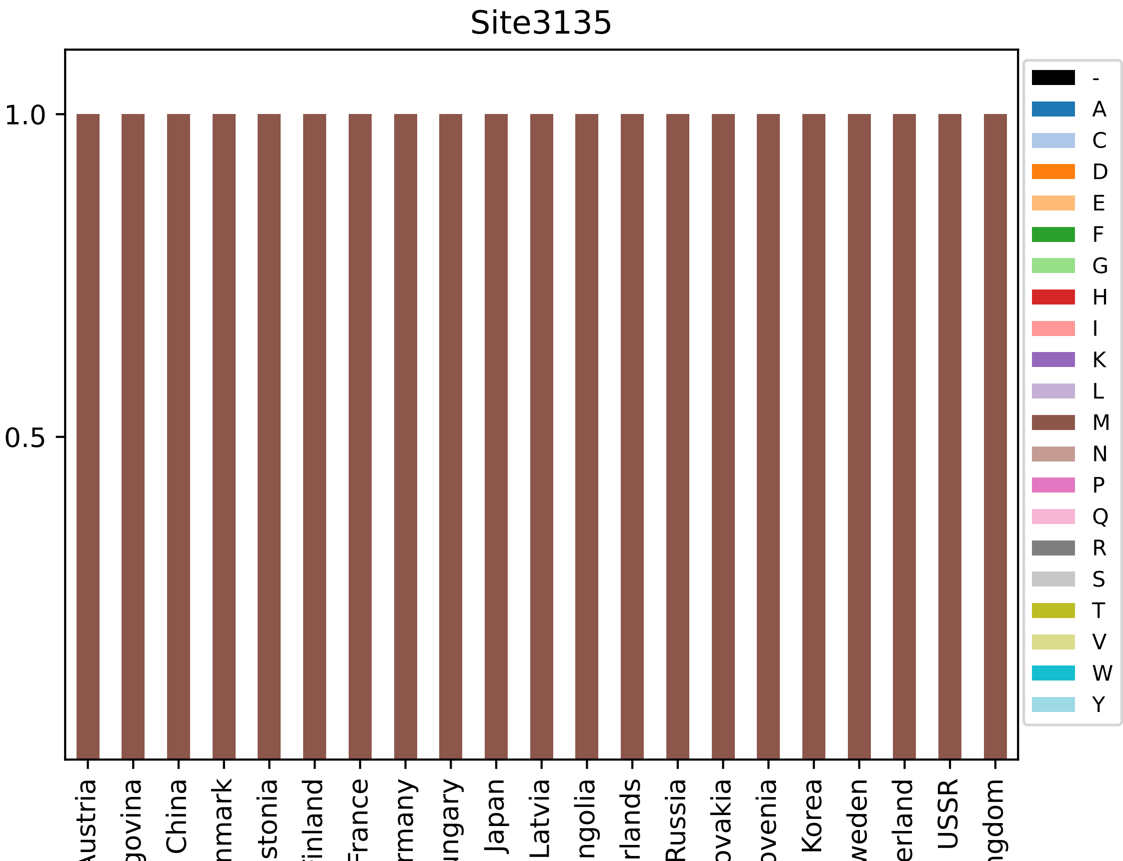
Site3135
1.0
0.5
-
A
C
D
E
F
G
H
I
K
L
M
N
P
Q
R
S
T
V
W
Y
China
Japan
Latvia
Russia
Korea
USSR

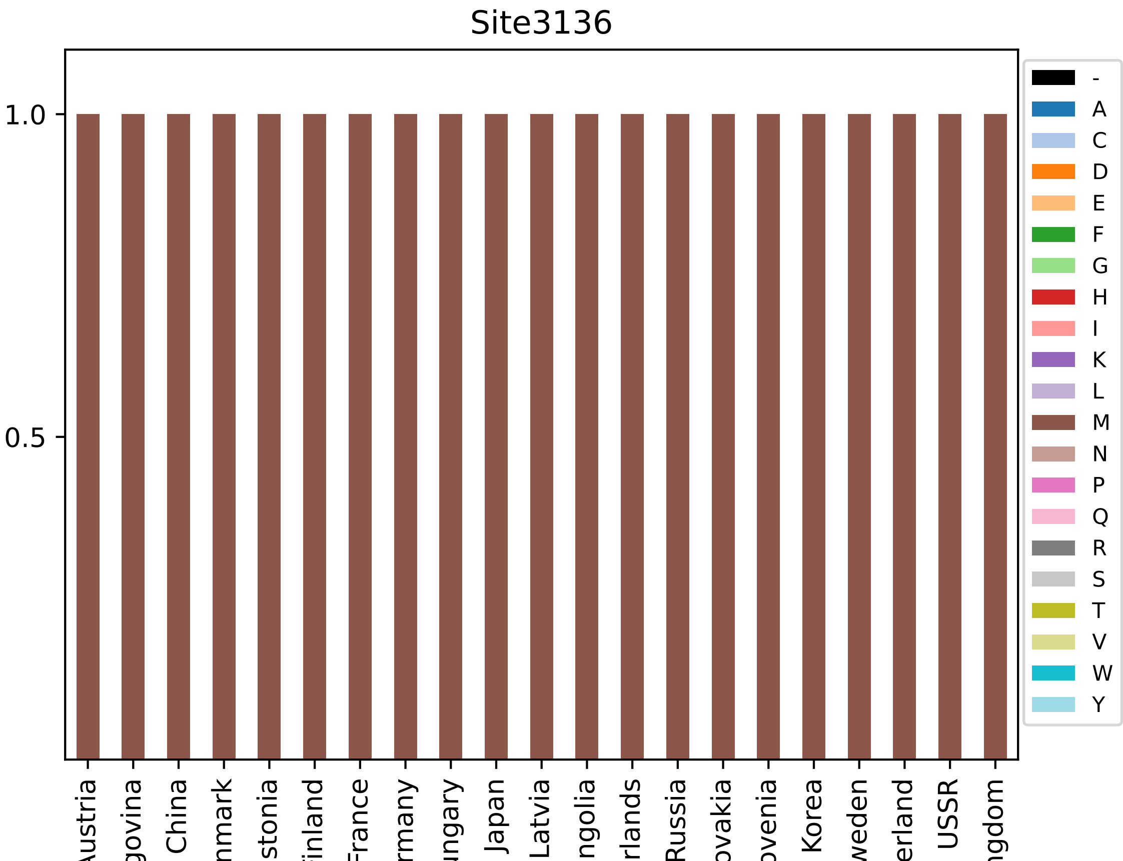
Site3136
1.0
0.5
-
A
C
D
E
F
G
H
I
K
L
M
N
P
Q
R
S
T
V
W
Y
Austria
govina
China
nmark
stonia
inland
France
rmany
ungary
Japan
Latvia
ngolia
rlands
Russia
ovakia
ovenia
Korea
weden
erland
USSR
ngdom

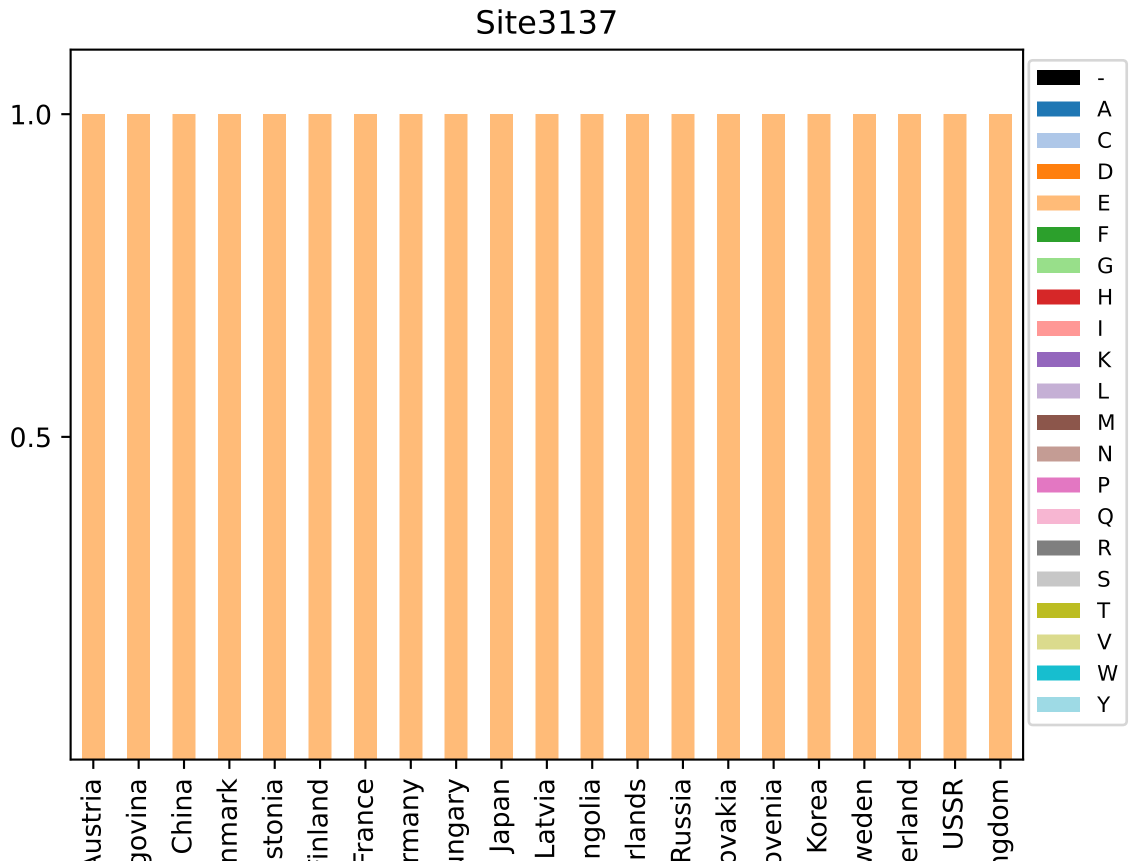

Site3137
1.0
0.5
-
A
C
D
E
F
G
H
I
K
L
M
N
P
Q
R
S
T
V
W
Y
Austria
govina
China
nmark
stonia
Finland
France
rmany
ungary
Japan
Latvia
ngolia
rlands
Russia
ovakia
ovenia
Korea
weden
erland
USSR
ngdom

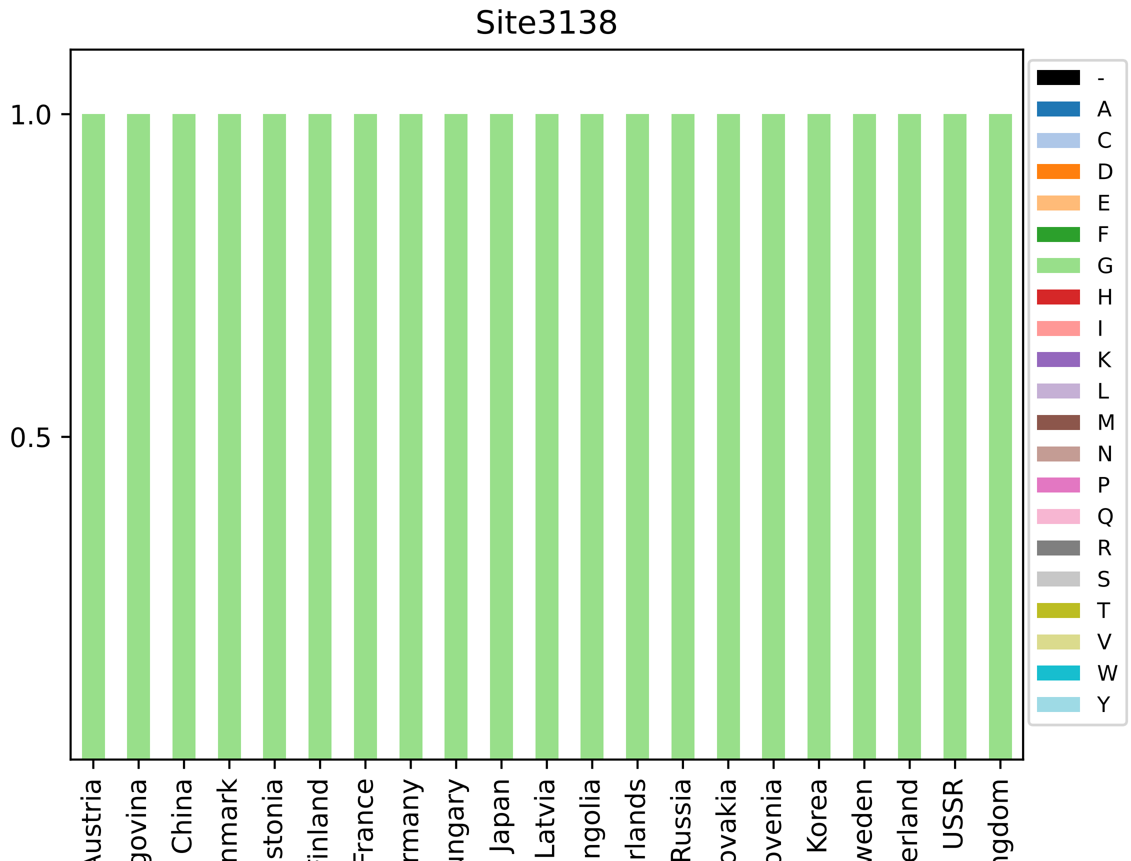

Site3138
1.0
0.5
-
A
C
D
E
F
G
H
I
K
L
M
N
P
Q
R
S
T
V
W
Y
Austria
China
Japan
Latvia
Russia
Korea
USSR

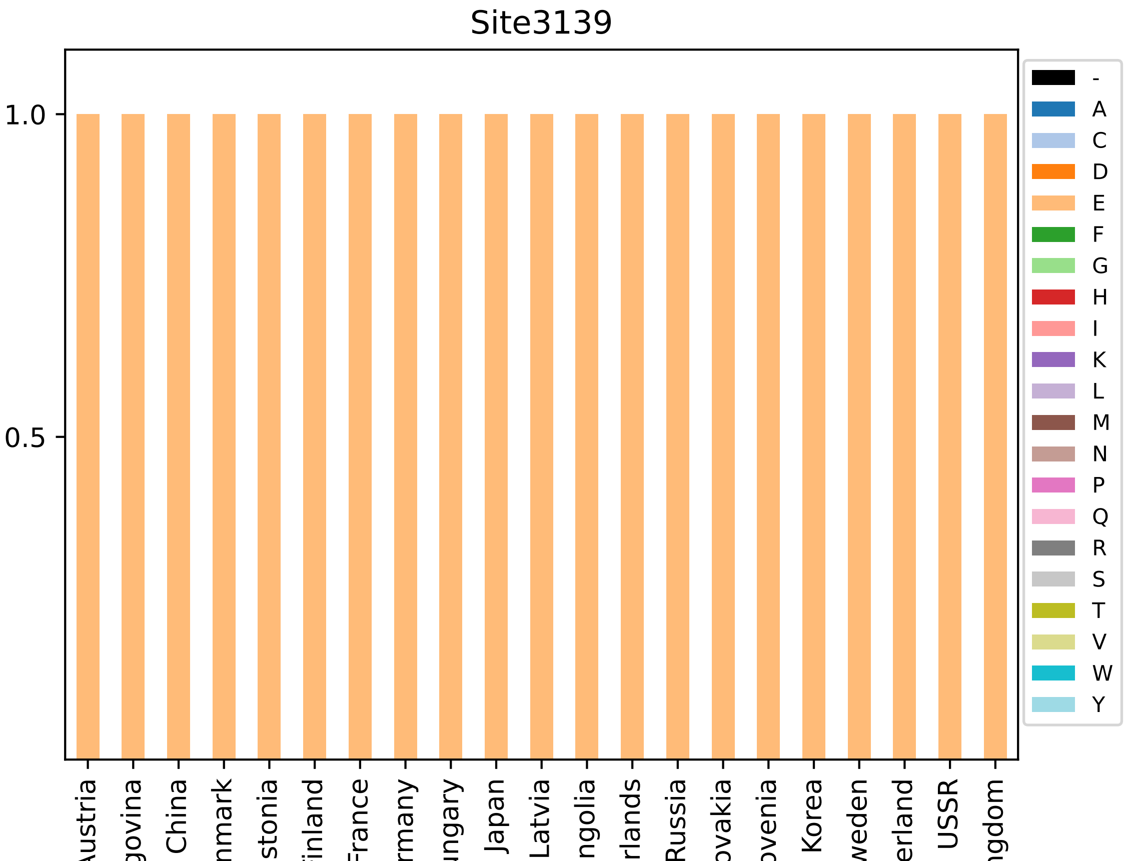
Site3139
1.0
0.5
China
Japan
Latvia
Russia
Korea
USSR
-
A
C
D
E
F
G
H
I
K
L
M
N
P
Q
R
S
T
V
W
Y

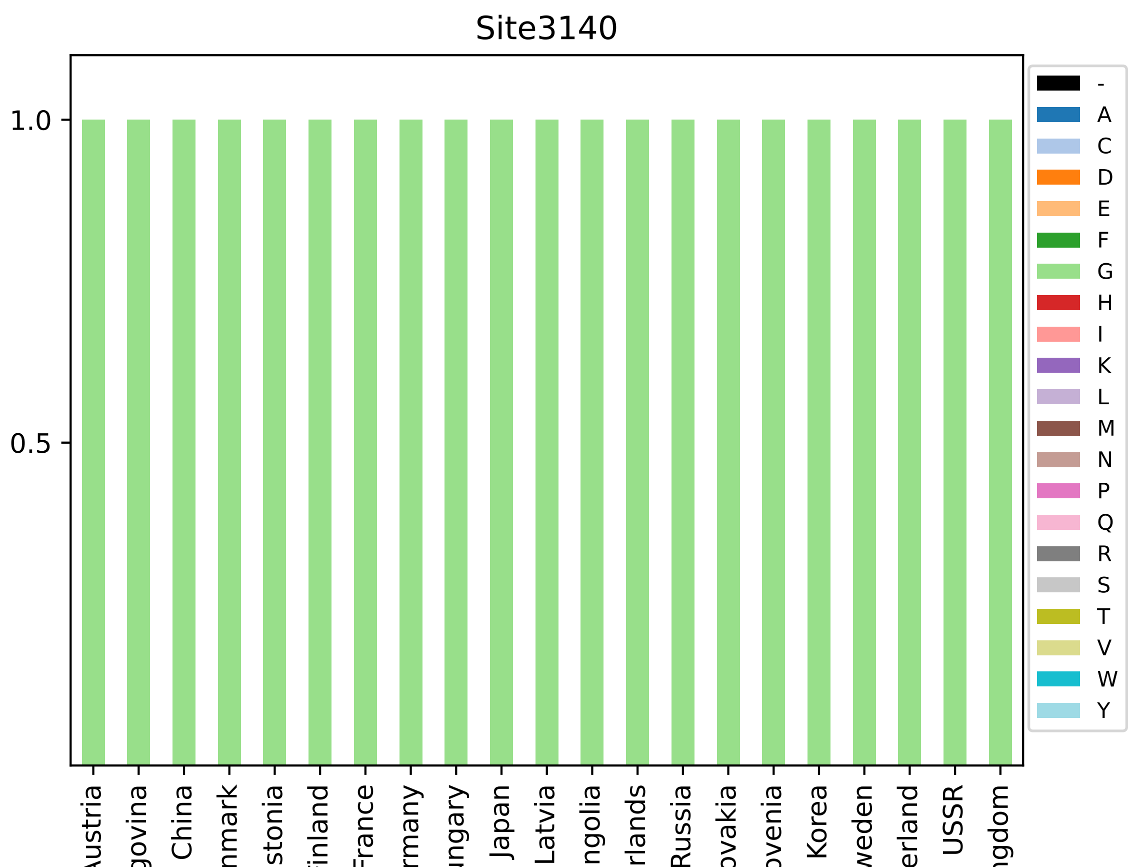
Site3140
1.0
0.5
-
A
C
D
E
F
G
H
I
K
L
M
N
P
Q
R
S
T
V
W
Y
Austria
govina
China
nmark
stonia
inland
France
rmany
ungary
Japan
Latvia
ngolia
rlands
Russia
ovakia
ovenia
Korea
weden
erland
USSR
ngdom

Site3141

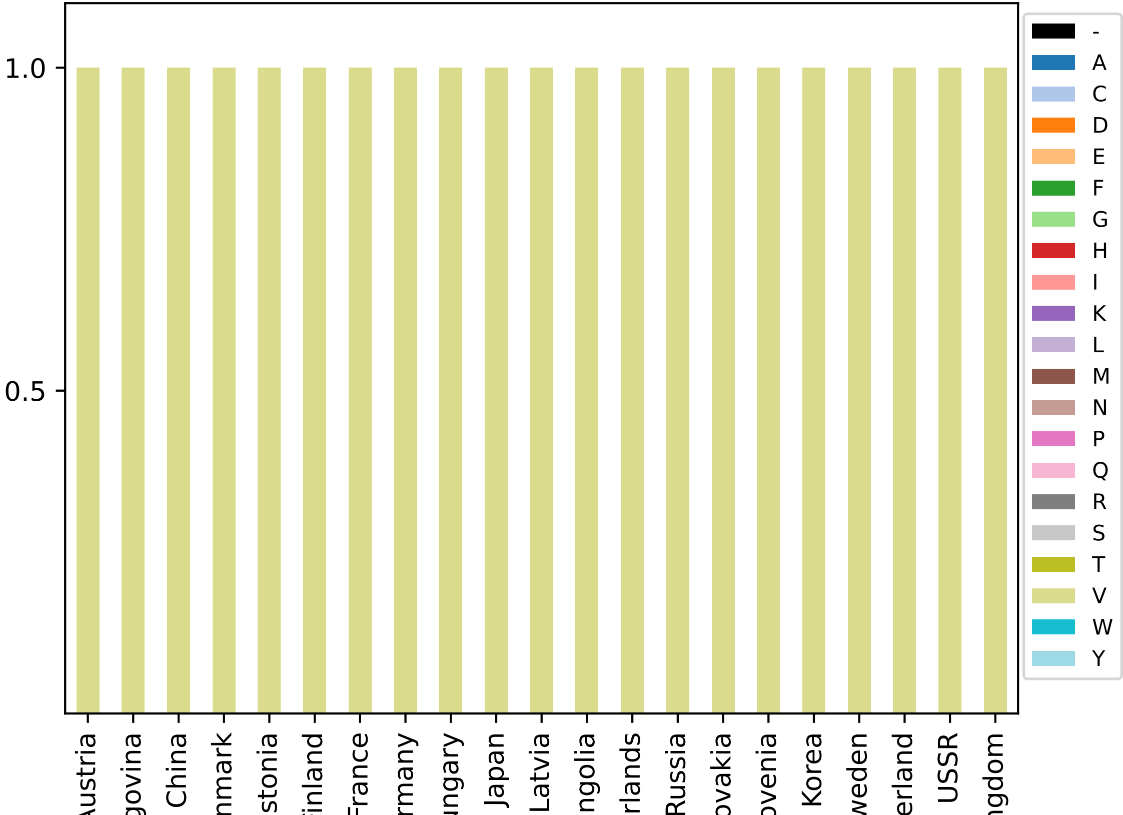

1.0
0.5
-
A
C
D
E
F
G
H
I
K
L
M
N
P
Q
R
S
T
V
W
Y
Austria
govina
China
nmark
stonia
Finland
France
rmany
ungary
Japan
Latvia
ngolia
rlands
Russia
ovakia
ovenia
Korea
weden
erland
USSR
ngdom

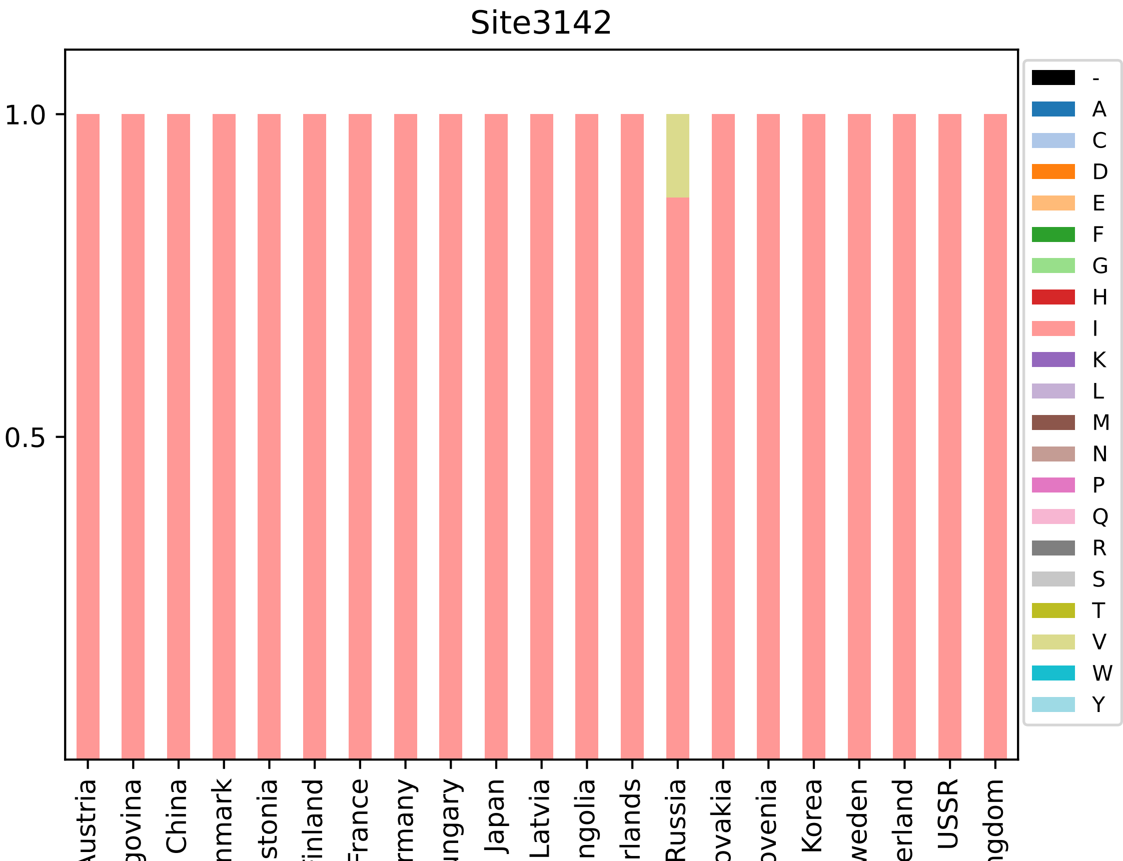
Site3142
1.0
0.5
-
A
C
D
E
F
G
H
I
K
L
M
N
P
Q
R
S
T
V
W
Y
China
Japan
Latvia
Russia
Korea
USSR

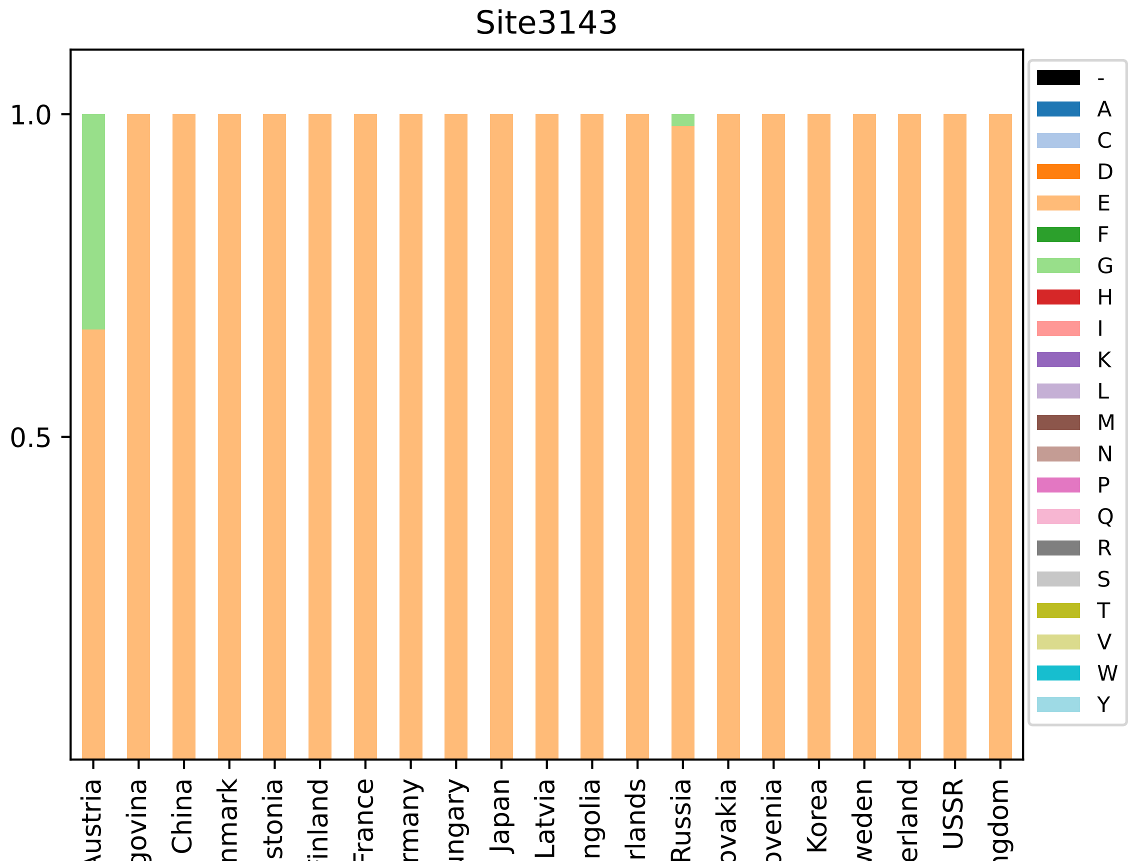

Site3143
1.0
0.5
-
A
C
D
E
F
G
H
I
K
L
M
N
P
Q
R
S
T
V
W
Y
China
Japan
Latvia
Russia
Korea
USSR

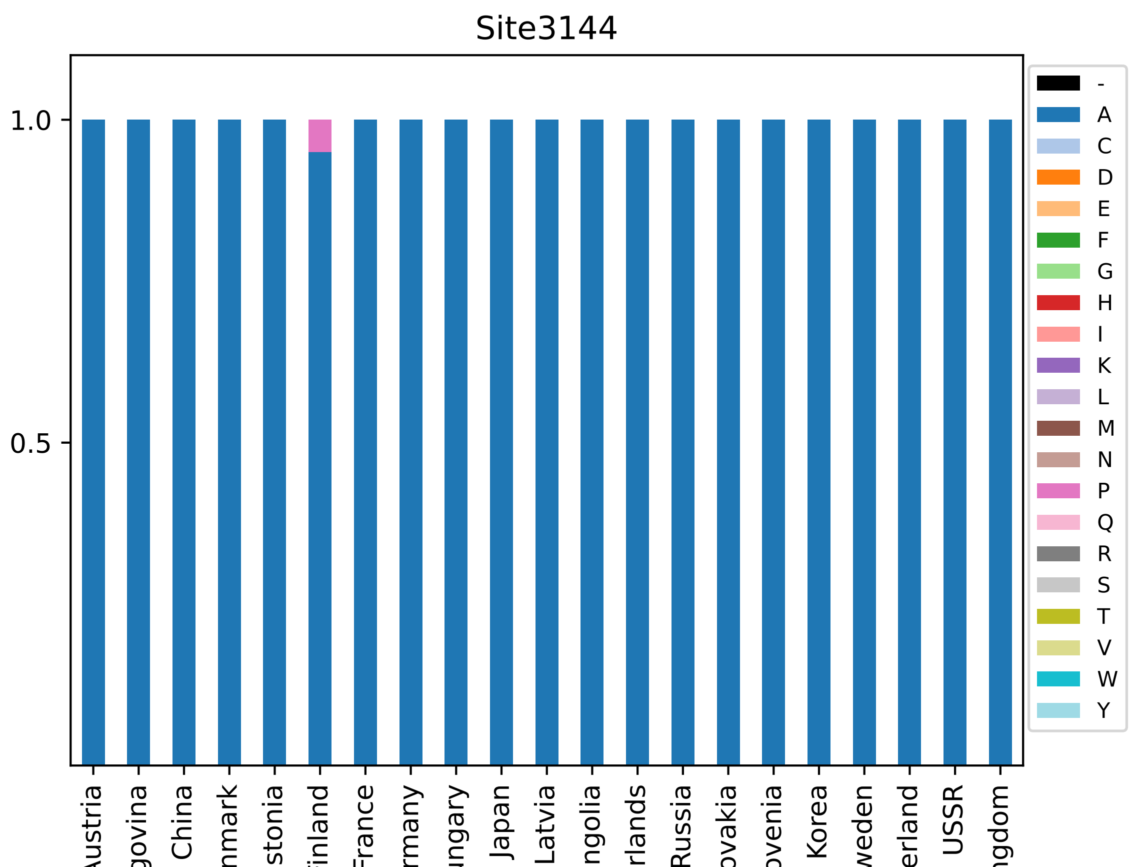

Site3144
1.0
0.5
-
A
C
D
E
F
G
H
I
K
L
M
N
P
Q
R
S
T
V
W
Y
Austria
govina
China
nmark
stonia
Finland
France
rmany
ungary
Japan
Latvia
ngolia
rlands
Russia
ovakia
ovenia
Korea
weden
erland
USSR
ngdom

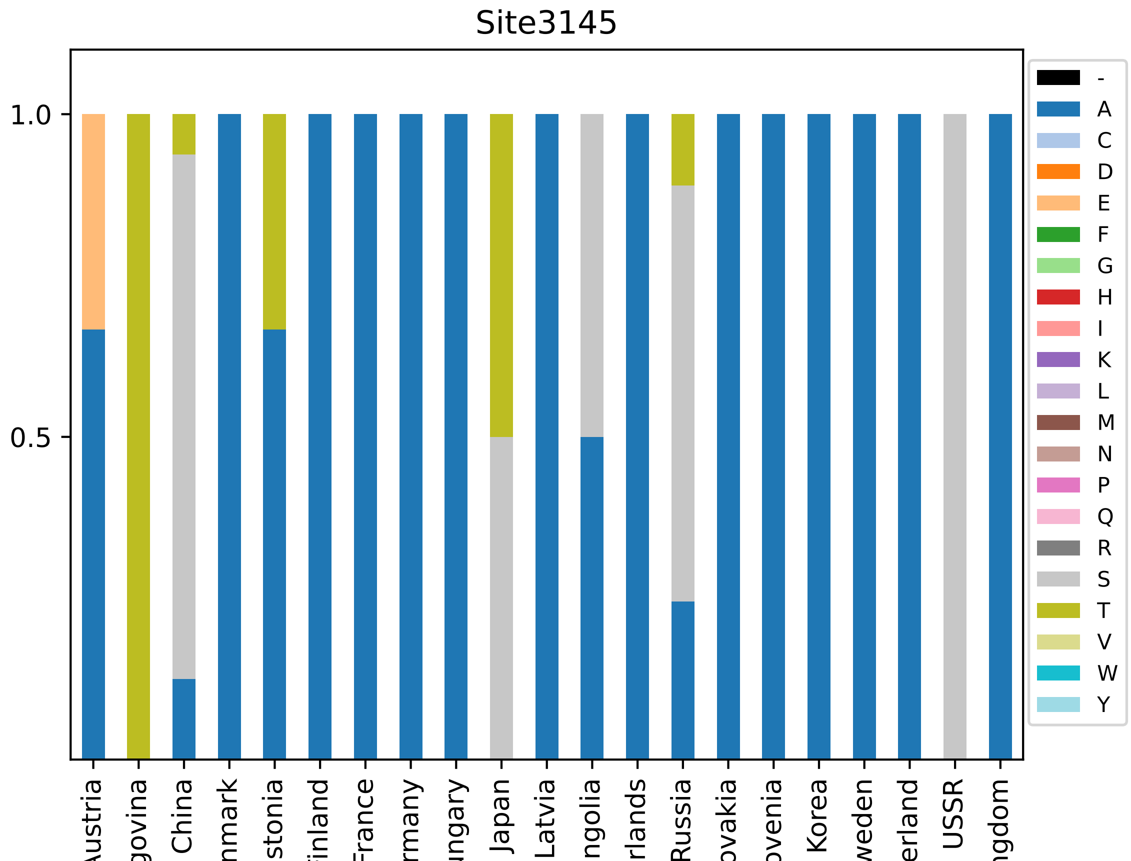

Site3145
1.0
0.5
-
A
C
D
E
F
G
H
I
K
L
M
N
P
Q
R
S
T
V
W
Y
Austria
govina
China
nmark
stonia
Finland
France
rmany
ungary
Japan
Latvia
ngolia
rlands
Russia
ovakia
ovenia
Korea
weden
erland
USSR
ngdom

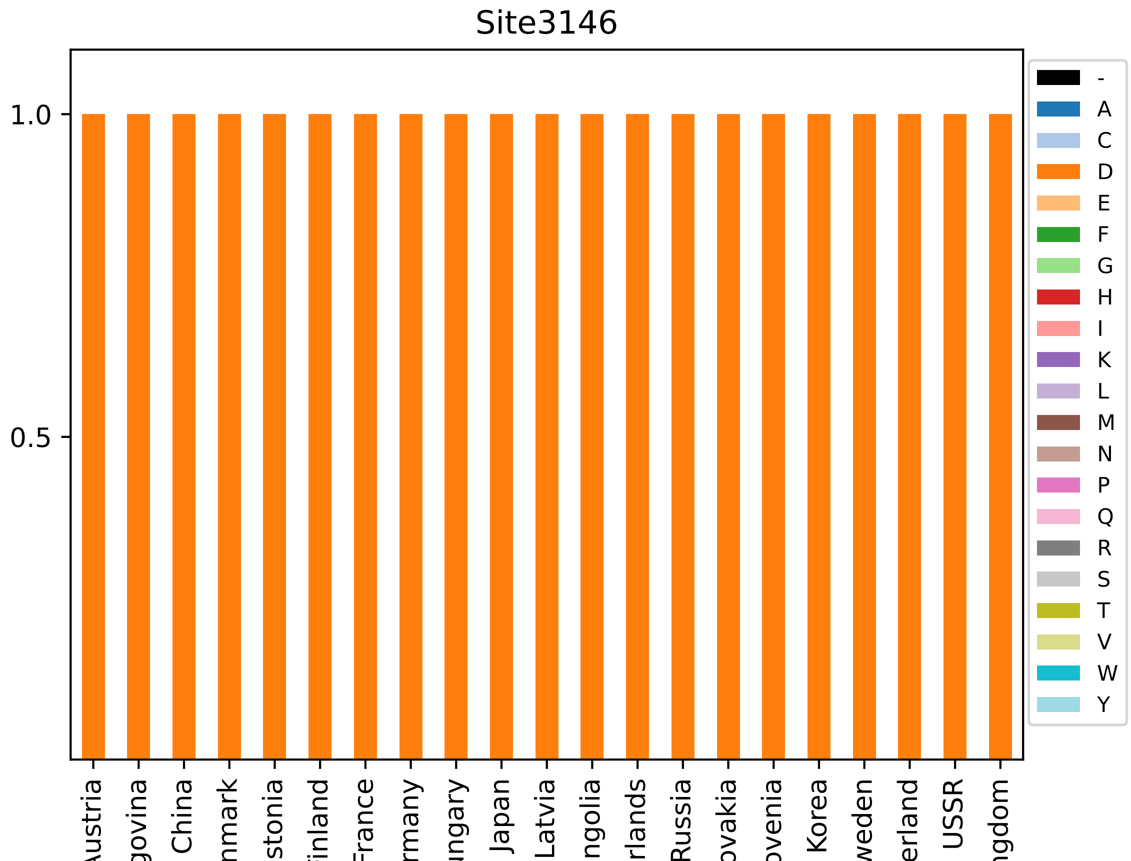
Site3146
1.0
0.5
-
A
C
D
E
F
G
H
I
K
L
M
N
P
Q
R
S
T
V
W
Y
China
Japan
Latvia
Russia
Korea
USSR

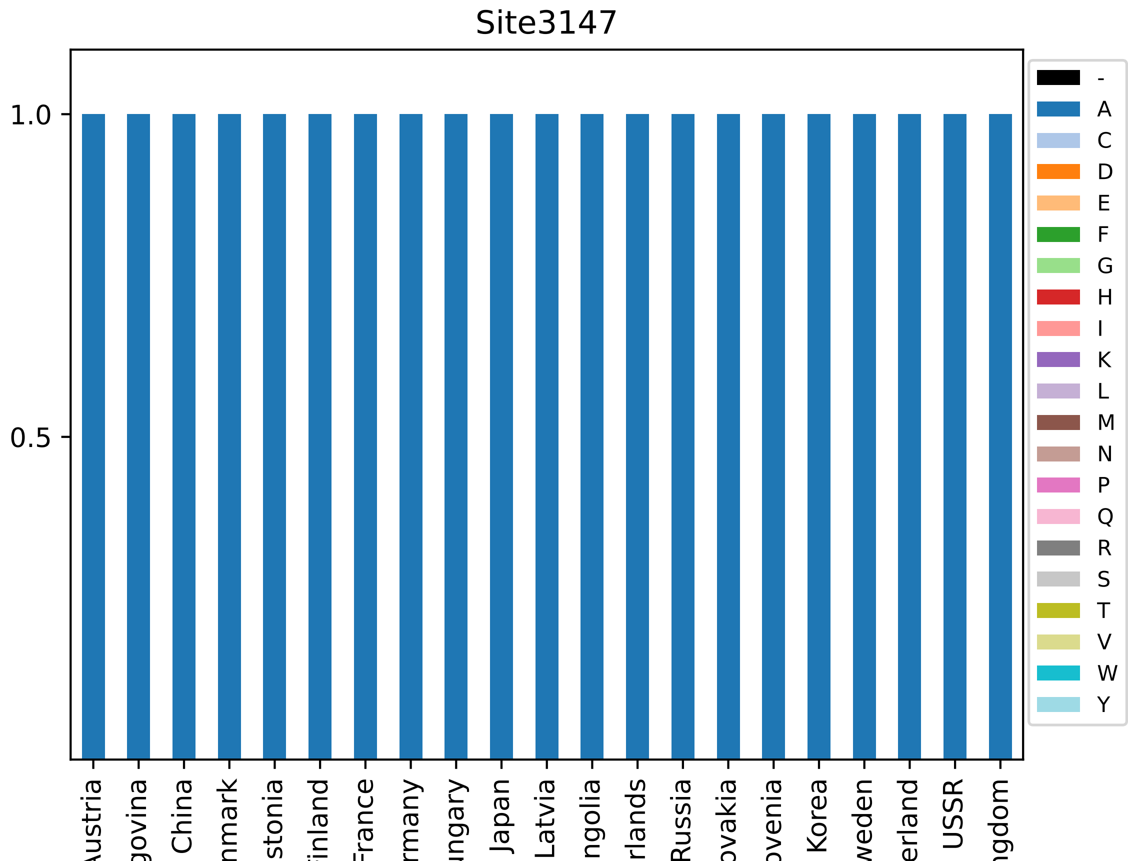

Site3147
1.0
0.5
-
A
C
D
E
F
G
H
I
K
L
M
N
P
Q
R
S
T
V
W
Y
China
Japan
Latvia
Russia
Korea
USSR

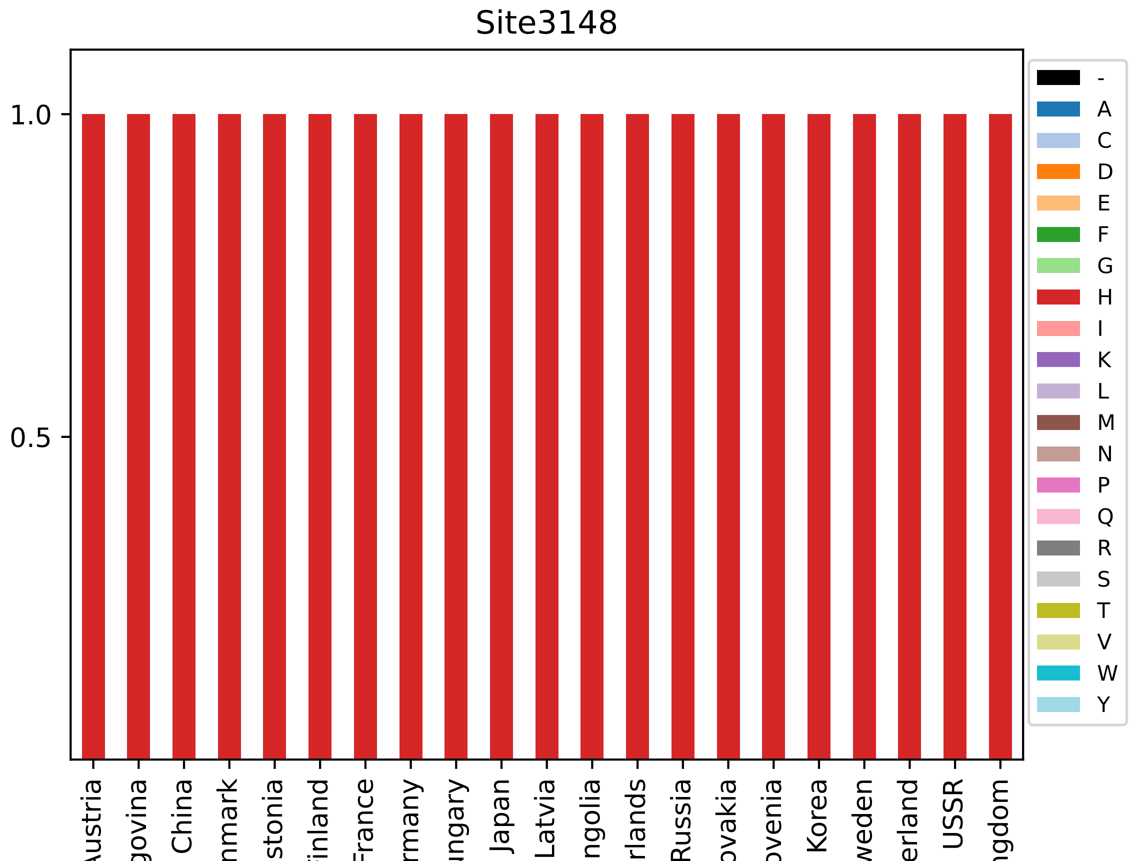
Site3148
1.0
0.5
Austria
govina
China
nmark
stonia
Finland
France
rmany
ungary
Japan
Latvia
ngolia
rlands
Russia
ovakia
ovenia
Korea
weden
erland
USSR
ngdom
-
A
C
D
E
F
G
H
I
K
L
M
N
P
Q
R
S
T
V
W
Y

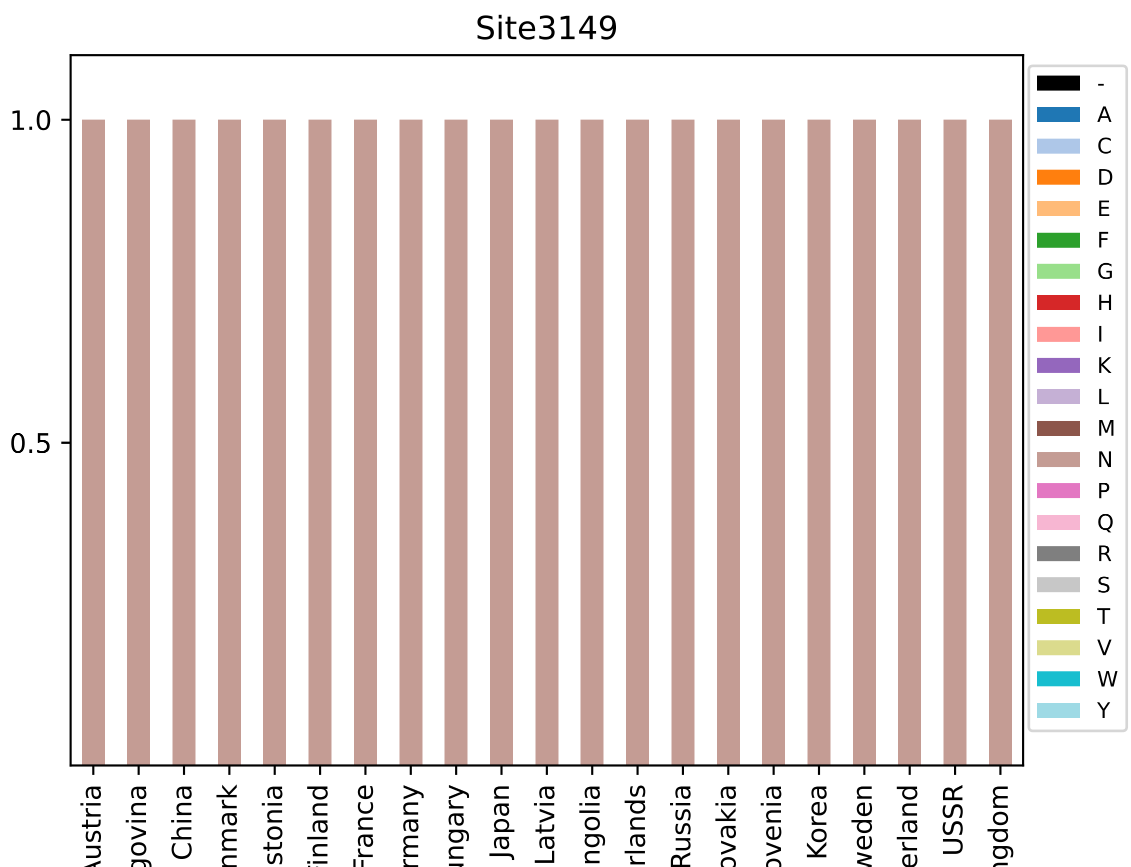
Site3149
1.0
0.5
ustria
govina
China
nmark
stonia
inland
rance
rmany
ungary
Japan
Latvia
ngolia
rlands
Russia
ovakia
ovenia
Korea
weden
erland
USSR
ngdom
-
A
C
D
E
F
G
H
I
K
L
M
N
P
Q
R
S
T
V
W
Y

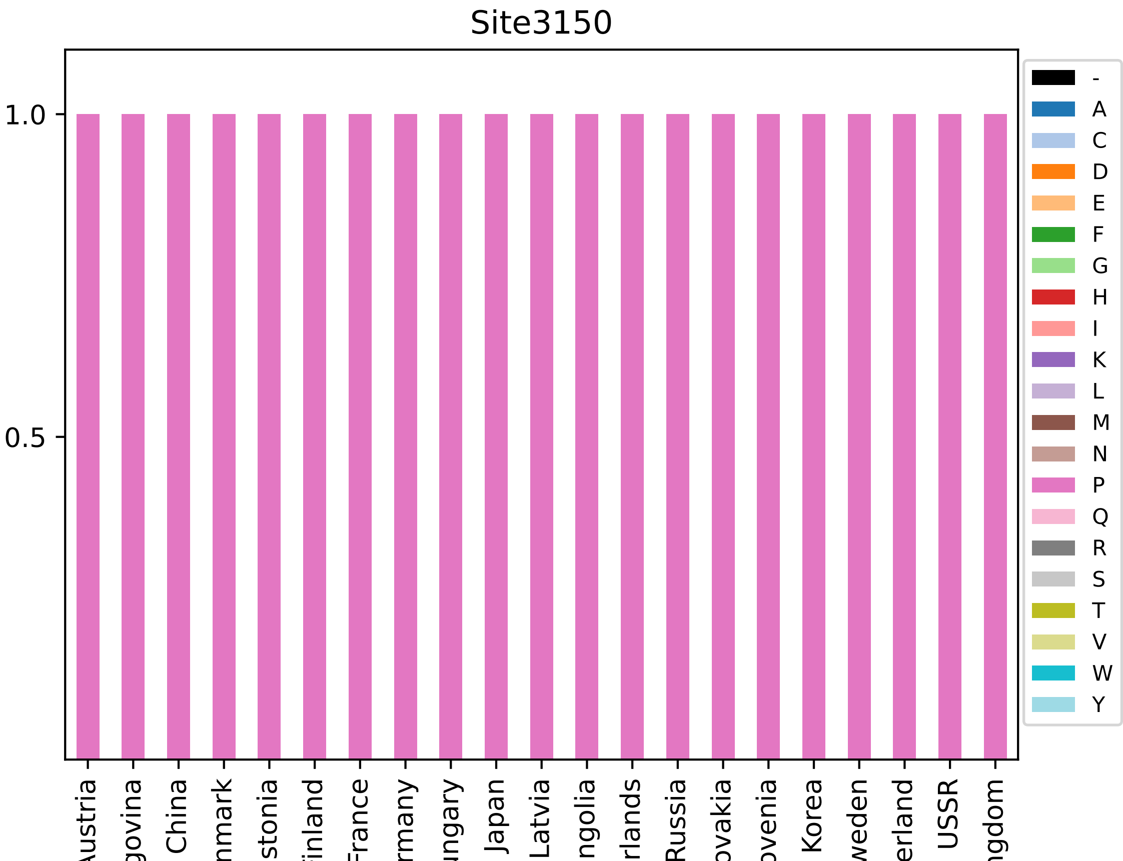
Site3150
1.0
0.5
-
A
C
D
E
F
G
H
I
K
L
M
N
P
Q
R
S
T
V
W
Y
China
Japan
Latvia
Russia
Korea
USSR

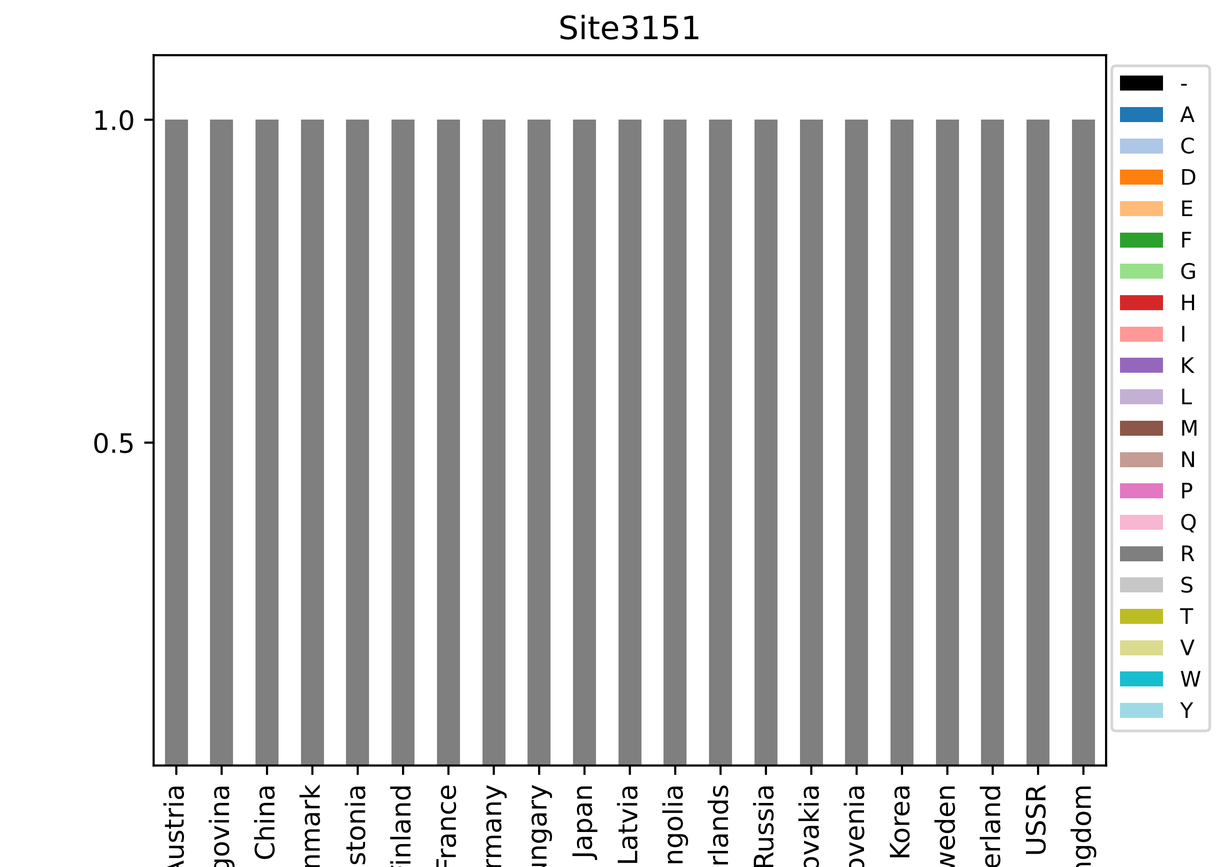
Site3151
1.0
0.5
-
A
C
D
E
F
G
H
I
K
L
M
N
P
Q
R
S
T
V
W
Y
Austria
govina
China
nmark
stonia
Finland
France
rmany
ungary
Japan
Latvia
ngolia
rlands
Russia
ovakia
ovenia
Korea
weden
erland
USSR
ngdom

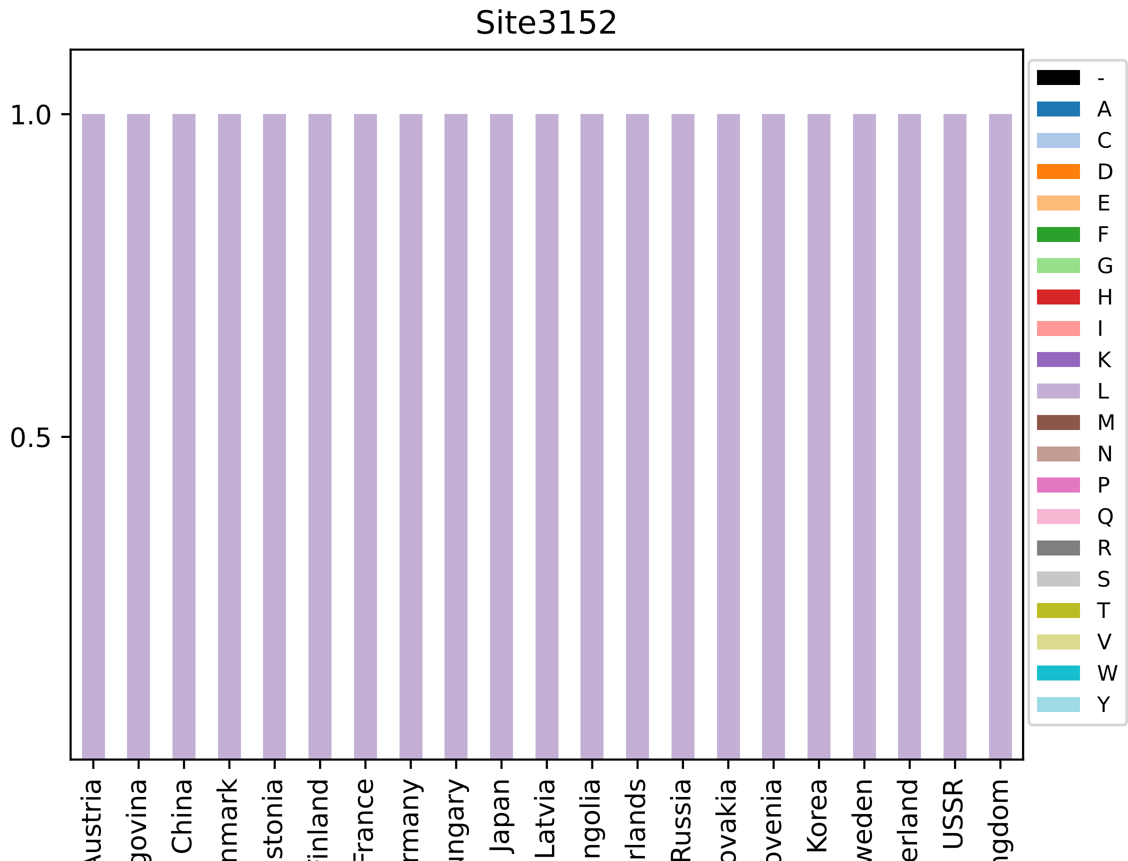

Site3152
1.0
0.5
Austria
govina
China
nmark
stonia
Finland
France
rmany
ungary
Japan
Latvia
ngolia
rlands
Russia
ovakia
ovenia
Korea
weden
erland
USSR
ngdom
-
A
C
D
E
F
G
H
I
K
L
M
N
P
Q
R
S
T
V
W
Y

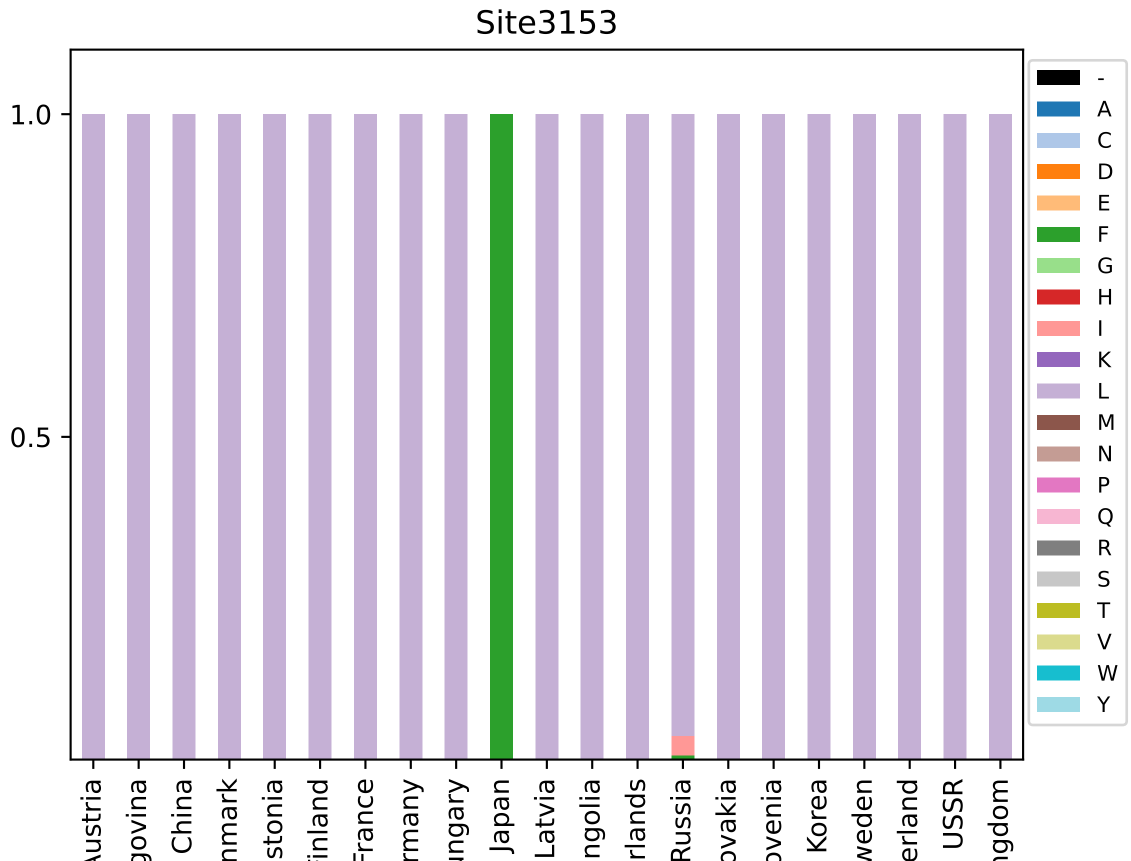
Site3153
1.0
0.5
-
A
C
D
E
F
G
H
I
K
L
M
N
P
Q
R
S
T
V
W
Y
Austria
govina
China
nmark
stonia
inland
France
rmany
ungary
Japan
Latvia
ngolia
rlands
Russia
ovakia
ovenia
Korea
weden
erland
USSR
ngdom

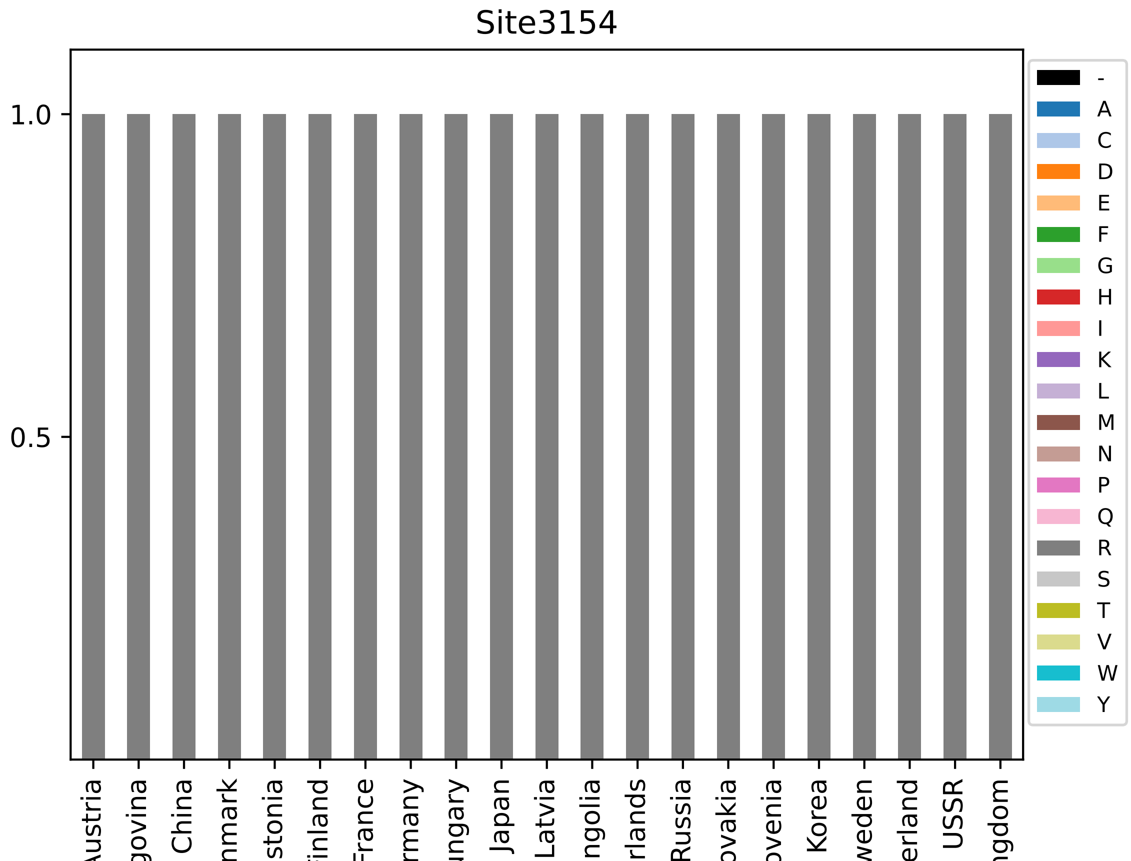

Site3154
1.0
0.5
-
A
C
D
E
F
G
H
I
K
L
M
N
P
Q
R
S
T
V
W
Y
China
Japan
Latvia
Russia
Korea
USSR

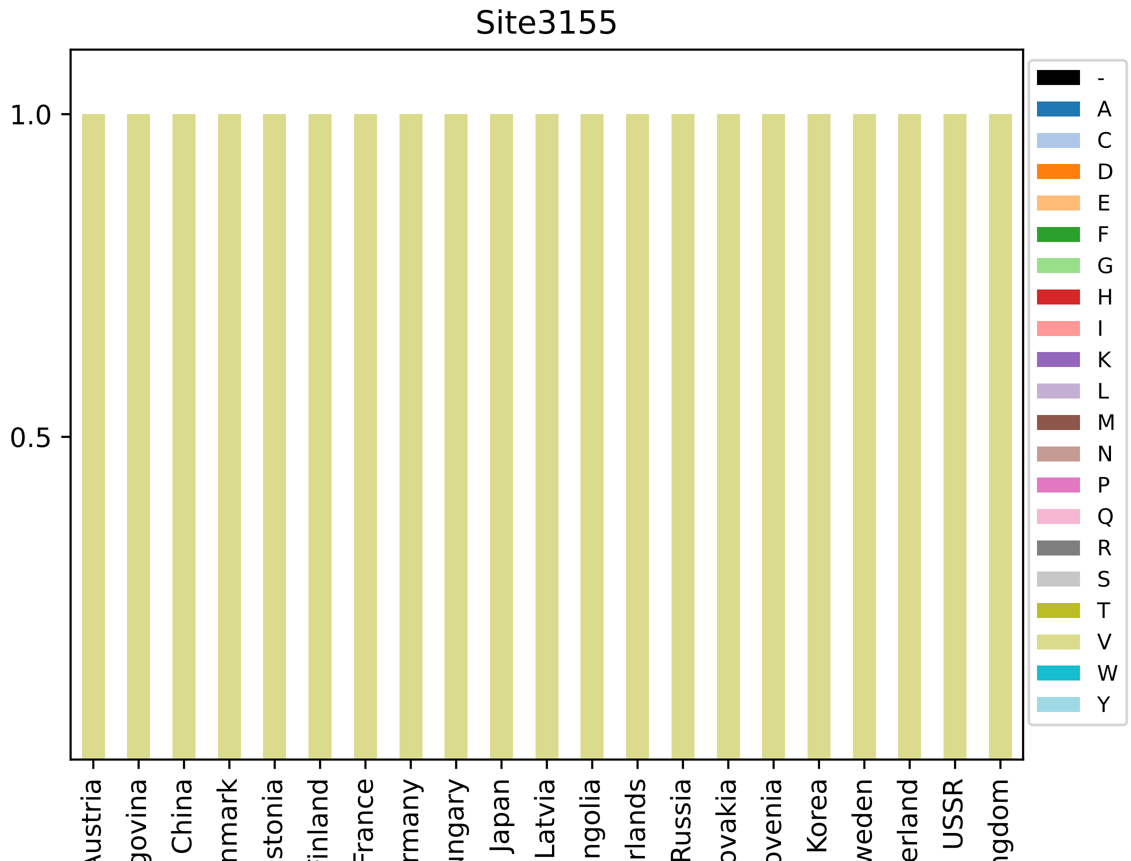

Site3155
1.0
0.5
-
A
C
D
E
F
G
H
I
K
L
M
N
P
Q
R
S
T
V
W
Y
China
Japan
Latvia
Russia
Korea
USSR

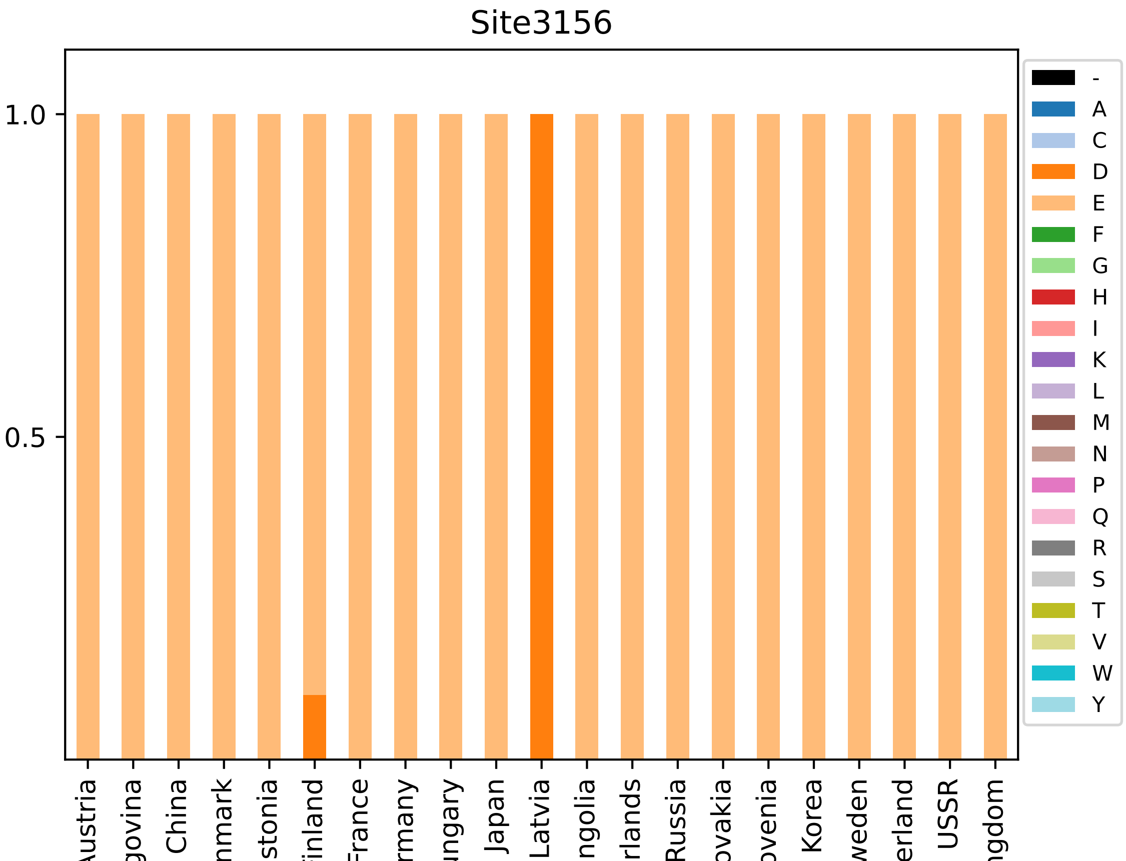
Site3156
1.0
0.5
-
A
C
D
E
F
G
H
I
K
L
M
N
P
Q
R
S
T
V
W
Y
China
Japan
Latvia
Russia
Korea
USSR

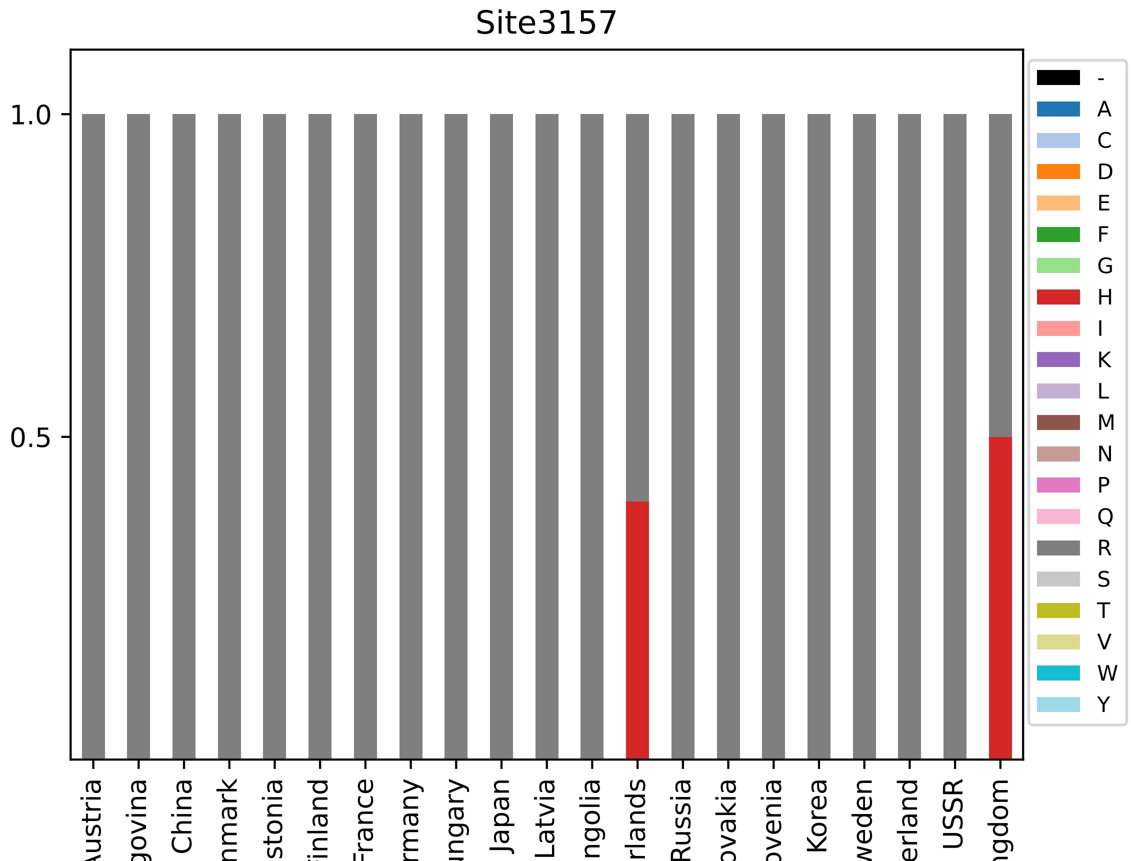

Site3157
1.0
0.5
-
A
C
D
E
F
G
H
I
K
L
M
N
P
Q
R
S
T
V
W
Y
Austria
govina
China
nmark
stonia
inland
France
rmany
ungary
Japan
Latvia
ngolia
rlands
Russia
ovakia
ovenia
Korea
weden
erland
USSR
ngdom

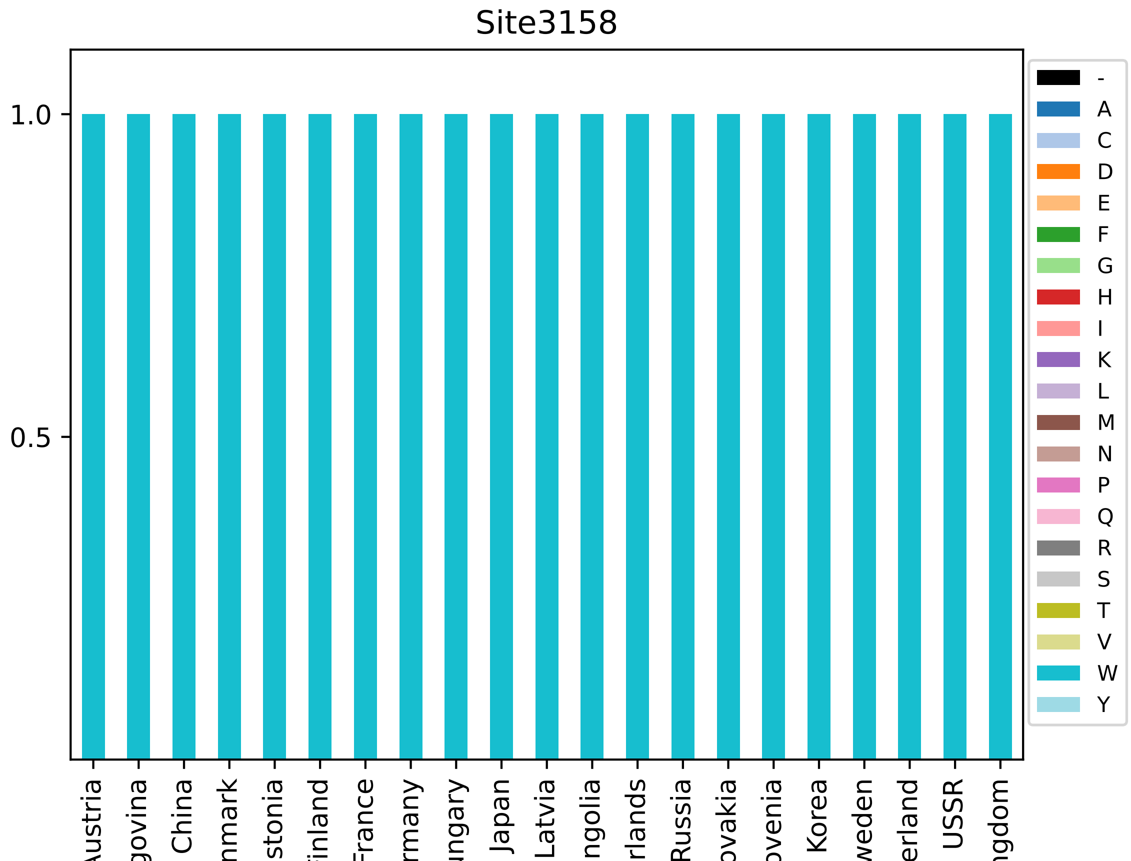
Site3158
1.0
0.5
-
A
C
D
E
F
G
H
I
K
L
M
N
P
Q
R
S
T
V
W
Y
China
Japan
Latvia
Russia
Korea
USSR

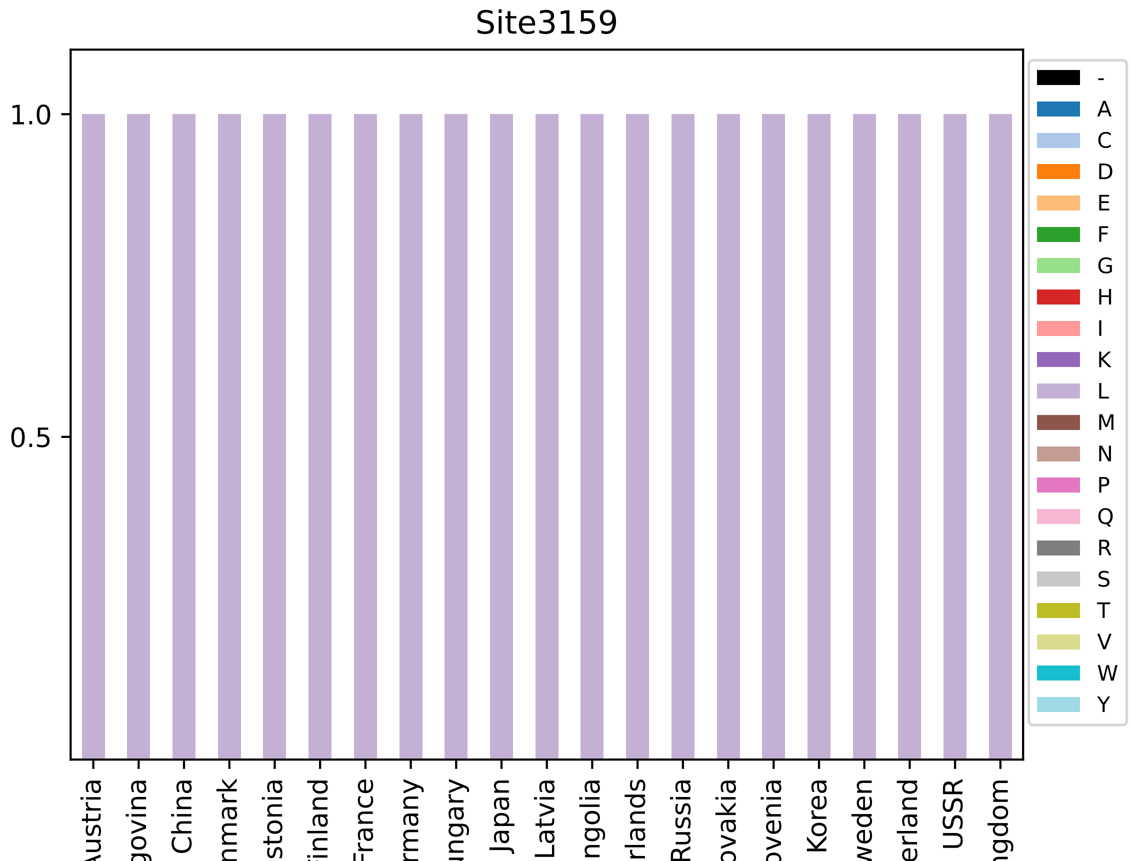
Site3159
1.0
0.5
-
A
C
D
E
F
G
H
I
K
L
M
N
P
Q
R
S
T
V
W
Y
Austria
govina
China
nmark
stonia
Finland
France
rmany
ungary
Japan
Latvia
ngolia
rlands
Russia
ovakia
ovenia
Korea
weden
erland
USSR
ngdom

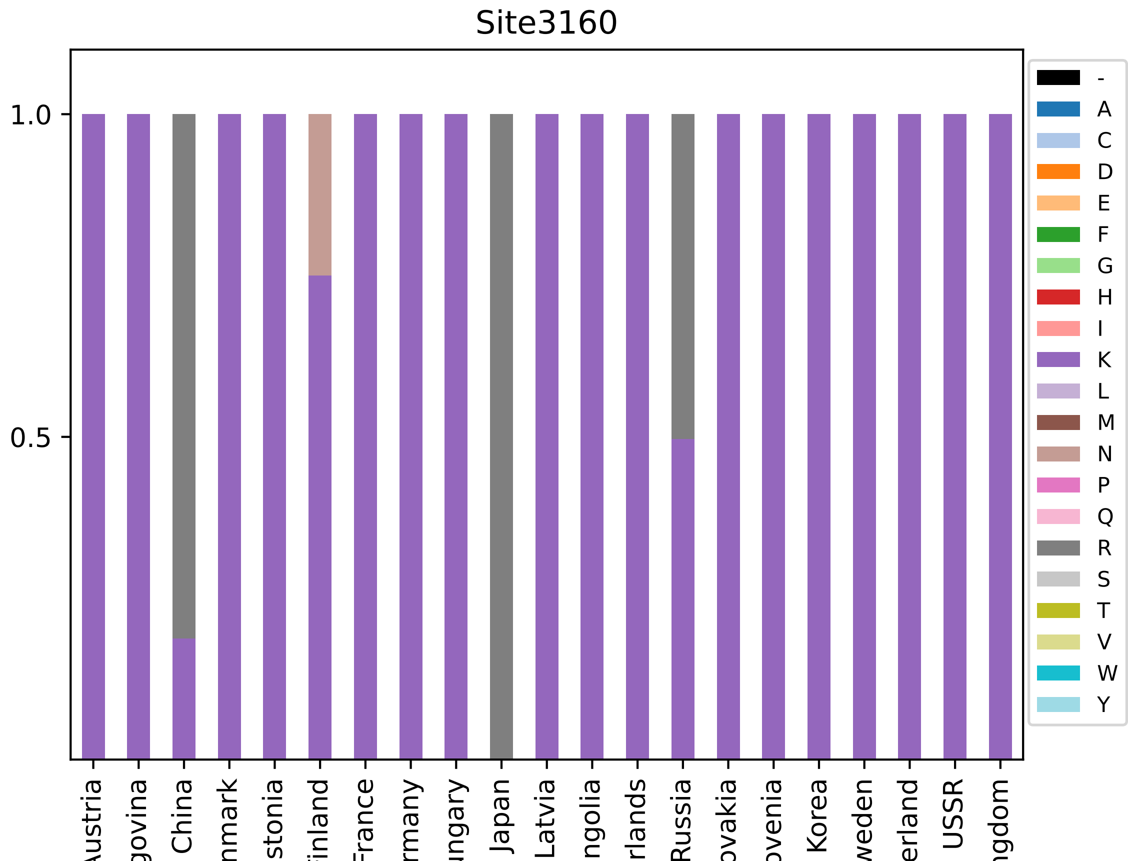

Site3160
1.0
0.5
-
A
C
D
E
F
G
H
I
K
L
M
N
P
Q
R
S
T
V
W
Y
Austria
govina
China
nmark
stonia
inland
France
rmany
ungary
Japan
Latvia
ngolia
rlands
Russia
ovakia
ovenia
Korea
weden
erland
USSR
ngdom

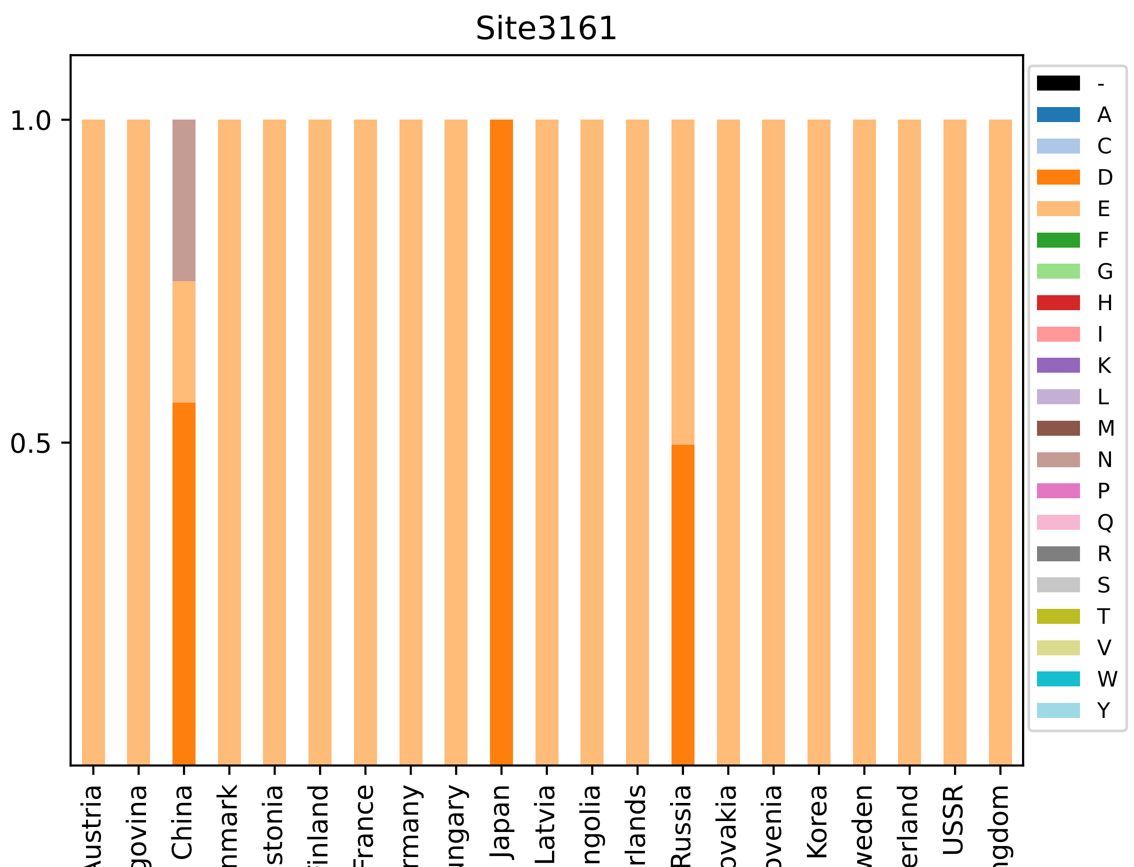

Site3161
1.0
0.5
-
A
C
D
E
F
G
H
I
K
L
M
N
P
Q
R
S
T
V
W
Y
China
Japan
Latvia
Russia
Korea
USSR

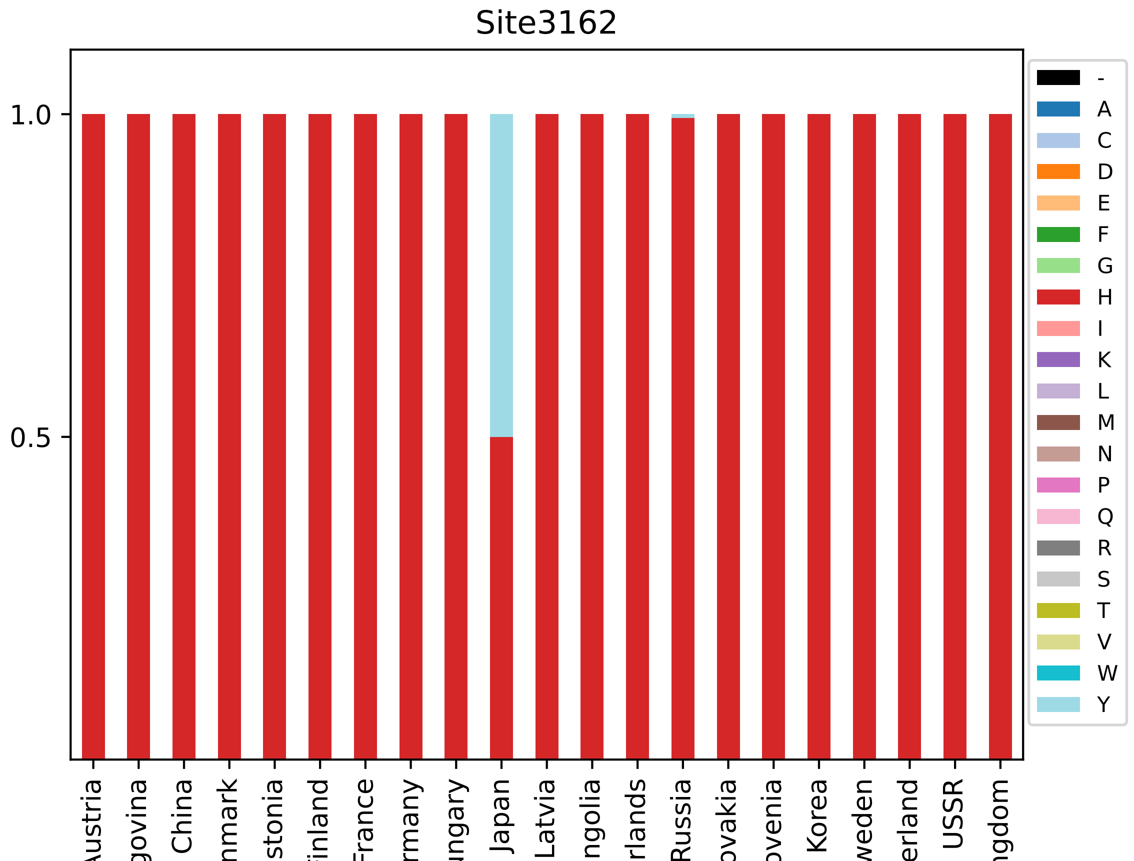
Site3162
1.0
0.5
-
A
C
D
E
F
G
H
I
K
L
M
N
P
Q
R
S
T
V
W
Y
Austria
China
Japan
Latvia
Russia
Korea
USSR

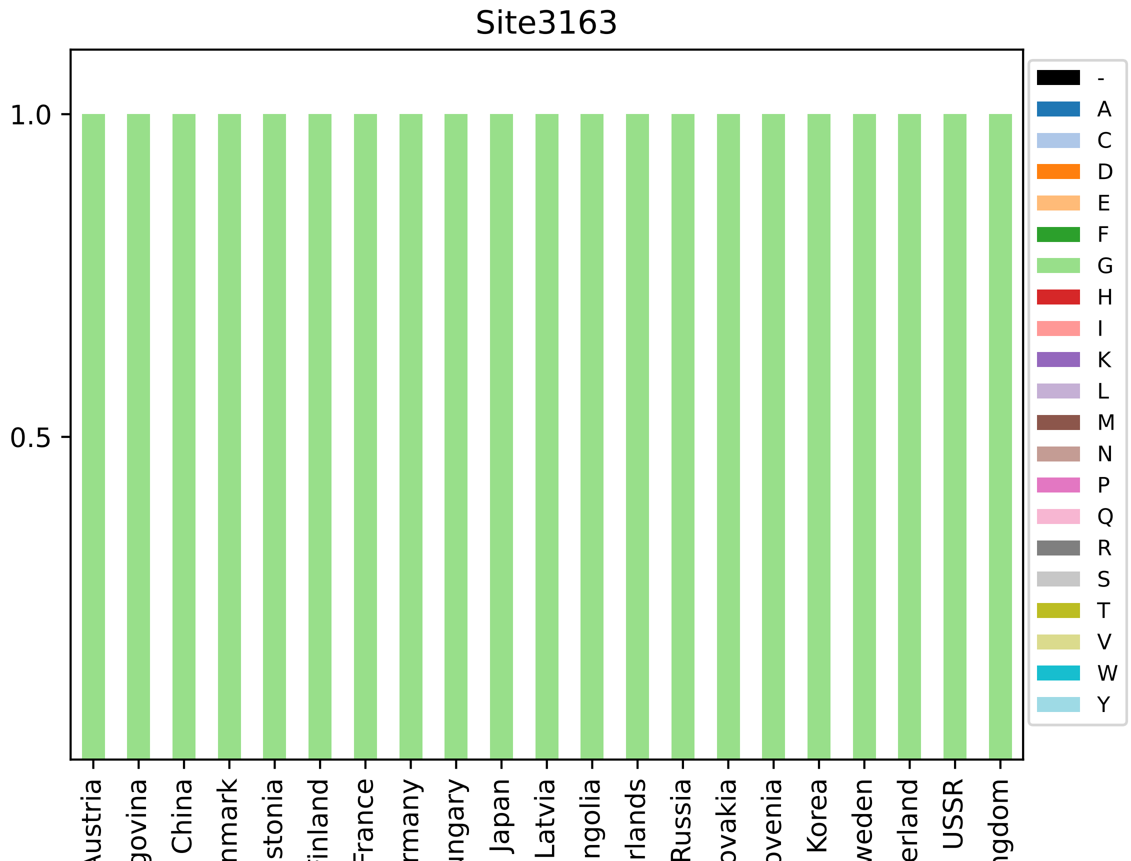
Site3163
1.0
0.5
-
A
C
D
E
F
G
H
I
K
L
M
N
P
Q
R
S
T
V
W
Y
China
Japan
Latvia
Russia
Korea
USSR

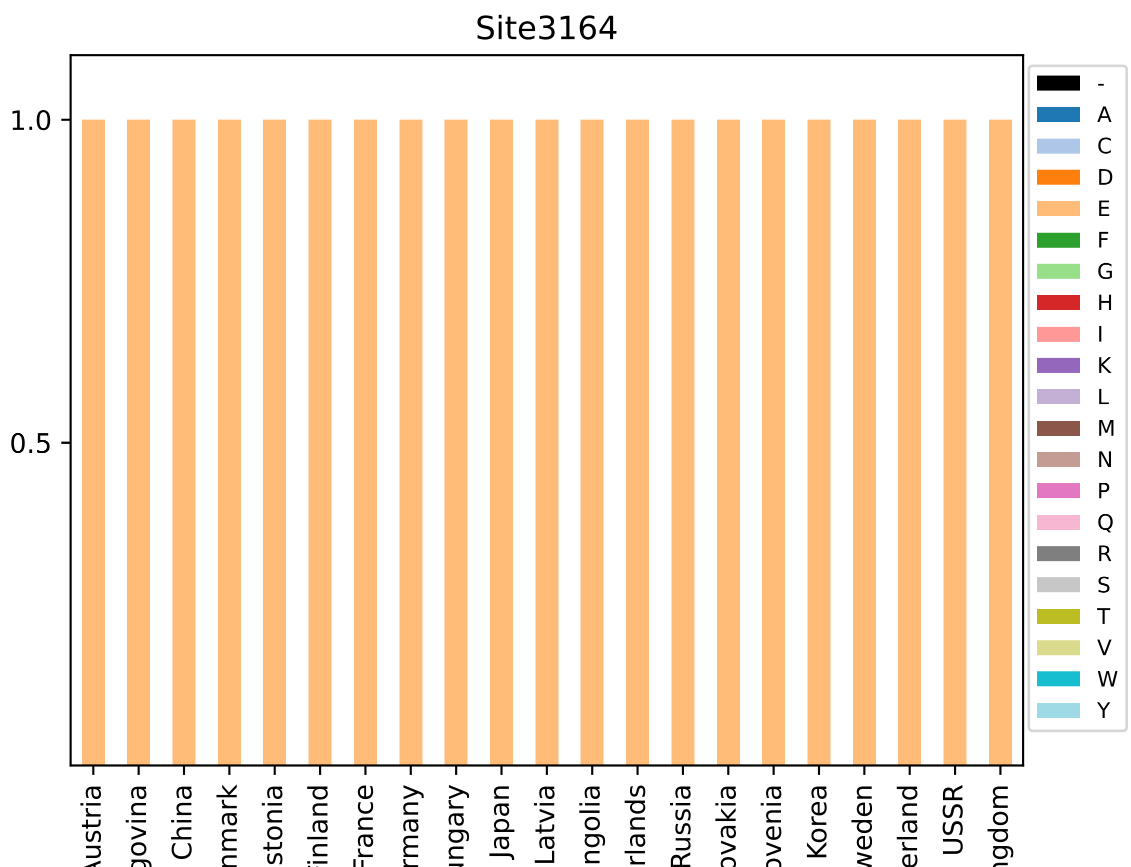
Site3164
1.0
0.5
-
A
C
D
E
F
G
H
I
K
L
M
N
P
Q
R
S
T
V
W
Y
Austria
China
Japan
Latvia
Russia
Korea
USSR

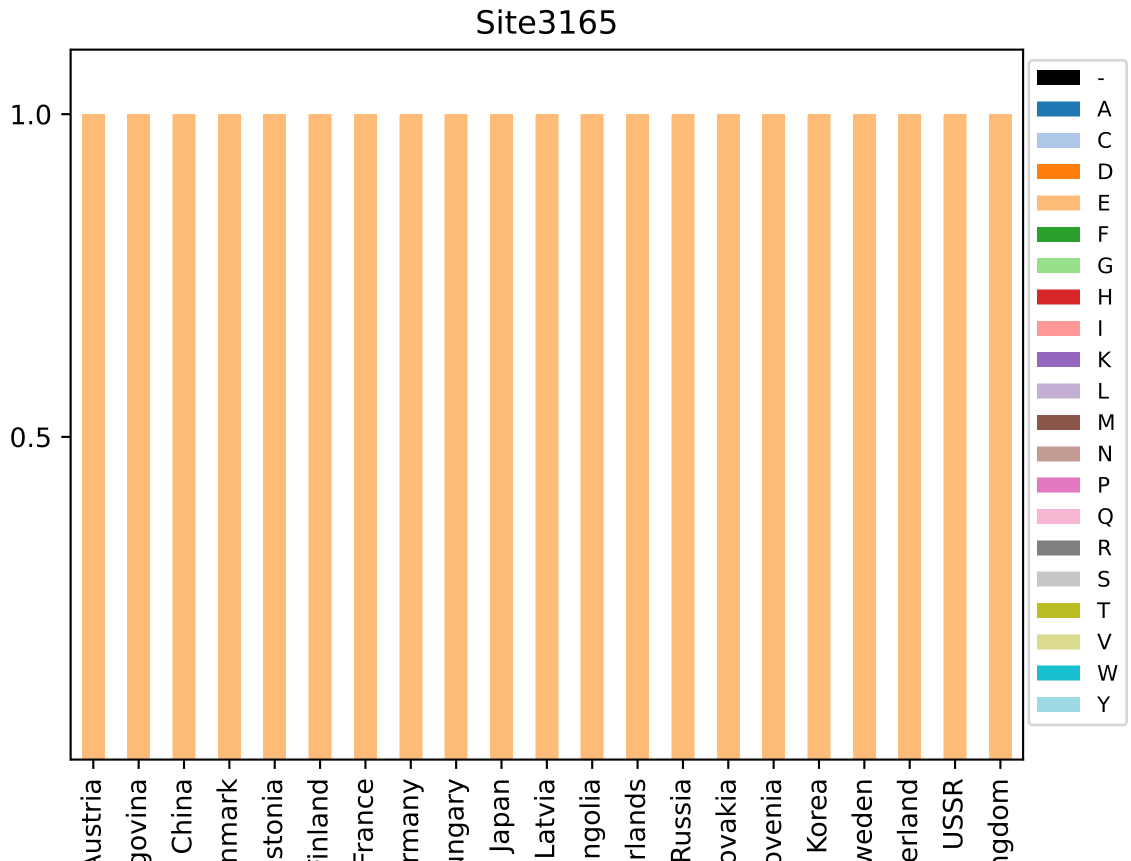
Site3165
1.0
0.5
-
A
C
D
E
F
G
H
I
K
L
M
N
P
Q
R
S
T
V
W
Y
China
Japan
Latvia
Russia
Korea
USSR

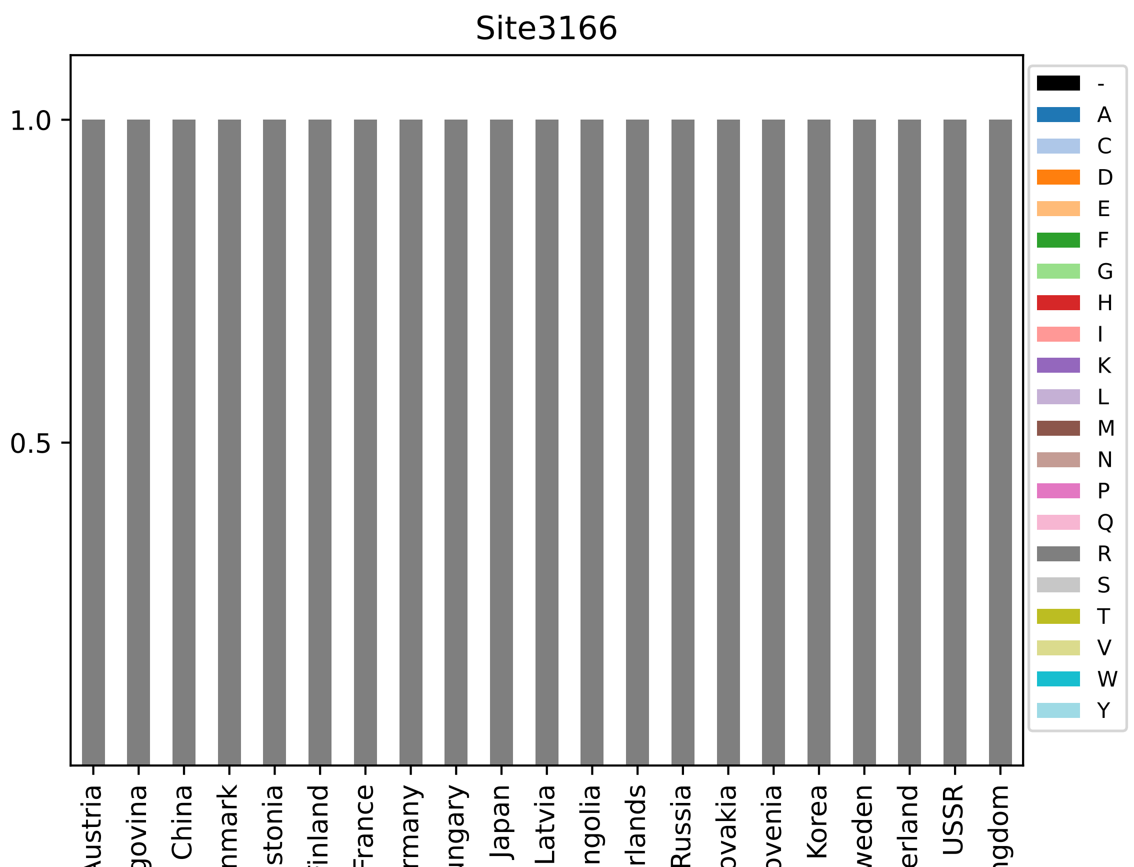

Site3166
1.0
0.5
-
A
C
D
E
F
G
H
I
K
L
M
N
P
Q
R
S
T
V
W
Y
Austria
govina
China
nmark
stonia
inland
France
rmany
ungary
Japan
Latvia
ngolia
rlands
Russia
ovakia
ovenia
Korea
weden
erland
USSR
ngdom

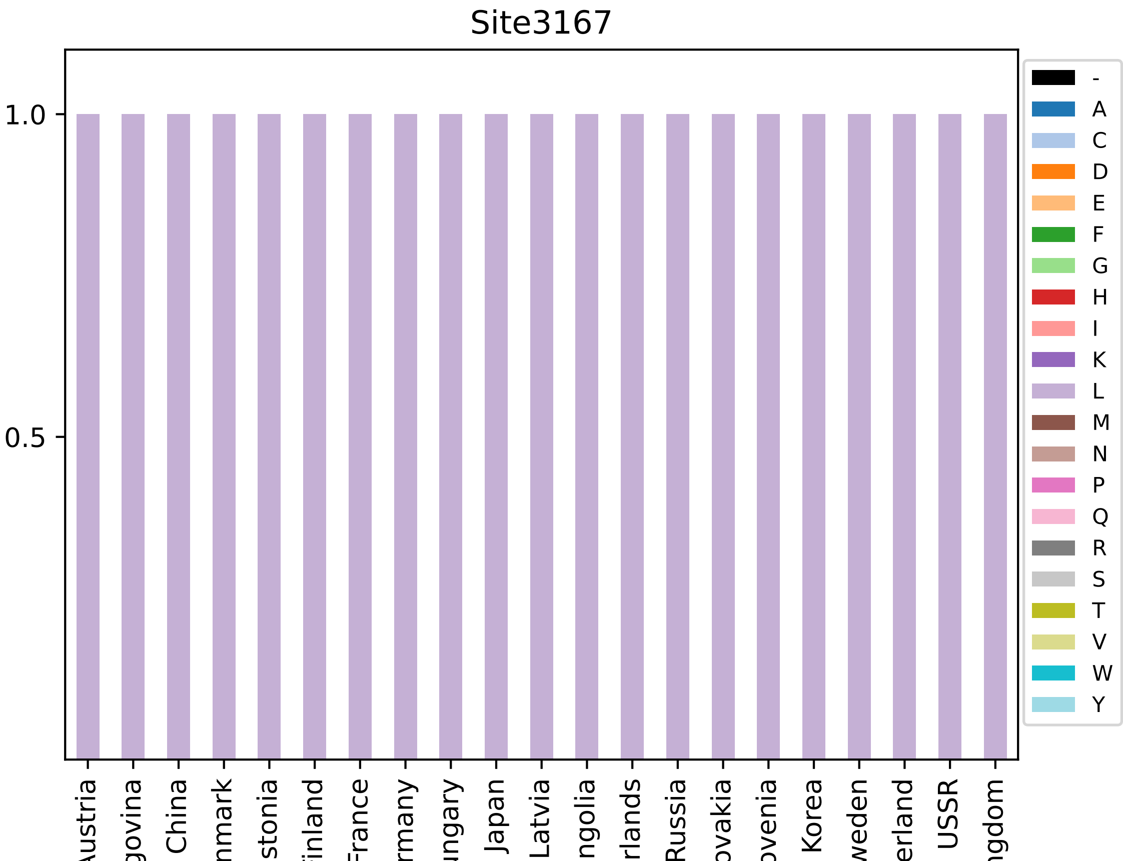

Site3167
1.0
0.5
-
A
C
D
E
F
G
H
I
K
L
M
N
P
Q
R
S
T
V
W
Y
China
Japan
Latvia
Russia
Korea
USSR

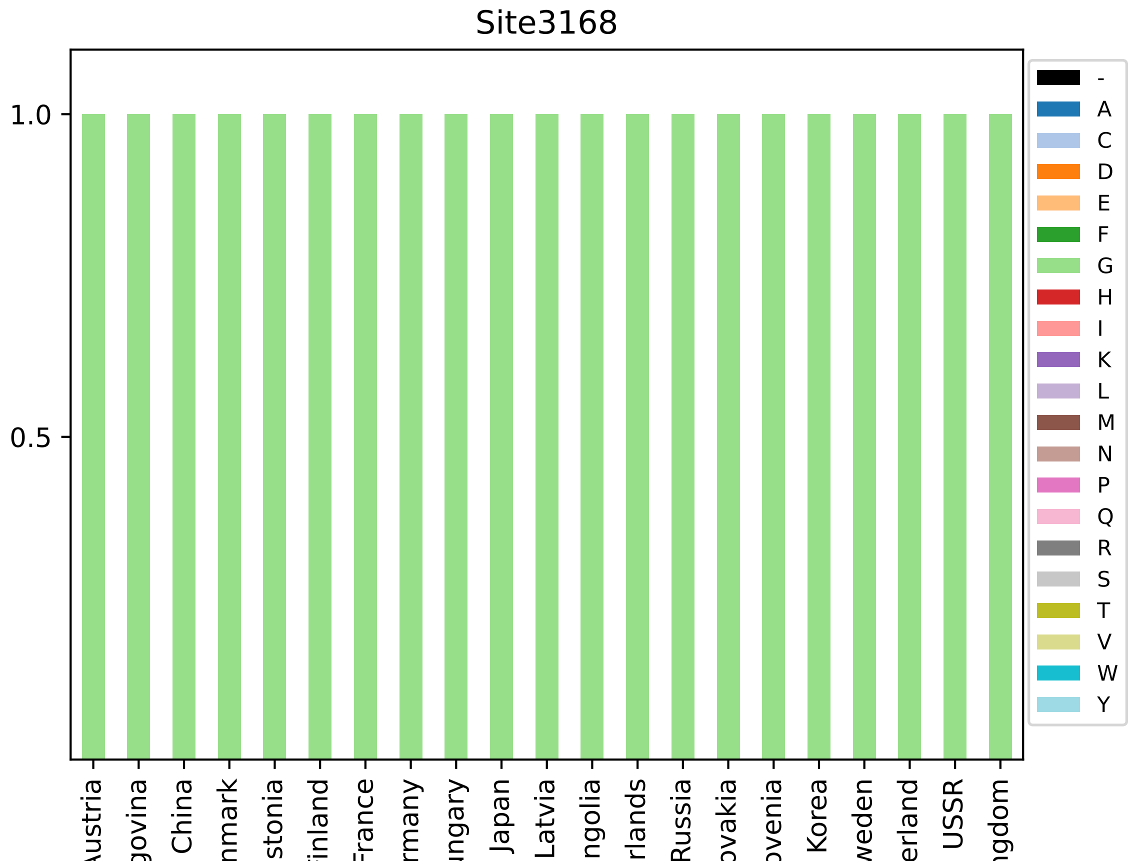
Site3168
1.0
0.5
-
A
C
D
E
F
G
H
I
K
L
M
N
P
Q
R
S
T
V
W
Y
China
Japan
Latvia
Russia
Korea
USSR

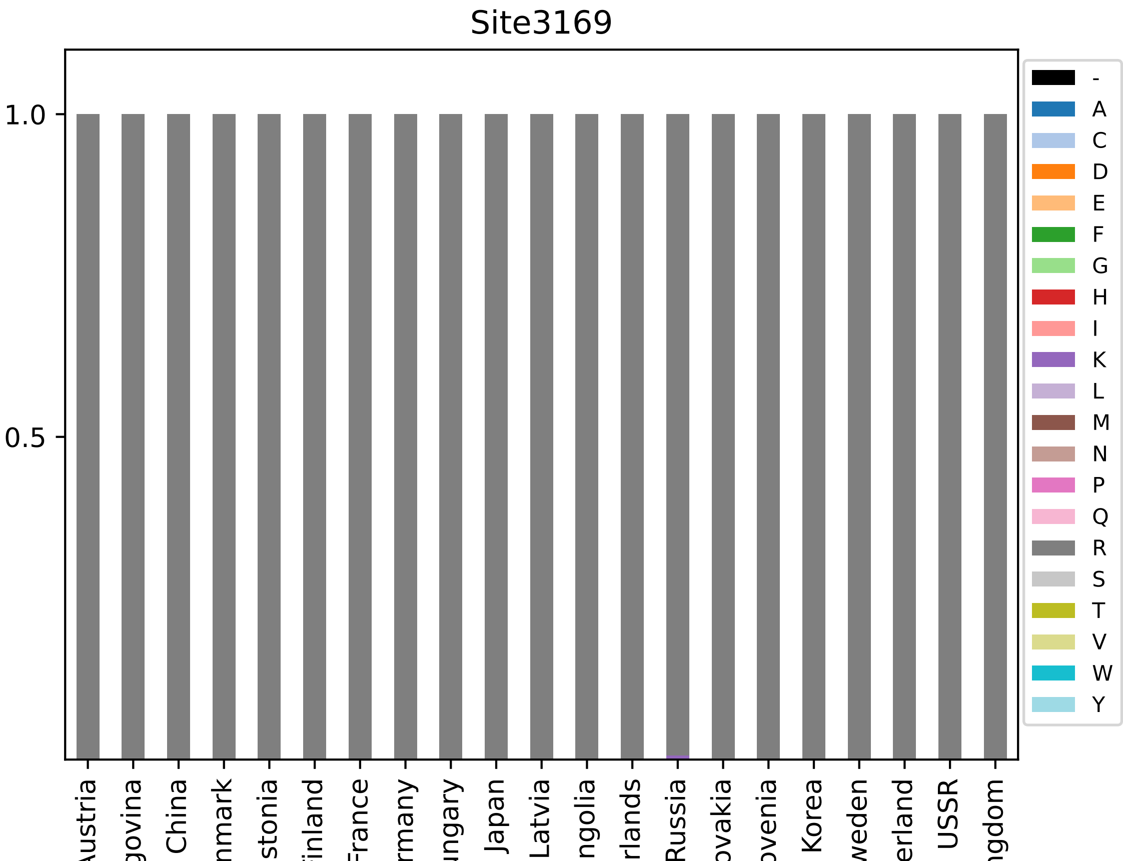
Site3169
1.0
0.5
-
A
C
D
E
F
G
H
I
K
L
M
N
P
Q
R
S
T
V
W
Y
Austria
govina
China
nmark
stonia
Finland
France
rmany
ungary
Japan
Latvia
ngolia
rlands
Russia
ovakia
ovenia
Korea
weden
erland
USSR
ngdom

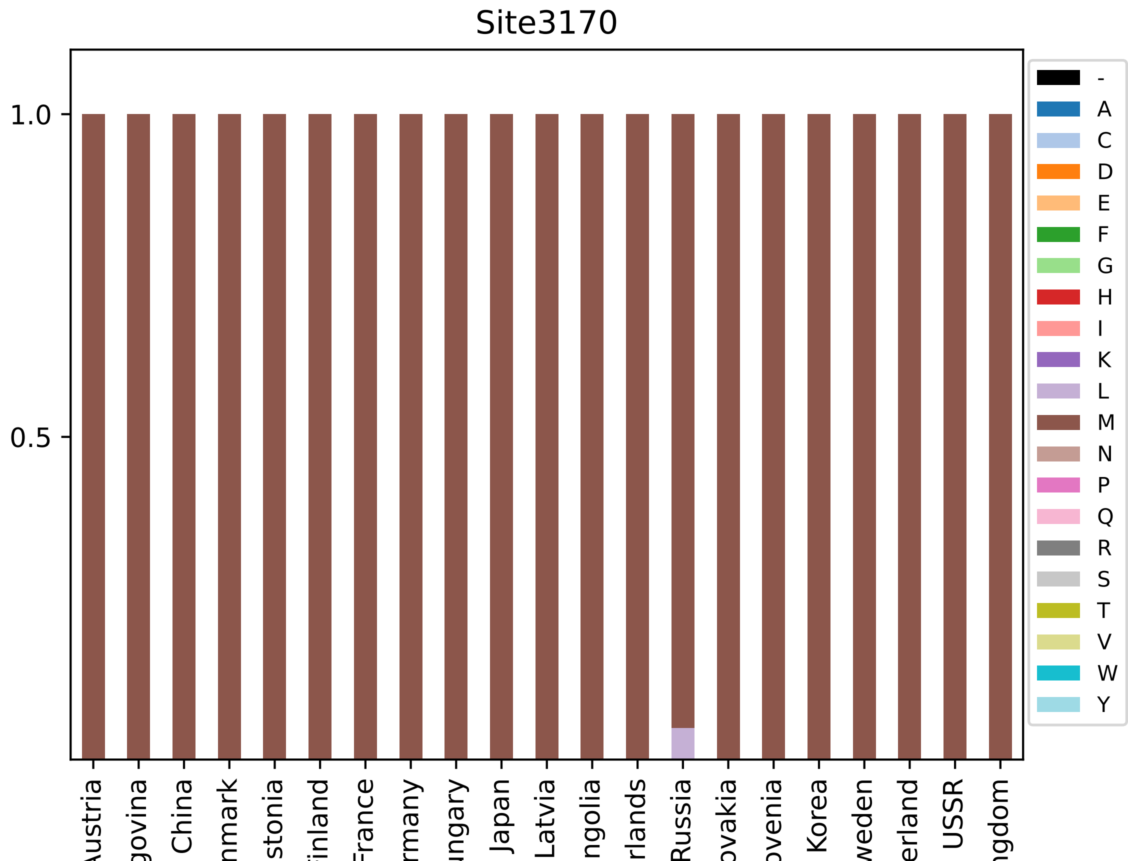
Site3170
1.0
0.5
-
A
C
D
E
F
G
H
I
K
L
M
N
P
Q
R
S
T
V
W
Y
China
Japan
Latvia
Russia
Korea
USSR

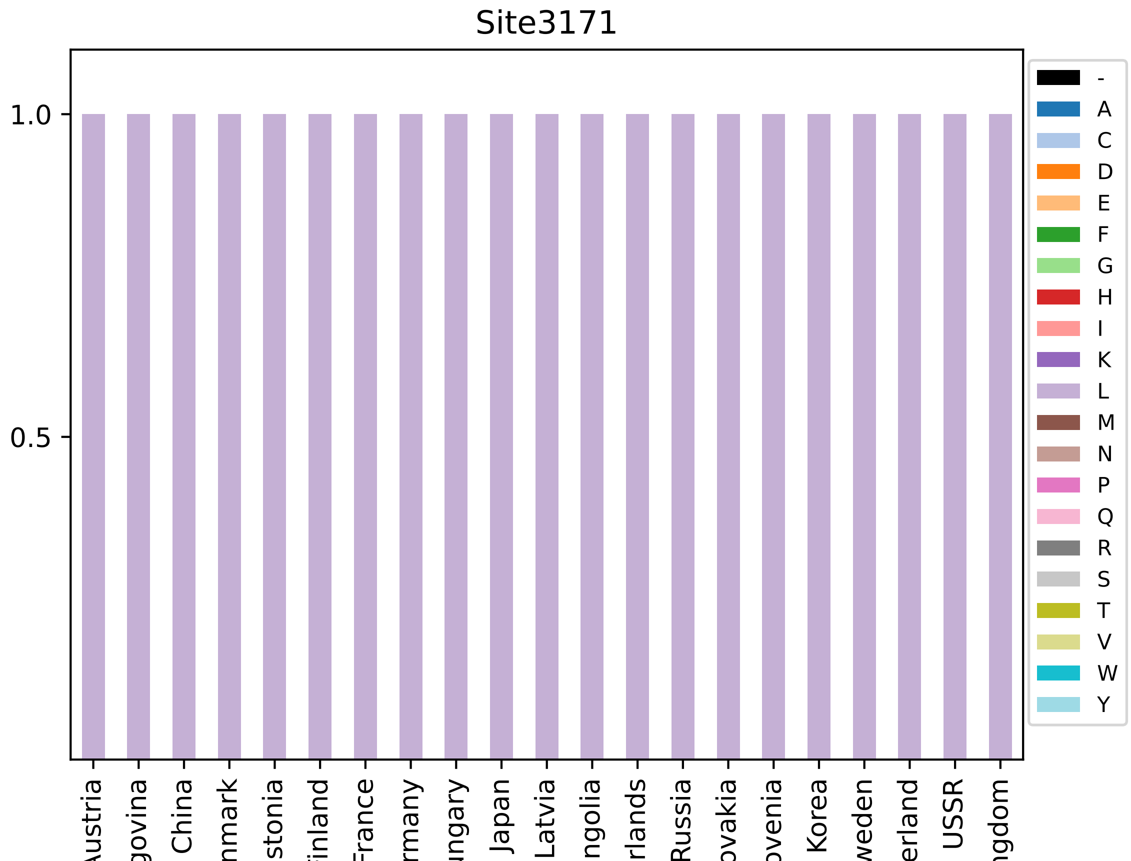

Site3171
1.0
0.5
-
A
C
D
E
F
G
H
I
K
L
M
N
P
Q
R
S
T
V
W
Y
China
Japan
Latvia
Russia
Korea
USSR

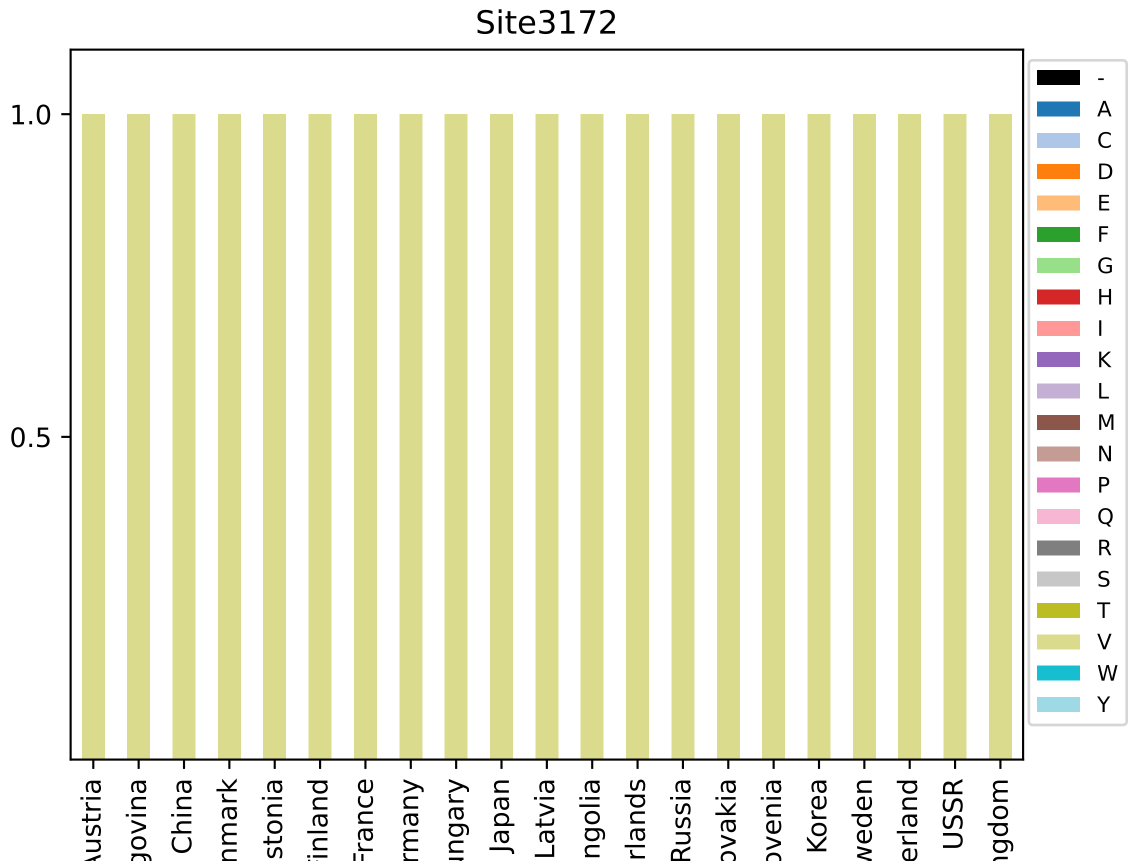
Site3172
1.0
0.5
-
A
C
D
E
F
G
H
I
K
L
M
N
P
Q
R
S
T
V
W
Y
China
Japan
Latvia
Russia
Korea
USSR

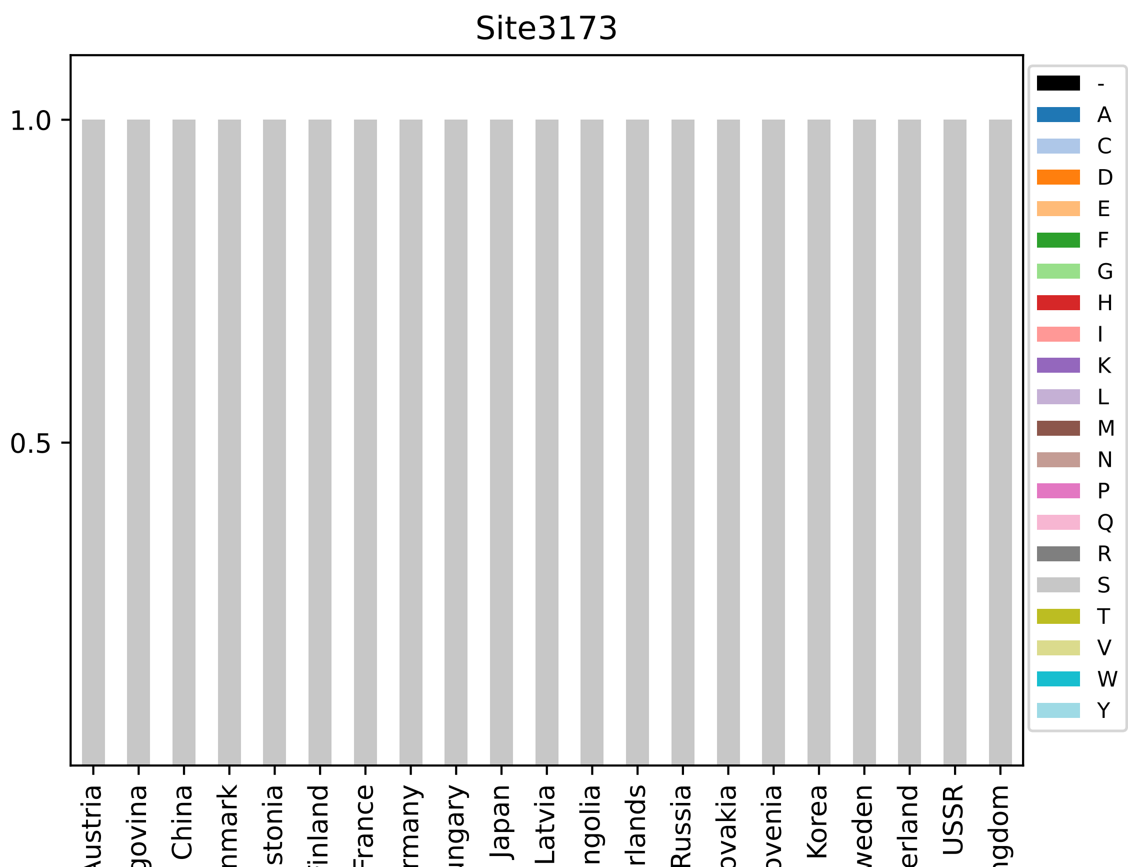

Site3173
1.0
0.5
-
A
C
D
E
F
G
H
I
K
L
M
N
P
Q
R
S
T
V
W
Y
Austria
govina
China
nmark
stonia
Finland
France
rmany
ungary
Japan
Latvia
ngolia
rlands
Russia
ovakia
ovenia
Korea
weden
erland
USSR
ngdom

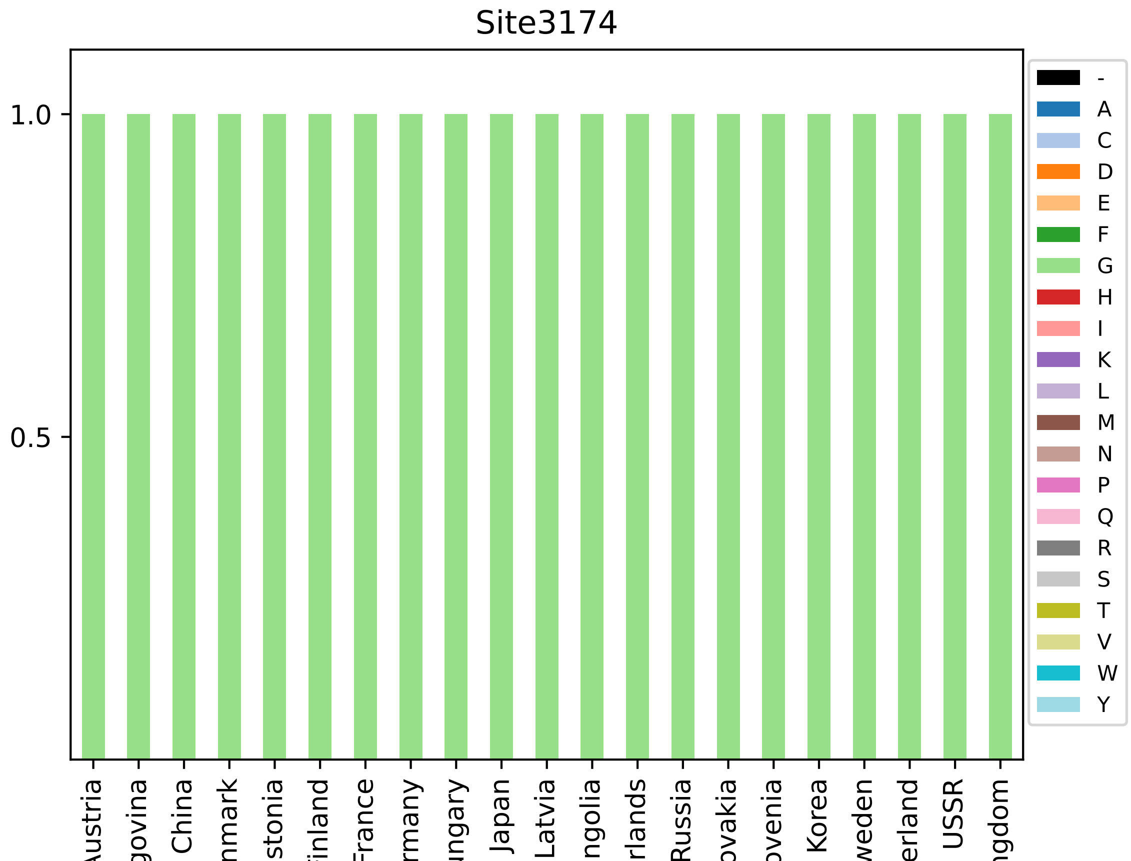
Site3174
1.0
0.5
Austria
govina
China
nmark
stonia
Finland
France
rmany
ungary
Japan
Latvia
ngolia
rlands
Russia
ovakia
ovenia
Korea
weden
erland
USSR
ngdom
-
A
C
D
E
F
G
H
I
K
L
M
N
P
Q
R
S
T
V
W
Y

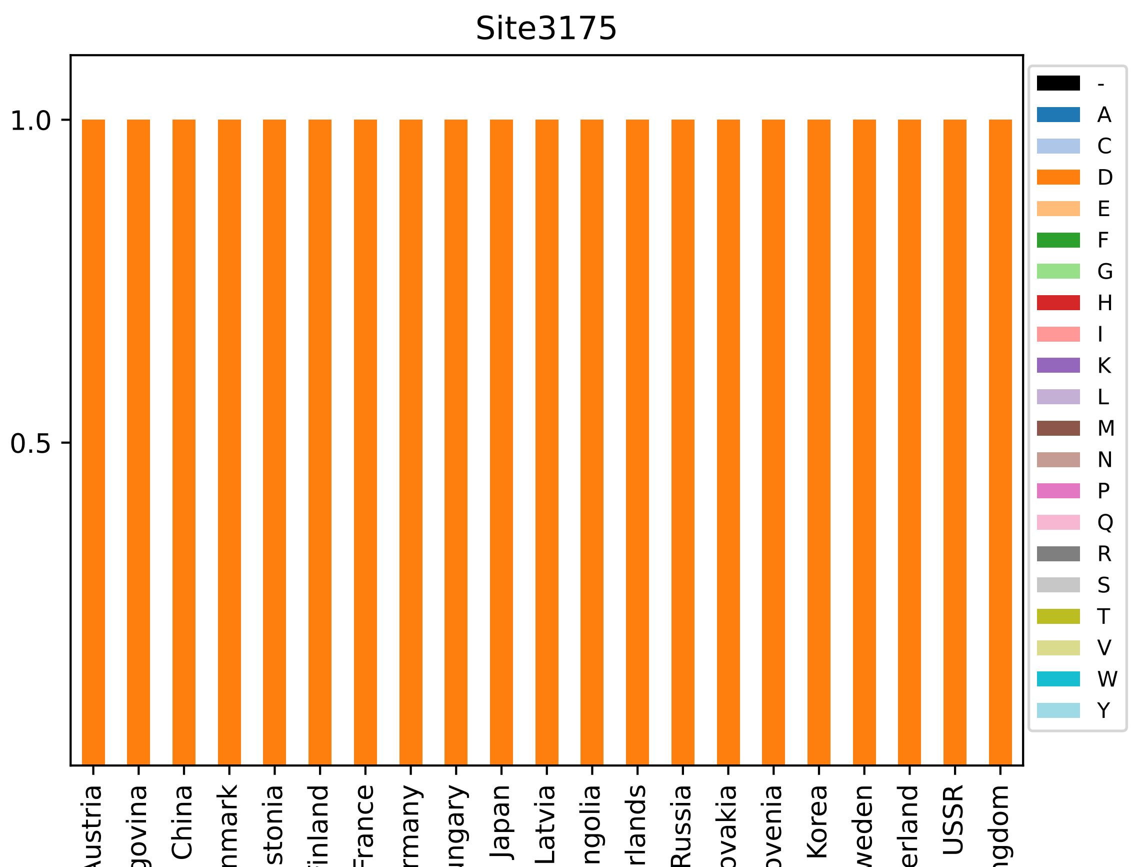

Site3175
1.0
0.5
-
A
C
D
E
F
G
H
I
K
L
M
N
P
Q
R
S
T
V
W
Y
China
Japan
Latvia
Russia
Korea
USSR

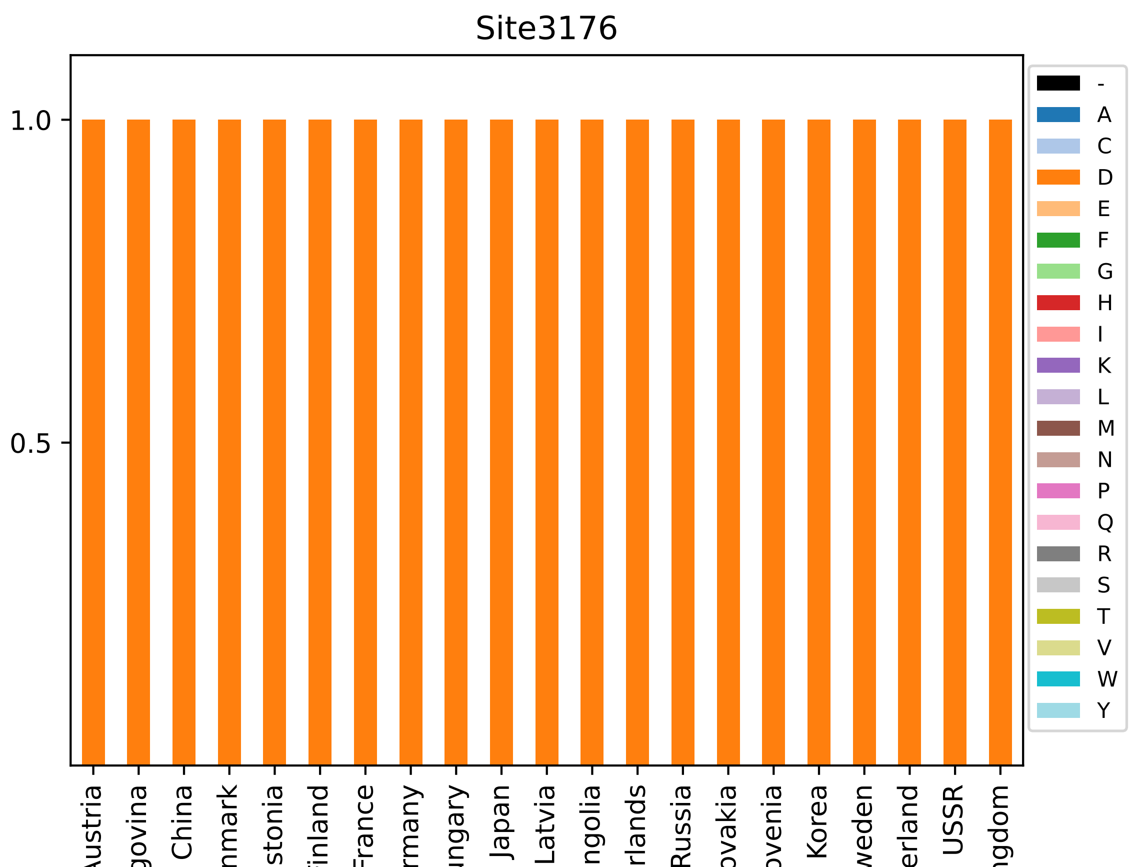

Site3176
1.0
0.5
-
A
C
D
E
F
G
H
I
K
L
M
N
P
Q
R
S
T
V
W
Y
Austria
govina
China
nmark
stonia
inland
France
rmany
ungary
Japan
Latvia
ngolia
rlands
Russia
ovakia
ovenia
Korea
weden
erland
USSR
ngdom

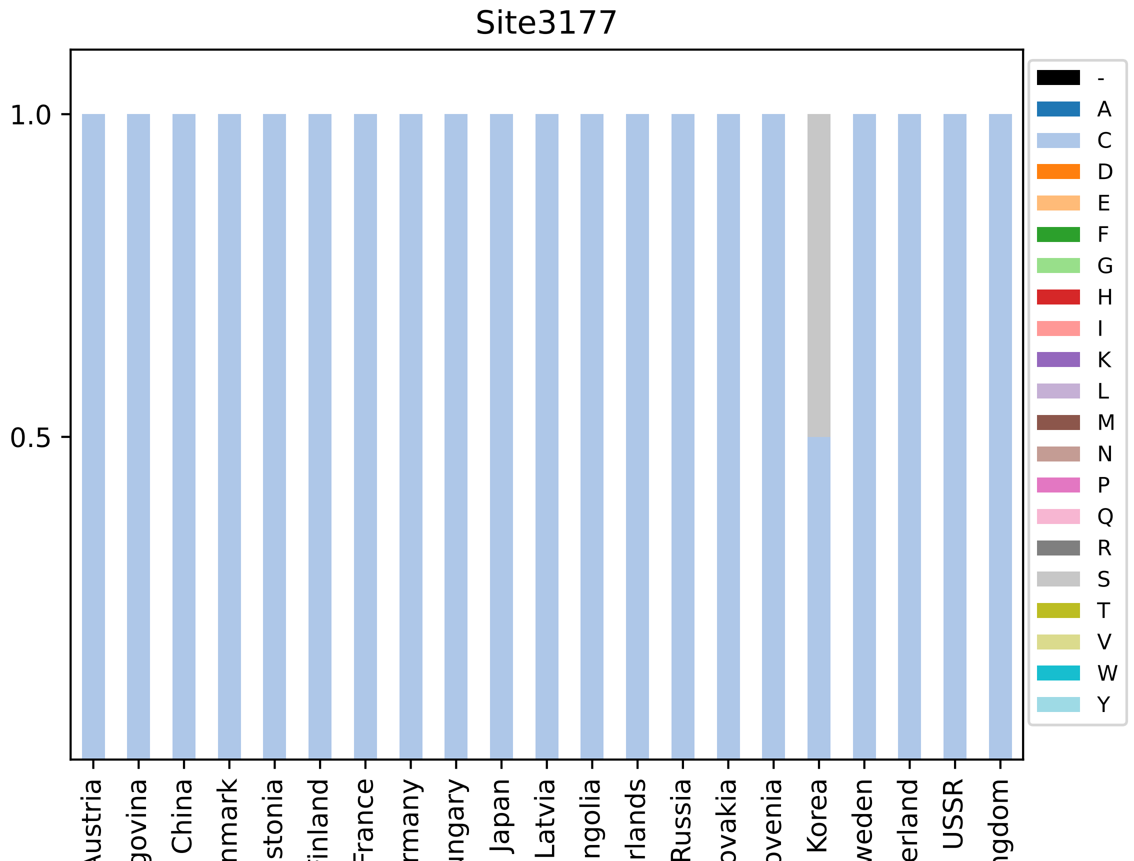
Site3177
1.0
0.5
-
A
C
D
E
F
G
H
I
K
L
M
N
P
Q
R
S
T
V
W
Y
Austria
China
Finland
France
Japan
Latvia
Russia
Korea
USSR

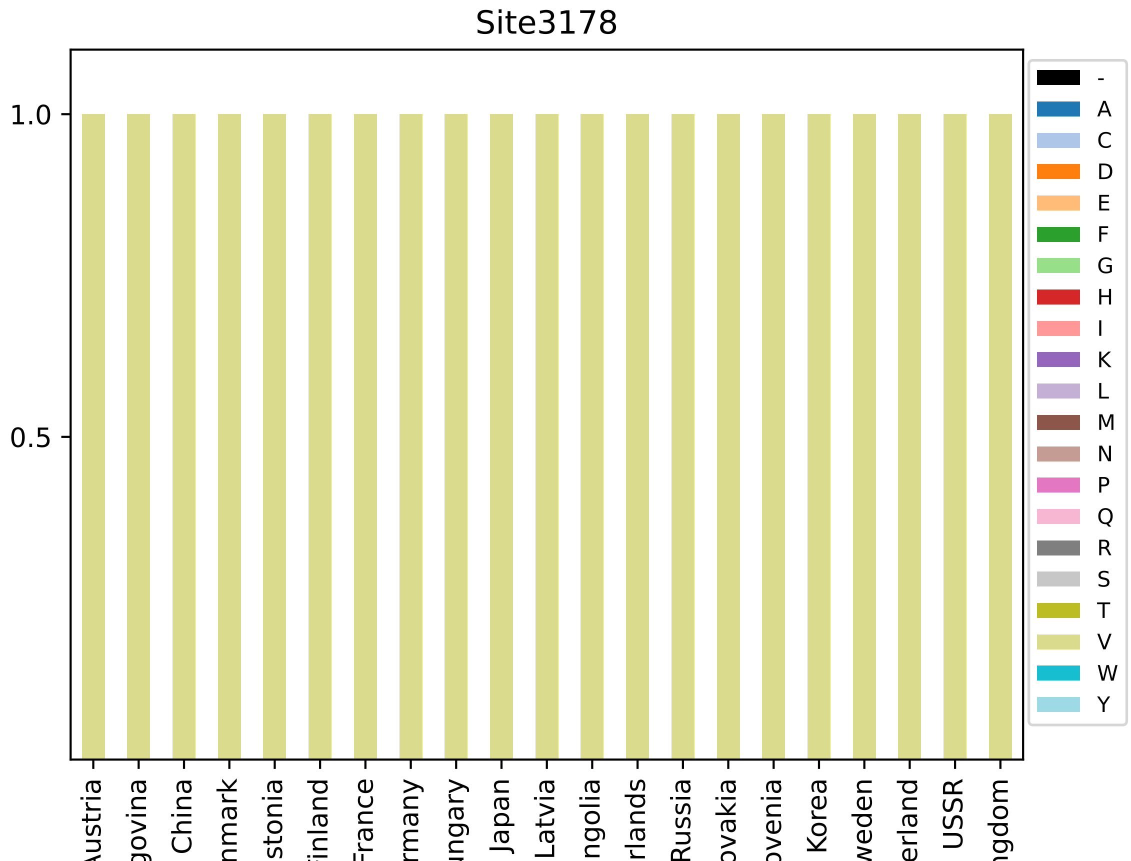

Site3178
1.0
0.5
Austria
govina
China
nmark
stonia
Finland
France
rmany
ungary
Japan
Latvia
ngolia
rlands
Russia
ovakia
ovenia
Korea
weden
erland
USSR
ngdom
-
A
C
D
E
F
G
H
I
K
L
M
N
P
Q
R
S
T
V
W
Y

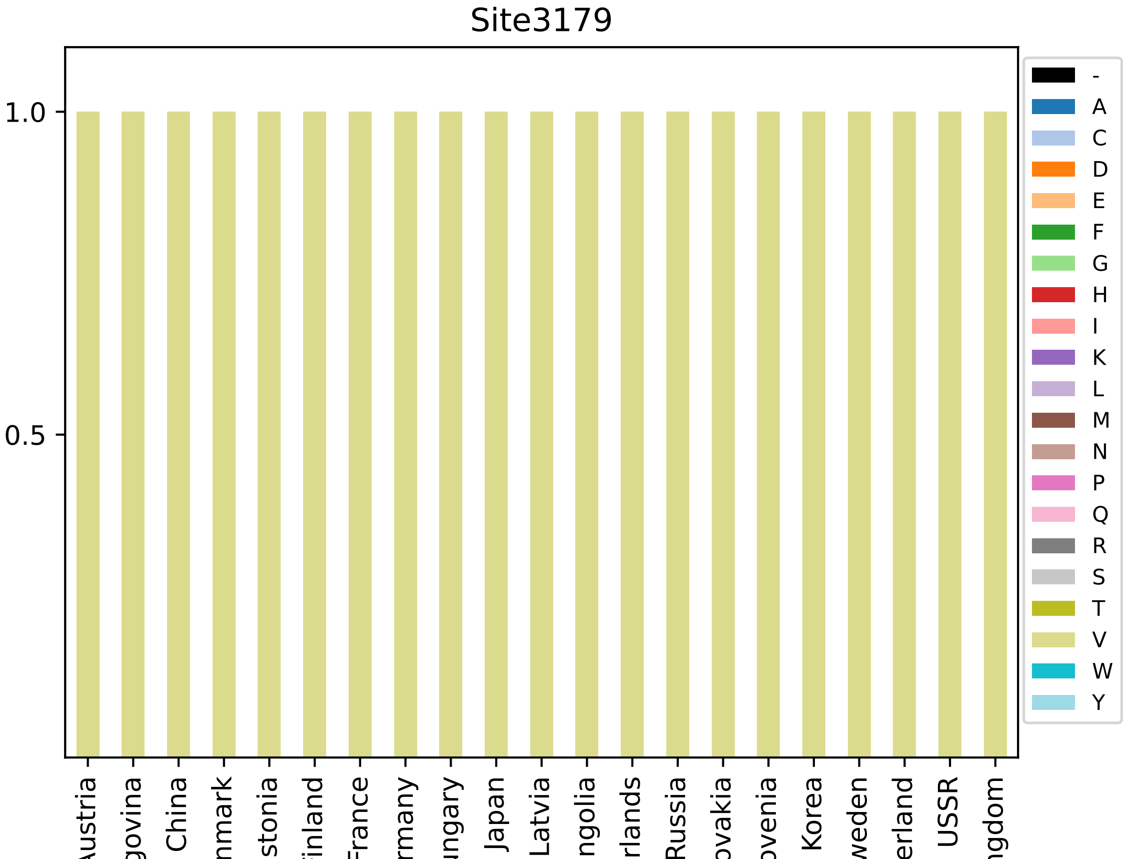
Site3179
1.0
0.5
-
A
C
D
E
F
G
H
I
K
L
M
N
P
Q
R
S
T
V
W
Y
Austria
China
Japan
Latvia
Russia
Korea
USSR

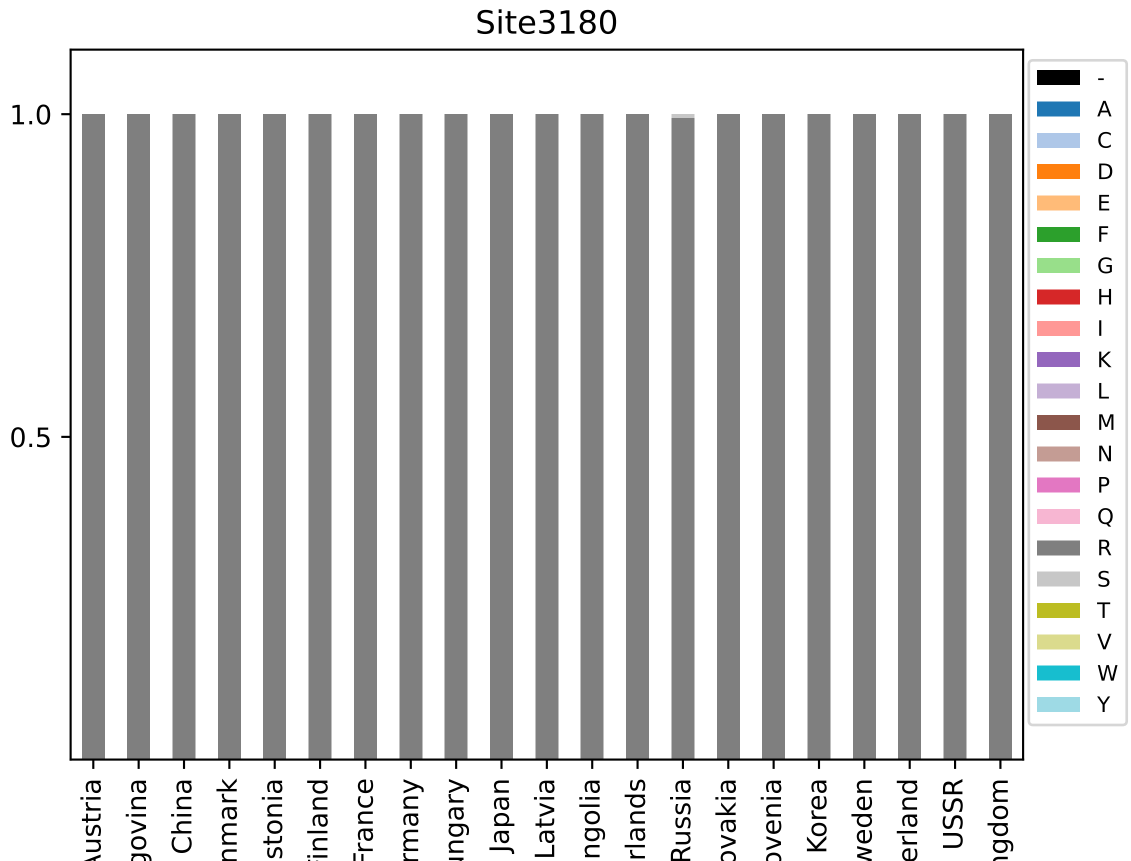
Site3180
1.0
0.5
-
A
C
D
E
F
G
H
I
K
L
M
N
P
Q
R
S
T
V
W
Y
Austria
China
Japan
Latvia
Russia
Korea
USSR

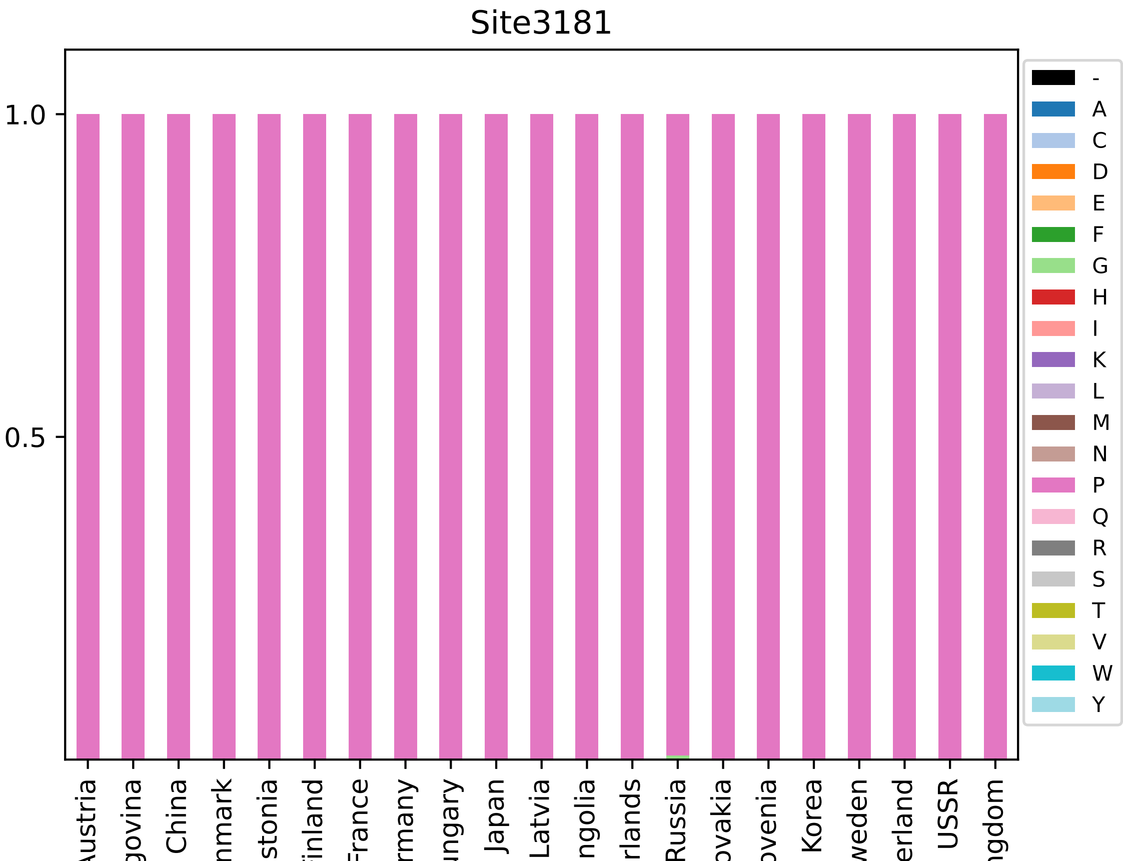

Site3181
1.0
0.5
-
A
C
D
E
F
G
H
I
K
L
M
N
P
Q
R
S
T
V
W
Y
China
Japan
Latvia
Russia
Korea
USSR

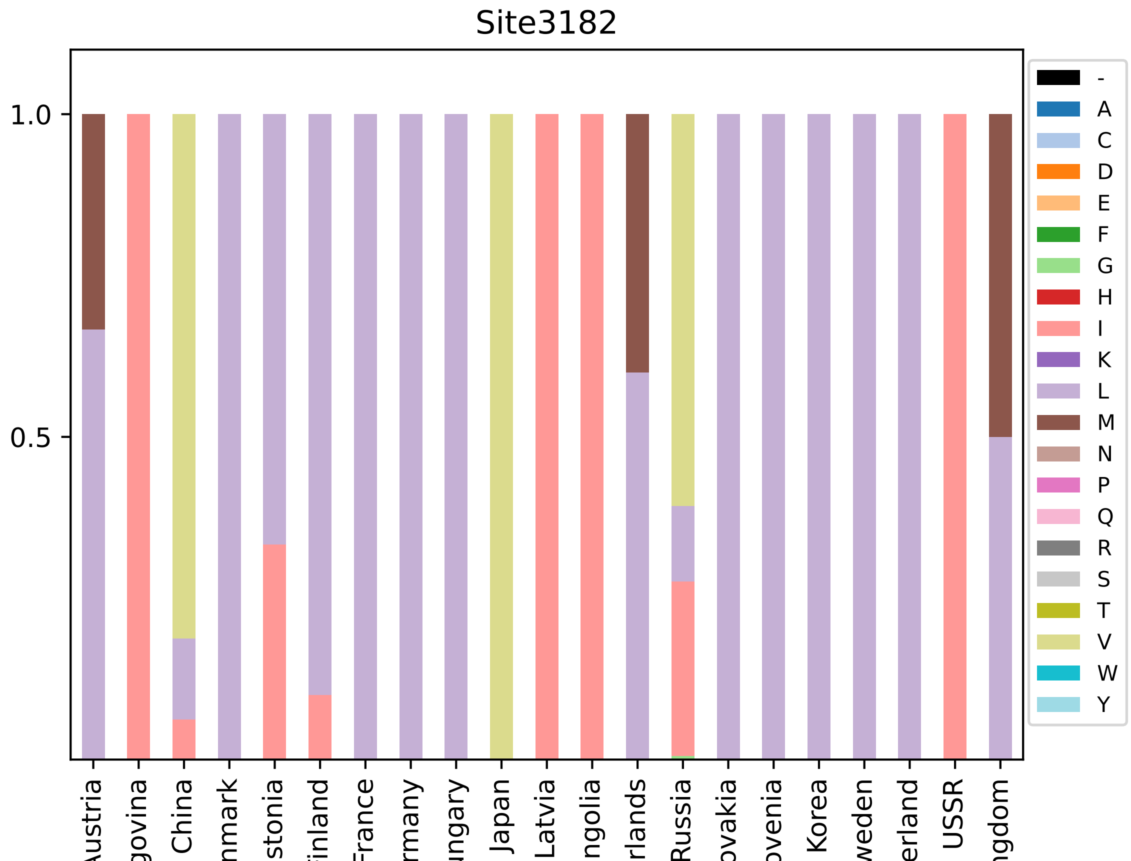

Site3182
1.0
0.5
-
A
C
D
E
F
G
H
I
K
L
M
N
P
Q
R
S
T
V
W
Y
Austria
govina
China
nmark
stonia
Finland
France
rmany
ungary
Japan
Latvia
ngolia
rlands
Russia
ovakia
ovenia
Korea
weden
erland
USSR
ngdom

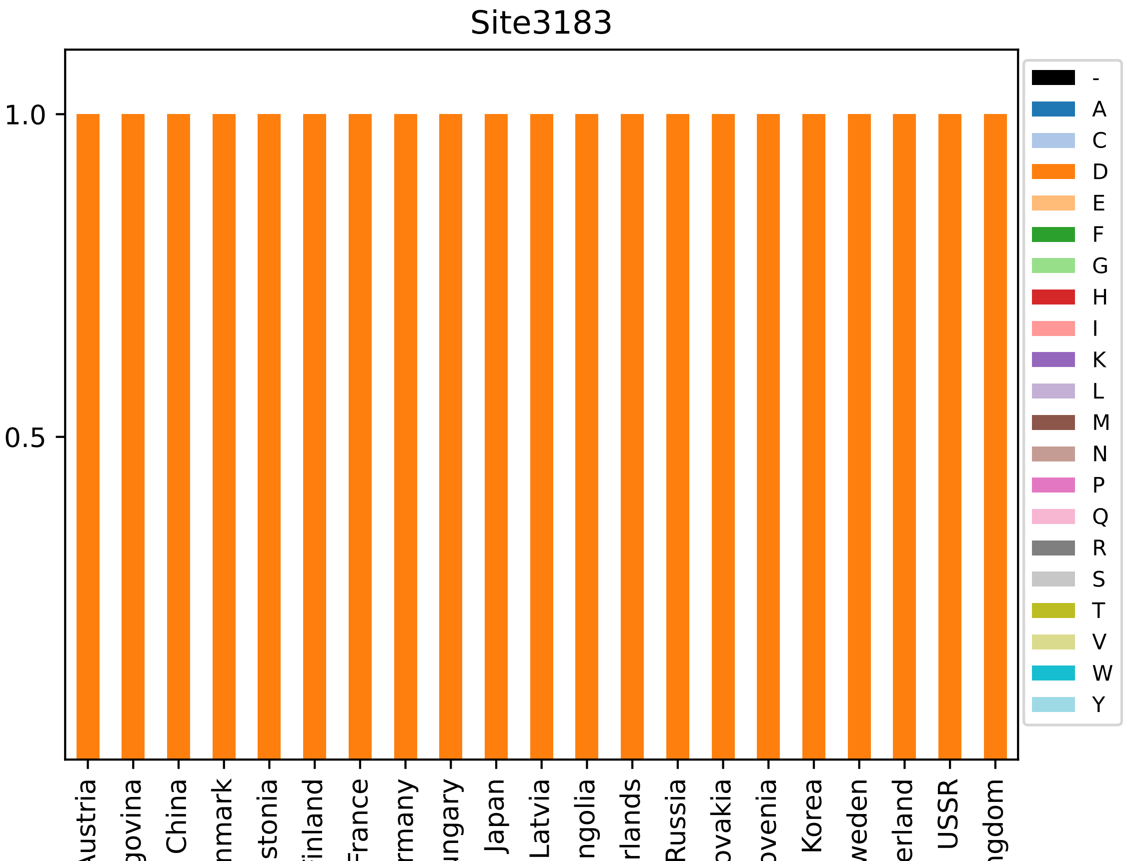
Site3183
1.0
0.5
-
A
C
D
E
F
G
H
I
K
L
M
N
P
Q
R
S
T
V
W
Y
China
Japan
Latvia
Russia
Korea
USSR

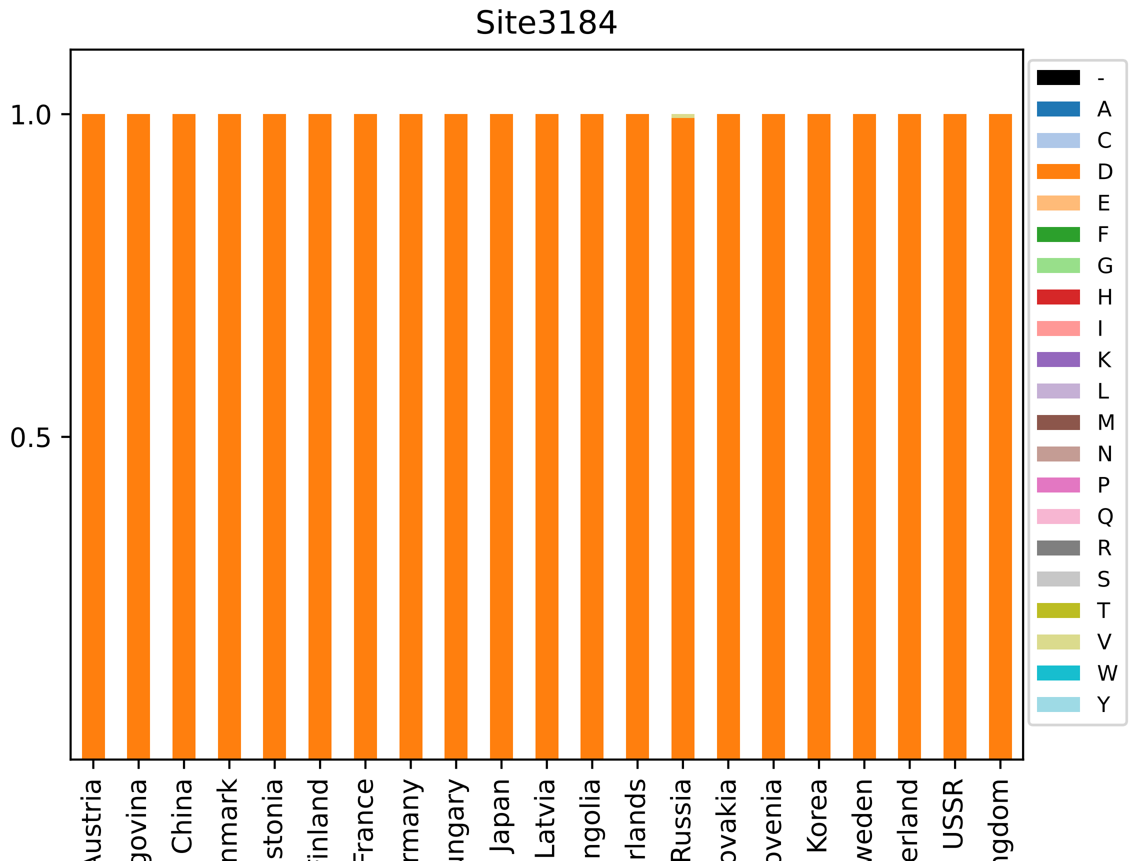

Site3184
1.0
0.5
-
A
C
D
E
F
G
H
I
K
L
M
N
P
Q
R
S
T
V
W
Y
China
Japan
Latvia
Russia
Korea
USSR

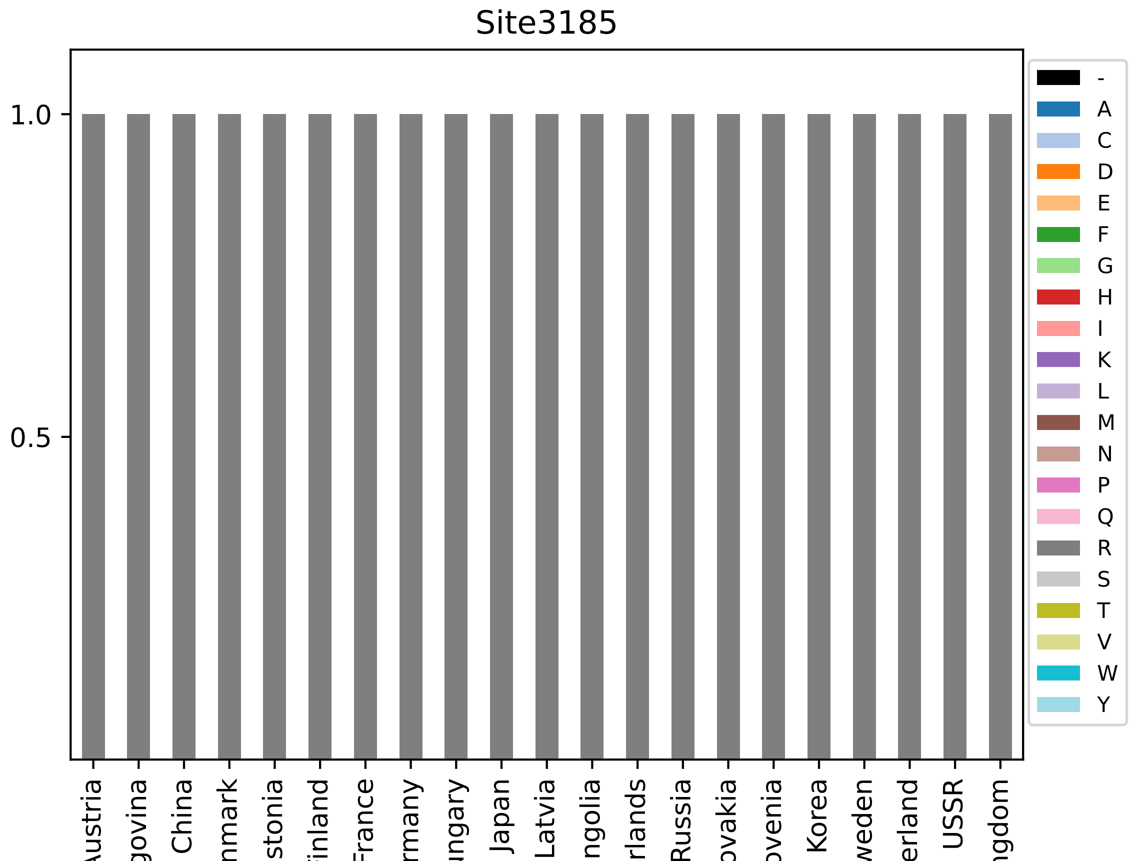
Site3185
1.0
0.5
-
A
C
D
E
F
G
H
I
K
L
M
N
P
Q
R
S
T
V
W
Y
Austria
govina
China
nmark
stonia
inland
France
rmany
ungary
Japan
Latvia
ngolia
rlands
Russia
ovakia
ovenia
Korea
weden
erland
USSR
ngdom

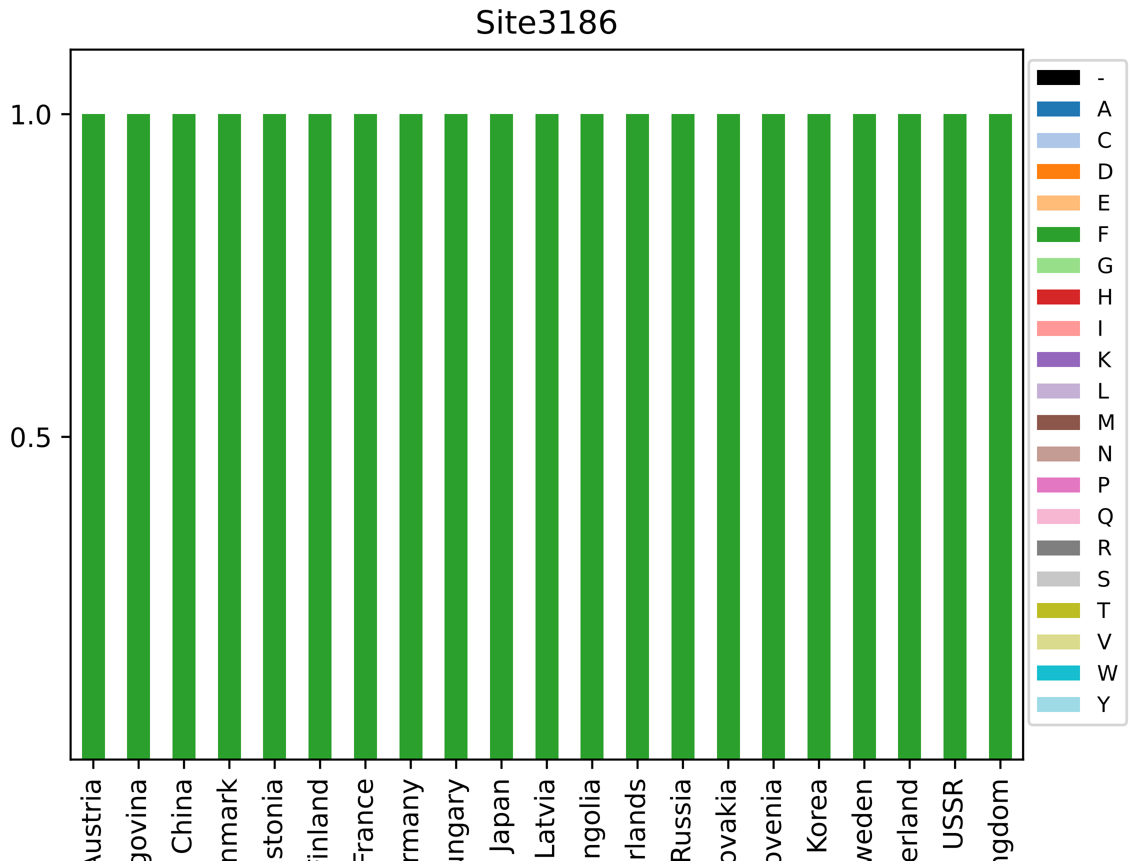
Site3186
1.0
0.5
China
Japan
Latvia
Russia
Korea
USSR
-
A
C
D
E
F
G
H
I
K
L
M
N
P
Q
R
S
T
V
W
Y

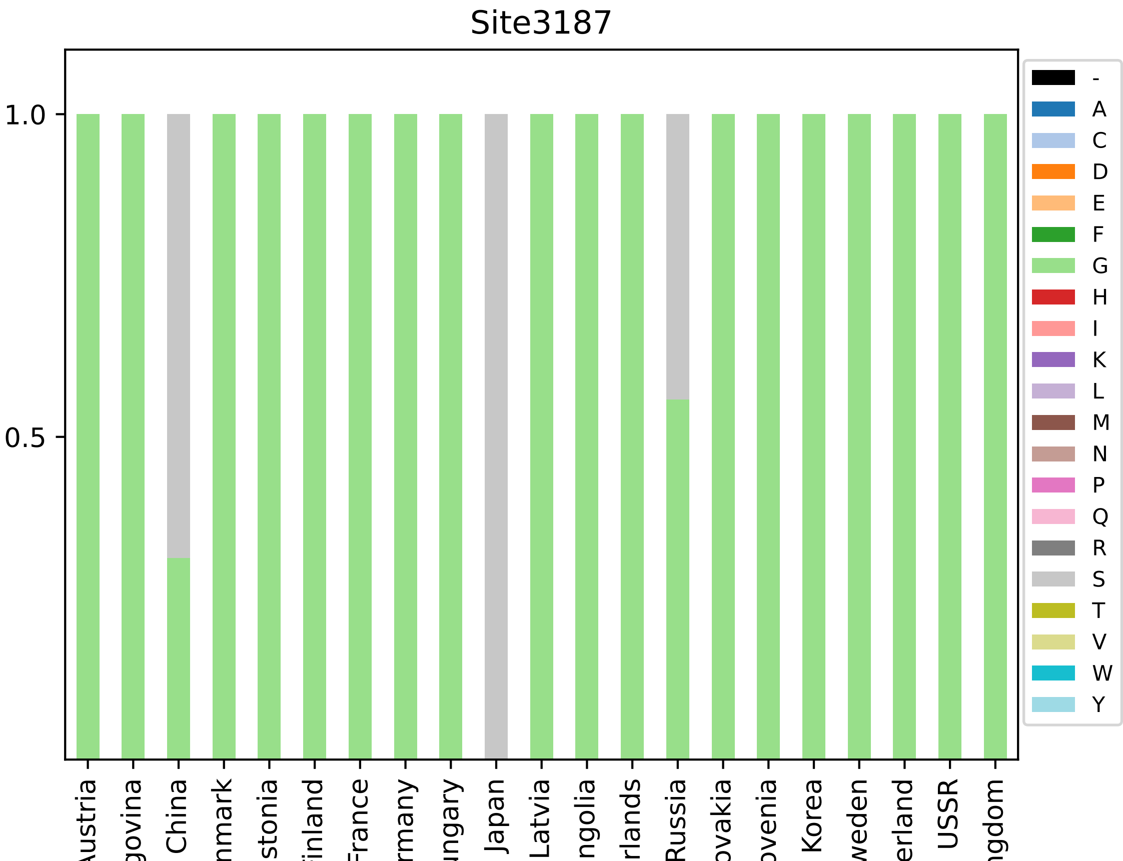

Site3187
1.0
0.5
-
A
C
D
E
F
G
H
I
K
L
M
N
P
Q
R
S
T
V
W
Y
Austria
govina
China
nmark
stonia
inland
France
rmany
ungary
Japan
Latvia
ngolia
rlands
Russia
ovakia
ovenia
Korea
weden
erland
USSR
ngdom

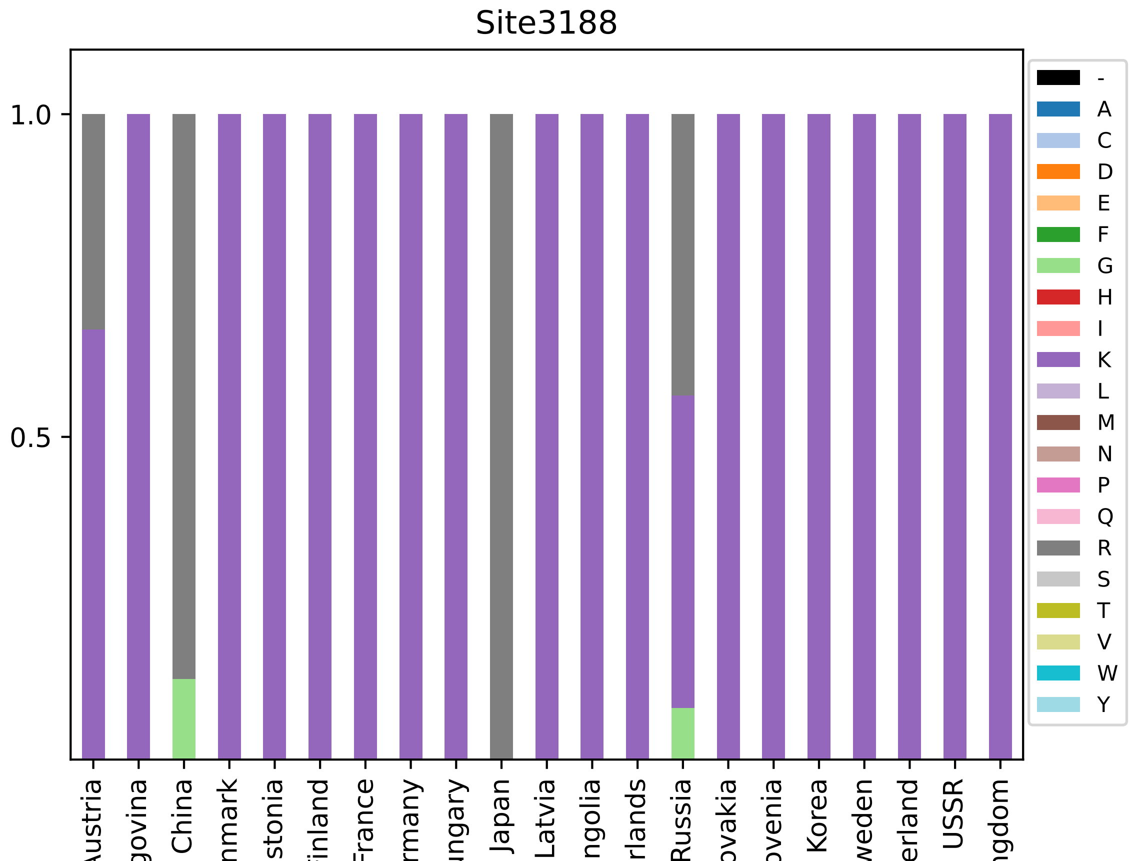

Site3188
1.0
0.5
-
A
C
D
E
F
G
H
I
K
L
M
N
P
Q
R
S
T
V
W
Y
China
Japan
Latvia
Russia
Korea
USSR

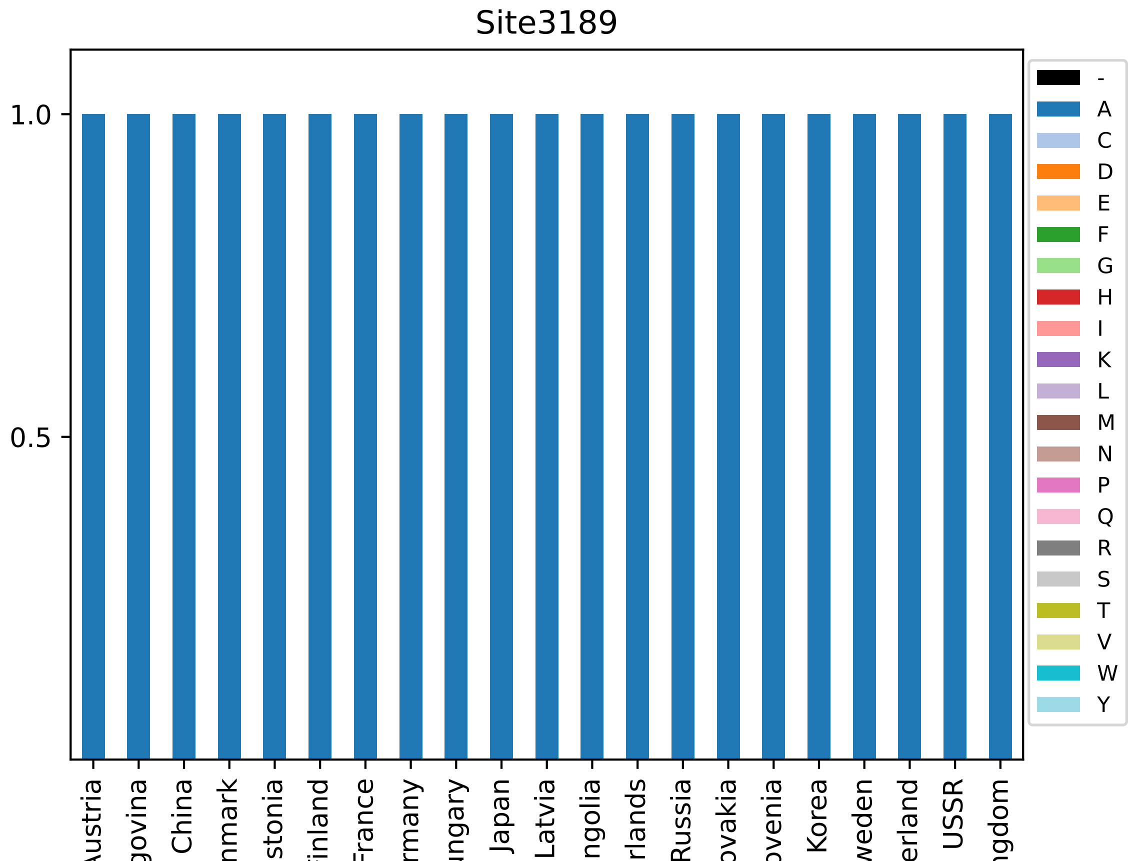

Site3189
1.0
0.5
-
A
C
D
E
F
G
H
I
K
L
M
N
P
Q
R
S
T
V
W
Y
China
Japan
Latvia
Russia
Korea
USSR

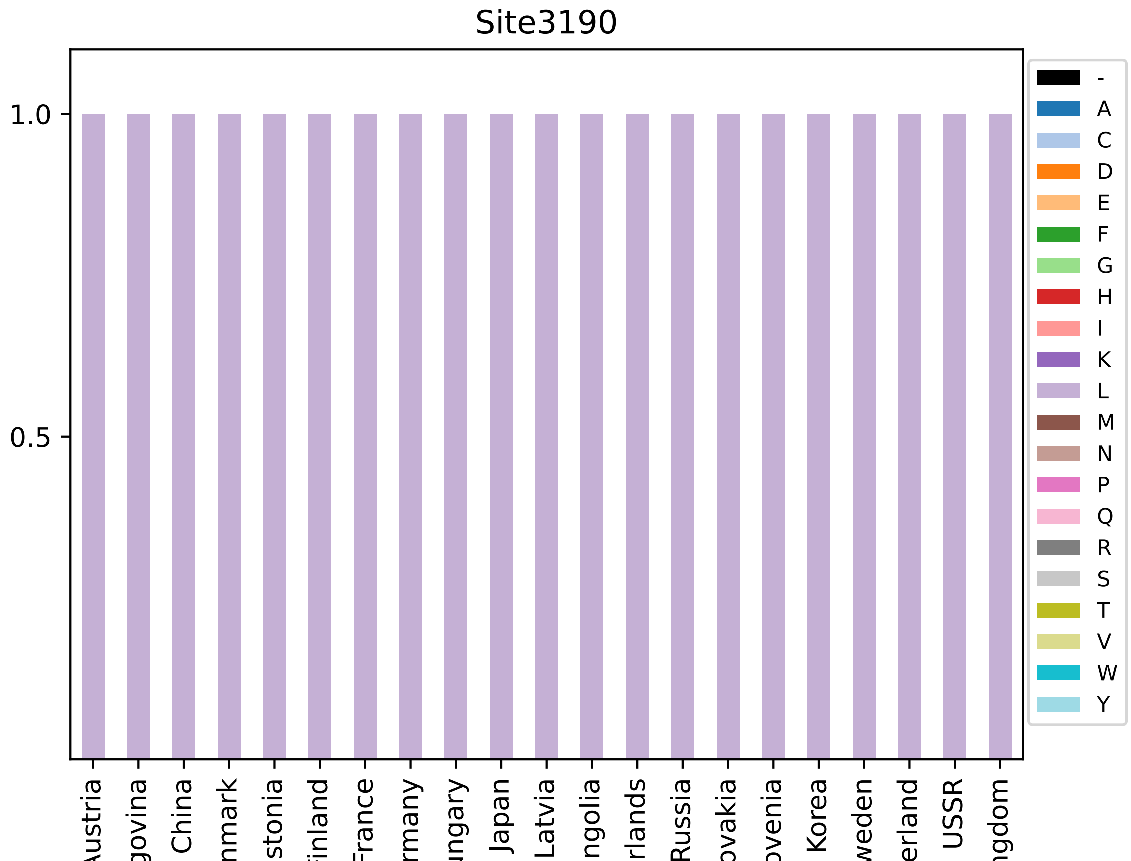

Site3190
1.0
0.5
China
Japan
Latvia
Russia
Korea
USSR
-
A
C
D
E
F
G
H
I
K
L
M
N
P
Q
R
S
T
V
W
Y

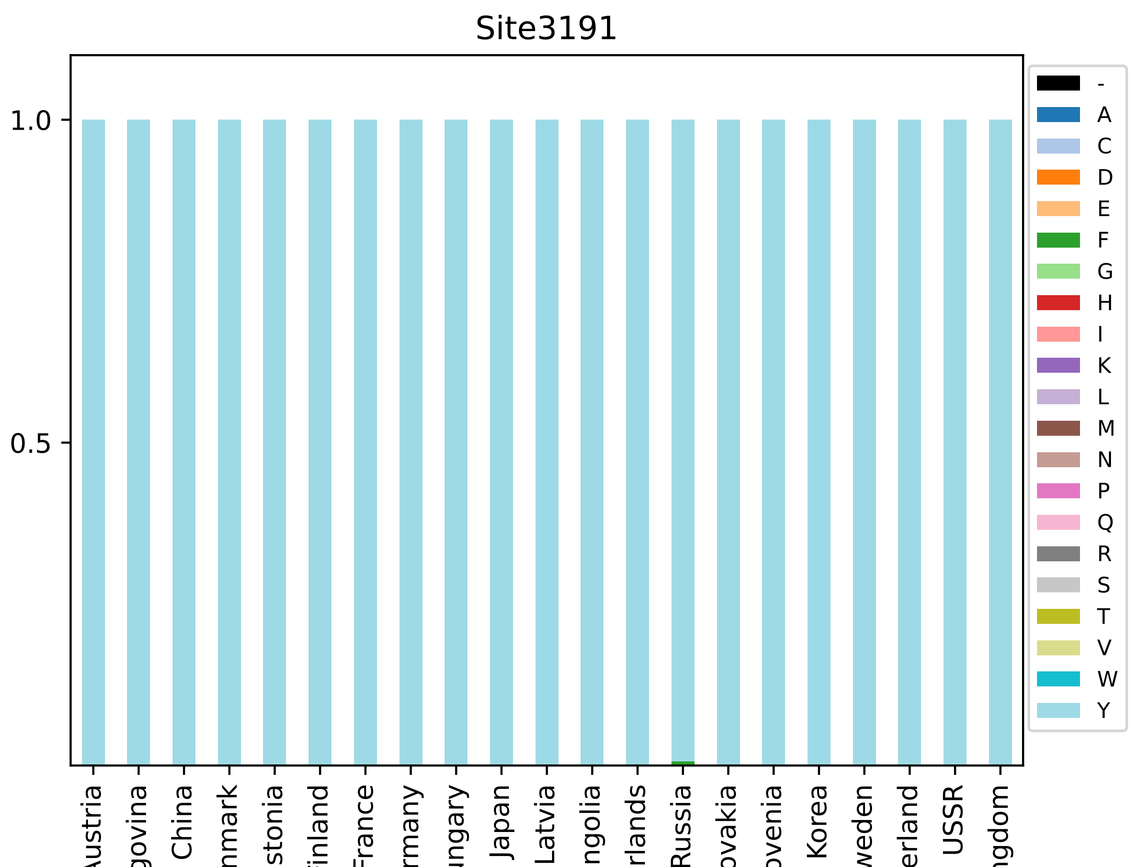

Site3191
1.0
0.5
-
A
C
D
E
F
G
H
I
K
L
M
N
P
Q
R
S
T
V
W
Y
Austria
govina
China
nmark
stonia
inland
France
rmany
ungary
Japan
Latvia
ngolia
rlands
Russia
ovakia
ovenia
Korea
weden
erland
USSR
ngdom

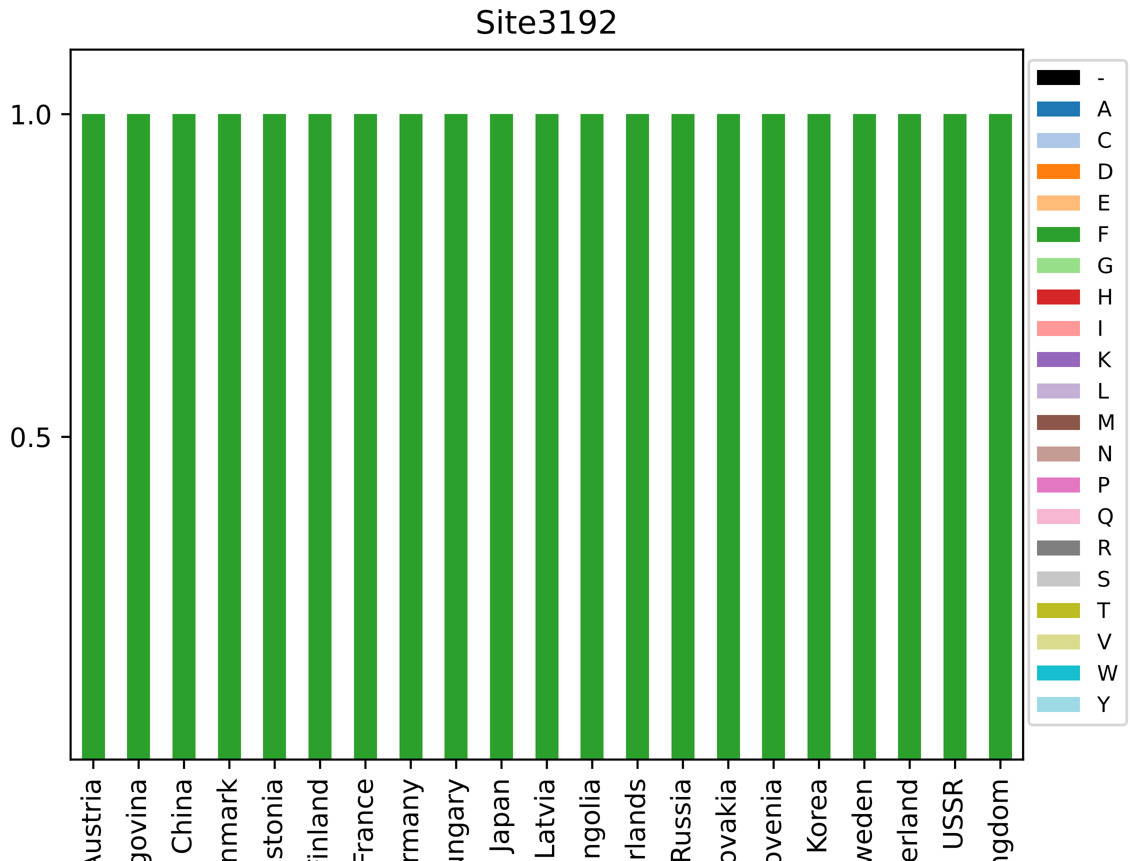
Site3192
1.0
0.5
-
A
C
D
E
F
G
H
I
K
L
M
N
P
Q
R
S
T
V
W
Y
Austria
China
Japan
Latvia
Russia
Korea
USSR

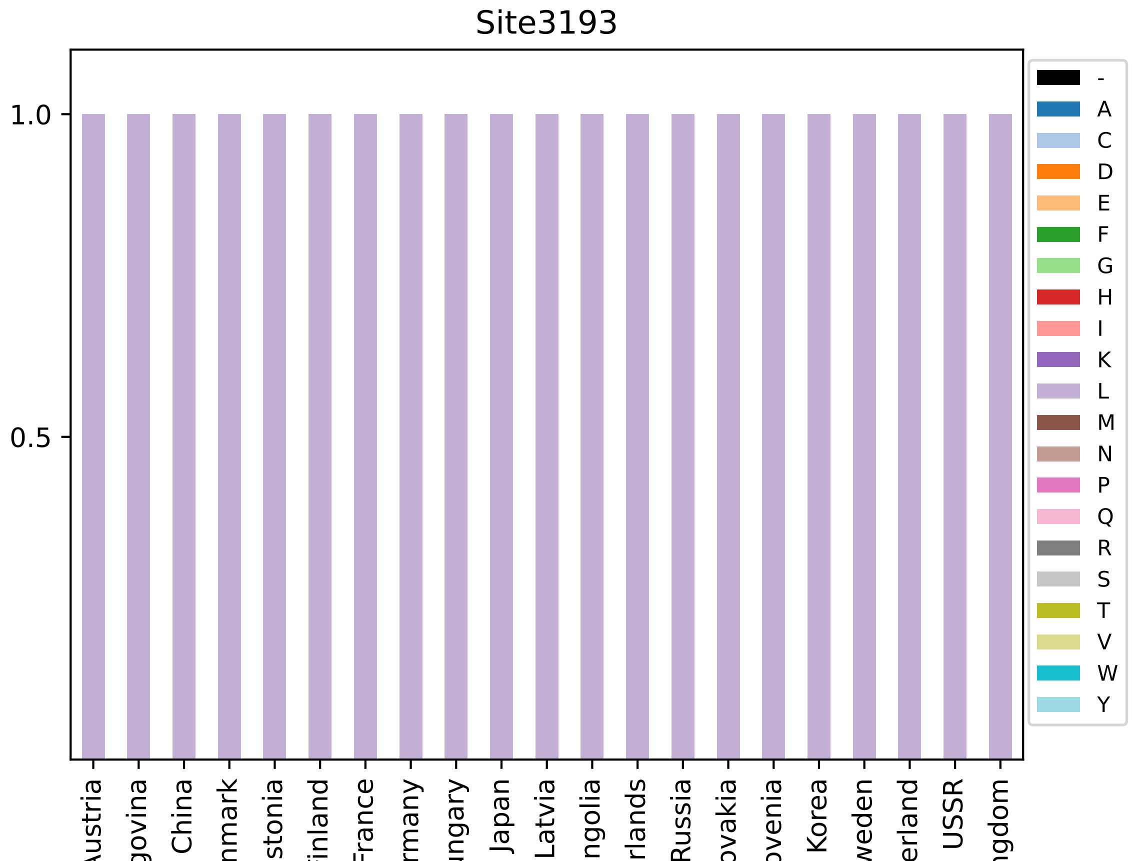

Site3193
1.0
0.5
Austria
govina
China
nmark
stonia
Finland
France
rmany
ungary
Japan
Latvia
ngolia
rlands
Russia
ovakia
ovenia
Korea
weden
erland
USSR
ngdom
-
A
C
D
E
F
G
H
I
K
L
M
N
P
Q
R
S
T
V
W
Y

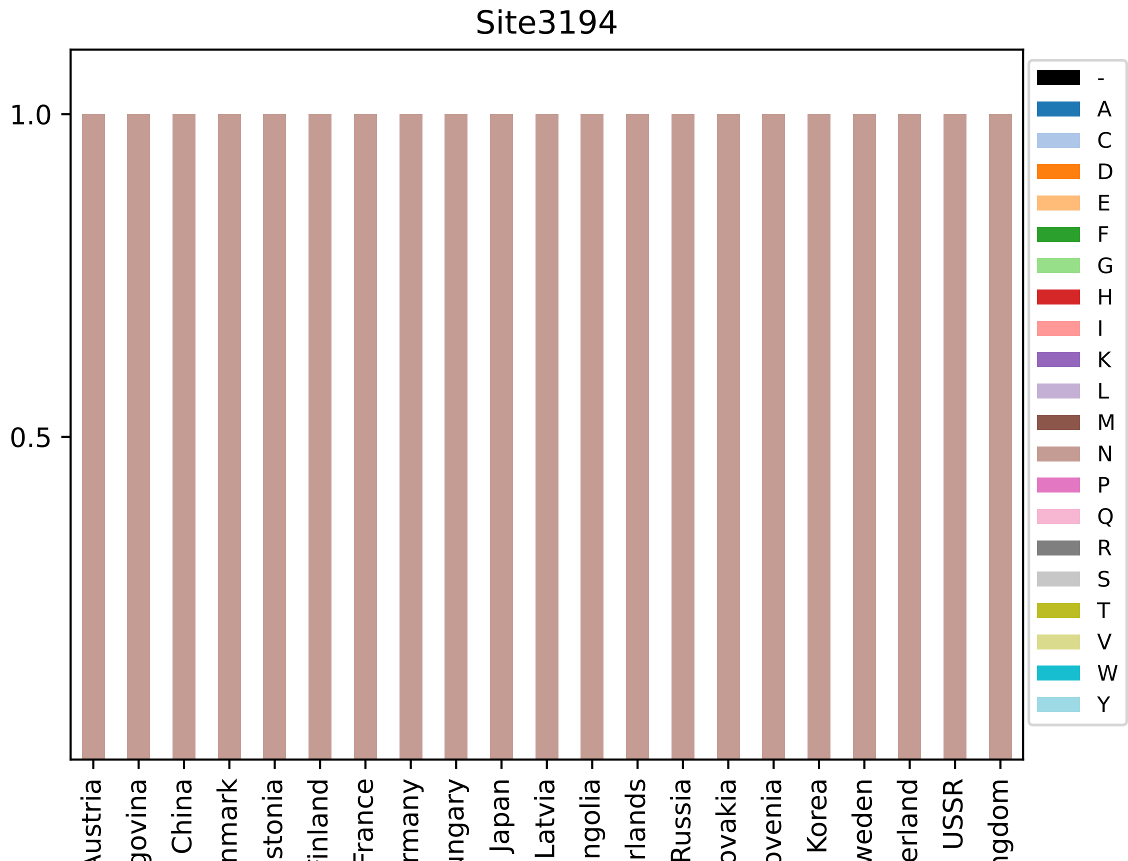
Site3194
1.0
0.5
Austria
govina
China
nmark
stonia
Finland
France
rmany
ungary
Japan
Latvia
ngolia
rlands
Russia
ovakia
ovenia
Korea
weden
erland
USSR
ngdom
-
A
C
D
E
F
G
H
I
K
L
M
N
P
Q
R
S
T
V
W
Y

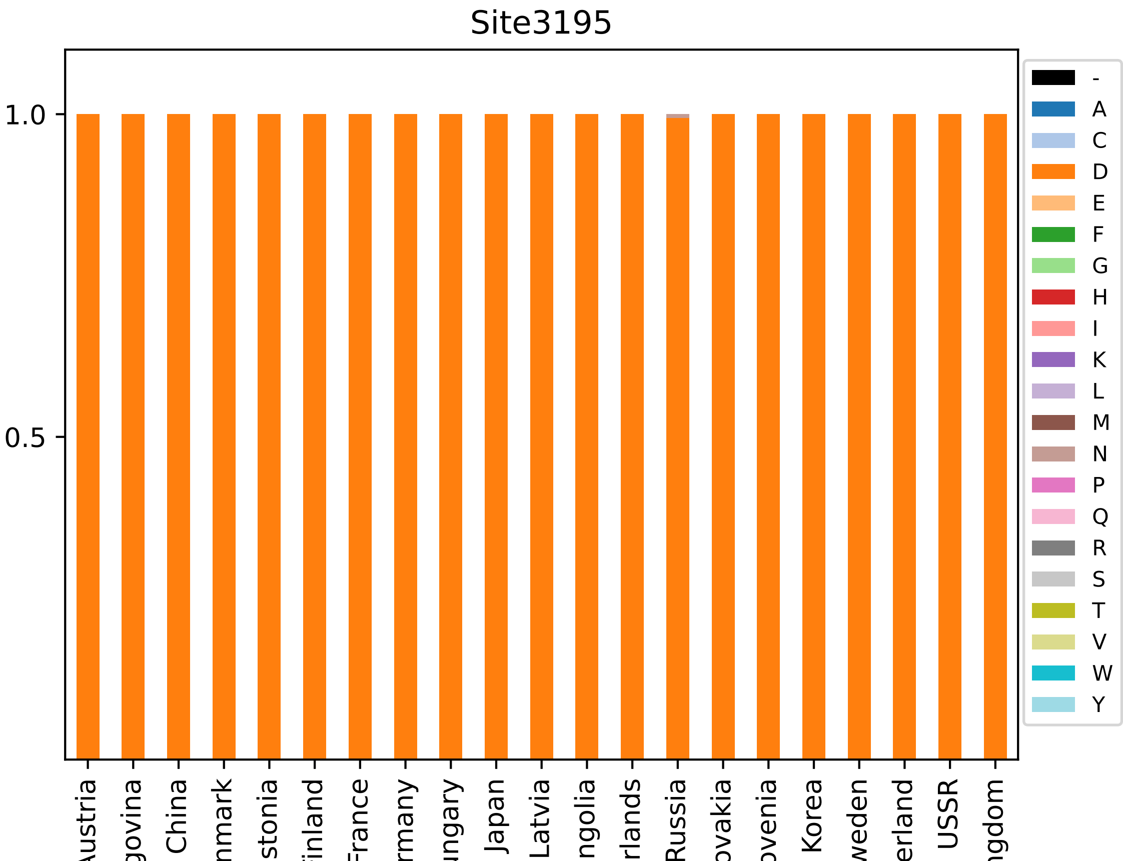
Site3195
1.0
0.5
-
A
C
D
E
F
G
H
I
K
L
M
N
P
Q
R
S
T
V
W
Y
China
Japan
Latvia
Russia
Korea
USSR

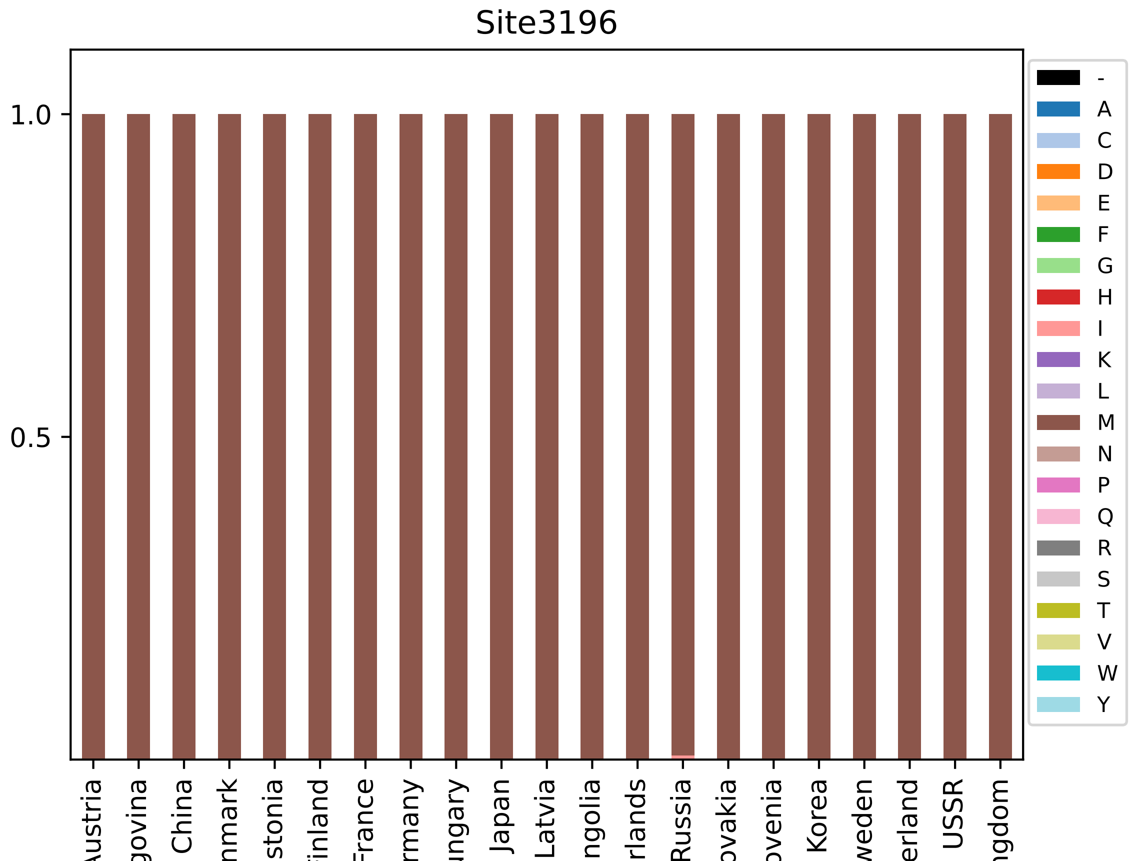
Site3196
1.0
0.5
-
A
C
D
E
F
G
H
I
K
L
M
N
P
Q
R
S
T
V
W
Y
China
Finland
France
Japan
Latvia
Russia
Korea
USSR

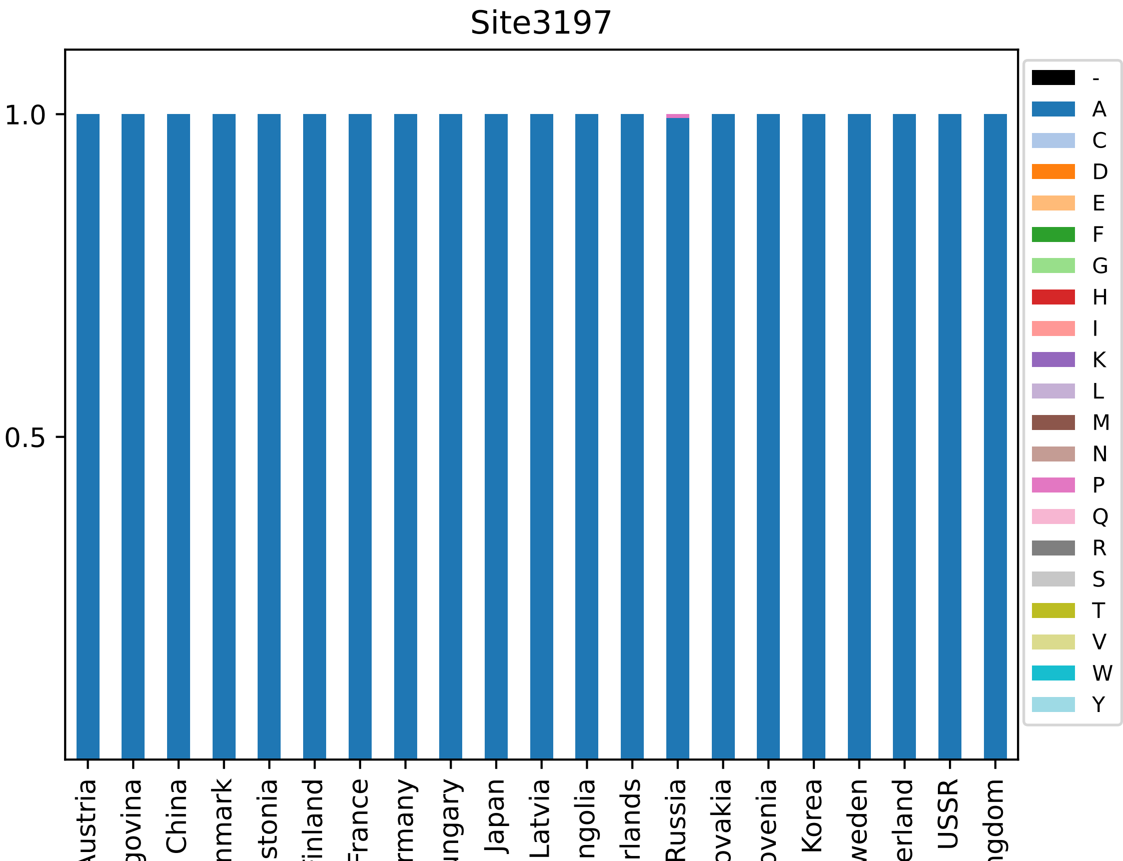

Site3197
1.0
0.5
-
A
C
D
E
F
G
H
I
K
L
M
N
P
Q
R
S
T
V
W
Y
China
Japan
Latvia
Russia
Korea
USSR

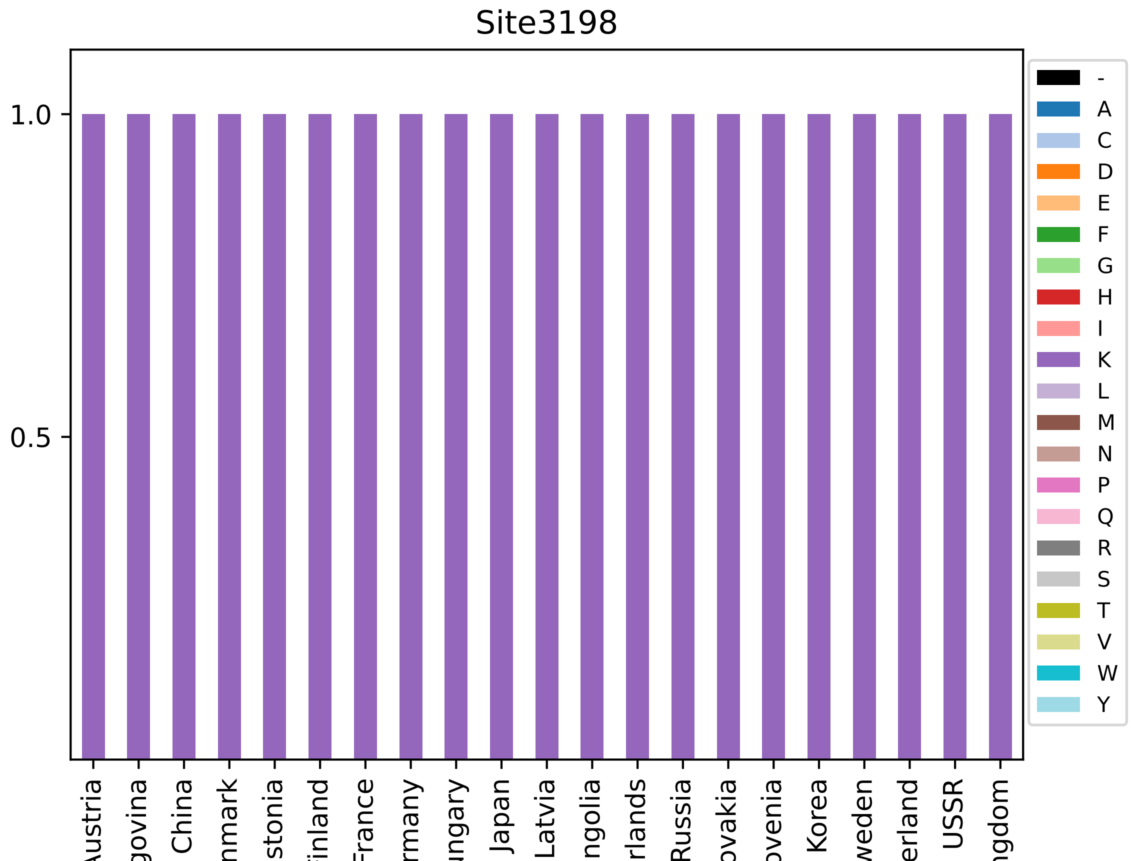

Site3198
1.0
0.5
-
A
C
D
E
F
G
H
I
K
L
M
N
P
Q
R
S
T
V
W
Y
China
Japan
Latvia
Russia
Korea
USSR

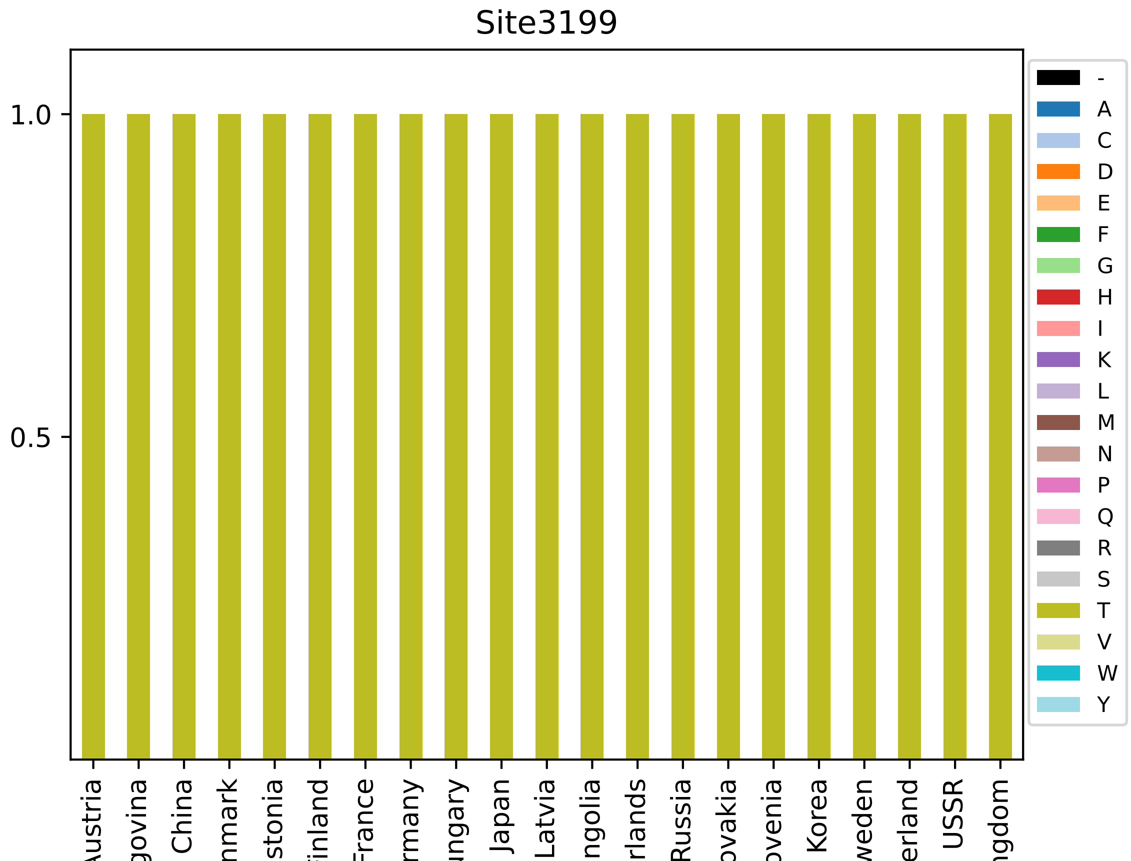
Site3199
1.0
0.5
-
A
C
D
E
F
G
H
I
K
L
M
N
P
Q
R
S
T
V
W
Y
China
Japan
Latvia
Russia
Korea
USSR

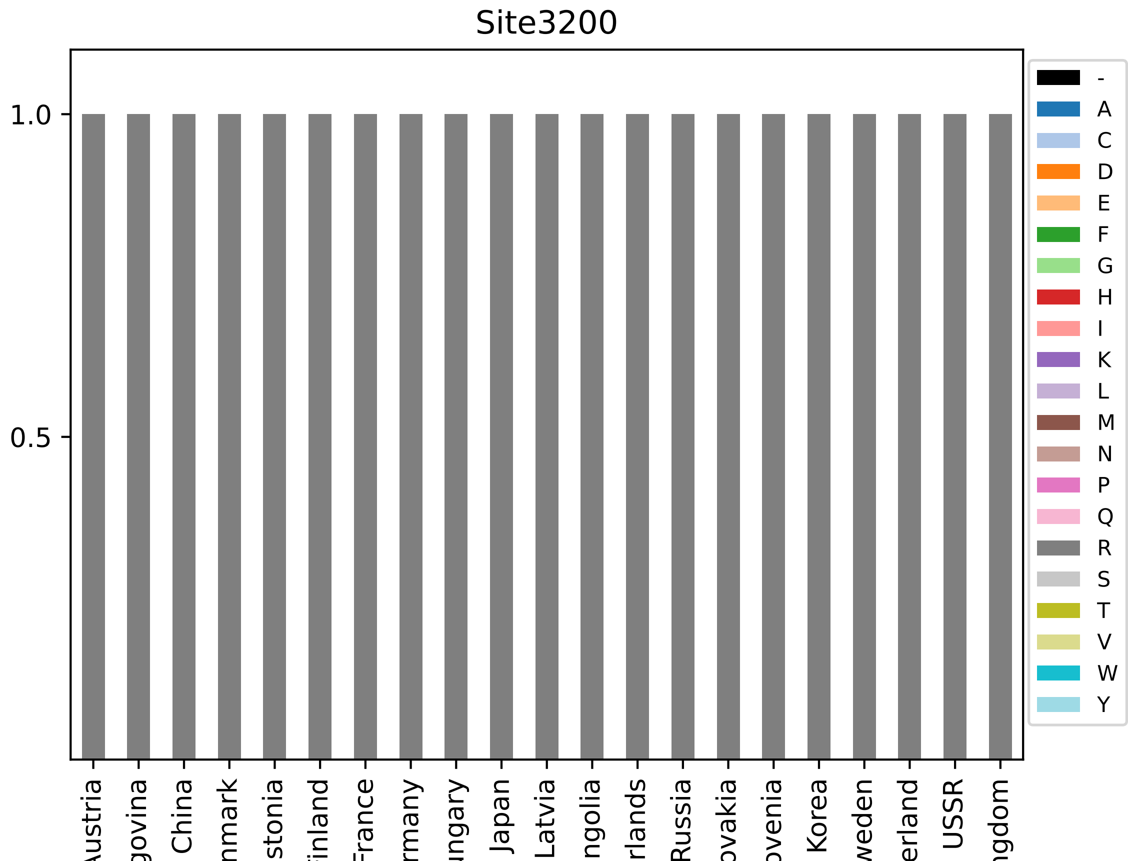

Site3200
1.0
0.5
-
A
C
D
E
F
G
H
I
K
L
M
N
P
Q
R
S
T
V
W
Y
Austria
govina
China
nmark
stonia
inland
France
rmany
ungary
Japan
Latvia
ngolia
rlands
Russia
ovakia
ovenia
Korea
weden
erland
USSR
ngdom

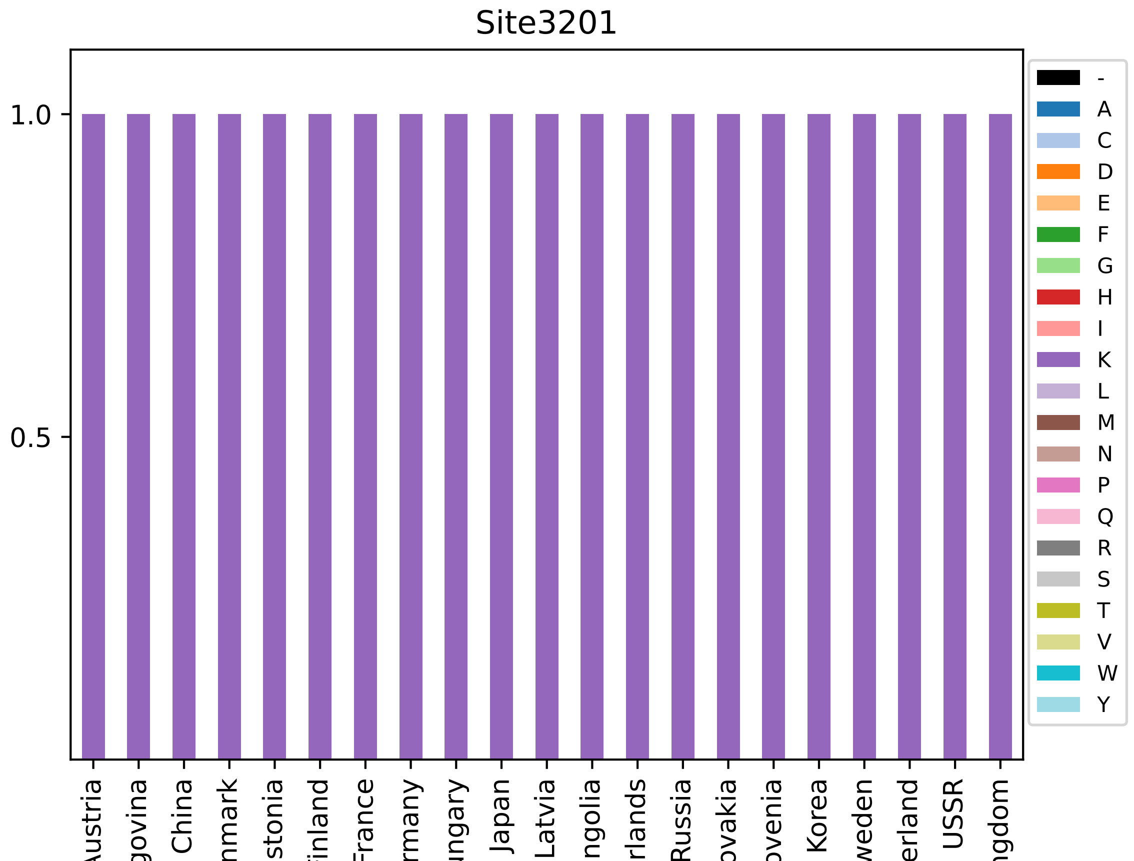

Site3201
1.0
0.5
-
A
C
D
E
F
G
H
I
K
L
M
N
P
Q
R
S
T
V
W
Y
China
Japan
Latvia
Russia
Korea
USSR

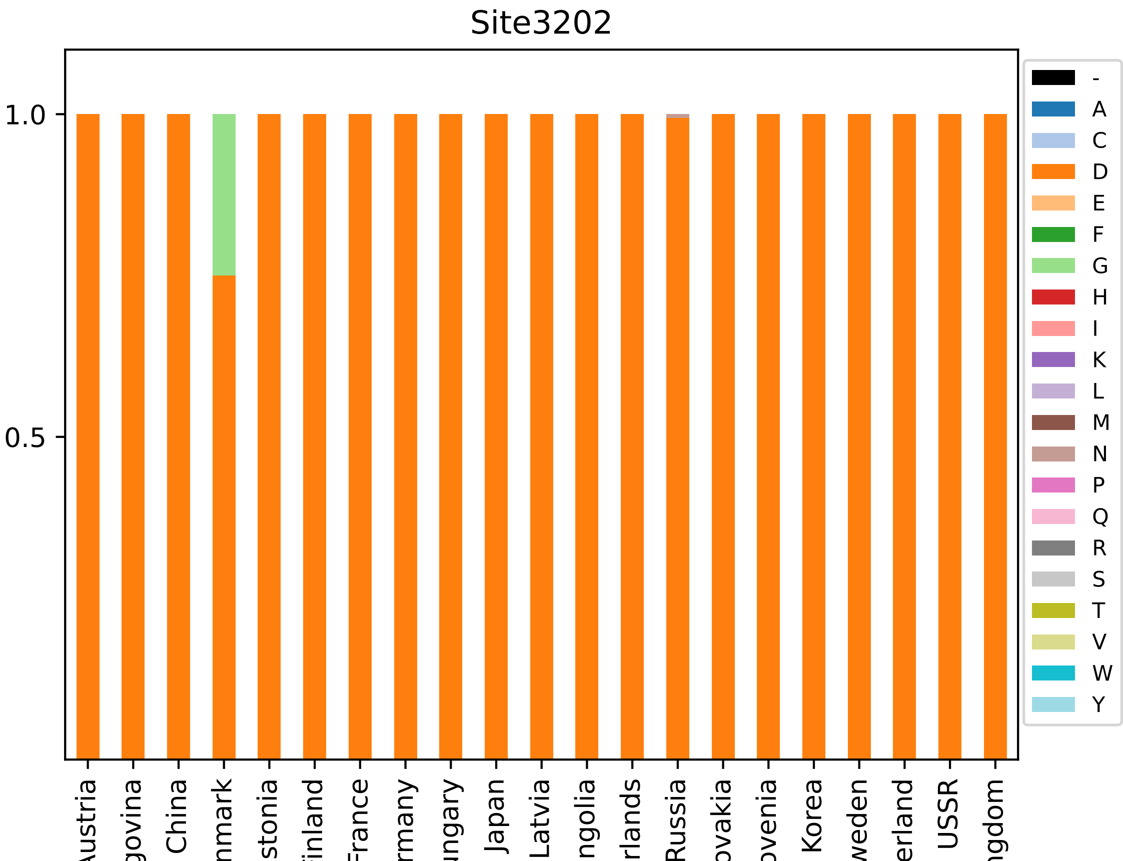

Site3202
1.0
0.5
-
A
C
D
E
F
G
H
I
K
L
M
N
P
Q
R
S
T
V
W
Y
China
Japan
Latvia
Russia
Korea
USSR

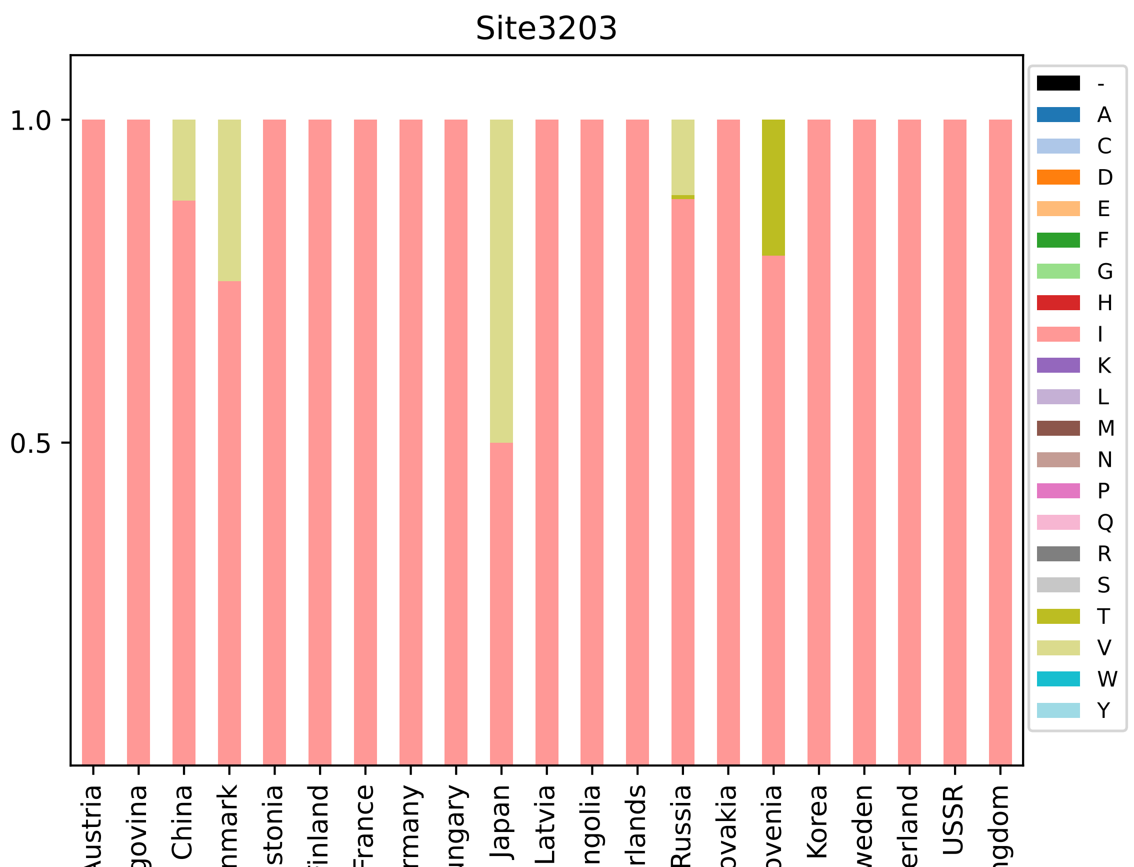

Site3203
1.0
0.5
-
A
C
D
E
F
G
H
I
K
L
M
N
P
Q
R
S
T
V
W
Y
China
Japan
Latvia
Russia
Korea
USSR

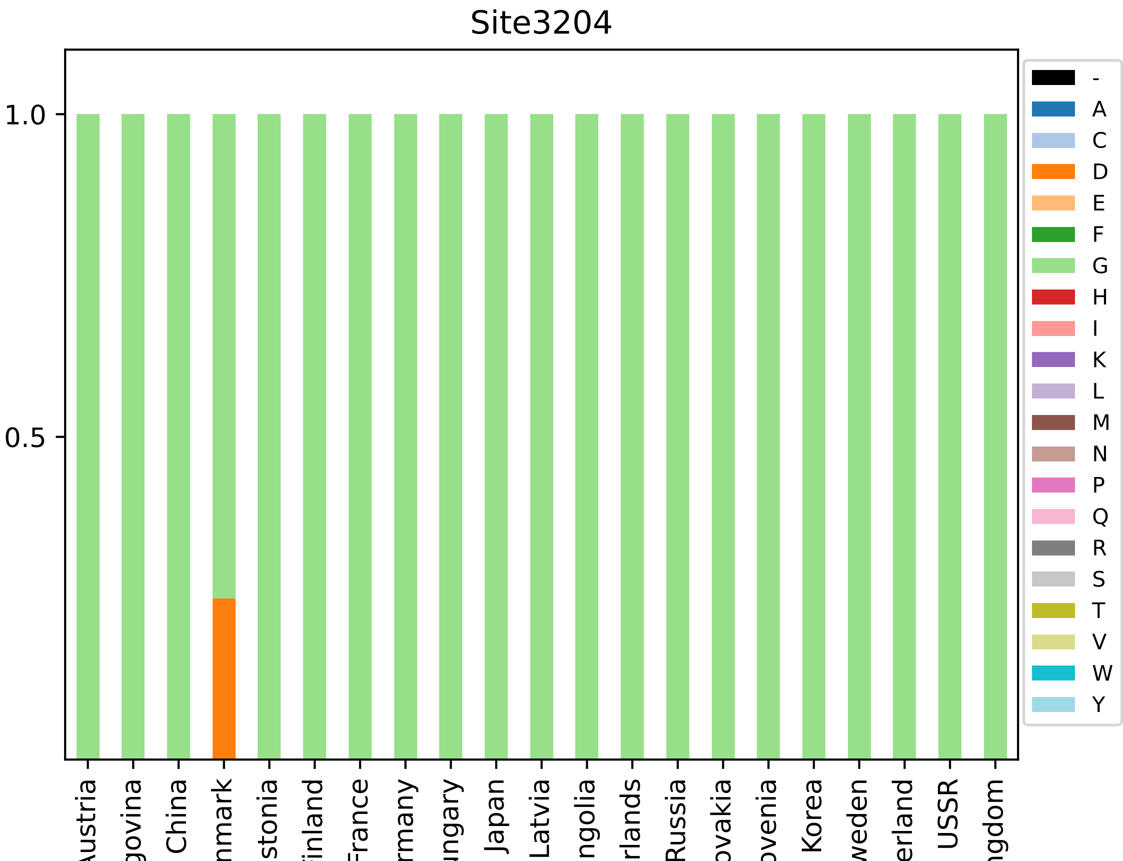
Site3204
1.0
0.5
-
A
C
D
E
F
G
H
I
K
L
M
N
P
Q
R
S
T
V
W
Y
Austria
govina
China
nmark
stonia
Finland
France
rmany
ungary
Japan
Latvia
ngolia
rlands
Russia
ovakia
ovenia
Korea
weden
erland
USSR
ngdom

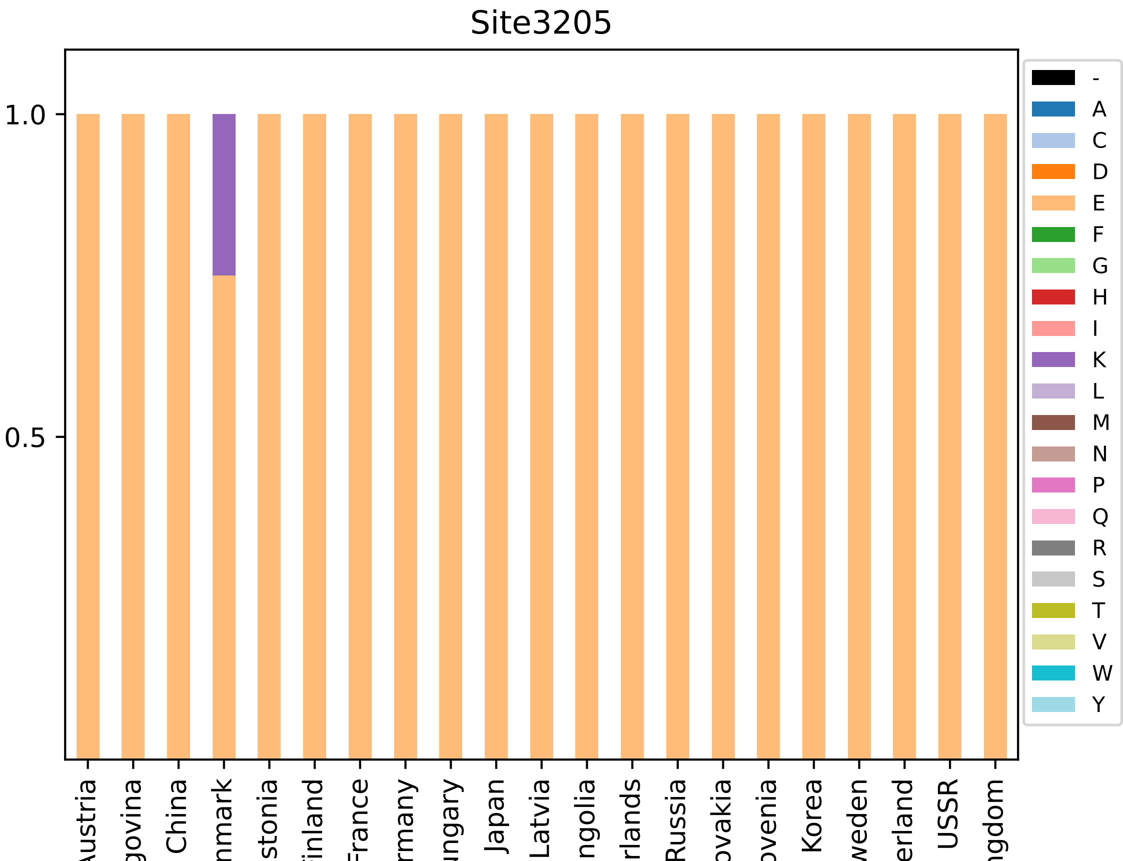
Site3205
1.0
0.5
-
A
C
D
E
F
G
H
I
K
L
M
N
P
Q
R
S
T
V
W
Y
China
Japan
Latvia
Russia
Korea
USSR

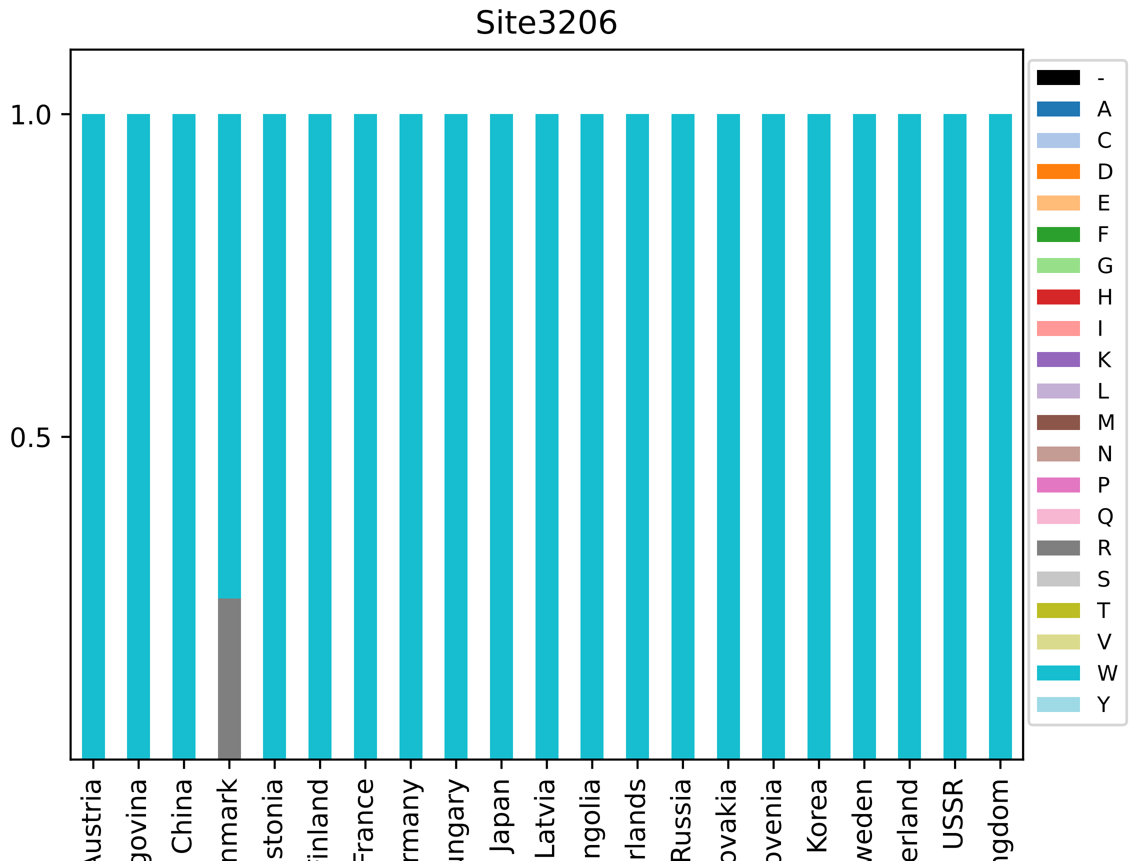

Site3206
1.0
0.5
-
A
C
D
E
F
G
H
I
K
L
M
N
P
Q
R
S
T
V
W
Y
China
Japan
Latvia
Russia
Korea
USSR

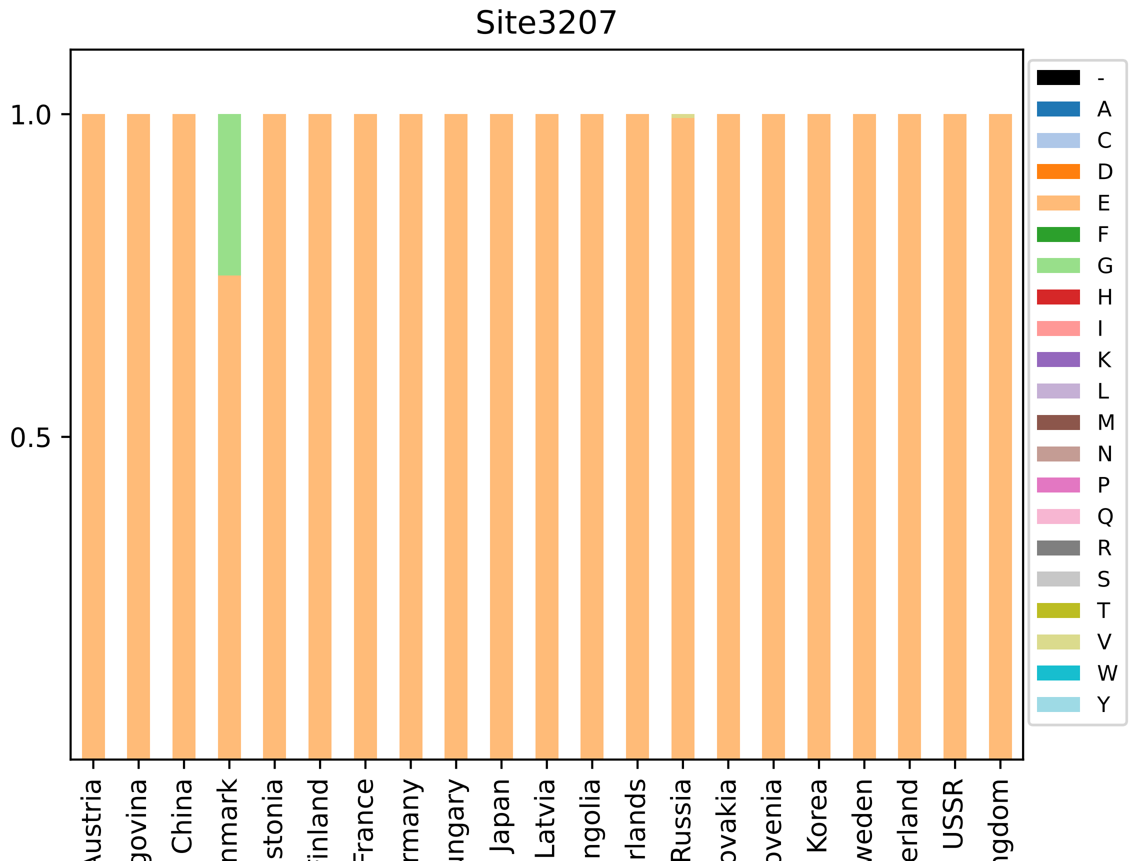

Site3207
1.0
0.5
-
A
C
D
E
F
G
H
I
K
L
M
N
P
Q
R
S
T
V
W
Y
Austria
govina
China
nmark
stonia
Finland
France
rmany
ungary
Japan
Latvia
ngolia
rlands
Russia
ovakia
ovenia
Korea
weden
erland
USSR
ngdom

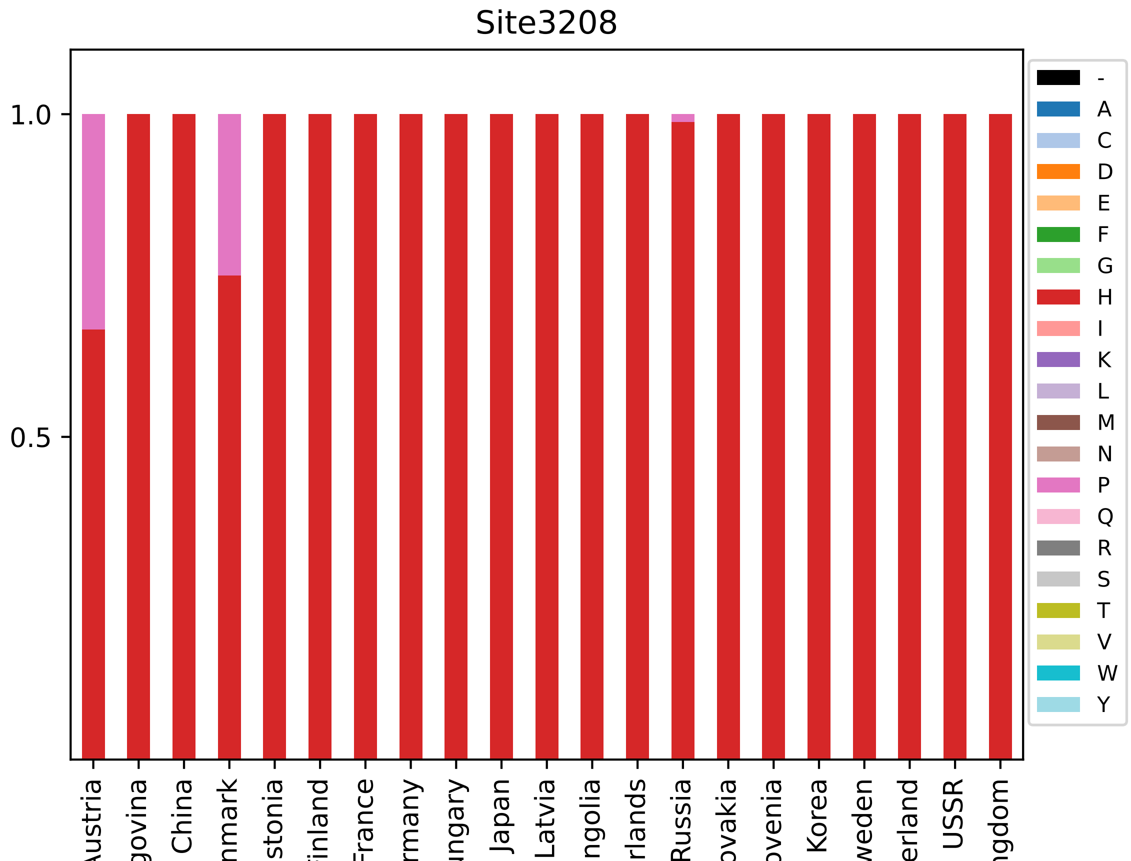
Site3208
1.0
0.5
-
A
C
D
E
F
G
H
I
K
L
M
N
P
Q
R
S
T
V
W
Y
China
Japan
Latvia
Russia
Korea
USSR

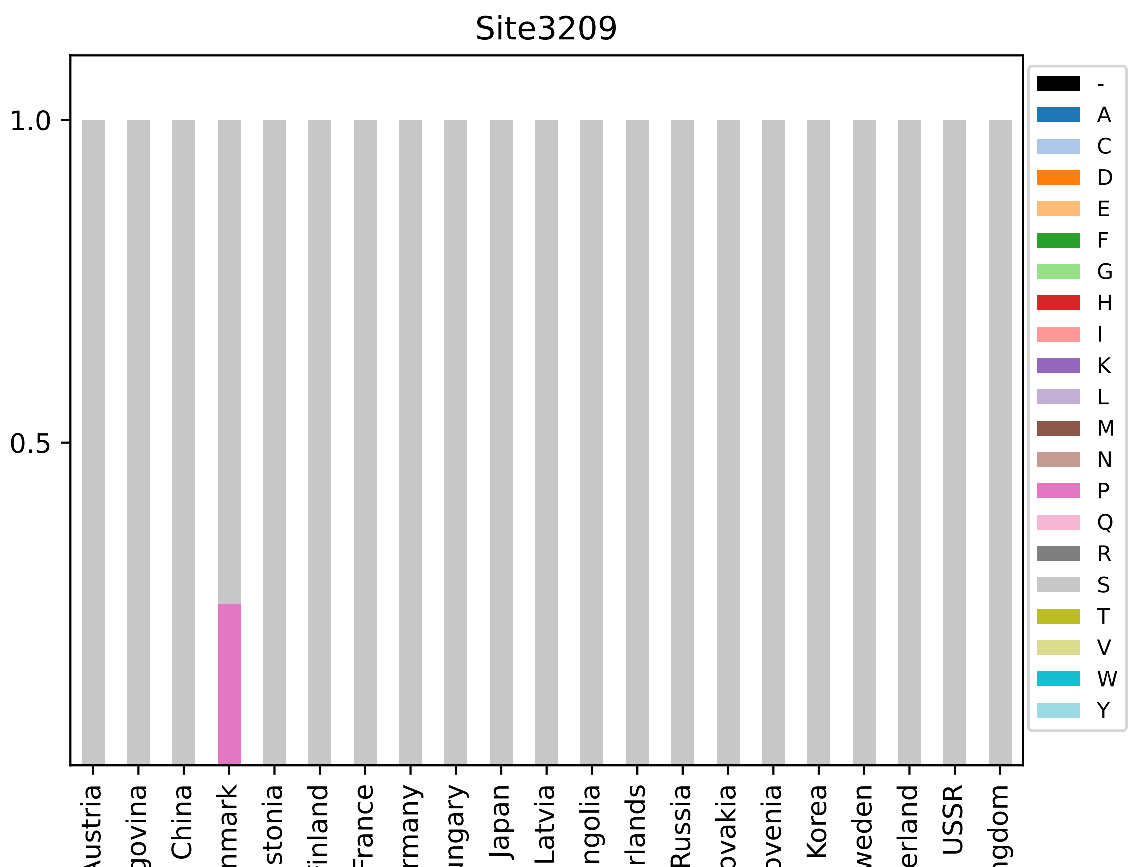

Site3209
1.0
0.5
Austria
govina
China
nmark
stonia
Finland
France
rmany
ungary
Japan
Latvia
ngolia
rlands
Russia
ovakia
ovenia
Korea
weden
erland
USSR
ngdom
-
A
C
D
E
F
G
H
I
K
L
M
N
P
Q
R
S
T
V
W
Y

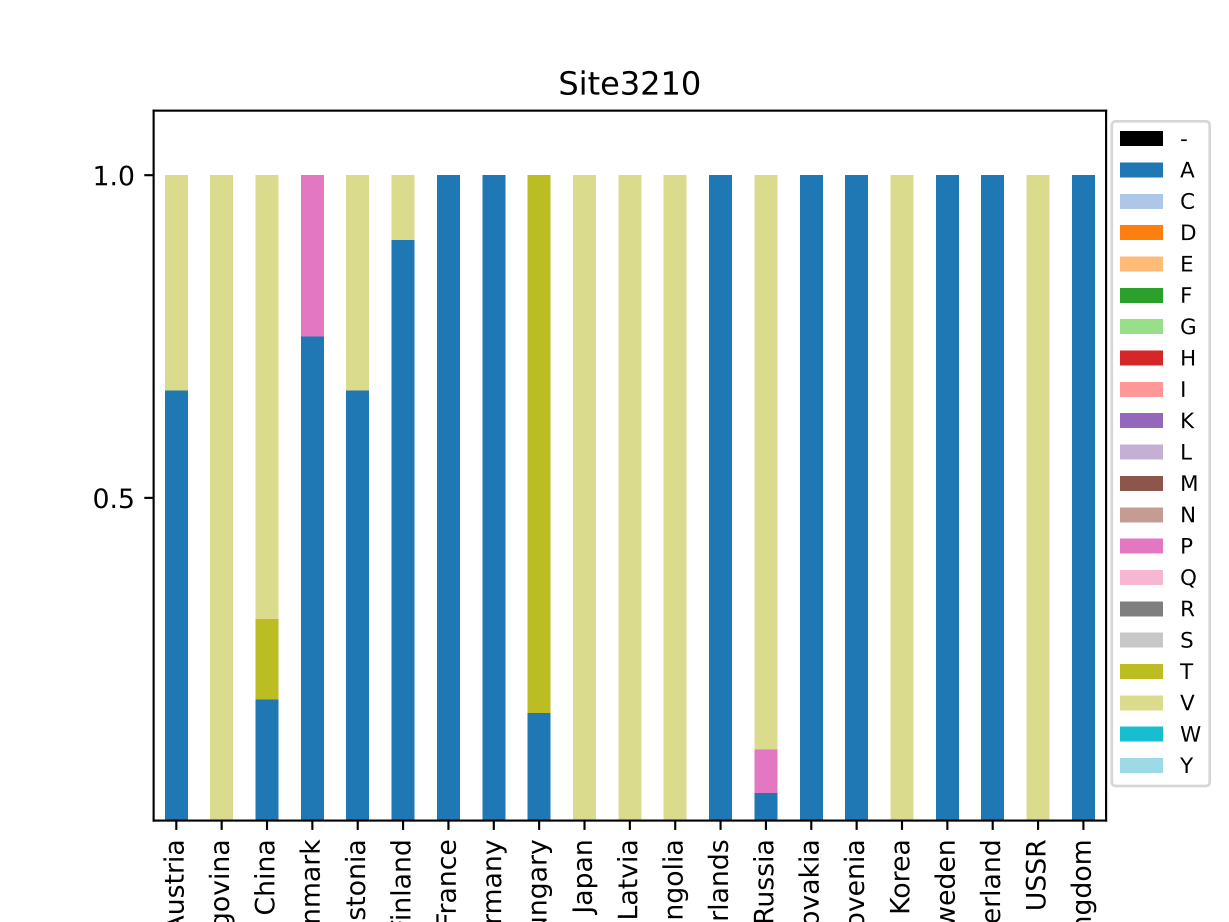

Site3210
1.0
0.5
-
A
C
D
E
F
G
H
I
K
L
M
N
P
Q
R
S
T
V
W
Y
China
Japan
Latvia
Russia
Korea
USSR

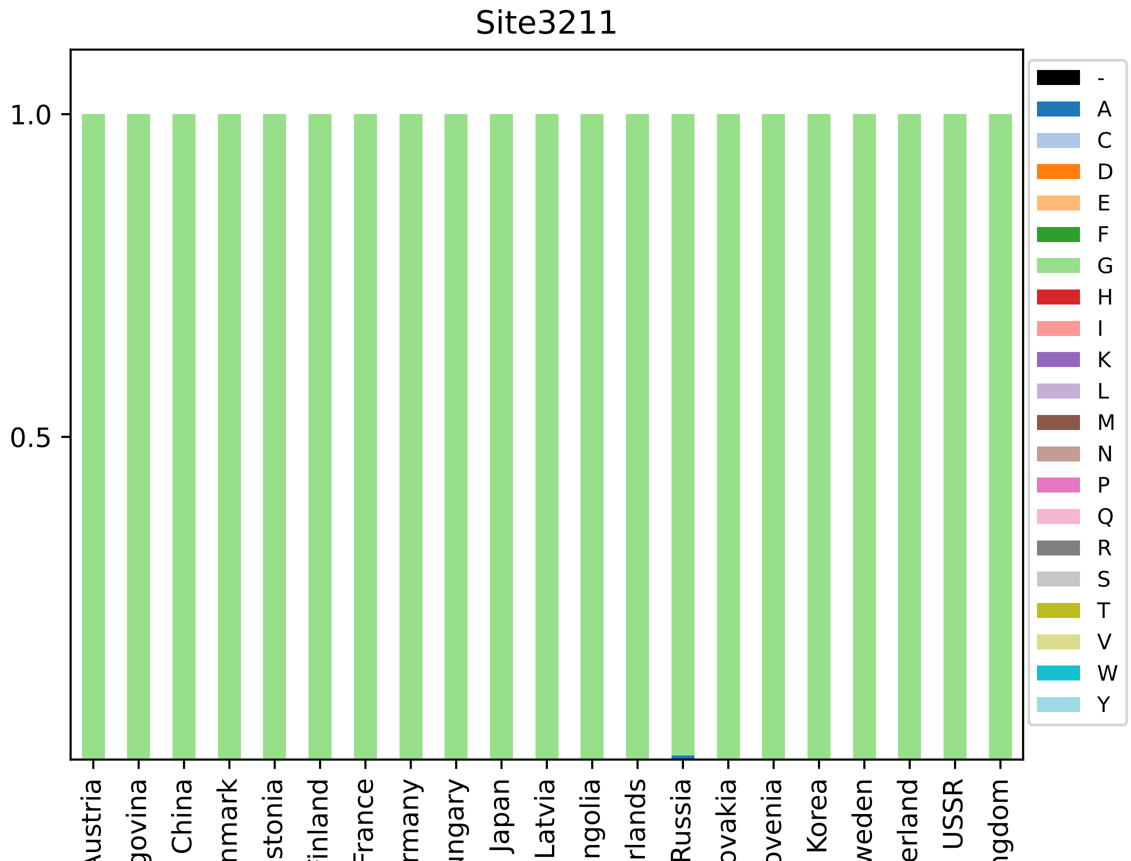

Site3211
1.0
0.5
-
A
C
D
E
F
G
H
I
K
L
M
N
P
Q
R
S
T
V
W
Y
Austria
govina
China
nmark
stonia
Finland
France
rmany
ungary
Japan
Latvia
ngolia
rlands
Russia
ovakia
ovenia
Korea
weden
erland
USSR
ngdom

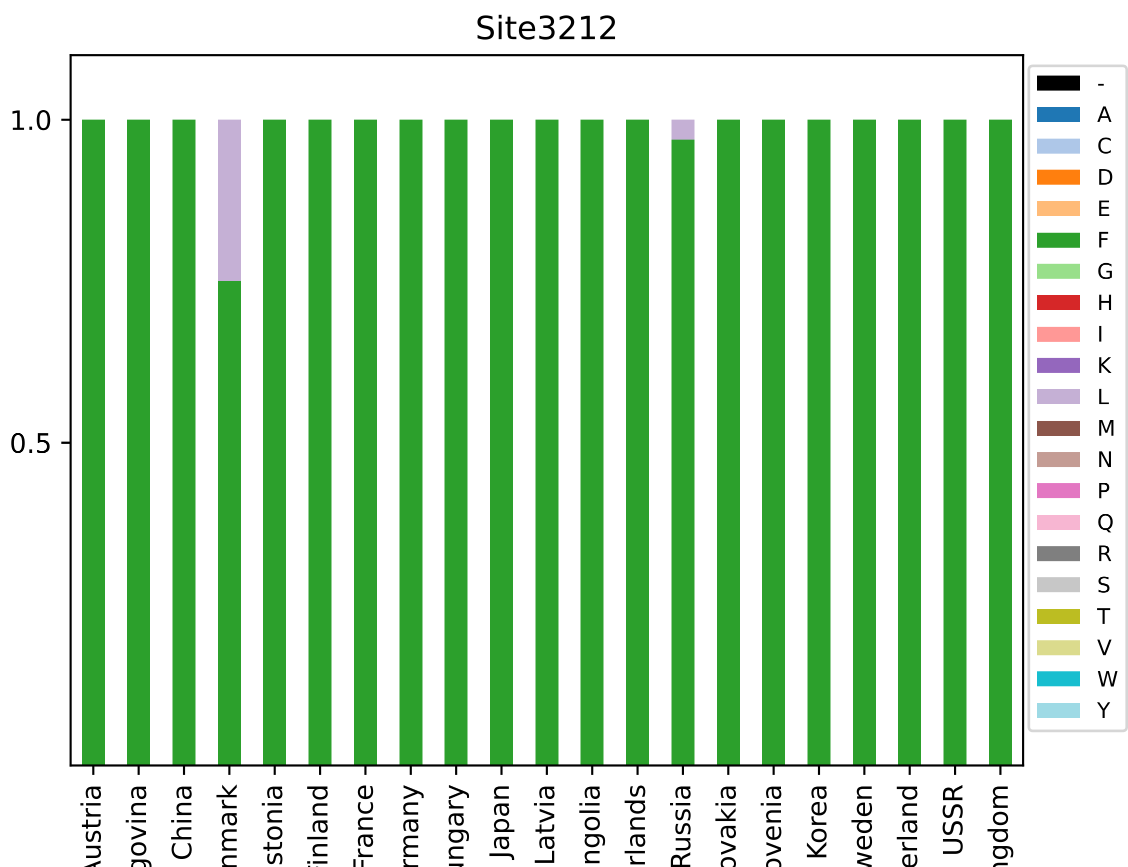
Site3212
1.0
0.5
-
A
C
D
E
F
G
H
I
K
L
M
N
P
Q
R
S
T
V
W
Y
Austria
China
Estonia
Finland
France
Hungary
Japan
Latvia
Russia
Korea
Sweden
USSR

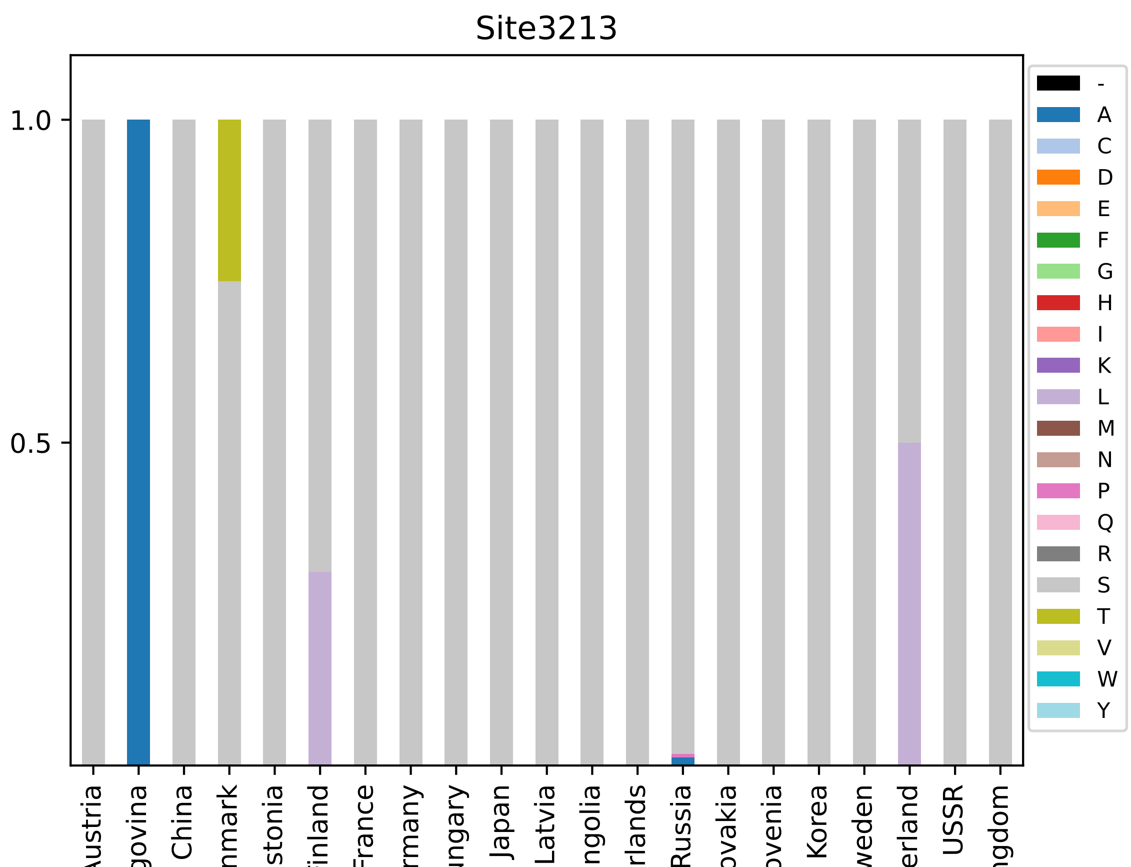
Site3213
1.0
0.5
-
A
C
D
E
F
G
H
I
K
L
M
N
P
Q
R
S
T
V
W
Y
China
Japan
Latvia
Russia
Korea
USSR

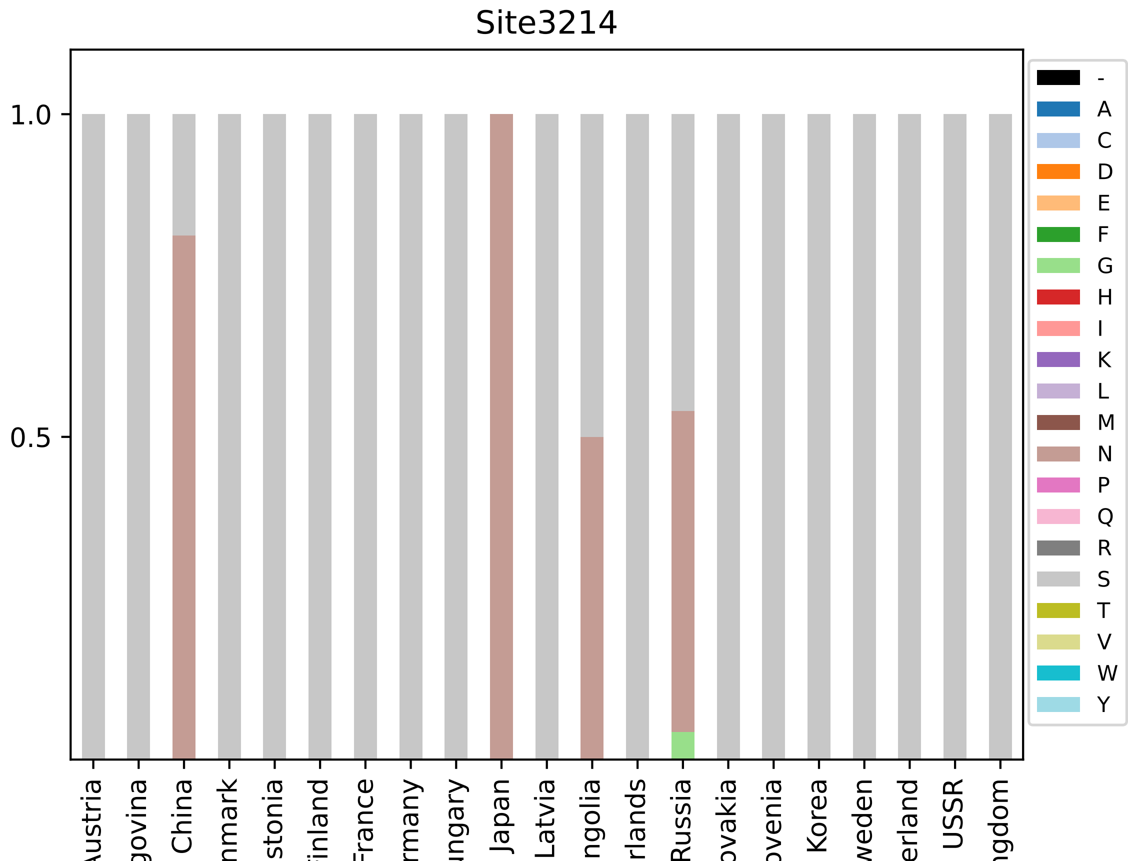

Site3214
1.0
0.5
-
A
C
D
E
F
G
H
I
K
L
M
N
P
Q
R
S
T
V
W
Y
Austria
govina
China
nmark
stonia
Finland
France
rmany
ungary
Japan
Latvia
ngolia
rlands
Russia
ovakia
ovenia
Korea
weden
erland
USSR
ngdom

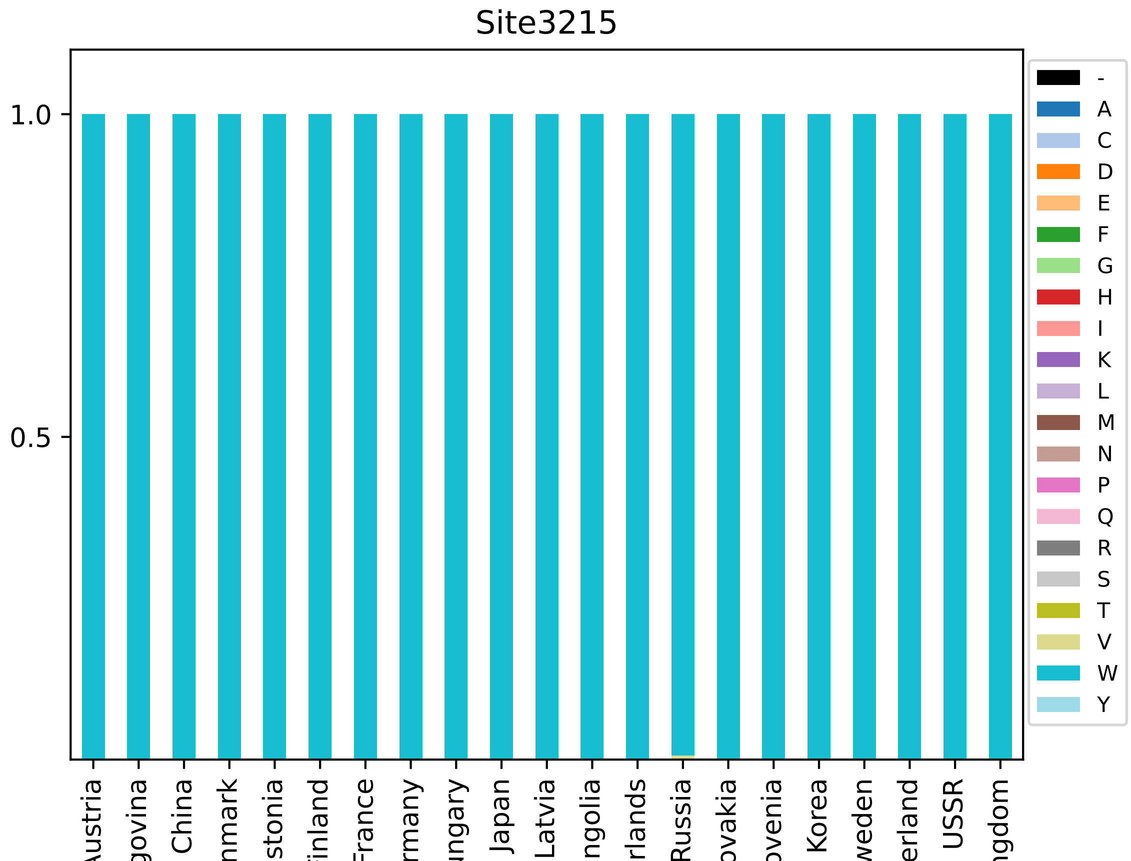
Site3215
1.0
0.5
-
A
C
D
E
F
G
H
I
K
L
M
N
P
Q
R
S
T
V
W
Y
Austria
China
Finland
France
Japan
Latvia
Russia
Korea
USSR

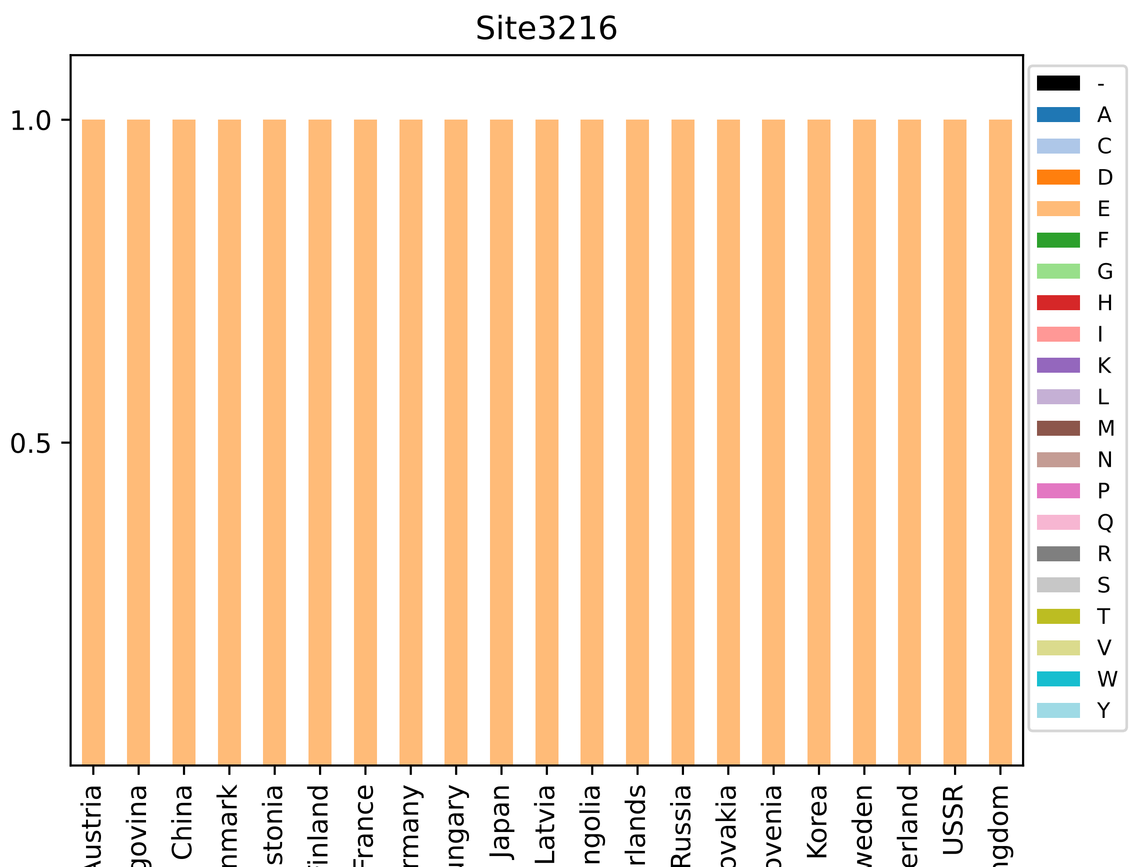

Site3216
1.0
0.5
-
A
C
D
E
F
G
H
I
K
L
M
N
P
Q
R
S
T
V
W
Y
China
Japan
Latvia
Russia
Korea
USSR

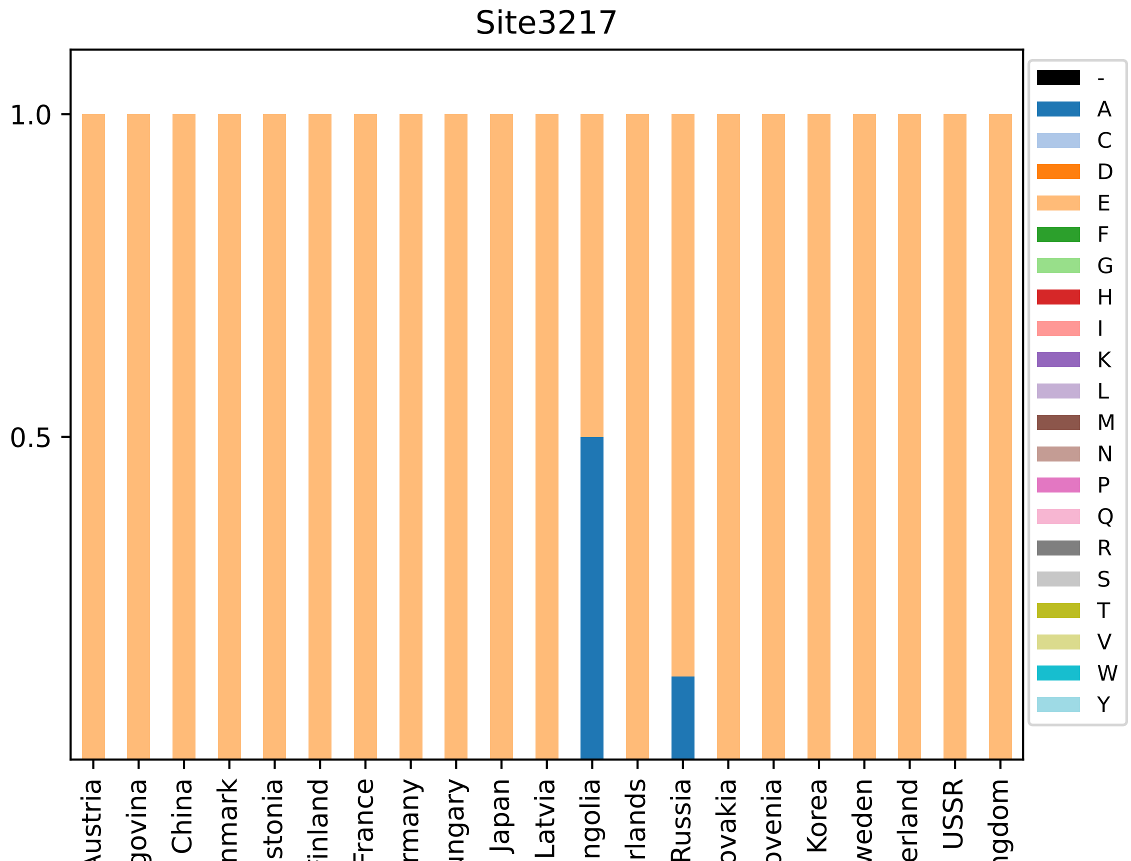

Site3217
1.0
0.5
-
A
C
D
E
F
G
H
I
K
L
M
N
P
Q
R
S
T
V
W
Y
China
Japan
Latvia
Russia
Korea
USSR

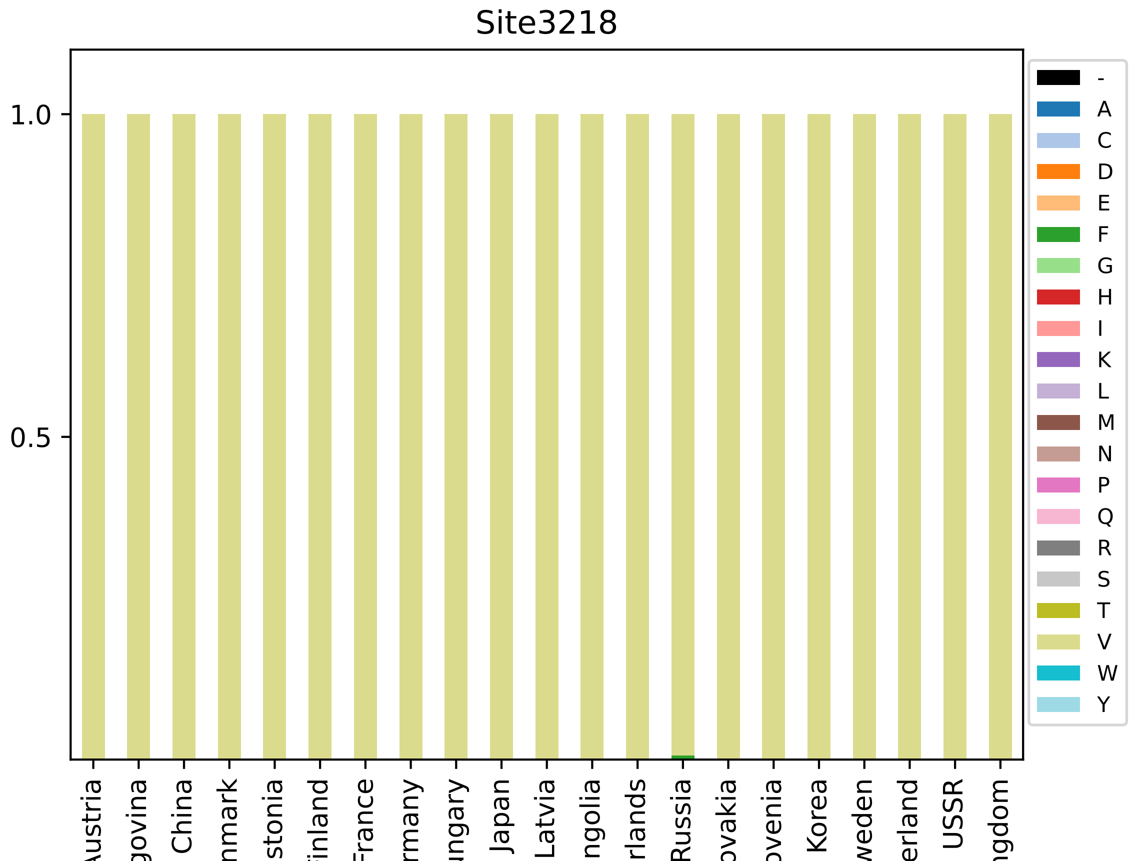

Site3218
1.0
0.5
-
A
C
D
E
F
G
H
I
K
L
M
N
P
Q
R
S
T
V
W
Y
Austria
govina
China
nmark
stonia
Finland
France
rmany
ungary
Japan
Latvia
ngolia
rlands
Russia
ovakia
ovenia
Korea
weden
erland
USSR
ngdom

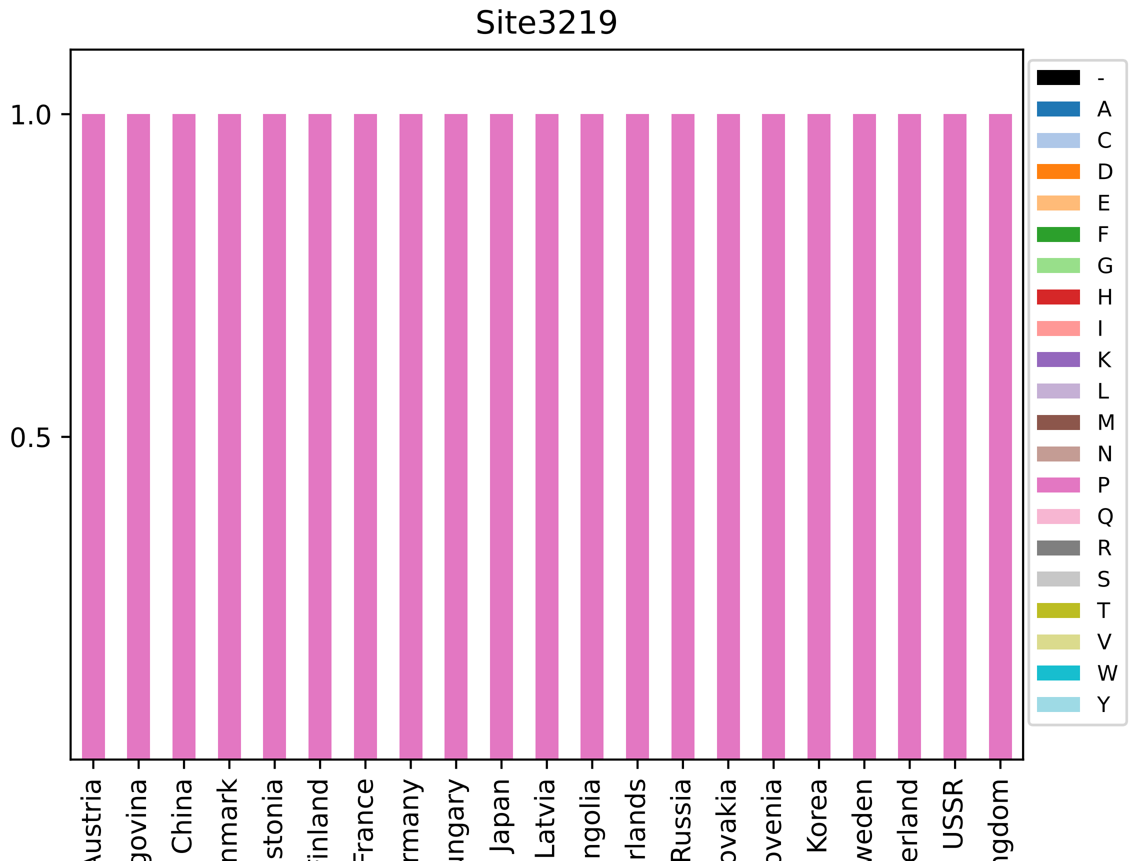
Site3219
1.0
0.5
-
A
C
D
E
F
G
H
I
K
L
M
N
P
Q
R
S
T
V
W
Y
China
Japan
Latvia
Russia
Korea
USSR

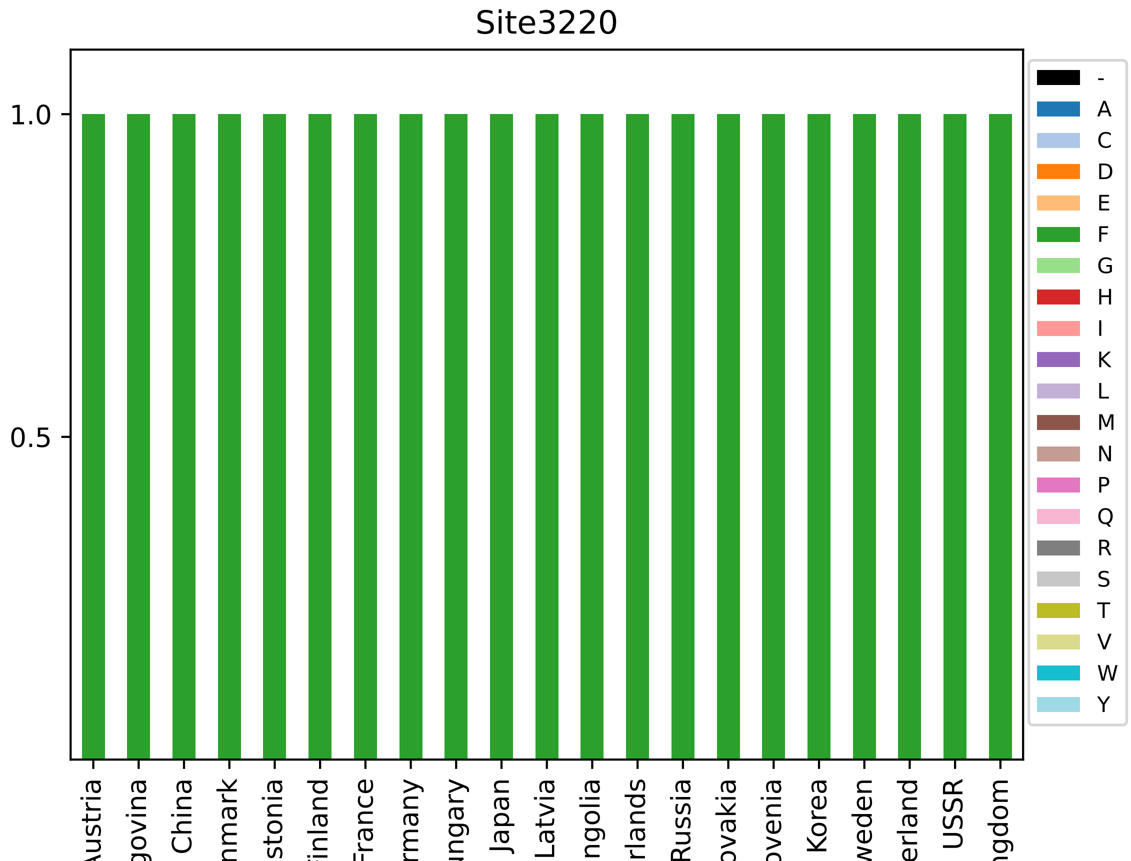

Site3220
1.0
0.5
-
A
C
D
E
F
G
H
I
K
L
M
N
P
Q
R
S
T
V
W
Y
China
Japan
Latvia
Korea
USSR

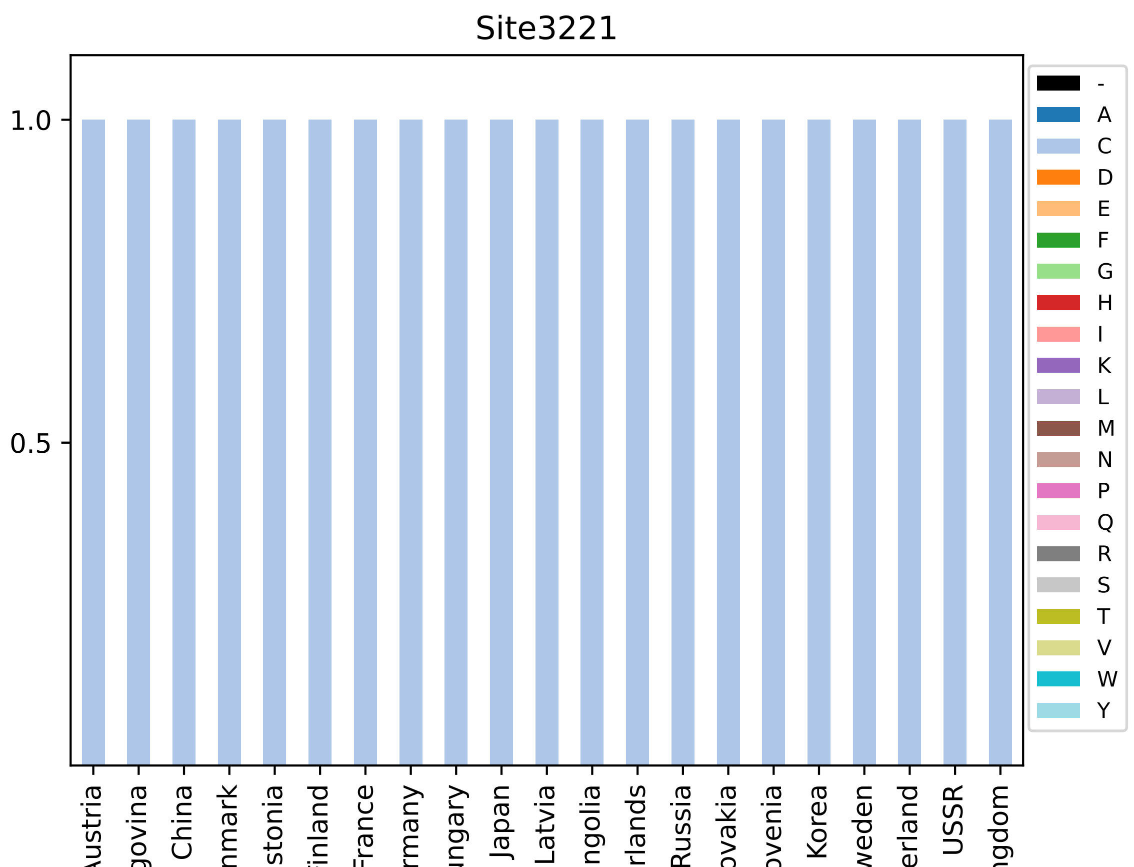
Site3221
1.0
0.5
-
A
C
D
E
F
G
H
I
K
L
M
N
P
Q
R
S
T
V
W
Y
Austria
govina
China
nmark
stonia
inland
France
rmany
ungary
Japan
Latvia
ngolia
rlands
Russia
ovakia
ovenia
Korea
weden
erland
USSR
ngdom

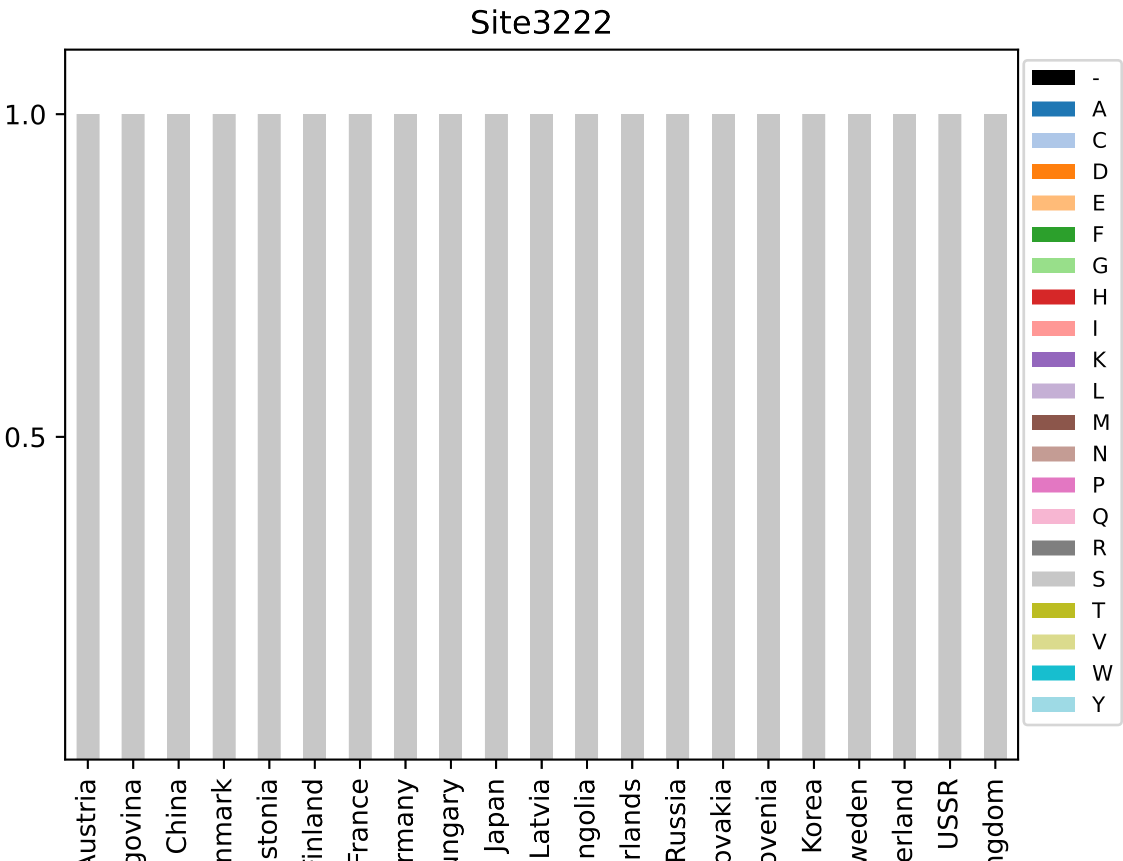
Site3222
1.0
0.5
-
A
C
D
E
F
G
H
I
K
L
M
N
P
Q
R
S
T
V
W
Y
Austria
China
Japan
Latvia
Russia
Korea
USSR

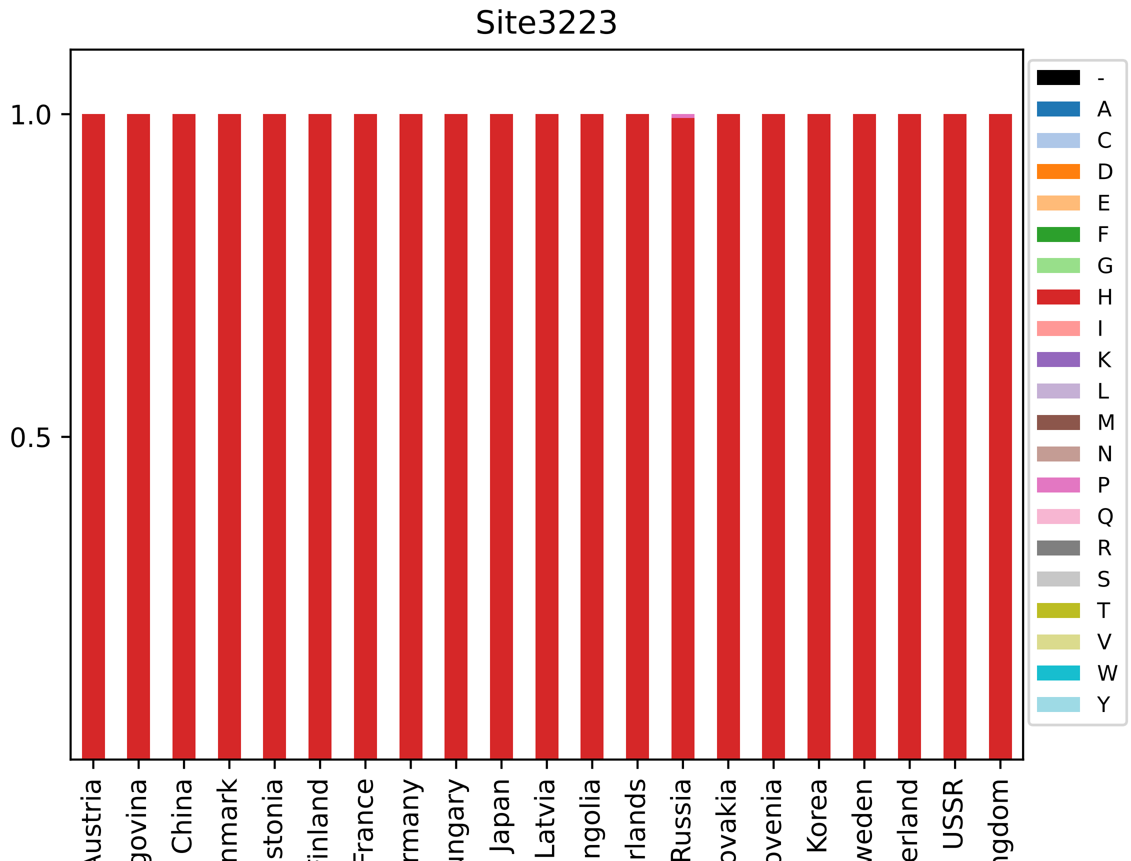
Site3223
1.0
0.5
-
A
C
D
E
F
G
H
I
K
L
M
N
P
Q
R
S
T
V
W
Y
China
Japan
Latvia
Russia
Korea
USSR

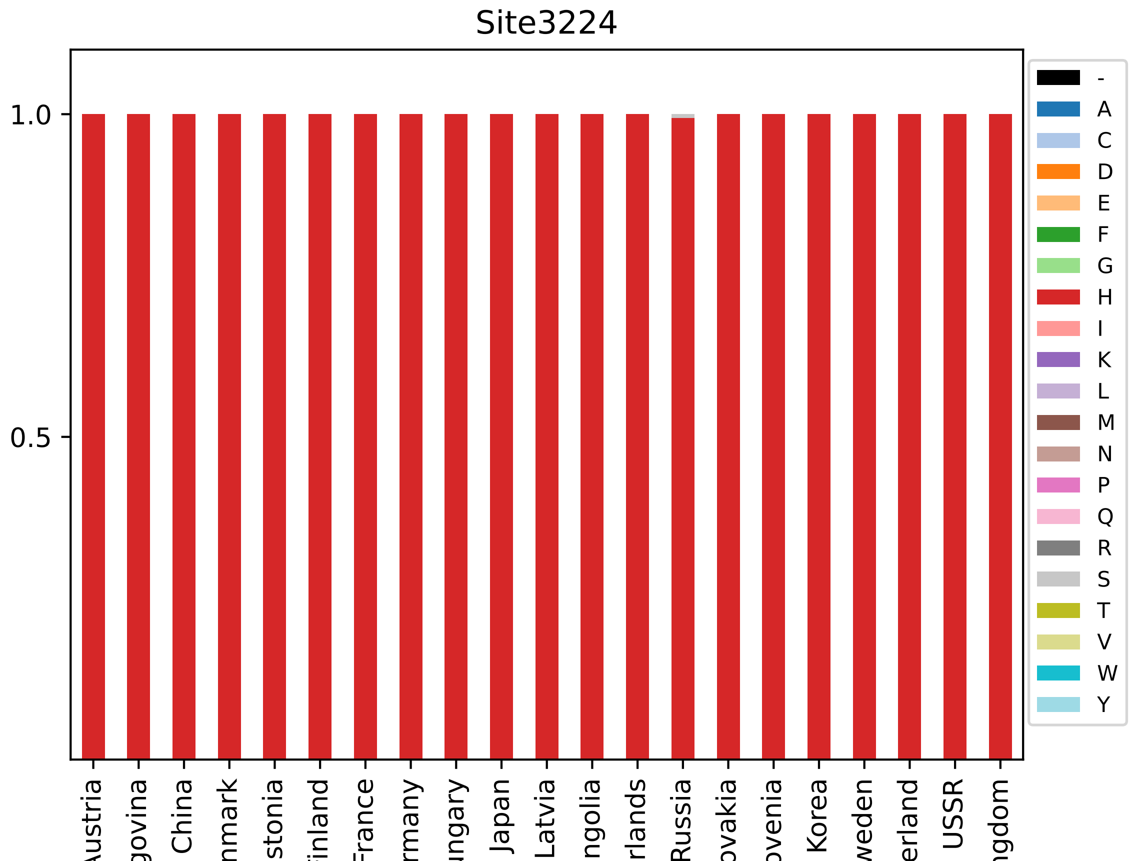

Site3224
1.0
0.5
-
A
C
D
E
F
G
H
I
K
L
M
N
P
Q
R
S
T
V
W
Y
China
Japan
Latvia
Russia
Korea
USSR

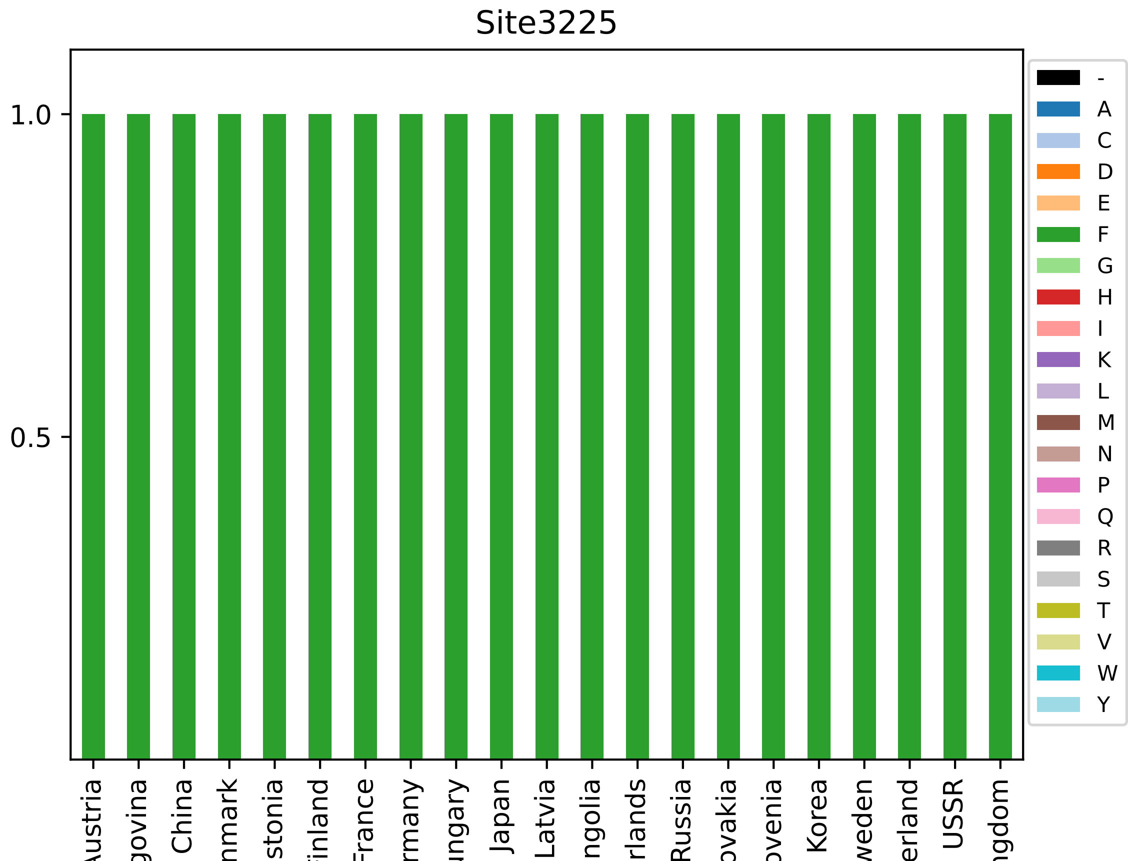

Site3225
1.0
0.5
-
A
C
D
E
F
G
H
I
K
L
M
N
P
Q
R
S
T
V
W
Y
Austria
China
Japan
Latvia
Russia
Korea
USSR

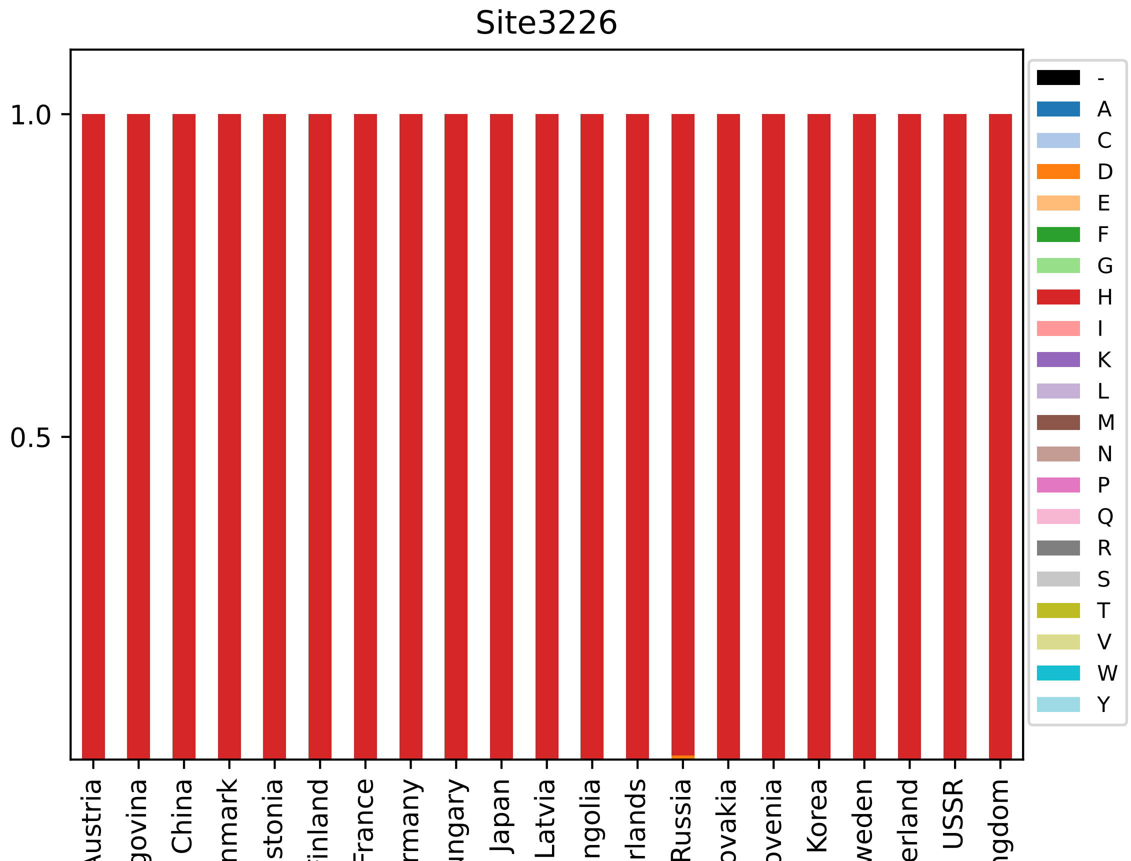

Site3226
1.0
0.5
-
A
C
D
E
F
G
H
I
K
L
M
N
P
Q
R
S
T
V
W
Y
Austria
China
Japan
Latvia
Russia
Korea
USSR

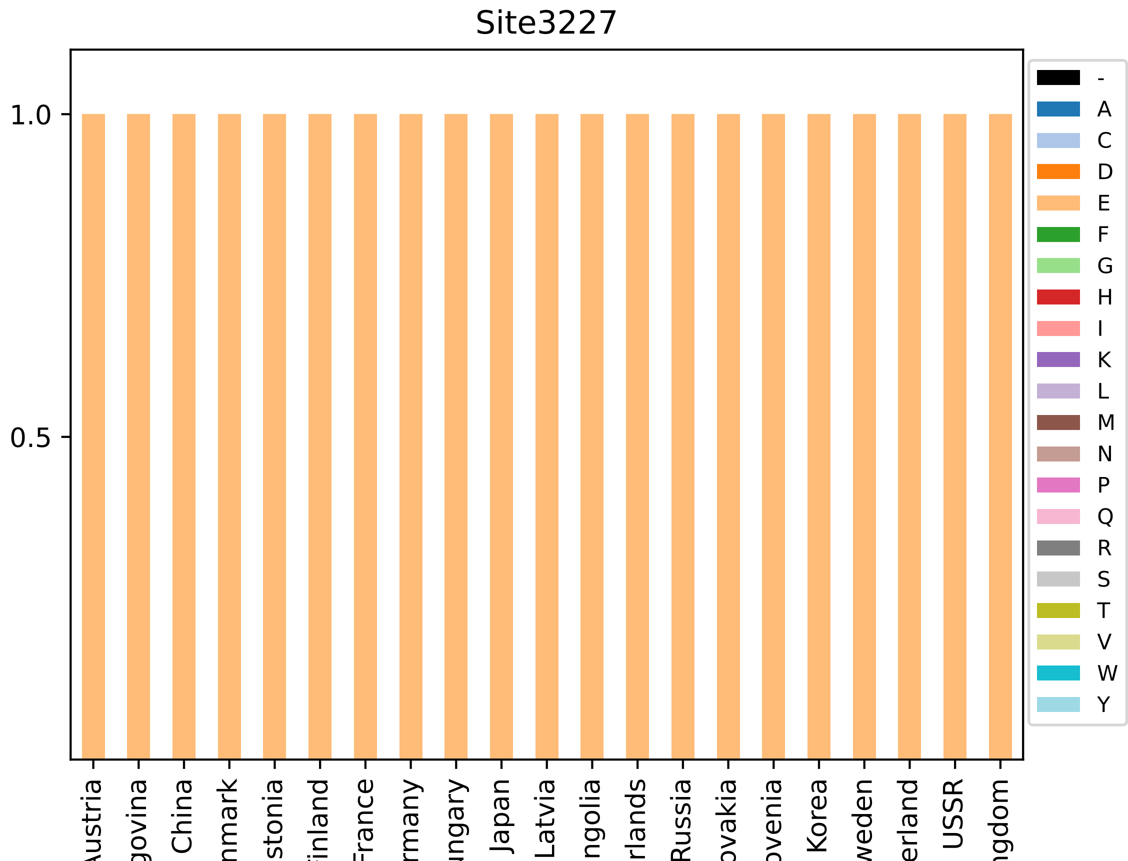

Site3227
1.0
0.5
-
A
C
D
E
F
G
H
I
K
L
M
N
P
Q
R
S
T
V
W
Y
Austria
China
Japan
Latvia
Russia
Korea
USSR

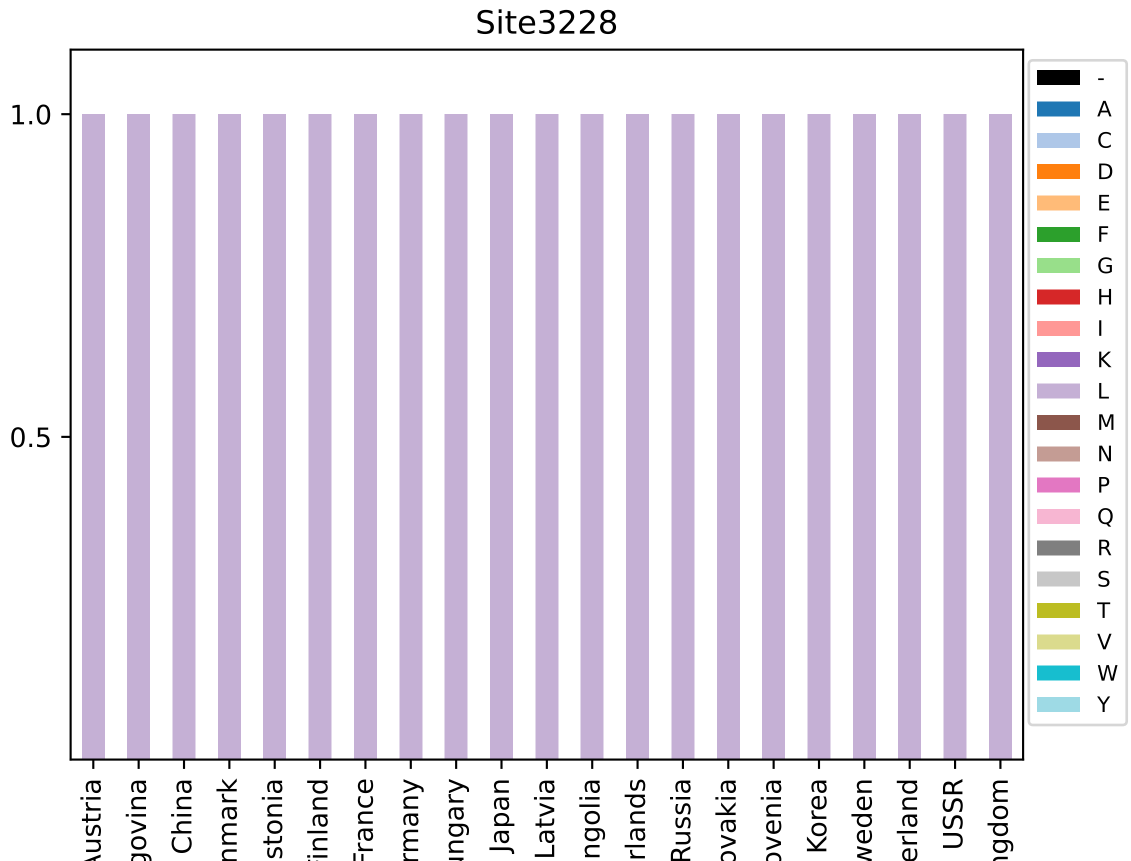

Site3228
1.0
0.5
-
A
C
D
E
F
G
H
I
K
L
M
N
P
Q
R
S
T
V
W
Y
Austria
govina
China
nmark
stonia
Finland
France
rmany
ungary
Japan
Latvia
ngolia
rlands
Russia
ovakia
ovenia
Korea
weden
erland
USSR
ngdom

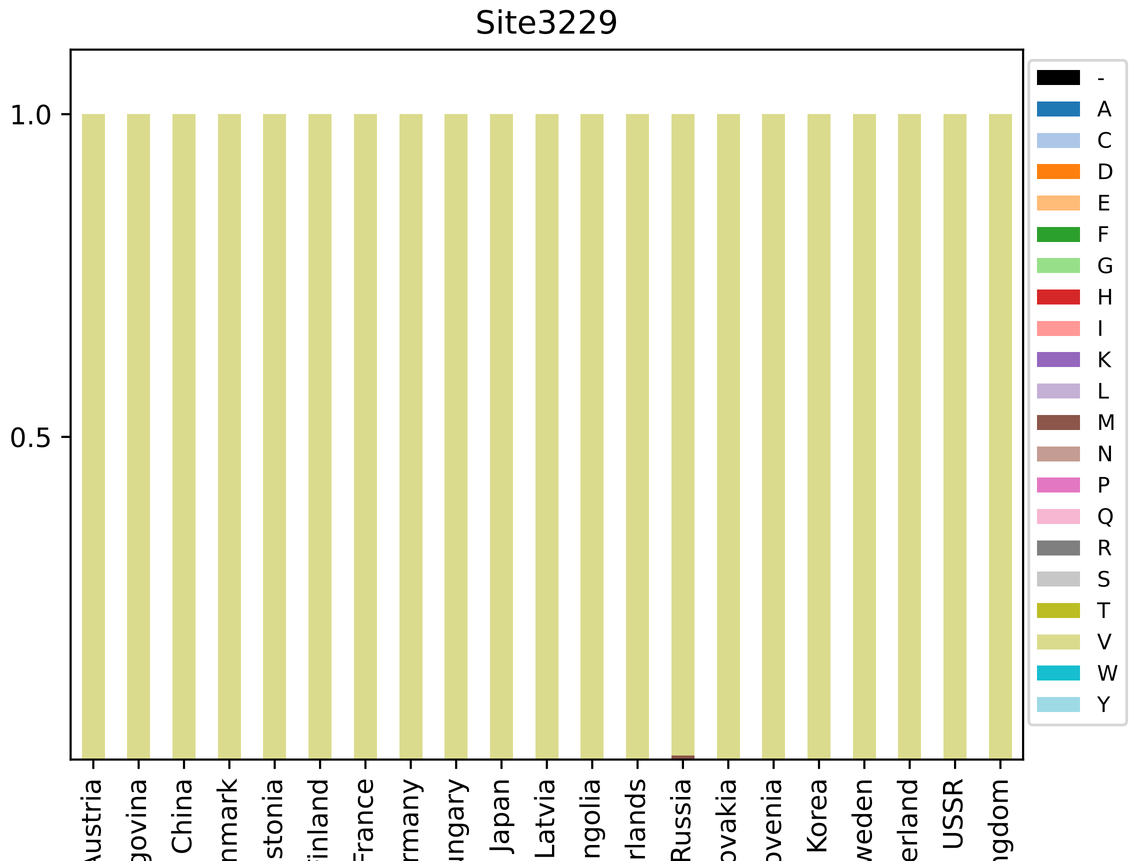
Site3229
1.0
0.5
-
A
C
D
E
F
G
H
I
K
L
M
N
P
Q
R
S
T
V
W
Y
Austria
govina
China
nmark
stonia
Finland
France
rmany
ungary
Japan
Latvia
ngolia
rlands
Russia
ovakia
ovenia
Korea
weden
erland
USSR
ngdom

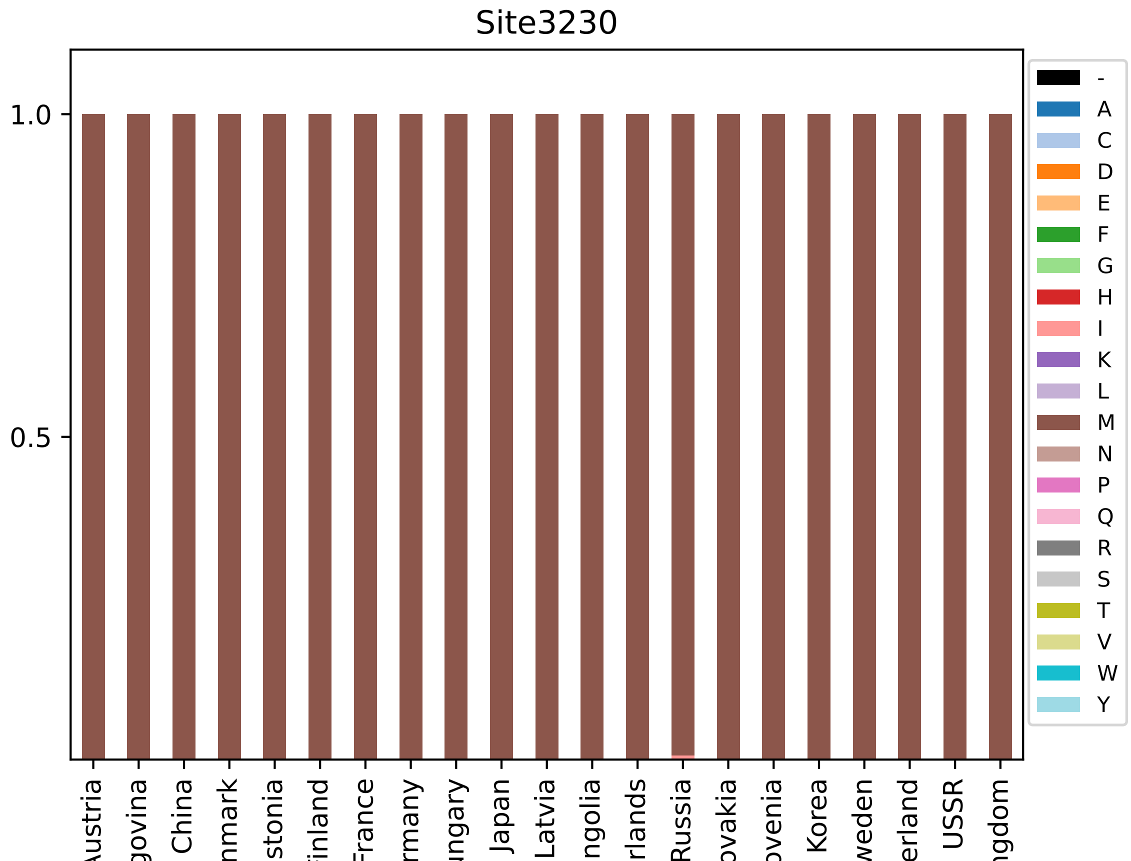

Site3230
1.0
0.5
-
A
C
D
E
F
G
H
I
K
L
M
N
P
Q
R
S
T
V
W
Y
Austria
govina
China
nmark
stonia
Finland
France
rmany
ungary
Japan
Latvia
ngolia
rlands
Russia
ovakia
ovenia
Korea
weden
erland
USSR
ngdom

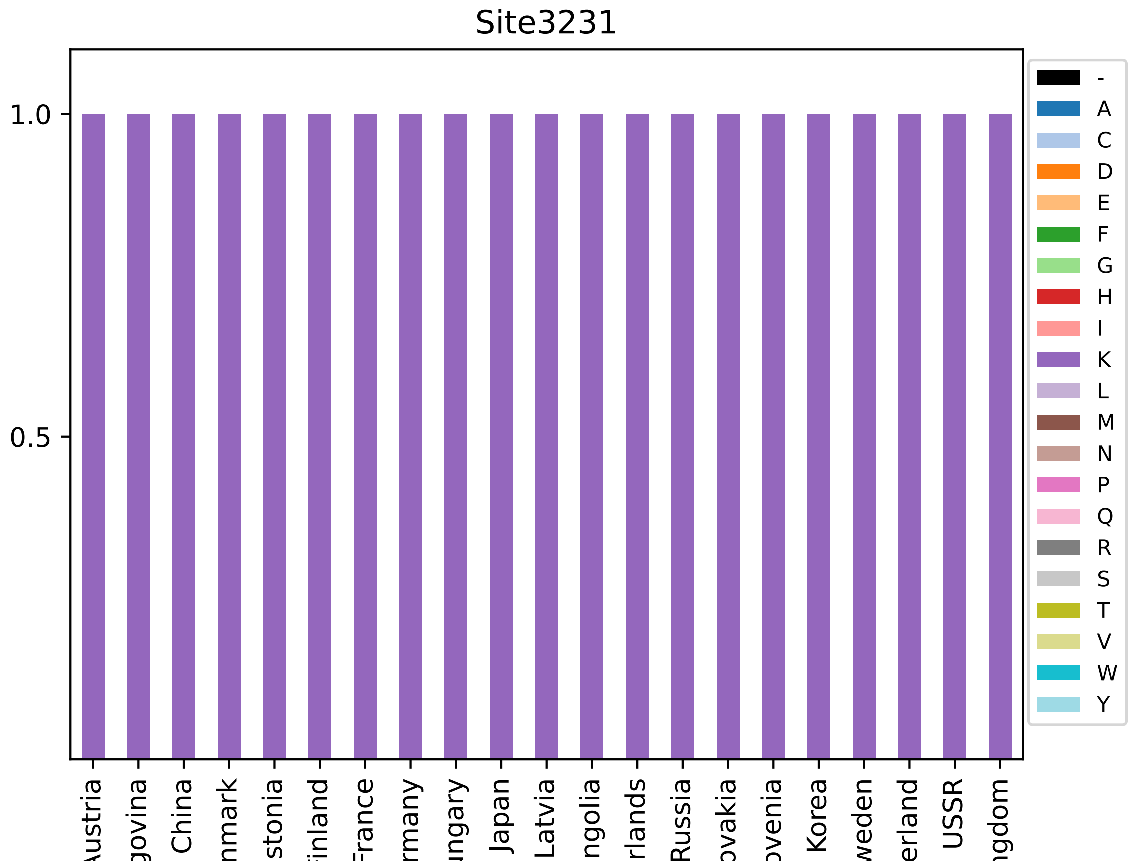

Site3231
1.0
0.5
-
A
C
D
E
F
G
H
I
K
L
M
N
P
Q
R
S
T
V
W
Y
China
Japan
Latvia
Russia
Korea
USSR

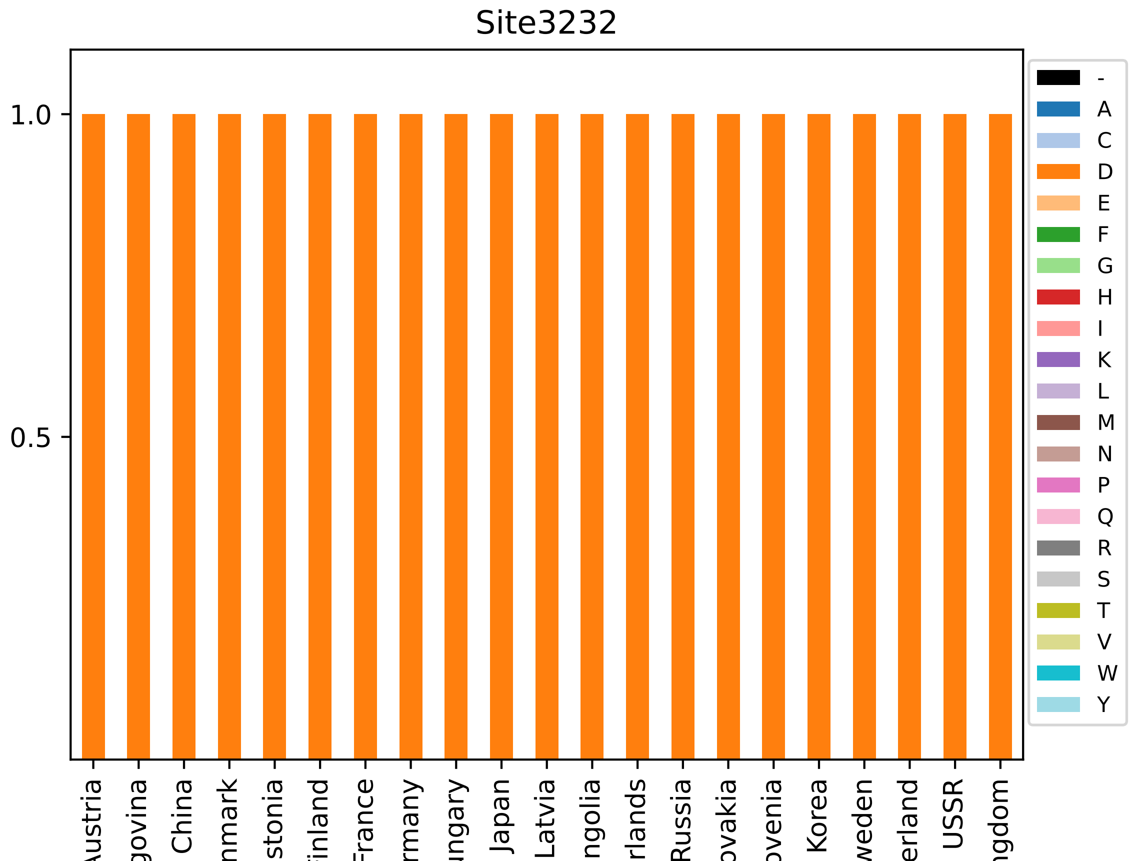
Site3232
1.0
0.5
-
A
C
D
E
F
G
H
I
K
L
M
N
P
Q
R
S
T
V
W
Y
Austria
govina
China
nmark
stonia
Finland
France
rmany
ungary
Japan
Latvia
ngolia
rlands
Russia
ovakia
ovenia
Korea
weden
erland
USSR
ngdom

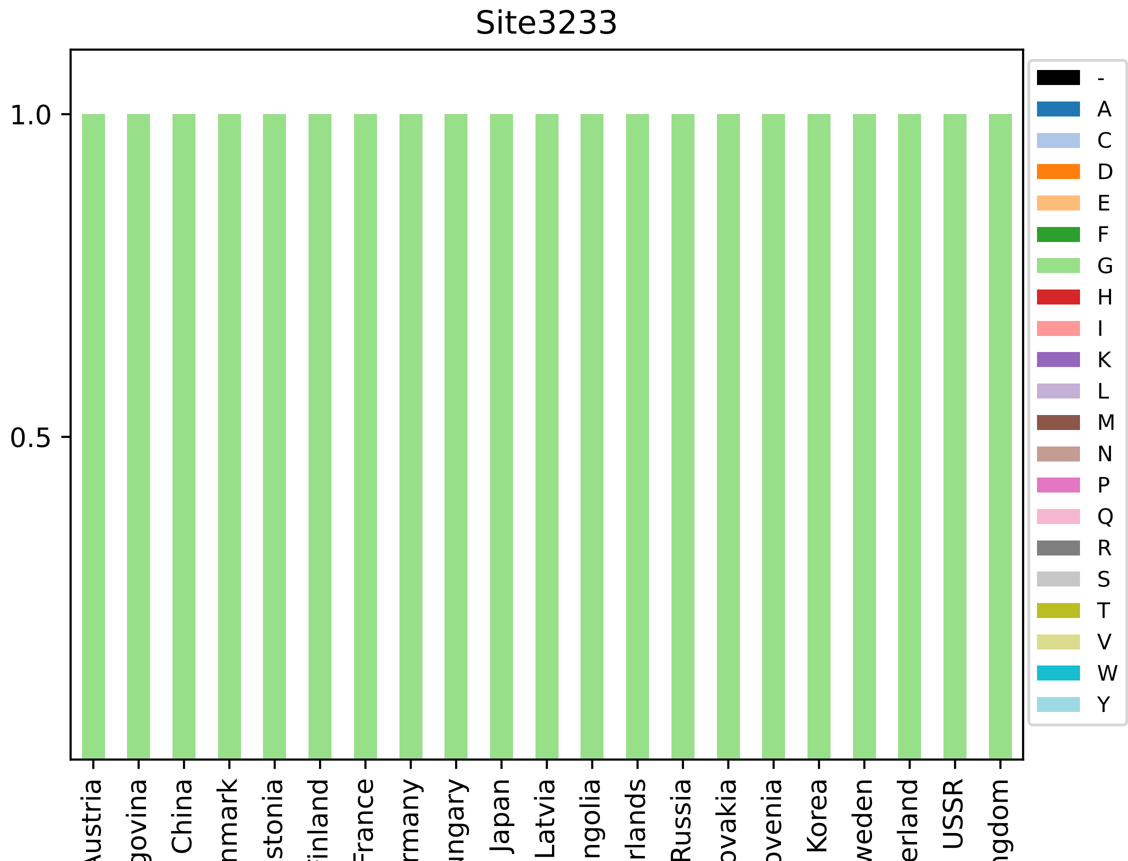
Site3233
1.0
0.5
China
Japan
Latvia
Russia
Korea
USSR
-
A
C
D
E
F
G
H
I
K
L
M
N
P
Q
R
S
T
V
W
Y

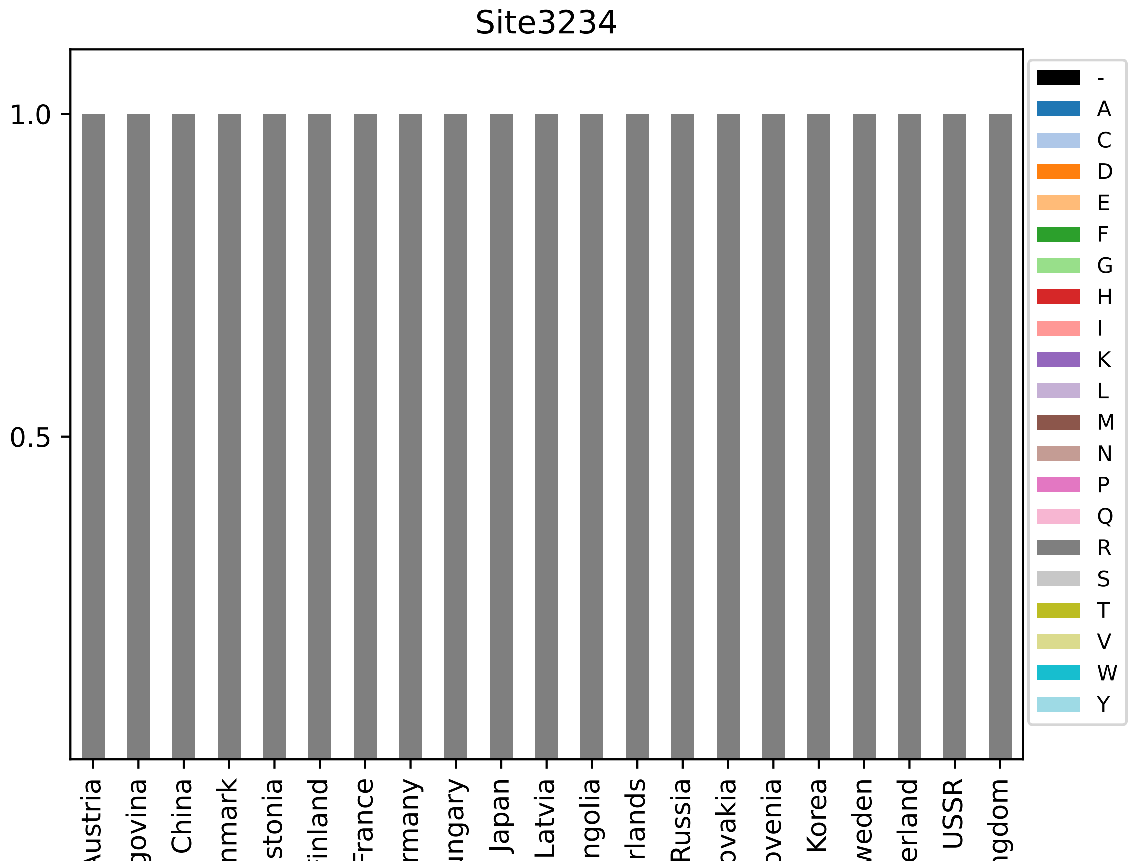
Site3234
1.0
0.5
-
A
C
D
E
F
G
H
I
K
L
M
N
P
Q
R
S
T
V
W
Y
Austria
govina
China
nmark
stonia
Finland
France
rmany
ungary
Japan
Latvia
ngolia
rlands
Russia
ovakia
ovenia
Korea
weden
erland
USSR
ngdom

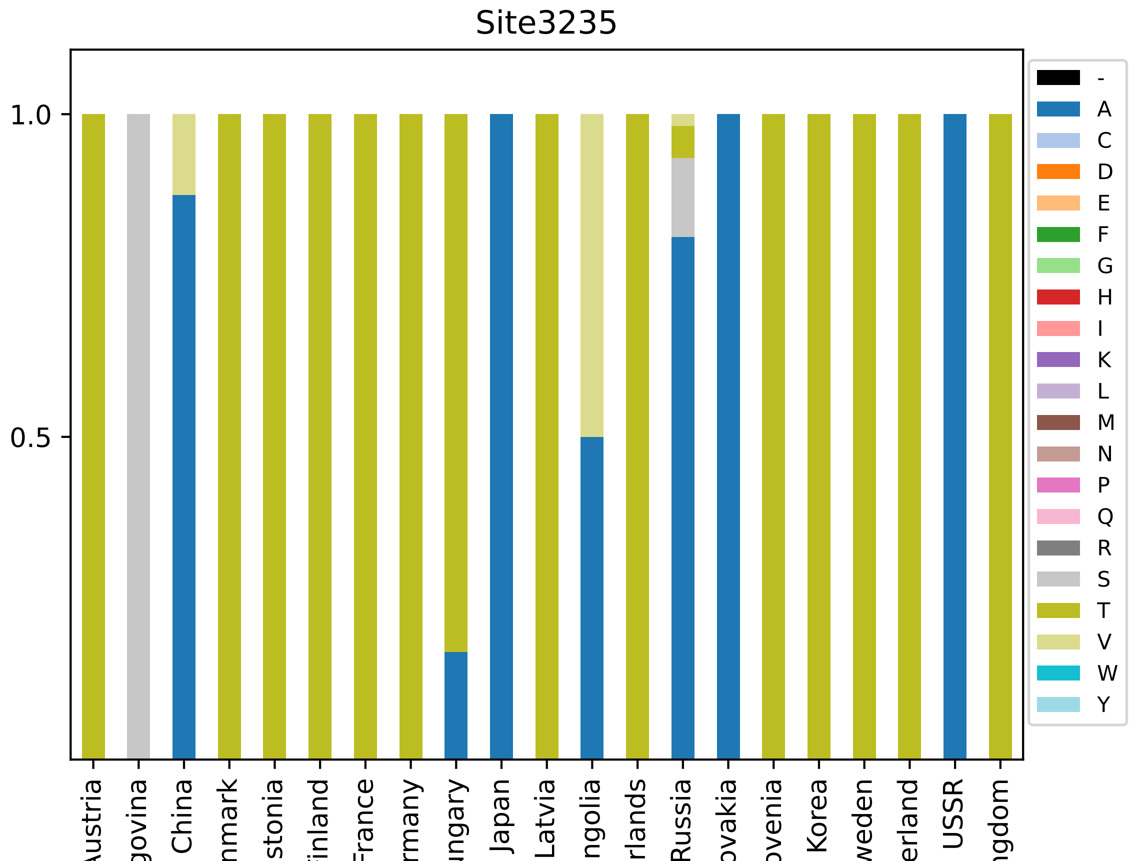

Site3235
1.0
0.5
-
A
C
D
E
F
G
H
I
K
L
M
N
P
Q
R
S
T
V
W
Y
China
Japan
Latvia
Russia
Korea
USSR

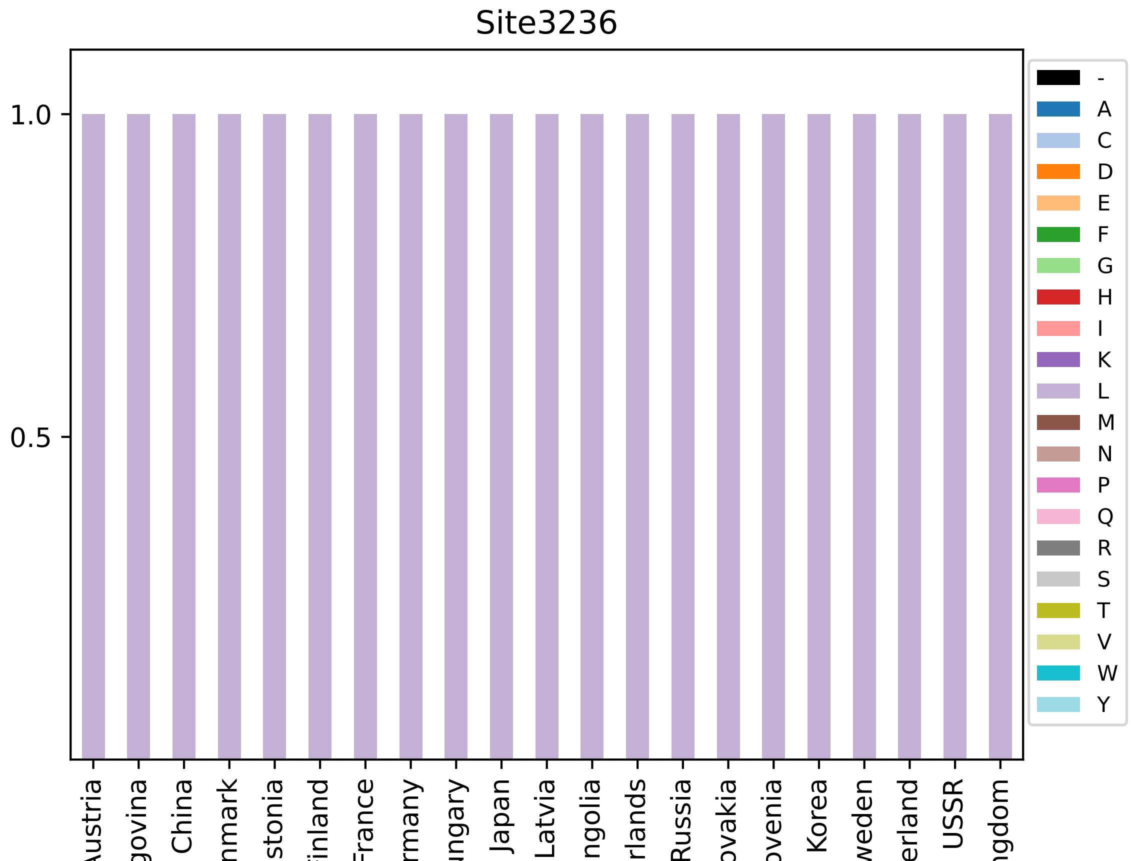
Site3236
1.0
0.5
-
A
C
D
E
F
G
H
I
K
L
M
N
P
Q
R
S
T
V
W
Y
China
Japan
Latvia
Korea
USSR

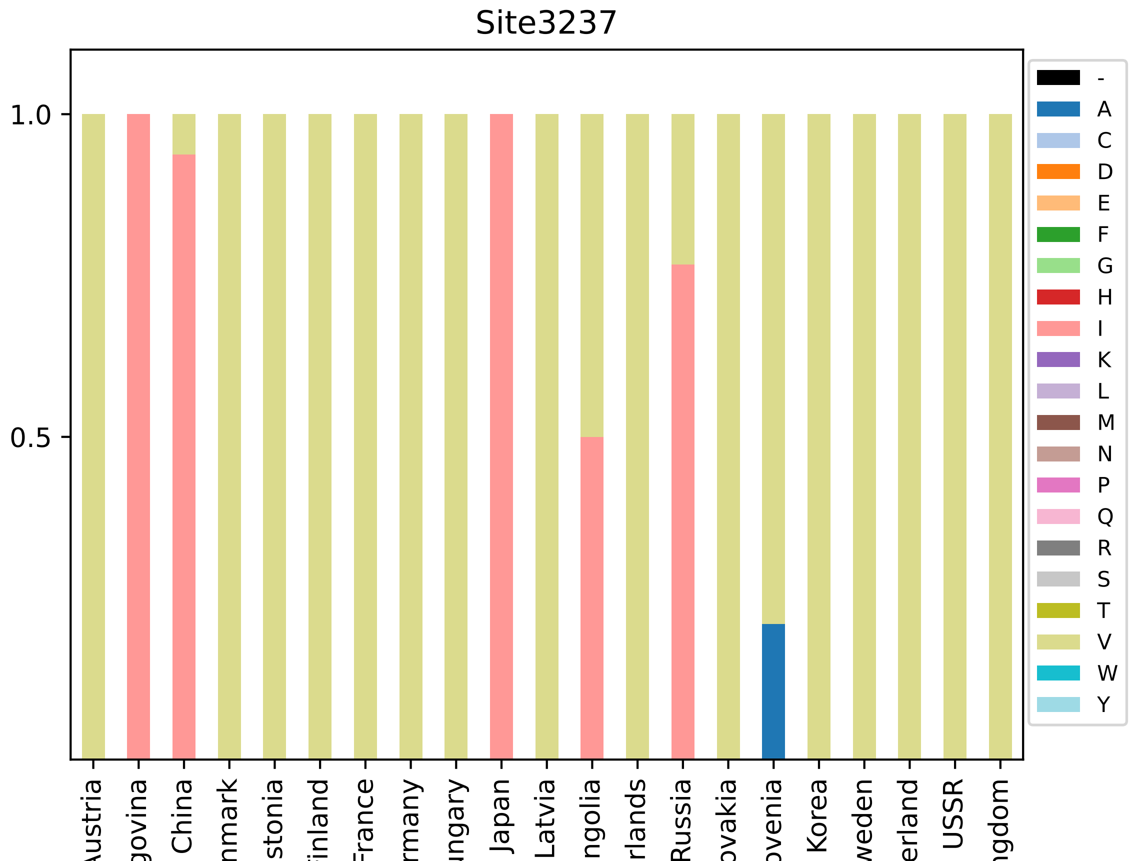

Site3237
1.0
0.5
-
A
C
D
E
F
G
H
I
K
L
M
N
P
Q
R
S
T
V
W
Y
Austria
govina
China
nmark
stonia
Finland
France
rmany
ungary
Japan
Latvia
ngolia
rlands
Russia
ovakia
ovenia
Korea
weden
erland
USSR
ngdom

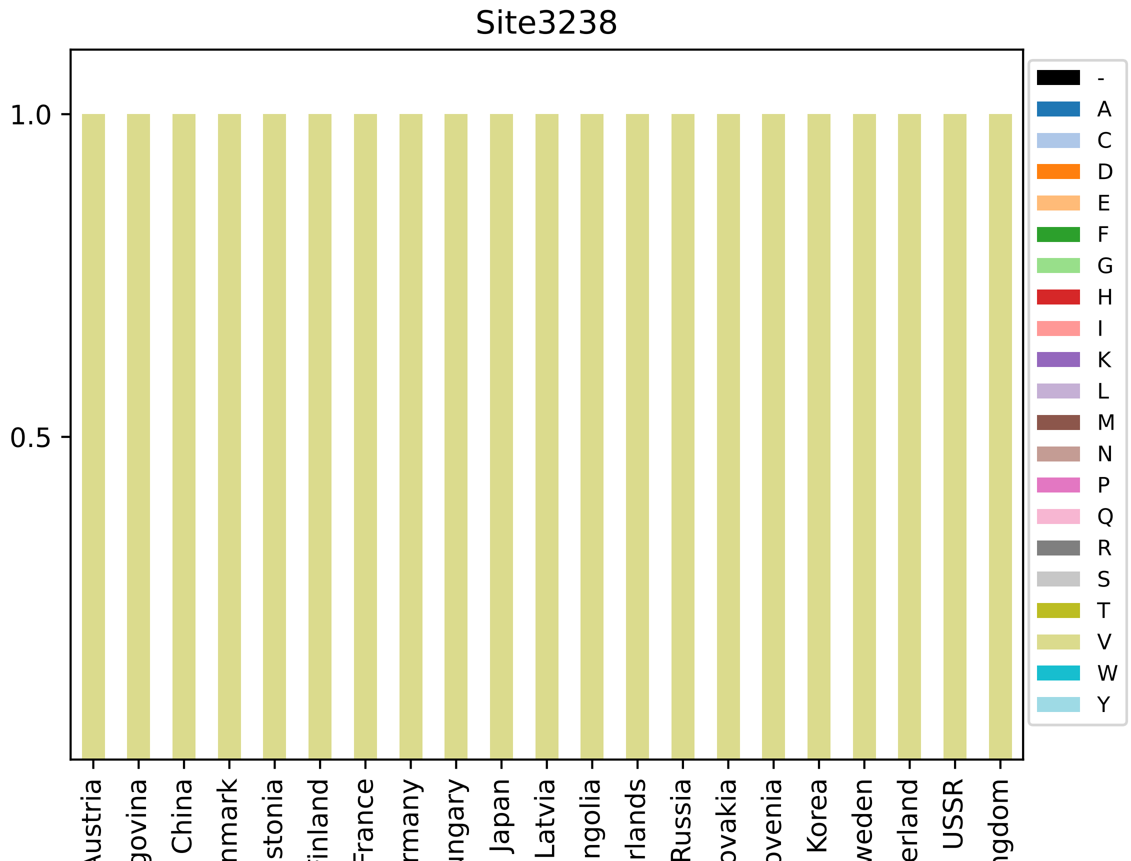
Site3238
1.0
0.5
-
A
C
D
E
F
G
H
I
K
L
M
N
P
Q
R
S
T
V
W
Y
Austria
govina
China
nmark
stonia
Finland
France
rmany
ungary
Japan
Latvia
ngolia
rlands
Russia
ovakia
ovenia
Korea
weden
erland
USSR
ngdom

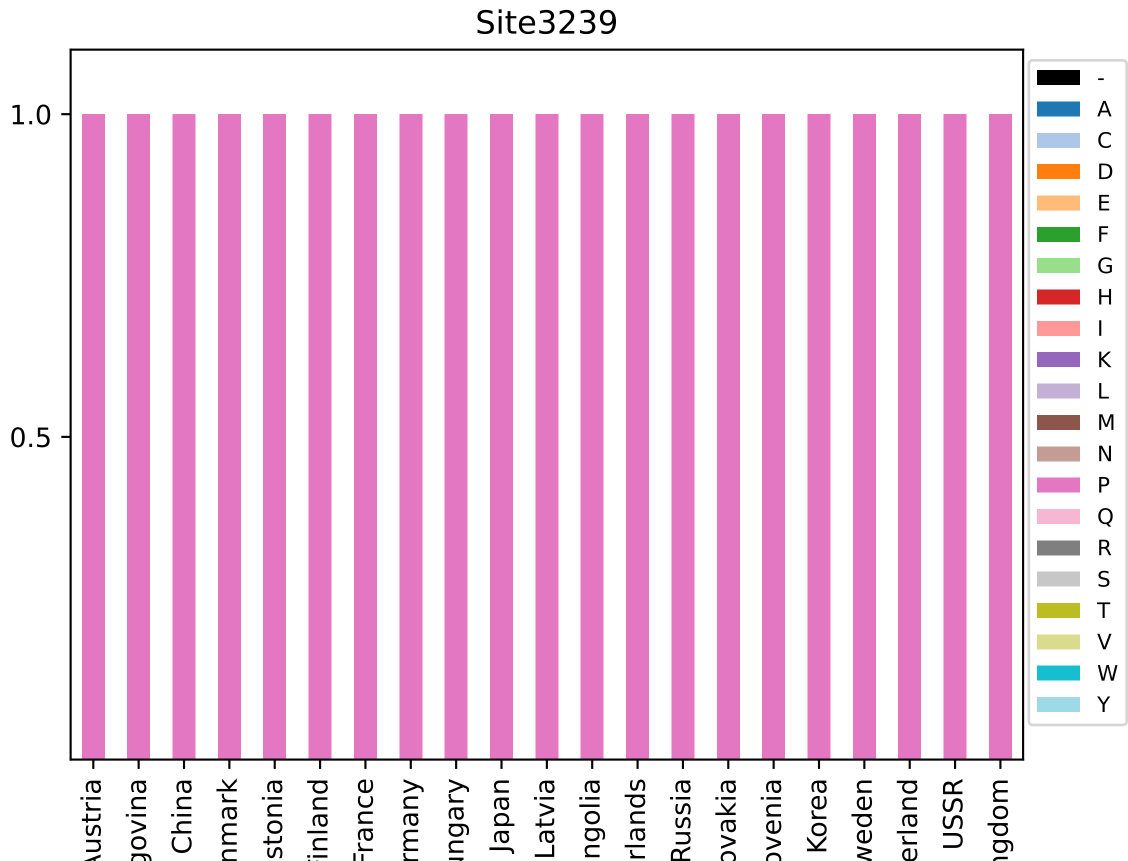

Site3239
1.0
0.5
-
A
C
D
E
F
G
H
I
K
L
M
N
P
Q
R
S
T
V
W
Y
China
Japan
Latvia
Russia
Korea
USSR

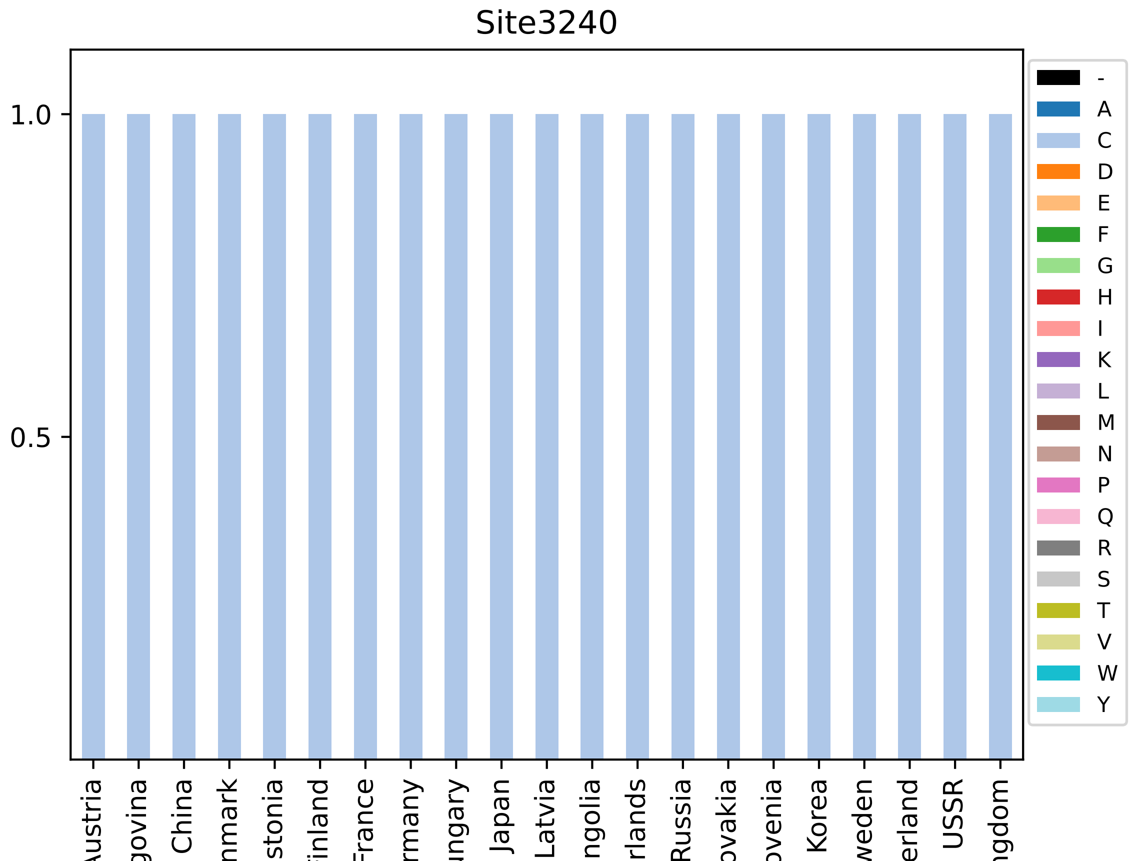
Site3240
1.0
0.5
-
A
C
D
E
F
G
H
I
K
L
M
N
P
Q
R
S
T
V
W
Y
China
Japan
Latvia
Russia
Korea
USSR

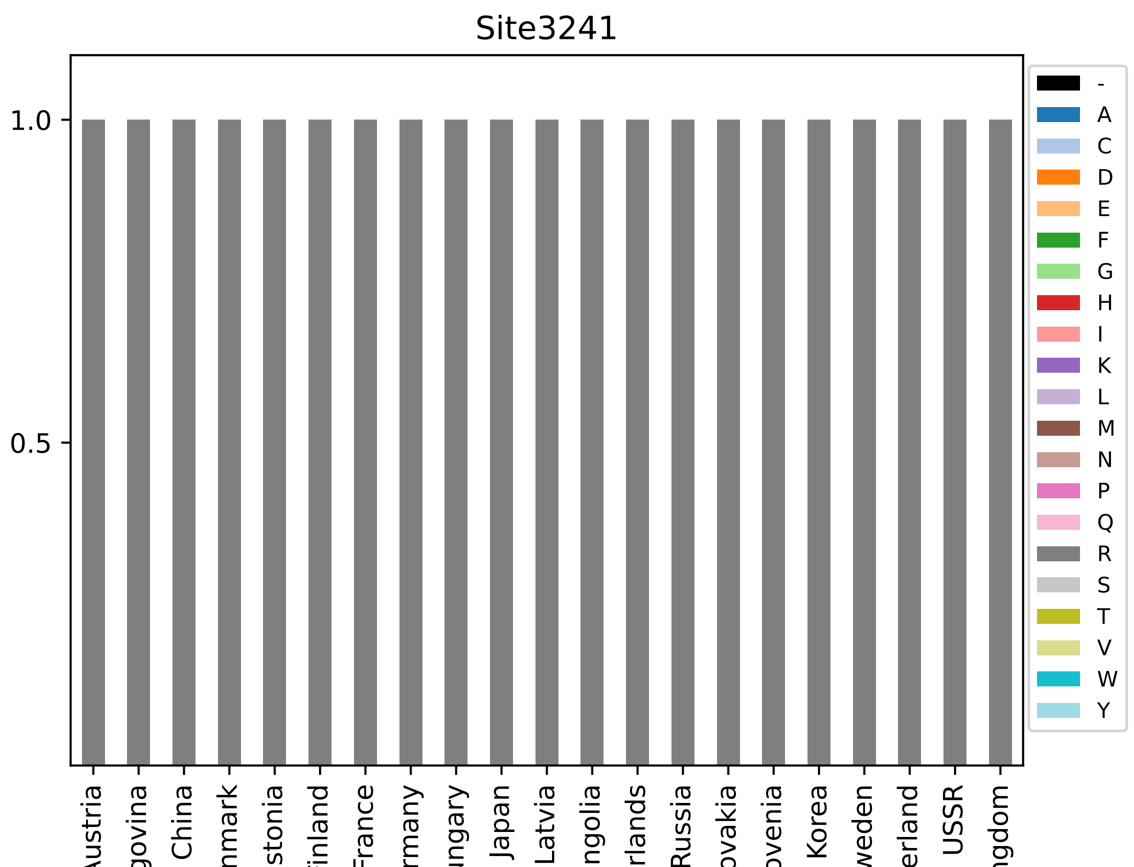
Site3241
1.0
0.5
-
A
C
D
E
F
G
H
I
K
L
M
N
P
Q
R
S
T
V
W
Y
Austria
China
Japan
Latvia
Russia
Korea
USSR

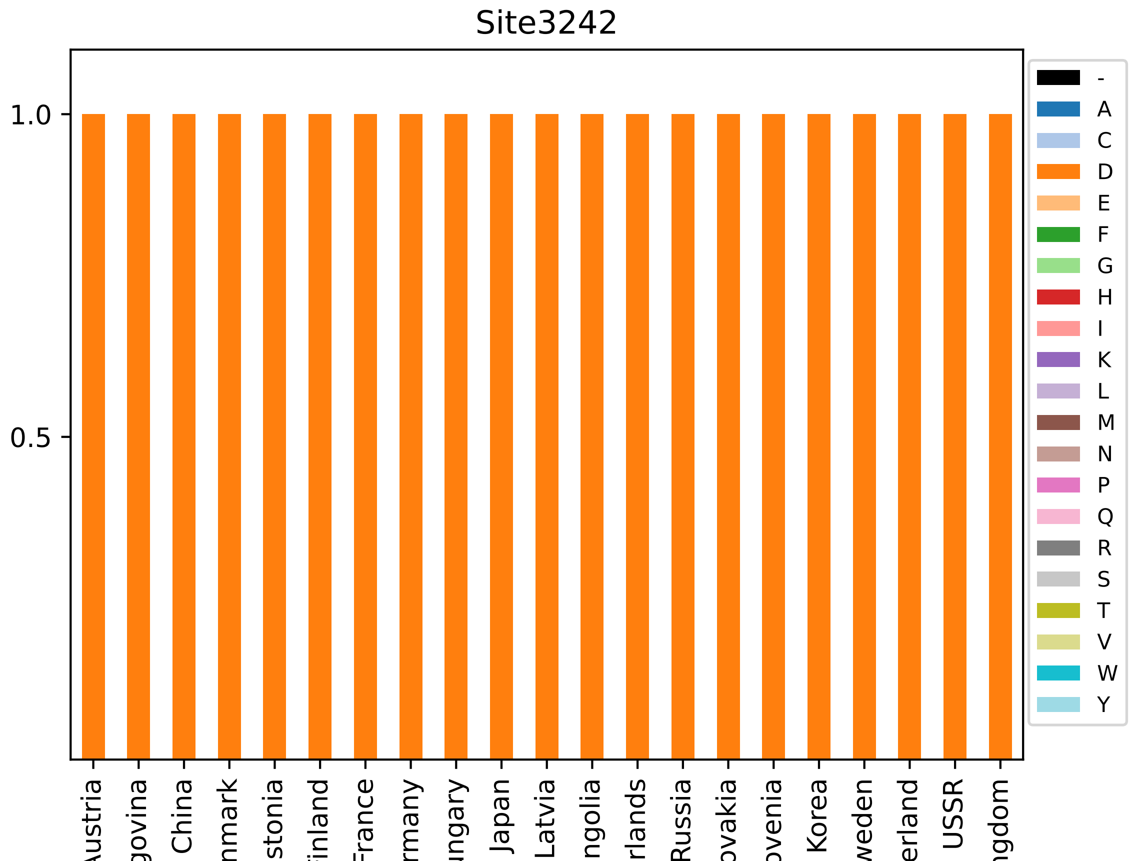
Site3242
1.0
0.5
-
A
C
D
E
F
G
H
I
K
L
M
N
P
Q
R
S
T
V
W
Y
China
Japan
Latvia
Russia
Korea
USSR

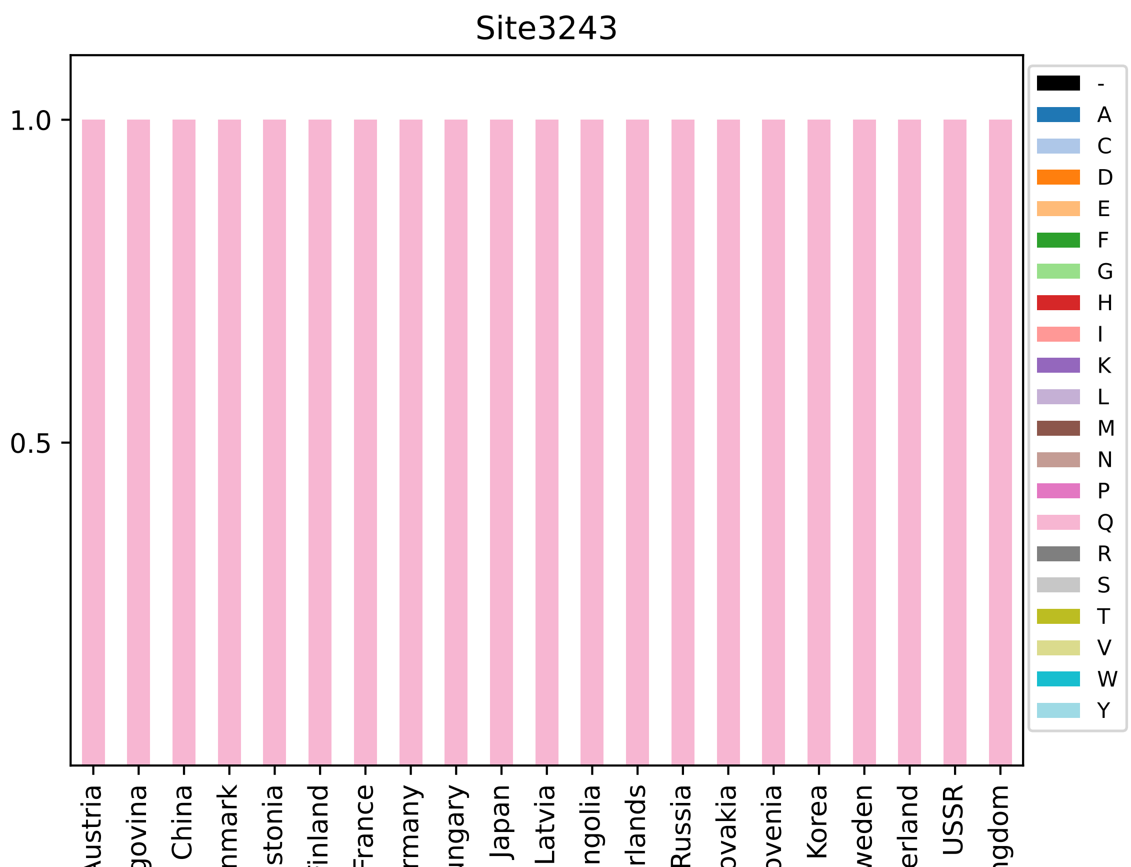
Site3243
1.0
0.5
-
A
C
D
E
F
G
H
I
K
L
M
N
P
Q
R
S
T
V
W
Y
China
Japan
Latvia
Russia
Korea
USSR

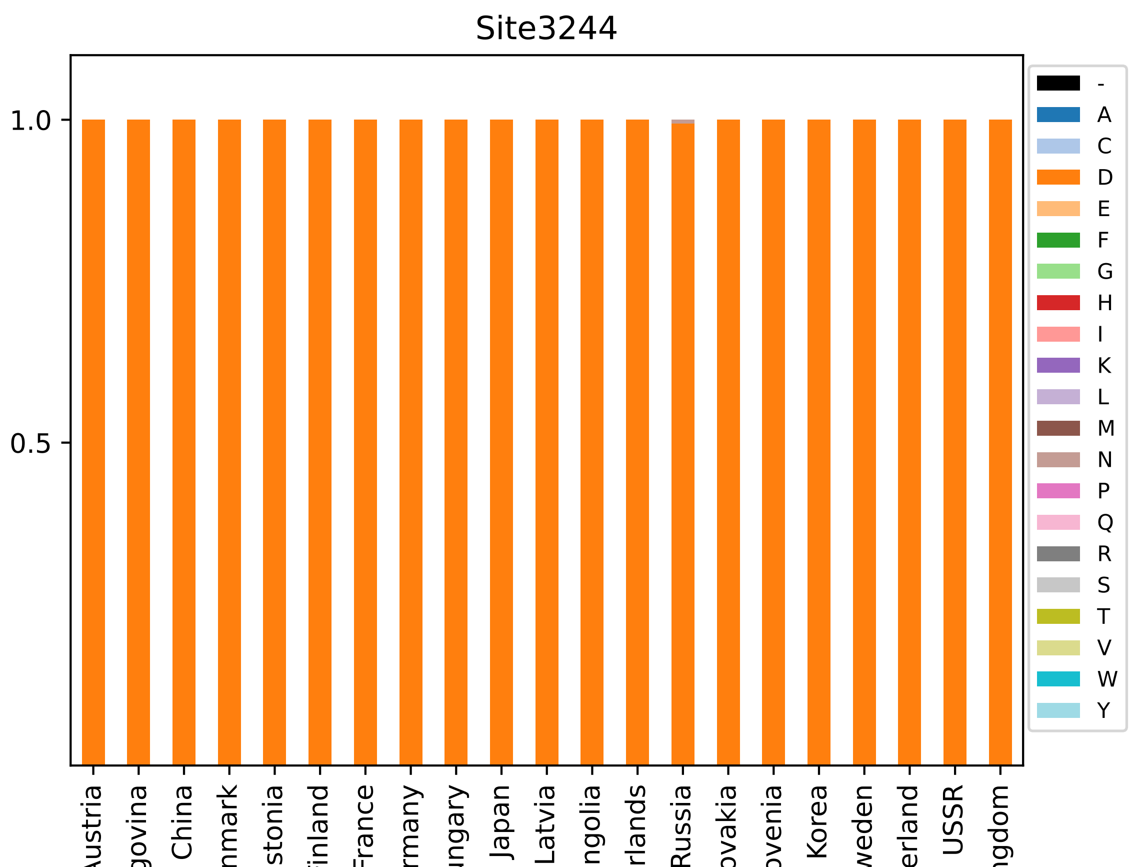

Site3244
1.0
0.5
-
A
C
D
E
F
G
H
I
K
L
M
N
P
Q
R
S
T
V
W
Y
Austria
govina
China
nmark
stonia
Finland
France
rmany
ungary
Japan
Latvia
ngolia
rlands
Russia
ovakia
ovenia
Korea
weden
erland
USSR
ngdom

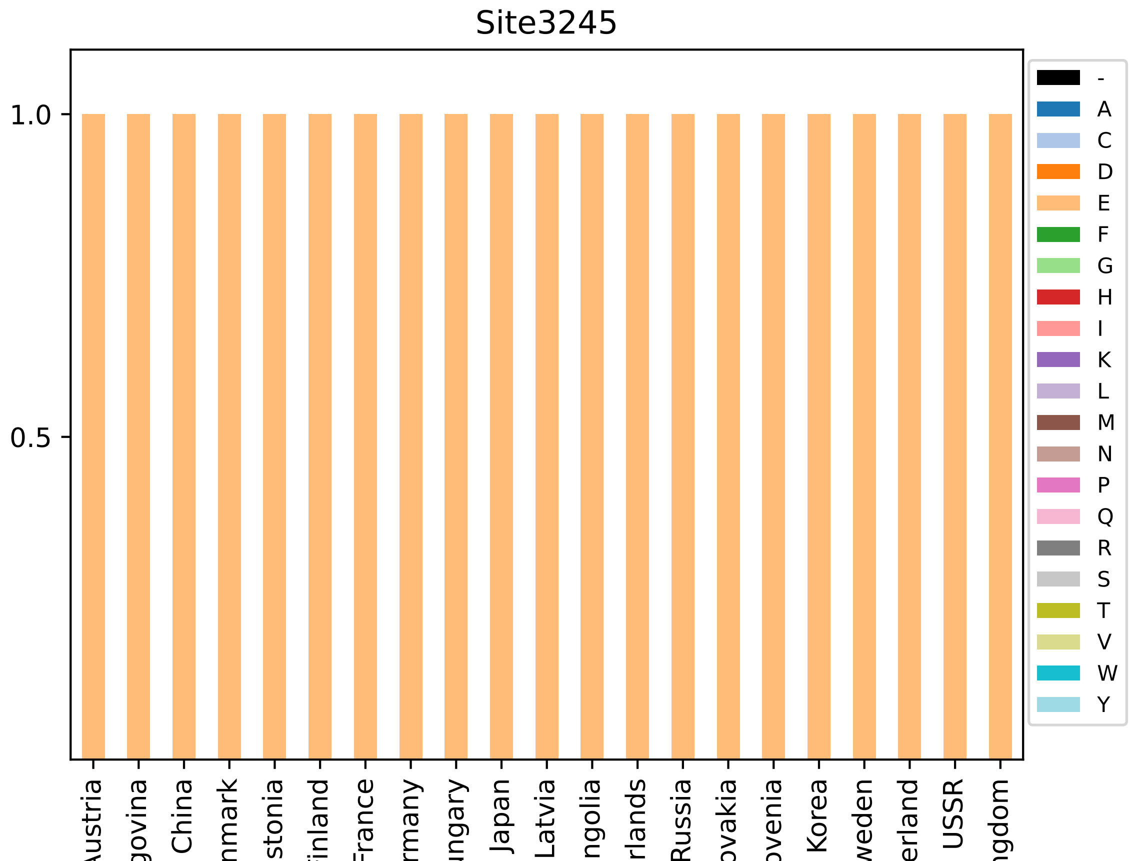

Site3245
1.0
0.5
China
Japan
Latvia
Russia
Korea
USSR
-
A
C
D
E
F
G
H
I
K
L
M
N
P
Q
R
S
T
V
W
Y

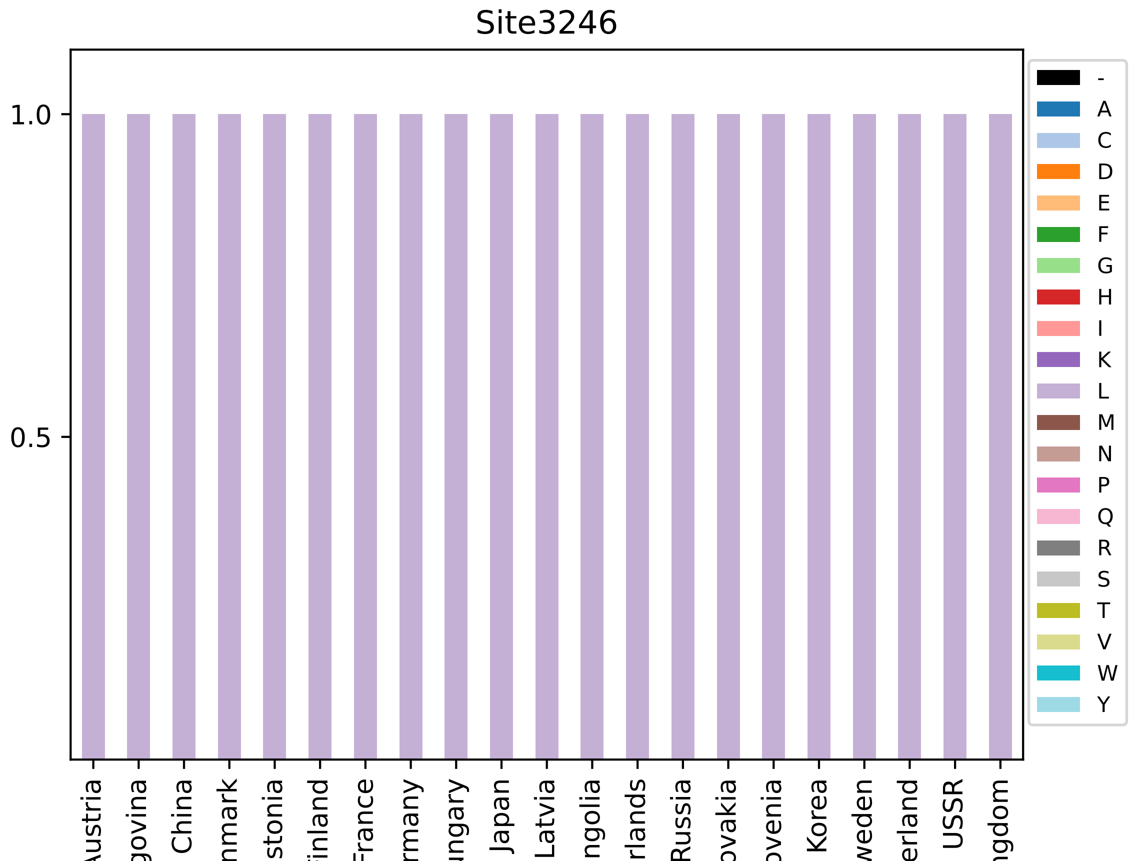

Site3246
1.0
0.5
-
A
C
D
E
F
G
H
I
K
L
M
N
P
Q
R
S
T
V
W
Y
China
Japan
Latvia
Russia
Korea
USSR

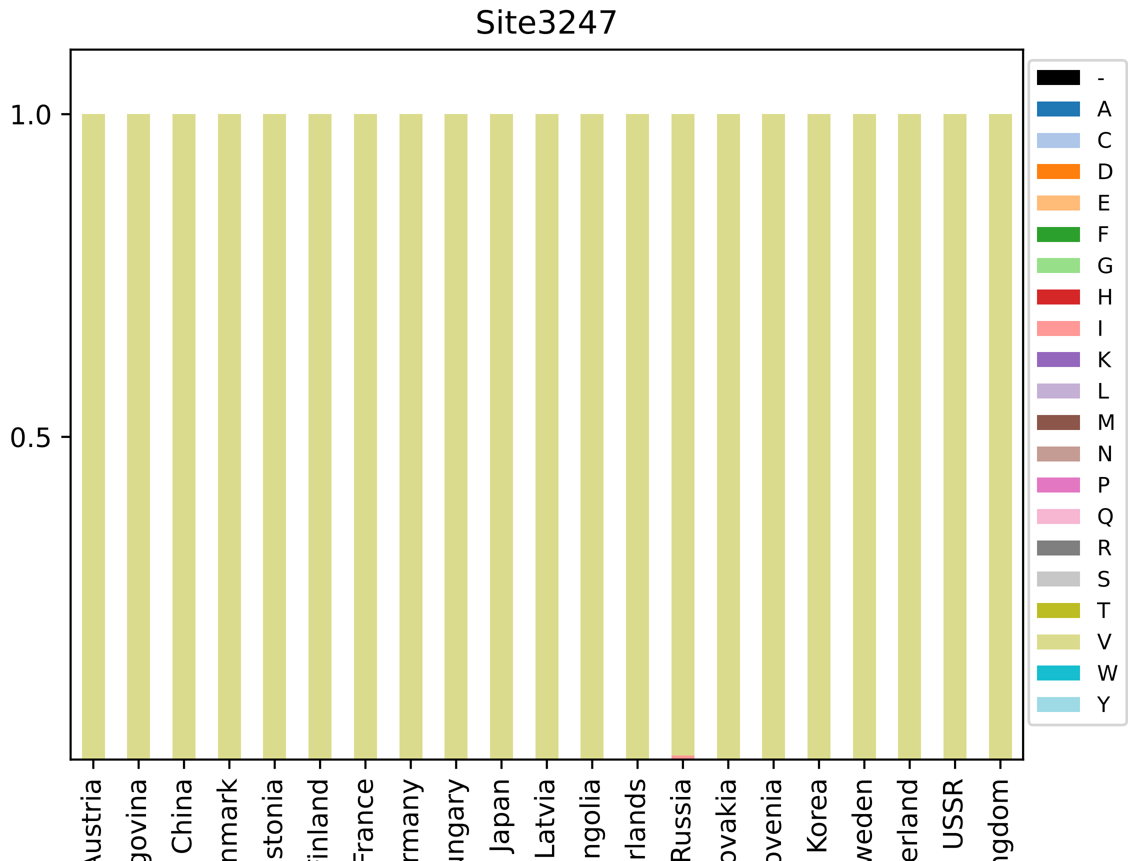

Site3247
1.0
0.5
-
A
C
D
E
F
G
H
I
K
L
M
N
P
Q
R
S
T
V
W
Y
Austria
govina
China
nmark
stonia
Finland
France
rmany
ungary
Japan
Latvia
ngolia
rlands
Russia
ovakia
ovenia
Korea
weden
erland
USSR
ngdom

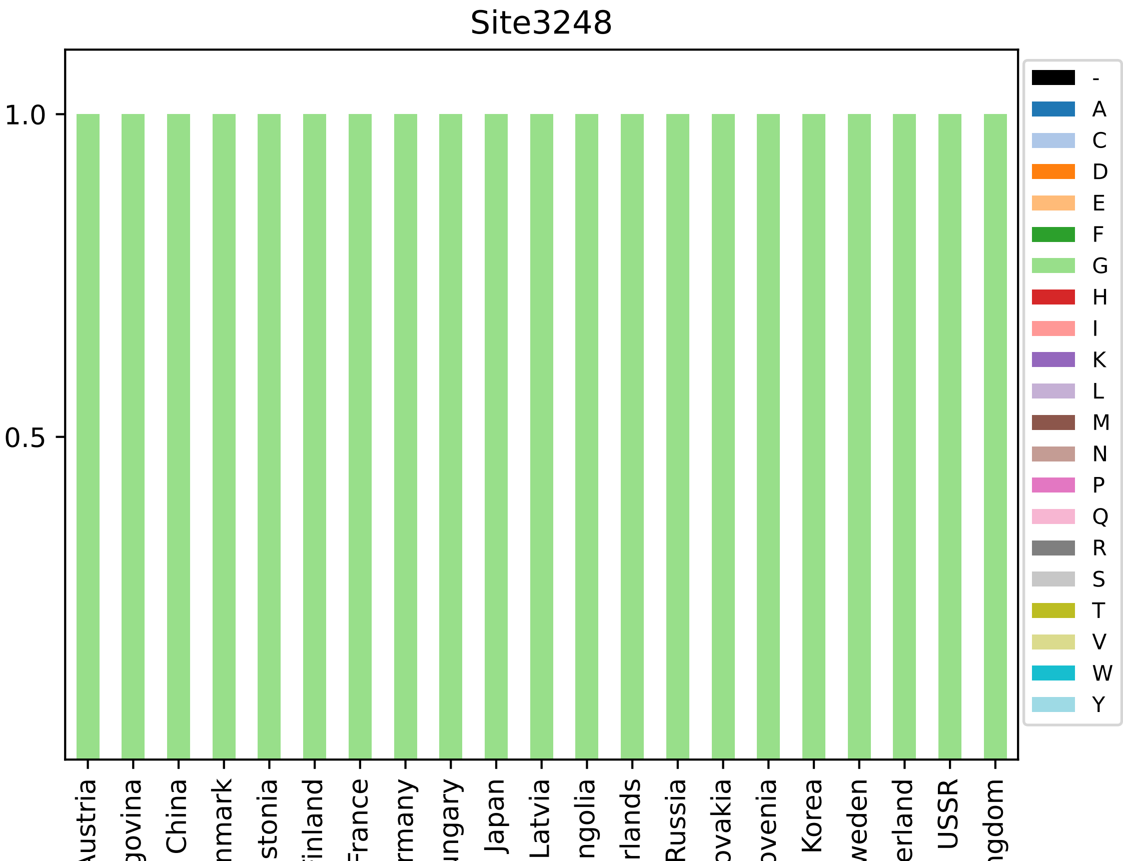

Site3248
1.0
0.5
-
A
C
D
E
F
G
H
I
K
L
M
N
P
Q
R
S
T
V
W
Y
China
Japan
Latvia
Russia
Korea
USSR

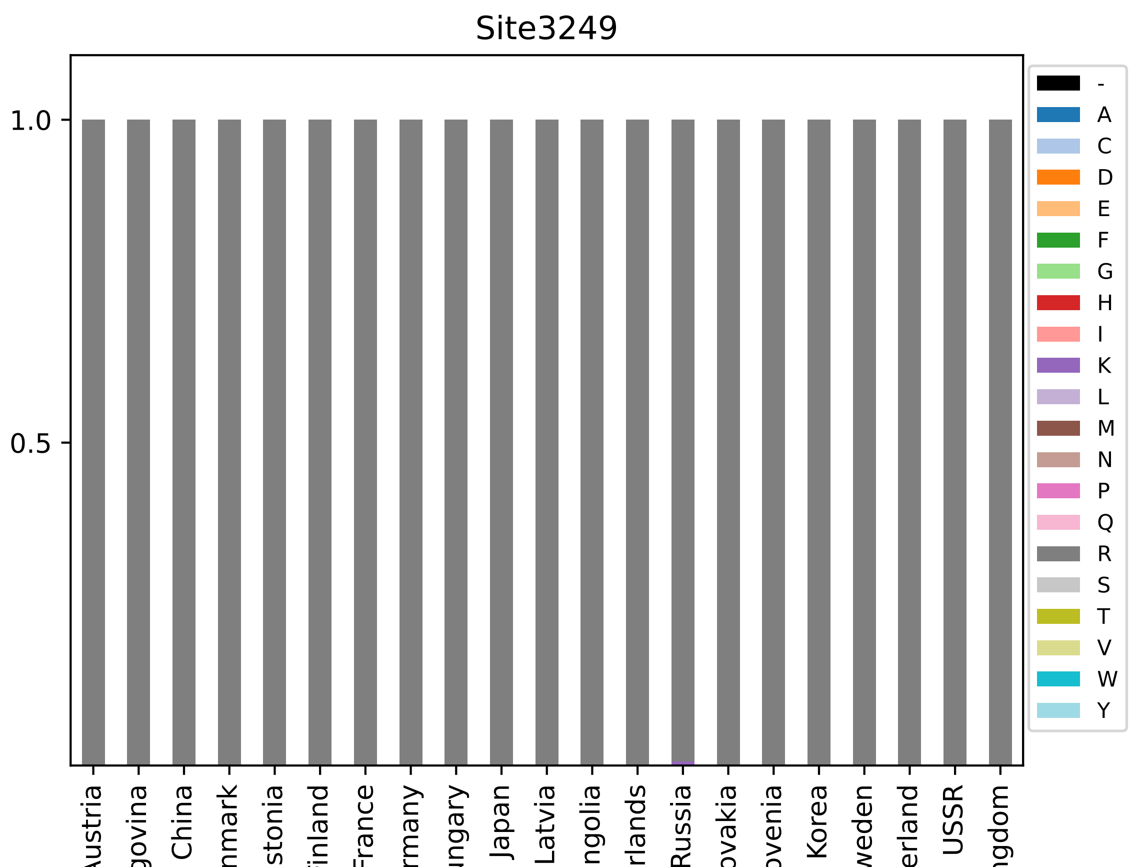

Site3249
1.0
0.5
-
A
C
D
E
F
G
H
I
K
L
M
N
P
Q
R
S
T
V
W
Y
Austria
govina
China
nmark
stonia
inland
France
rmany
ungary
Japan
Latvia
ngolia
rlands
Russia
ovakia
ovenia
Korea
weden
erland
USSR
ngdom

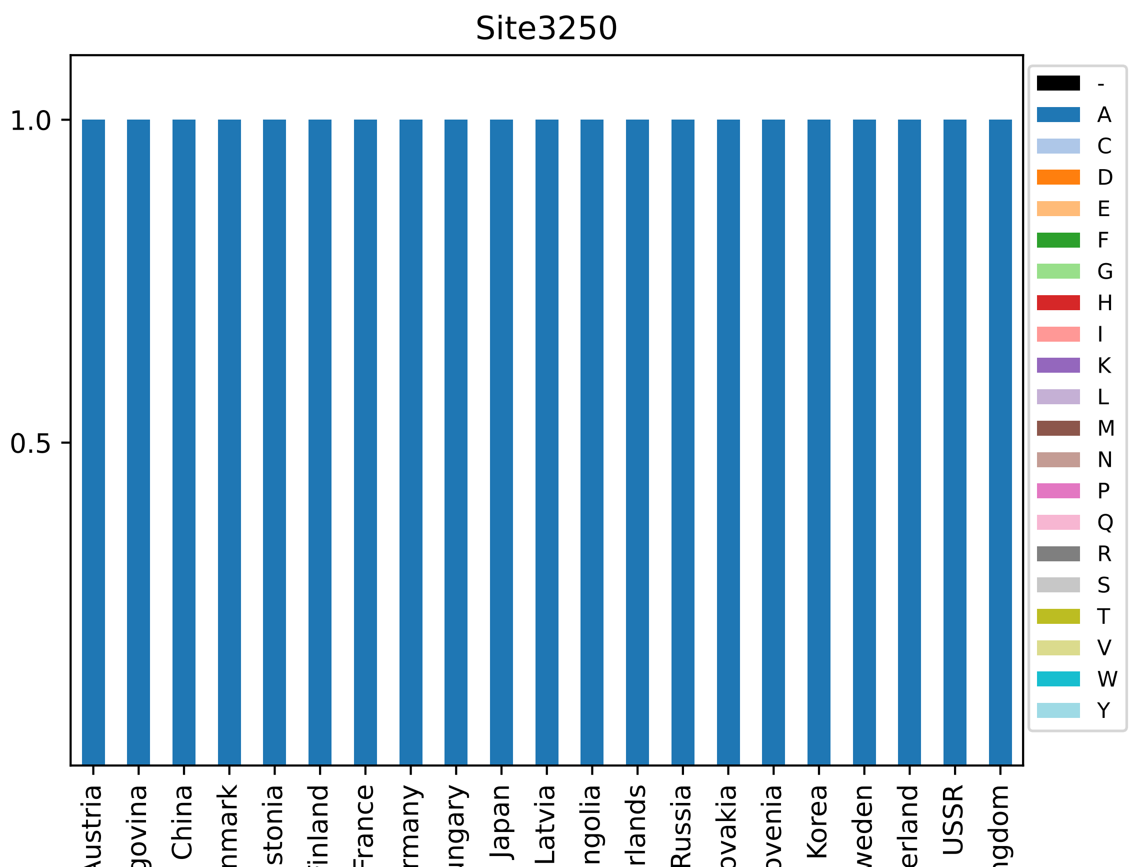

Site3250
1.0
0.5
-
A
C
D
E
F
G
H
I
K
L
M
N
P
Q
R
S
T
V
W
Y
China
Japan
Latvia
Russia
Korea
USSR

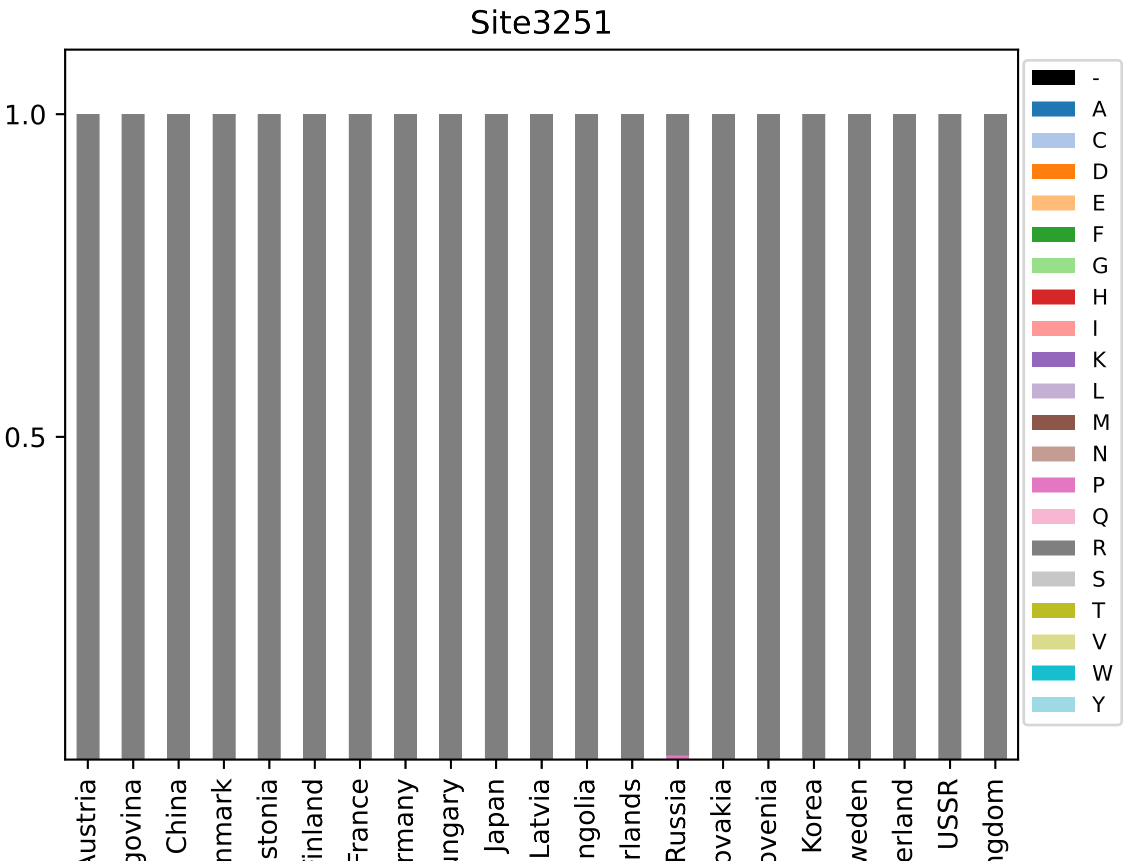

Site3251
1.0
0.5
-
A
C
D
E
F
G
H
I
K
L
M
N
P
Q
R
S
T
V
W
Y
Austria
govina
China
nmark
stonia
inland
France
rmany
ungary
Japan
Latvia
ngolia
rlands
Russia
ovakia
ovenia
Korea
weden
erland
USSR
ngdom

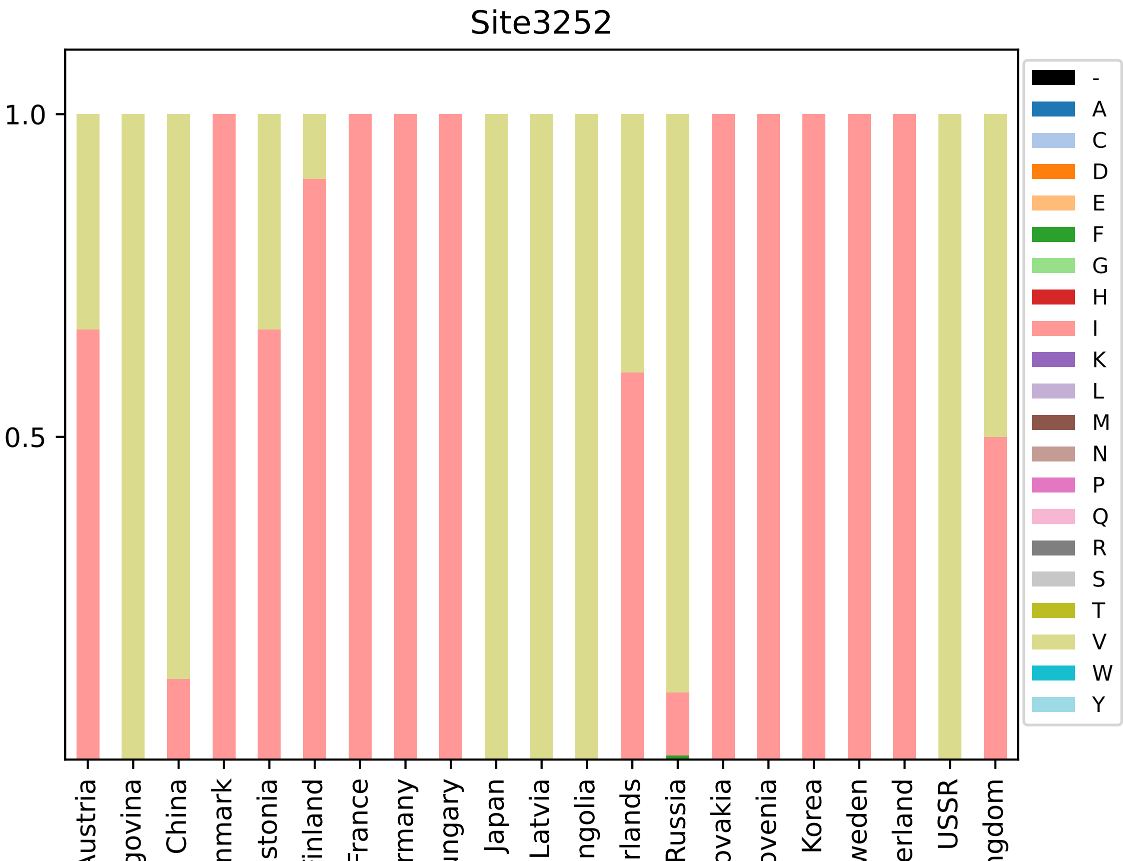
Site3252
1.0
0.5
-
A
C
D
E
F
G
H
I
K
L
M
N
P
Q
R
S
T
V
W
Y
China
Japan
Latvia
Russia
Korea
USSR

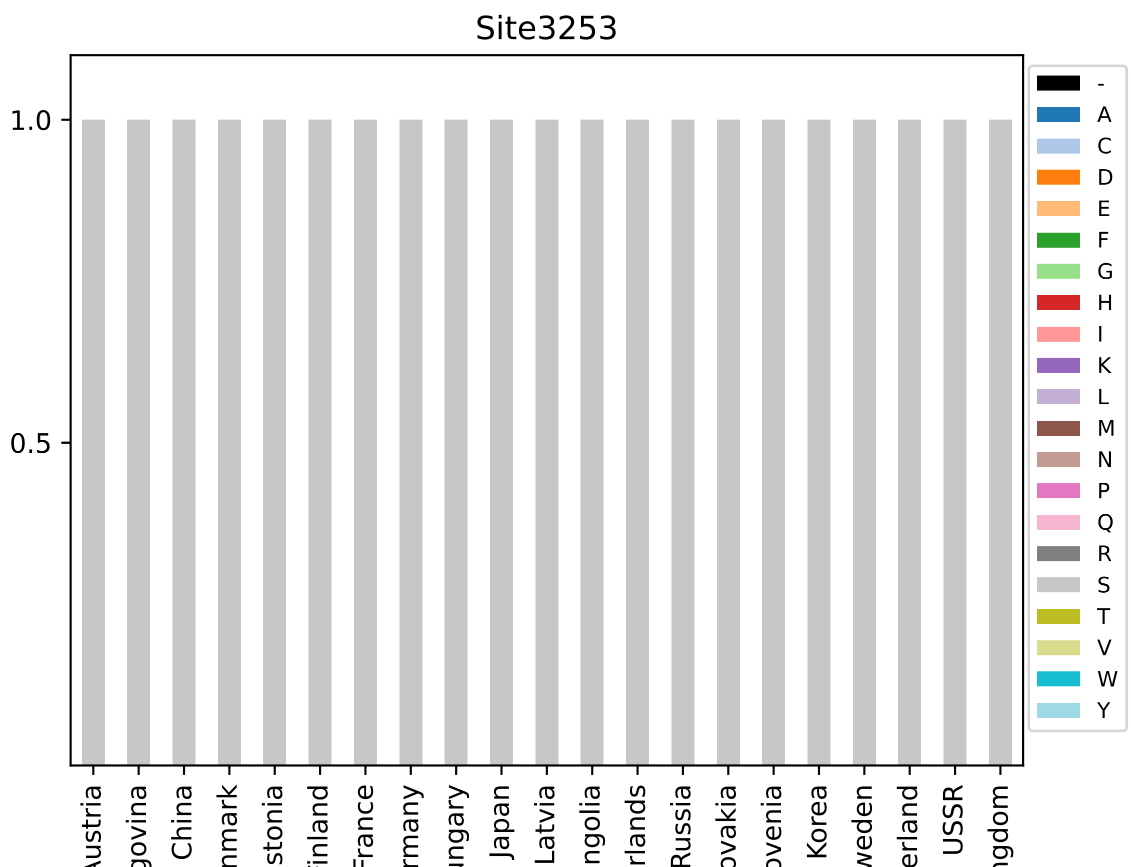
Site3253
1.0
0.5
-
A
C
D
E
F
G
H
I
K
L
M
N
P
Q
R
S
T
V
W
Y
Austria
govina
China
nmark
stonia
Finland
France
rmany
ungary
Japan
Latvia
ngolia
rlands
Russia
ovakia
ovenia
Korea
weden
erland
USSR
ngdom

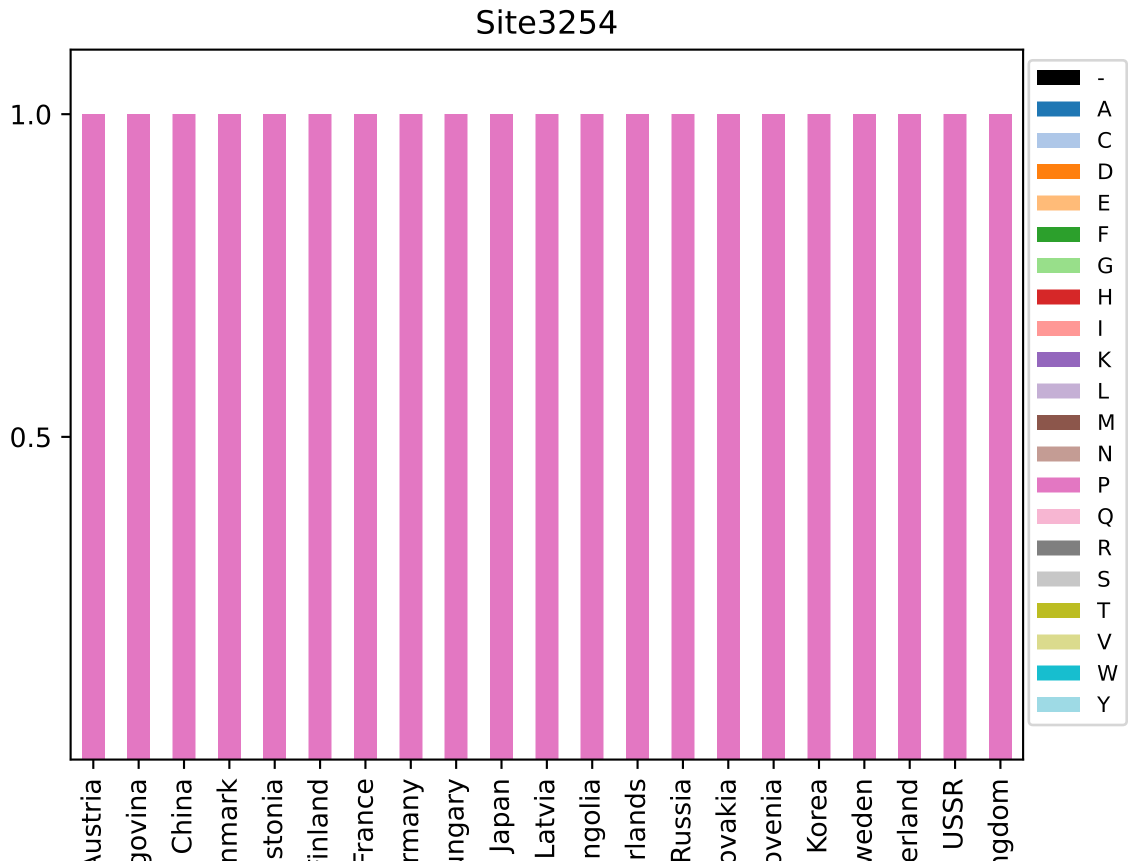
Site3254
1.0
0.5
-
A
C
D
E
F
G
H
I
K
L
M
N
P
Q
R
S
T
V
W
Y
China
Japan
Latvia
Russia
Korea
USSR

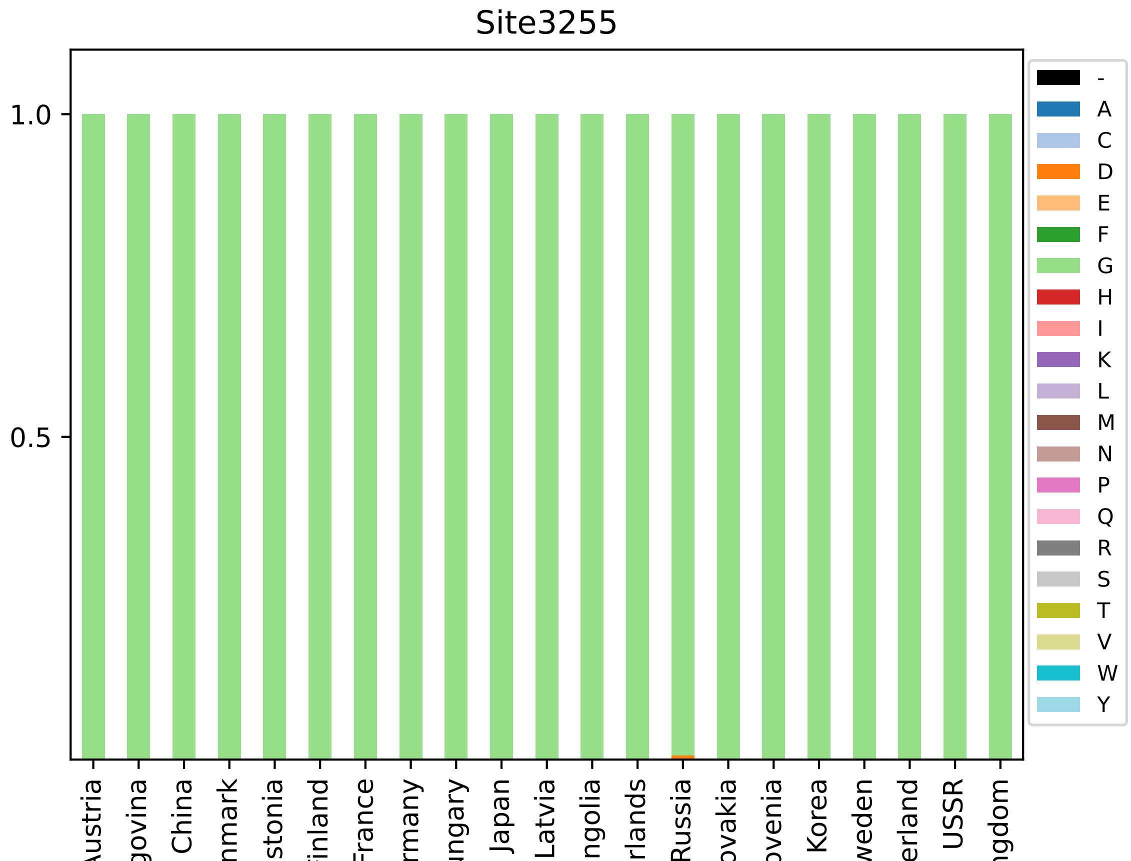
Site3255
1.0
0.5
-
A
C
D
E
F
G
H
I
K
L
M
N
P
Q
R
S
T
V
W
Y
China
Japan
Latvia
Russia
Korea
USSR

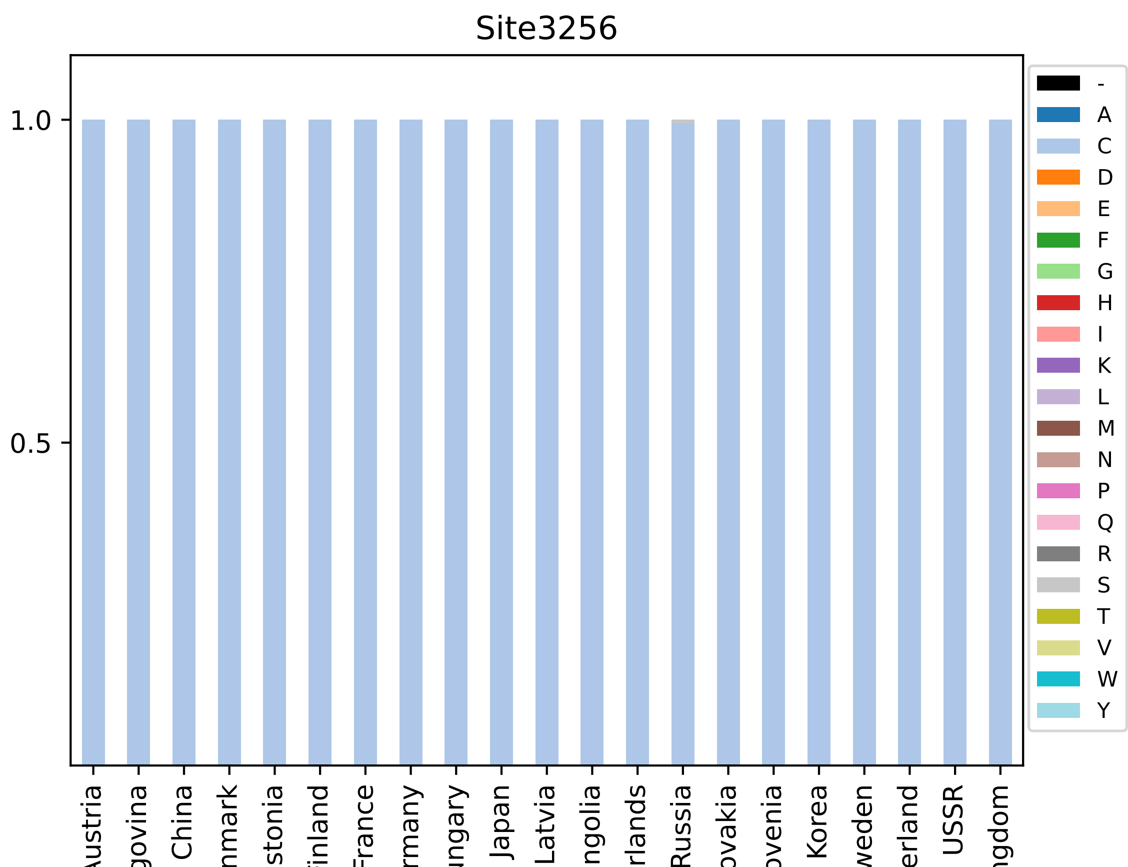

Site3256
1.0
0.5
-
A
C
D
E
F
G
H
I
K
L
M
N
P
Q
R
S
T
V
W
Y
Austria
govina
China
nmark
stonia
Finland
France
rmany
ungary
Japan
Latvia
ngolia
rlands
Russia
ovakia
ovenia
Korea
weden
erland
USSR
ngdom

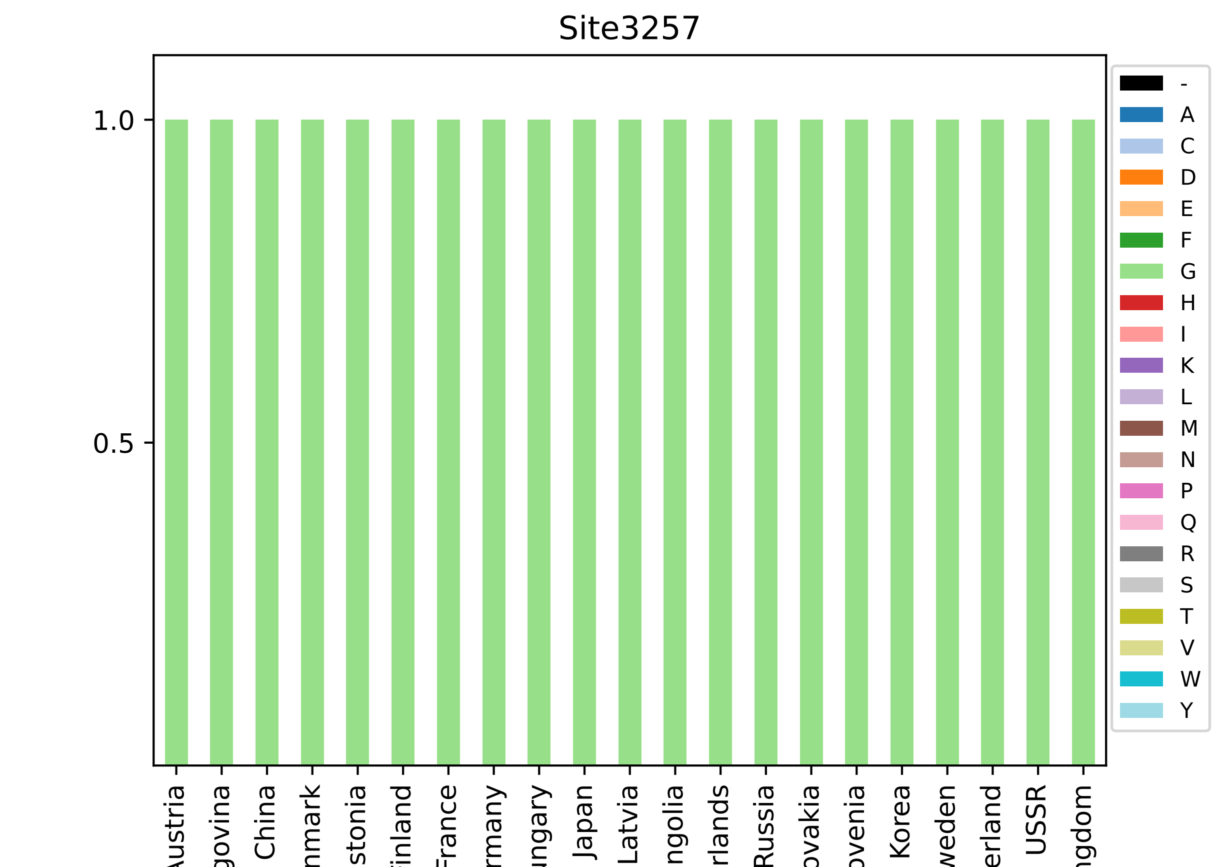
Site3257
1.0
0.5
-
A
C
D
E
F
G
H
I
K
L
M
N
P
Q
R
S
T
V
W
Y
Austria
govina
China
nmark
stonia
inland
France
rmany
ungary
Japan
Latvia
ngolia
rlands
Russia
ovakia
ovenia
Korea
weden
erland
USSR
ngdom

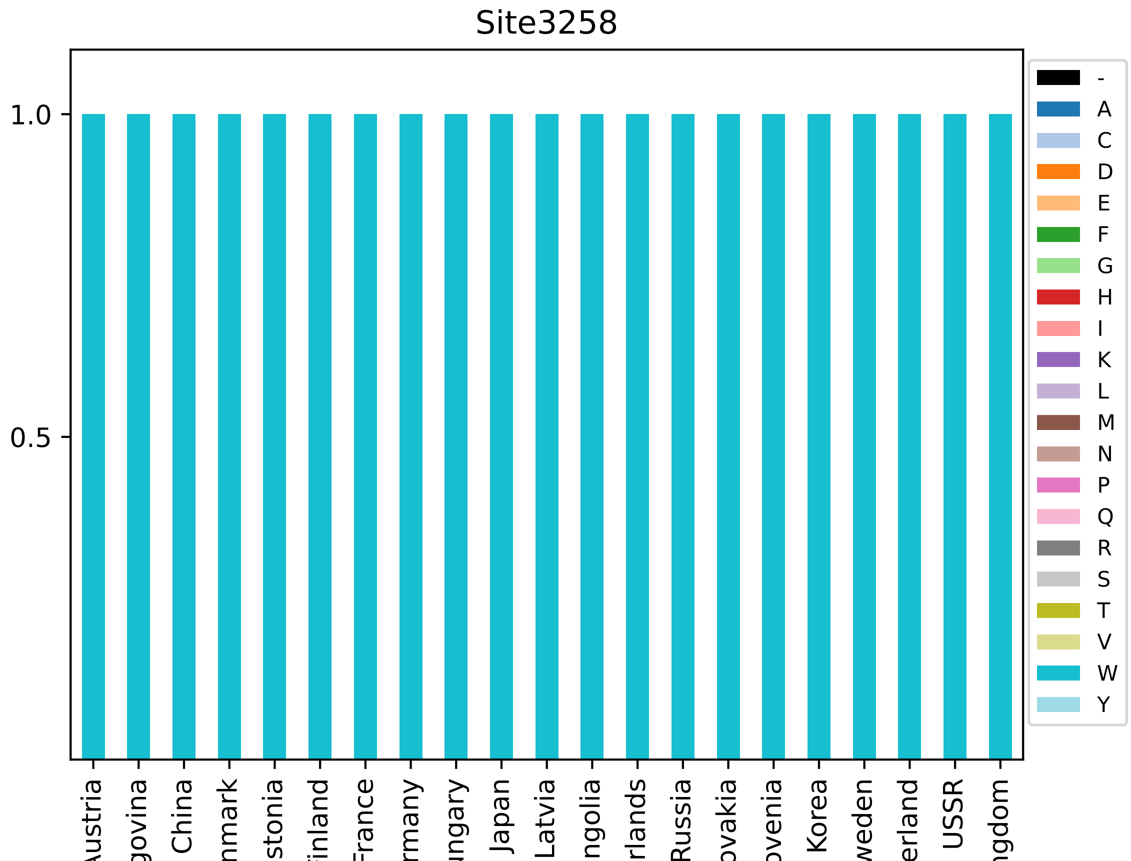

Site3258
1.0
0.5
-
A
C
D
E
F
G
H
I
K
L
M
N
P
Q
R
S
T
V
W
Y
China
Japan
Latvia
Russia
Korea
USSR

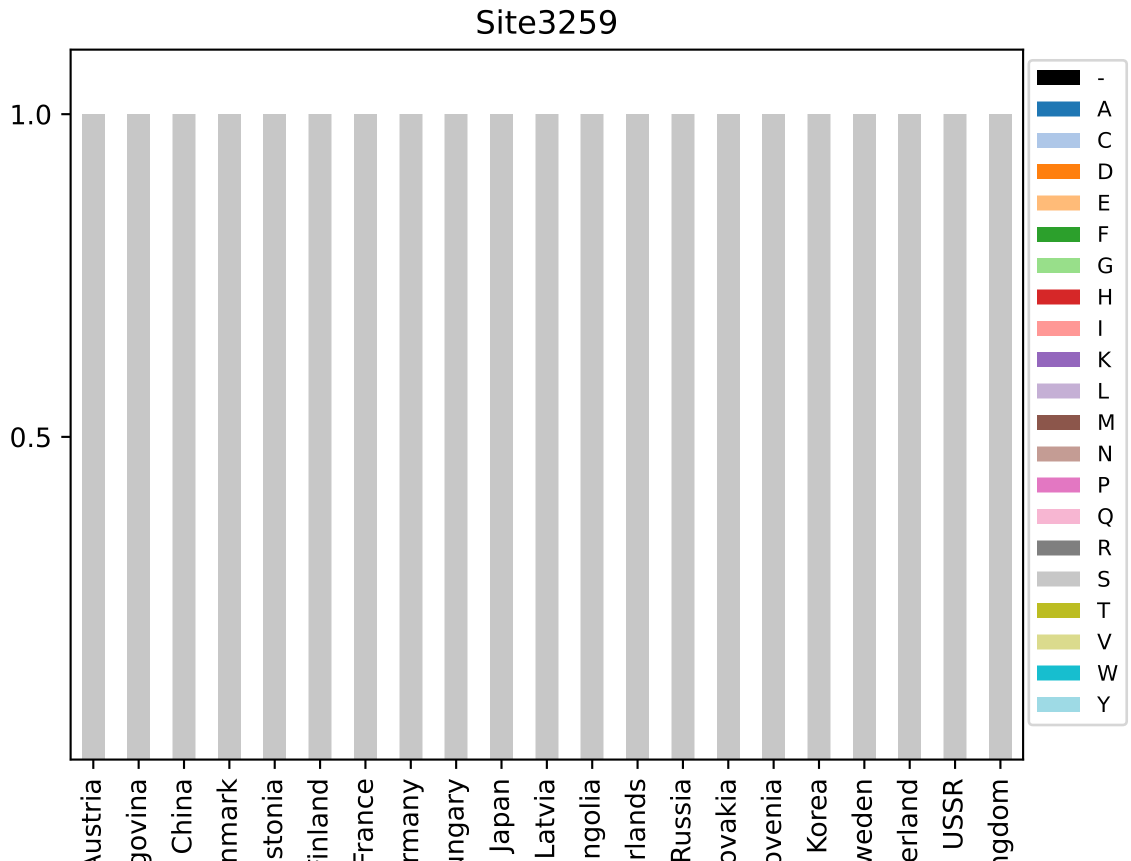

Site3259
1.0
0.5
-
A
C
D
E
F
G
H
I
K
L
M
N
P
Q
R
S
T
V
W
Y
China
Japan
Latvia
Russia
Korea
USSR

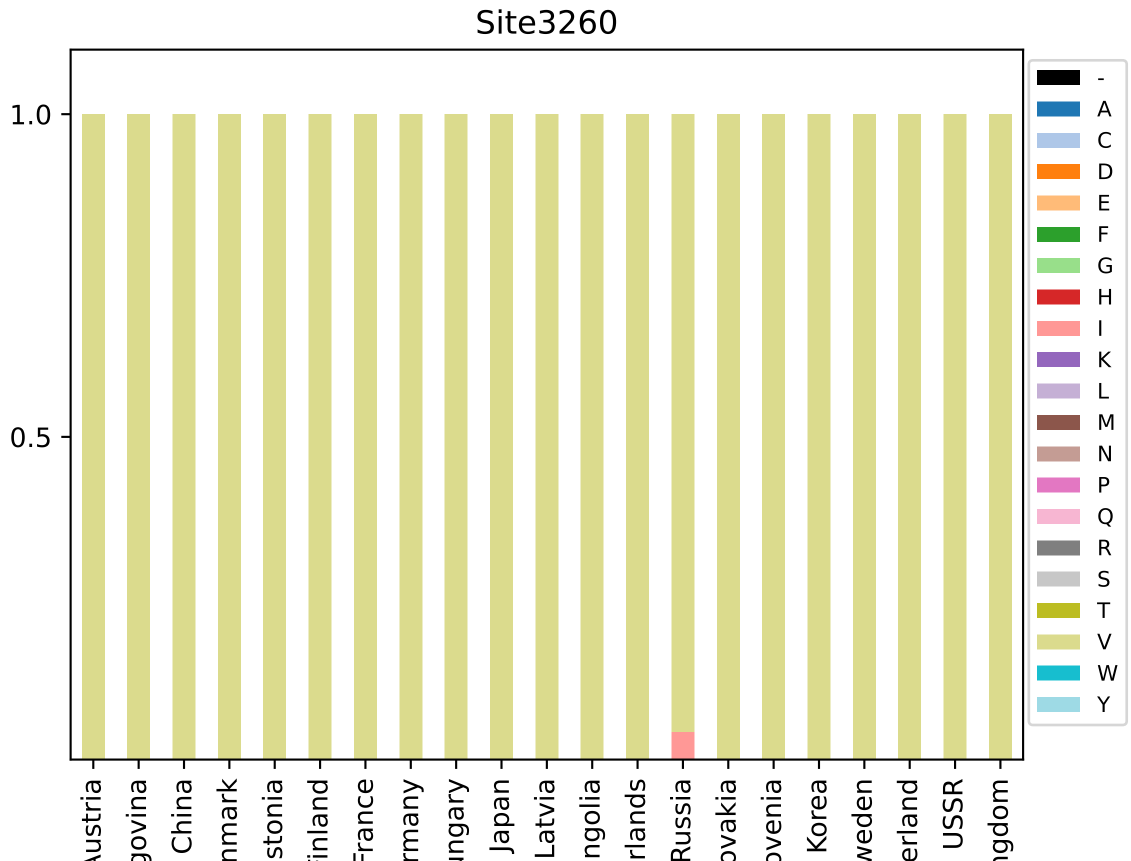
Site3260
1.0
0.5
-
A
C
D
E
F
G
H
I
K
L
M
N
P
Q
R
S
T
V
W
Y
Austria
govina
China
nmark
stonia
Finland
France
rmany
ungary
Japan
Latvia
ngolia
rlands
Russia
ovakia
ovenia
Korea
weden
erland
USSR
ngdom

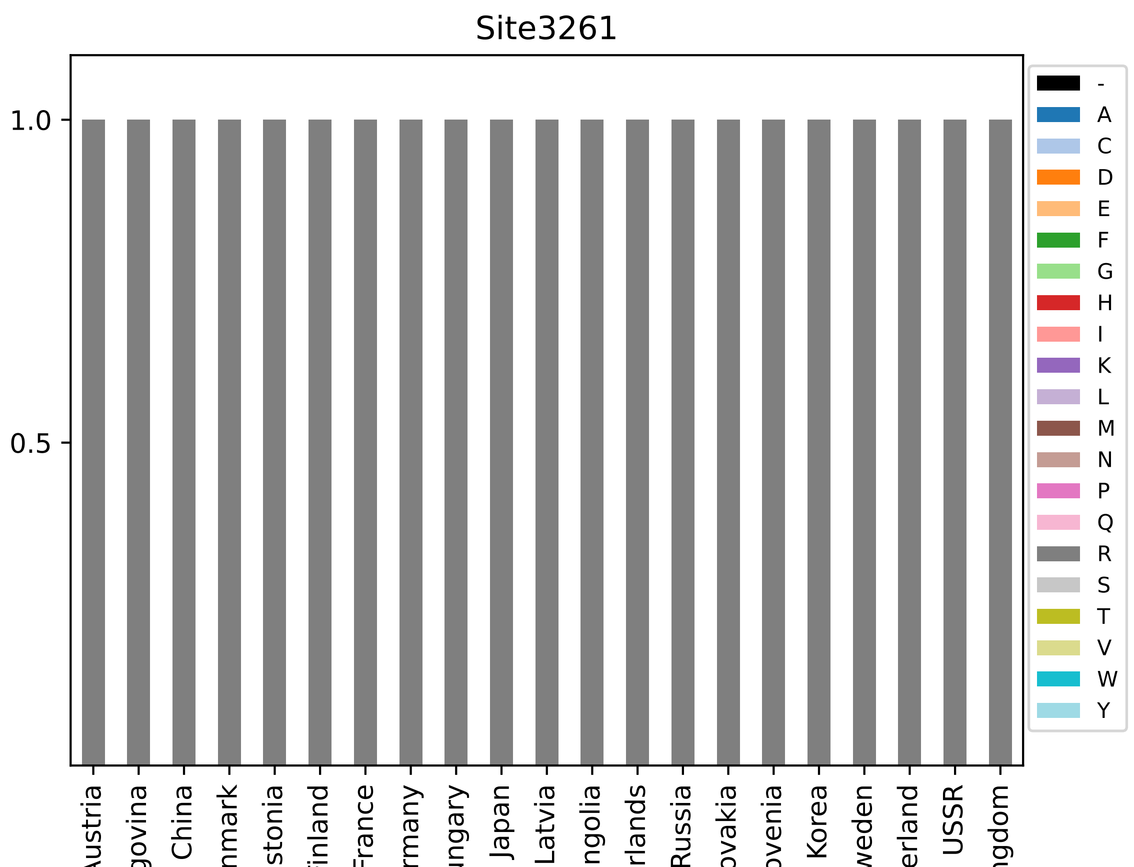

Site3261
1.0
0.5
-
A
C
D
E
F
G
H
I
K
L
M
N
P
Q
R
S
T
V
W
Y
Austria
govina
China
nmark
stonia
inland
France
rmany
ungary
Japan
Latvia
ngolia
rlands
Russia
ovakia
ovenia
Korea
weden
erland
USSR
ngdom

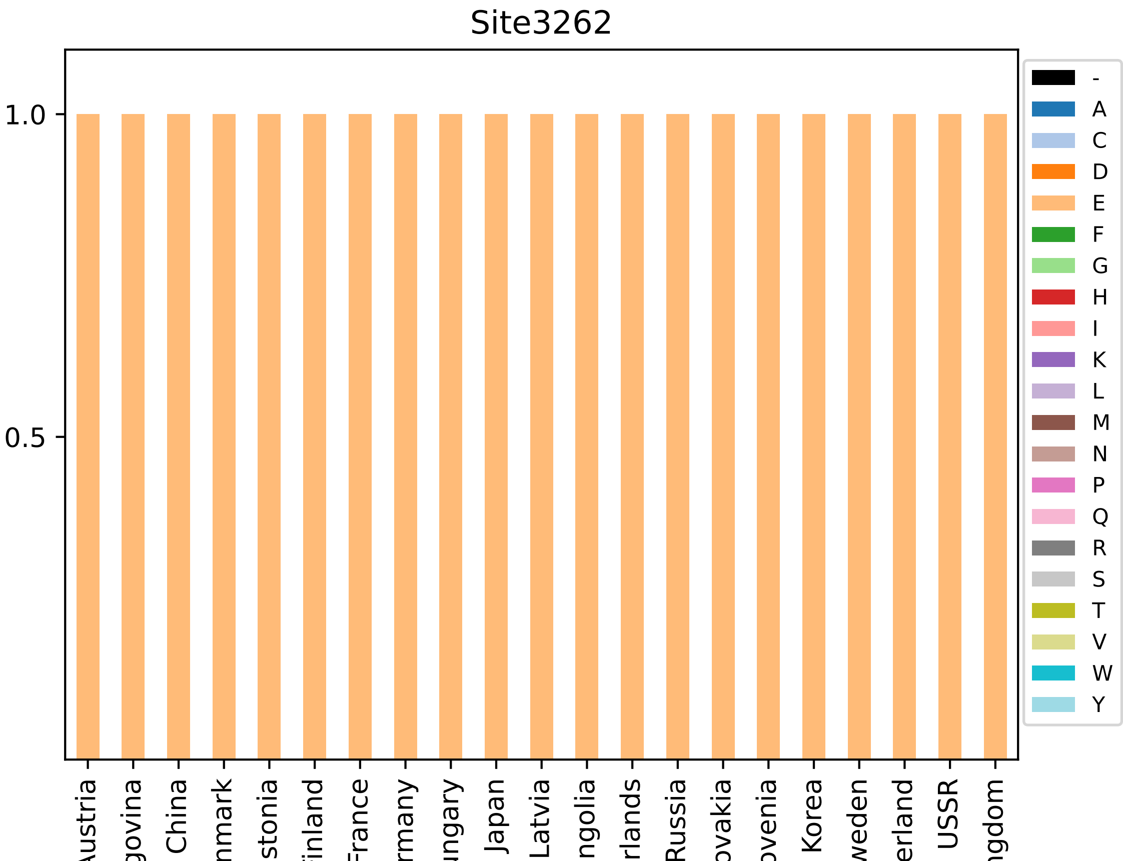
Site3262
1.0
0.5
-
A
C
D
E
F
G
H
I
K
L
M
N
P
Q
R
S
T
V
W
Y
China
Japan
Latvia
Russia
Korea
USSR

Site3263

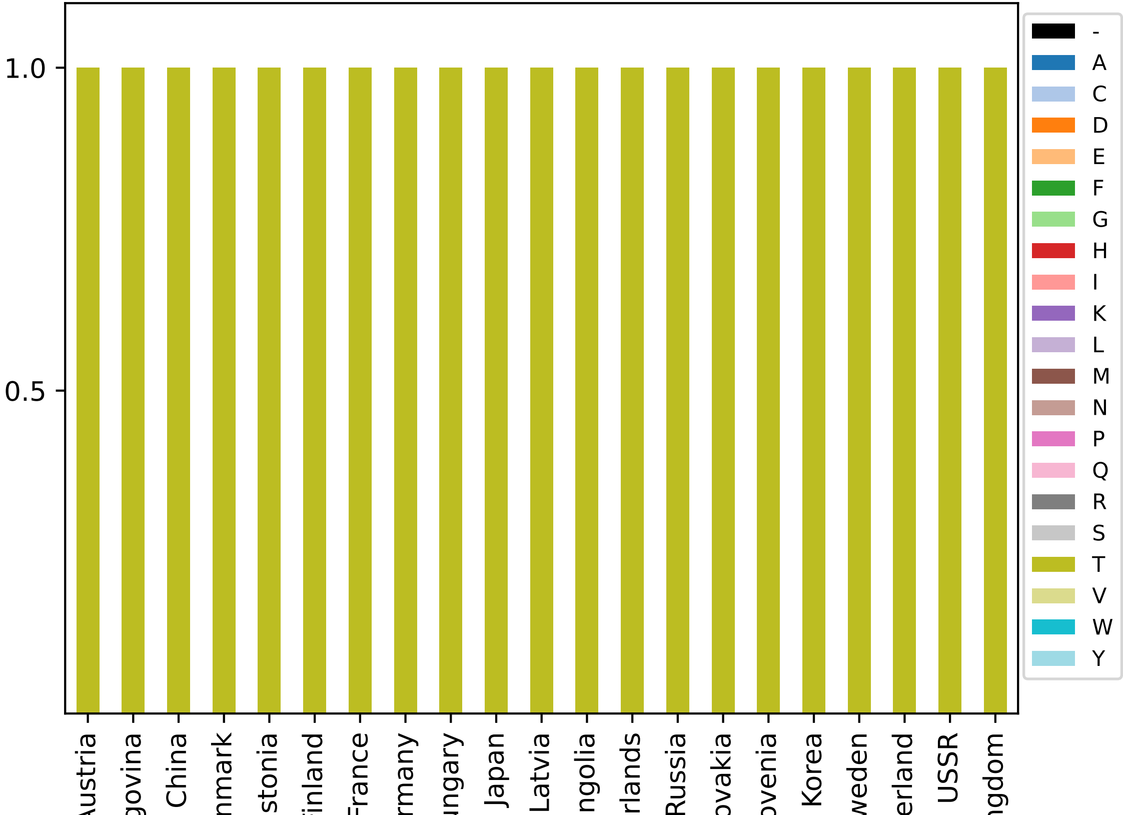

1.0
0.5
ustria
govina
China
nmark
stonia
inland
rance
rmany
ungary
Japan
Latvia
ngolia
rlands
Russia
ovakia
ovenia
Korea
weden
erland
USSR
ngdom
-
A
C
D
E
F
G
H
I
K
L
M
N
P
Q
R
S
T
V
W
Y

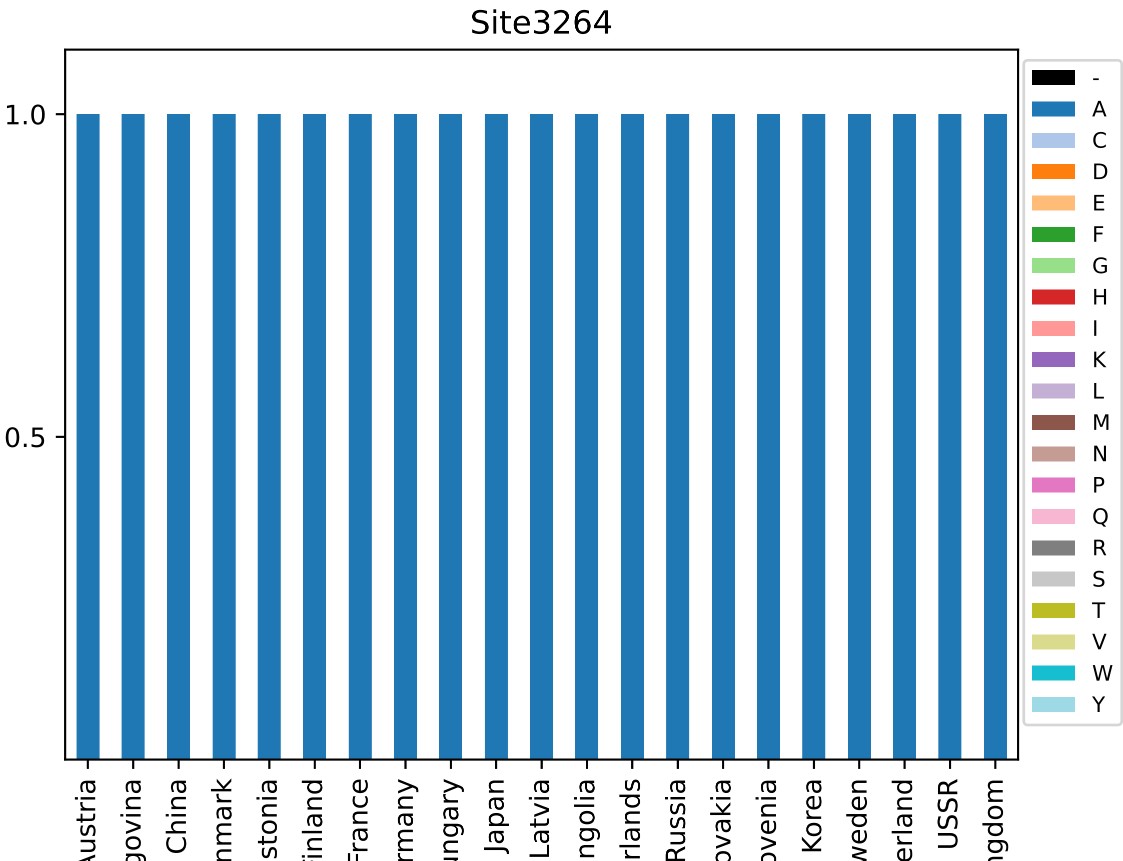
Site3264
1.0
0.5
-
A
C
D
E
F
G
H
I
K
L
M
N
P
Q
R
S
T
V
W
Y
ustria
govina
China
nmark
stonia
inland
rance
rmany
ungary
Japan
Latvia
ngolia
rlands
Russia
ovakia
ovenia
Korea
weden
erland
USSR
ngdom

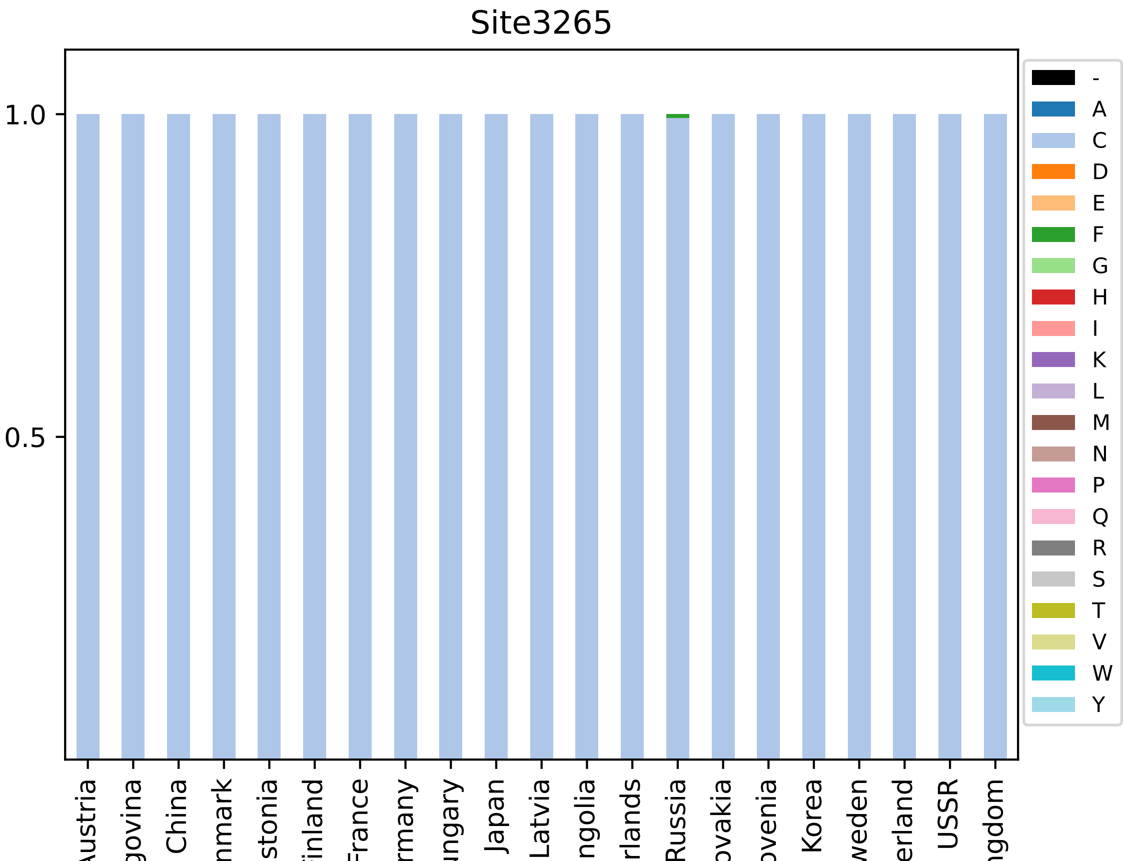

Site3265
1.0
0.5
-
A
C
D
E
F
G
H
I
K
L
M
N
P
Q
R
S
T
V
W
Y
Austria
China
Finland
France
Japan
Latvia
Russia
Korea
USSR

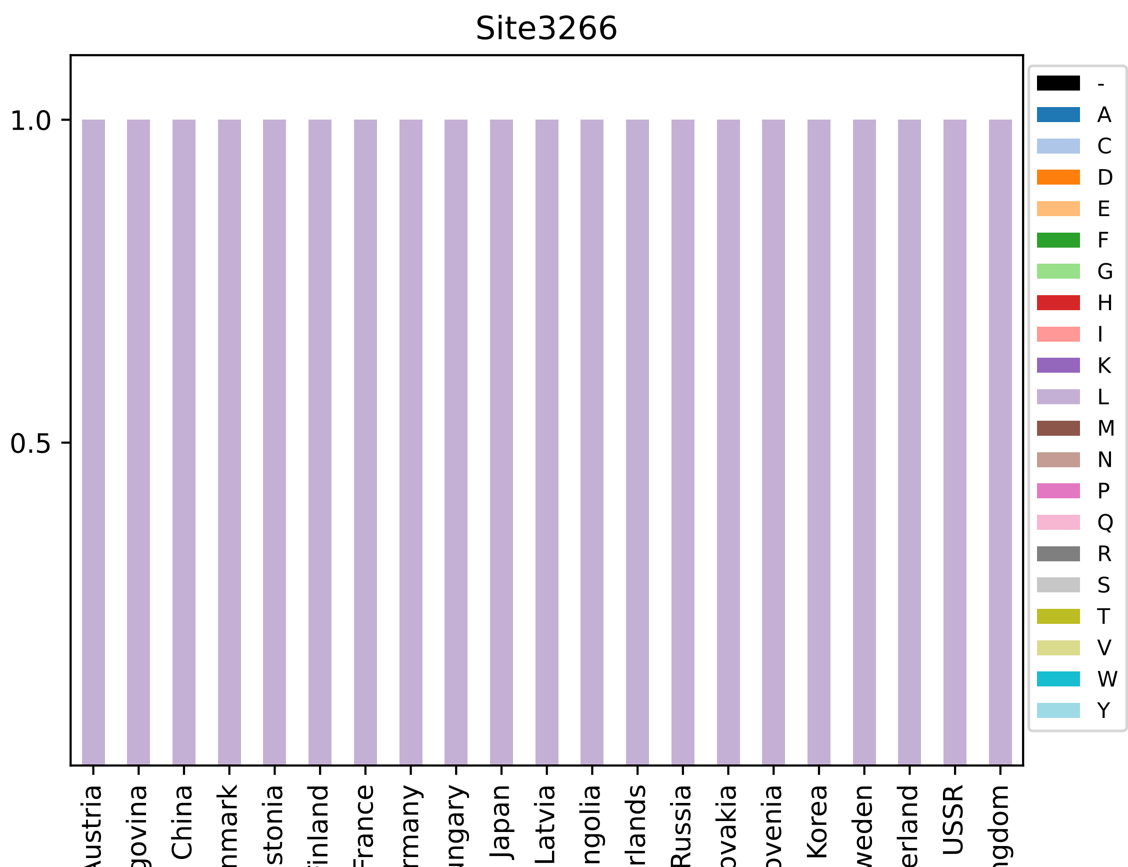
Site3266
1.0
0.5
-
A
C
D
E
F
G
H
I
K
L
M
N
P
Q
R
S
T
V
W
Y
China
Japan
Latvia
Russia
Korea
USSR

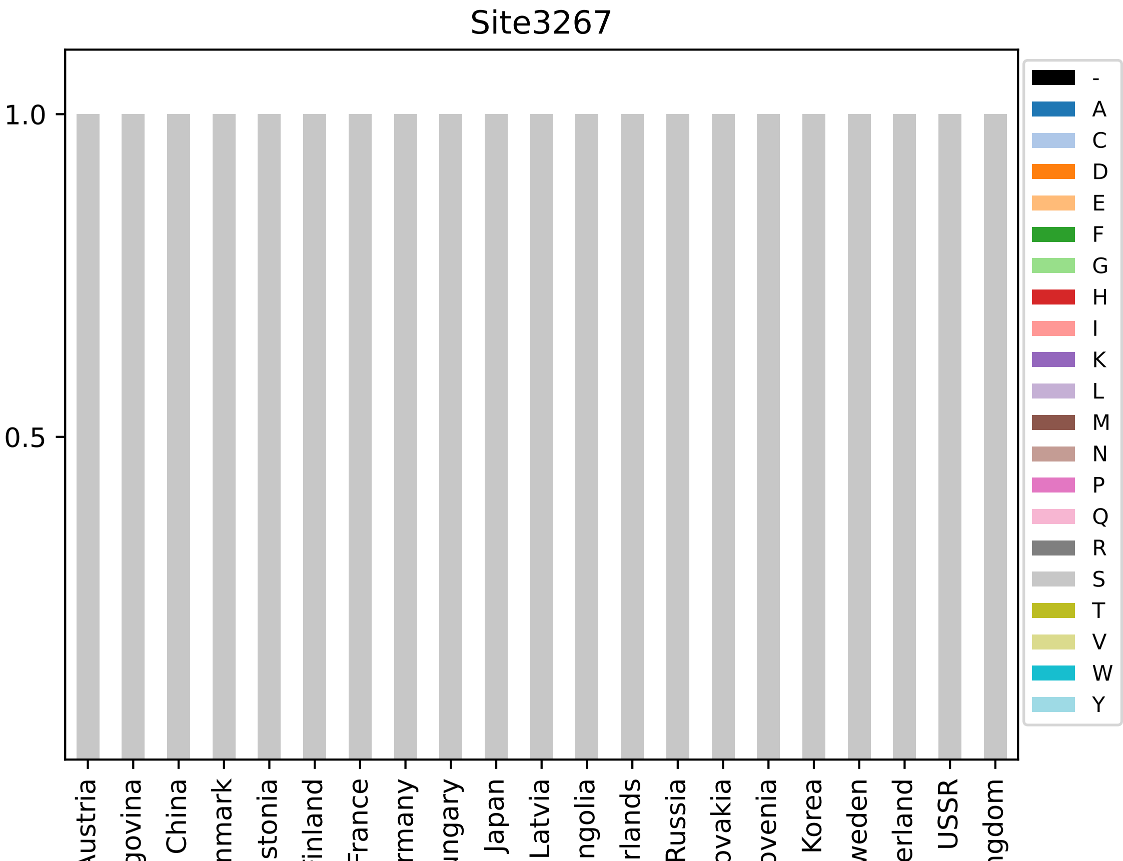

Site3267
1.0
0.5
-
A
C
D
E
F
G
H
I
K
L
M
N
P
Q
R
S
T
V
W
Y
China
Japan
Latvia
Russia
Korea
USSR

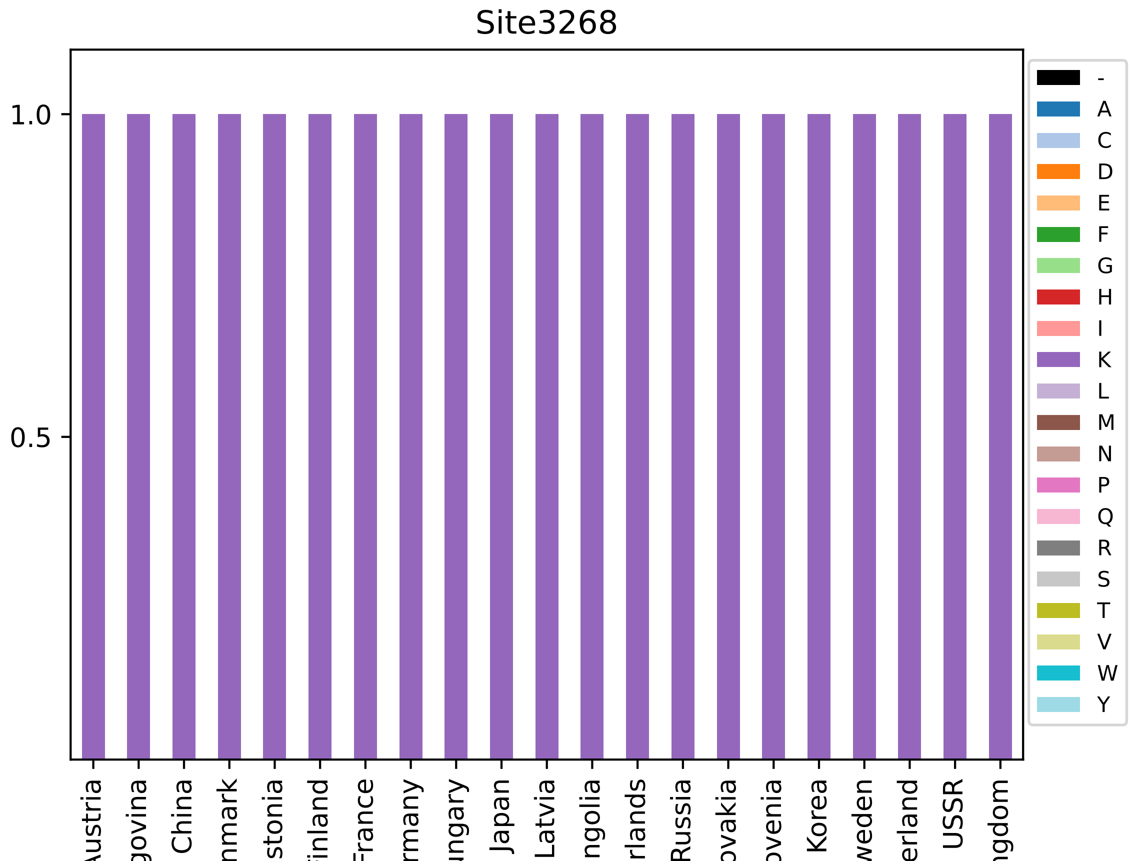

Site3268
1.0
0.5
-
A
C
D
E
F
G
H
I
K
L
M
N
P
Q
R
S
T
V
W
Y
Austria
govina
China
nmark
stonia
inland
France
rmany
ungary
Japan
Latvia
ngolia
rlands
Russia
ovakia
ovenia
Korea
weden
erland
USSR
ngdom

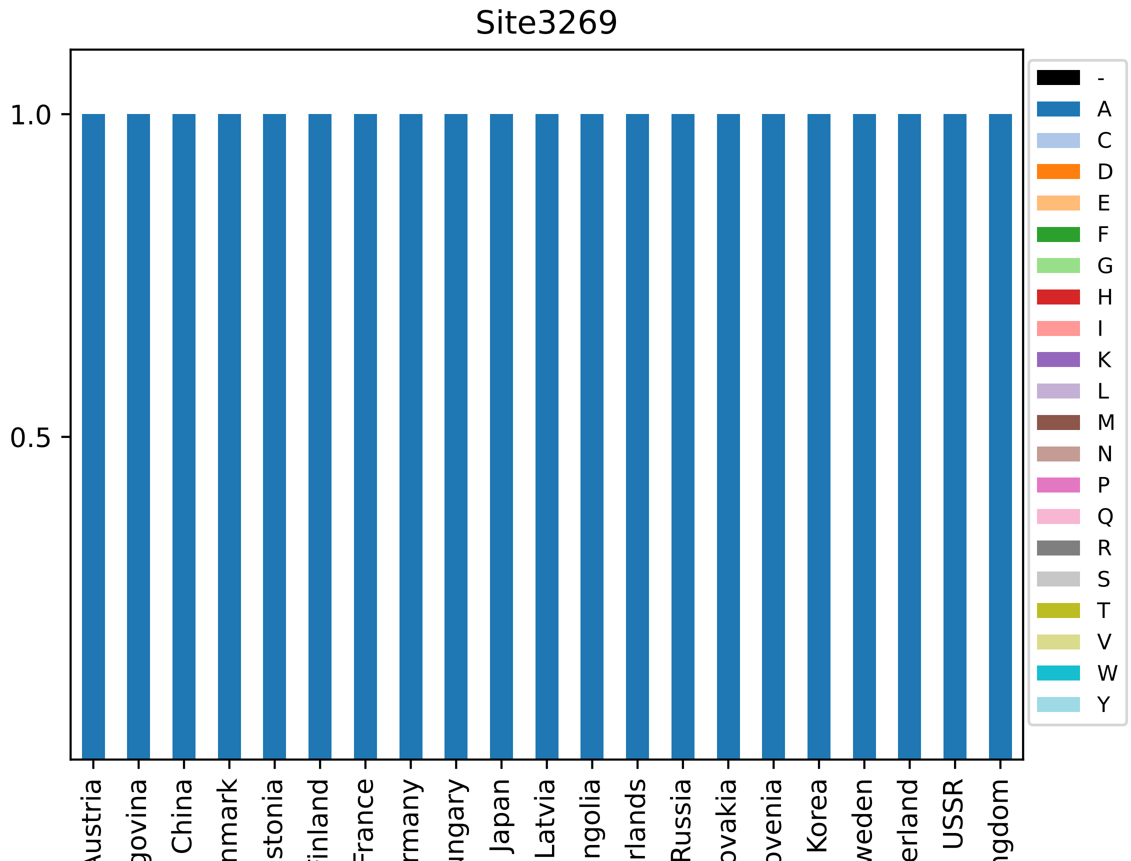
Site3269
1.0
0.5
China
Japan
Latvia
Russia
Korea
USSR
-
A
C
D
E
F
G
H
I
K
L
M
N
P
Q
R
S
T
V
W
Y

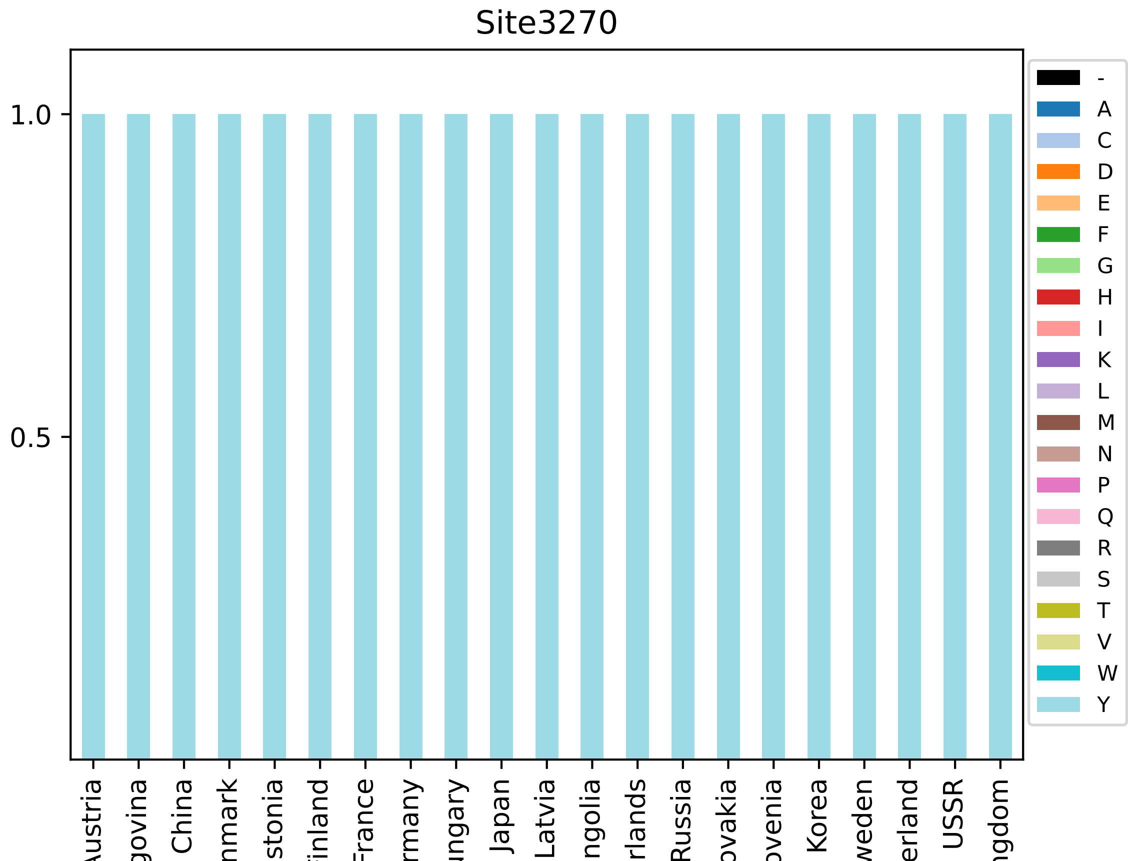
Site3270
1.0
0.5
-
A
C
D
E
F
G
H
I
K
L
M
N
P
Q
R
S
T
V
W
Y
Austria
govina
China
nmark
stonia
Finland
France
rmany
ungary
Japan
Latvia
ngolia
rlands
Russia
ovakia
ovenia
Korea
weden
erland
USSR
ngdom

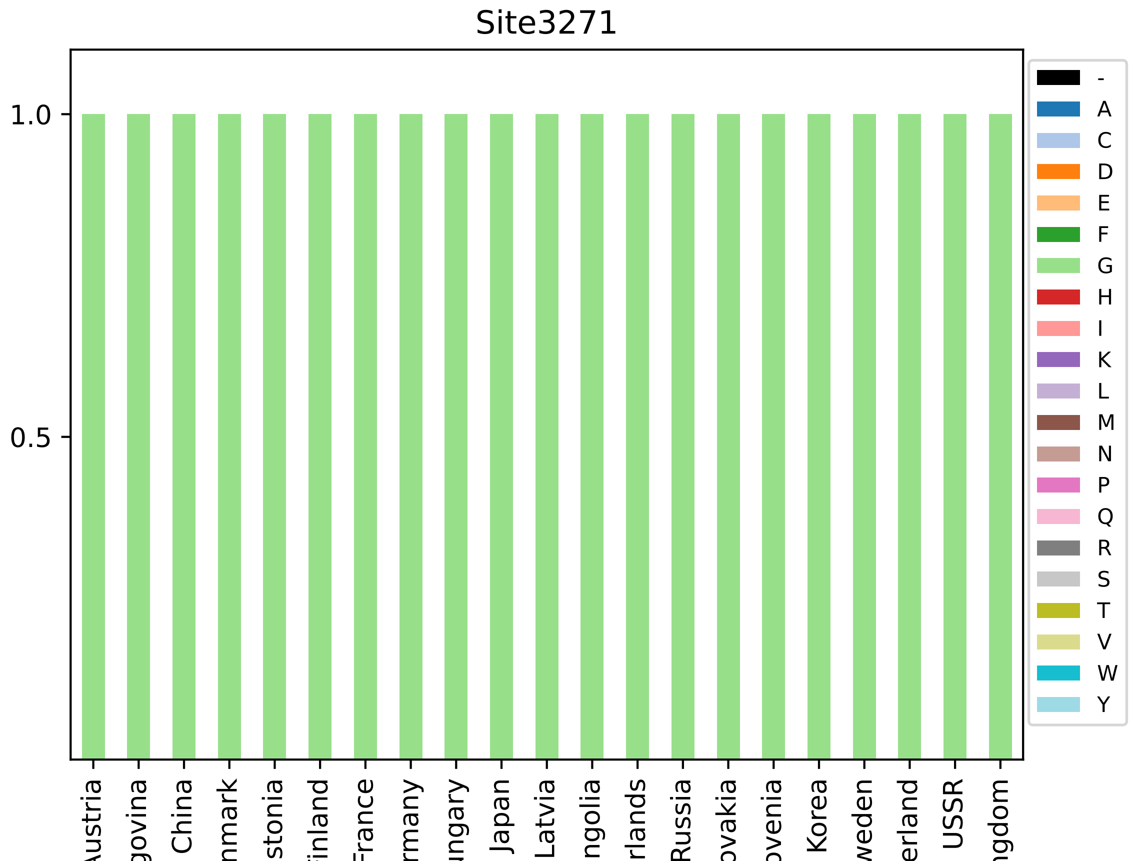

Site3271
1.0
0.5
China
Japan
Latvia
Russia
Korea
USSR
-
A
C
D
E
F
G
H
I
K
L
M
N
P
Q
R
S
T
V
W
Y

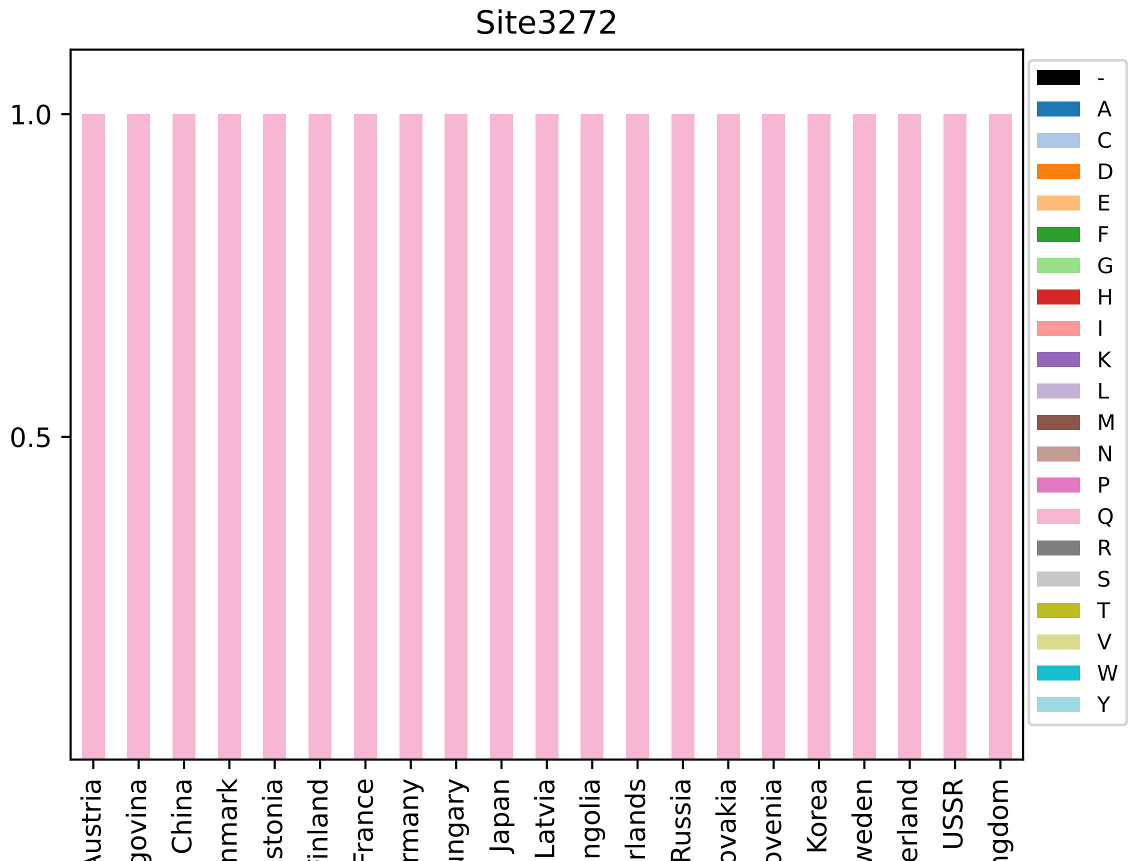

Site3272
1.0
0.5
-
A
C
D
E
F
G
H
I
K
L
M
N
P
Q
R
S
T
V
W
Y
Austria
govina
China
nmark
stonia
Finland
France
rmany
ungary
Japan
Latvia
ngolia
rlands
Russia
ovakia
ovenia
Korea
weden
erland
USSR
ngdom

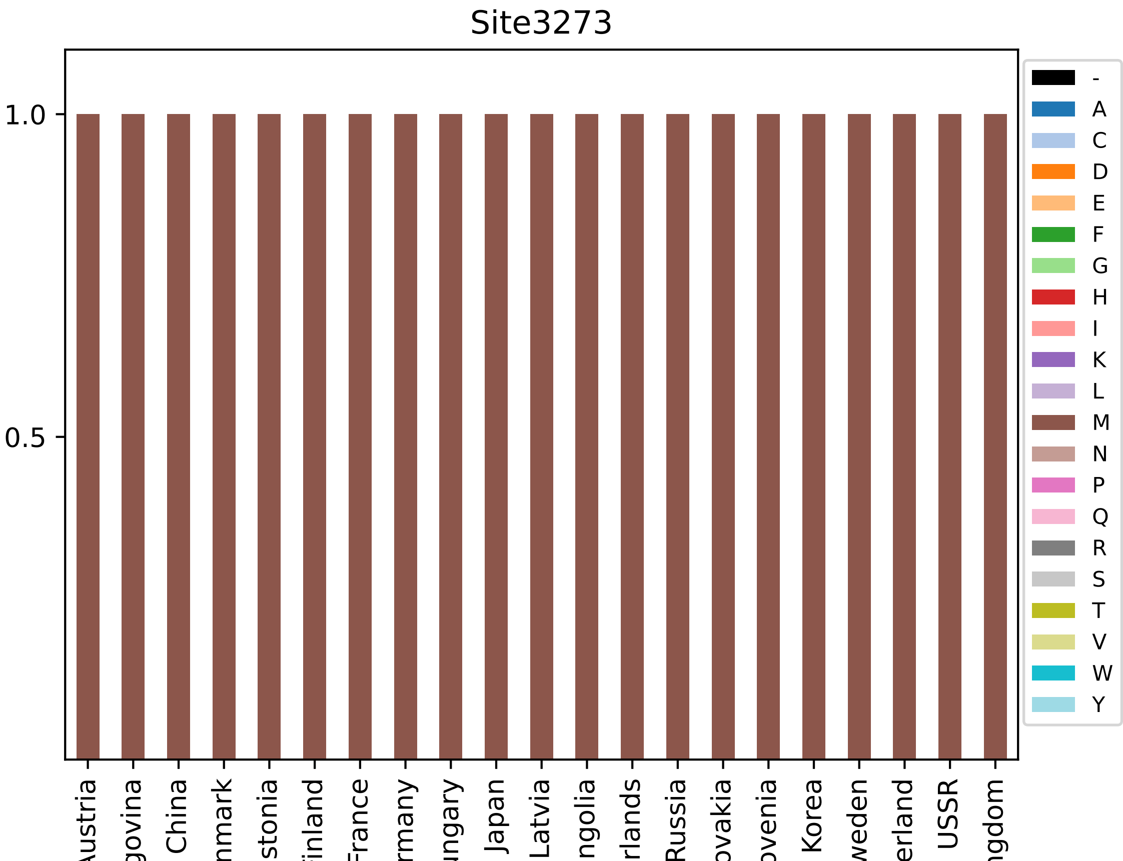

Site3273
1.0
0.5
China
Japan
Latvia
Russia
Korea
USSR
-
A
C
D
E
F
G
H
I
K
L
M
N
P
Q
R
S
T
V
W
Y

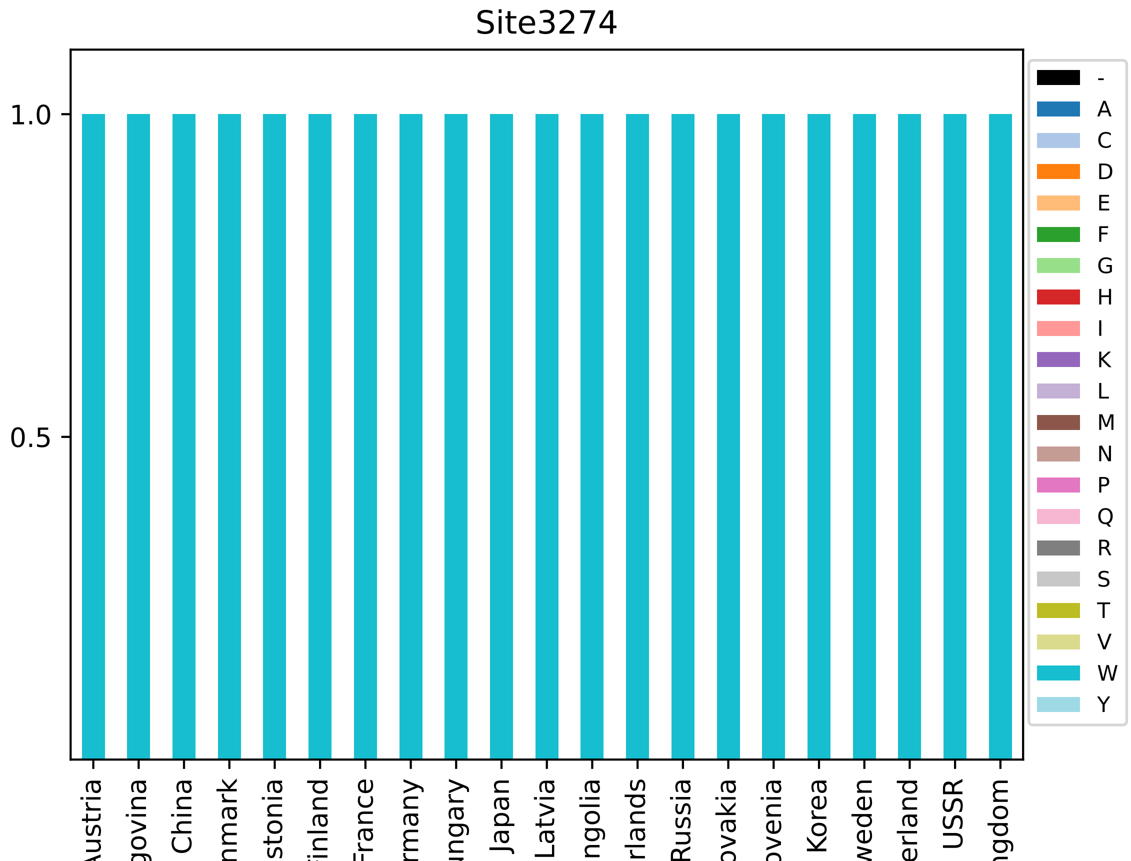
Site3274
1.0
0.5
-
A
C
D
E
F
G
H
I
K
L
M
N
P
Q
R
S
T
V
W
Y
Austria
China
Japan
Latvia
Russia
Korea
USSR

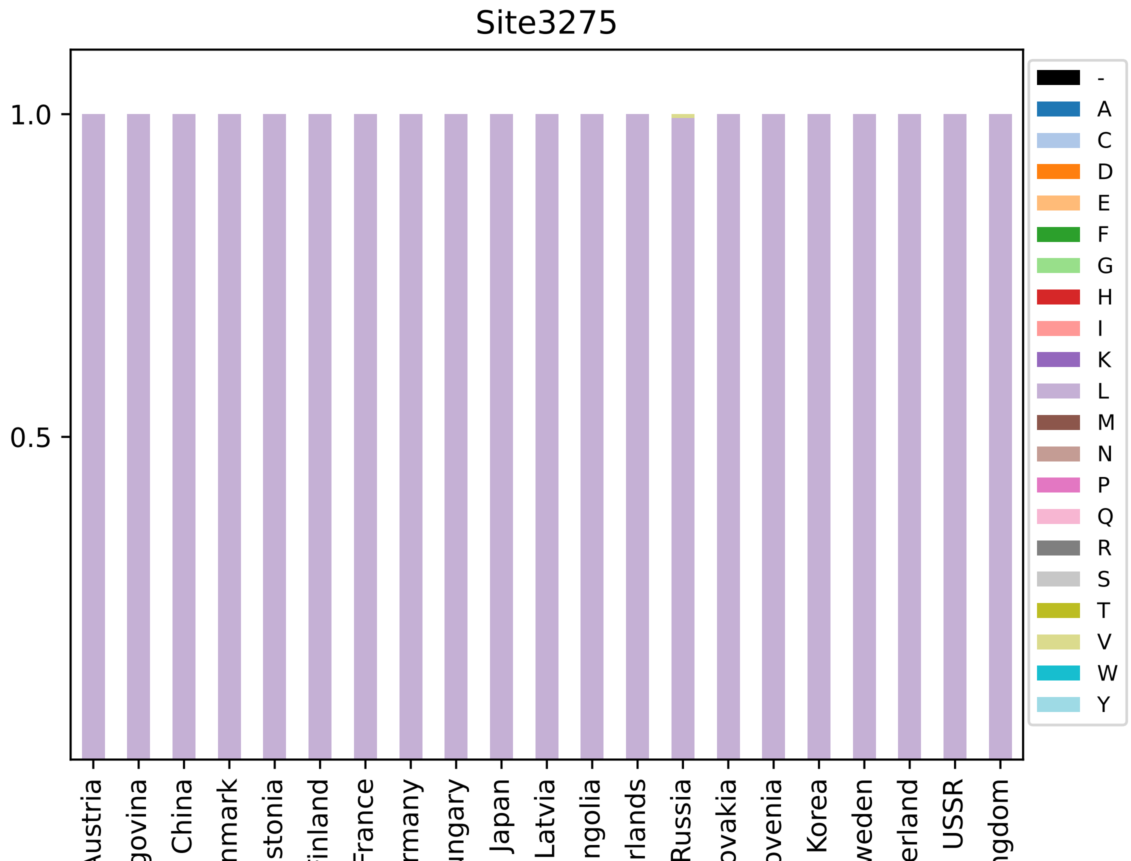

Site3275
1.0
0.5
-
A
C
D
E
F
G
H
I
K
L
M
N
P
Q
R
S
T
V
W
Y
Austria
China
Japan
Latvia
Russia
Korea
USSR

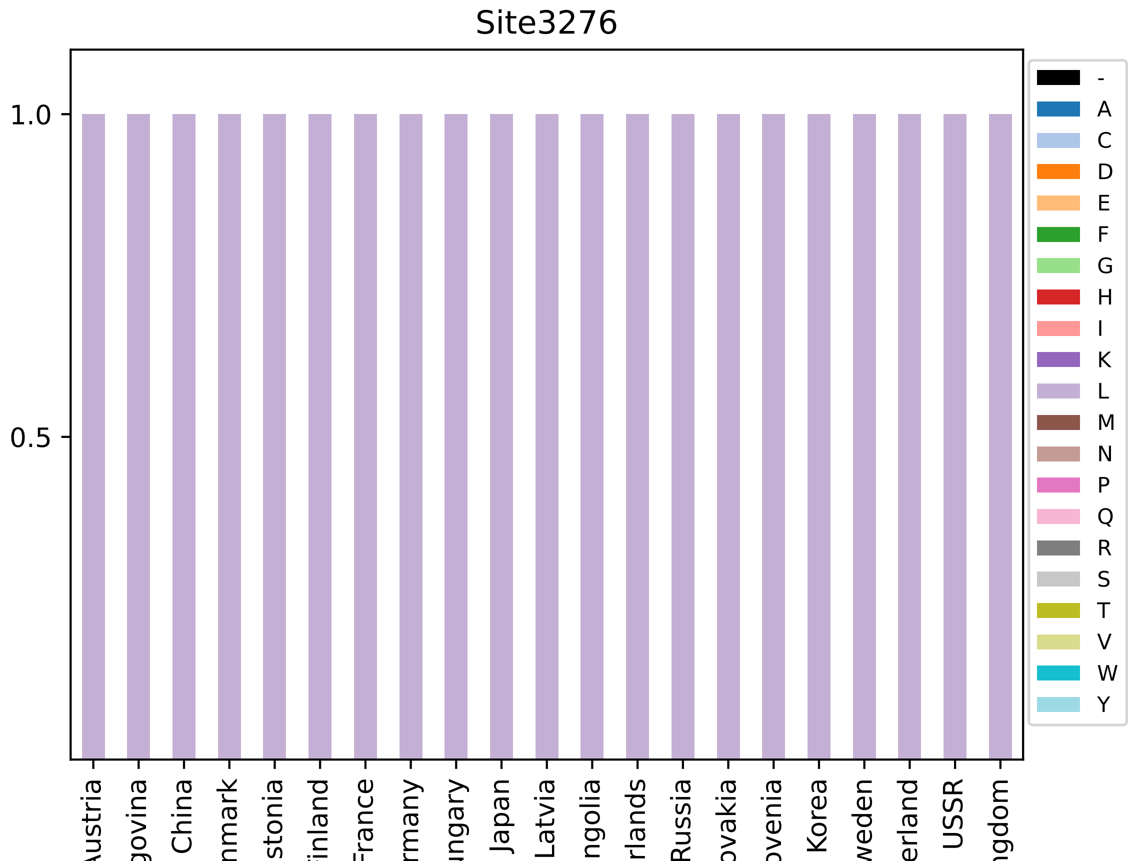
Site3276
1.0
0.5
-
A
C
D
E
F
G
H
I
K
L
M
N
P
Q
R
S
T
V
W
Y
China
Japan
Latvia
Russia
Korea
USSR

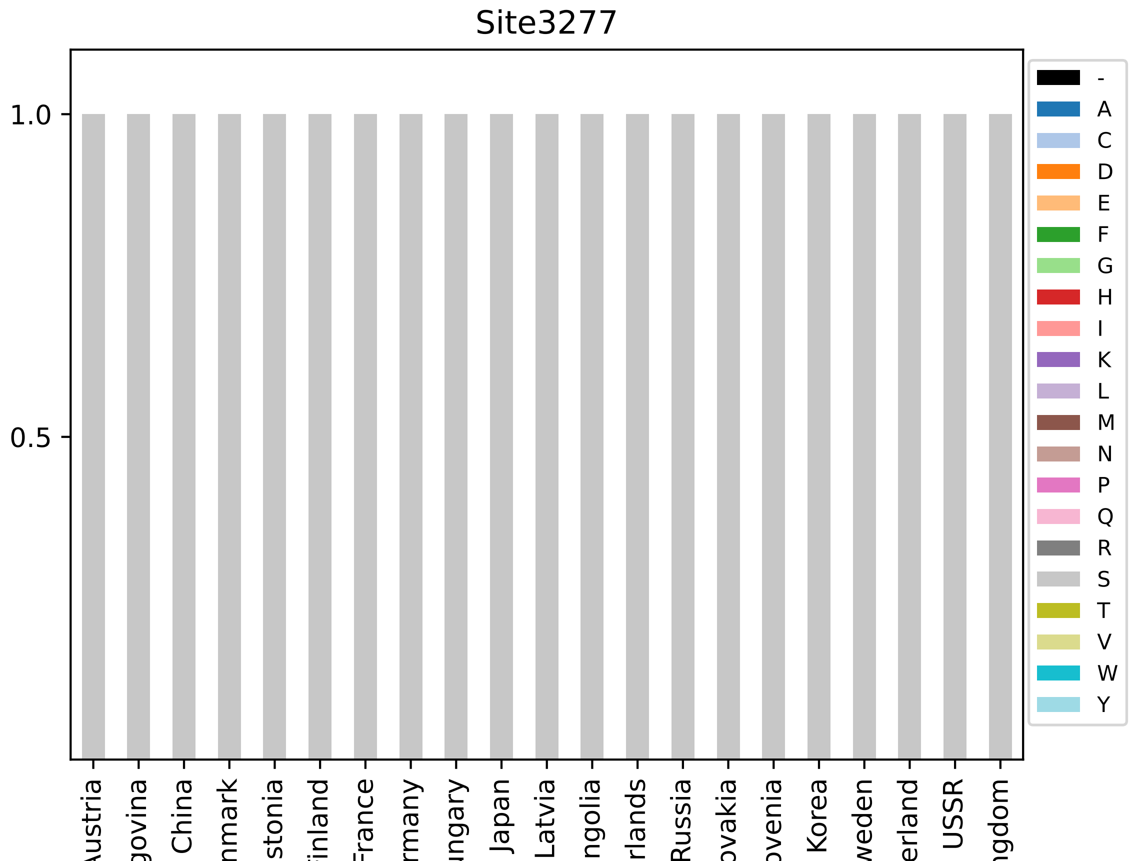
Site3277
1.0
0.5
-
A
C
D
E
F
G
H
I
K
L
M
N
P
Q
R
S
T
V
W
Y
Austria
govina
China
nmark
stonia
Finland
France
rmany
ungary
Japan
Latvia
ngolia
rlands
Russia
ovakia
ovenia
Korea
weden
erland
USSR
ngdom

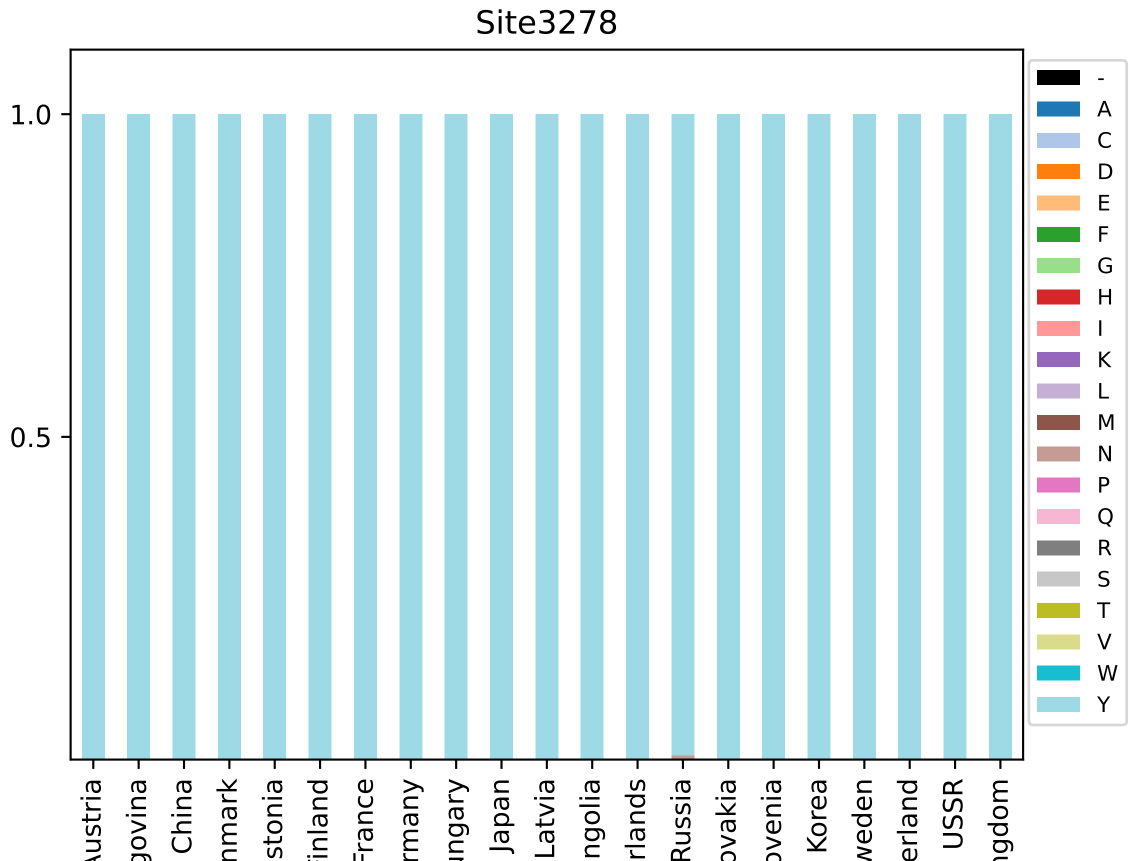

Site3278
1.0
0.5
-
A
C
D
E
F
G
H
I
K
L
M
N
P
Q
R
S
T
V
W
Y
Austria
China
Finland
France
Japan
Latvia
Russia
Korea
USSR

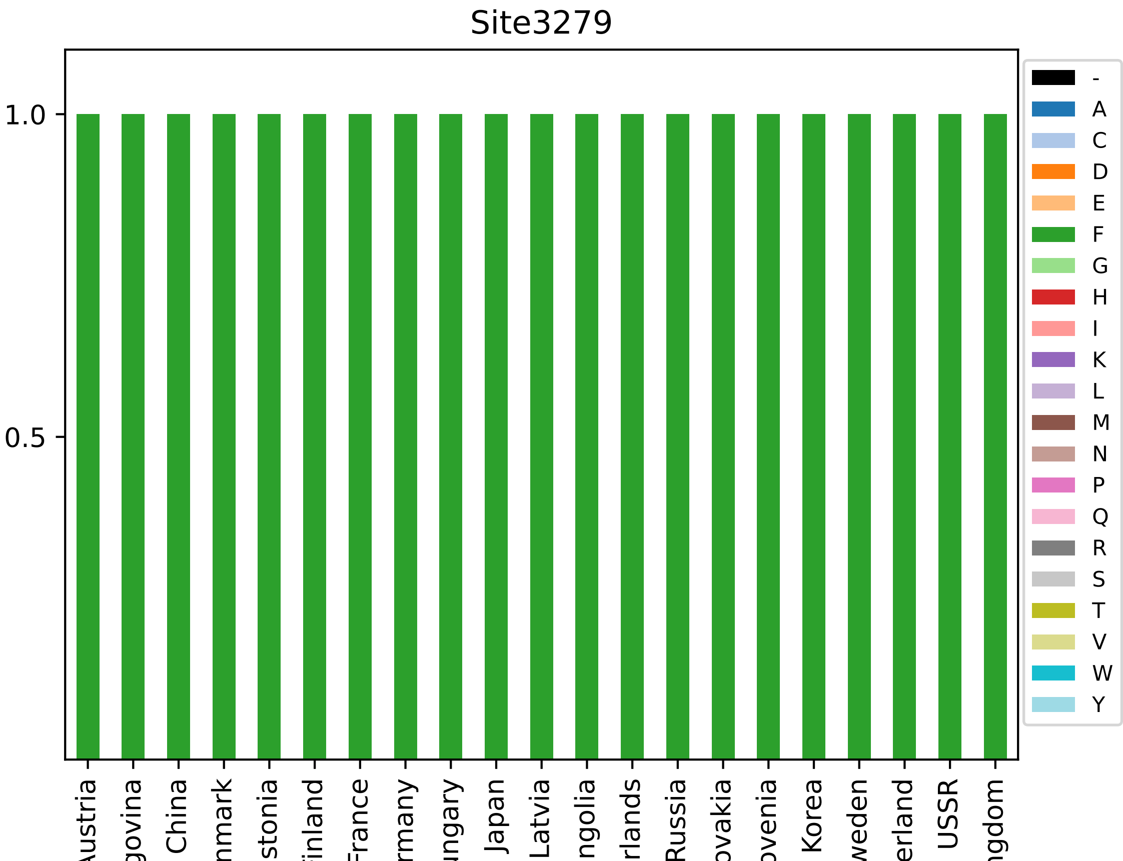
Site3279
1.0
0.5
-
A
C
D
E
F
G
H
I
K
L
M
N
P
Q
R
S
T
V
W
Y
Austria
govina
China
nmark
stonia
Finland
France
rmany
ungary
Japan
Latvia
ngolia
rlands
Russia
ovakia
ovenia
Korea
weden
erland
USSR
ngdom

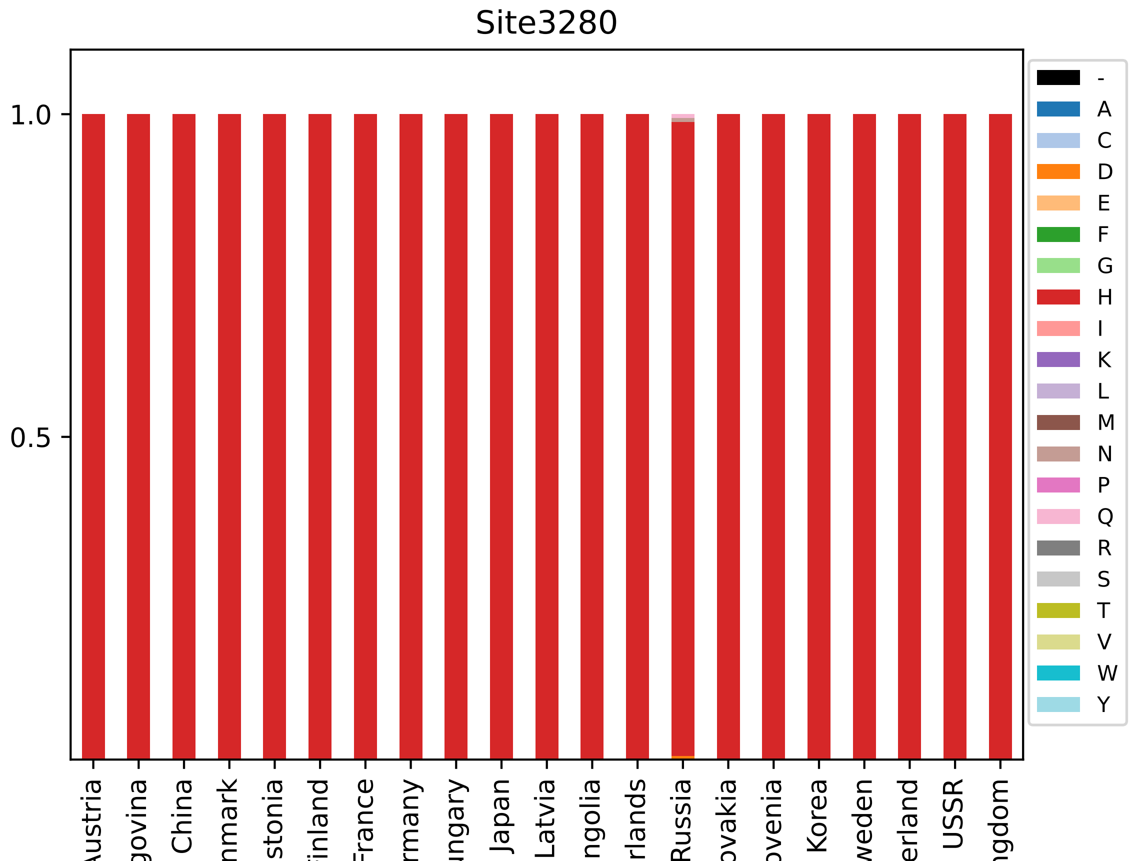
Site3280
1.0
0.5
-
A
C
D
E
F
G
H
I
K
L
M
N
P
Q
R
S
T
V
W
Y
Austria
China
Japan
Latvia
Russia
Korea
USSR

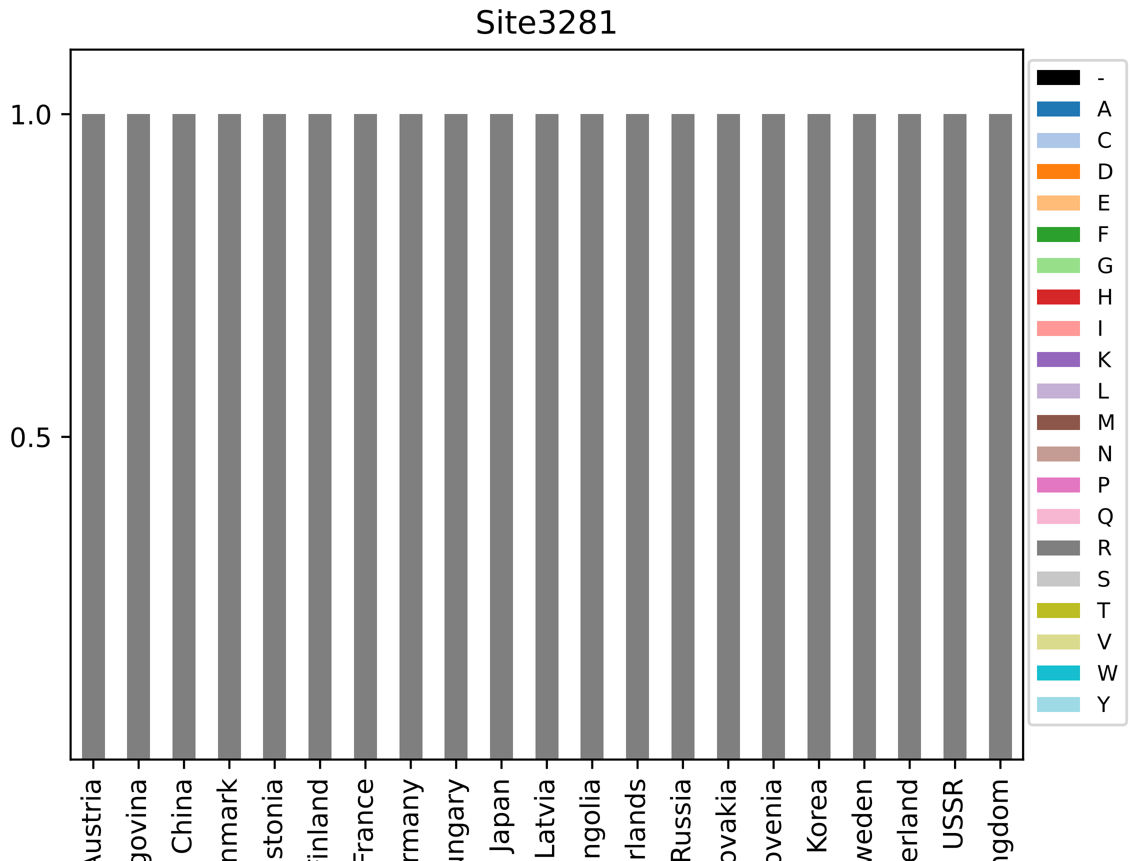
Site3281
1.0
0.5
-
A
C
D
E
F
G
H
I
K
L
M
N
P
Q
R
S
T
V
W
Y
China
Japan
Latvia
Russia
Korea
USSR

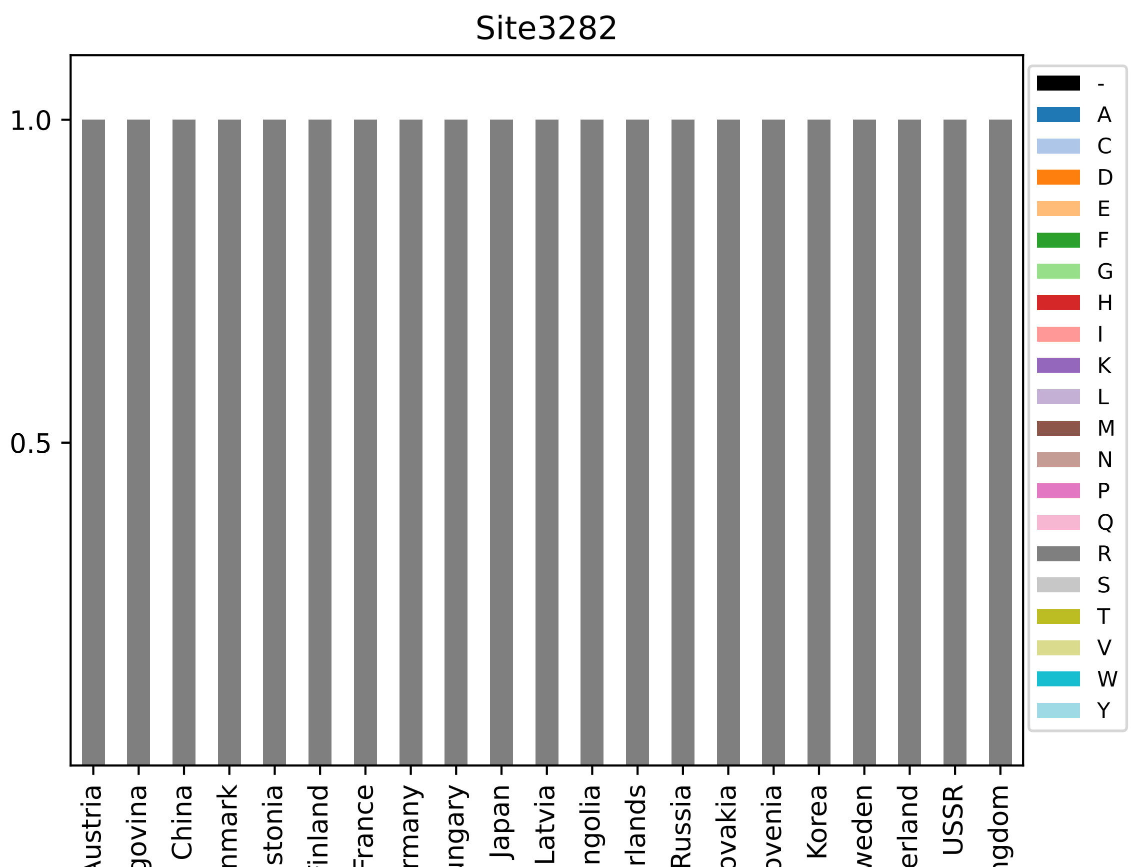
Site3282
1.0
0.5
China
Japan
Latvia
Russia
Korea
USSR
-
A
C
D
E
F
G
H
I
K
L
M
N
P
Q
R
S
T
V
W
Y

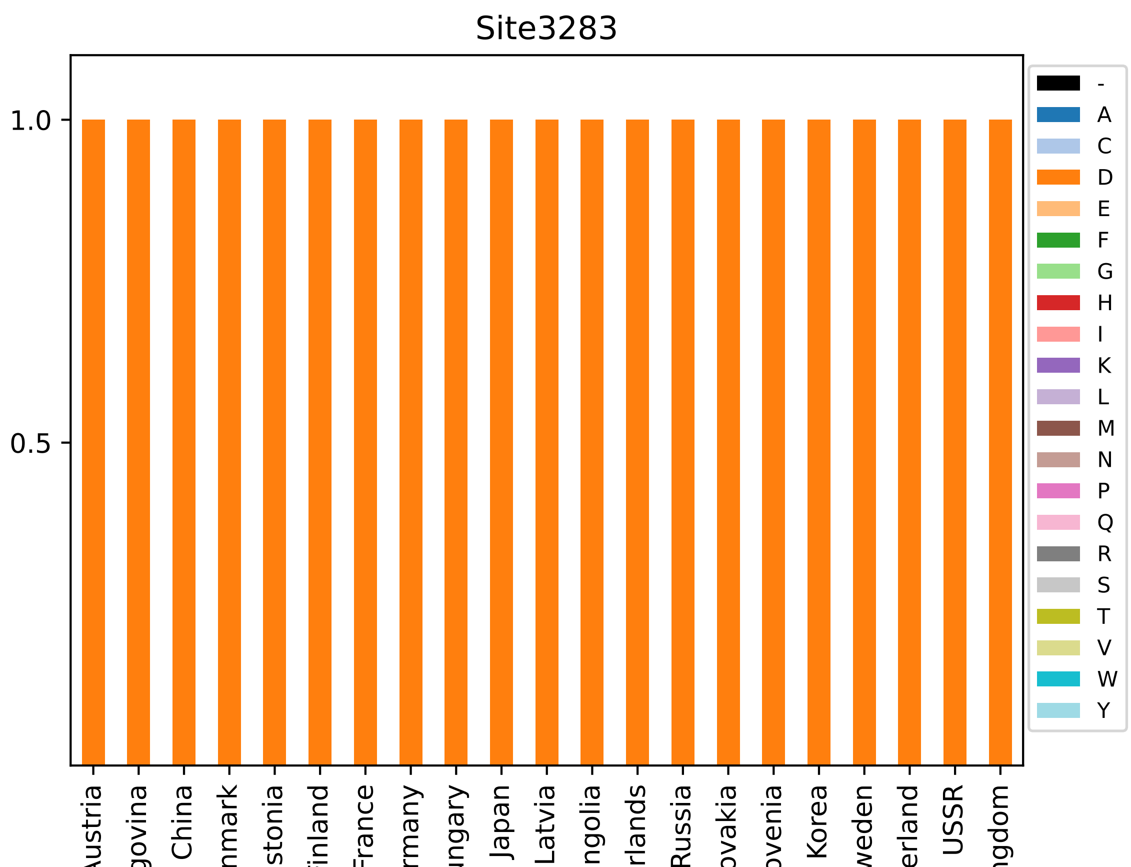
Site3283
1.0
0.5
-
A
C
D
E
F
G
H
I
K
L
M
N
P
Q
R
S
T
V
W
Y
China
Japan
Latvia
Russia
Korea
USSR

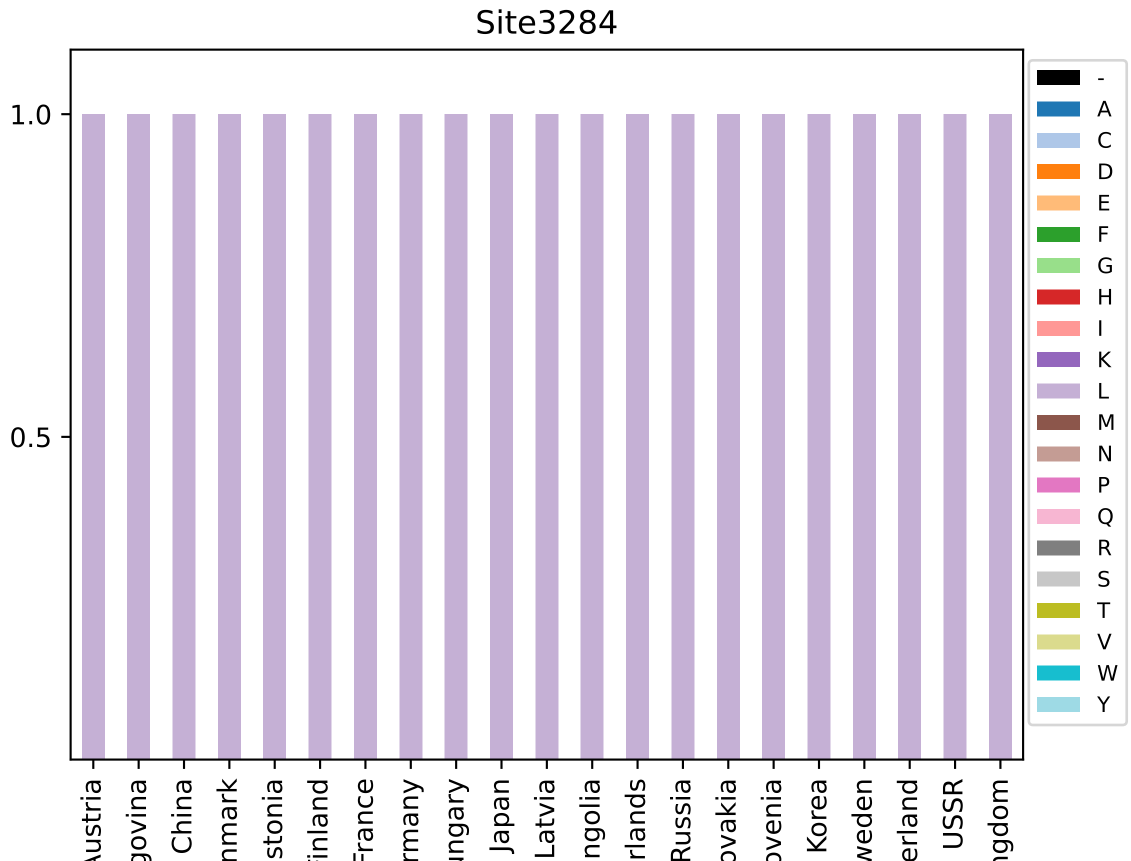
Site3284
1.0
0.5
-
A
C
D
E
F
G
H
I
K
L
M
N
P
Q
R
S
T
V
W
Y
Austria
govina
China
nmark
stonia
Finland
France
rmany
ungary
Japan
Latvia
ngolia
rlands
Russia
ovakia
ovenia
Korea
weden
erland
USSR
ngdom

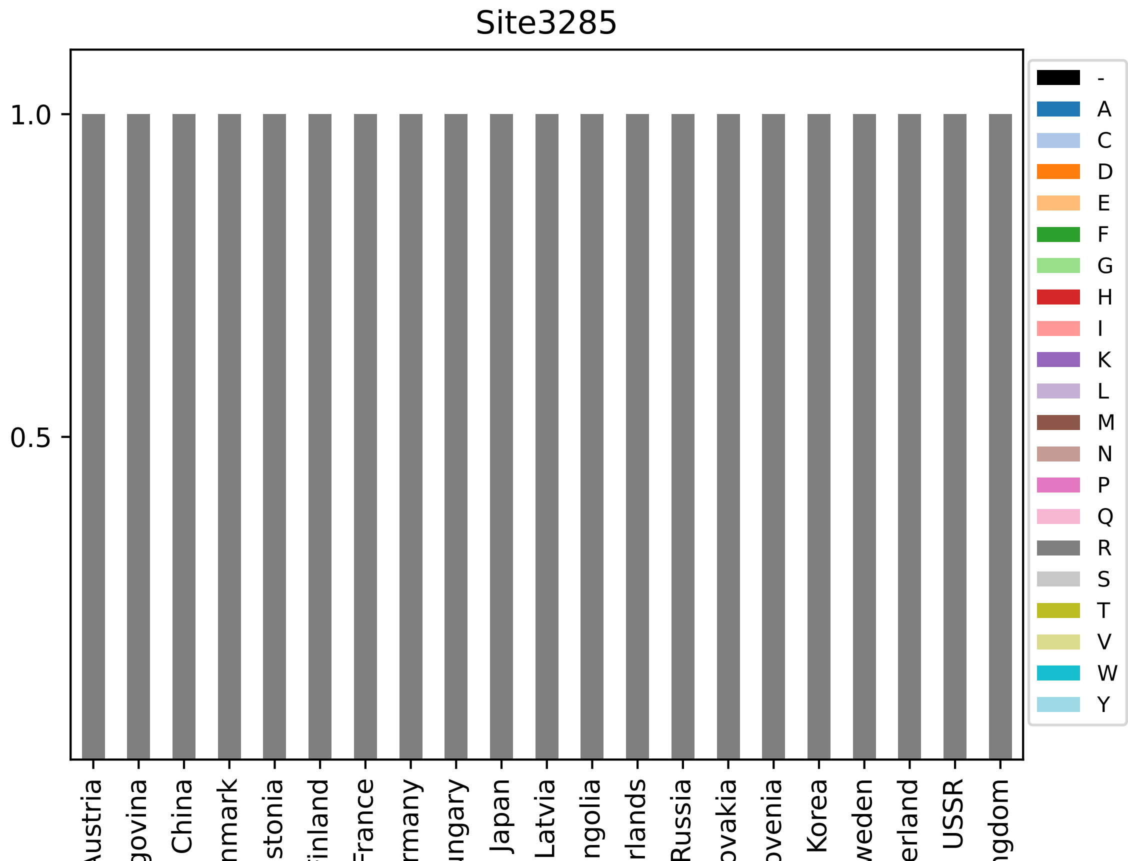
Site3285
1.0
0.5
-
A
C
D
E
F
G
H
I
K
L
M
N
P
Q
R
S
T
V
W
Y
China
Japan
Latvia
Russia
Korea
USSR

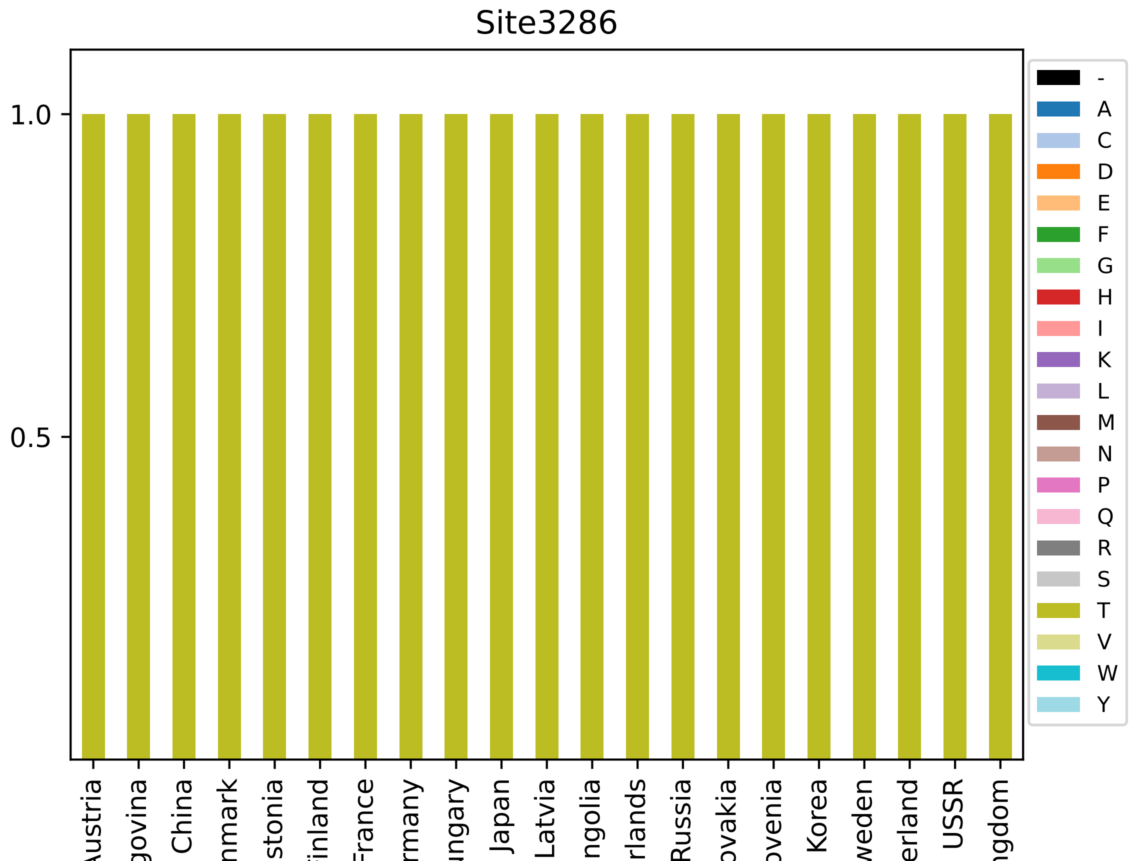
Site3286
1.0
0.5
-
A
C
D
E
F
G
H
I
K
L
M
N
P
Q
R
S
T
V
W
Y
China
Japan
Latvia
Russia
Korea
USSR

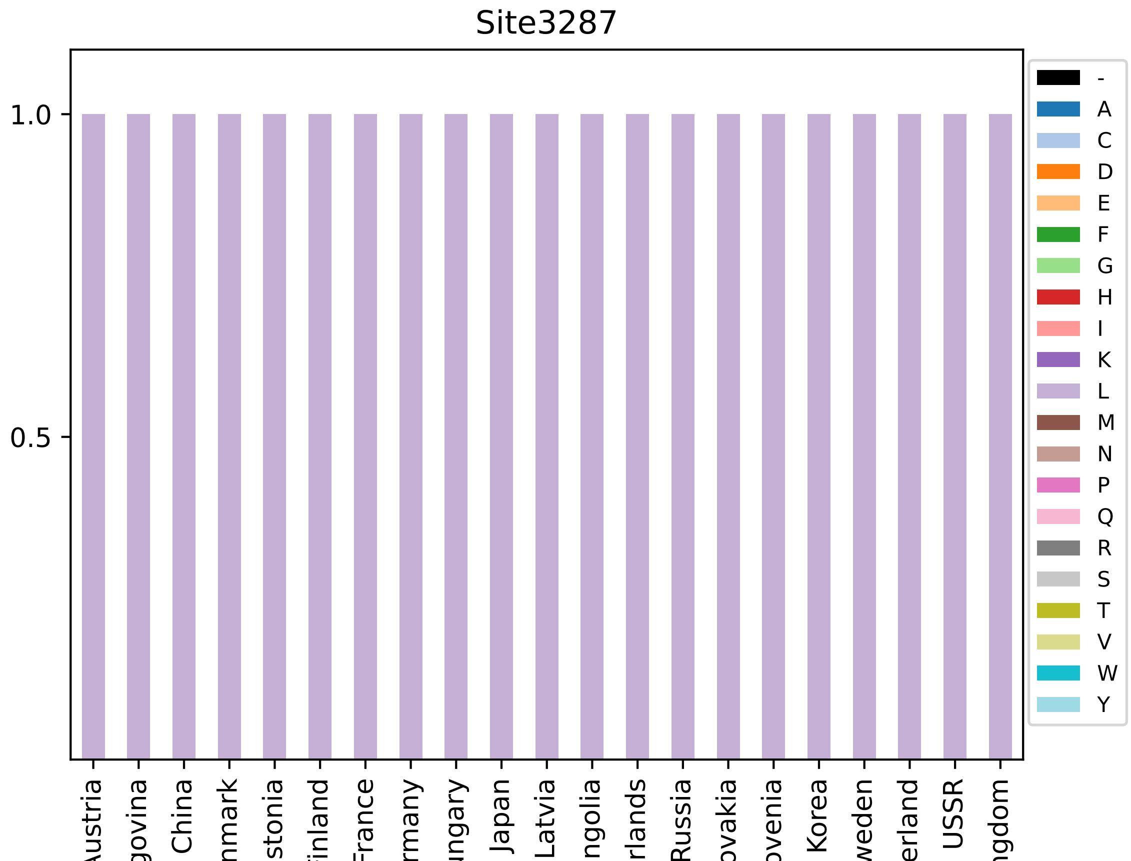
Site3287
1.0
0.5
-
A
C
D
E
F
G
H
I
K
L
M
N
P
Q
R
S
T
V
W
Y
China
Japan
Latvia
Russia
Korea
USSR

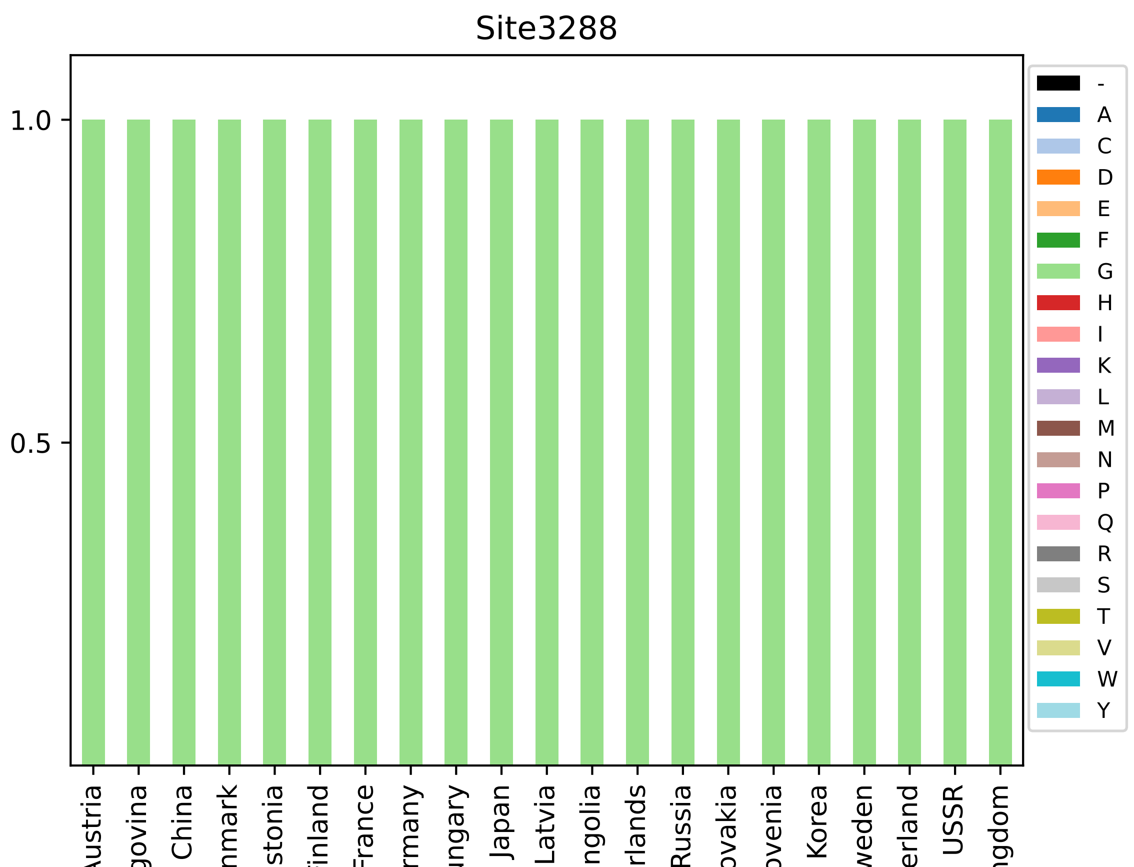

Site3288
1.0
0.5
-
A
C
D
E
F
G
H
I
K
L
M
N
P
Q
R
S
T
V
W
Y
China
Japan
Latvia
Russia
Korea
USSR

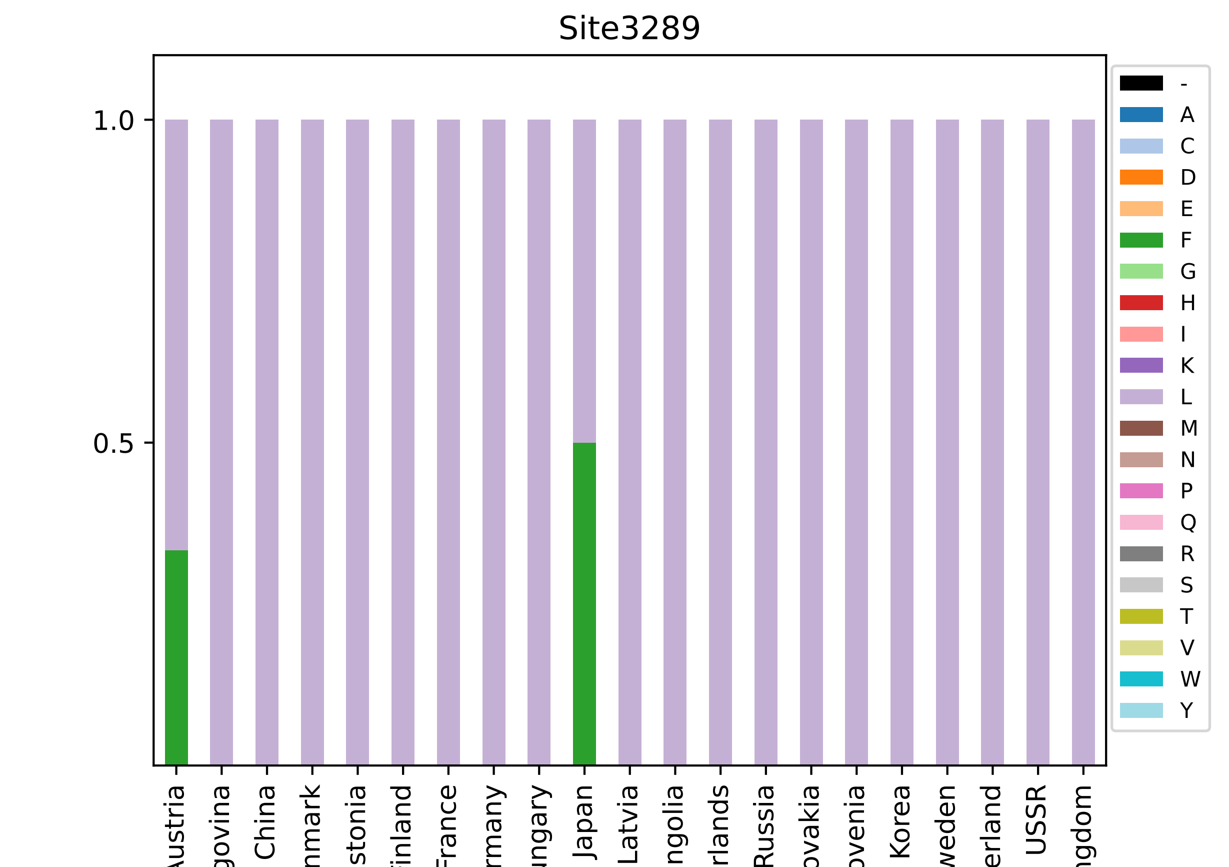

Site3289
1.0
0.5
-
A
C
D
E
F
G
H
I
K
L
M
N
P
Q
R
S
T
V
W
Y
Austria
China
Estonia
Finland
France
Japan
Latvia
Russia
Korea
USSR

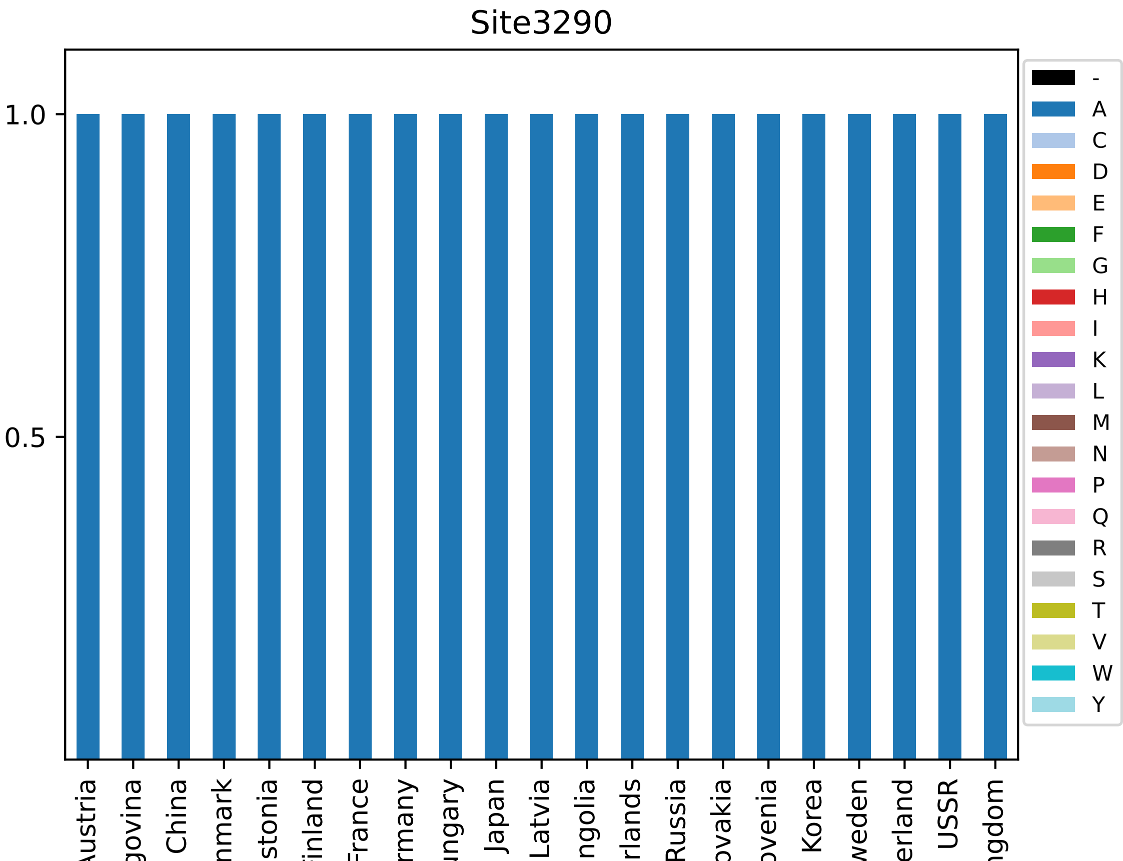

Site3290
1.0
0.5
China
Japan
Latvia
Russia
Korea
USSR
-
A
C
D
E
F
G
H
I
K
L
M
N
P
Q
R
S
T
V
W
Y

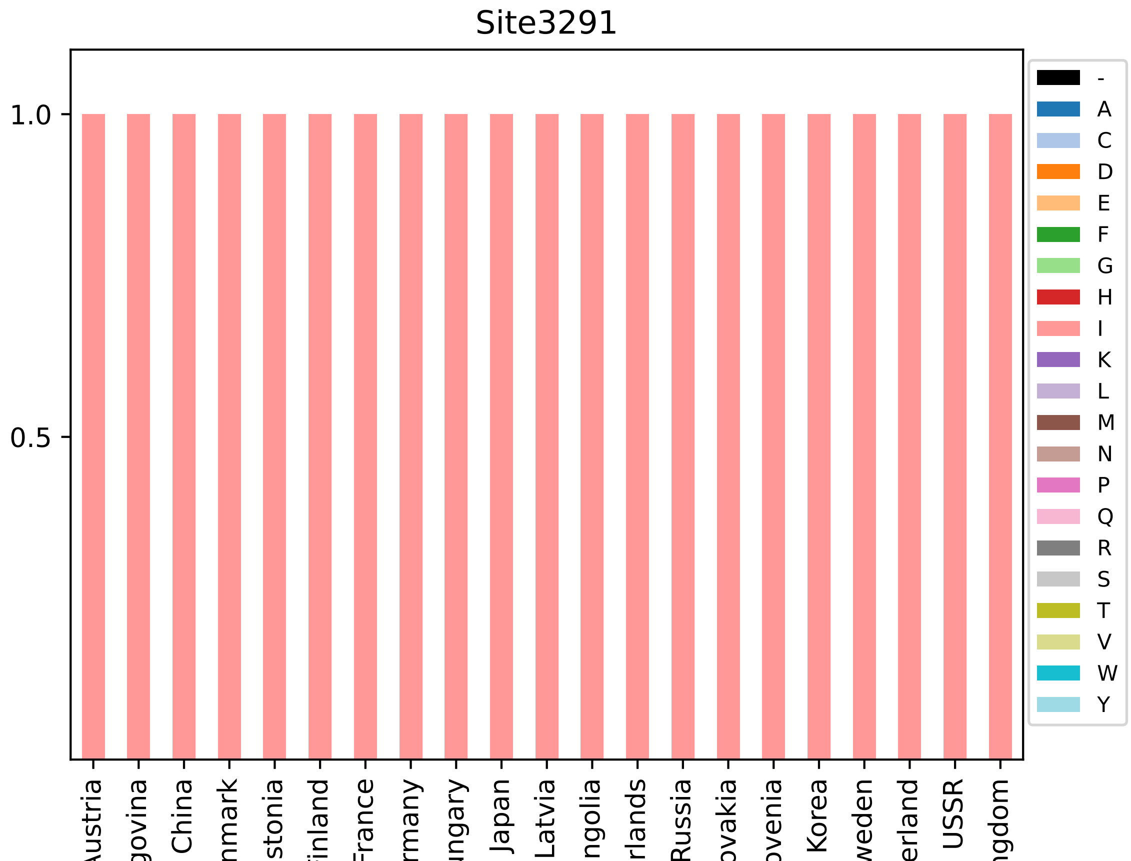
Site3291
1.0
0.5
Austria
govina
China
nmark
stonia
Finland
France
rmany
ungary
Japan
Latvia
ngolia
rlands
Russia
ovakia
ovenia
Korea
weden
erland
USSR
ngdom
-
A
C
D
E
F
G
H
I
K
L
M
N
P
Q
R
S
T
V
W
Y

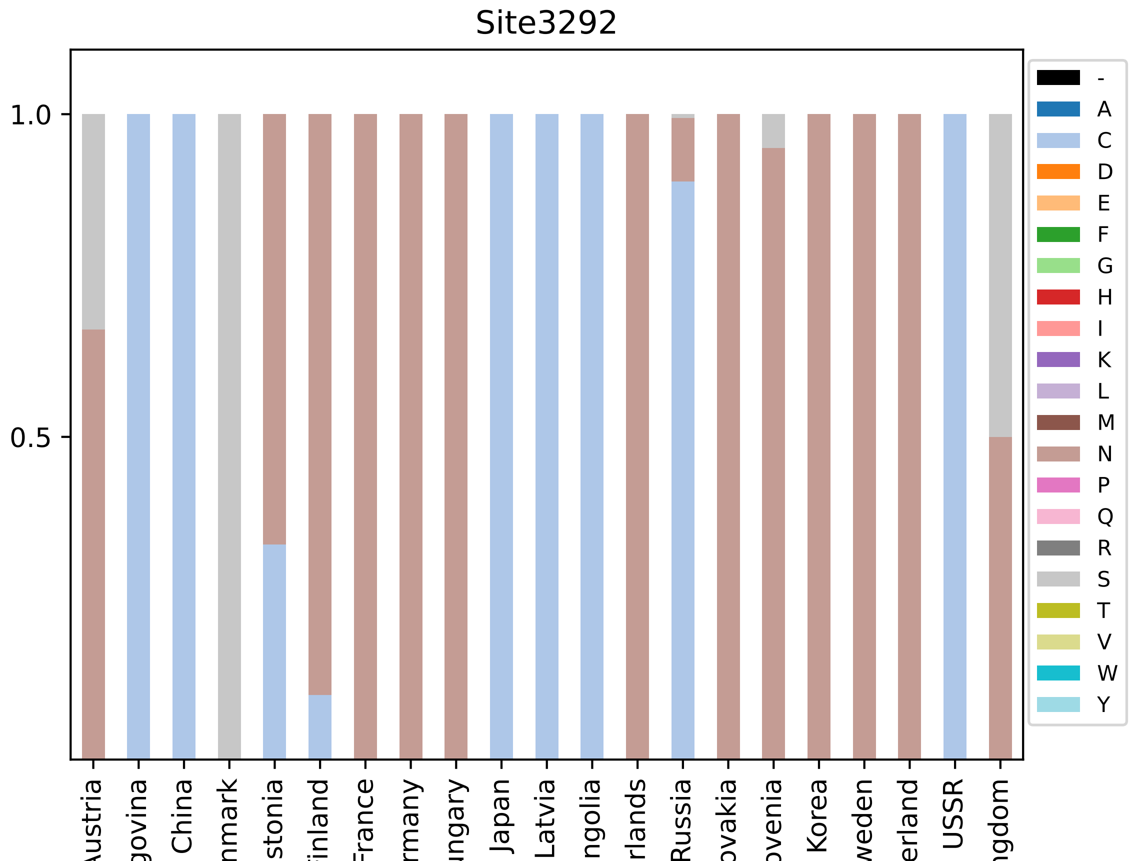

Site3292
1.0
0.5
-
A
C
D
E
F
G
H
I
K
L
M
N
P
Q
R
S
T
V
W
Y
China
Japan
Latvia
Russia
Korea
USSR

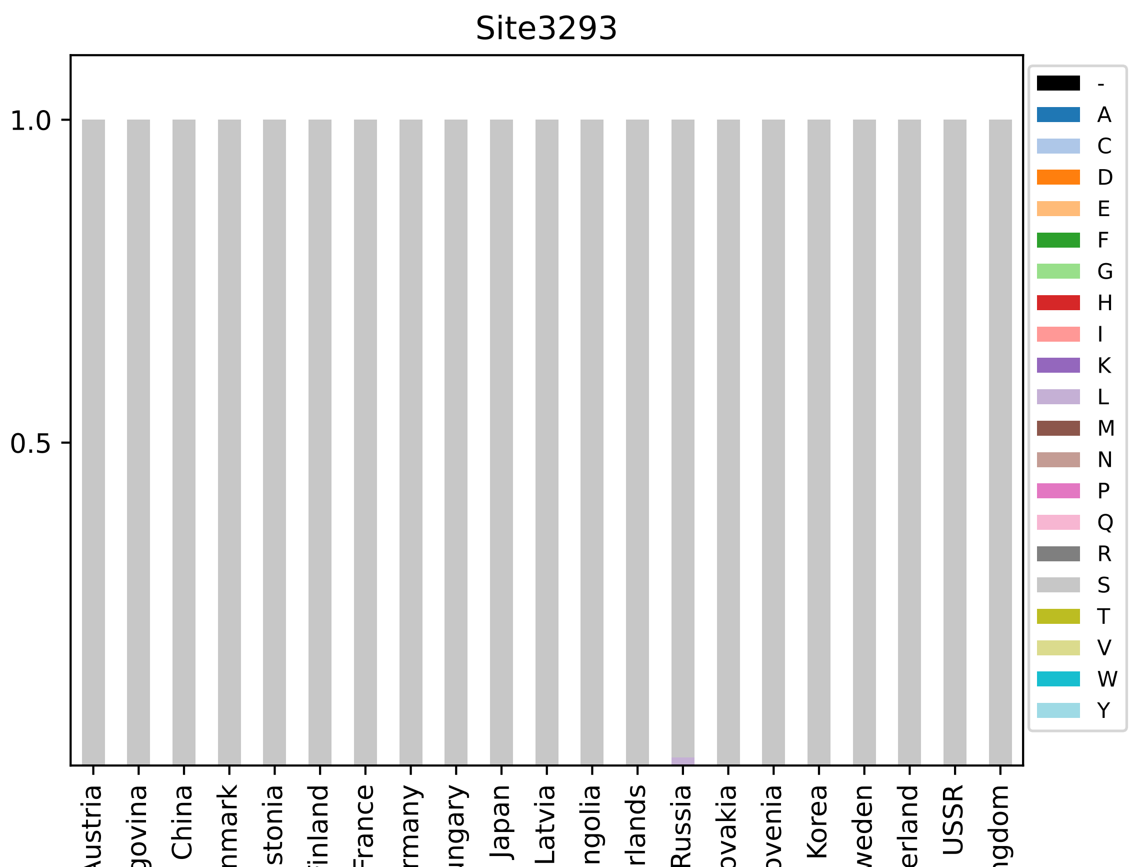

Site3293
1.0
0.5
-
A
C
D
E
F
G
H
I
K
L
M
N
P
Q
R
S
T
V
W
Y
Austria
govina
China
nmark
stonia
Finland
France
rmany
ungary
Japan
Latvia
ngolia
rlands
Russia
ovakia
ovenia
Korea
weden
erland
USSR
ngdom

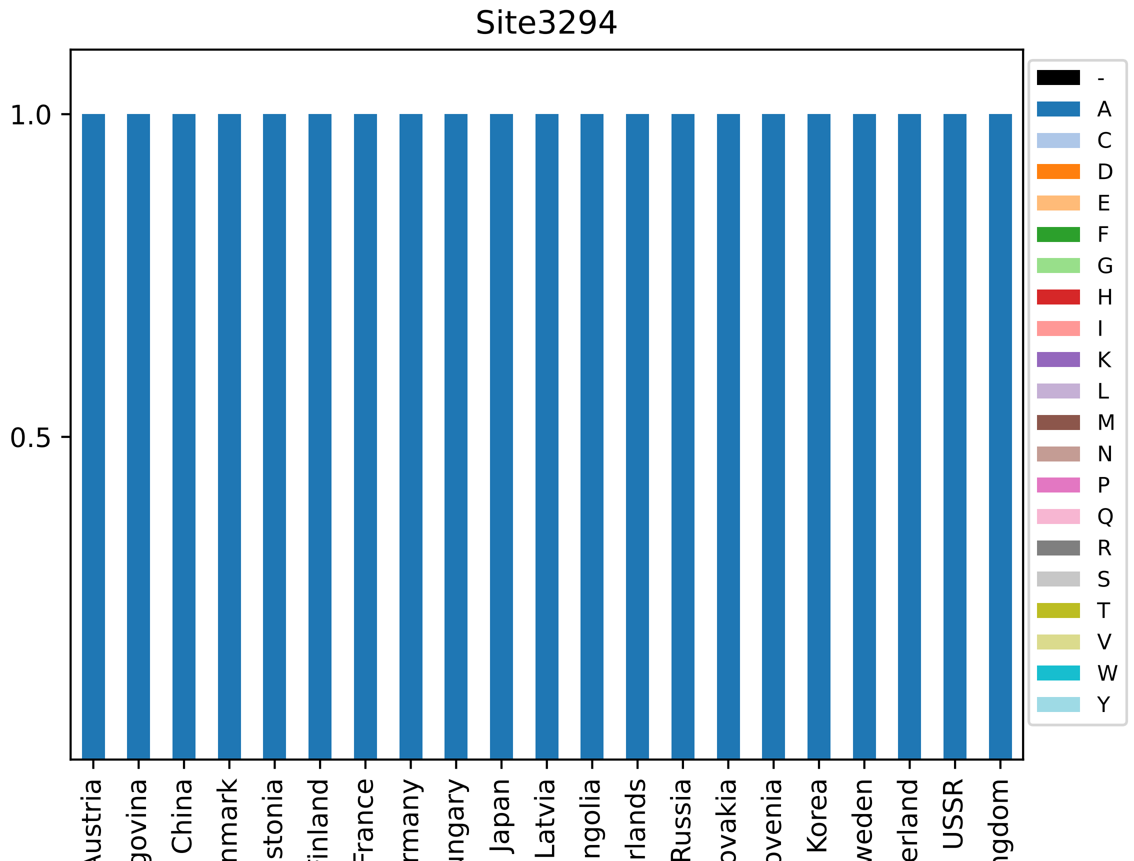
Site3294
1.0
0.5
-
A
C
D
E
F
G
H
I
K
L
M
N
P
Q
R
S
T
V
W
Y
China
Japan
Latvia
Russia
Korea
USSR

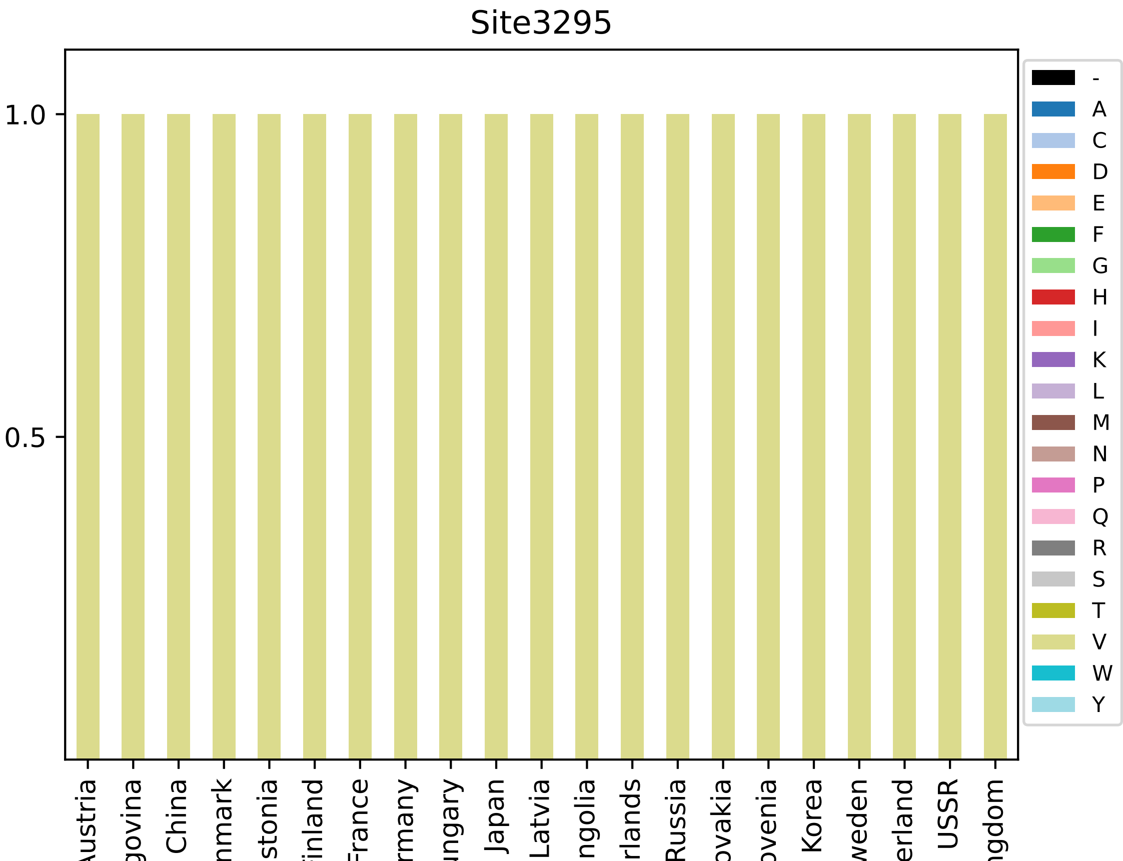

Site3295
1.0
0.5
-
A
C
D
E
F
G
H
I
K
L
M
N
P
Q
R
S
T
V
W
Y
Austria
govina
China
nmark
stonia
Finland
France
rmany
ungary
Japan
Latvia
ngolia
rlands
Russia
ovakia
ovenia
Korea
weden
erland
USSR
ngdom

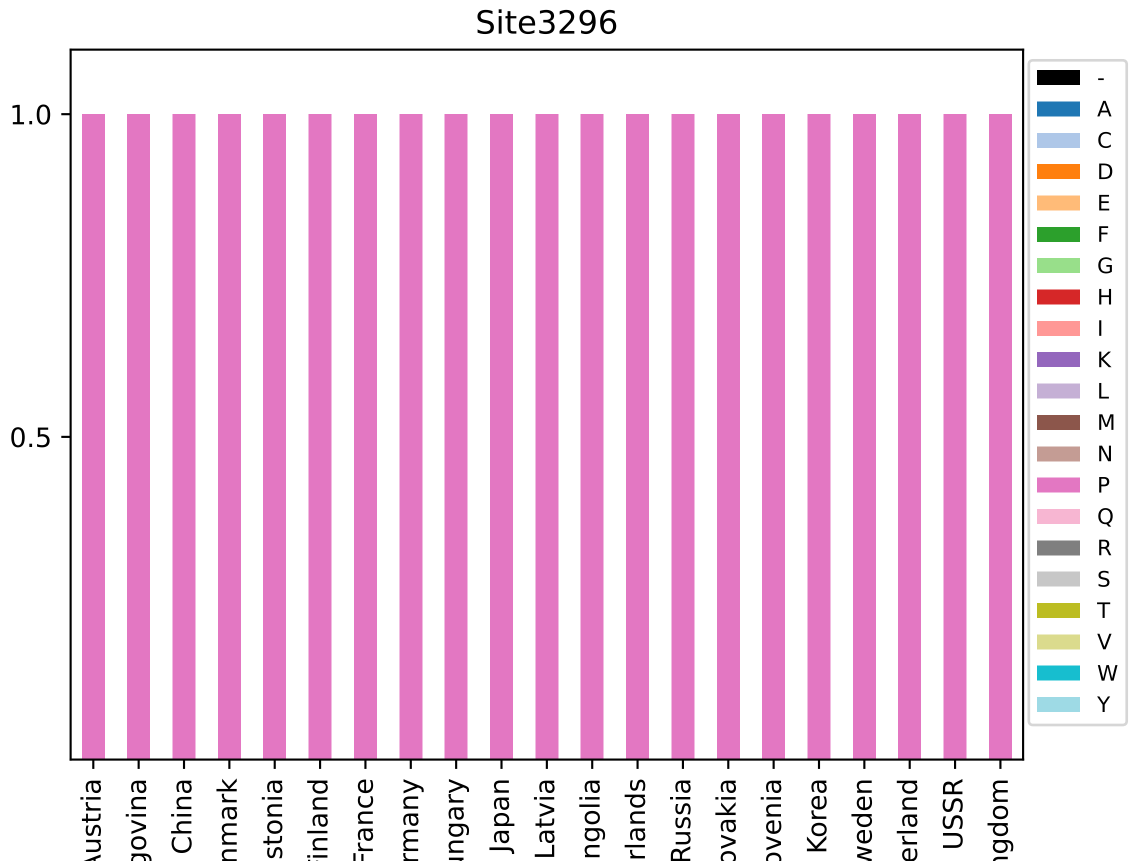
Site3296
1.0
0.5
-
A
C
D
E
F
G
H
I
K
L
M
N
P
Q
R
S
T
V
W
Y
China
Japan
Latvia
Russia
Korea
USSR

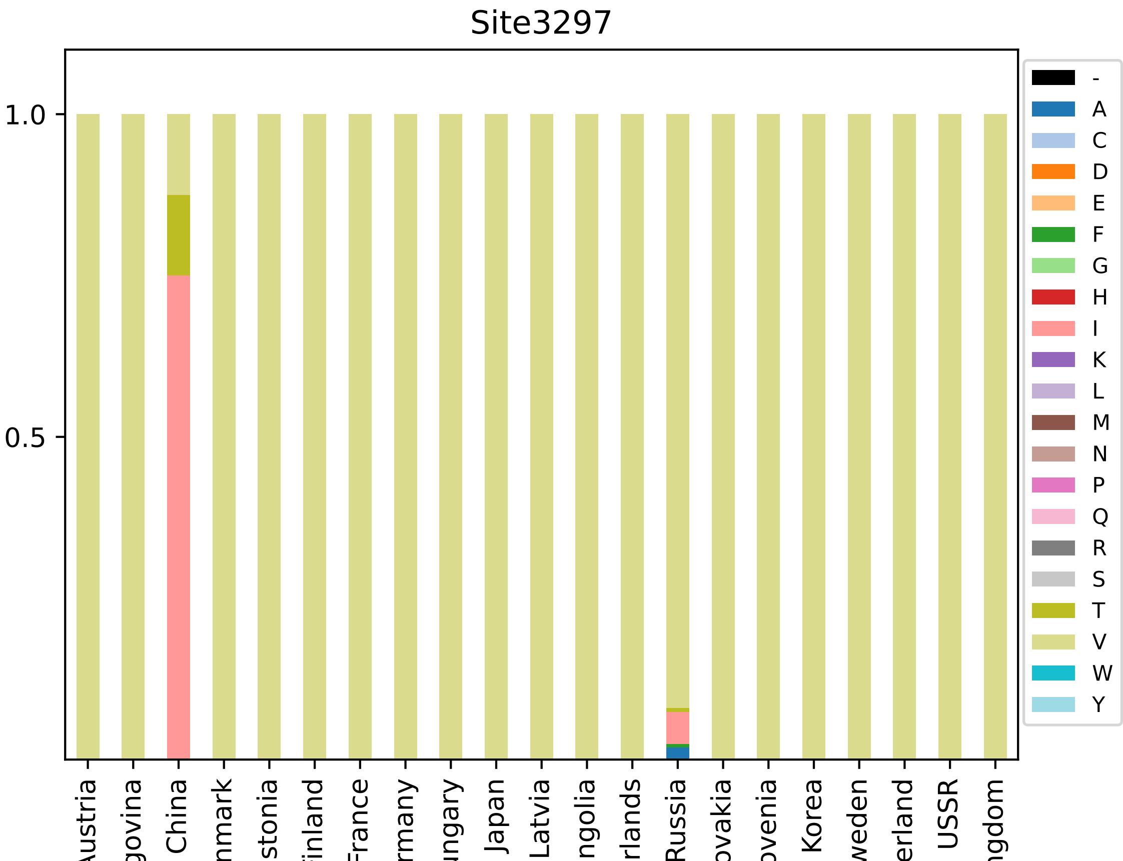
Site3297
1.0
0.5
-
A
C
D
E
F
G
H
I
K
L
M
N
P
Q
R
S
T
V
W
Y
China
Japan
Latvia
Russia
Korea
USSR

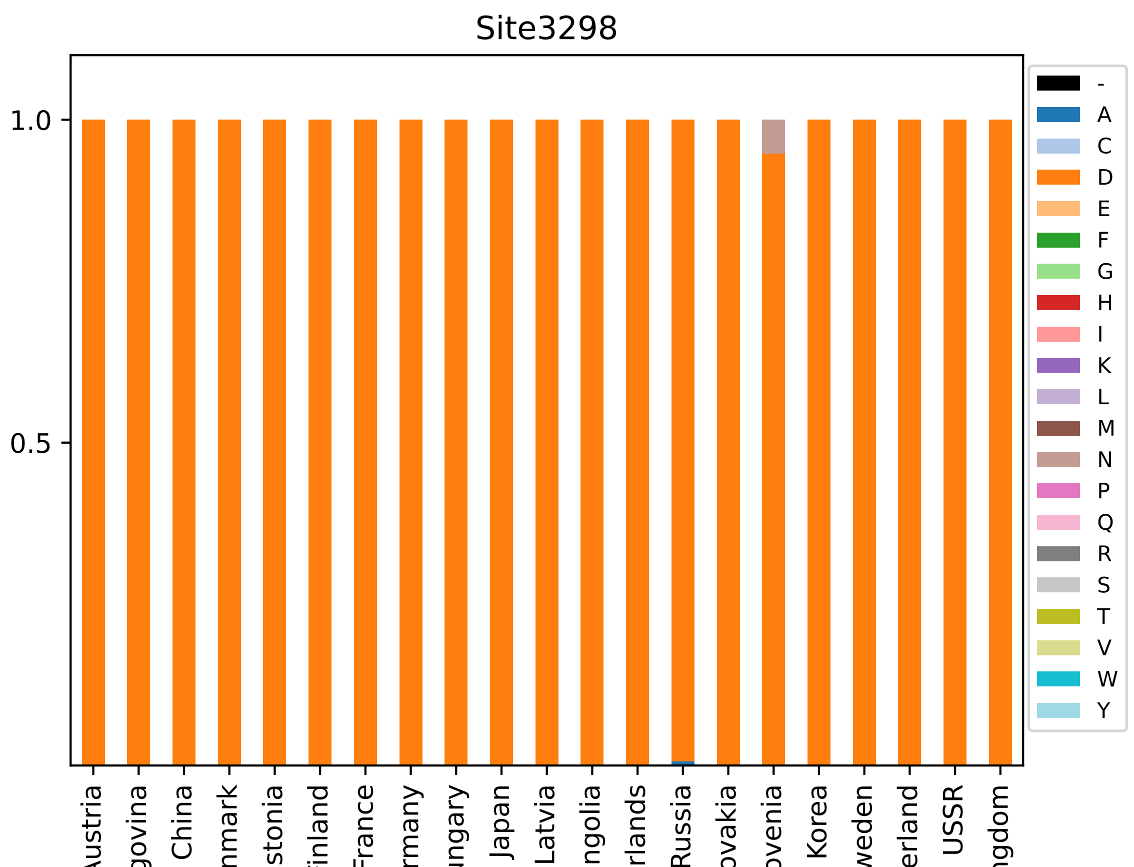

Site3298
1.0
0.5
-
A
C
D
E
F
G
H
I
K
L
M
N
P
Q
R
S
T
V
W
Y
Austria
govina
China
nmark
stonia
Finland
France
rmany
ungary
Japan
Latvia
ngolia
rlands
Russia
ovakia
ovenia
Korea
weden
erland
USSR
ngdom

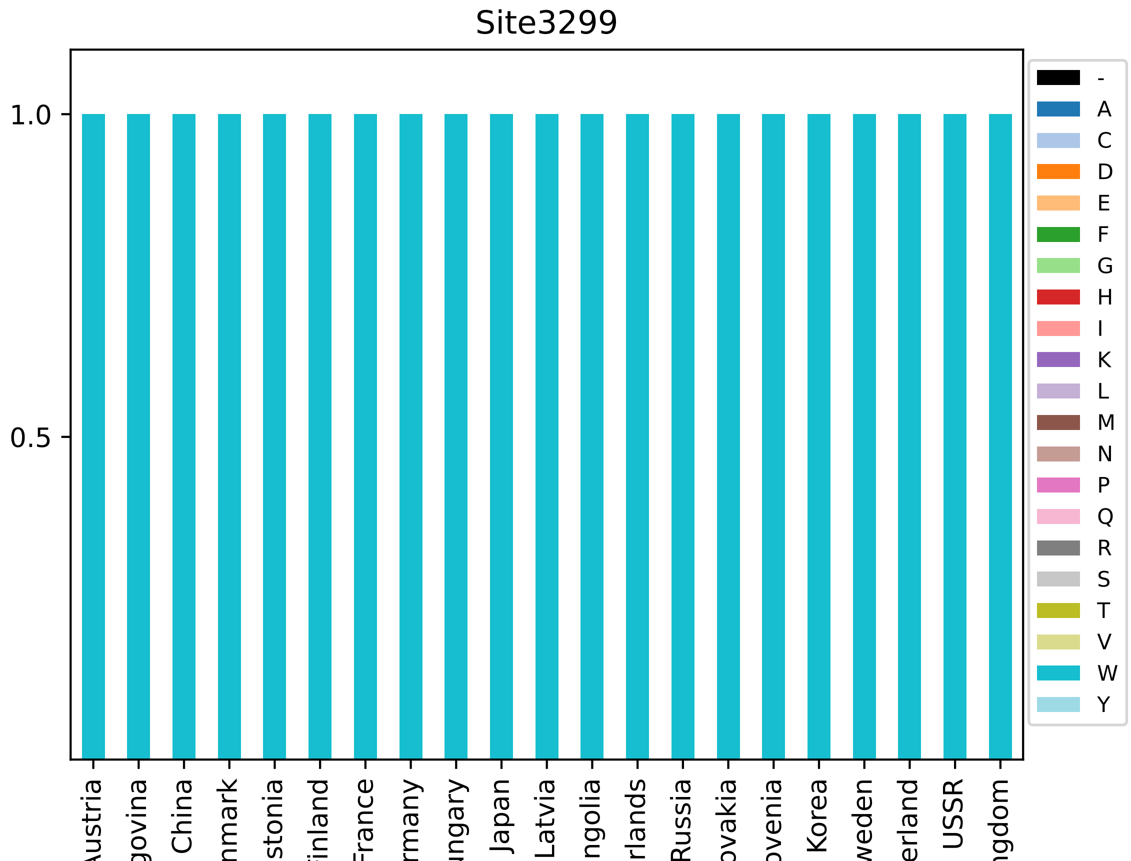

Site3299
1.0
0.5
-
A
C
D
E
F
G
H
I
K
L
M
N
P
Q
R
S
T
V
W
Y
Austria
govina
China
nmark
stonia
inland
France
rmany
ungary
Japan
Latvia
ngolia
rlands
Russia
ovakia
ovenia
Korea
weden
erland
USSR
ngdom

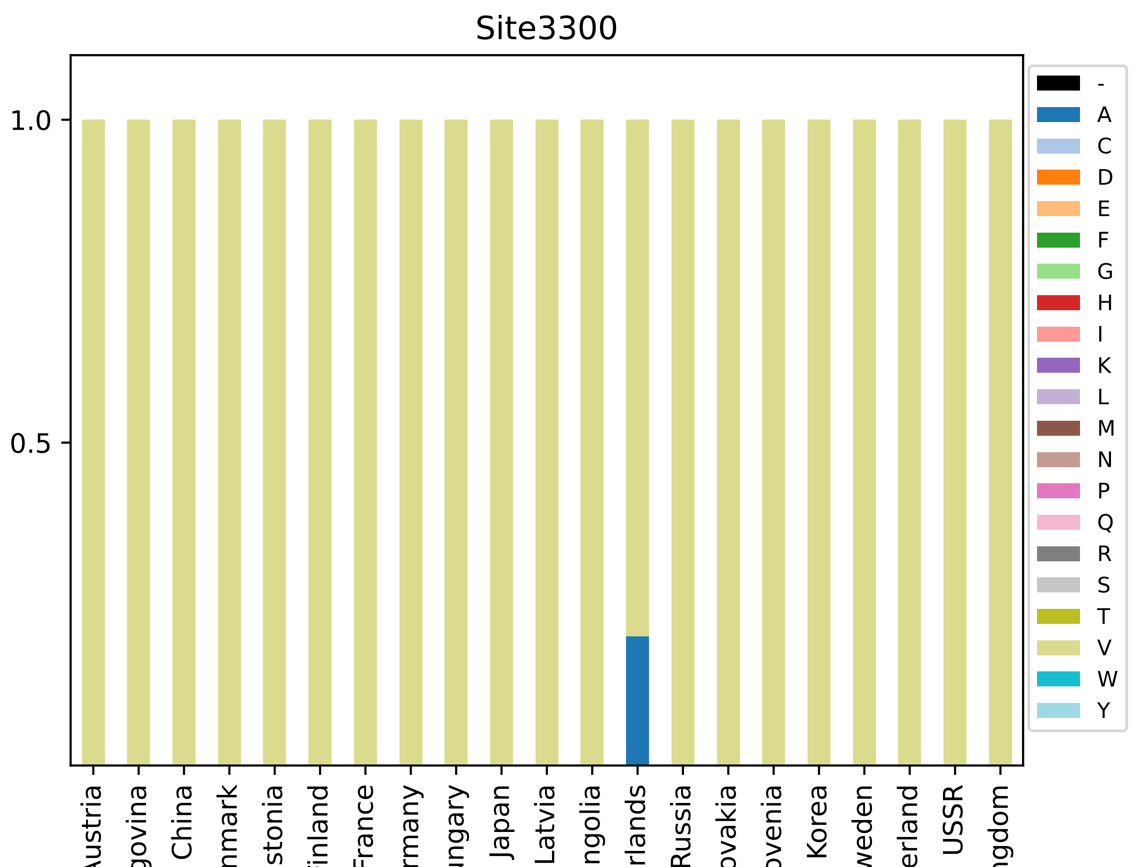
Site3300
1.0
0.5
-
A
C
D
E
F
G
H
I
K
L
M
N
P
Q
R
S
T
V
W
Y
Austria
China
Japan
Latvia
Russia
Korea
USSR

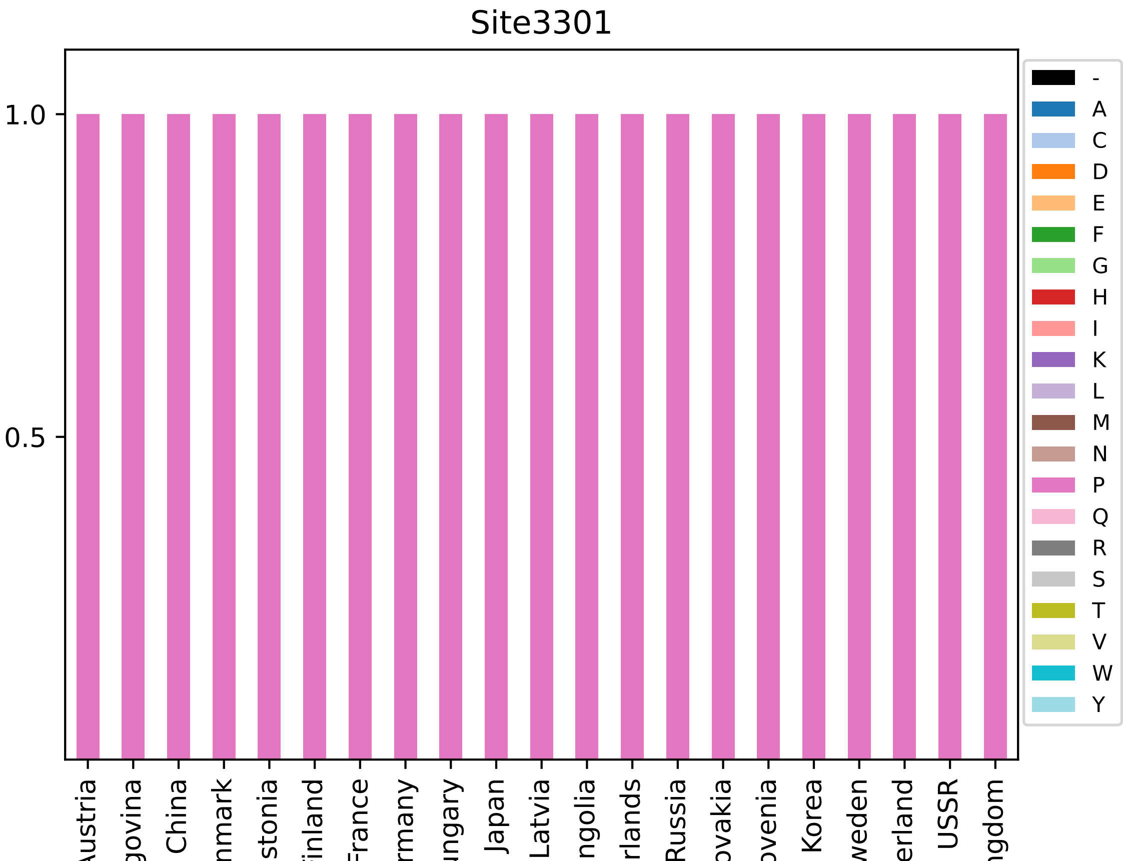
Site3301
1.0
0.5
-
A
C
D
E
F
G
H
I
K
L
M
N
P
Q
R
S
T
V
W
Y
China
Japan
Latvia
Russia
Korea
USSR

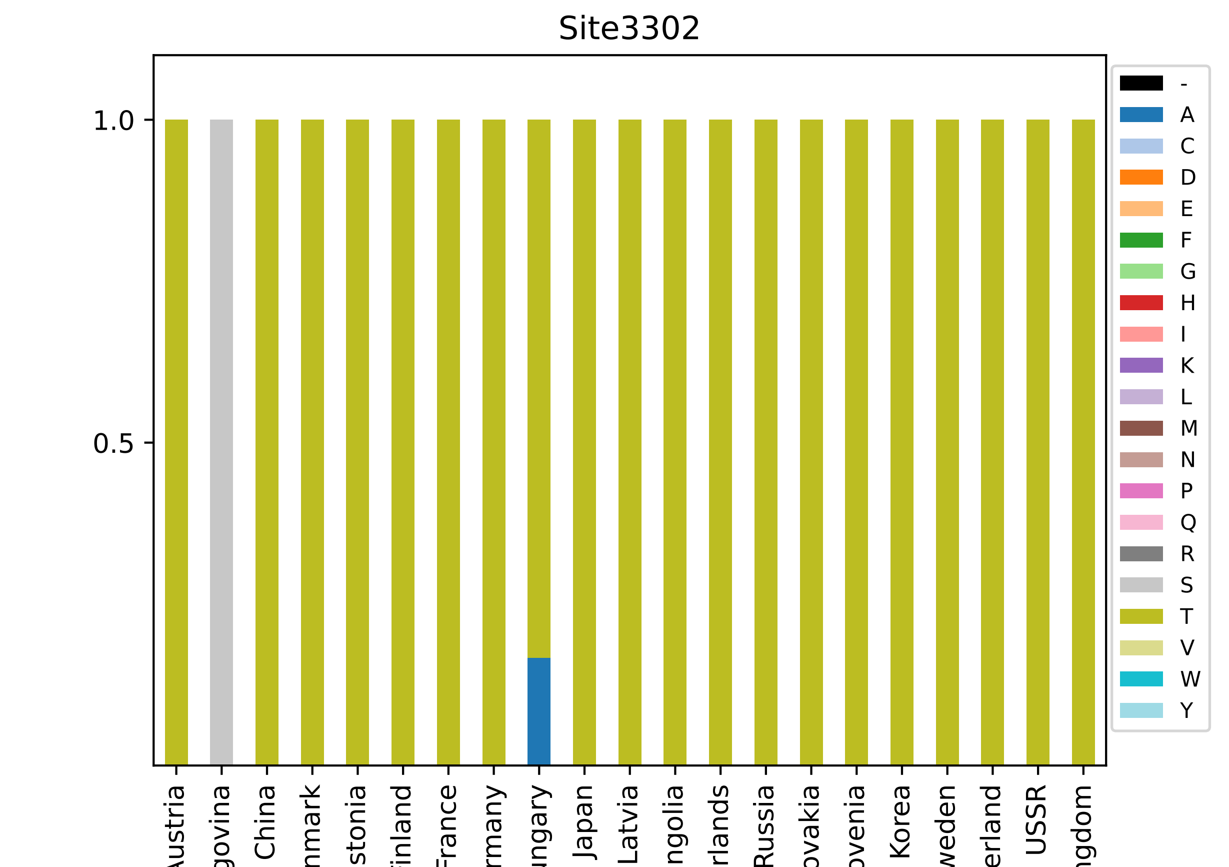

Site3302
1.0
0.5
-
A
C
D
E
F
G
H
I
K
L
M
N
P
Q
R
S
T
V
W
Y
China
Japan
Latvia
Russia
Korea
USSR

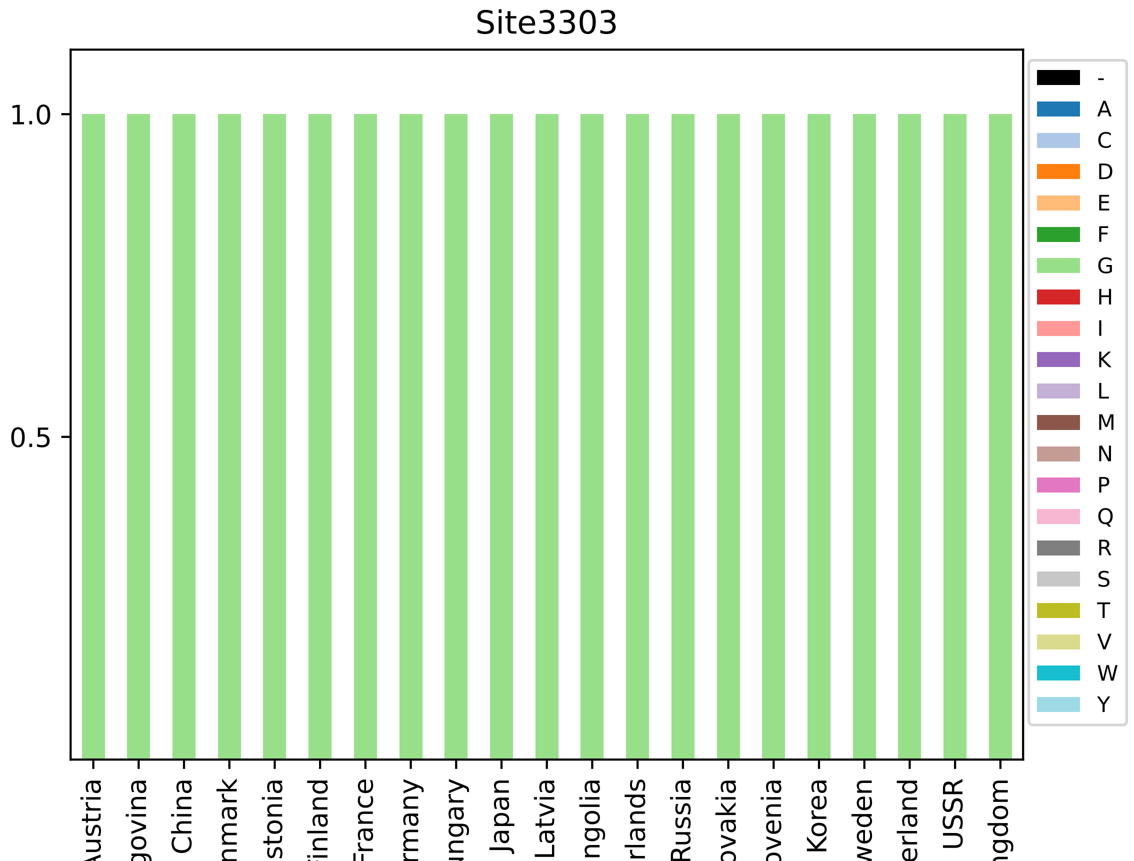
Site3303
1.0
0.5
-
A
C
D
E
F
G
H
I
K
L
M
N
P
Q
R
S
T
V
W
Y
China
Japan
Latvia
Russia
Korea
USSR

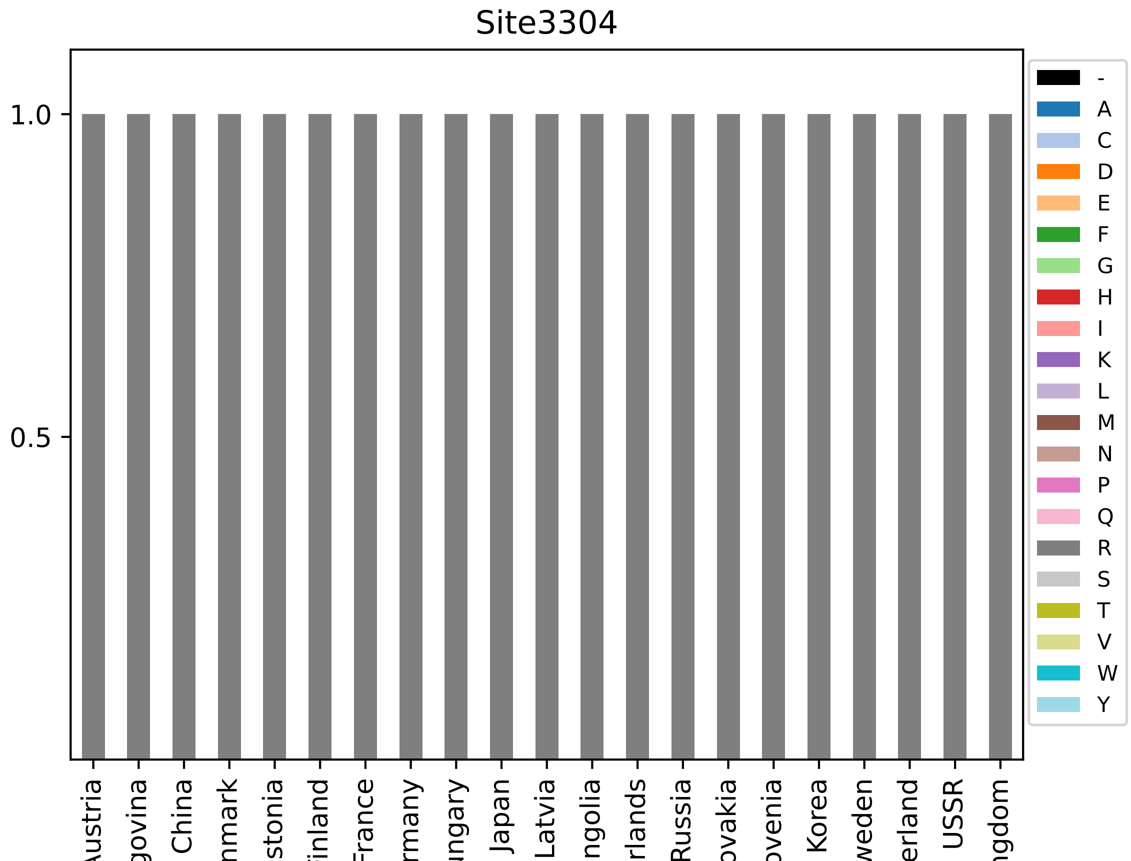
Site3304
1.0
0.5
-
A
C
D
E
F
G
H
I
K
L
M
N
P
Q
R
S
T
V
W
Y
China
Japan
Latvia
Russia
Korea
USSR

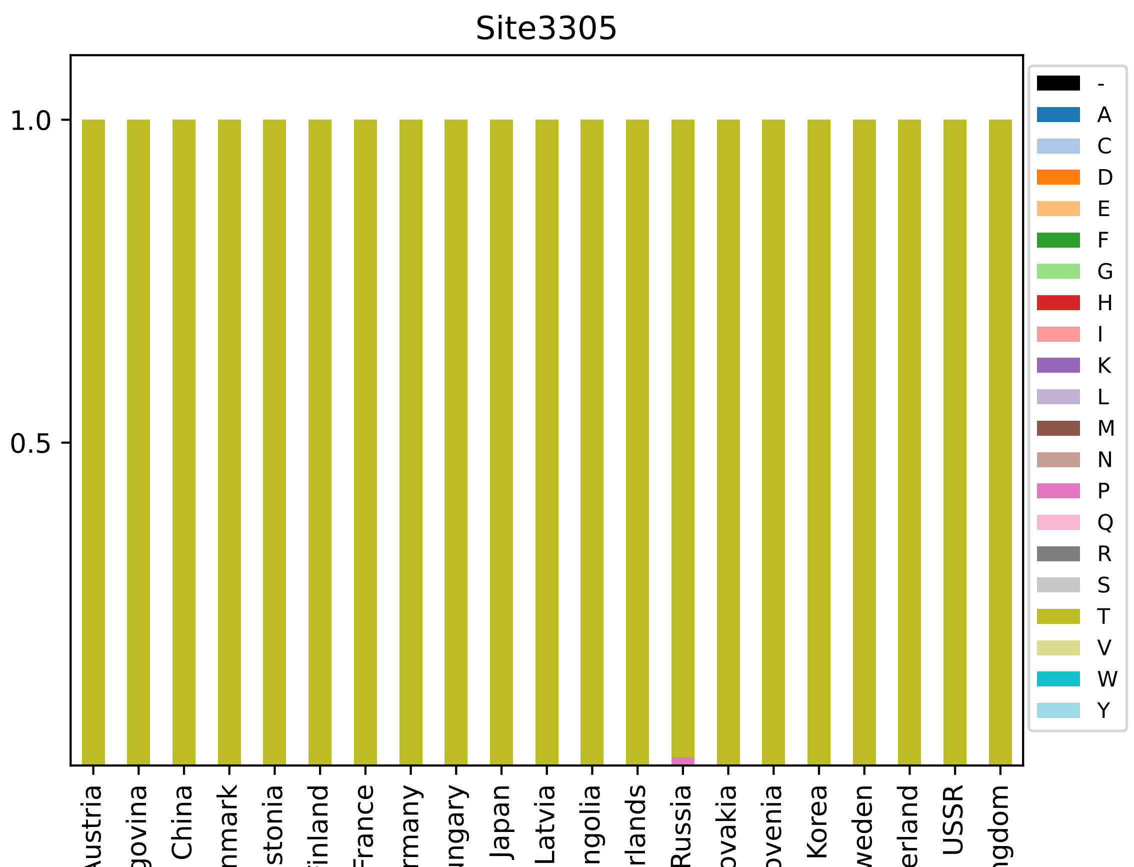

Site3305
1.0
0.5
-
A
C
D
E
F
G
H
I
K
L
M
N
P
Q
R
S
T
V
W
Y
Austria
govina
China
nmark
stonia
Finland
France
rmany
ungary
Japan
Latvia
ngolia
rlands
Russia
ovakia
ovenia
Korea
weden
erland
USSR
ngdom

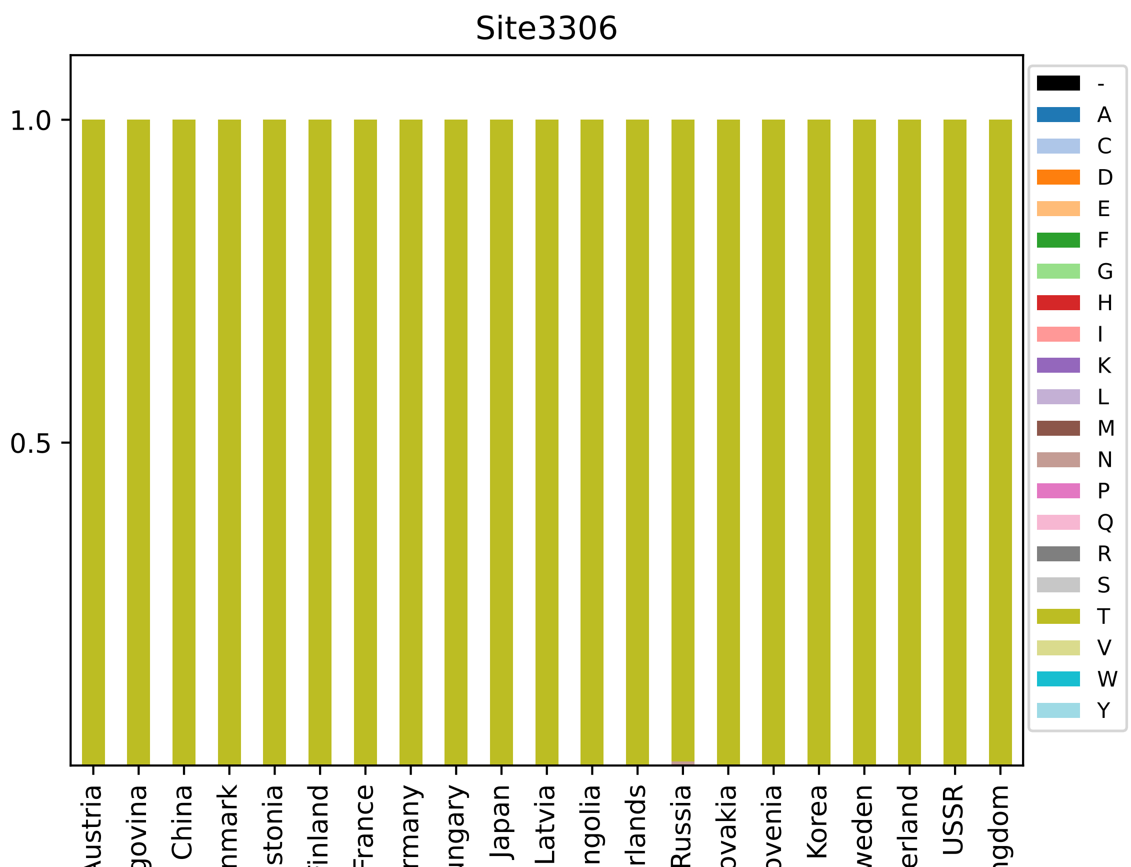
Site3306
1.0
0.5
-
A
C
D
E
F
G
H
I
K
L
M
N
P
Q
R
S
T
V
W
Y
Austria
govina
China
nmark
stonia
inland
France
rmany
ungary
Japan
Latvia
ngolia
rlands
Russia
ovakia
ovenia
Korea
weden
erland
USSR
ngdom

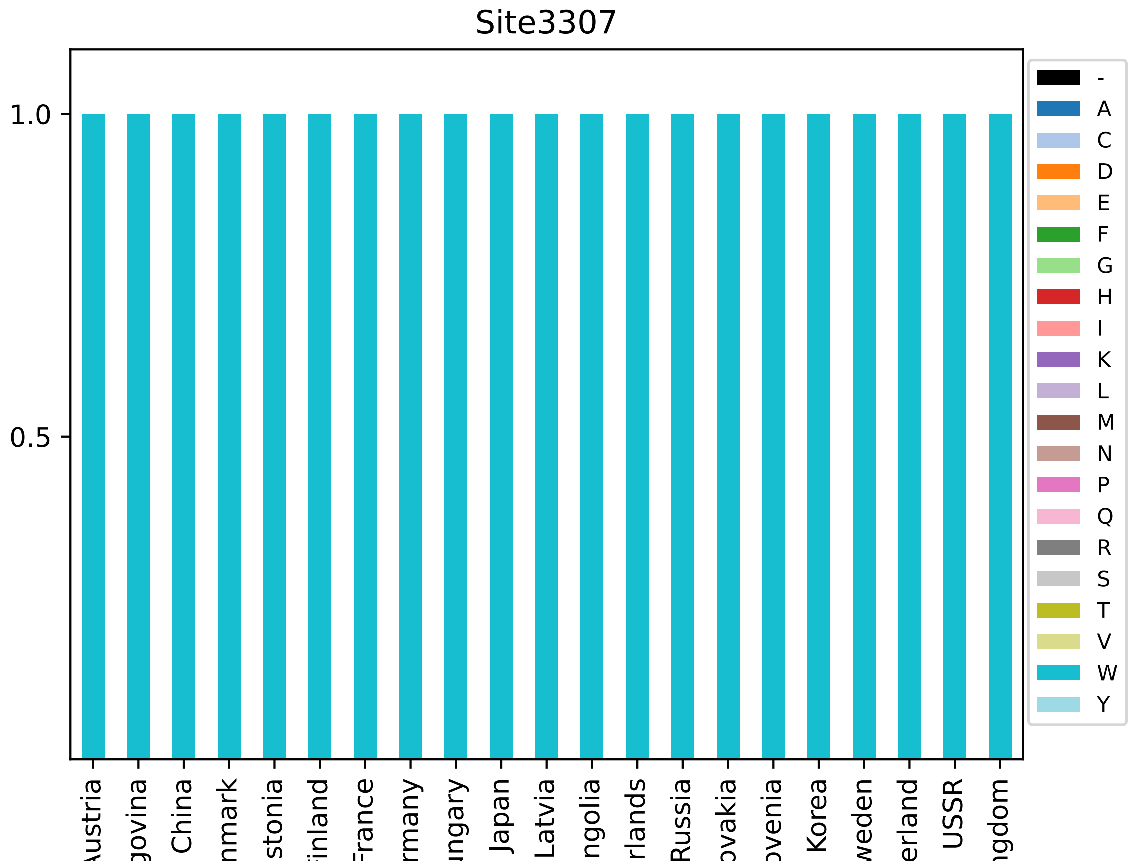

Site3307
1.0
0.5
-
A
C
D
E
F
G
H
I
K
L
M
N
P
Q
R
S
T
V
W
Y
China
Japan
Latvia
Russia
Korea
USSR

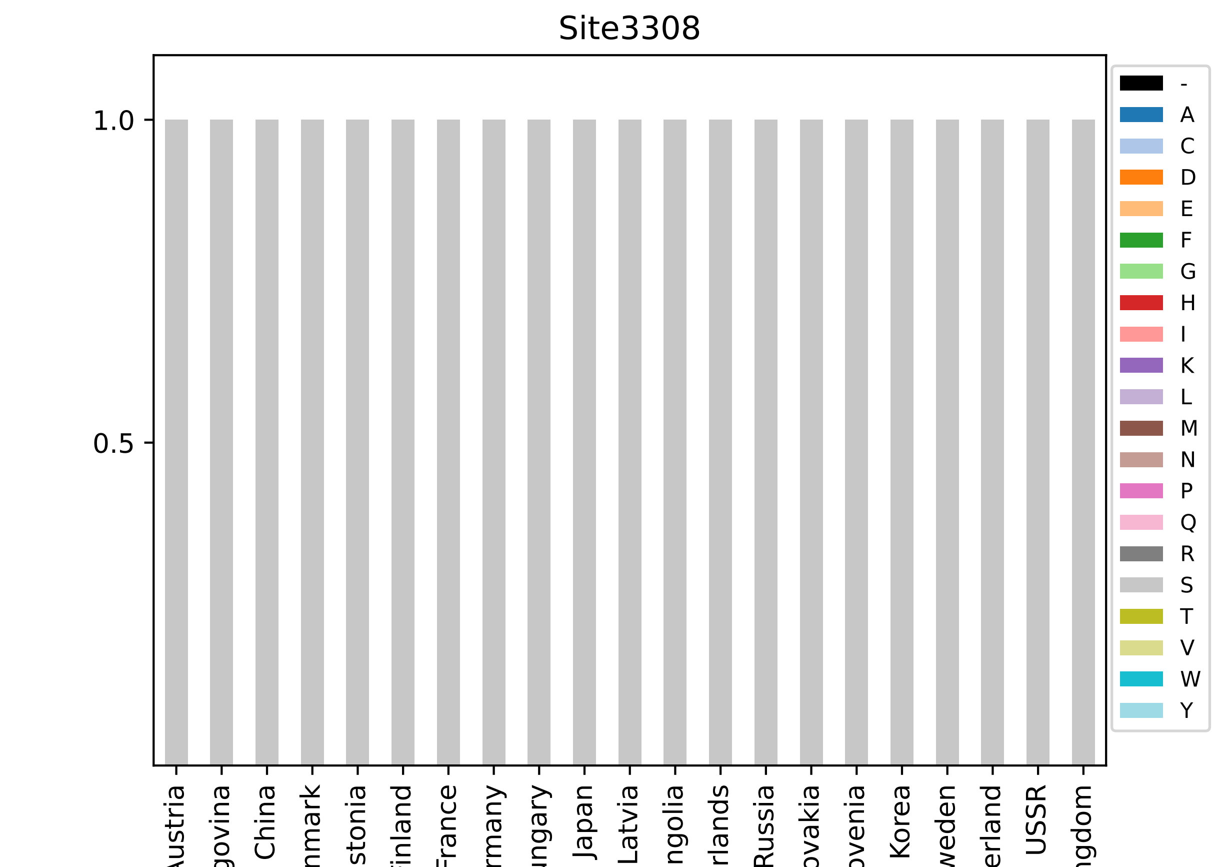

Site3308
1.0
0.5
-
A
C
D
E
F
G
H
I
K
L
M
N
P
Q
R
S
T
V
W
Y
Austria
govina
China
nmark
stonia
Finland
France
rmany
ungary
Japan
Latvia
ngolia
rlands
Russia
ovakia
ovenia
Korea
weden
erland
USSR
ngdom

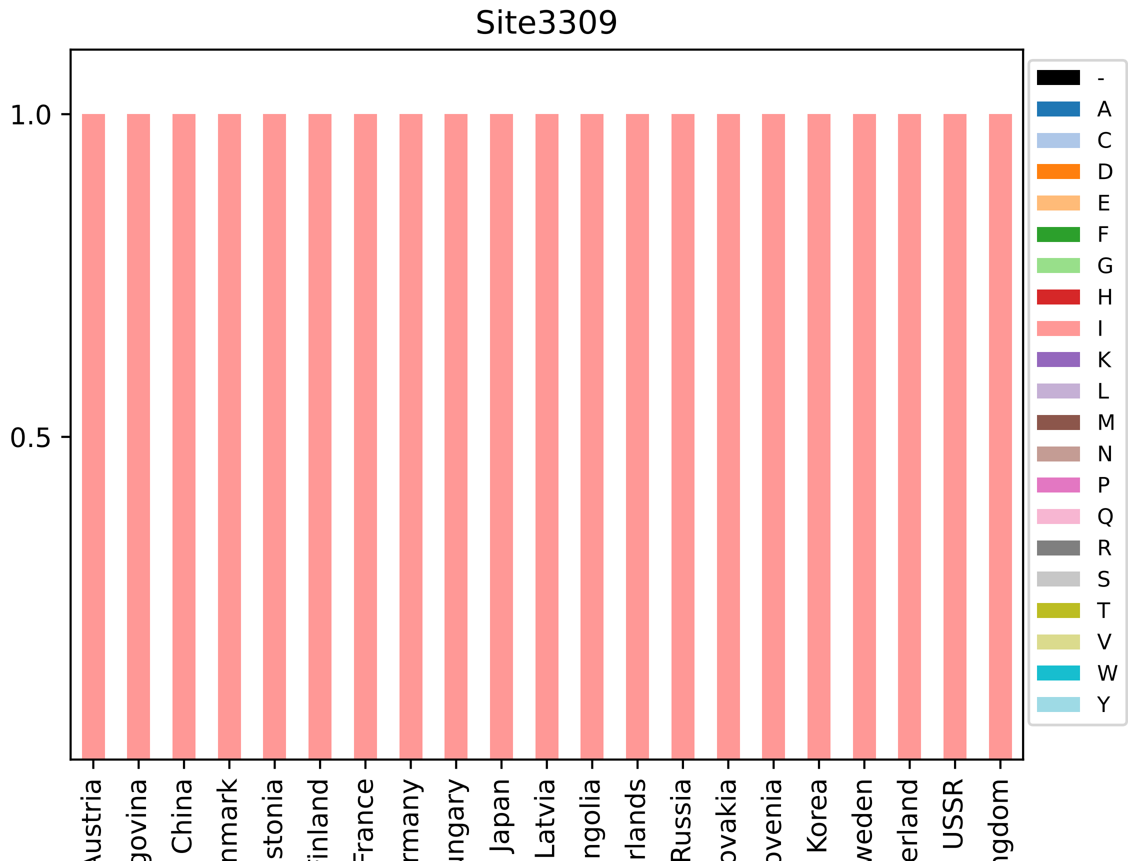

Site3309
1.0
0.5
Austria
govina
China
nmark
stonia
Finland
France
rmany
ungary
Japan
Latvia
ngolia
rlands
Russia
ovakia
ovenia
Korea
weden
erland
USSR
ngdom
-
A
C
D
E
F
G
H
I
K
L
M
N
P
Q
R
S
T
V
W
Y

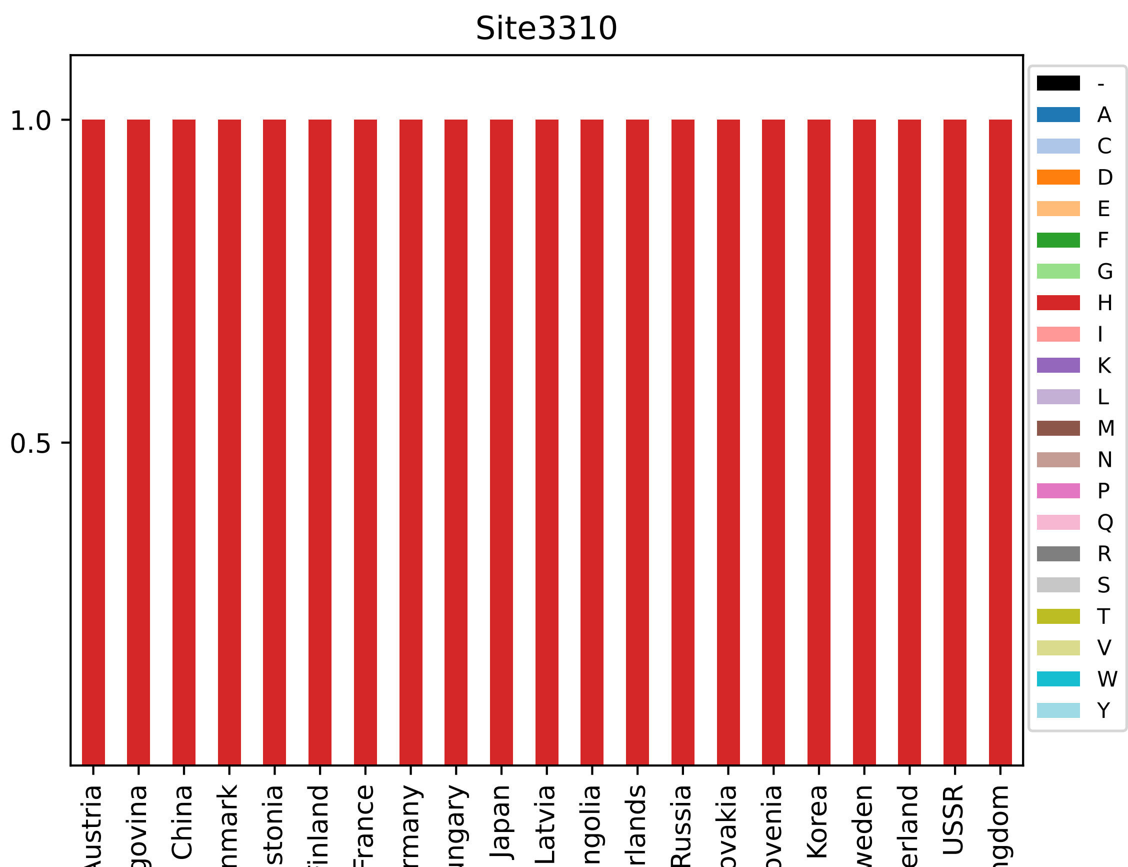
Site3310
1.0
0.5
-
A
C
D
E
F
G
H
I
K
L
M
N
P
Q
R
S
T
V
W
Y
China
Japan
Latvia
Russia
Korea
USSR

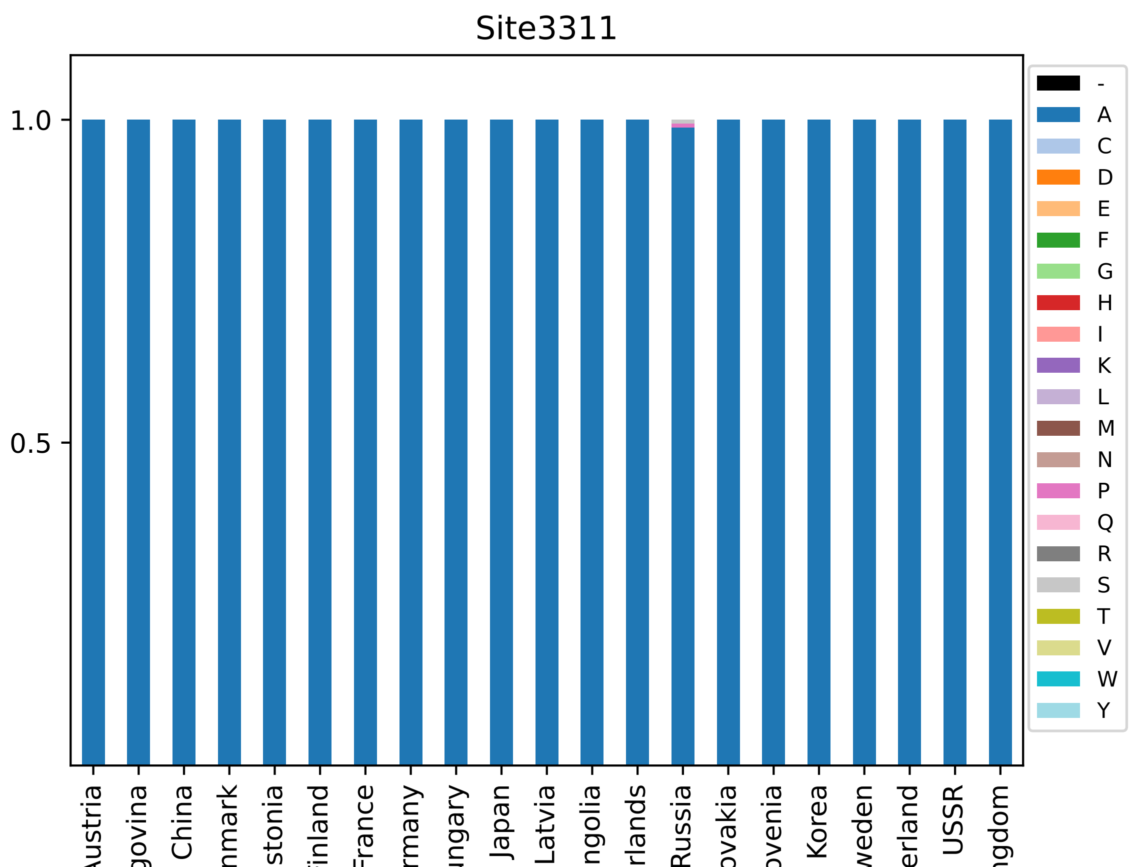

Site3311
1.0
0.5
-
A
C
D
E
F
G
H
I
K
L
M
N
P
Q
R
S
T
V
W
Y
Austria
govina
China
nmark
stonia
inland
France
rmany
ungary
Japan
Latvia
ngolia
rlands
Russia
ovakia
ovenia
Korea
weden
erland
USSR
ngdom

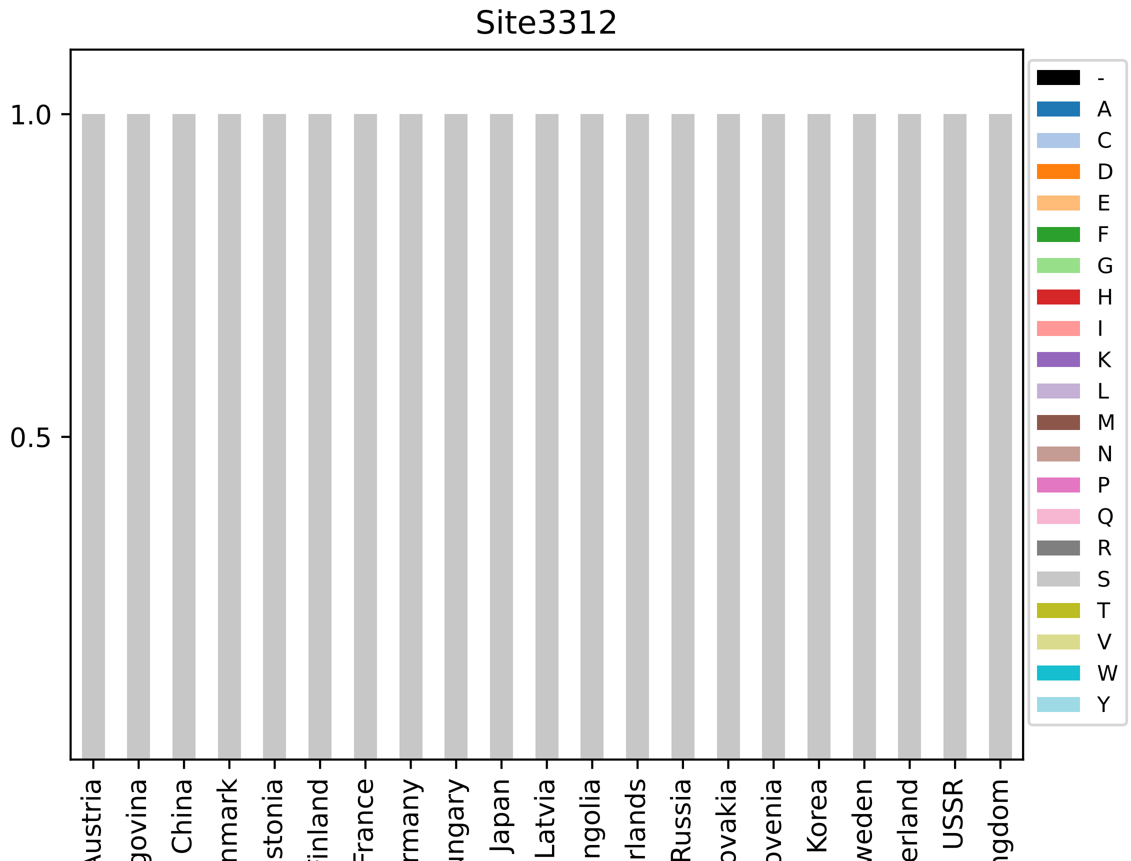
Site3312
1.0
0.5
-
A
C
D
E
F
G
H
I
K
L
M
N
P
Q
R
S
T
V
W
Y
Austria
govina
China
nmark
stonia
Finland
France
rmany
ungary
Japan
Latvia
ngolia
rlands
Russia
ovakia
ovenia
Korea
weden
erland
USSR
ngdom

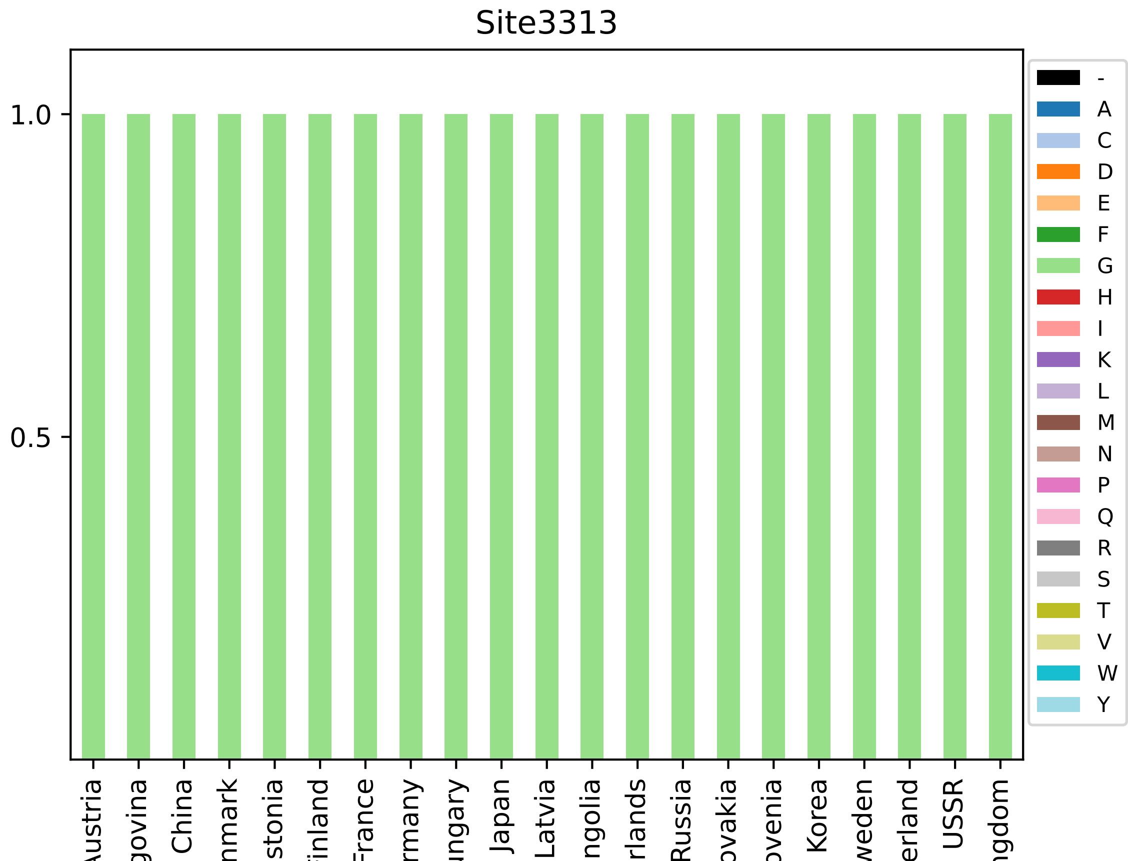
Site3313
1.0
0.5
-
A
C
D
E
F
G
H
I
K
L
M
N
P
Q
R
S
T
V
W
Y
China
Japan
Latvia
Russia
Korea
USSR

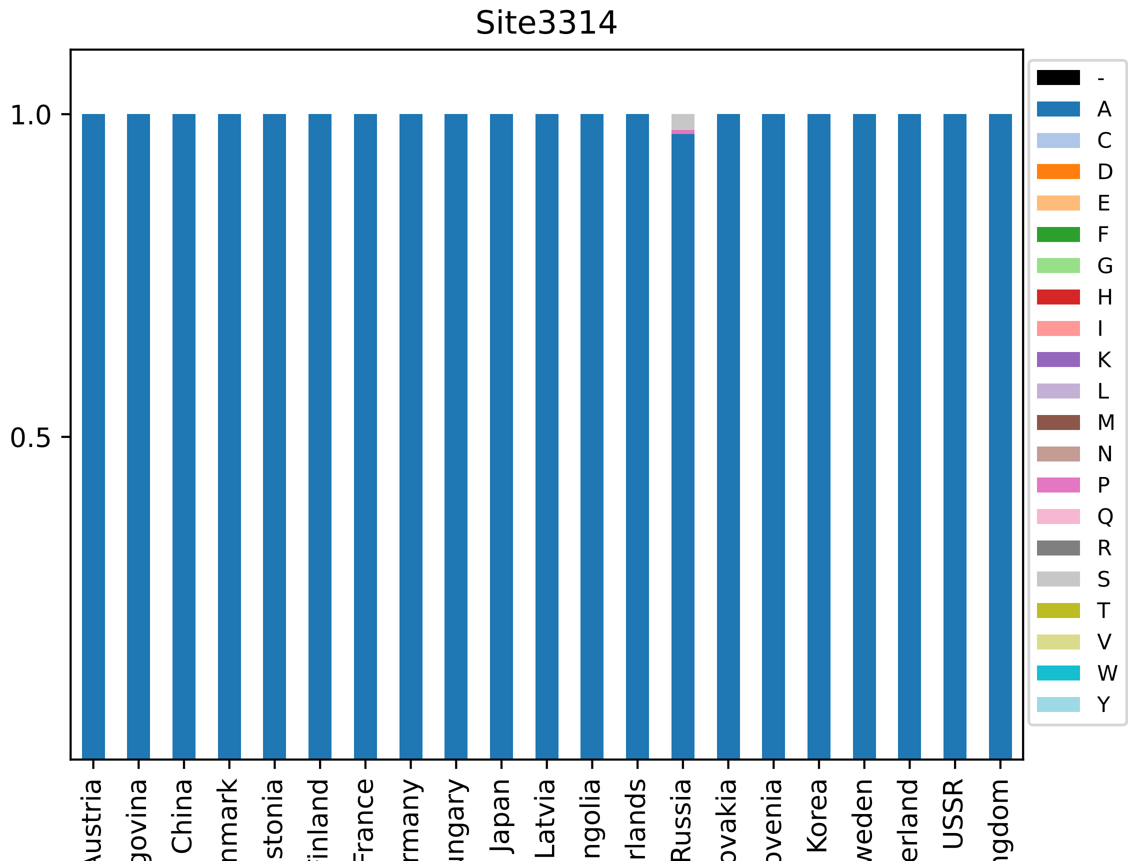
Site3314
1.0
0.5
-
A
C
D
E
F
G
H
I
K
L
M
N
P
Q
R
S
T
V
W
Y
ustria
govina
China
nmark
stonia
inland
rance
rmany
ungary
Japan
Latvia
ngolia
rlands
Russia
ovakia
ovenia
Korea
weden
erland
USSR
ngdom

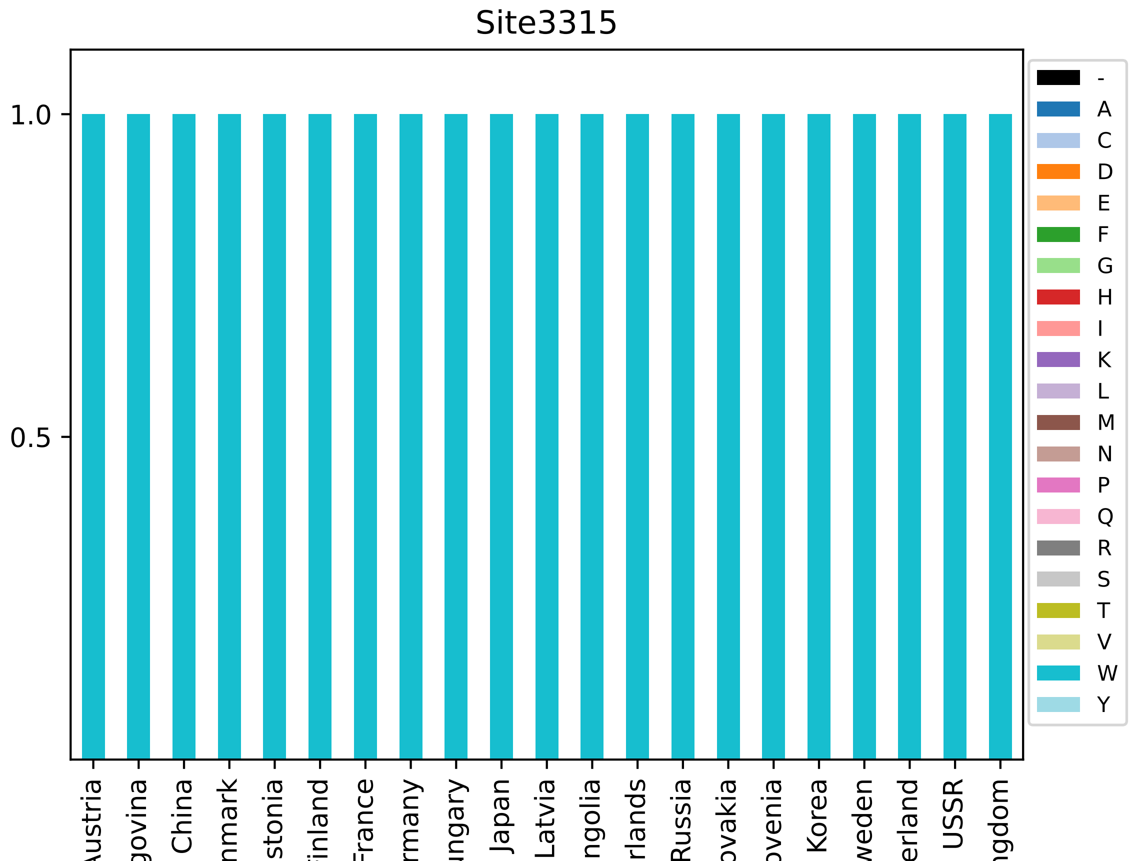
Site3315
1.0
0.5
-
A
C
D
E
F
G
H
I
K
L
M
N
P
Q
R
S
T
V
W
Y
Austria
govina
China
nmark
stonia
inland
France
rmany
ungary
Japan
Latvia
ngolia
rlands
Russia
ovakia
ovenia
Korea
weden
erland
USSR
ngdom

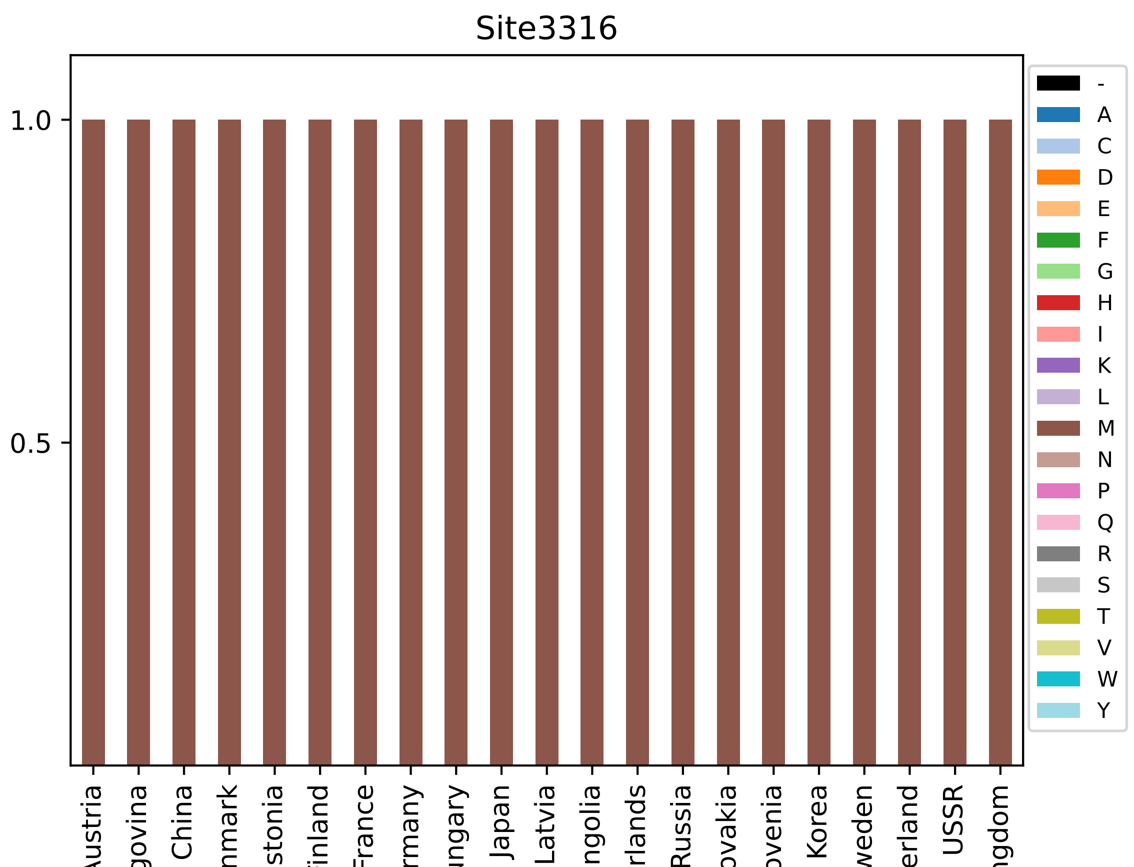

Site3316
1.0
0.5
-
A
C
D
E
F
G
H
I
K
L
M
N
P
Q
R
S
T
V
W
Y
Austria
China
Japan
Latvia
Russia
Korea
USSR

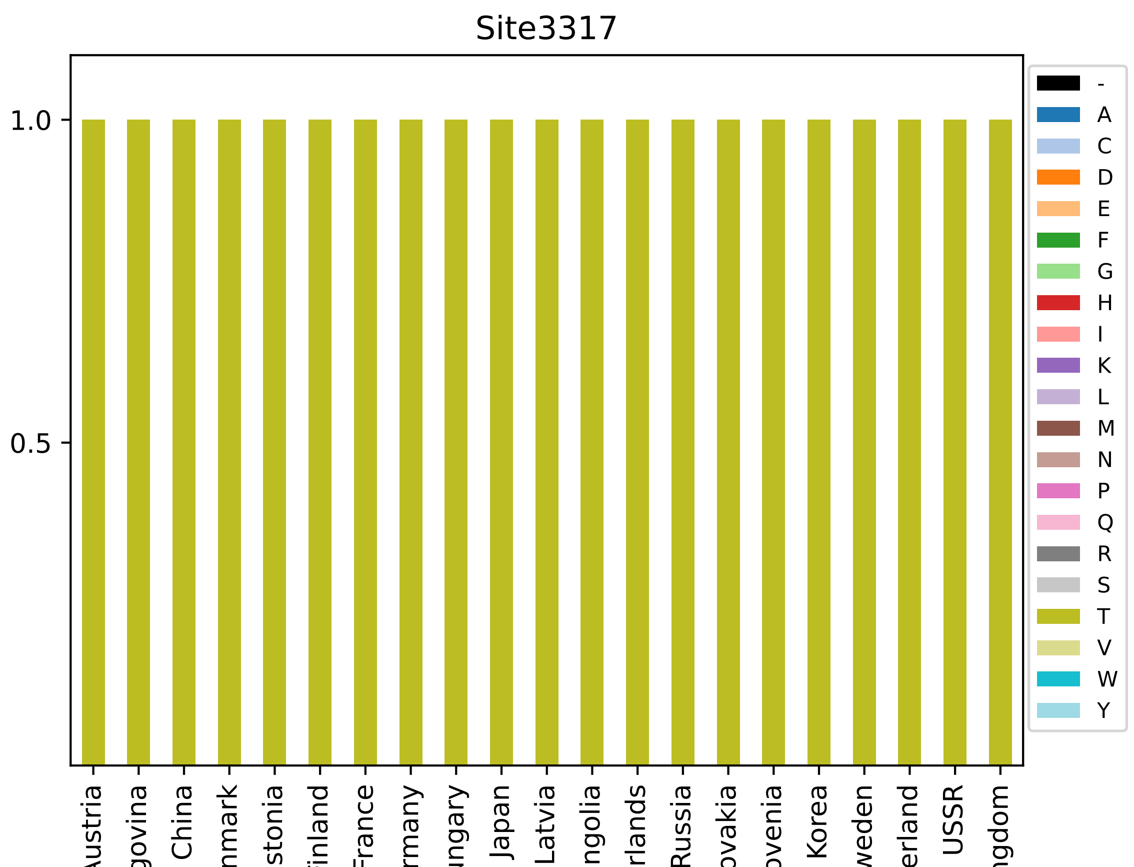
Site3317
1.0
0.5
-
A
C
D
E
F
G
H
I
K
L
M
N
P
Q
R
S
T
V
W
Y
Austria
govina
China
nmark
stonia
inland
France
rmany
ungary
Japan
Latvia
ngolia
rlands
Russia
ovakia
ovenia
Korea
weden
erland
USSR
ngdom

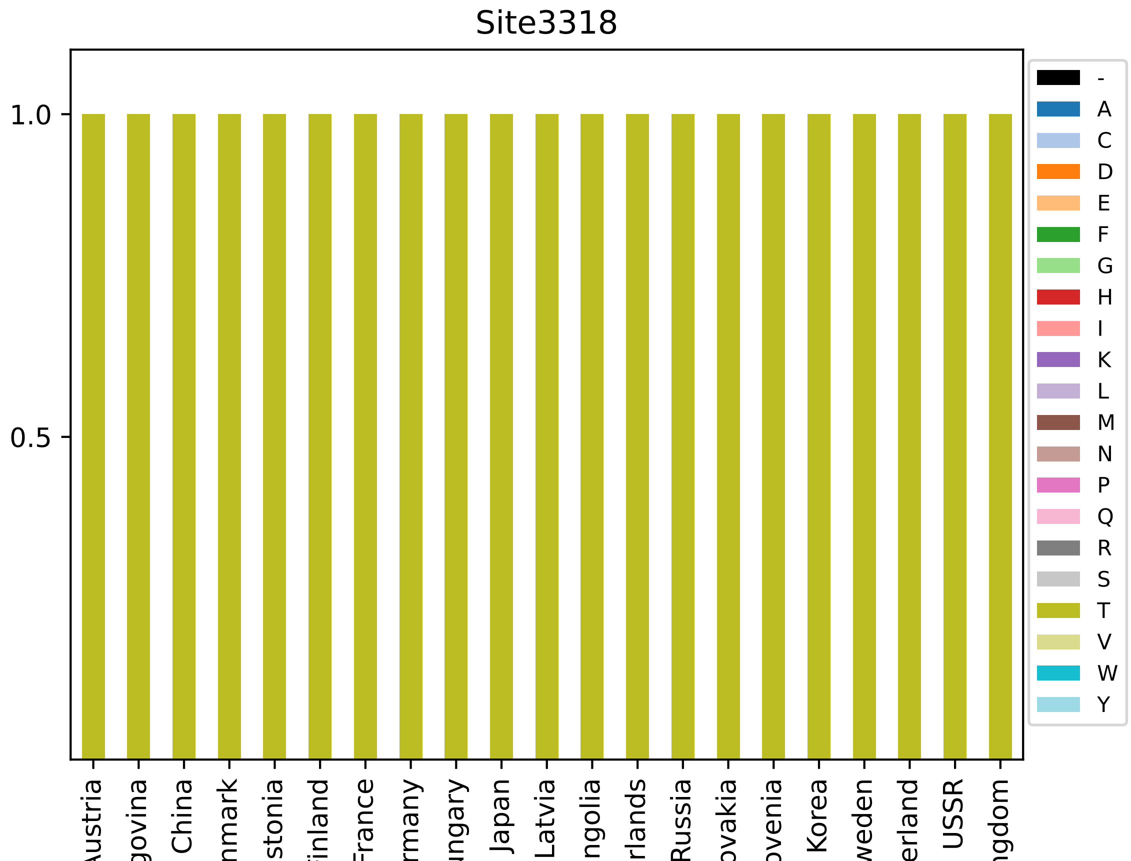

Site3318
1.0
0.5
-
A
C
D
E
F
G
H
I
K
L
M
N
P
Q
R
S
T
V
W
Y
China
Japan
Latvia
Russia
Korea
USSR

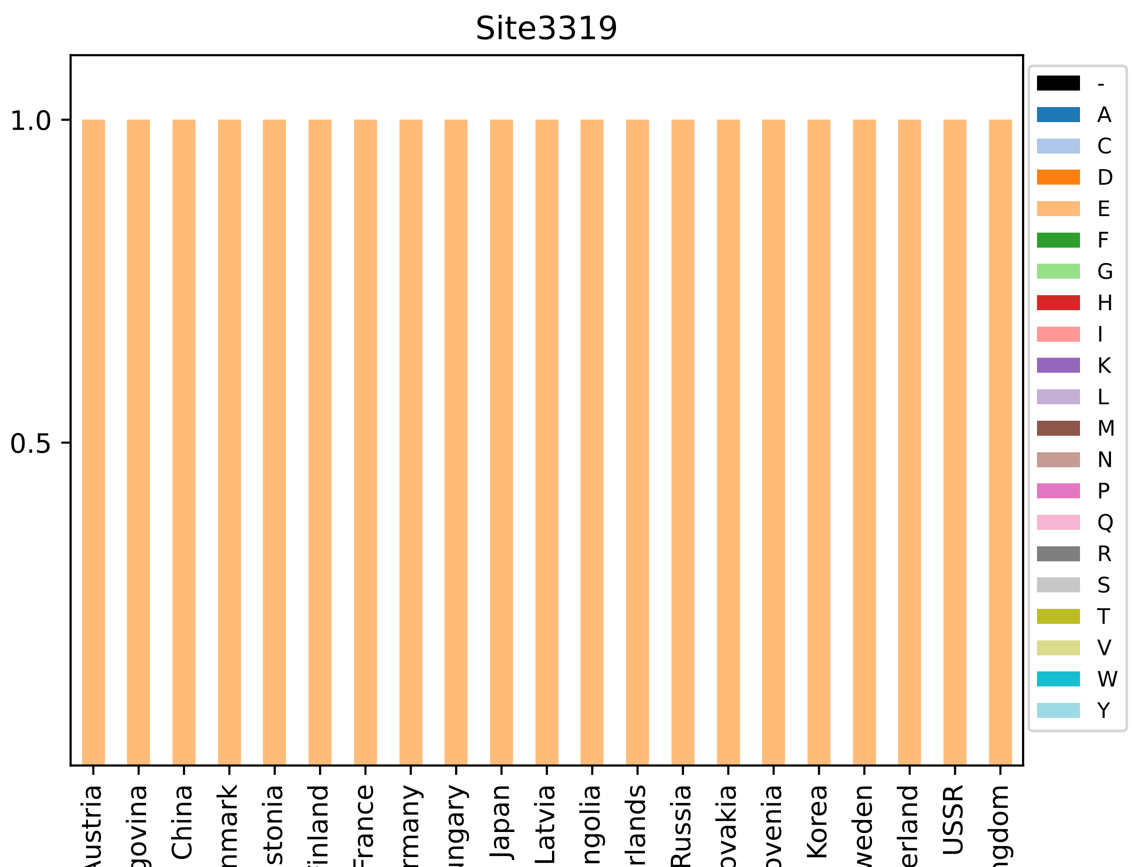

Site3319
1.0
0.5
-
A
C
D
E
F
G
H
I
K
L
M
N
P
Q
R
S
T
V
W
Y
Austria
govina
China
nmark
stonia
inland
France
rmany
ungary
Japan
Latvia
ngolia
rlands
Russia
ovakia
ovenia
Korea
weden
erland
USSR
ngdom

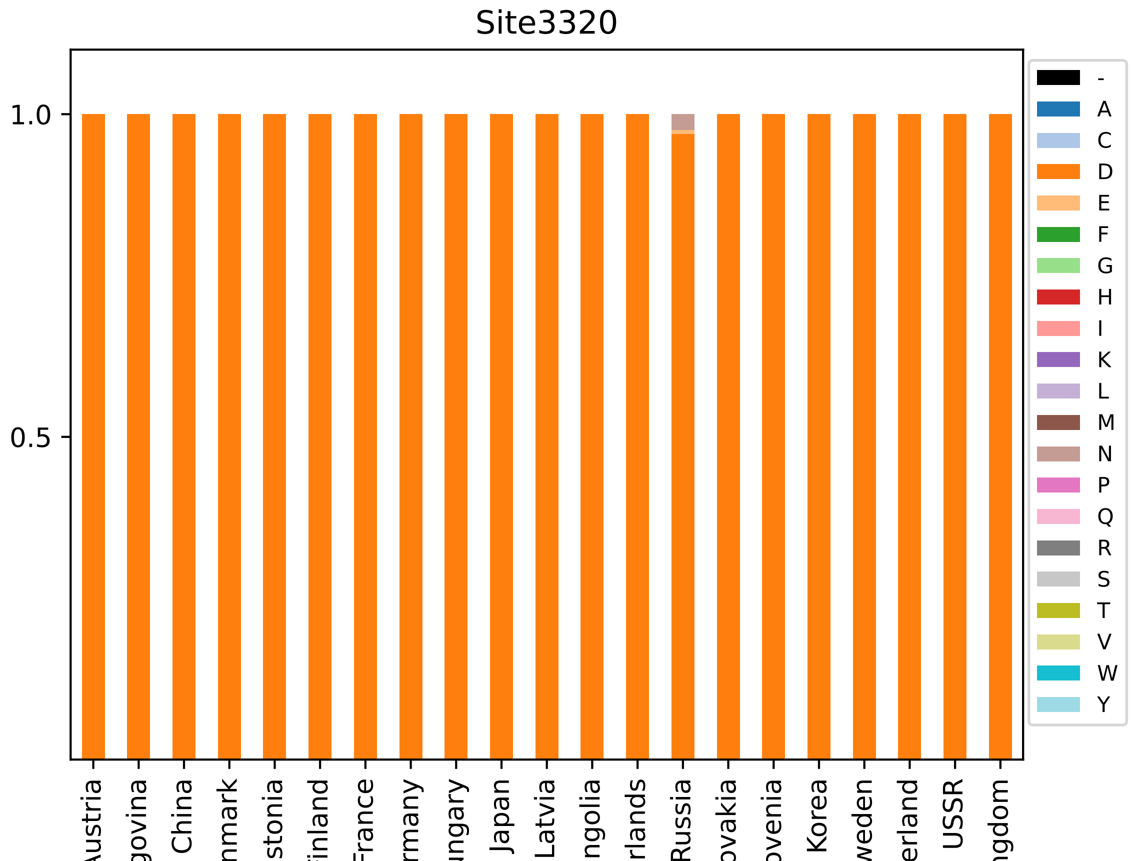

Site3320
1.0
0.5
-
A
C
D
E
F
G
H
I
K
L
M
N
P
Q
R
S
T
V
W
Y
Austria
govina
China
nmark
stonia
Finland
France
rmany
ungary
Japan
Latvia
ngolia
rlands
Russia
ovakia
ovenia
Korea
weden
erland
USSR
ngdom

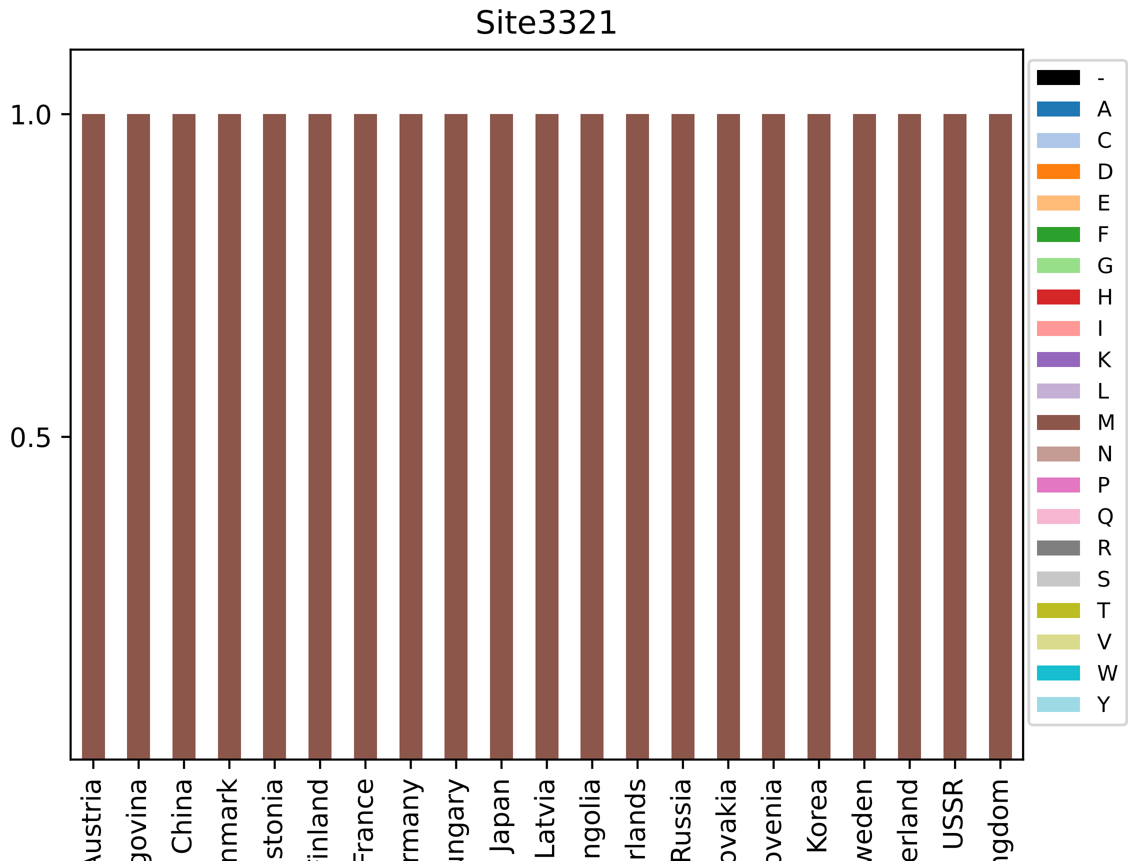

Site3321
1.0
0.5
-
A
C
D
E
F
G
H
I
K
L
M
N
P
Q
R
S
T
V
W
Y
Austria
govina
China
nmark
stonia
Finland
France
rmany
ungary
Japan
Latvia
ngolia
rlands
Russia
ovakia
ovenia
Korea
weden
erland
USSR
ngdom

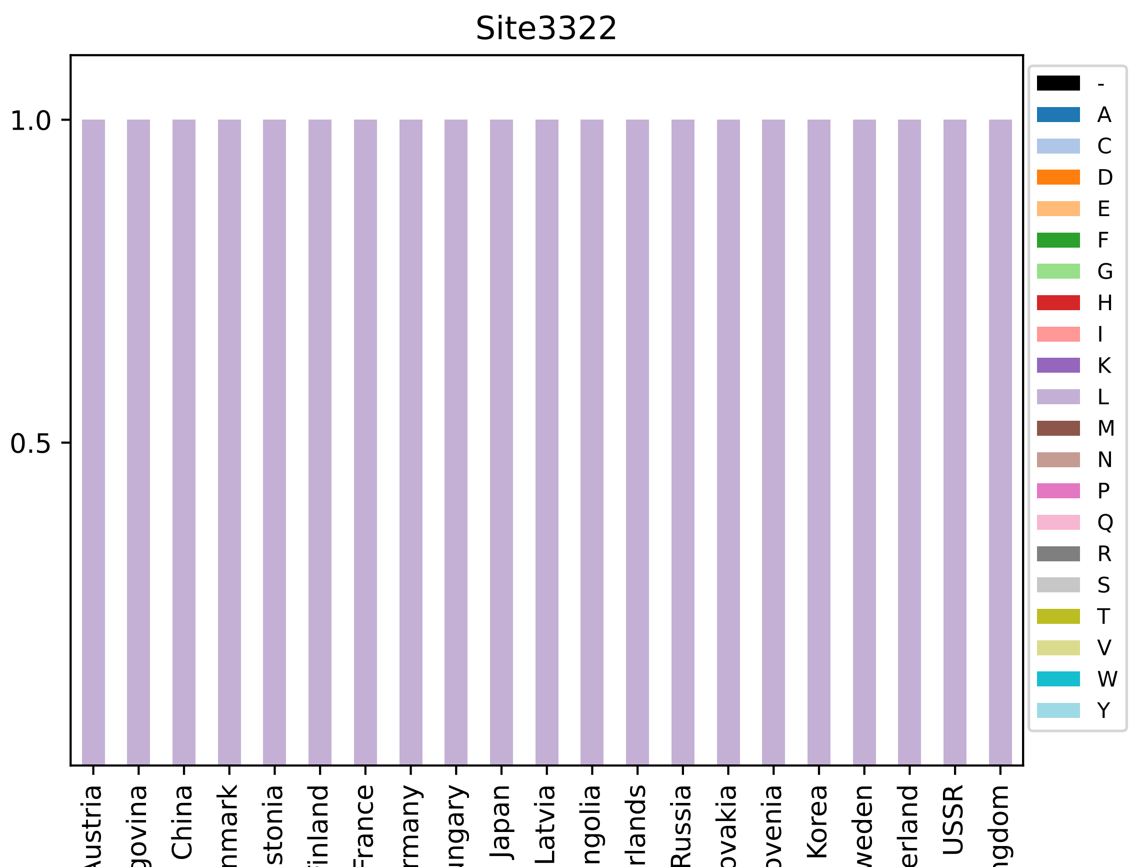

Site3322
1.0
0.5
-
A
C
D
E
F
G
H
I
K
L
M
N
P
Q
R
S
T
V
W
Y
China
Japan
Latvia
Russia
Korea
USSR

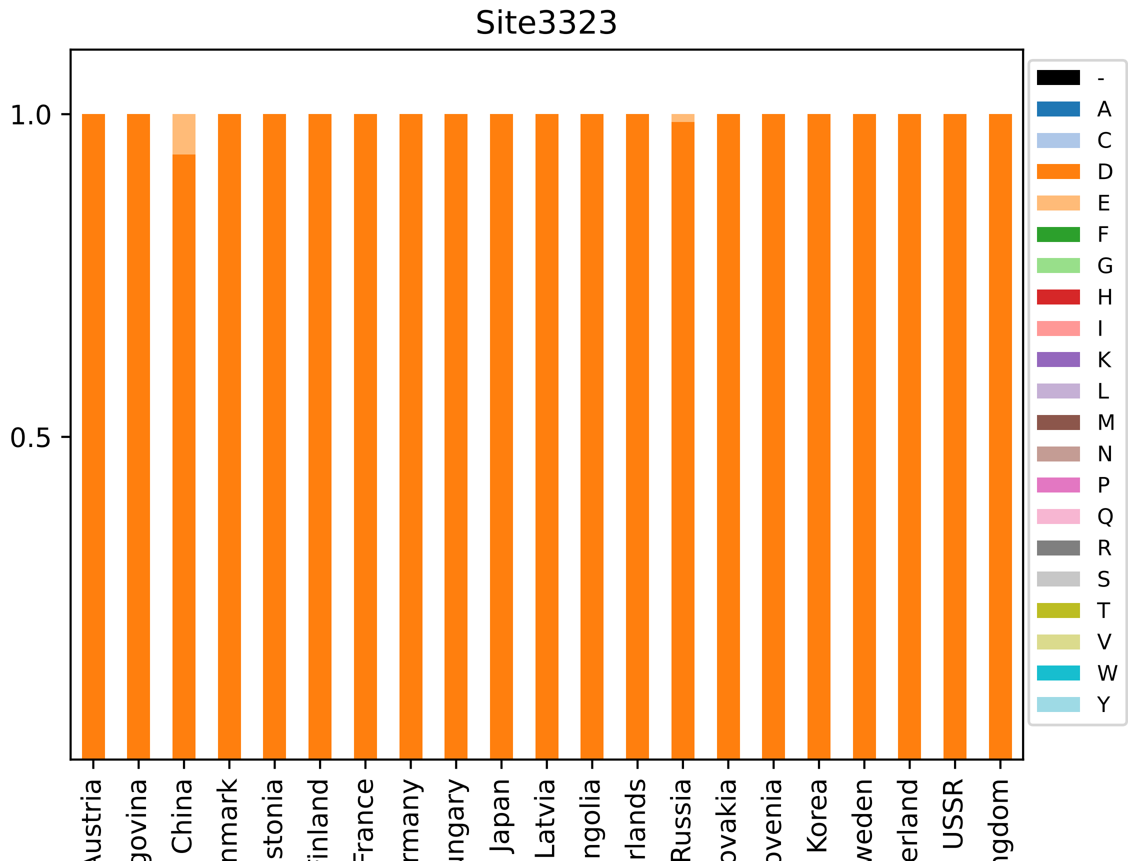
Site3323
1.0
0.5
-
A
C
D
E
F
G
H
I
K
L
M
N
P
Q
R
S
T
V
W
Y
Austria
govina
China
nmark
stonia
inland
France
rmany
ungary
Japan
Latvia
ngolia
rlands
Russia
ovakia
ovenia
Korea
weden
erland
USSR
ngdom

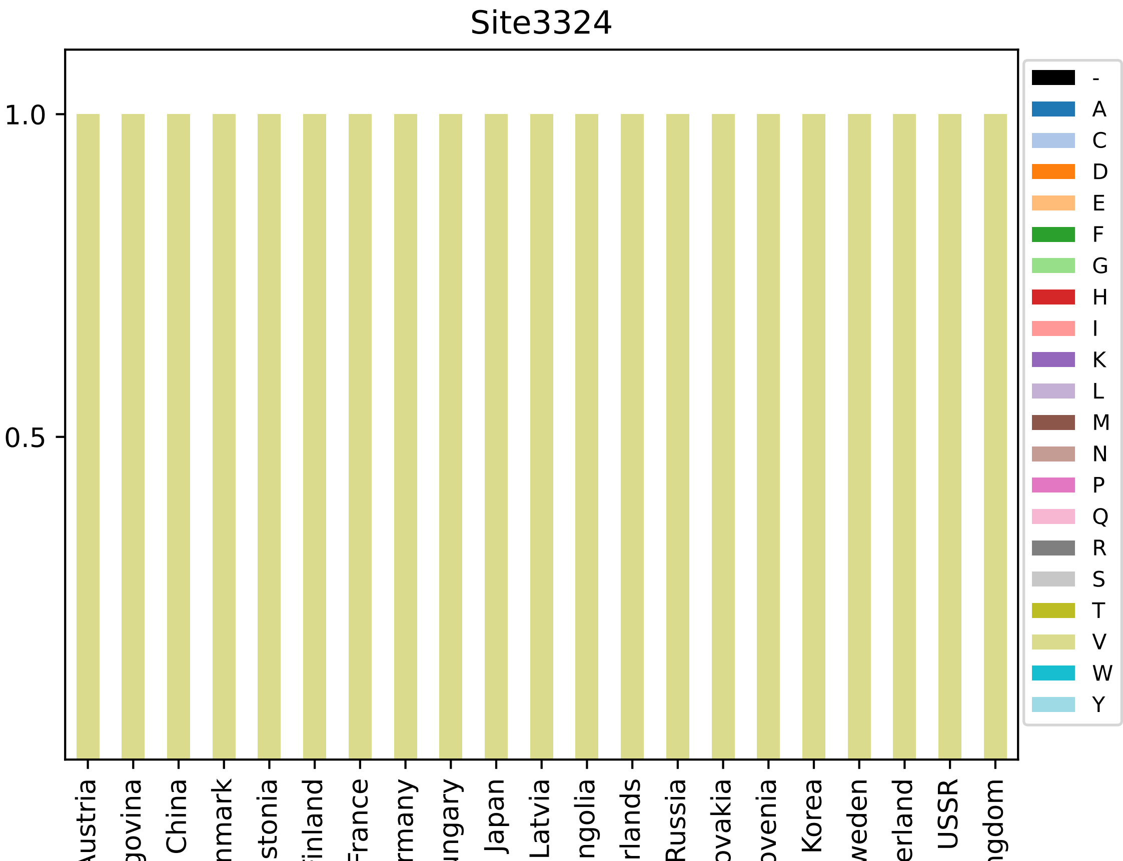
Site3324
1.0
0.5
-
A
C
D
E
F
G
H
I
K
L
M
N
P
Q
R
S
T
V
W
Y
China
Finland
Japan
Latvia
Russia
Korea
USSR

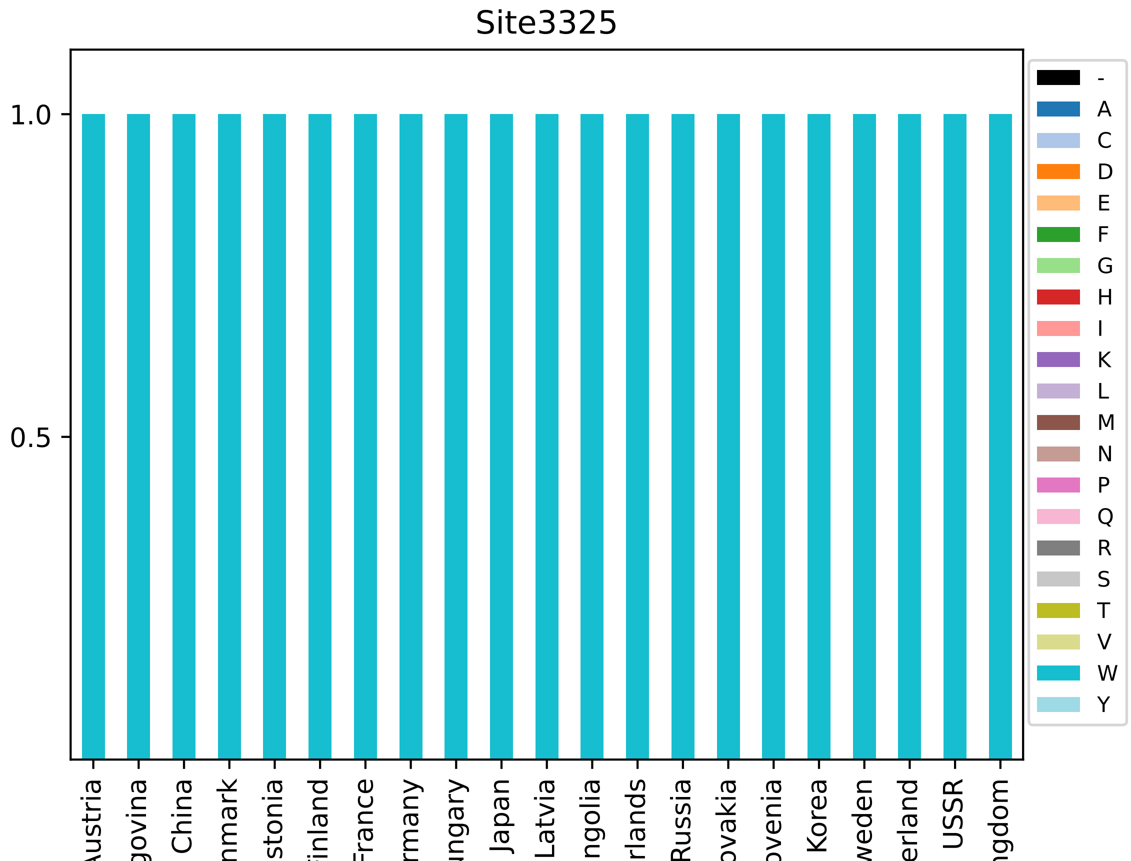

Site3325
1.0
0.5
-
A
C
D
E
F
G
H
I
K
L
M
N
P
Q
R
S
T
V
W
Y
Austria
China
Finland
France
Japan
Latvia
Russia
Korea
USSR

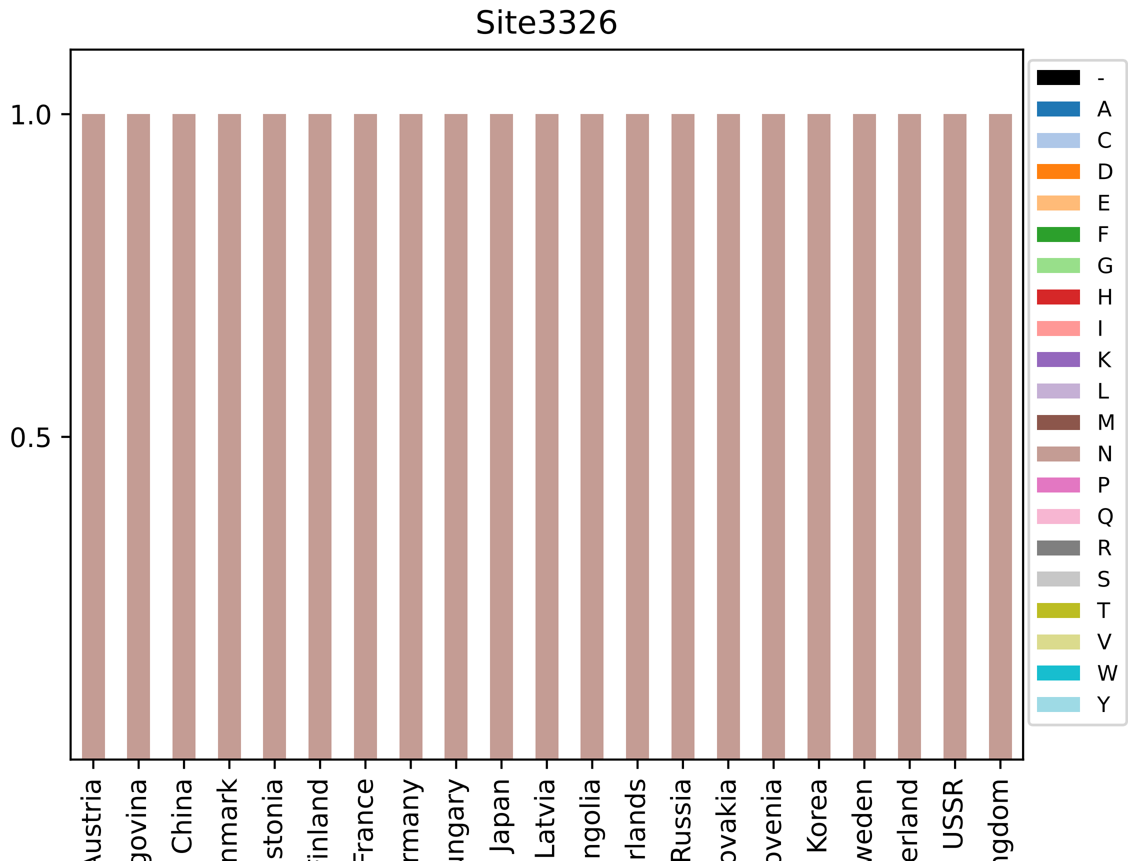
Site3326
1.0
0.5
-
A
C
D
E
F
G
H
I
K
L
M
N
P
Q
R
S
T
V
W
Y
Austria
China
Japan
Latvia
Russia
Korea
USSR

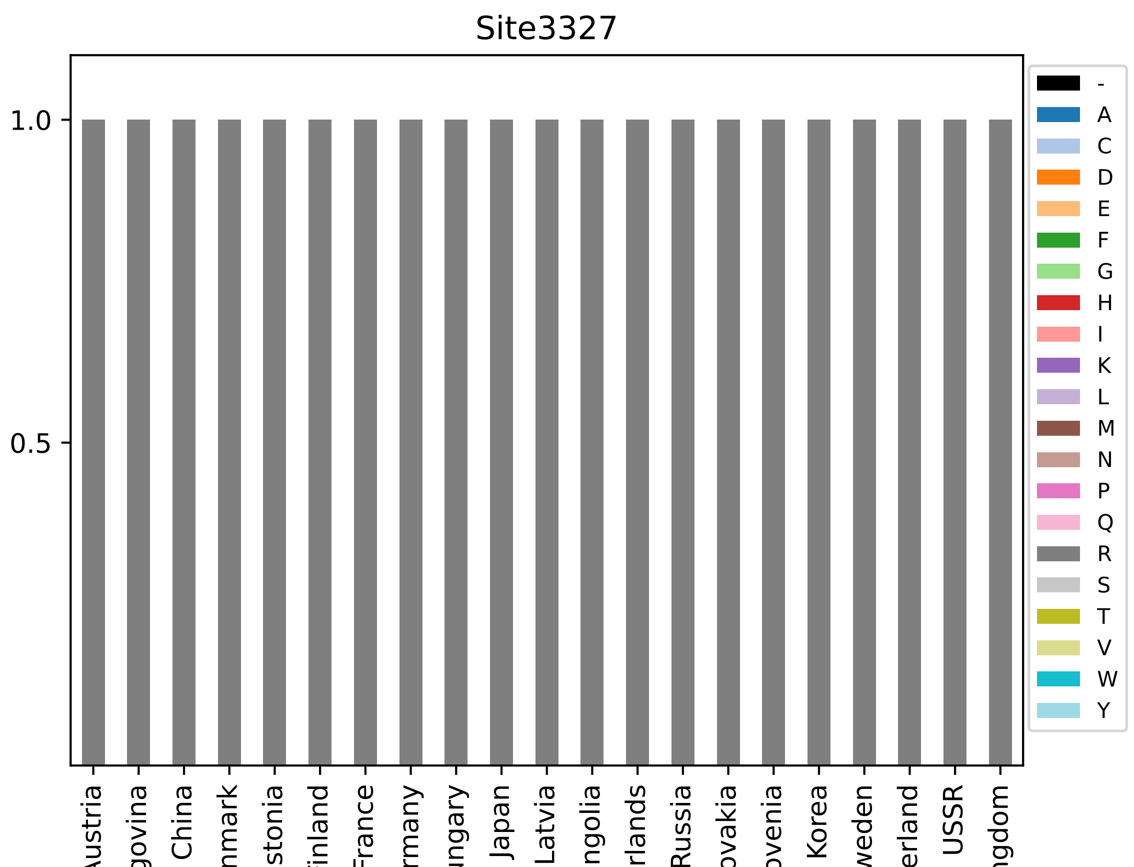

Site3327
1.0
0.5
Austria
govina
China
nmark
stonia
inland
France
rmany
ungary
Japan
Latvia
ngolia
rlands
Russia
ovakia
ovenia
Korea
weden
erland
USSR
ngdom
-
A
C
D
E
F
G
H
I
K
L
M
N
P
Q
R
S
T
V
W
Y

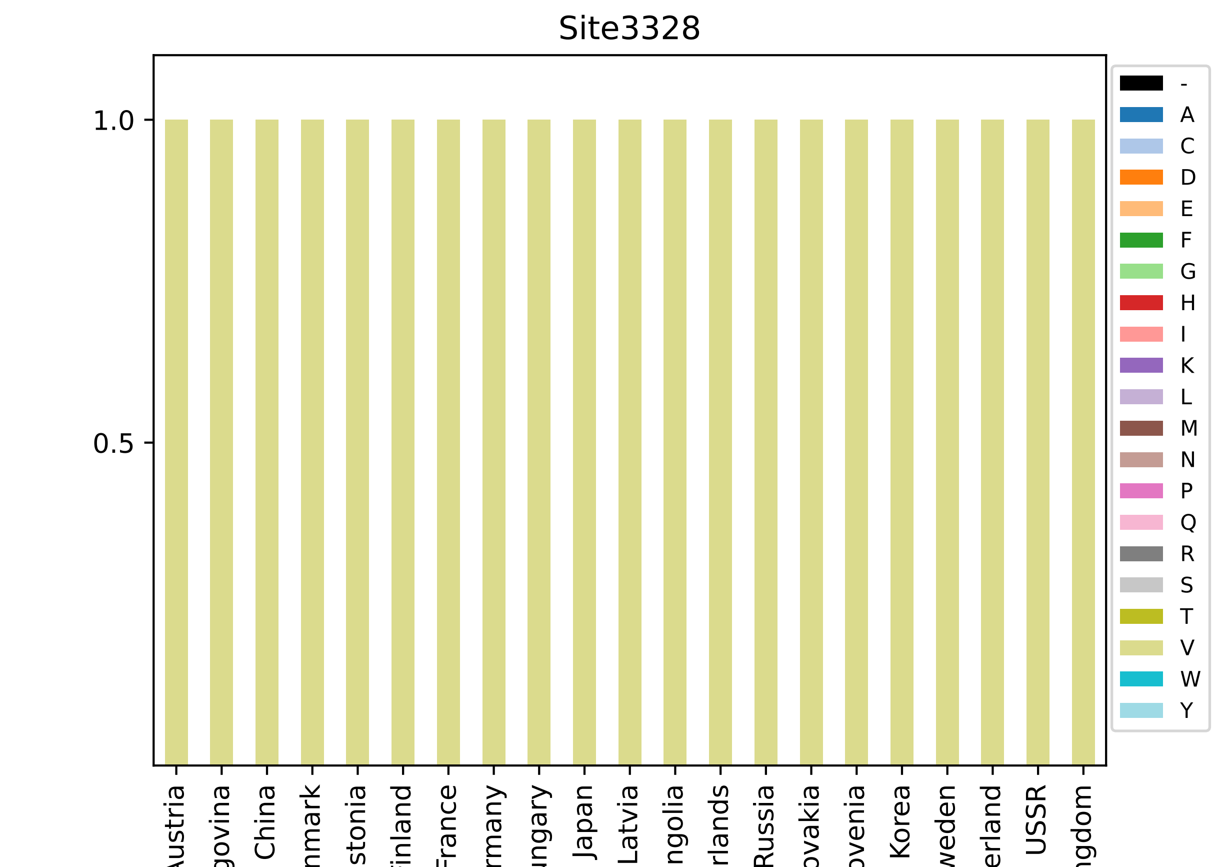
Site3328
1.0
0.5
-
A
C
D
E
F
G
H
I
K
L
M
N
P
Q
R
S
T
V
W
Y
China
Japan
Latvia
Russia
Korea
USSR

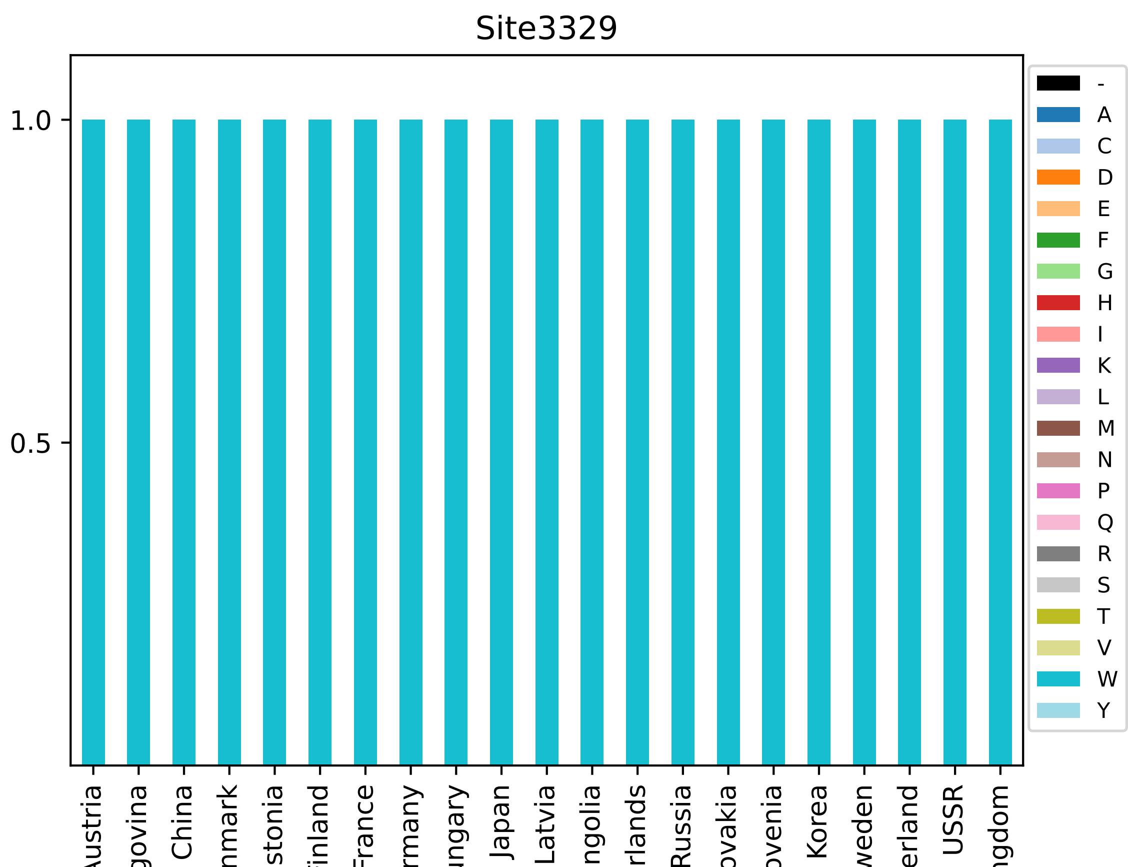
Site3329
1.0
0.5
-
A
C
D
E
F
G
H
I
K
L
M
N
P
Q
R
S
T
V
W
Y
China
Japan
Latvia
Russia
Korea
USSR

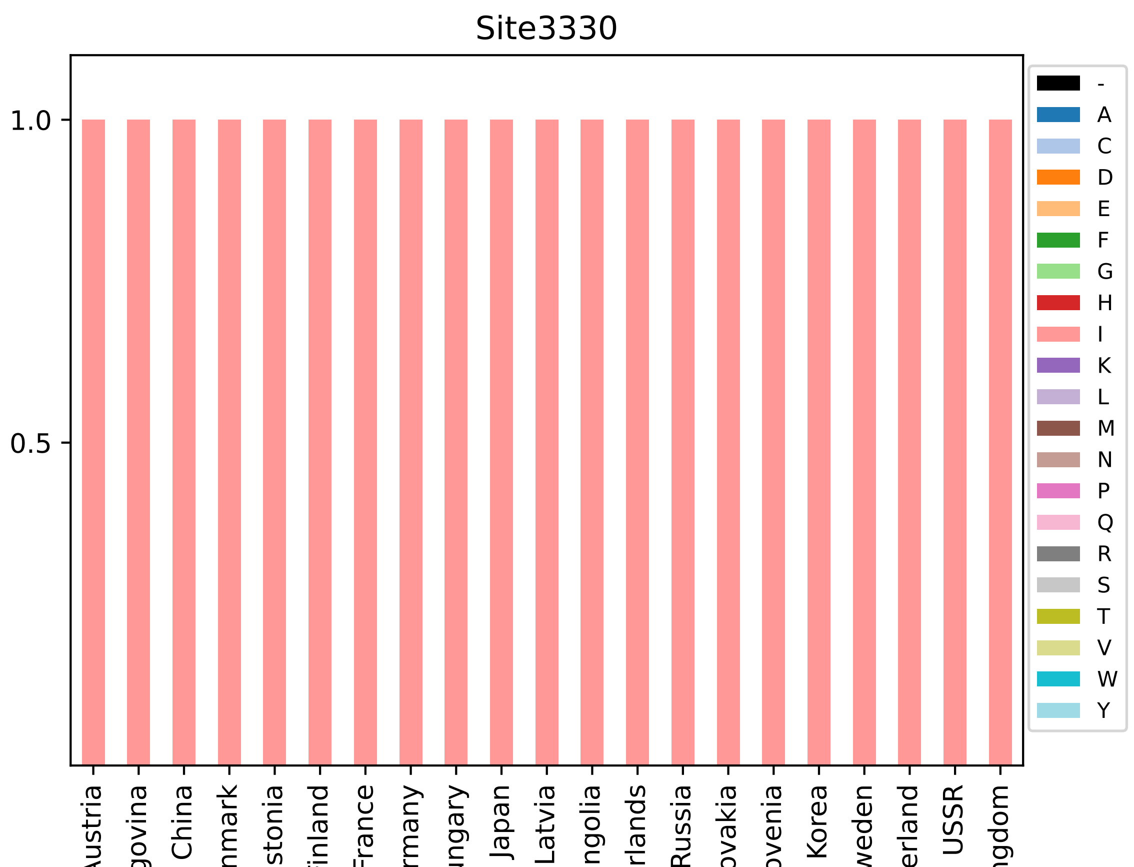

Site3330
1.0
0.5
-
A
C
D
E
F
G
H
I
K
L
M
N
P
Q
R
S
T
V
W
Y
China
Japan
Latvia
Russia
Korea
USSR

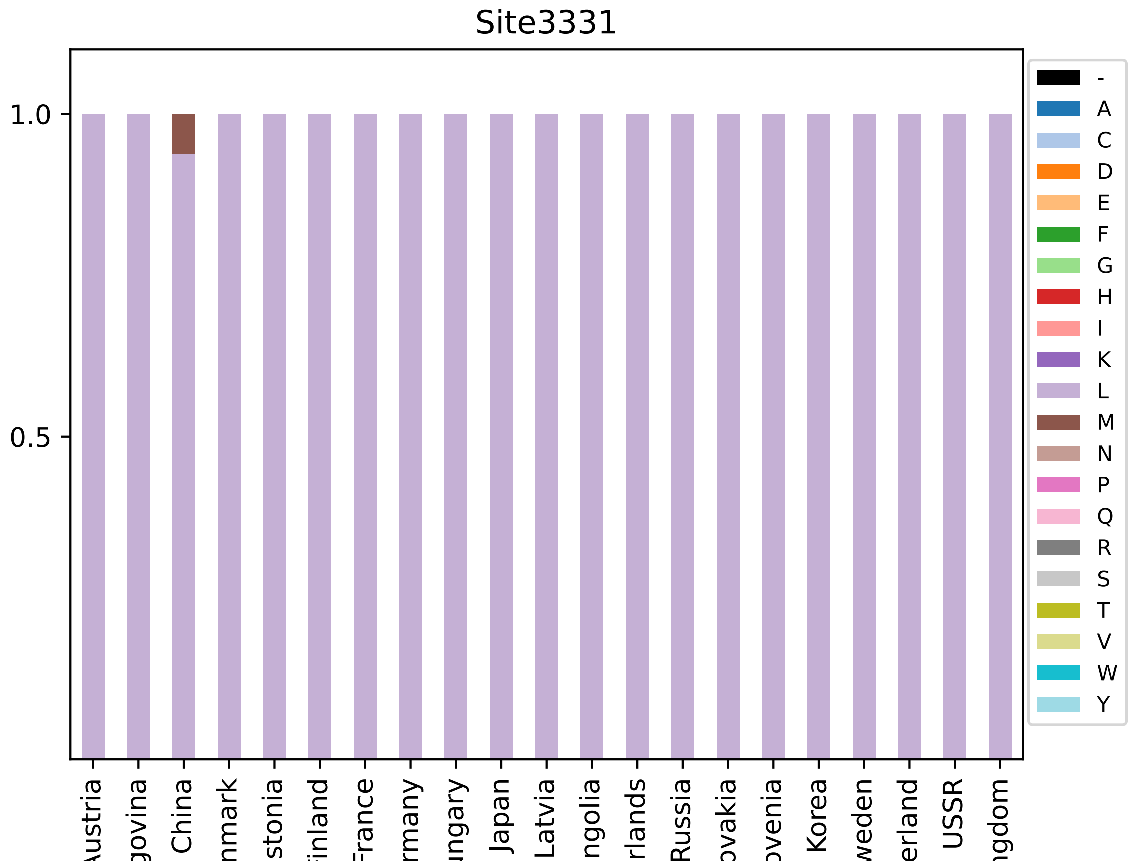

Site3331
1.0
0.5
-
A
C
D
E
F
G
H
I
K
L
M
N
P
Q
R
S
T
V
W
Y
China
Japan
Latvia
Russia
Korea
USSR

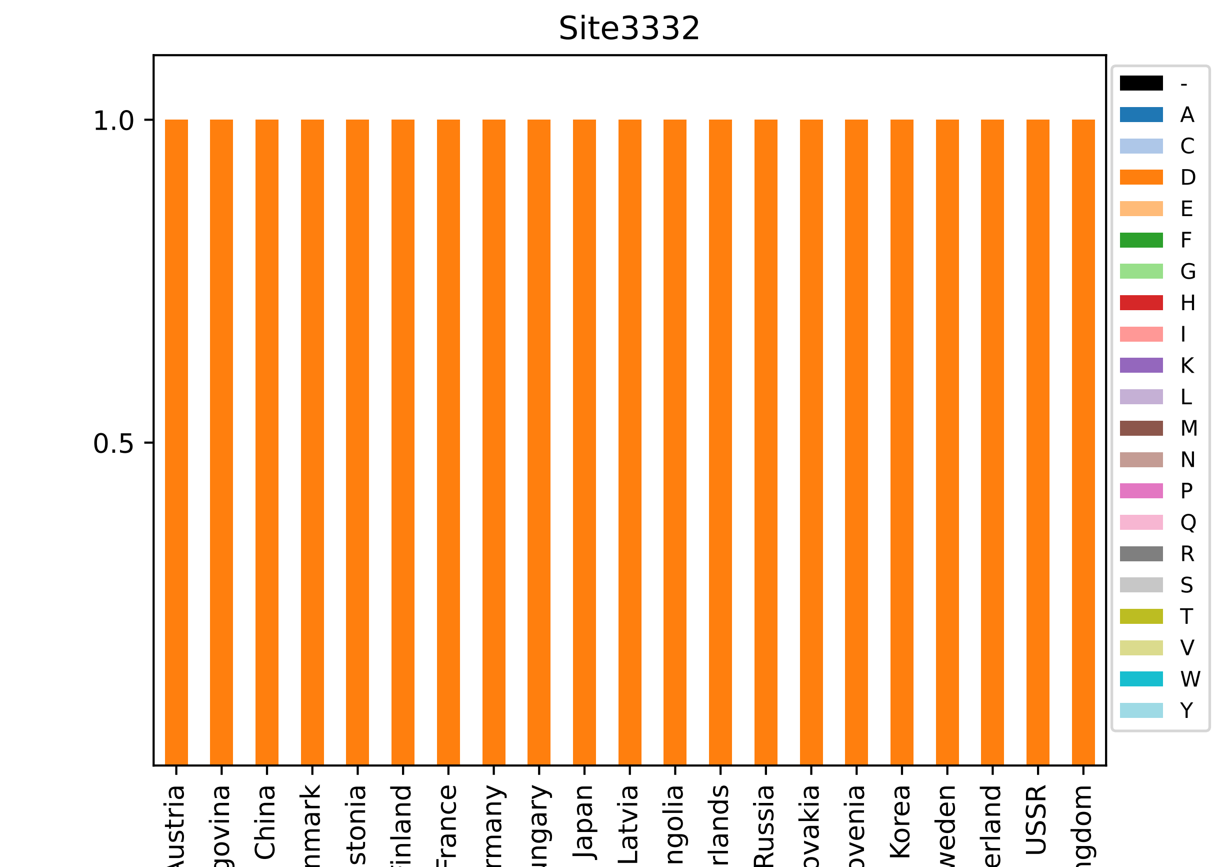
Site3332
1.0
0.5
-
A
C
D
E
F
G
H
I
K
L
M
N
P
Q
R
S
T
V
W
Y
Austria
govina
China
nmark
stonia
inland
France
rmany
ungary
Japan
Latvia
ngolia
rlands
Russia
ovakia
ovenia
Korea
weden
erland
USSR
ngdom

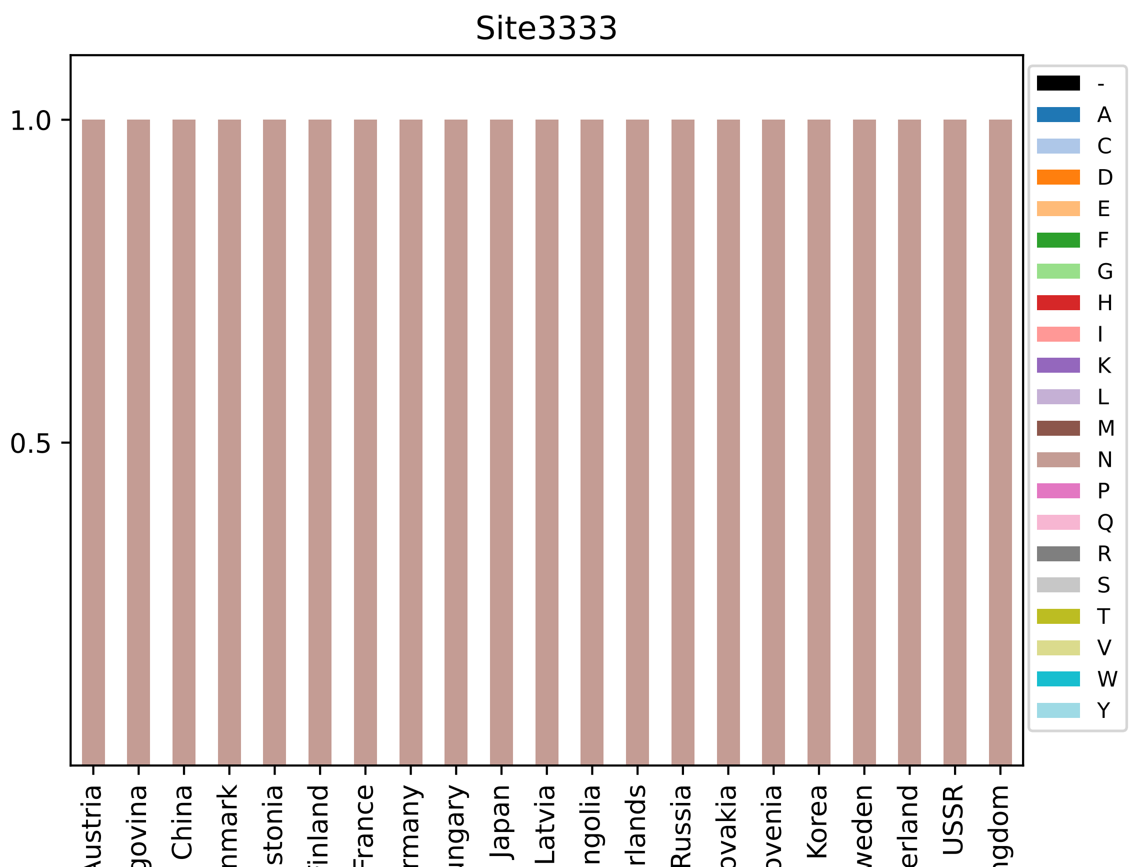

Site3333
1.0
0.5
-
A
C
D
E
F
G
H
I
K
L
M
N
P
Q
R
S
T
V
W
Y
Austria
govina
China
nmark
stonia
Finland
France
rmany
ungary
Japan
Latvia
ngolia
rlands
Russia
ovakia
ovenia
Korea
weden
erland
USSR
ngdom

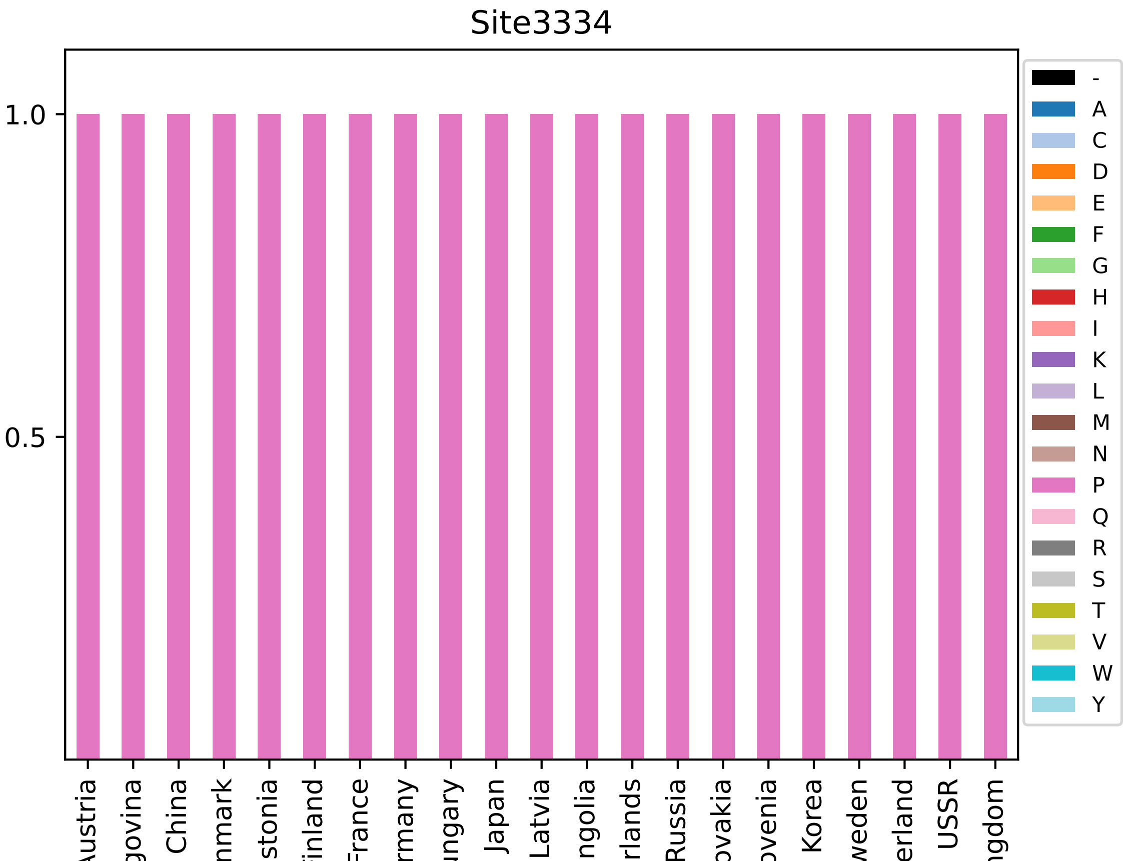
Site3334
1.0
0.5
-
A
C
D
E
F
G
H
I
K
L
M
N
P
Q
R
S
T
V
W
Y
China
Japan
Latvia
Russia
Korea
USSR

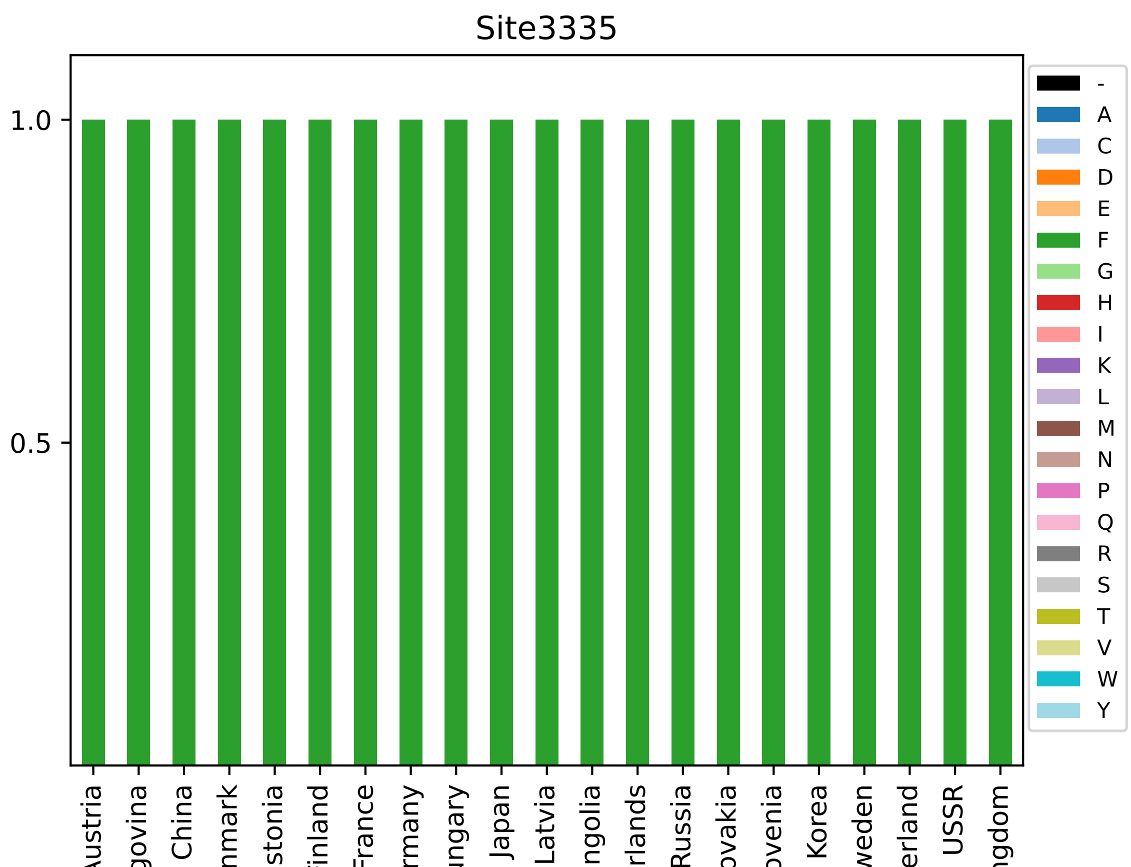

Site3335
1.0
0.5
-
A
C
D
E
F
G
H
I
K
L
M
N
P
Q
R
S
T
V
W
Y
Austria
govina
China
nmark
stonia
Finland
France
rmany
ungary
Japan
Latvia
ngolia
rlands
Russia
ovakia
ovenia
Korea
weden
erland
USSR
ngdom

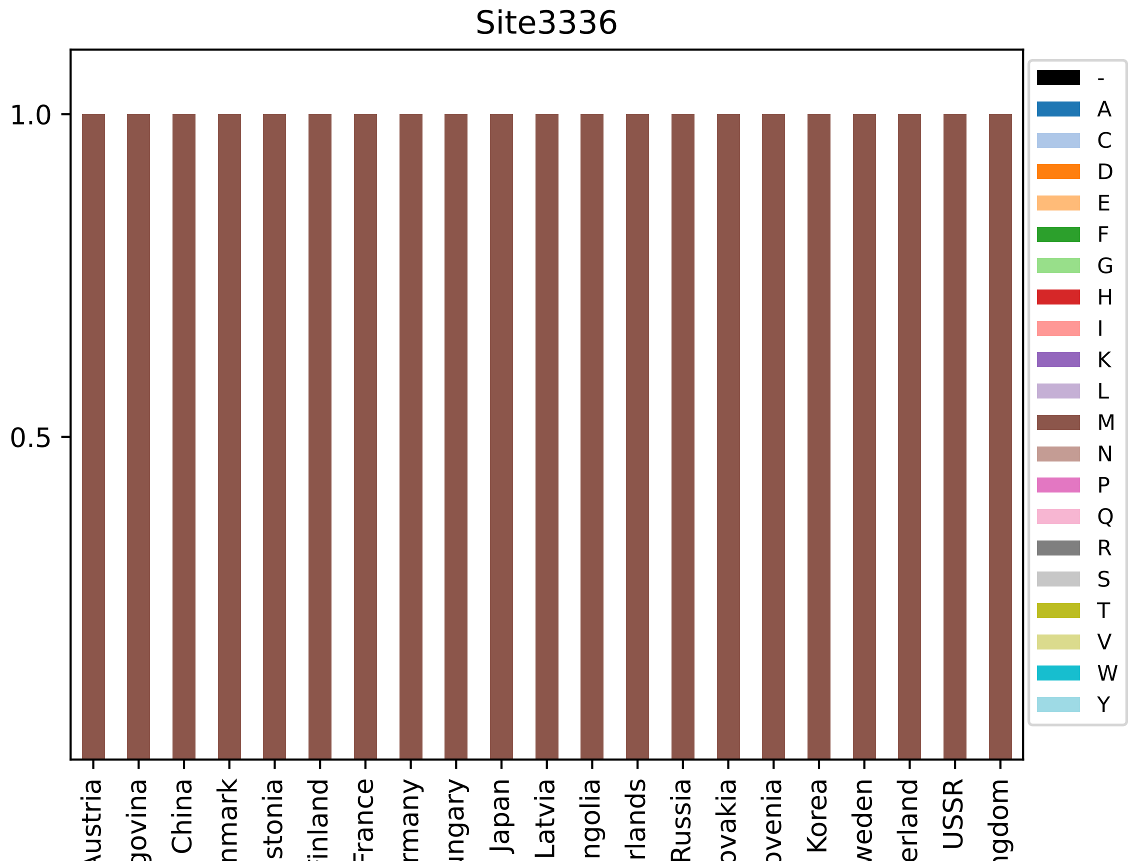
Site3336
1.0
0.5
-
A
C
D
E
F
G
H
I
K
L
M
N
P
Q
R
S
T
V
W
Y
China
Japan
Latvia
Russia
Korea
USSR

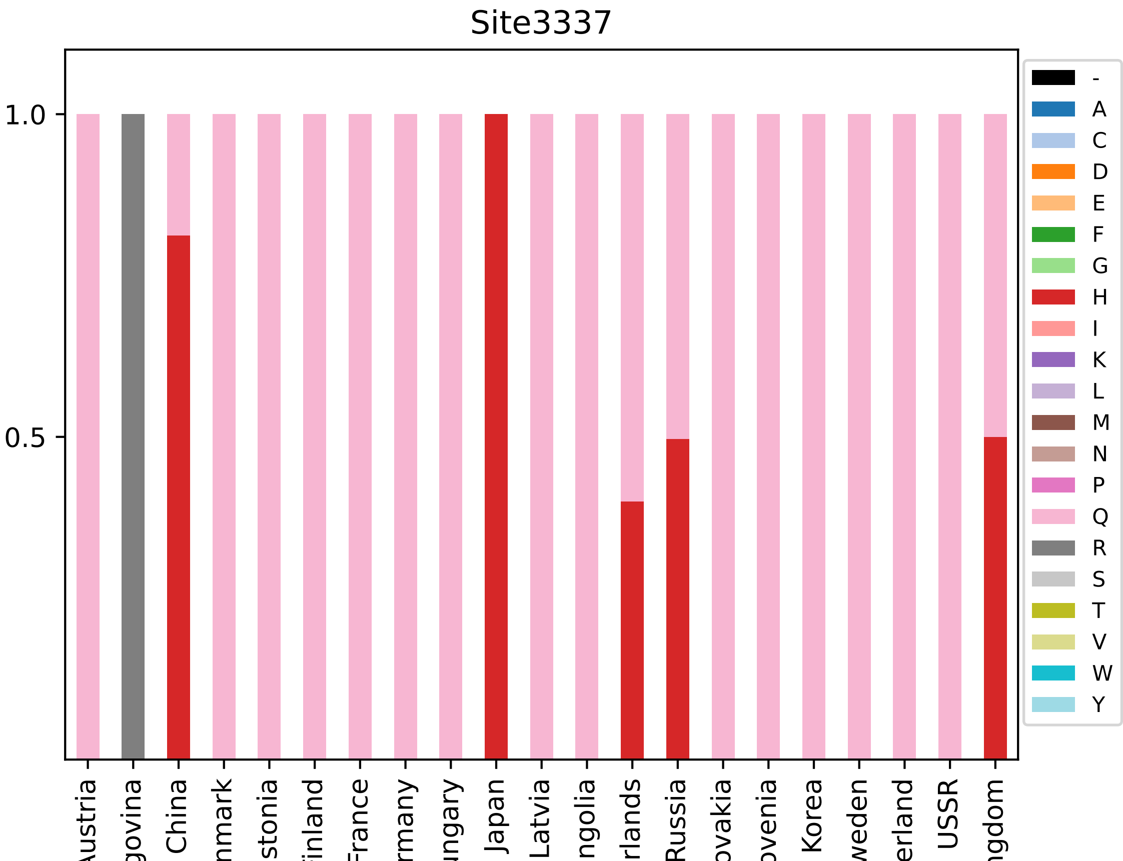
Site3337
1.0
0.5
-
A
C
D
E
F
G
H
I
K
L
M
N
P
Q
R
S
T
V
W
Y
Austria
China
Japan
Latvia
Russia
Korea
USSR

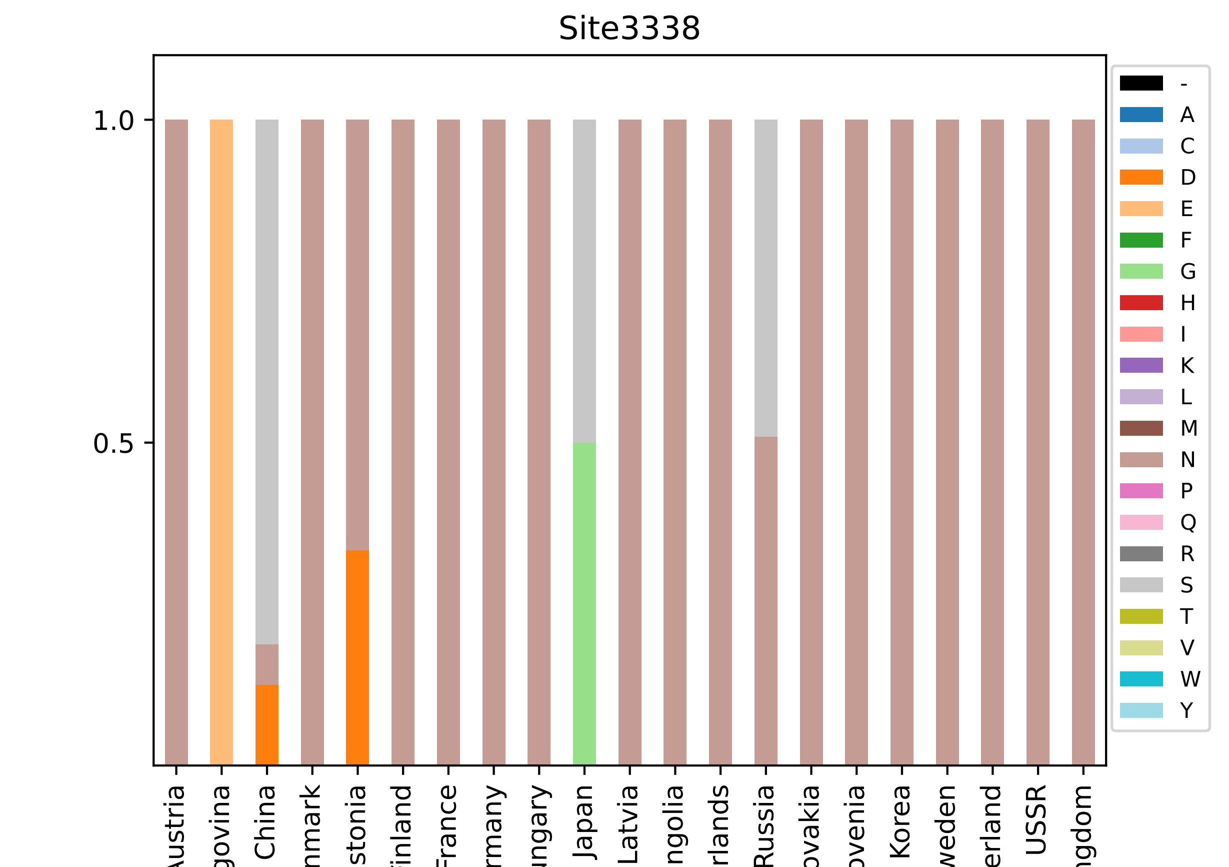

Site3338
1.0
0.5
-
A
C
D
E
F
G
H
I
K
L
M
N
P
Q
R
S
T
V
W
Y
Austria
govina
China
nmark
stonia
Finland
France
rmany
ungary
Japan
Latvia
ngolia
rlands
Russia
ovakia
ovenia
Korea
weden
erland
USSR
ngdom

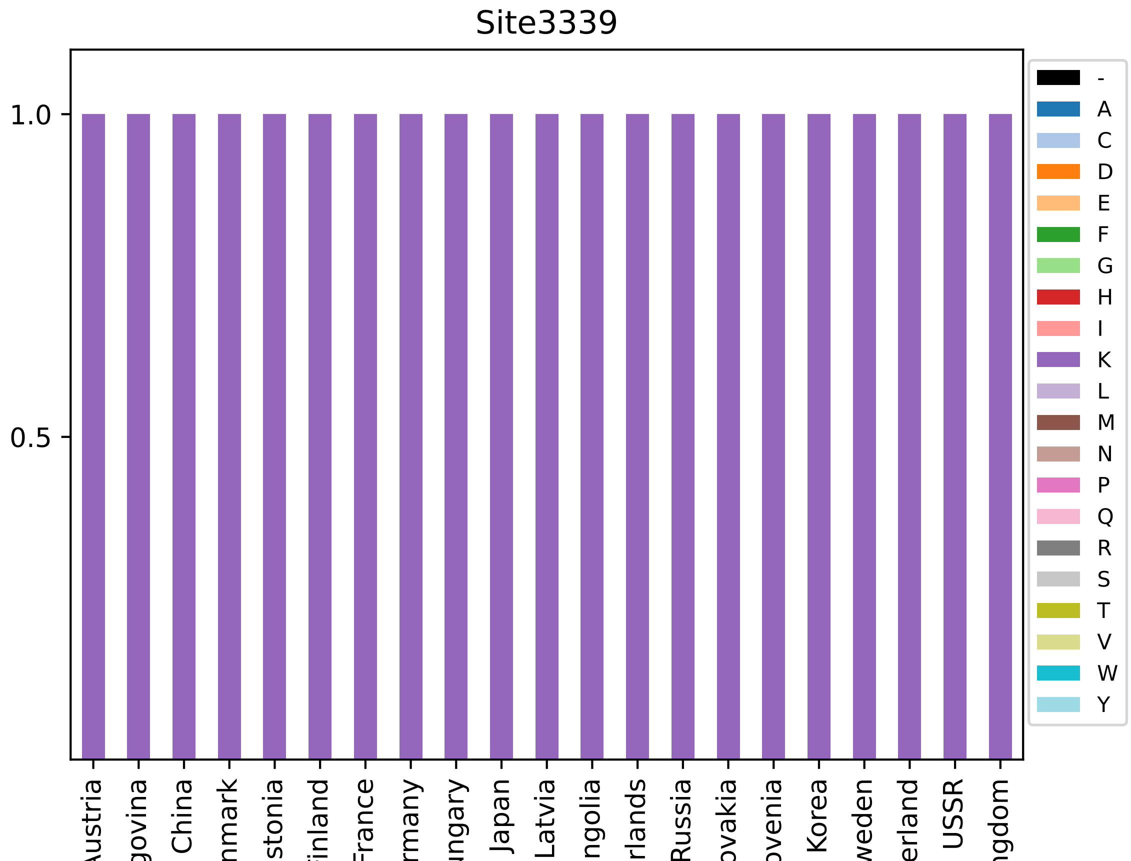

Site3339
1.0
0.5
-
A
C
D
E
F
G
H
I
K
L
M
N
P
Q
R
S
T
V
W
Y
China
Japan
Latvia
Russia
Korea
USSR

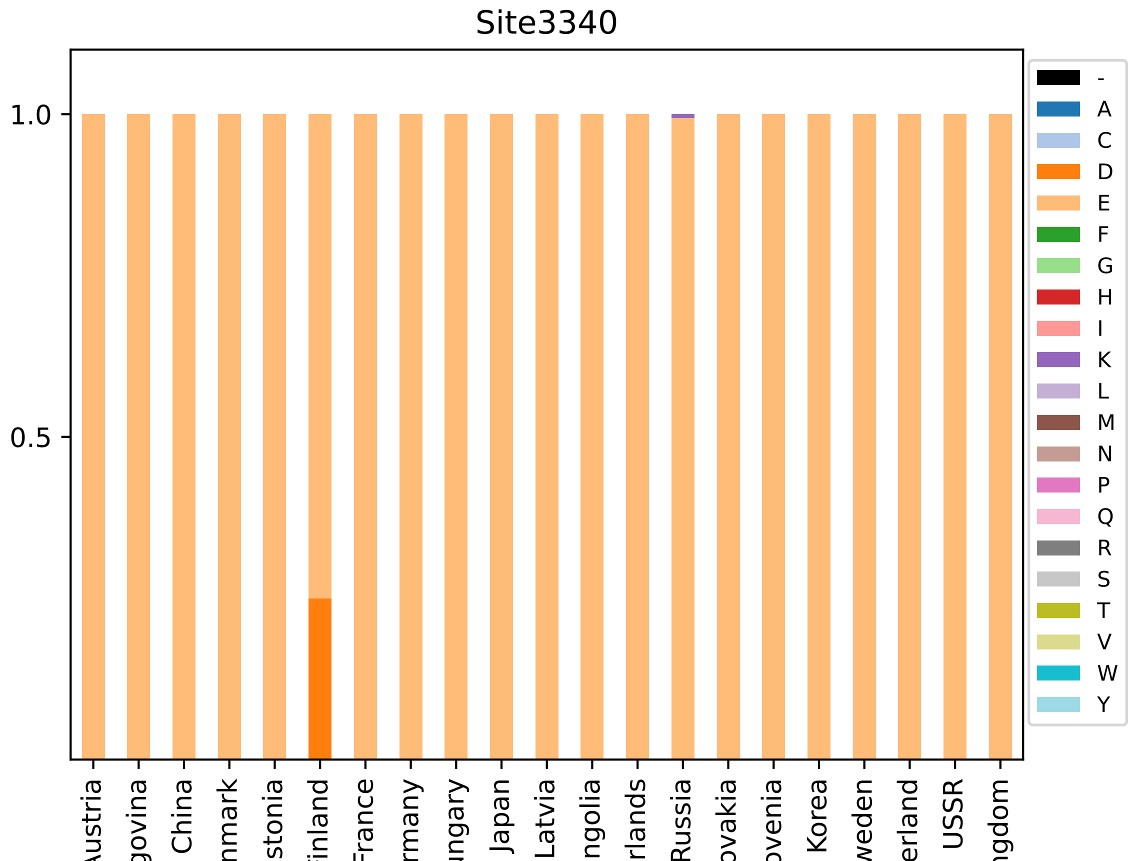
Site3340
1.0
0.5
-
A
C
D
E
F
G
H
I
K
L
M
N
P
Q
R
S
T
V
W
Y
China
Japan
Latvia
Russia
Korea
USSR

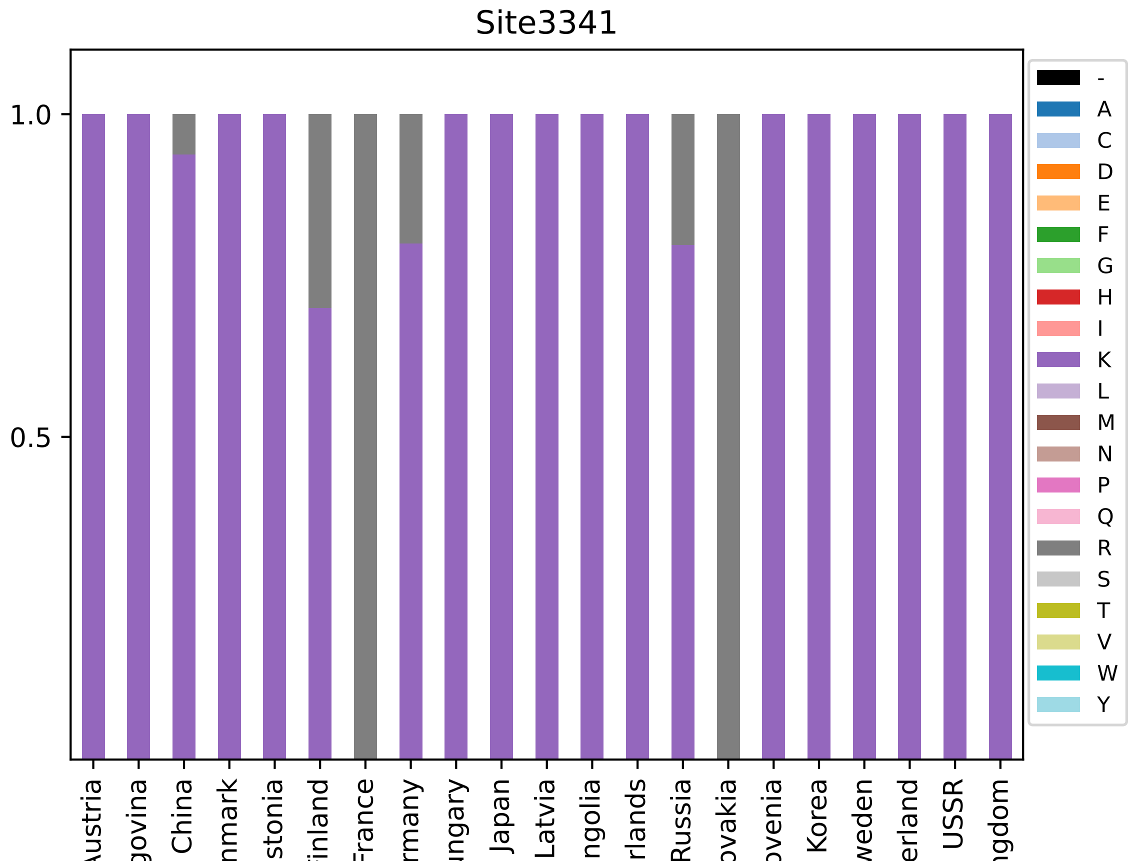

Site3341
1.0
0.5
-
A
C
D
E
F
G
H
I
K
L
M
N
P
Q
R
S
T
V
W
Y
China
Japan
Latvia
Russia
Korea
USSR

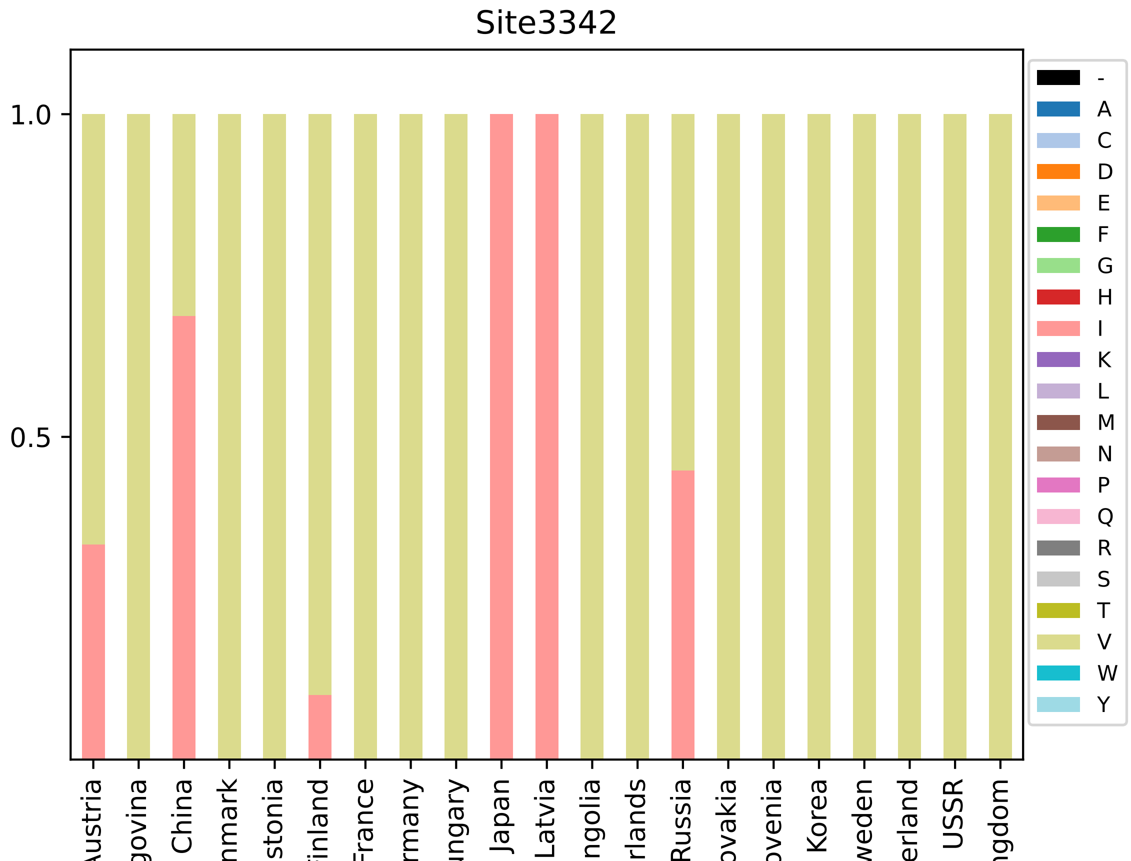
Site3342
1.0
0.5
-
A
C
D
E
F
G
H
I
K
L
M
N
P
Q
R
S
T
V
W
Y
Austria
govina
China
nmark
stonia
Finland
France
rmany
ungary
Japan
Latvia
ngolia
rlands
Russia
ovakia
ovenia
Korea
weden
erland
USSR
ngdom

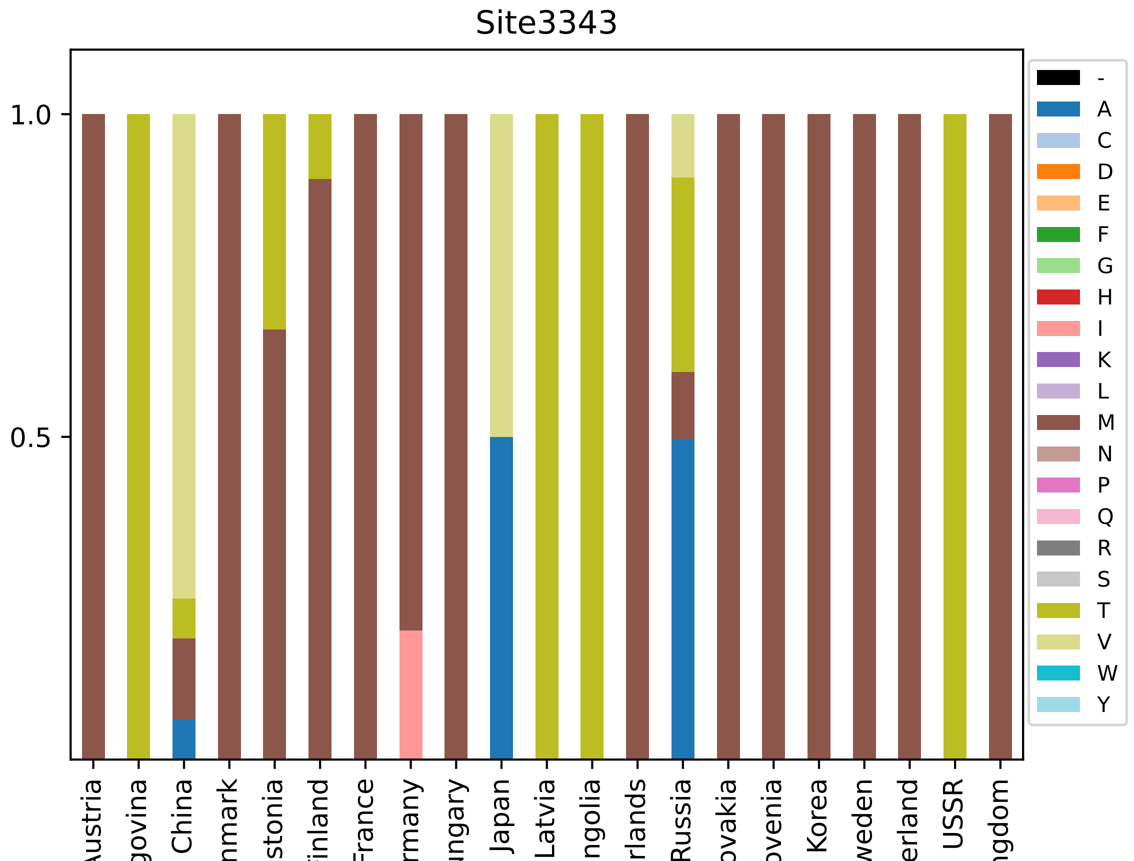

Site3343
1.0
0.5
-
A
C
D
E
F
G
H
I
K
L
M
N
P
Q
R
S
T
V
W
Y
China
Finland
France
Japan
Latvia
Russia
Korea
USSR

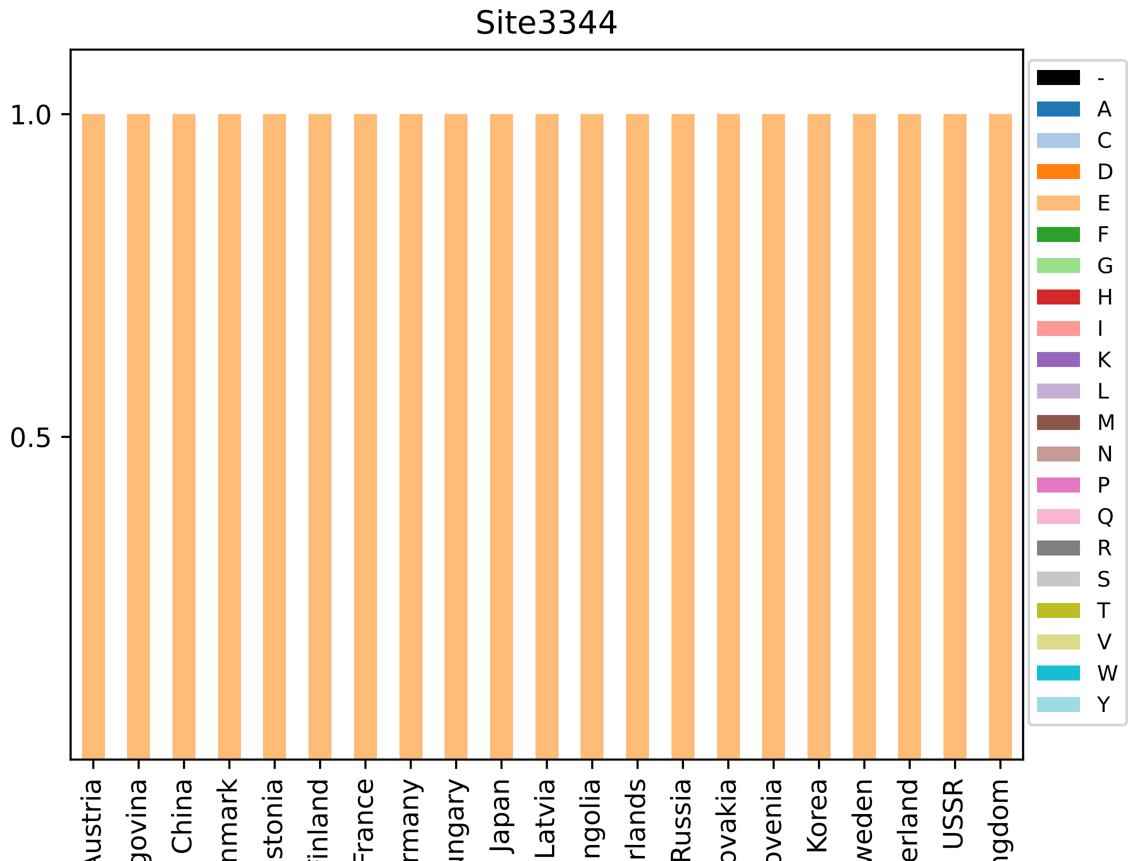
Site3344
1.0
0.5
-
A
C
D
E
F
G
H
I
K
L
M
N
P
Q
R
S
T
V
W
Y
China
Japan
Latvia
Russia
Korea
USSR

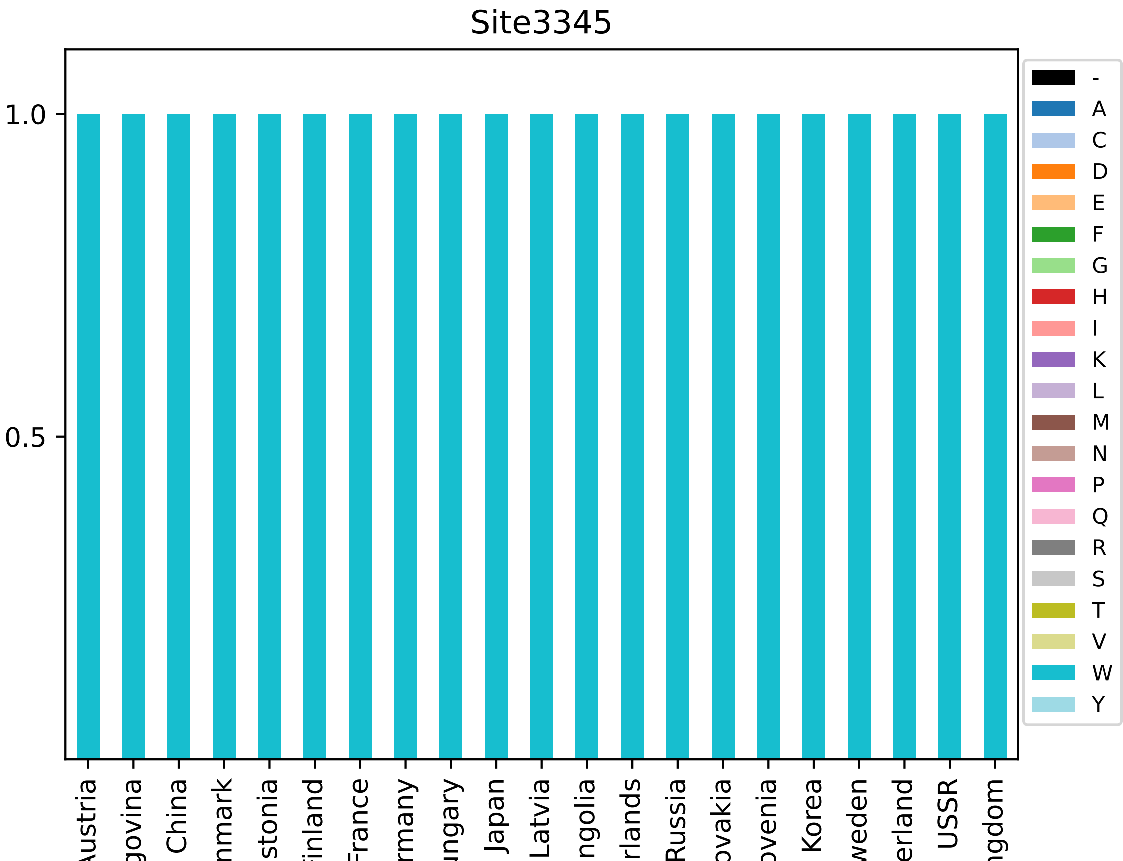

Site3345
1.0
0.5
-
A
C
D
E
F
G
H
I
K
L
M
N
P
Q
R
S
T
V
W
Y
China
Japan
Latvia
Russia
Korea
USSR

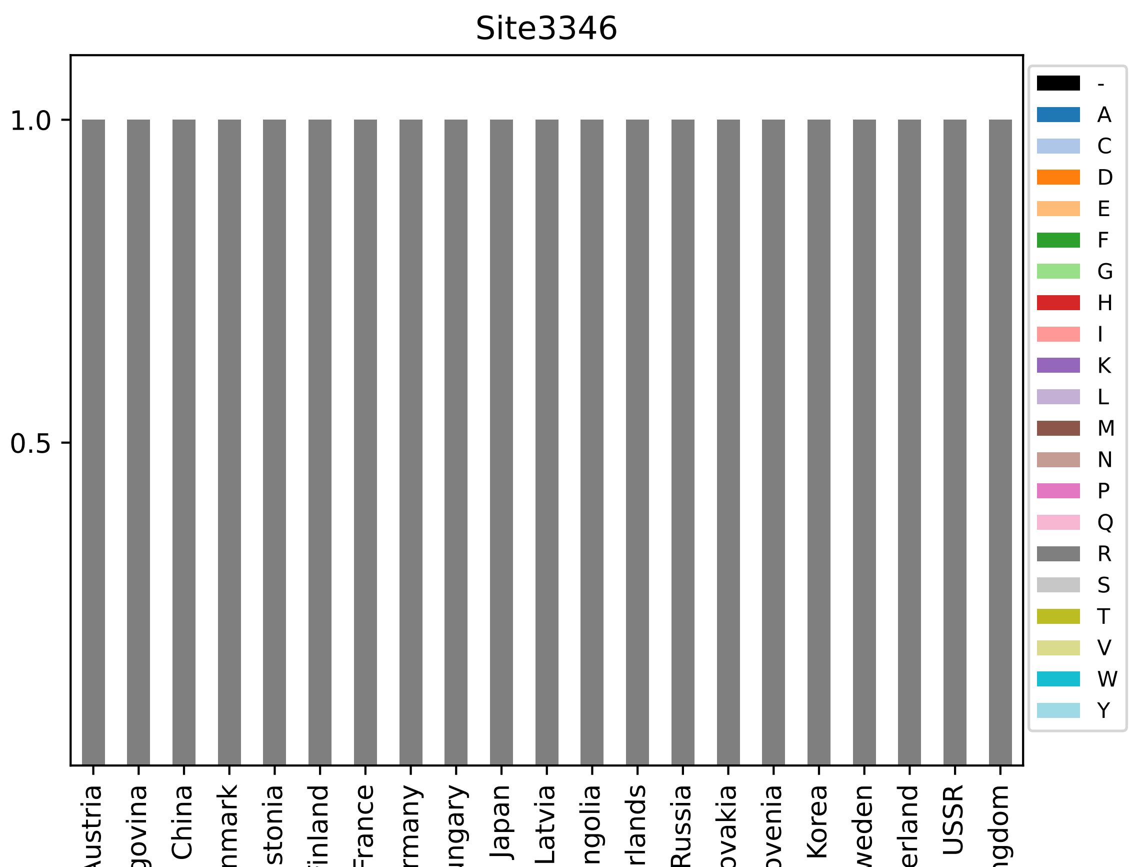
Site3346
1.0
0.5
-
A
C
D
E
F
G
H
I
K
L
M
N
P
Q
R
S
T
V
W
Y
China
Japan
Latvia
Russia
Korea
USSR

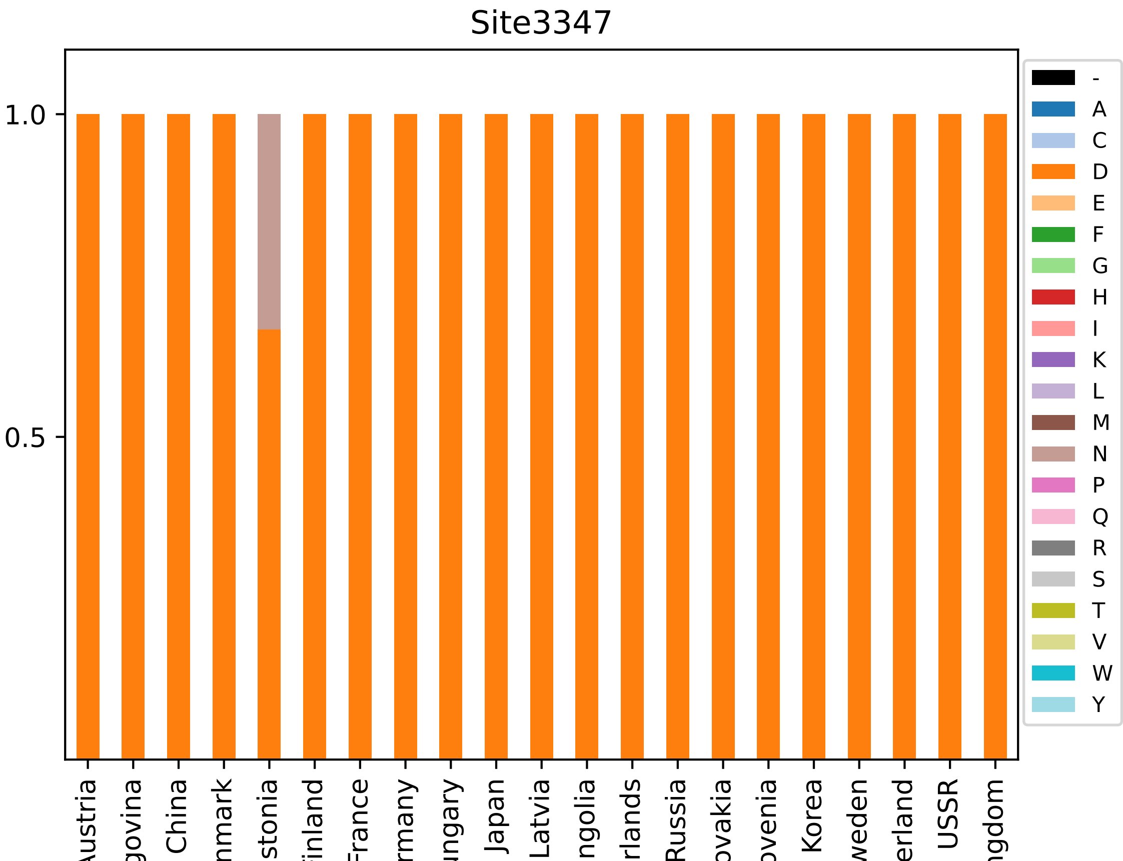
Site3347
1.0
0.5
-
A
C
D
E
F
G
H
I
K
L
M
N
P
Q
R
S
T
V
W
Y
Austria
govina
China
nmark
stonia
Finland
France
rmany
ungary
Japan
Latvia
ngolia
rlands
Russia
ovakia
ovenia
Korea
weden
erland
USSR
ngdom

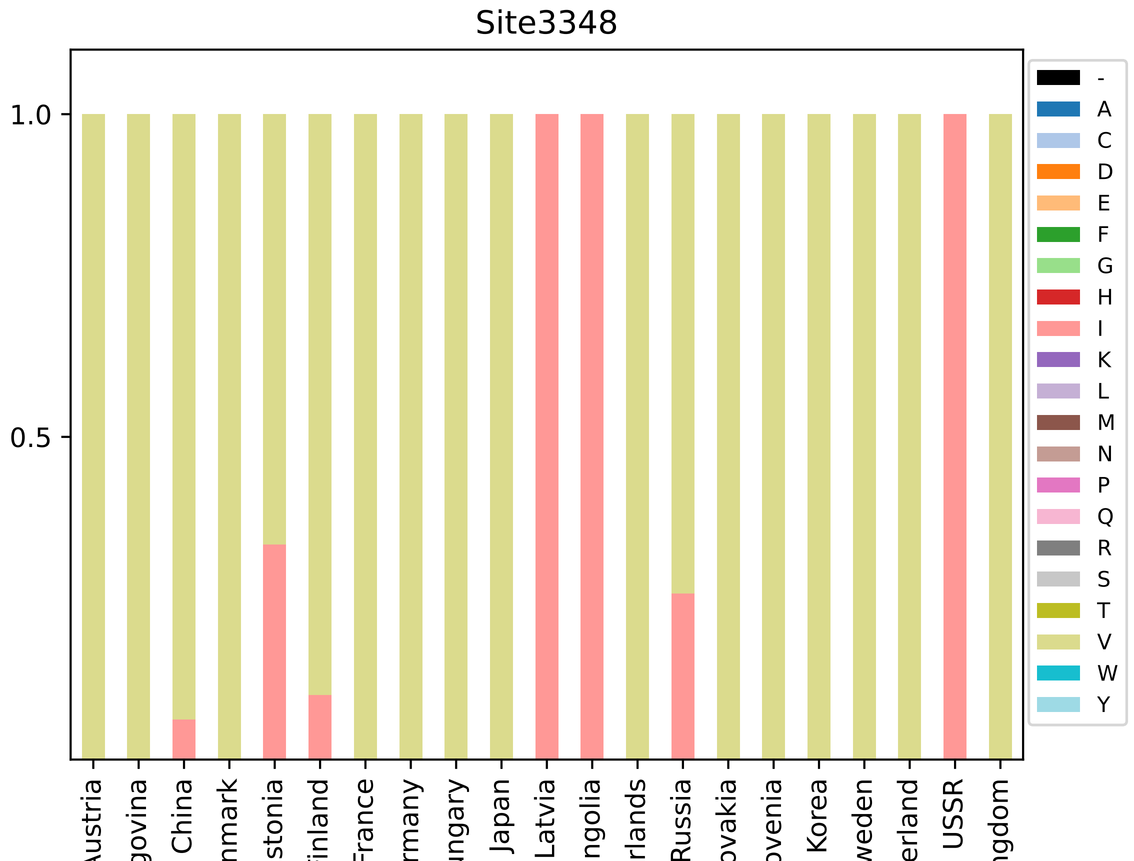

Site3348
1.0
0.5
China
Japan
Latvia
Russia
Korea
USSR
-
A
C
D
E
F
G
H
I
K
L
M
N
P
Q
R
S
T
V
W
Y

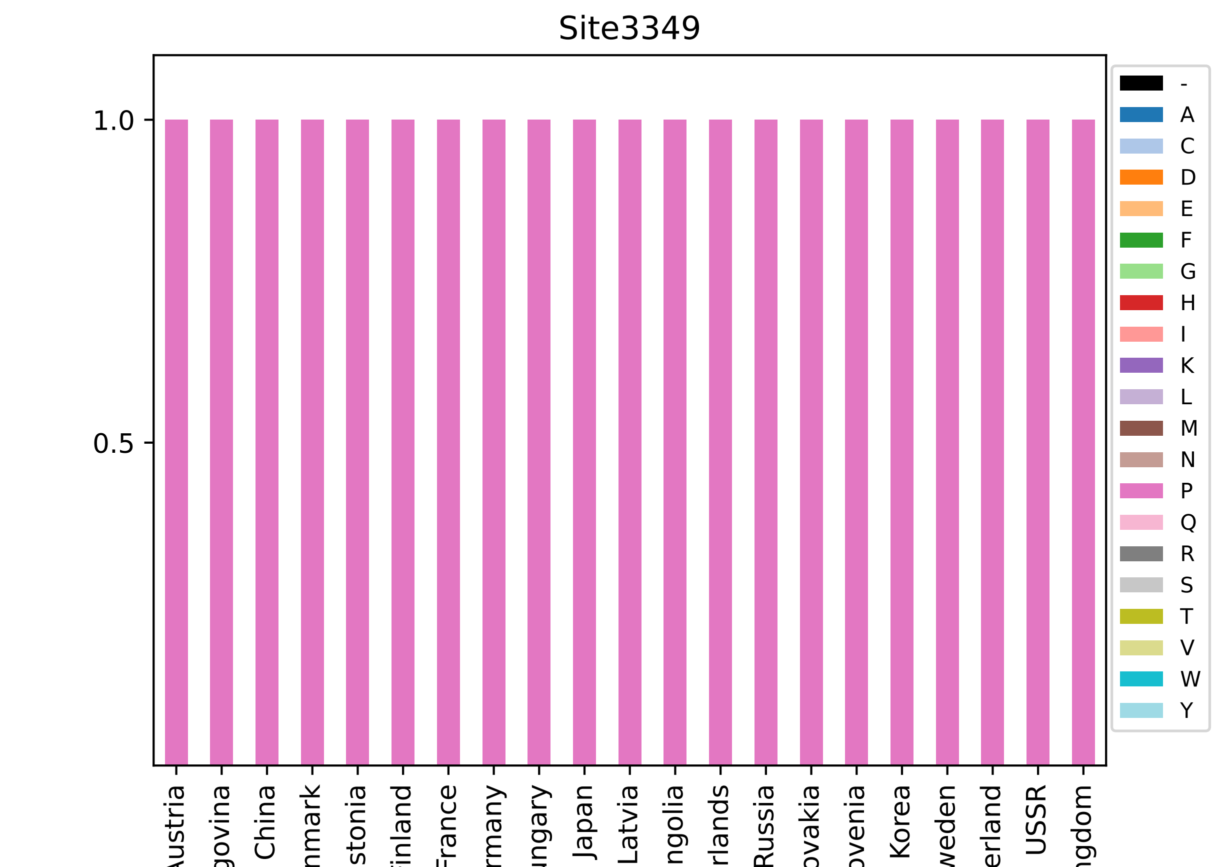
Site3349
1.0
0.5
-
A
C
D
E
F
G
H
I
K
L
M
N
P
Q
R
S
T
V
W
Y
Austria
govina
China
nmark
stonia
Finland
France
rmany
ungary
Japan
Latvia
ngolia
rlands
Russia
ovakia
ovenia
Korea
weden
erland
USSR
ngdom

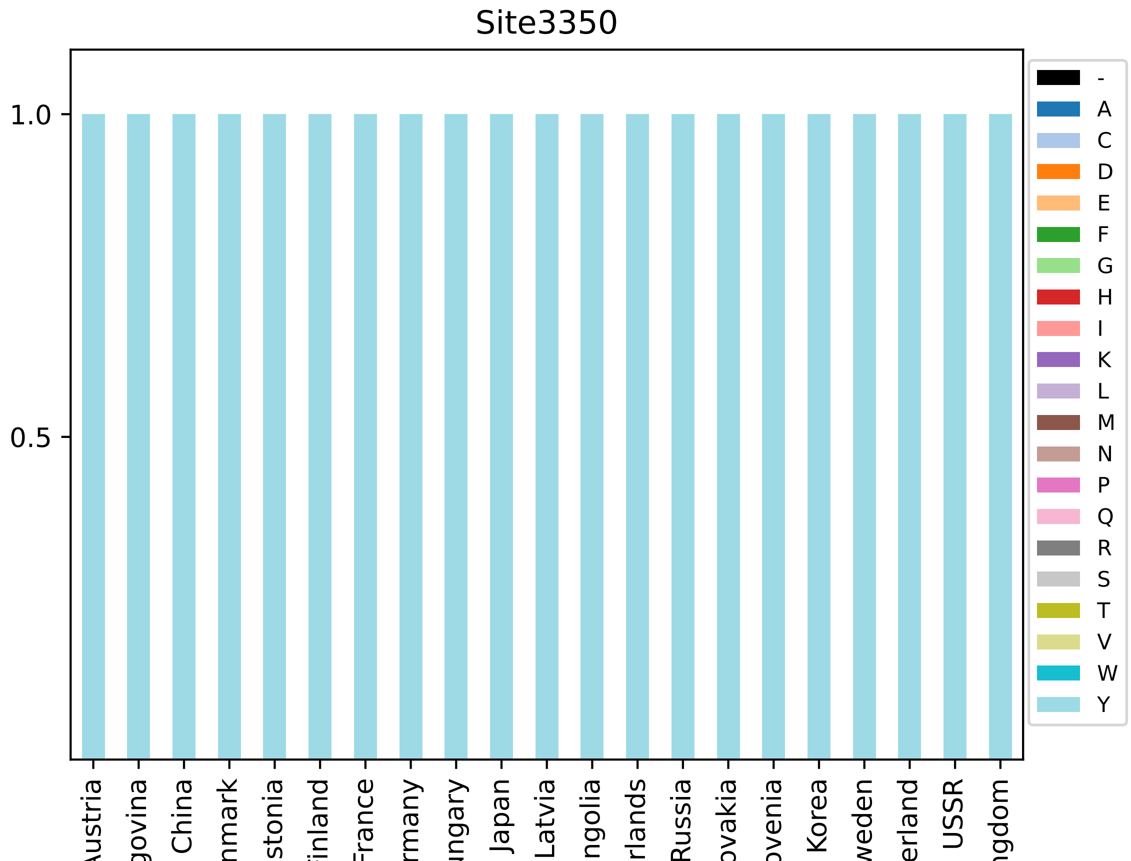

Site3350
1.0
0.5
-
A
C
D
E
F
G
H
I
K
L
M
N
P
Q
R
S
T
V
W
Y
Austria
govina
China
nmark
stonia
inland
France
rmany
ungary
Japan
Latvia
ngolia
rlands
Russia
ovakia
ovenia
Korea
weden
erland
USSR
ngdom

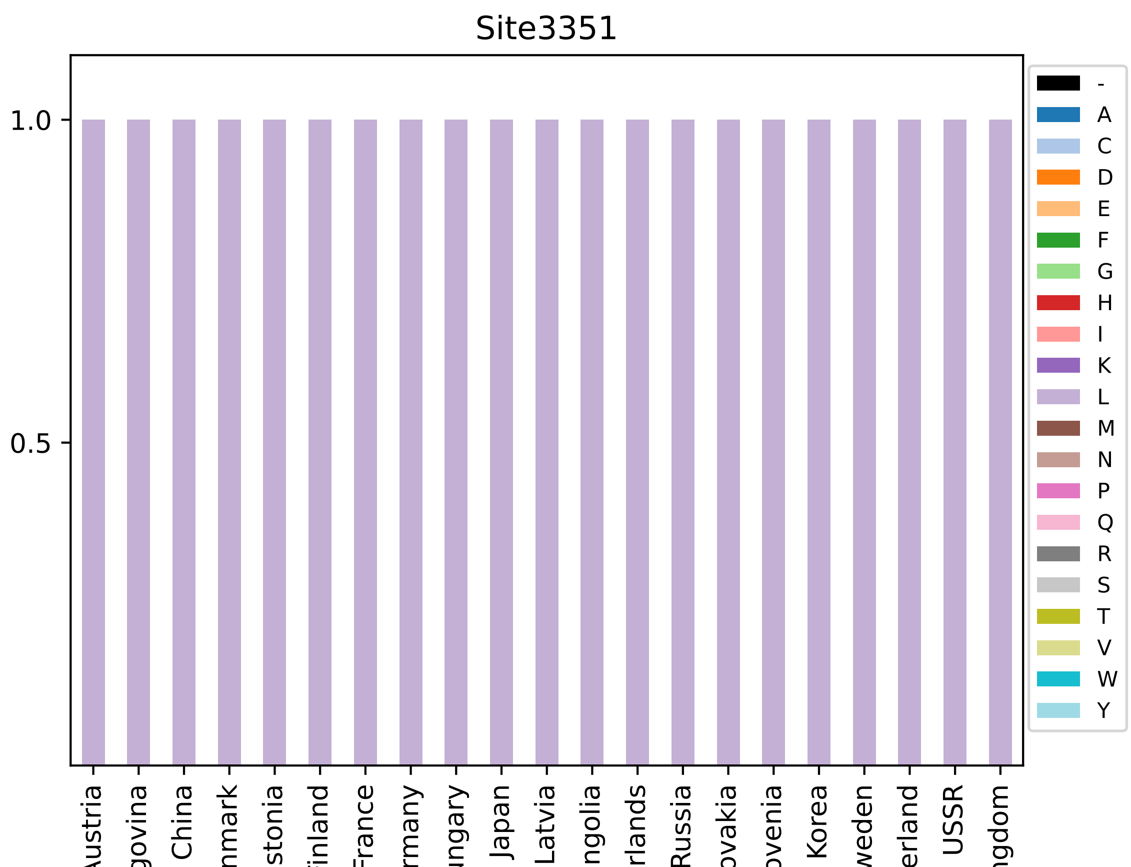

Site3351
1.0
0.5
-
A
C
D
E
F
G
H
I
K
L
M
N
P
Q
R
S
T
V
W
Y
China
Japan
Latvia
Russia
Korea
USSR

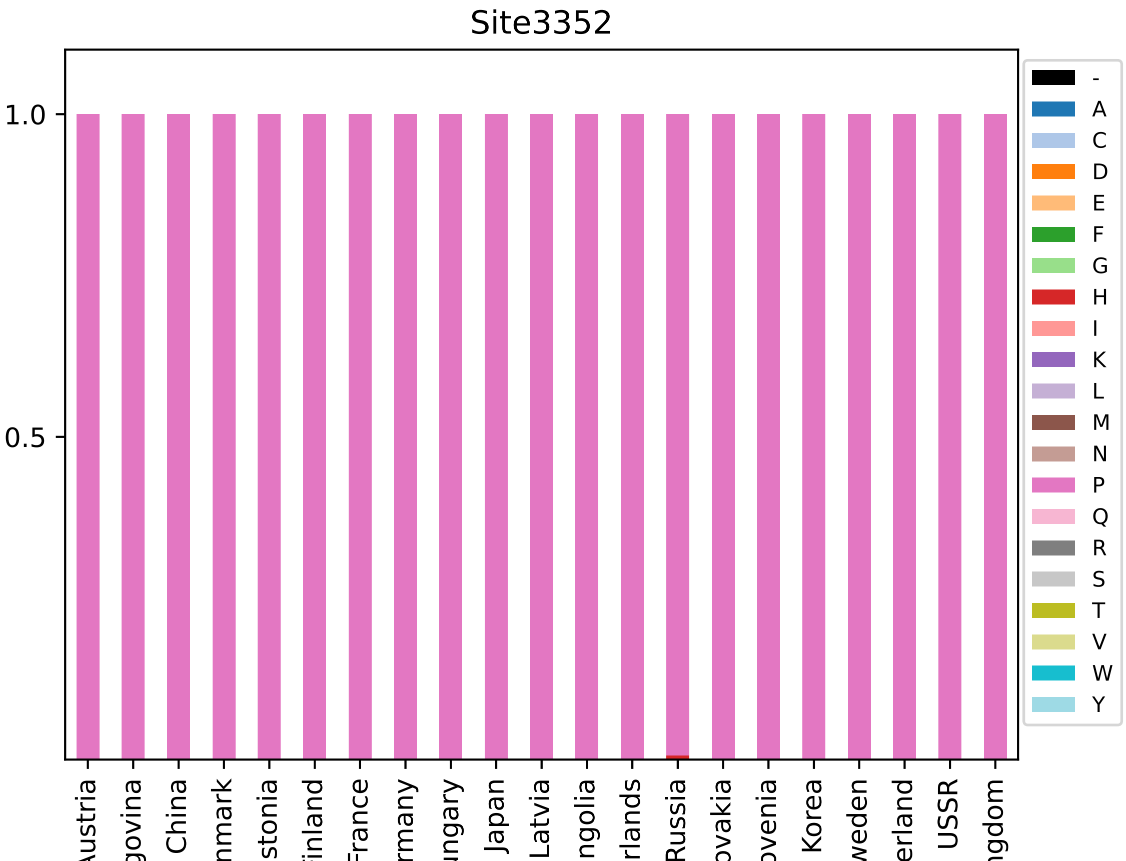

Site3352
1.0
0.5
-
A
C
D
E
F
G
H
I
K
L
M
N
P
Q
R
S
T
V
W
Y
Austria
China
Estonia
Finland
France
Japan
Latvia
Russia
Korea
USSR

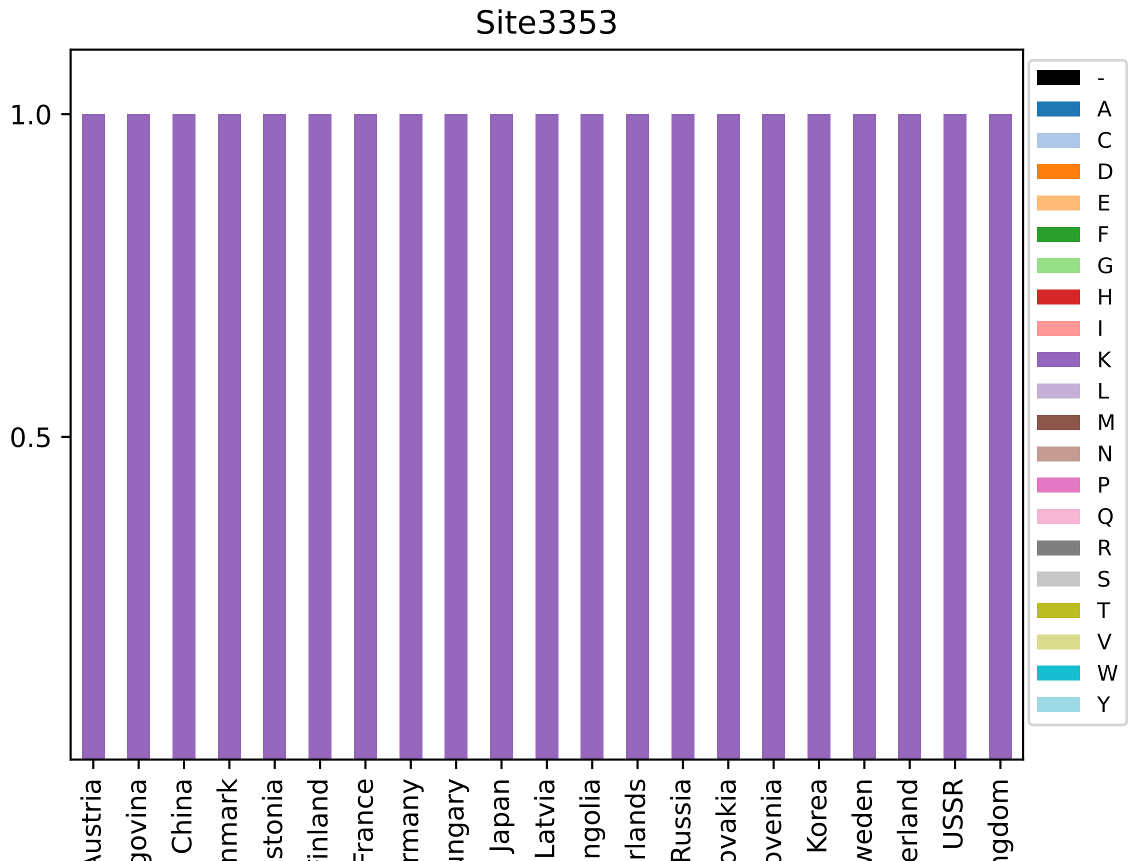
Site3353
1.0
0.5
-
A
C
D
E
F
G
H
I
K
L
M
N
P
Q
R
S
T
V
W
Y
Austria
China
Japan
Latvia
Russia
Korea
USSR

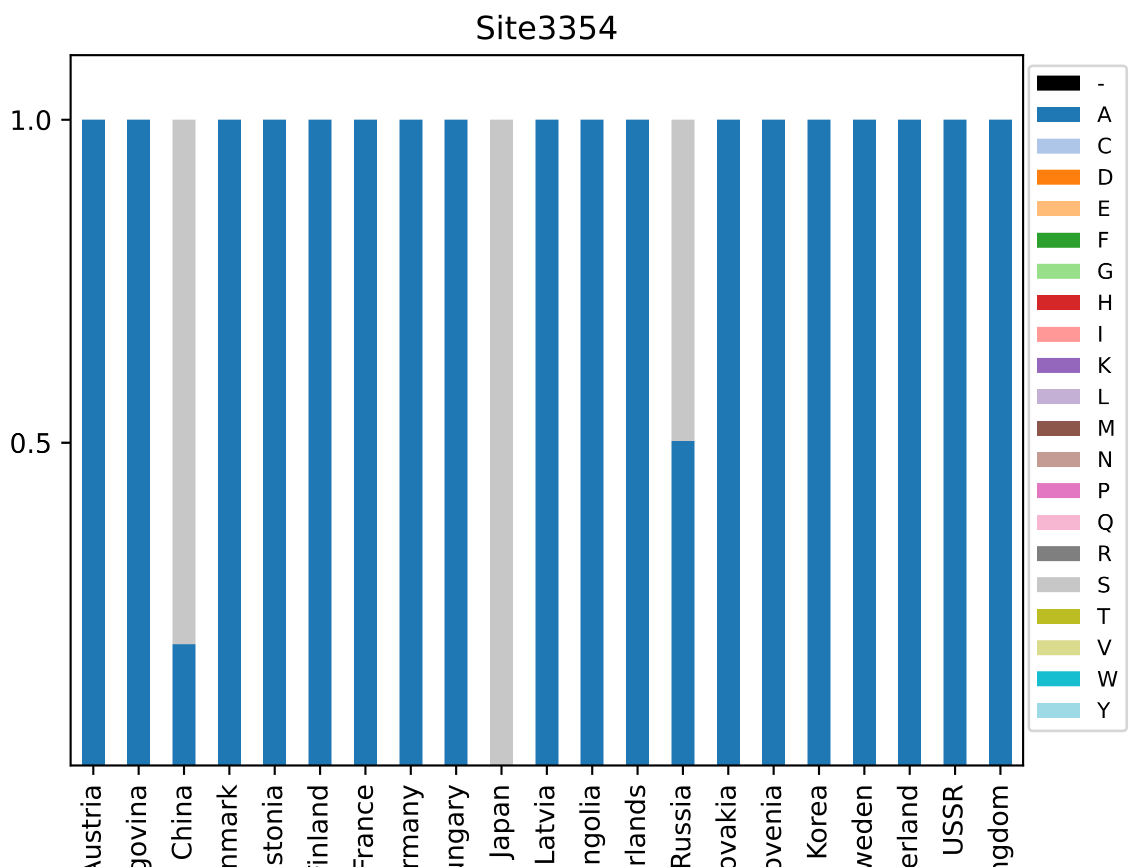
Site3354
1.0
0.5
-
A
C
D
E
F
G
H
I
K
L
M
N
P
Q
R
S
T
V
W
Y
China
France
Japan
Latvia
Russia
Korea
USSR

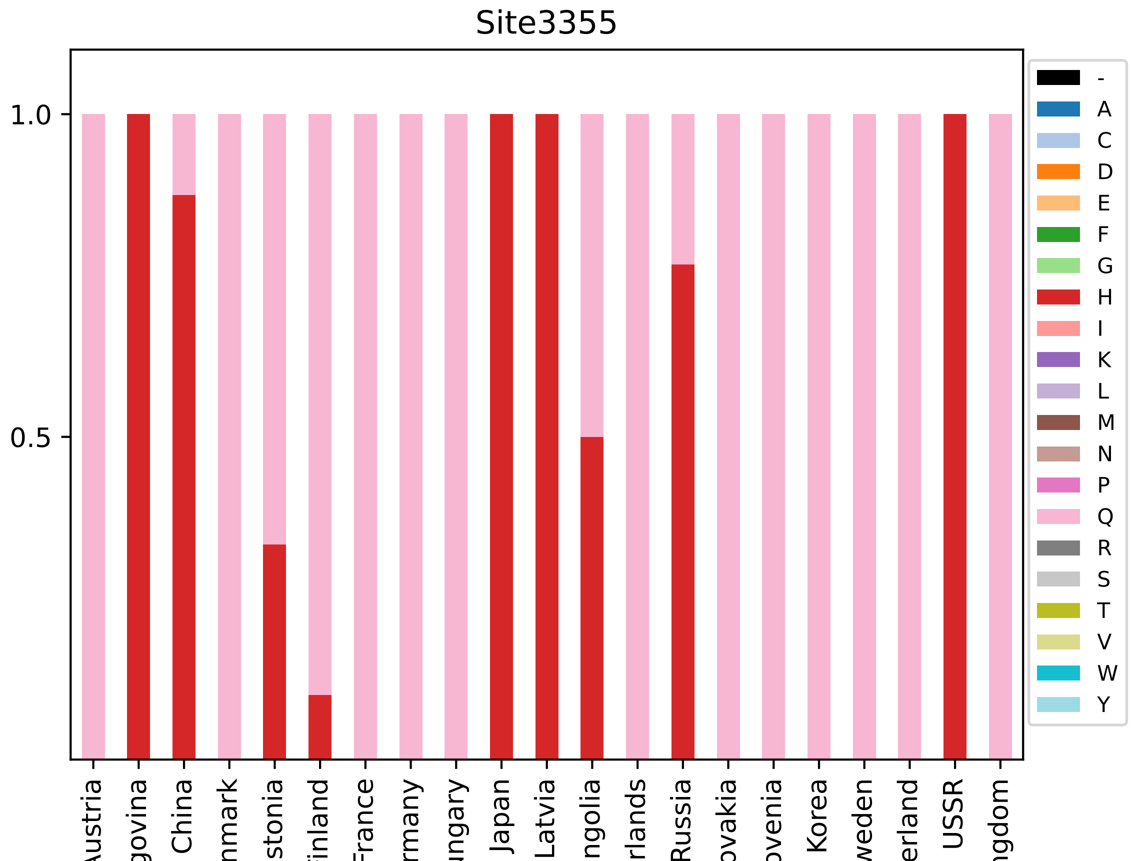

Site3355
1.0
0.5
-
A
C
D
E
F
G
H
I
K
L
M
N
P
Q
R
S
T
V
W
Y
China
Japan
Latvia
Russia
Korea
USSR

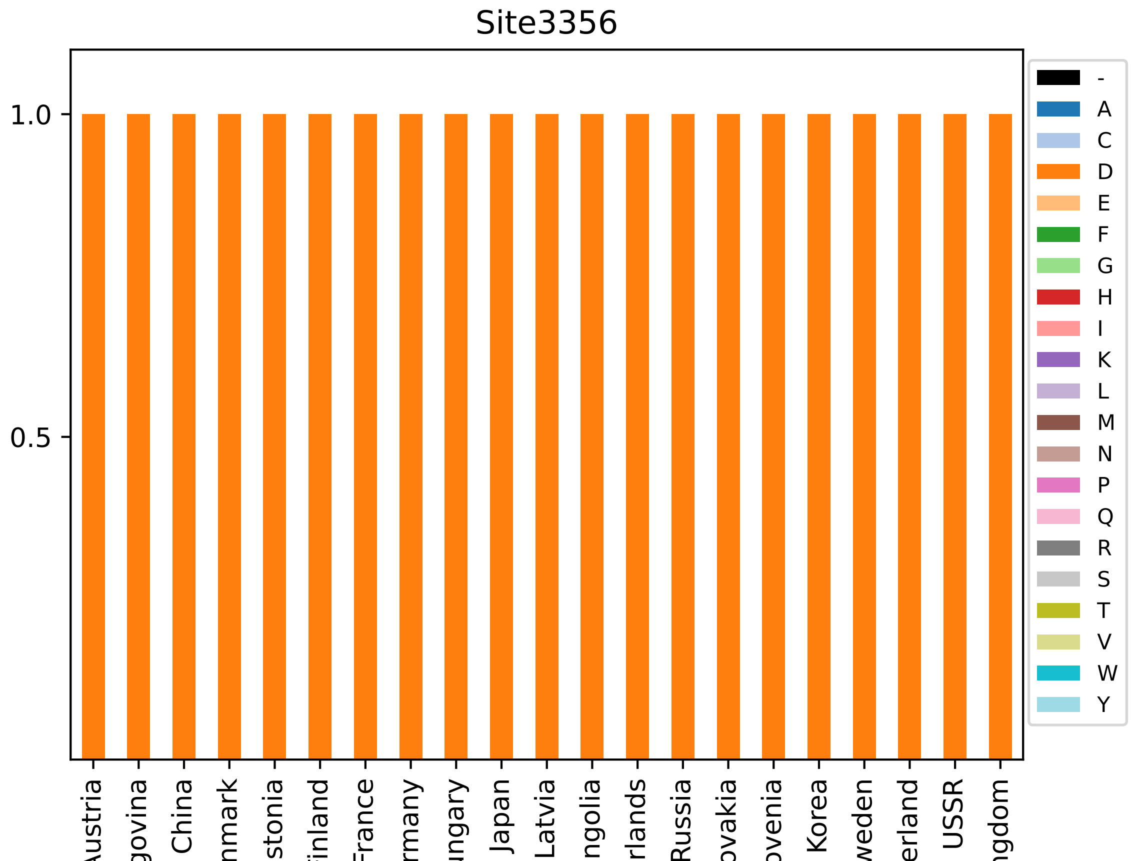

Site3356
1.0
0.5
-
A
C
D
E
F
G
H
I
K
L
M
N
P
Q
R
S
T
V
W
Y
Austria
govina
China
nmark
stonia
Finland
France
rmany
ungary
Japan
Latvia
ngolia
rlands
Russia
ovakia
ovenia
Korea
weden
erland
USSR
ngdom

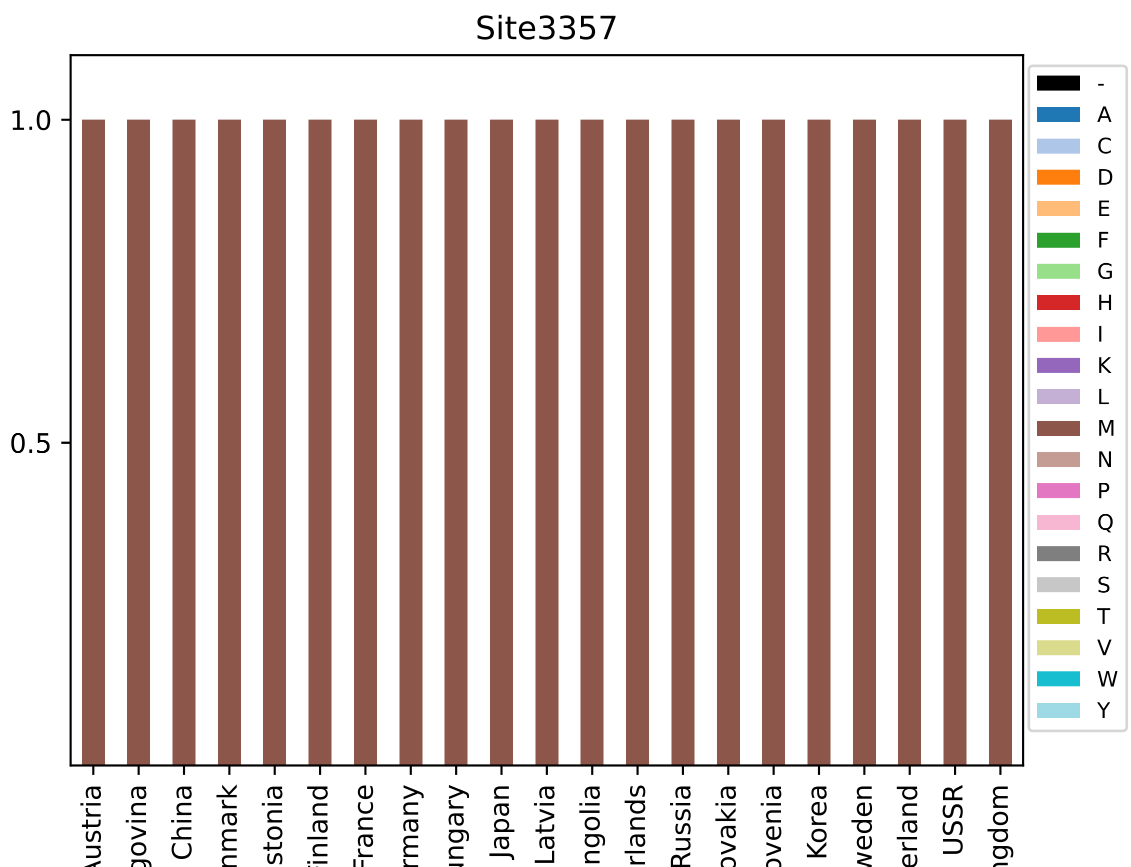

Site3357
1.0
0.5
-
A
C
D
E
F
G
H
I
K
L
M
N
P
Q
R
S
T
V
W
Y
Austria
govina
China
nmark
stonia
inland
France
rmany
ungary
Japan
Latvia
ngolia
rlands
Russia
ovakia
ovenia
Korea
weden
erland
USSR
ngdom

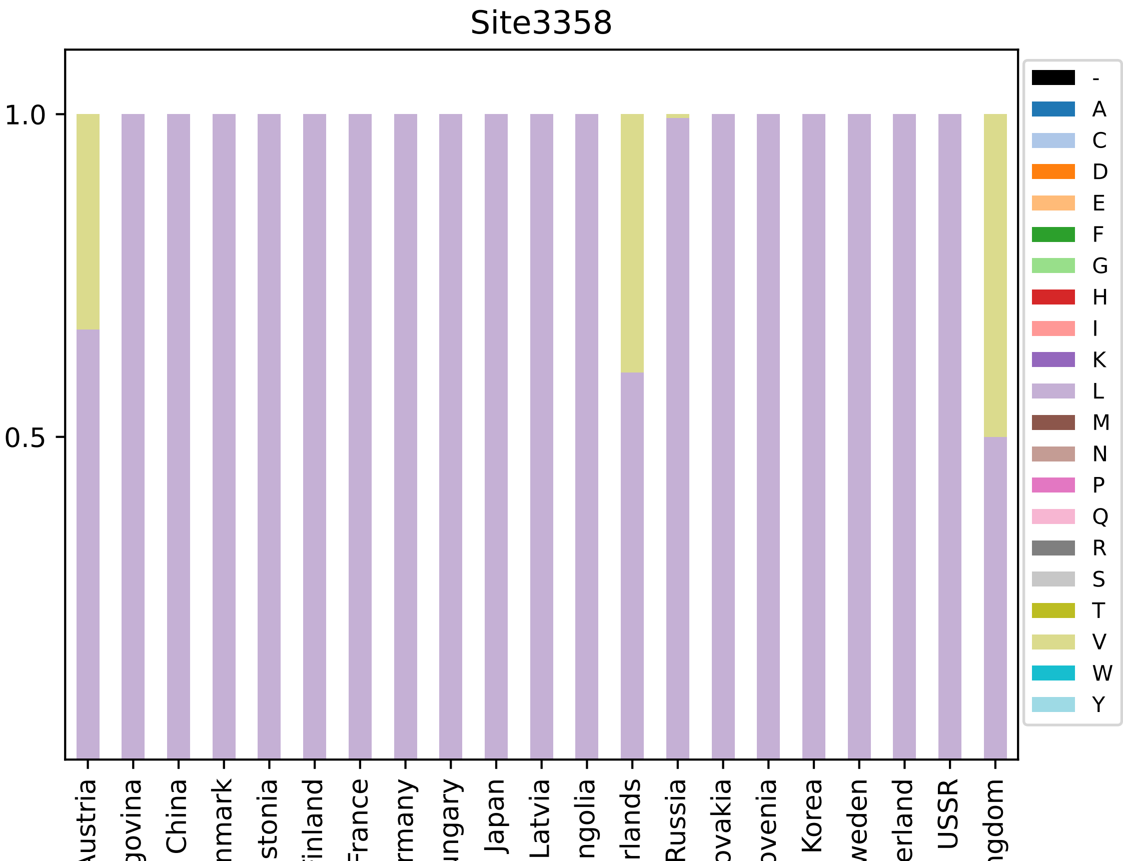

Site3358
1.0
0.5
-
A
C
D
E
F
G
H
I
K
L
M
N
P
Q
R
S
T
V
W
Y
China
Japan
Latvia
Russia
Korea
USSR

Site3359

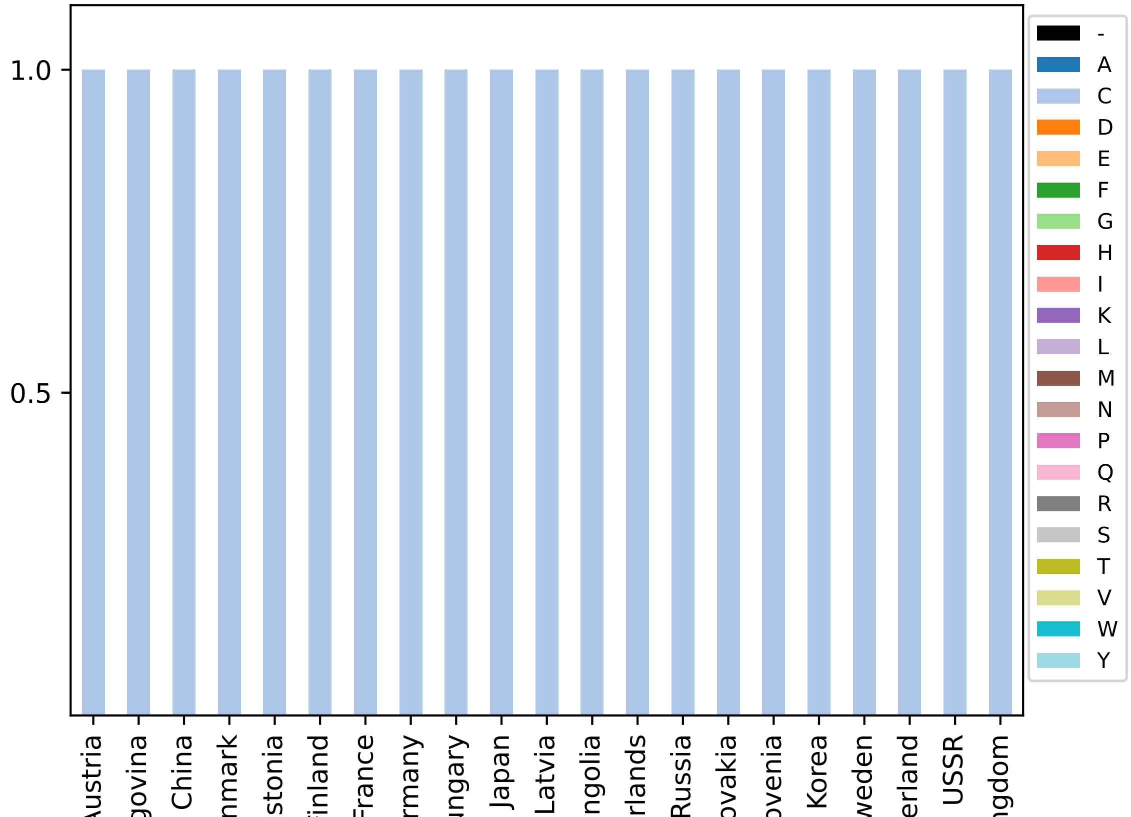
1.0
0.5
-
A
C
D
E
F
G
H
I
K
L
M
N
P
Q
R
S
T
V
W
Y
Austria
govina
China
nmark
stonia
Finland
France
rmany
ungary
Japan
Latvia
ngolia
rlands
Russia
ovakia
ovenia
Korea
weden
erland
USSR
ngdom

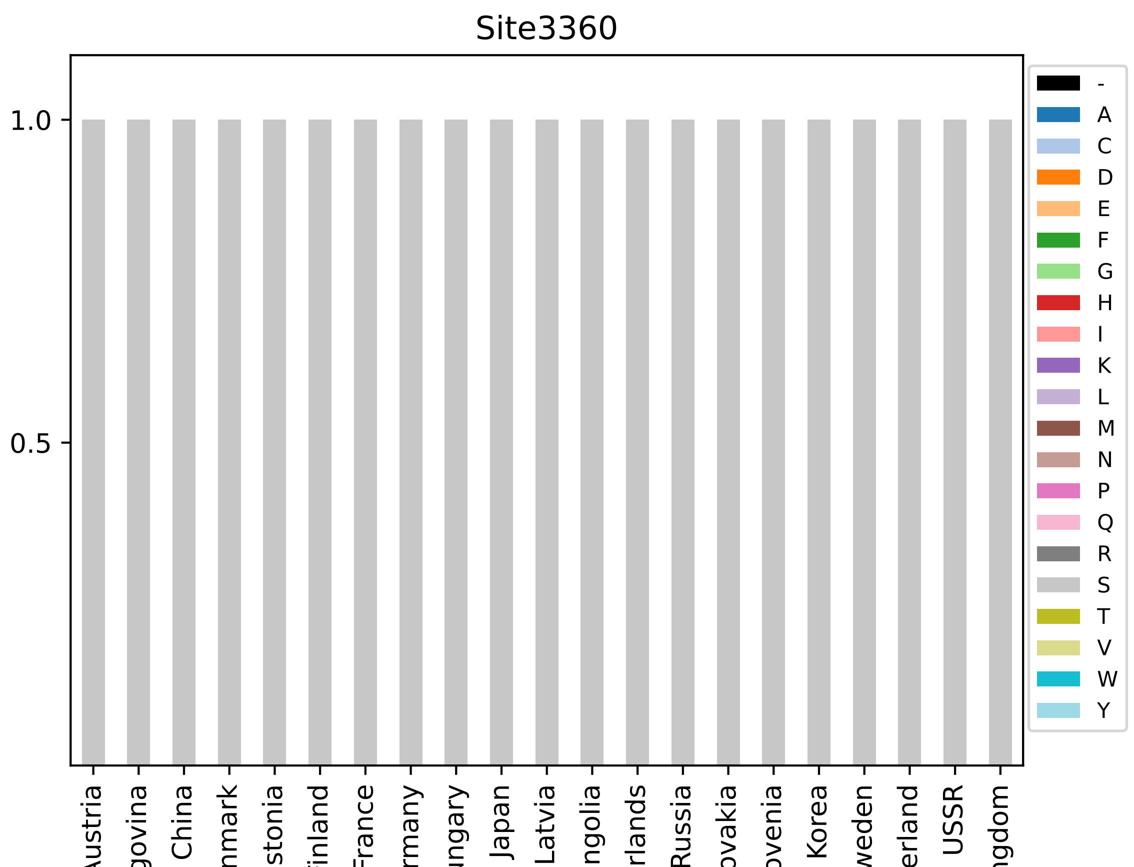
Site3360
1.0
0.5
-
A
C
D
E
F
G
H
I
K
L
M
N
P
Q
R
S
T
V
W
Y
Austria
China
Japan
Latvia
Russia
Korea
USSR

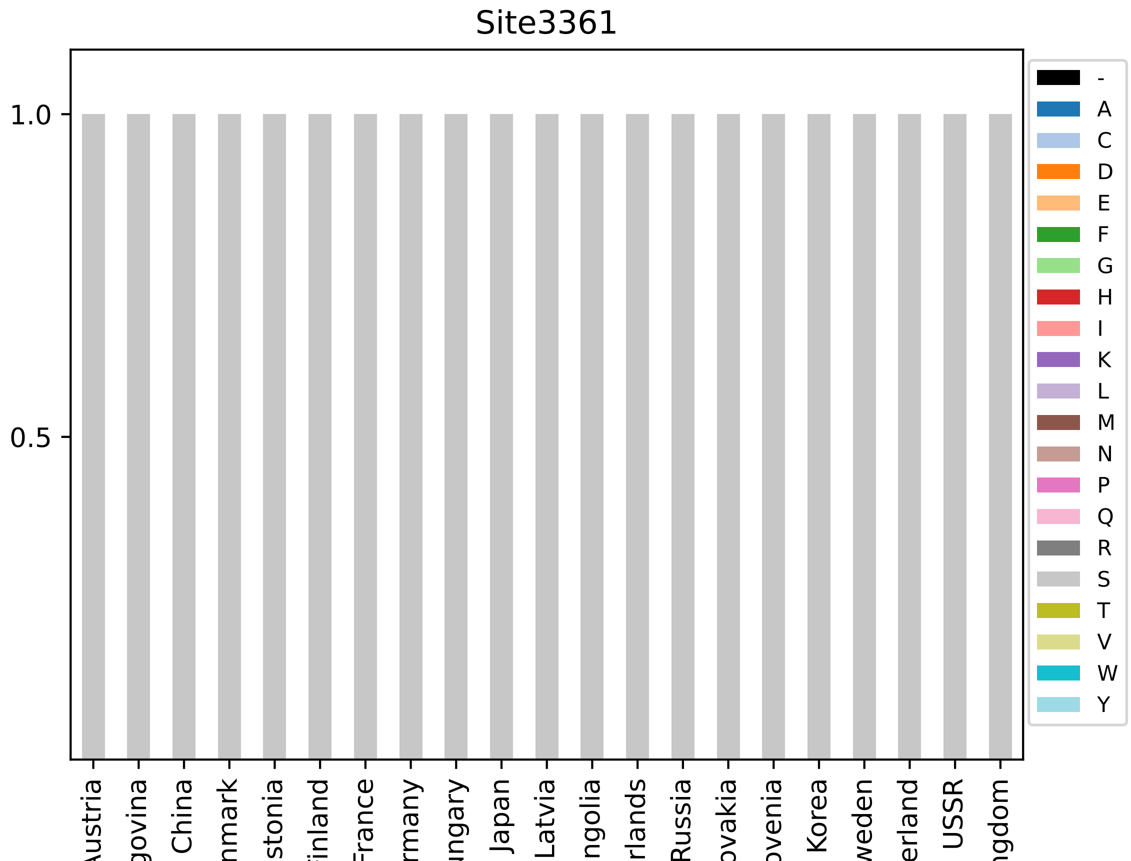
Site3361
1.0
0.5
-
A
C
D
E
F
G
H
I
K
L
M
N
P
Q
R
S
T
V
W
Y
Austria
govina
China
nmark
stonia
Finland
France
rmany
ungary
Japan
Latvia
ngolia
rlands
Russia
ovakia
ovenia
Korea
weden
erland
USSR
ngdom

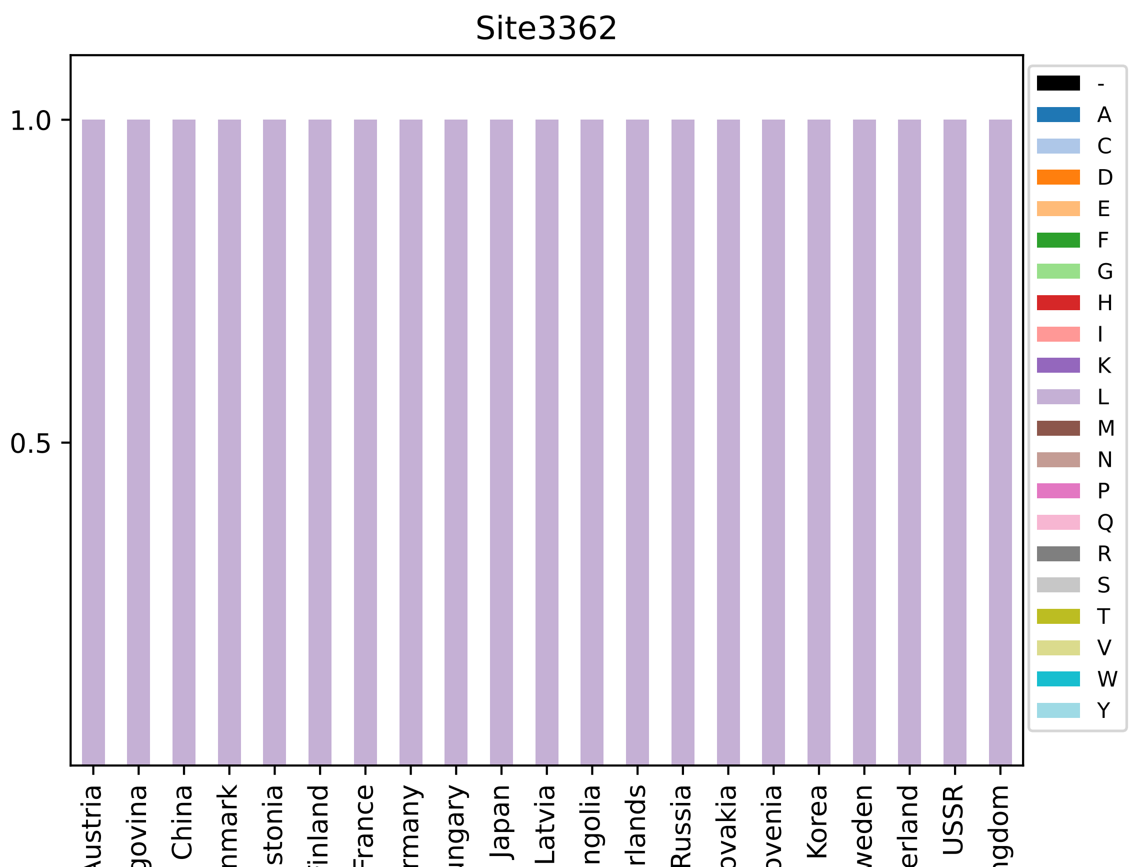

Site3362
1.0
0.5
-
A
C
D
E
F
G
H
I
K
L
M
N
P
Q
R
S
T
V
W
Y
Austria
govina
China
nmark
stonia
inland
France
rmany
ungary
Japan
Latvia
ngolia
rlands
Russia
ovakia
ovenia
Korea
weden
erland
USSR
ngdom

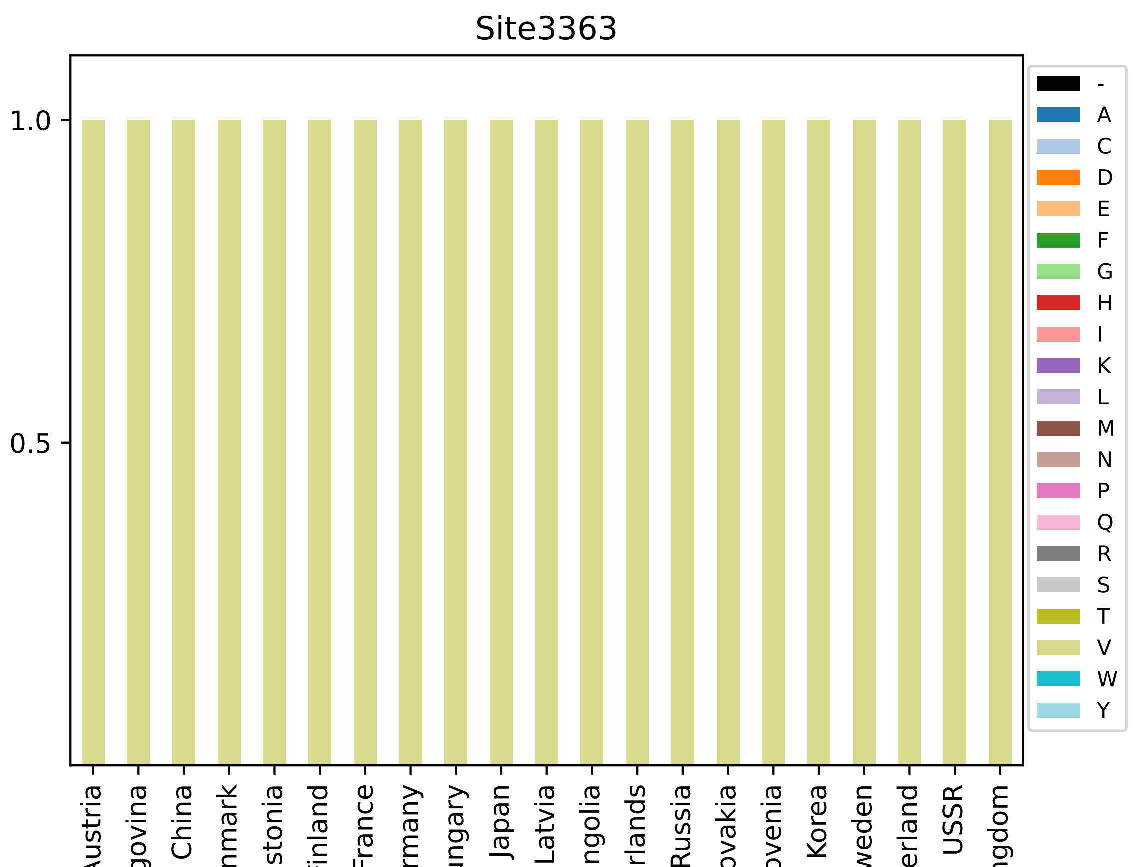
Site3363
1.0
0.5
Austria
govina
China
nmark
stonia
Finland
France
rmany
ungary
Japan
Latvia
ngolia
rlands
Russia
ovakia
ovenia
Korea
weden
erland
USSR
ngdom
-
A
C
D
E
F
G
H
I
K
L
M
N
P
Q
R
S
T
V
W
Y

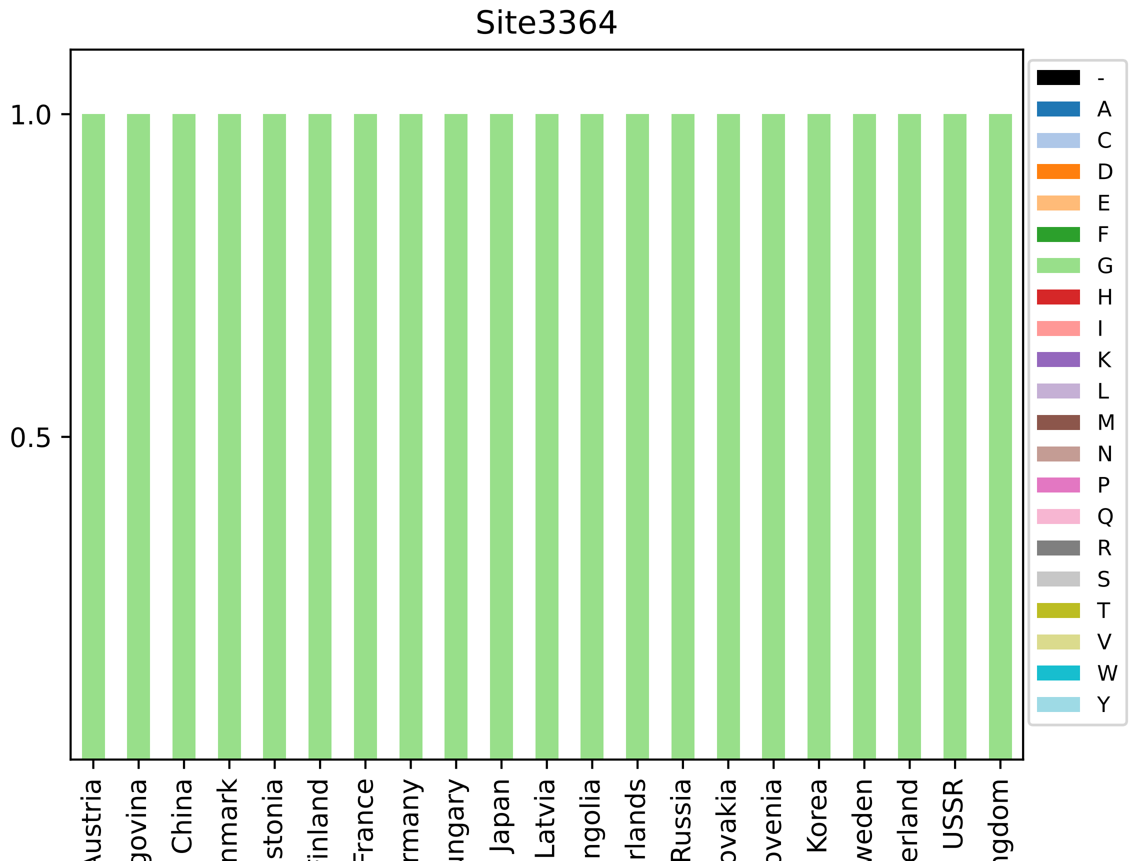

Site3364
1.0
0.5
-
A
C
D
E
F
G
H
I
K
L
M
N
P
Q
R
S
T
V
W
Y
China
Japan
Latvia
Russia
Korea
USSR

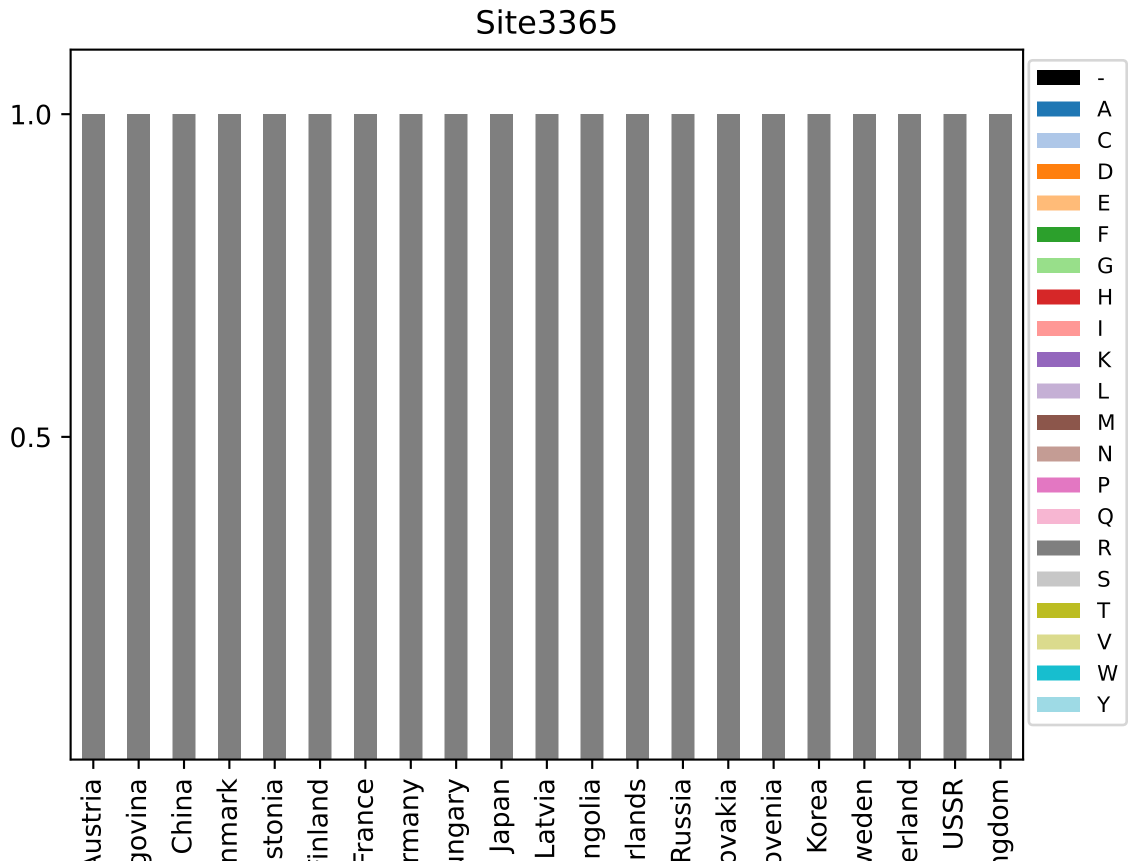

Site3365
1.0
0.5
-
A
C
D
E
F
G
H
I
K
L
M
N
P
Q
R
S
T
V
W
Y
Austria
govina
China
nmark
stonia
inland
France
rmany
ungary
Japan
Latvia
ngolia
rlands
Russia
ovakia
ovenia
Korea
weden
erland
USSR
ngdom

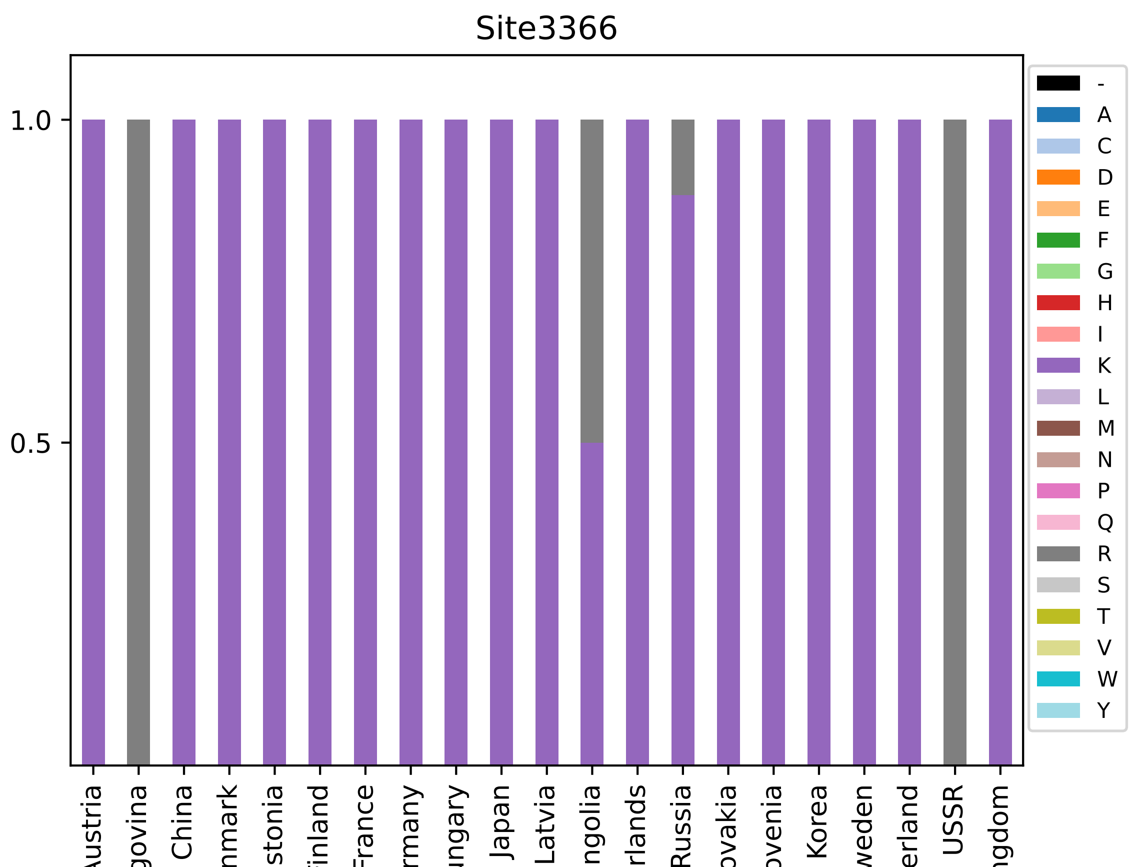
Site3366
1.0
0.5
-
A
C
D
E
F
G
H
I
K
L
M
N
P
Q
R
S
T
V
W
Y
Austria
China
Japan
Latvia
Russia
Korea
USSR

Site3367

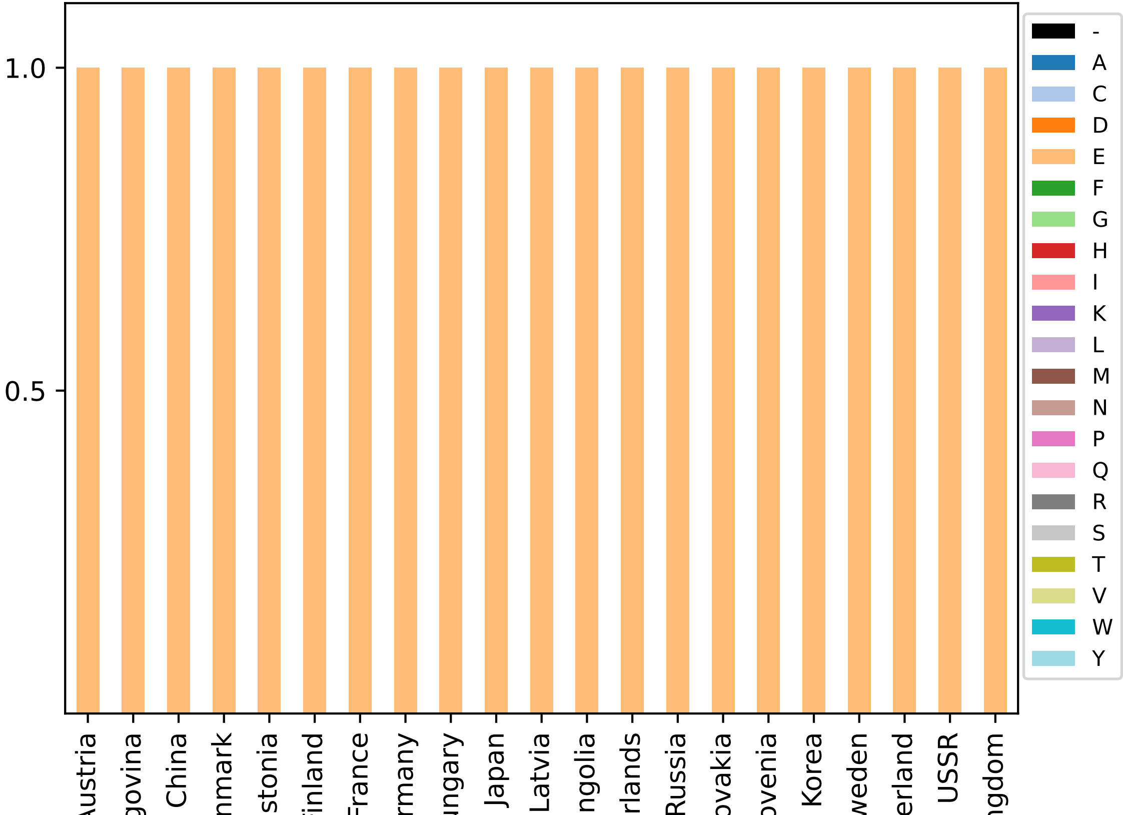

1.0
0.5
-
A
C
D
E
F
G
H
I
K
L
M
N
P
Q
R
S
T
V
W
Y
China
Japan
Latvia
Russia
Korea
USSR

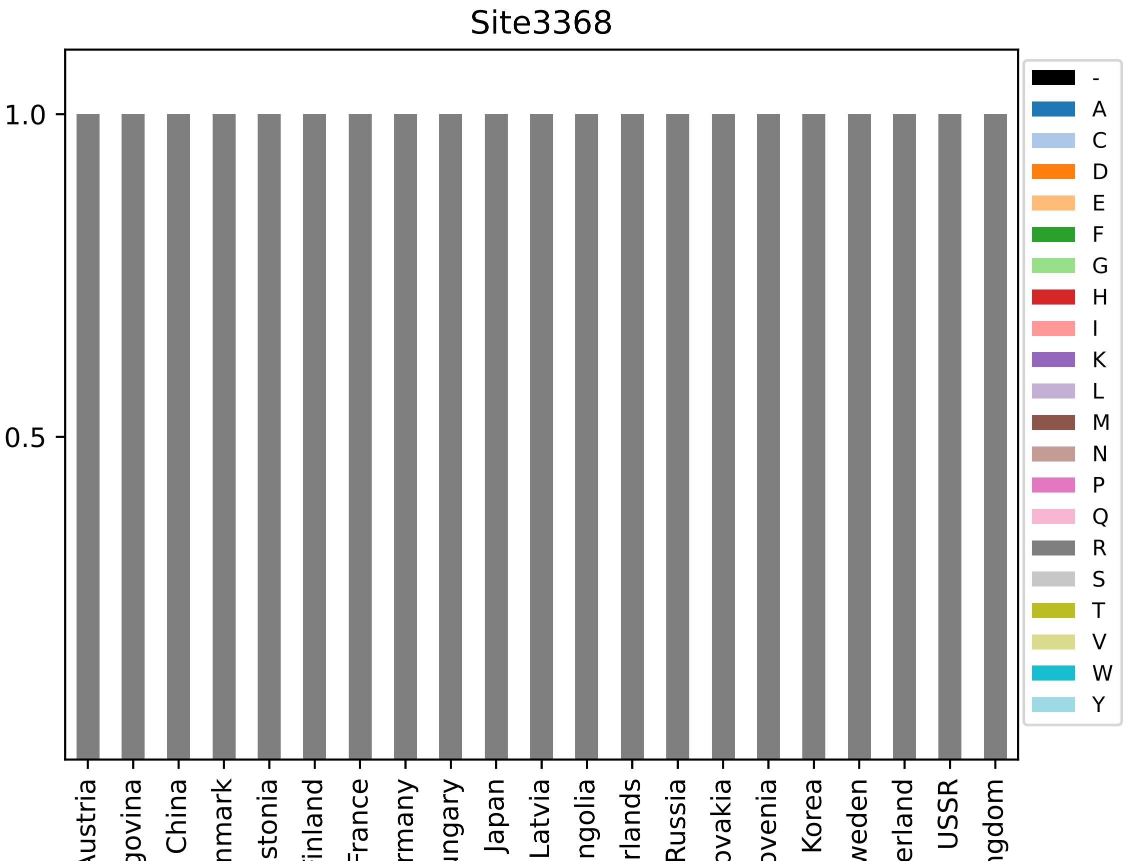
Site3368
1.0
0.5
-
A
C
D
E
F
G
H
I
K
L
M
N
P
Q
R
S
T
V
W
Y
China
Japan
Latvia
Russia
Korea
USSR

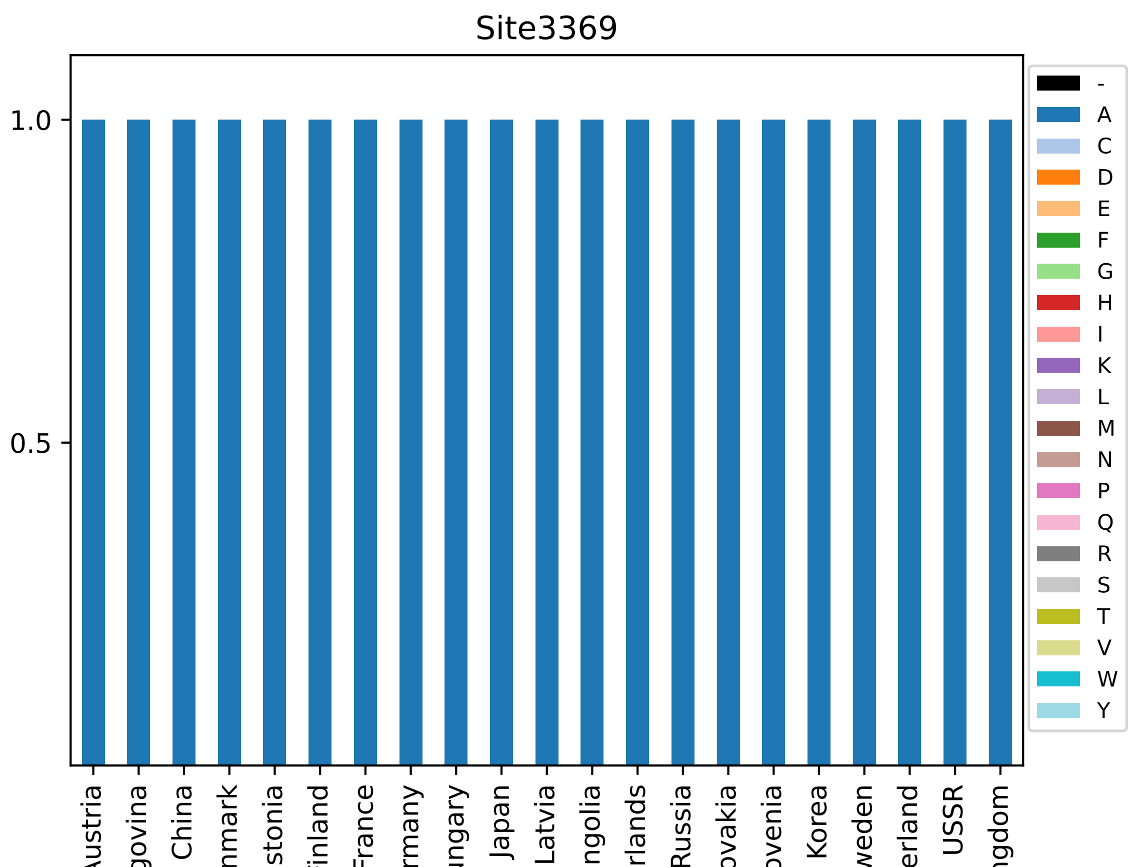

Site3369
1.0
0.5
-
A
C
D
E
F
G
H
I
K
L
M
N
P
Q
R
S
T
V
W
Y
China
Japan
Latvia
Russia
Korea
USSR

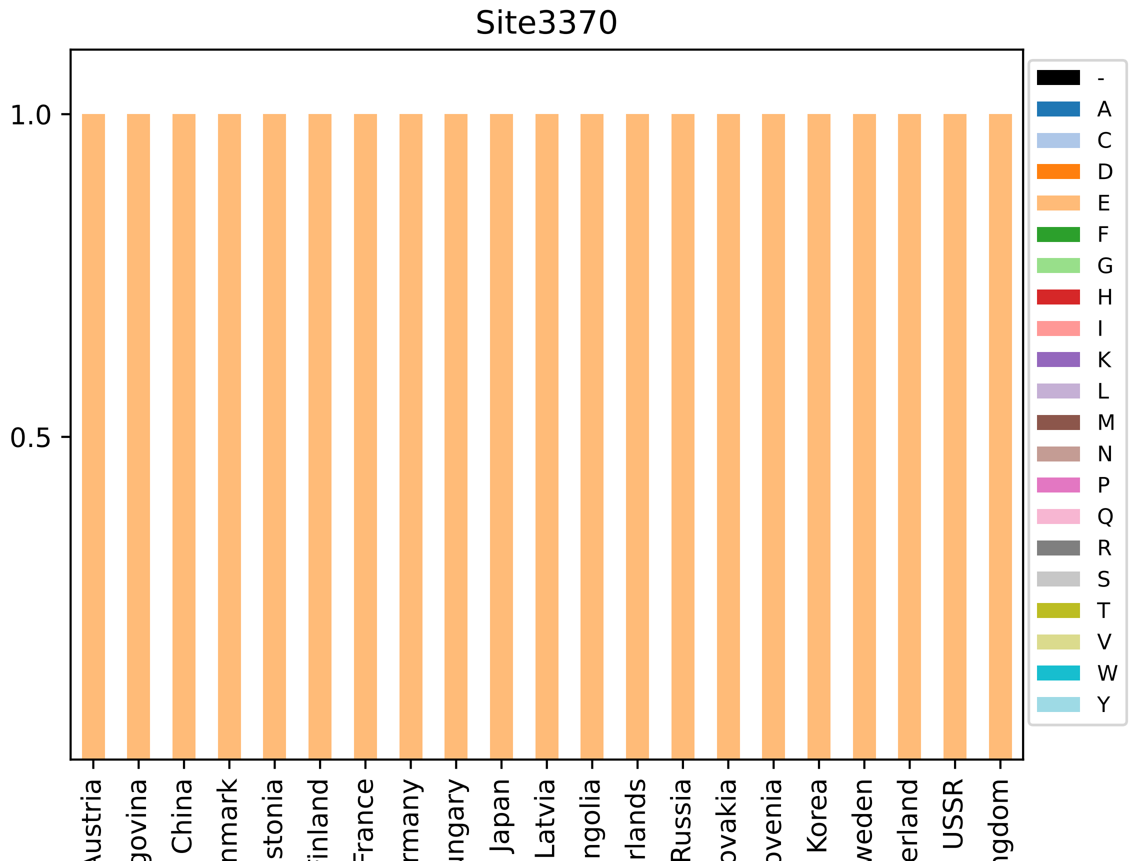
Site3370
1.0
0.5
-
A
C
D
E
F
G
H
I
K
L
M
N
P
Q
R
S
T
V
W
Y
Austria
China
Japan
Latvia
Russia
Korea
USSR

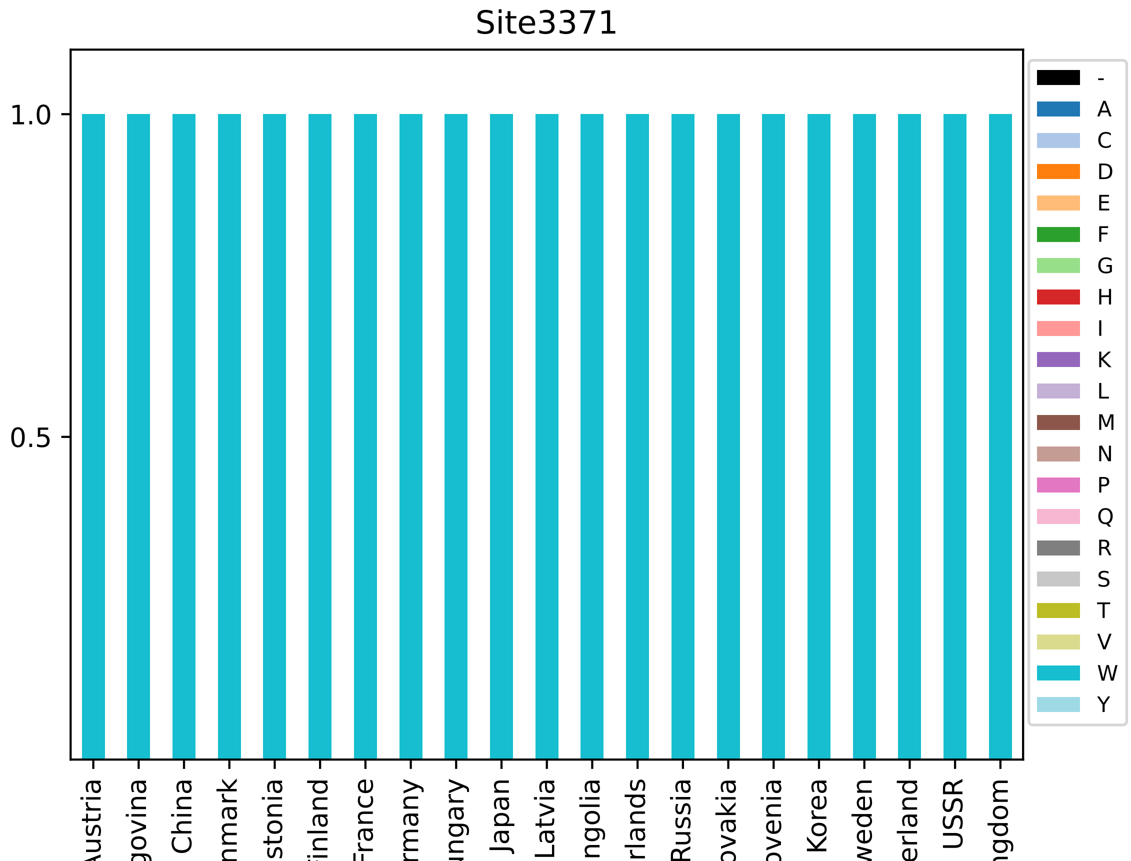
Site3371
1.0
0.5
-
A
C
D
E
F
G
H
I
K
L
M
N
P
Q
R
S
T
V
W
Y
Austria
govina
China
nmark
stonia
inland
France
rmany
ungary
Japan
Latvia
ngolia
rlands
Russia
ovakia
ovenia
Korea
weden
erland
USSR
ngdom

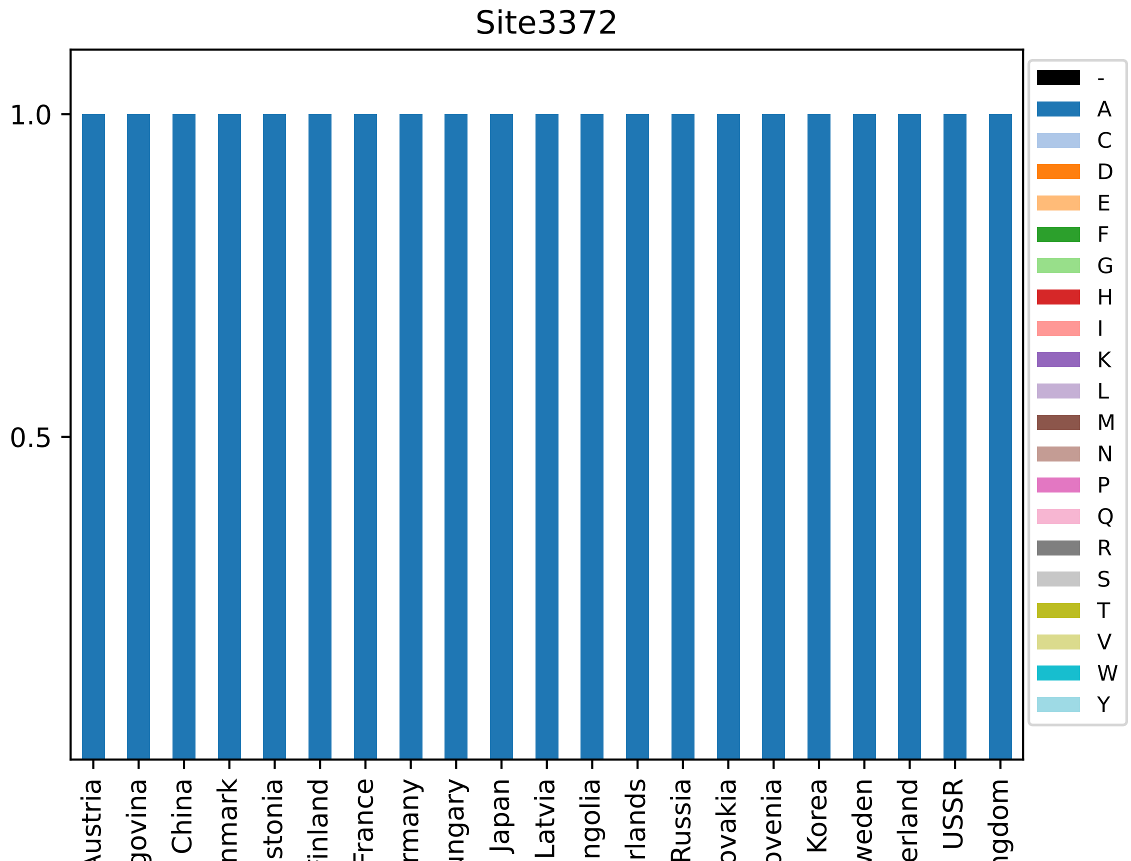
Site3372
1.0
0.5
-
A
C
D
E
F
G
H
I
K
L
M
N
P
Q
R
S
T
V
W
Y
China
Japan
Latvia
Russia
Korea
USSR

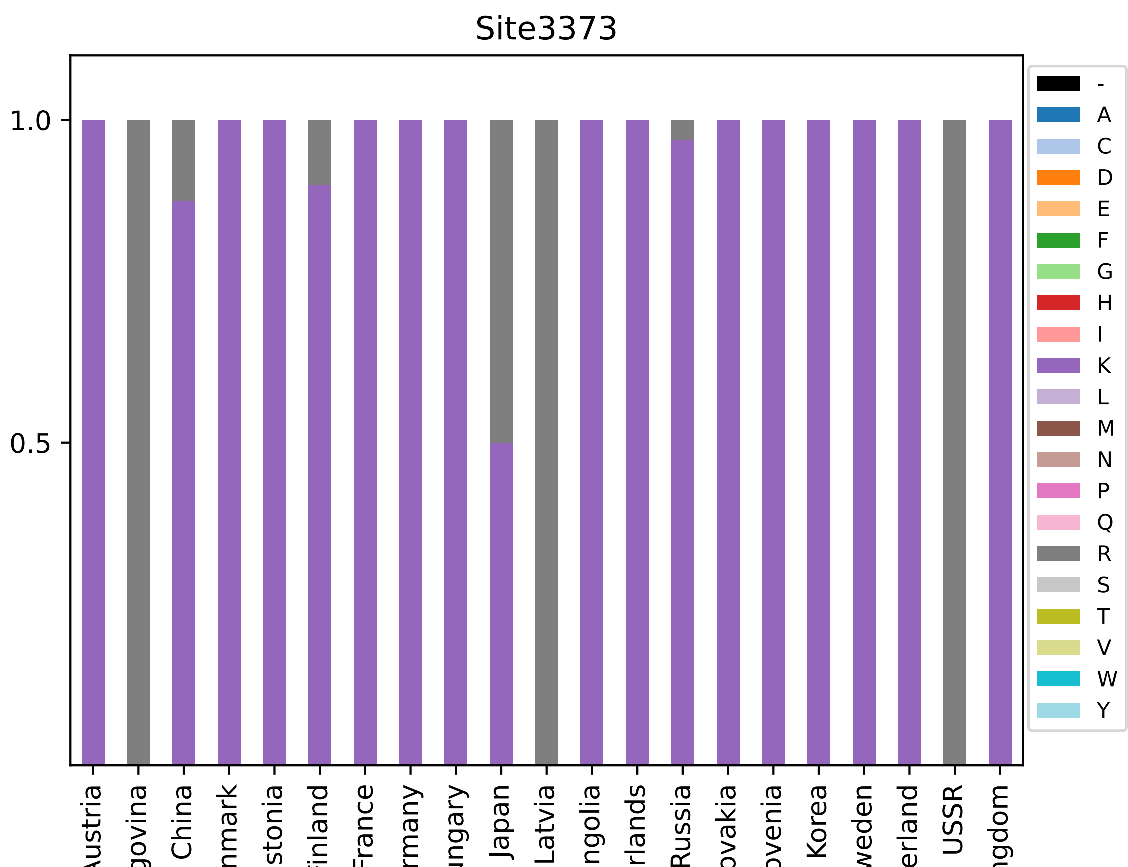

Site3373
1.0
0.5
Austria
govina
China
nmark
stonia
Finland
France
rmany
ungary
Japan
Latvia
ngolia
rlands
Russia
ovakia
ovenia
Korea
weden
erland
USSR
ngdom
-
A
C
D
E
F
G
H
I
K
L
M
N
P
Q
R
S
T
V
W
Y

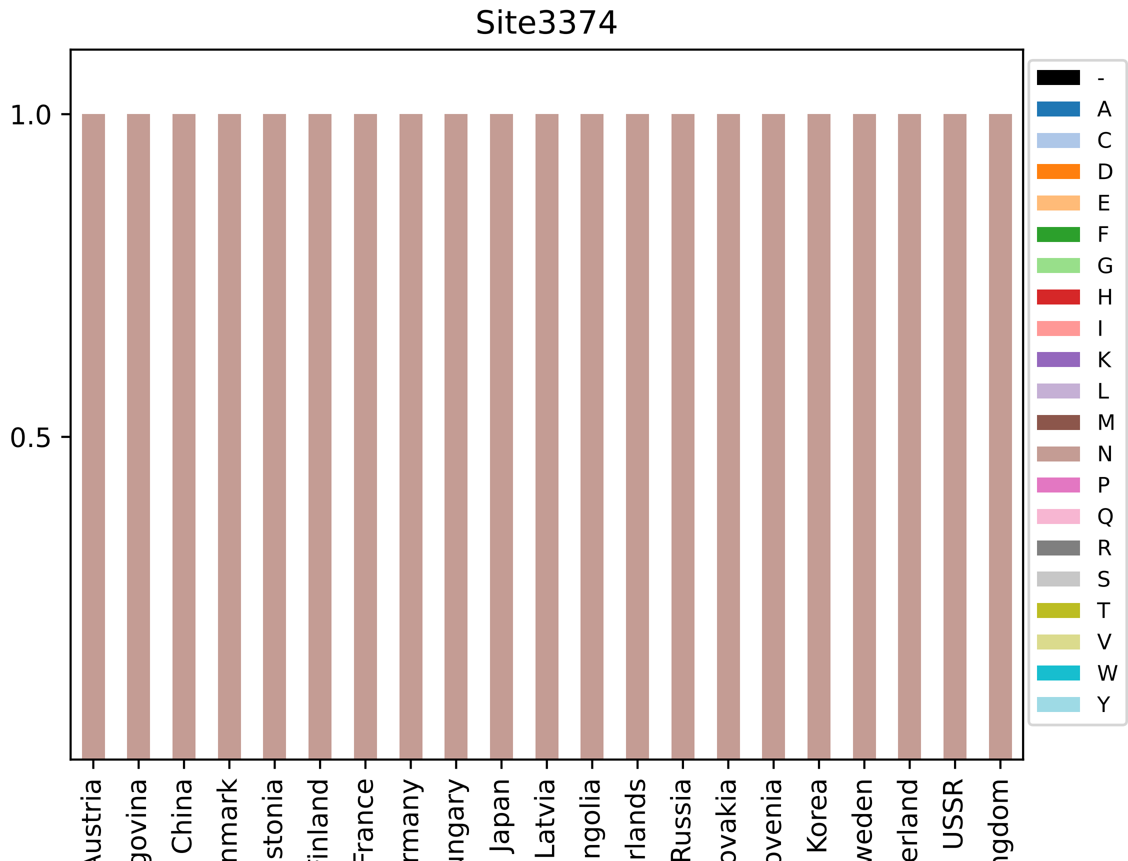

Site3374
1.0
0.5
-
A
C
D
E
F
G
H
I
K
L
M
N
P
Q
R
S
T
V
W
Y
China
Japan
Latvia
Russia
Korea
USSR

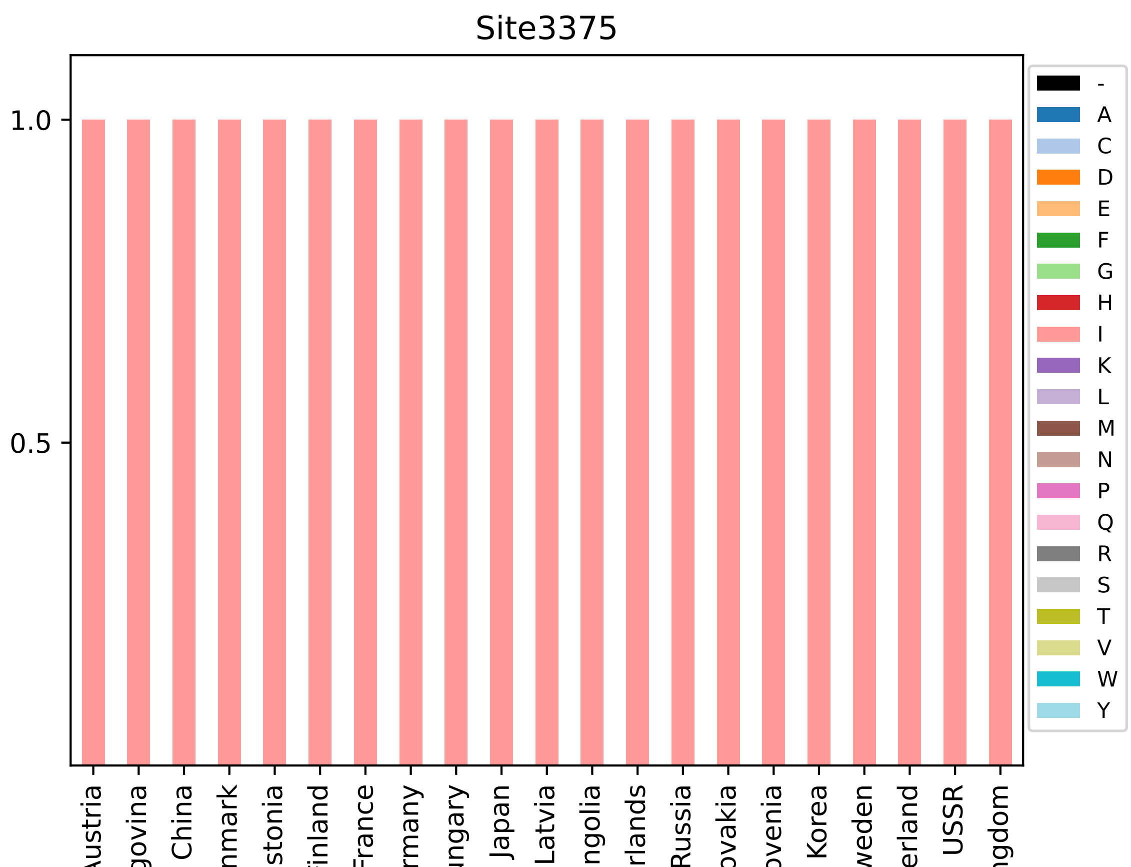

Site3375
1.0
0.5
-
A
C
D
E
F
G
H
I
K
L
M
N
P
Q
R
S
T
V
W
Y
Austria
govina
China
nmark
stonia
inland
France
rmany
ungary
Japan
Latvia
ngolia
rlands
Russia
ovakia
ovenia
Korea
weden
erland
USSR
ngdom

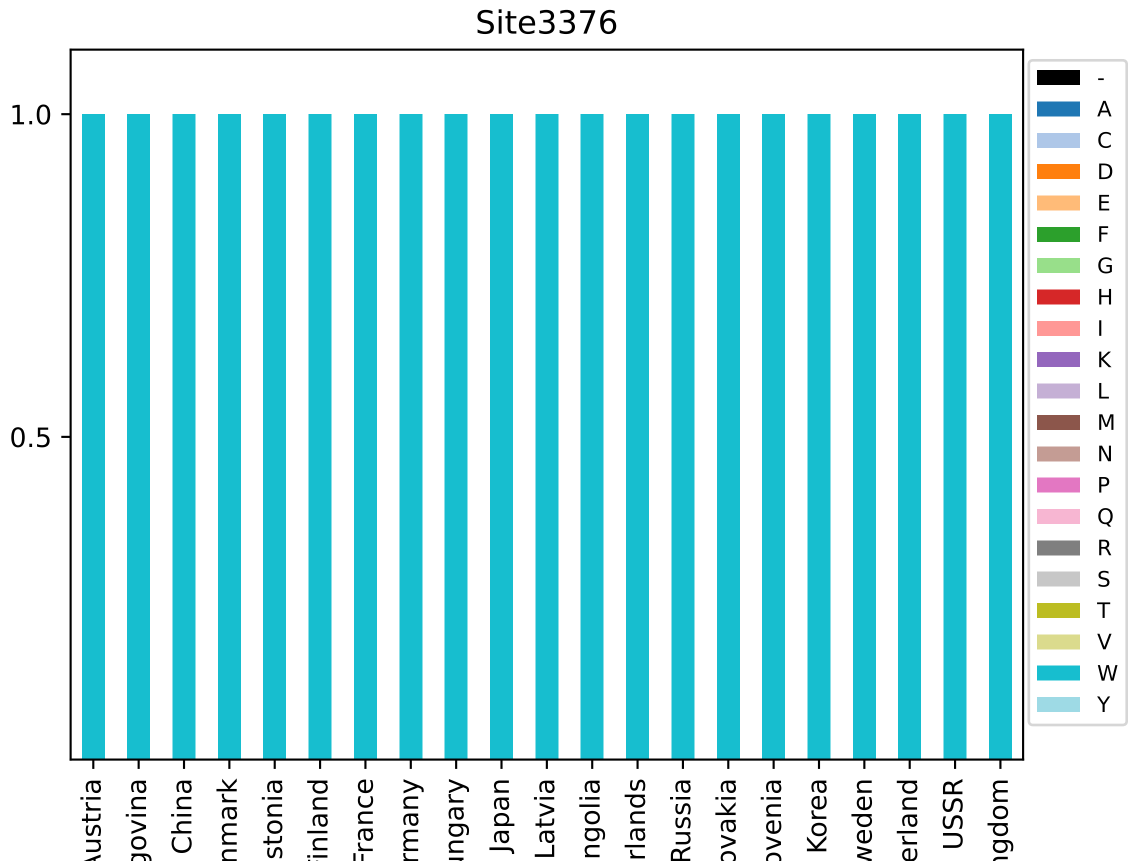
Site3376
1.0
0.5
-
A
C
D
E
F
G
H
I
K
L
M
N
P
Q
R
S
T
V
W
Y
China
Japan
Latvia
Russia
Korea
USSR

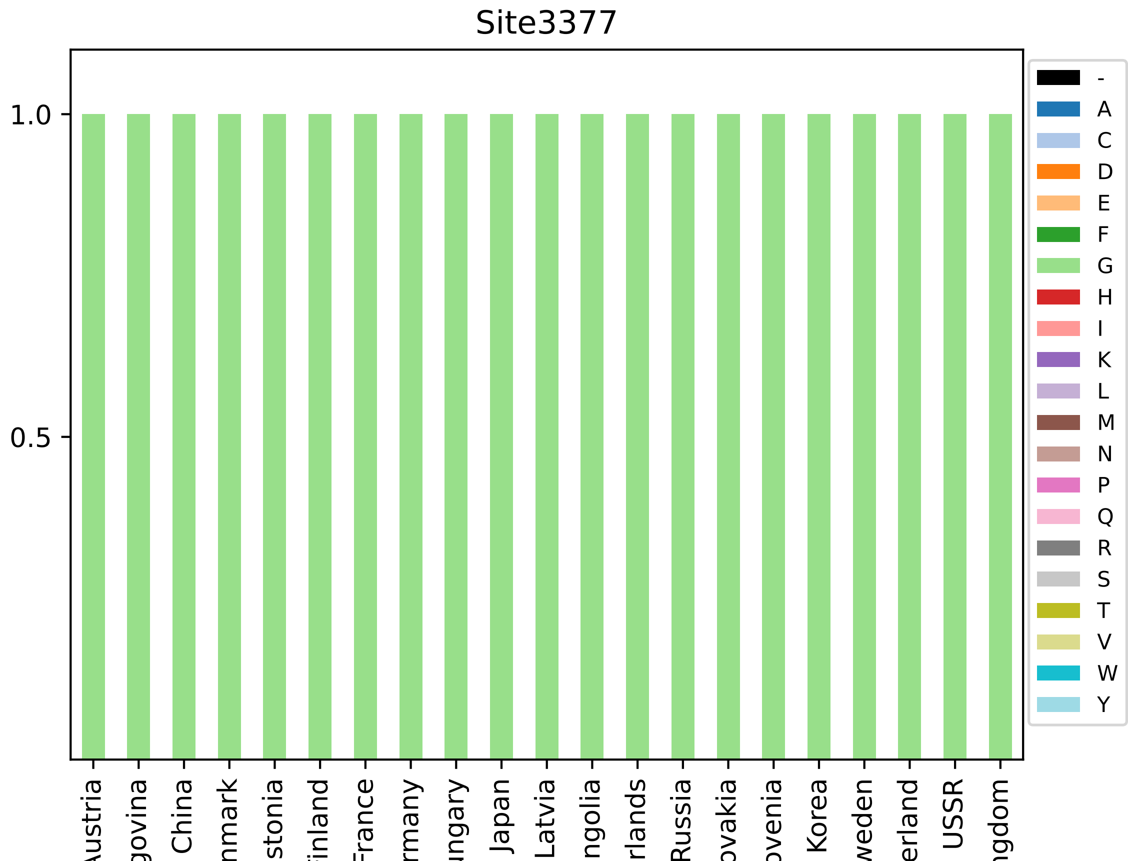

Site3377
1.0
0.5
-
A
C
D
E
F
G
H
I
K
L
M
N
P
Q
R
S
T
V
W
Y
Austria
govina
China
nmark
stonia
Finland
France
rmany
ungary
Japan
Latvia
ngolia
rlands
Russia
ovakia
ovenia
Korea
weden
erland
USSR
ngdom

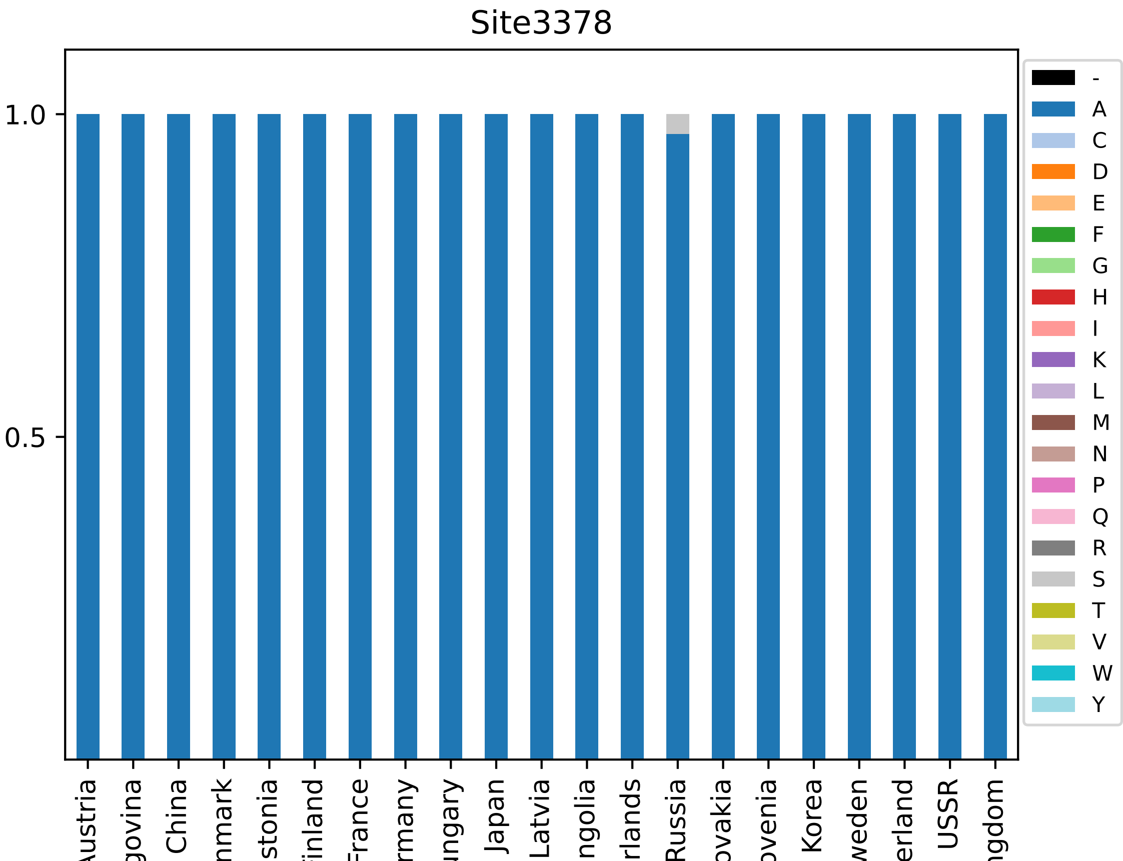

Site3378
1.0
0.5
-
A
C
D
E
F
G
H
I
K
L
M
N
P
Q
R
S
T
V
W
Y
China
Japan
Latvia
Russia
Korea
USSR

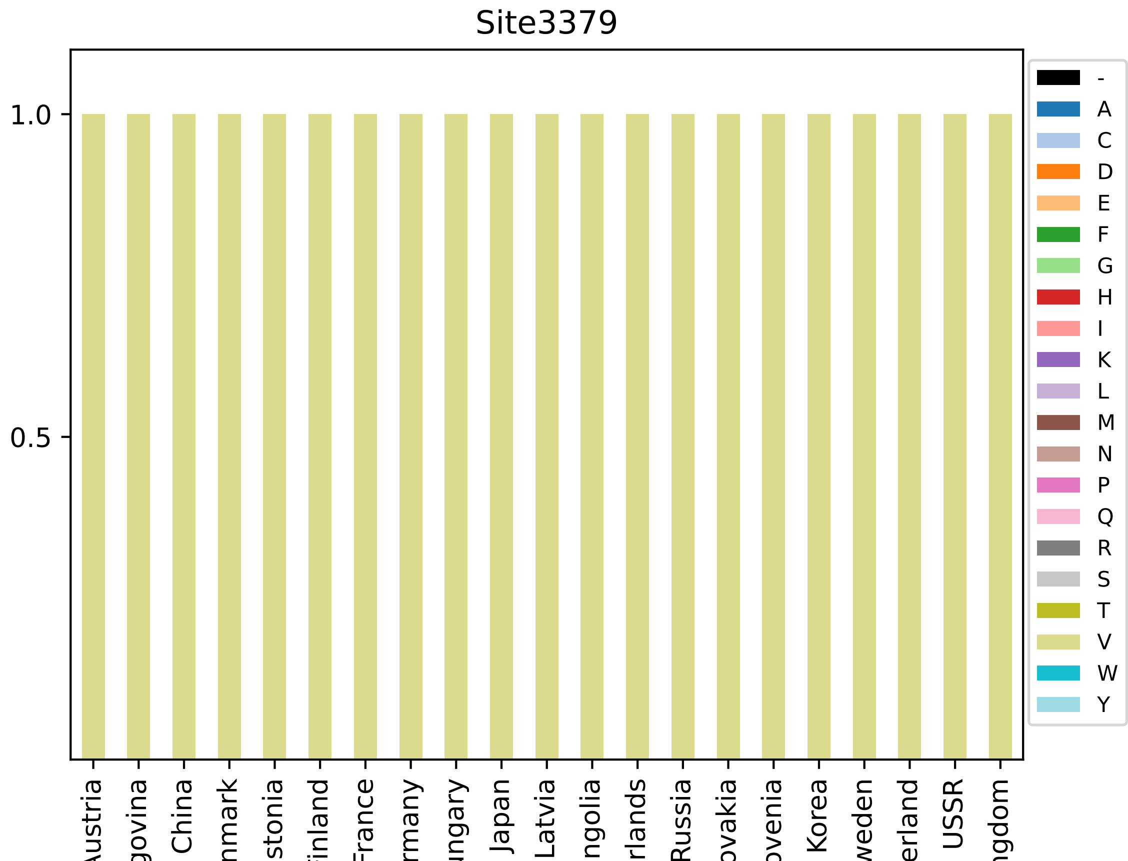
Site3379
1.0
0.5
-
A
C
D
E
F
G
H
I
K
L
M
N
P
Q
R
S
T
V
W
Y
Austria
China
Japan
Latvia
Russia
Korea
USSR

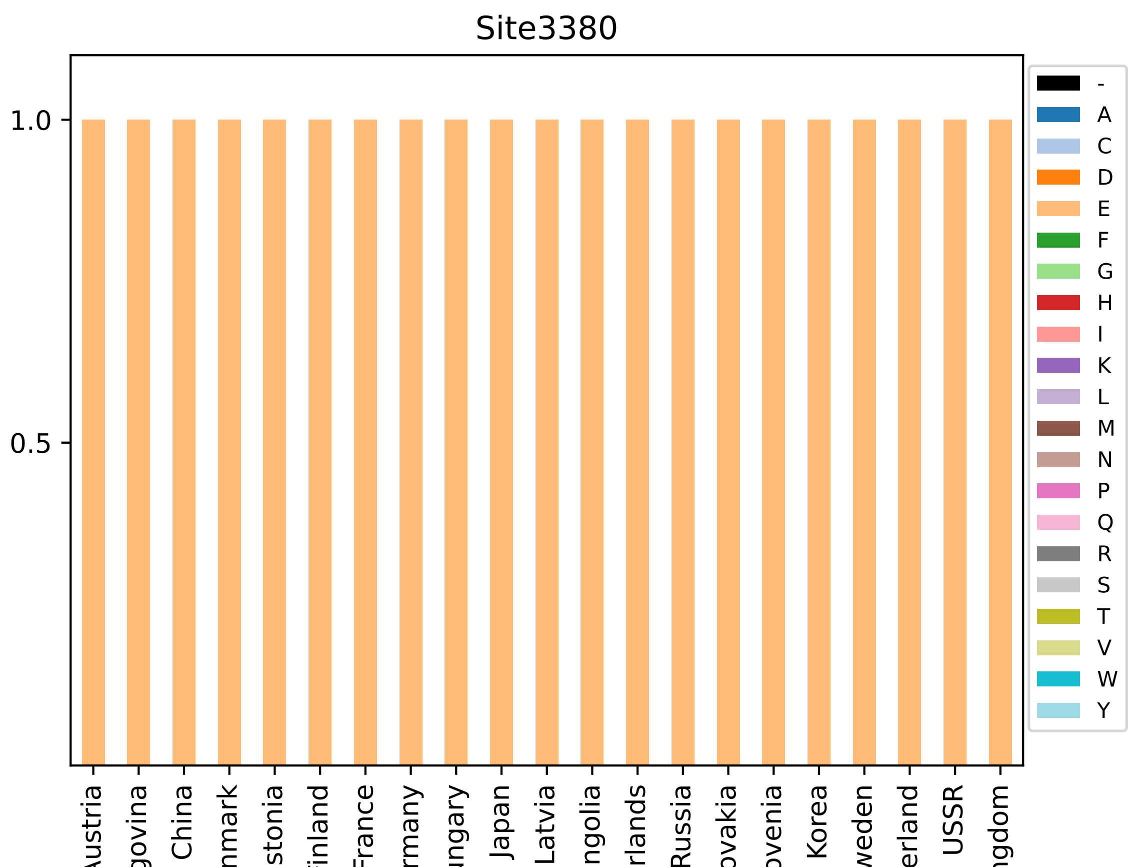
Site3380
1.0
0.5
-
A
C
D
E
F
G
H
I
K
L
M
N
P
Q
R
S
T
V
W
Y
China
Japan
Latvia
Russia
Korea
USSR

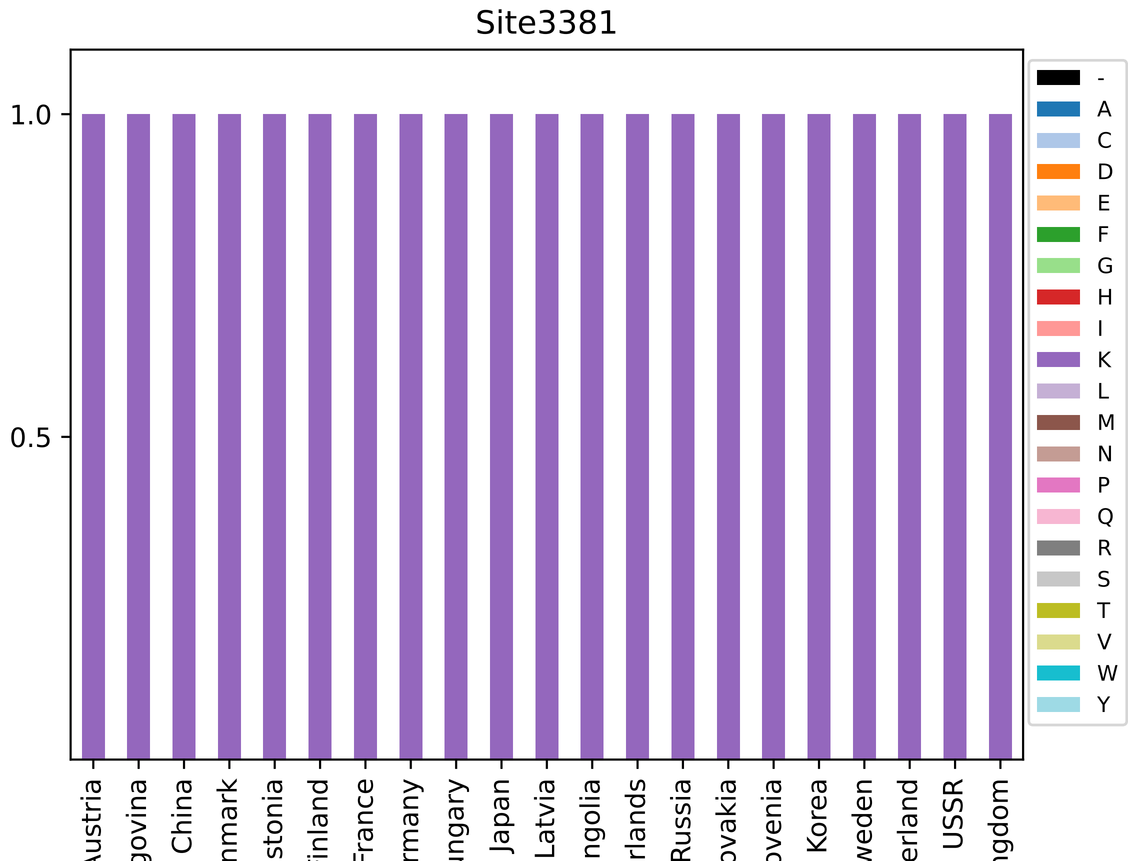

Site3381
1.0
0.5
-
A
C
D
E
F
G
H
I
K
L
M
N
P
Q
R
S
T
V
W
Y
Austria
govina
China
nmark
stonia
inland
France
rmany
ungary
Japan
Latvia
ngolia
rlands
Russia
ovakia
ovenia
Korea
weden
erland
USSR
ngdom

Site3382

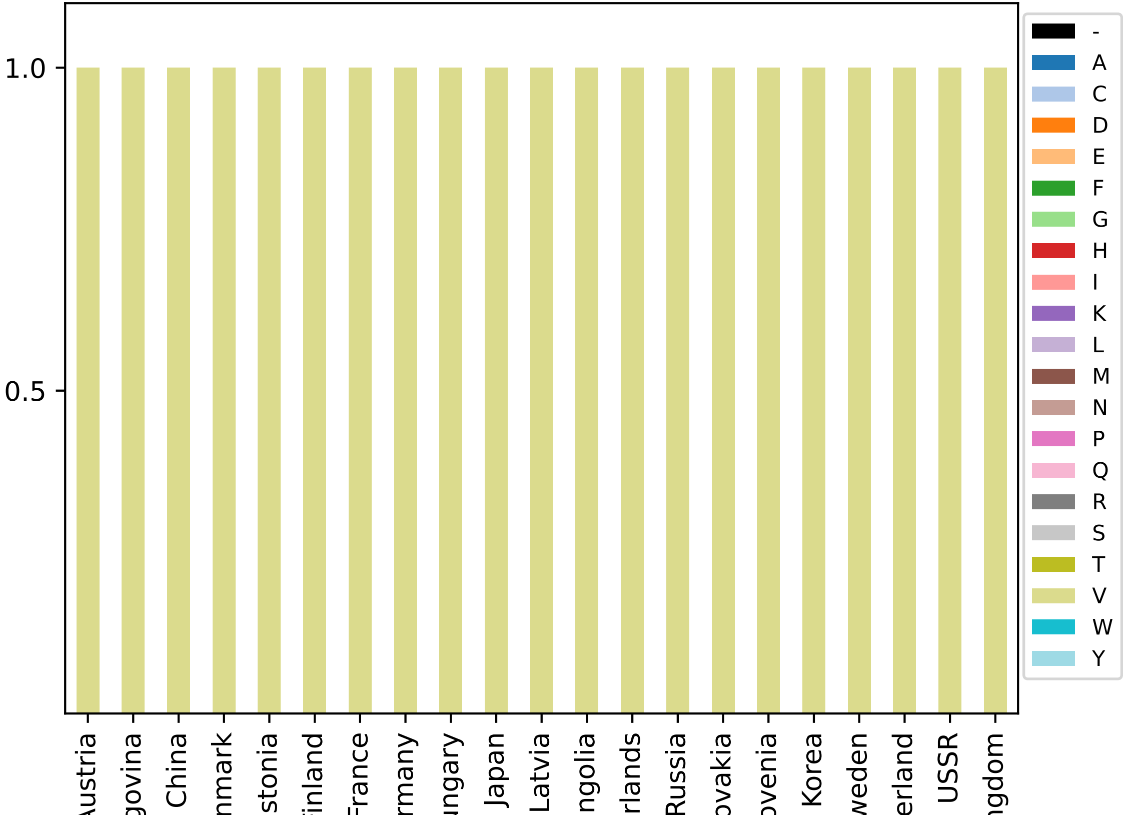
1.0
0.5
-
A
C
D
E
F
G
H
I
K
L
M
N
P
Q
R
S
T
V
W
Y
Austria
China
Japan
Latvia
Russia
Korea
USSR

Site3383

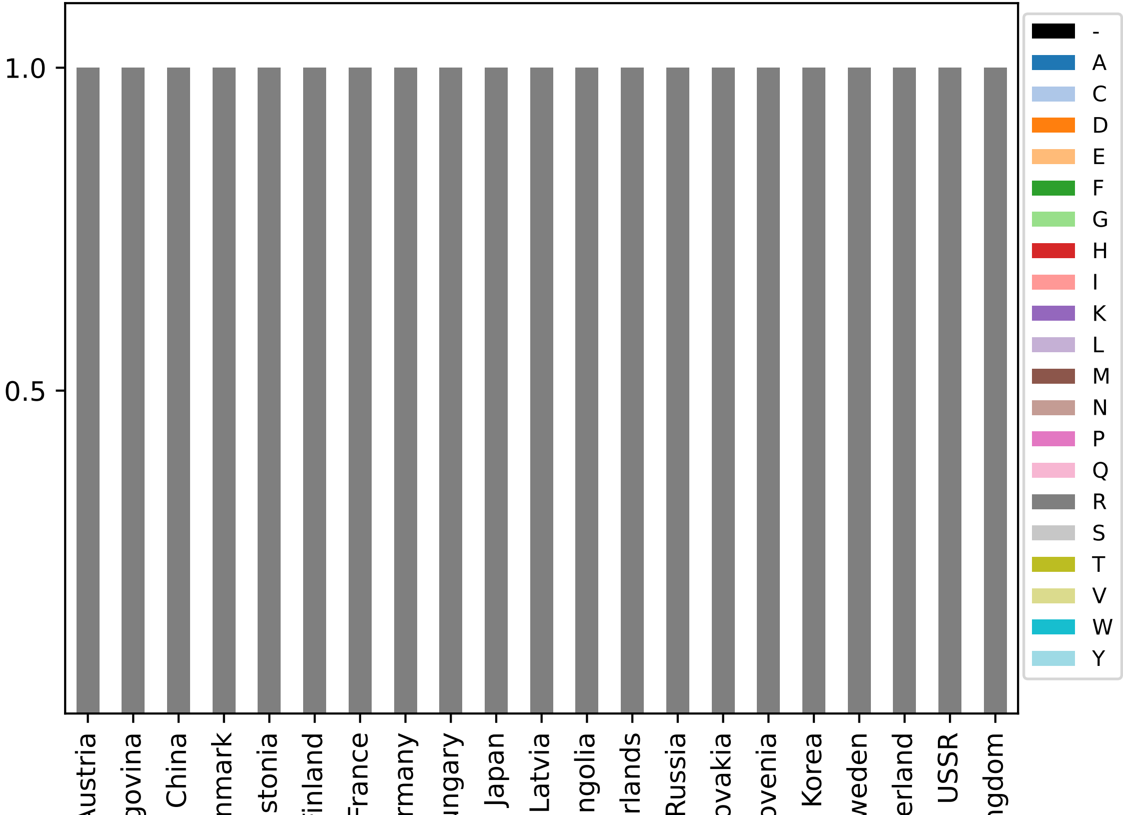

1.0
0.5
Austria
govina
China
nmark
stonia
Finland
France
rmany
ungary
Japan
Latvia
ngolia
rlands
Russia
ovakia
ovenia
Korea
weden
erland
USSR
ngdom
-
A
C
D
E
F
G
H
I
K
L
M
N
P
Q
R
S
T
V
W
Y

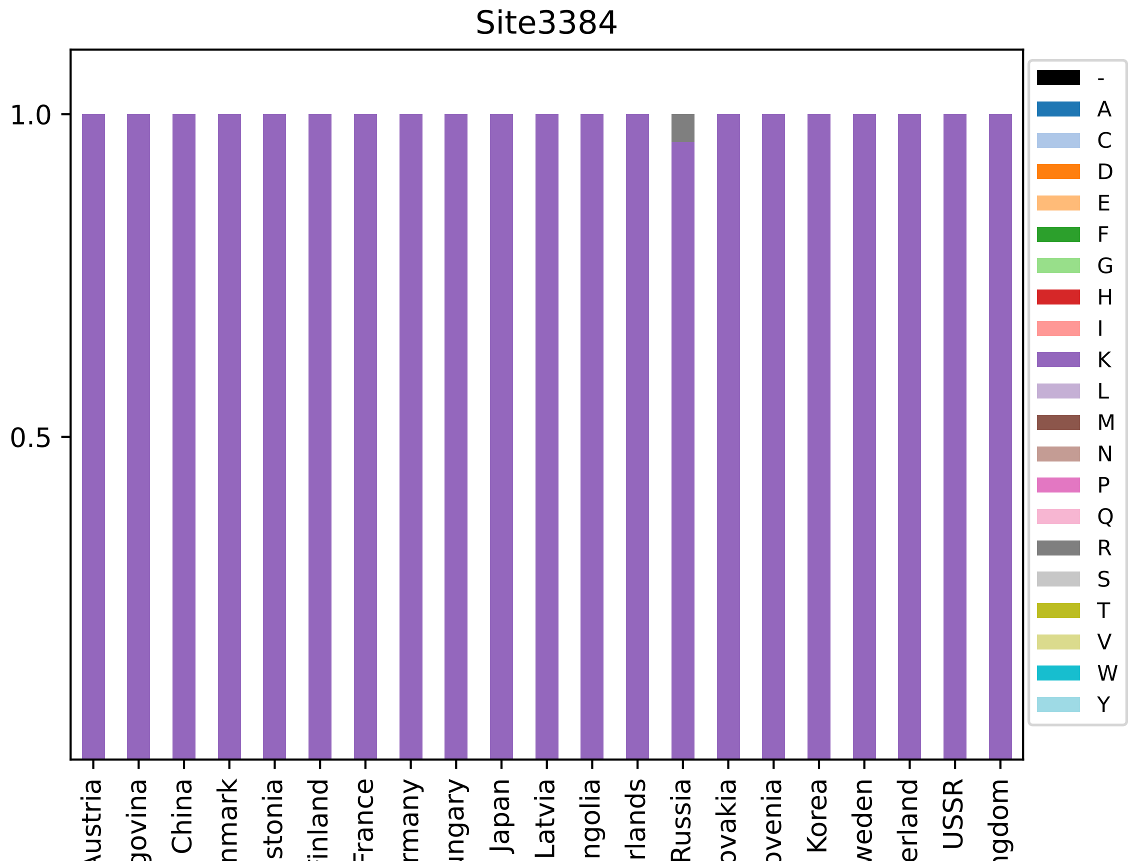
Site3384
1.0
0.5
-
A
C
D
E
F
G
H
I
K
L
M
N
P
Q
R
S
T
V
W
Y
China
Japan
Latvia
Russia
Korea
USSR

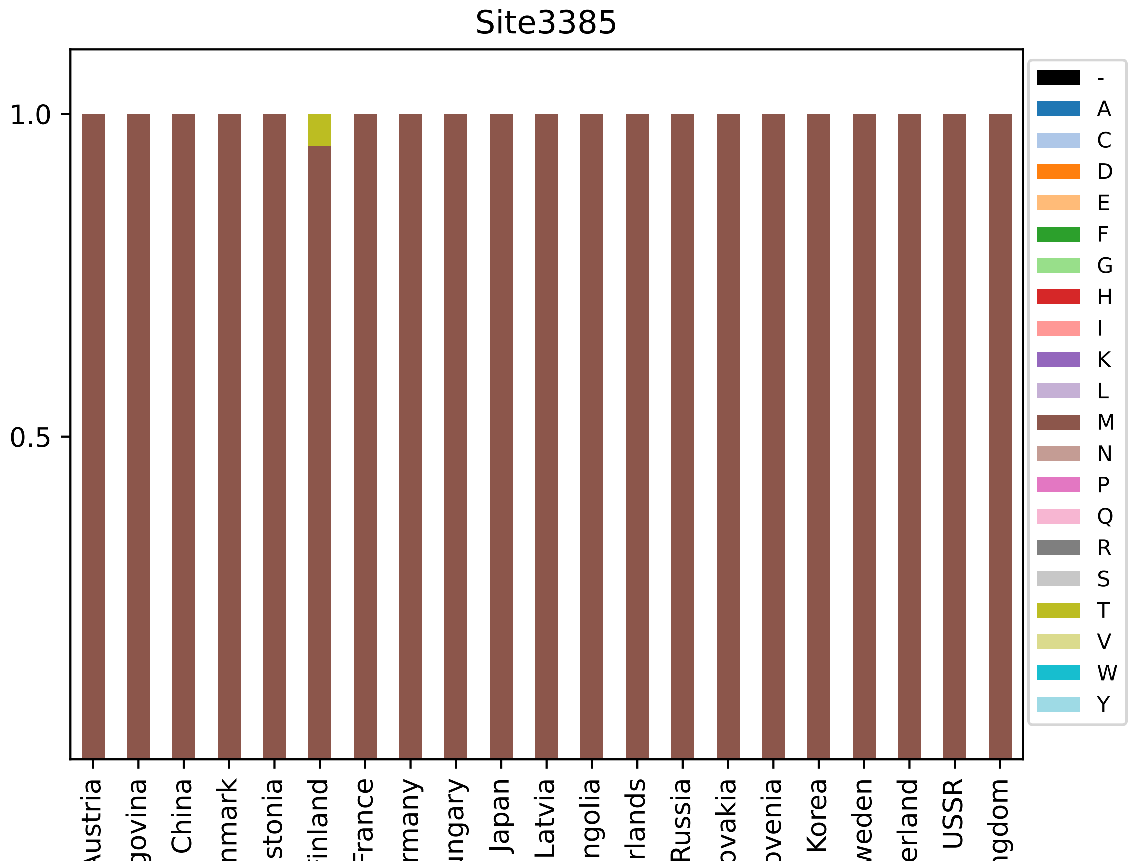
Site3385
1.0
0.5
-
A
C
D
E
F
G
H
I
K
L
M
N
P
Q
R
S
T
V
W
Y
Austria
govina
China
nmark
stonia
inland
France
rmany
ungary
Japan
Latvia
ngolia
rlands
Russia
ovakia
ovenia
Korea
weden
erland
USSR
ngdom

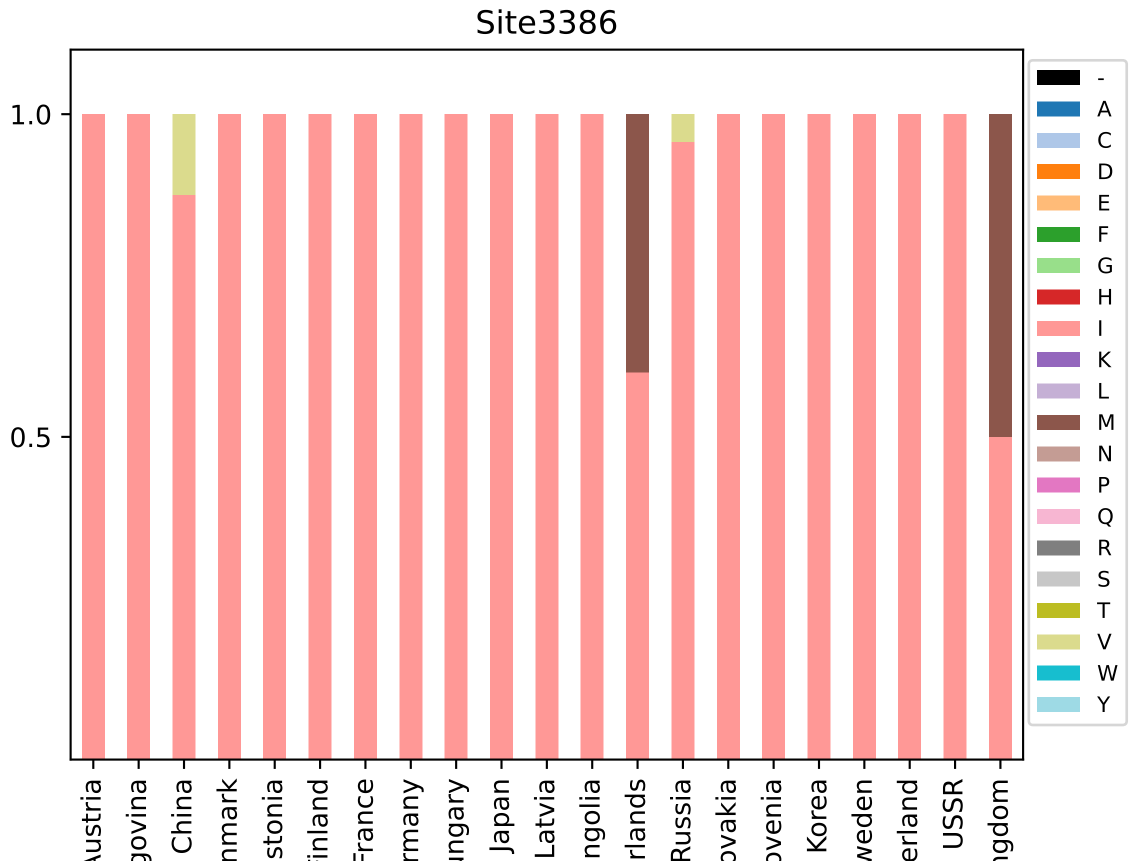

Site3386
1.0
0.5
-
A
C
D
E
F
G
H
I
K
L
M
N
P
Q
R
S
T
V
W
Y
China
Japan
Latvia
Russia
Korea
USSR

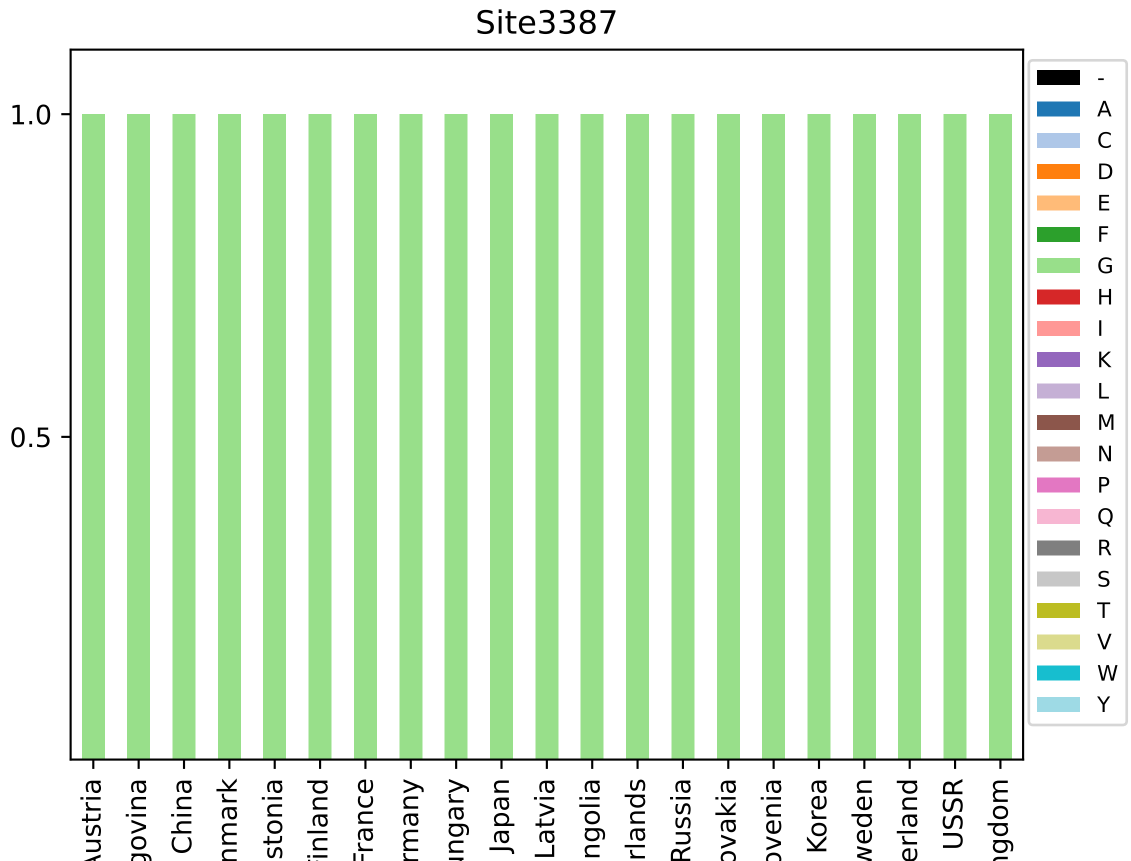
Site3387
1.0
0.5
-
A
C
D
E
F
G
H
I
K
L
M
N
P
Q
R
S
T
V
W
Y
China
Japan
Latvia
Russia
Korea
USSR

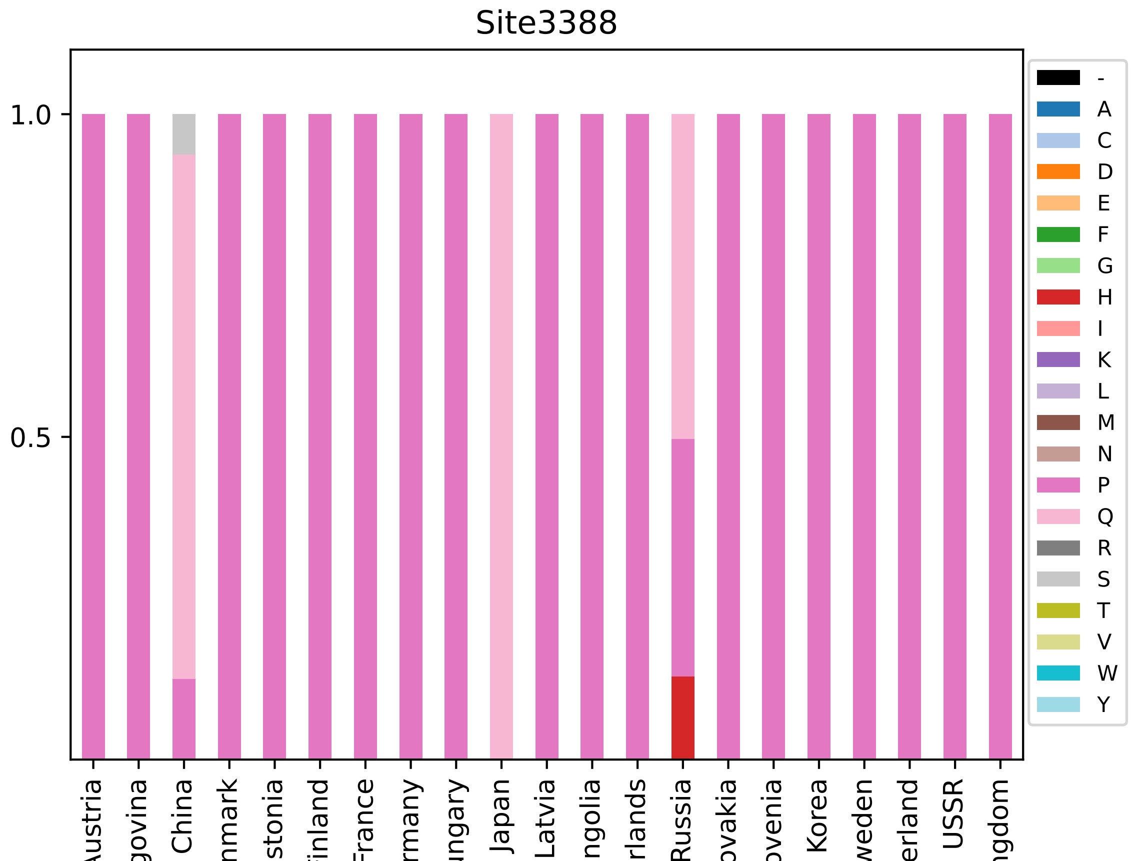
Site3388
1.0
0.5
-
A
C
D
E
F
G
H
I
K
L
M
N
P
Q
R
S
T
V
W
Y
China
Japan
Latvia
Russia
Korea
USSR

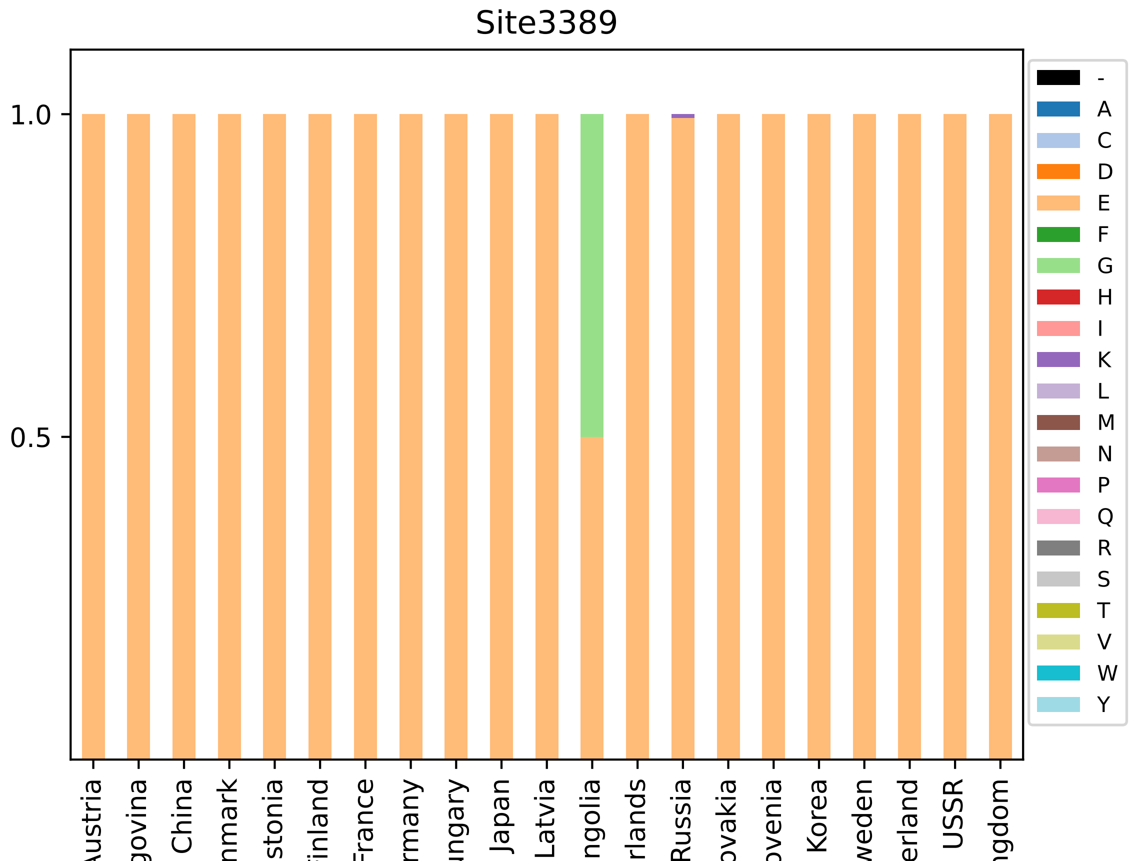

Site3389
1.0
0.5
-
A
C
D
E
F
G
H
I
K
L
M
N
P
Q
R
S
T
V
W
Y
Austria
govina
China
nmark
stonia
Finland
France
rmany
ungary
Japan
Latvia
ngolia
rlands
Russia
ovakia
ovenia
Korea
weden
erland
USSR
ngdom

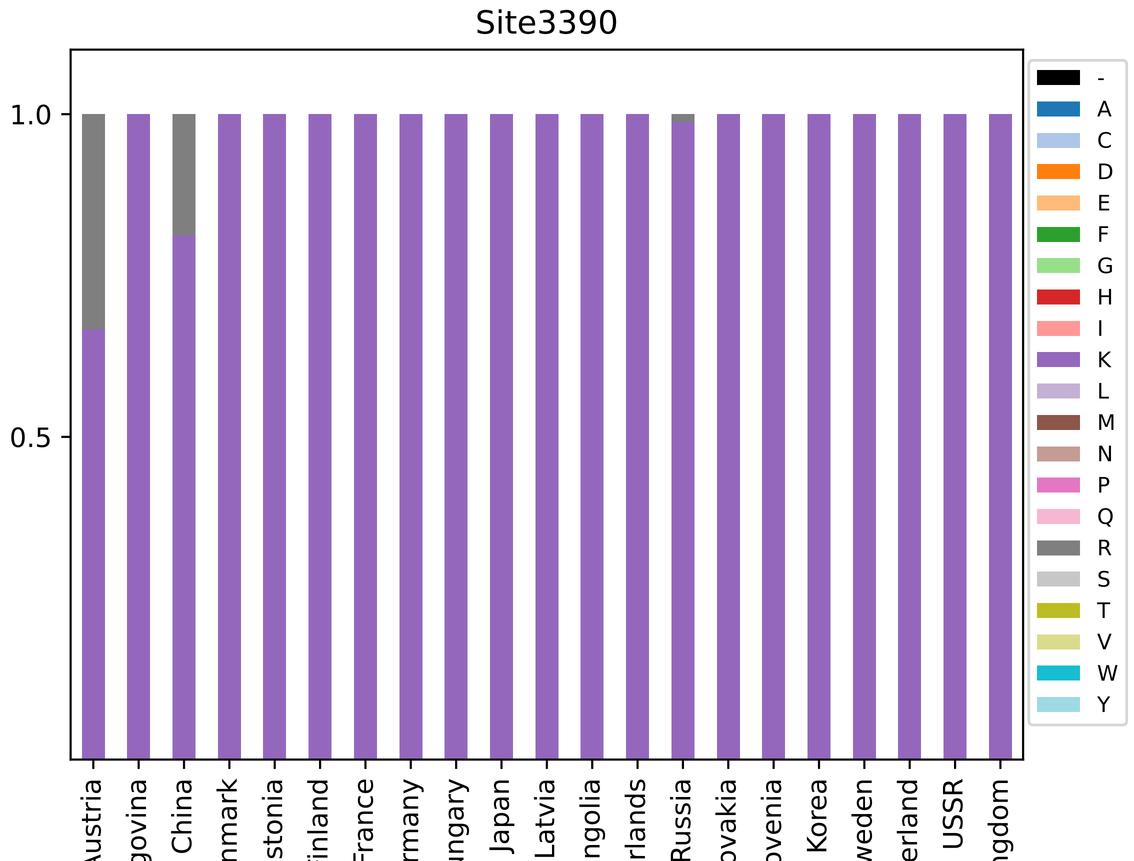

Site3390
1.0
0.5
-
A
C
D
E
F
G
H
I
K
L
M
N
P
Q
R
S
T
V
W
Y
China
Japan
Latvia
Russia
Korea
USSR

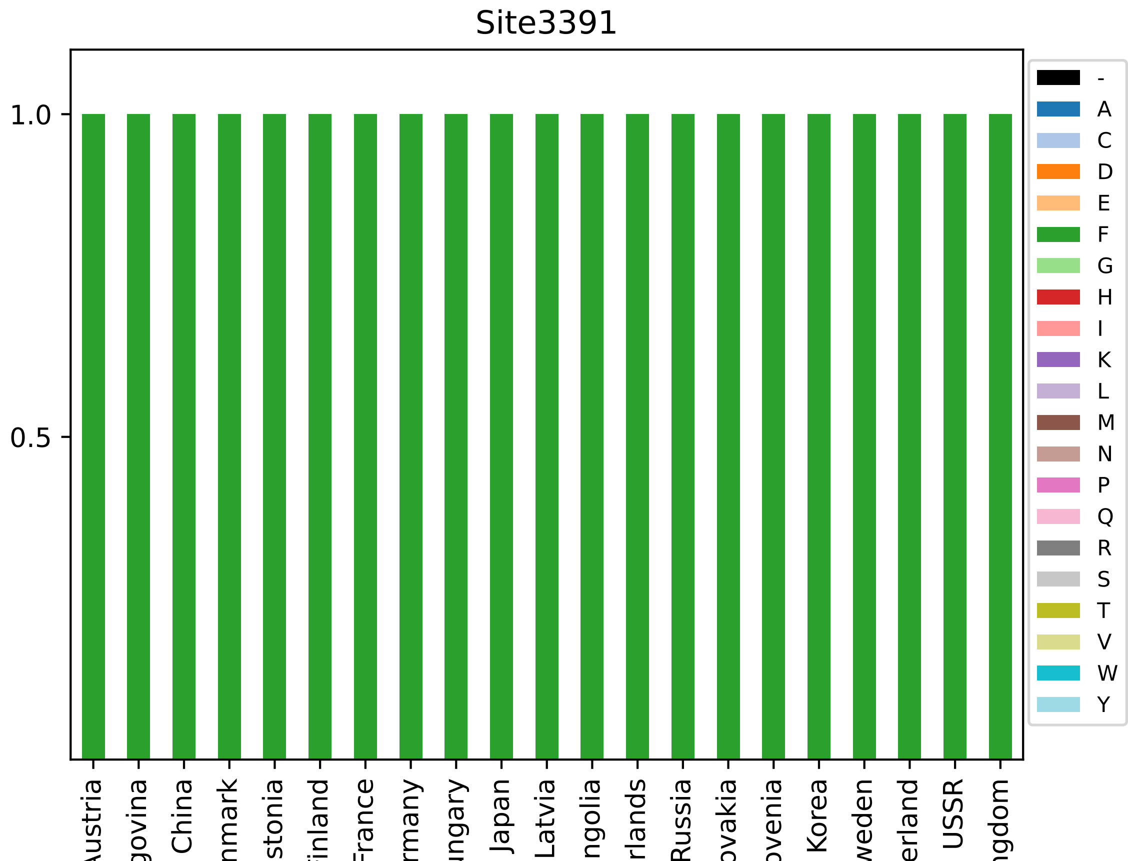
Site3391
1.0
0.5
-
A
C
D
E
F
G
H
I
K
L
M
N
P
Q
R
S
T
V
W
Y
China
Japan
Latvia
Russia
Korea
USSR

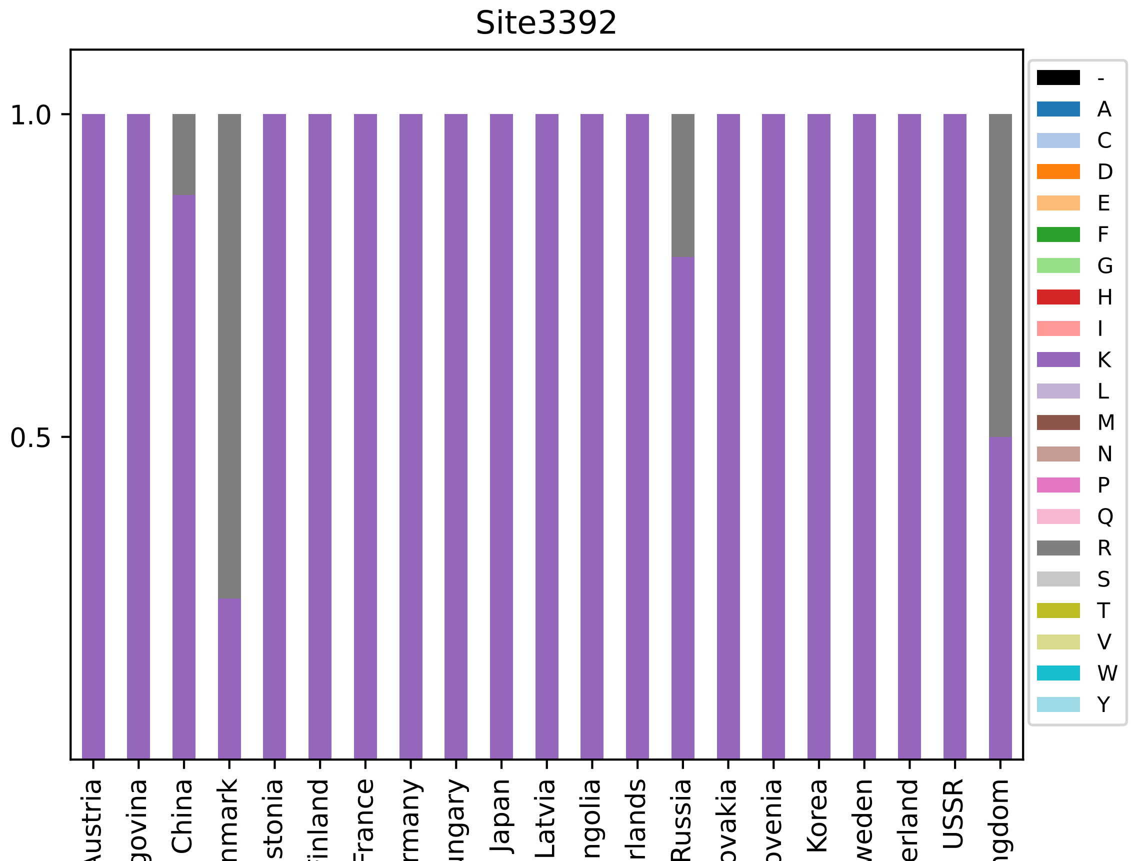
Site3392
1.0
0.5
-
A
C
D
E
F
G
H
I
K
L
M
N
P
Q
R
S
T
V
W
Y
Austria
China
stonia
Finland
France
rmany
ungary
Japan
Latvia
ngolia
rlands
Russia
ovakia
ovenia
Korea
weden
erland
USSR
ngdom

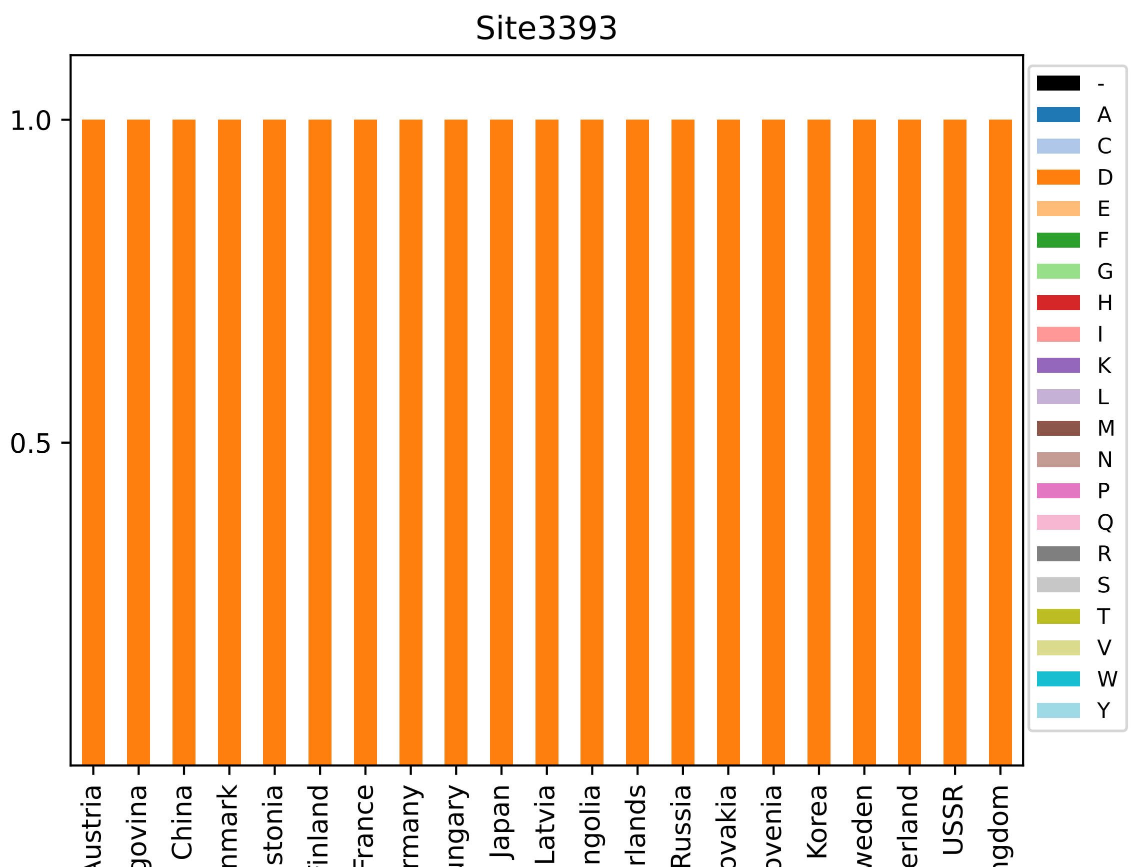

Site3393
1.0
0.5
-
A
C
D
E
F
G
H
I
K
L
M
N
P
Q
R
S
T
V
W
Y
China
Japan
Latvia
Russia
Korea
USSR

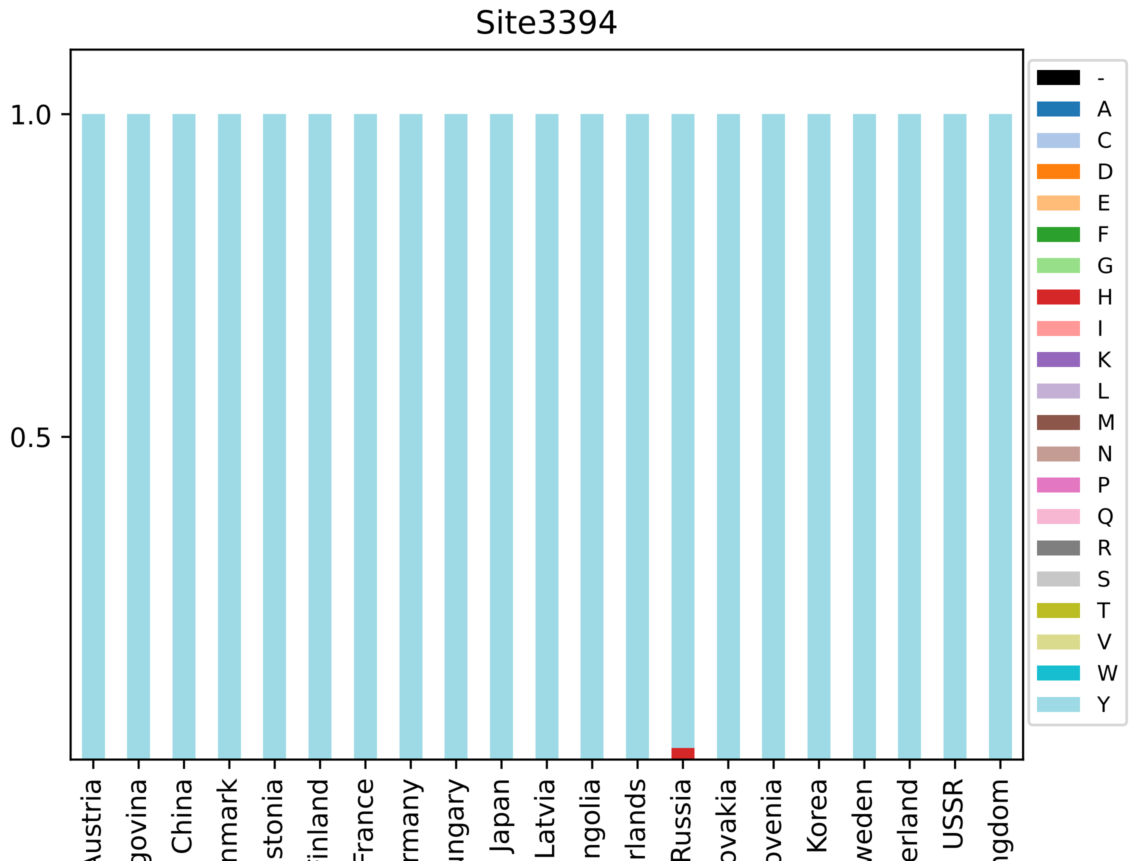

Site3394
1.0
0.5
Austria
govina
China
nmark
stonia
Finland
France
rmany
ungary
Japan
Latvia
ngolia
rlands
Russia
ovakia
ovenia
Korea
weden
erland
USSR
ngdom
-
A
C
D
E
F
G
H
I
K
L
M
N
P
Q
R
S
T
V
W
Y

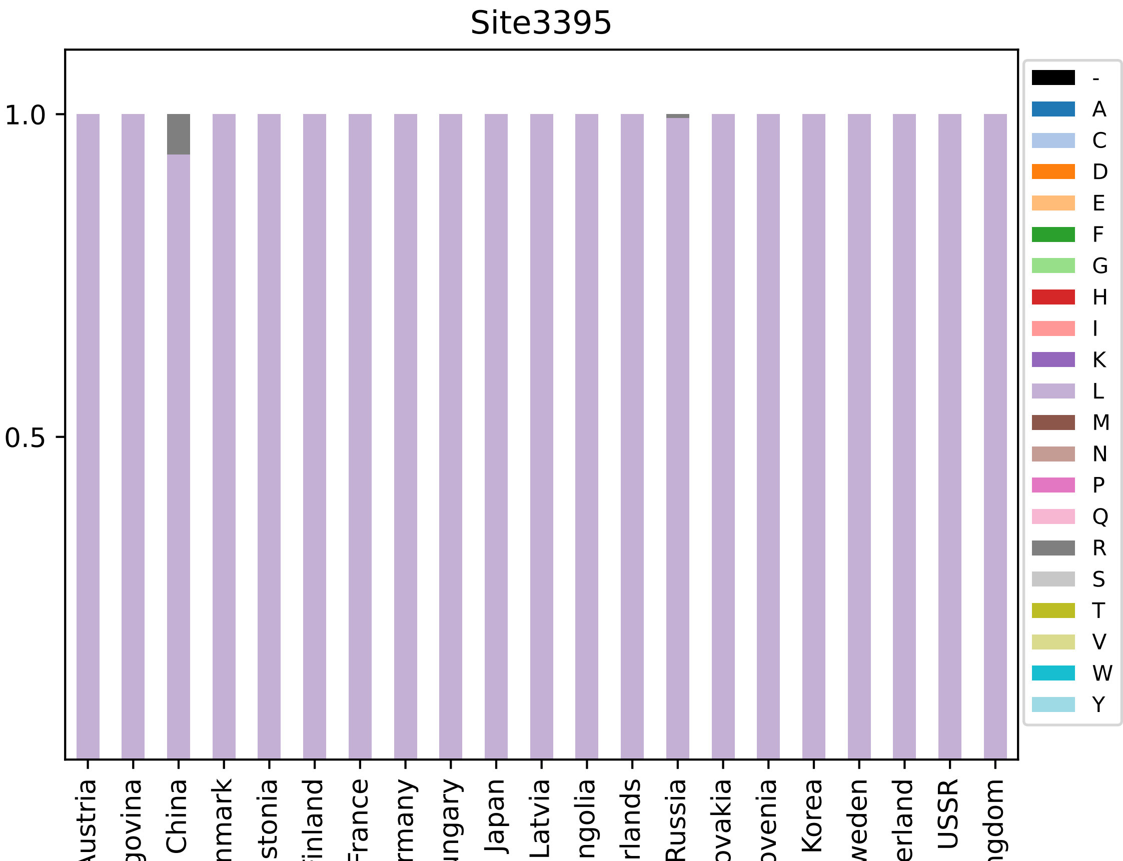
Site3395
1.0
0.5
-
A
C
D
E
F
G
H
I
K
L
M
N
P
Q
R
S
T
V
W
Y
Austria
govina
China
nmark
stonia
Finland
France
rmany
ungary
Japan
Latvia
ngolia
rlands
Russia
ovakia
ovenia
Korea
weden
erland
USSR
ngdom

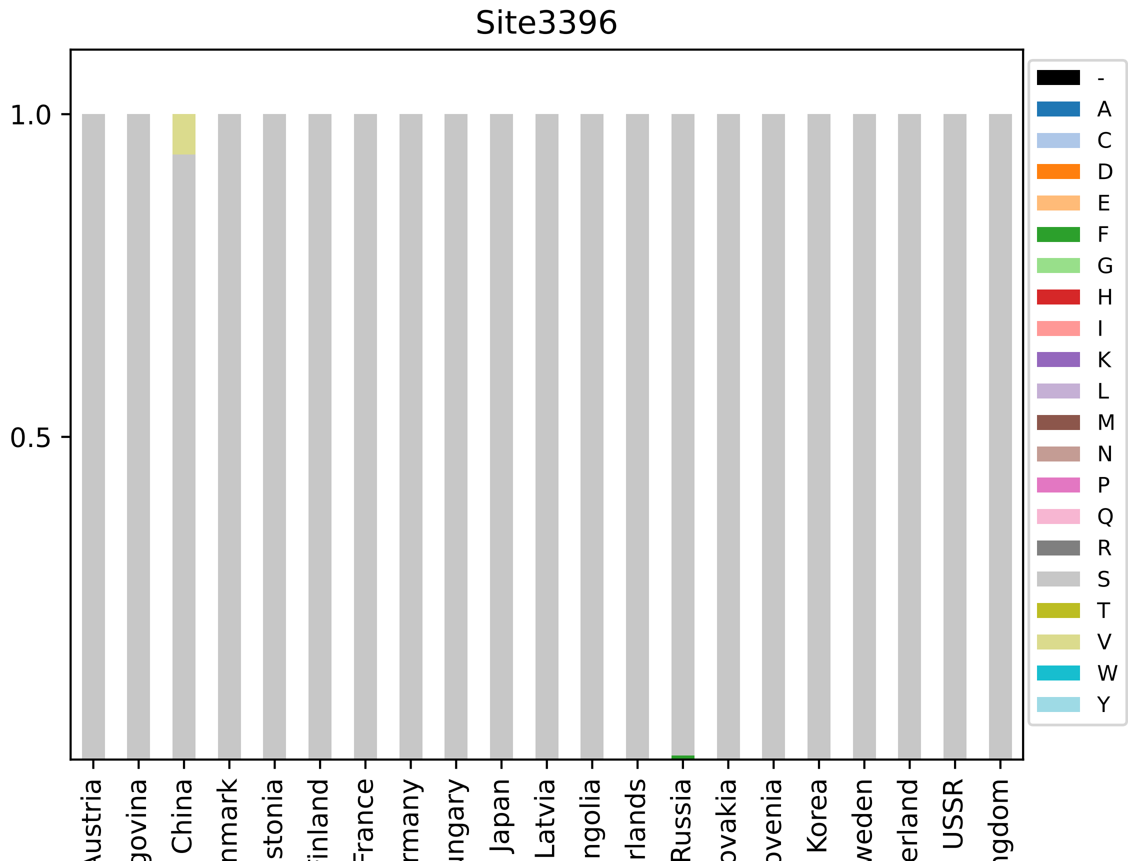

Site3396
1.0
0.5
-
A
C
D
E
F
G
H
I
K
L
M
N
P
Q
R
S
T
V
W
Y
Austria
govina
China
nmark
stonia
Finland
France
rmany
ungary
Japan
Latvia
ngolia
rlands
Russia
ovakia
ovenia
Korea
weden
erland
USSR
ngdom

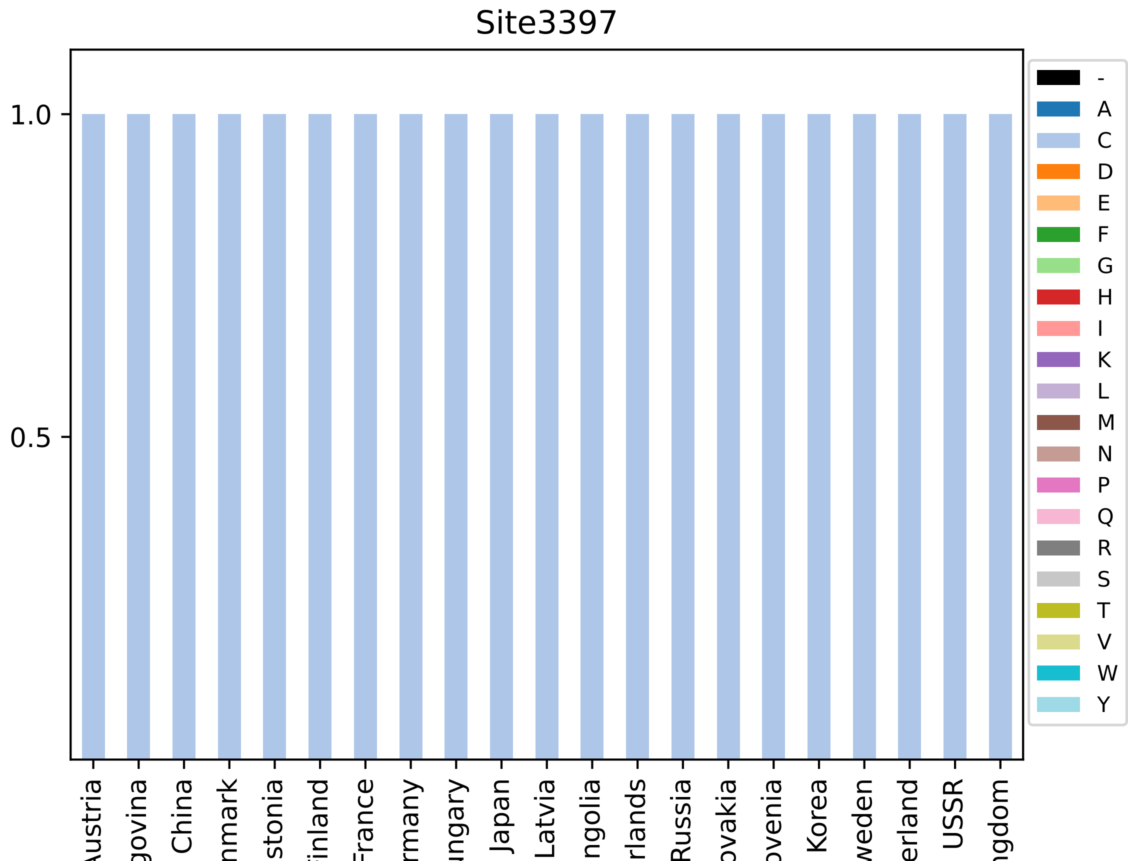
Site3397
1.0
0.5
-
A
C
D
E
F
G
H
I
K
L
M
N
P
Q
R
S
T
V
W
Y
China
Japan
Latvia
Russia
Korea
USSR

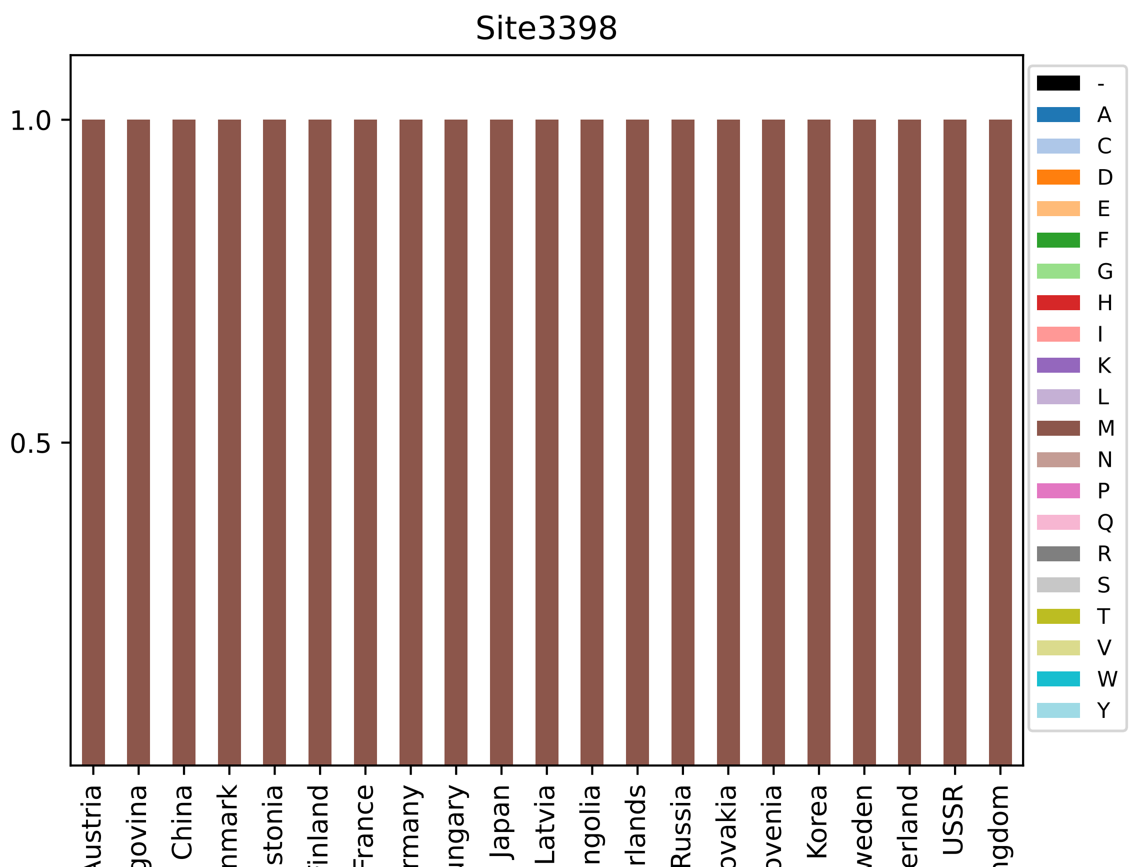

Site3398
1.0
0.5
-
A
C
D
E
F
G
H
I
K
L
M
N
P
Q
R
S
T
V
W
Y
Austria
govina
China
nmark
stonia
inland
France
rmany
ungary
Japan
Latvia
ngolia
rlands
Russia
ovakia
ovenia
Korea
weden
erland
USSR
ngdom

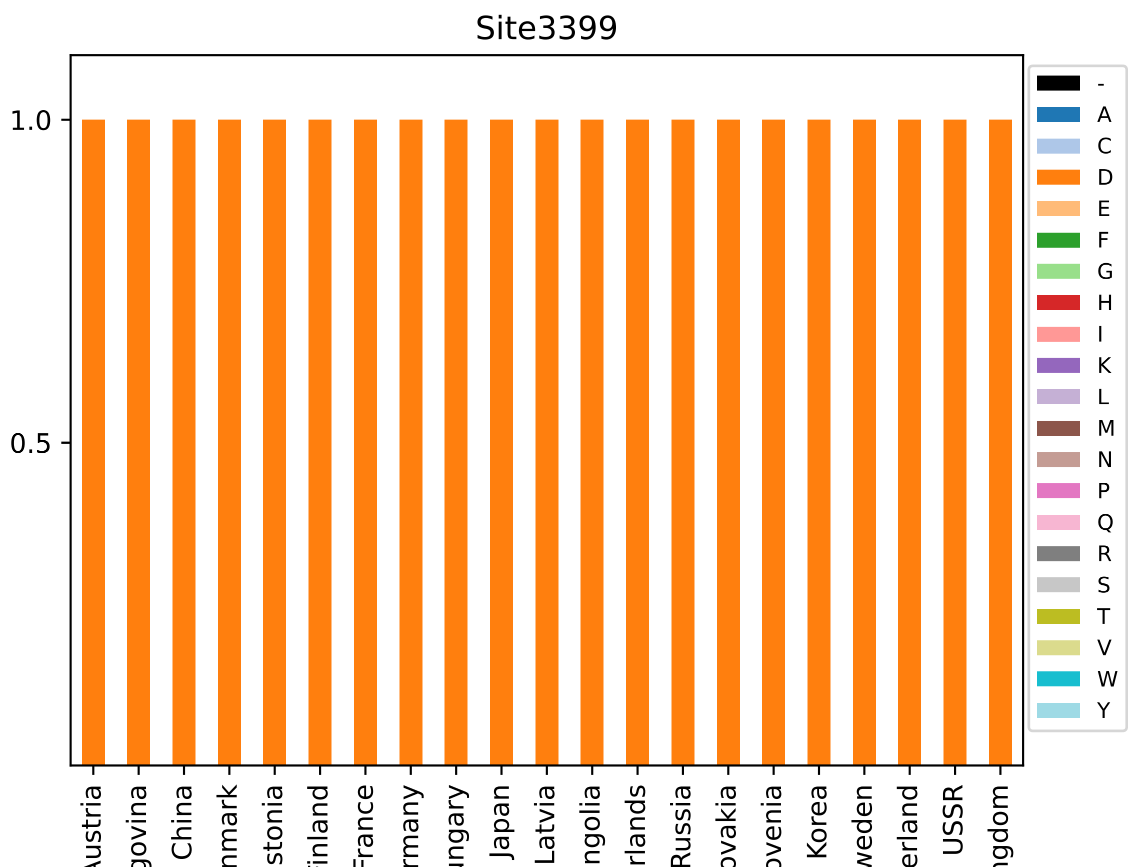
Site3399
1.0
0.5
-
A
C
D
E
F
G
H
I
K
L
M
N
P
Q
R
S
T
V
W
Y
China
Japan
Latvia
Russia
Korea
USSR

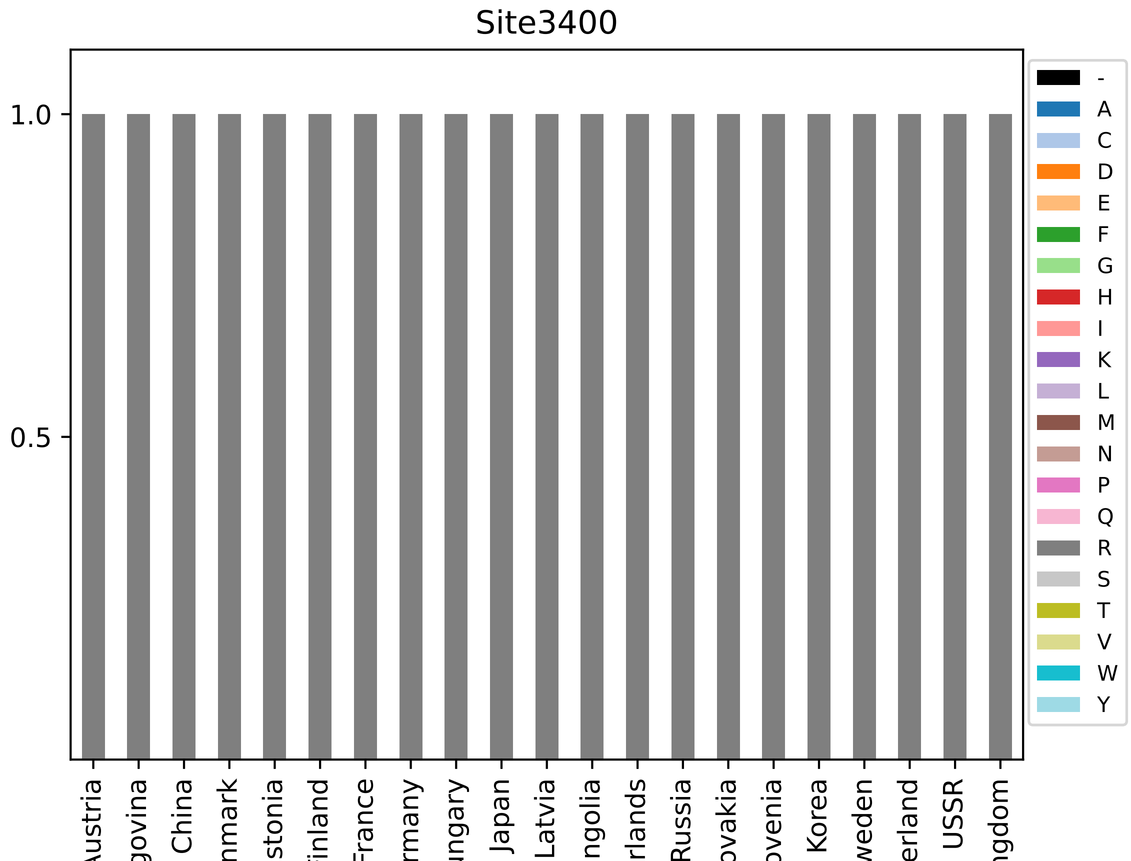
Site3400
1.0
0.5
-
A
C
D
E
F
G
H
I
K
L
M
N
P
Q
R
S
T
V
W
Y
Austria
govina
China
nmark
stonia
Finland
France
rmany
ungary
Japan
Latvia
ngolia
rlands
Russia
ovakia
ovenia
Korea
weden
erland
USSR
ngdom

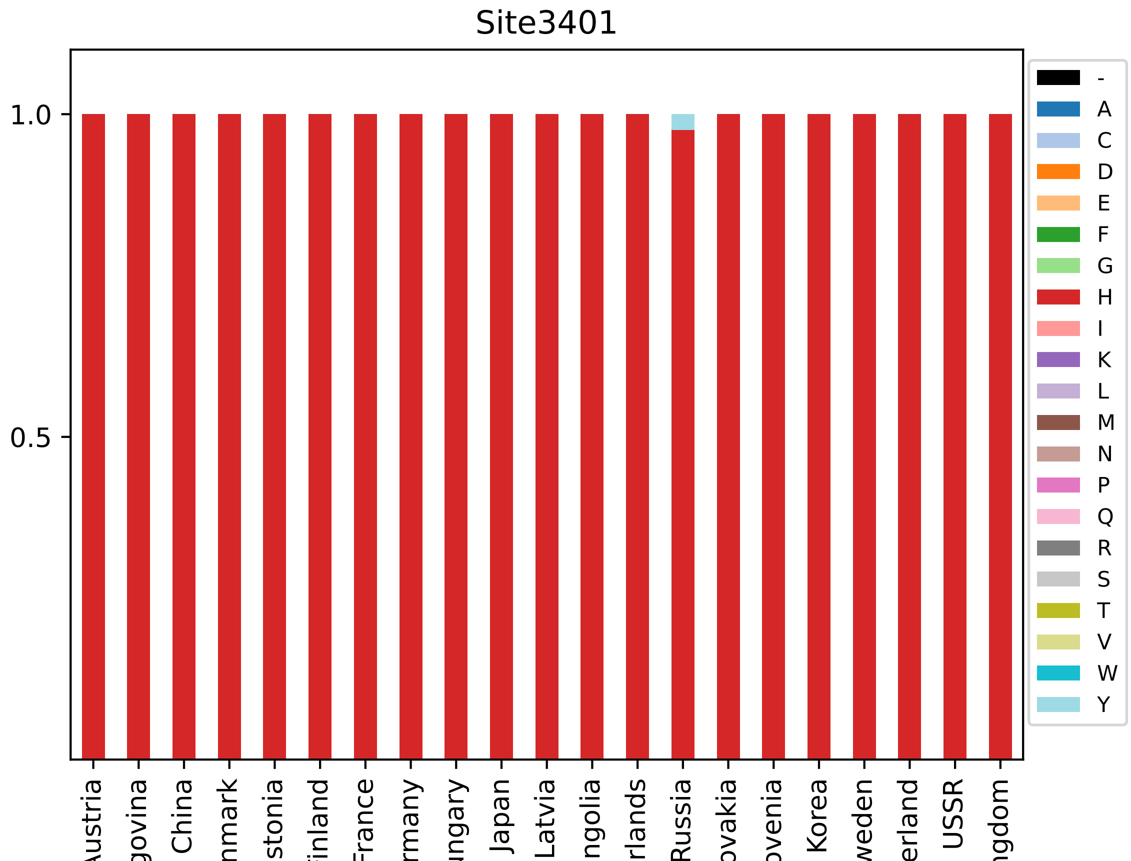

Site3401
1.0
0.5
-
A
C
D
E
F
G
H
I
K
L
M
N
P
Q
R
S
T
V
W
Y
China
Japan
Latvia
Russia
Korea
USSR

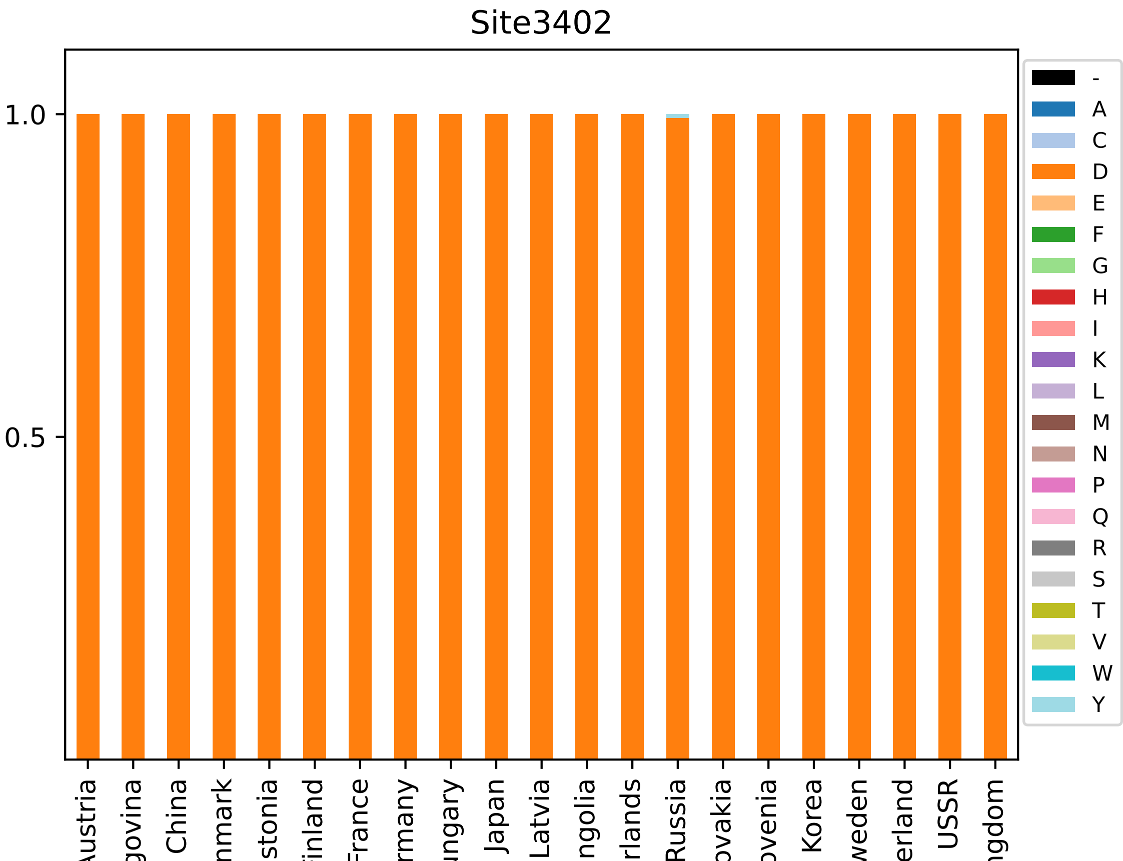

Site3402
1.0
0.5
-
A
C
D
E
F
G
H
I
K
L
M
N
P
Q
R
S
T
V
W
Y
Austria
govina
China
nmark
stonia
Finland
France
rmany
ungary
Japan
Latvia
ngolia
rlands
Russia
ovakia
ovenia
Korea
weden
erland
USSR
ngdom

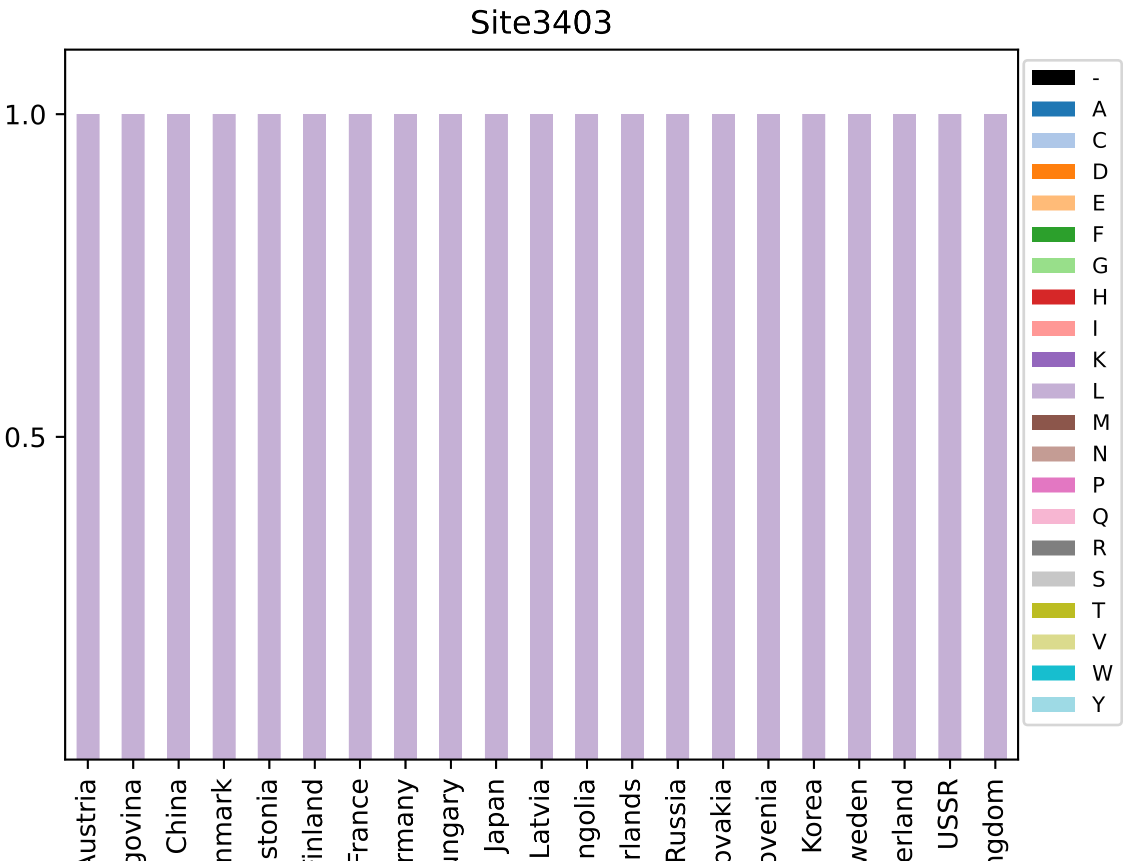

Site3403
1.0
0.5
-
A
C
D
E
F
G
H
I
K
L
M
N
P
Q
R
S
T
V
W
Y
China
Japan
Latvia
Russia
Korea
USSR

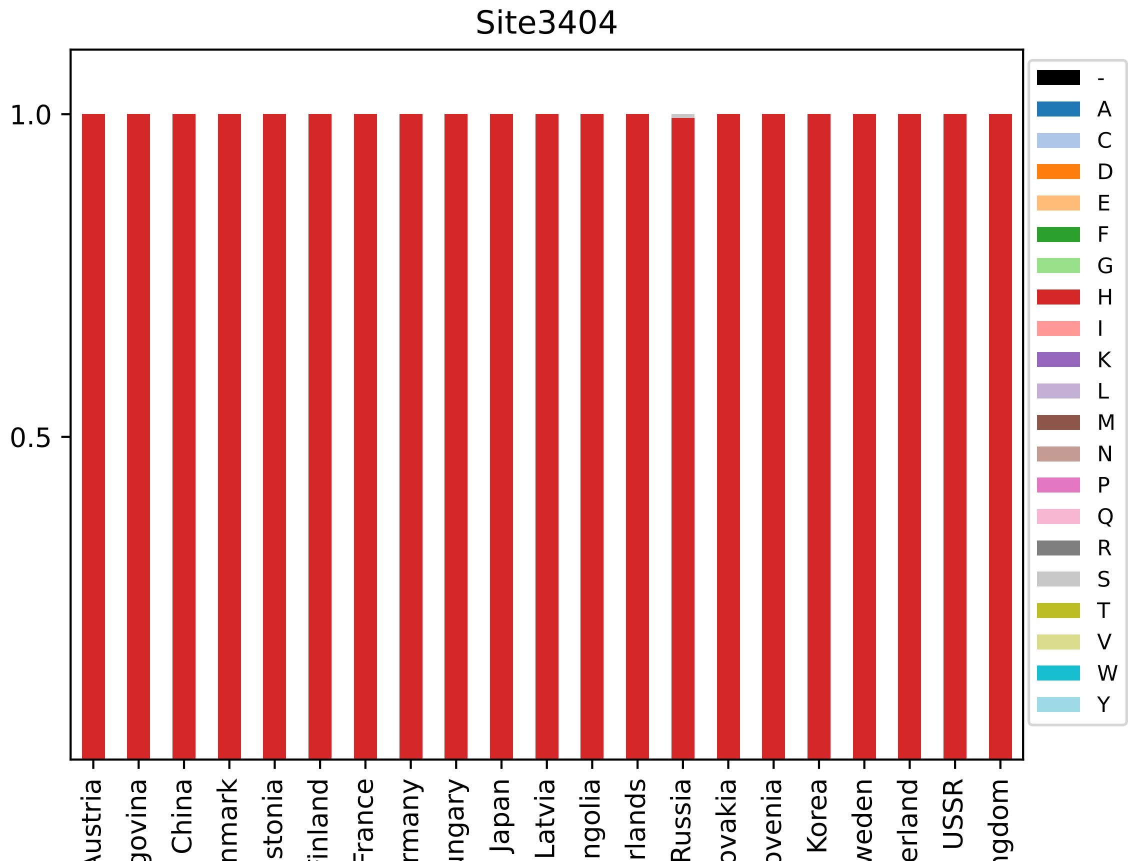
Site3404
1.0
0.5
-
A
C
D
E
F
G
H
I
K
L
M
N
P
Q
R
S
T
V
W
Y
China
Japan
Latvia
Russia
Korea
USSR

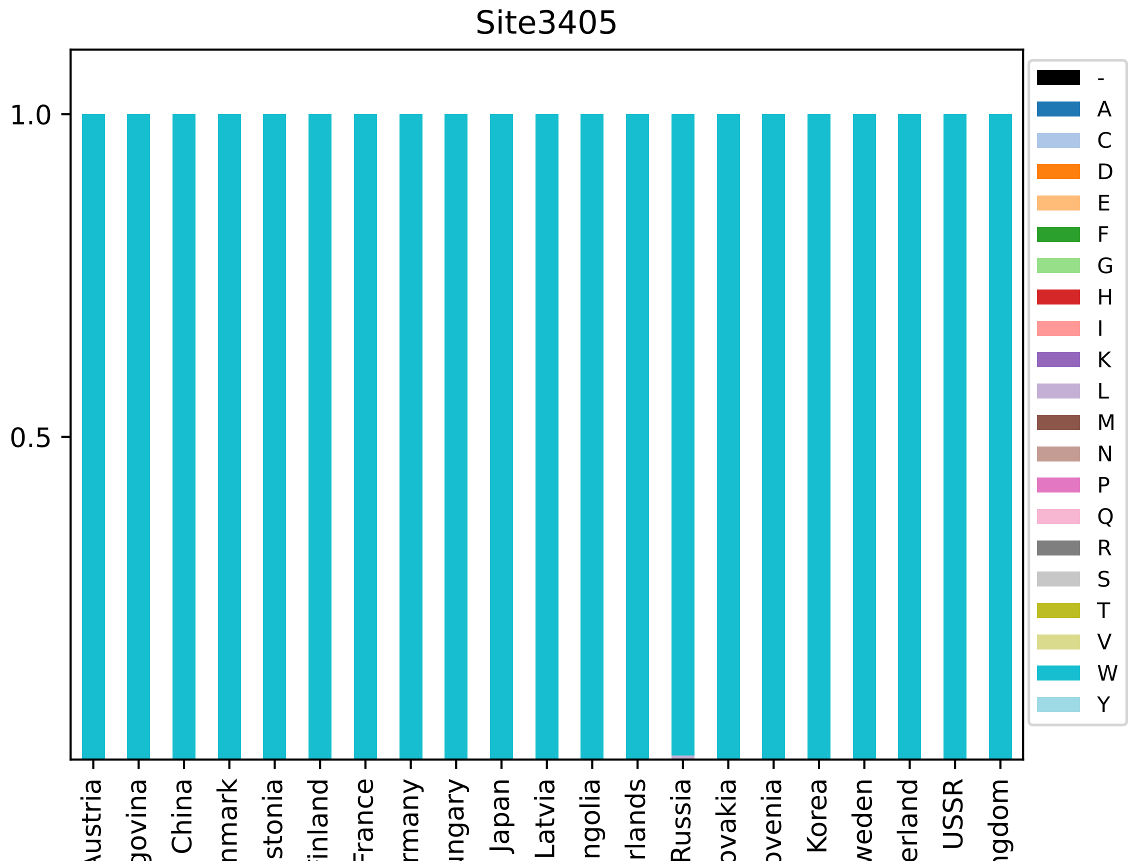
Site3405
1.0
0.5
-
A
C
D
E
F
G
H
I
K
L
M
N
P
Q
R
S
T
V
W
Y
Austria
govina
China
nmark
stonia
Finland
France
rmany
ungary
Japan
Latvia
ngolia
rlands
Russia
ovakia
ovenia
Korea
weden
erland
USSR
ngdom

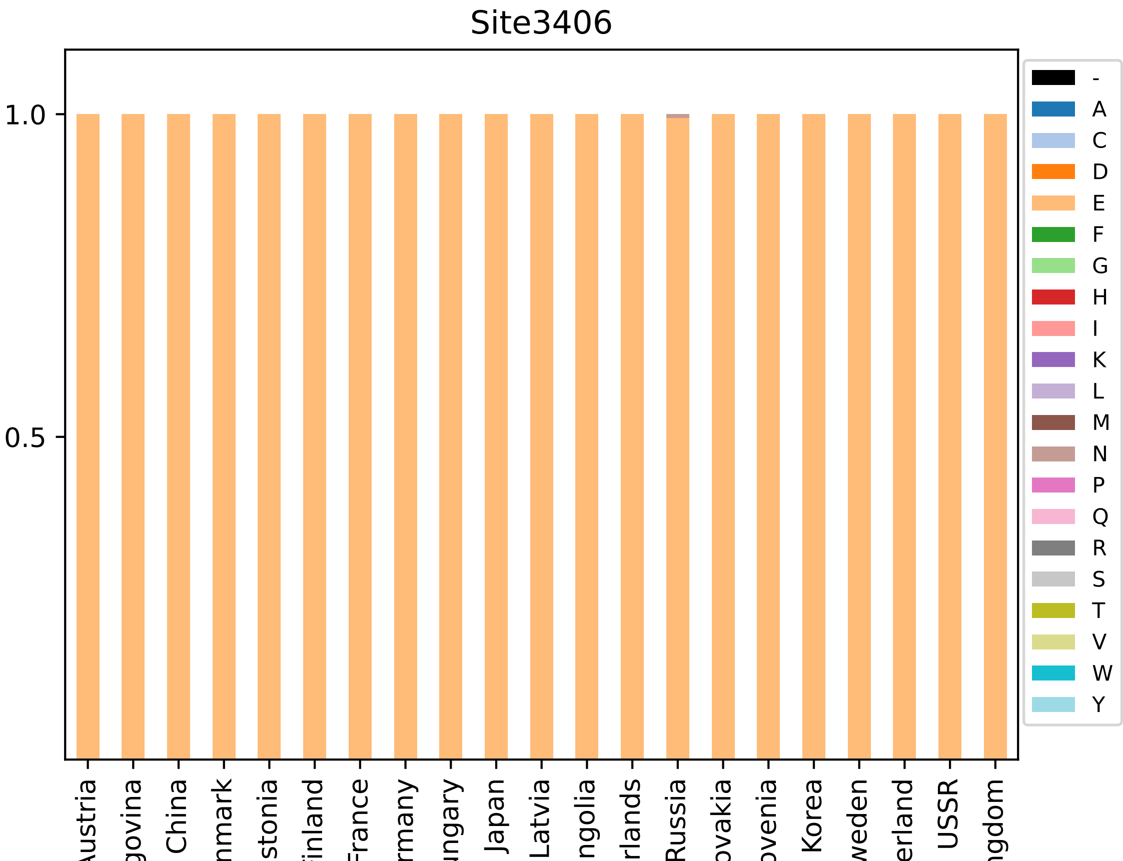

Site3406
1.0
0.5
-
A
C
D
E
F
G
H
I
K
L
M
N
P
Q
R
S
T
V
W
Y
Austria
gOVina
China
nmark
stonia
Finland
France
rmany
ungary
Japan
Latvia
ngolia
rlands
Russia
ovakia
ovenia
Korea
weden
erland
USSR
ngdom

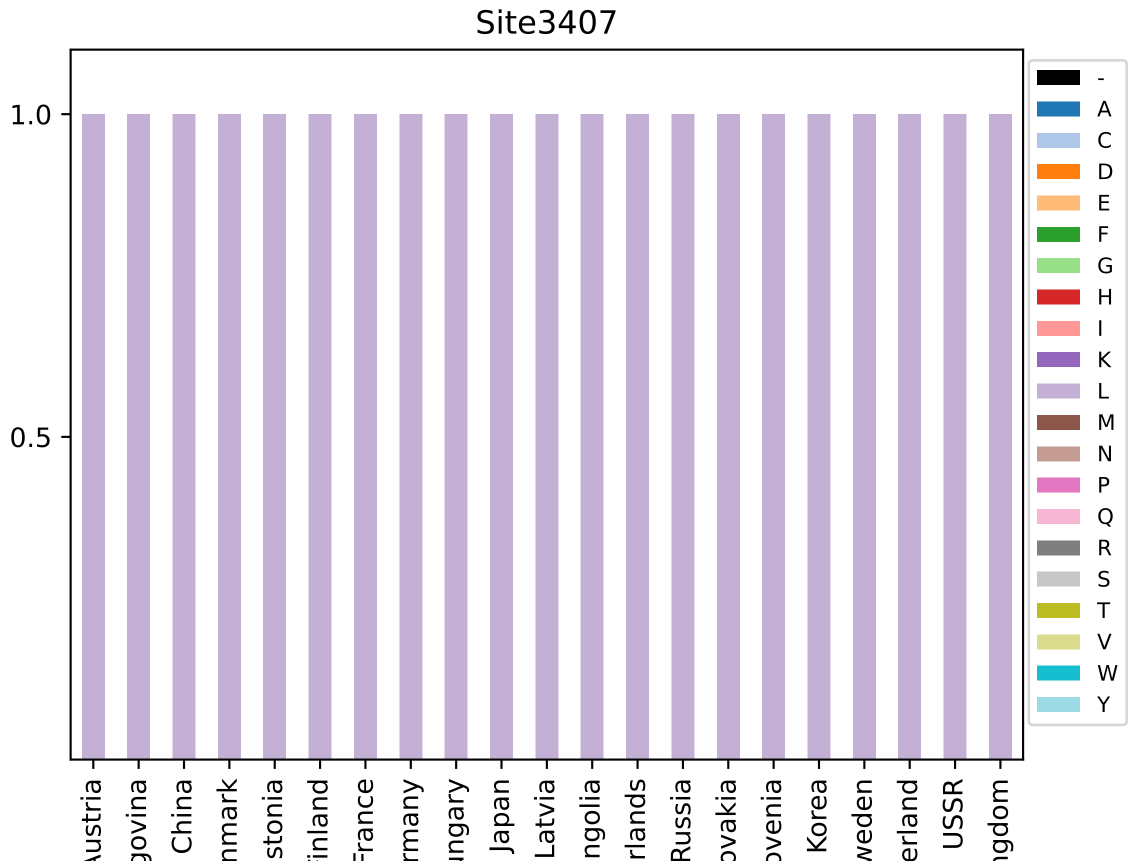
Site3407
1.0
0.5
-
A
C
D
E
F
G
H
I
K
L
M
N
P
Q
R
S
T
V
W
Y
China
Japan
Latvia
Russia
Korea
USSR

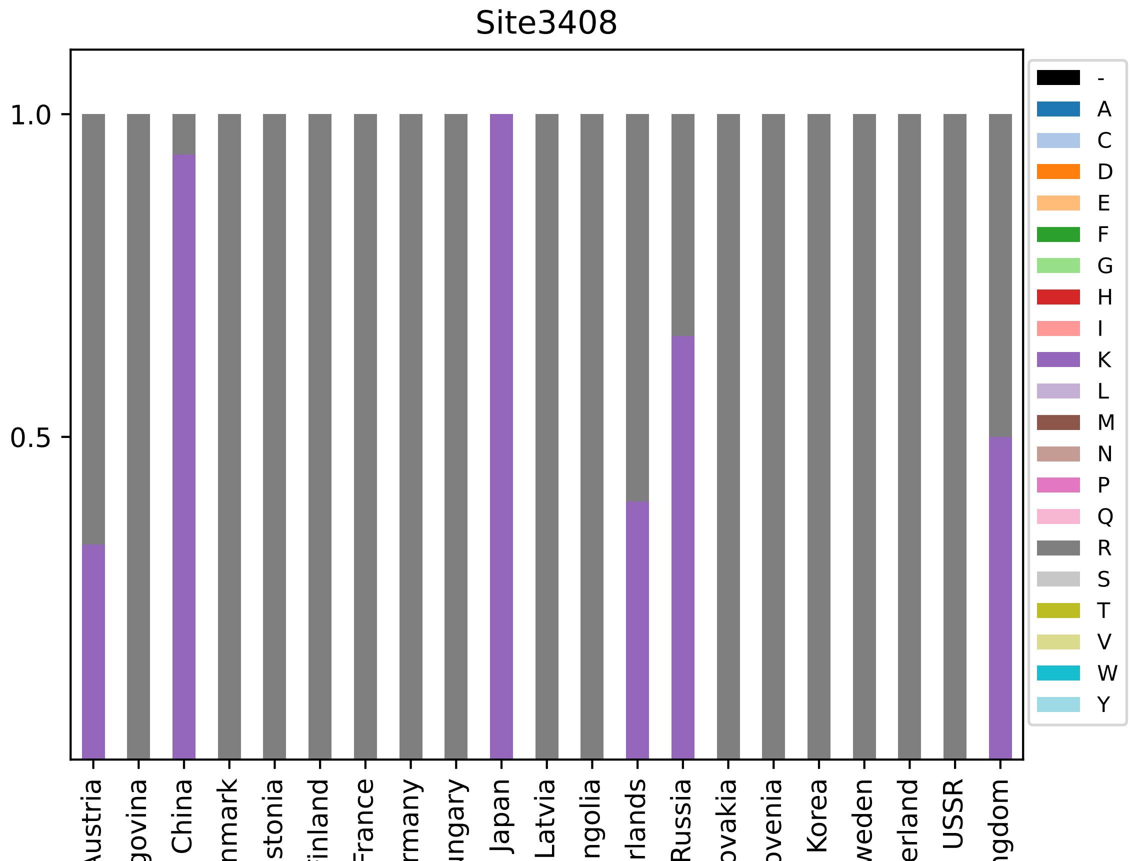

Site3408
1.0
0.5
-
A
C
D
E
F
G
H
I
K
L
M
N
P
Q
R
S
T
V
W
Y
China
Japan
Latvia
Russia
Korea
USSR

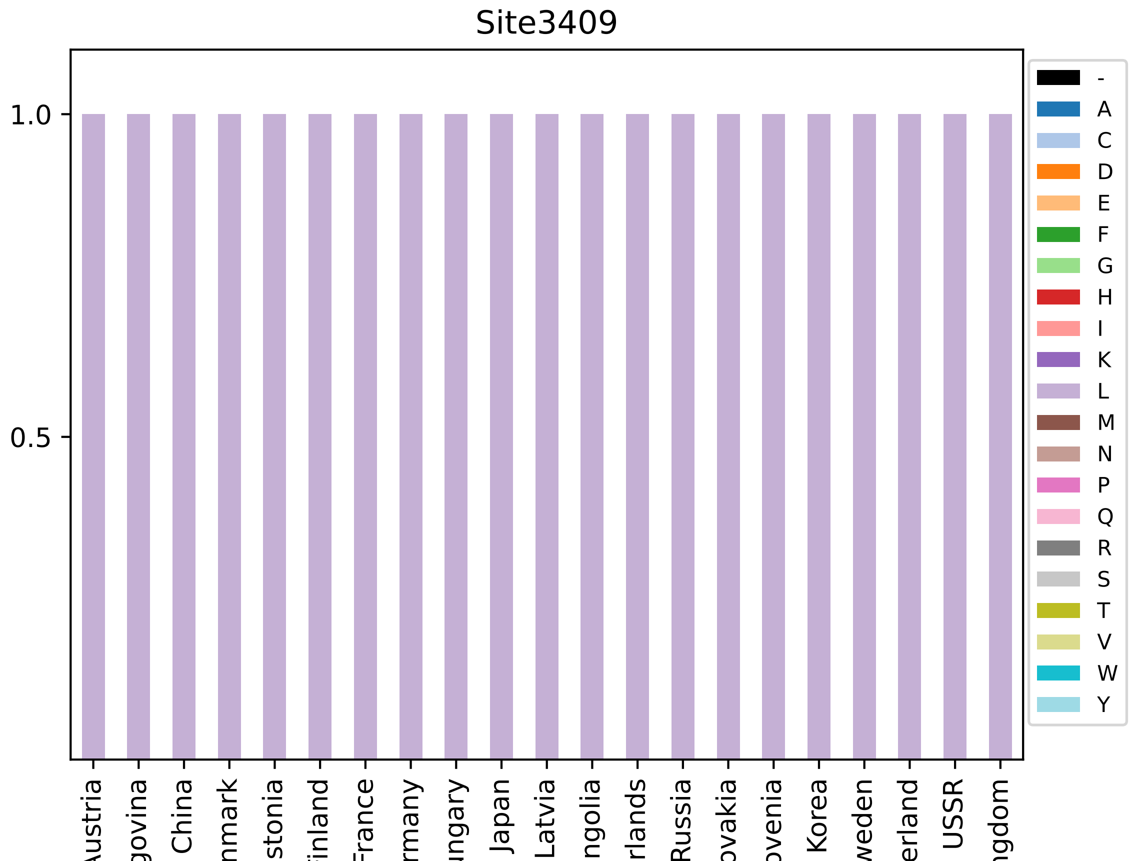
Site3409
1.0
0.5
-
A
C
D
E
F
G
H
I
K
L
M
N
P
Q
R
S
T
V
W
Y
China
Japan
Latvia
Russia
Korea
USSR

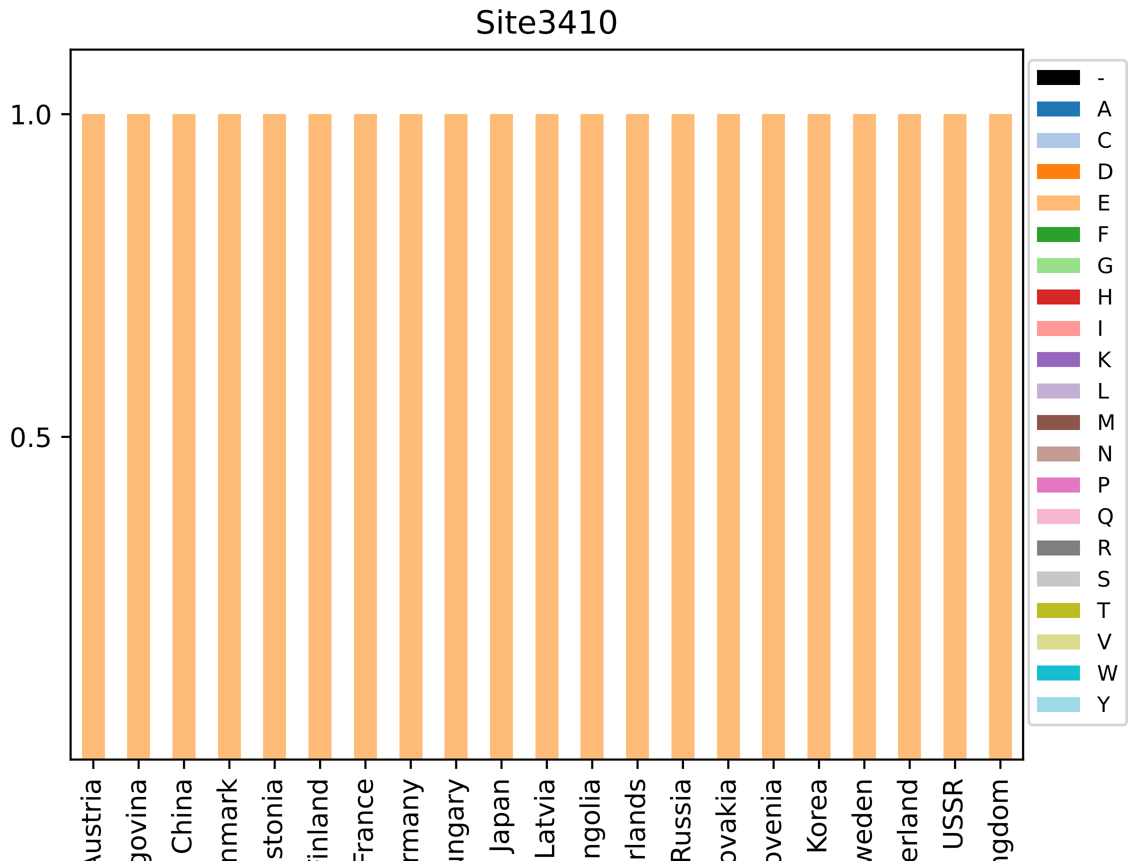
Site3410
1.0
0.5
-
A
C
D
E
F
G
H
I
K
L
M
N
P
Q
R
S
T
V
W
Y
China
Japan
Latvia
Russia
Korea
USSR

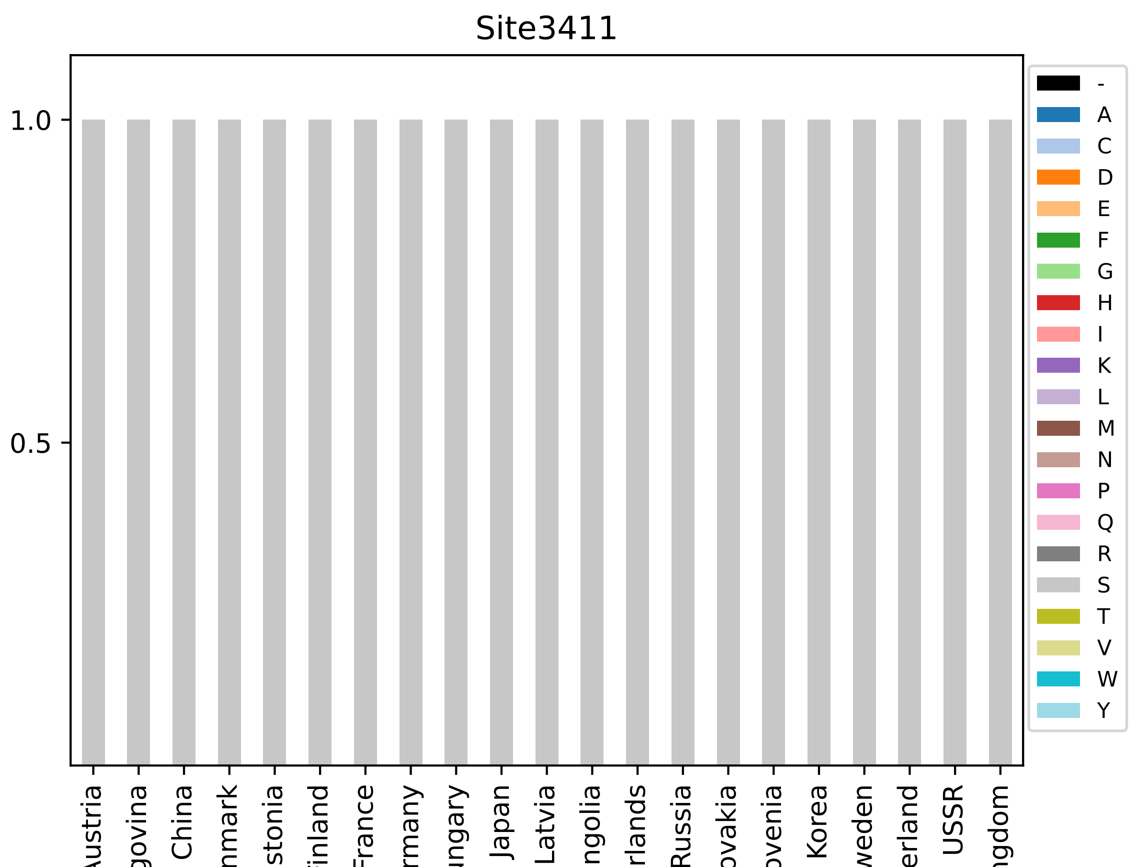
Site3411
1.0
0.5
-
A
C
D
E
F
G
H
I
K
L
M
N
P
Q
R
S
T
V
W
Y
Austria
China
Japan
Latvia
Russia
Korea
USSR

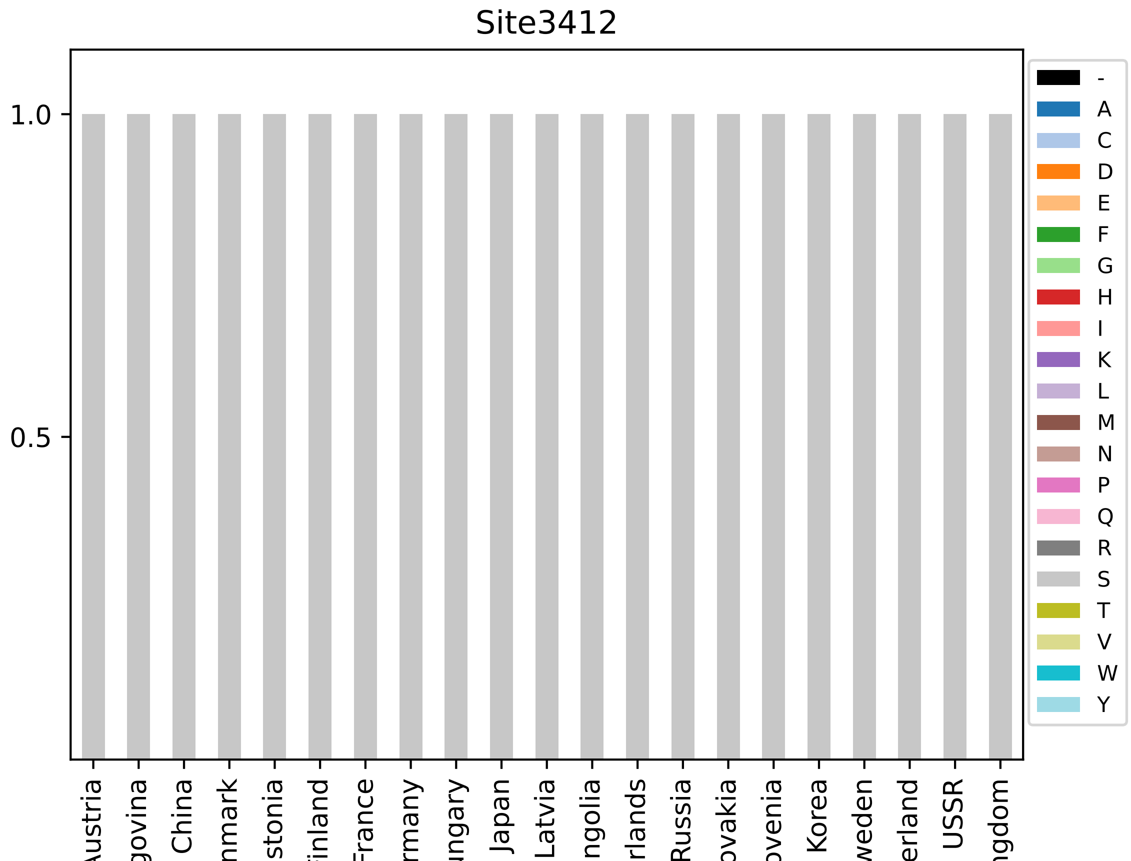
Site3412
1.0
0.5
China
Japan
Latvia
Russia
Korea
USSR
-
A
C
D
E
F
G
H
I
K
L
M
N
P
Q
R
S
T
V
W
Y

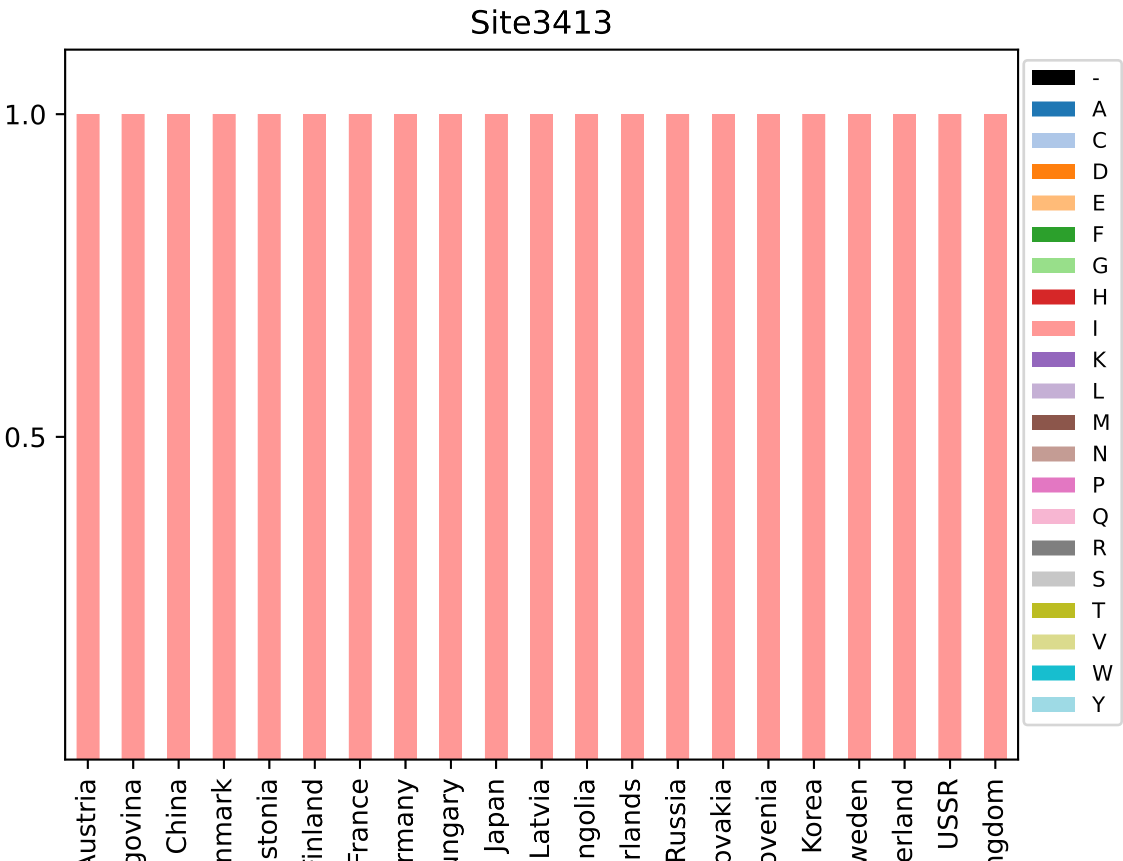
Site3413
1.0
0.5
-
A
C
D
E
F
G
H
I
K
L
M
N
P
Q
R
S
T
V
W
Y
China
Japan
Latvia
Russia
Korea
USSR

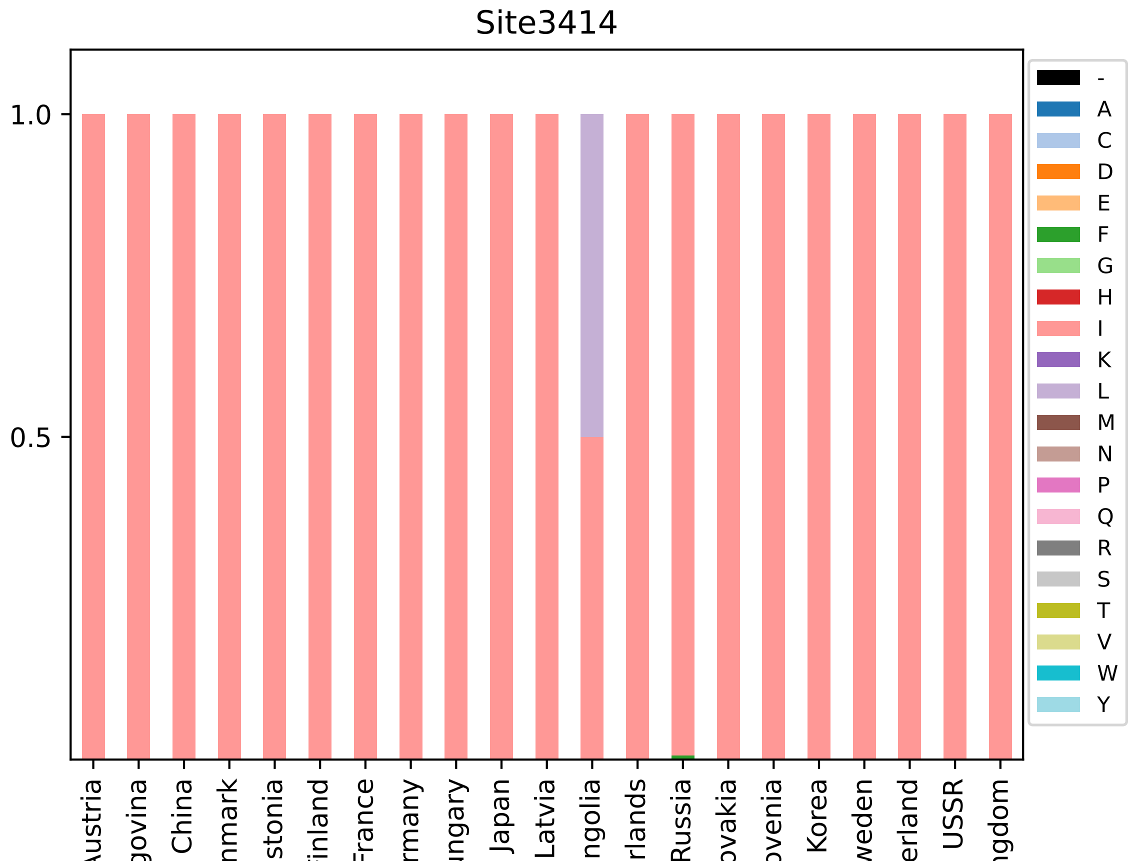

Site3414
1.0
0.5
-
A
C
D
E
F
G
H
I
K
L
M
N
P
Q
R
S
T
V
W
Y
Austria
China
Finland
France
Japan
Latvia
Russia
Korea
USSR

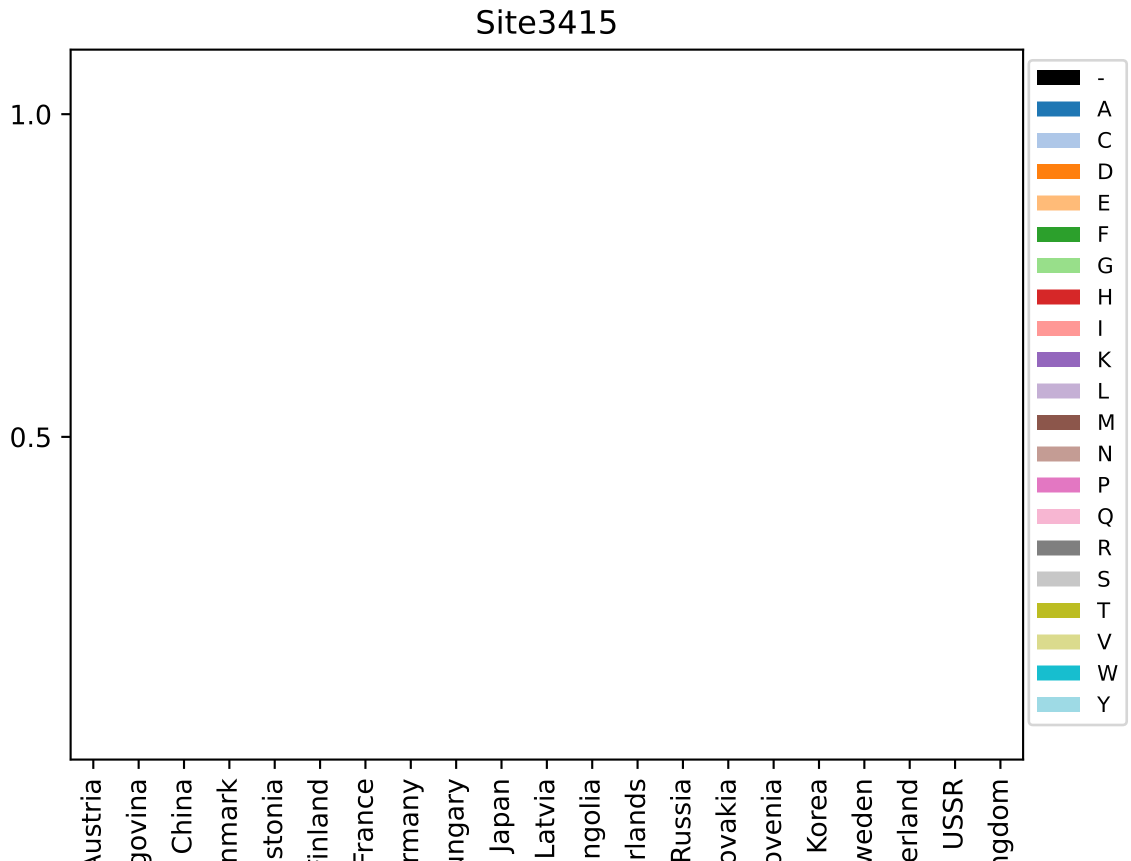
Site3415
1.0
0.5
-
A
C
D
E
F
G
H
I
K
L
M
N
P
Q
R
S
T
V
W
Y
China
Japan
Latvia
Russia
Korea
USSR
