# Supplementary material for: Evolutionary Patterns and Genotype-Specific Amino Acid Mutations of Tick-Borne Encephalitis Virus
Source: Int J Mol Sci. 2025 Jan 23;26(3):954. doi: 10.3390/ijms26030954 (PMC11817229; doi:10.3390/ijms26030954)
Supplement: Supplementary file 1 [file ijms-26-00954-s001.zip › ijms-3423653-supplementary/Supplementary Data S2.zip!/genotype/genotype_aa_mutation.pdf]

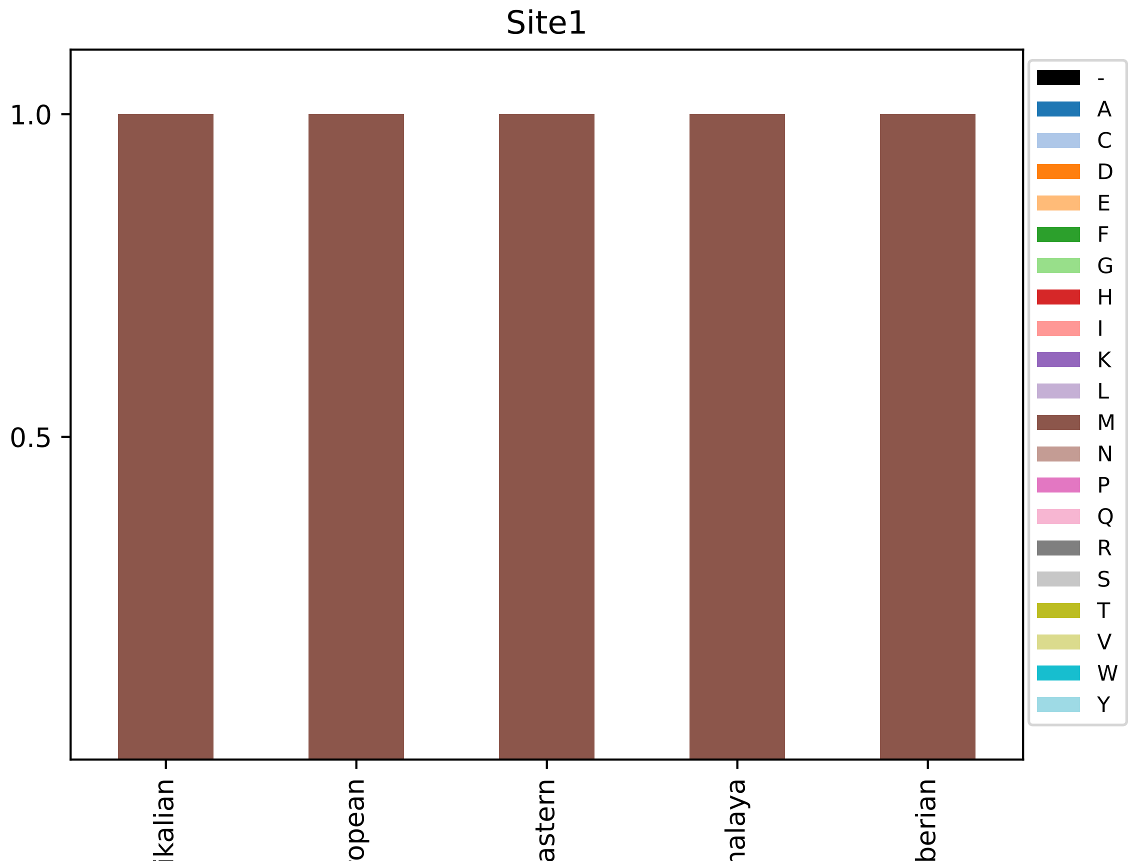
Site1
1.0
0.5
ikalian
ropean
astern
nalaya
berian
-
A
C
D
E
F
G
H
I
K
L
M
N
P
Q
R
S
T
V
W
Y

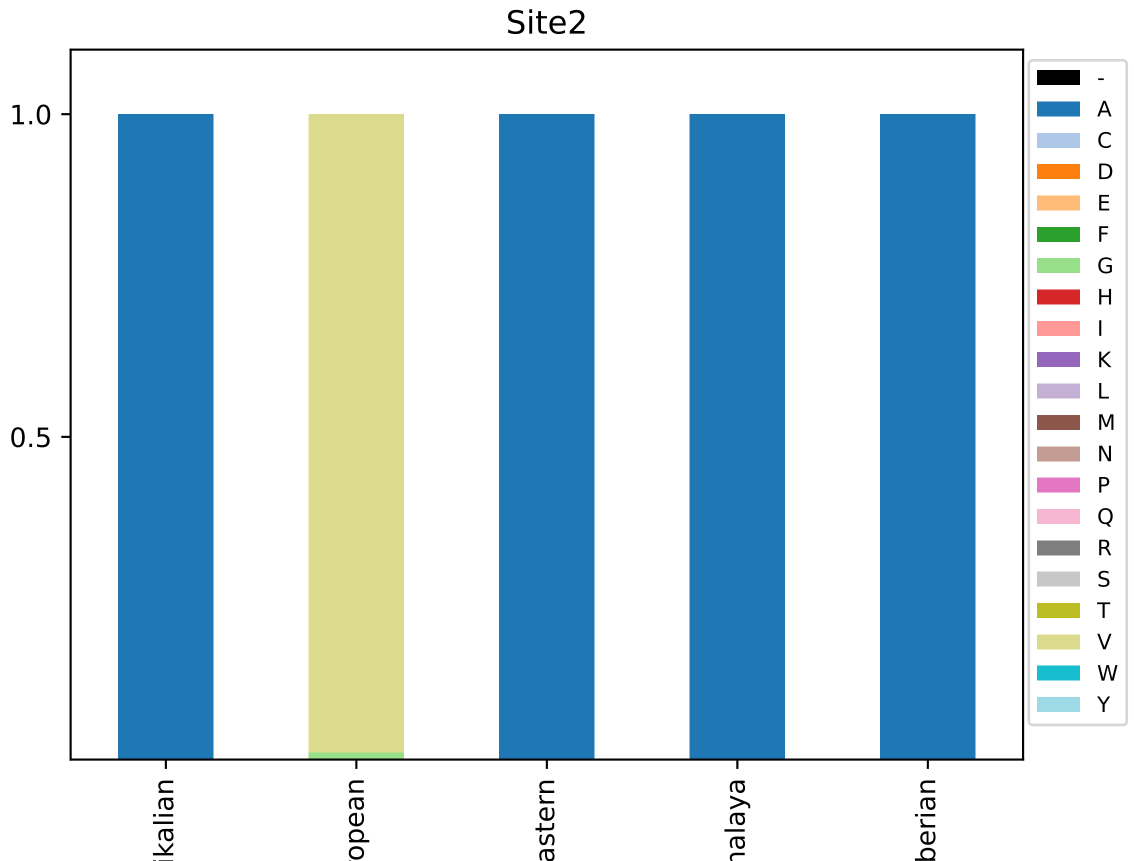
Site2
1.0
0.5
-
A
C
D
E
F
G
H
I
K
L
M
N
P
Q
R
S
T
V
W
Y
ikalian
ropean
astern
nalaya
berian

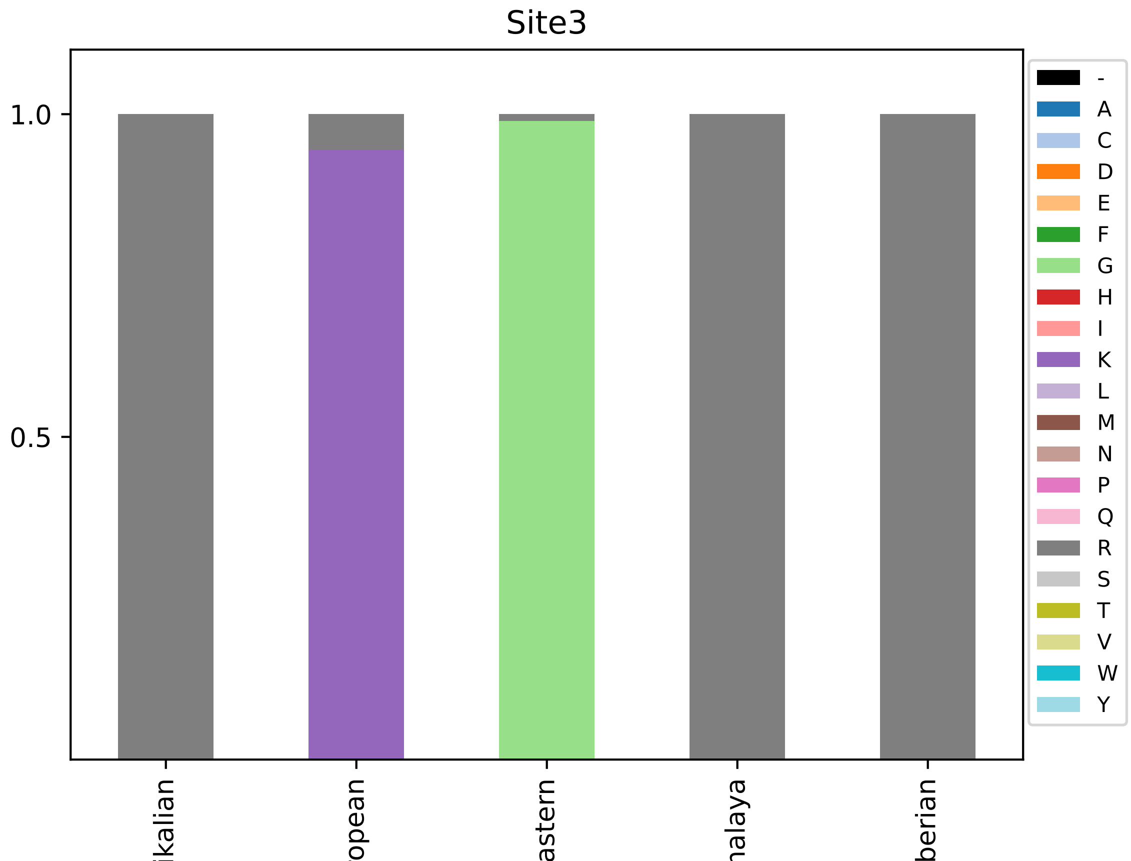
Site3
1.0
0.5
-
A
C
D
E
F
G
H
I
K
L
M
N
P
Q
R
S
T
V
W
Y
ikalian
opean
astern
nalaya
berian

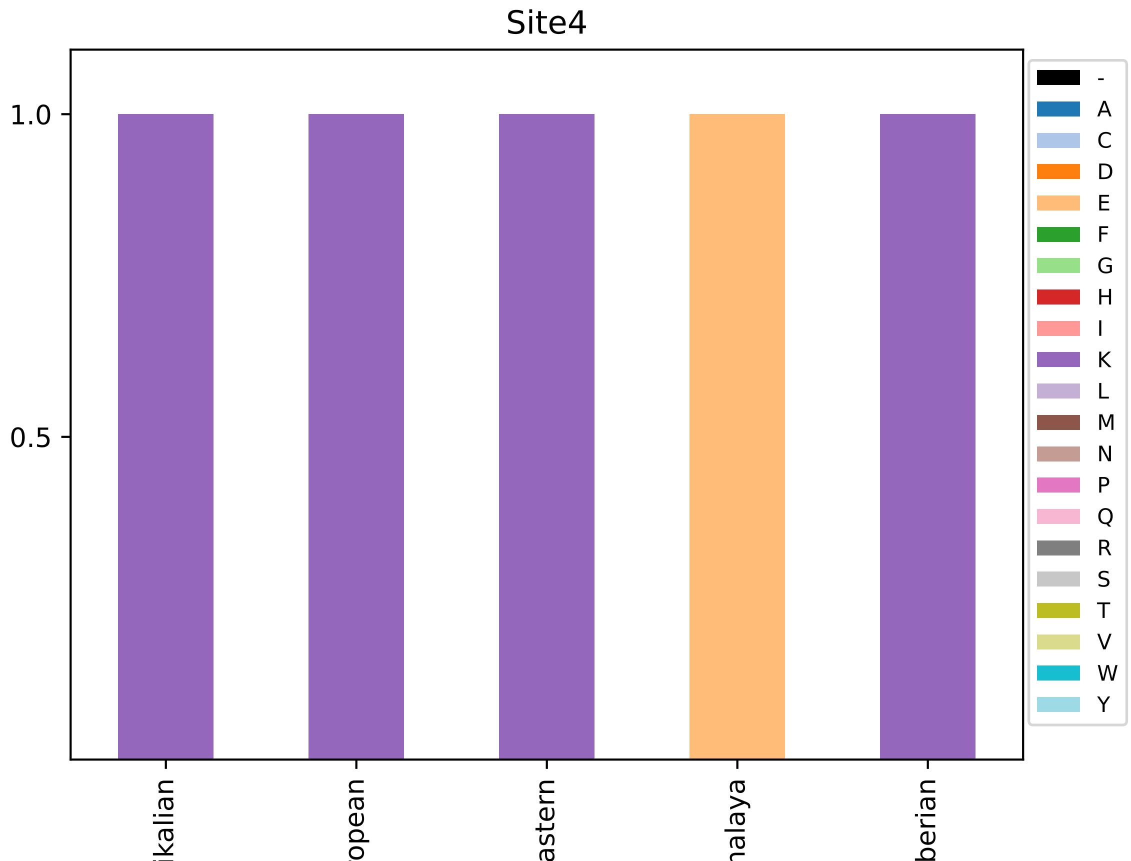
Site4
1.0
0.5
-
A
C
D
E
F
G
H
I
K
L
M
N
P
Q
R
S
T
V
W
Y

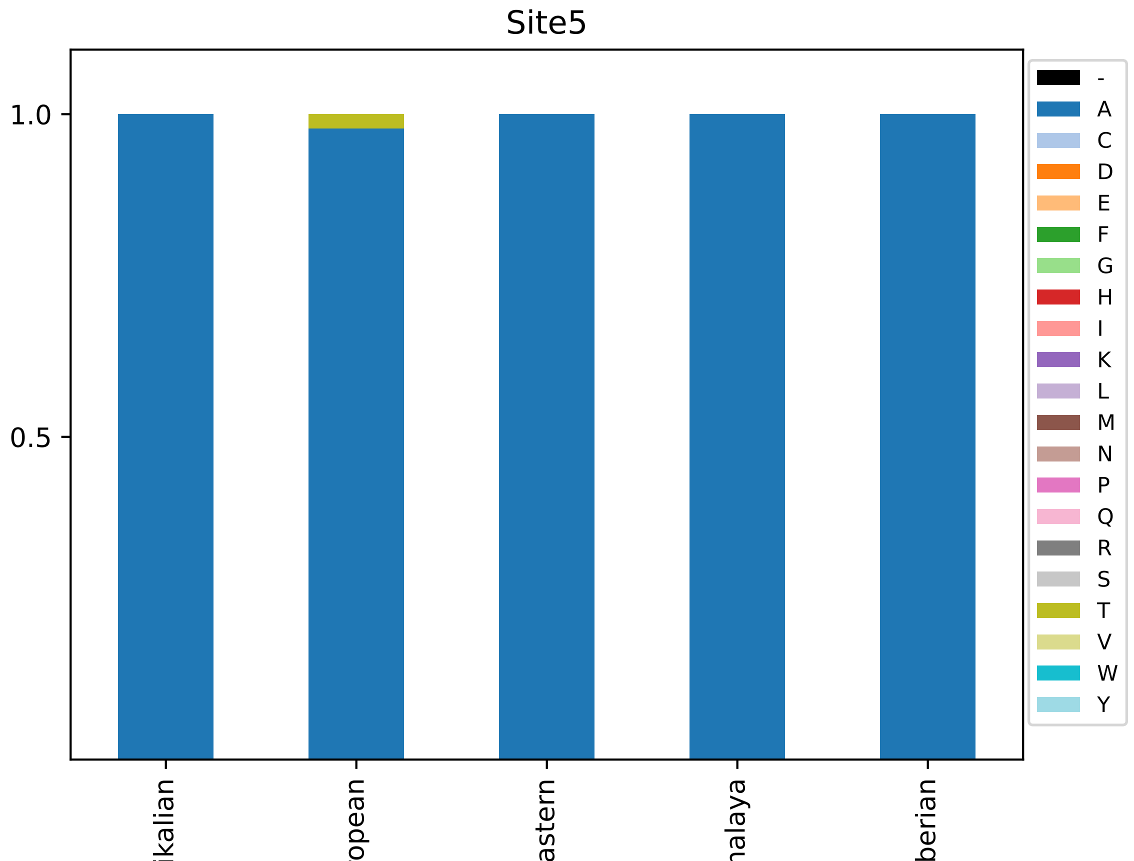
Site5
1.0
0.5
ikalian
opean
astern
nalaya
berian
-
A
C
D
E
F
G
H
I
K
L
M
N
P
Q
R
S
T
V
W
Y

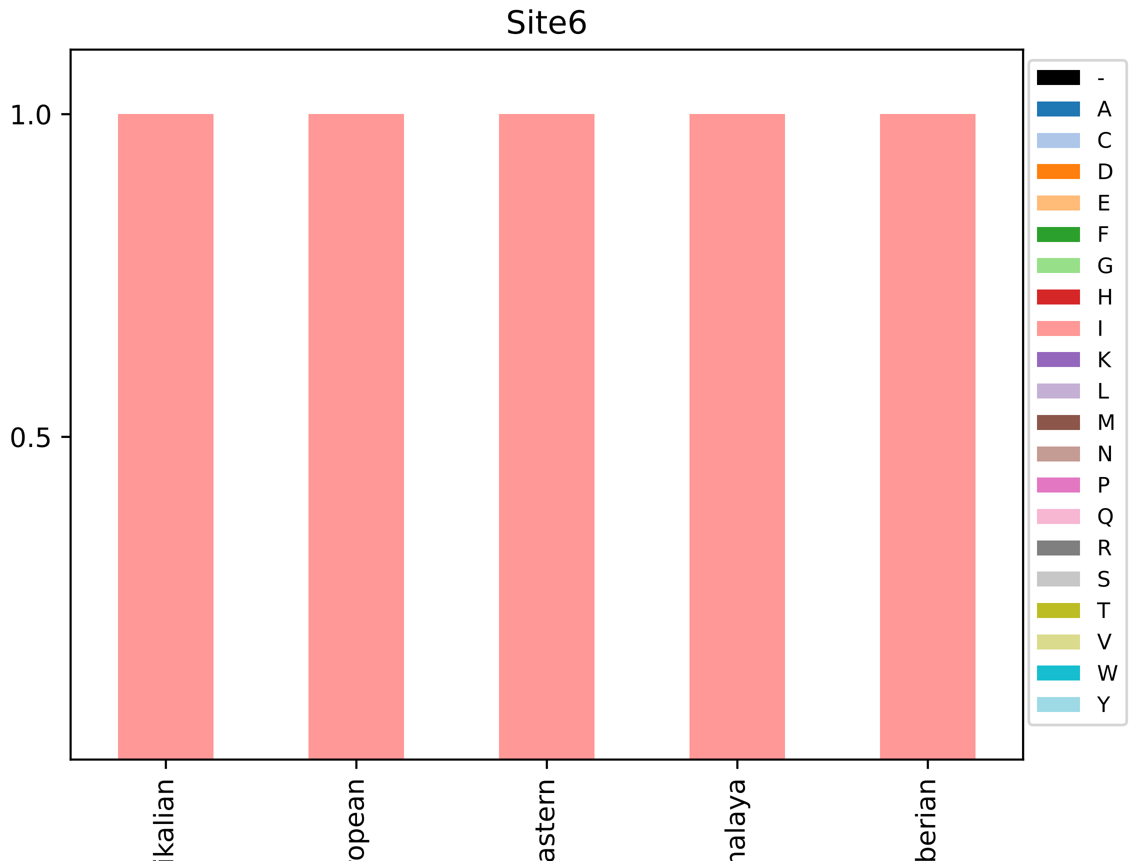

Site6
1.0
0.5
-
A
C
D
E
F
G
H
I
K
L
M
N
P
Q
R
S
T
V
W
Y
ikalian
opean
astern
nalaya
berian

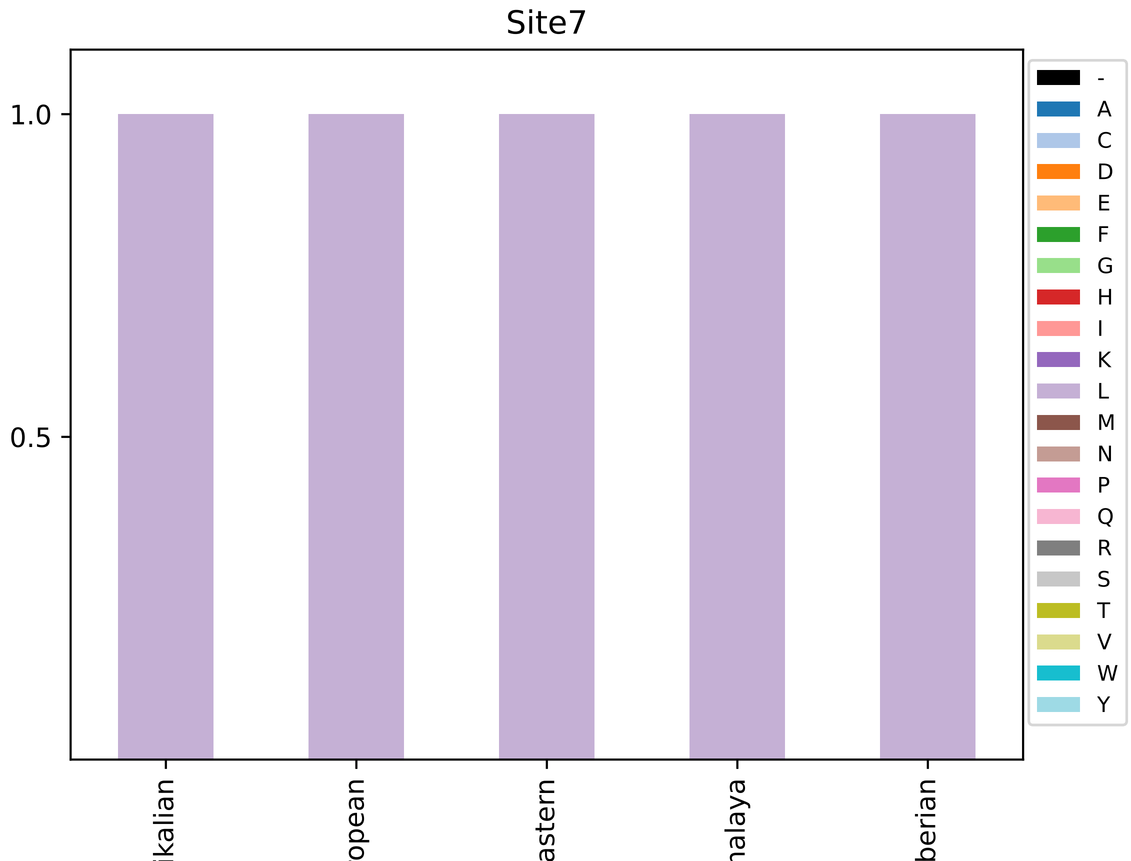

Site7
1.0
0.5
-
A
C
D
E
F
G
H
I
K
L
M
N
P
Q
R
S
T
V
W
Y
ikalian
opean
astern
nalaya
berian

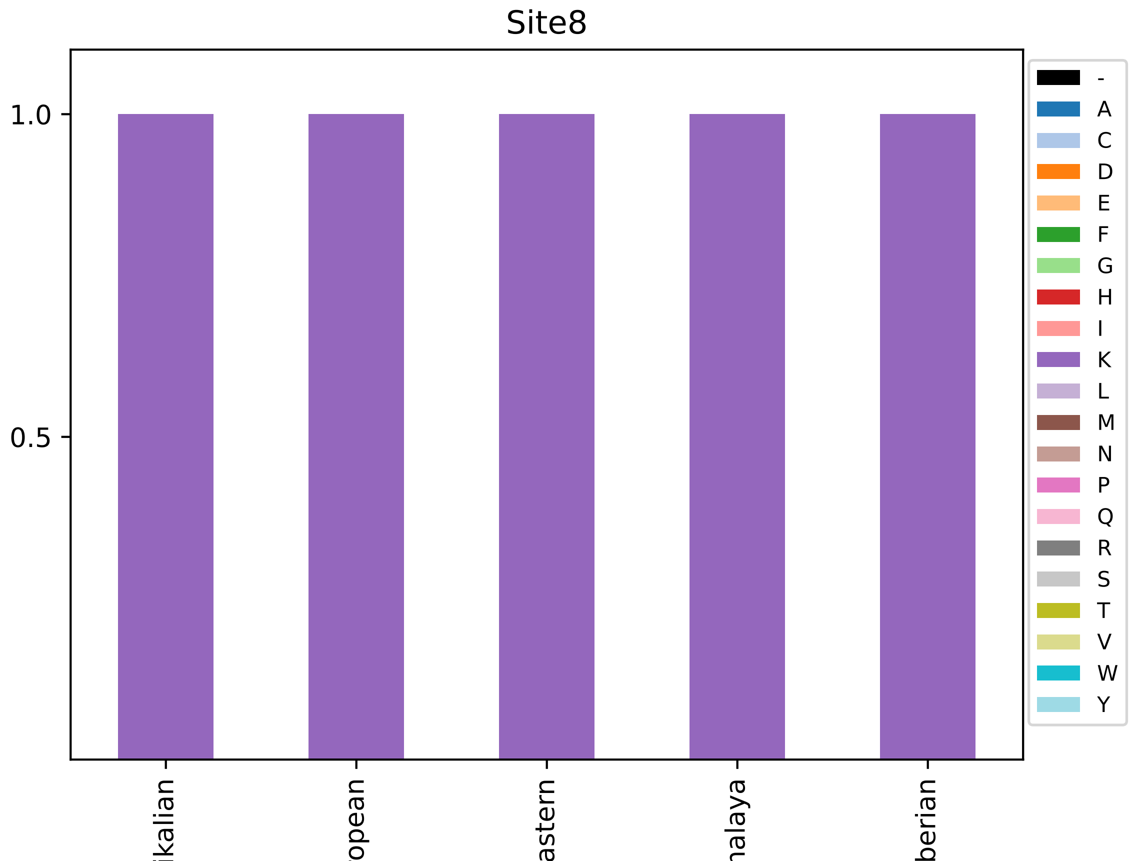
Site8
1.0
0.5
-
A
C
D
E
F
G
H
I
K
L
M
N
P
Q
R
S
T
V
W
Y

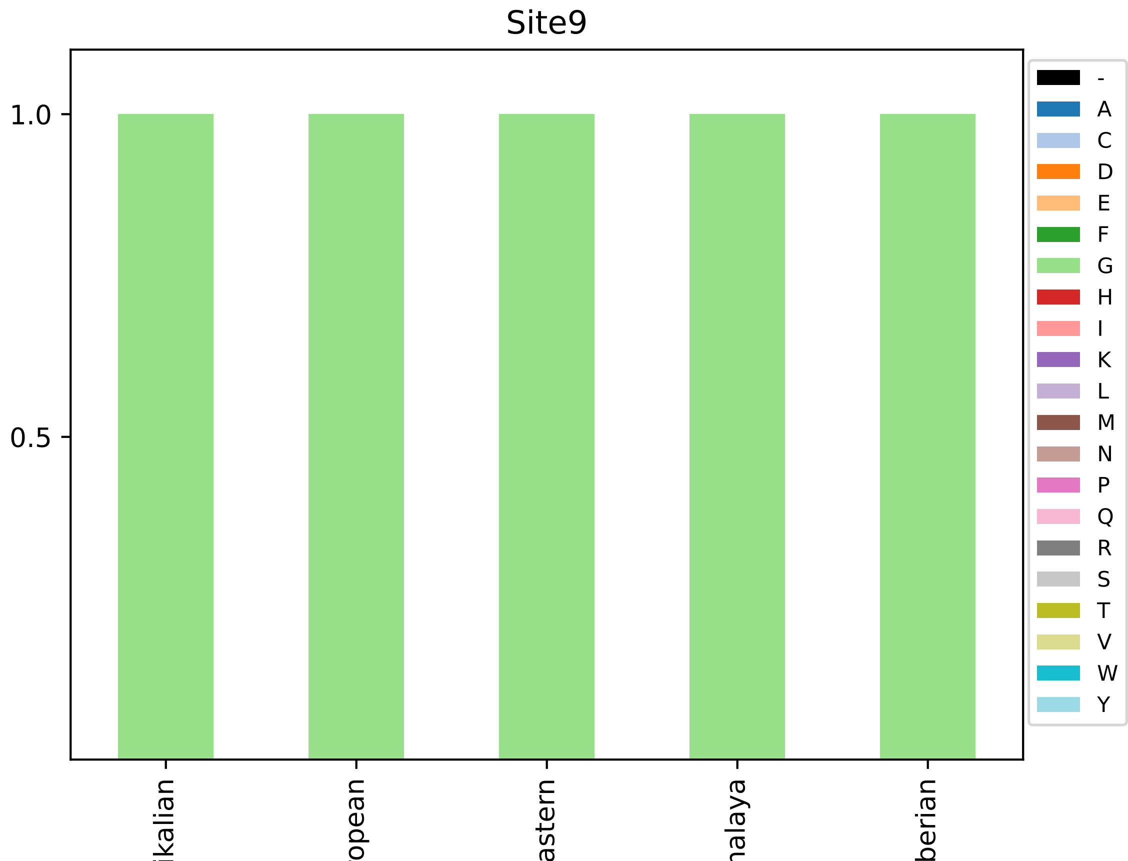
Site9
1.0
0.5
-
A
C
D
E
F
G
H
I
K
L
M
N
P
Q
R
S
T
V
W
Y
ikalian
opean
astern
nalaya
berian

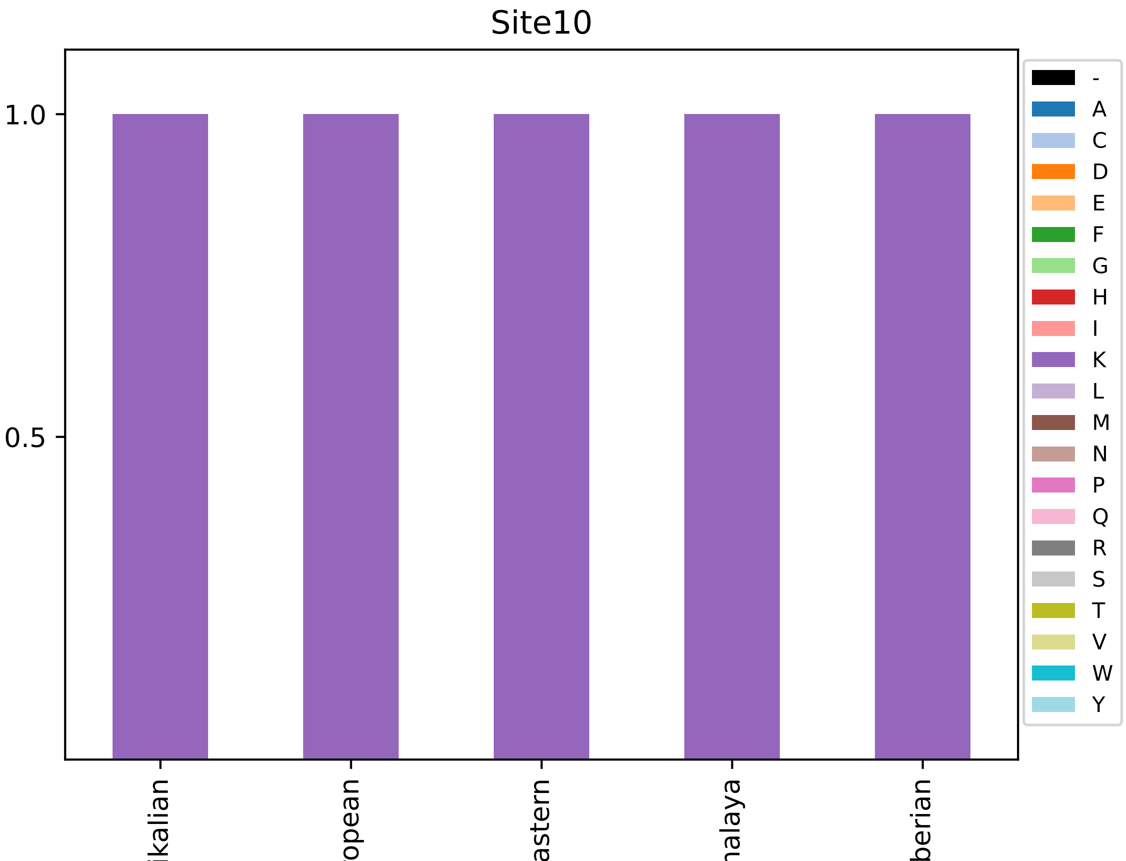
Site10
1.0
0.5
-
A
C
D
E
F
G
H
I
K
L
M
N
P
Q
R
S
T
V
W
Y
ikalian
opean
astern
nalaya
berian

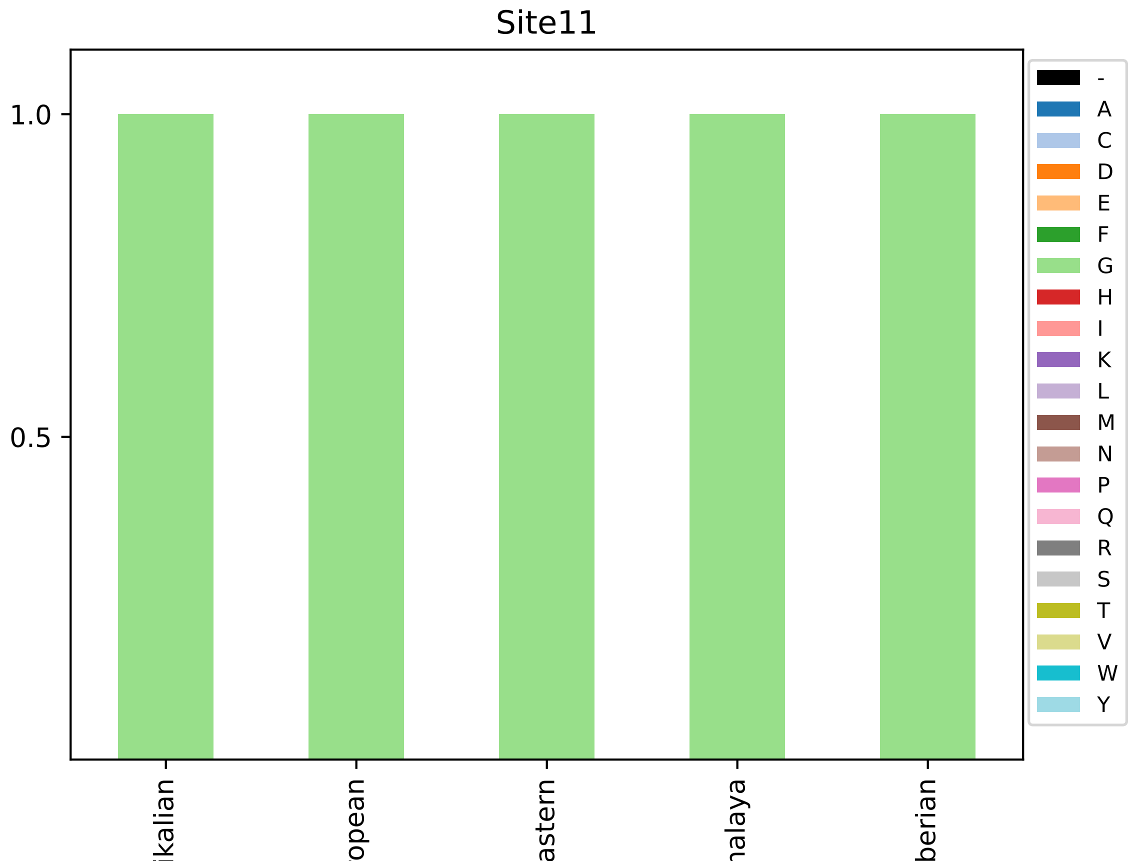
Site11
1.0
0.5
-
A
C
D
E
F
G
H
I
K
L
M
N
P
Q
R
S
T
V
W
Y
ikalian
opean
astern
nalaya
berian

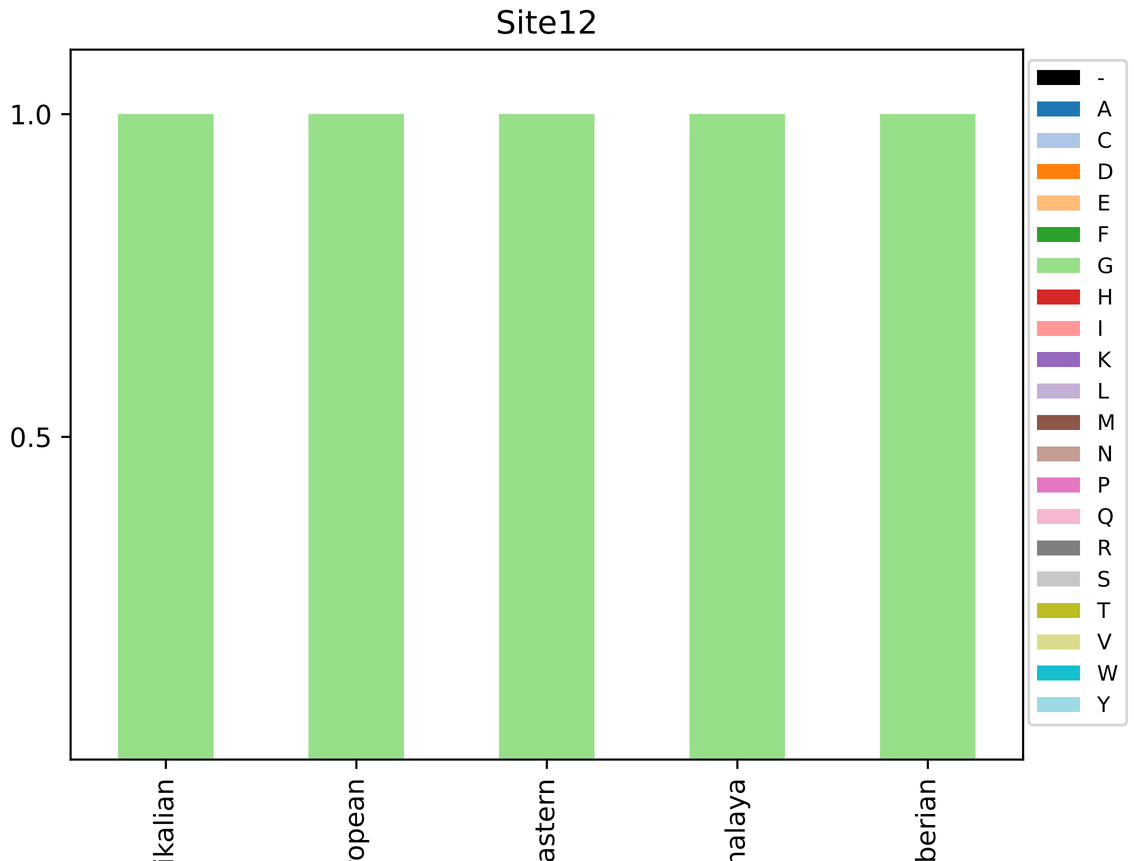

Site12
1.0
0.5
-
A
C
D
E
F
G
H
I
K
L
M
N
P
Q
R
S
T
V
W
Y

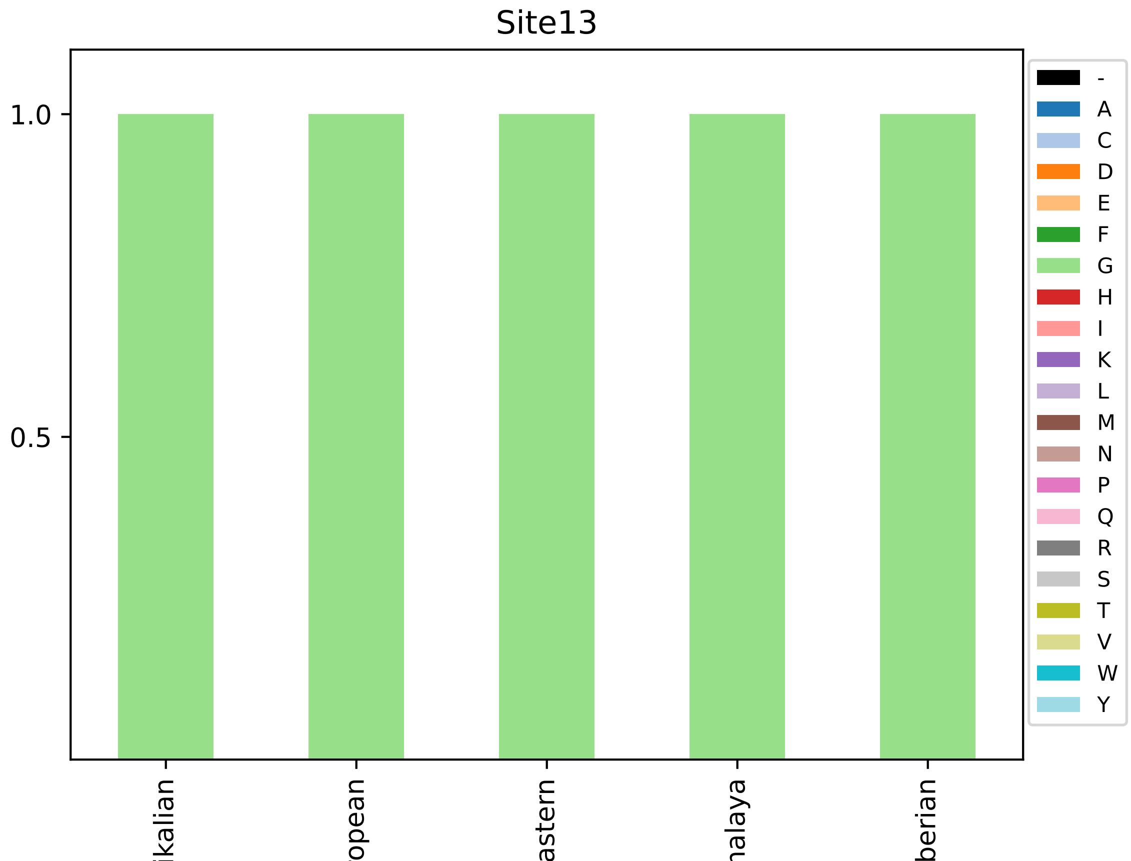

Site13
1.0
0.5
-
A
C
D
E
F
G
H
I
K
L
M
N
P
Q
R
S
T
V
W
Y
ikalian
ropean
astern
halaya
berian

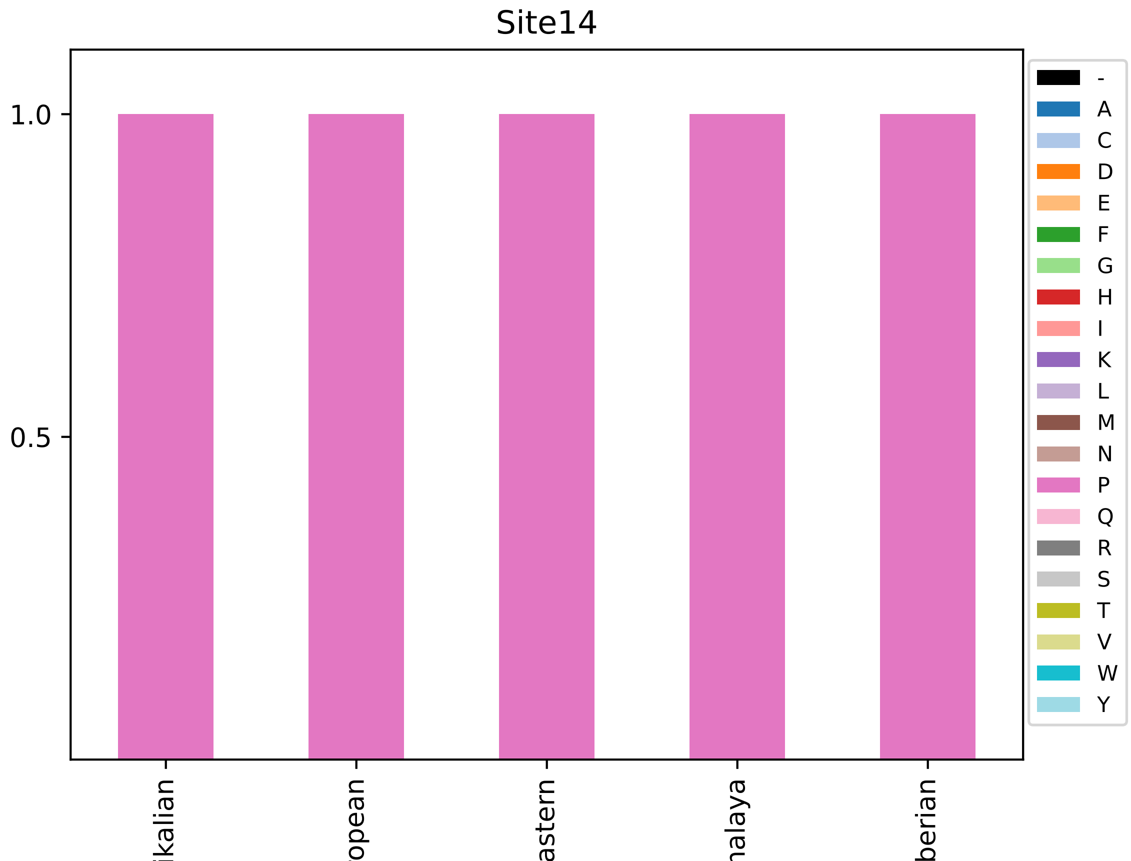
Site14
1.0
0.5
-
A
C
D
E
F
G
H
I
K
L
M
N
P
Q
R
S
T
V
W
Y
ikalian
opean
astern
nalaya
berian

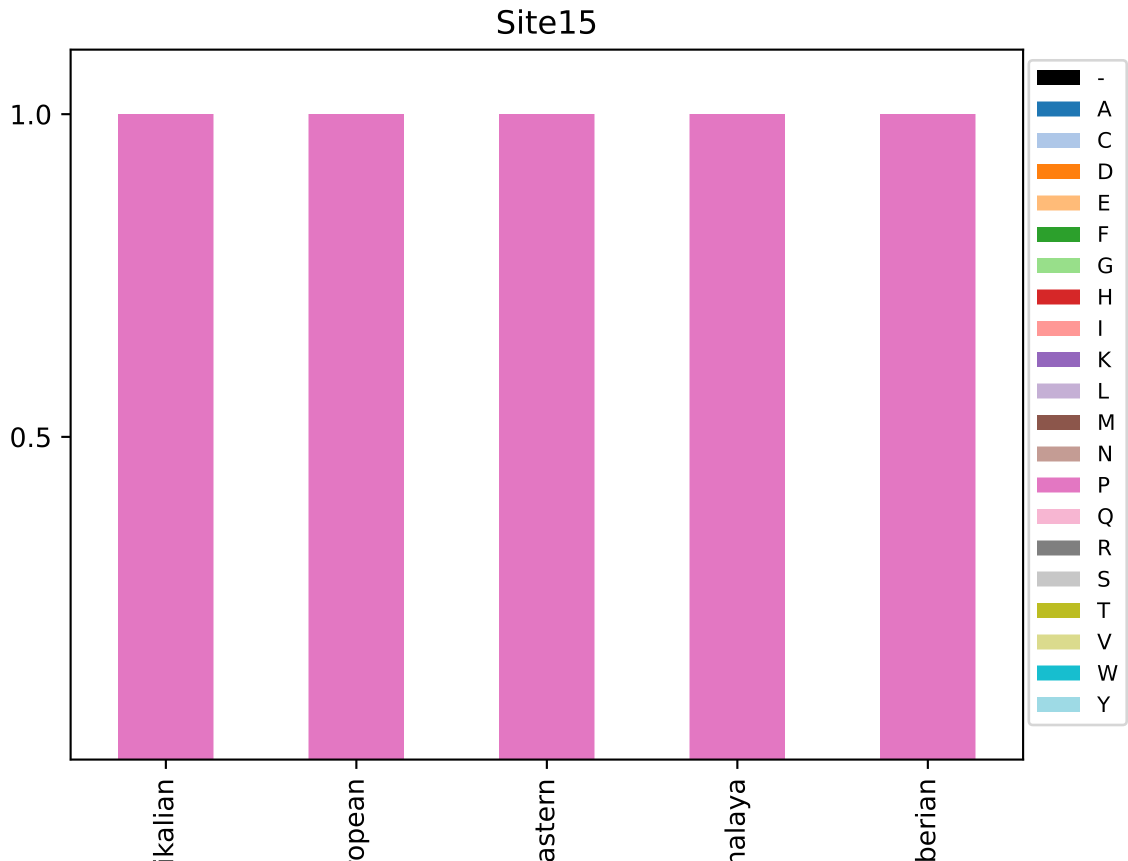

Site15
1.0
0.5
-
A
C
D
E
F
G
H
I
K
L
M
N
P
Q
R
S
T
V
W
Y
ikalian
opean
astern
nalaya
berian

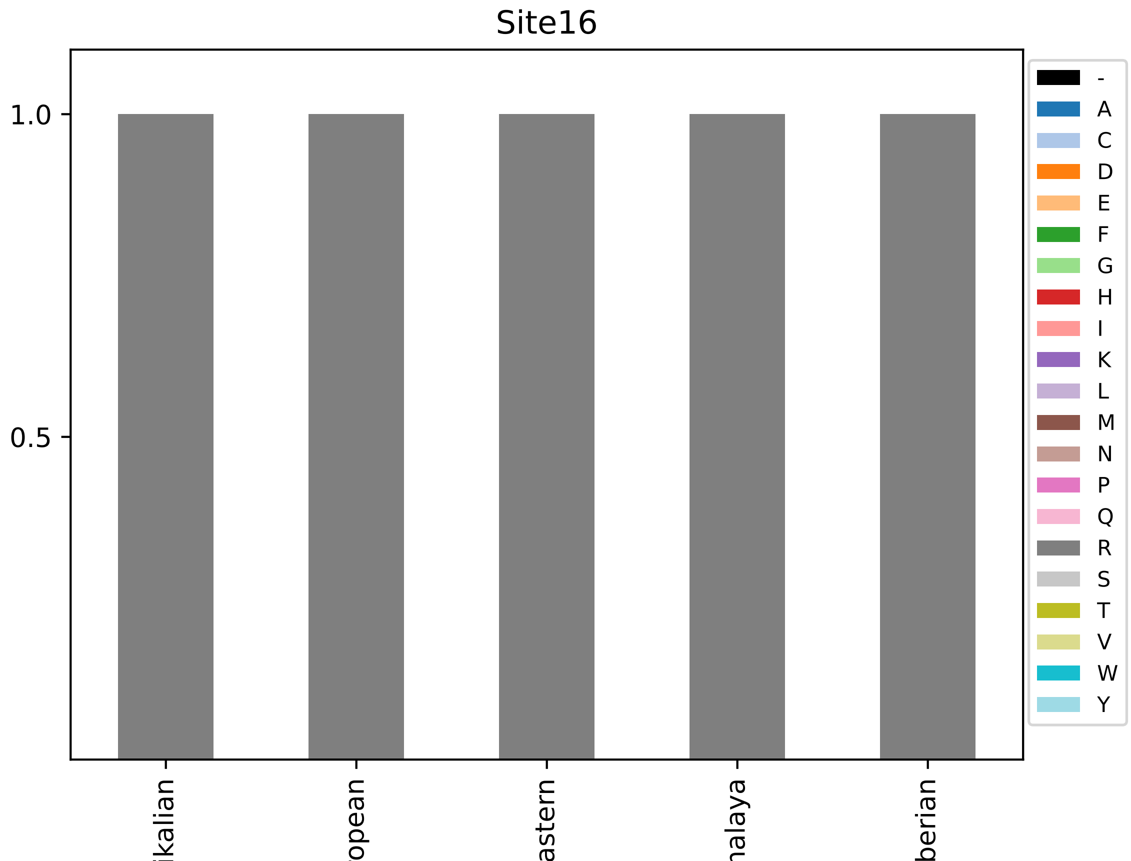

Site16
1.0
0.5
-
A
C
D
E
F
G
H
I
K
L
M
N
P
Q
R
S
T
V
W
Y
ikalian
opean
astern
nalaya
berian

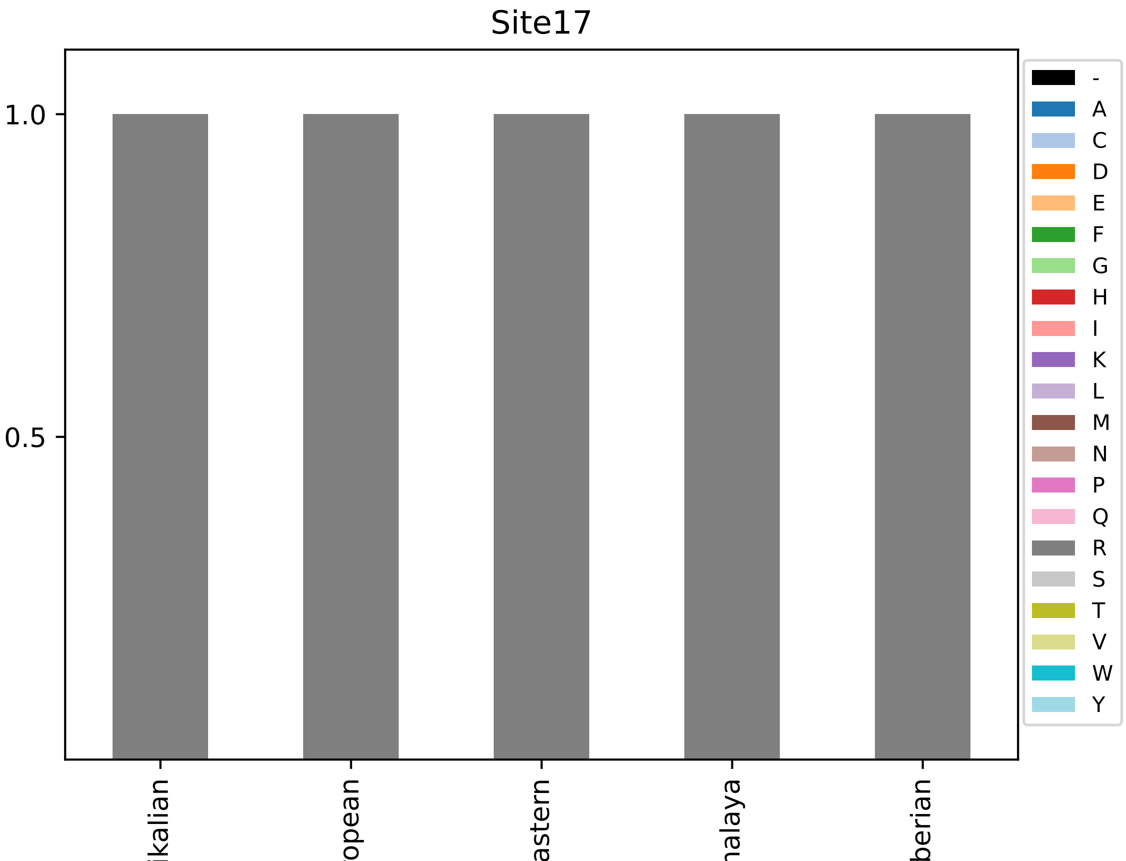

Site17
1.0
0.5
-
A
C
D
E
F
G
H
I
K
L
M
N
P
Q
R
S
T
V
W
Y
ikalian
opean
astern
nalaya
berian

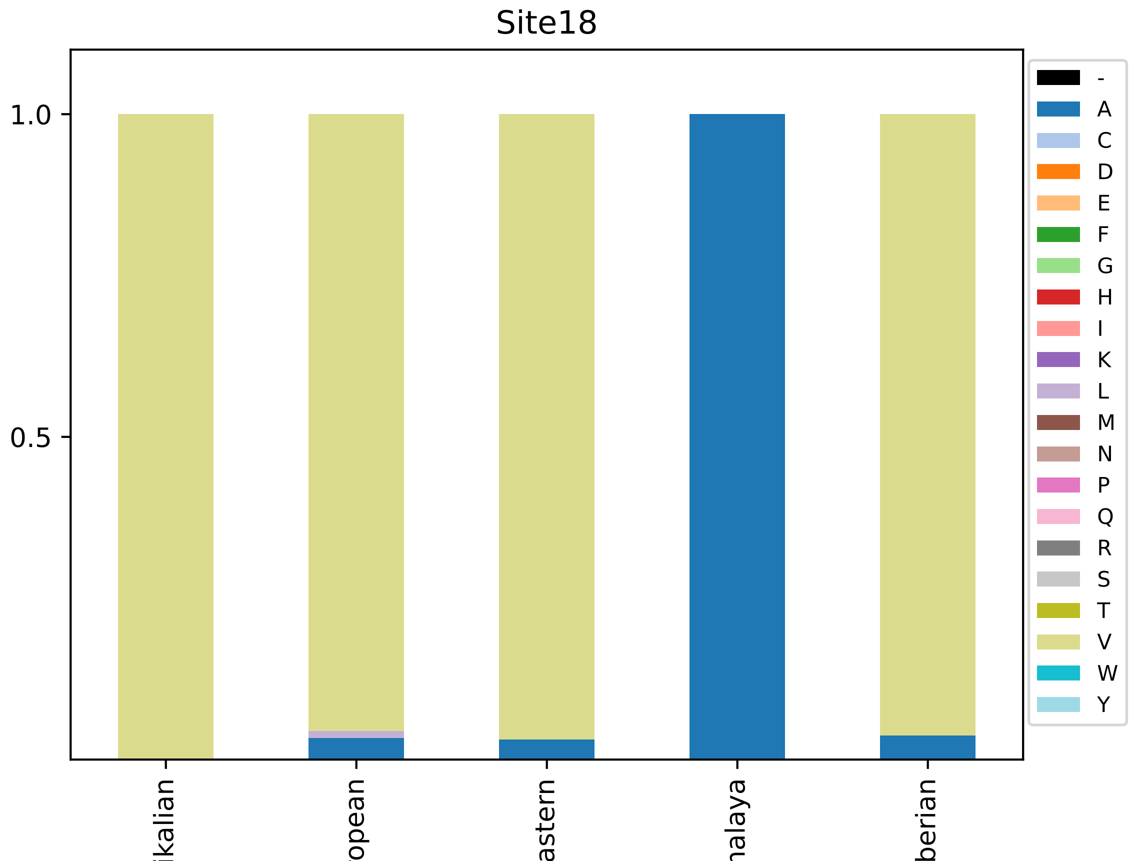

Site18
1.0
0.5
ikalian
opean
astern
nalaya
berian
-
A
C
D
E
F
G
H
I
K
L
M
N
P
Q
R
S
T
V
W
Y

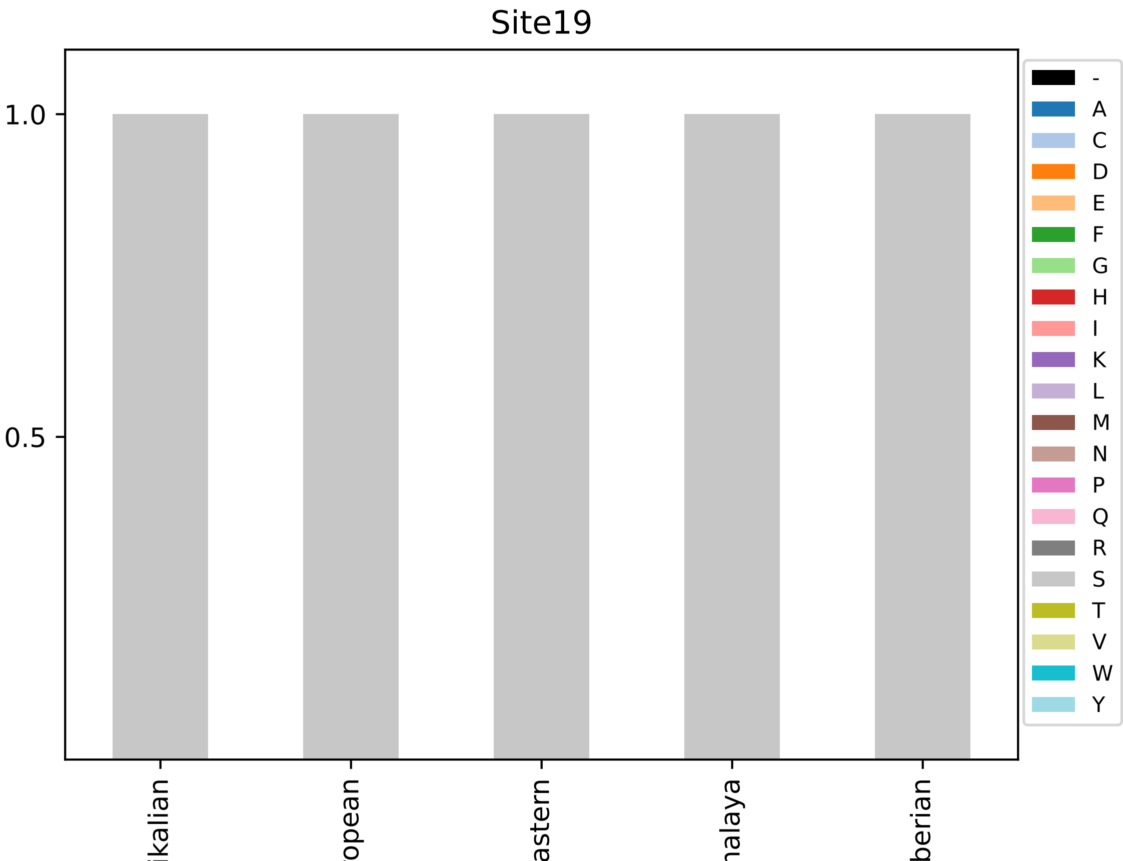

Site19
1.0
0.5
-
A
C
D
E
F
G
H
I
K
L
M
N
P
Q
R
S
T
V
W
Y
ikalian
opean
astern
nalaya
berian

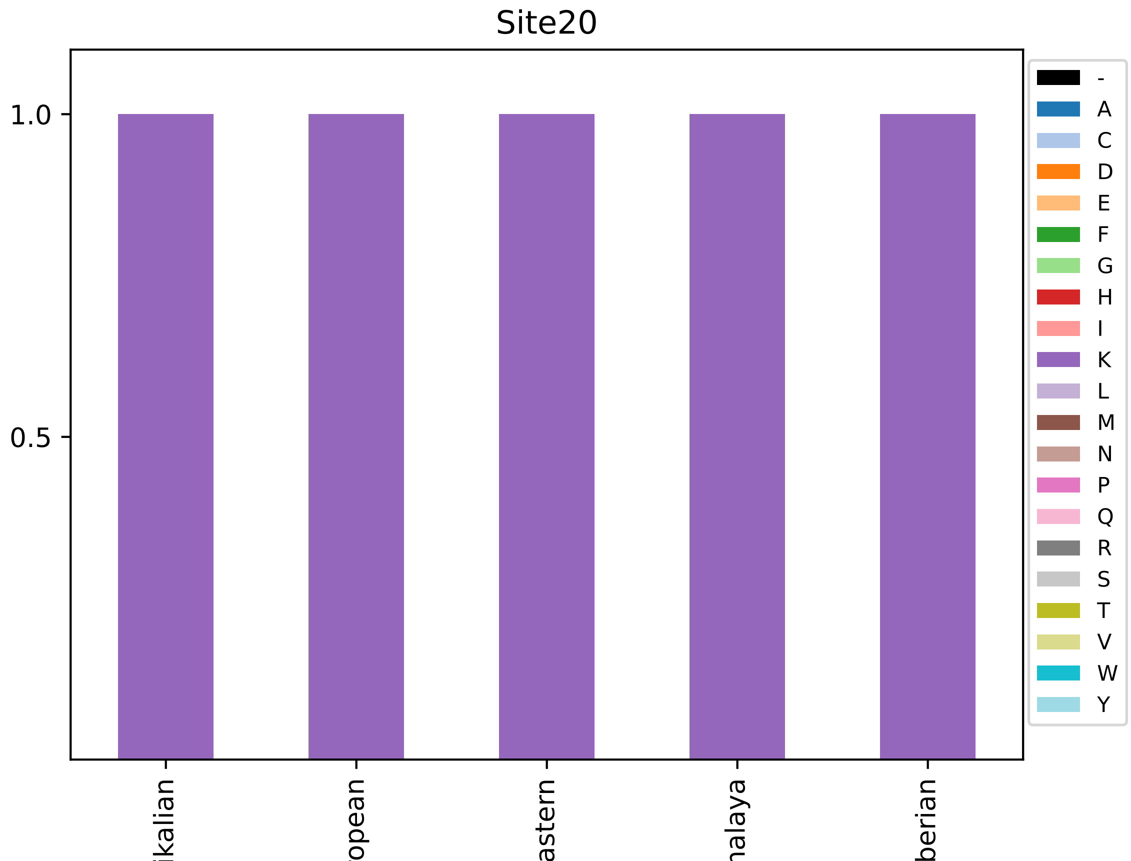

Site20
1.0
0.5
-
A
C
D
E
F
G
H
I
K
L
M
N
P
Q
R
S
T
V
W
Y
ikalian
opean
astern
nalaya
berian

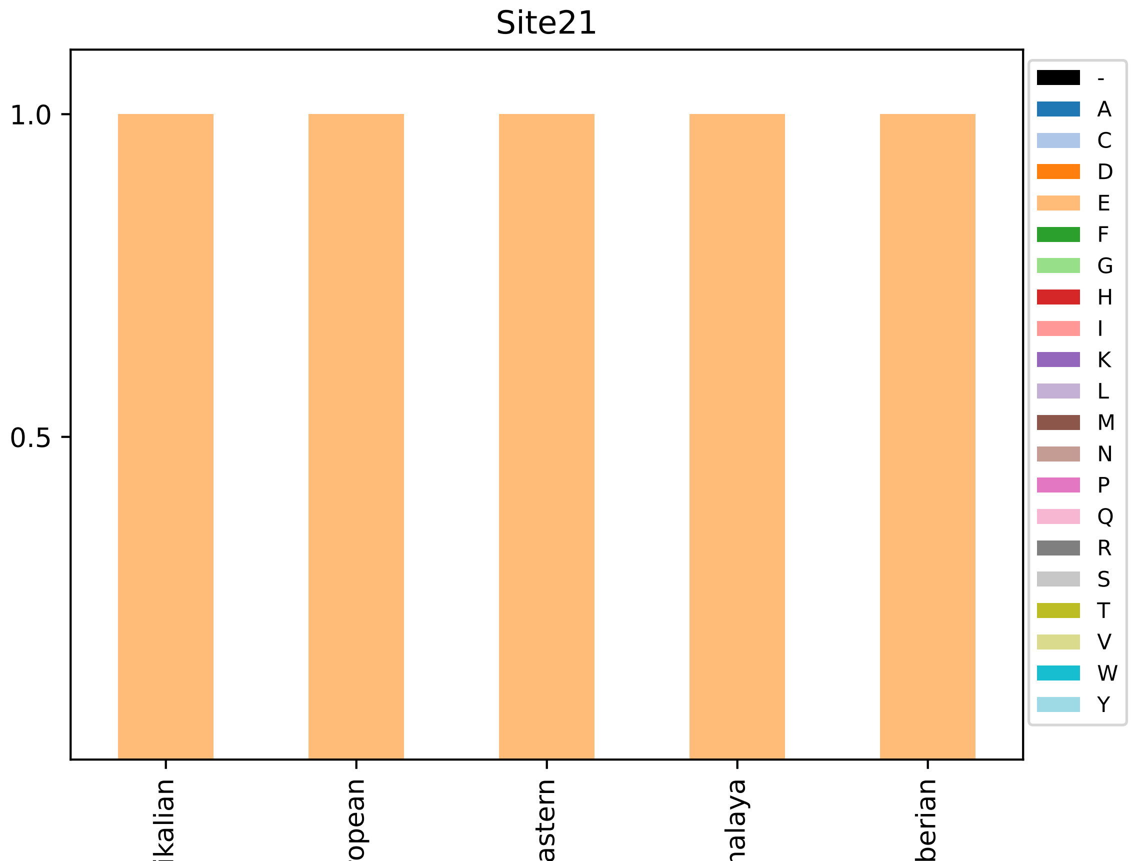
Site21
1.0
0.5
ikalian
opean
astern
nalaya
berian
-
A
C
D
E
F
G
H
I
K
L
M
N
P
Q
R
S
T
V
W
Y

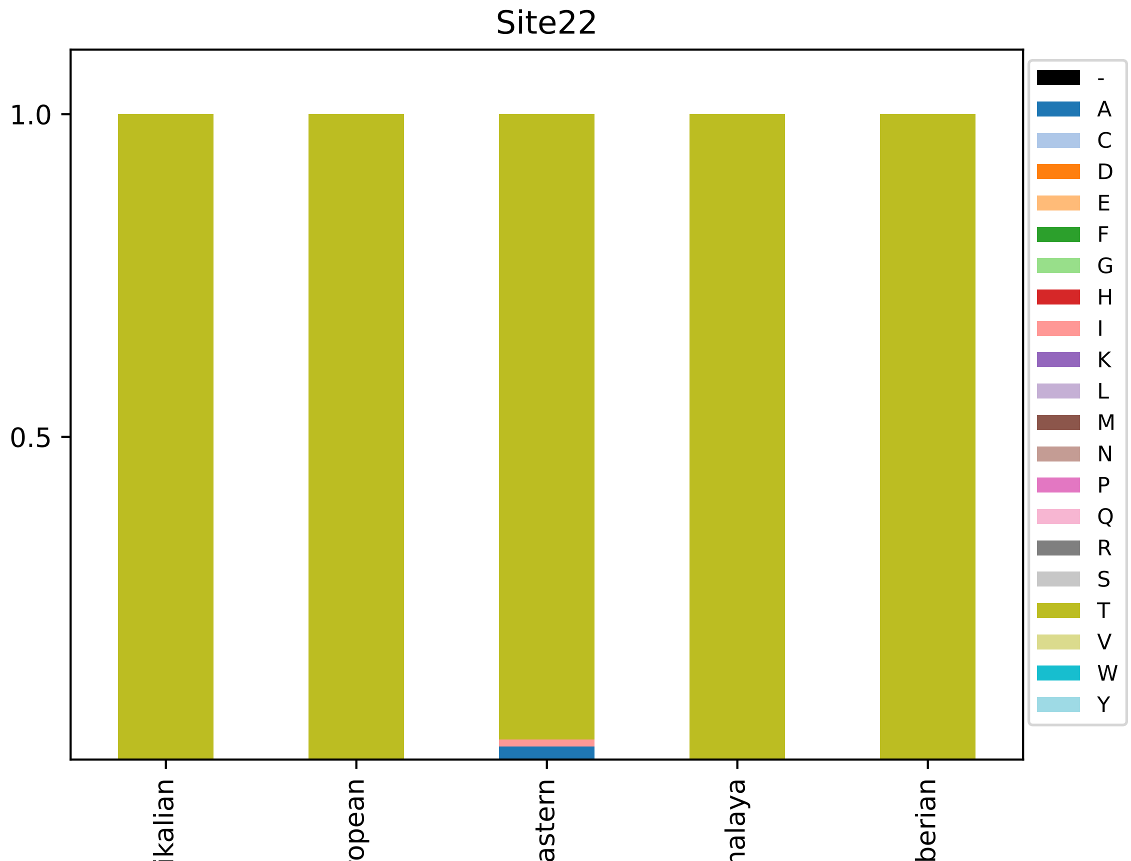
Site22
1.0
0.5
ikalian
opean
astern
nalaya
berian
-
A
C
D
E
F
G
H
I
K
L
M
N
P
Q
R
S
T
V
W
Y

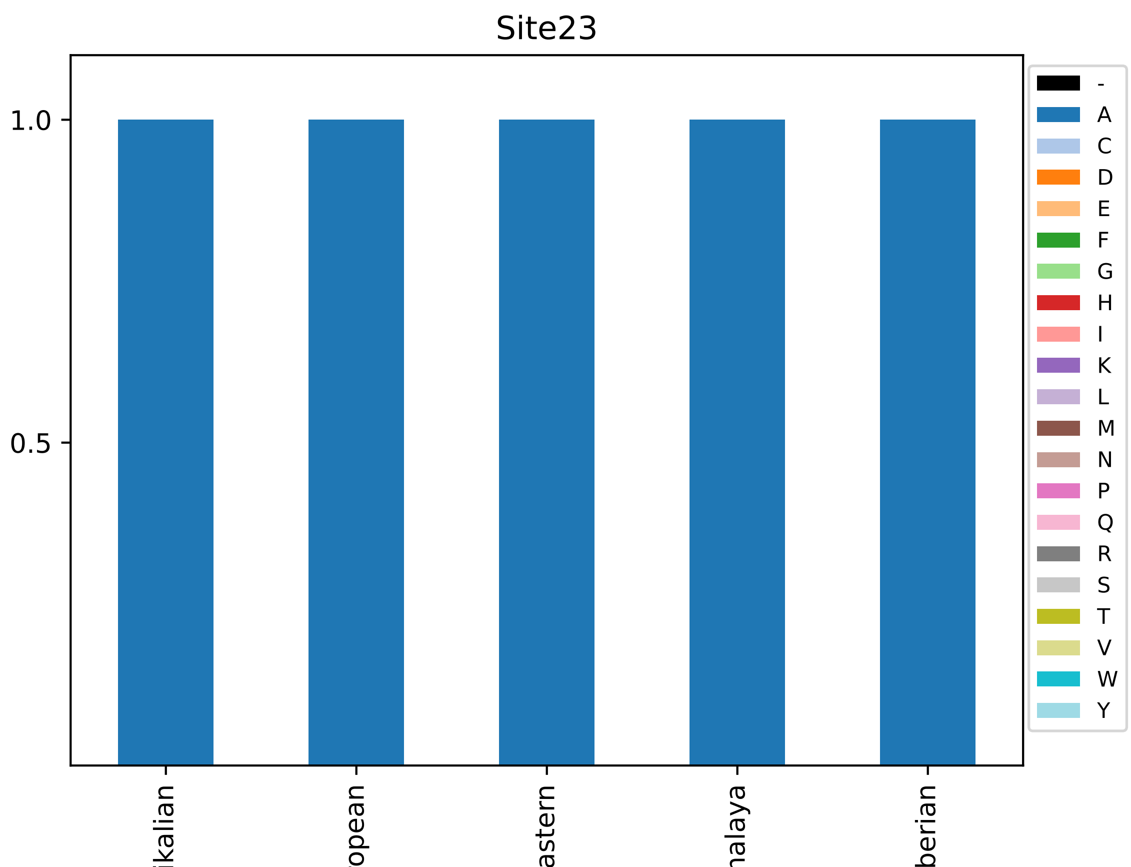
Site23
1.0
0.5
-
A
C
D
E
F
G
H
I
K
L
M
N
P
Q
R
S
T
V
W
Y
ikalian
opean
astern
nalaya
berian

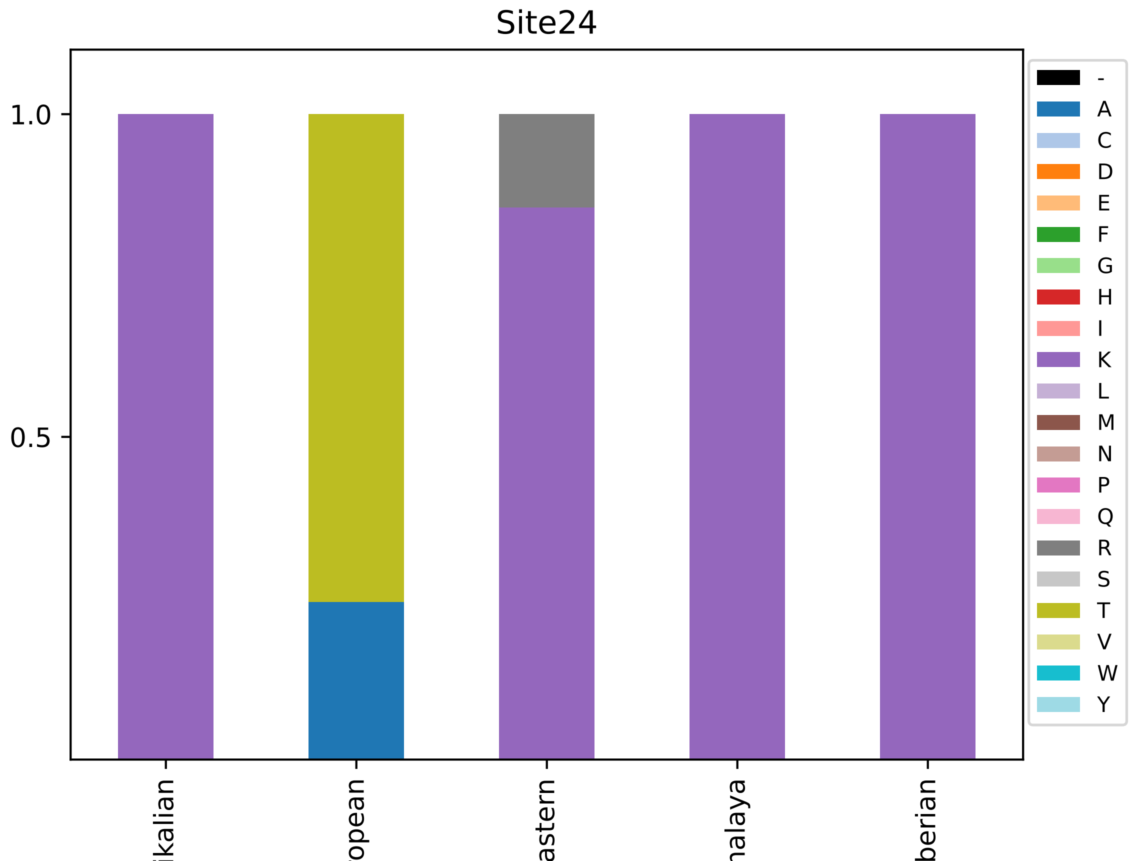

Site24
1.0
0.5
-
A
C
D
E
F
G
H
I
K
L
M
N
P
Q
R
S
T
V
W
Y
ikalian
opean
astern
nalaya
berian

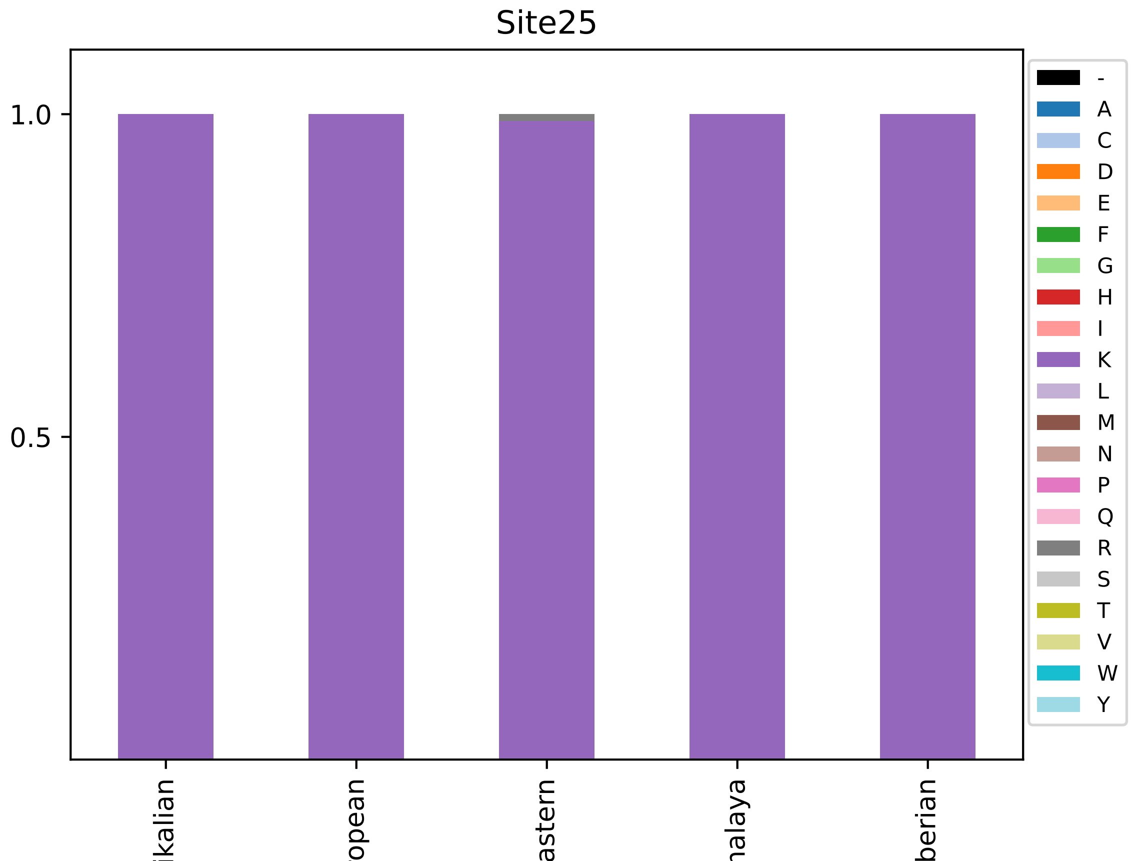
Site25
1.0
0.5
-
A
C
D
E
F
G
H
I
K
L
M
N
P
Q
R
S
T
V
W
Y
ikalian
opean
astern
nalaya
berian

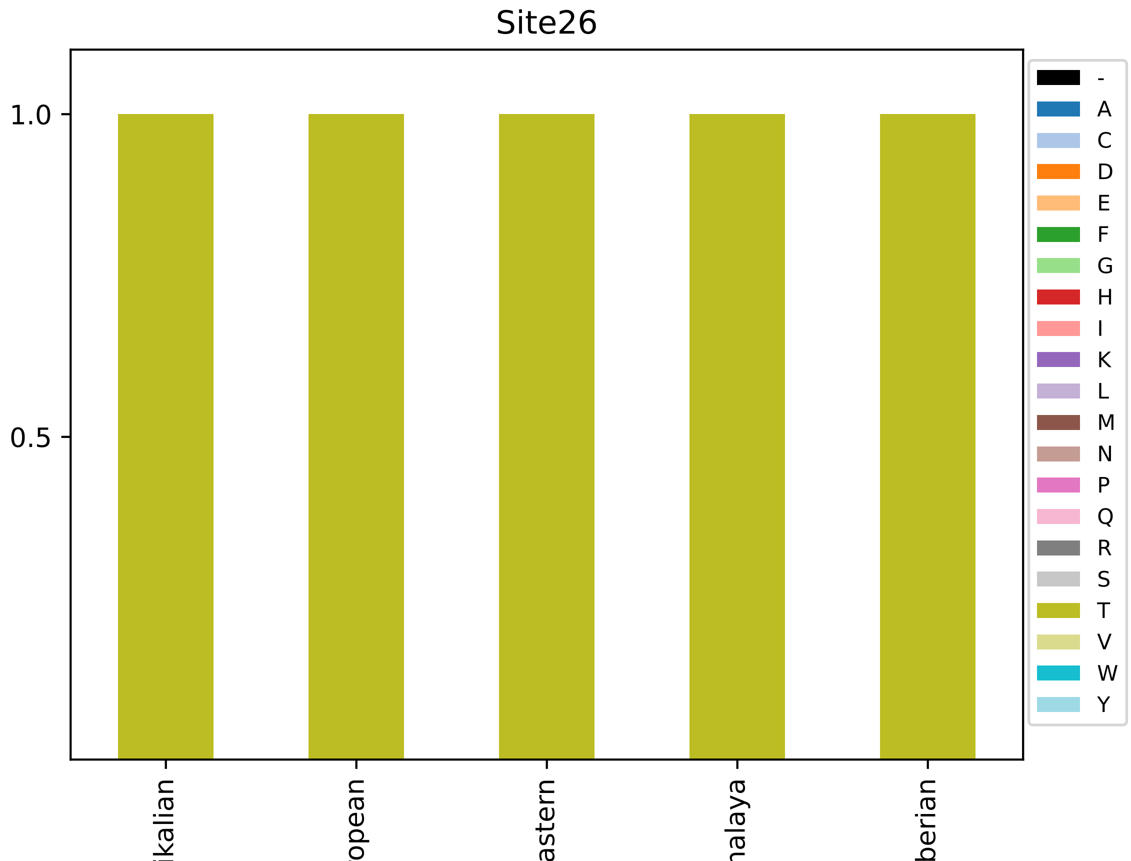
Site26
1.0
0.5
-
A
C
D
E
F
G
H
I
K
L
M
N
P
Q
R
S
T
V
W
Y
ikalian
opean
astern
nalaya
berian

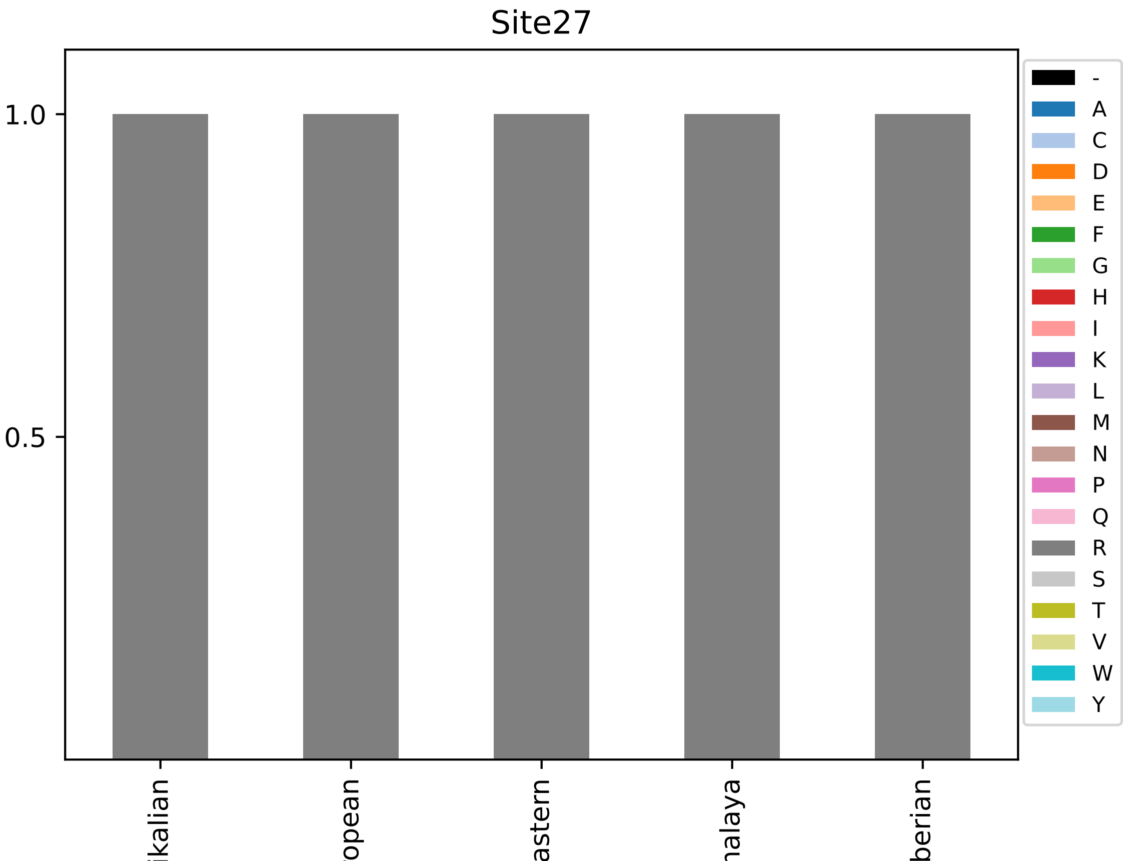
Site27
1.0
0.5
-
A
C
D
E
F
G
H
I
K
L
M
N
P
Q
R
S
T
V
W
Y
ikalian
opean
astern
nalaya
berian

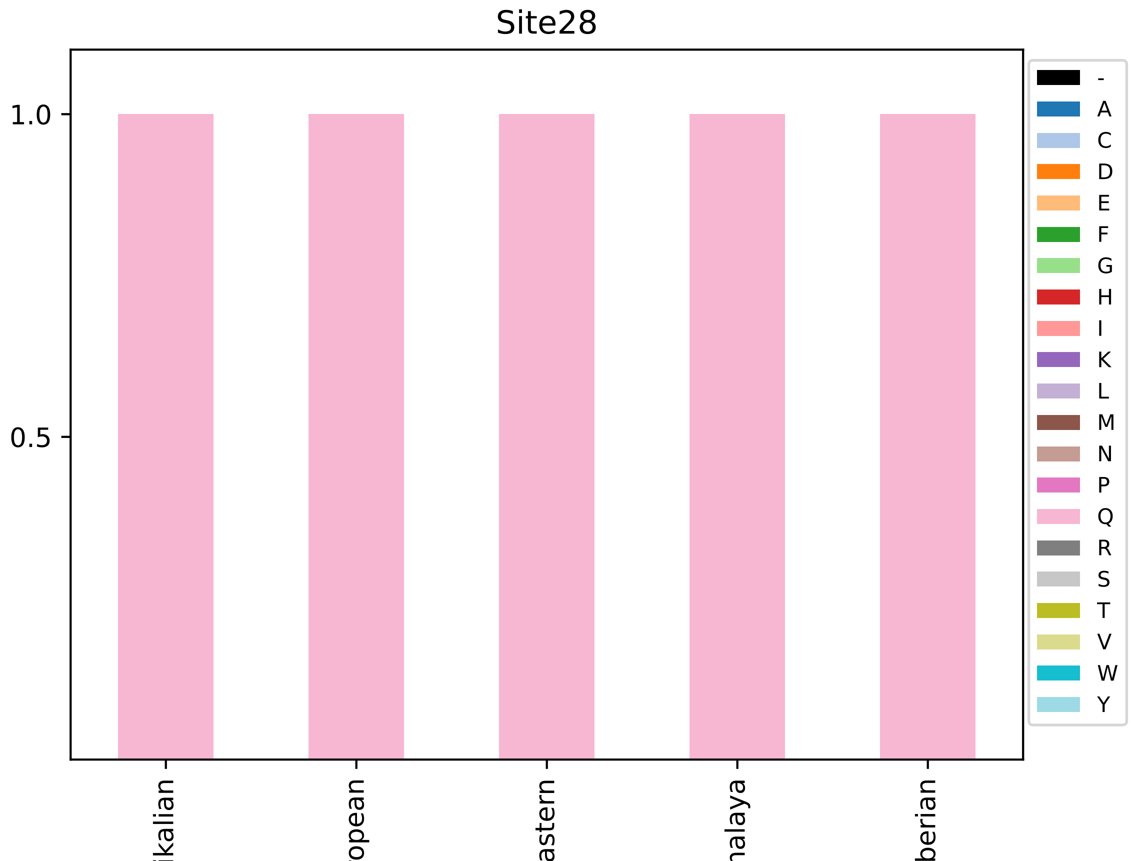
Site28
1.0
0.5
-
A
C
D
E
F
G
H
I
K
L
M
N
P
Q
R
S
T
V
W
Y
ikalian
opean
astern
nalaya
berian

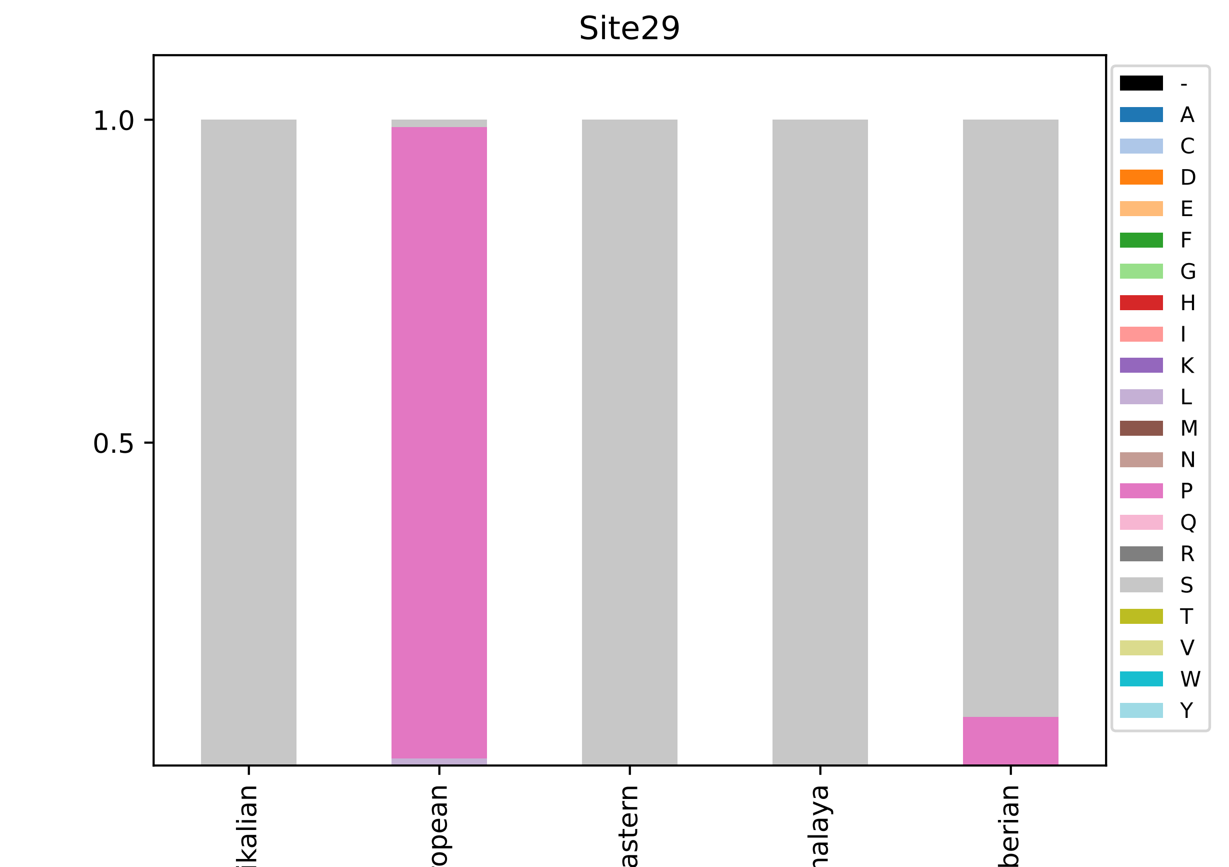
Site29
1.0
0.5
-
A
C
D
E
F
G
H
I
K
L
M
N
P
Q
R
S
T
V
W
Y
ikalian
opean
astern
nalaya
berian

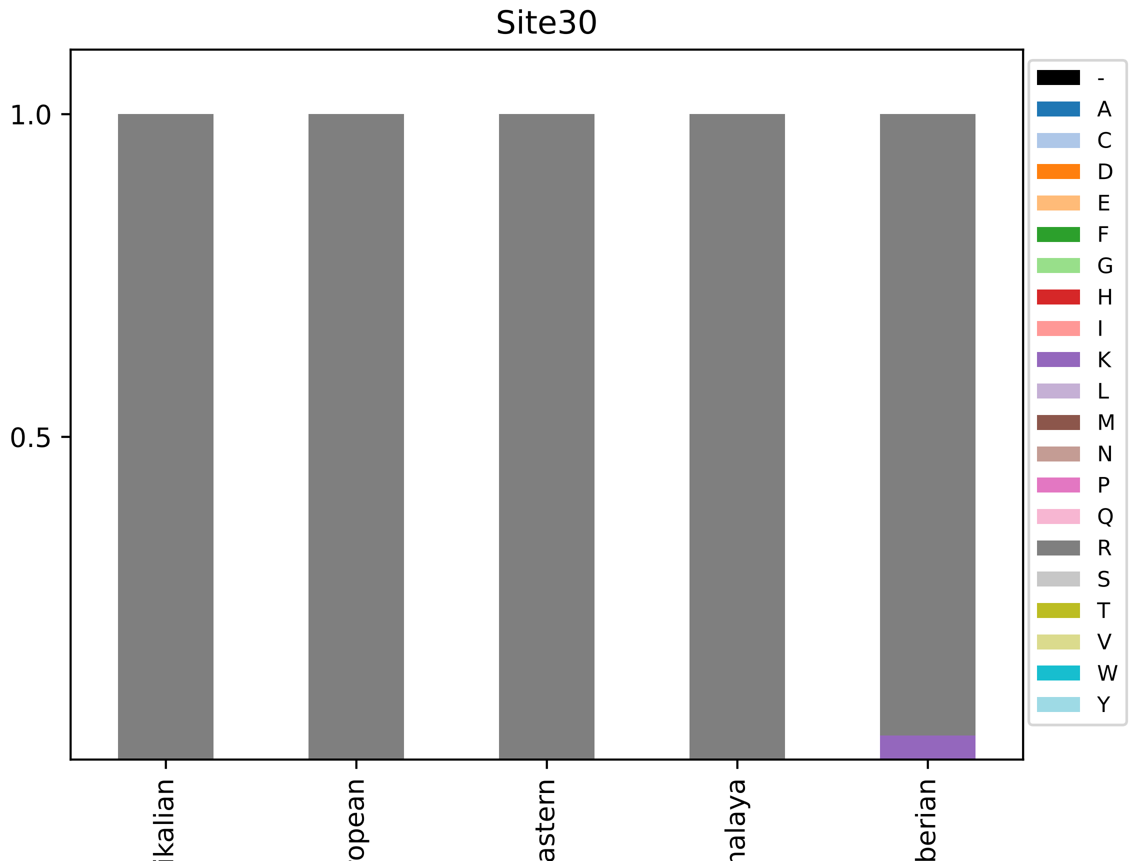
Site30
1.0
0.5
-
A
C
D
E
F
G
H
I
K
L
M
N
P
Q
R
S
T
V
W
Y
ikalian
opean
astern
nalaya
berian

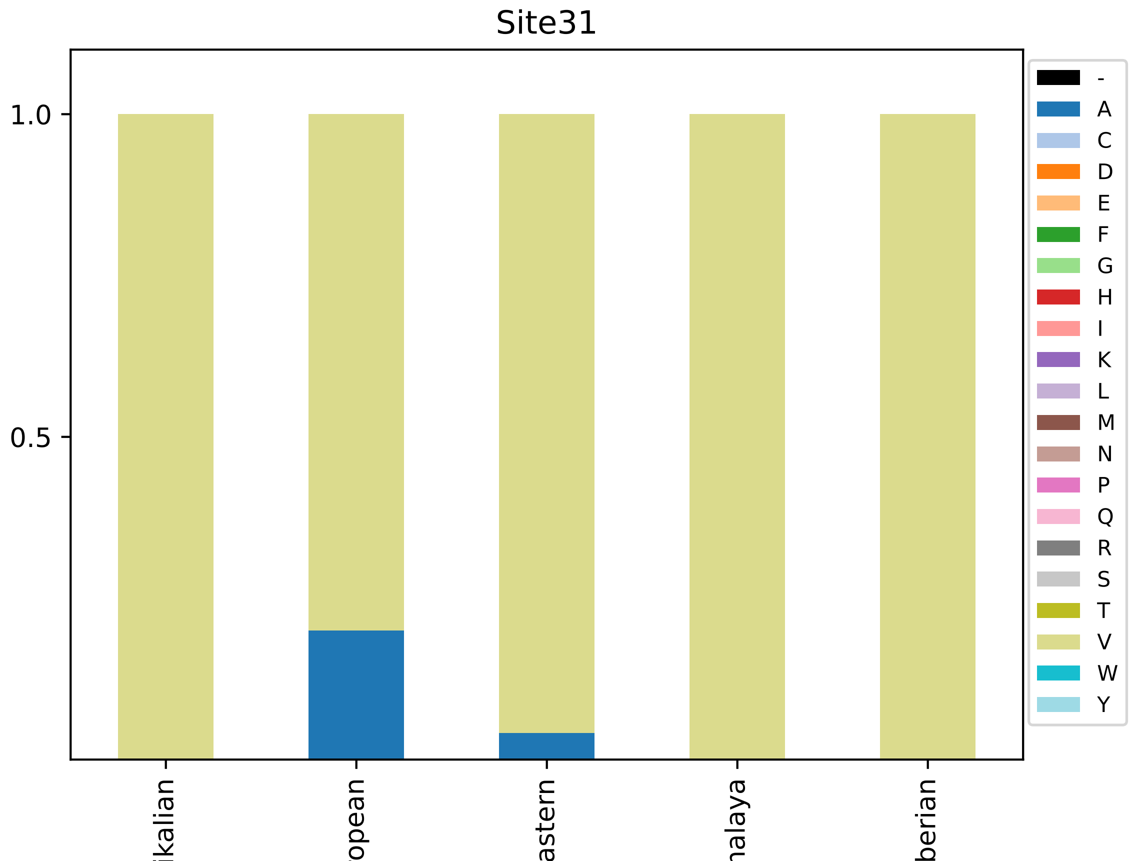
Site31
1.0
0.5
-
A
C
D
E
F
G
H
I
K
L
M
N
P
Q
R
S
T
V
W
Y
ikalian
opean
astern
nalaya
berian

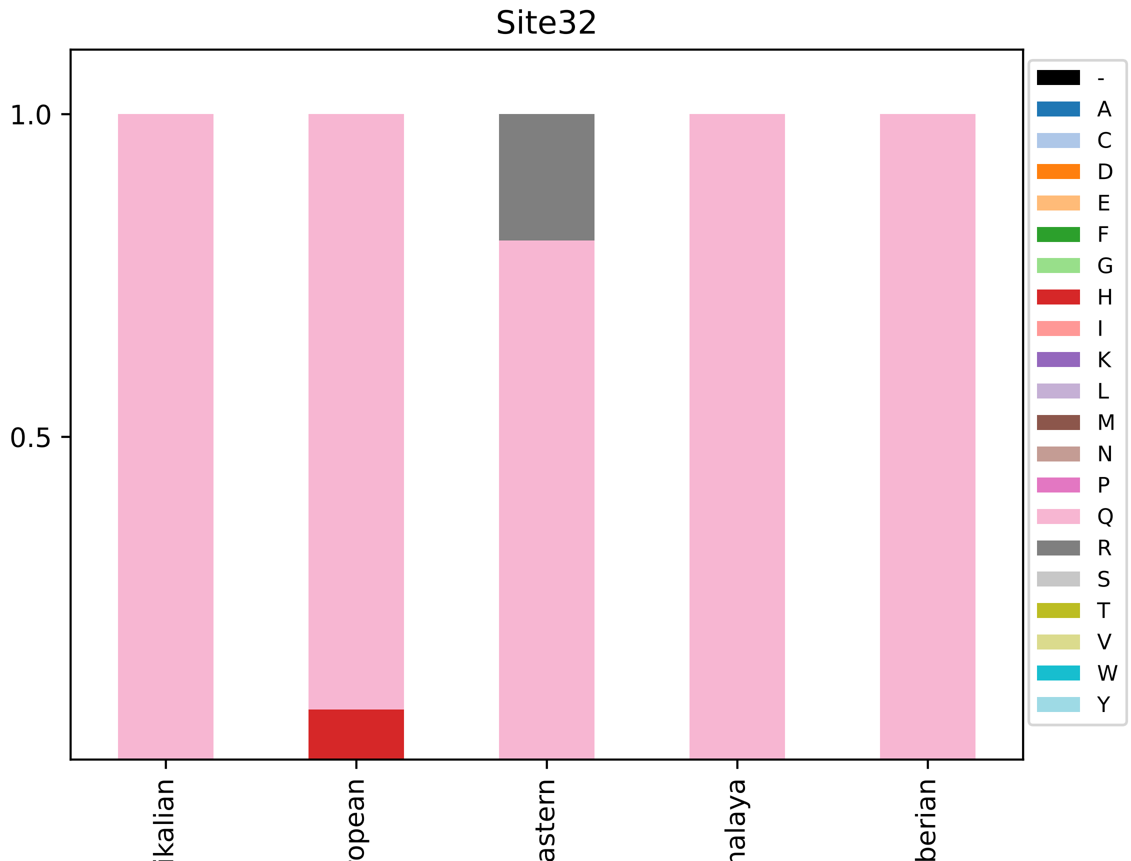
Site32
1.0
0.5
ikalian
opean
astern
nalaya
berian
-
A
C
D
E
F
G
H
I
K
L
M
N
P
Q
R
S
T
V
W
Y

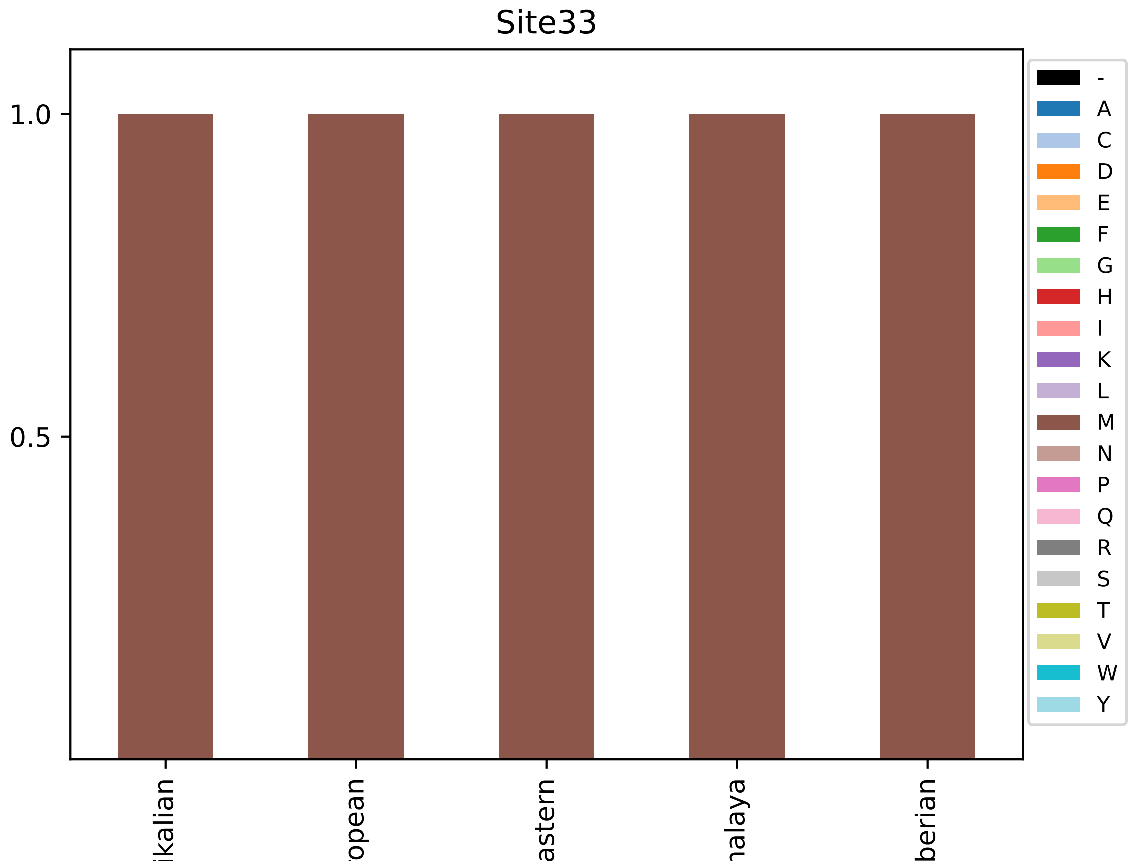
Site33
1.0
0.5
-
A
C
D
E
F
G
H
I
K
L
M
N
P
Q
R
S
T
V
W
Y
ikalian
opean
astern
nalaya
berian

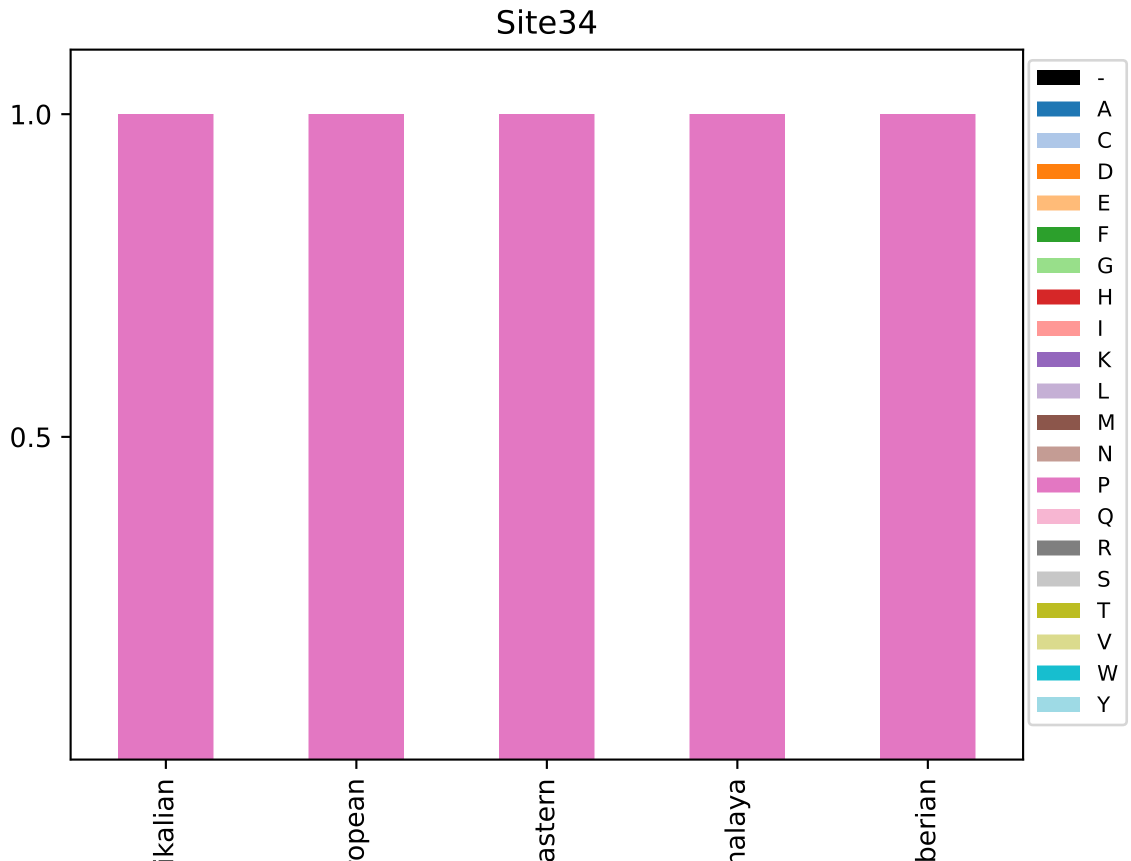
Site34
1.0
0.5
-
A
C
D
E
F
G
H
I
K
L
M
N
P
Q
R
S
T
V
W
Y
ikalian
opean
astern
nalaya
berian

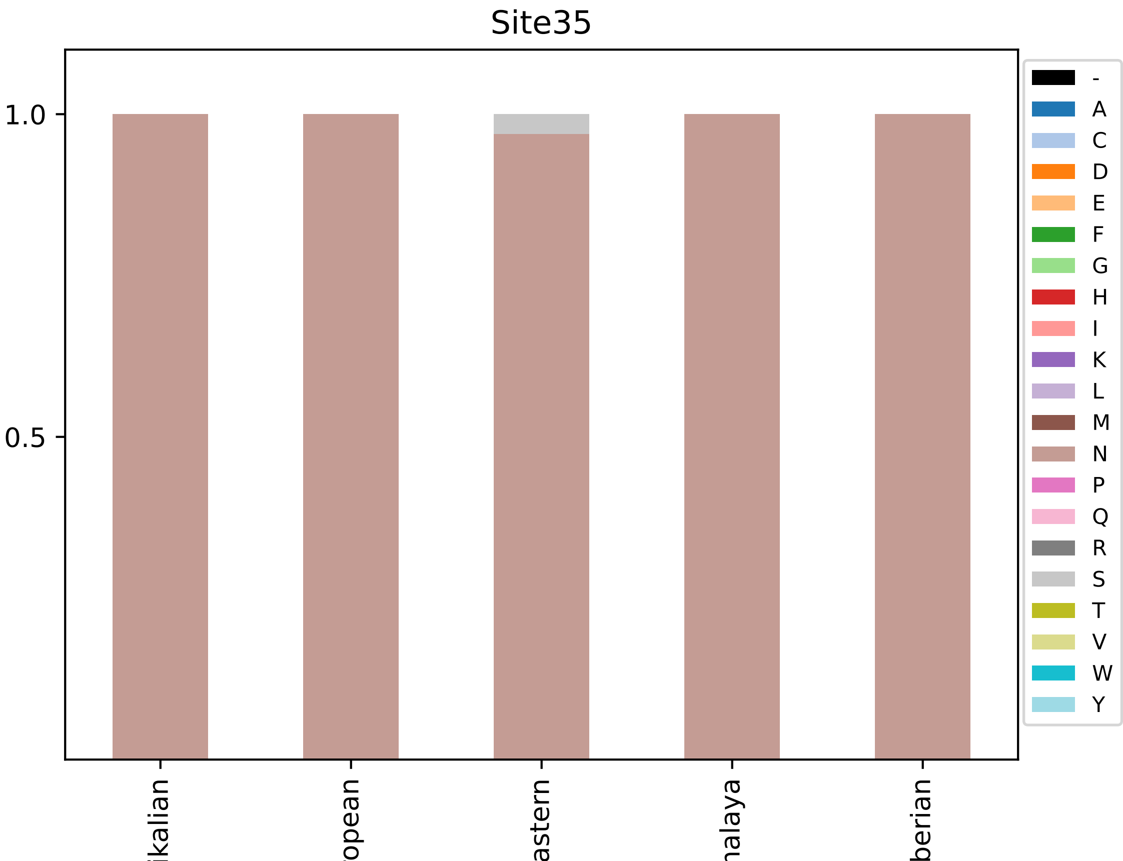

Site35
1.0
0.5
-
A
C
D
E
F
G
H
I
K
L
M
N
P
Q
R
S
T
V
W
Y

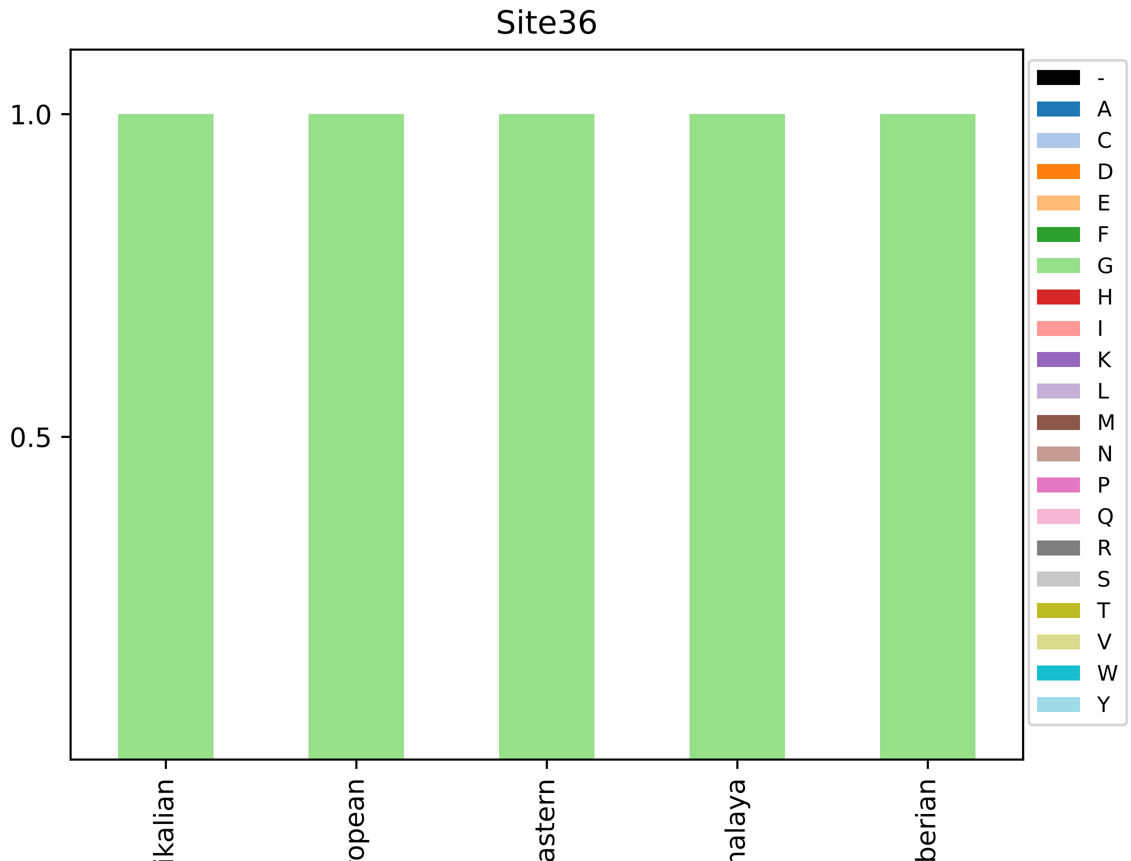

Site36
1.0
0.5
-
A
C
D
E
F
G
H
I
K
L
M
N
P
Q
R
S
T
V
W
Y
ikalian
opean
astern
nalaya
berian

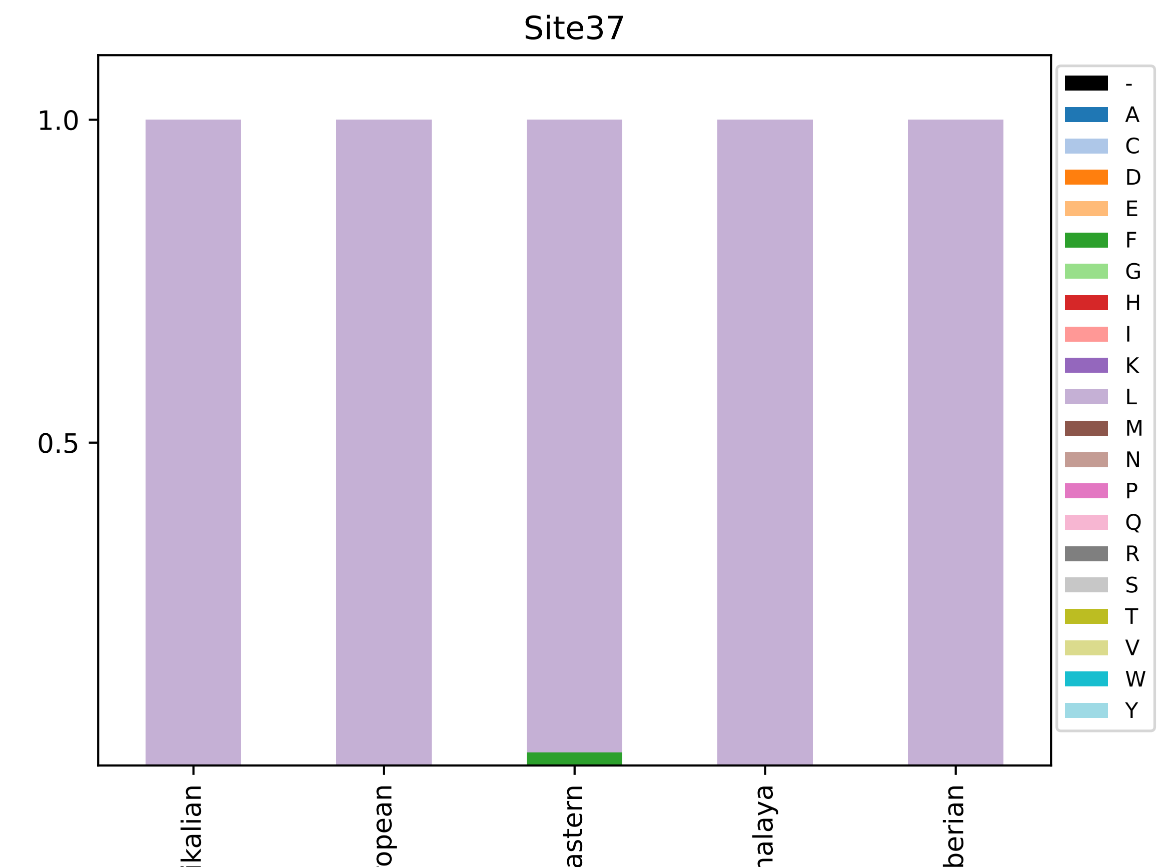
Site37
1.0
0.5
-
A
C
D
E
F
G
H
I
K
L
M
N
P
Q
R
S
T
V
W
Y
ikalian
opean
astern
nalaya
berian

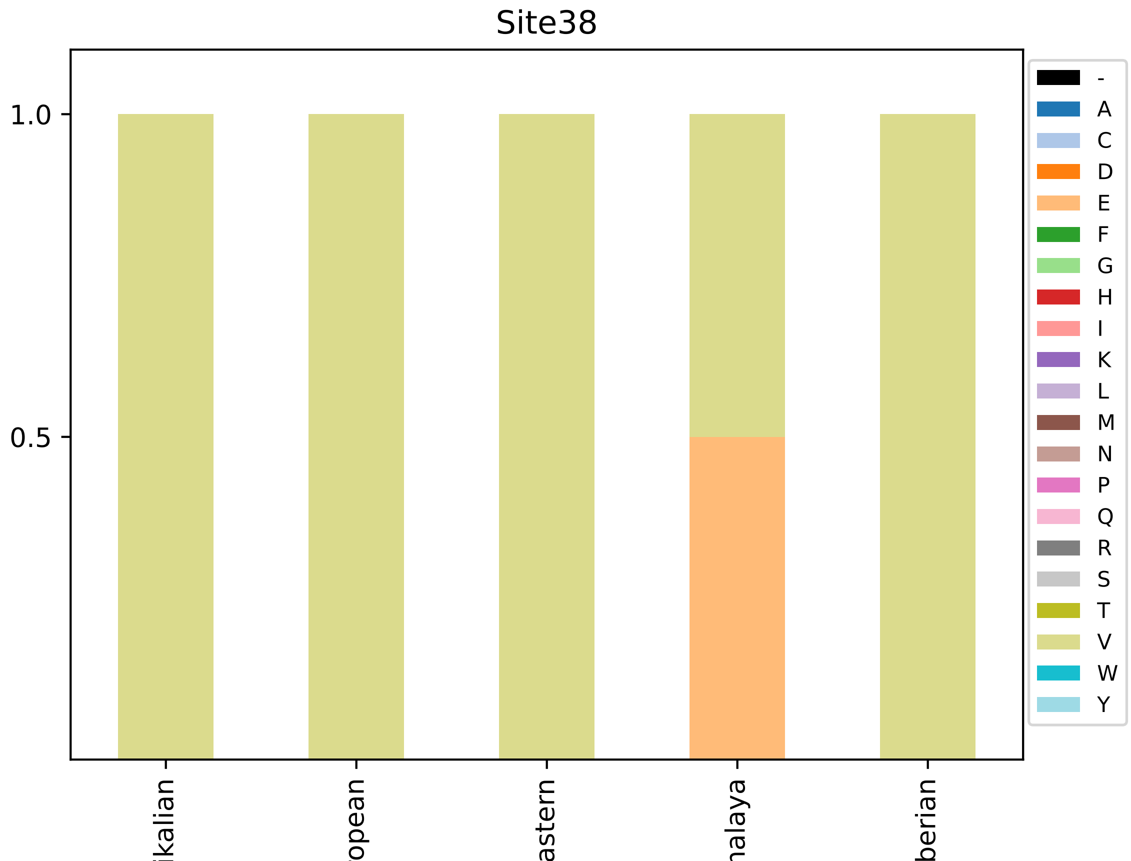

Site38
1.0
0.5
ikalian
ropean
astern
nalaya
berian
-
A
C
D
E
F
G
H
I
K
L
M
N
P
Q
R
S
T
V
W
Y

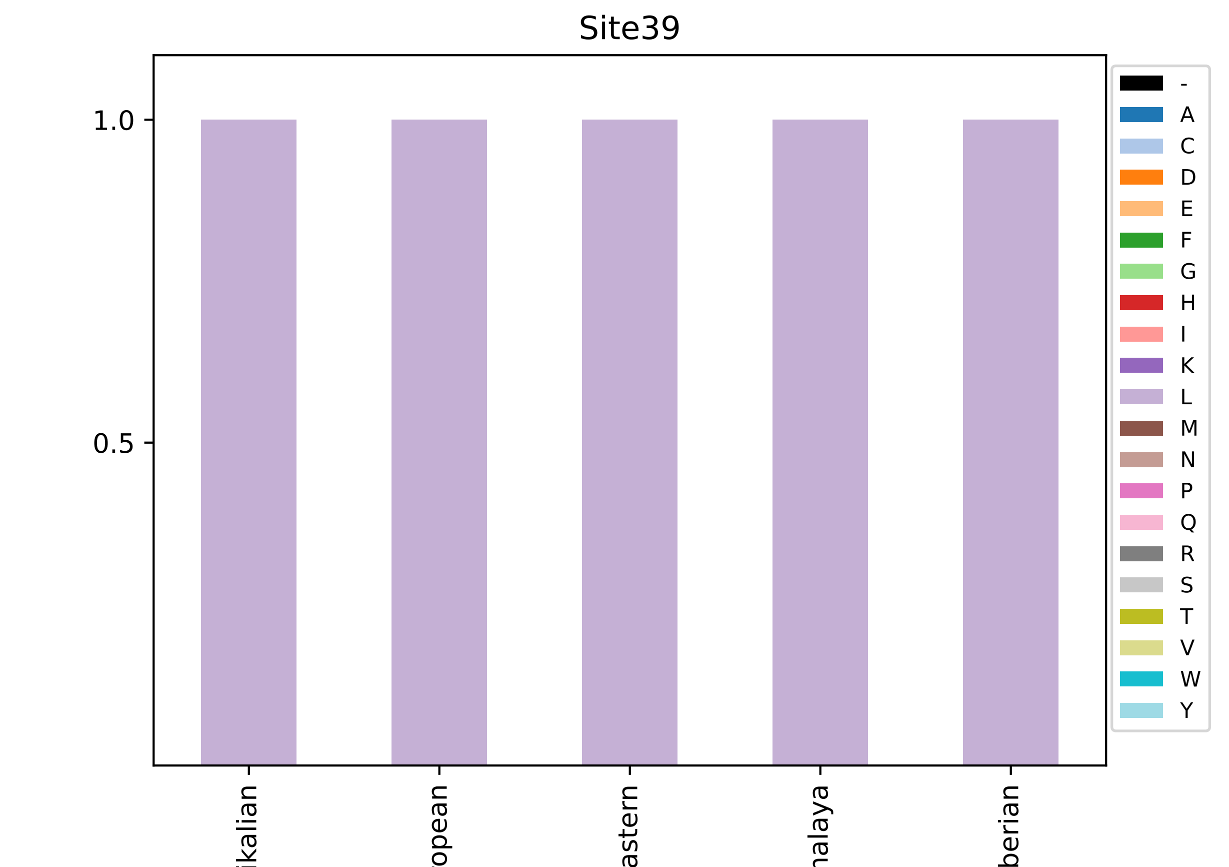
Site39
1.0
0.5
-
A
C
D
E
F
G
H
I
K
L
M
N
P
Q
R
S
T
V
W
Y
ikalian
opean
astern
nalaya
berian

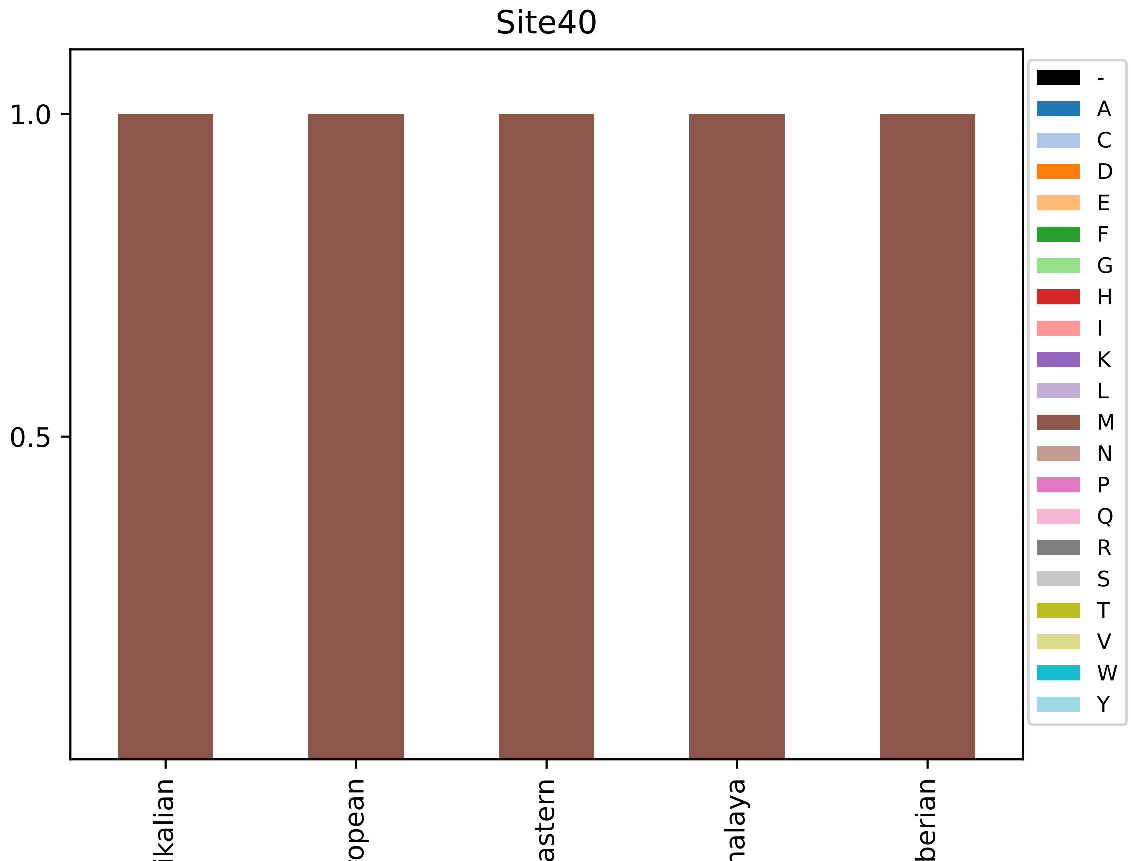
Site40
1.0
0.5
ikalian
opean
astern
nalaya
berian
-
A
C
D
E
F
G
H
I
K
L
M
N
P
Q
R
S
T
V
W
Y

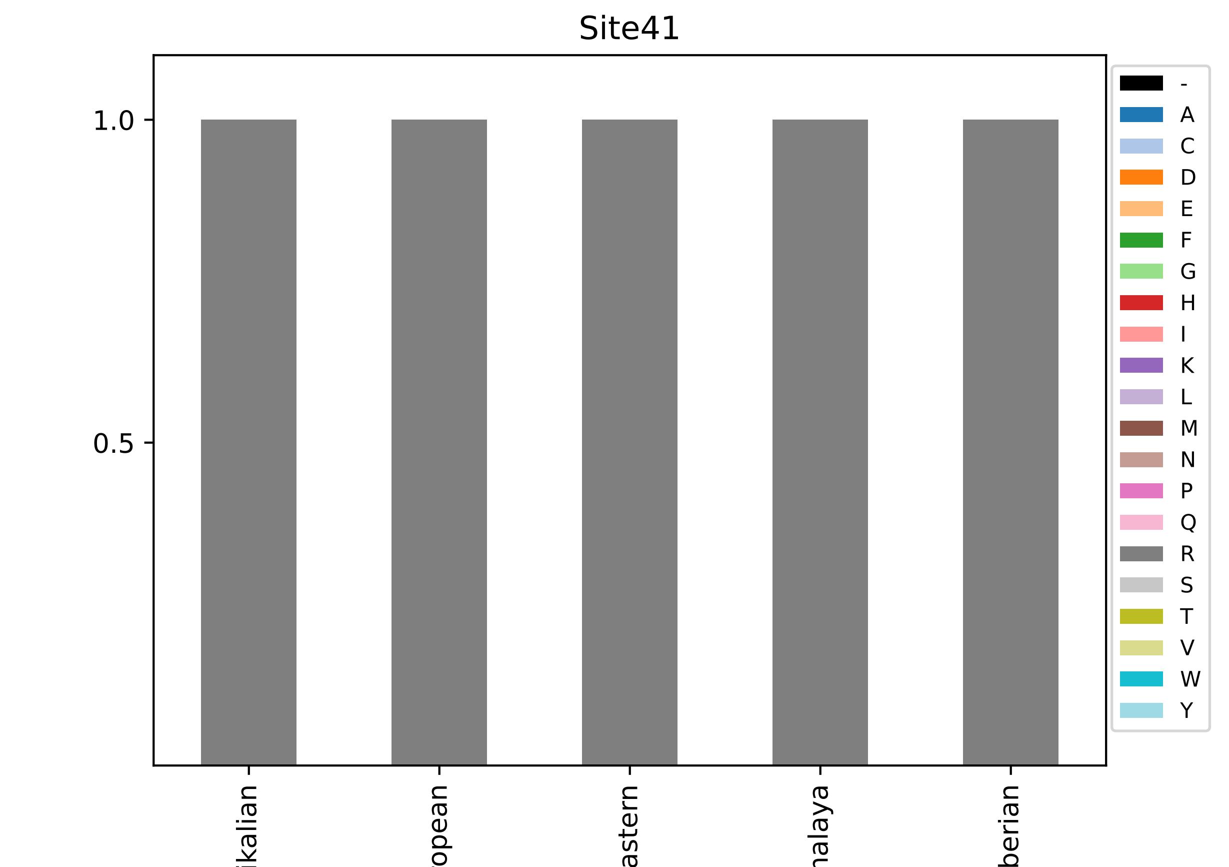
Site41
1.0
0.5
ikalian
opean
astern
nalaya
berian
-
A
C
D
E
F
G
H
I
K
L
M
N
P
Q
R
S
T
V
W
Y

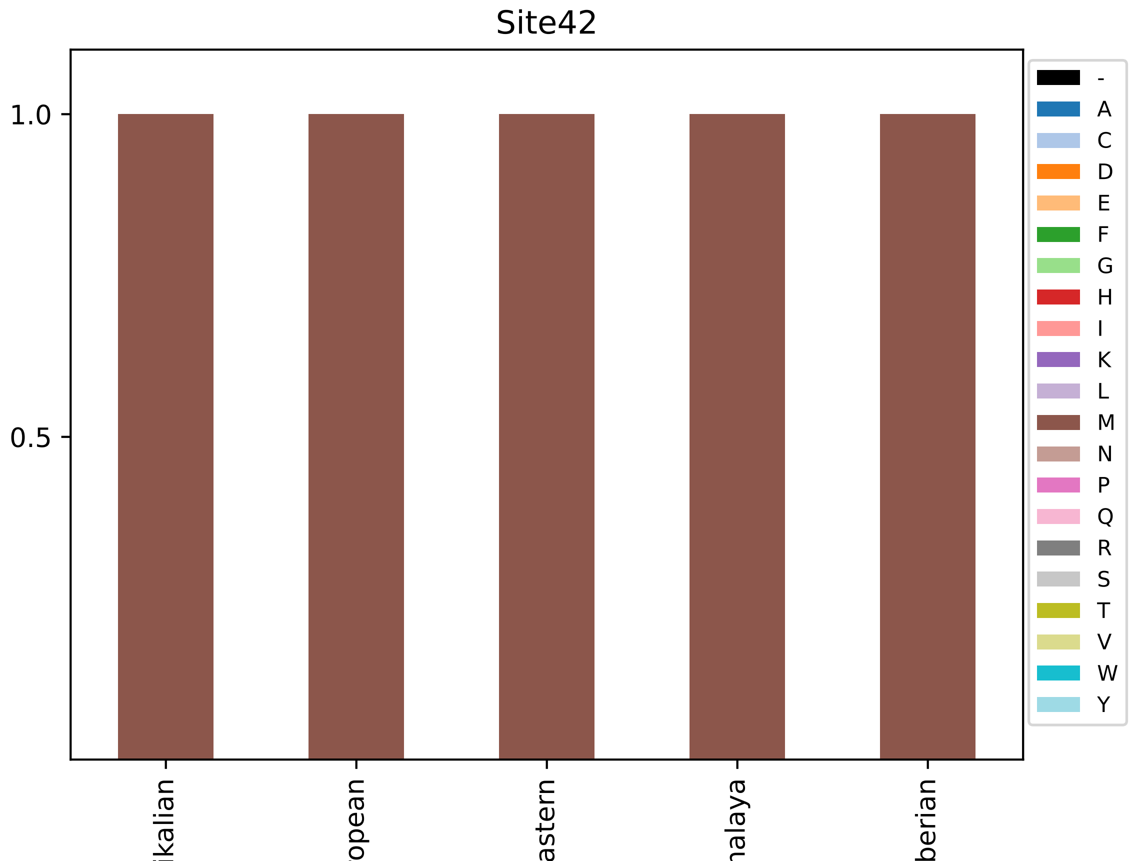

Site42
1.0
0.5
ikalian
ropean
astern
nalaya
berian
-
A
C
D
E
F
G
H
I
K
L
M
N
P
Q
R
S
T
V
W
Y

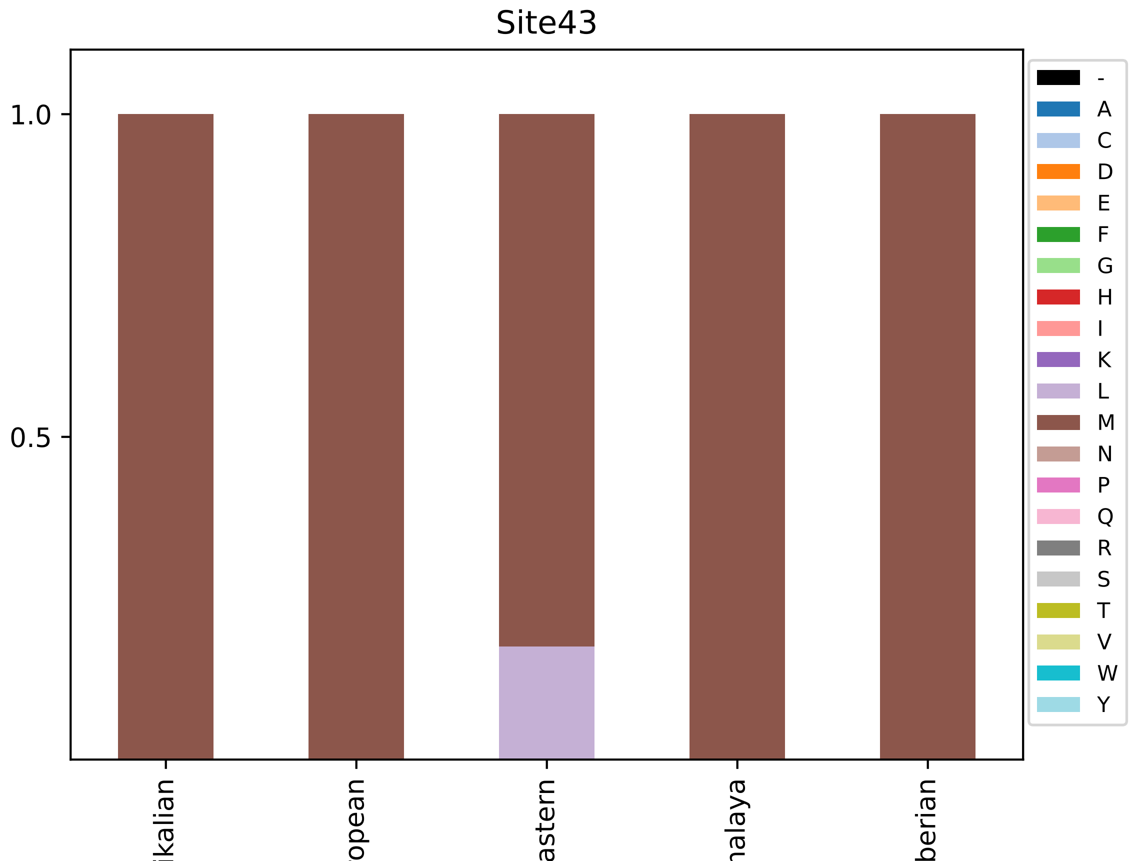

Site43
1.0
0.5
-
A
C
D
E
F
G
H
I
K
L
M
N
P
Q
R
S
T
V
W
Y
ikalian
opean
astern
nalaya
berian

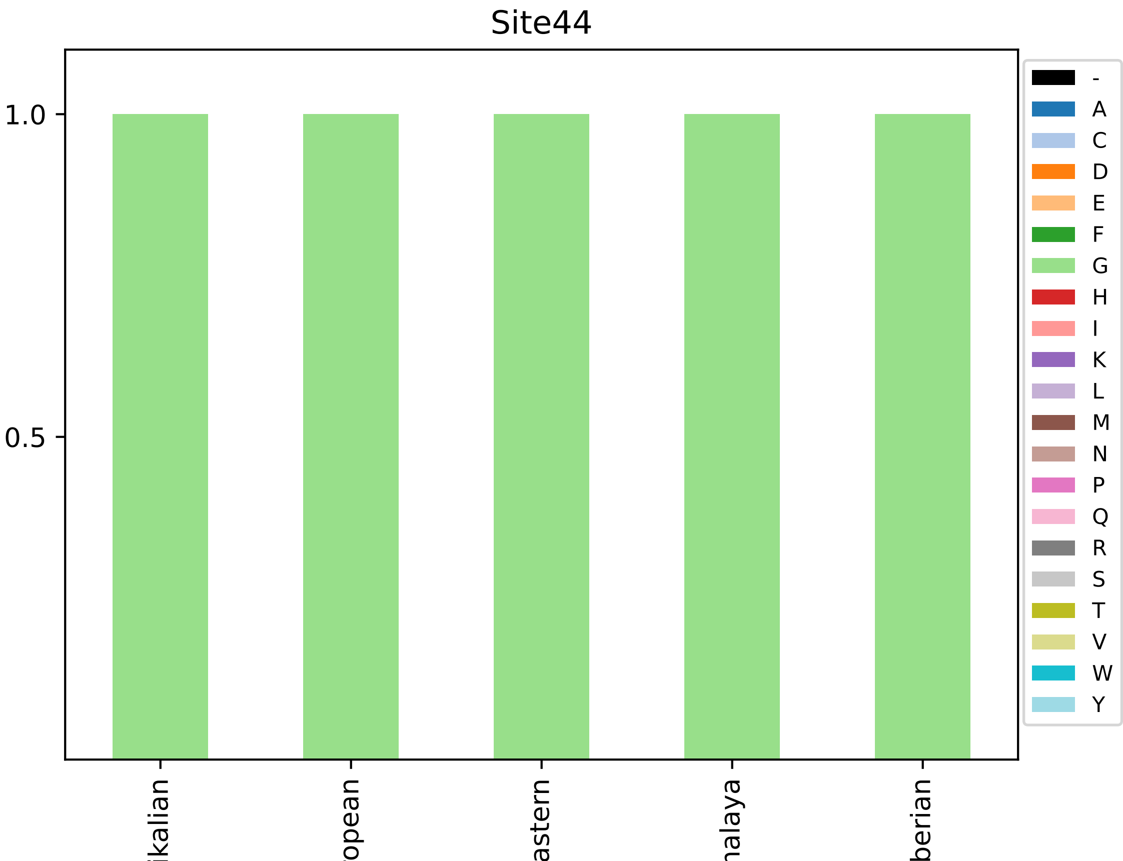
Site44
1.0
0.5
-
A
C
D
E
F
G
H
I
K
L
M
N
P
Q
R
S
T
V
W
Y
ikalian
opean
astern
nalaya
berian

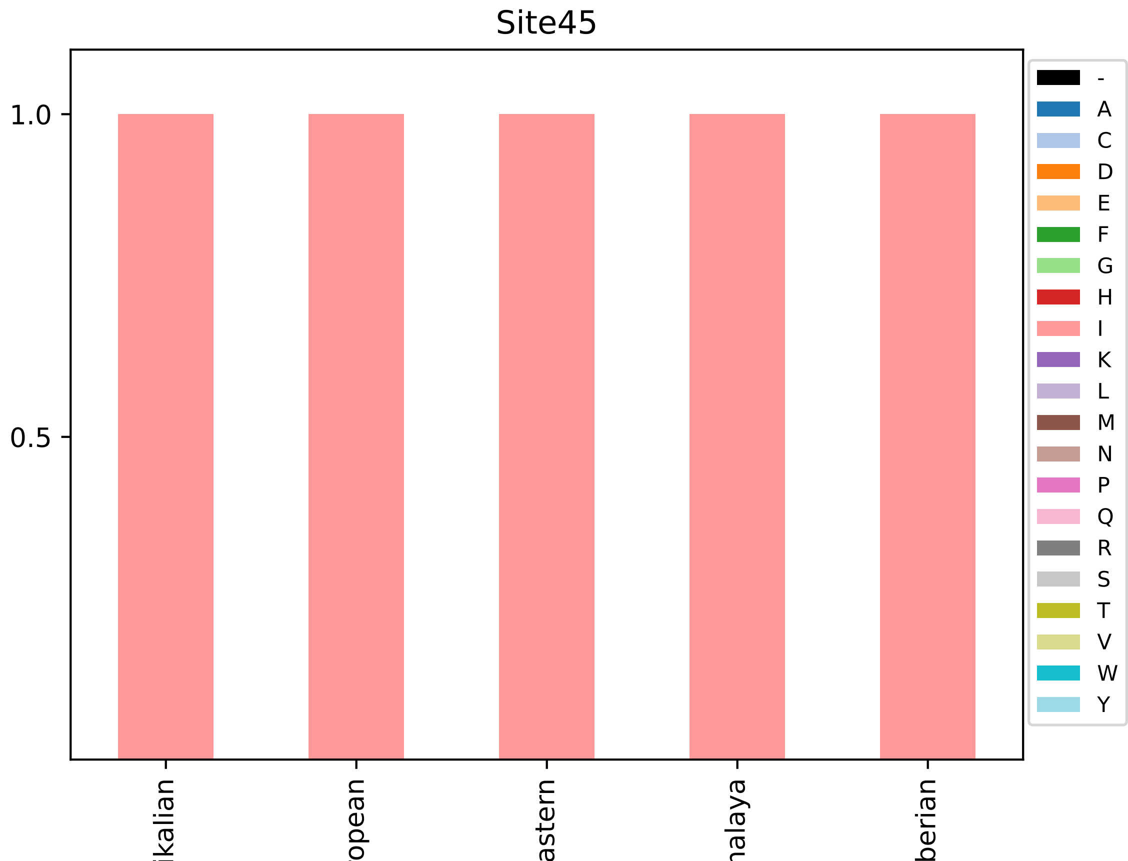

Site45
1.0
0.5
ikalian
opean
astern
nalaya
berian
-
A
C
D
E
F
G
H
I
K
L
M
N
P
Q
R
S
T
V
W
Y

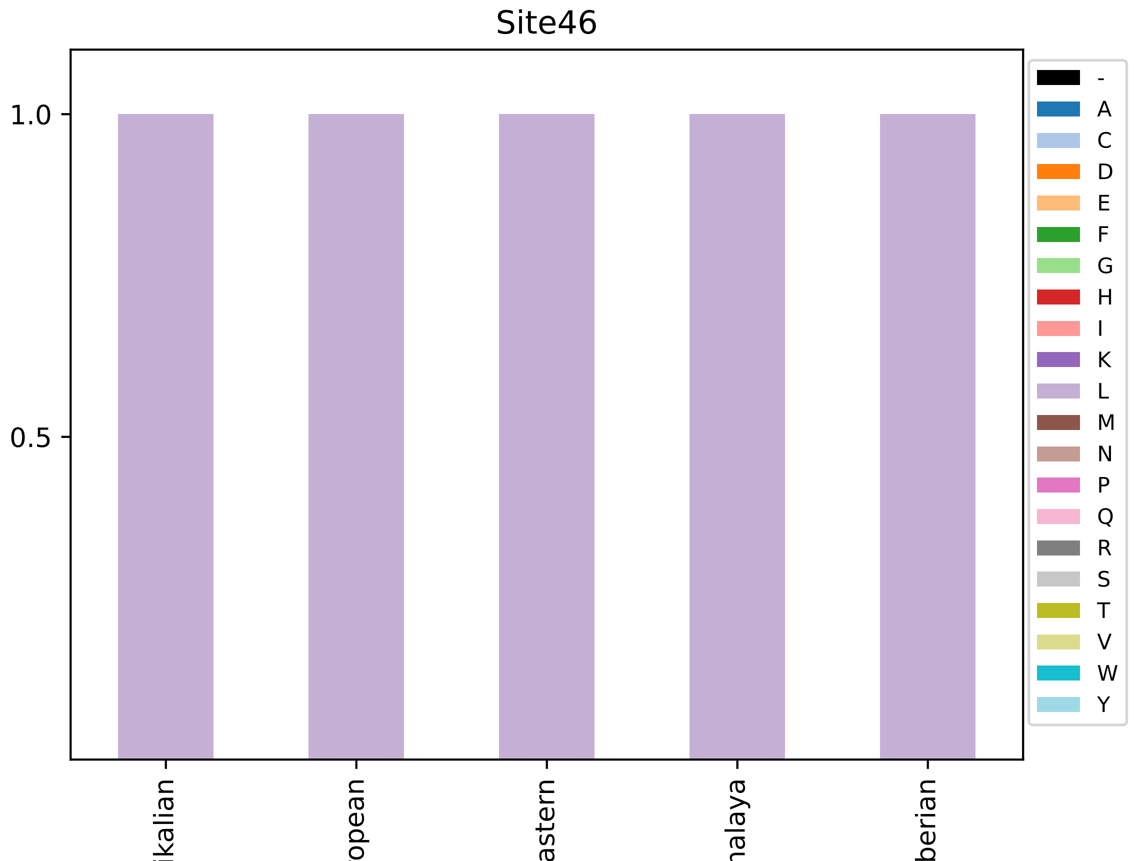
Site46
1.0
0.5
-
A
C
D
E
F
G
H
I
K
L
M
N
P
Q
R
S
T
V
W
Y

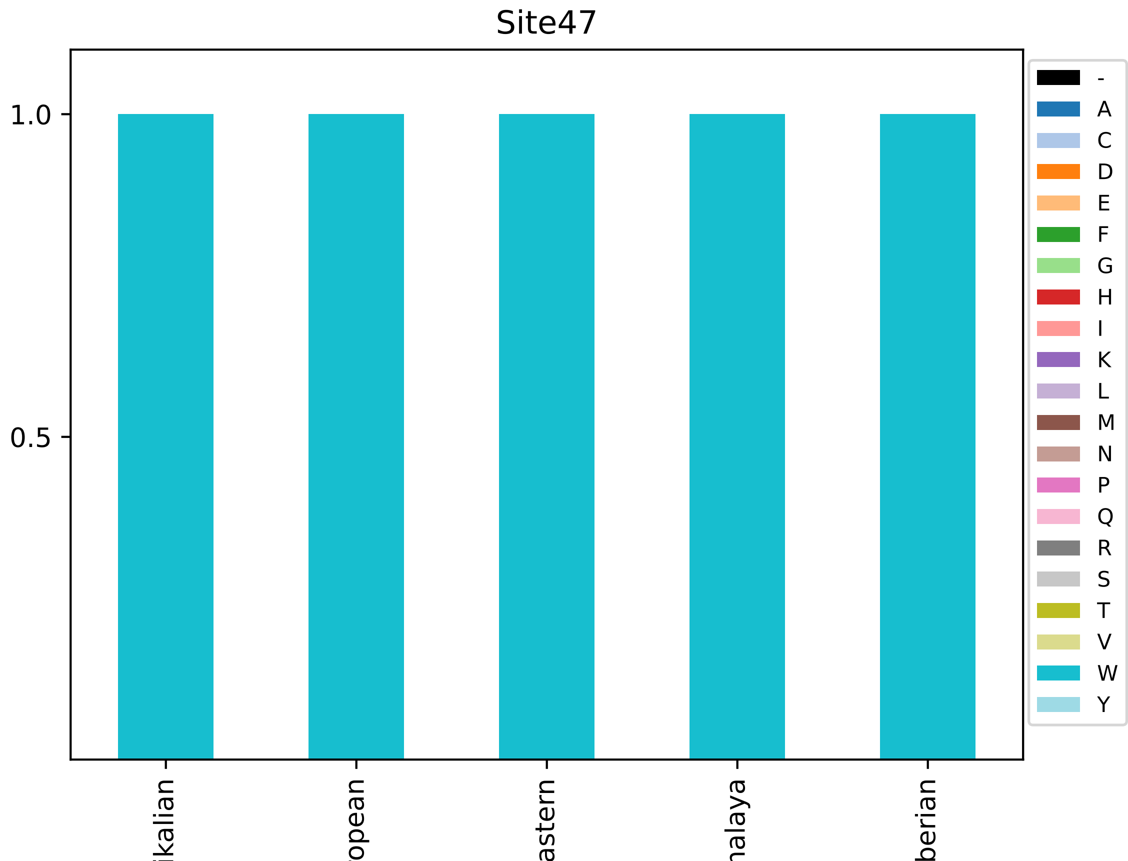

Site47
1.0
0.5
-
A
C
D
E
F
G
H
I
K
L
M
N
P
Q
R
S
T
V
W
Y
ikalian
ropean
astern
nalaya
berian

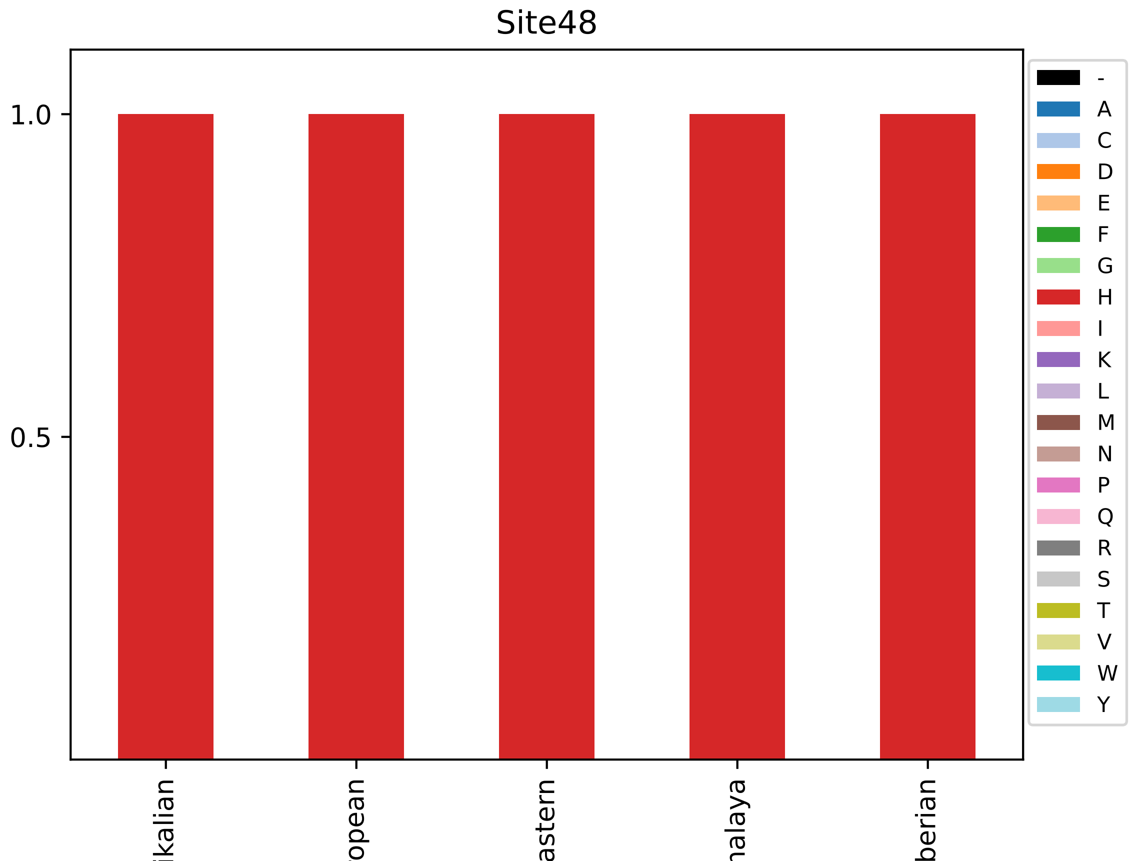
Site48
1.0
0.5
ikalian
opean
astern
nalaya
berian
-
A
C
D
E
F
G
H
I
K
L
M
N
P
Q
R
S
T
V
W
Y

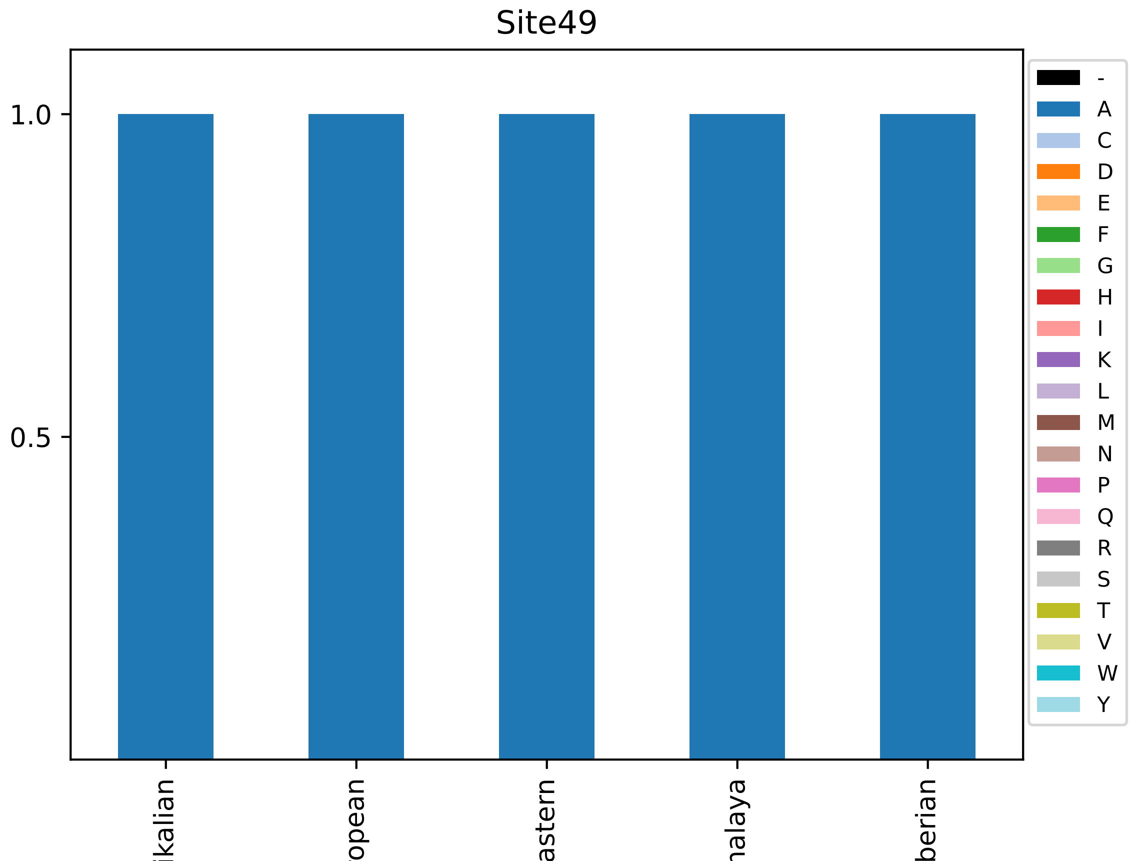

Site49
1.0
0.5
-
A
C
D
E
F
G
H
I
K
L
M
N
P
Q
R
S
T
V
W
Y

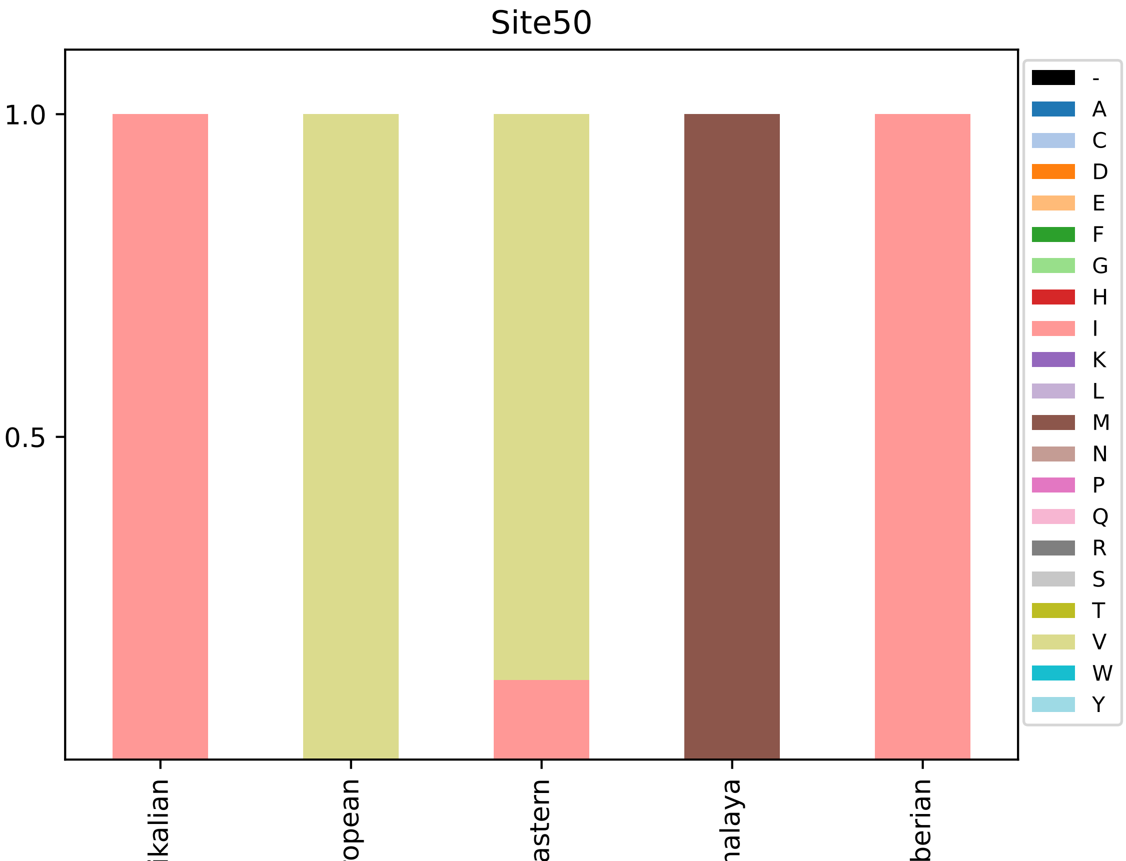
Site50
1.0
0.5
-
A
C
D
E
F
G
H
I
K
L
M
N
P
Q
R
S
T
V
W
Y
ikalian
opean
astern
nalaya
berian

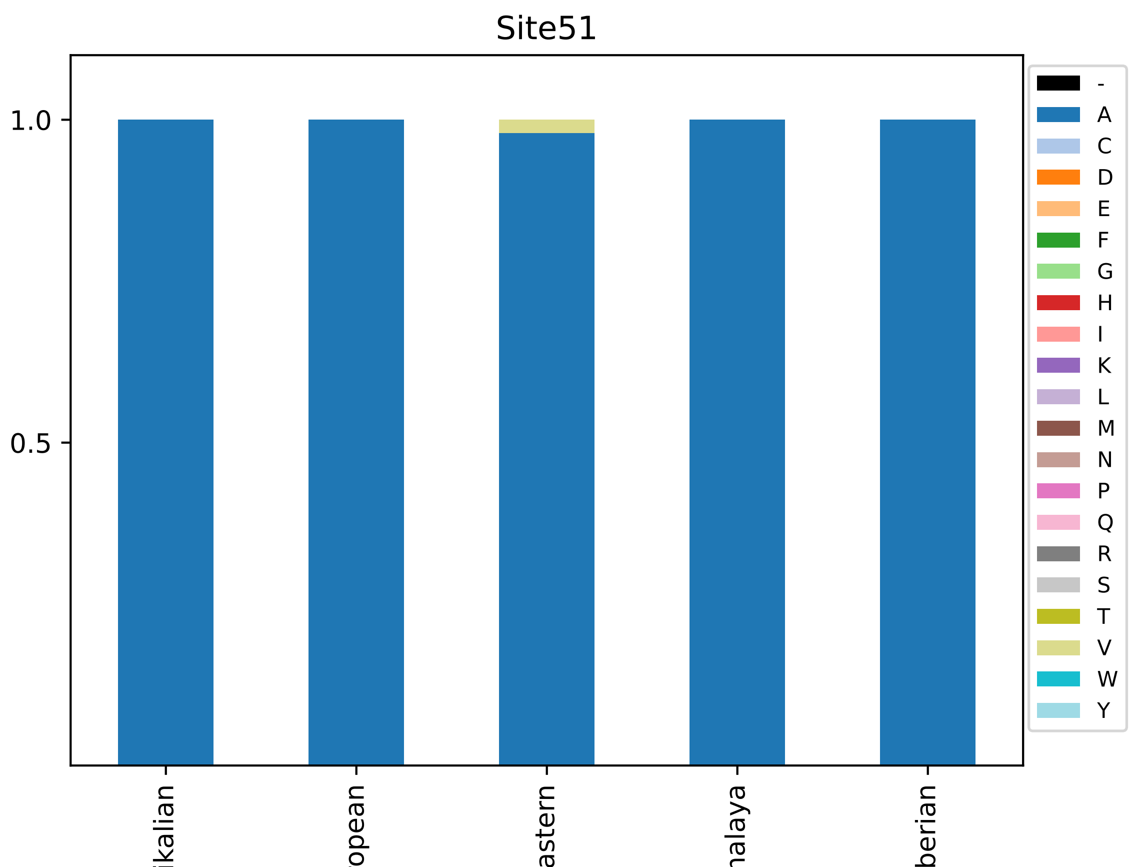
Site51
1.0
0.5
-
A
C
D
E
F
G
H
I
K
L
M
N
P
Q
R
S
T
V
W
Y
ikalian
ropean
astern
nalaya
berian

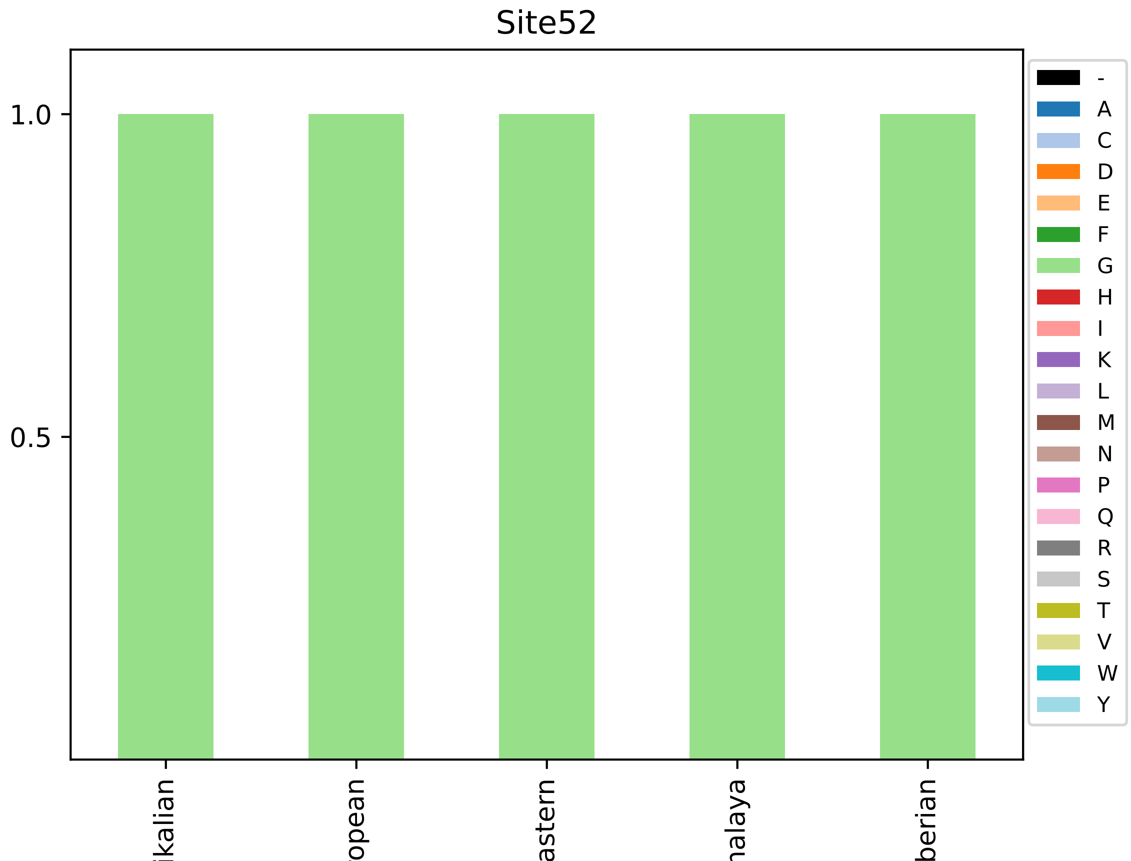
Site52
1.0
0.5
-
A
C
D
E
F
G
H
I
K
L
M
N
P
Q
R
S
T
V
W
Y
ikalian
opean
astern
nalaya
berian

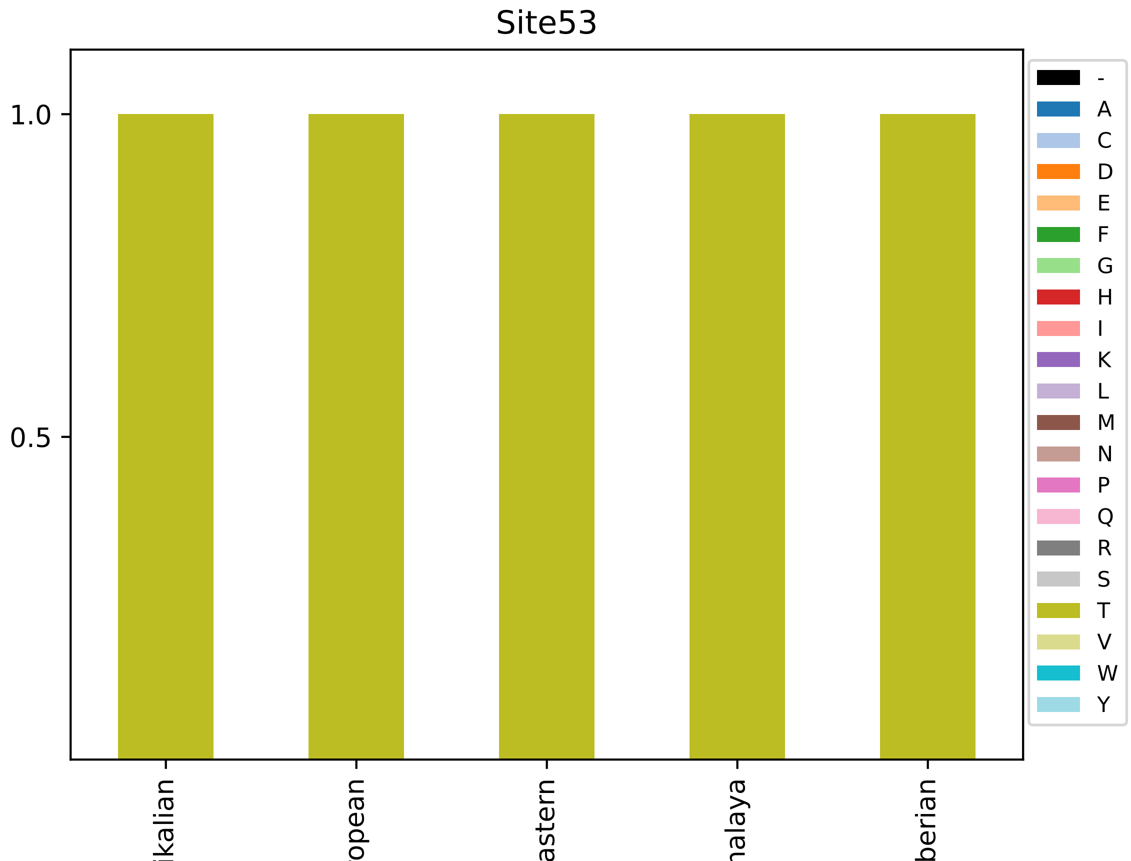

Site53
1.0
0.5
-
A
C
D
E
F
G
H
I
K
L
M
N
P
Q
R
S
T
V
W
Y
ikalian
opean
astern
nalaya
berian

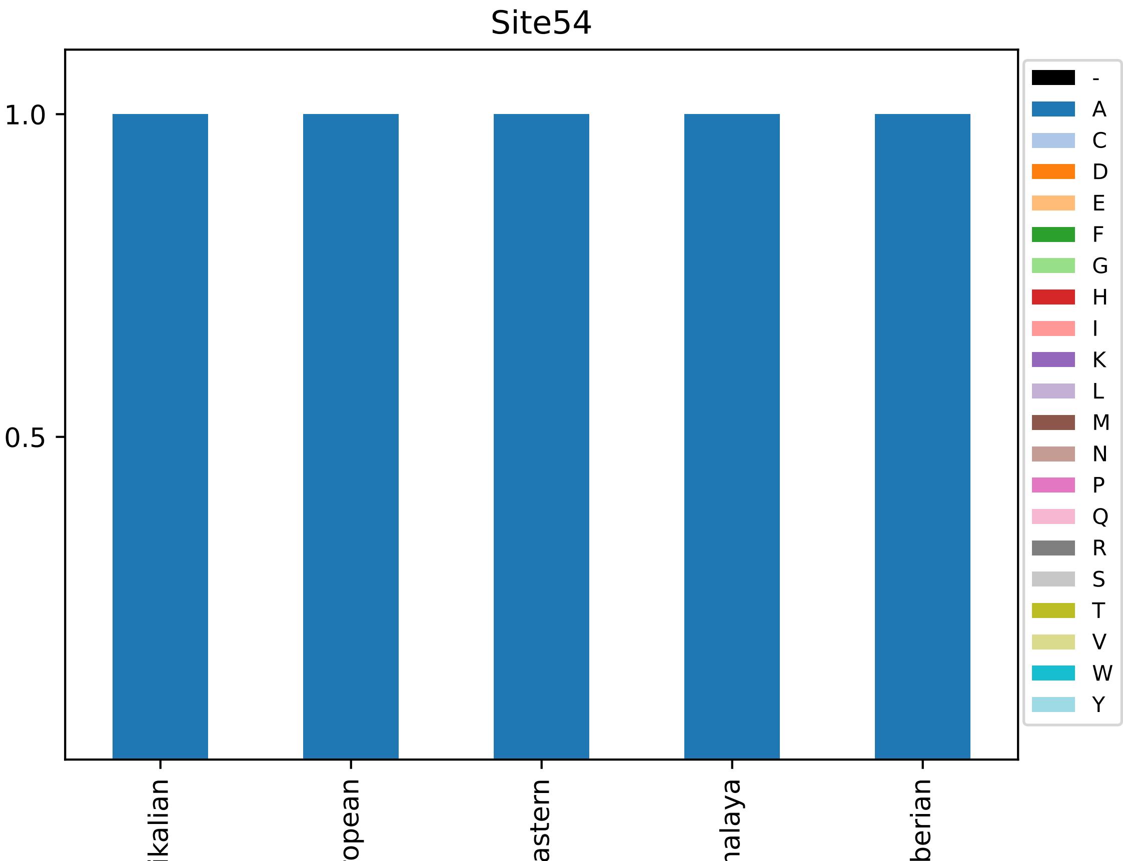
Site54
1.0
0.5
-
A
C
D
E
F
G
H
I
K
L
M
N
P
Q
R
S
T
V
W
Y

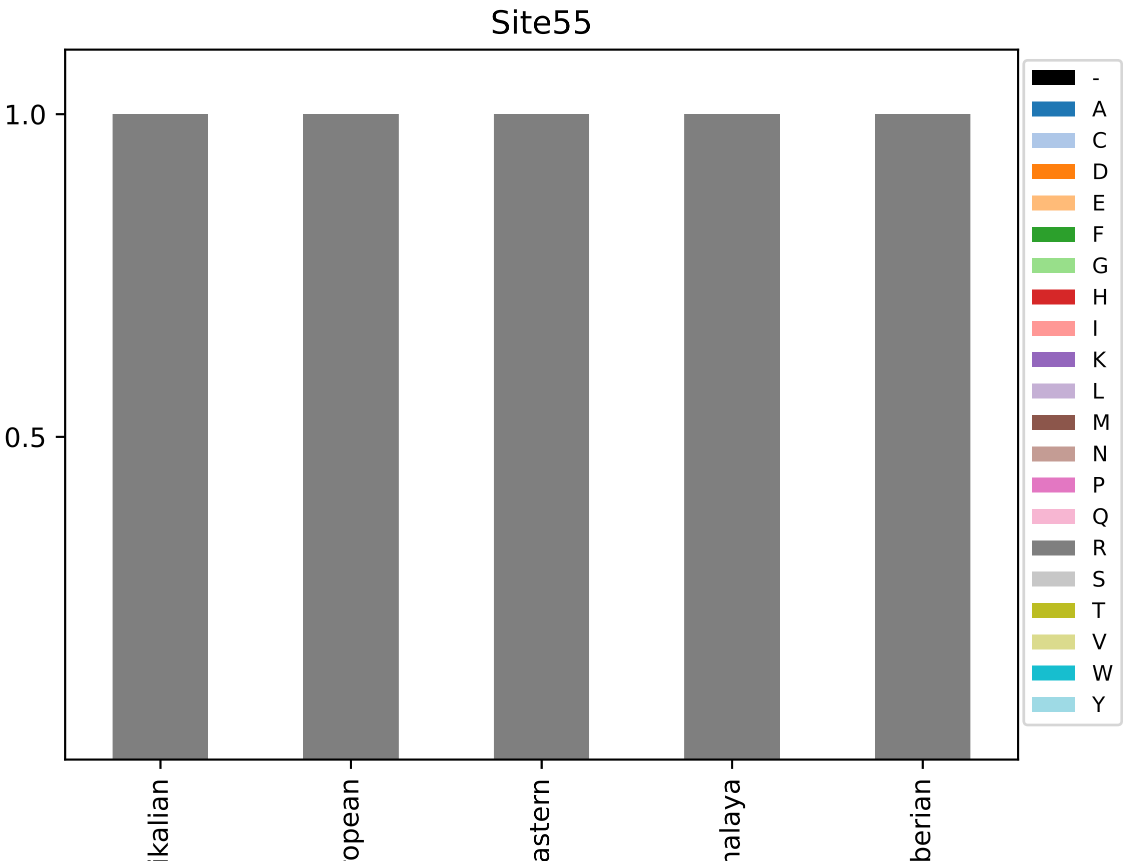
Site55
1.0
0.5
-
A
C
D
E
F
G
H
I
K
L
M
N
P
Q
R
S
T
V
W
Y

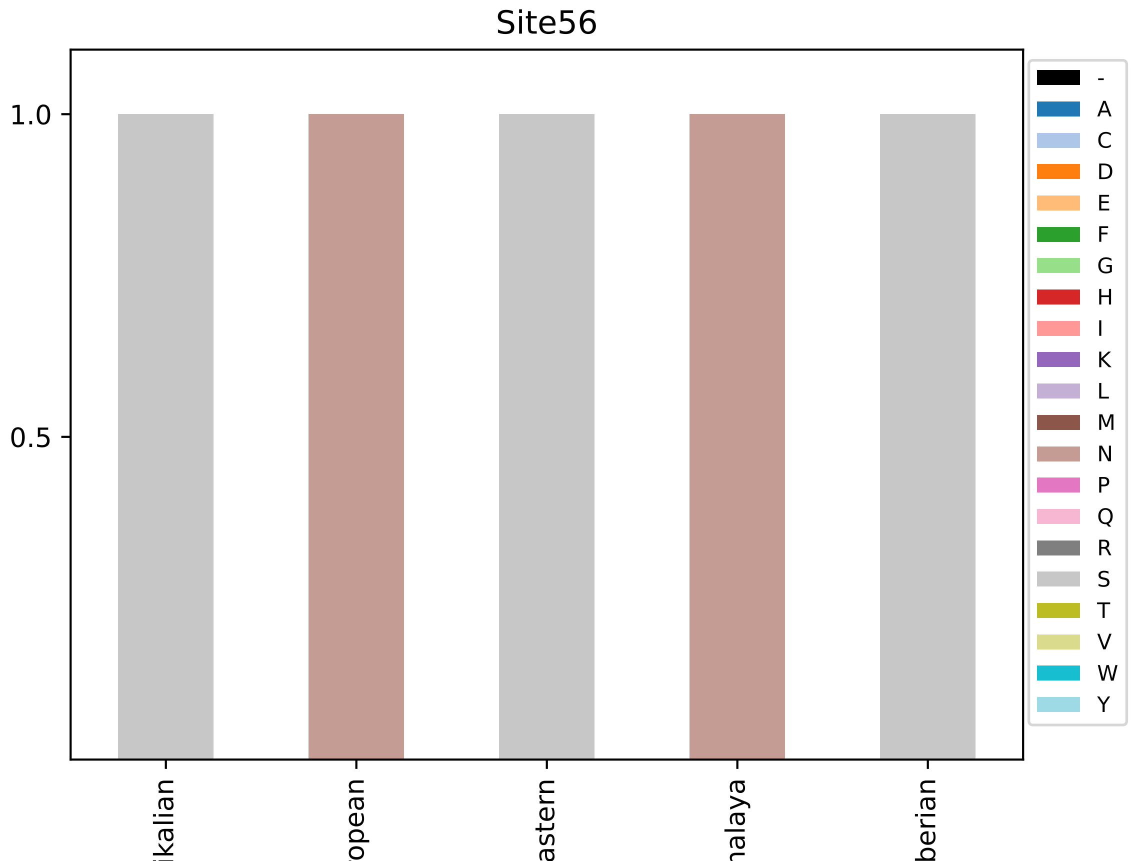
Site56
1.0
0.5
-
A
C
D
E
F
G
H
I
K
L
M
N
P
Q
R
S
T
V
W
Y
ikalian
opean
astern
nalaya
berian

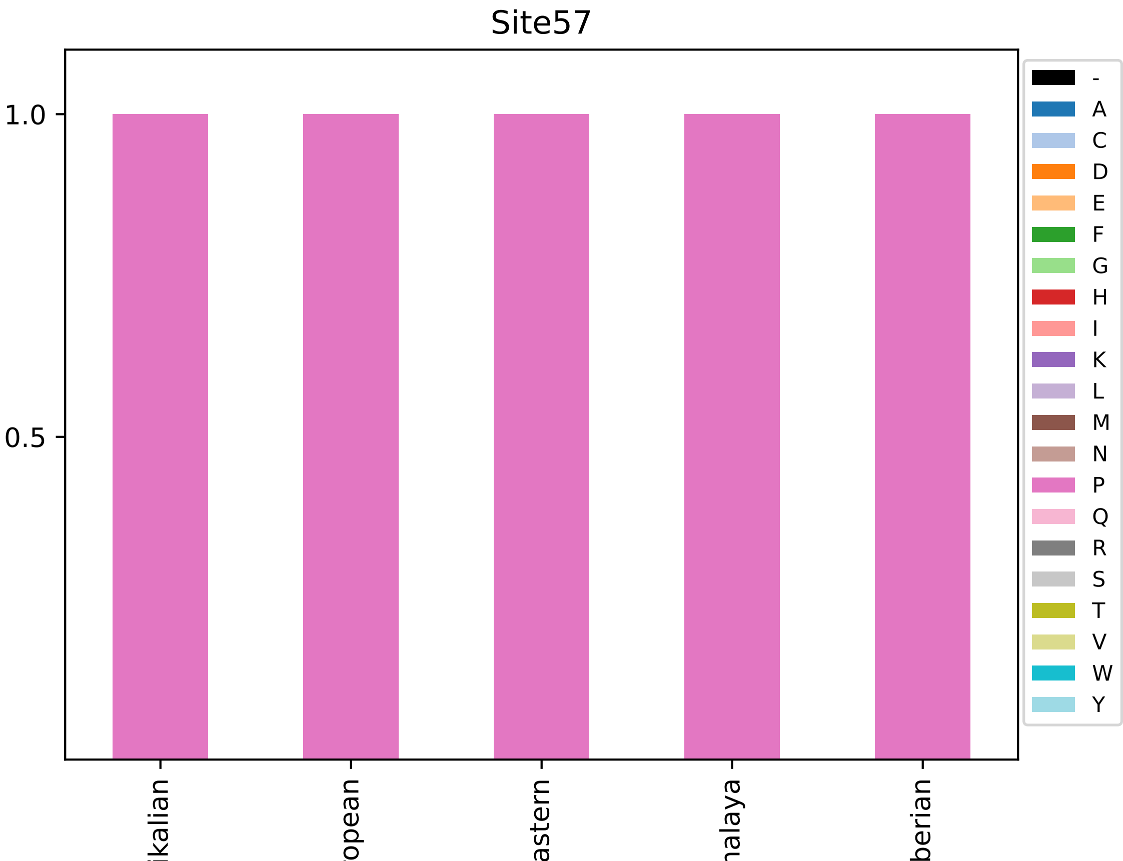

Site57
1.0
0.5
-
A
C
D
E
F
G
H
I
K
L
M
N
P
Q
R
S
T
V
W
Y
ikalian
opean
astern
nalaya
berian

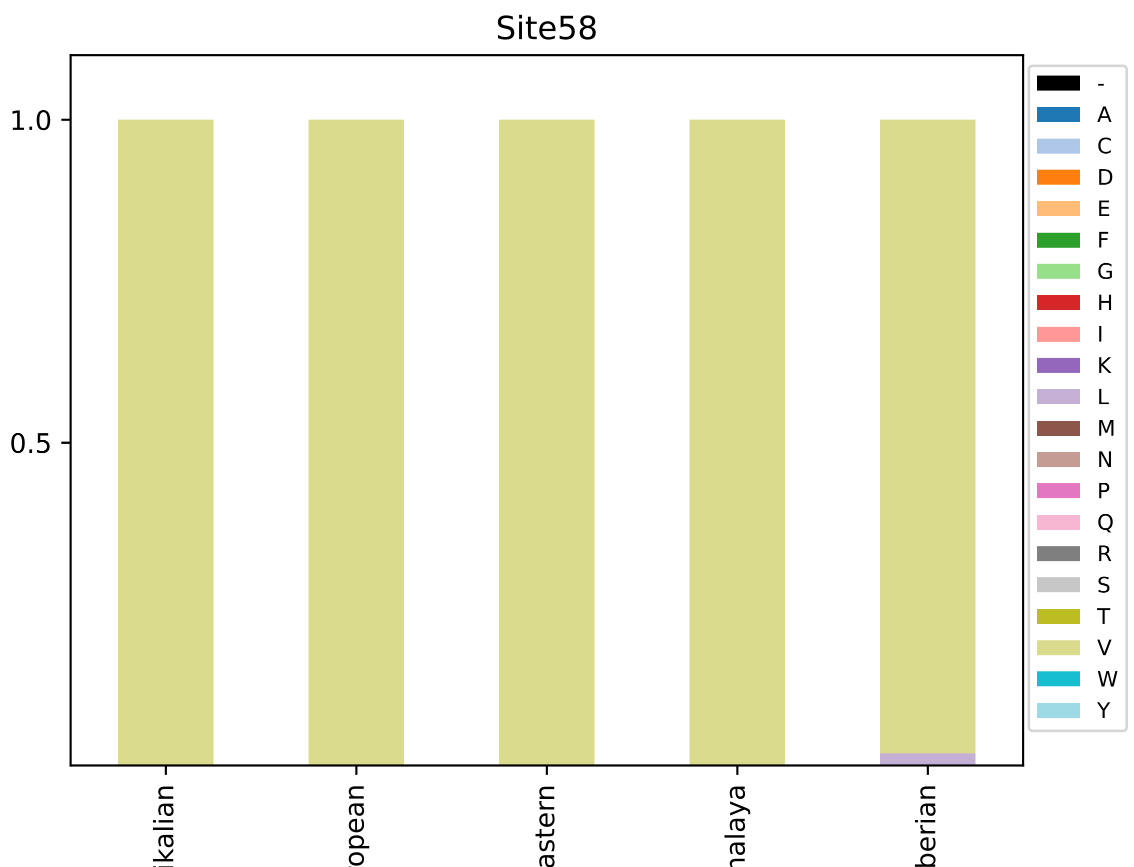
Site58
1.0
0.5
ikalian
opean
astern
nalaya
berian
-
A
C
D
E
F
G
H
I
K
L
M
N
P
Q
R
S
T
V
W
Y

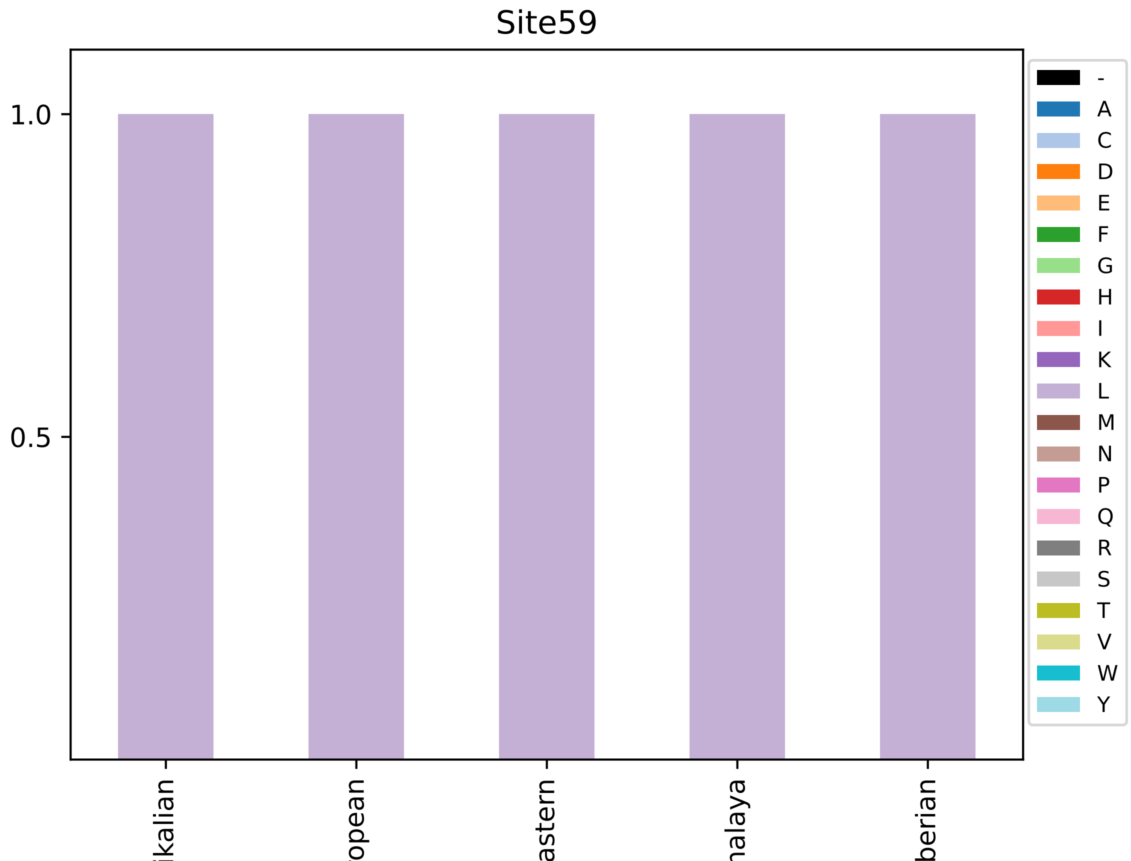
Site59
1.0
0.5
-
A
C
D
E
F
G
H
I
K
L
M
N
P
Q
R
S
T
V
W
Y
ikalian
opean
astern
halaya
berian

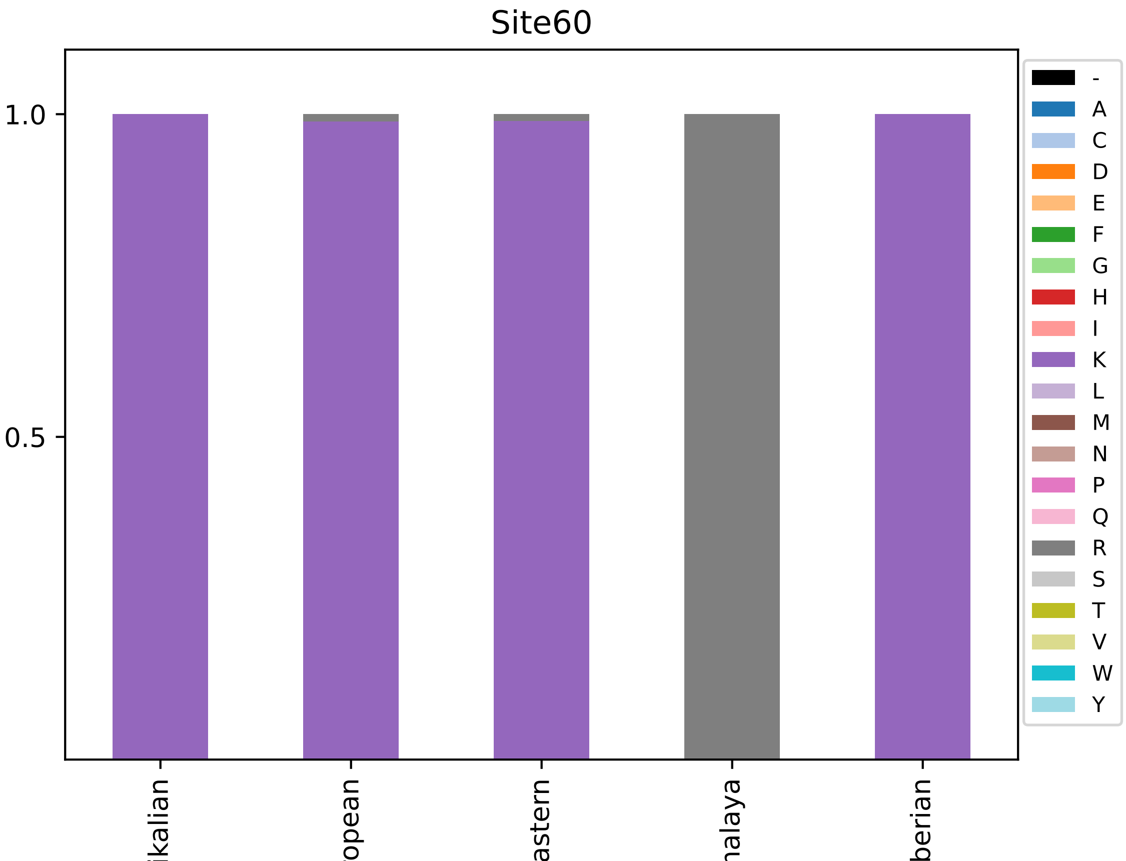

Site60
1.0
0.5
-
A
C
D
E
F
G
H
I
K
L
M
N
P
Q
R
S
T
V
W
Y
ikalian
opean
astern
nalaya
berian

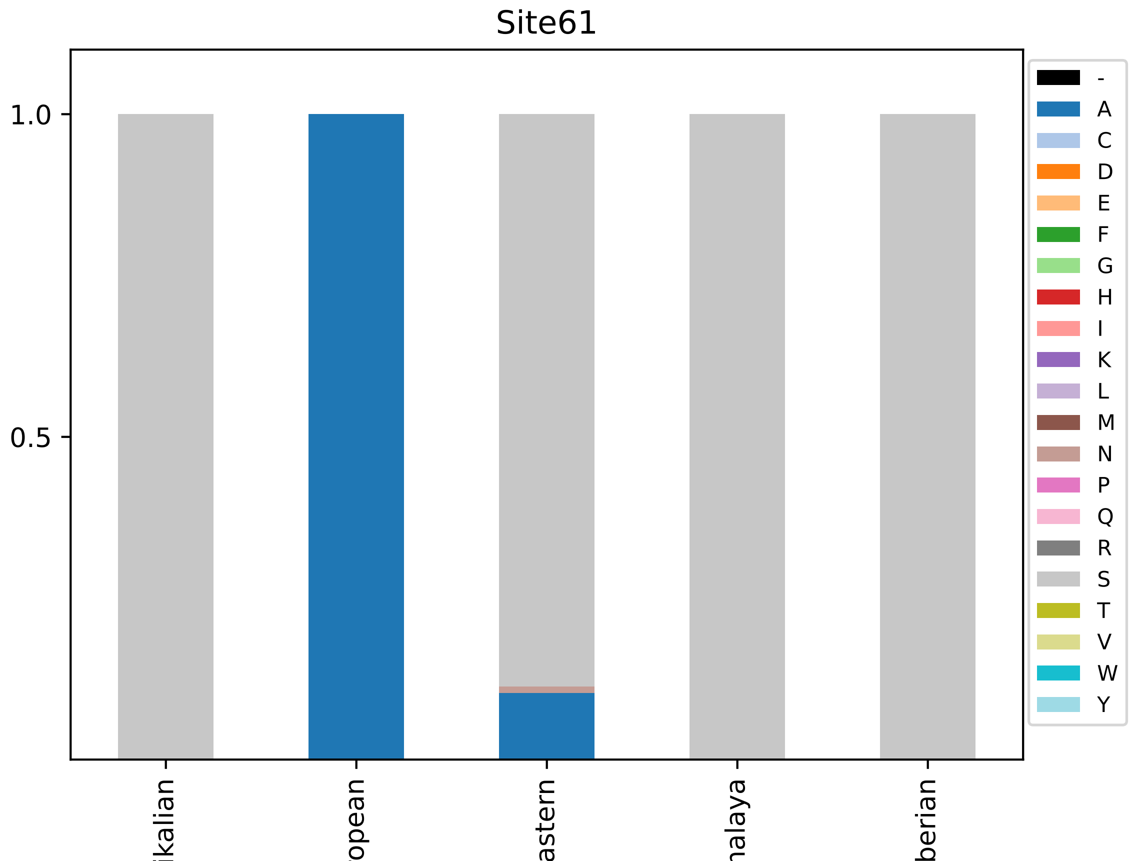
Site61
1.0
0.5
-
A
C
D
E
F
G
H
I
K
L
M
N
P
Q
R
S
T
V
W
Y
ikalian
opean
astern
nalaya
berian

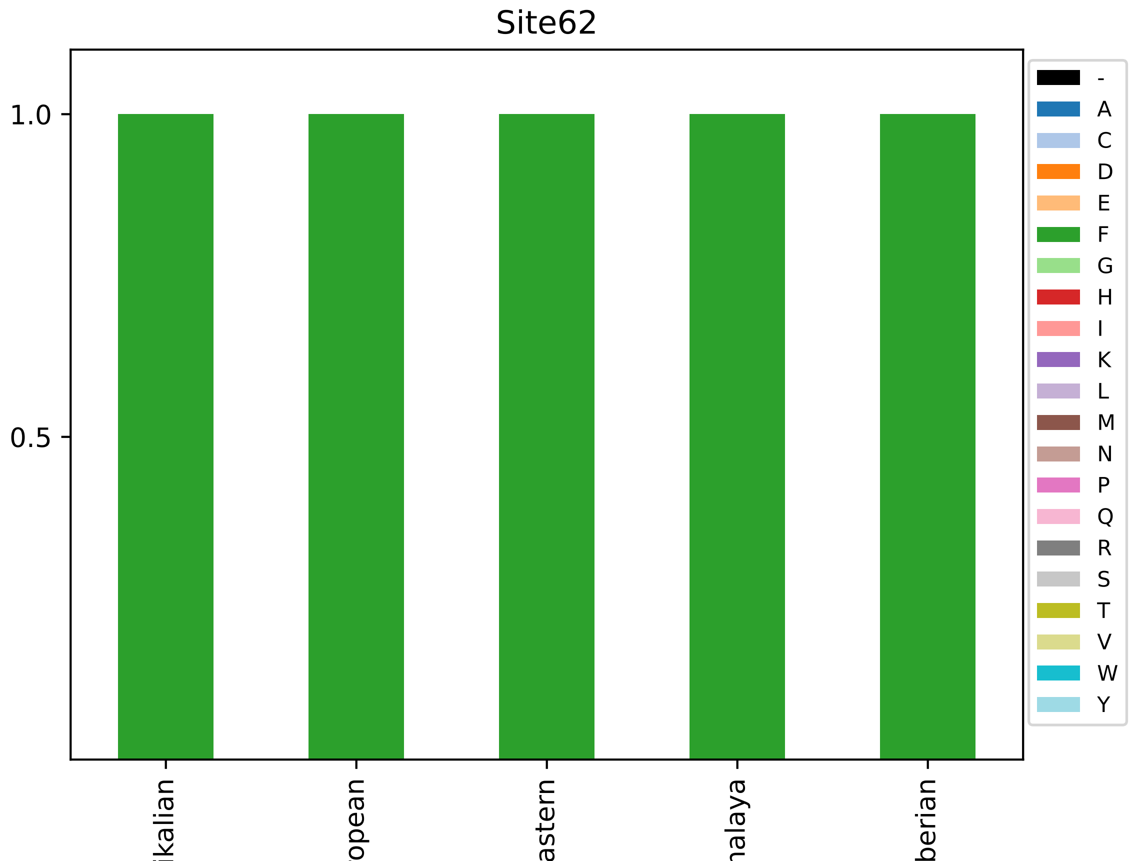
Site62
1.0
0.5
-
A
C
D
E
F
G
H
I
K
L
M
N
P
Q
R
S
T
V
W
Y
ikalian
opean
astern
nalaya
berian

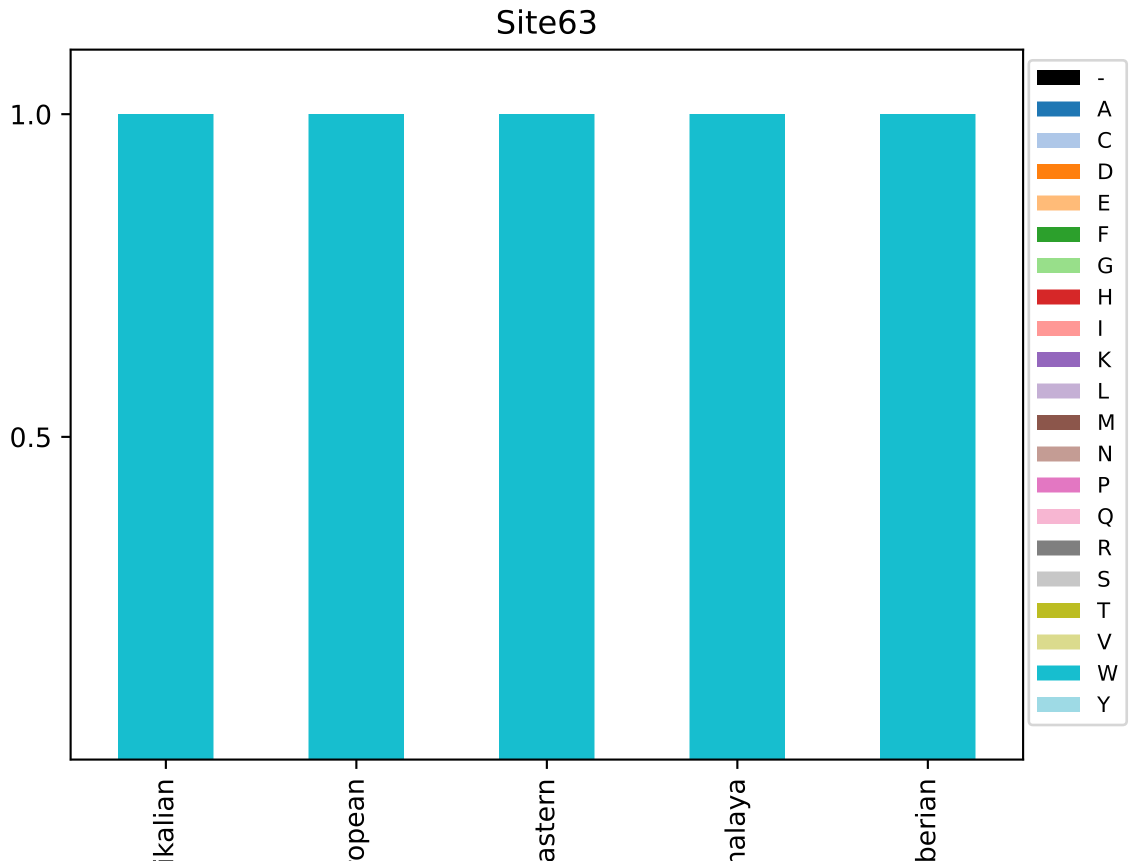

Site63
1.0
0.5
-
A
C
D
E
F
G
H
I
K
L
M
N
P
Q
R
S
T
V
W
Y
ikalian
opean
astern
nalaya
berian

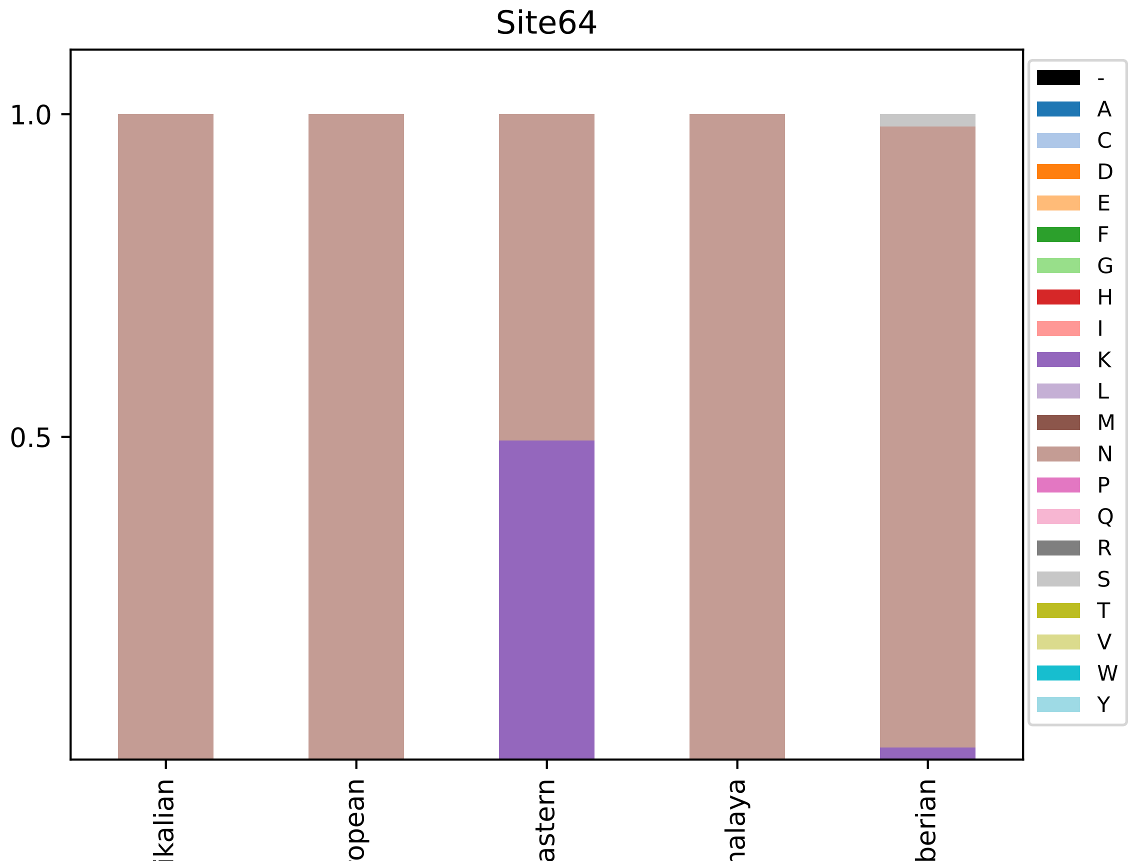

Site64
1.0
0.5
-
A
C
D
E
F
G
H
I
K
L
M
N
P
Q
R
S
T
V
W
Y
ikalian
opean
astern
nalaya
berian

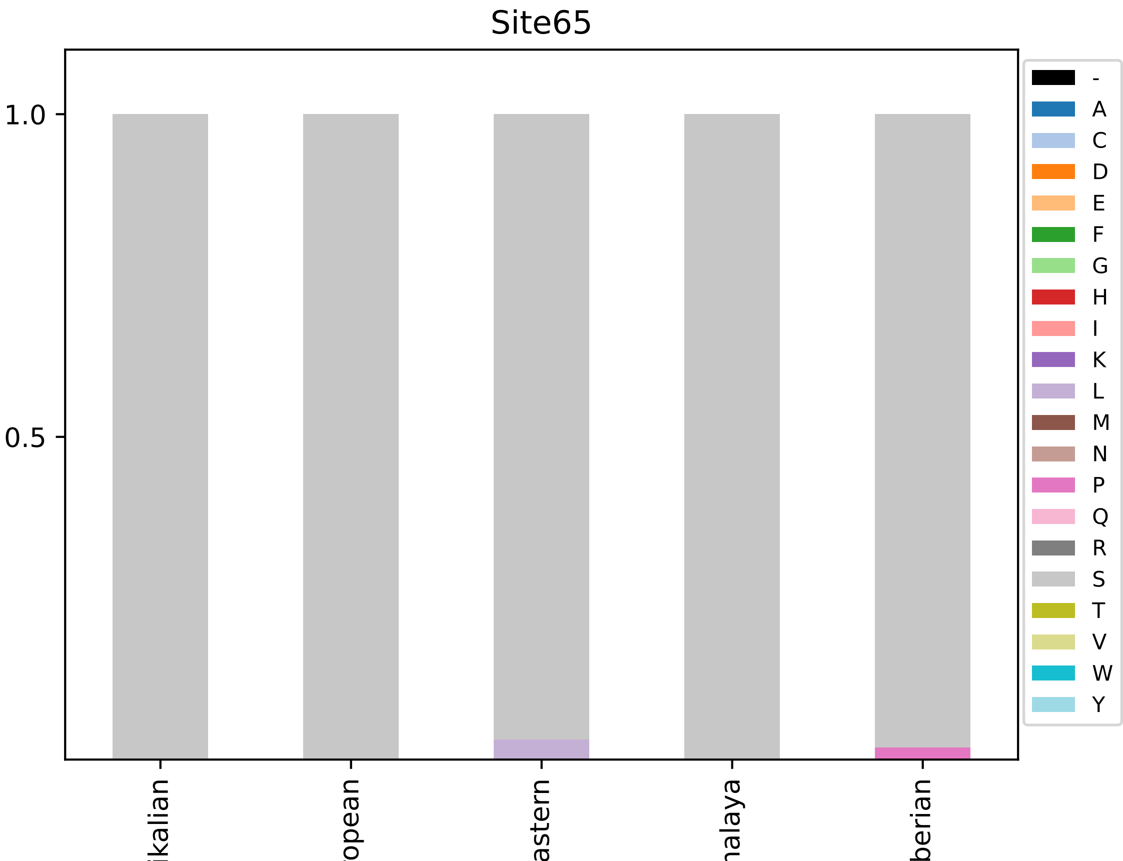
Site65
1.0
0.5
-
A
C
D
E
F
G
H
I
K
L
M
N
P
Q
R
S
T
V
W
Y
ikalian
opean
astern
nalaya
berian

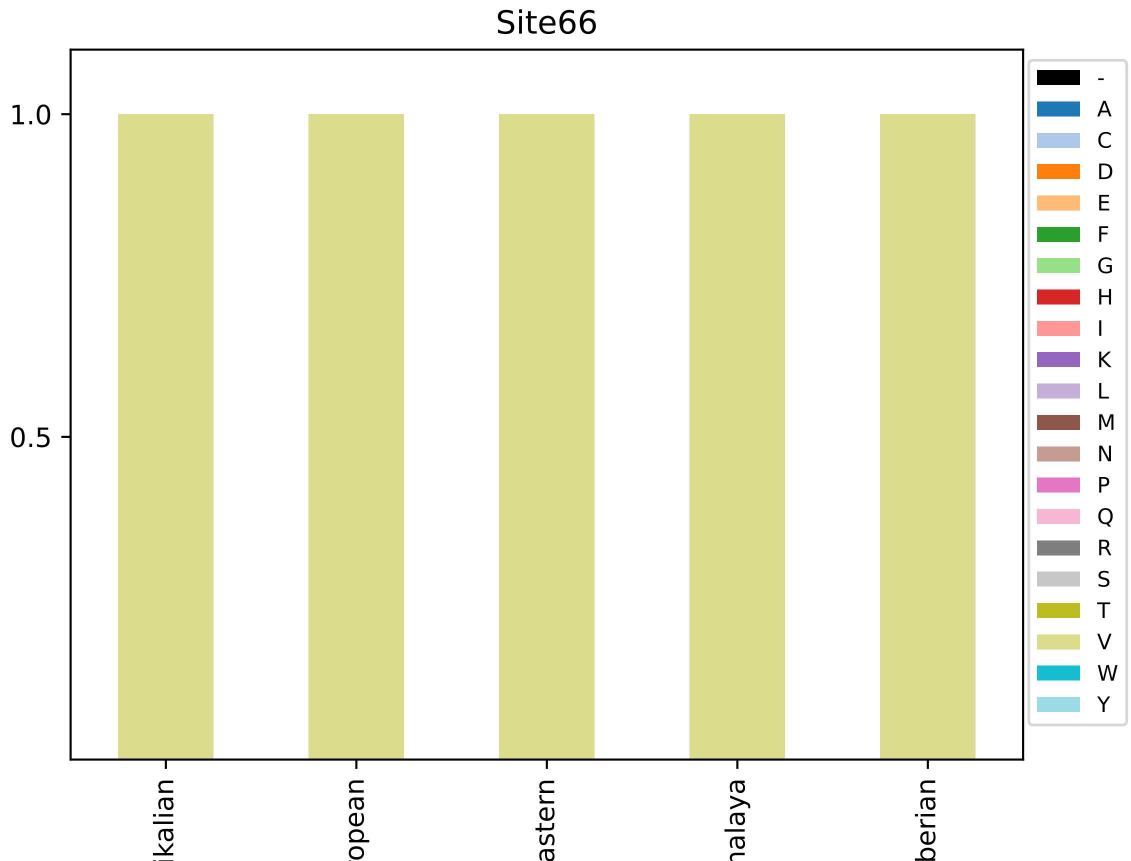
Site66
1.0
0.5
-
A
C
D
E
F
G
H
I
K
L
M
N
P
Q
R
S
T
V
W
Y
ikalian
opean
astern
halaya
berian

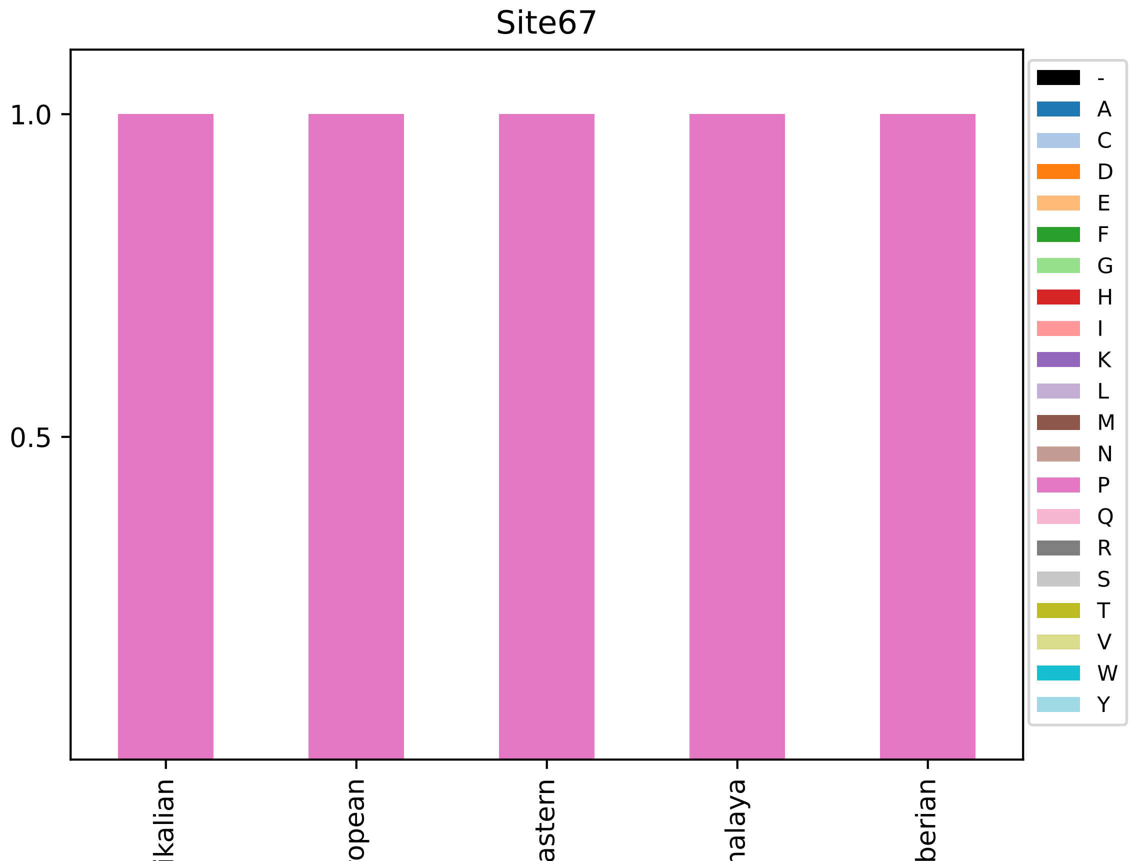
Site67
1.0
0.5
-
A
C
D
E
F
G
H
I
K
L
M
N
P
Q
R
S
T
V
W
Y
ikalian
opean
astern
nalaya
berian

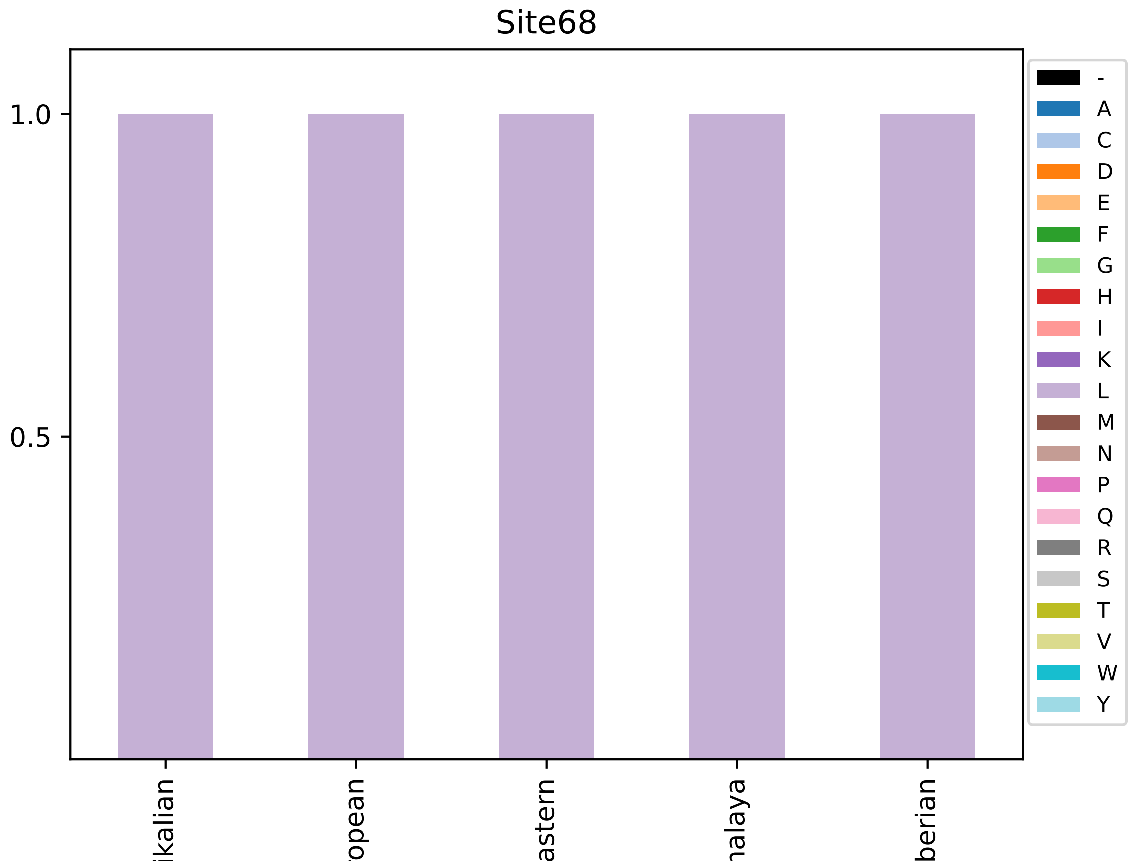

Site68
1.0
0.5
-
A
C
D
E
F
G
H
I
K
L
M
N
P
Q
R
S
T
V
W
Y
ikalian
opean
astern
nalaya
berian

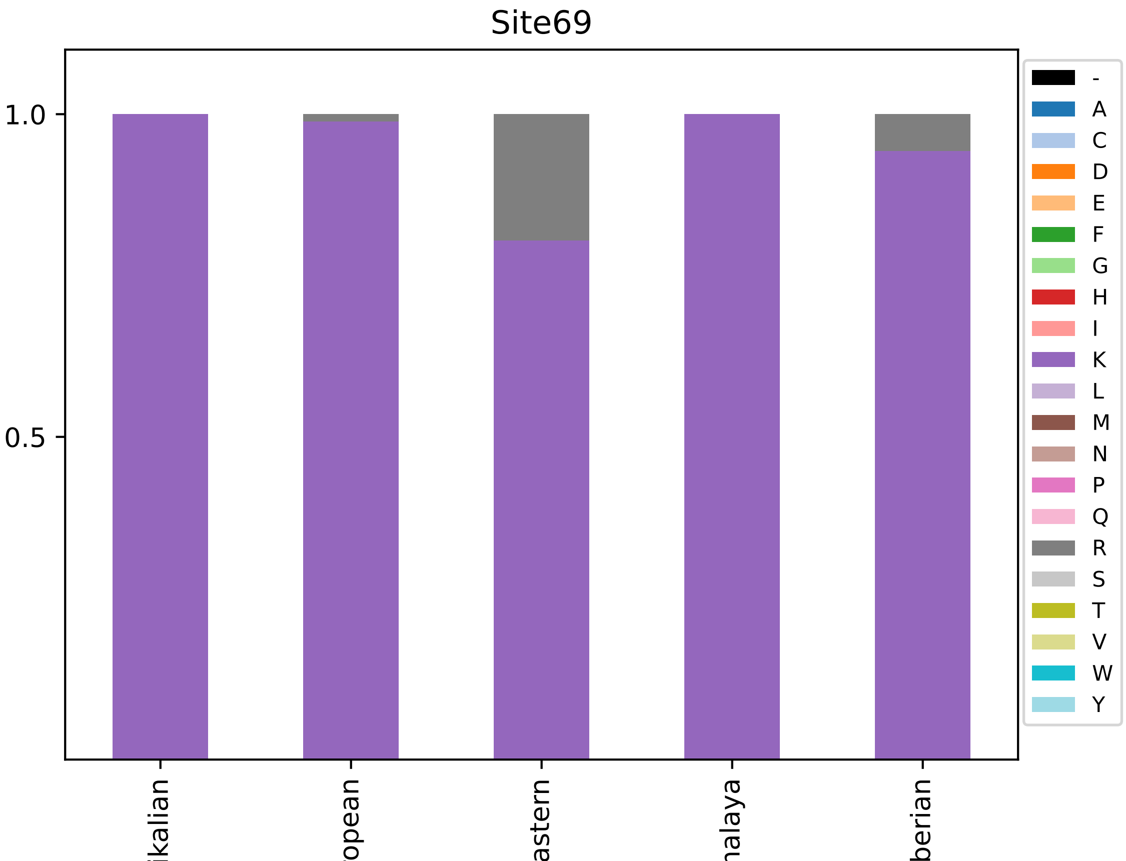

Site69
1.0
0.5
-
A
C
D
E
F
G
H
I
K
L
M
N
P
Q
R
S
T
V
W
Y

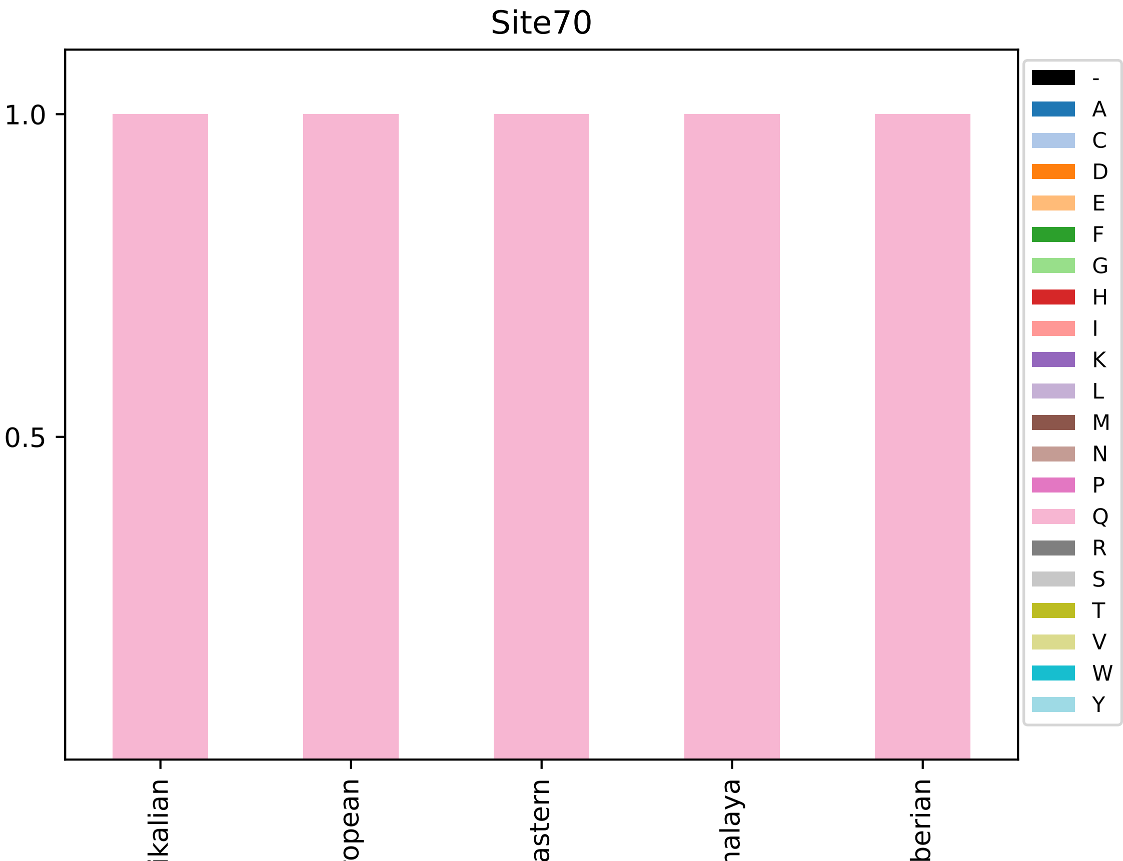
Site70
1.0
0.5
-
A
C
D
E
F
G
H
I
K
L
M
N
P
Q
R
S
T
V
W
Y
ikalian
opean
astern
nalaya
berian

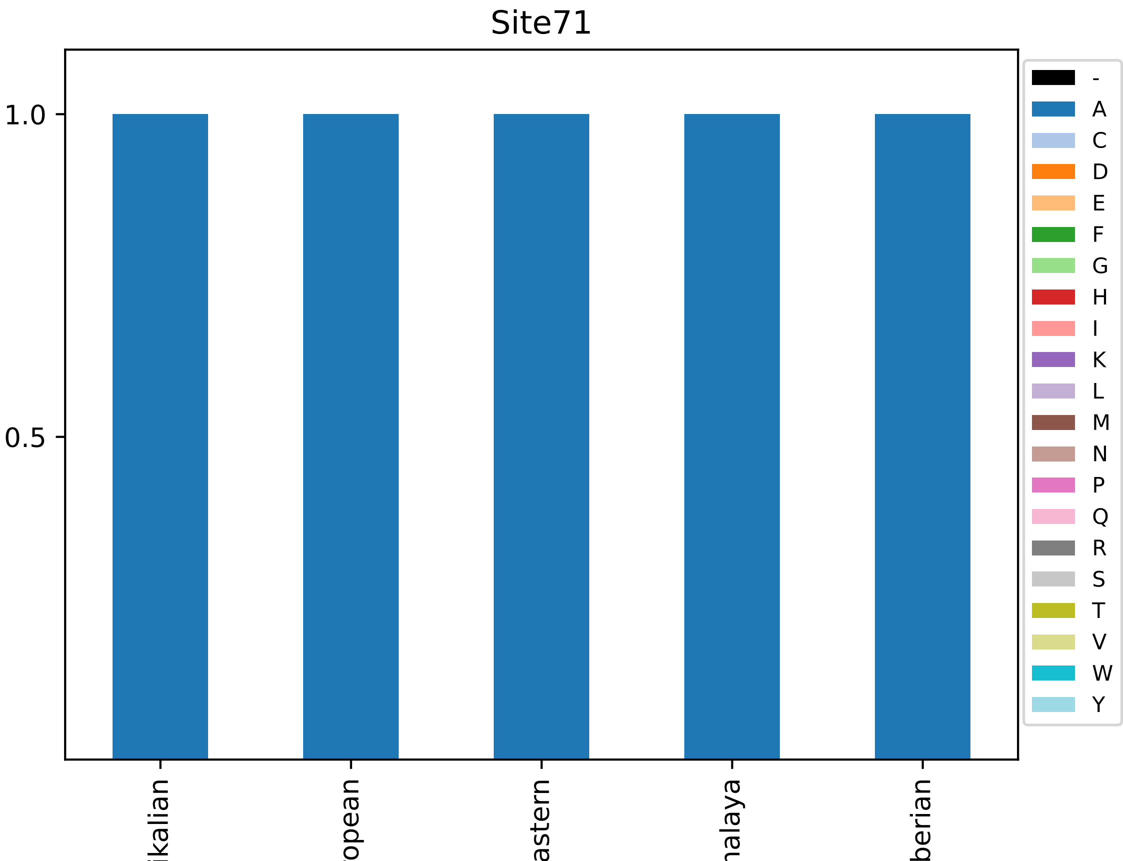

Site71
1.0
0.5
-
A
C
D
E
F
G
H
I
K
L
M
N
P
Q
R
S
T
V
W
Y
ikalian
opean
astern
nalaya
berian

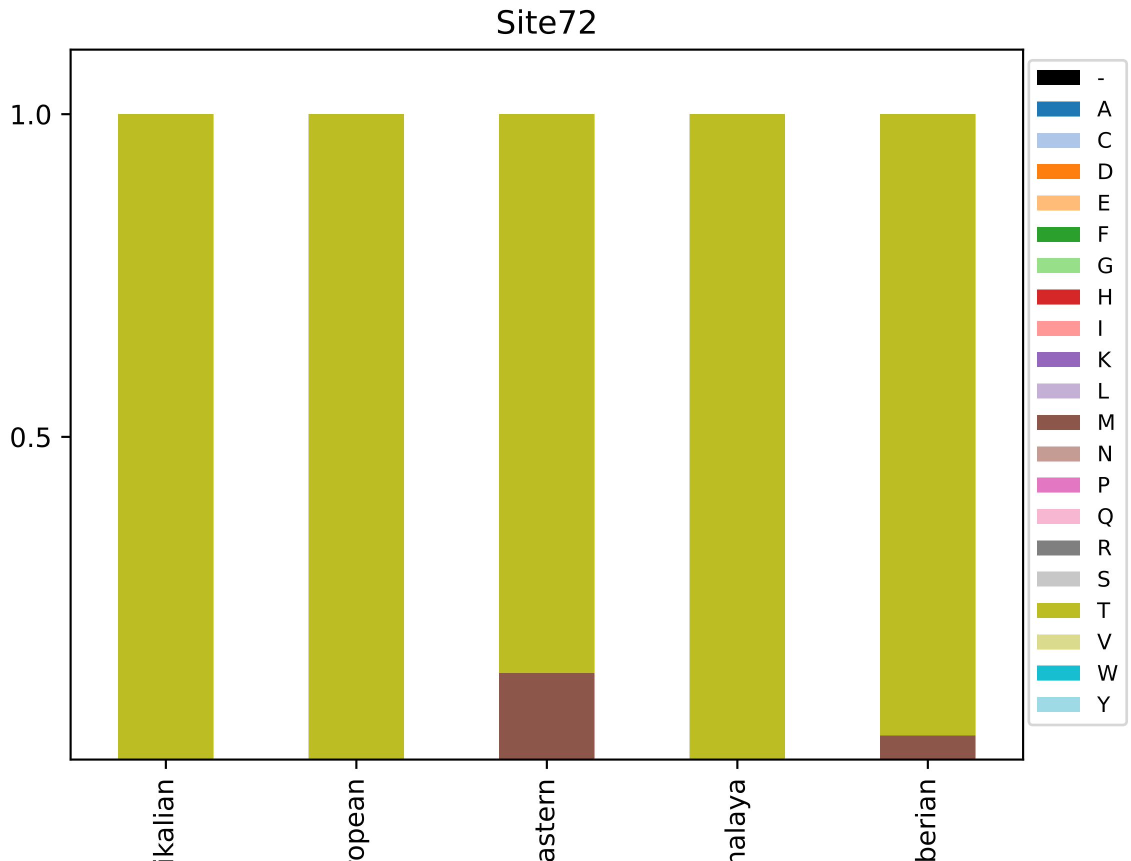
Site72
1.0
0.5
-
A
C
D
E
F
G
H
I
K
L
M
N
P
Q
R
S
T
V
W
Y
ikalian
opean
astern
nalaya
berian

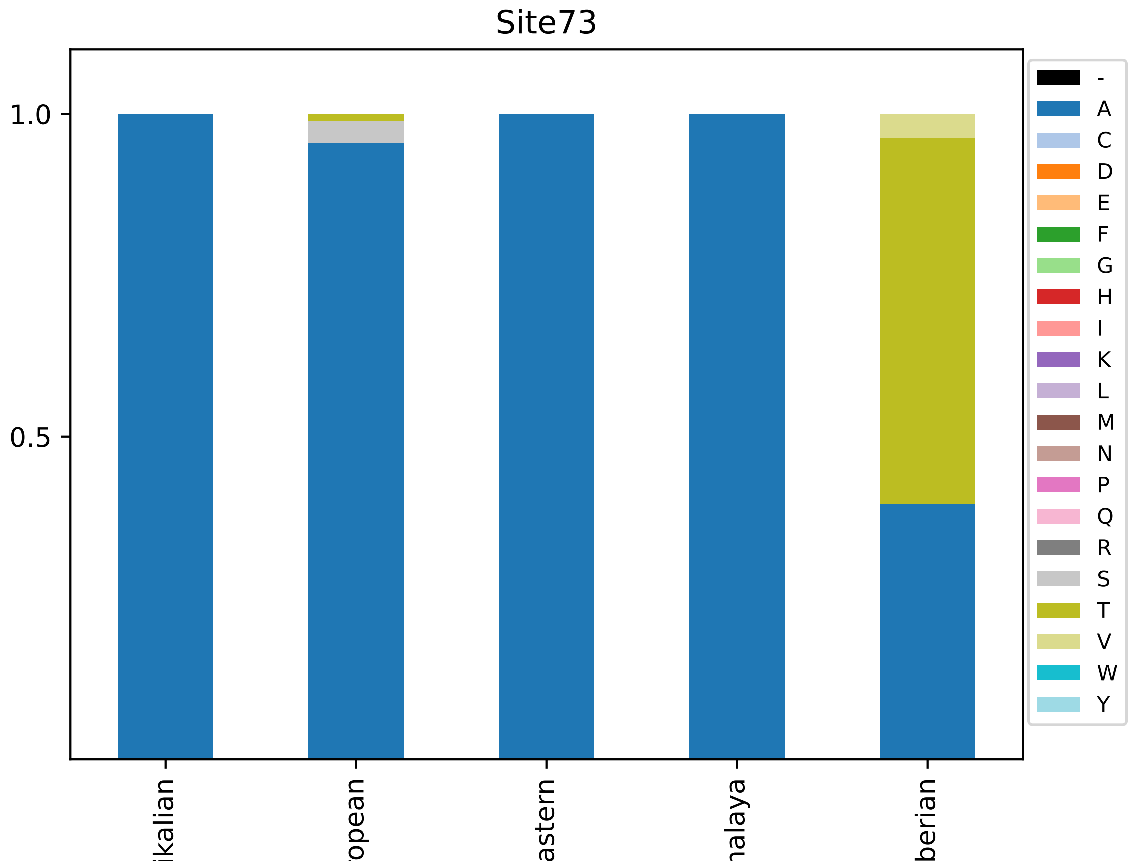
Site73
1.0
0.5
-
A
C
D
E
F
G
H
I
K
L
M
N
P
Q
R
S
T
V
W
Y
ikalian
ropean
astern
nalaya
berian

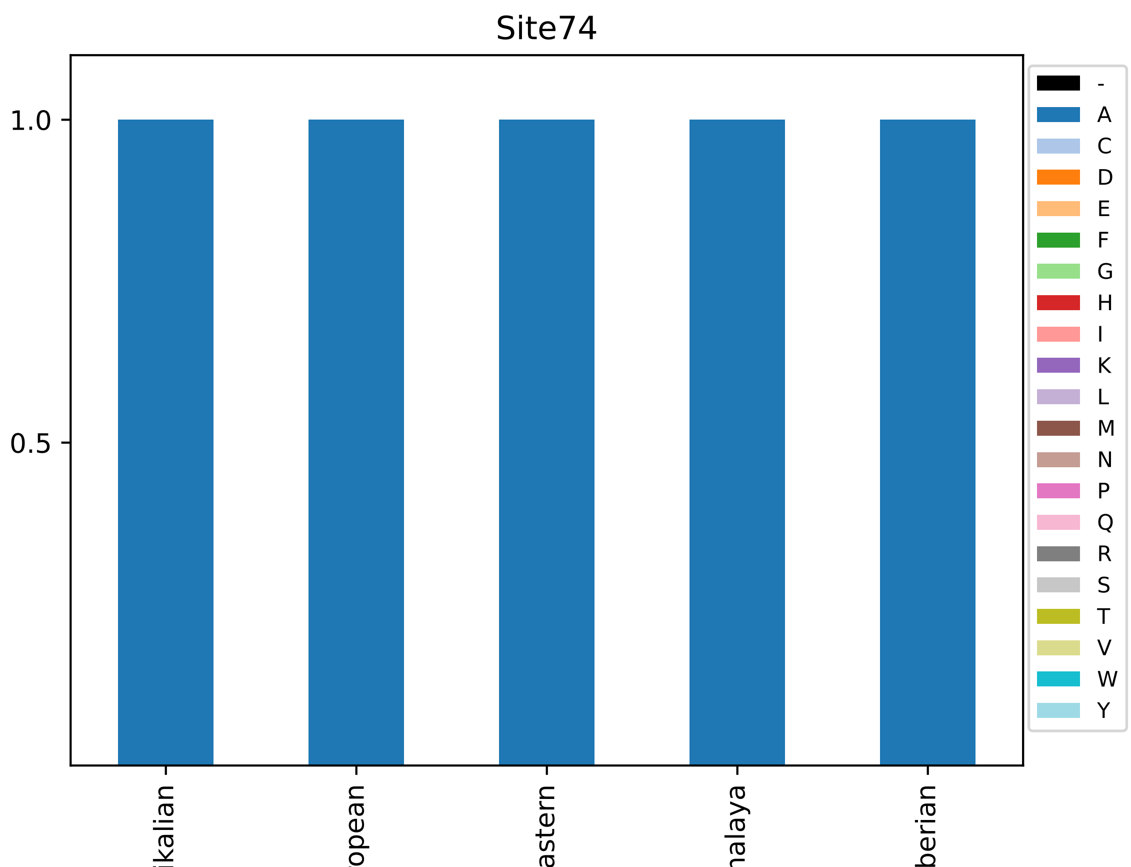
Site74
1.0
0.5
-
A
C
D
E
F
G
H
I
K
L
M
N
P
Q
R
S
T
V
W
Y
ikalian
opean
astern
nalaya
berian

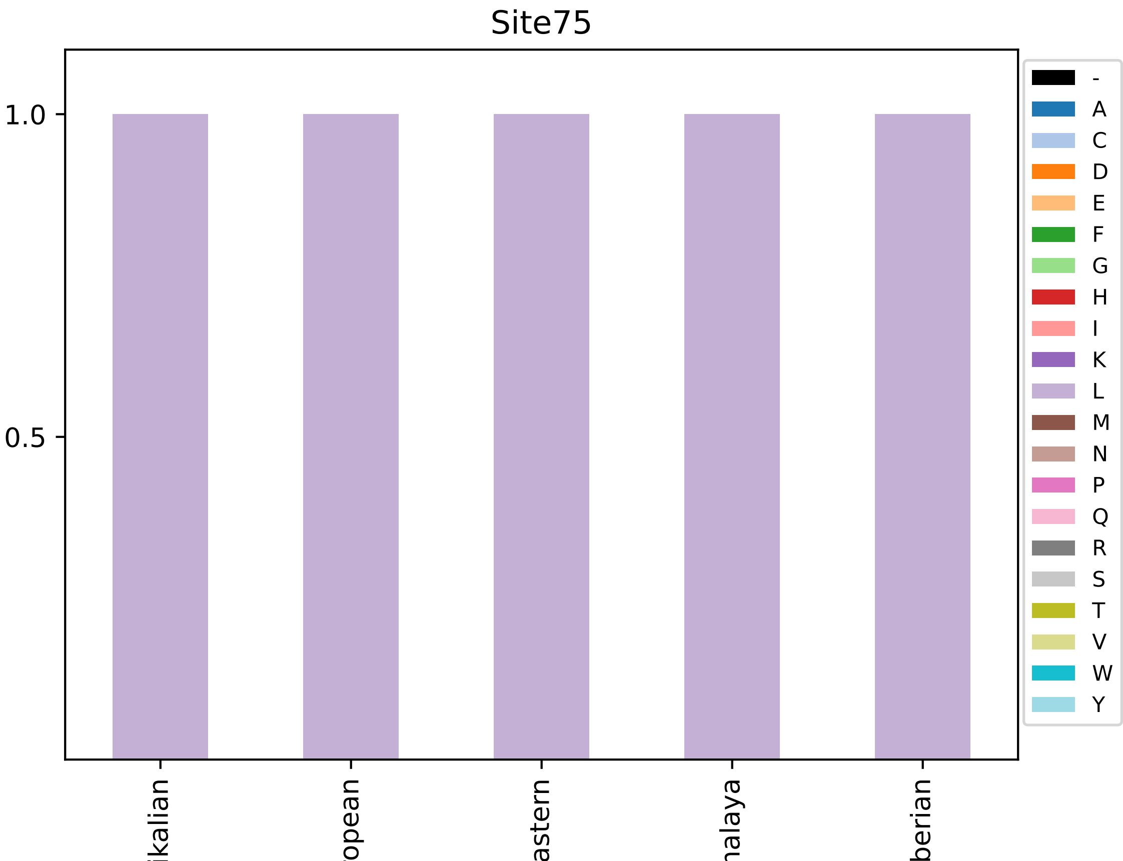

Site75
1.0
0.5
-
A
C
D
E
F
G
H
I
K
L
M
N
P
Q
R
S
T
V
W
Y
ikalian
opean
astern
nalaya
berian

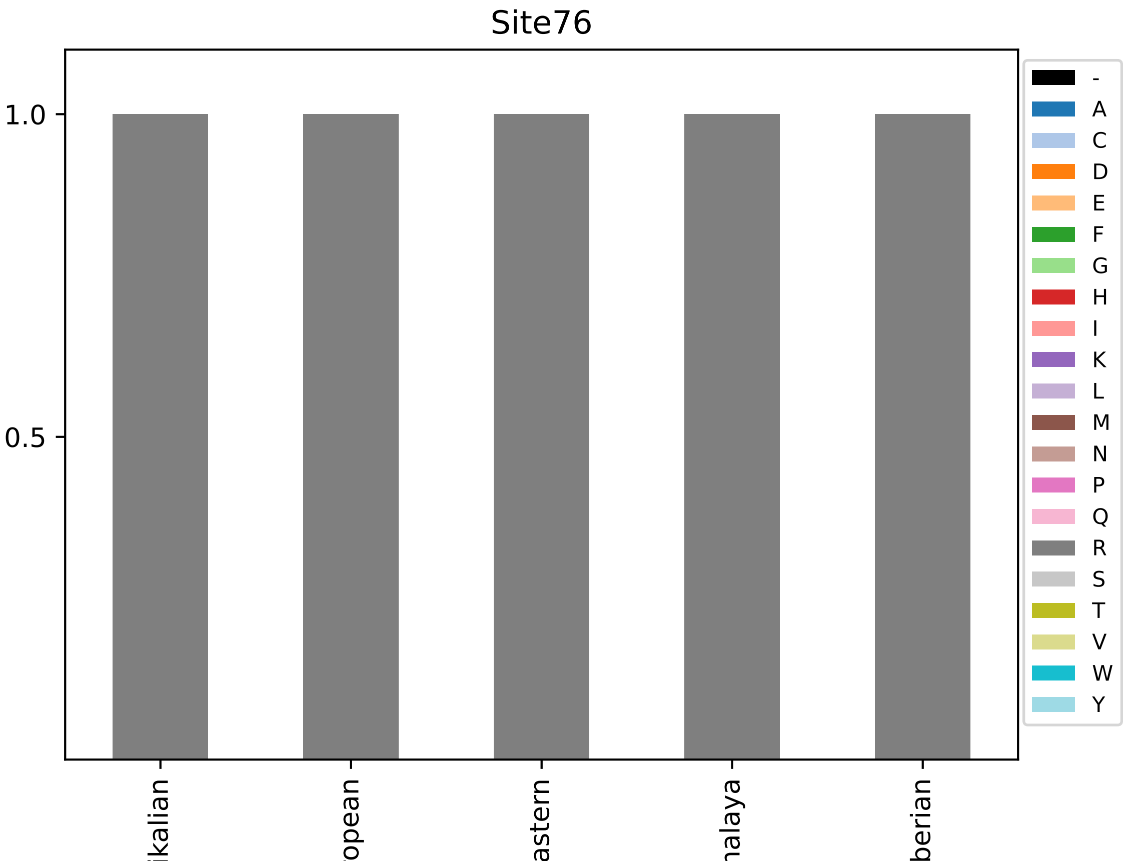
Site76
1.0
0.5
-
A
C
D
E
F
G
H
I
K
L
M
N
P
Q
R
S
T
V
W
Y
ikalian
opean
astern
nalaya
berian

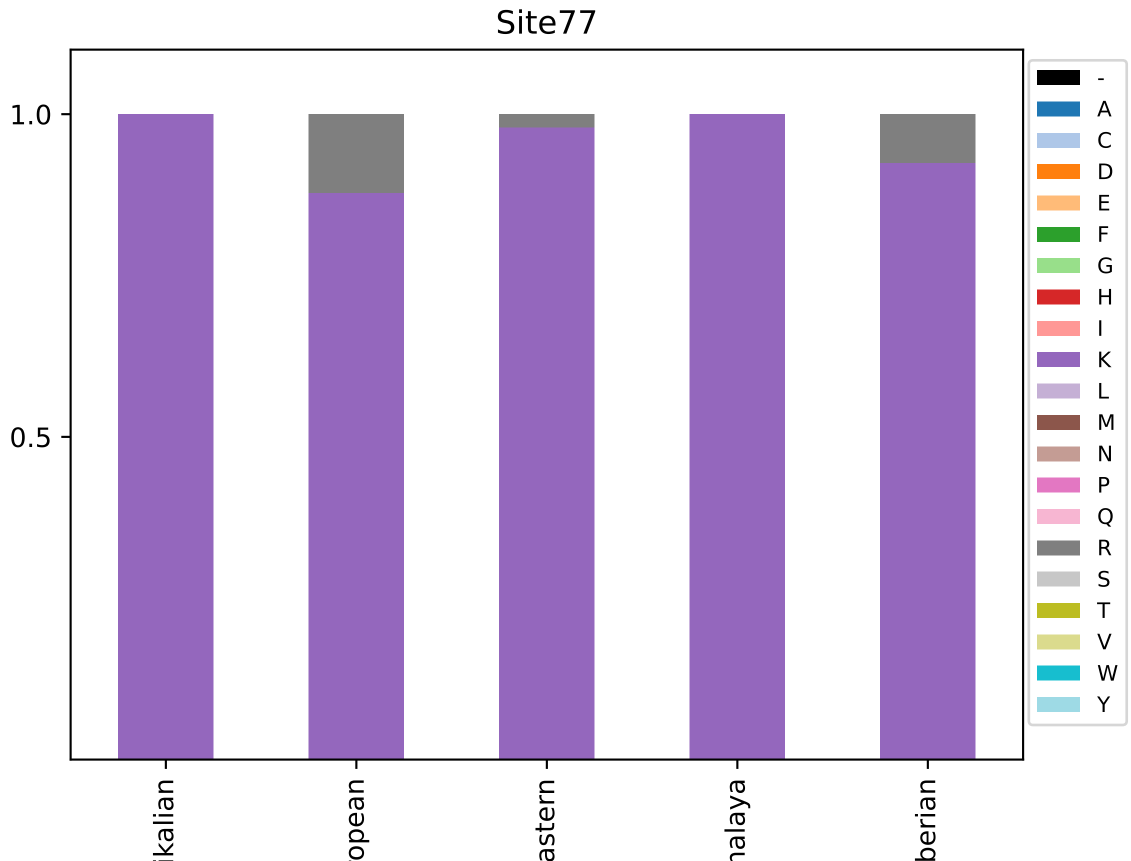
Site77
1.0
0.5
-
A
C
D
E
F
G
H
I
K
L
M
N
P
Q
R
S
T
V
W
Y
ikalian
ropean
astern
nalaya
berian

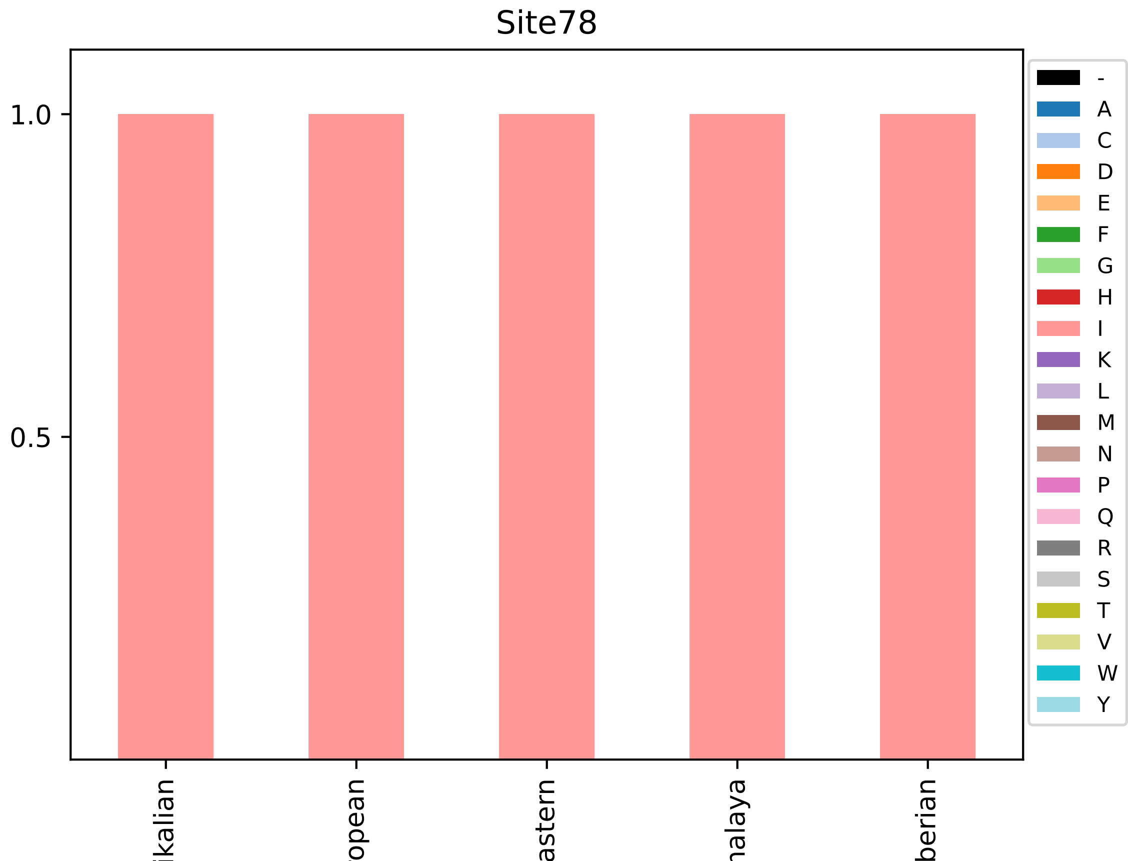

Site78
1.0
0.5
-
A
C
D
E
F
G
H
I
K
L
M
N
P
Q
R
S
T
V
W
Y
ikalian
opean
astern
nalaya
berian

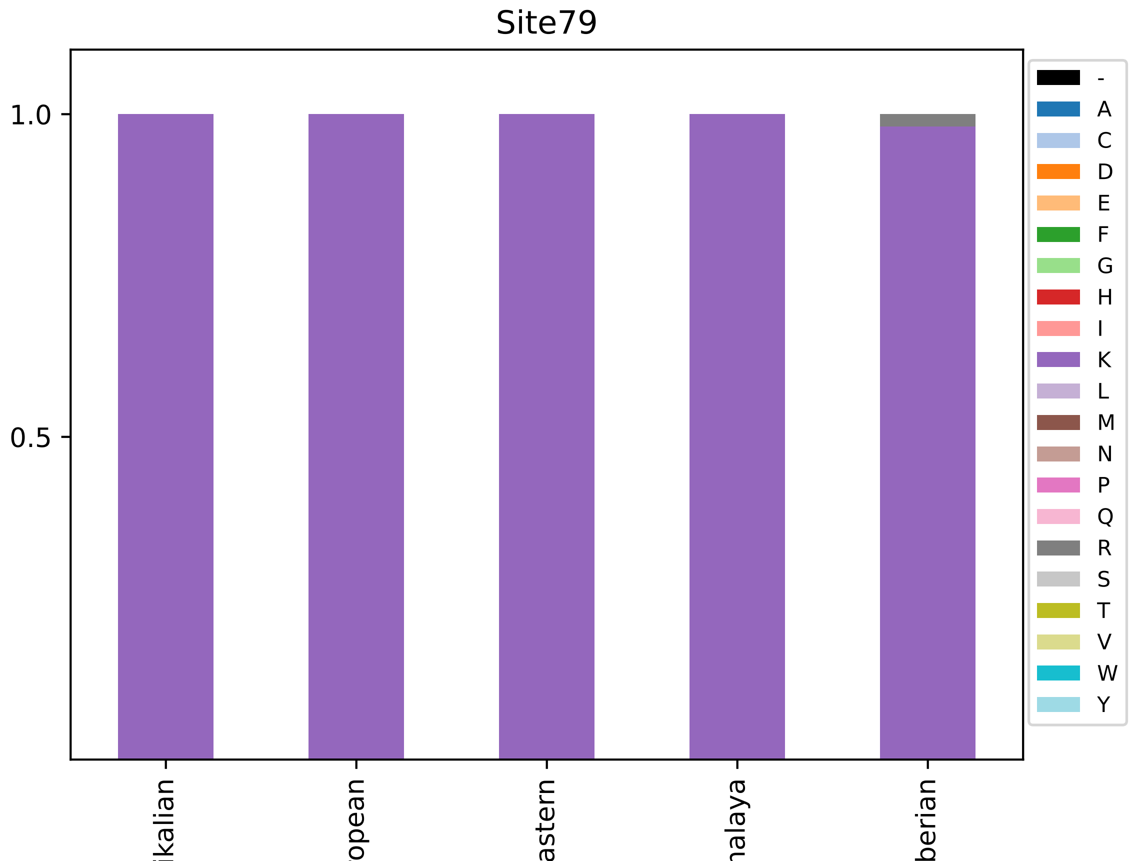

Site79
1.0
0.5
-
A
C
D
E
F
G
H
I
K
L
M
N
P
Q
R
S
T
V
W
Y
ikalian
opean
astern
nalaya
berian

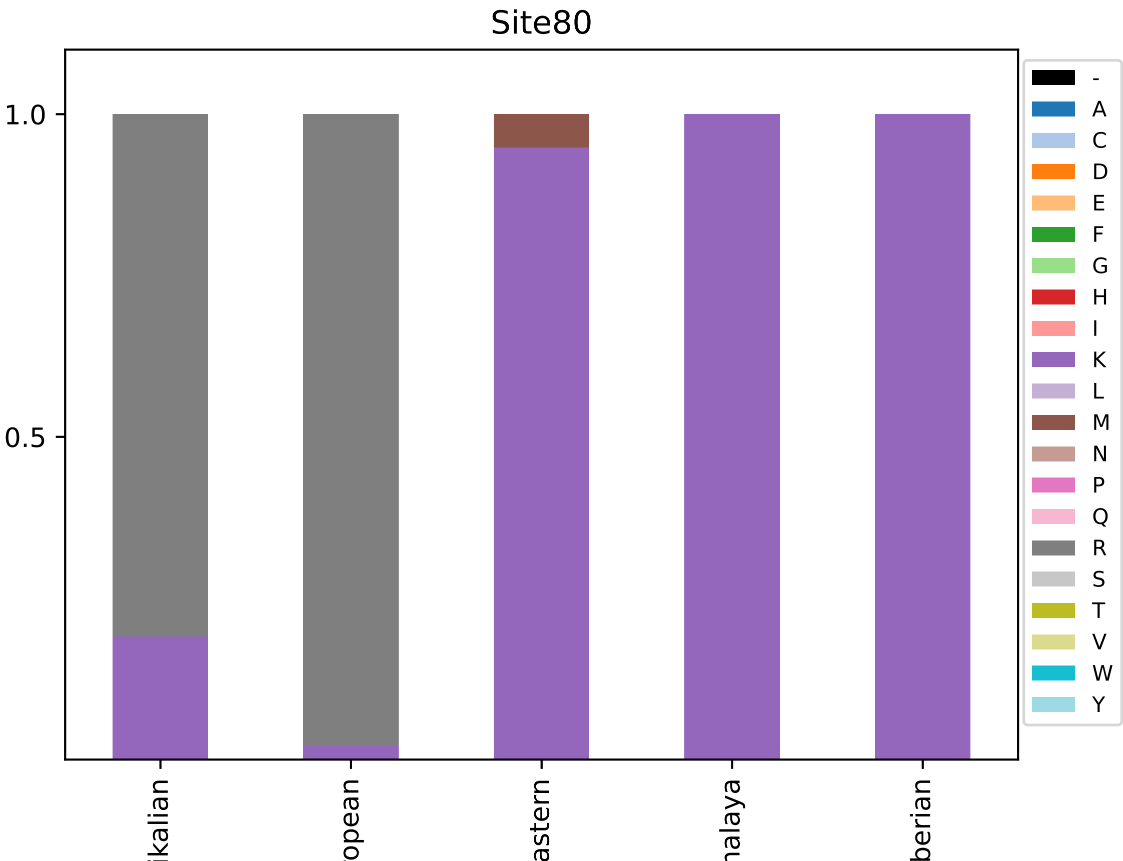
Site80
1.0
0.5
-
A
C
D
E
F
G
H
I
K
L
M
N
P
Q
R
S
T
V
W
Y
ikalian
opean
astern
nalaya
berian

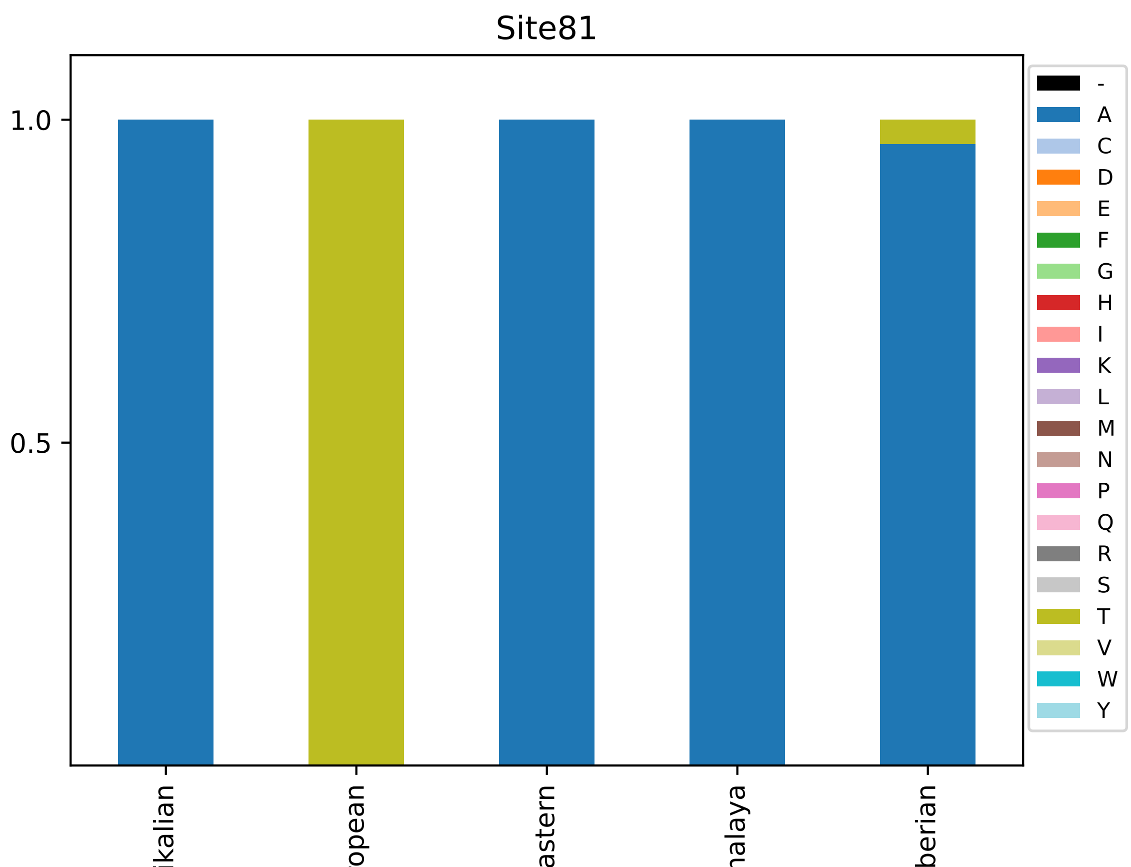

Site81
1.0
0.5
-
A
C
D
E
F
G
H
I
K
L
M
N
P
Q
R
S
T
V
W
Y
ikalian
opean
astern
nalaya
berian

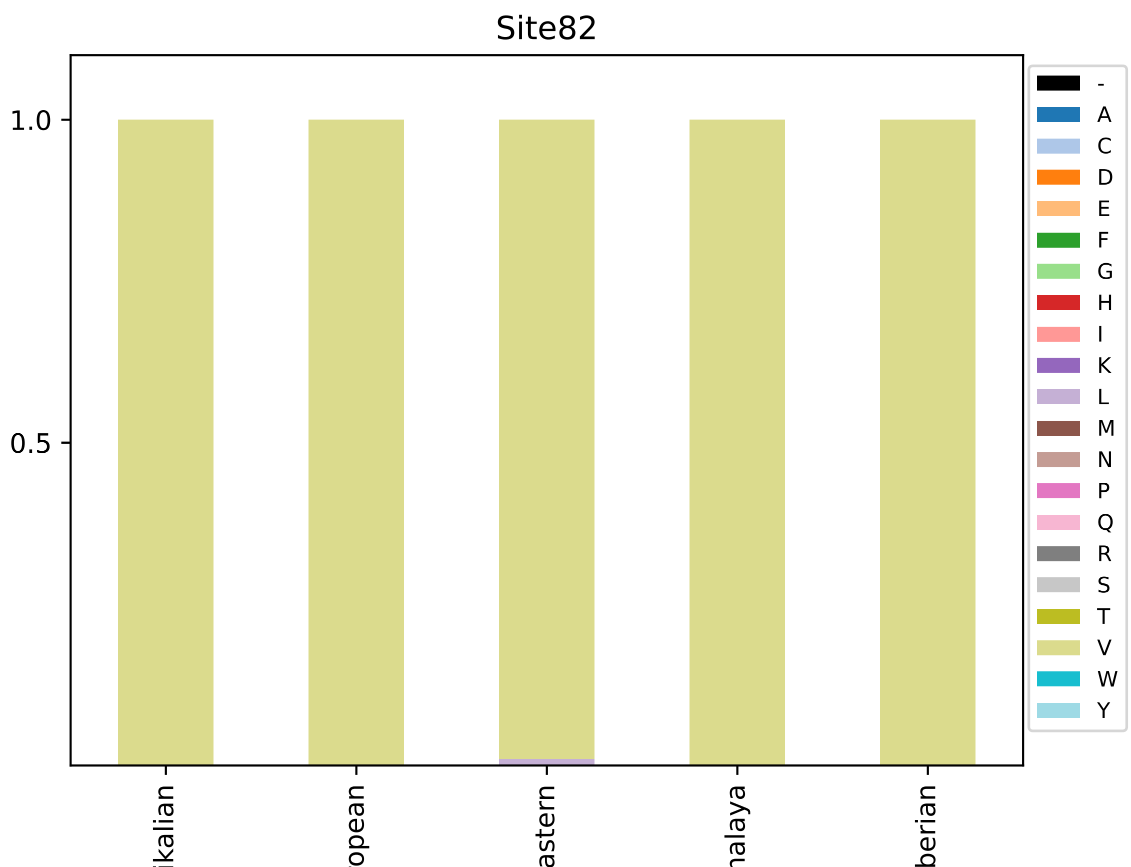

Site82
1.0
0.5
-
A
C
D
E
F
G
H
I
K
L
M
N
P
Q
R
S
T
V
W
Y
ikalian
opean
astern
nalaya
berian

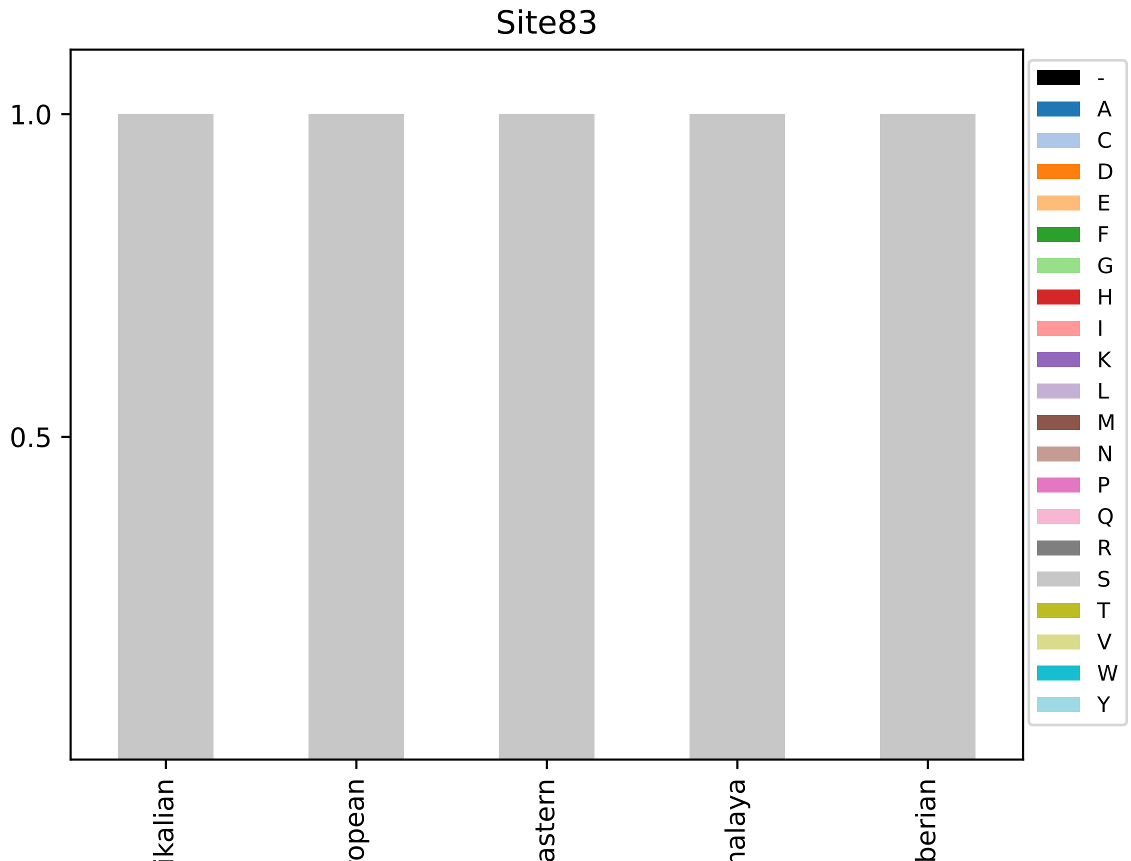
Site83
1.0
0.5
-
A
C
D
E
F
G
H
I
K
L
M
N
P
Q
R
S
T
V
W
Y
ikalian
opean
astern
nalaya
berian

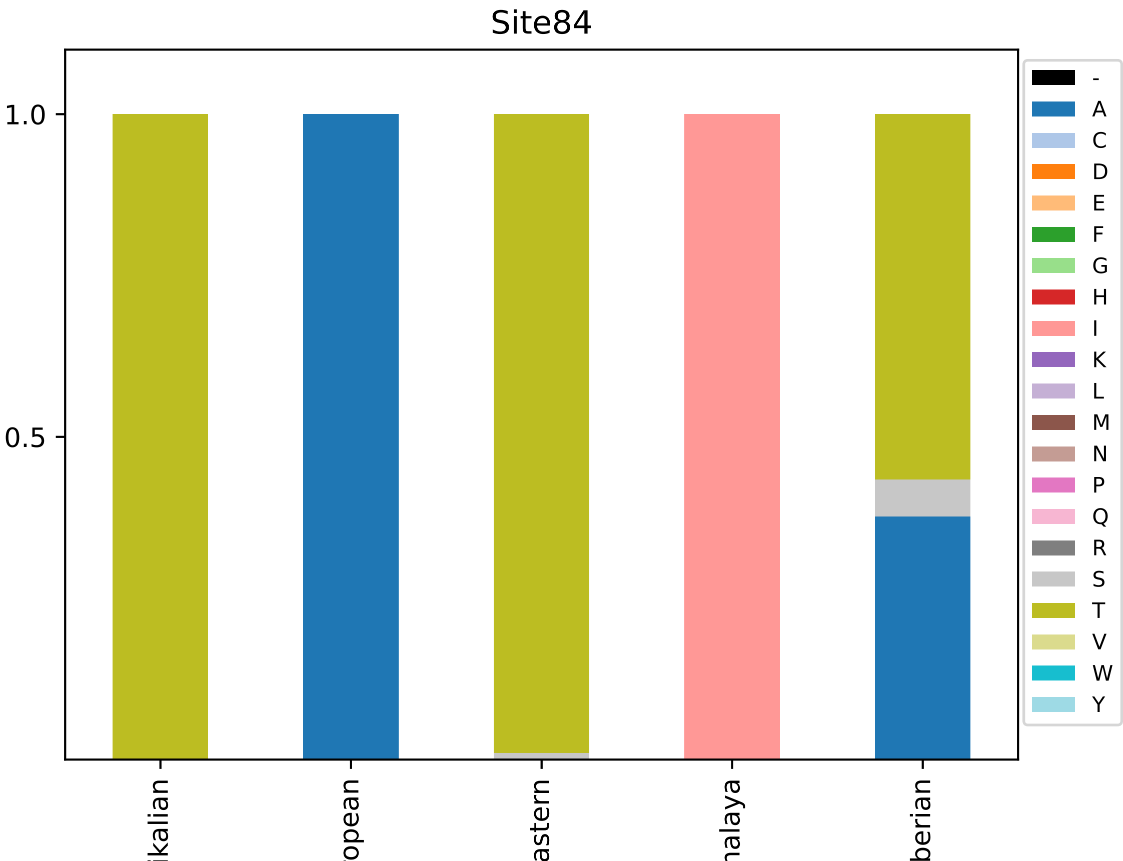

Site84
1.0
0.5
-
A
C
D
E
F
G
H
I
K
L
M
N
P
Q
R
S
T
V
W
Y
ikalian
opean
astern
nalaya
berian

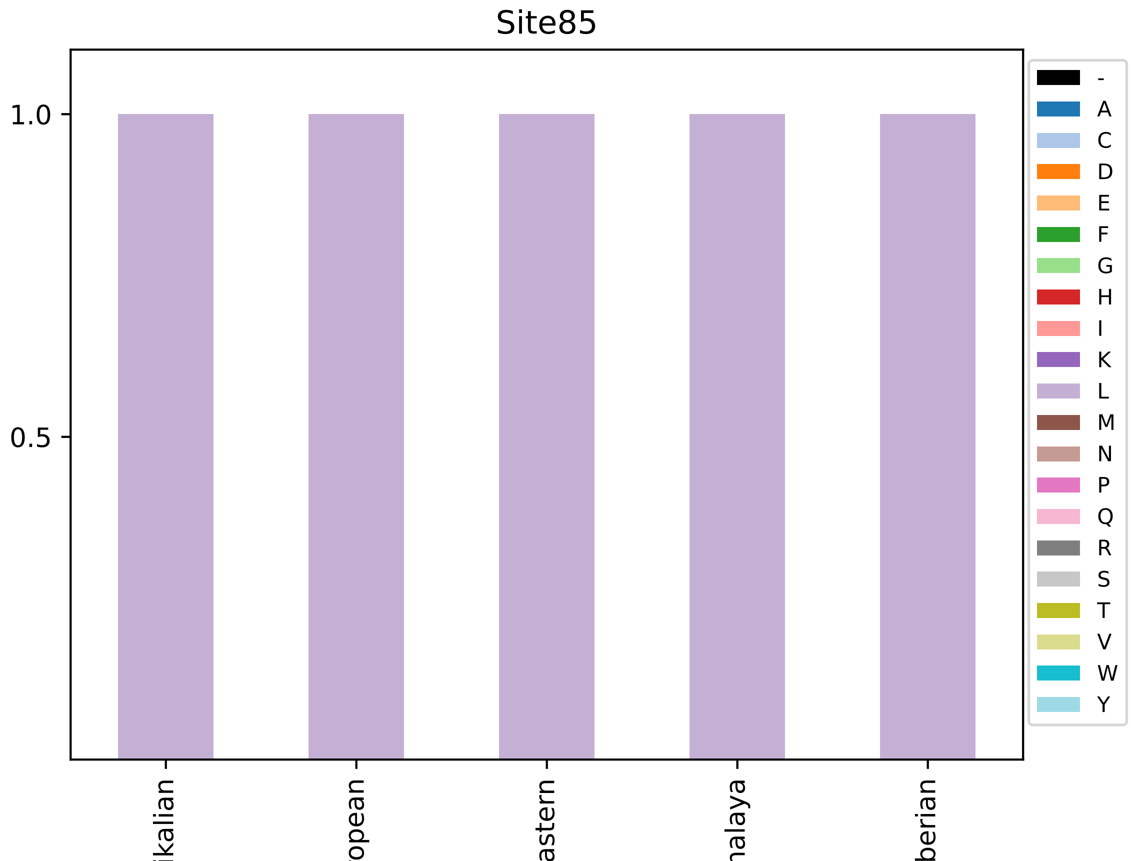
Site85
1.0
0.5
-
A
C
D
E
F
G
H
I
K
L
M
N
P
Q
R
S
T
V
W
Y

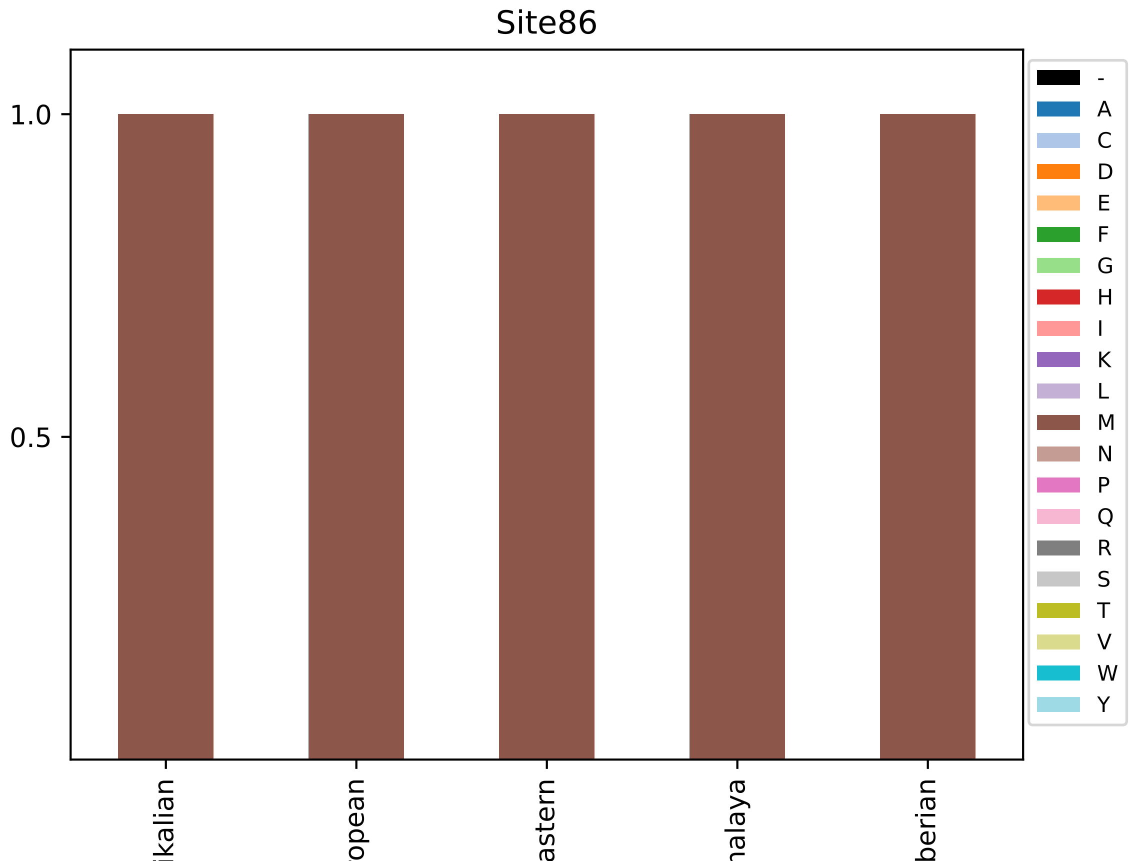
Site86
1.0
0.5
ikalian
opean
astern
nalaya
berian
-
A
C
D
E
F
G
H
I
K
L
M
N
P
Q
R
S
T
V
W
Y

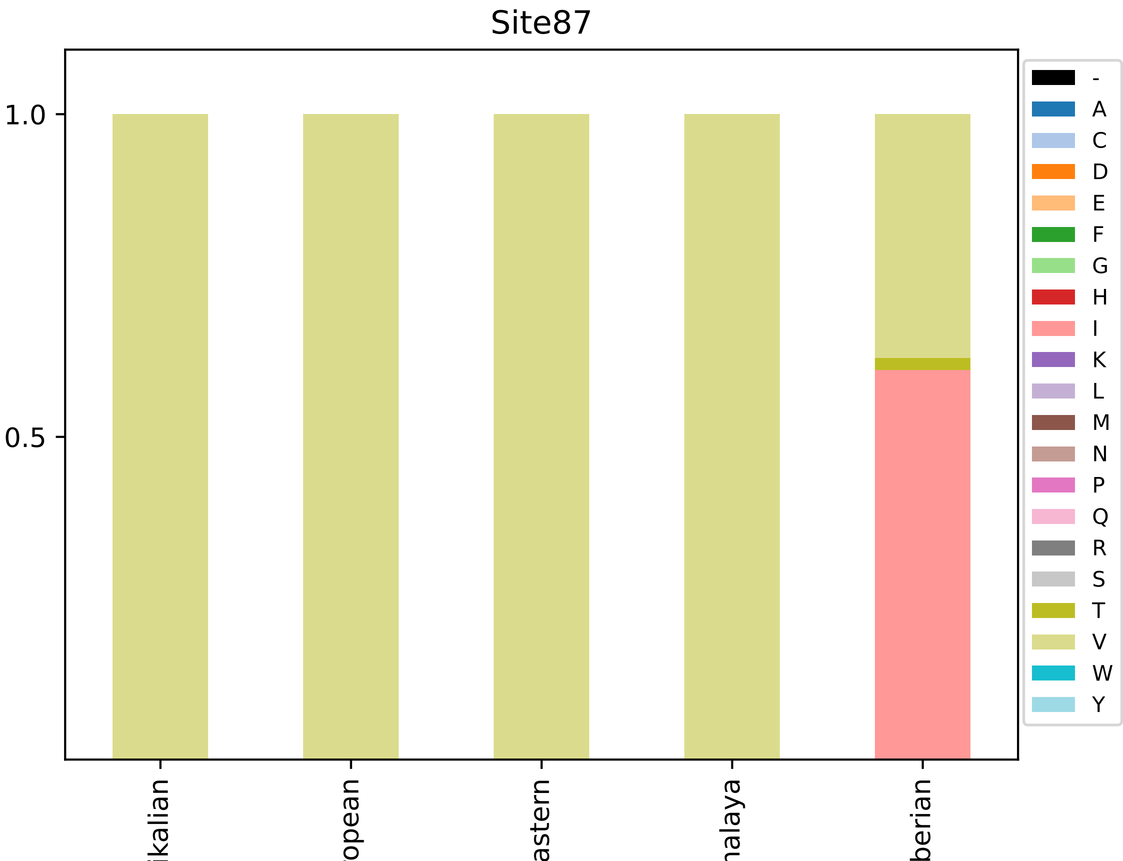
Site87
1.0
0.5
-
A
C
D
E
F
G
H
I
K
L
M
N
P
Q
R
S
T
V
W
Y

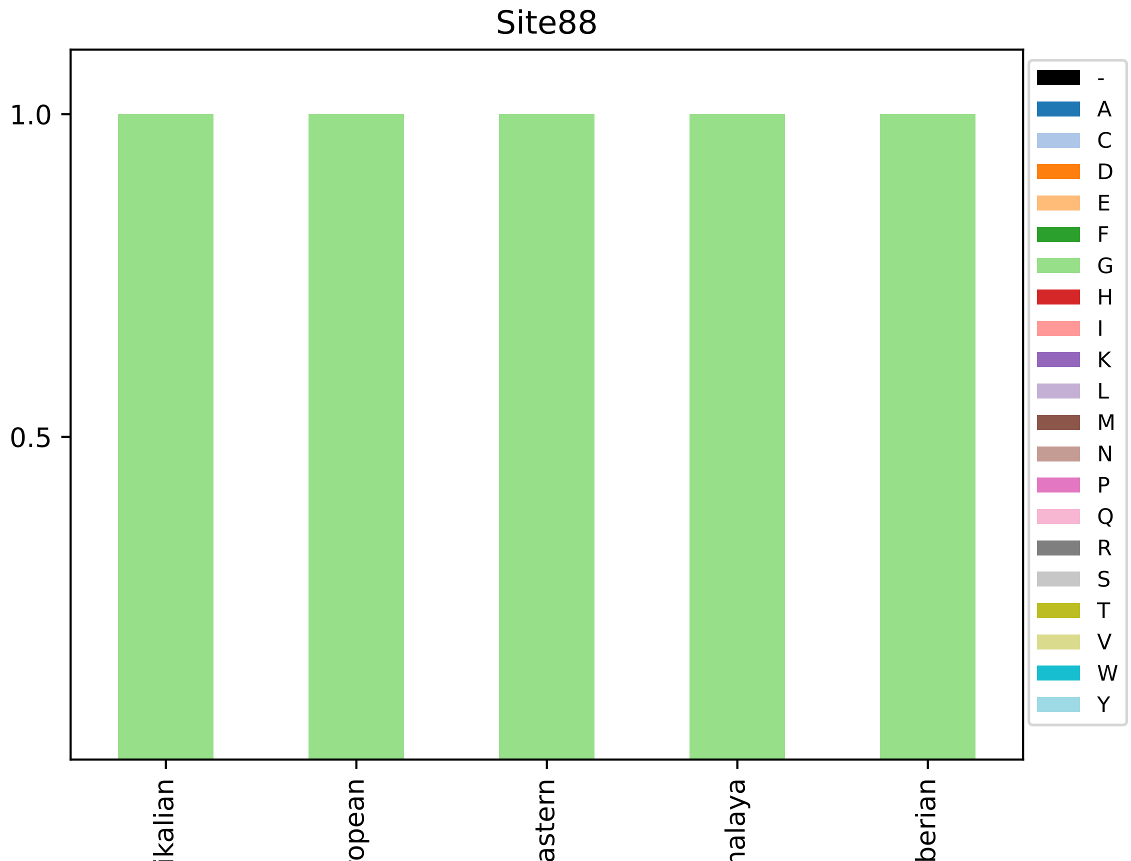

Site88
1.0
0.5
-
A
C
D
E
F
G
H
I
K
L
M
N
P
Q
R
S
T
V
W
Y
ikalian
opean
astern
nalaya
berian

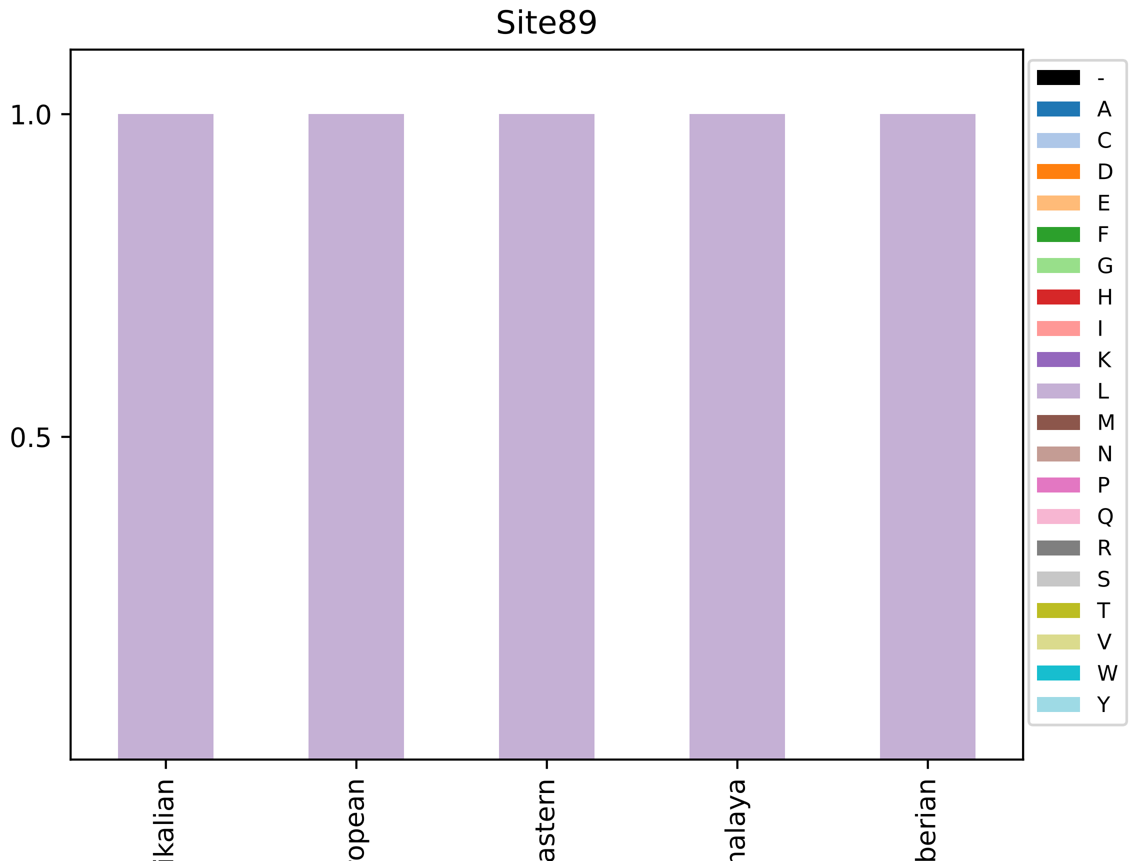
Site89
1.0
0.5
-
A
C
D
E
F
G
H
I
K
L
M
N
P
Q
R
S
T
V
W
Y
ikalian
opean
astern
nalaya
berian

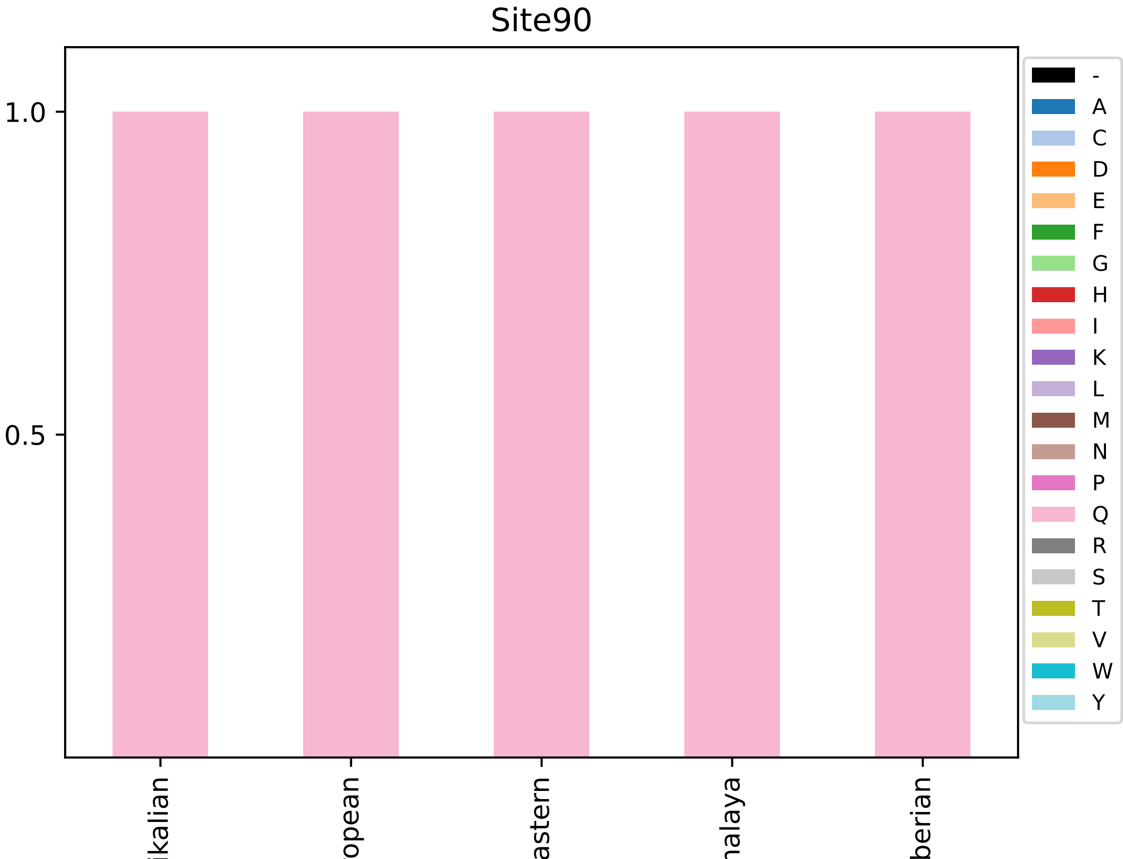
Site90
1.0
0.5
-
A
C
D
E
F
G
H
I
K
L
M
N
P
Q
R
S
T
V
W
Y
ikalian
opean
astern
nalaya
berian

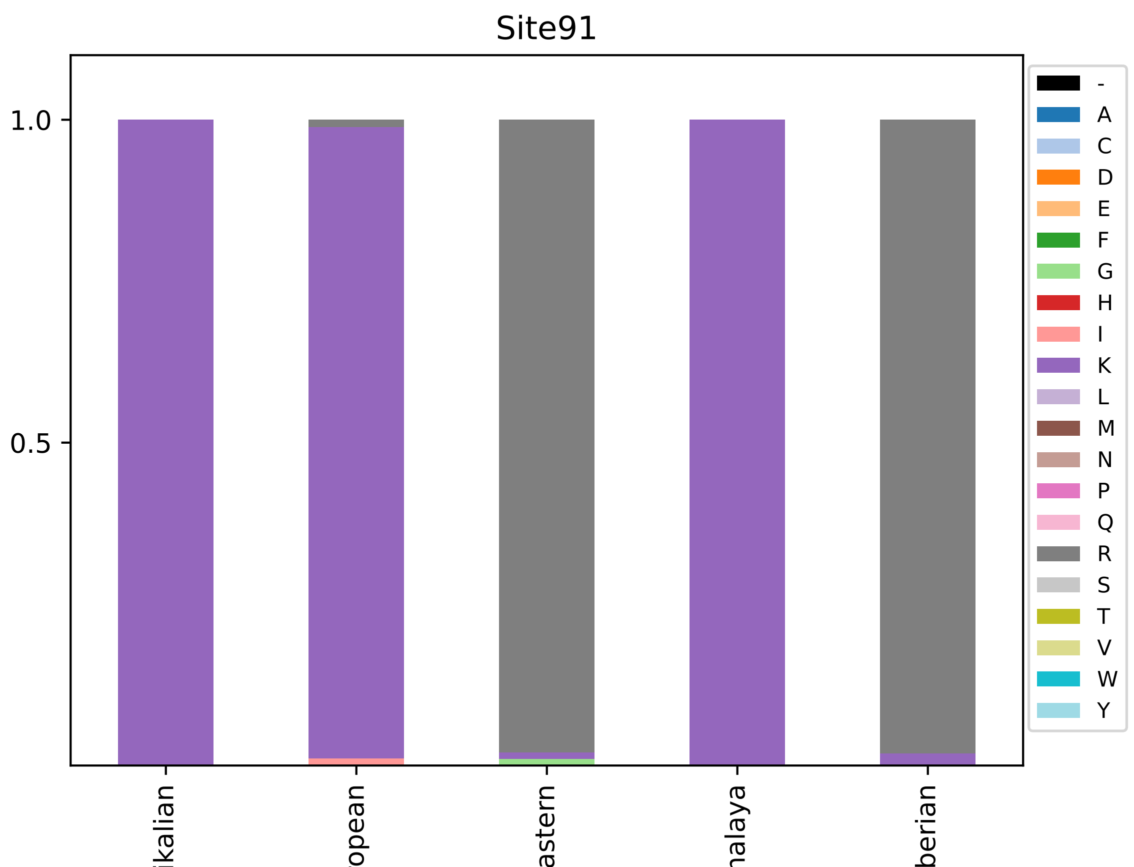

Site91
1.0
0.5
-
A
C
D
E
F
G
H
I
K
L
M
N
P
Q
R
S
T
V
W
Y
ikalian
opean
astern
nalaya
berian

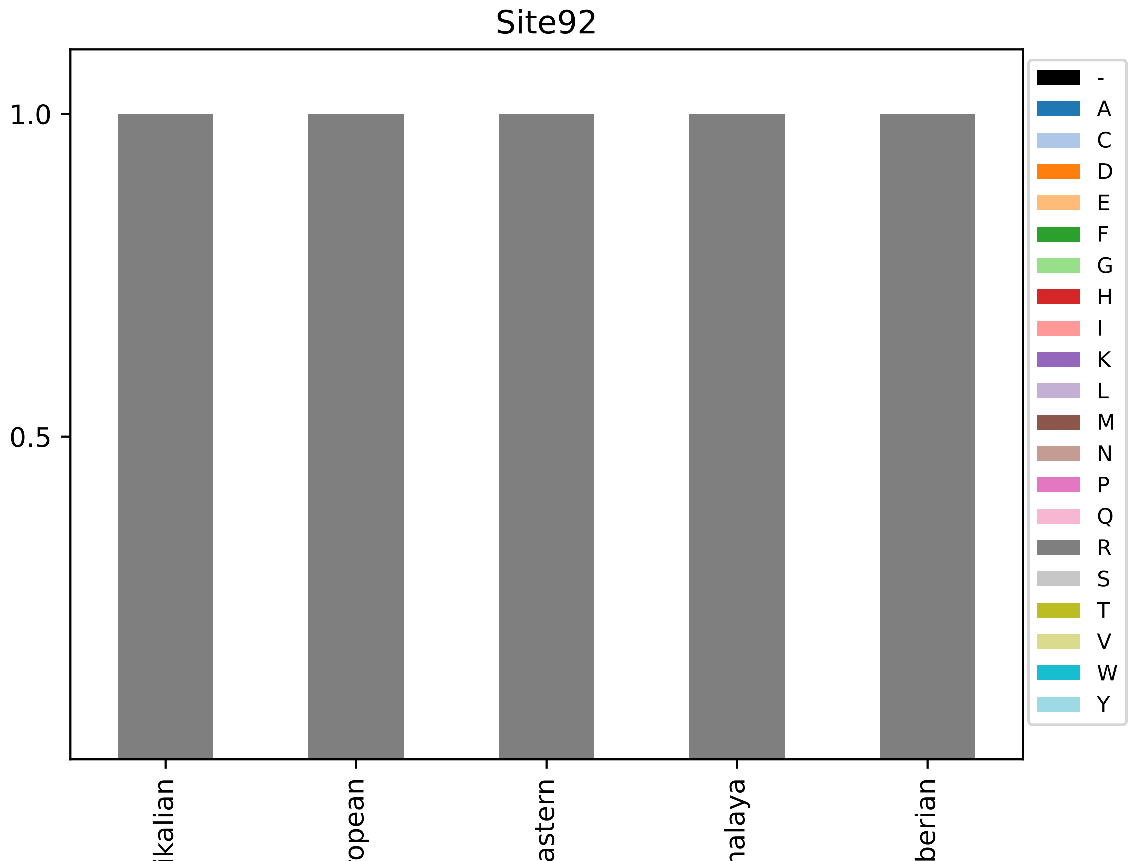

Site92
1.0
0.5
-
A
C
D
E
F
G
H
I
K
L
M
N
P
Q
R
S
T
V
W
Y
ikalian
opean
astern
nalaya
berian

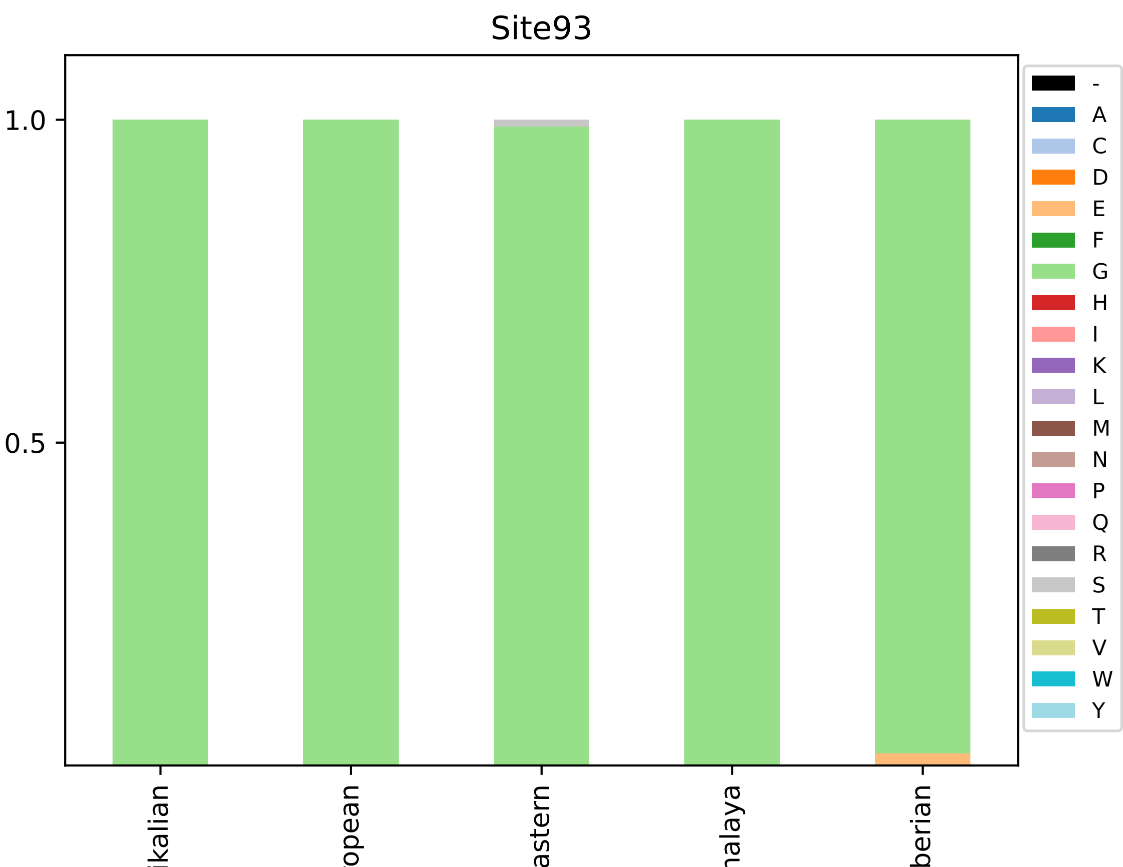

Site93
1.0
0.5
-
A
C
D
E
F
G
H
I
K
L
M
N
P
Q
R
S
T
V
W
Y
ikalian
opean
astern
nalaya
berian

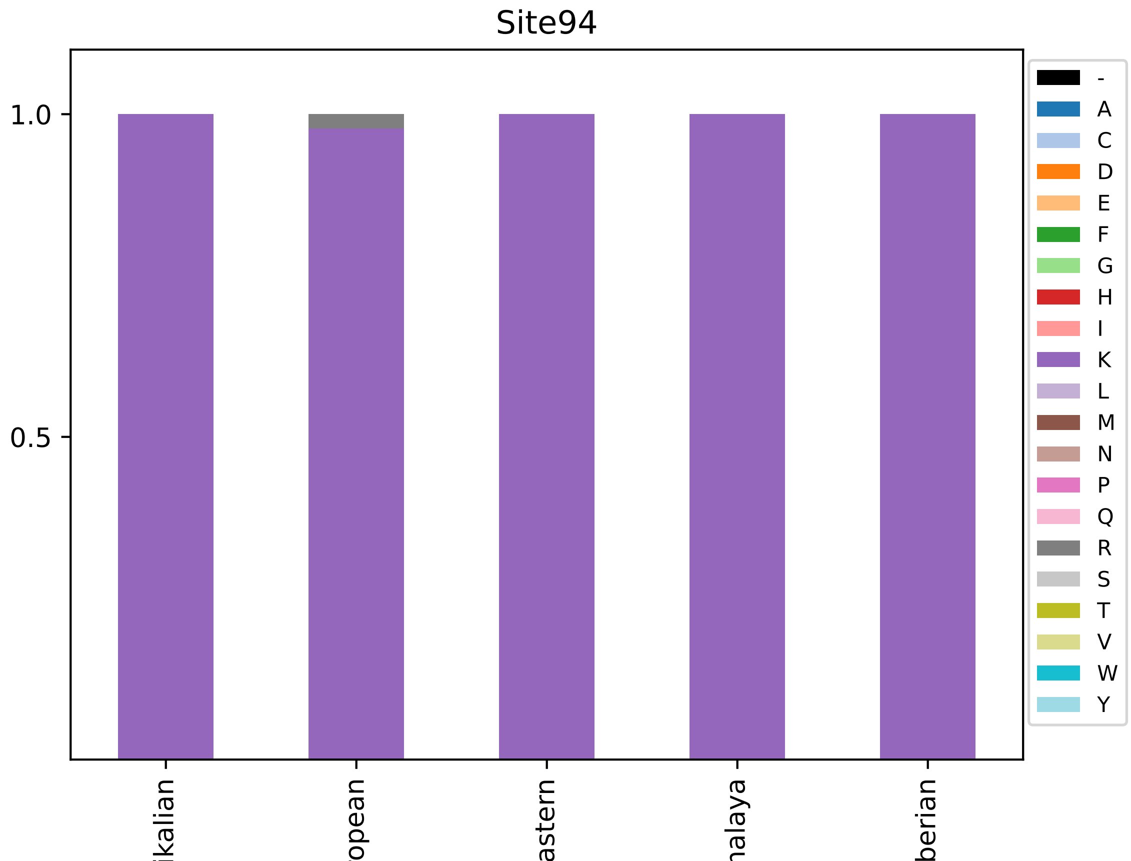

Site94
1.0
0.5
-
A
C
D
E
F
G
H
I
K
L
M
N
P
Q
R
S
T
V
W
Y
ikalian
opean
astern
nalaya
berian

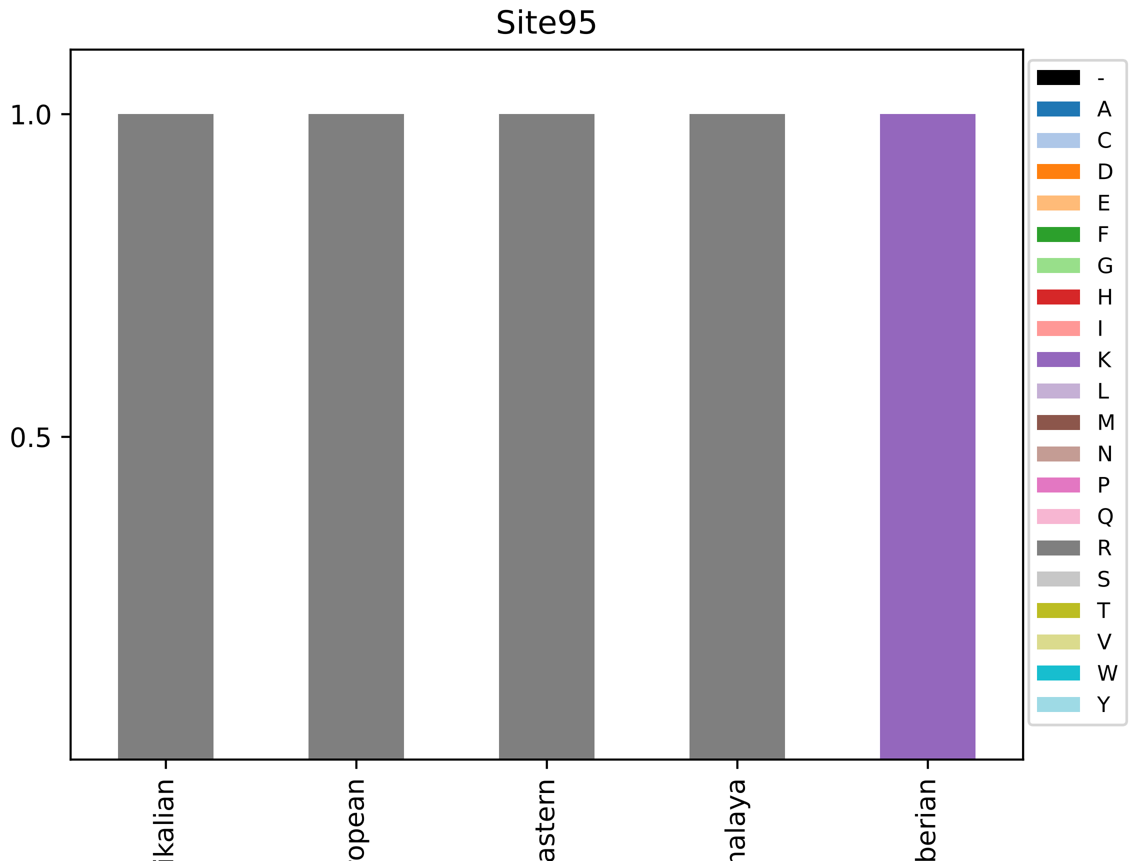

Site95
1.0
0.5
ikalian
opean
astern
nalaya
berian
-
A
C
D
E
F
G
H
I
K
L
M
N
P
Q
R
S
T
V
W
Y

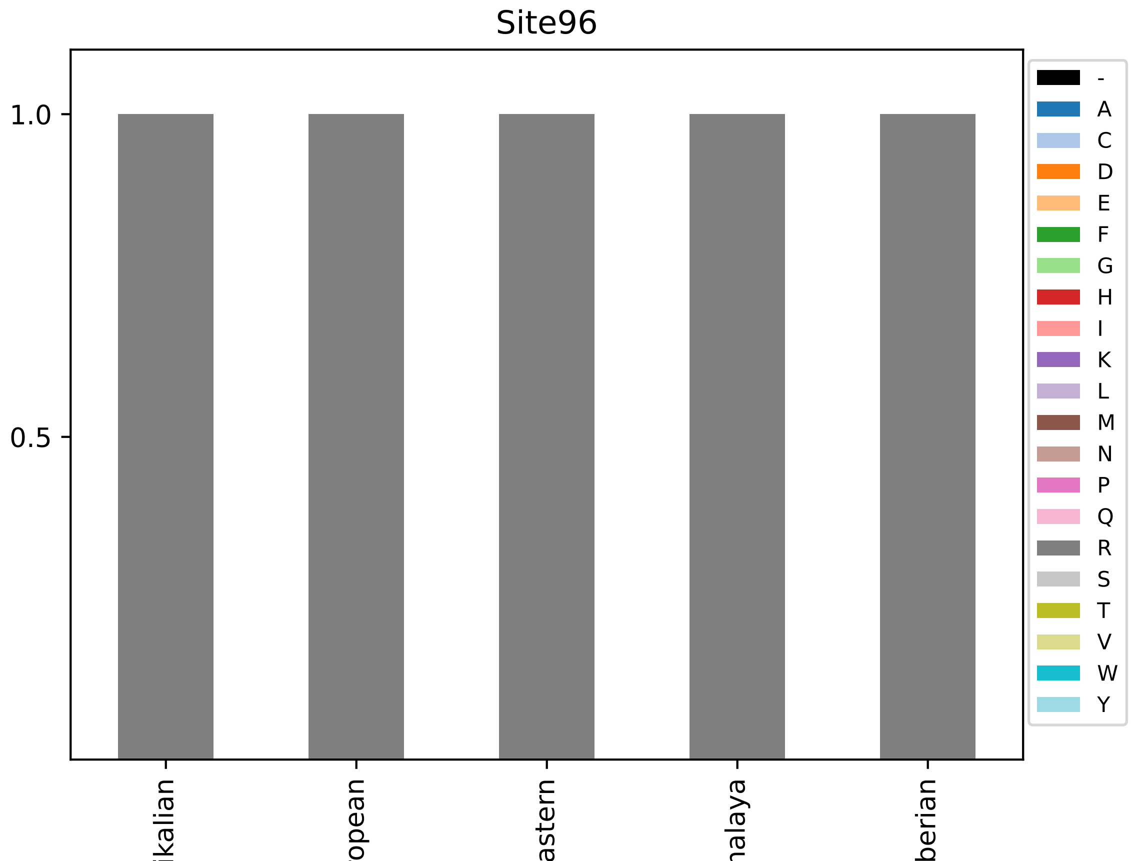
Site96
1.0
0.5
-
A
C
D
E
F
G
H
I
K
L
M
N
P
Q
R
S
T
V
W
Y
ikalian
opean
astern
nalaya
berian

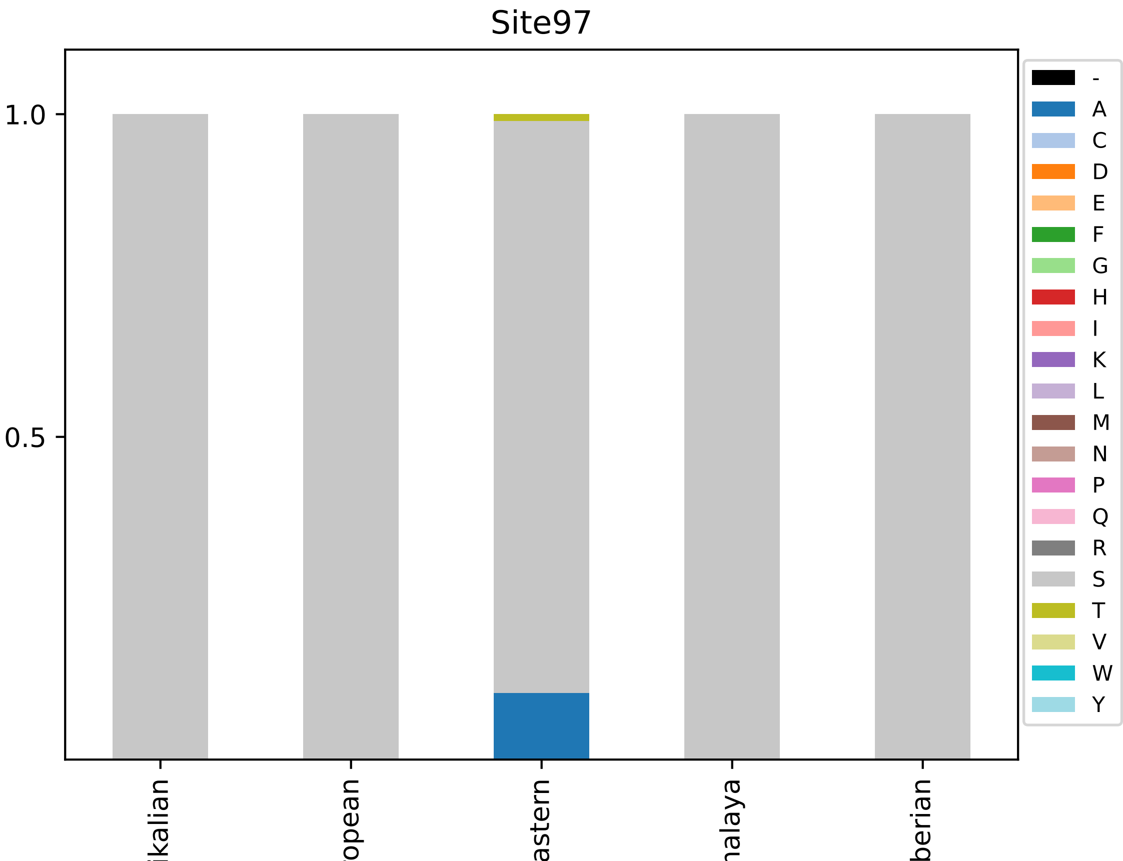
Site97
1.0
0.5
-
A
C
D
E
F
G
H
I
K
L
M
N
P
Q
R
S
T
V
W
Y
ikalian
opean
astern
nalaya
berian

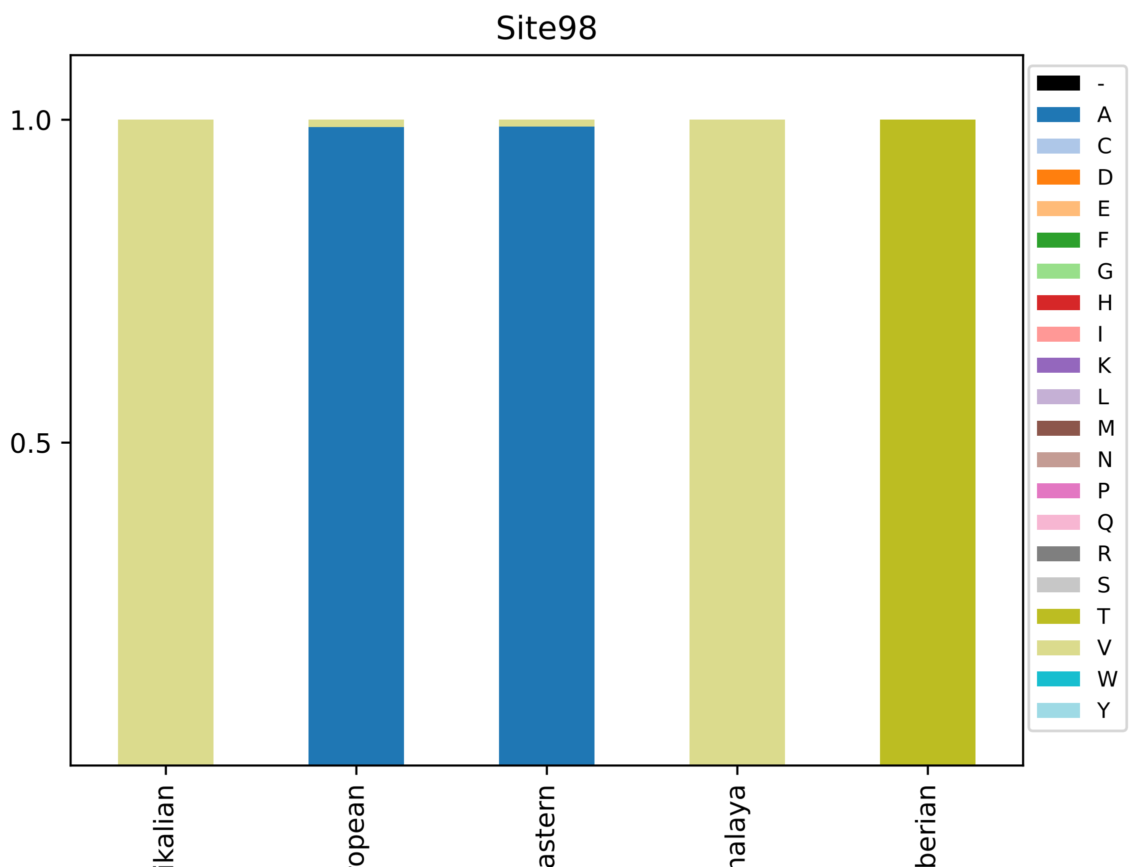
Site98
1.0
0.5
-
A
C
D
E
F
G
H
I
K
L
M
N
P
Q
R
S
T
V
W
Y
ikalian
opean
astern
nalaya
berian

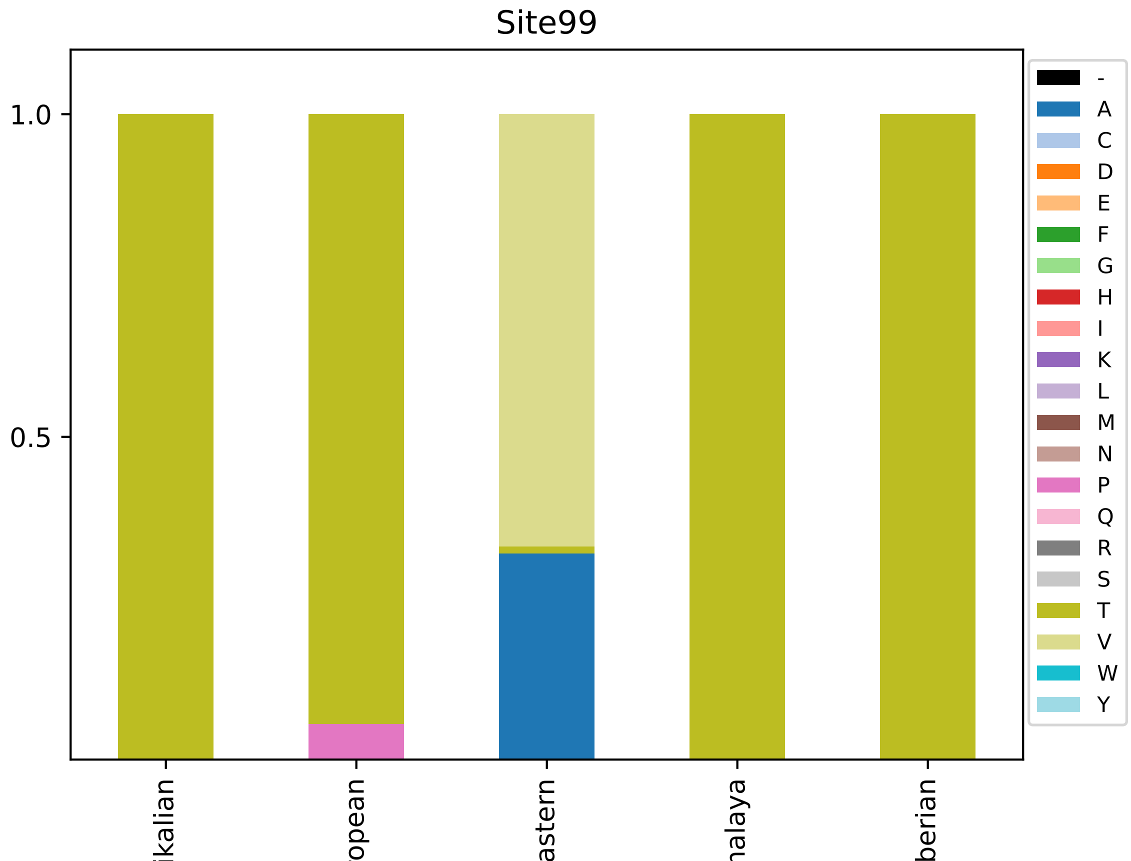
Site99
1.0
0.5
ikalian
opean
astern
nalaya
berian
-
A
C
D
E
F
G
H
I
K
L
M
N
P
Q
R
S
T
V
W
Y

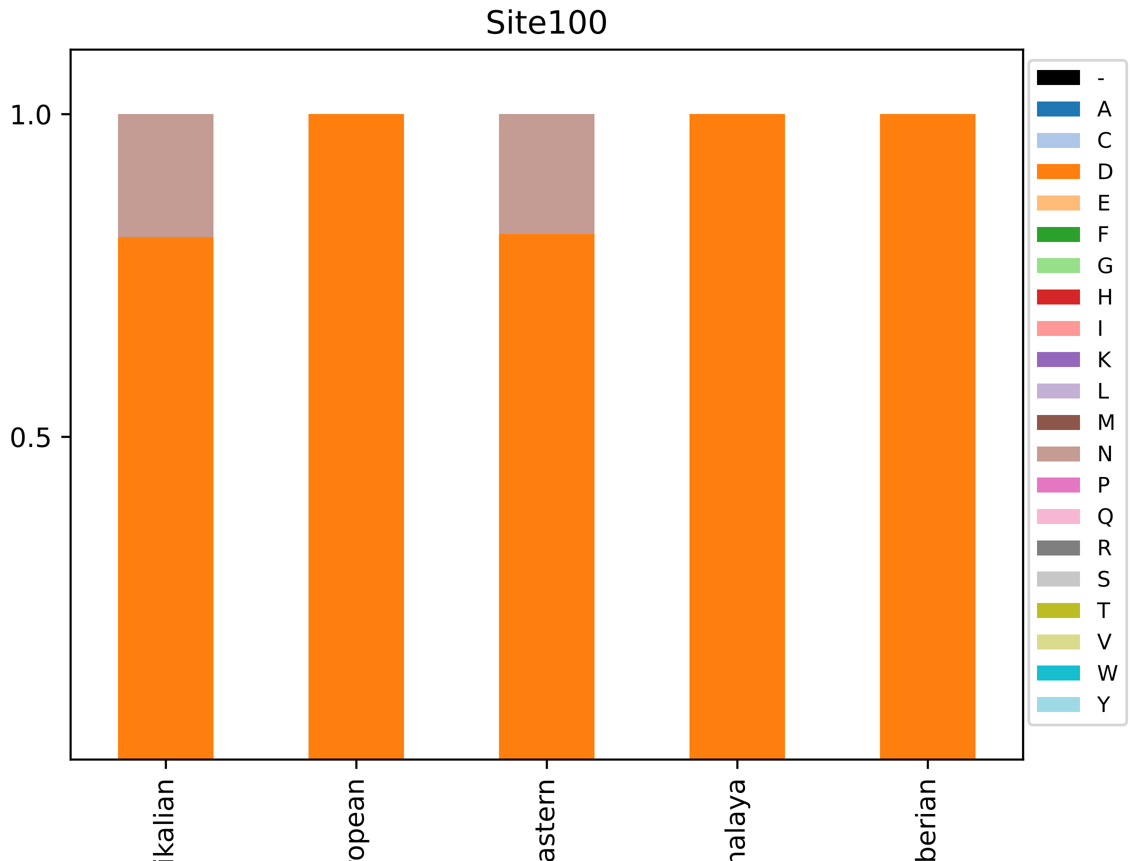
Site100
1.0
0.5
-
A
C
D
E
F
G
H
I
K
L
M
N
P
Q
R
S
T
V
W
Y
ikalian
opean
astern
halaya
berian

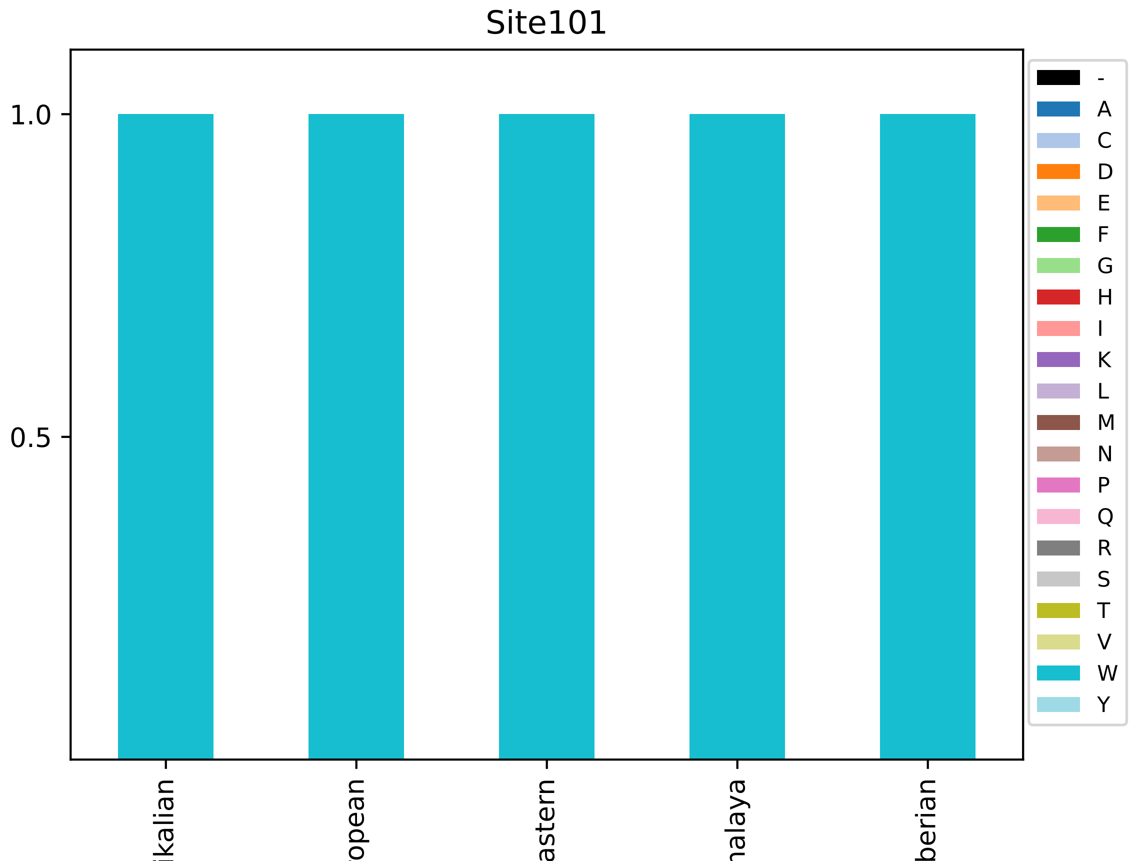

Site101
1.0
0.5
-
A
C
D
E
F
G
H
I
K
L
M
N
P
Q
R
S
T
V
W
Y
ikalian
opean
astern
nalaya
berian

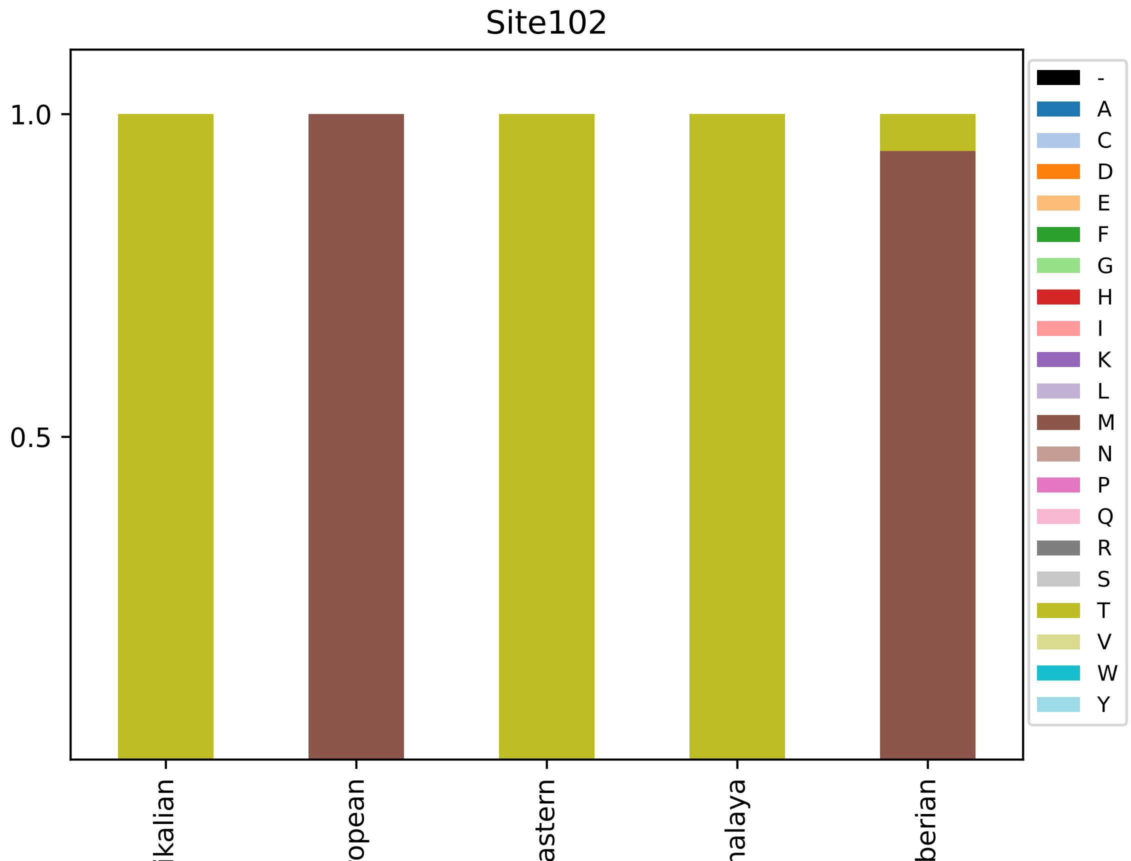
Site102
1.0
0.5
-
A
C
D
E
F
G
H
I
K
L
M
N
P
Q
R
S
T
V
W
Y
ikalian
opean
astern
nalaya
berian

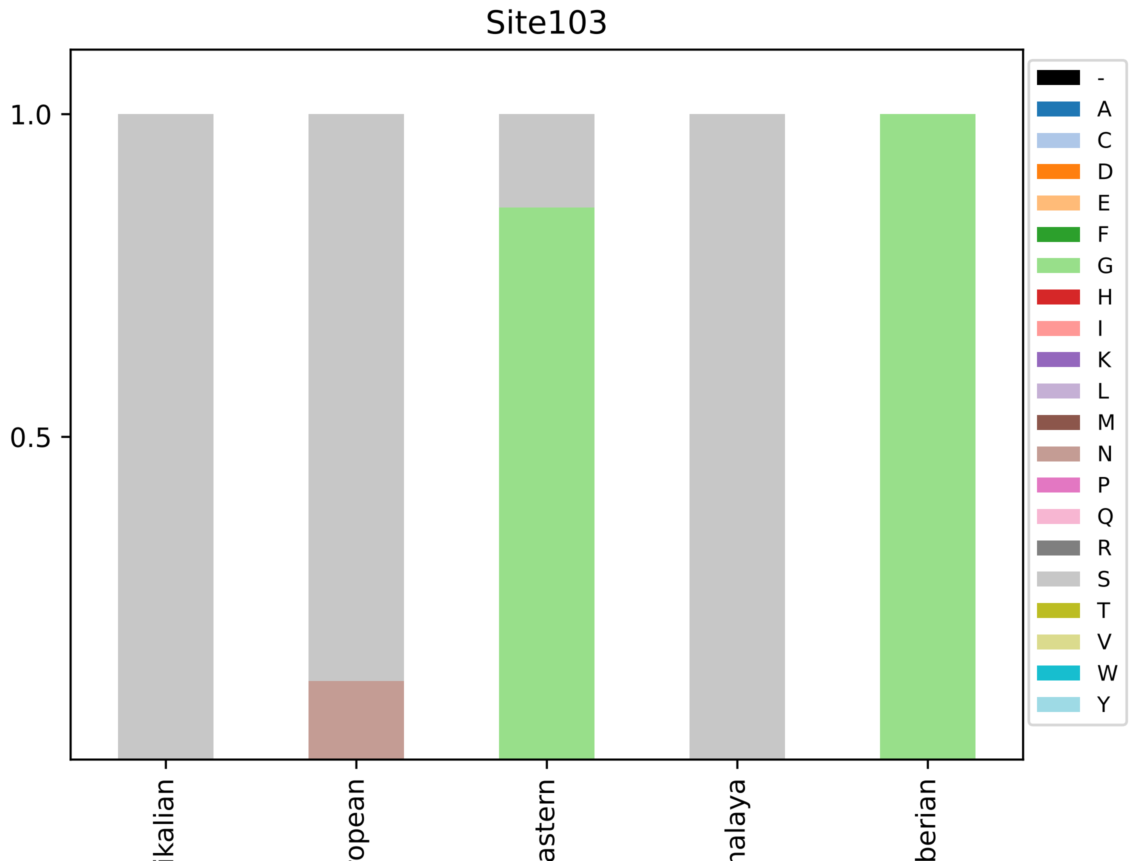

Site103
1.0
0.5
-
A
C
D
E
F
G
H
I
K
L
M
N
P
Q
R
S
T
V
W
Y
ikalian
opean
astern
nalaya
berian

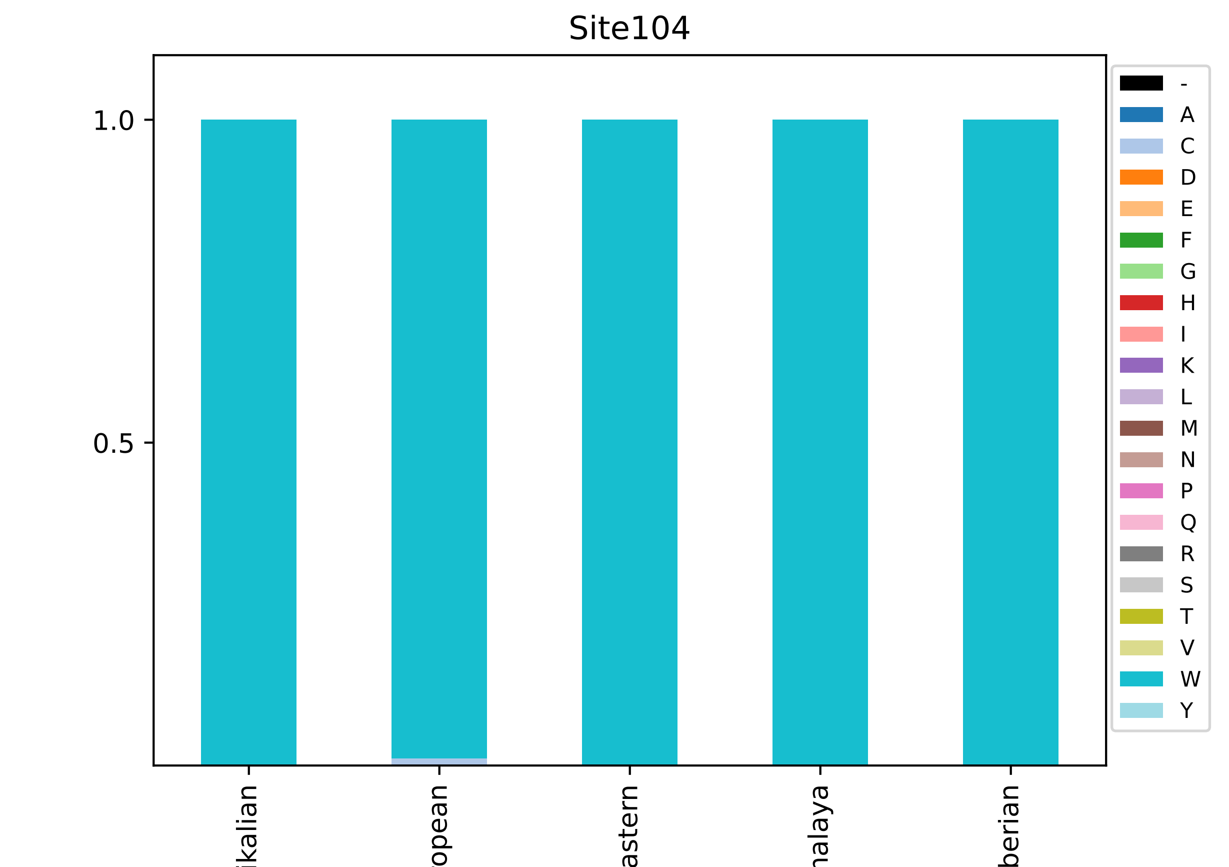
Site104
1.0
0.5
-
A
C
D
E
F
G
H
I
K
L
M
N
P
Q
R
S
T
V
W
Y
ikalian
opean
astern
nalaya
berian

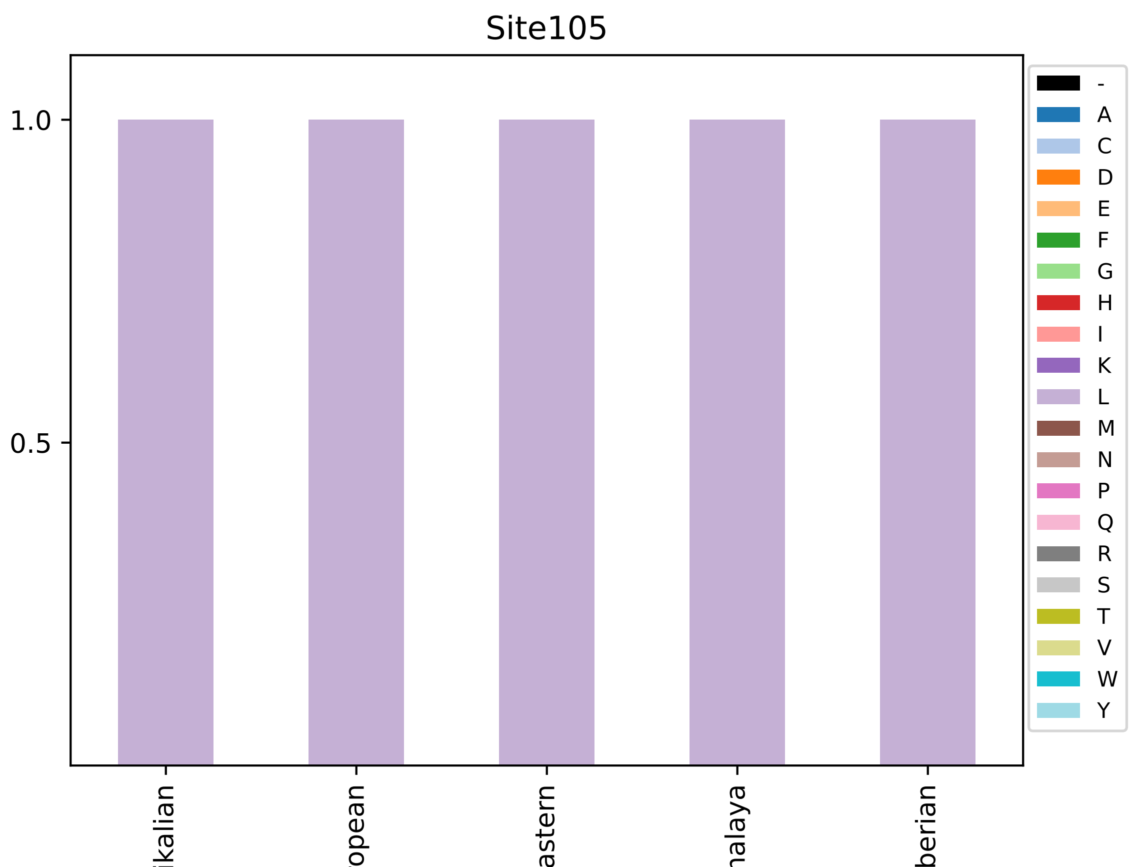
Site105
1.0
0.5
-
A
C
D
E
F
G
H
I
K
L
M
N
P
Q
R
S
T
V
W
Y
ikalian
opean
astern
nalaya
berian

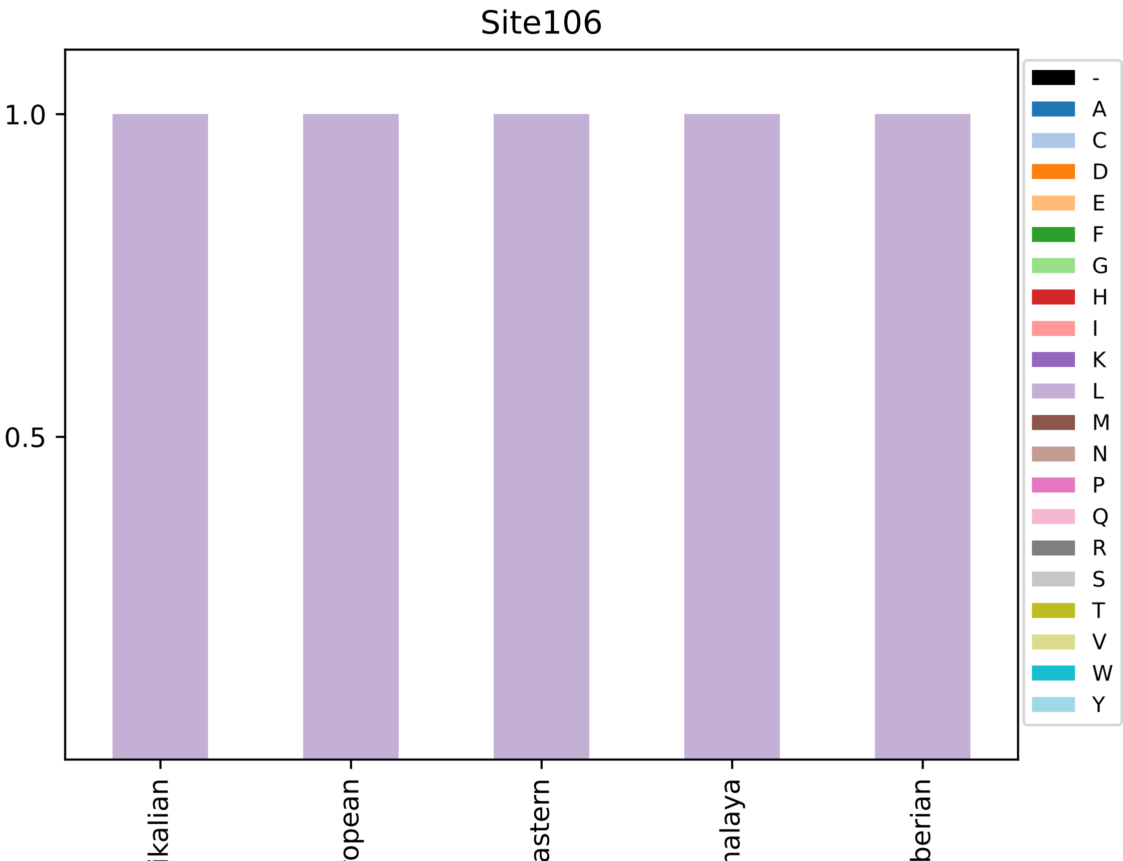
Site106
1.0
0.5
-
A
C
D
E
F
G
H
I
K
L
M
N
P
Q
R
S
T
V
W
Y
ikalian
opean
astern
nalaya
berian

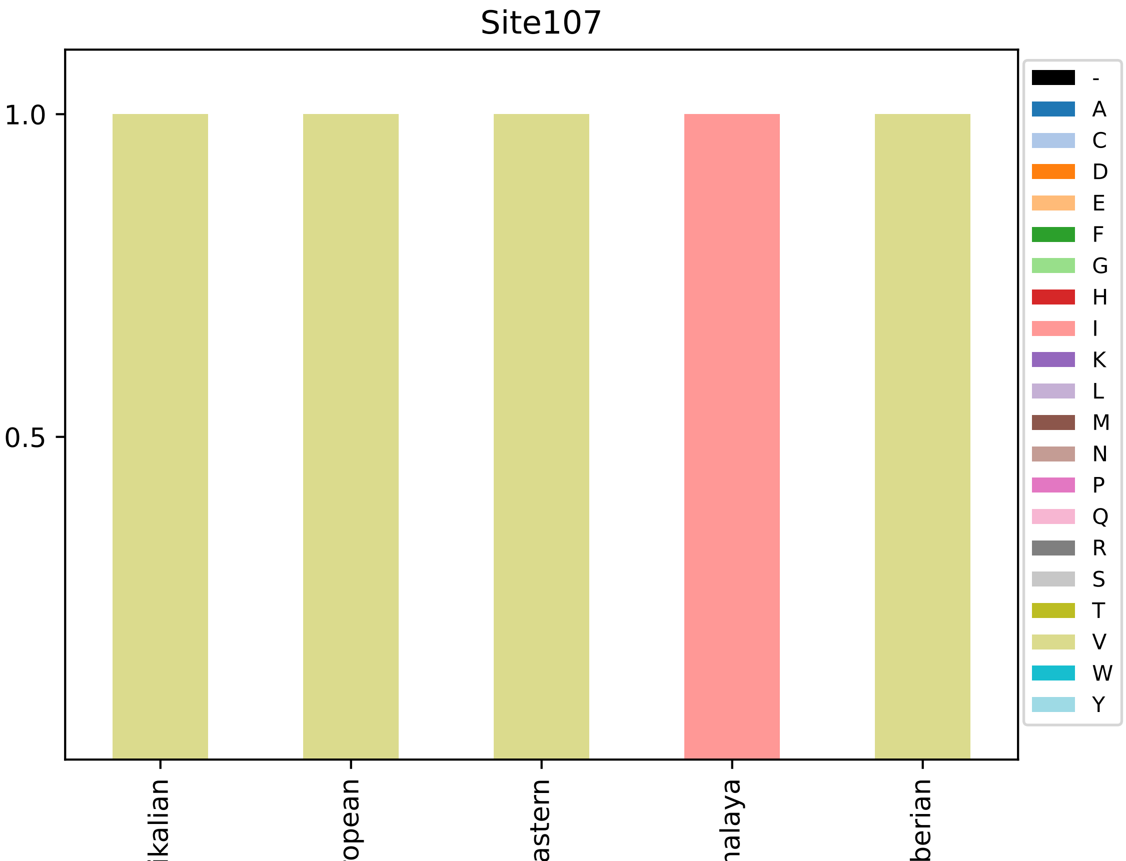

Site107
1.0
0.5
-
A
C
D
E
F
G
H
I
K
L
M
N
P
Q
R
S
T
V
W
Y
ikalian
opean
astern
nalaya
berian

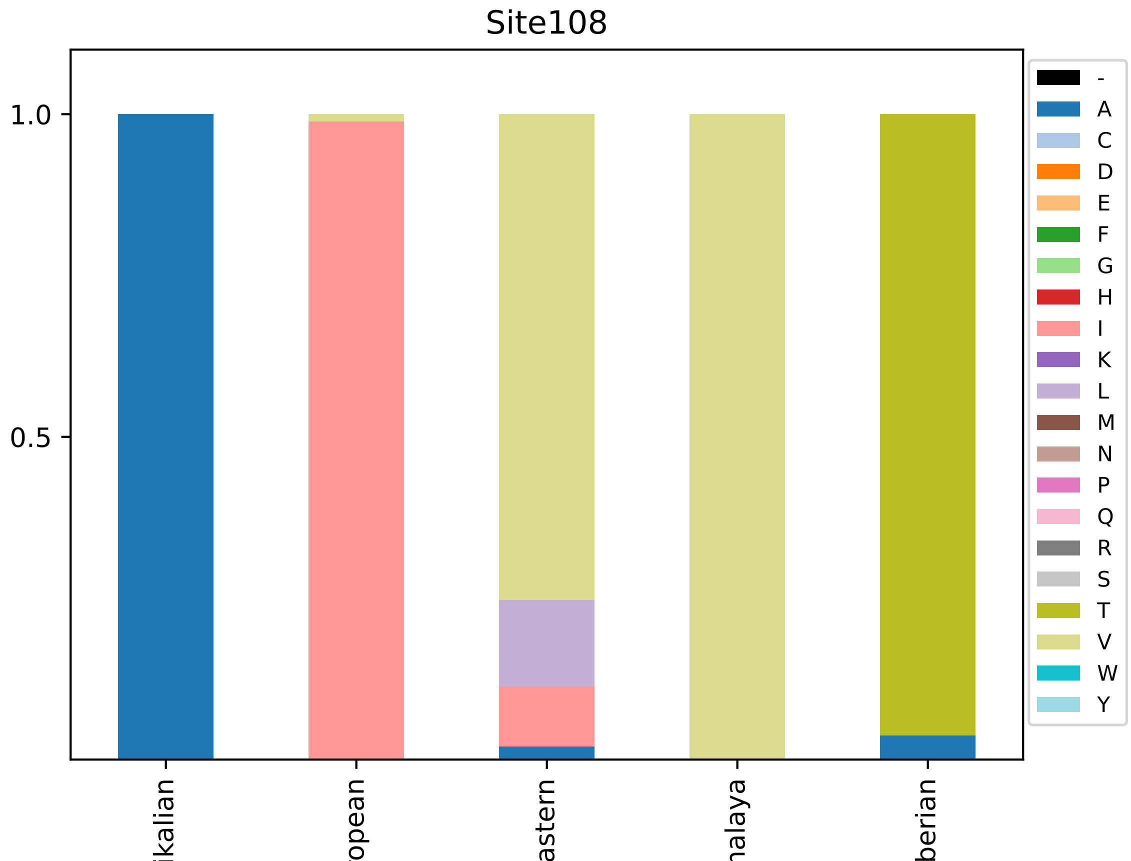

Site108
1.0
0.5
ikalian
opean
astern
nalaya
berian
-
A
C
D
E
F
G
H
I
K
L
M
N
P
Q
R
S
T
V
W
Y

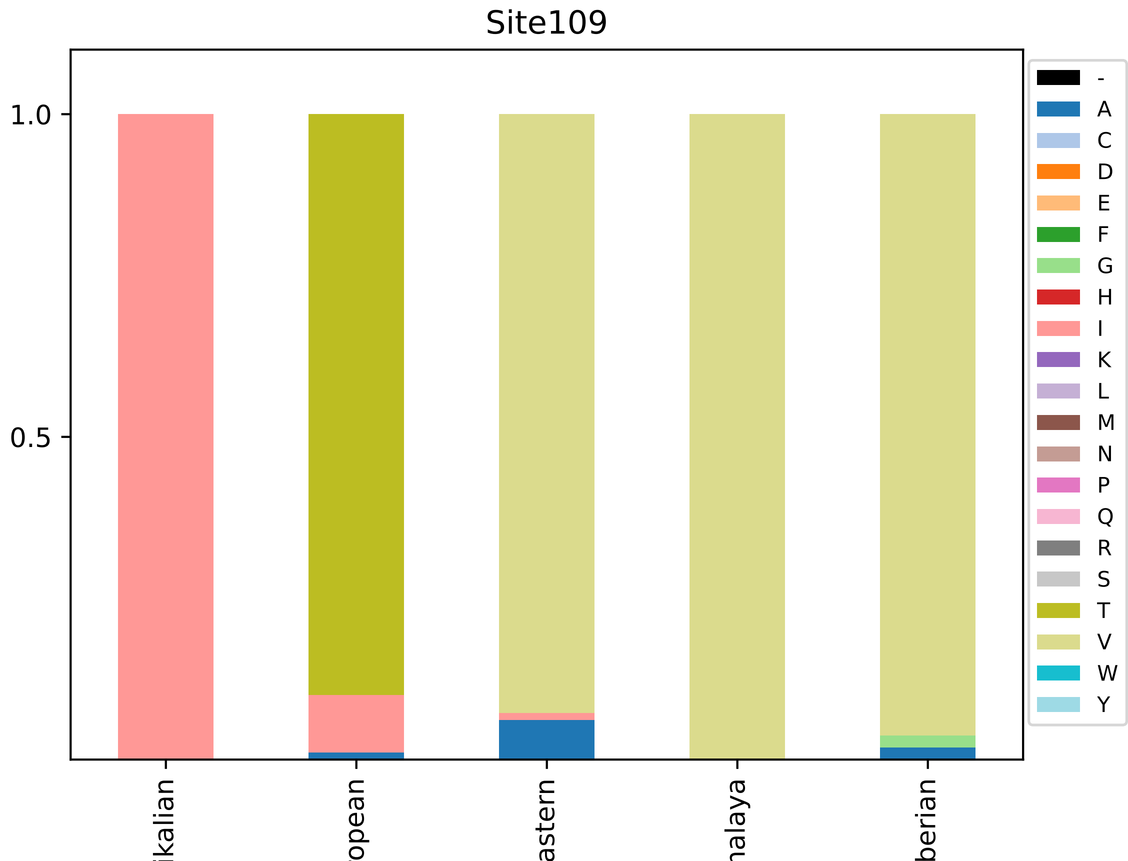
Site109
1.0
0.5
ikalian
ropean
astern
nalaya
berian
-
A
C
D
E
F
G
H
I
K
L
M
N
P
Q
R
S
T
V
W
Y

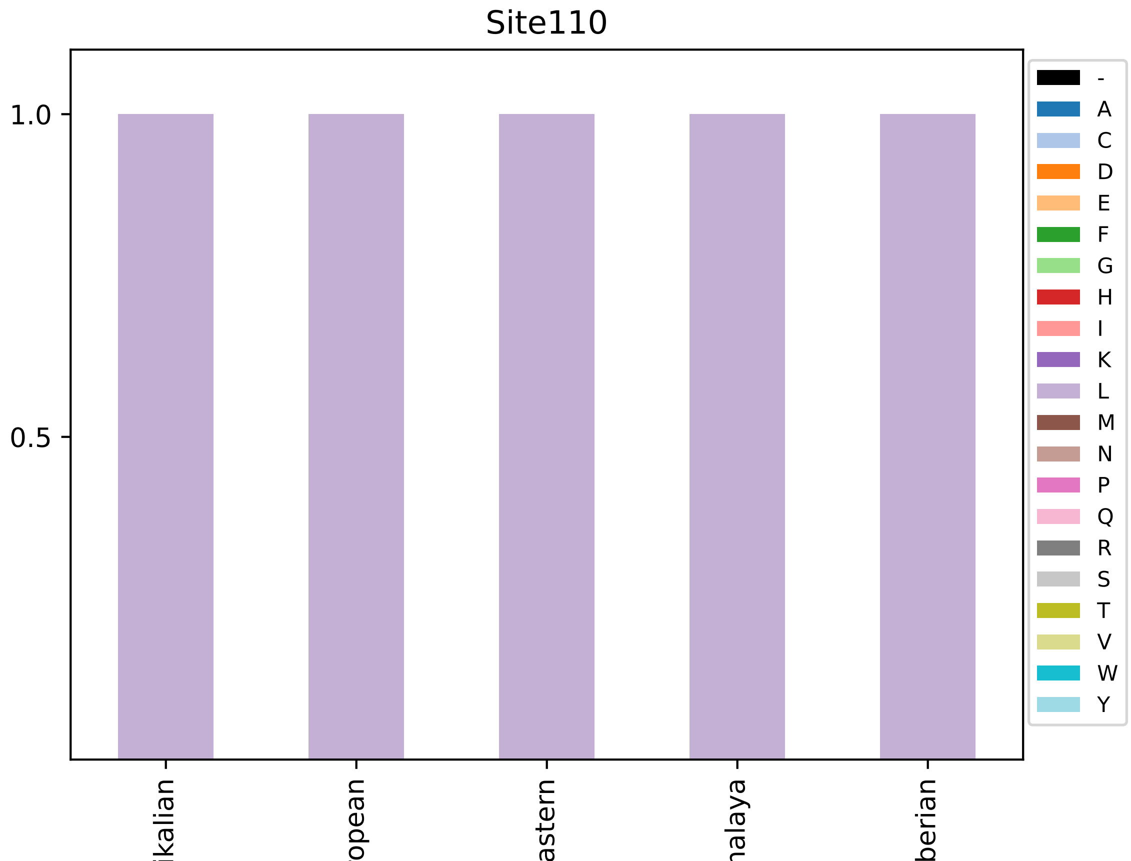

Site110
1.0
0.5
-
A
C
D
E
F
G
H
I
K
L
M
N
P
Q
R
S
T
V
W
Y
ikalian
opean
astern
nalaya
berian

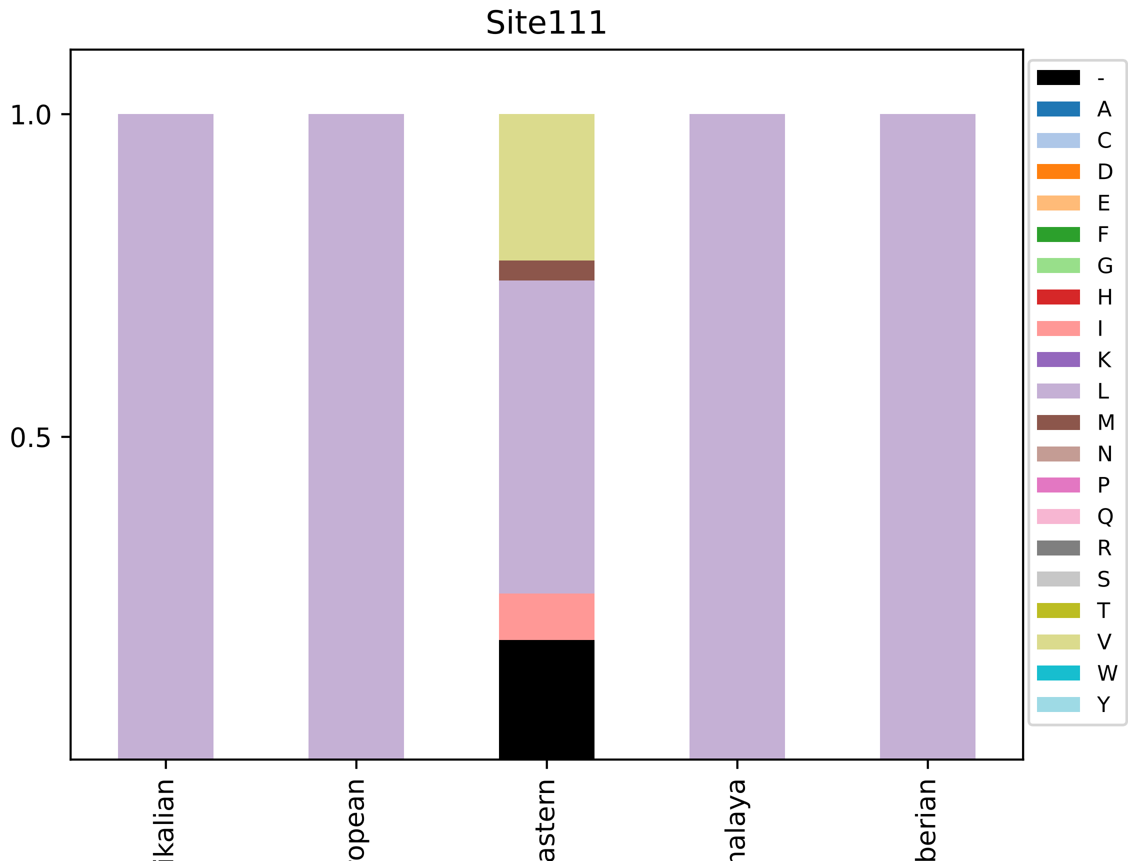

Site111
1.0
0.5
-
A
C
D
E
F
G
H
I
K
L
M
N
P
Q
R
S
T
V
W
Y
ikalian
ropean
astern
nalaya
berian

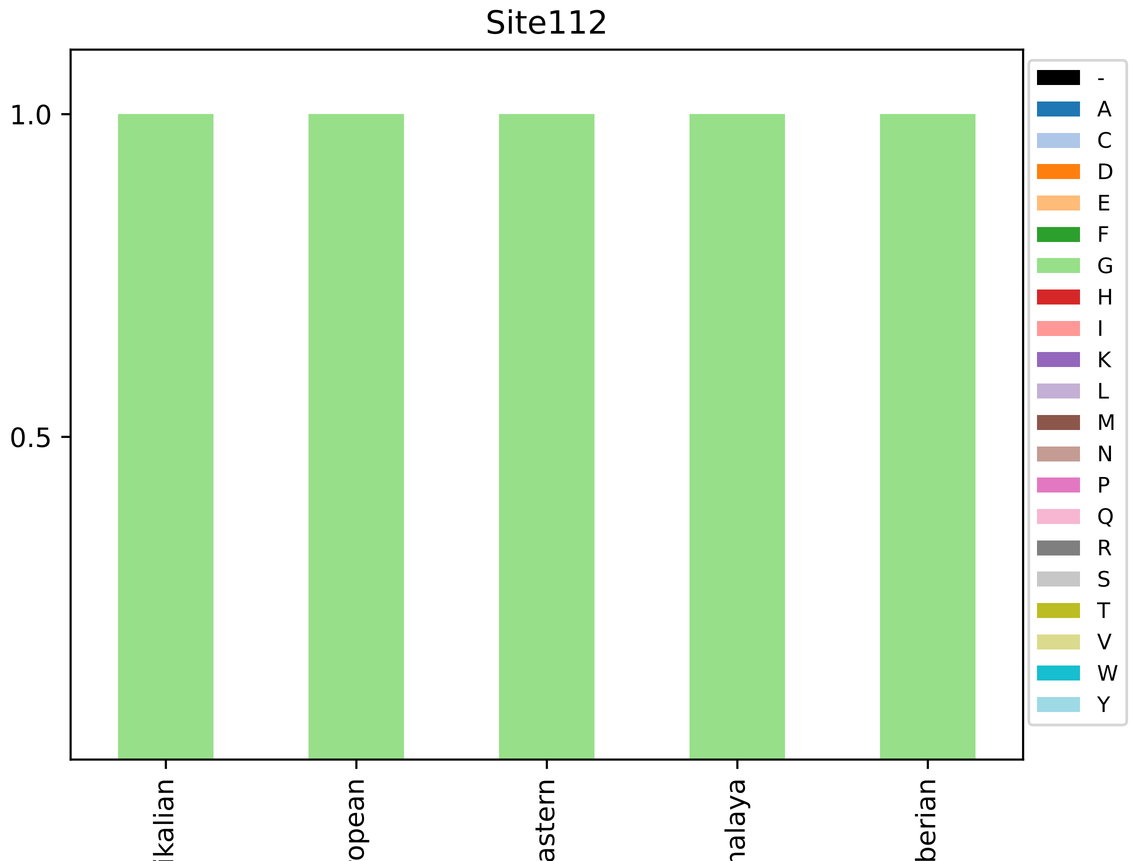

Site112
1.0
0.5
-
A
C
D
E
F
G
H
I
K
L
M
N
P
Q
R
S
T
V
W
Y

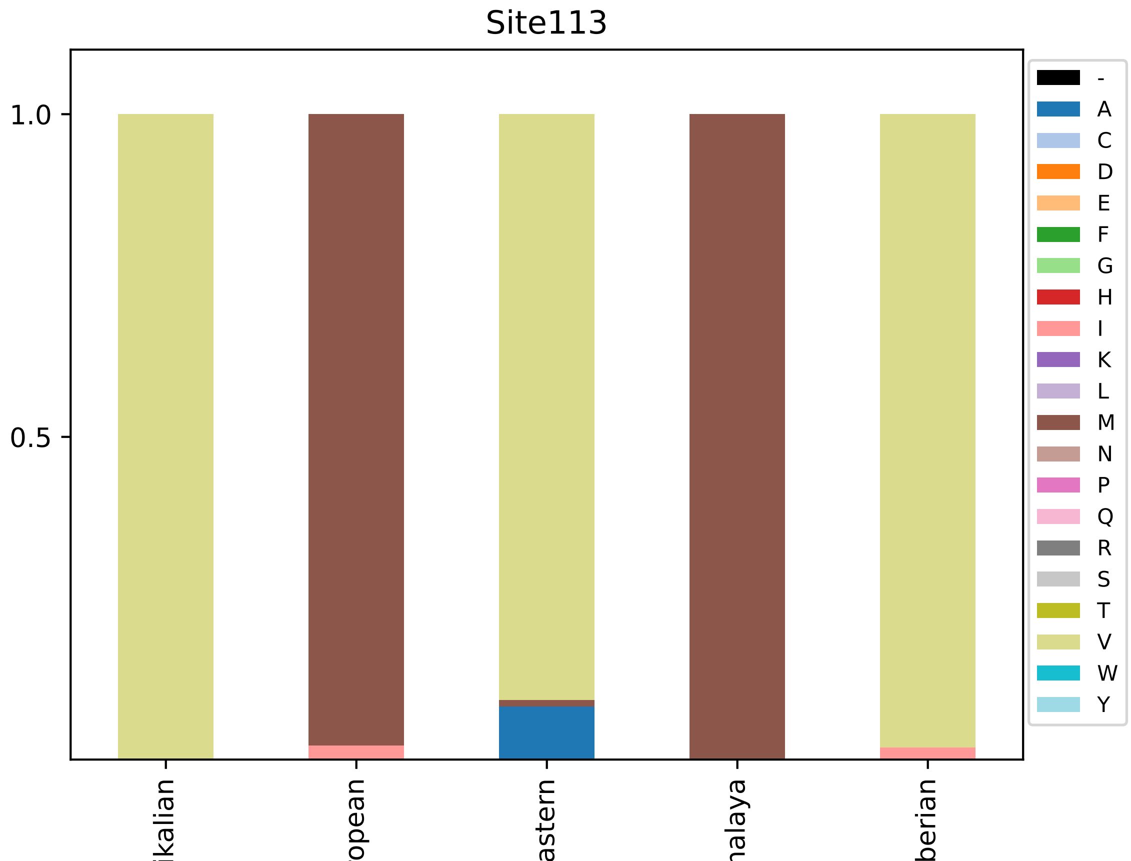
Site113
1.0
0.5
-
A
C
D
E
F
G
H
I
K
L
M
N
P
Q
R
S
T
V
W
Y
ikalian
opean
astern
nalaya
berian

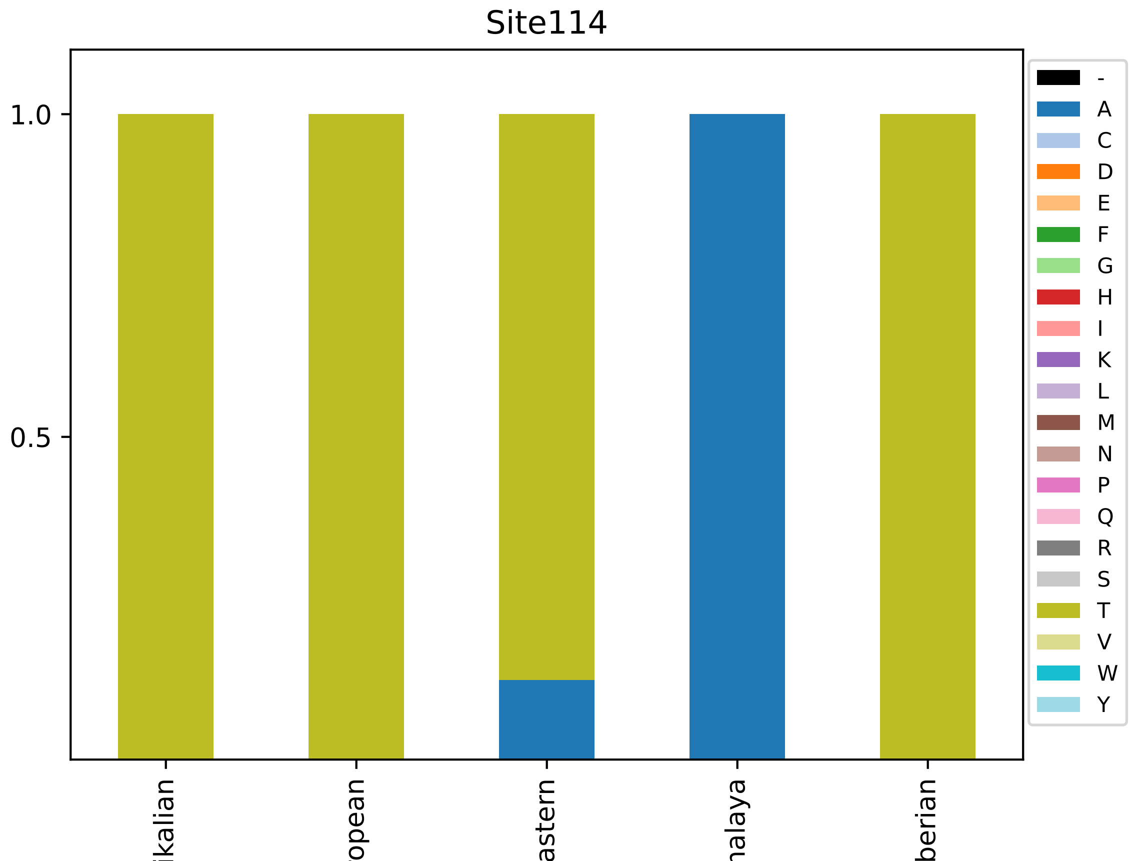
Site114
1.0
0.5
-
A
C
D
E
F
G
H
I
K
L
M
N
P
Q
R
S
T
V
W
Y
ikalian
opean
astern
nalaya
berian

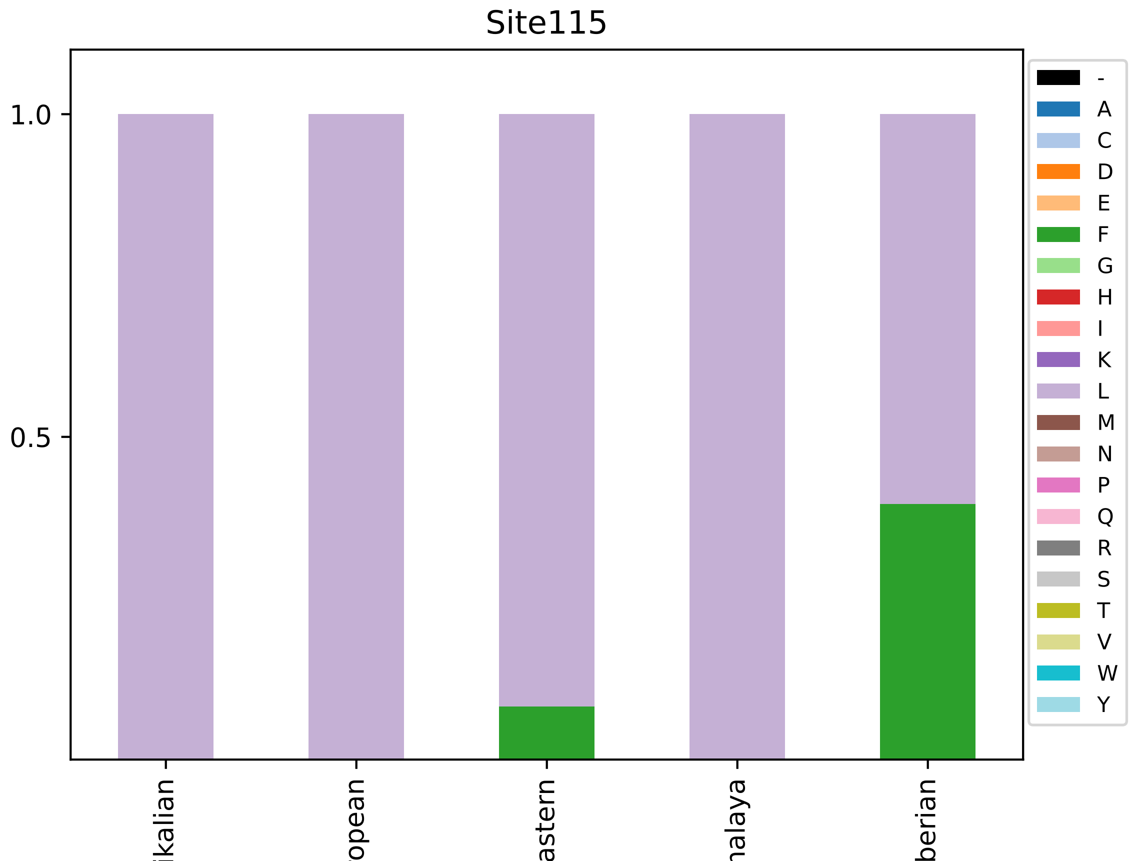

Site115
1.0
0.5
-
A
C
D
E
F
G
H
I
K
L
M
N
P
Q
R
S
T
V
W
Y
ikalian
opean
astern
nalaya
berian

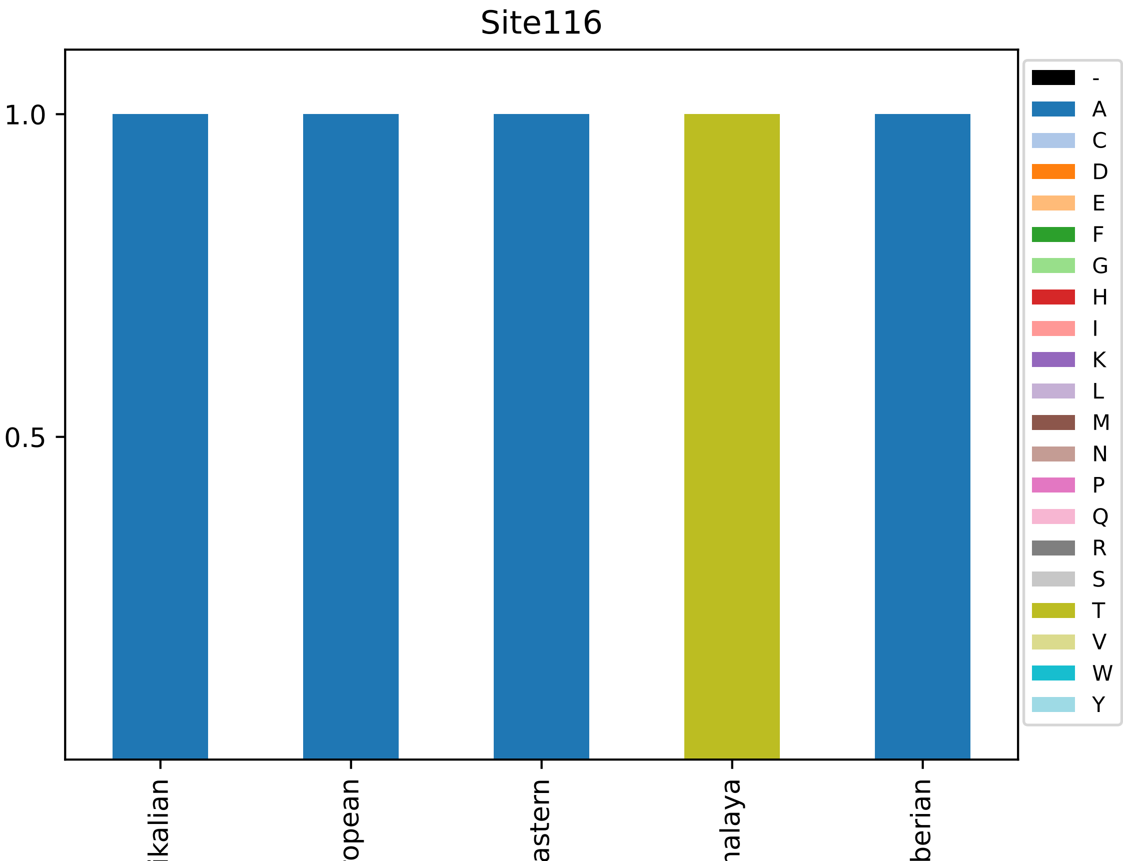

Site116
1.0
0.5
-
A
C
D
E
F
G
H
I
K
L
M
N
P
Q
R
S
T
V
W
Y
ikalian
ropean
astern
nalaya
berian

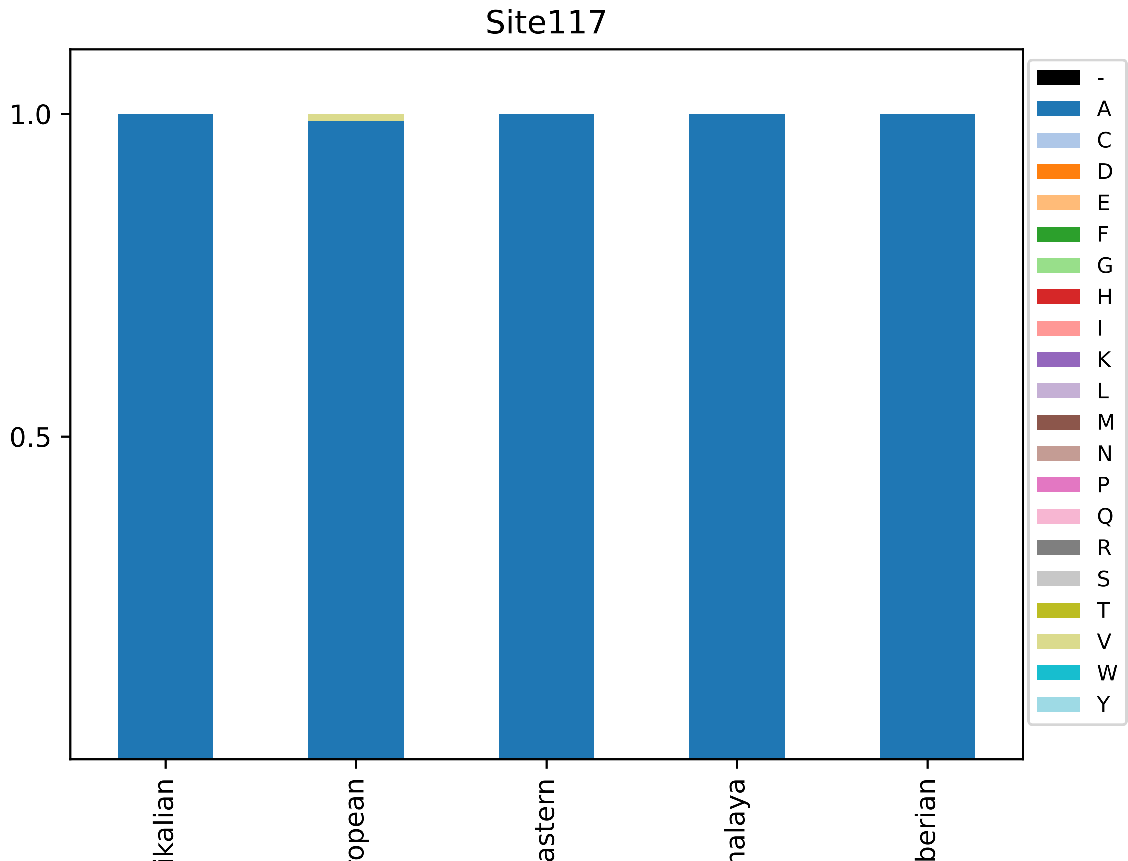
Site117
1.0
0.5
-
A
C
D
E
F
G
H
I
K
L
M
N
P
Q
R
S
T
V
W
Y
ikalian
opean
astern
nalaya
berian

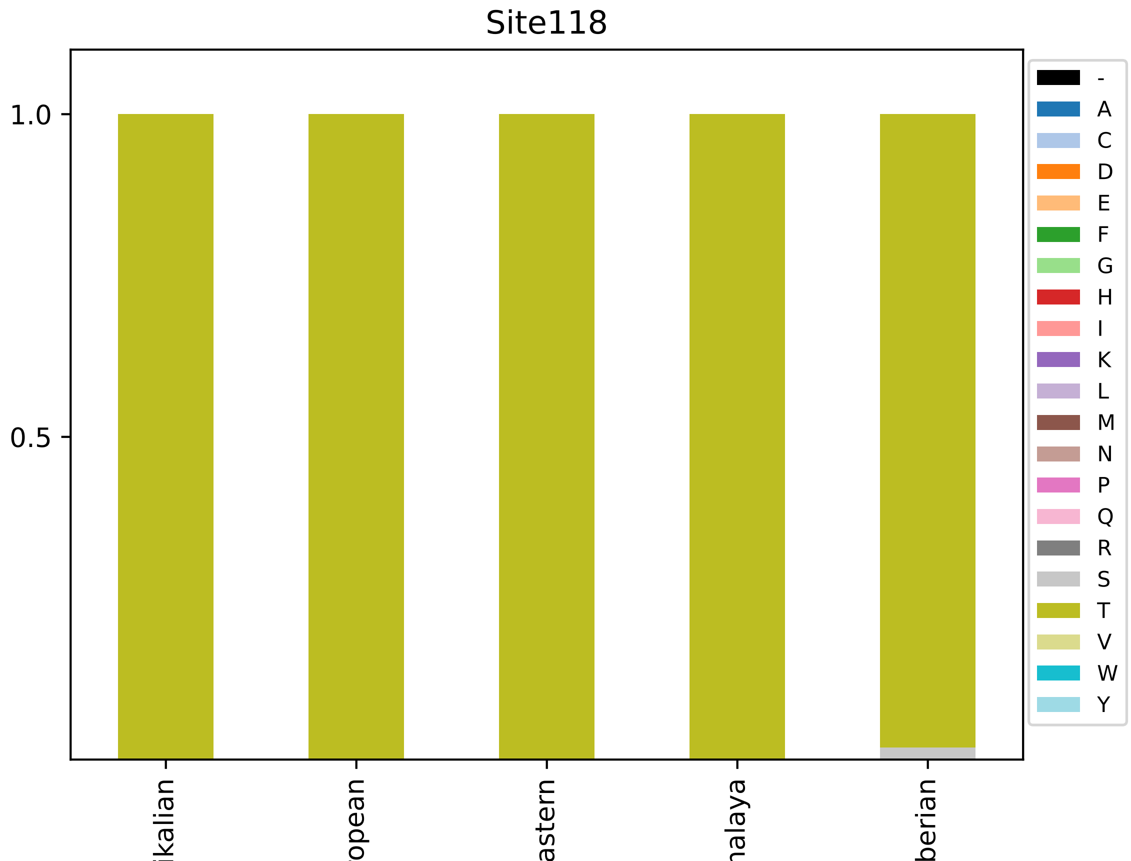
Site118
1.0
0.5
-
A
C
D
E
F
G
H
I
K
L
M
N
P
Q
R
S
T
V
W
Y
ikalian
ropean
astern
nalaya
berian

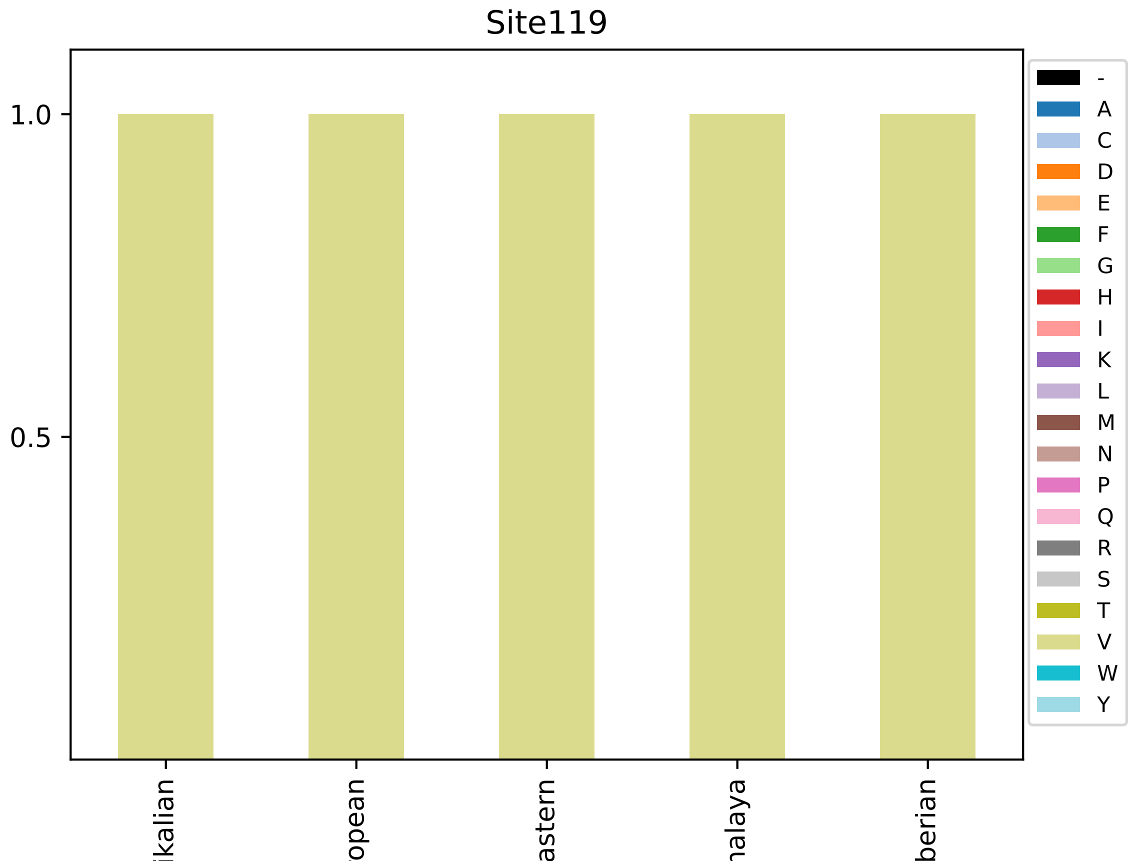
Site119
1.0
0.5
-
A
C
D
E
F
G
H
I
K
L
M
N
P
Q
R
S
T
V
W
Y
ikalian
opean
astern
nalaya
berian

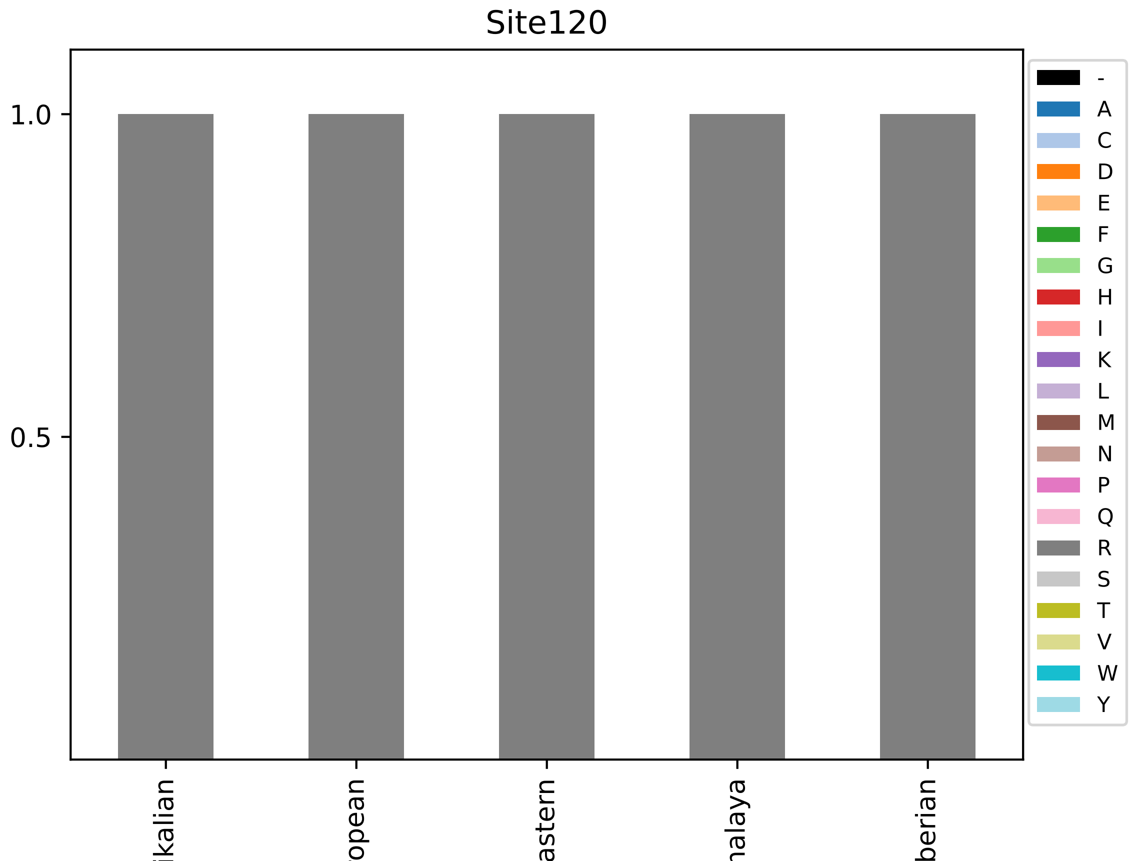
Site120
1.0
0.5
-
A
C
D
E
F
G
H
I
K
L
M
N
P
Q
R
S
T
V
W
Y
ikalian
opean
astern
nalaya
berian

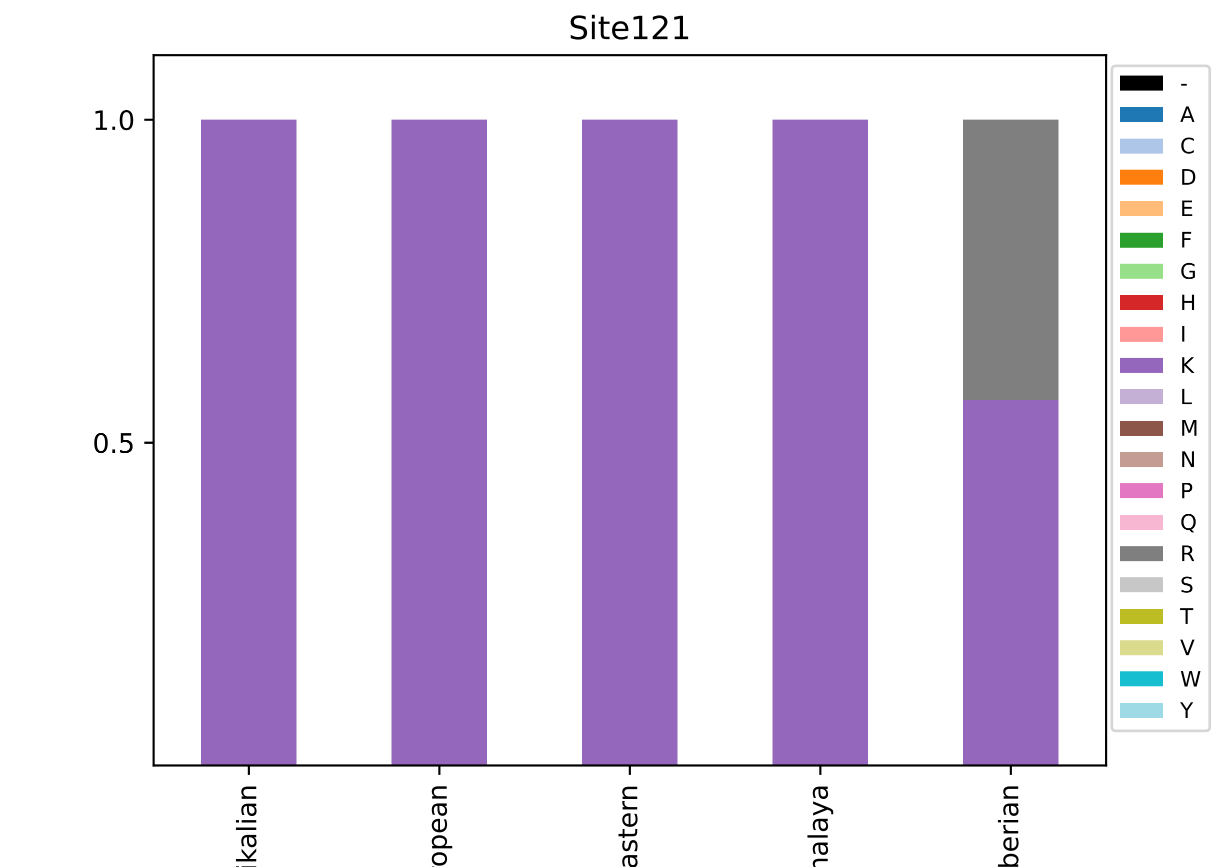
Site121
1.0
0.5
-
A
C
D
E
F
G
H
I
K
L
M
N
P
Q
R
S
T
V
W
Y
ikalian
opean
astern
nalaya
berian

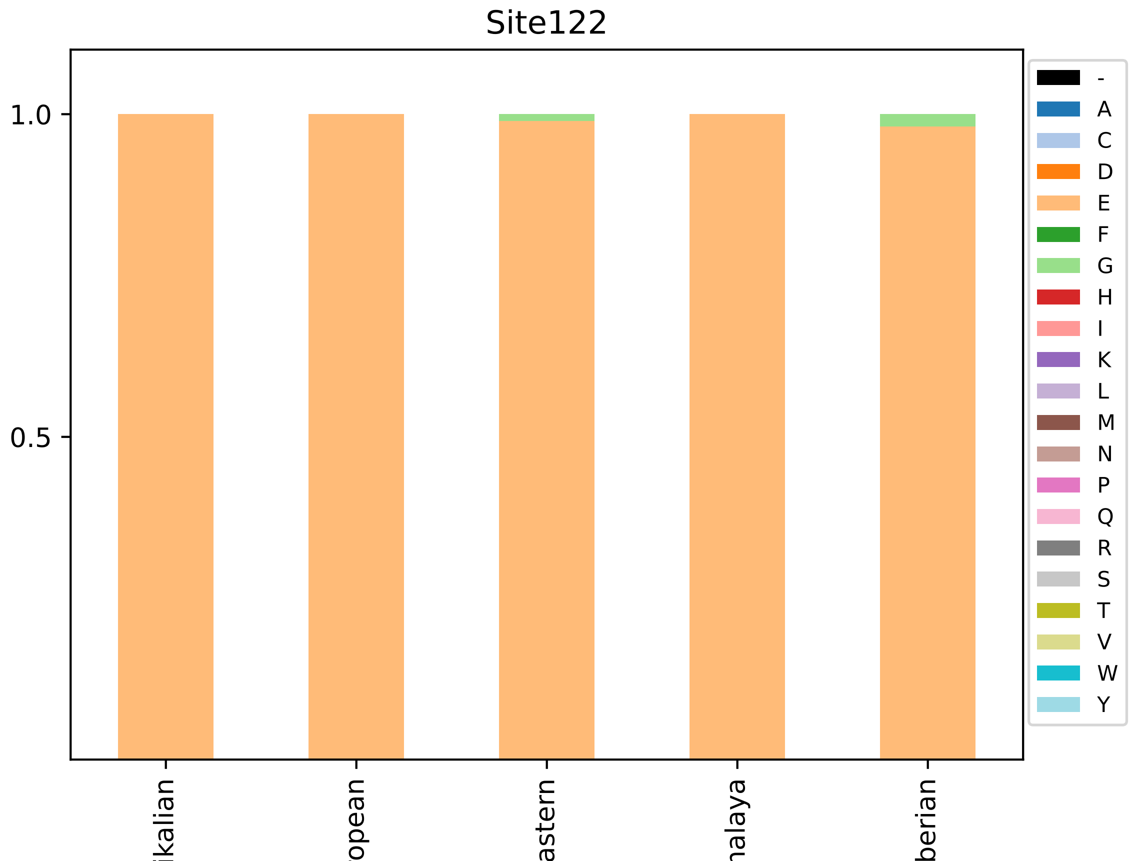

Site122
1.0
0.5
-
A
C
D
E
F
G
H
I
K
L
M
N
P
Q
R
S
T
V
W
Y
ikalian
opean
astern
nalaya
berian

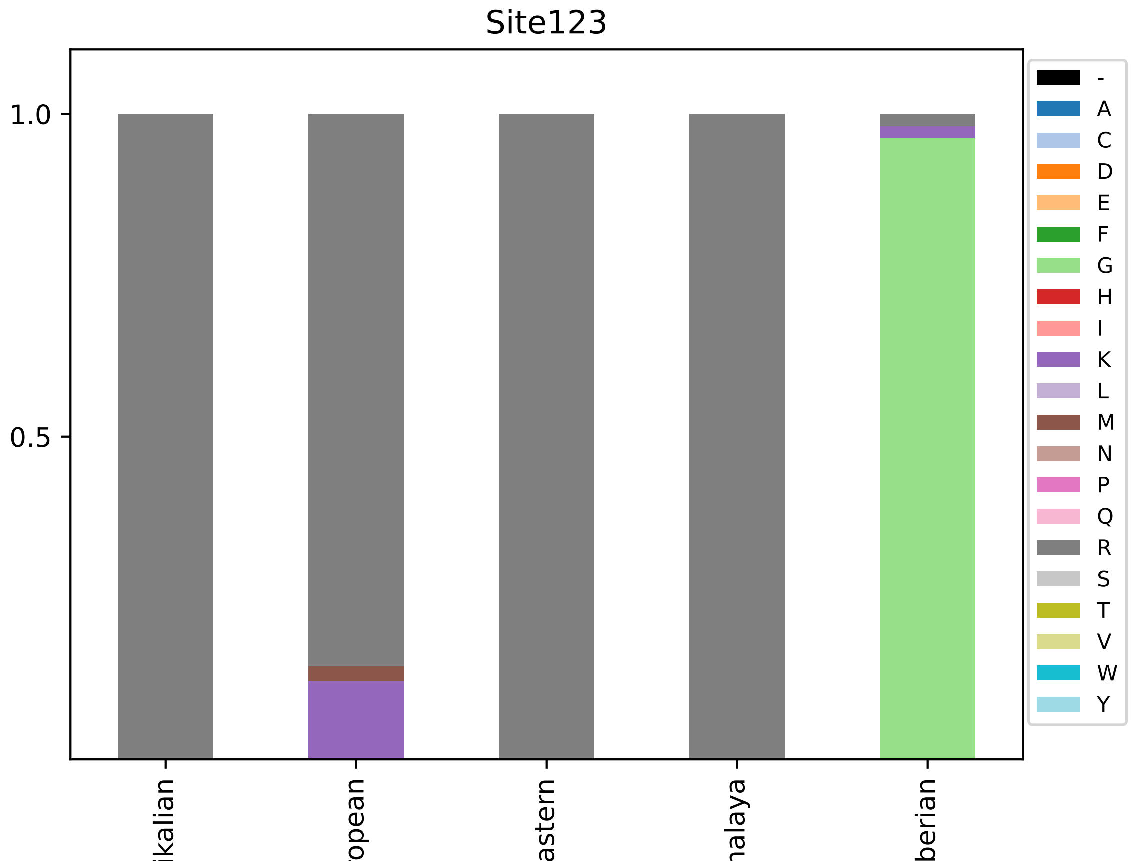
Site123
1.0
0.5
-
A
C
D
E
F
G
H
I
K
L
M
N
P
Q
R
S
T
V
W
Y
ikalian
opean
astern
nalaya
berian

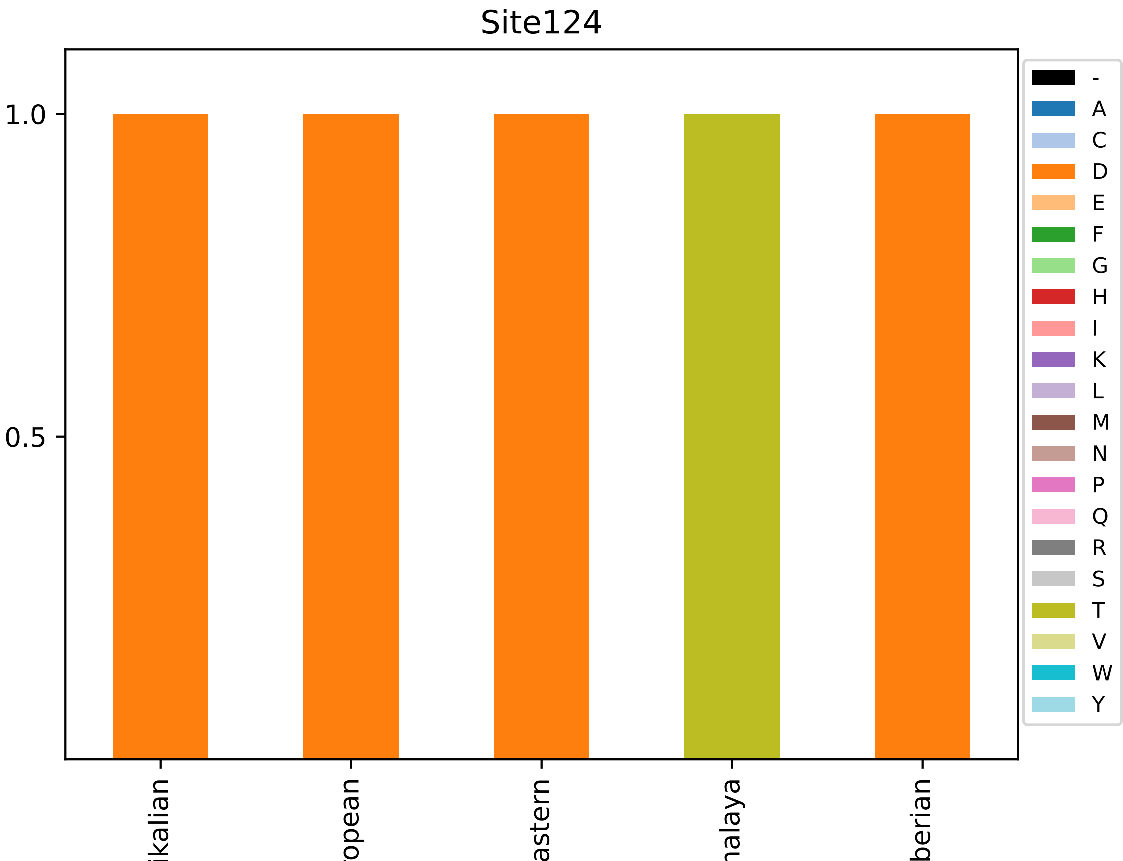
Site124
1.0
0.5
-
A
C
D
E
F
G
H
I
K
L
M
N
P
Q
R
S
T
V
W
Y

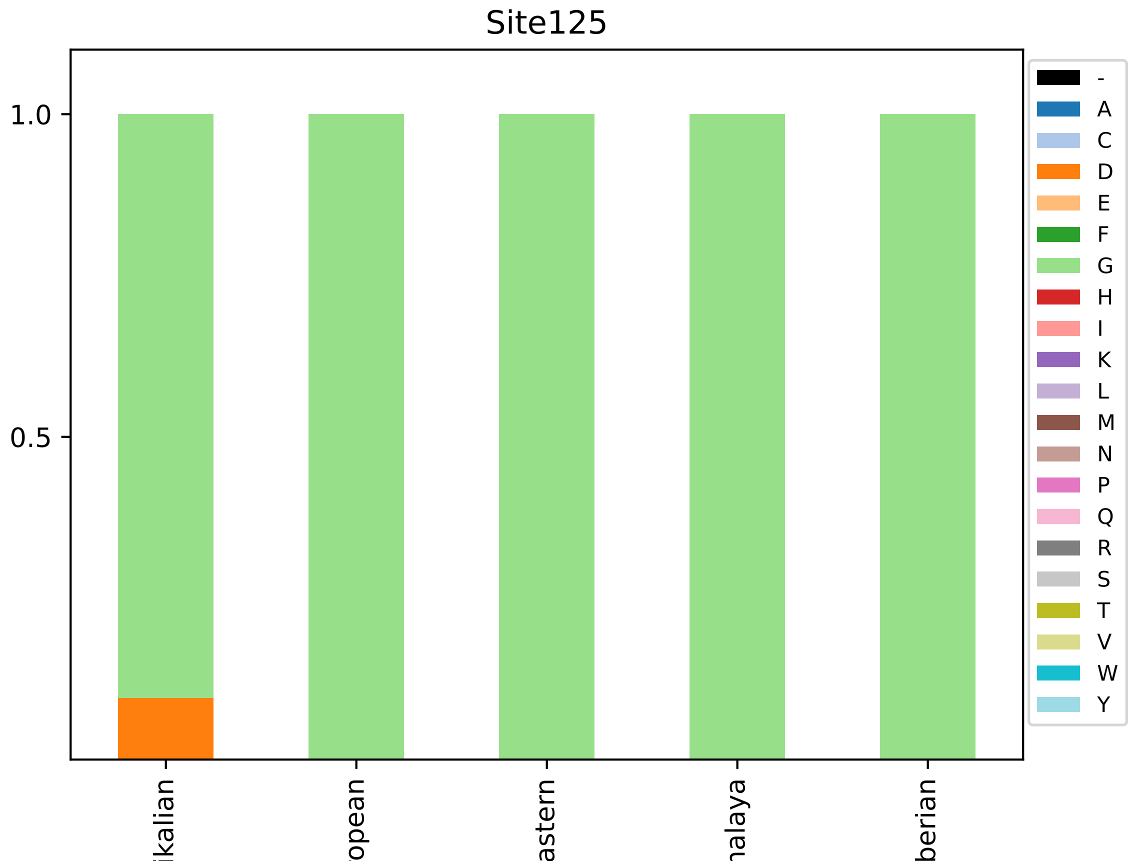

Site125
1.0
0.5
-
A
C
D
E
F
G
H
I
K
L
M
N
P
Q
R
S
T
V
W
Y
ikalian
opean
astern
nalaya
berian

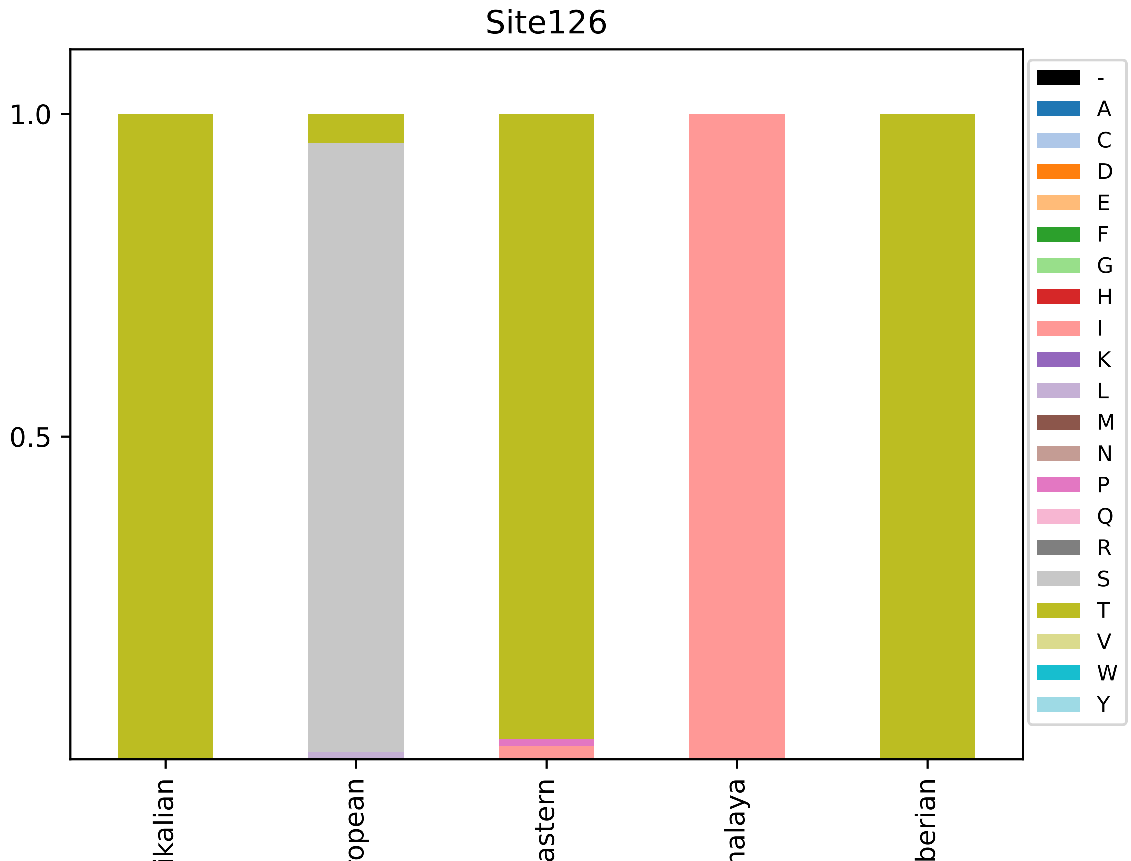
Site126
1.0
0.5
-
A
C
D
E
F
G
H
I
K
L
M
N
P
Q
R
S
T
V
W
Y
ikalian
ropean
astern
nalaya
berian

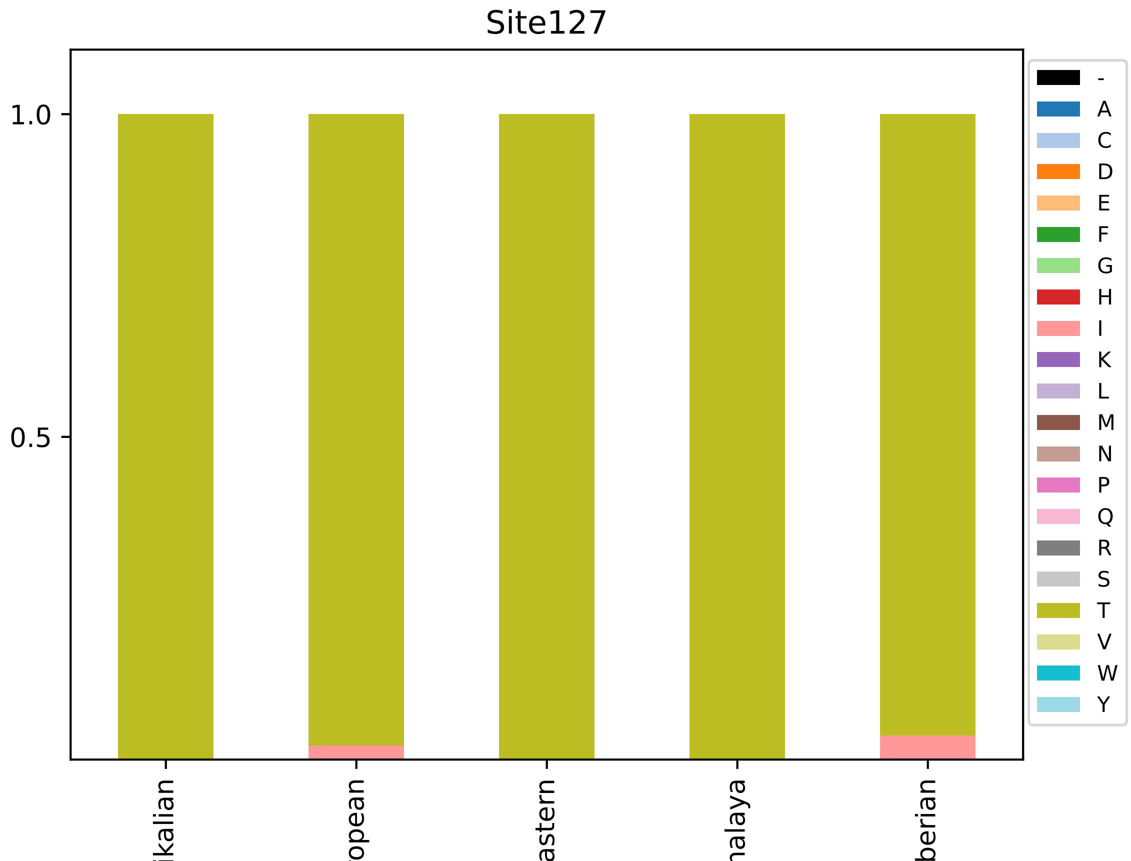

Site127
1.0
0.5
ikalian
opean
astern
nalaya
berian
-
A
C
D
E
F
G
H
I
K
L
M
N
P
Q
R
S
T
V
W
Y

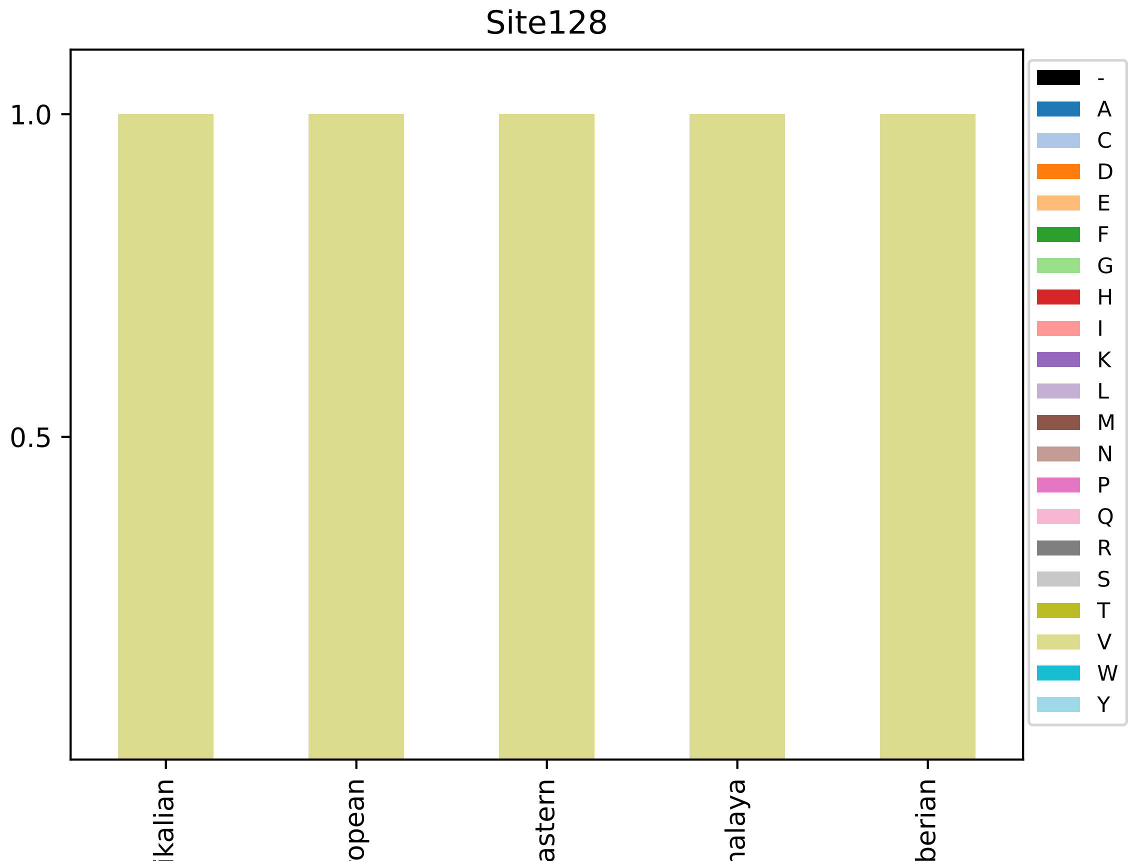

Site128
1.0
0.5
-
A
C
D
E
F
G
H
I
K
L
M
N
P
Q
R
S
T
V
W
Y
ikalian
opean
astern
nalaya
berian

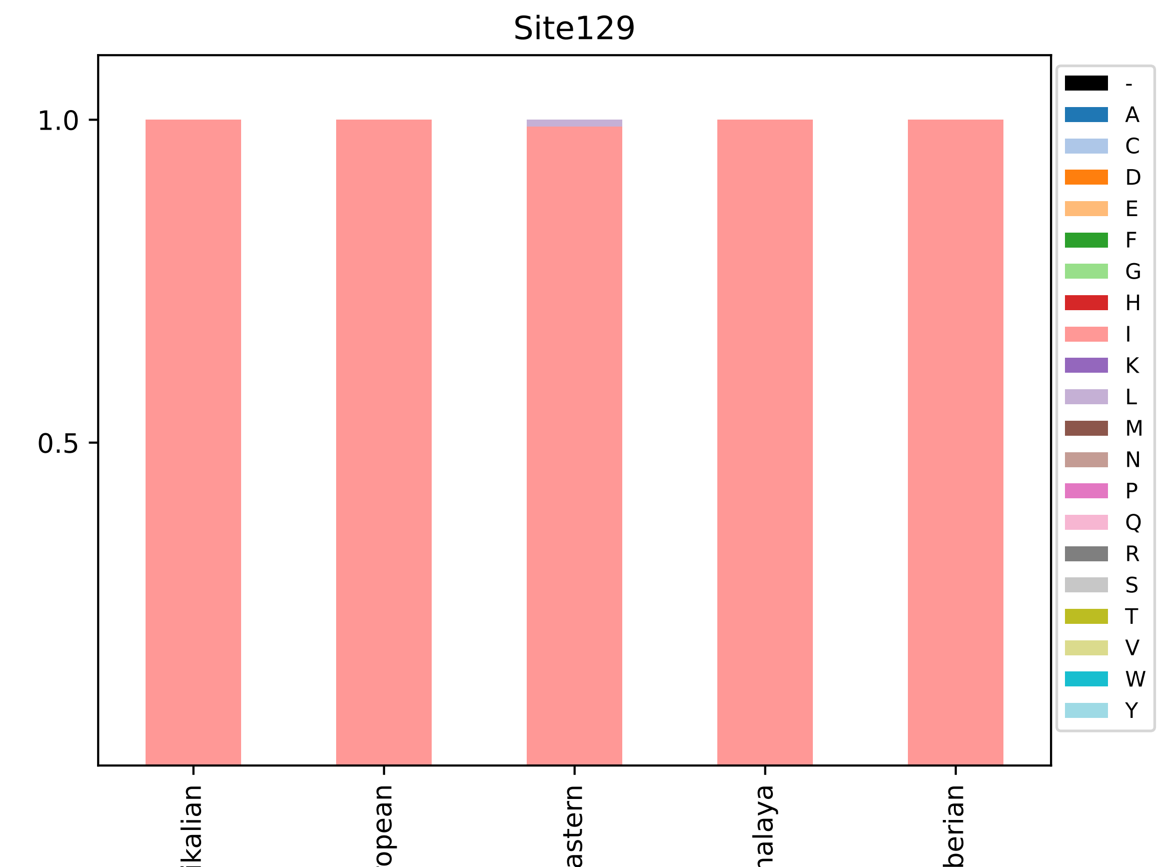
Site129
1.0
0.5
ikalian
opean
astern
nalaya
berian
-
A
C
D
E
F
G
H
I
K
L
M
N
P
Q
R
S
T
V
W
Y

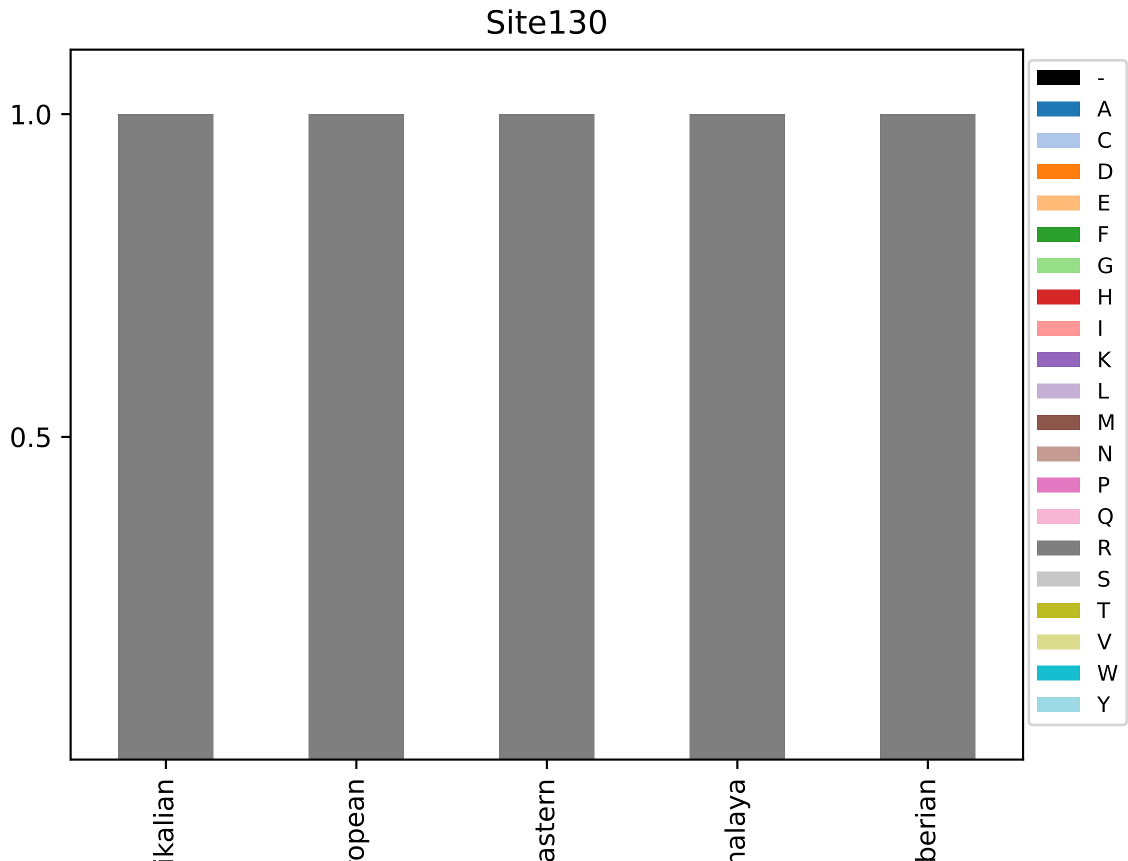
Site130
1.0
0.5
-
A
C
D
E
F
G
H
I
K
L
M
N
P
Q
R
S
T
V
W
Y
ikalian
opean
astern
nalaya
berian

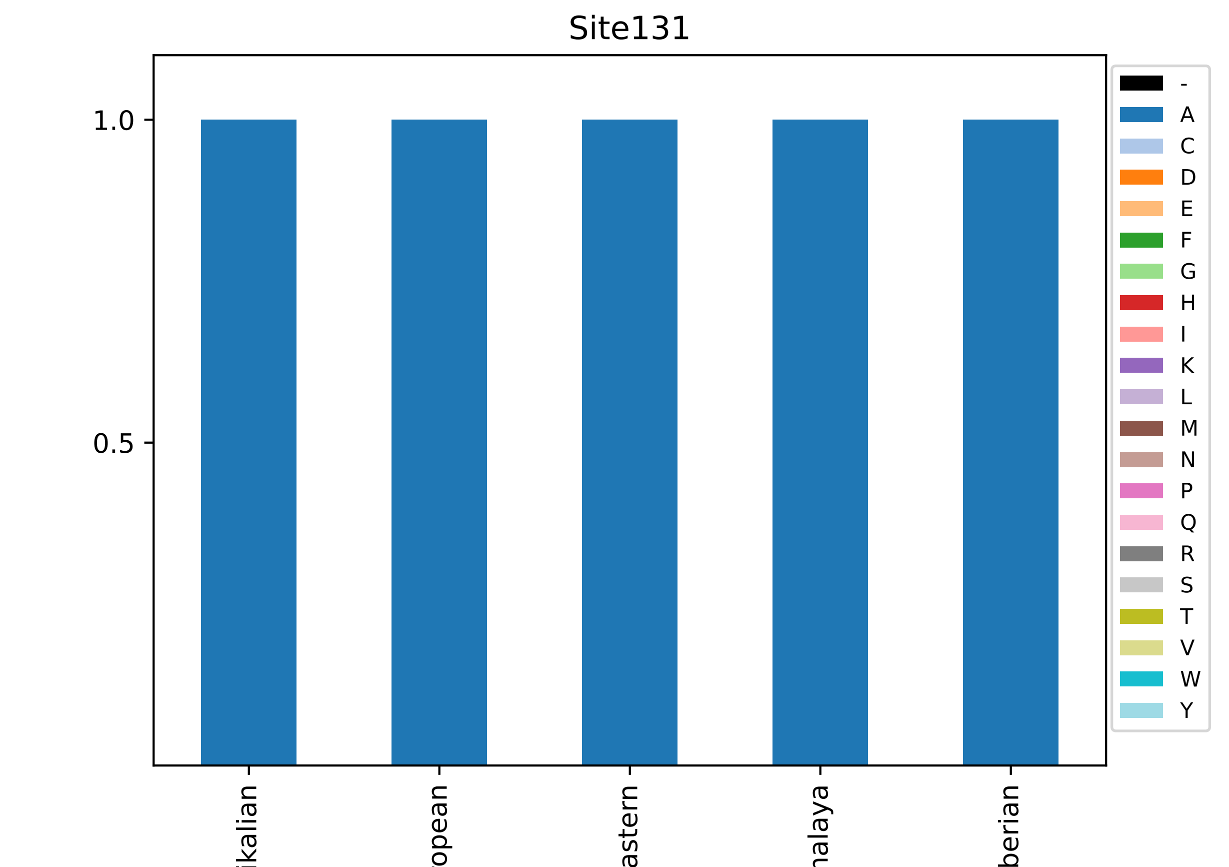
Site131
1.0
0.5
-
A
C
D
E
F
G
H
I
K
L
M
N
P
Q
R
S
T
V
W
Y
ikalian
ropean
astern
nalaya
berian

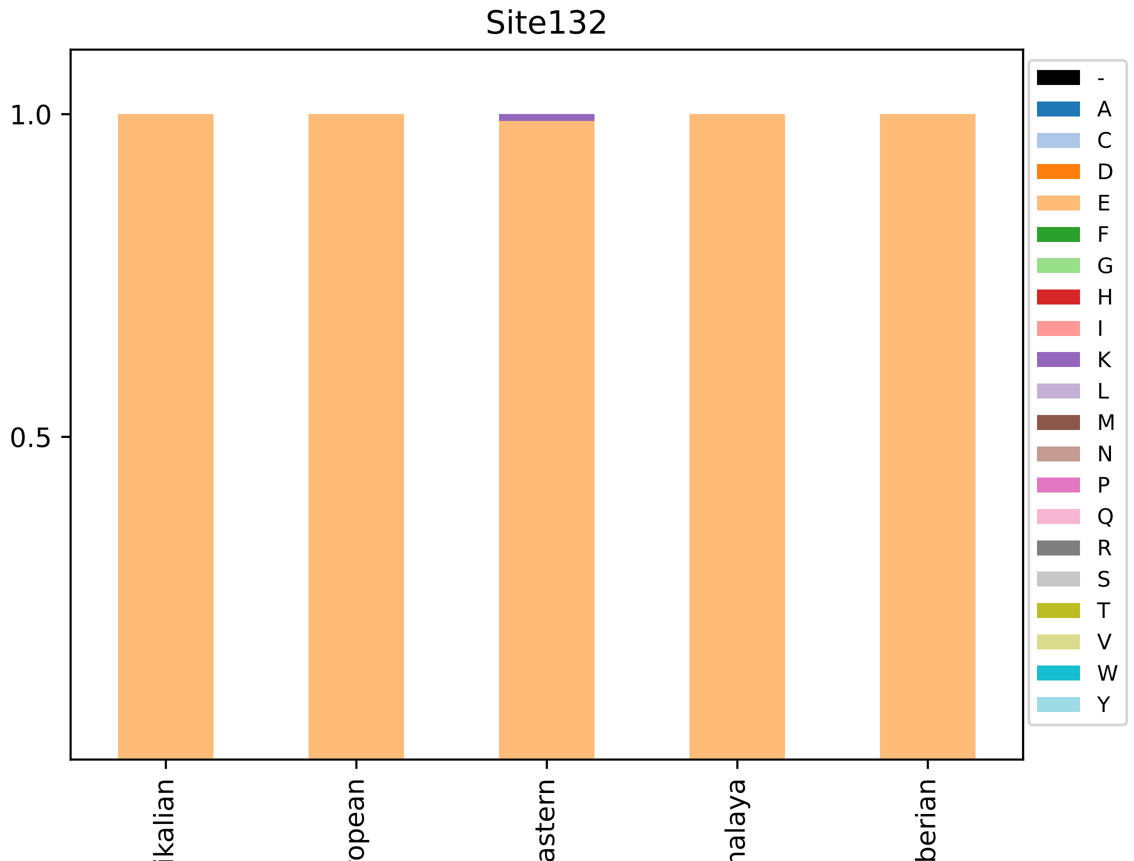

Site132
1.0
0.5
-
A
C
D
E
F
G
H
I
K
L
M
N
P
Q
R
S
T
V
W
Y
ikalian
opean
astern
nalaya
berian

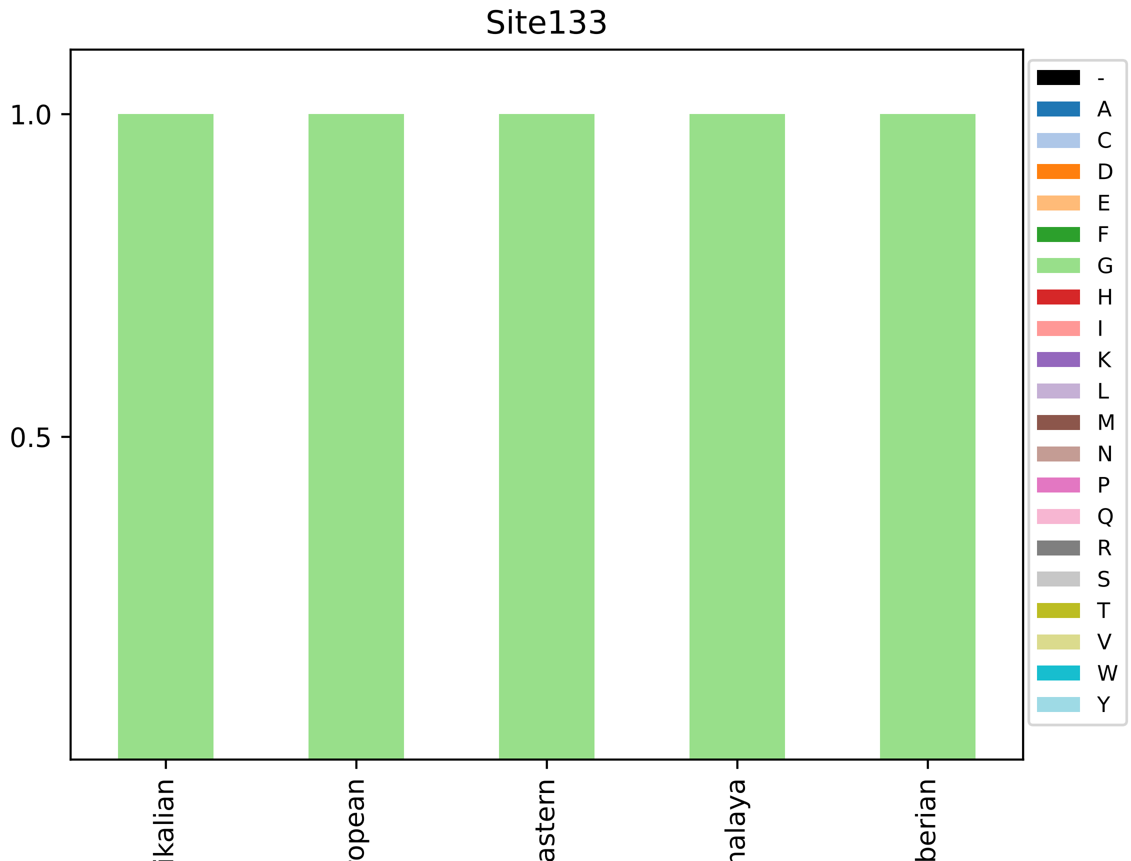
Site133
1.0
0.5
ikalian
opean
astern
nalaya
berian
-
A
C
D
E
F
G
H
I
K
L
M
N
P
Q
R
S
T
V
W
Y

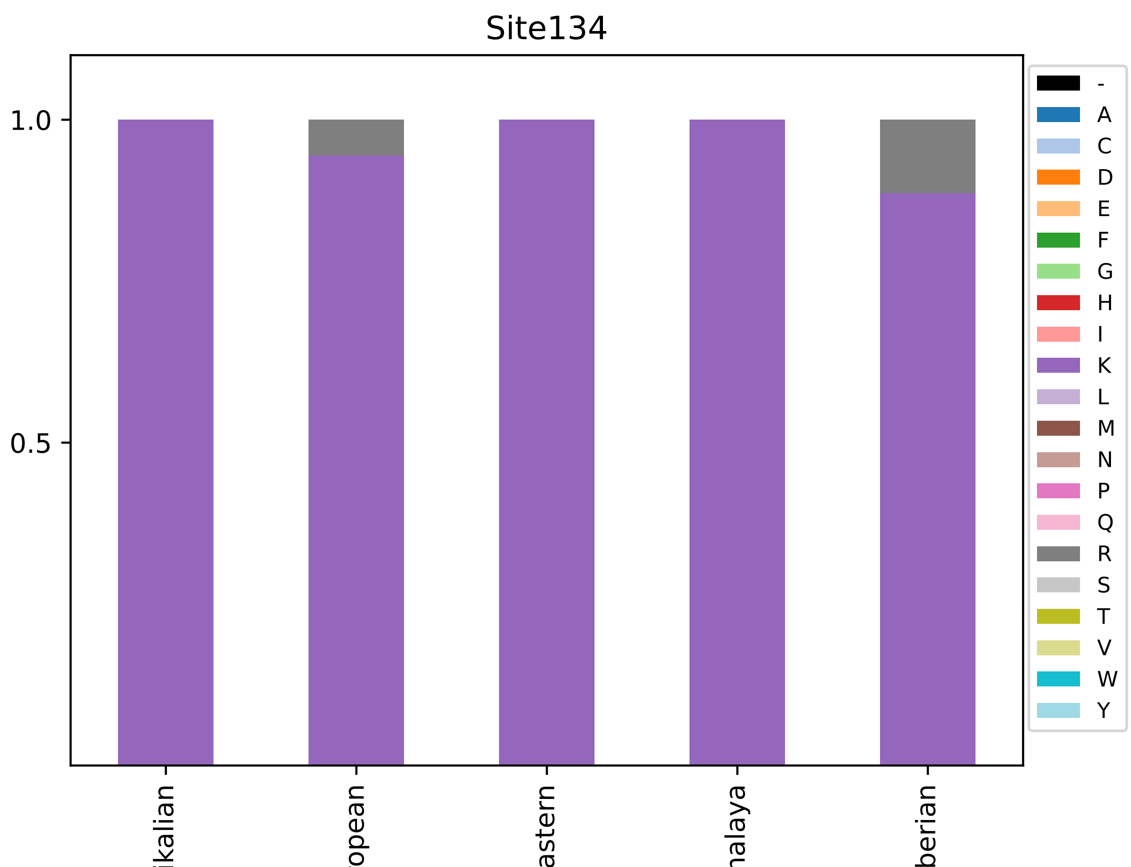

Site134
1.0
0.5
-
A
C
D
E
F
G
H
I
K
L
M
N
P
Q
R
S
T
V
W
Y
ikalian
opean
astern
nalaya
berian

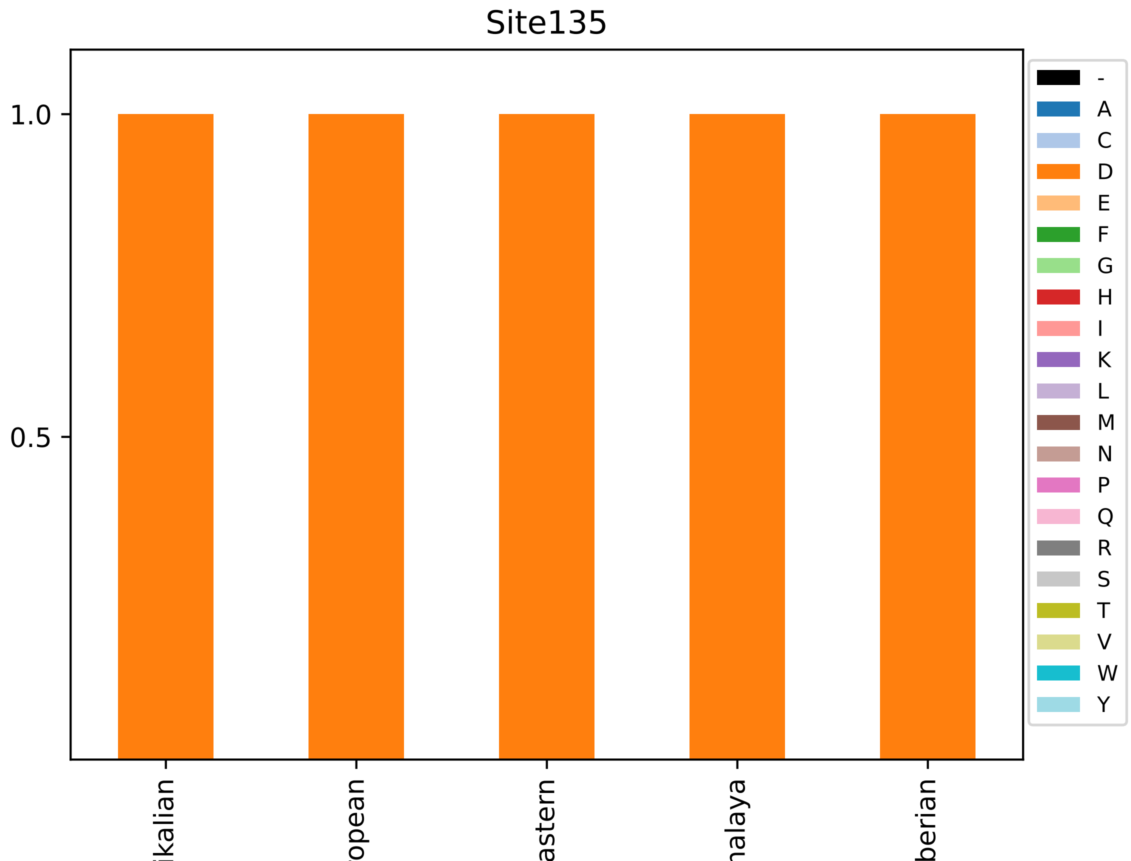
Site135
1.0
0.5
-
A
C
D
E
F
G
H
I
K
L
M
N
P
Q
R
S
T
V
W
Y
ikalian
opean
astern
nalaya
berian

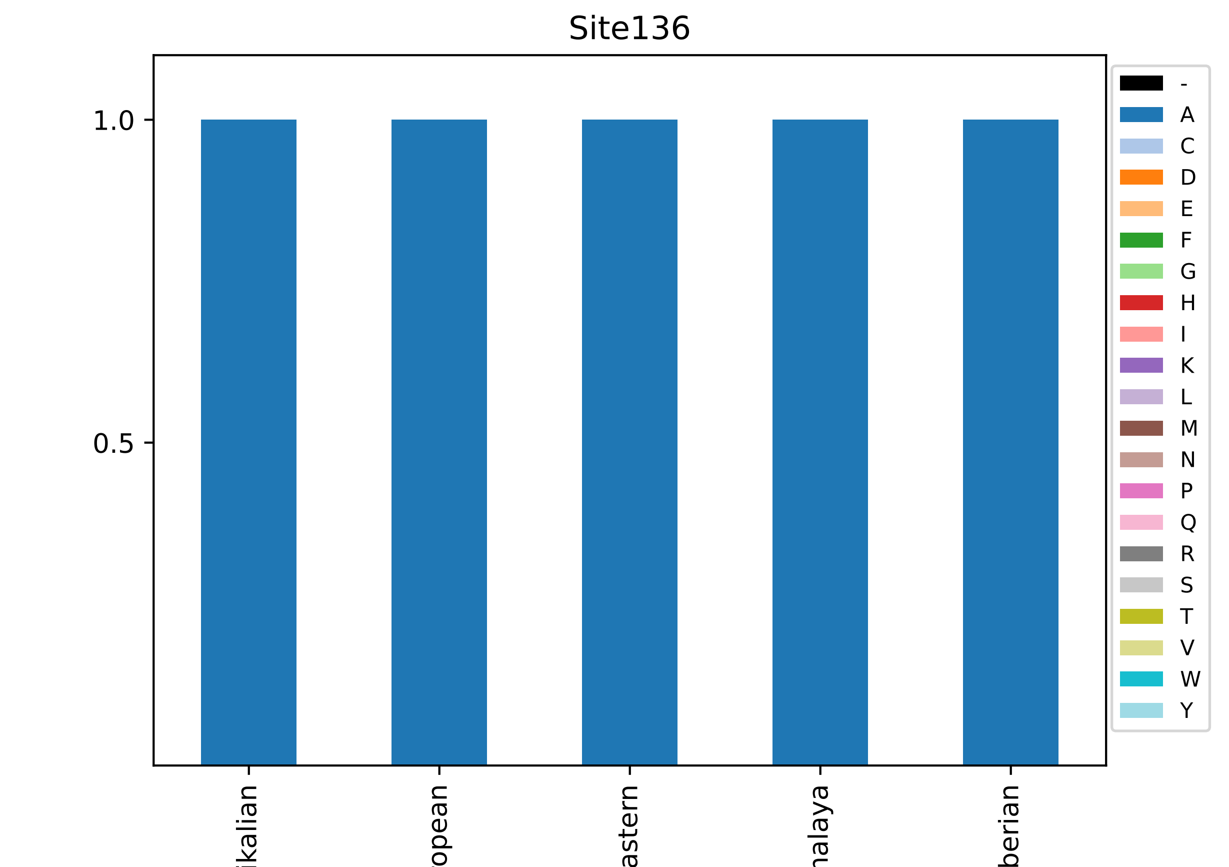
Site136
1.0
0.5
-
A
C
D
E
F
G
H
I
K
L
M
N
P
Q
R
S
T
V
W
Y
ikalian
opean
astern
nalaya
berian

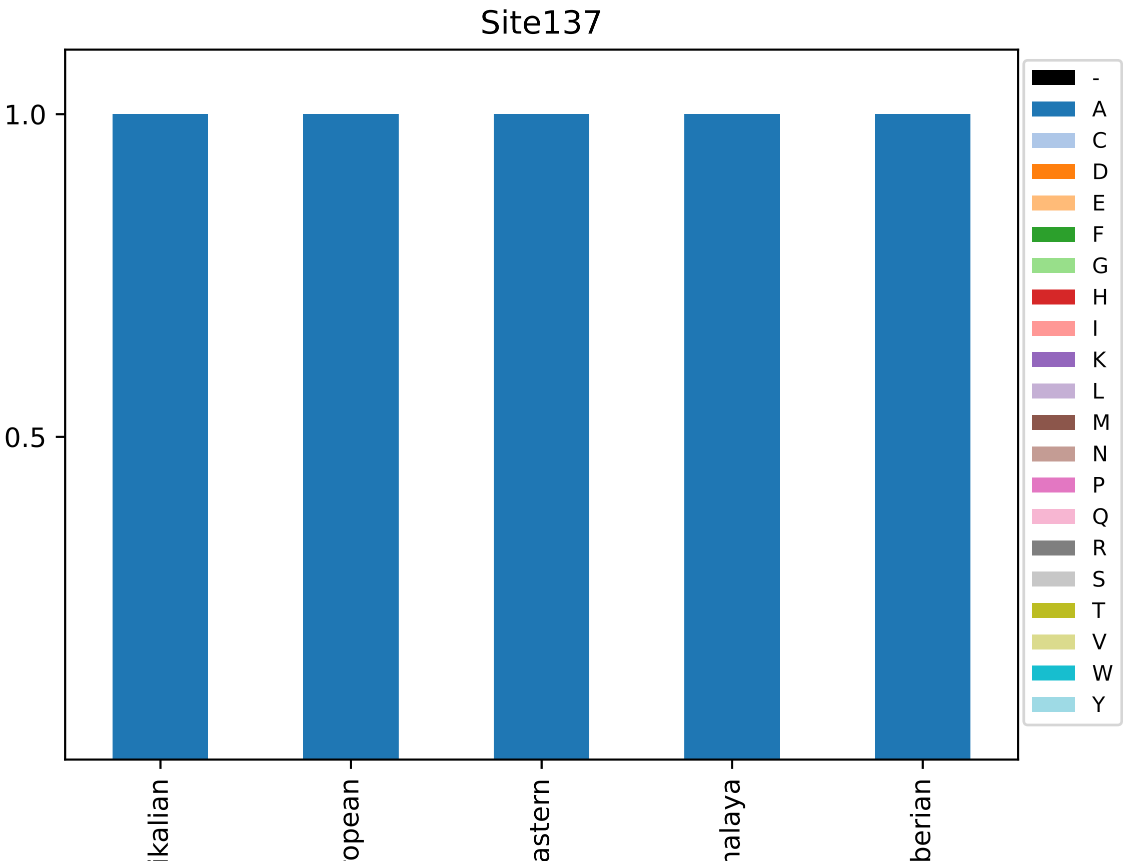
Site137
1.0
0.5
-
A
C
D
E
F
G
H
I
K
L
M
N
P
Q
R
S
T
V
W
Y

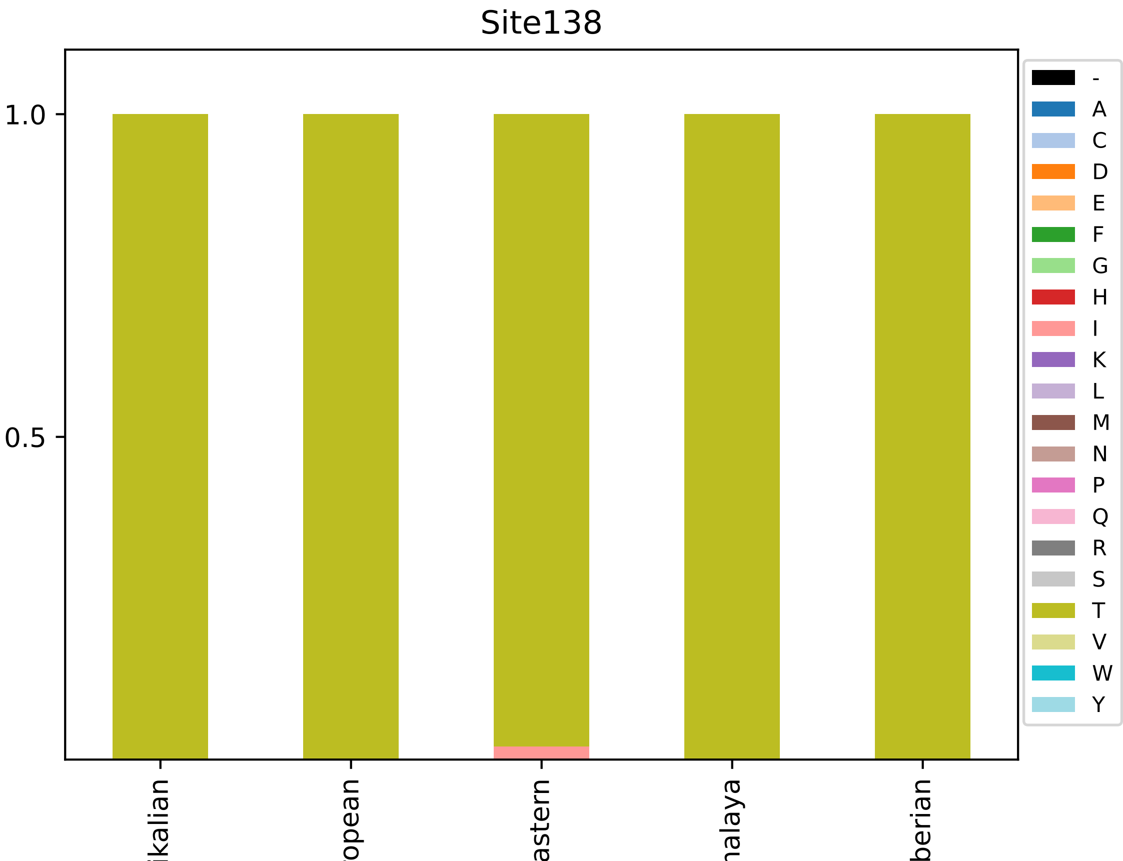

Site138
1.0
0.5
-
A
C
D
E
F
G
H
I
K
L
M
N
P
Q
R
S
T
V
W
Y
ikalian
ropean
astern
nalaya
berian

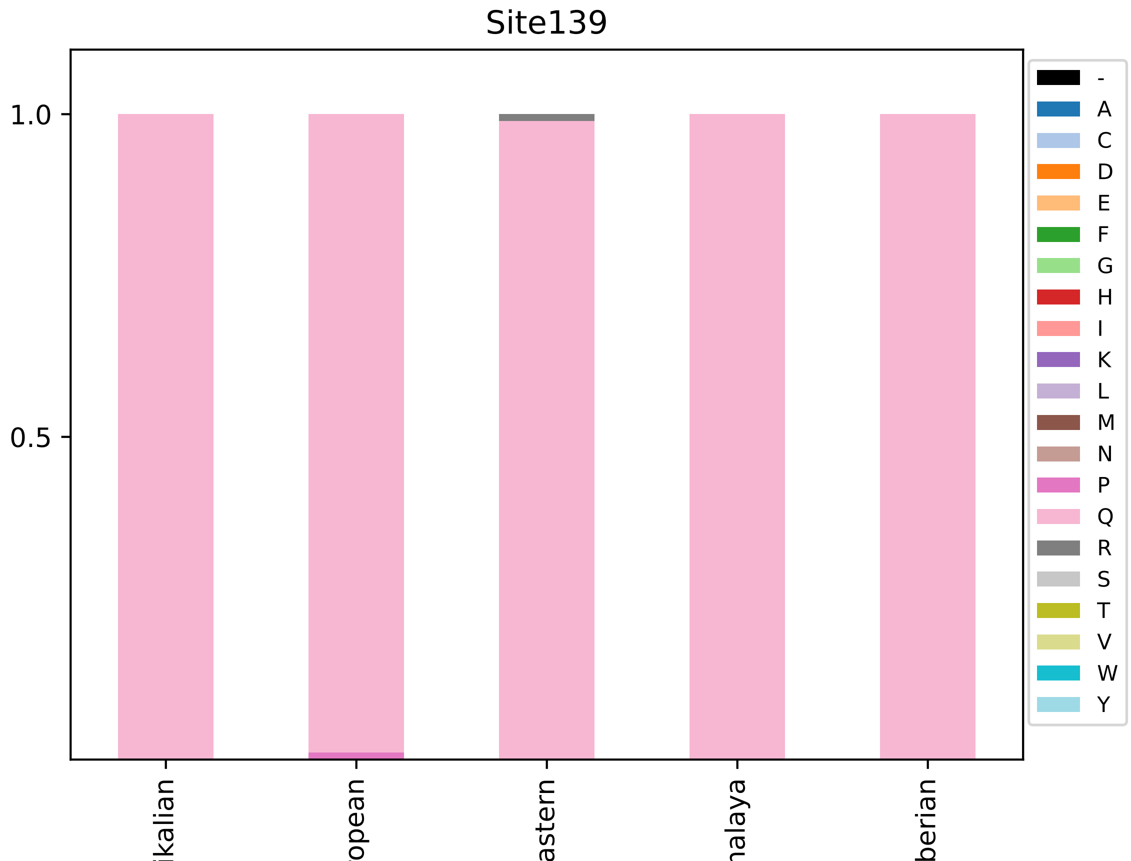
Site139
1.0
0.5
ikalian
opean
astern
nalaya
berian
-
A
C
D
E
F
G
H
I
K
L
M
N
P
Q
R
S
T
V
W
Y

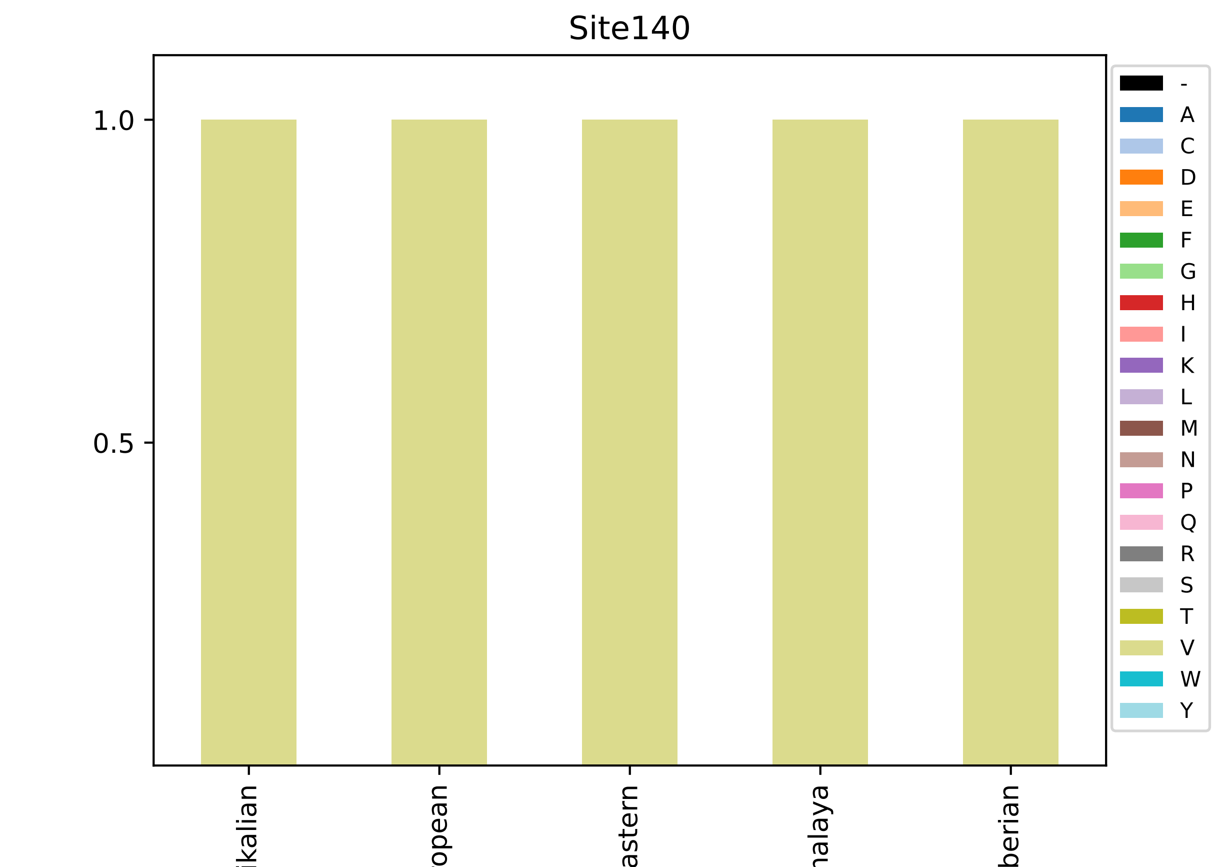

Site140
1.0
0.5
-
A
C
D
E
F
G
H
I
K
L
M
N
P
Q
R
S
T
V
W
Y
ikalian
opean
astern
nalaya
berian

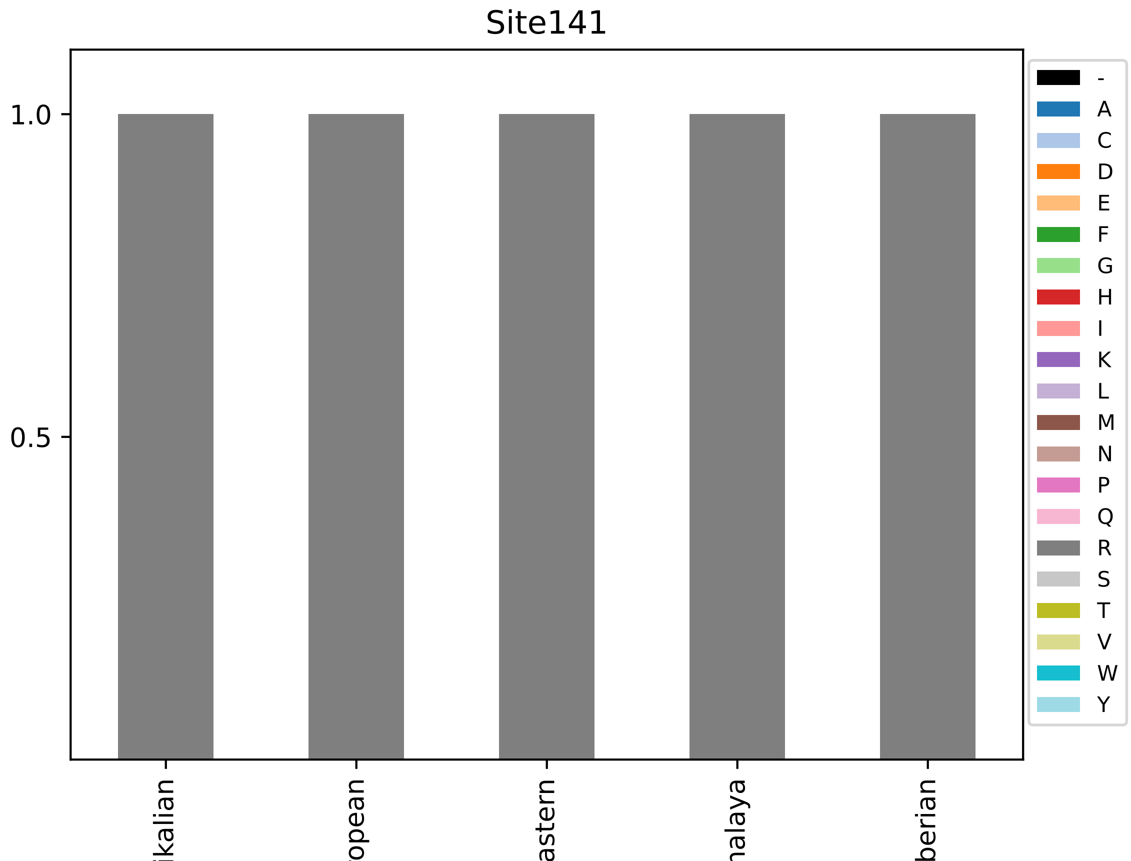

Site141
1.0
0.5
-
A
C
D
E
F
G
H
I
K
L
M
N
P
Q
R
S
T
V
W
Y
ikalian
opean
astern
nalaya
berian

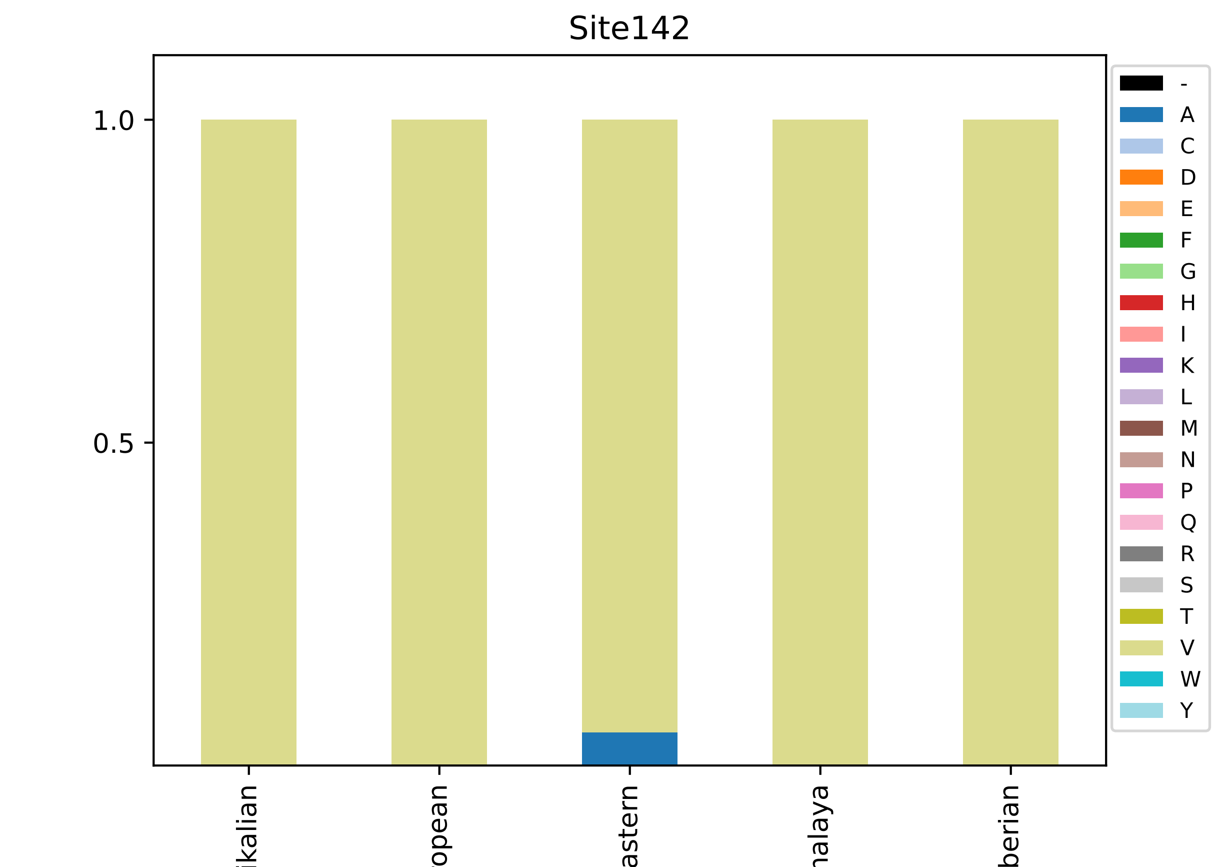

Site142
1.0
0.5
-
A
C
D
E
F
G
H
I
K
L
M
N
P
Q
R
S
T
V
W
Y

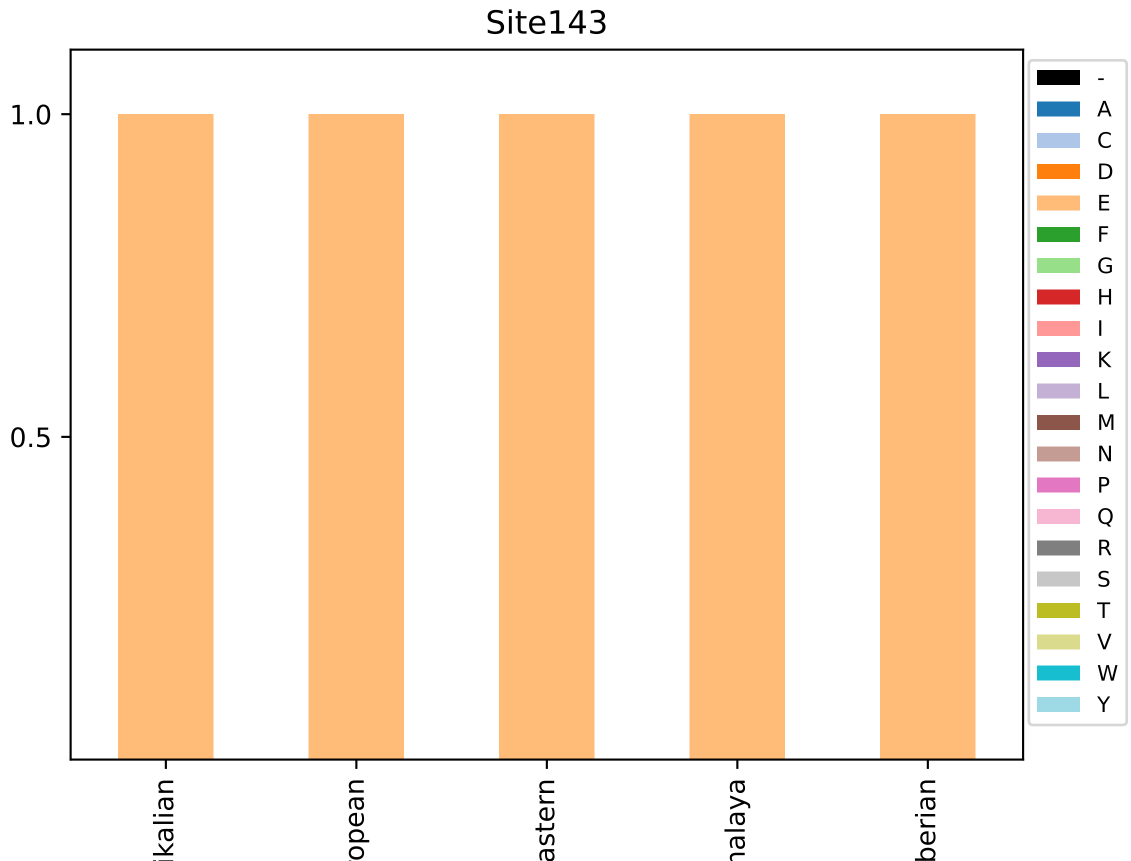
Site143
1.0
0.5
-
A
C
D
E
F
G
H
I
K
L
M
N
P
Q
R
S
T
V
W
Y
ikalian
opean
astern
nalaya
berian

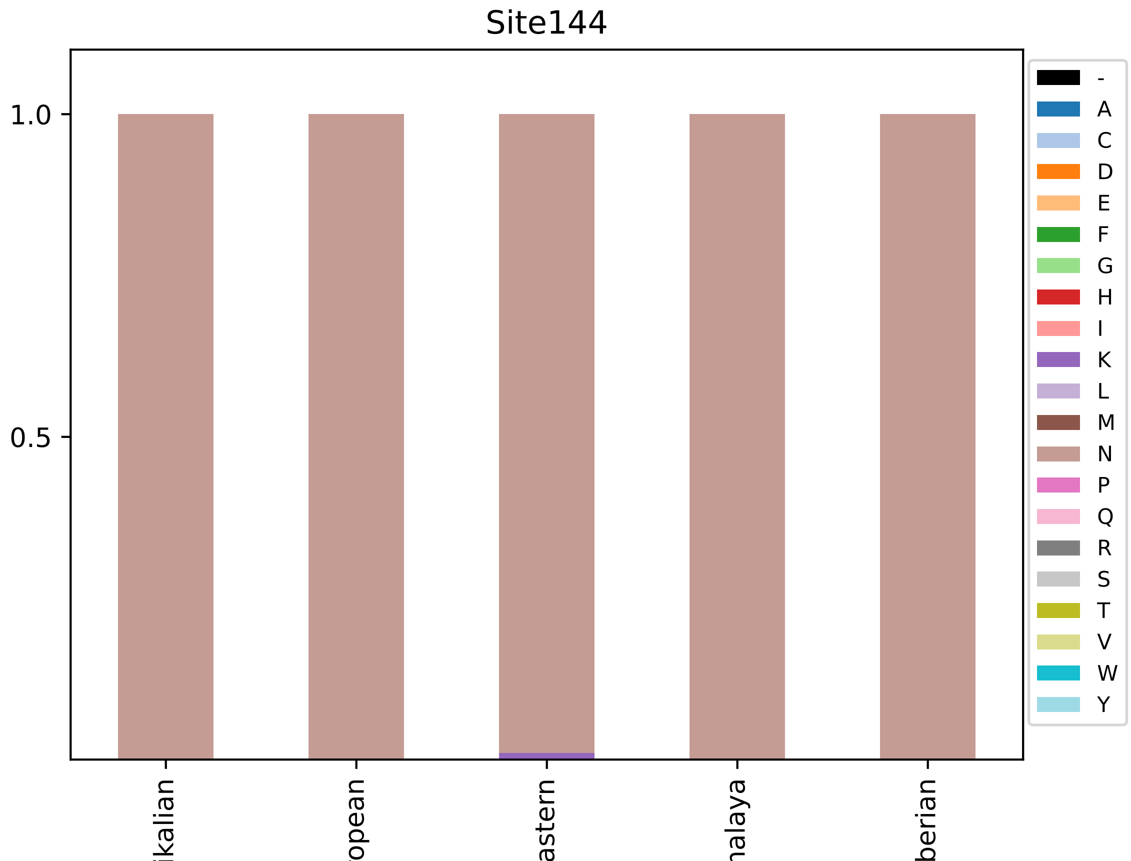

Site144
1.0
0.5
-
A
C
D
E
F
G
H
I
K
L
M
N
P
Q
R
S
T
V
W
Y
ikalian
opean
astern
nalaya
berian

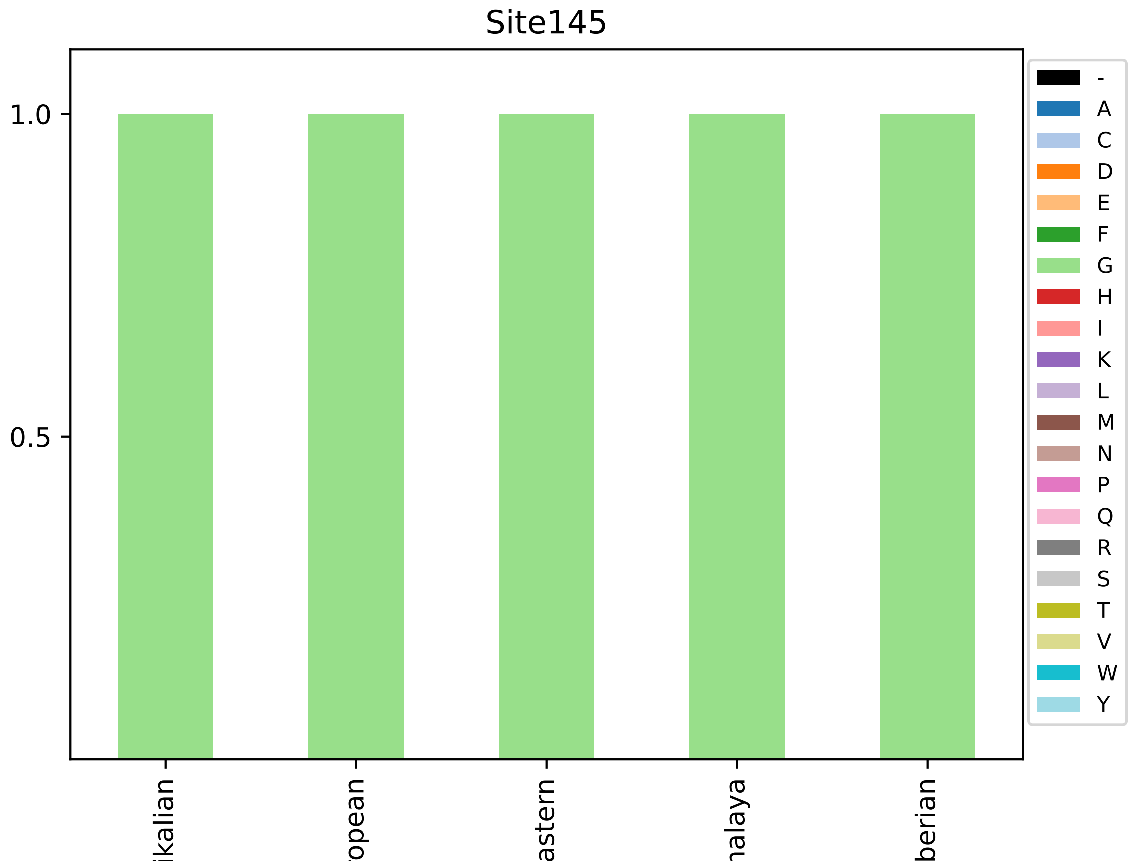
Site145
1.0
0.5
-
A
C
D
E
F
G
H
I
K
L
M
N
P
Q
R
S
T
V
W
Y
ikalian
opean
astern
nalaya
berian

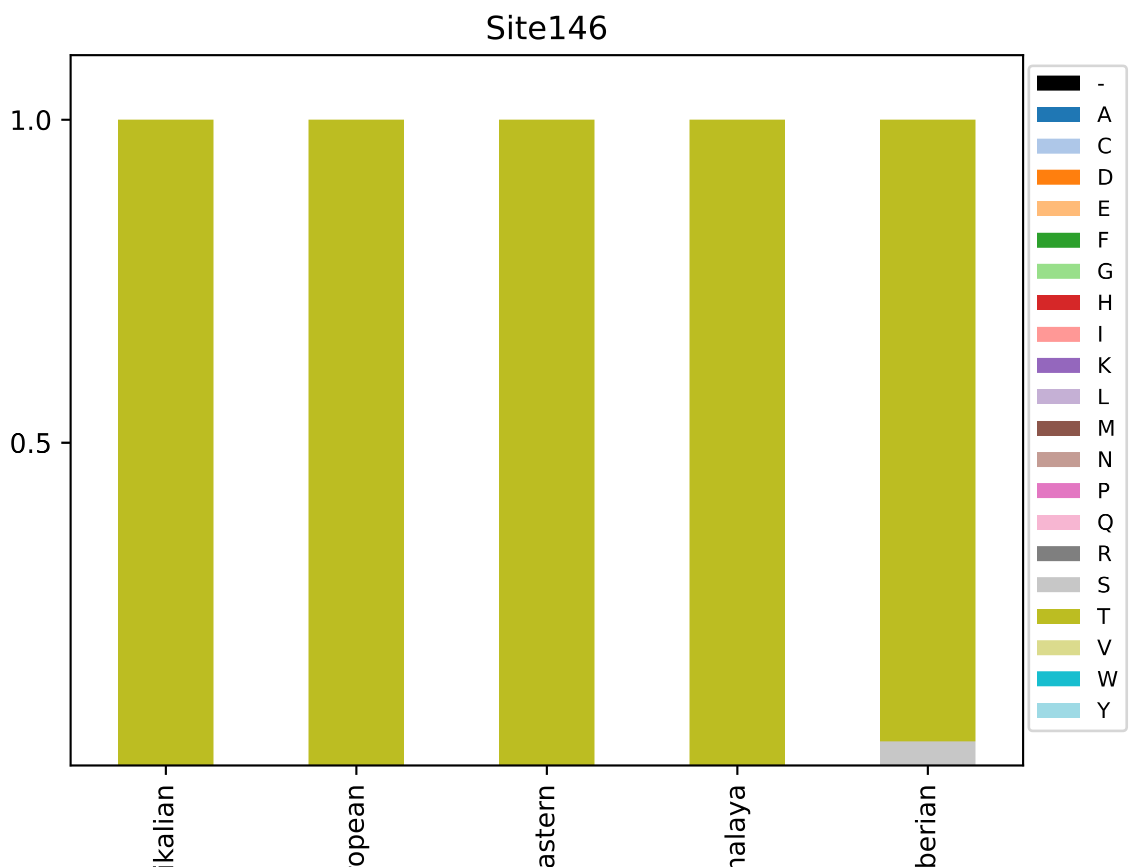
Site146
1.0
0.5
-
A
C
D
E
F
G
H
I
K
L
M
N
P
Q
R
S
T
V
W
Y
ikalian
opean
astern
nalaya
berian

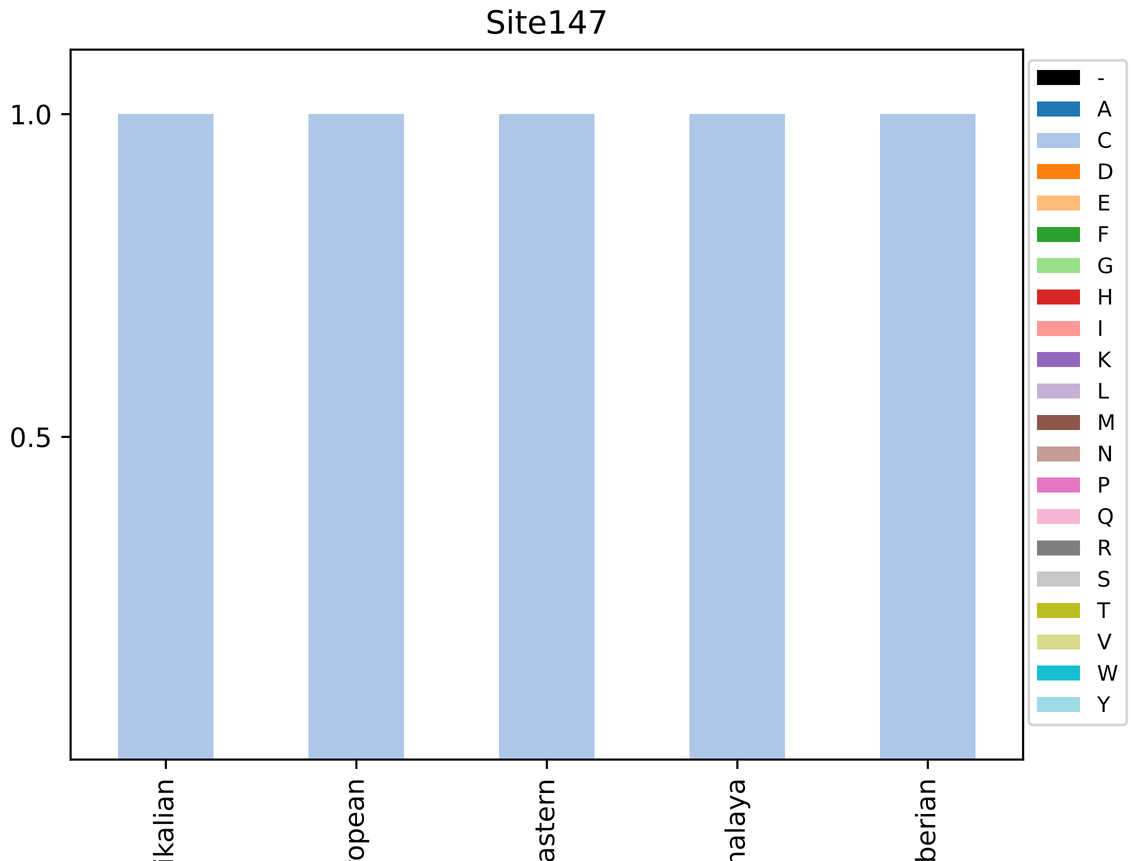
Site147
1.0
0.5
-
A
C
D
E
F
G
H
I
K
L
M
N
P
Q
R
S
T
V
W
Y
ikalian
opean
astern
nalaya
berian

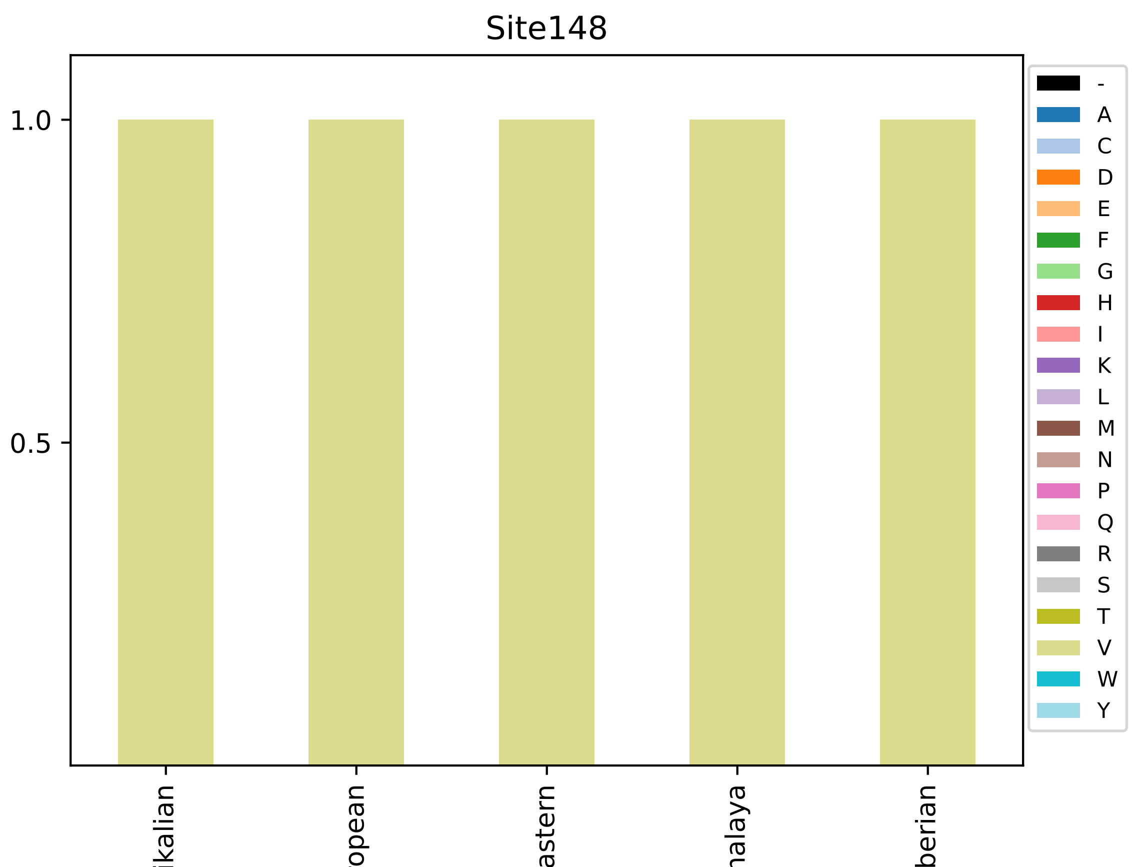

Site148
1.0
0.5
-
A
C
D
E
F
G
H
I
K
L
M
N
P
Q
R
S
T
V
W
Y
ikalian
opean
astern
nalaya
berian

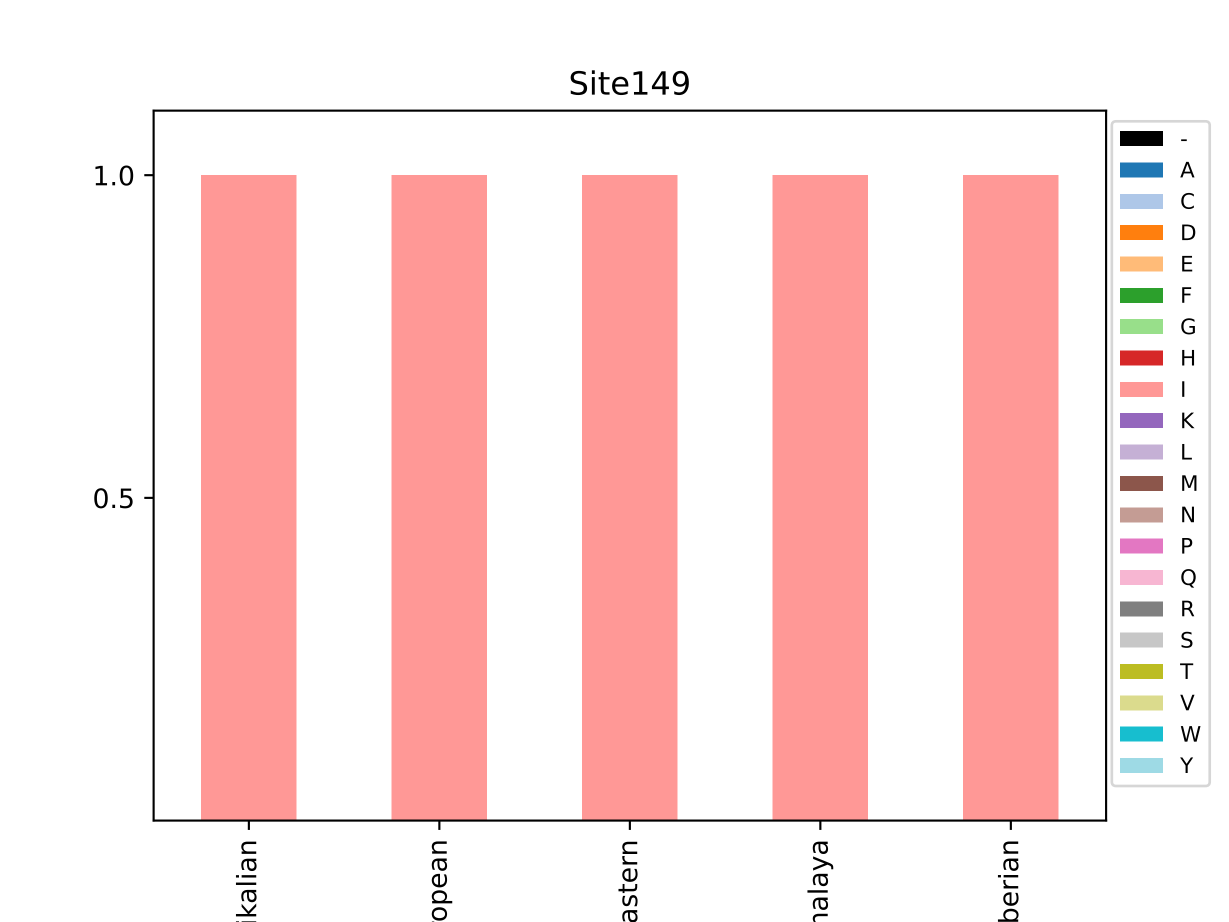
Site149
1.0
0.5
-
A
C
D
E
F
G
H
I
K
L
M
N
P
Q
R
S
T
V
W
Y
ikalian
opean
astern
nalaya
berian

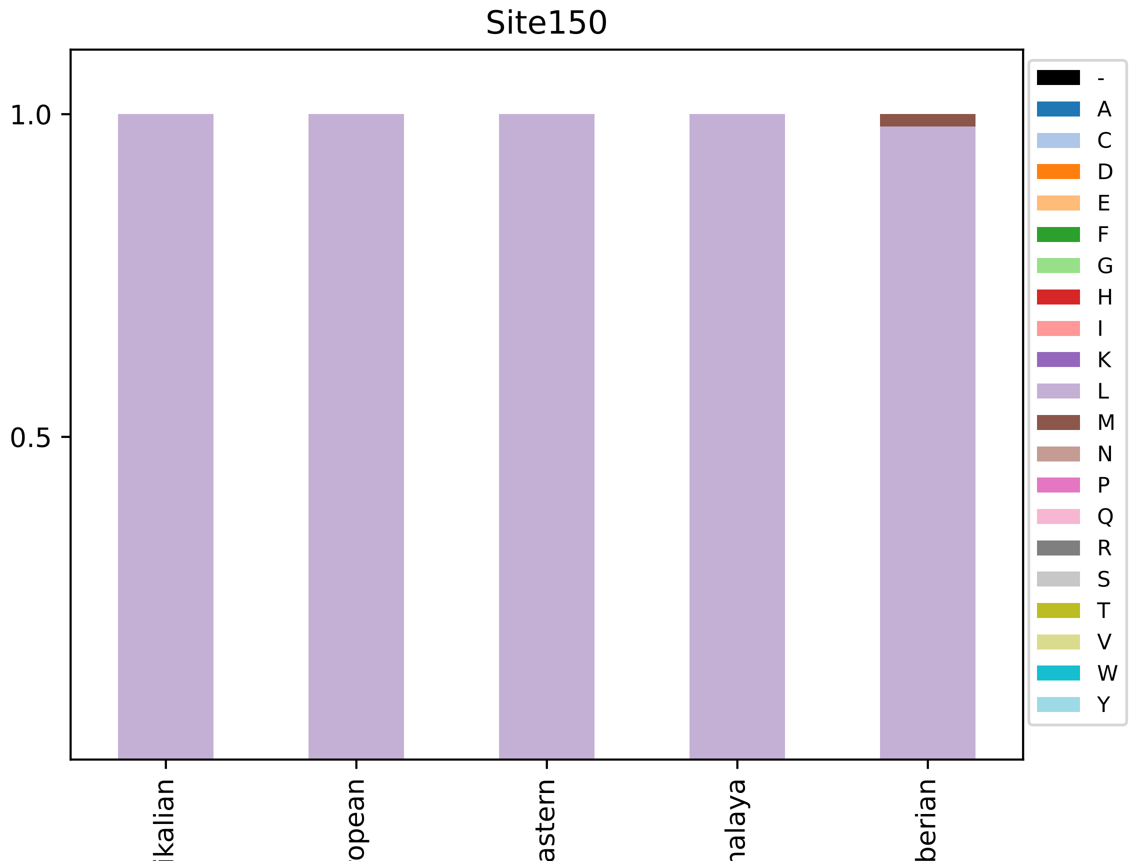

Site150
1.0
0.5
-
A
C
D
E
F
G
H
I
K
L
M
N
P
Q
R
S
T
V
W
Y
ikalian
opean
astern
nalaya
berian

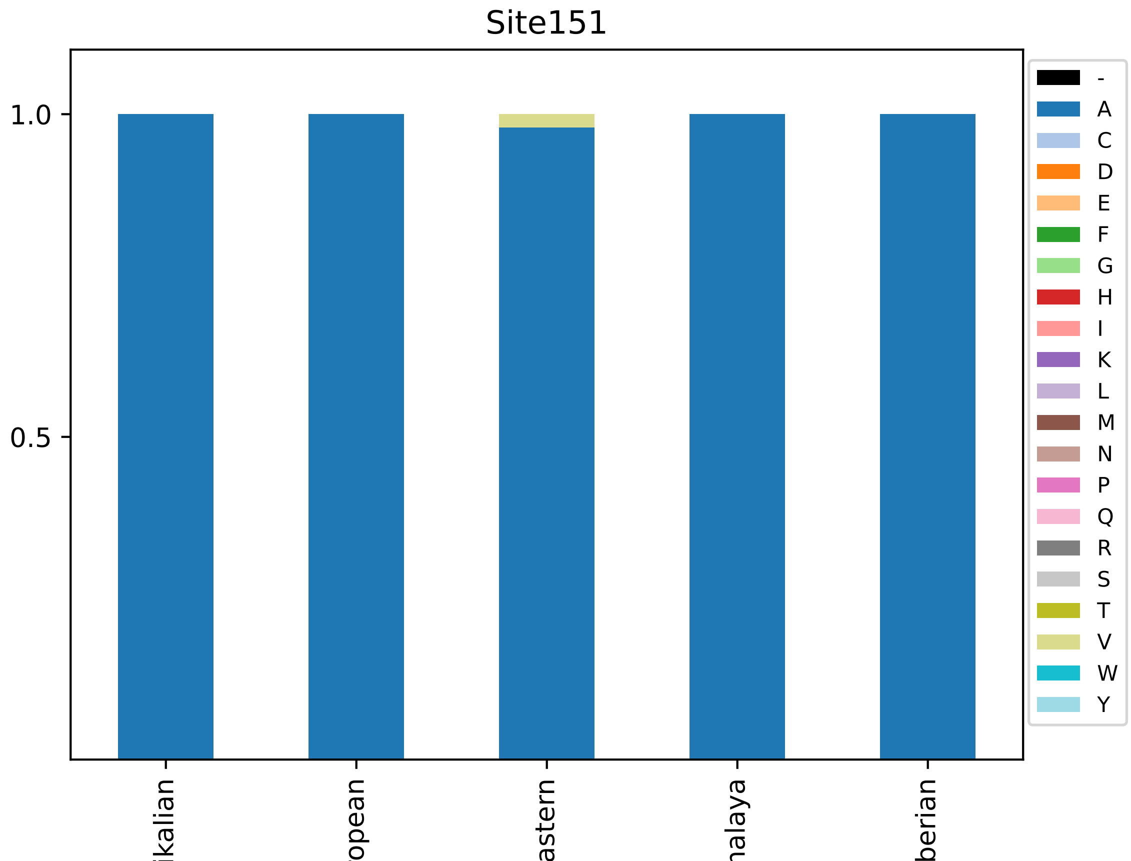
Site151
1.0
0.5
-
A
C
D
E
F
G
H
I
K
L
M
N
P
Q
R
S
T
V
W
Y
ikalian
opean
astern
nalaya
berian

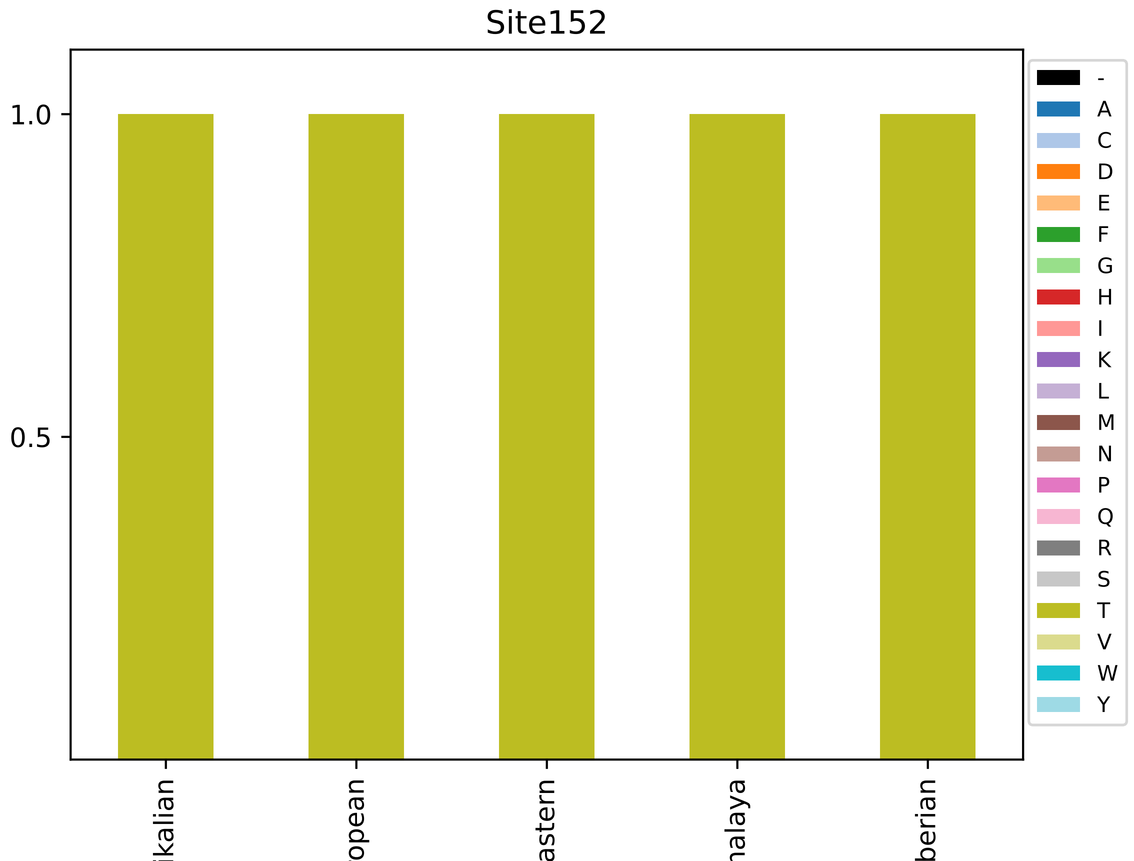

Site152
1.0
0.5
-
A
C
D
E
F
G
H
I
K
L
M
N
P
Q
R
S
T
V
W
Y
ikalian
opean
astern
nalaya
berian

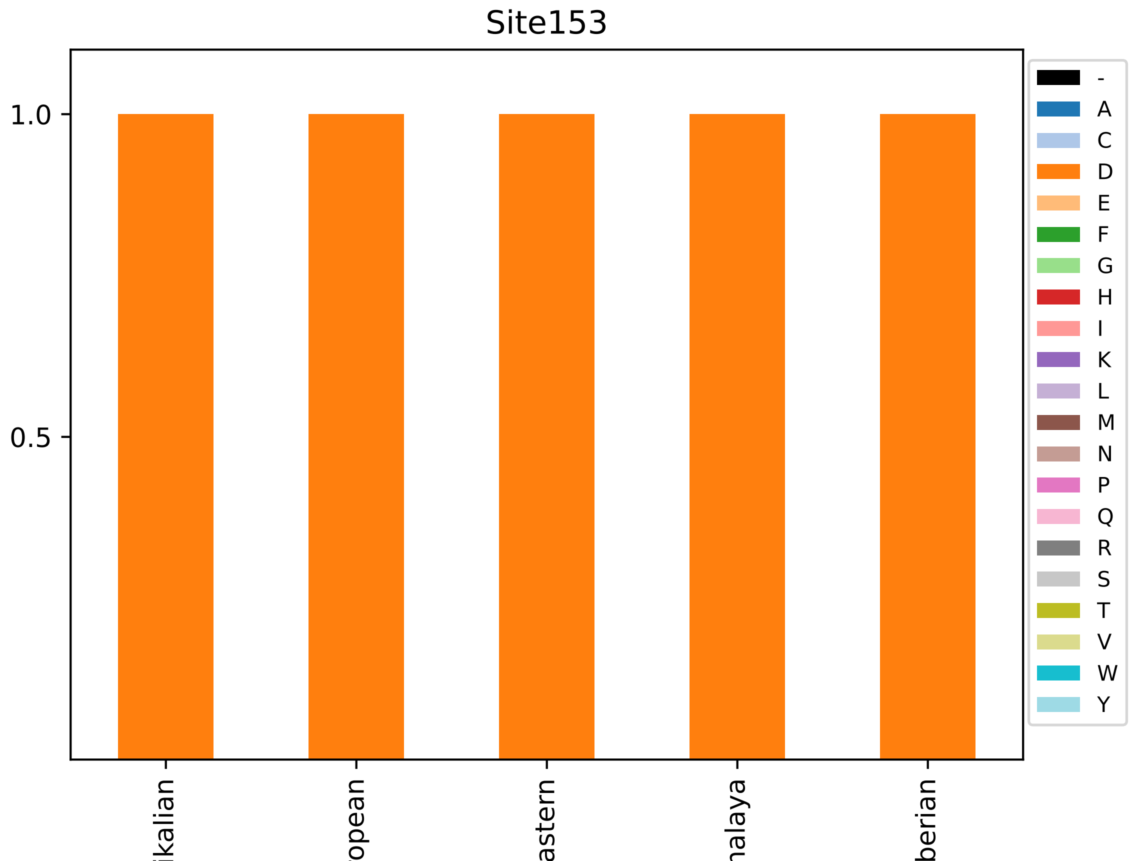

Site153
1.0
0.5
-
A
C
D
E
F
G
H
I
K
L
M
N
P
Q
R
S
T
V
W
Y
ikalian
ropean
astern
nalaya
berian

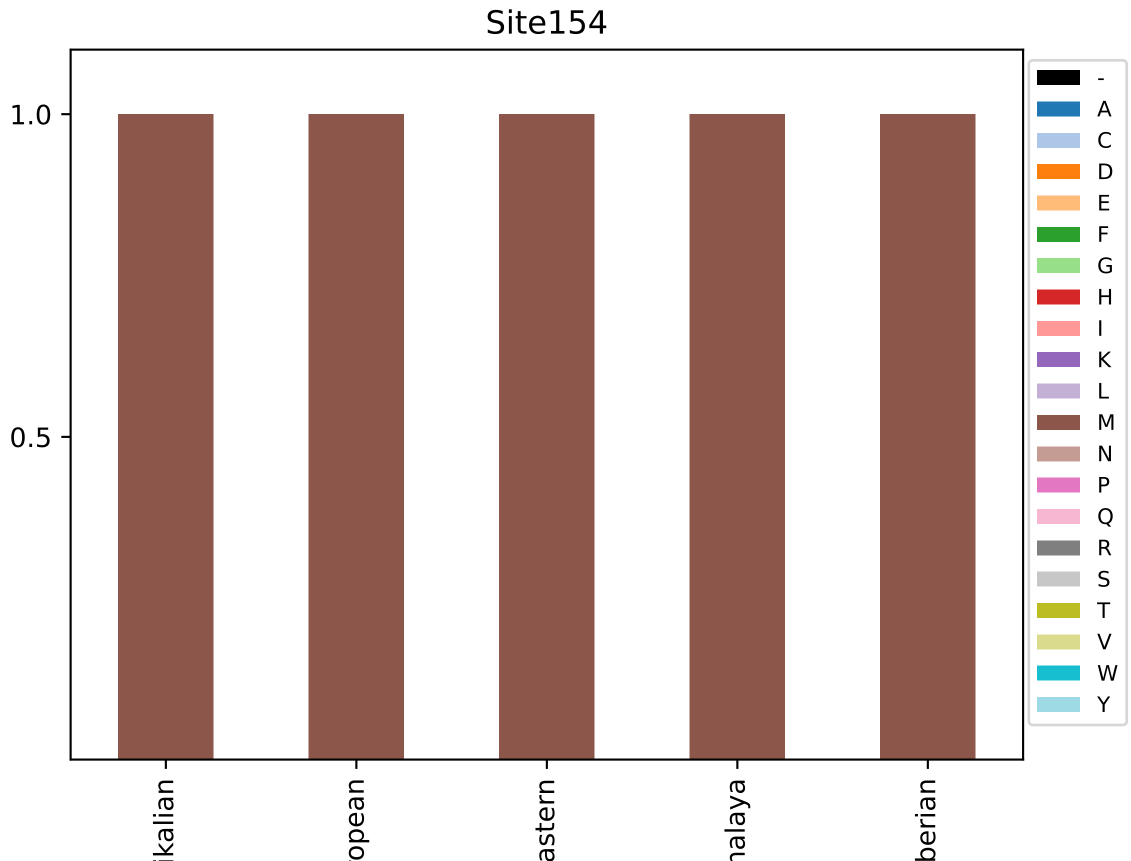
Site154
1.0
0.5
-
A
C
D
E
F
G
H
I
K
L
M
N
P
Q
R
S
T
V
W
Y
ikalian
opean
astern
nalaya
berian

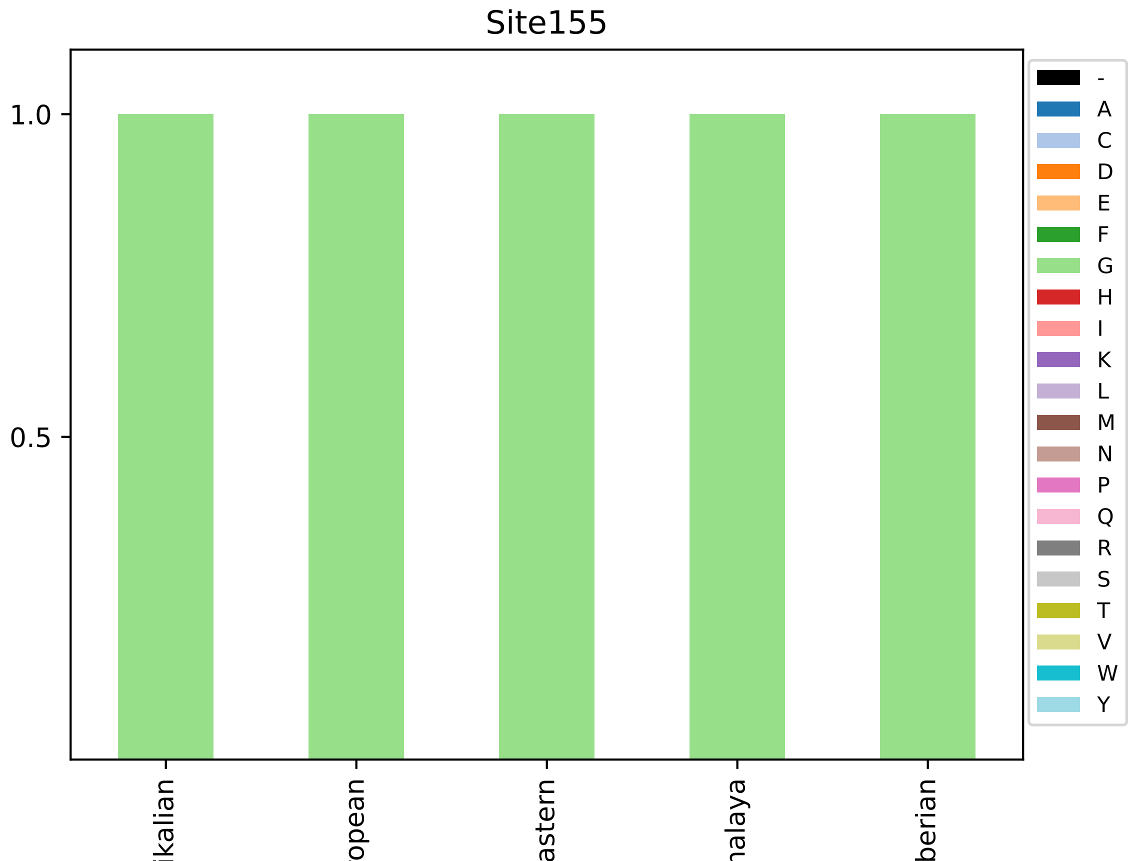
Site155
1.0
0.5
ikalian
opean
astern
nalaya
berian
-
A
C
D
E
F
G
H
I
K
L
M
N
P
Q
R
S
T
V
W
Y

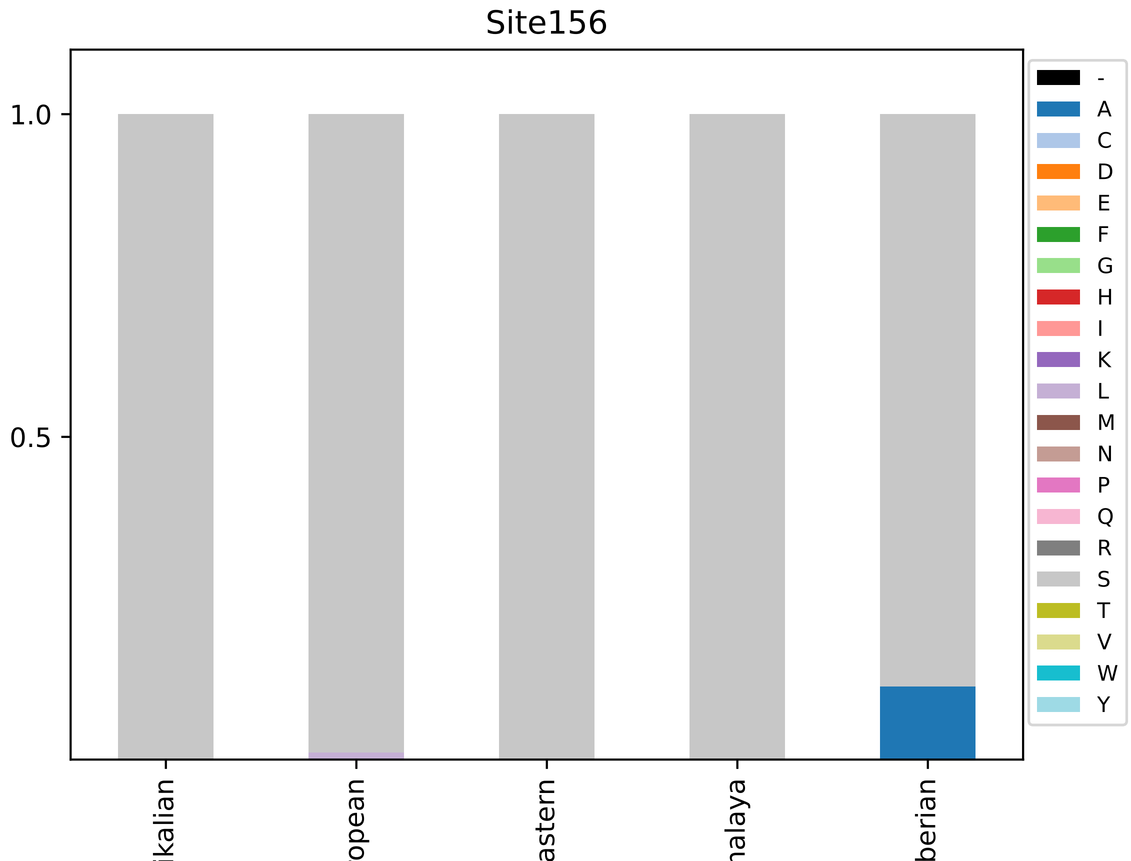

Site156
1.0
0.5
-
A
C
D
E
F
G
H
I
K
L
M
N
P
Q
R
S
T
V
W
Y
ikalian
opean
astern
nalaya
berian

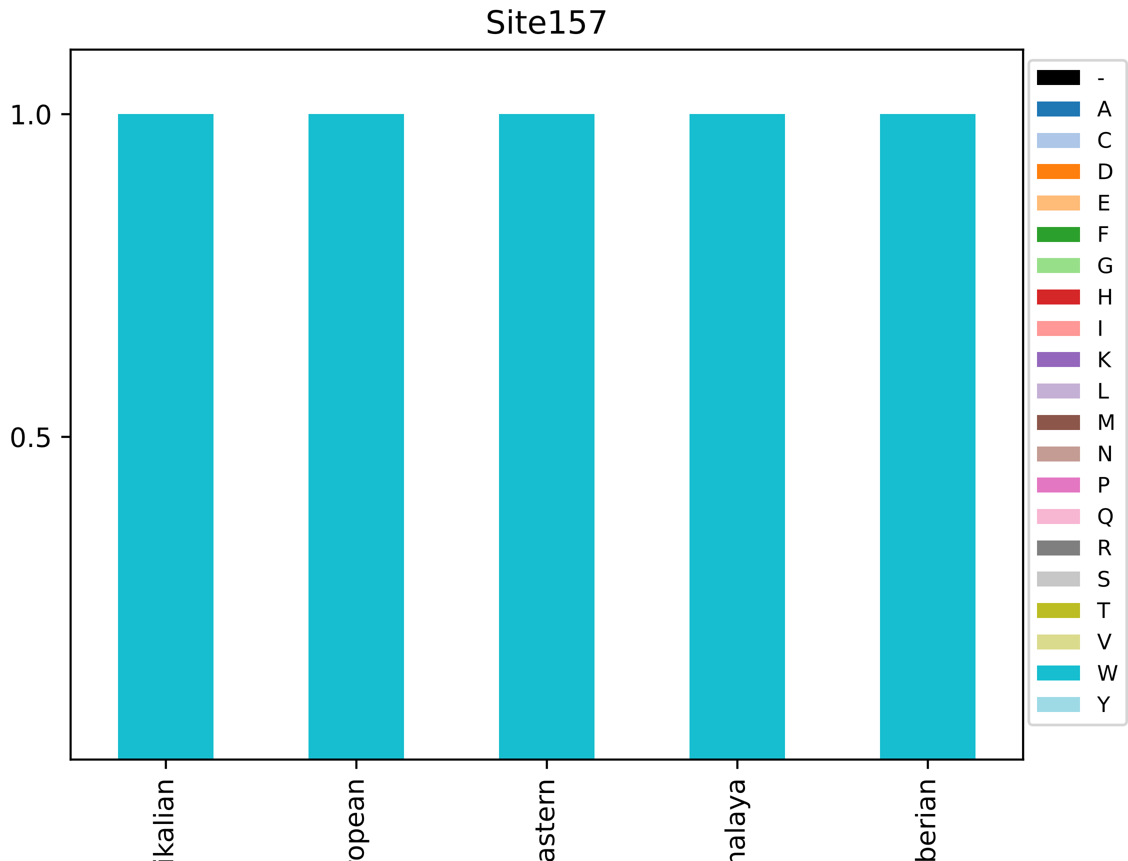
Site157
1.0
0.5
-
A
C
D
E
F
G
H
I
K
L
M
N
P
Q
R
S
T
V
W
Y
ikalian
opean
astern
nalaya
berian

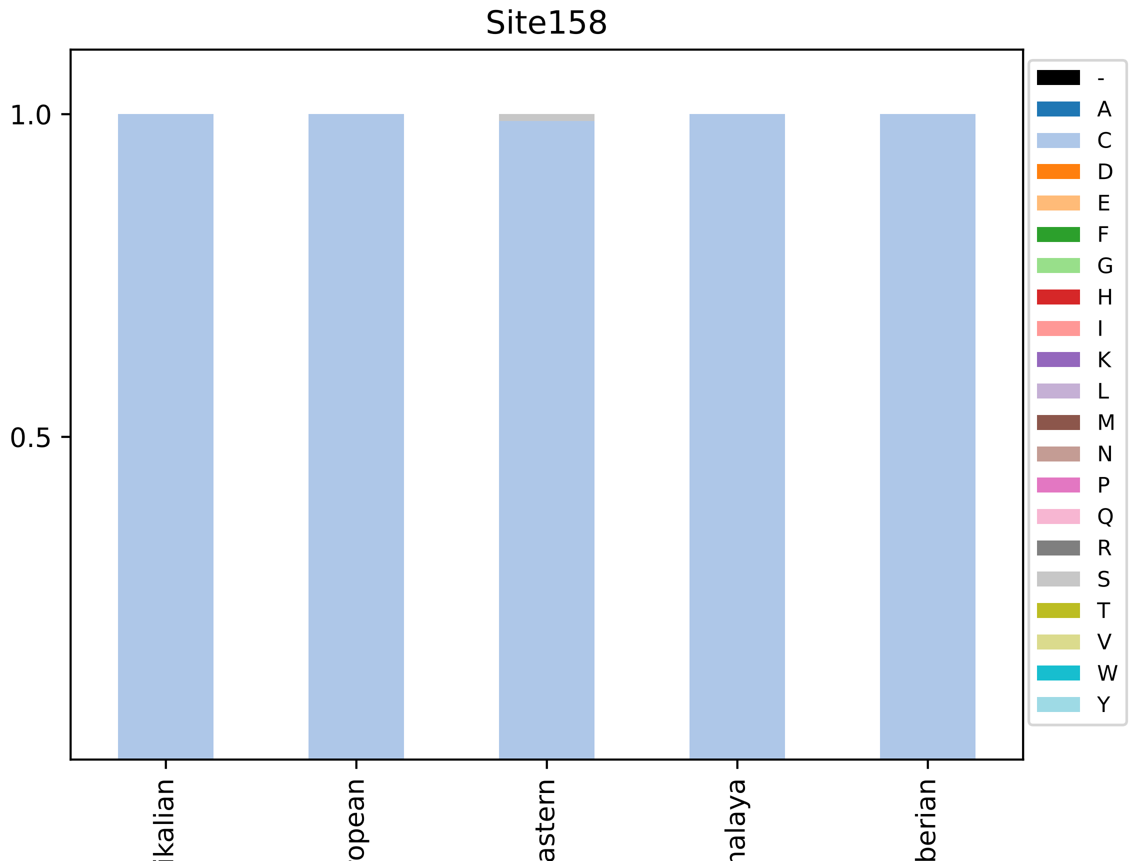
Site158
1.0
0.5
-
A
C
D
E
F
G
H
I
K
L
M
N
P
Q
R
S
T
V
W
Y
ikalian
opean
astern
nalaya
berian

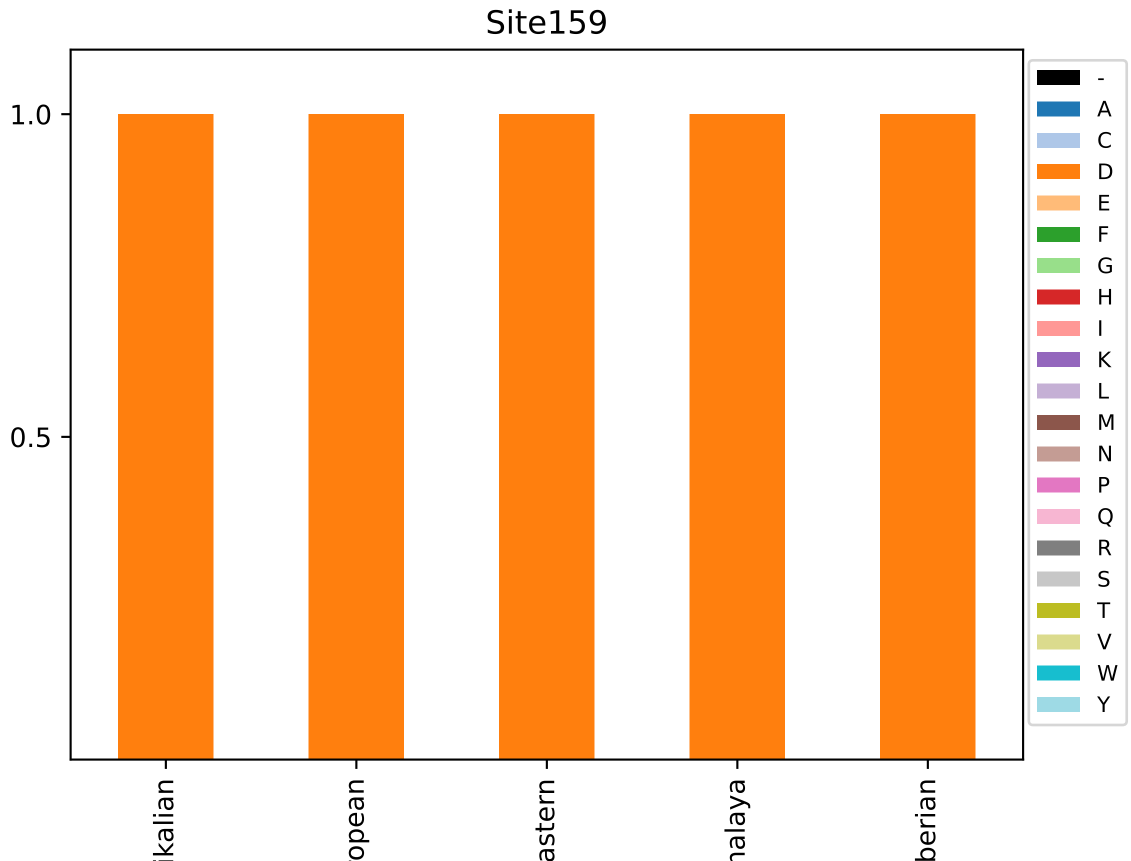
Site159
1.0
0.5
-
A
C
D
E
F
G
H
I
K
L
M
N
P
Q
R
S
T
V
W
Y
ikalian
opean
astern
nalaya
berian

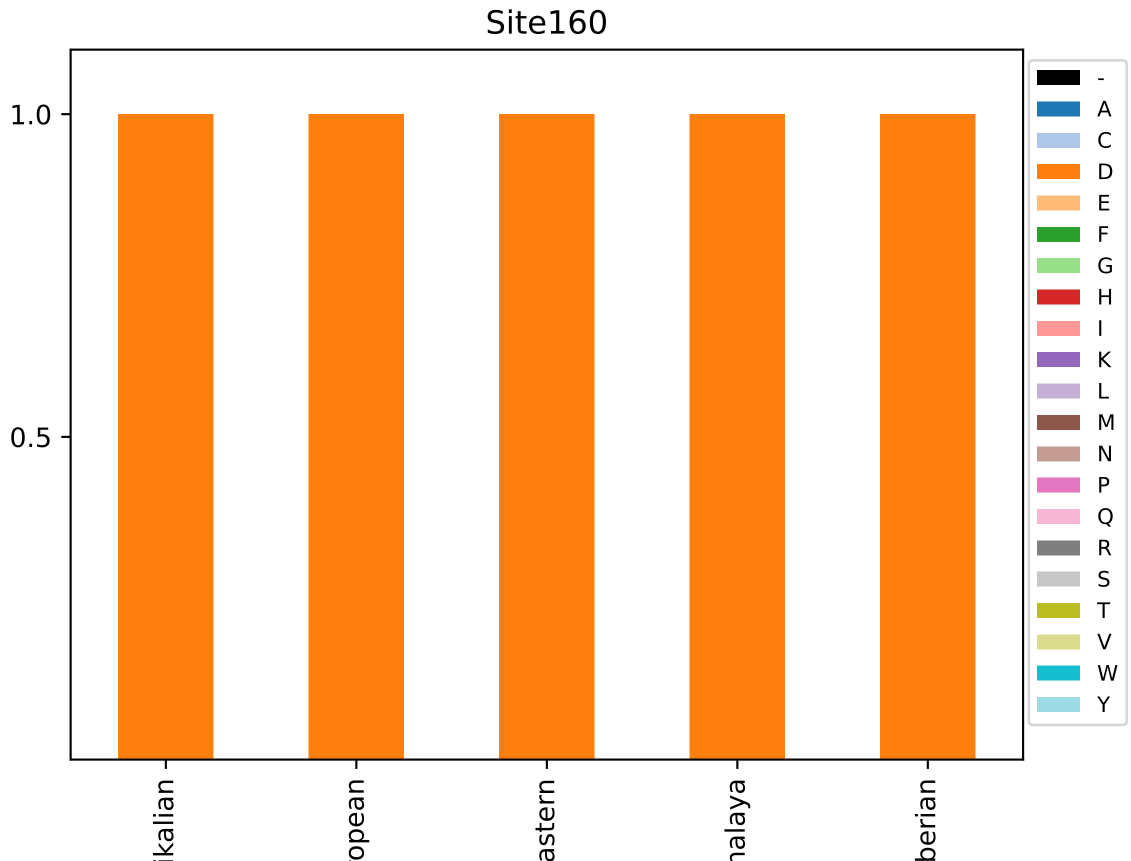
Site160
1.0
0.5
ikalian
opean
astern
nalaya
berian
-
A
C
D
E
F
G
H
I
K
L
M
N
P
Q
R
S
T
V
W
Y

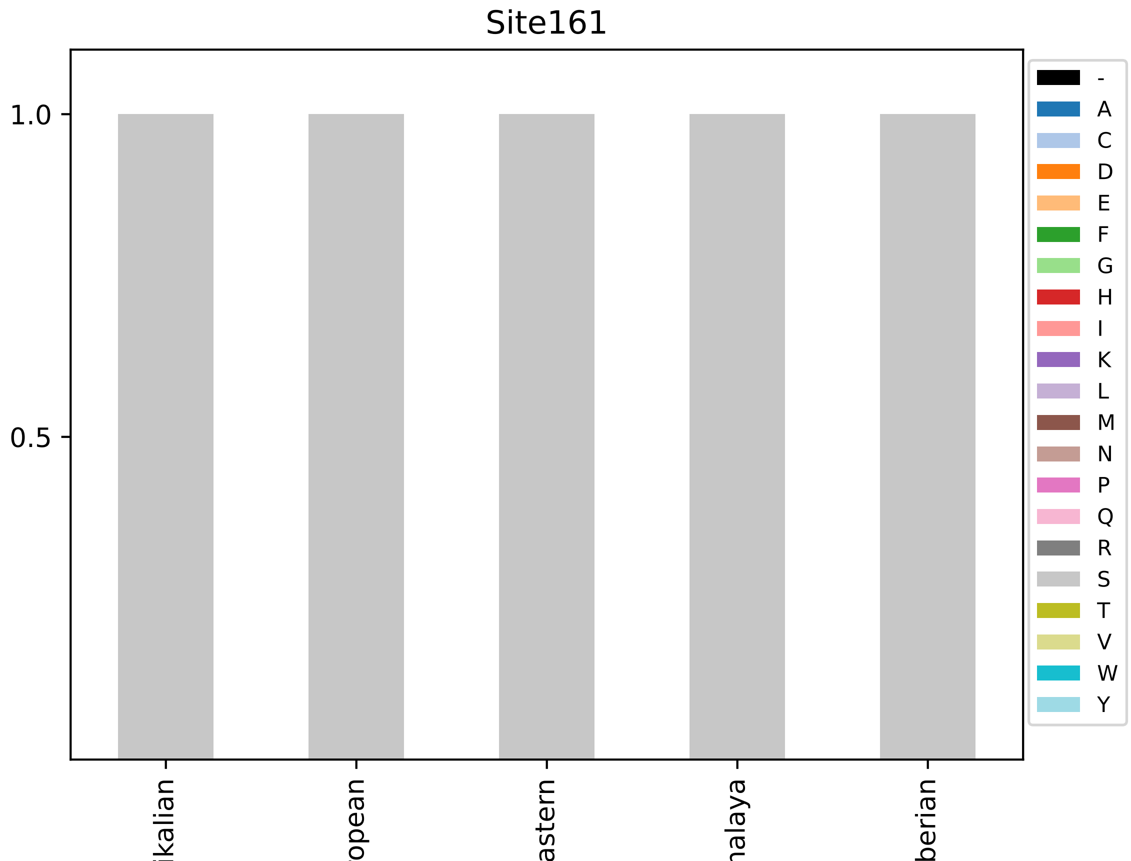
Site161
1.0
0.5
-
A
C
D
E
F
G
H
I
K
L
M
N
P
Q
R
S
T
V
W
Y

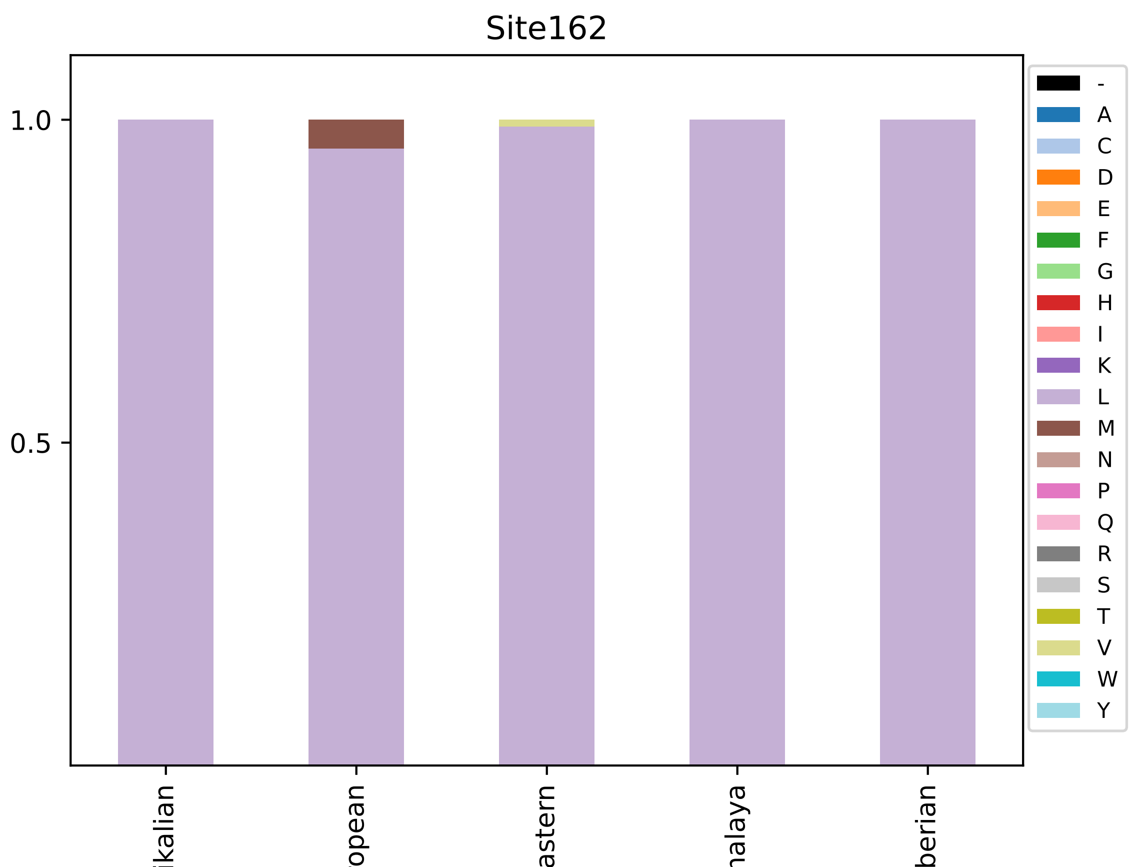
Site162
1.0
0.5
ikalian
opean
astern
nalaya
berian
-
A
C
D
E
F
G
H
I
K
L
M
N
P
Q
R
S
T
V
W
Y

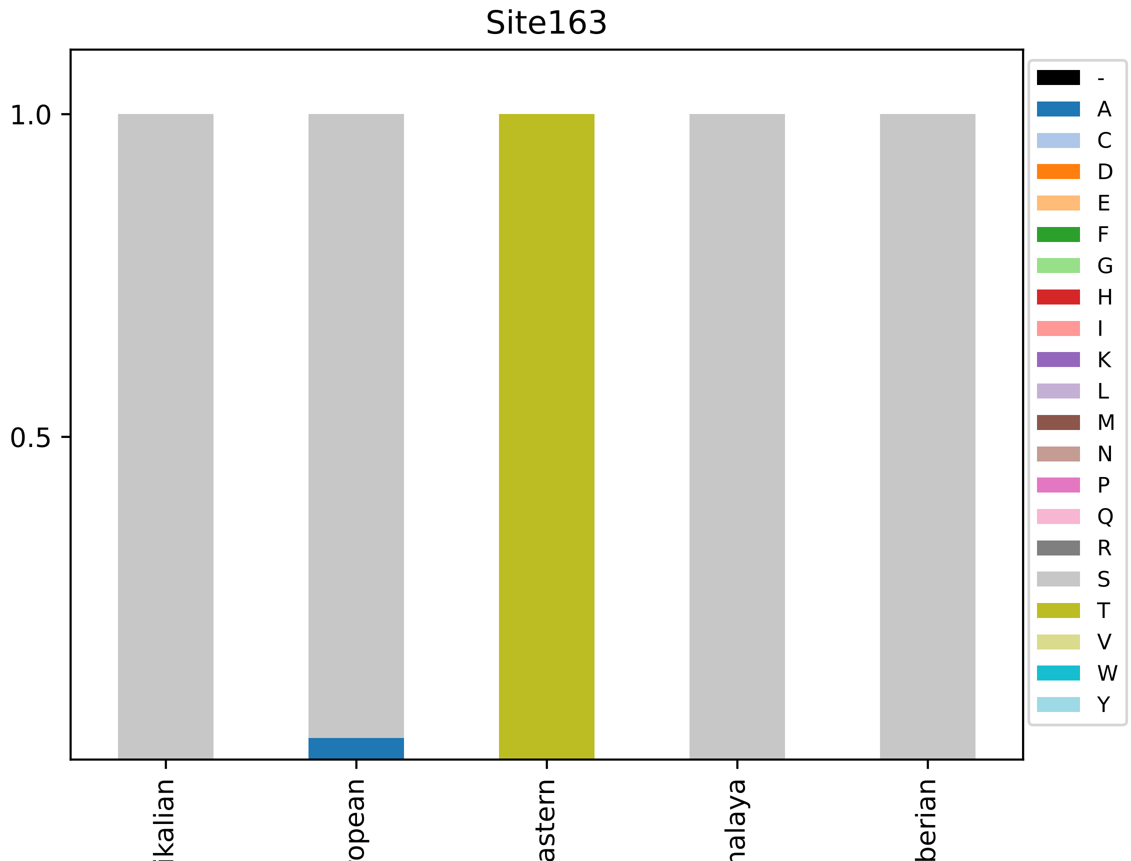
Site163
1.0
0.5
-
A
C
D
E
F
G
H
I
K
L
M
N
P
Q
R
S
T
V
W
Y
ikalian
opean
astern
nalaya
berian

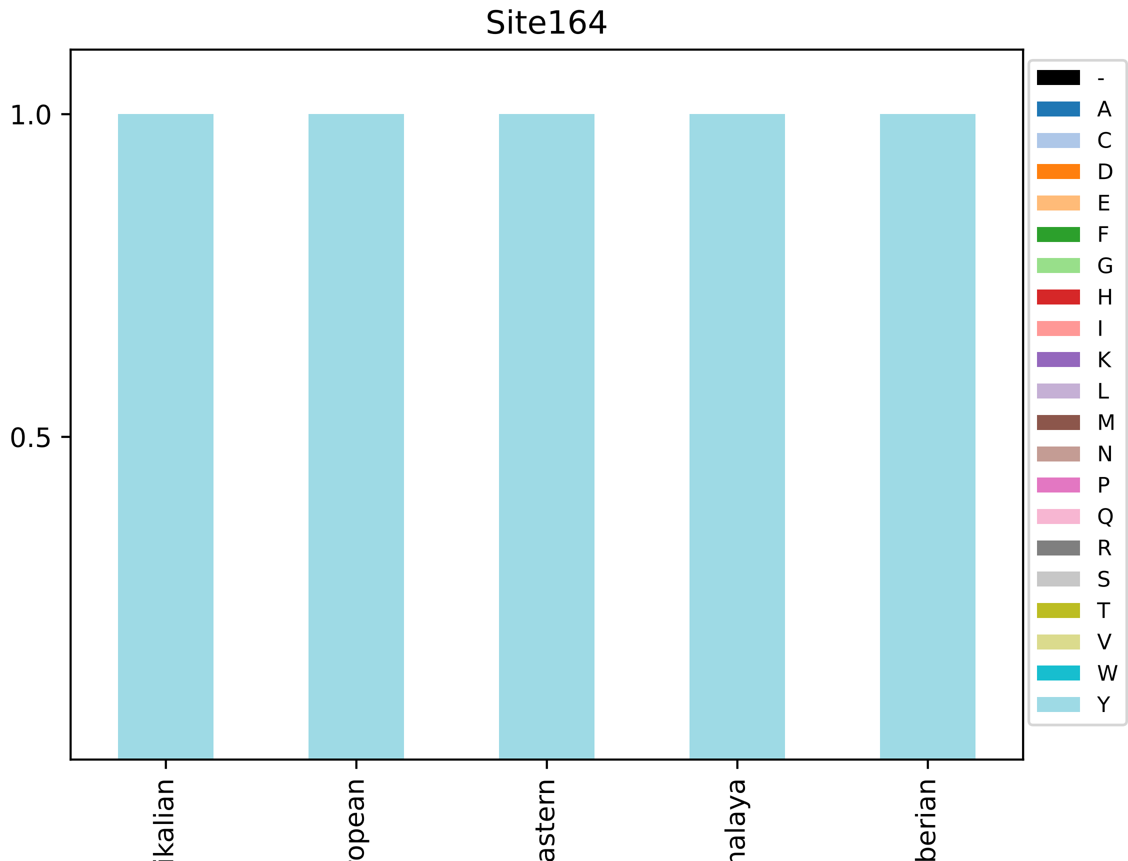
Site164
1.0
0.5
-
A
C
D
E
F
G
H
I
K
L
M
N
P
Q
R
S
T
V
W
Y
ikalian
opean
astern
nalaya
berian

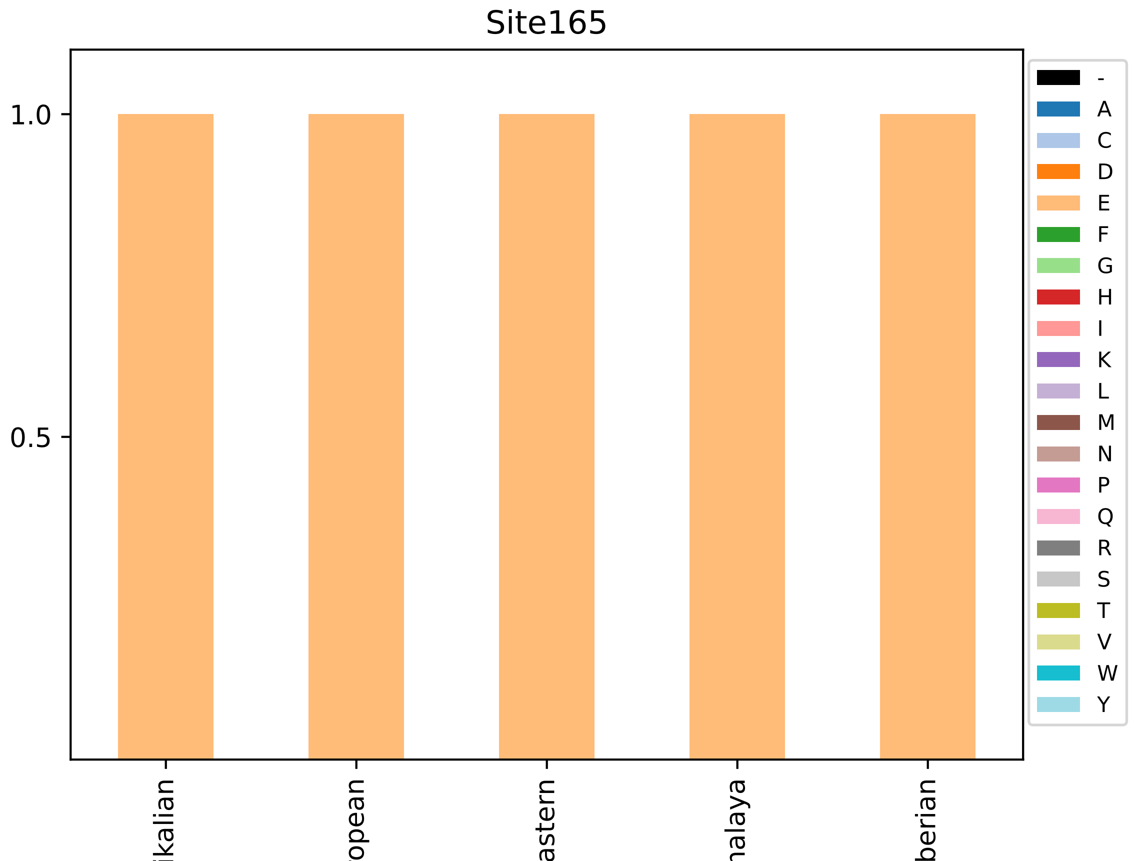

Site165
1.0
0.5
-
A
C
D
E
F
G
H
I
K
L
M
N
P
Q
R
S
T
V
W
Y
ikalian
opean
astern
nalaya
berian

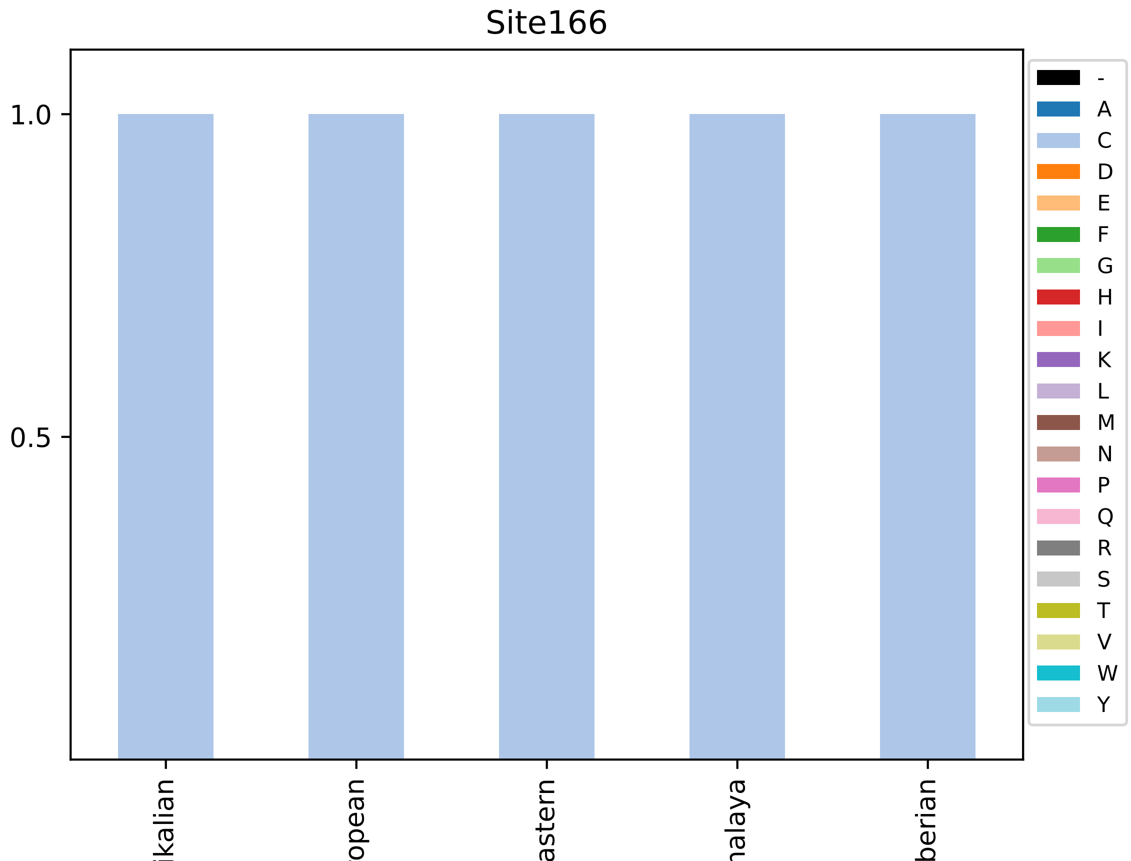
Site166
1.0
0.5
-
A
C
D
E
F
G
H
I
K
L
M
N
P
Q
R
S
T
V
W
Y
ikalian
opean
astern
nalaya
berian

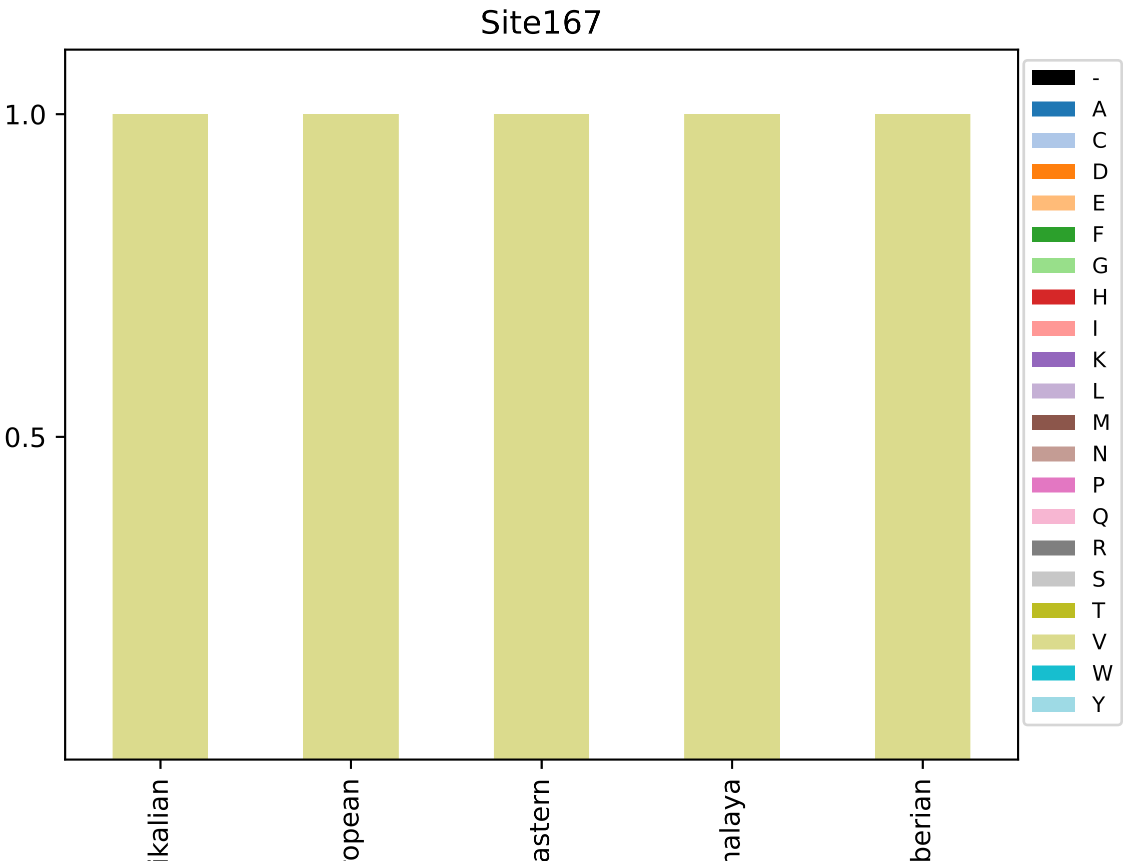

Site167
1.0
0.5
-
A
C
D
E
F
G
H
I
K
L
M
N
P
Q
R
S
T
V
W
Y
ikalian
opean
astern
nalaya
berian

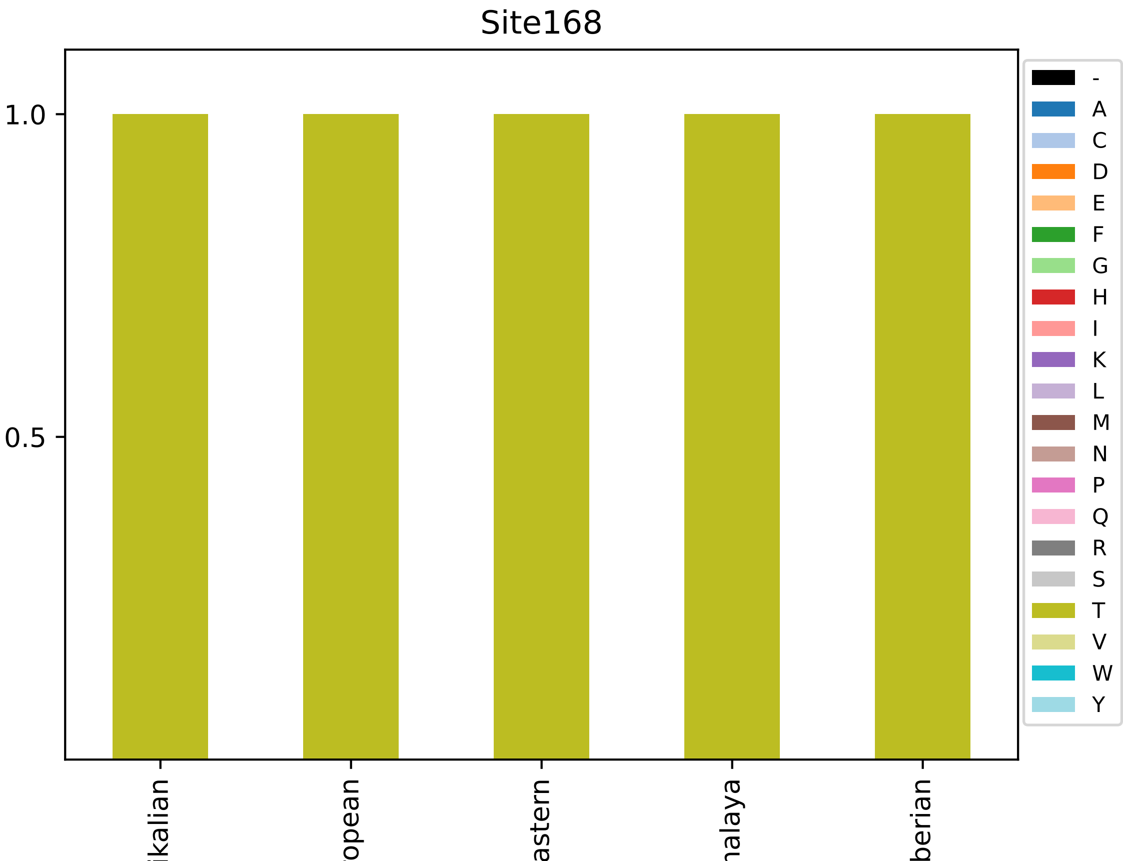
Site168
1.0
0.5
-
A
C
D
E
F
G
H
I
K
L
M
N
P
Q
R
S
T
V
W
Y
ikalian
opean
astern
nalaya
berian

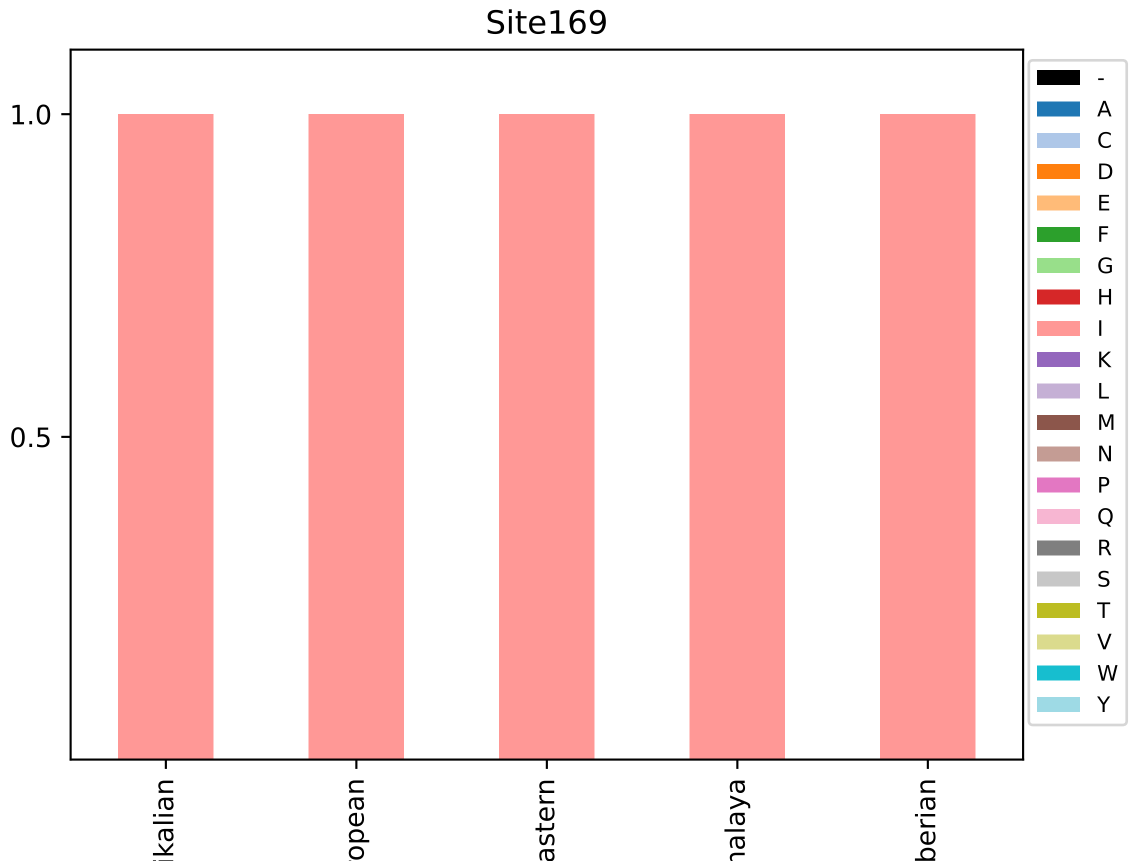
Site169
1.0
0.5
-
A
C
D
E
F
G
H
I
K
L
M
N
P
Q
R
S
T
V
W
Y
ikalian
opean
astern
nalaya
berian

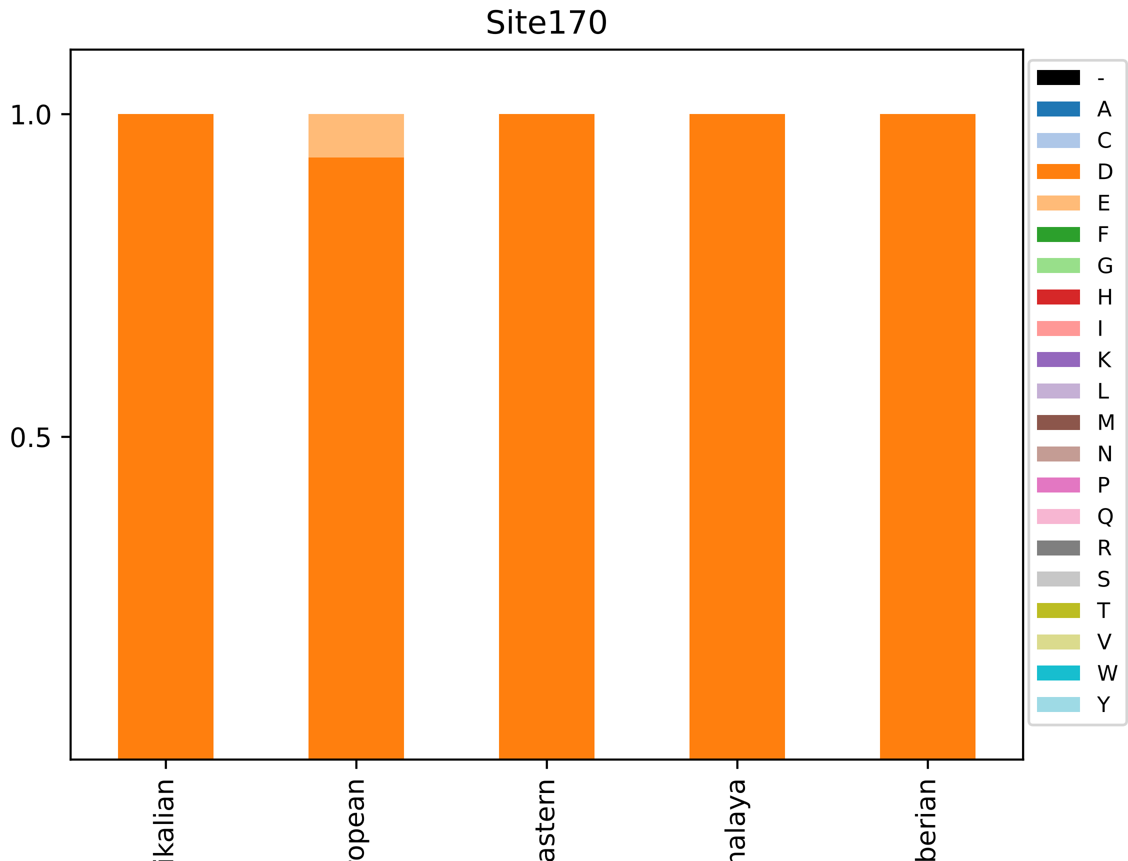
Site170
1.0
0.5
-
A
C
D
E
F
G
H
I
K
L
M
N
P
Q
R
S
T
V
W
Y
ikalian
opean
astern
nalaya
berian

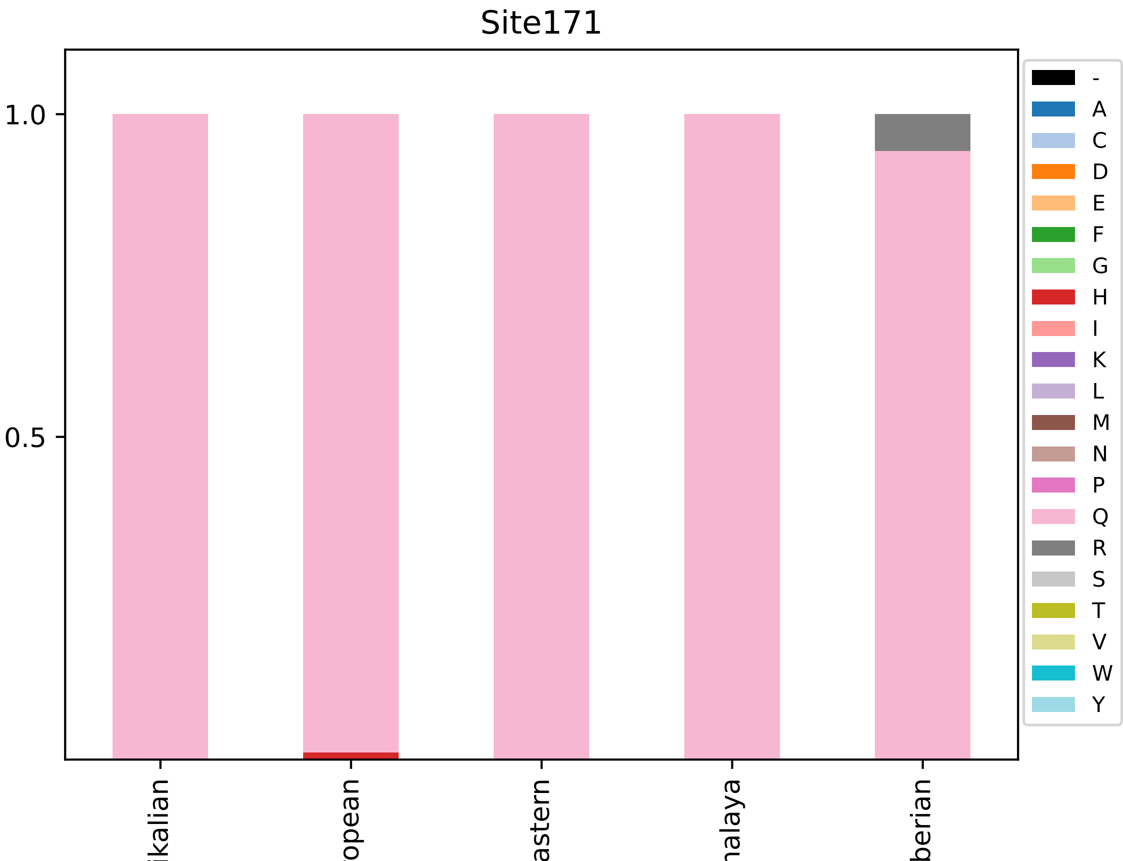
Site171
1.0
0.5
ikalian
ropean
astern
nalaya
berian
-
A
C
D
E
F
G
H
I
K
L
M
N
P
Q
R
S
T
V
W
Y

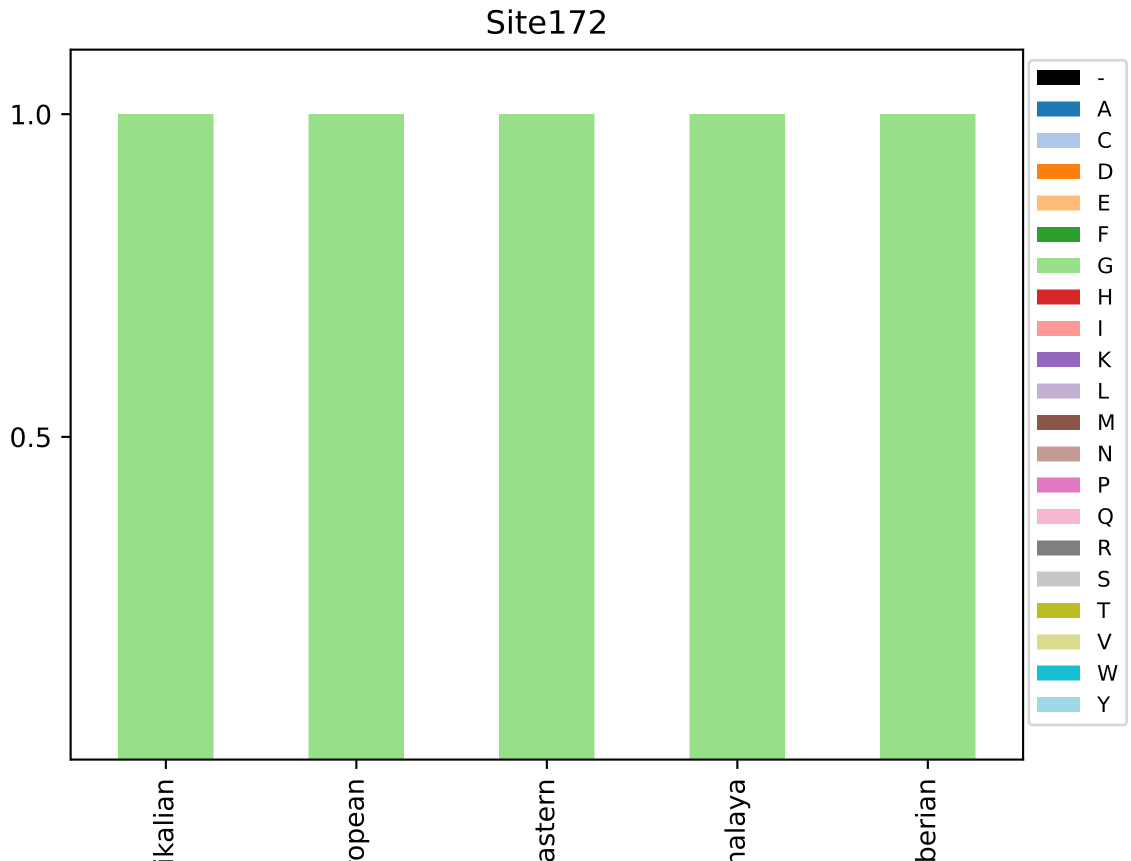

Site172
1.0
0.5
-
A
C
D
E
F
G
H
I
K
L
M
N
P
Q
R
S
T
V
W
Y
ikalian
opean
astern
nalaya
berian

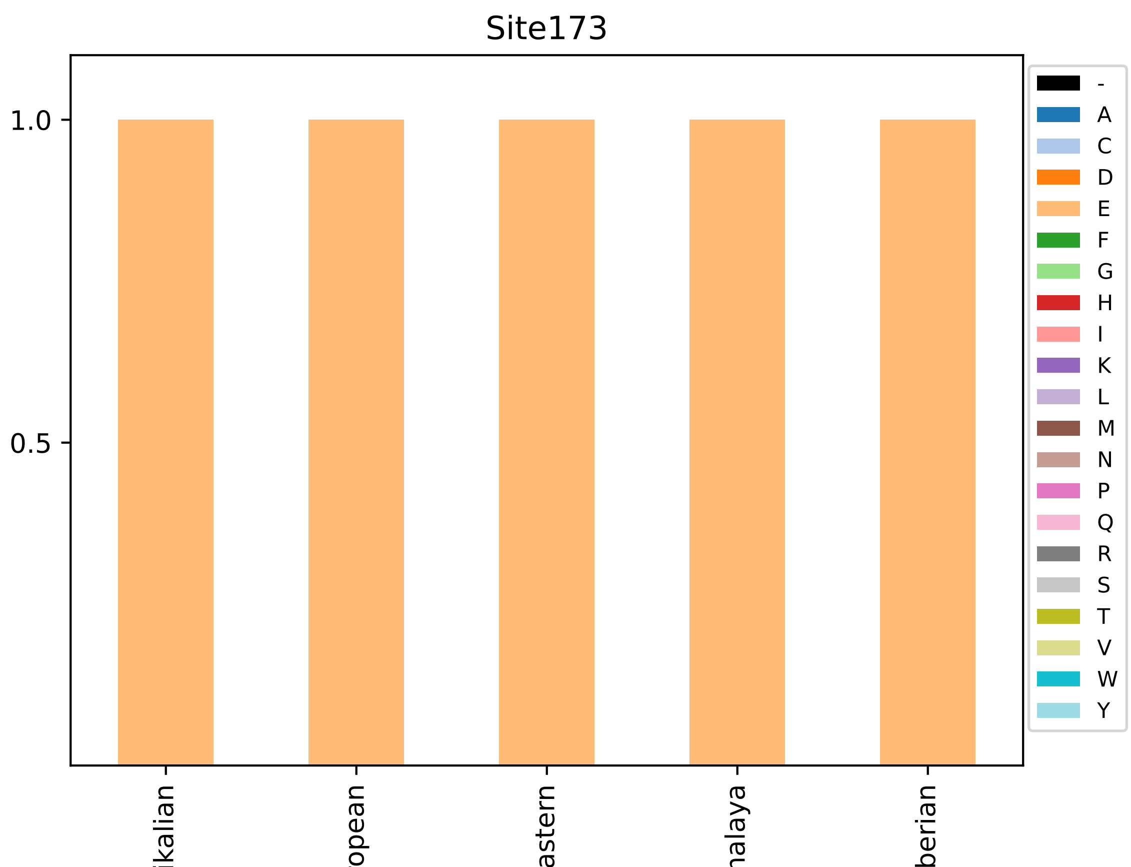

Site173
1.0
0.5
-
A
C
D
E
F
G
H
I
K
L
M
N
P
Q
R
S
T
V
W
Y
ikalian
opean
astern
nalaya
berian

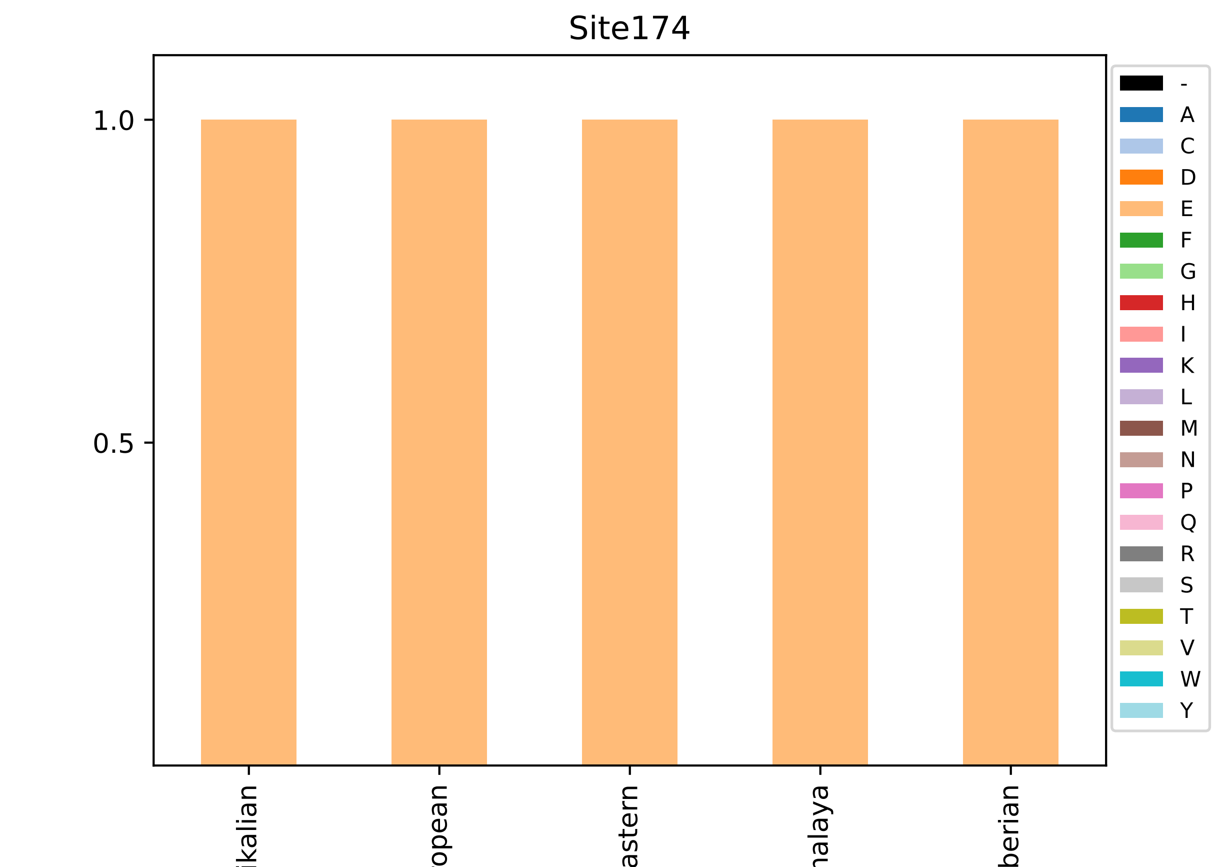

Site174
1.0
0.5
-
A
C
D
E
F
G
H
I
K
L
M
N
P
Q
R
S
T
V
W
Y
ikalian
opean
astern
nalaya
berian

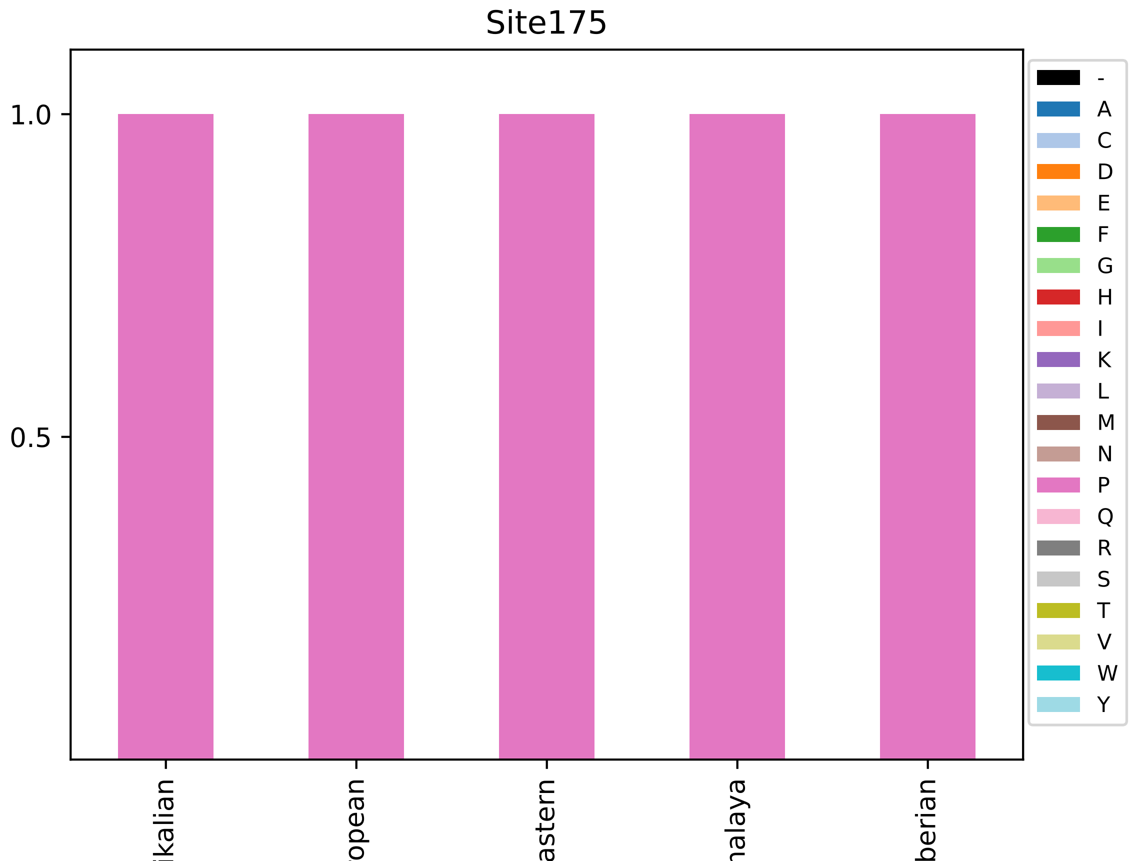
Site175
1.0
0.5
-
A
C
D
E
F
G
H
I
K
L
M
N
P
Q
R
S
T
V
W
Y
ikalian
opean
astern
nalaya
berian

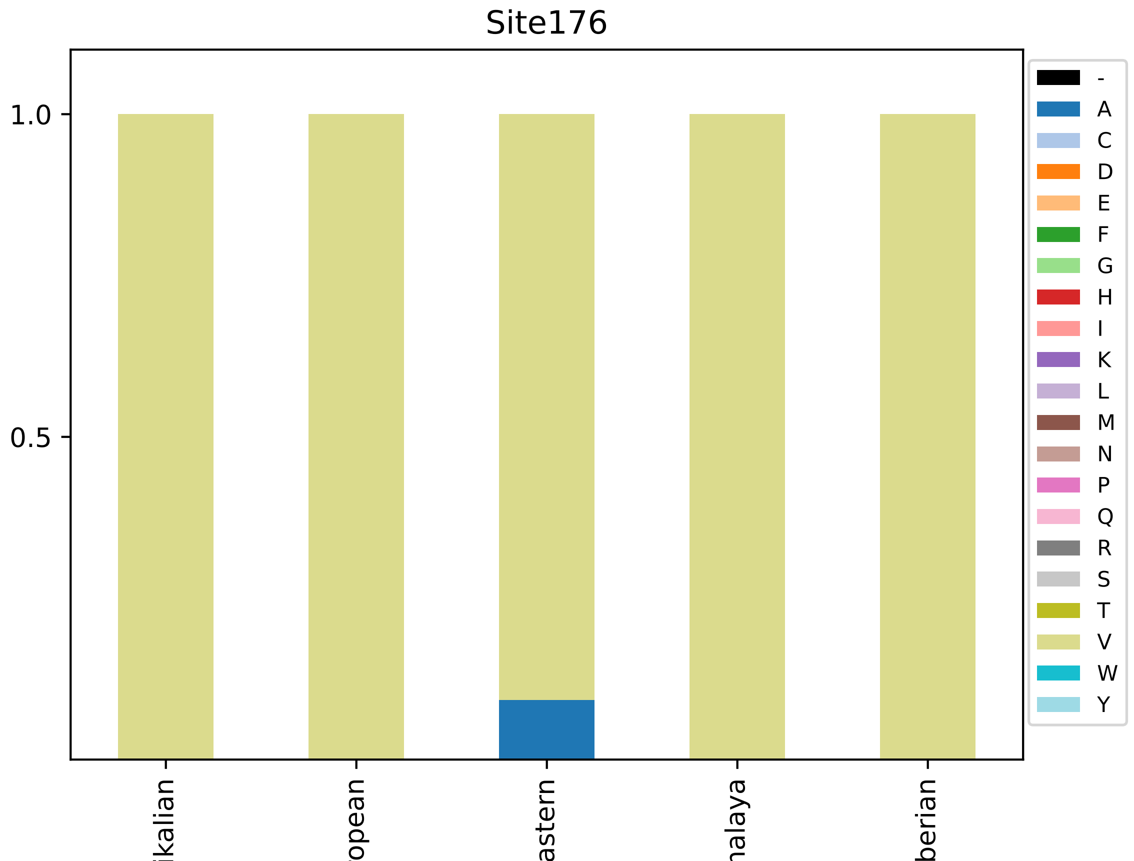
Site176
1.0
0.5
-
A
C
D
E
F
G
H
I
K
L
M
N
P
Q
R
S
T
V
W
Y
ikalian
opean
astern
nalaya
berian

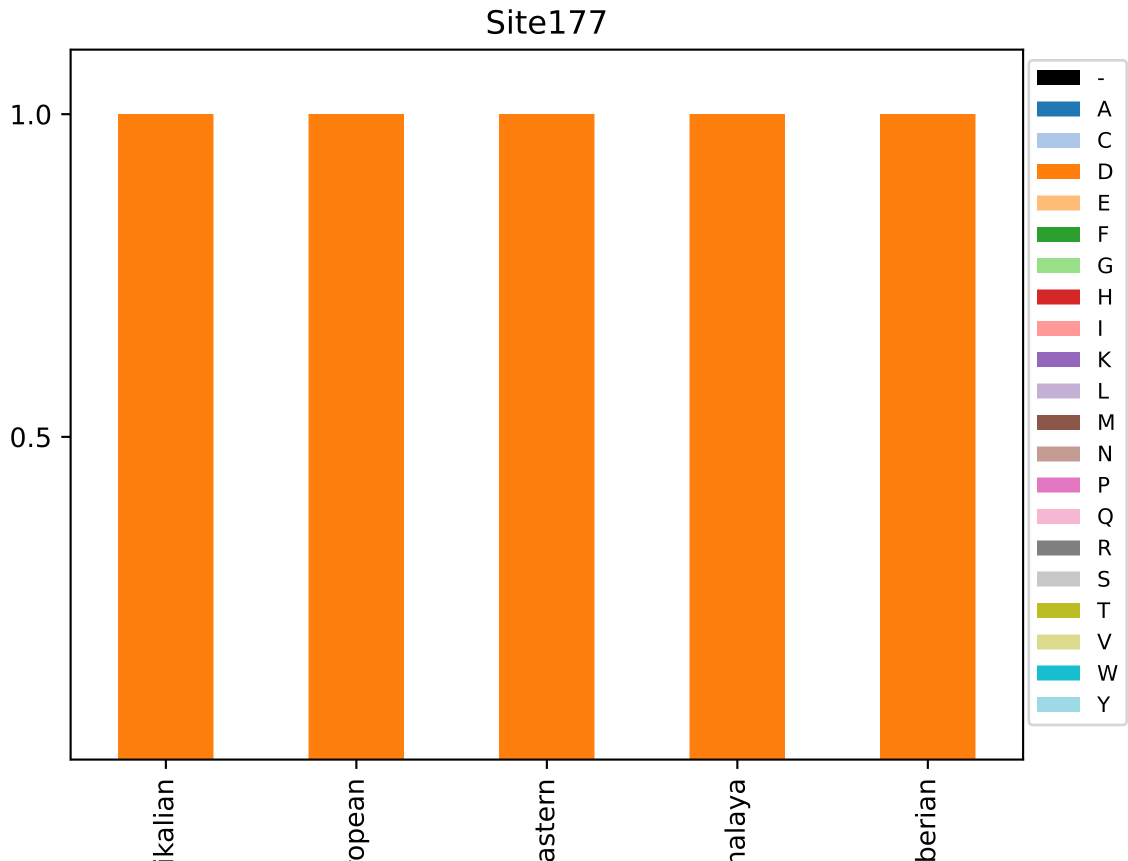

Site177
1.0
0.5
-
A
C
D
E
F
G
H
I
K
L
M
N
P
Q
R
S
T
V
W
Y
ikalian
opean
astern
nalaya
berian

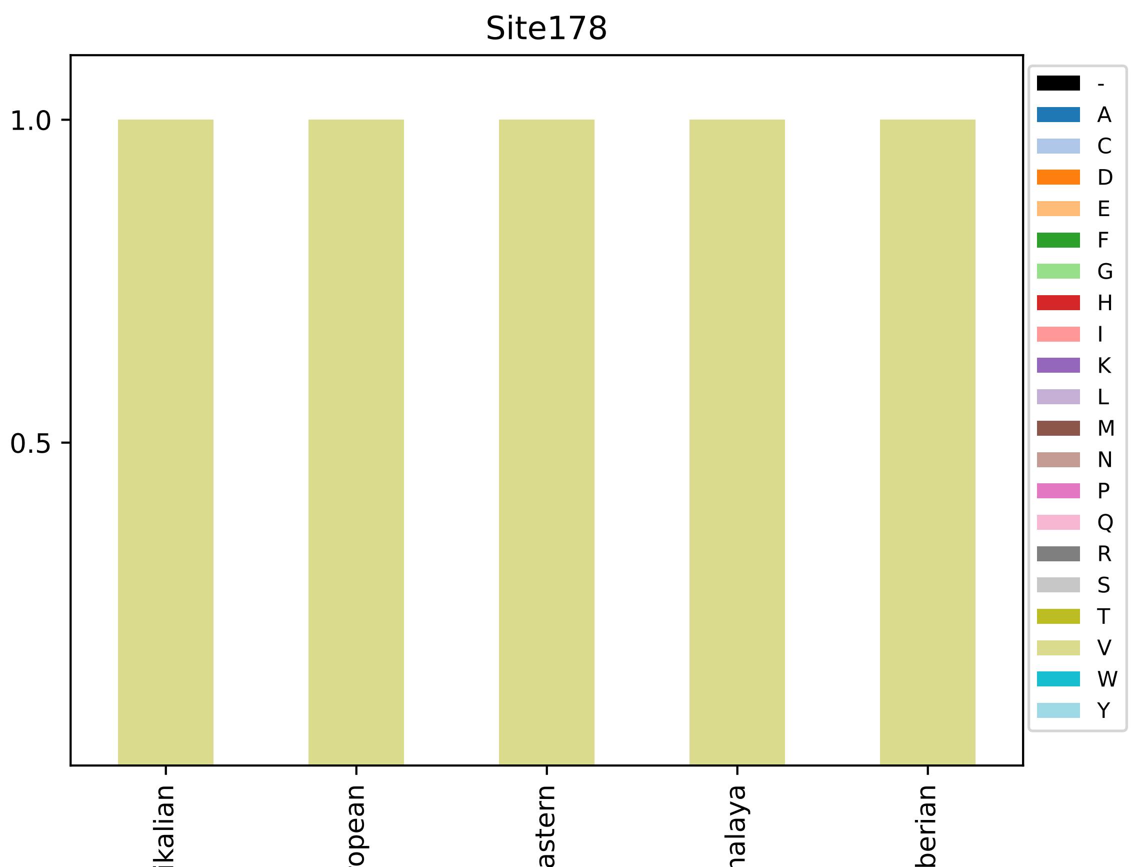

Site178
1.0
0.5
-
A
C
D
E
F
G
H
I
K
L
M
N
P
Q
R
S
T
V
W
Y
ikalian
opean
astern
nalaya
berian

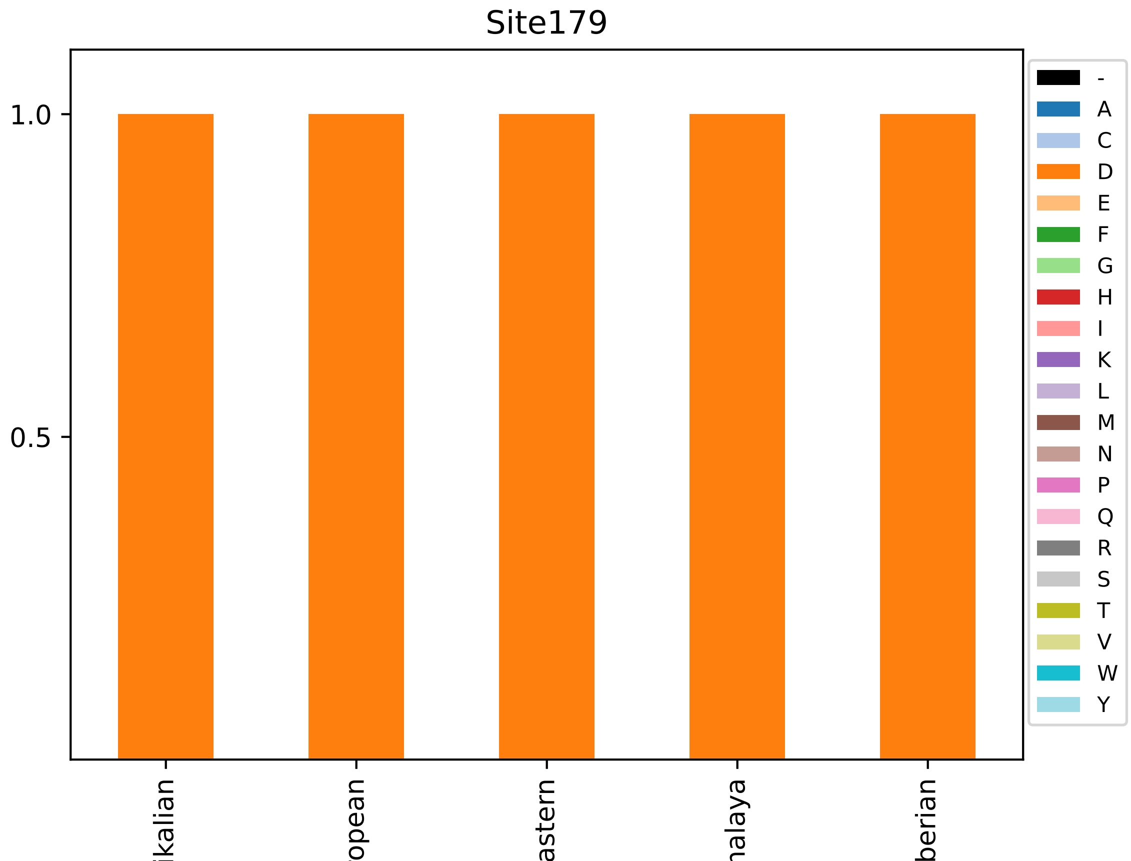
Site179
1.0
0.5
-
A
C
D
E
F
G
H
I
K
L
M
N
P
Q
R
S
T
V
W
Y

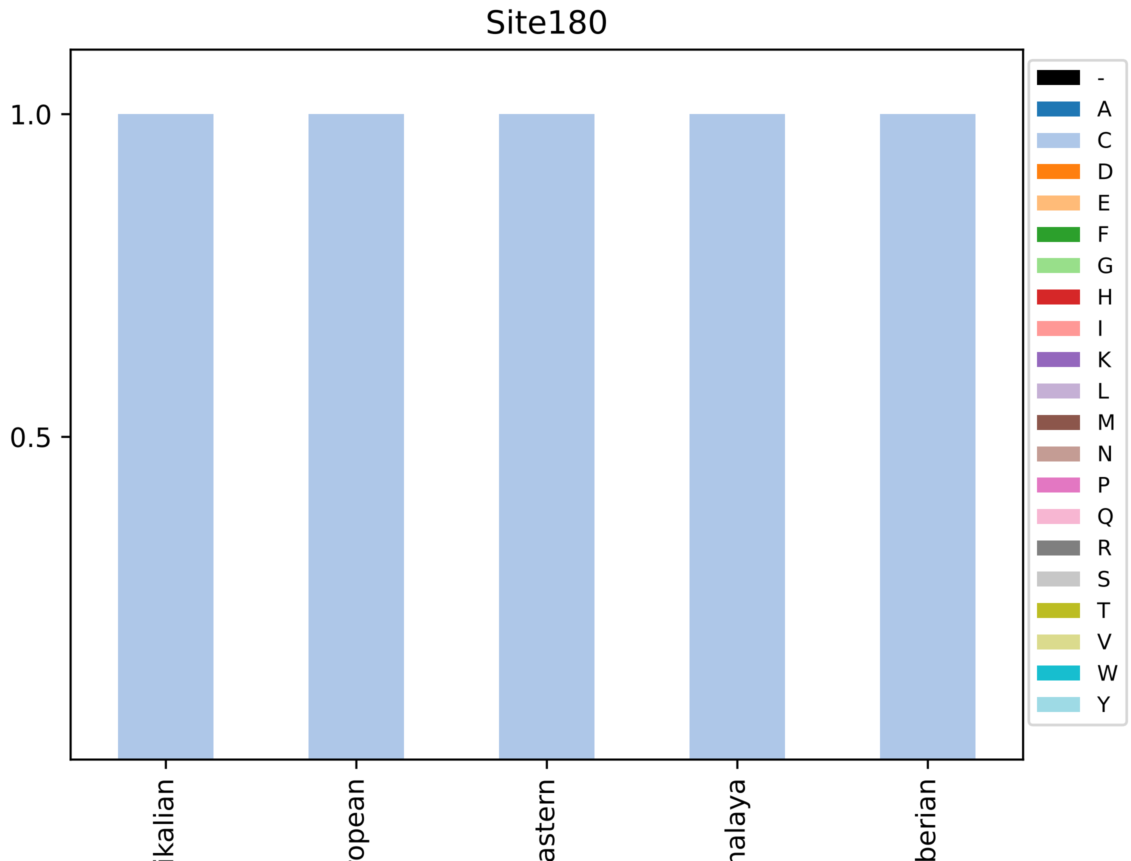
Site180
1.0
0.5
-
A
C
D
E
F
G
H
I
K
L
M
N
P
Q
R
S
T
V
W
Y
ikalian
opean
astern
nalaya
berian

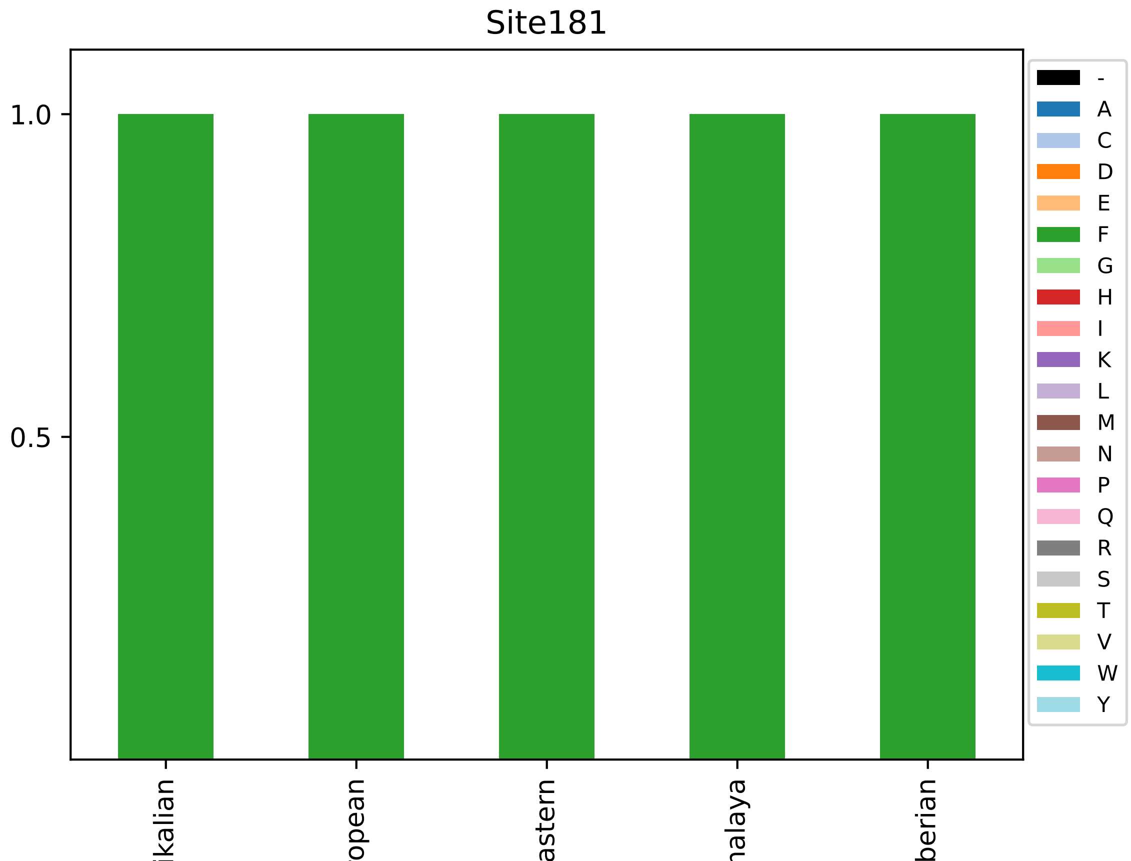

Site181
1.0
0.5
ikalian
opean
astern
nalaya
berian
-
A
C
D
E
F
G
H
I
K
L
M
N
P
Q
R
S
T
V
W
Y

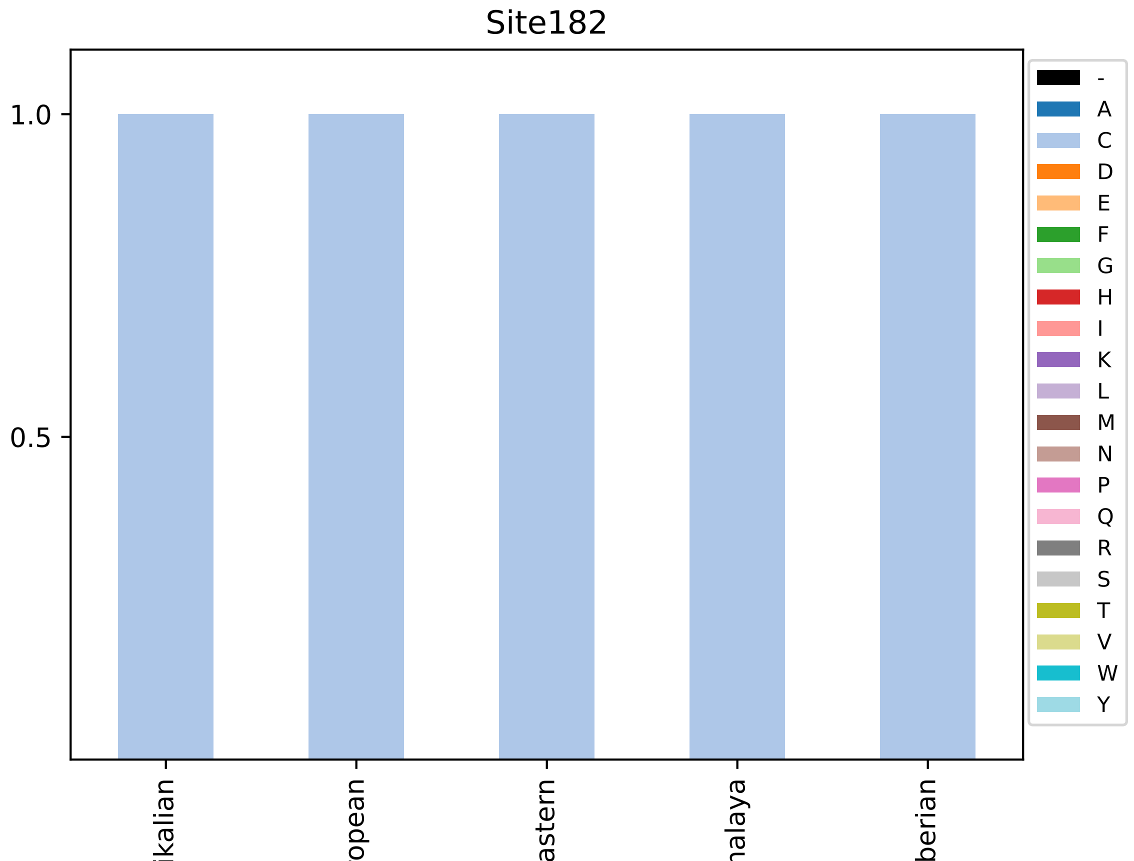

Site182
1.0
0.5
-
A
C
D
E
F
G
H
I
K
L
M
N
P
Q
R
S
T
V
W
Y
ikalian
opean
astern
nalaya
berian

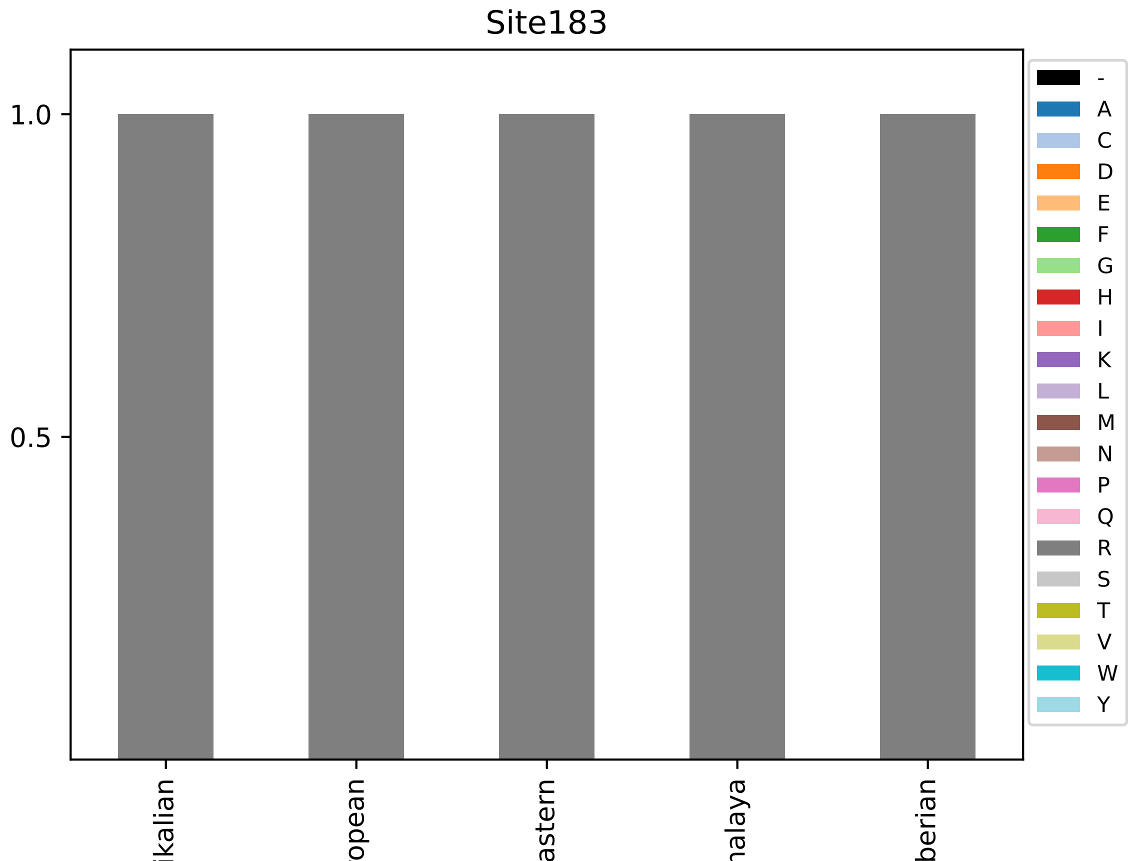
Site183
1.0
0.5
-
A
C
D
E
F
G
H
I
K
L
M
N
P
Q
R
S
T
V
W
Y
ikalian
opean
astern
nalaya
berian

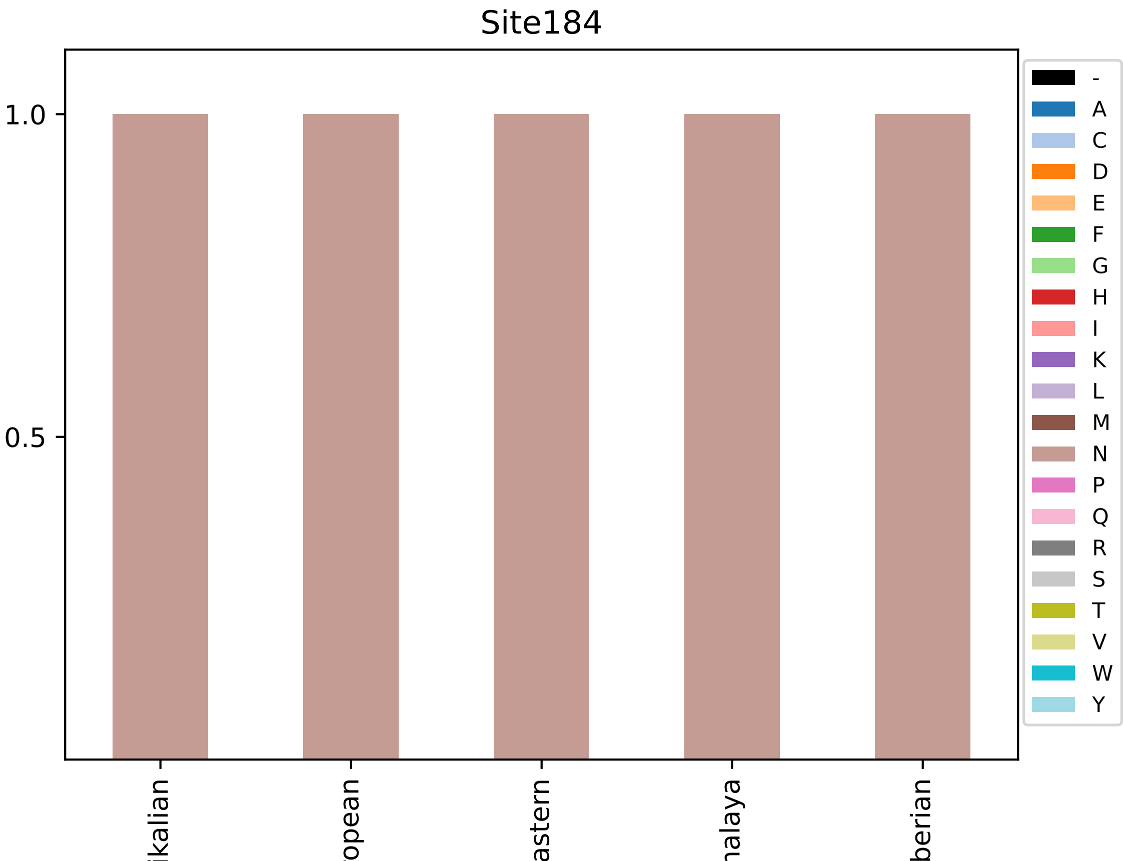

Site184
1.0
0.5
-
A
C
D
E
F
G
H
I
K
L
M
N
P
Q
R
S
T
V
W
Y
ikalian
ropean
astern
nalaya
berian

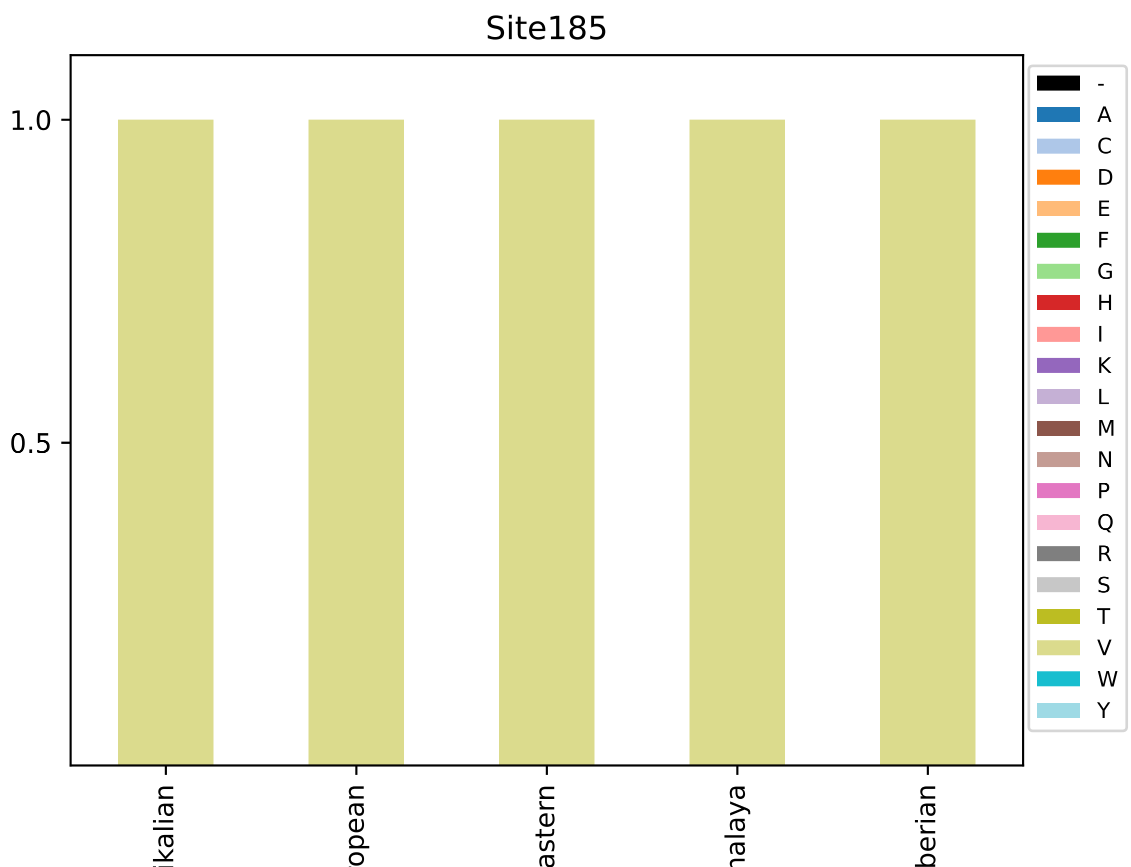
Site185
1.0
0.5
-
A
C
D
E
F
G
H
I
K
L
M
N
P
Q
R
S
T
V
W
Y
ikalian
opean
astern
nalaya
berian

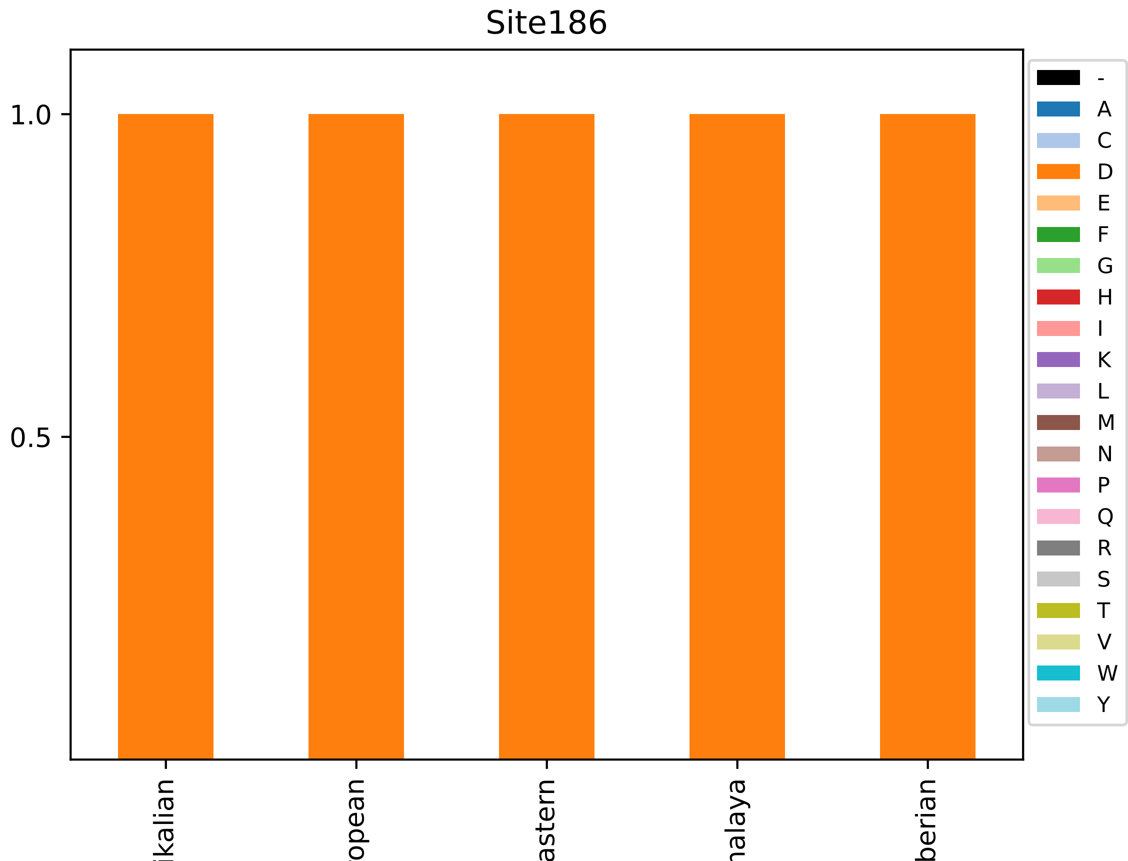

Site186
1.0
0.5
-
A
C
D
E
F
G
H
I
K
L
M
N
P
Q
R
S
T
V
W
Y
ikalian
opean
astern
nalaya
berian

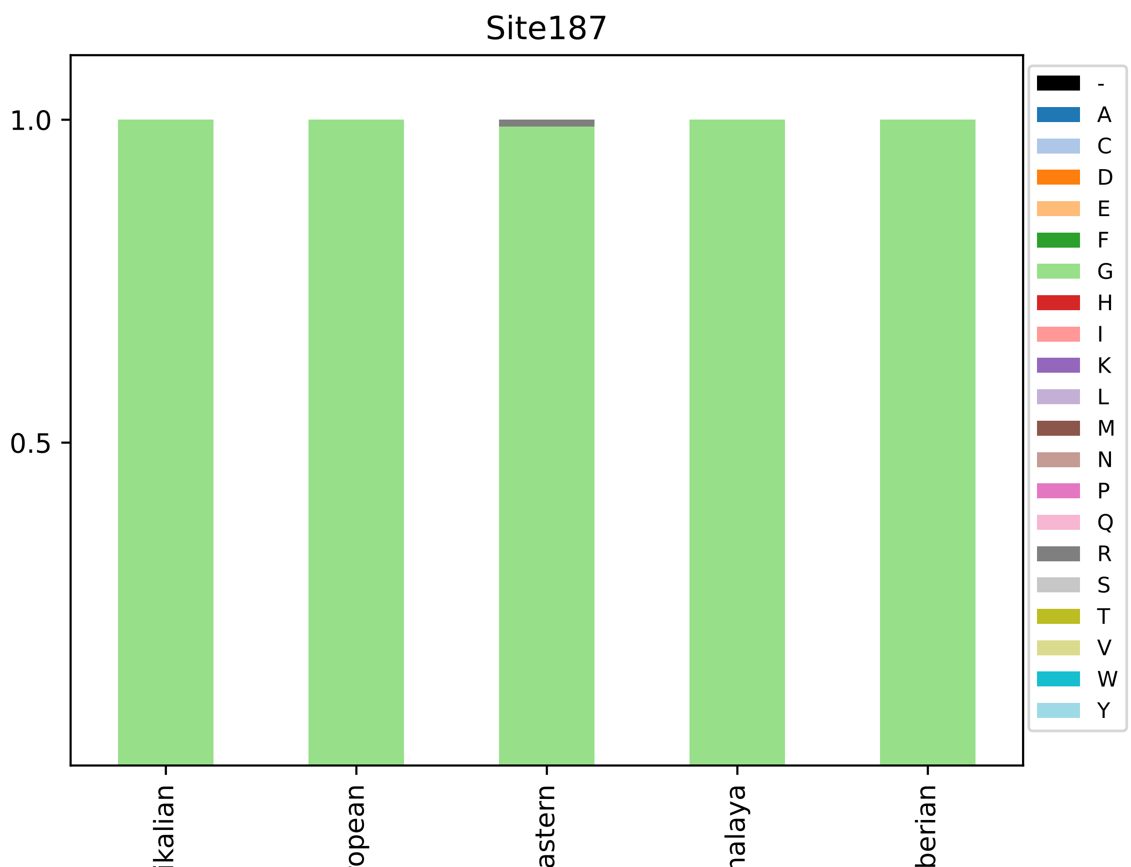

Site187
1.0
0.5
-
A
C
D
E
F
G
H
I
K
L
M
N
P
Q
R
S
T
V
W
Y
ikalian
opean
astern
nalaya
berian

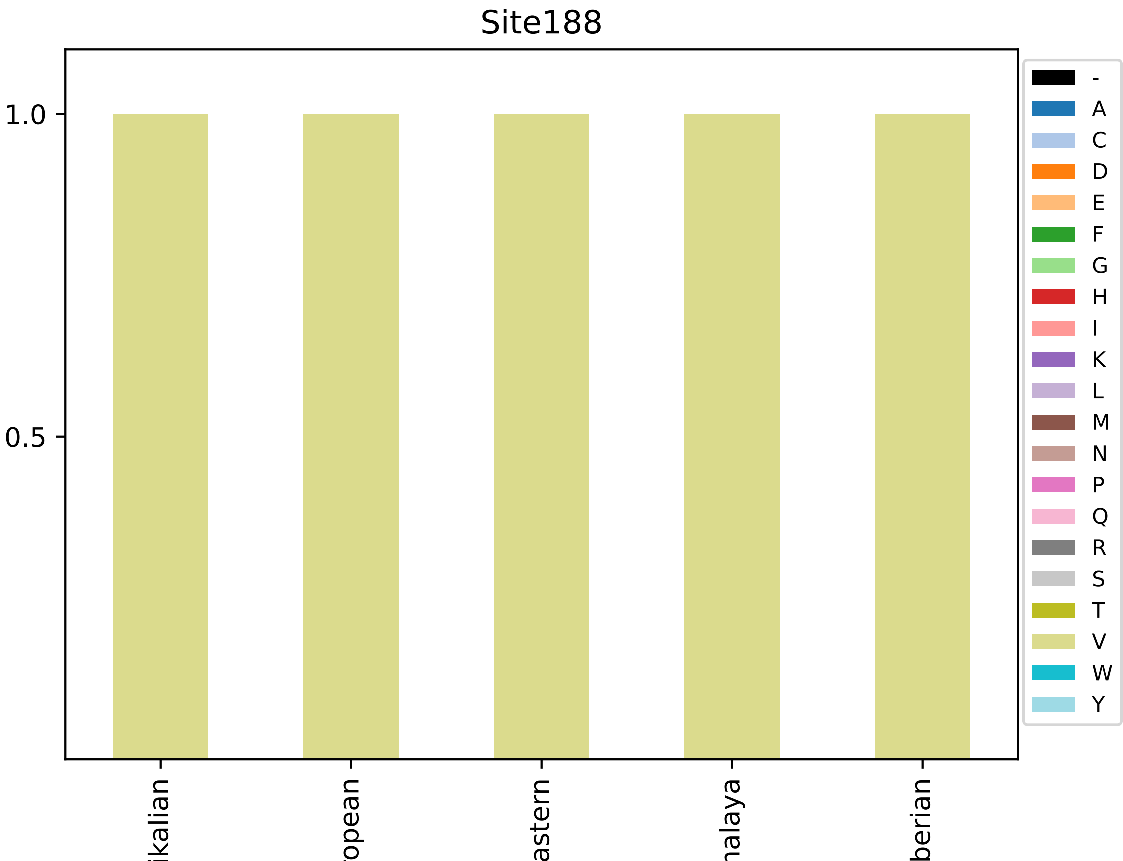
Site188
1.0
0.5
-
A
C
D
E
F
G
H
I
K
L
M
N
P
Q
R
S
T
V
W
Y

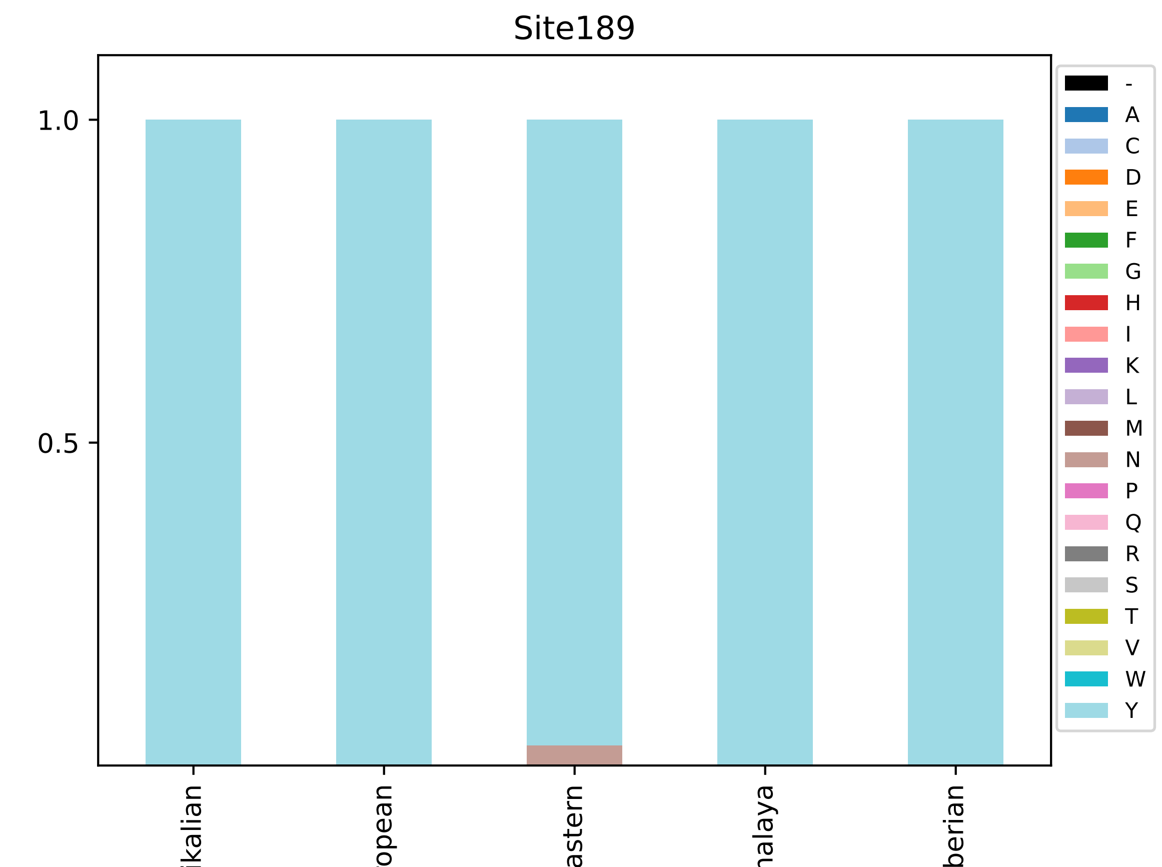
Site189
1.0
0.5
-
A
C
D
E
F
G
H
I
K
L
M
N
P
Q
R
S
T
V
W
Y
ikalian
opean
astern
nalaya
berian

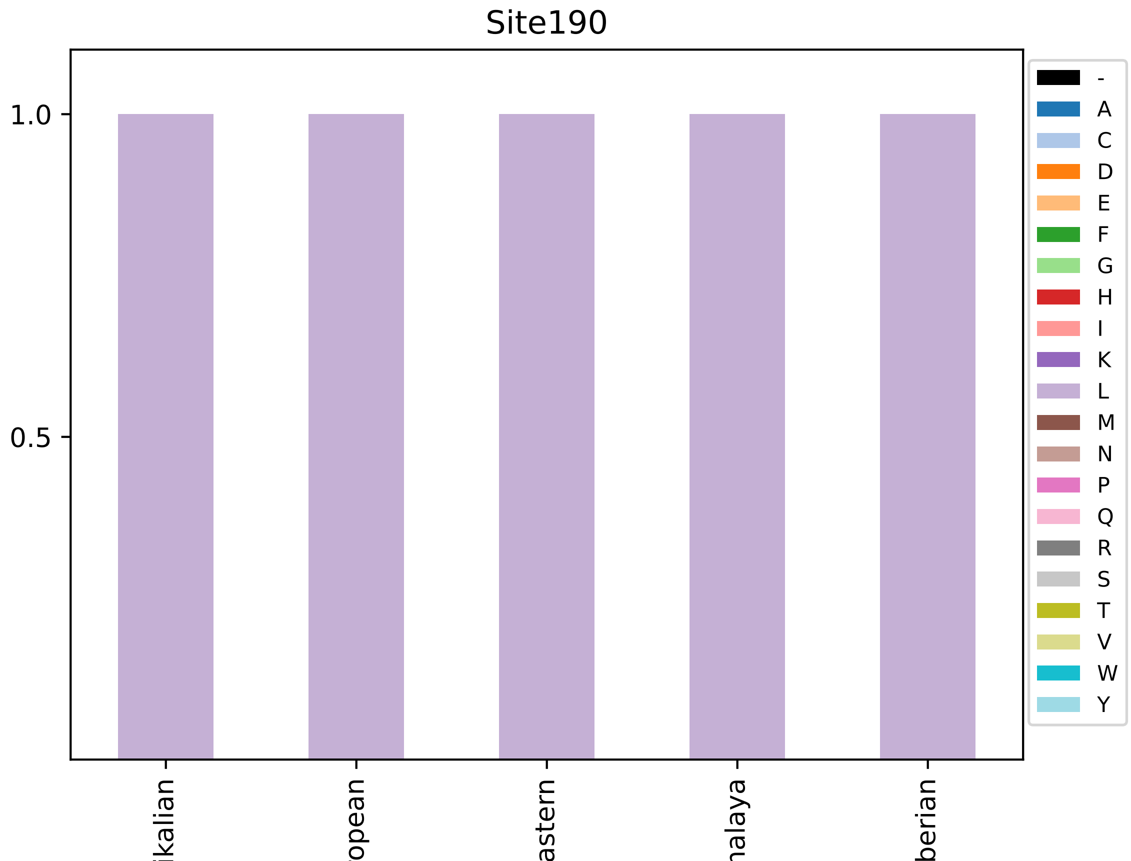
Site190
1.0
0.5
-
A
C
D
E
F
G
H
I
K
L
M
N
P
Q
R
S
T
V
W
Y
ikalian
opean
astern
nalaya
berian

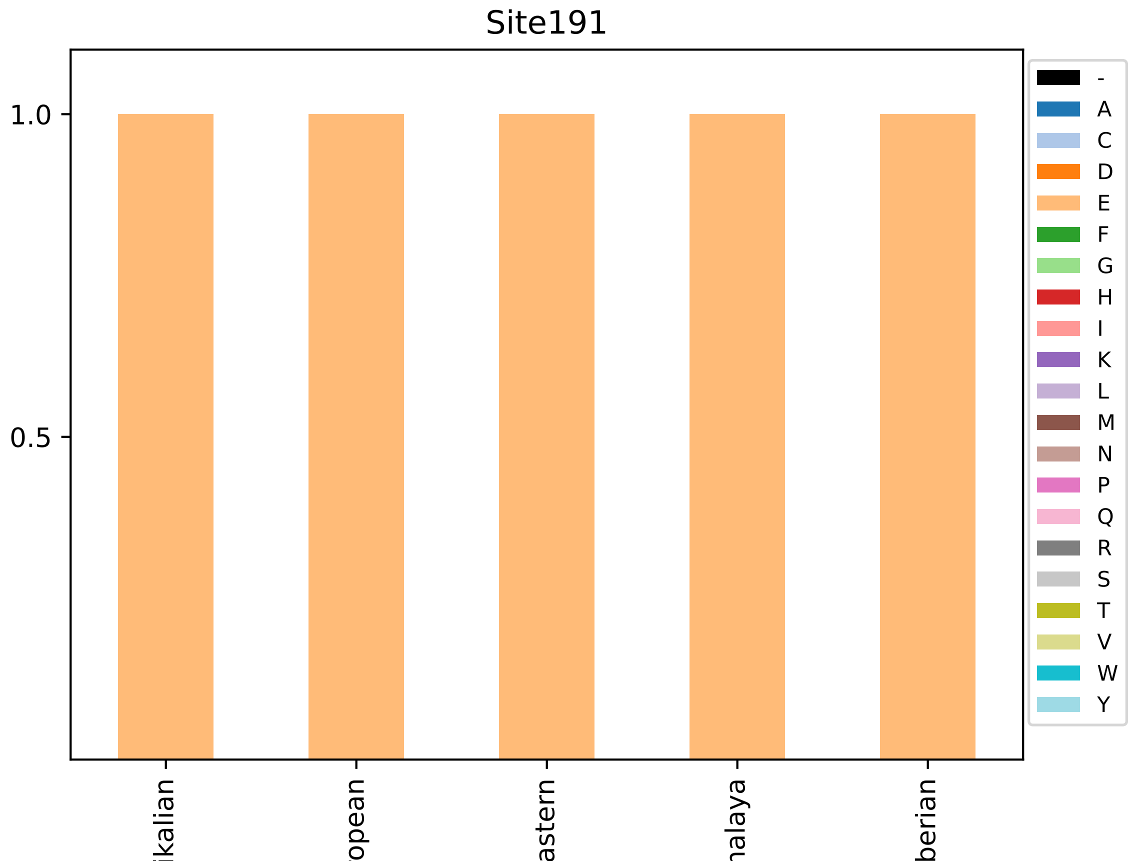
Site191
1.0
0.5
-
A
C
D
E
F
G
H
I
K
L
M
N
P
Q
R
S
T
V
W
Y
ikalian
opean
astern
nalaya
berian

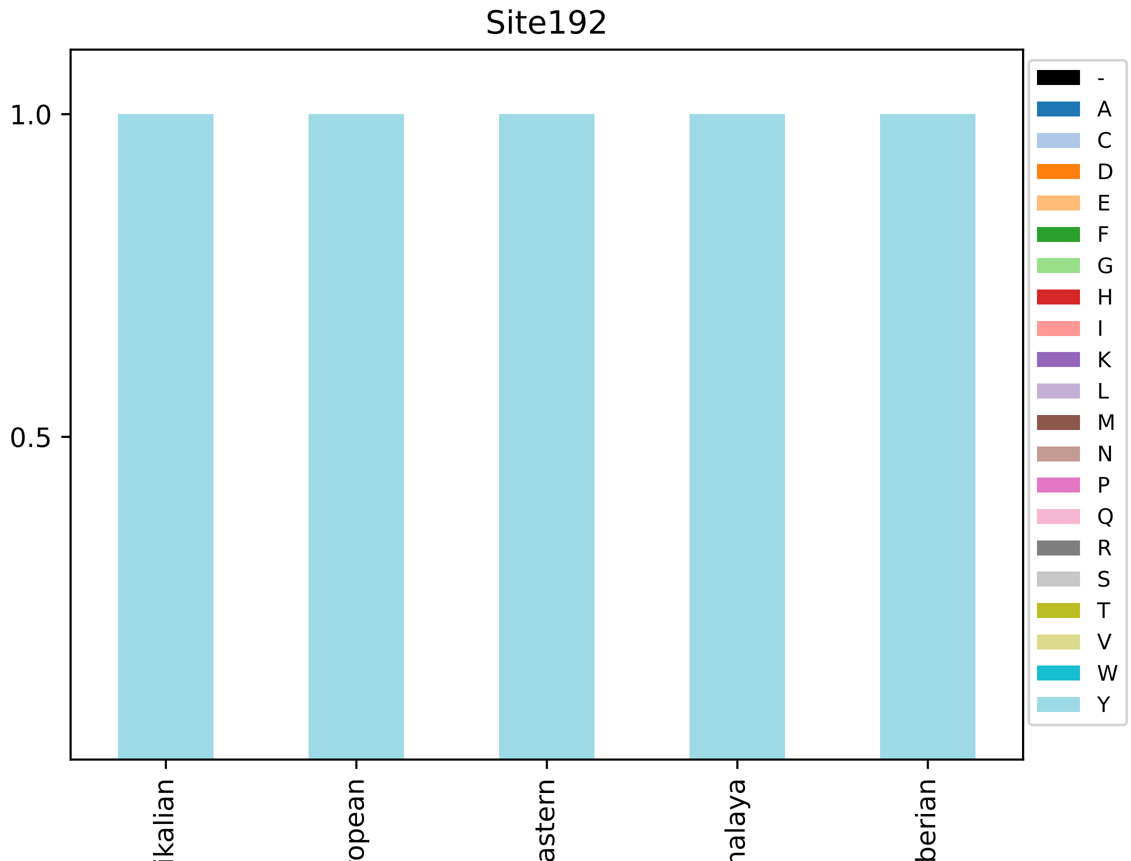

Site192
1.0
0.5
-
A
C
D
E
F
G
H
I
K
L
M
N
P
Q
R
S
T
V
W
Y
ikalian
opean
astern
halaya
berian

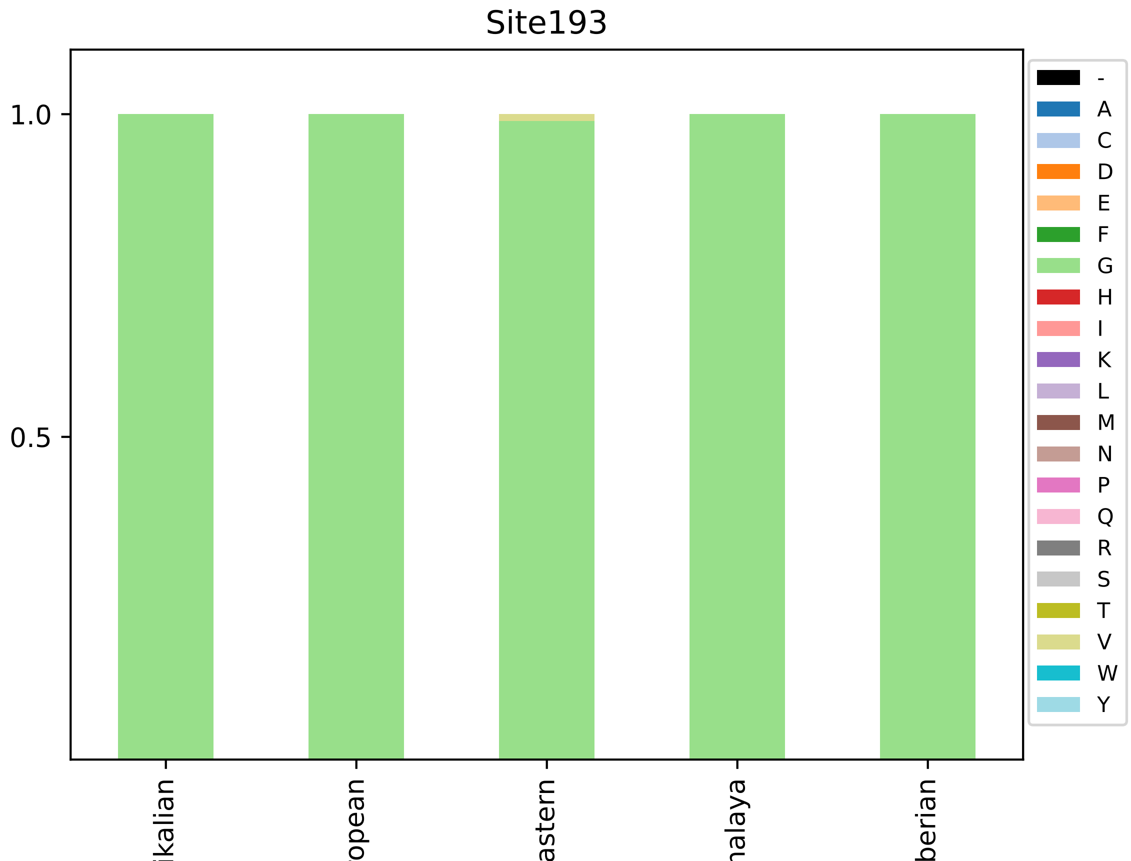

Site193
1.0
0.5
-
A
C
D
E
F
G
H
I
K
L
M
N
P
Q
R
S
T
V
W
Y
ikalian
opean
astern
nalaya
berian

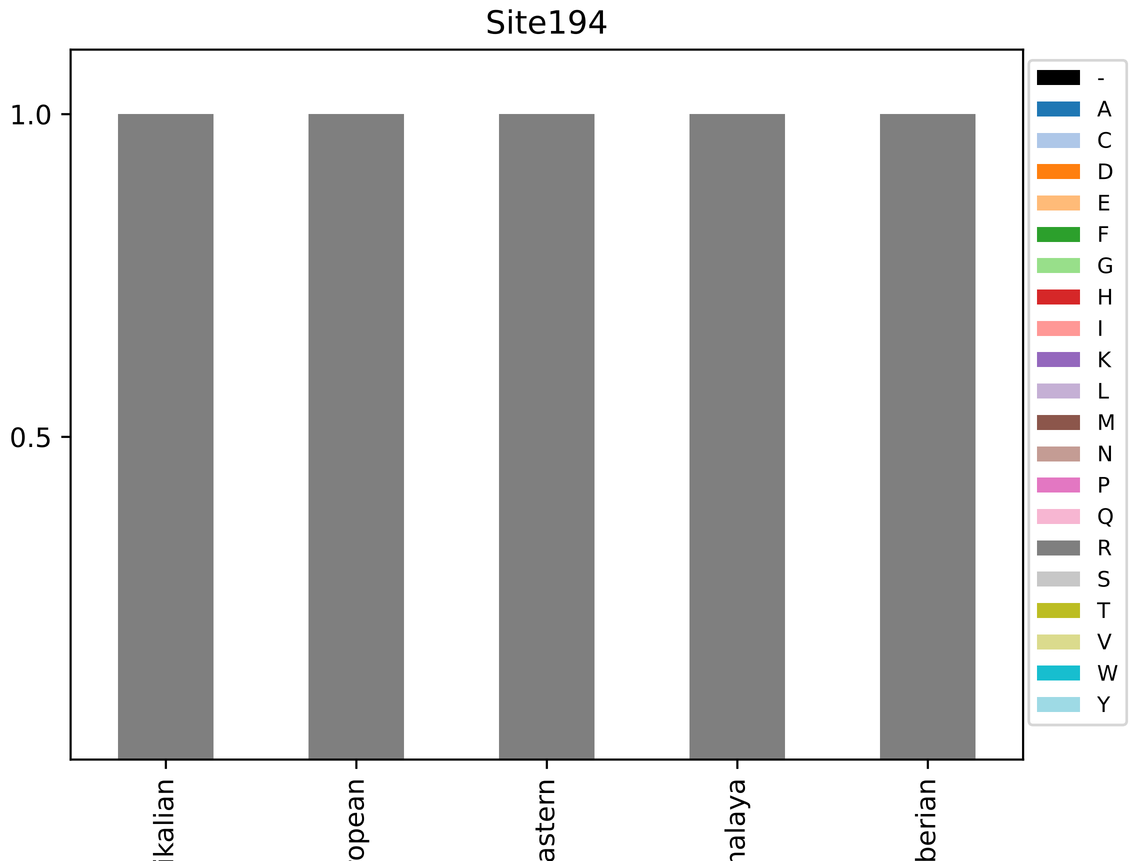
Site194
1.0
0.5
-
A
C
D
E
F
G
H
I
K
L
M
N
P
Q
R
S
T
V
W
Y
ikalian
opean
astern
nalaya
berian

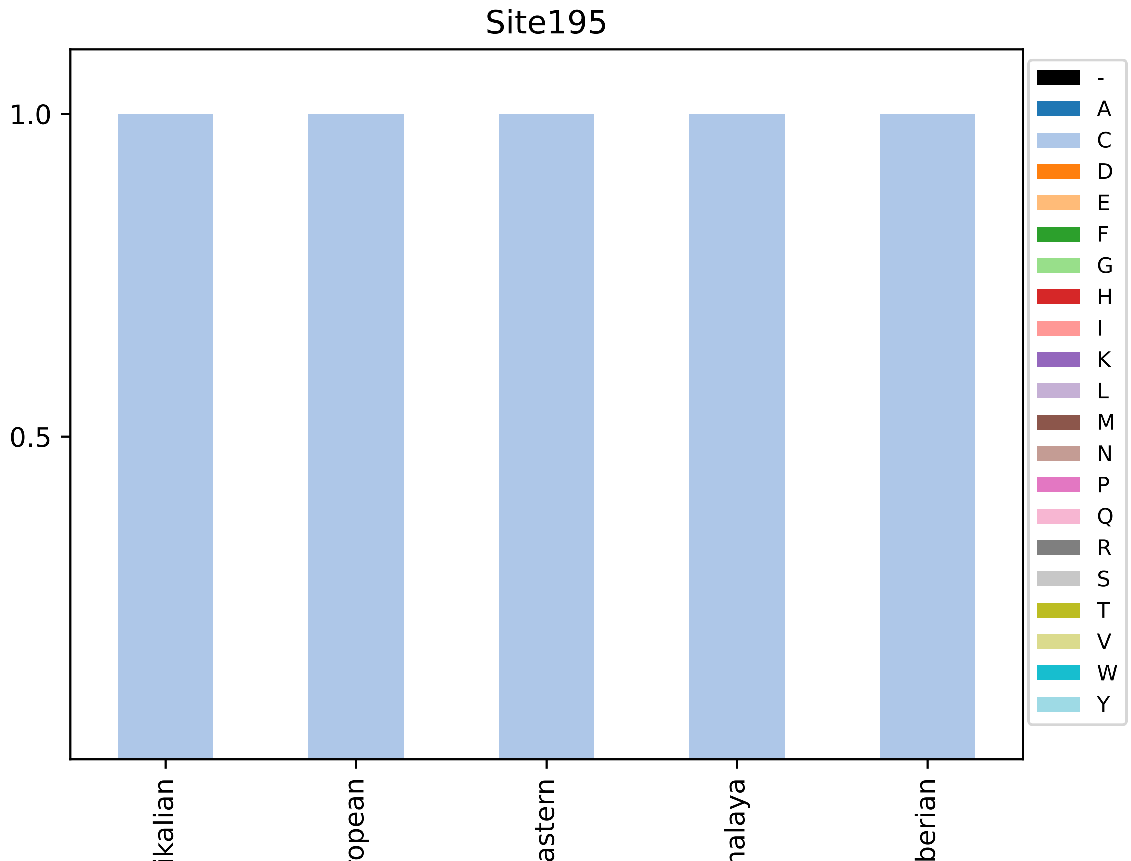

Site195
1.0
0.5
-
A
C
D
E
F
G
H
I
K
L
M
N
P
Q
R
S
T
V
W
Y

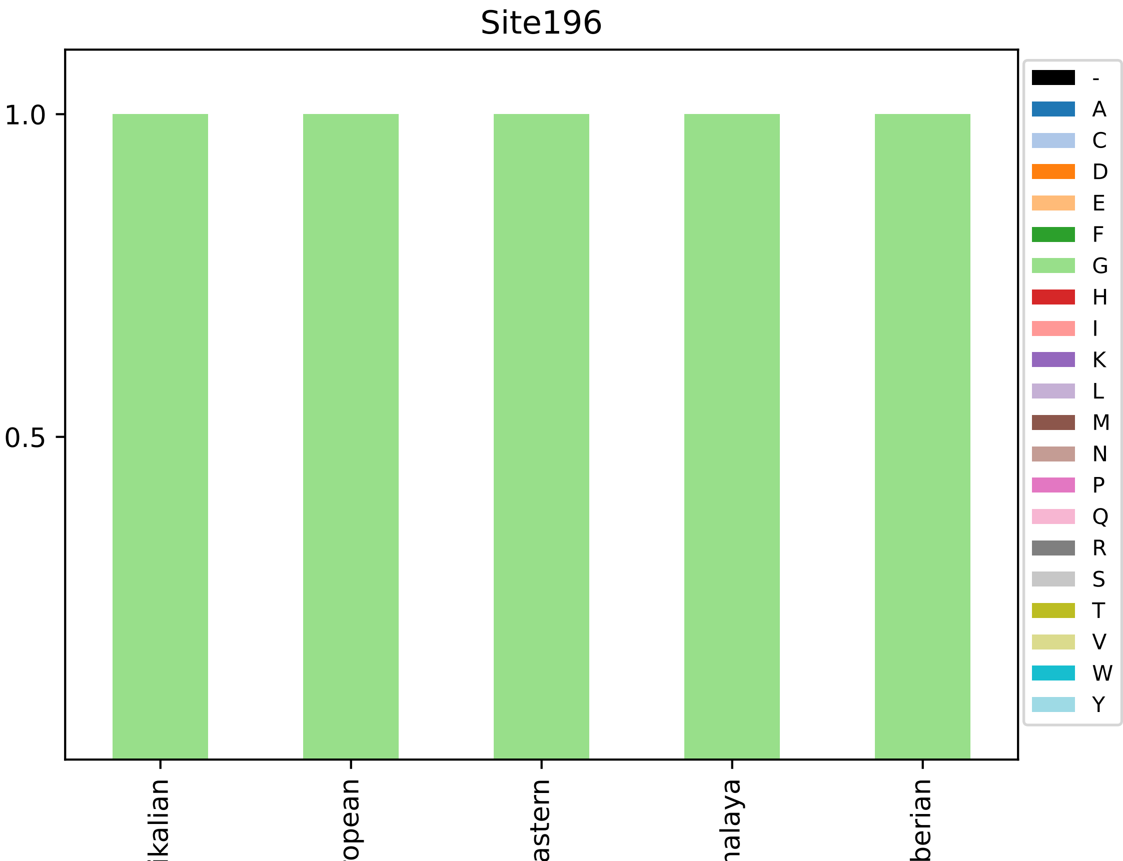
Site196
1.0
0.5
-
A
C
D
E
F
G
H
I
K
L
M
N
P
Q
R
S
T
V
W
Y
ikalian
opean
astern
nalaya
berian

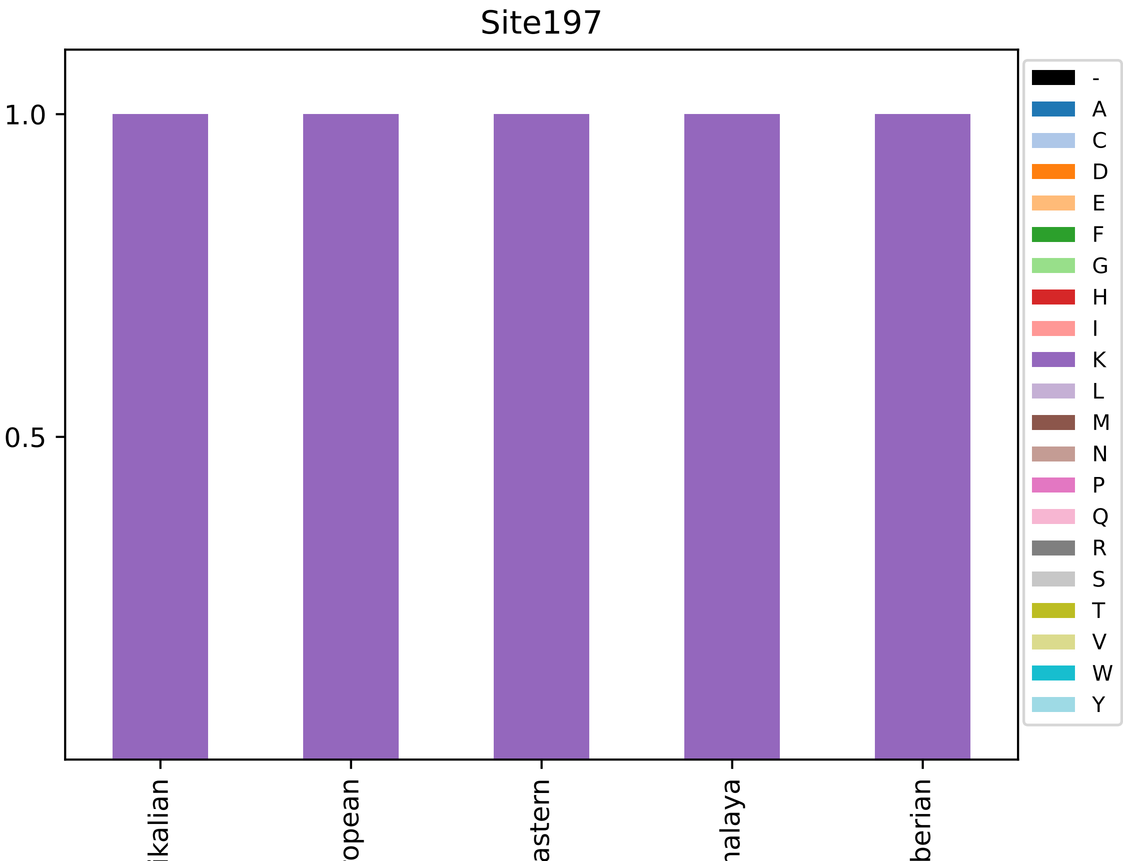

Site197
1.0
0.5
-
A
C
D
E
F
G
H
I
K
L
M
N
P
Q
R
S
T
V
W
Y
ikalian
opean
astern
nalaya
berian

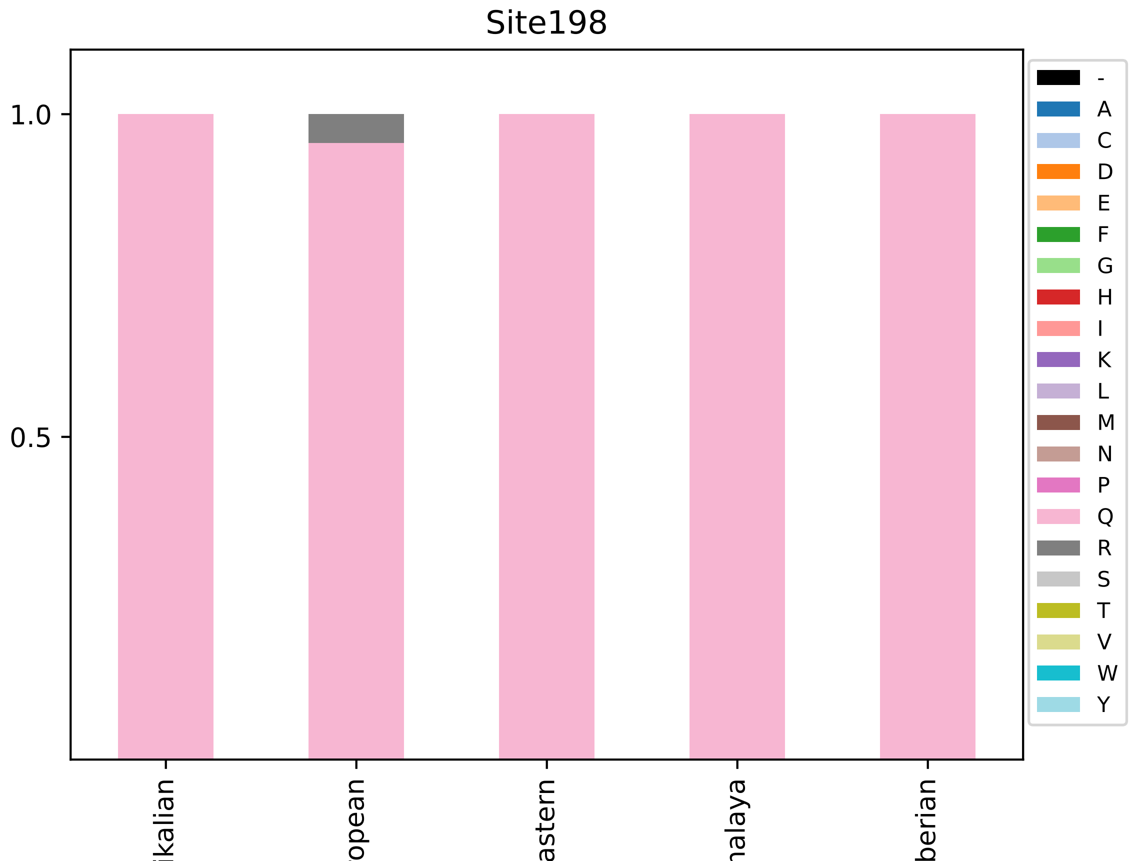
Site198
1.0
0.5
-
A
C
D
E
F
G
H
I
K
L
M
N
P
Q
R
S
T
V
W
Y
ikalian
opean
astern
nalaya
berian

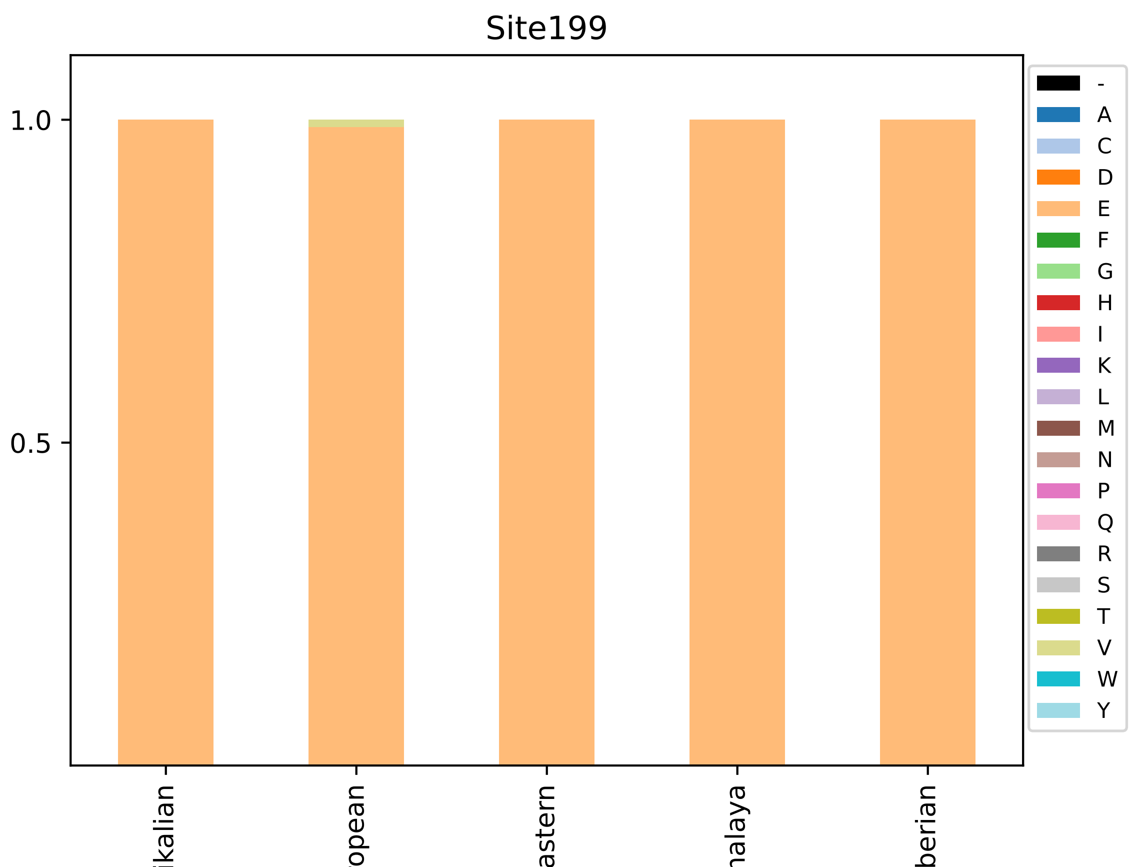
Site199
1.0
0.5
-
A
C
D
E
F
G
H
I
K
L
M
N
P
Q
R
S
T
V
W
Y
ikalian
opean
astern
nalaya
berian

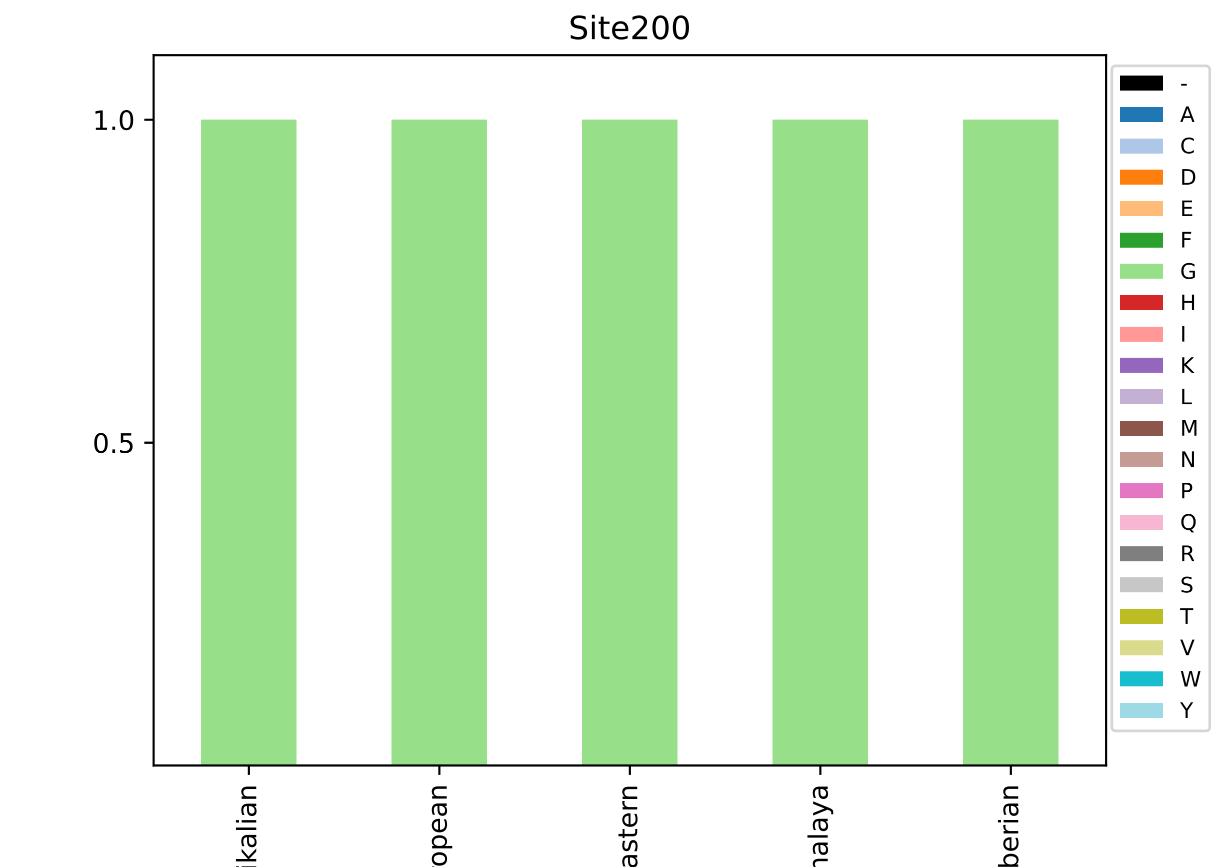
Site200
1.0
0.5
-
A
C
D
E
F
G
H
I
K
L
M
N
P
Q
R
S
T
V
W
Y
ikalian
opean
astern
nalaya
berian

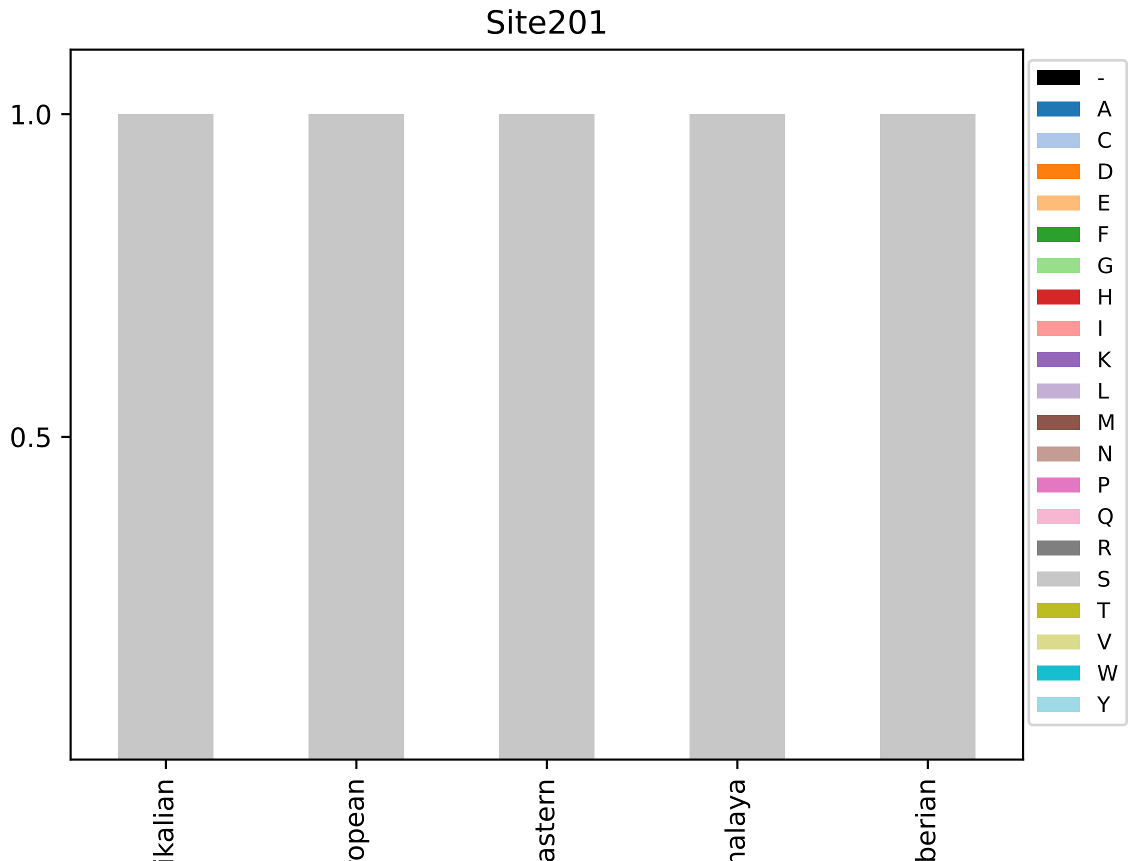
Site201
1.0
0.5
-
A
C
D
E
F
G
H
I
K
L
M
N
P
Q
R
S
T
V
W
Y
ikalian
opean
astern
nalaya
berian

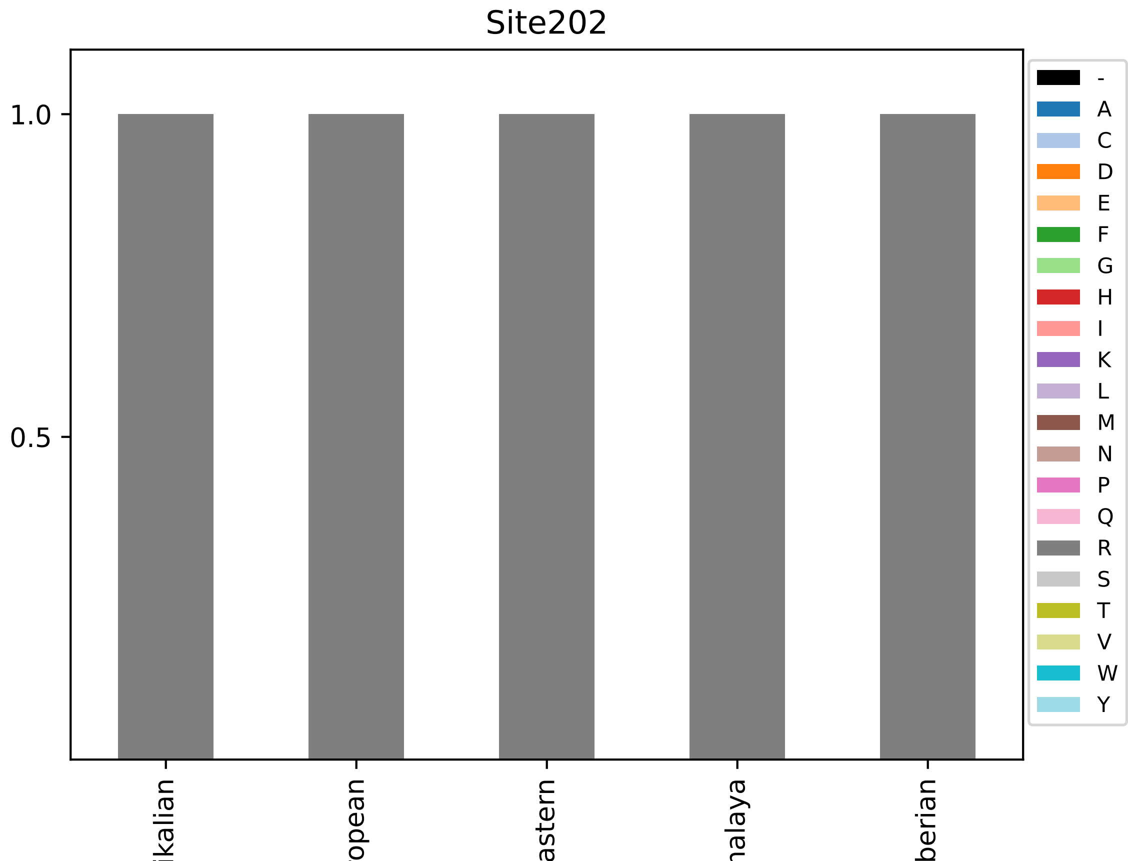
Site202
1.0
0.5
-
A
C
D
E
F
G
H
I
K
L
M
N
P
Q
R
S
T
V
W
Y
ikalian
opean
astern
nalaya
berian

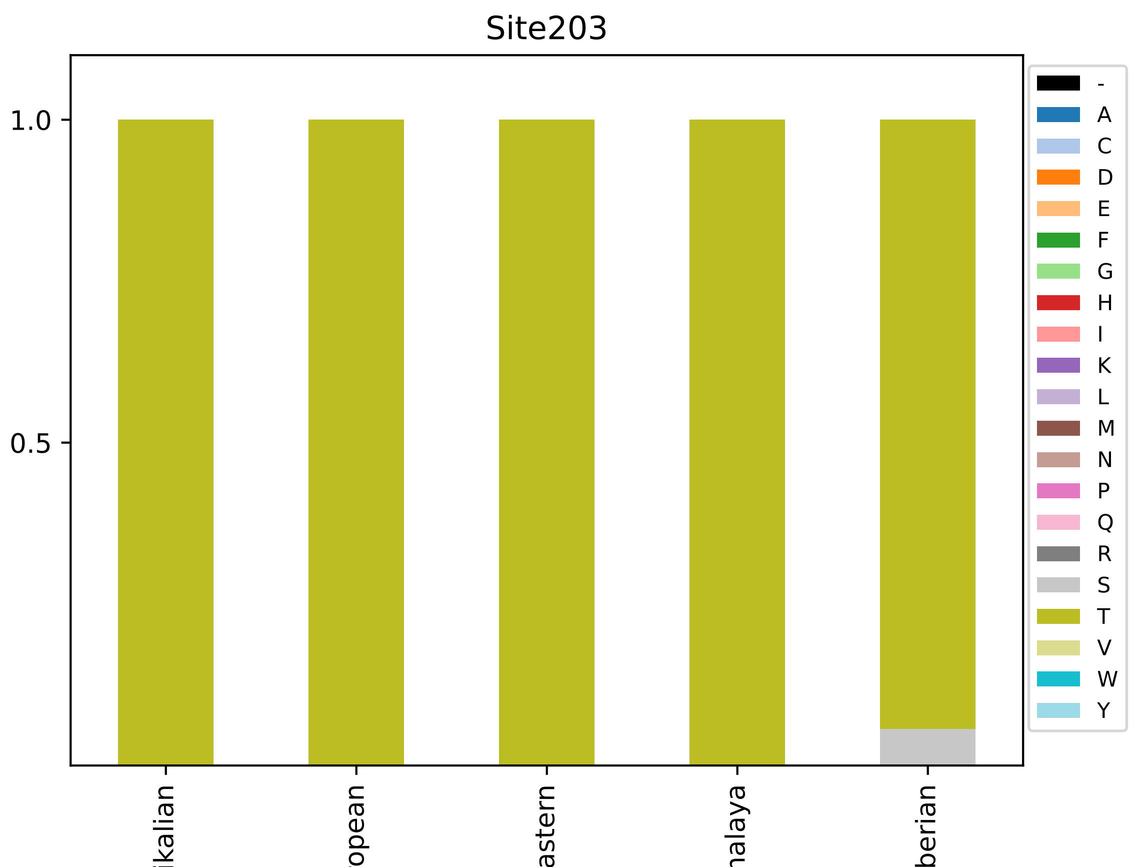

Site203
1.0
0.5
-
A
C
D
E
F
G
H
I
K
L
M
N
P
Q
R
S
T
V
W
Y
ikalian
ropean
astern
nalaya
berian

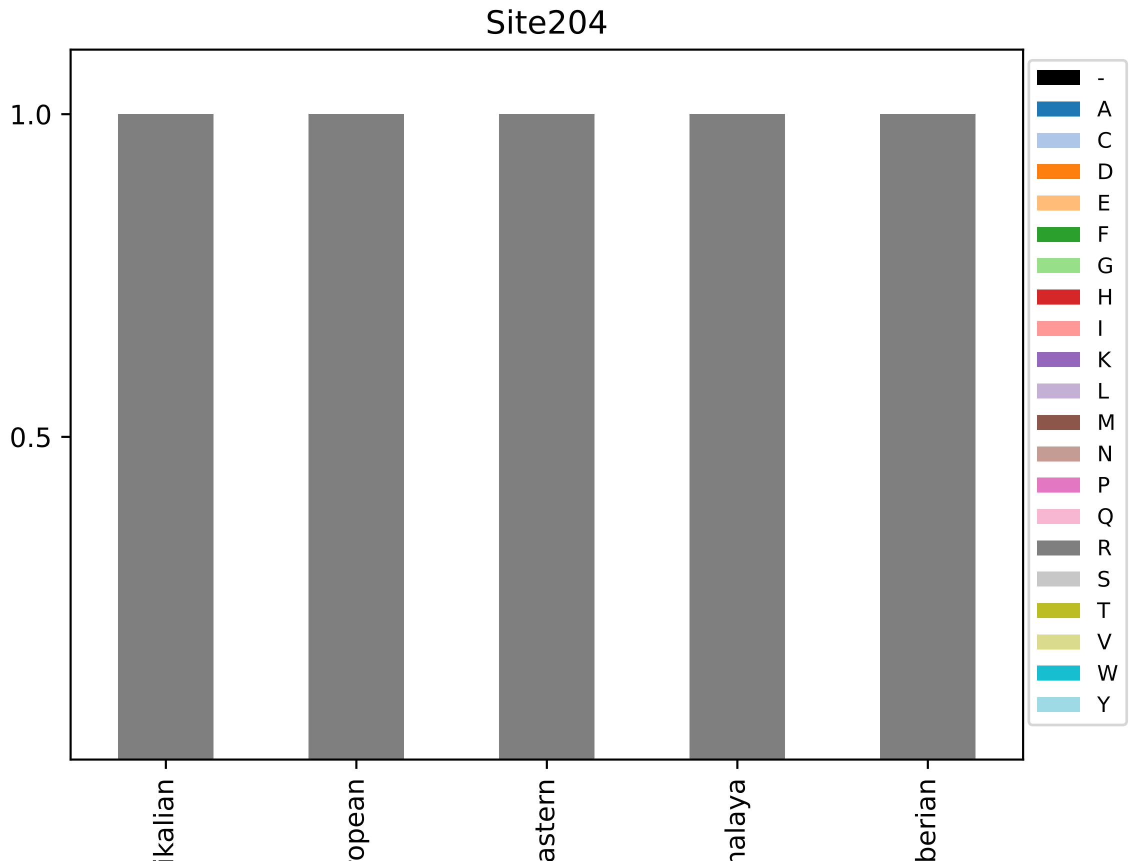
Site204
1.0
0.5
-
A
C
D
E
F
G
H
I
K
L
M
N
P
Q
R
S
T
V
W
Y
ikalian
opean
astern
nalaya
berian

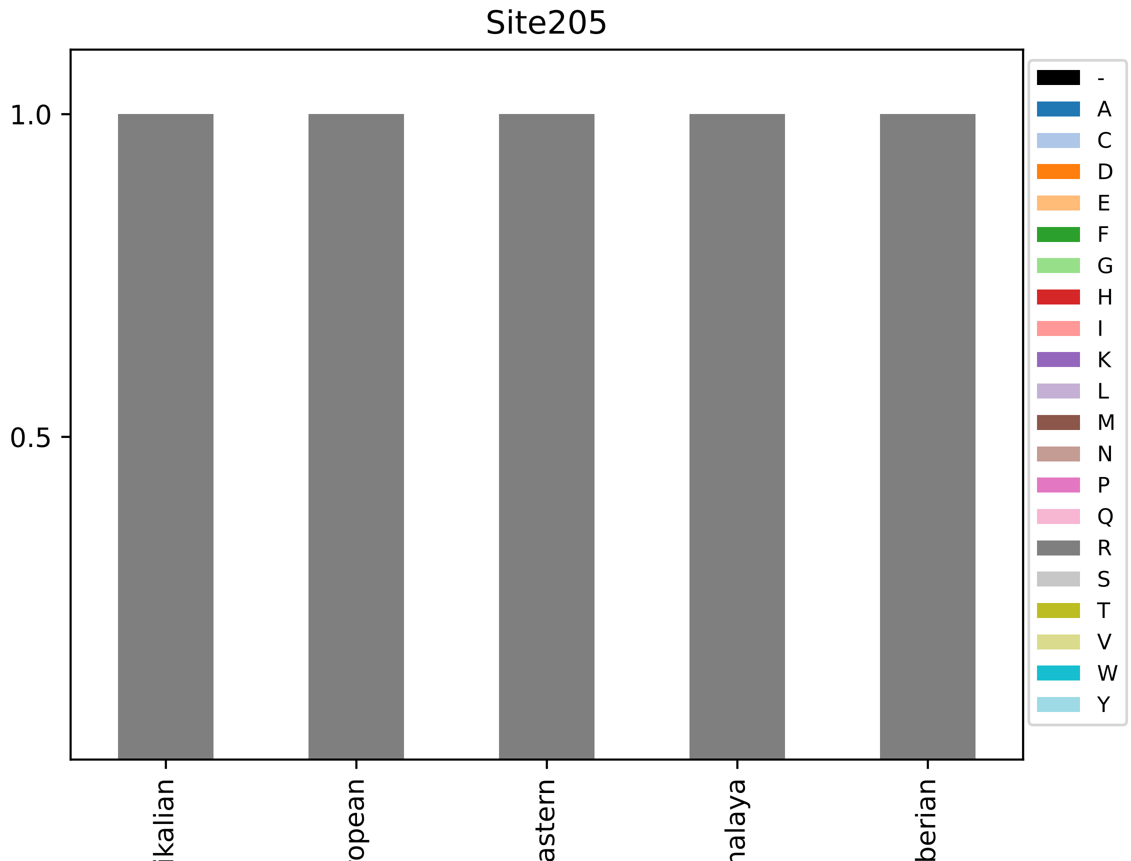

Site205
1.0
0.5
-
A
C
D
E
F
G
H
I
K
L
M
N
P
Q
R
S
T
V
W
Y
ikalian
opean
astern
nalaya
berian

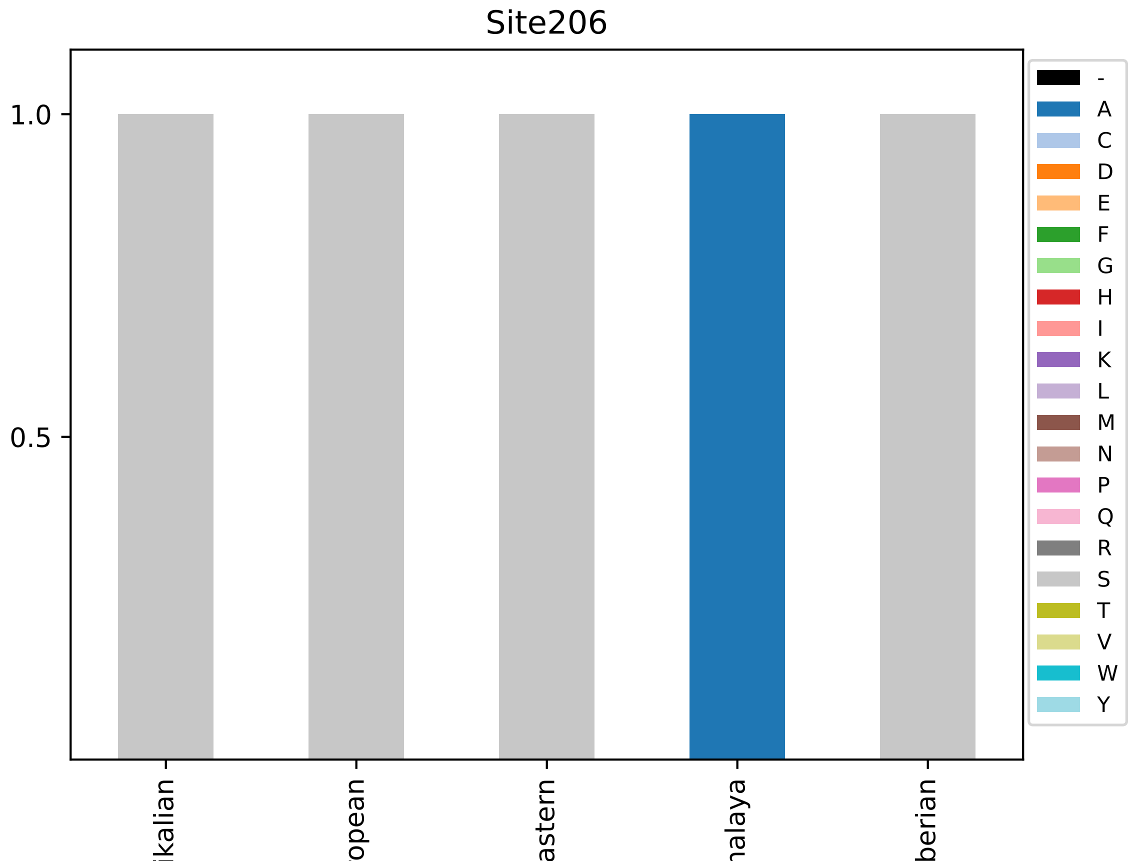
Site206
1.0
0.5
-
A
C
D
E
F
G
H
I
K
L
M
N
P
Q
R
S
T
V
W
Y
ikalian
opean
astern
nalaya
berian

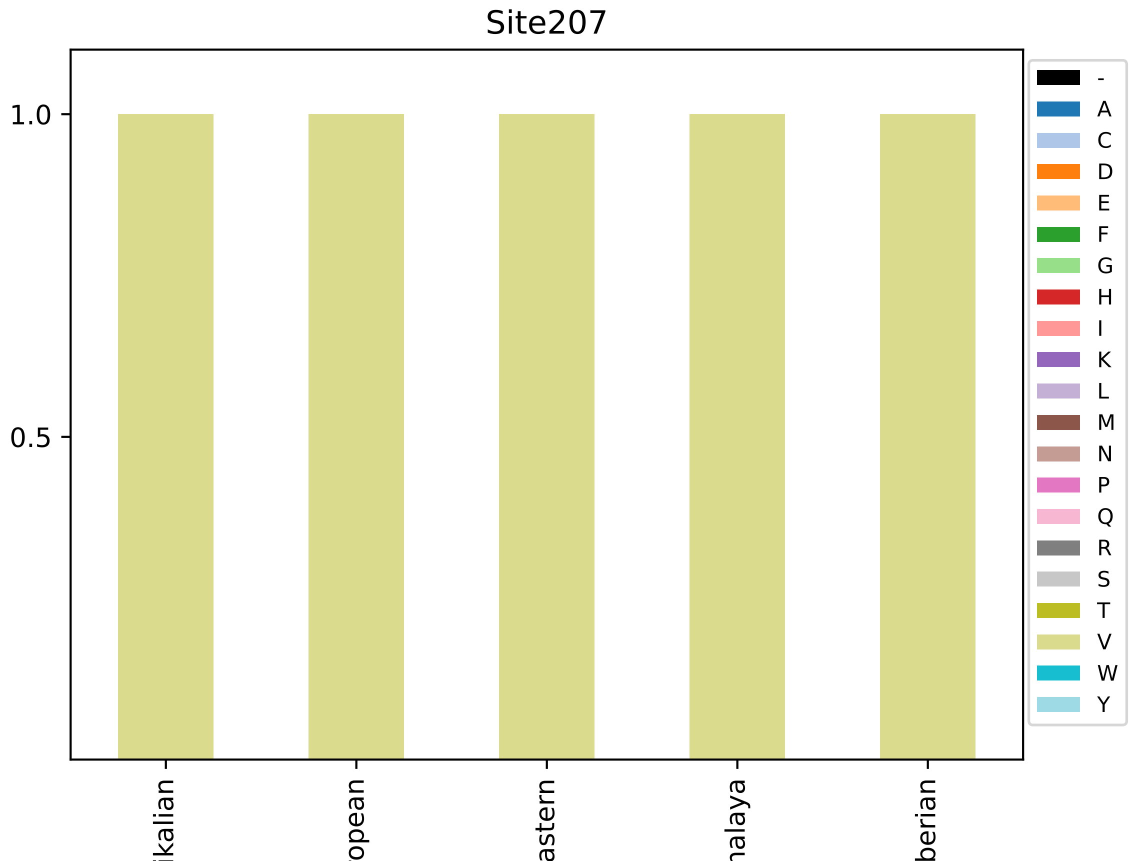
Site207
1.0
0.5
-
A
C
D
E
F
G
H
I
K
L
M
N
P
Q
R
S
T
V
W
Y
ikalian
opean
astern
nalaya
berian

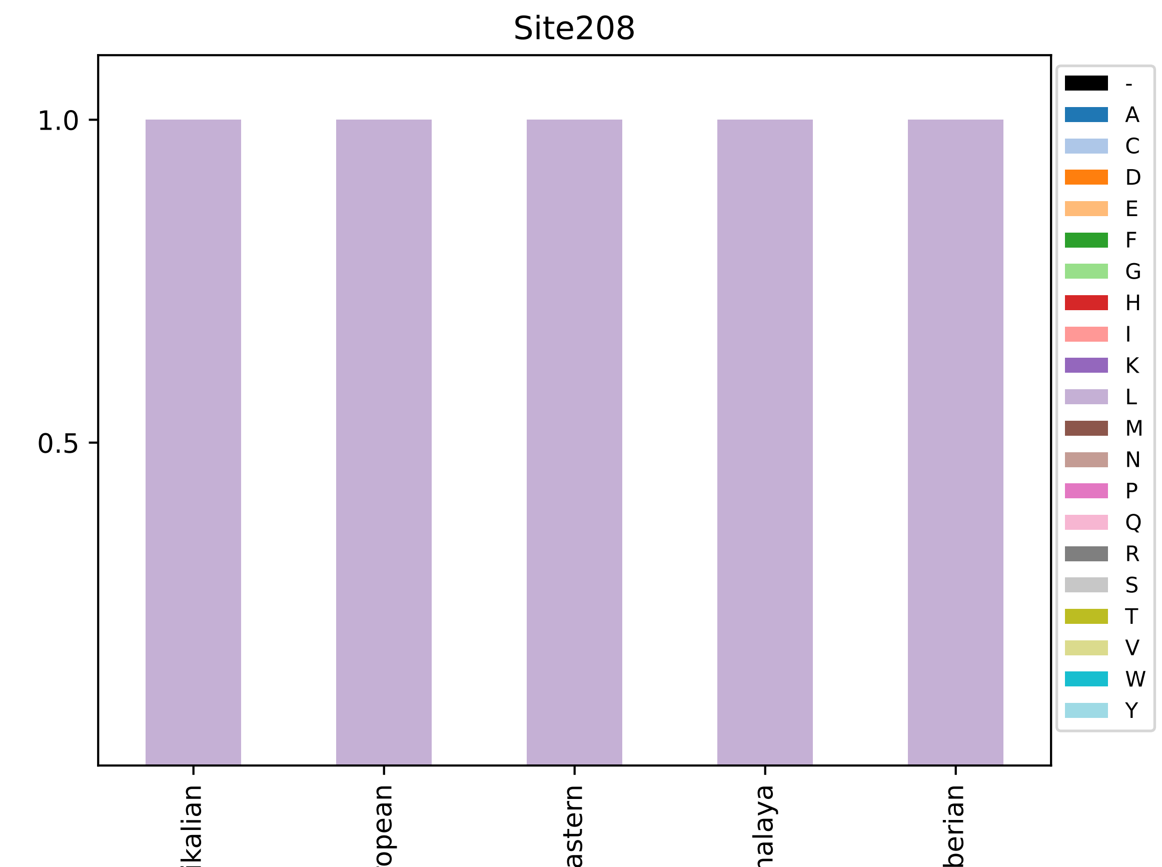
Site208
1.0
0.5
-
A
C
D
E
F
G
H
I
K
L
M
N
P
Q
R
S
T
V
W
Y
ikalian
opean
astern
nalaya
berian

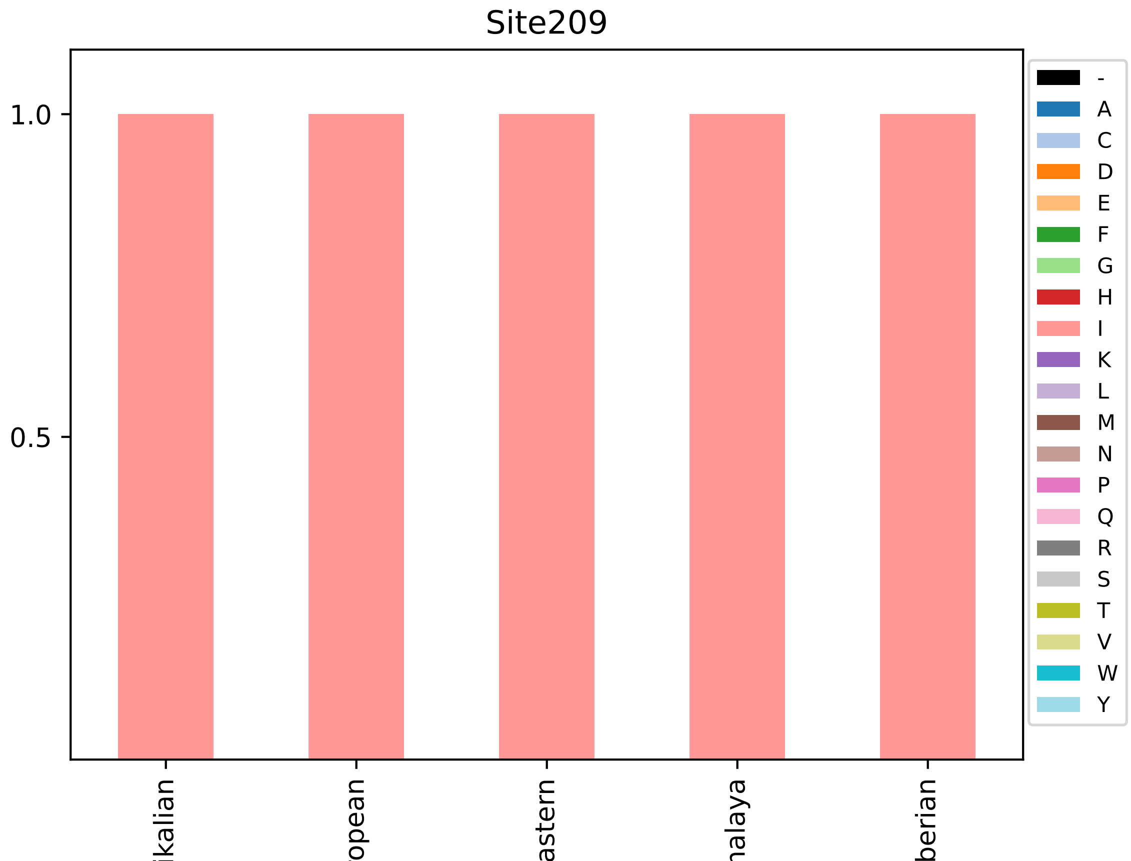
Site209
1.0
0.5
-
A
C
D
E
F
G
H
I
K
L
M
N
P
Q
R
S
T
V
W
Y
ikalian
opean
astern
nalaya
berian

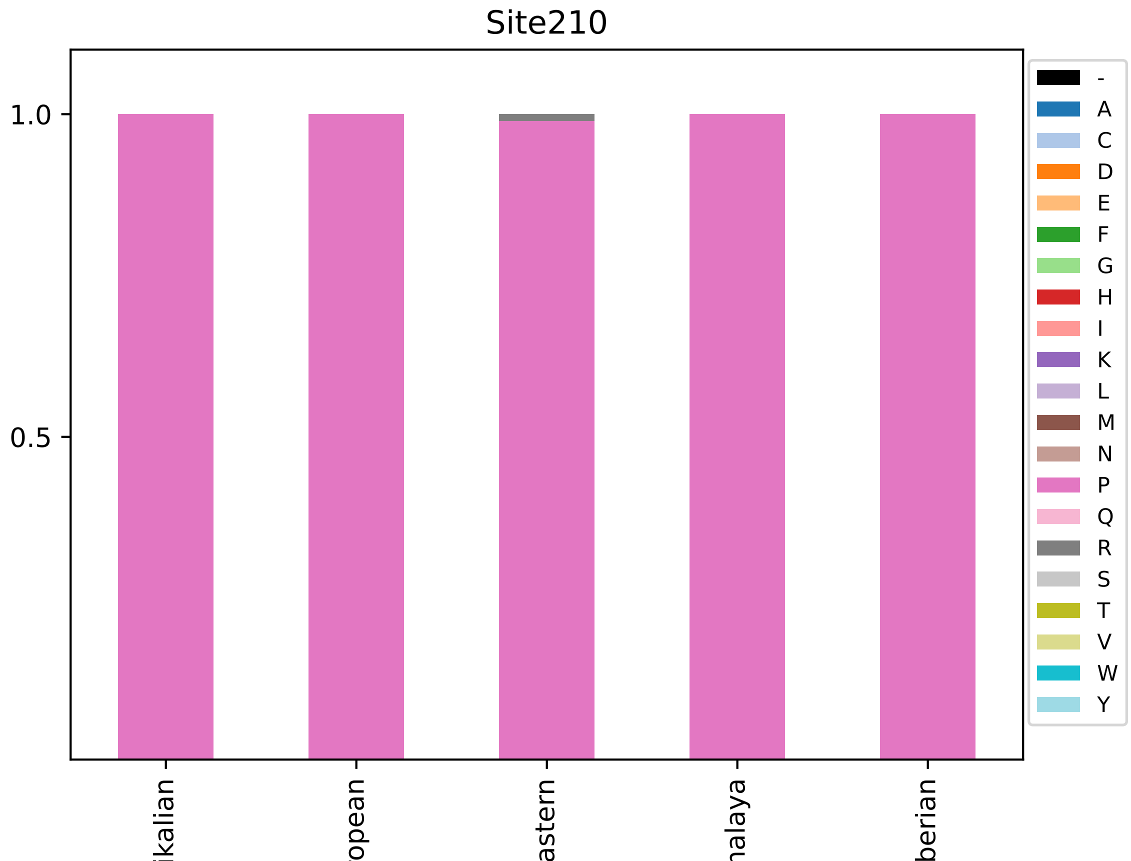
Site210
1.0
0.5
-
A
C
D
E
F
G
H
I
K
L
M
N
P
Q
R
S
T
V
W
Y
ikalian
opean
astern
nalaya
berian

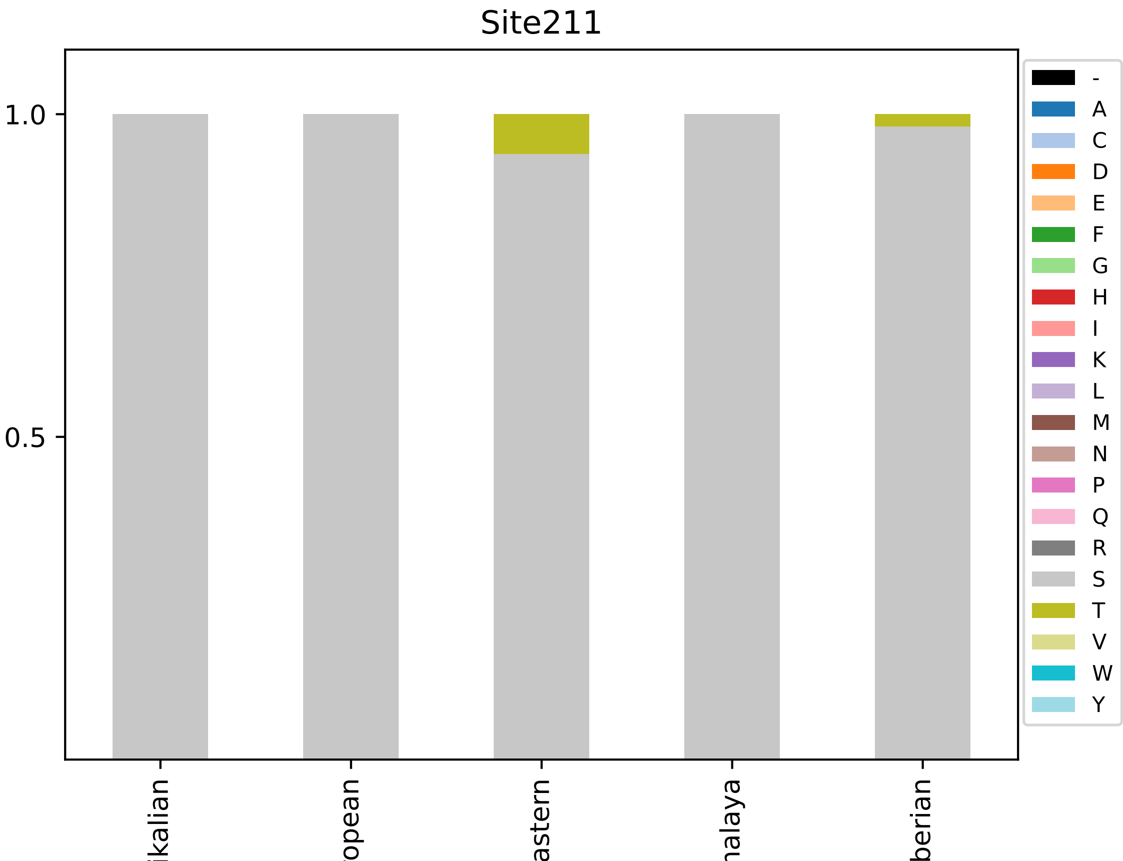
Site211
1.0
0.5
-
A
C
D
E
F
G
H
I
K
L
M
N
P
Q
R
S
T
V
W
Y

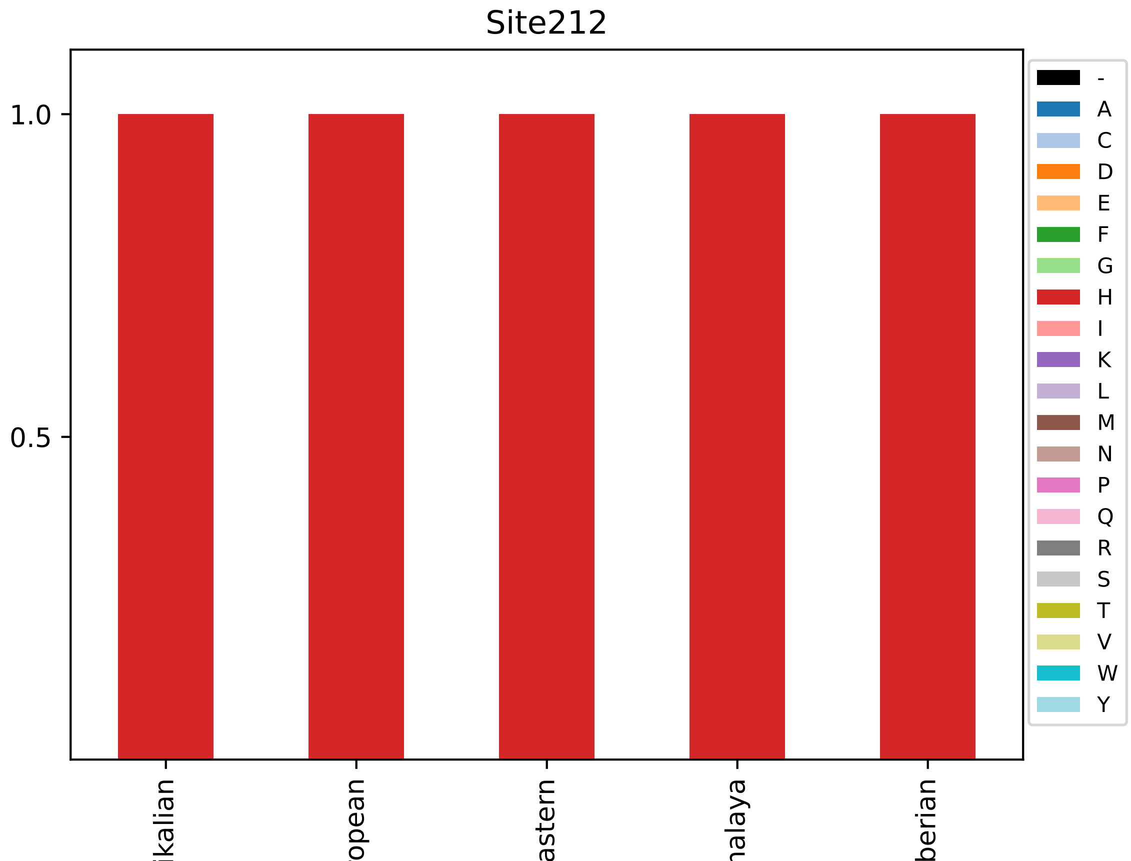

Site212
1.0
0.5
-
A
C
D
E
F
G
H
I
K
L
M
N
P
Q
R
S
T
V
W
Y

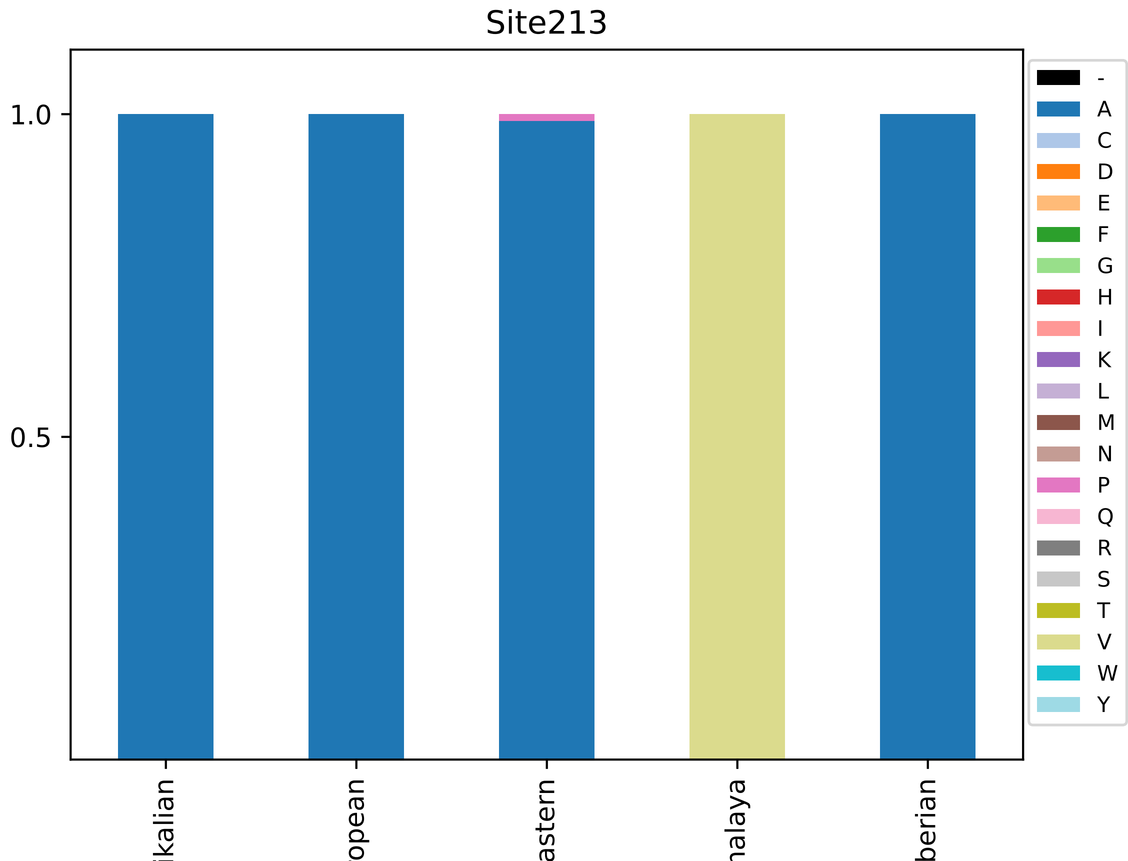

Site213
1.0
0.5
-
A
C
D
E
F
G
H
I
K
L
M
N
P
Q
R
S
T
V
W
Y
ikalian
opean
astern
nalaya
berian

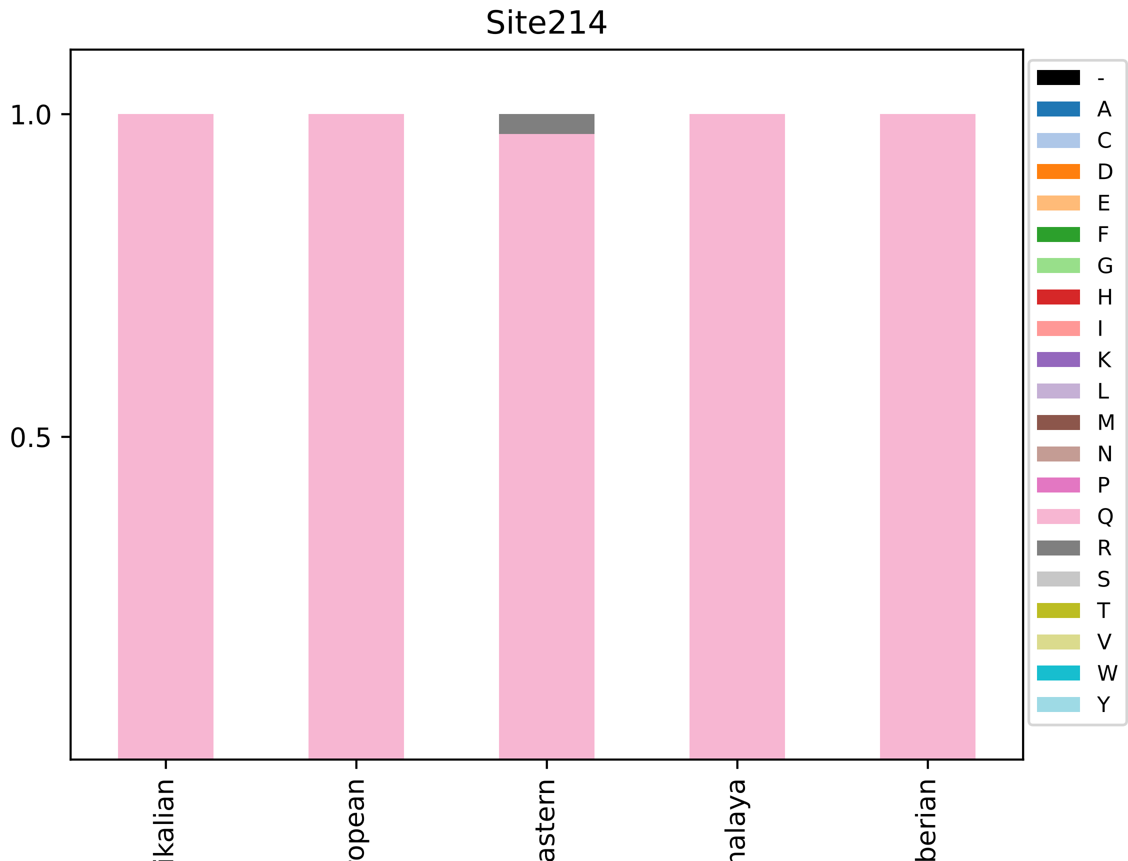

Site214
1.0
0.5
-
A
C
D
E
F
G
H
I
K
L
M
N
P
Q
R
S
T
V
W
Y
ikalian
opean
astern
nalaya
berian

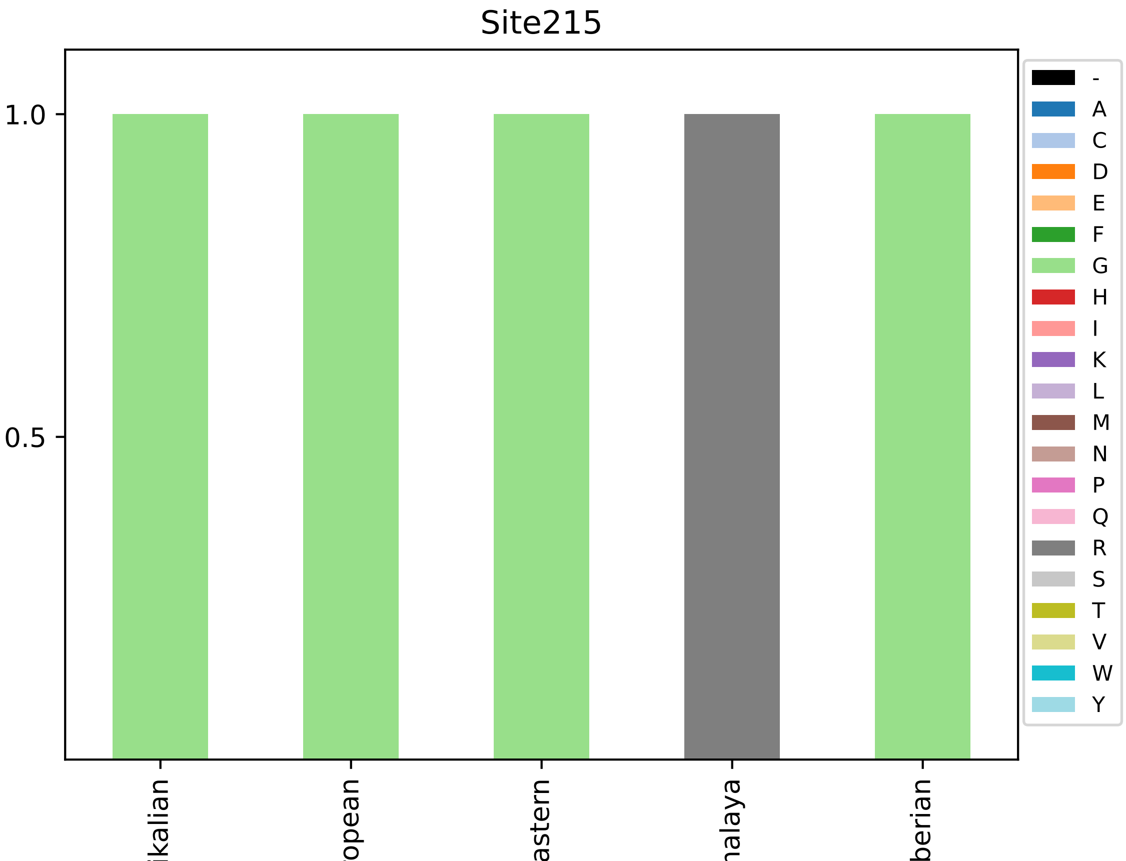
Site215
1.0
0.5
-
A
C
D
E
F
G
H
I
K
L
M
N
P
Q
R
S
T
V
W
Y
ikalian
opean
astern
nalaya
berian

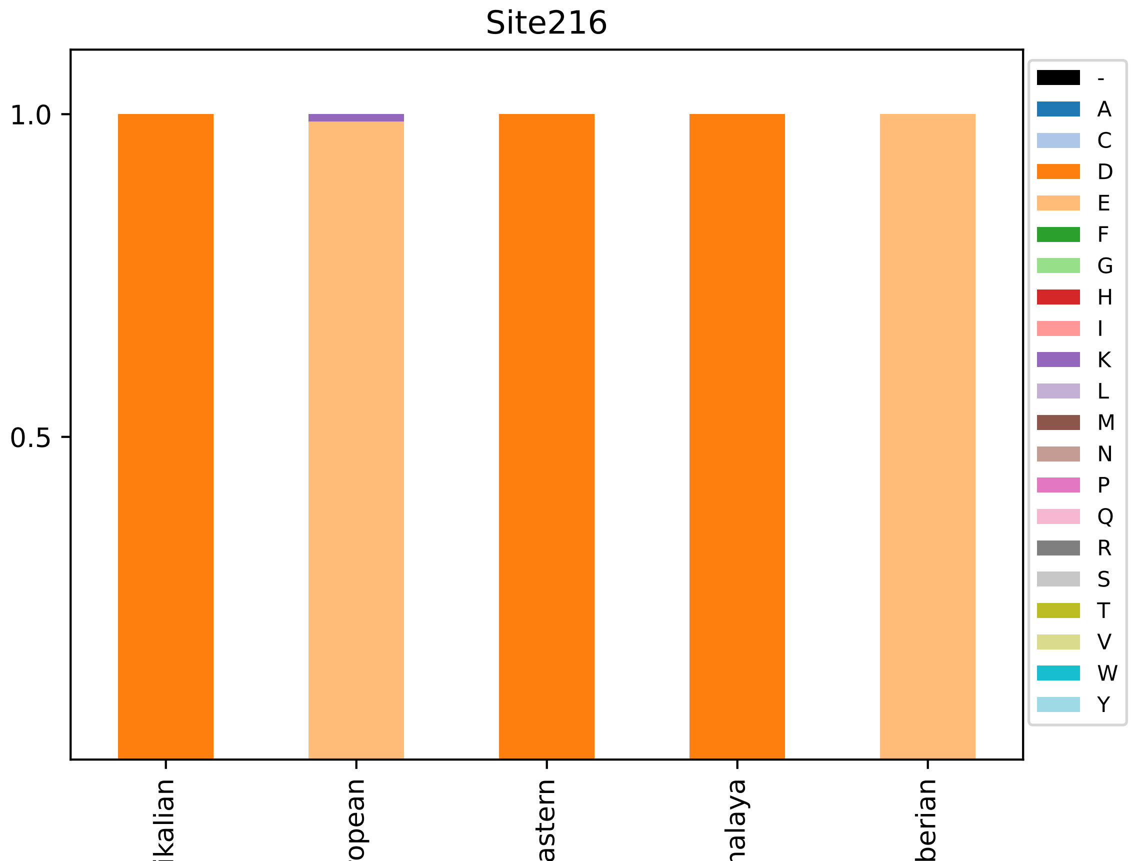

Site216
1.0
0.5
-
A
C
D
E
F
G
H
I
K
L
M
N
P
Q
R
S
T
V
W
Y
ikalian
opean
astern
nalaya
berian

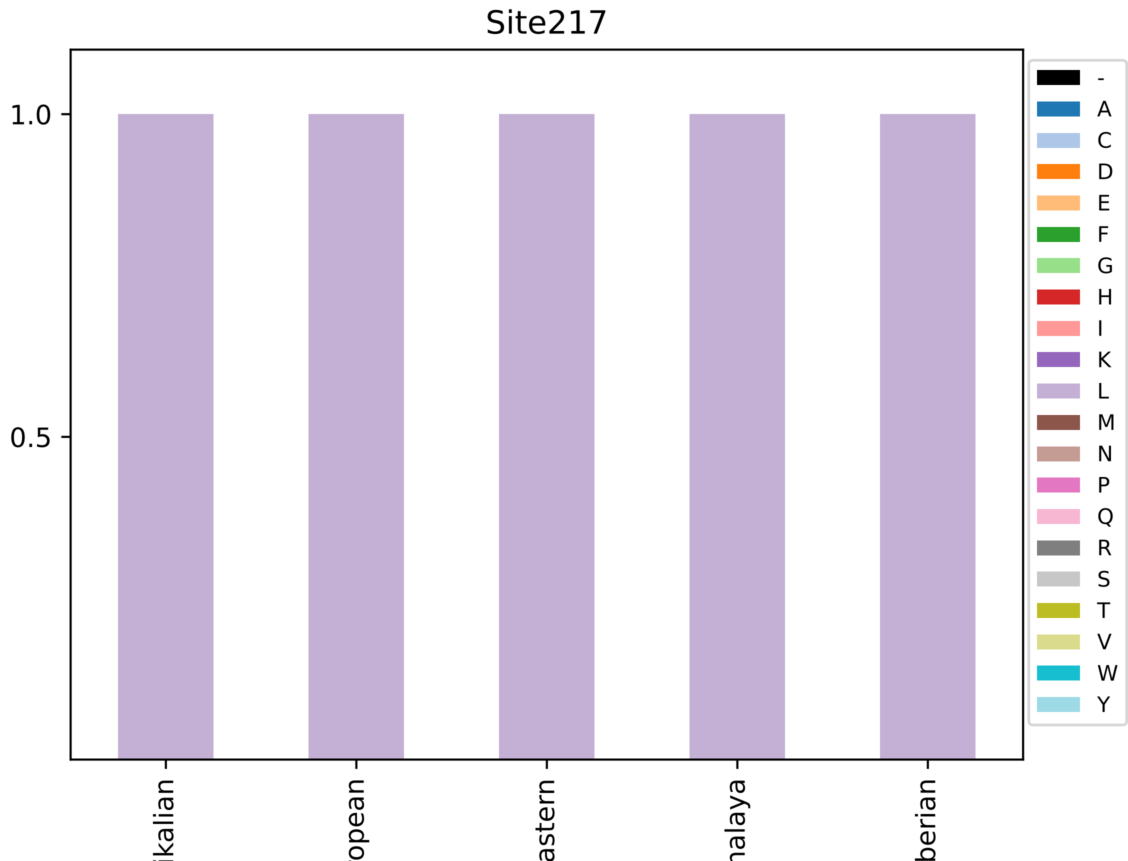

Site217
1.0
0.5
ikalian
opean
astern
nalaya
berian
-
A
C
D
E
F
G
H
I
K
L
M
N
P
Q
R
S
T
V
W
Y

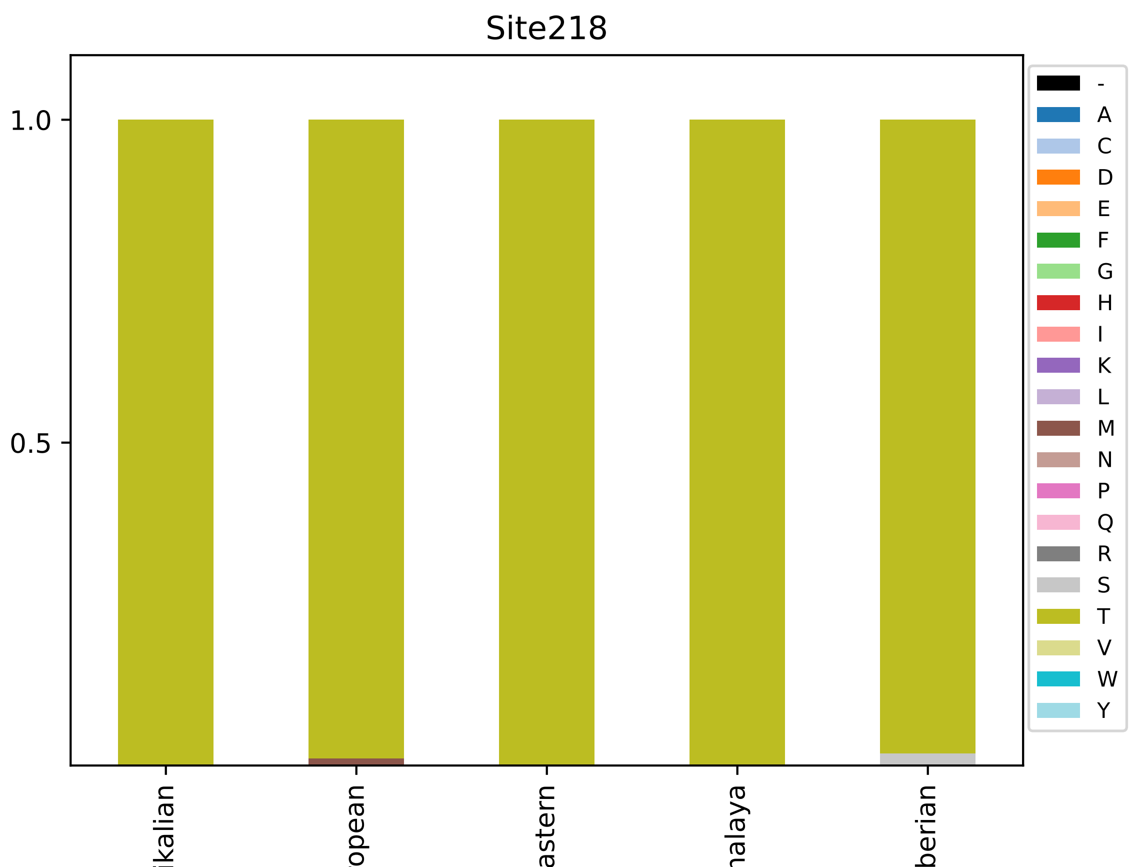
Site218
1.0
0.5
-
A
C
D
E
F
G
H
I
K
L
M
N
P
Q
R
S
T
V
W
Y
ikalian
opean
astern
nalaya
berian

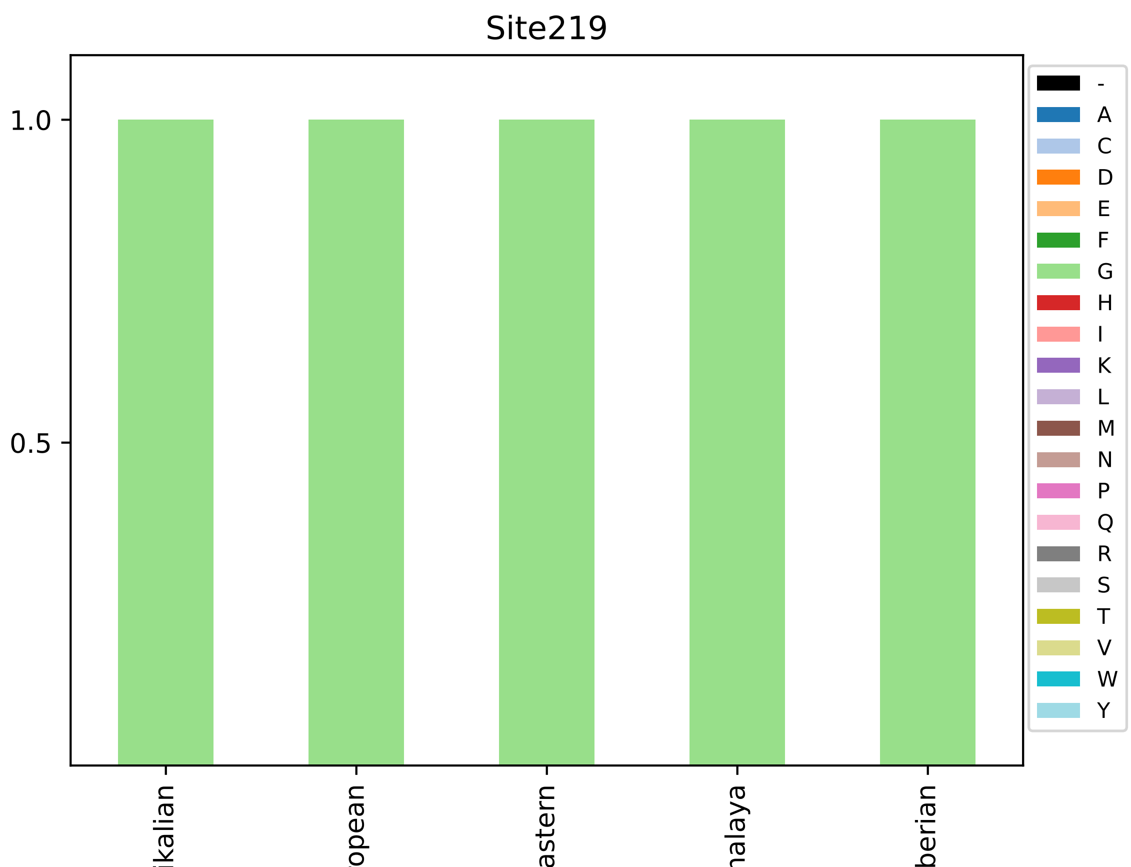
Site219
1.0
0.5
ikalian
opean
astern
nalaya
berian
-
A
C
D
E
F
G
H
I
K
L
M
N
P
Q
R
S
T
V
W
Y

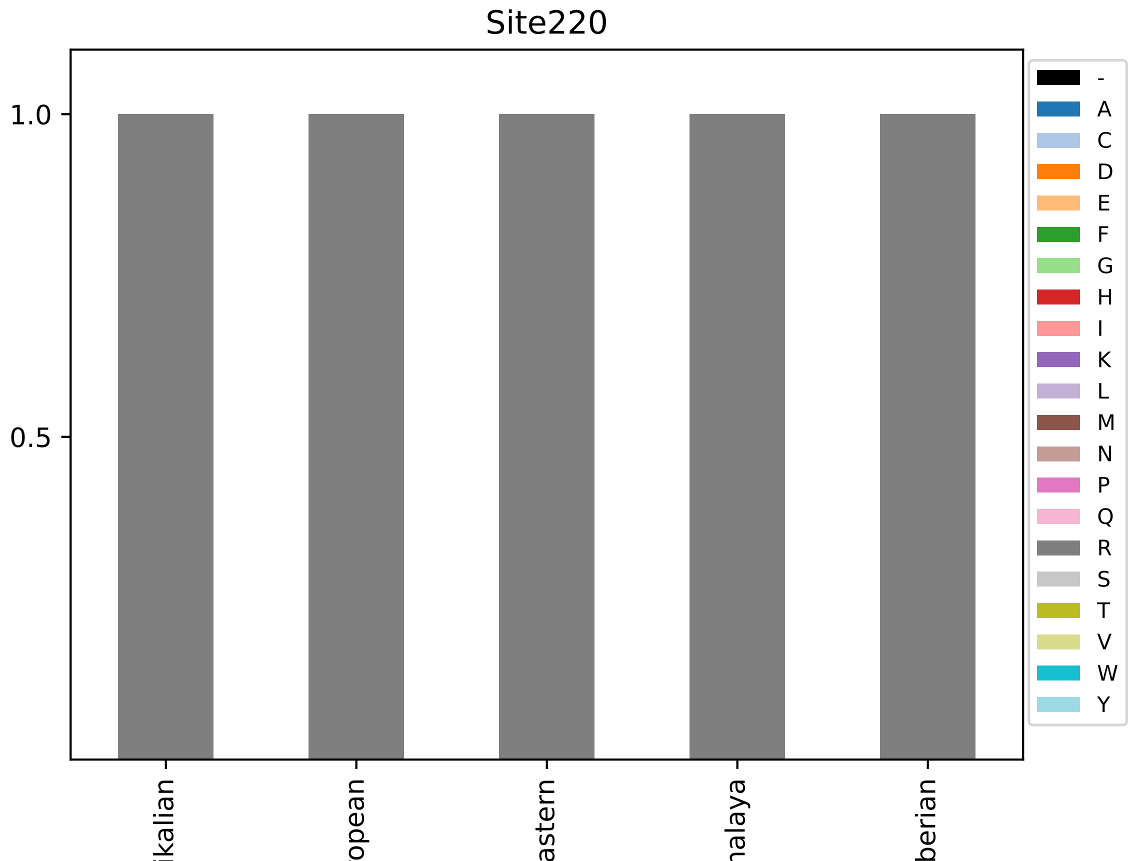

Site220
1.0
0.5
ikalian
opean
astern
nalaya
berian
-
A
C
D
E
F
G
H
I
K
L
M
N
P
Q
R
S
T
V
W
Y

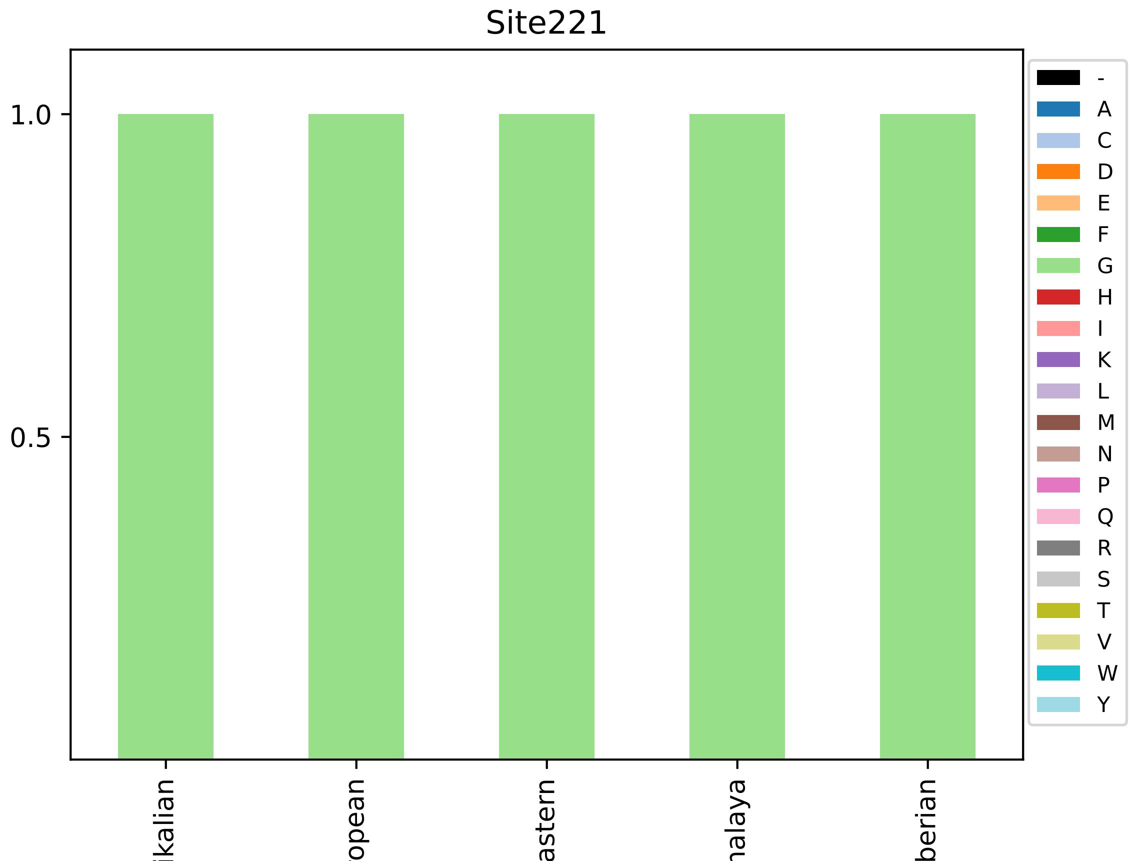

Site221
1.0
0.5
-
A
C
D
E
F
G
H
I
K
L
M
N
P
Q
R
S
T
V
W
Y
ikalian
opean
astern
nalaya
berian

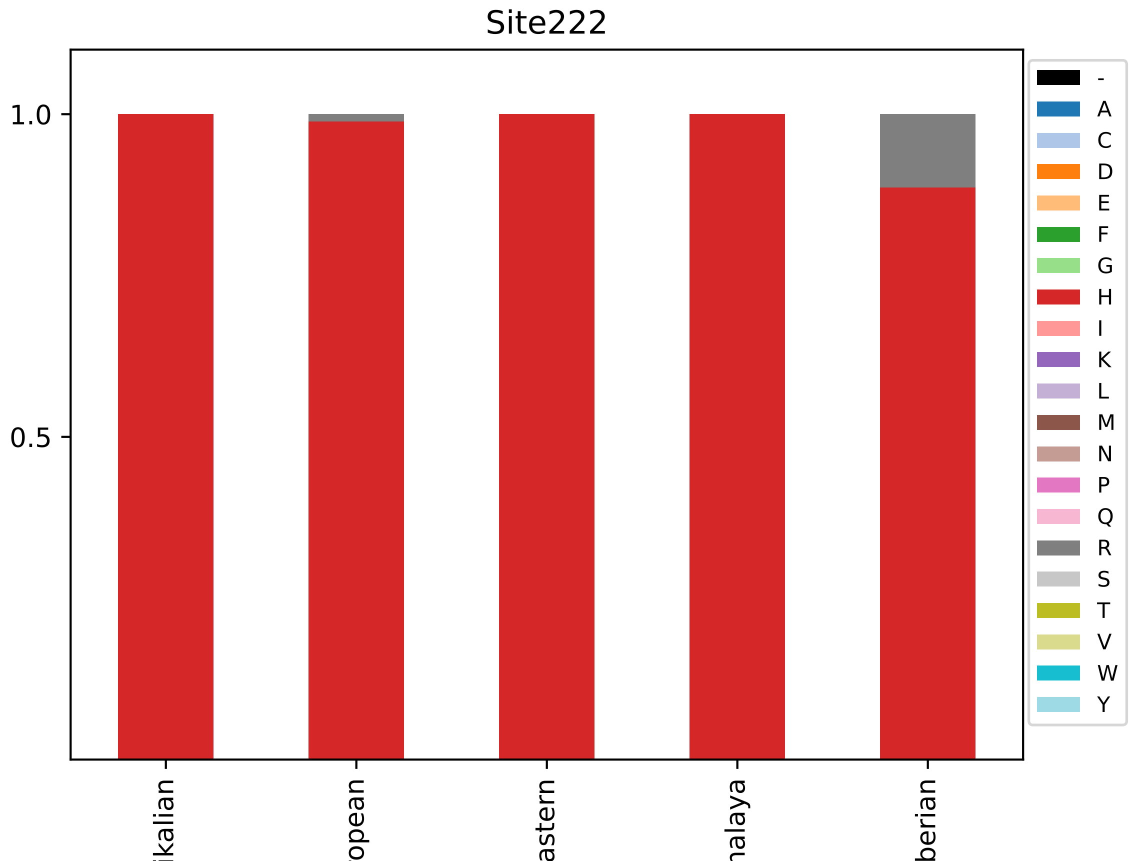
Site222
1.0
0.5
ikalian
opean
astern
nalaya
berian
-
A
C
D
E
F
G
H
I
K
L
M
N
P
Q
R
S
T
V
W
Y

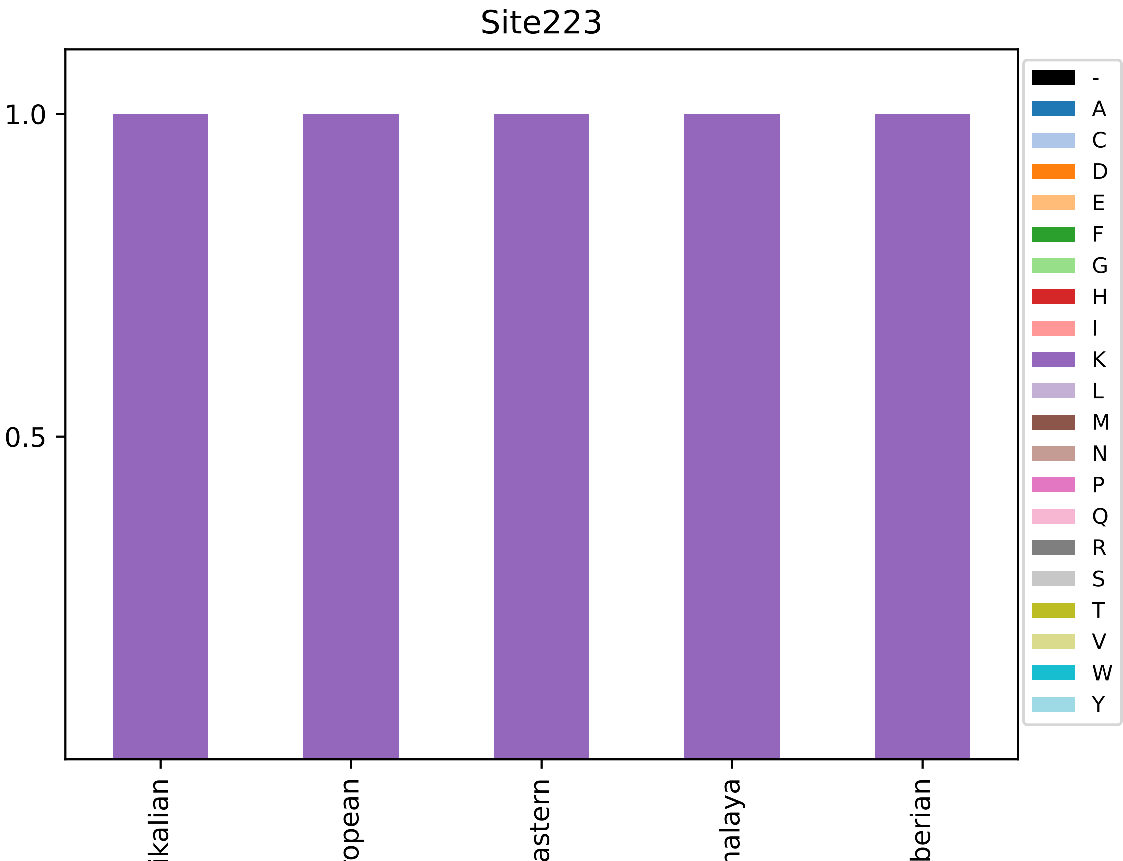
Site223
1.0
0.5
-
A
C
D
E
F
G
H
I
K
L
M
N
P
Q
R
S
T
V
W
Y
ikalian
opean
astern
nalaya
berian

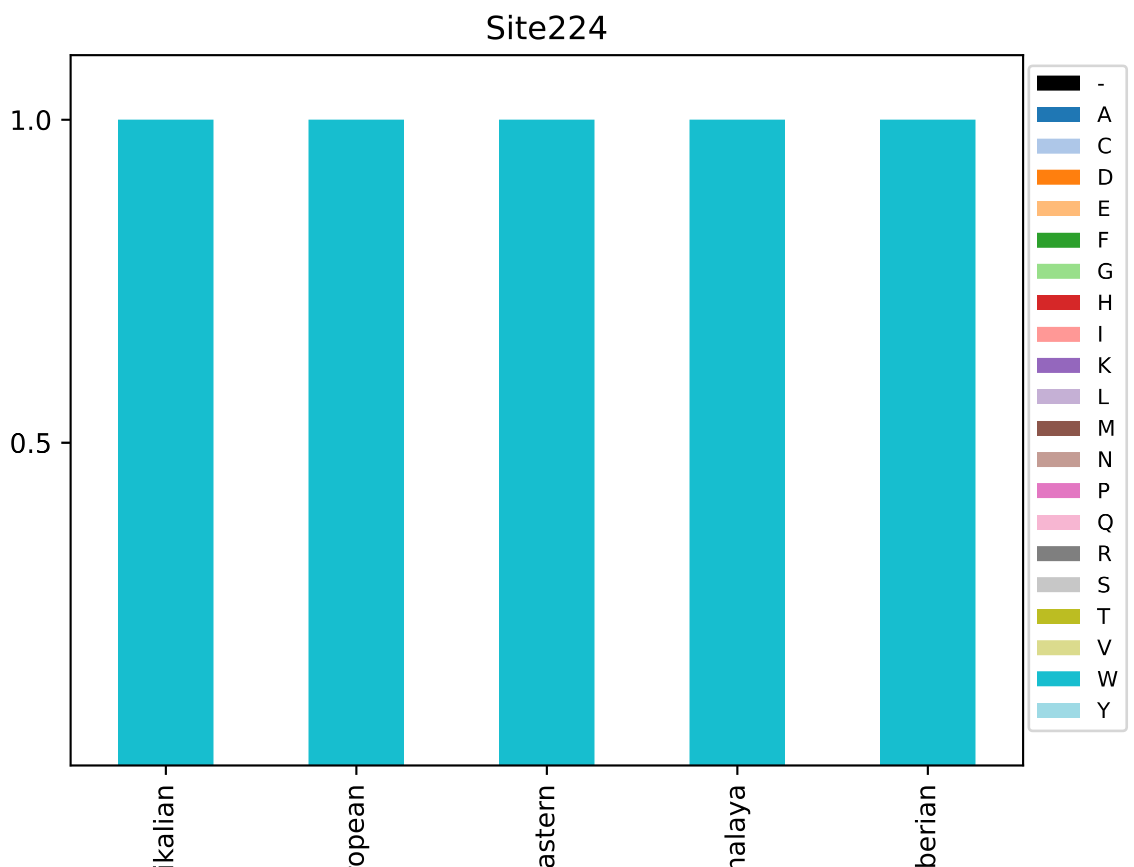
Site224
1.0
0.5
-
A
C
D
E
F
G
H
I
K
L
M
N
P
Q
R
S
T
V
W
Y
ikalian
opean
astern
nalaya
berian

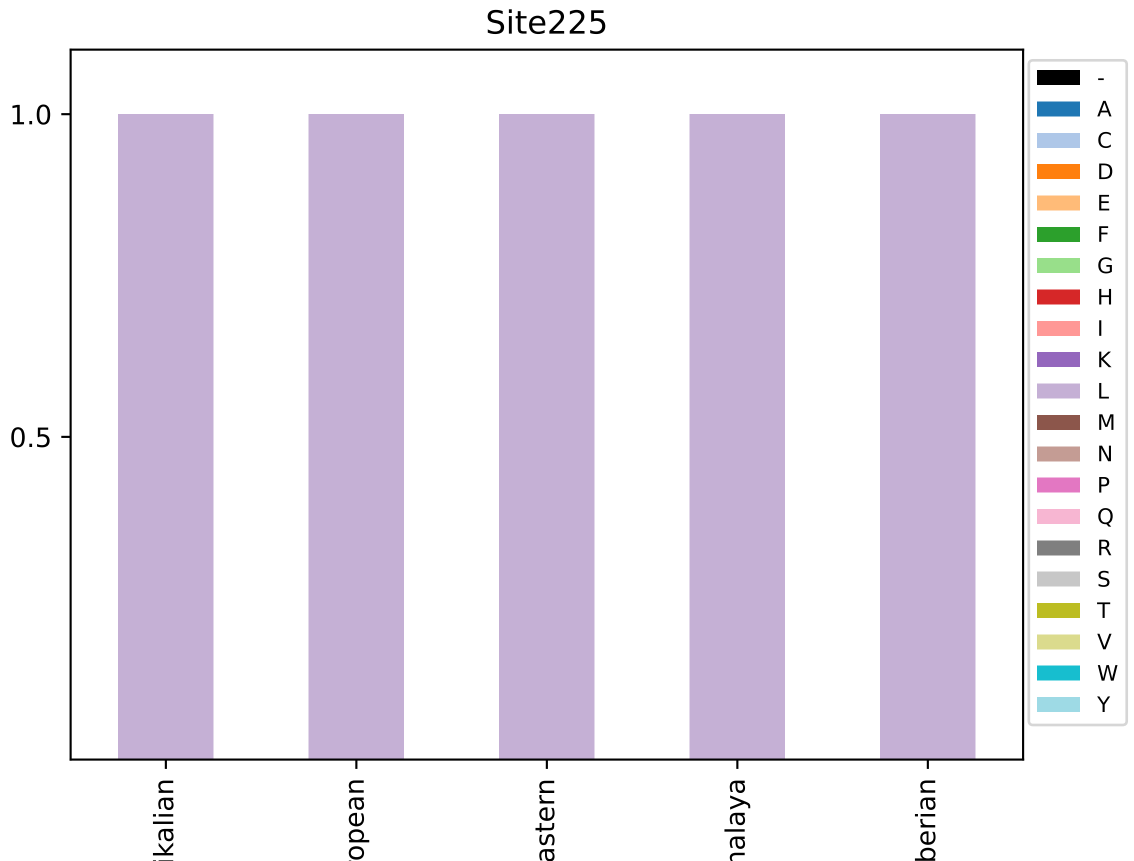
Site225
1.0
0.5
-
A
C
D
E
F
G
H
I
K
L
M
N
P
Q
R
S
T
V
W
Y
ikalian
opean
astern
nalaya
berian

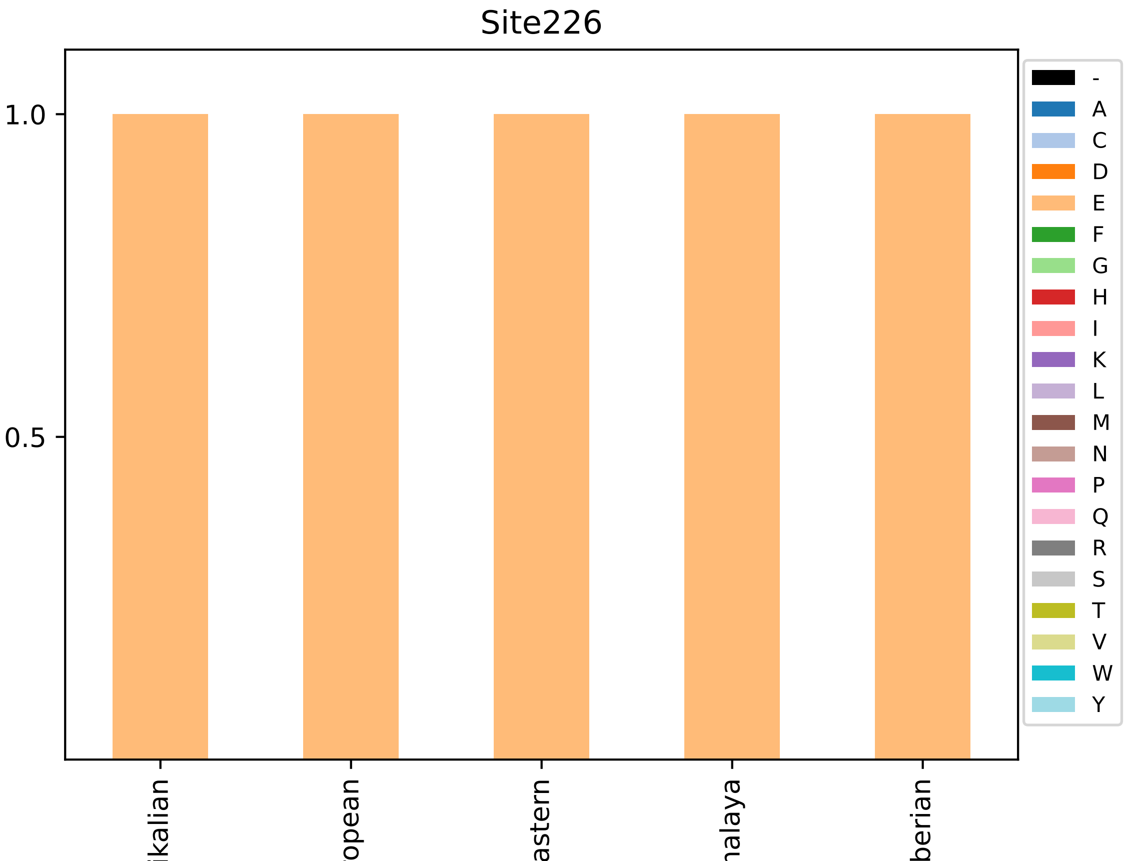
Site226
1.0
0.5
-
A
C
D
E
F
G
H
I
K
L
M
N
P
Q
R
S
T
V
W
Y
ikalian
opean
astern
nalaya
berian

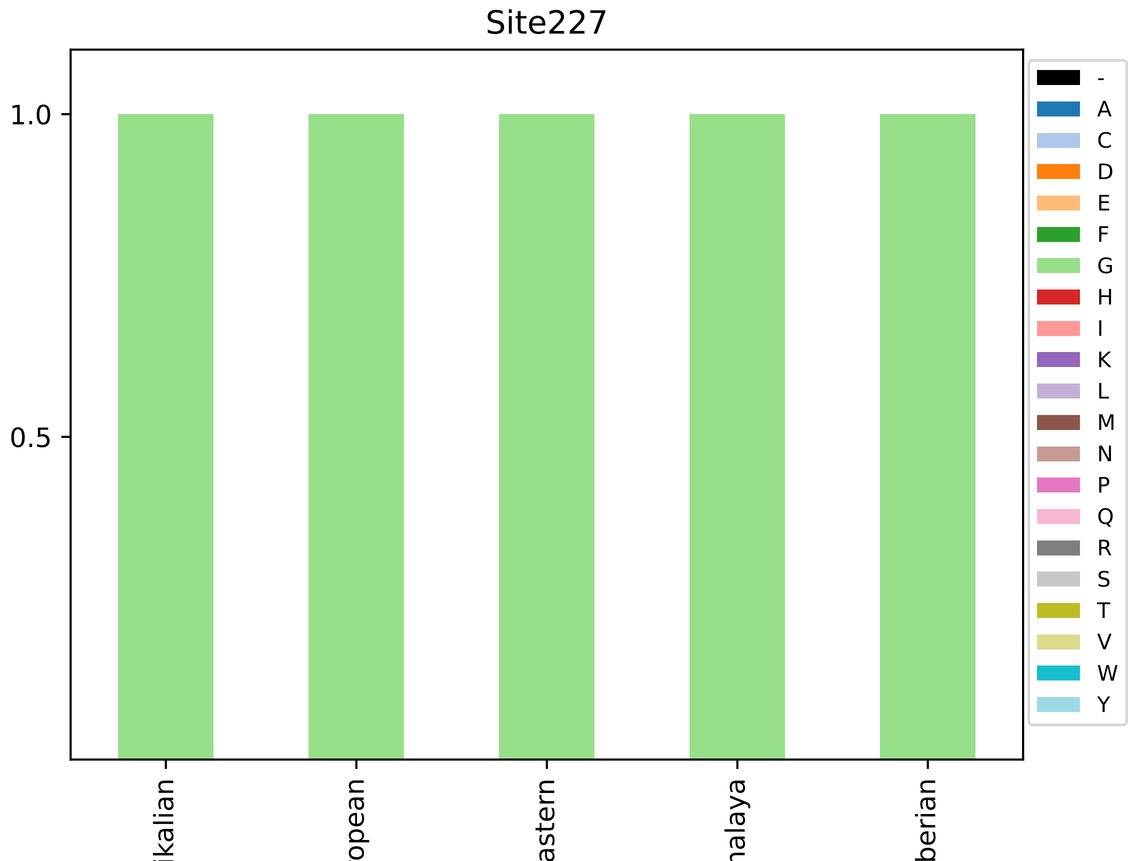
Site227
1.0
0.5
-
A
C
D
E
F
G
H
I
K
L
M
N
P
Q
R
S
T
V
W
Y

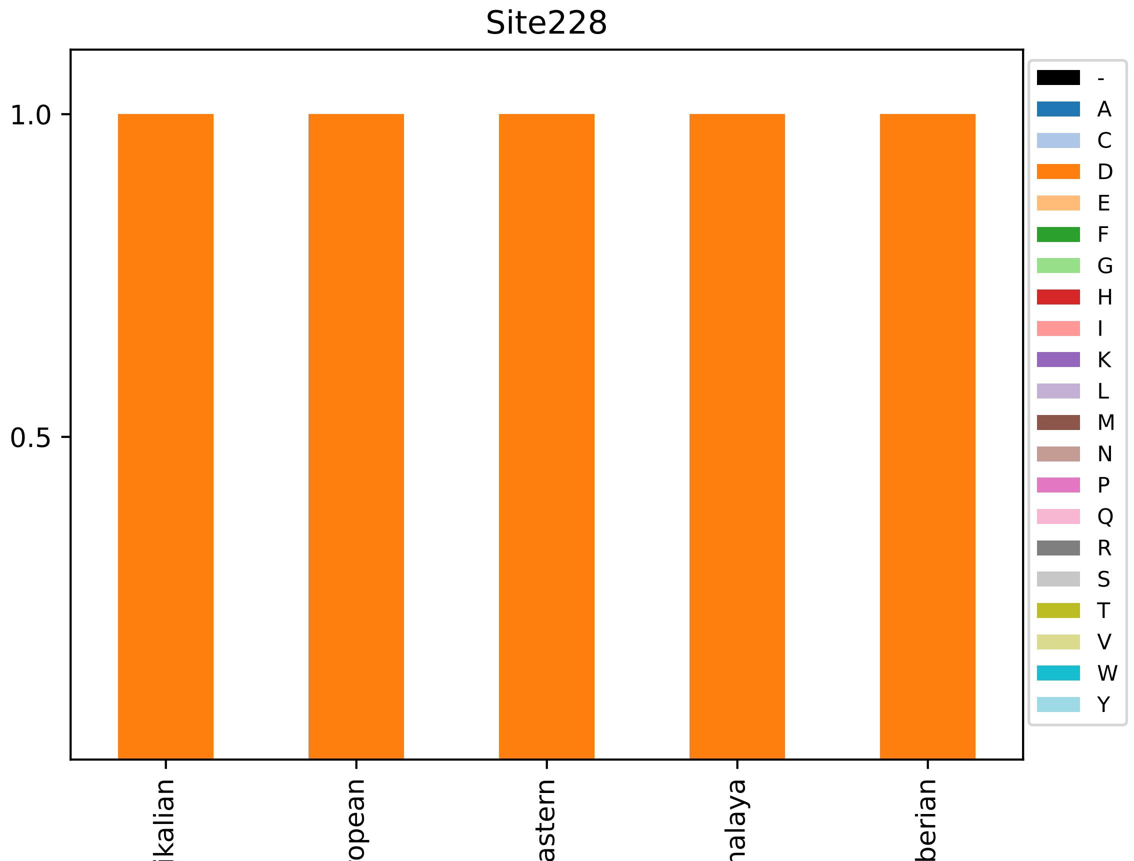

Site228
1.0
0.5
-
A
C
D
E
F
G
H
I
K
L
M
N
P
Q
R
S
T
V
W
Y
ikalian
opean
astern
nalaya
berian

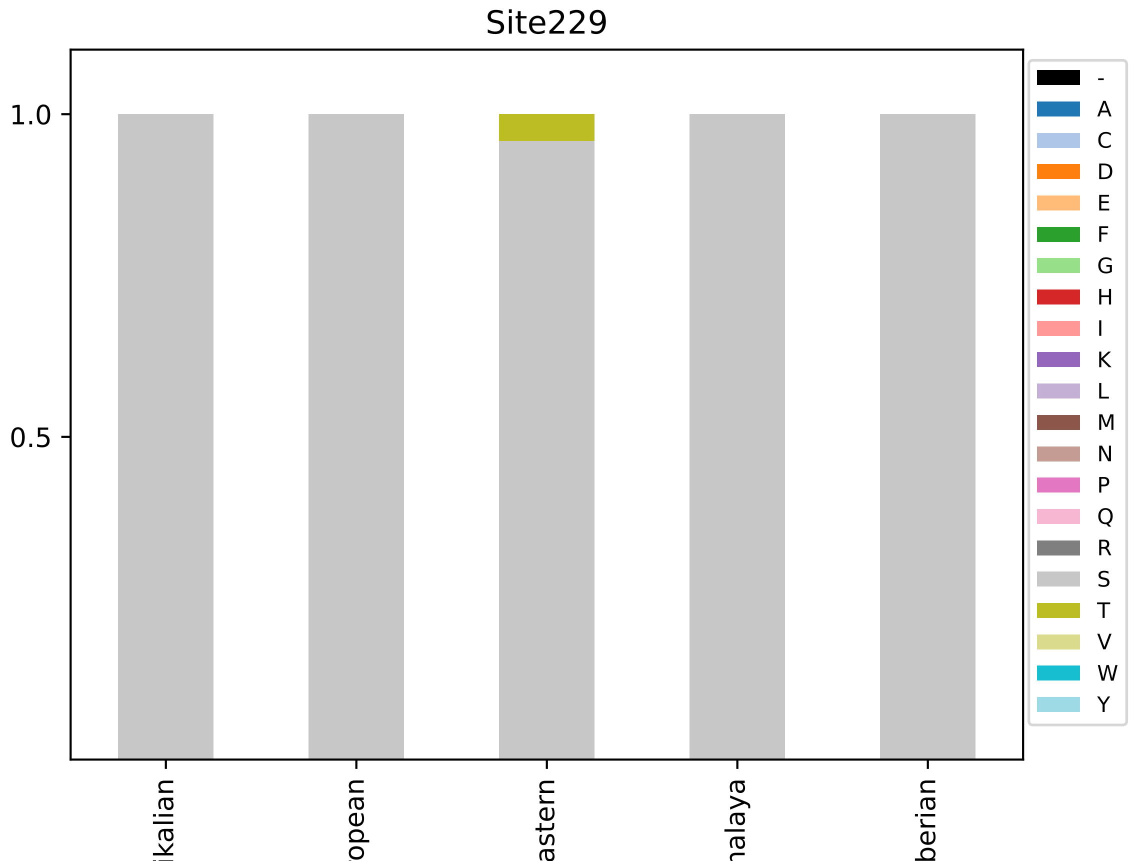

Site229
1.0
0.5
-
A
C
D
E
F
G
H
I
K
L
M
N
P
Q
R
S
T
V
W
Y
ikalian
opean
astern
nalaya
berian

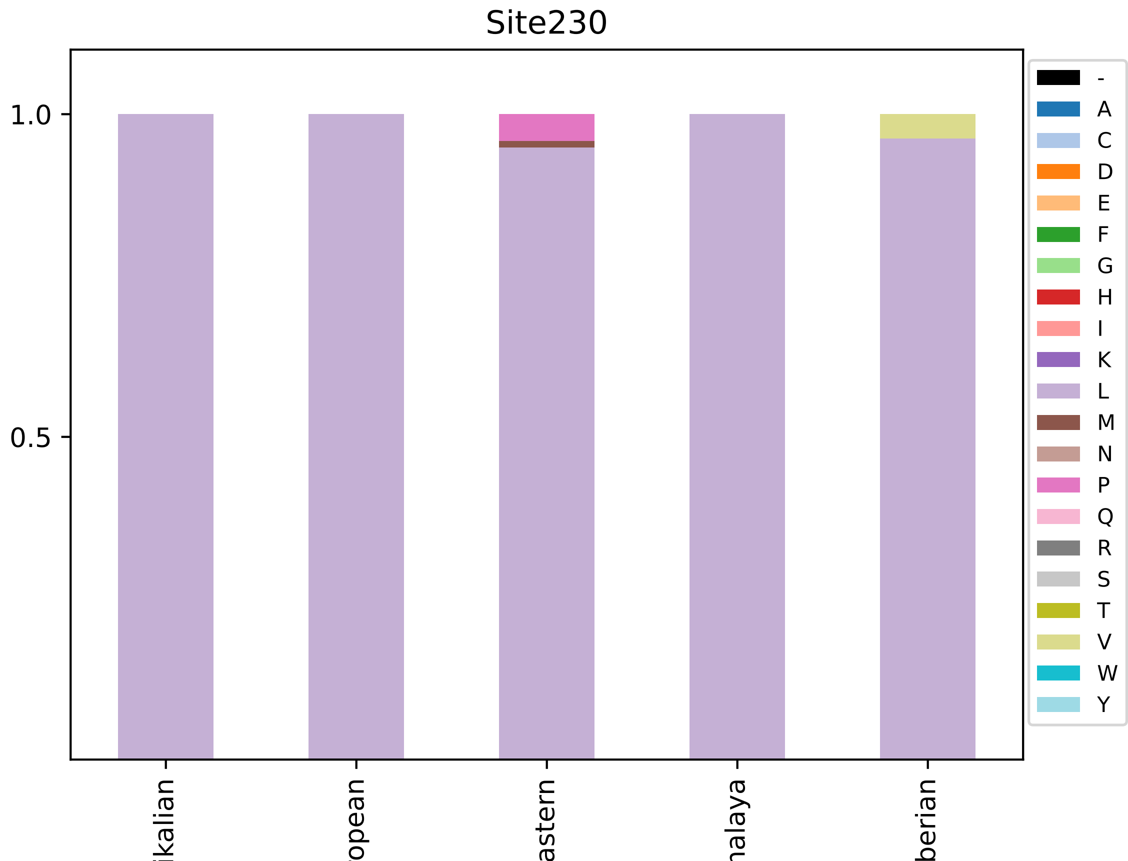
Site230
1.0
0.5
-
A
C
D
E
F
G
H
I
K
L
M
N
P
Q
R
S
T
V
W
Y
ikalian
opean
astern
nalaya
berian

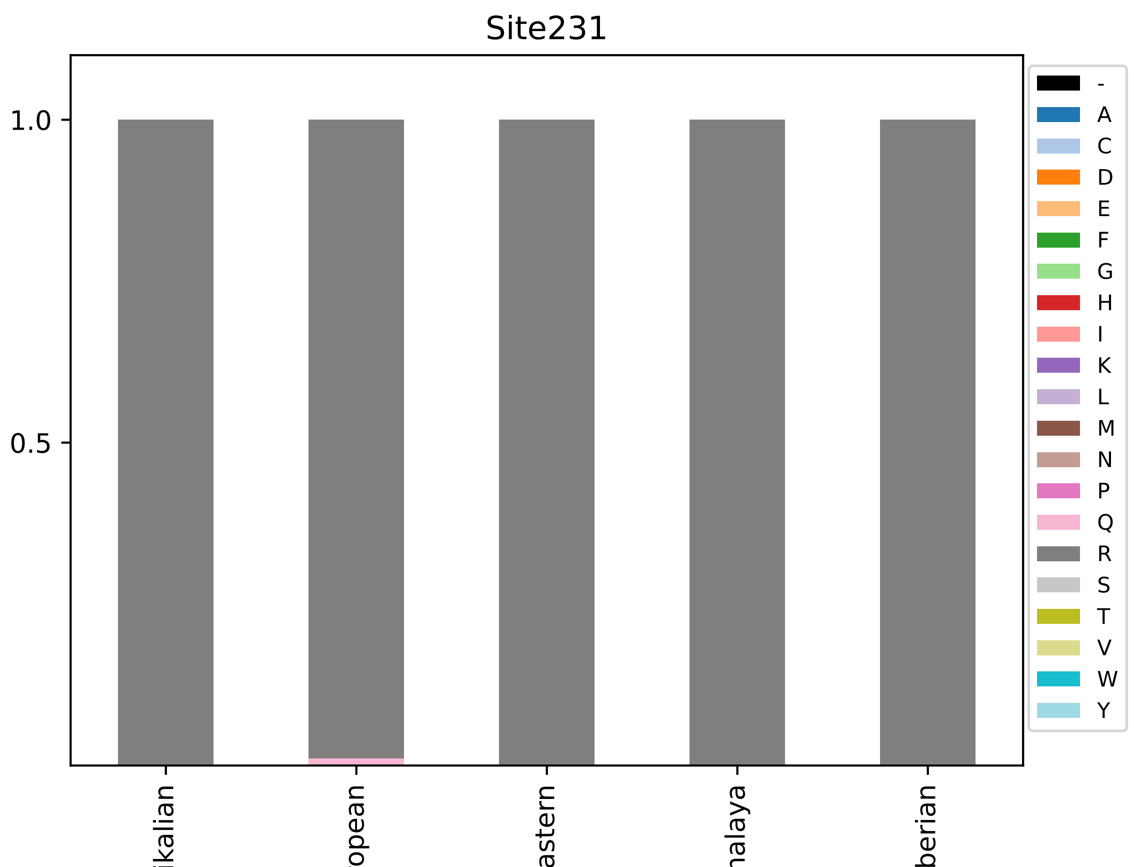

Site231
1.0
0.5
-
A
C
D
E
F
G
H
I
K
L
M
N
P
Q
R
S
T
V
W
Y
ikalian
opean
astern
nalaya
berian

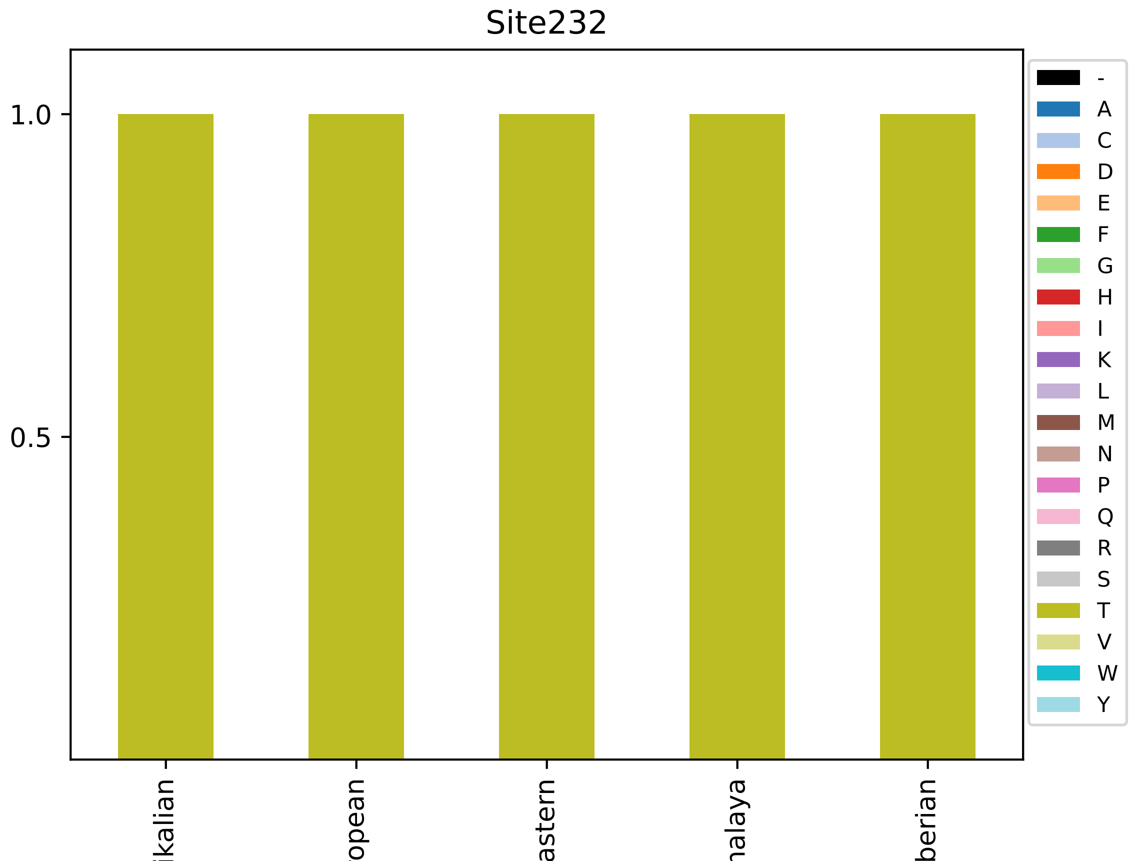

Site232
1.0
0.5
-
A
C
D
E
F
G
H
I
K
L
M
N
P
Q
R
S
T
V
W
Y
ikalian
opean
astern
nalaya
berian

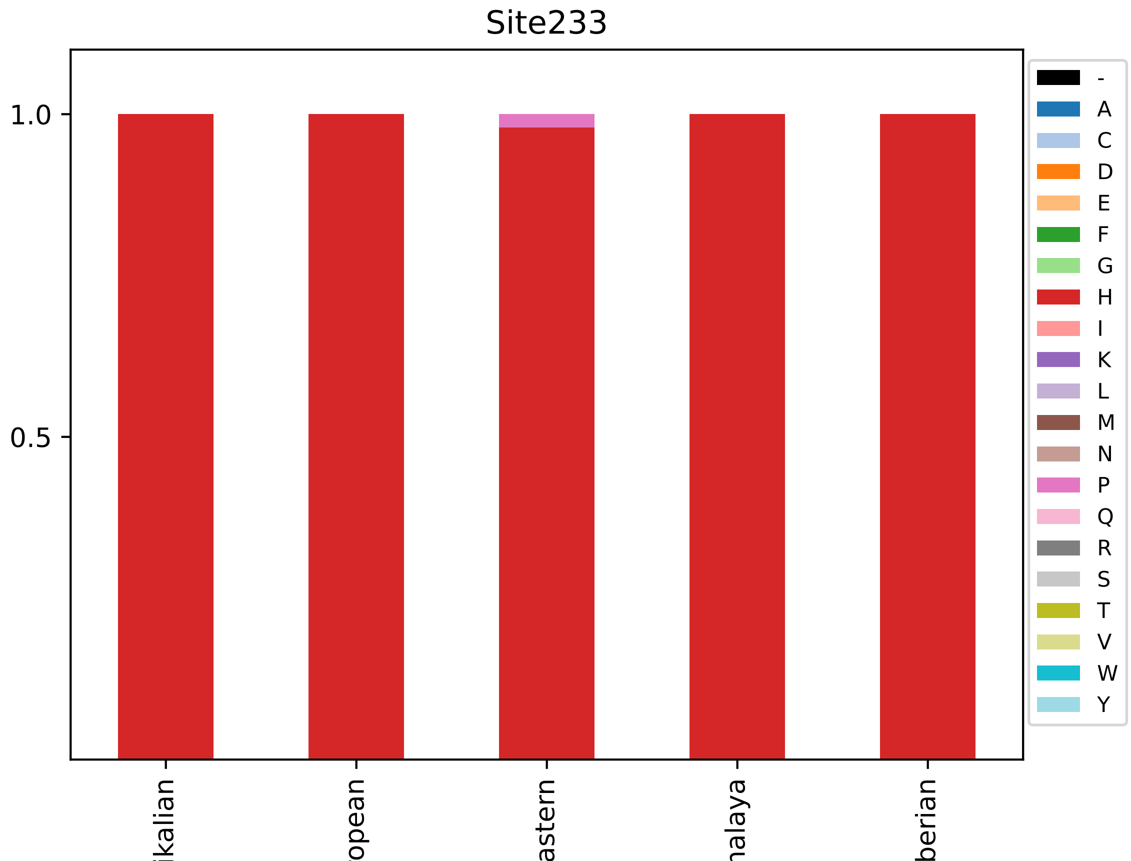
Site233
1.0
0.5
-
A
C
D
E
F
G
H
I
K
L
M
N
P
Q
R
S
T
V
W
Y
ikalian
opean
astern
nalaya
berian

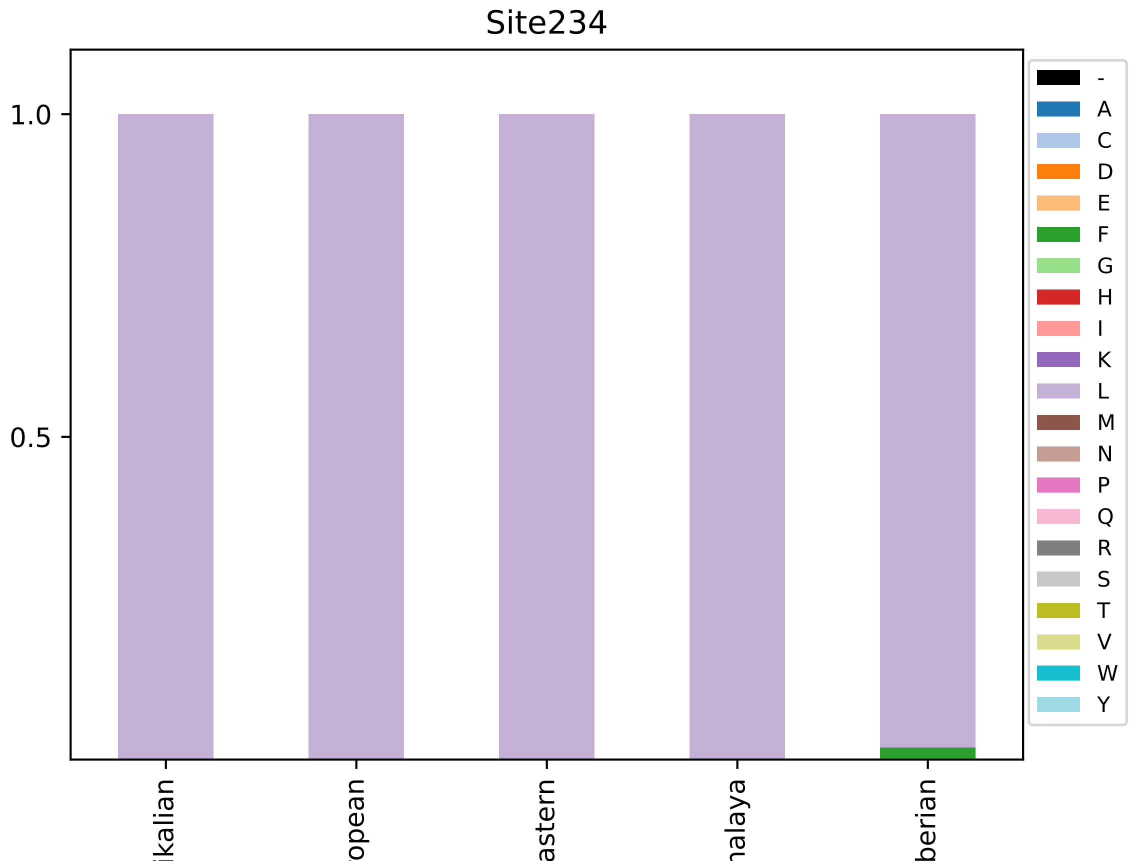
Site234
1.0
0.5
-
A
C
D
E
F
G
H
I
K
L
M
N
P
Q
R
S
T
V
W
Y
ikalian
opean
astern
nalaya
berian

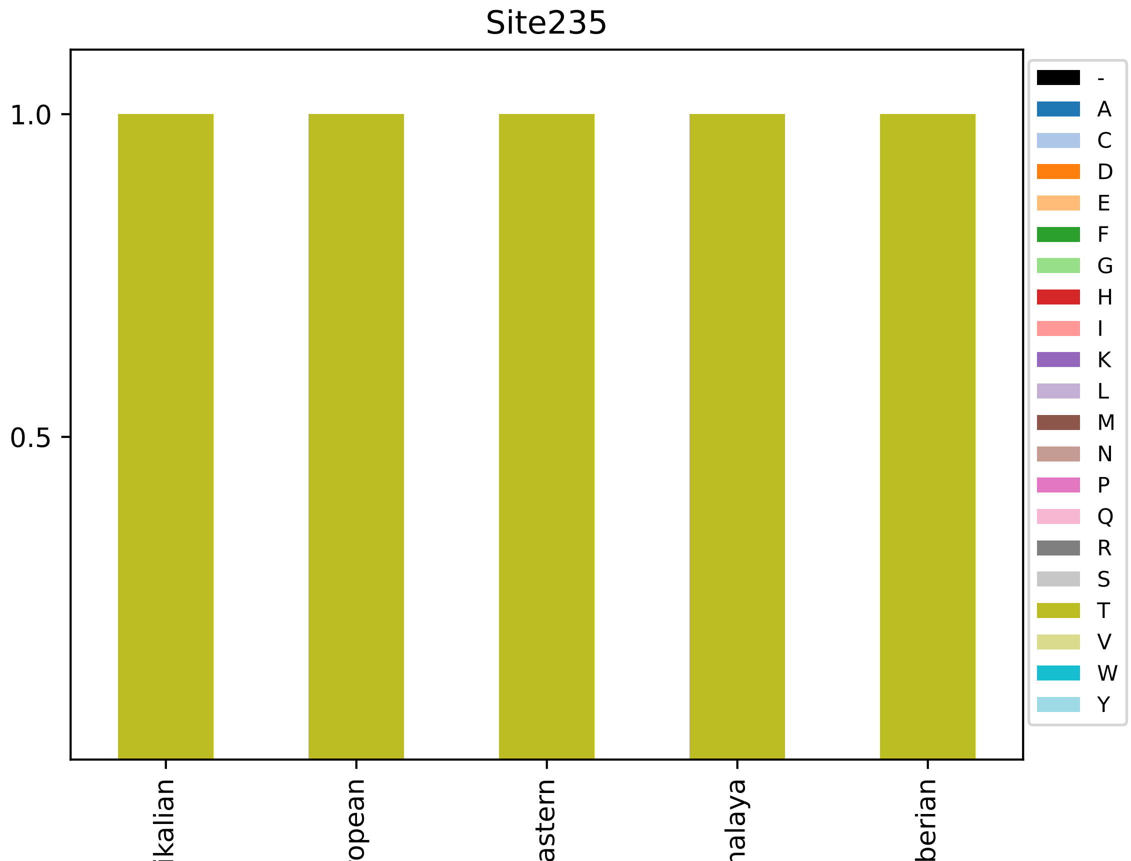

Site235
1.0
0.5
ikalian
opean
astern
nalaya
berian
-
A
C
D
E
F
G
H
I
K
L
M
N
P
Q
R
S
T
V
W
Y

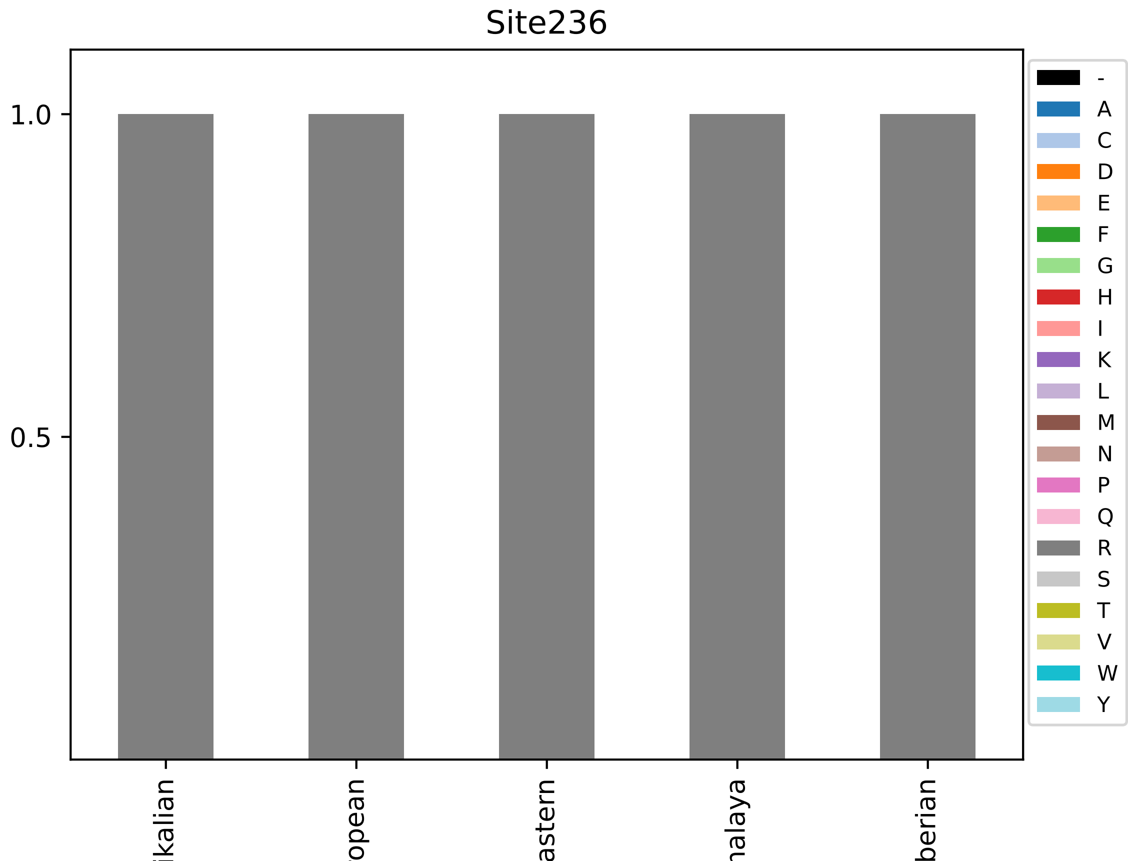
Site236
1.0
0.5
-
A
C
D
E
F
G
H
I
K
L
M
N
P
Q
R
S
T
V
W
Y
ikalian
opean
astern
nalaya
berian

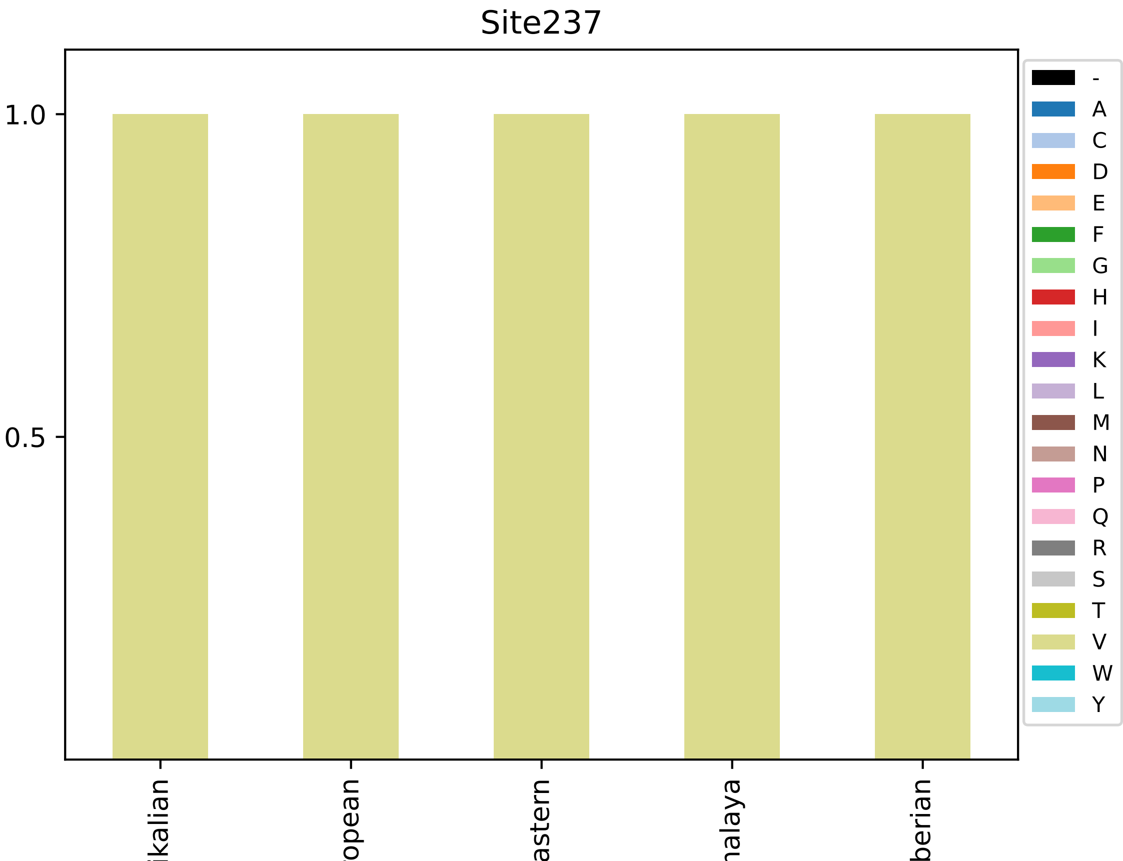

Site237
1.0
0.5
-
A
C
D
E
F
G
H
I
K
L
M
N
P
Q
R
S
T
V
W
Y
ikalian
opean
astern
nalaya
berian

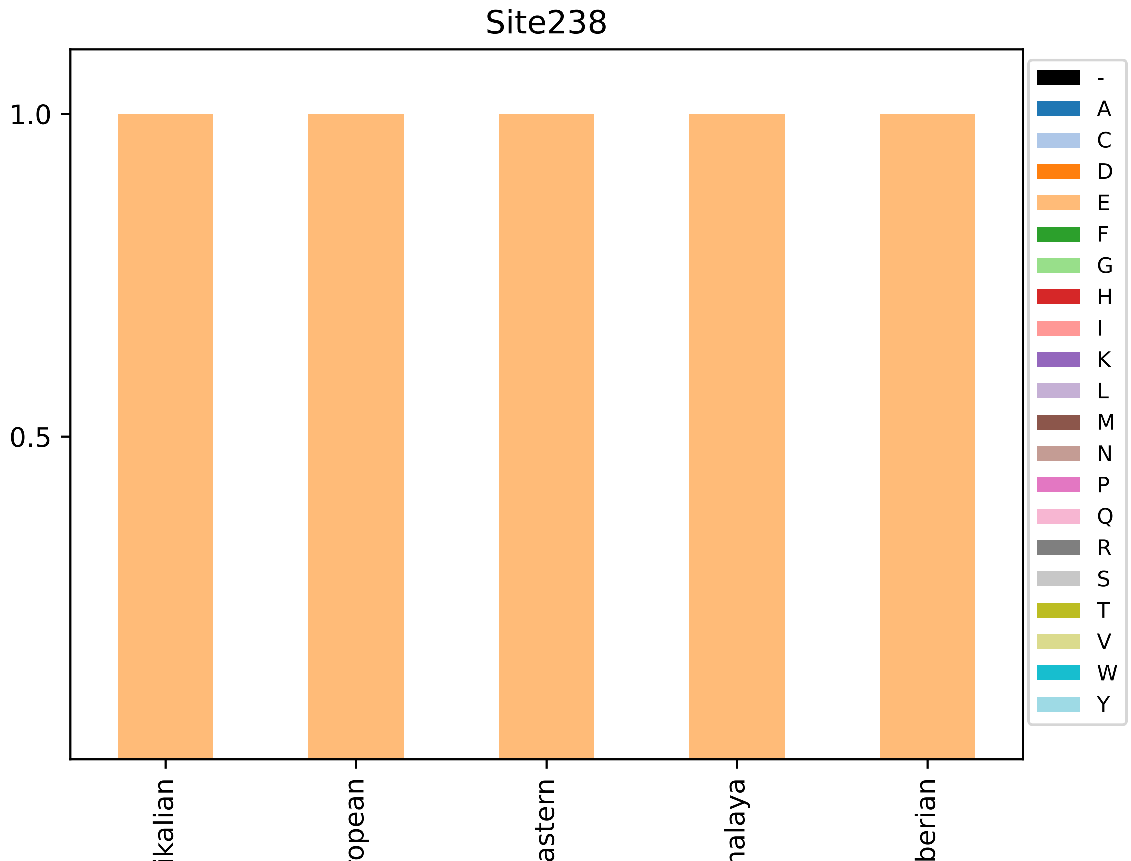
Site238
1.0
0.5
-
A
C
D
E
F
G
H
I
K
L
M
N
P
Q
R
S
T
V
W
Y
ikalian
opean
astern
nalaya
berian

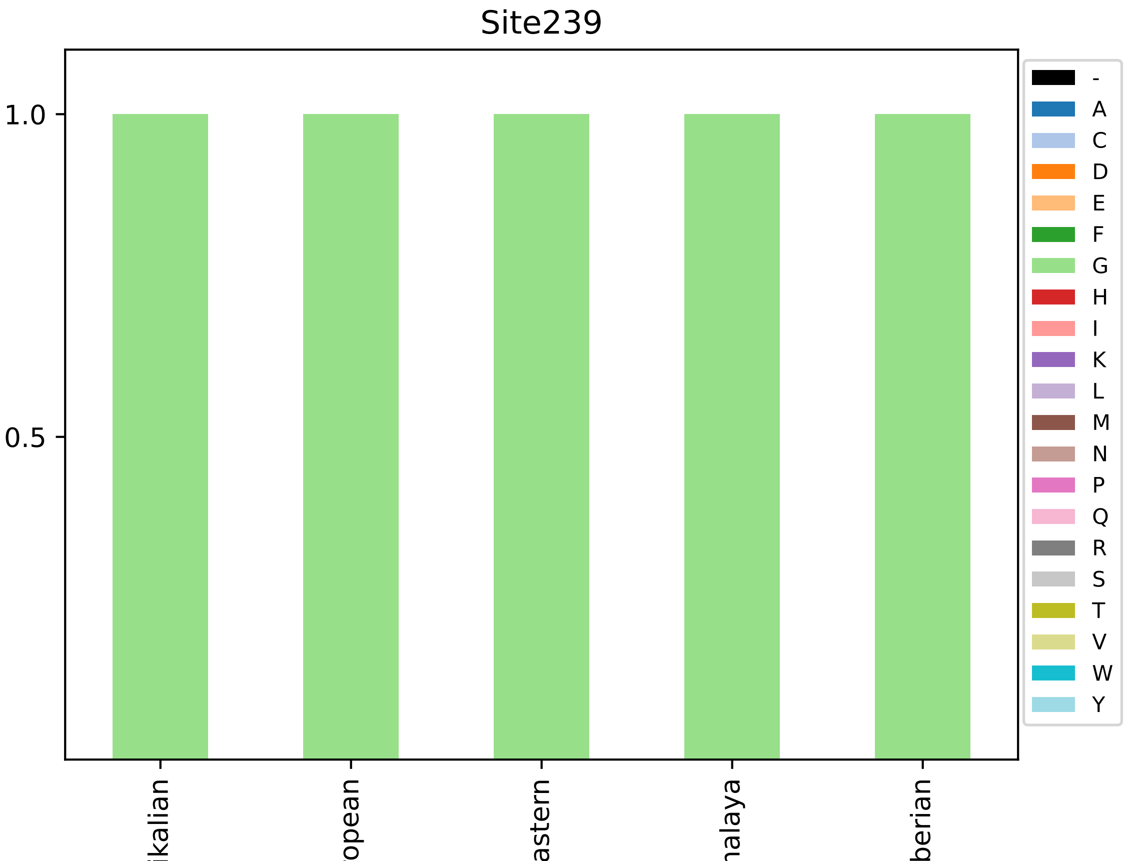

Site239
1.0
0.5
-
A
C
D
E
F
G
H
I
K
L
M
N
P
Q
R
S
T
V
W
Y

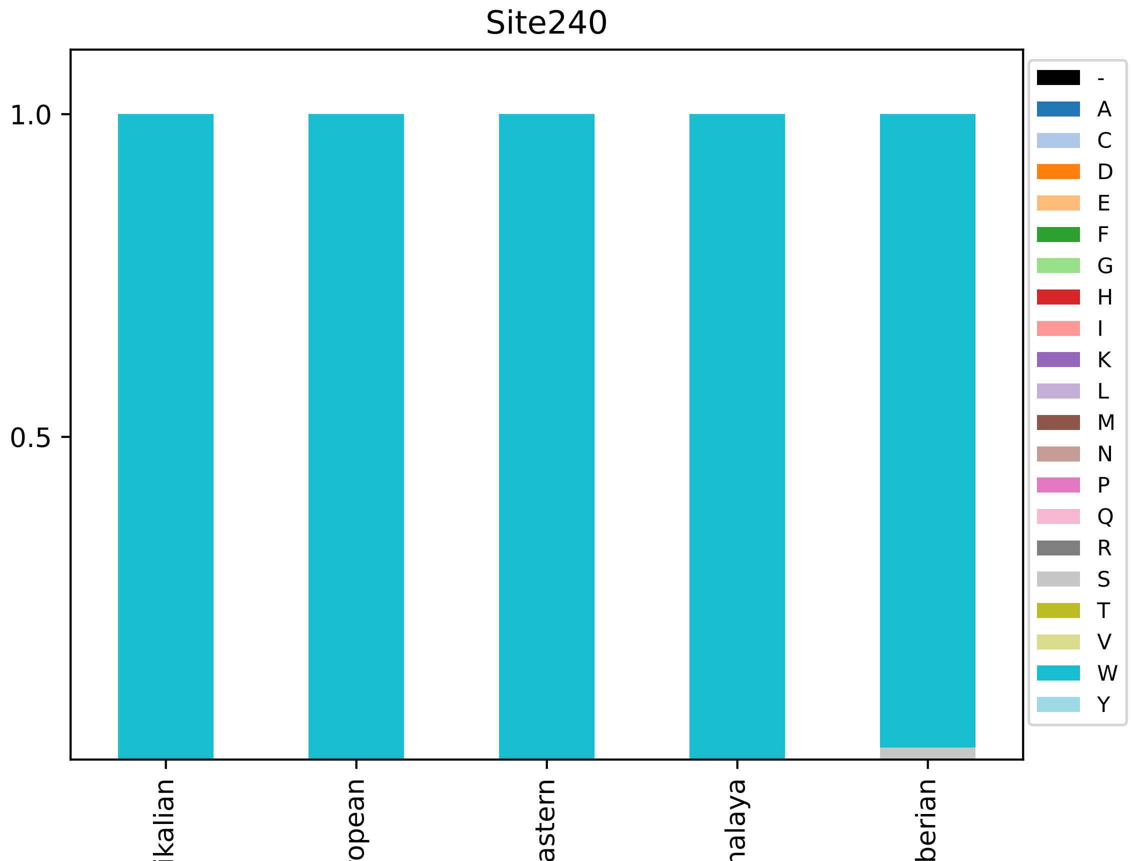
Site240
1.0
0.5
-
A
C
D
E
F
G
H
I
K
L
M
N
P
Q
R
S
T
V
W
Y
ikalian
opean
astern
nalaya
berian

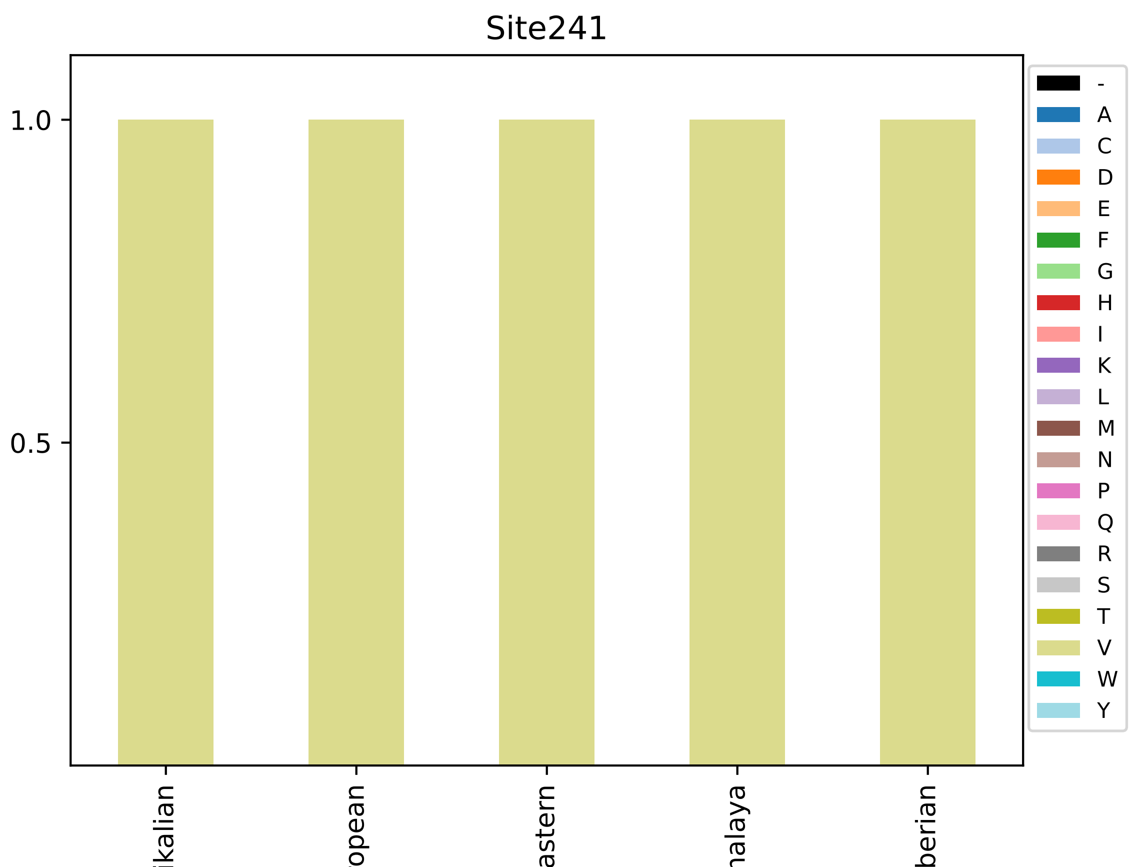
Site241
1.0
0.5
-
A
C
D
E
F
G
H
I
K
L
M
N
P
Q
R
S
T
V
W
Y
ikalian
opean
astern
nalaya
berian

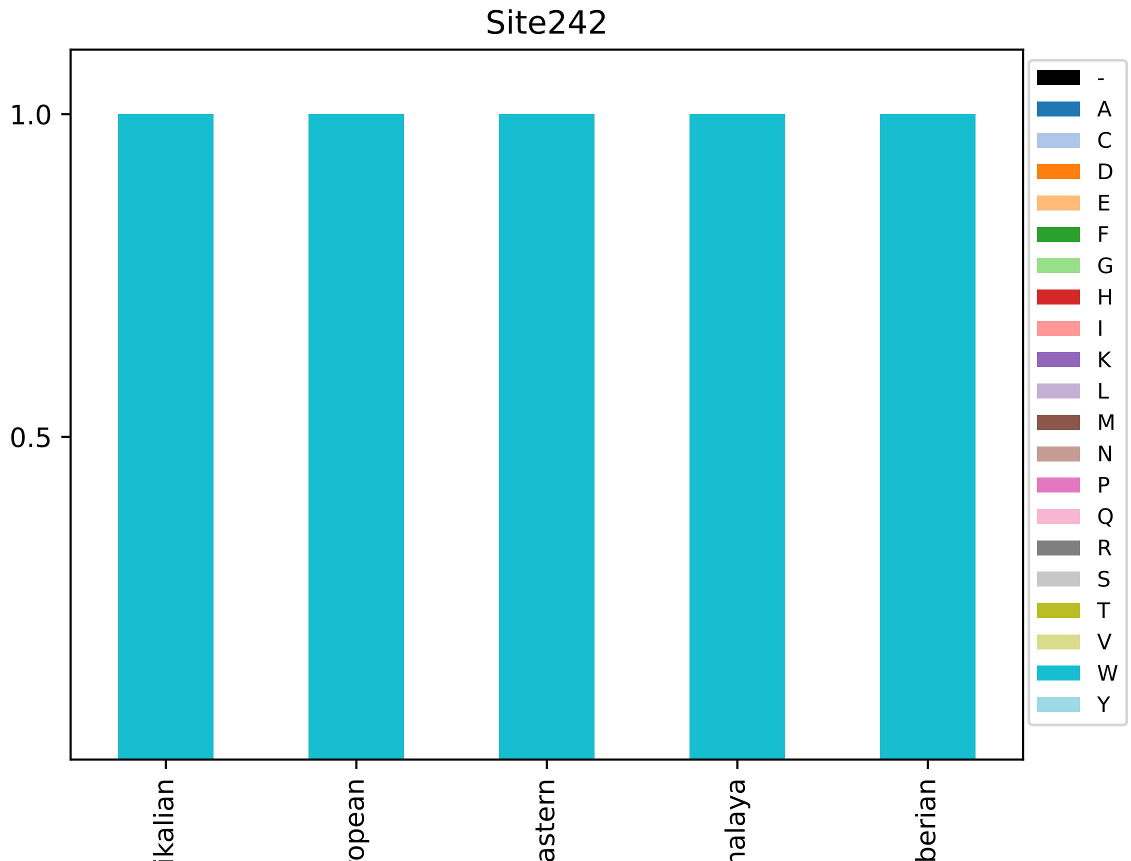

Site242
1.0
0.5
-
A
C
D
E
F
G
H
I
K
L
M
N
P
Q
R
S
T
V
W
Y
ikalian
opean
astern
nalaya
berian

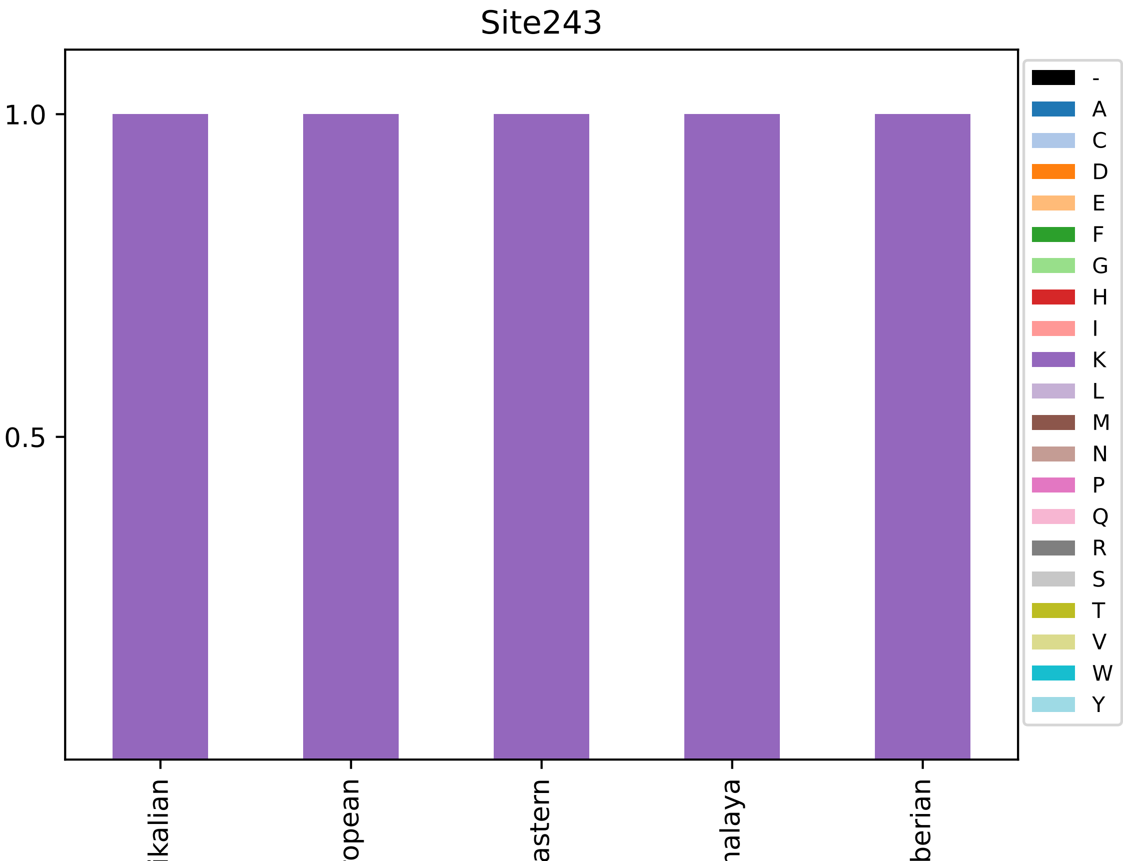

Site243
1.0
0.5
-
A
C
D
E
F
G
H
I
K
L
M
N
P
Q
R
S
T
V
W
Y

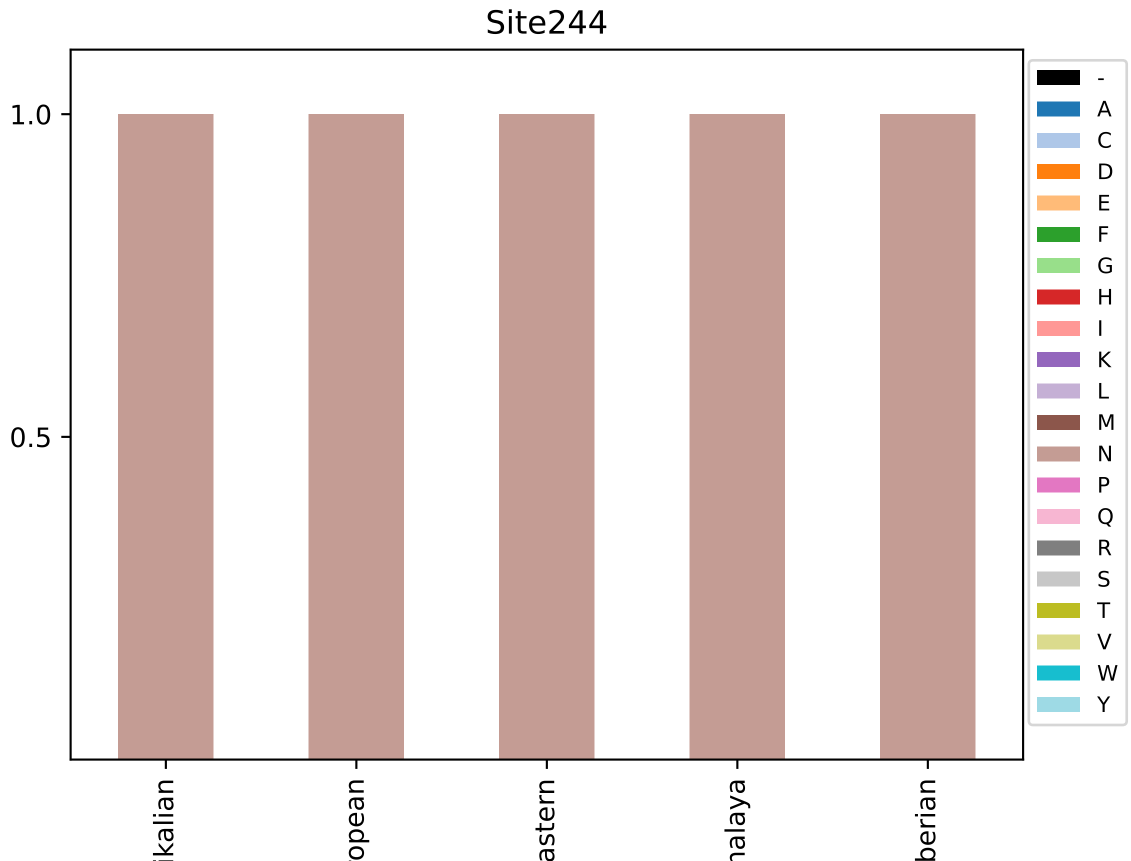

Site244
1.0
0.5
-
A
C
D
E
F
G
H
I
K
L
M
N
P
Q
R
S
T
V
W
Y
ikalian
opean
astern
nalaya
berian

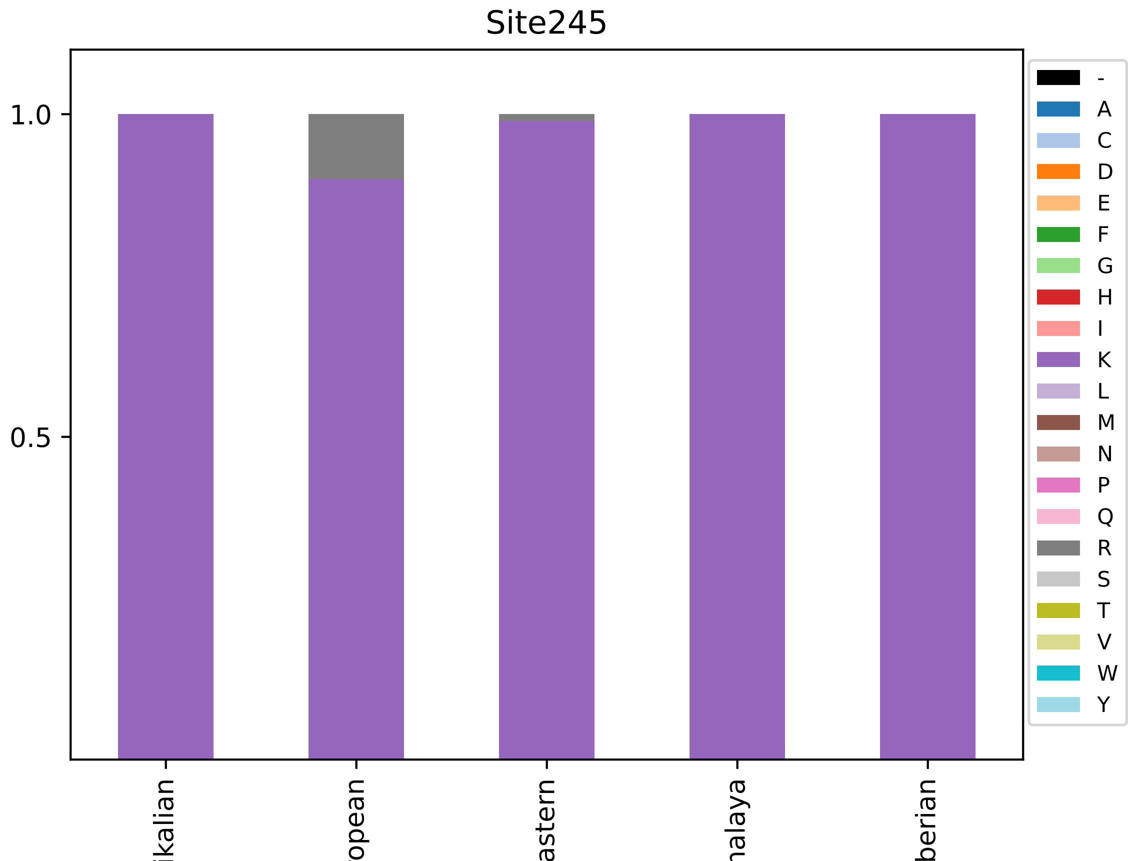
Site245
1.0
0.5
-
A
C
D
E
F
G
H
I
K
L
M
N
P
Q
R
S
T
V
W
Y
ikalian
opean
astern
nalaya
berian

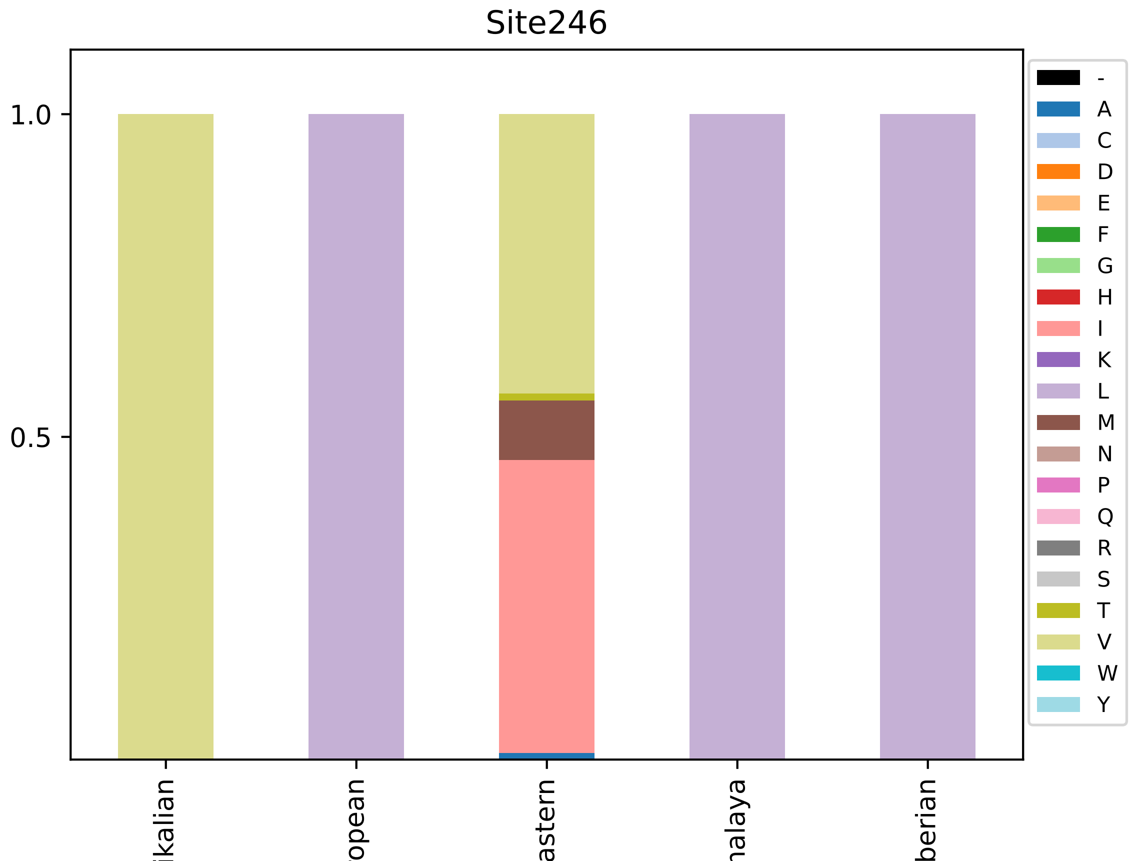

Site246
1.0
0.5
-
A
C
D
E
F
G
H
I
K
L
M
N
P
Q
R
S
T
V
W
Y
ikalian
opean
astern
halaya
berian

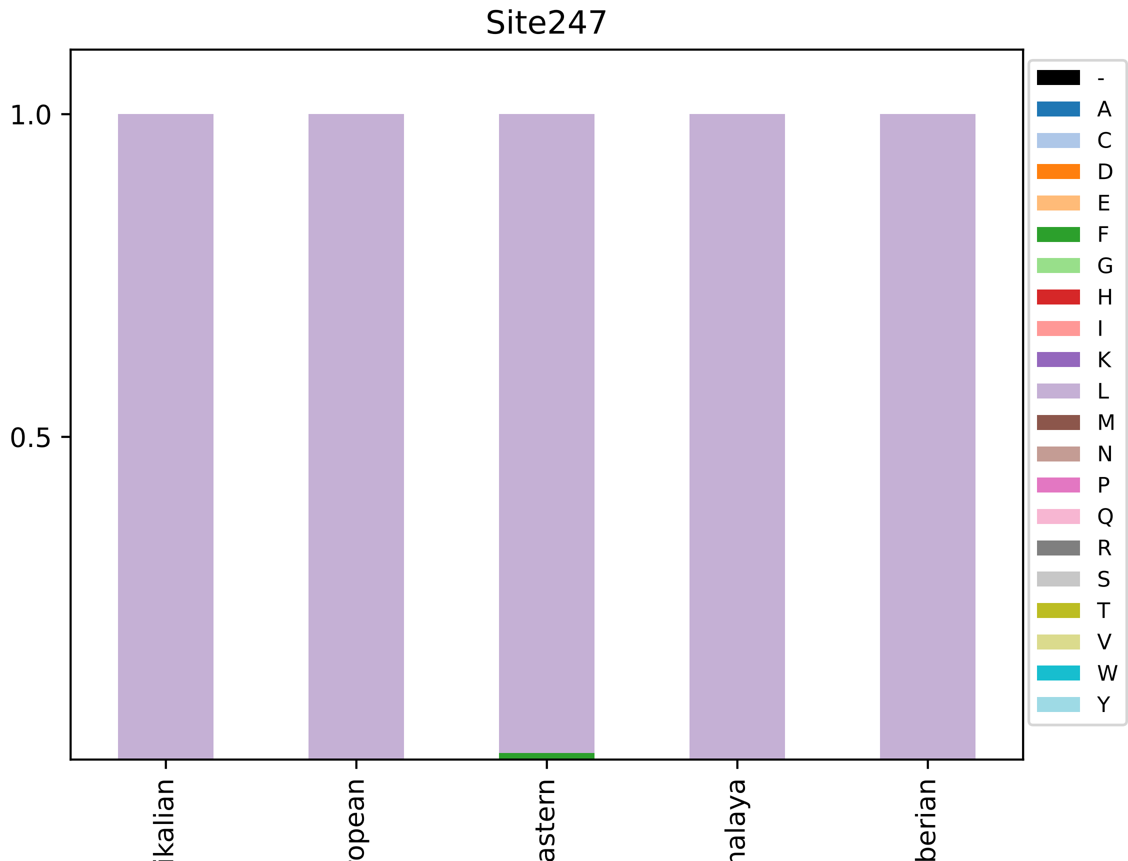
Site247
1.0
0.5
-
A
C
D
E
F
G
H
I
K
L
M
N
P
Q
R
S
T
V
W
Y
ikalian
opean
astern
nalaya
berian

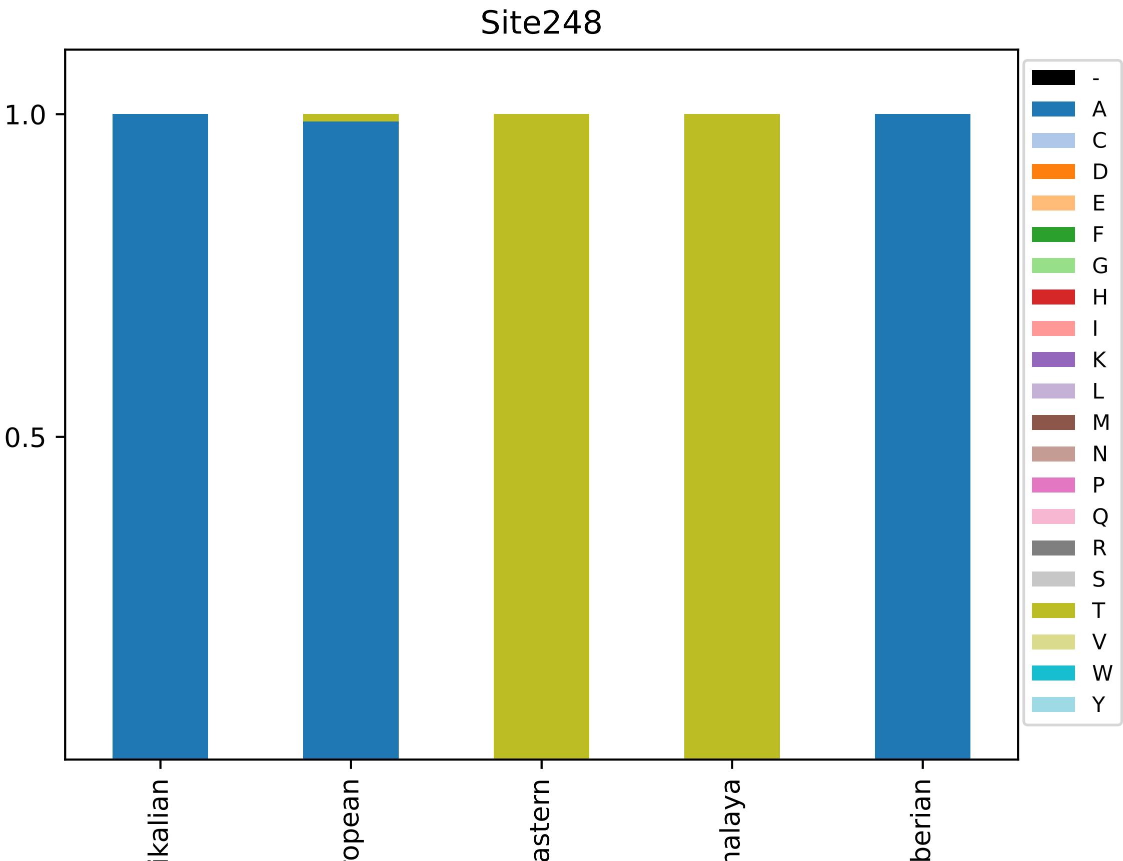
Site248
1.0
0.5
-
A
C
D
E
F
G
H
I
K
L
M
N
P
Q
R
S
T
V
W
Y
ikalian
opean
astern
nalaya
berian

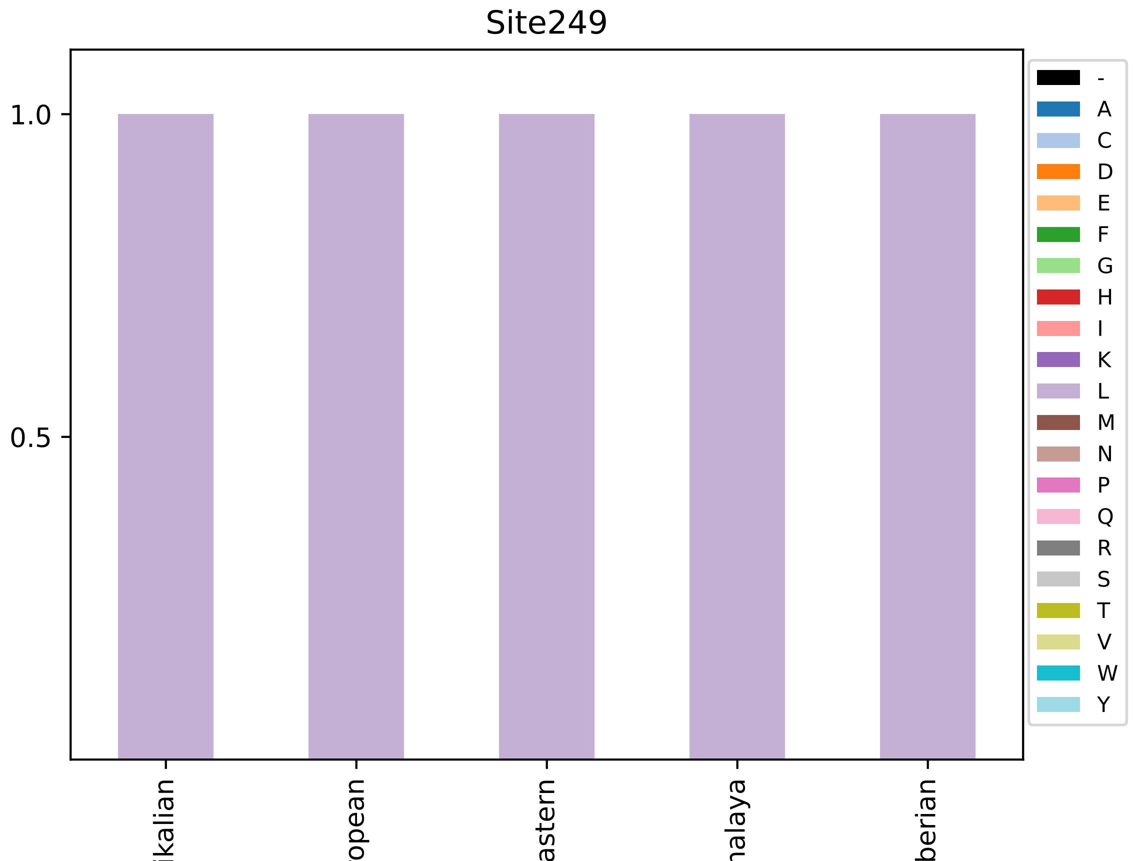
Site249
1.0
0.5
-
A
C
D
E
F
G
H
I
K
L
M
N
P
Q
R
S
T
V
W
Y
ikalian
opean
astern
nalaya
berian

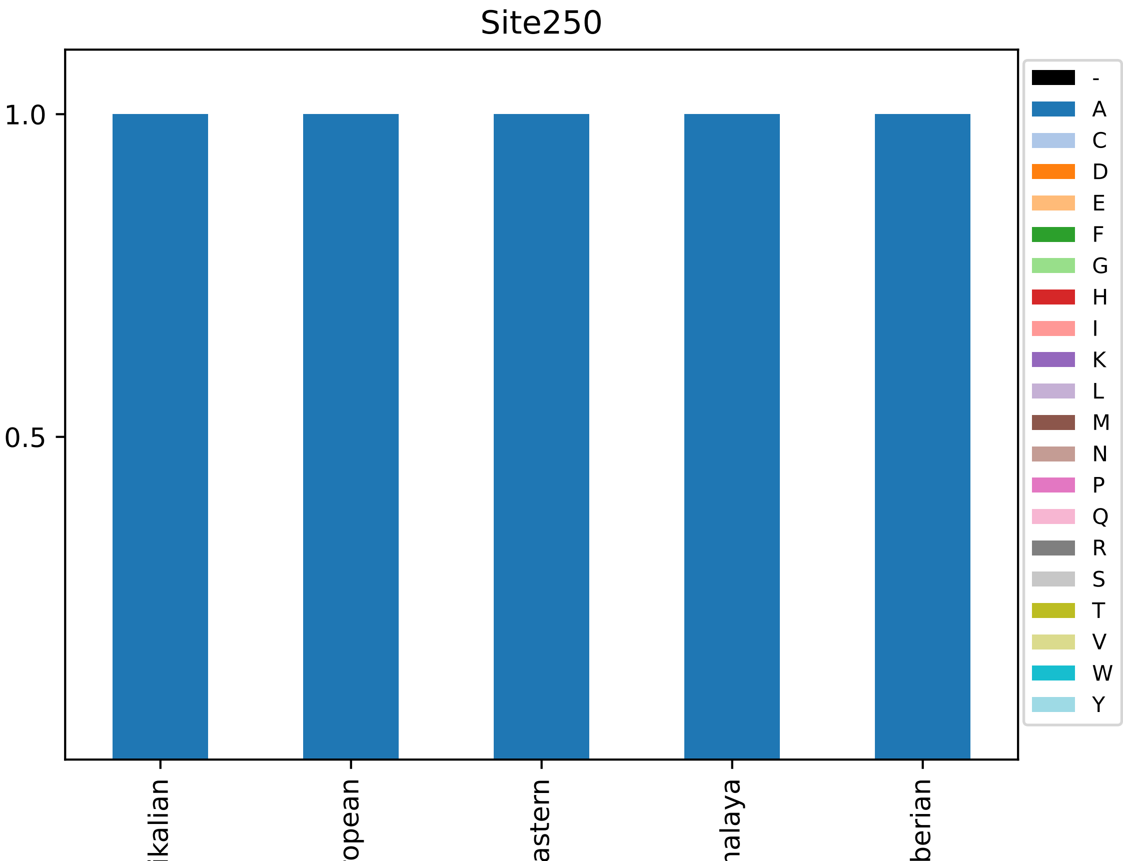

Site250
1.0
0.5
-
A
C
D
E
F
G
H
I
K
L
M
N
P
Q
R
S
T
V
W
Y
ikalian
opean
astern
nalaya
berian

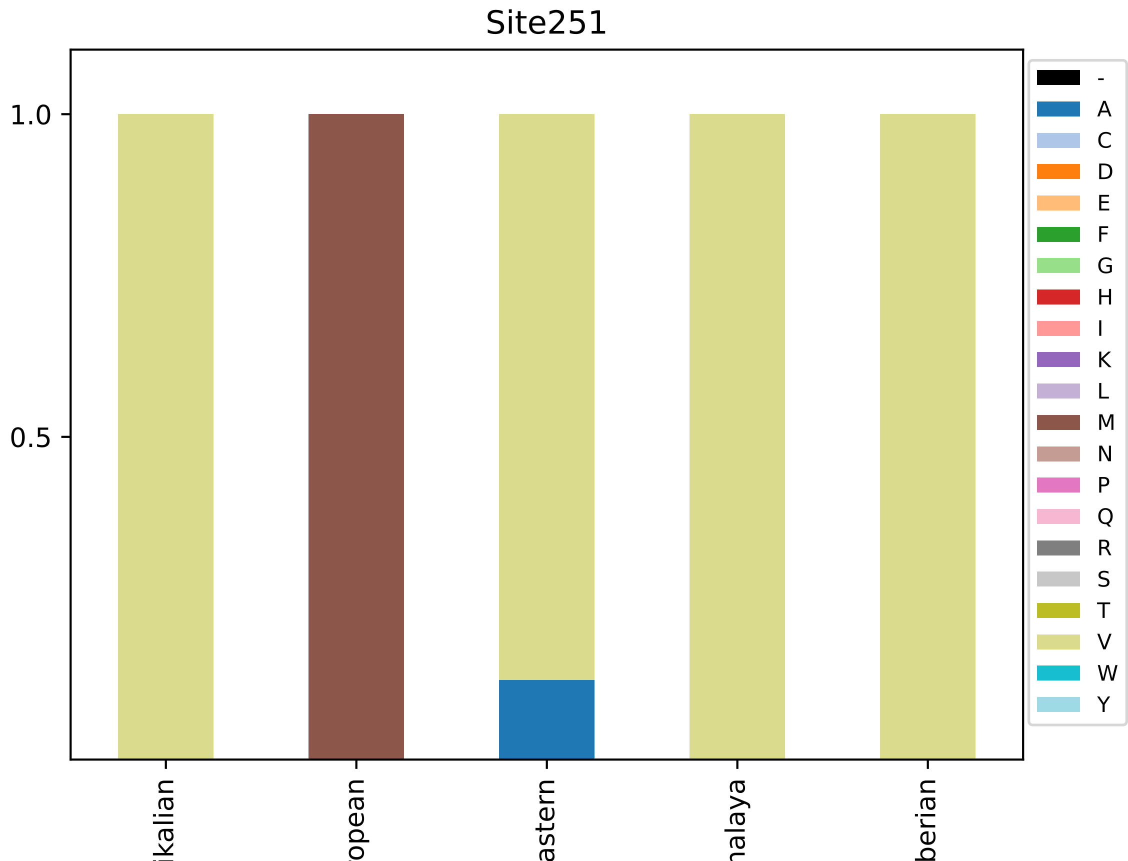
Site251
1.0
0.5
-
A
C
D
E
F
G
H
I
K
L
M
N
P
Q
R
S
T
V
W
Y
ikalian
opean
astern
nalaya
berian

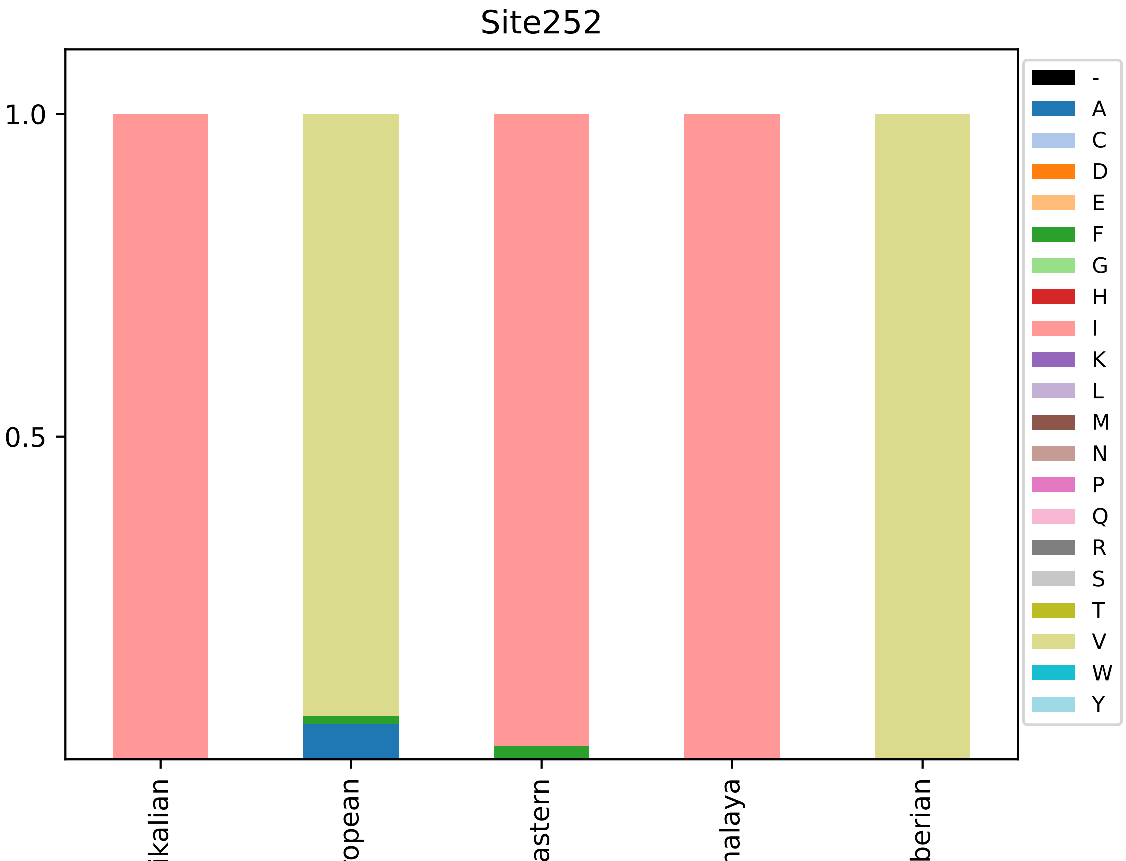
Site252
1.0
0.5
-
A
C
D
E
F
G
H
I
K
L
M
N
P
Q
R
S
T
V
W
Y
ikalian
opean
astern
nalaya
berian

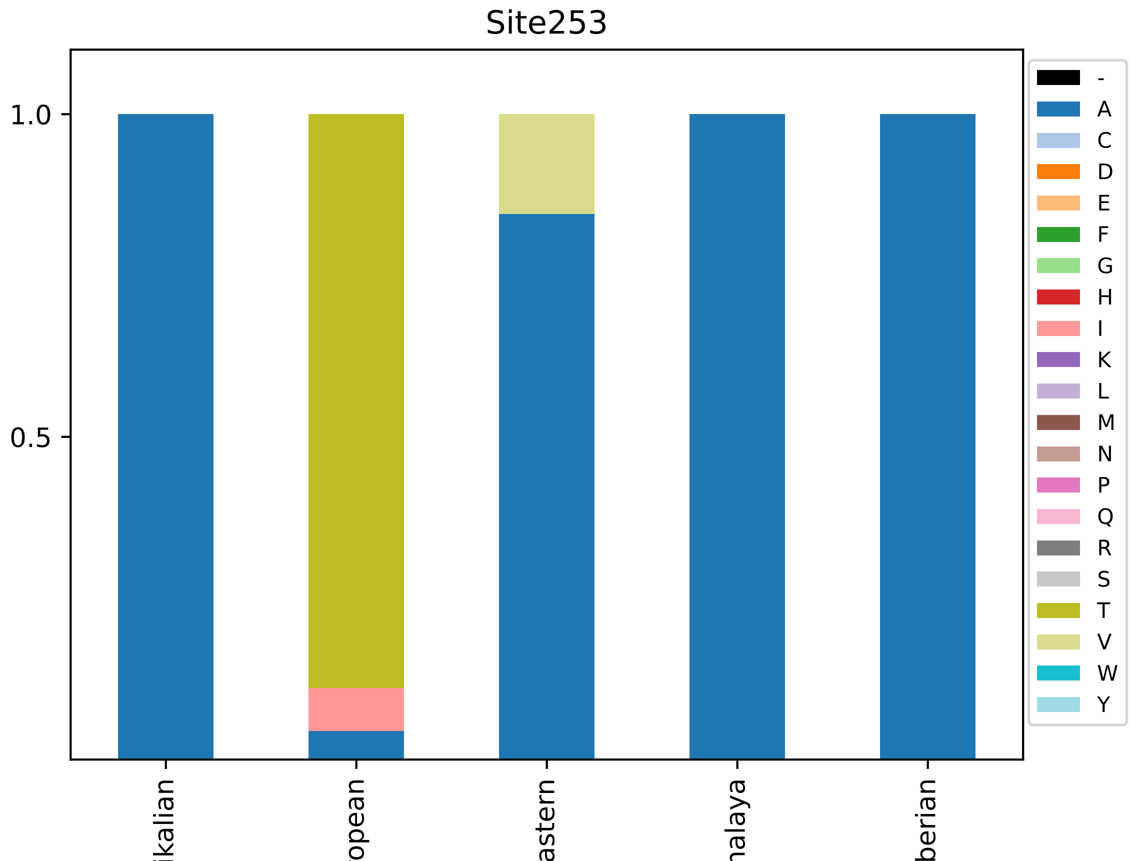
Site253
1.0
0.5
-
A
C
D
E
F
G
H
I
K
L
M
N
P
Q
R
S
T
V
W
Y
ikalian
opean
astern
nalaya
berian

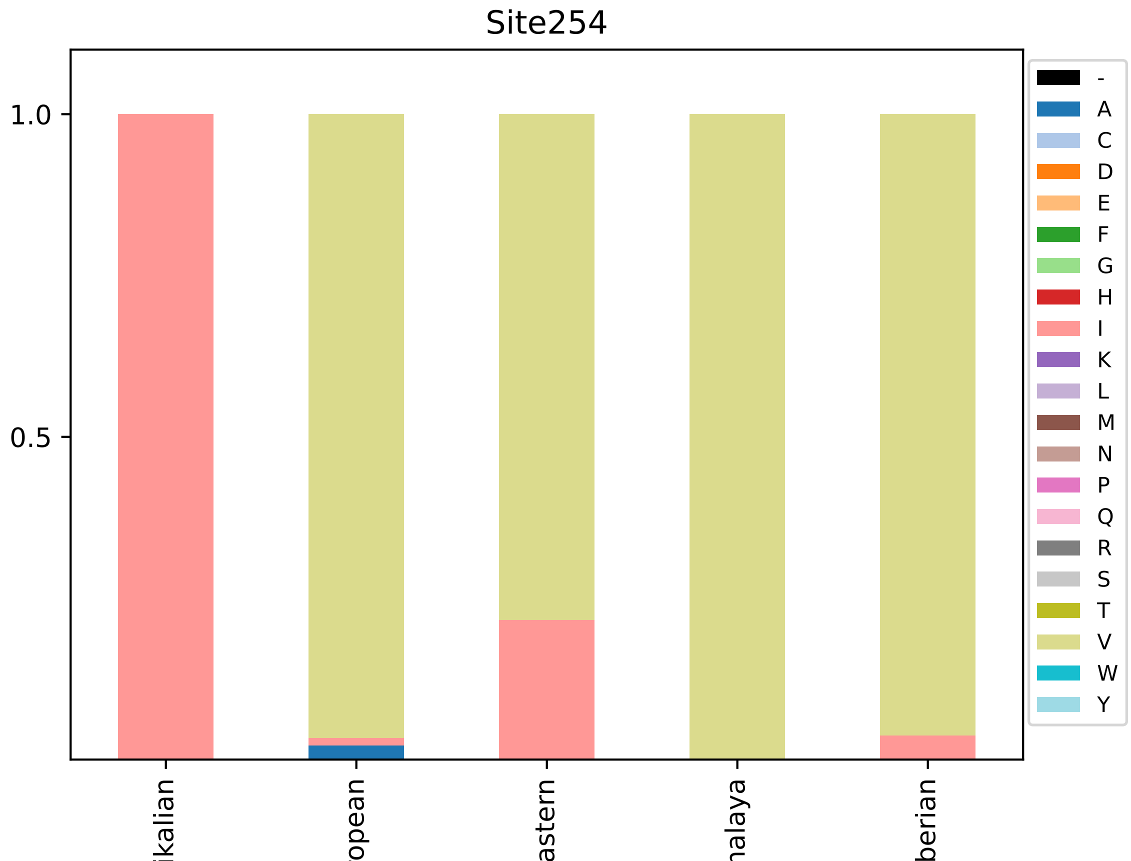
Site254
1.0
0.5
-
A
C
D
E
F
G
H
I
K
L
M
N
P
Q
R
S
T
V
W
Y
ikalian
opean
astern
nalaya
berian

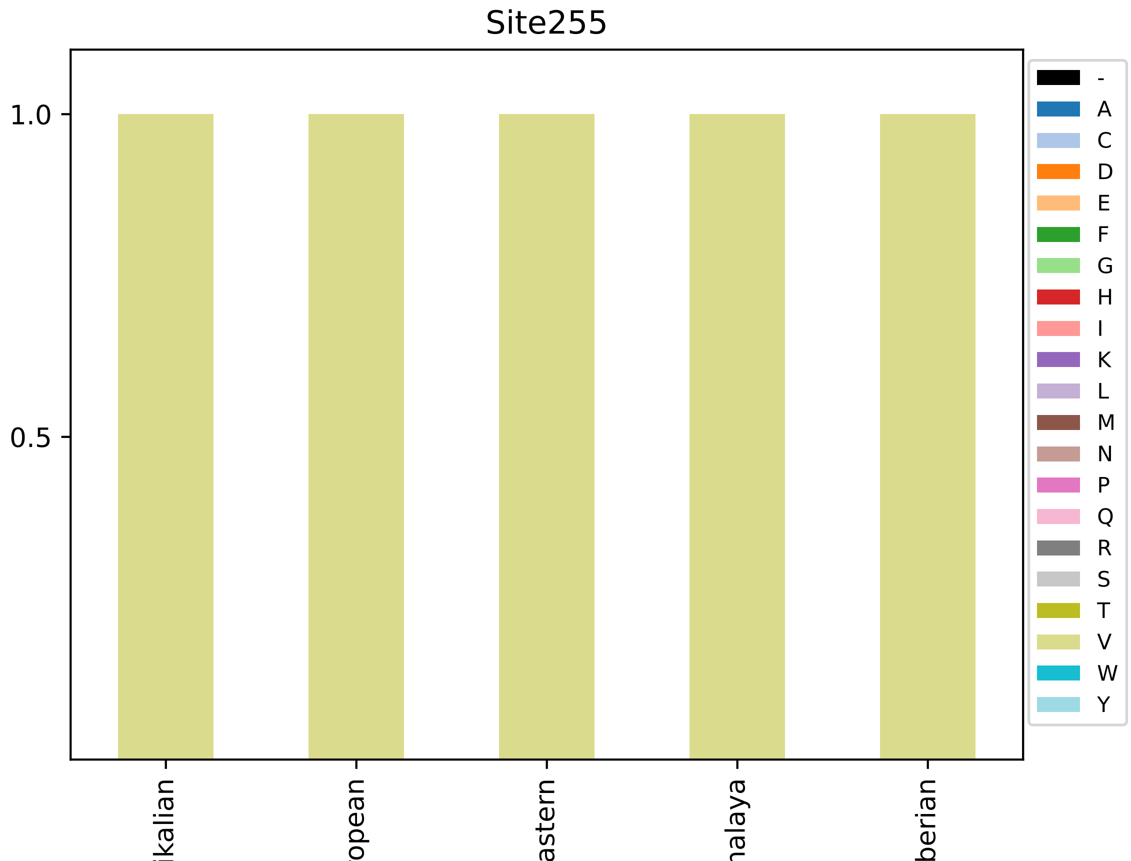
Site255
1.0
0.5
-
A
C
D
E
F
G
H
I
K
L
M
N
P
Q
R
S
T
V
W
Y
ikalian
opean
astern
nalaya
berian

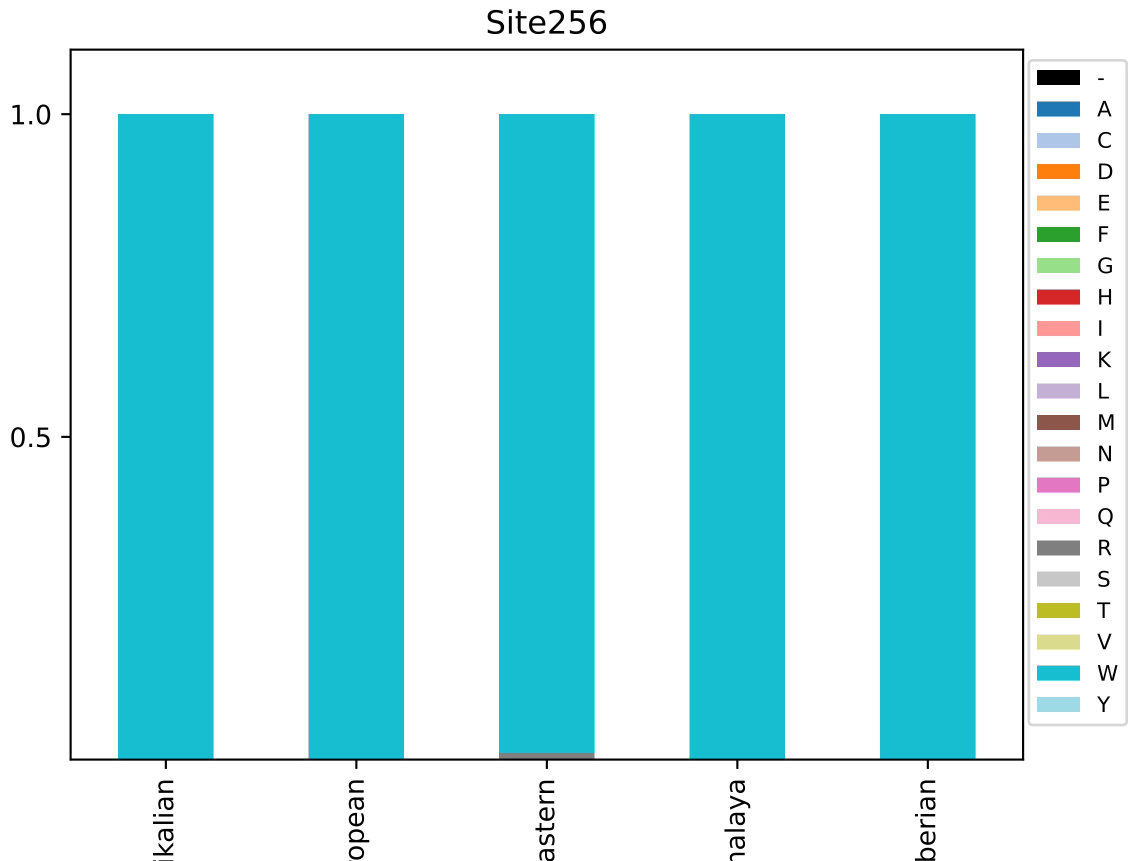
Site256
1.0
0.5
-
A
C
D
E
F
G
H
I
K
L
M
N
P
Q
R
S
T
V
W
Y
ikalian
opean
astern
nalaya
berian

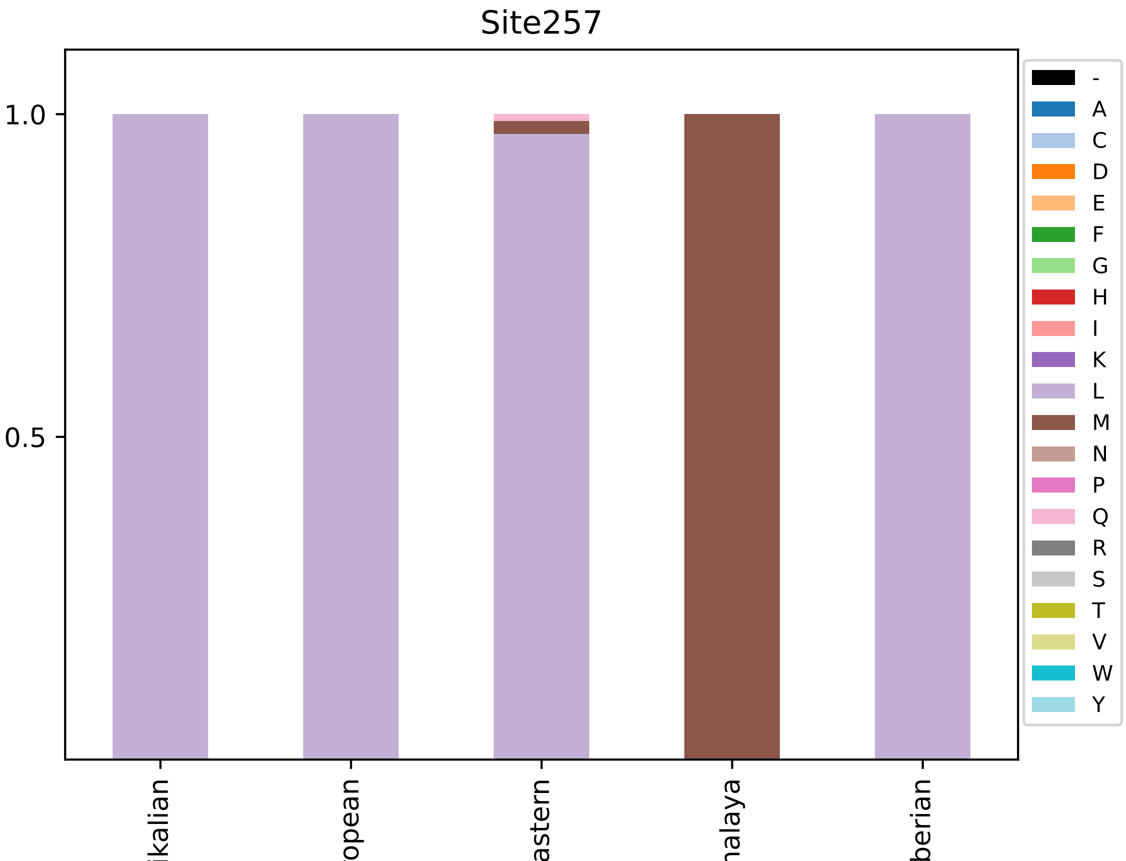
Site257
1.0
0.5
-
A
C
D
E
F
G
H
I
K
L
M
N
P
Q
R
S
T
V
W
Y
ikalian
opean
astern
nalaya
berian

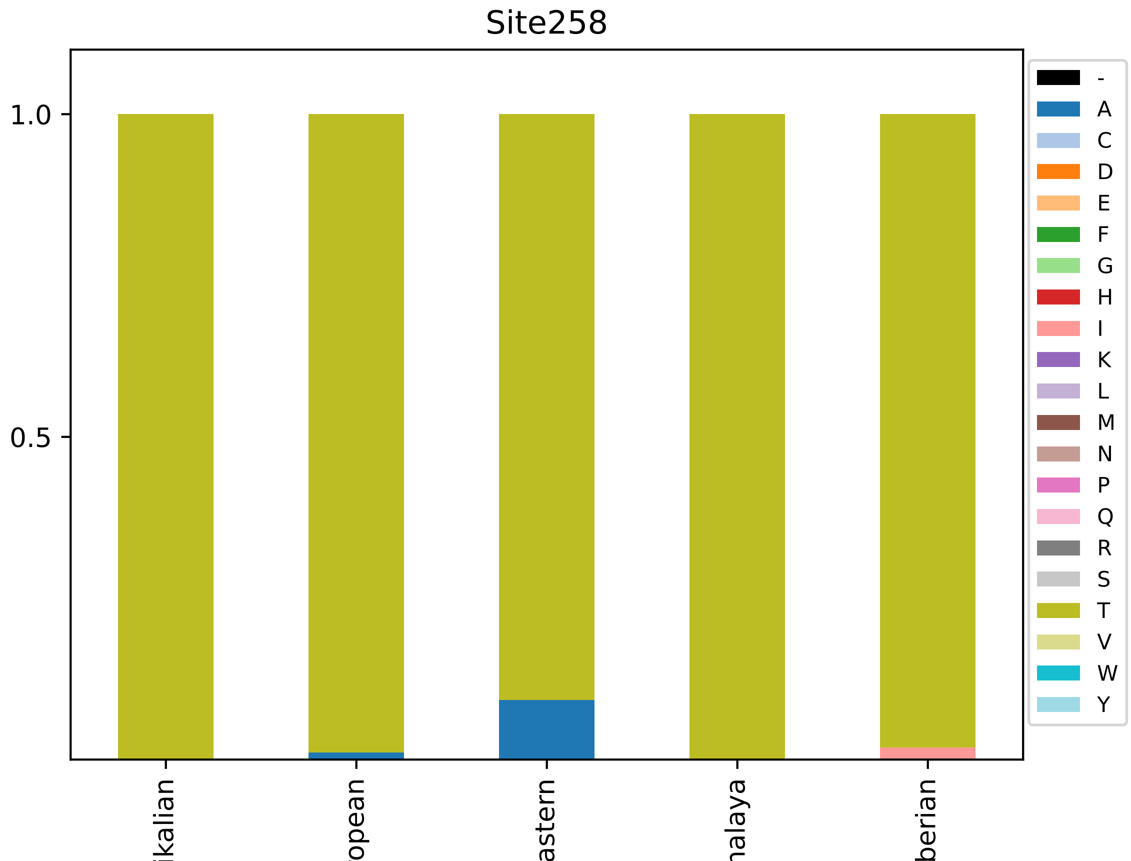
Site258
1.0
0.5
-
A
C
D
E
F
G
H
I
K
L
M
N
P
Q
R
S
T
V
W
Y
ikalian
ropean
astern
nalaya
berian

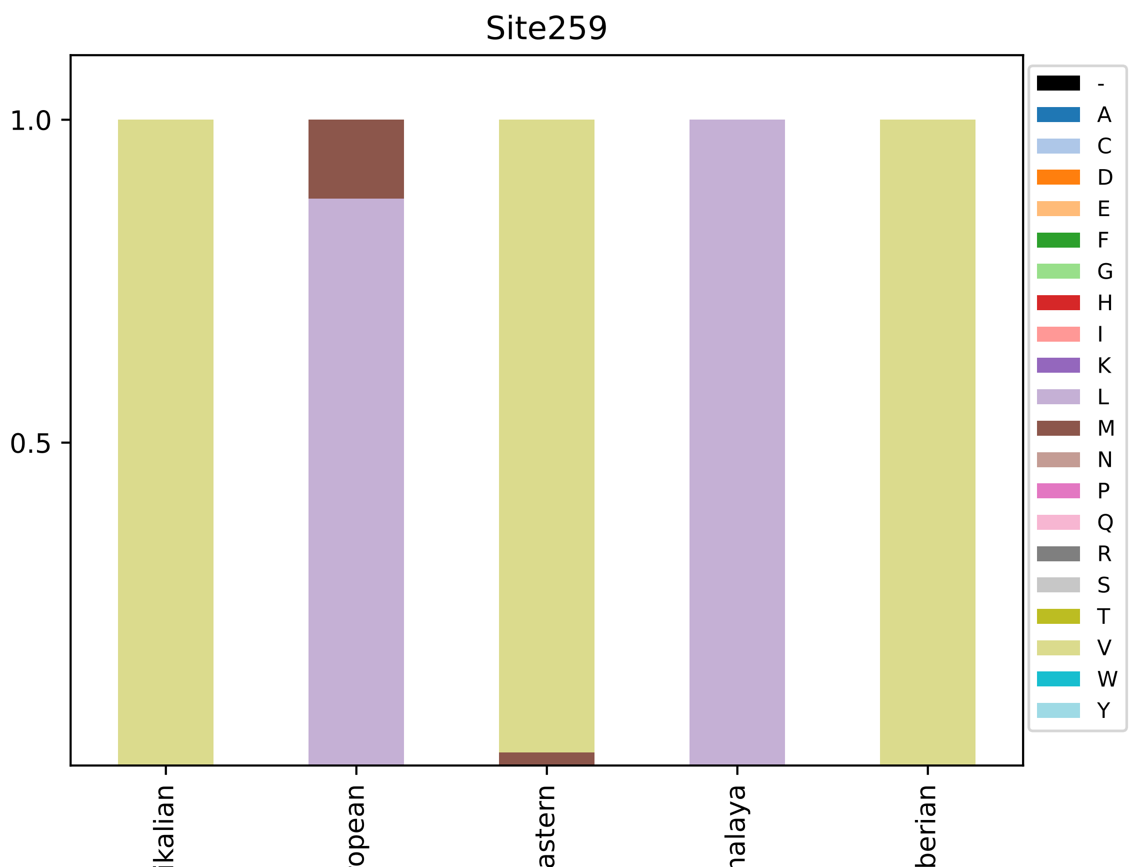
Site259
1.0
0.5
-
A
C
D
E
F
G
H
I
K
L
M
N
P
Q
R
S
T
V
W
Y
ikalian
opean
astern
nalaya
berian

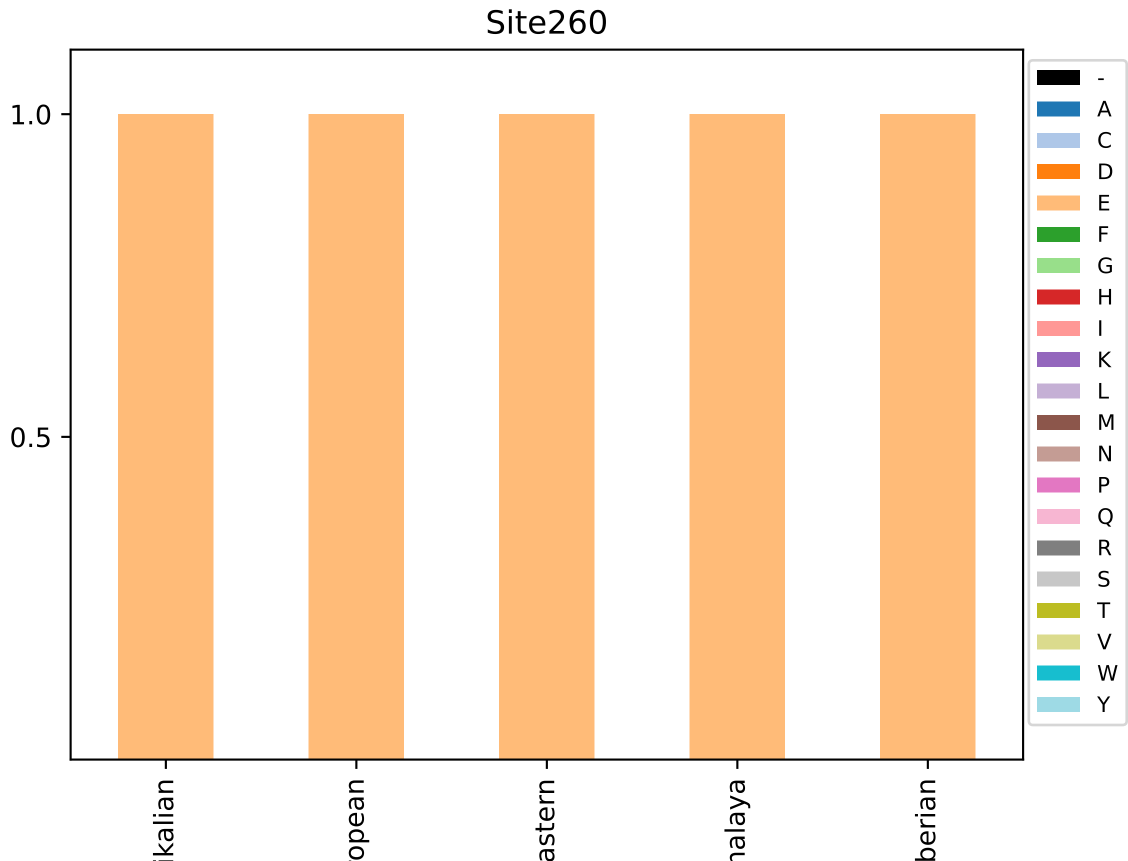

Site260
1.0
0.5
-
A
C
D
E
F
G
H
I
K
L
M
N
P
Q
R
S
T
V
W
Y
ikalian
opean
astern
nalaya
berian

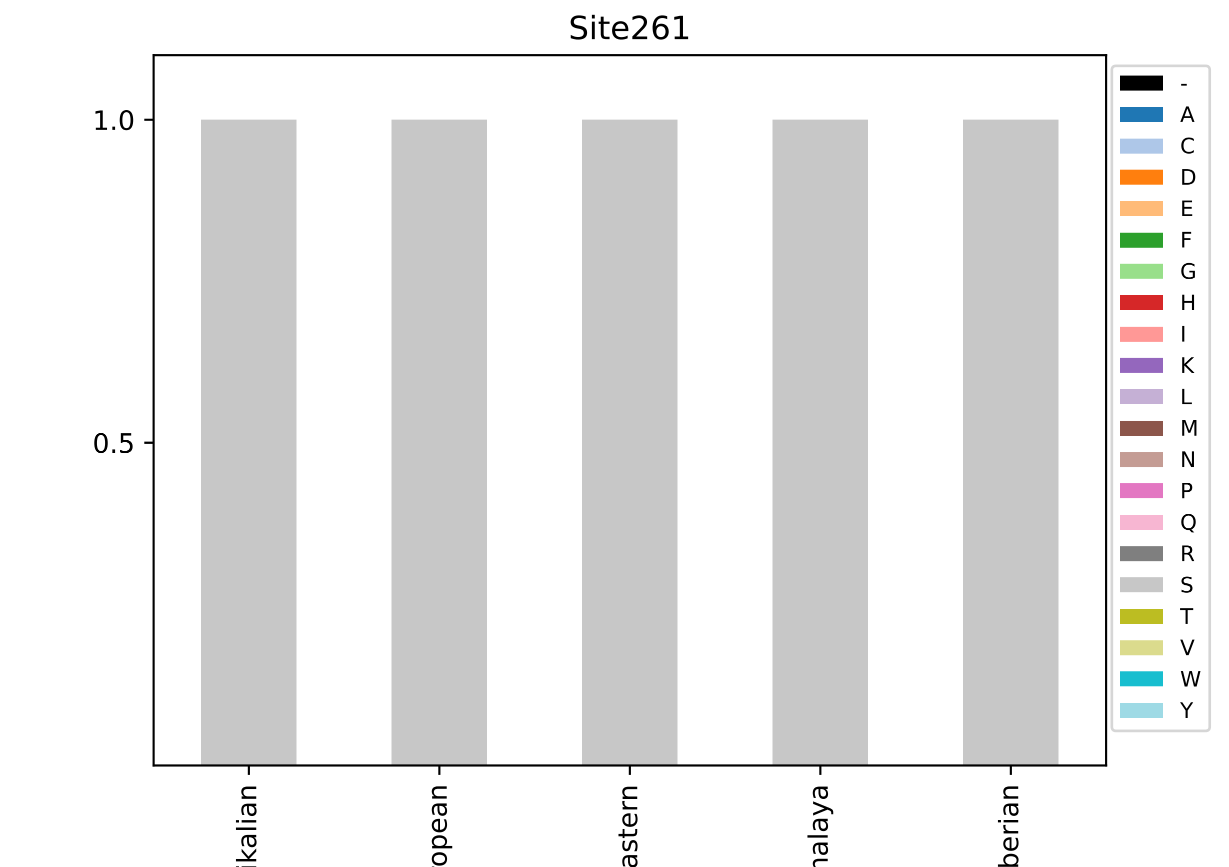
Site261
1.0
0.5
-
A
C
D
E
F
G
H
I
K
L
M
N
P
Q
R
S
T
V
W
Y

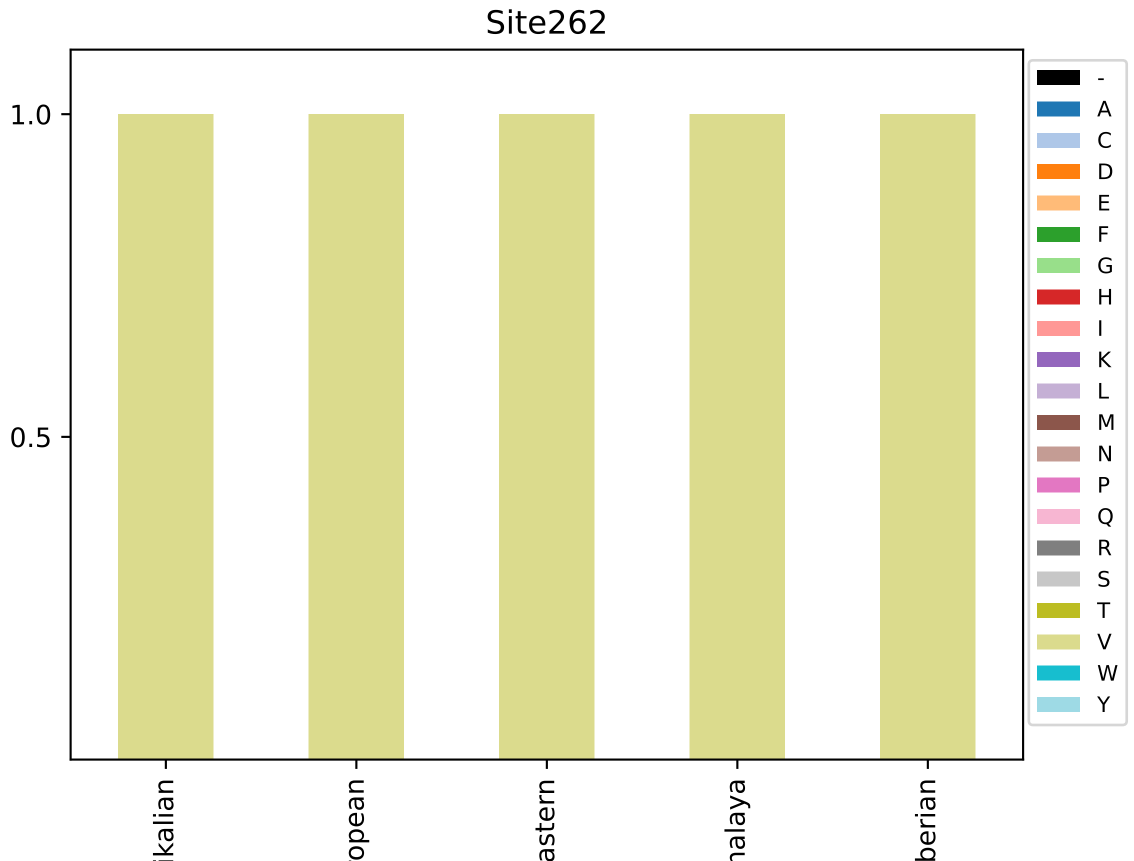

Site262
1.0
0.5
-
A
C
D
E
F
G
H
I
K
L
M
N
P
Q
R
S
T
V
W
Y
ikalian
opean
astern
nalaya
berian

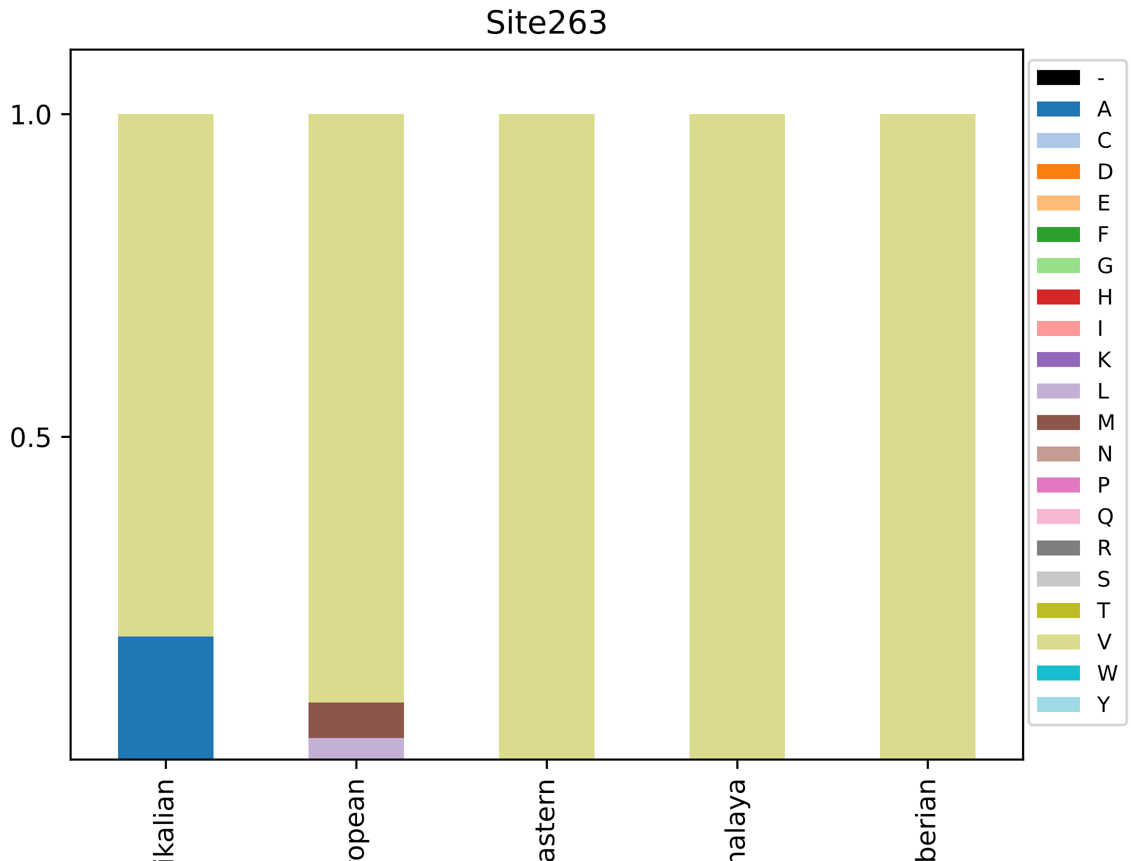
Site263
1.0
0.5
-
A
C
D
E
F
G
H
I
K
L
M
N
P
Q
R
S
T
V
W
Y
ikalian
opean
astern
nalaya
berian

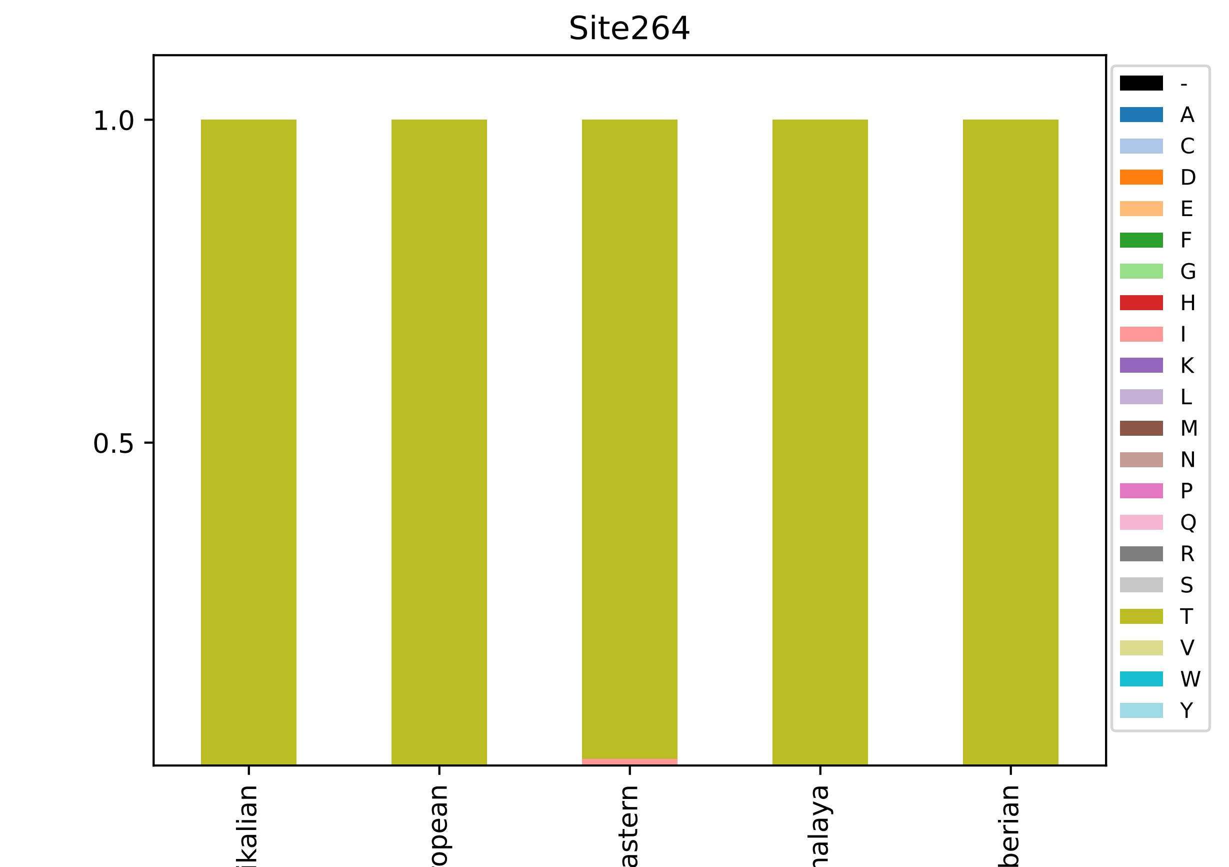

Site264
1.0
0.5
-
A
C
D
E
F
G
H
I
K
L
M
N
P
Q
R
S
T
V
W
Y
ikalian
opean
astern
nalaya
berian

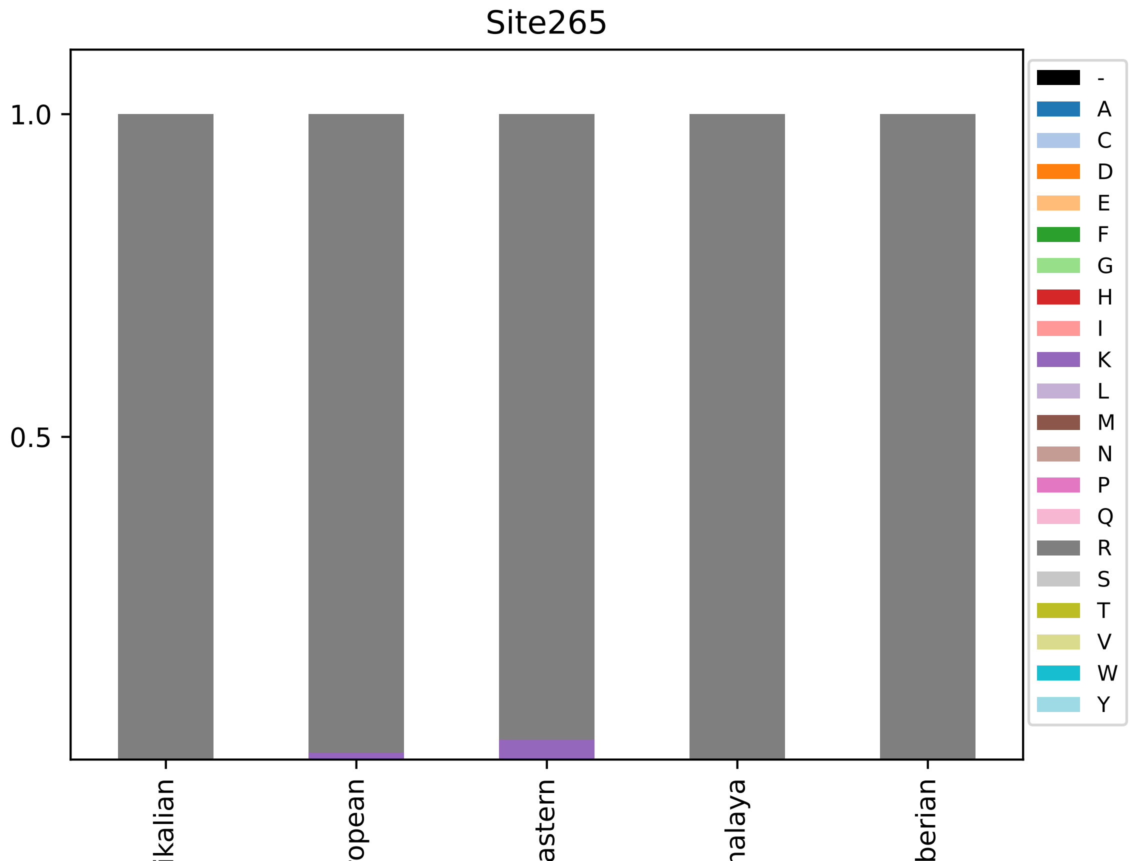

Site265
1.0
0.5
-
A
C
D
E
F
G
H
I
K
L
M
N
P
Q
R
S
T
V
W
Y

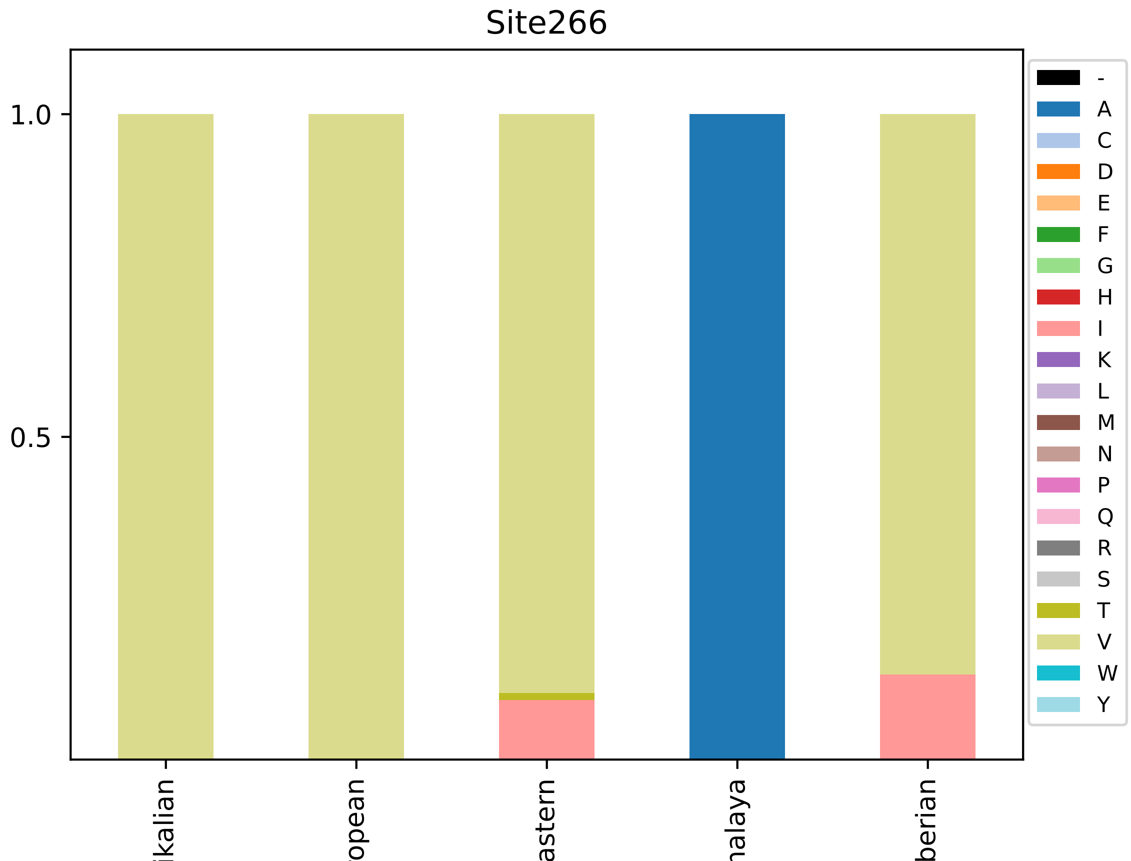

Site266
1.0
0.5
-
A
C
D
E
F
G
H
I
K
L
M
N
P
Q
R
S
T
V
W
Y
ikalian
opean
astern
nalaya
berian

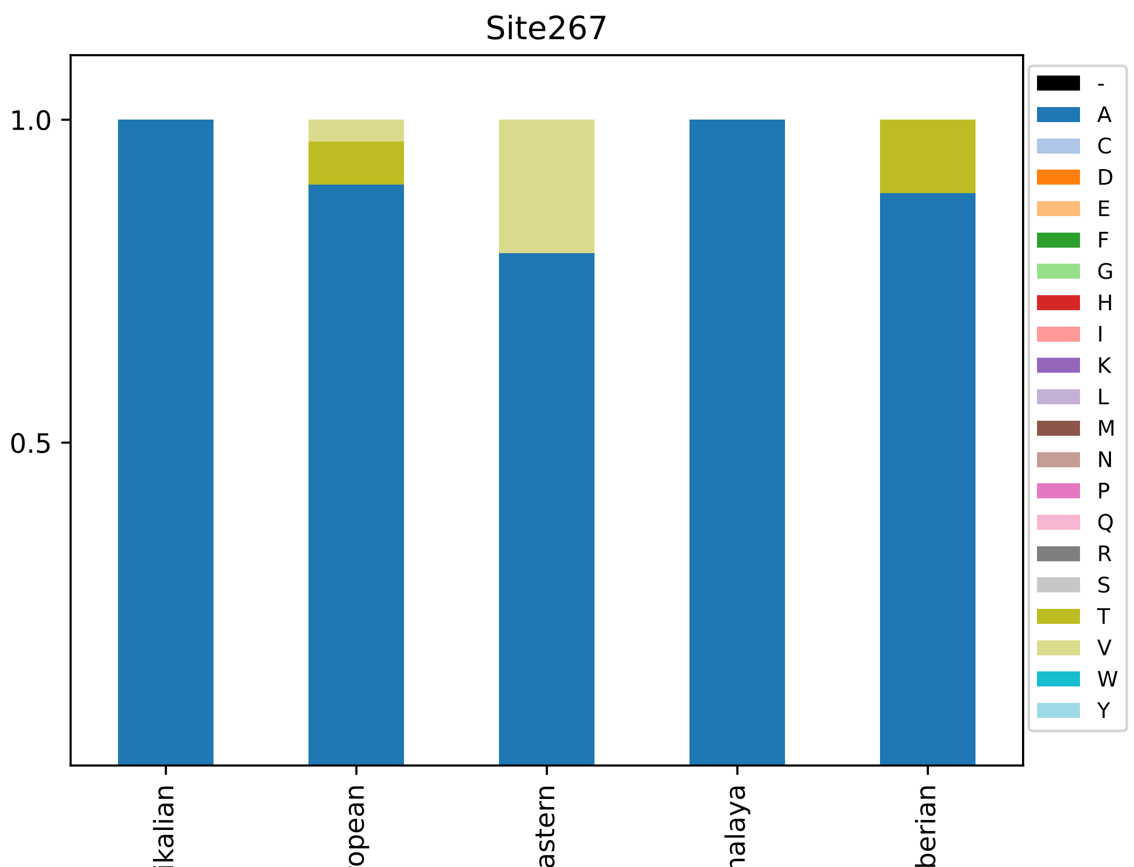

Site267
1.0
0.5
-
A
C
D
E
F
G
H
I
K
L
M
N
P
Q
R
S
T
V
W
Y
ikalian
opean
astern
nalaya
berian

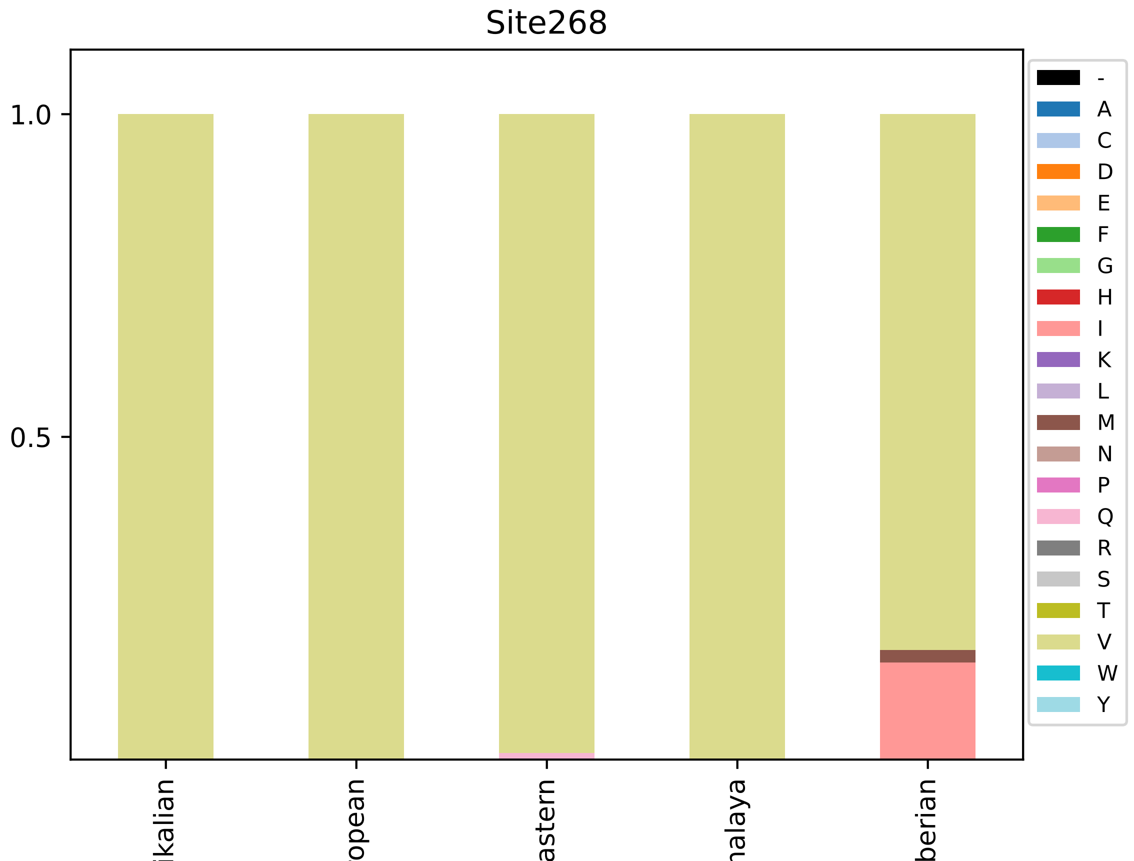
Site268
1.0
0.5
ikalian
opean
astern
nalaya
berian
-
A
C
D
E
F
G
H
I
K
L
M
N
P
Q
R
S
T
V
W
Y

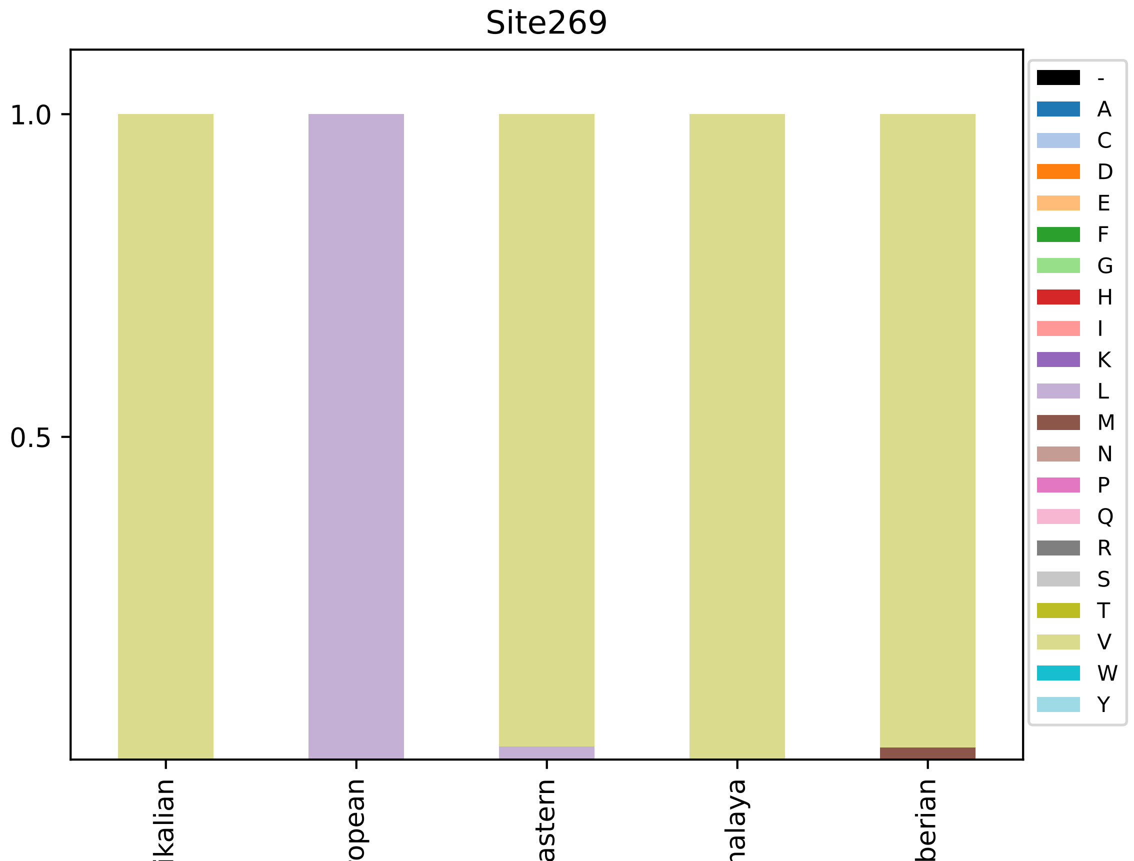

Site269
1.0
0.5
ikalian
opean
astern
nalaya
berian
-
A
C
D
E
F
G
H
I
K
L
M
N
P
Q
R
S
T
V
W
Y

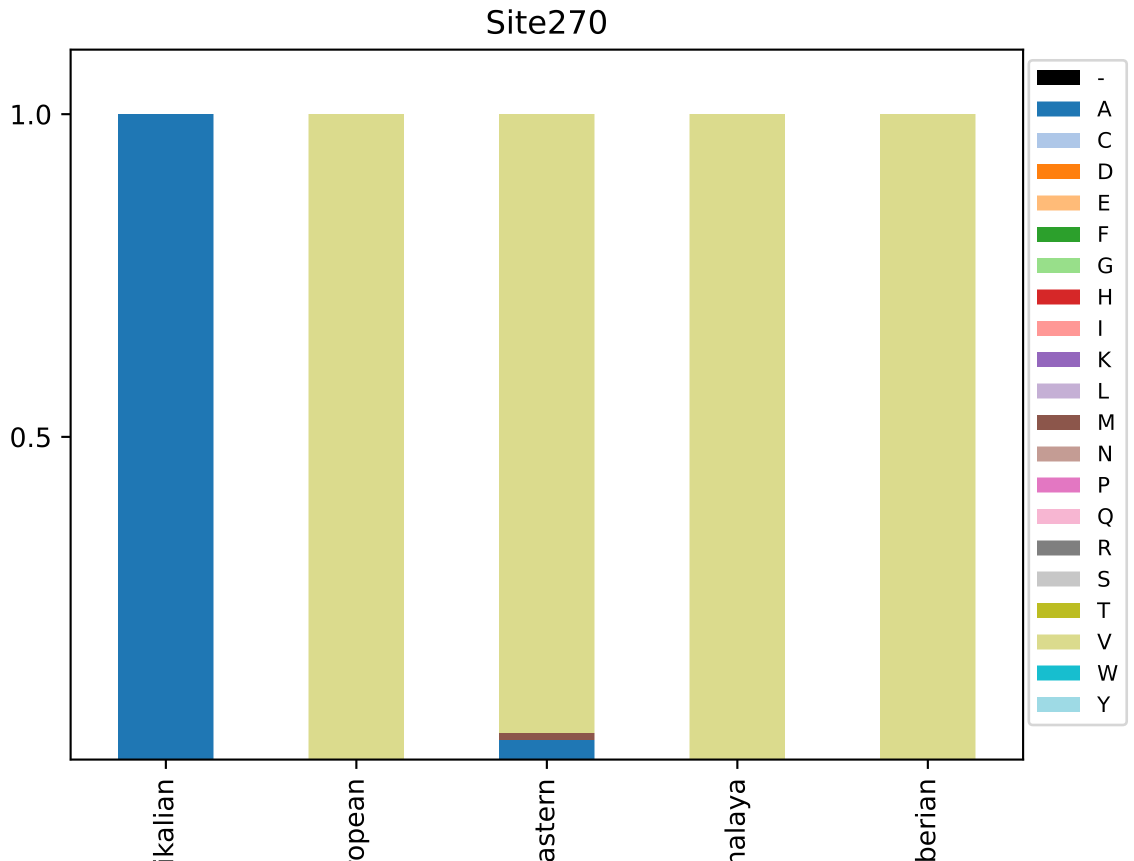

Site270
1.0
0.5
-
A
C
D
E
F
G
H
I
K
L
M
N
P
Q
R
S
T
V
W
Y
ikalian
ropean
astern
nalaya
berian

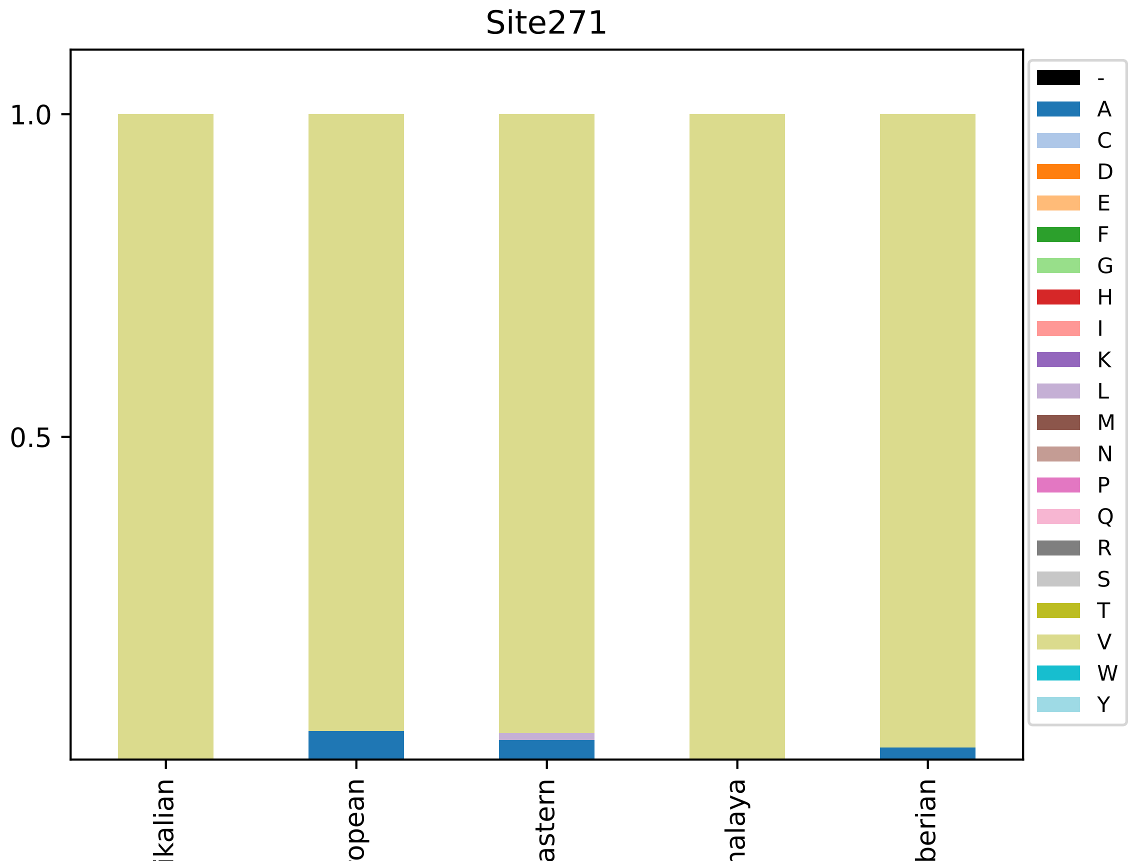

Site271
1.0
0.5
ikalian
ropean
astern
nalaya
berian
-
A
C
D
E
F
G
H
I
K
L
M
N
P
Q
R
S
T
V
W
Y

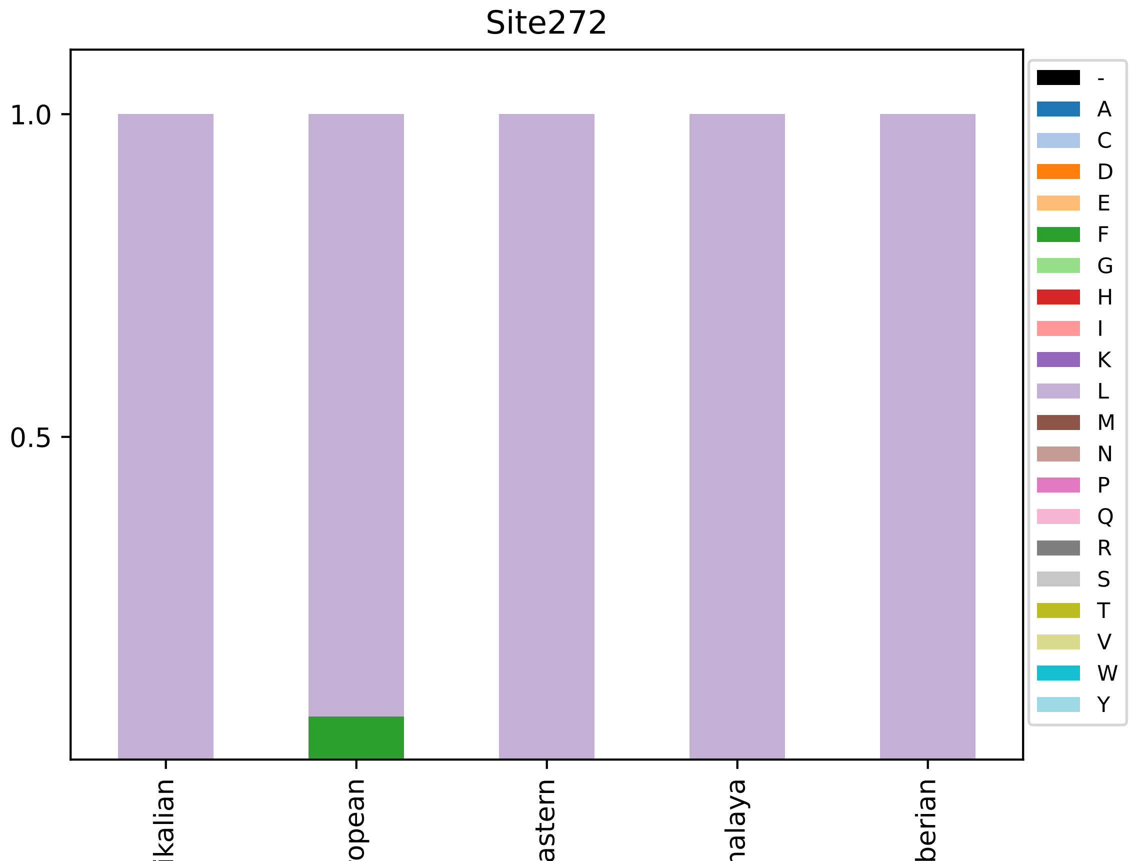

Site272
1.0
0.5
-
A
C
D
E
F
G
H
I
K
L
M
N
P
Q
R
S
T
V
W
Y
ikalian
opean
astern
nalaya
berian

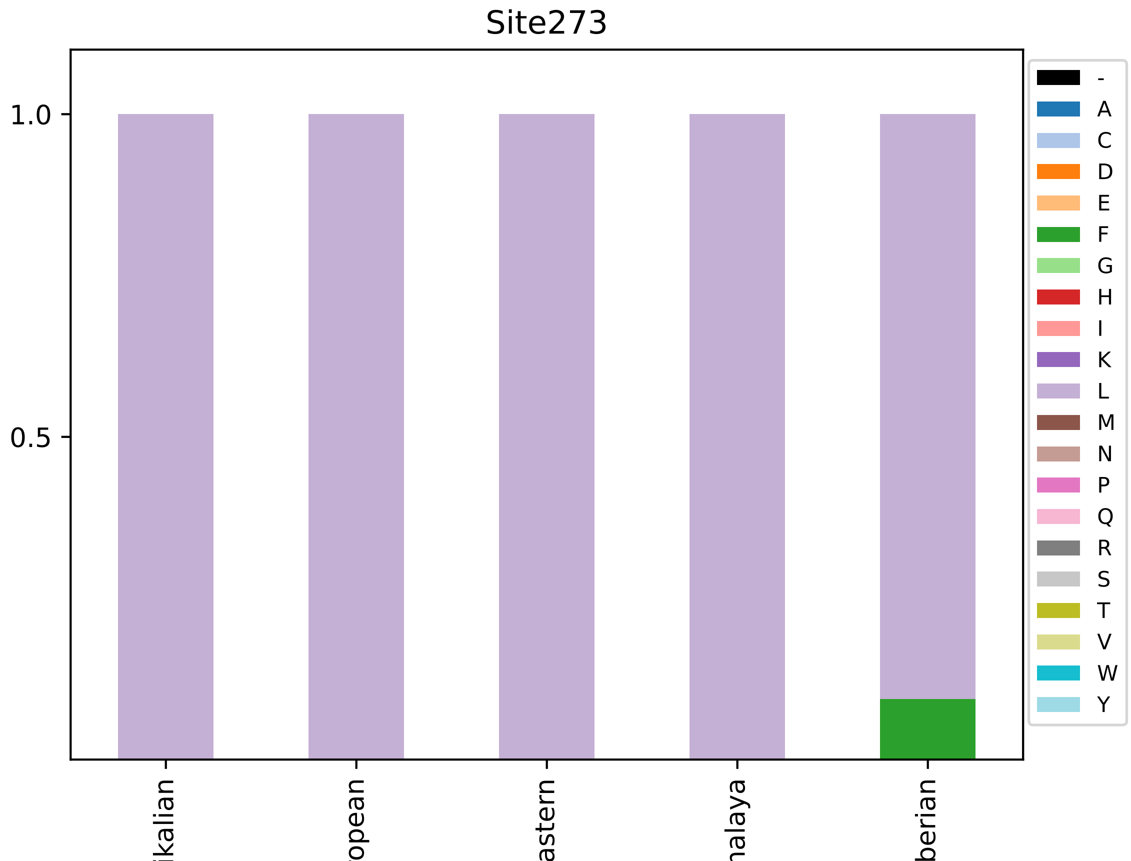

Site273
1.0
0.5
-
A
C
D
E
F
G
H
I
K
L
M
N
P
Q
R
S
T
V
W
Y
ikalian
opean
astern
nalaya
berian

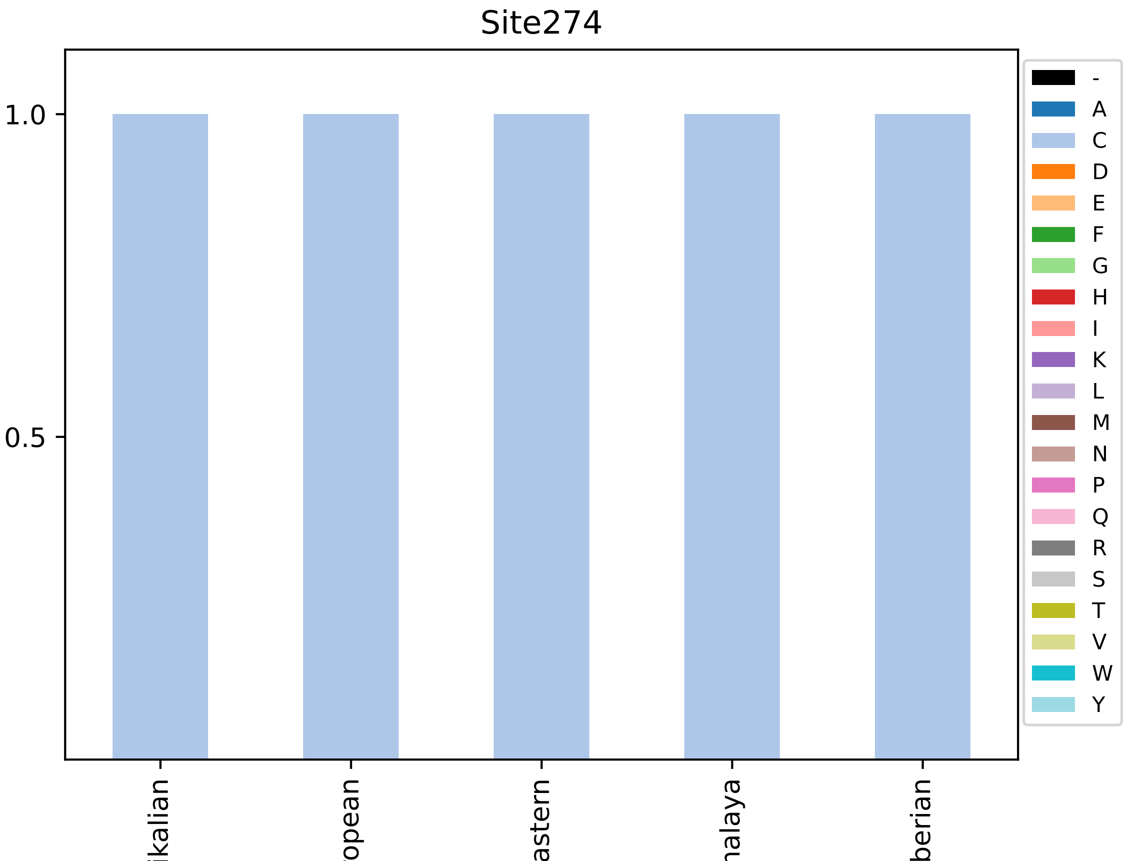
Site274
1.0
0.5
-
A
C
D
E
F
G
H
I
K
L
M
N
P
Q
R
S
T
V
W
Y
ikalian
opean
astern
nalaya
berian

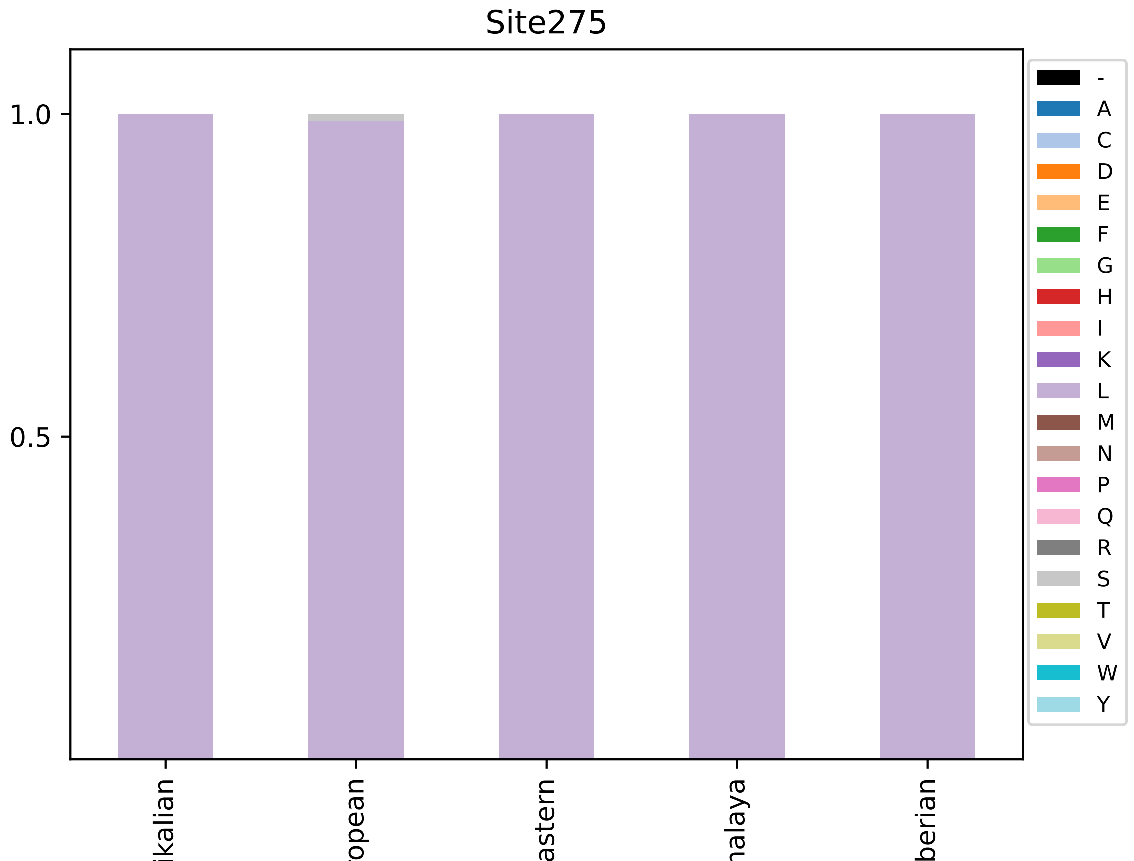

Site275
1.0
0.5
-
A
C
D
E
F
G
H
I
K
L
M
N
P
Q
R
S
T
V
W
Y
ikalian
opean
astern
nalaya
berian

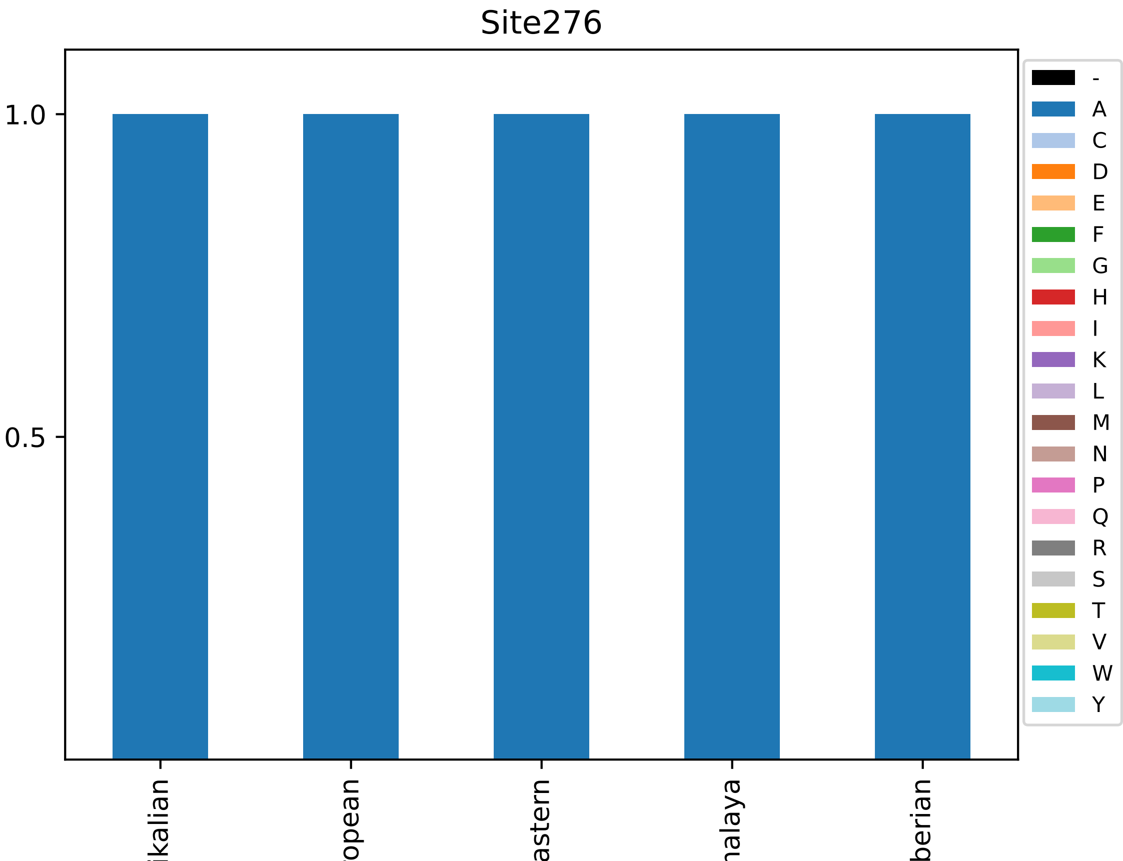

Site276
1.0
0.5
-
A
C
D
E
F
G
H
I
K
L
M
N
P
Q
R
S
T
V
W
Y
ikalian
opean
astern
nalaya
berian

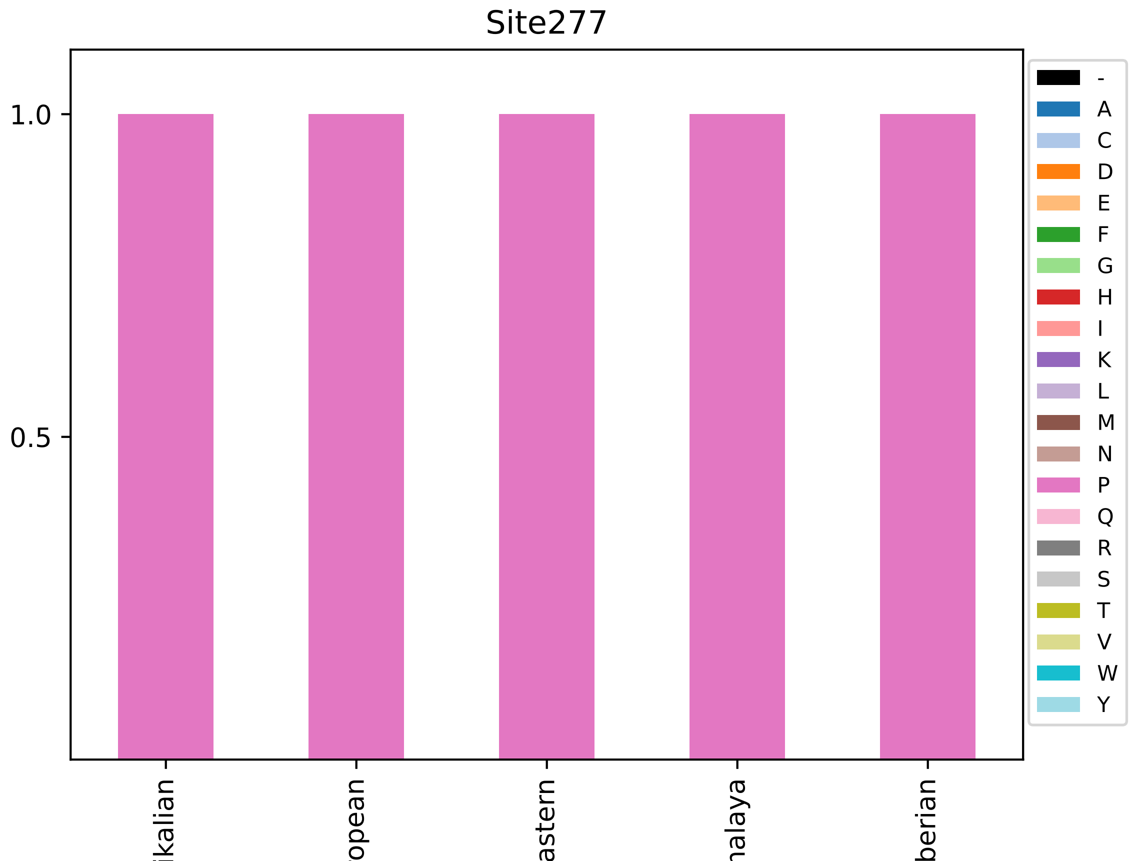

Site277
1.0
0.5
-
A
C
D
E
F
G
H
I
K
L
M
N
P
Q
R
S
T
V
W
Y
ikalian
opean
astern
nalaya
berian

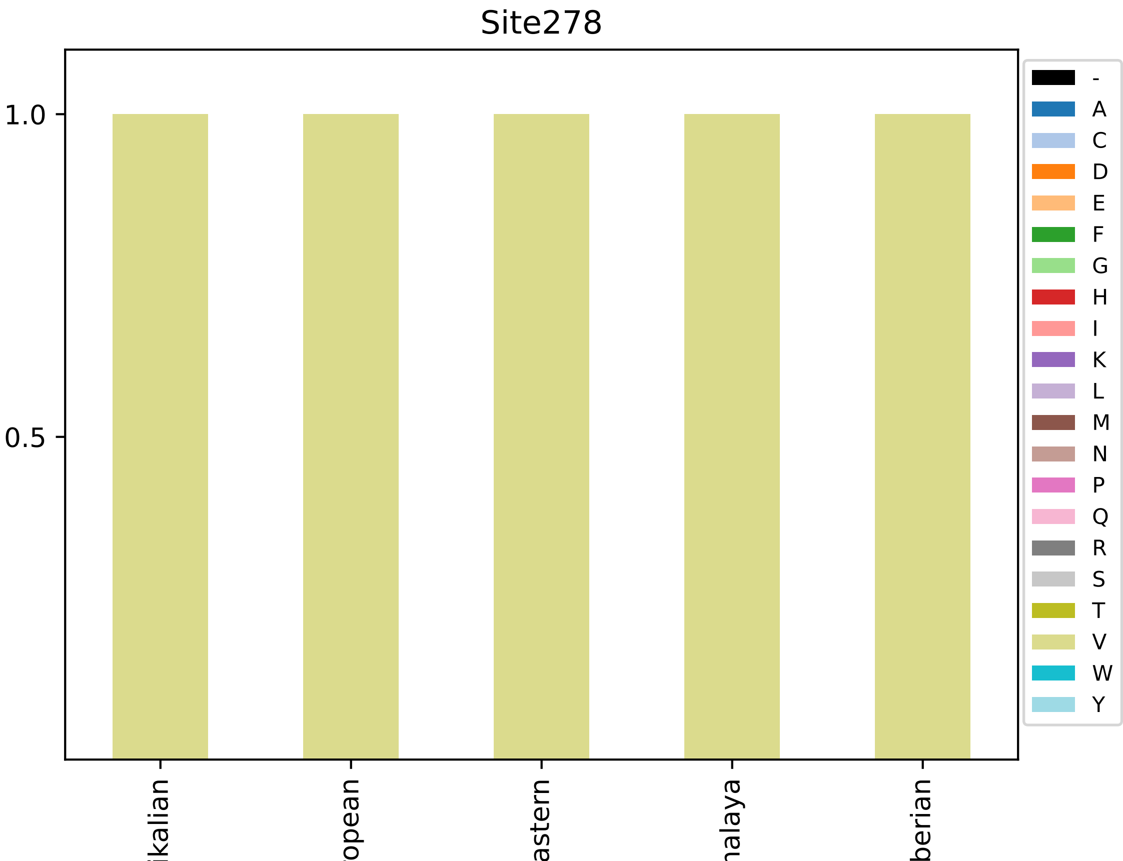

Site278
1.0
0.5
-
A
C
D
E
F
G
H
I
K
L
M
N
P
Q
R
S
T
V
W
Y
ikalian
opean
astern
nalaya
berian

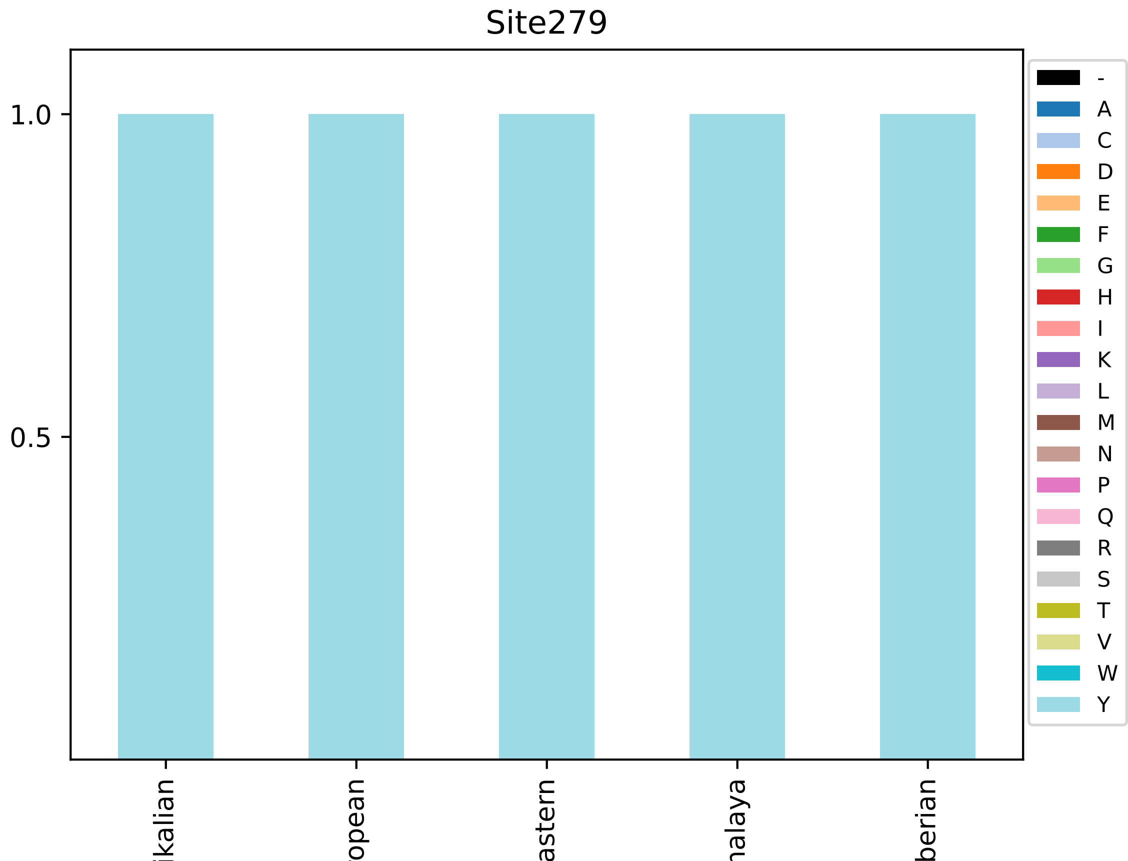
Site279
1.0
0.5
-
A
C
D
E
F
G
H
I
K
L
M
N
P
Q
R
S
T
V
W
Y

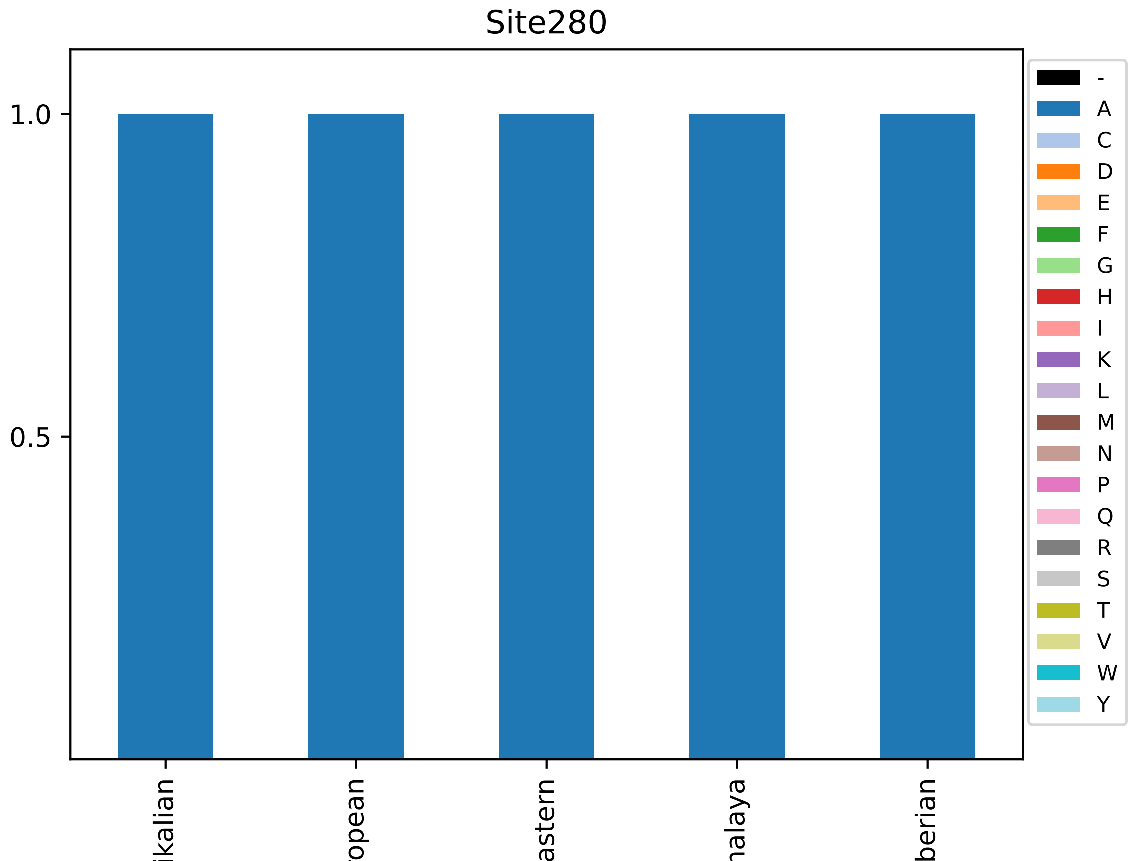
Site280
1.0
0.5
ikalian
opean
astern
nalaya
berian
-
A
C
D
E
F
G
H
I
K
L
M
N
P
Q
R
S
T
V
W
Y

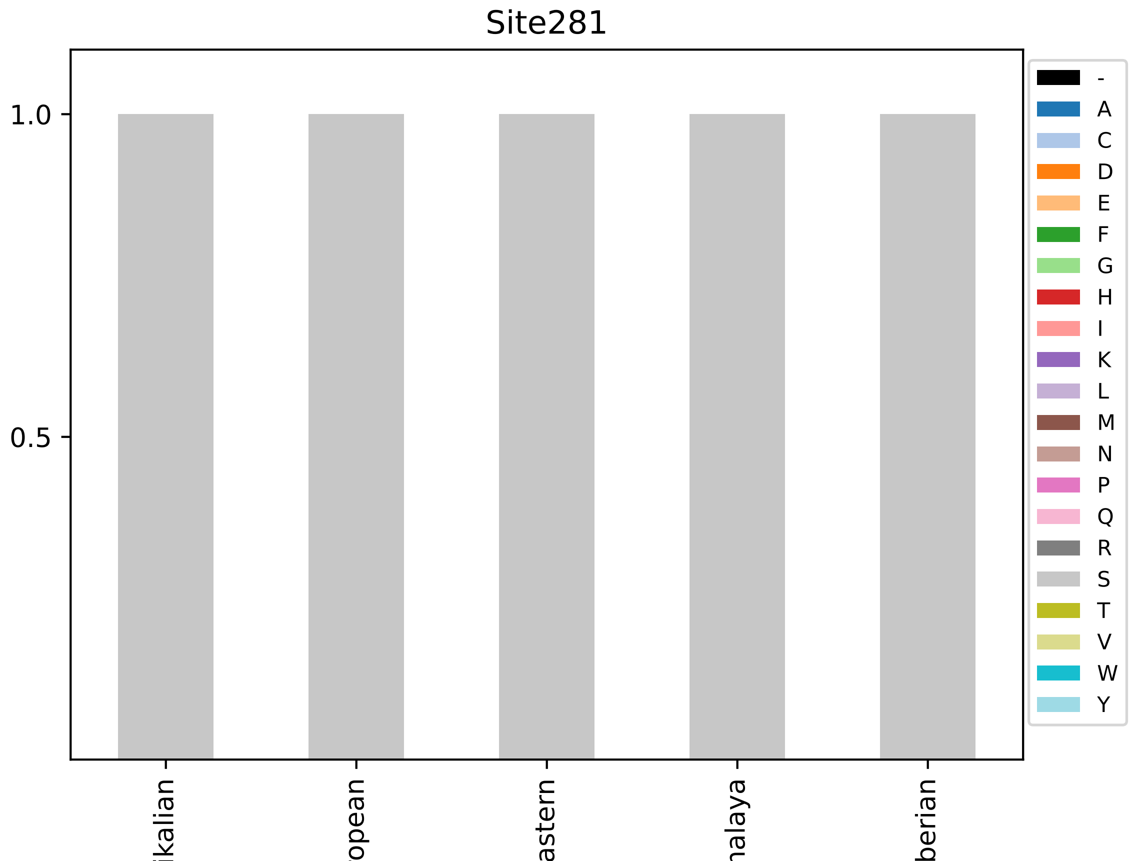
Site281
1.0
0.5
-
A
C
D
E
F
G
H
I
K
L
M
N
P
Q
R
S
T
V
W
Y
ikalian
opean
astern
nalaya
berian

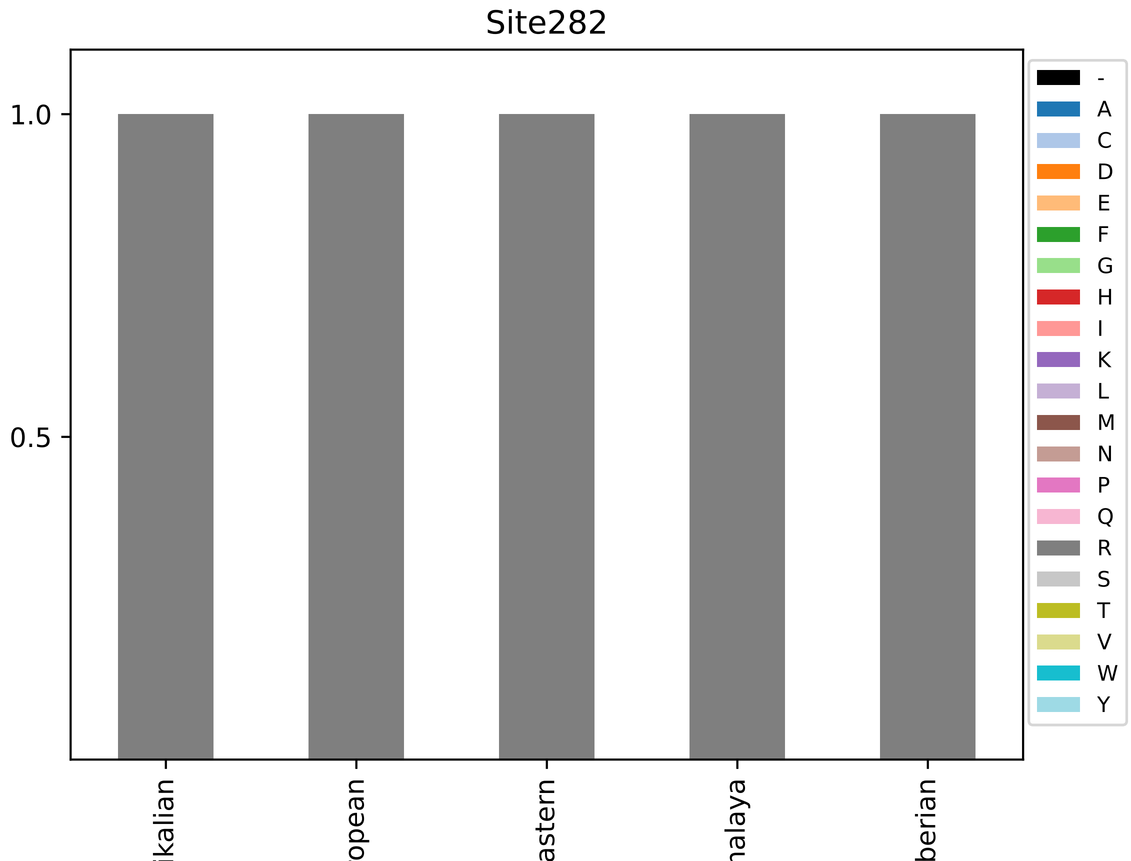
Site282
1.0
0.5
ikalian
opean
astern
nalaya
berian
-
A
C
D
E
F
G
H
I
K
L
M
N
P
Q
R
S
T
V
W
Y

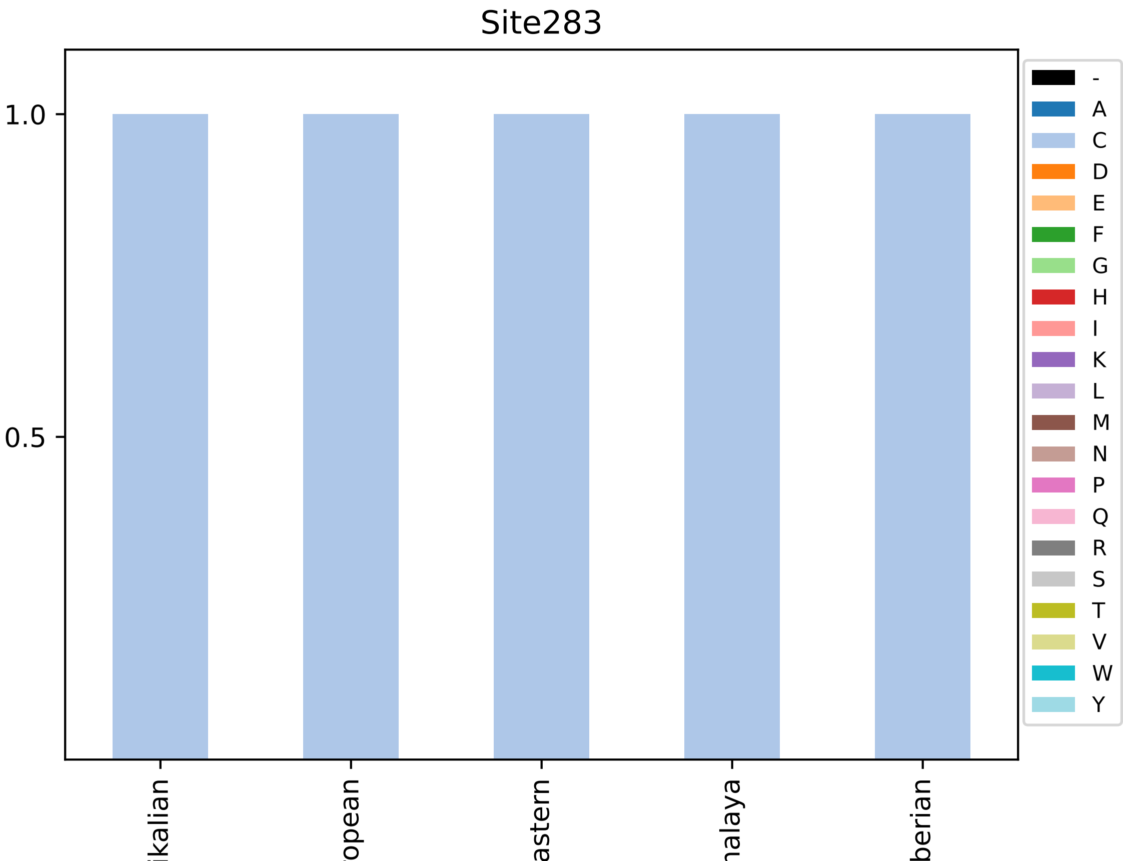

Site283
1.0
0.5
-
A
C
D
E
F
G
H
I
K
L
M
N
P
Q
R
S
T
V
W
Y
ikalian
opean
astern
nalaya
berian

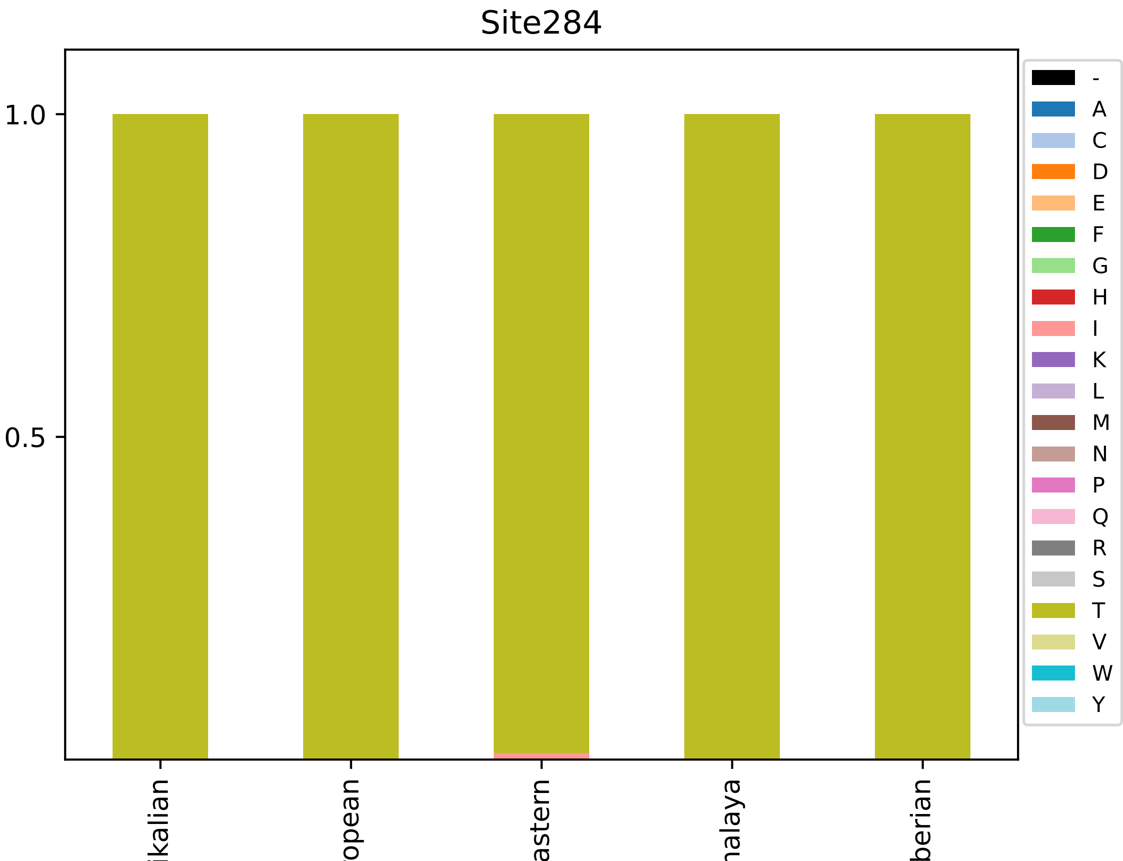

Site284
1.0
0.5
-
A
C
D
E
F
G
H
I
K
L
M
N
P
Q
R
S
T
V
W
Y
ikalian
opean
astern
nalaya
berian

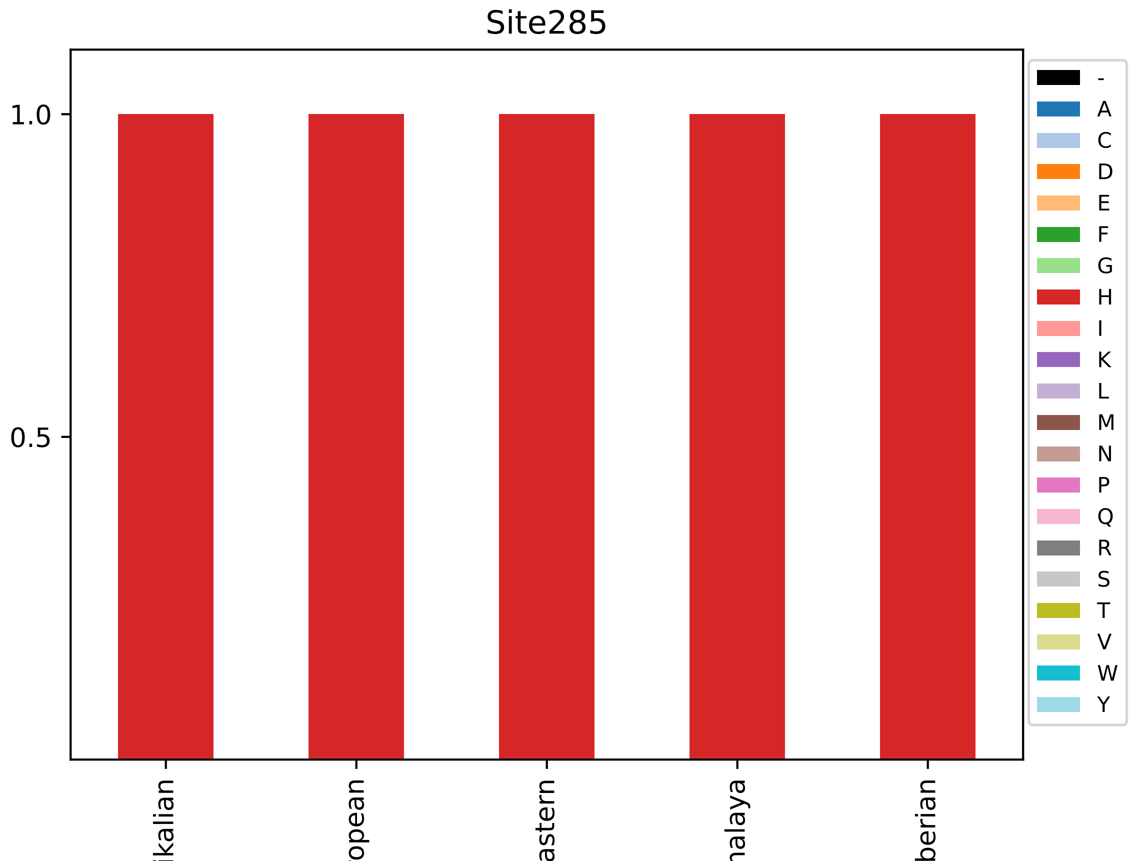
Site285
1.0
0.5
-
A
C
D
E
F
G
H
I
K
L
M
N
P
Q
R
S
T
V
W
Y
ikalian
opean
astern
nalaya
berian

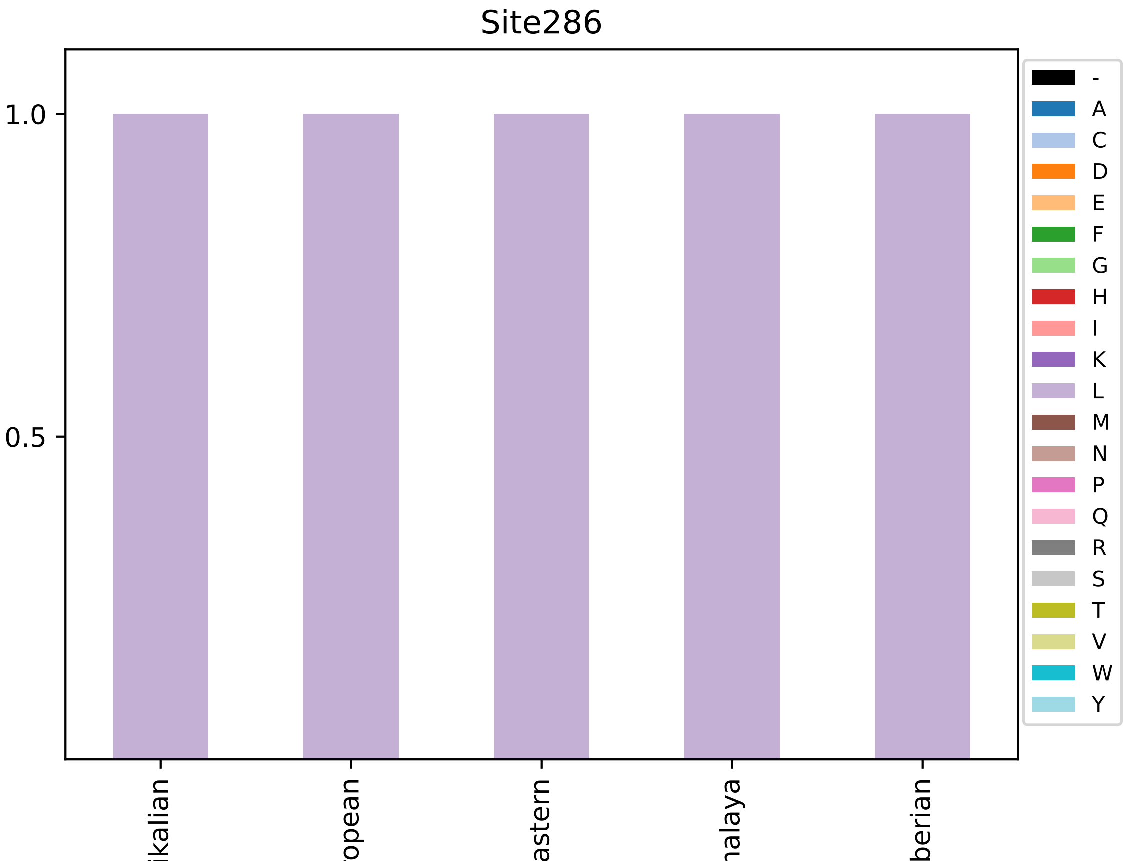
Site286
1.0
0.5
ikalian
opean
astern
nalaya
berian
-
A
C
D
E
F
G
H
I
K
L
M
N
P
Q
R
S
T
V
W
Y

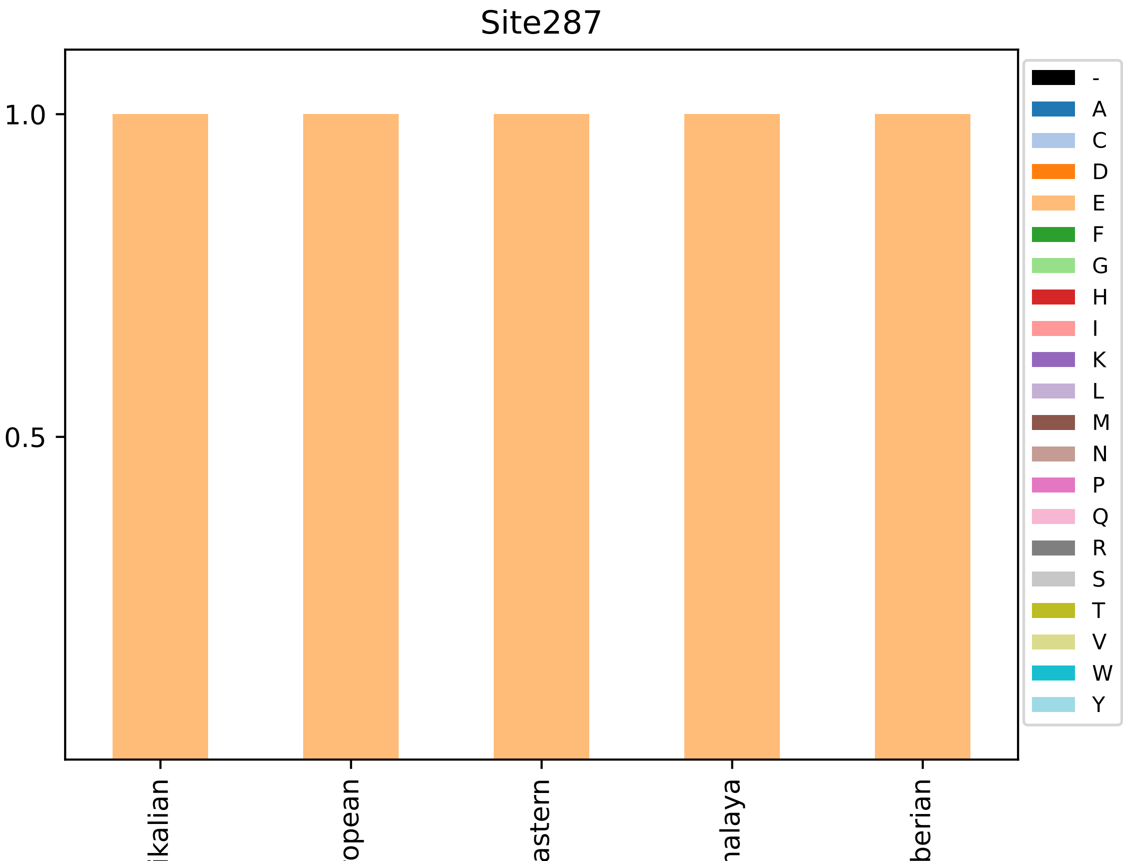
Site287
1.0
0.5
-
A
C
D
E
F
G
H
I
K
L
M
N
P
Q
R
S
T
V
W
Y
ikalian
opean
astern
nalaya
berian

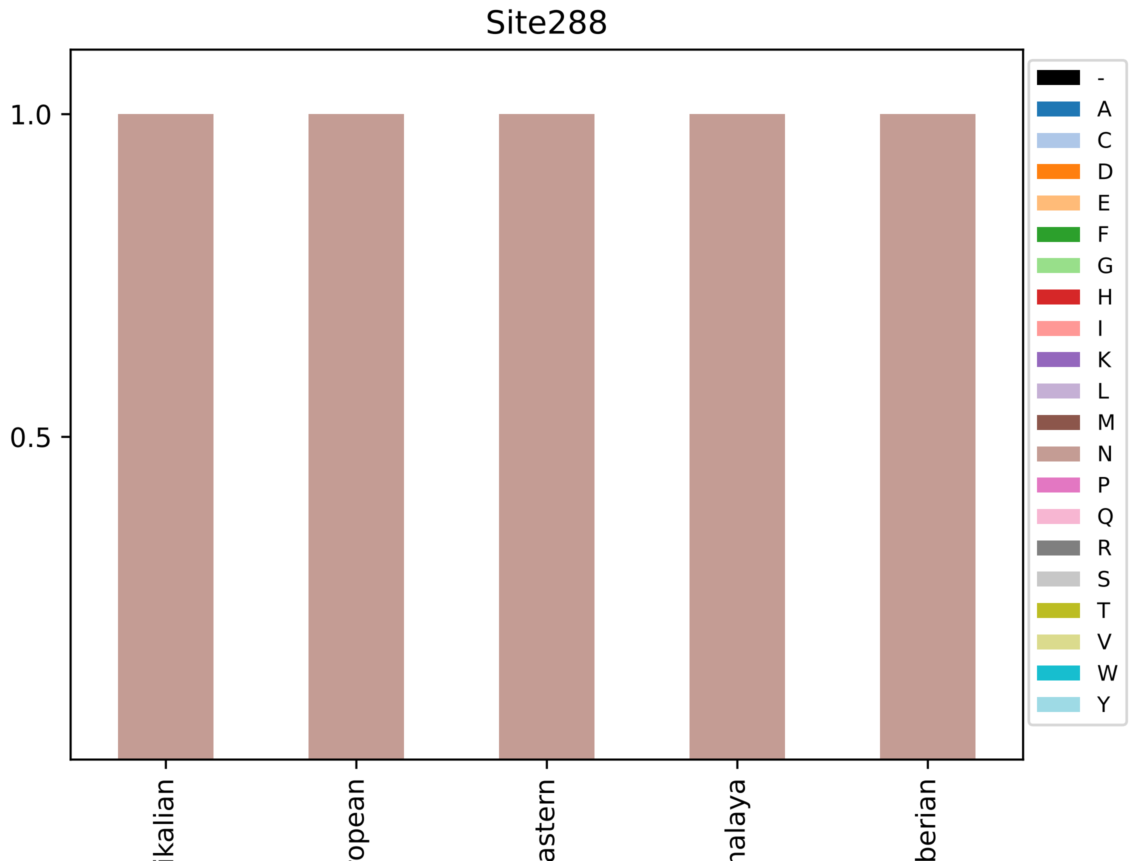

Site288
1.0
0.5
ikalian
opean
astern
nalaya
berian
-
A
C
D
E
F
G
H
I
K
L
M
N
P
Q
R
S
T
V
W
Y

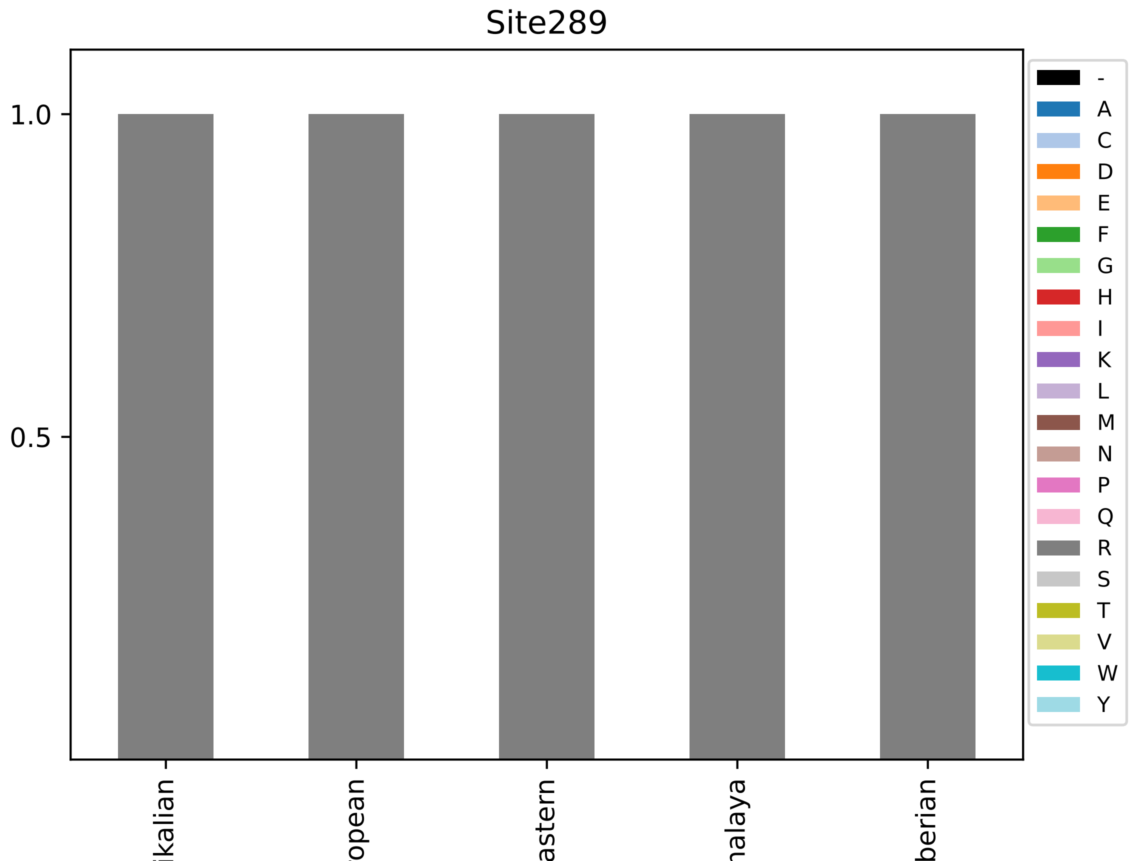

Site289
1.0
0.5
-
A
C
D
E
F
G
H
I
K
L
M
N
P
Q
R
S
T
V
W
Y
ikalian
opean
astern
nalaya
berian

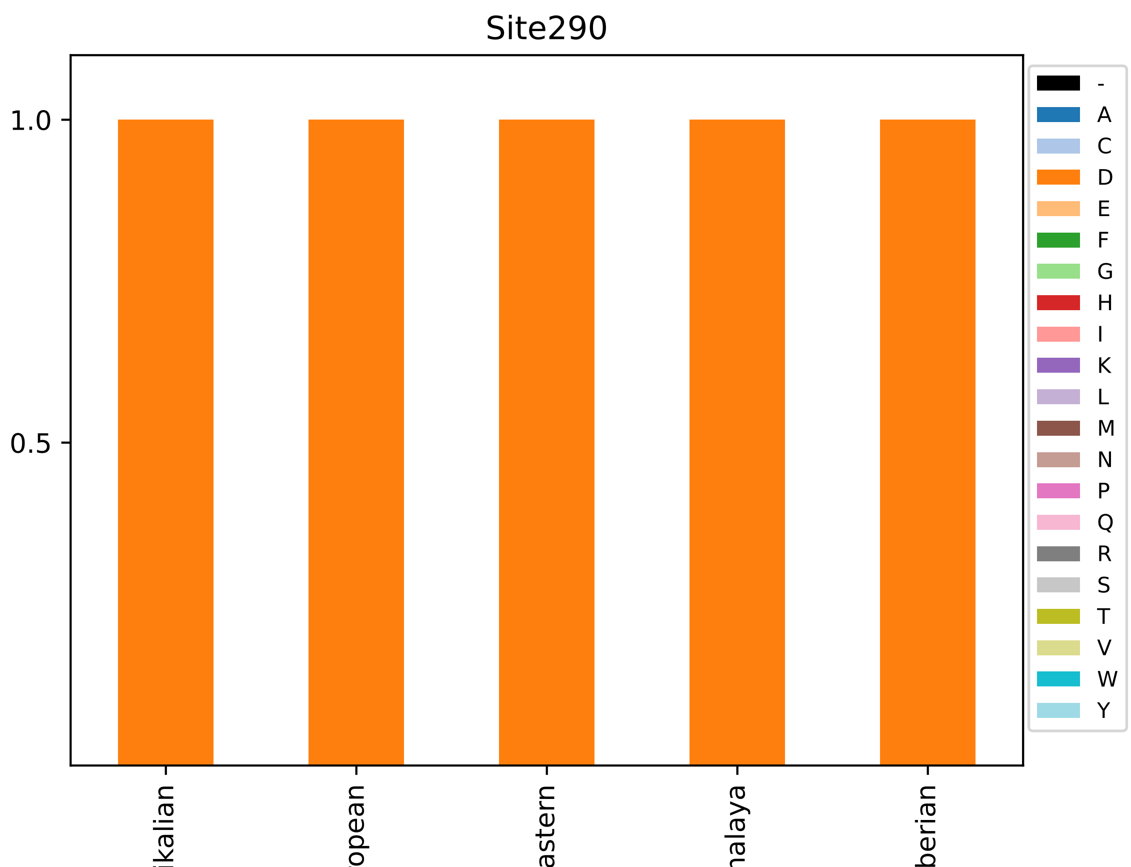

Site290
1.0
0.5
-
A
C
D
E
F
G
H
I
K
L
M
N
P
Q
R
S
T
V
W
Y
ikalian
opean
astern
nalaya
berian

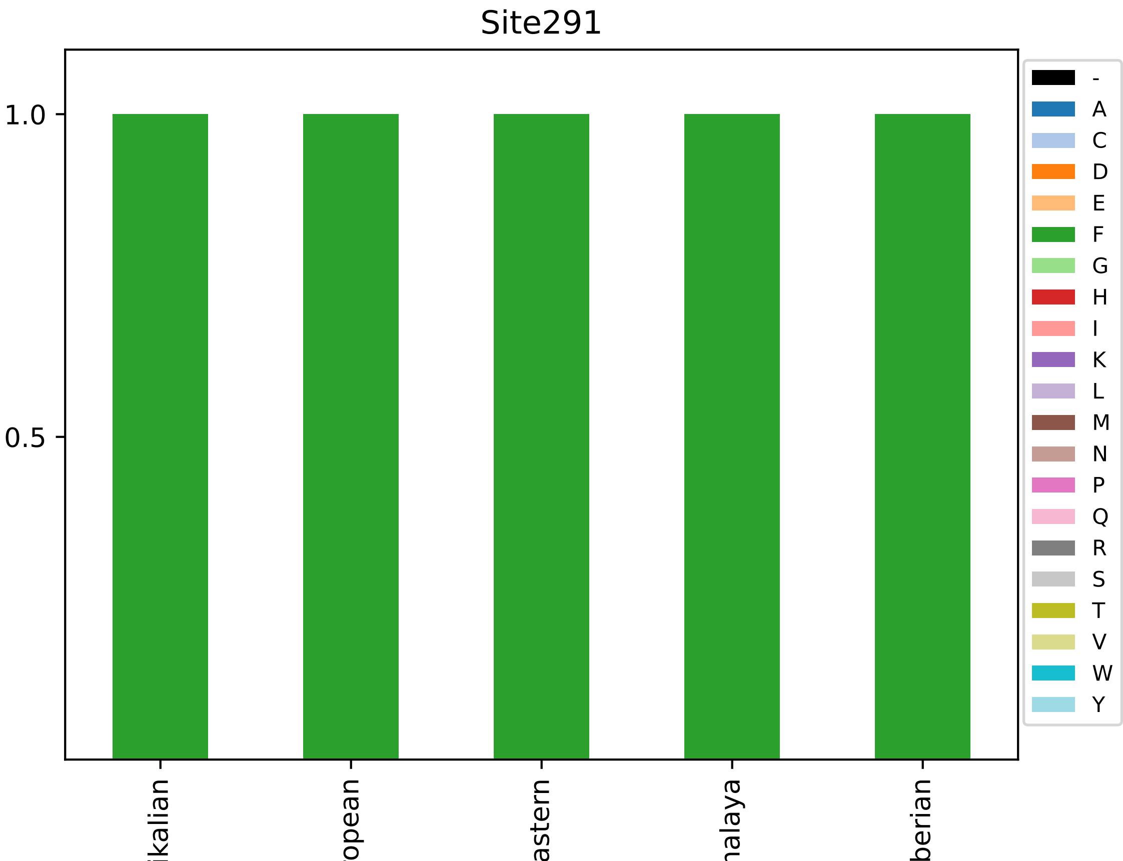

Site291
1.0
0.5
-
A
C
D
E
F
G
H
I
K
L
M
N
P
Q
R
S
T
V
W
Y
ikalian
opean
astern
nalaya
berian

Site292

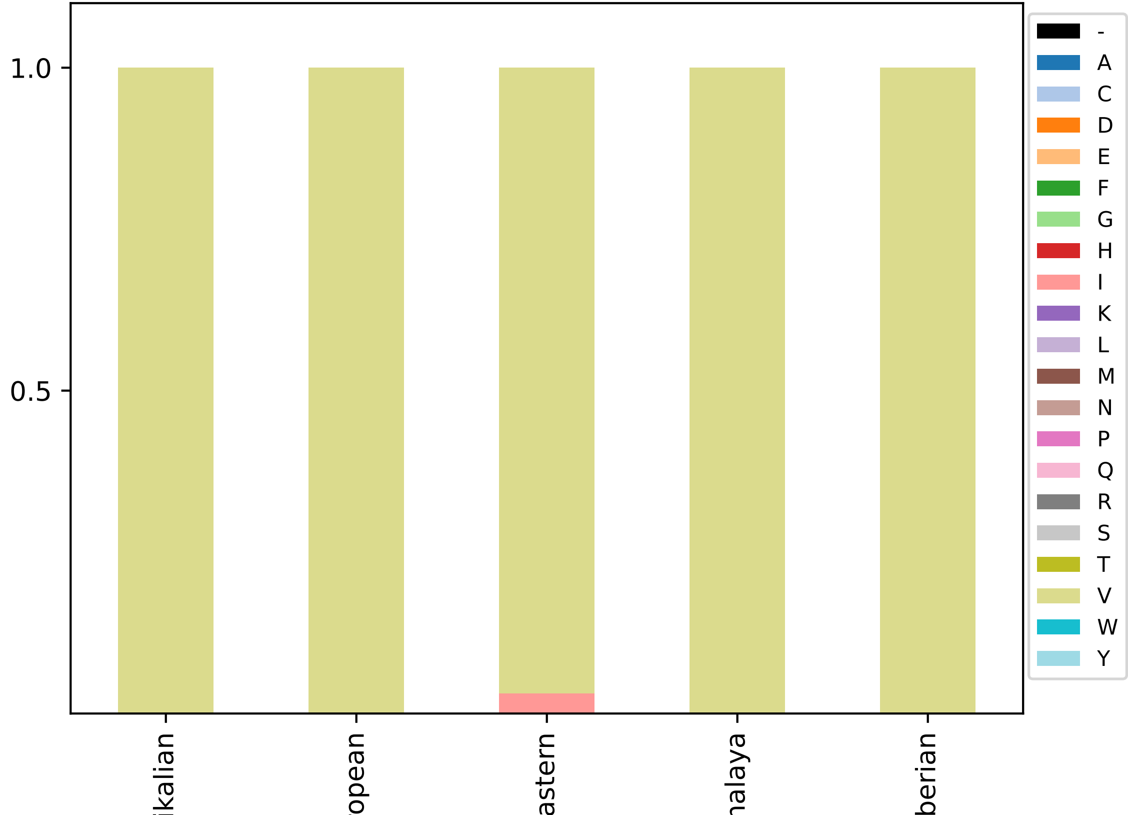

1.0
0.5
-
A
C
D
E
F
G
H
I
K
L
M
N
P
Q
R
S
T
V
W
Y
ikalian
opean
astern
nalaya
berian

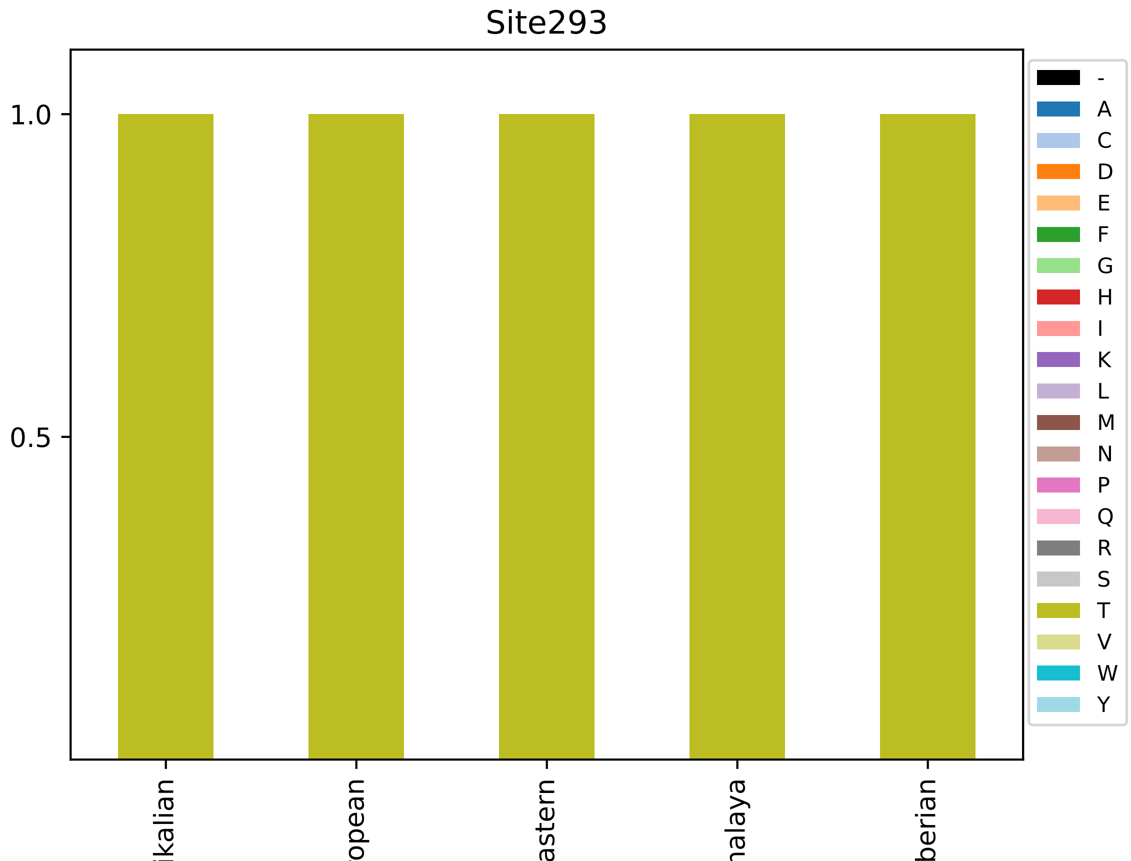
Site293
1.0
0.5
-
A
C
D
E
F
G
H
I
K
L
M
N
P
Q
R
S
T
V
W
Y
ikalian
opean
astern
nalaya
berian

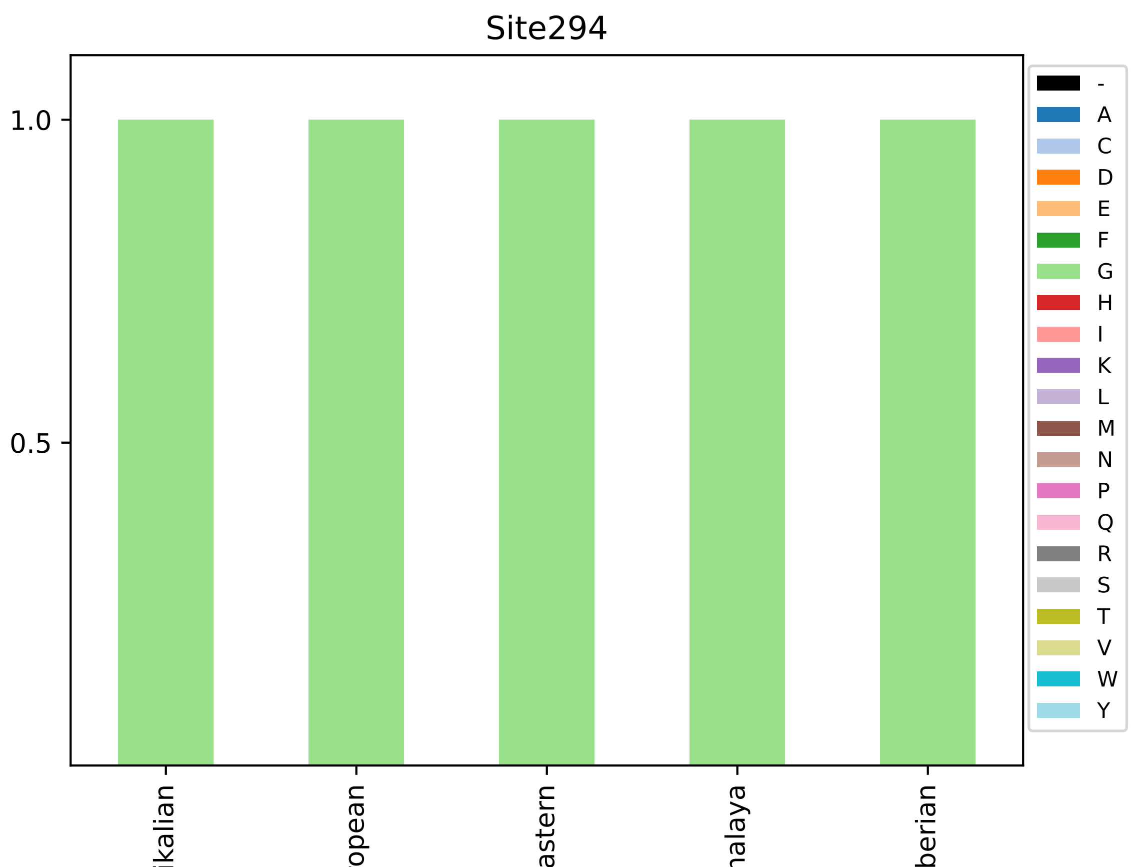
Site294
1.0
0.5
-
A
C
D
E
F
G
H
I
K
L
M
N
P
Q
R
S
T
V
W
Y
ikalian
opean
astern
nalaya
berian

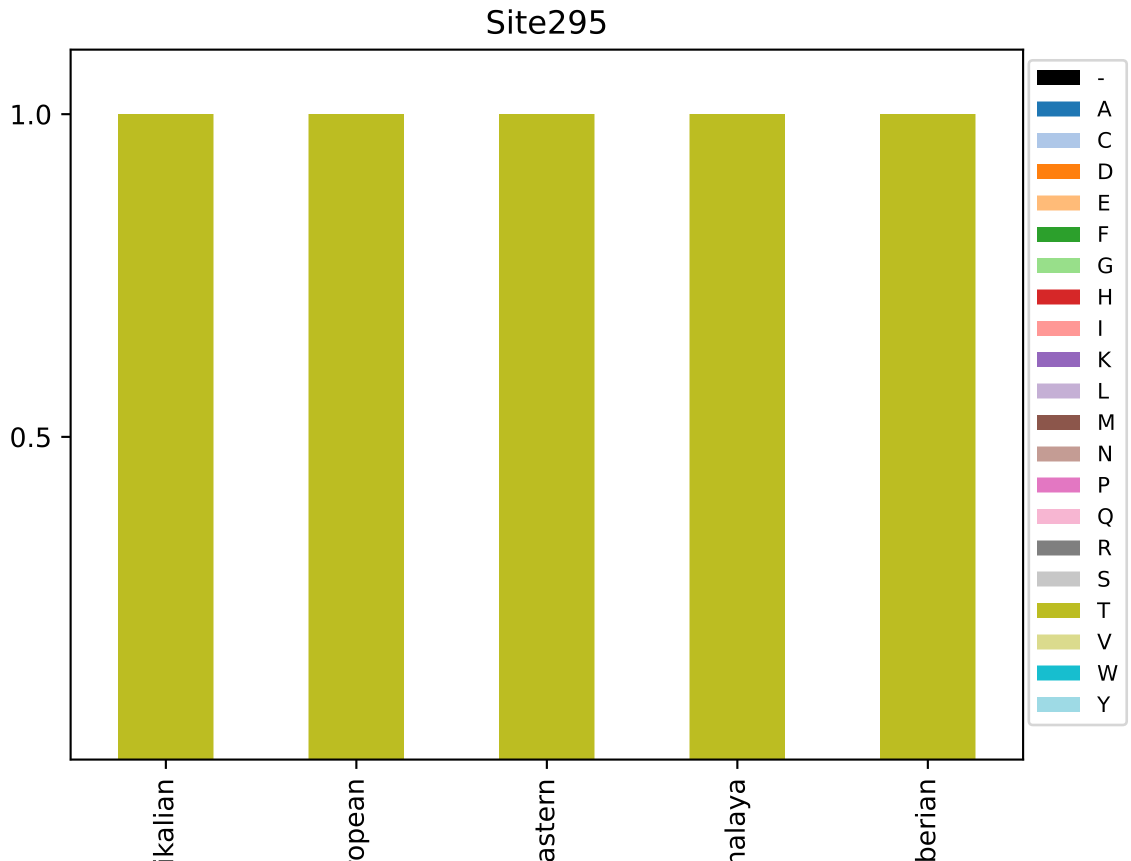
Site295
1.0
0.5
-
A
C
D
E
F
G
H
I
K
L
M
N
P
Q
R
S
T
V
W
Y
ikalian
opean
astern
nalaya
berian

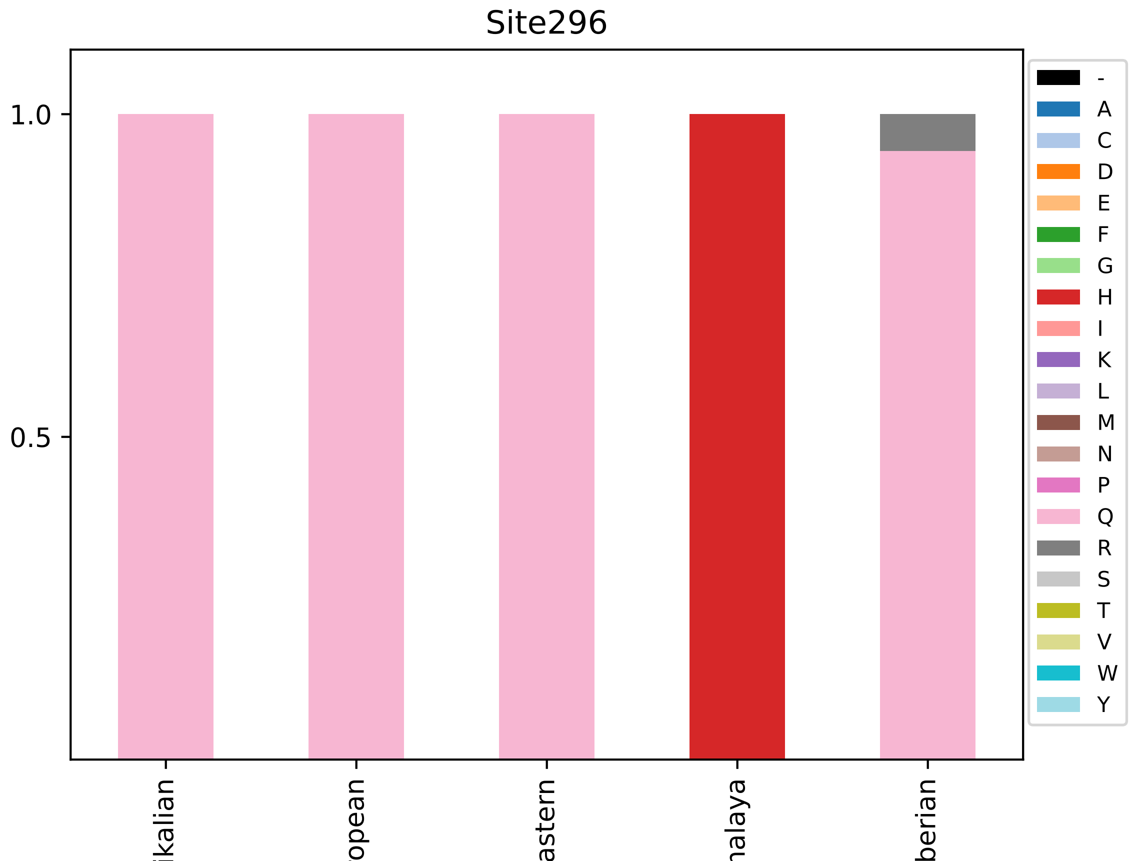
Site296
1.0
0.5
-
A
C
D
E
F
G
H
I
K
L
M
N
P
Q
R
S
T
V
W
Y
ikalian
opean
astern
nalaya
berian

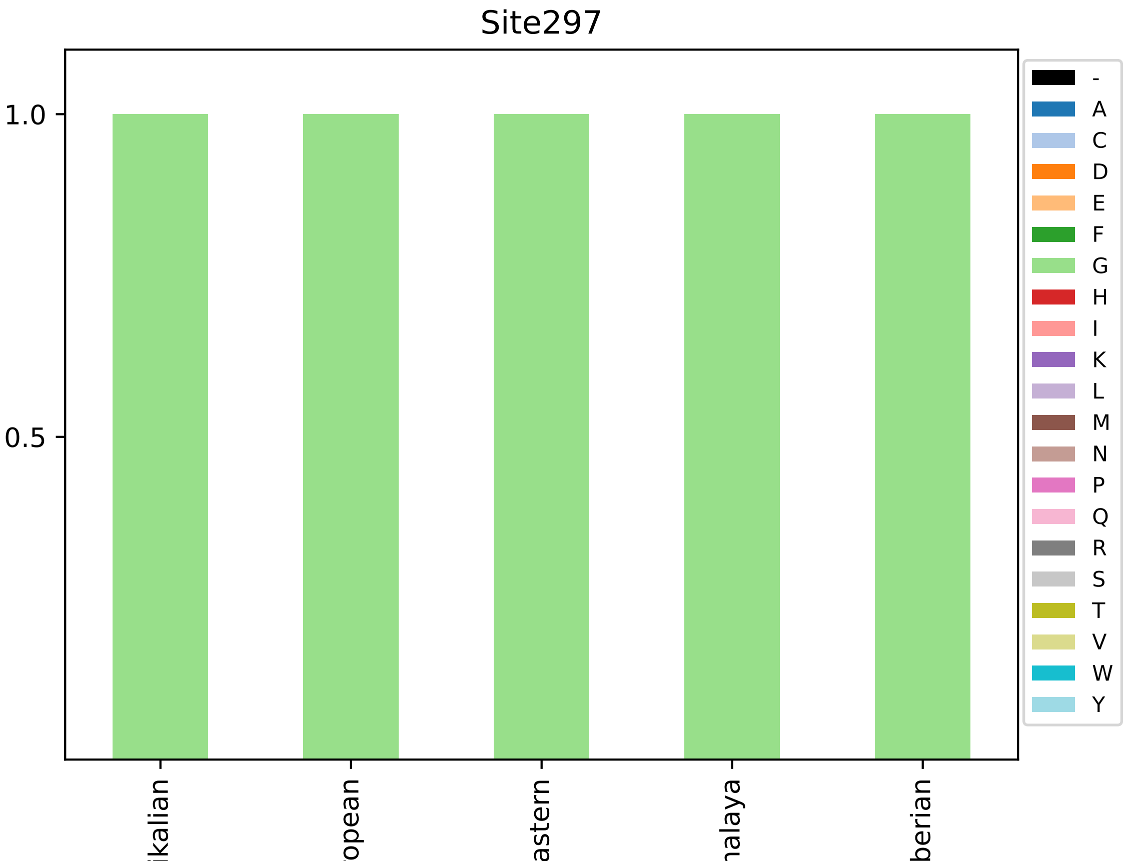

Site297
1.0
0.5
-
A
C
D
E
F
G
H
I
K
L
M
N
P
Q
R
S
T
V
W
Y
ikalian
opean
astern
nalaya
berian

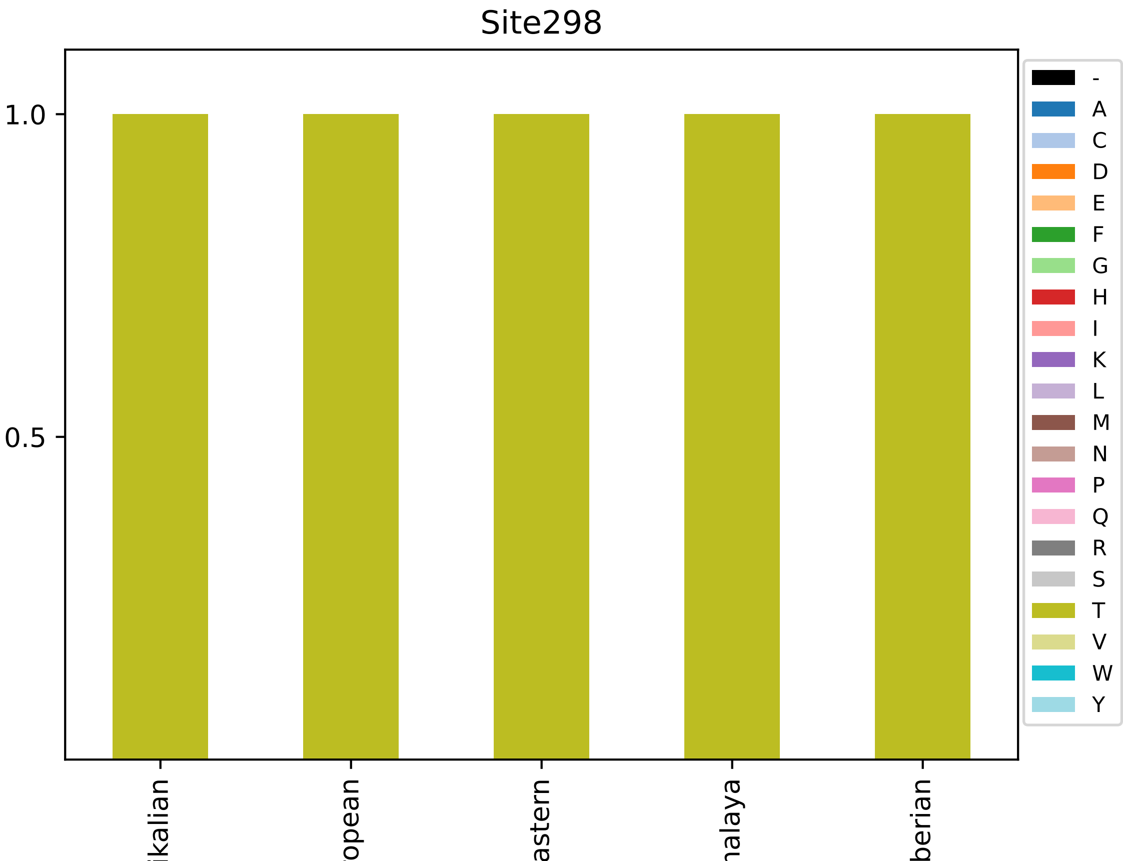
Site298
1.0
0.5
-
A
C
D
E
F
G
H
I
K
L
M
N
P
Q
R
S
T
V
W
Y
ikalian
opean
astern
nalaya
berian

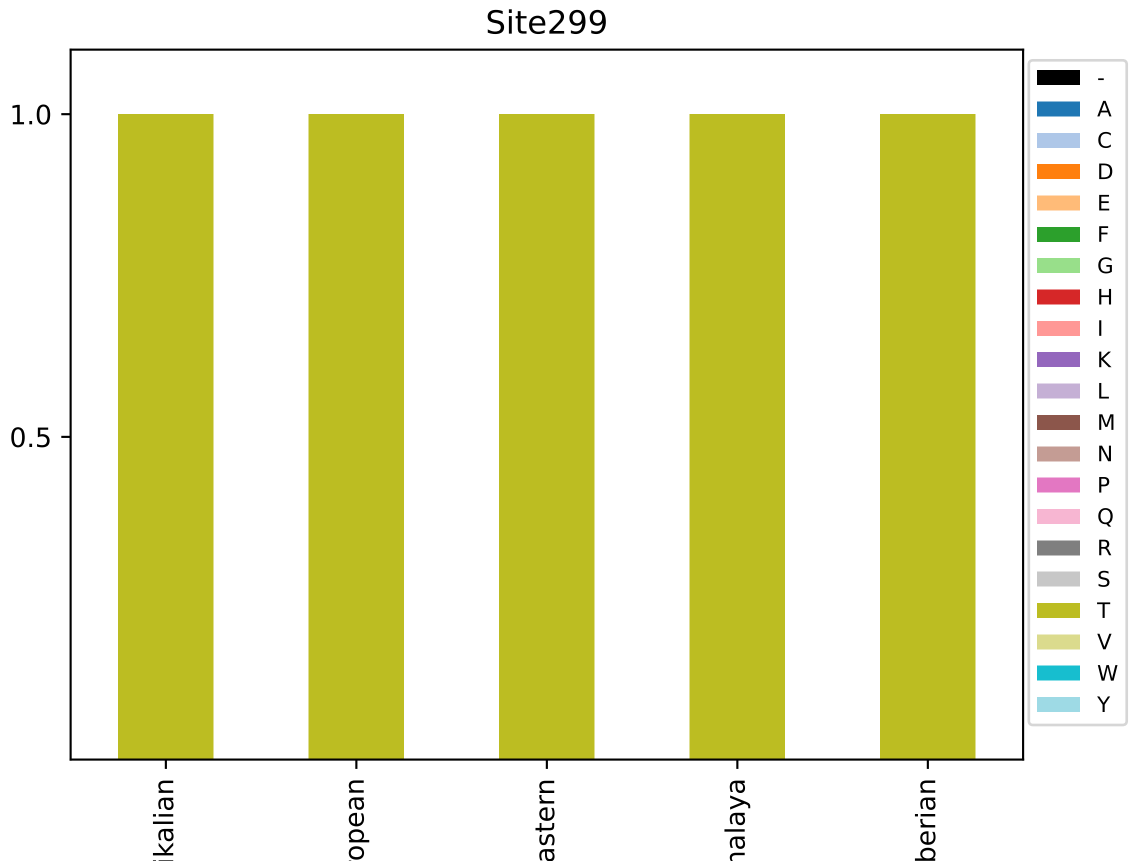

Site299
1.0
0.5
-
A
C
D
E
F
G
H
I
K
L
M
N
P
Q
R
S
T
V
W
Y
ikalian
opean
astern
nalaya
berian

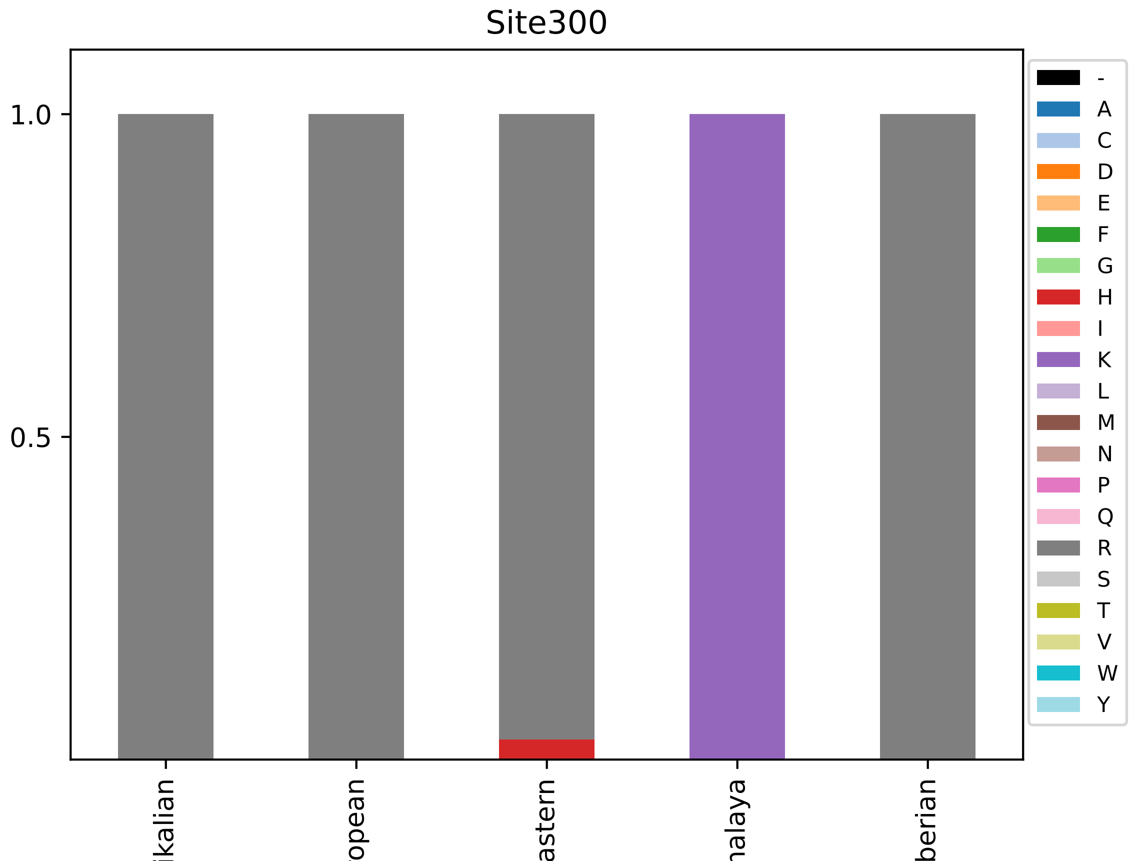
Site300
1.0
0.5
-
A
C
D
E
F
G
H
I
K
L
M
N
P
Q
R
S
T
V
W
Y
ikalian
ropean
astern
nalaya
berian

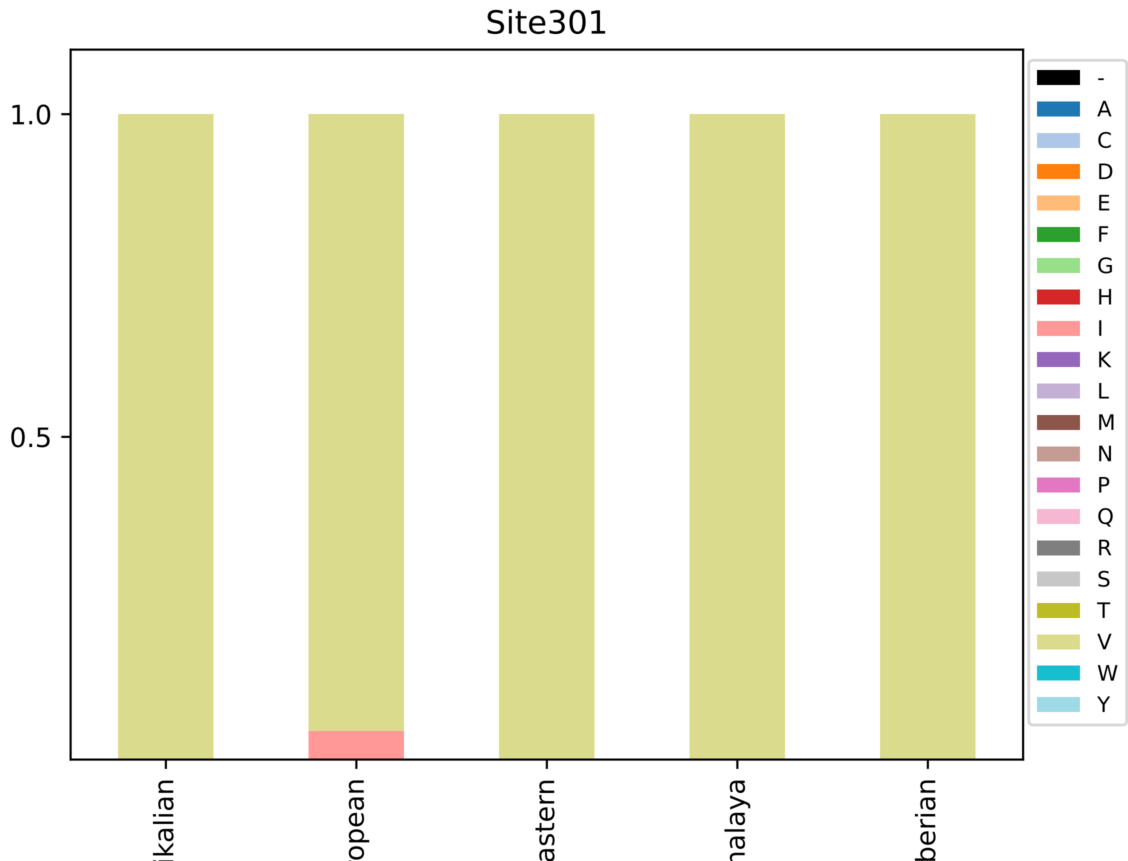

Site301
1.0
0.5
ikalian
opean
astern
nalaya
berian
-
A
C
D
E
F
G
H
I
K
L
M
N
P
Q
R
S
T
V
W
Y

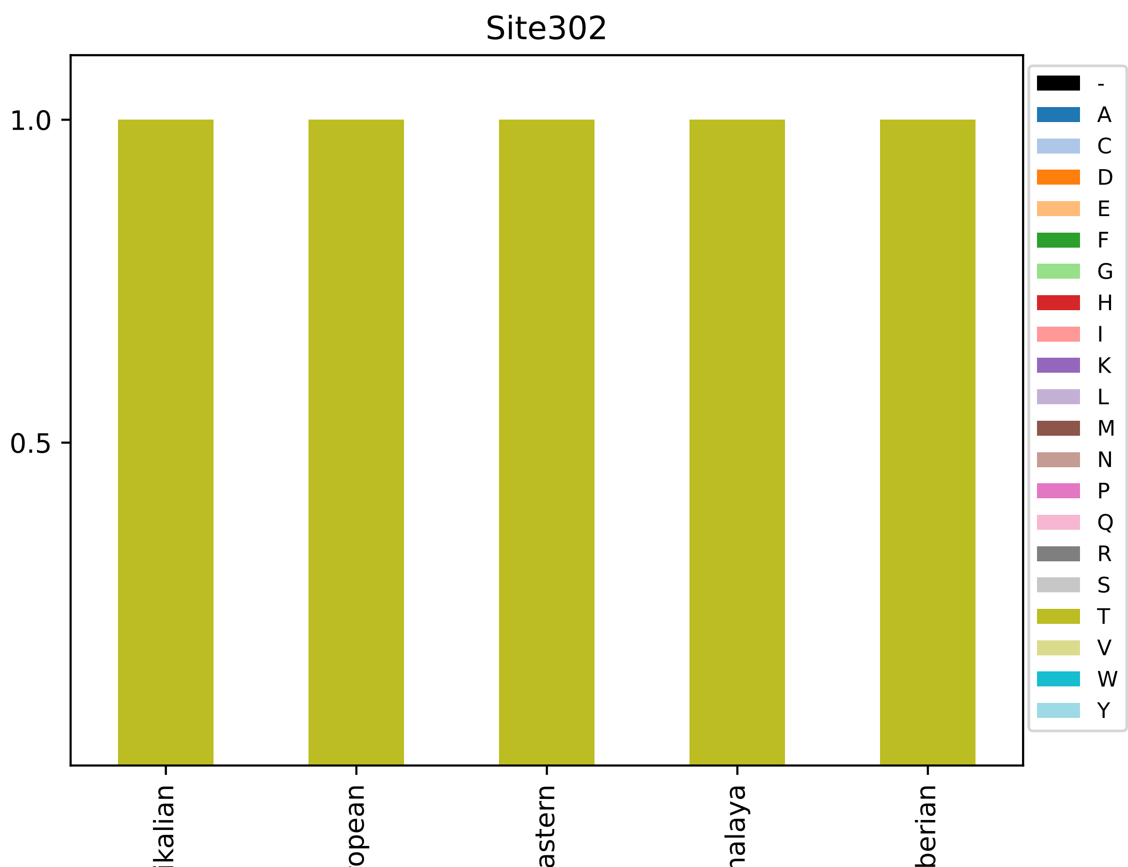
Site302
1.0
0.5
-
A
C
D
E
F
G
H
I
K
L
M
N
P
Q
R
S
T
V
W
Y
ikalian
opean
astern
nalaya
berian

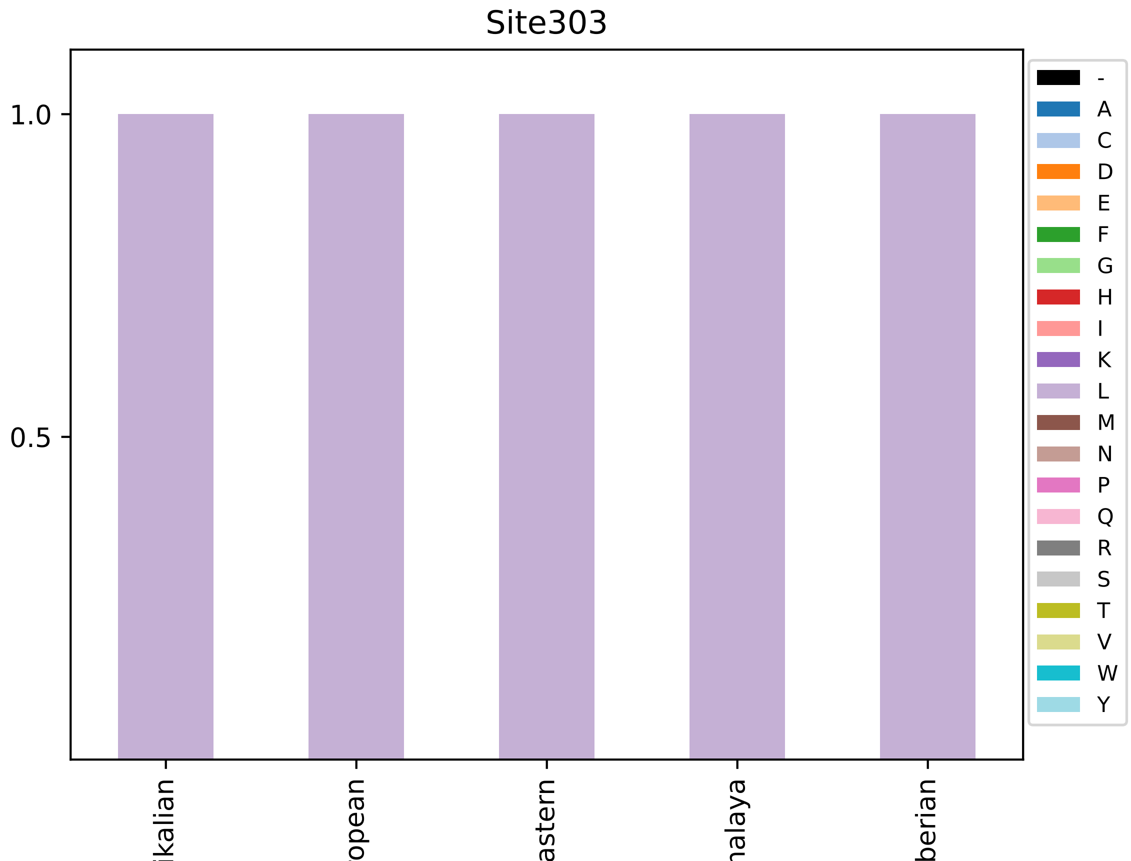

Site303
1.0
0.5
ikalian
opean
astern
nalaya
berian
-
A
C
D
E
F
G
H
I
K
L
M
N
P
Q
R
S
T
V
W
Y

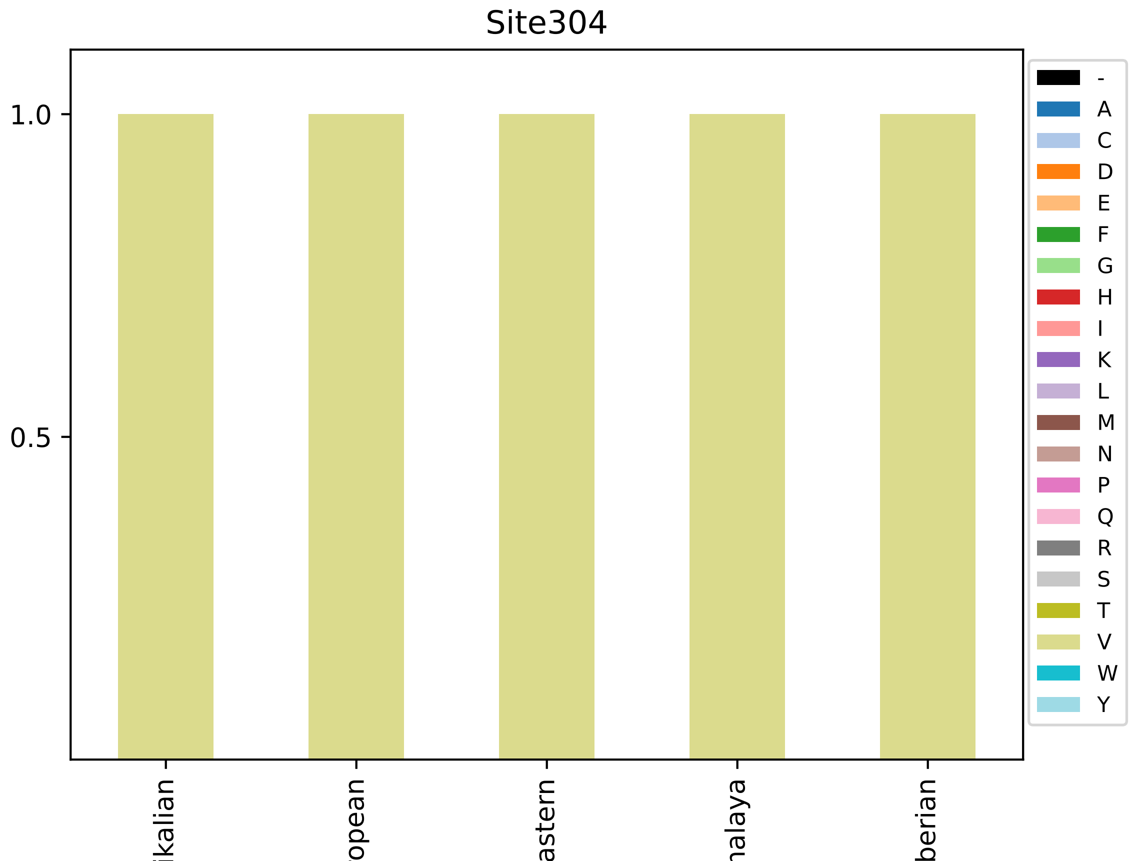
Site304
1.0
0.5
-
A
C
D
E
F
G
H
I
K
L
M
N
P
Q
R
S
T
V
W
Y
ikalian
opean
astern
halaya
berian

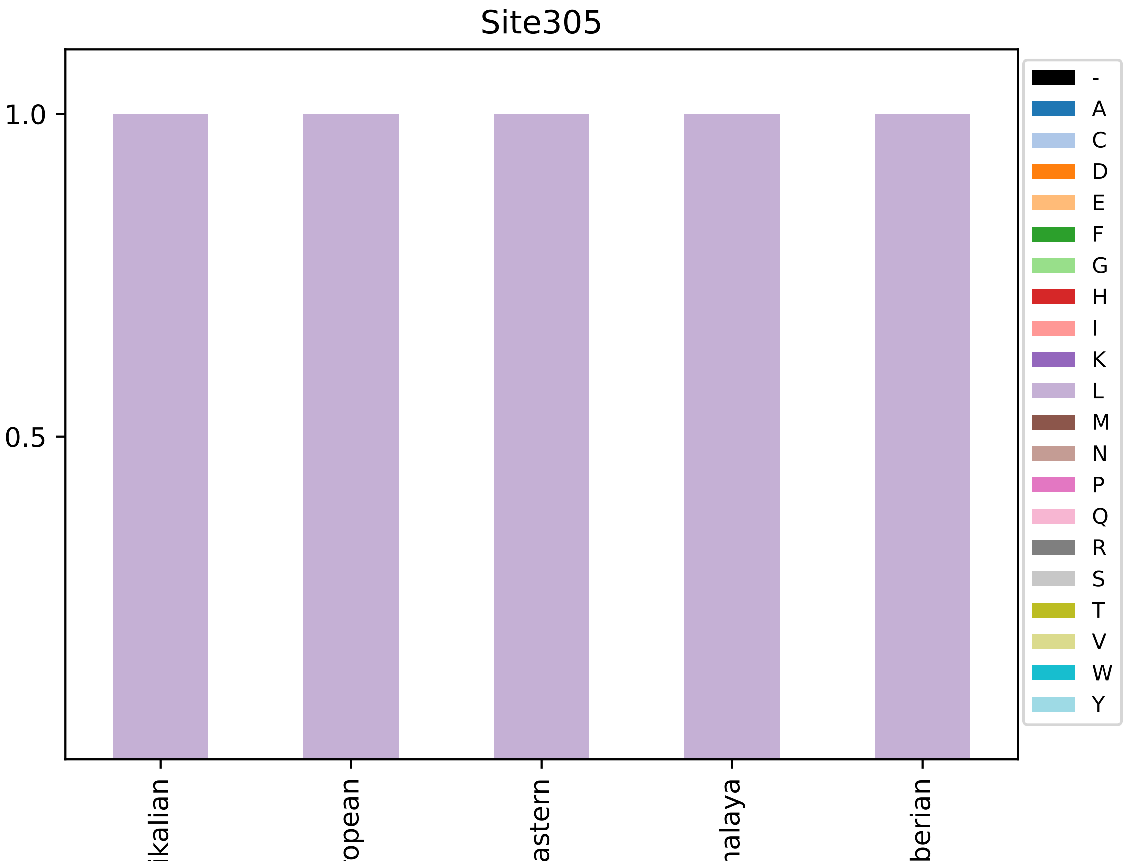
Site305
1.0
0.5
-
A
C
D
E
F
G
H
I
K
L
M
N
P
Q
R
S
T
V
W
Y
ikalian
ropean
astern
nalaya
berian

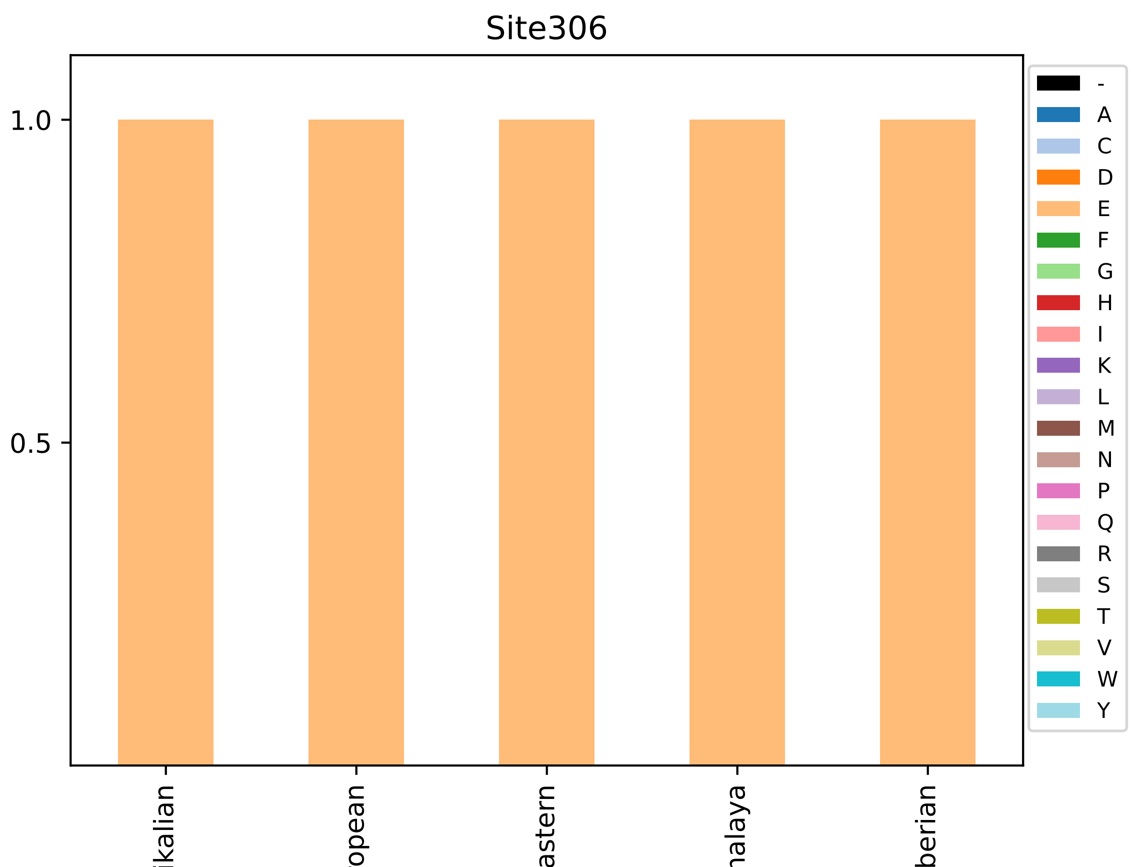
Site306
1.0
0.5
-
A
C
D
E
F
G
H
I
K
L
M
N
P
Q
R
S
T
V
W
Y
ikalian
opean
astern
nalaya
berian

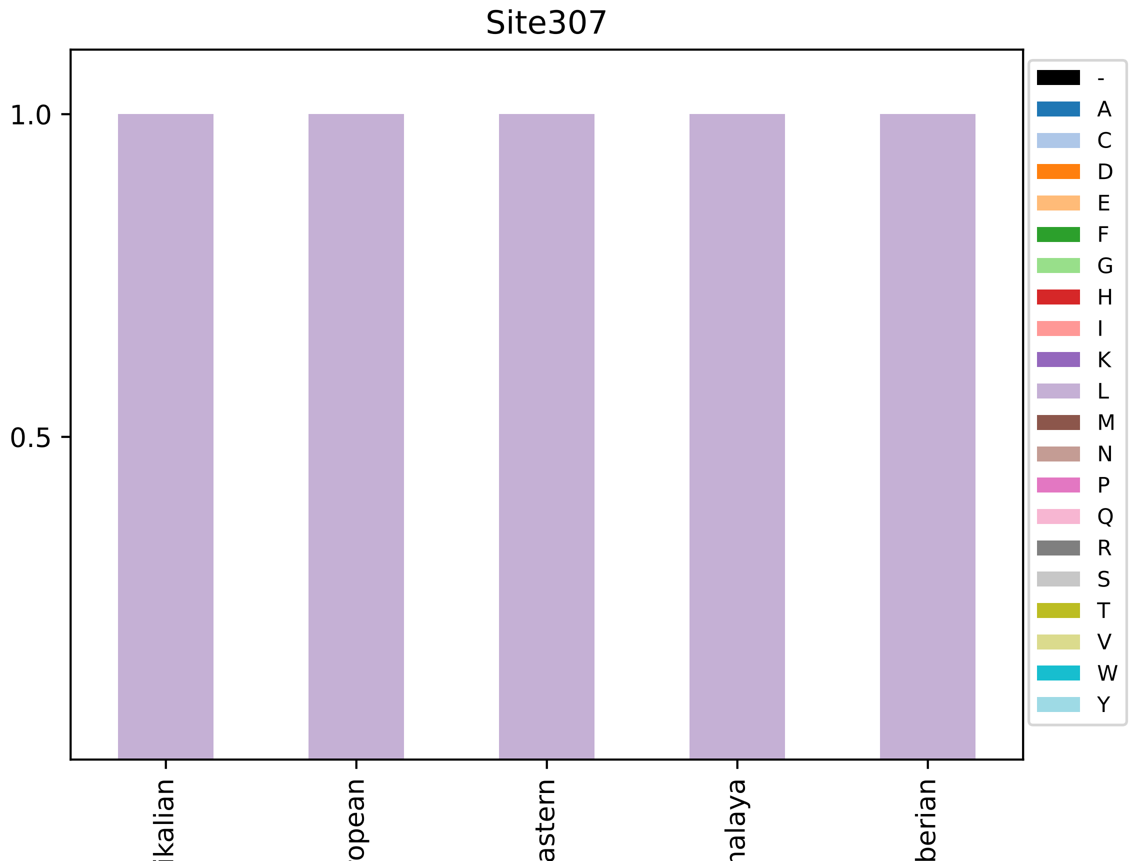

Site307
1.0
0.5
-
A
C
D
E
F
G
H
I
K
L
M
N
P
Q
R
S
T
V
W
Y
ikalian
opean
astern
nalaya
berian

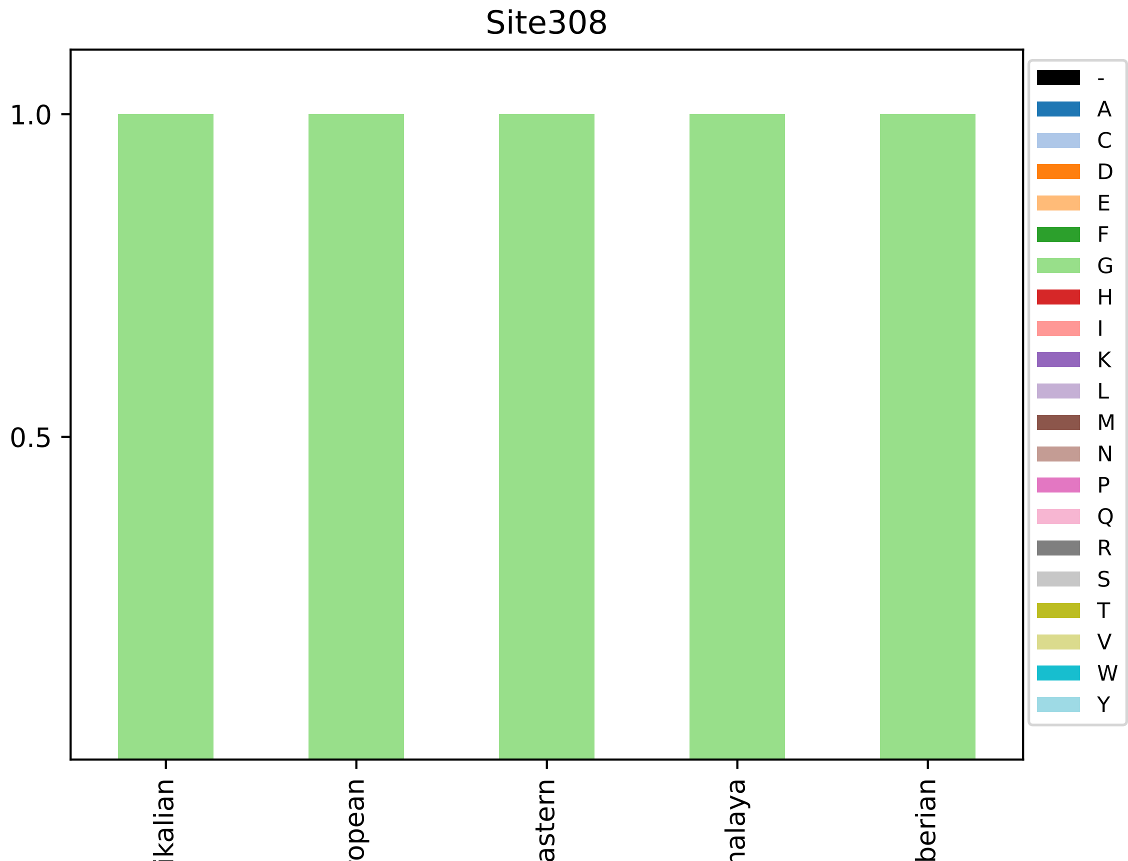

Site308
1.0
0.5
-
A
C
D
E
F
G
H
I
K
L
M
N
P
Q
R
S
T
V
W
Y
ikalian
opean
astern
nalaya
berian

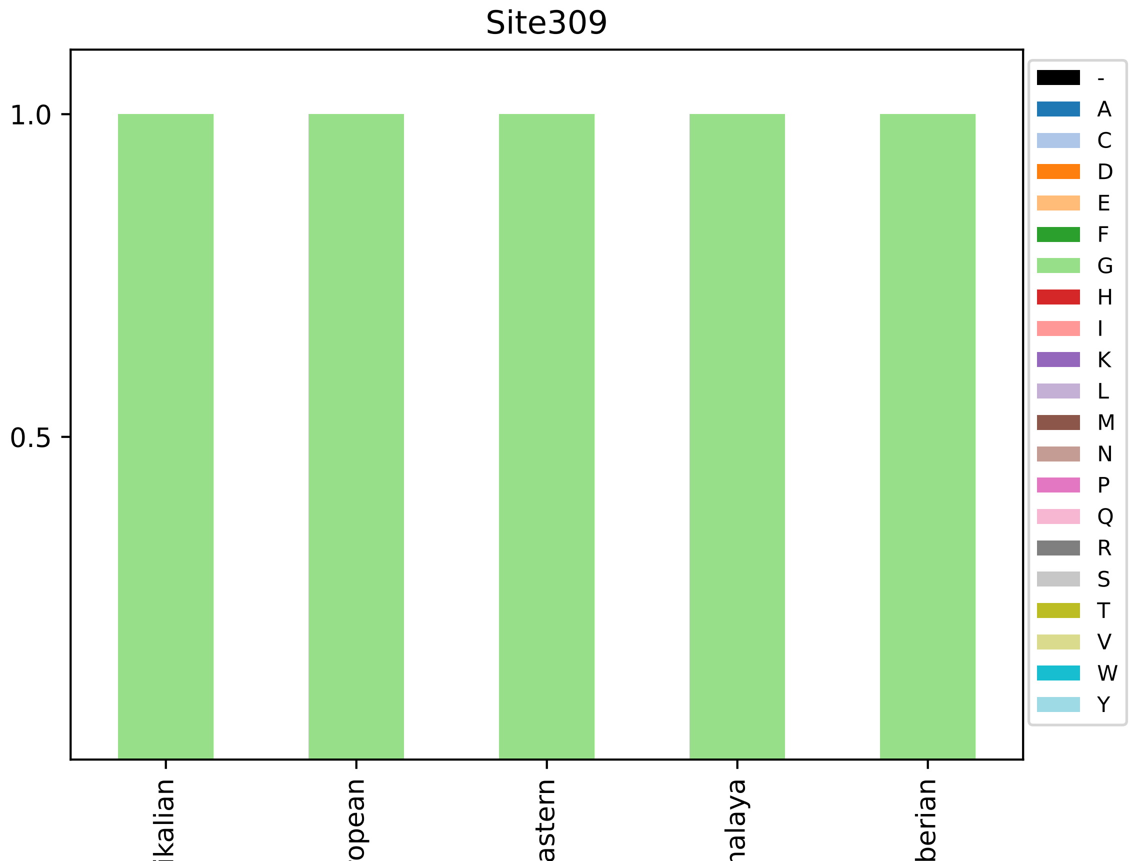
Site309
1.0
0.5
-
A
C
D
E
F
G
H
I
K
L
M
N
P
Q
R
S
T
V
W
Y
ikalian
opean
astern
nalaya
berian

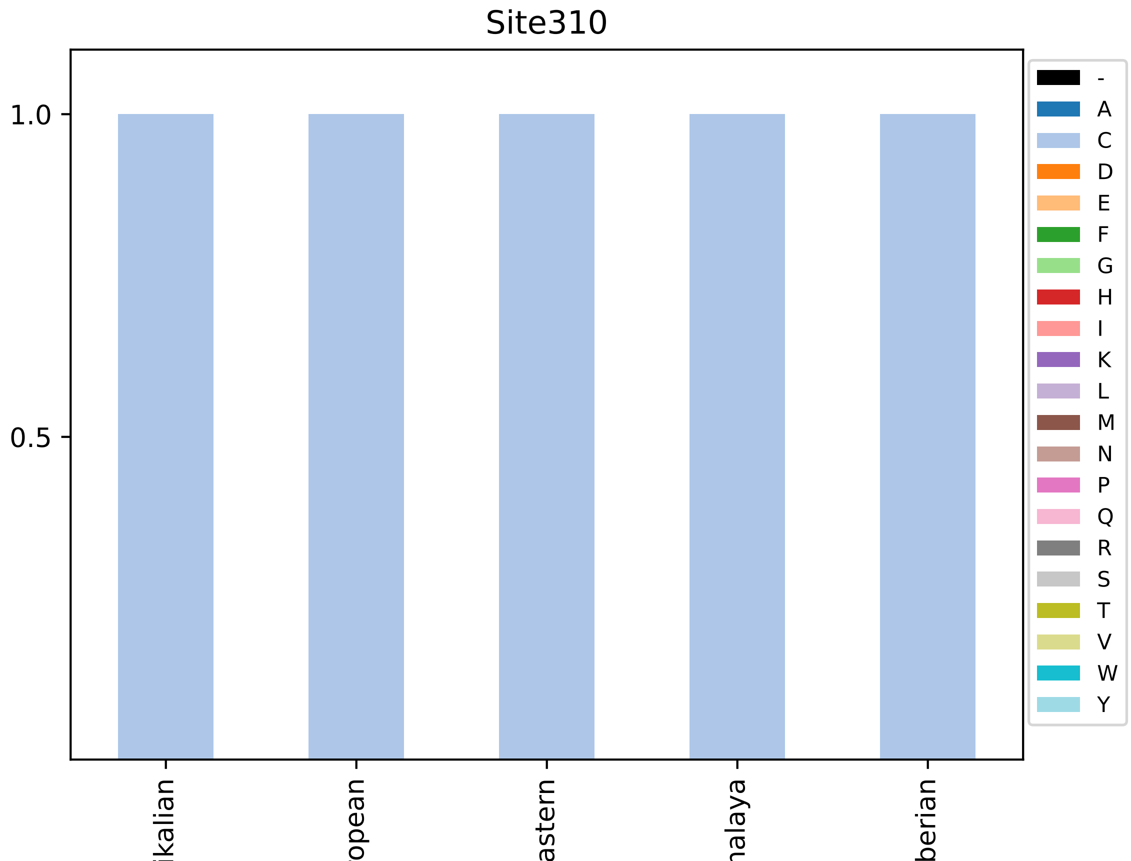

Site310
1.0
0.5
-
A
C
D
E
F
G
H
I
K
L
M
N
P
Q
R
S
T
V
W
Y
ikalian
opean
astern
nalaya
berian

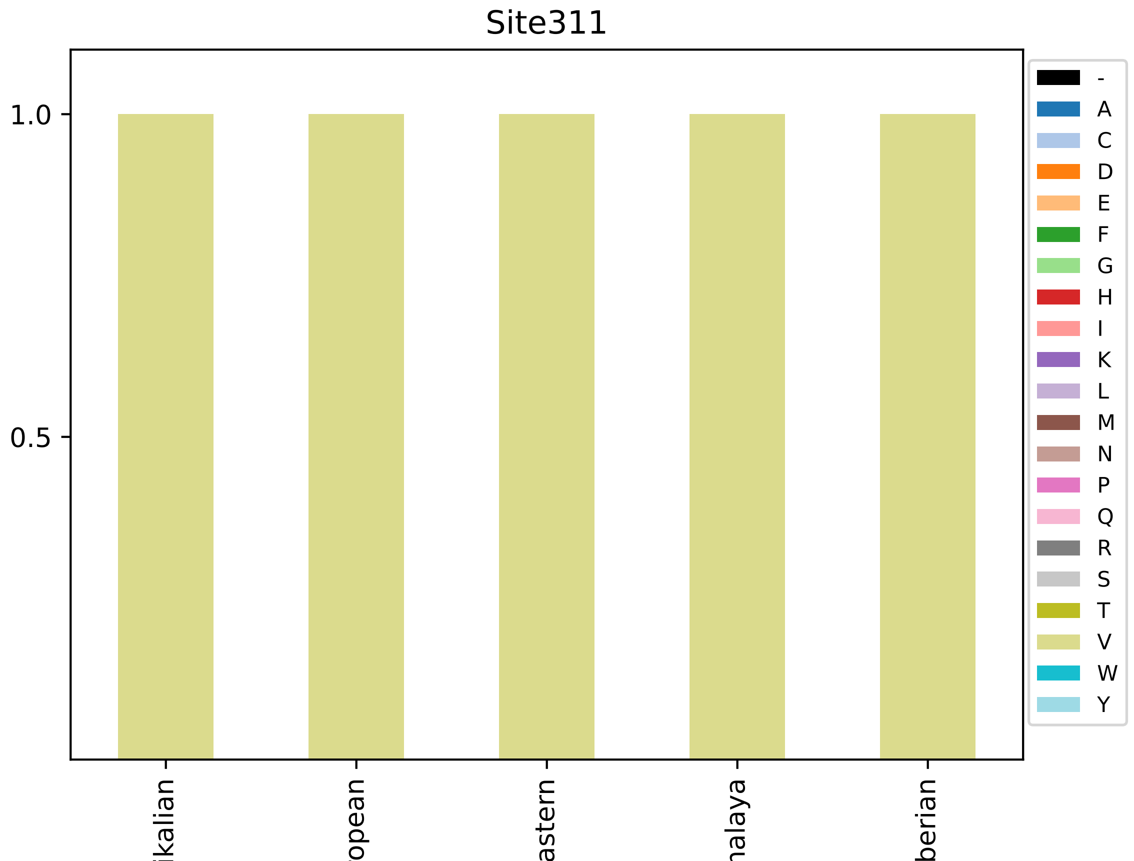

Site311
1.0
0.5
-
A
C
D
E
F
G
H
I
K
L
M
N
P
Q
R
S
T
V
W
Y
ikalian
opean
astern
halaya
berian

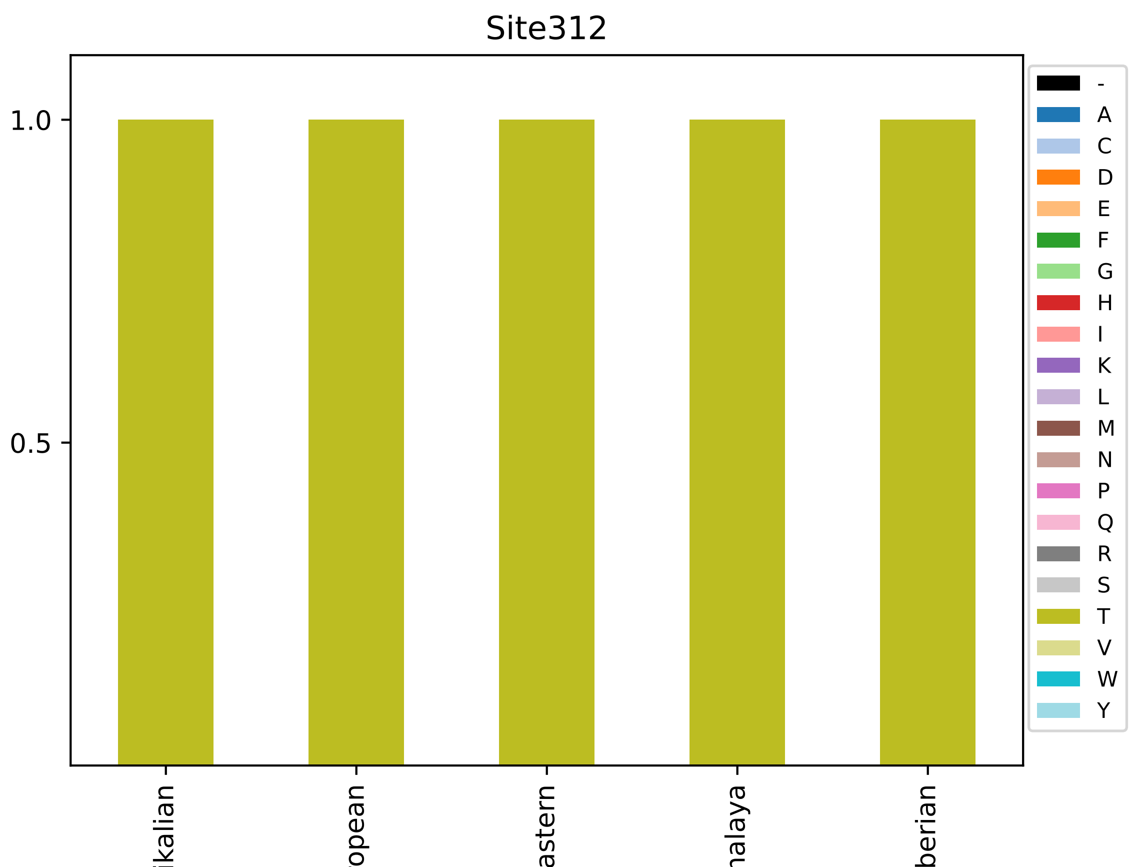
Site312
1.0
0.5
-
A
C
D
E
F
G
H
I
K
L
M
N
P
Q
R
S
T
V
W
Y
ikalian
opean
astern
nalaya
berian

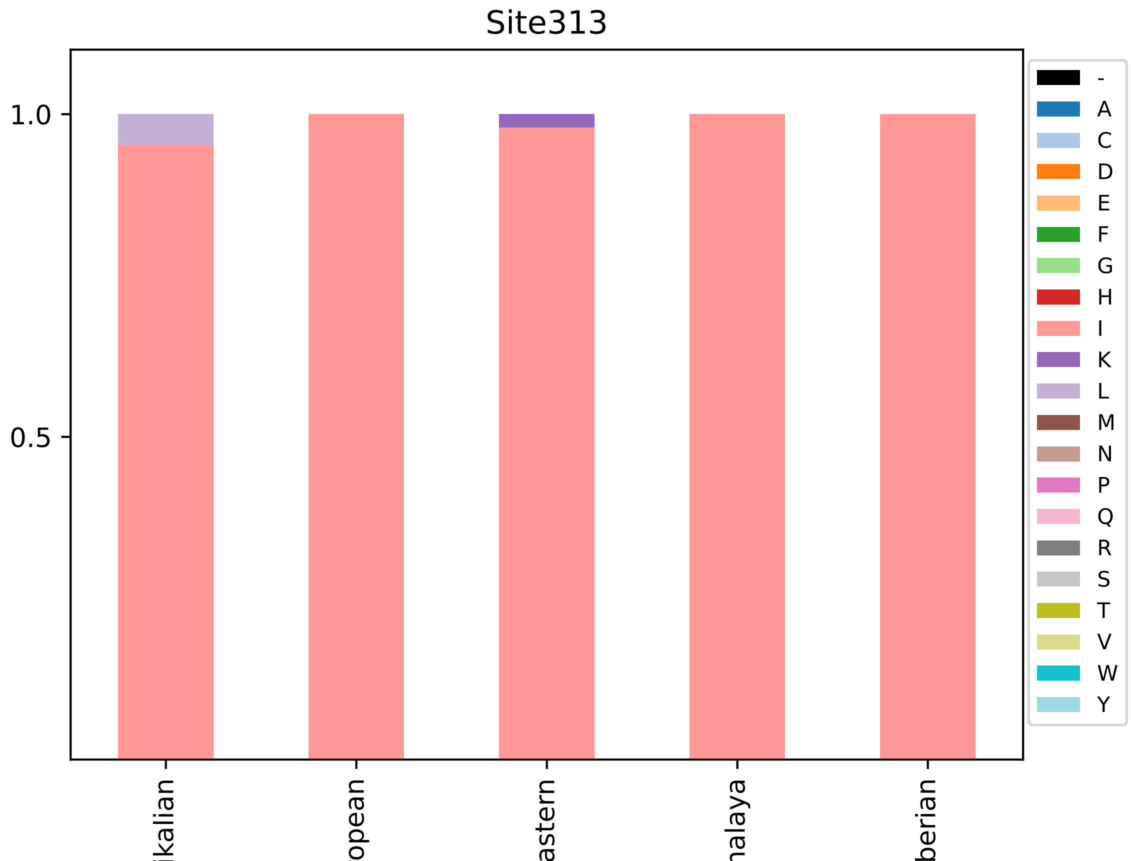
Site313
1.0
0.5
-
A
C
D
E
F
G
H
I
K
L
M
N
P
Q
R
S
T
V
W
Y
ikalian
opean
astern
nalaya
berian

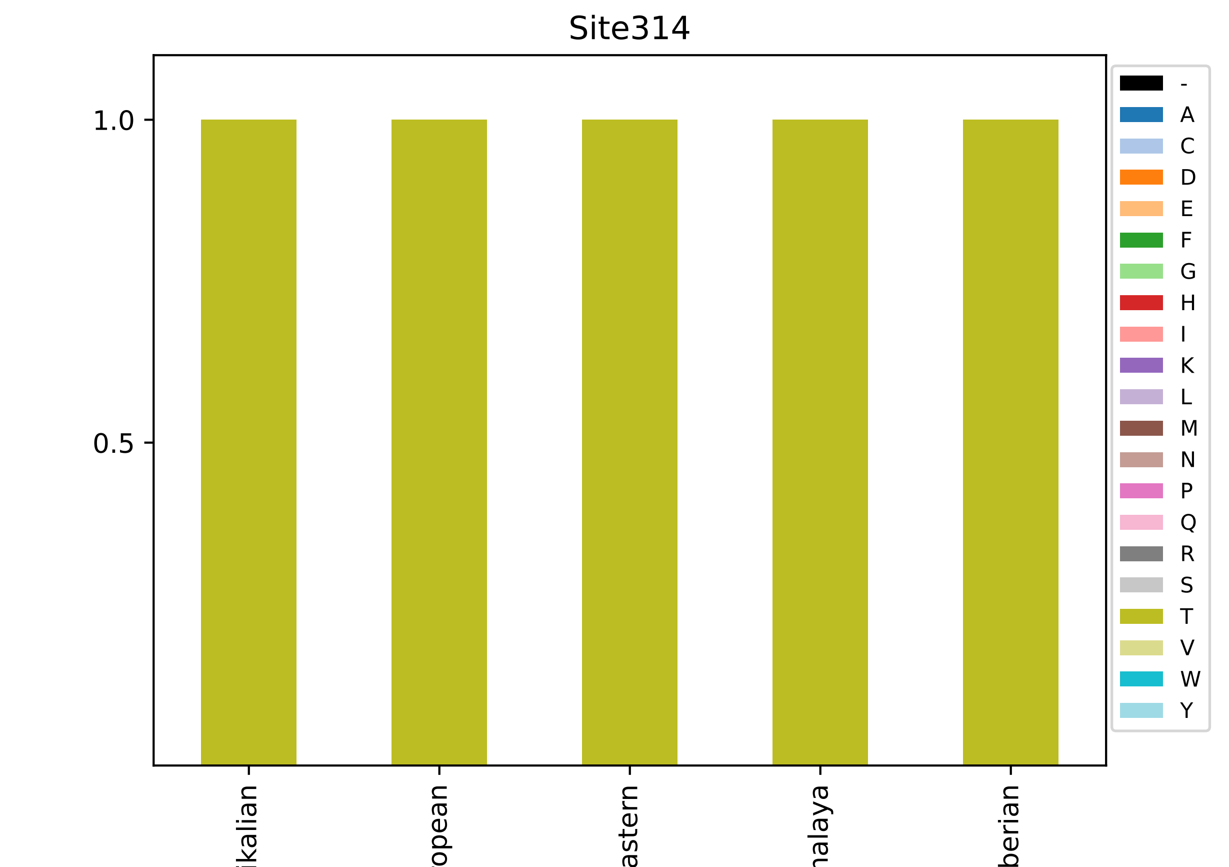
Site314
1.0
0.5
-
A
C
D
E
F
G
H
I
K
L
M
N
P
Q
R
S
T
V
W
Y
ikalian
opean
astern
nalaya
berian

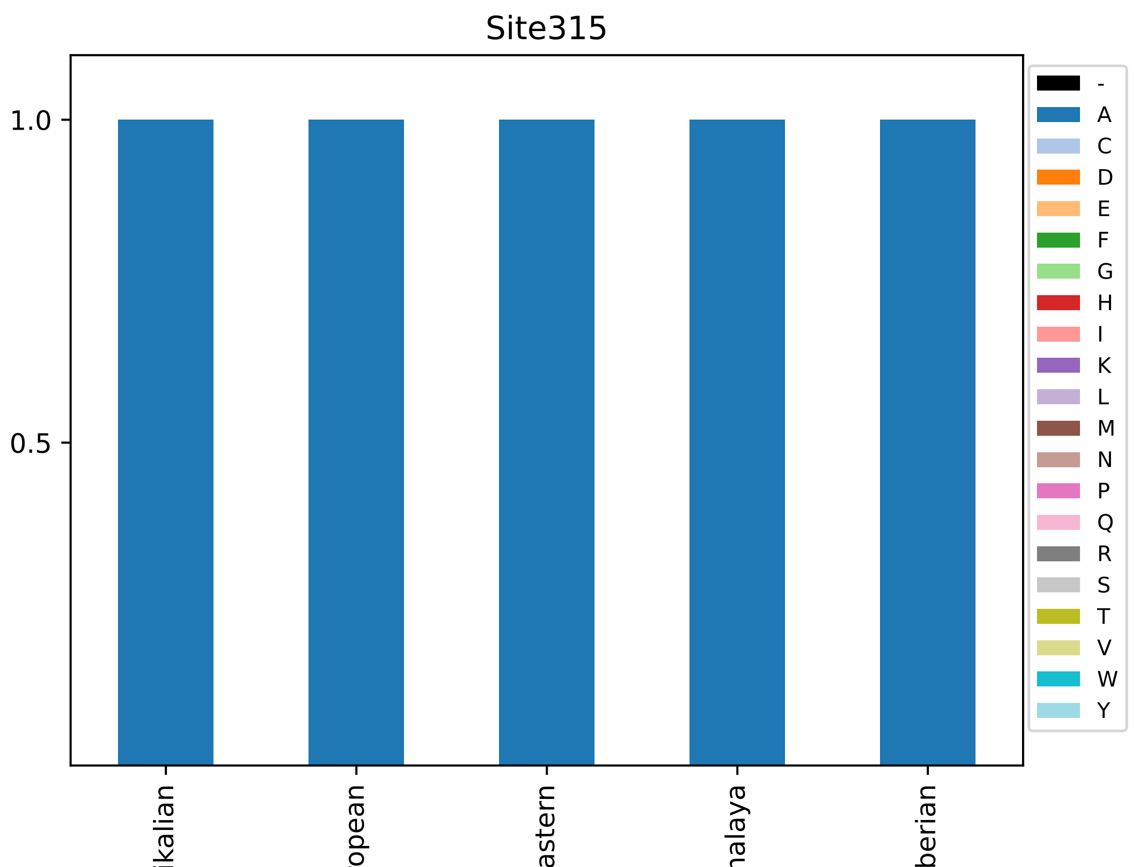

Site315
1.0
0.5
-
A
C
D
E
F
G
H
I
K
L
M
N
P
Q
R
S
T
V
W
Y
ikalian
opean
astern
nalaya
berian

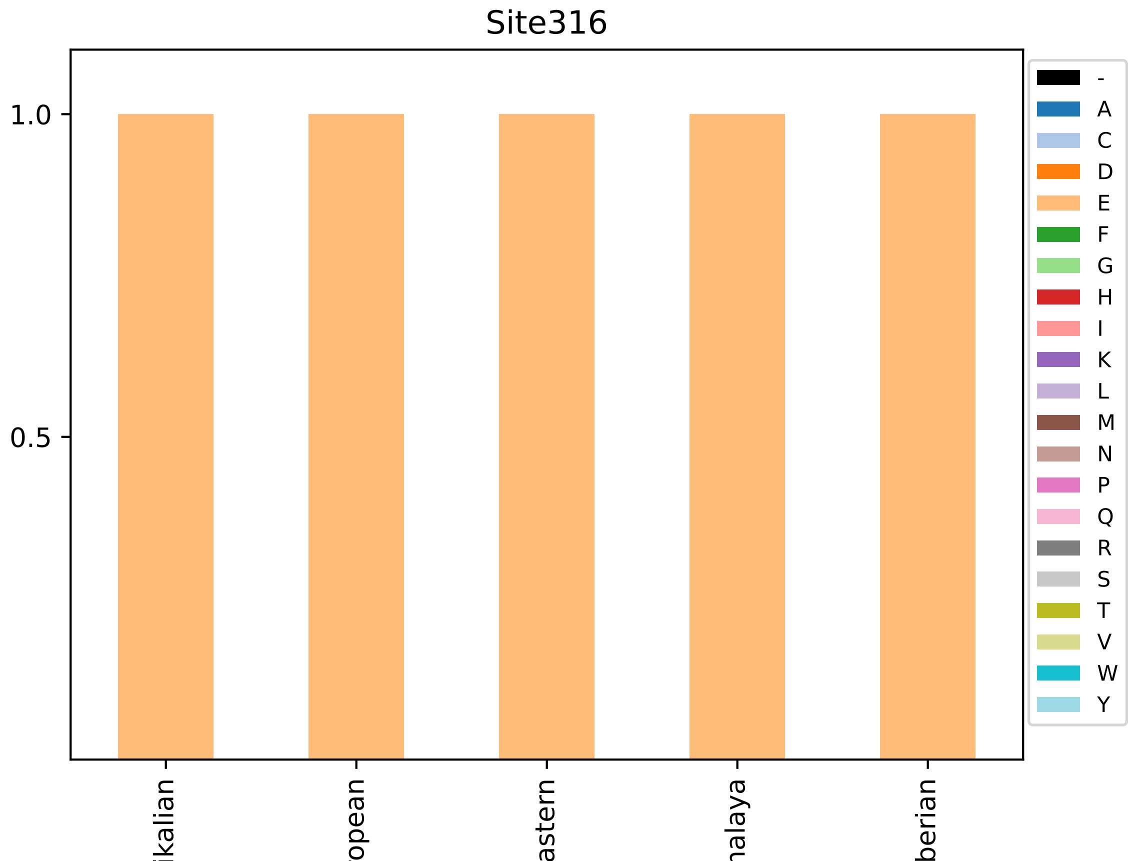

Site316
1.0
0.5
-
A
C
D
E
F
G
H
I
K
L
M
N
P
Q
R
S
T
V
W
Y
ikalian
opean
astern
nalaya
berian

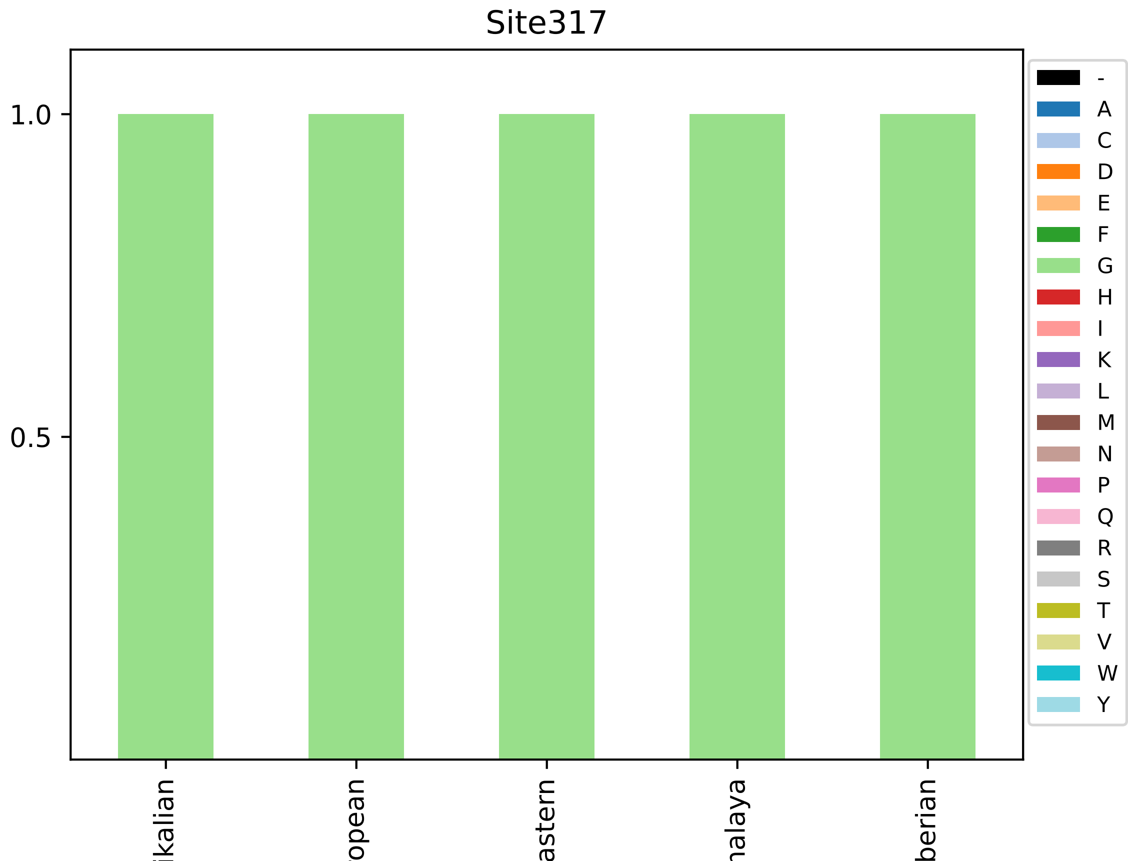

Site317
1.0
0.5
-
A
C
D
E
F
G
H
I
K
L
M
N
P
Q
R
S
T
V
W
Y
ikalian
opean
astern
nalaya
berian

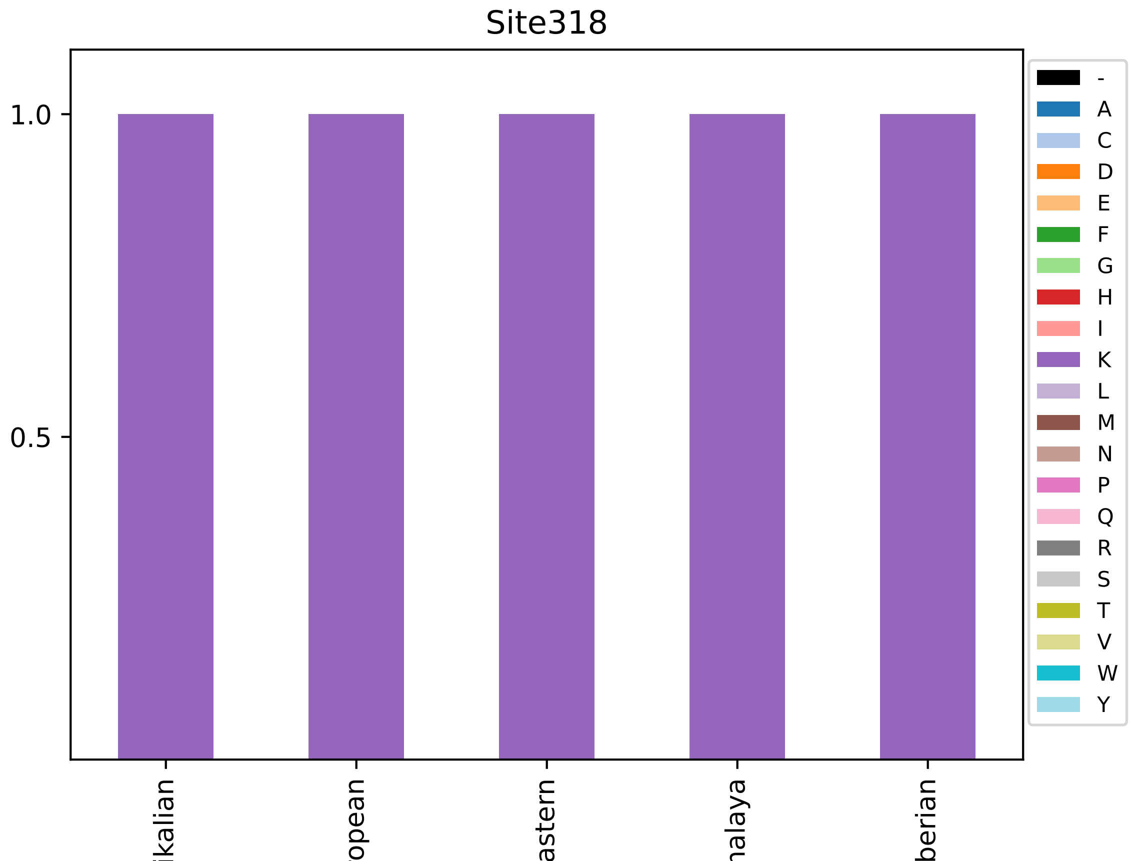
Site318
1.0
0.5
-
A
C
D
E
F
G
H
I
K
L
M
N
P
Q
R
S
T
V
W
Y
ikalian
ropean
astern
nalaya
berian

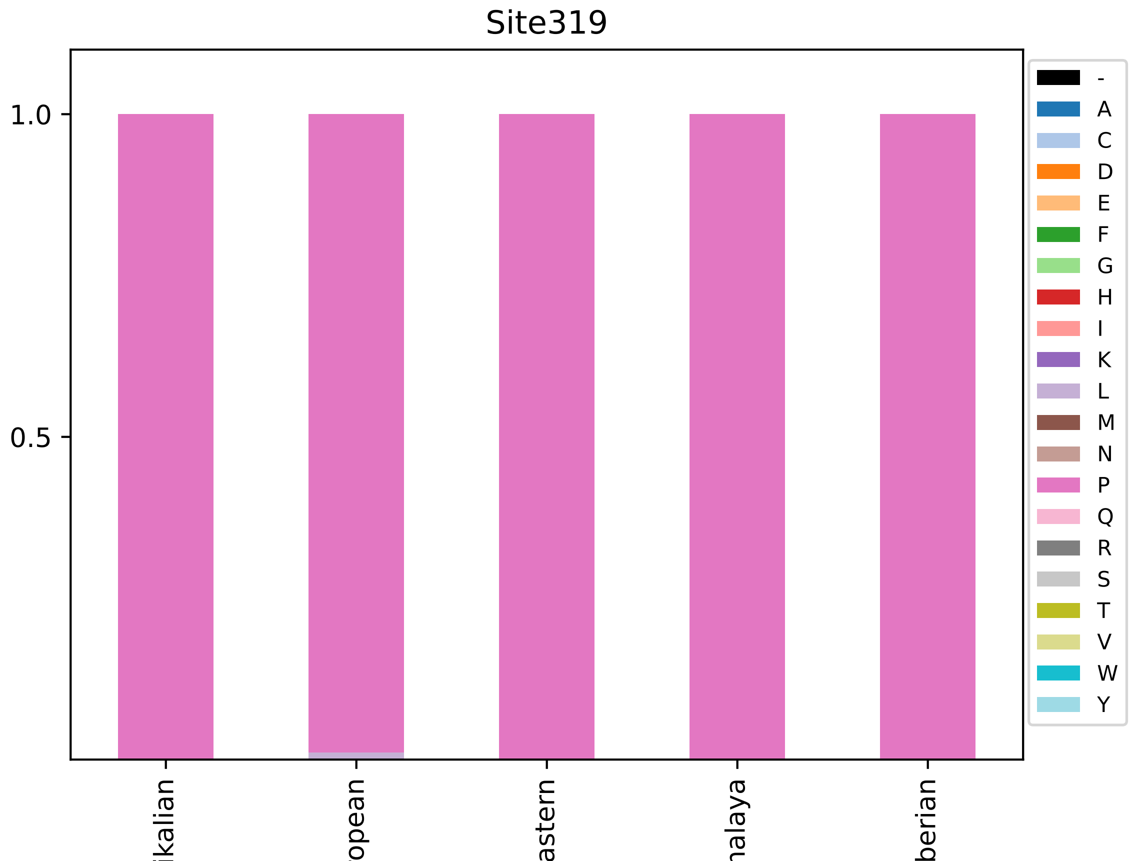
Site319
1.0
0.5
ikalian
opean
astern
nalaya
berian
-
A
C
D
E
F
G
H
I
K
L
M
N
P
Q
R
S
T
V
W
Y

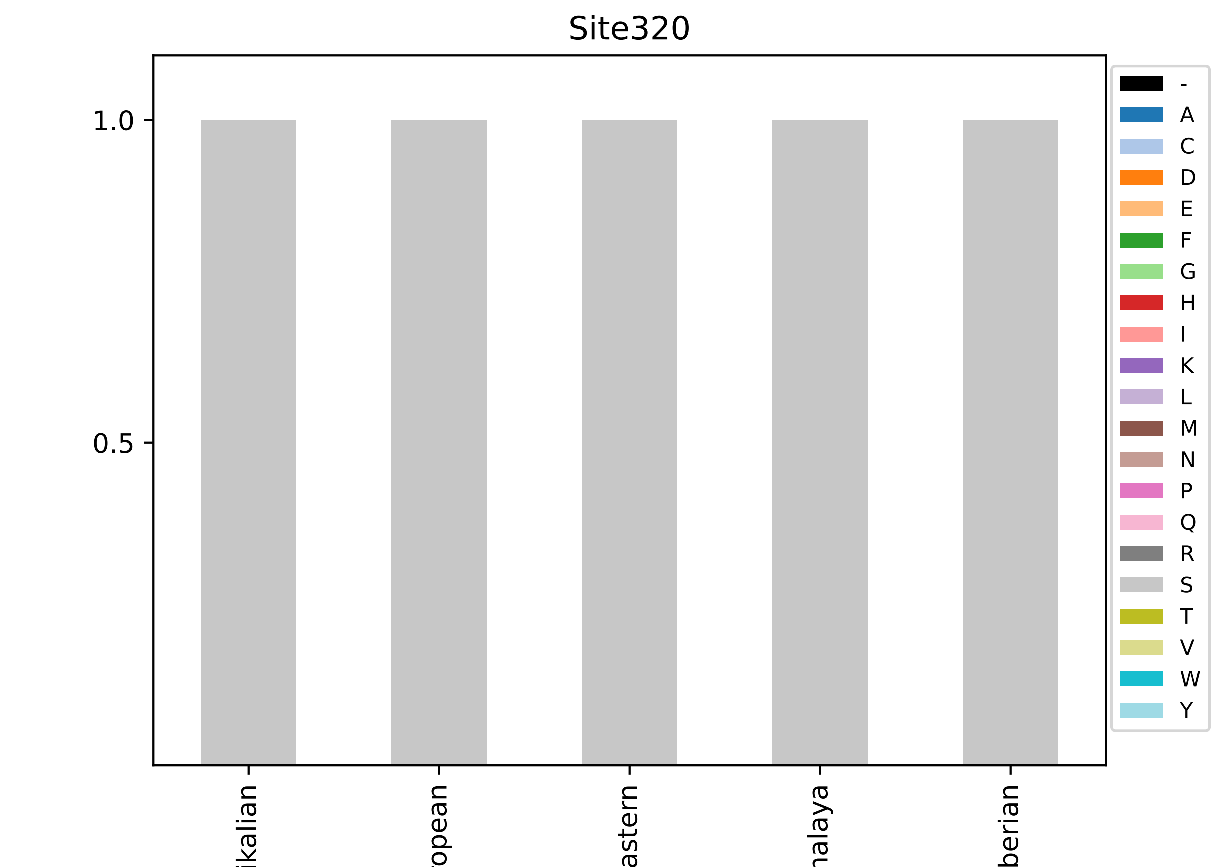

Site320
1.0
0.5
-
A
C
D
E
F
G
H
I
K
L
M
N
P
Q
R
S
T
V
W
Y
ikalian
opean
astern
nalaya
berian

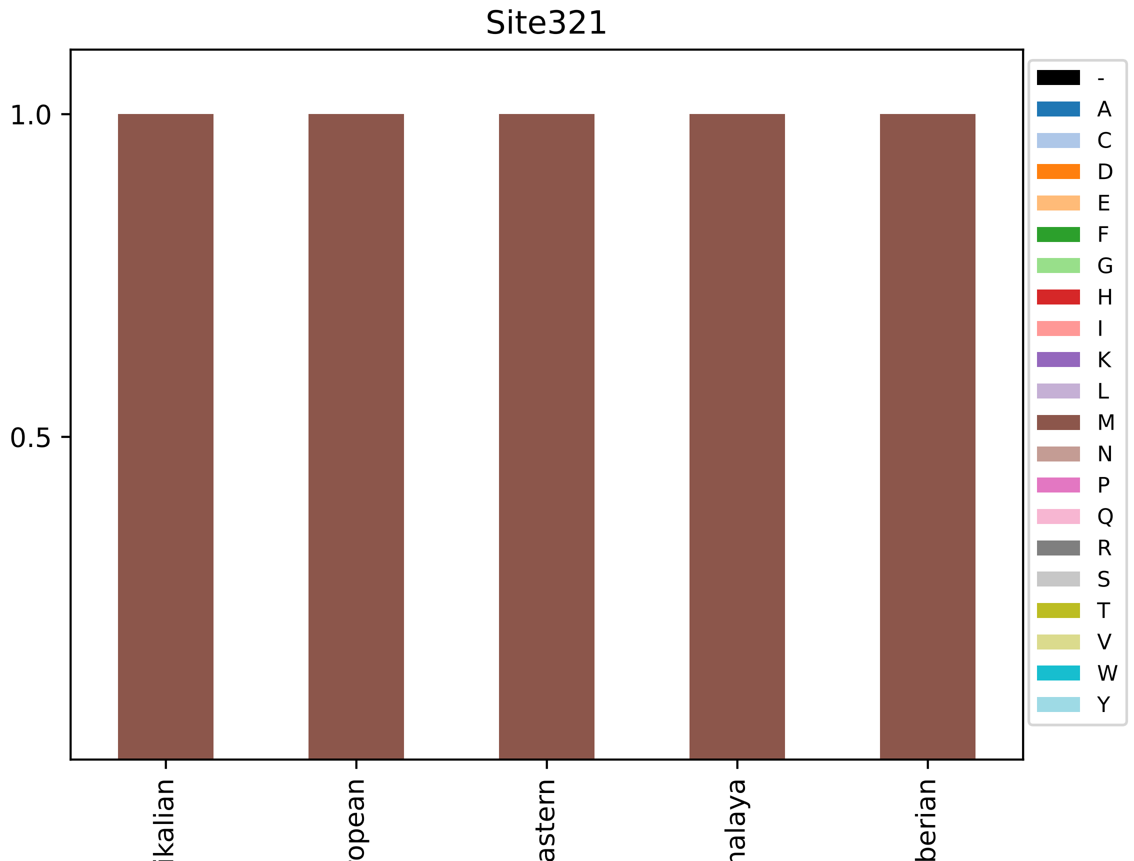
Site321
1.0
0.5
-
A
C
D
E
F
G
H
I
K
L
M
N
P
Q
R
S
T
V
W
Y

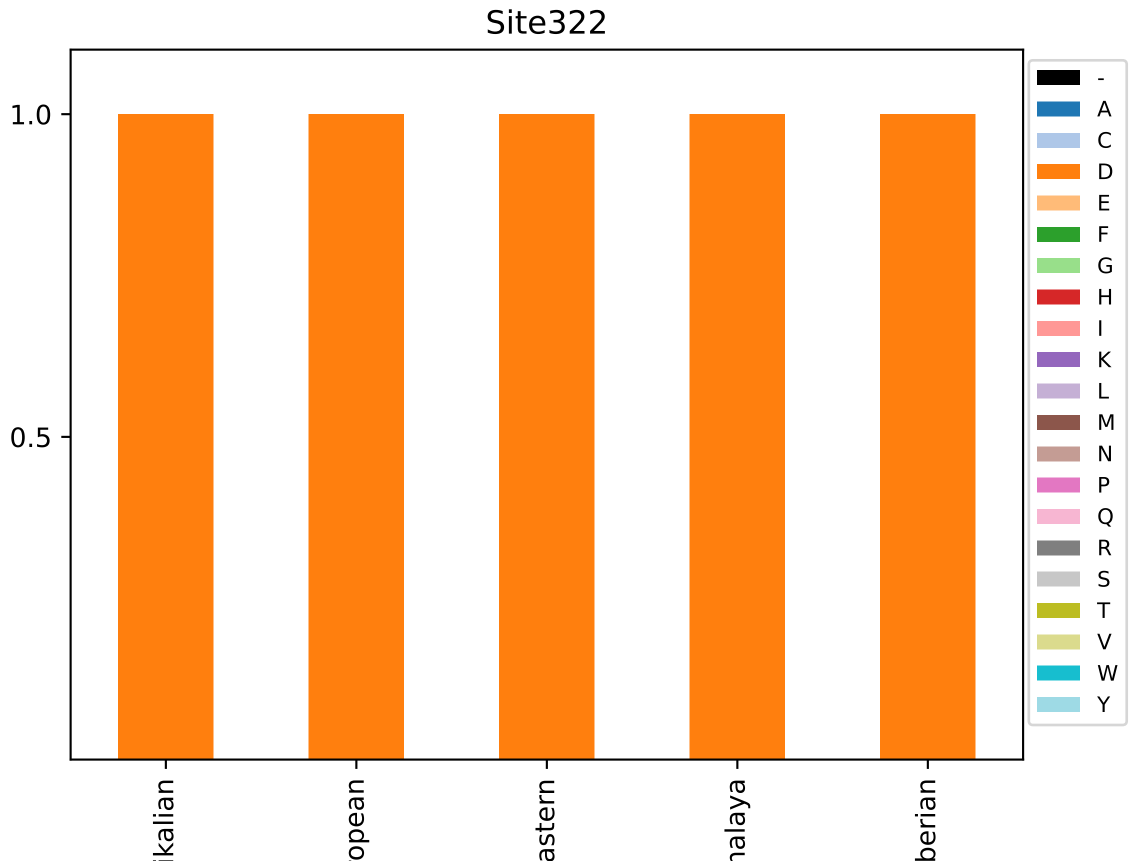

Site322
1.0
0.5
-
A
C
D
E
F
G
H
I
K
L
M
N
P
Q
R
S
T
V
W
Y
ikalian
opean
astern
nalaya
berian

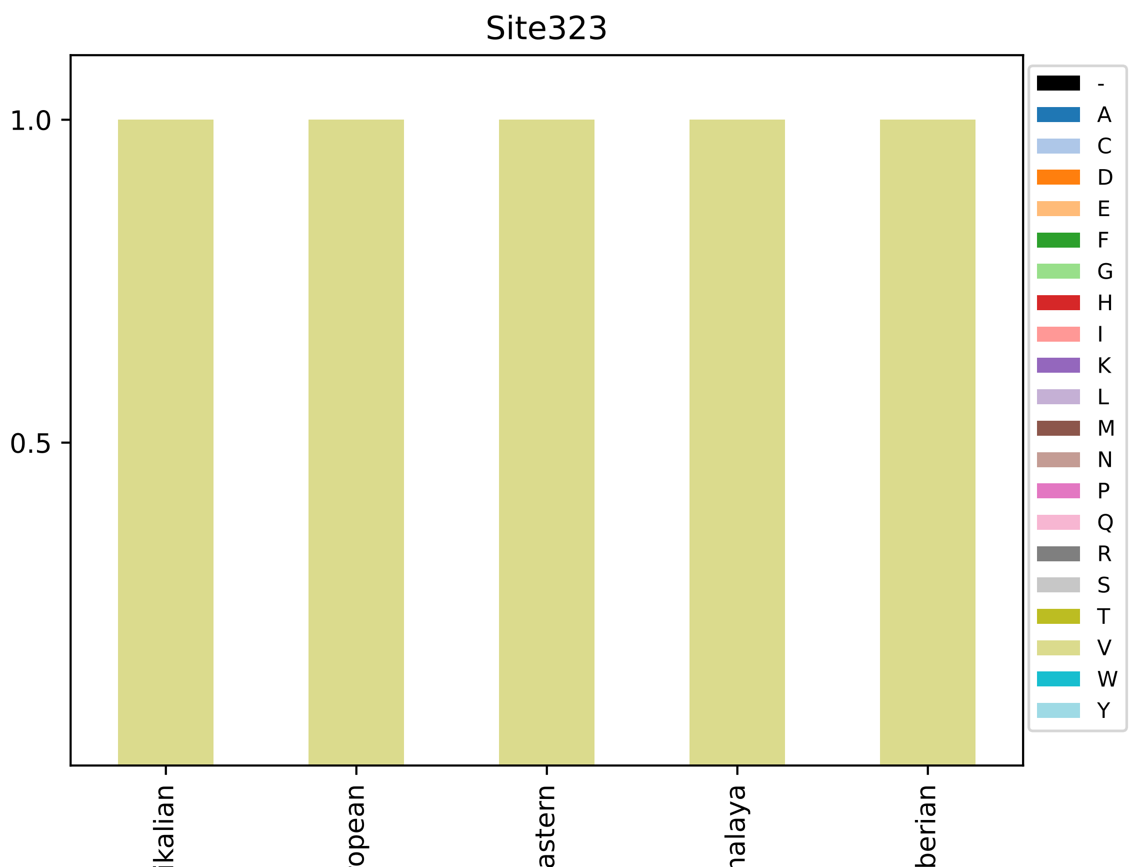

Site323
1.0
0.5
-
A
C
D
E
F
G
H
I
K
L
M
N
P
Q
R
S
T
V
W
Y
ikalian
opean
astern
nalaya
berian

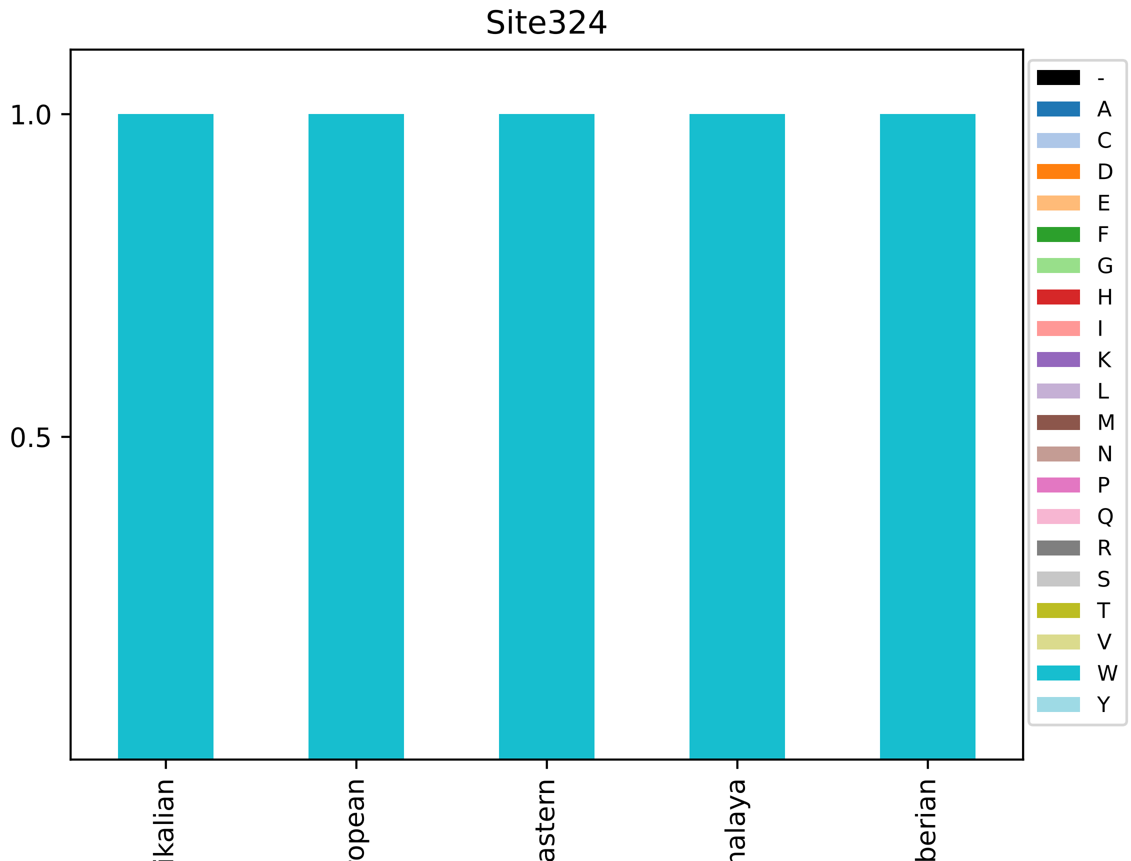

Site324
1.0
0.5
-
A
C
D
E
F
G
H
I
K
L
M
N
P
Q
R
S
T
V
W
Y
ikalian
opean
astern
nalaya
berian

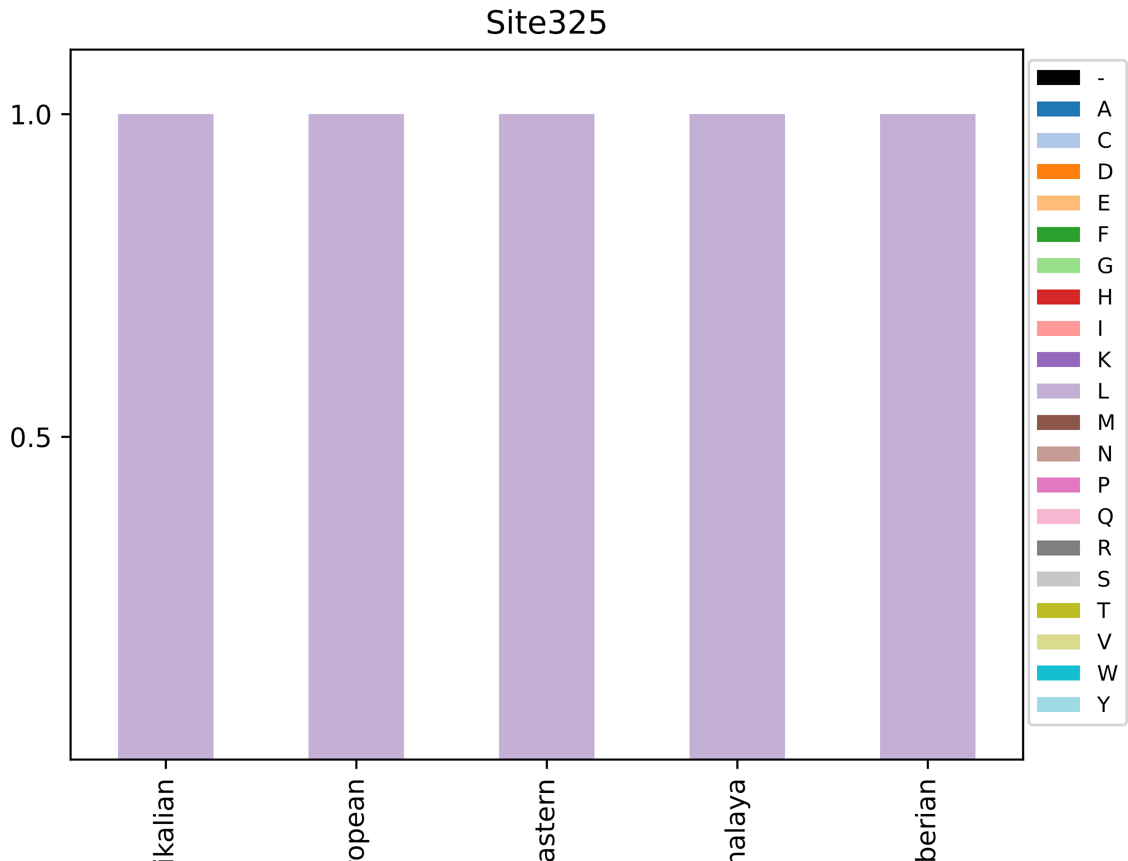

Site325
1.0
0.5
-
A
C
D
E
F
G
H
I
K
L
M
N
P
Q
R
S
T
V
W
Y
ikalian
opean
astern
nalaya
berian

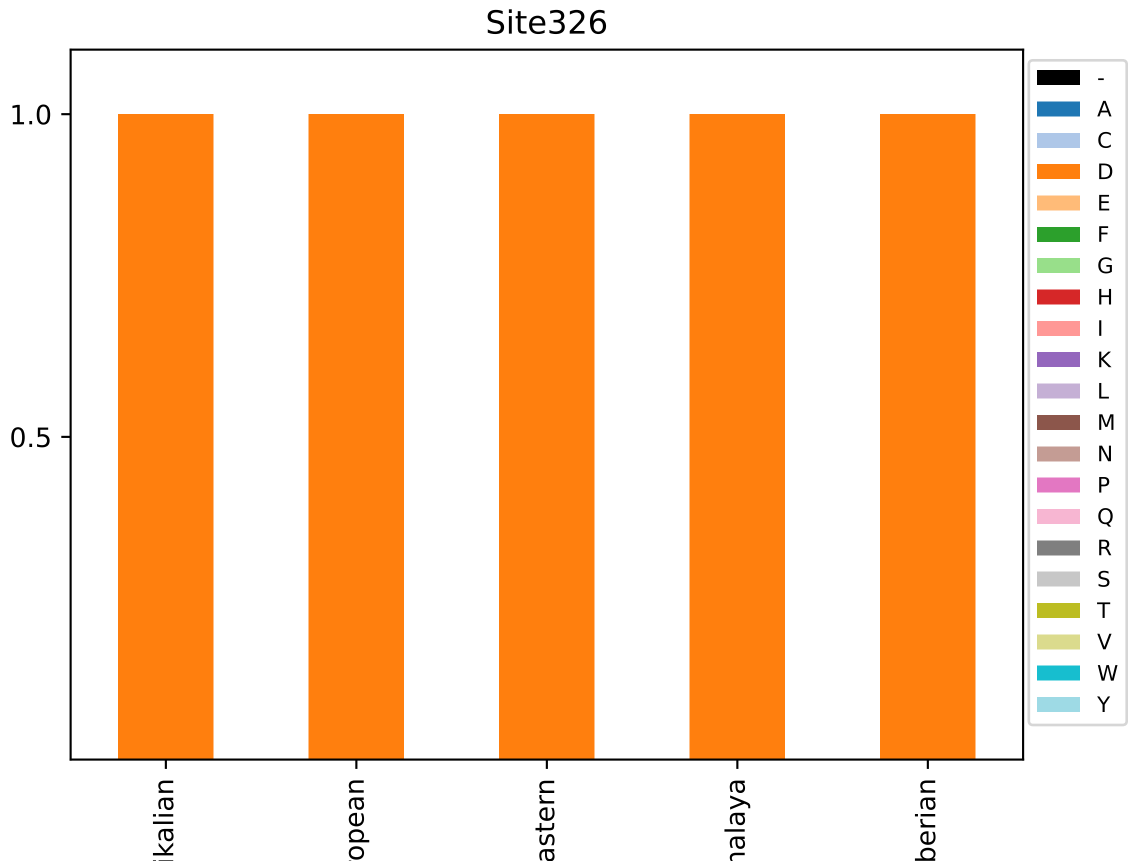
Site326
1.0
0.5
ikalian
opean
astern
nalaya
berian
-
A
C
D
E
F
G
H
I
K
L
M
N
P
Q
R
S
T
V
W
Y

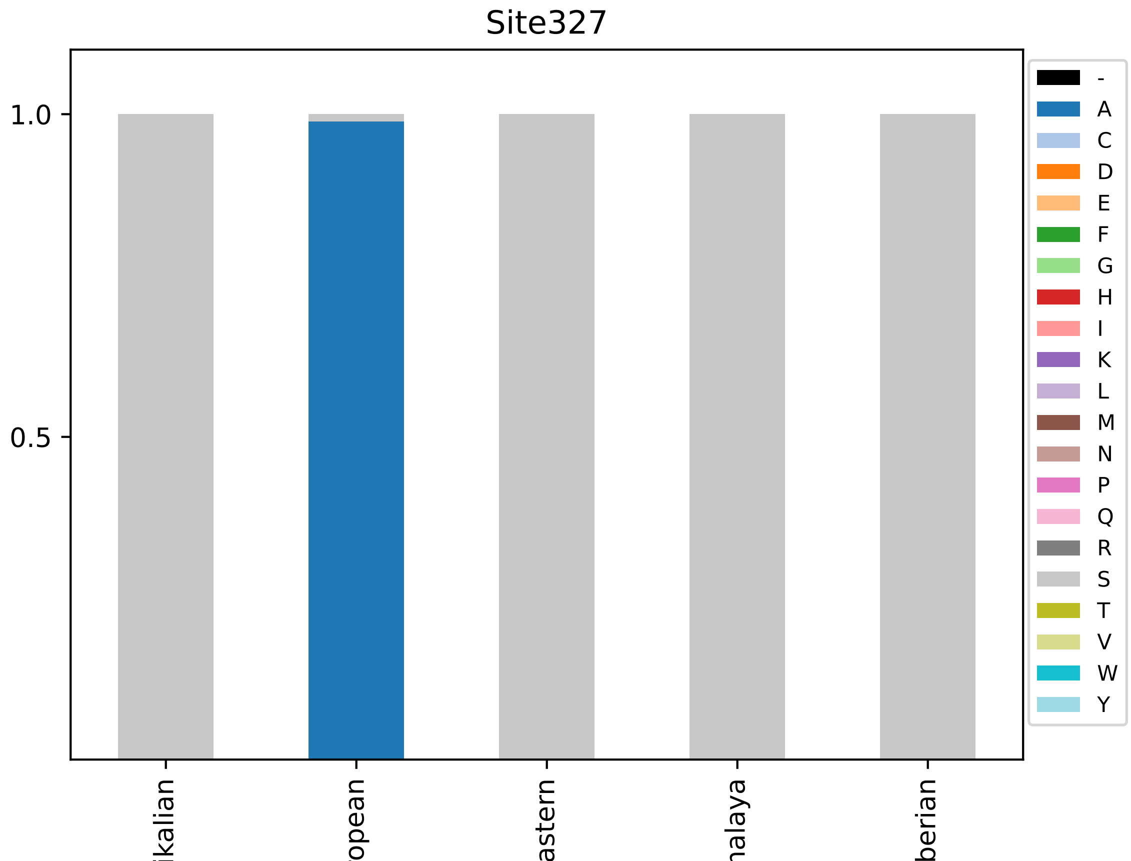
Site327
1.0
0.5
-
A
C
D
E
F
G
H
I
K
L
M
N
P
Q
R
S
T
V
W
Y
ikalian
opean
astern
nalaya
berian

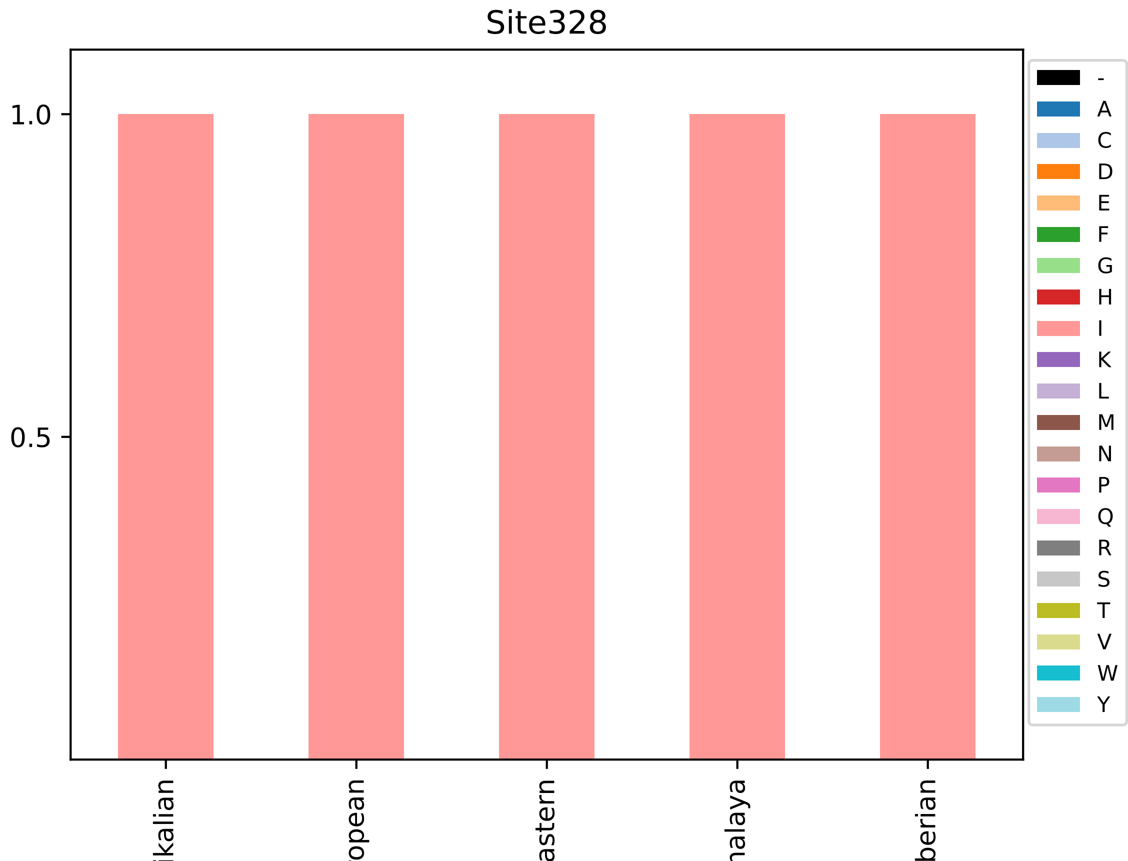
Site328
1.0
0.5
-
A
C
D
E
F
G
H
I
K
L
M
N
P
Q
R
S
T
V
W
Y
ikalian
opean
astern
nalaya
berian

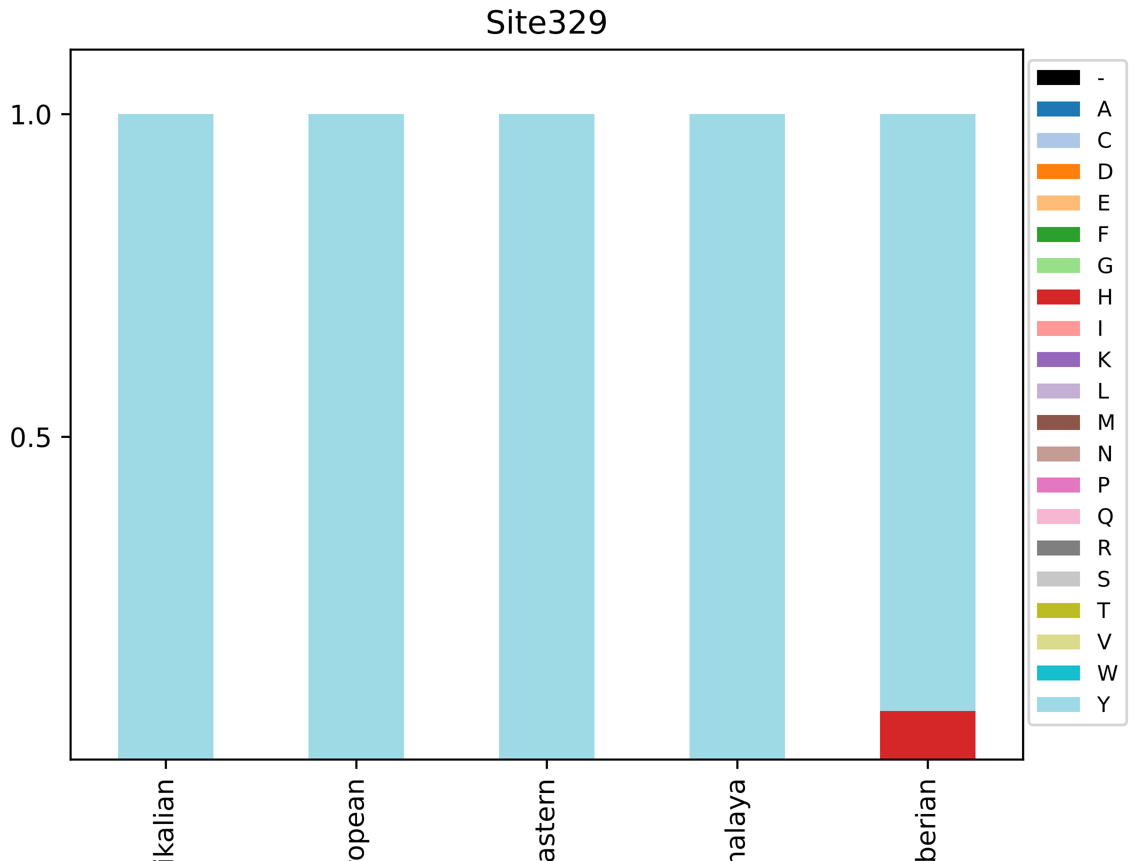

Site329
1.0
0.5
-
A
C
D
E
F
G
H
I
K
L
M
N
P
Q
R
S
T
V
W
Y
ikalian
ropean
astern
nalaya
berian

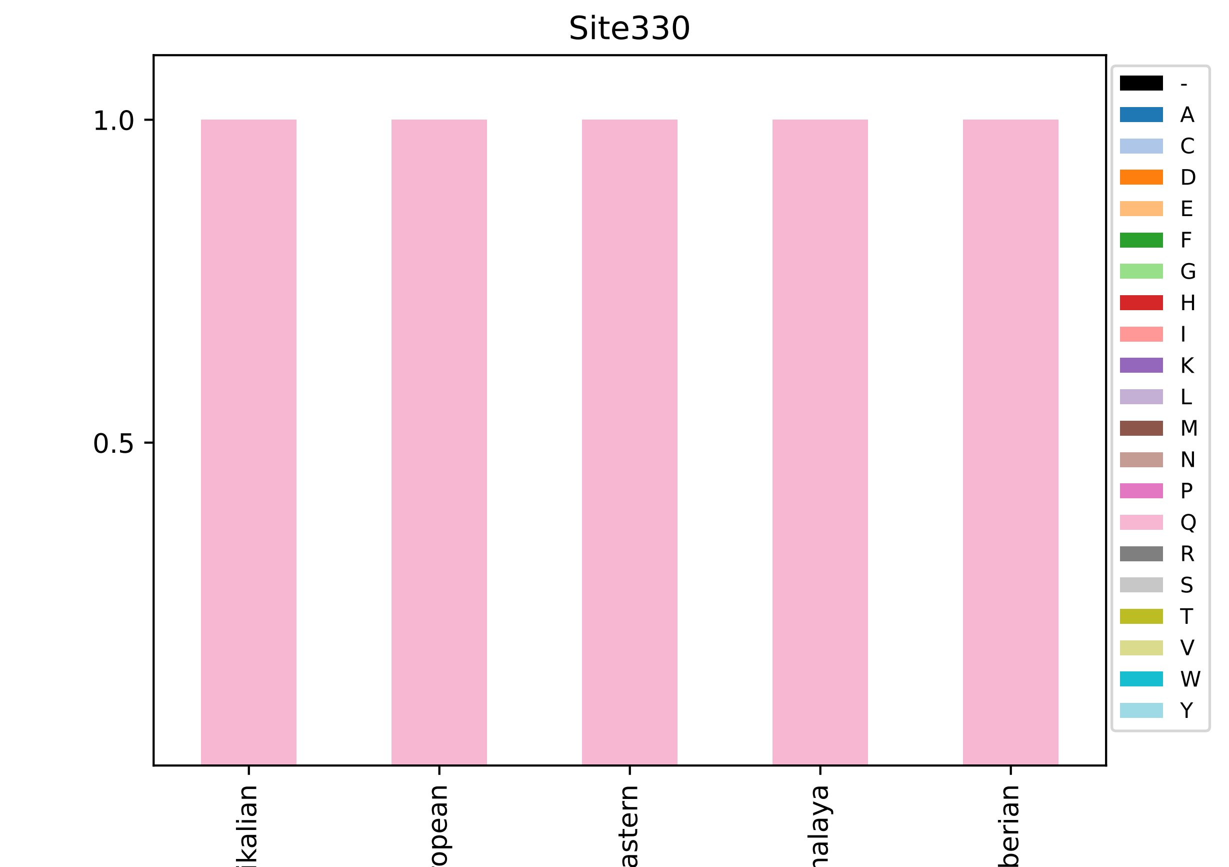
Site330
1.0
0.5
-
A
C
D
E
F
G
H
I
K
L
M
N
P
Q
R
S
T
V
W
Y
ikalian
opean
astern
nalaya
berian

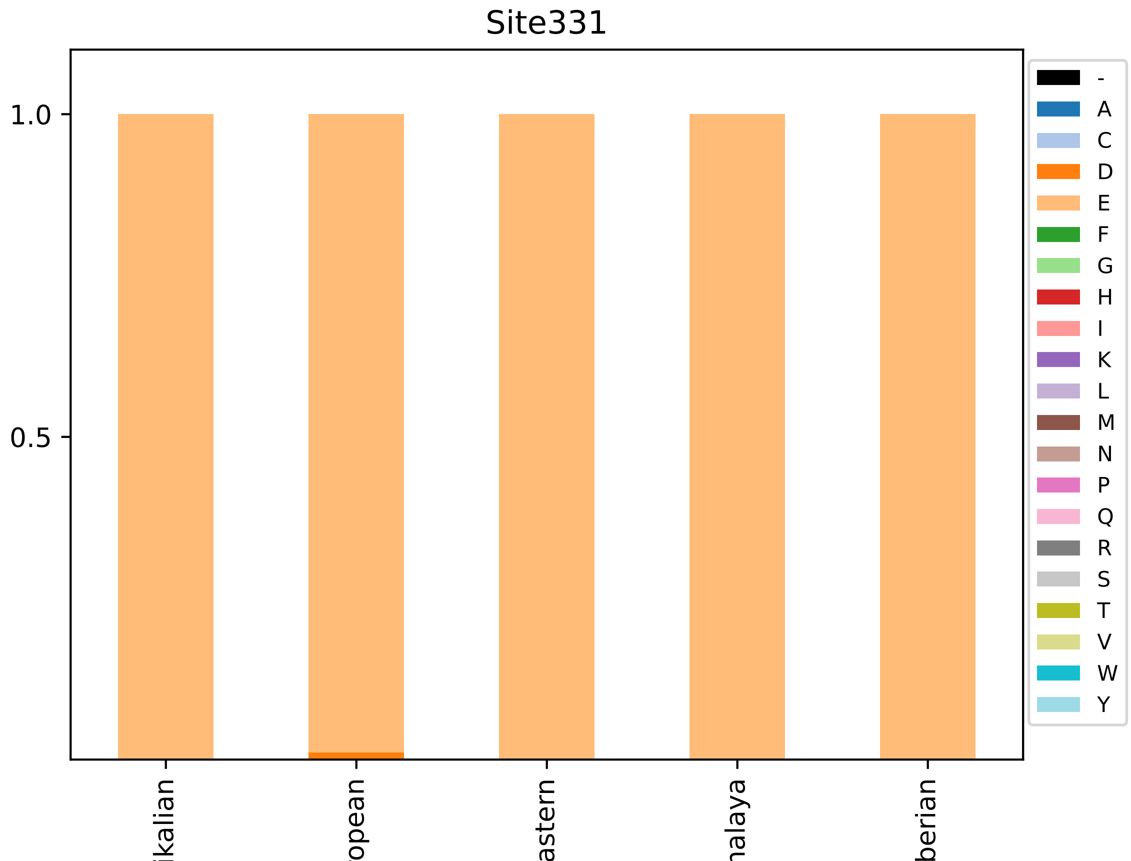
Site331
1.0
0.5
-
A
C
D
E
F
G
H
I
K
L
M
N
P
Q
R
S
T
V
W
Y
ikalian
opean
astern
nalaya
berian

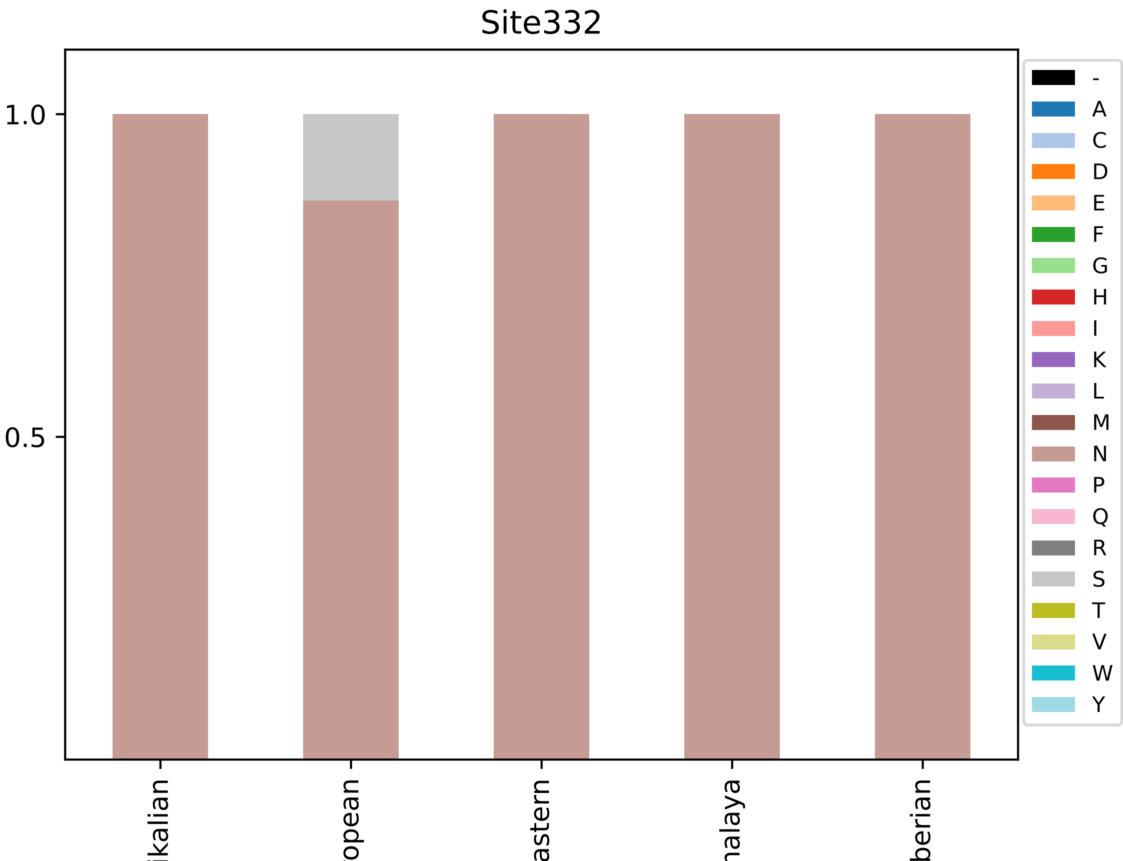

Site332
1.0
0.5
-
A
C
D
E
F
G
H
I
K
L
M
N
P
Q
R
S
T
V
W
Y
ikalian
opean
astern
nalaya
berian

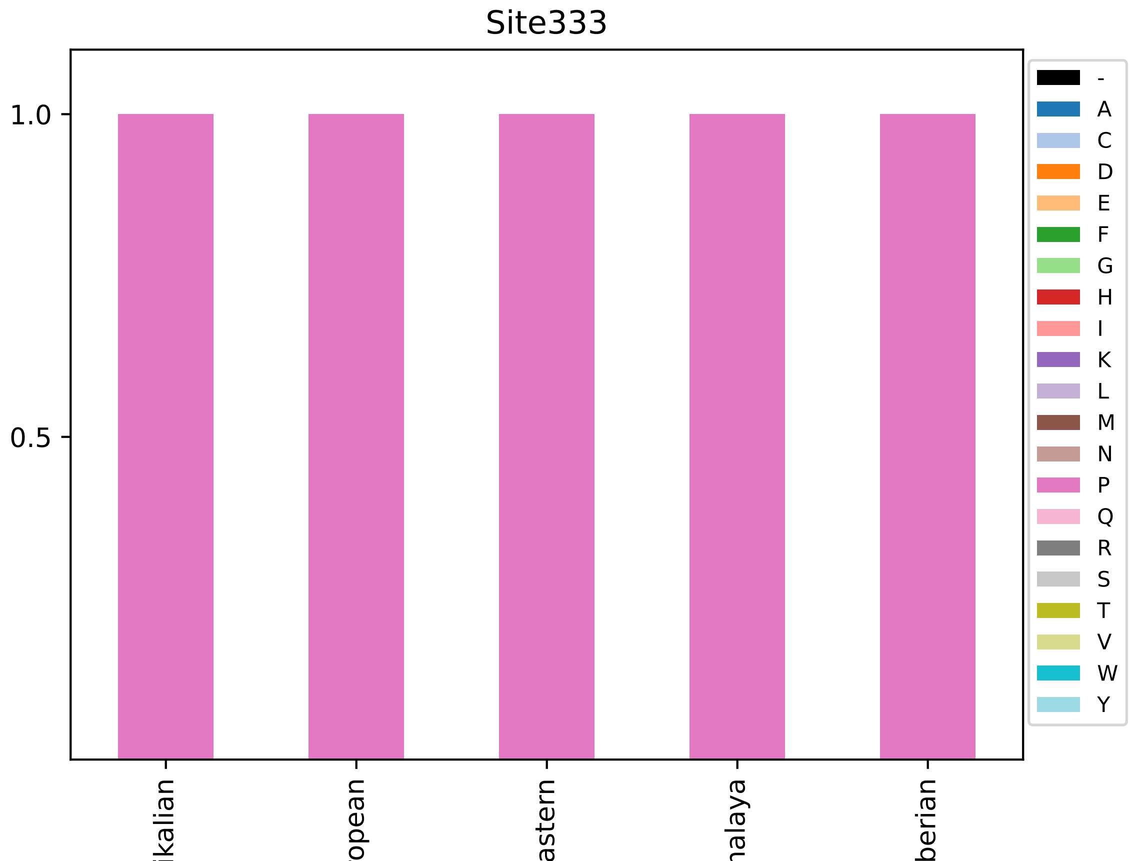
Site333
1.0
0.5
ikalian
opean
astern
nalaya
berian
-
A
C
D
E
F
G
H
I
K
L
M
N
P
Q
R
S
T
V
W
Y

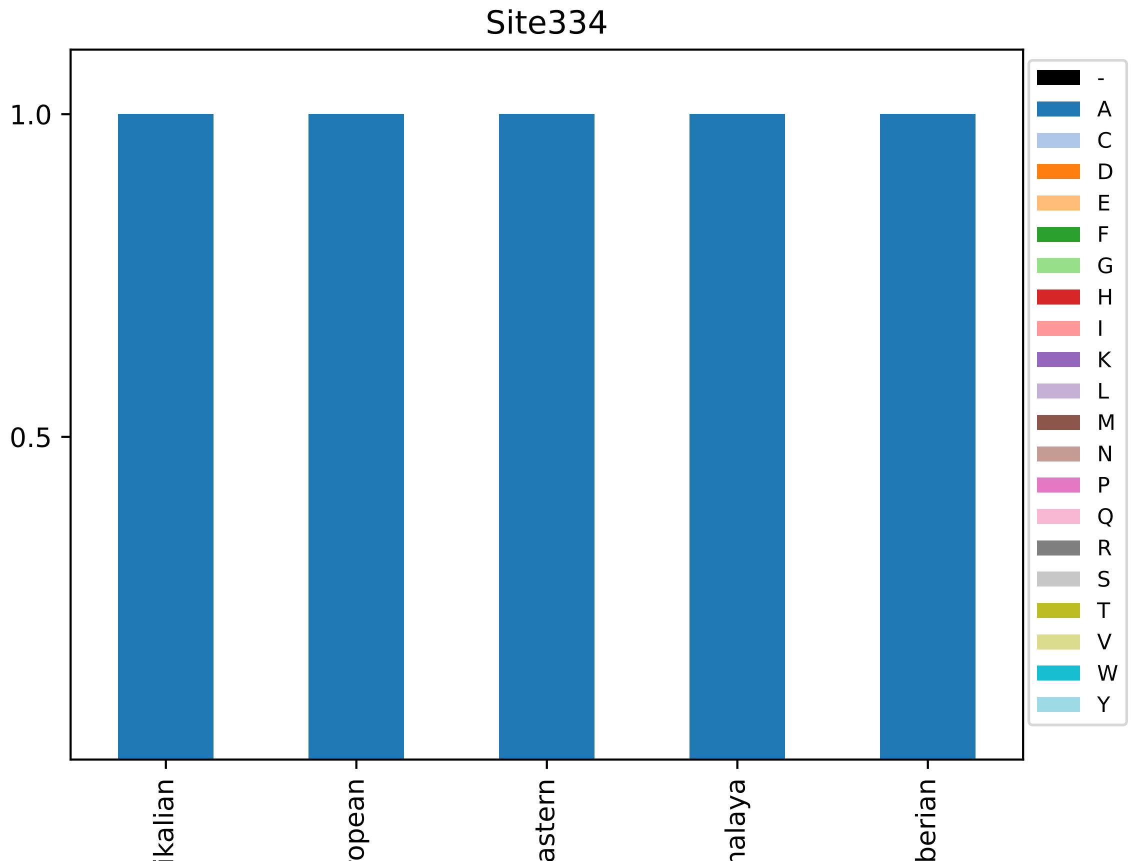

Site334
1.0
0.5
-
A
C
D
E
F
G
H
I
K
L
M
N
P
Q
R
S
T
V
W
Y

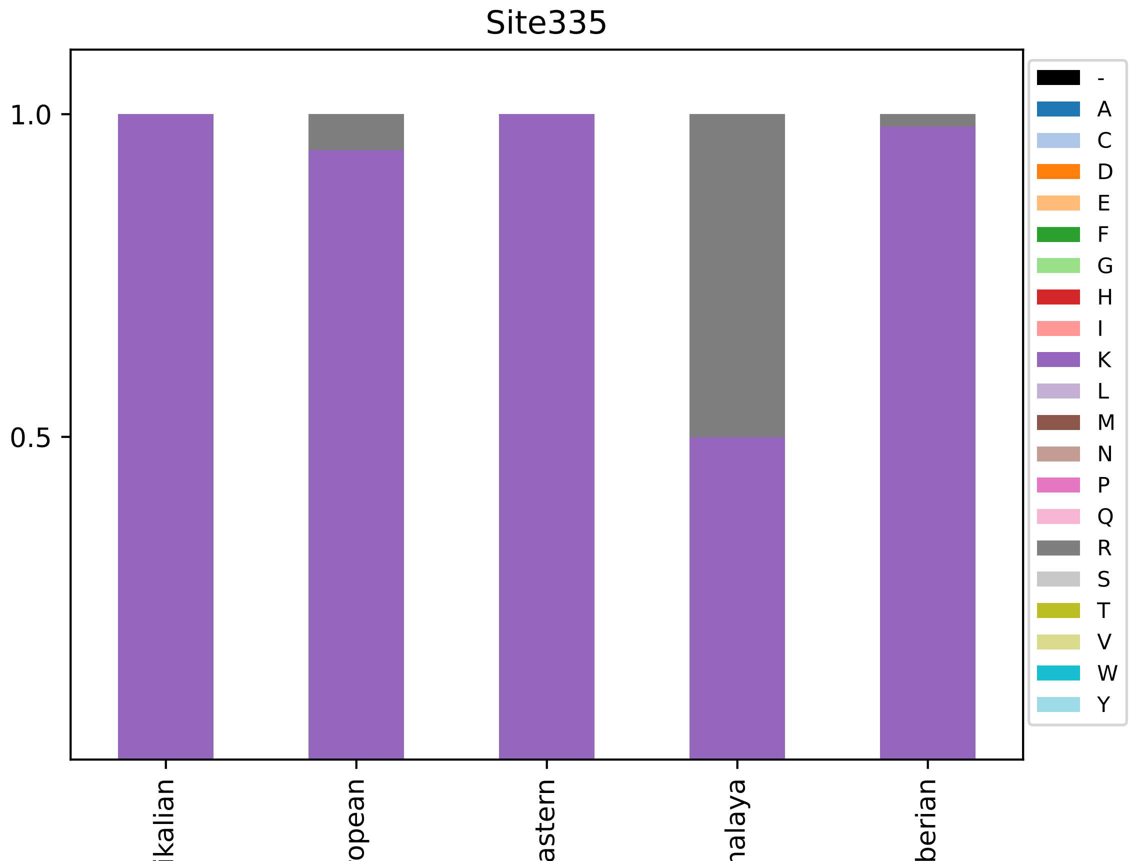

Site335
1.0
0.5
ikalian
opean
astern
nalaya
berian
-
A
C
D
E
F
G
H
I
K
L
M
N
P
Q
R
S
T
V
W
Y

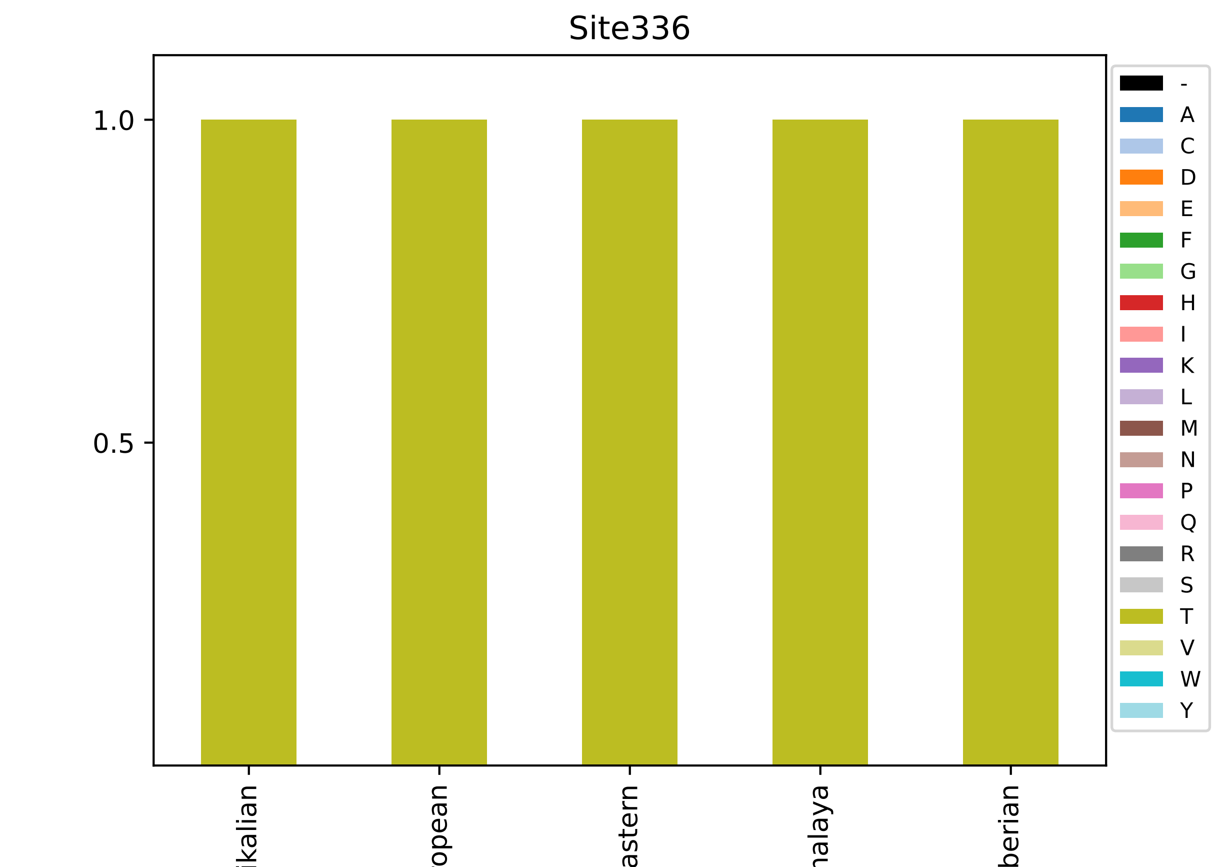
Site336
1.0
0.5
-
A
C
D
E
F
G
H
I
K
L
M
N
P
Q
R
S
T
V
W
Y
ikalian
ropean
astern
nalaya
berian

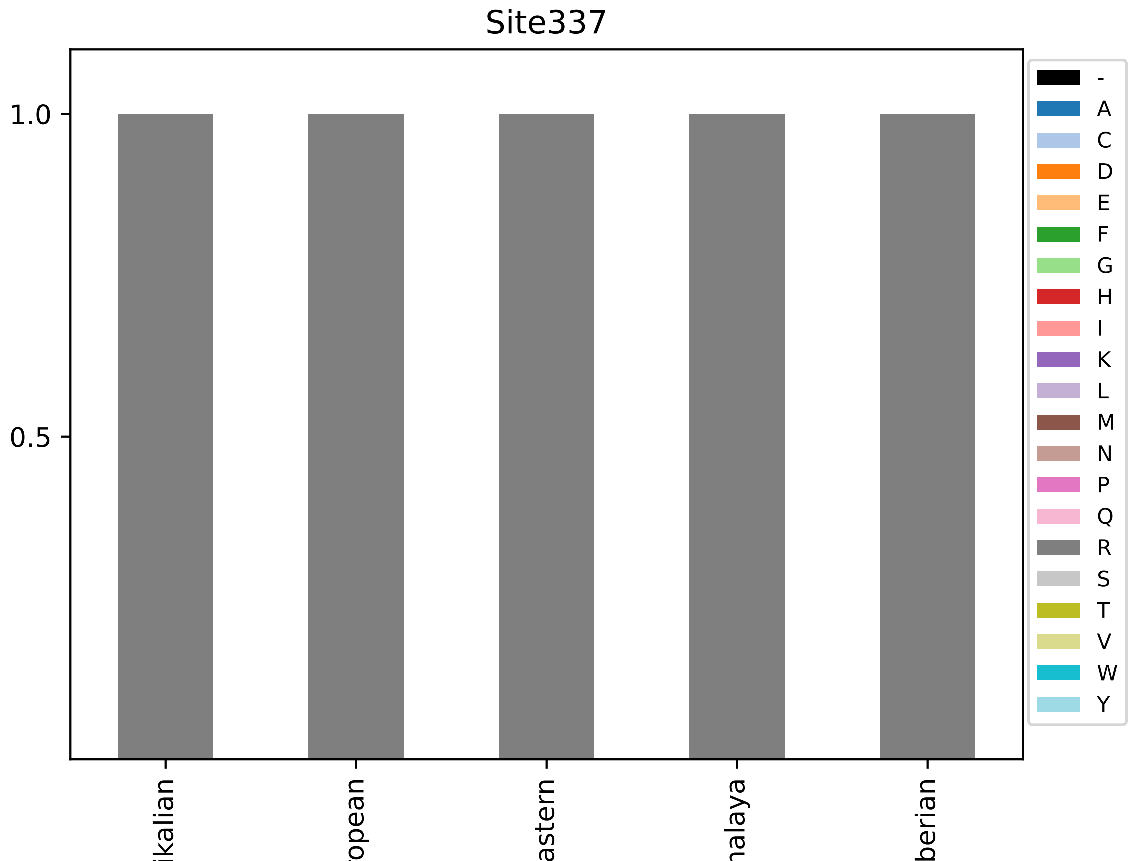
Site337
1.0
0.5
ikalian
opean
astern
nalaya
berian
-
A
C
D
E
F
G
H
I
K
L
M
N
P
Q
R
S
T
V
W
Y

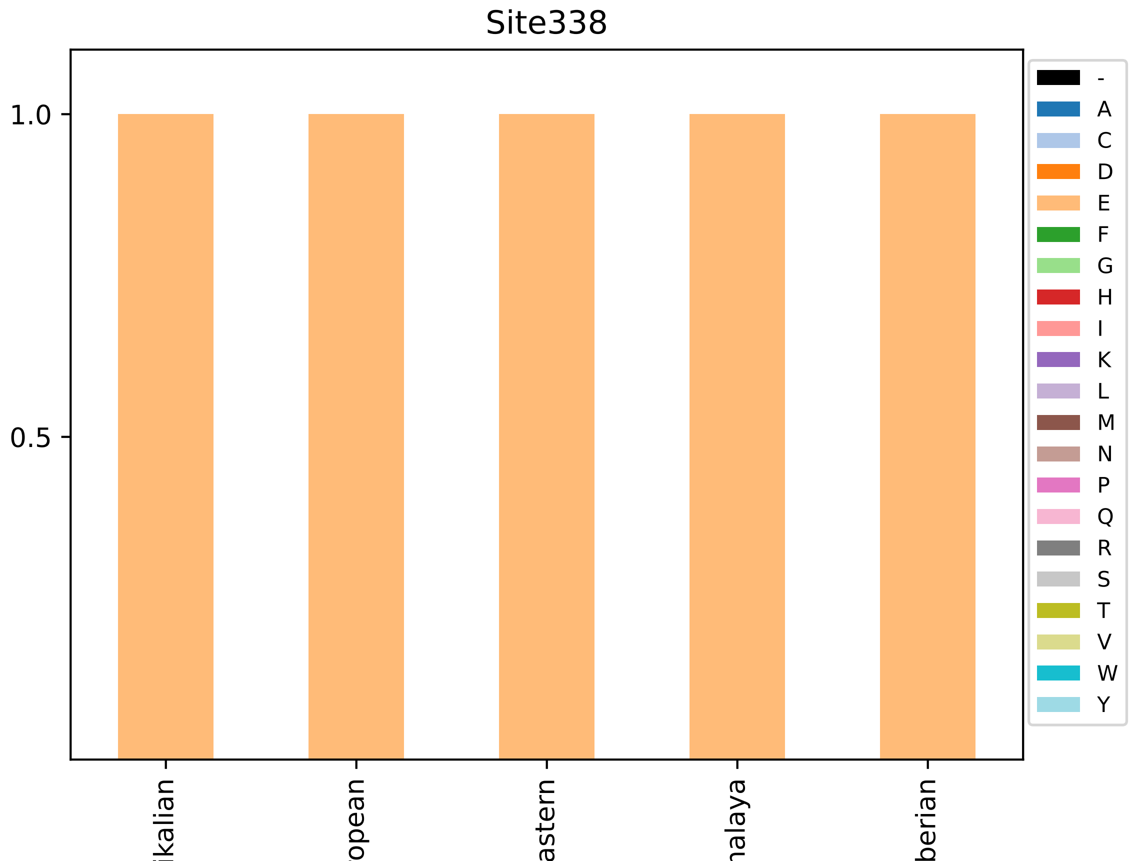

Site338
1.0
0.5
-
A
C
D
E
F
G
H
I
K
L
M
N
P
Q
R
S
T
V
W
Y
ikalian
ropean
astern
nalaya
berian

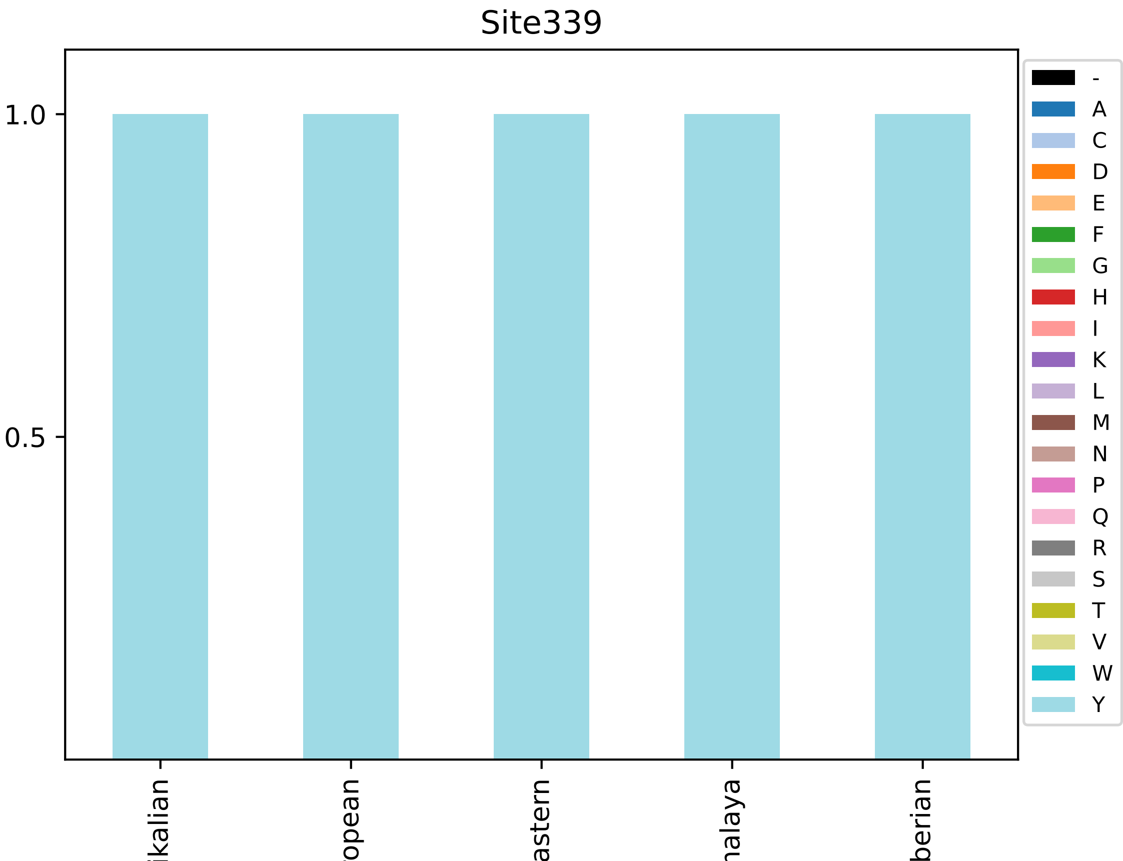

Site339
1.0
0.5
-
A
C
D
E
F
G
H
I
K
L
M
N
P
Q
R
S
T
V
W
Y
ikalian
opean
astern
nalaya
berian

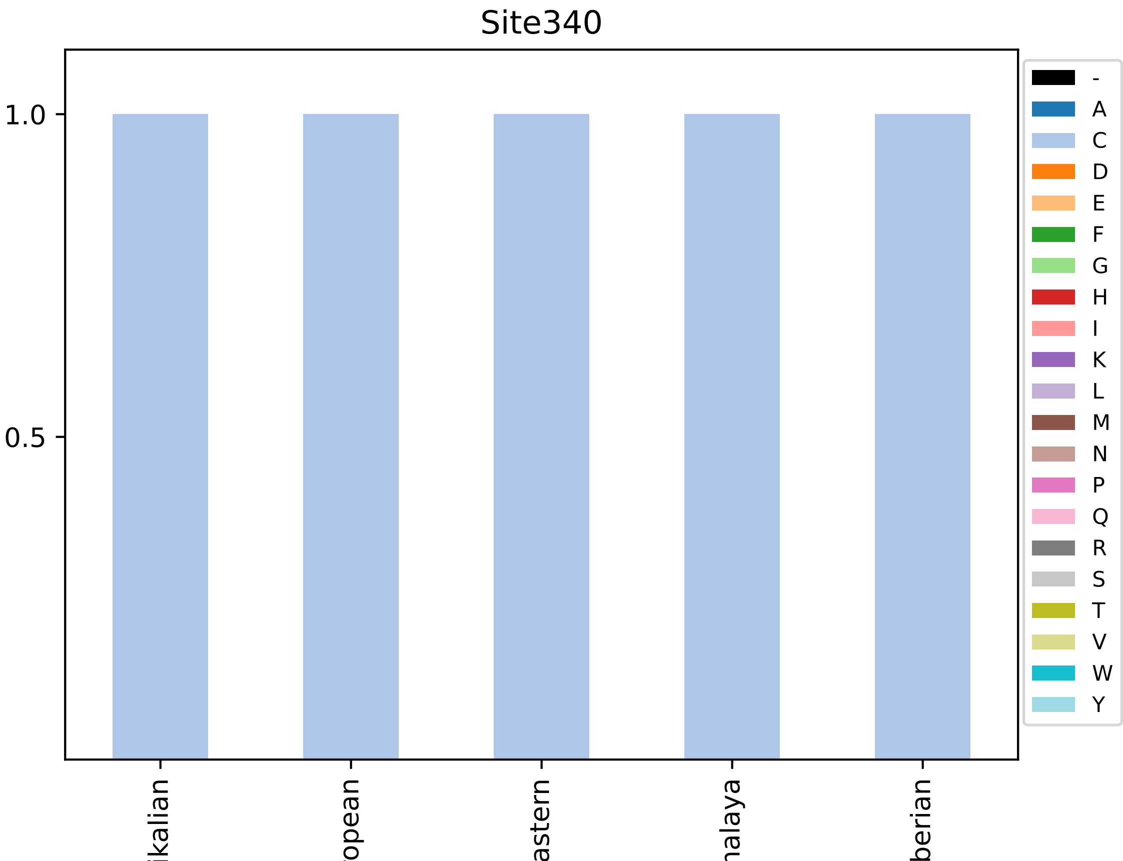
Site340
1.0
0.5
ikalian
opean
astern
nalaya
berian
-
A
C
D
E
F
G
H
I
K
L
M
N
P
Q
R
S
T
V
W
Y

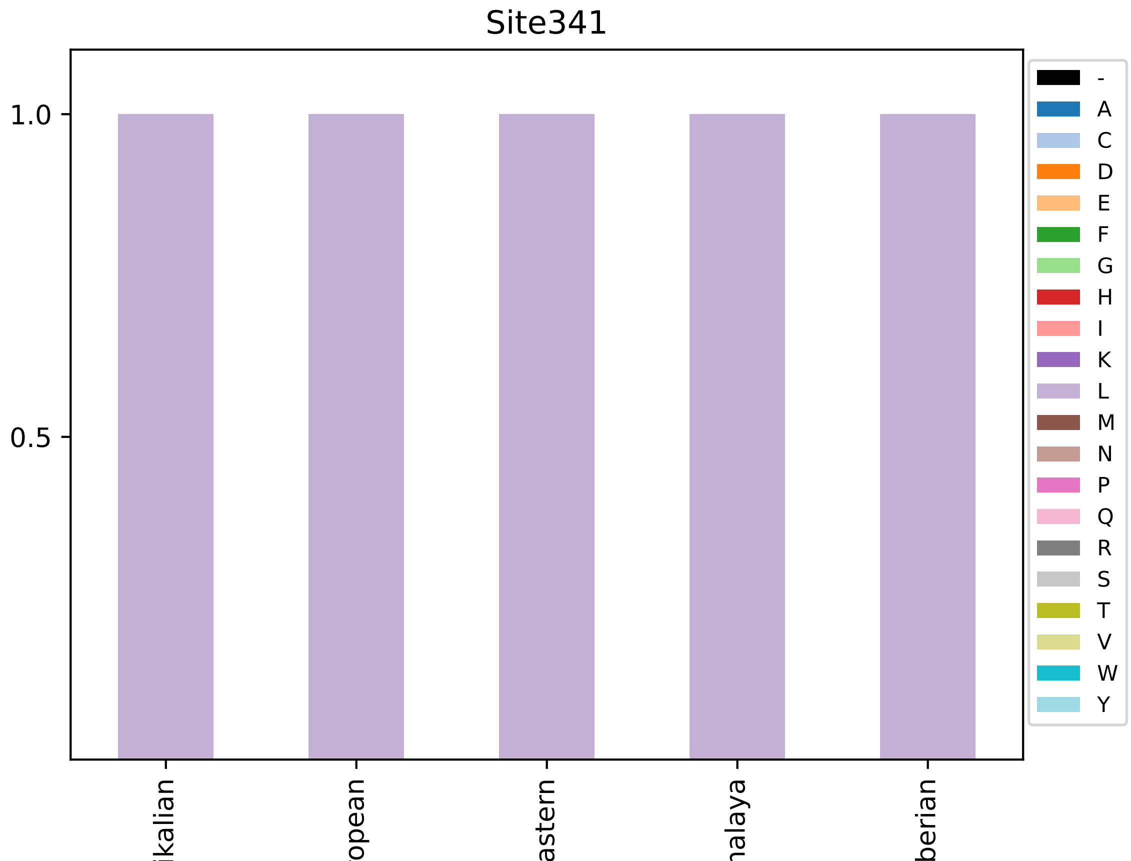
Site341
1.0
0.5
-
A
C
D
E
F
G
H
I
K
L
M
N
P
Q
R
S
T
V
W
Y
ikalian
opean
astern
nalaya
berian

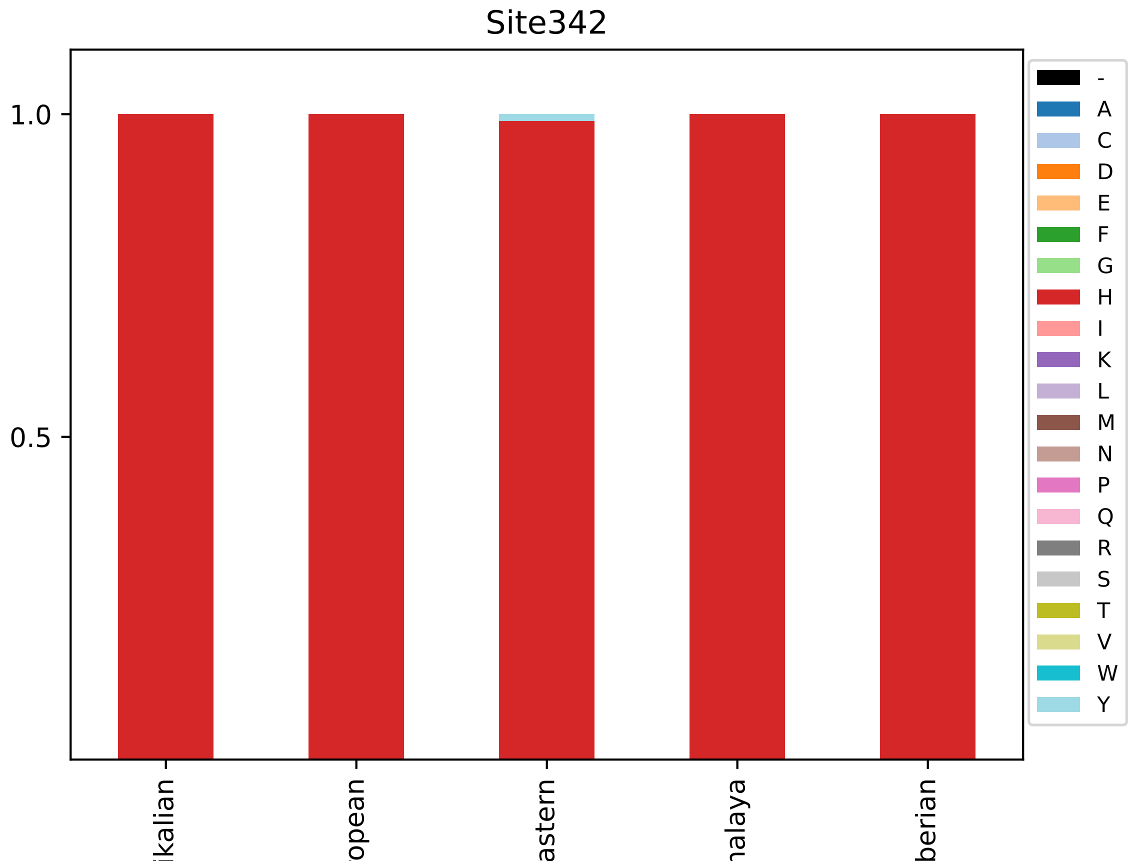

Site342
1.0
0.5
-
A
C
D
E
F
G
H
I
K
L
M
N
P
Q
R
S
T
V
W
Y
ikalian
opean
astern
nalaya
berian

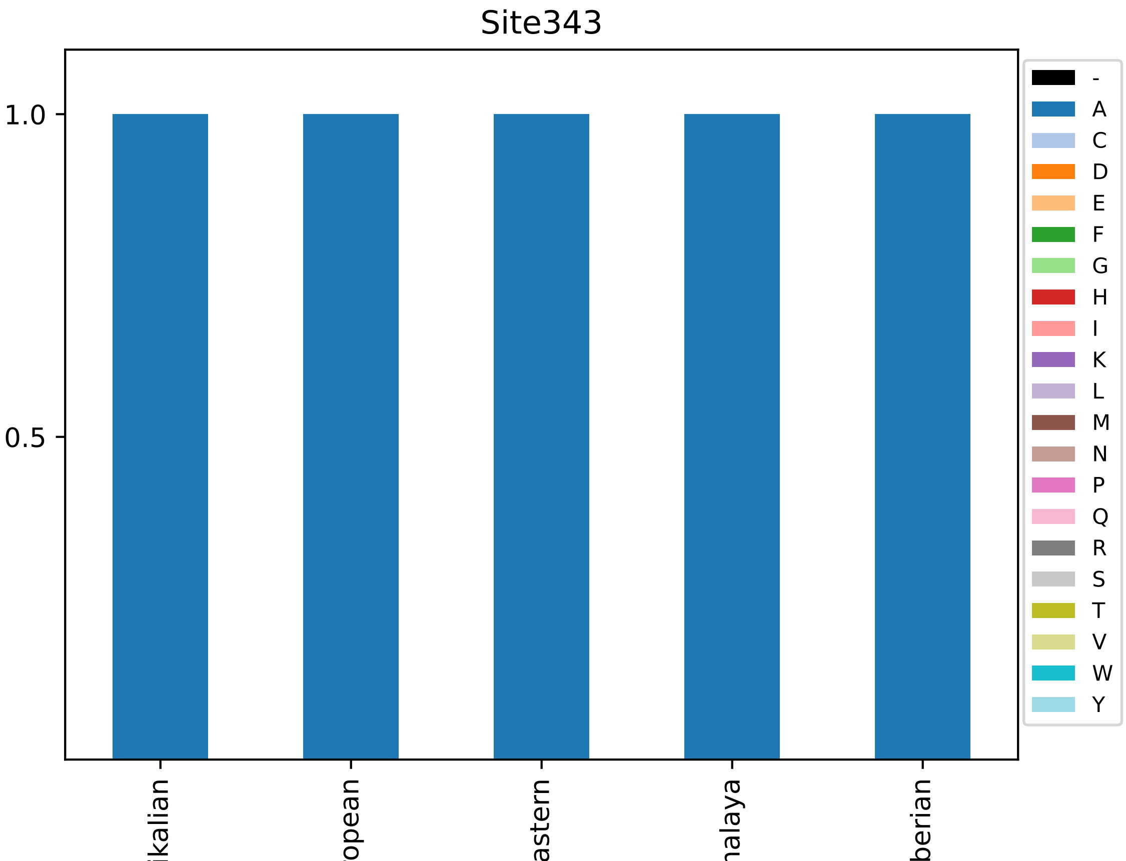
Site343
1.0
0.5
-
A
C
D
E
F
G
H
I
K
L
M
N
P
Q
R
S
T
V
W
Y
ikalian
opean
astern
nalaya
berian

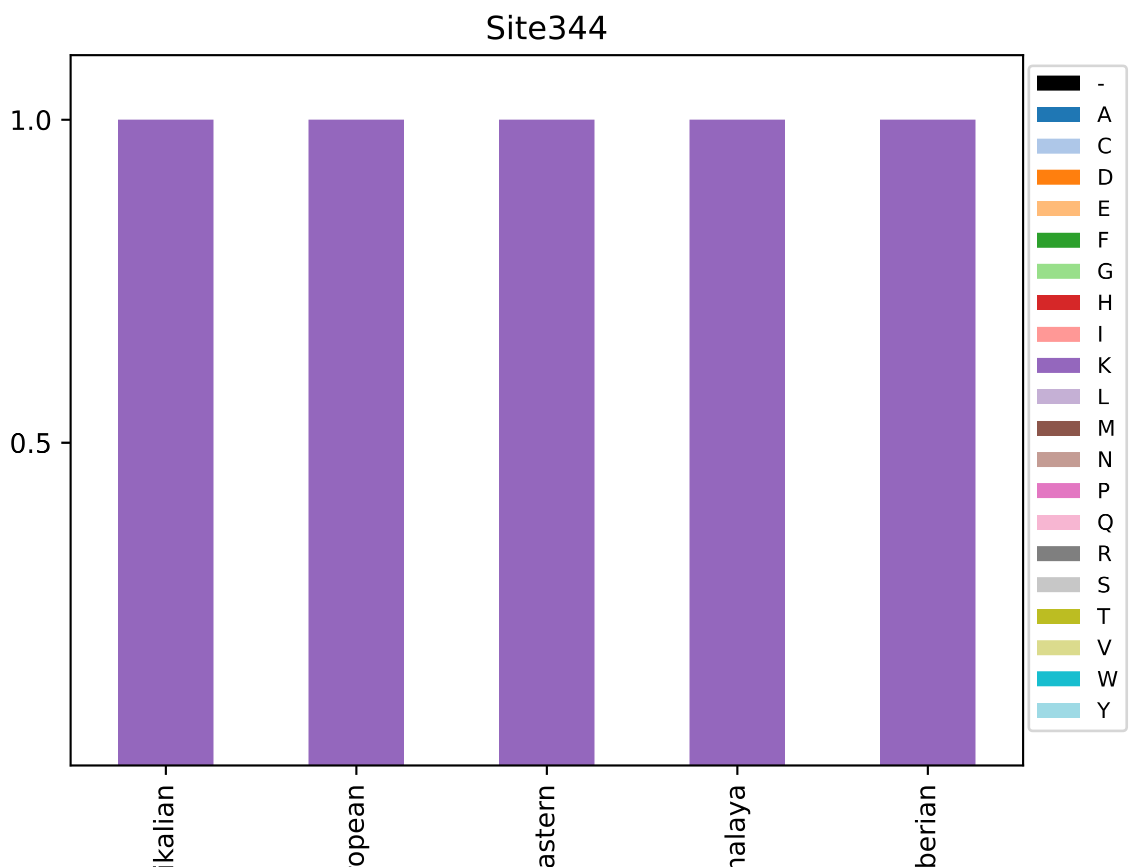

Site344
1.0
0.5
-
A
C
D
E
F
G
H
I
K
L
M
N
P
Q
R
S
T
V
W
Y
ikalian
opean
astern
nalaya
berian

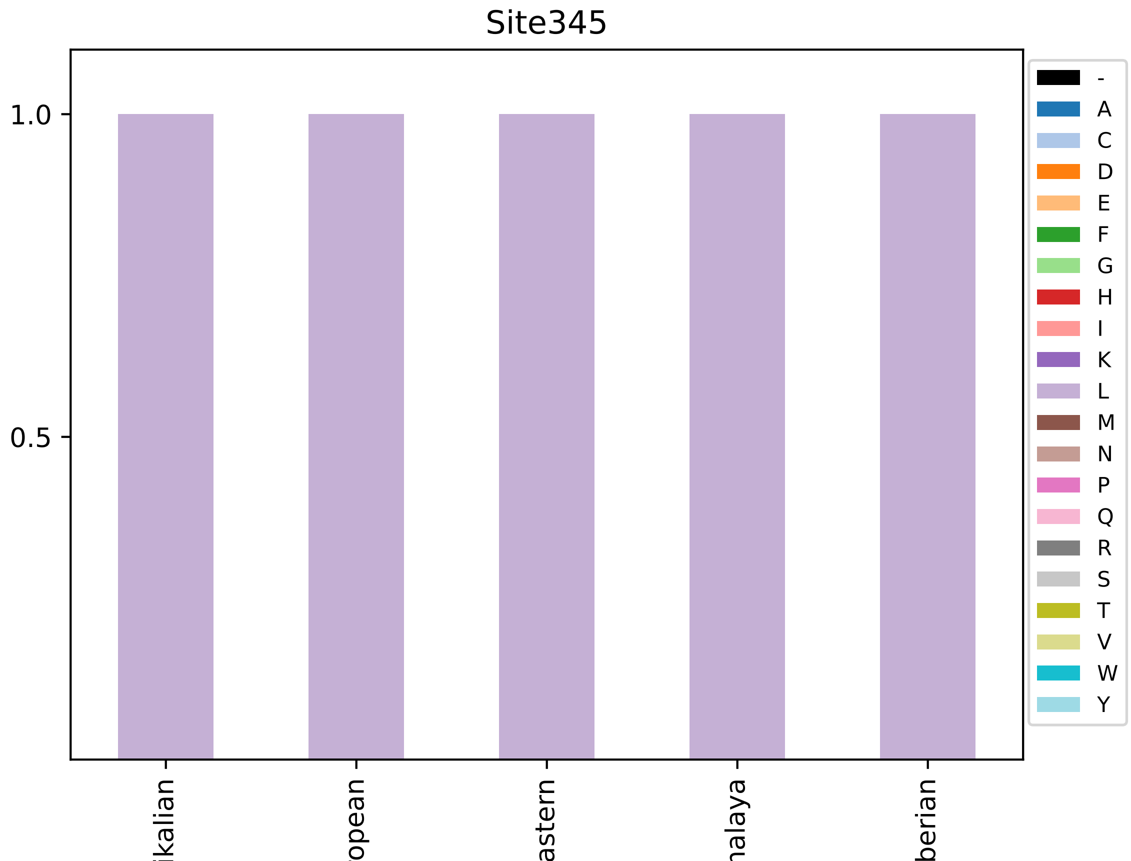
Site345
1.0
0.5
-
A
C
D
E
F
G
H
I
K
L
M
N
P
Q
R
S
T
V
W
Y
ikalian
opean
astern
nalaya
berian

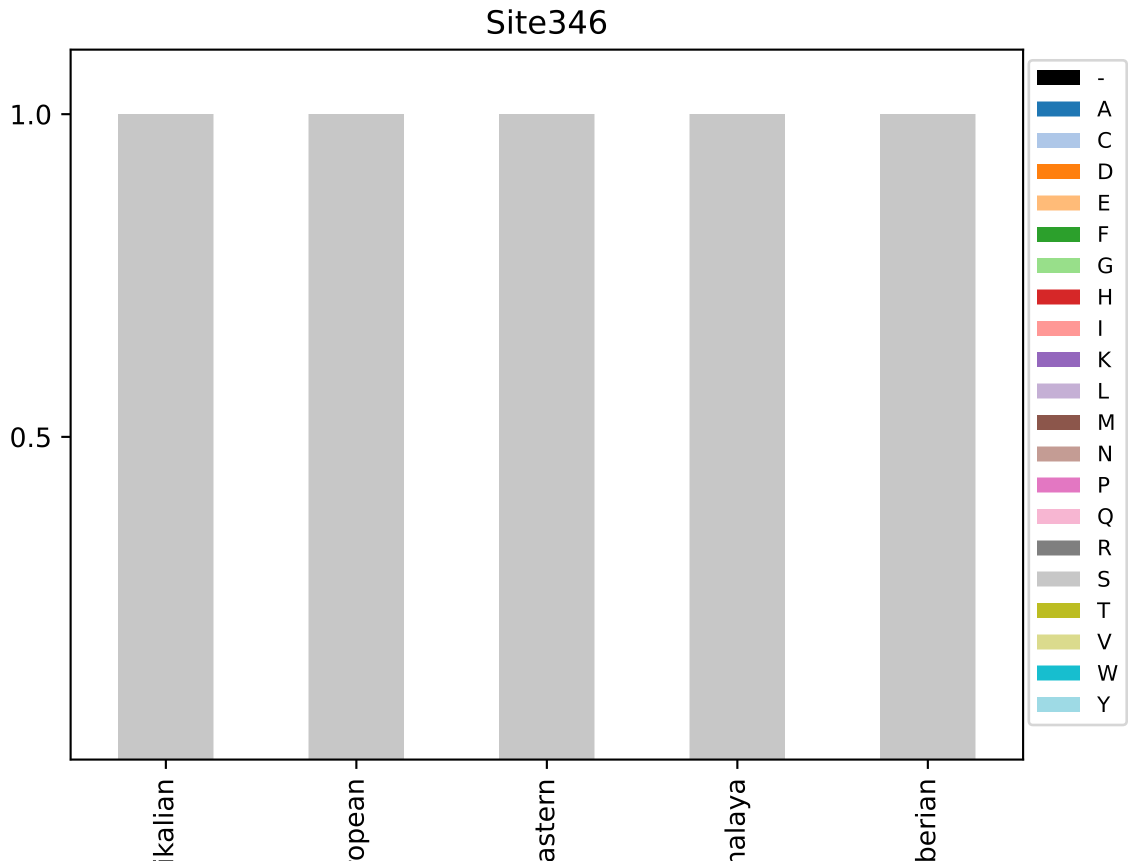
Site346
1.0
0.5
-
A
C
D
E
F
G
H
I
K
L
M
N
P
Q
R
S
T
V
W
Y
ikalian
opean
astern
nalaya
berian

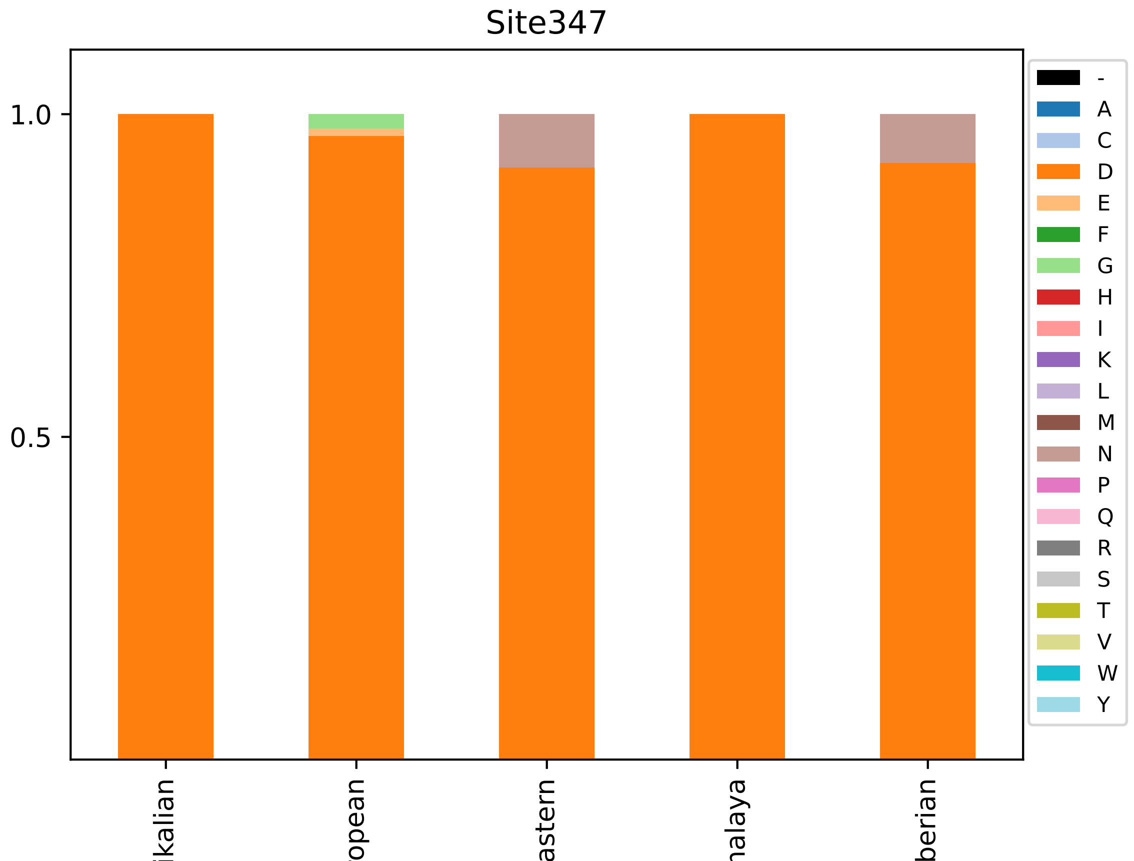

Site347
1.0
0.5
-
A
C
D
E
F
G
H
I
K
L
M
N
P
Q
R
S
T
V
W
Y

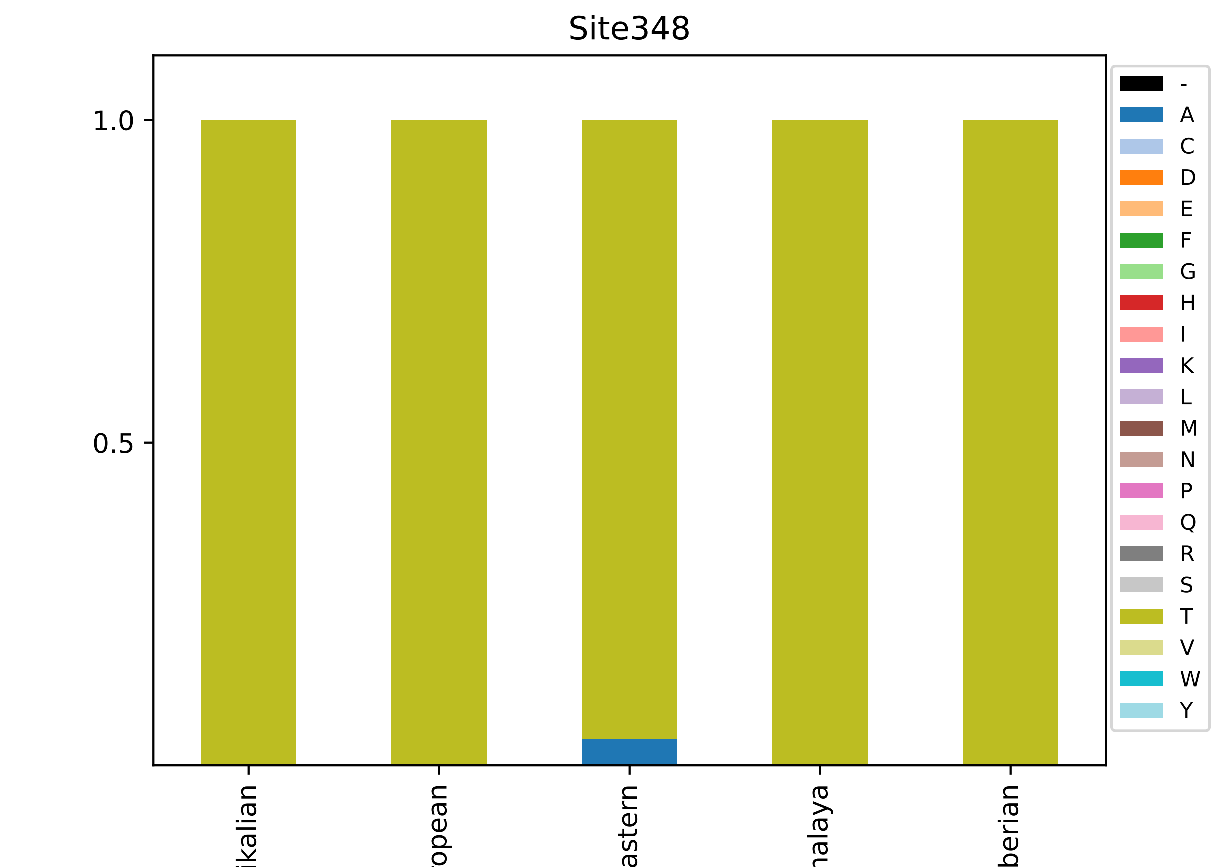

Site348
1.0
0.5
-
A
C
D
E
F
G
H
I
K
L
M
N
P
Q
R
S
T
V
W
Y
ikalian
opean
astern
nalaya
berian

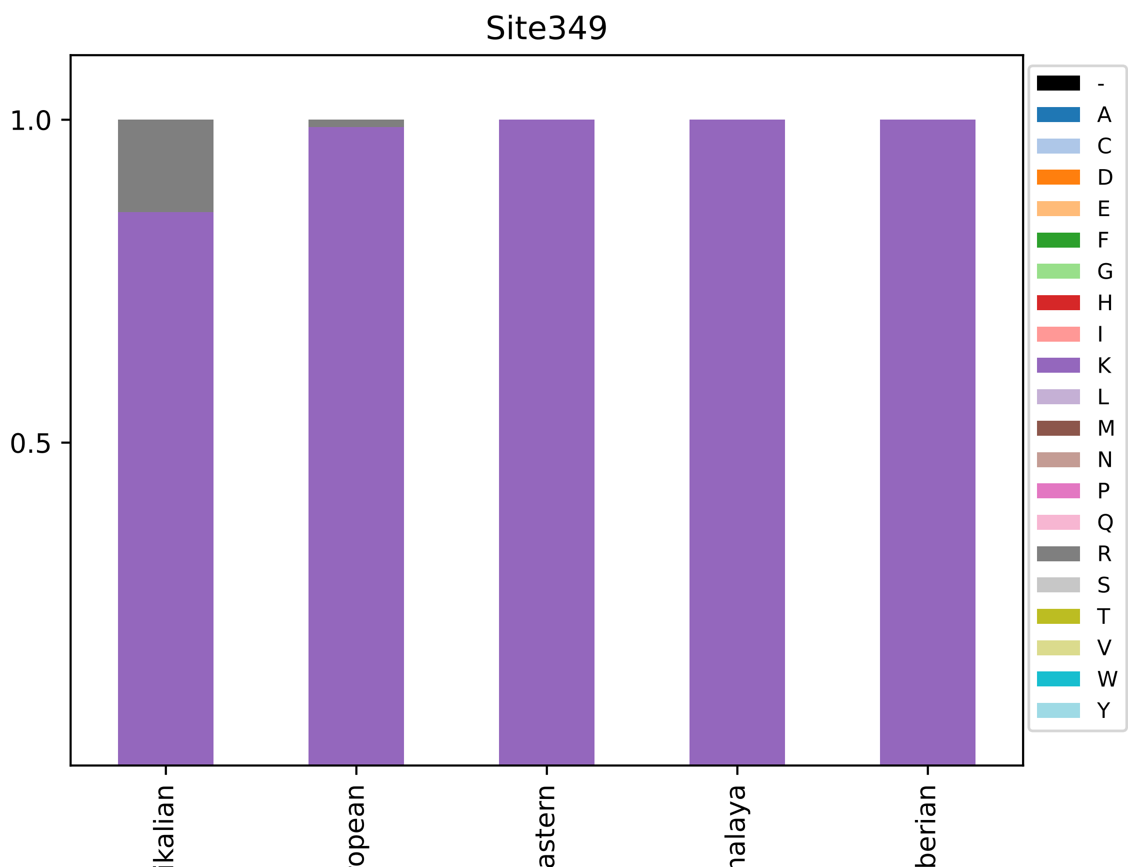
Site349
1.0
0.5
-
A
C
D
E
F
G
H
I
K
L
M
N
P
Q
R
S
T
V
W
Y
ikalian
opean
astern
nalaya
berian

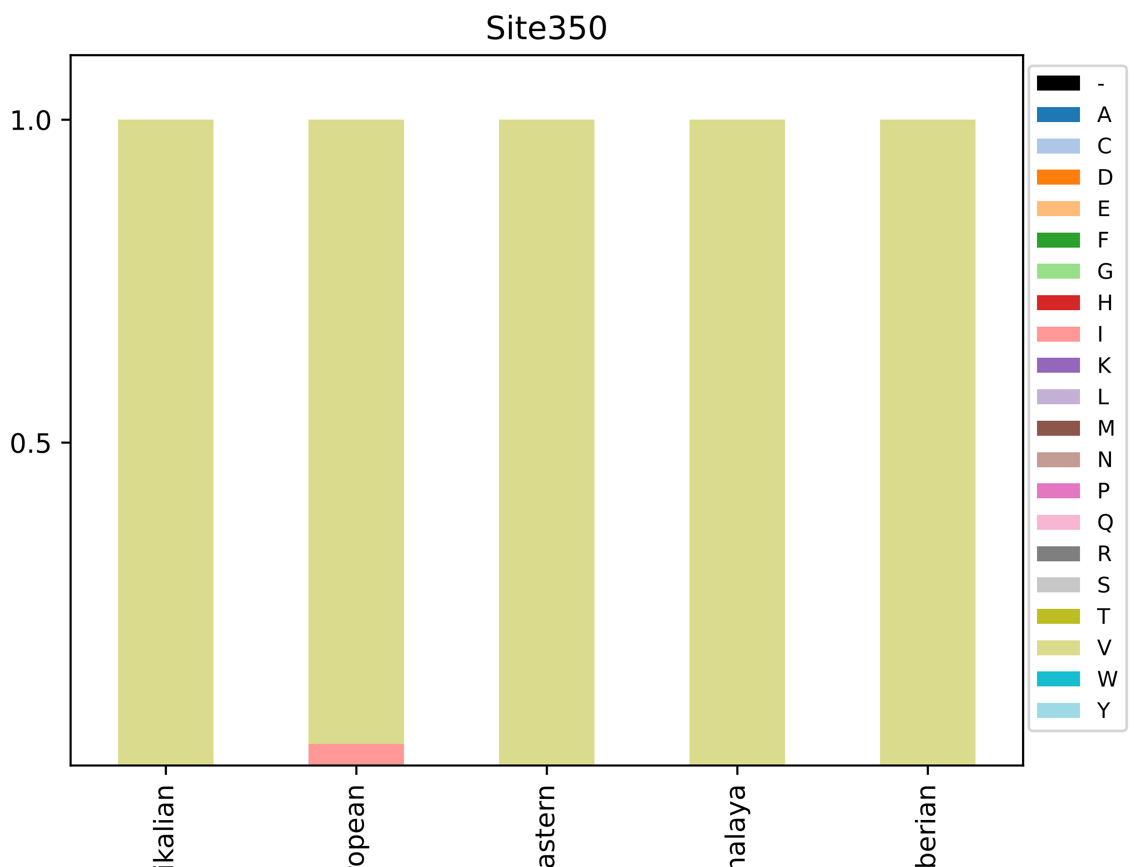
Site350
1.0
0.5
-
A
C
D
E
F
G
H
I
K
L
M
N
P
Q
R
S
T
V
W
Y
ikalian
opean
astern
nalaya
berian

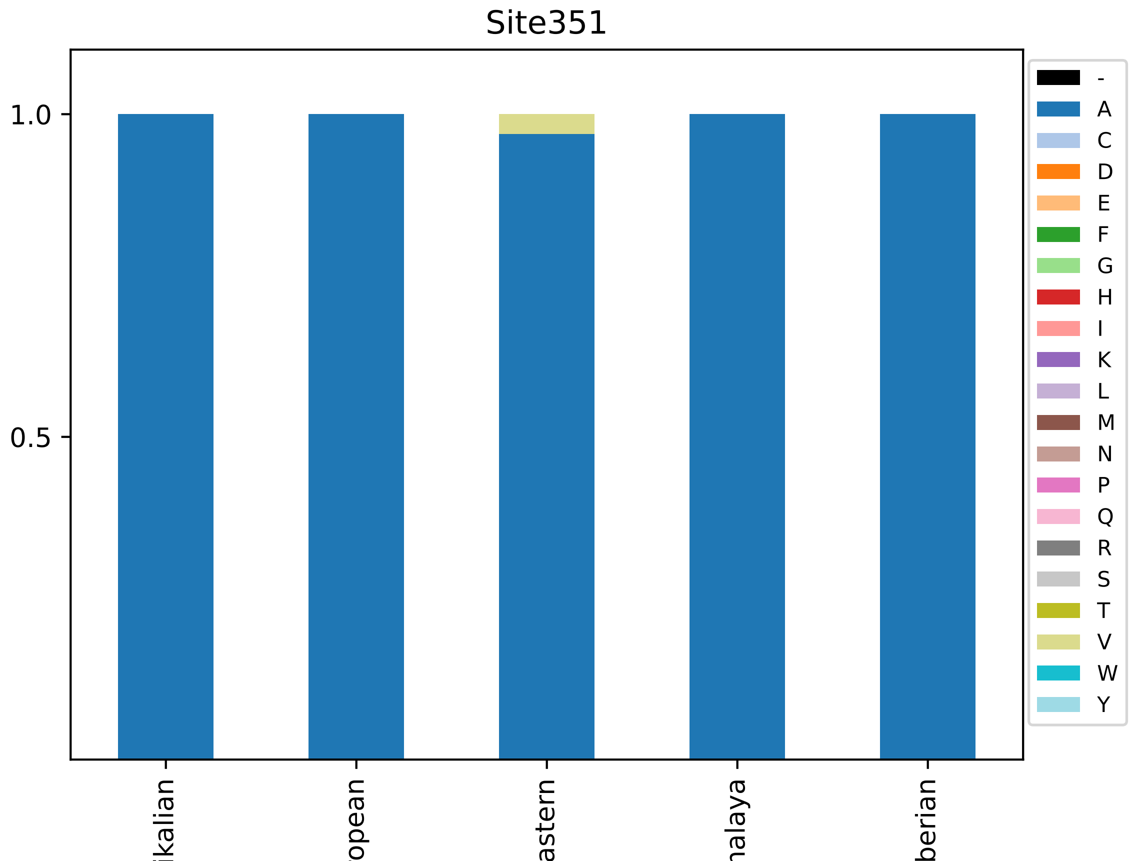
Site351
1.0
0.5
-
A
C
D
E
F
G
H
I
K
L
M
N
P
Q
R
S
T
V
W
Y
ikalian
opean
astern
nalaya
berian

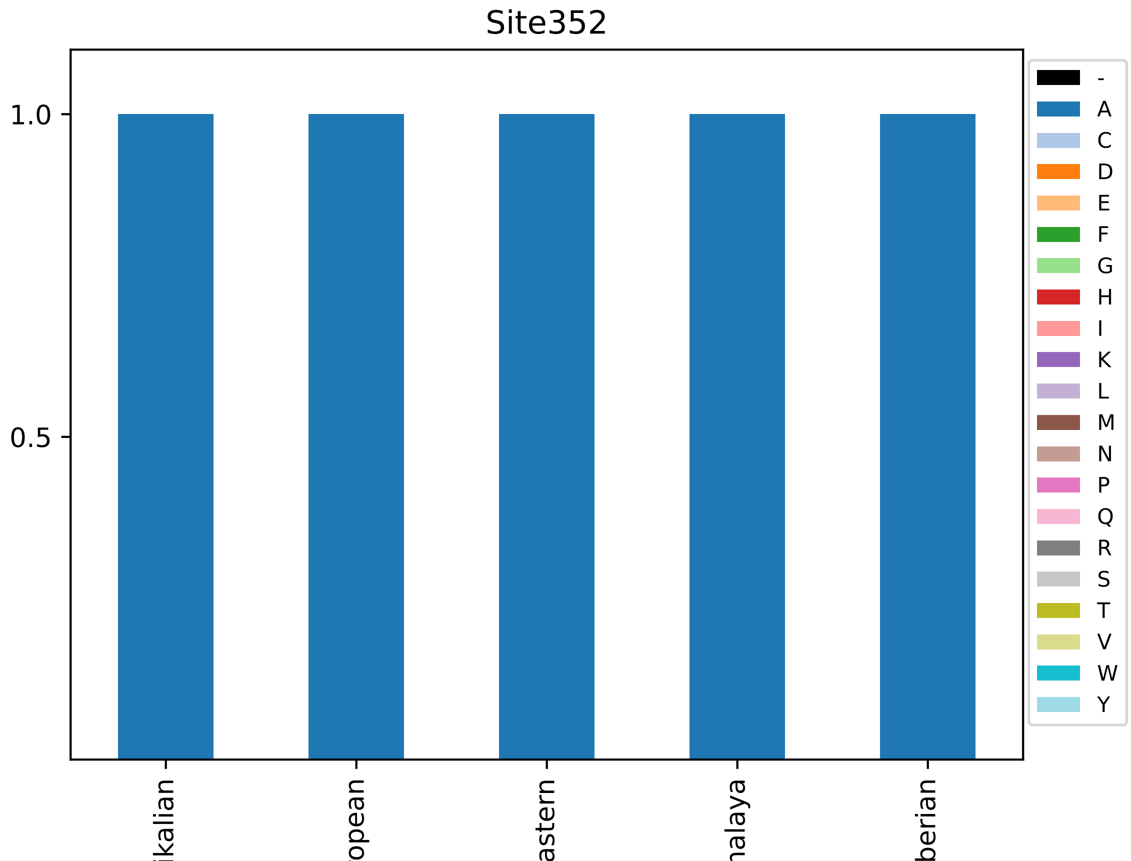
Site352
1.0
0.5
-
A
C
D
E
F
G
H
I
K
L
M
N
P
Q
R
S
T
V
W
Y
ikalian
opean
astern
nalaya
berian

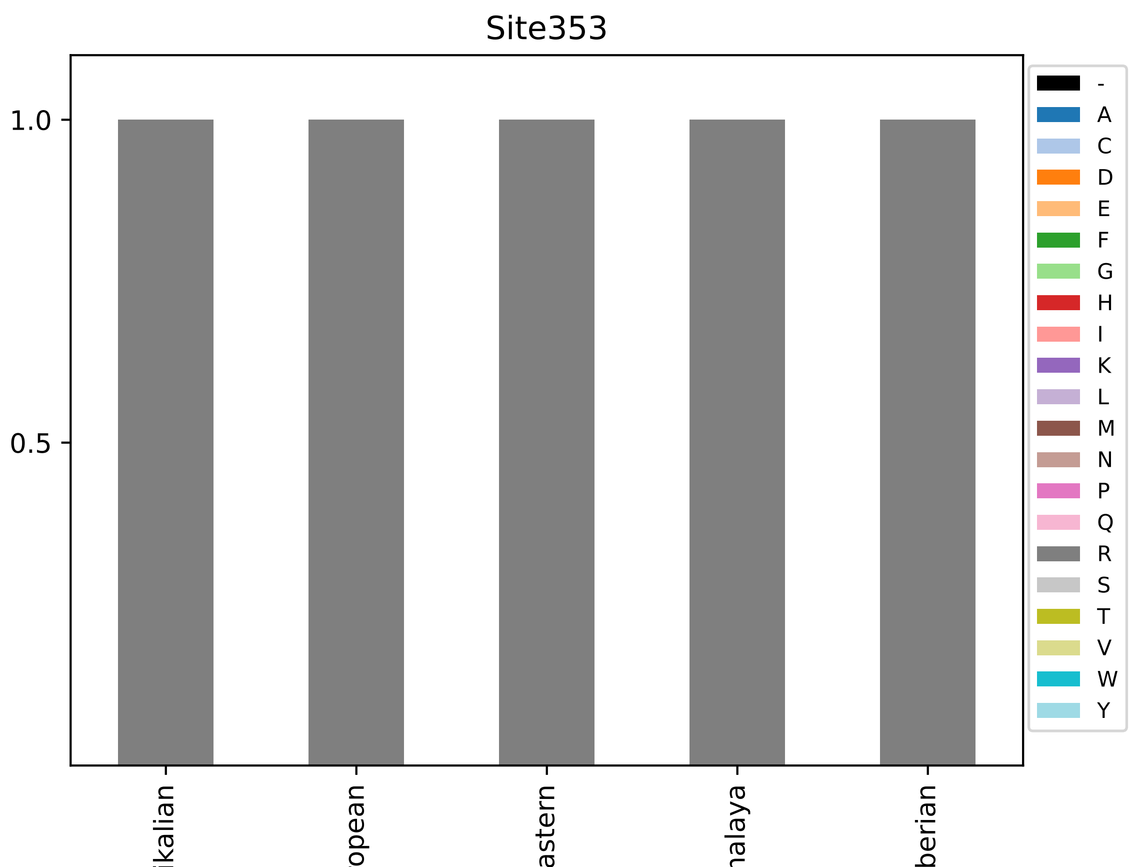

Site353
1.0
0.5
-
A
C
D
E
F
G
H
I
K
L
M
N
P
Q
R
S
T
V
W
Y
ikalian
opean
astern
nalaya
berian

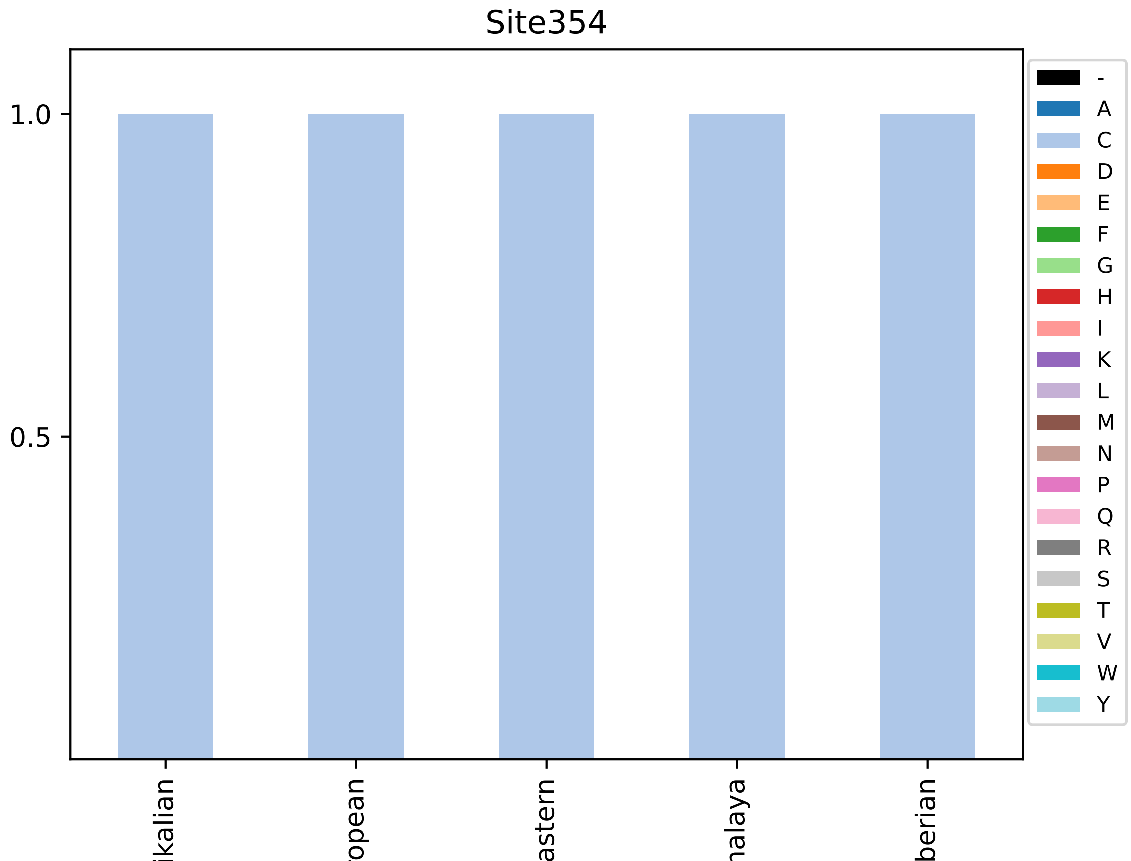
Site354
1.0
0.5
-
A
C
D
E
F
G
H
I
K
L
M
N
P
Q
R
S
T
V
W
Y
ikalian
opean
astern
nalaya
berian

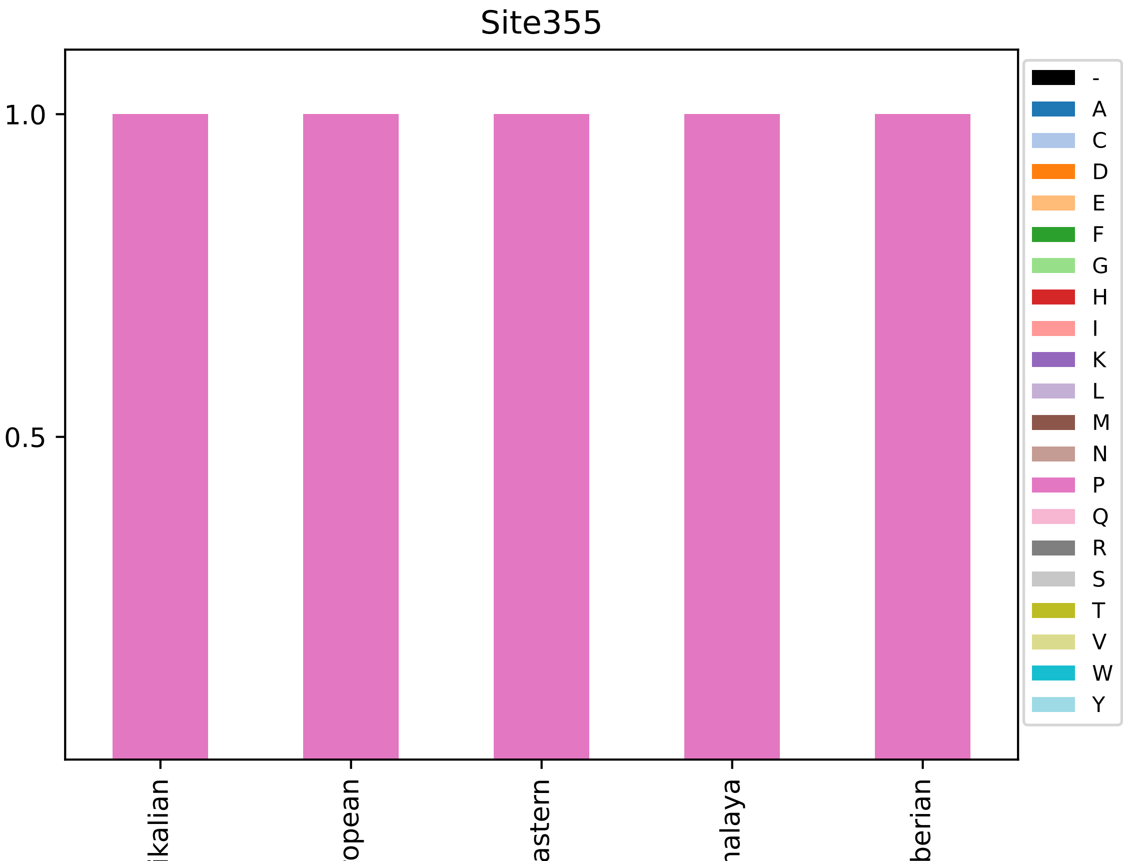
Site355
1.0
0.5
-
A
C
D
E
F
G
H
I
K
L
M
N
P
Q
R
S
T
V
W
Y
ikalian
opean
astern
nalaya
berian

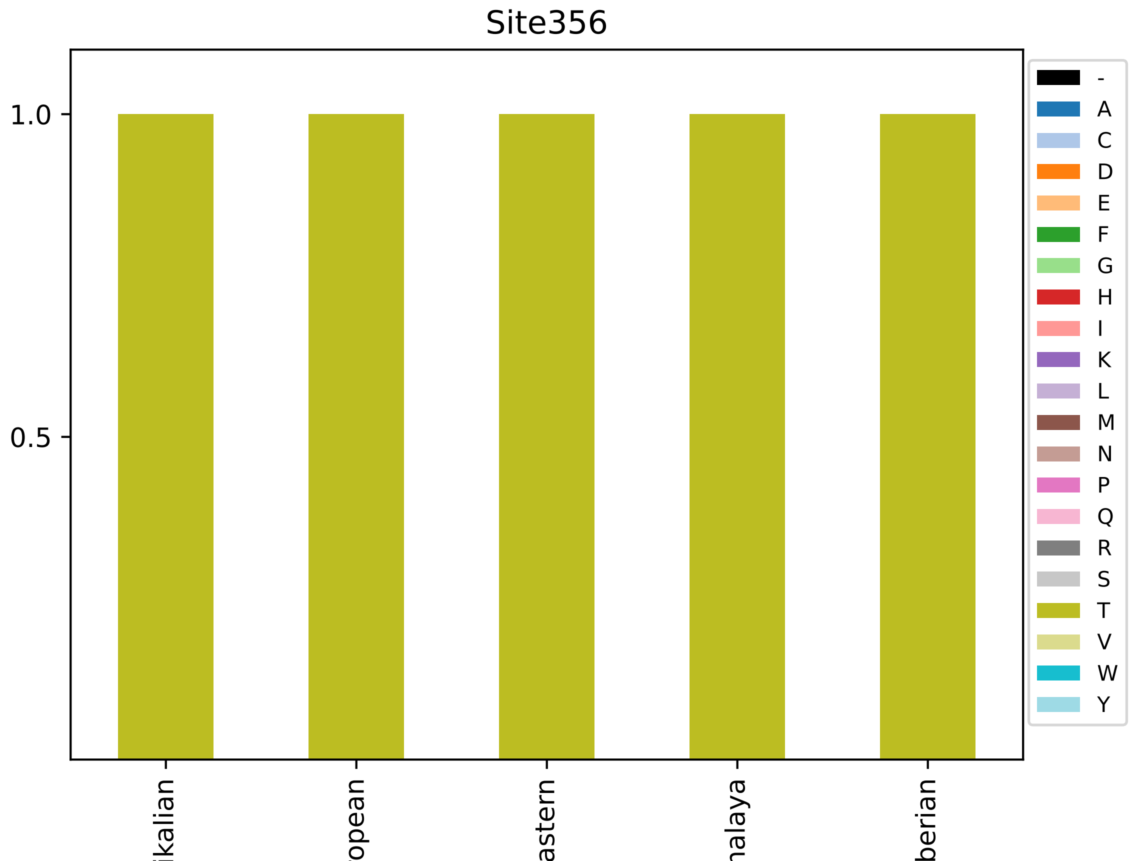

Site356
1.0
0.5
-
A
C
D
E
F
G
H
I
K
L
M
N
P
Q
R
S
T
V
W
Y
ikalian
opean
astern
nalaya
berian

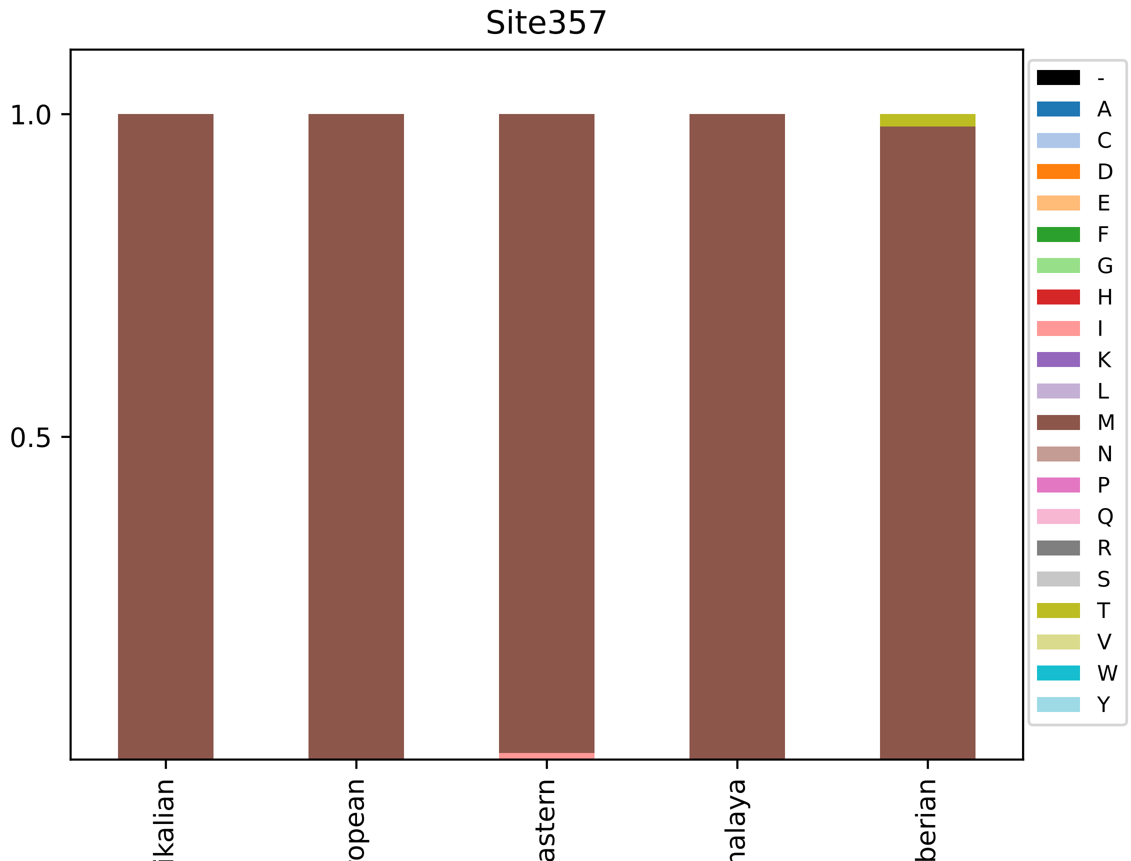
Site357
1.0
0.5
ikalian
opean
astern
nalaya
berian
-
A
C
D
E
F
G
H
I
K
L
M
N
P
Q
R
S
T
V
W
Y

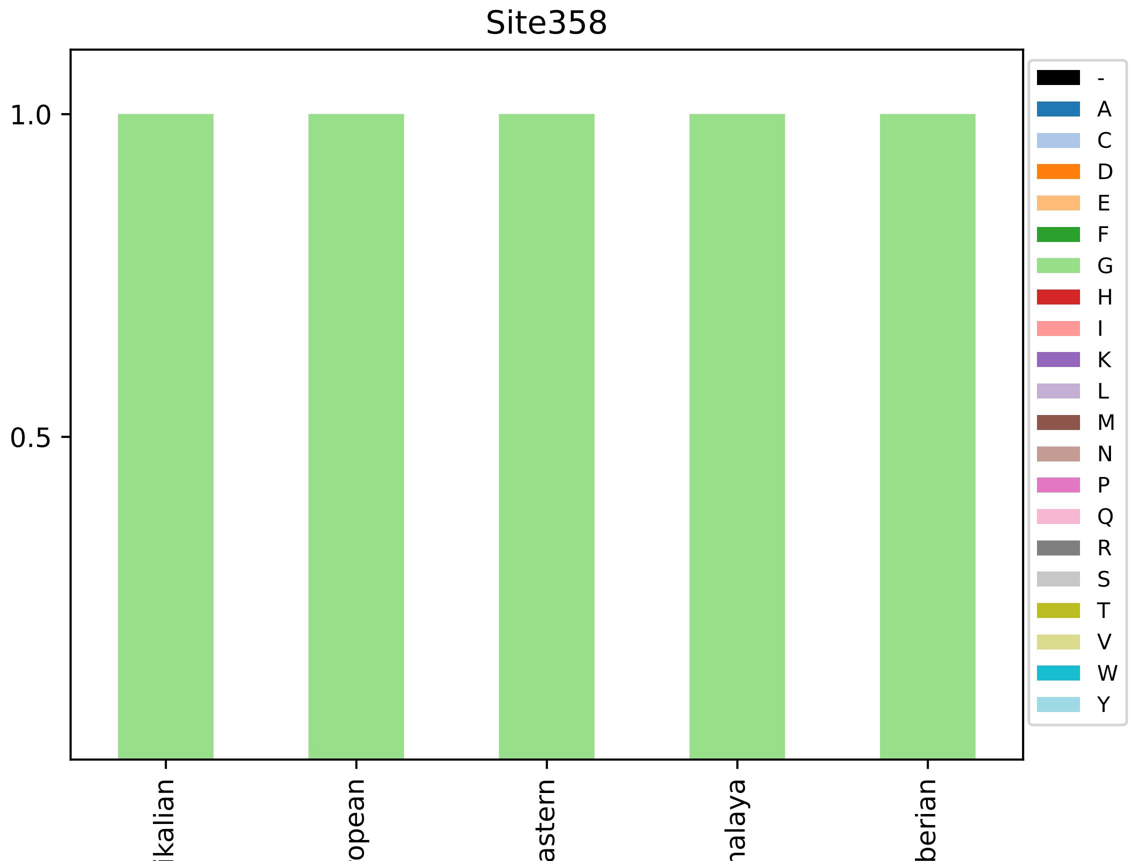

Site358
1.0
0.5
-
A
C
D
E
F
G
H
I
K
L
M
N
P
Q
R
S
T
V
W
Y
ikalian
opean
astern
nalaya
berian

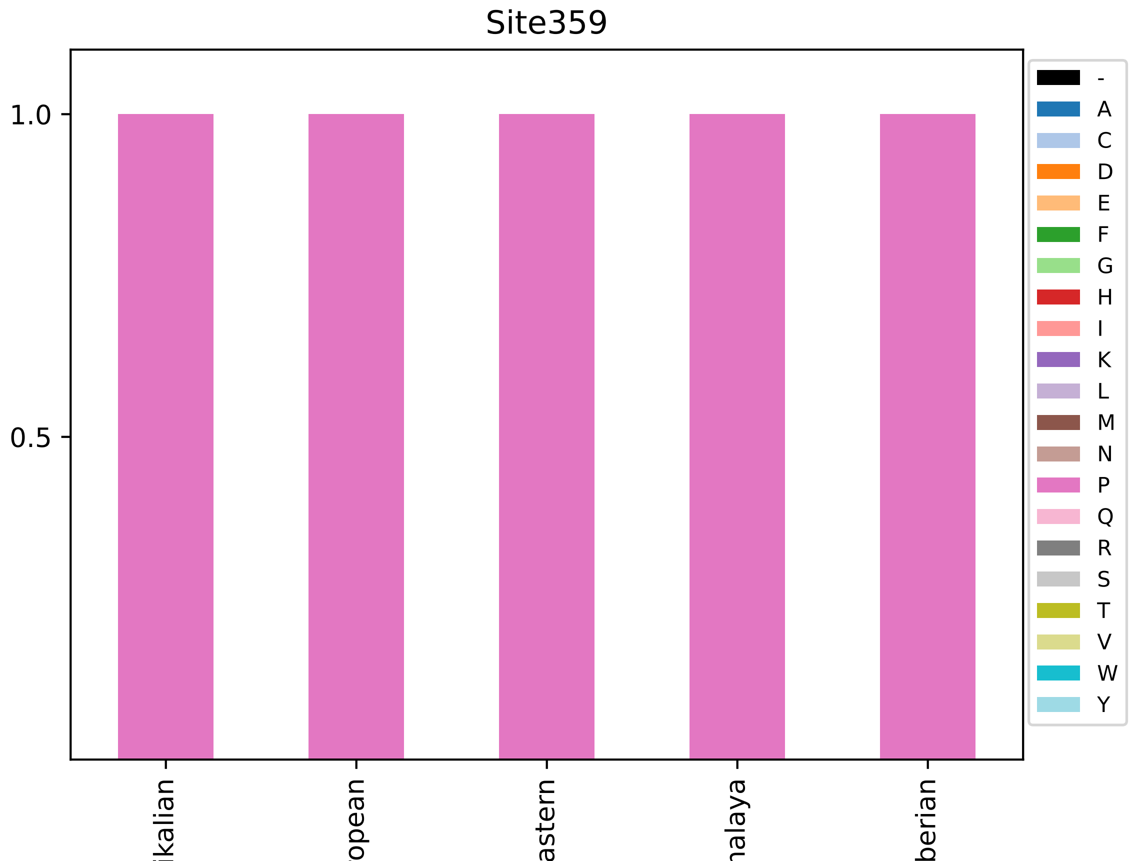

Site359
1.0
0.5
-
A
C
D
E
F
G
H
I
K
L
M
N
P
Q
R
S
T
V
W
Y
ikalian
opean
astern
nalaya
berian

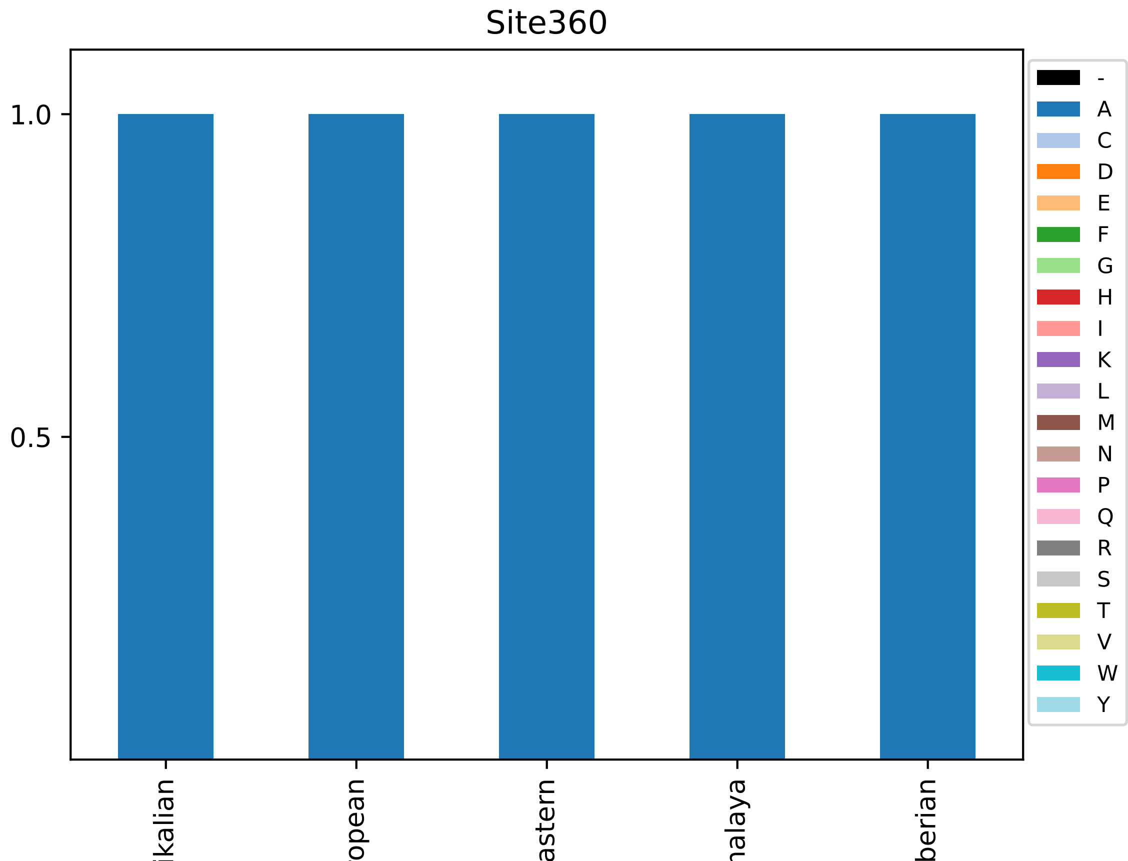
Site360
1.0
0.5
-
A
C
D
E
F
G
H
I
K
L
M
N
P
Q
R
S
T
V
W
Y

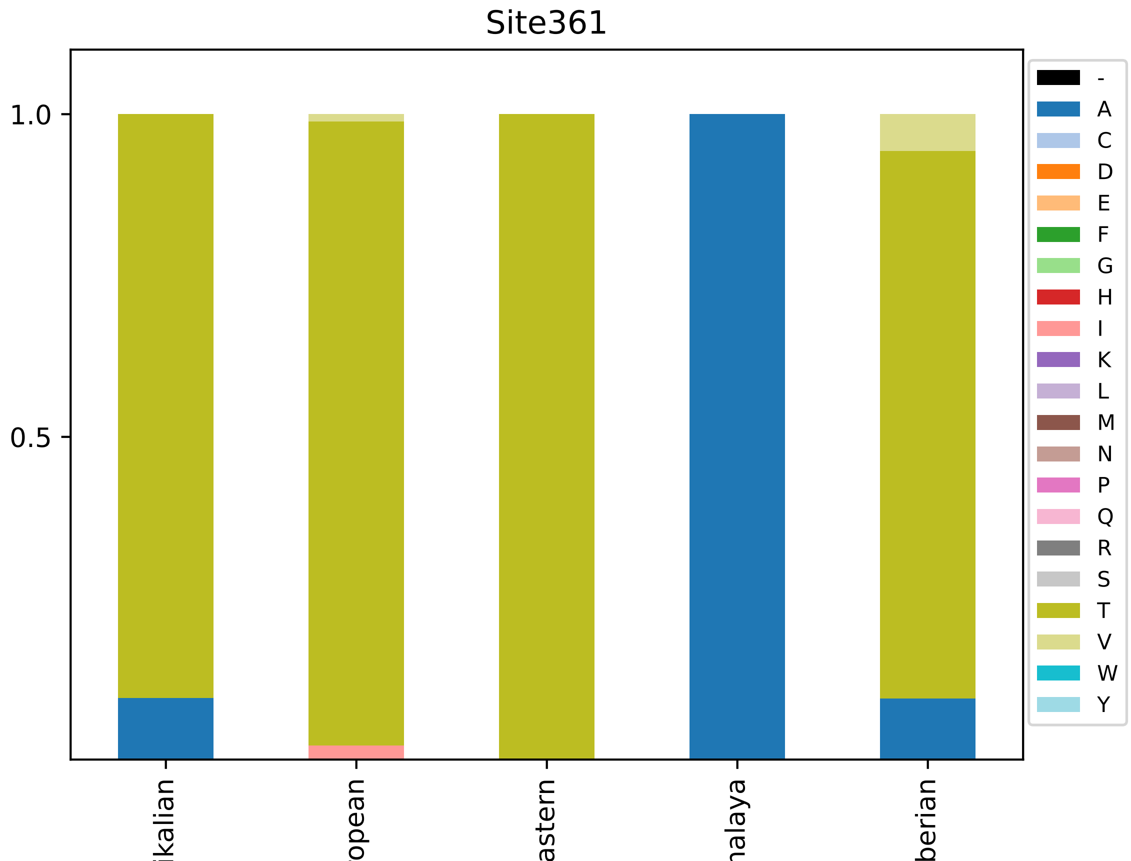
Site361
1.0
0.5
-
A
C
D
E
F
G
H
I
K
L
M
N
P
Q
R
S
T
V
W
Y
ikalian
opean
astern
nalaya
berian

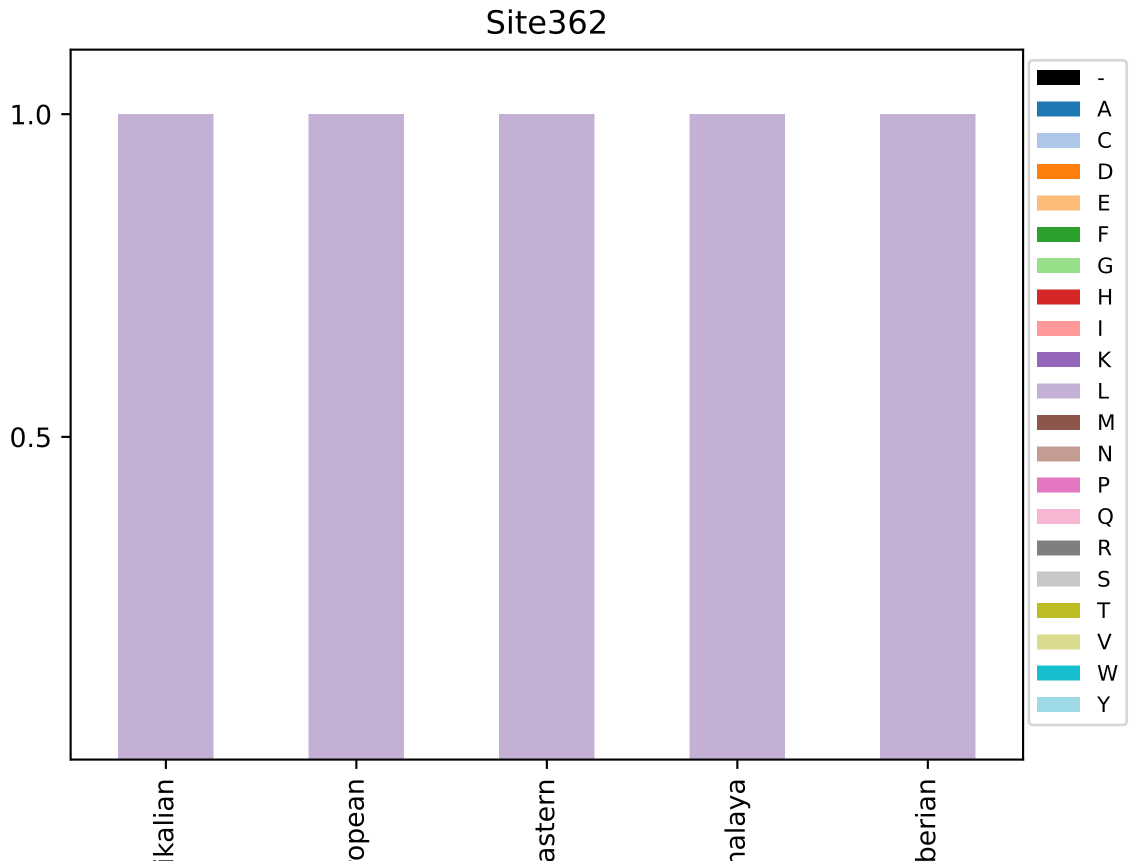
Site362
1.0
0.5
-
A
C
D
E
F
G
H
I
K
L
M
N
P
Q
R
S
T
V
W
Y
ikalian
opean
astern
nalaya
berian

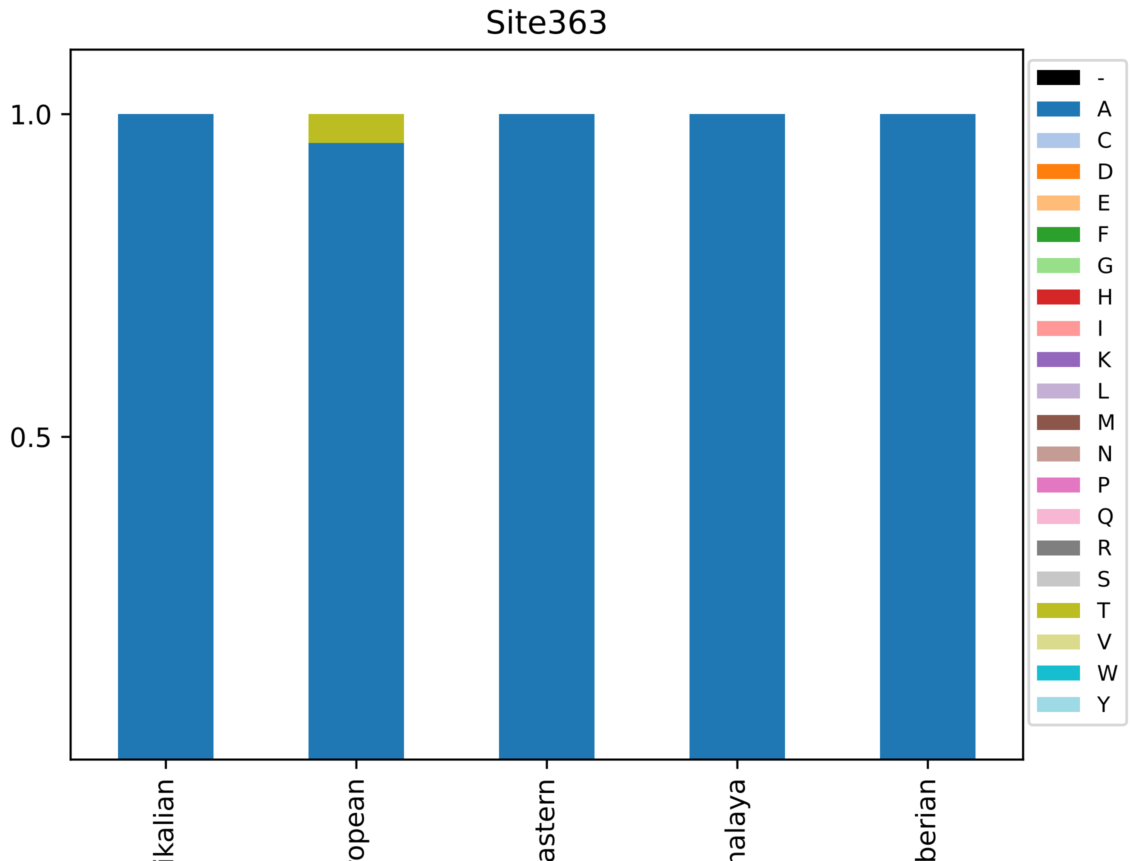
Site363
1.0
0.5
-
A
C
D
E
F
G
H
I
K
L
M
N
P
Q
R
S
T
V
W
Y
ikalian
opean
astern
nalaya
berian

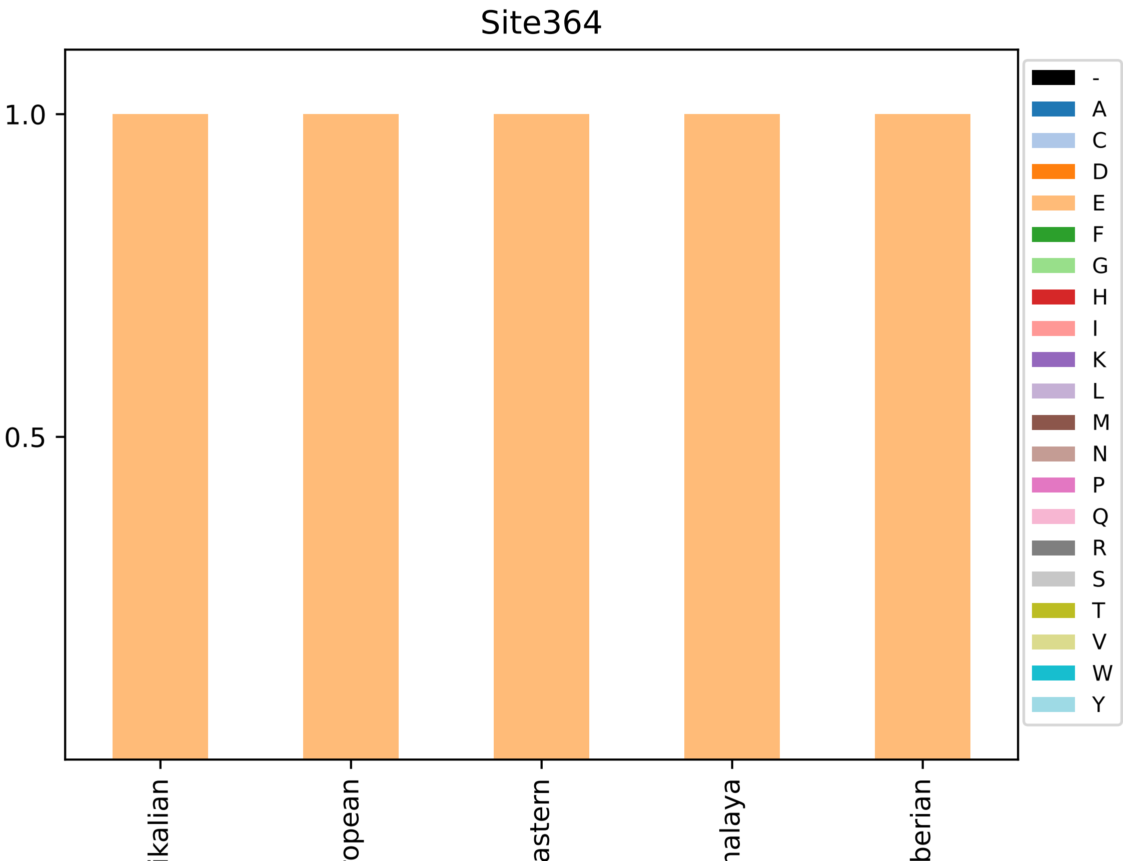
Site364
1.0
0.5
-
A
C
D
E
F
G
H
I
K
L
M
N
P
Q
R
S
T
V
W
Y
ikalian
opean
astern
nalaya
berian

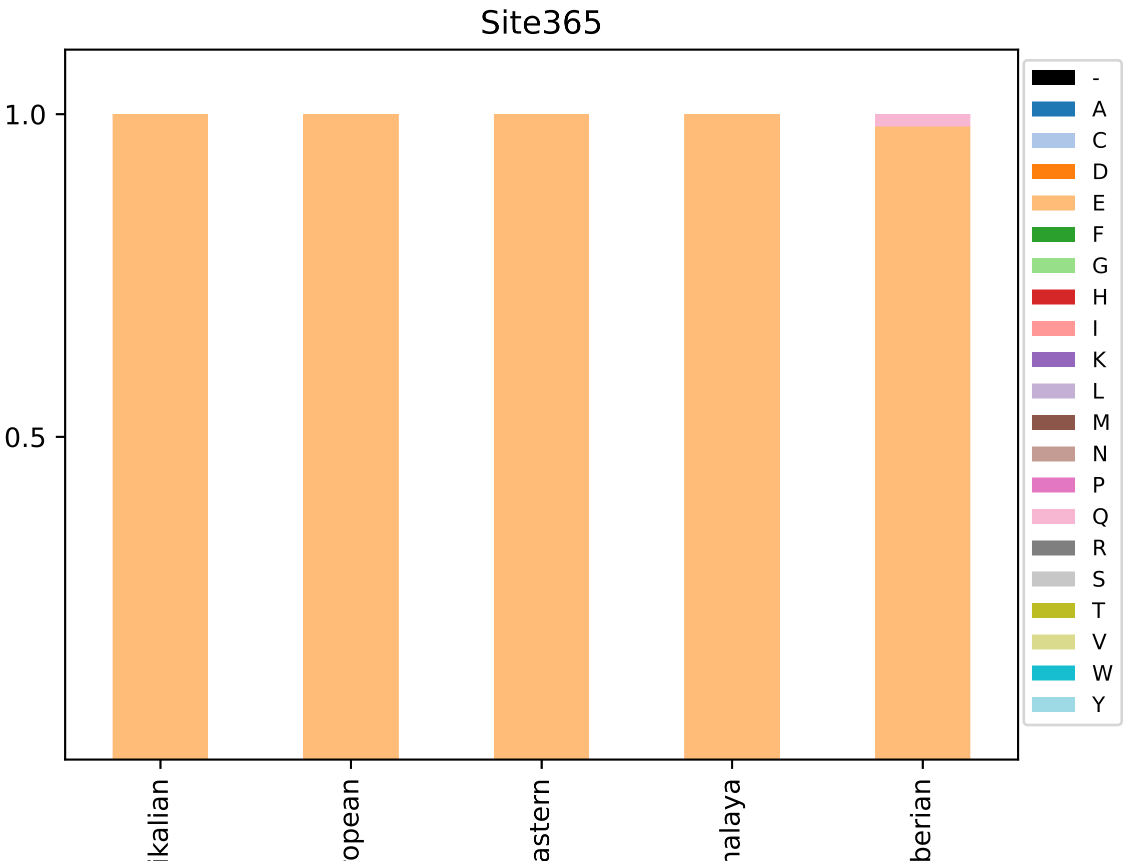
Site365
1.0
0.5
ikalian
opean
astern
nalaya
berian
-
A
C
D
E
F
G
H
I
K
L
M
N
P
Q
R
S
T
V
W
Y

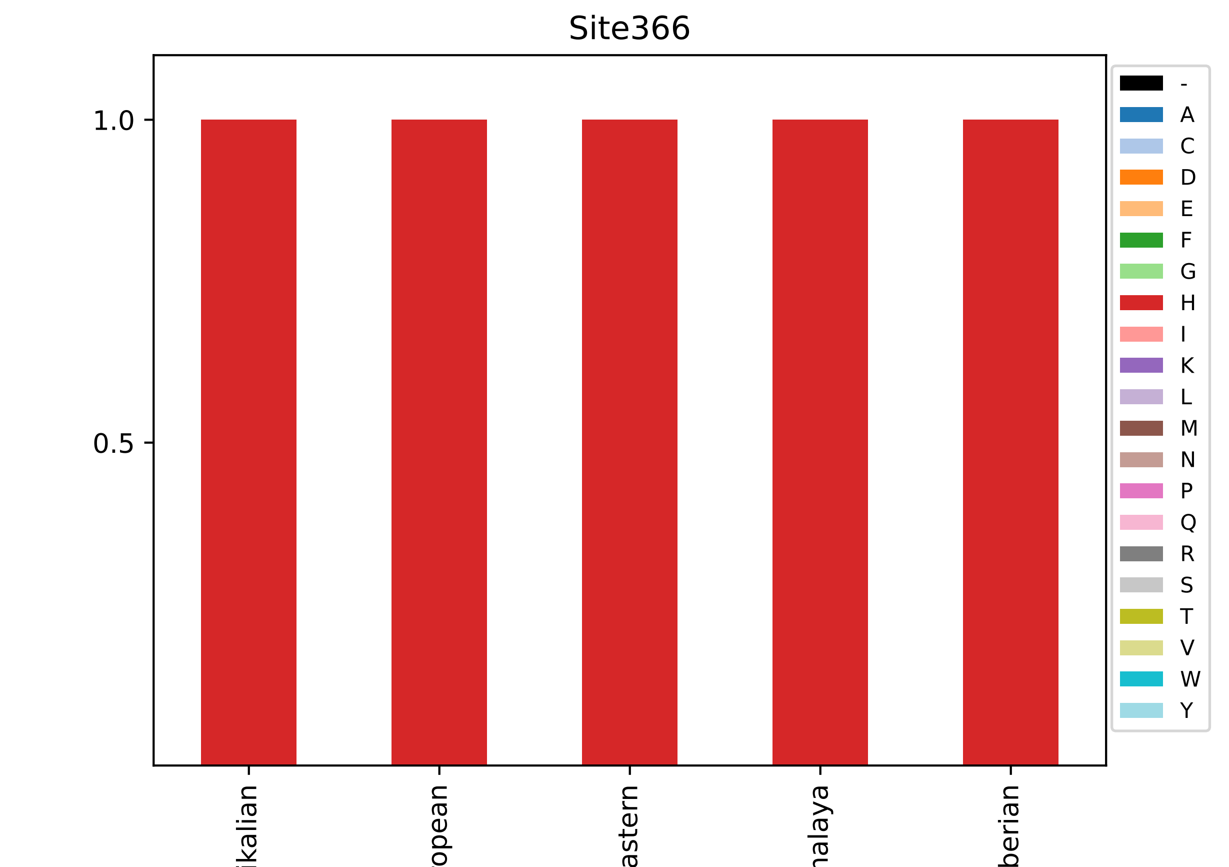
Site366
1.0
0.5
-
A
C
D
E
F
G
H
I
K
L
M
N
P
Q
R
S
T
V
W
Y
ikalian
opean
astern
nalaya
berian

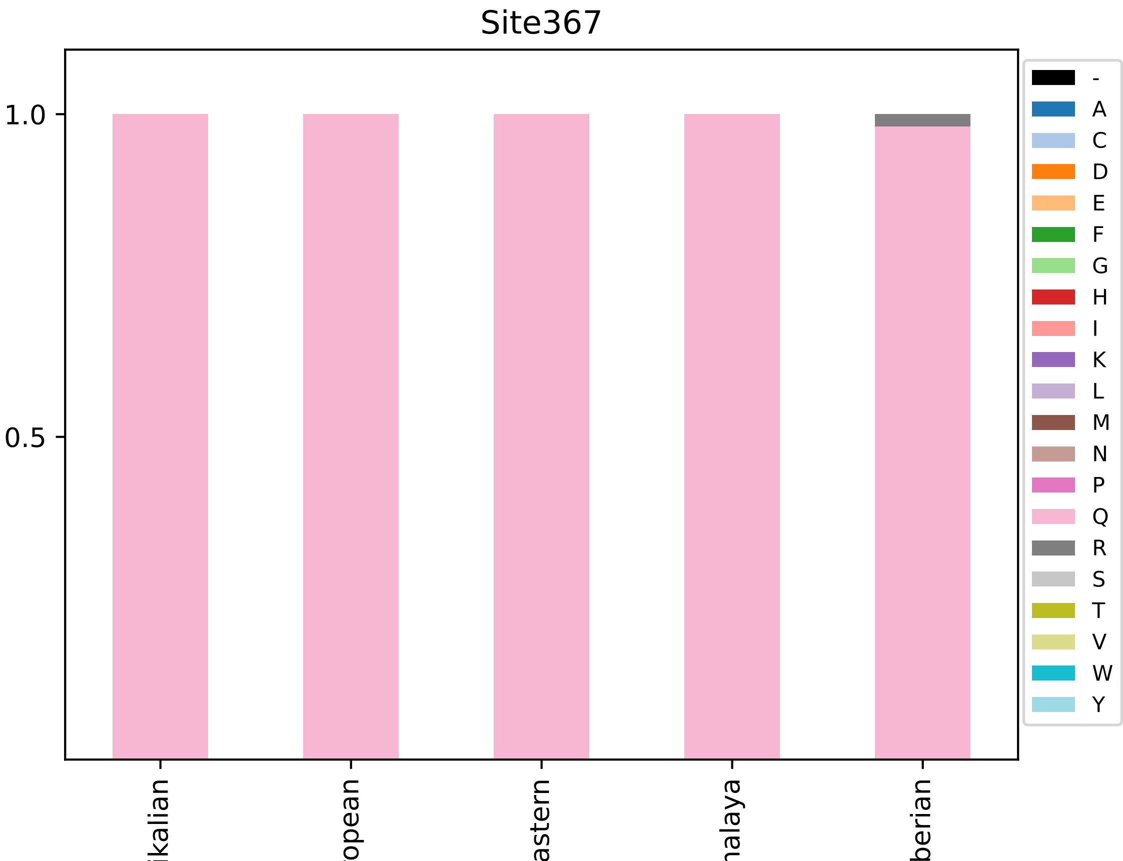

Site367
1.0
0.5
-
A
C
D
E
F
G
H
I
K
L
M
N
P
Q
R
S
T
V
W
Y
ikalian
opean
astern
nalaya
berian

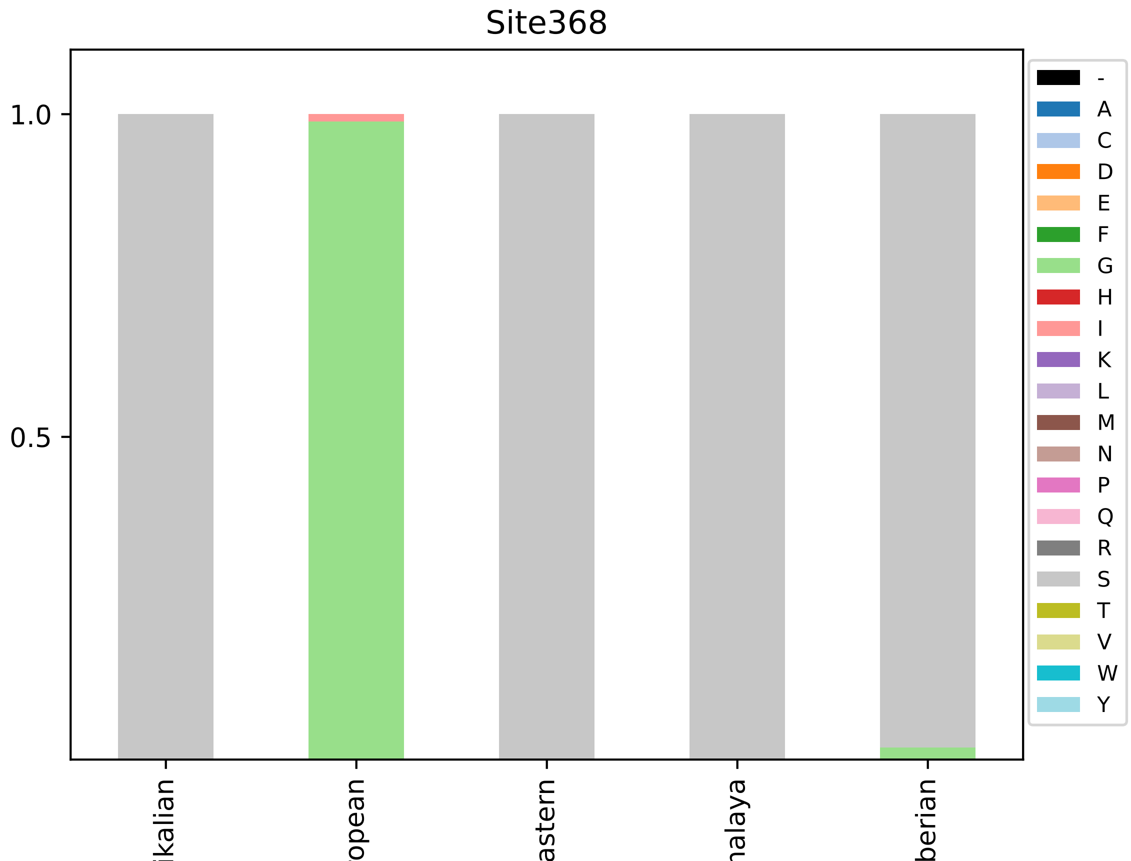
Site368
1.0
0.5
-
A
C
D
E
F
G
H
I
K
L
M
N
P
Q
R
S
T
V
W
Y
ikalian
opean
astern
nalaya
berian

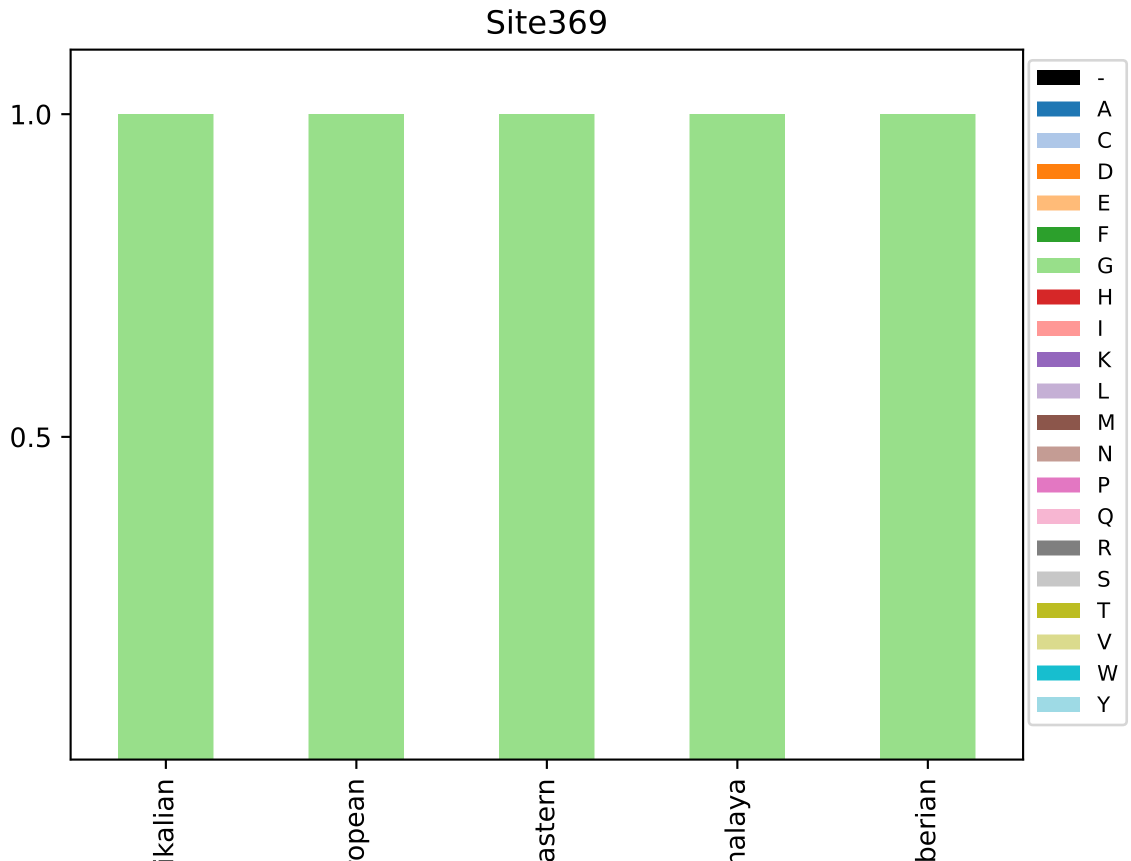
Site369
1.0
0.5
ikalian
opean
astern
nalaya
berian
-
A
C
D
E
F
G
H
I
K
L
M
N
P
Q
R
S
T
V
W
Y

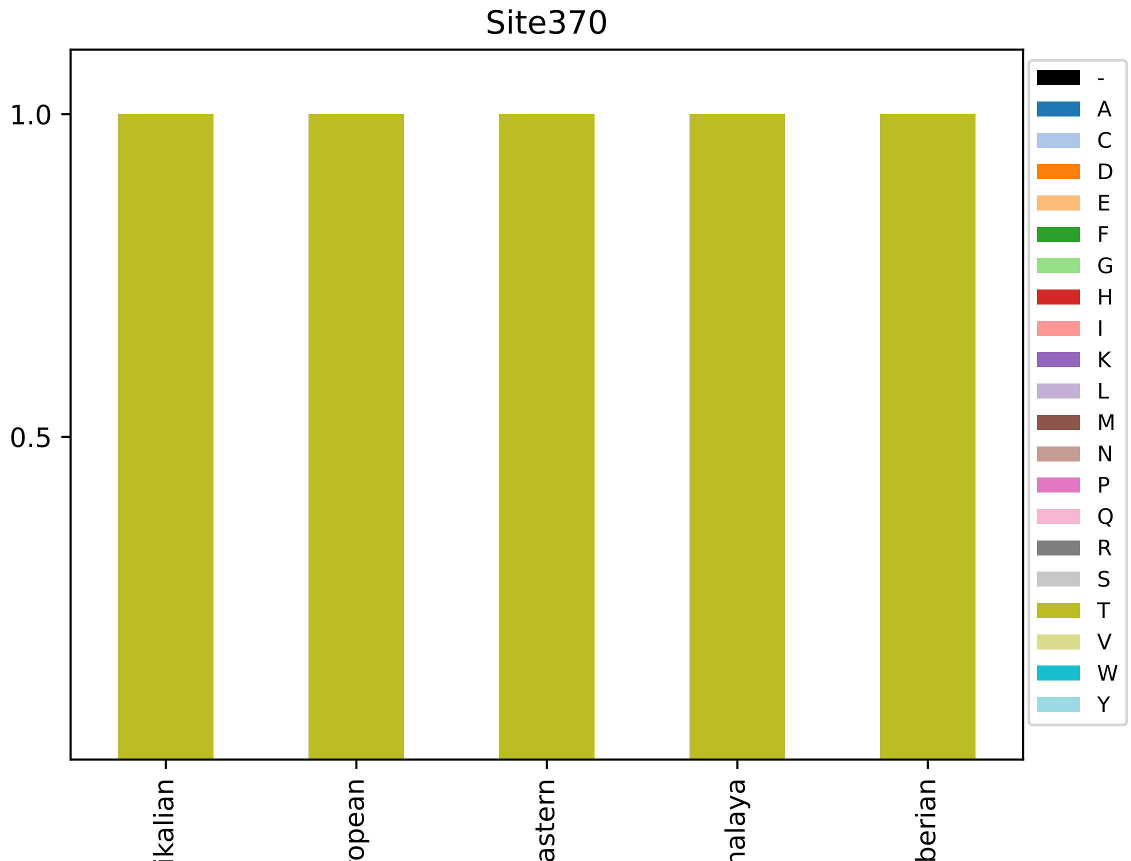
Site370
1.0
0.5
-
A
C
D
E
F
G
H
I
K
L
M
N
P
Q
R
S
T
V
W
Y
ikalian
opean
astern
nalaya
berian

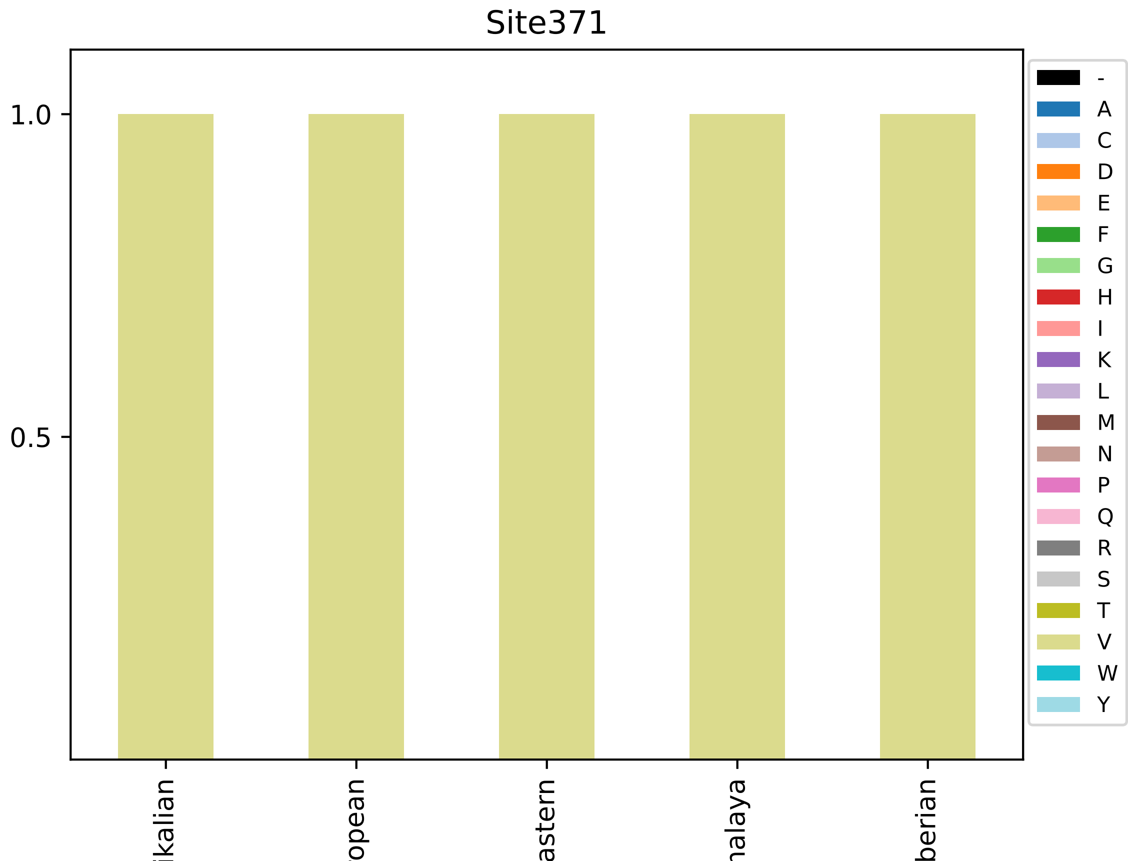

Site371
1.0
0.5
-
A
C
D
E
F
G
H
I
K
L
M
N
P
Q
R
S
T
V
W
Y
ikalian
ropean
astern
nalaya
berian

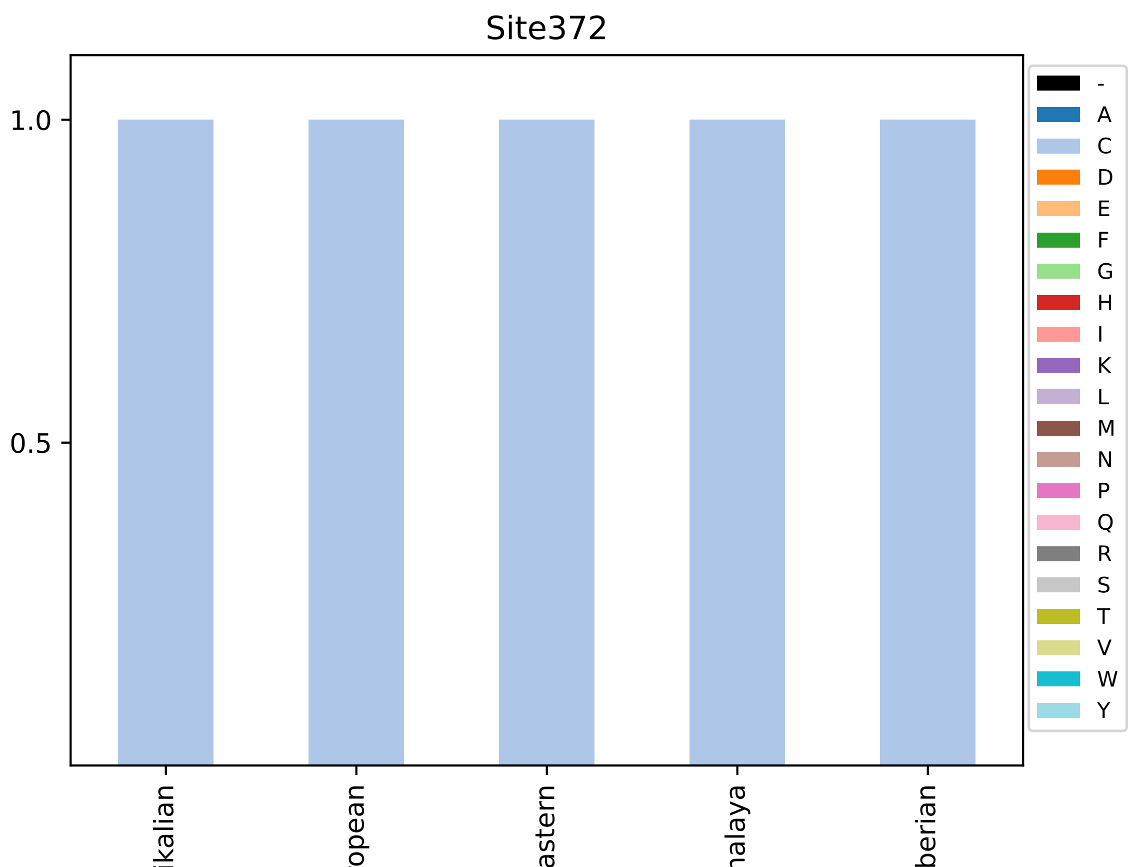
Site372
1.0
0.5
-
A
C
D
E
F
G
H
I
K
L
M
N
P
Q
R
S
T
V
W
Y
ikalian
opean
astern
halaya
berian

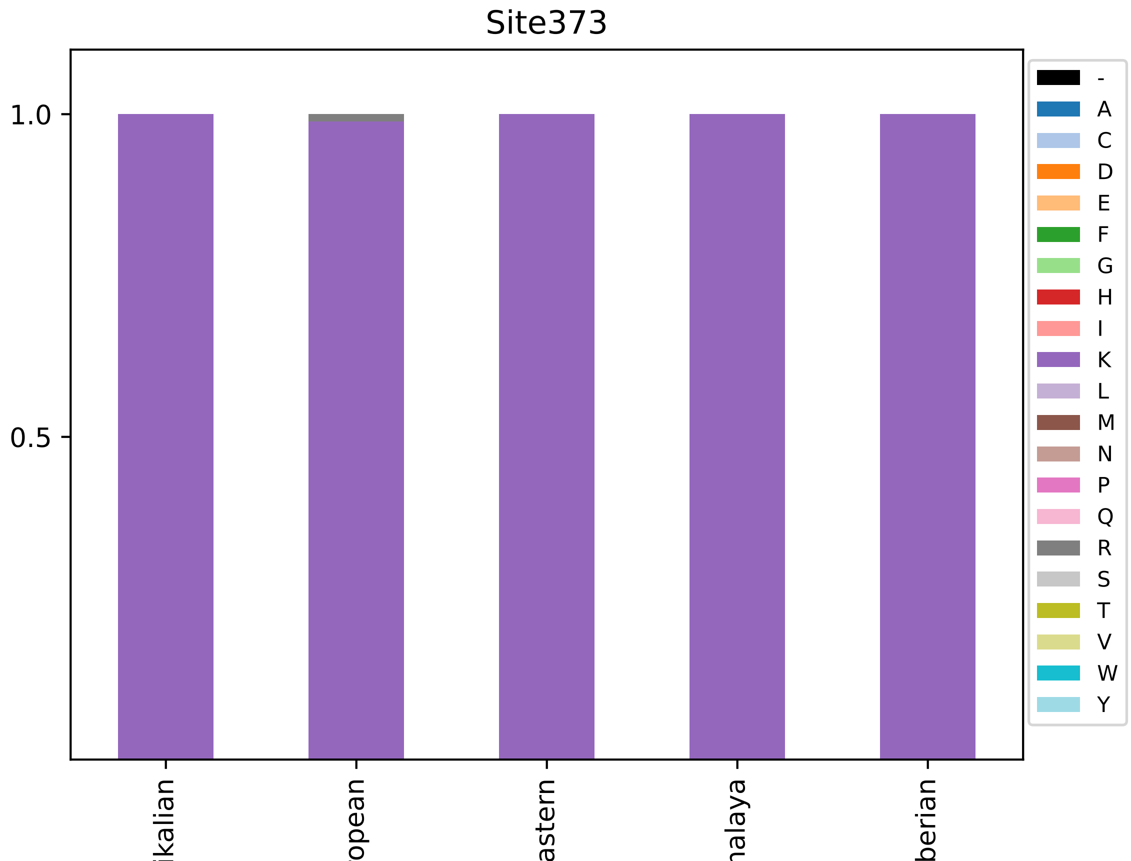
Site373
1.0
0.5
-
A
C
D
E
F
G
H
I
K
L
M
N
P
Q
R
S
T
V
W
Y

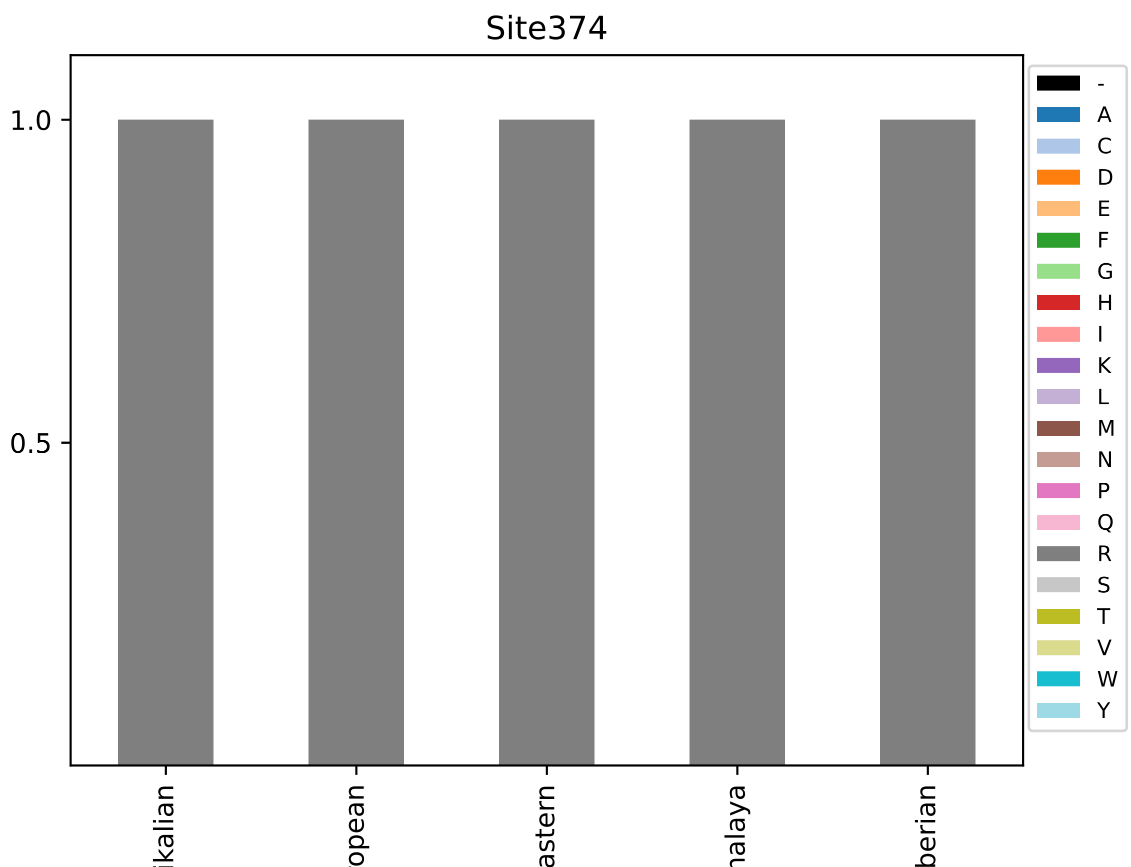

Site374
1.0
0.5
-
A
C
D
E
F
G
H
I
K
L
M
N
P
Q
R
S
T
V
W
Y
ikalian
opean
astern
nalaya
berian

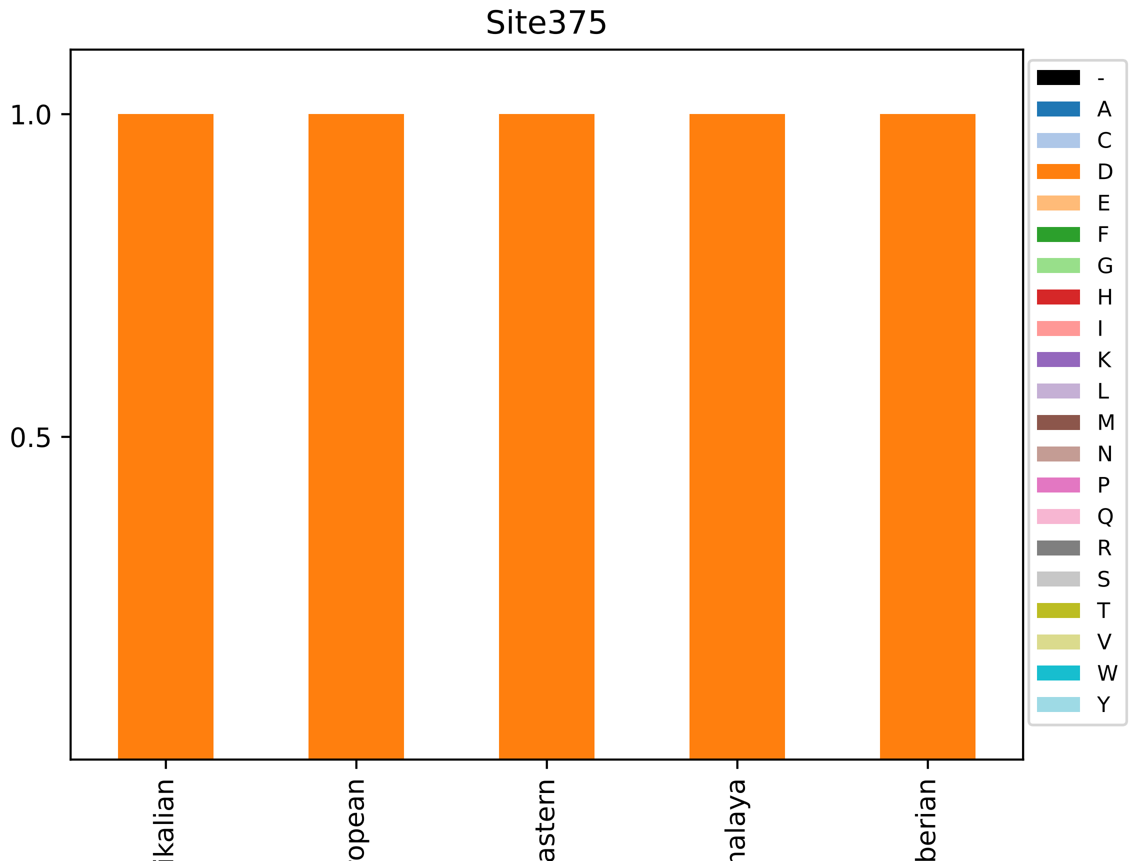
Site375
1.0
0.5
-
A
C
D
E
F
G
H
I
K
L
M
N
P
Q
R
S
T
V
W
Y
ikalian
opean
astern
nalaya
berian

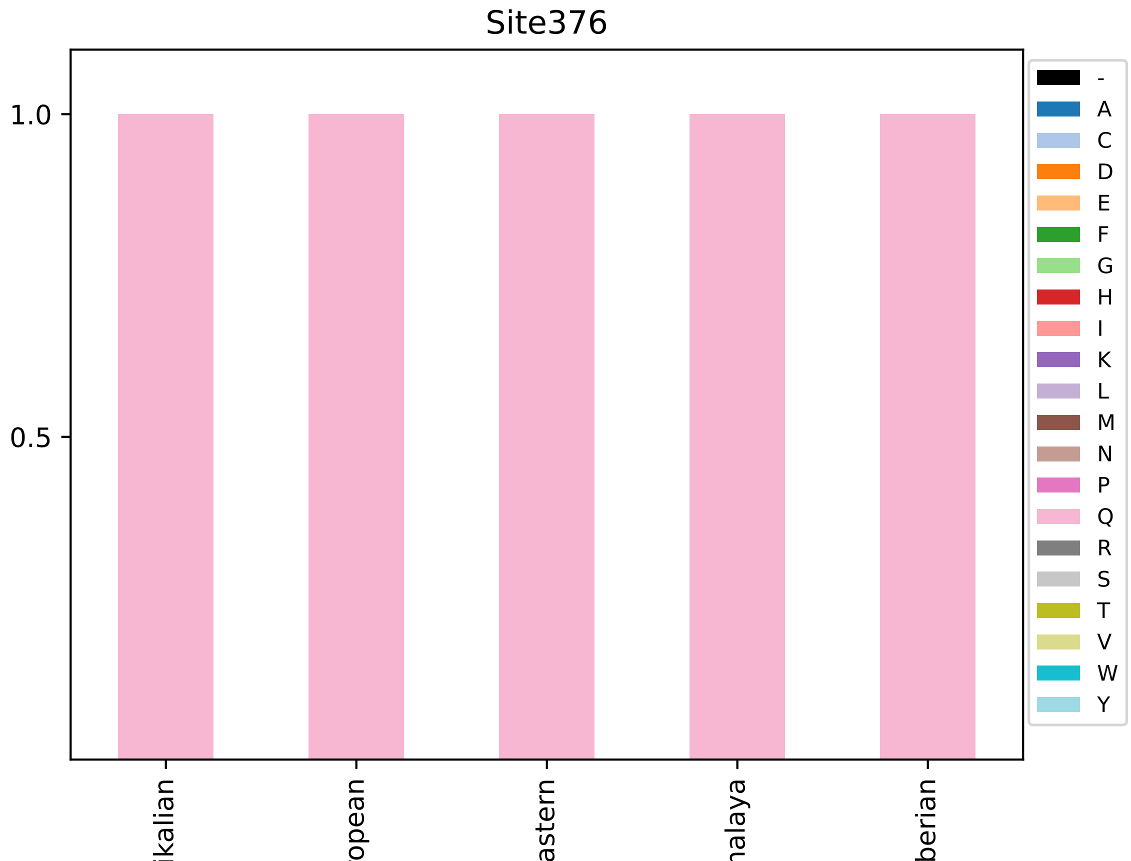
Site376
1.0
0.5
-
A
C
D
E
F
G
H
I
K
L
M
N
P
Q
R
S
T
V
W
Y
ikalian
opean
astern
nalaya
berian

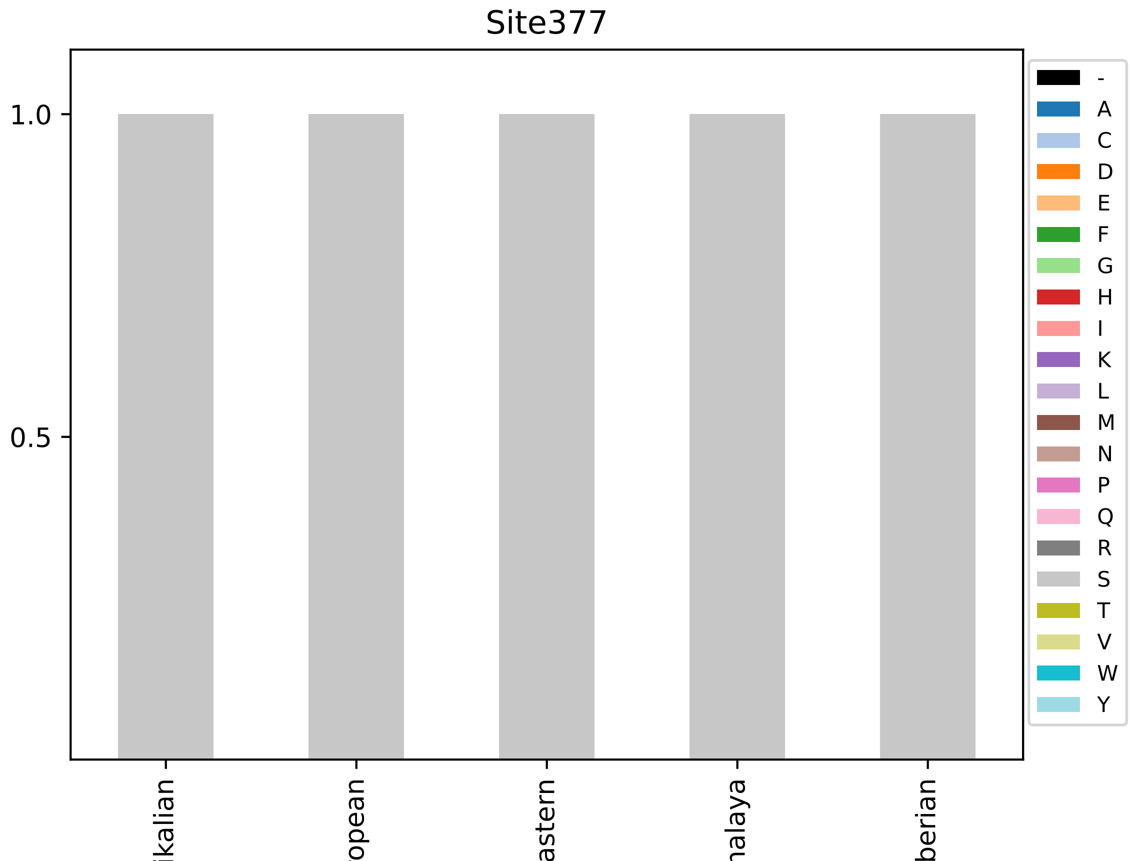
Site377
1.0
0.5
-
A
C
D
E
F
G
H
I
K
L
M
N
P
Q
R
S
T
V
W
Y
ikalian
opean
astern
nalaya
berian

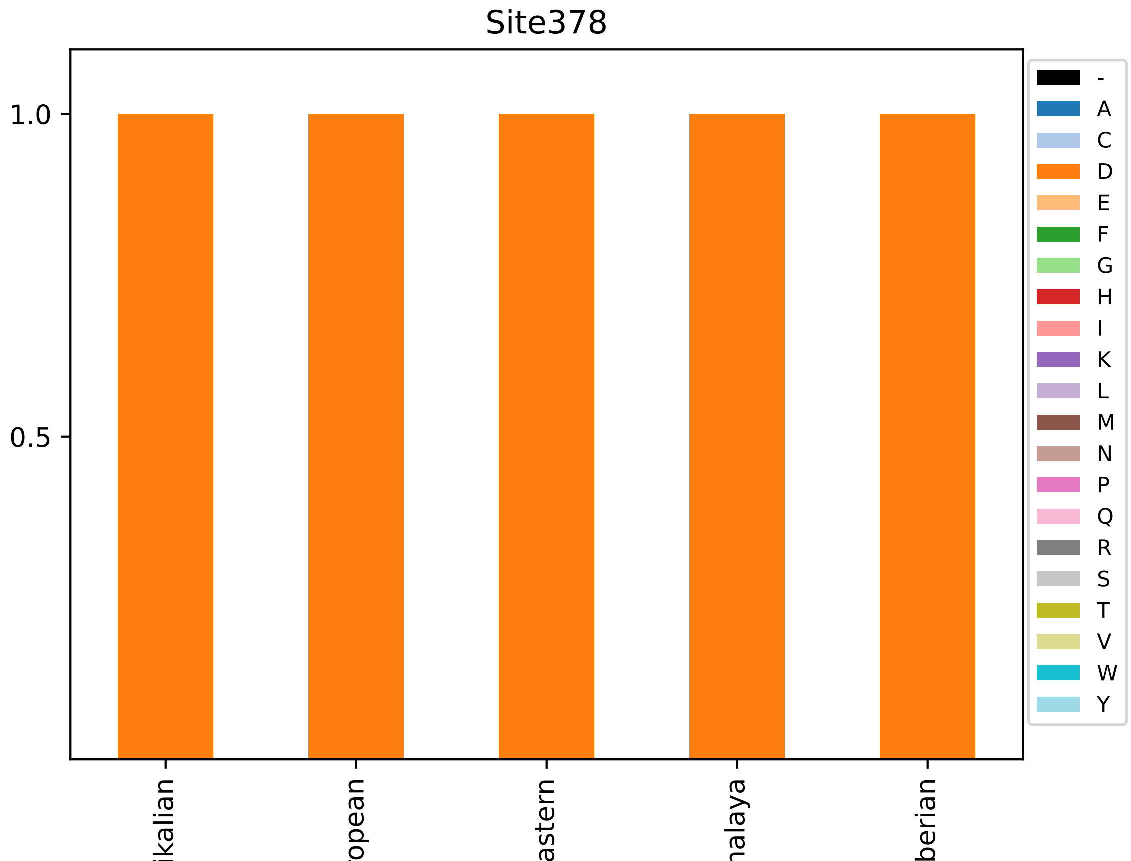

Site378
1.0
0.5
-
A
C
D
E
F
G
H
I
K
L
M
N
P
Q
R
S
T
V
W
Y
ikalian
opean
astern
nalaya
berian

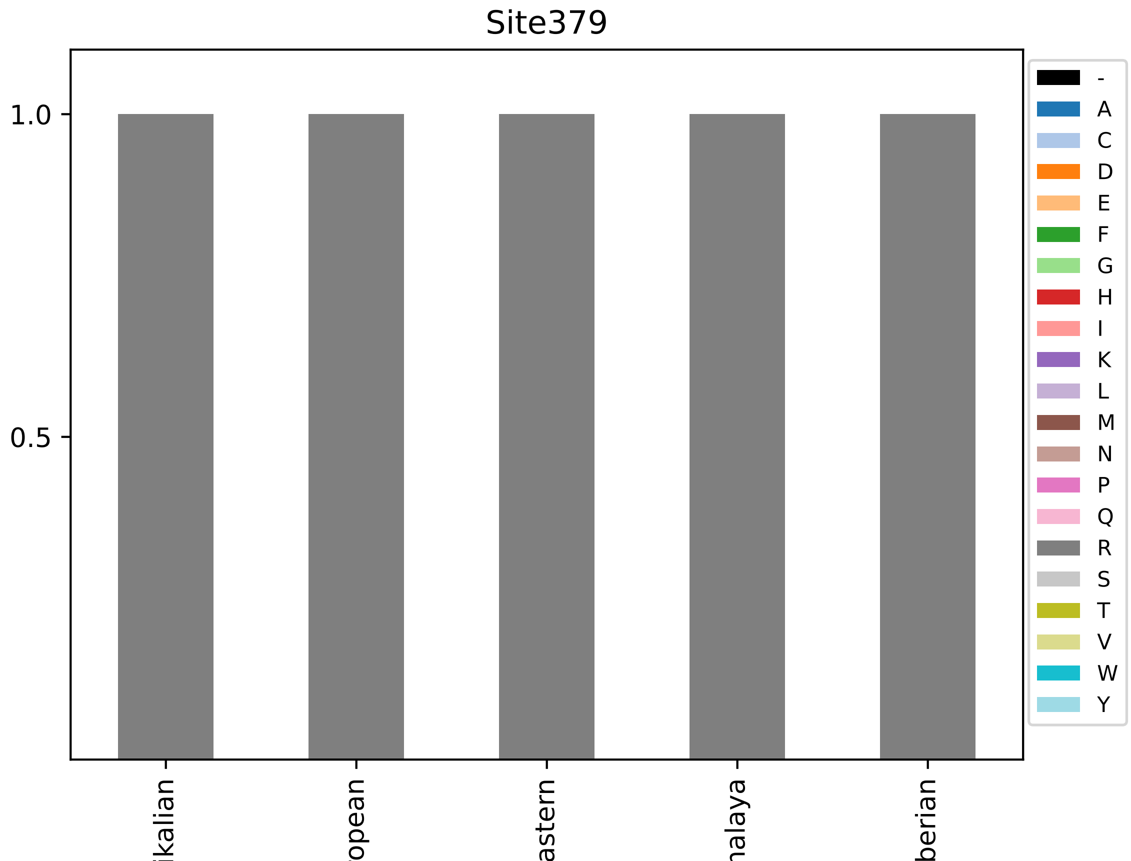
Site379
1.0
0.5
-
A
C
D
E
F
G
H
I
K
L
M
N
P
Q
R
S
T
V
W
Y
ikalian
opean
astern
nalaya
berian

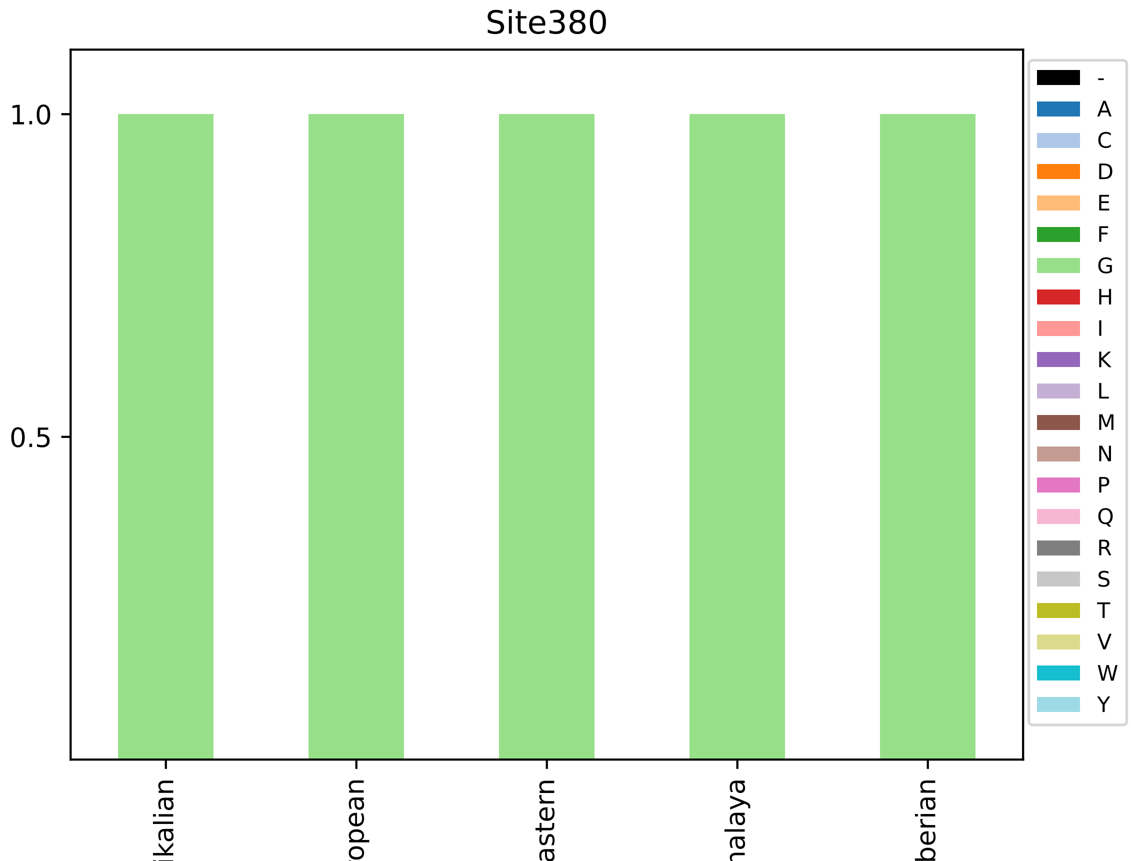

Site380
1.0
0.5
-
A
C
D
E
F
G
H
I
K
L
M
N
P
Q
R
S
T
V
W
Y
ikalian
opean
astern
nalaya
berian

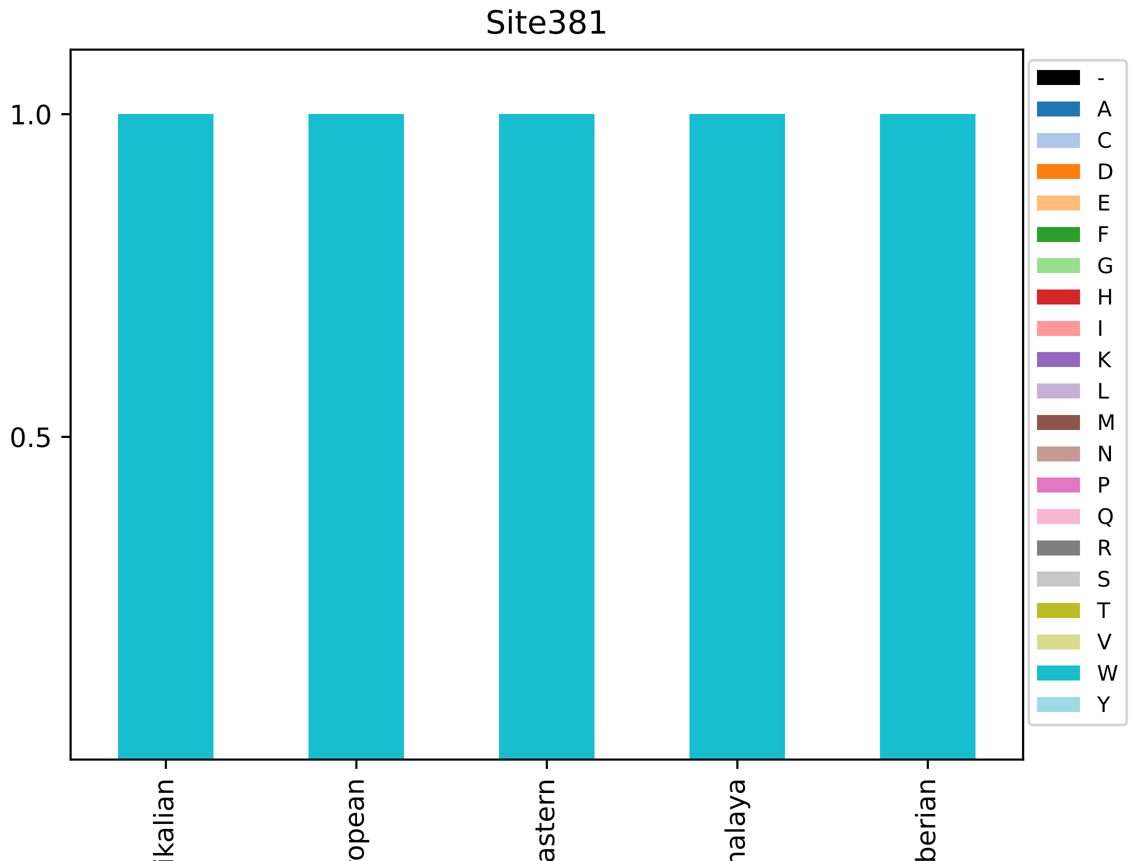

Site381
1.0
0.5
-
A
C
D
E
F
G
H
I
K
L
M
N
P
Q
R
S
T
V
W
Y
ikalian
opean
astern
nalaya
berian

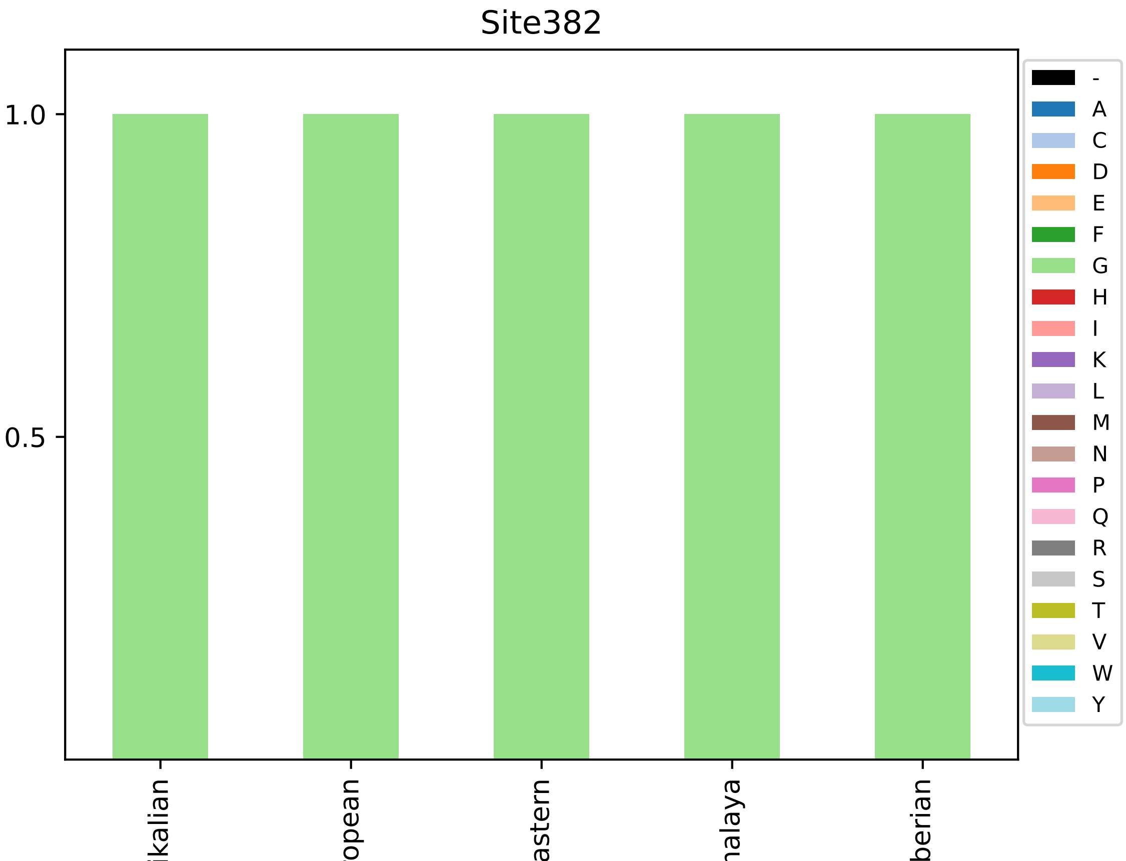
Site382
1.0
0.5
-
A
C
D
E
F
G
H
I
K
L
M
N
P
Q
R
S
T
V
W
Y
ikalian
opean
astern
nalaya
berian

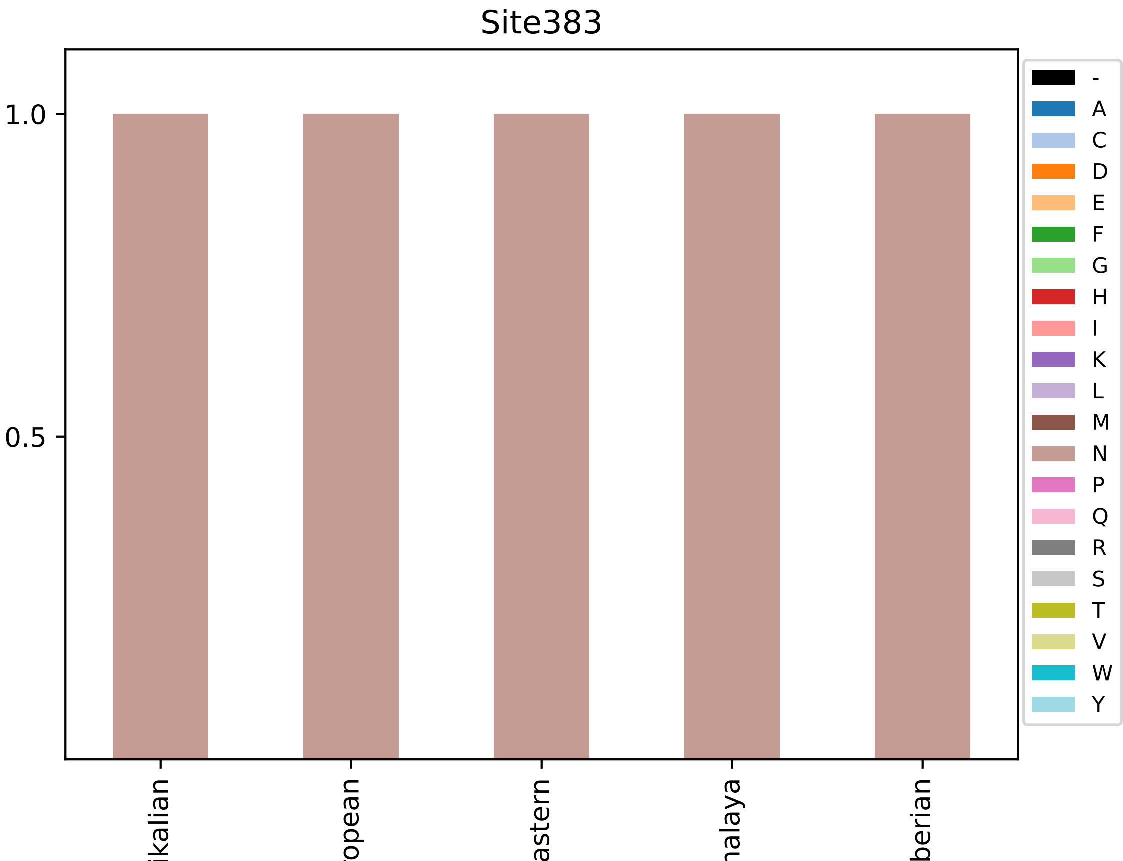
Site383
1.0
0.5
-
A
C
D
E
F
G
H
I
K
L
M
N
P
Q
R
S
T
V
W
Y
ikalian
opean
astern
nalaya
berian

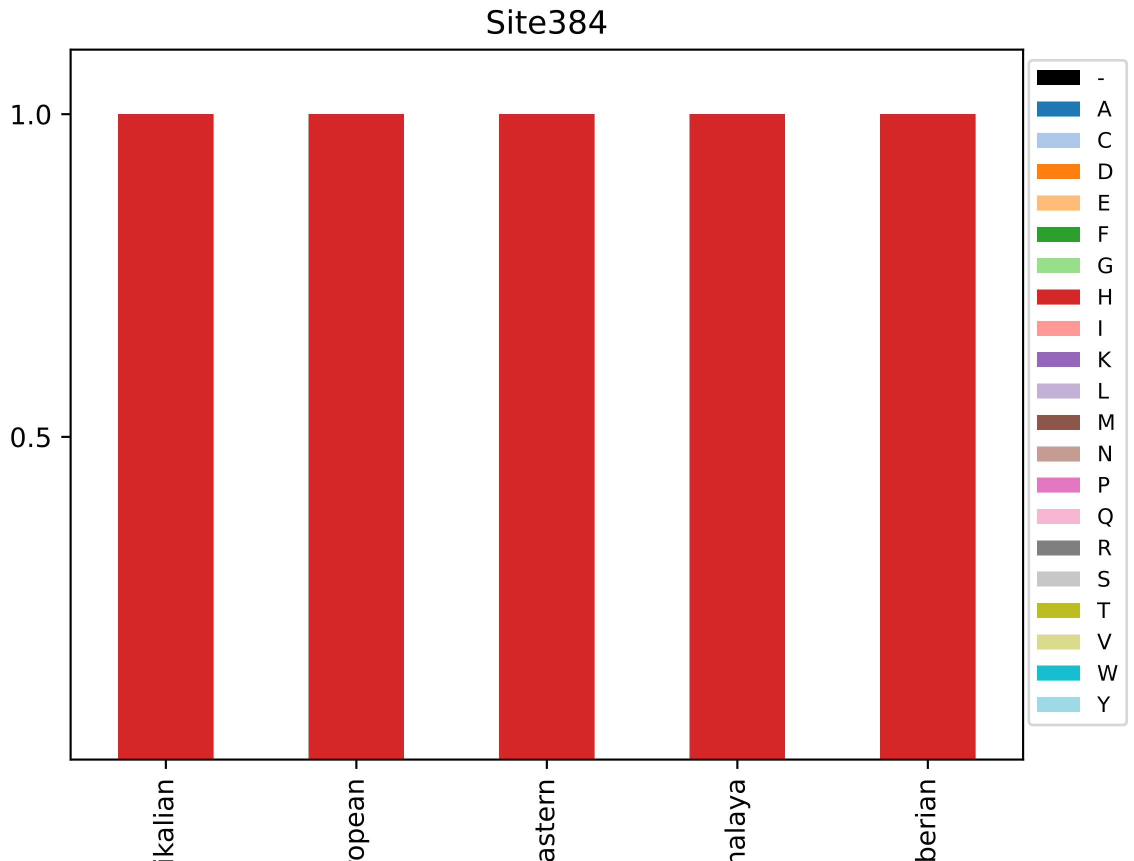

Site384
1.0
0.5
-
A
C
D
E
F
G
H
I
K
L
M
N
P
Q
R
S
T
V
W
Y
ikalian
ropean
astern
nalaya
berian

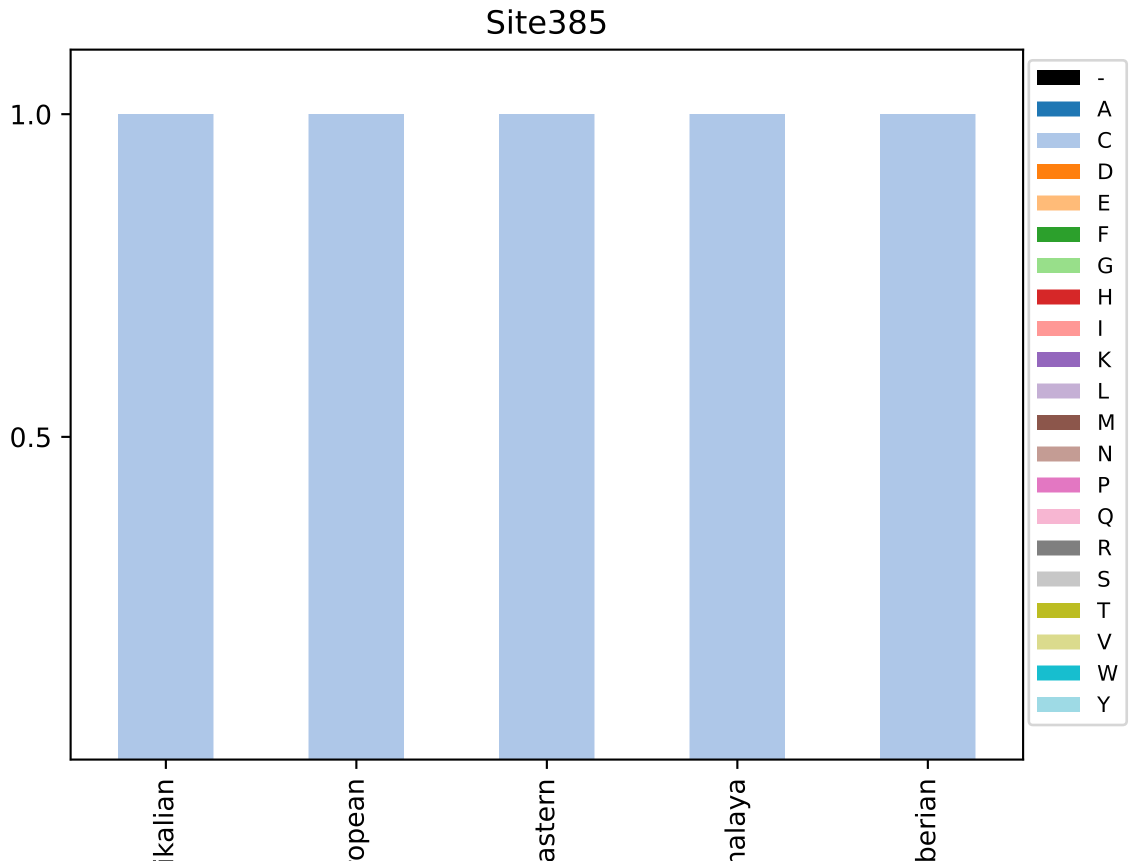
Site385
1.0
0.5
-
A
C
D
E
F
G
H
I
K
L
M
N
P
Q
R
S
T
V
W
Y
ikalian
ropean
astern
nalaya
berian

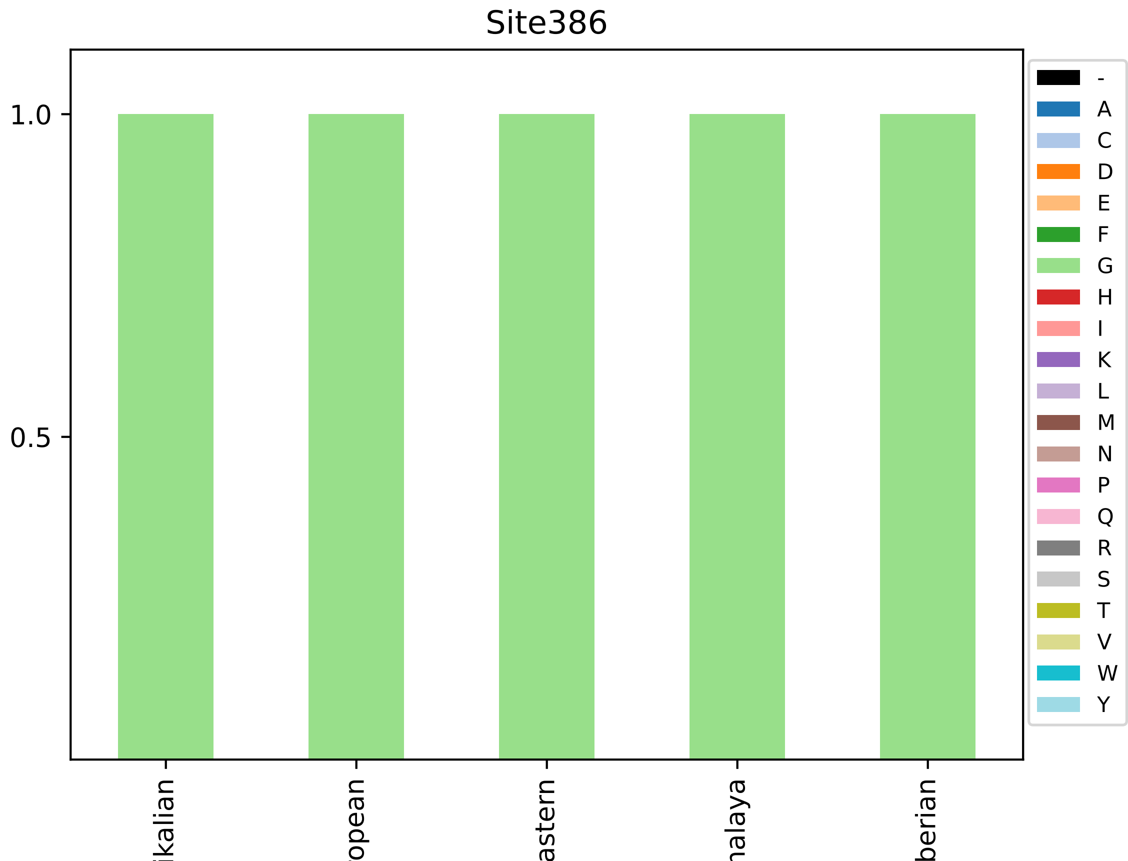

Site386
1.0
0.5
-
A
C
D
E
F
G
H
I
K
L
M
N
P
Q
R
S
T
V
W
Y
ikalian
opean
astern
nalaya
berian

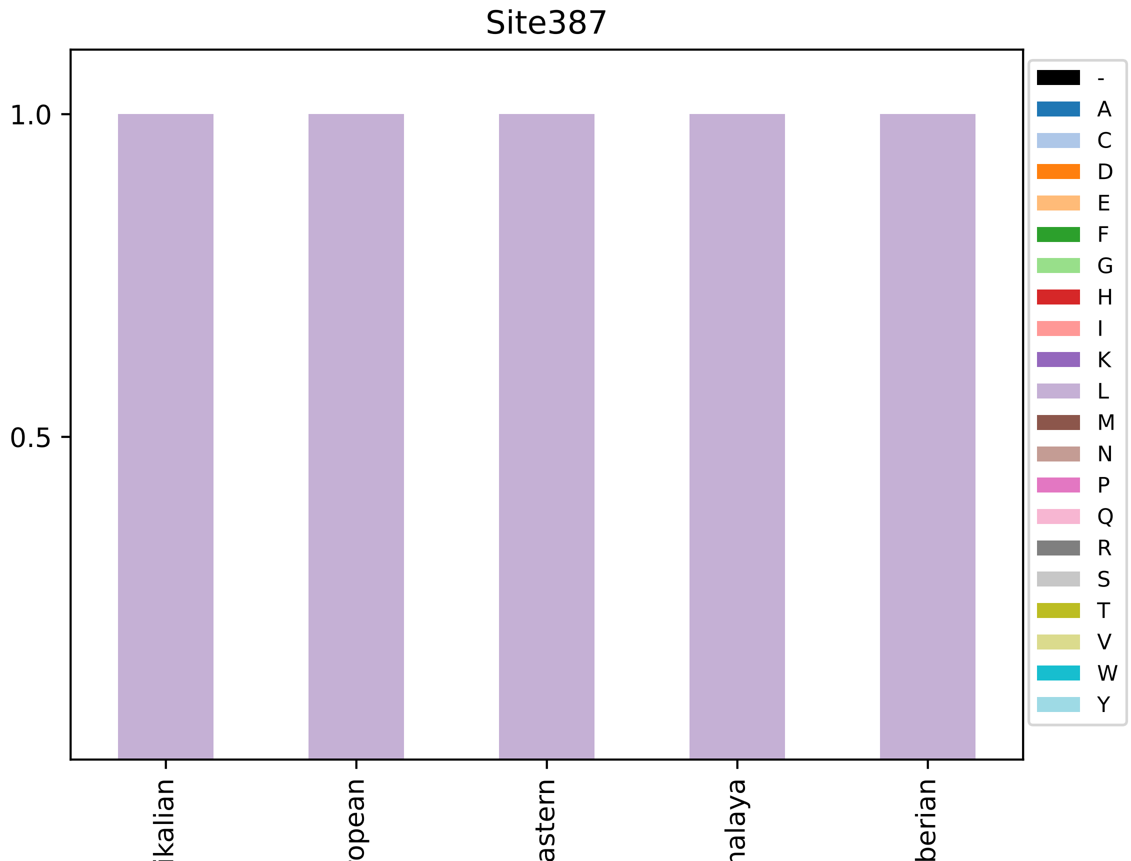

Site387
1.0
0.5
-
A
C
D
E
F
G
H
I
K
L
M
N
P
Q
R
S
T
V
W
Y
ikalian
opean
astern
nalaya
berian

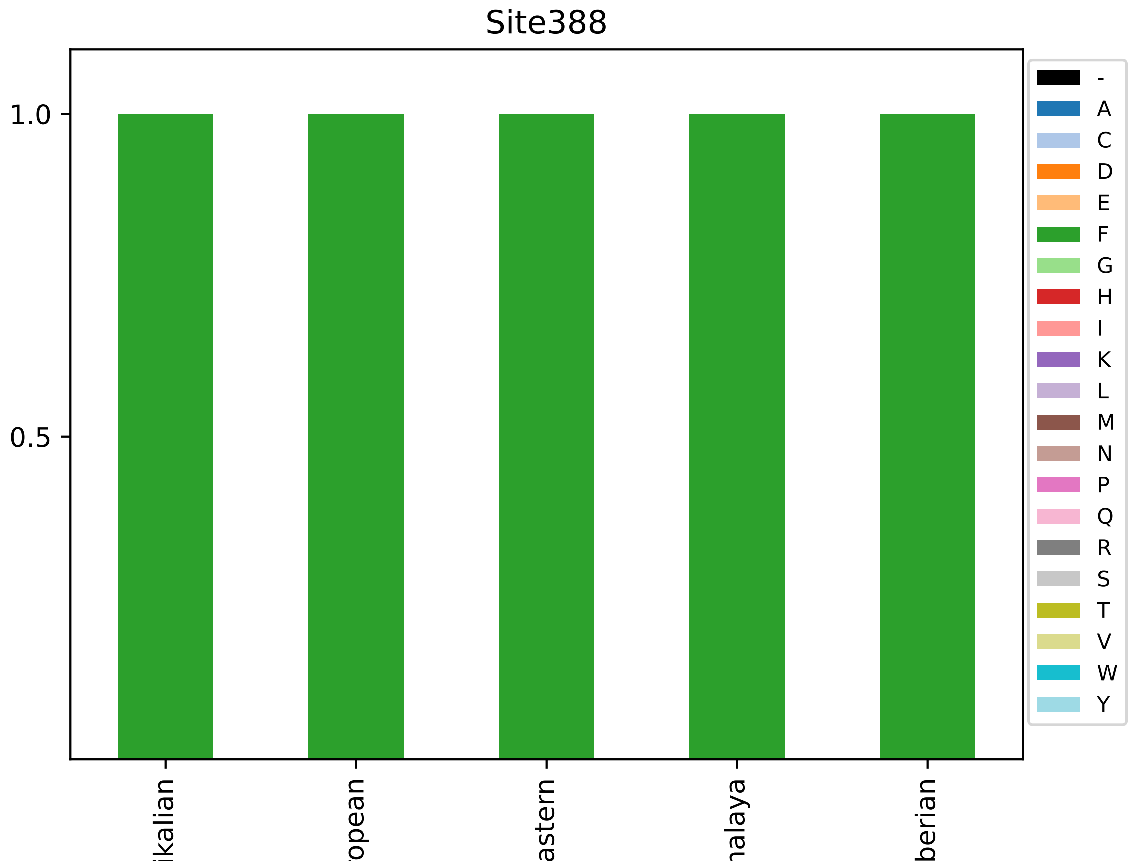

Site388
1.0
0.5
-
A
C
D
E
F
G
H
I
K
L
M
N
P
Q
R
S
T
V
W
Y

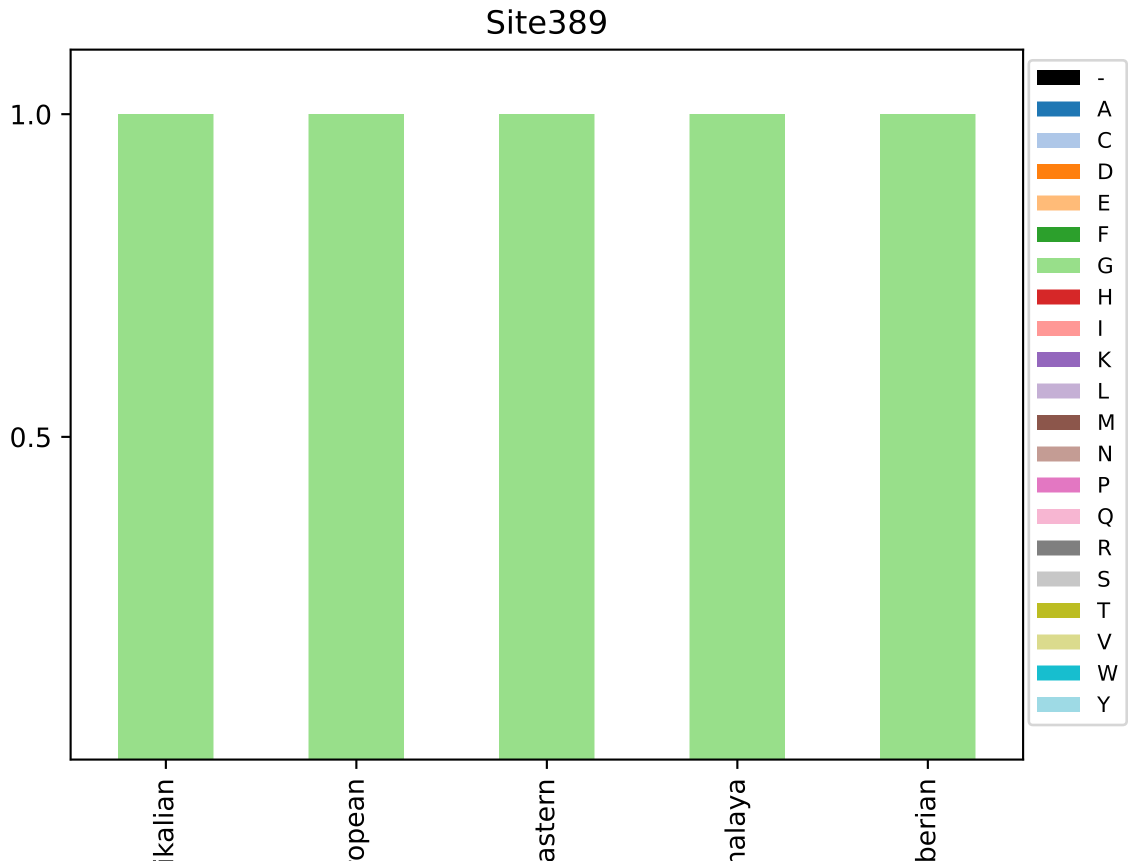
Site389
1.0
0.5
-
A
C
D
E
F
G
H
I
K
L
M
N
P
Q
R
S
T
V
W
Y
ikalian
opean
astern
nalaya
berian

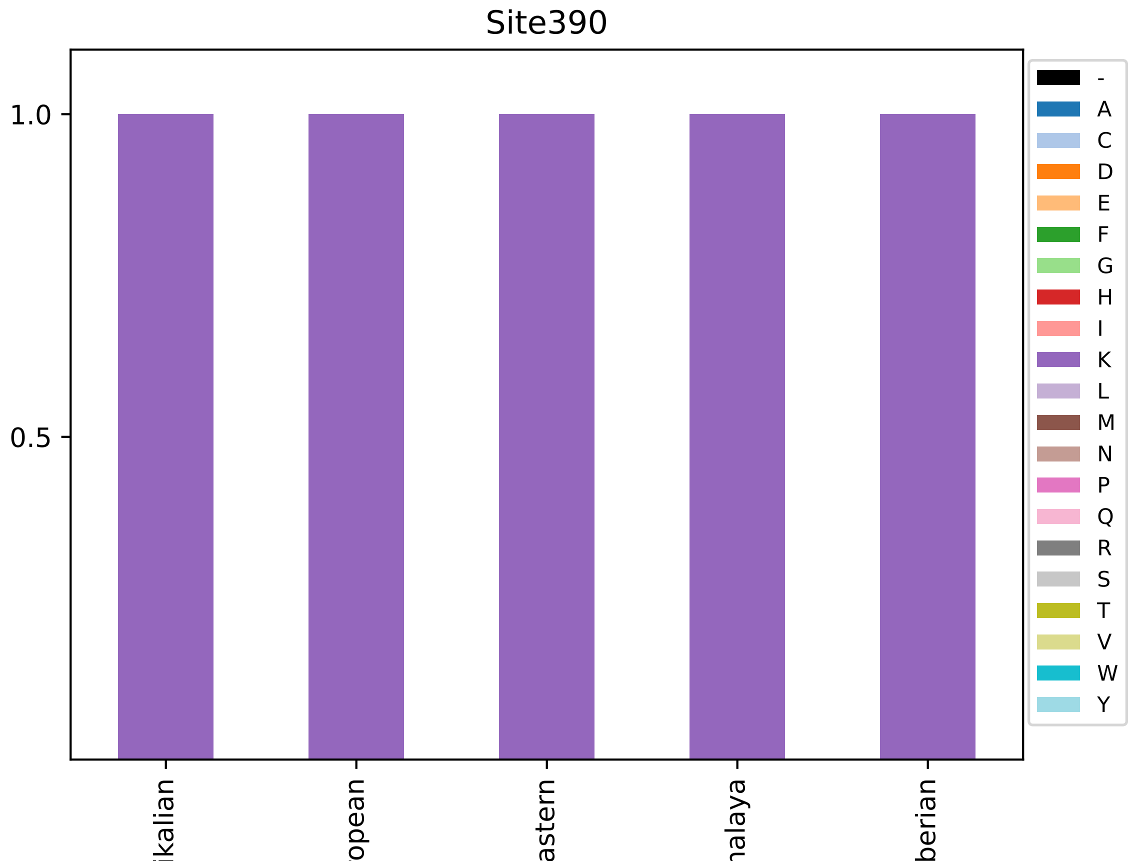

Site390
1.0
0.5
-
A
C
D
E
F
G
H
I
K
L
M
N
P
Q
R
S
T
V
W
Y
ikalian
opean
astern
nalaya
berian

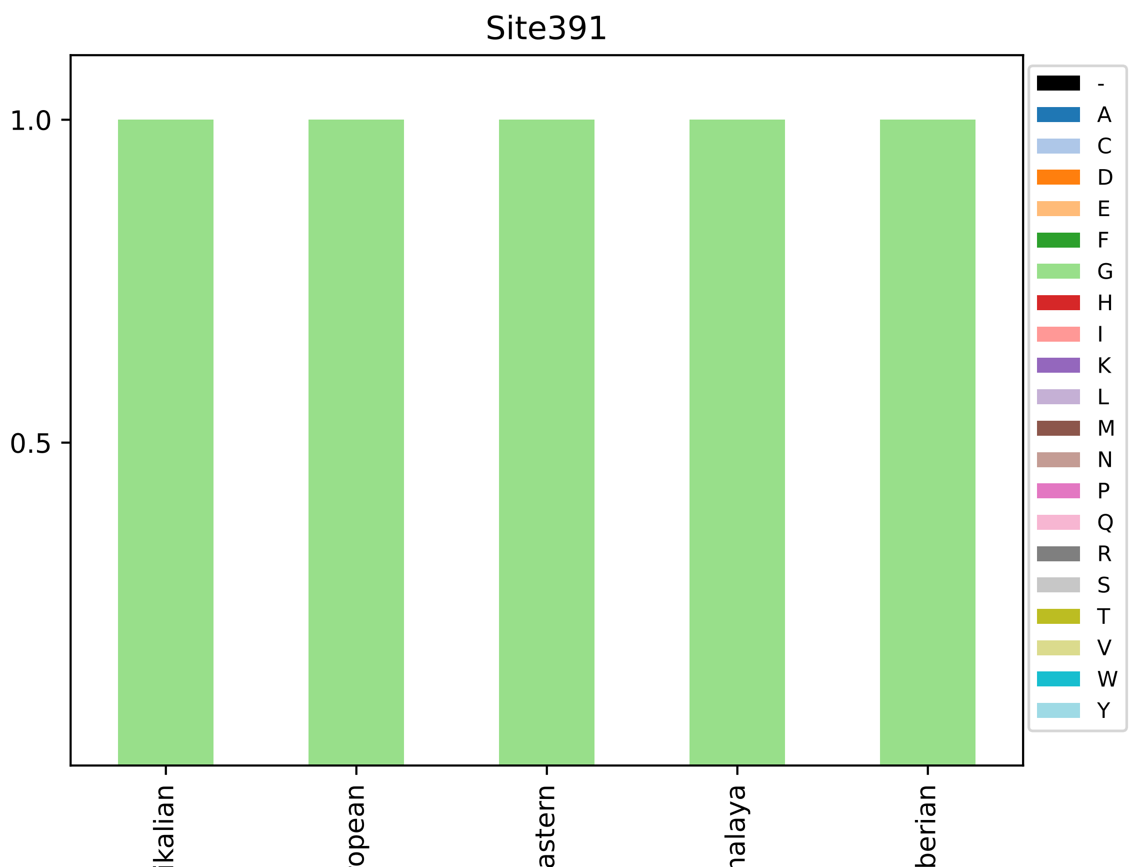

Site391
1.0
0.5
-
A
C
D
E
F
G
H
I
K
L
M
N
P
Q
R
S
T
V
W
Y
ikalian
opean
astern
nalaya
berian

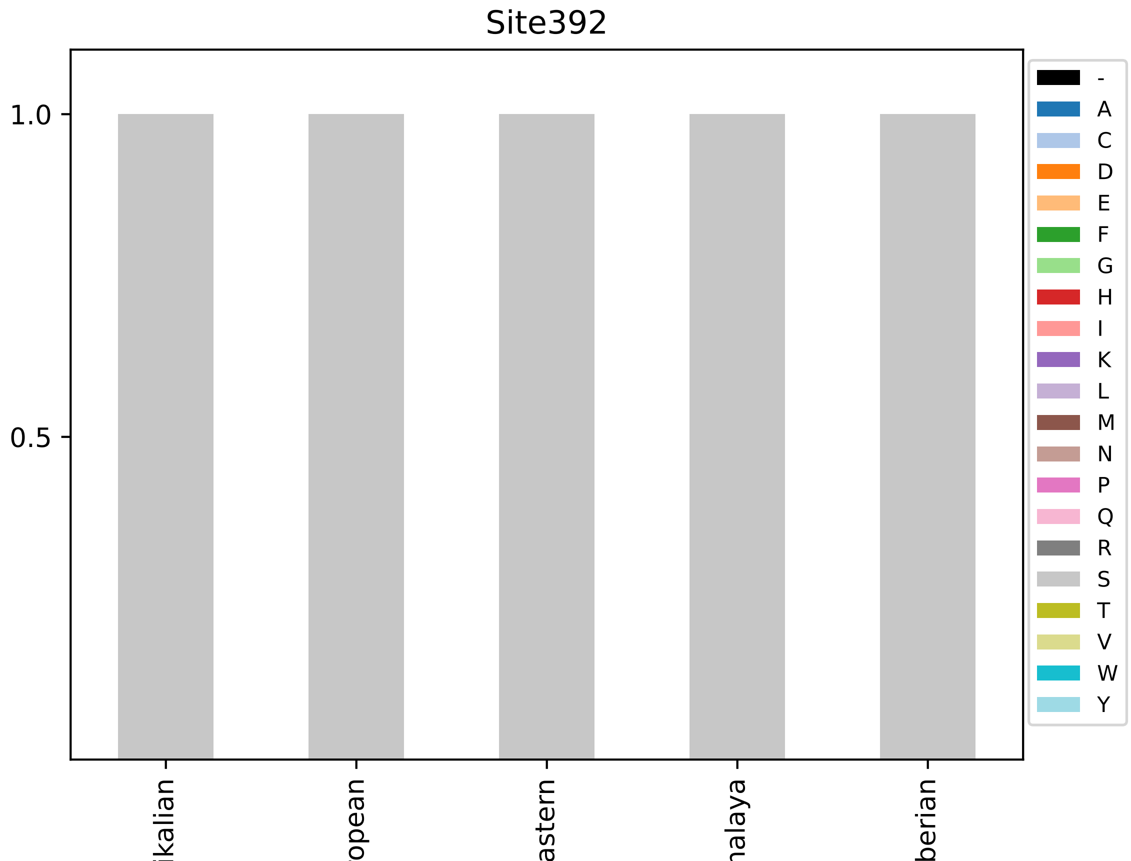

Site392
1.0
0.5
-
A
C
D
E
F
G
H
I
K
L
M
N
P
Q
R
S
T
V
W
Y
ikalian
opean
astern
nalaya
berian

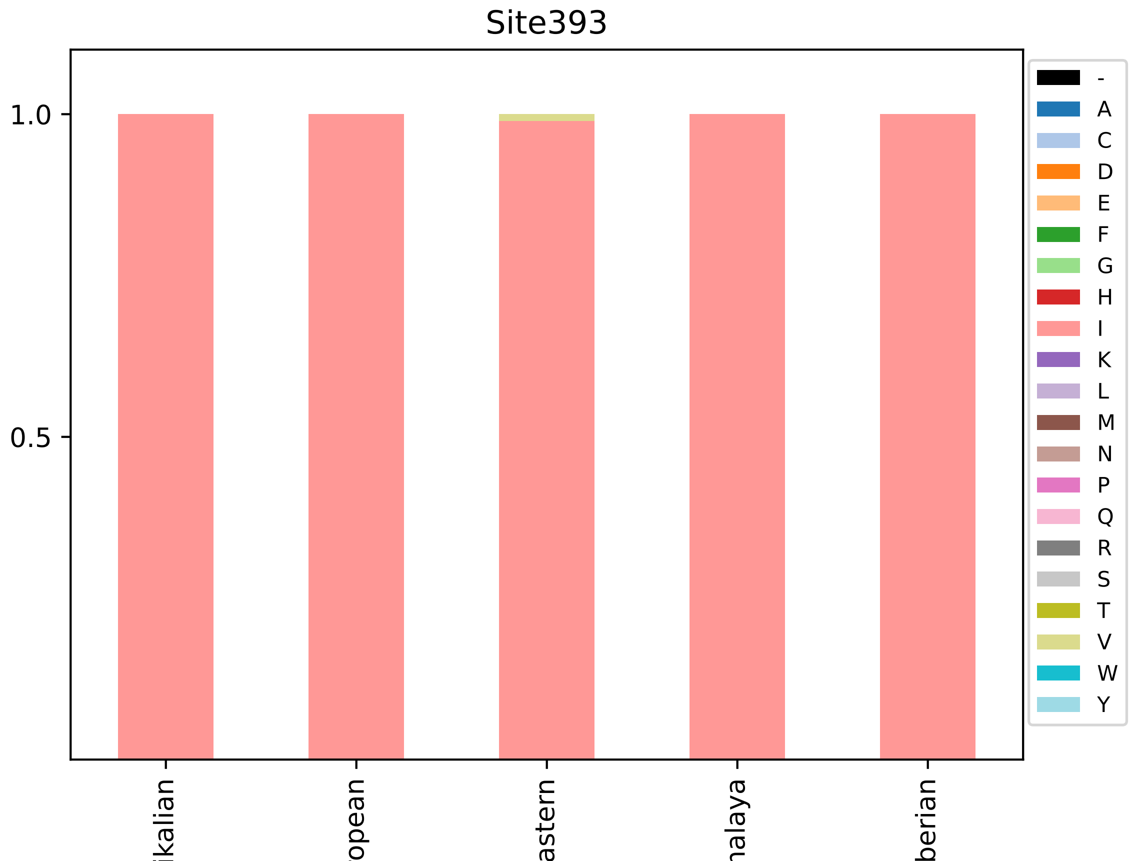
Site393
1.0
0.5
-
A
C
D
E
F
G
H
I
K
L
M
N
P
Q
R
S
T
V
W
Y
ikalian
opean
astern
nalaya
berian

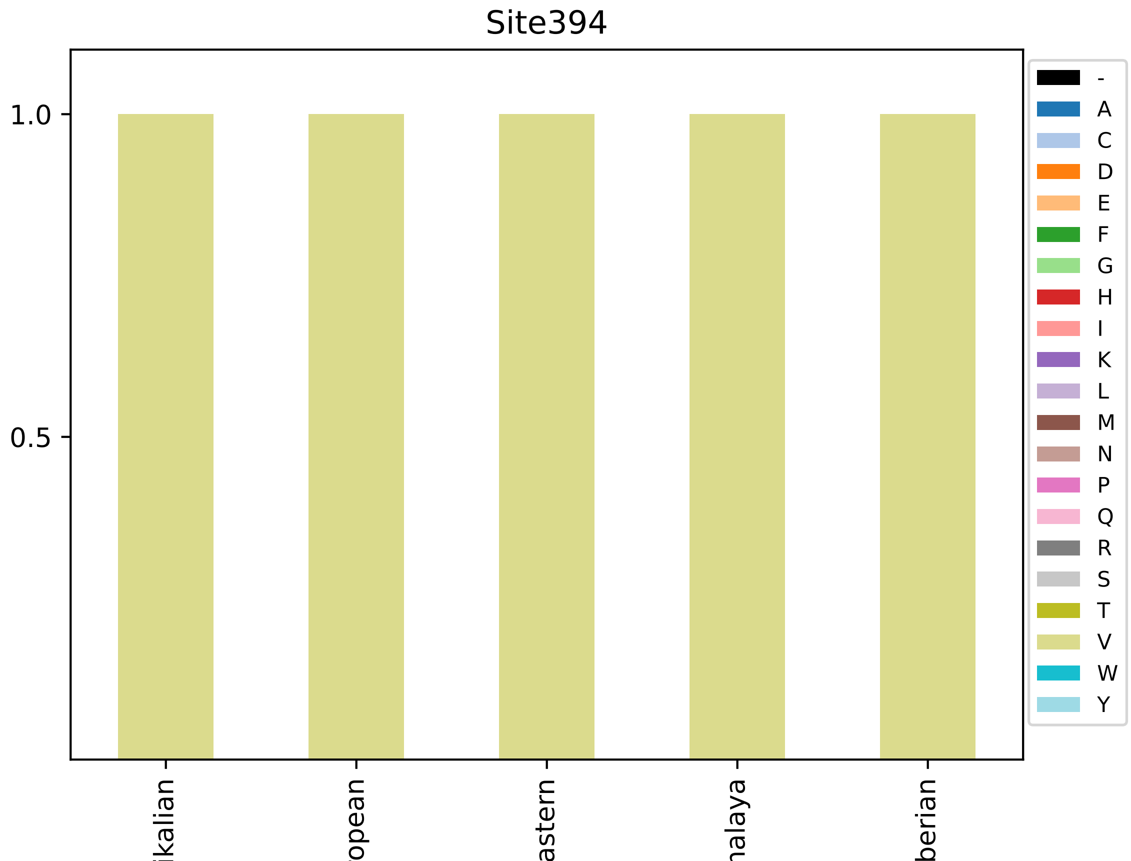
Site394
1.0
0.5
-
A
C
D
E
F
G
H
I
K
L
M
N
P
Q
R
S
T
V
W
Y
ikalian
opean
astern
nalaya
berian

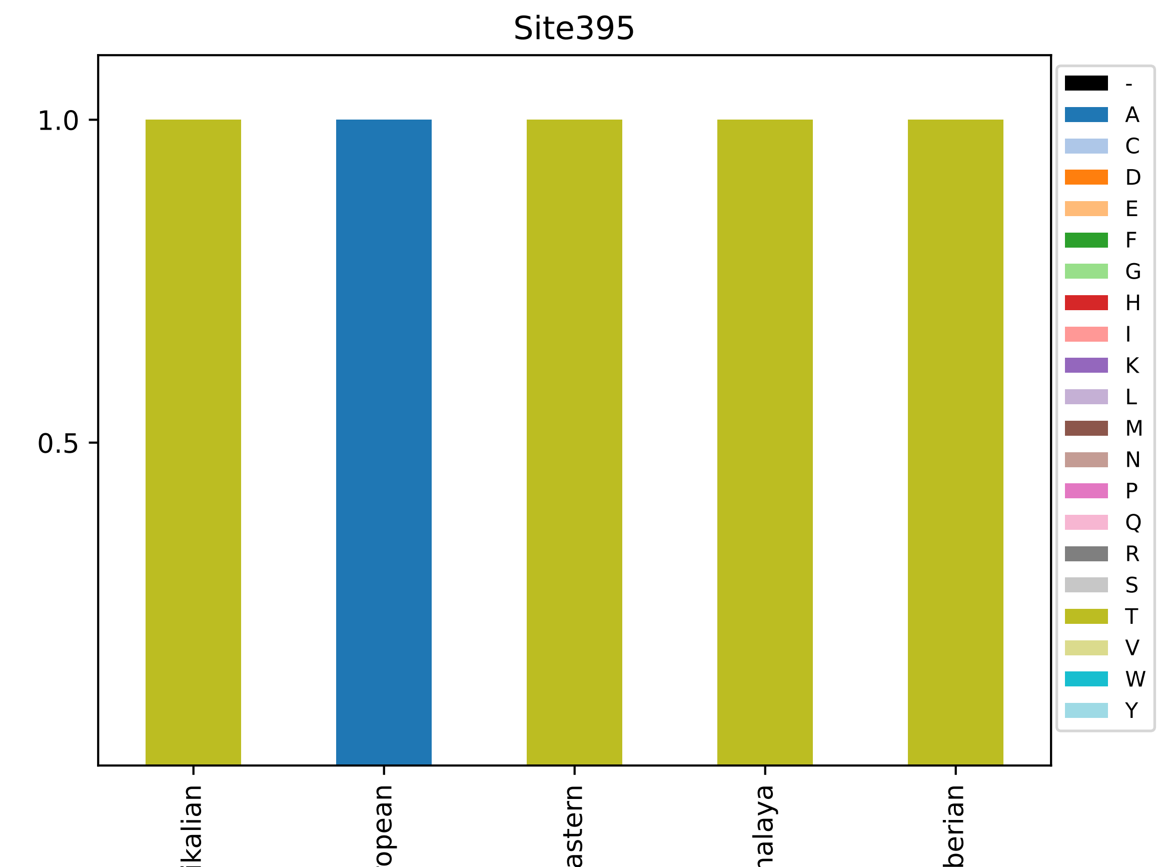
Site395
1.0
0.5
ikalian
opean
astern
nalaya
berian
-
A
C
D
E
F
G
H
I
K
L
M
N
P
Q
R
S
T
V
W
Y

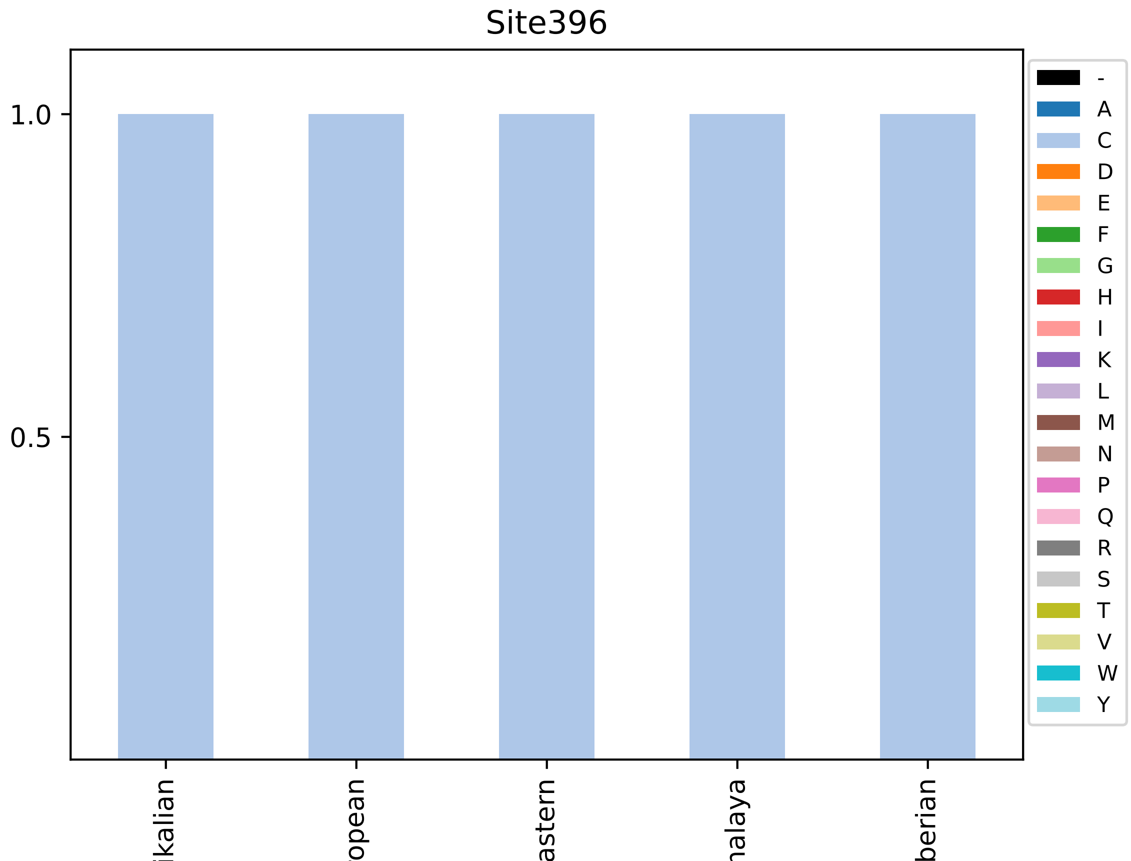

Site396
1.0
0.5
-
A
C
D
E
F
G
H
I
K
L
M
N
P
Q
R
S
T
V
W
Y
ikalian
opean
astern
nalaya
berian

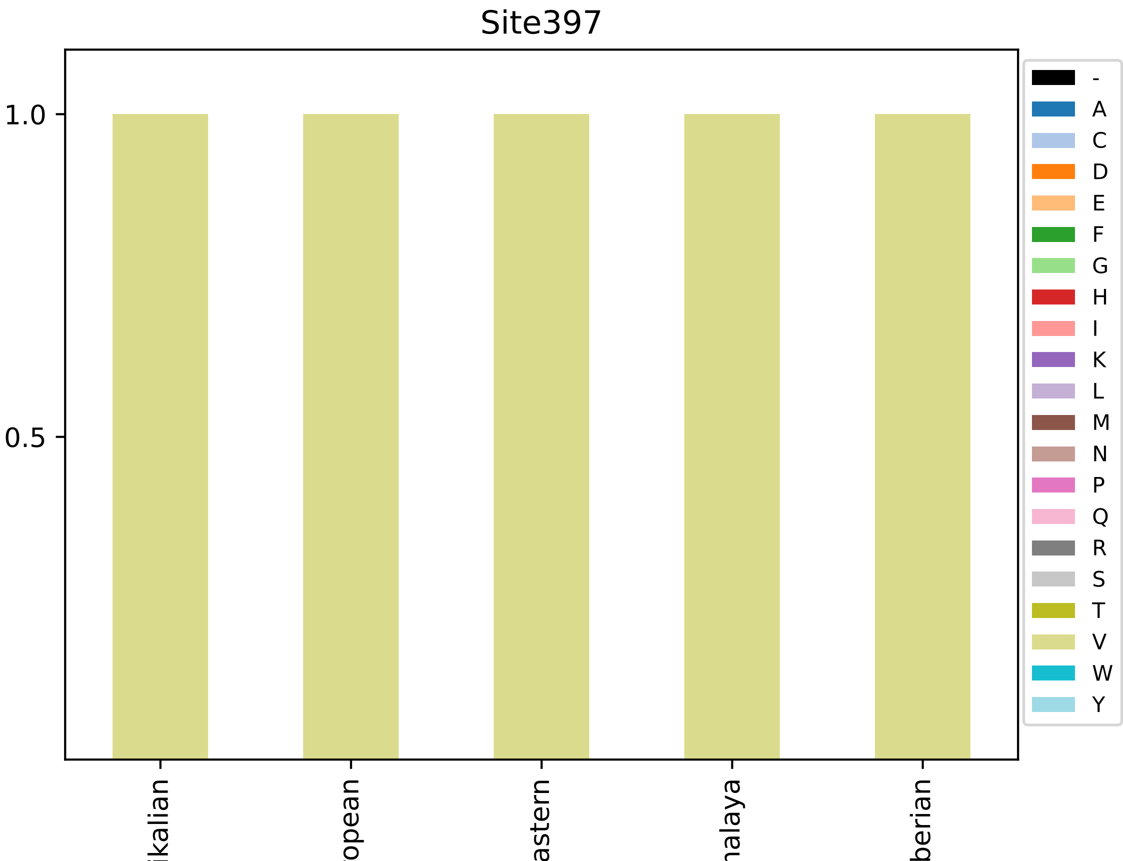
Site397
1.0
0.5
-
A
C
D
E
F
G
H
I
K
L
M
N
P
Q
R
S
T
V
W
Y
ikalian
ropean
astern
nalaya
berian

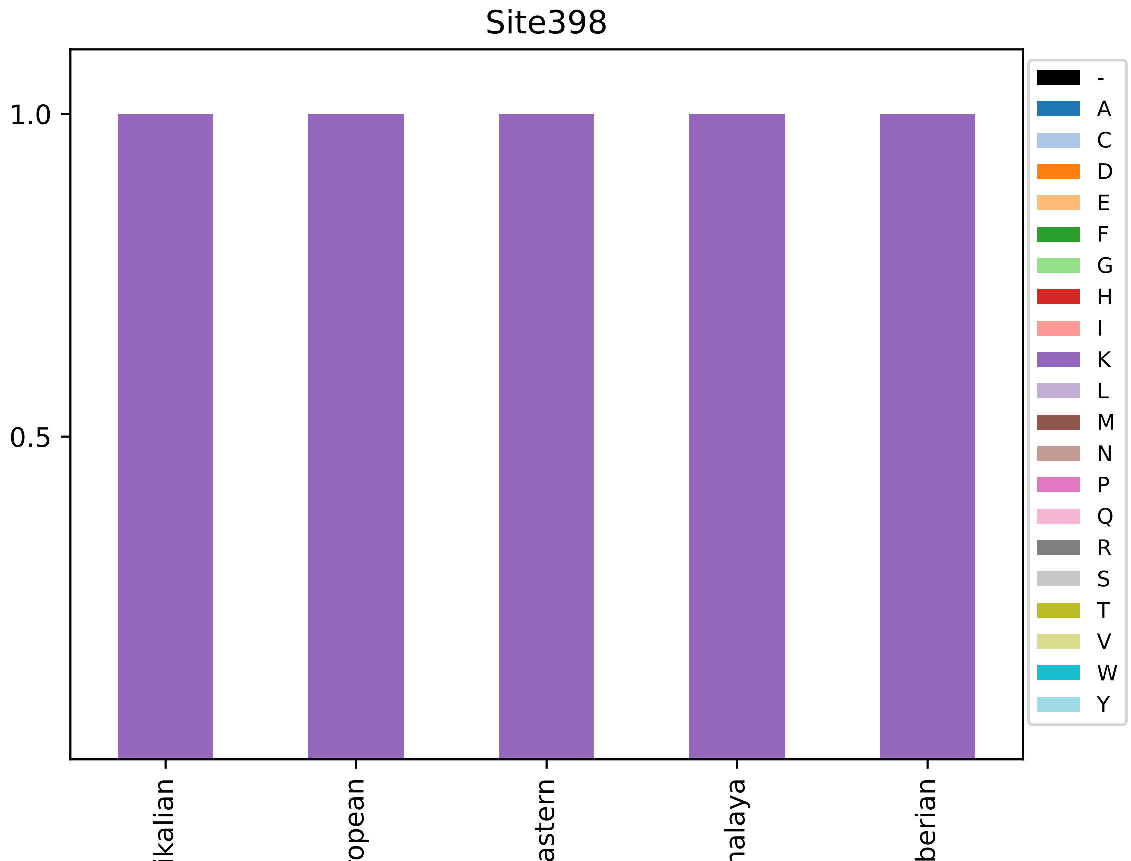

Site398
1.0
0.5
-
A
C
D
E
F
G
H
I
K
L
M
N
P
Q
R
S
T
V
W
Y

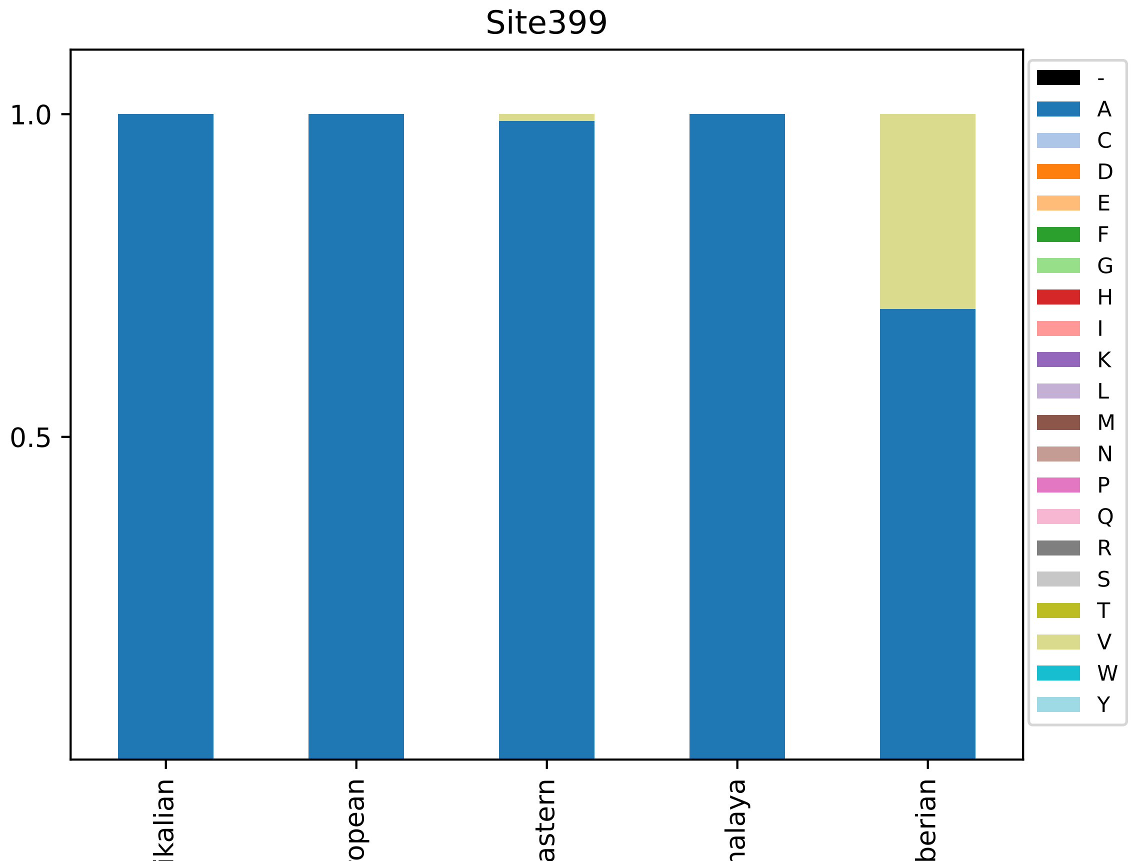
Site399
1.0
0.5
-
A
C
D
E
F
G
H
I
K
L
M
N
P
Q
R
S
T
V
W
Y
ikalian
opean
astern
nalaya
berian

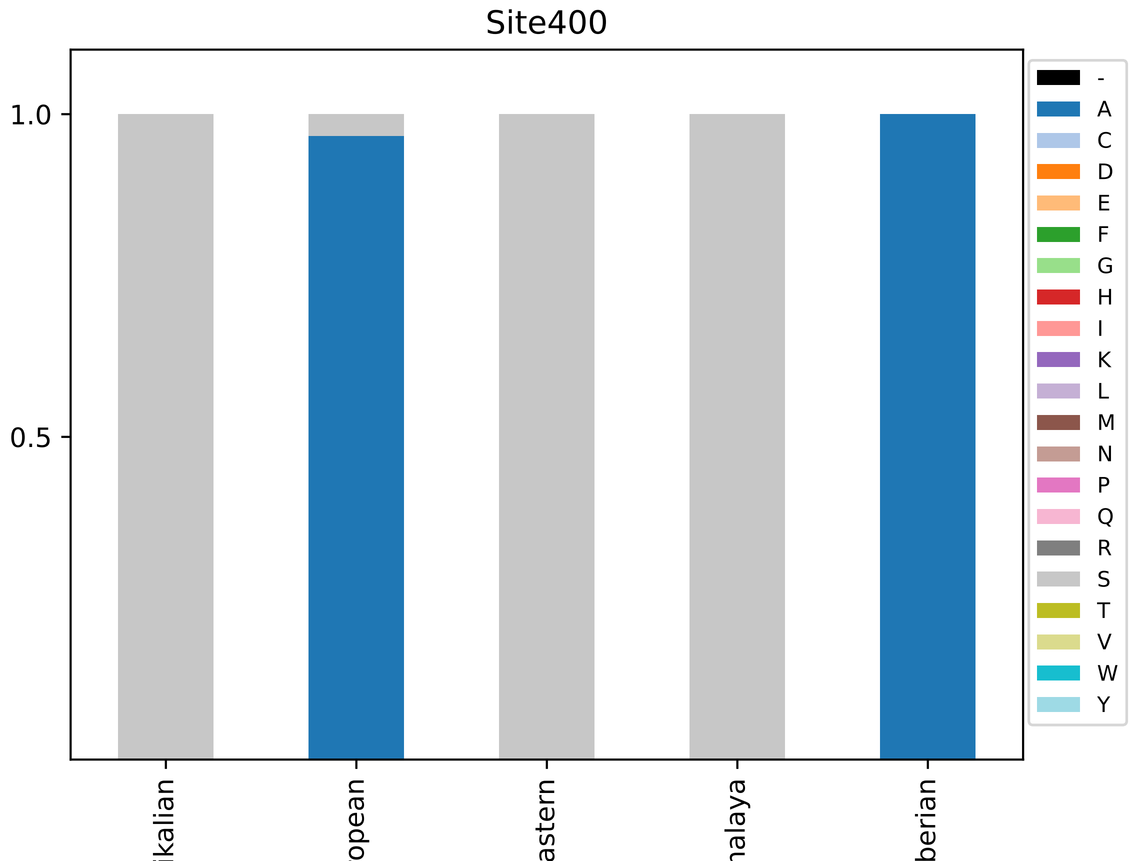
Site400
1.0
0.5
-
A
C
D
E
F
G
H
I
K
L
M
N
P
Q
R
S
T
V
W
Y
ikalian
opean
astern
nalaya
berian

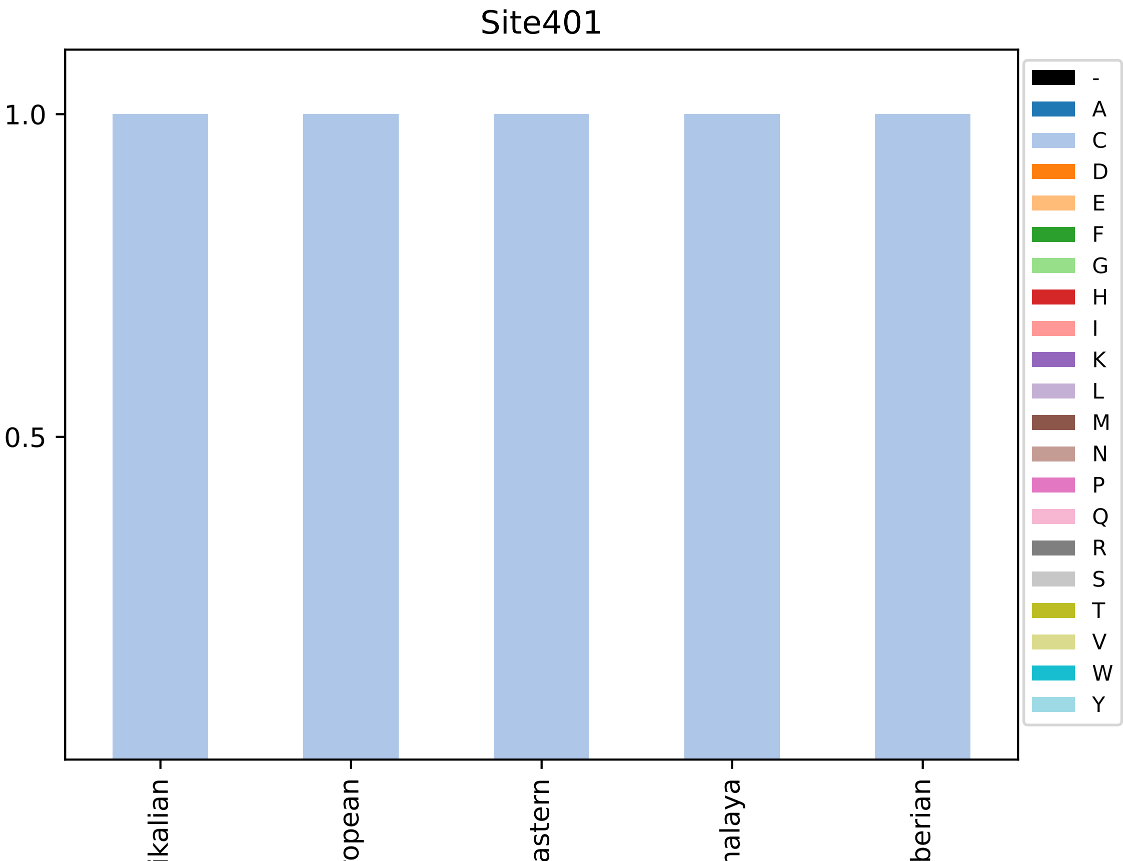

Site401
1.0
0.5
-
A
C
D
E
F
G
H
I
K
L
M
N
P
Q
R
S
T
V
W
Y
ikalian
opean
astern
nalaya
berian

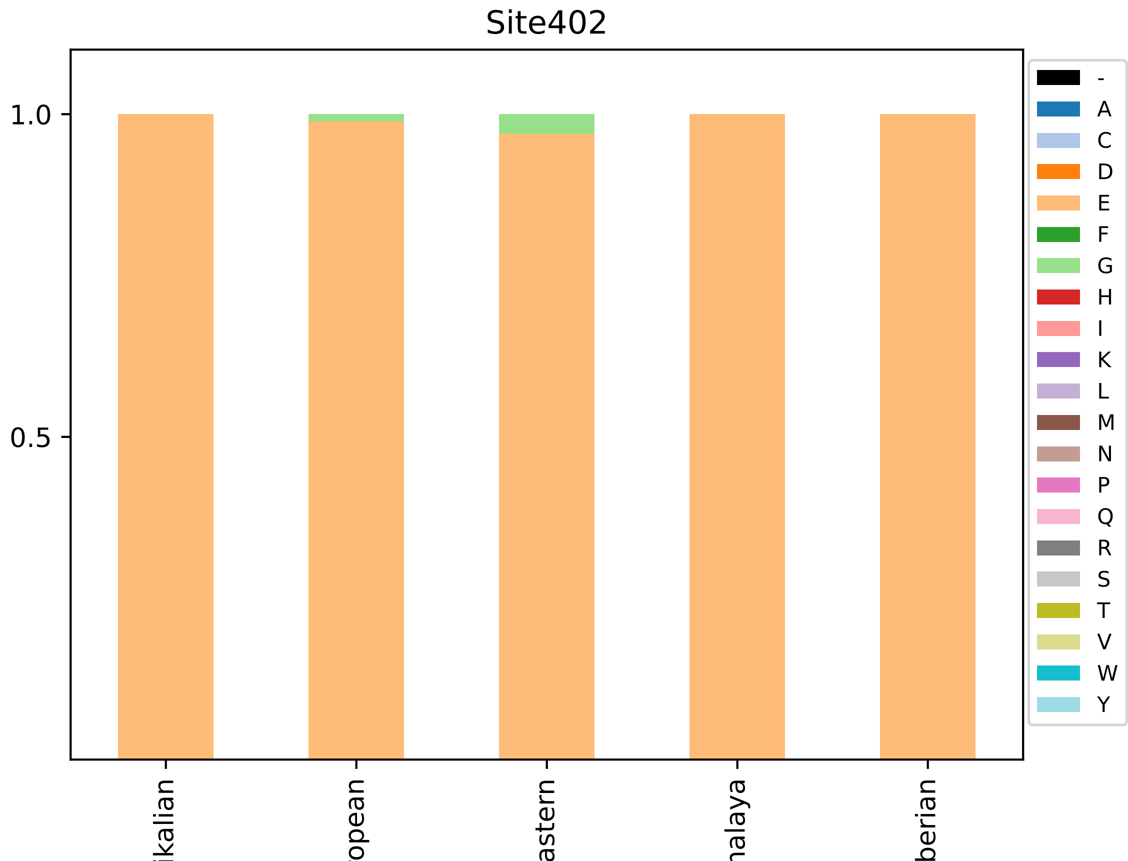

Site402
1.0
0.5
-
A
C
D
E
F
G
H
I
K
L
M
N
P
Q
R
S
T
V
W
Y
ikalian
opean
astern
nalaya
berian

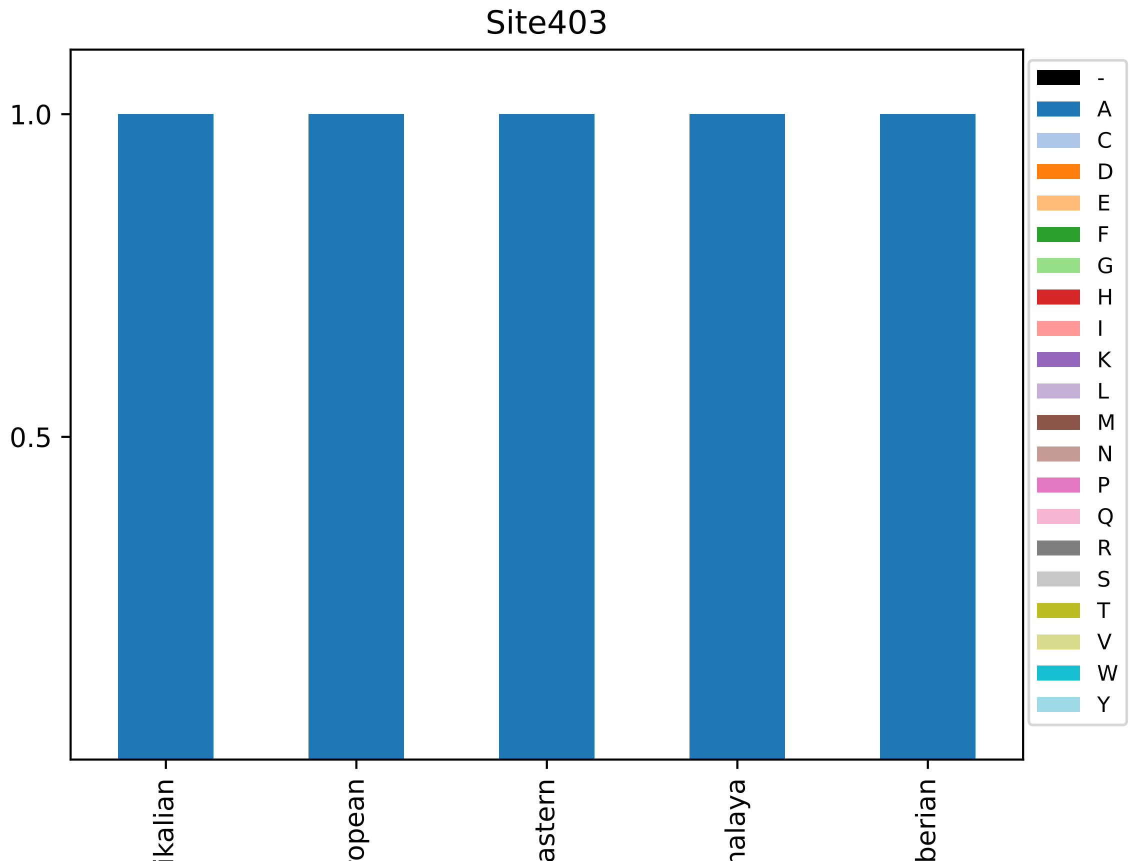

Site403
1.0
0.5
-
A
C
D
E
F
G
H
I
K
L
M
N
P
Q
R
S
T
V
W
Y
ikalian
opean
astern
nalaya
berian

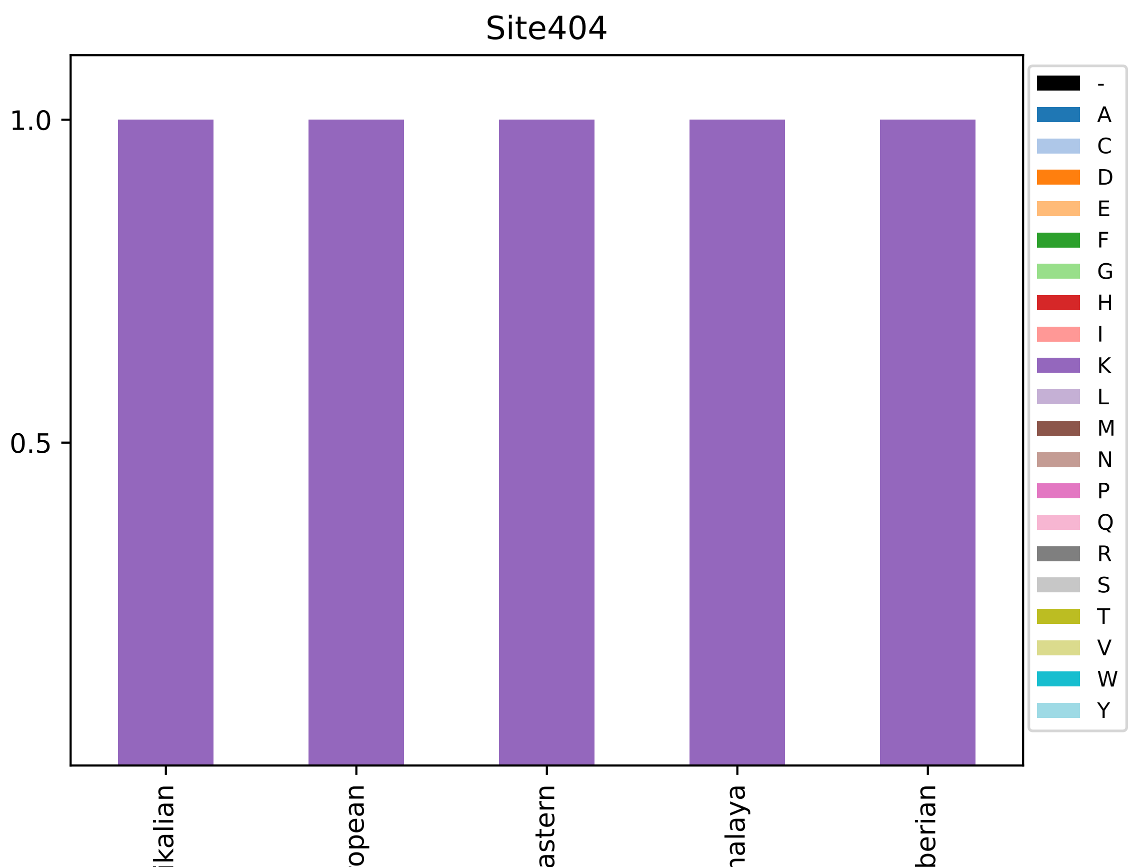

Site404
1.0
0.5
-
A
C
D
E
F
G
H
I
K
L
M
N
P
Q
R
S
T
V
W
Y
ikalian
opean
astern
nalaya
berian

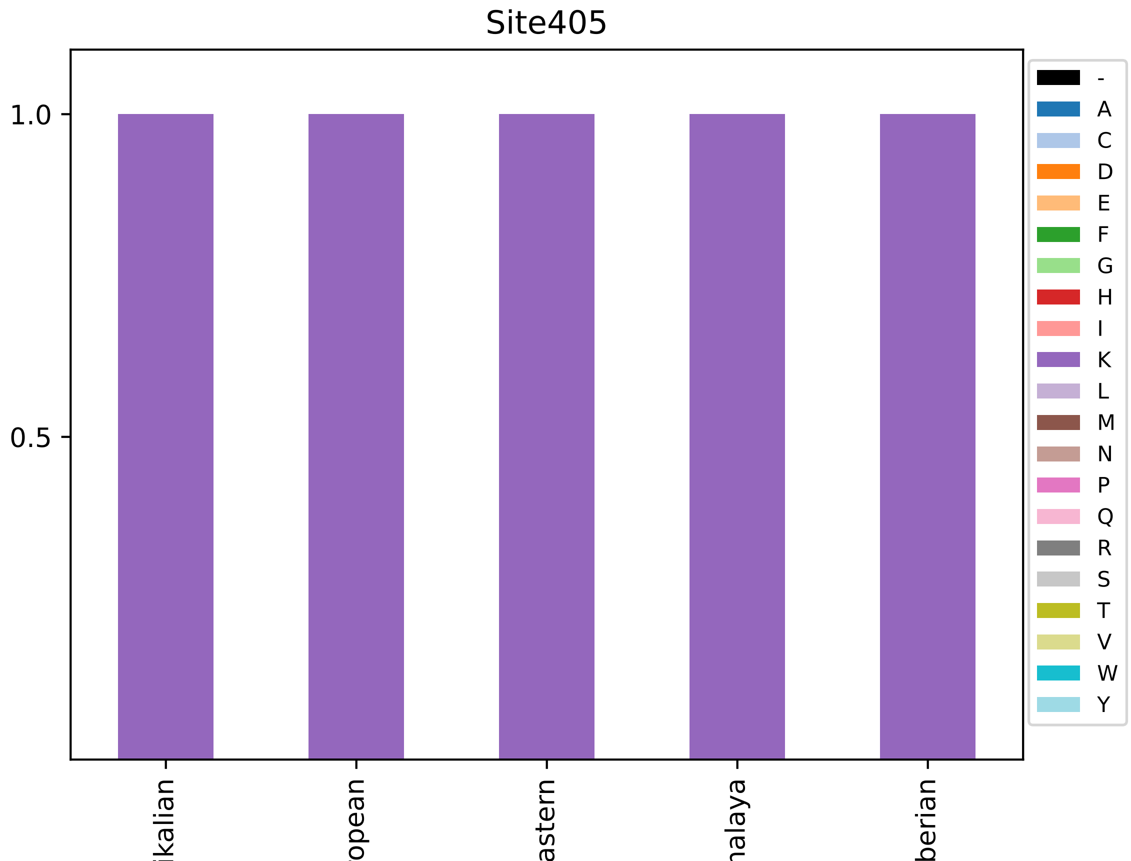

Site405
1.0
0.5
-
A
C
D
E
F
G
H
I
K
L
M
N
P
Q
R
S
T
V
W
Y
ikalian
opean
astern
nalaya
berian

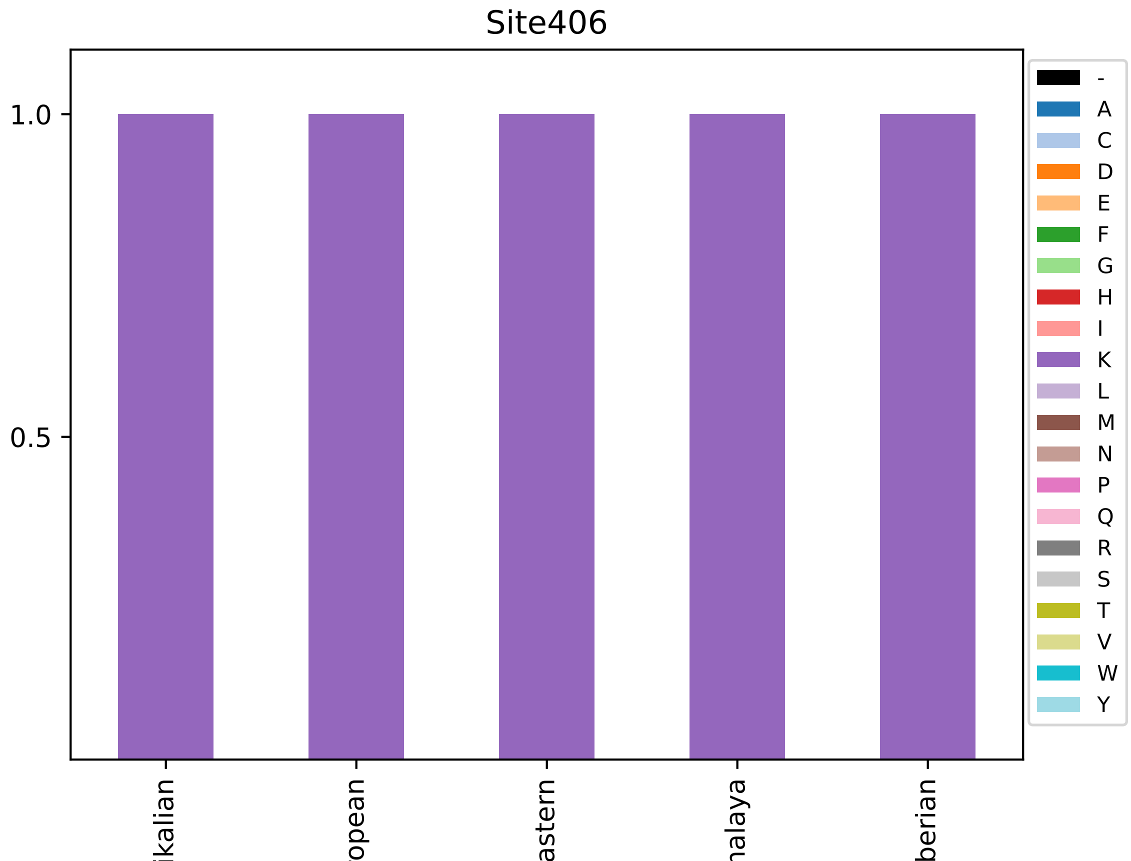
Site406
1.0
0.5
-
A
C
D
E
F
G
H
I
K
L
M
N
P
Q
R
S
T
V
W
Y
ikalian
opean
astern
nalaya
berian

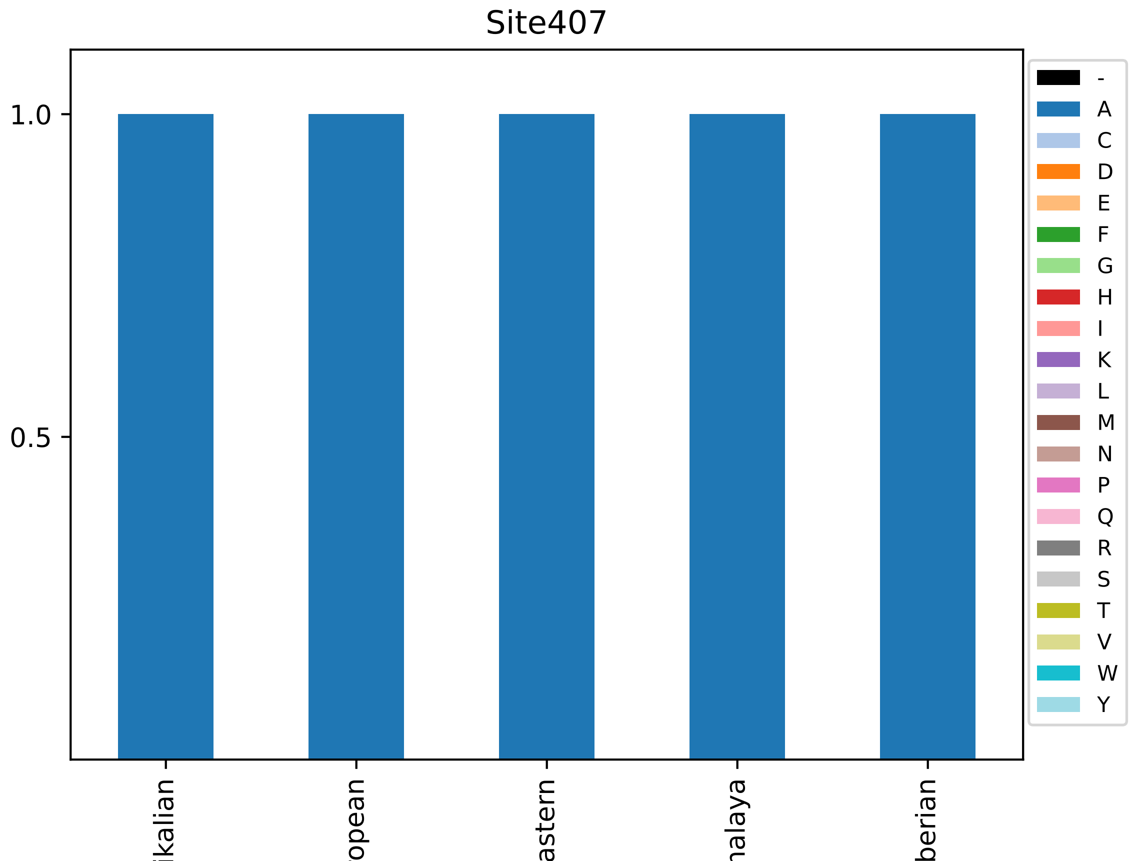

Site407
1.0
0.5
-
A
C
D
E
F
G
H
I
K
L
M
N
P
Q
R
S
T
V
W
Y
ikalian
opean
astern
nalaya
berian

Site408

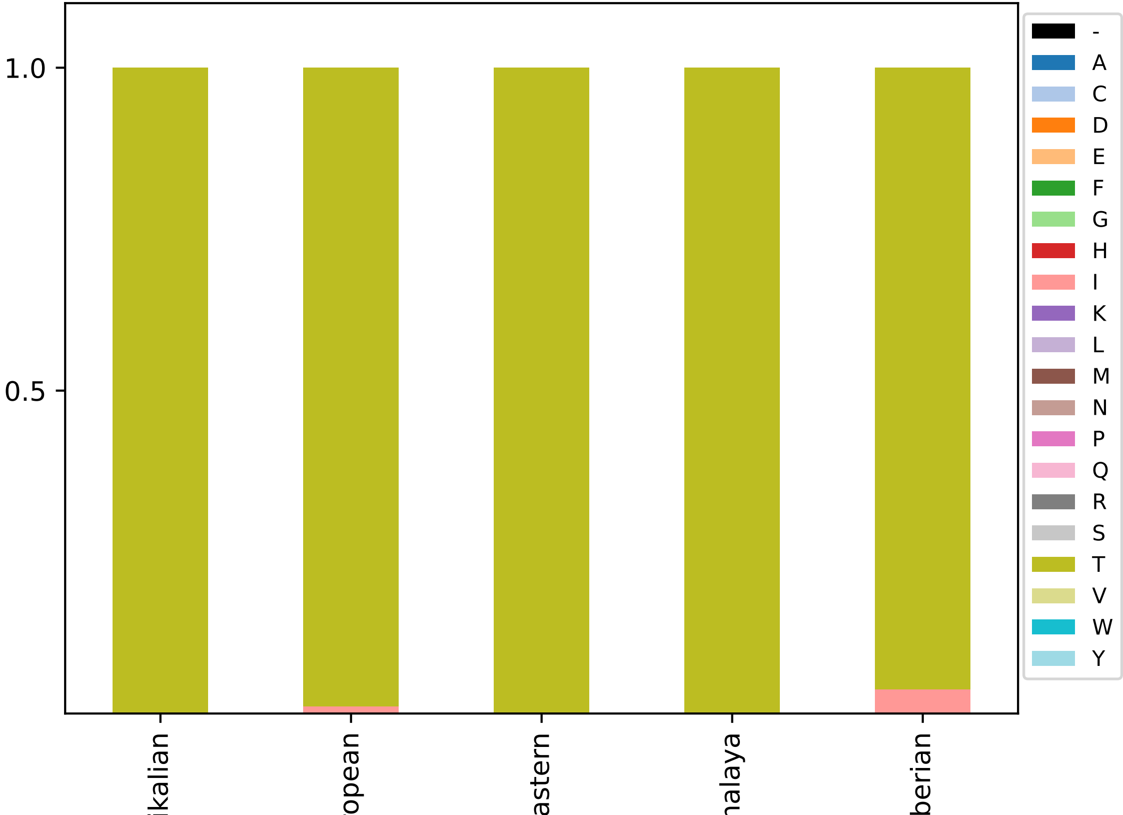
1.0
0.5
-
A
C
D
E
F
G
H
I
K
L
M
N
P
Q
R
S
T
V
W
Y
ikalian
opean
astern
nalaya
berian

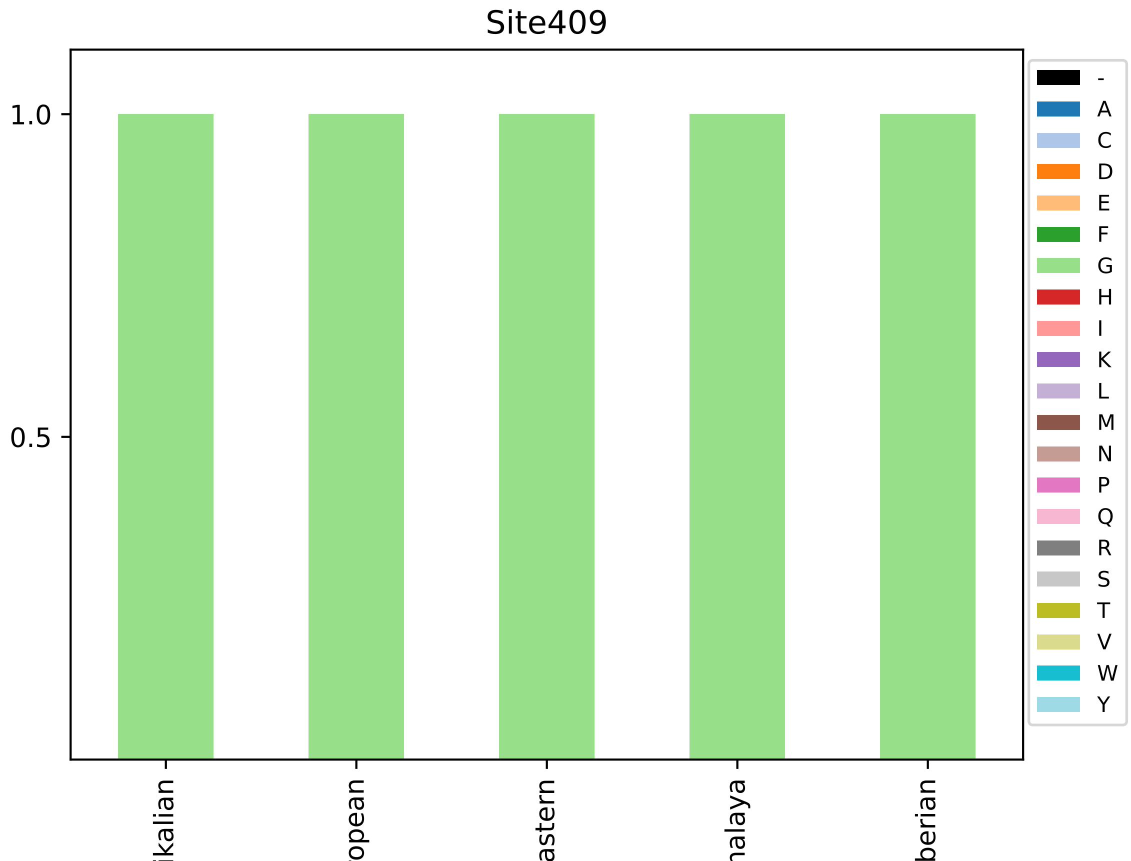

Site409
1.0
0.5
-
A
C
D
E
F
G
H
I
K
L
M
N
P
Q
R
S
T
V
W
Y
ikalian
opean
astern
nalaya
berian

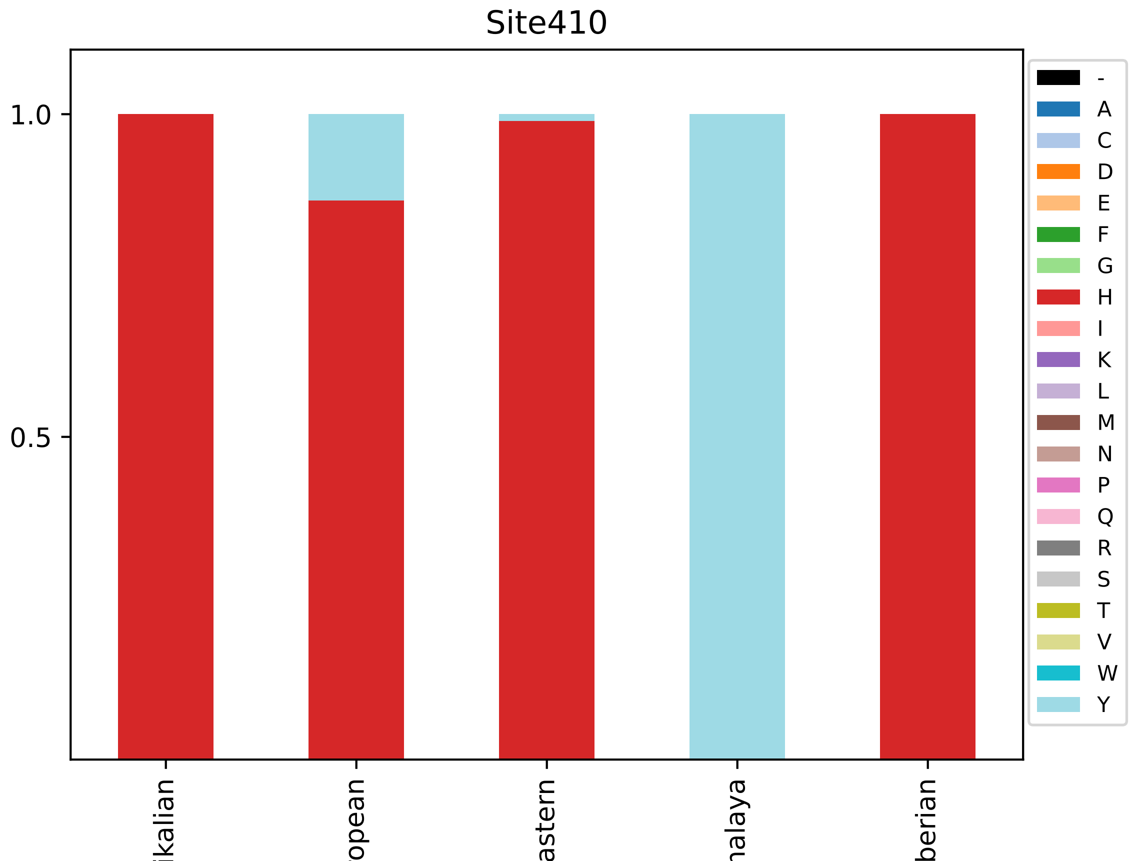
Site410
1.0
0.5
-
A
C
D
E
F
G
H
I
K
L
M
N
P
Q
R
S
T
V
W
Y
ikalian
opean
astern
nalaya
berian

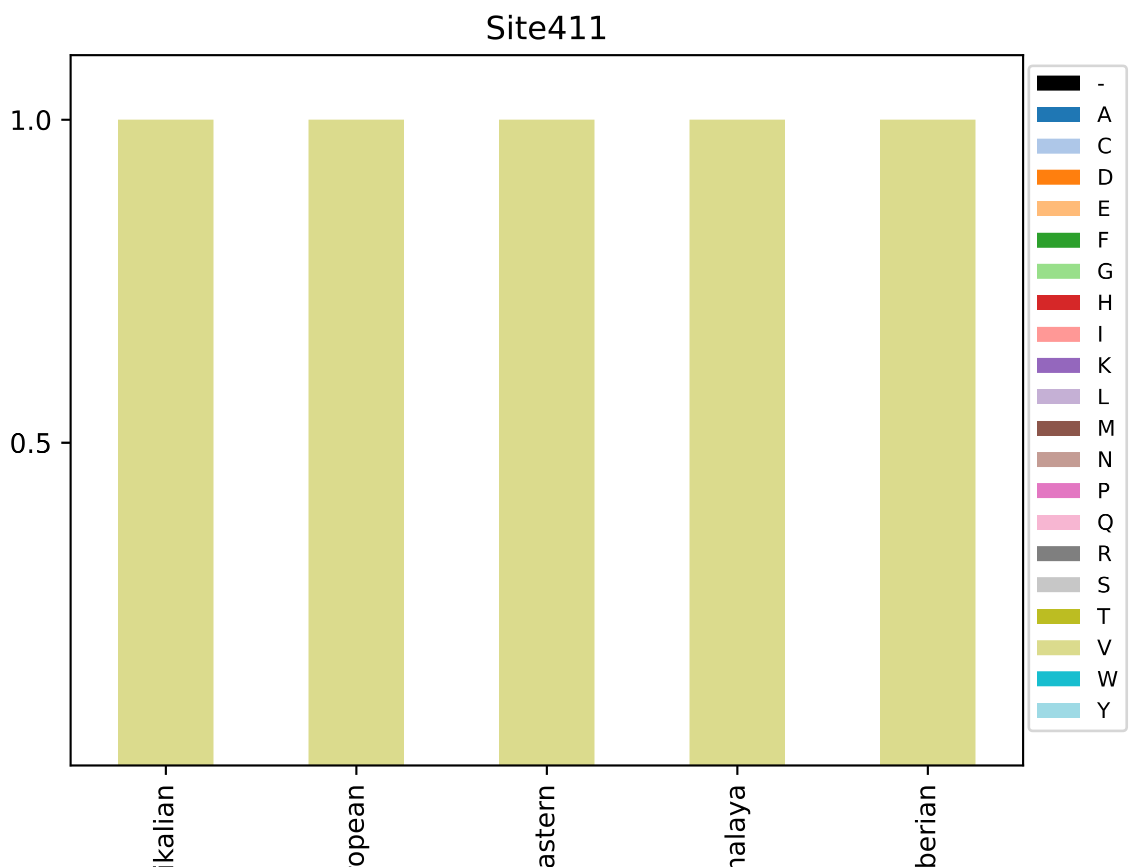

Site411
1.0
0.5
ikalian
opean
astern
nalaya
berian
-
A
C
D
E
F
G
H
I
K
L
M
N
P
Q
R
S
T
V
W
Y

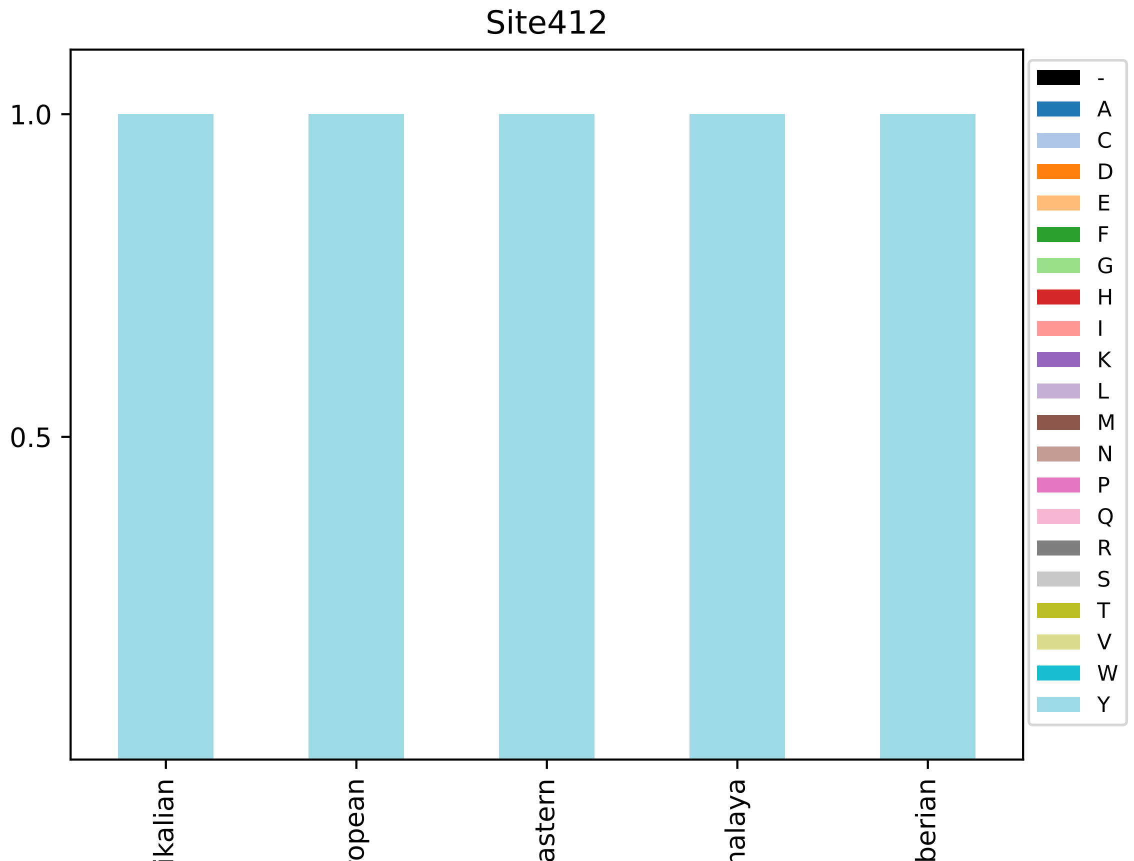
Site412
1.0
0.5
-
A
C
D
E
F
G
H
I
K
L
M
N
P
Q
R
S
T
V
W
Y
ikalian
opean
astern
nalaya
berian

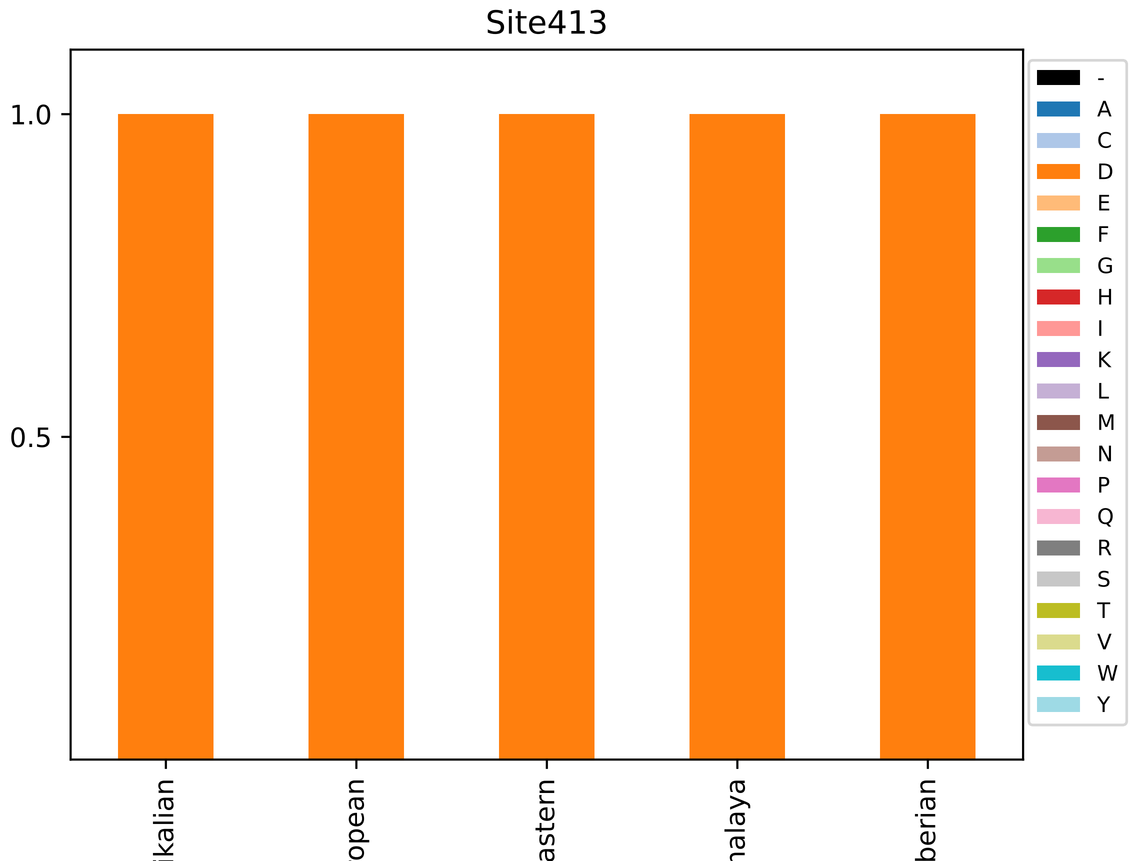
Site413
1.0
0.5
-
A
C
D
E
F
G
H
I
K
L
M
N
P
Q
R
S
T
V
W
Y
ikalian
opean
astern
nalaya
berian

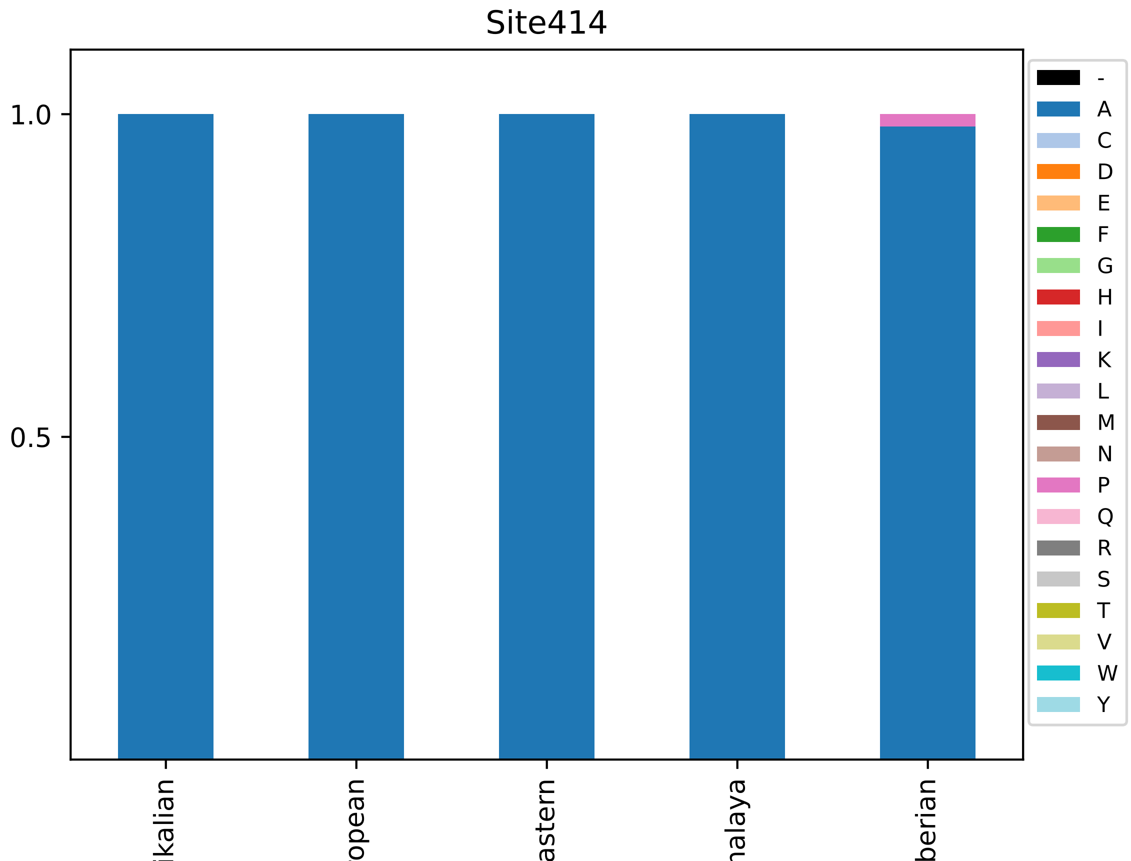
Site414
1.0
0.5
-
A
C
D
E
F
G
H
I
K
L
M
N
P
Q
R
S
T
V
W
Y
ikalian
opean
astern
nalaya
berian

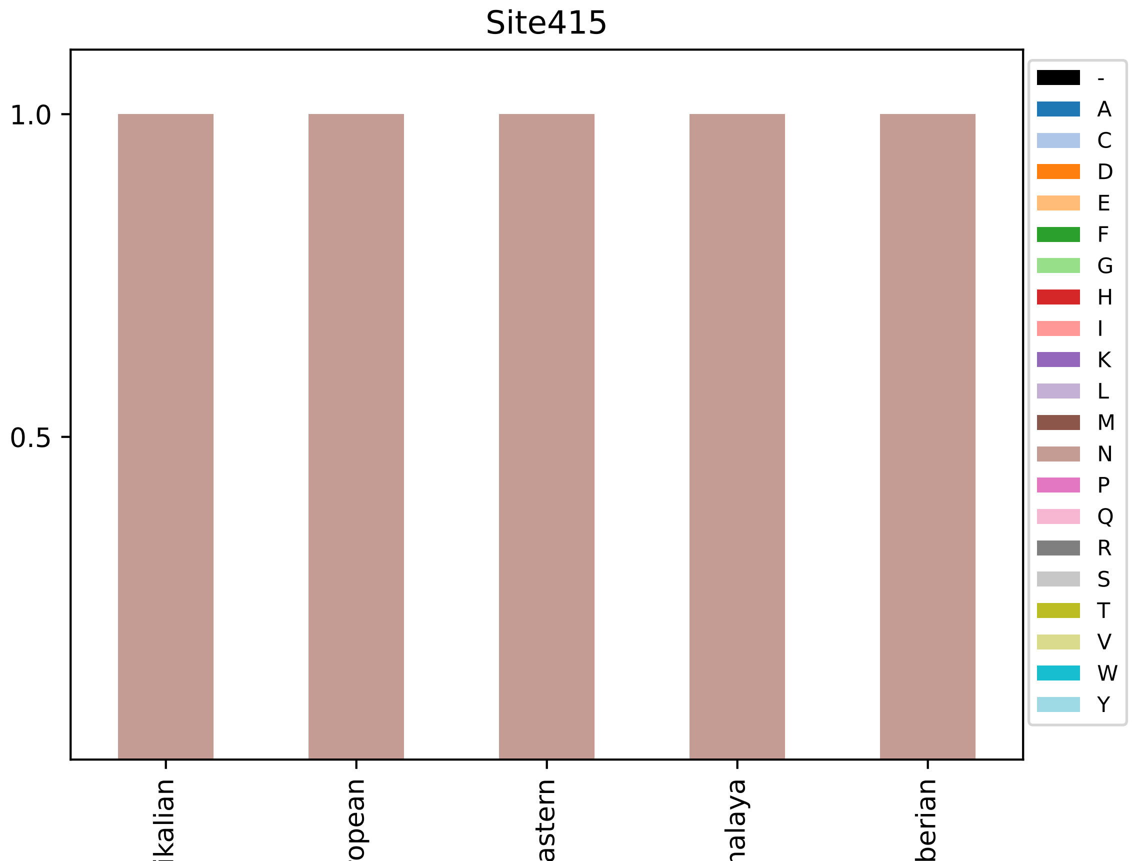
Site415
1.0
0.5
-
A
C
D
E
F
G
H
I
K
L
M
N
P
Q
R
S
T
V
W
Y
ikalian
opean
astern
nalaya
berian

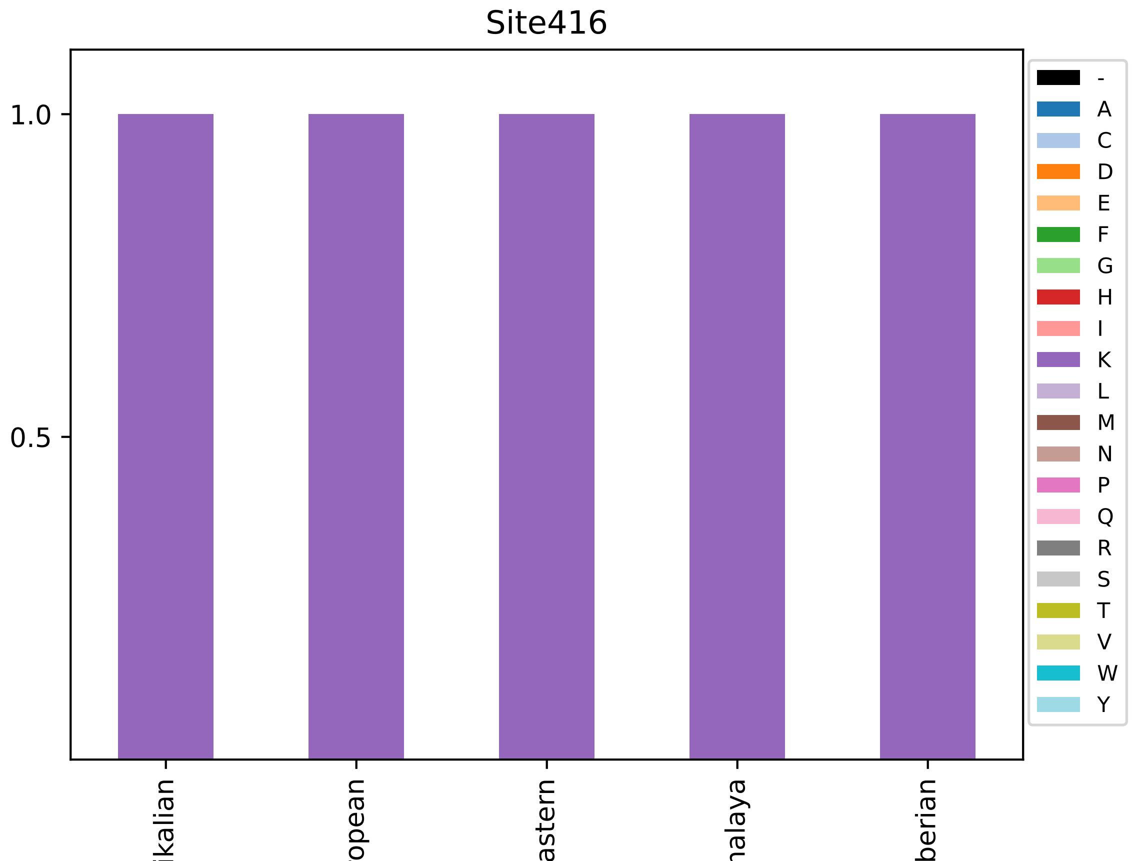

Site416
1.0
0.5
-
A
C
D
E
F
G
H
I
K
L
M
N
P
Q
R
S
T
V
W
Y
ikalian
opean
astern
nalaya
berian

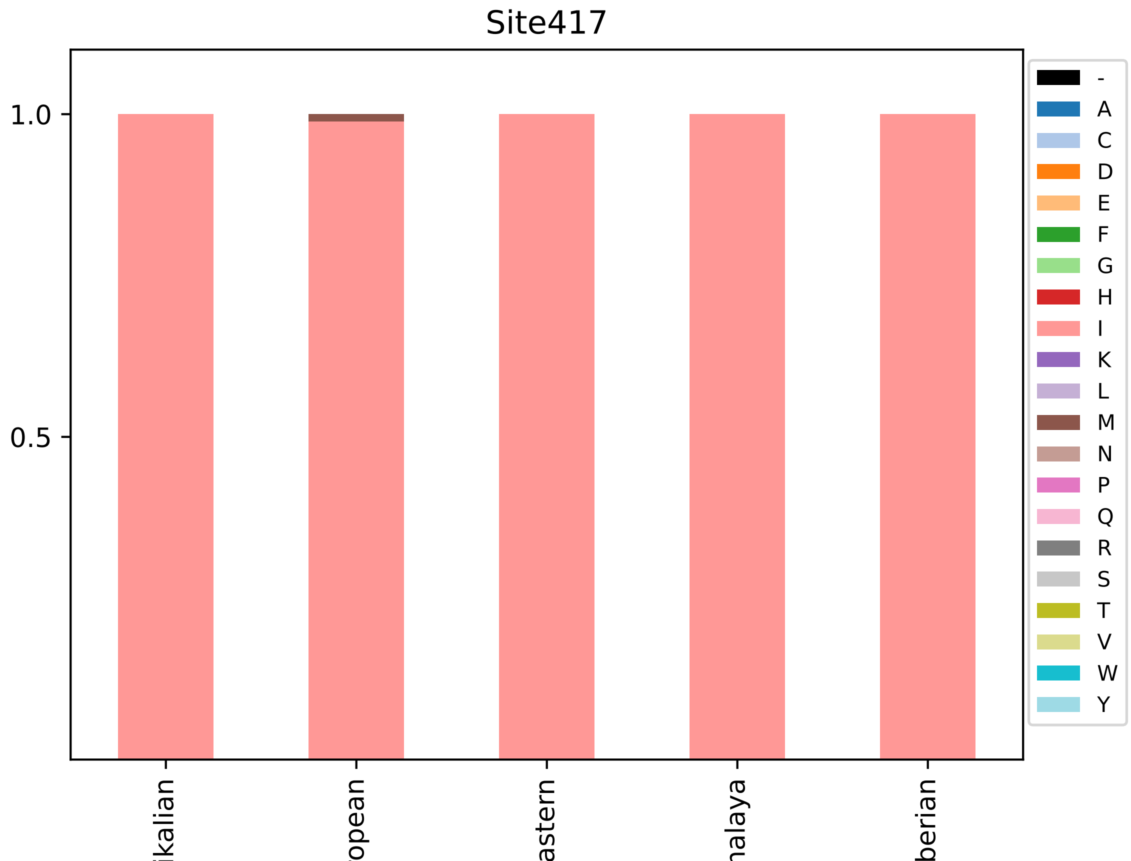
Site417
1.0
0.5
-
A
C
D
E
F
G
H
I
K
L
M
N
P
Q
R
S
T
V
W
Y
ikalian
opean
astern
nalaya
berian

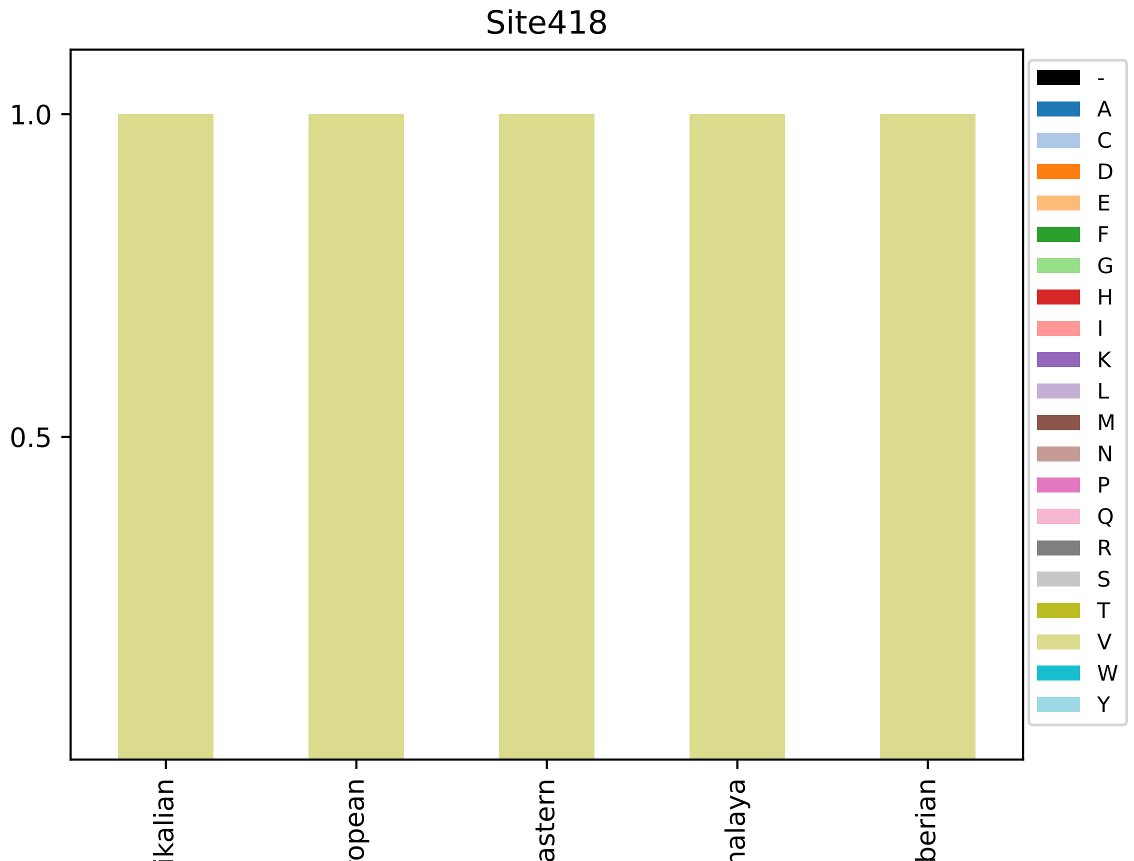
Site418
1.0
0.5
-
A
C
D
E
F
G
H
I
K
L
M
N
P
Q
R
S
T
V
W
Y
ikalian
opean
astern
nalaya
berian

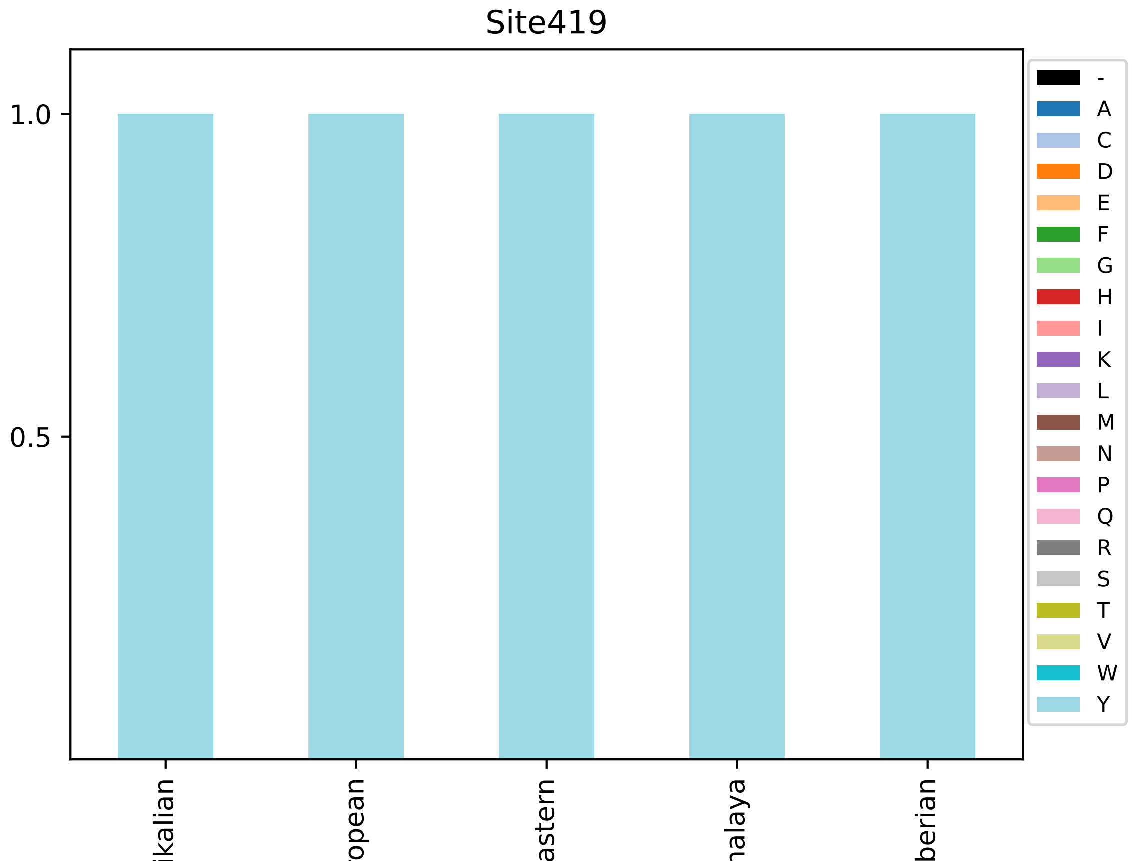

Site419
1.0
0.5
-
A
C
D
E
F
G
H
I
K
L
M
N
P
Q
R
S
T
V
W
Y
ikalian
opean
astern
nalaya
berian

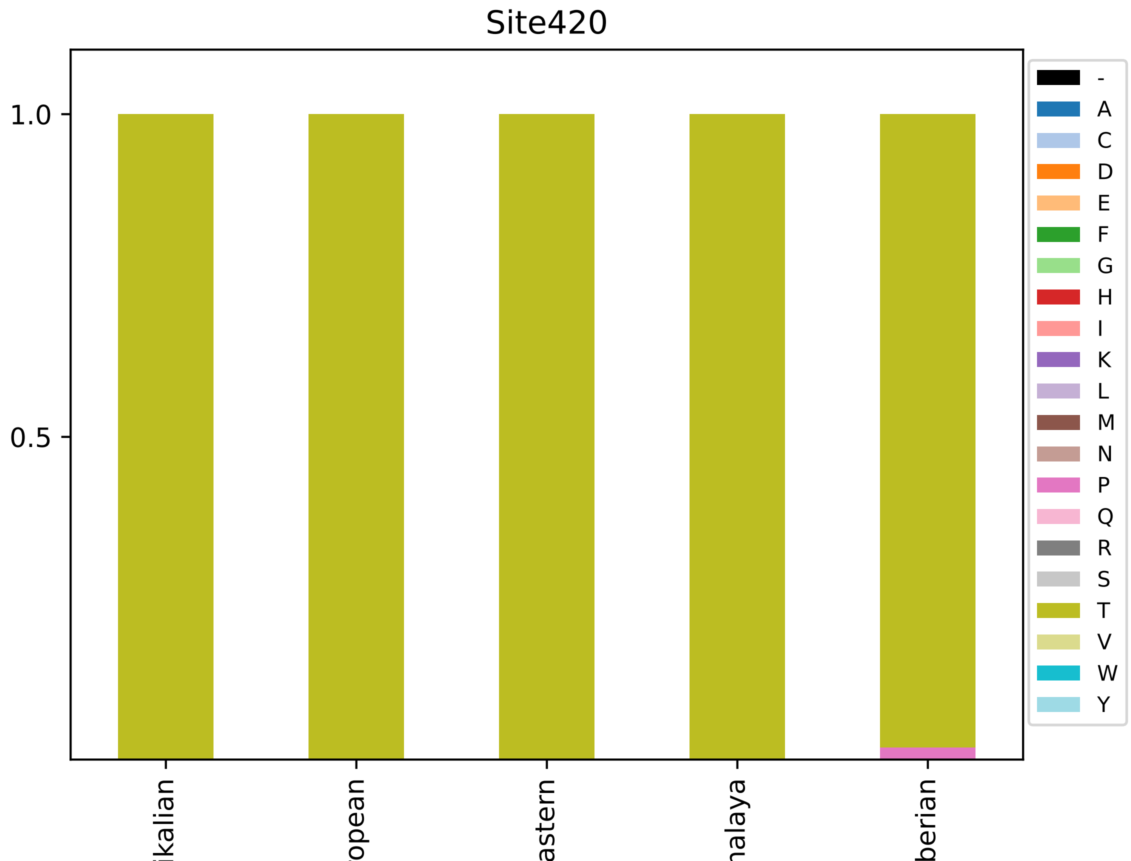

Site420
1.0
0.5
-
A
C
D
E
F
G
H
I
K
L
M
N
P
Q
R
S
T
V
W
Y
ikalian
opean
astern
nalaya
berian

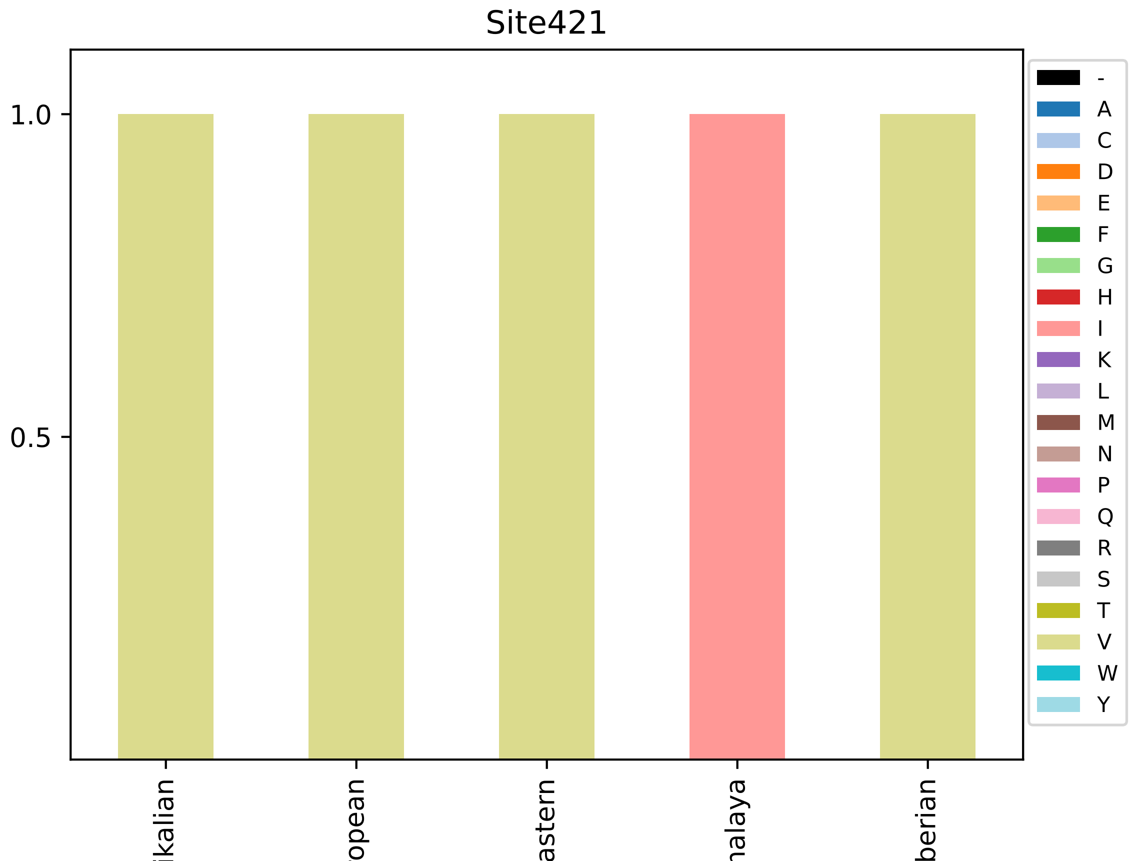

Site421
1.0
0.5
-
A
C
D
E
F
G
H
I
K
L
M
N
P
Q
R
S
T
V
W
Y
ikalian
opean
astern
nalaya
berian

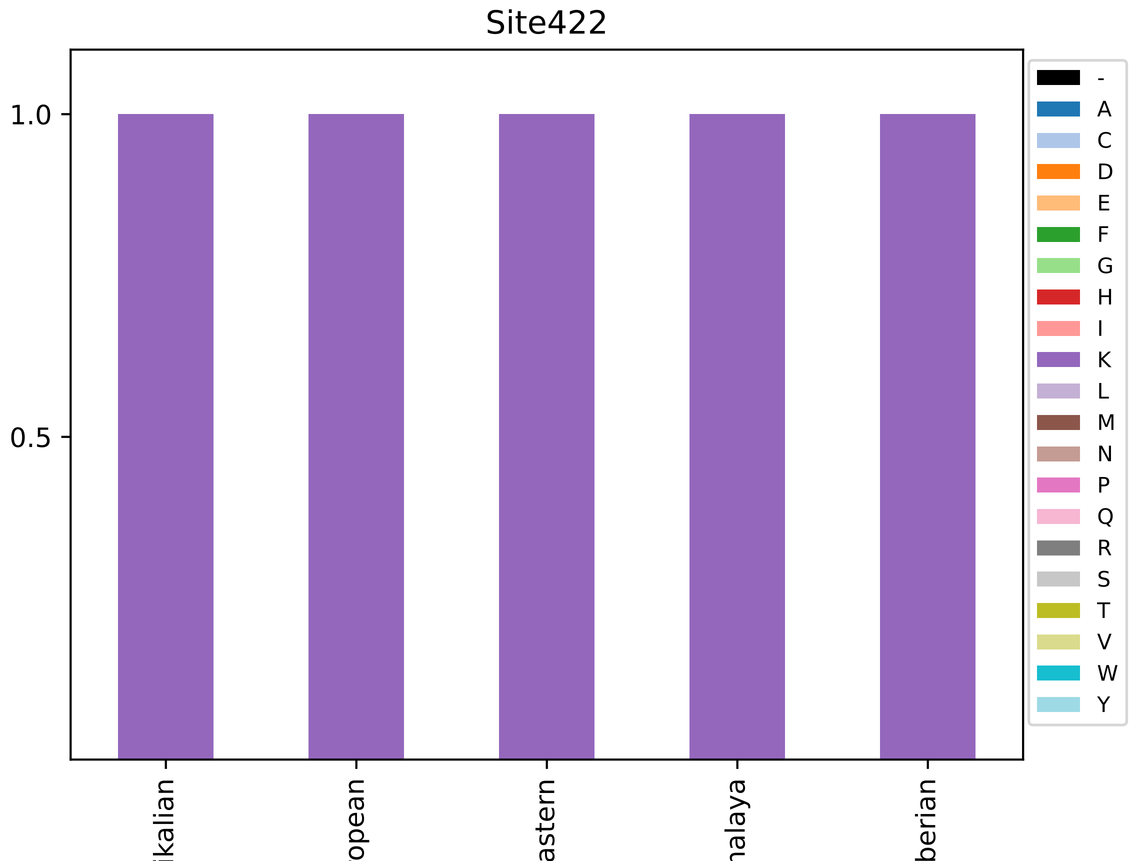
Site422
1.0
0.5
-
A
C
D
E
F
G
H
I
K
L
M
N
P
Q
R
S
T
V
W
Y
ikalian
opean
astern
nalaya
berian

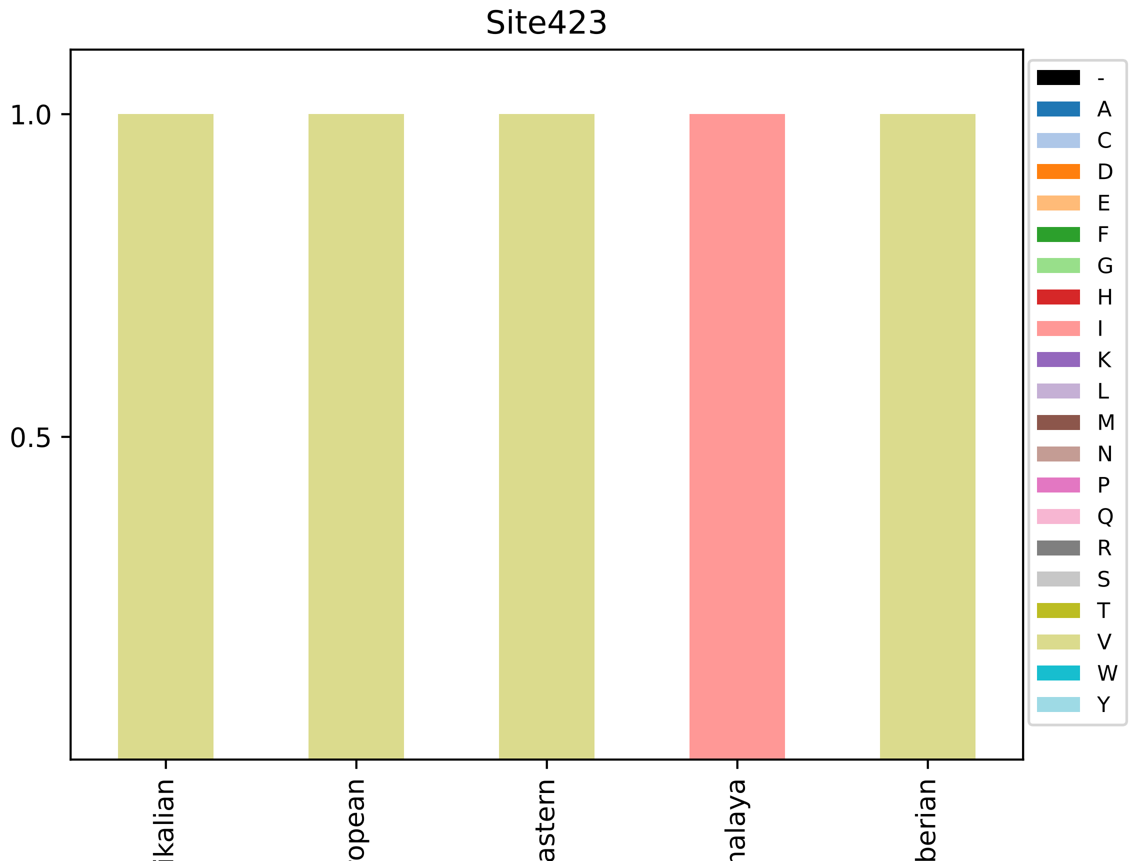
Site423
1.0
0.5
ikalian
opean
astern
nalaya
berian
-
A
C
D
E
F
G
H
I
K
L
M
N
P
Q
R
S
T
V
W
Y

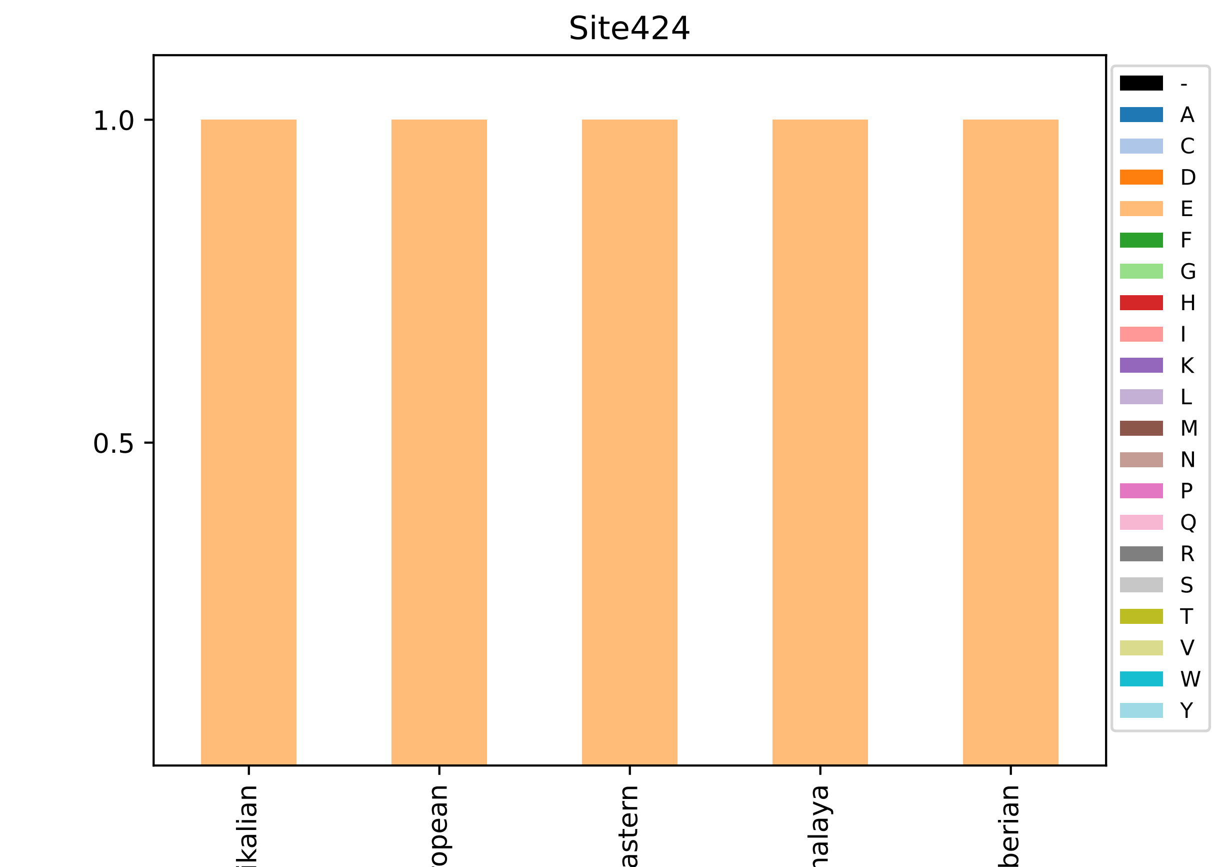

Site424
1.0
0.5
-
A
C
D
E
F
G
H
I
K
L
M
N
P
Q
R
S
T
V
W
Y
ikalian
opean
astern
nalaya
berian

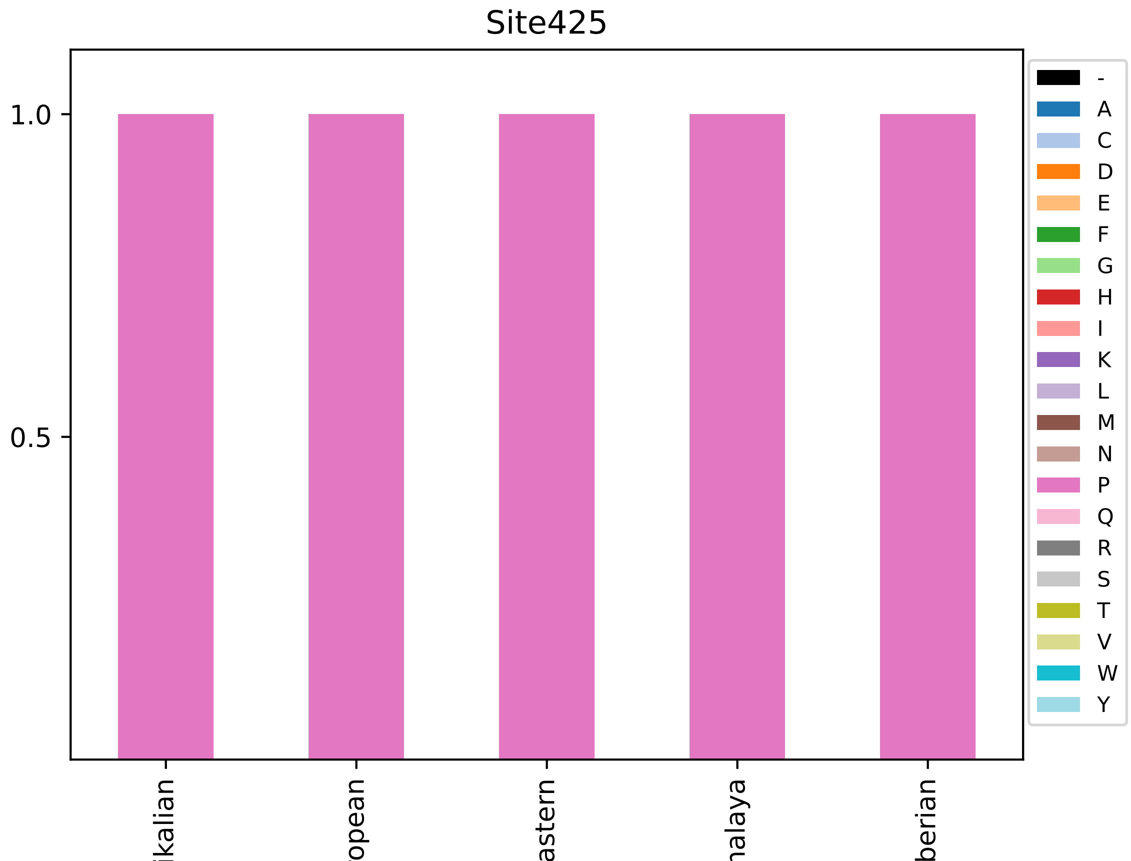
Site425
1.0
0.5
-
A
C
D
E
F
G
H
I
K
L
M
N
P
Q
R
S
T
V
W
Y
ikalian
opean
astern
nalaya
berian

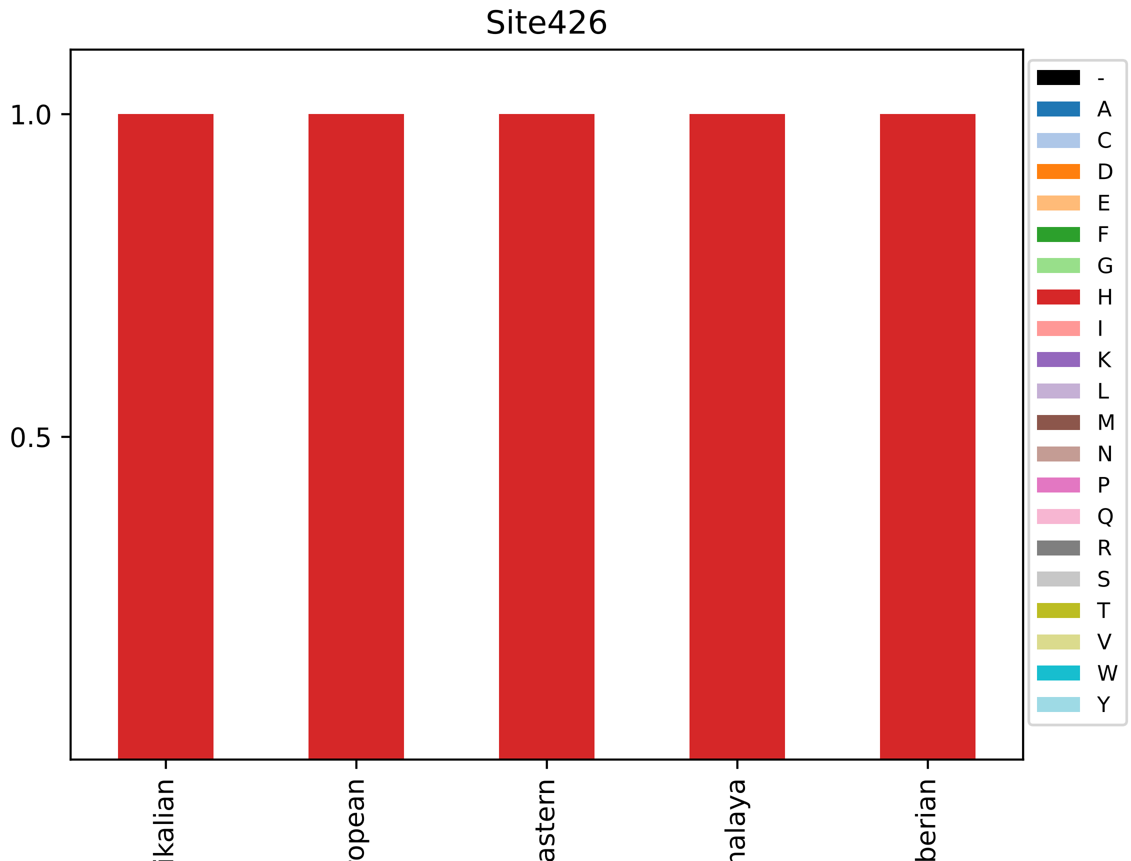
Site426
1.0
0.5
-
A
C
D
E
F
G
H
I
K
L
M
N
P
Q
R
S
T
V
W
Y
ikalian
opean
astern
nalaya
berian

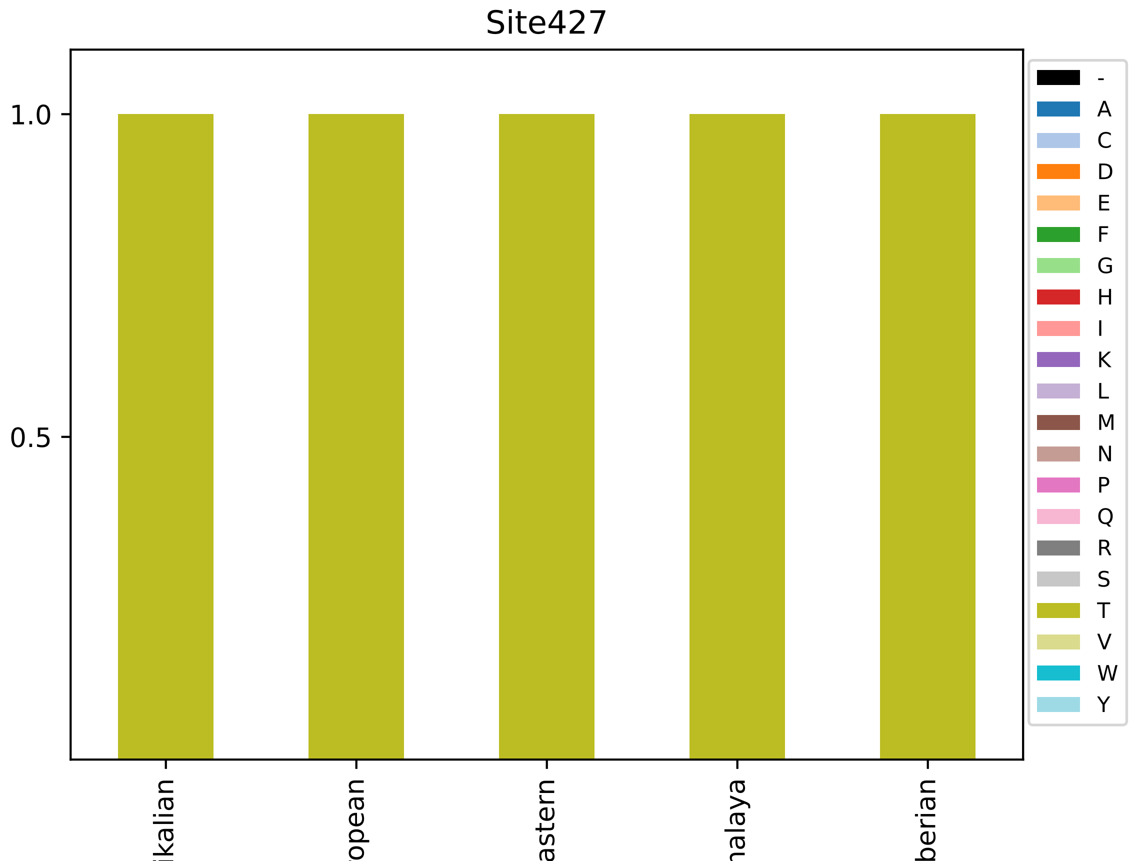

Site427
1.0
0.5
-
A
C
D
E
F
G
H
I
K
L
M
N
P
Q
R
S
T
V
W
Y
ikalian
opean
astern
nalaya
berian

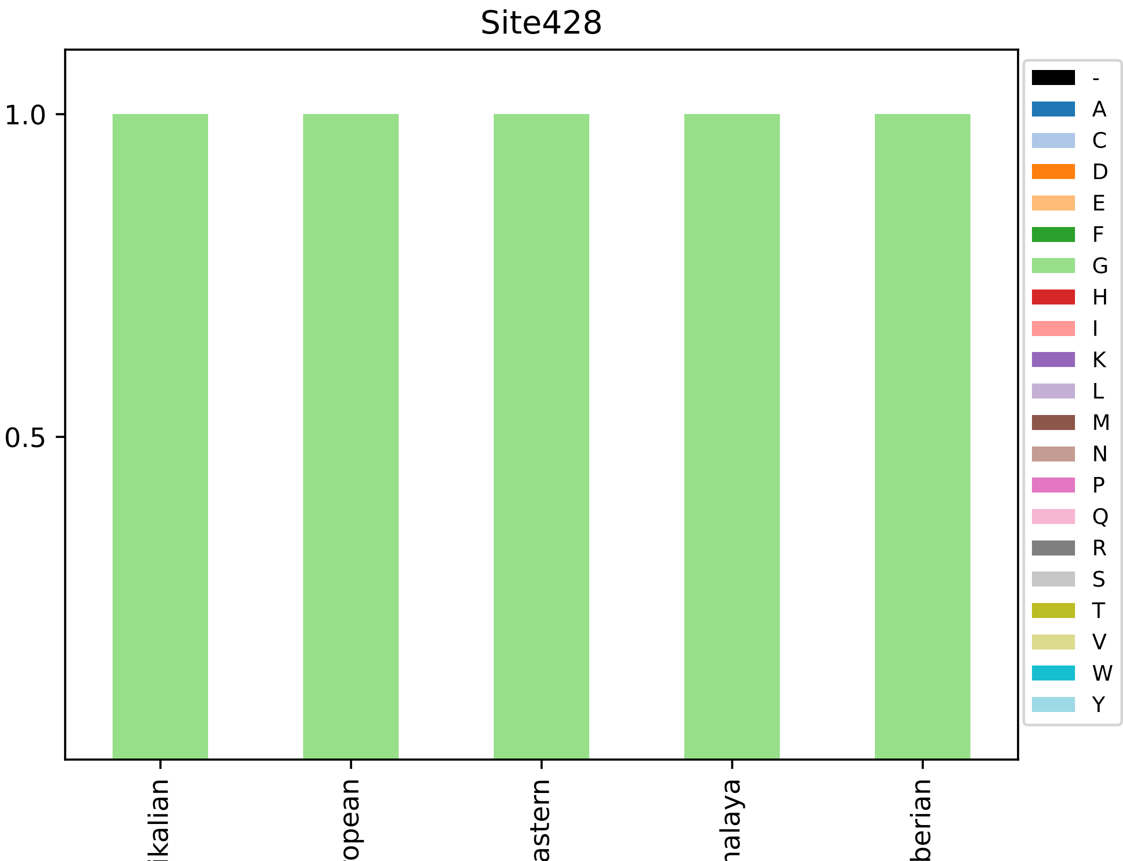

Site428
1.0
0.5
ikalian
opean
astern
nalaya
berian
-
A
C
D
E
F
G
H
I
K
L
M
N
P
Q
R
S
T
V
W
Y

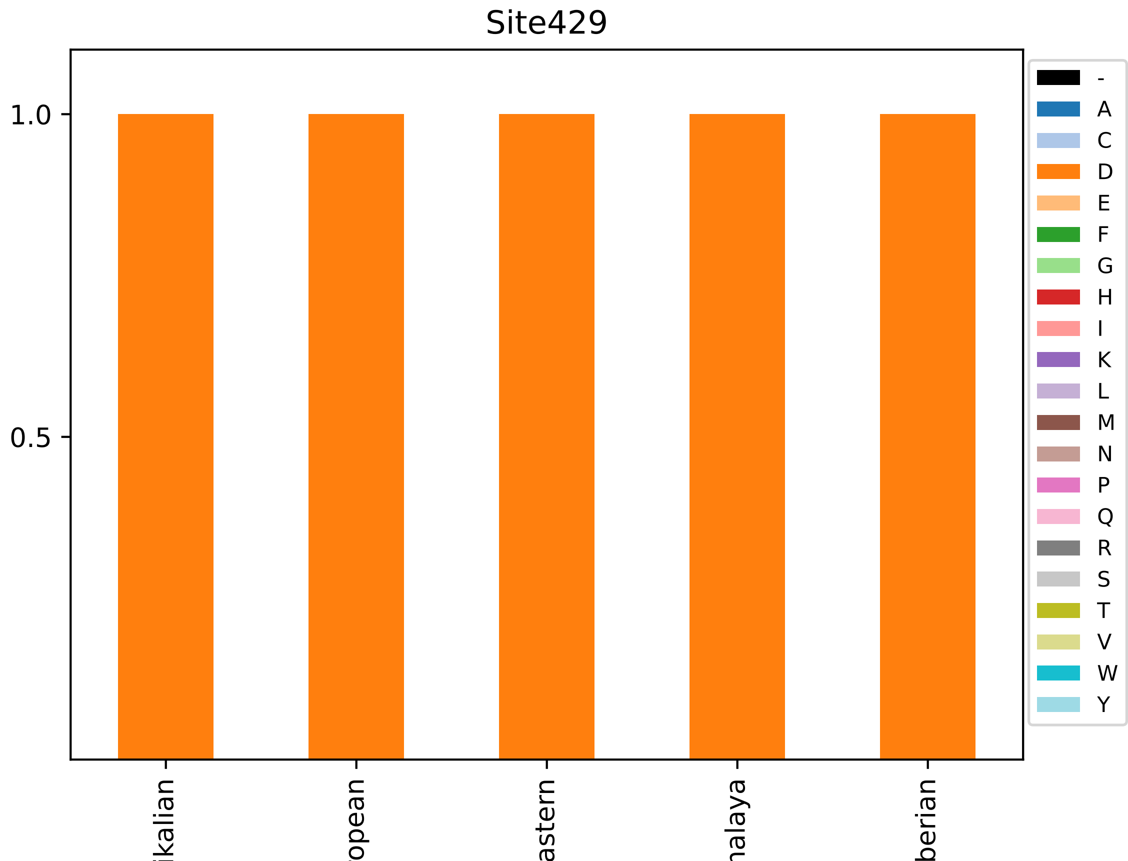

Site429
1.0
0.5
-
A
C
D
E
F
G
H
I
K
L
M
N
P
Q
R
S
T
V
W
Y

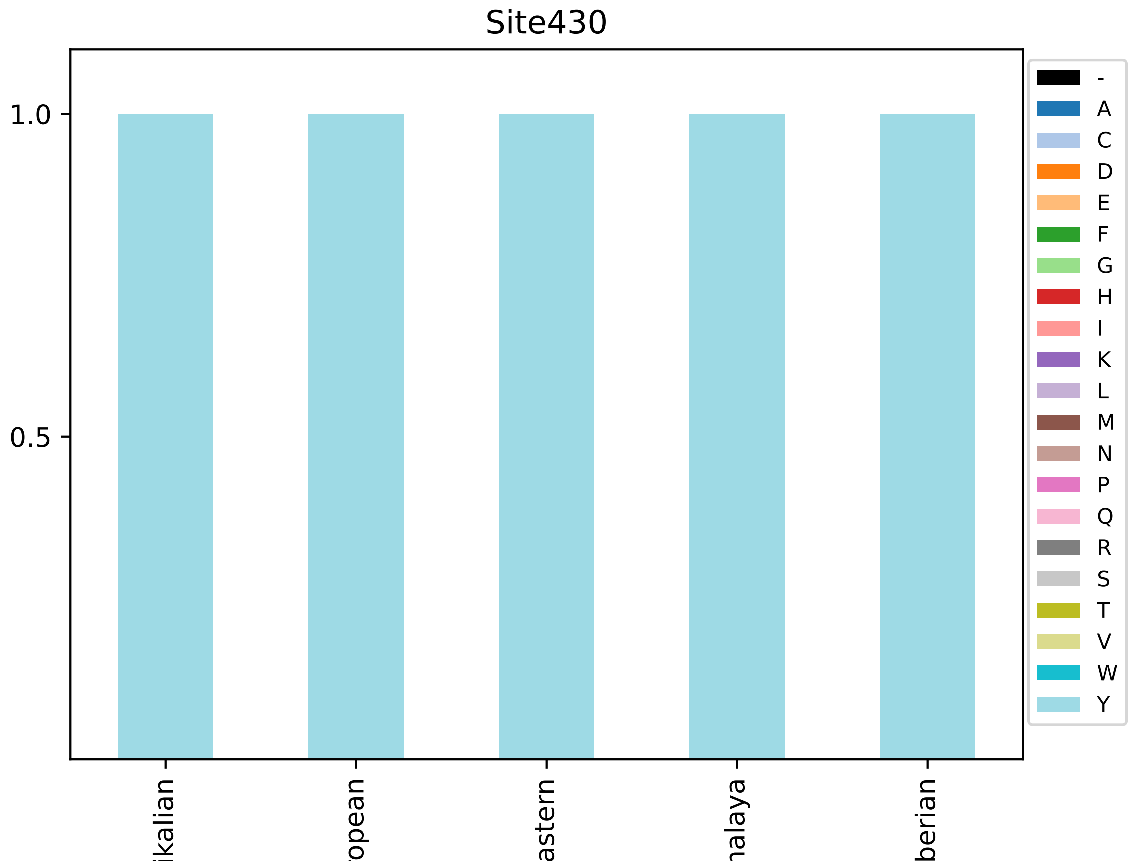

Site430
1.0
0.5
-
A
C
D
E
F
G
H
I
K
L
M
N
P
Q
R
S
T
V
W
Y
ikalian
opean
astern
nalaya
berian

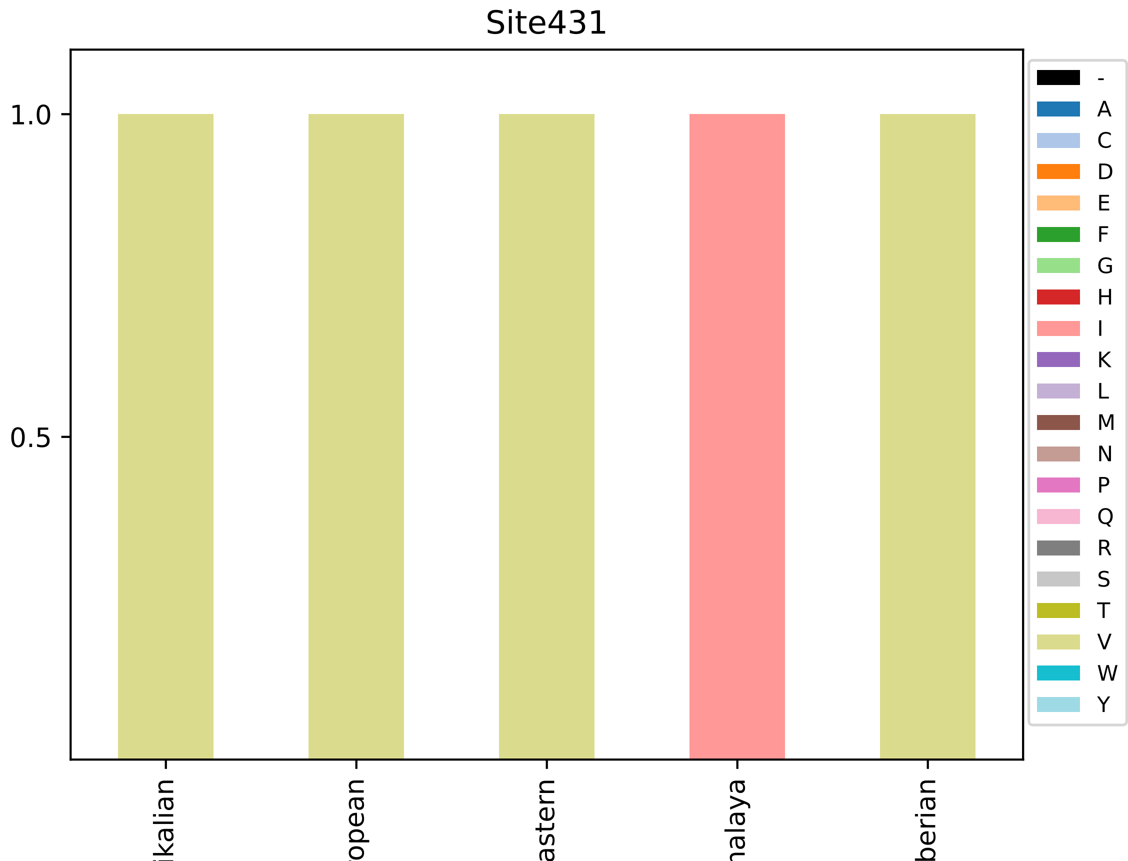

Site431
1.0
0.5
-
A
C
D
E
F
G
H
I
K
L
M
N
P
Q
R
S
T
V
W
Y
ikalian
opean
astern
nalaya
berian

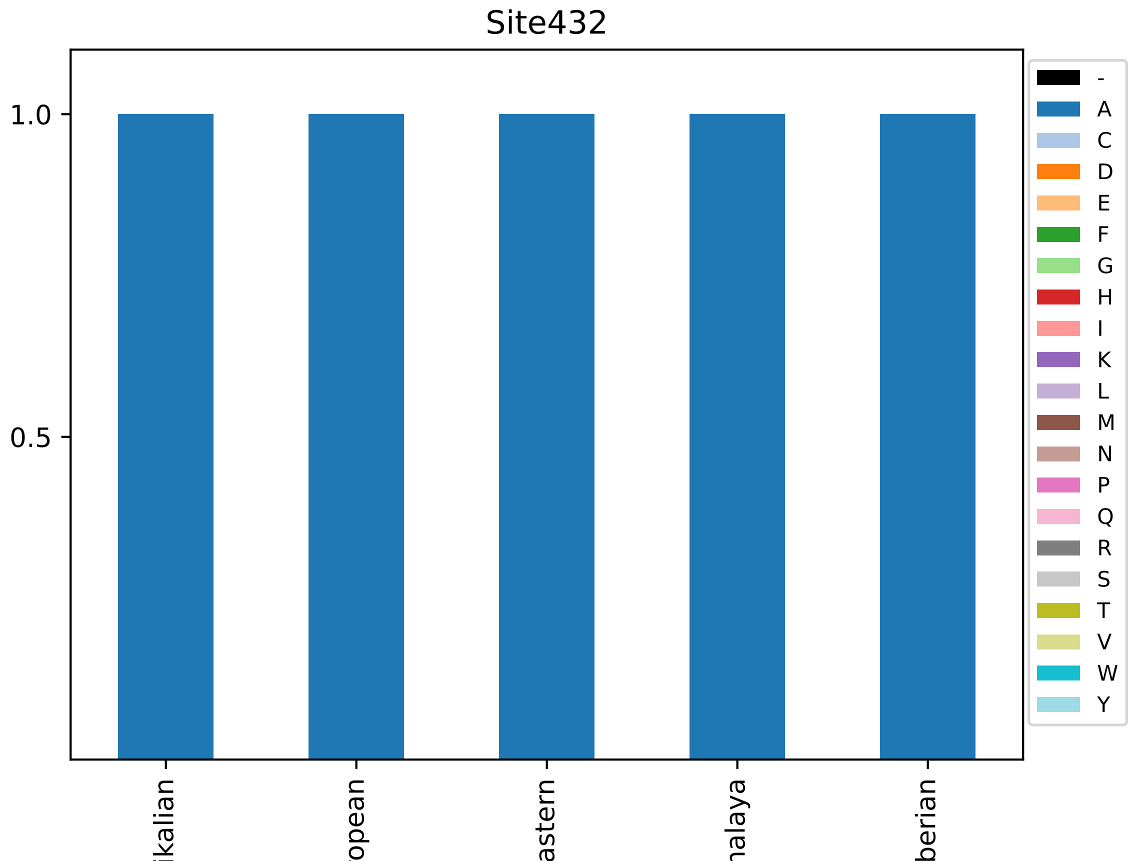
Site432
1.0
0.5
-
A
C
D
E
F
G
H
I
K
L
M
N
P
Q
R
S
T
V
W
Y
ikalian
opean
astern
nalaya
berian

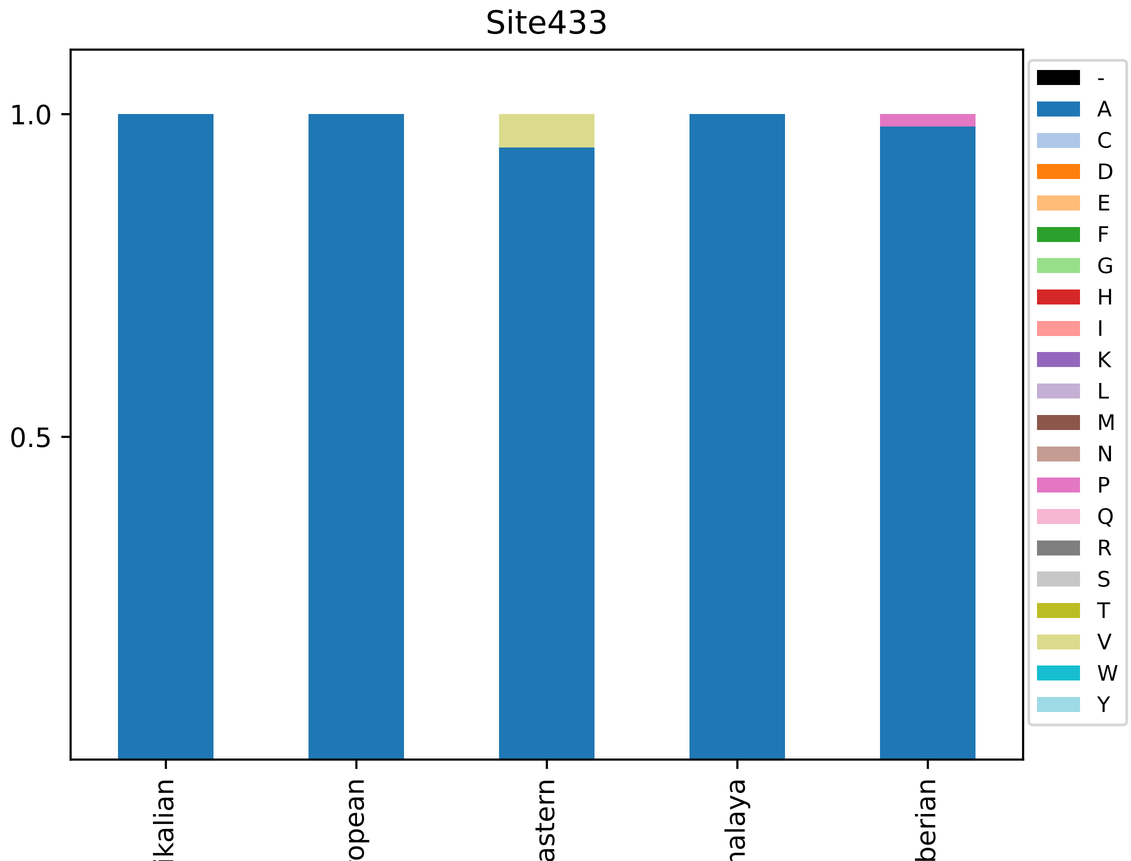
Site433
1.0
0.5
-
A
C
D
E
F
G
H
I
K
L
M
N
P
Q
R
S
T
V
W
Y
ikalian
opean
astern
nalaya
berian

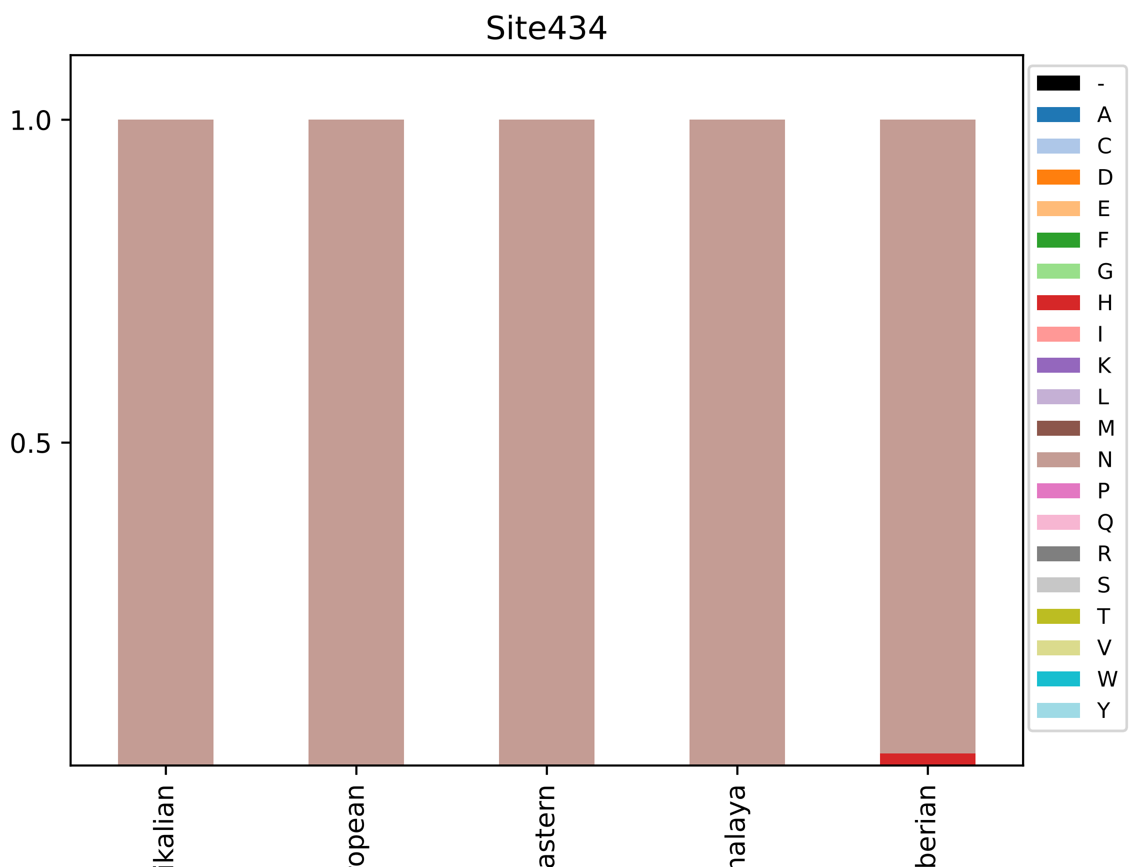

Site434
1.0
0.5
-
A
C
D
E
F
G
H
I
K
L
M
N
P
Q
R
S
T
V
W
Y
ikalian
opean
astern
nalaya
berian

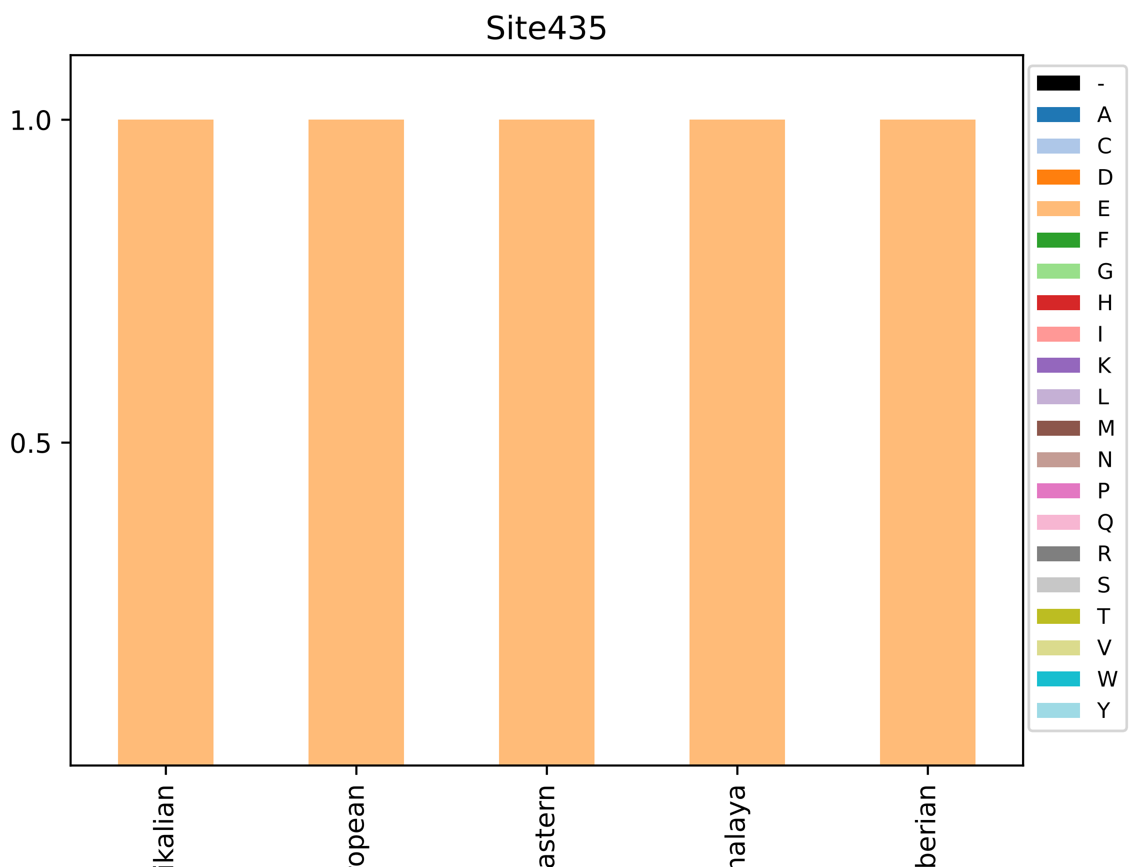
Site435
1.0
0.5
-
A
C
D
E
F
G
H
I
K
L
M
N
P
Q
R
S
T
V
W
Y
ikalian
opean
astern
nalaya
berian

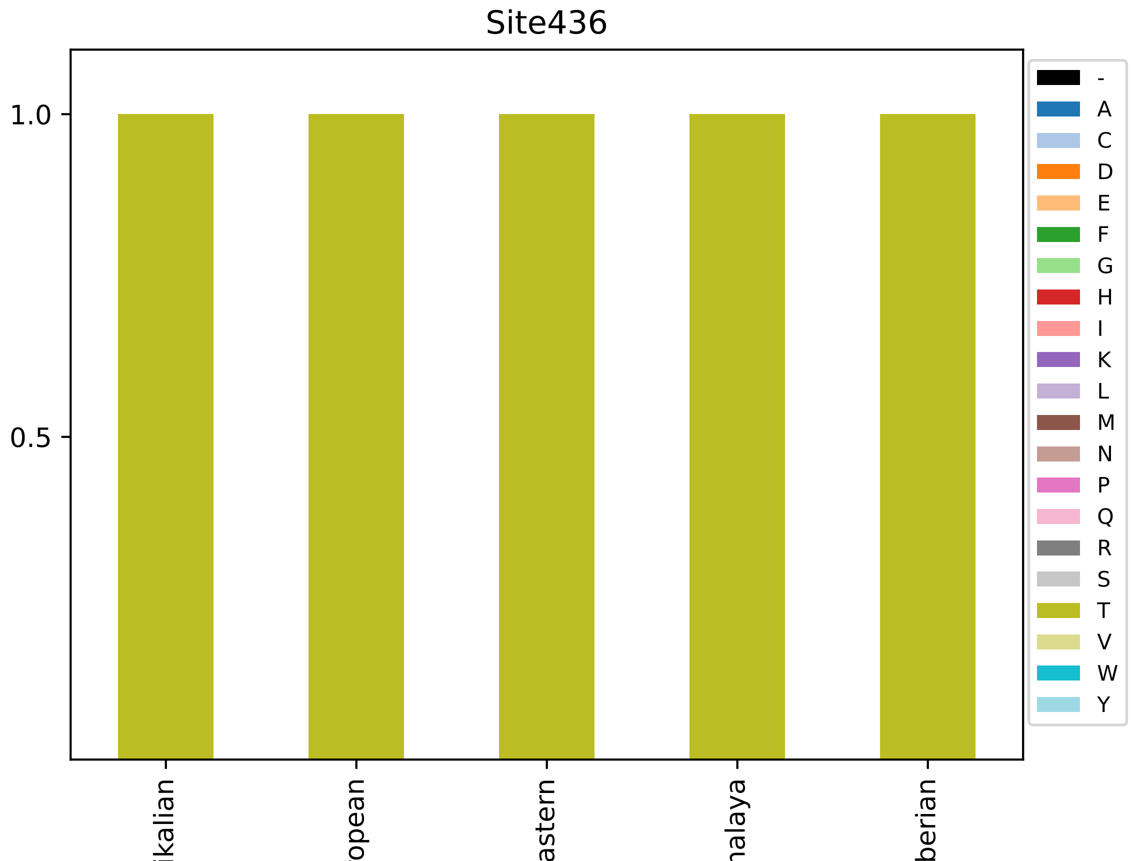

Site436
1.0
0.5
-
A
C
D
E
F
G
H
I
K
L
M
N
P
Q
R
S
T
V
W
Y
ikalian
ropean
astern
nalaya
berian

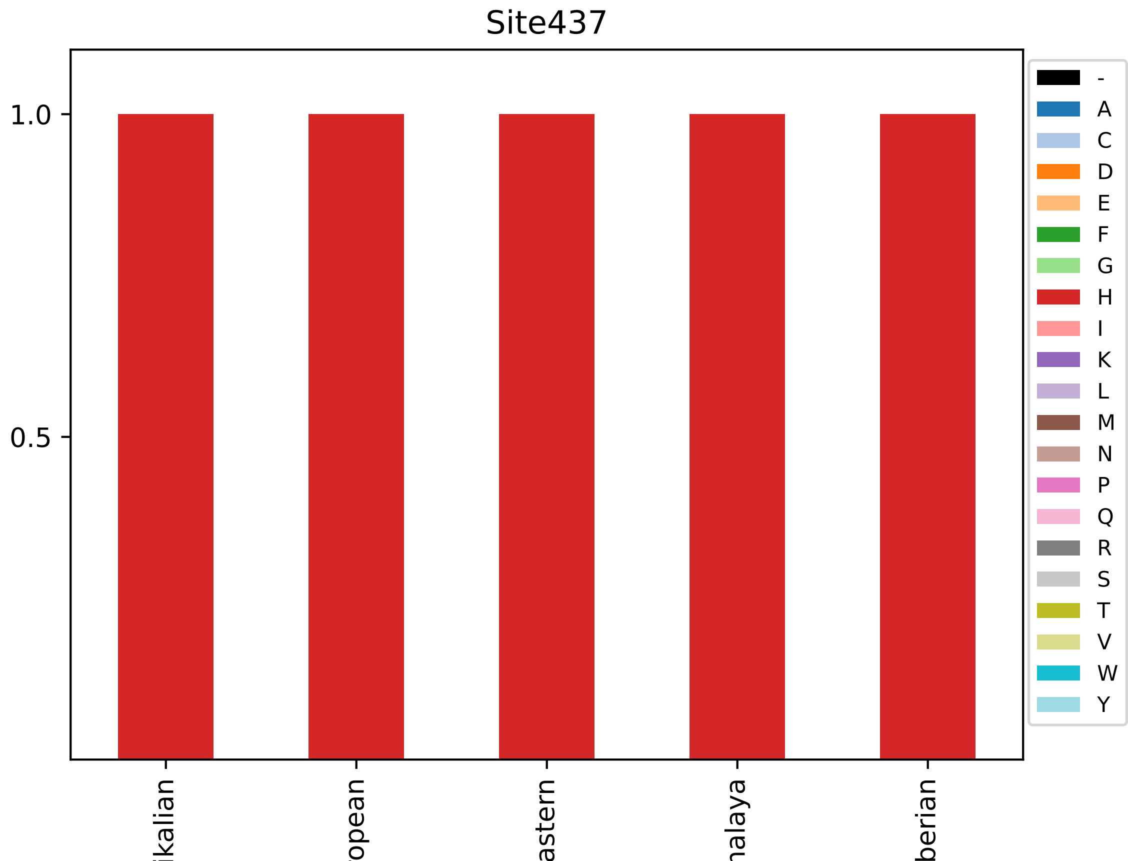
Site437
1.0
0.5
-
A
C
D
E
F
G
H
I
K
L
M
N
P
Q
R
S
T
V
W
Y
ikalian
opean
astern
nalaya
berian

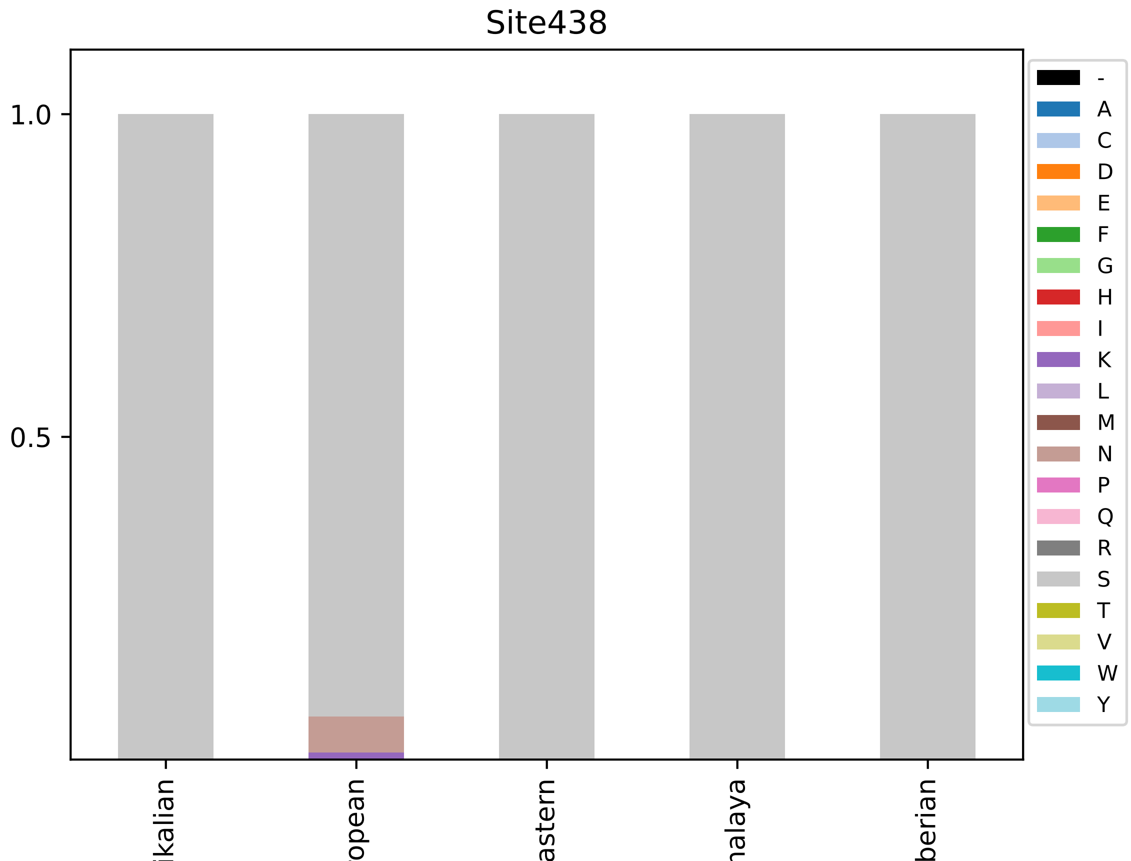
Site438
1.0
0.5
-
A
C
D
E
F
G
H
I
K
L
M
N
P
Q
R
S
T
V
W
Y
ikalian
opean
astern
nalaya
berian

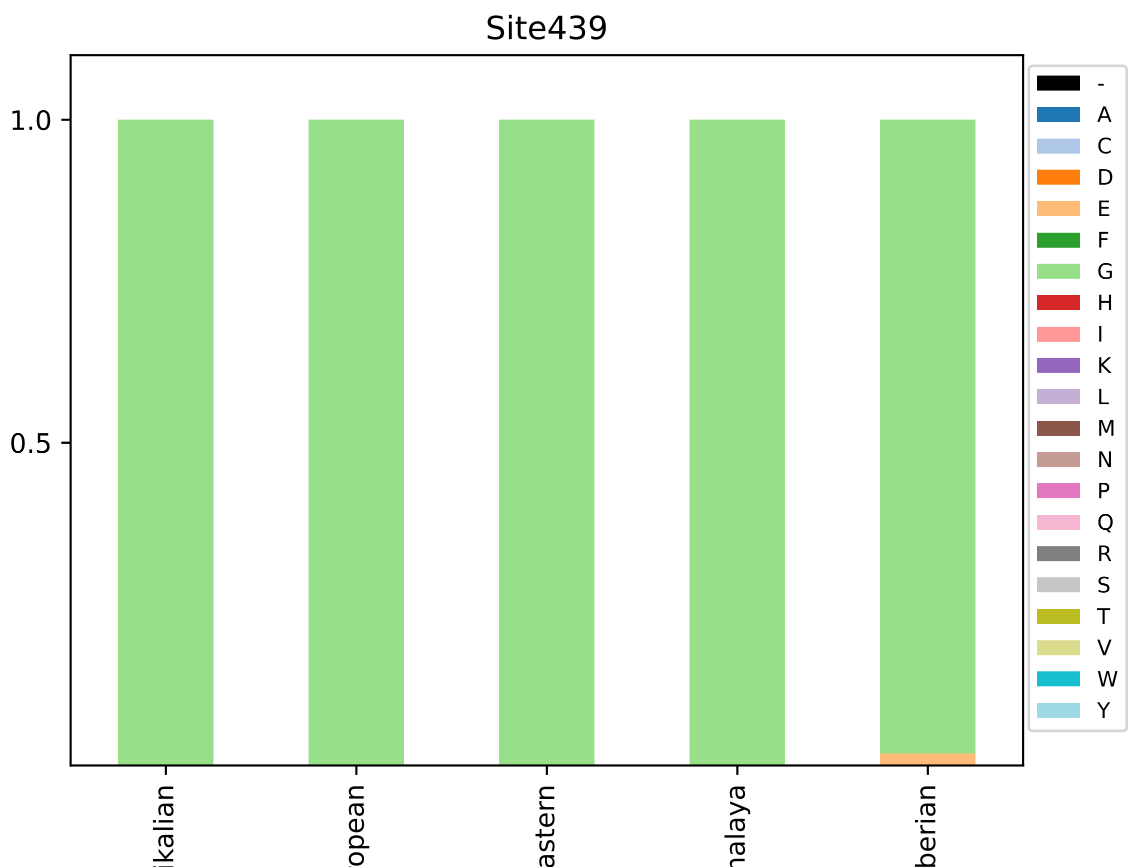
Site439
1.0
0.5
-
A
C
D
E
F
G
H
I
K
L
M
N
P
Q
R
S
T
V
W
Y
ikalian
opean
astern
nalaya
berian

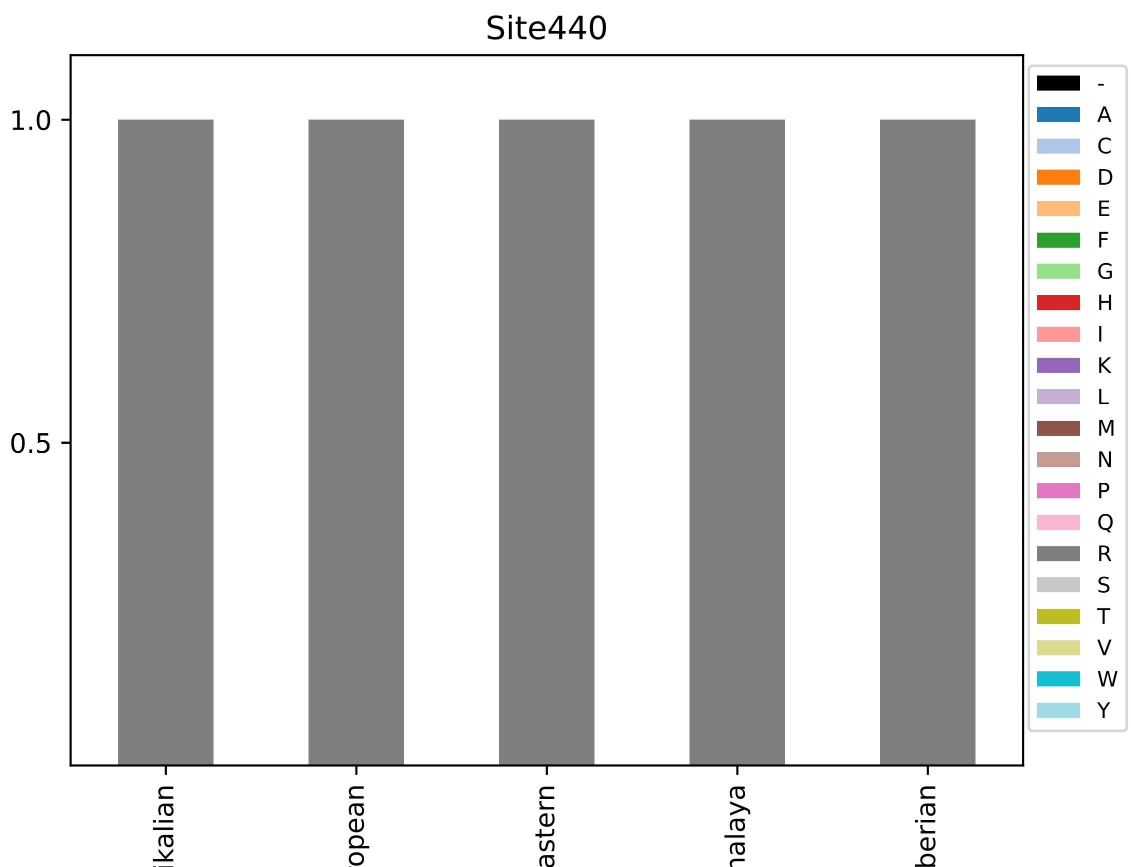
Site440
1.0
0.5
-
A
C
D
E
F
G
H
I
K
L
M
N
P
Q
R
S
T
V
W
Y
ikalian
opean
astern
nalaya
berian

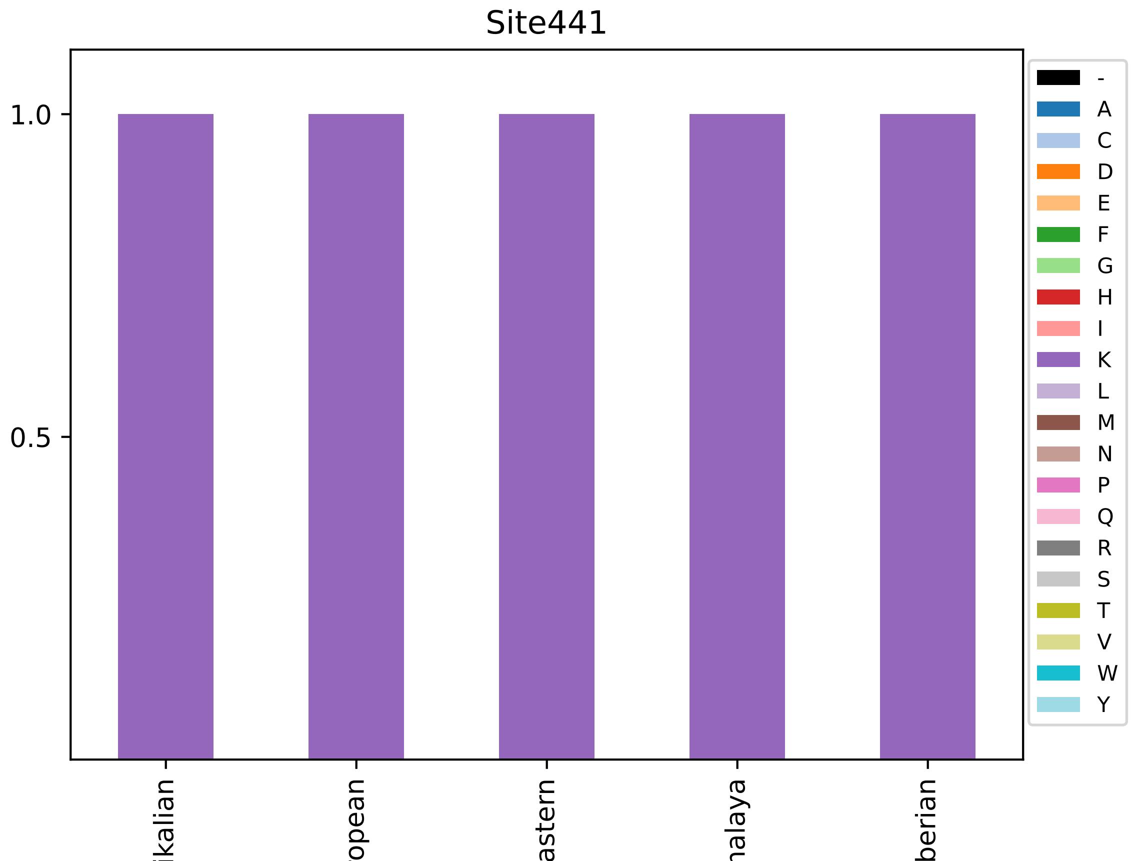
Site441
1.0
0.5
-
A
C
D
E
F
G
H
I
K
L
M
N
P
Q
R
S
T
V
W
Y
ikalian
opean
astern
nalaya
berian

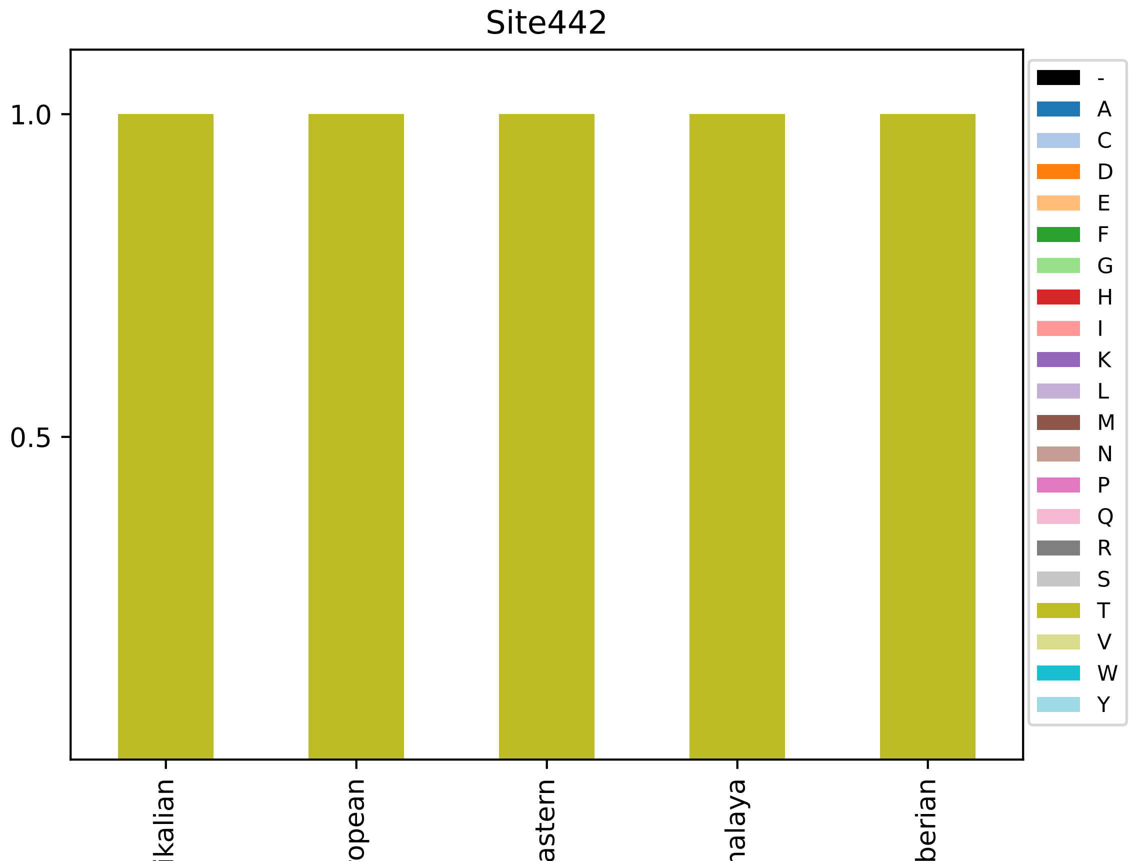
Site442
1.0
0.5
ikalian
opean
astern
nalaya
berian
-
A
C
D
E
F
G
H
I
K
L
M
N
P
Q
R
S
T
V
W
Y

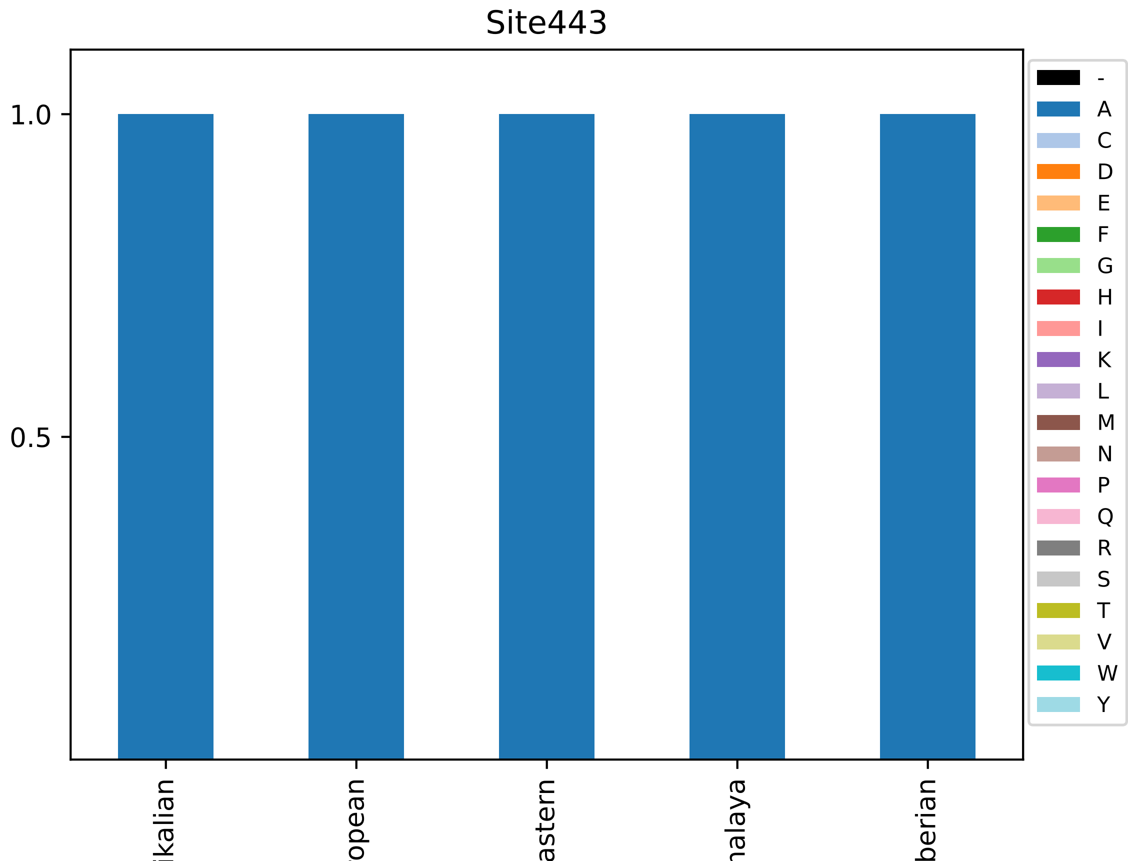
Site443
1.0
0.5
-
A
C
D
E
F
G
H
I
K
L
M
N
P
Q
R
S
T
V
W
Y
ikalian
opean
astern
nalaya
berian

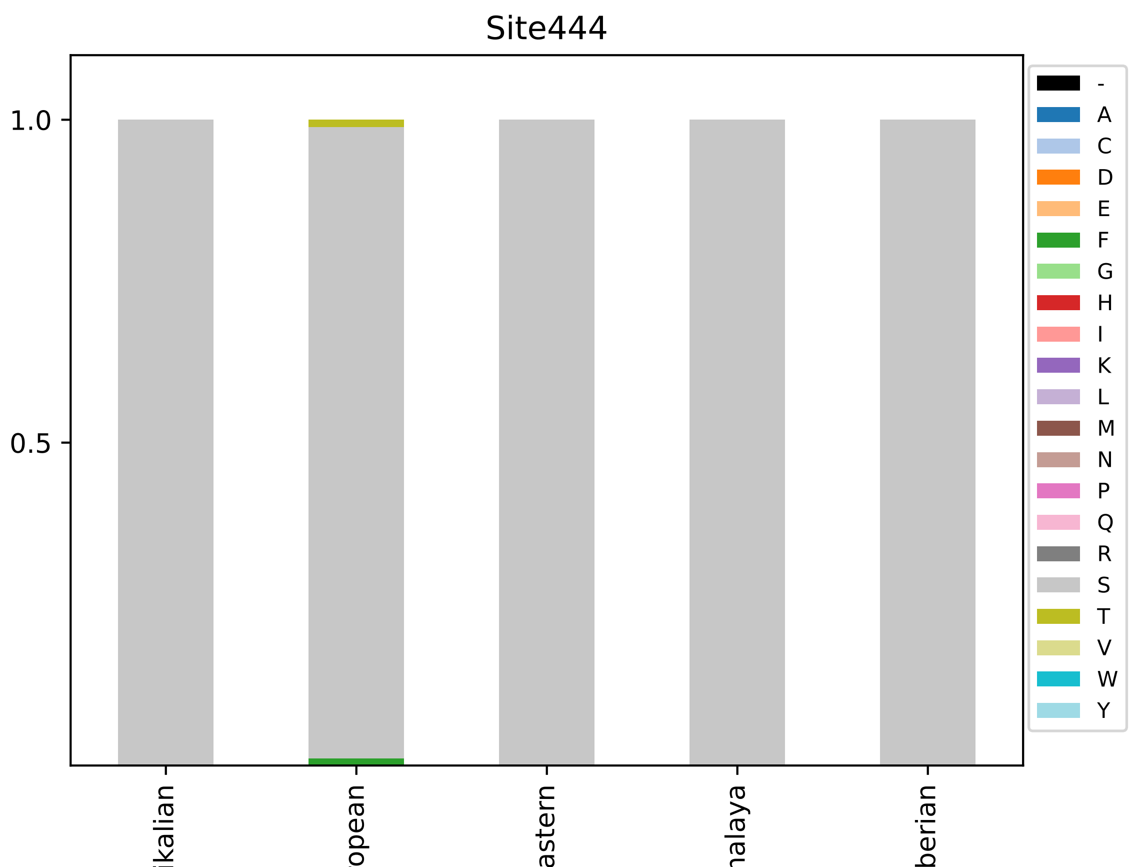
Site444
1.0
0.5
-
A
C
D
E
F
G
H
I
K
L
M
N
P
Q
R
S
T
V
W
Y
ikalian
opean
astern
nalaya
berian

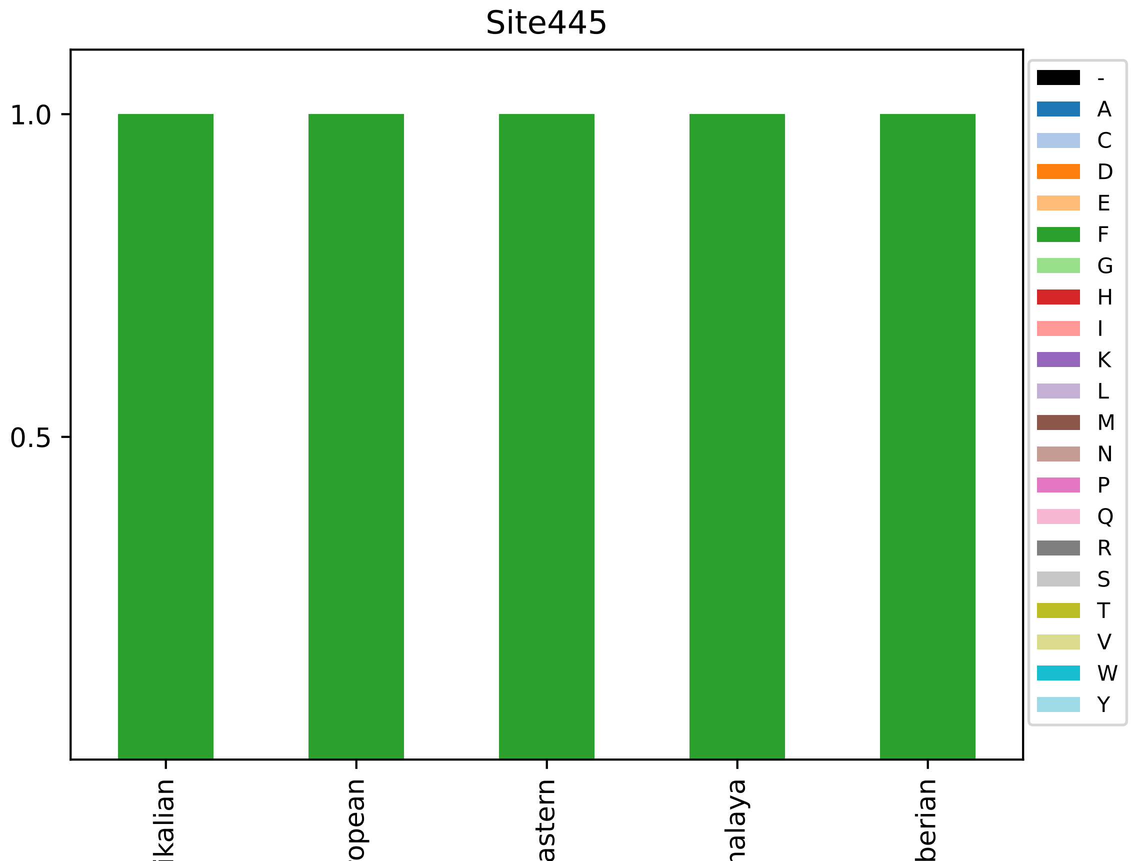

Site445
1.0
0.5
-
A
C
D
E
F
G
H
I
K
L
M
N
P
Q
R
S
T
V
W
Y
ikalian
opean
astern
nalaya
berian

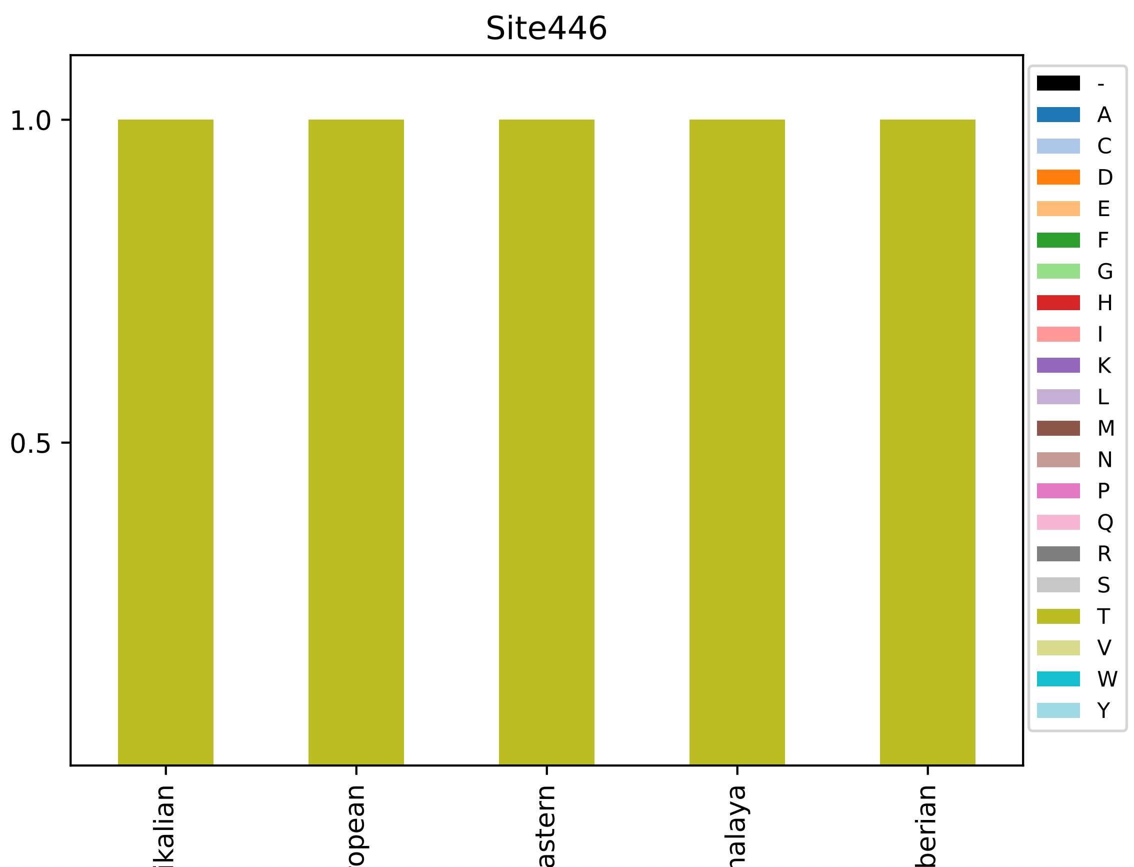
Site446
1.0
0.5
-
A
C
D
E
F
G
H
I
K
L
M
N
P
Q
R
S
T
V
W
Y
ikalian
opean
astern
nalaya
berian

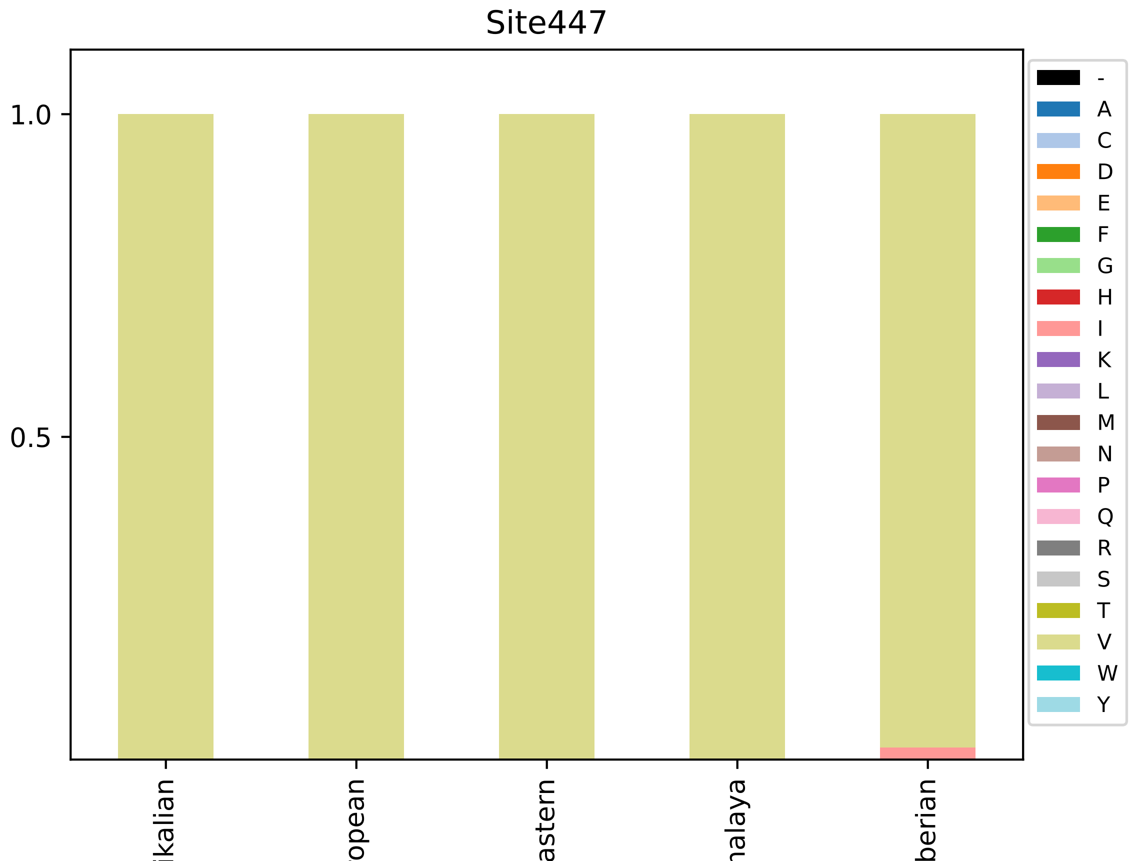
Site447
1.0
0.5
ikalian
opean
astern
nalaya
berian
-
A
C
D
E
F
G
H
I
K
L
M
N
P
Q
R
S
T
V
W
Y

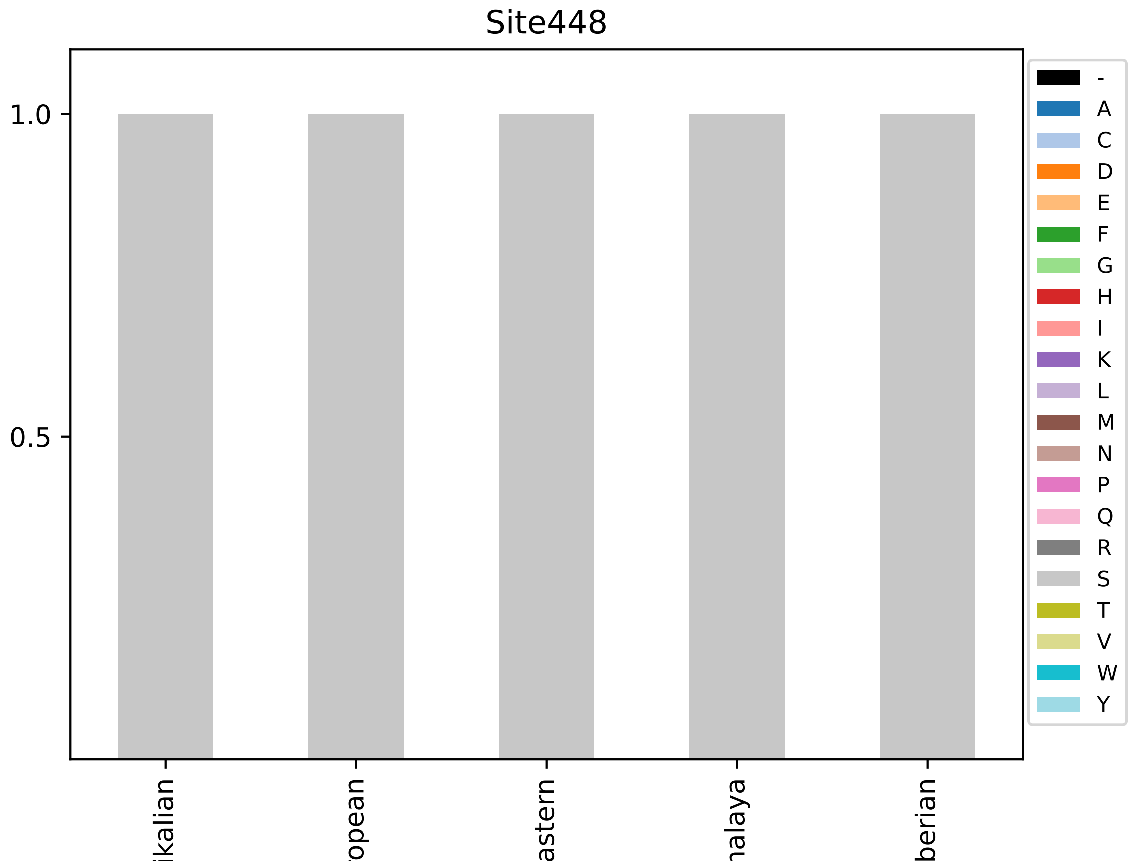

Site448
1.0
0.5
-
A
C
D
E
F
G
H
I
K
L
M
N
P
Q
R
S
T
V
W
Y
ikalian
opean
astern
nalaya
berian

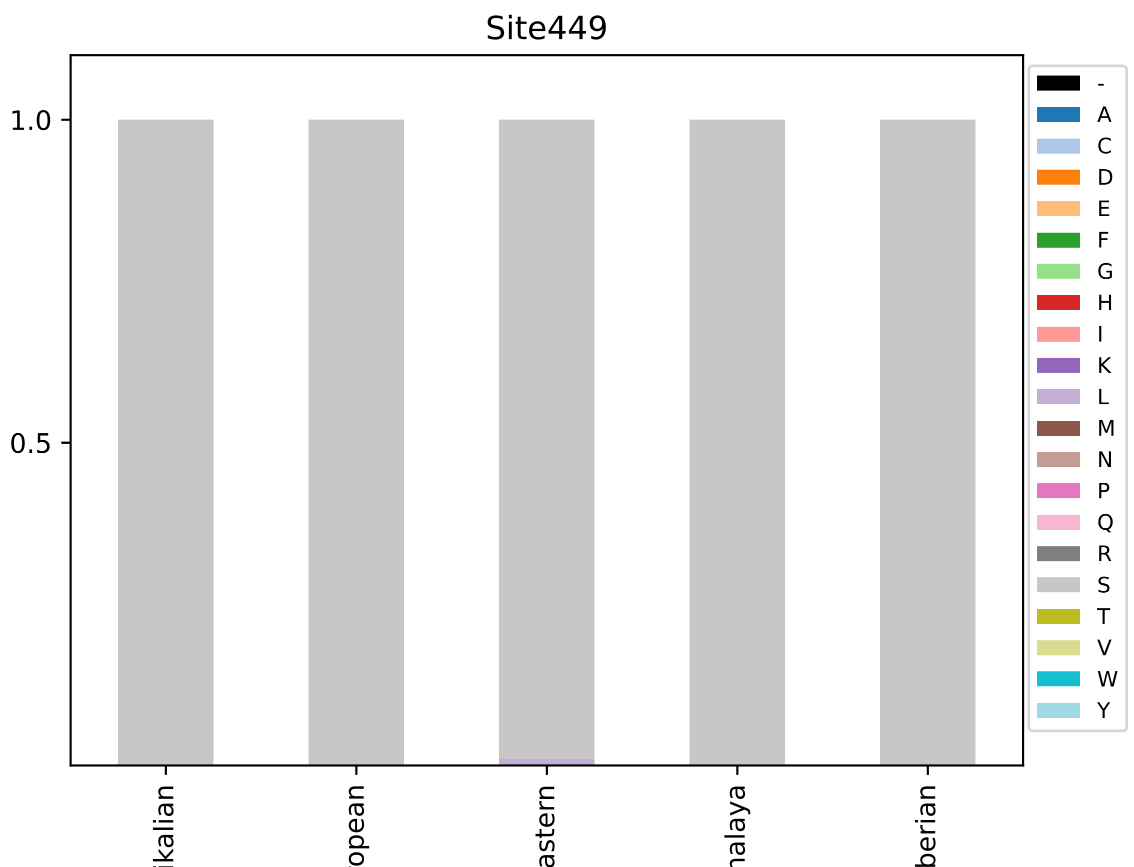
Site449
1.0
0.5
ikalian
opean
astern
nalaya
berian
-
A
C
D
E
F
G
H
I
K
L
M
N
P
Q
R
S
T
V
W
Y

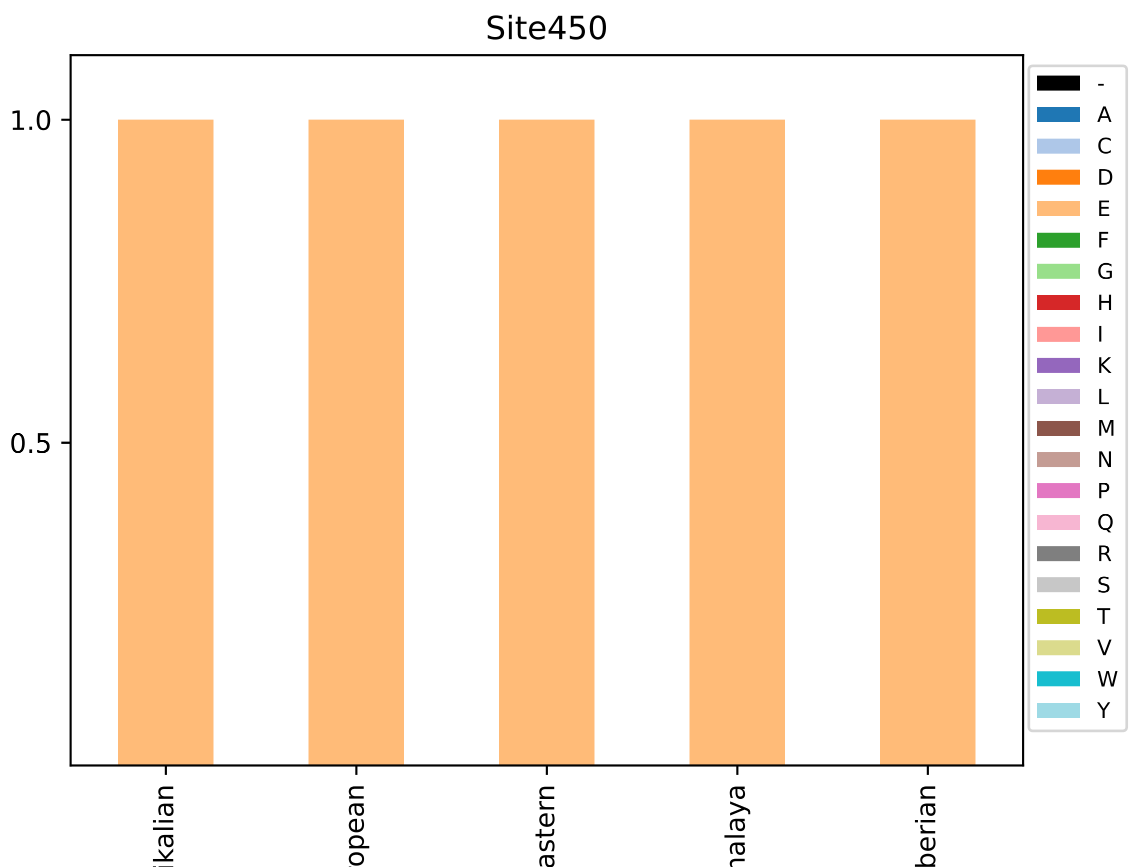
Site450
1.0
0.5
-
A
C
D
E
F
G
H
I
K
L
M
N
P
Q
R
S
T
V
W
Y
ikalian
opean
astern
nalaya
berian

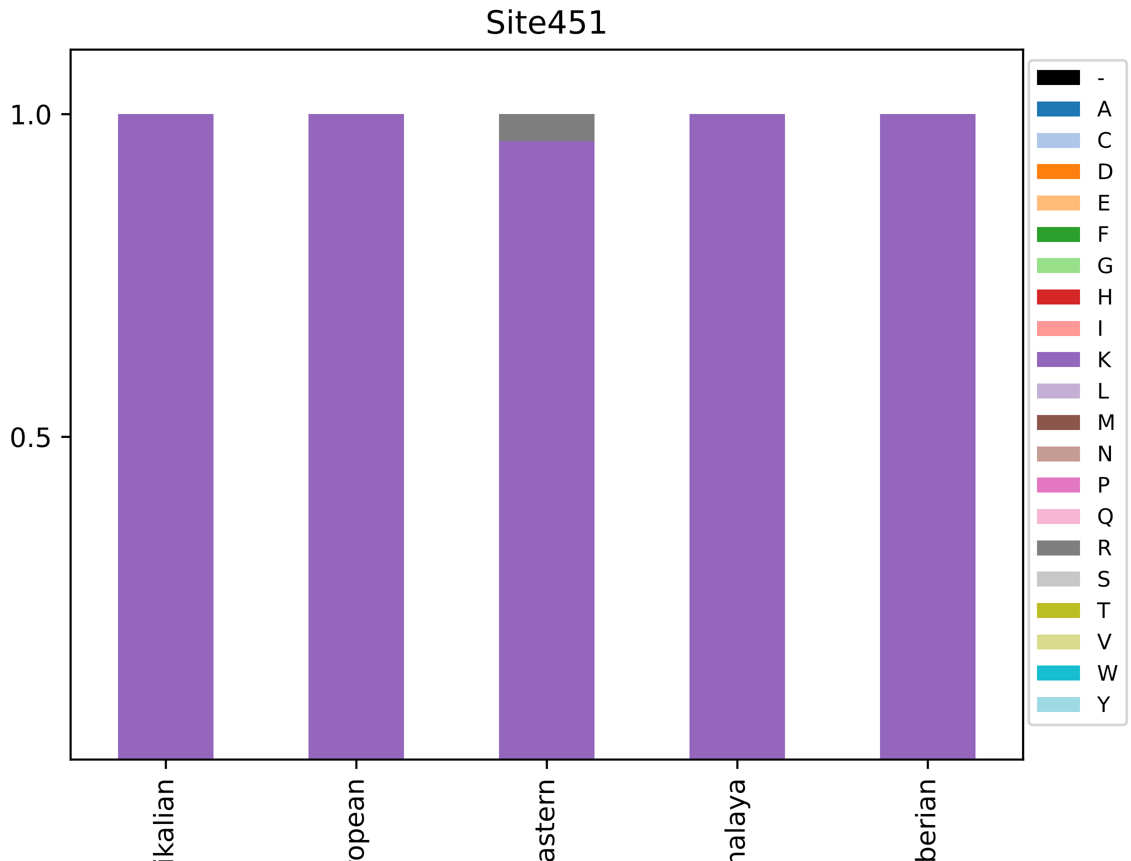
Site451
1.0
0.5
-
A
C
D
E
F
G
H
I
K
L
M
N
P
Q
R
S
T
V
W
Y
ikalian
opean
astern
nalaya
berian

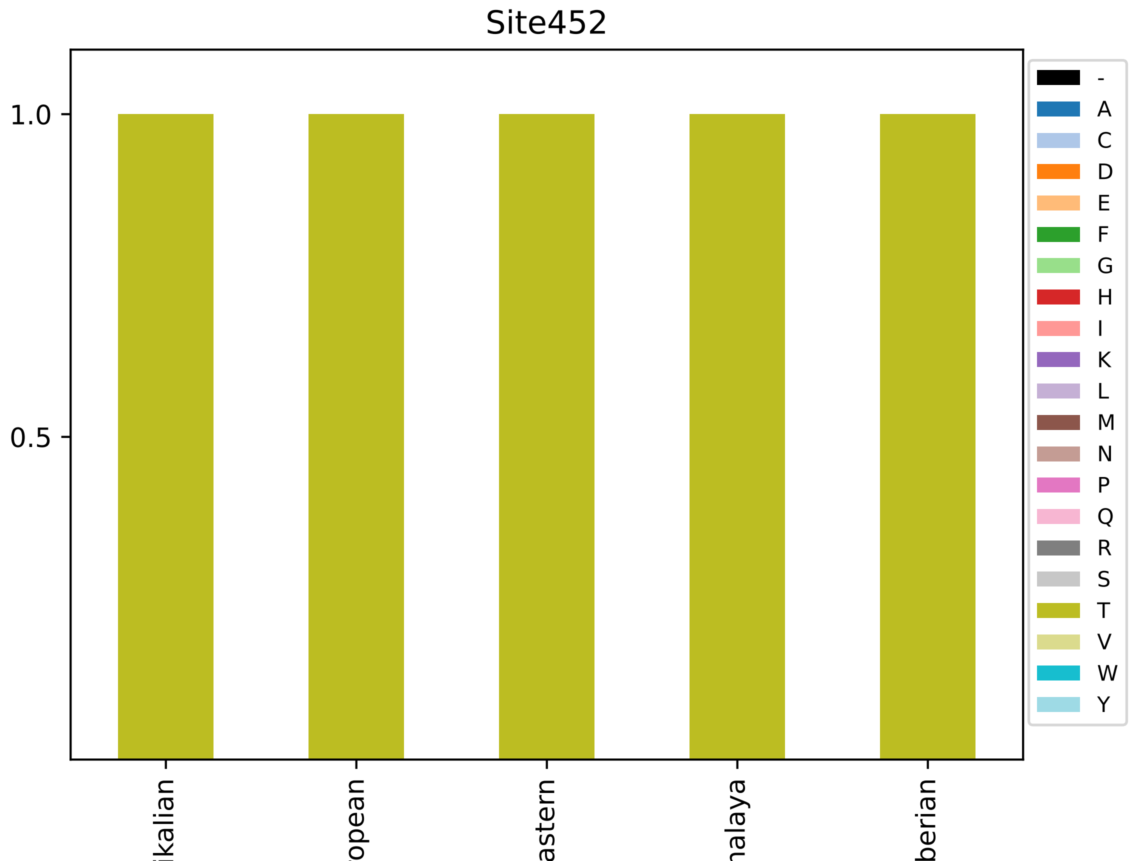

Site452
1.0
0.5
-
A
C
D
E
F
G
H
I
K
L
M
N
P
Q
R
S
T
V
W
Y
ikalian
opean
astern
nalaya
berian

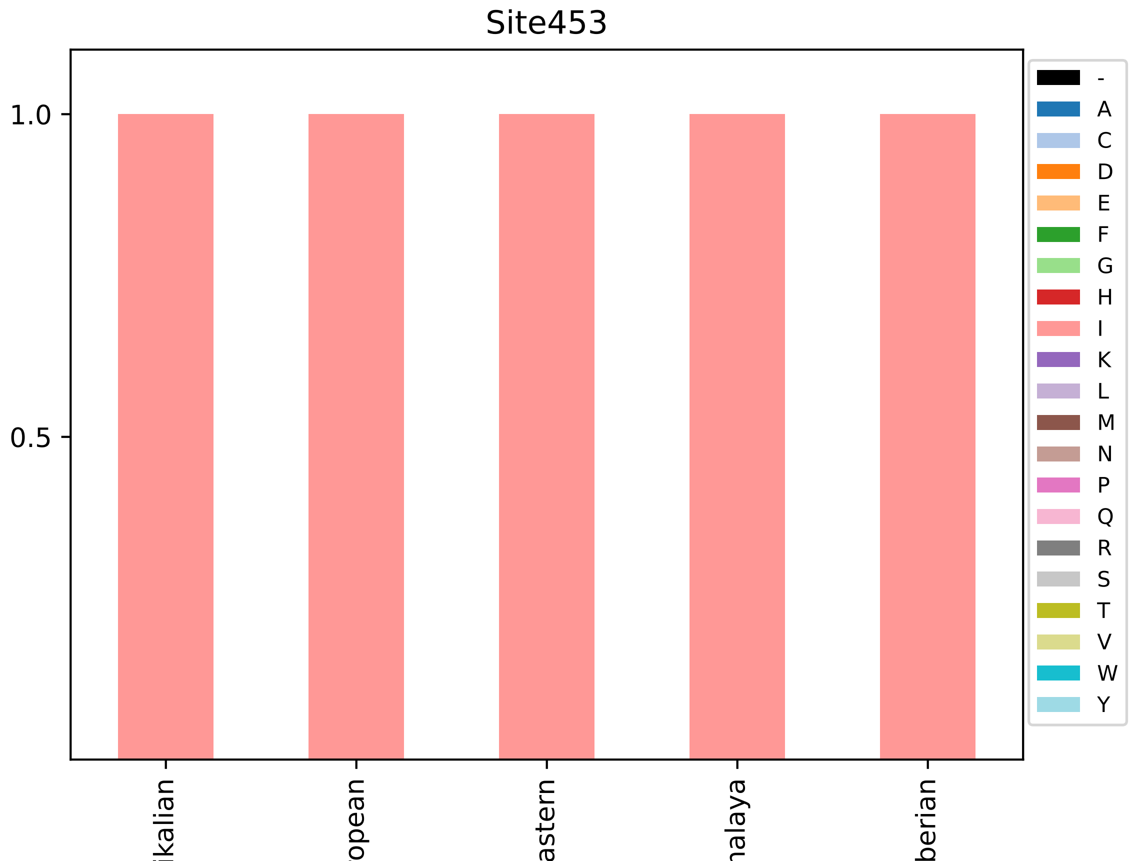

Site453
1.0
0.5
-
A
C
D
E
F
G
H
I
K
L
M
N
P
Q
R
S
T
V
W
Y
ikalian
opean
astern
nalaya
berian

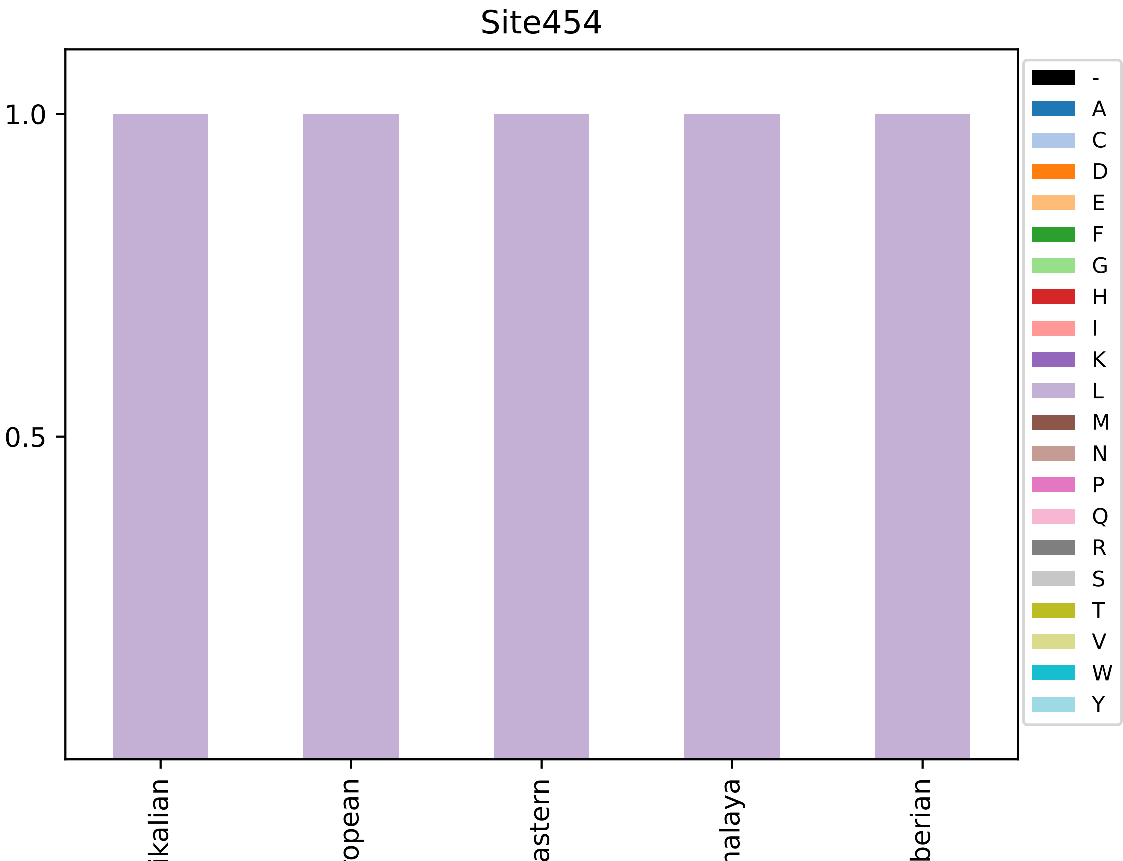

Site454
1.0
0.5
-
A
C
D
E
F
G
H
I
K
L
M
N
P
Q
R
S
T
V
W
Y
ikalian
ropean
astern
nalaya
berian

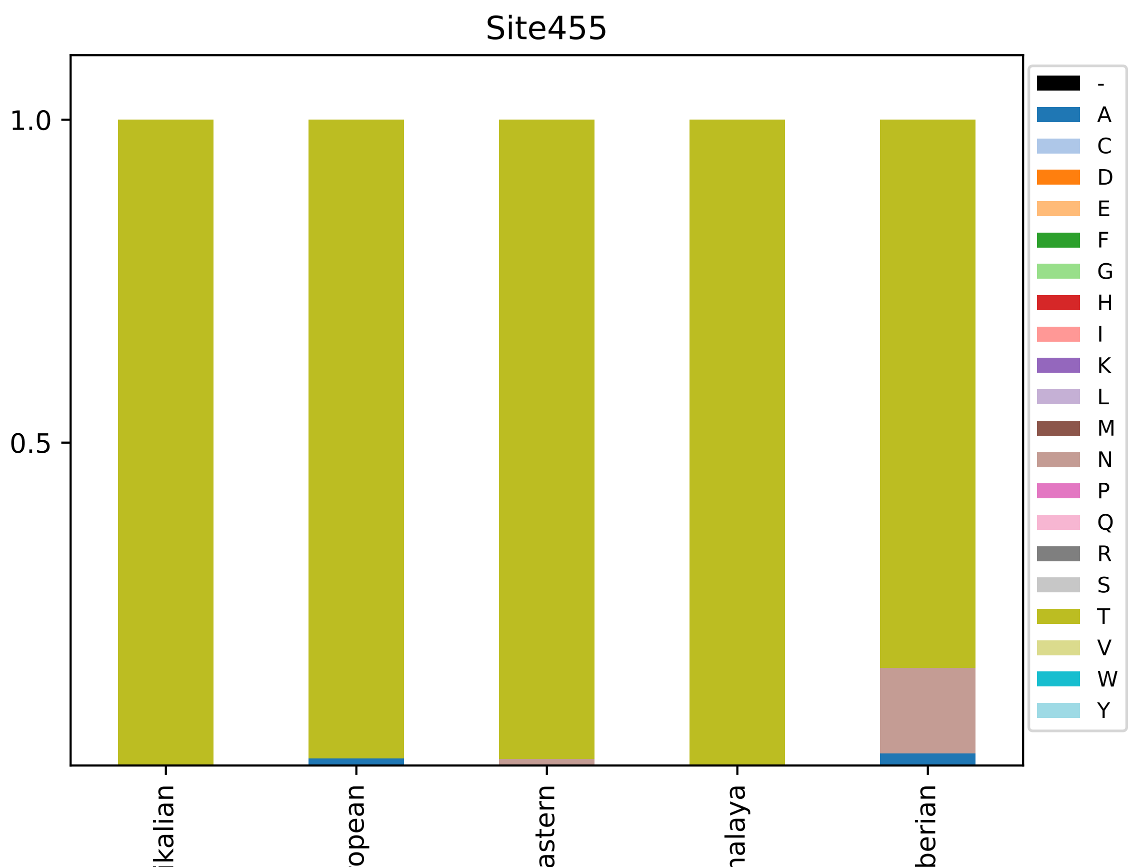

Site455
1.0
0.5
-
A
C
D
E
F
G
H
I
K
L
M
N
P
Q
R
S
T
V
W
Y
ikalian
opean
astern
nalaya
berian

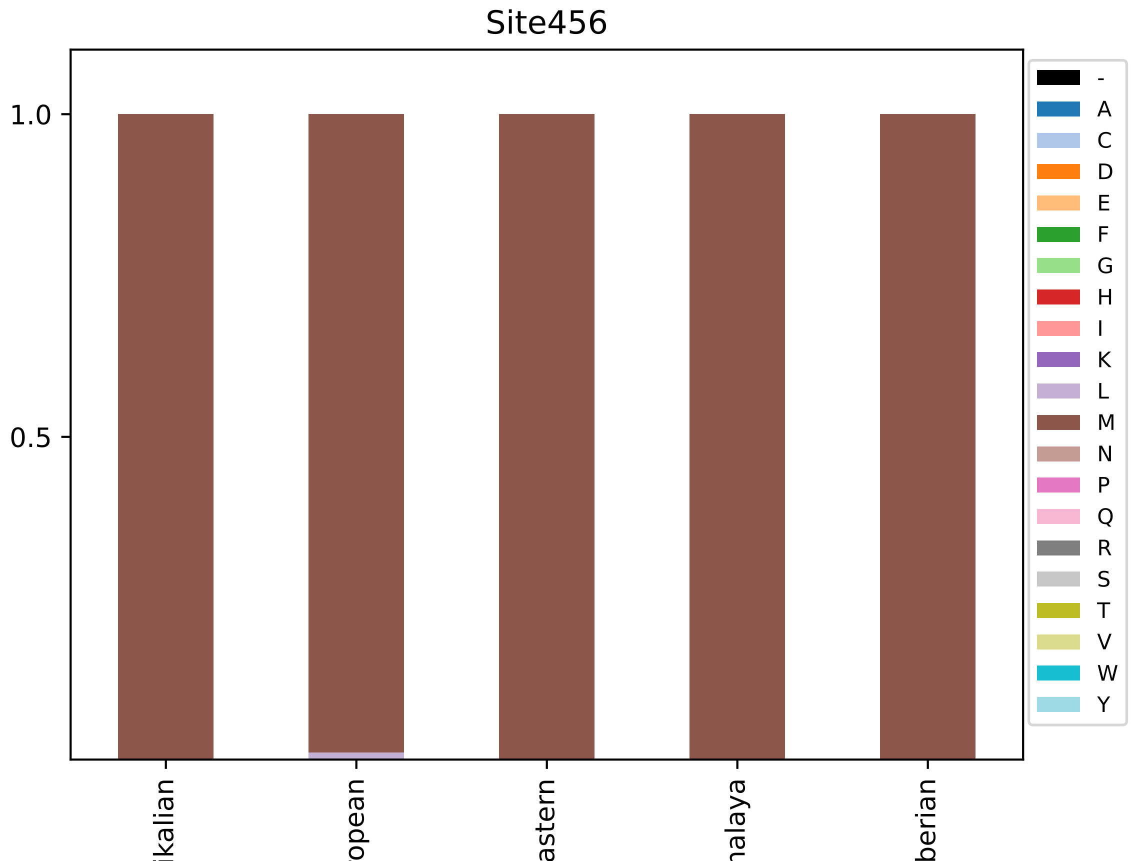
Site456
1.0
0.5
ikalian
opean
astern
nalaya
berian
-
A
C
D
E
F
G
H
I
K
L
M
N
P
Q
R
S
T
V
W
Y

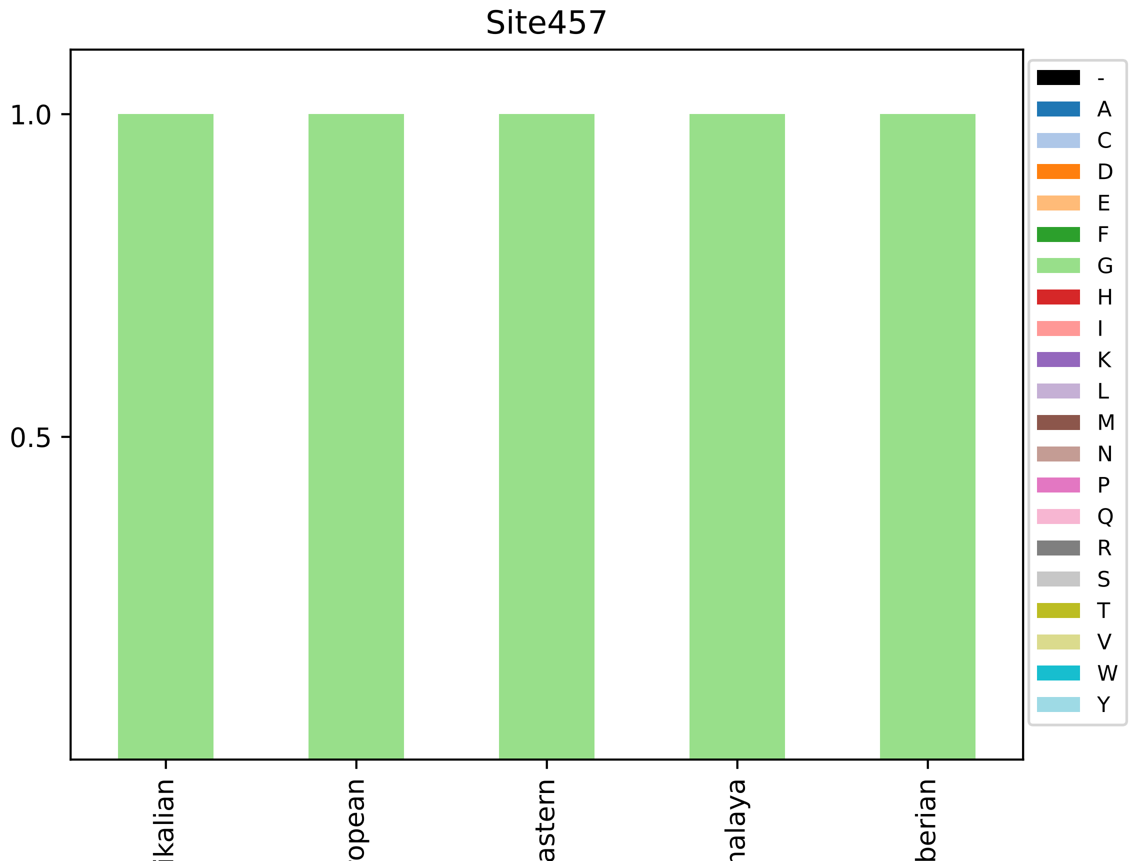
Site457
1.0
0.5
-
A
C
D
E
F
G
H
I
K
L
M
N
P
Q
R
S
T
V
W
Y
ikalian
opean
astern
nalaya
berian

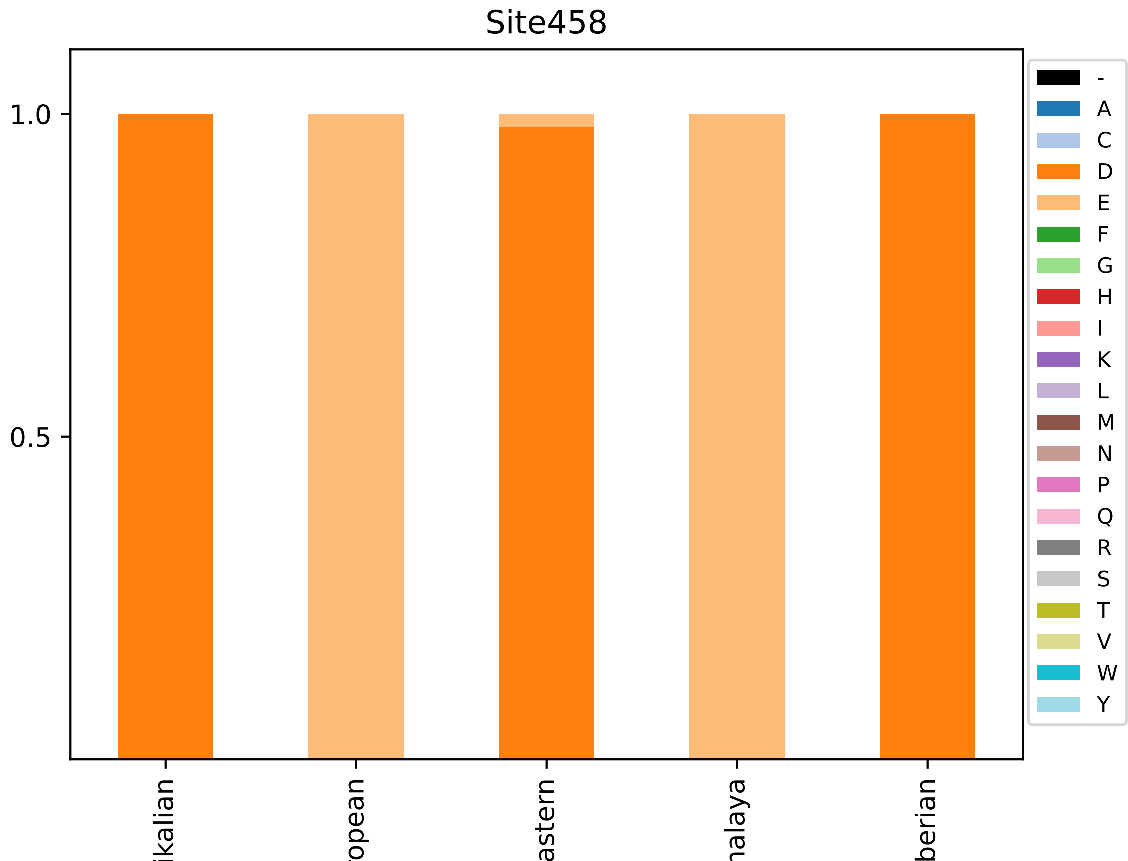

Site458
1.0
0.5
-
A
C
D
E
F
G
H
I
K
L
M
N
P
Q
R
S
T
V
W
Y
ikalian
opean
astern
nalaya
berian

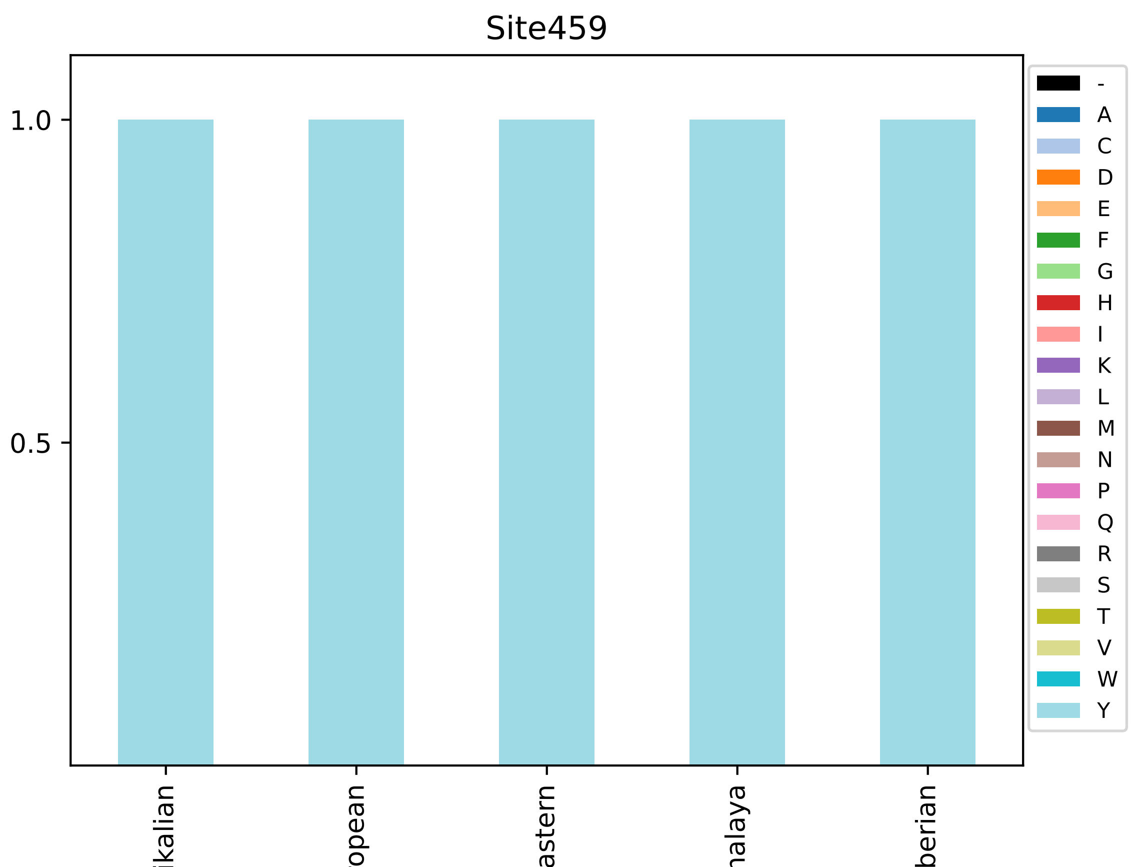

Site459
1.0
0.5
-
A
C
D
E
F
G
H
I
K
L
M
N
P
Q
R
S
T
V
W
Y
ikalian
opean
astern
nalaya
berian

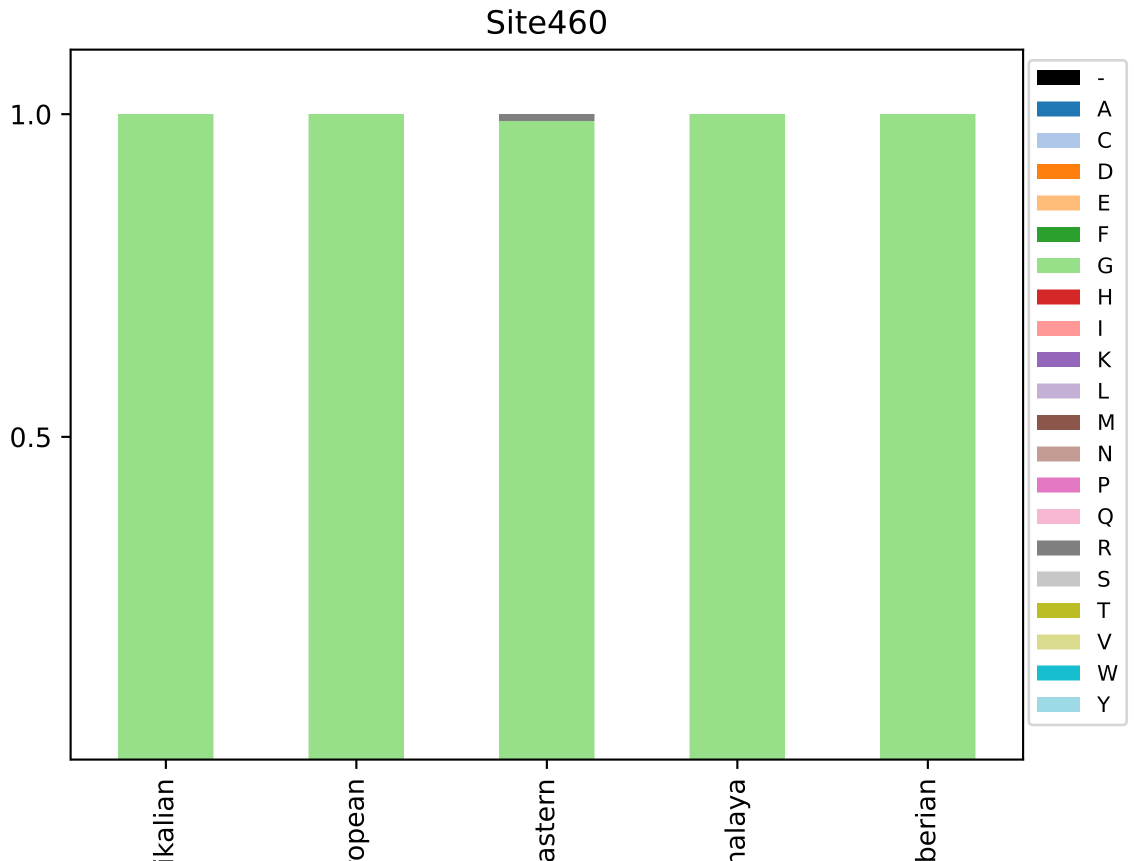
Site460
1.0
0.5
-
A
C
D
E
F
G
H
I
K
L
M
N
P
Q
R
S
T
V
W
Y
ikalian
opean
astern
nalaya
berian

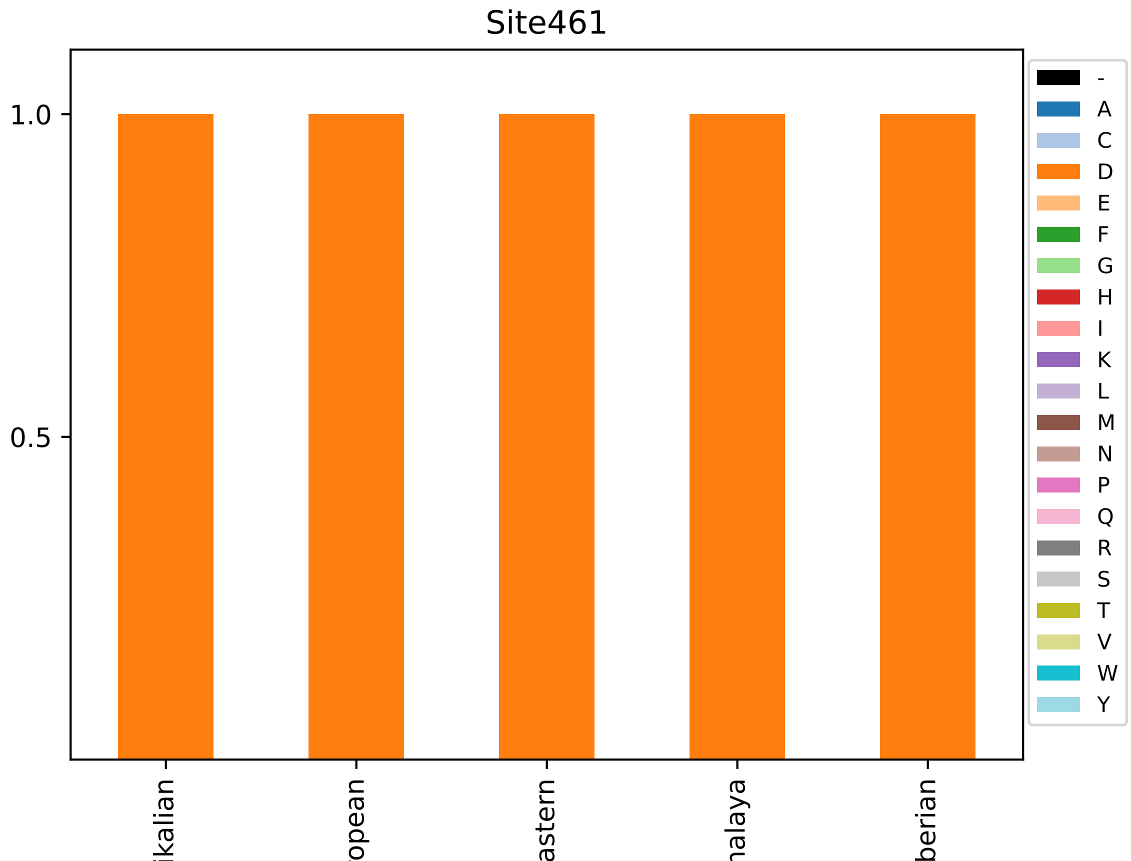
Site461
1.0
0.5
-
A
C
D
E
F
G
H
I
K
L
M
N
P
Q
R
S
T
V
W
Y

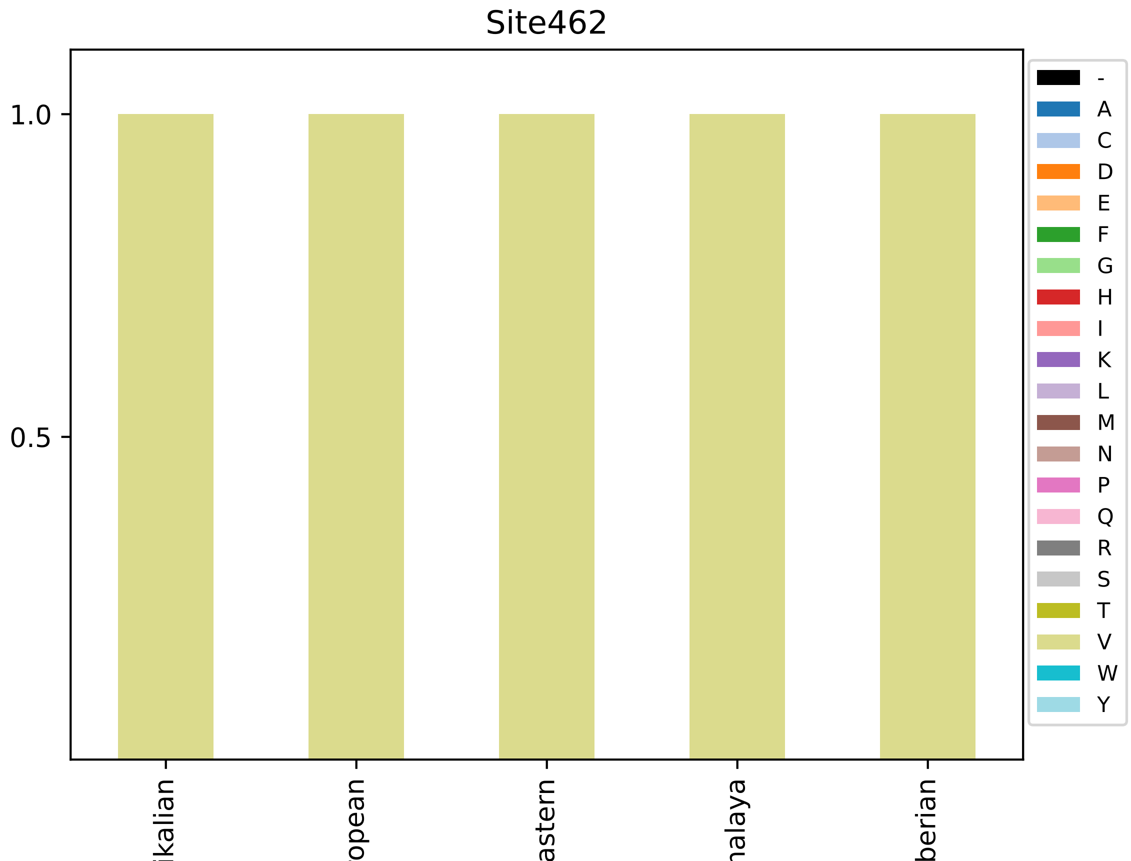

Site462
1.0
0.5
-
A
C
D
E
F
G
H
I
K
L
M
N
P
Q
R
S
T
V
W
Y
ikalian
opean
astern
nalaya
berian

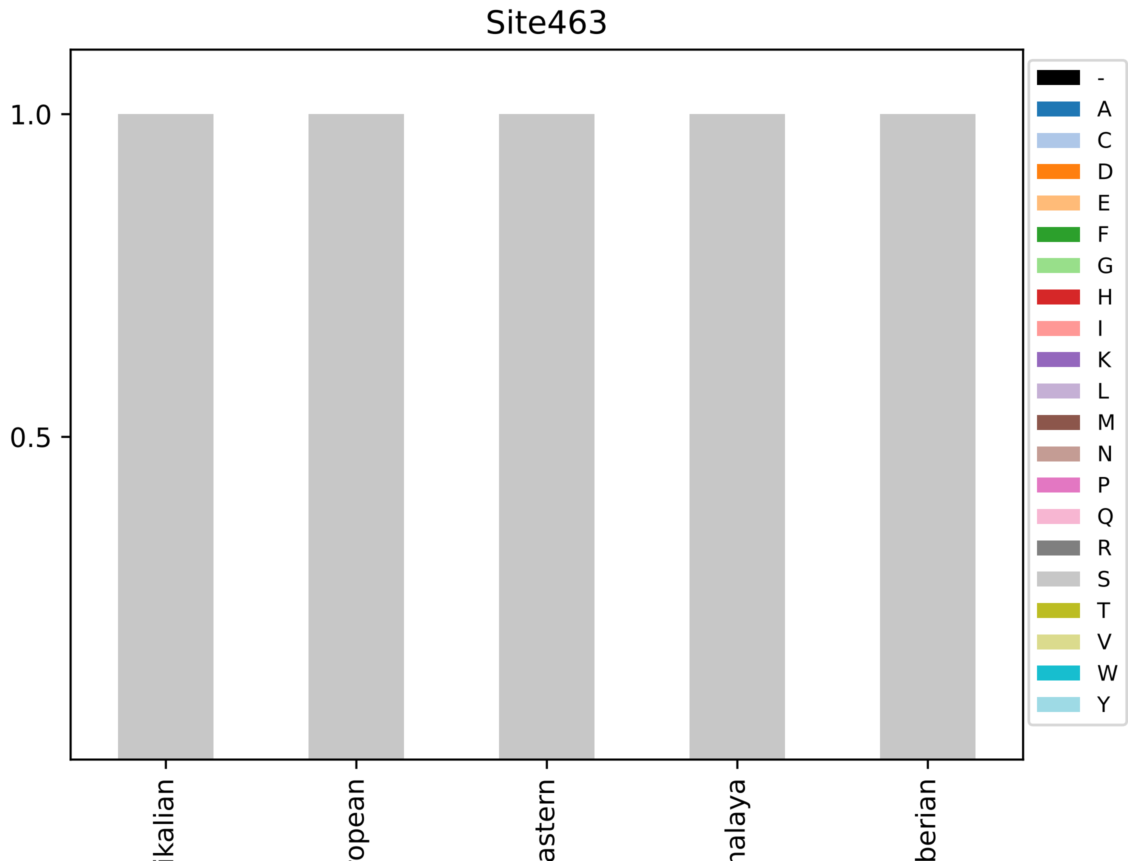

Site463
1.0
0.5
-
A
C
D
E
F
G
H
I
K
L
M
N
P
Q
R
S
T
V
W
Y
ikalian
opean
astern
nalaya
berian

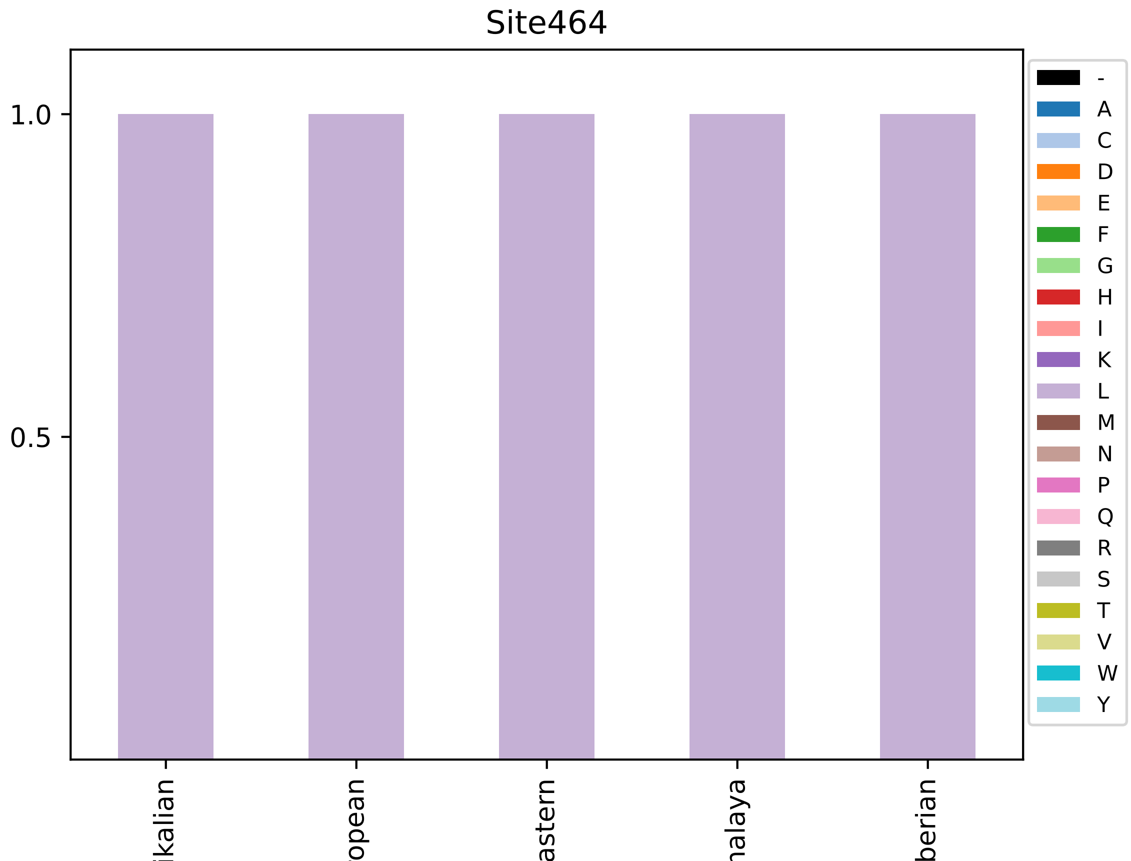

Site464
1.0
0.5
-
A
C
D
E
F
G
H
I
K
L
M
N
P
Q
R
S
T
V
W
Y

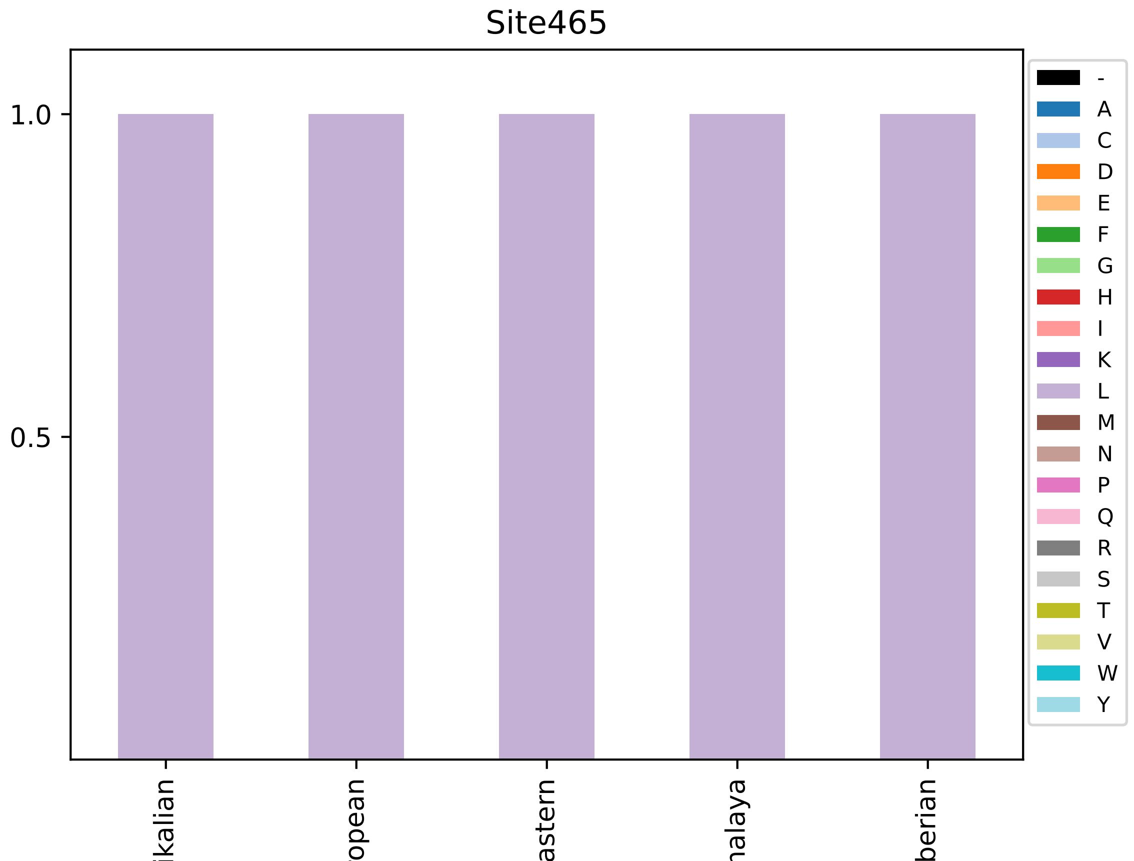

Site465
1.0
0.5
-
A
C
D
E
F
G
H
I
K
L
M
N
P
Q
R
S
T
V
W
Y
ikalian
opean
astern
nalaya
berian

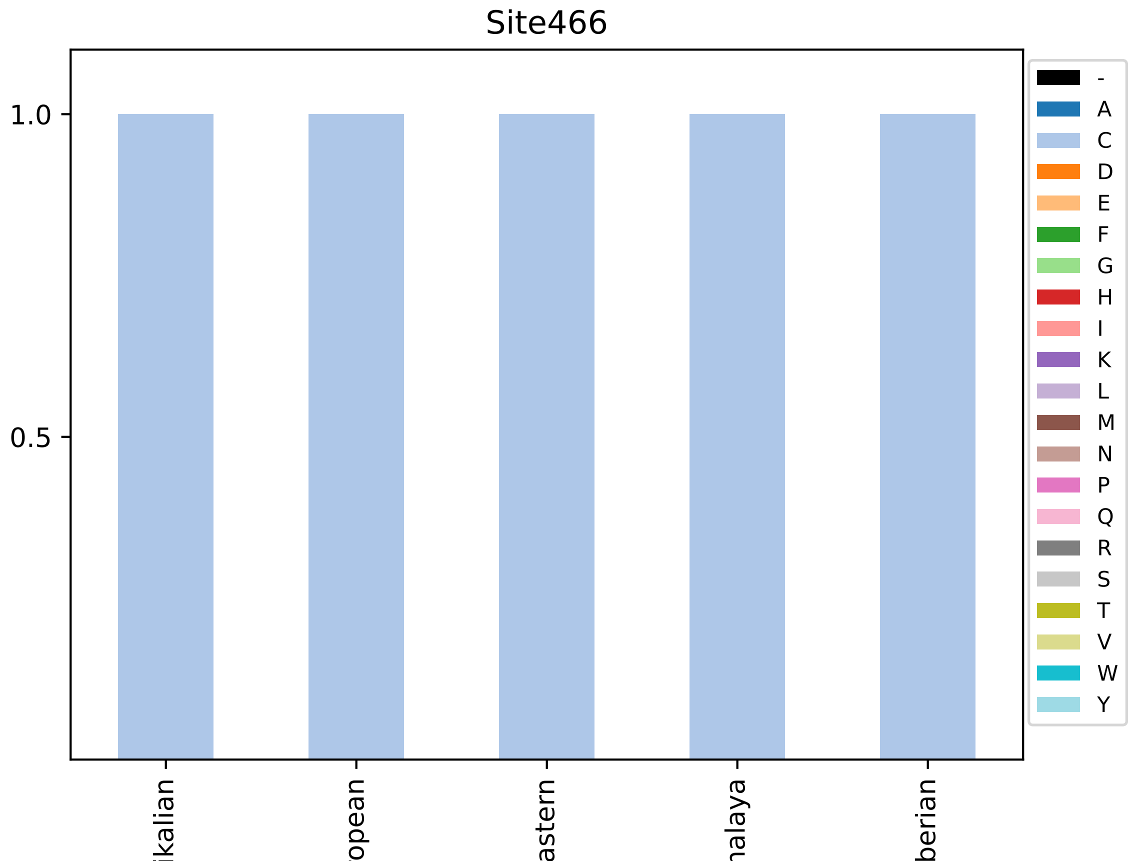
Site466
1.0
0.5
-
A
C
D
E
F
G
H
I
K
L
M
N
P
Q
R
S
T
V
W
Y
ikalian
opean
astern
nalaya
berian

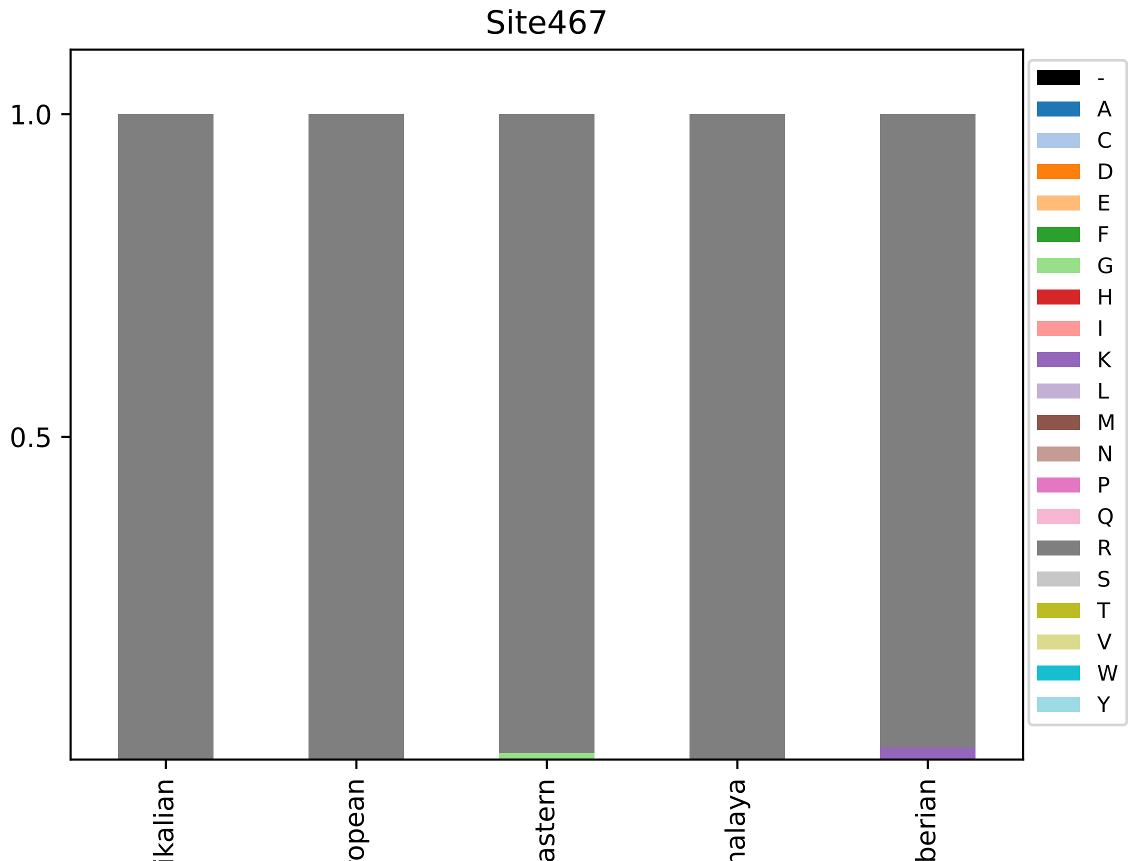
Site467
1.0
0.5
-
A
C
D
E
F
G
H
I
K
L
M
N
P
Q
R
S
T
V
W
Y
ikalian
opean
astern
nalaya
berian

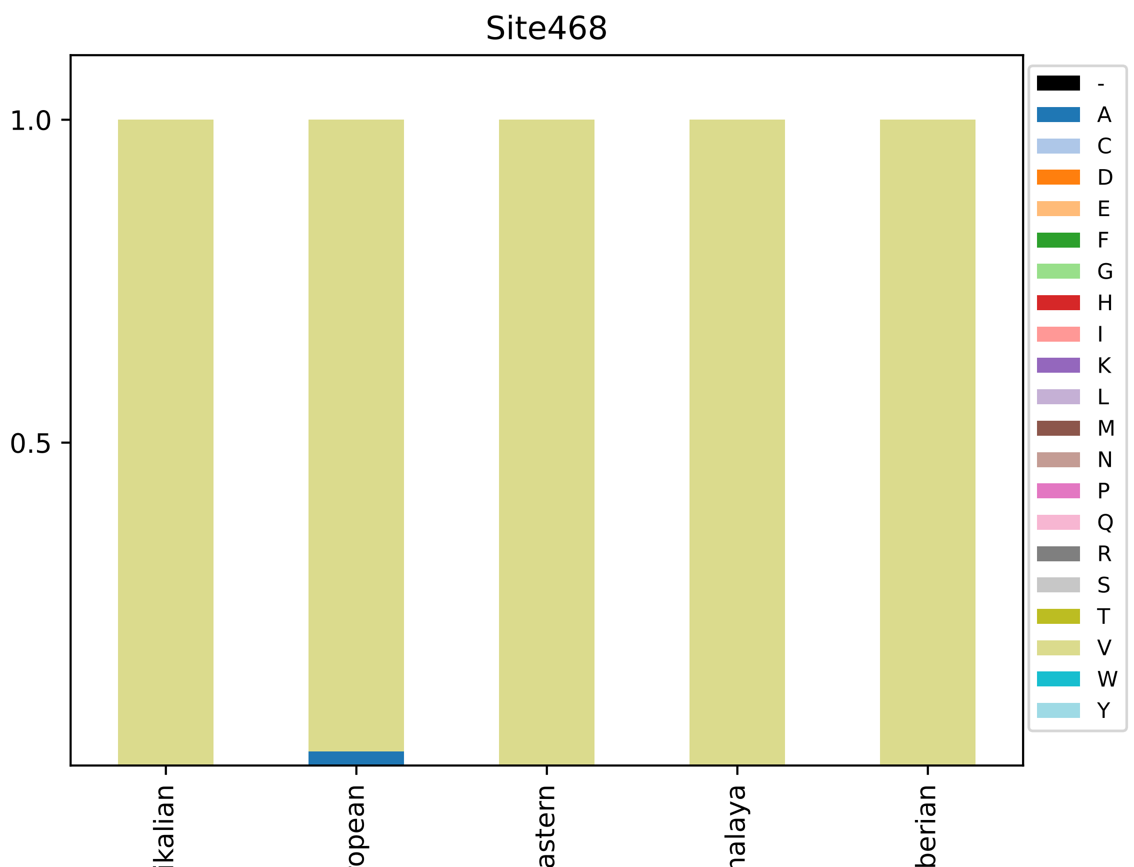
Site468
1.0
0.5
ikalian
ropean
astern
nalaya
berian
-
A
C
D
E
F
G
H
I
K
L
M
N
P
Q
R
S
T
V
W
Y

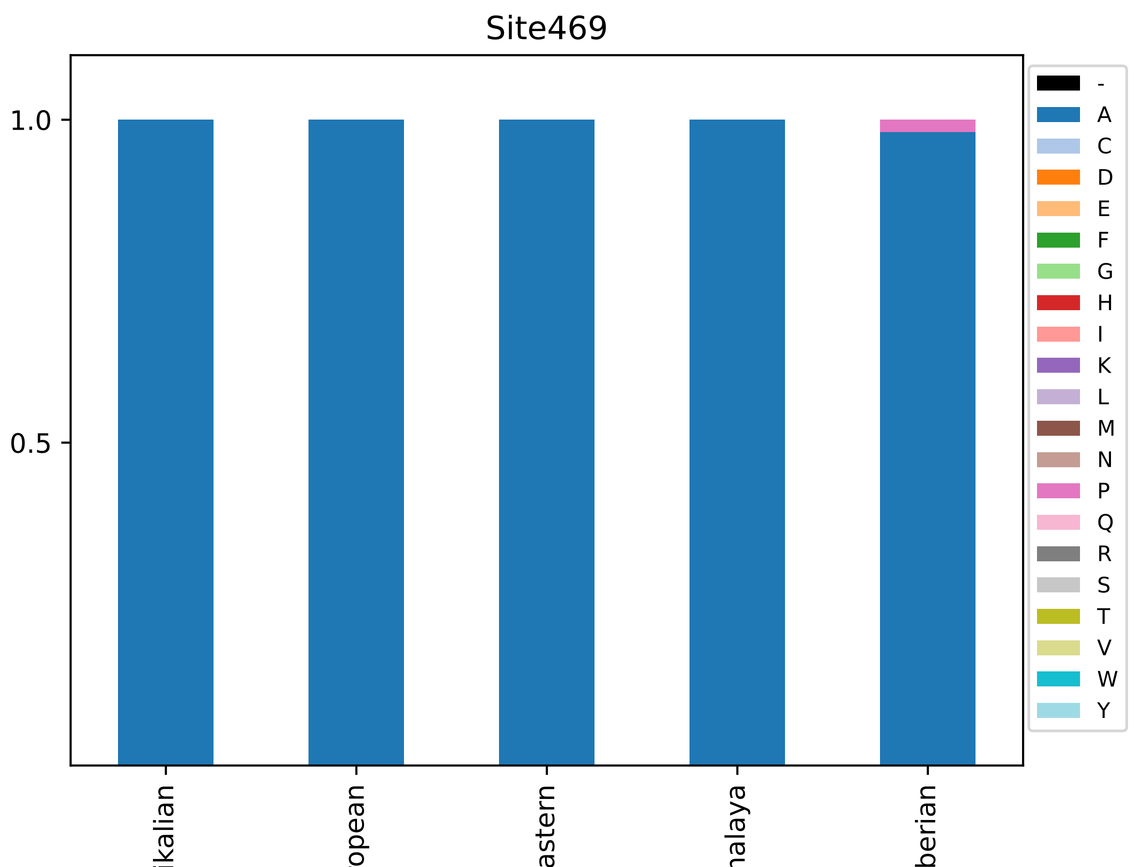
Site469
1.0
0.5
-
A
C
D
E
F
G
H
I
K
L
M
N
P
Q
R
S
T
V
W
Y
ikalian
opean
astern
nalaya
berian

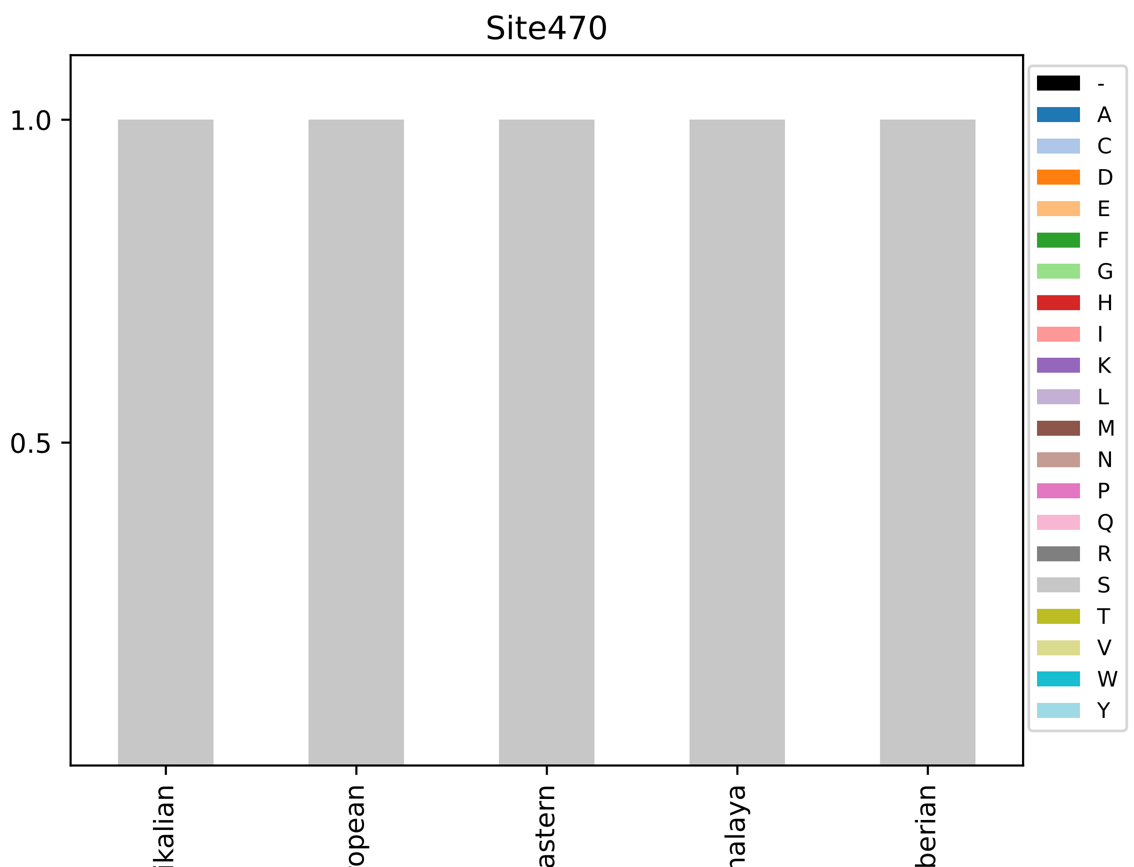

Site470
1.0
0.5
-
A
C
D
E
F
G
H
I
K
L
M
N
P
Q
R
S
T
V
W
Y
ikalian
opean
astern
nalaya
berian

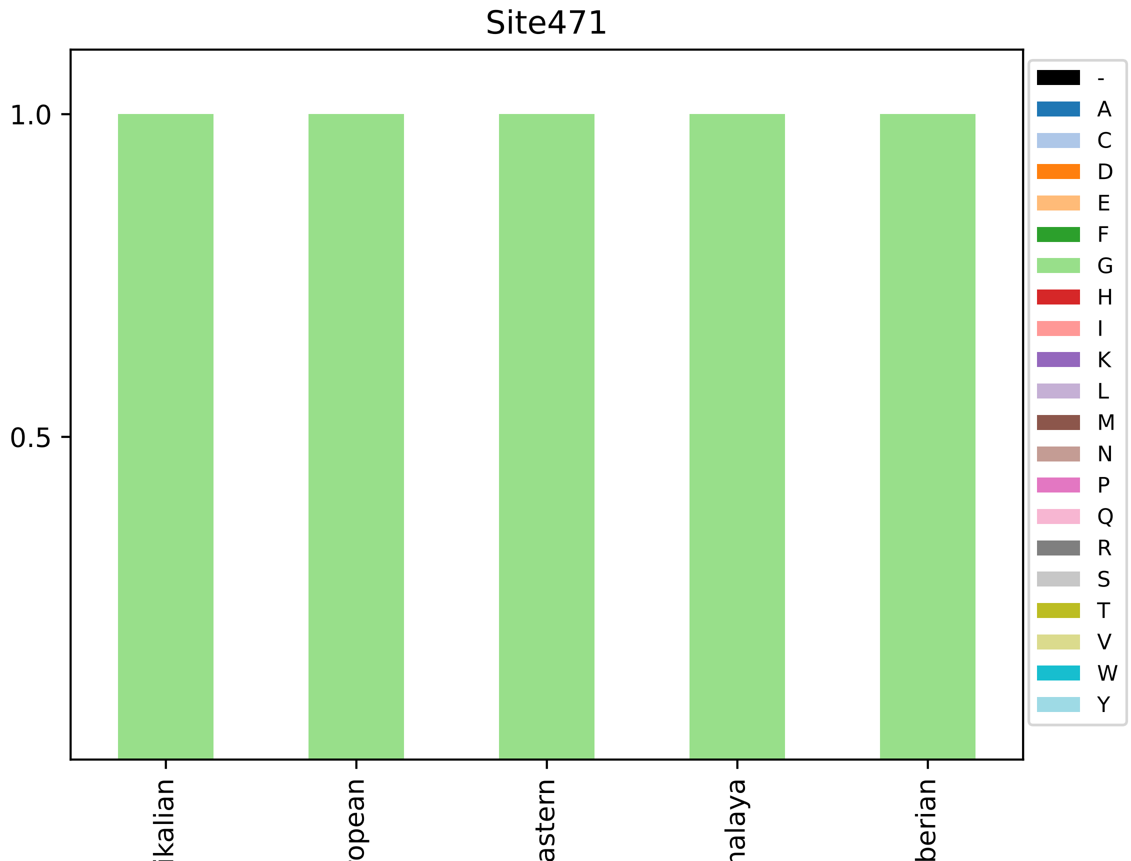
Site471
1.0
0.5
-
A
C
D
E
F
G
H
I
K
L
M
N
P
Q
R
S
T
V
W
Y
ikalian
opean
astern
nalaya
berian

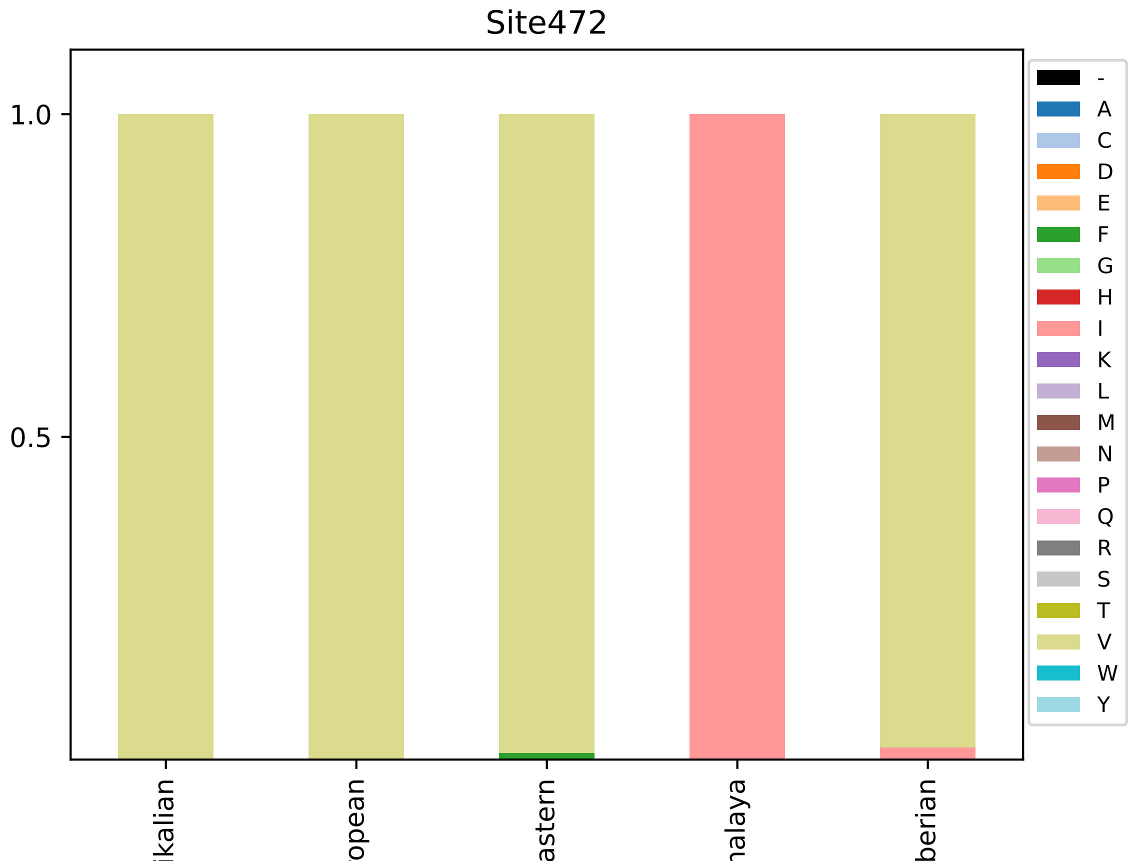
Site472
1.0
0.5
-
A
C
D
E
F
G
H
I
K
L
M
N
P
Q
R
S
T
V
W
Y
ikalian
opean
astern
nalaya
berian

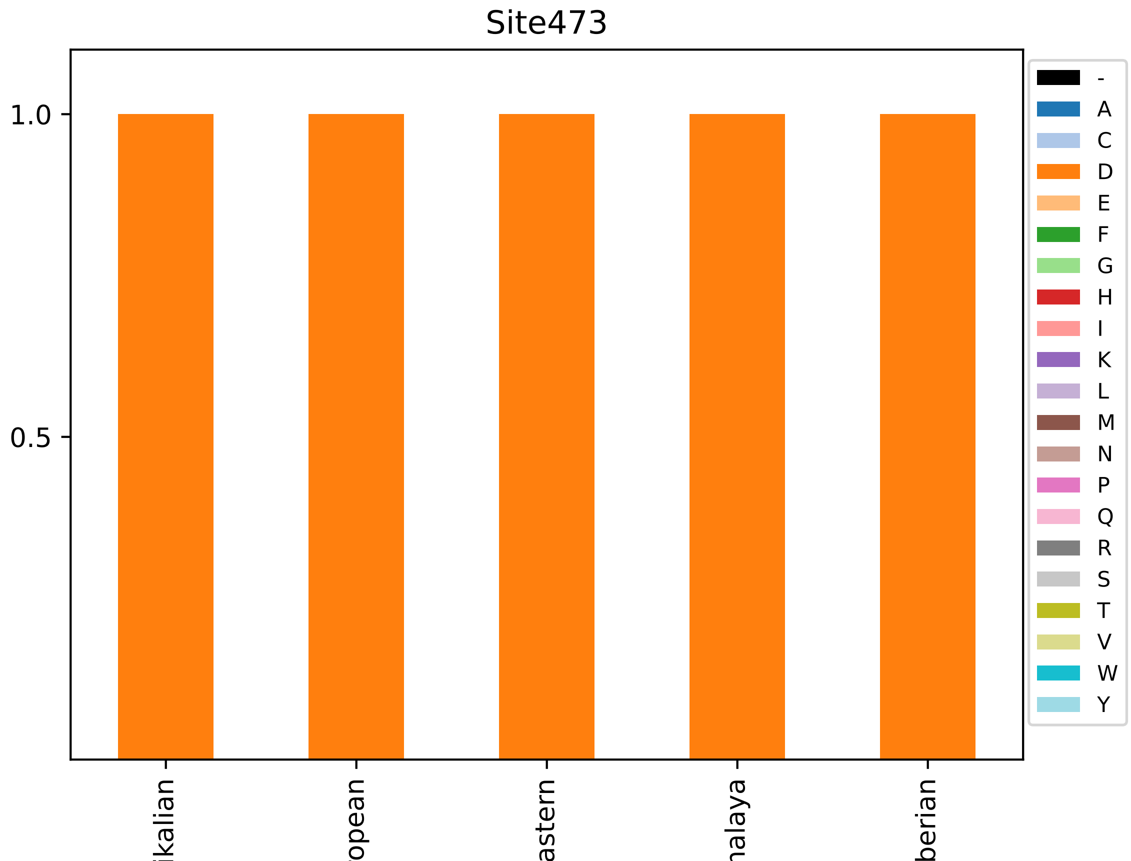

Site473
1.0
0.5
-
A
C
D
E
F
G
H
I
K
L
M
N
P
Q
R
S
T
V
W
Y
ikalian
ropean
astern
nalaya
berian

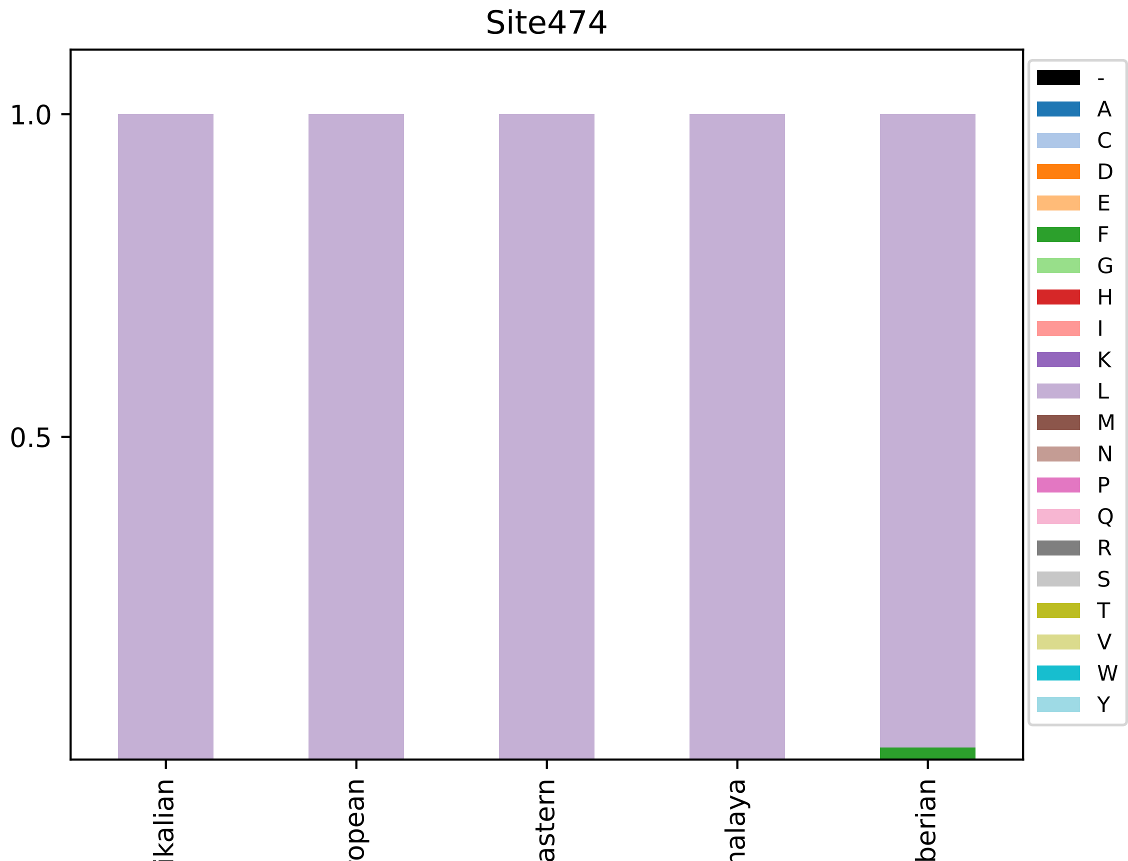

Site474
1.0
0.5
-
A
C
D
E
F
G
H
I
K
L
M
N
P
Q
R
S
T
V
W
Y
ikalian
opean
astern
nalaya
berian

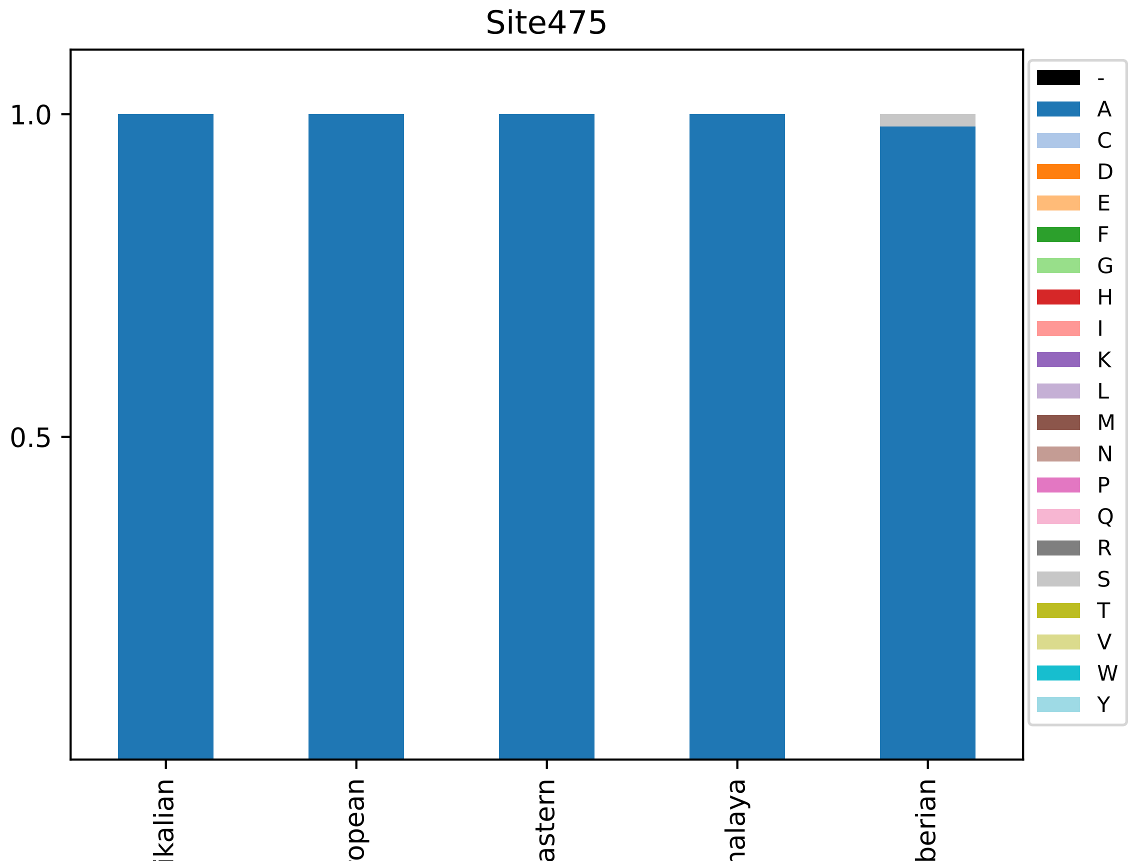
Site475
1.0
0.5
-
A
C
D
E
F
G
H
I
K
L
M
N
P
Q
R
S
T
V
W
Y
ikalian
opean
astern
nalaya
berian

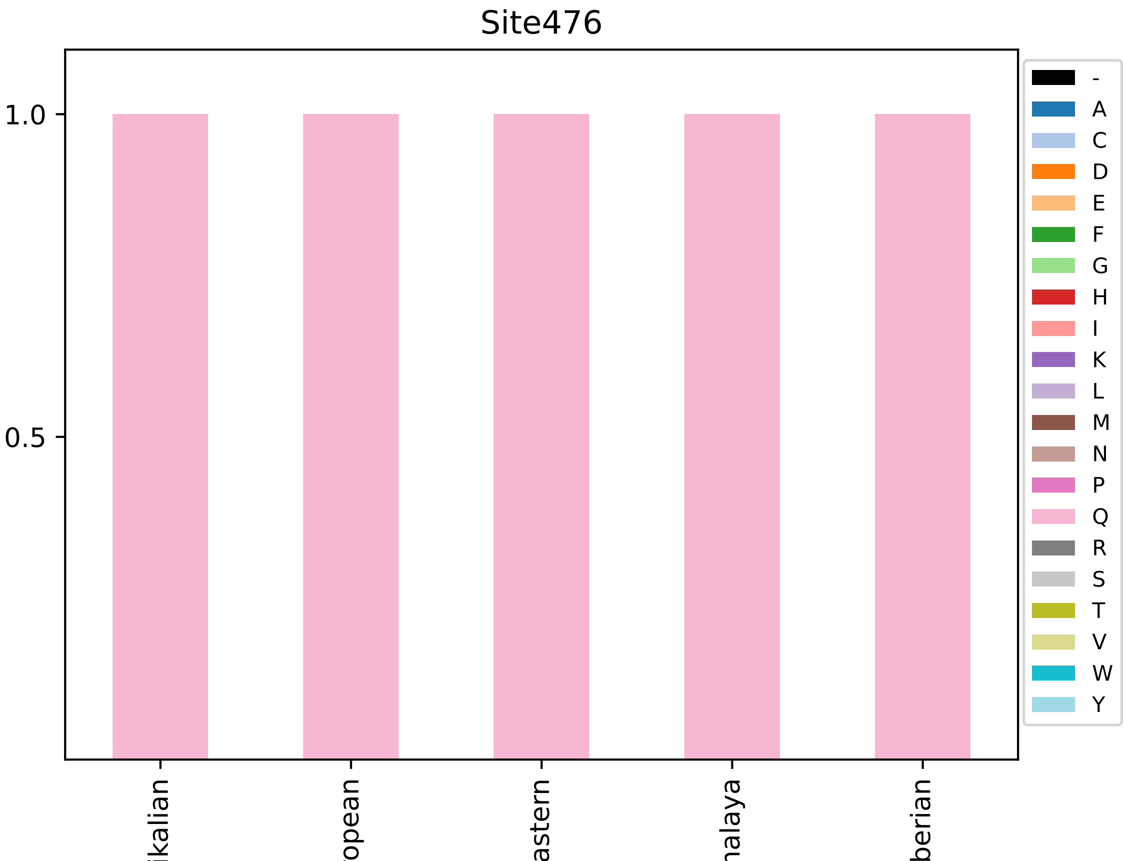
Site476
1.0
0.5
-
A
C
D
E
F
G
H
I
K
L
M
N
P
Q
R
S
T
V
W
Y
ikalian
ropean
astern
nalaya
berian

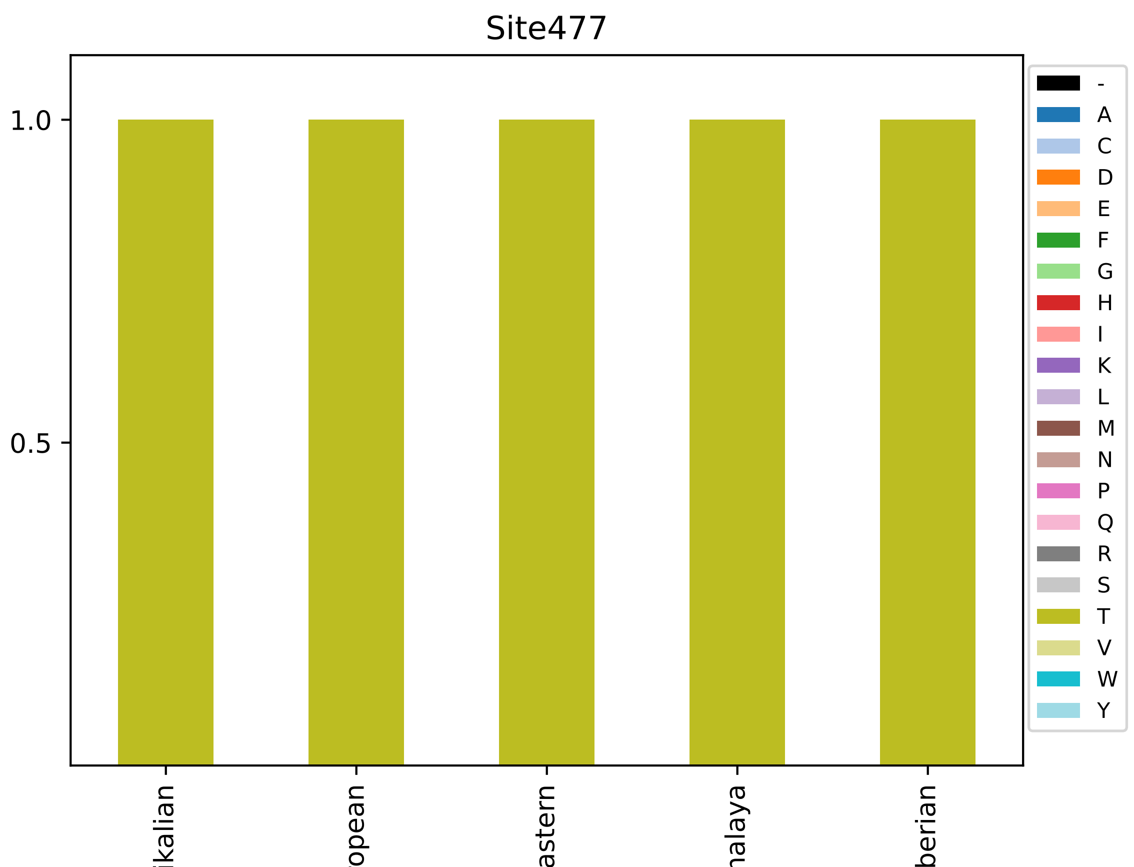
Site477
1.0
0.5
-
A
C
D
E
F
G
H
I
K
L
M
N
P
Q
R
S
T
V
W
Y
ikalian
opean
astern
nalaya
berian

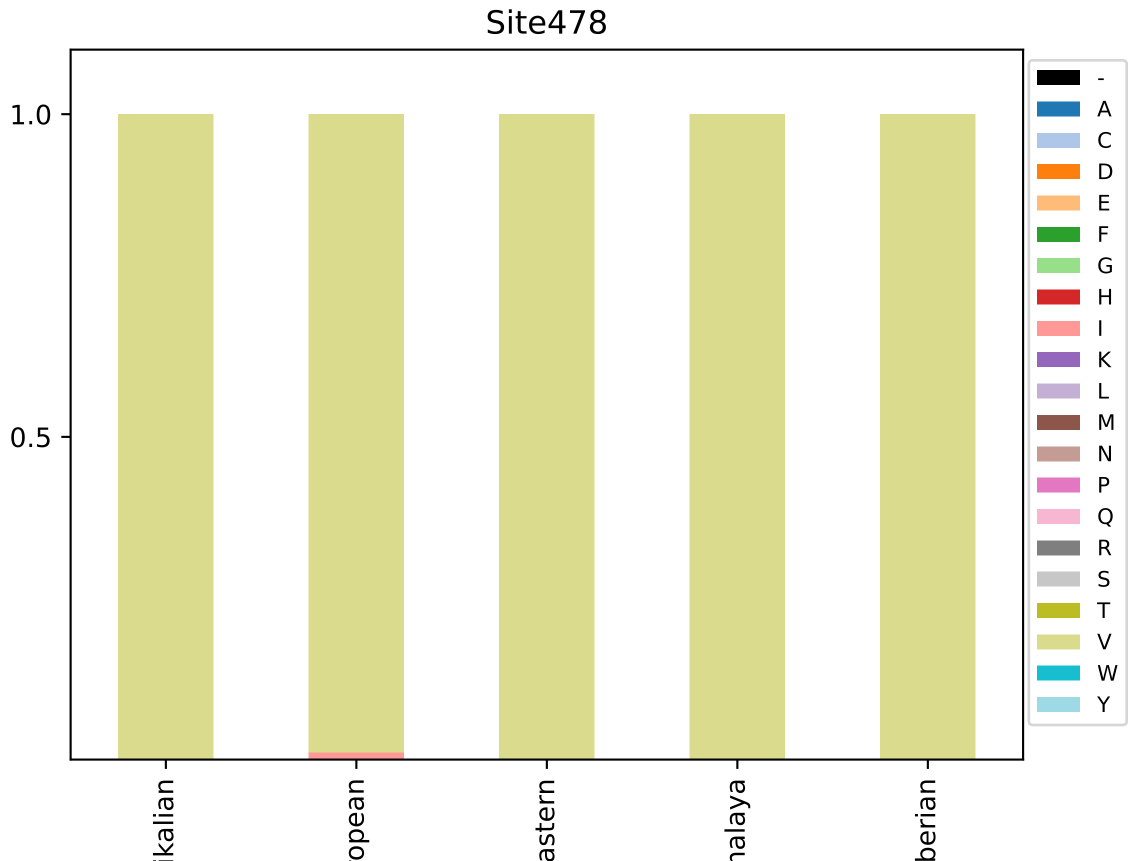
Site478
1.0
0.5
-
A
C
D
E
F
G
H
I
K
L
M
N
P
Q
R
S
T
V
W
Y
ikalian
opean
astern
nalaya
berian

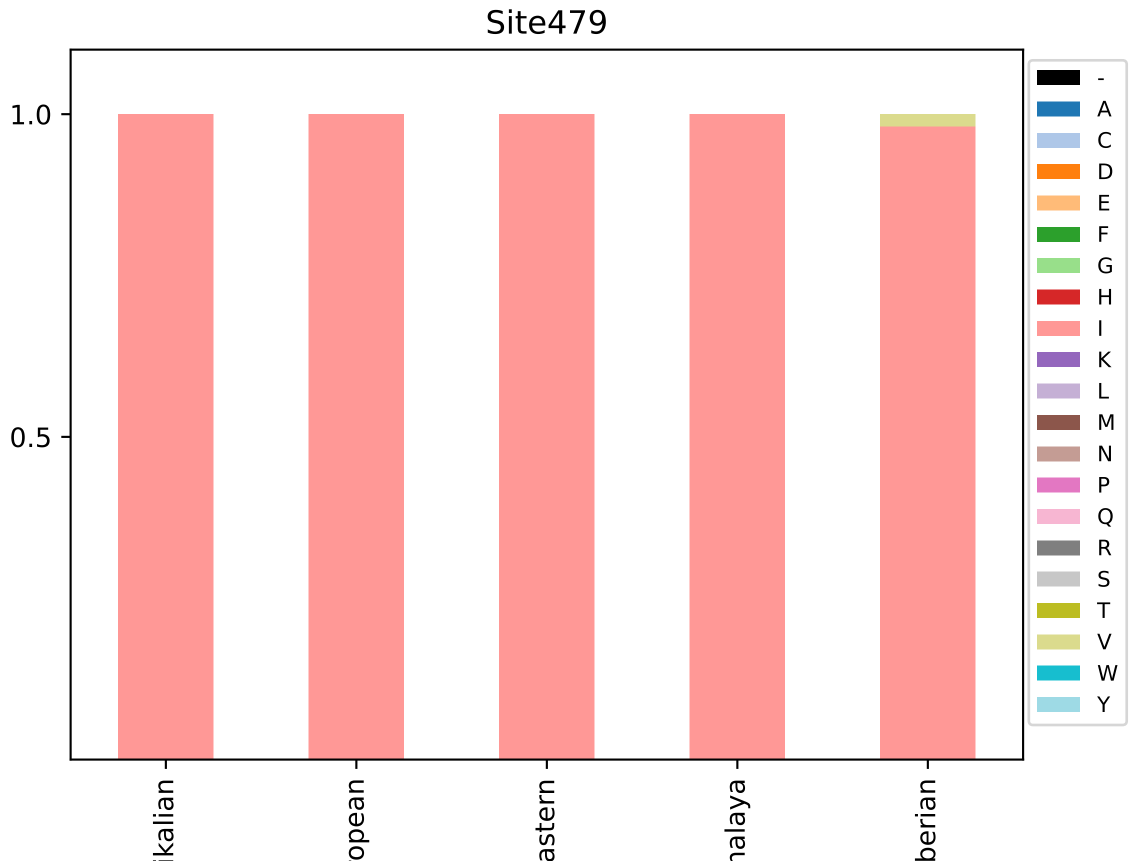

Site479
1.0
0.5
ikalian
opean
astern
nalaya
berian
-
A
C
D
E
F
G
H
I
K
L
M
N
P
Q
R
S
T
V
W
Y

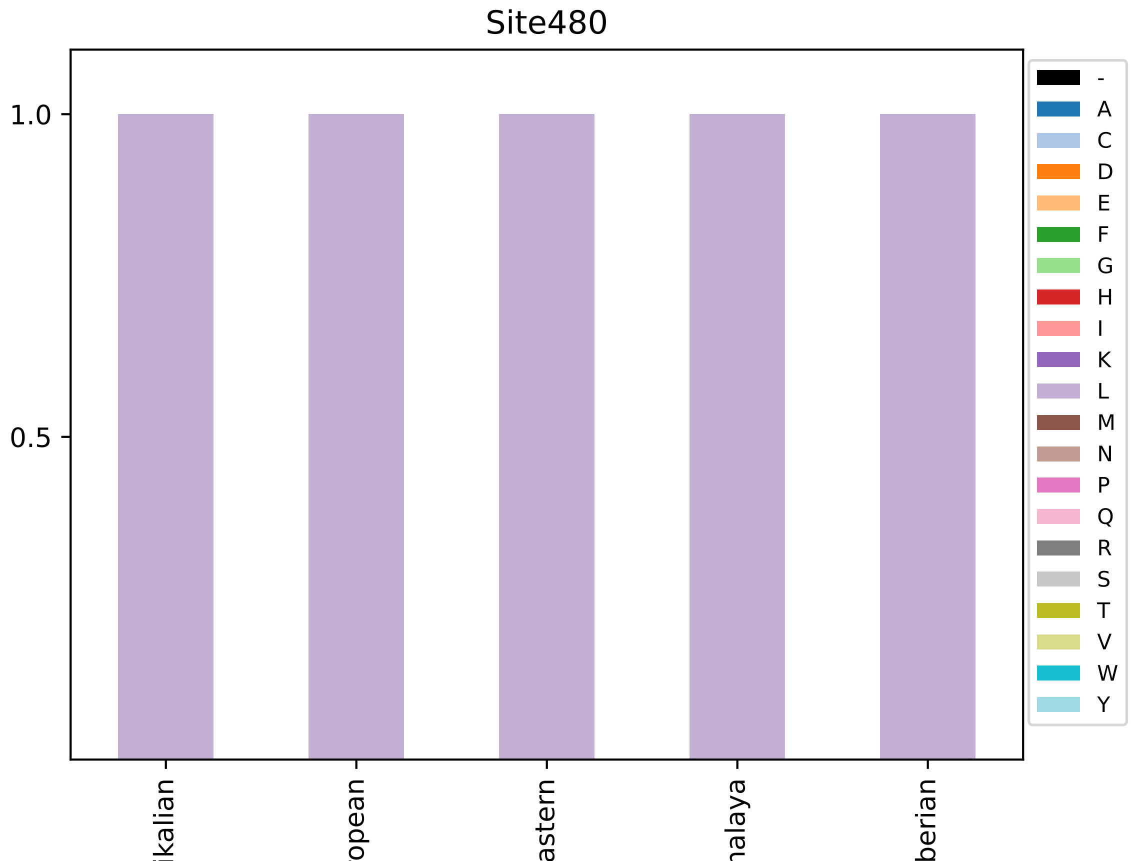

Site480
1.0
0.5
-
A
C
D
E
F
G
H
I
K
L
M
N
P
Q
R
S
T
V
W
Y

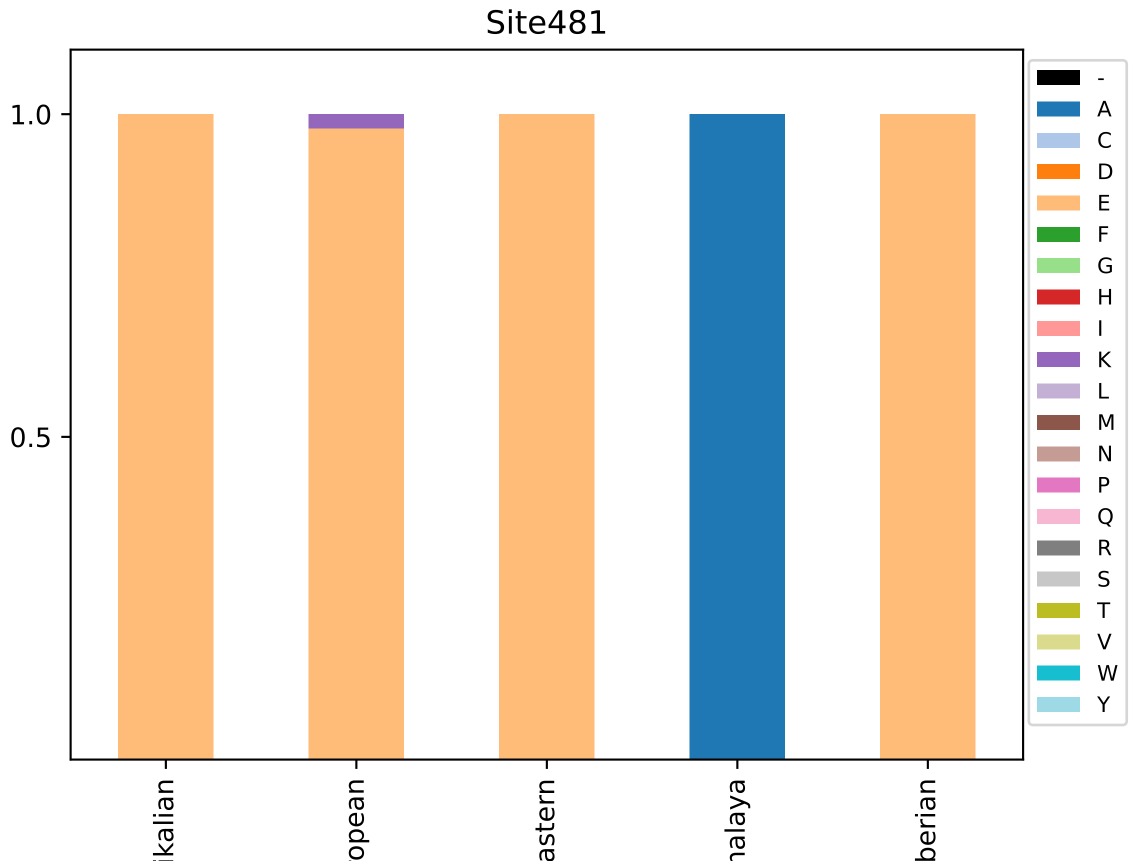

Site481
1.0
0.5
-
A
C
D
E
F
G
H
I
K
L
M
N
P
Q
R
S
T
V
W
Y
ikalian
opean
astern
nalaya
berian

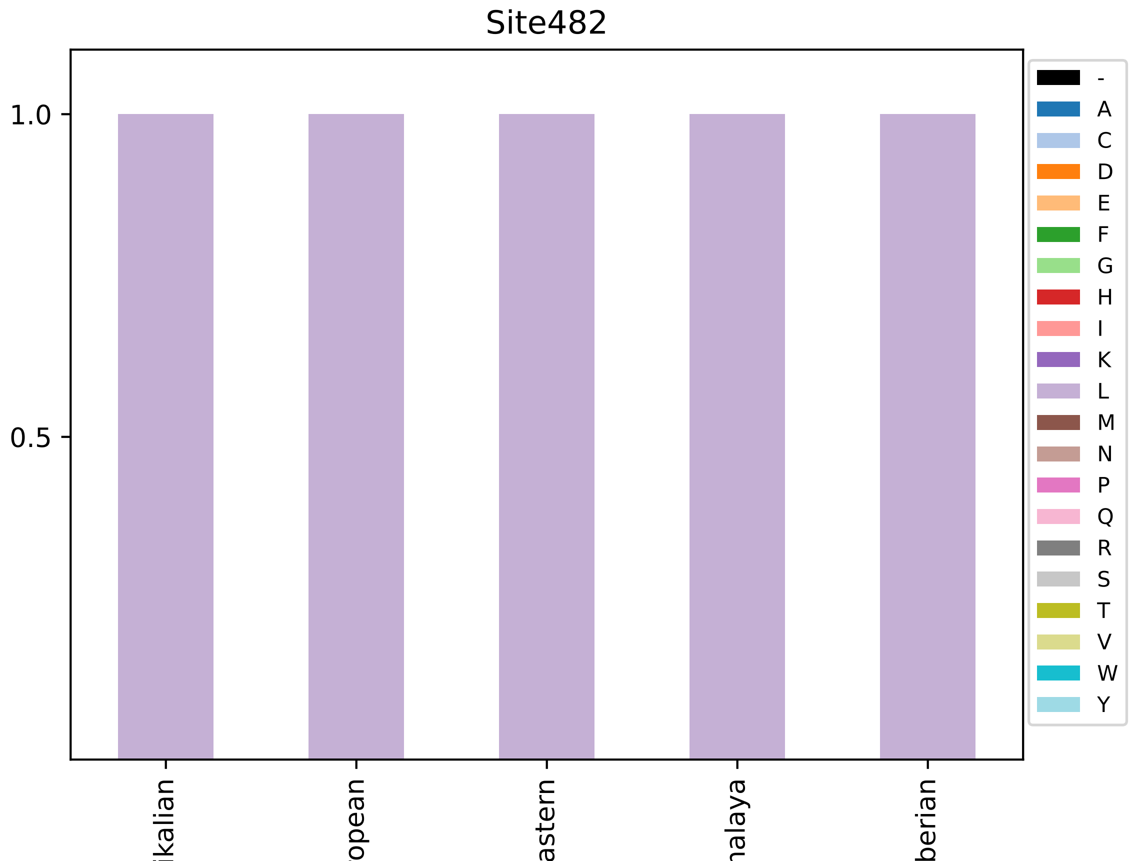
Site482
1.0
0.5
-
A
C
D
E
F
G
H
I
K
L
M
N
P
Q
R
S
T
V
W
Y
ikalian
opean
astern
nalaya
berian

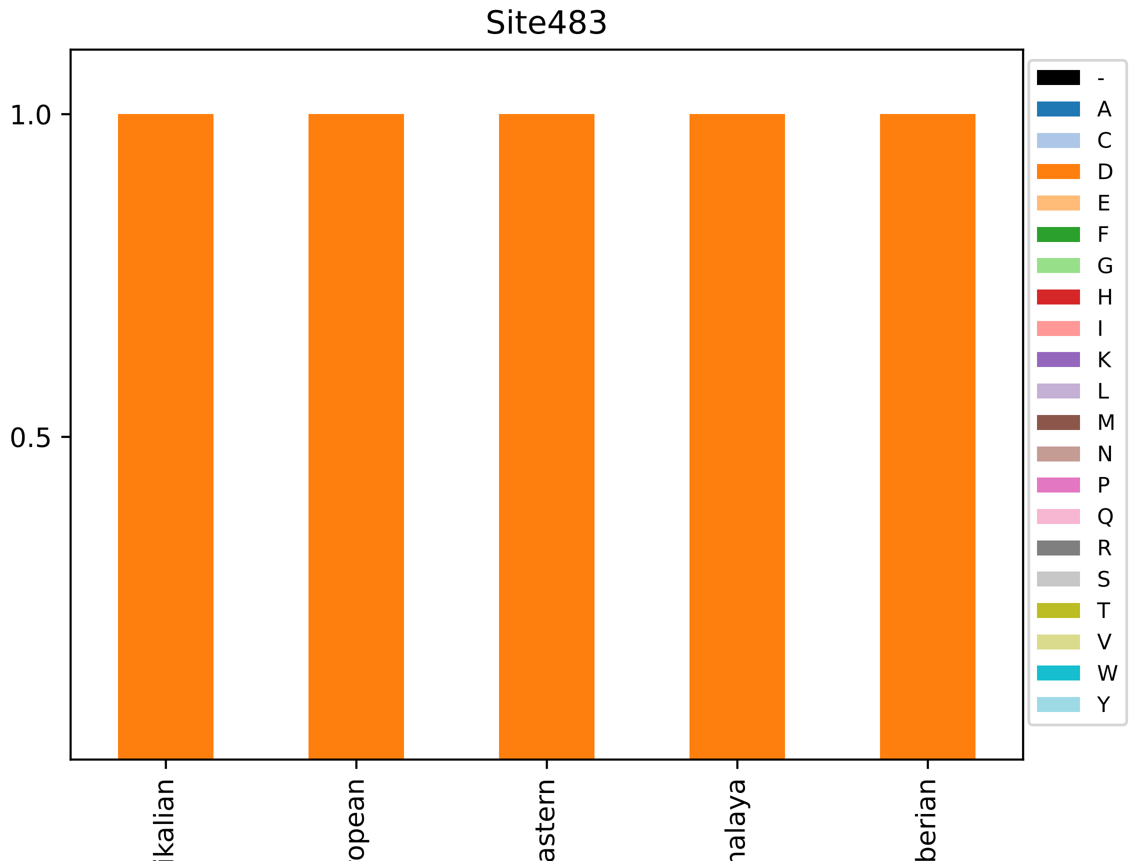
Site483
1.0
0.5
-
A
C
D
E
F
G
H
I
K
L
M
N
P
Q
R
S
T
V
W
Y
ikalian
opean
astern
nalaya
berian

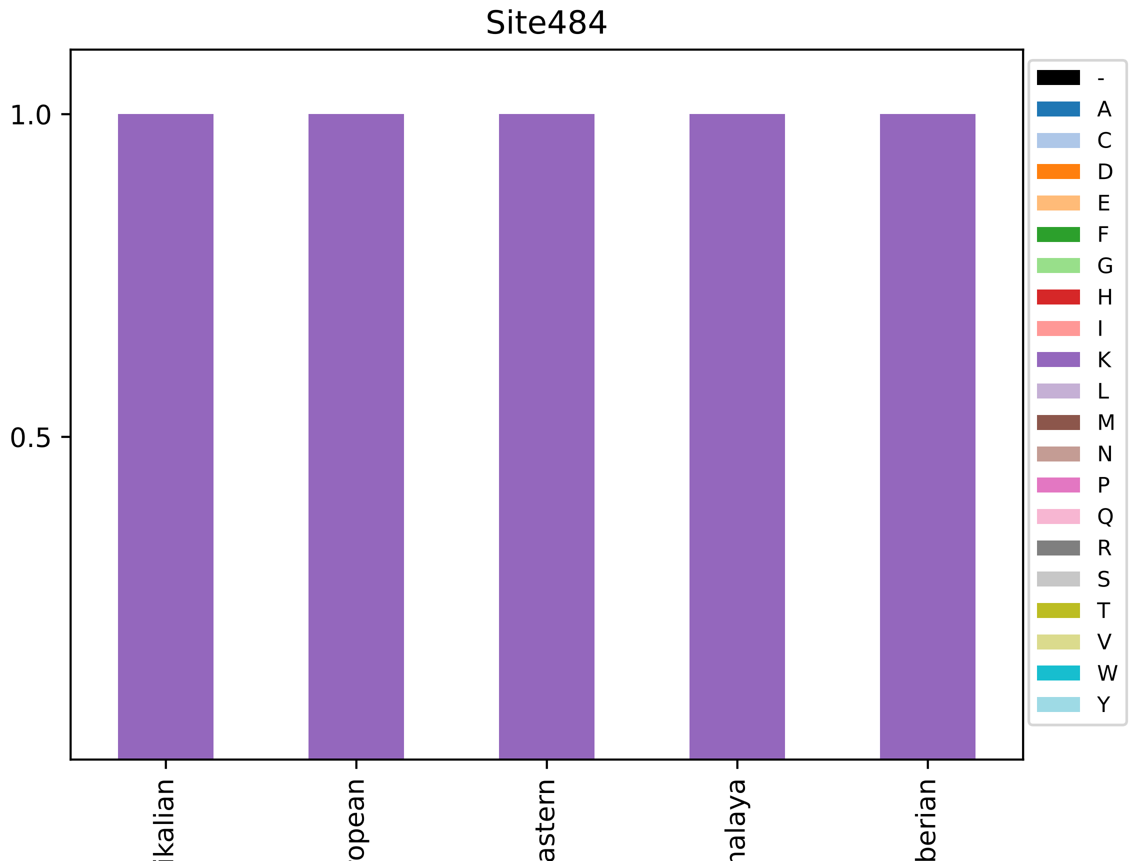

Site484
1.0
0.5
-
A
C
D
E
F
G
H
I
K
L
M
N
P
Q
R
S
T
V
W
Y
ikalian
opean
astern
nalaya
berian

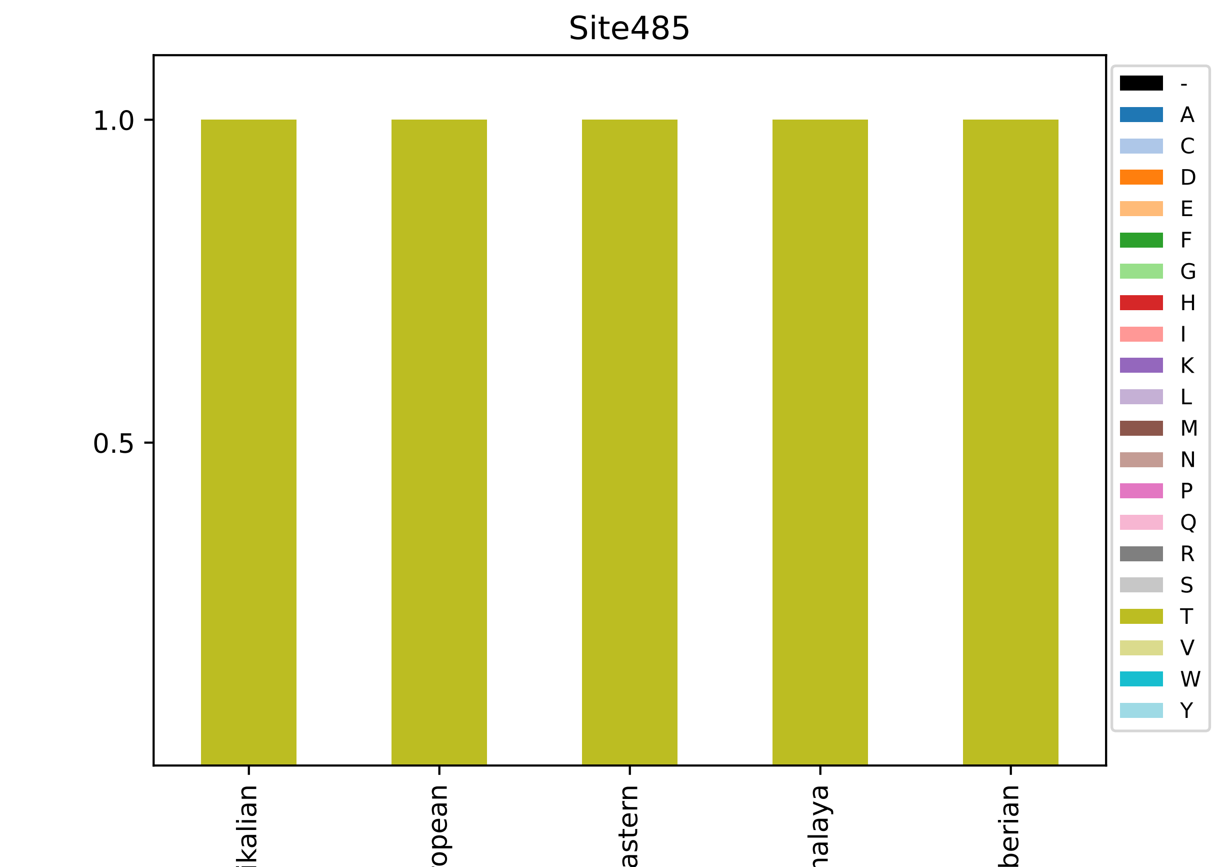

Site485
1.0
0.5
-
A
C
D
E
F
G
H
I
K
L
M
N
P
Q
R
S
T
V
W
Y

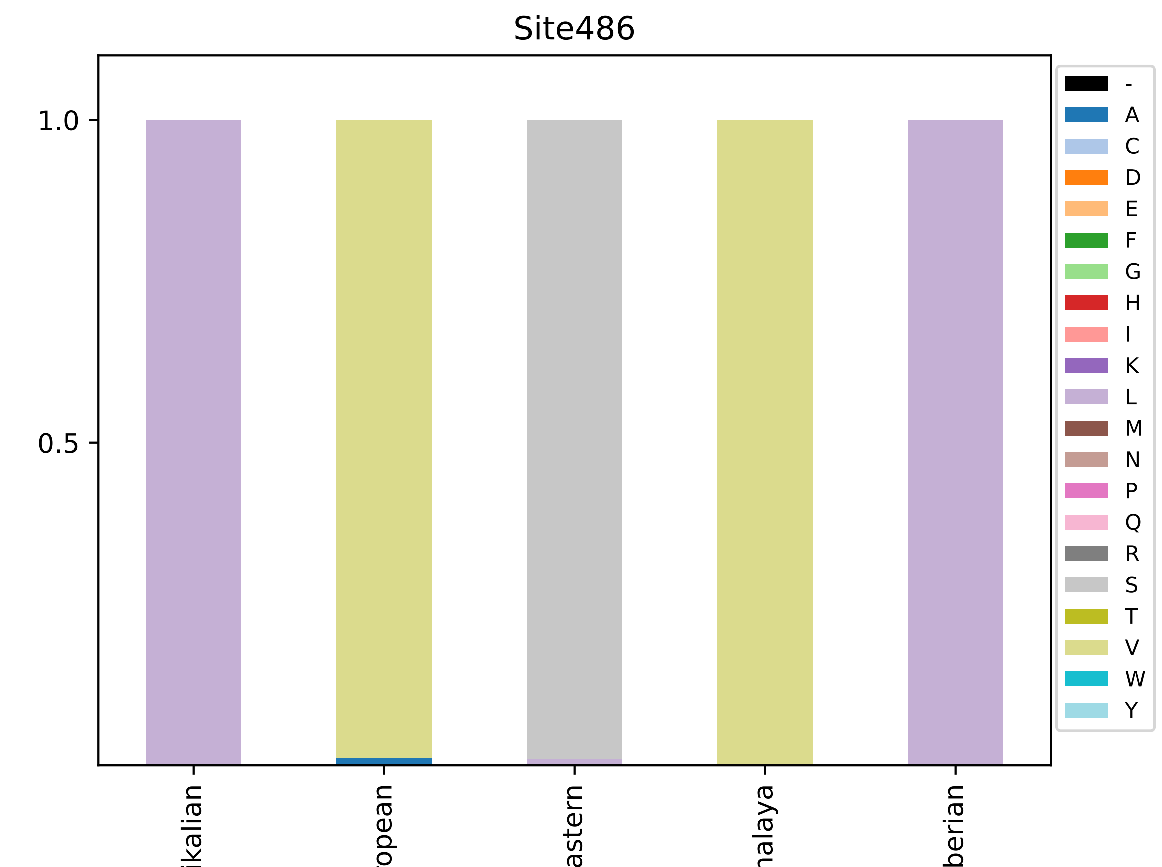

Site486
1.0
0.5
-
A
C
D
E
F
G
H
I
K
L
M
N
P
Q
R
S
T
V
W
Y
ikalian
opean
astern
nalaya
berian

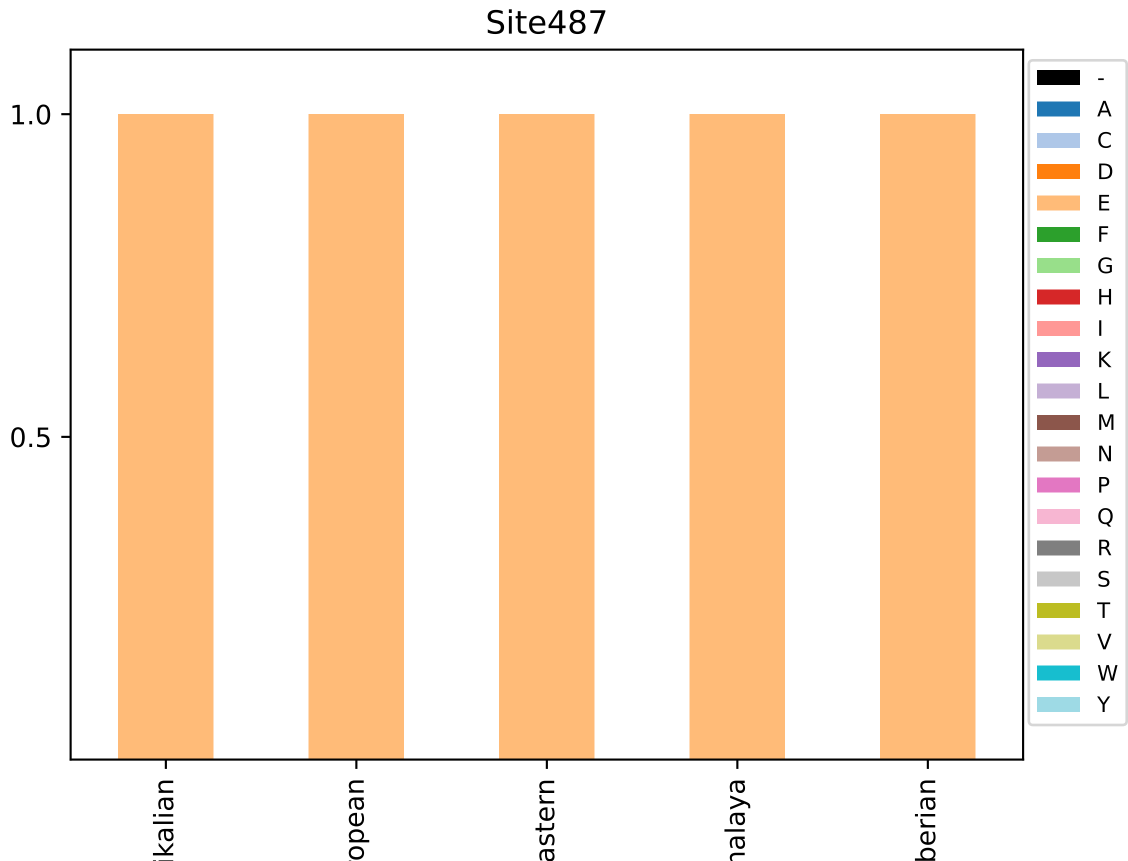

Site487
1.0
0.5
-
A
C
D
E
F
G
H
I
K
L
M
N
P
Q
R
S
T
V
W
Y
ikalian
opean
astern
nalaya
berian

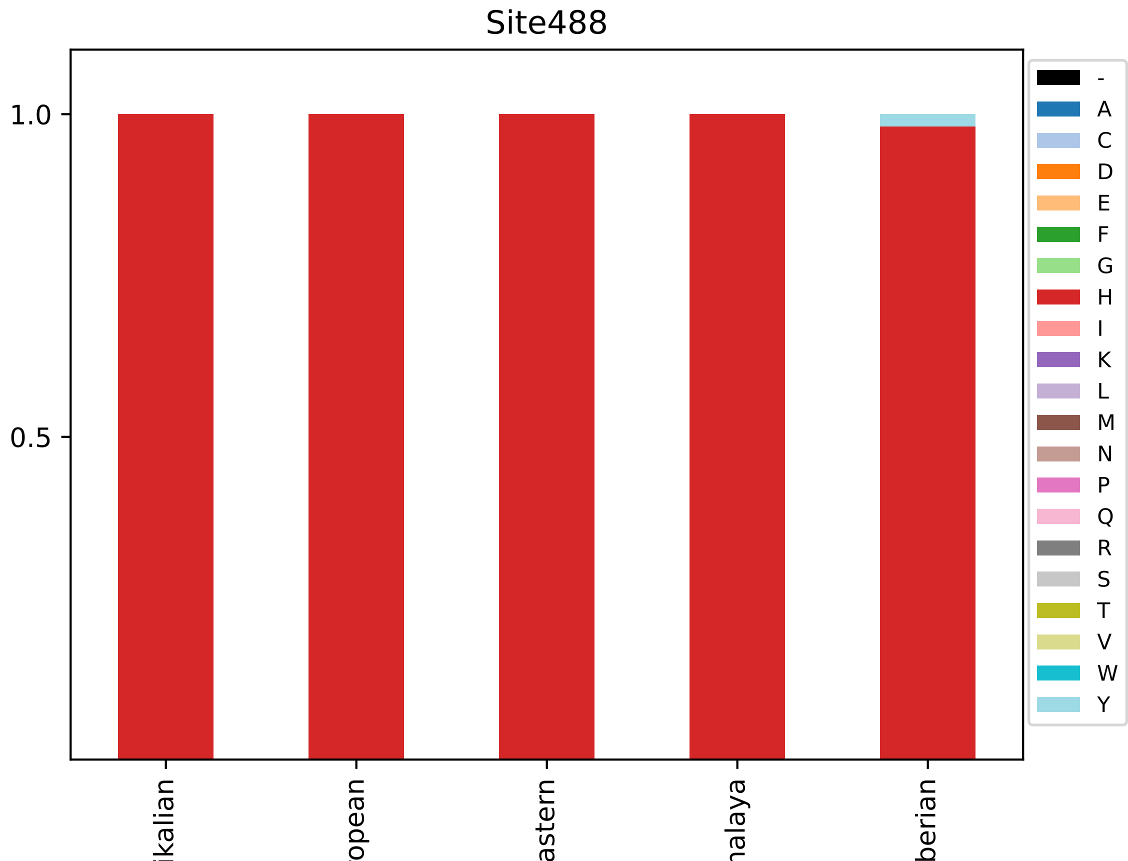
Site488
1.0
0.5
-
A
C
D
E
F
G
H
I
K
L
M
N
P
Q
R
S
T
V
W
Y
ikalian
opean
astern
nalaya
berian

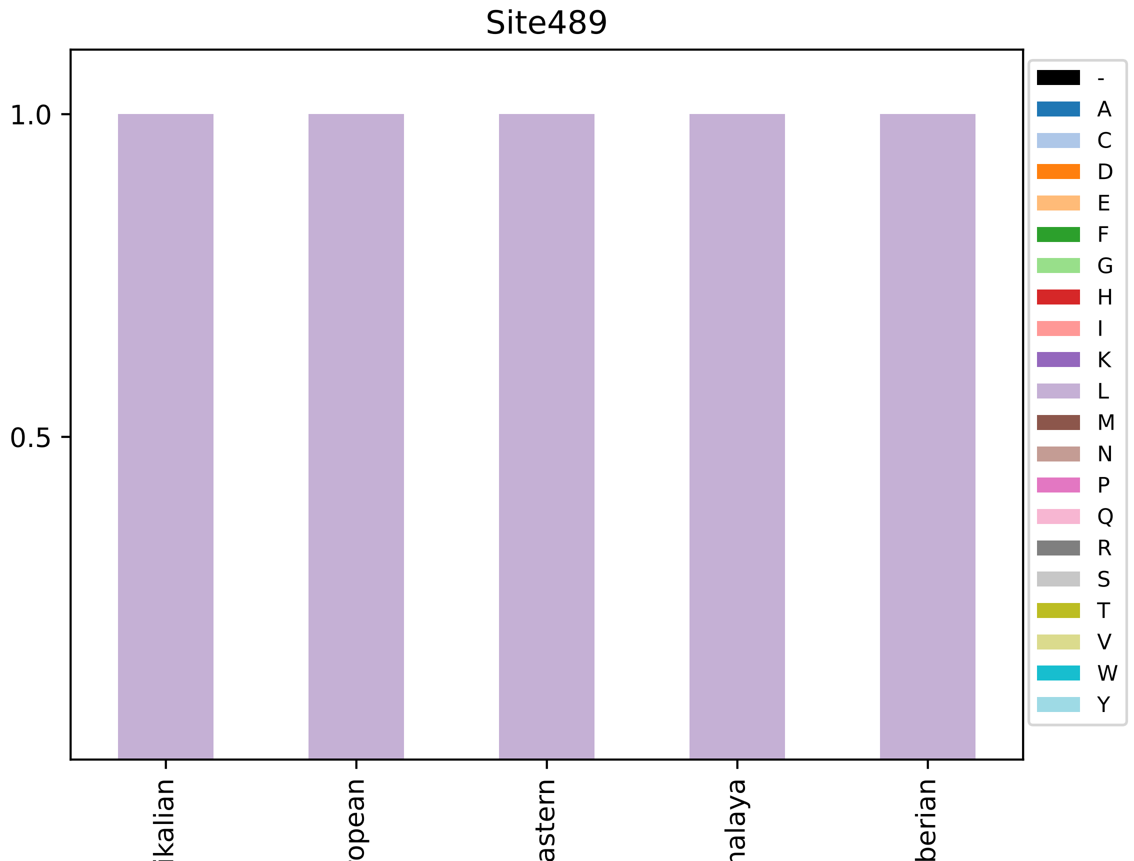
Site489
1.0
0.5
-
A
C
D
E
F
G
H
I
K
L
M
N
P
Q
R
S
T
V
W
Y
ikalian
opean
astern
nalaya
berian

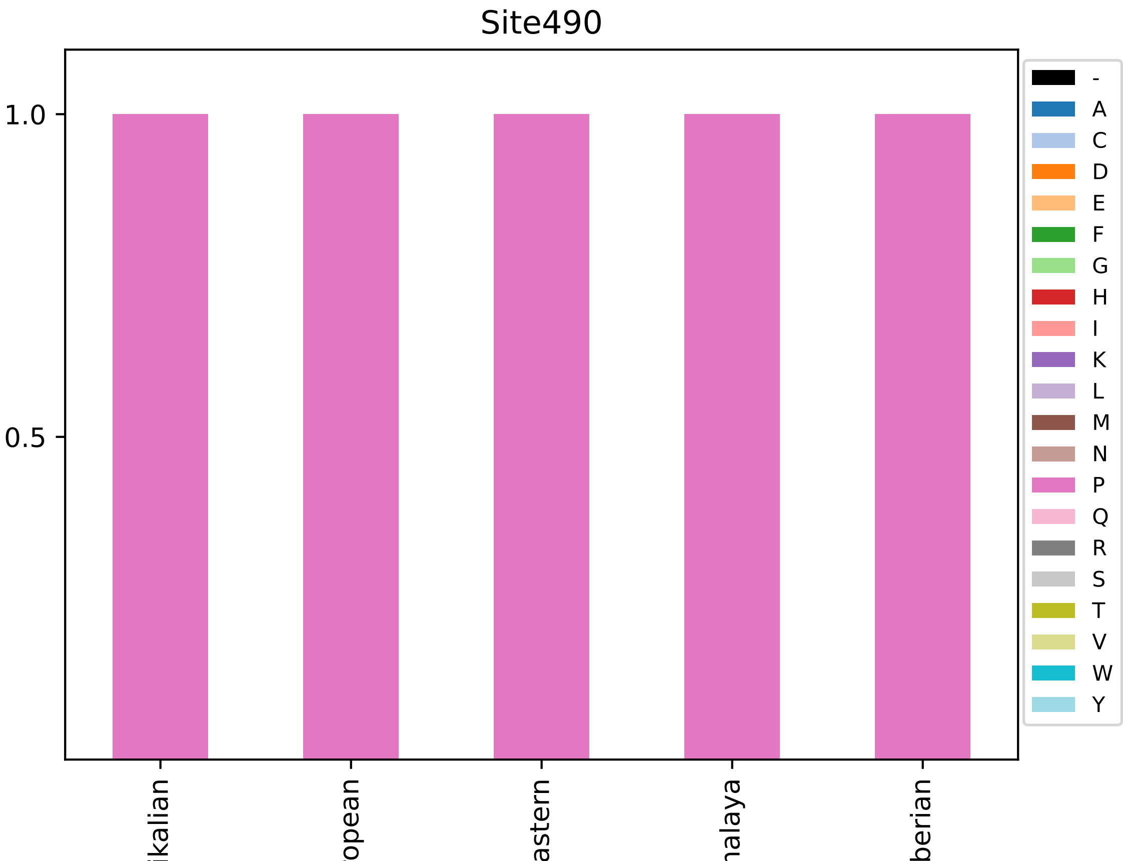
Site490
1.0
0.5
-
A
C
D
E
F
G
H
I
K
L
M
N
P
Q
R
S
T
V
W
Y
ikalian
opean
astern
nalaya
berian

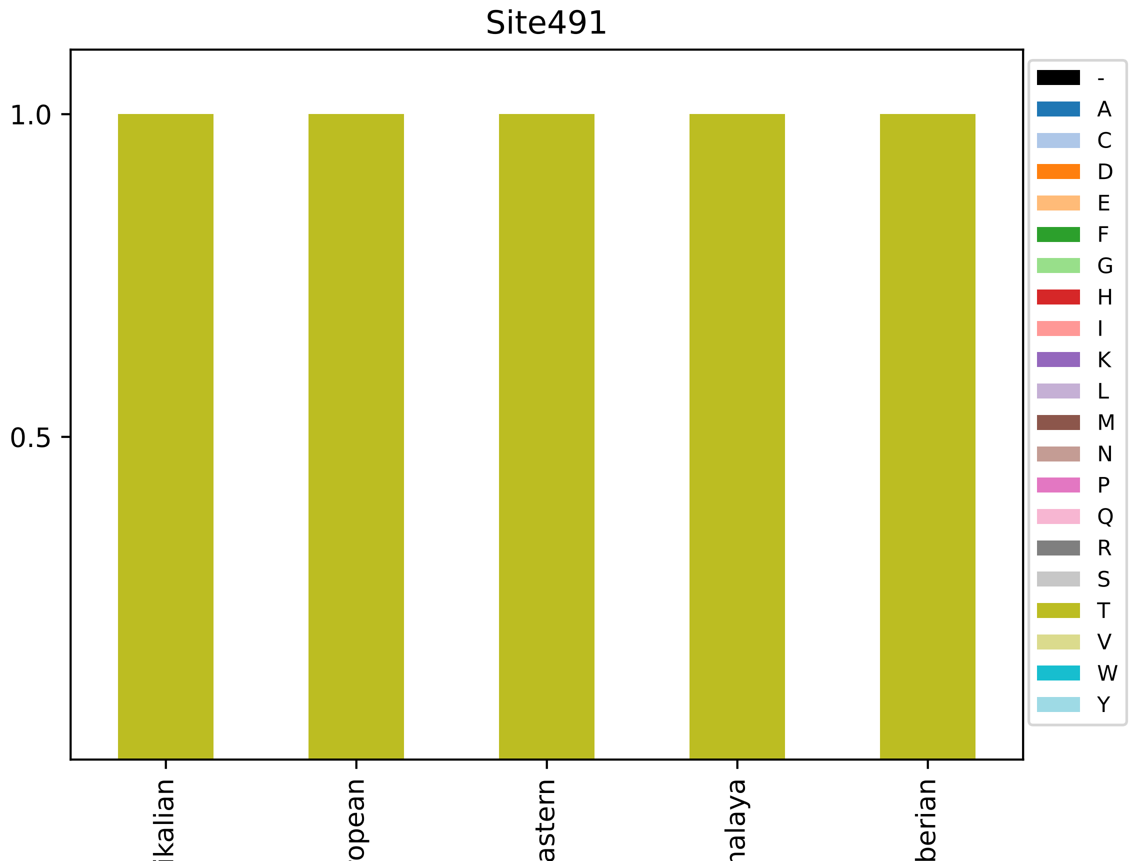
Site491
1.0
0.5
-
A
C
D
E
F
G
H
I
K
L
M
N
P
Q
R
S
T
V
W
Y
ikalian
ropean
astern
nalaya
berian

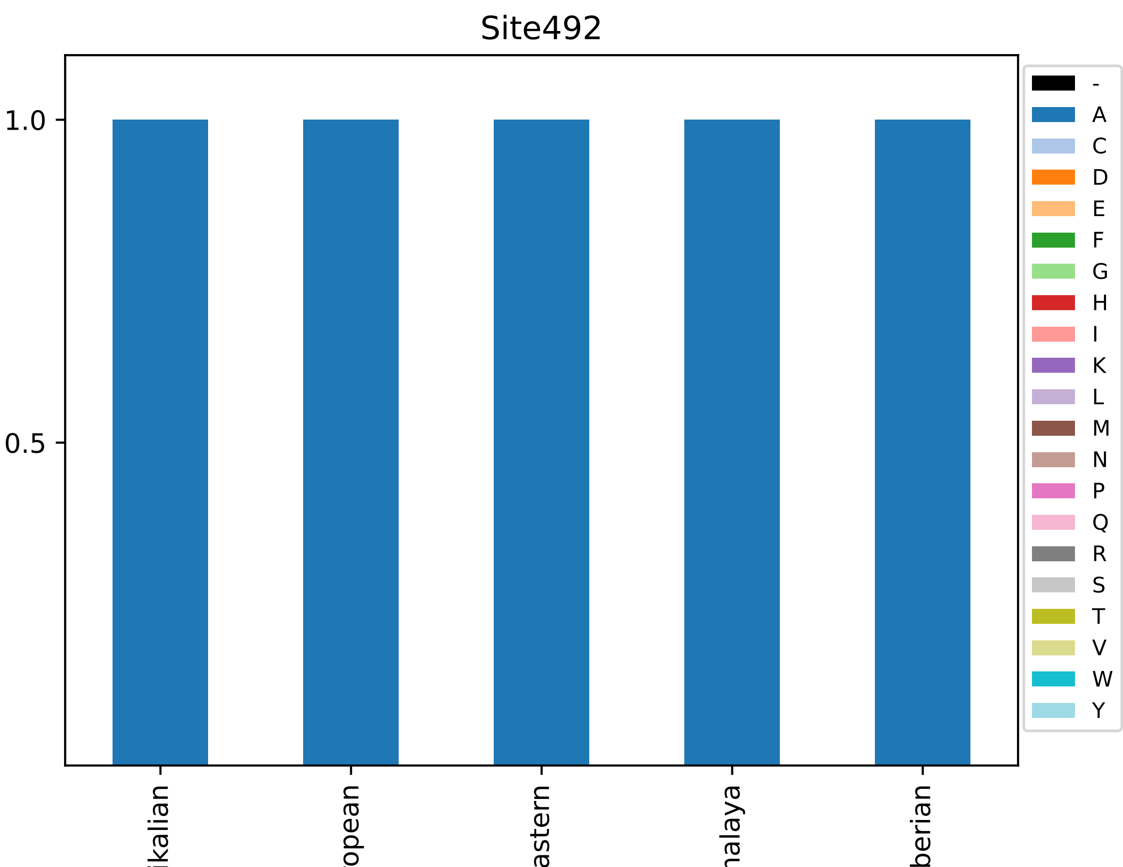

Site492
1.0
0.5
-
A
C
D
E
F
G
H
I
K
L
M
N
P
Q
R
S
T
V
W
Y

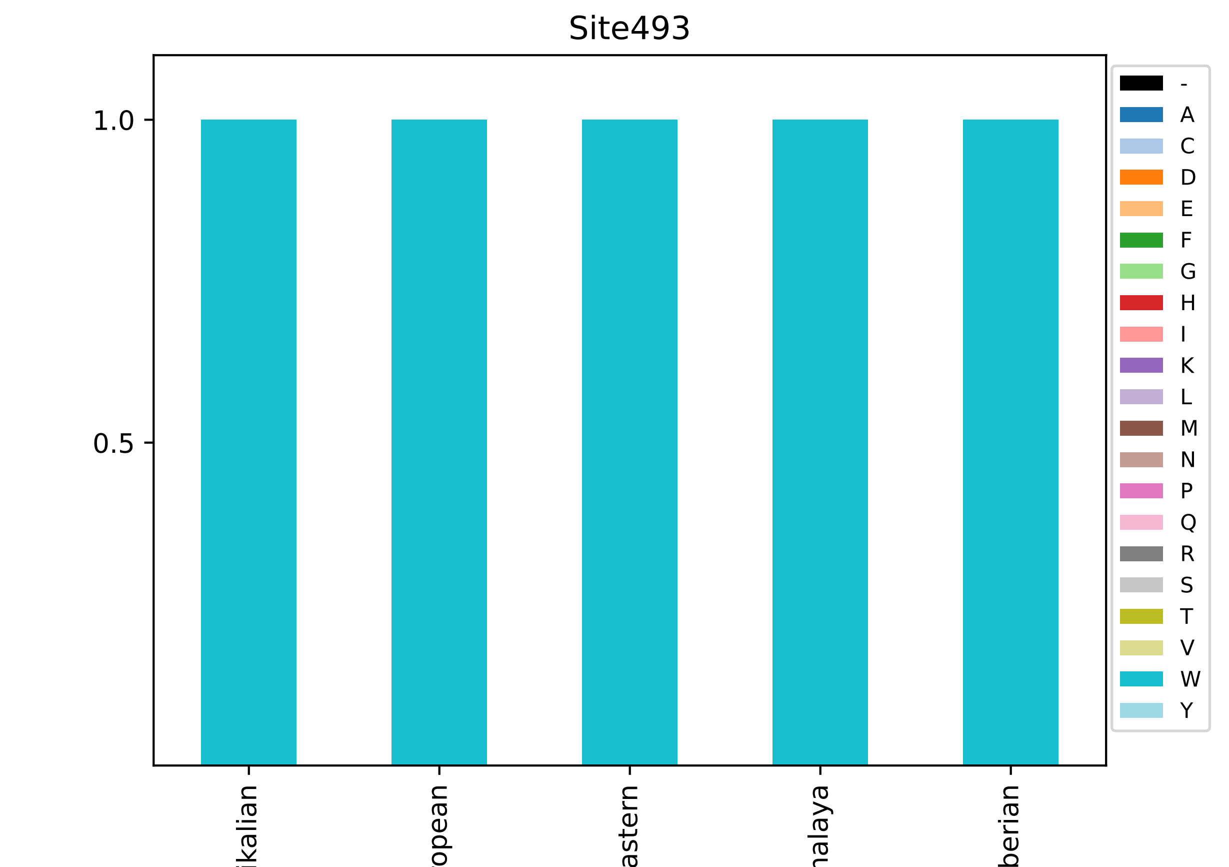

Site493
1.0
0.5
-
A
C
D
E
F
G
H
I
K
L
M
N
P
Q
R
S
T
V
W
Y
ikalian
opean
astern
nalaya
berian

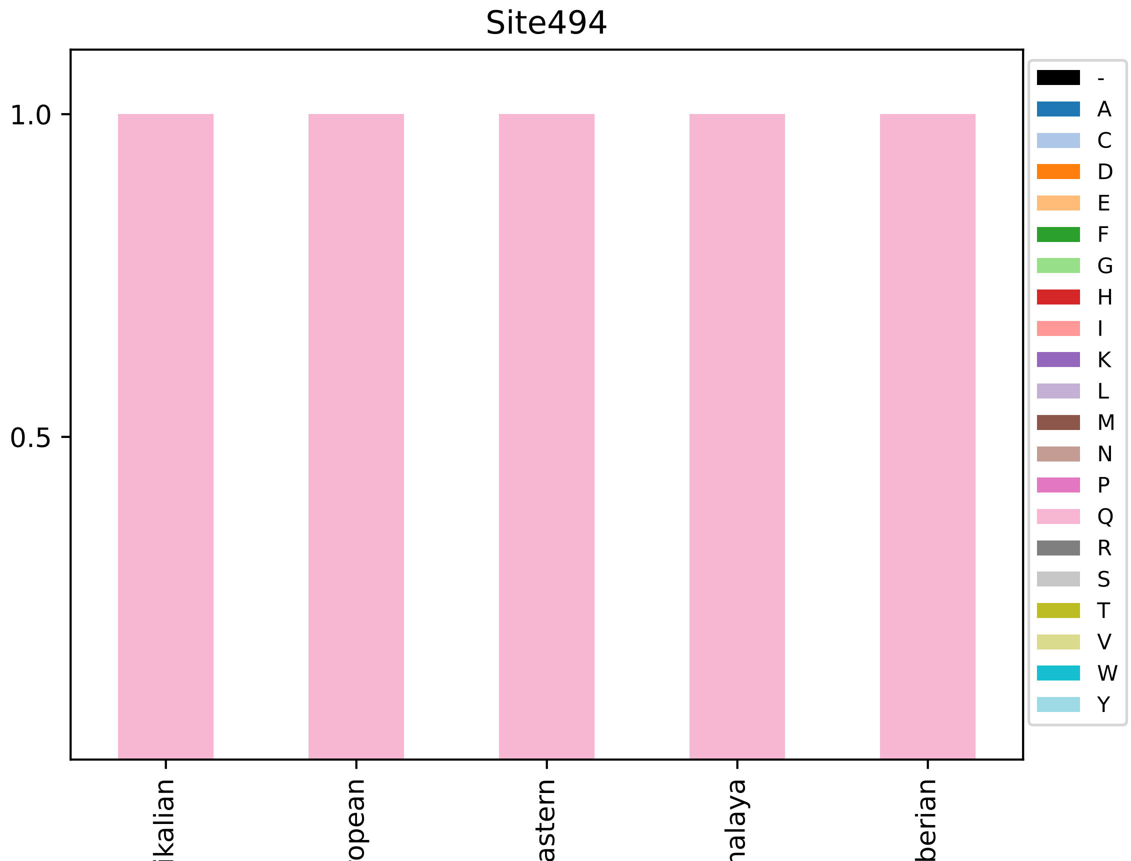

Site494
1.0
0.5
-
A
C
D
E
F
G
H
I
K
L
M
N
P
Q
R
S
T
V
W
Y
ikalian
opean
astern
nalaya
berian

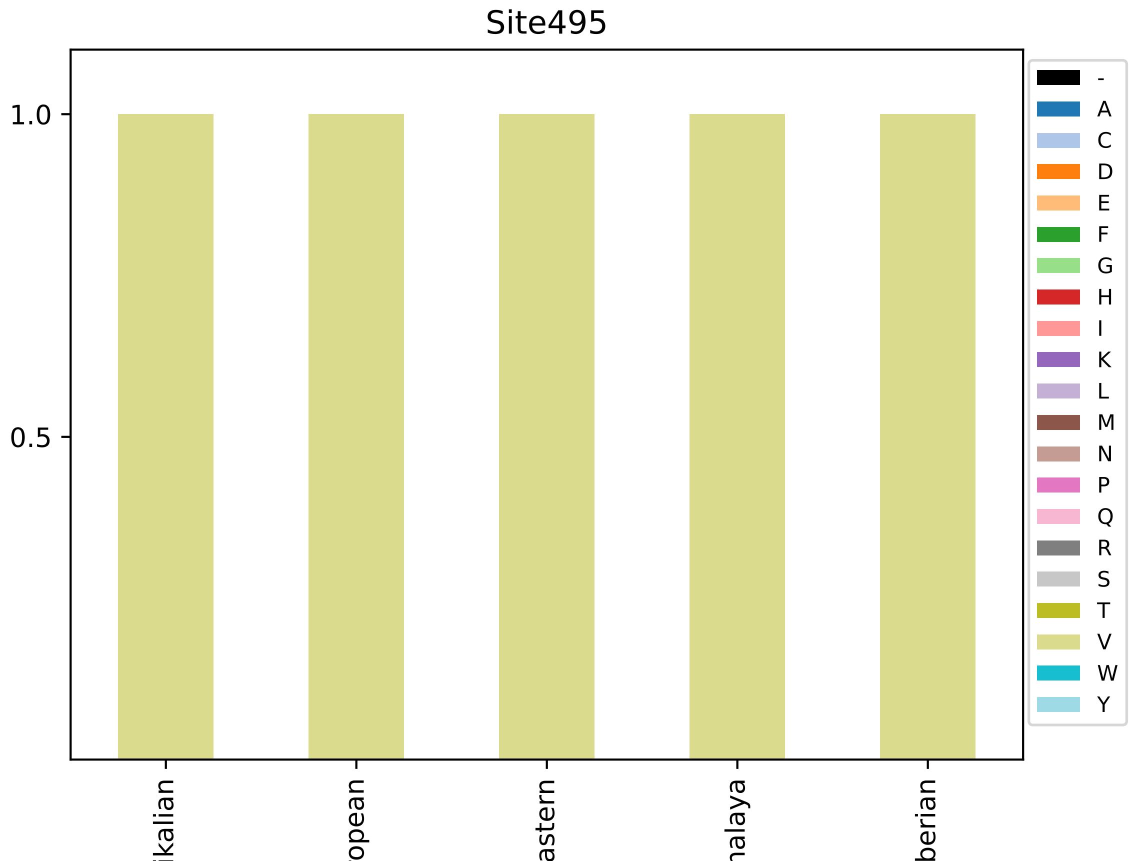
Site495
1.0
0.5
-
A
C
D
E
F
G
H
I
K
L
M
N
P
Q
R
S
T
V
W
Y
ikalian
opean
astern
nalaya
berian

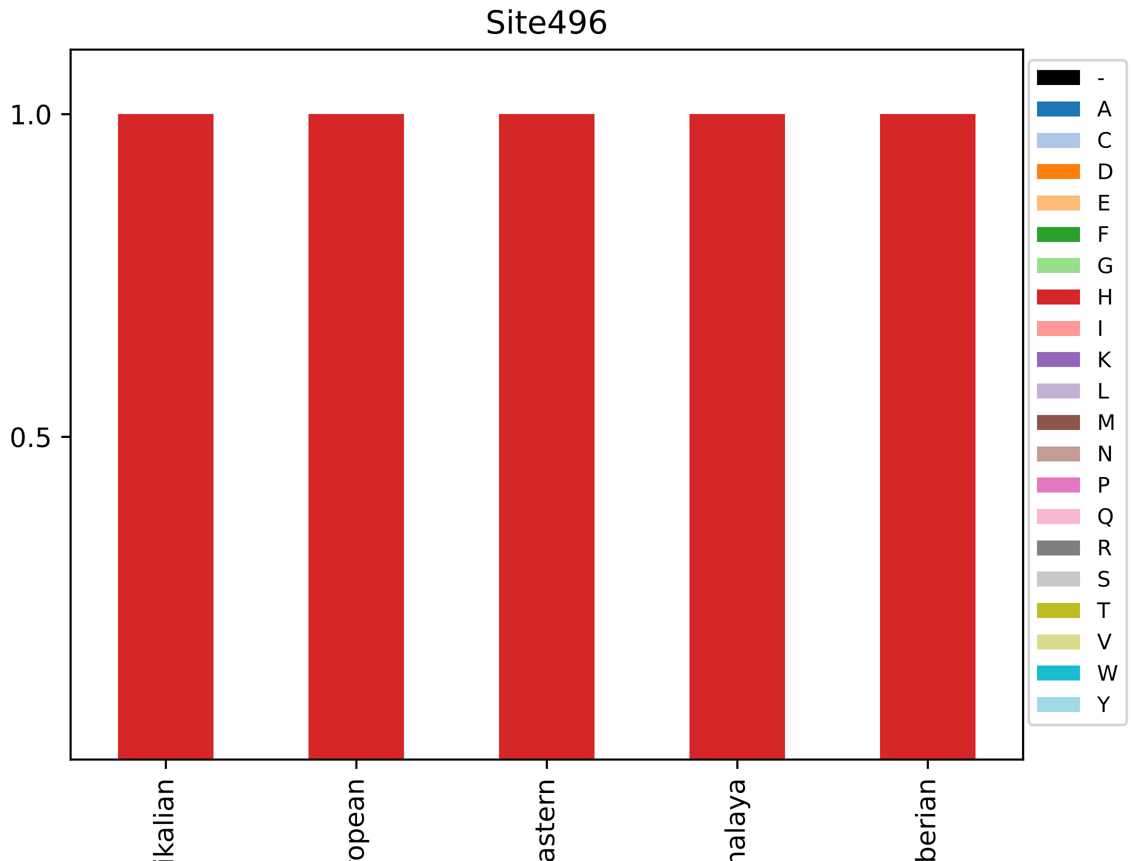
Site496
1.0
0.5
-
A
C
D
E
F
G
H
I
K
L
M
N
P
Q
R
S
T
V
W
Y
ikalian
opean
astern
nalaya
berian

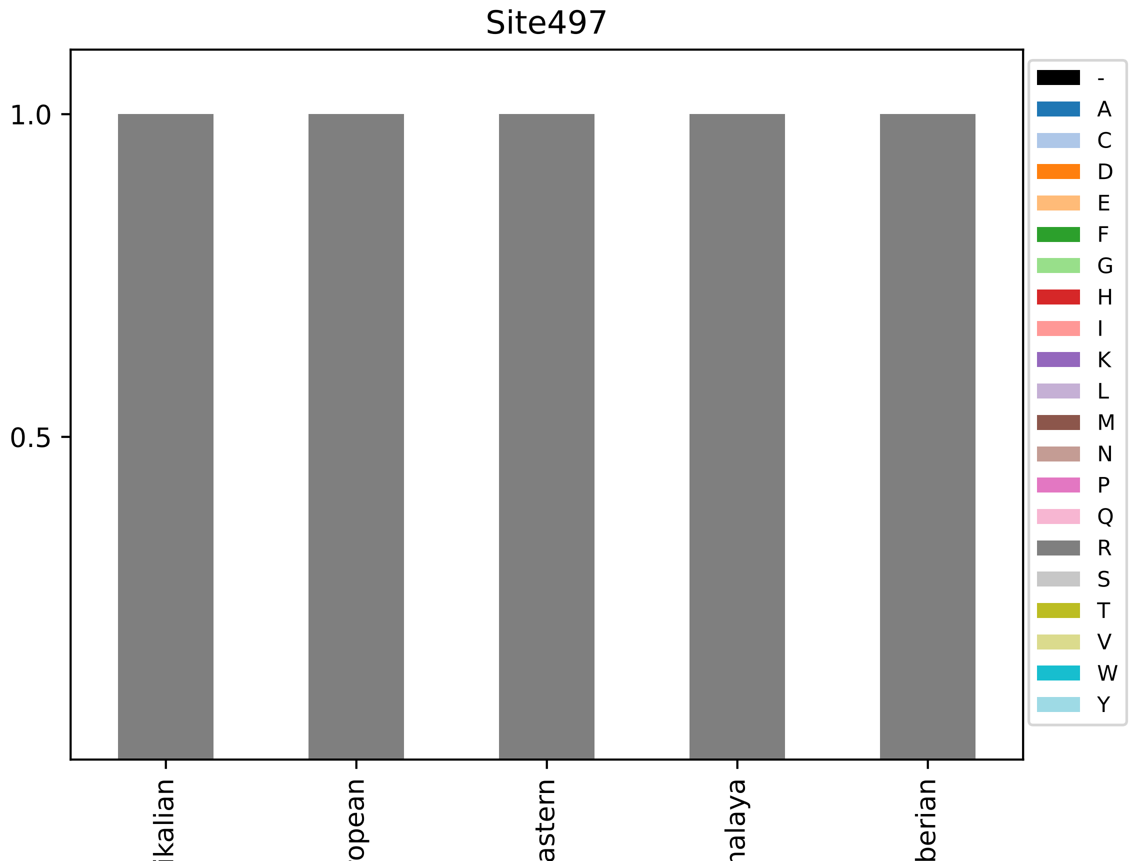

Site497
1.0
0.5
-
A
C
D
E
F
G
H
I
K
L
M
N
P
Q
R
S
T
V
W
Y
ikalian
opean
astern
nalaya
berian

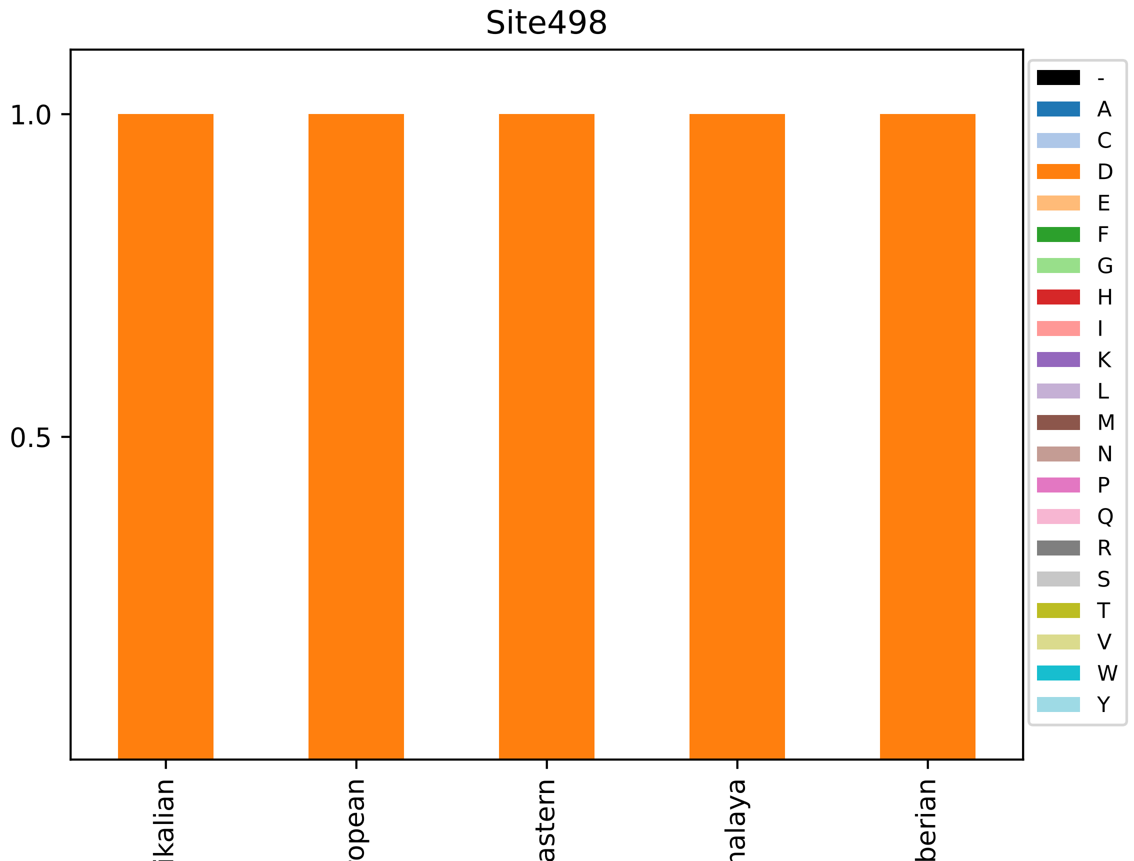
Site498
1.0
0.5
-
A
C
D
E
F
G
H
I
K
L
M
N
P
Q
R
S
T
V
W
Y
ikalian
opean
astern
nalaya
berian

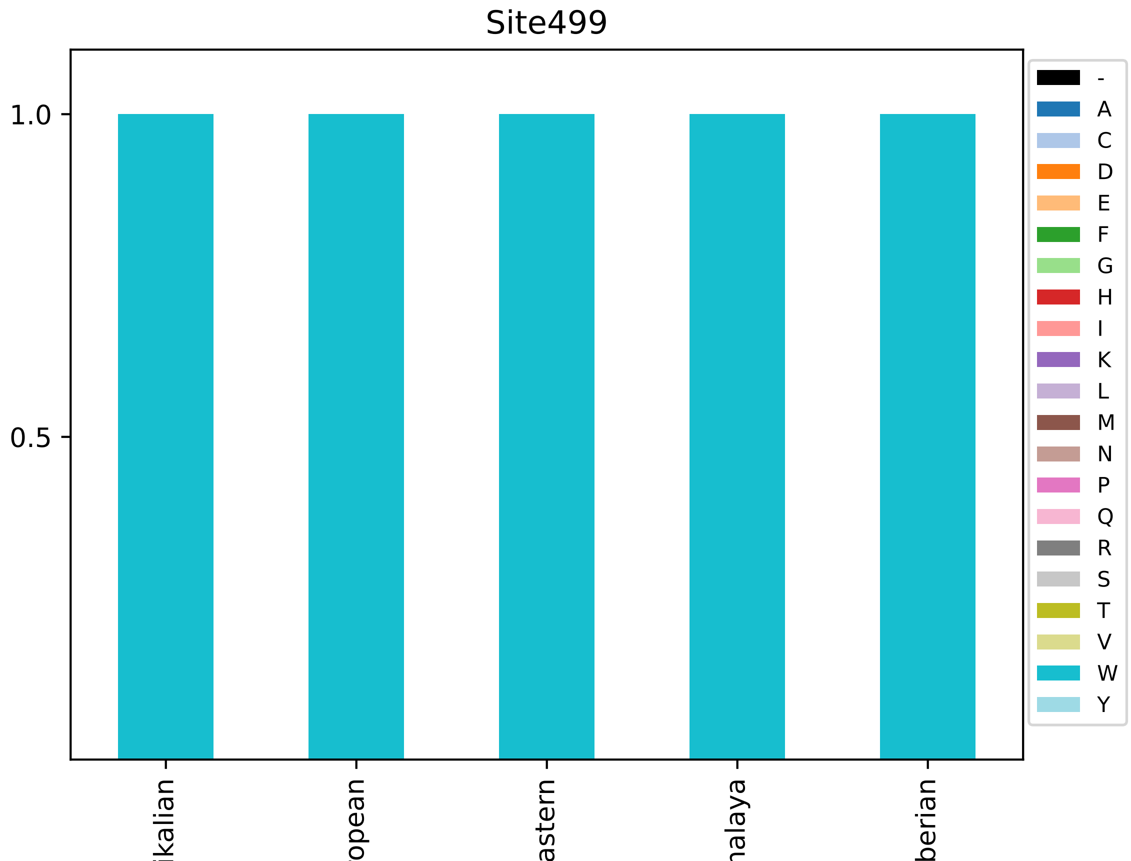

Site499
1.0
0.5
-
A
C
D
E
F
G
H
I
K
L
M
N
P
Q
R
S
T
V
W
Y

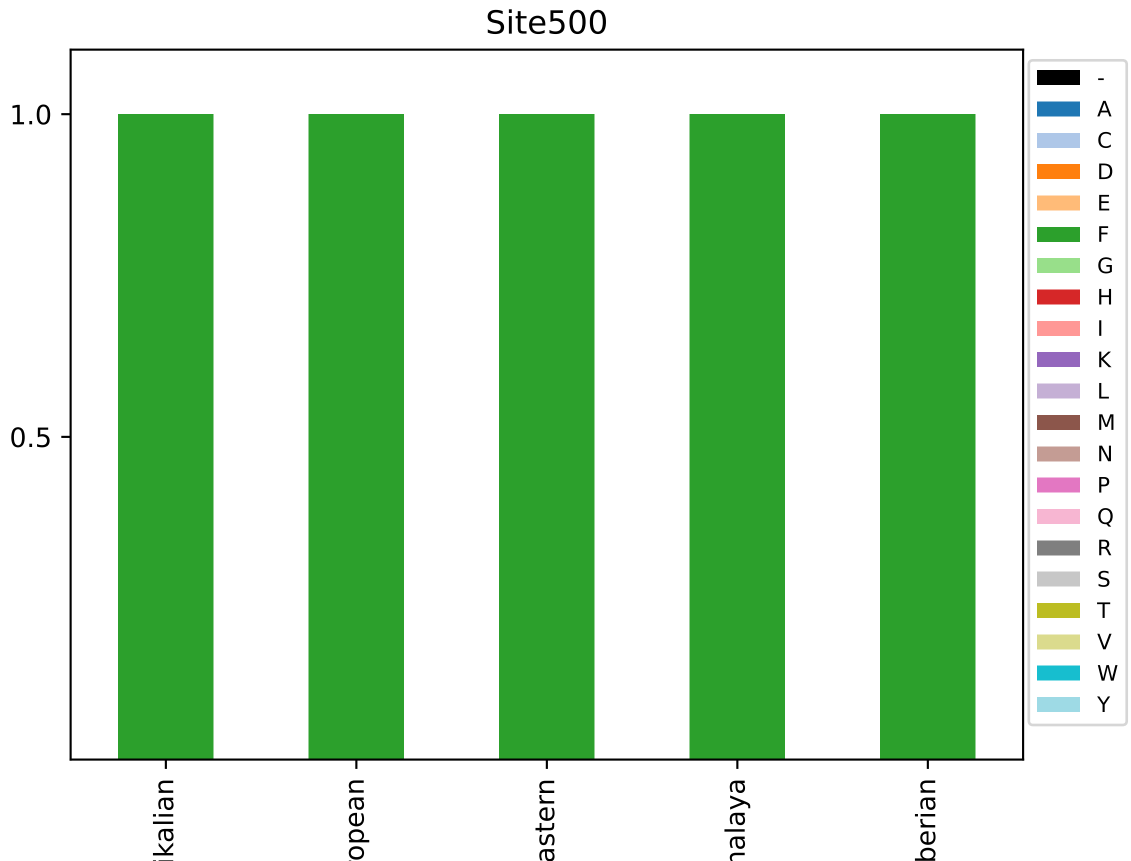
Site500
1.0
0.5
ikalian
opean
astern
nalaya
berian
-
A
C
D
E
F
G
H
I
K
L
M
N
P
Q
R
S
T
V
W
Y

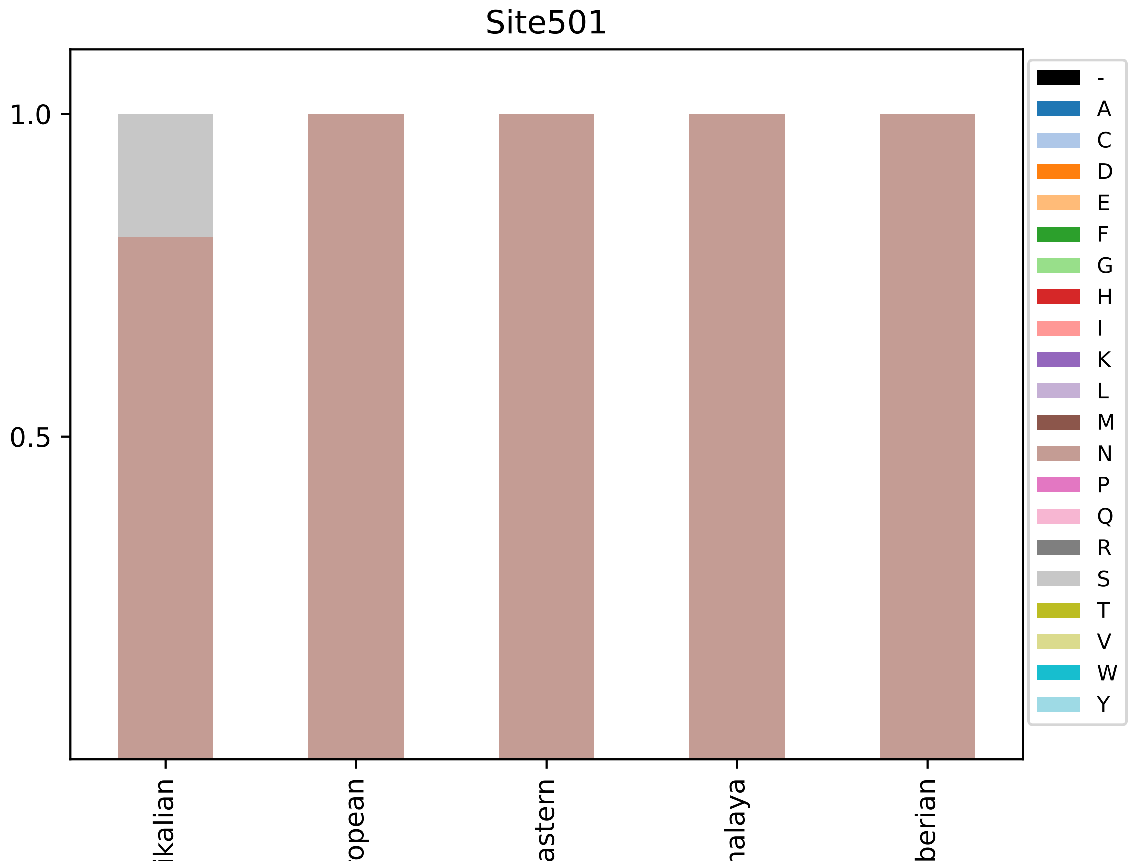
Site501
1.0
0.5
-
A
C
D
E
F
G
H
I
K
L
M
N
P
Q
R
S
T
V
W
Y
ikalian
opean
astern
nalaya
berian

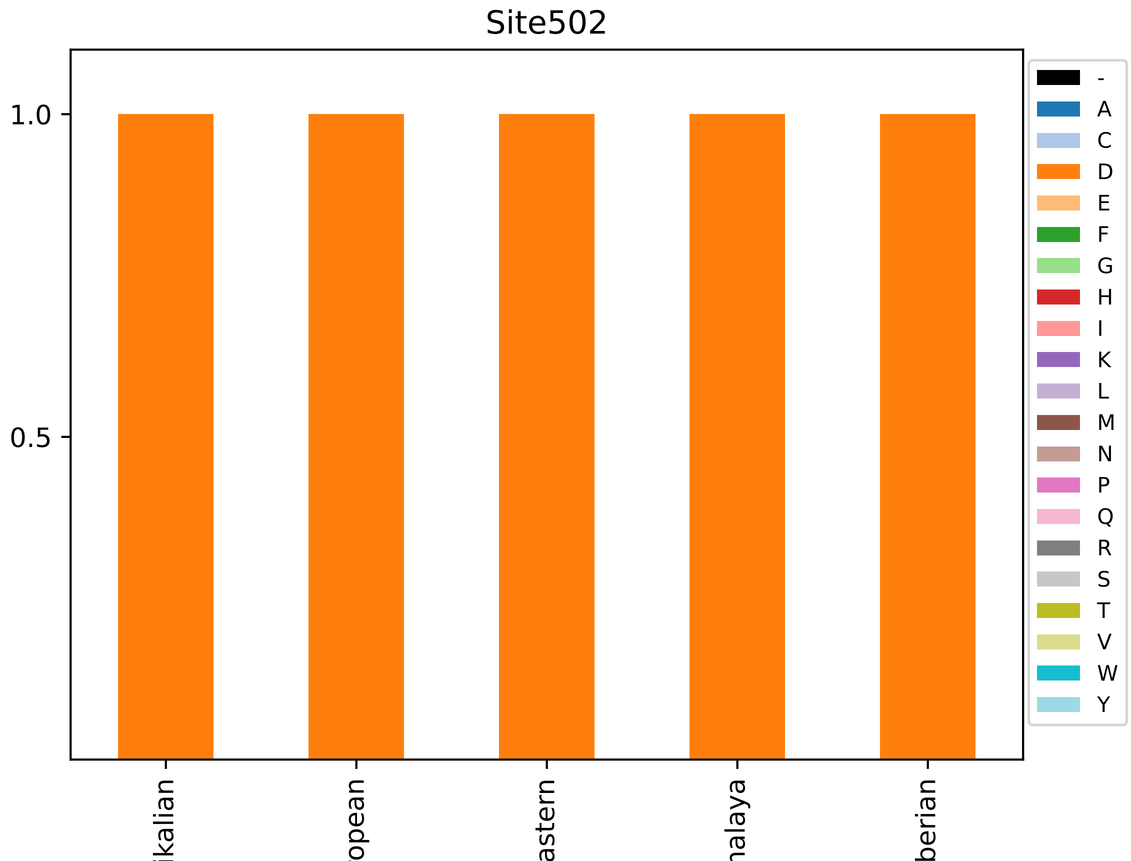
Site502
1.0
0.5
-
A
C
D
E
F
G
H
I
K
L
M
N
P
Q
R
S
T
V
W
Y
ikalian
opean
astern
nalaya
berian

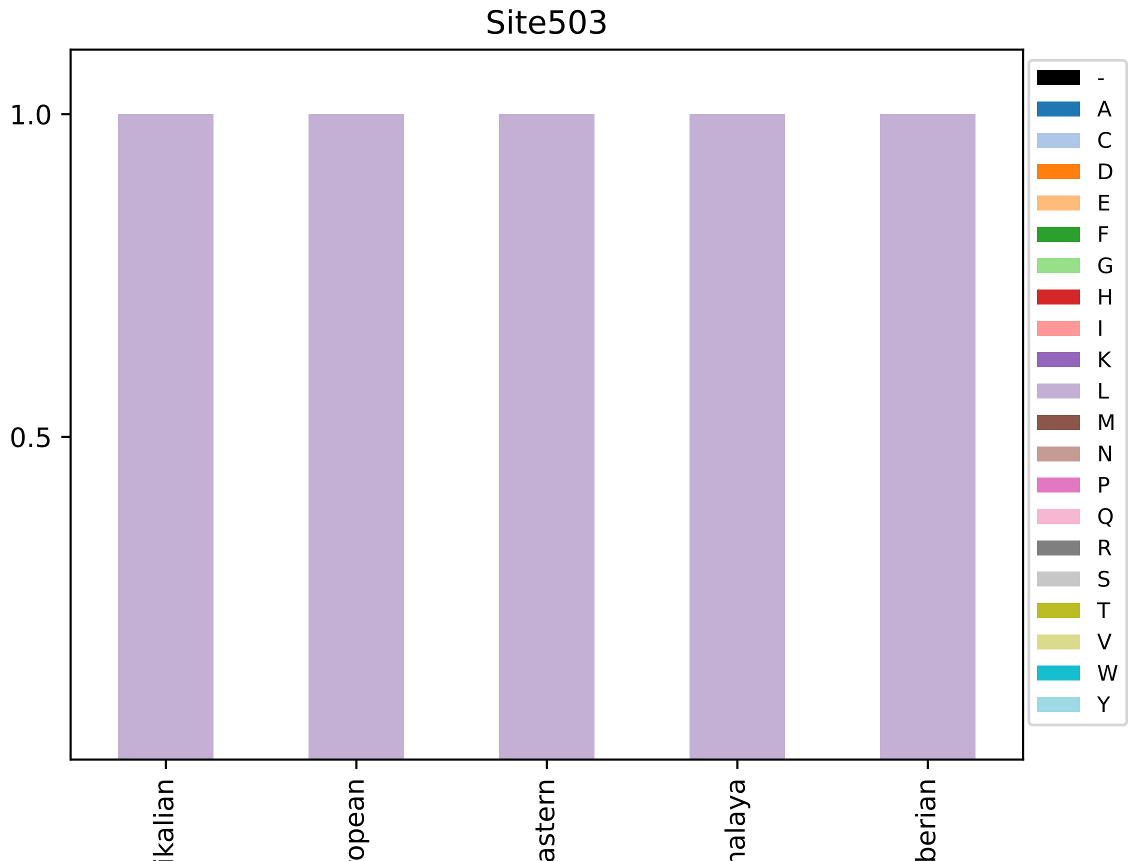
Site503
1.0
0.5
-
A
C
D
E
F
G
H
I
K
L
M
N
P
Q
R
S
T
V
W
Y

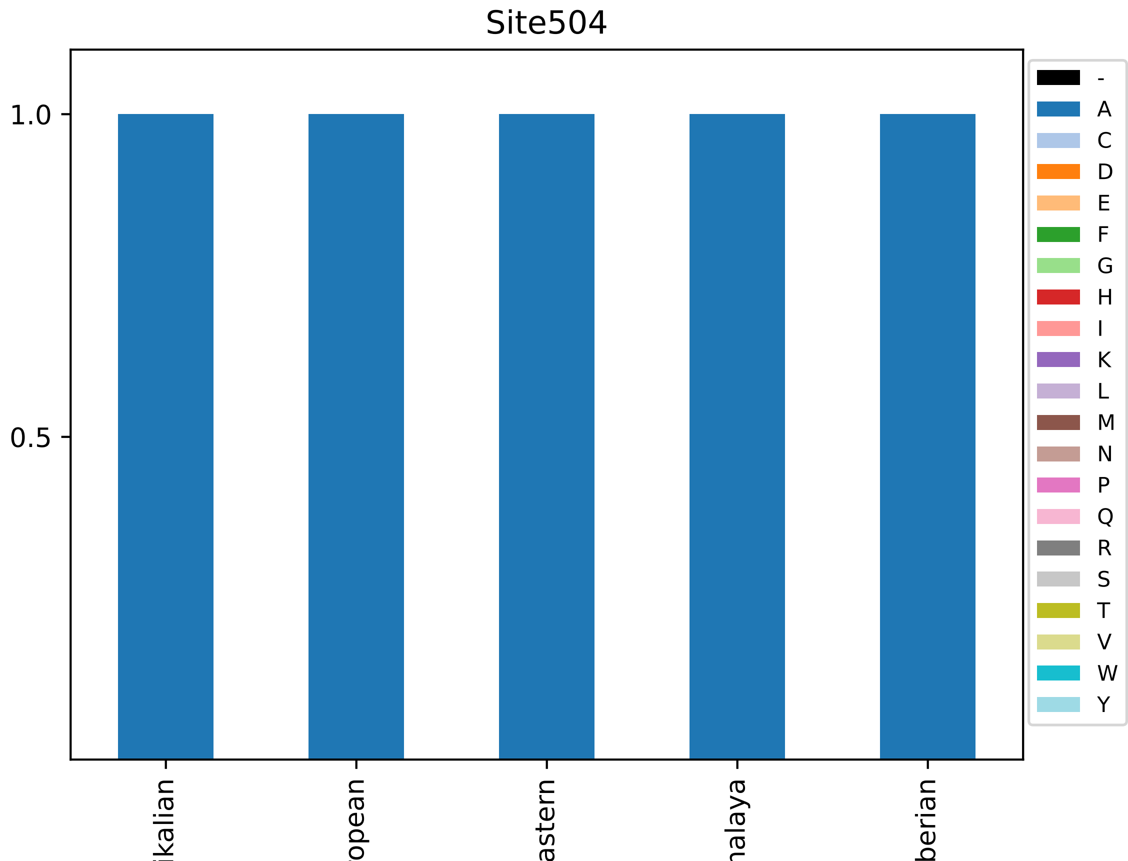
Site504
1.0
0.5
-
A
C
D
E
F
G
H
I
K
L
M
N
P
Q
R
S
T
V
W
Y
ikalian
opean
astern
nalaya
berian

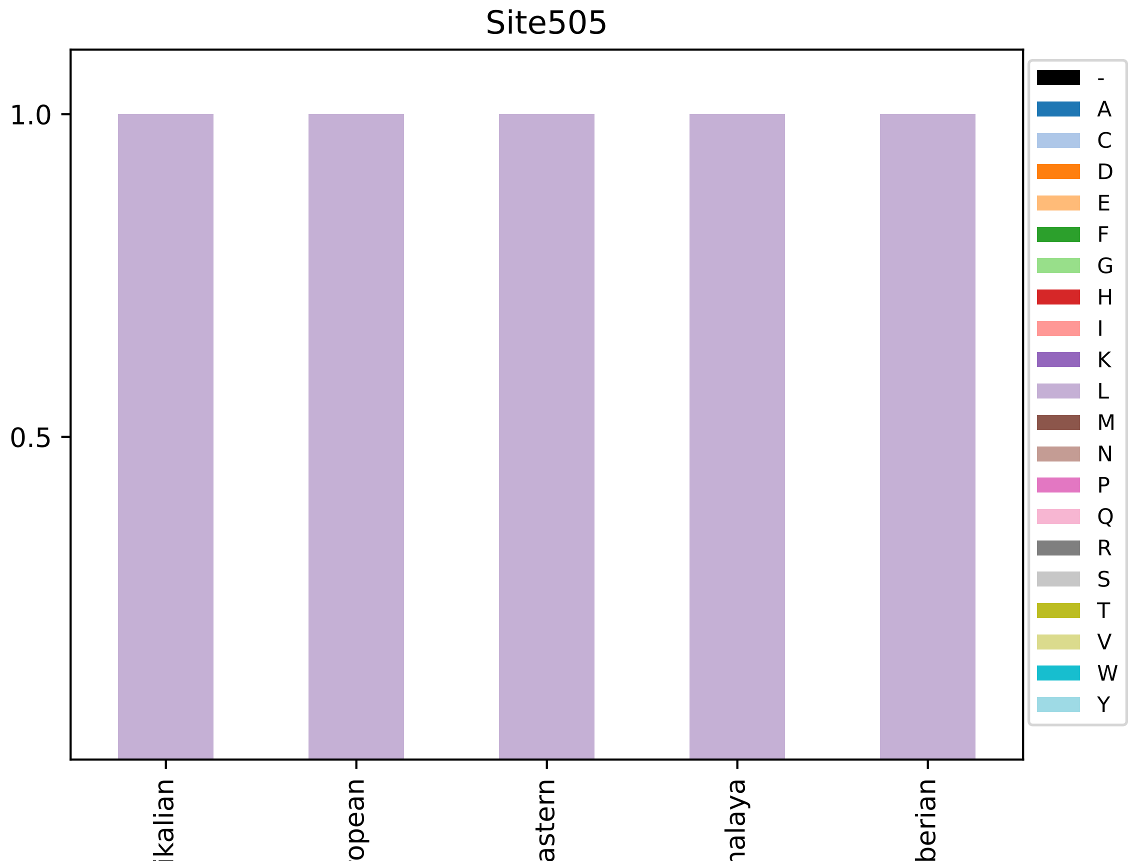
Site505
1.0
0.5
-
A
C
D
E
F
G
H
I
K
L
M
N
P
Q
R
S
T
V
W
Y
ikalian
opean
astern
nalaya
berian

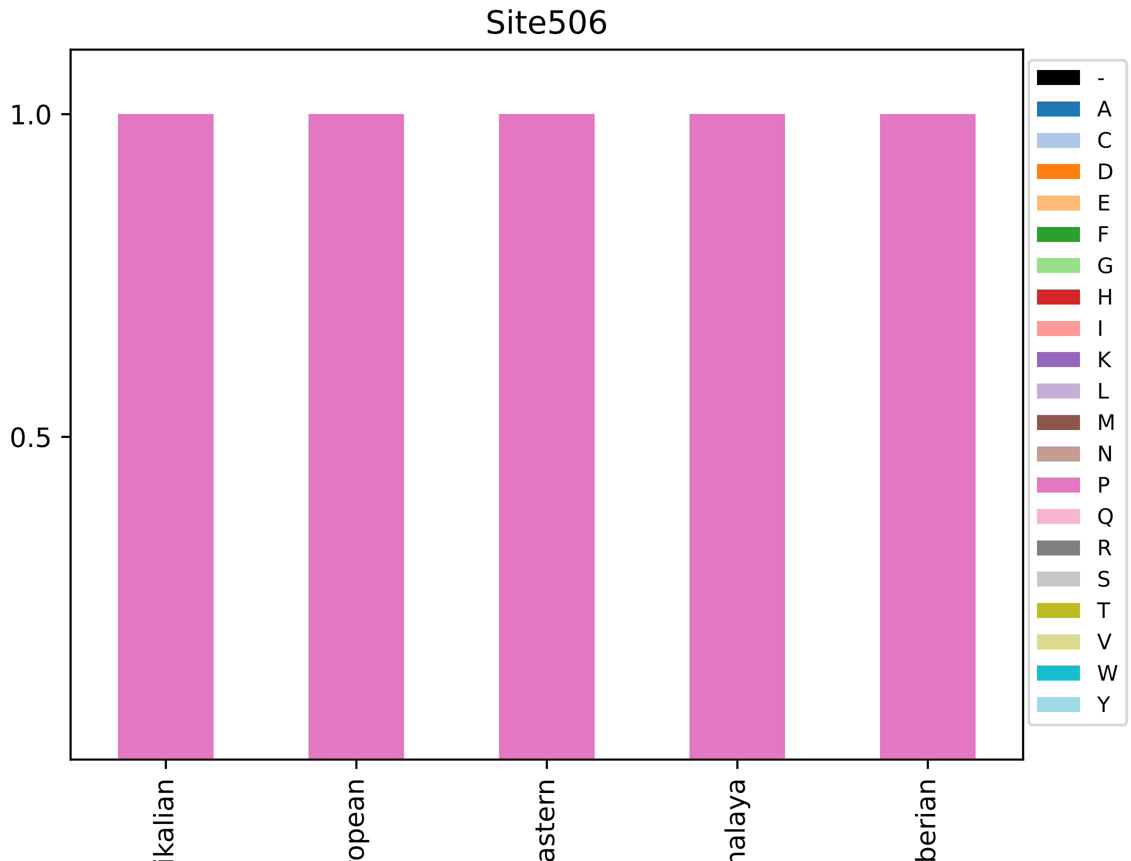

Site506
1.0
0.5
-
A
C
D
E
F
G
H
I
K
L
M
N
P
Q
R
S
T
V
W
Y

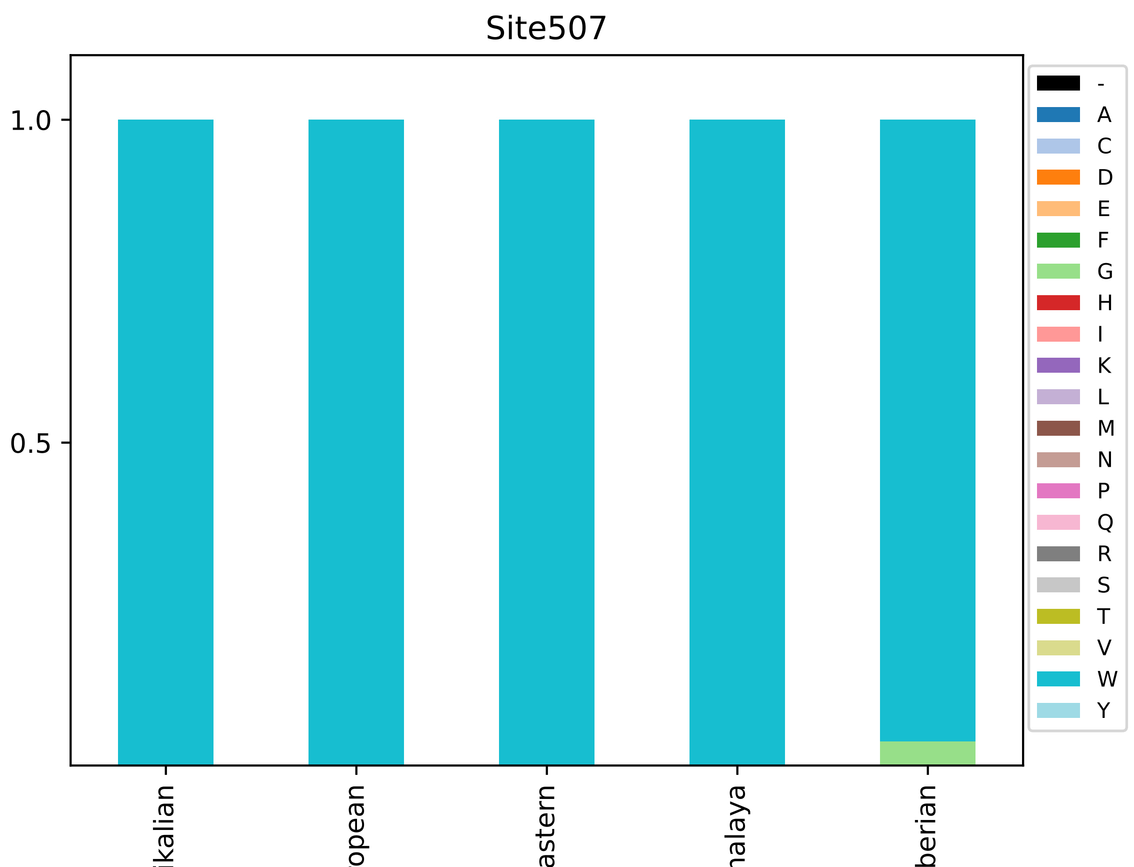

Site507
1.0
0.5
-
A
C
D
E
F
G
H
I
K
L
M
N
P
Q
R
S
T
V
W
Y
ikalian
opean
astern
nalaya
berian

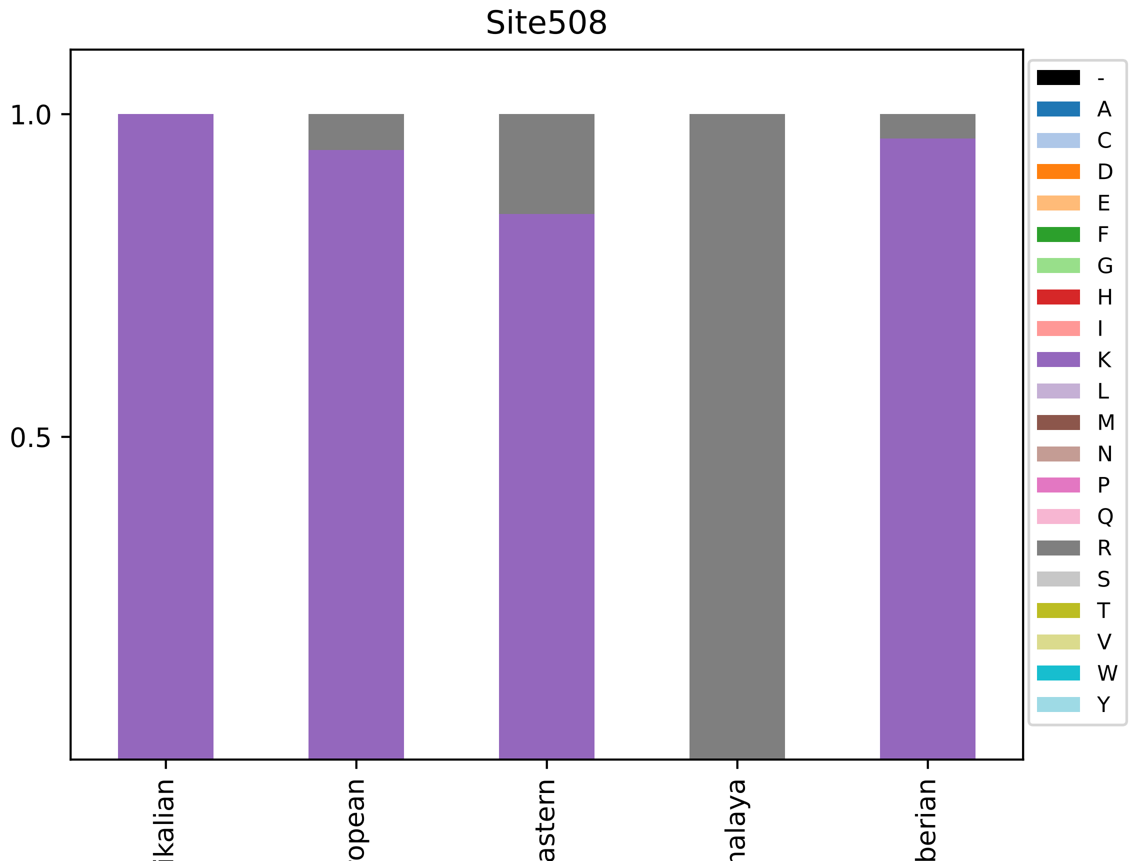
Site508
1.0
0.5
-
A
C
D
E
F
G
H
I
K
L
M
N
P
Q
R
S
T
V
W
Y
ikalian
opean
astern
nalaya
berian

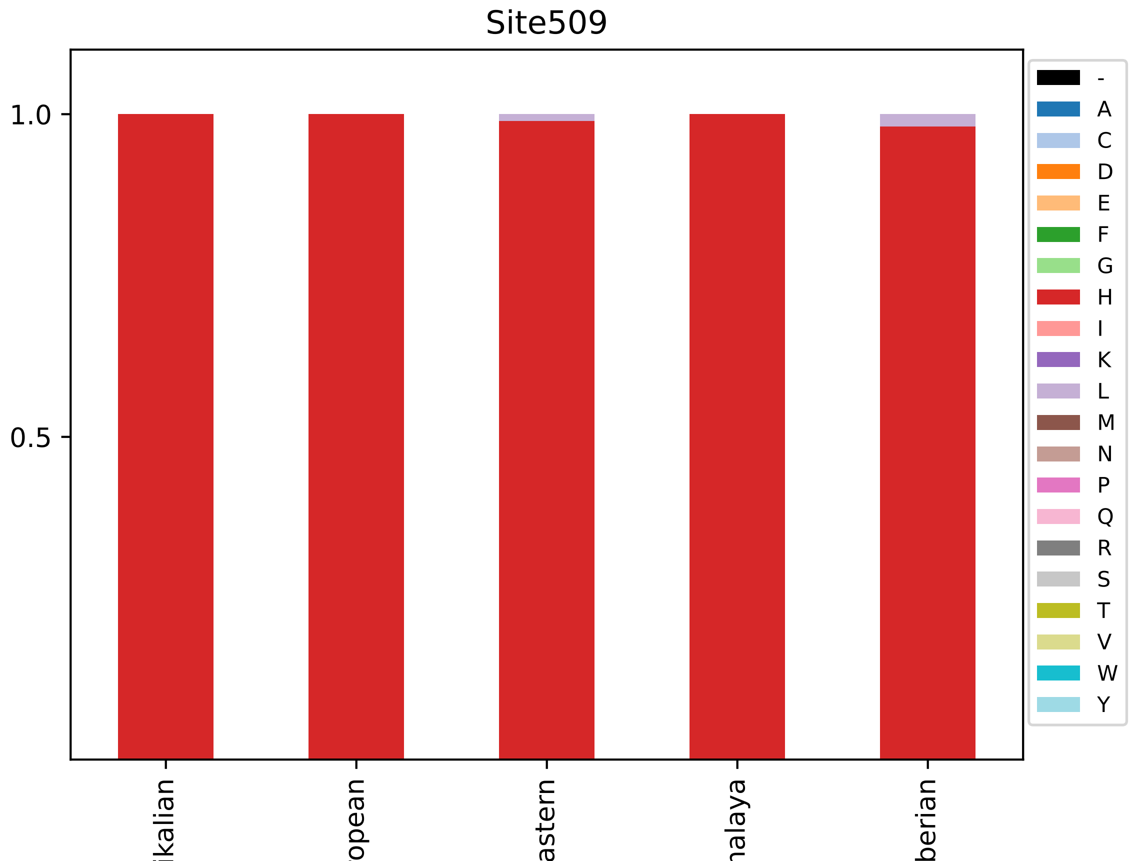
Site509
1.0
0.5
ikalian
opean
astern
nalaya
berian
-
A
C
D
E
F
G
H
I
K
L
M
N
P
Q
R
S
T
V
W
Y

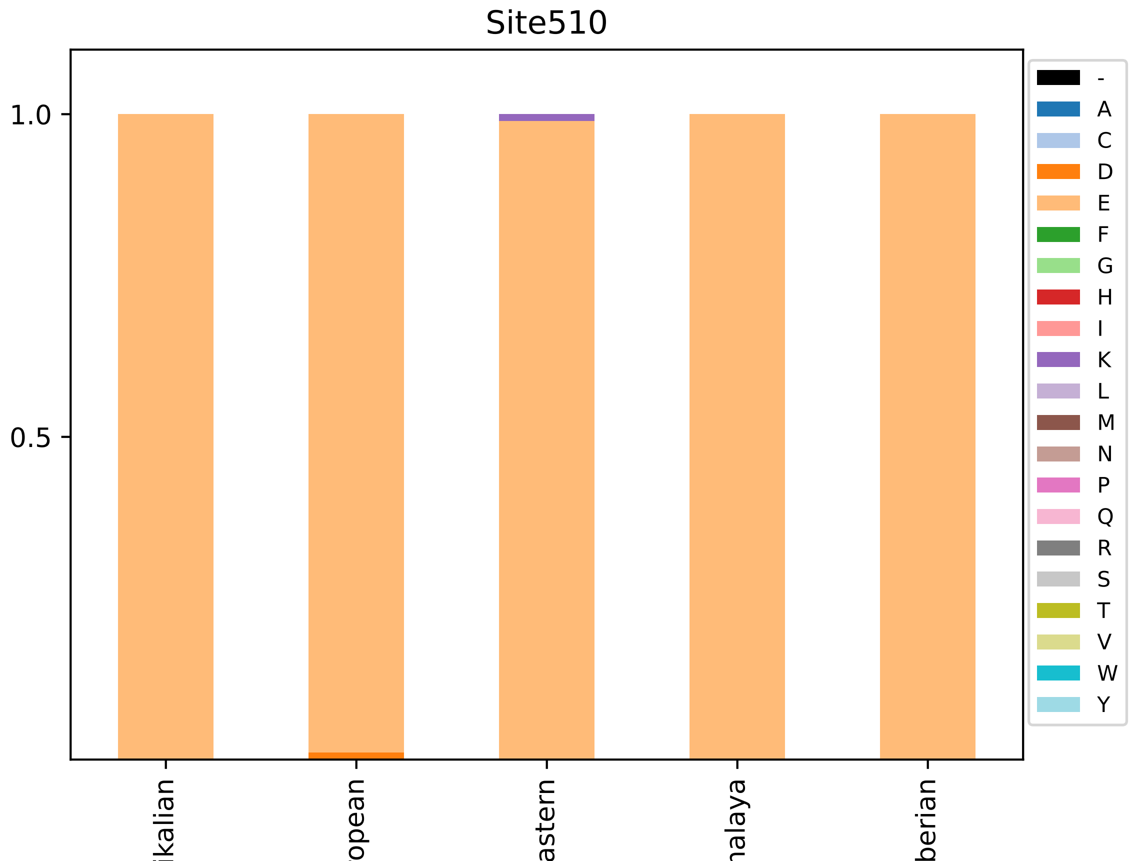

Site510
1.0
0.5
-
A
C
D
E
F
G
H
I
K
L
M
N
P
Q
R
S
T
V
W
Y

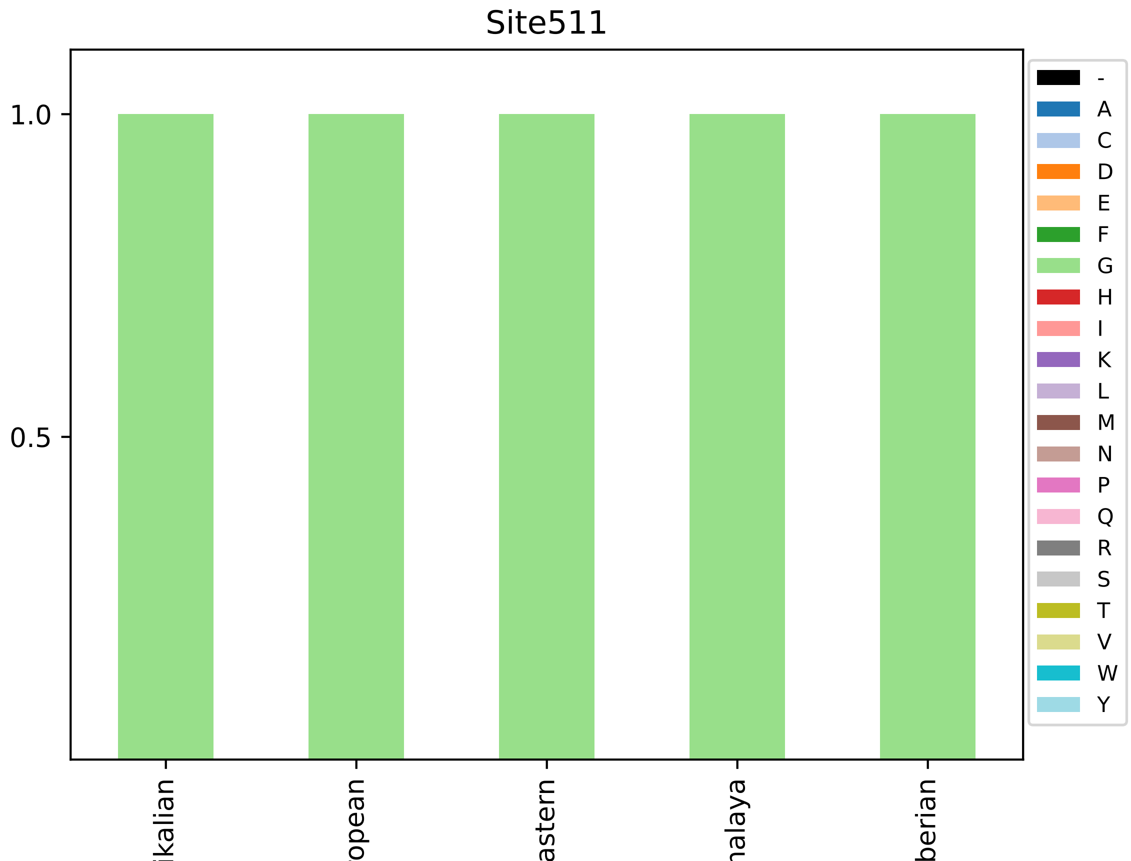

Site511
1.0
0.5
-
A
C
D
E
F
G
H
I
K
L
M
N
P
Q
R
S
T
V
W
Y
ikalian
opean
astern
nalaya
berian

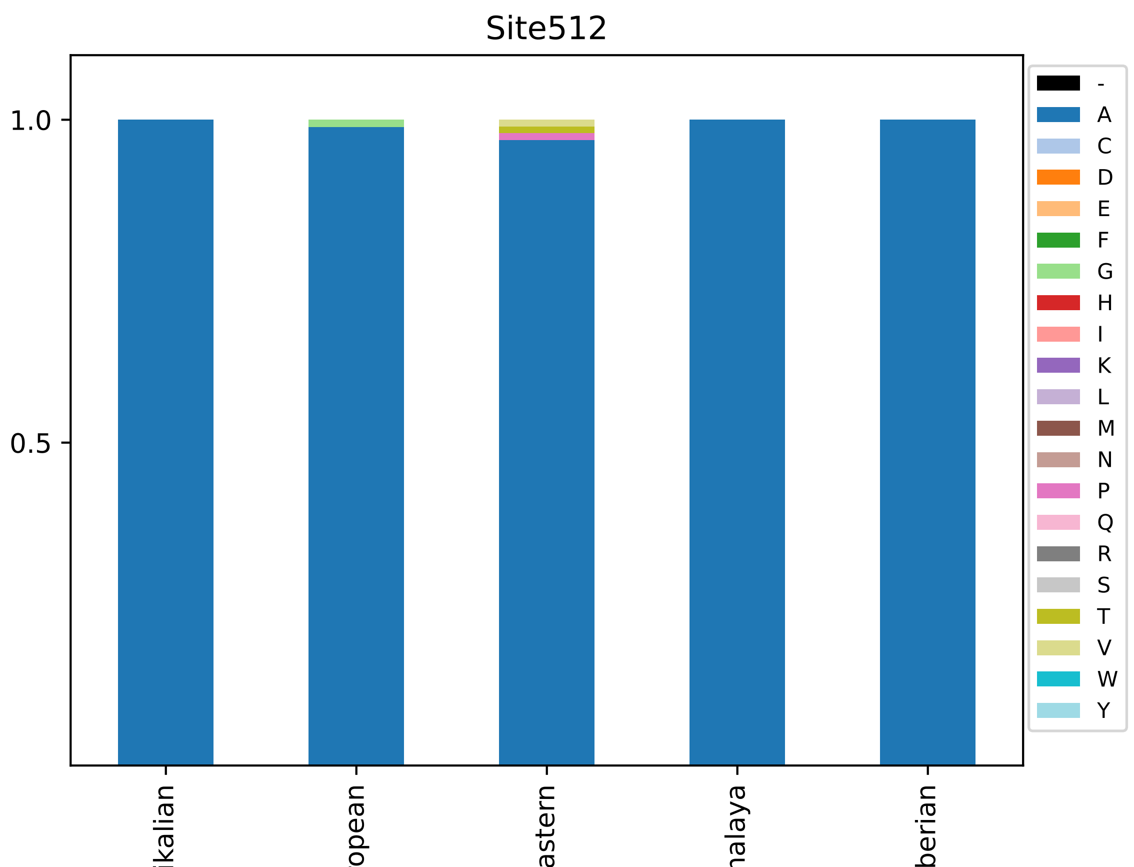
Site512
1.0
0.5
-
A
C
D
E
F
G
H
I
K
L
M
N
P
Q
R
S
T
V
W
Y
ikalian
opean
astern
nalaya
berian

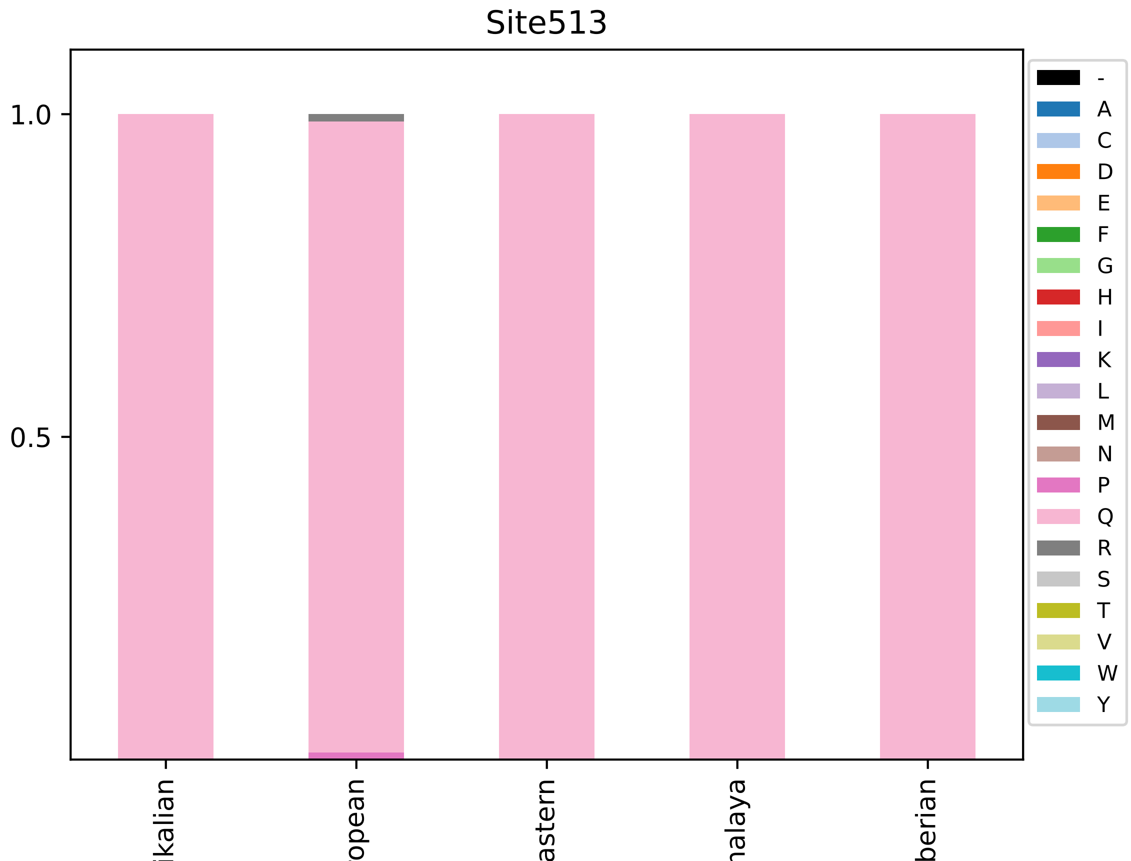
Site513
1.0
0.5
-
A
C
D
E
F
G
H
I
K
L
M
N
P
Q
R
S
T
V
W
Y
ikalian
opean
astern
nalaya
berian

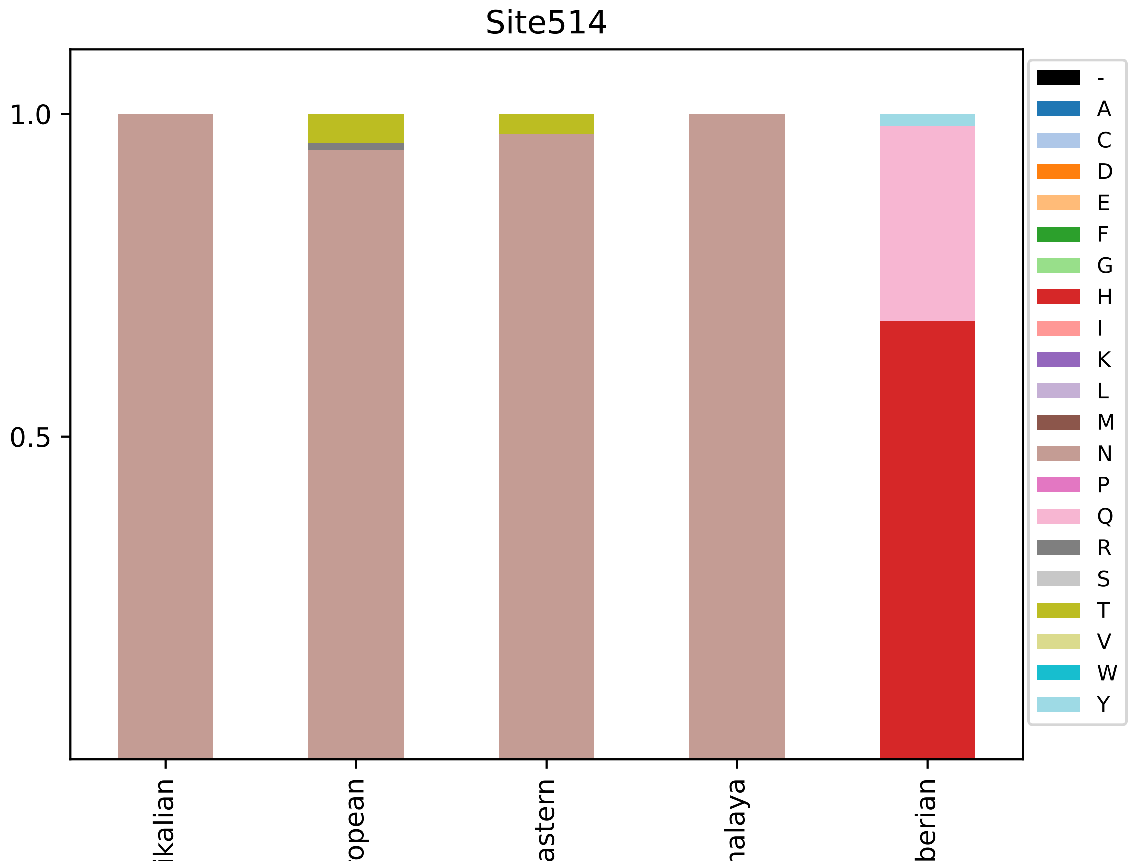

Site514
1.0
0.5
-
A
C
D
E
F
G
H
I
K
L
M
N
P
Q
R
S
T
V
W
Y
ikalian
opean
astern
nalaya
berian

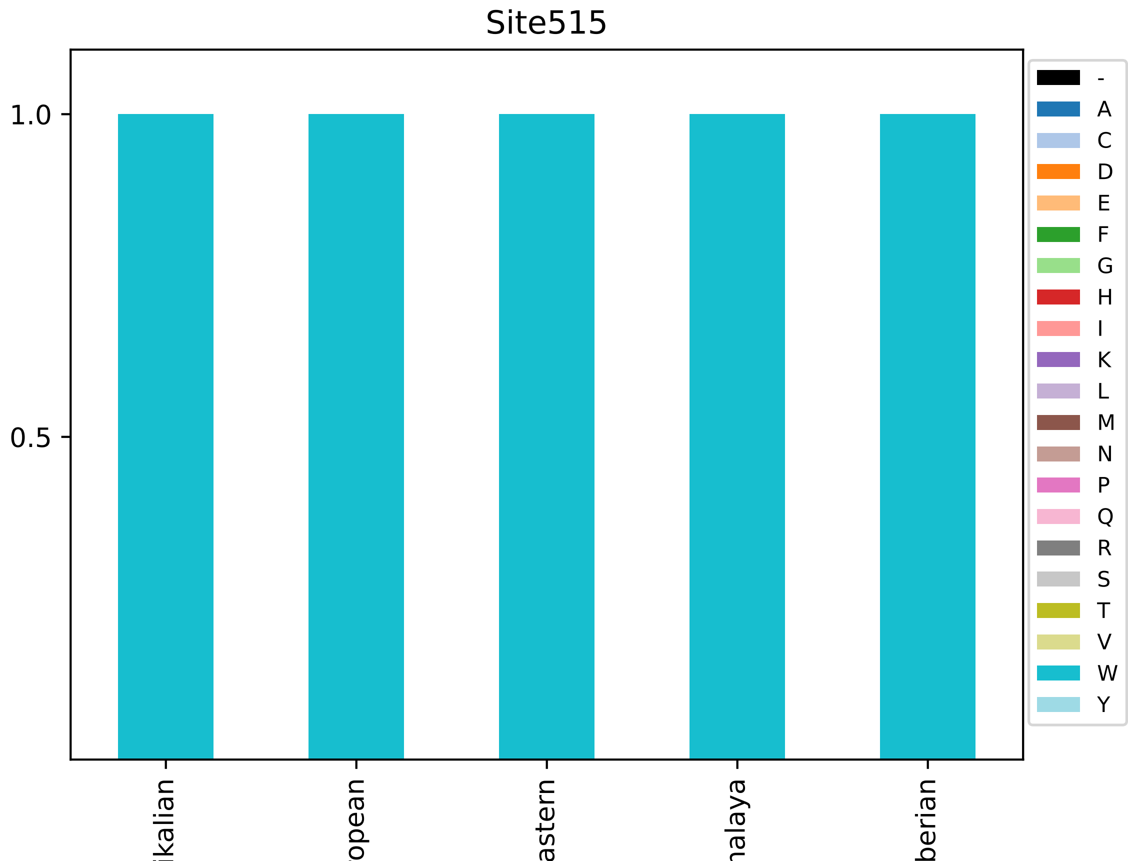

Site515
1.0
0.5
-
A
C
D
E
F
G
H
I
K
L
M
N
P
Q
R
S
T
V
W
Y
ikalian
opean
astern
nalaya
berian

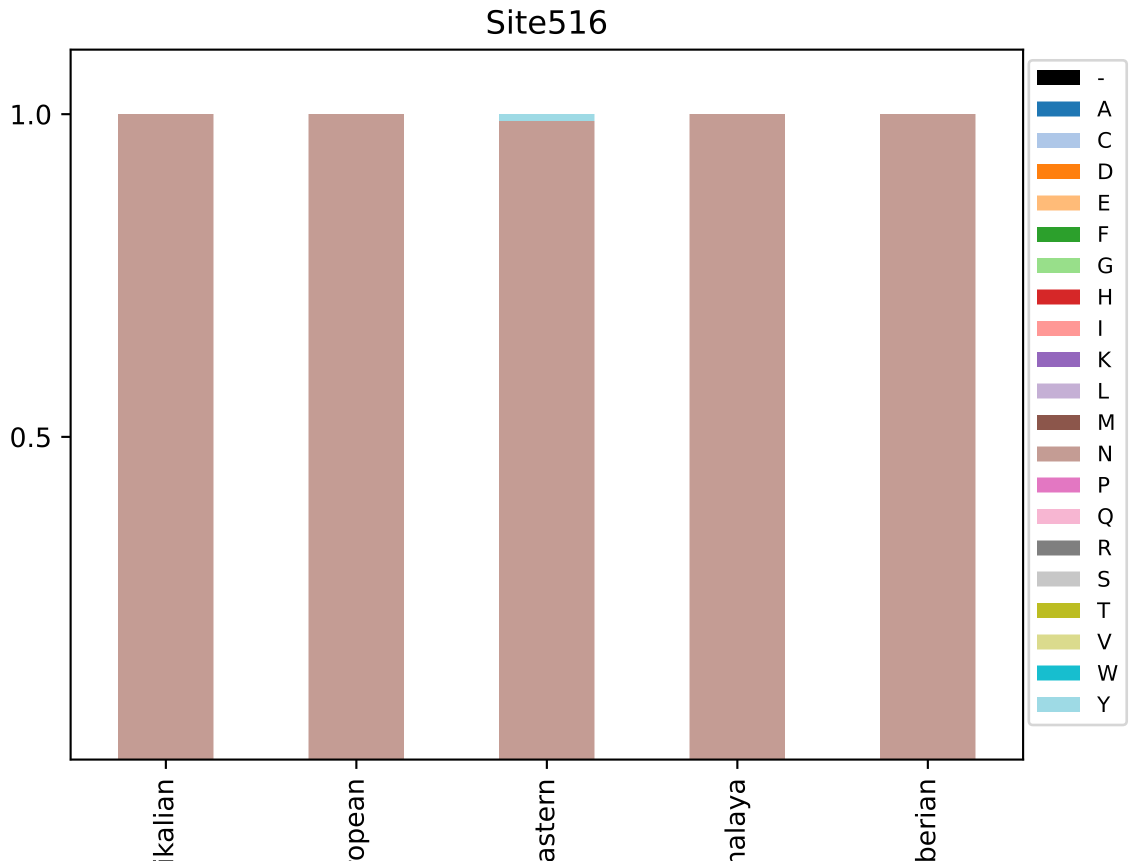
Site516
1.0
0.5
ikalian
opean
astern
halaya
berian
-
A
C
D
E
F
G
H
I
K
L
M
N
P
Q
R
S
T
V
W
Y

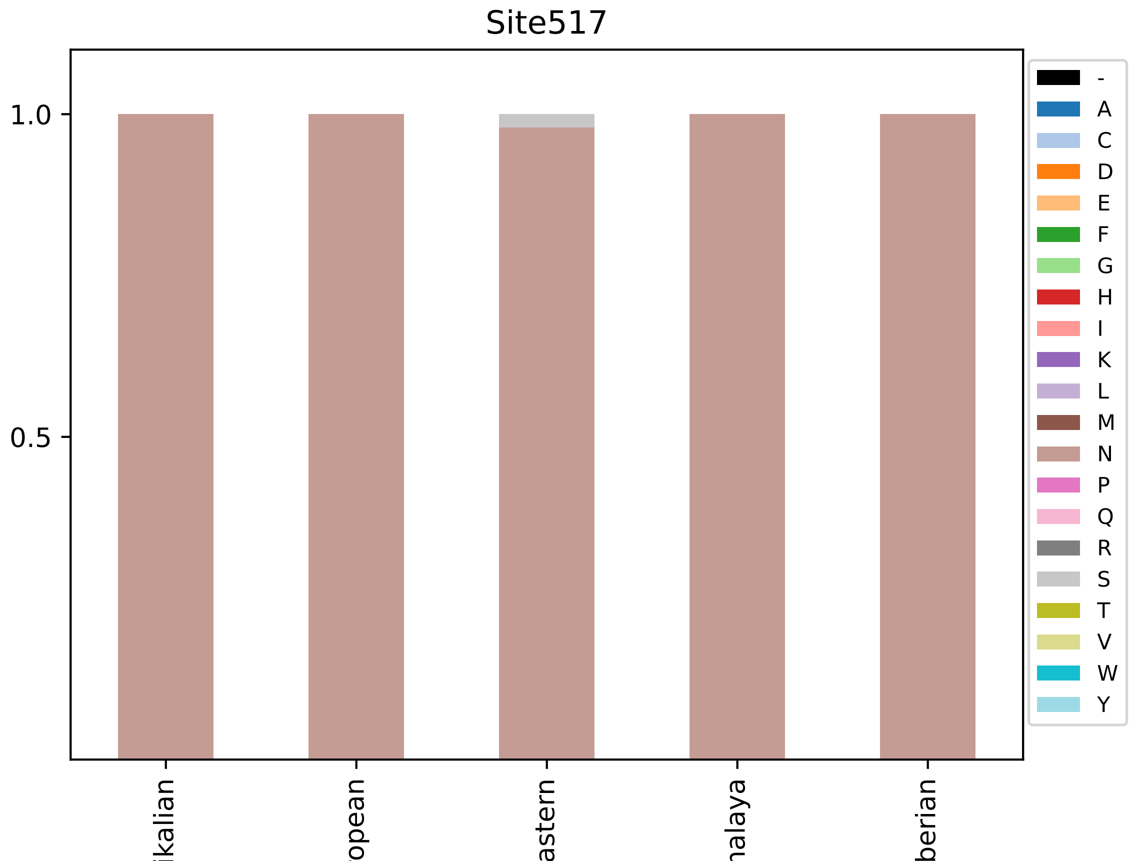

Site517
1.0
0.5
-
A
C
D
E
F
G
H
I
K
L
M
N
P
Q
R
S
T
V
W
Y

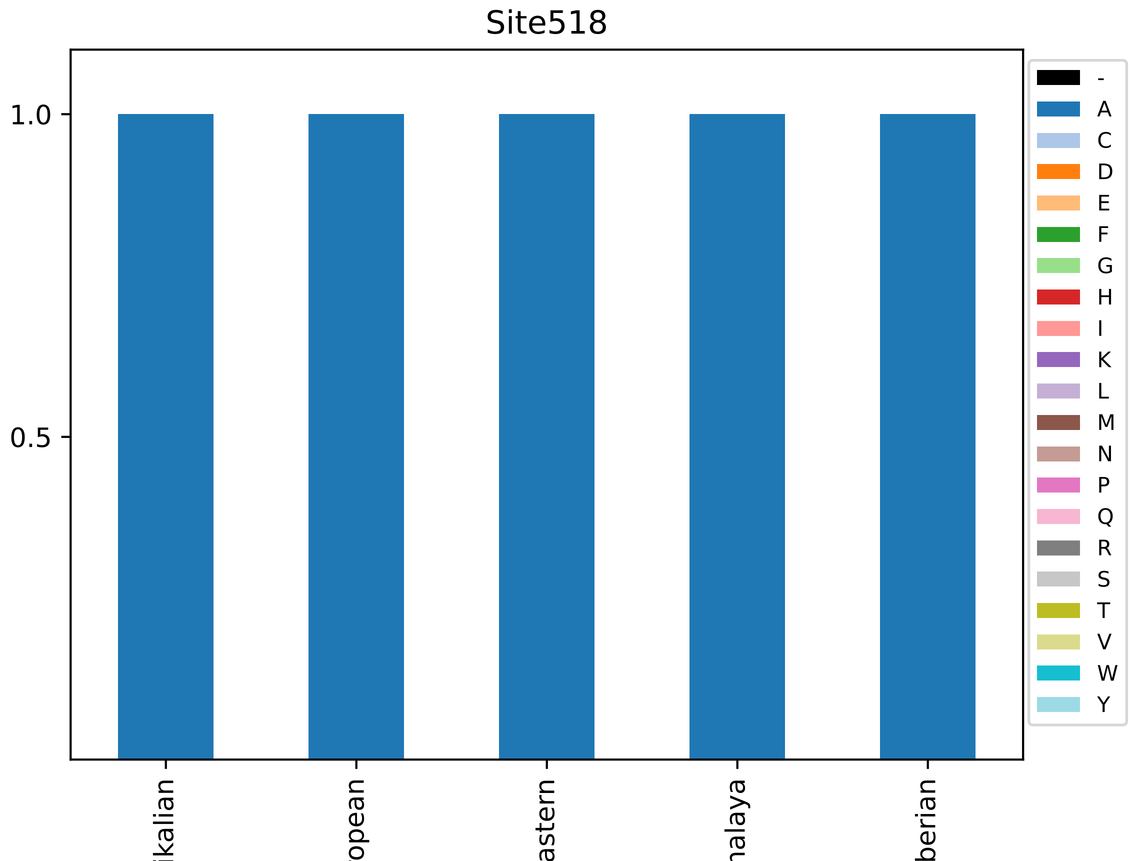
Site518
1.0
0.5
-
A
C
D
E
F
G
H
I
K
L
M
N
P
Q
R
S
T
V
W
Y
ikalian
opean
astern
nalaya
berian

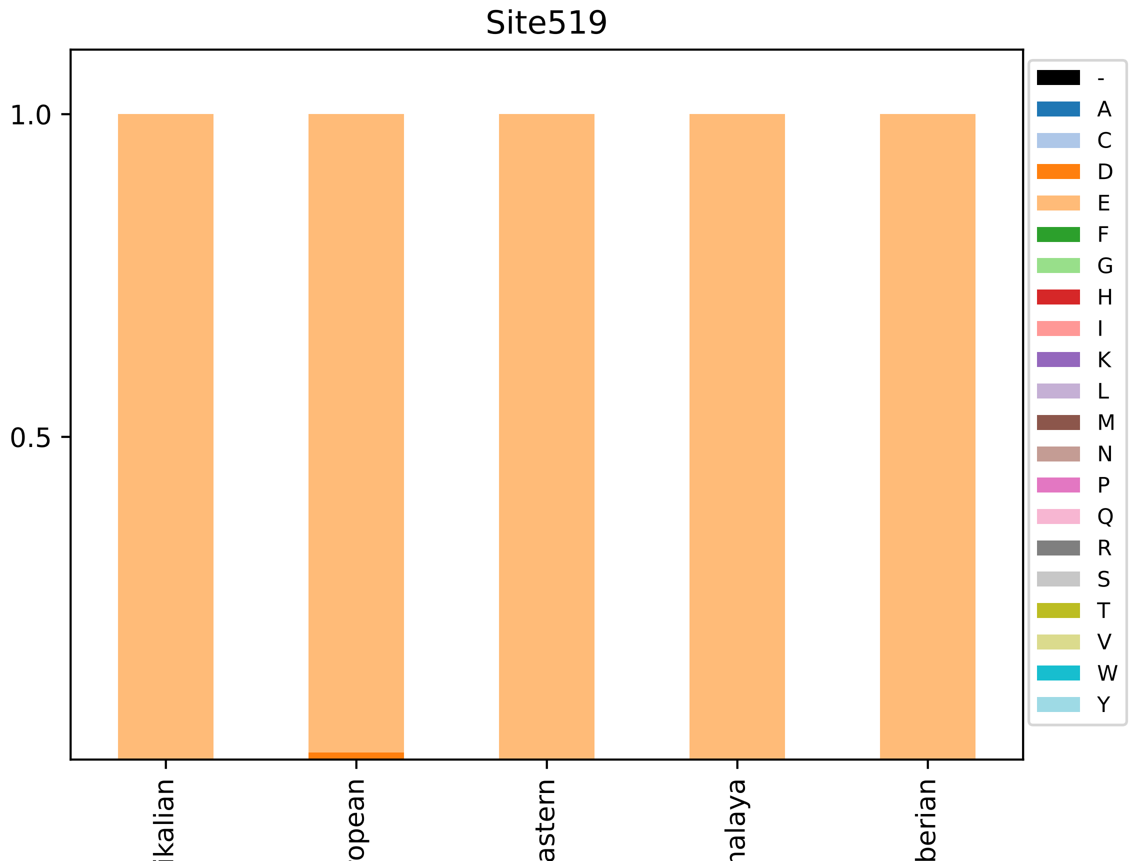
Site519
1.0
0.5
-
A
C
D
E
F
G
H
I
K
L
M
N
P
Q
R
S
T
V
W
Y
ikalian
opean
astern
nalaya
berian

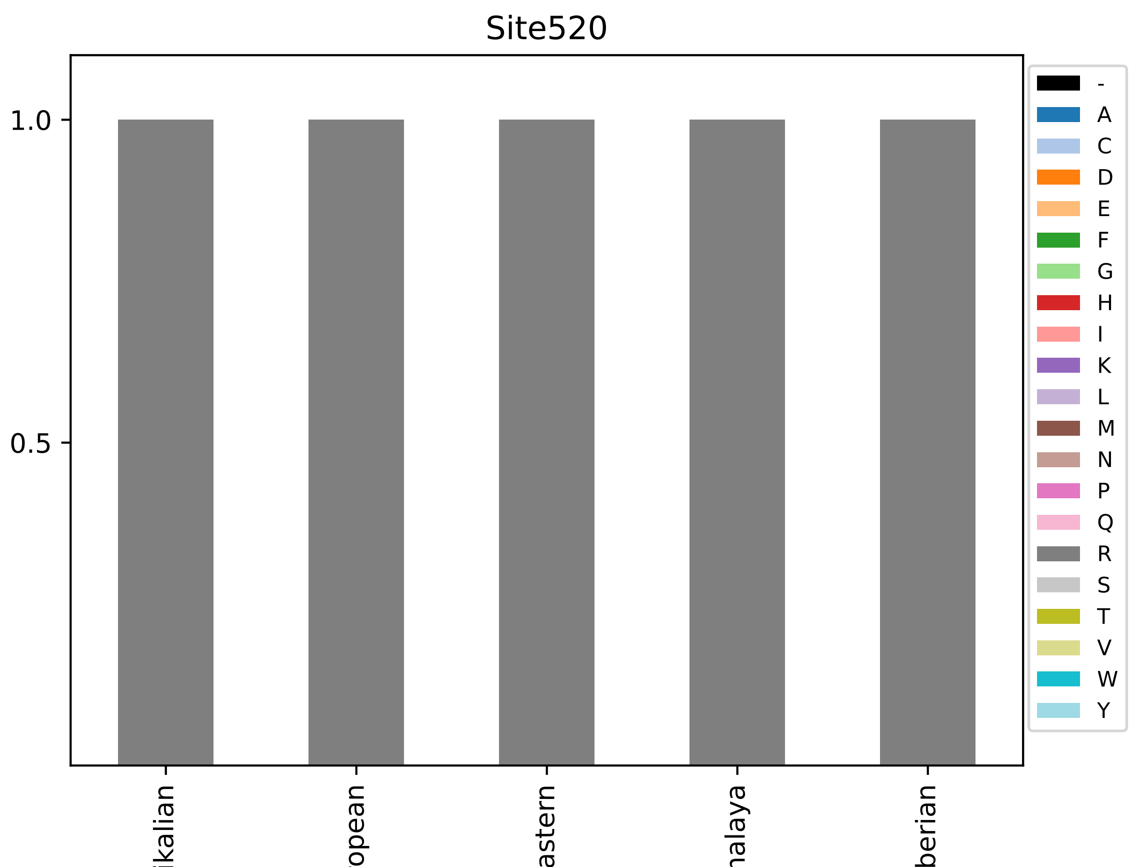

Site520
1.0
0.5
-
A
C
D
E
F
G
H
I
K
L
M
N
P
Q
R
S
T
V
W
Y
ikalian
opean
astern
nalaya
berian

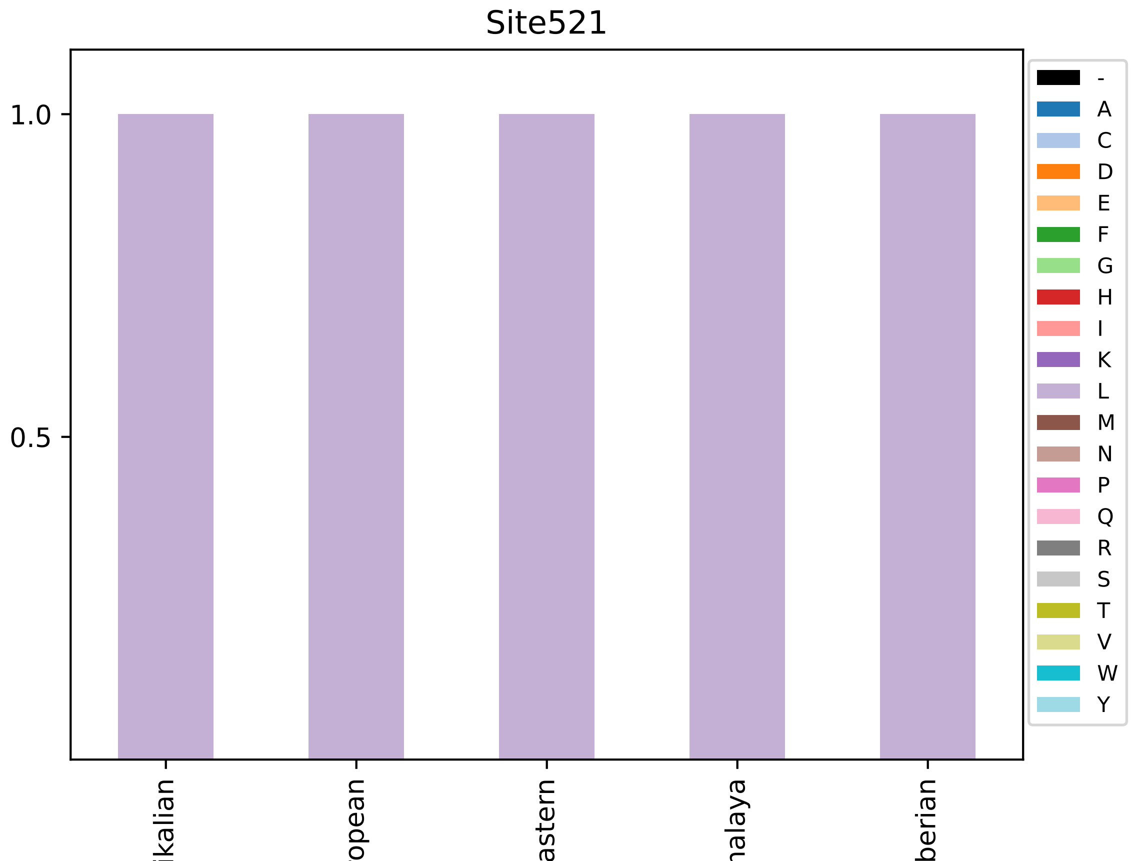
Site521
1.0
0.5
-
A
C
D
E
F
G
H
I
K
L
M
N
P
Q
R
S
T
V
W
Y
ikalian
opean
astern
nalaya
berian

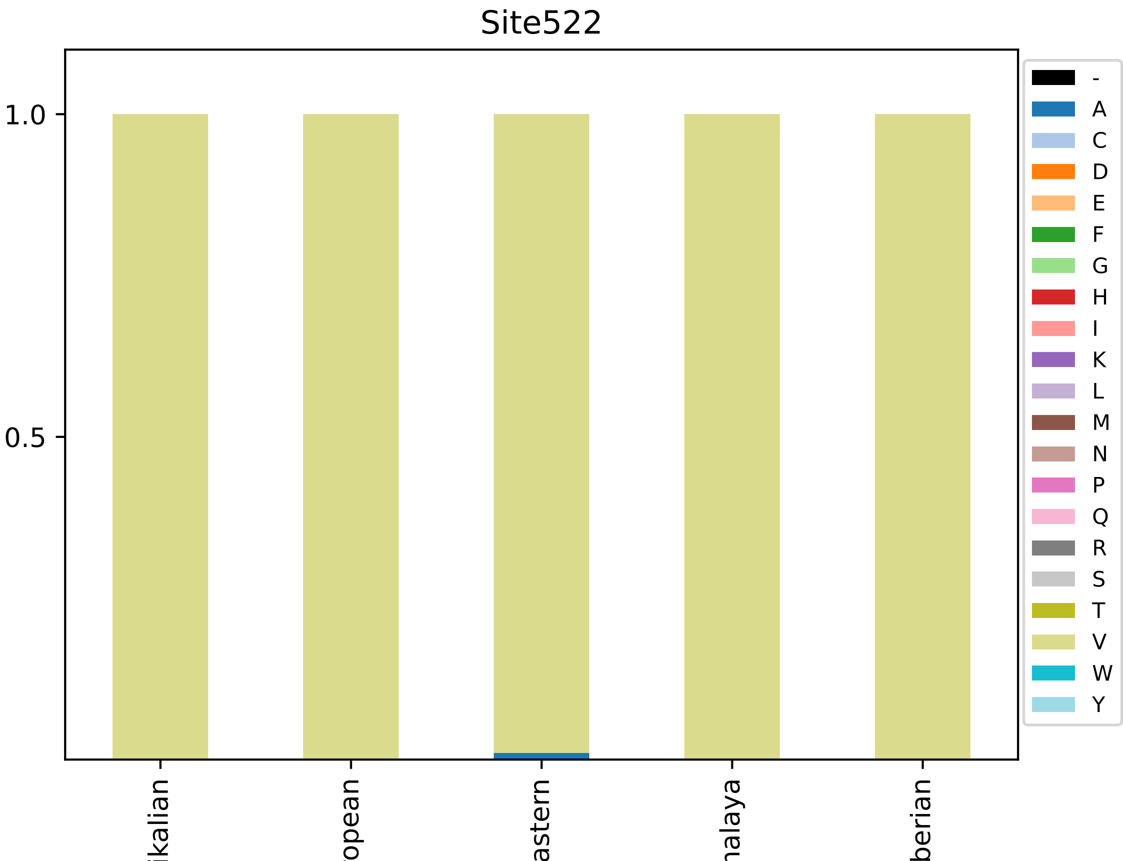

Site522
1.0
0.5
-
A
C
D
E
F
G
H
I
K
L
M
N
P
Q
R
S
T
V
W
Y
ikalian
opean
astern
nalaya
berian

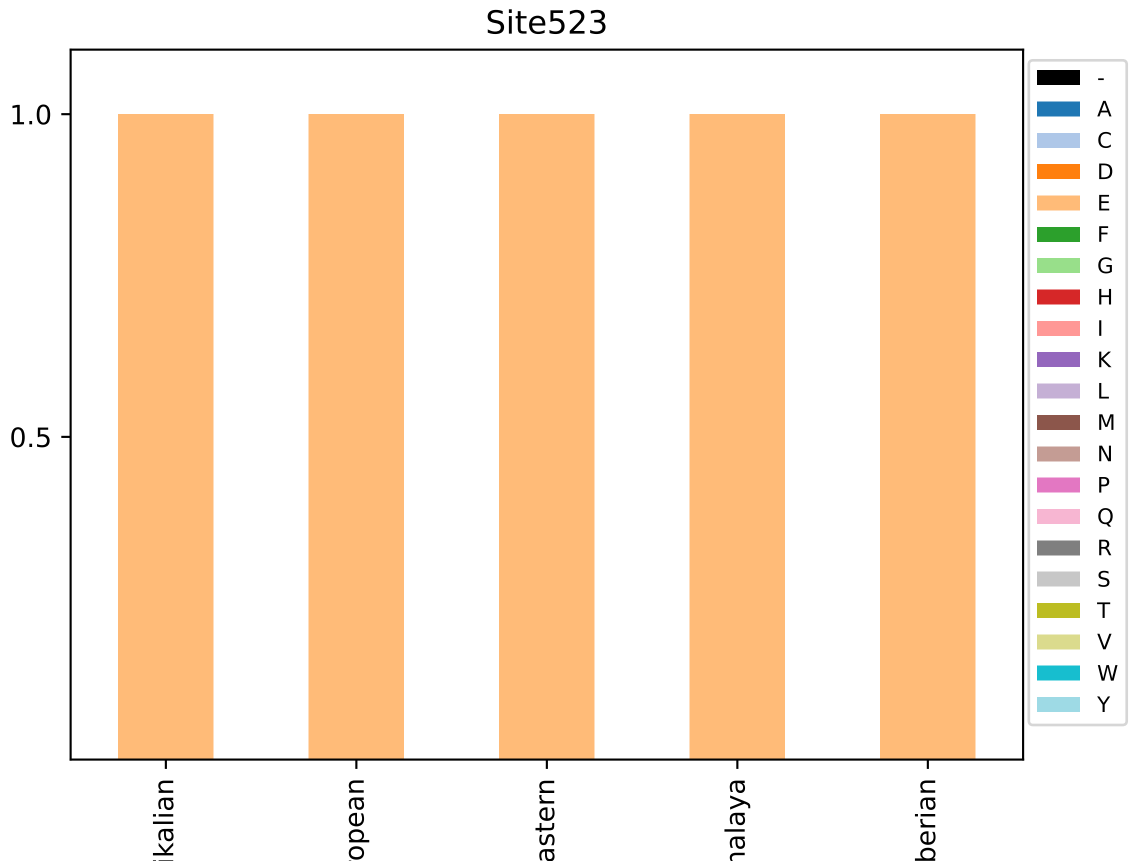

Site523
1.0
0.5
-
A
C
D
E
F
G
H
I
K
L
M
N
P
Q
R
S
T
V
W
Y
ikalian
opean
astern
nalaya
berian

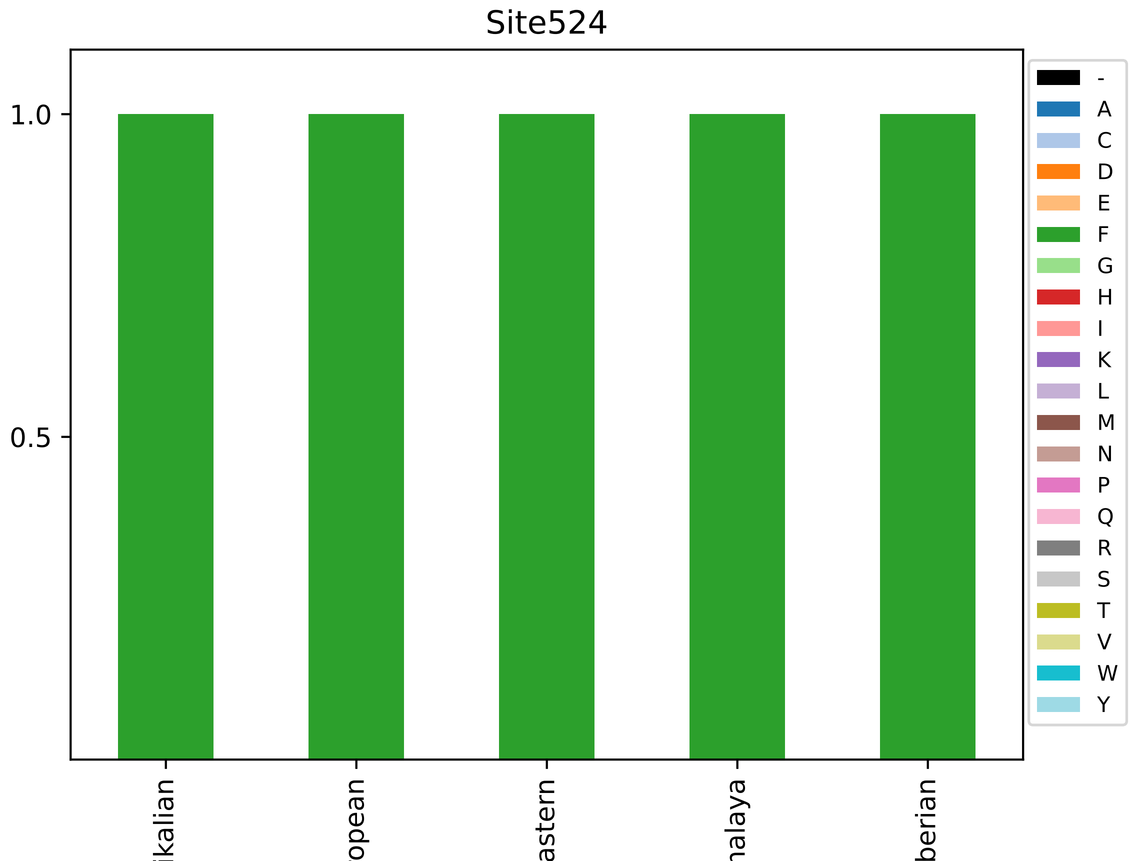
Site524
1.0
0.5
-
A
C
D
E
F
G
H
I
K
L
M
N
P
Q
R
S
T
V
W
Y
ikalian
opean
astern
nalaya
berian

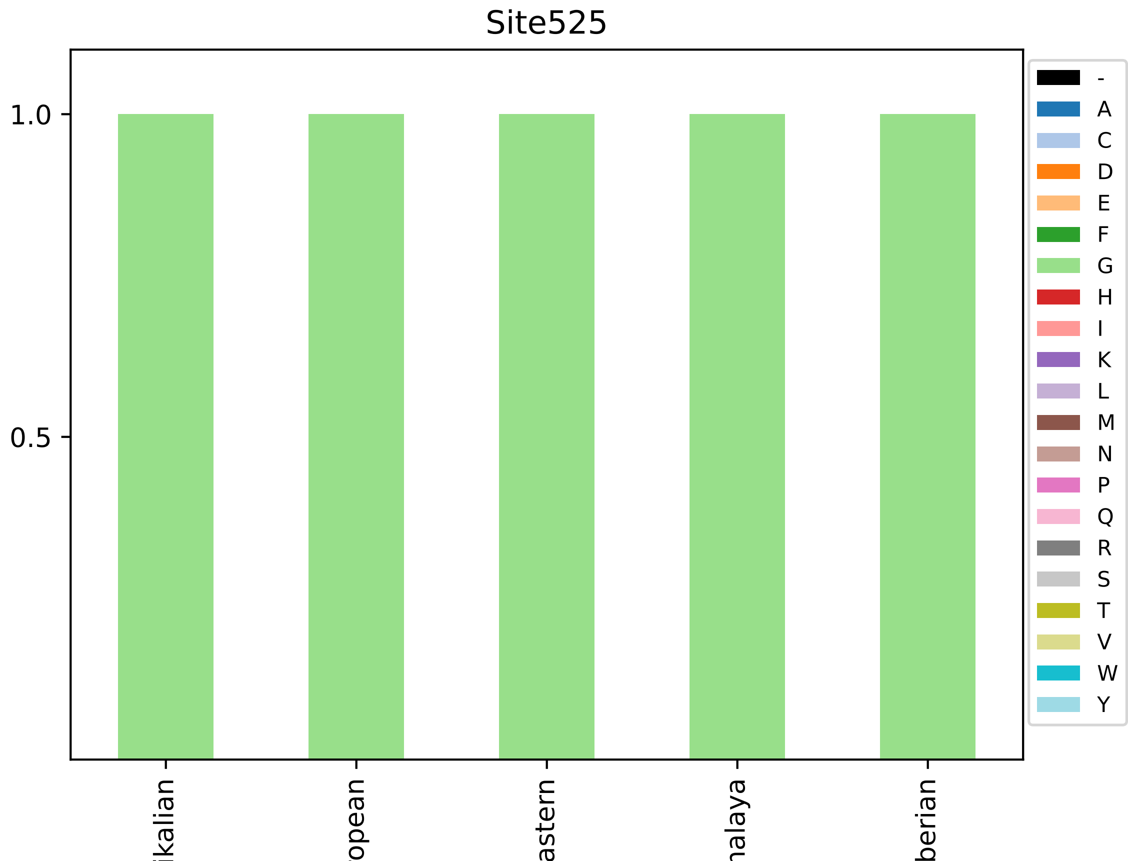

Site525
1.0
0.5
-
A
C
D
E
F
G
H
I
K
L
M
N
P
Q
R
S
T
V
W
Y
ikalian
opean
astern
nalaya
berian

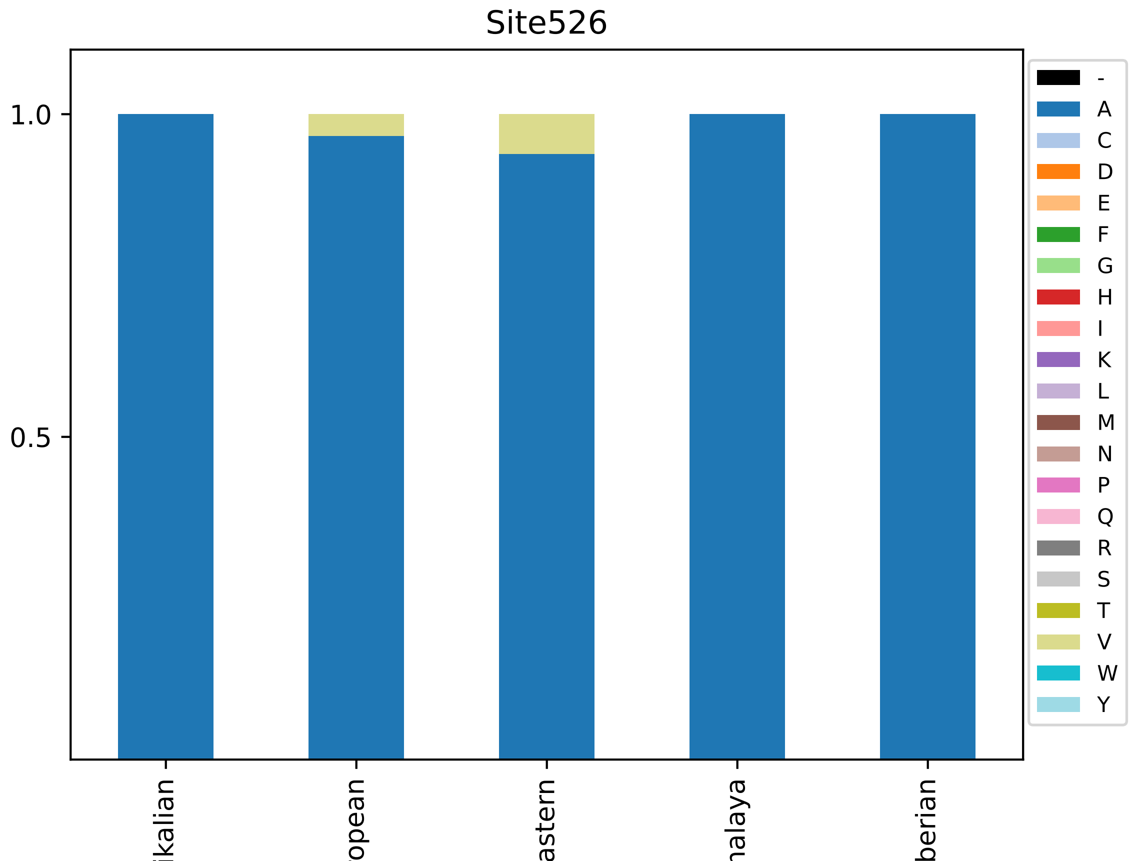
Site526
1.0
0.5
ikalian
opean
astern
nalaya
berian
-
A
C
D
E
F
G
H
I
K
L
M
N
P
Q
R
S
T
V
W
Y

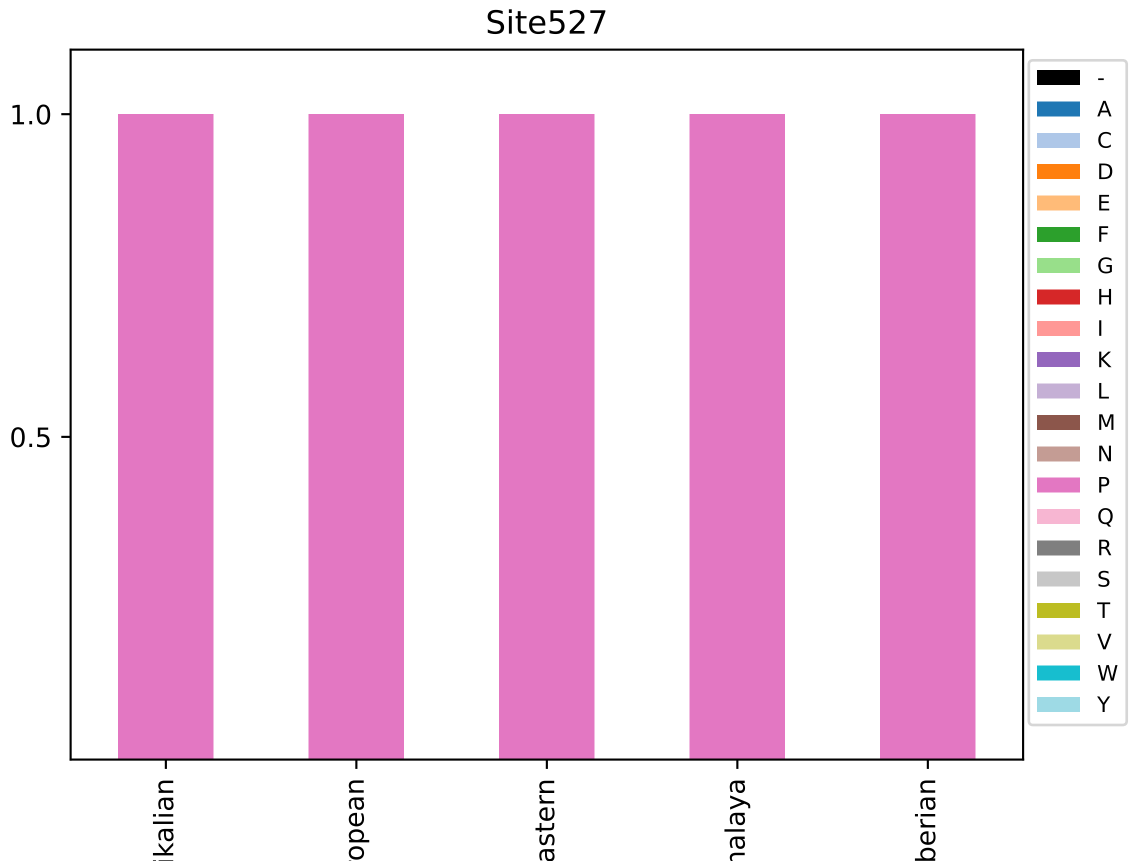

Site527
1.0
0.5
-
A
C
D
E
F
G
H
I
K
L
M
N
P
Q
R
S
T
V
W
Y
ikalian
opean
astern
nalaya
berian

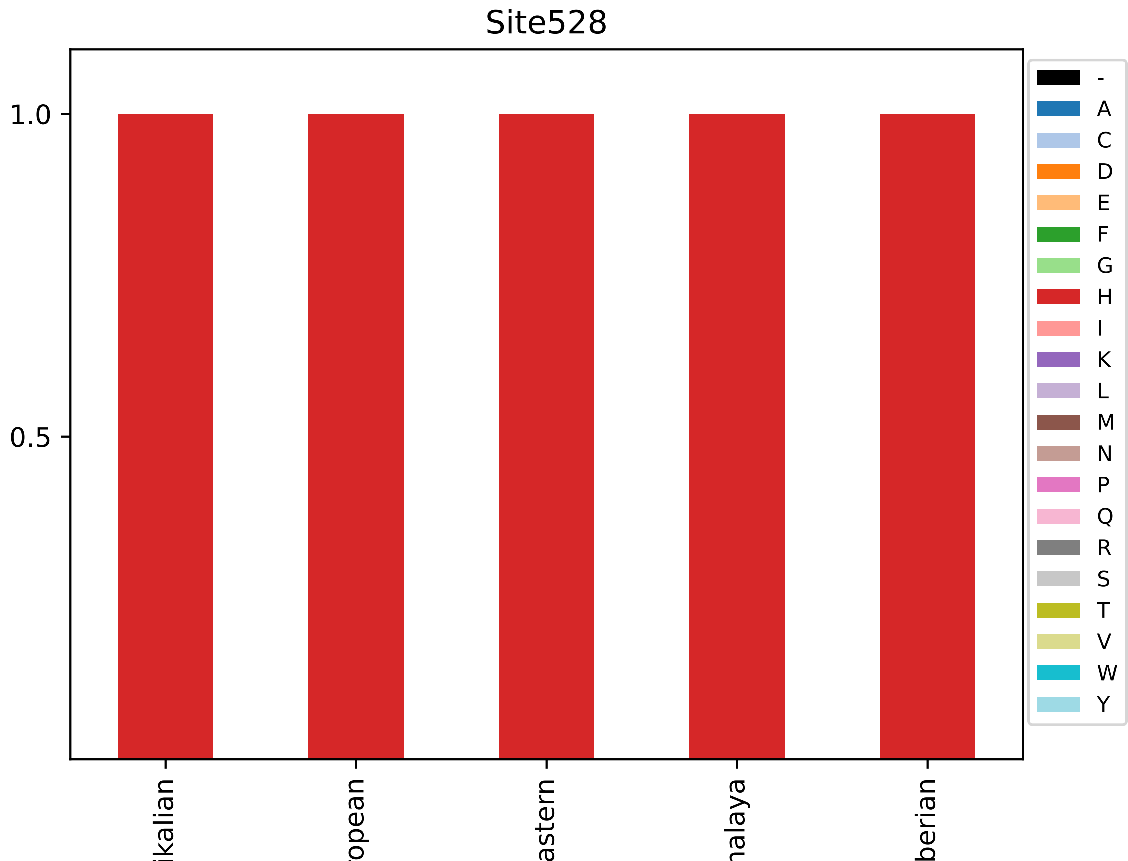

Site528
1.0
0.5
-
A
C
D
E
F
G
H
I
K
L
M
N
P
Q
R
S
T
V
W
Y

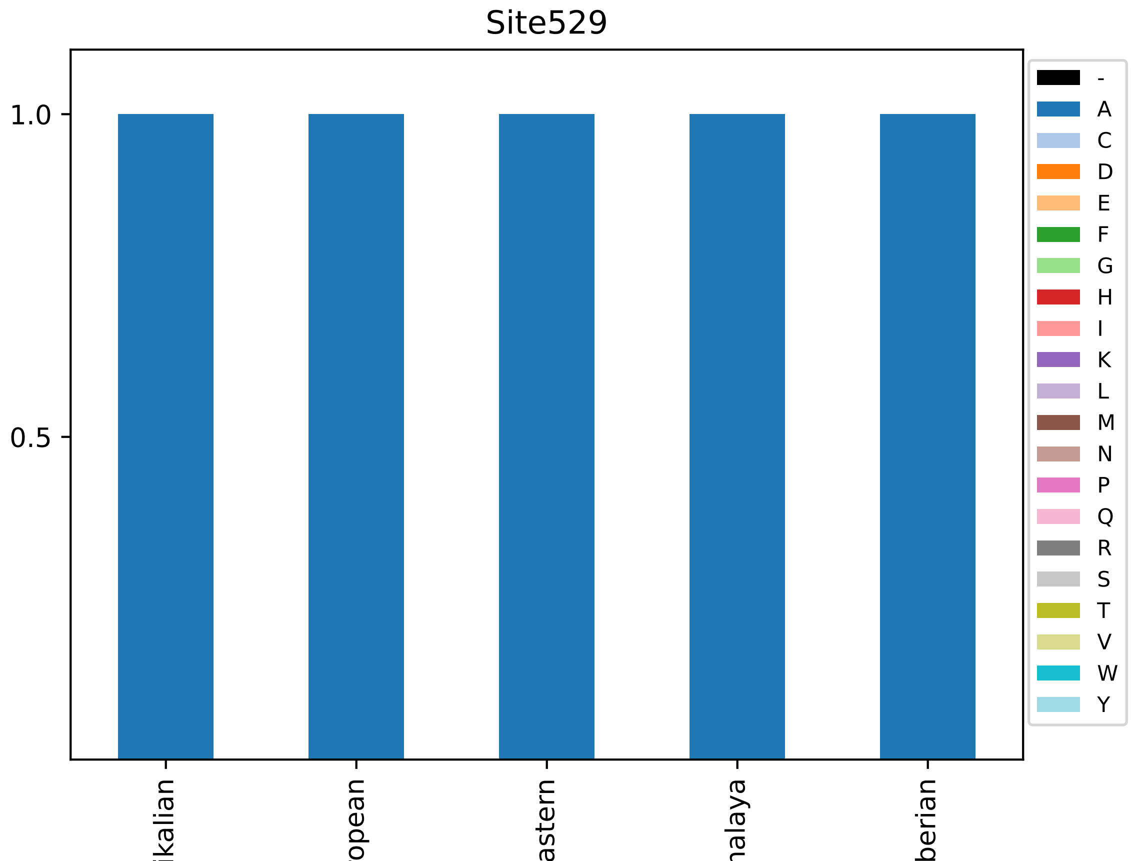
Site529
1.0
0.5
-
A
C
D
E
F
G
H
I
K
L
M
N
P
Q
R
S
T
V
W
Y
ikalian
opean
astern
nalaya
berian

Site530

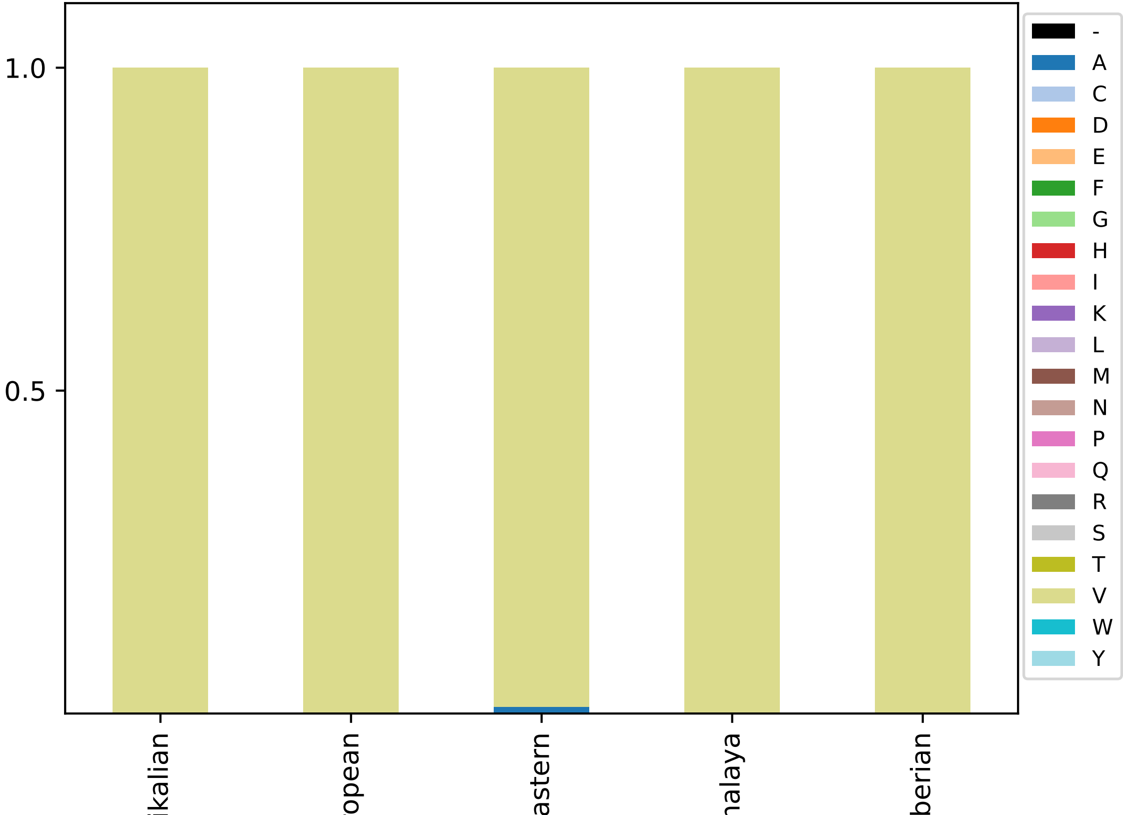

1.0
0.5
-
A
C
D
E
F
G
H
I
K
L
M
N
P
Q
R
S
T
V
W
Y
ikalian
opean
astern
nalaya
berian

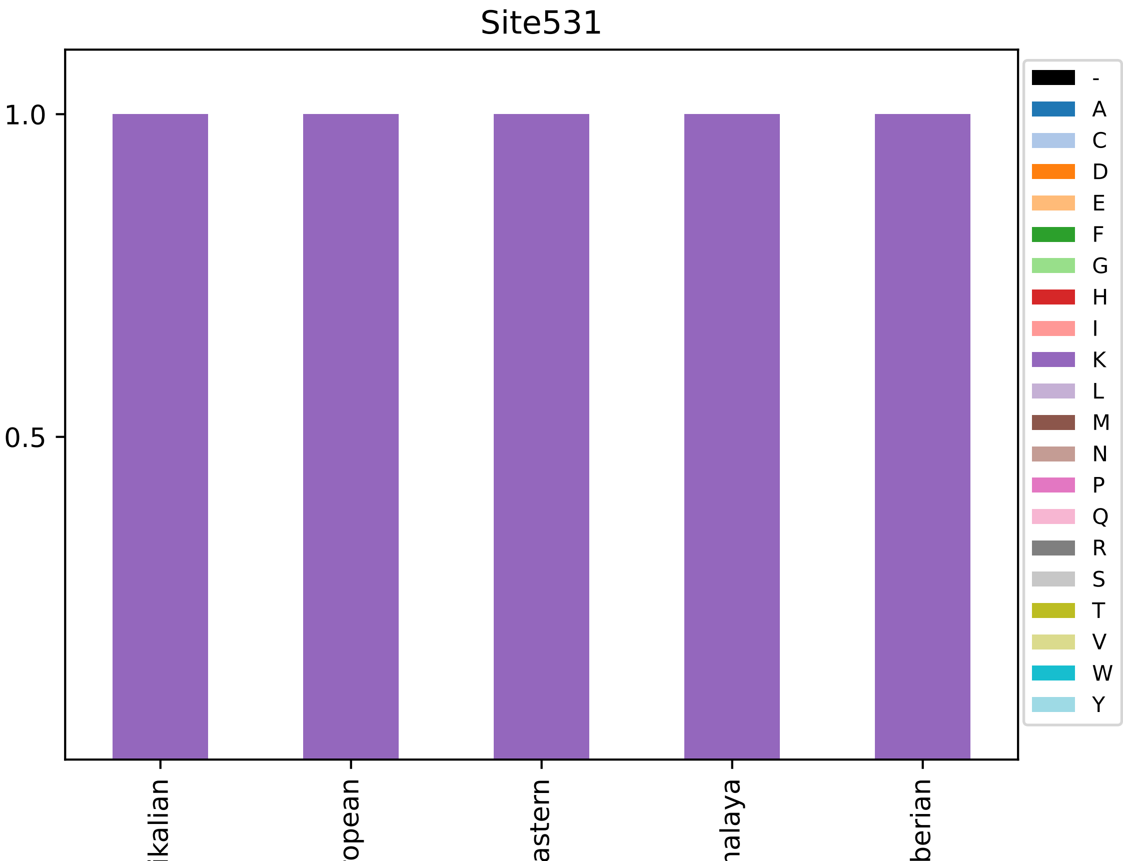

Site531
1.0
0.5
-
A
C
D
E
F
G
H
I
K
L
M
N
P
Q
R
S
T
V
W
Y
ikalian
opean
astern
nalaya
berian

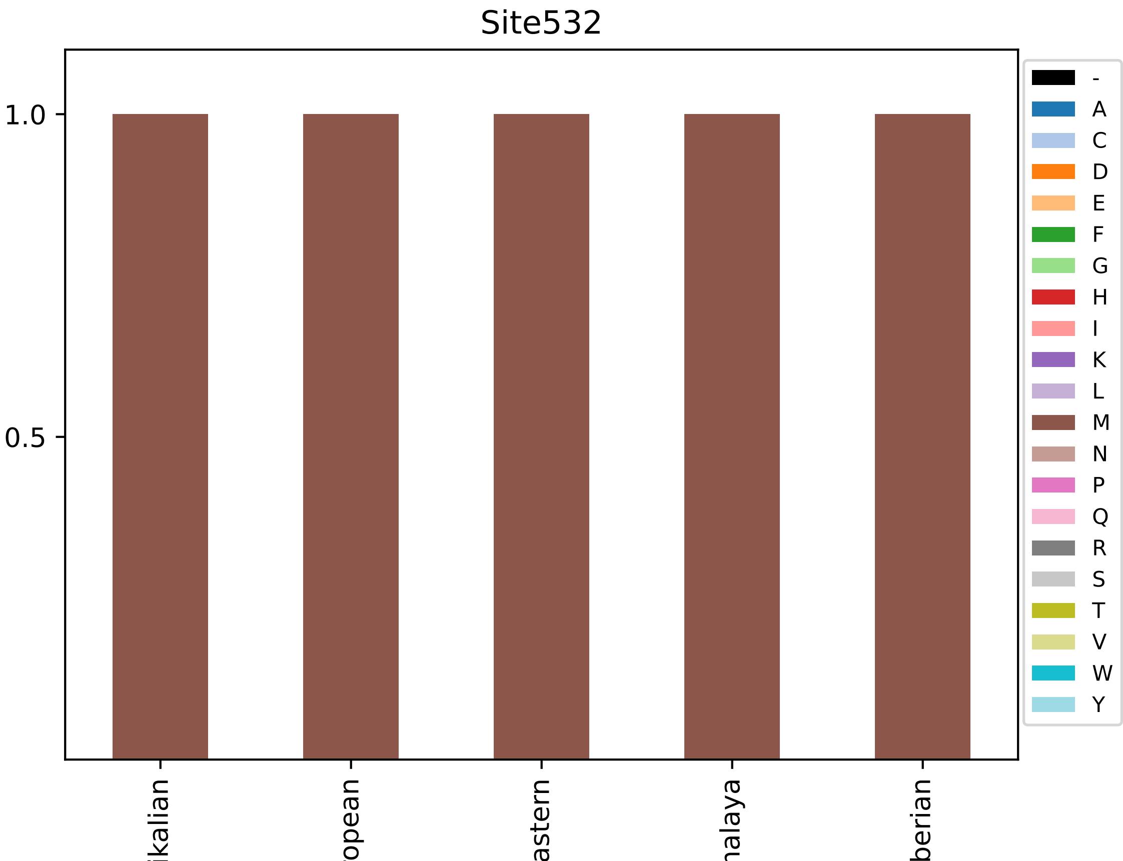

Site532
1.0
0.5
-
A
C
D
E
F
G
H
I
K
L
M
N
P
Q
R
S
T
V
W
Y
ikalian
opean
astern
nalaya
berian

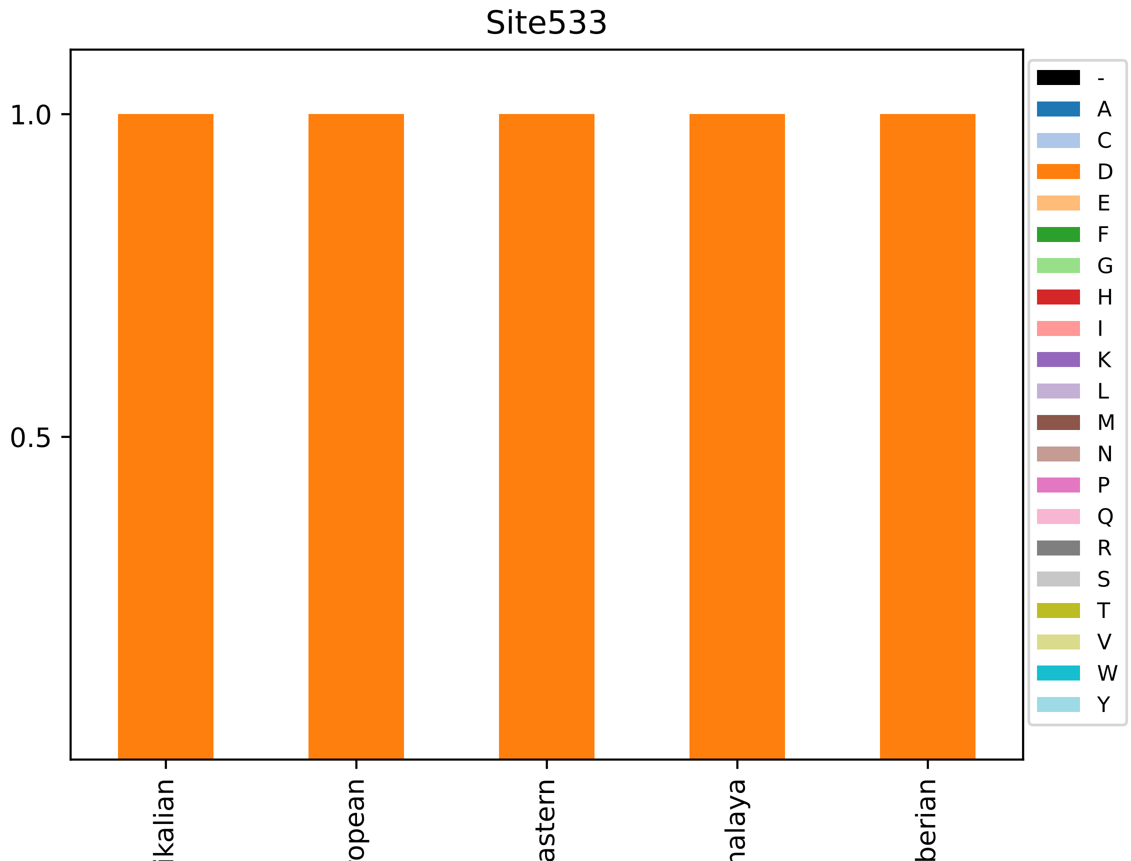

Site533
1.0
0.5
-
A
C
D
E
F
G
H
I
K
L
M
N
P
Q
R
S
T
V
W
Y
ikalian
opean
astern
nalaya
berian

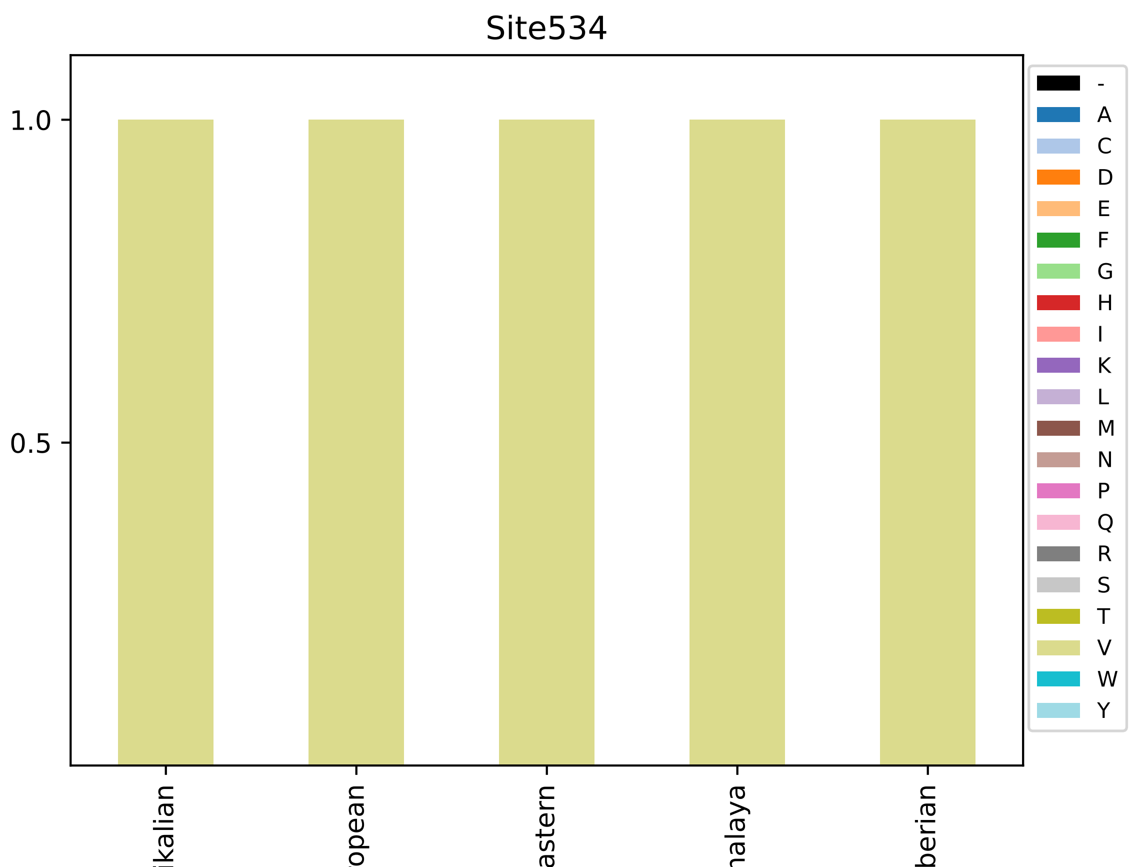
Site534
1.0
0.5
-
A
C
D
E
F
G
H
I
K
L
M
N
P
Q
R
S
T
V
W
Y
ikalian
opean
astern
nalaya
berian

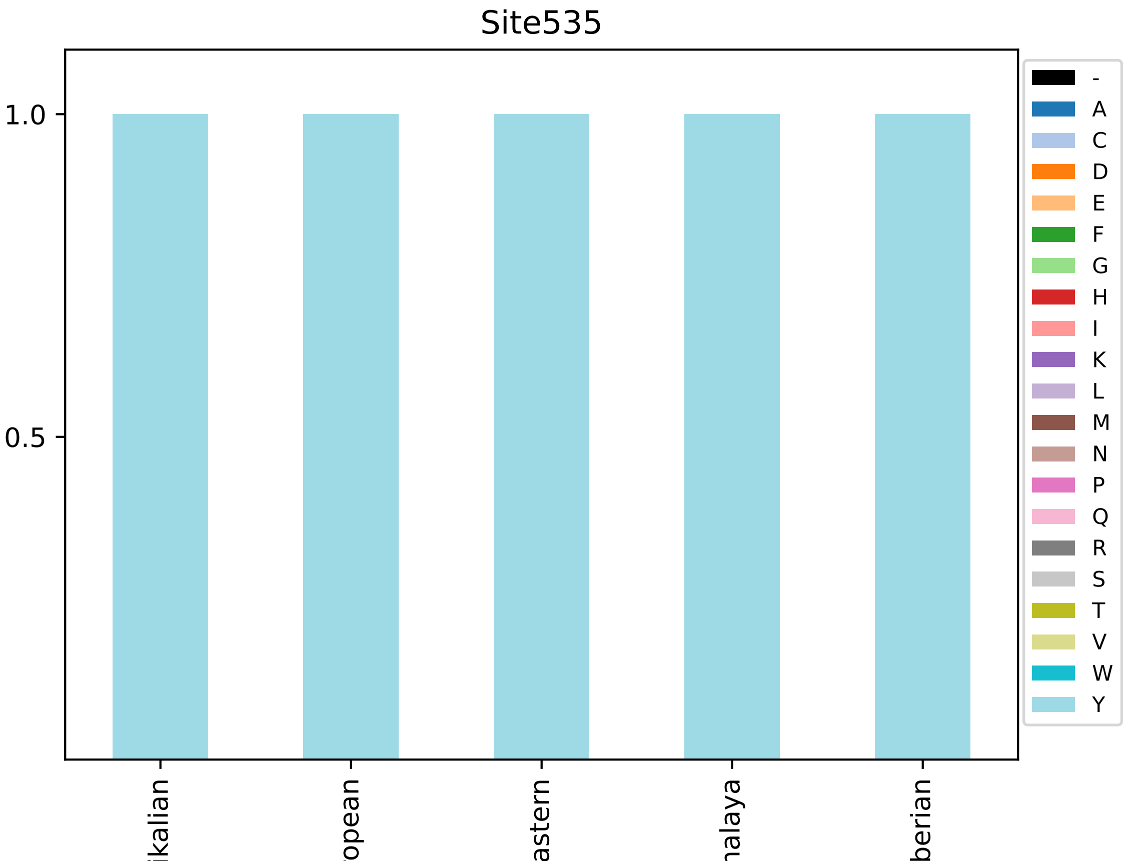
Site535
1.0
0.5
ikalian
opean
astern
nalaya
berian
-
A
C
D
E
F
G
H
I
K
L
M
N
P
Q
R
S
T
V
W
Y

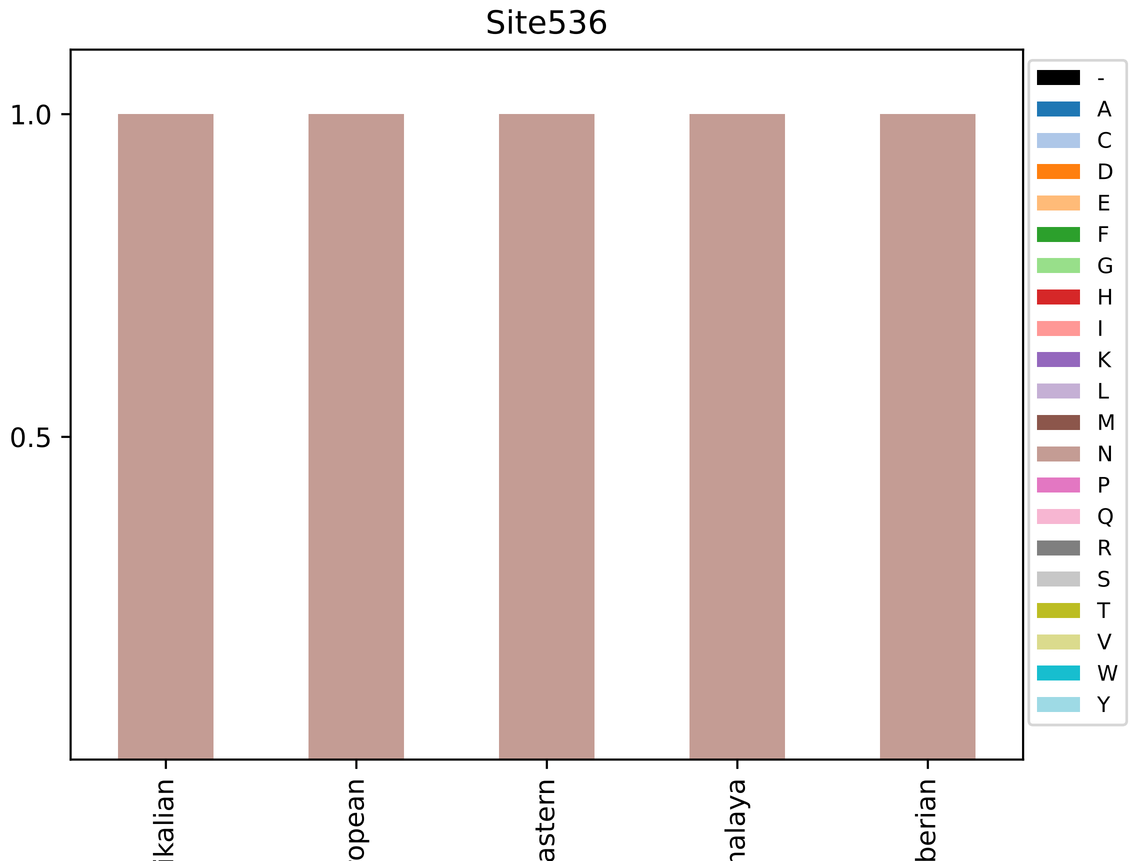
Site536
1.0
0.5
-
A
C
D
E
F
G
H
I
K
L
M
N
P
Q
R
S
T
V
W
Y
ikalian
opean
astern
nalaya
berian

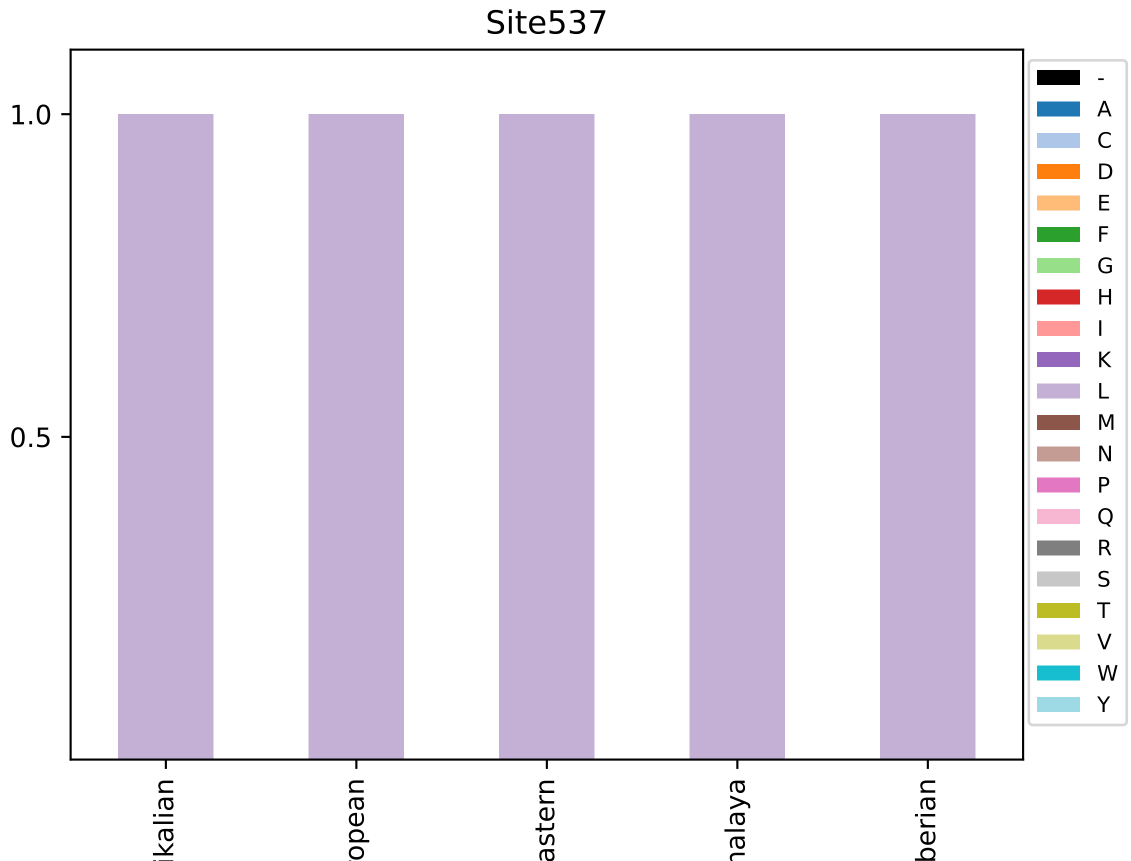
Site537
1.0
0.5
-
A
C
D
E
F
G
H
I
K
L
M
N
P
Q
R
S
T
V
W
Y
ikalian
opean
astern
halaya
berian

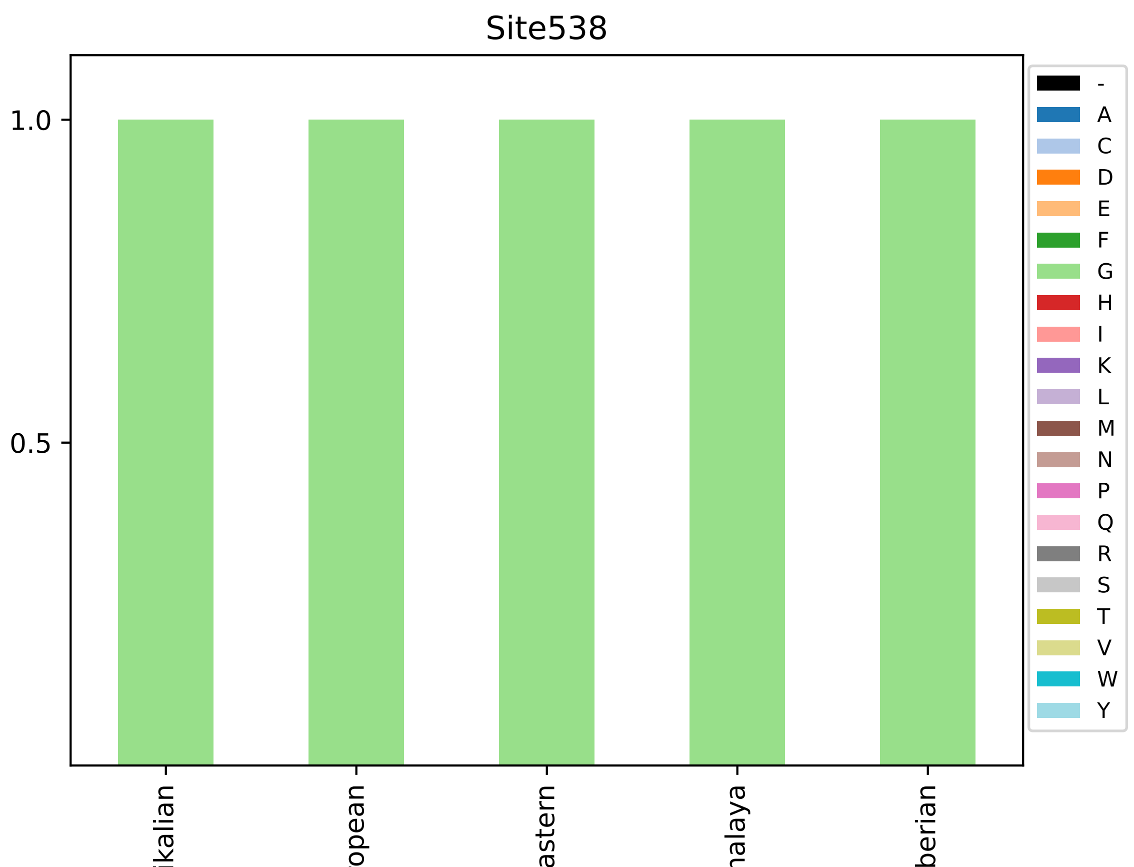
Site538
1.0
0.5
-
A
C
D
E
F
G
H
I
K
L
M
N
P
Q
R
S
T
V
W
Y
ikalian
opean
astern
nalaya
berian

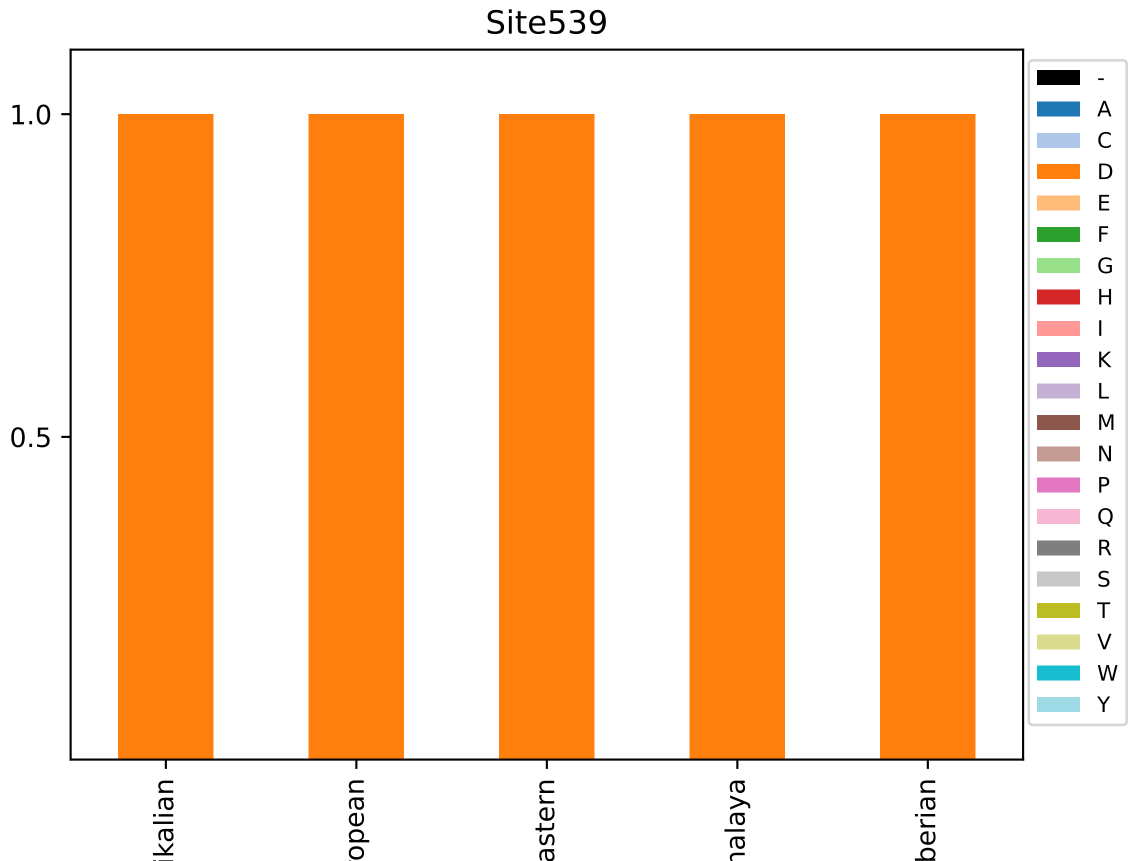
Site539
1.0
0.5
-
A
C
D
E
F
G
H
I
K
L
M
N
P
Q
R
S
T
V
W
Y
ikalian
opean
astern
nalaya
berian

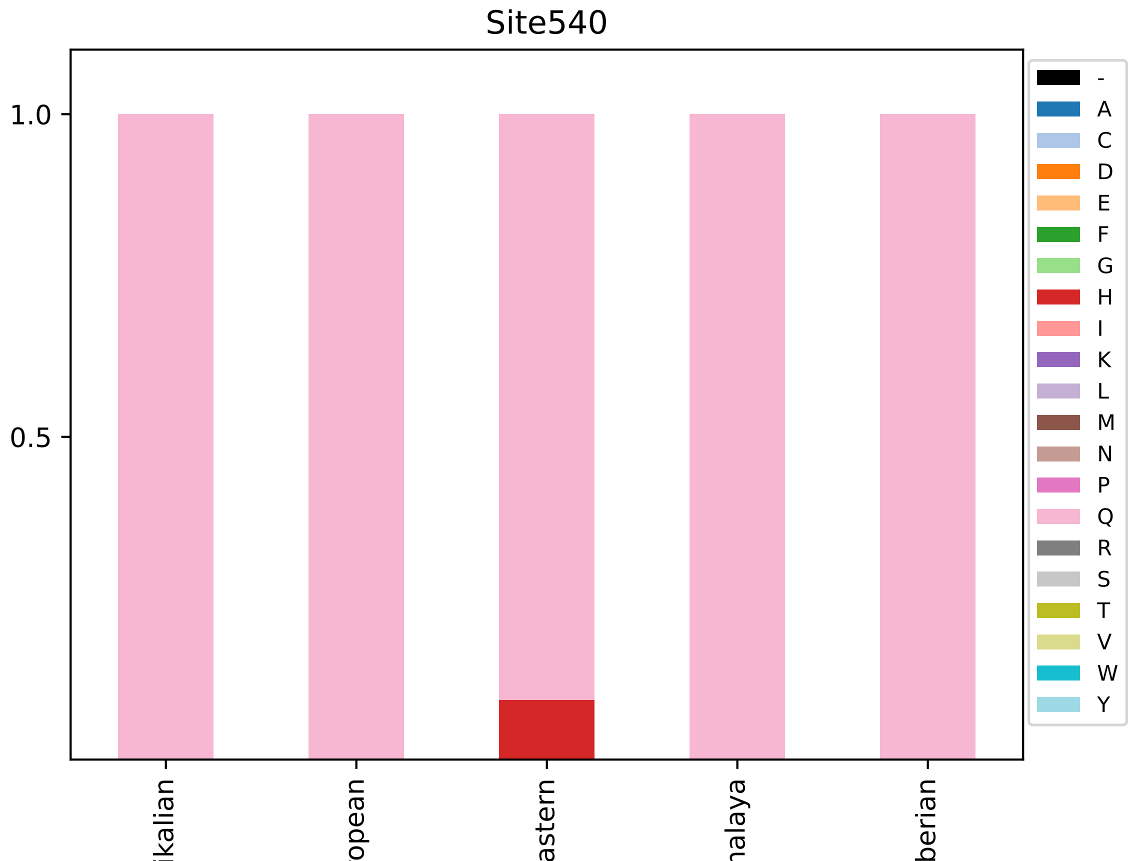
Site540
1.0
0.5
-
A
C
D
E
F
G
H
I
K
L
M
N
P
Q
R
S
T
V
W
Y
ikalian
opean
astern
nalaya
berian

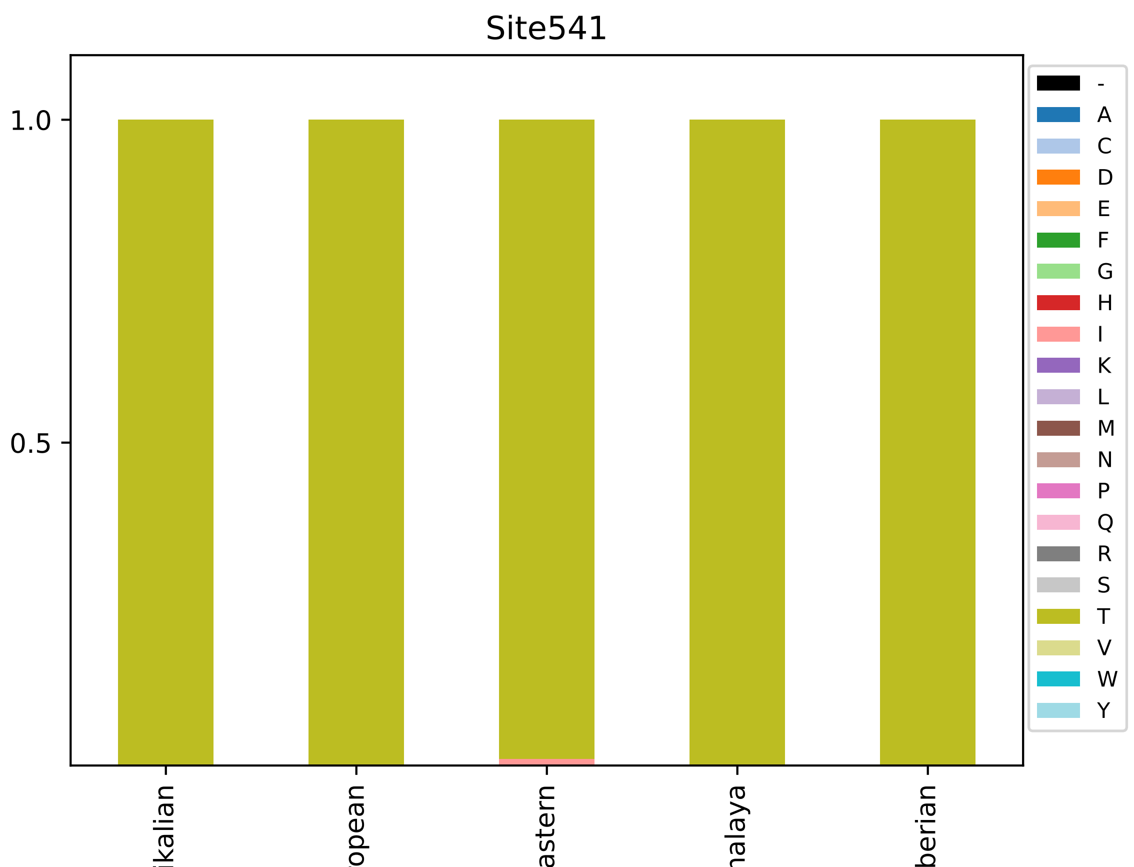

Site541
1.0
0.5
-
A
C
D
E
F
G
H
I
K
L
M
N
P
Q
R
S
T
V
W
Y
ikalian
opean
astern
nalaya
berian

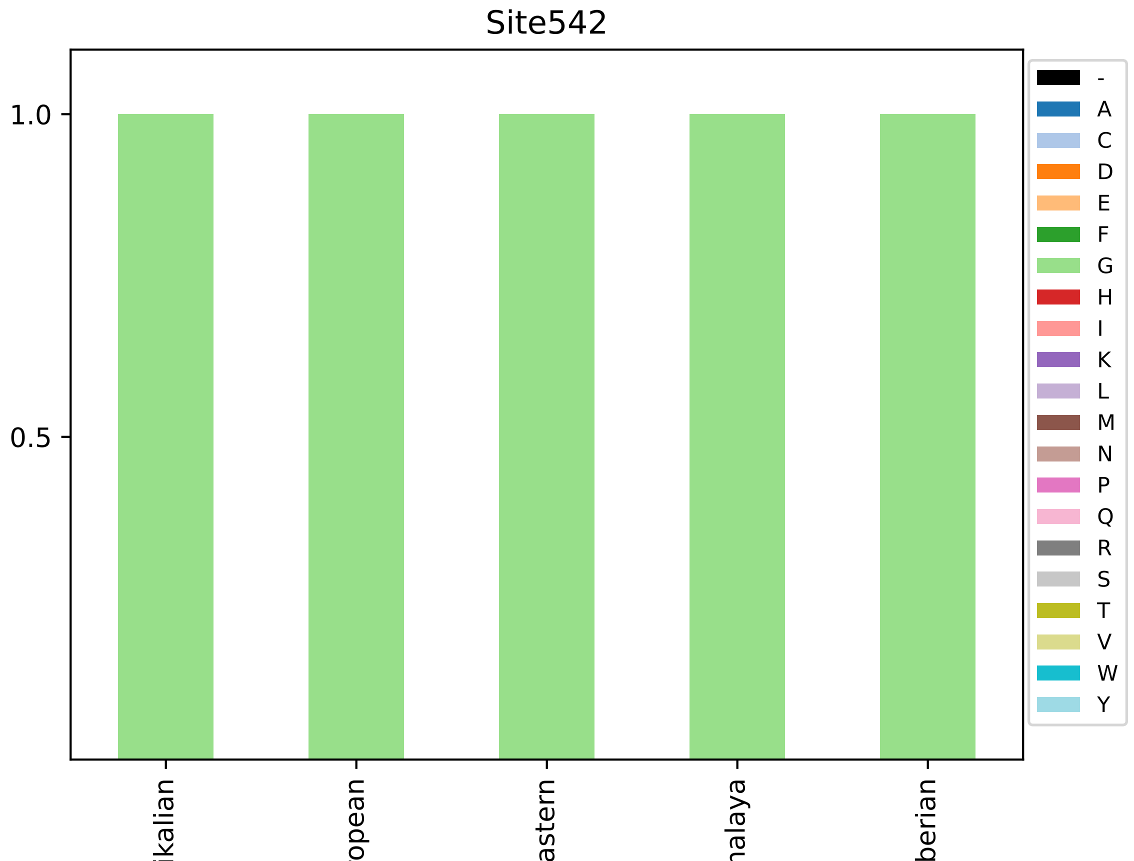

Site542
1.0
0.5
-
A
C
D
E
F
G
H
I
K
L
M
N
P
Q
R
S
T
V
W
Y
ikalian
opean
astern
nalaya
berian

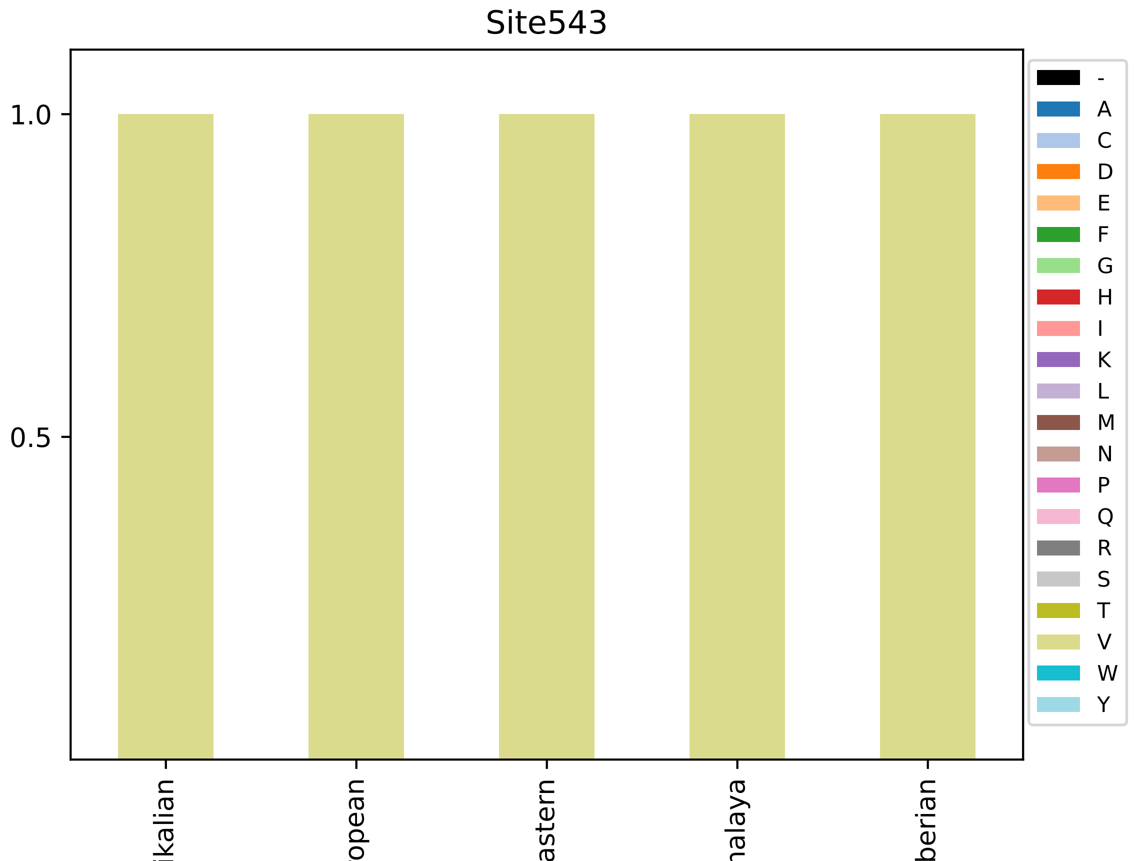

Site543
1.0
0.5
-
A
C
D
E
F
G
H
I
K
L
M
N
P
Q
R
S
T
V
W
Y
ikalian
opean
astern
nalaya
berian

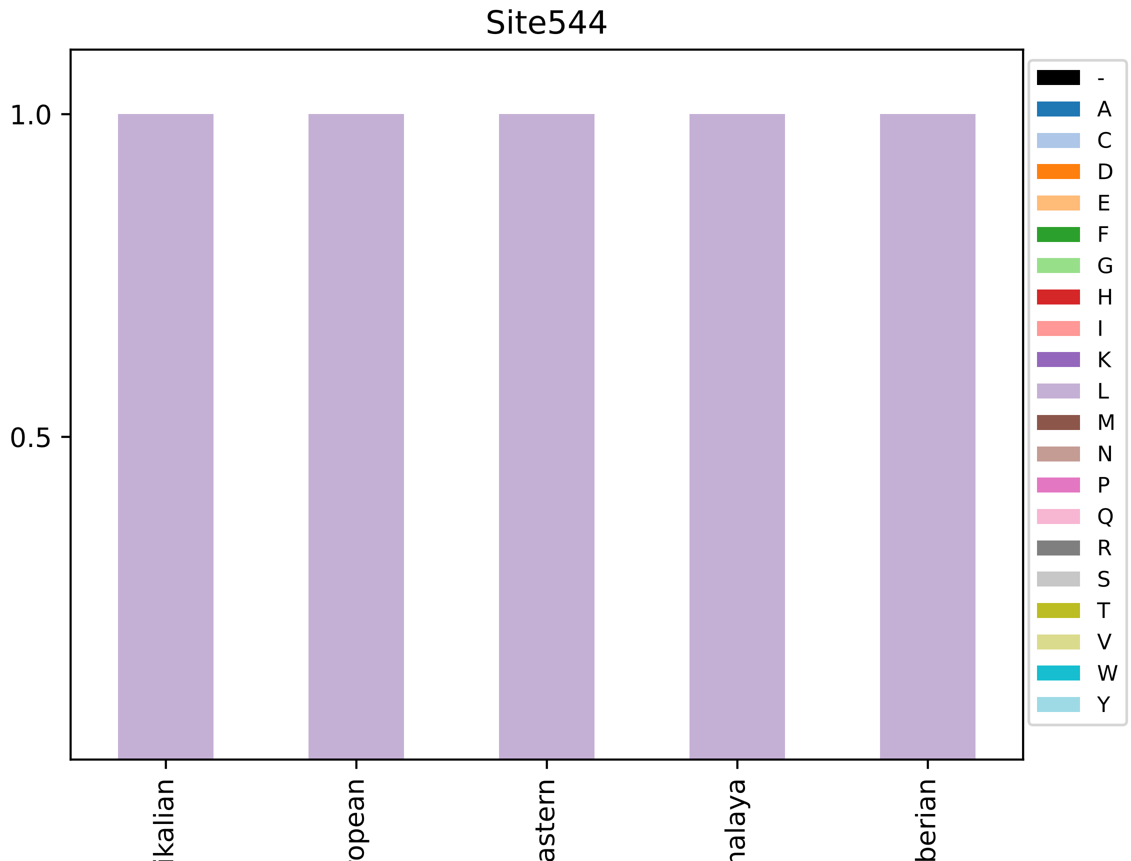
Site544
1.0
0.5
-
A
C
D
E
F
G
H
I
K
L
M
N
P
Q
R
S
T
V
W
Y
ikalian
opean
astern
nalaya
berian

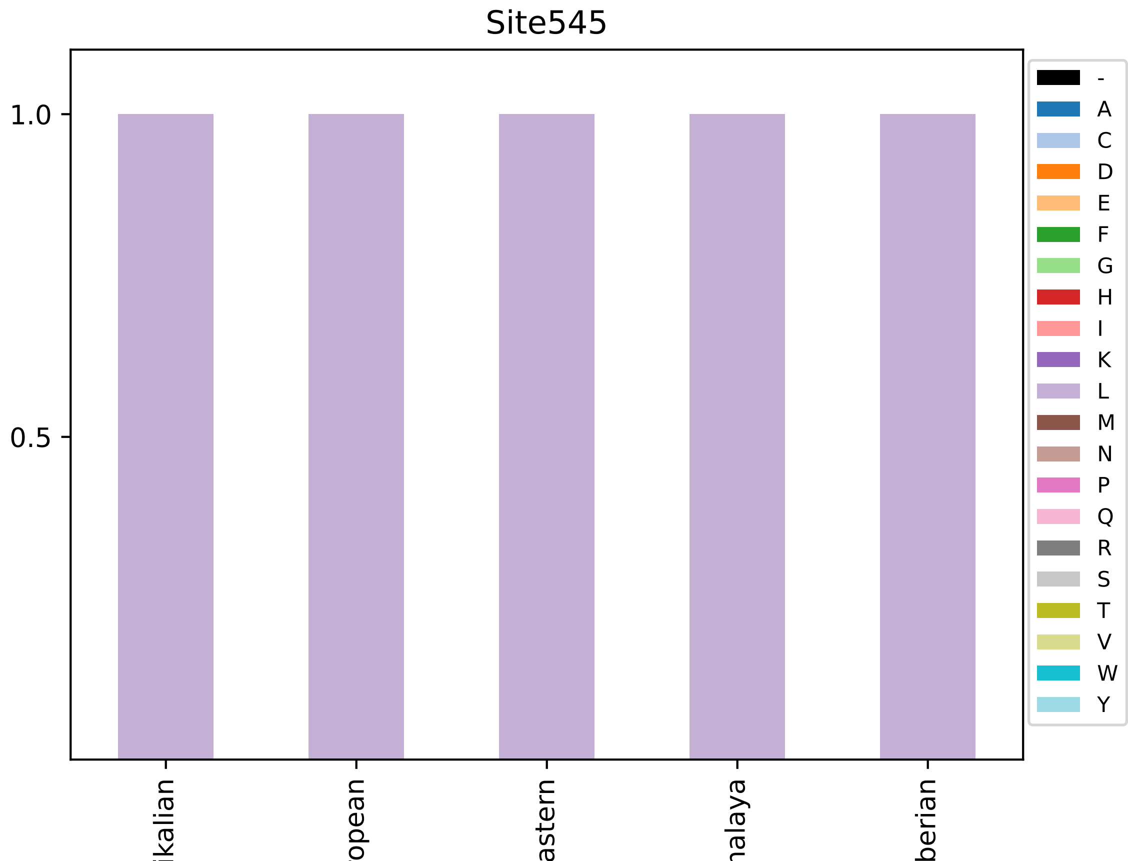
Site545
1.0
0.5
-
A
C
D
E
F
G
H
I
K
L
M
N
P
Q
R
S
T
V
W
Y
ikalian
opean
astern
nalaya
berian

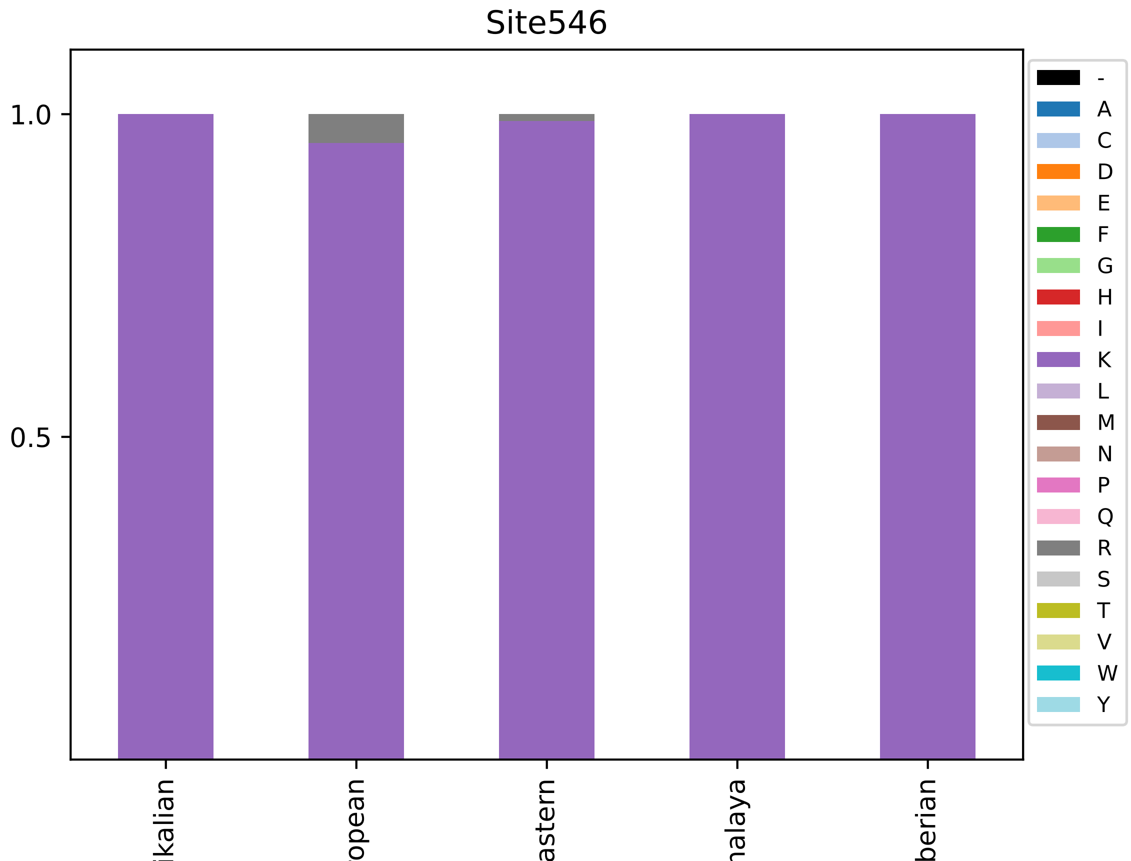
Site546
1.0
0.5
ikalian
opean
astern
nalaya
berian
-
A
C
D
E
F
G
H
I
K
L
M
N
P
Q
R
S
T
V
W
Y

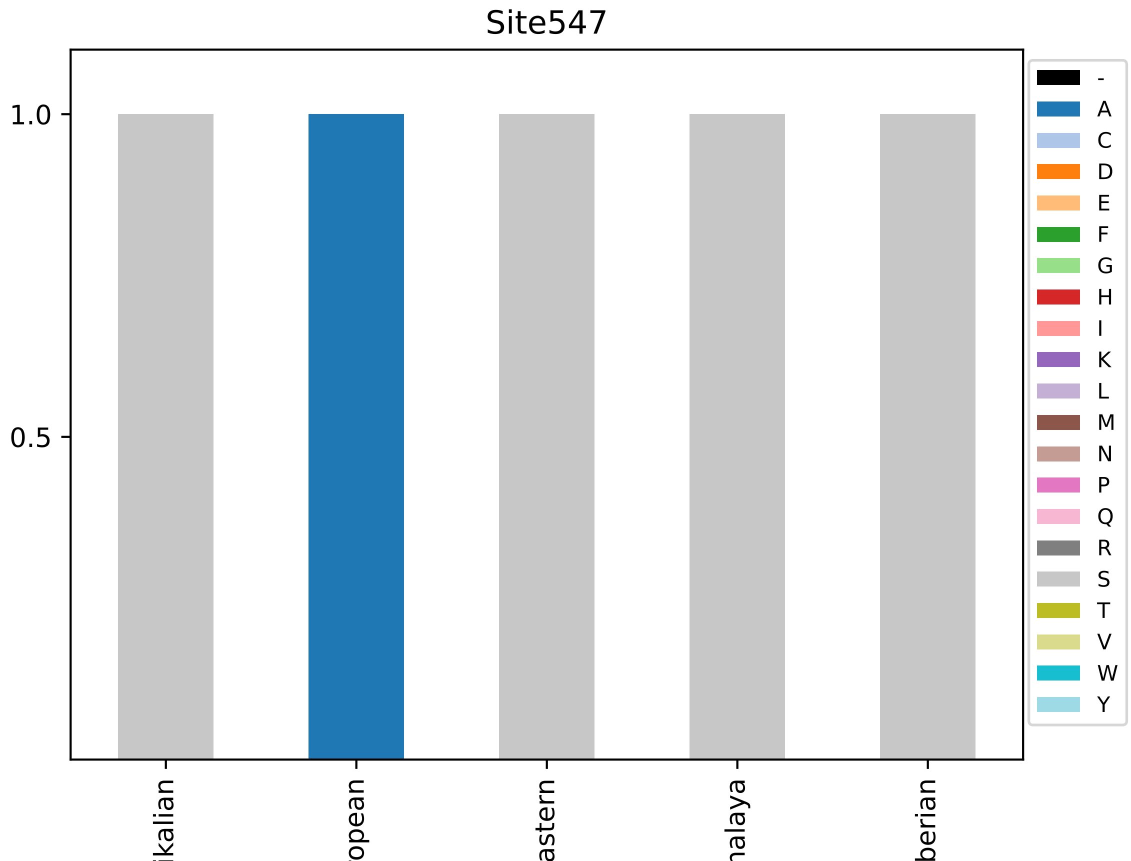
Site547
1.0
0.5
-
A
C
D
E
F
G
H
I
K
L
M
N
P
Q
R
S
T
V
W
Y
ikalian
ropean
astern
nalaya
berian

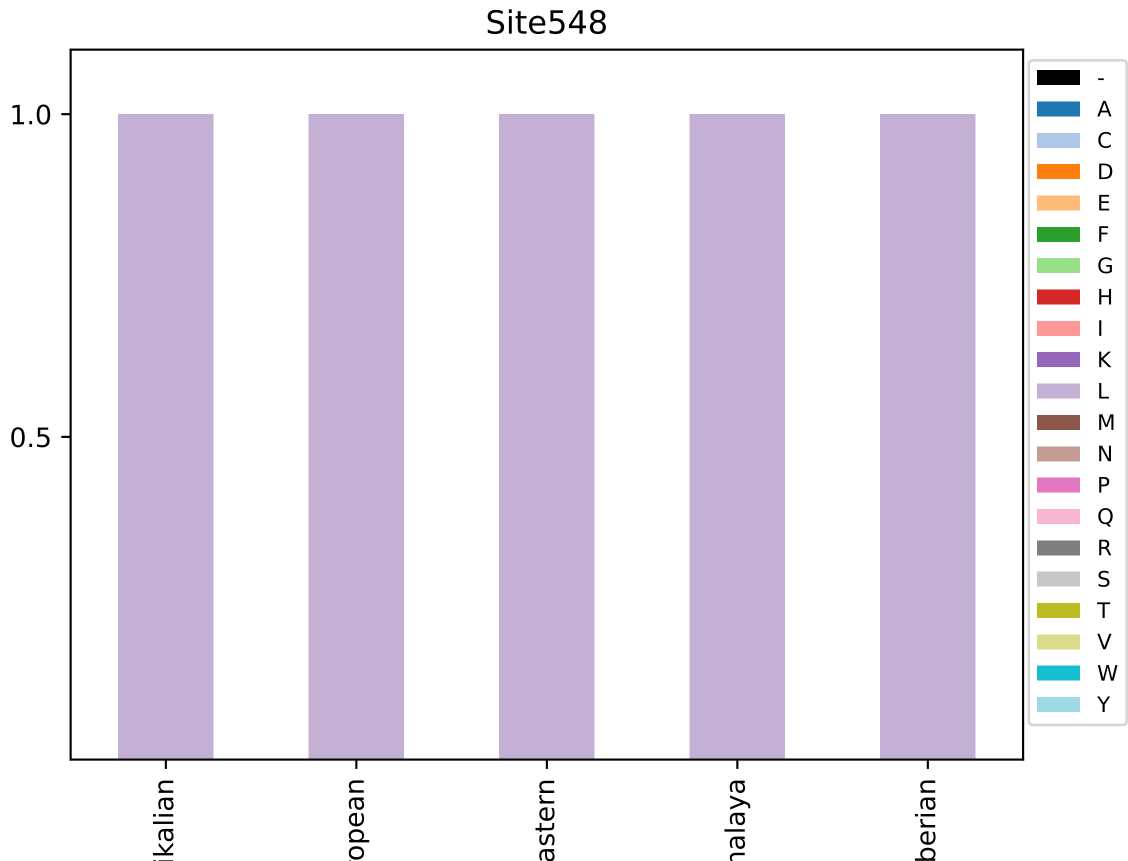
Site548
1.0
0.5
-
A
C
D
E
F
G
H
I
K
L
M
N
P
Q
R
S
T
V
W
Y
ikalian
opean
astern
nalaya
berian

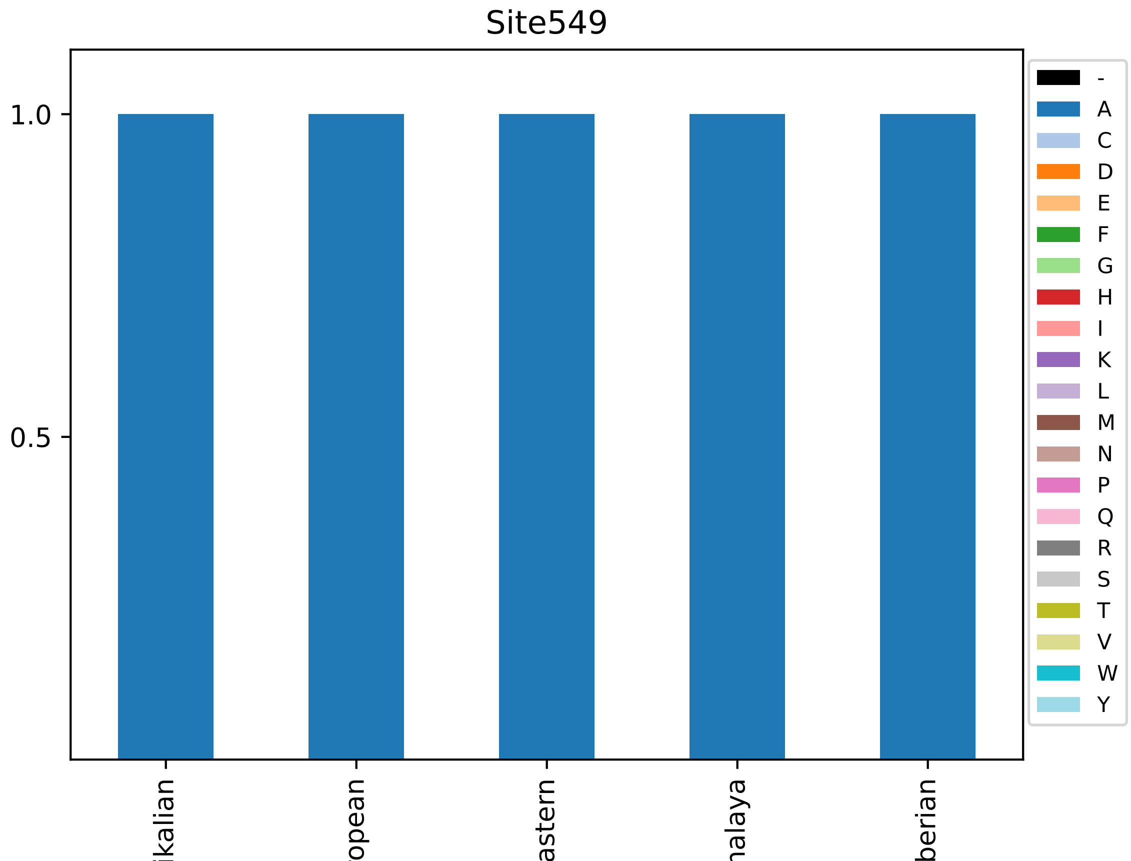
Site549
1.0
0.5
-
A
C
D
E
F
G
H
I
K
L
M
N
P
Q
R
S
T
V
W
Y
ikalian
opean
astern
nalaya
berian

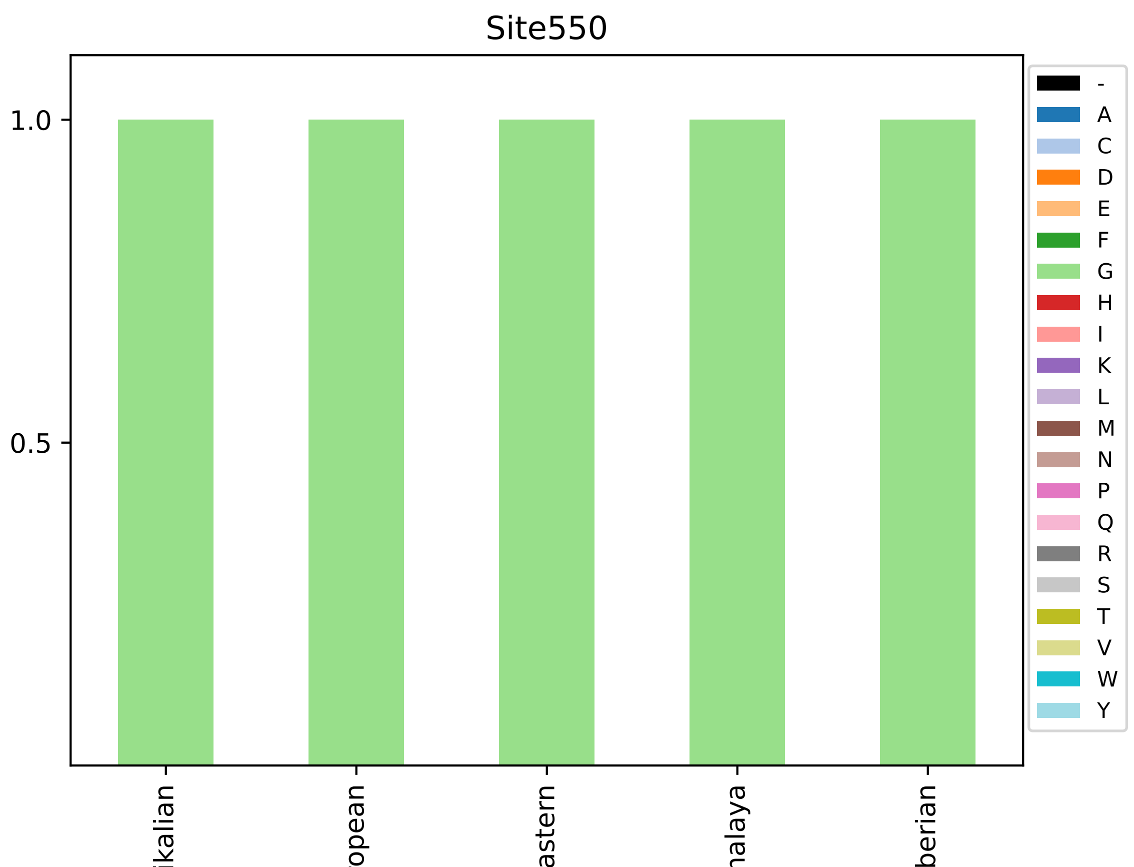
Site550
1.0
0.5
-
A
C
D
E
F
G
H
I
K
L
M
N
P
Q
R
S
T
V
W
Y
ikalian
opean
astern
nalaya
berian

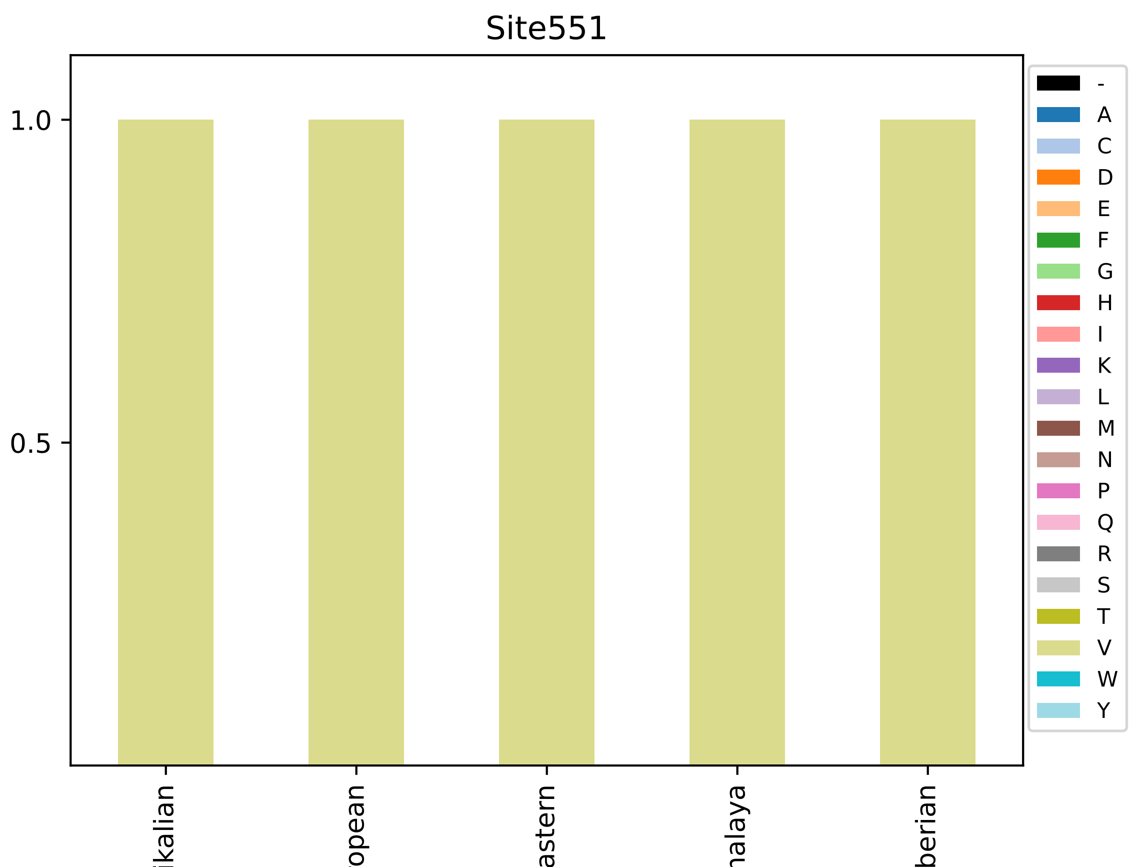
Site551
1.0
0.5
-
A
C
D
E
F
G
H
I
K
L
M
N
P
Q
R
S
T
V
W
Y
ikalian
ropean
astern
nalaya
berian

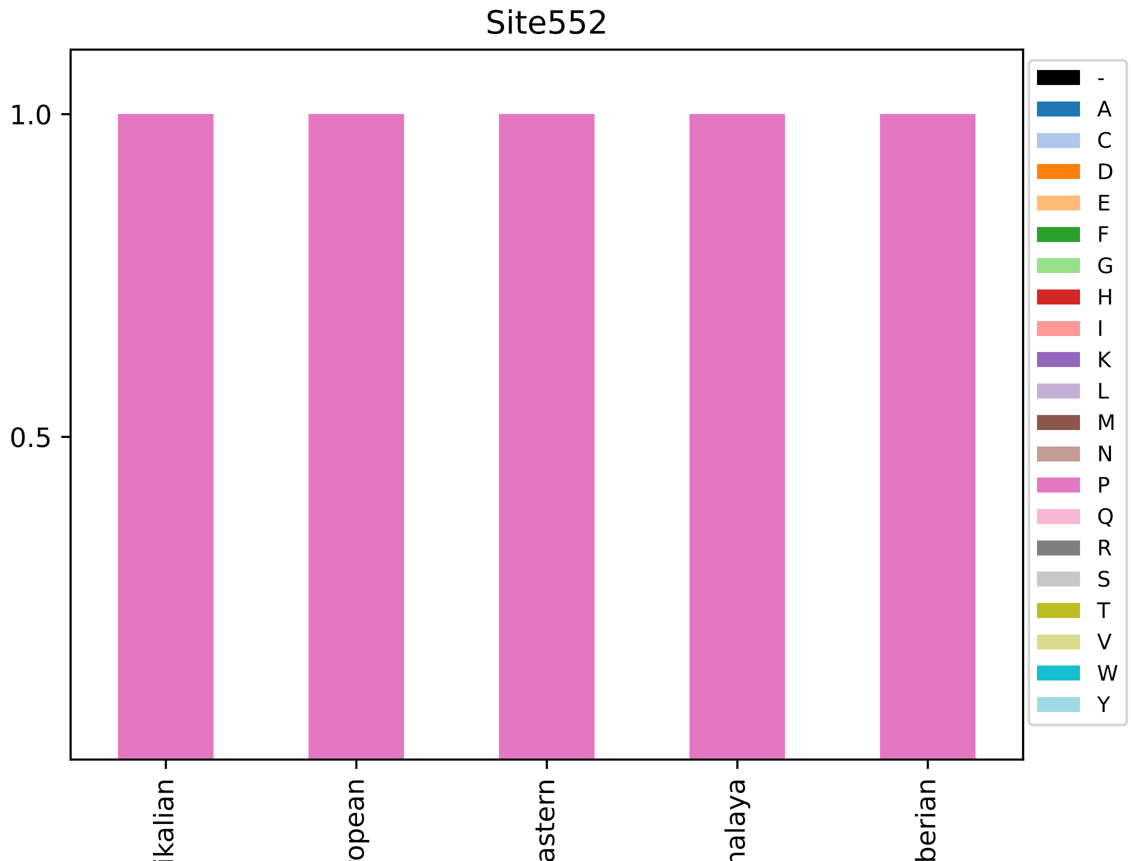
Site552
1.0
0.5
-
A
C
D
E
F
G
H
I
K
L
M
N
P
Q
R
S
T
V
W
Y
ikalian
opean
astern
nalaya
berian

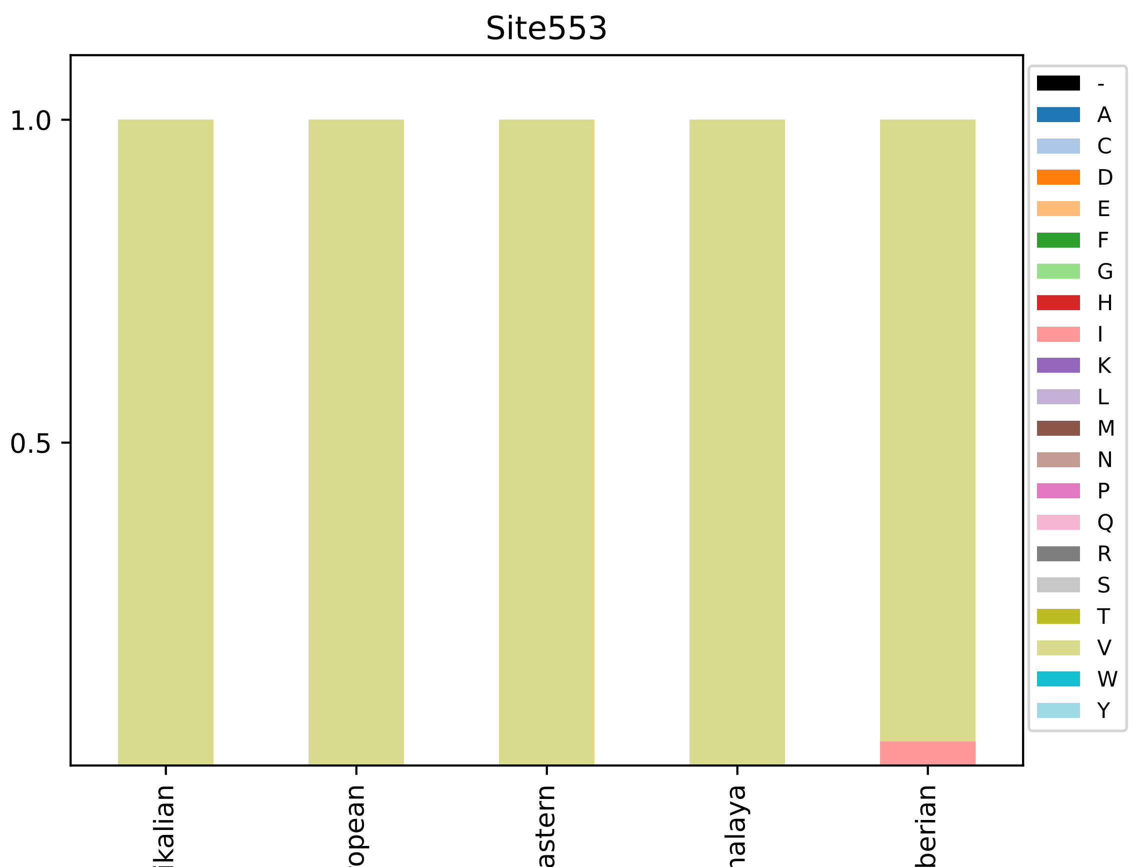

Site553
1.0
0.5
-
A
C
D
E
F
G
H
I
K
L
M
N
P
Q
R
S
T
V
W
Y
ikalian
opean
astern
nalaya
berian

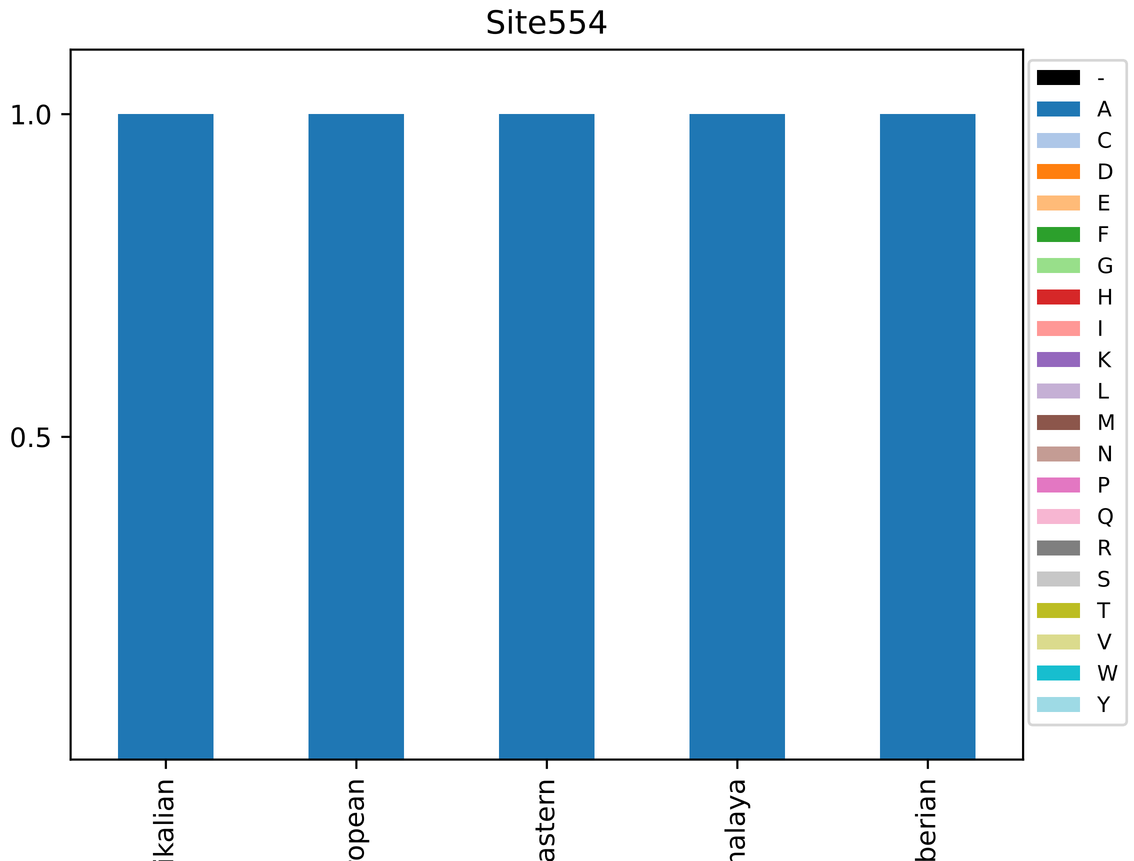
Site554
1.0
0.5
-
A
C
D
E
F
G
H
I
K
L
M
N
P
Q
R
S
T
V
W
Y
ikalian
opean
astern
nalaya
berian

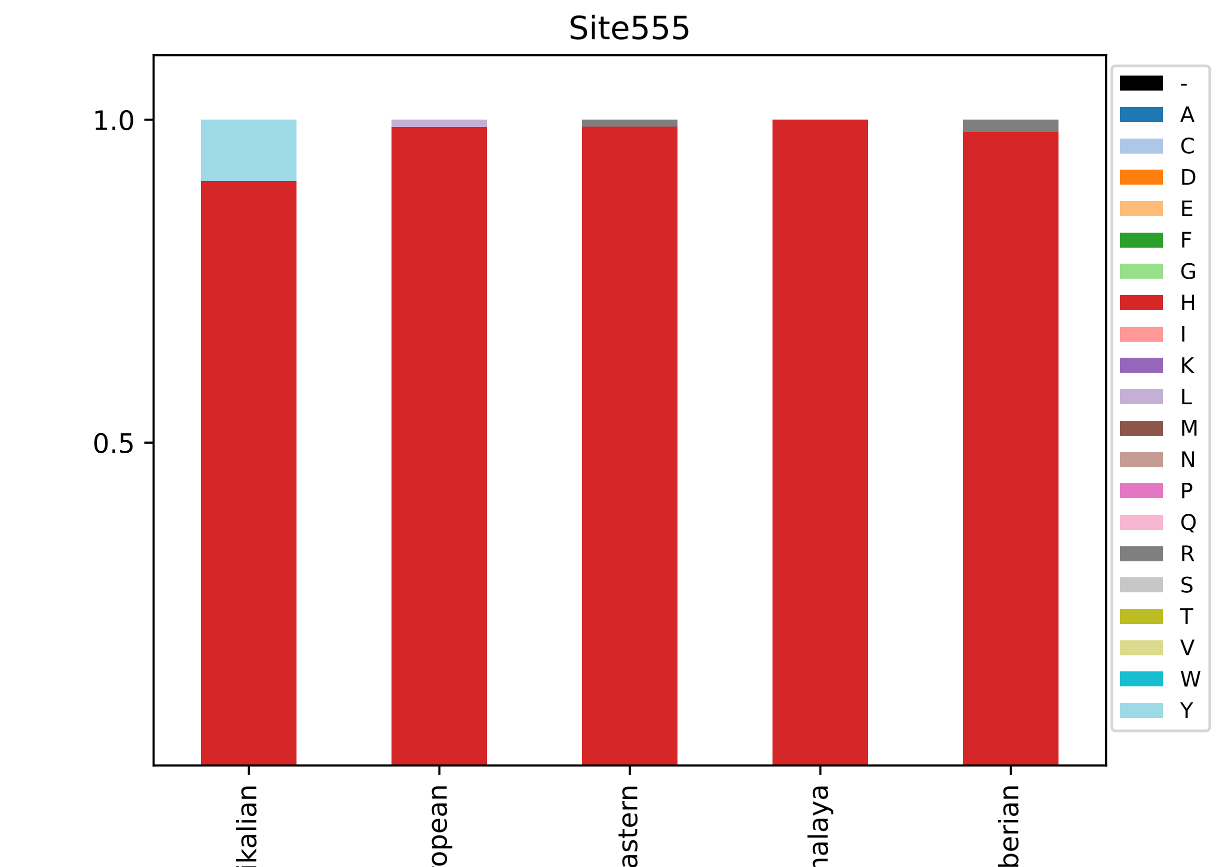
Site555
1.0
0.5
-
A
C
D
E
F
G
H
I
K
L
M
N
P
Q
R
S
T
V
W
Y
ikalian
opean
astern
nalaya
berian

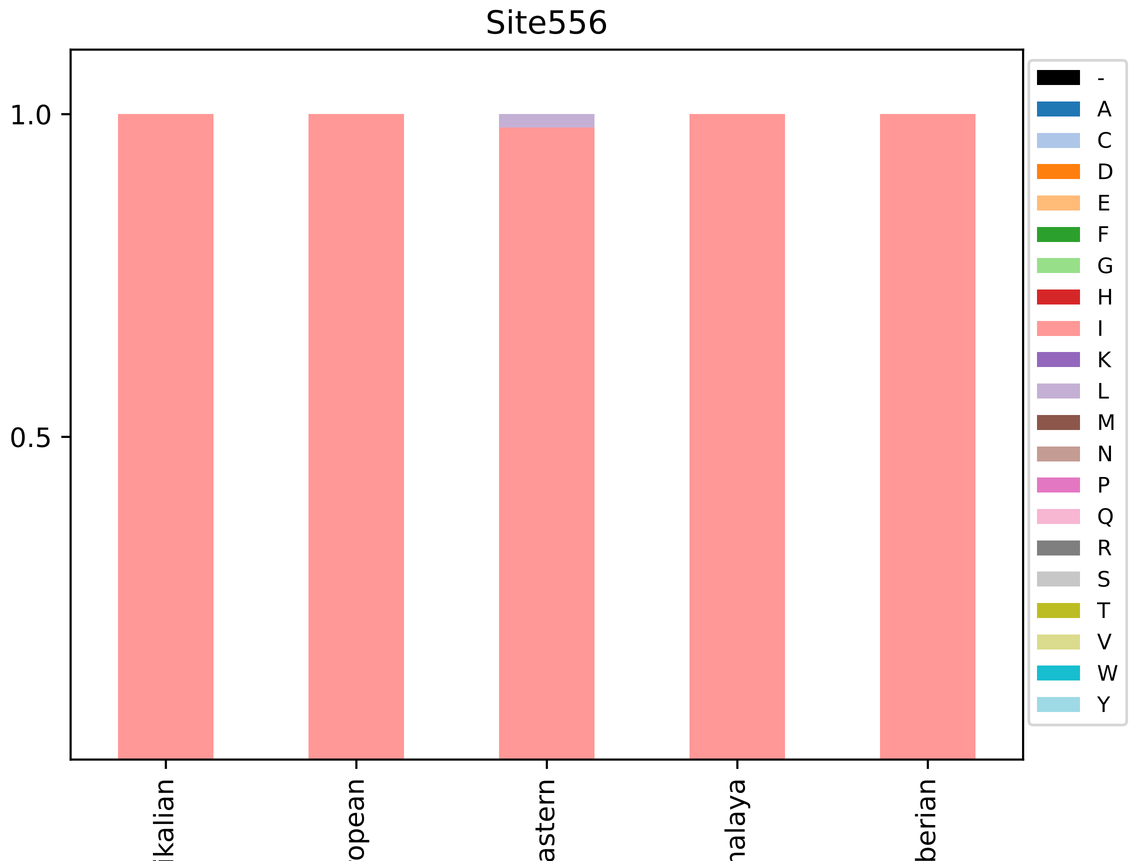
Site556
1.0
0.5
-
A
C
D
E
F
G
H
I
K
L
M
N
P
Q
R
S
T
V
W
Y
ikalian
ropean
astern
nalaya
berian

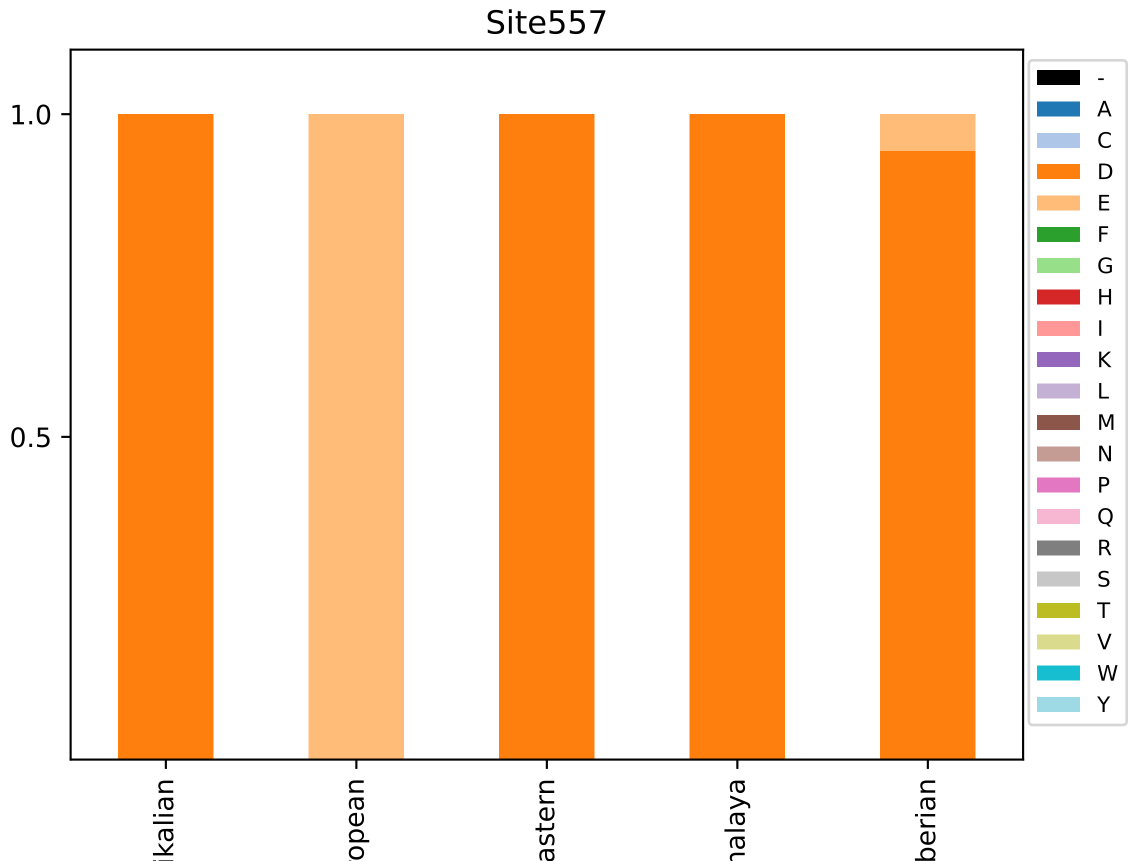

Site557
1.0
0.5
-
A
C
D
E
F
G
H
I
K
L
M
N
P
Q
R
S
T
V
W
Y
ikalian
opean
astern
nalaya
berian

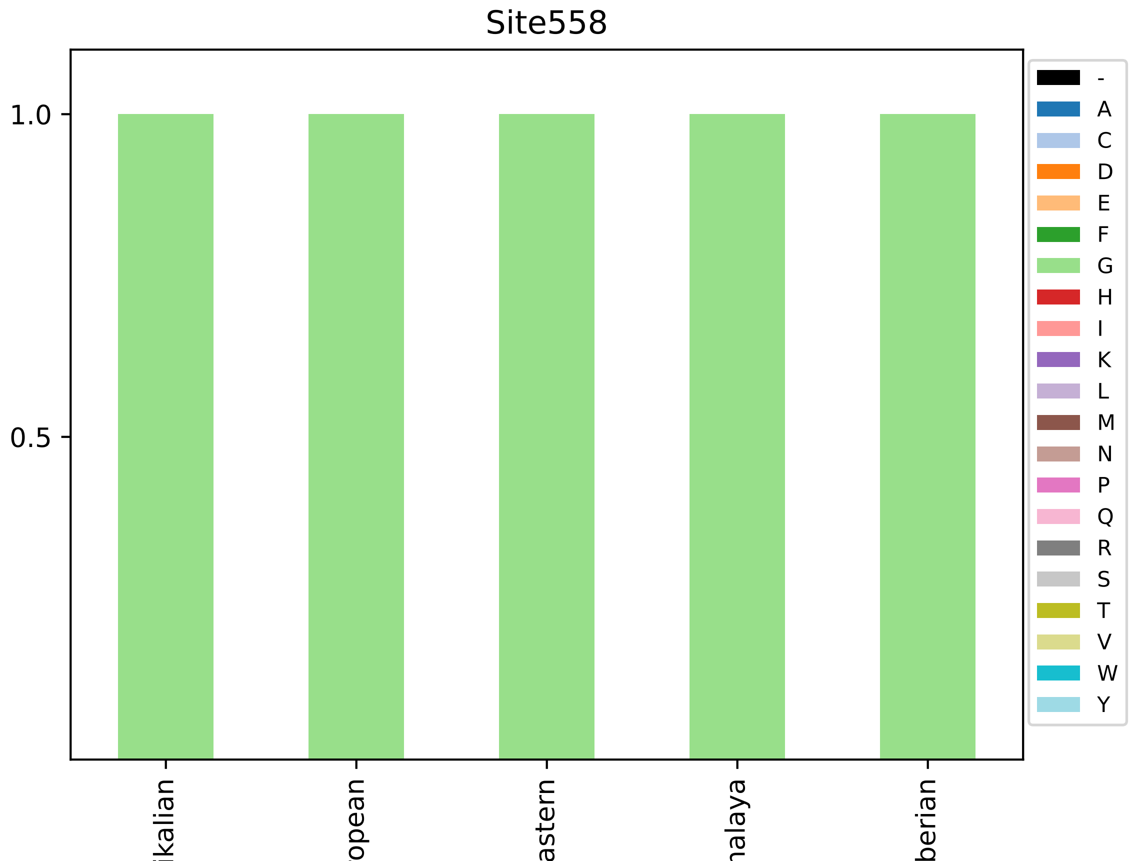

Site558
1.0
0.5
ikalian
opean
astern
nalaya
berian
-
A
C
D
E
F
G
H
I
K
L
M
N
P
Q
R
S
T
V
W
Y

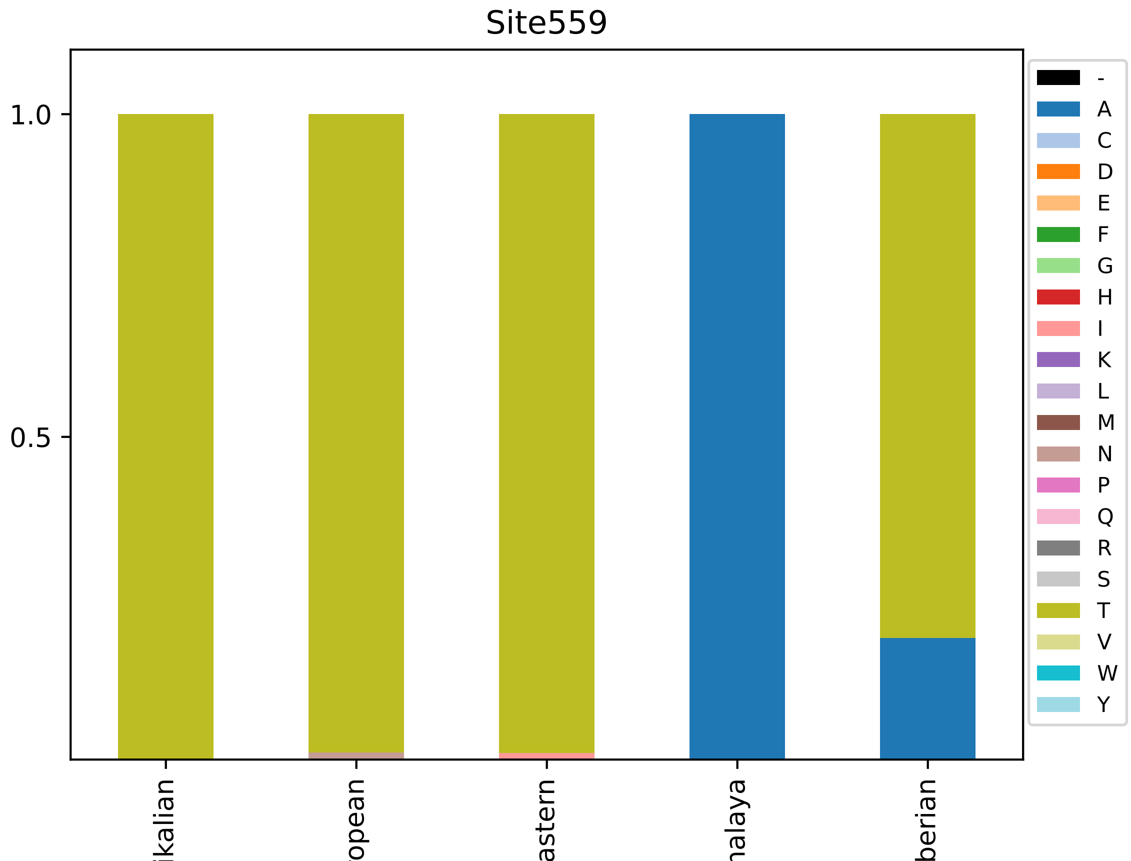

Site559
1.0
0.5
-
A
C
D
E
F
G
H
I
K
L
M
N
P
Q
R
S
T
V
W
Y
ikalian
opean
astern
nalaya
berian

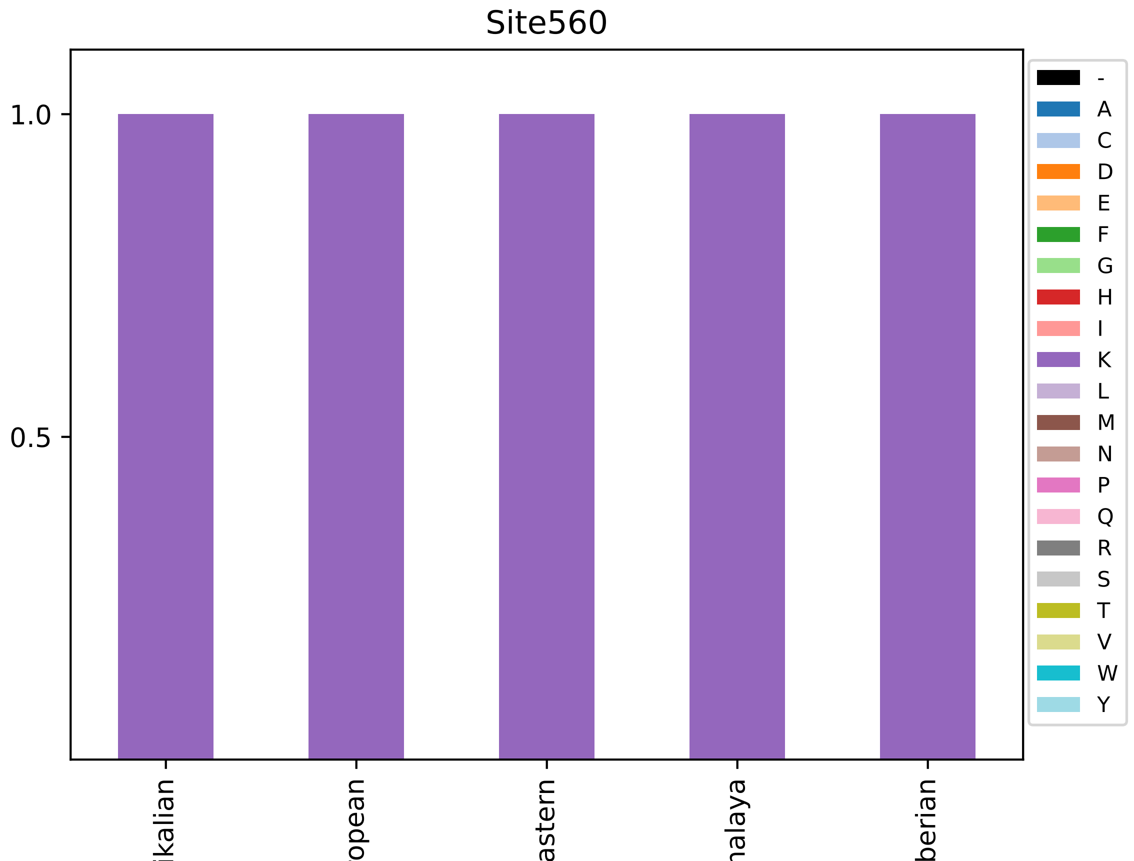

Site560
1.0
0.5
-
A
C
D
E
F
G
H
I
K
L
M
N
P
Q
R
S
T
V
W
Y

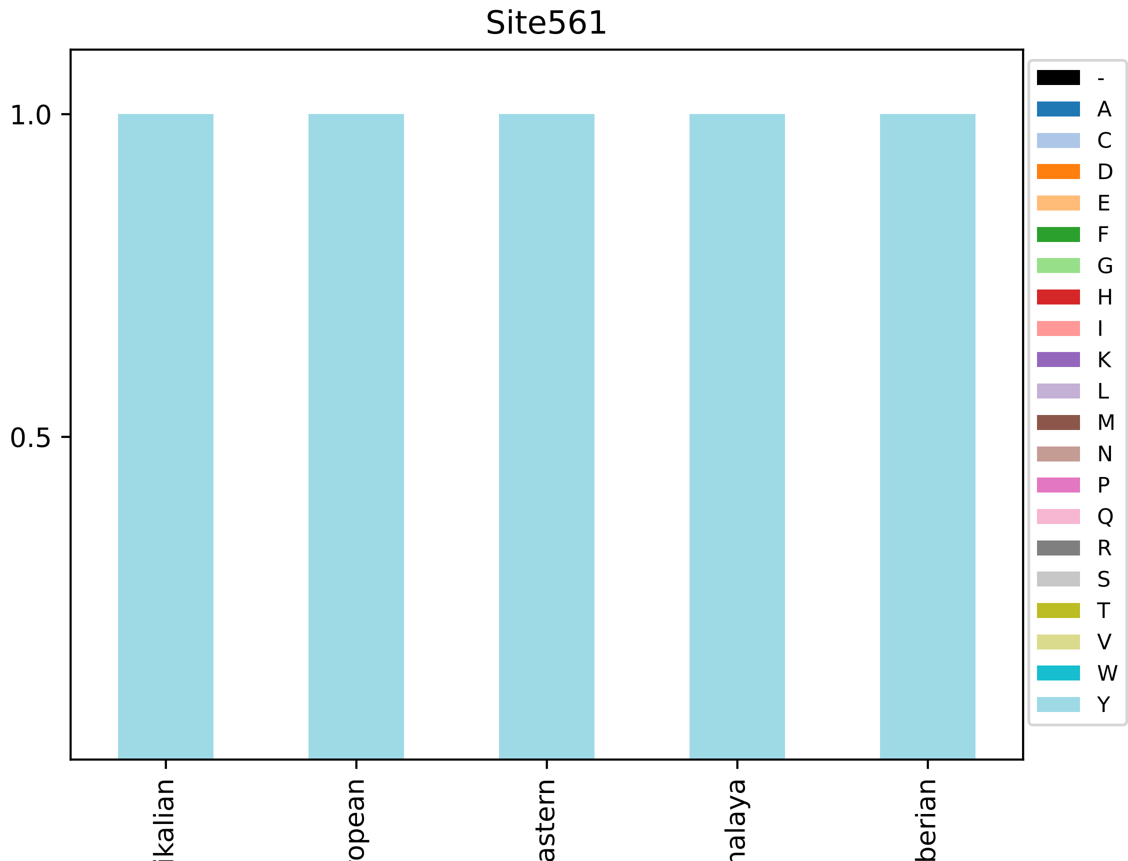
Site561
1.0
0.5
-
A
C
D
E
F
G
H
I
K
L
M
N
P
Q
R
S
T
V
W
Y
ikalian
opean
astern
halaya
berian

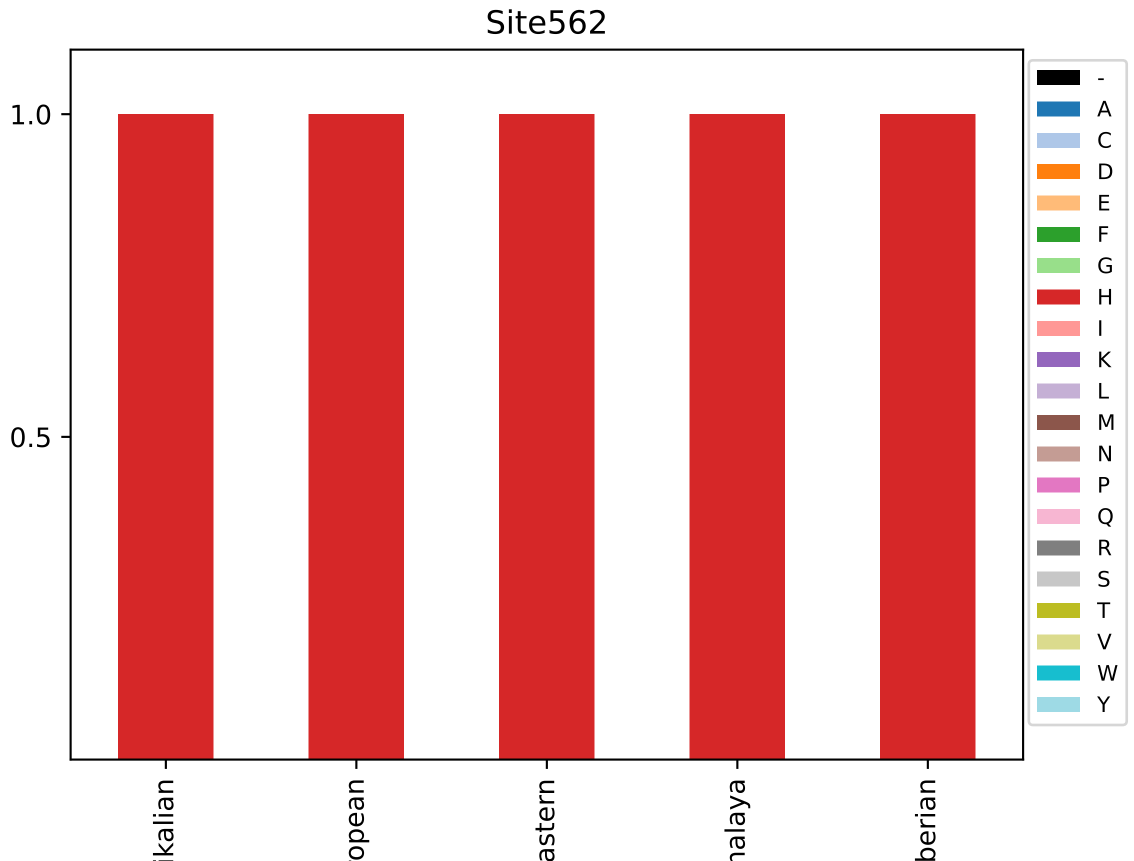

Site562
1.0
0.5
-
A
C
D
E
F
G
H
I
K
L
M
N
P
Q
R
S
T
V
W
Y

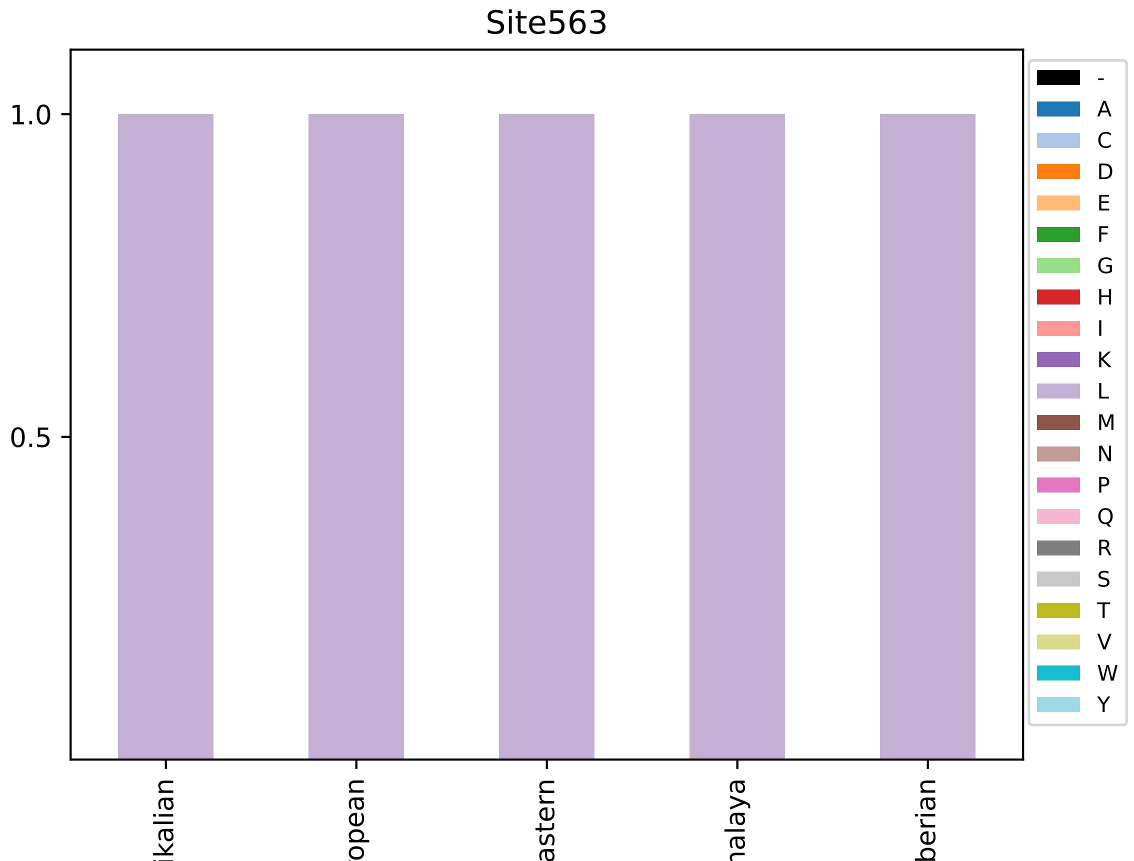
Site563
1.0
0.5
-
A
C
D
E
F
G
H
I
K
L
M
N
P
Q
R
S
T
V
W
Y
ikalian
opean
astern
nalaya
berian

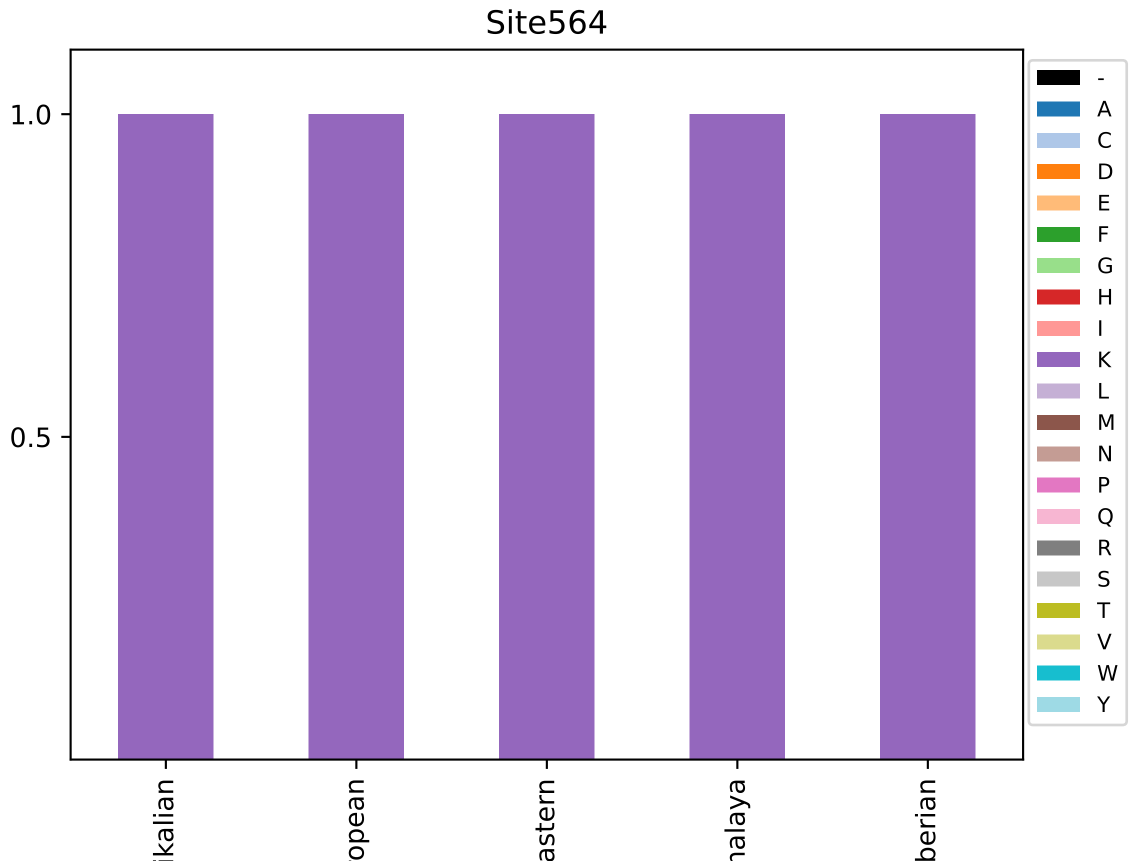

Site564
1.0
0.5
-
A
C
D
E
F
G
H
I
K
L
M
N
P
Q
R
S
T
V
W
Y
ikalian
opean
astern
nalaya
berian

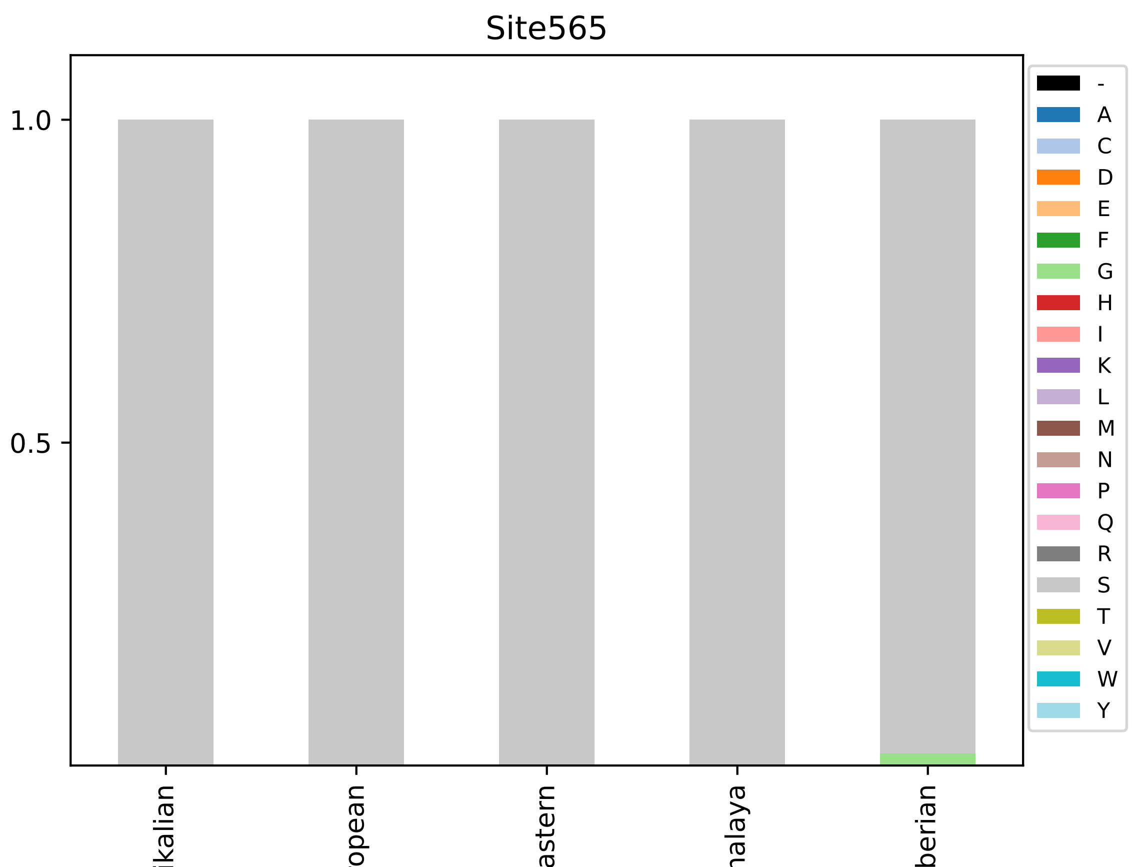

Site565
1.0
0.5
-
A
C
D
E
F
G
H
I
K
L
M
N
P
Q
R
S
T
V
W
Y
ikalian
opean
astern
nalaya
berian

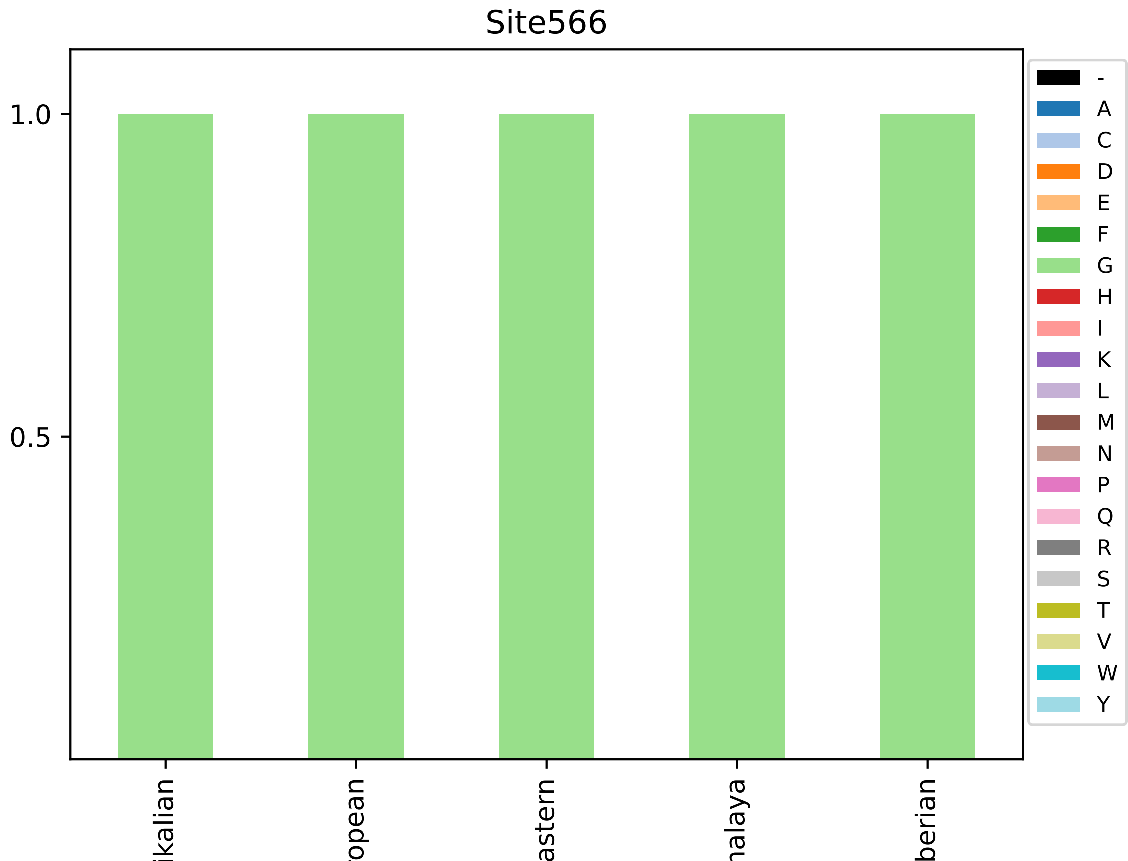

Site566
1.0
0.5
-
A
C
D
E
F
G
H
I
K
L
M
N
P
Q
R
S
T
V
W
Y
ikalian
opean
astern
nalaya
berian

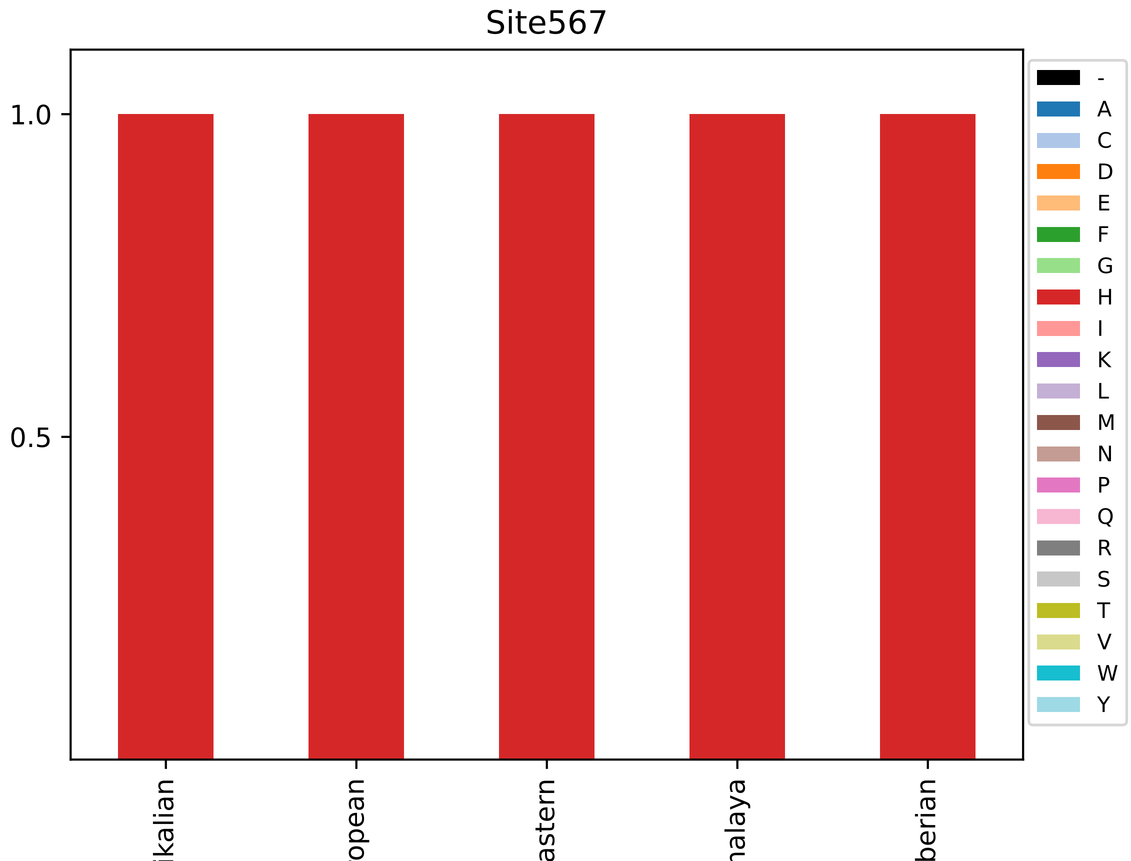
Site567
1.0
0.5
-
A
C
D
E
F
G
H
I
K
L
M
N
P
Q
R
S
T
V
W
Y
ikalian
opean
astern
nalaya
berian

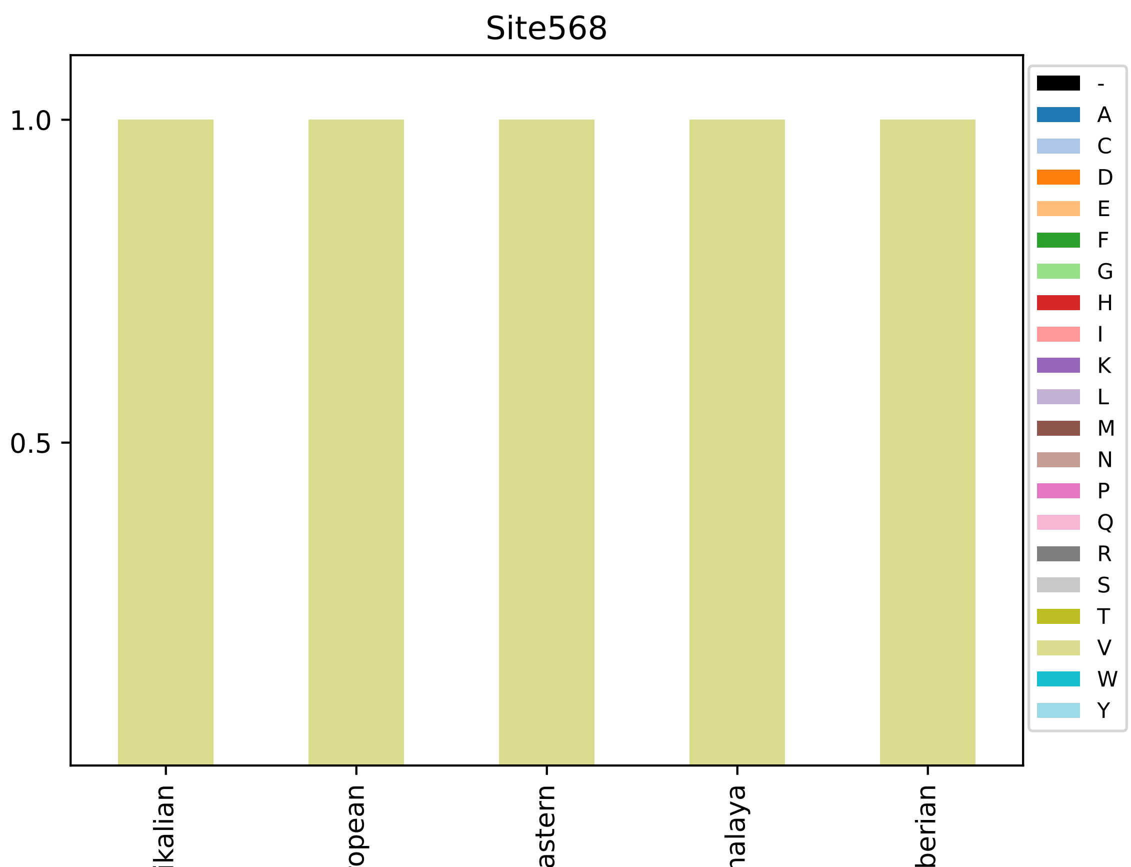

Site568
1.0
0.5
-
A
C
D
E
F
G
H
I
K
L
M
N
P
Q
R
S
T
V
W
Y

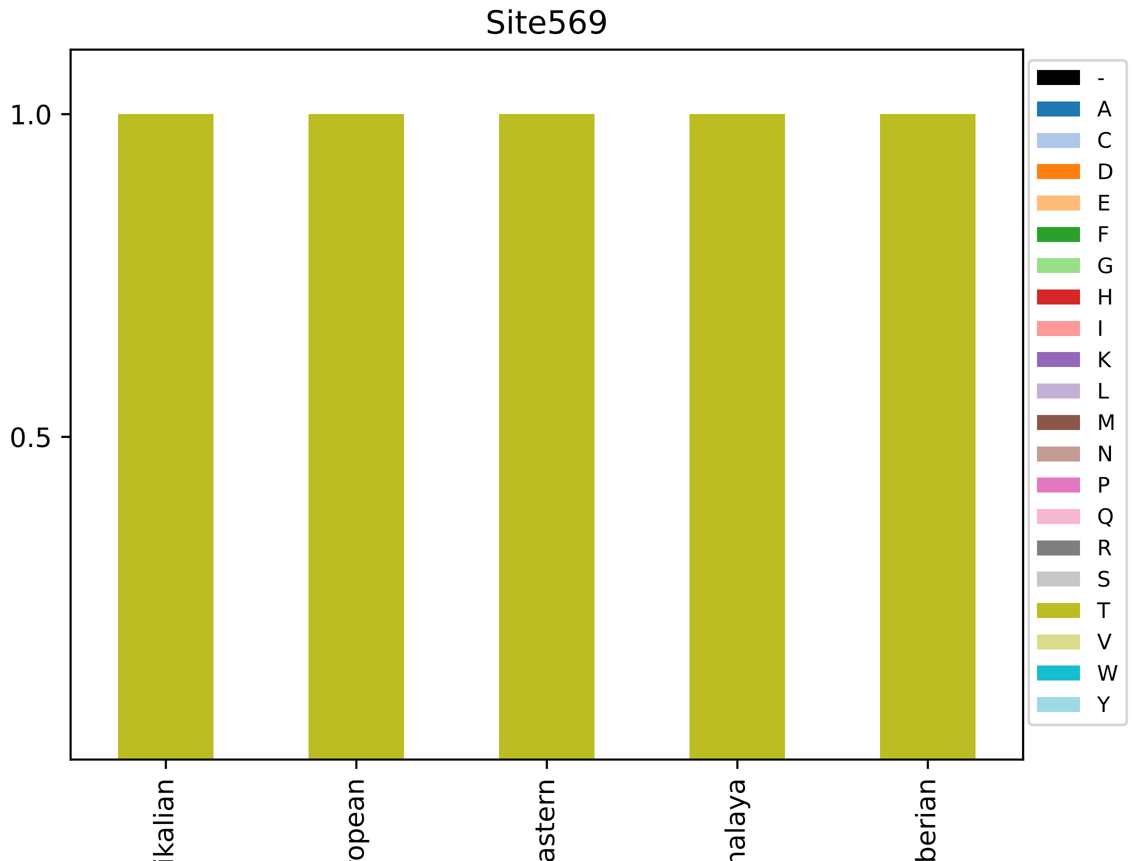
Site569
1.0
0.5
-
A
C
D
E
F
G
H
I
K
L
M
N
P
Q
R
S
T
V
W
Y
ikalian
opean
astern
nalaya
berian

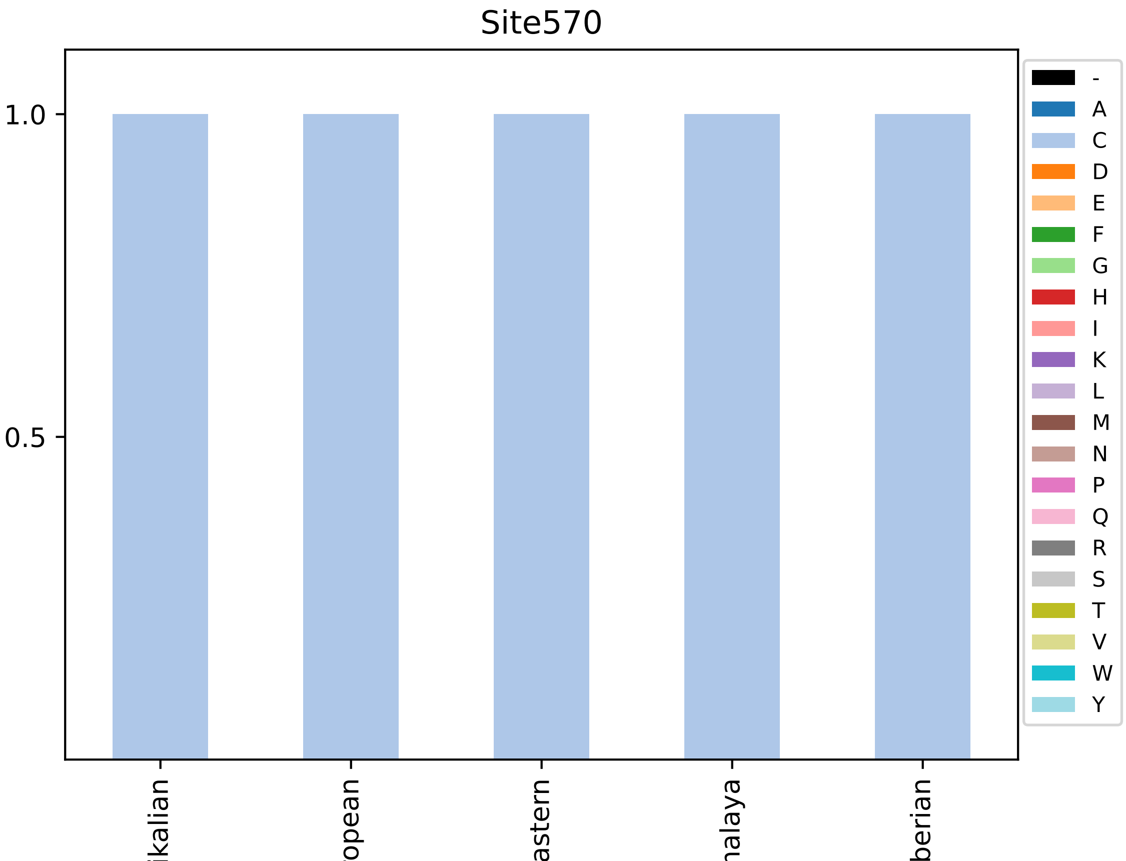
Site570
1.0
0.5
ikalian
opean
astern
nalaya
berian
-
A
C
D
E
F
G
H
I
K
L
M
N
P
Q
R
S
T
V
W
Y

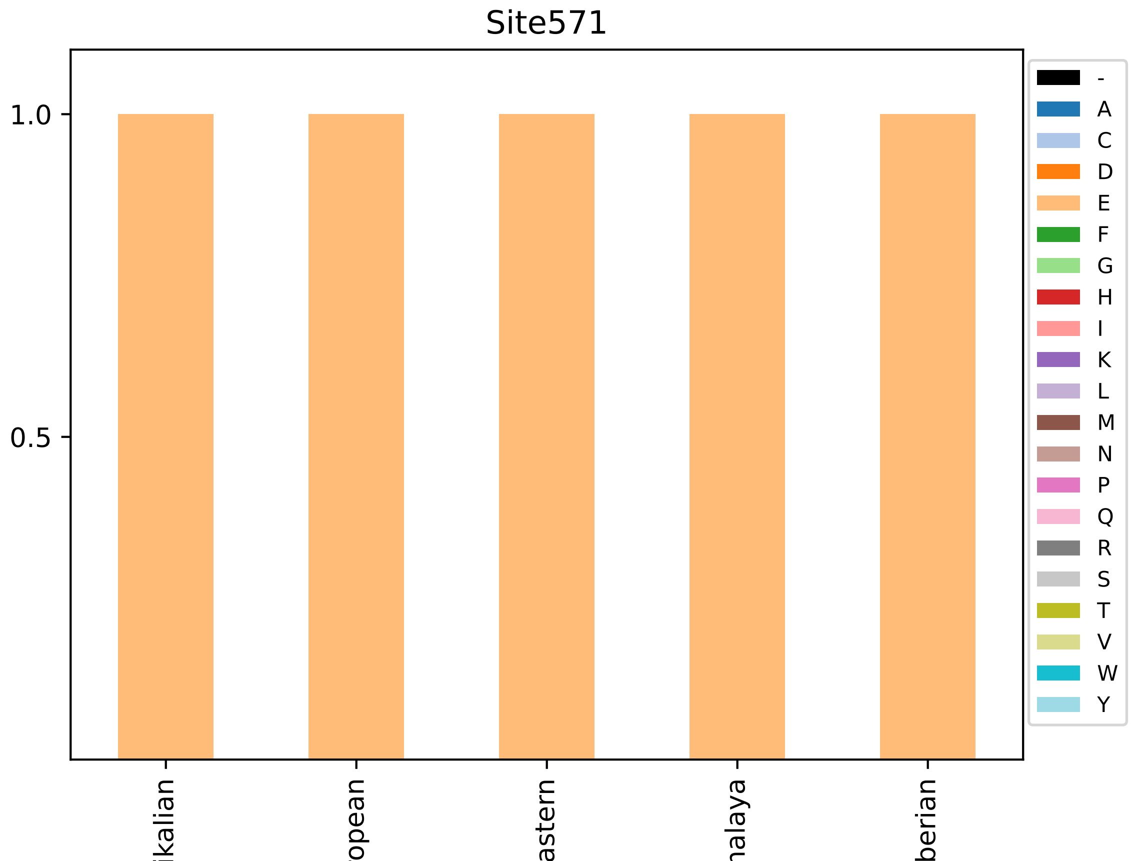
Site571
1.0
0.5
-
A
C
D
E
F
G
H
I
K
L
M
N
P
Q
R
S
T
V
W
Y
ikalian
opean
astern
nalaya
berian

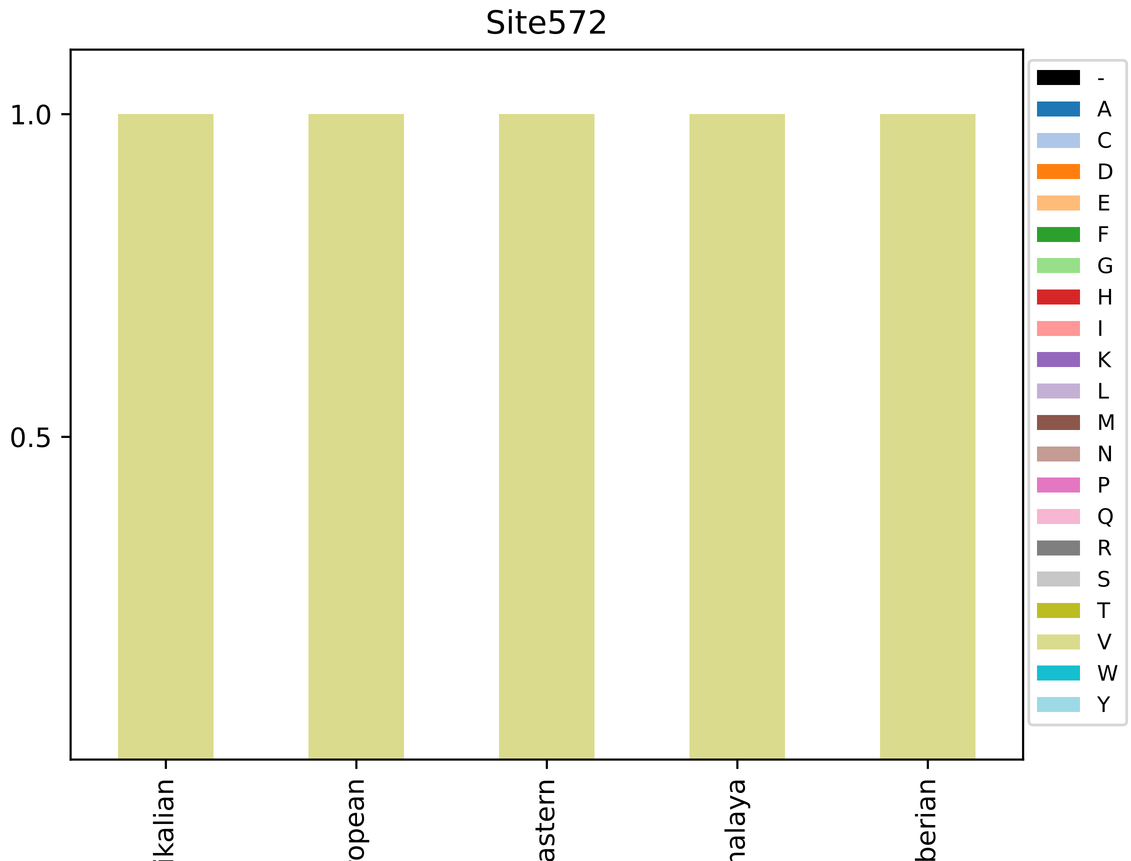
Site572
1.0
0.5
-
A
C
D
E
F
G
H
I
K
L
M
N
P
Q
R
S
T
V
W
Y
ikalian
opean
astern
nalaya
berian

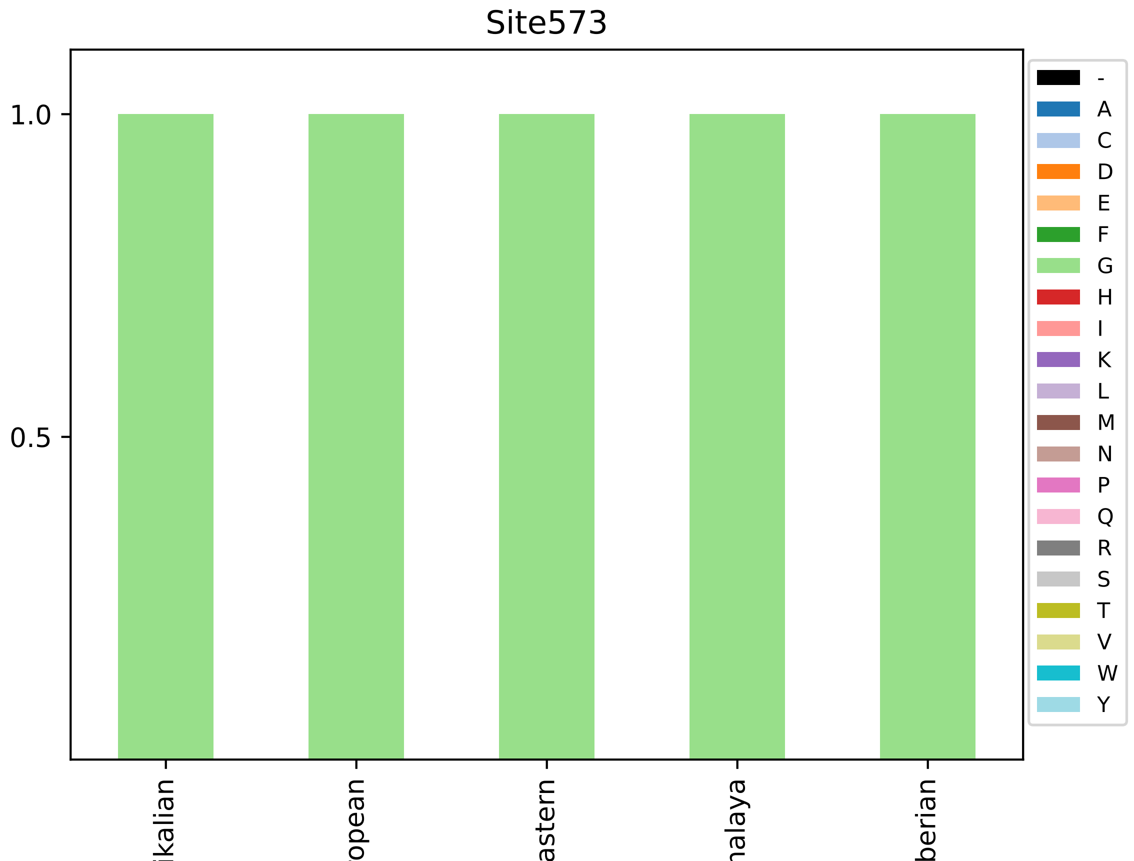

Site573
1.0
0.5
-
A
C
D
E
F
G
H
I
K
L
M
N
P
Q
R
S
T
V
W
Y
ikalian
opean
astern
nalaya
berian

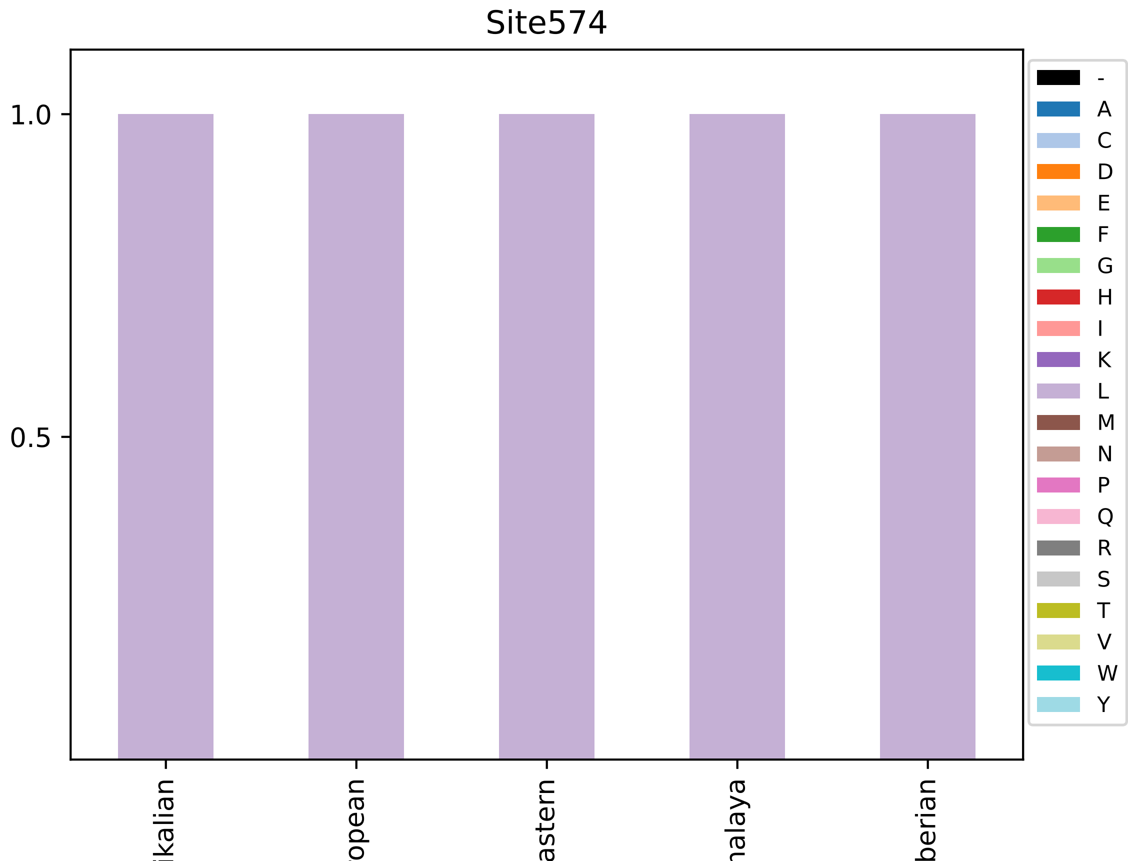

Site574
1.0
0.5
ikalian
opean
astern
nalaya
berian
-
A
C
D
E
F
G
H
I
K
L
M
N
P
Q
R
S
T
V
W
Y

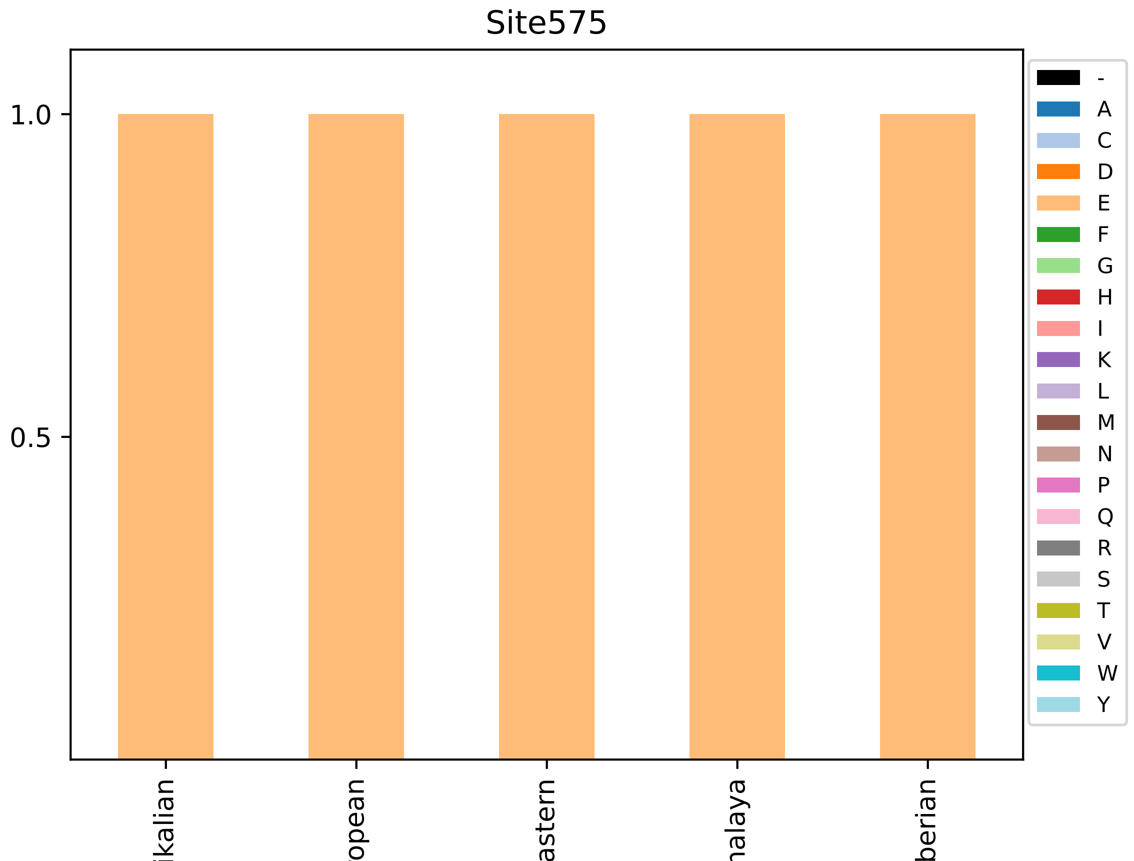
Site575
1.0
0.5
-
A
C
D
E
F
G
H
I
K
L
M
N
P
Q
R
S
T
V
W
Y
ikalian
opean
astern
nalaya
berian

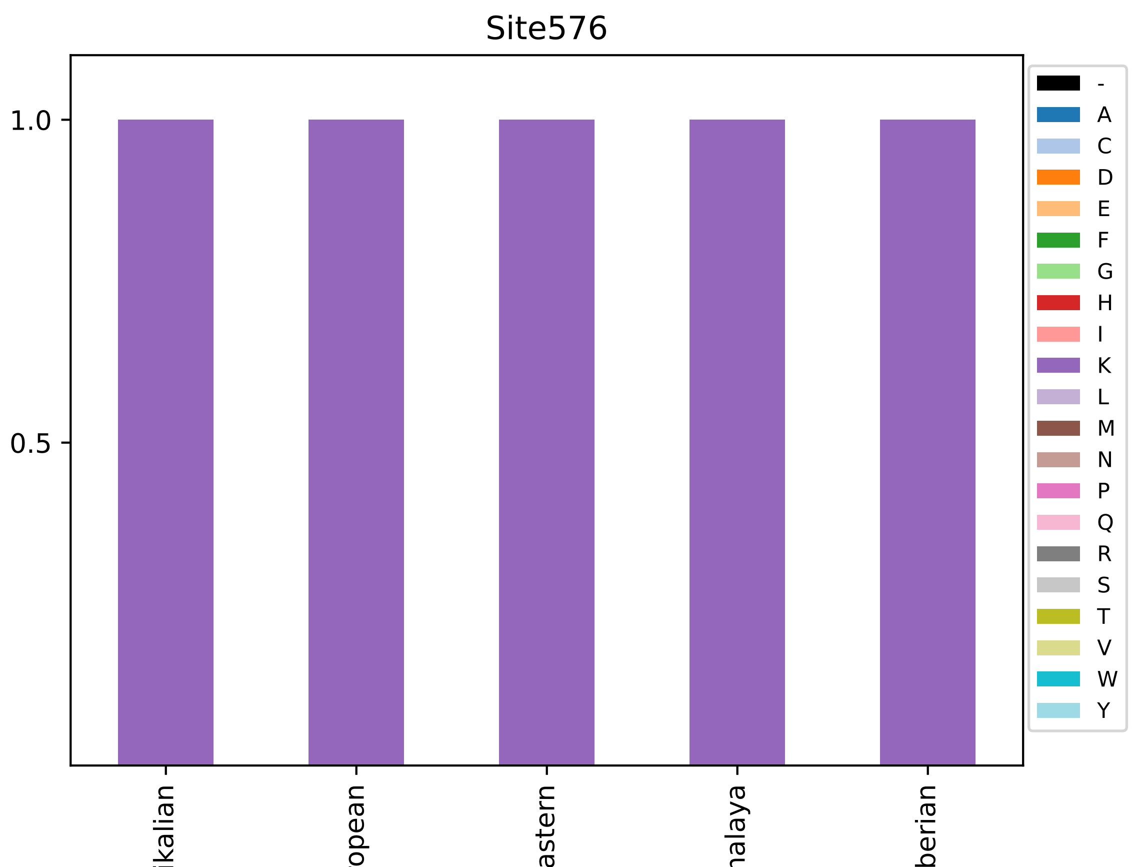

Site576
1.0
0.5
-
A
C
D
E
F
G
H
I
K
L
M
N
P
Q
R
S
T
V
W
Y
ikalian
opean
astern
nalaya
berian

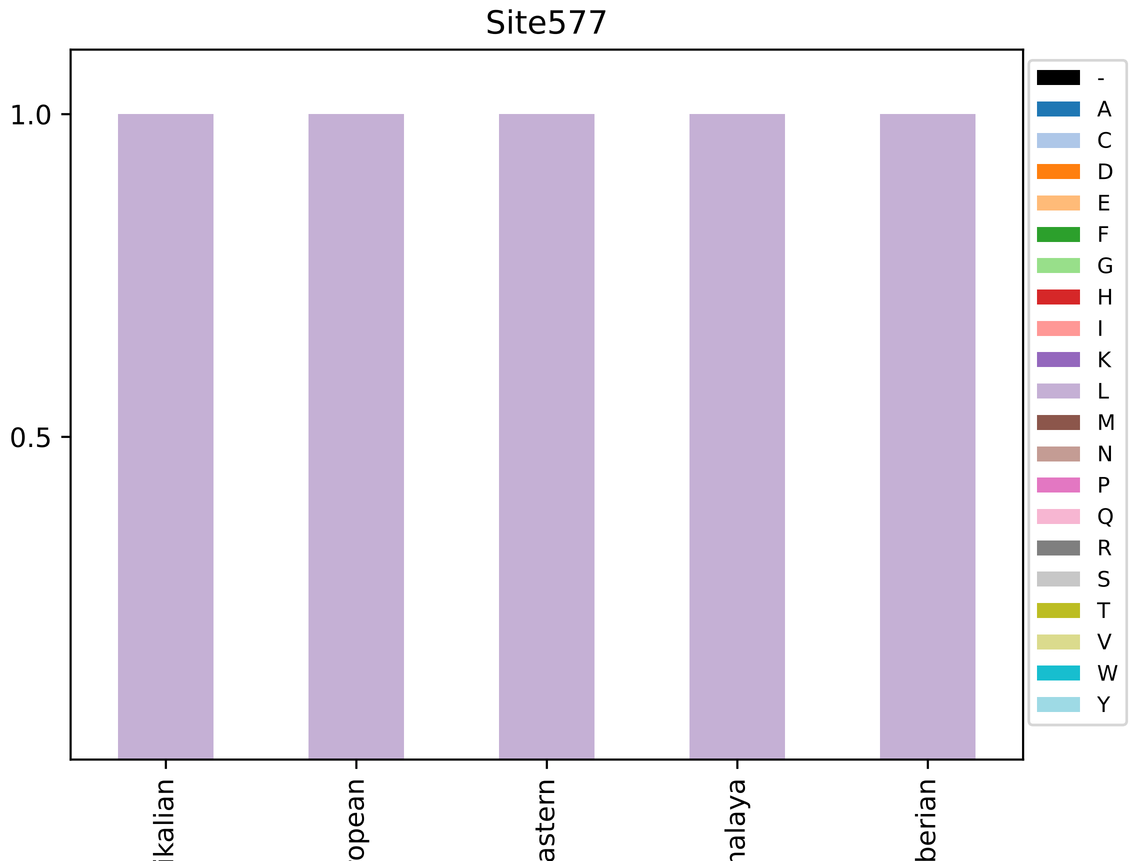

Site577
1.0
0.5
-
A
C
D
E
F
G
H
I
K
L
M
N
P
Q
R
S
T
V
W
Y

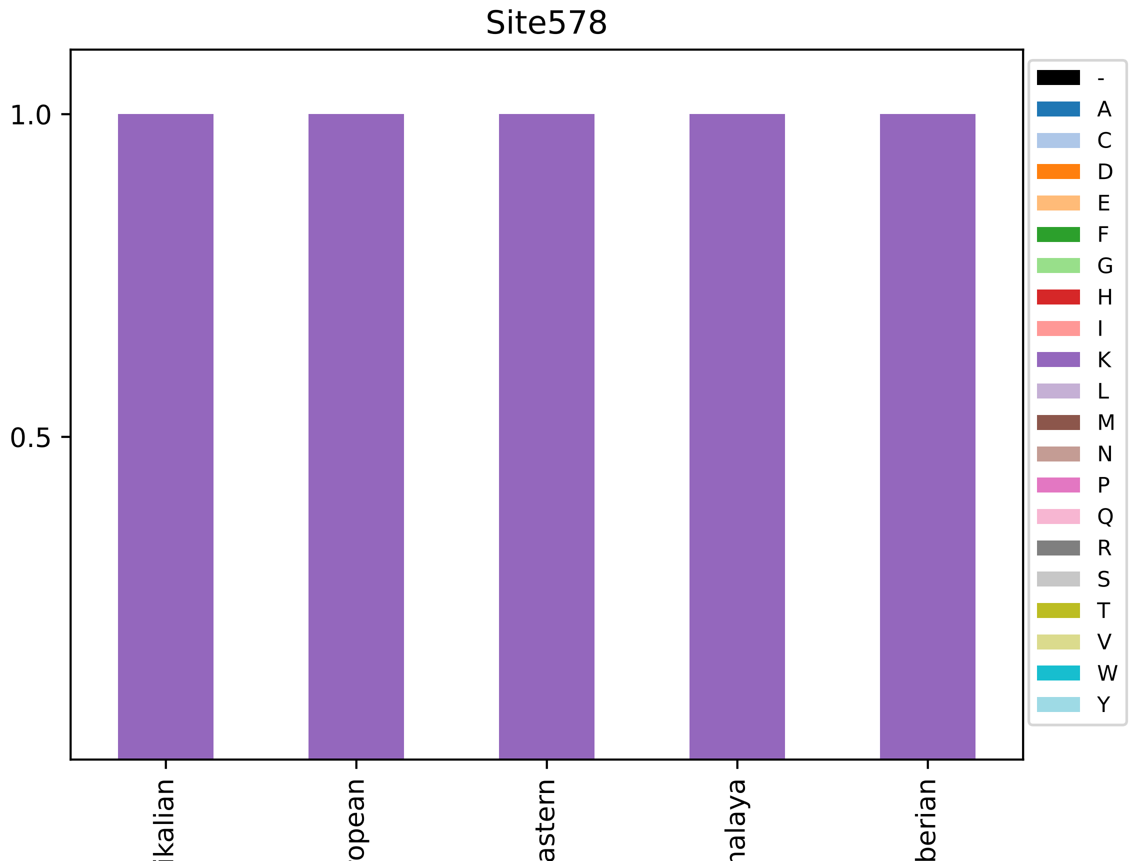

Site578
1.0
0.5
-
A
C
D
E
F
G
H
I
K
L
M
N
P
Q
R
S
T
V
W
Y
ikalian
opean
astern
nalaya
berian

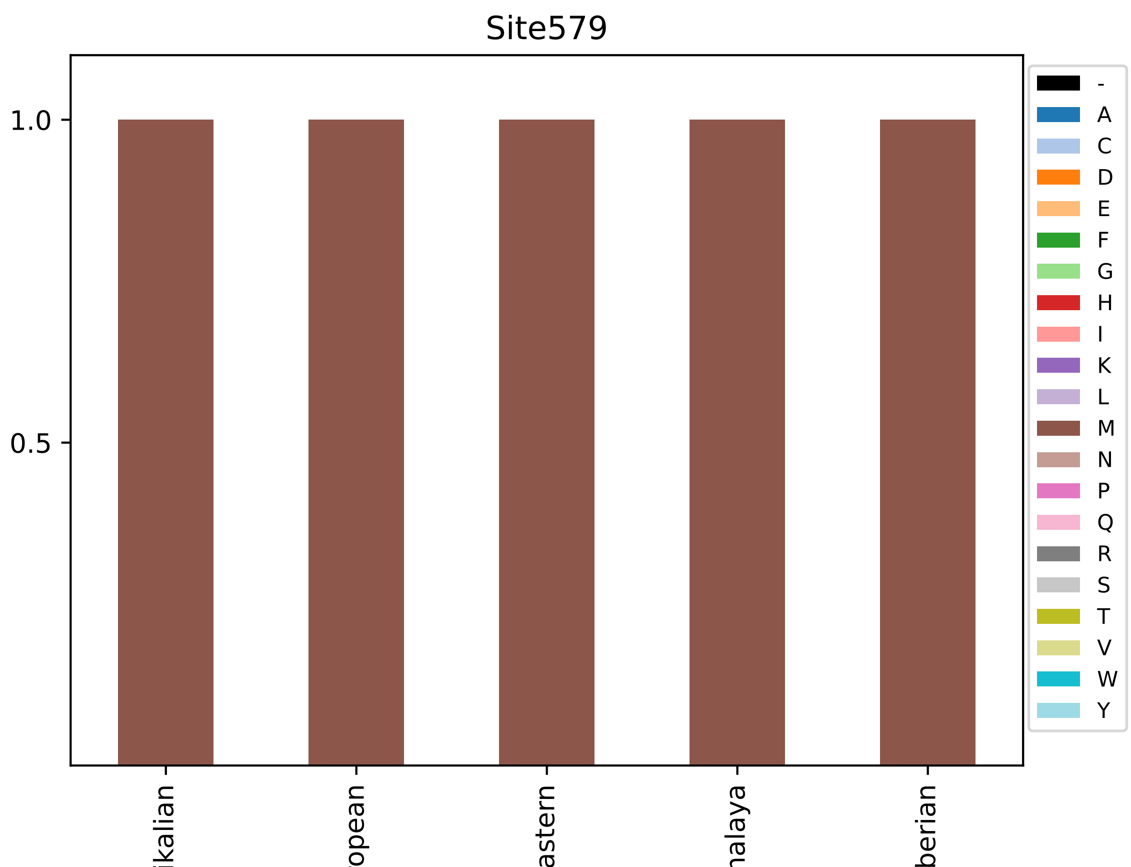

Site579
1.0
0.5
-
A
C
D
E
F
G
H
I
K
L
M
N
P
Q
R
S
T
V
W
Y
ikalian
opean
astern
nalaya
berian

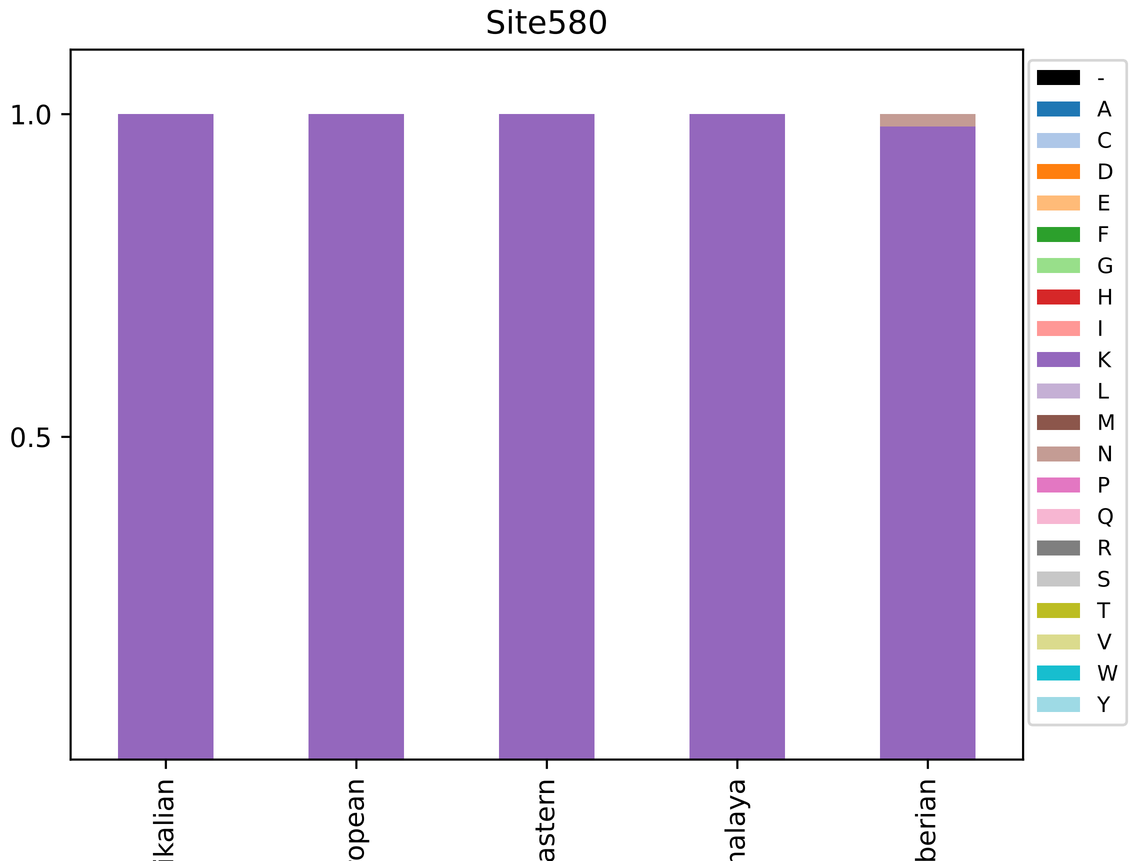
Site580
1.0
0.5
-
A
C
D
E
F
G
H
I
K
L
M
N
P
Q
R
S
T
V
W
Y
ikalian
opean
astern
nalaya
berian

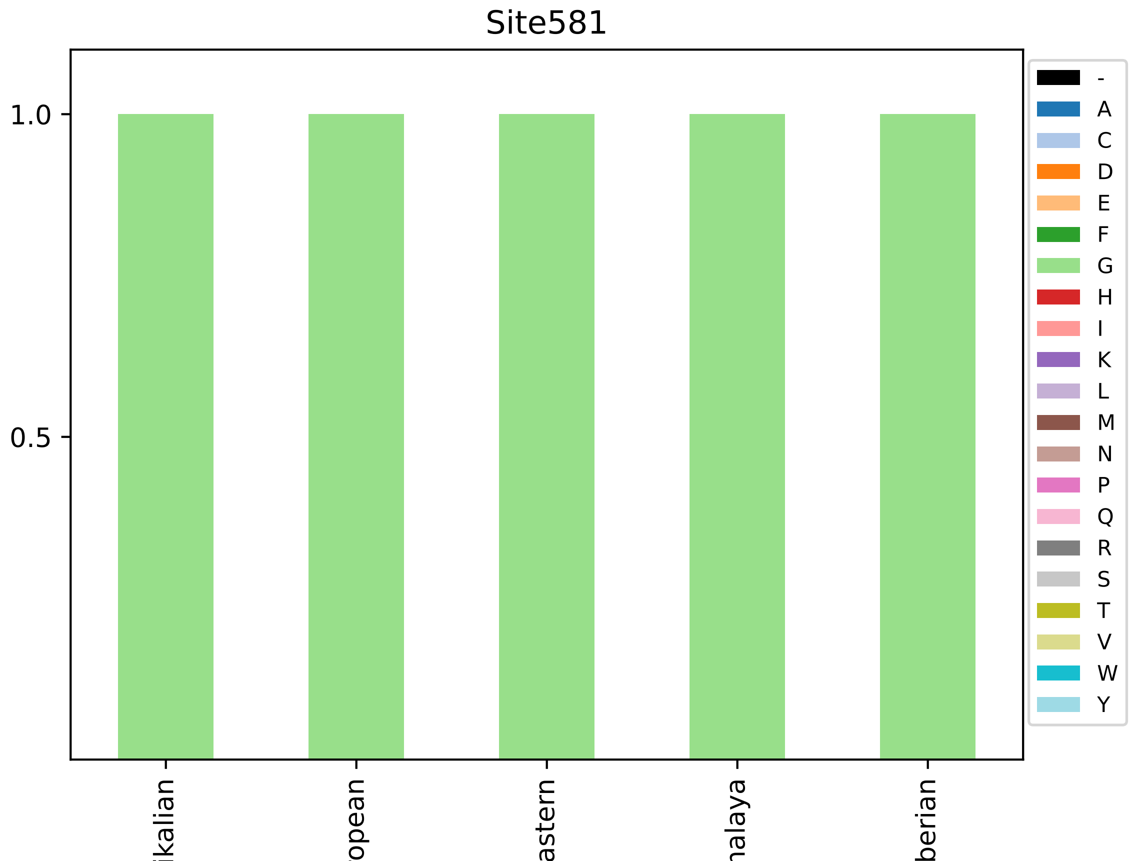

Site581
1.0
0.5
-
A
C
D
E
F
G
H
I
K
L
M
N
P
Q
R
S
T
V
W
Y
ikalian
opean
astern
nalaya
berian

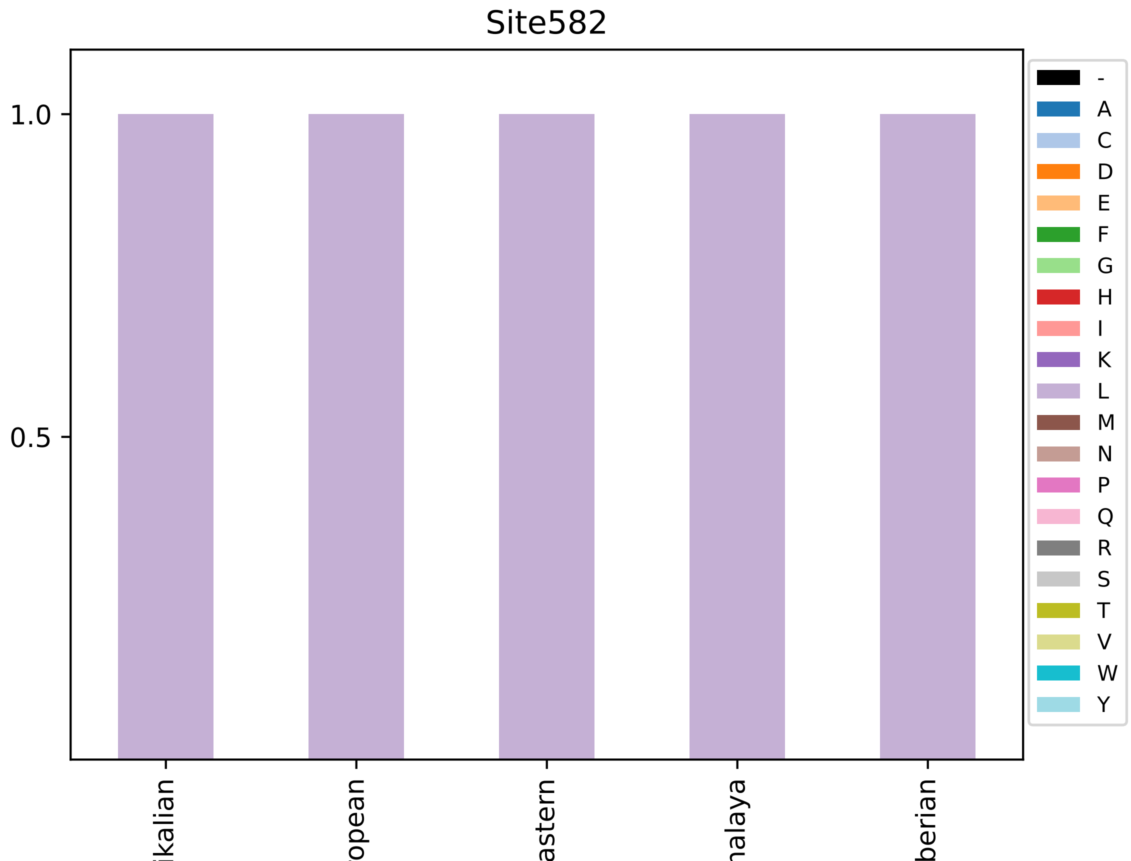
Site582
1.0
0.5
-
A
C
D
E
F
G
H
I
K
L
M
N
P
Q
R
S
T
V
W
Y
ikalian
opean
astern
nalaya
berian

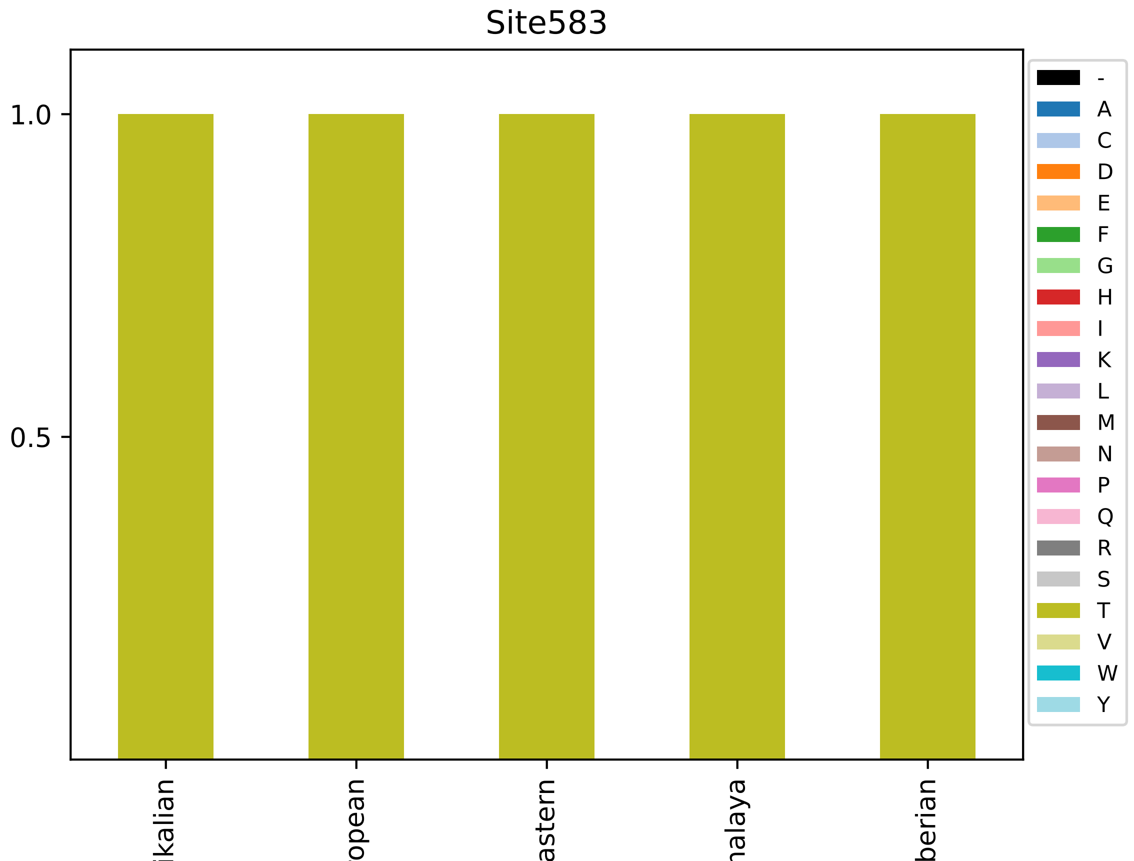
Site583
1.0
0.5
-
A
C
D
E
F
G
H
I
K
L
M
N
P
Q
R
S
T
V
W
Y
ikalian
opean
astern
nalaya
berian

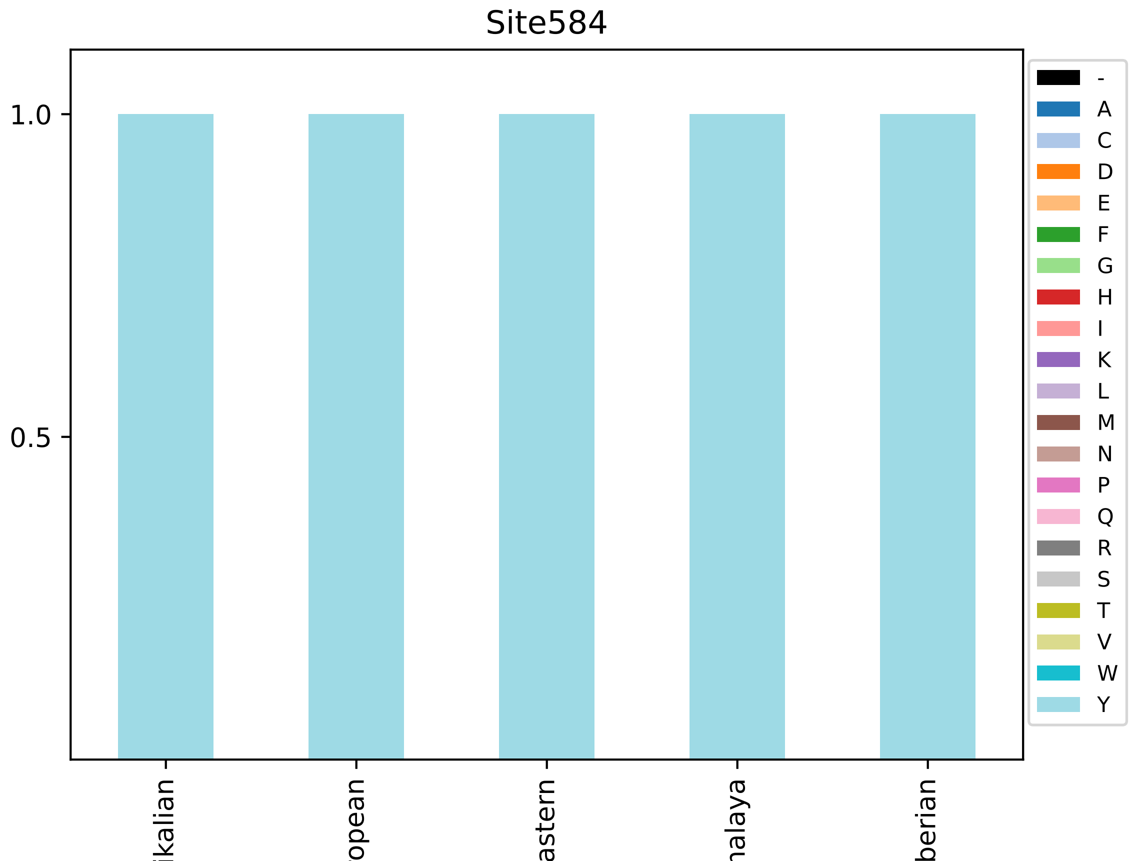
Site584
1.0
0.5
-
A
C
D
E
F
G
H
I
K
L
M
N
P
Q
R
S
T
V
W
Y
ikalian
opean
astern
nalaya
berian

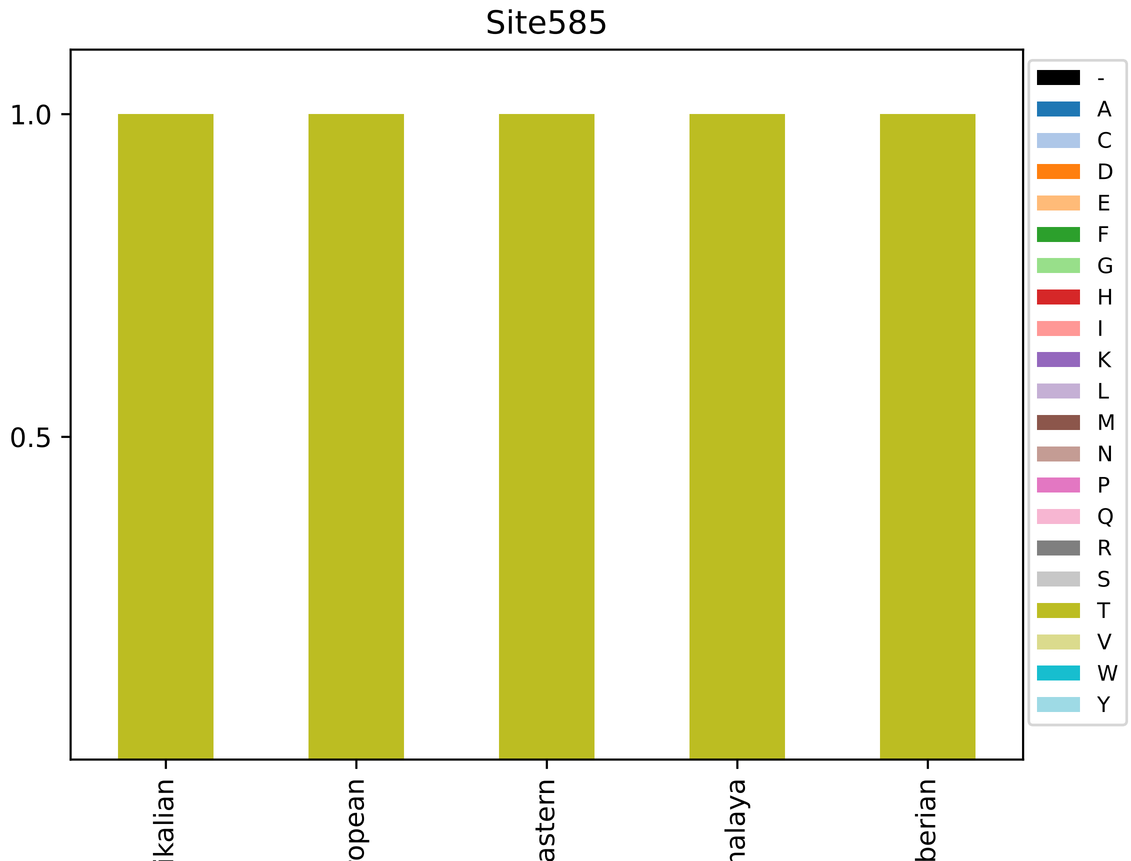
Site585
1.0
0.5
-
A
C
D
E
F
G
H
I
K
L
M
N
P
Q
R
S
T
V
W
Y
ikalian
opean
astern
nalaya
berian

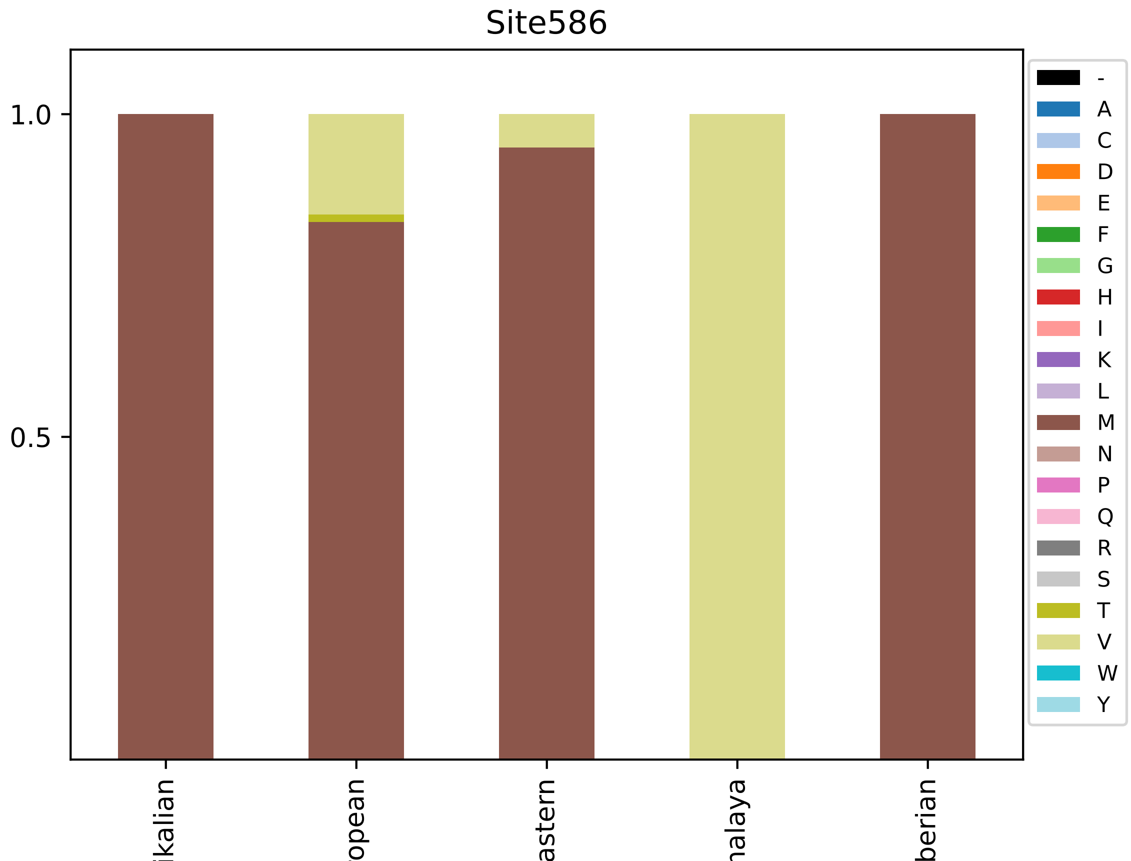
Site586
1.0
0.5
-
A
C
D
E
F
G
H
I
K
L
M
N
P
Q
R
S
T
V
W
Y
ikalian
opean
astern
nalaya
berian

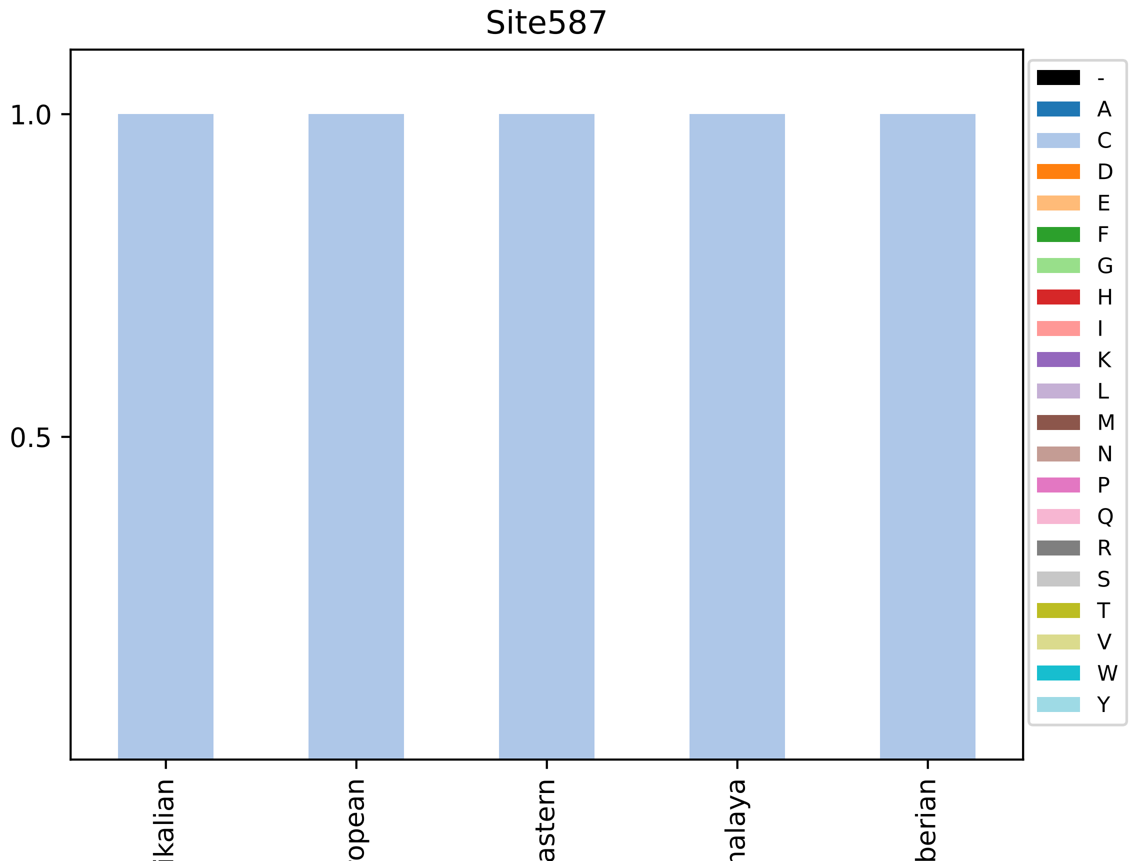

Site587
1.0
0.5
ikalian
opean
astern
nalaya
berian
-
A
C
D
E
F
G
H
I
K
L
M
N
P
Q
R
S
T
V
W
Y

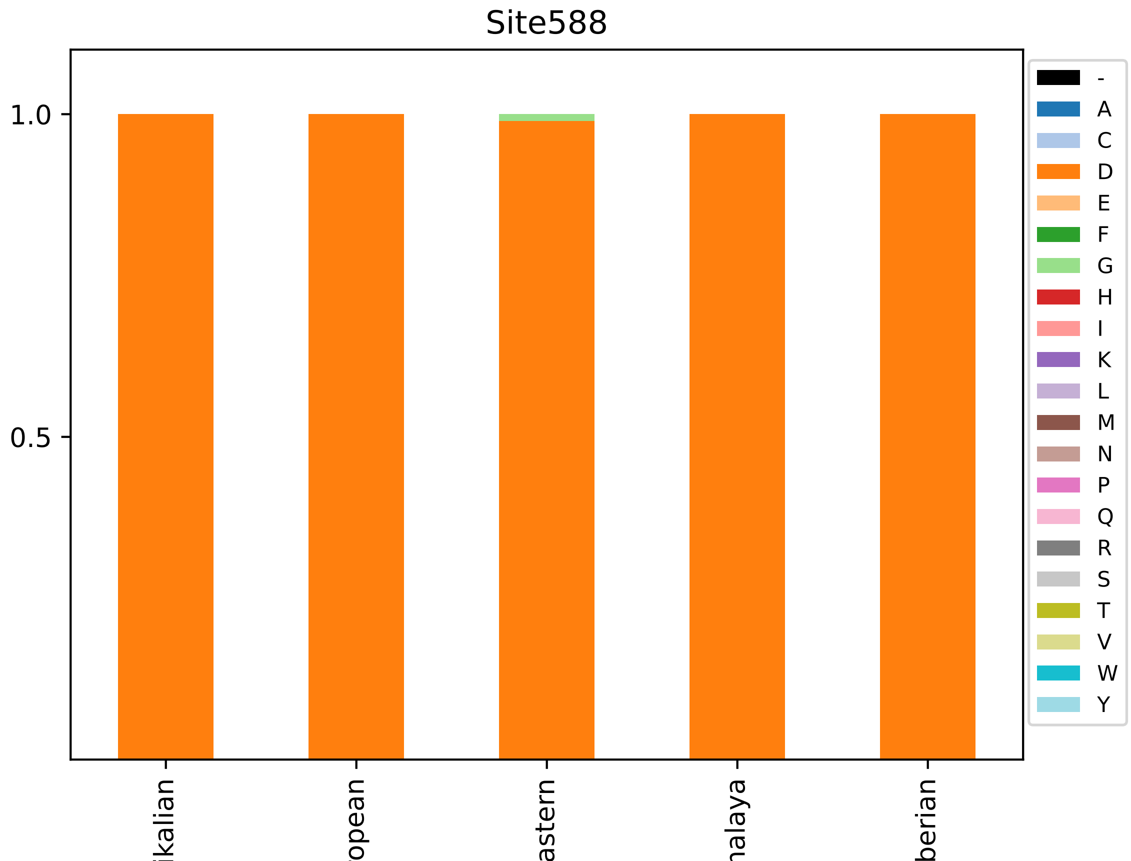
Site588
1.0
0.5
-
A
C
D
E
F
G
H
I
K
L
M
N
P
Q
R
S
T
V
W
Y
ikalian
opean
astern
nalaya
berian

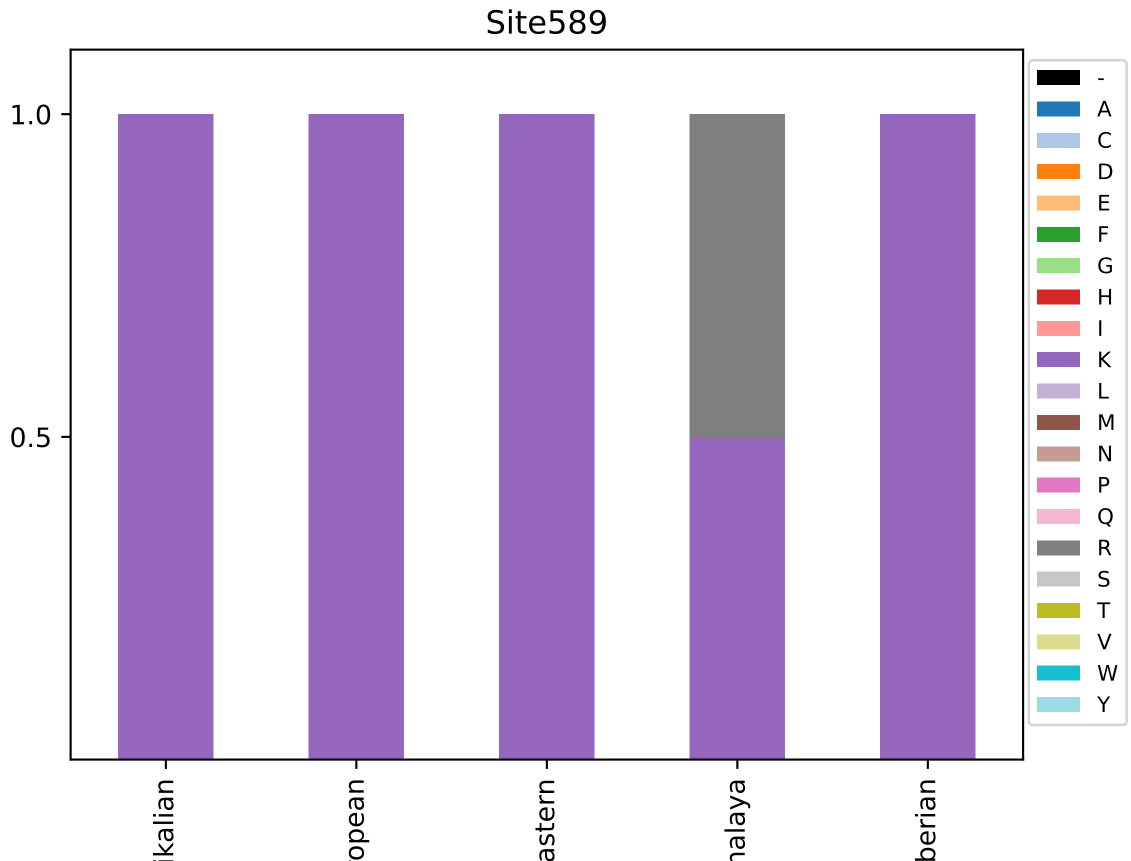

Site589
1.0
0.5
-
A
C
D
E
F
G
H
I
K
L
M
N
P
Q
R
S
T
V
W
Y
ikalian
opean
astern
nalaya
berian

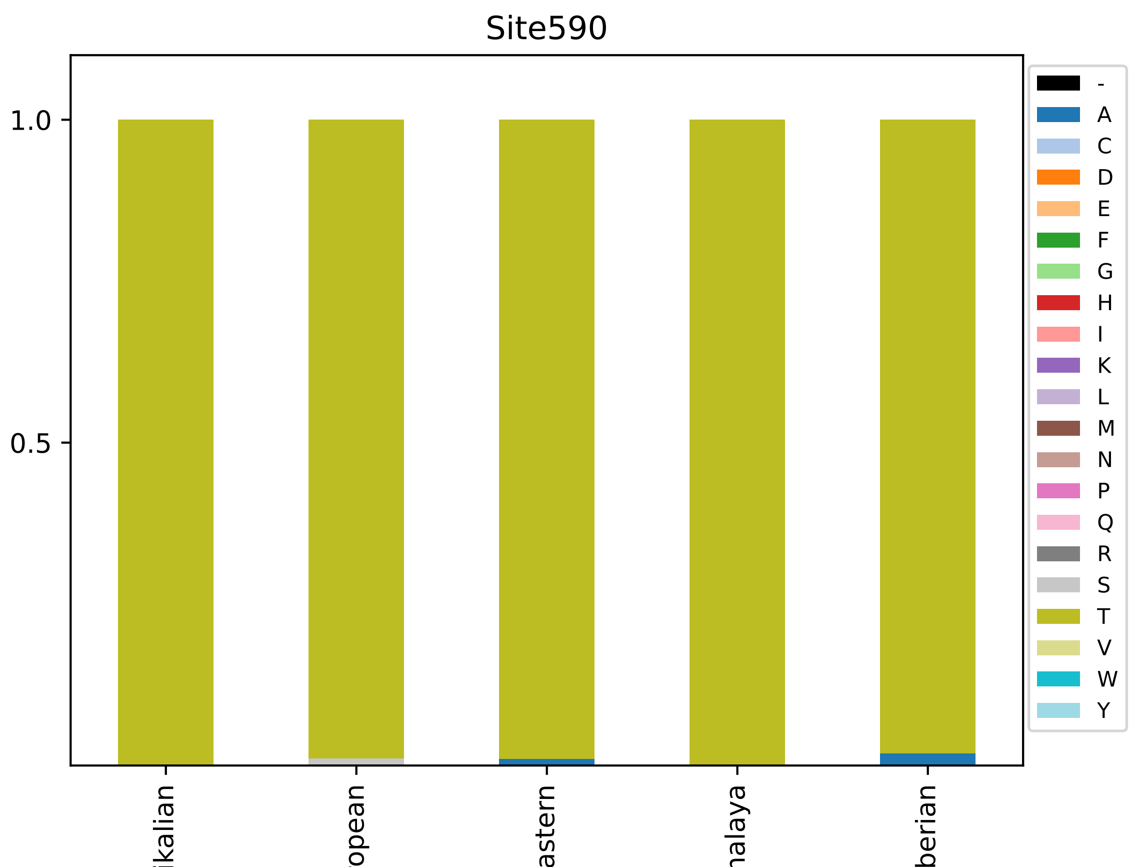

Site590
1.0
0.5
ikalian
opean
astern
nalaya
berian
-
A
C
D
E
F
G
H
I
K
L
M
N
P
Q
R
S
T
V
W
Y

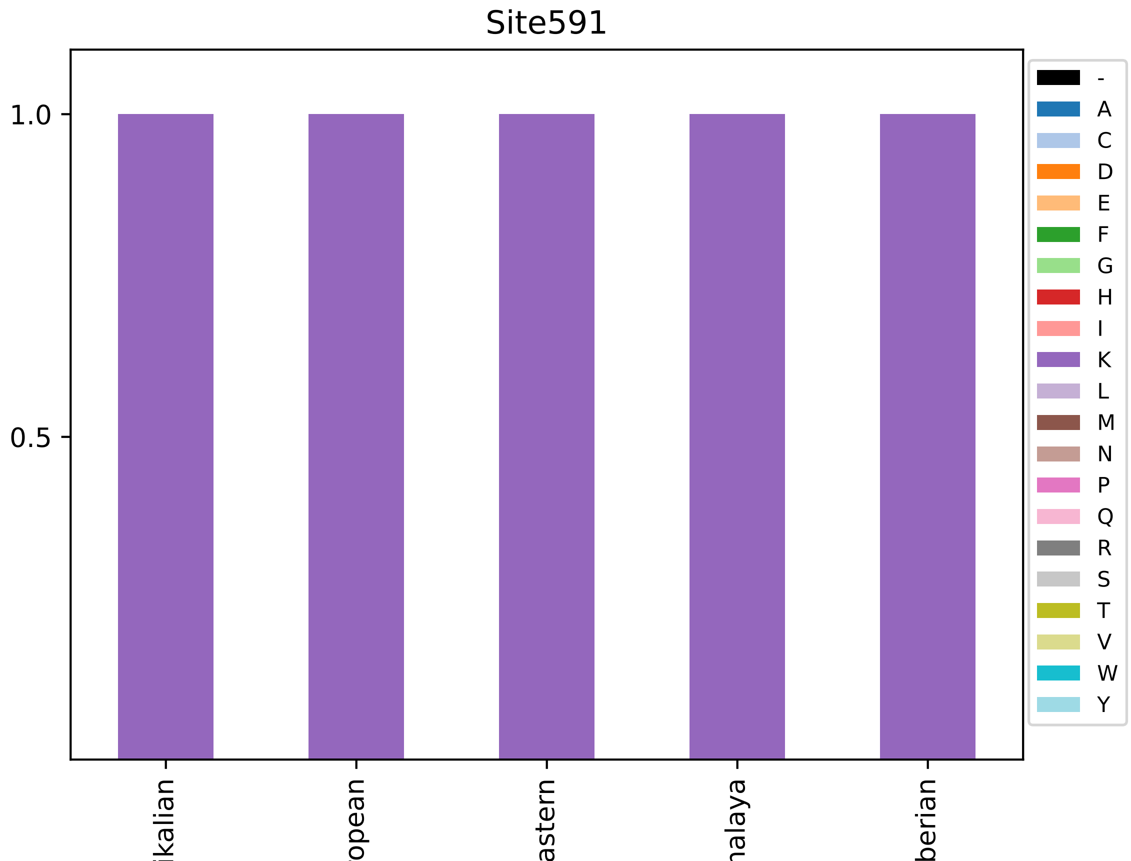

Site591
1.0
0.5
-
A
C
D
E
F
G
H
I
K
L
M
N
P
Q
R
S
T
V
W
Y
ikalian
opean
astern
nalaya
berian

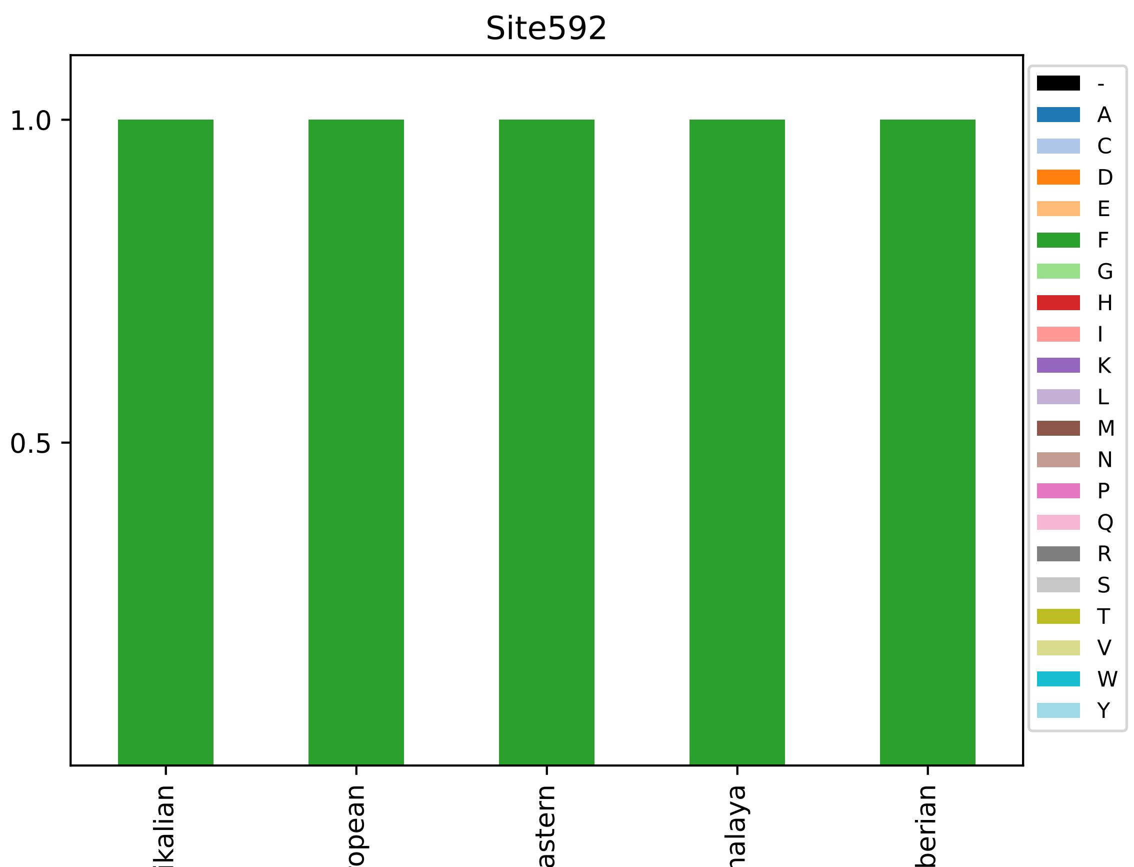
Site592
1.0
0.5
-
A
C
D
E
F
G
H
I
K
L
M
N
P
Q
R
S
T
V
W
Y
ikalian
ropean
astern
nalaya
berian

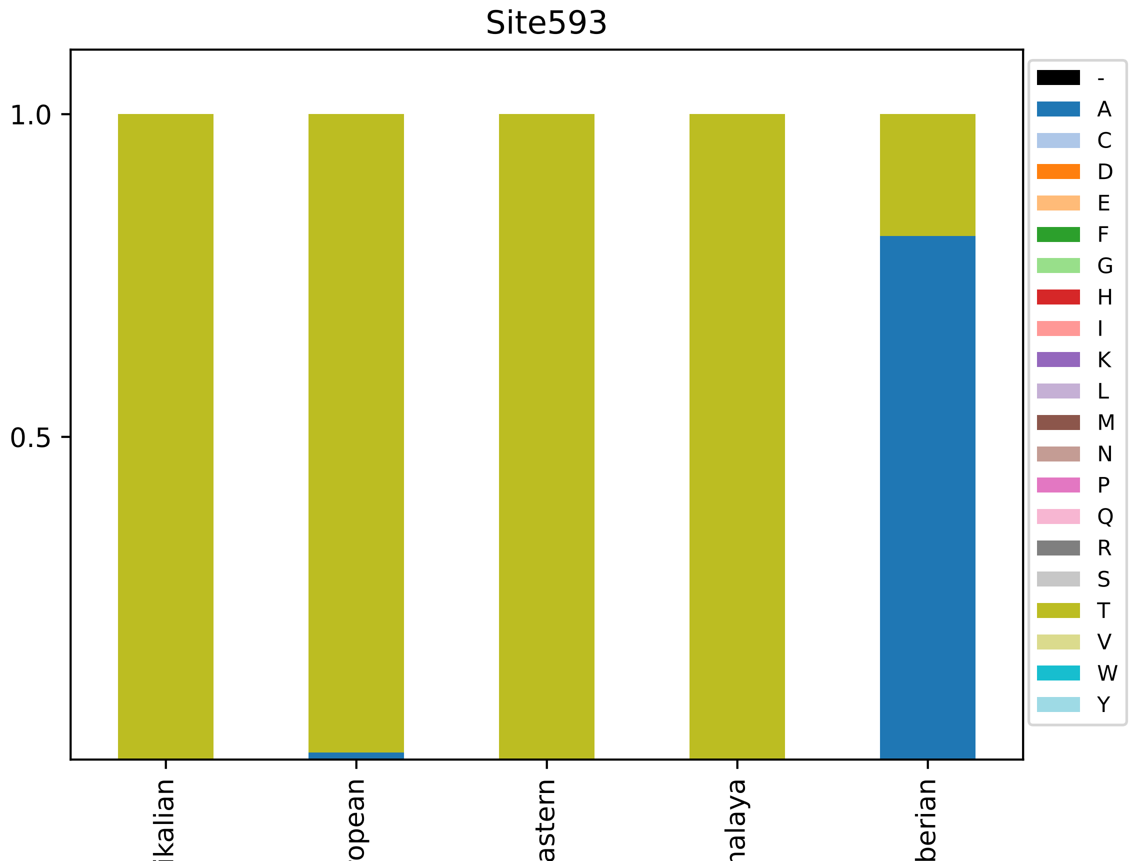
Site593
1.0
0.5
ikalian
opean
astern
nalaya
berian
-
A
C
D
E
F
G
H
I
K
L
M
N
P
Q
R
S
T
V
W
Y

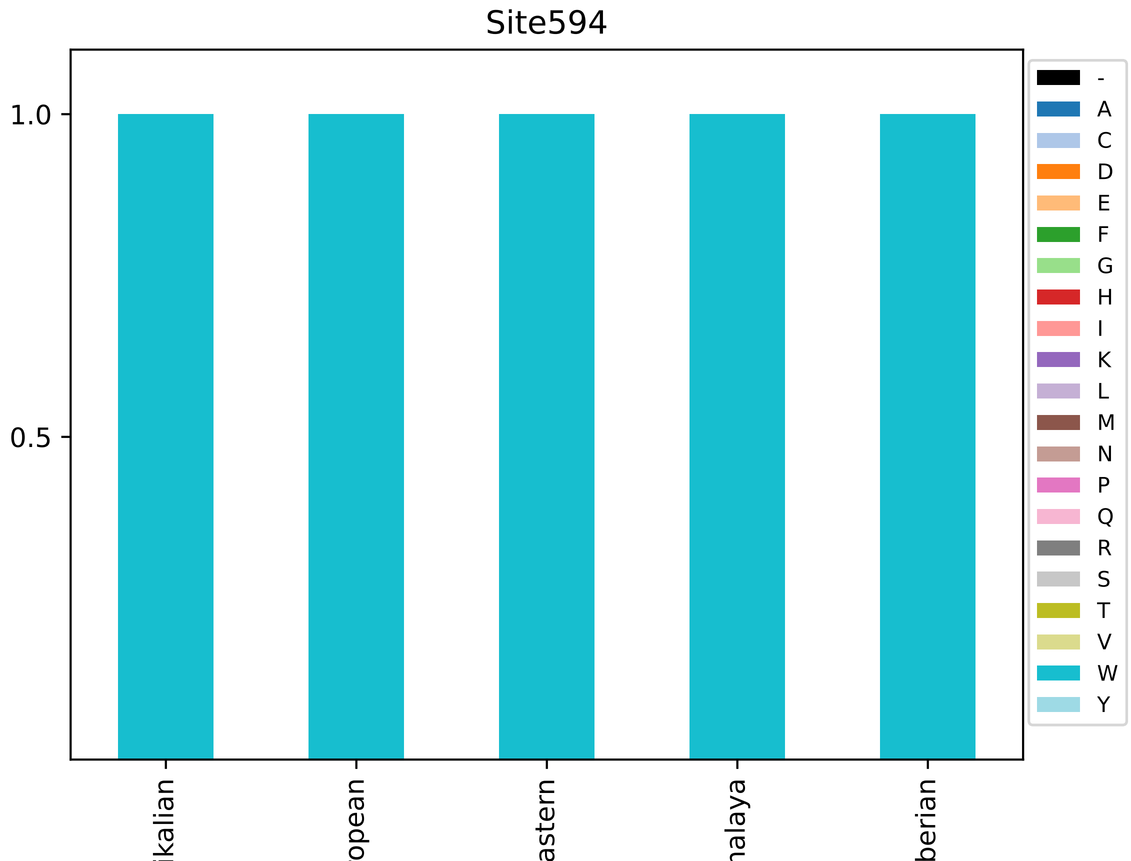

Site594
1.0
0.5
ikalian
opean
astern
nalaya
berian
-
A
C
D
E
F
G
H
I
K
L
M
N
P
Q
R
S
T
V
W
Y

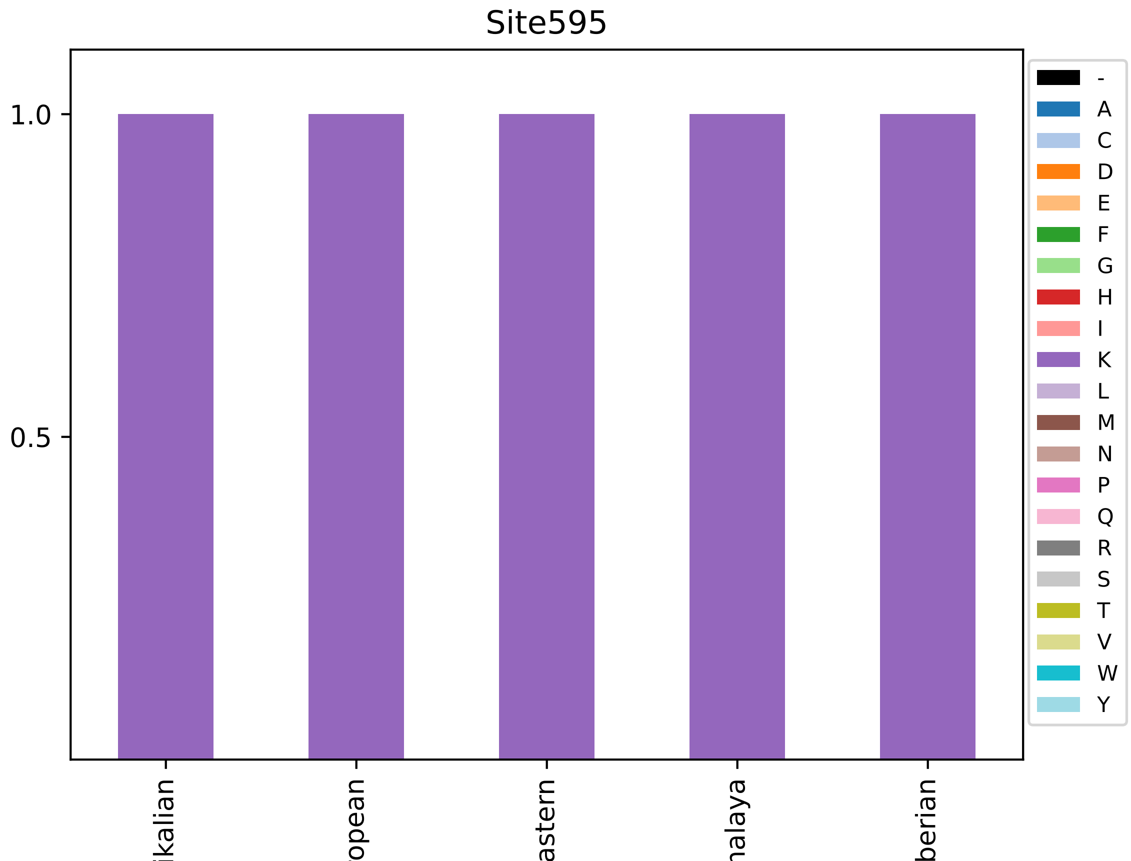
Site595
1.0
0.5
-
A
C
D
E
F
G
H
I
K
L
M
N
P
Q
R
S
T
V
W
Y

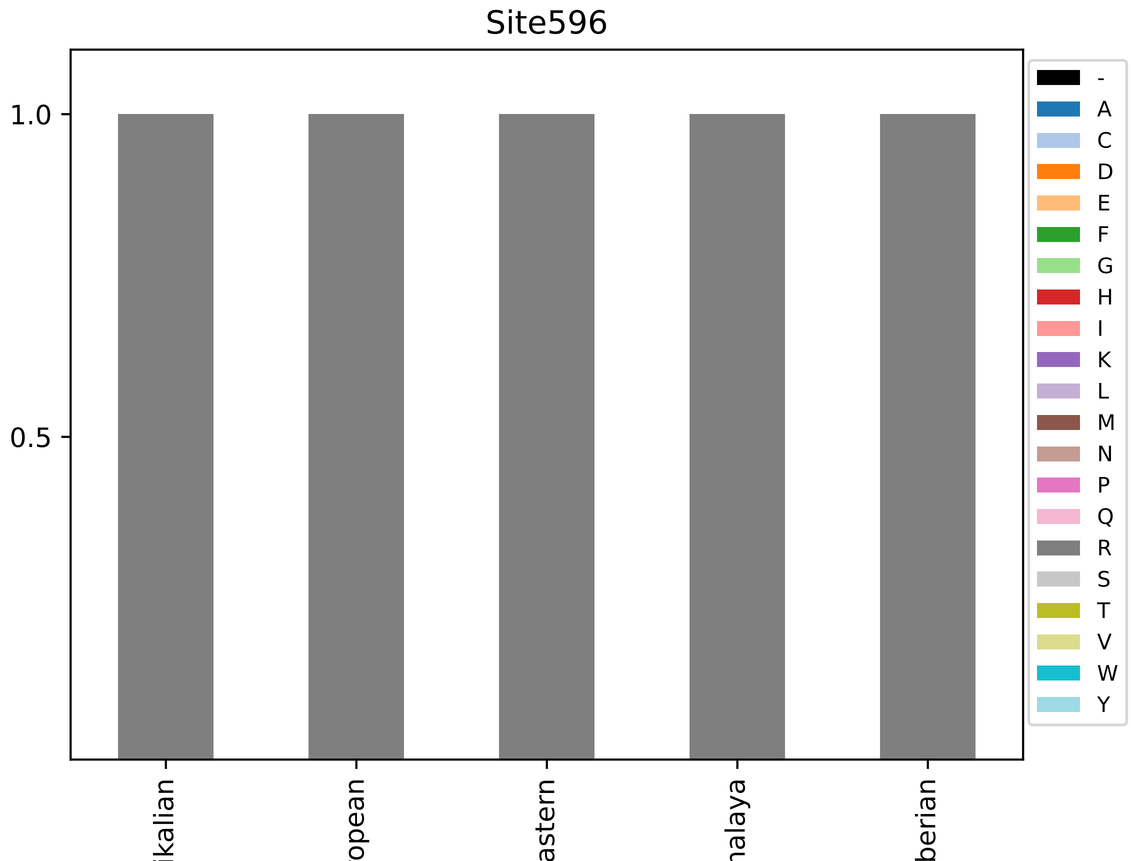
Site596
1.0
0.5
-
A
C
D
E
F
G
H
I
K
L
M
N
P
Q
R
S
T
V
W
Y
ikalian
opean
astern
nalaya
berian

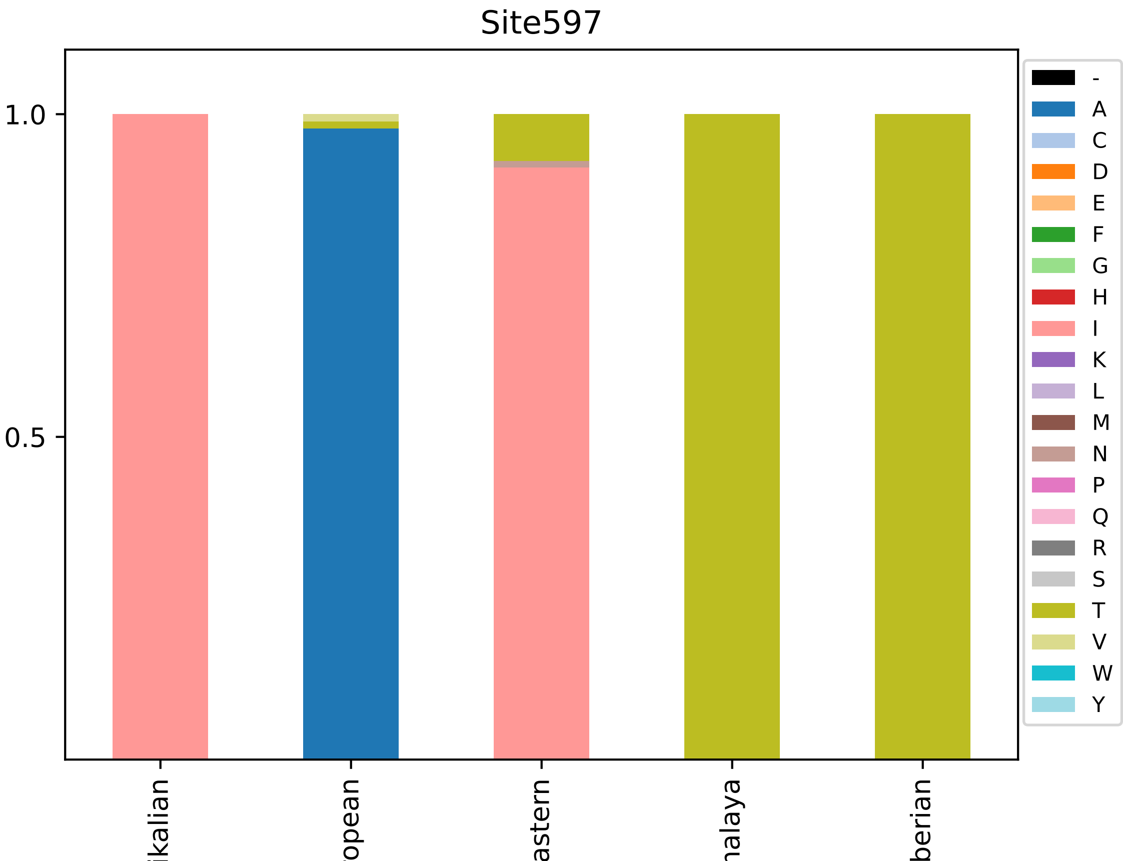
Site597
1.0
0.5
-
A
C
D
E
F
G
H
I
K
L
M
N
P
Q
R
S
T
V
W
Y
ikalian
opean
astern
nalaya
berian

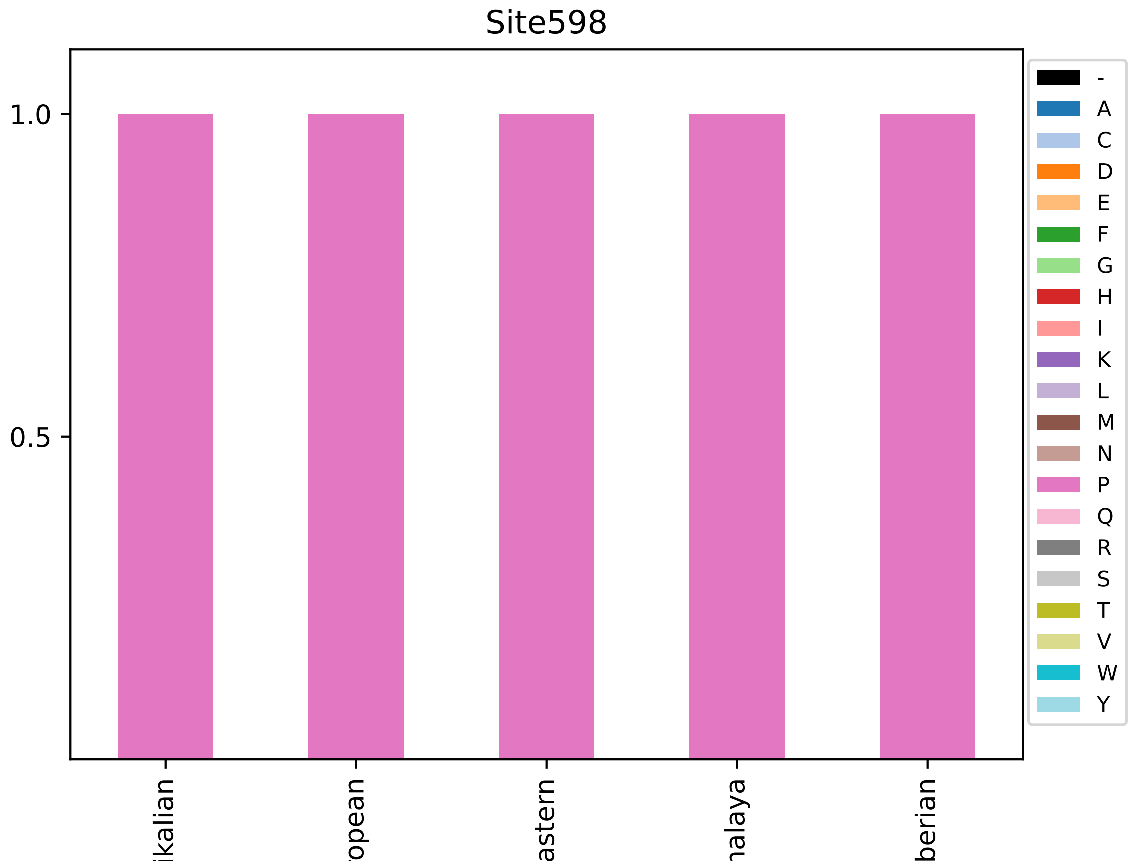
Site598
1.0
0.5
-
A
C
D
E
F
G
H
I
K
L
M
N
P
Q
R
S
T
V
W
Y
ikalian
opean
astern
nalaya
berian

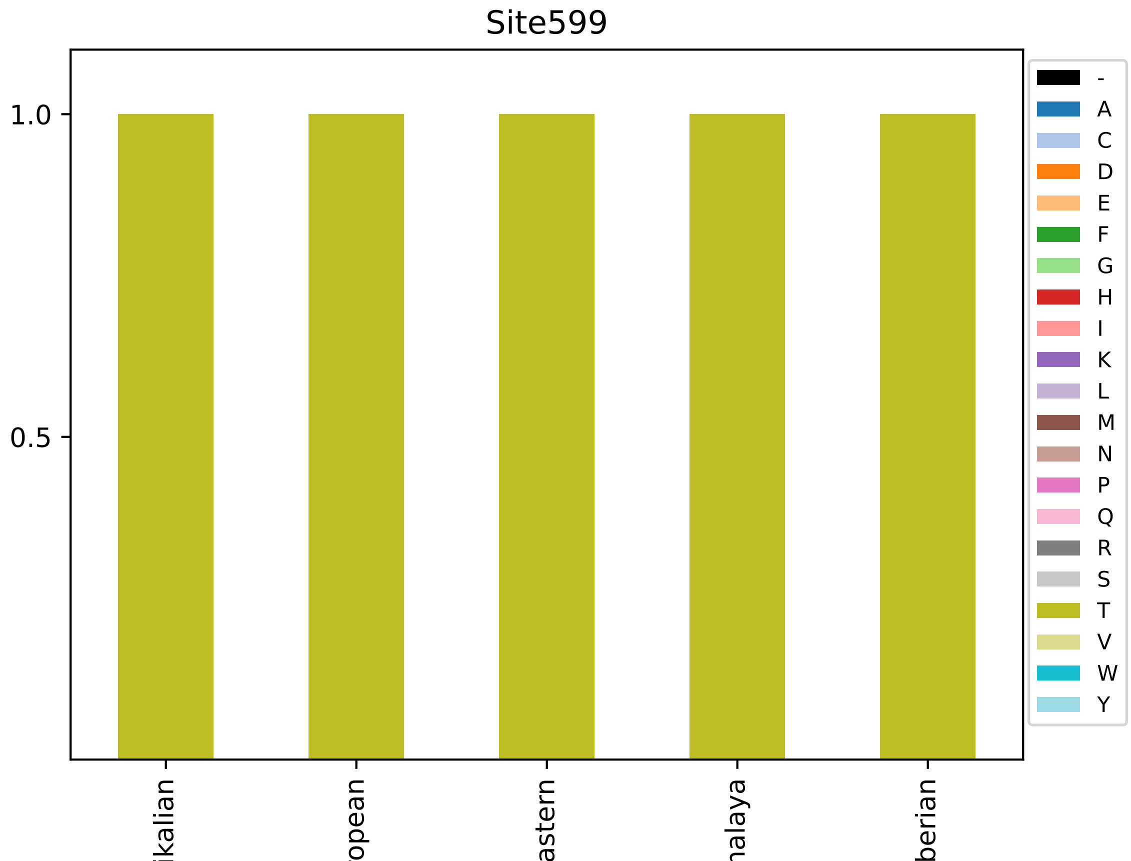

Site599
1.0
0.5
-
A
C
D
E
F
G
H
I
K
L
M
N
P
Q
R
S
T
V
W
Y
ikalian
opean
astern
nalaya
berian

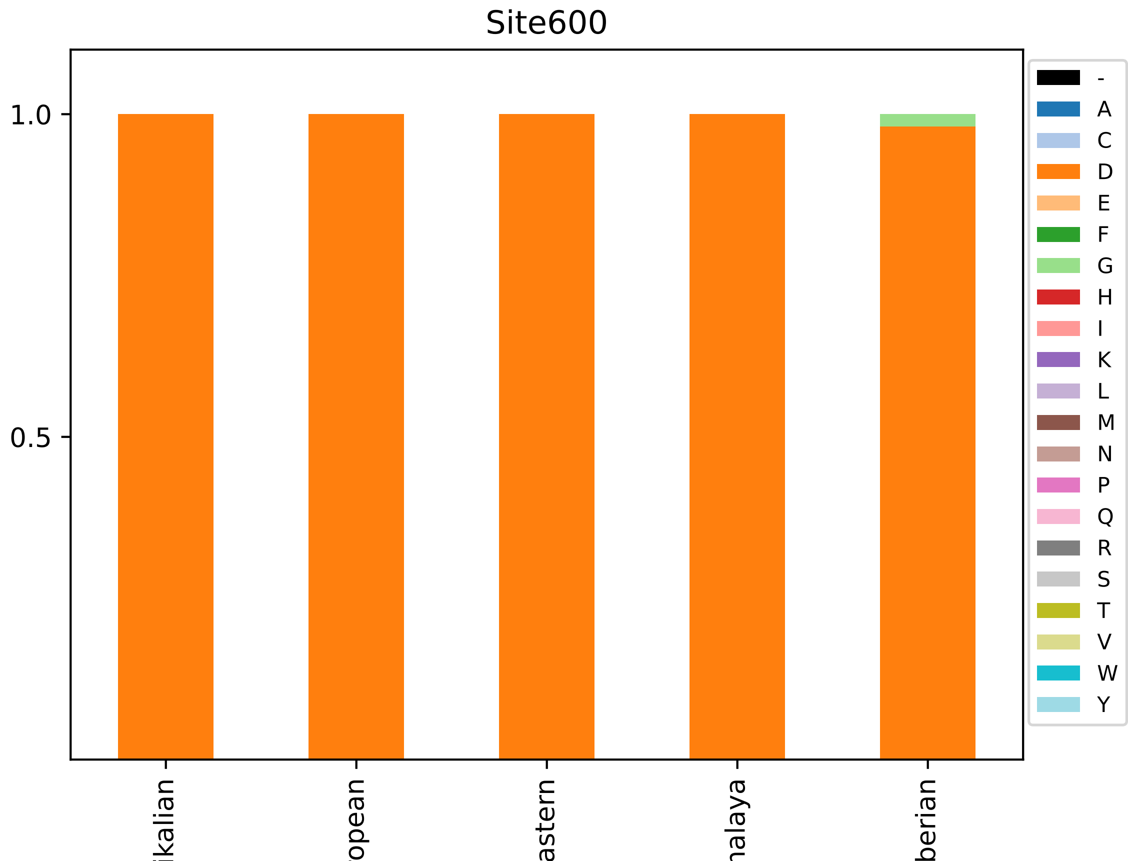
Site600
1.0
0.5
-
A
C
D
E
F
G
H
I
K
L
M
N
P
Q
R
S
T
V
W
Y
ikalian
opean
astern
nalaya
berian

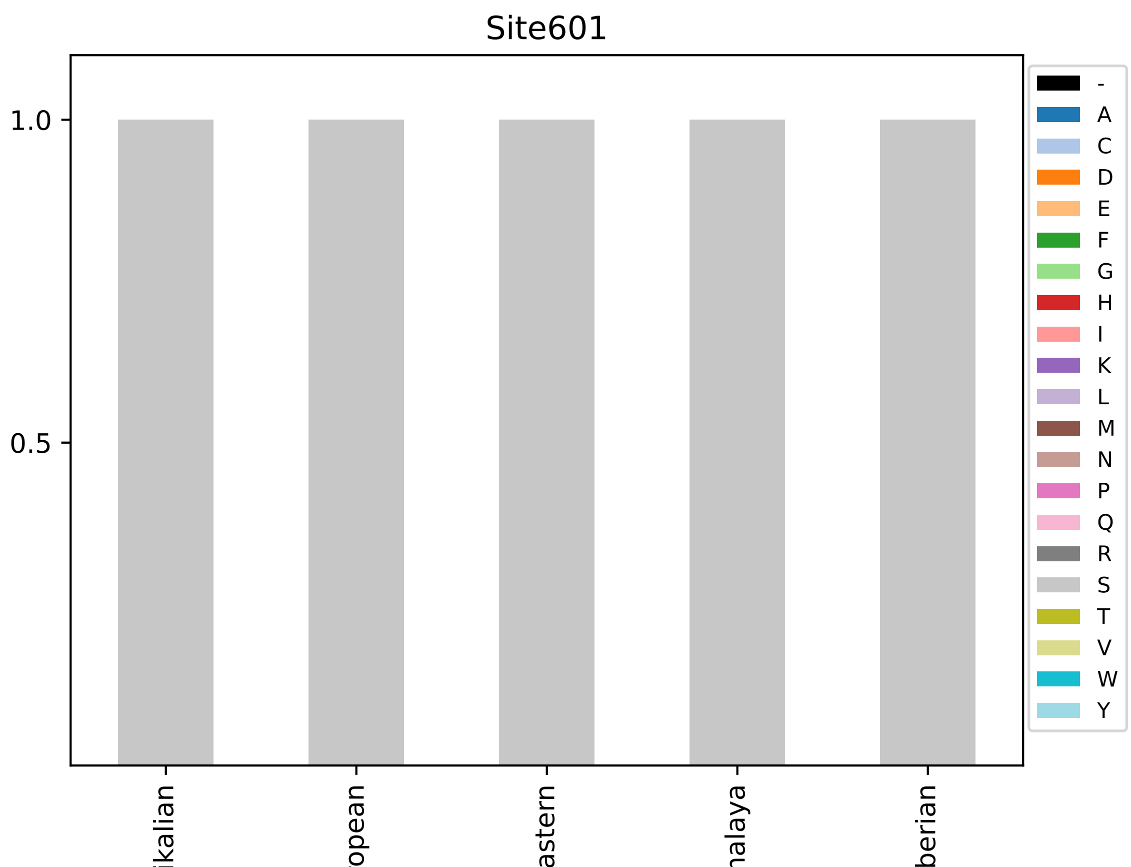
Site601
1.0
0.5
-
A
C
D
E
F
G
H
I
K
L
M
N
P
Q
R
S
T
V
W
Y
ikalian
ropean
astern
nalaya
berian

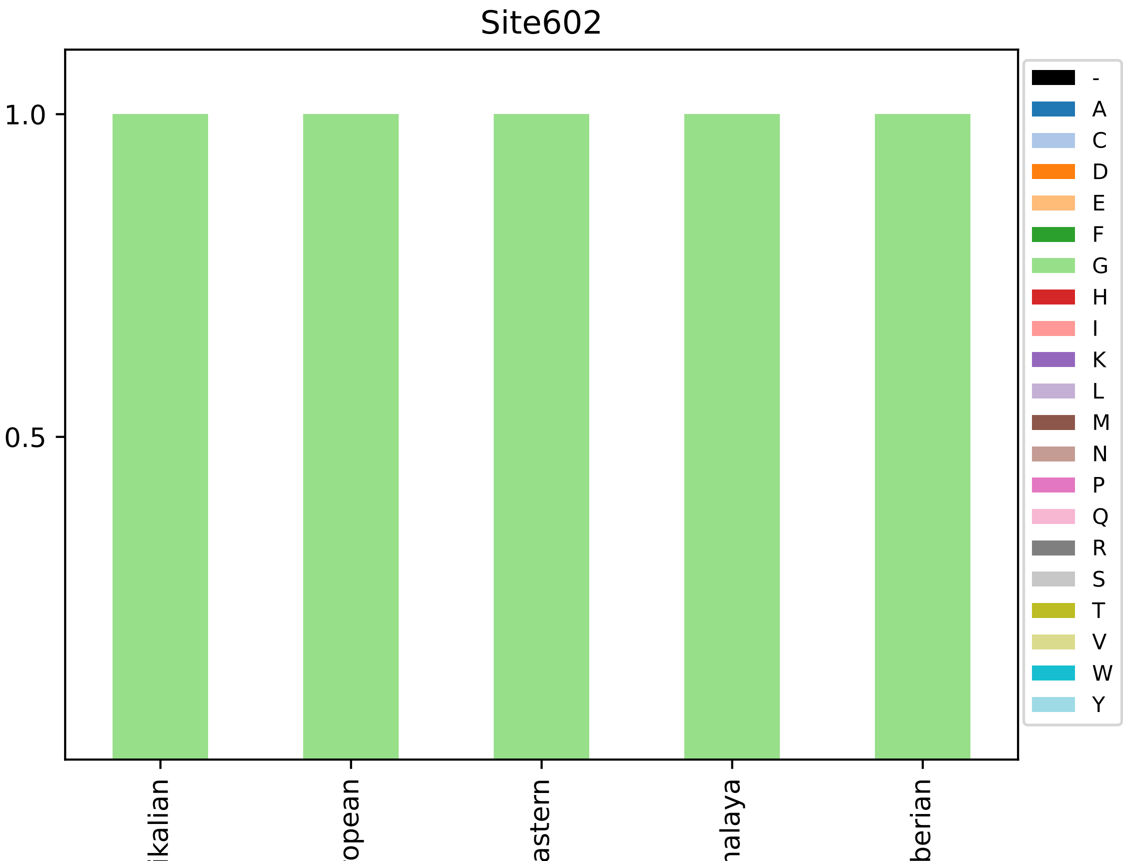

Site602
1.0
0.5
ikalian
opean
astern
nalaya
berian
-
A
C
D
E
F
G
H
I
K
L
M
N
P
Q
R
S
T
V
W
Y

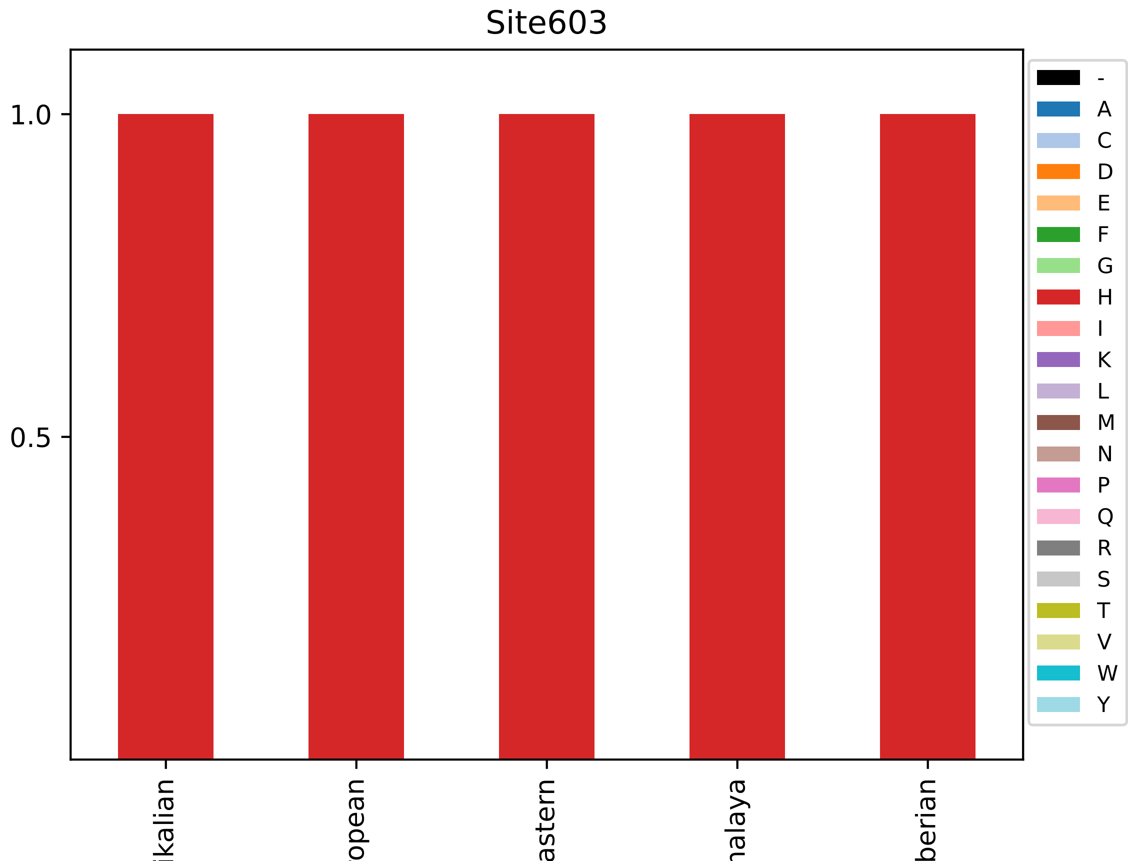
Site603
1.0
0.5
-
A
C
D
E
F
G
H
I
K
L
M
N
P
Q
R
S
T
V
W
Y
ikalian
opean
astern
nalaya
berian

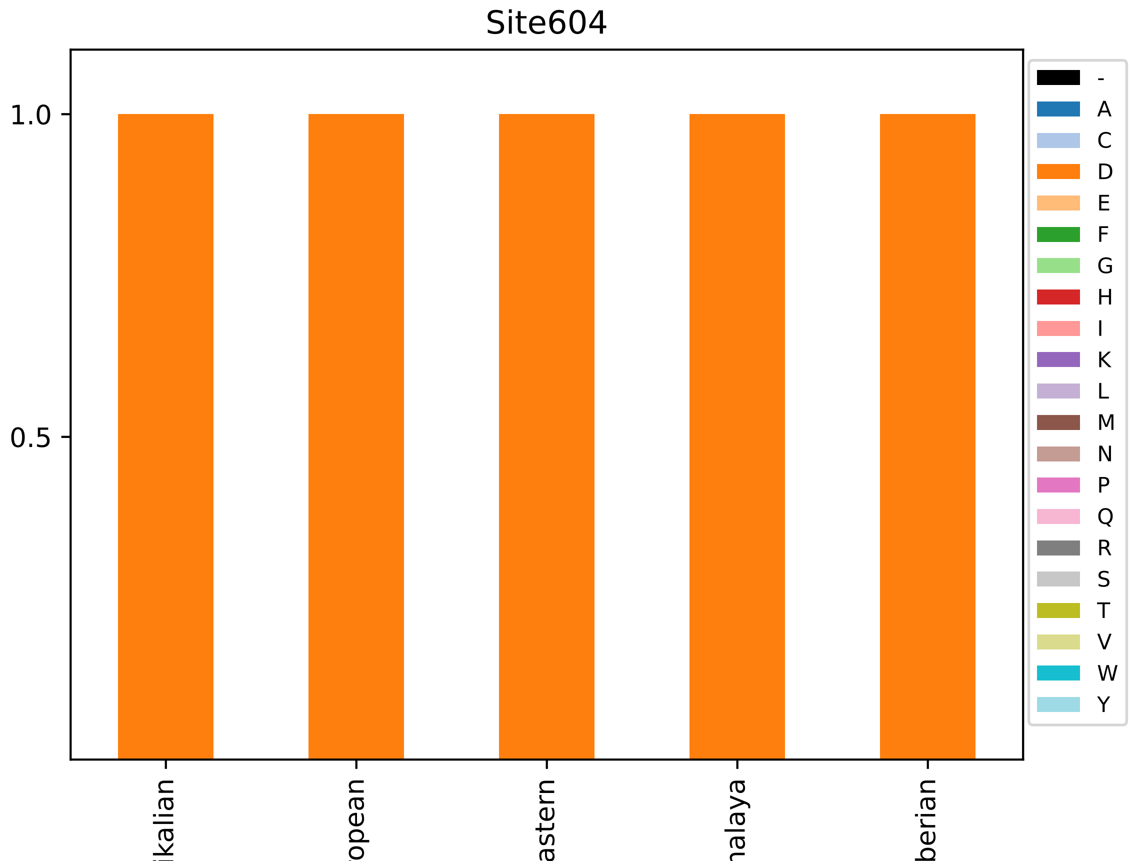

Site604
1.0
0.5
-
A
C
D
E
F
G
H
I
K
L
M
N
P
Q
R
S
T
V
W
Y
ikalian
opean
astern
nalaya
berian

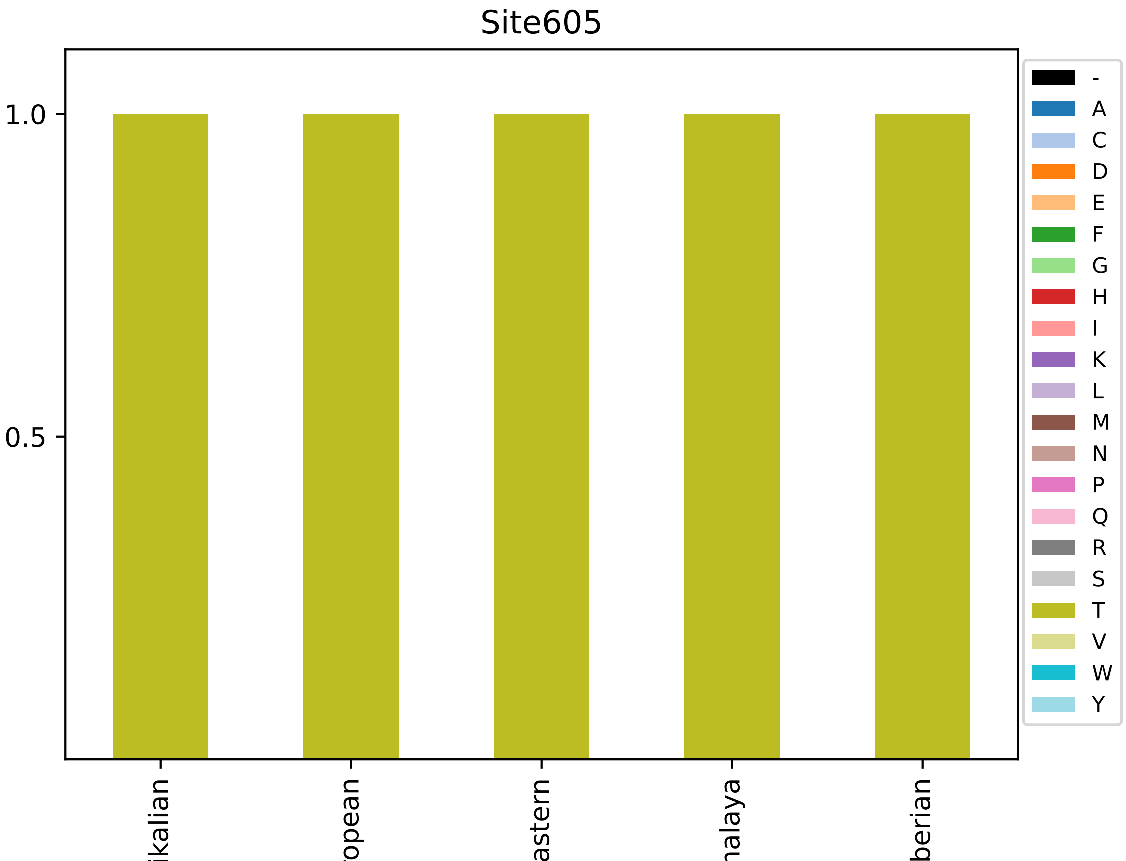
Site605
1.0
0.5
-
A
C
D
E
F
G
H
I
K
L
M
N
P
Q
R
S
T
V
W
Y
ikalian
opean
astern
nalaya
berian

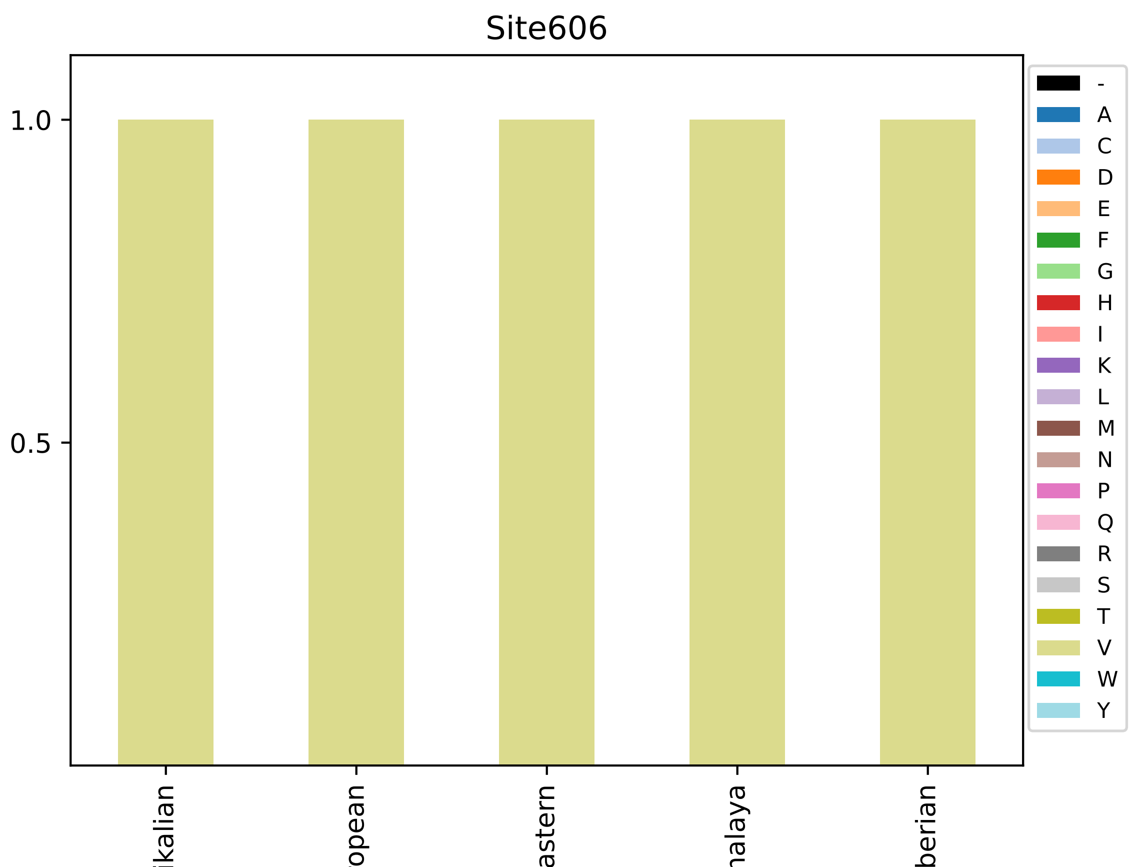
Site606
1.0
0.5
-
A
C
D
E
F
G
H
I
K
L
M
N
P
Q
R
S
T
V
W
Y
ikalian
opean
astern
nalaya
berian

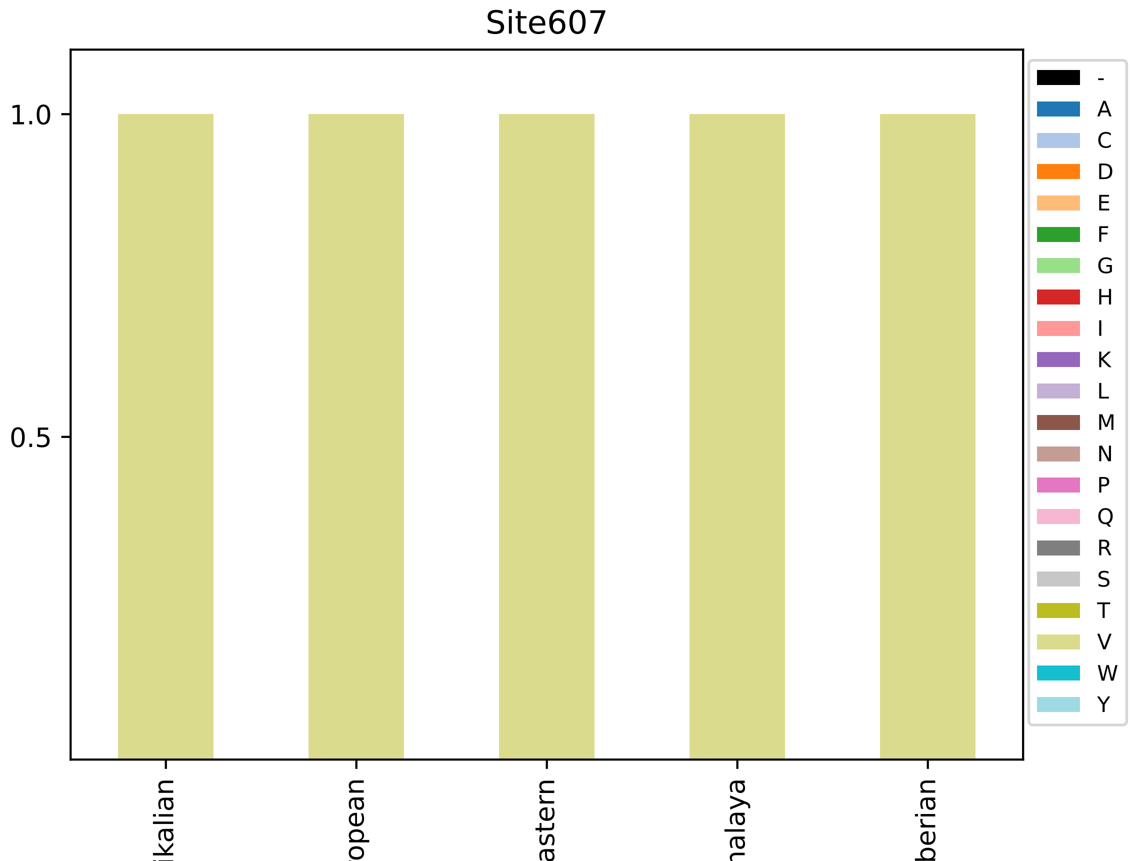

Site607
1.0
0.5
-
A
C
D
E
F
G
H
I
K
L
M
N
P
Q
R
S
T
V
W
Y
ikalian
opean
astern
nalaya
berian

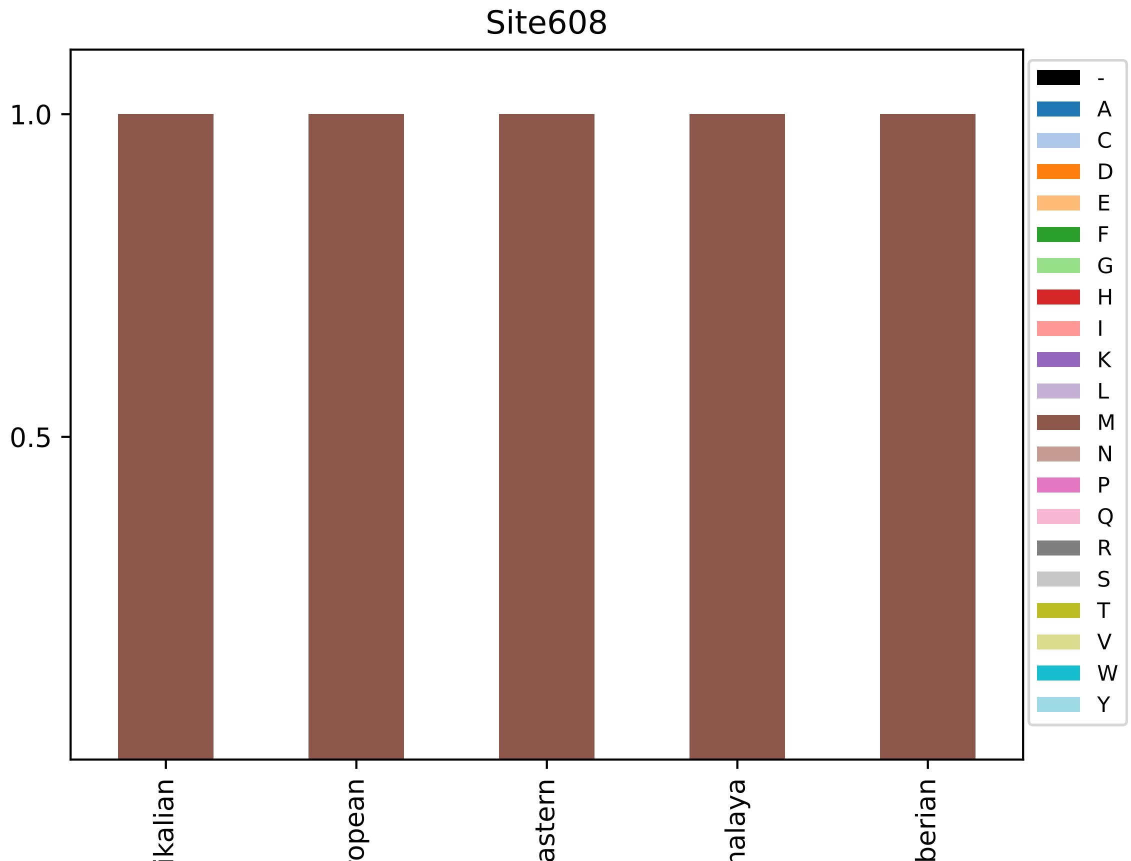
Site608
1.0
0.5
-
A
C
D
E
F
G
H
I
K
L
M
N
P
Q
R
S
T
V
W
Y
ikalian
opean
astern
nalaya
berian

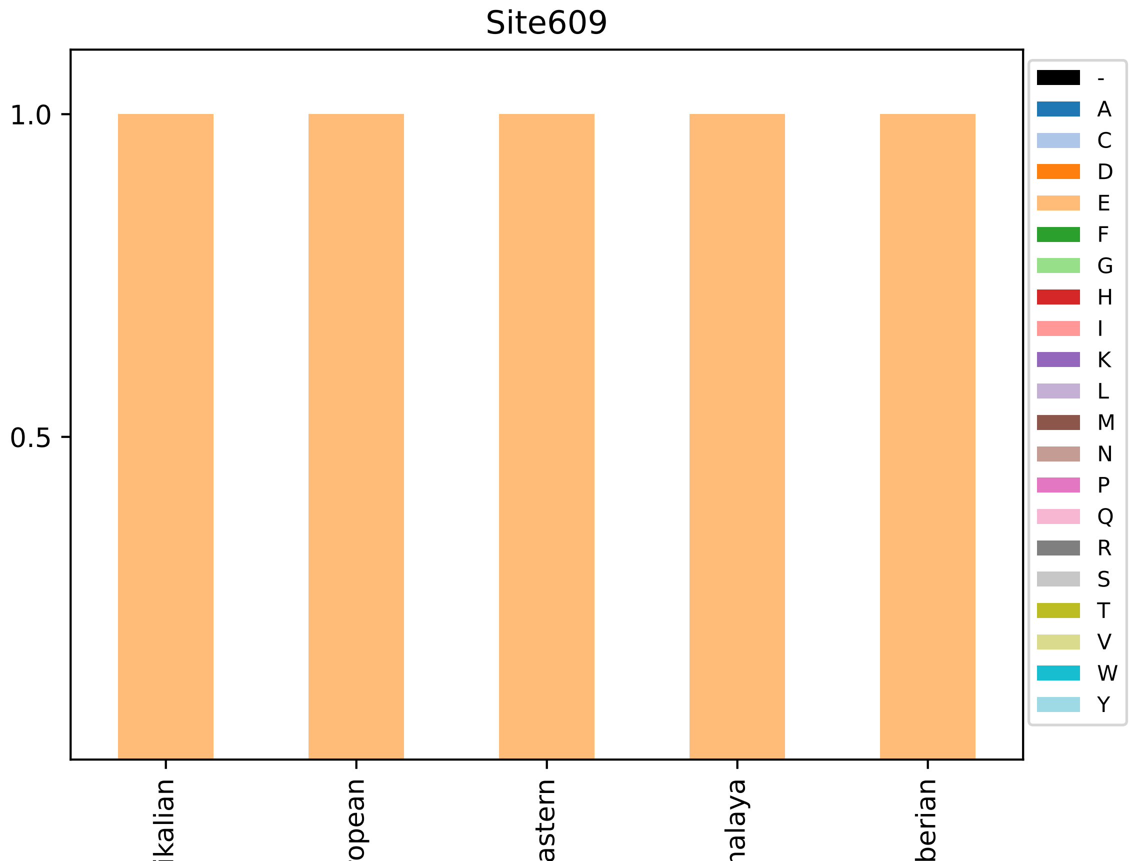
Site609
1.0
0.5
-
A
C
D
E
F
G
H
I
K
L
M
N
P
Q
R
S
T
V
W
Y
ikalian
opean
astern
nalaya
berian

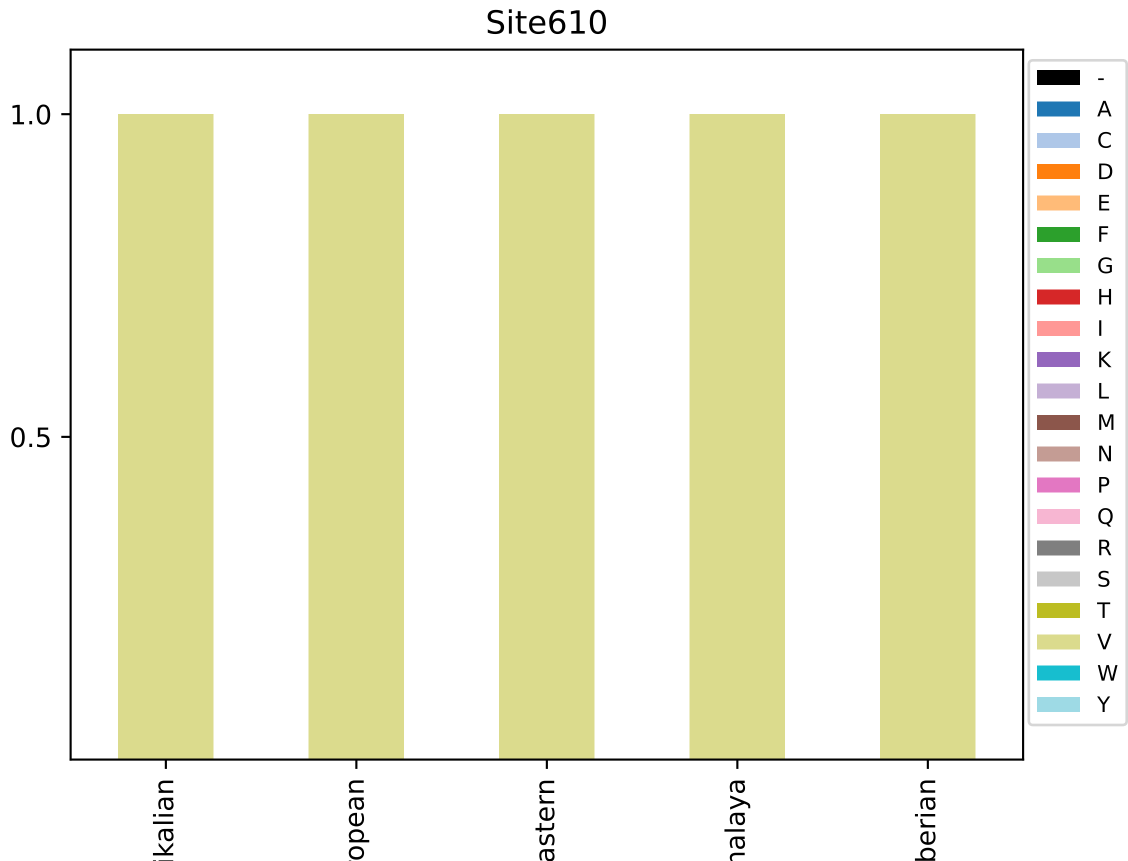
Site610
1.0
0.5
-
A
C
D
E
F
G
H
I
K
L
M
N
P
Q
R
S
T
V
W
Y
ikalian
opean
astern
nalaya
berian

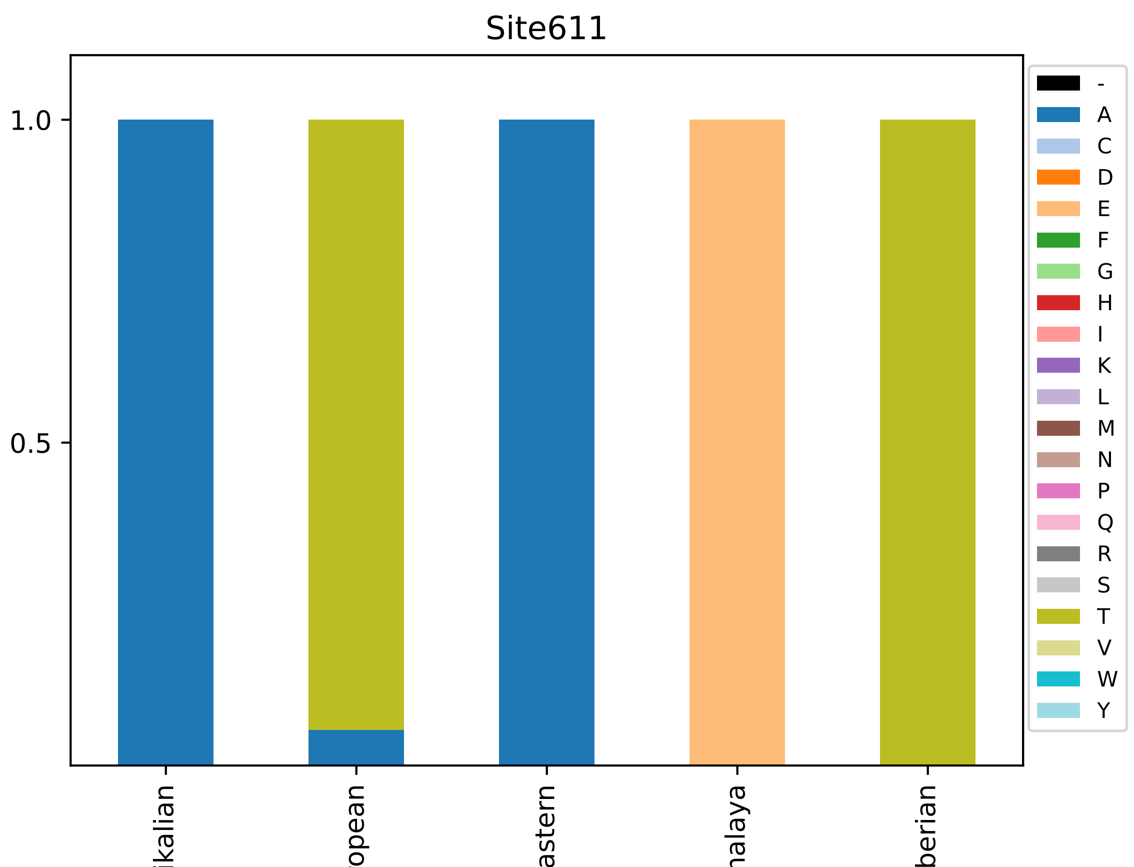
Site611
1.0
0.5
-
A
C
D
E
F
G
H
I
K
L
M
N
P
Q
R
S
T
V
W
Y
ikalian
opean
astern
nalaya
berian

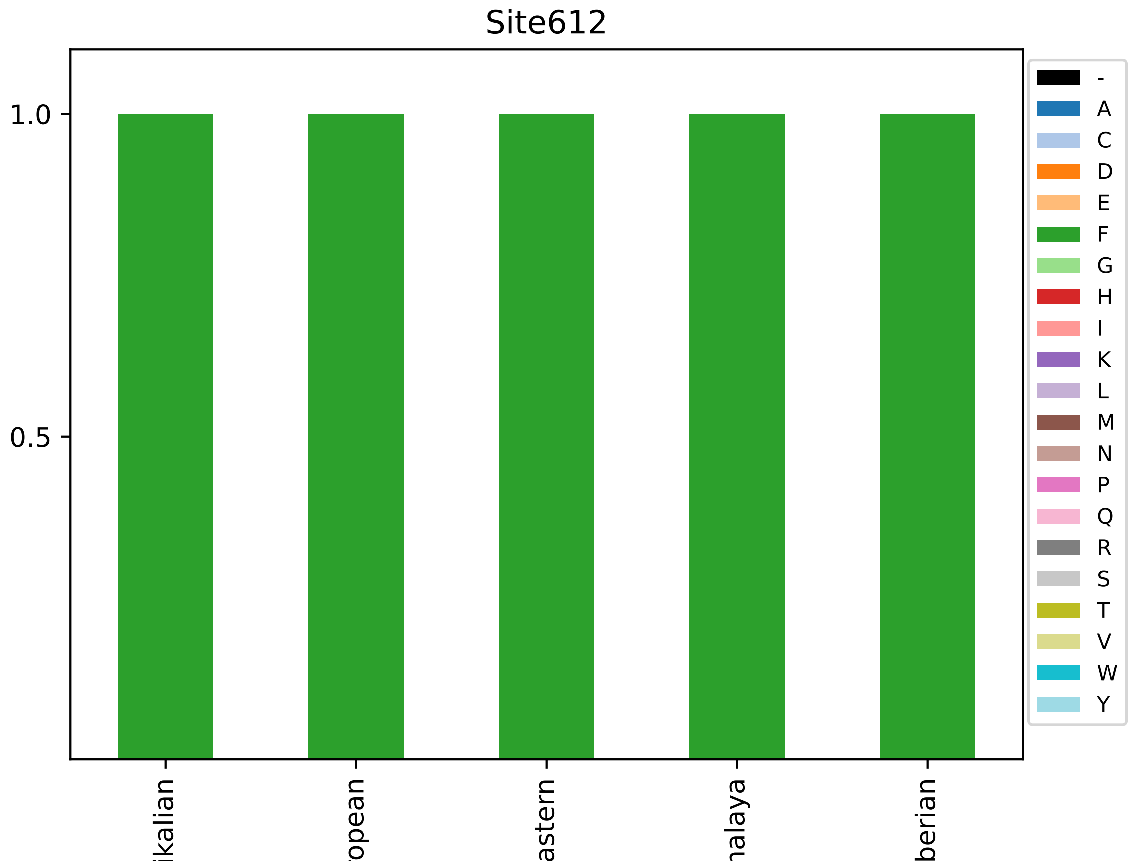
Site612
1.0
0.5
ikalian
opean
astern
nalaya
berian
-
A
C
D
E
F
G
H
I
K
L
M
N
P
Q
R
S
T
V
W
Y

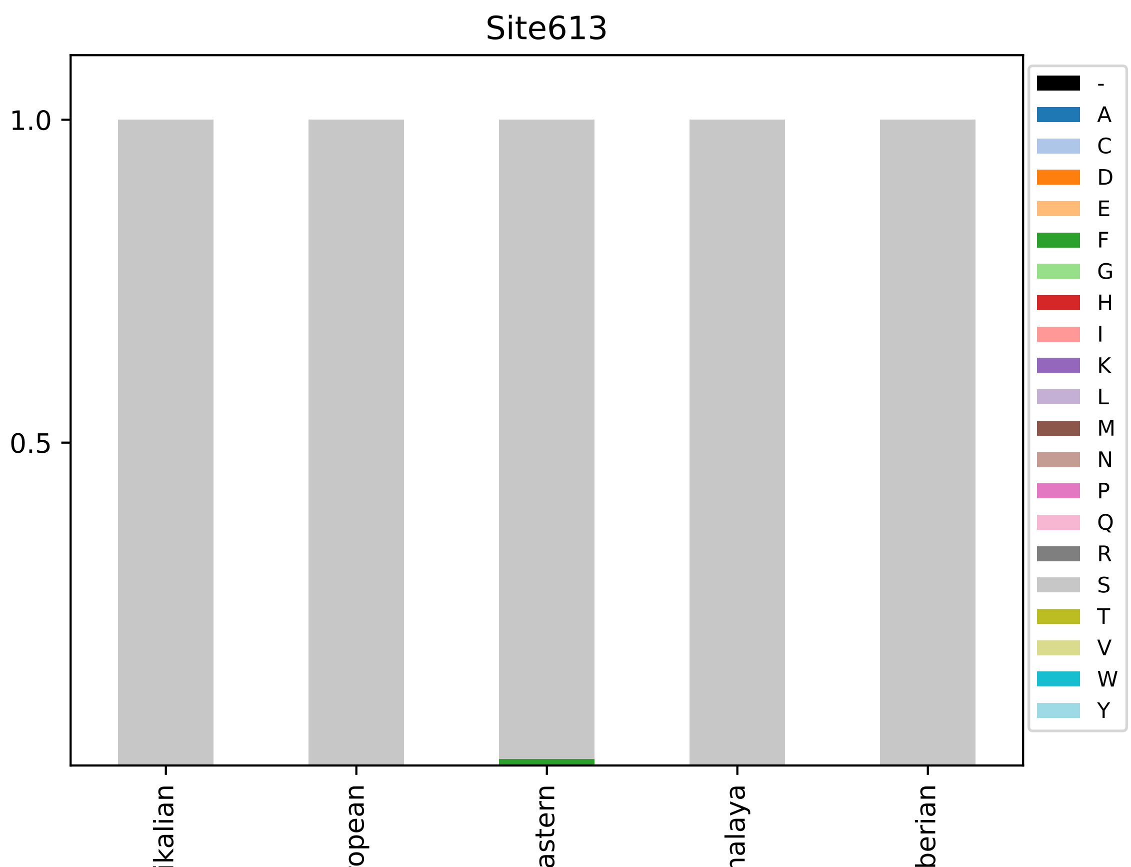

Site613
1.0
0.5
-
A
C
D
E
F
G
H
I
K
L
M
N
P
Q
R
S
T
V
W
Y
ikalian
opean
astern
nalaya
berian

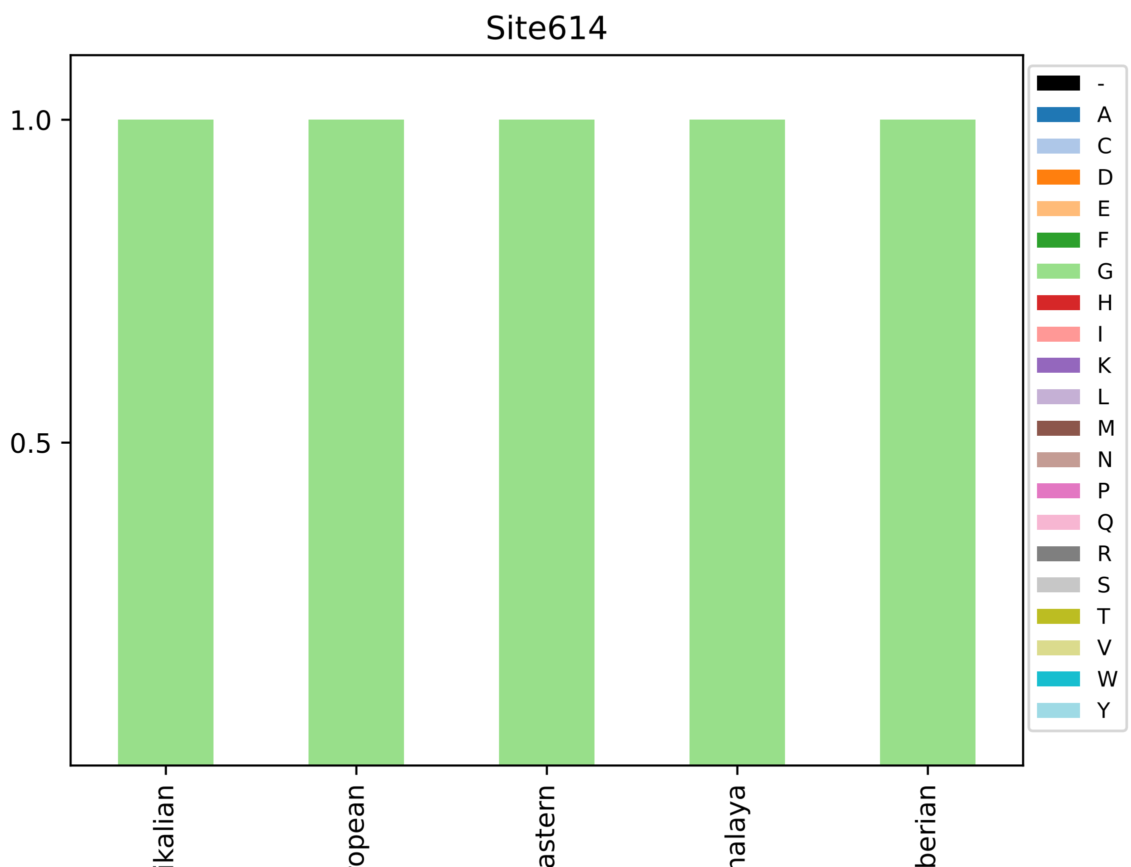
Site614
1.0
0.5
-
A
C
D
E
F
G
H
I
K
L
M
N
P
Q
R
S
T
V
W
Y
ikalian
opean
astern
nalaya
berian

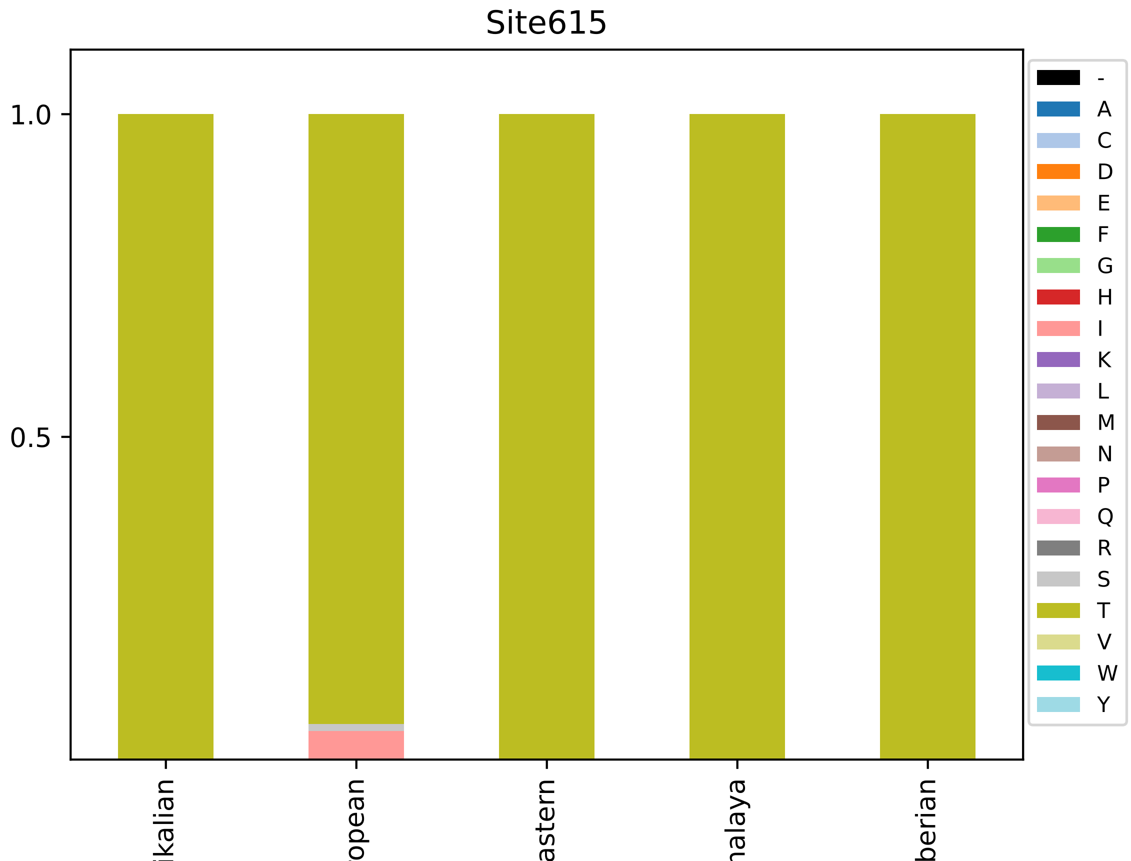
Site615
1.0
0.5
-
A
C
D
E
F
G
H
I
K
L
M
N
P
Q
R
S
T
V
W
Y

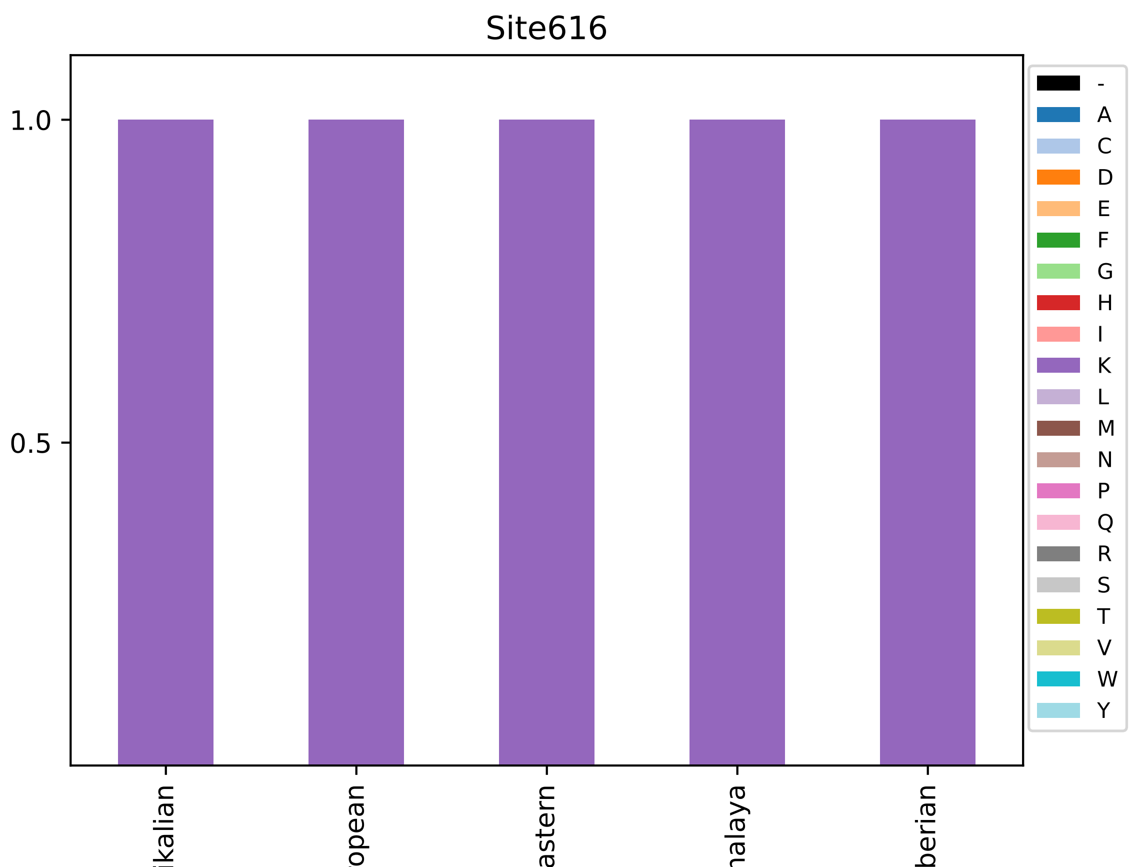

Site616
1.0
0.5
-
A
C
D
E
F
G
H
I
K
L
M
N
P
Q
R
S
T
V
W
Y
ikalian
opean
astern
nalaya
berian

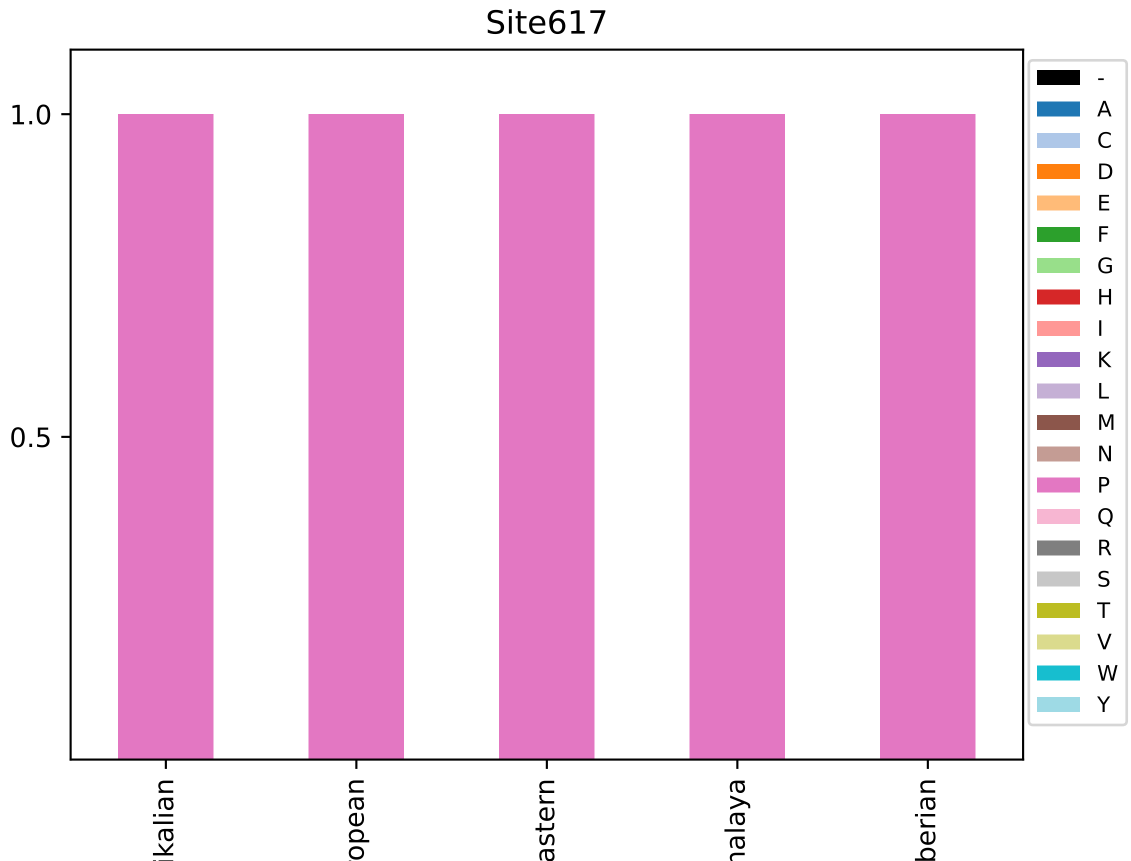
Site617
1.0
0.5
-
A
C
D
E
F
G
H
I
K
L
M
N
P
Q
R
S
T
V
W
Y
ikalian
opean
astern
nalaya
berian

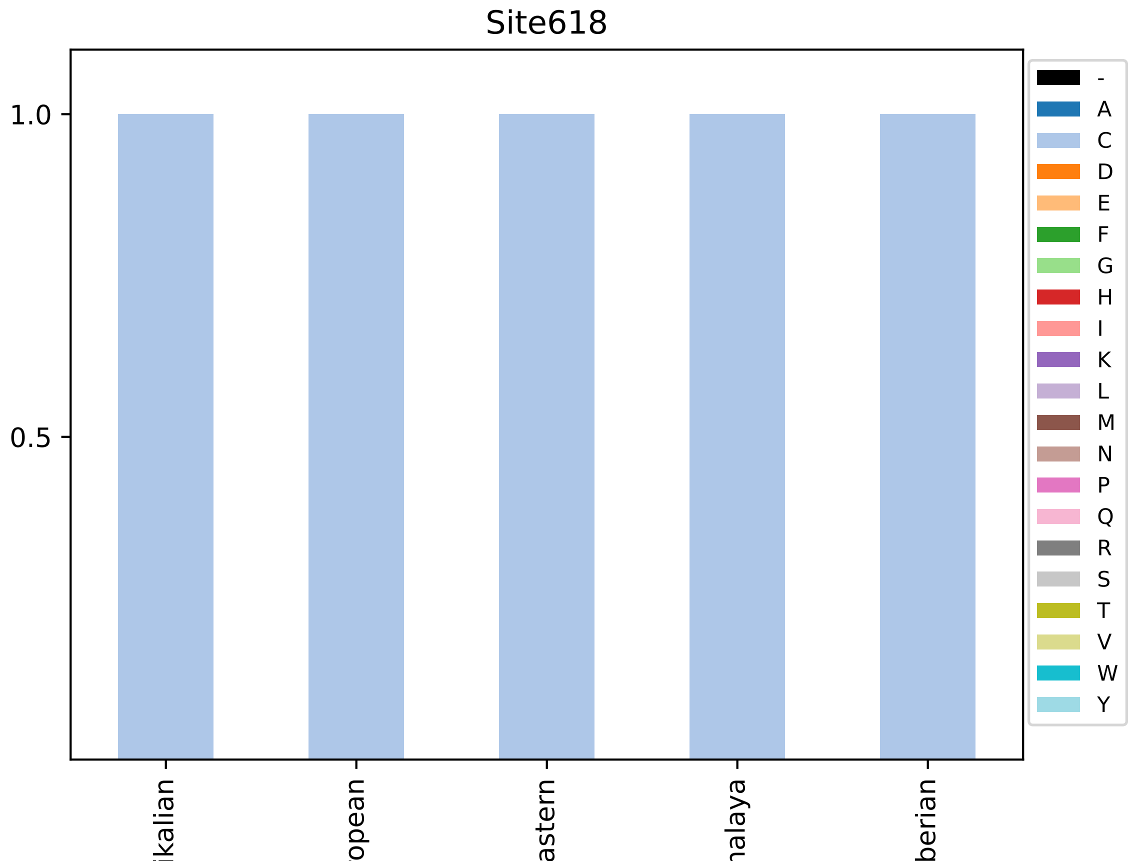
Site618
1.0
0.5
-
A
C
D
E
F
G
H
I
K
L
M
N
P
Q
R
S
T
V
W
Y
ikalian
opean
astern
nalaya
berian

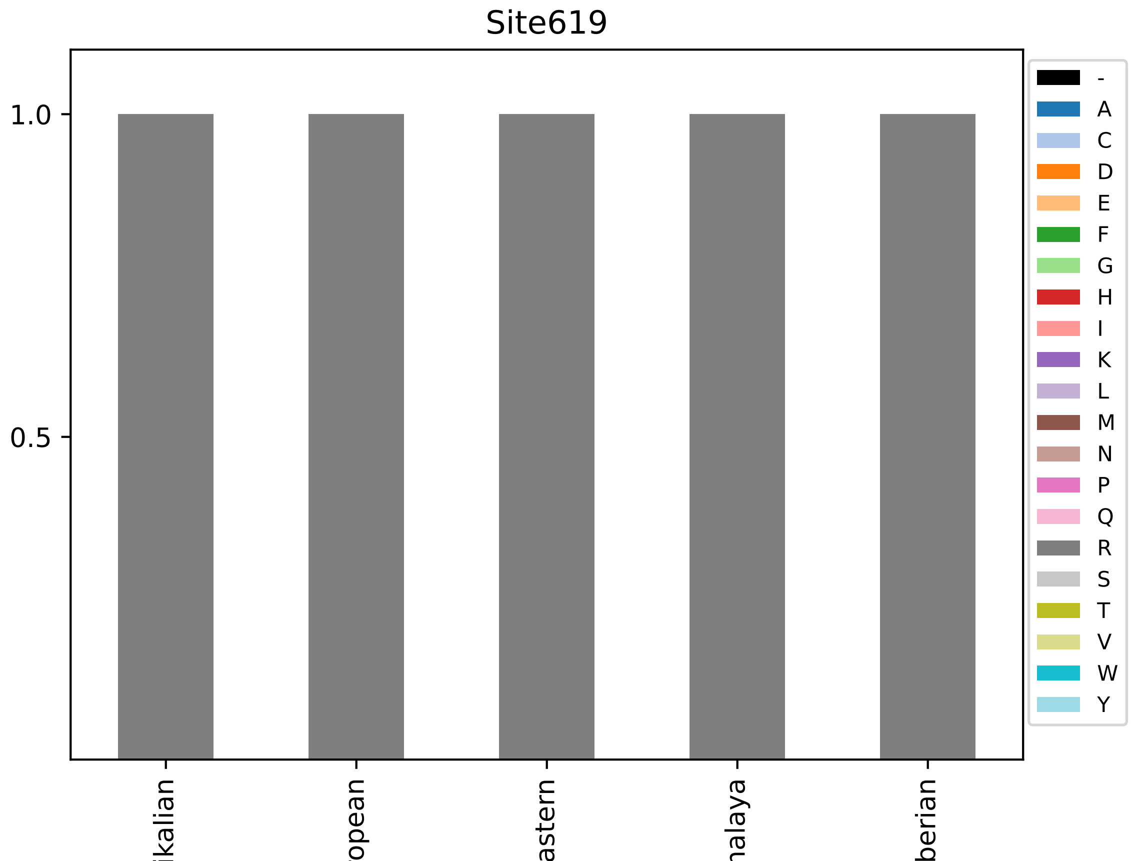
Site619
1.0
0.5
-
A
C
D
E
F
G
H
I
K
L
M
N
P
Q
R
S
T
V
W
Y
ikalian
opean
astern
nalaya
berian

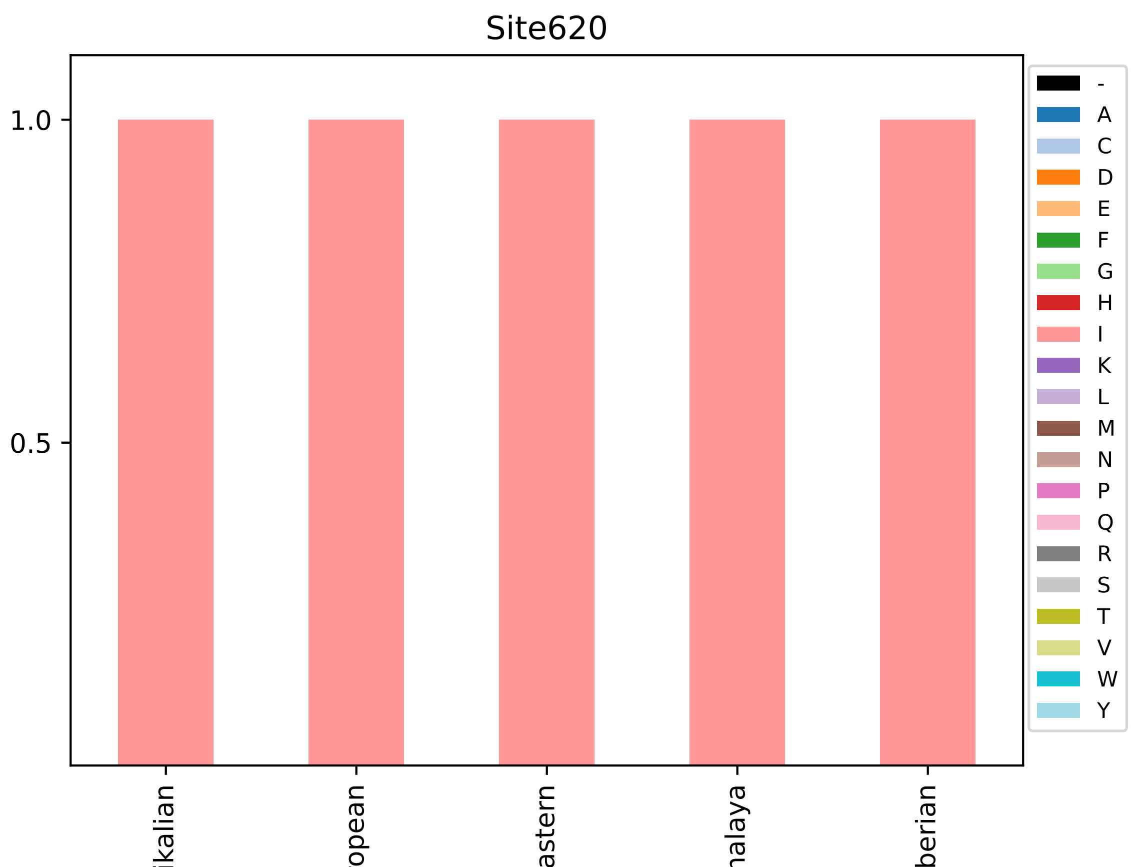
Site620
1.0
0.5
-
A
C
D
E
F
G
H
I
K
L
M
N
P
Q
R
S
T
V
W
Y
ikalian
opean
astern
nalaya
berian

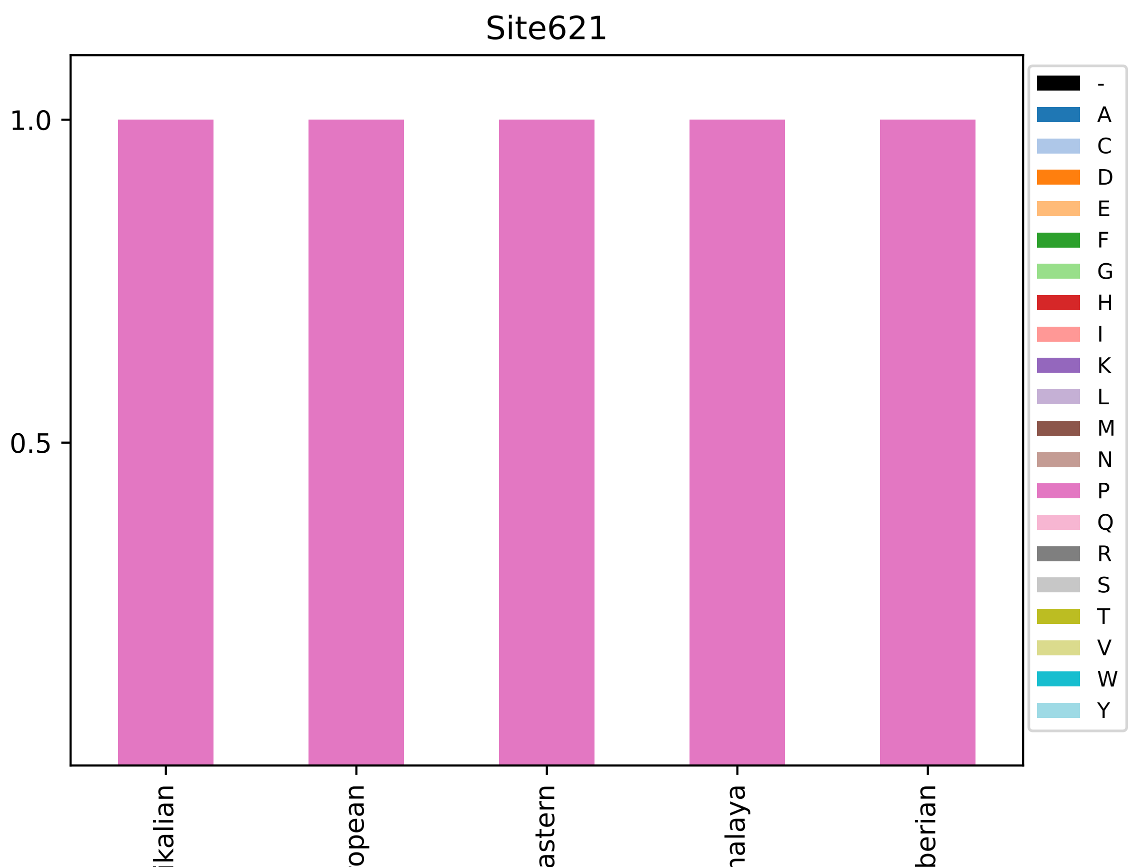
Site621
1.0
0.5
-
A
C
D
E
F
G
H
I
K
L
M
N
P
Q
R
S
T
V
W
Y
ikalian
opean
astern
nalaya
berian

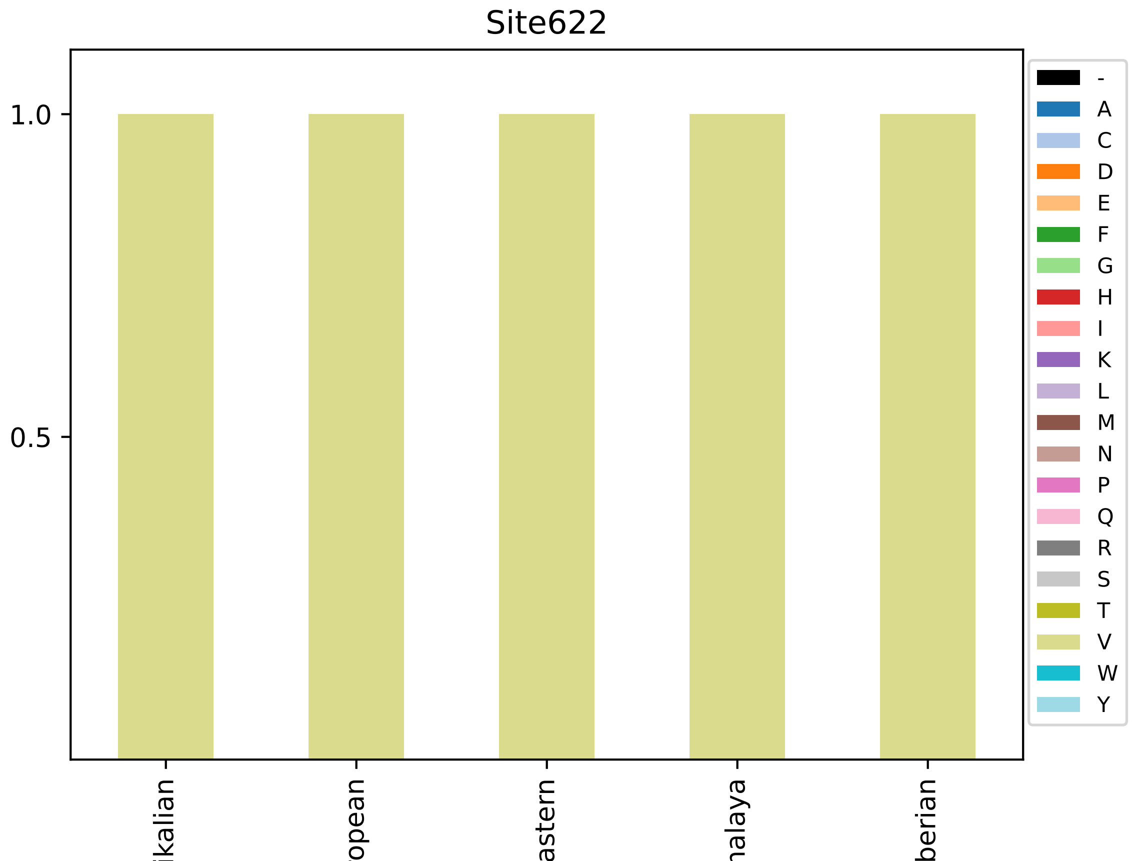

Site622
1.0
0.5
-
A
C
D
E
F
G
H
I
K
L
M
N
P
Q
R
S
T
V
W
Y
ikalian
opean
astern
nalaya
berian

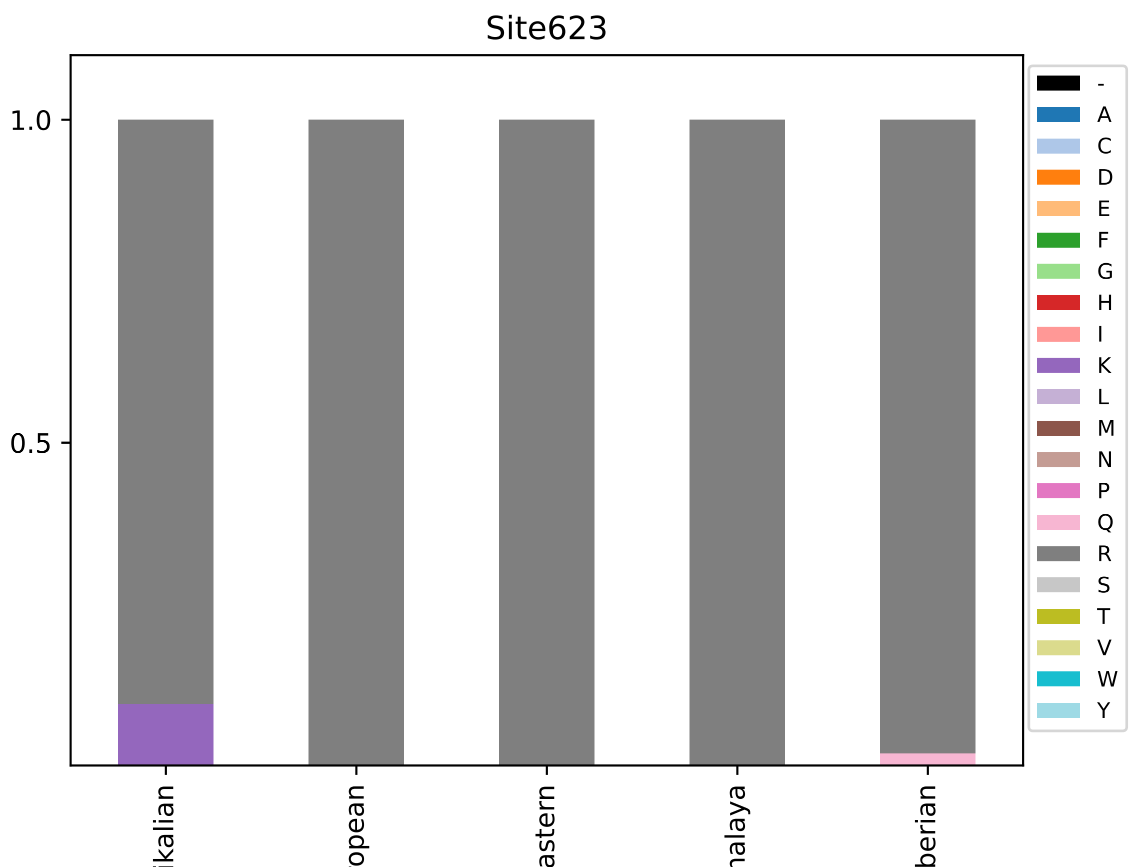

Site623
1.0
0.5
-
A
C
D
E
F
G
H
I
K
L
M
N
P
Q
R
S
T
V
W
Y
ikalian
opean
astern
nalaya
berian

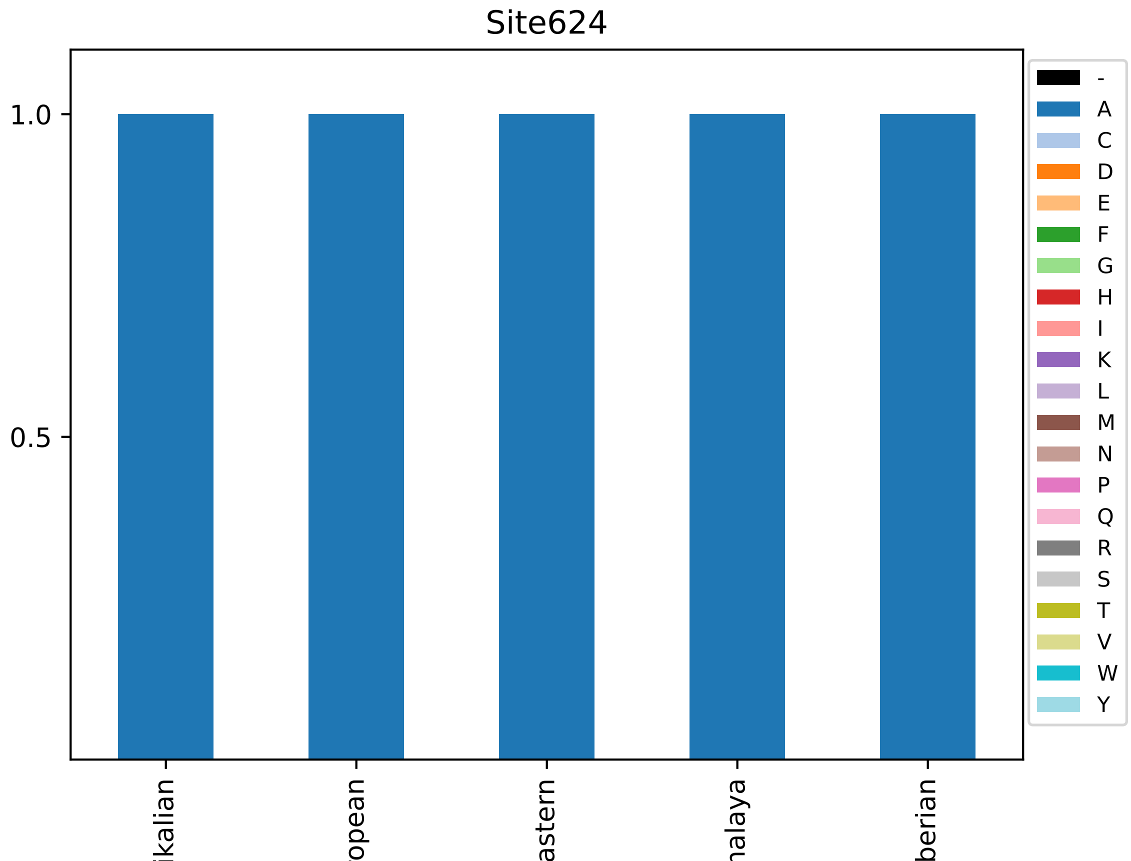
Site624
1.0
0.5
-
A
C
D
E
F
G
H
I
K
L
M
N
P
Q
R
S
T
V
W
Y
ikalian
opean
astern
nalaya
berian

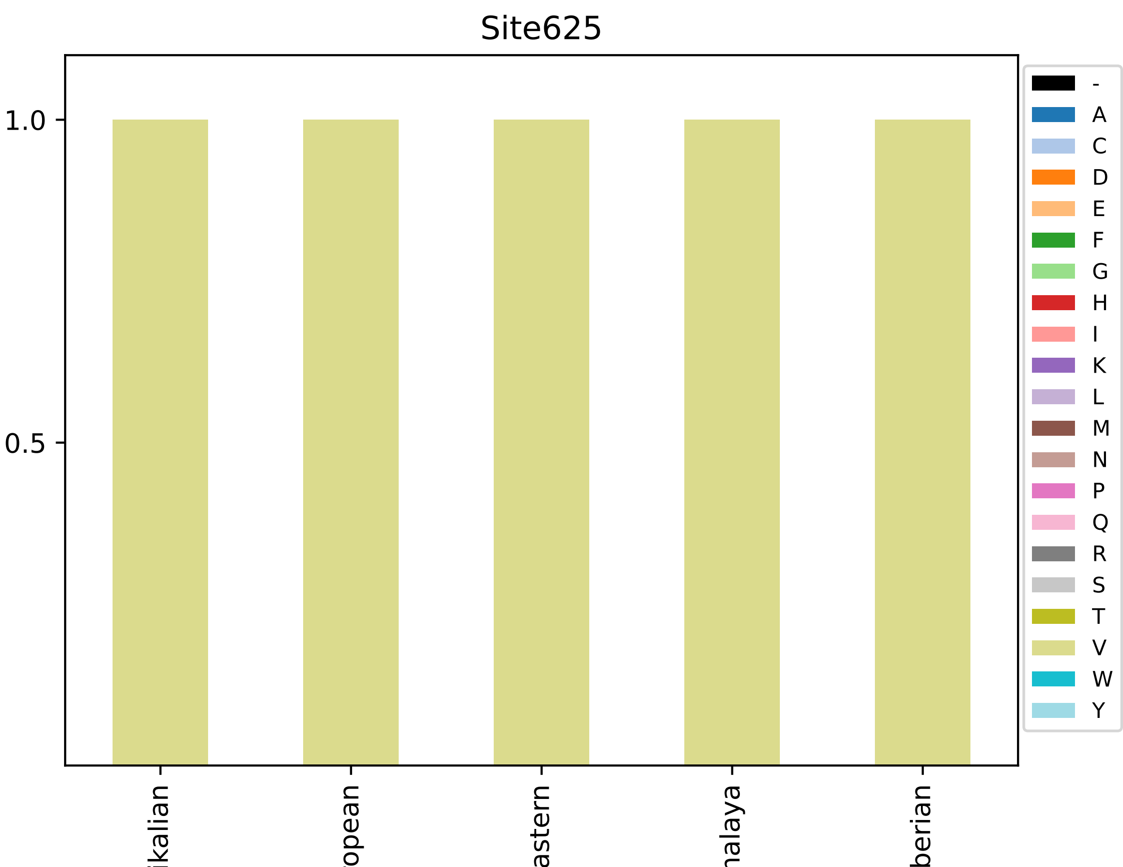

Site625
1.0
0.5
-
A
C
D
E
F
G
H
I
K
L
M
N
P
Q
R
S
T
V
W
Y
ikalian
opean
astern
nalaya
berian

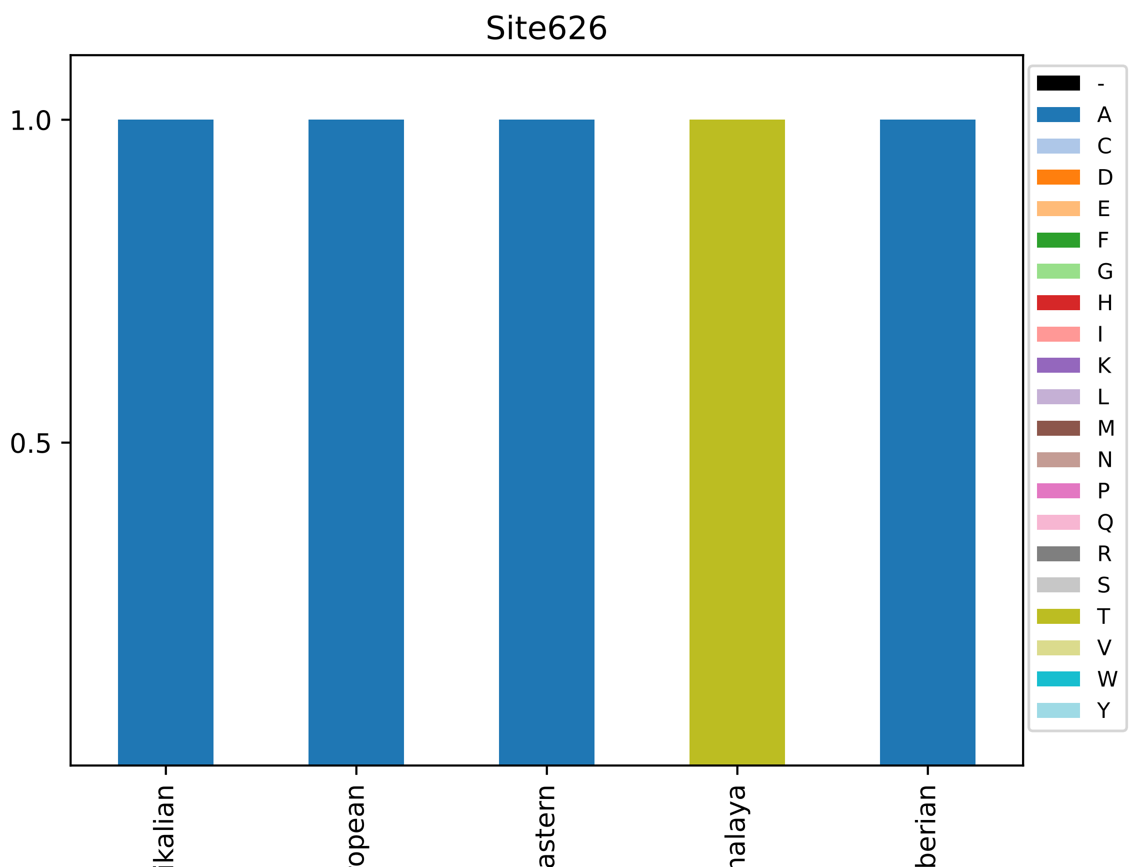
Site626
1.0
0.5
-
A
C
D
E
F
G
H
I
K
L
M
N
P
Q
R
S
T
V
W
Y
ikalian
opean
astern
halaya
berian

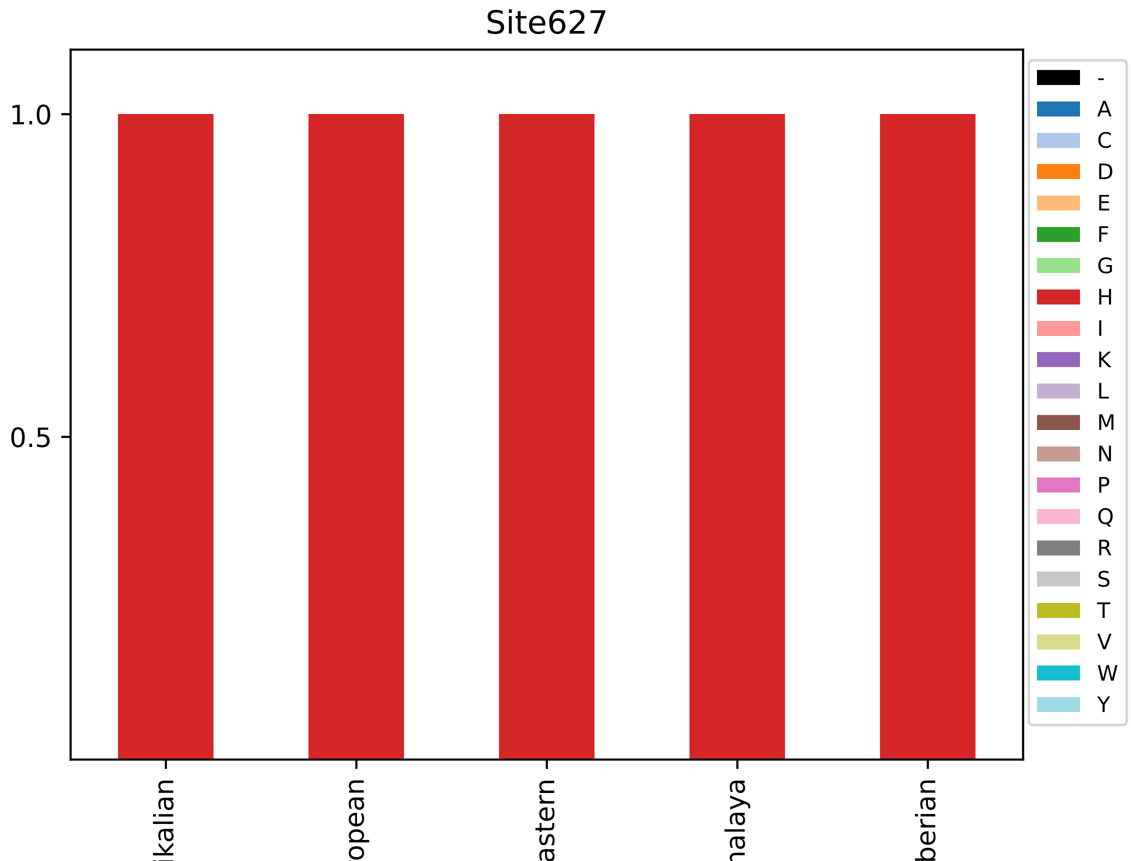

Site627
1.0
0.5
ikalian
opean
astern
nalaya
berian
-
A
C
D
E
F
G
H
I
K
L
M
N
P
Q
R
S
T
V
W
Y

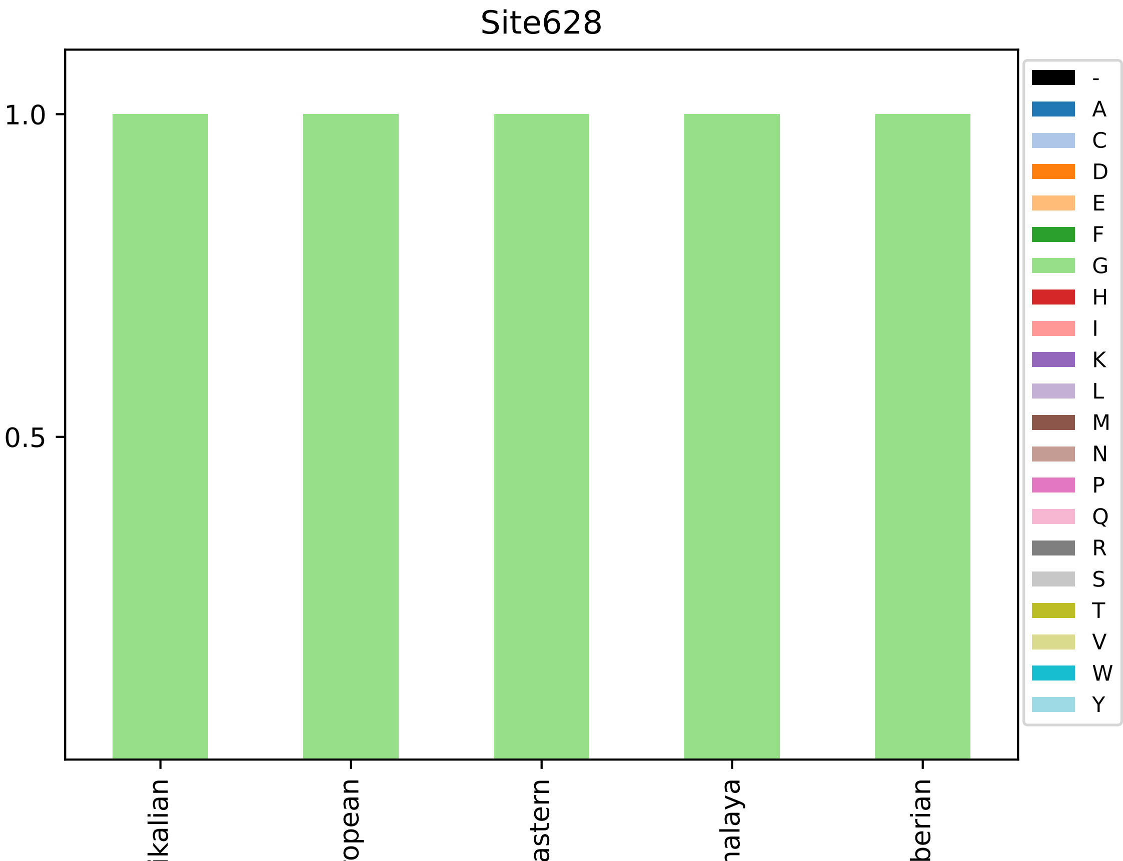

Site628
1.0
0.5
ikalian
opean
astern
nalaya
berian
-
A
C
D
E
F
G
H
I
K
L
M
N
P
Q
R
S
T
V
W
Y

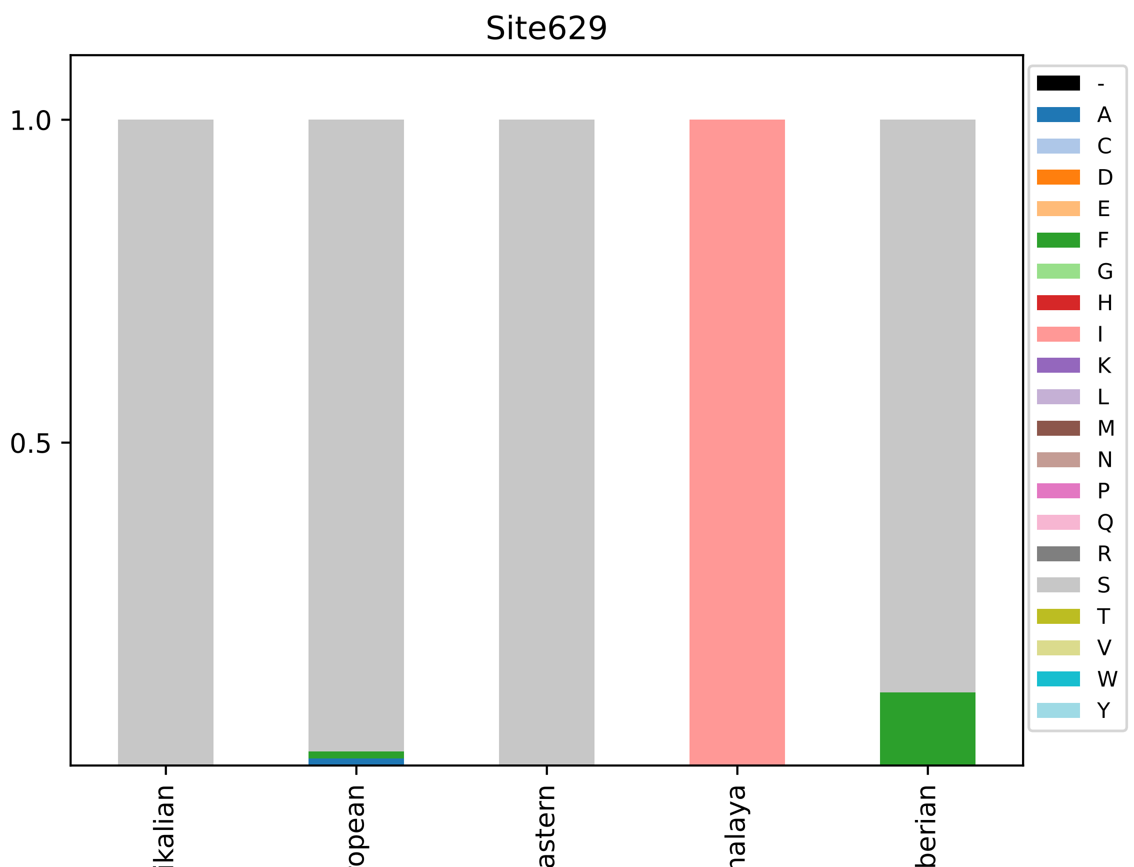

Site629
1.0
0.5
-
A
C
D
E
F
G
H
I
K
L
M
N
P
Q
R
S
T
V
W
Y
ikalian
opean
astern
nalaya
berian

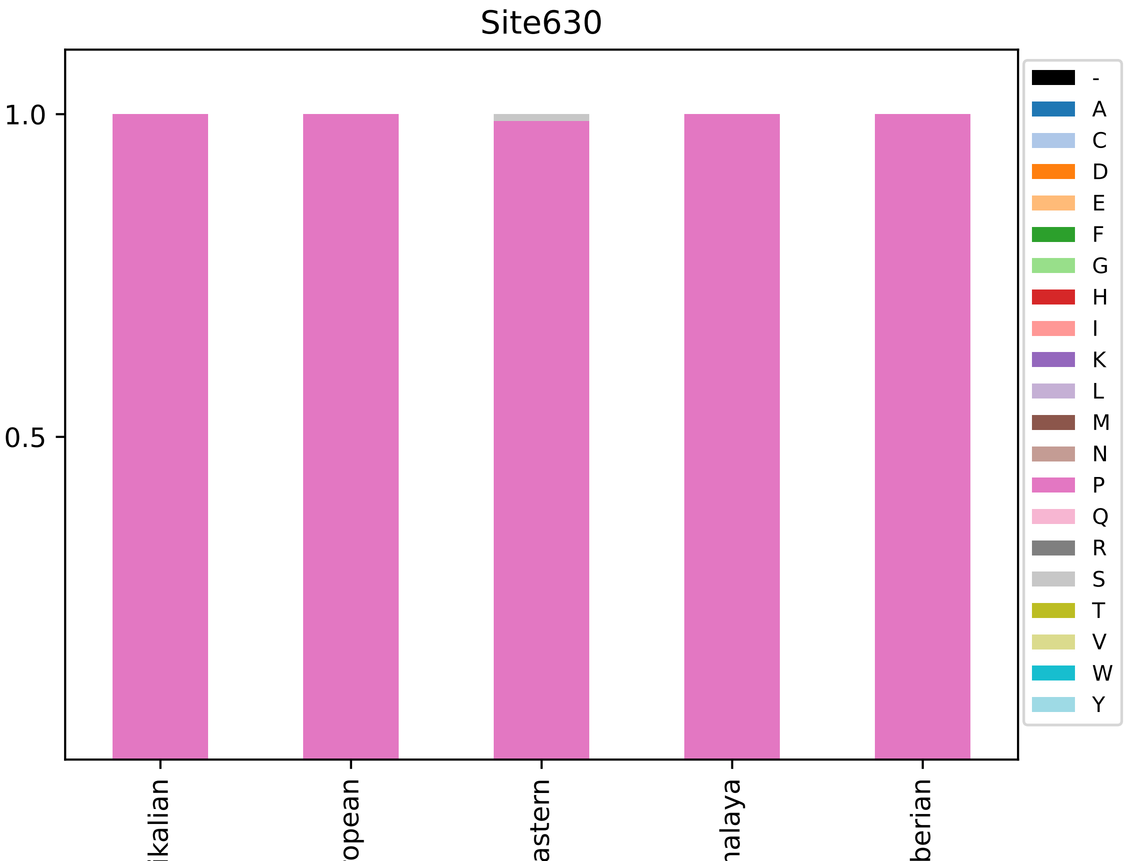
Site630
1.0
0.5
-
A
C
D
E
F
G
H
I
K
L
M
N
P
Q
R
S
T
V
W
Y

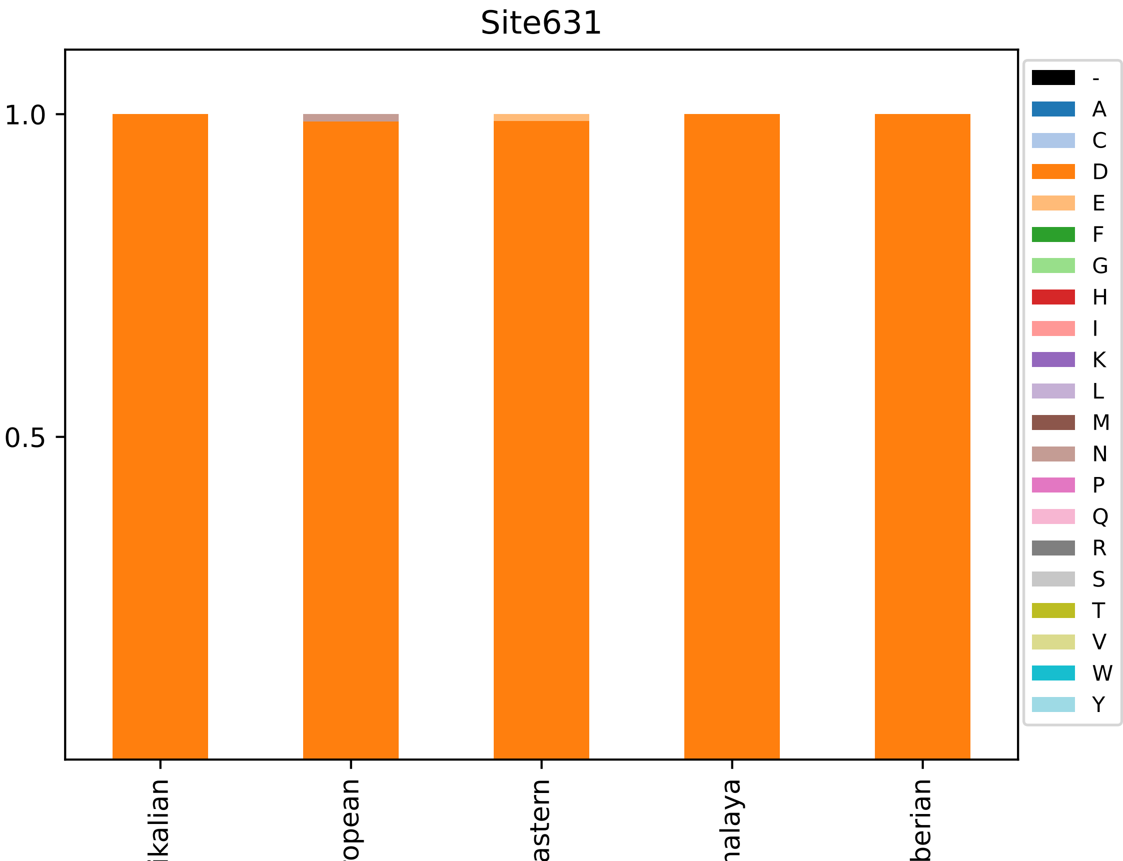
Site631
1.0
0.5
-
A
C
D
E
F
G
H
I
K
L
M
N
P
Q
R
S
T
V
W
Y
ikalian
opean
astern
nalaya
berian

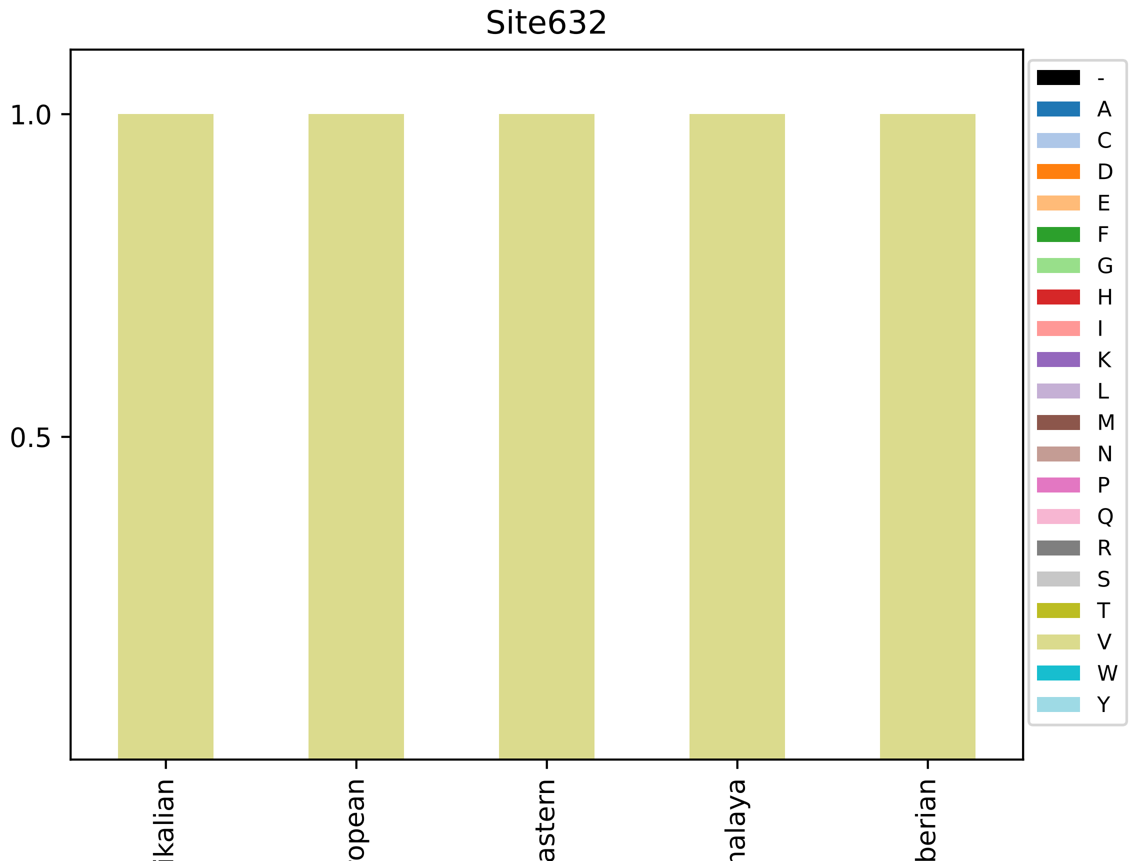
Site632
1.0
0.5
-
A
C
D
E
F
G
H
I
K
L
M
N
P
Q
R
S
T
V
W
Y
ikalian
opean
astern
nalaya
berian

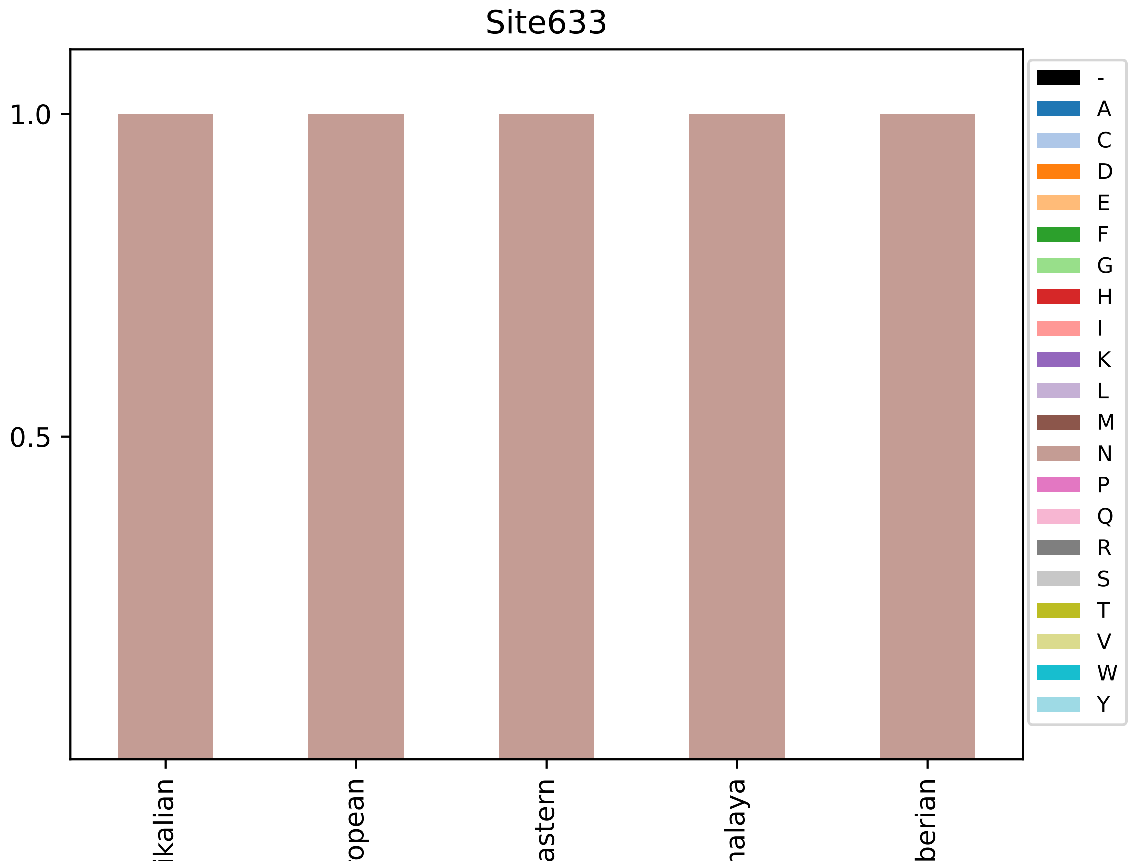

Site633
1.0
0.5
-
A
C
D
E
F
G
H
I
K
L
M
N
P
Q
R
S
T
V
W
Y
ikalian
opean
astern
nalaya
berian

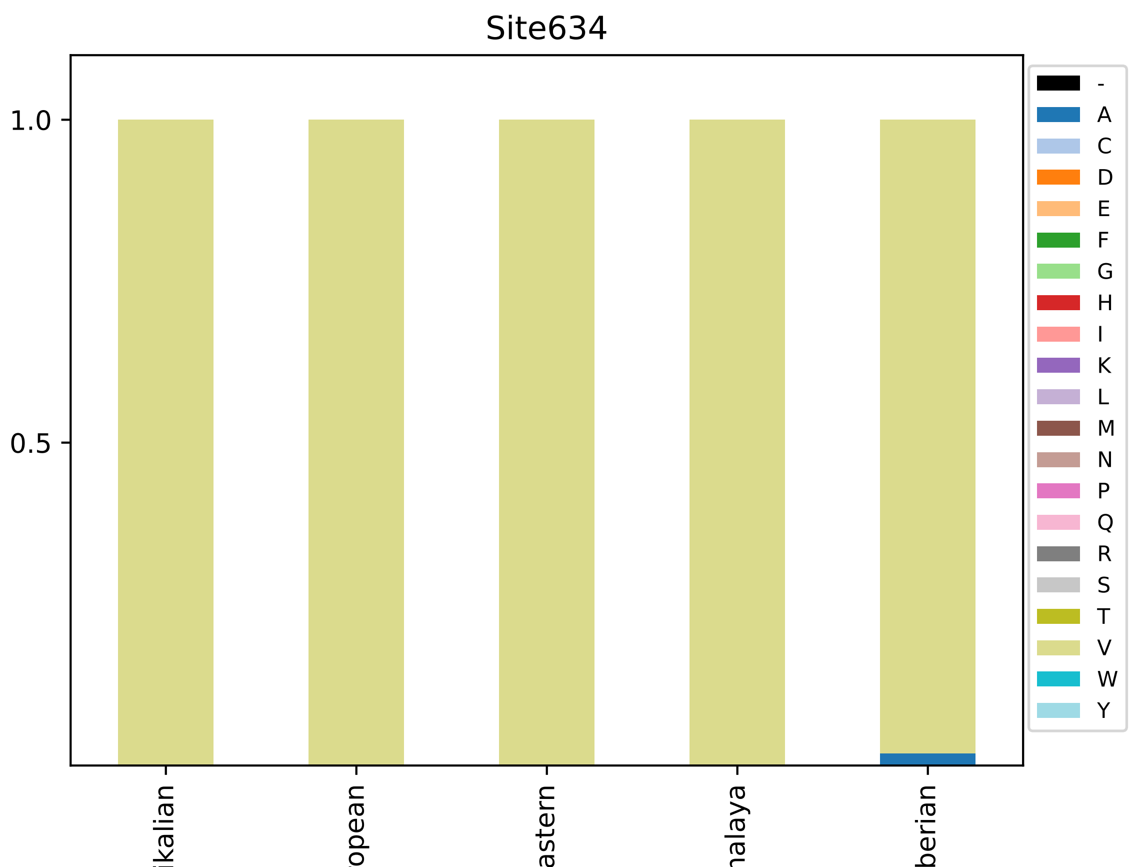

Site634
1.0
0.5
-
A
C
D
E
F
G
H
I
K
L
M
N
P
Q
R
S
T
V
W
Y
ikalian
opean
astern
nalaya
berian

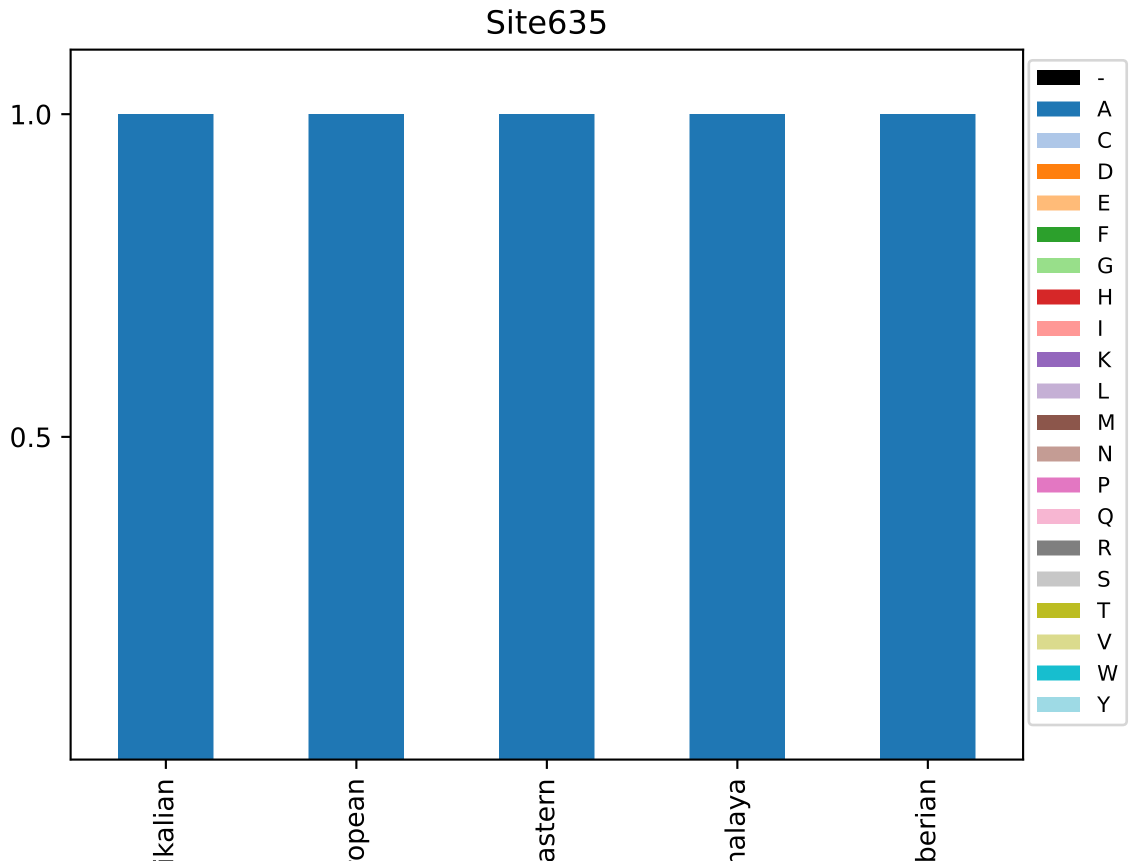

Site635
1.0
0.5
-
A
C
D
E
F
G
H
I
K
L
M
N
P
Q
R
S
T
V
W
Y
ikalian
opean
astern
nalaya
berian

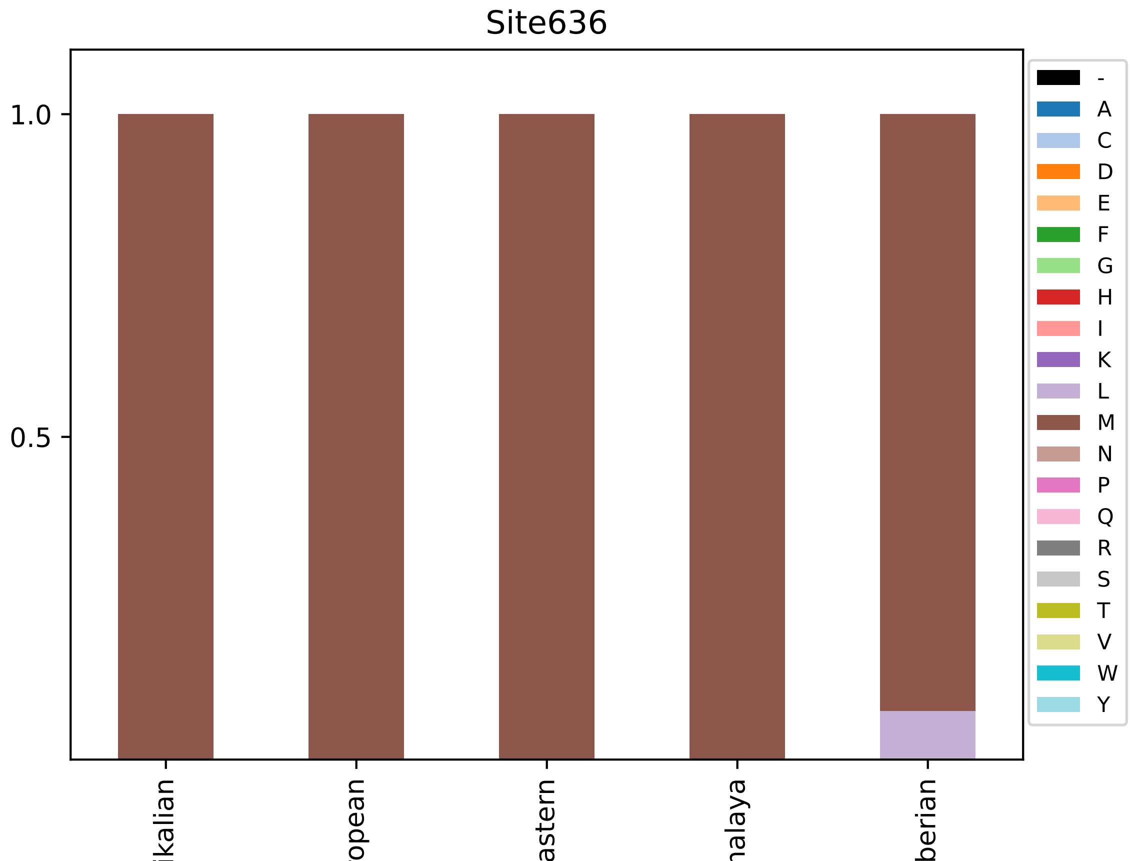

Site636
1.0
0.5
-
A
C
D
E
F
G
H
I
K
L
M
N
P
Q
R
S
T
V
W
Y
ikalian
opean
astern
nalaya
berian

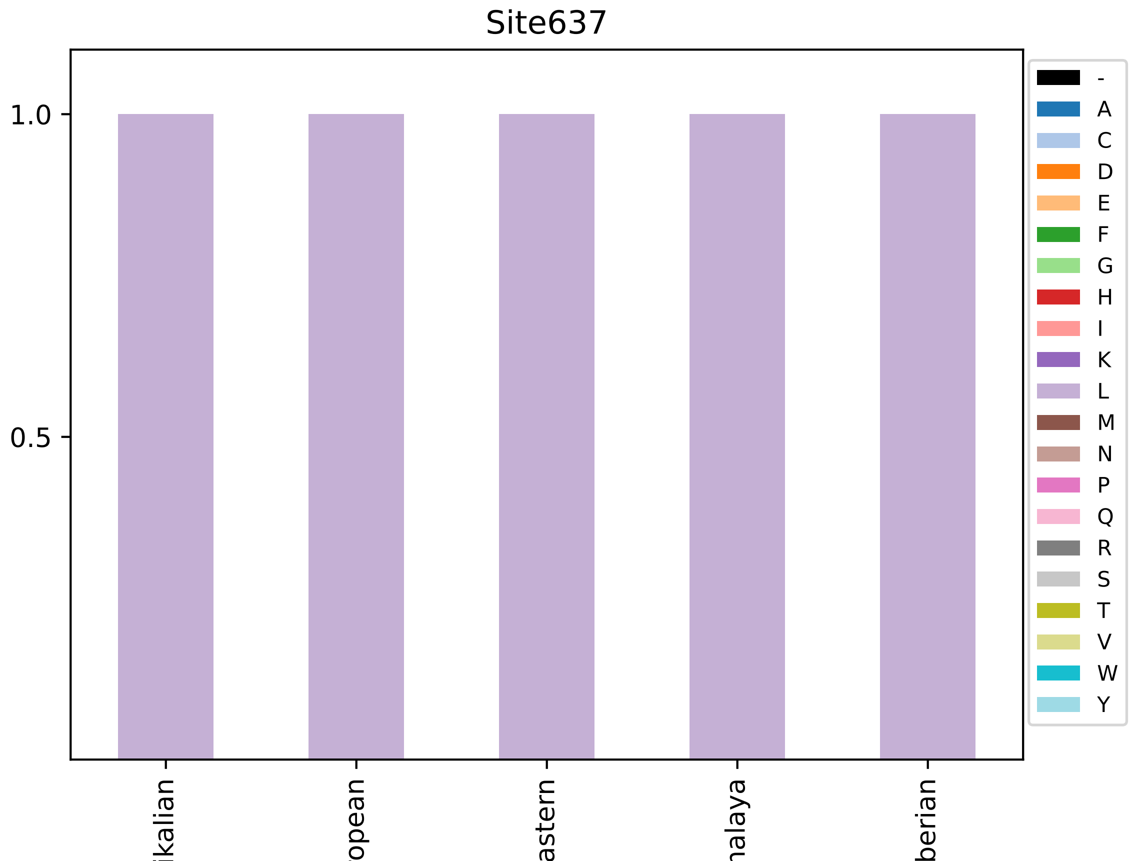

Site637
1.0
0.5
-
A
C
D
E
F
G
H
I
K
L
M
N
P
Q
R
S
T
V
W
Y

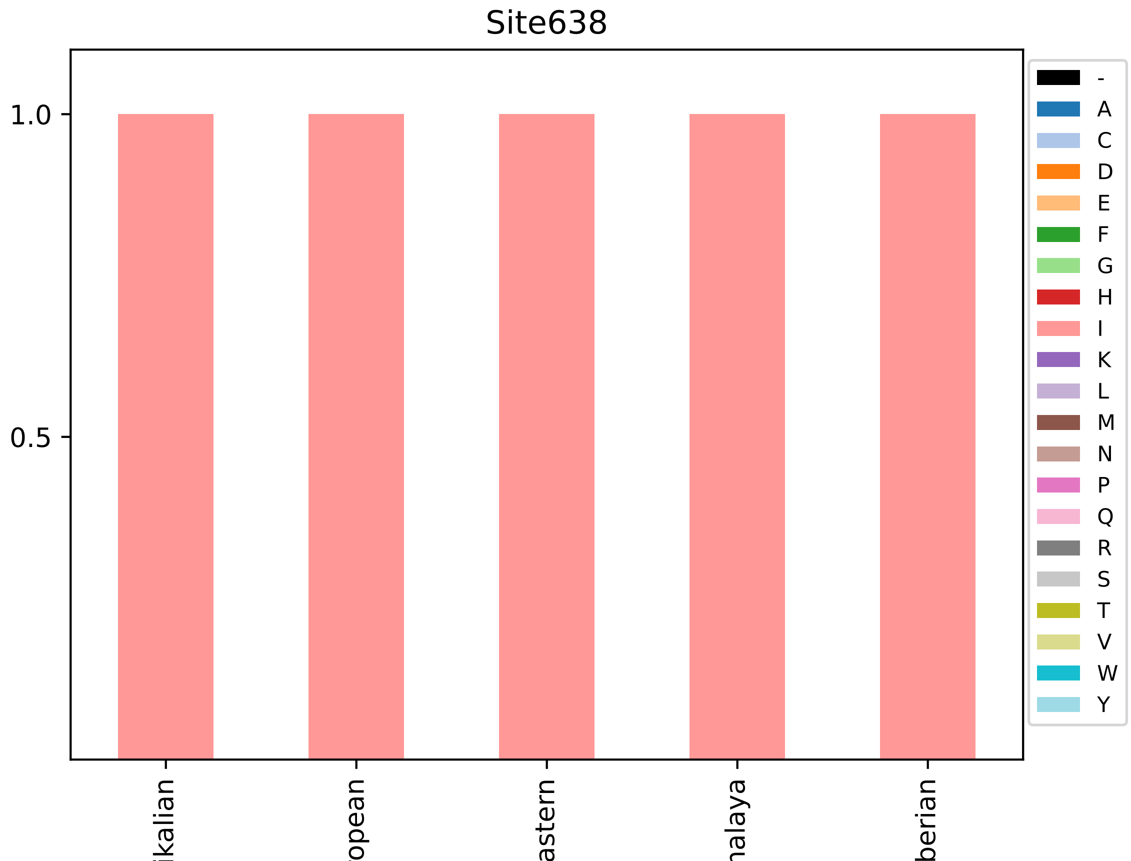

Site638
1.0
0.5
-
A
C
D
E
F
G
H
I
K
L
M
N
P
Q
R
S
T
V
W
Y
ikalian
ropean
astern
nalaya
berian

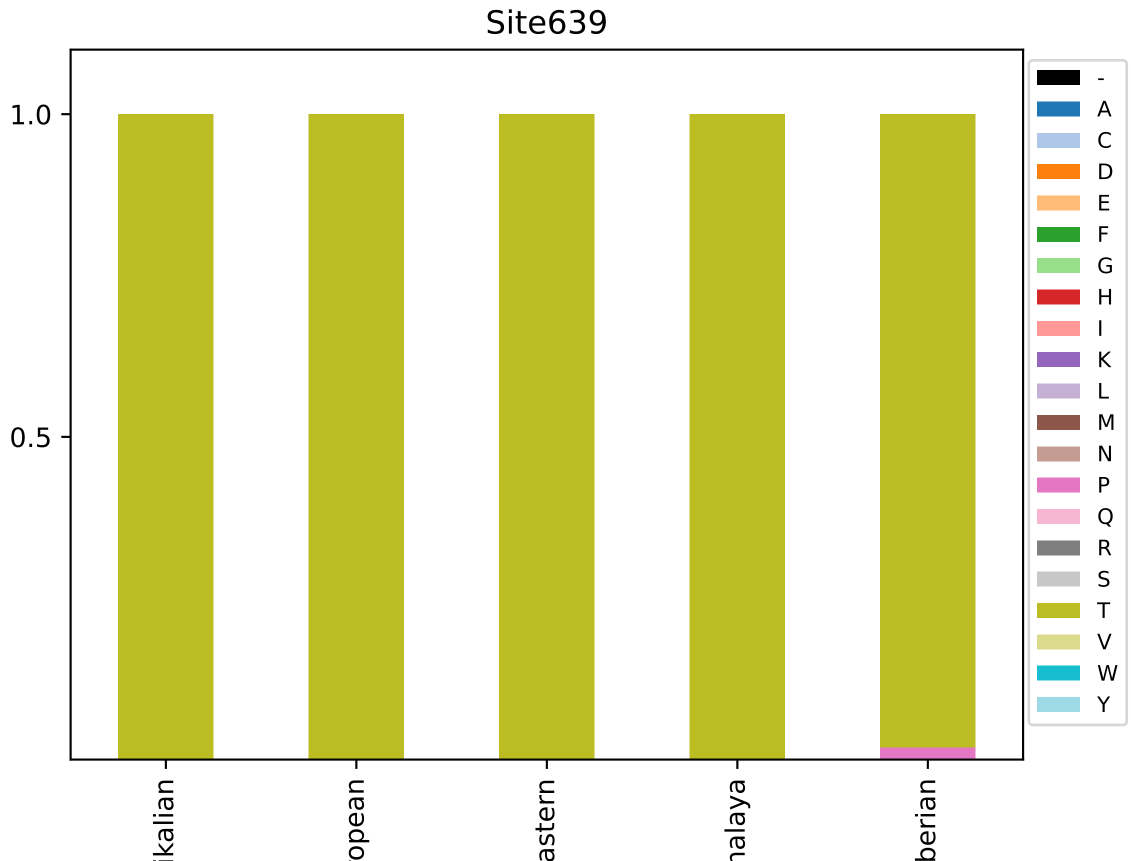
Site639
1.0
0.5
-
A
C
D
E
F
G
H
I
K
L
M
N
P
Q
R
S
T
V
W
Y
ikalian
opean
astern
nalaya
berian

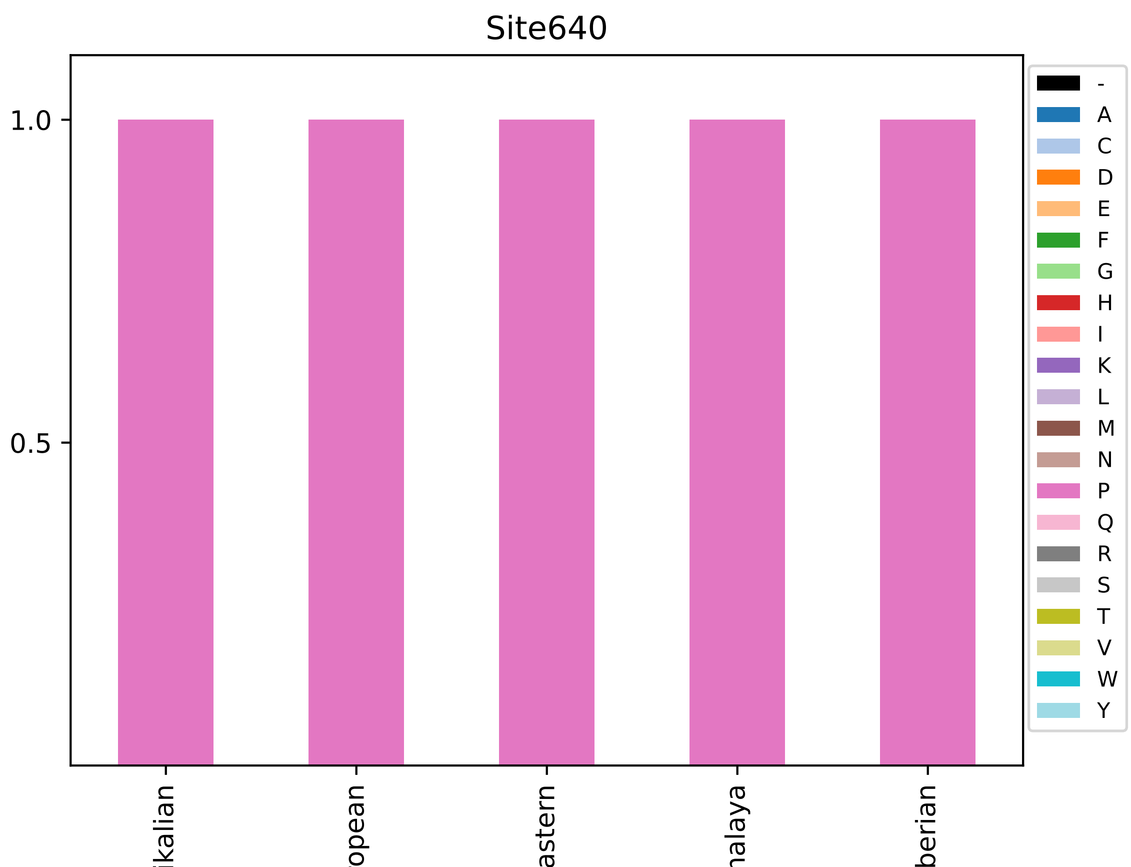
Site640
1.0
0.5
-
A
C
D
E
F
G
H
I
K
L
M
N
P
Q
R
S
T
V
W
Y
ikalian
opean
astern
nalaya
berian

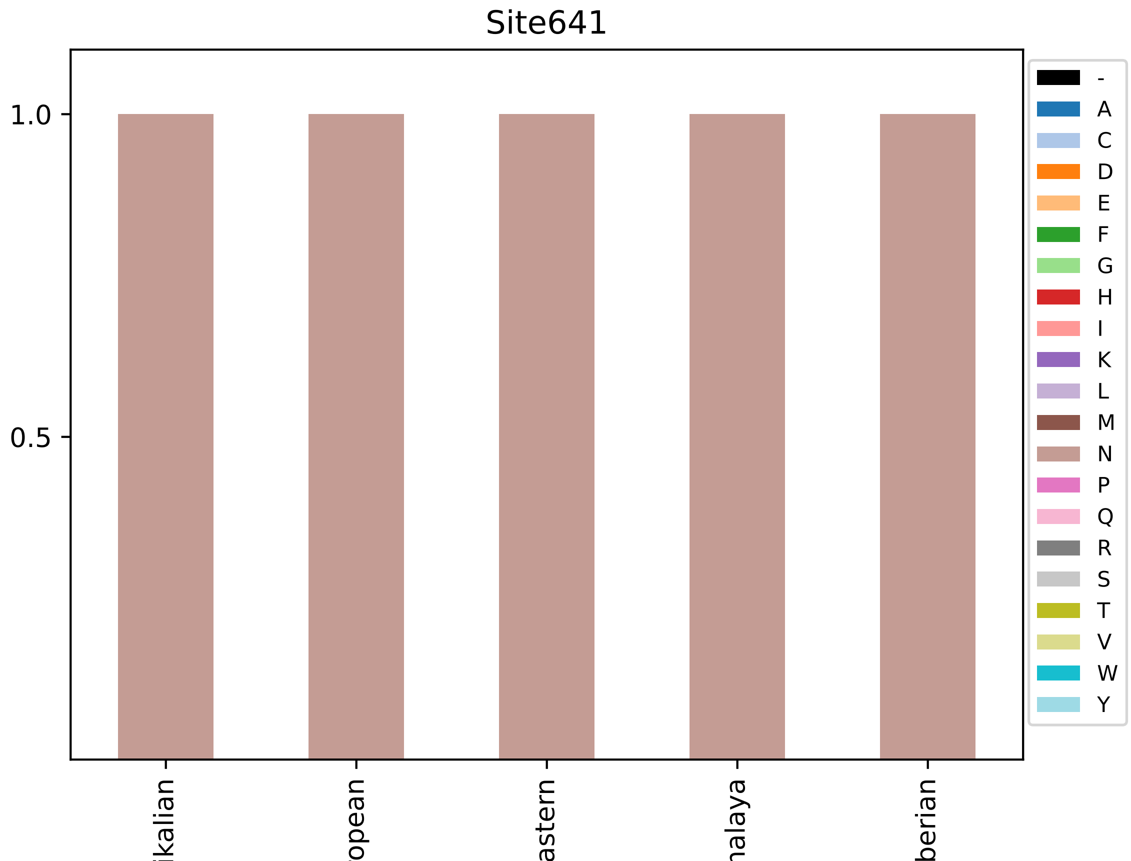
Site641
1.0
0.5
-
A
C
D
E
F
G
H
I
K
L
M
N
P
Q
R
S
T
V
W
Y
ikalian
opean
astern
nalaya
berian

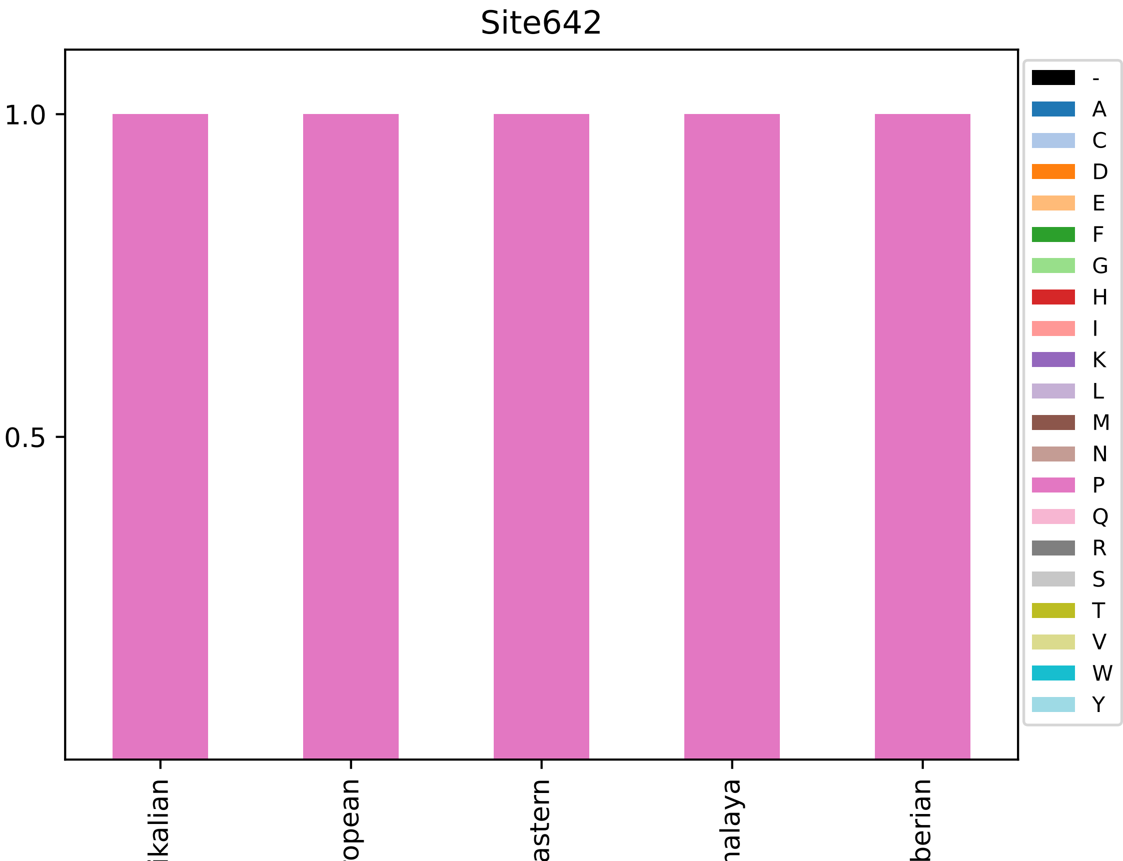

Site642
1.0
0.5
-
A
C
D
E
F
G
H
I
K
L
M
N
P
Q
R
S
T
V
W
Y

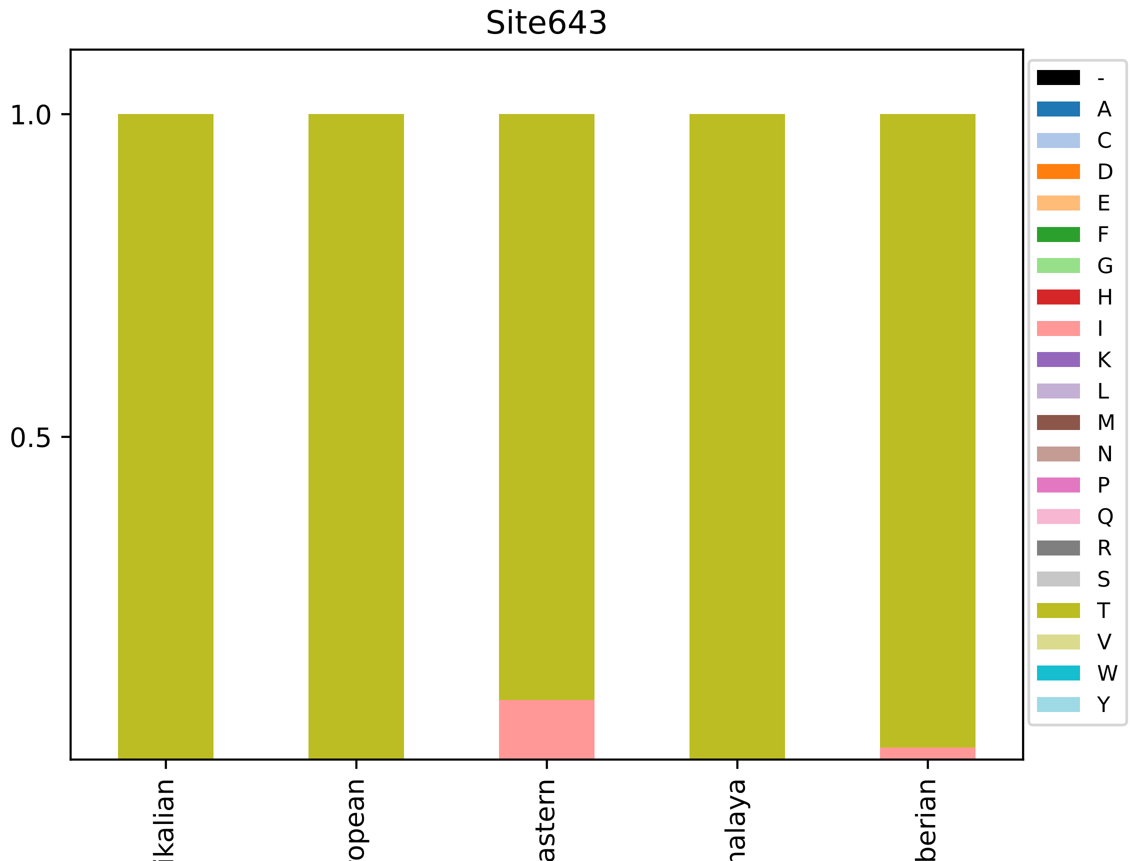

Site643
1.0
0.5
-
A
C
D
E
F
G
H
I
K
L
M
N
P
Q
R
S
T
V
W
Y
ikalian
opean
astern
nalaya
berian

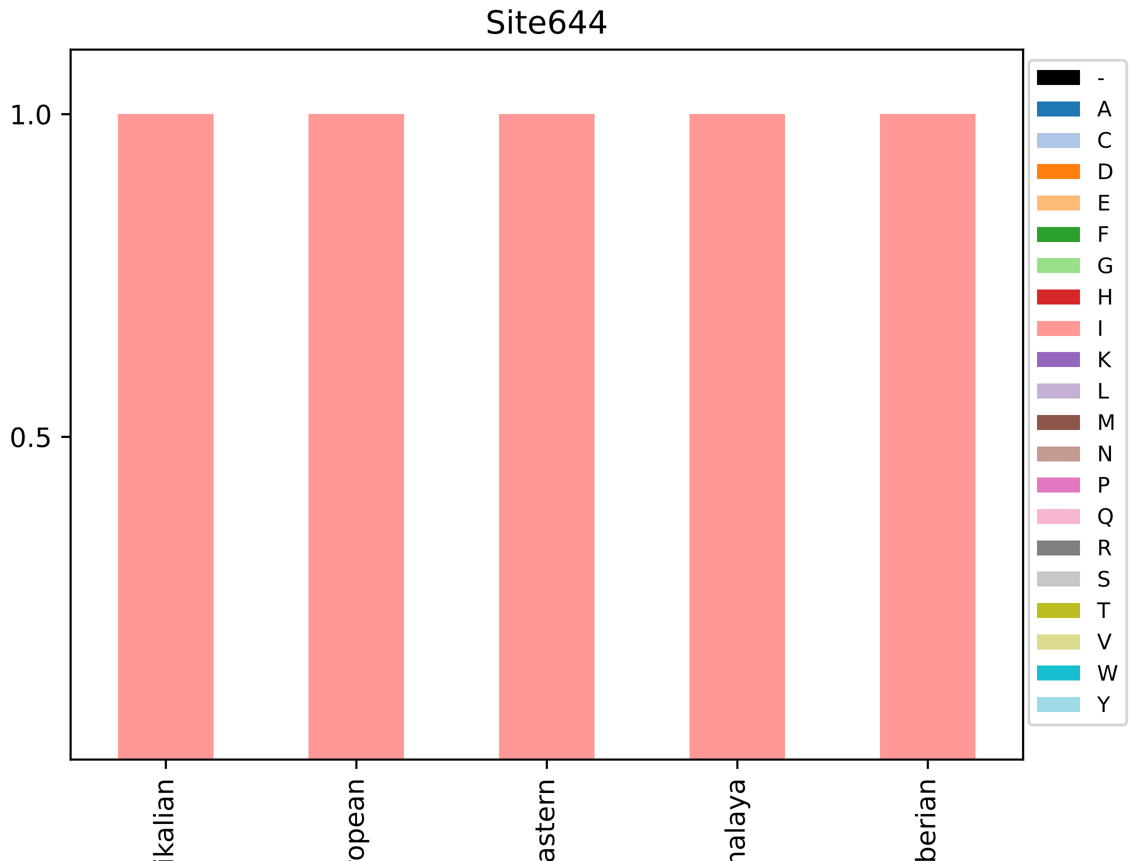
Site644
1.0
0.5
-
A
C
D
E
F
G
H
I
K
L
M
N
P
Q
R
S
T
V
W
Y
ikalian
opean
astern
nalaya
berian

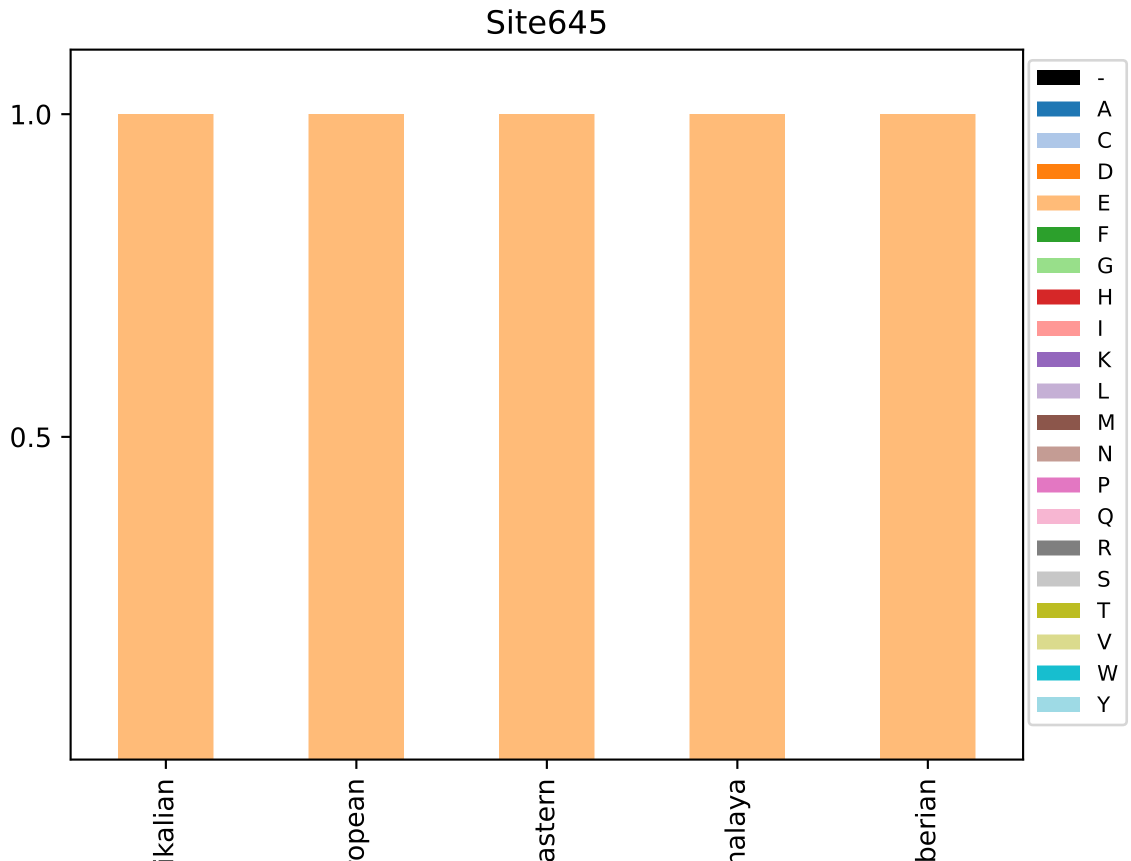
Site645
1.0
0.5
-
A
C
D
E
F
G
H
I
K
L
M
N
P
Q
R
S
T
V
W
Y
ikalian
opean
astern
nalaya
berian

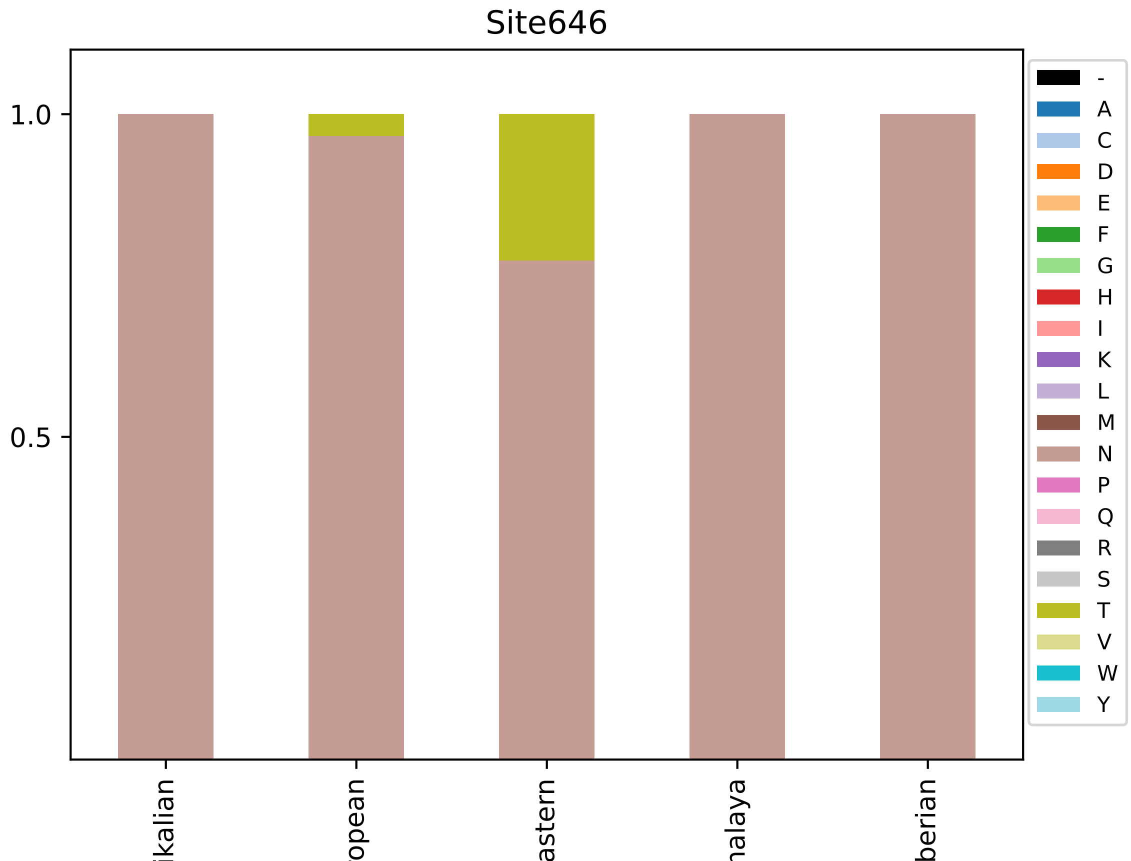
Site646
1.0
0.5
-
A
C
D
E
F
G
H
I
K
L
M
N
P
Q
R
S
T
V
W
Y
ikalian
opean
astern
nalaya
berian

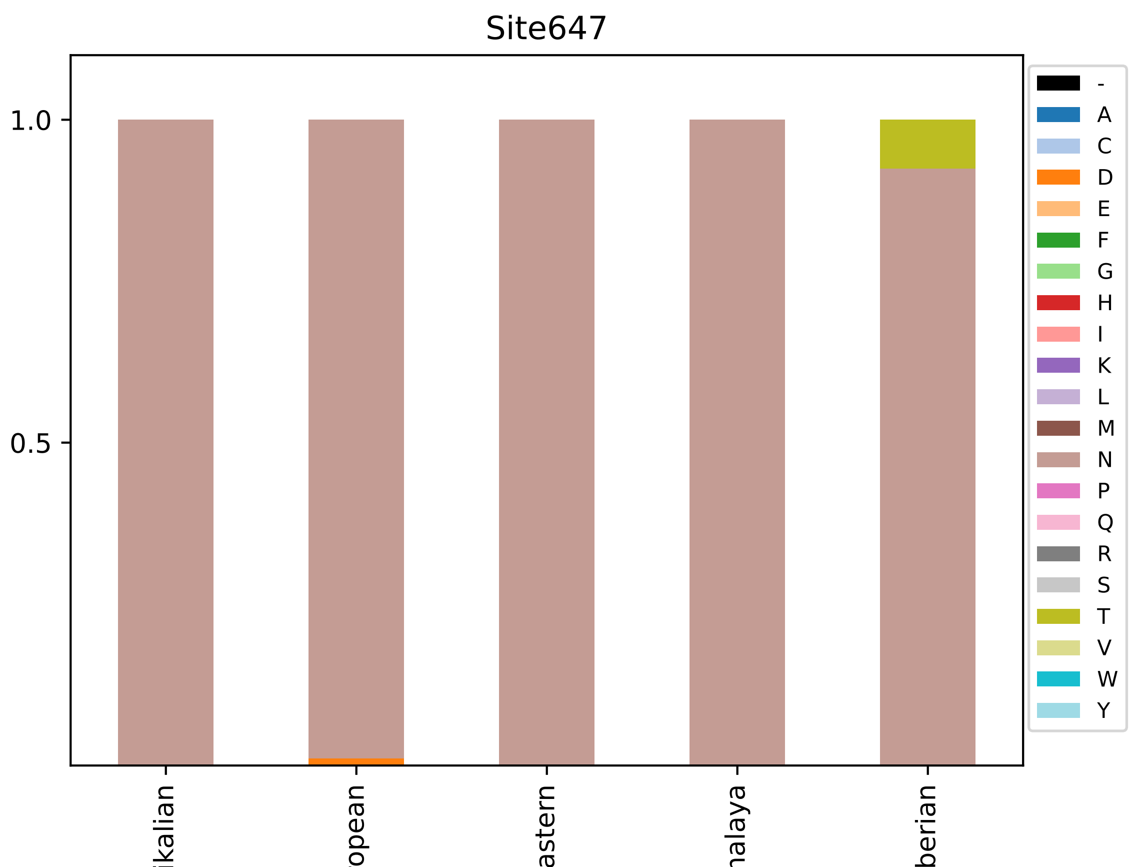
Site647
1.0
0.5
-
A
C
D
E
F
G
H
I
K
L
M
N
P
Q
R
S
T
V
W
Y
ikalian
opean
astern
nalaya
berian

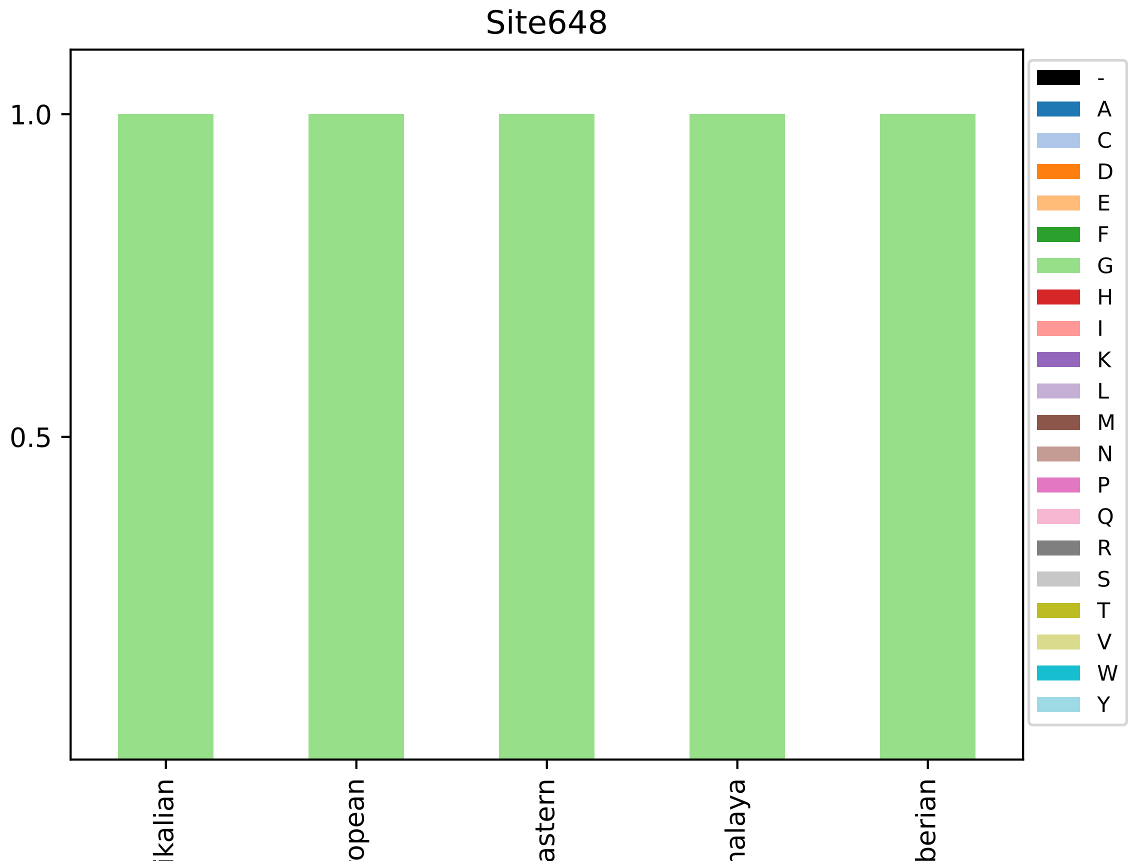

Site648
1.0
0.5
-
A
C
D
E
F
G
H
I
K
L
M
N
P
Q
R
S
T
V
W
Y
ikalian
opean
astern
nalaya
berian

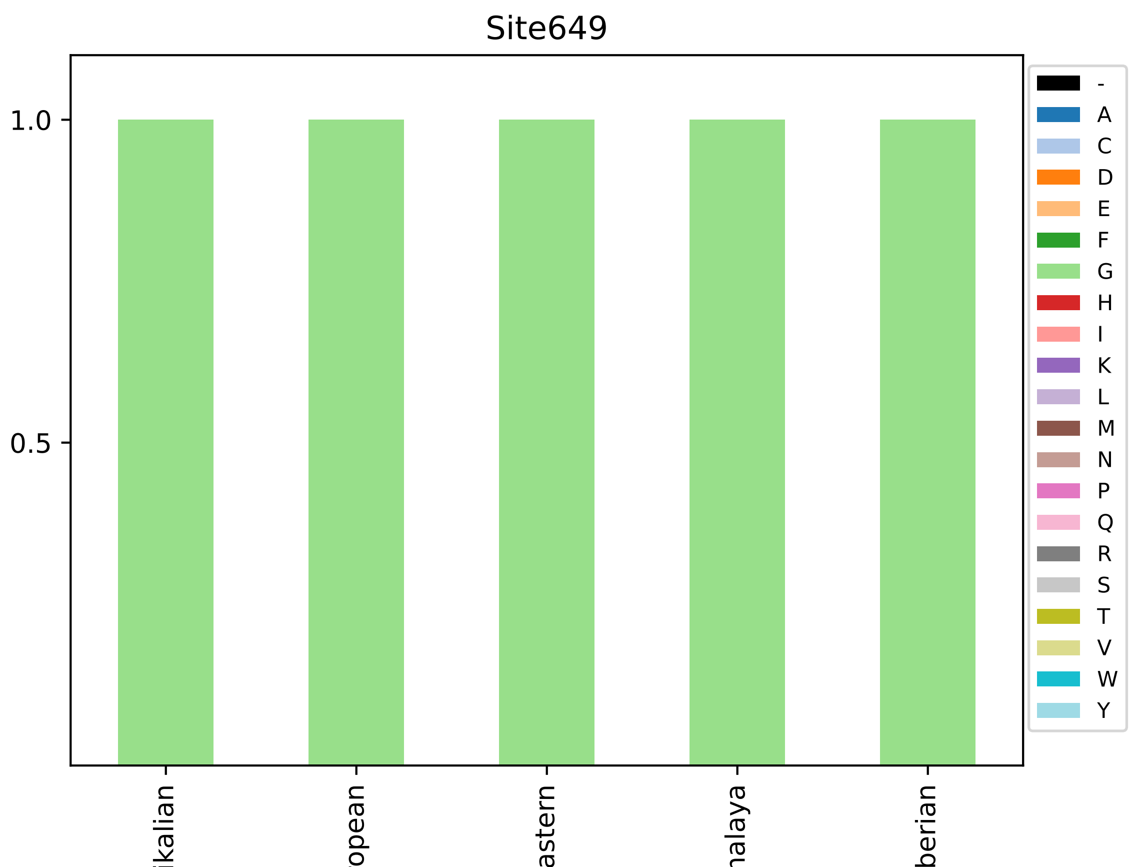

Site649
1.0
0.5
-
A
C
D
E
F
G
H
I
K
L
M
N
P
Q
R
S
T
V
W
Y
ikalian
opean
astern
nalaya
berian

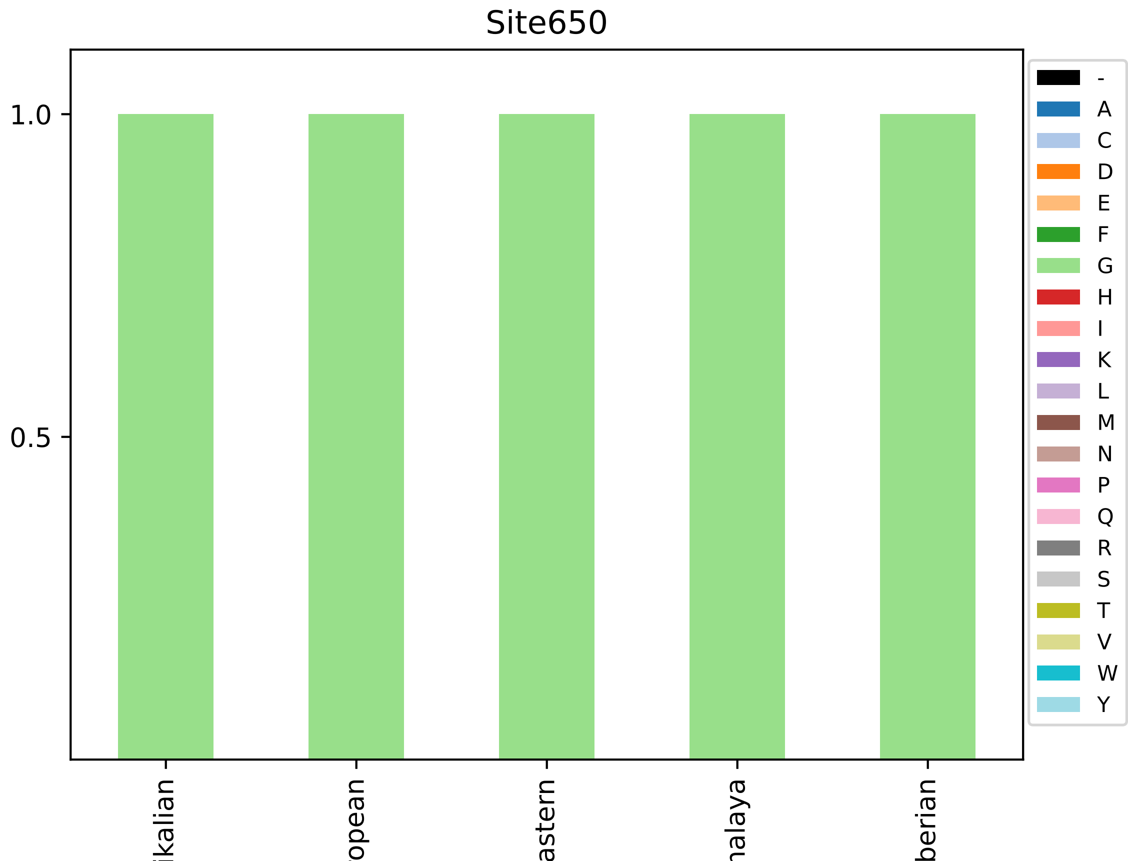

Site650
1.0
0.5
-
A
C
D
E
F
G
H
I
K
L
M
N
P
Q
R
S
T
V
W
Y
ikalian
opean
astern
nalaya
berian

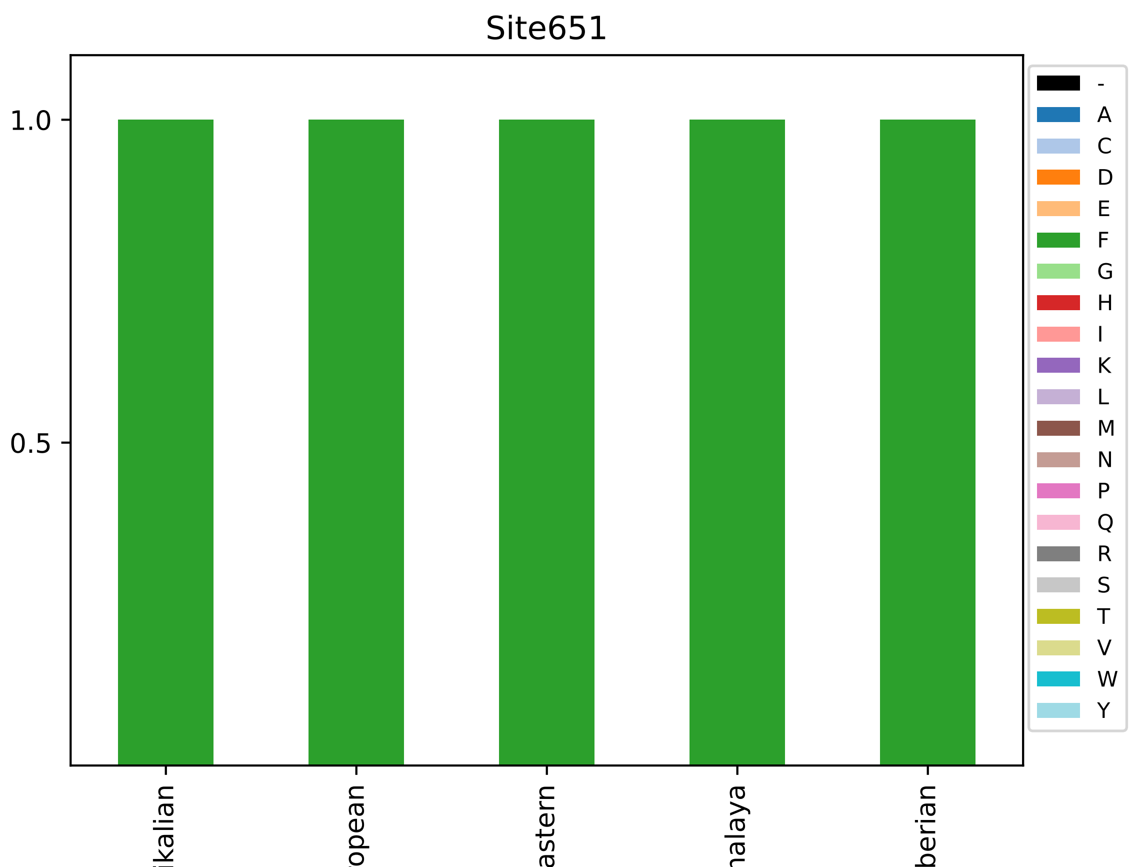
Site651
1.0
0.5
ikalian
ropean
astern
nalaya
berian
-
A
C
D
E
F
G
H
I
K
L
M
N
P
Q
R
S
T
V
W
Y

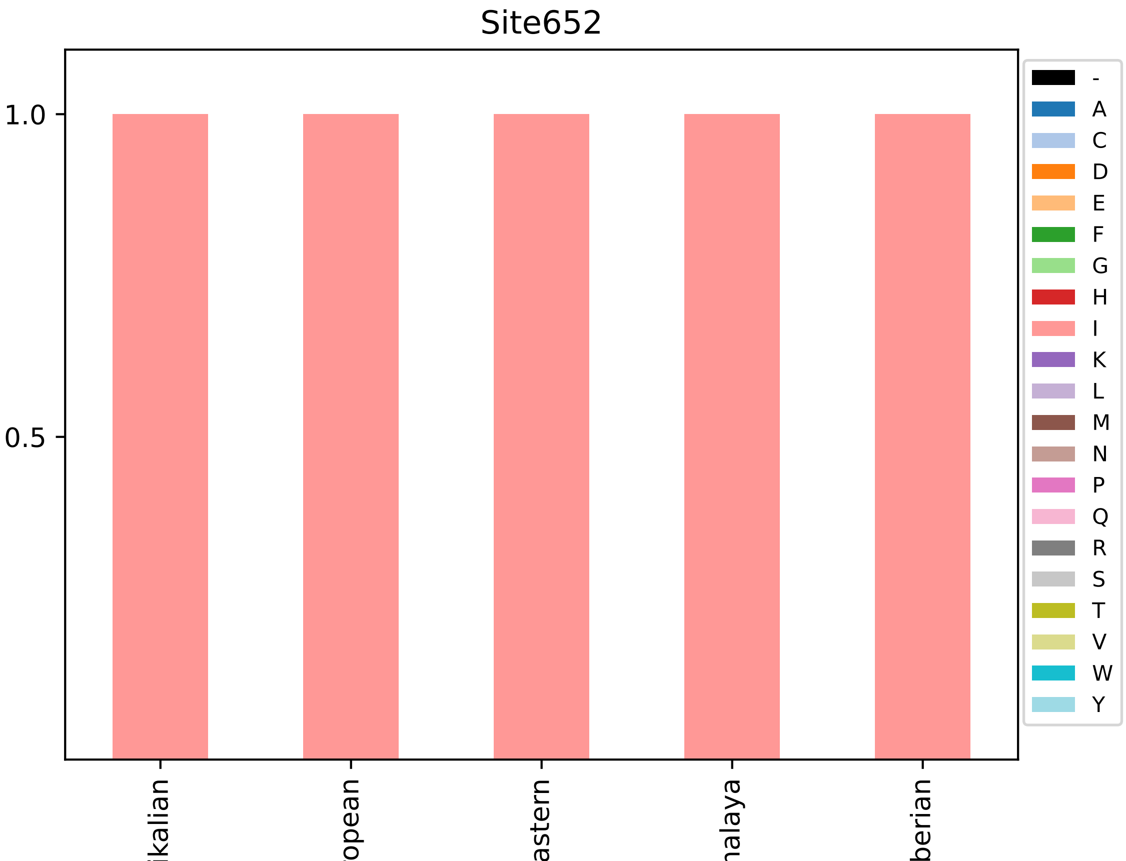

Site652
1.0
0.5
-
A
C
D
E
F
G
H
I
K
L
M
N
P
Q
R
S
T
V
W
Y
ikalian
opean
astern
nalaya
berian

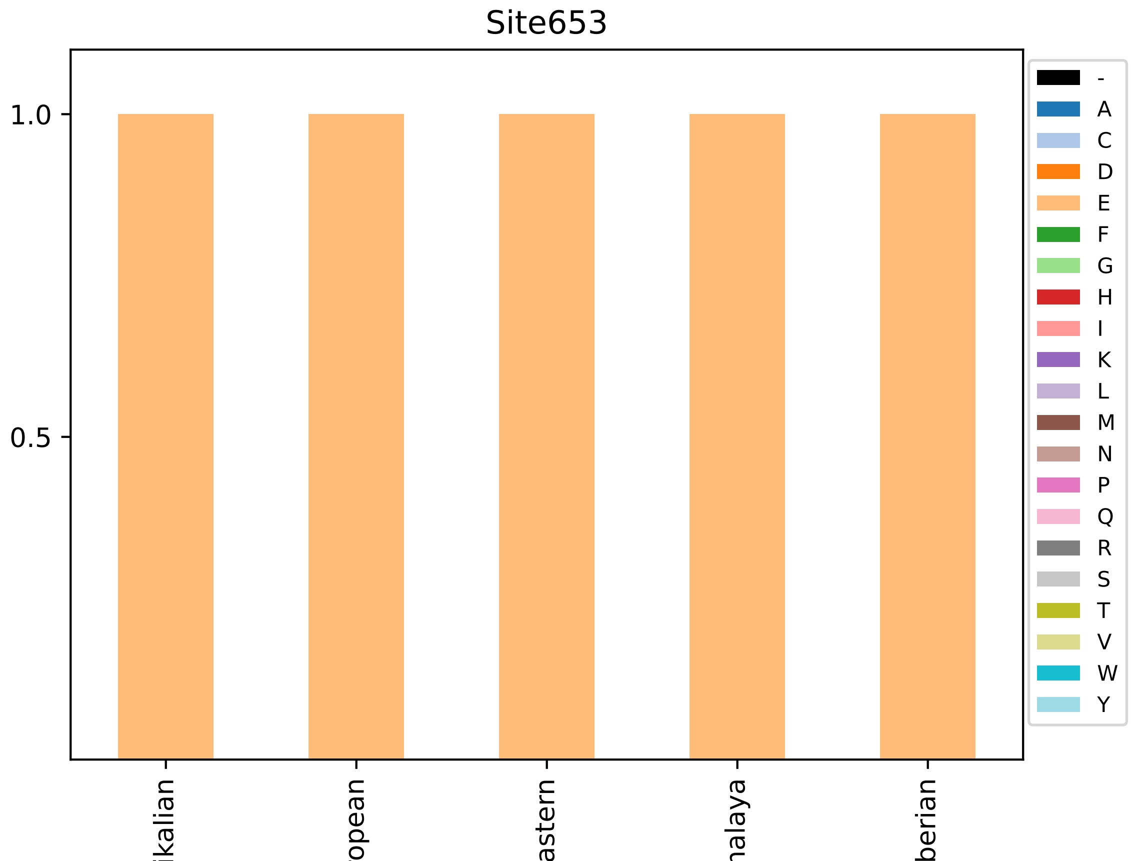
Site653
1.0
0.5
-
A
C
D
E
F
G
H
I
K
L
M
N
P
Q
R
S
T
V
W
Y

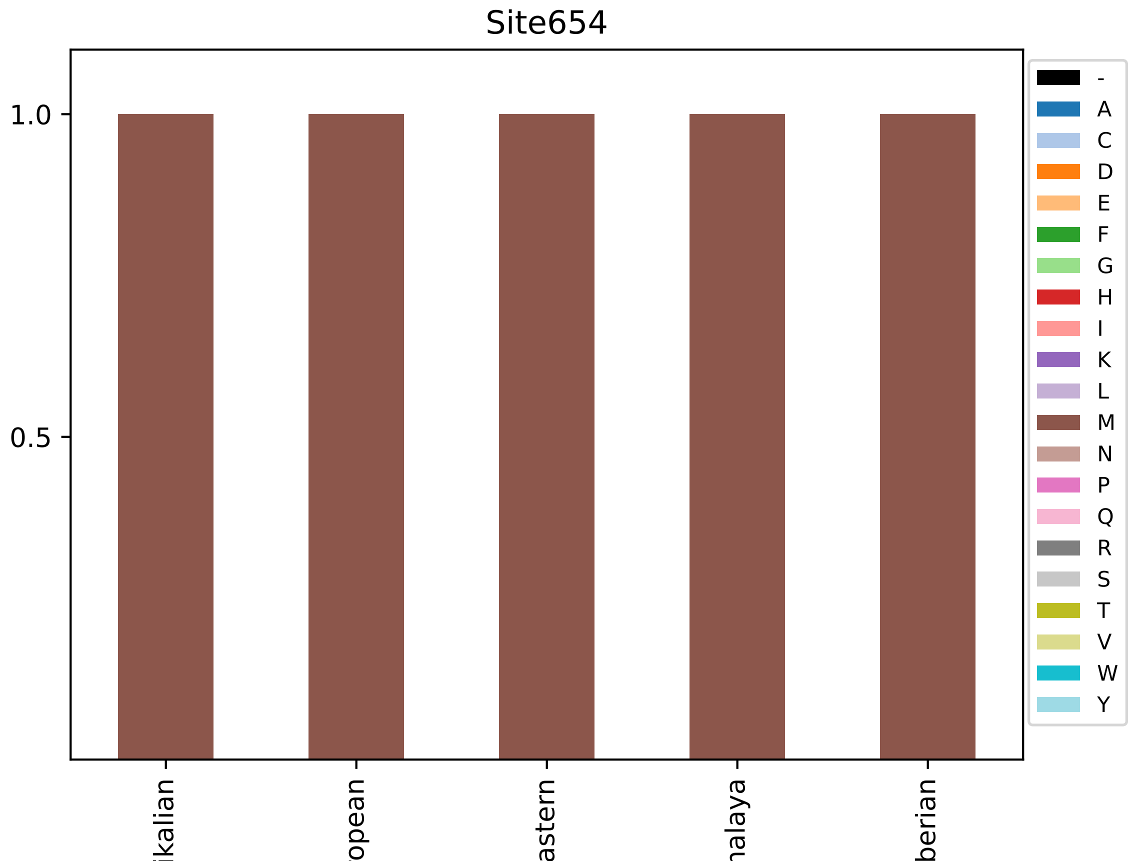
Site654
1.0
0.5
ikalian
opean
astern
nalaya
berian
-
A
C
D
E
F
G
H
I
K
L
M
N
P
Q
R
S
T
V
W
Y

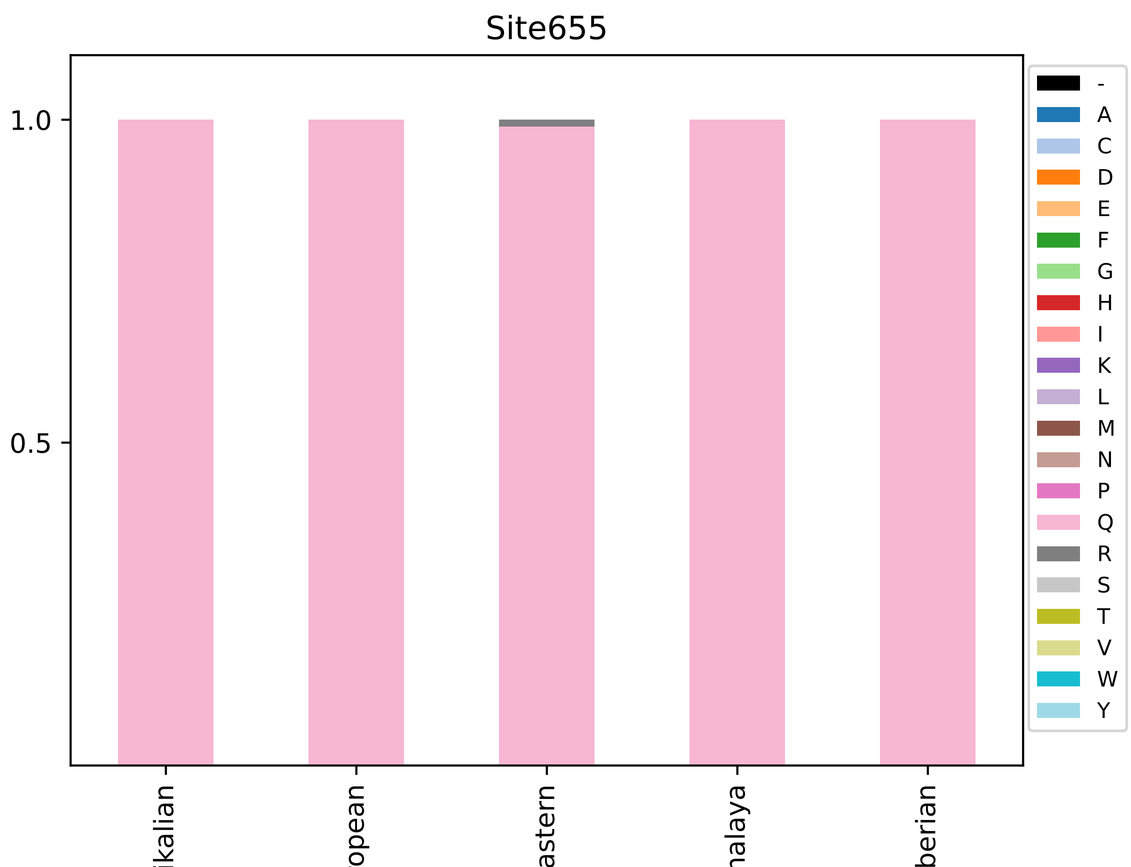
Site655
1.0
0.5
-
A
C
D
E
F
G
H
I
K
L
M
N
P
Q
R
S
T
V
W
Y
ikalian
opean
astern
nalaya
berian

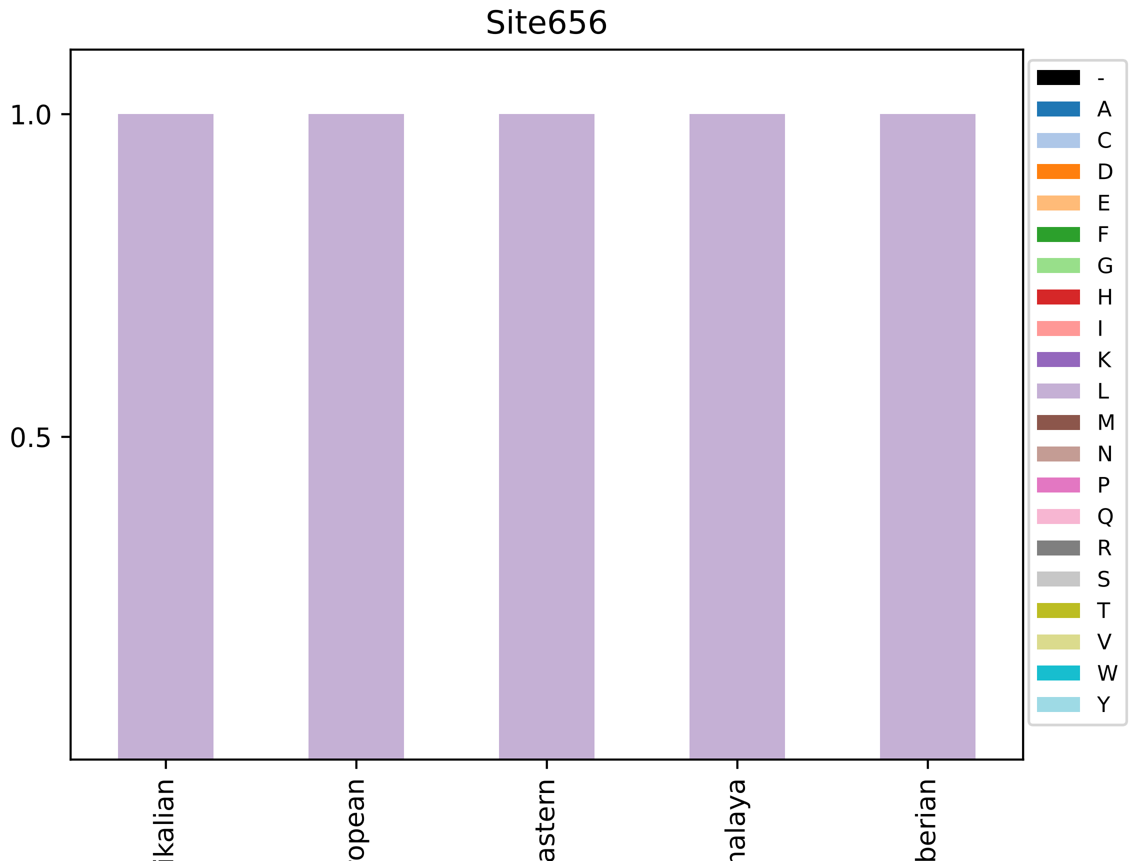

Site656
1.0
0.5
-
A
C
D
E
F
G
H
I
K
L
M
N
P
Q
R
S
T
V
W
Y
ikalian
opean
astern
nalaya
berian

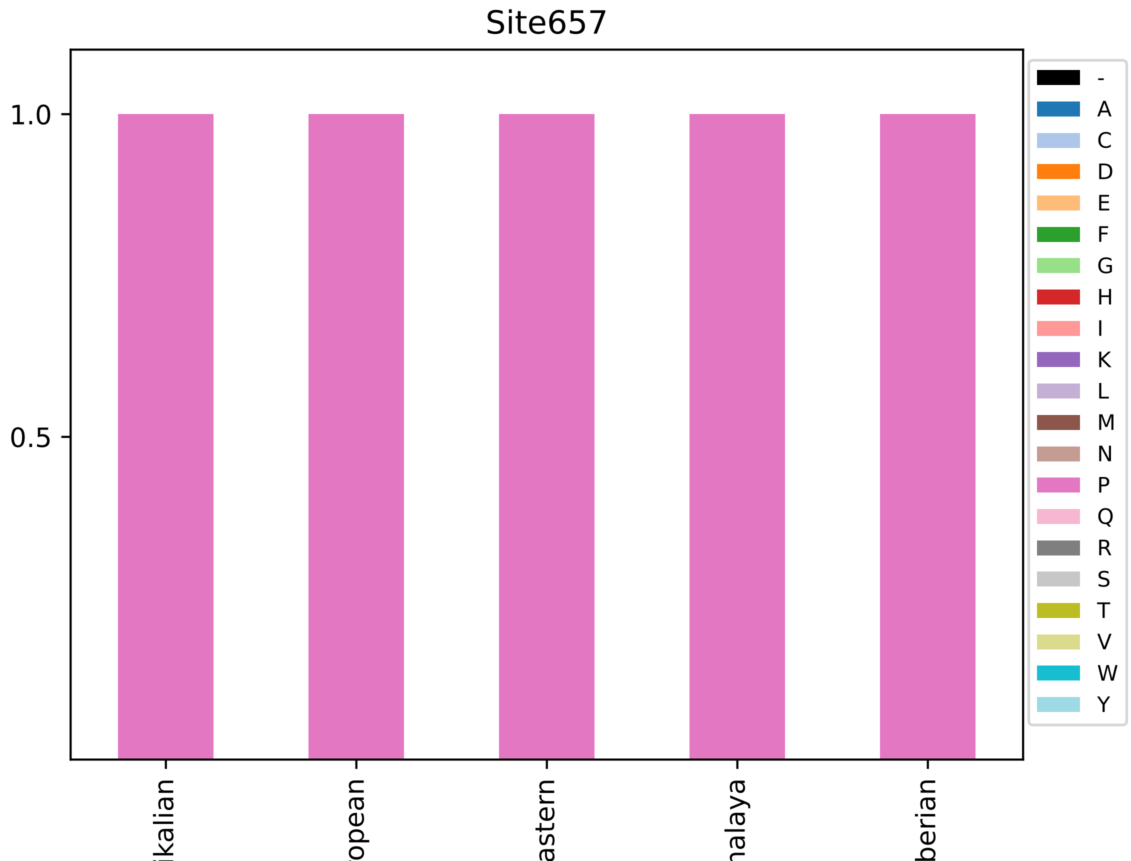
Site657
1.0
0.5
-
A
C
D
E
F
G
H
I
K
L
M
N
P
Q
R
S
T
V
W
Y
ikalian
opean
astern
nalaya
berian

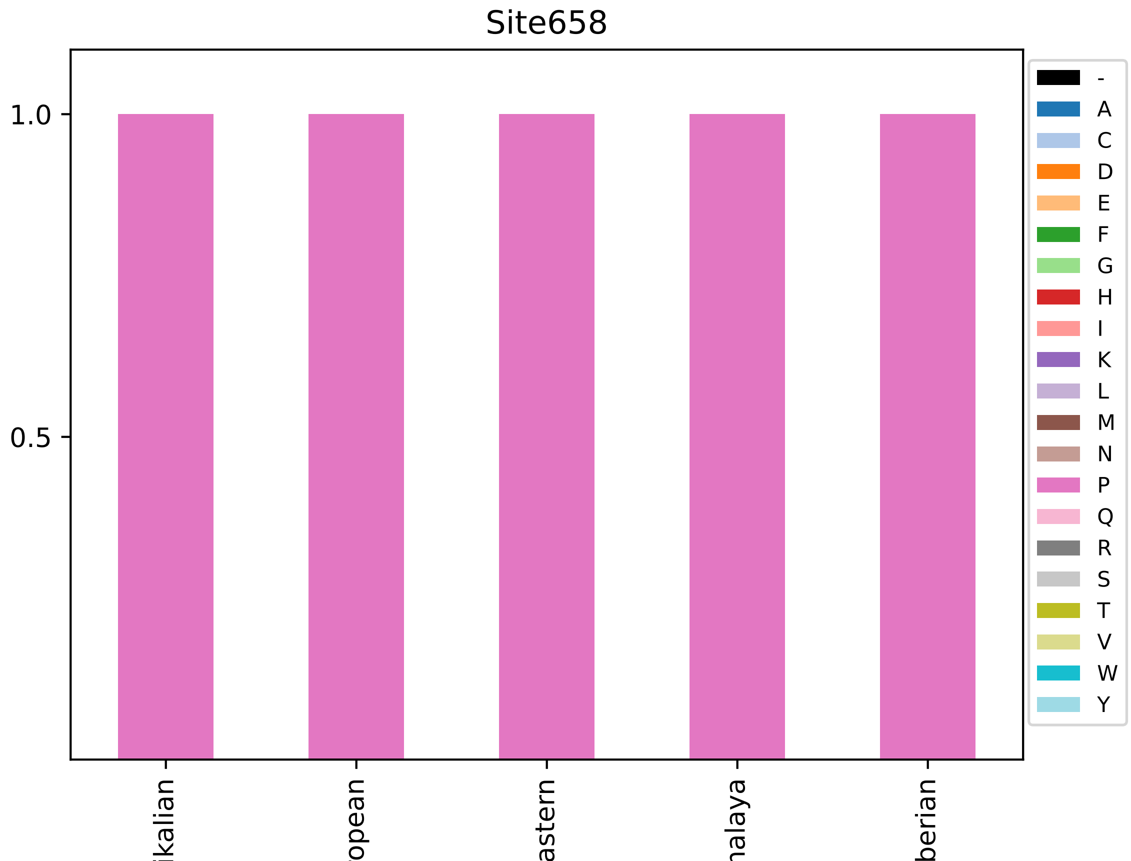
Site658
1.0
0.5
-
A
C
D
E
F
G
H
I
K
L
M
N
P
Q
R
S
T
V
W
Y
ikalian
opean
astern
nalaya
berian

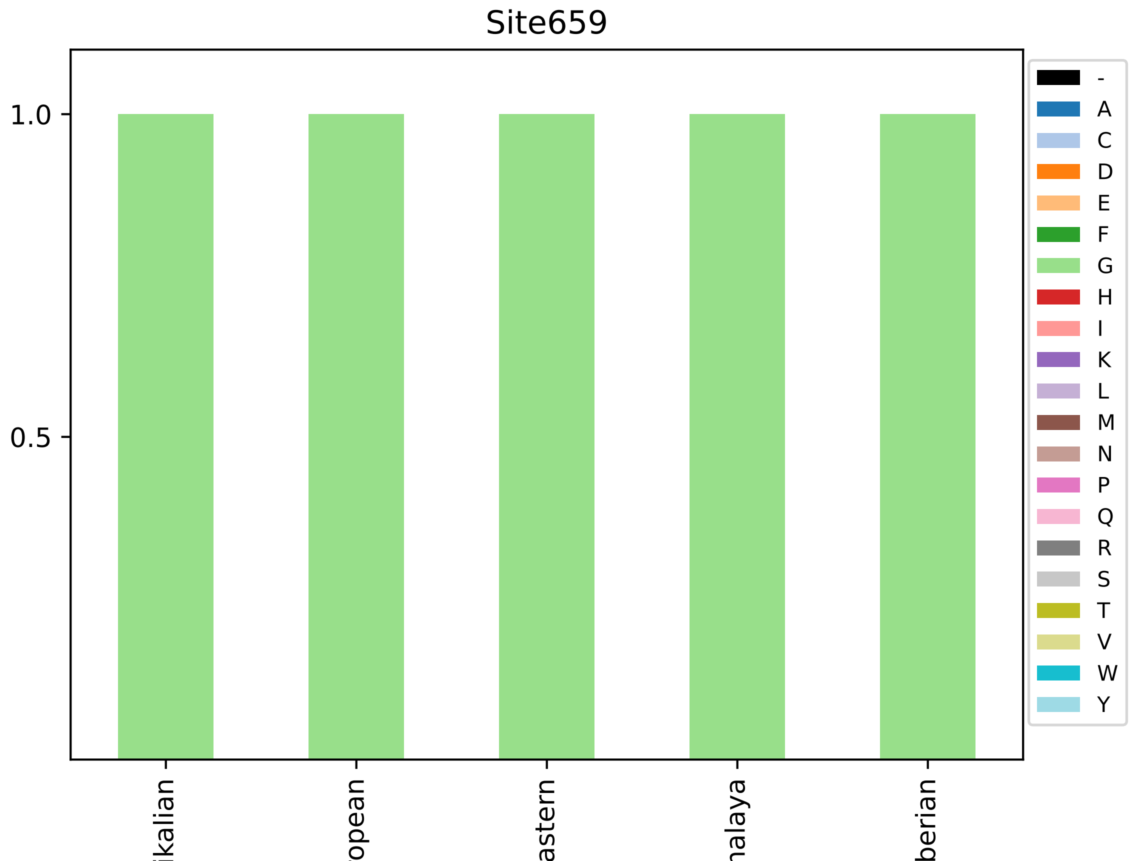

Site659
1.0
0.5
ikalian
opean
astern
nalaya
berian
-
A
C
D
E
F
G
H
I
K
L
M
N
P
Q
R
S
T
V
W
Y

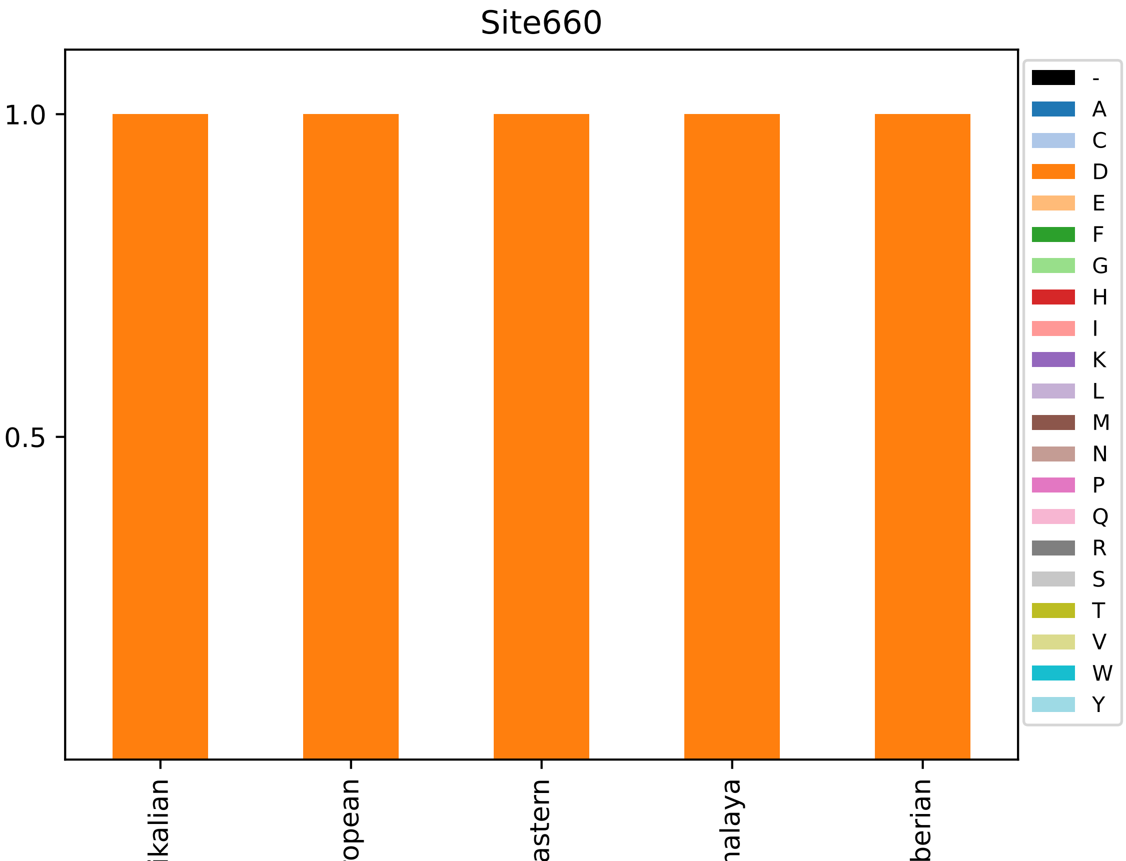
Site660
1.0
0.5
-
A
C
D
E
F
G
H
I
K
L
M
N
P
Q
R
S
T
V
W
Y
ikalian
opean
astern
nalaya
berian

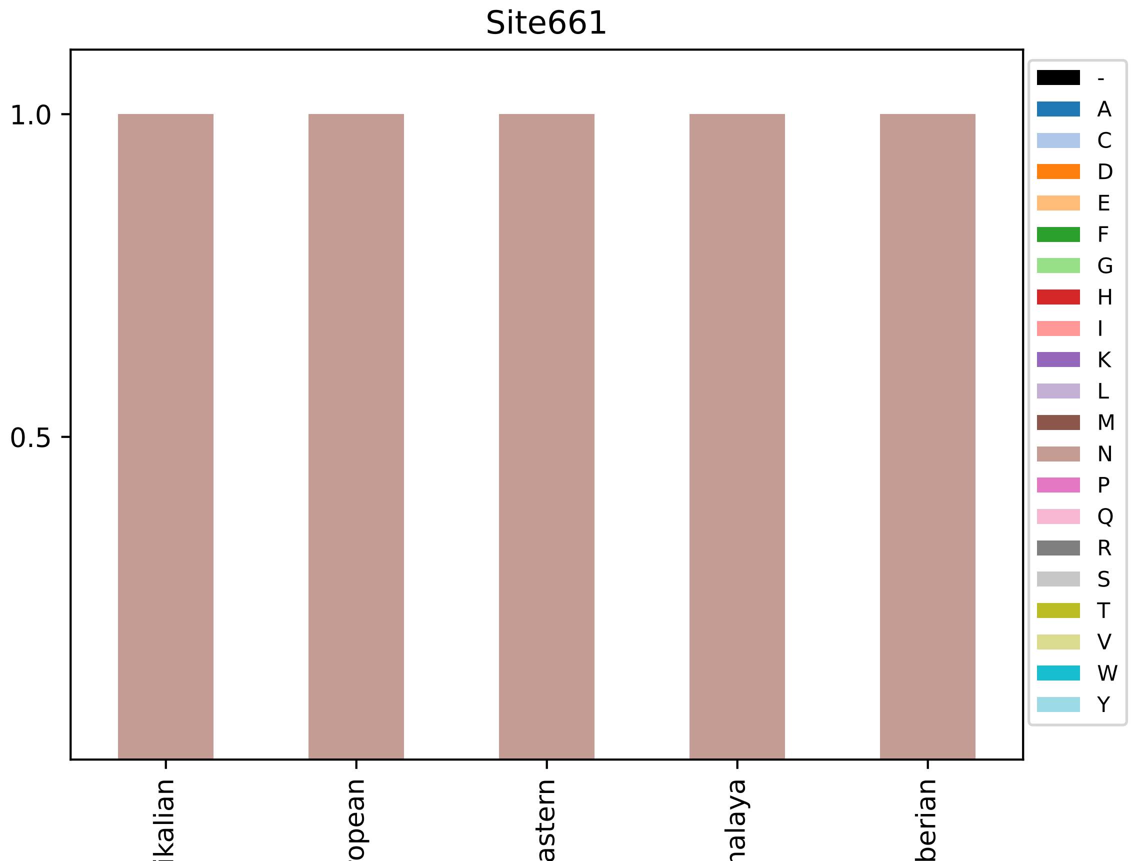
Site661
1.0
0.5
-
A
C
D
E
F
G
H
I
K
L
M
N
P
Q
R
S
T
V
W
Y
ikalian
opean
astern
nalaya
berian

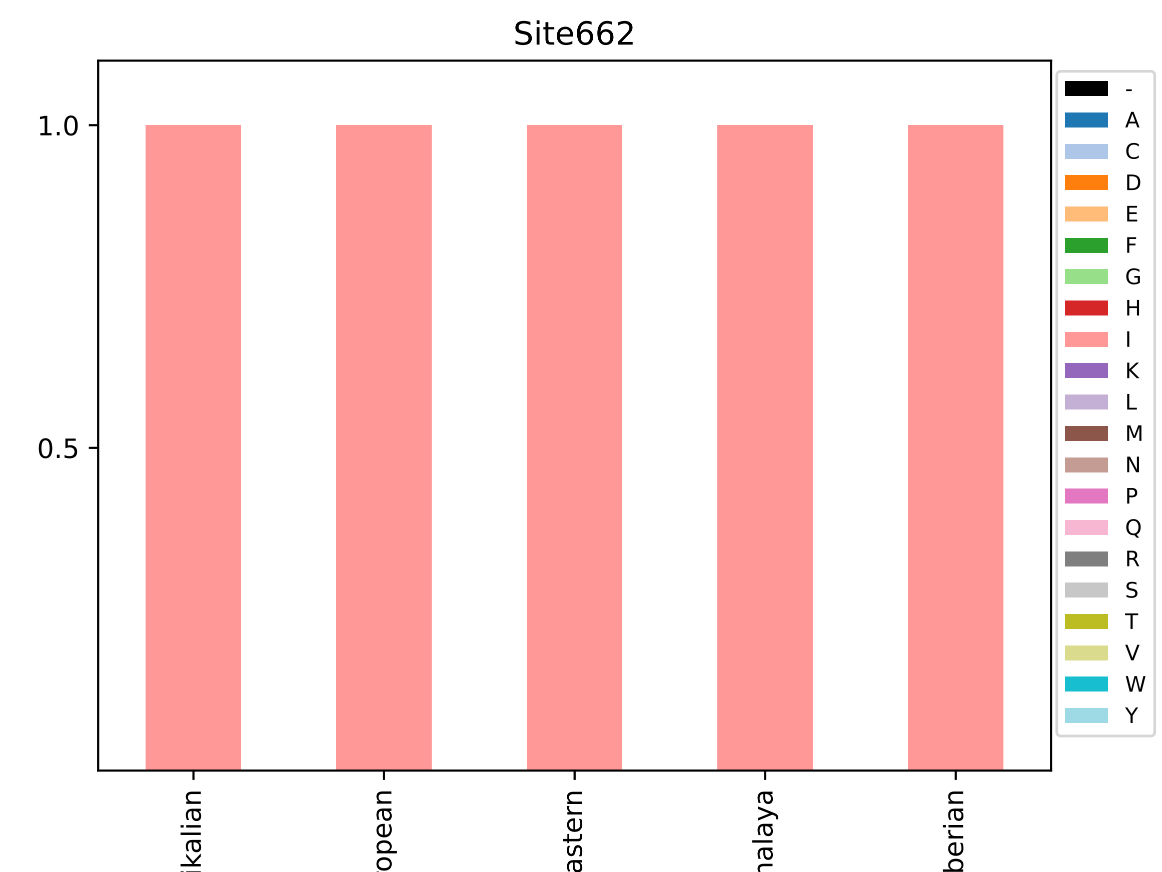
Site662
1.0
0.5
ikalian
opean
astern
nalaya
berian
-
A
C
D
E
F
G
H
I
K
L
M
N
P
Q
R
S
T
V
W
Y

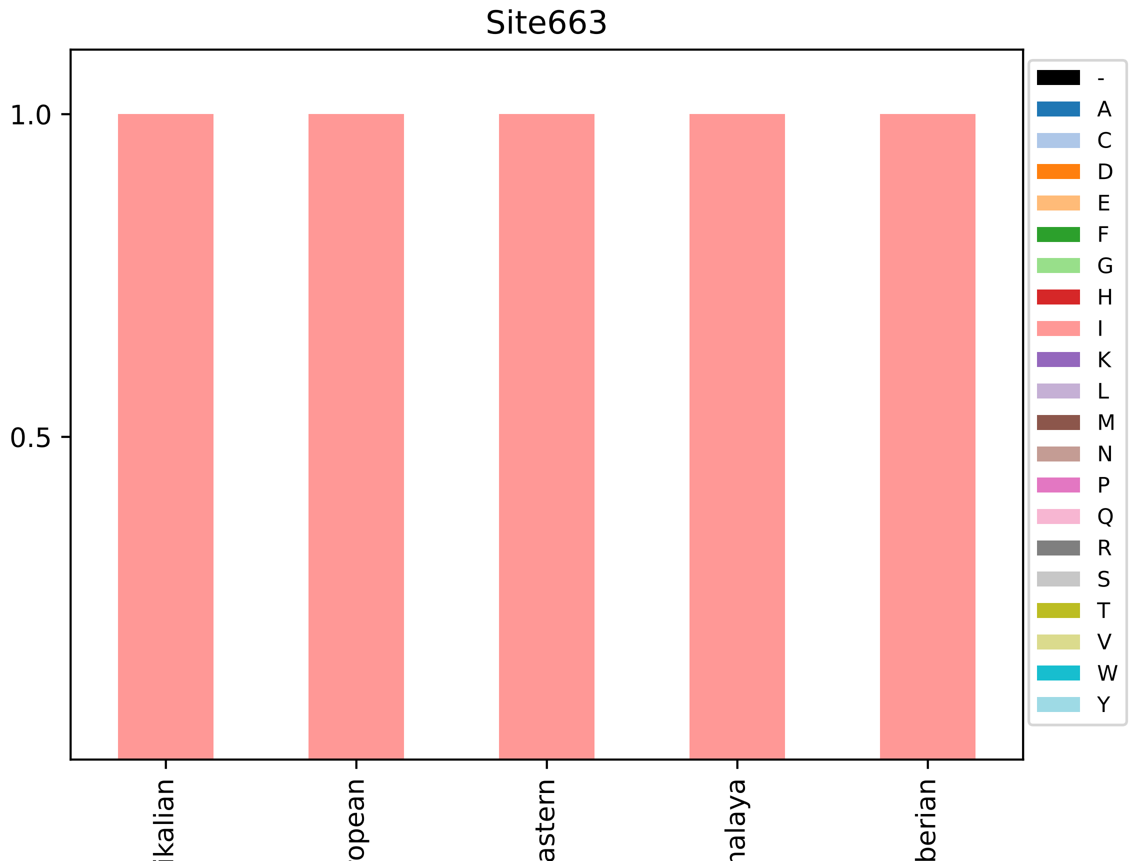

Site663
1.0
0.5
-
A
C
D
E
F
G
H
I
K
L
M
N
P
Q
R
S
T
V
W
Y
ikalian
opean
astern
nalaya
berian

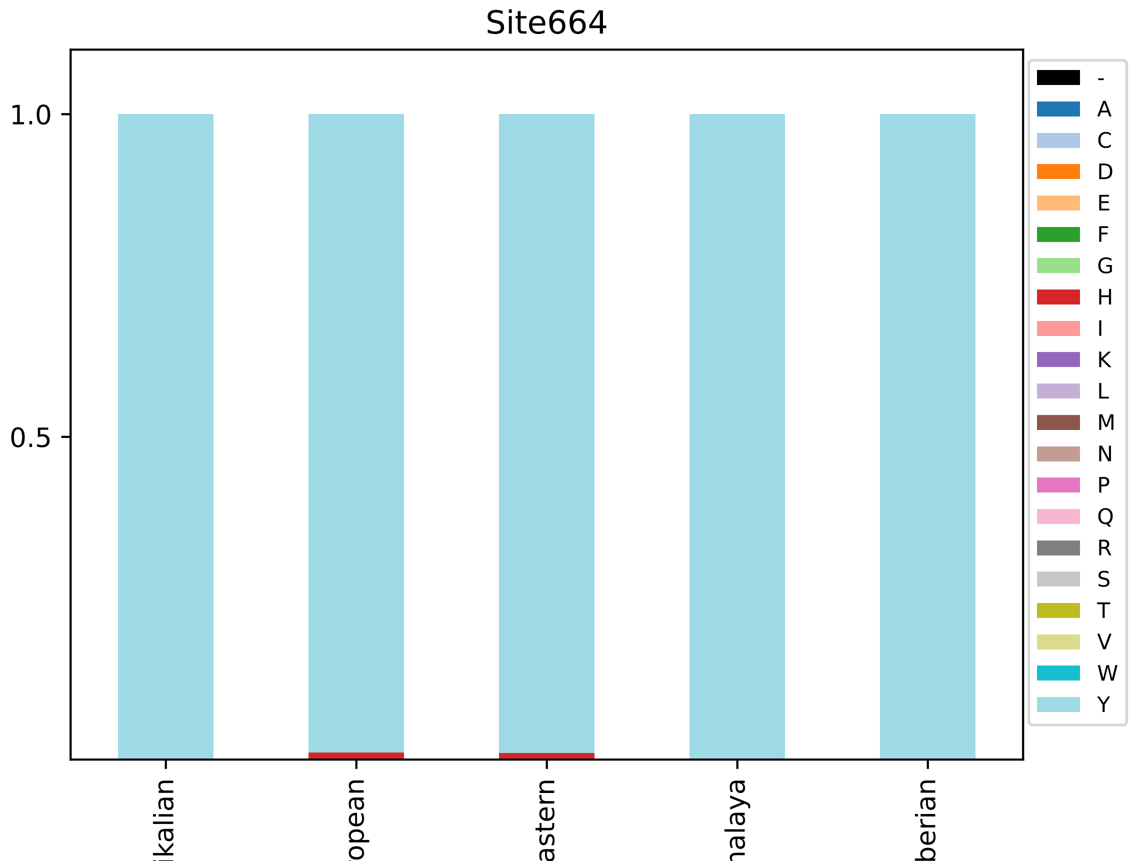
Site664
1.0
0.5
-
A
C
D
E
F
G
H
I
K
L
M
N
P
Q
R
S
T
V
W
Y
ikalian
opean
astern
nalaya
berian

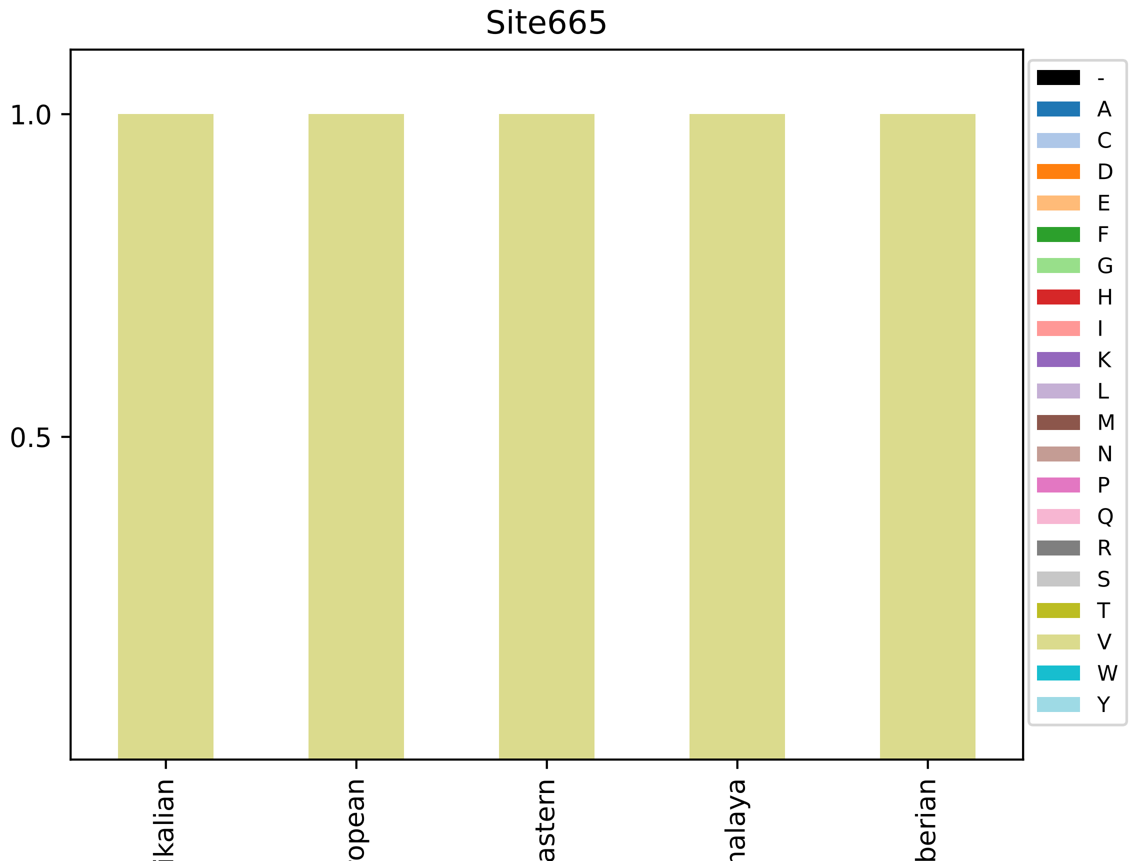

Site665
1.0
0.5
-
A
C
D
E
F
G
H
I
K
L
M
N
P
Q
R
S
T
V
W
Y
ikalian
opean
astern
nalaya
berian

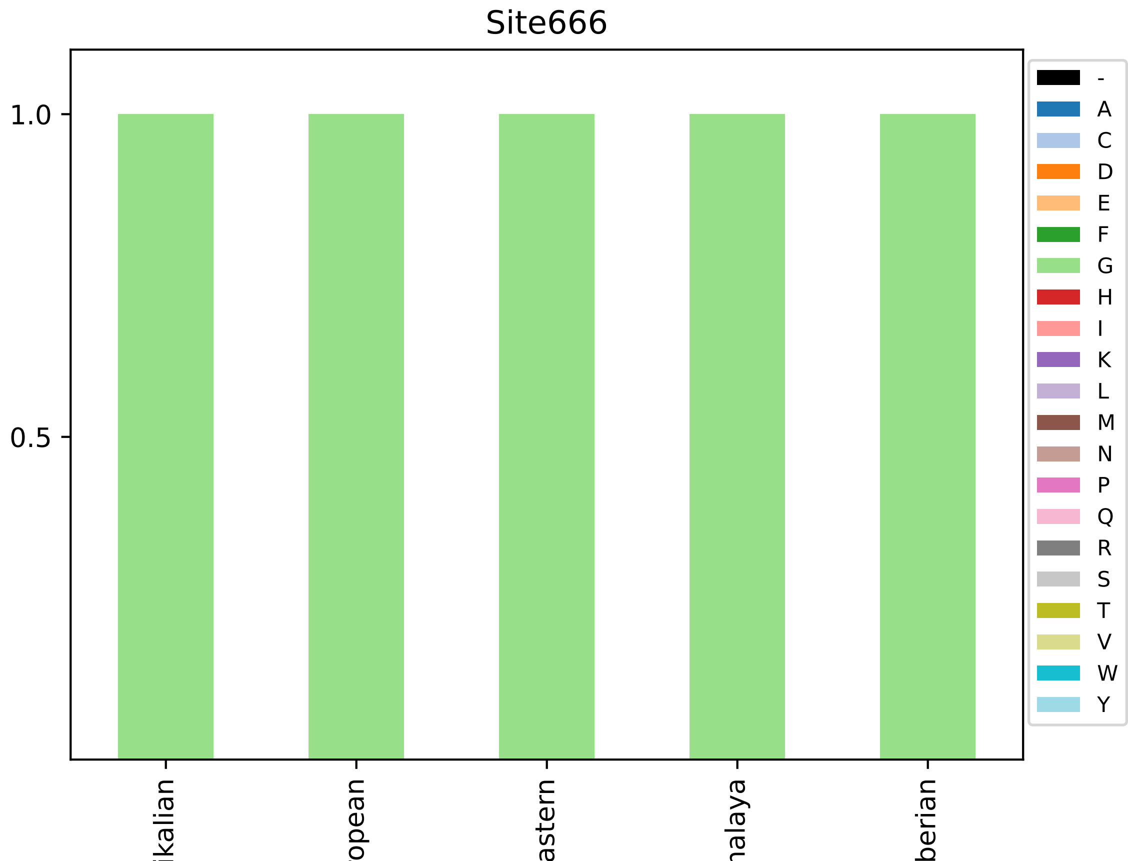

Site666
1.0
0.5
-
A
C
D
E
F
G
H
I
K
L
M
N
P
Q
R
S
T
V
W
Y
ikalian
opean
astern
nalaya
berian

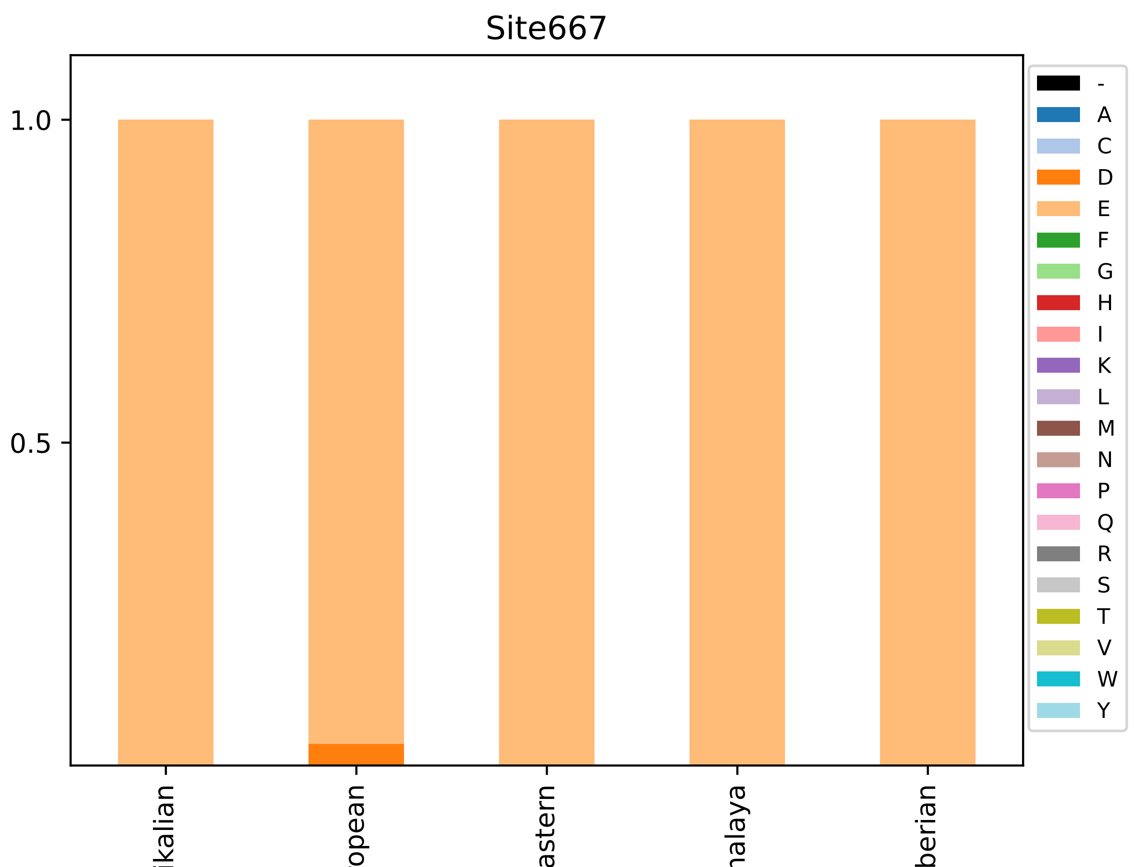
Site667
1.0
0.5
-
A
C
D
E
F
G
H
I
K
L
M
N
P
Q
R
S
T
V
W
Y
ikalian
opean
astern
nalaya
berian

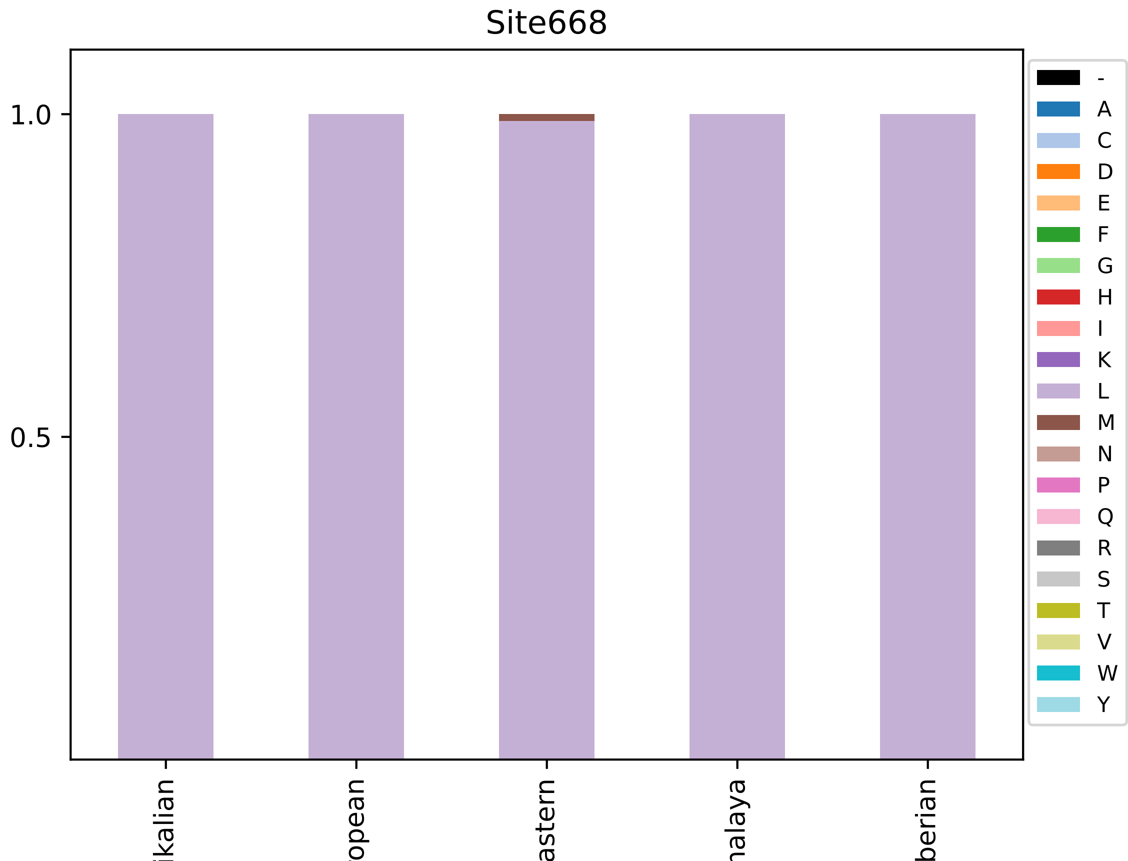

Site668
1.0
0.5
ikalian
opean
astern
nalaya
berian
-
A
C
D
E
F
G
H
I
K
L
M
N
P
Q
R
S
T
V
W
Y

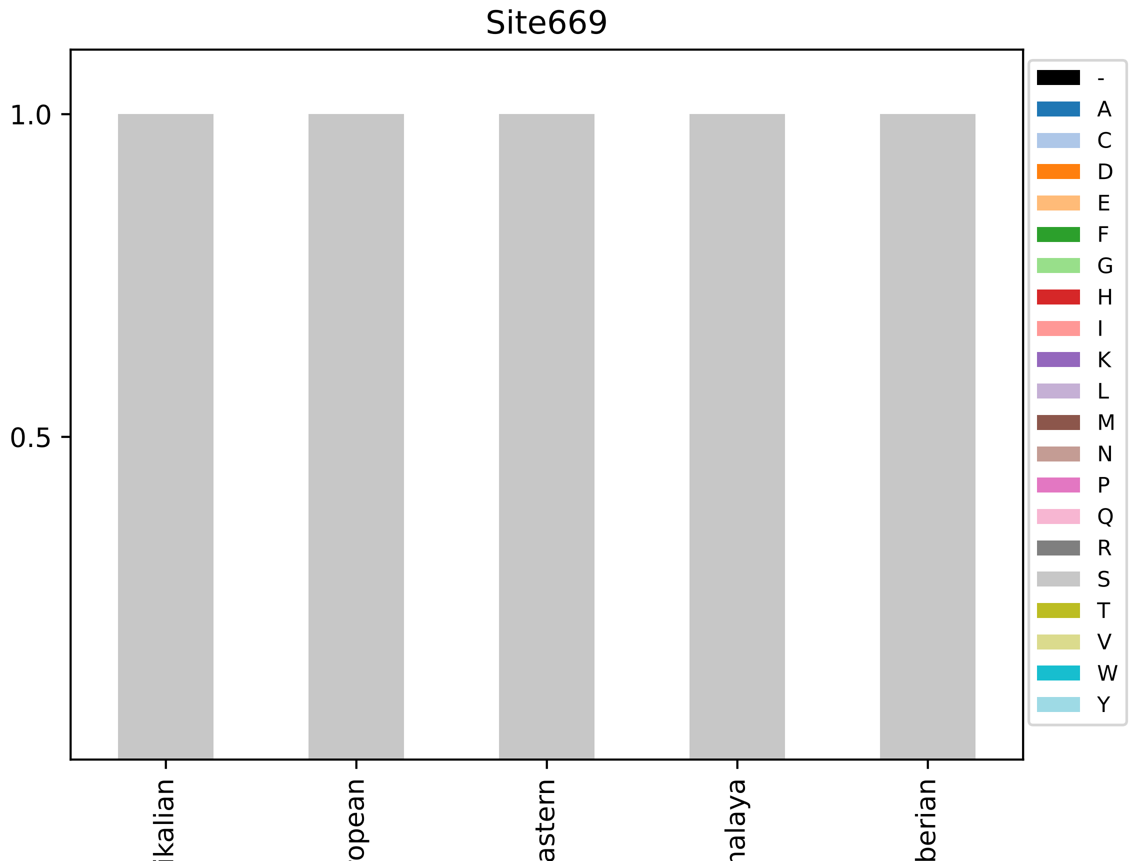

Site669
1.0
0.5
-
A
C
D
E
F
G
H
I
K
L
M
N
P
Q
R
S
T
V
W
Y
ikalian
ropean
astern
nalaya
berian

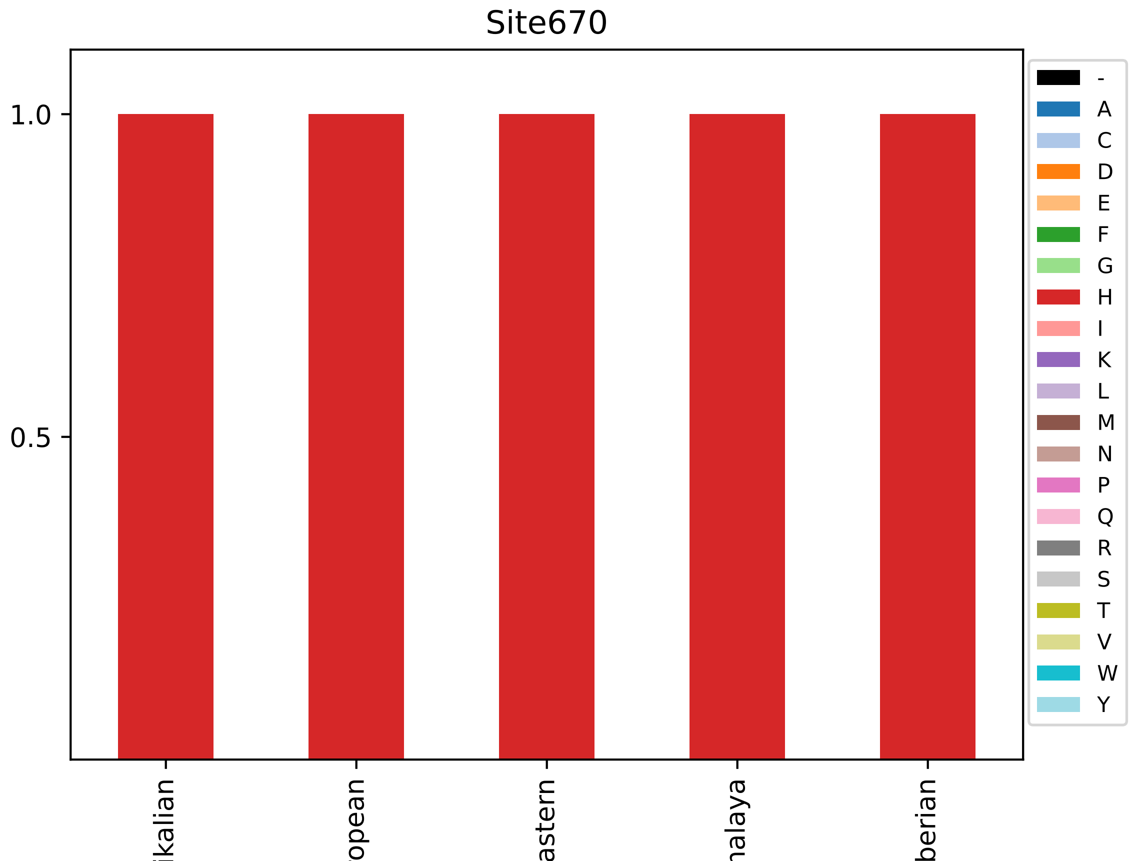

Site670
1.0
0.5
-
A
C
D
E
F
G
H
I
K
L
M
N
P
Q
R
S
T
V
W
Y
ikalian
opean
astern
nalaya
berian

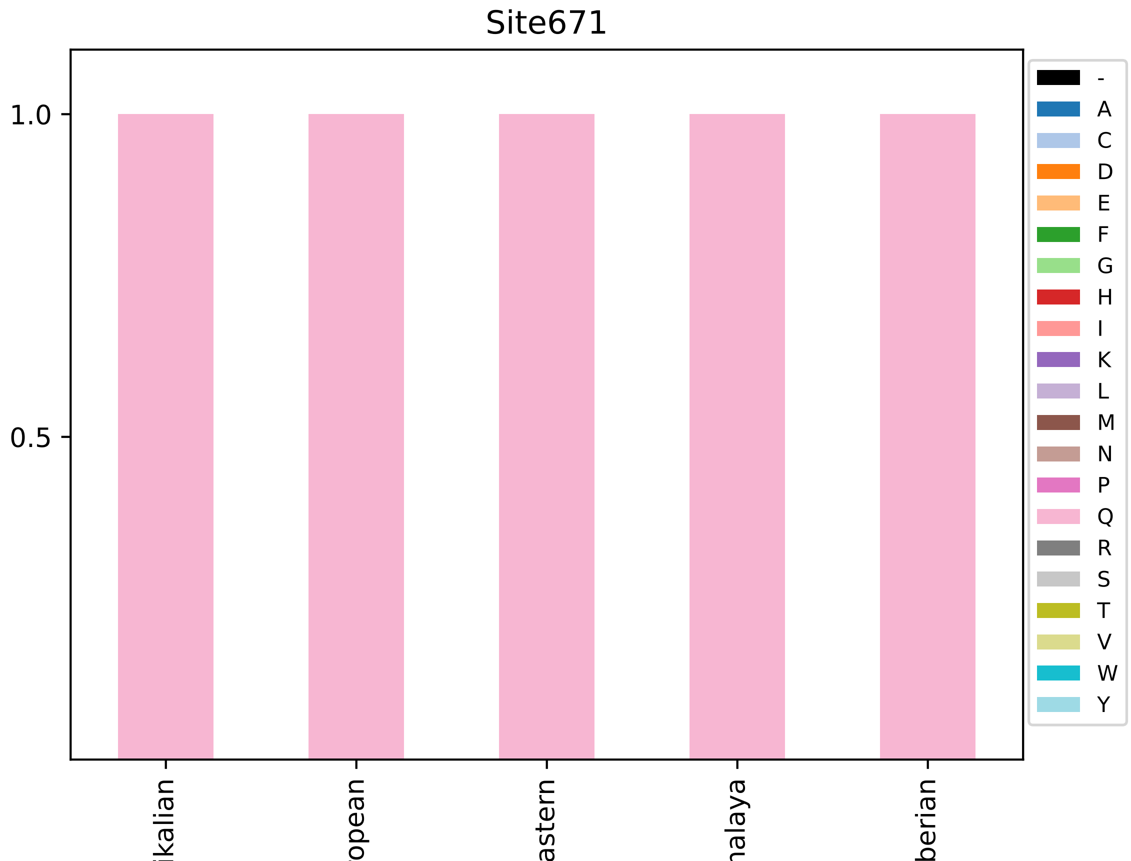
Site671
1.0
0.5
-
A
C
D
E
F
G
H
I
K
L
M
N
P
Q
R
S
T
V
W
Y
ikalian
opean
astern
nalaya
berian

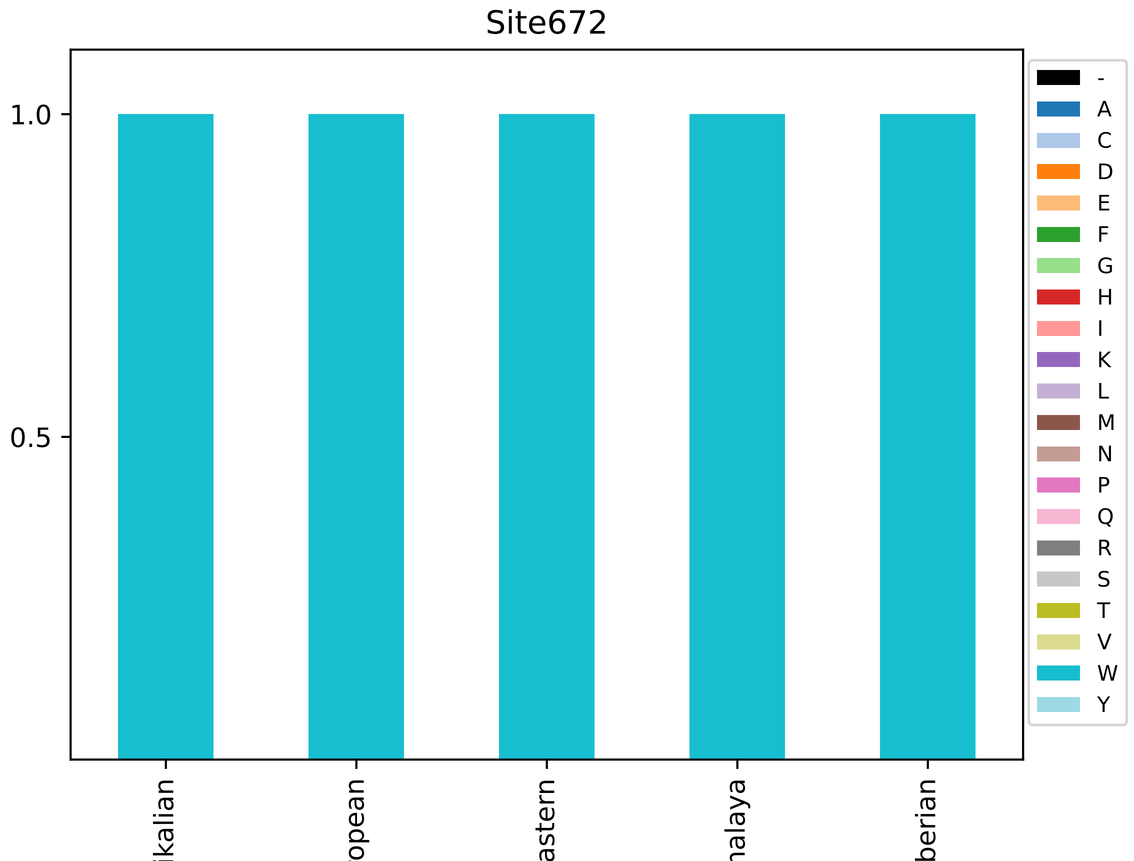

Site672
1.0
0.5
-
A
C
D
E
F
G
H
I
K
L
M
N
P
Q
R
S
T
V
W
Y
ikalian
opean
astern
nalaya
berian

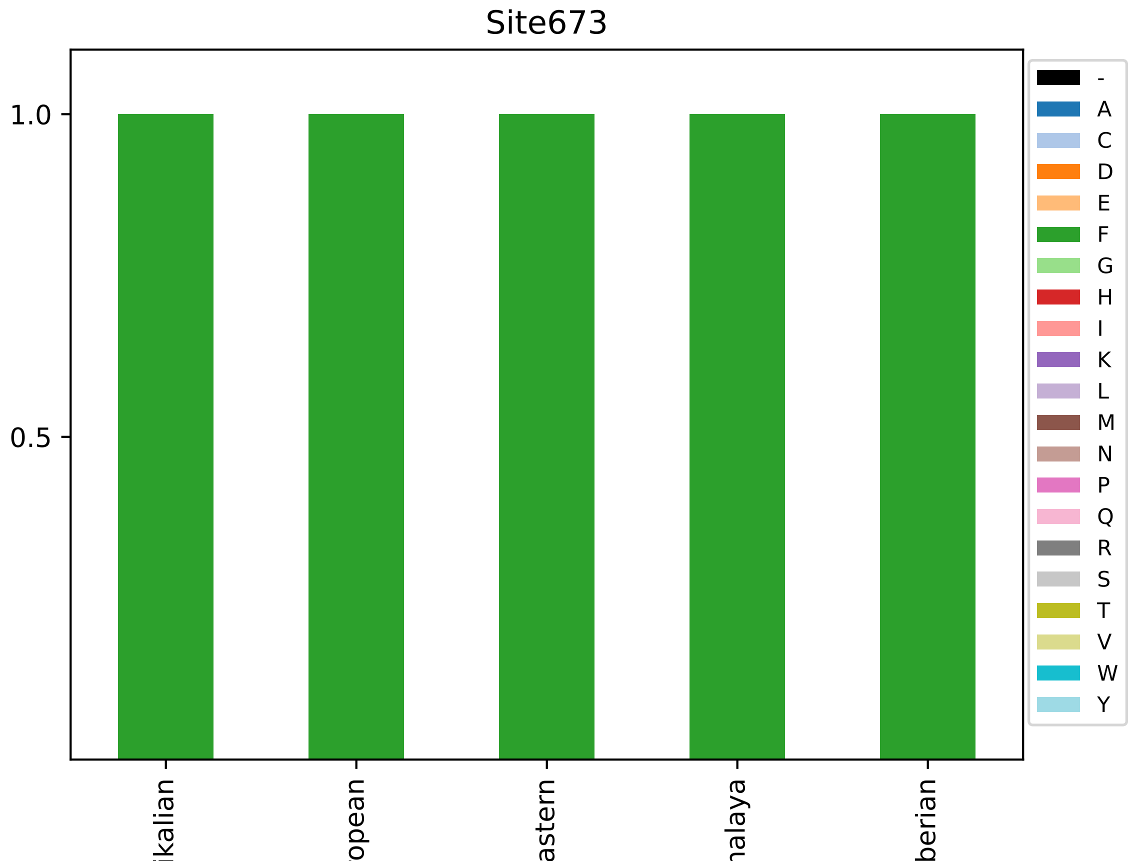

Site673
1.0
0.5
ikalian
opean
astern
nalaya
berian
-
A
C
D
E
F
G
H
I
K
L
M
N
P
Q
R
S
T
V
W
Y

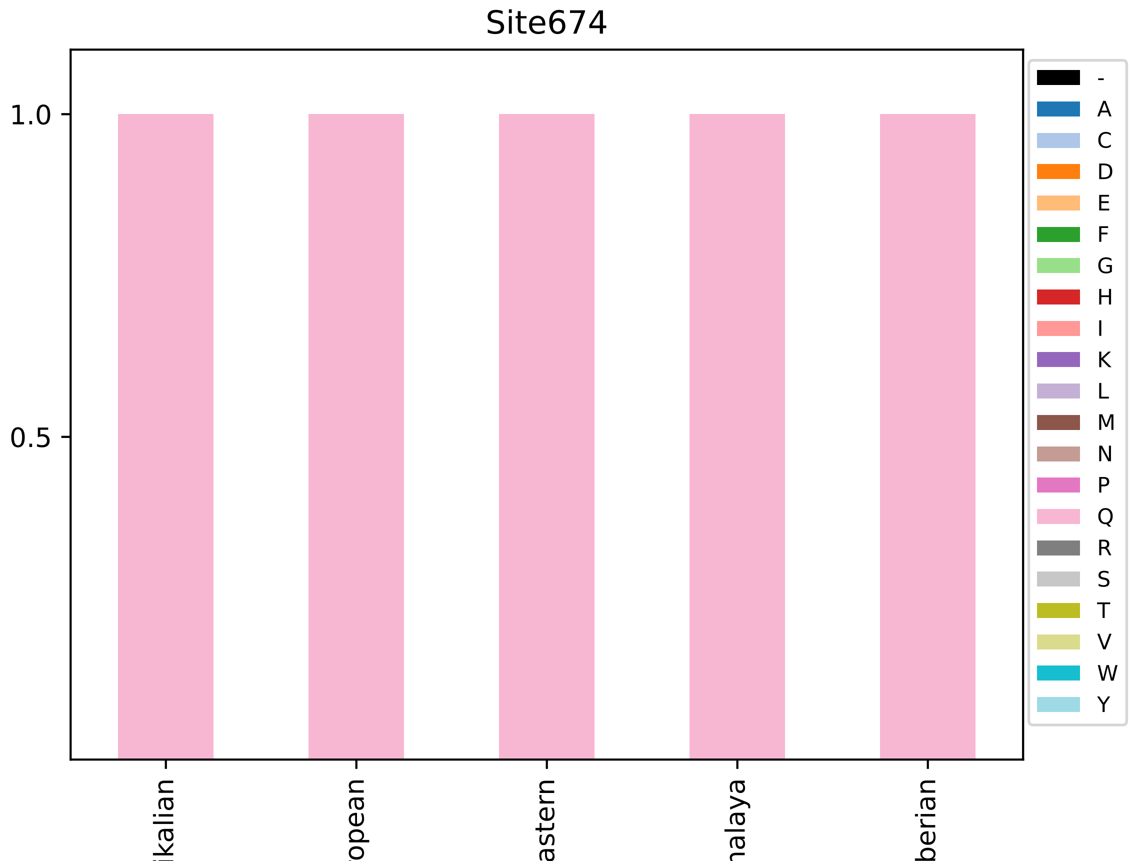

Site674
1.0
0.5
-
A
C
D
E
F
G
H
I
K
L
M
N
P
Q
R
S
T
V
W
Y
ikalian
opean
astern
nalaya
berian

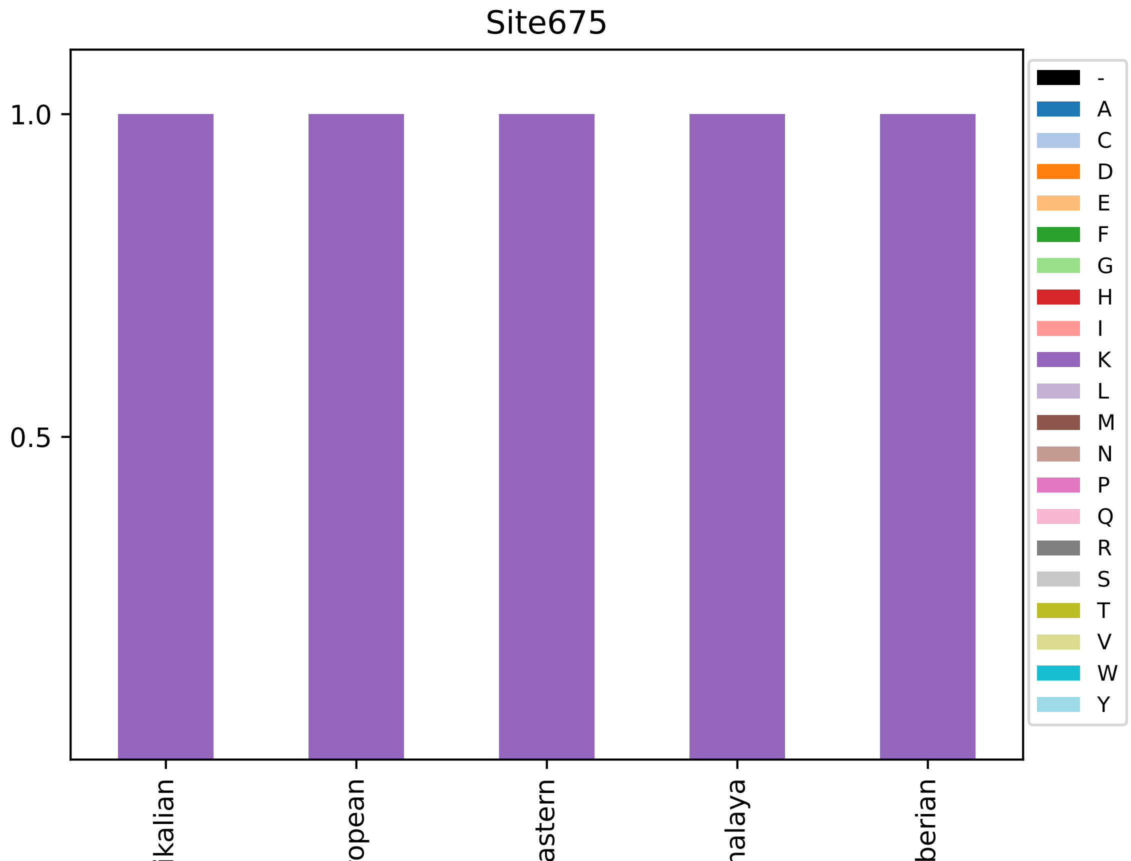
Site675
1.0
0.5
-
A
C
D
E
F
G
H
I
K
L
M
N
P
Q
R
S
T
V
W
Y
ikalian
opean
astern
nalaya
berian

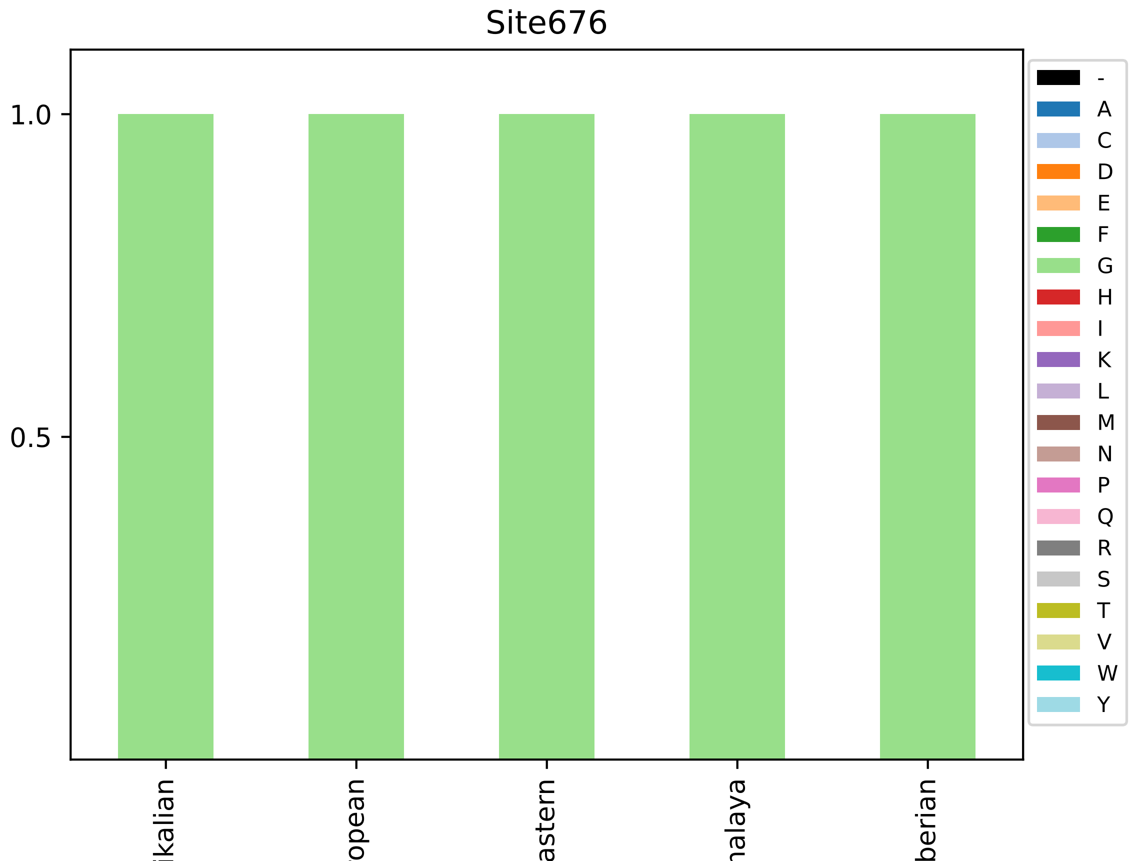
Site676
1.0
0.5
-
A
C
D
E
F
G
H
I
K
L
M
N
P
Q
R
S
T
V
W
Y
ikalian
opean
astern
nalaya
berian

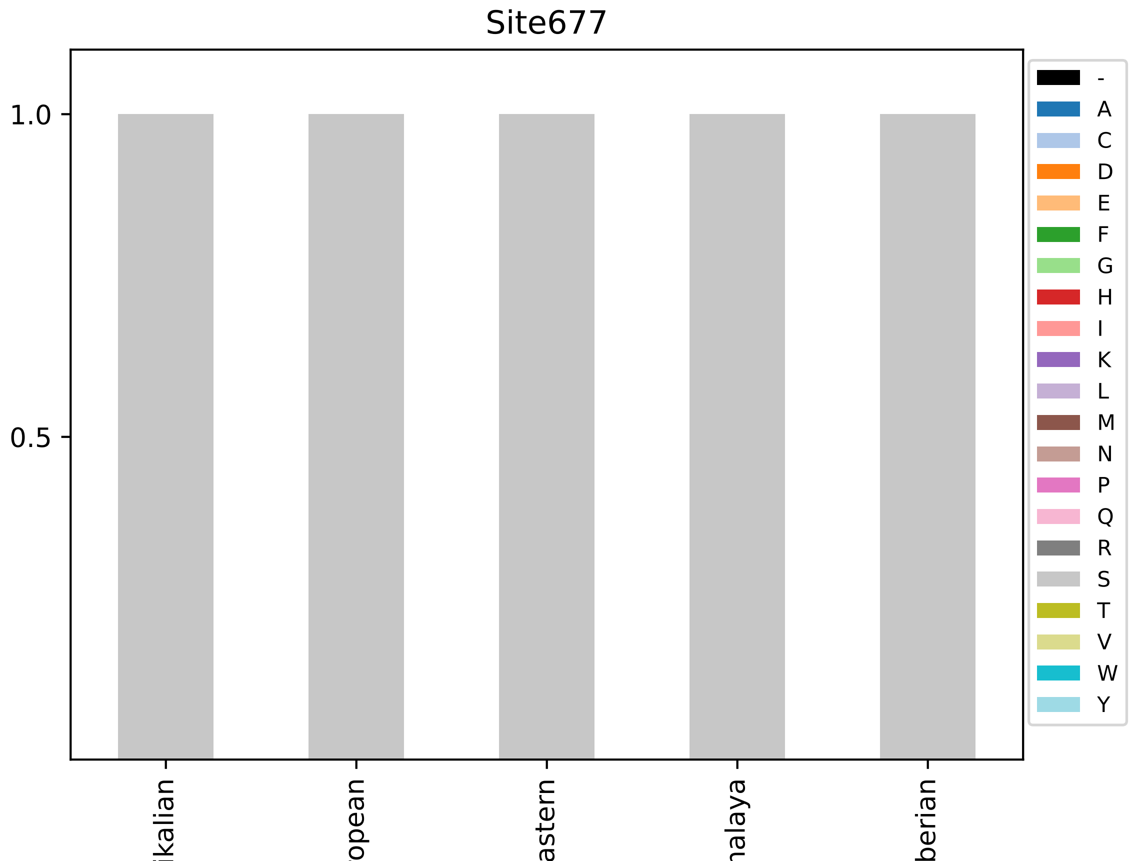
Site677
1.0
0.5
ikalian
opean
astern
nalaya
berian
-
A
C
D
E
F
G
H
I
K
L
M
N
P
Q
R
S
T
V
W
Y

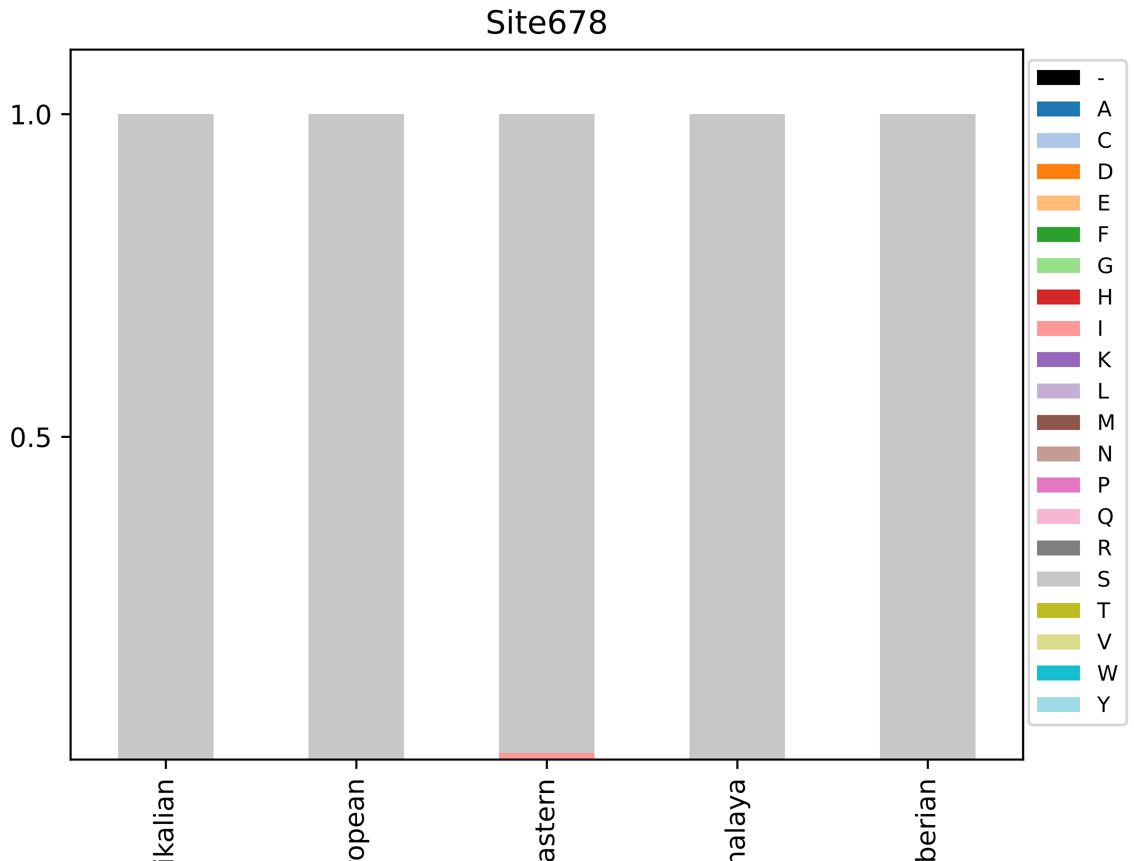

Site678
1.0
0.5
-
A
C
D
E
F
G
H
I
K
L
M
N
P
Q
R
S
T
V
W
Y
ikalian
opean
astern
nalaya
berian

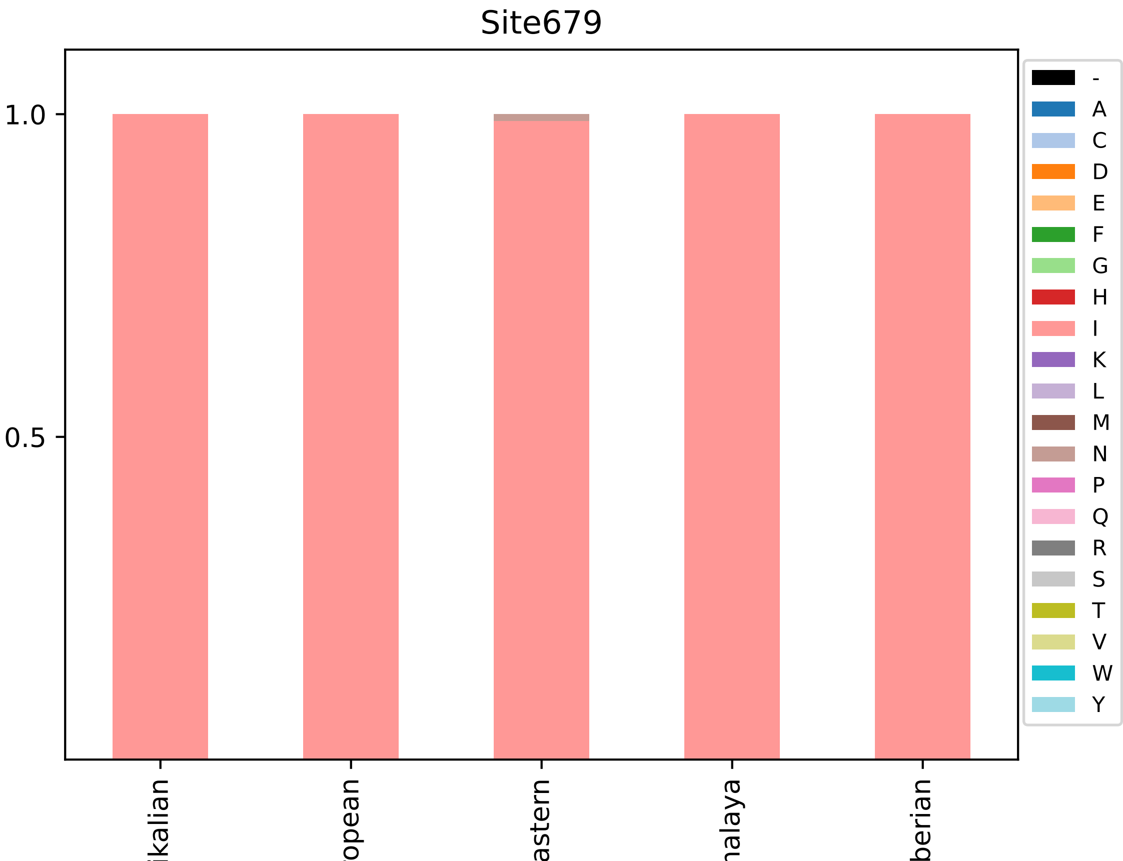
Site679
1.0
0.5
-
A
C
D
E
F
G
H
I
K
L
M
N
P
Q
R
S
T
V
W
Y
ikalian
ropean
astern
nalaya
berian

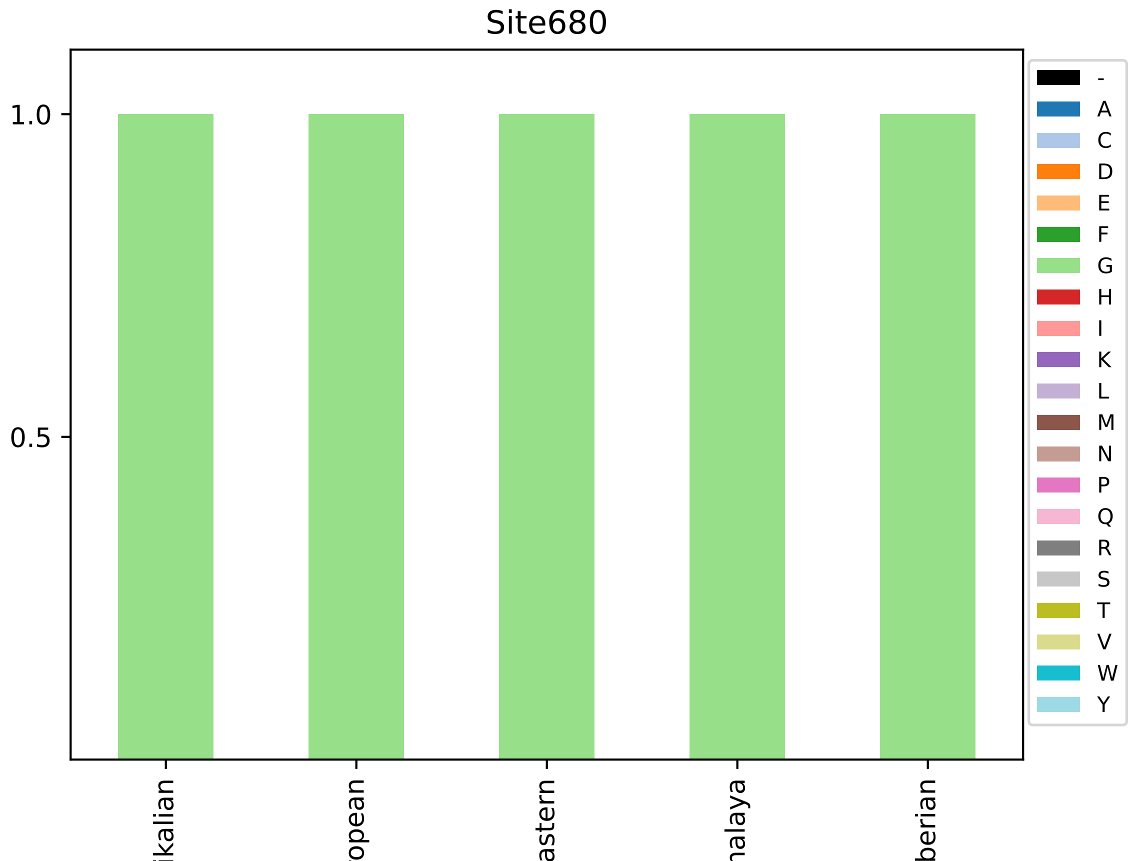

Site680
1.0
0.5
ikalian
ropean
astern
nalaya
berian
-
A
C
D
E
F
G
H
I
K
L
M
N
P
Q
R
S
T
V
W
Y

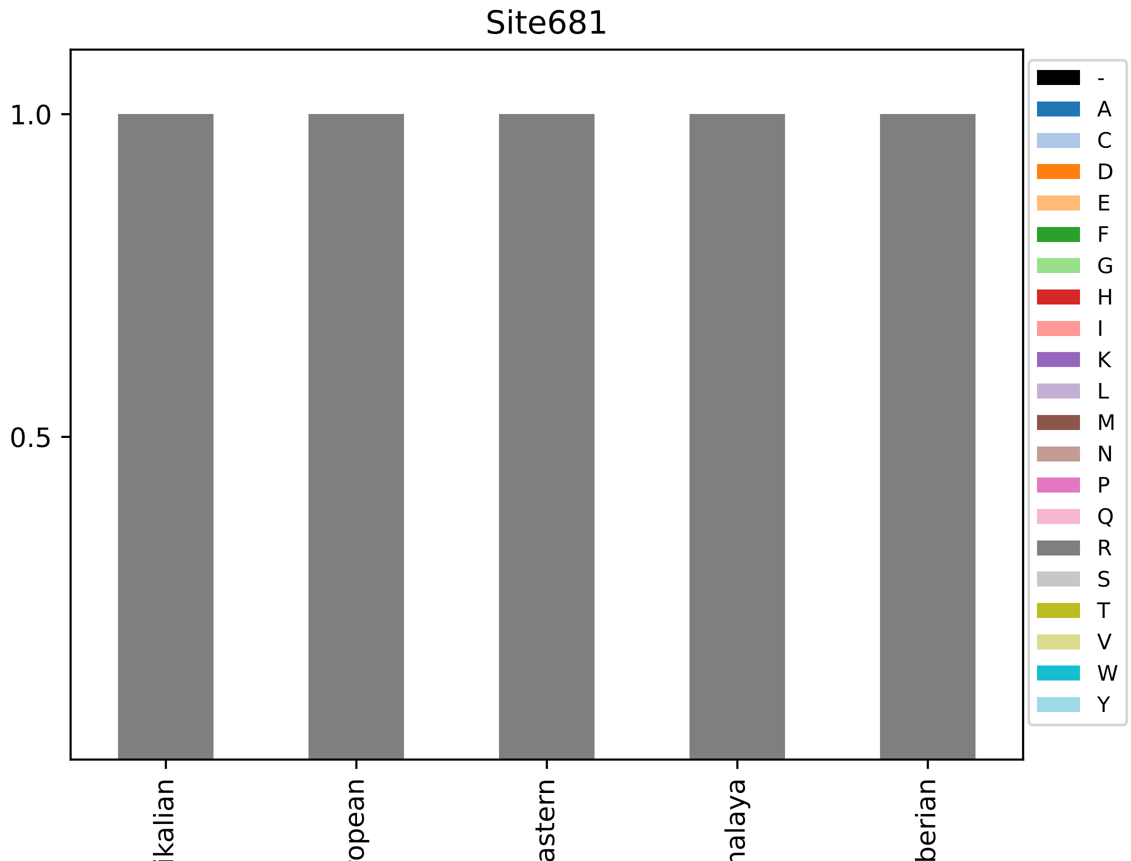
Site681
1.0
0.5
-
A
C
D
E
F
G
H
I
K
L
M
N
P
Q
R
S
T
V
W
Y
ikalian
opean
astern
nalaya
berian

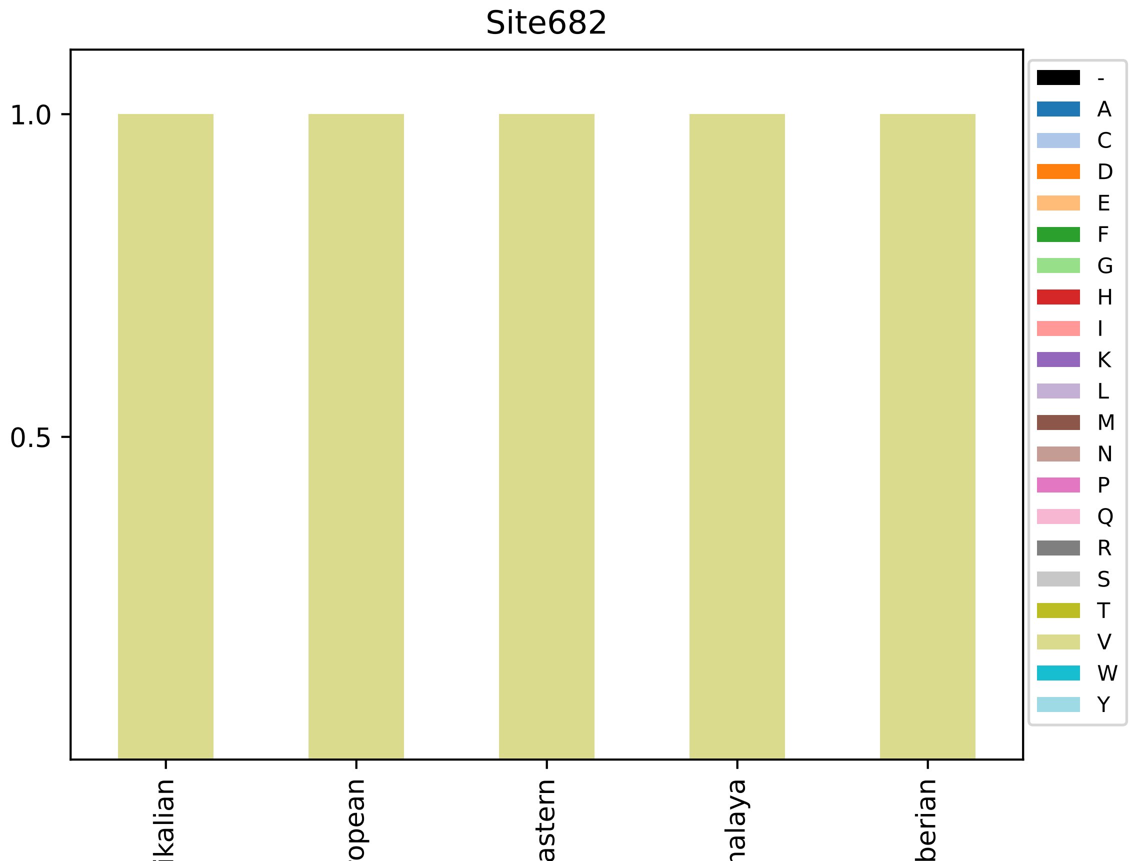

Site682
1.0
0.5
-
A
C
D
E
F
G
H
I
K
L
M
N
P
Q
R
S
T
V
W
Y

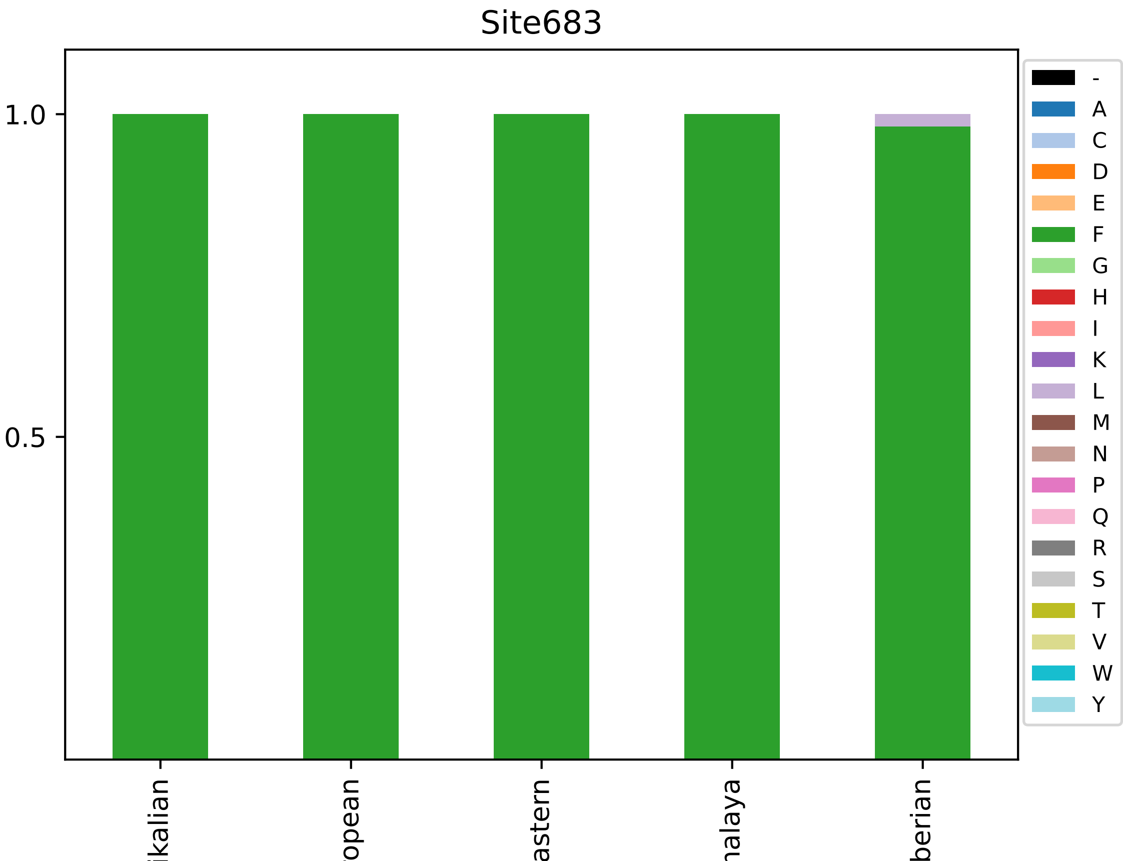

Site683
1.0
0.5
-
A
C
D
E
F
G
H
I
K
L
M
N
P
Q
R
S
T
V
W
Y
ikalian
opean
astern
nalaya
berian

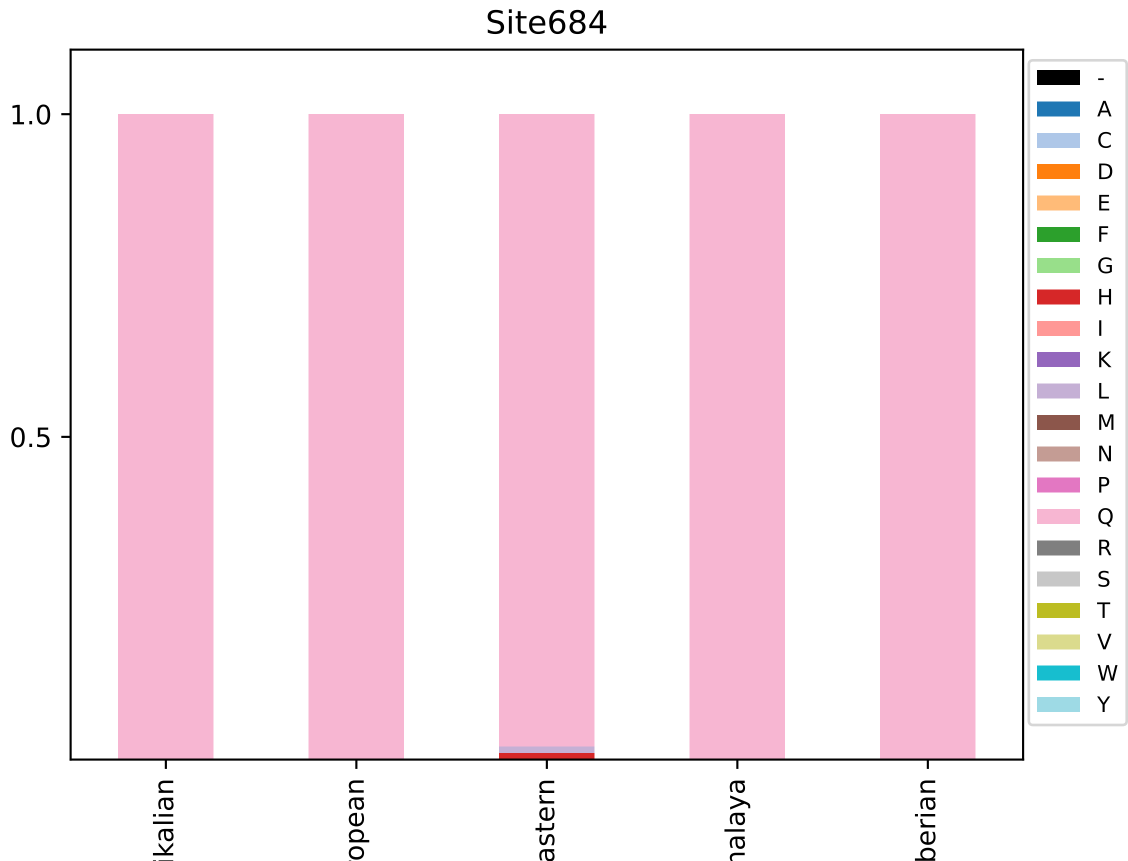

Site684
1.0
0.5
-
A
C
D
E
F
G
H
I
K
L
M
N
P
Q
R
S
T
V
W
Y
ikalian
opean
astern
nalaya
berian

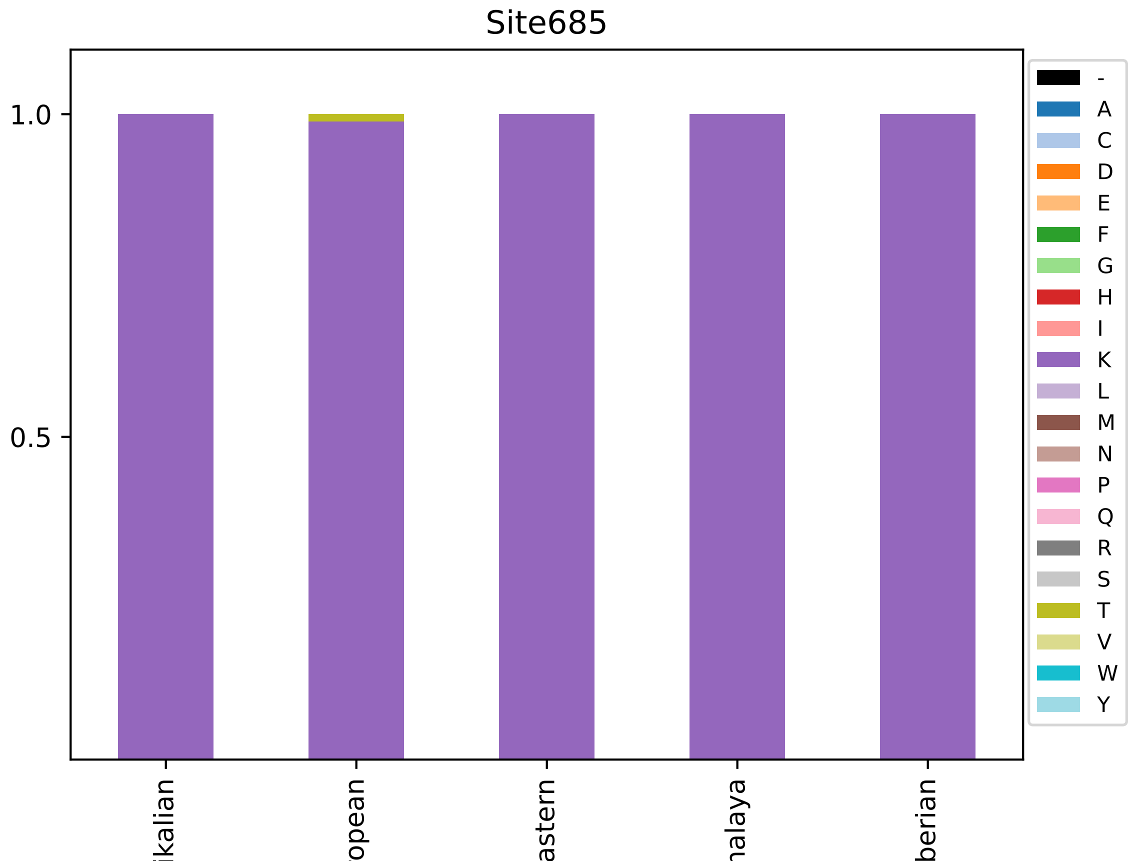
Site685
1.0
0.5
-
A
C
D
E
F
G
H
I
K
L
M
N
P
Q
R
S
T
V
W
Y
ikalian
opean
astern
nalaya
berian

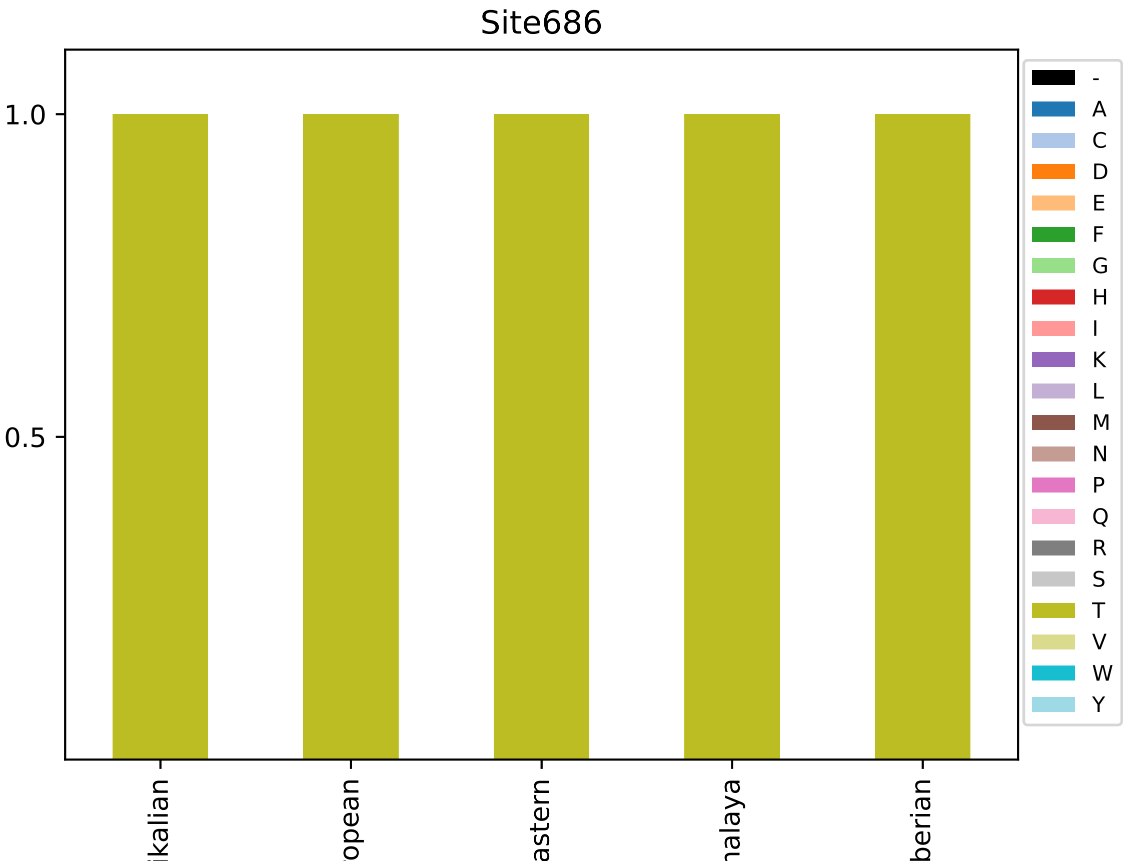
Site686
1.0
0.5
-
A
C
D
E
F
G
H
I
K
L
M
N
P
Q
R
S
T
V
W
Y
ikalian
opean
astern
nalaya
berian

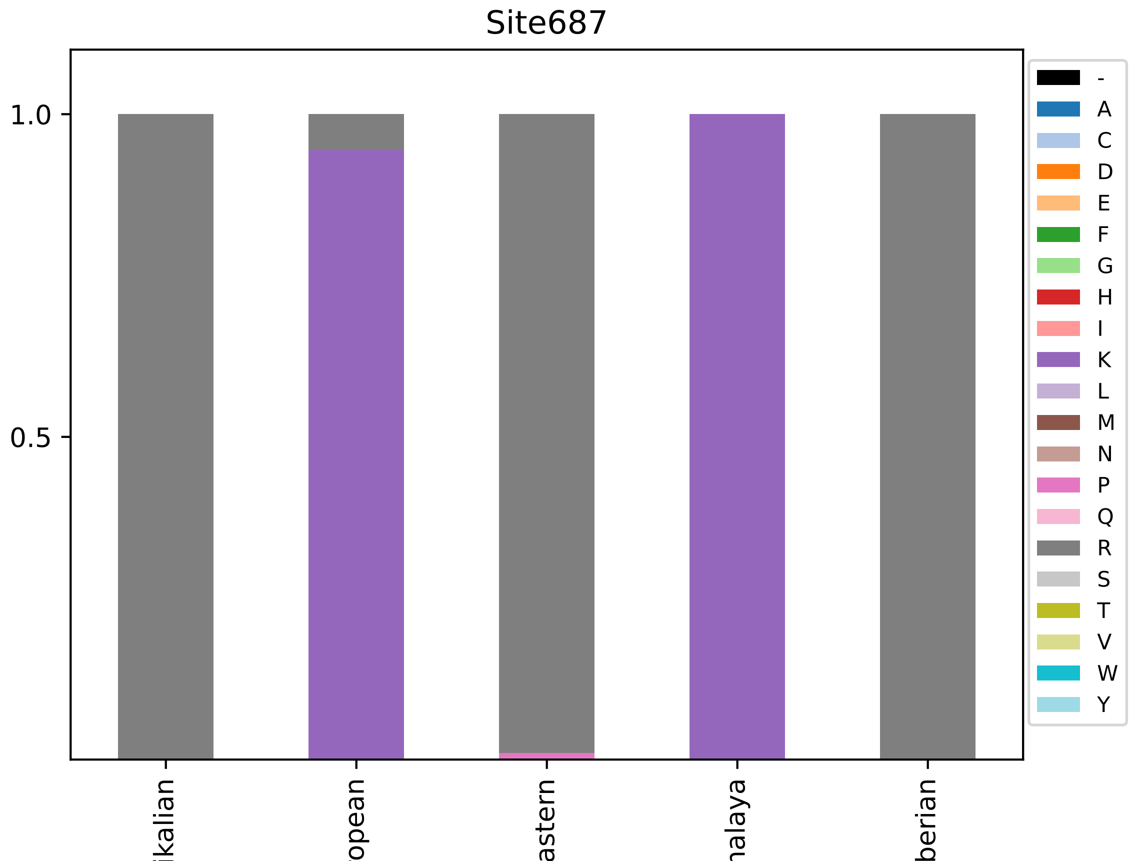

Site687
1.0
0.5
-
A
C
D
E
F
G
H
I
K
L
M
N
P
Q
R
S
T
V
W
Y
ikalian
opean
astern
nalaya
berian

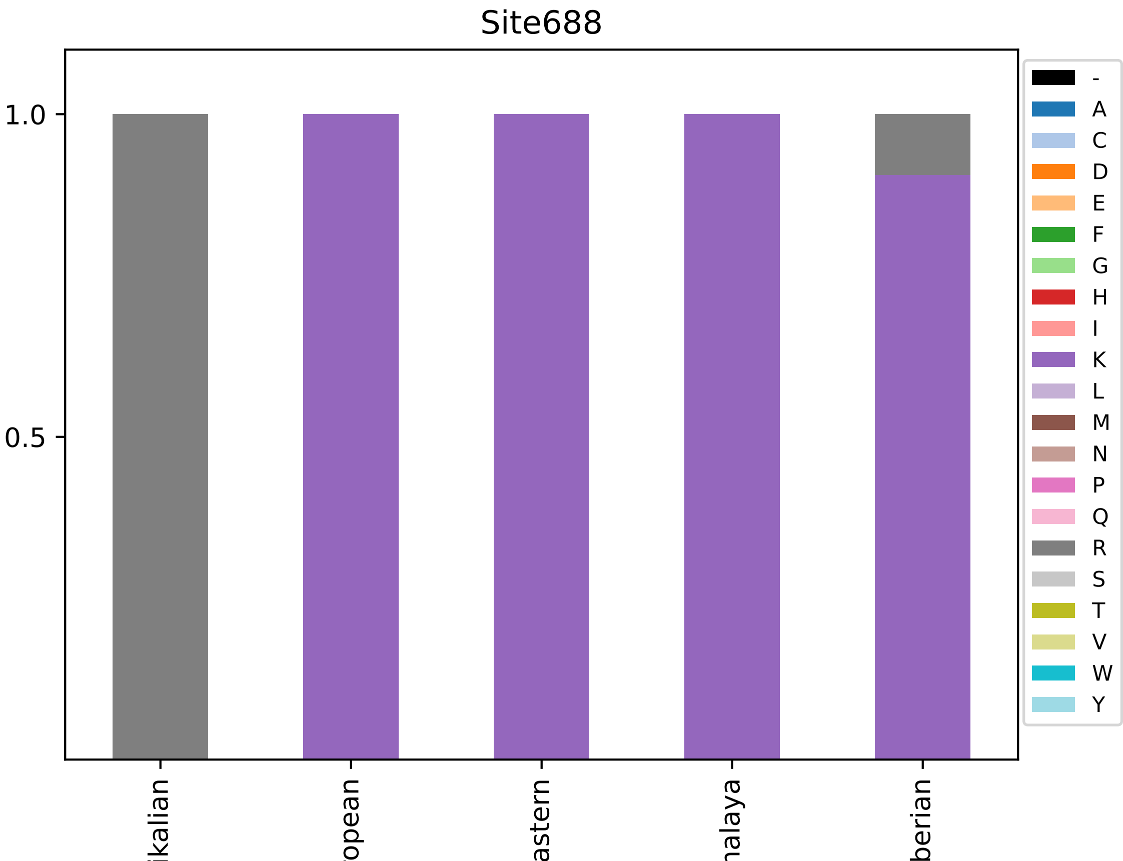
Site688
1.0
0.5
-
A
C
D
E
F
G
H
I
K
L
M
N
P
Q
R
S
T
V
W
Y
ikalian
opean
astern
nalaya
berian

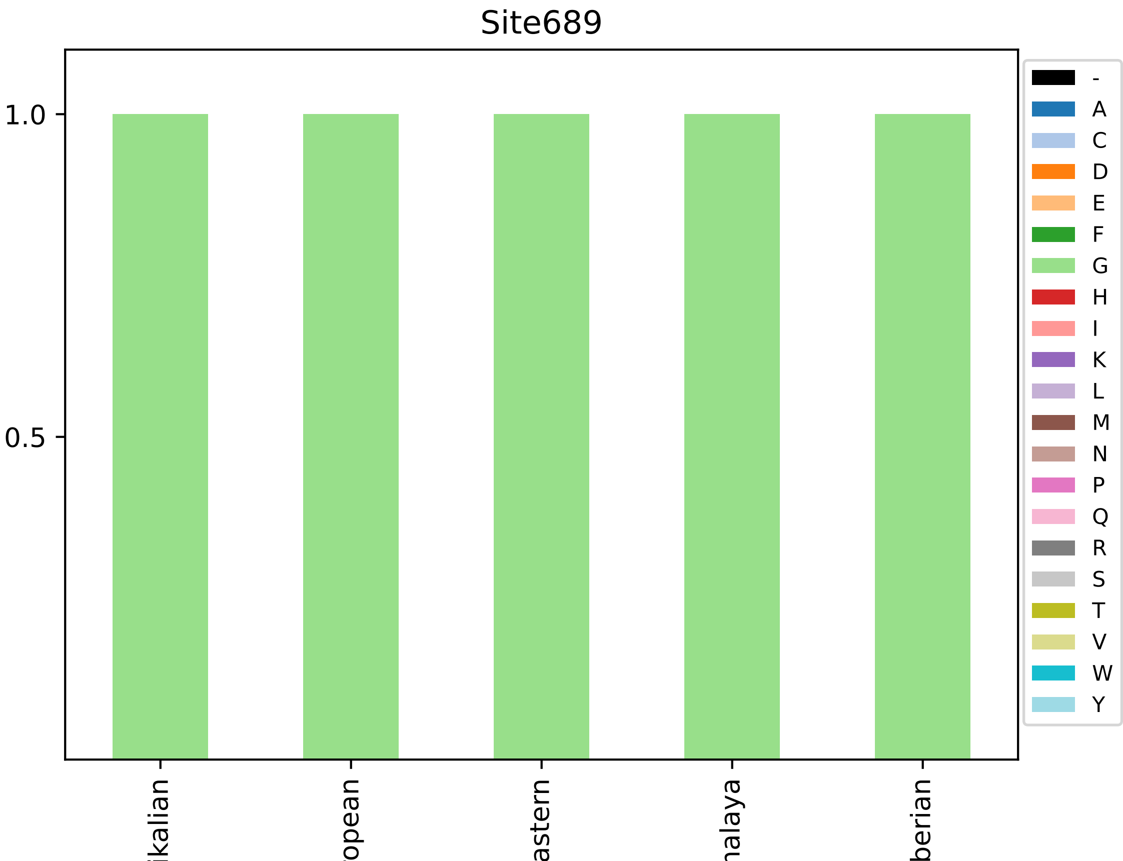
Site689
1.0
0.5
ikalian
opean
astern
nalaya
berian
-
A
C
D
E
F
G
H
I
K
L
M
N
P
Q
R
S
T
V
W
Y

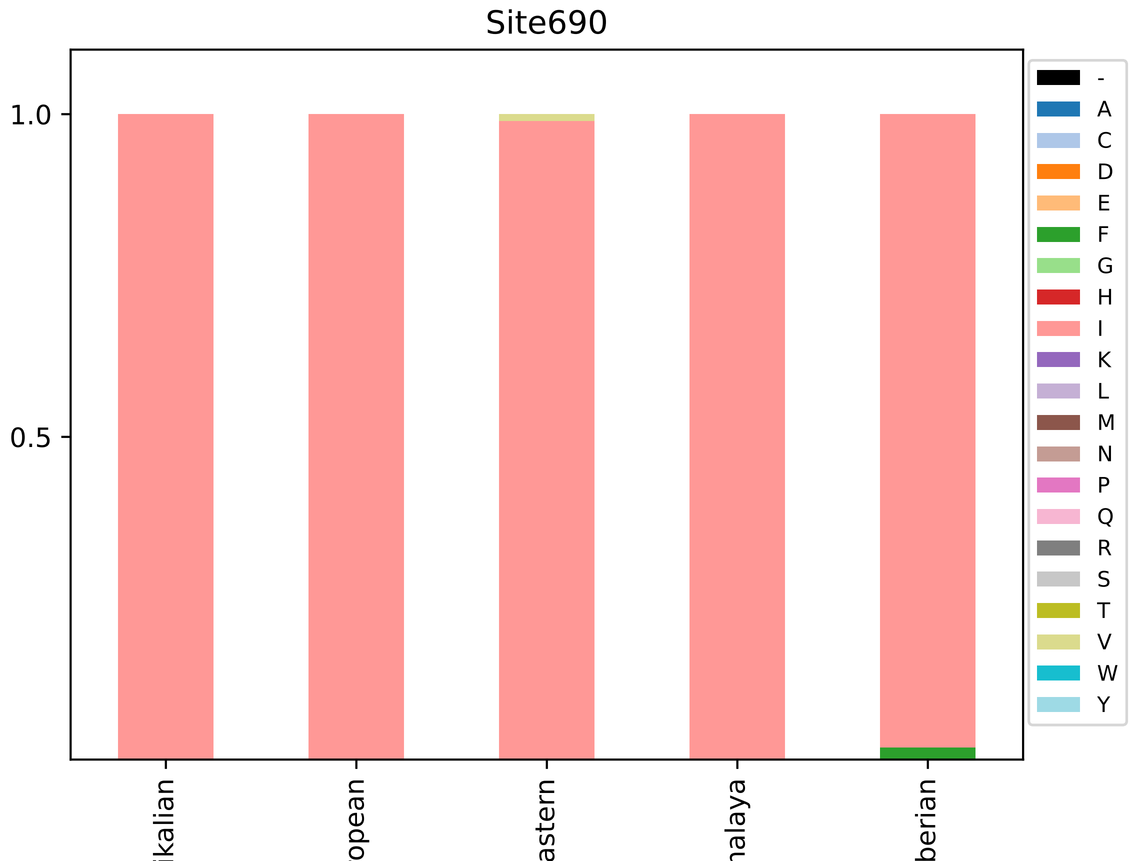
Site690
1.0
0.5
-
A
C
D
E
F
G
H
I
K
L
M
N
P
Q
R
S
T
V
W
Y
ikalian
opean
astern
nalaya
berian

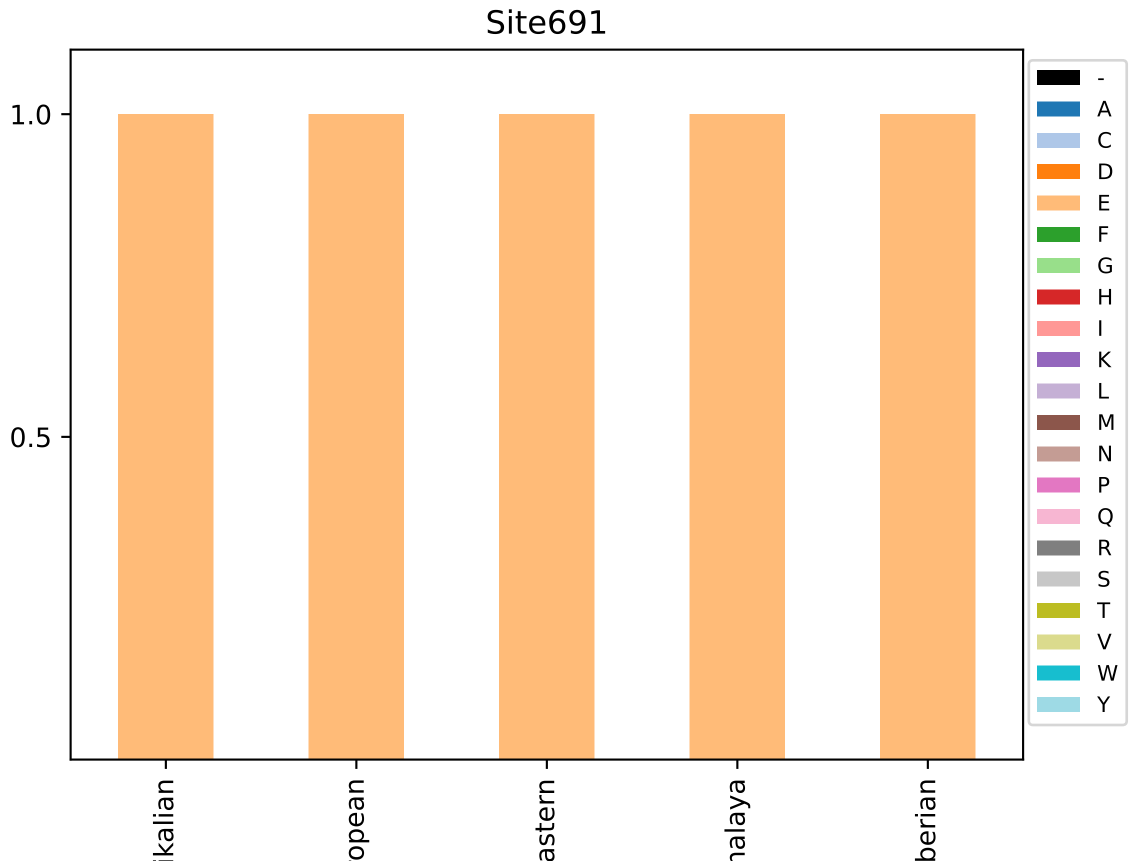

Site691
1.0
0.5
-
A
C
D
E
F
G
H
I
K
L
M
N
P
Q
R
S
T
V
W
Y
ikalian
opean
astern
nalaya
berian

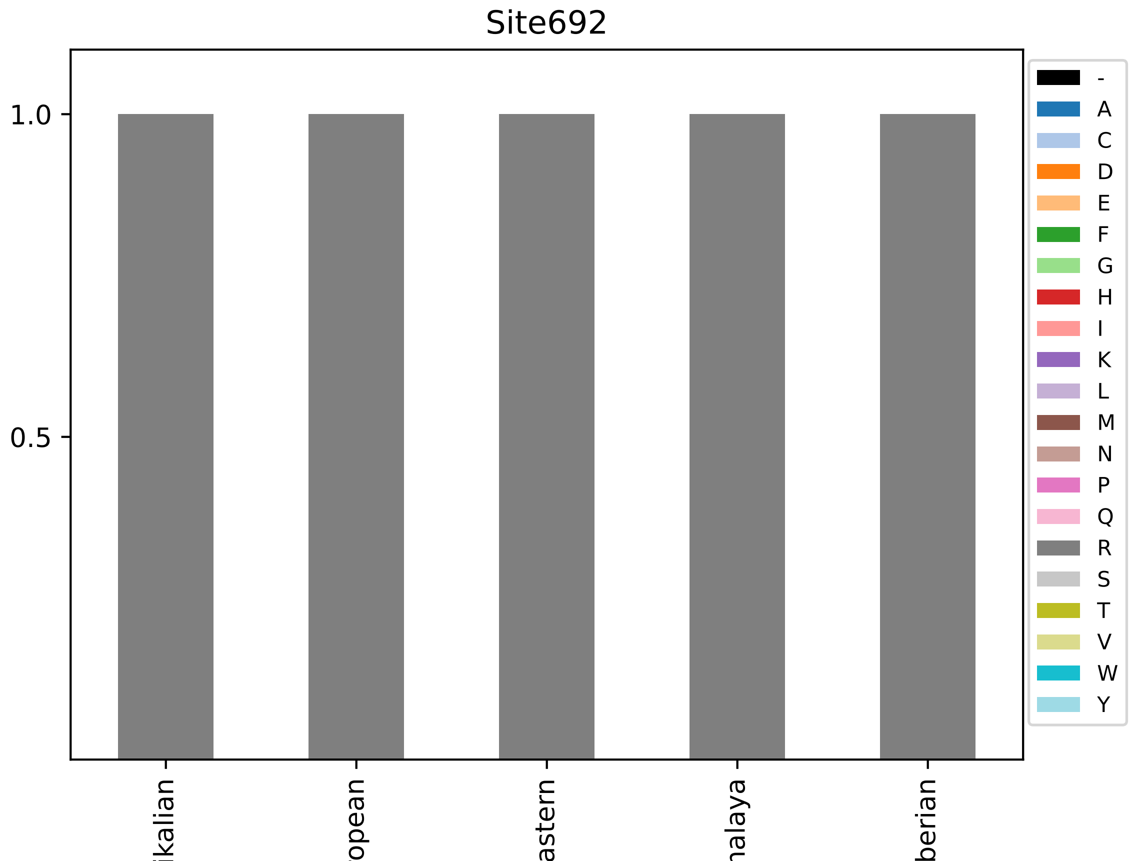

Site692
1.0
0.5
ikalian
opean
astern
nalaya
berian
-
A
C
D
E
F
G
H
I
K
L
M
N
P
Q
R
S
T
V
W
Y

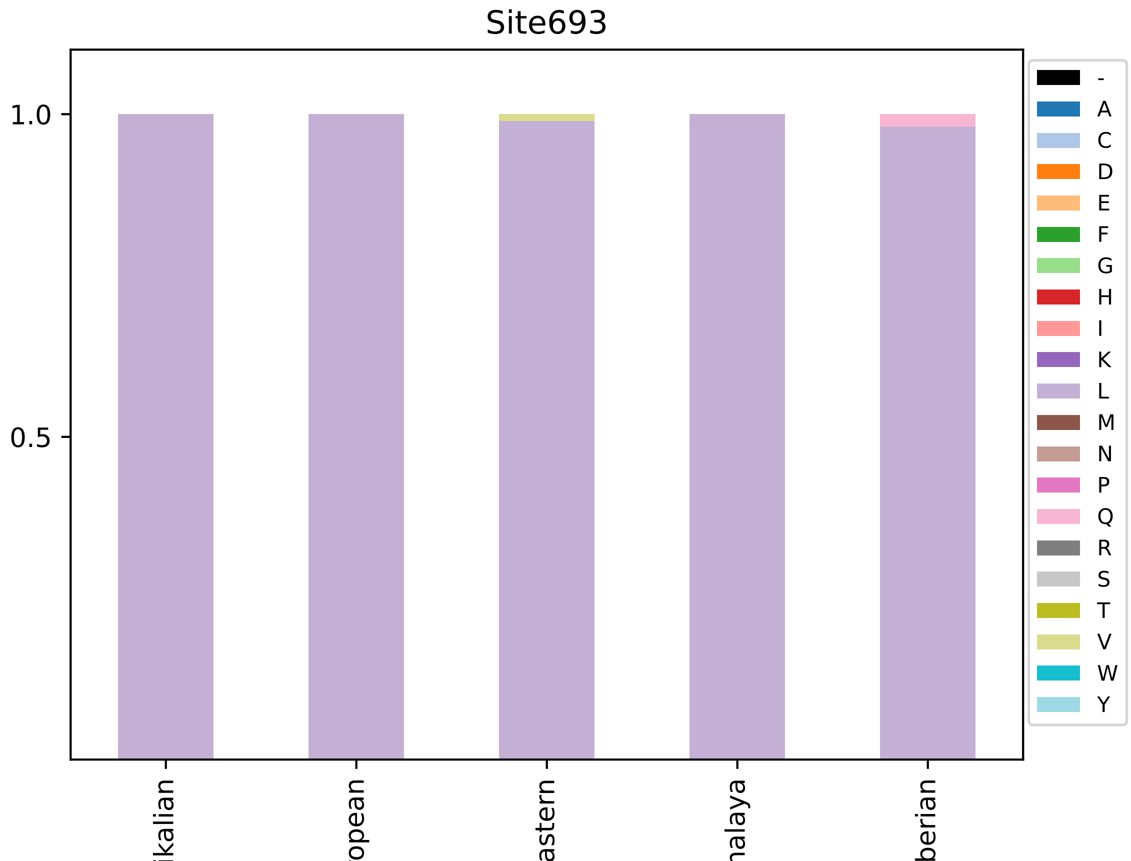

Site693
1.0
0.5
-
A
C
D
E
F
G
H
I
K
L
M
N
P
Q
R
S
T
V
W
Y
ikalian
opean
astern
nalaya
berian

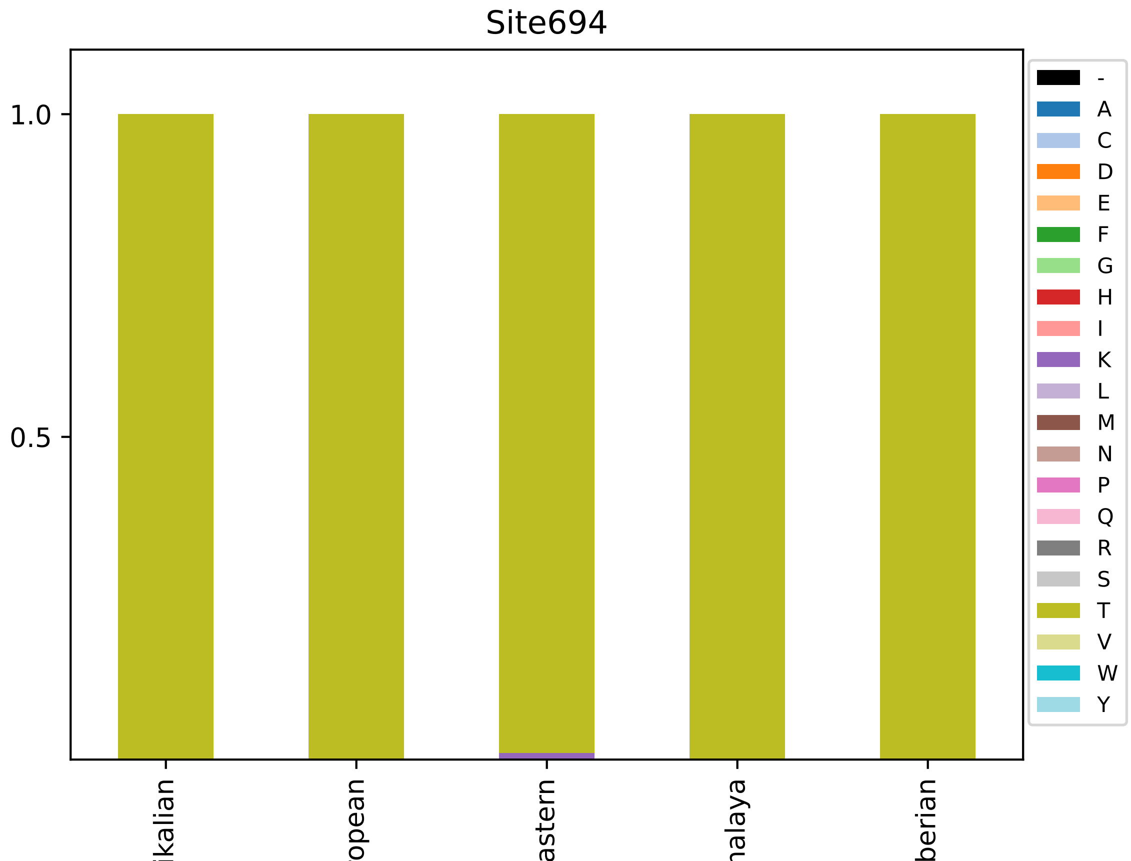
Site694
1.0
0.5
ikalian
opean
astern
nalaya
berian
-
A
C
D
E
F
G
H
I
K
L
M
N
P
Q
R
S
T
V
W
Y

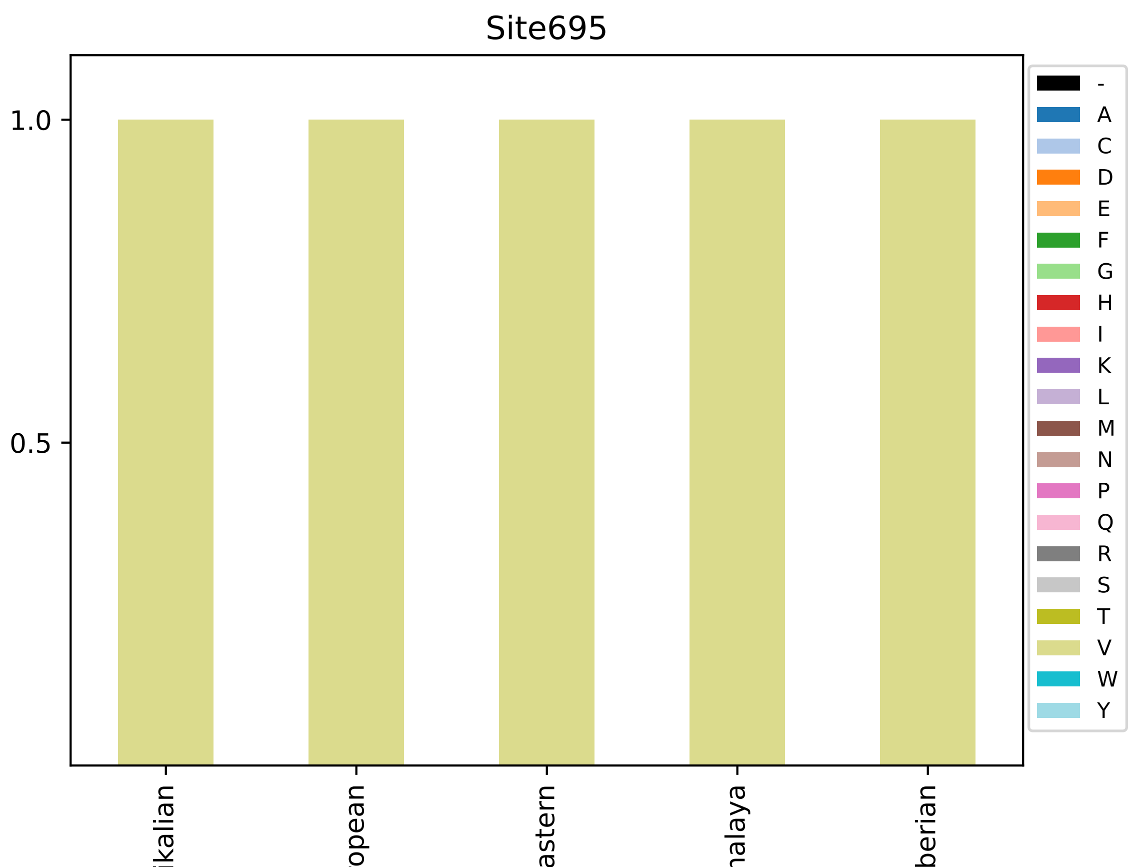
Site695
1.0
0.5
ikalian
opean
astern
nalaya
berian
-
A
C
D
E
F
G
H
I
K
L
M
N
P
Q
R
S
T
V
W
Y

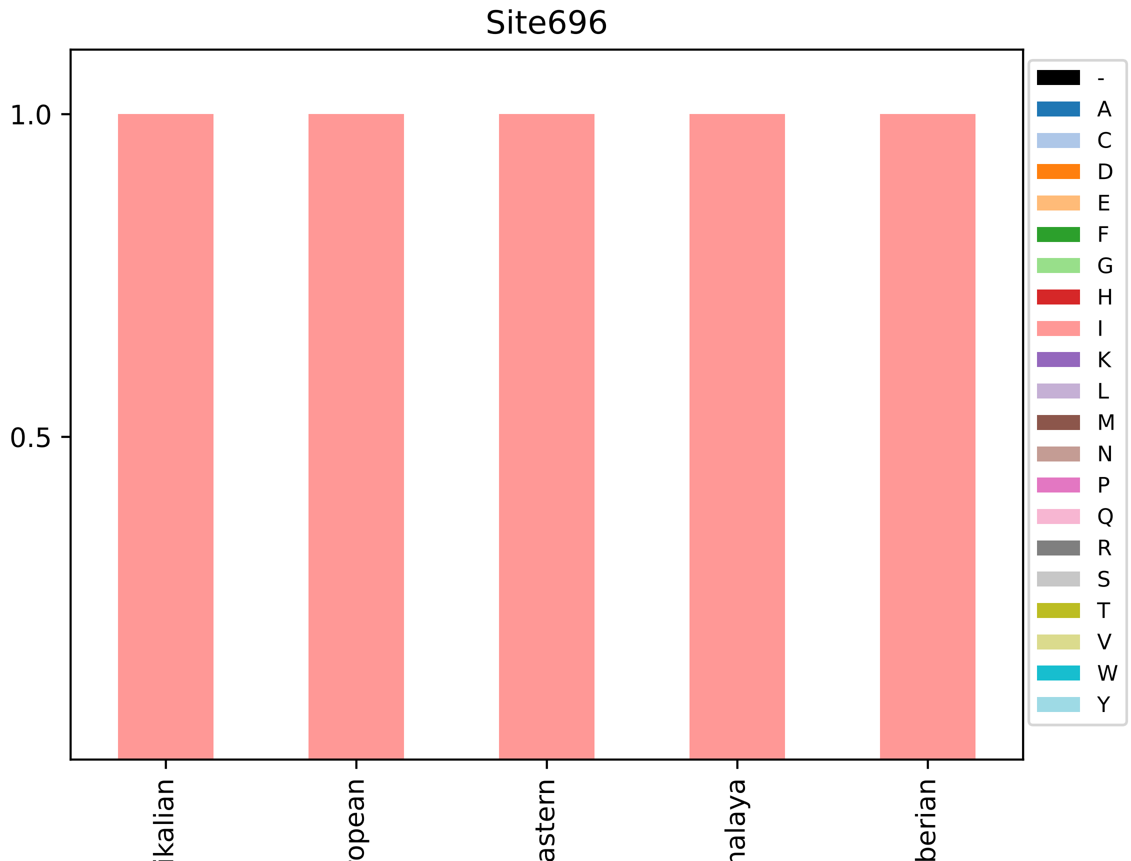
Site696
1.0
0.5
-
A
C
D
E
F
G
H
I
K
L
M
N
P
Q
R
S
T
V
W
Y
ikalian
opean
astern
nalaya
berian

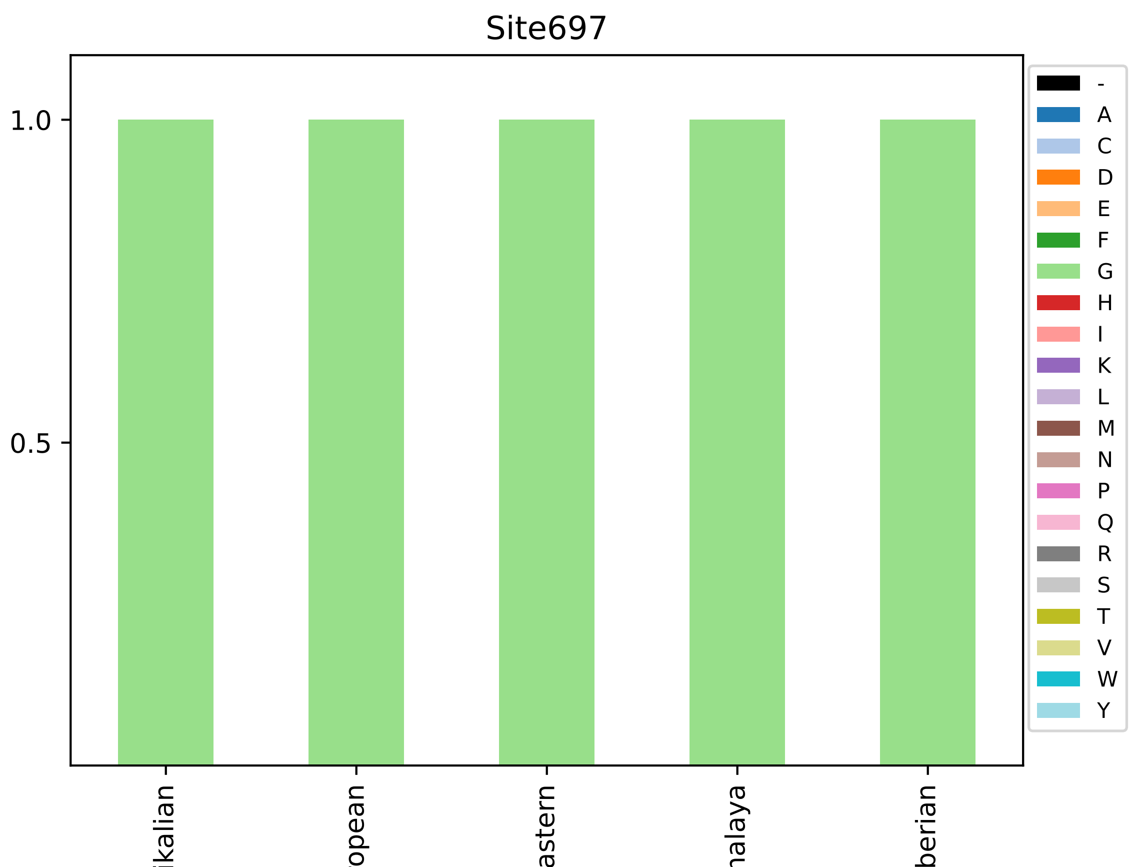

Site697
1.0
0.5
-
A
C
D
E
F
G
H
I
K
L
M
N
P
Q
R
S
T
V
W
Y
ikalian
opean
astern
nalaya
berian

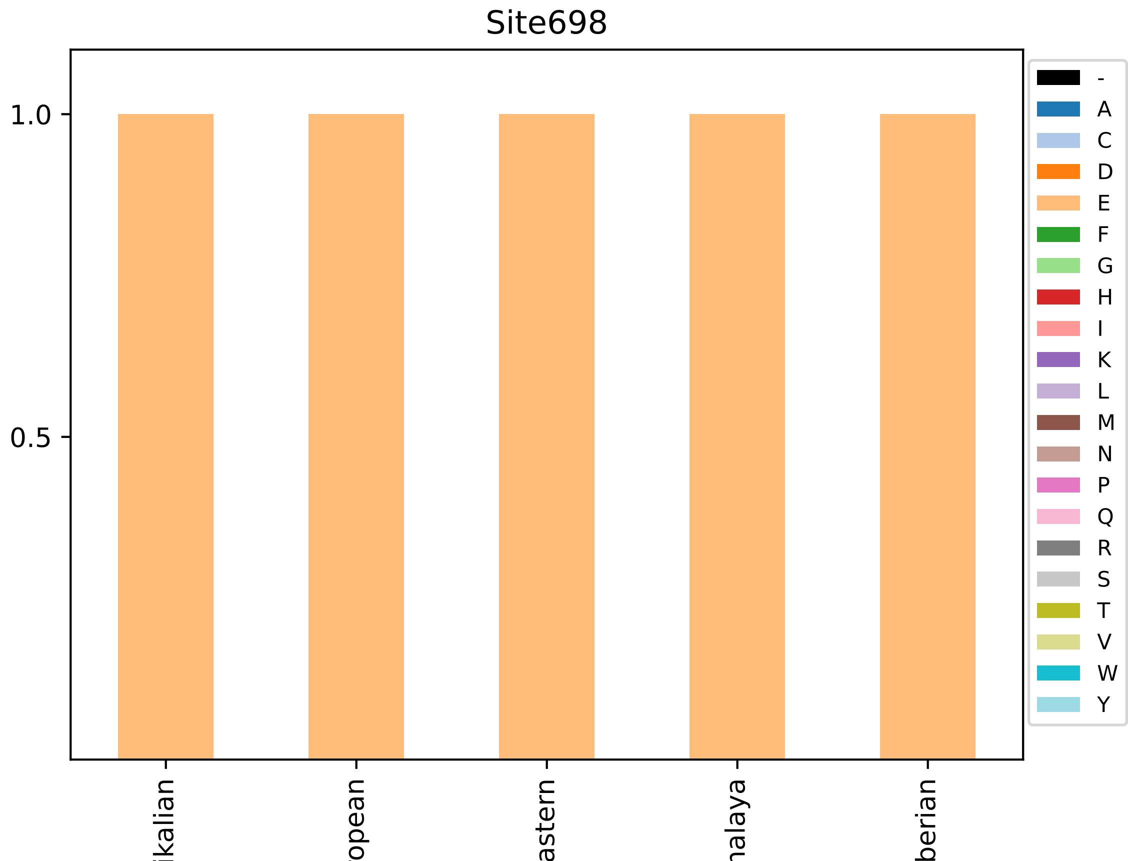
Site698
1.0
0.5
ikalian
opean
astern
nalaya
berian
-
A
C
D
E
F
G
H
I
K
L
M
N
P
Q
R
S
T
V
W
Y

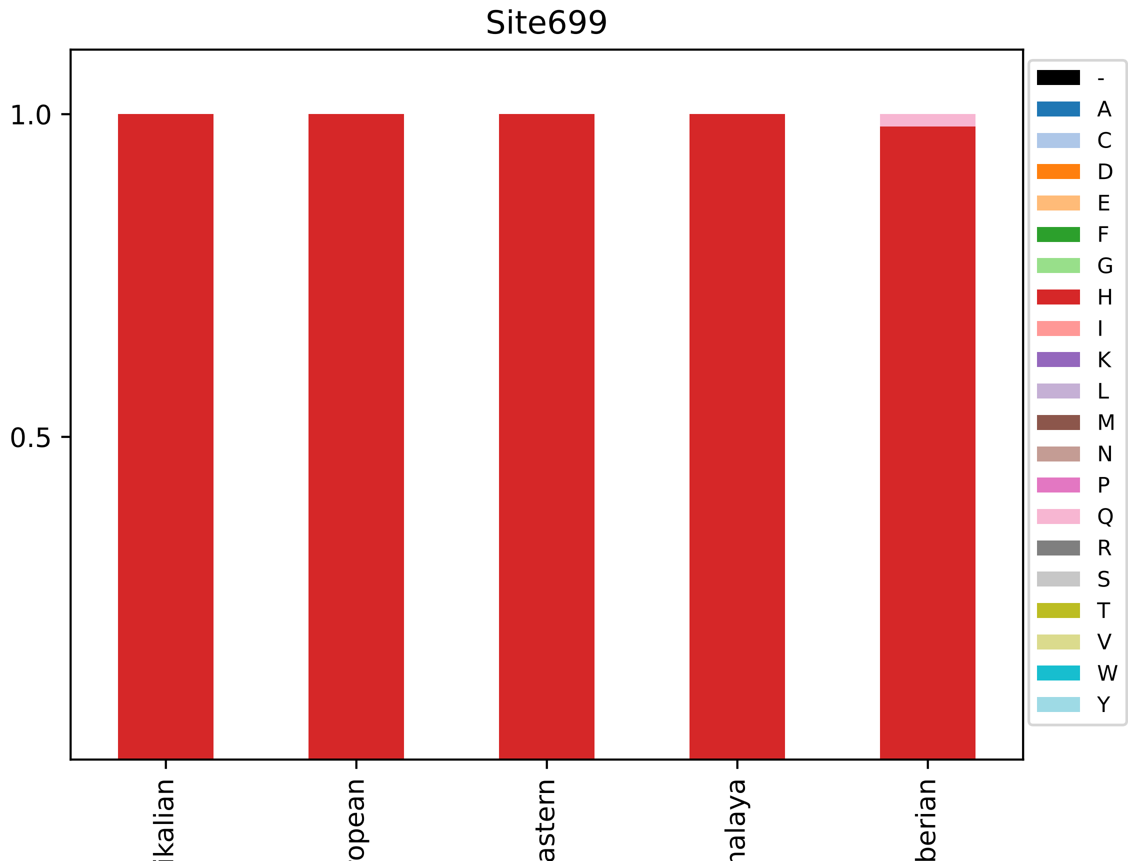
Site699
1.0
0.5
-
A
C
D
E
F
G
H
I
K
L
M
N
P
Q
R
S
T
V
W
Y
ikalian
opean
astern
nalaya
berian

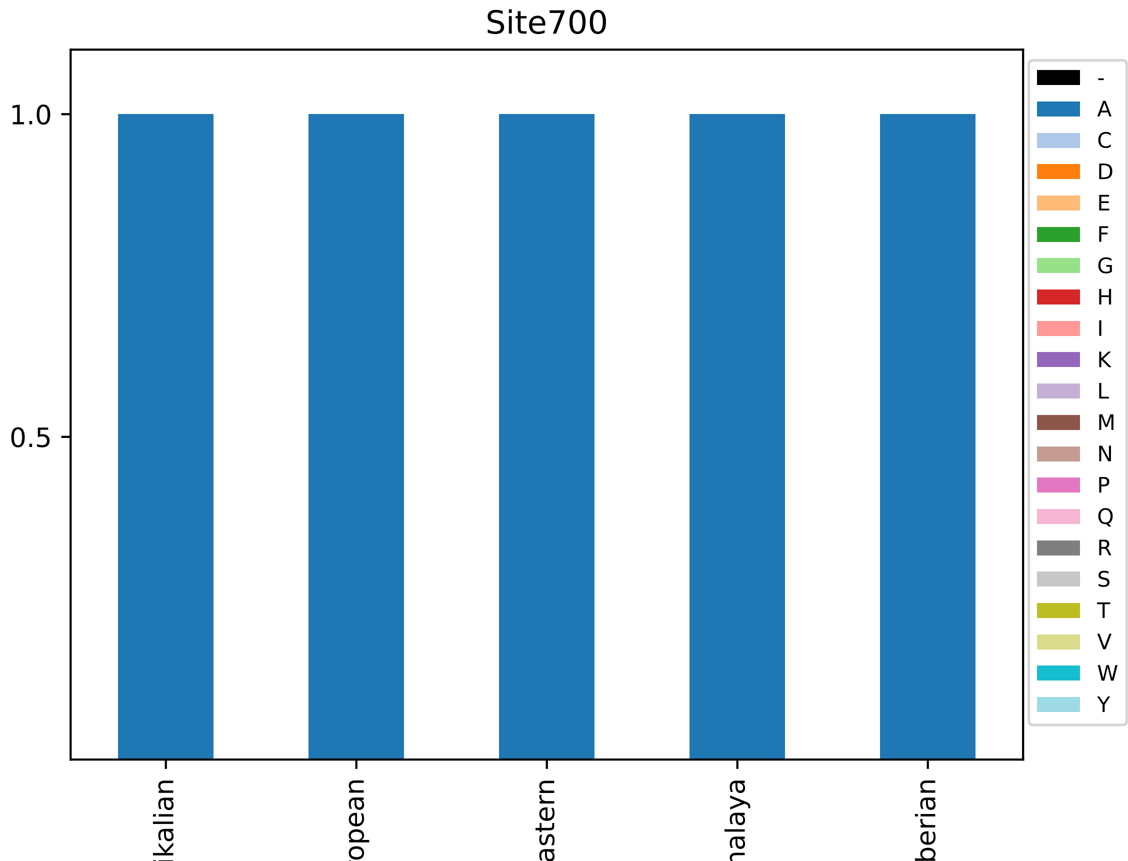
Site700
1.0
0.5
-
A
C
D
E
F
G
H
I
K
L
M
N
P
Q
R
S
T
V
W
Y
ikalian
opean
astern
nalaya
berian

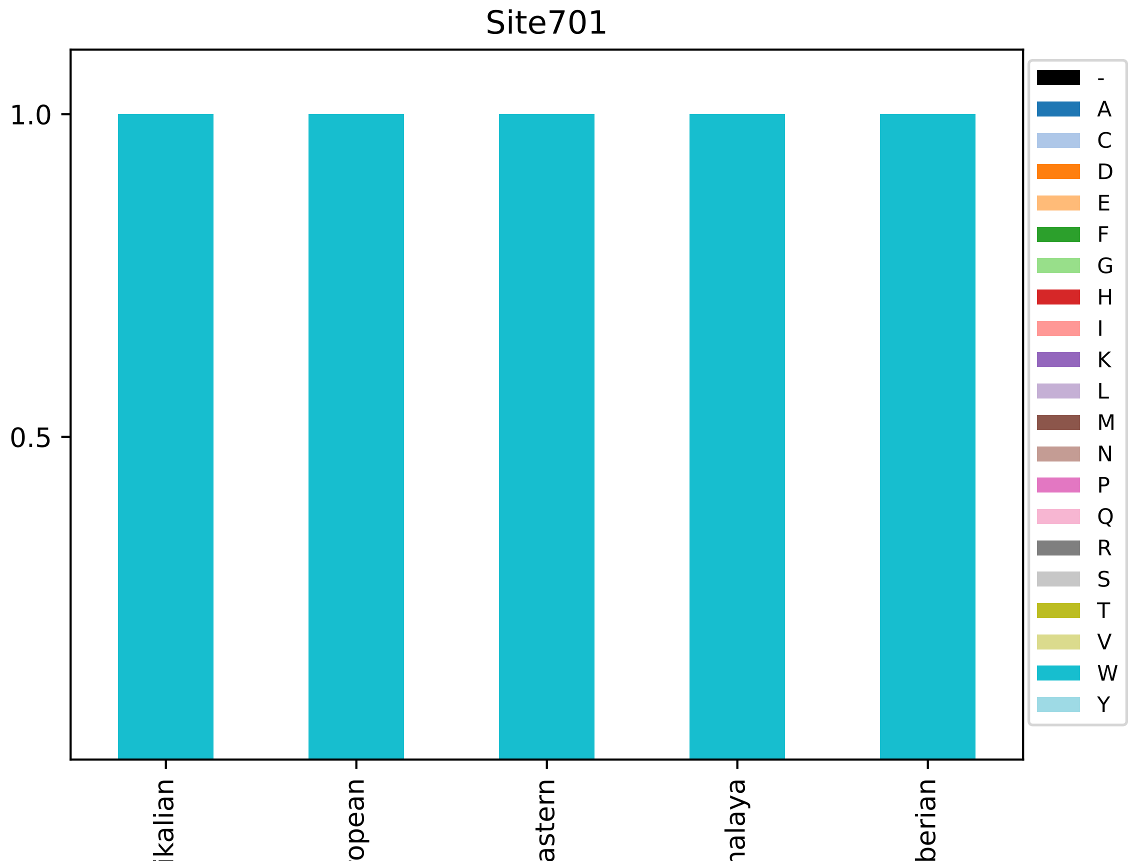
Site701
1.0
0.5
-
A
C
D
E
F
G
H
I
K
L
M
N
P
Q
R
S
T
V
W
Y
ikalian
opean
astern
nalaya
berian

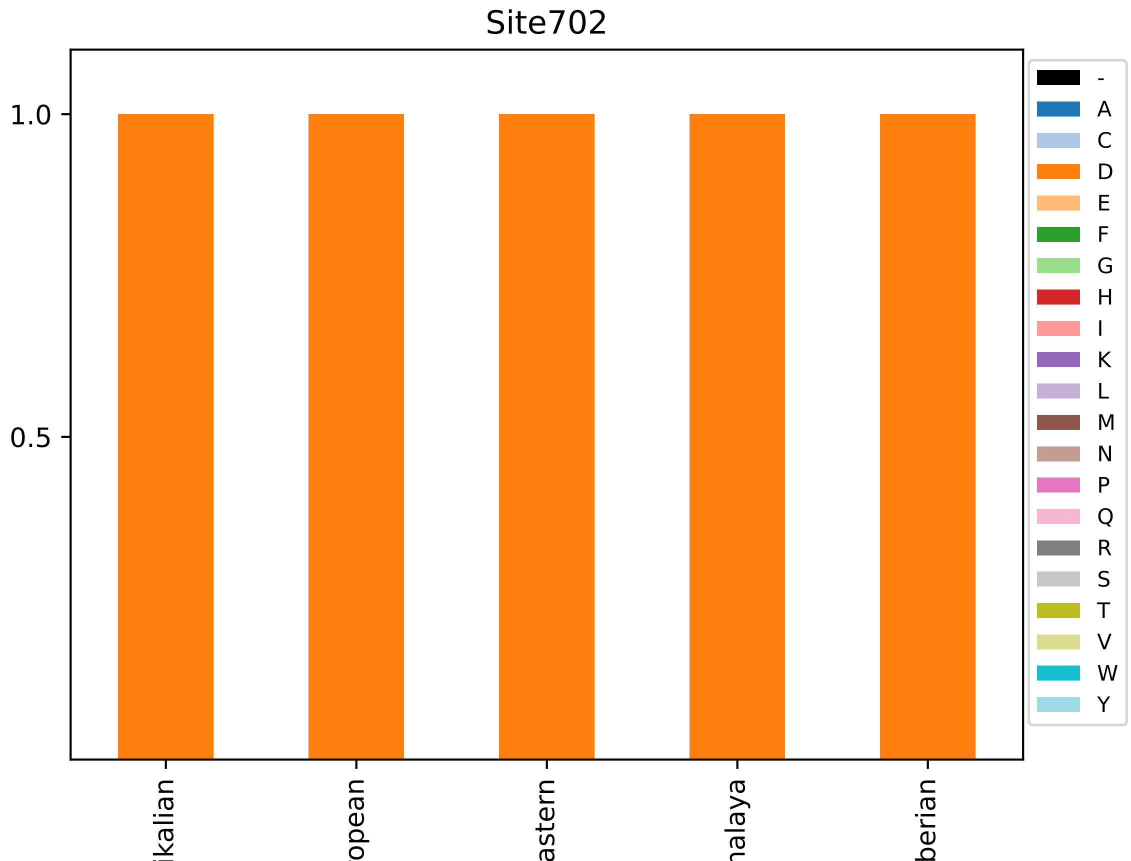
Site702
1.0
0.5
-
A
C
D
E
F
G
H
I
K
L
M
N
P
Q
R
S
T
V
W
Y
ikalian
opean
astern
nalaya
berian

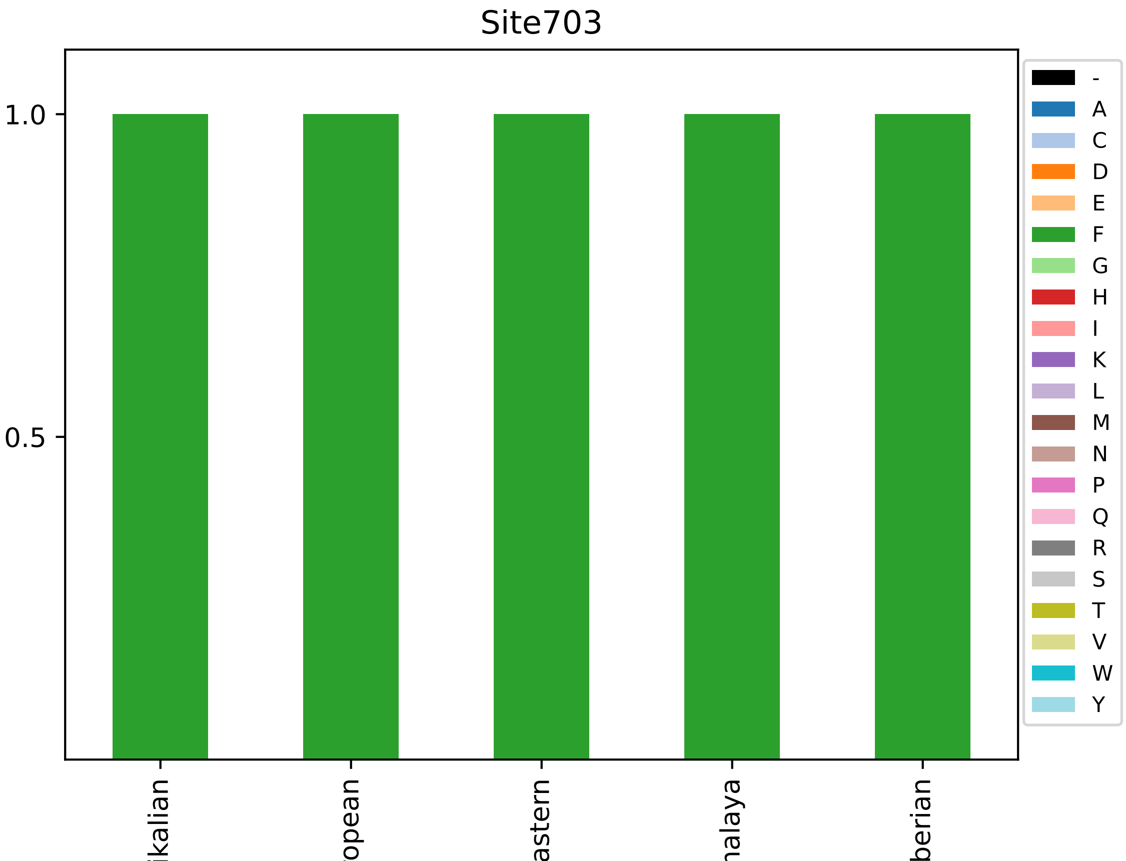
Site703
1.0
0.5
-
A
C
D
E
F
G
H
I
K
L
M
N
P
Q
R
S
T
V
W
Y
ikalian
opean
astern
nalaya
berian

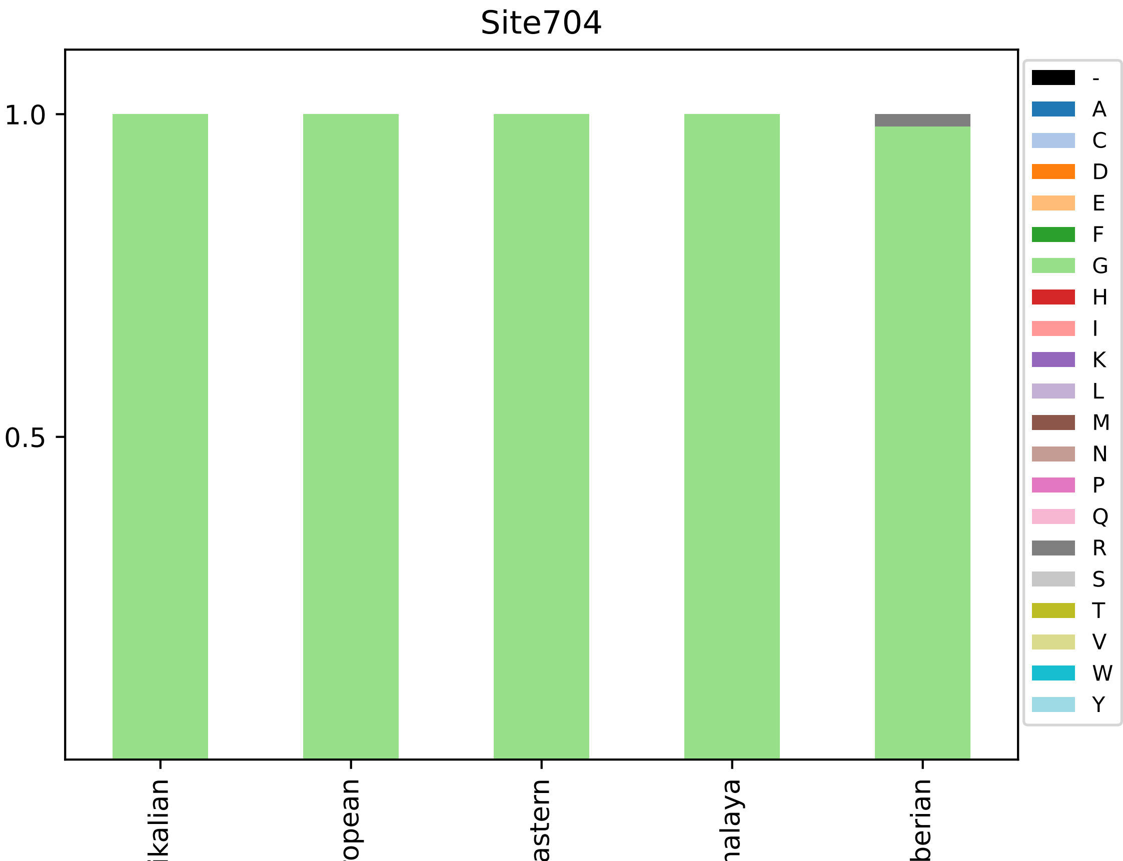

Site704
1.0
0.5
-
A
C
D
E
F
G
H
I
K
L
M
N
P
Q
R
S
T
V
W
Y
ikalian
opean
astern
alaya
berian

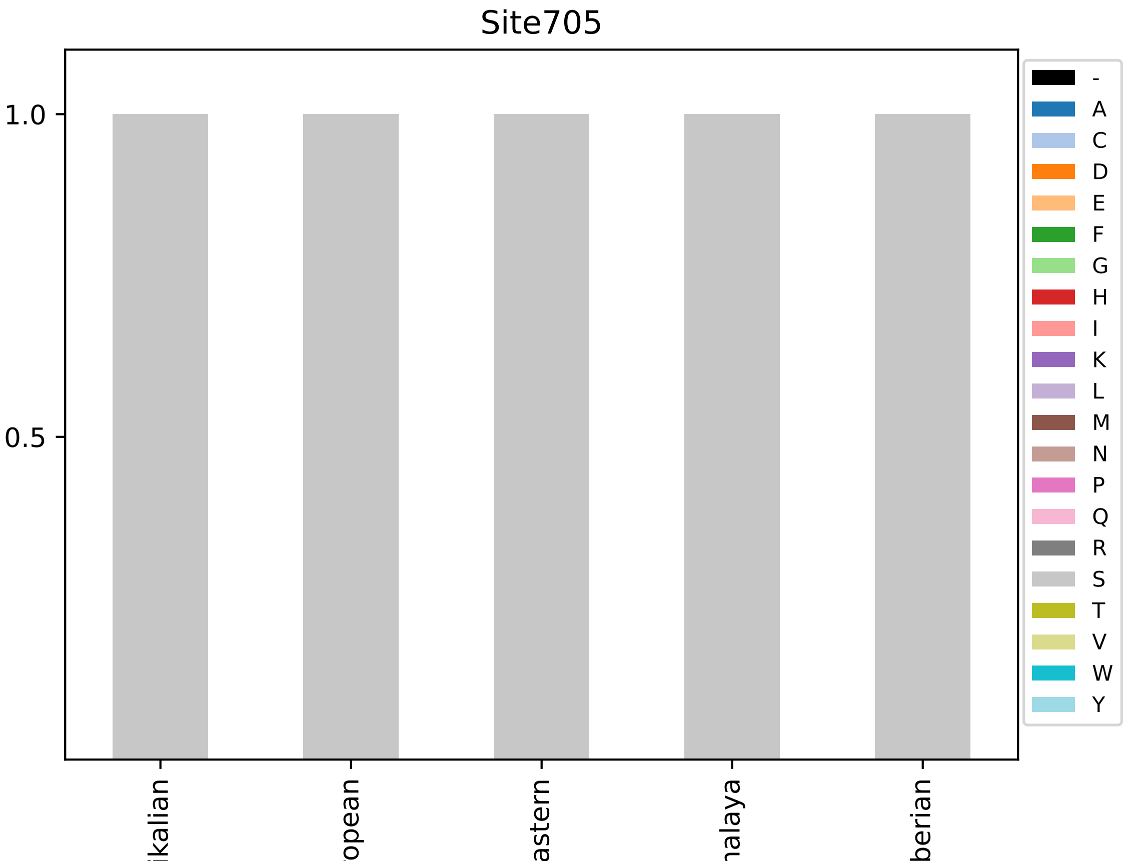

Site705
1.0
0.5
-
A
C
D
E
F
G
H
I
K
L
M
N
P
Q
R
S
T
V
W
Y
ikalian
ropean
astern
nalaya
berian

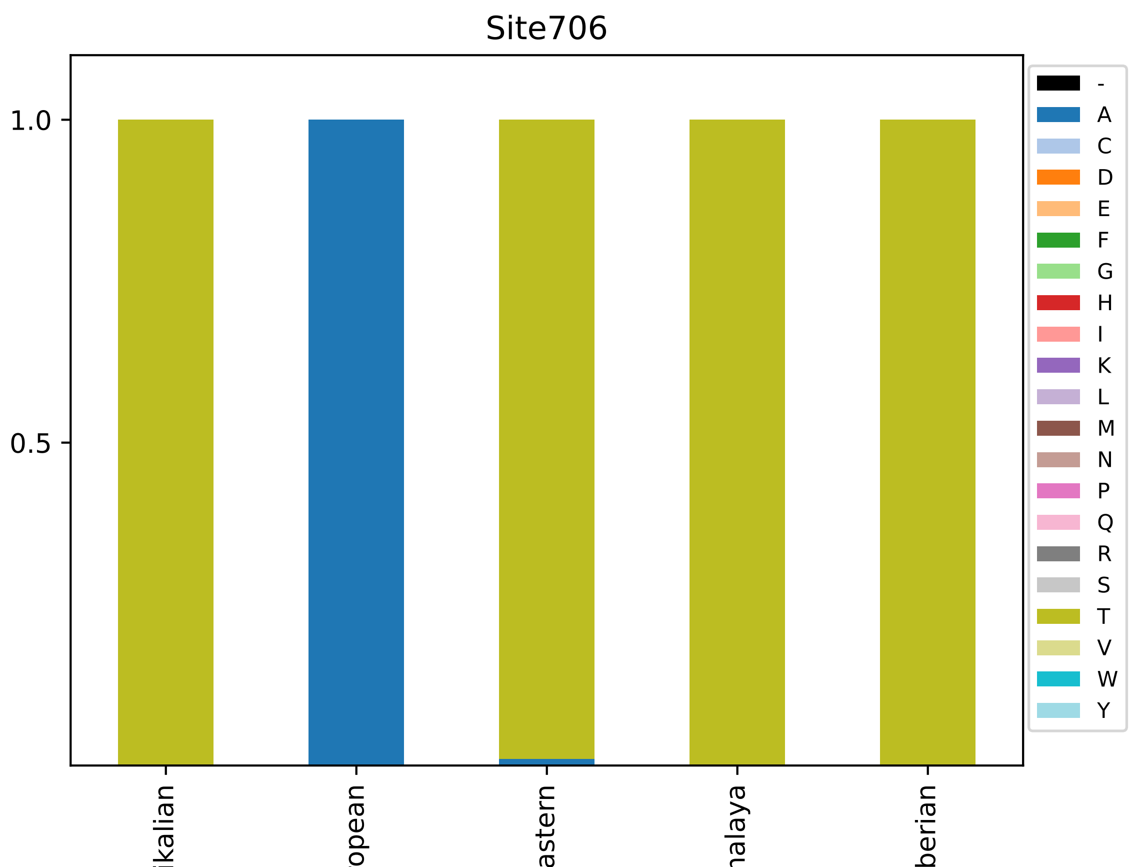

Site706
1.0
0.5
ikalian
opean
astern
nalaya
berian
-
A
C
D
E
F
G
H
I
K
L
M
N
P
Q
R
S
T
V
W
Y

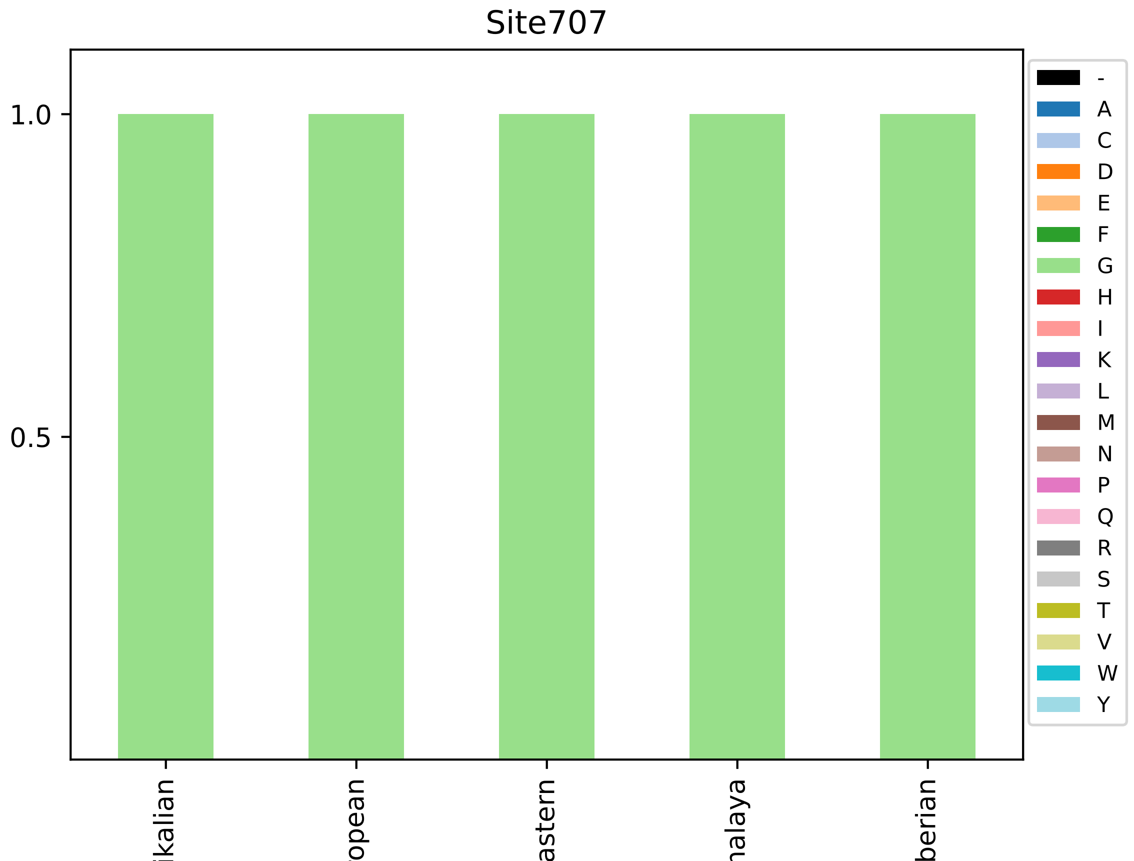
Site707
1.0
0.5
ikalian
opean
astern
nalaya
berian
-
A
C
D
E
F
G
H
I
K
L
M
N
P
Q
R
S
T
V
W
Y

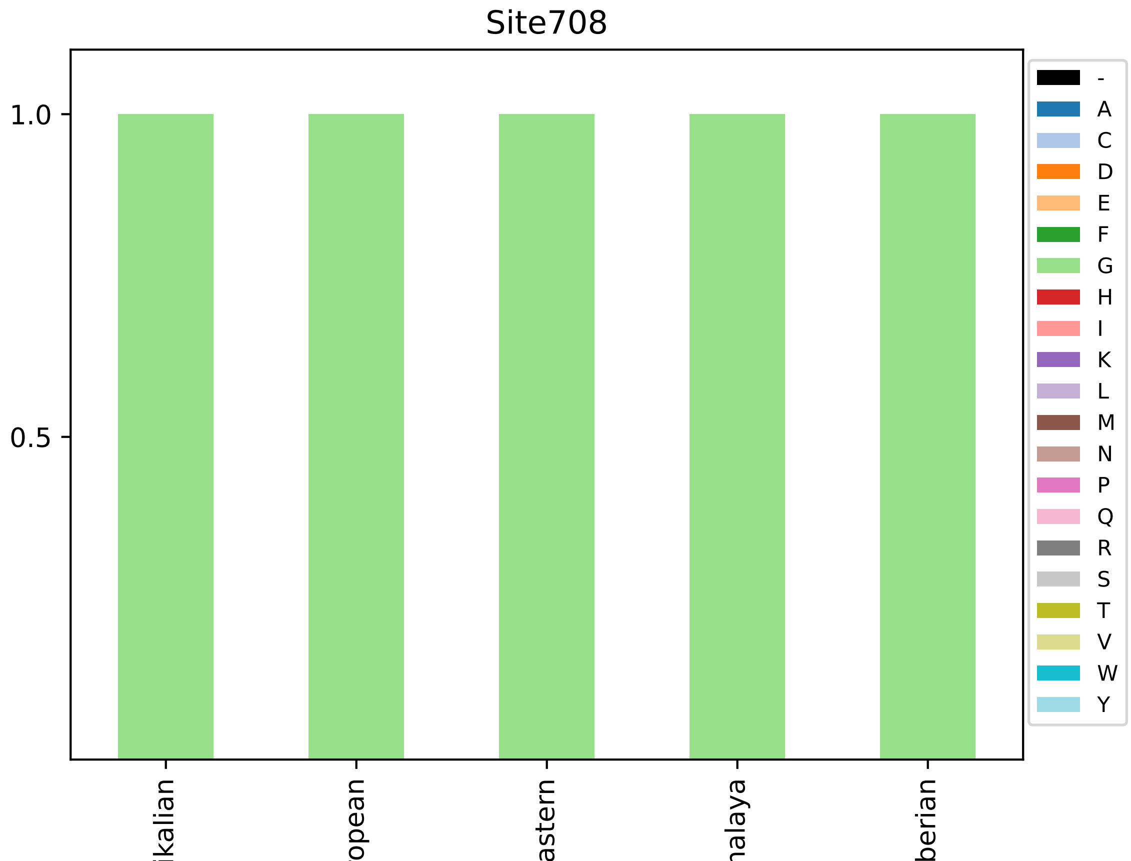

Site708
1.0
0.5
ikalian
opean
astern
nalaya
berian
-
A
C
D
E
F
G
H
I
K
L
M
N
P
Q
R
S
T
V
W
Y

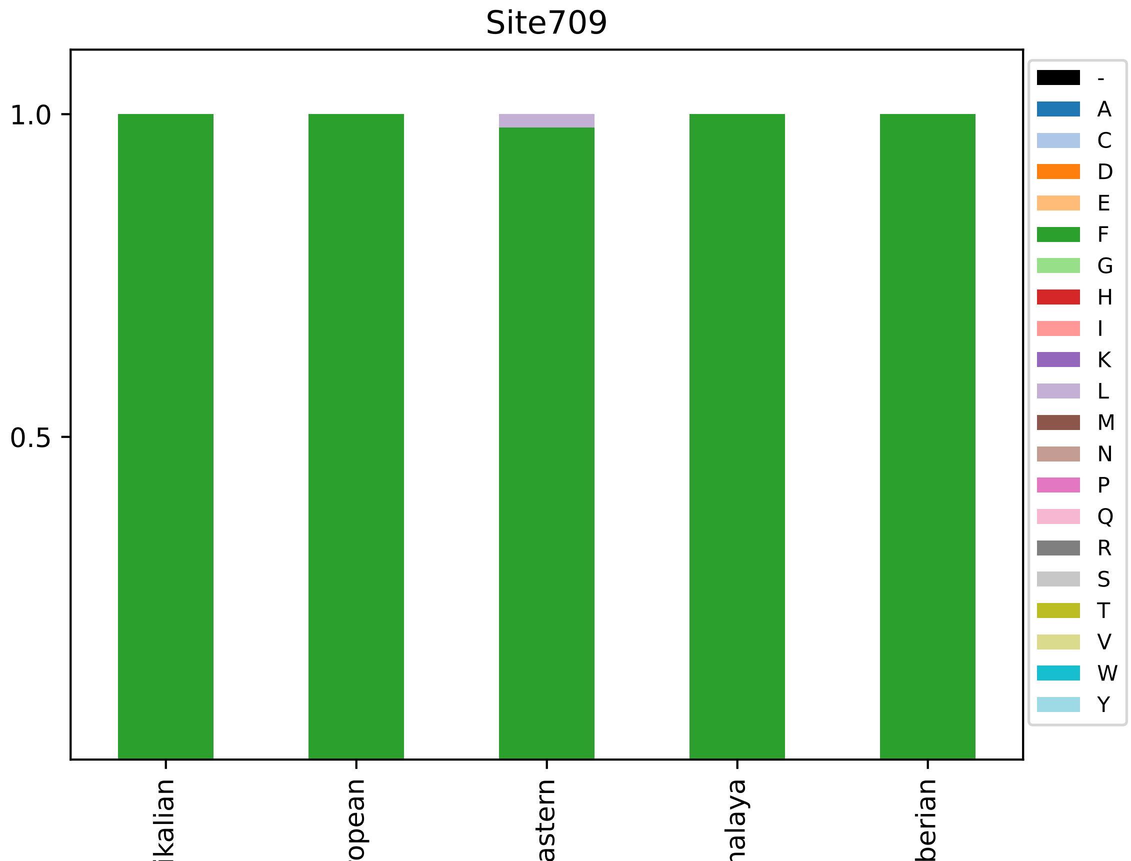

Site709
1.0
0.5
ikalian
opean
astern
nalaya
berian
-
A
C
D
E
F
G
H
I
K
L
M
N
P
Q
R
S
T
V
W
Y

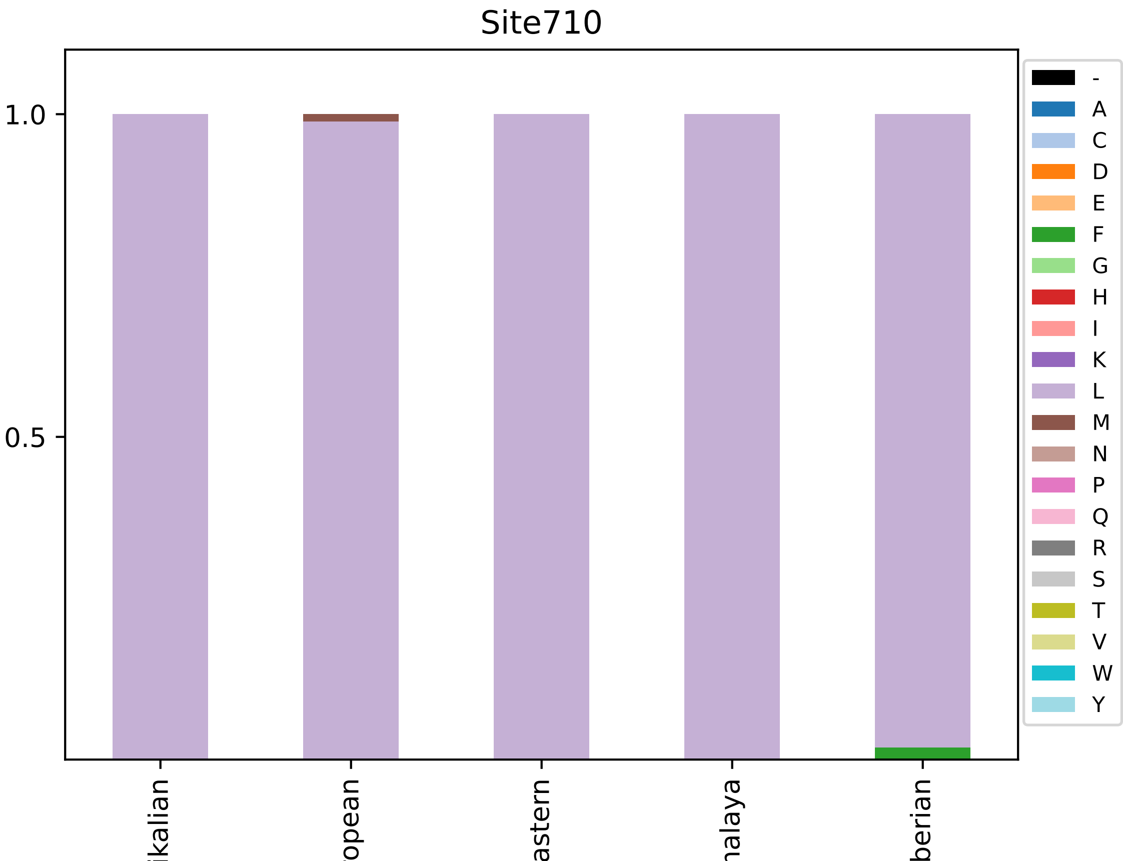
Site710
1.0
0.5
-
A
C
D
E
F
G
H
I
K
L
M
N
P
Q
R
S
T
V
W
Y

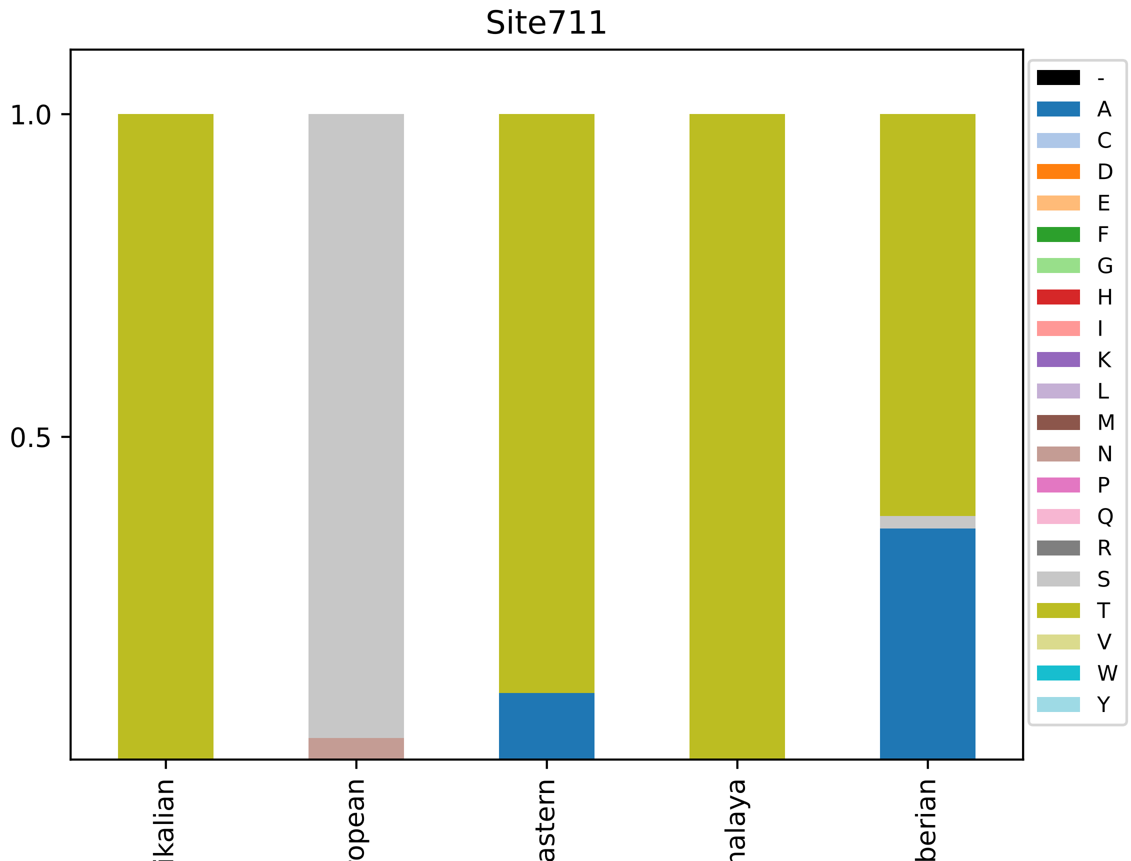
Site711
1.0
0.5
-
A
C
D
E
F
G
H
I
K
L
M
N
P
Q
R
S
T
V
W
Y
ikalian
opean
astern
nalaya
berian

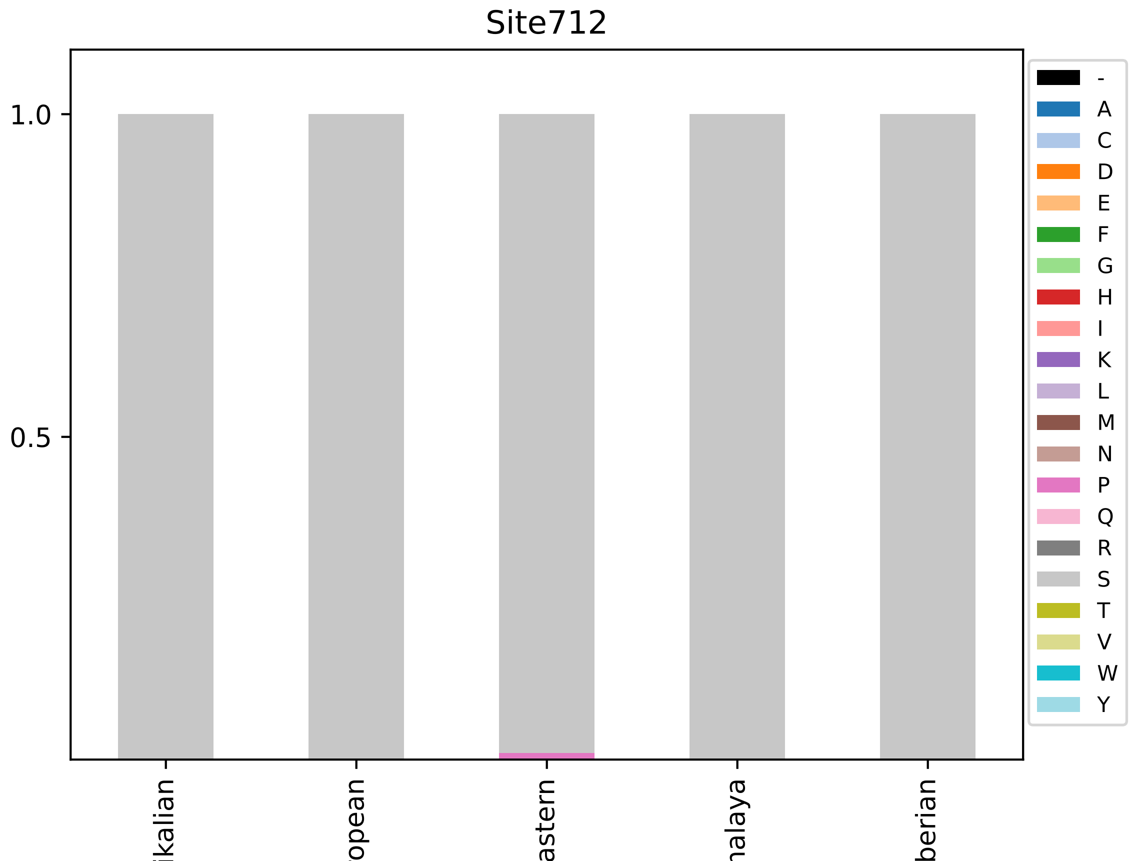

Site712
1.0
0.5
-
A
C
D
E
F
G
H
I
K
L
M
N
P
Q
R
S
T
V
W
Y
ikalian
opean
astern
nalaya
berian

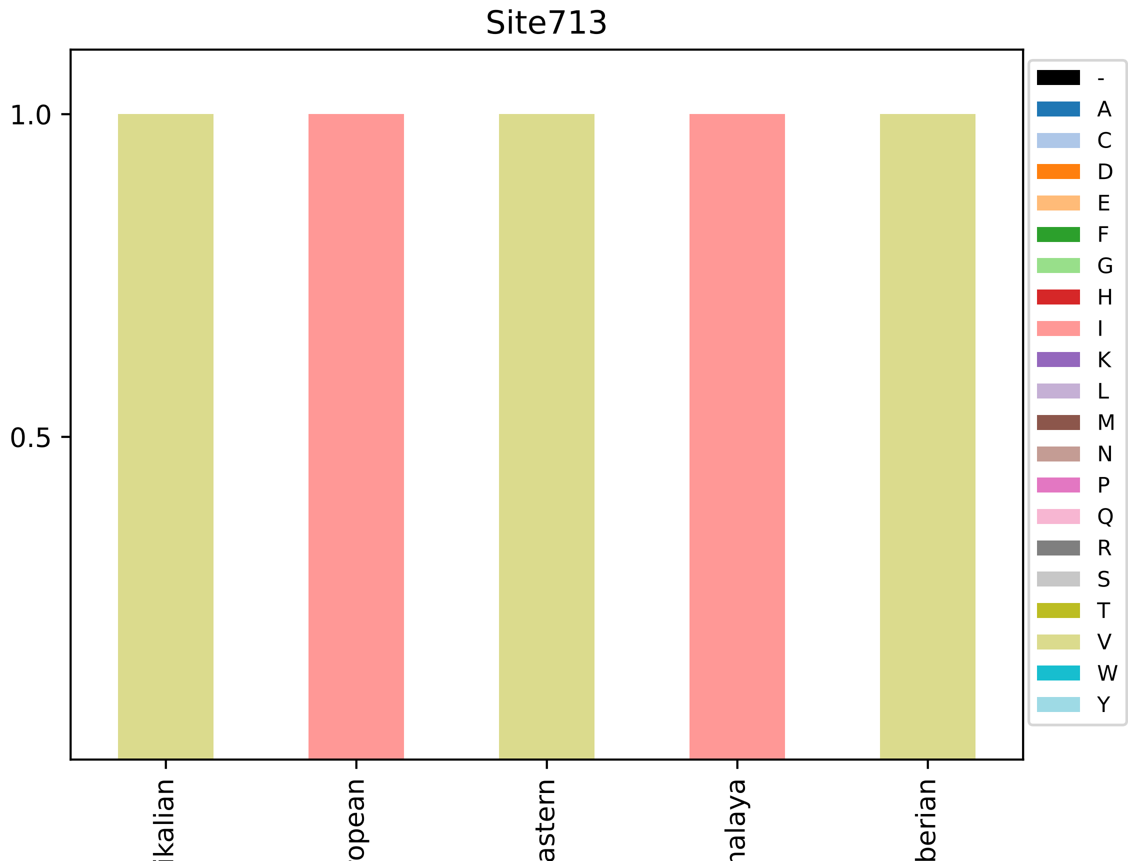

Site713
1.0
0.5
ikalian
opean
astern
nalaya
berian
-
A
C
D
E
F
G
H
I
K
L
M
N
P
Q
R
S
T
V
W
Y

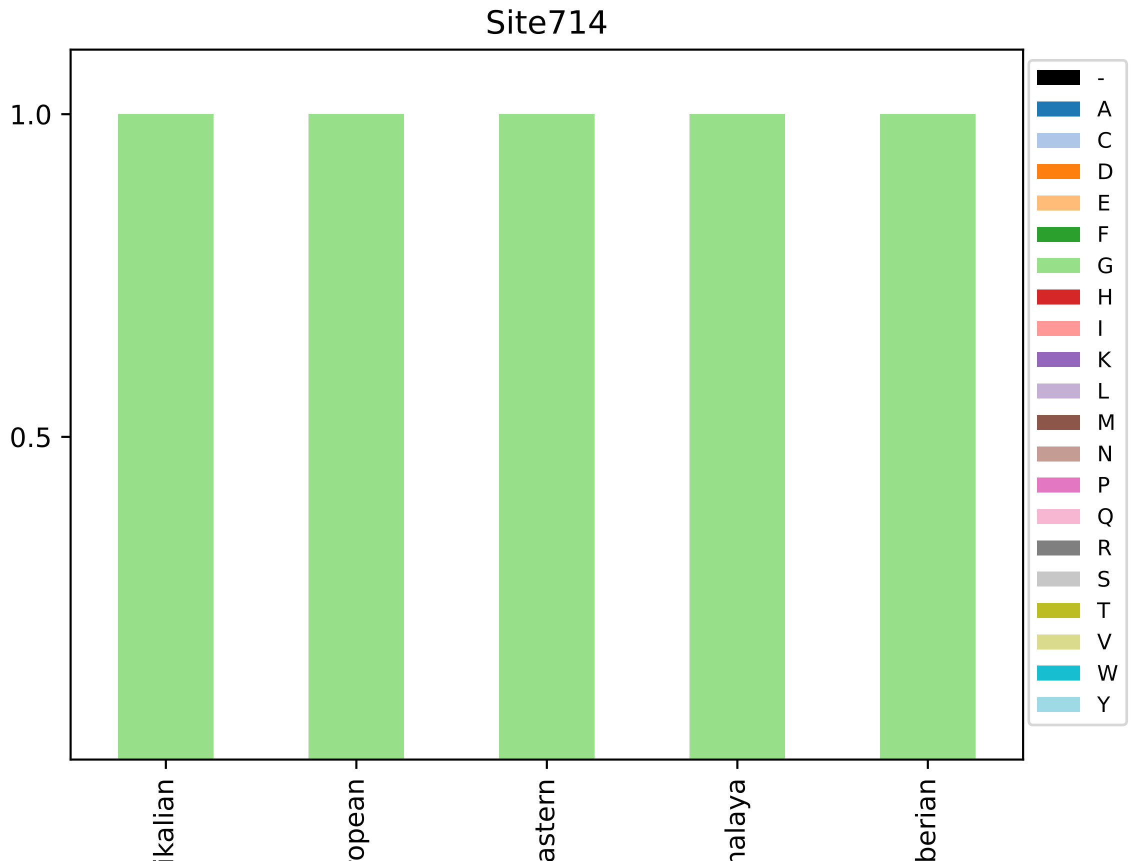

Site714
1.0
0.5
-
A
C
D
E
F
G
H
I
K
L
M
N
P
Q
R
S
T
V
W
Y
ikalian
ropean
astern
nalaya
berian

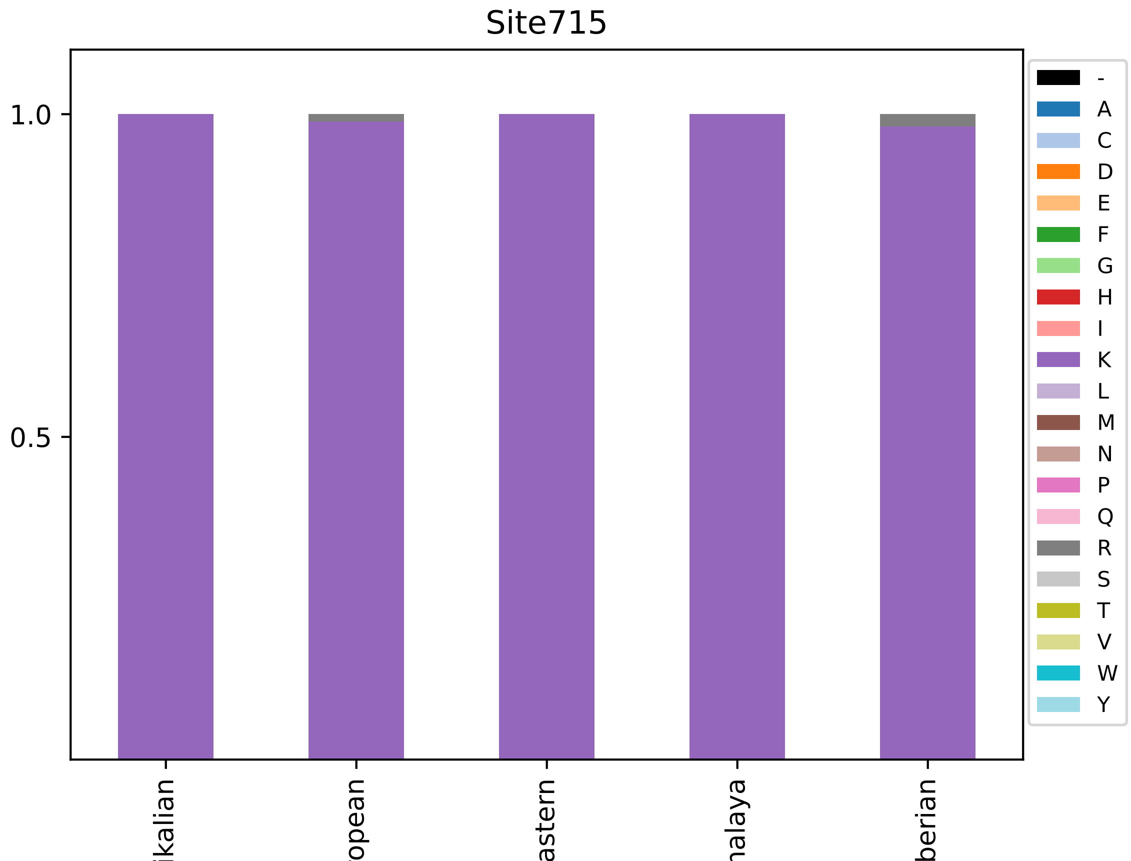
Site715
1.0
0.5
-
A
C
D
E
F
G
H
I
K
L
M
N
P
Q
R
S
T
V
W
Y
ikalian
opean
astern
nalaya
berian

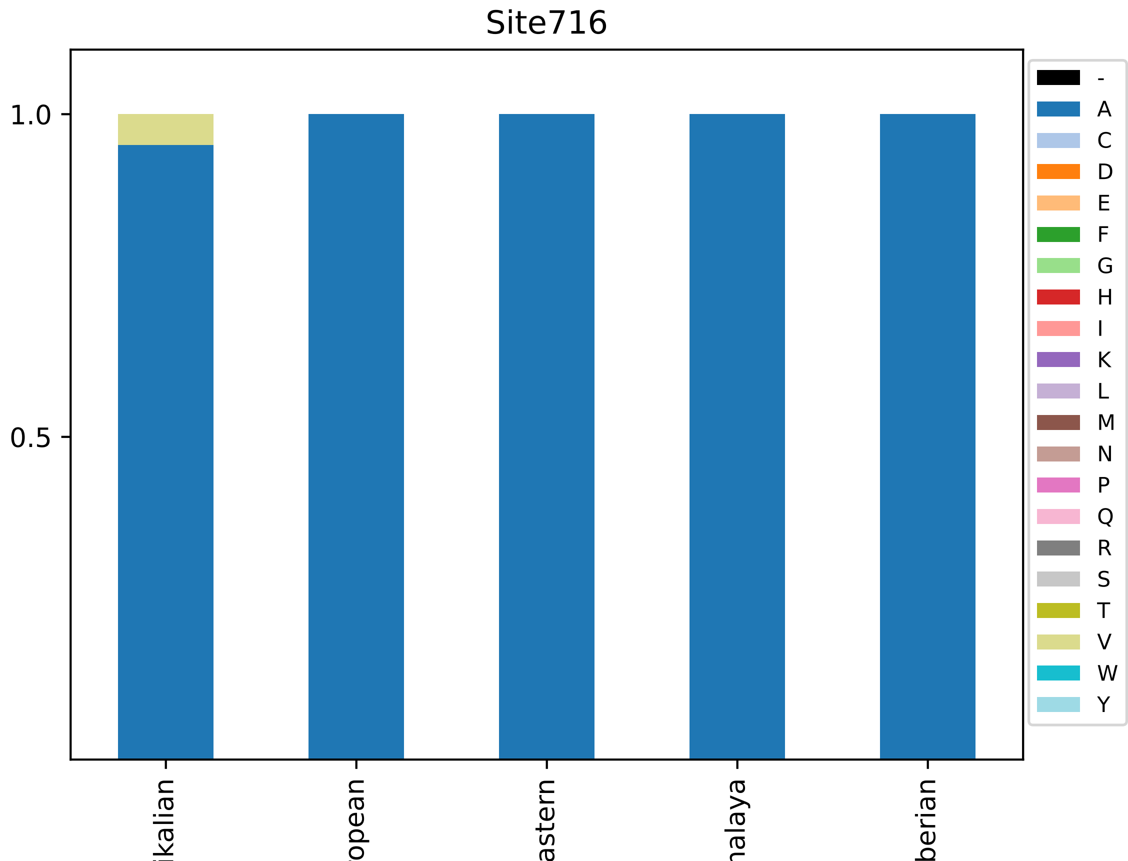
Site716
1.0
0.5
-
A
C
D
E
F
G
H
I
K
L
M
N
P
Q
R
S
T
V
W
Y
ikalian
opean
astern
nalaya
berian

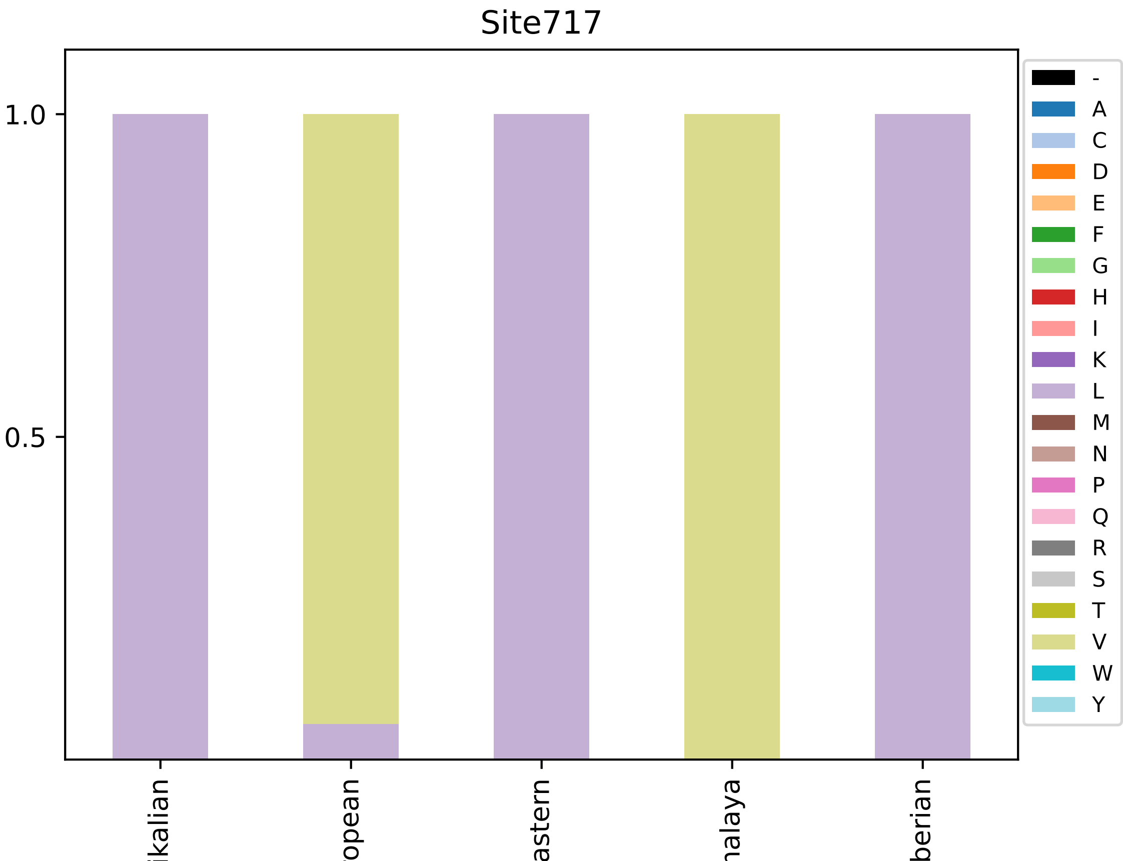

Site717
1.0
0.5
-
A
C
D
E
F
G
H
I
K
L
M
N
P
Q
R
S
T
V
W
Y
ikalian
opean
astern
nalaya
berian

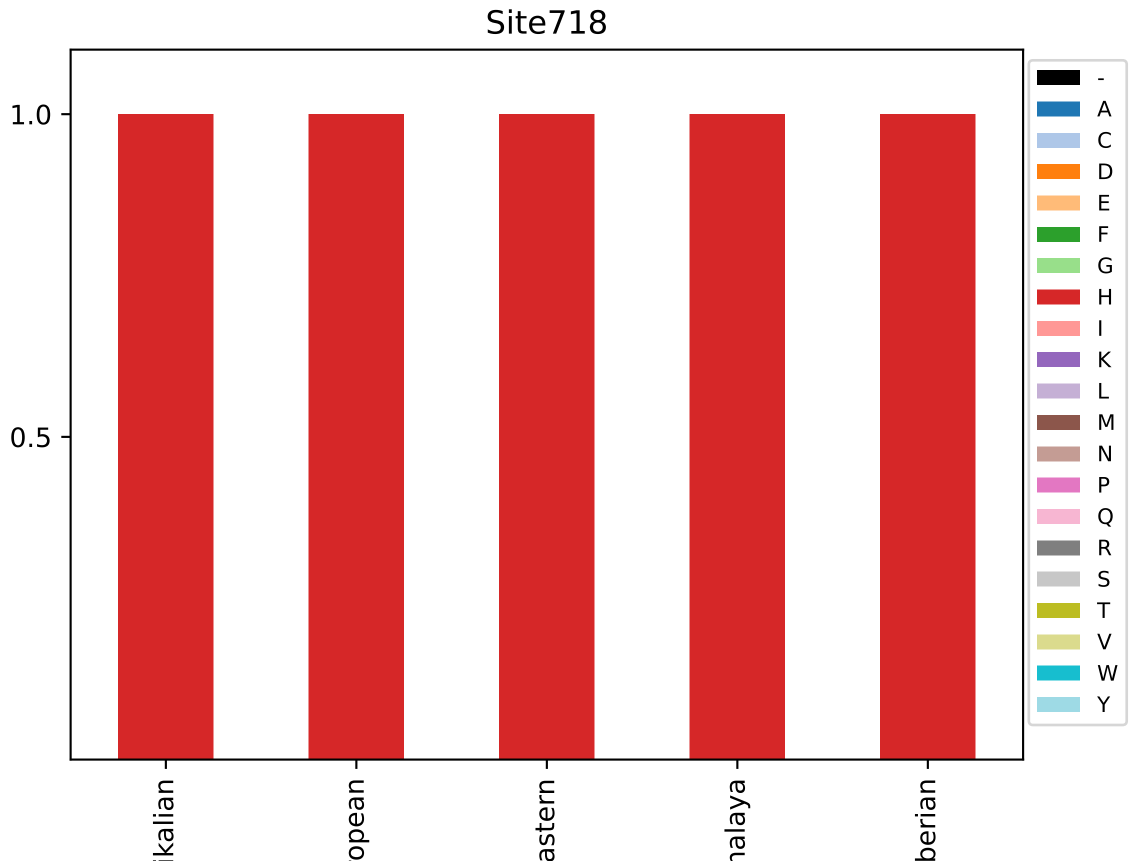
Site718
1.0
0.5
-
A
C
D
E
F
G
H
I
K
L
M
N
P
Q
R
S
T
V
W
Y
ikalian
opean
astern
nalaya
berian

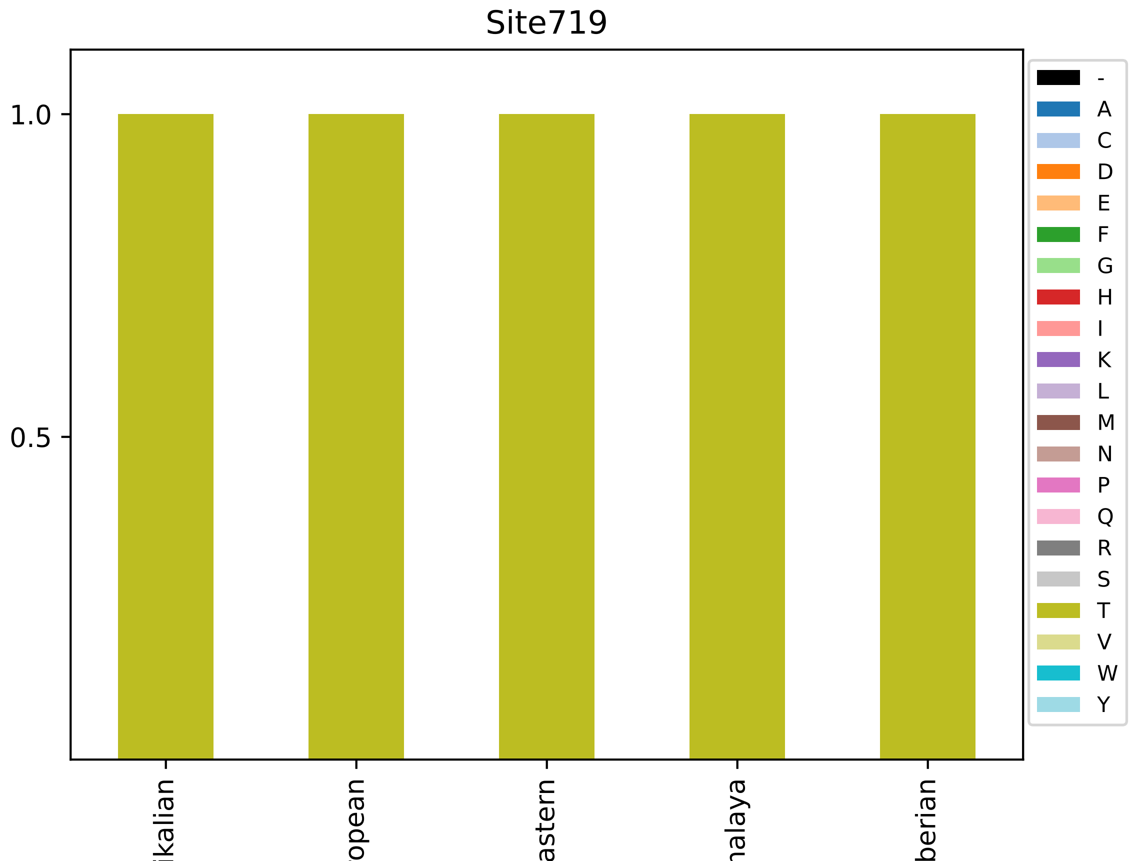
Site719
1.0
0.5
-
A
C
D
E
F
G
H
I
K
L
M
N
P
Q
R
S
T
V
W
Y
ikalian
ropean
astern
nalaya
berian

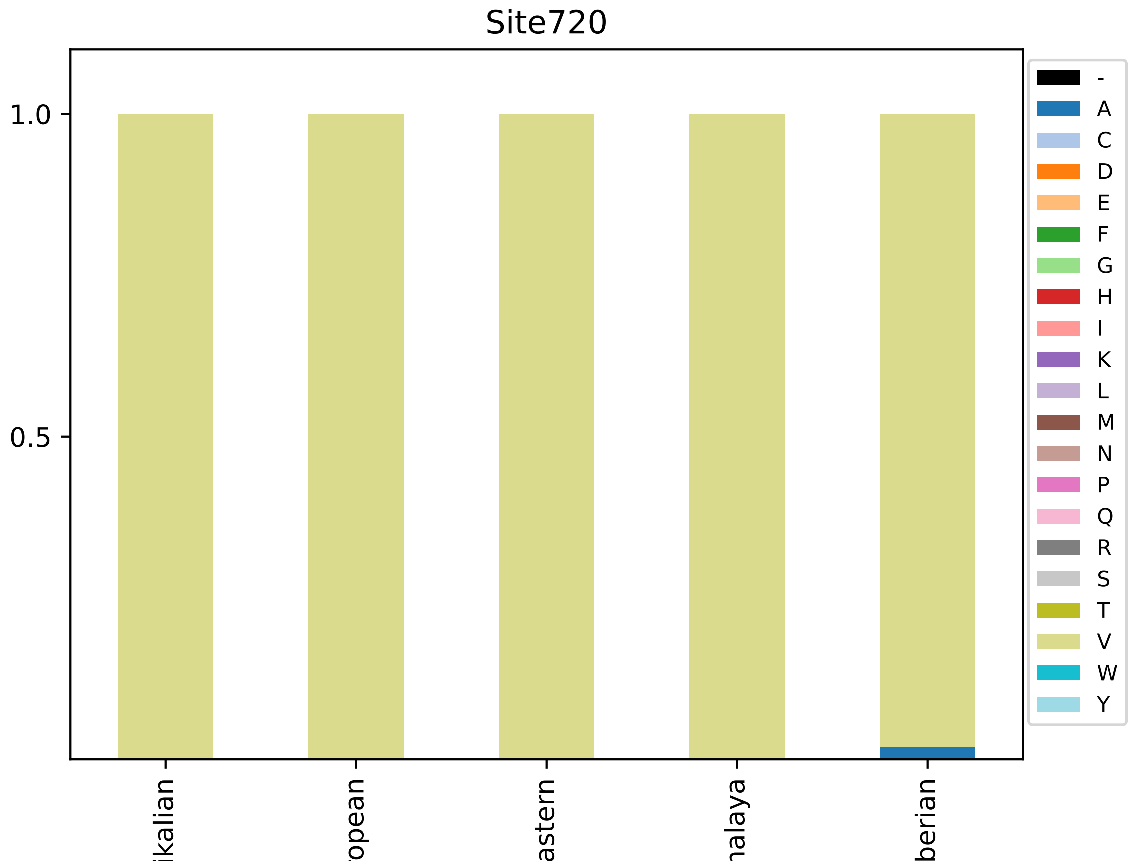

Site720
1.0
0.5
-
A
C
D
E
F
G
H
I
K
L
M
N
P
Q
R
S
T
V
W
Y
ikalian
opean
astern
nalaya
berian

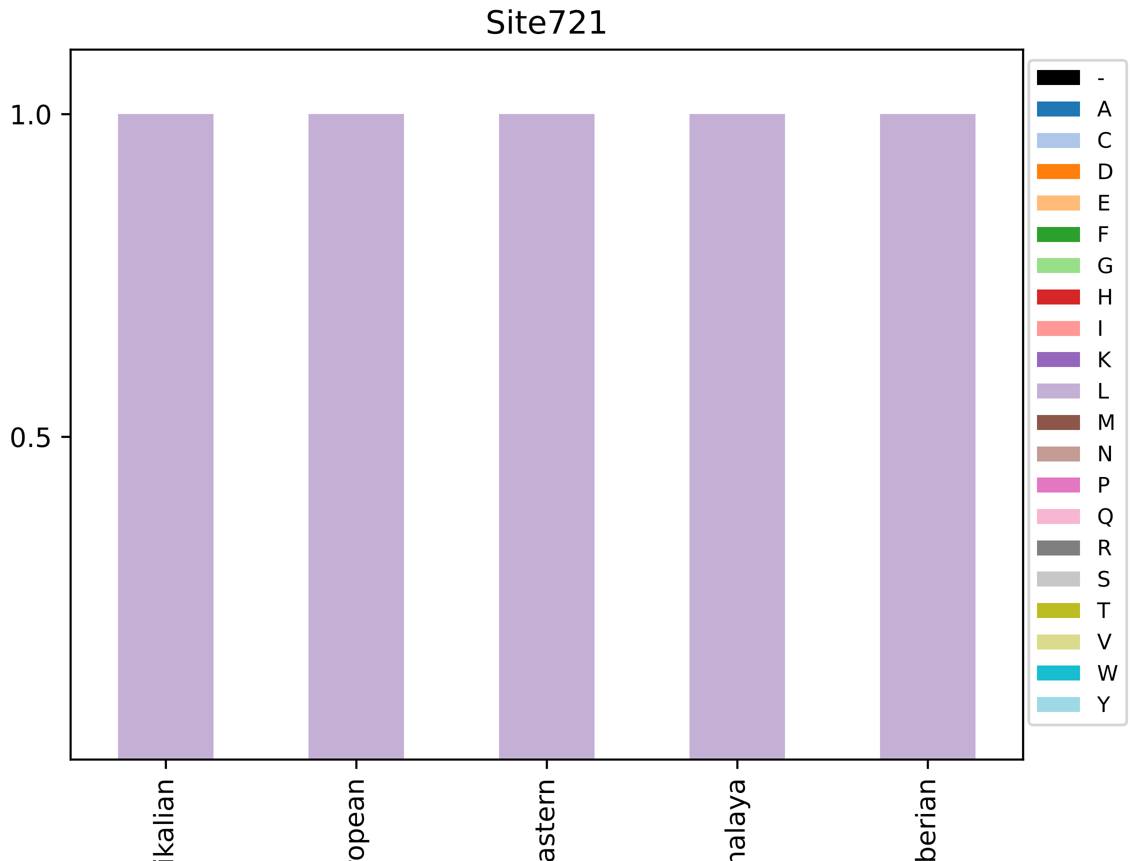

Site721
1.0
0.5
-
A
C
D
E
F
G
H
I
K
L
M
N
P
Q
R
S
T
V
W
Y
ikalian
opean
astern
nalaya
berian

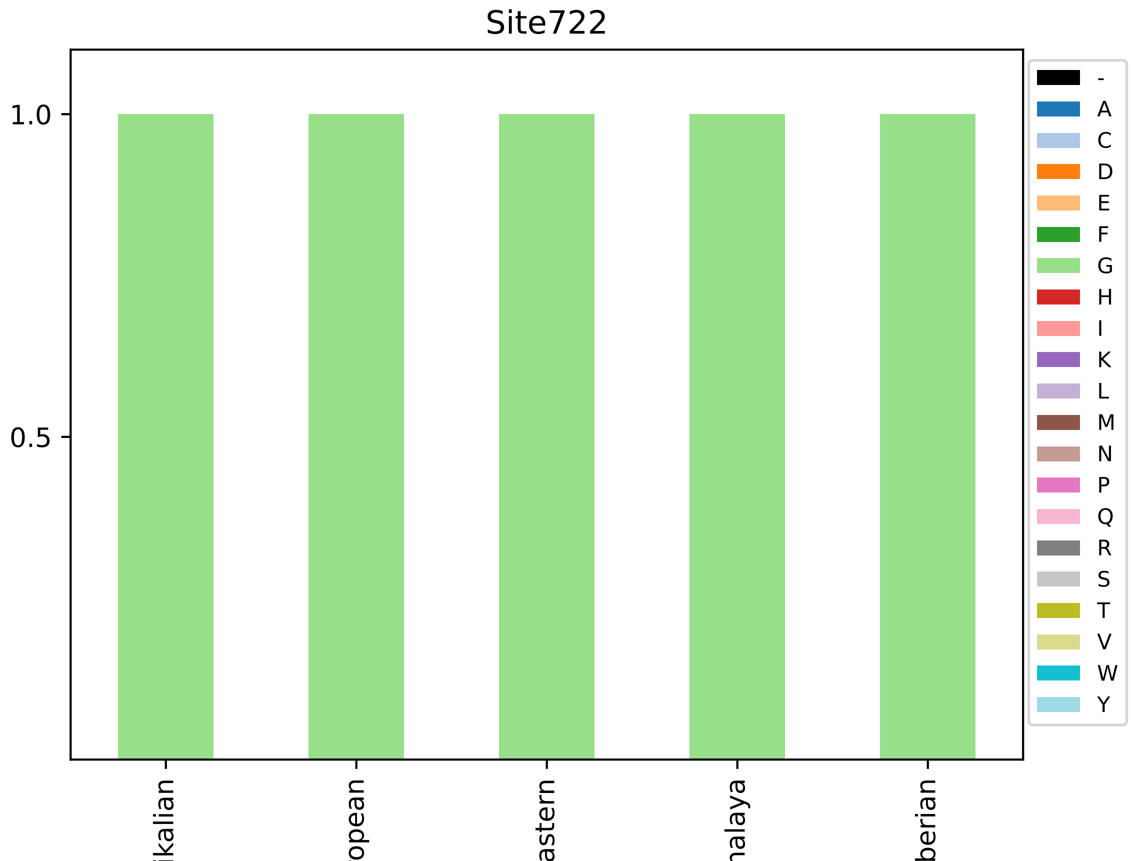
Site722
1.0
0.5
ikalian
opean
astern
nalaya
berian
-
A
C
D
E
F
G
H
I
K
L
M
N
P
Q
R
S
T
V
W
Y

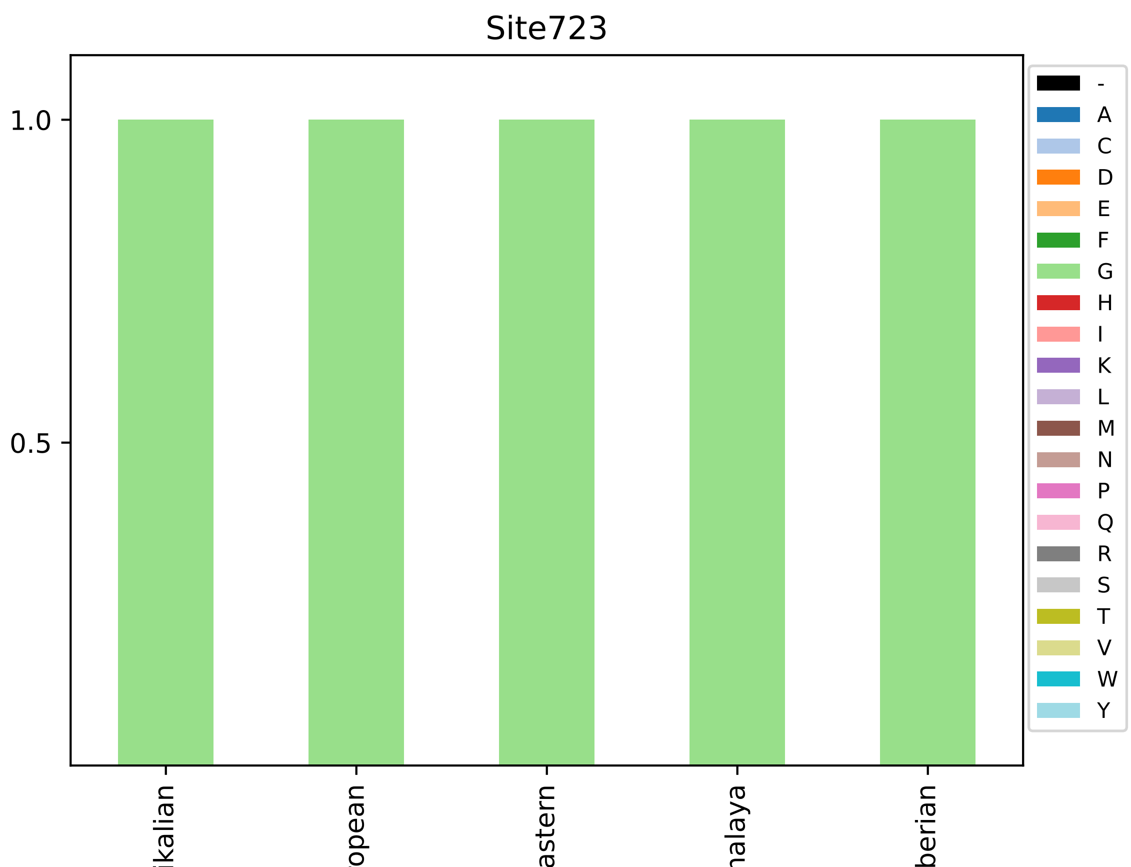
Site723
1.0
0.5
-
A
C
D
E
F
G
H
I
K
L
M
N
P
Q
R
S
T
V
W
Y
ikalian
opean
astern
nalaya
berian

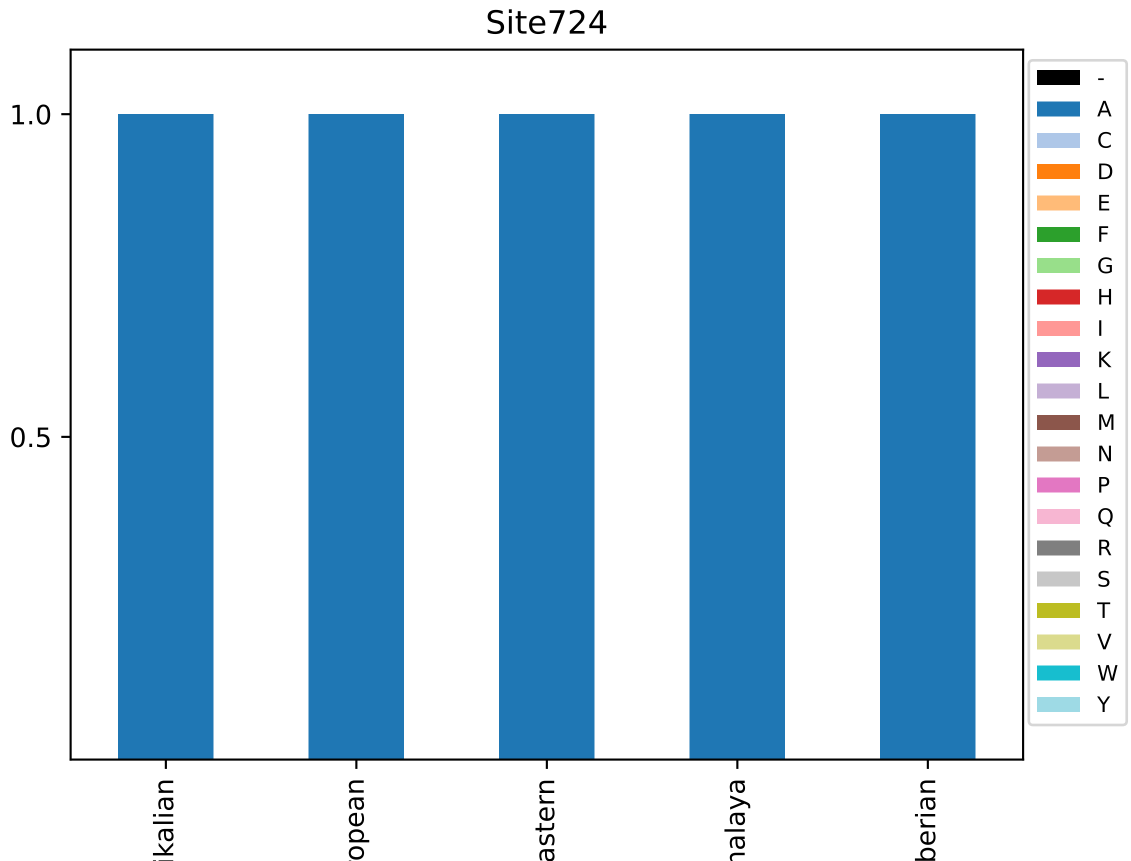

Site724
1.0
0.5
-
A
C
D
E
F
G
H
I
K
L
M
N
P
Q
R
S
T
V
W
Y
ikalian
opean
astern
nalaya
berian

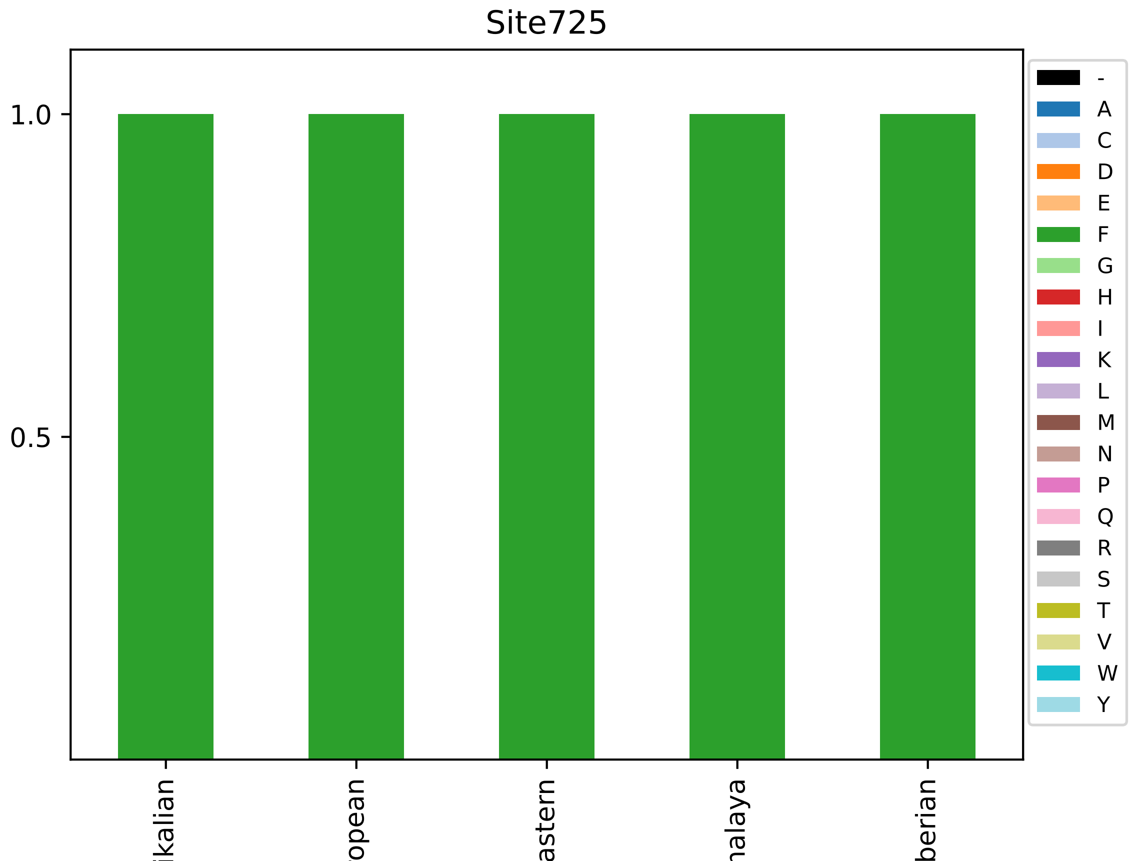
Site725
1.0
0.5
-
A
C
D
E
F
G
H
I
K
L
M
N
P
Q
R
S
T
V
W
Y
ikalian
ropean
astern
nalaya
berian

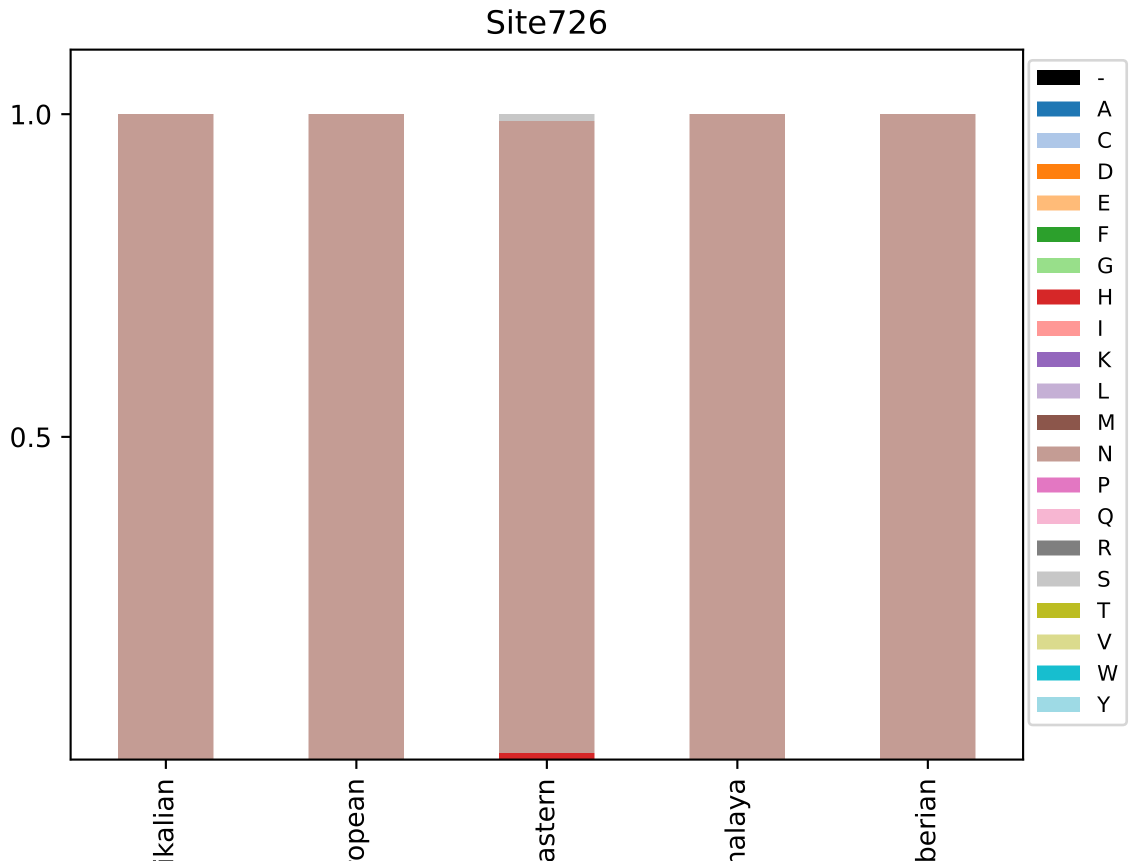

Site726
1.0
0.5
-
A
C
D
E
F
G
H
I
K
L
M
N
P
Q
R
S
T
V
W
Y
ikalian
opean
astern
nalaya
berian

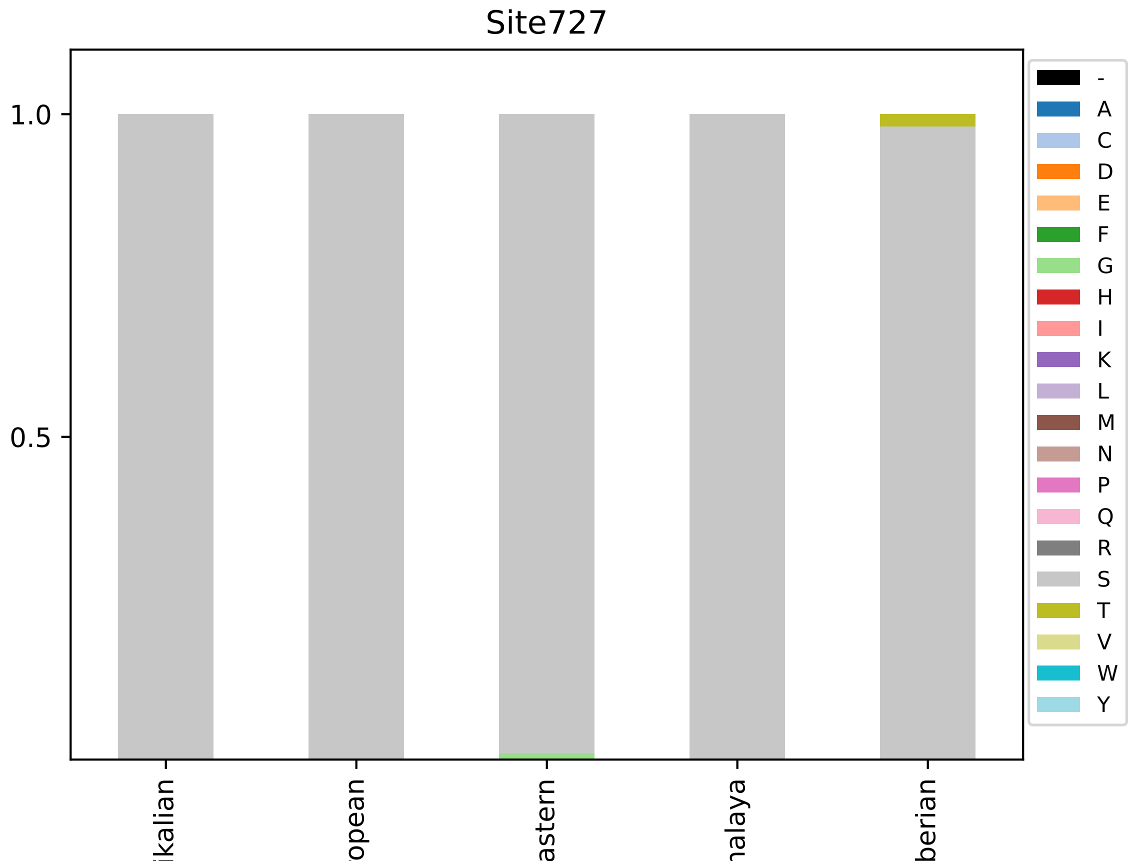

Site727
1.0
0.5
ikalian
opean
astern
nalaya
berian
-
A
C
D
E
F
G
H
I
K
L
M
N
P
Q
R
S
T
V
W
Y

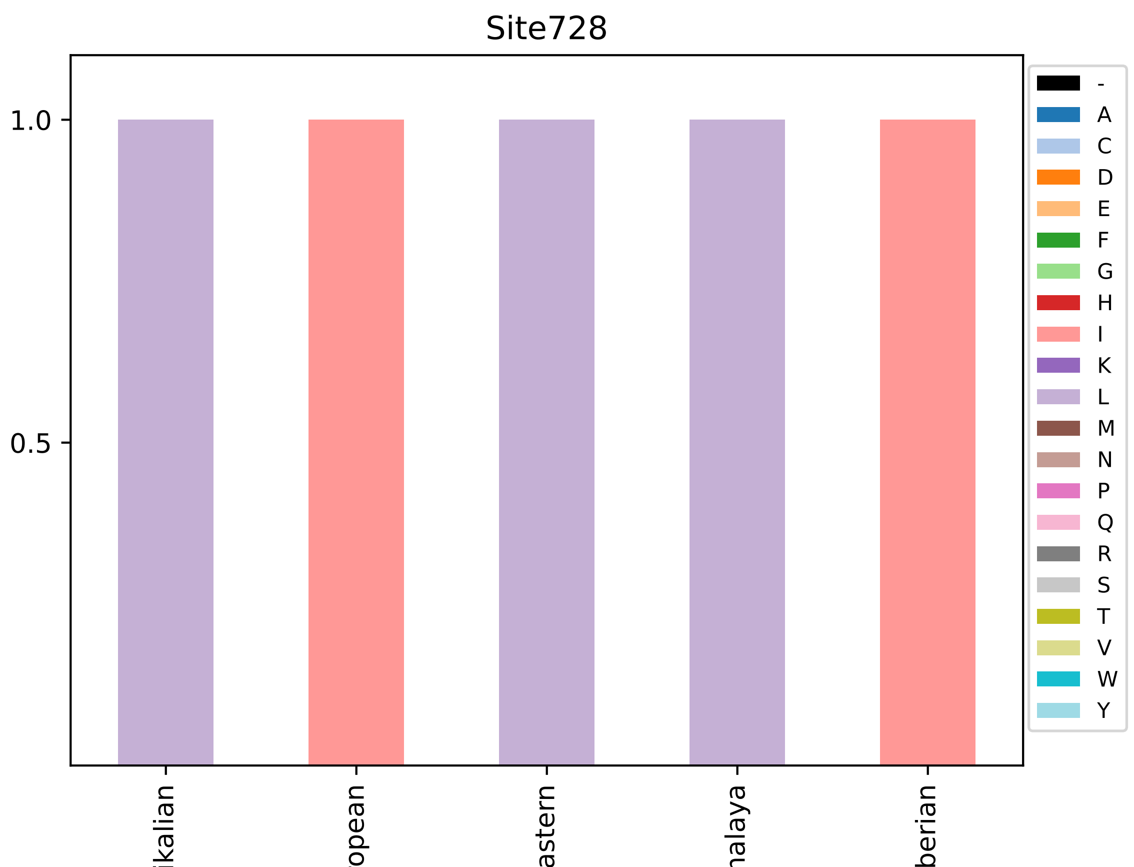
Site728
1.0
0.5
-
A
C
D
E
F
G
H
I
K
L
M
N
P
Q
R
S
T
V
W
Y
ikalian
opean
astern
nalaya
berian

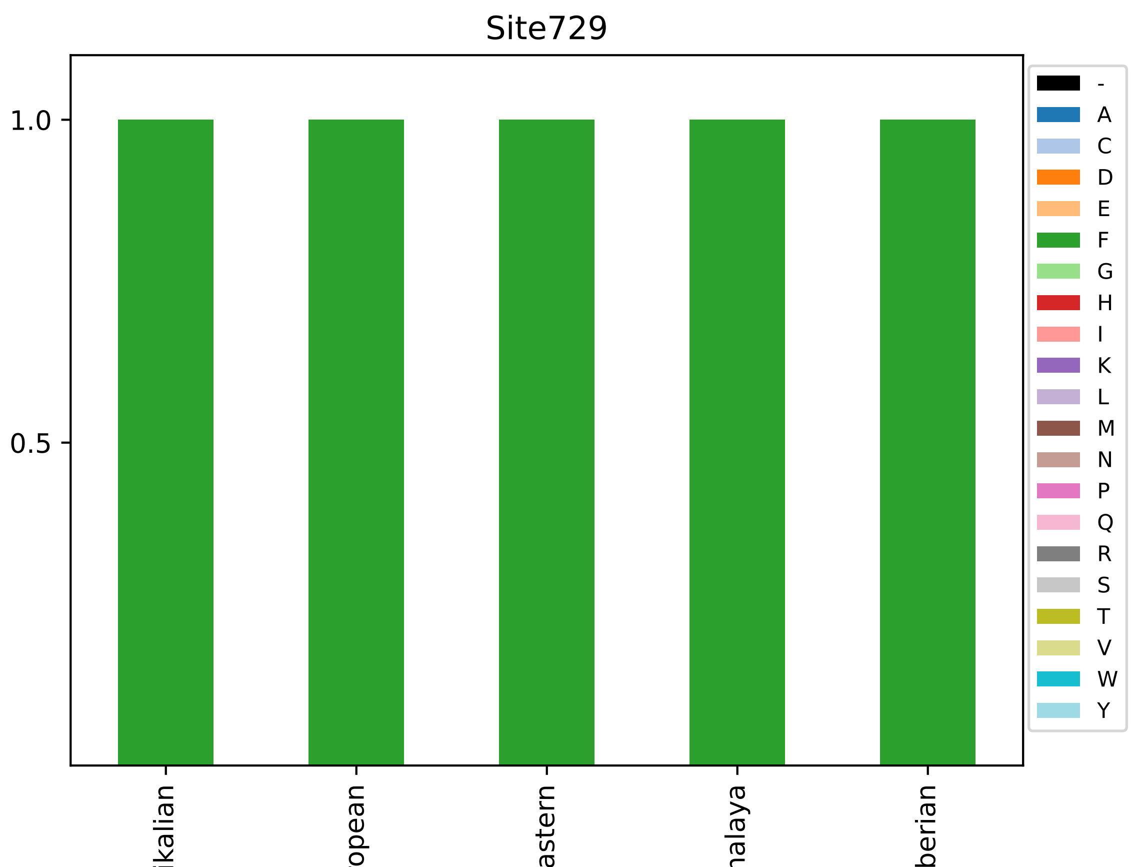

Site729
1.0
0.5
-
A
C
D
E
F
G
H
I
K
L
M
N
P
Q
R
S
T
V
W
Y
ikalian
ropean
astern
nalaya
berian

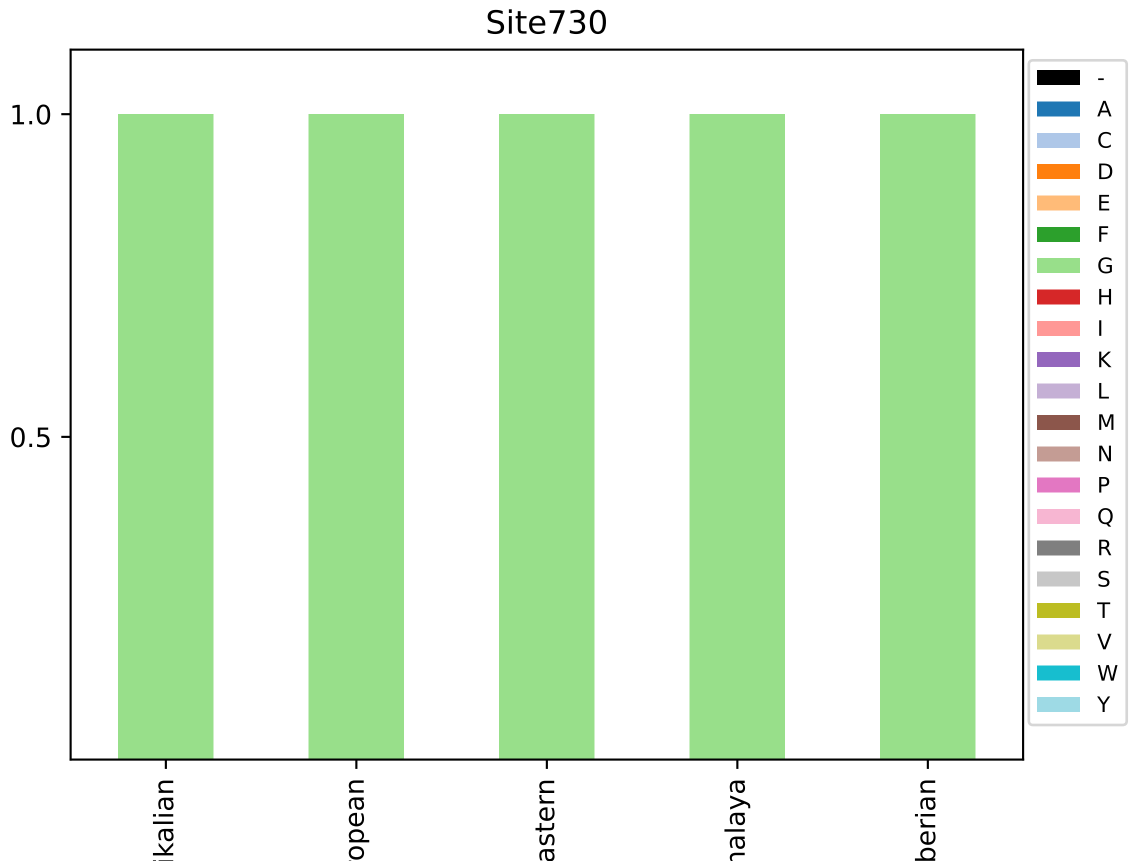
Site730
1.0
0.5
-
A
C
D
E
F
G
H
I
K
L
M
N
P
Q
R
S
T
V
W
Y
ikalian
opean
astern
nalaya
berian

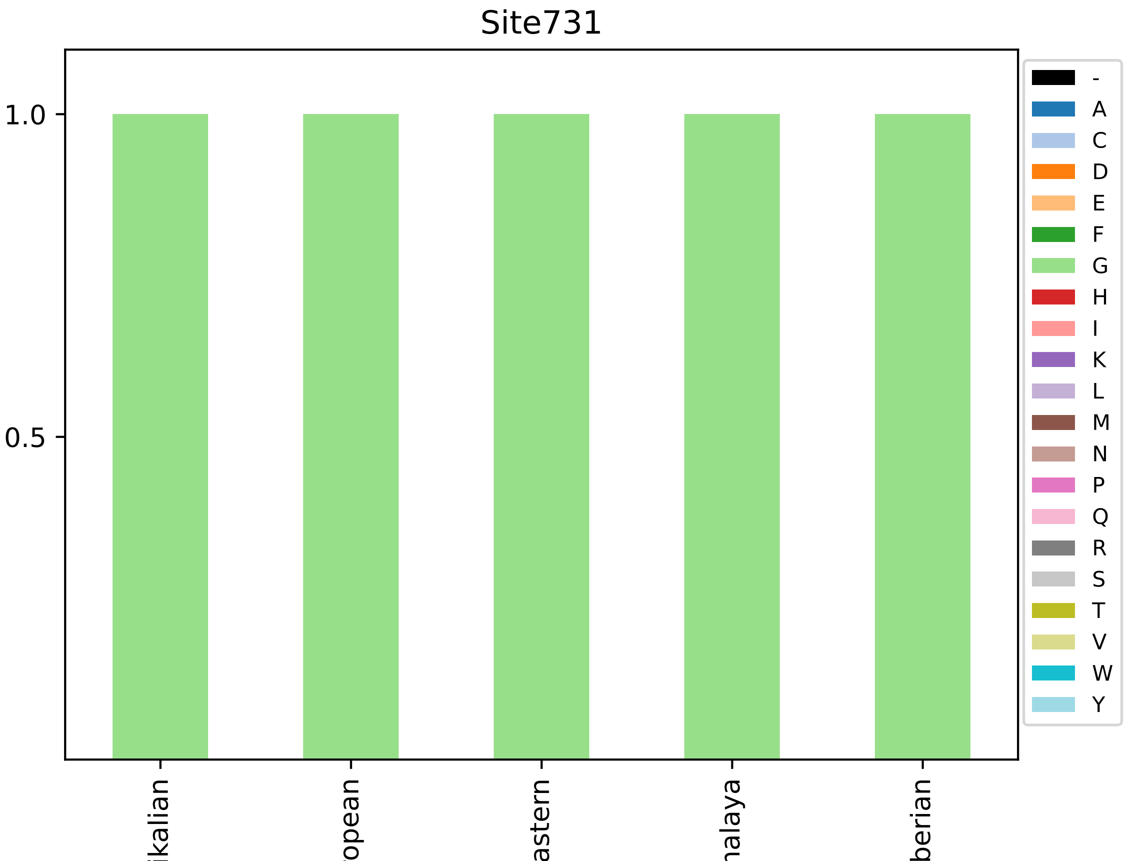

Site731
1.0
0.5
-
A
C
D
E
F
G
H
I
K
L
M
N
P
Q
R
S
T
V
W
Y
ikalian
opean
astern
nalaya
berian

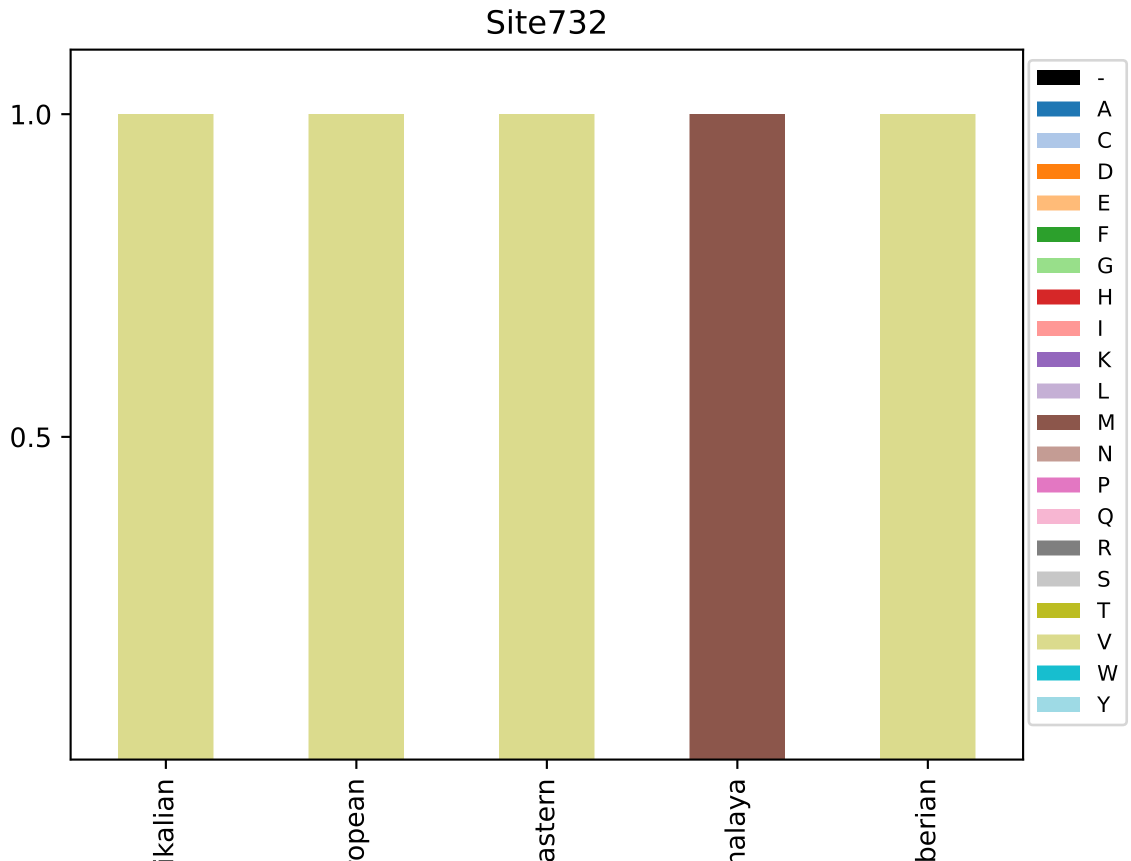

Site732
1.0
0.5
ikalian
opean
astern
nalaya
berian
-
A
C
D
E
F
G
H
I
K
L
M
N
P
Q
R
S
T
V
W
Y

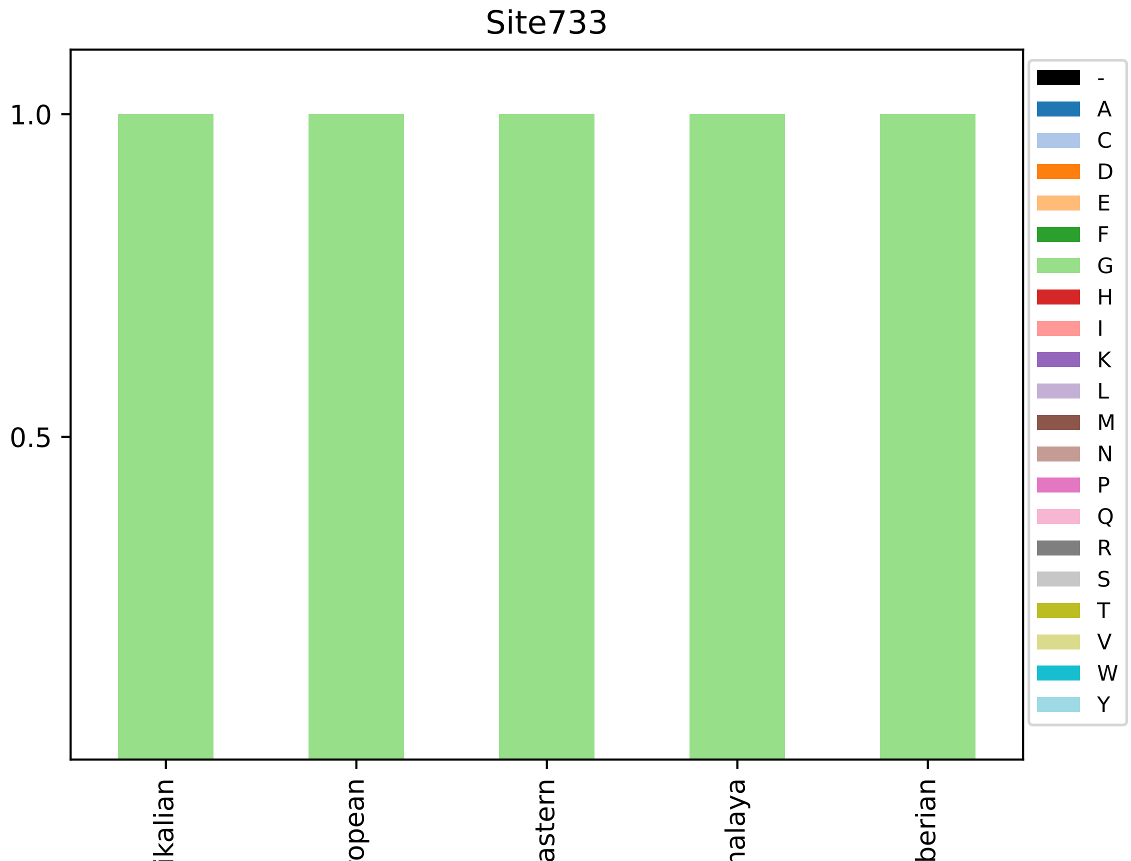

Site733
1.0
0.5
-
A
C
D
E
F
G
H
I
K
L
M
N
P
Q
R
S
T
V
W
Y
ikalian
opean
astern
nalaya
berian

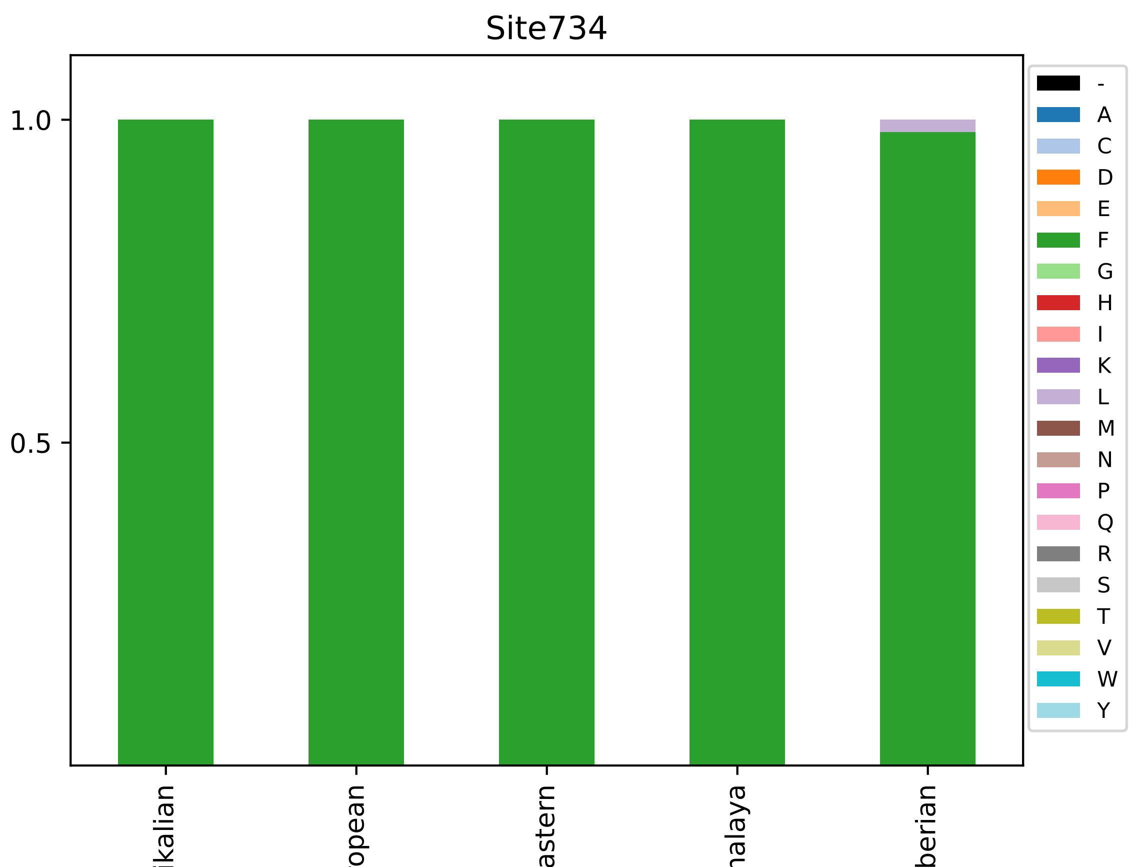
Site734
1.0
0.5
-
A
C
D
E
F
G
H
I
K
L
M
N
P
Q
R
S
T
V
W
Y
ikalian
opean
astern
nalaya
berian

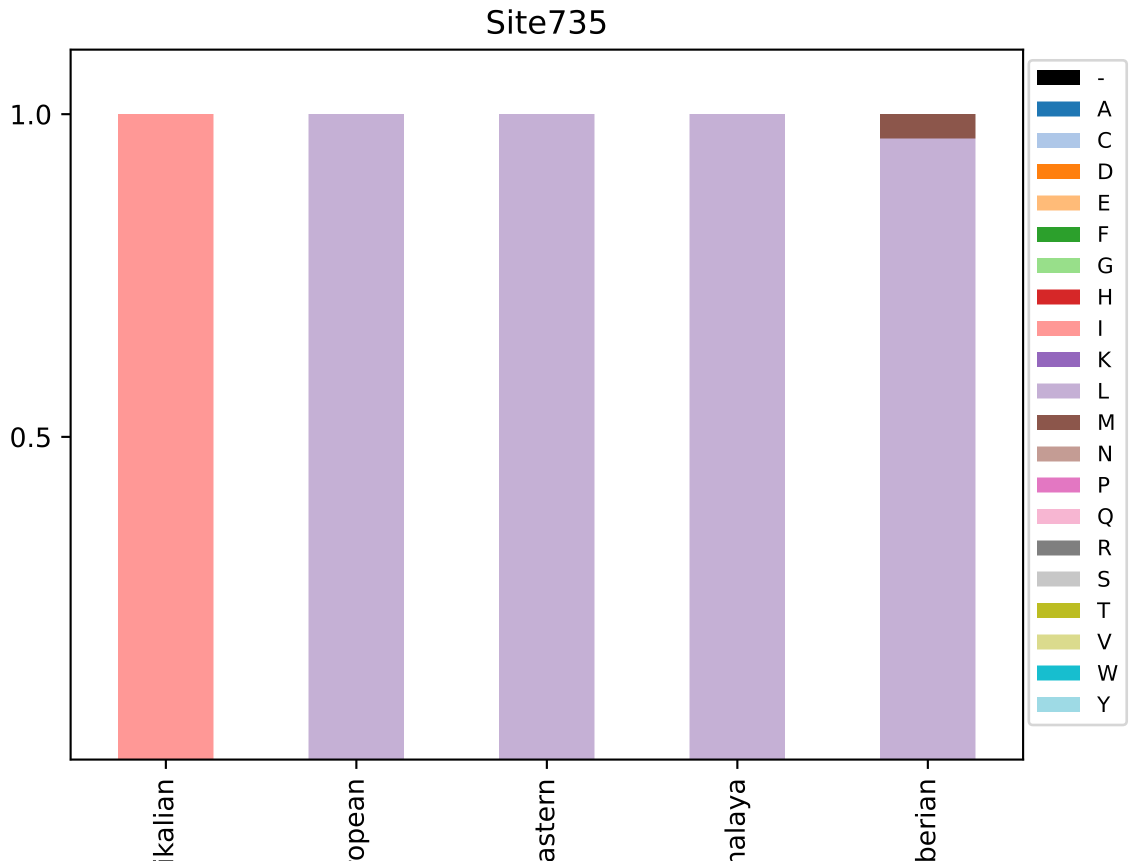
Site735
1.0
0.5
-
A
C
D
E
F
G
H
I
K
L
M
N
P
Q
R
S
T
V
W
Y
ikalian
opean
astern
nalaya
berian

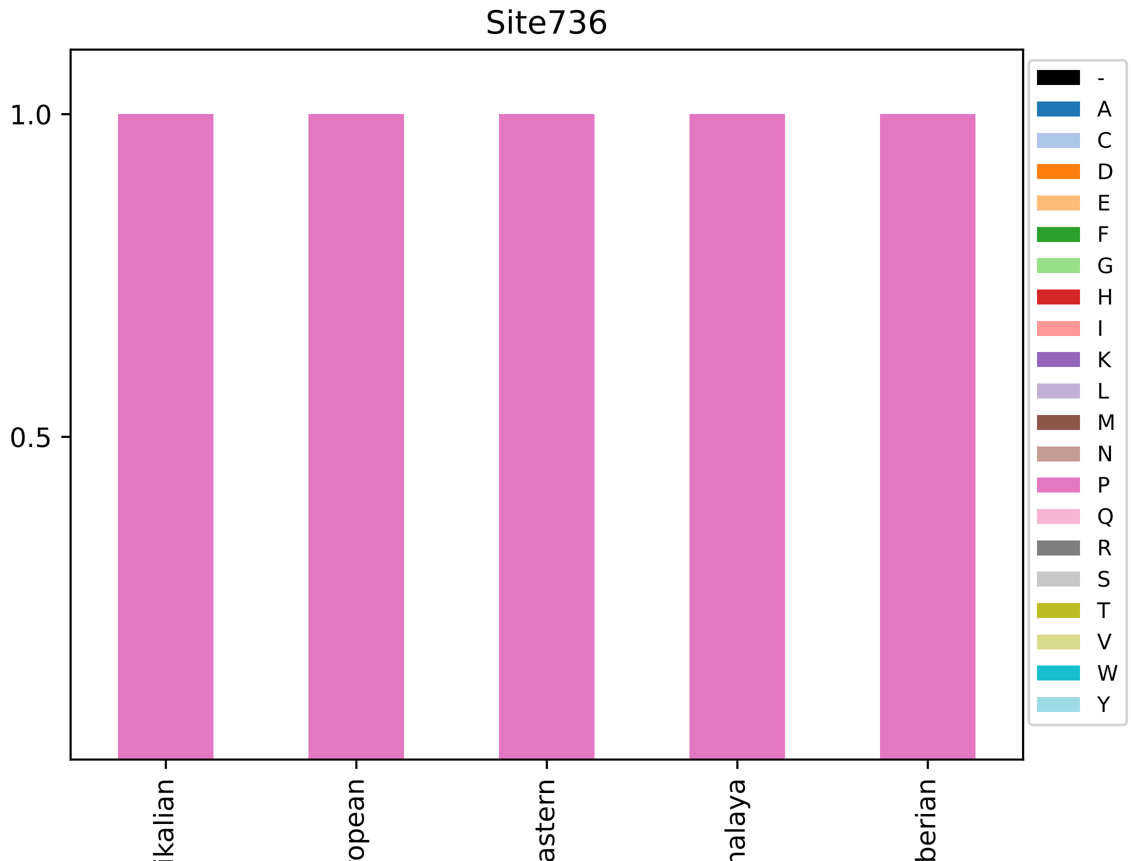
Site736
1.0
0.5
-
A
C
D
E
F
G
H
I
K
L
M
N
P
Q
R
S
T
V
W
Y
ikalian
opean
astern
nalaya
berian

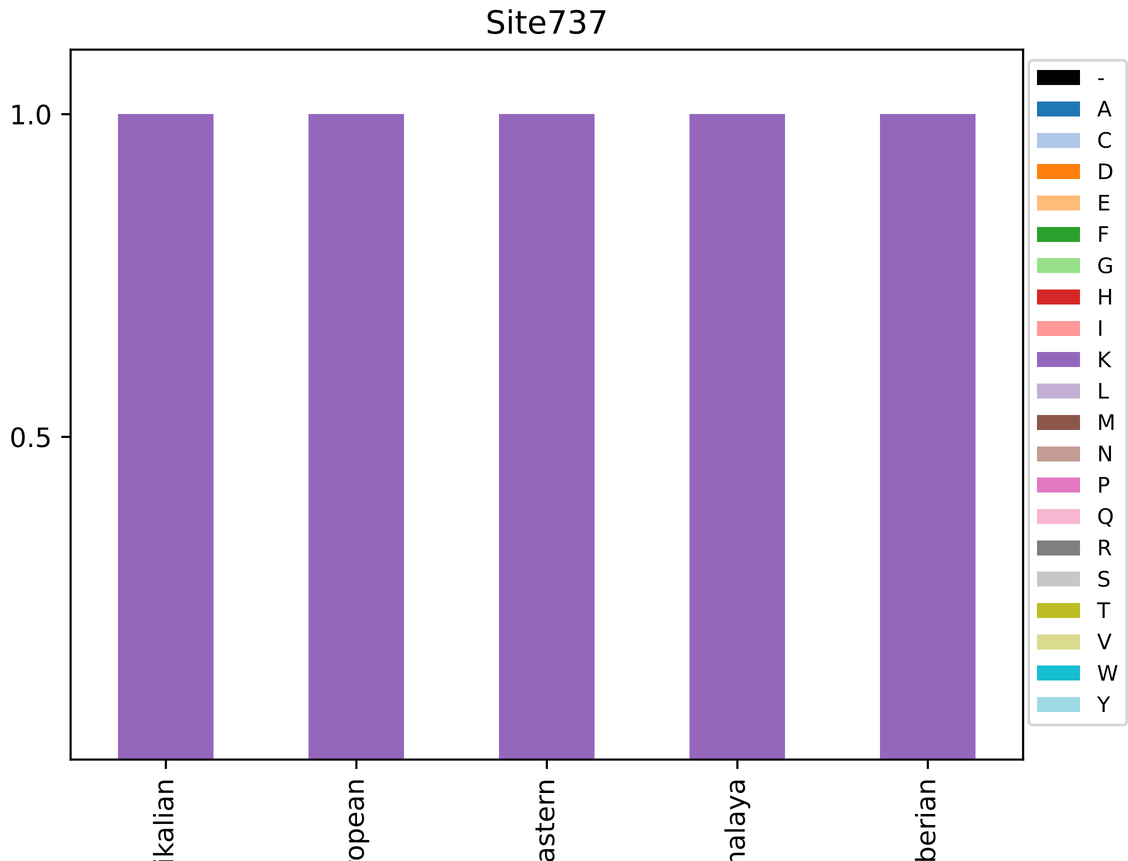

Site737
1.0
0.5
-
A
C
D
E
F
G
H
I
K
L
M
N
P
Q
R
S
T
V
W
Y

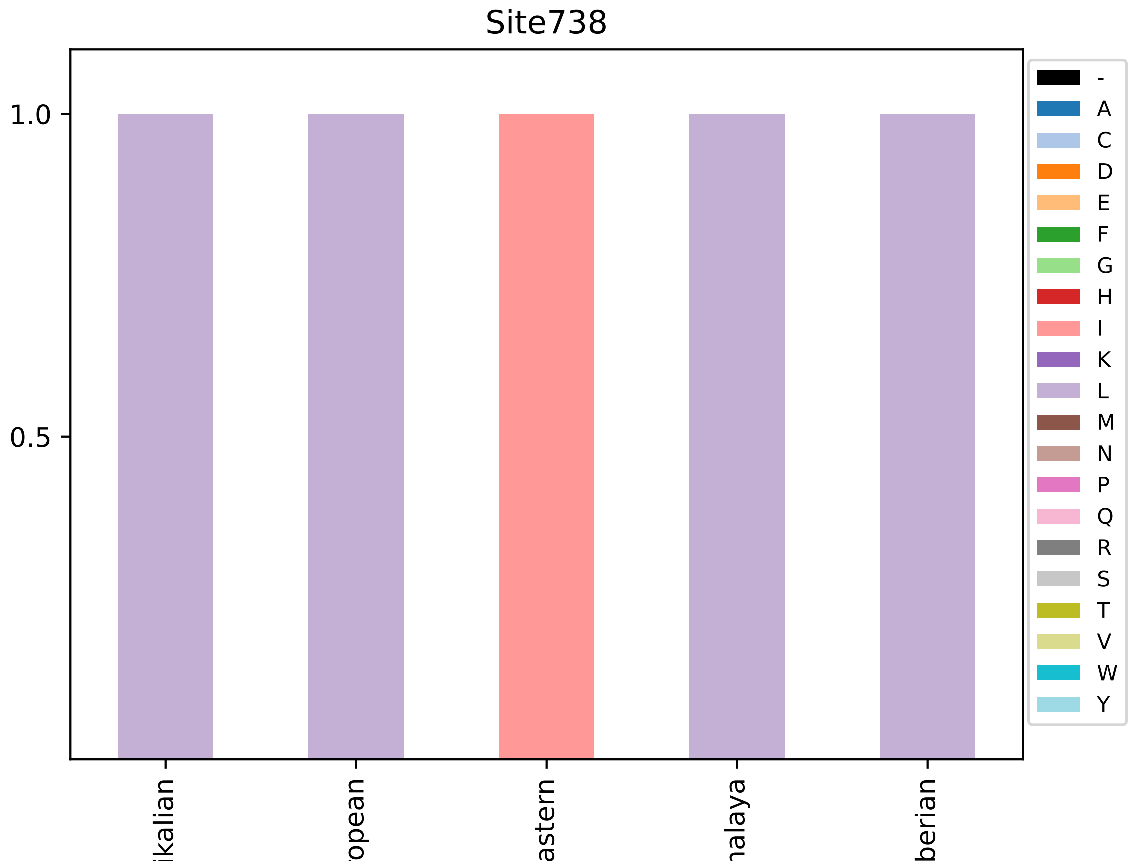
Site738
1.0
0.5
-
A
C
D
E
F
G
H
I
K
L
M
N
P
Q
R
S
T
V
W
Y

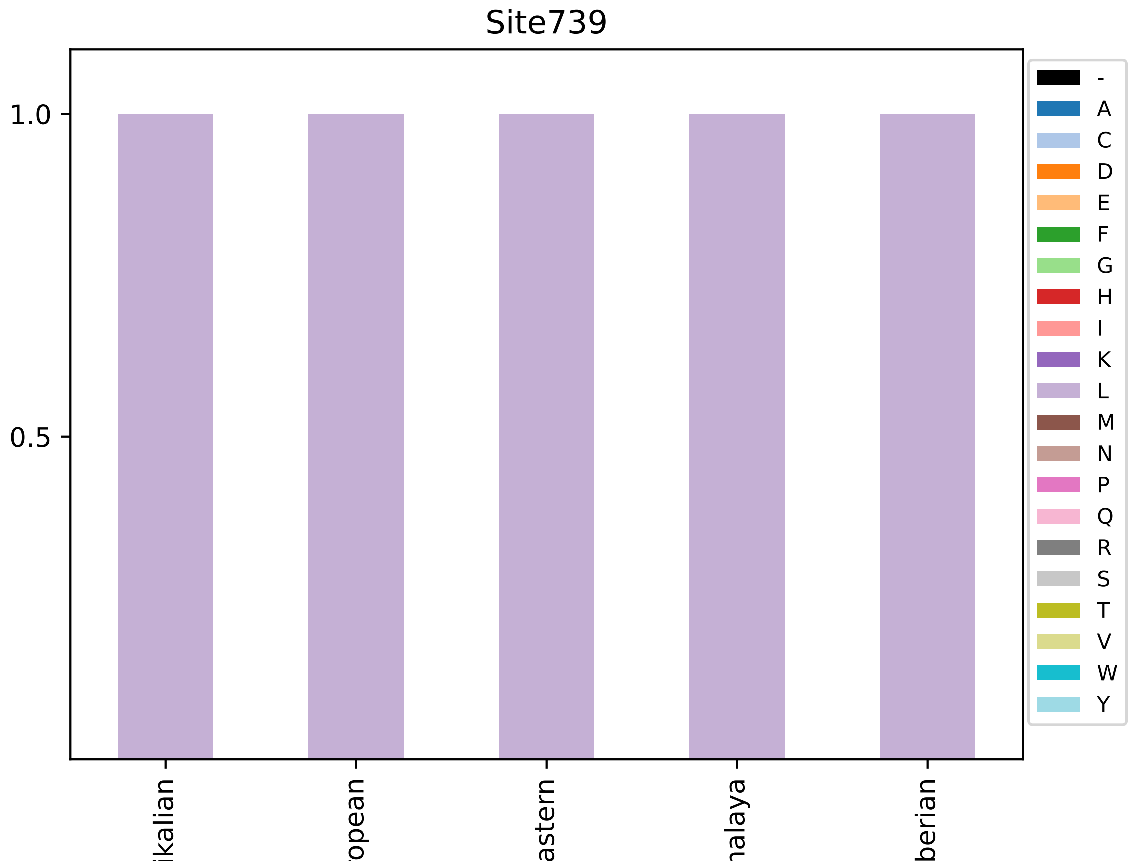
Site739
1.0
0.5
-
A
C
D
E
F
G
H
I
K
L
M
N
P
Q
R
S
T
V
W
Y
ikalian
opean
astern
nalaya
berian

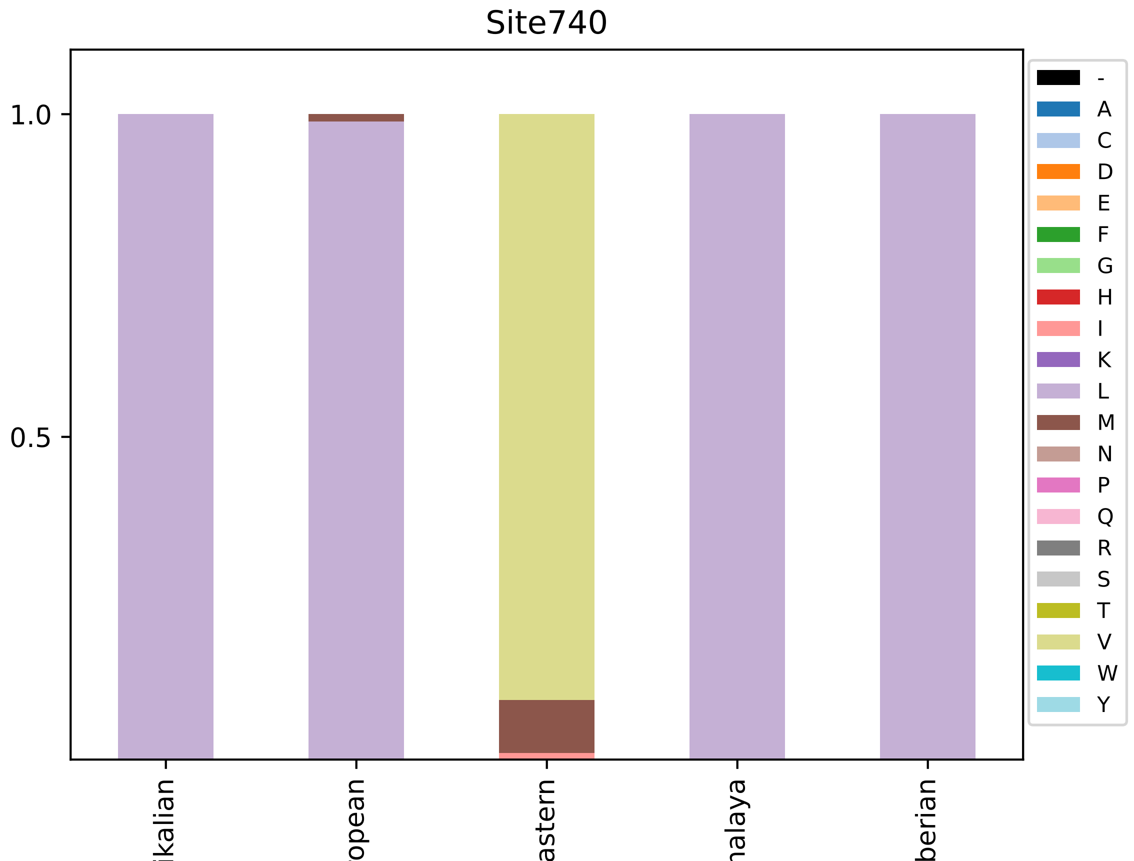
Site740
1.0
0.5
-
A
C
D
E
F
G
H
I
K
L
M
N
P
Q
R
S
T
V
W
Y
ikalian
opean
astern
nalaya
berian

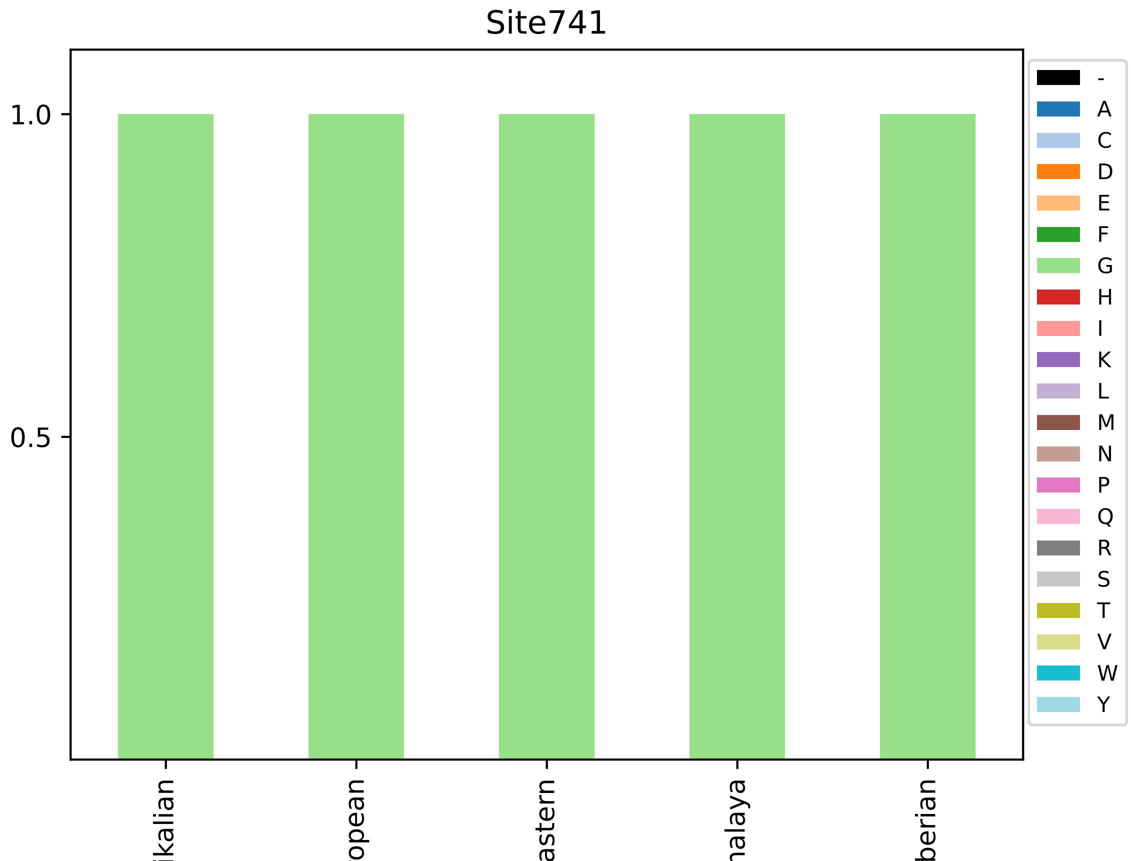
Site741
1.0
0.5
-
A
C
D
E
F
G
H
I
K
L
M
N
P
Q
R
S
T
V
W
Y
ikalian
opean
astern
nalaya
berian

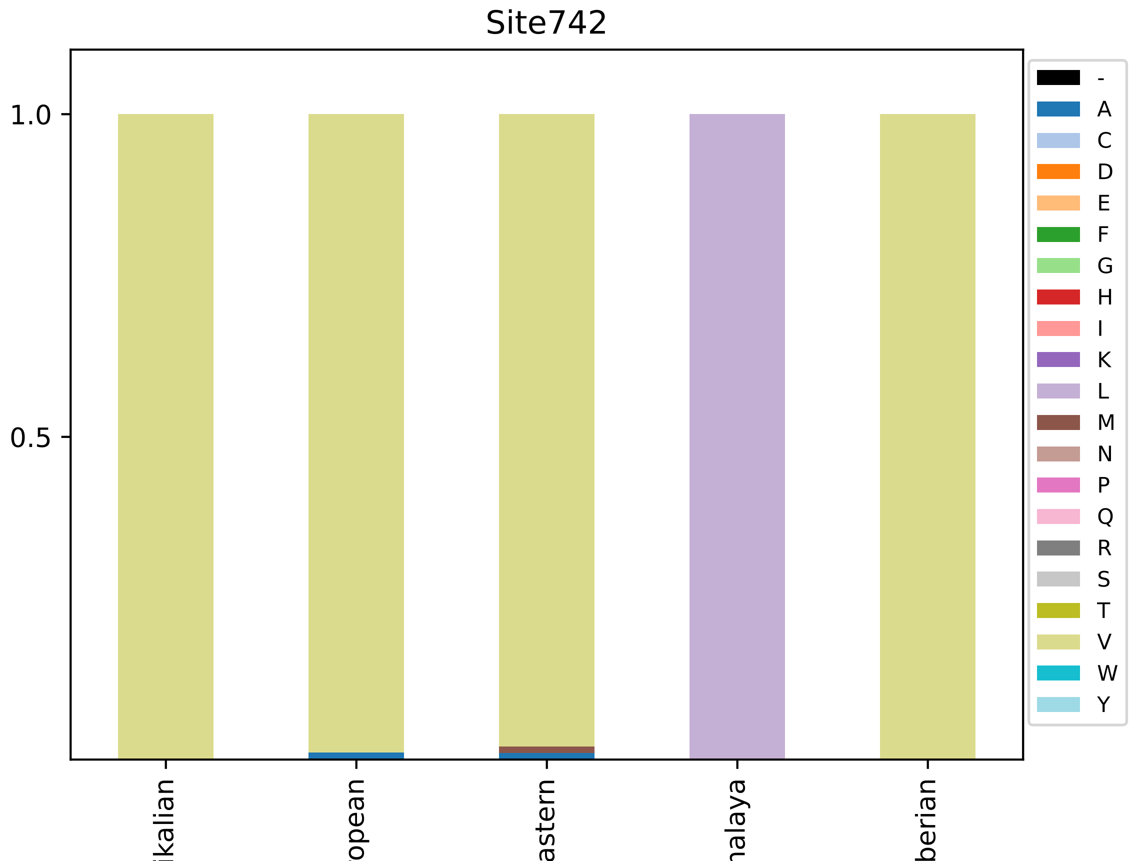
Site742
1.0
0.5
-
A
C
D
E
F
G
H
I
K
L
M
N
P
Q
R
S
T
V
W
Y

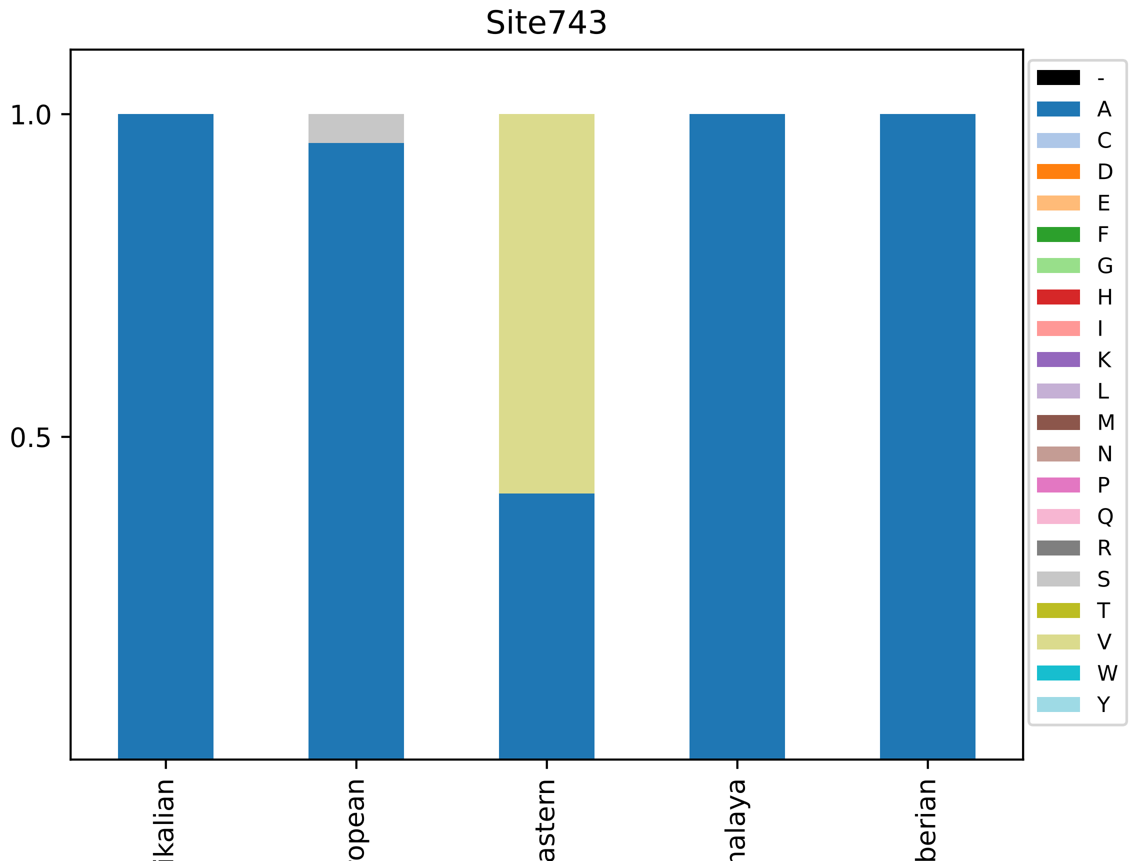
Site743
1.0
0.5
-
A
C
D
E
F
G
H
I
K
L
M
N
P
Q
R
S
T
V
W
Y
ikalian
opean
astern
nalaya
berian

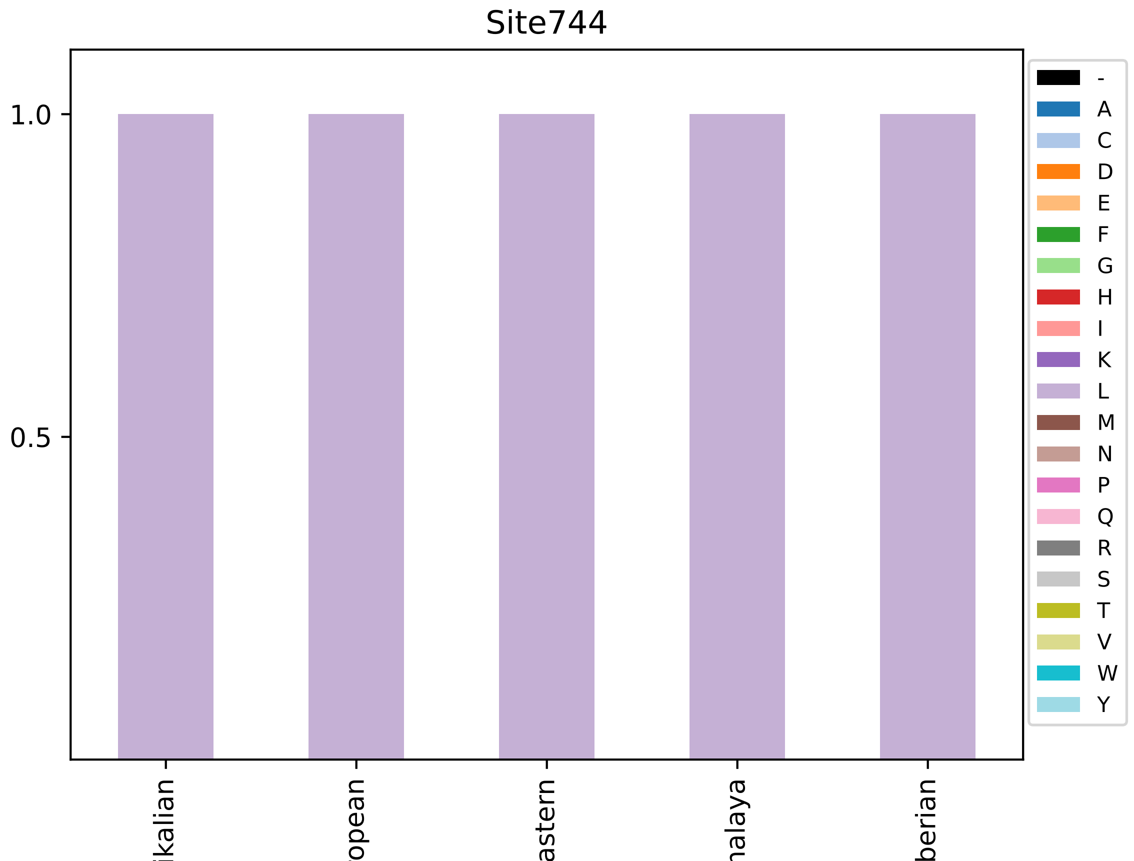

Site744
1.0
0.5
-
A
C
D
E
F
G
H
I
K
L
M
N
P
Q
R
S
T
V
W
Y
ikalian
opean
astern
nalaya
berian

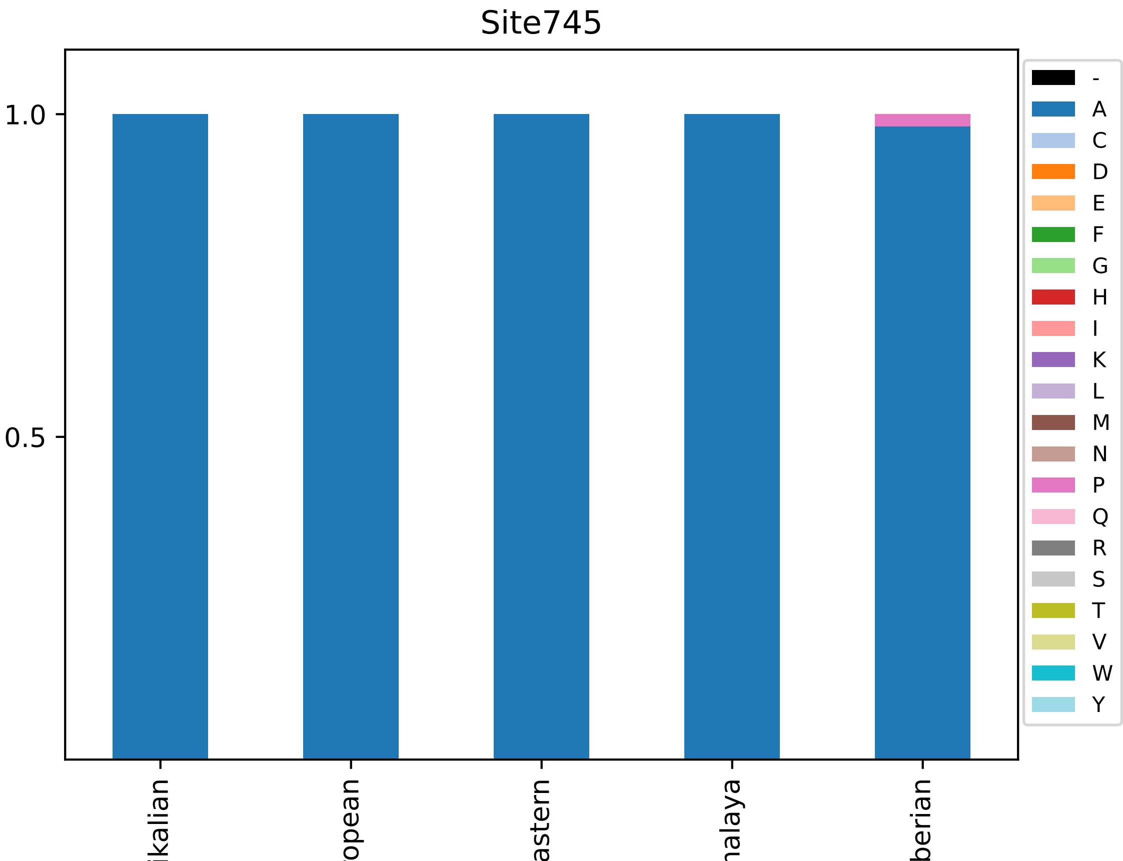
Site745
1.0
0.5
-
A
C
D
E
F
G
H
I
K
L
M
N
P
Q
R
S
T
V
W
Y
ikalian
opean
astern
nalaya
berian

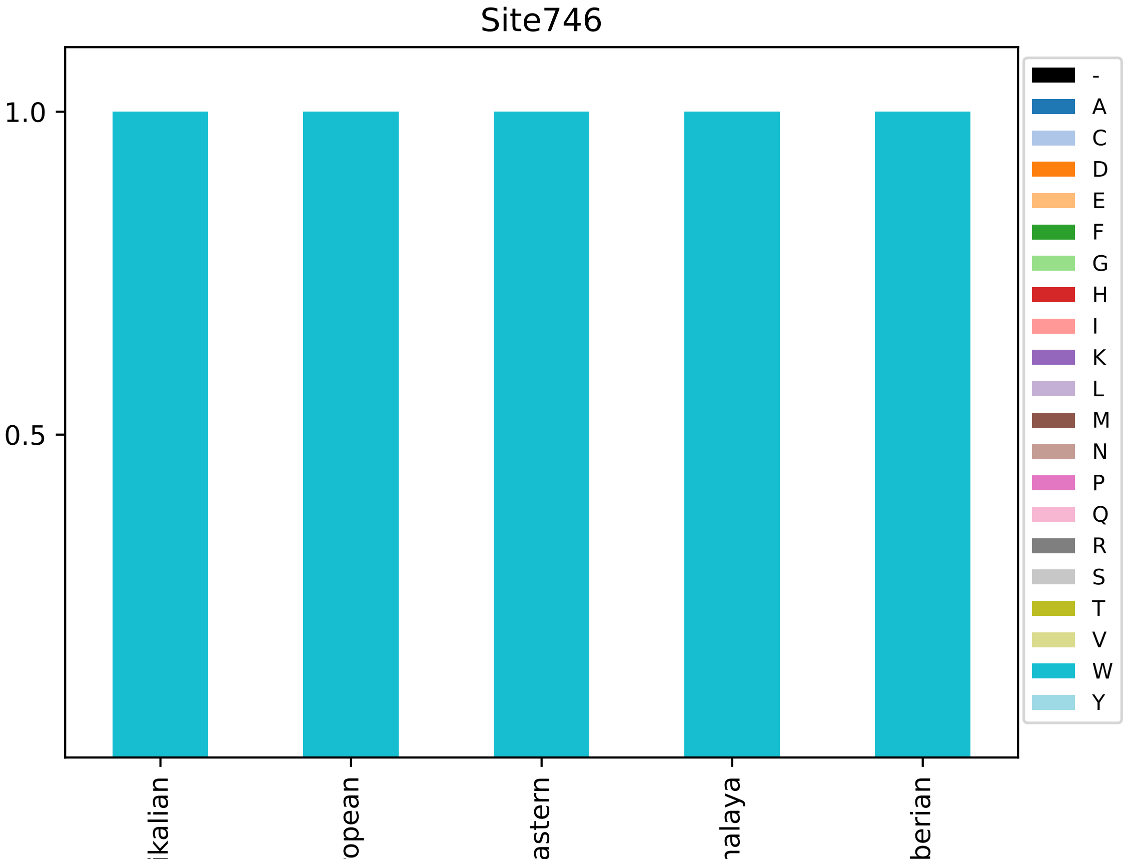
Site746
1.0
0.5
-
A
C
D
E
F
G
H
I
K
L
M
N
P
Q
R
S
T
V
W
Y
ikalian
opean
astern
nalaya
berian

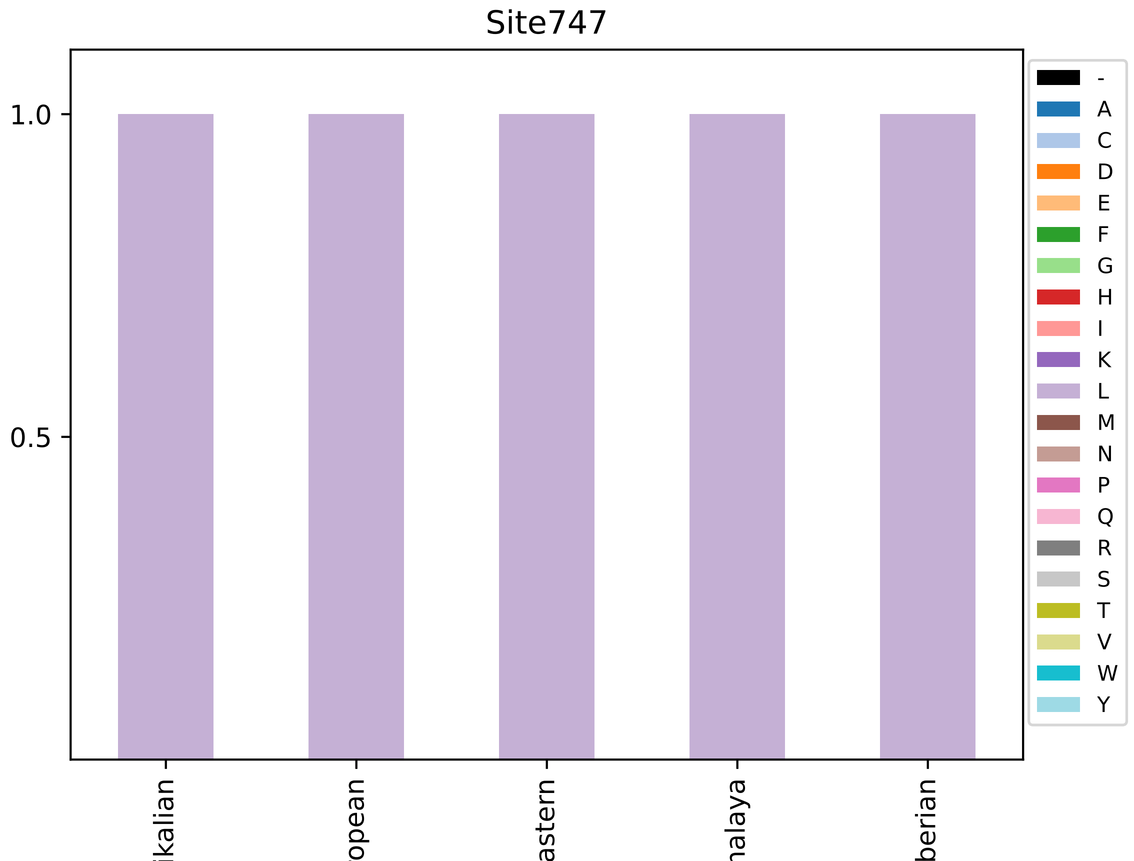
Site747
1.0
0.5
-
A
C
D
E
F
G
H
I
K
L
M
N
P
Q
R
S
T
V
W
Y
ikalian
opean
astern
nalaya
berian

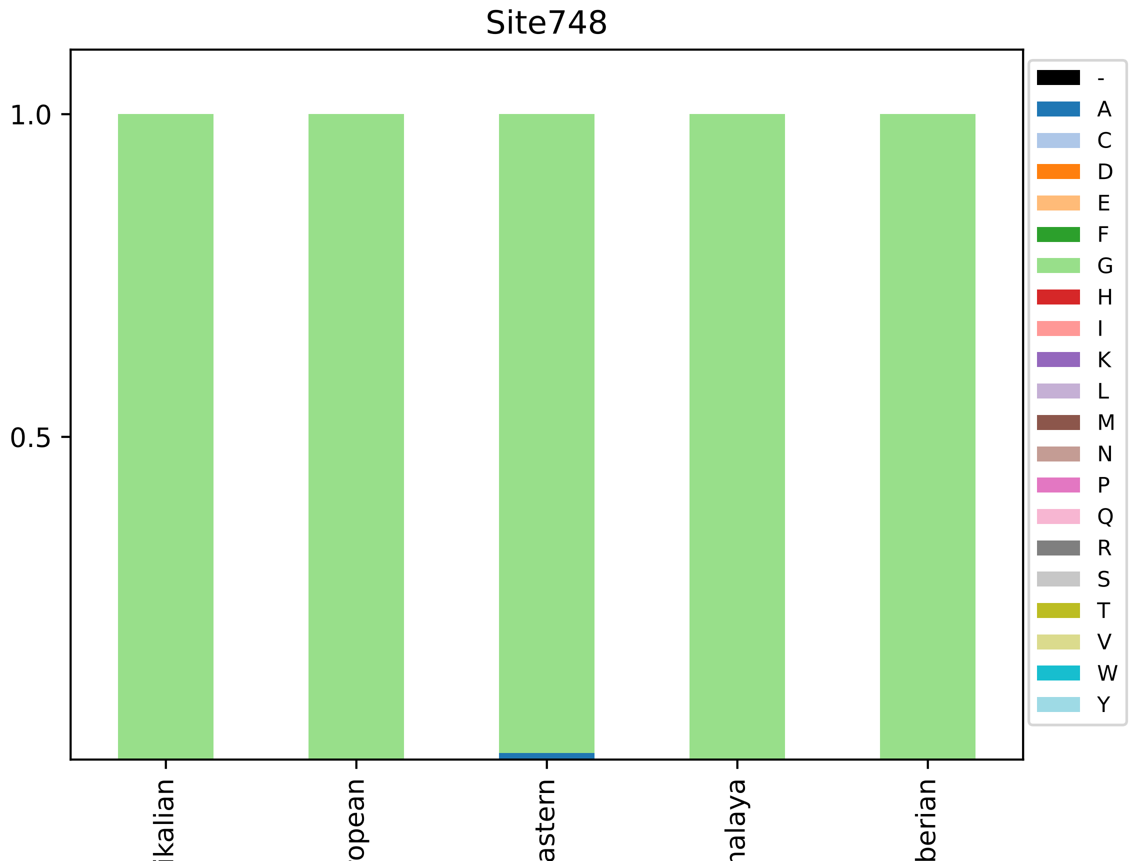
Site748
1.0
0.5
-
A
C
D
E
F
G
H
I
K
L
M
N
P
Q
R
S
T
V
W
Y

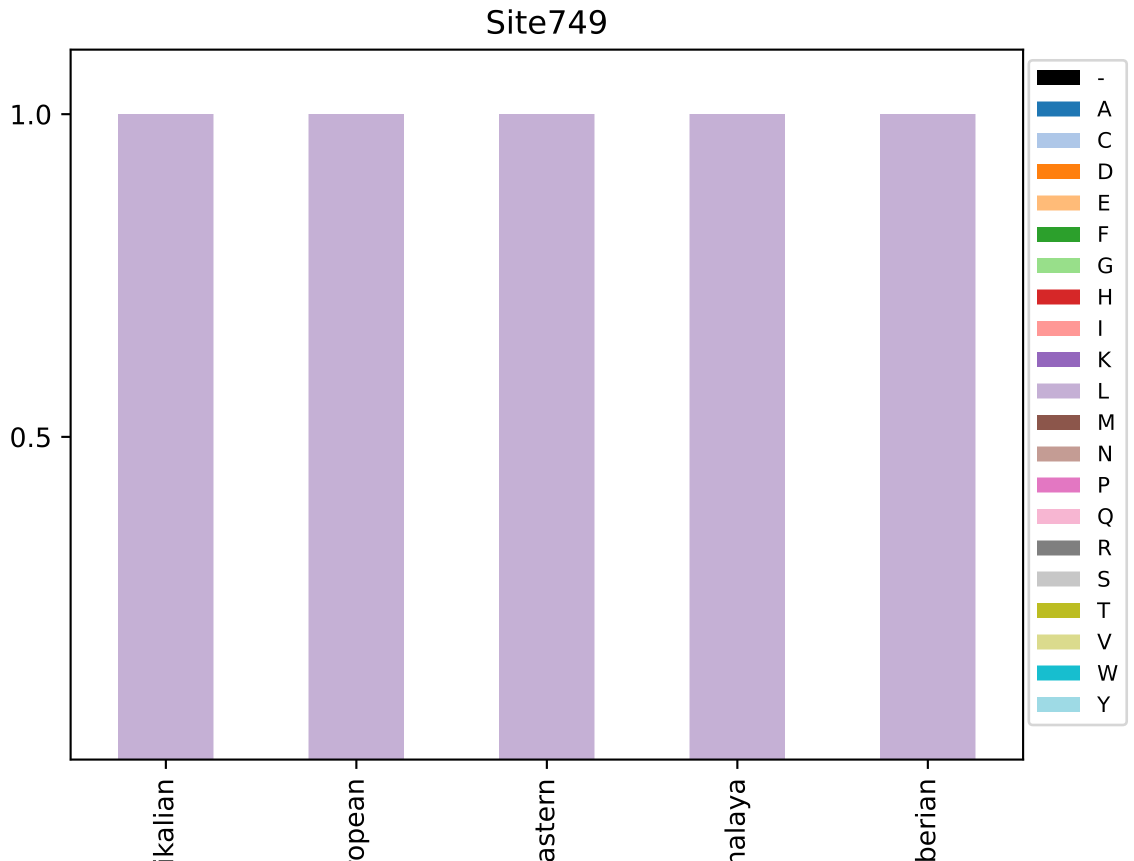

Site749
1.0
0.5
-
A
C
D
E
F
G
H
I
K
L
M
N
P
Q
R
S
T
V
W
Y

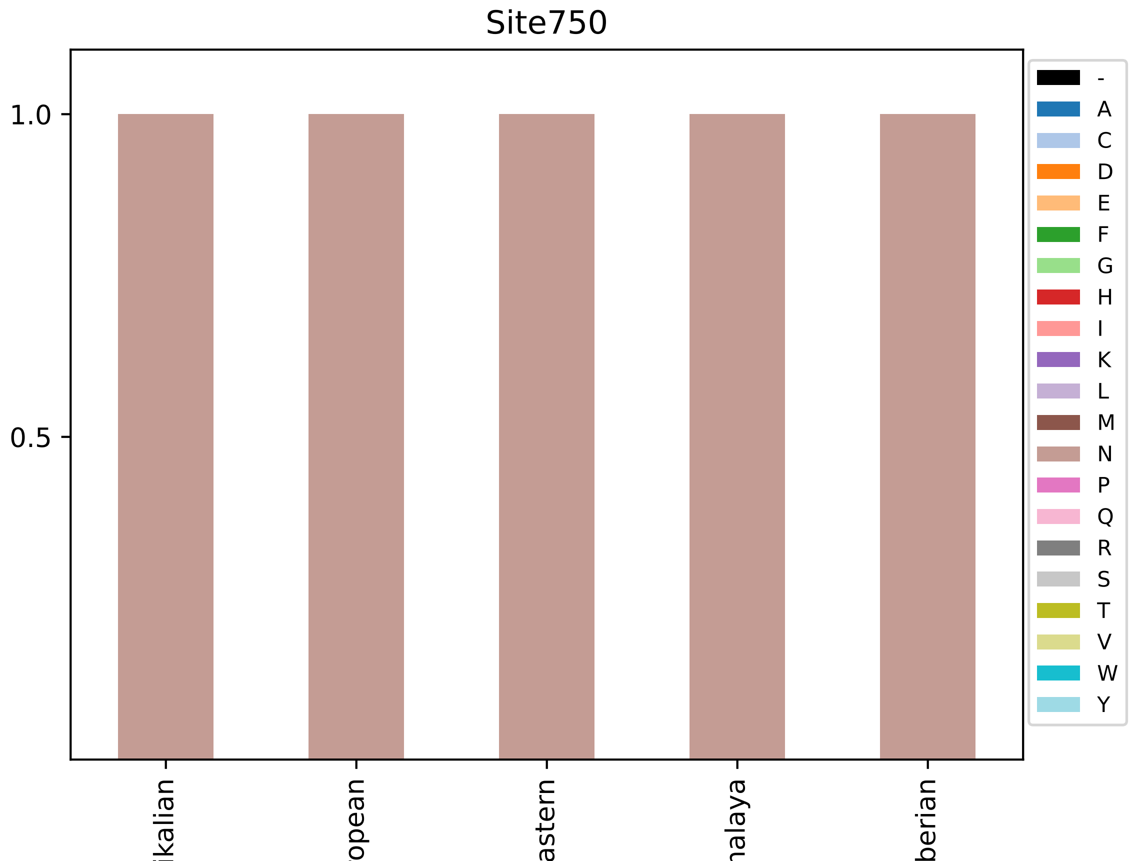
Site750
1.0
0.5
-
A
C
D
E
F
G
H
I
K
L
M
N
P
Q
R
S
T
V
W
Y
ikalian
opean
astern
nalaya
berian

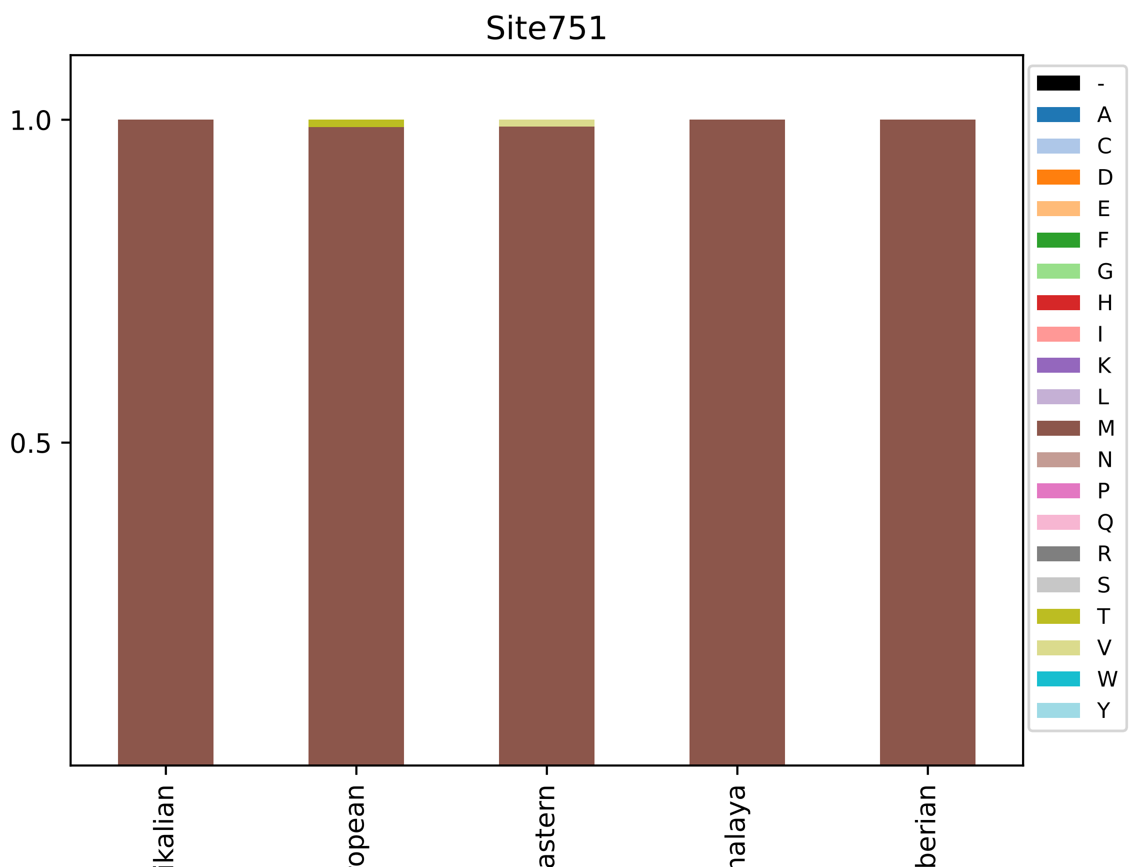

Site751
1.0
0.5
-
A
C
D
E
F
G
H
I
K
L
M
N
P
Q
R
S
T
V
W
Y
ikalian
opean
astern
nalaya
berian

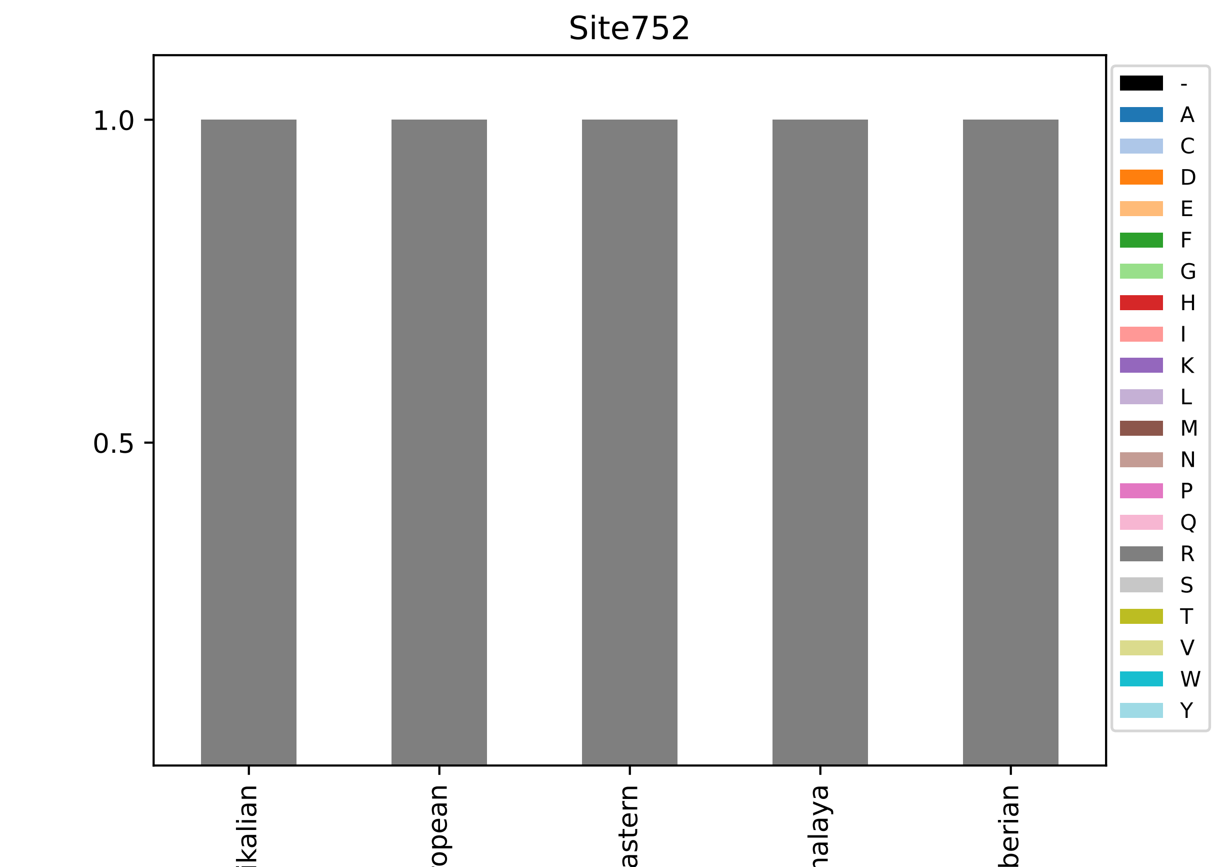

Site752
1.0
0.5
-
A
C
D
E
F
G
H
I
K
L
M
N
P
Q
R
S
T
V
W
Y
ikalian
opean
astern
nalaya
berian

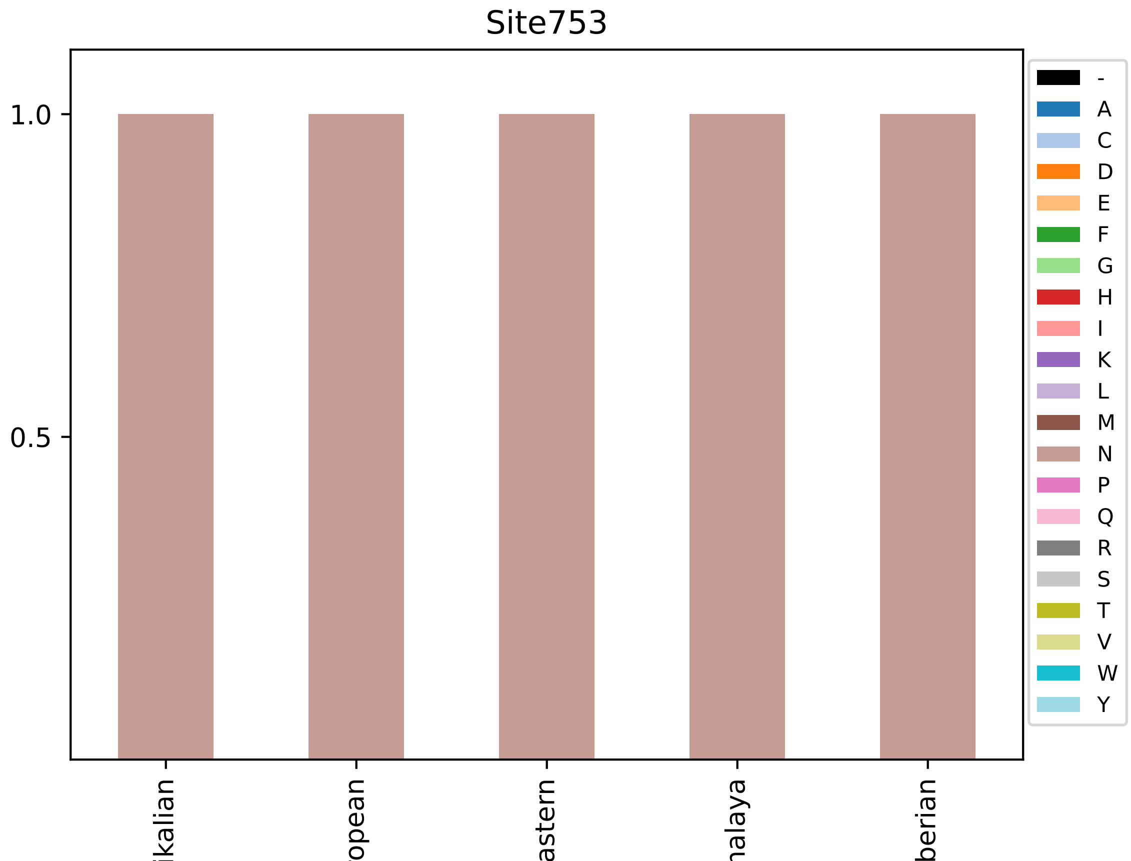
Site753
1.0
0.5
ikalian
opean
astern
nalaya
berian
-
A
C
D
E
F
G
H
I
K
L
M
N
P
Q
R
S
T
V
W
Y

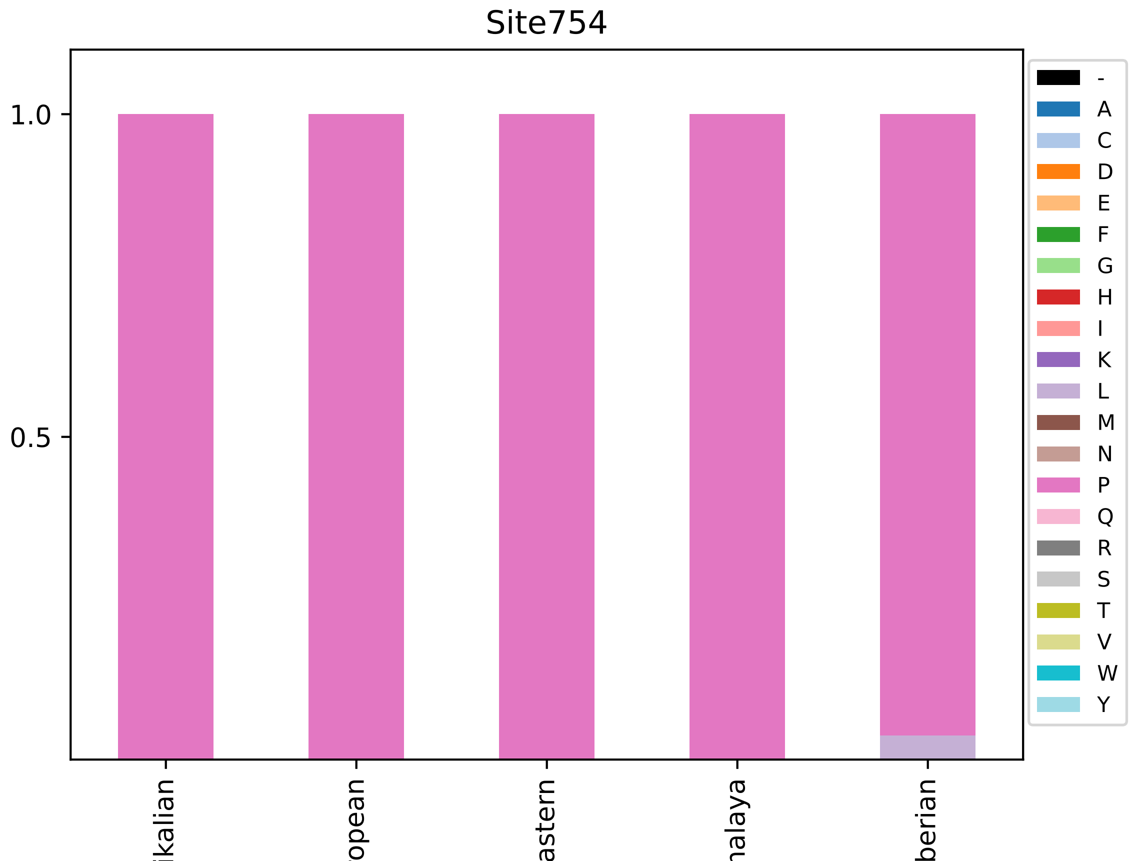
Site754
1.0
0.5
-
A
C
D
E
F
G
H
I
K
L
M
N
P
Q
R
S
T
V
W
Y

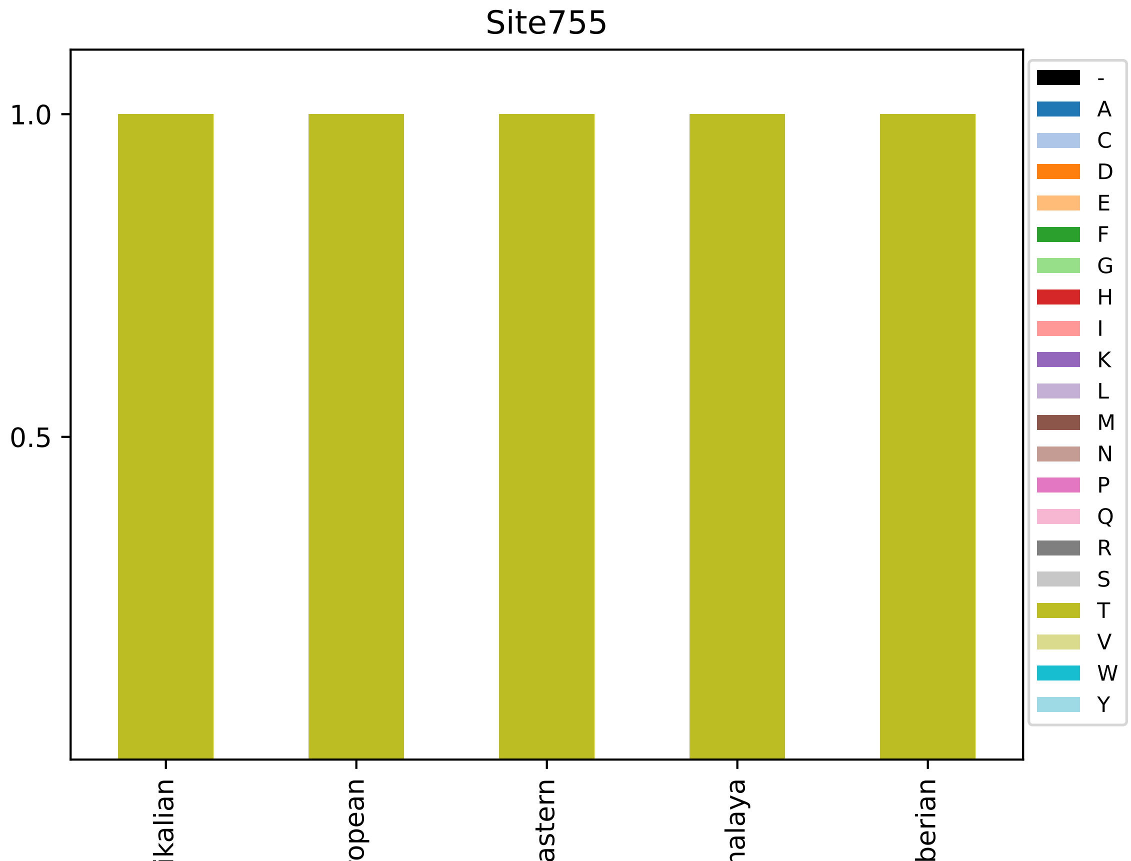
Site755
1.0
0.5
-
A
C
D
E
F
G
H
I
K
L
M
N
P
Q
R
S
T
V
W
Y
ikalian
opean
astern
nalaya
berian

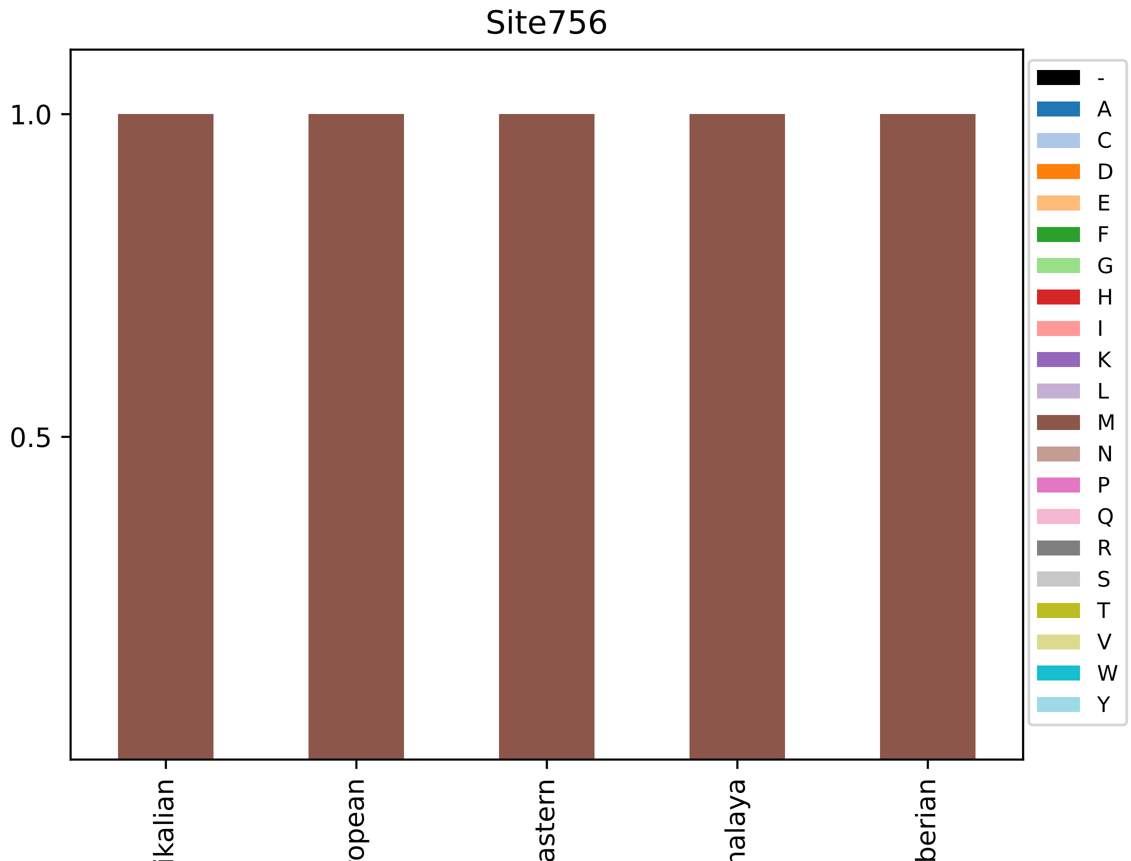
Site756
1.0
0.5
-
A
C
D
E
F
G
H
I
K
L
M
N
P
Q
R
S
T
V
W
Y
ikalian
opean
astern
nalaya
berian

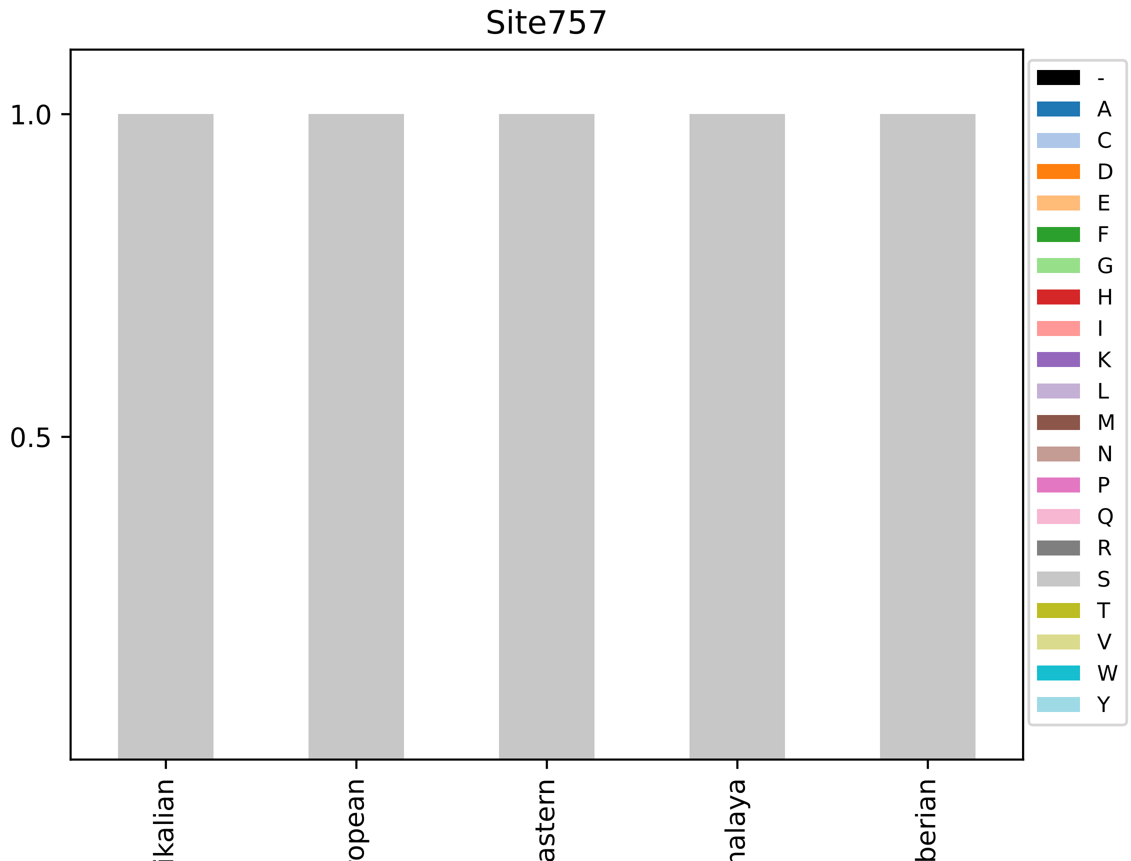
Site757
1.0
0.5
-
A
C
D
E
F
G
H
I
K
L
M
N
P
Q
R
S
T
V
W
Y
ikalian
opean
astern
nalaya
berian

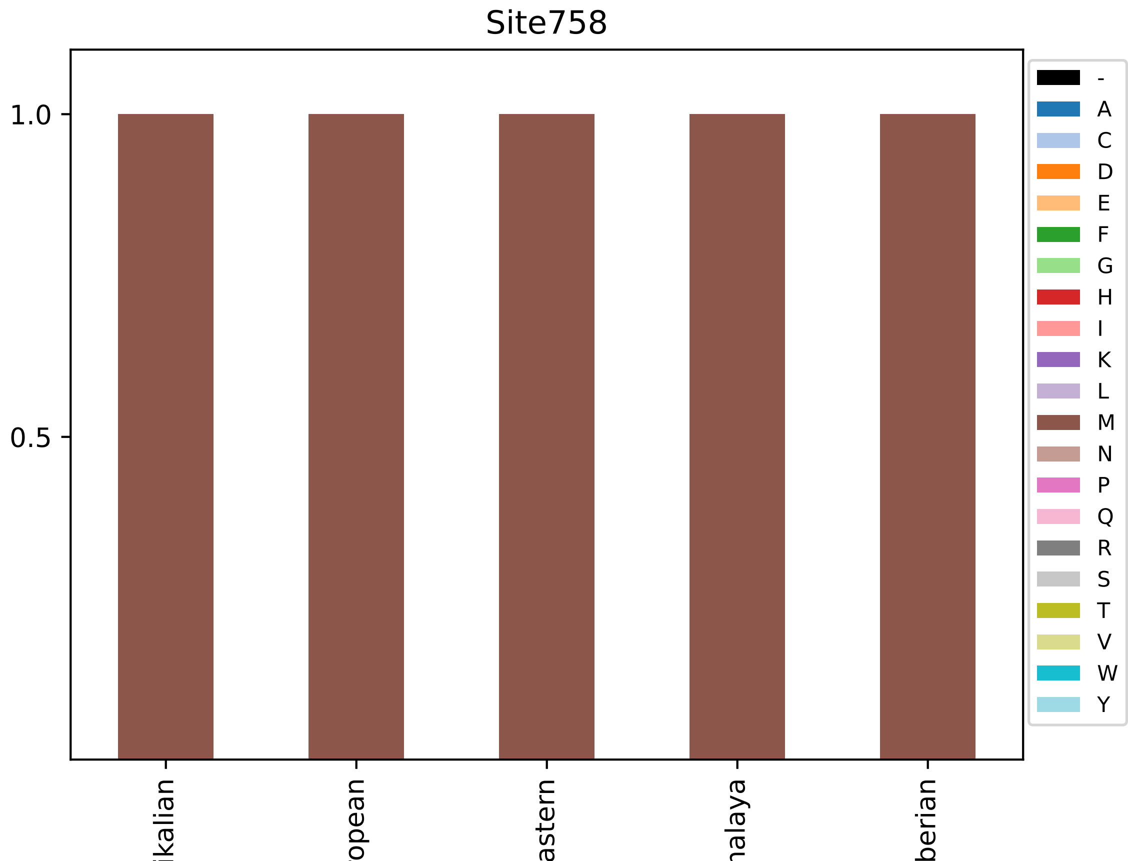

Site758
1.0
0.5
ikalian
opean
astern
nalaya
berian
-
A
C
D
E
F
G
H
I
K
L
M
N
P
Q
R
S
T
V
W
Y

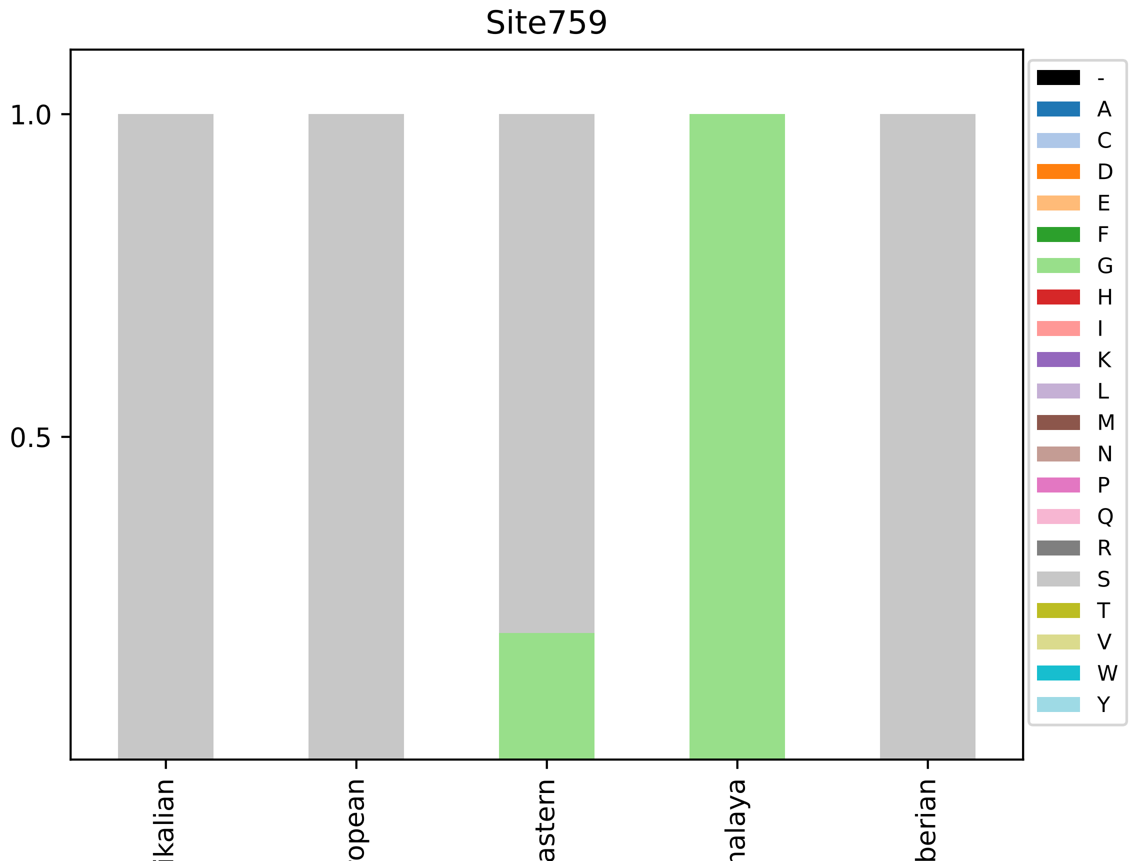
Site759
1.0
0.5
-
A
C
D
E
F
G
H
I
K
L
M
N
P
Q
R
S
T
V
W
Y
ikalian
opean
astern
nalaya
berian

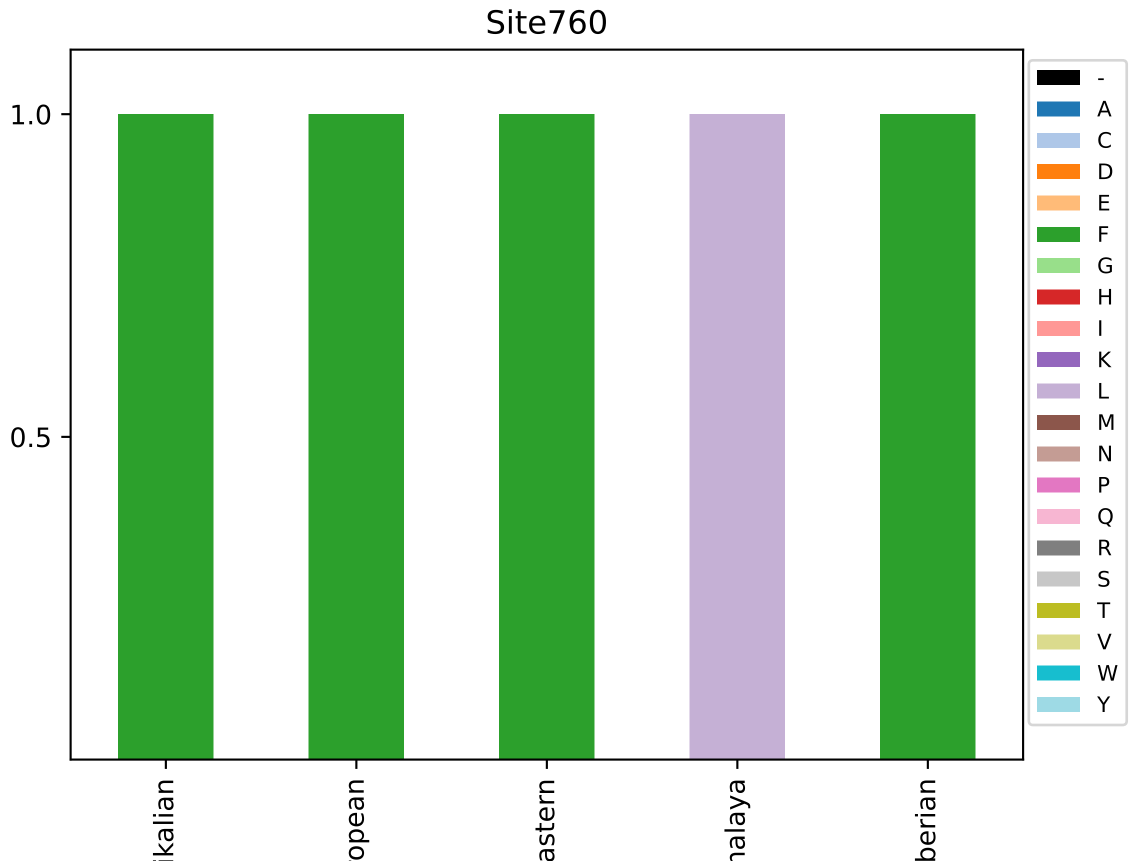

Site760
1.0
0.5
-
A
C
D
E
F
G
H
I
K
L
M
N
P
Q
R
S
T
V
W
Y
ikalian
opean
astern
nalaya
berian

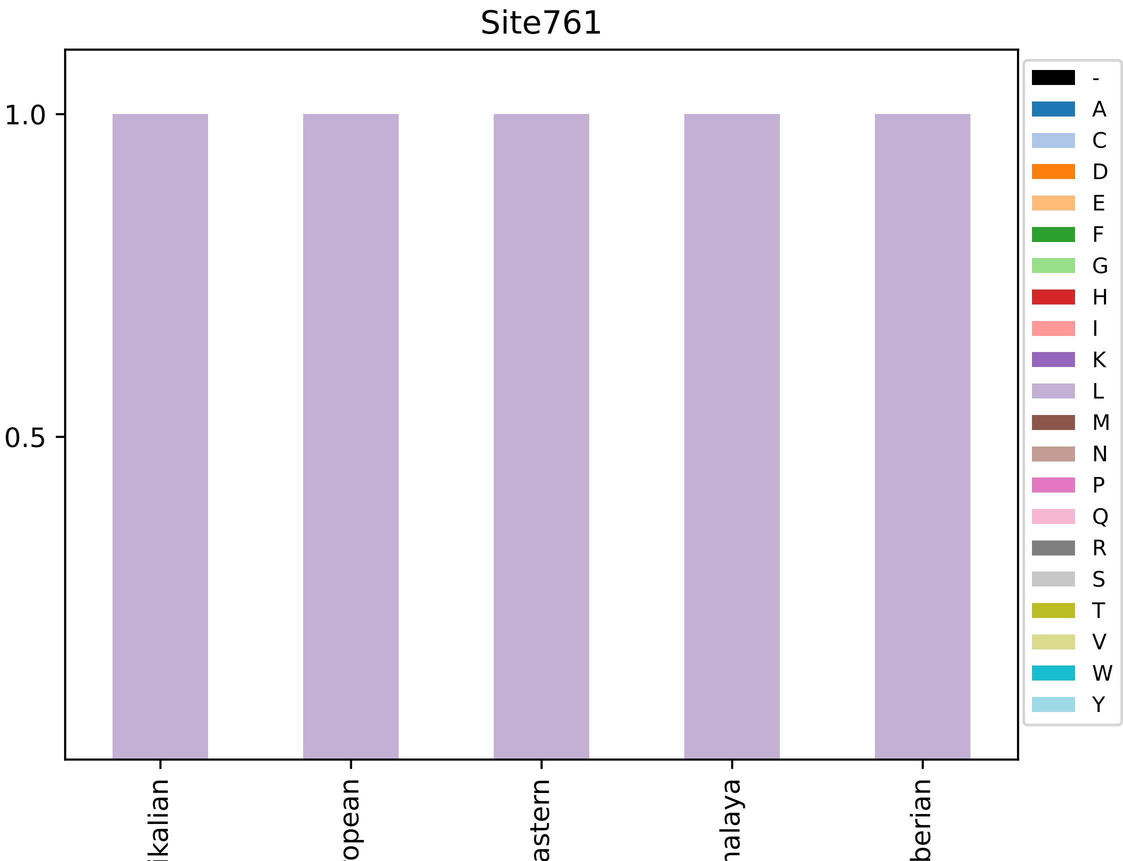

Site761
1.0
0.5
-
A
C
D
E
F
G
H
I
K
L
M
N
P
Q
R
S
T
V
W
Y

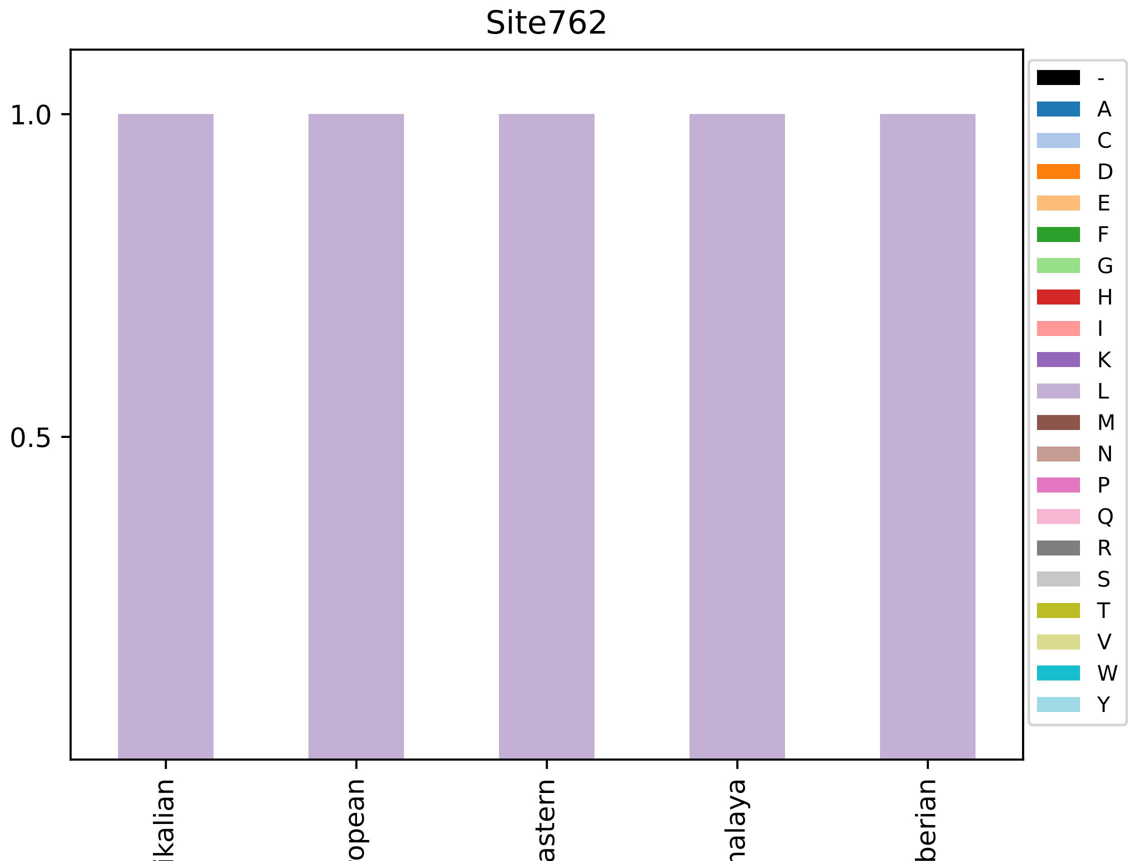
Site762
1.0
0.5
-
A
C
D
E
F
G
H
I
K
L
M
N
P
Q
R
S
T
V
W
Y
ikalian
opean
astern
nalaya
berian

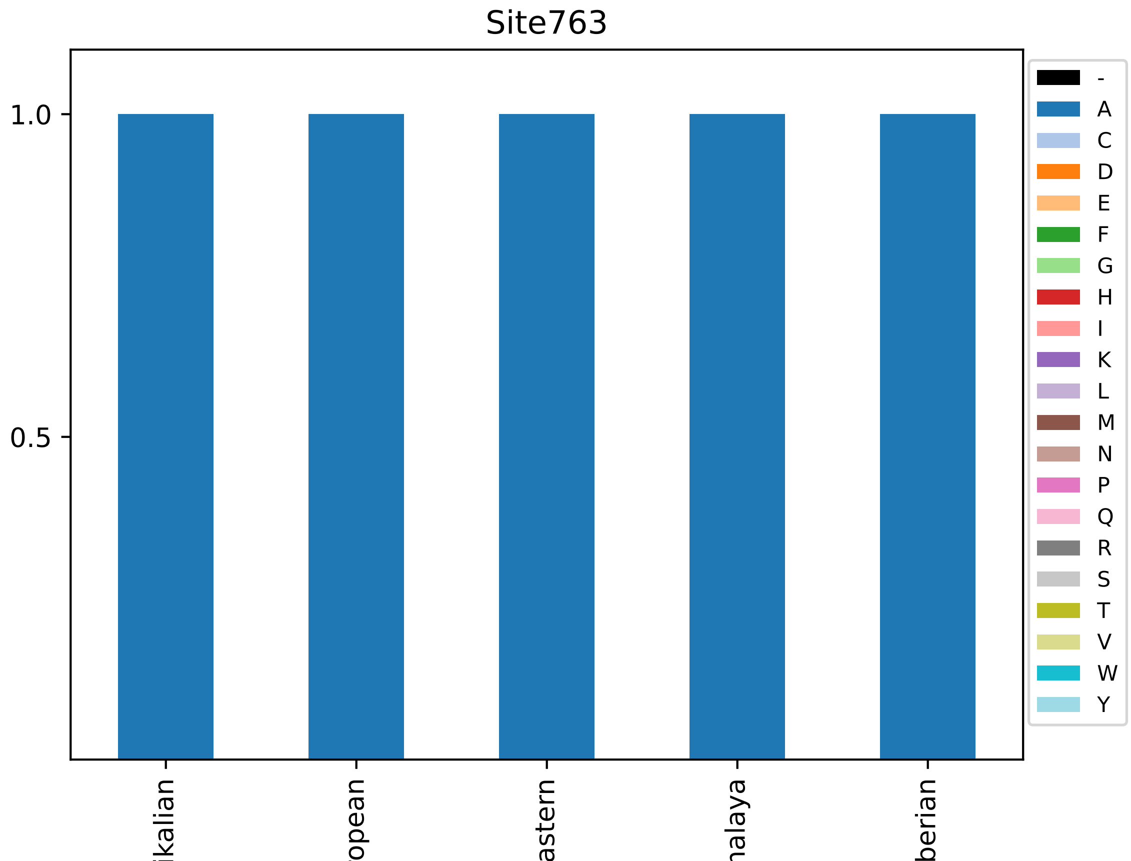

Site763
1.0
0.5
-
A
C
D
E
F
G
H
I
K
L
M
N
P
Q
R
S
T
V
W
Y
ikalian
opean
astern
nalaya
berian

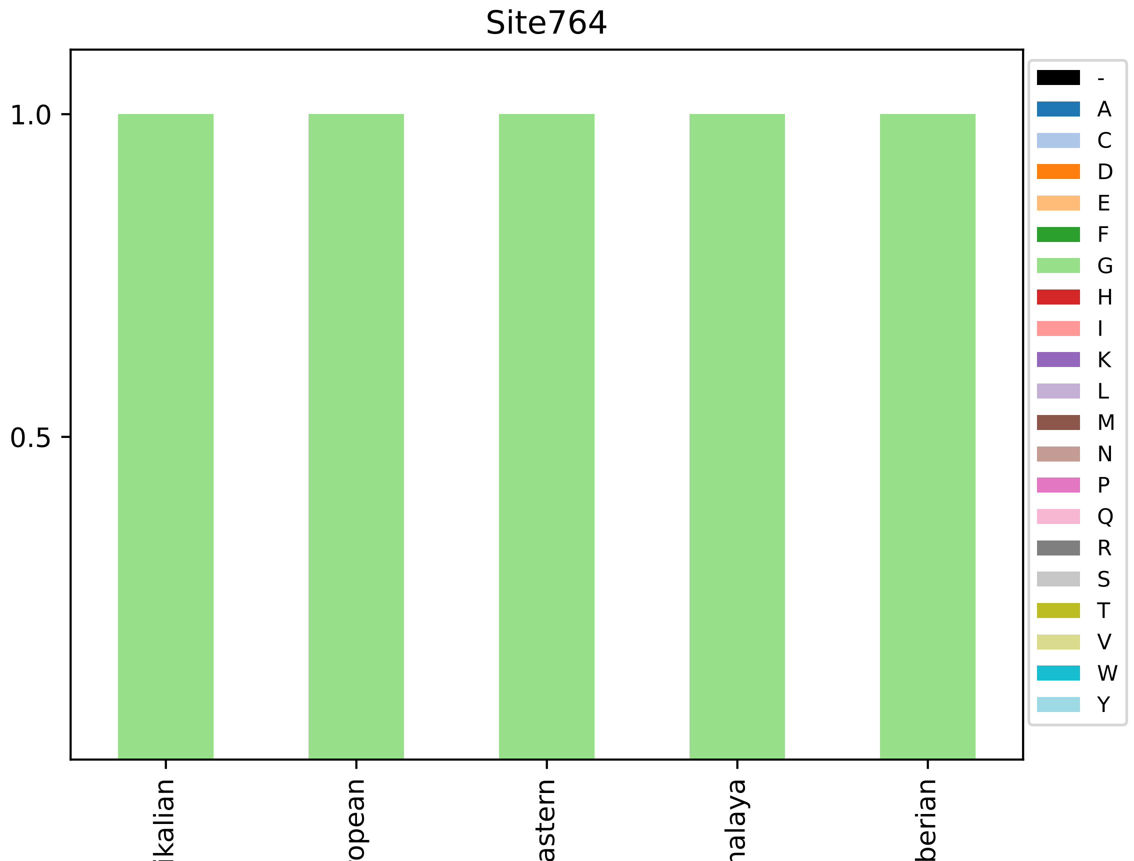
Site764
1.0
0.5
-
A
C
D
E
F
G
H
I
K
L
M
N
P
Q
R
S
T
V
W
Y
ikalian
opean
astern
nalaya
berian

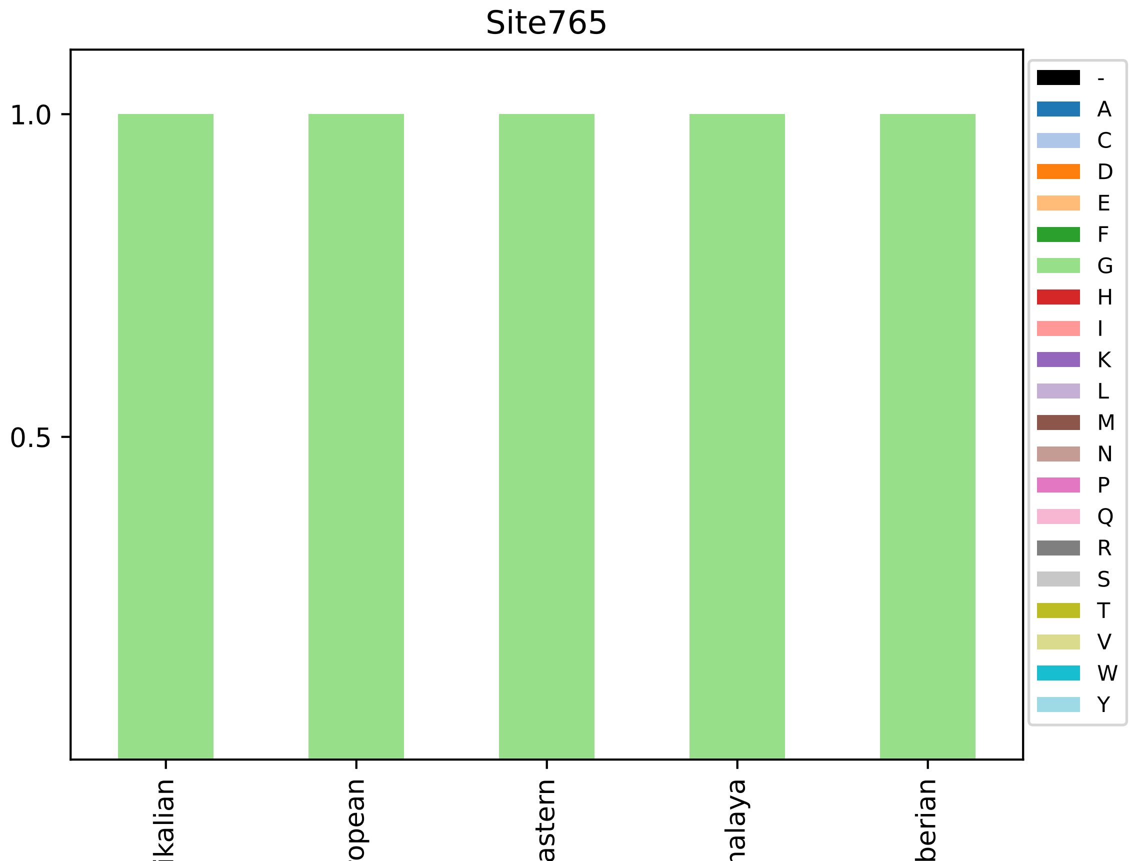
Site765
1.0
0.5
ikalian
opean
astern
nalaya
berian
-
A
C
D
E
F
G
H
I
K
L
M
N
P
Q
R
S
T
V
W
Y

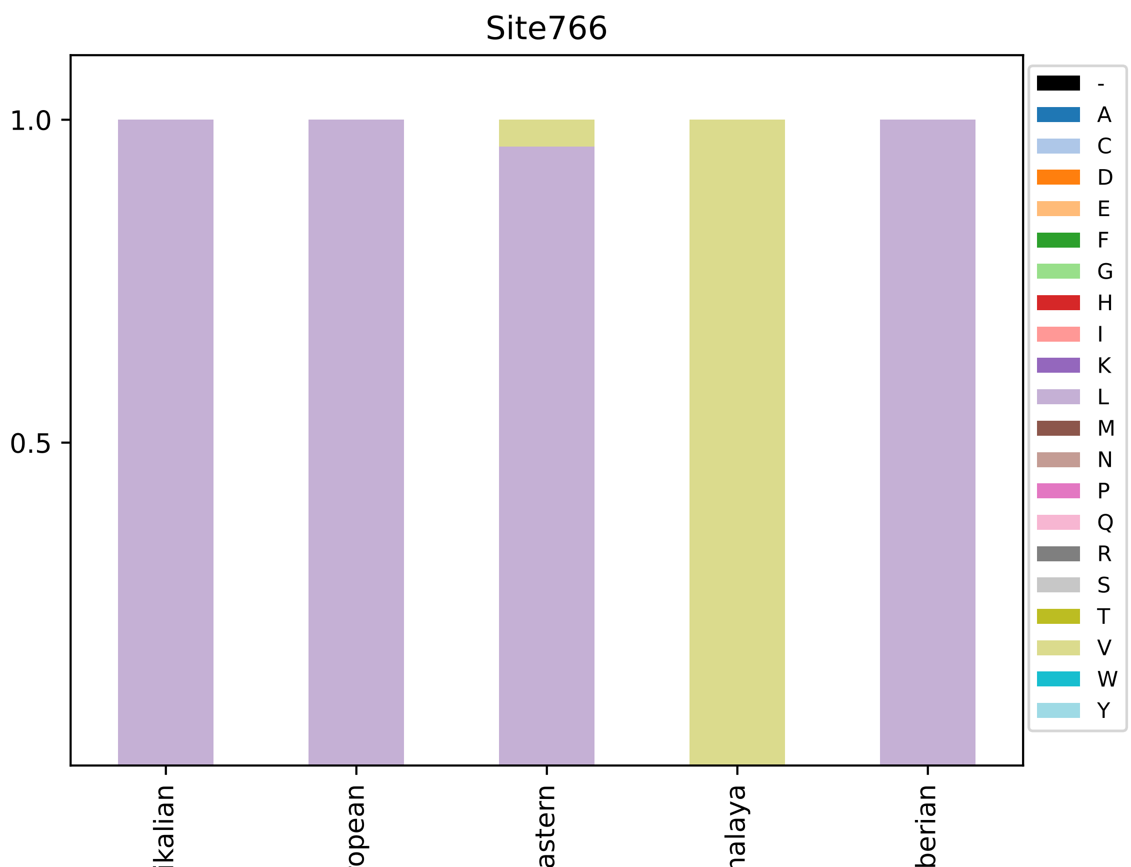
Site766
1.0
0.5
-
A
C
D
E
F
G
H
I
K
L
M
N
P
Q
R
S
T
V
W
Y
ikalian
ropean
astern
nalaya
berian

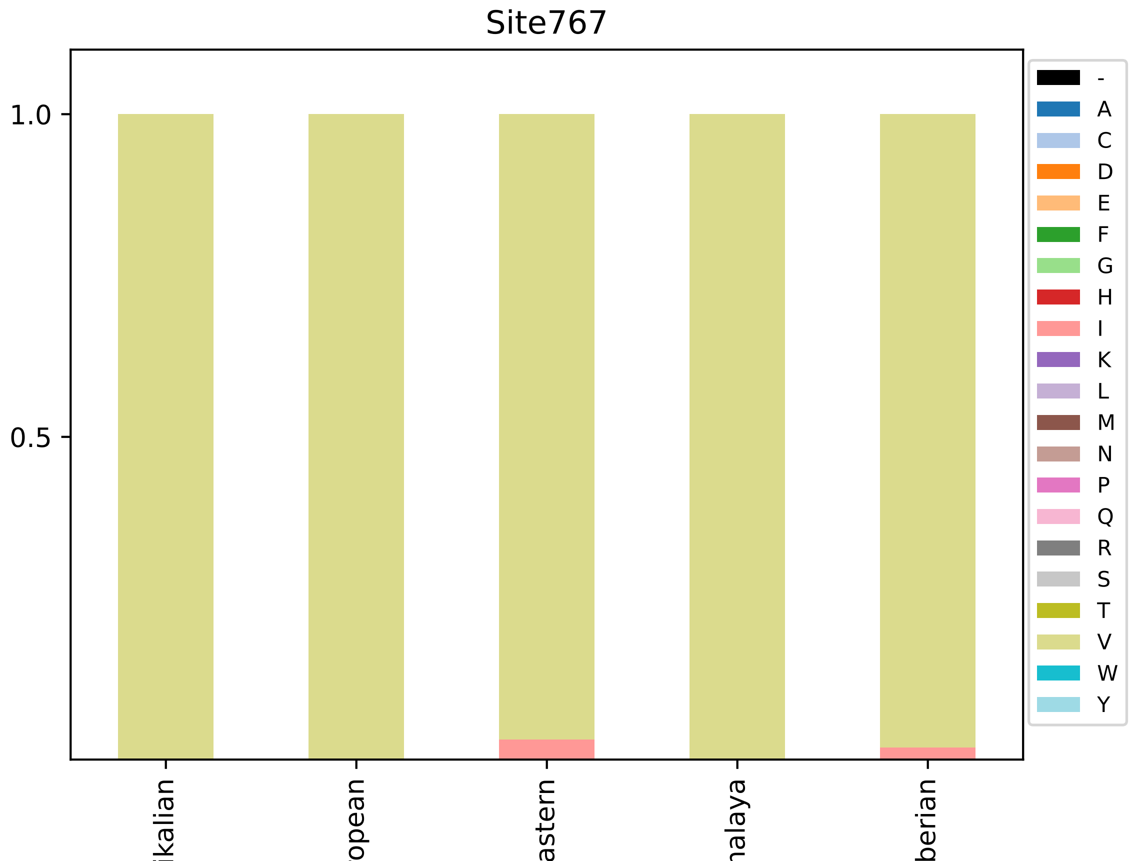
Site767
1.0
0.5
-
A
C
D
E
F
G
H
I
K
L
M
N
P
Q
R
S
T
V
W
Y
ikalian
opean
astern
nalaya
berian

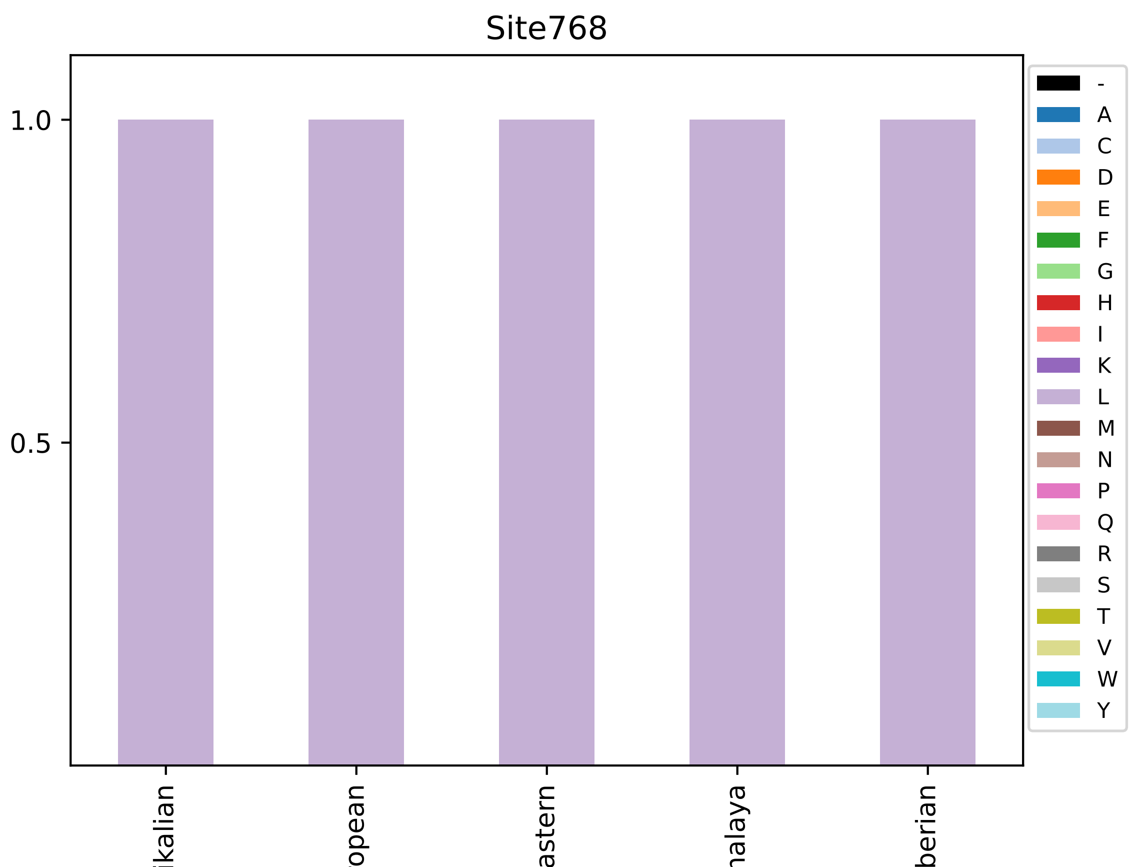
Site768
1.0
0.5
-
A
C
D
E
F
G
H
I
K
L
M
N
P
Q
R
S
T
V
W
Y
ikalian
opean
astern
nalaya
berian

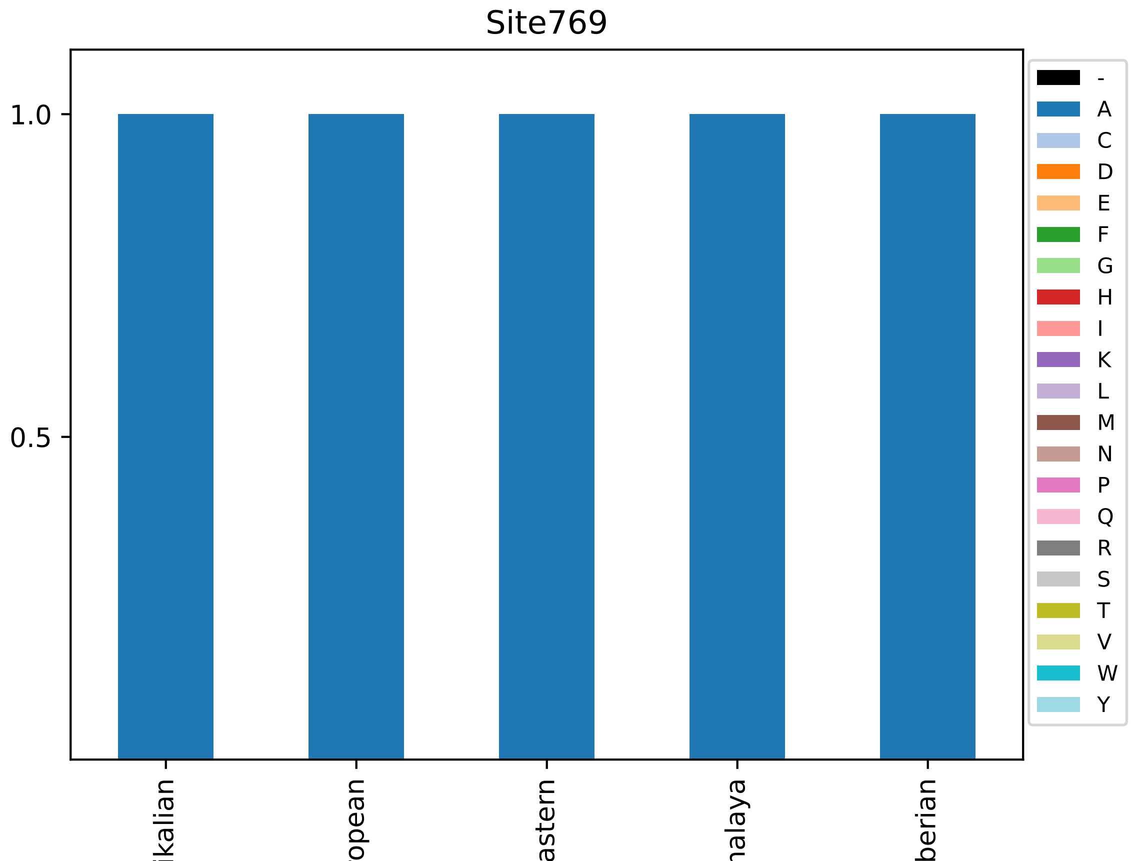

Site769
1.0
0.5
-
A
C
D
E
F
G
H
I
K
L
M
N
P
Q
R
S
T
V
W
Y
ikalian
opean
astern
nalaya
berian

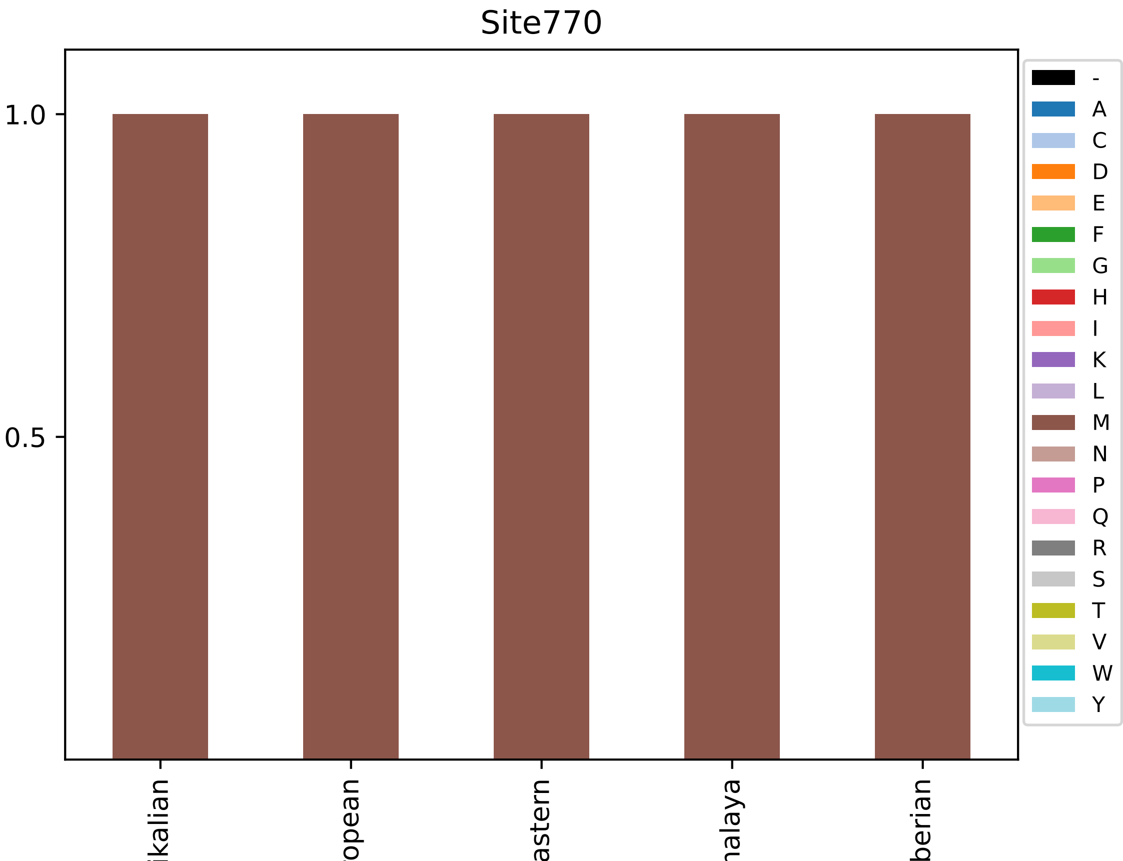

Site770
1.0
0.5
-
A
C
D
E
F
G
H
I
K
L
M
N
P
Q
R
S
T
V
W
Y
ikalian
opean
astern
nalaya
berian

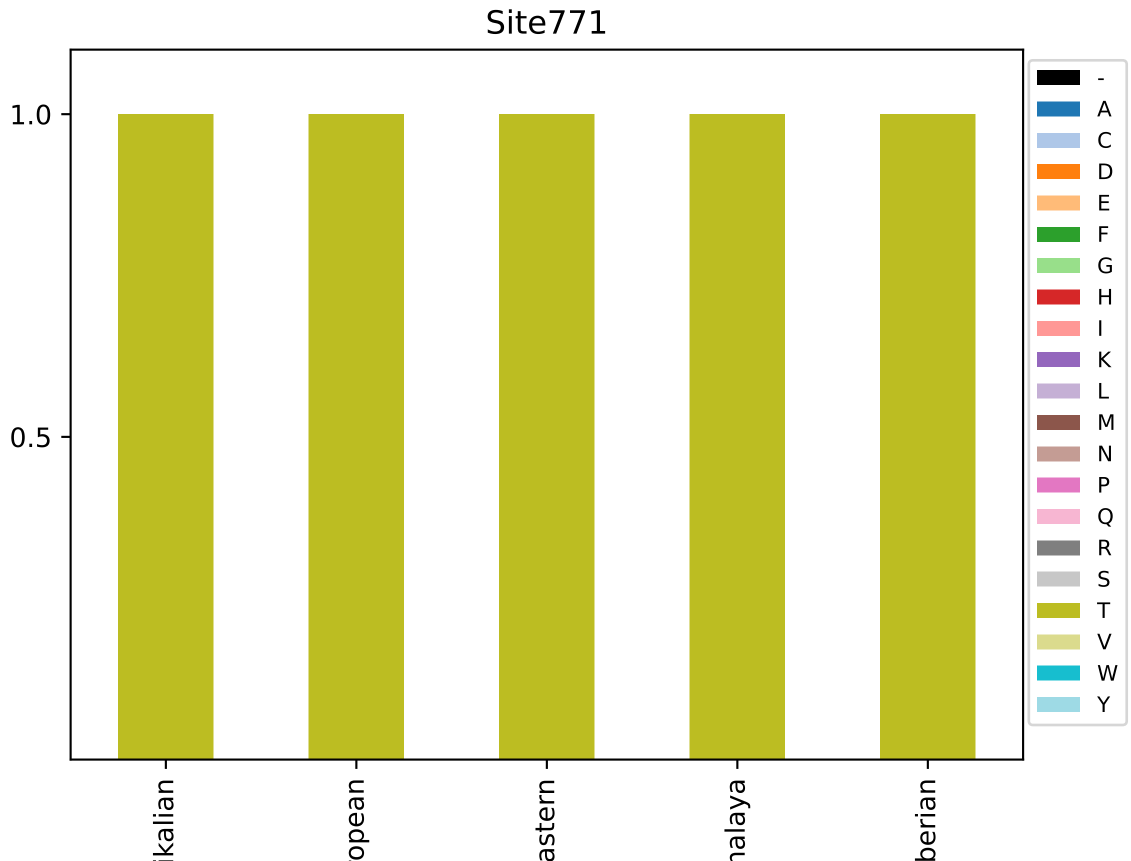

Site771
1.0
0.5
-
A
C
D
E
F
G
H
I
K
L
M
N
P
Q
R
S
T
V
W
Y
ikalian
opean
astern
nalaya
berian

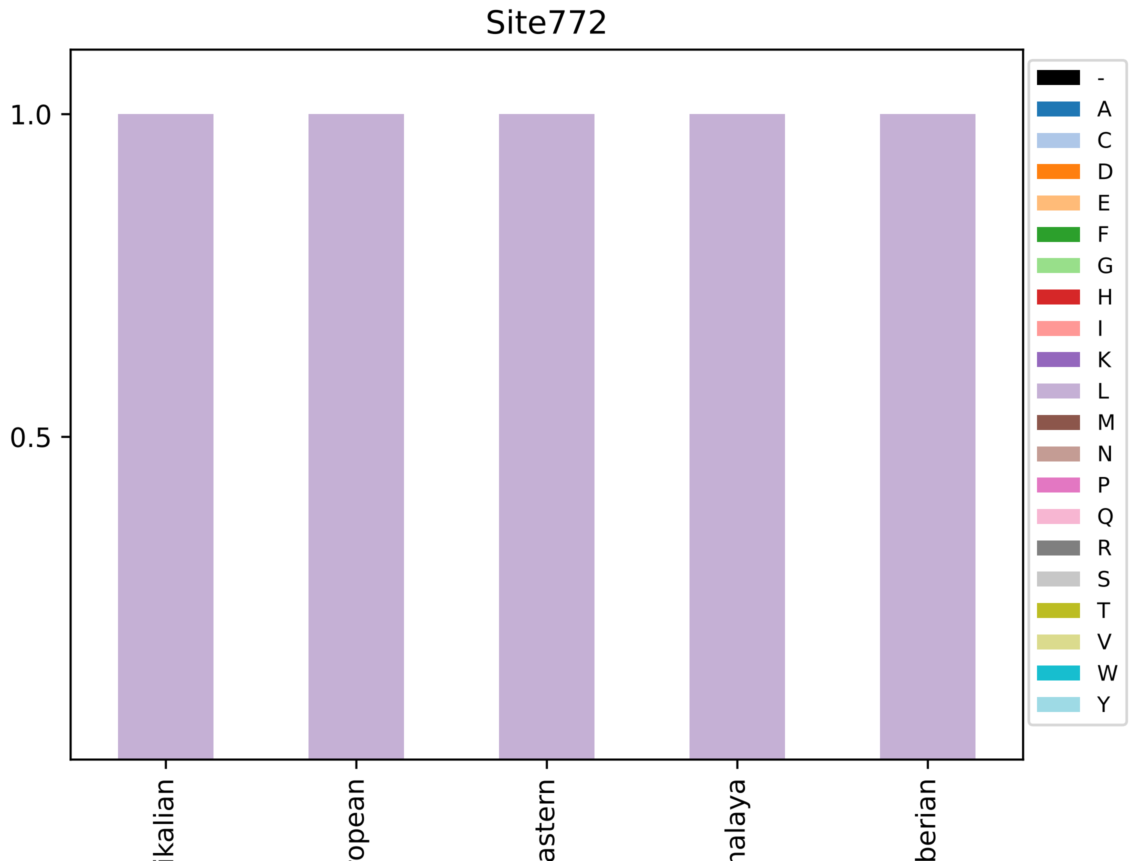

Site772
1.0
0.5
-
A
C
D
E
F
G
H
I
K
L
M
N
P
Q
R
S
T
V
W
Y
ikalian
opean
astern
nalaya
berian

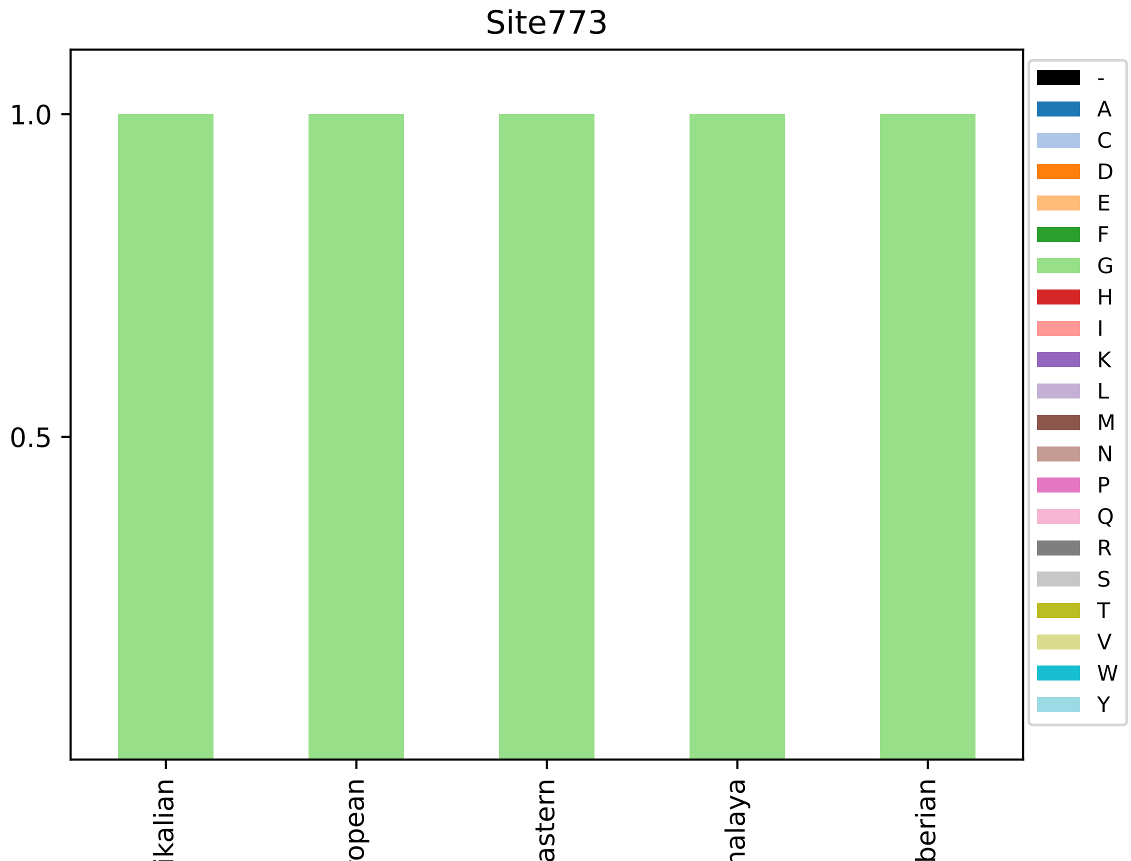
Site773
1.0
0.5
-
A
C
D
E
F
G
H
I
K
L
M
N
P
Q
R
S
T
V
W
Y
ikalian
opean
astern
nalaya
berian

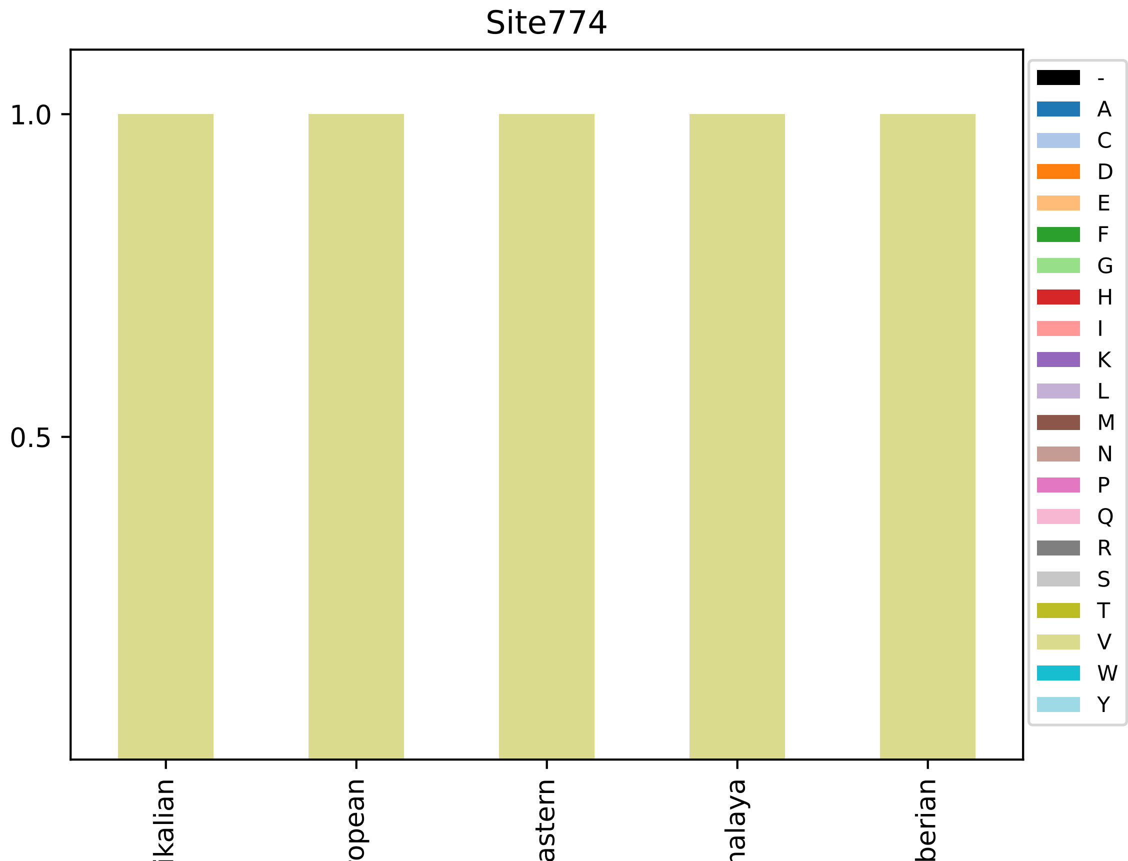

Site774
1.0
0.5
ikalian
ropean
astern
nalaya
berian
-
A
C
D
E
F
G
H
I
K
L
M
N
P
Q
R
S
T
V
W
Y

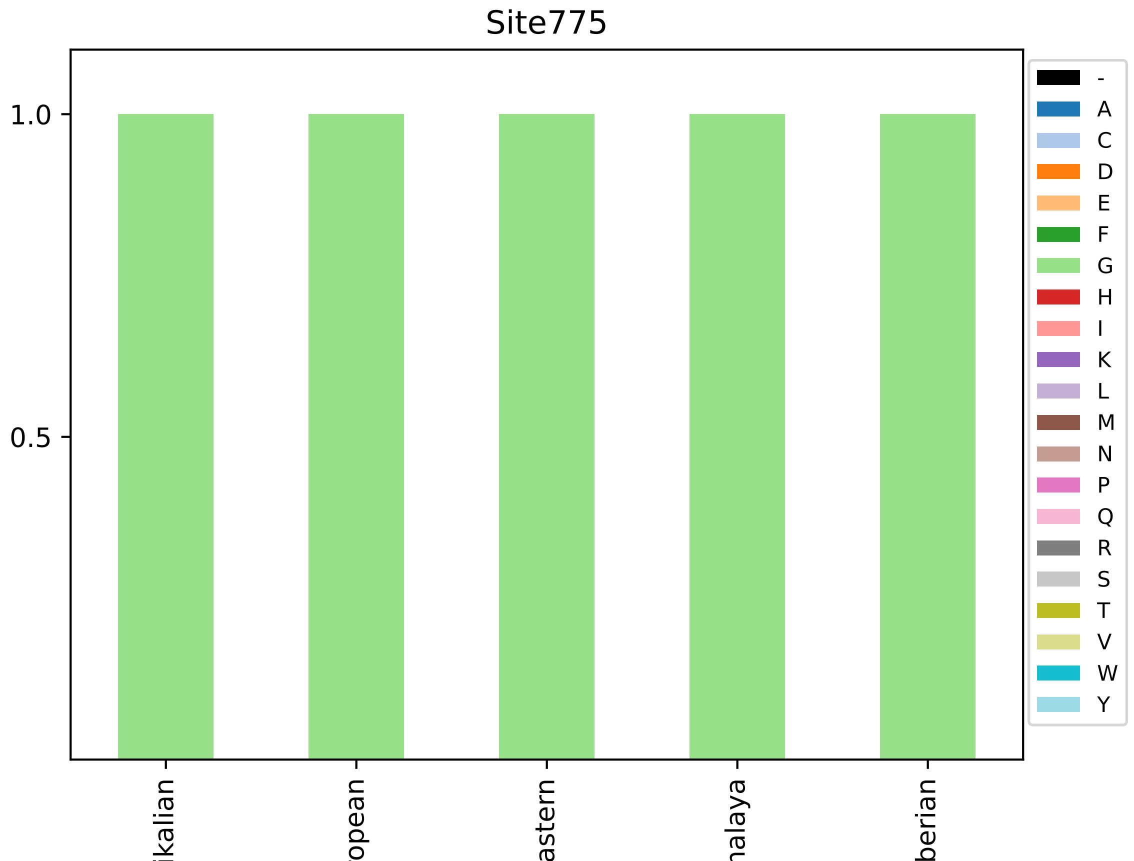

Site775
1.0
0.5
-
A
C
D
E
F
G
H
I
K
L
M
N
P
Q
R
S
T
V
W
Y
ikalian
opean
astern
nalaya
berian

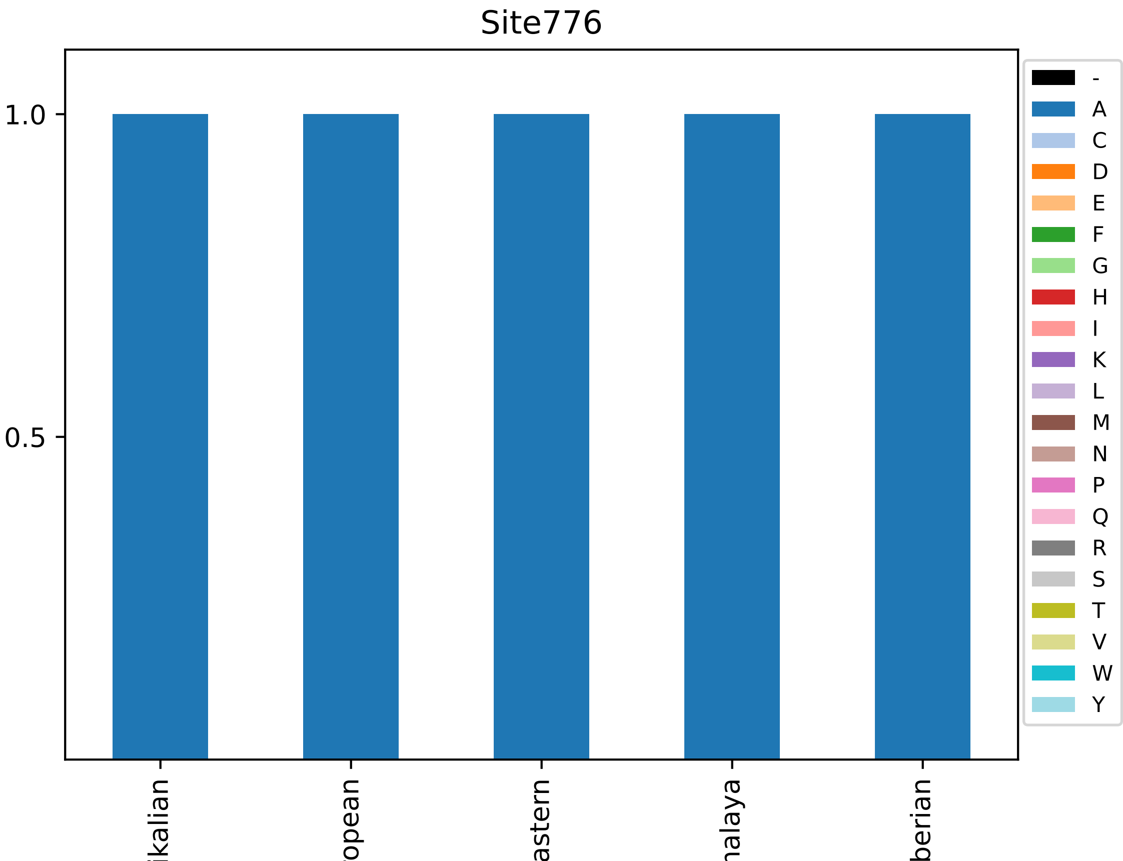

Site776
1.0
0.5
ikalian
opean
astern
halaya
berian
-
A
C
D
E
F
G
H
I
K
L
M
N
P
Q
R
S
T
V
W
Y

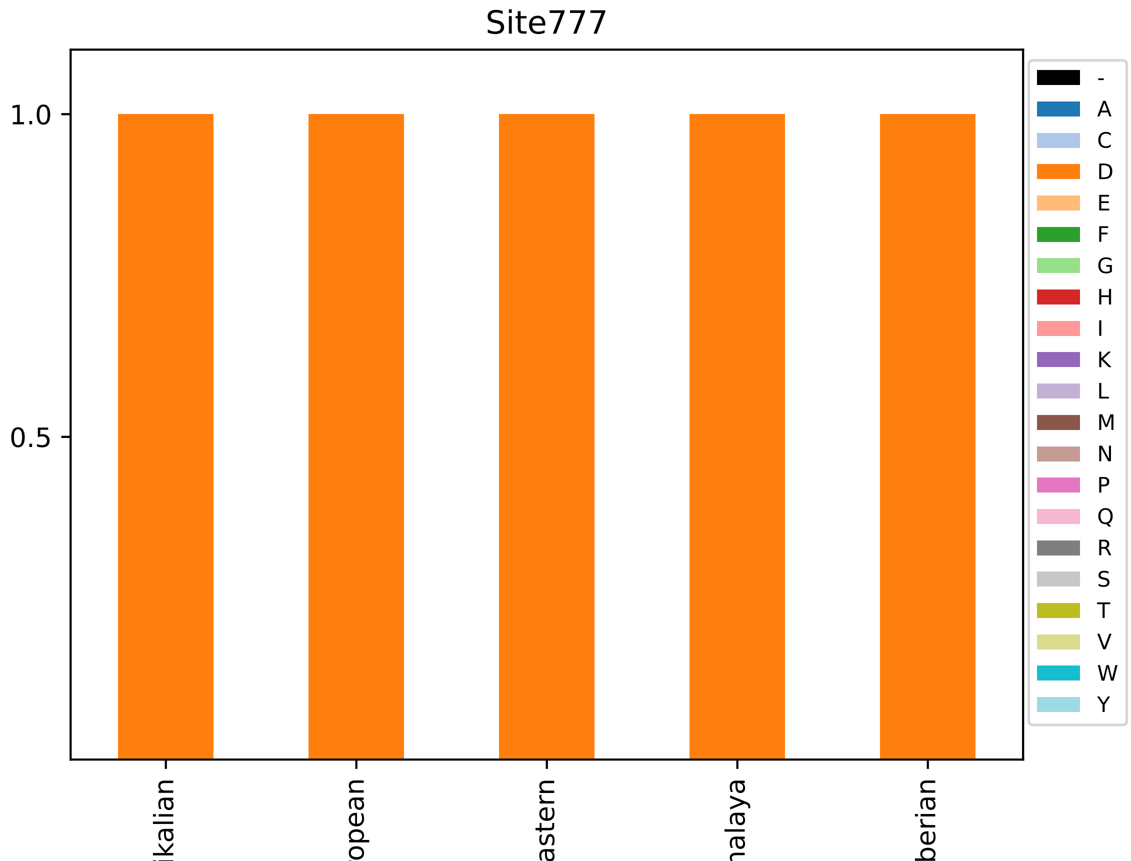
Site777
1.0
0.5
-
A
C
D
E
F
G
H
I
K
L
M
N
P
Q
R
S
T
V
W
Y
ikalian
opean
astern
nalaya
berian

Site778

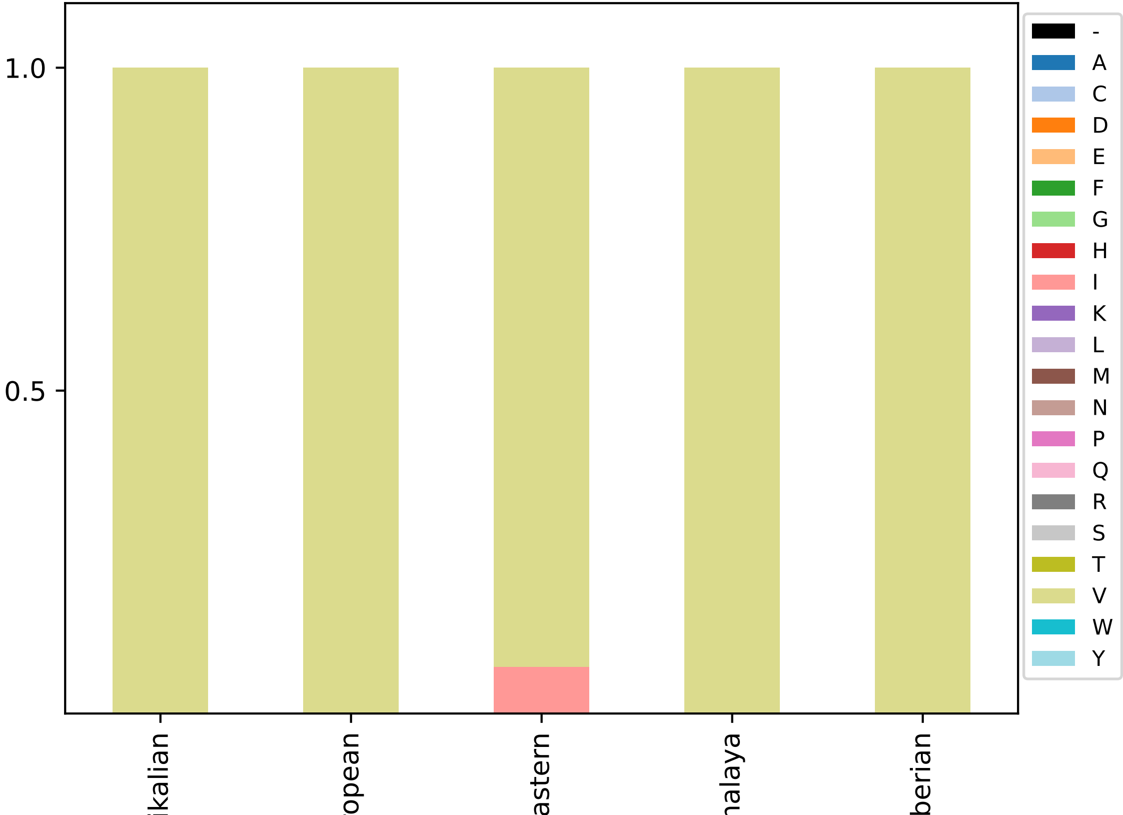

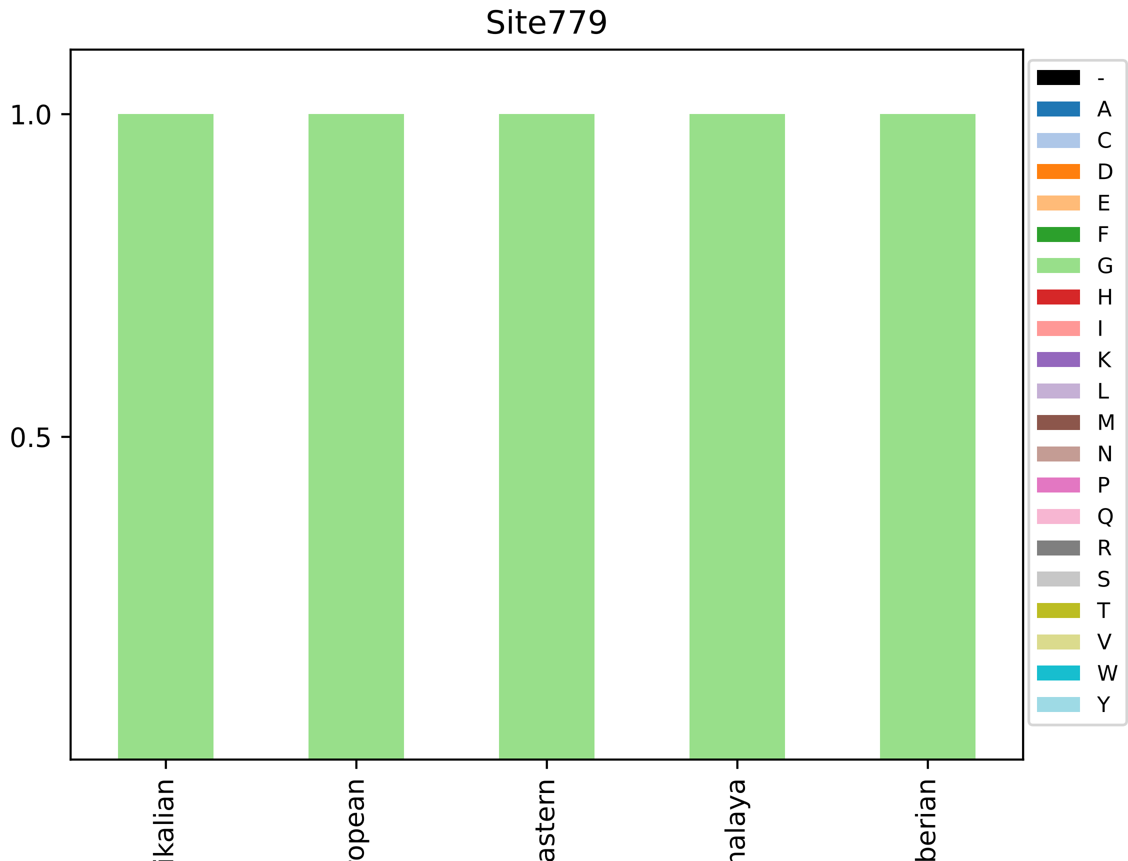

Site779
1.0
0.5
-
A
C
D
E
F
G
H
I
K
L
M
N
P
Q
R
S
T
V
W
Y
ikalian
opean
astern
nalaya
berian

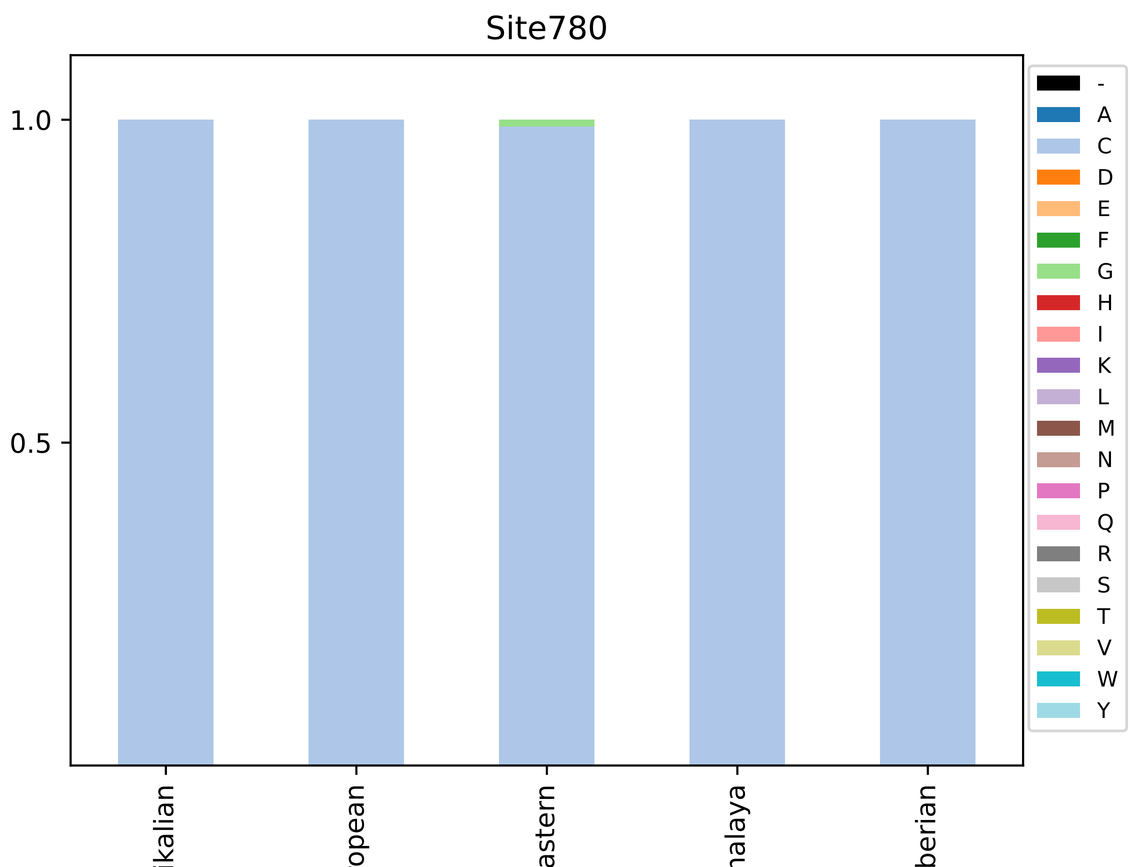
Site780
1.0
0.5
-
A
C
D
E
F
G
H
I
K
L
M
N
P
Q
R
S
T
V
W
Y
ikalian
opean
astern
nalaya
berian

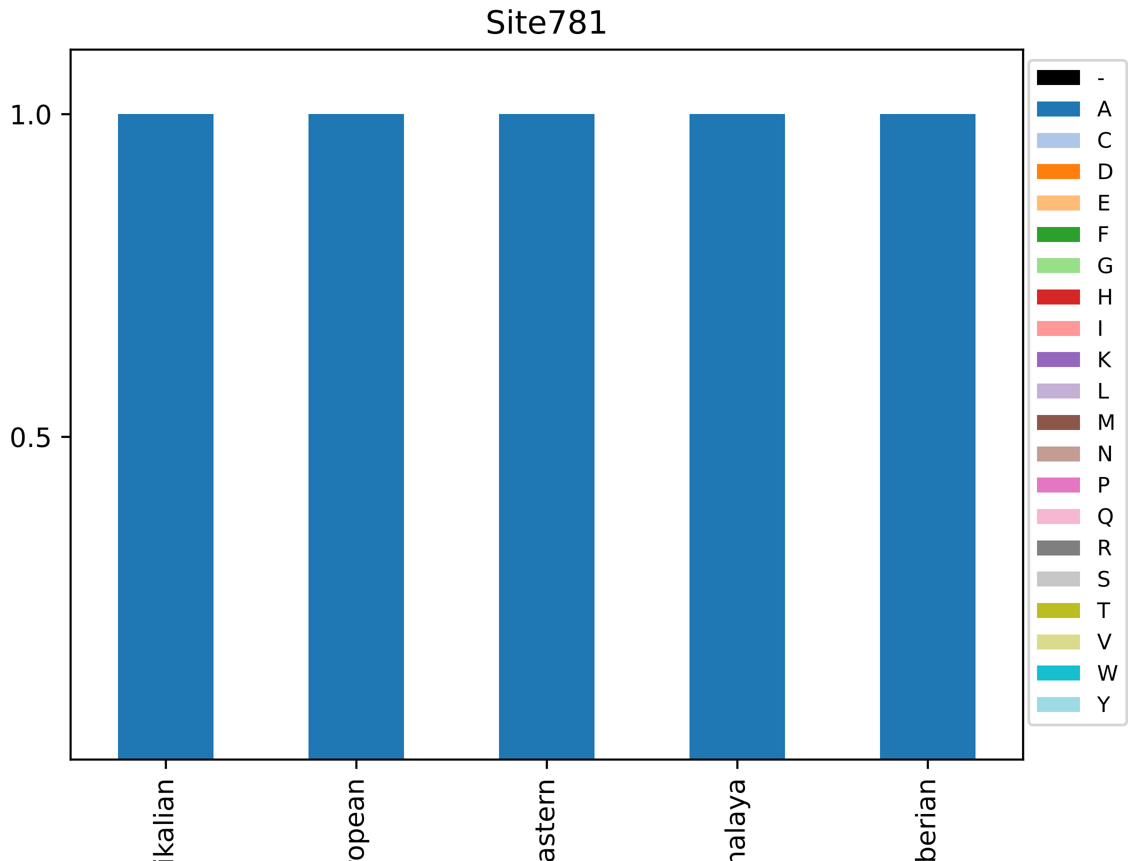

Site781
1.0
0.5
-
A
C
D
E
F
G
H
I
K
L
M
N
P
Q
R
S
T
V
W
Y
ikalian
opean
astern
nalaya
berian

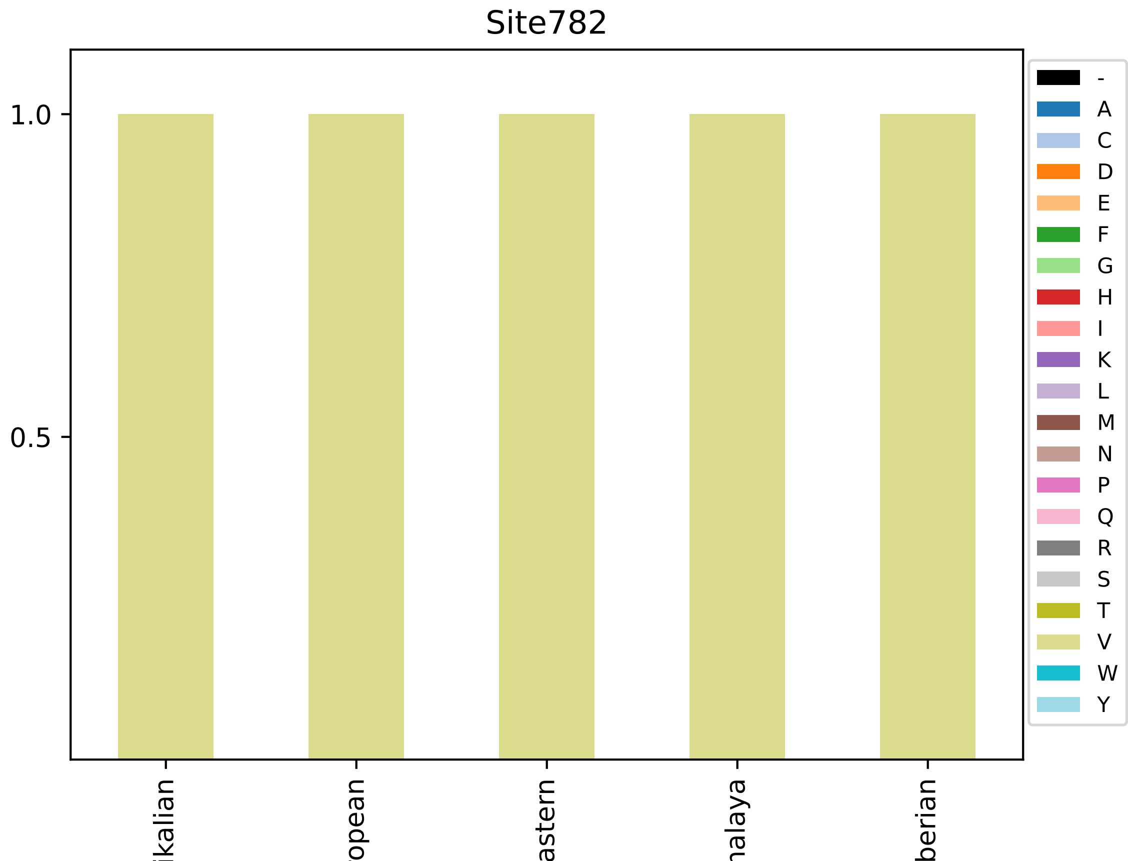
Site782
1.0
0.5
-
A
C
D
E
F
G
H
I
K
L
M
N
P
Q
R
S
T
V
W
Y
ikalian
opean
astern
nalaya
berian

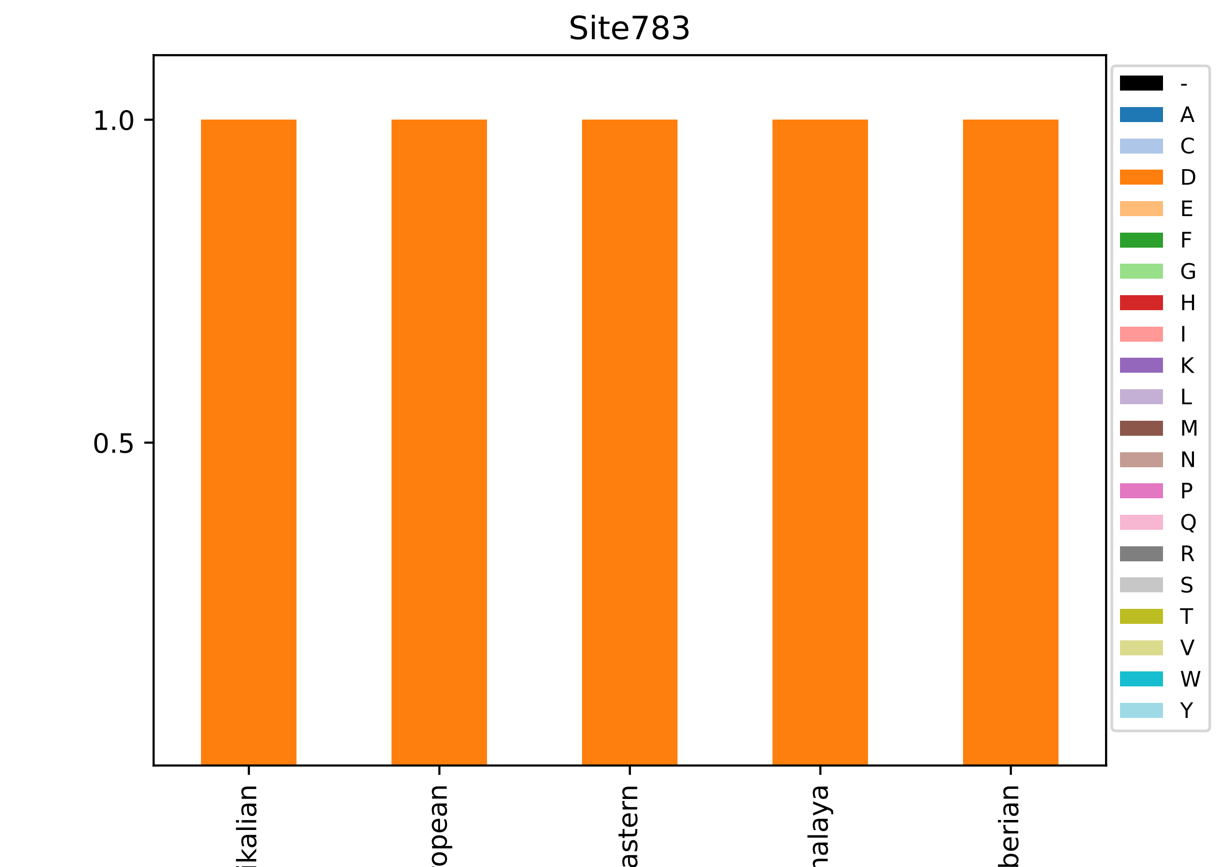

Site783
1.0
0.5
ikalian
opean
astern
nalaya
berian
-
A
C
D
E
F
G
H
I
K
L
M
N
P
Q
R
S
T
V
W
Y

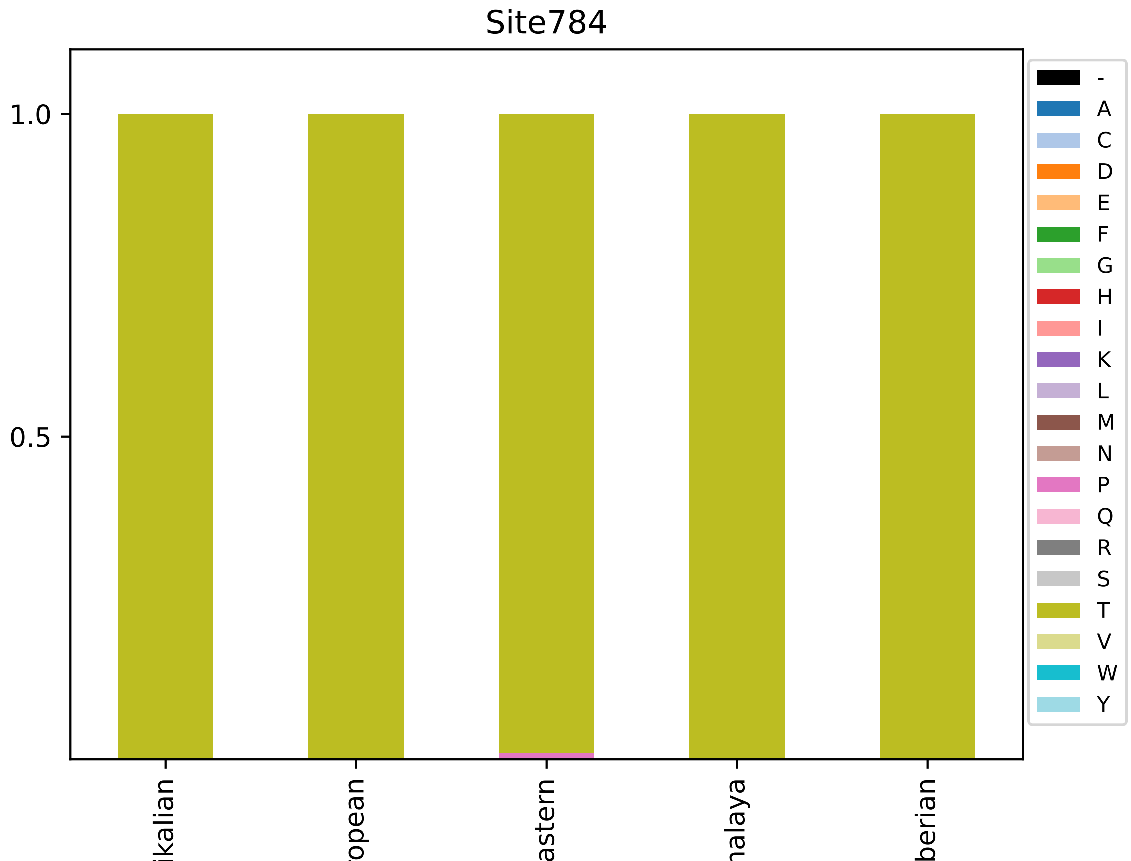
Site784
1.0
0.5
ikalian
opean
astern
nalaya
berian
-
A
C
D
E
F
G
H
I
K
L
M
N
P
Q
R
S
T
V
W
Y

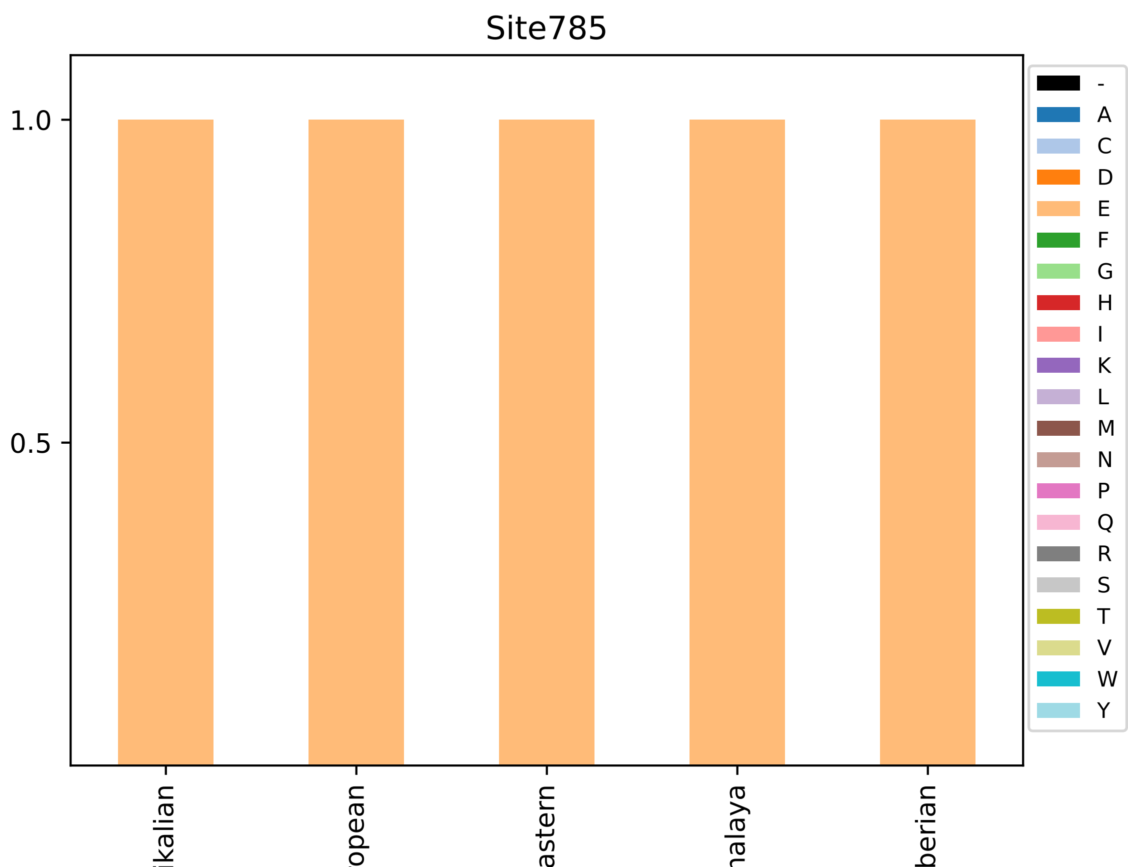

Site785
1.0
0.5
ikalian
opean
astern
nalaya
berian
-
A
C
D
E
F
G
H
I
K
L
M
N
P
Q
R
S
T
V
W
Y

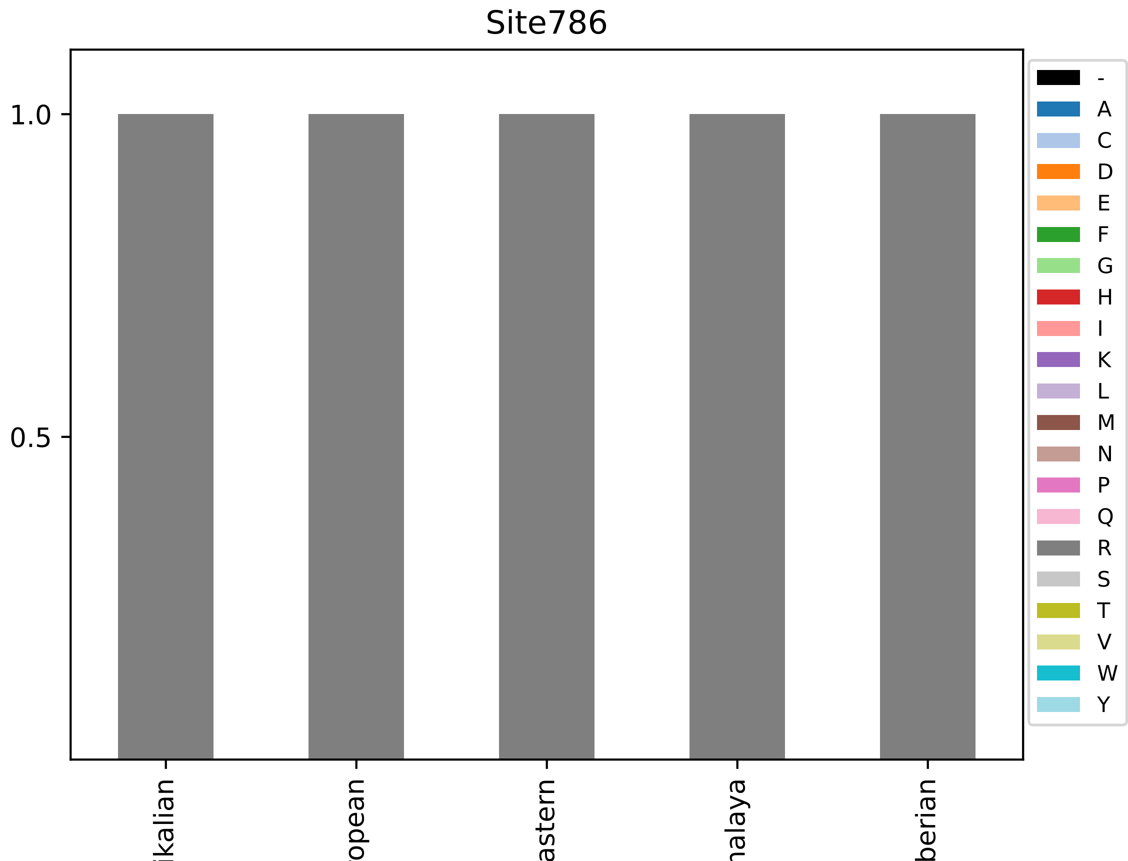

Site786
1.0
0.5
-
A
C
D
E
F
G
H
I
K
L
M
N
P
Q
R
S
T
V
W
Y
ikalian
opean
astern
nalaya
berian

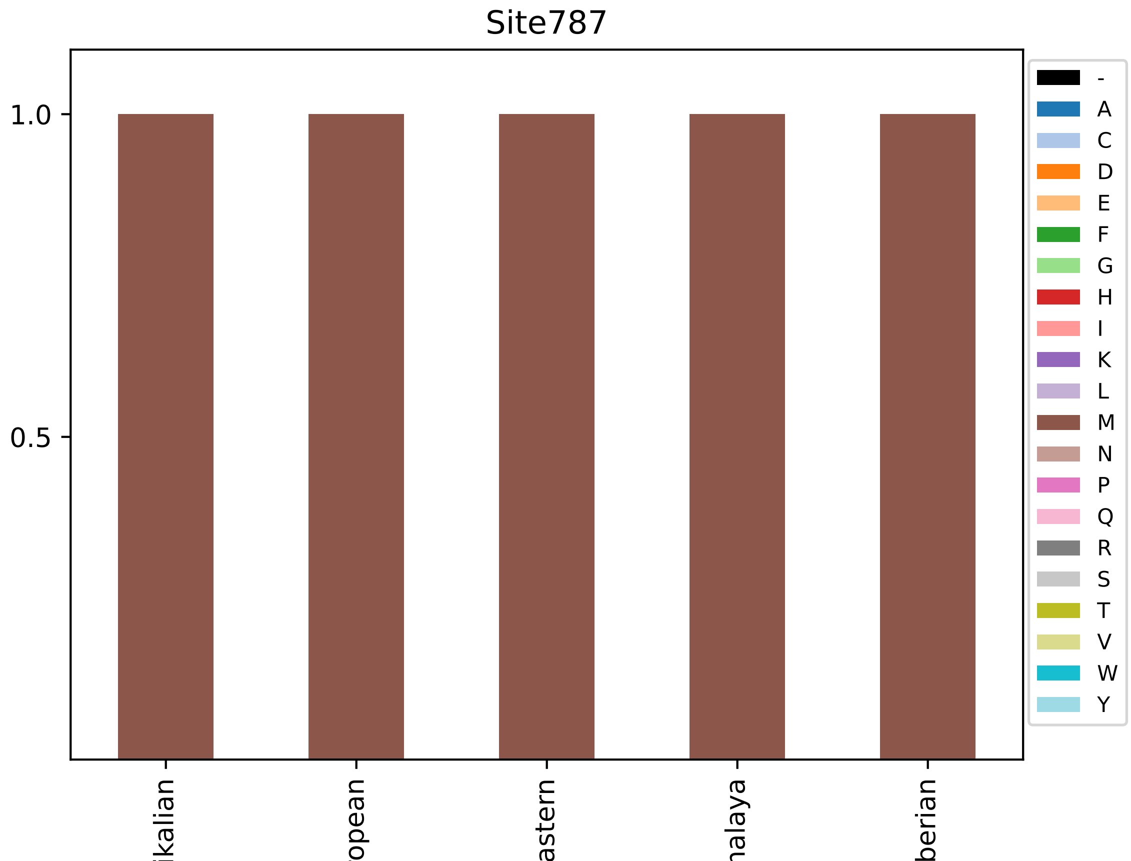
Site787
1.0
0.5
-
A
C
D
E
F
G
H
I
K
L
M
N
P
Q
R
S
T
V
W
Y
ikalian
opean
astern
nalaya
berian

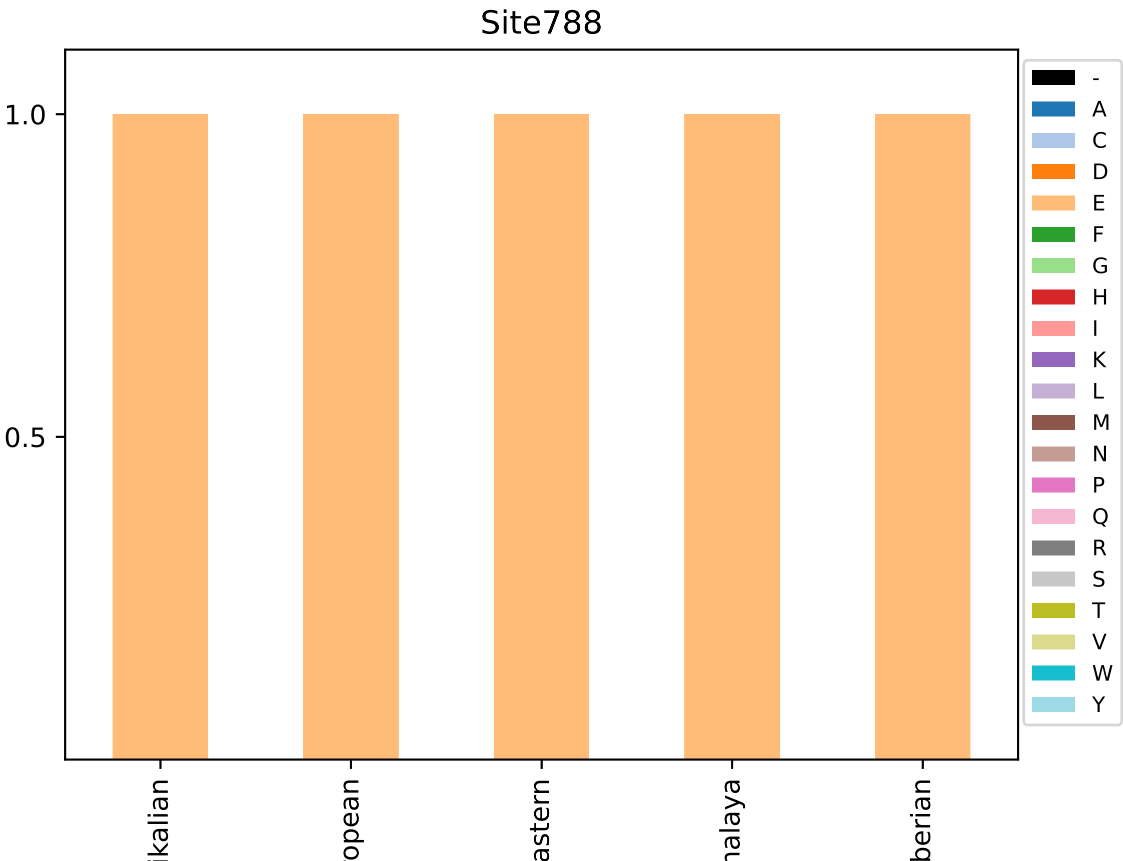
Site788
1.0
0.5
-
A
C
D
E
F
G
H
I
K
L
M
N
P
Q
R
S
T
V
W
Y
ikalian
opean
astern
nalaya
berian

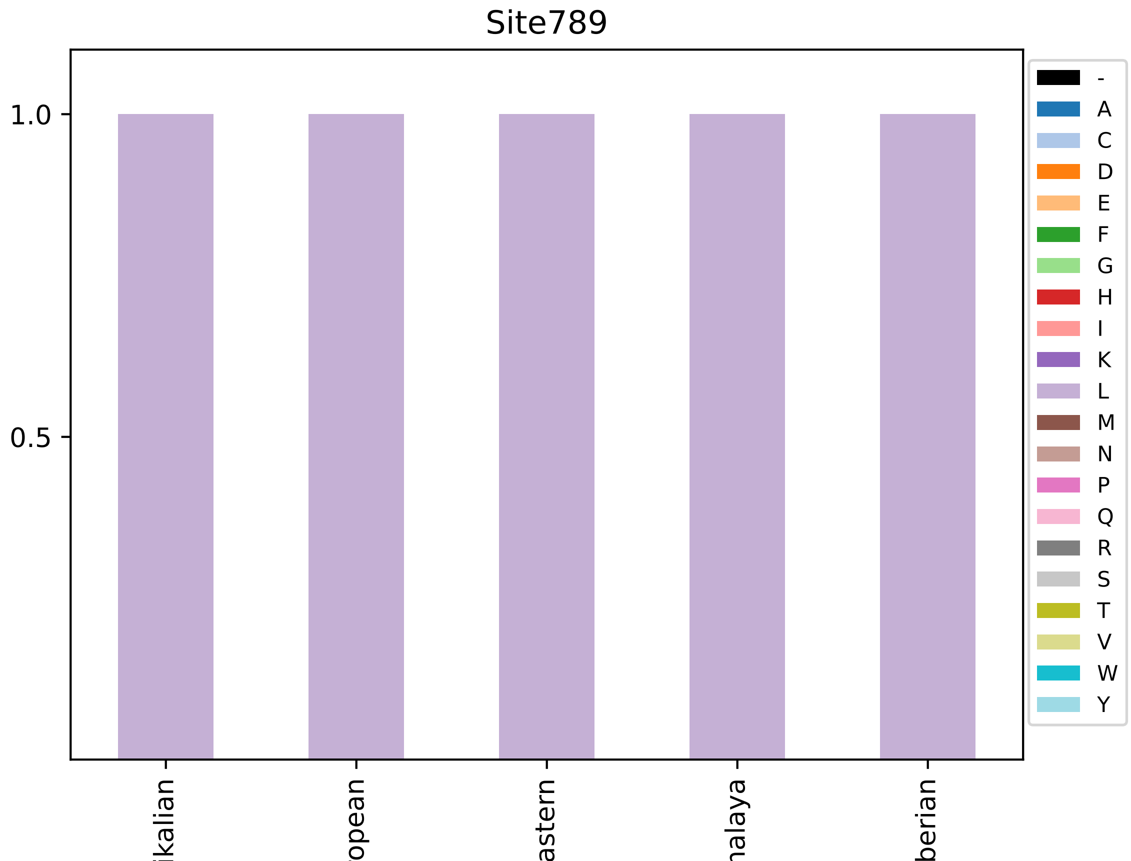
Site789
1.0
0.5
ikalian
opean
astern
nalaya
berian
-
A
C
D
E
F
G
H
I
K
L
M
N
P
Q
R
S
T
V
W
Y

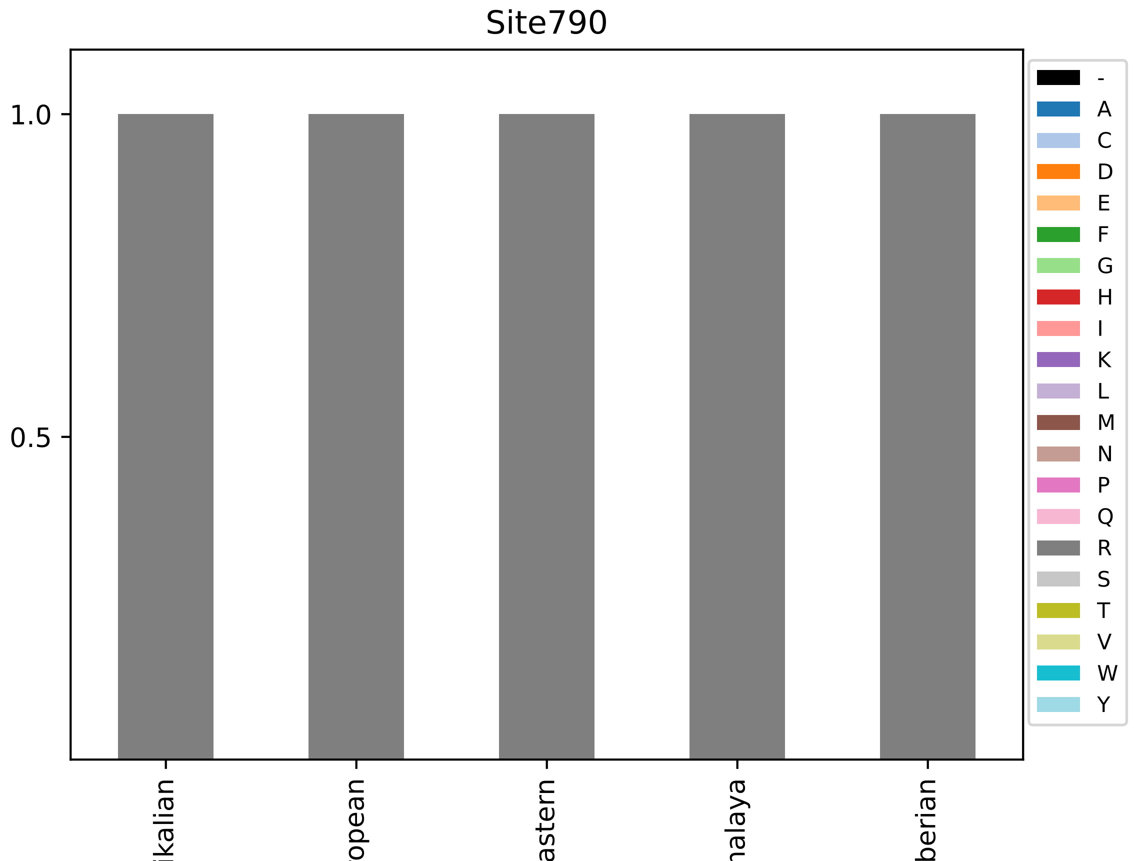
Site790
1.0
0.5
-
A
C
D
E
F
G
H
I
K
L
M
N
P
Q
R
S
T
V
W
Y
ikalian
opean
astern
nalaya
berian

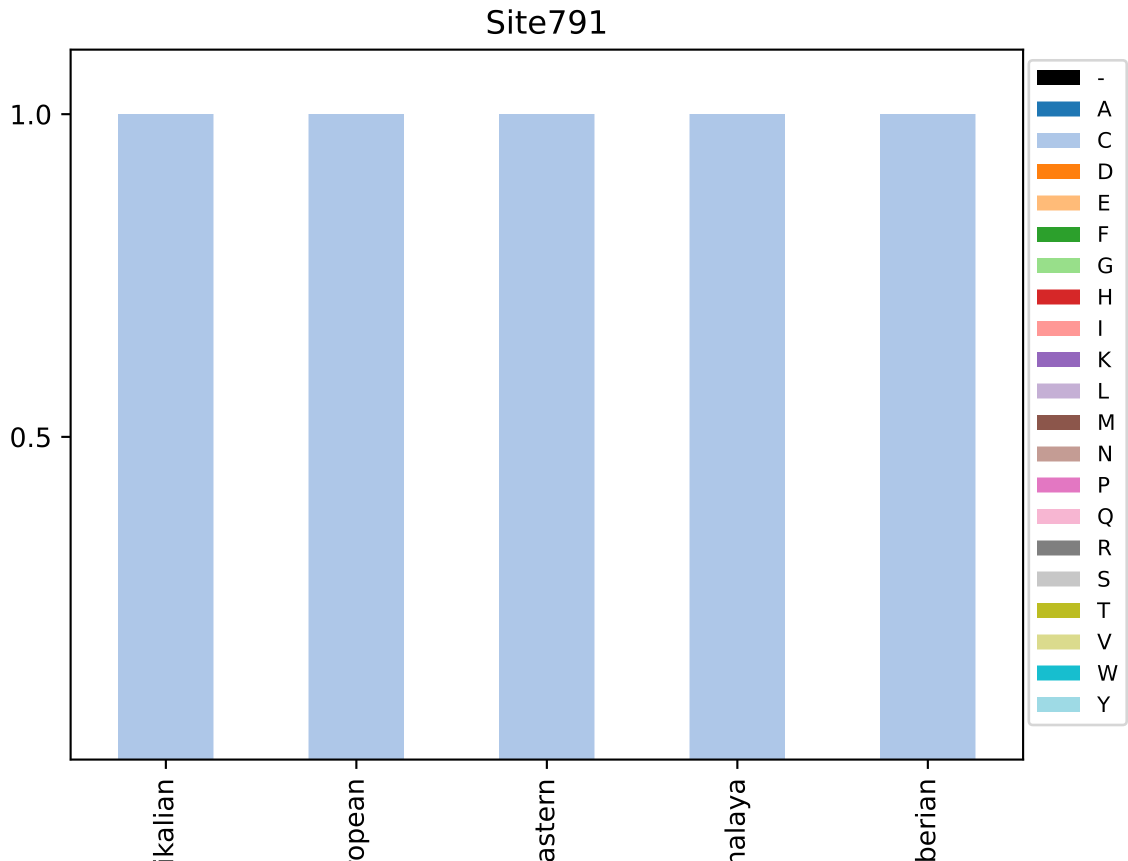

Site791
1.0
0.5
ikalian
opean
astern
nalaya
berian
-
A
C
D
E
F
G
H
I
K
L
M
N
P
Q
R
S
T
V
W
Y

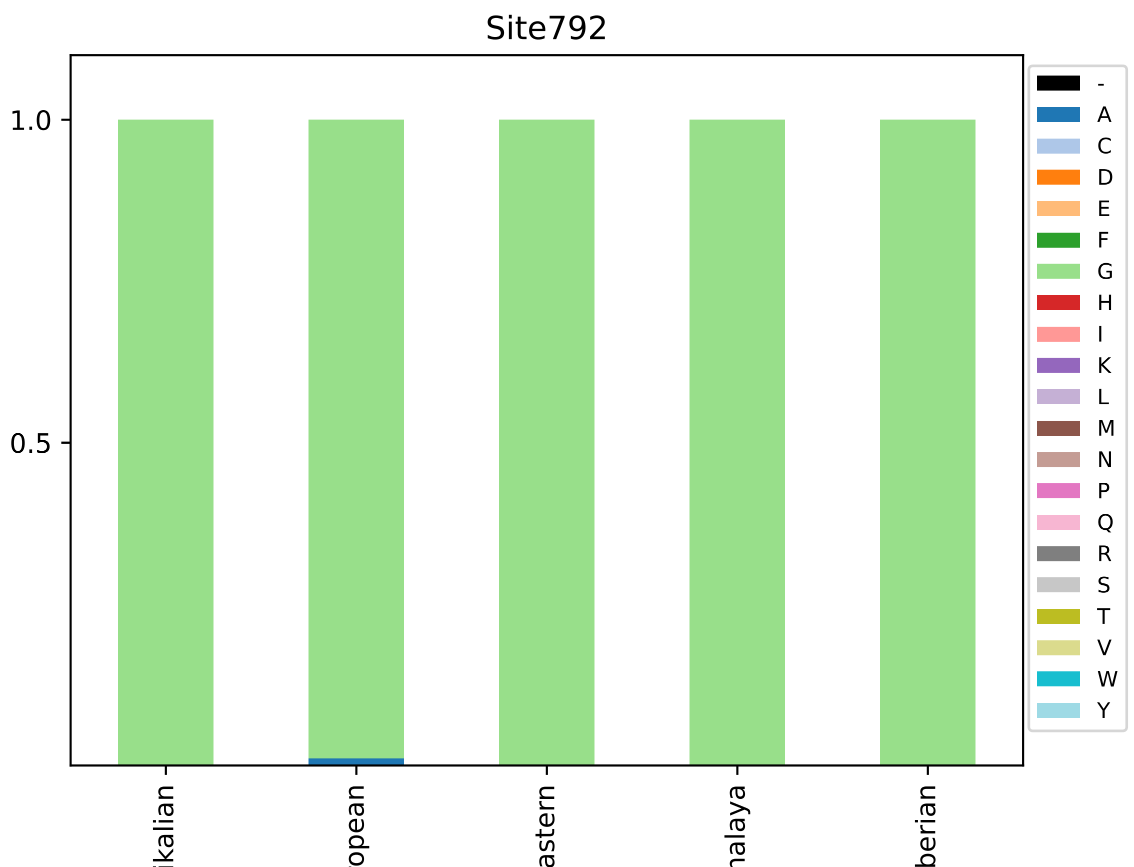
Site792
1.0
0.5
-
A
C
D
E
F
G
H
I
K
L
M
N
P
Q
R
S
T
V
W
Y
ikalian
opean
astern
nalaya
berian

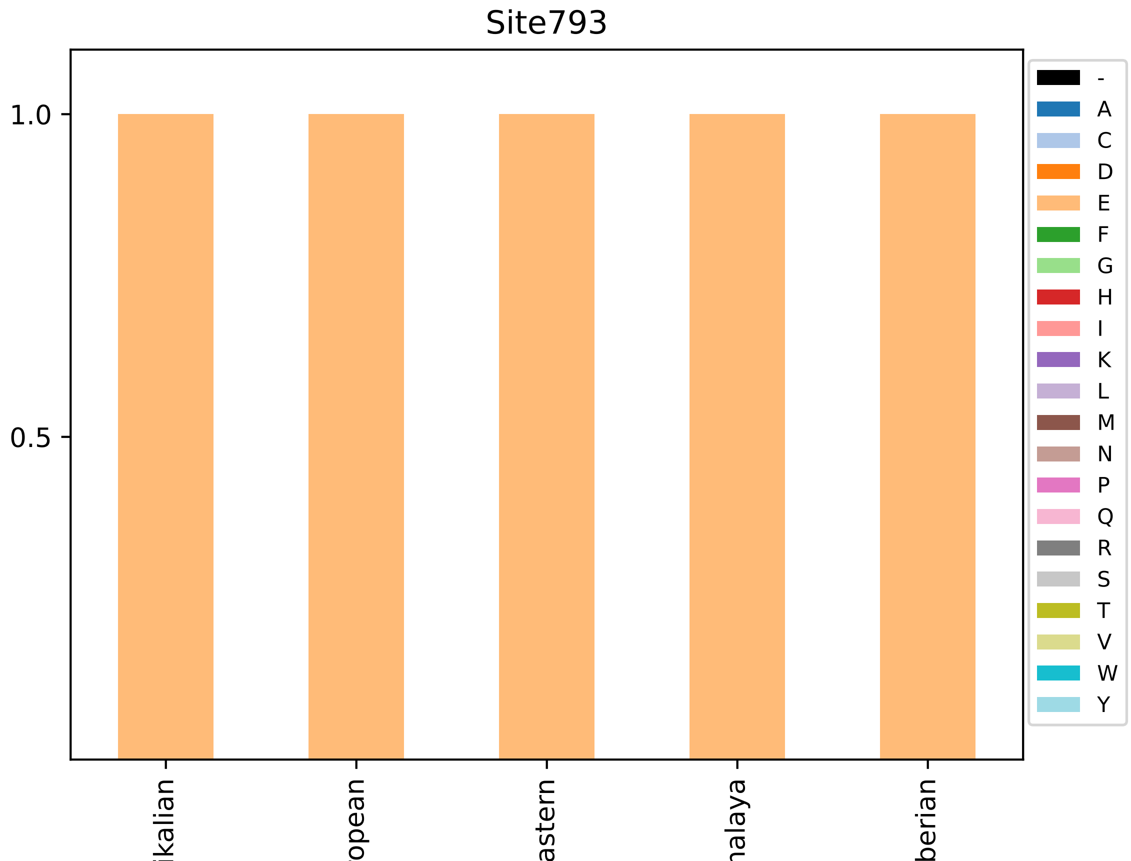

Site793
1.0
0.5
ikalian
opean
astern
nalaya
berian
-
A
C
D
E
F
G
H
I
K
L
M
N
P
Q
R
S
T
V
W
Y

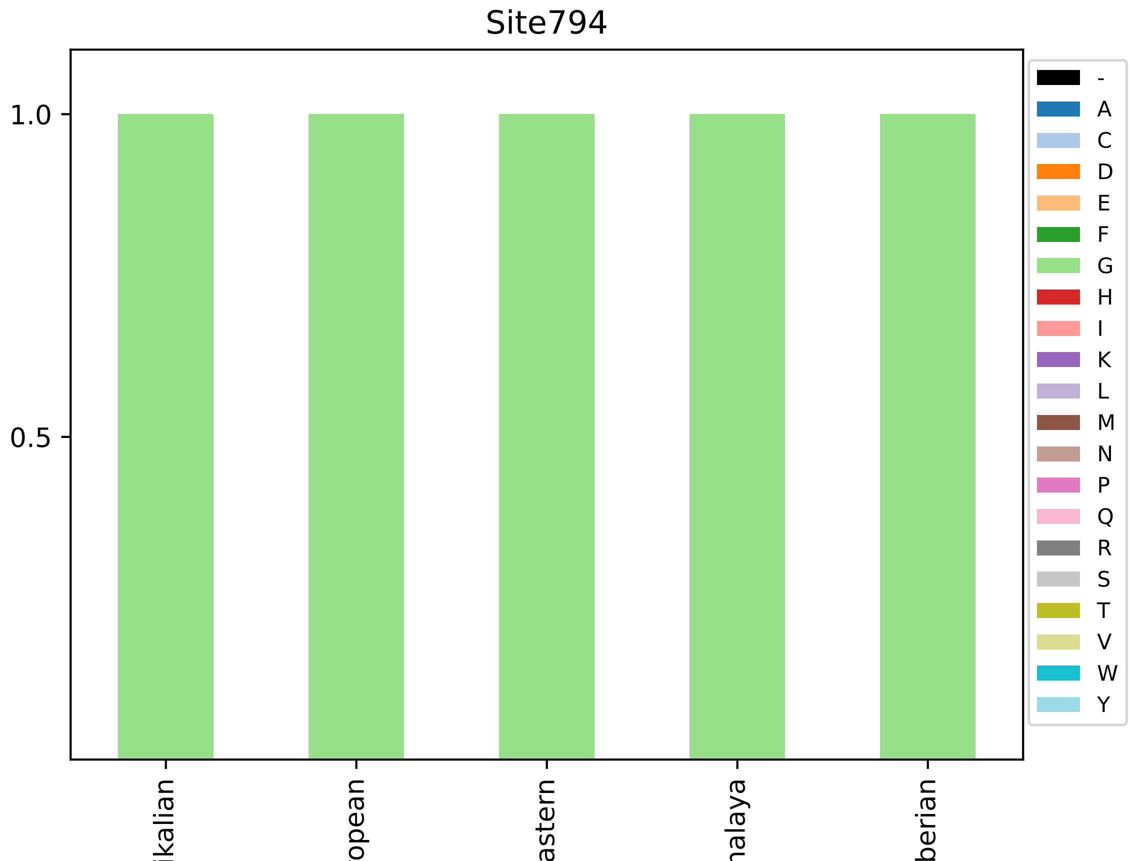
Site794
1.0
0.5
ikalian
opean
astern
nalaya
berian
-
A
C
D
E
F
G
H
I
K
L
M
N
P
Q
R
S
T
V
W
Y

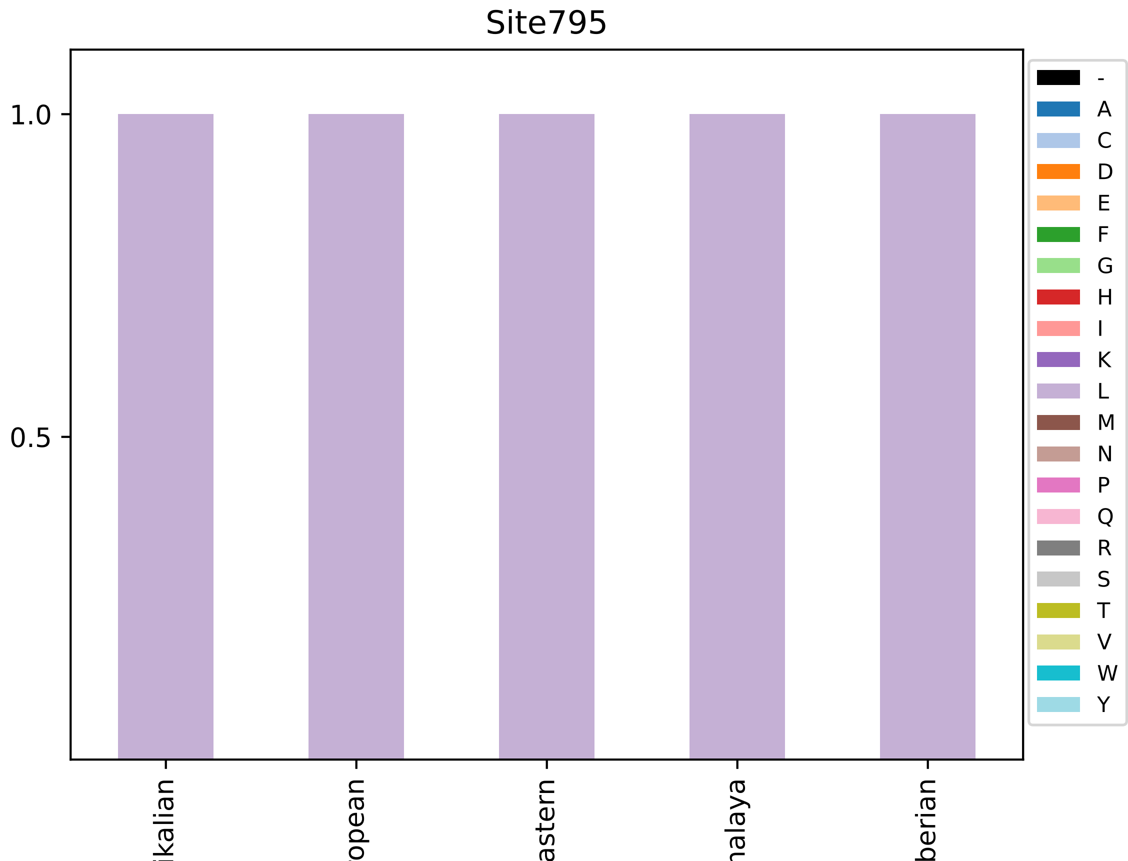
Site795
1.0
0.5
-
A
C
D
E
F
G
H
I
K
L
M
N
P
Q
R
S
T
V
W
Y
ikalian
opean
astern
nalaya
berian

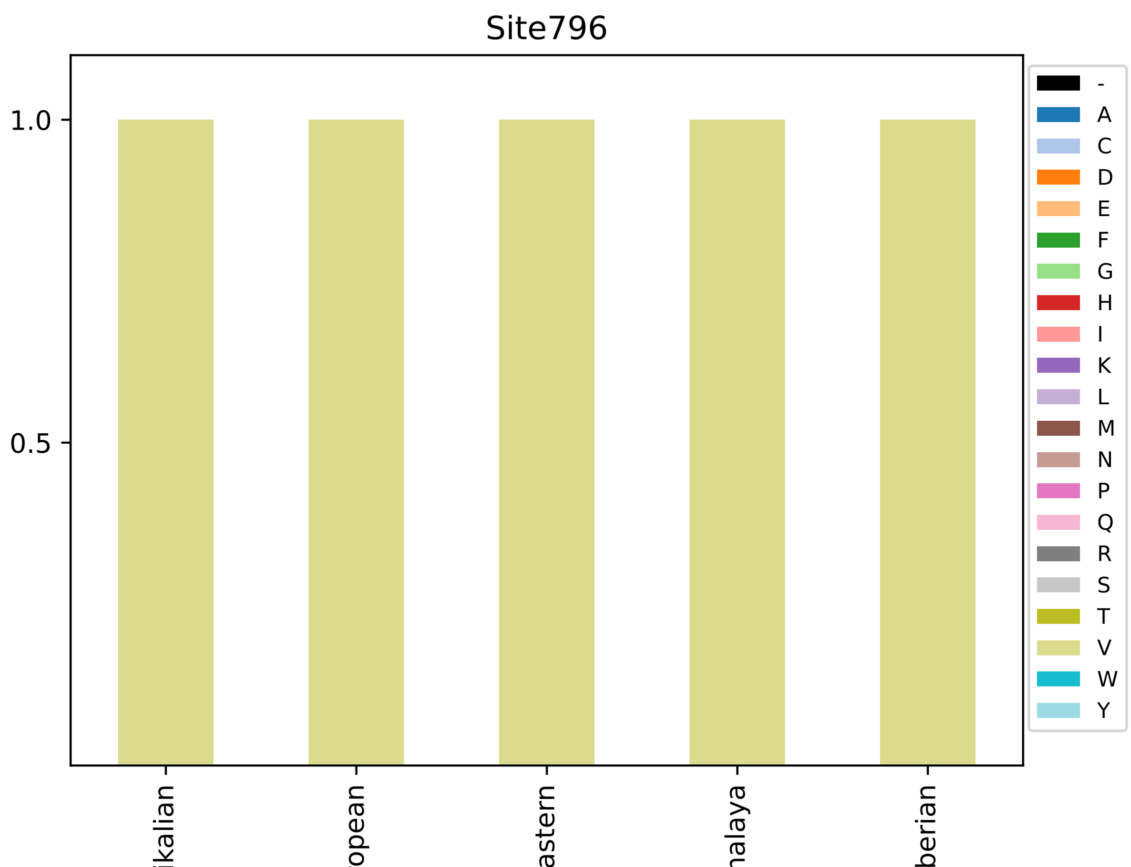

Site796
1.0
0.5
ikalian
ropean
astern
nalaya
berian
-
A
C
D
E
F
G
H
I
K
L
M
N
P
Q
R
S
T
V
W
Y

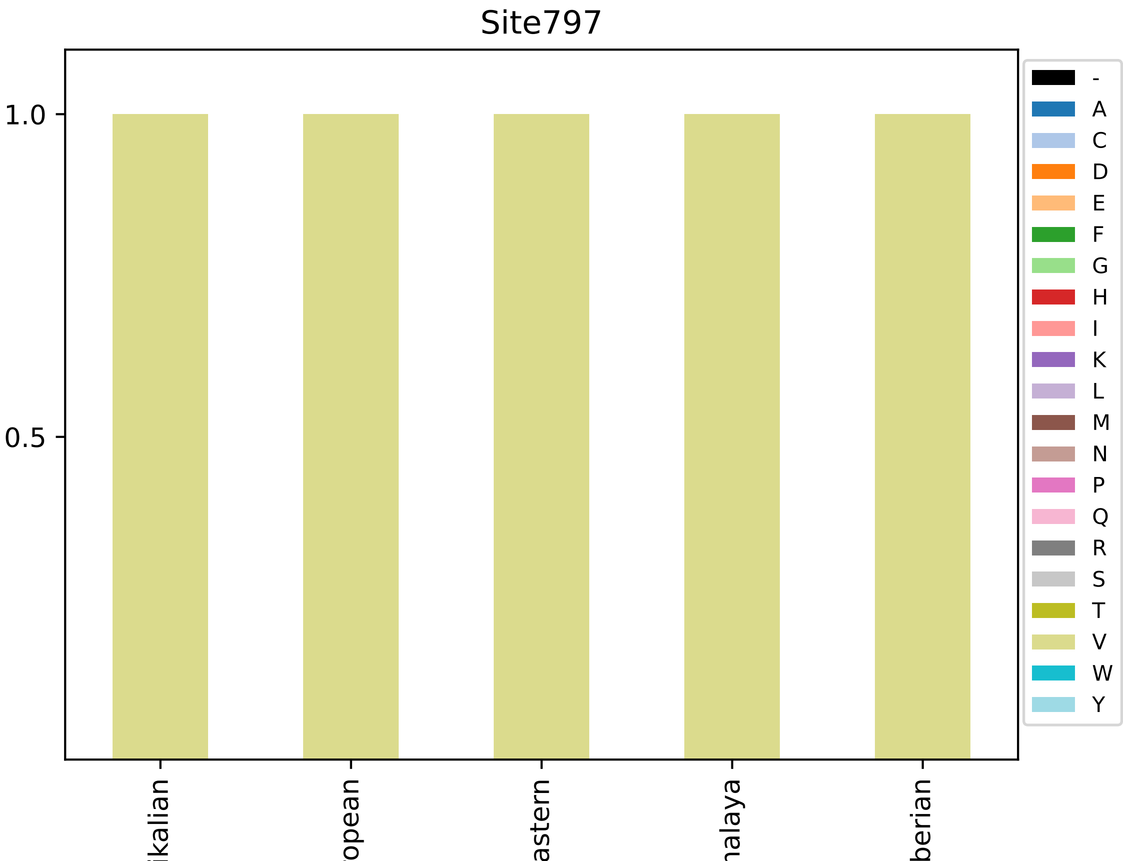
Site797
1.0
0.5
-
A
C
D
E
F
G
H
I
K
L
M
N
P
Q
R
S
T
V
W
Y
ikalian
opean
astern
nalaya
berian

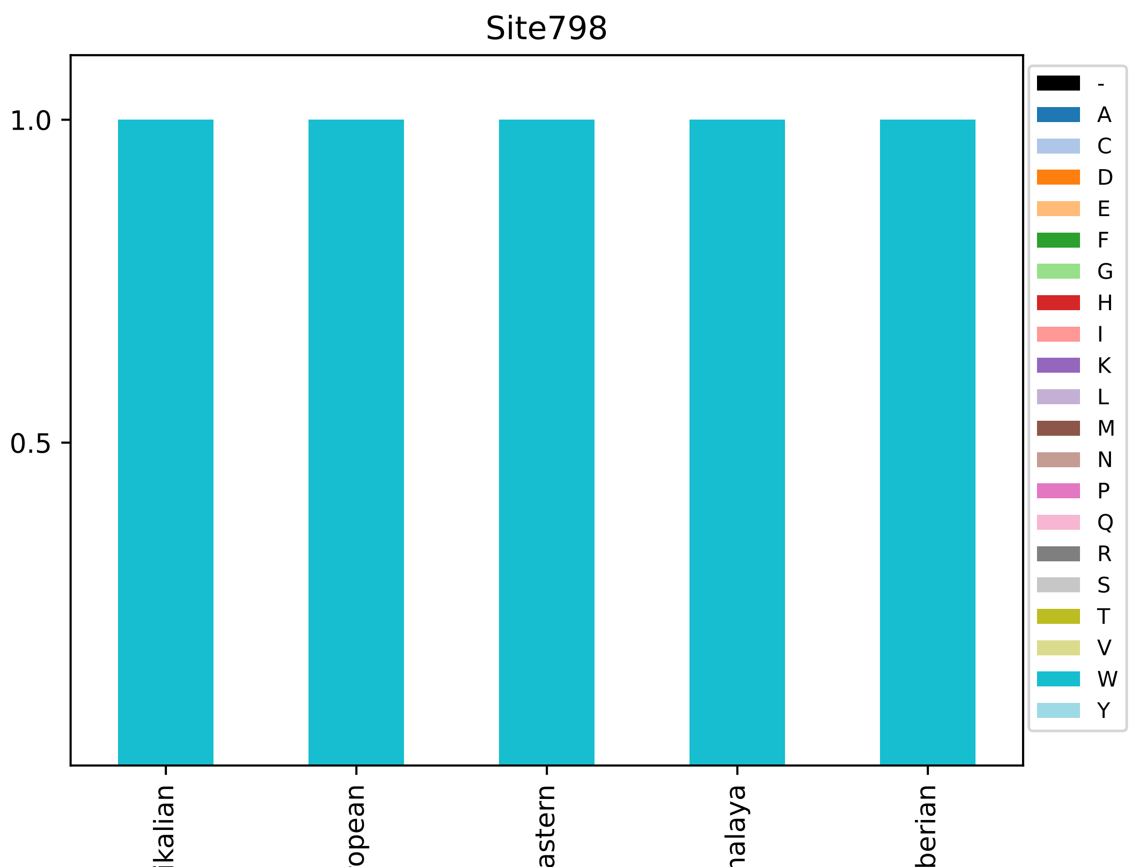

Site798
1.0
0.5
-
A
C
D
E
F
G
H
I
K
L
M
N
P
Q
R
S
T
V
W
Y

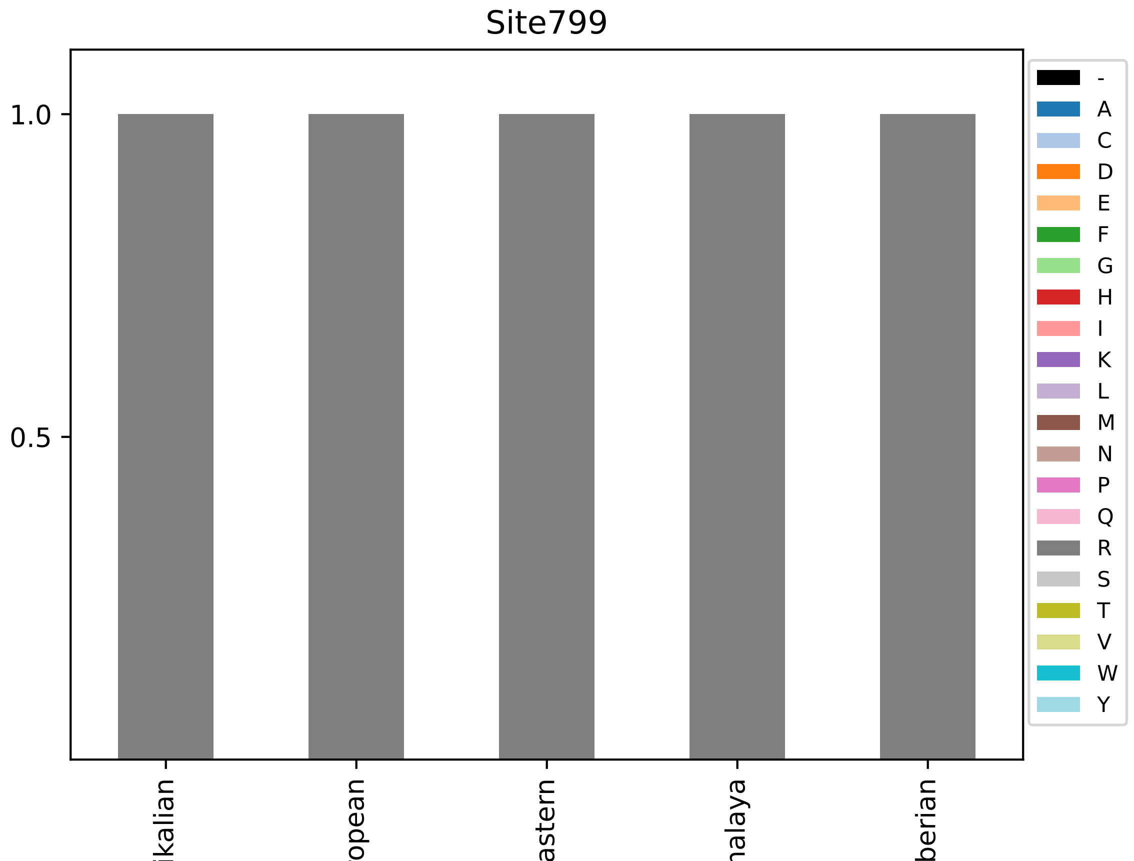
Site799
1.0
0.5
-
A
C
D
E
F
G
H
I
K
L
M
N
P
Q
R
S
T
V
W
Y
ikalian
ropean
astern
nalaya
berian

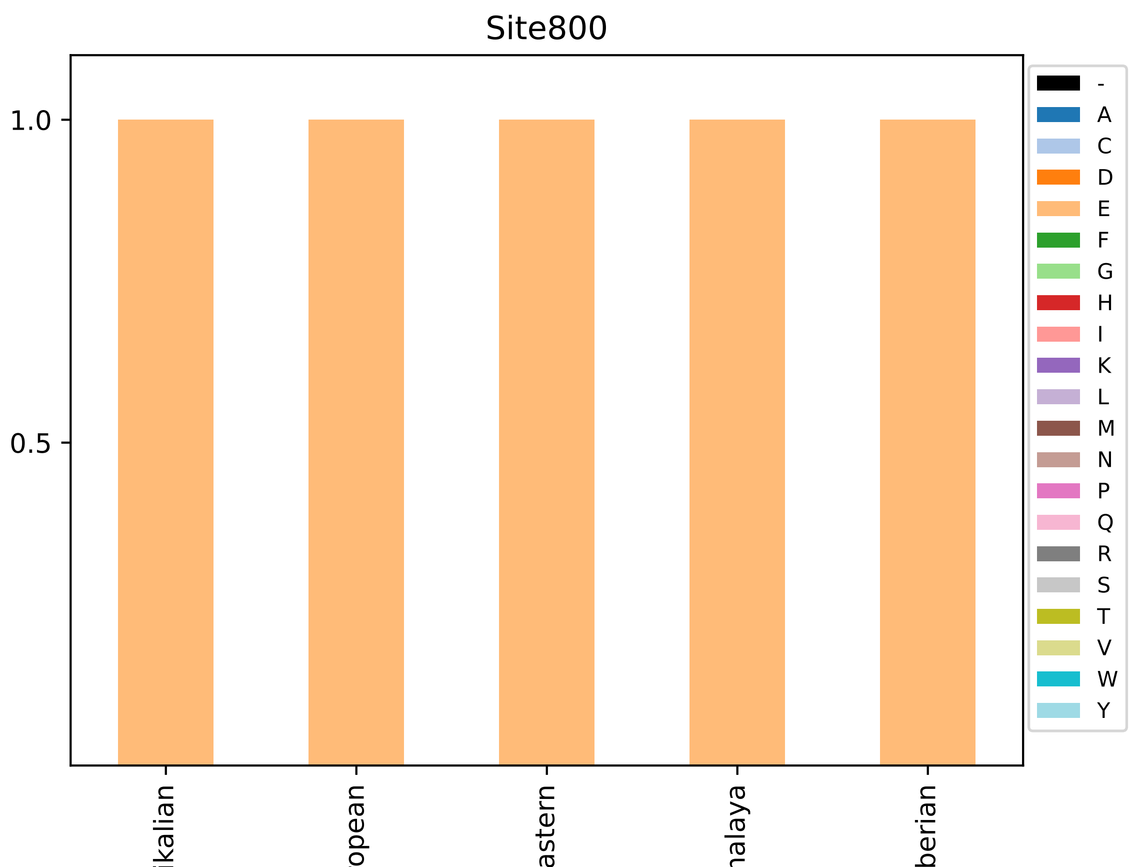

Site800
1.0
0.5
-
A
C
D
E
F
G
H
I
K
L
M
N
P
Q
R
S
T
V
W
Y
ikalian
opean
astern
nalaya
berian

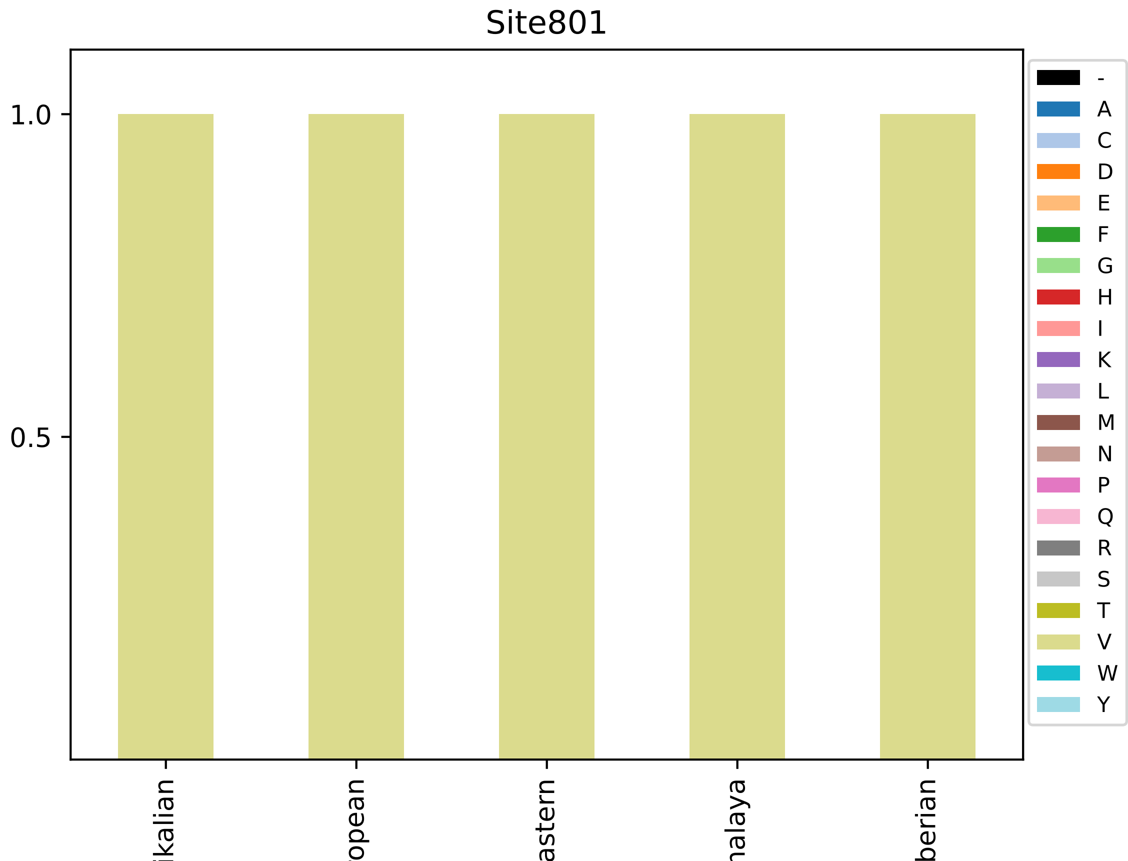

Site801
1.0
0.5
-
A
C
D
E
F
G
H
I
K
L
M
N
P
Q
R
S
T
V
W
Y
ikalian
opean
astern
nalaya
berian

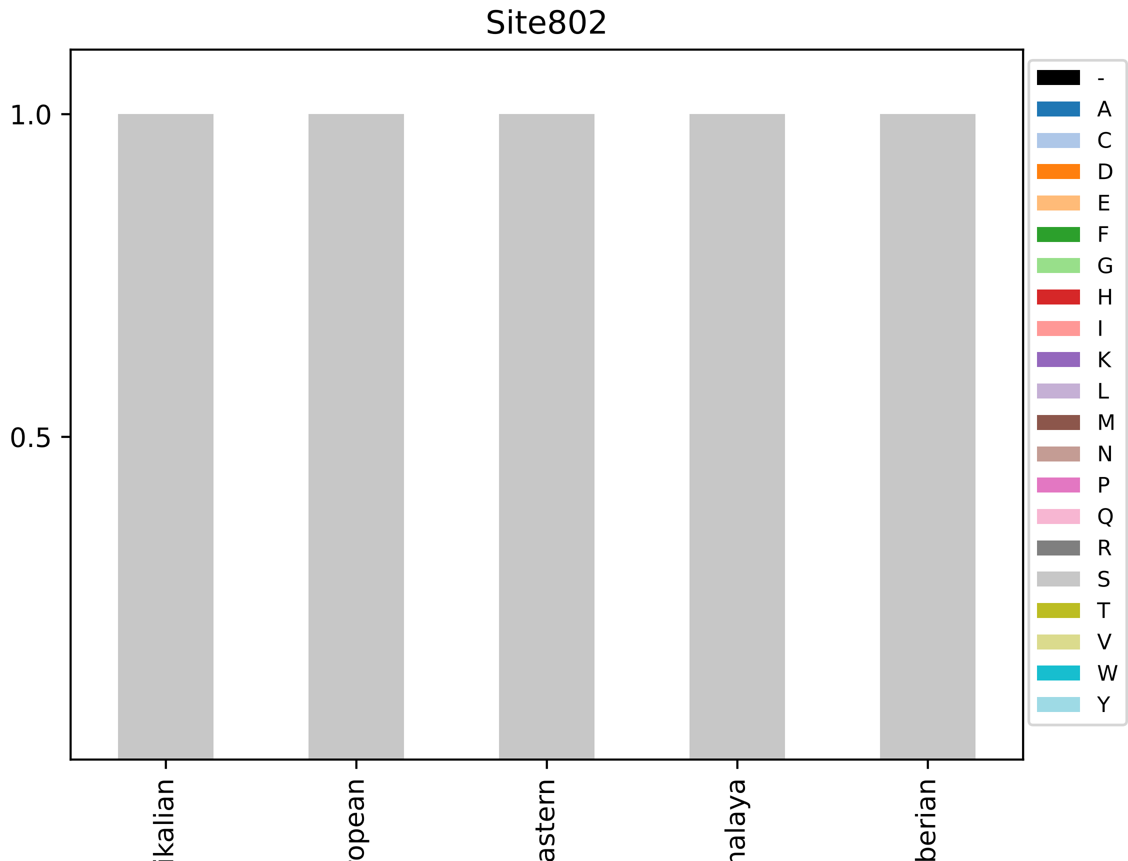
Site802
1.0
0.5
-
A
C
D
E
F
G
H
I
K
L
M
N
P
Q
R
S
T
V
W
Y
ikalian
opean
astern
nalaya
berian

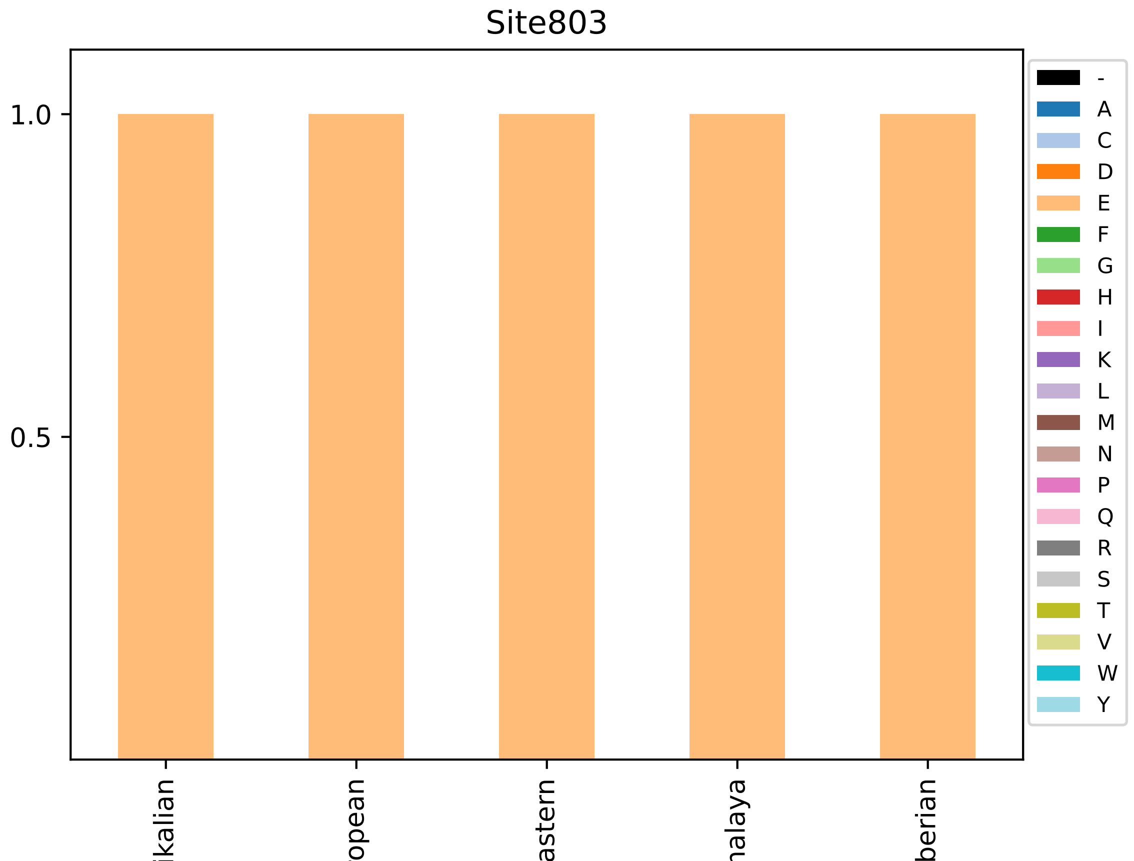
Site803
1.0
0.5
ikalian
opean
astern
nalaya
berian
-
A
C
D
E
F
G
H
I
K
L
M
N
P
Q
R
S
T
V
W
Y

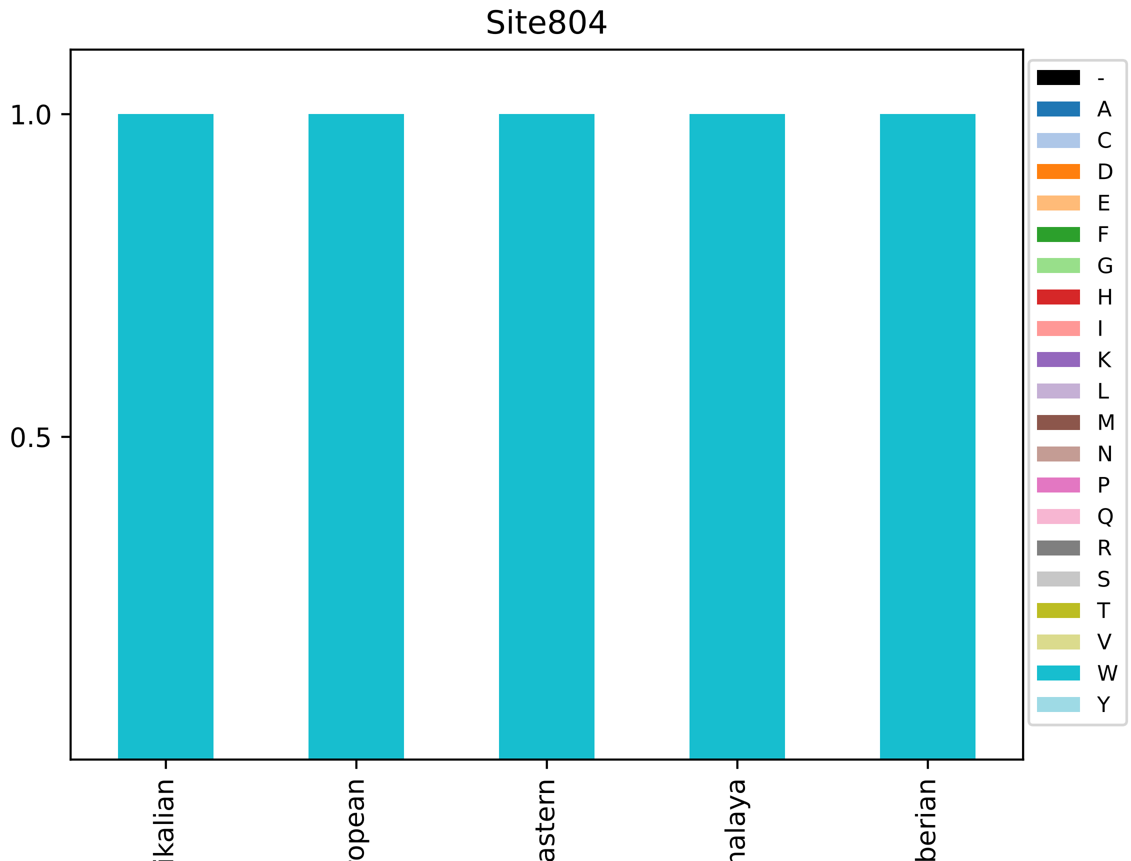

Site804
1.0
0.5
ikalian
opean
astern
nalaya
berian
-
A
C
D
E
F
G
H
I
K
L
M
N
P
Q
R
S
T
V
W
Y

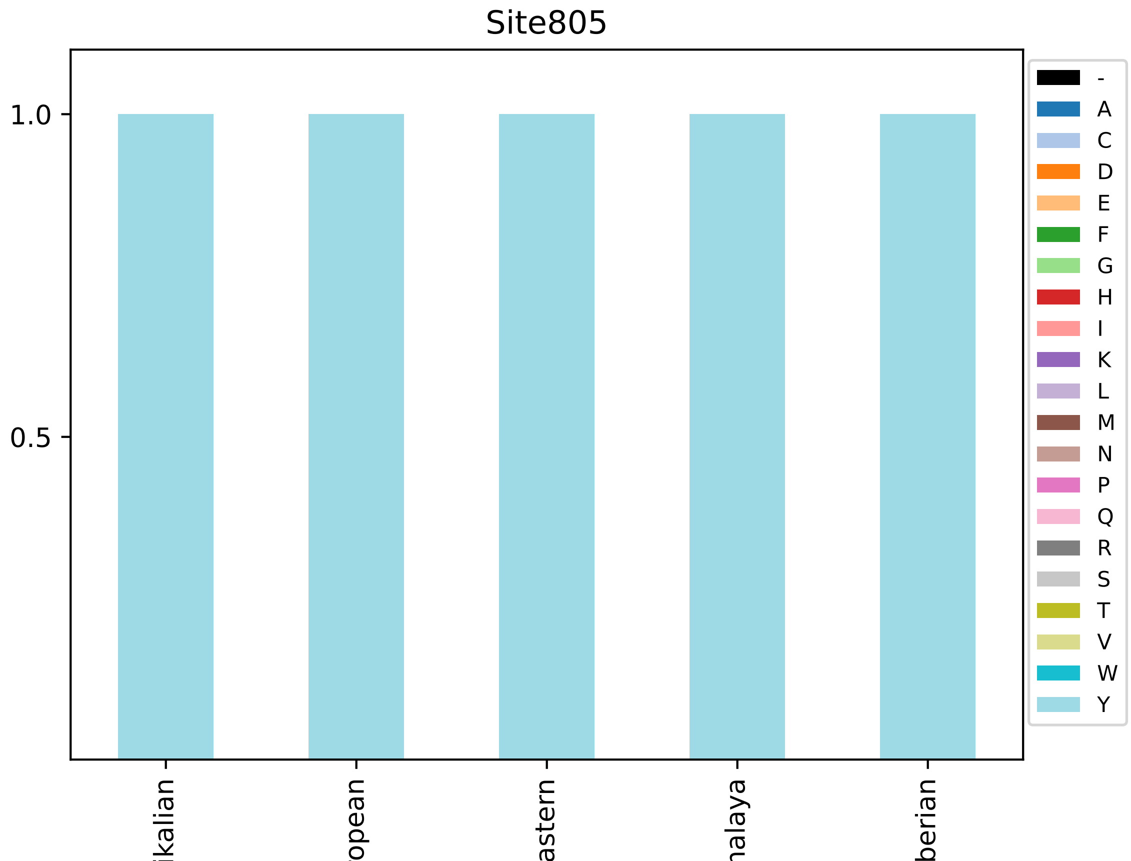

Site805
1.0
0.5
-
A
C
D
E
F
G
H
I
K
L
M
N
P
Q
R
S
T
V
W
Y
ikalian
opean
astern
nalaya
berian

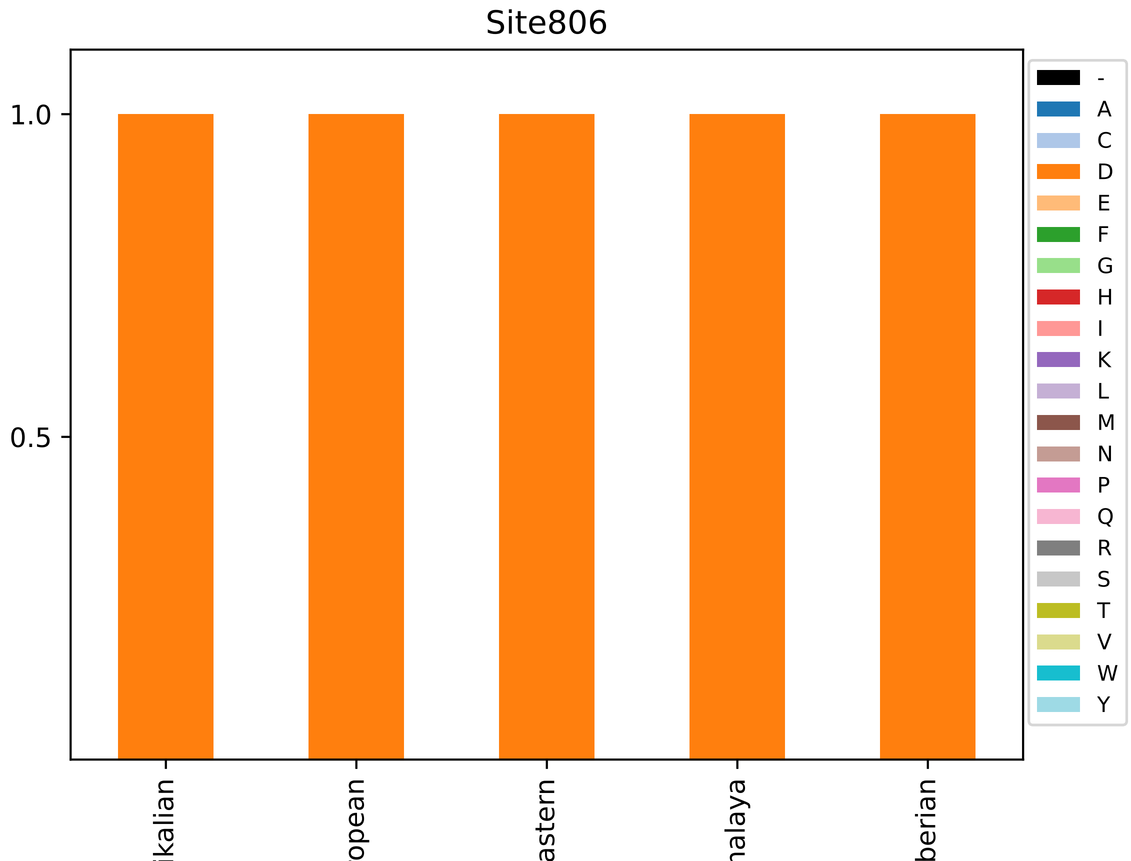
Site806
1.0
0.5
-
A
C
D
E
F
G
H
I
K
L
M
N
P
Q
R
S
T
V
W
Y
ikalian
opean
astern
nalaya
berian

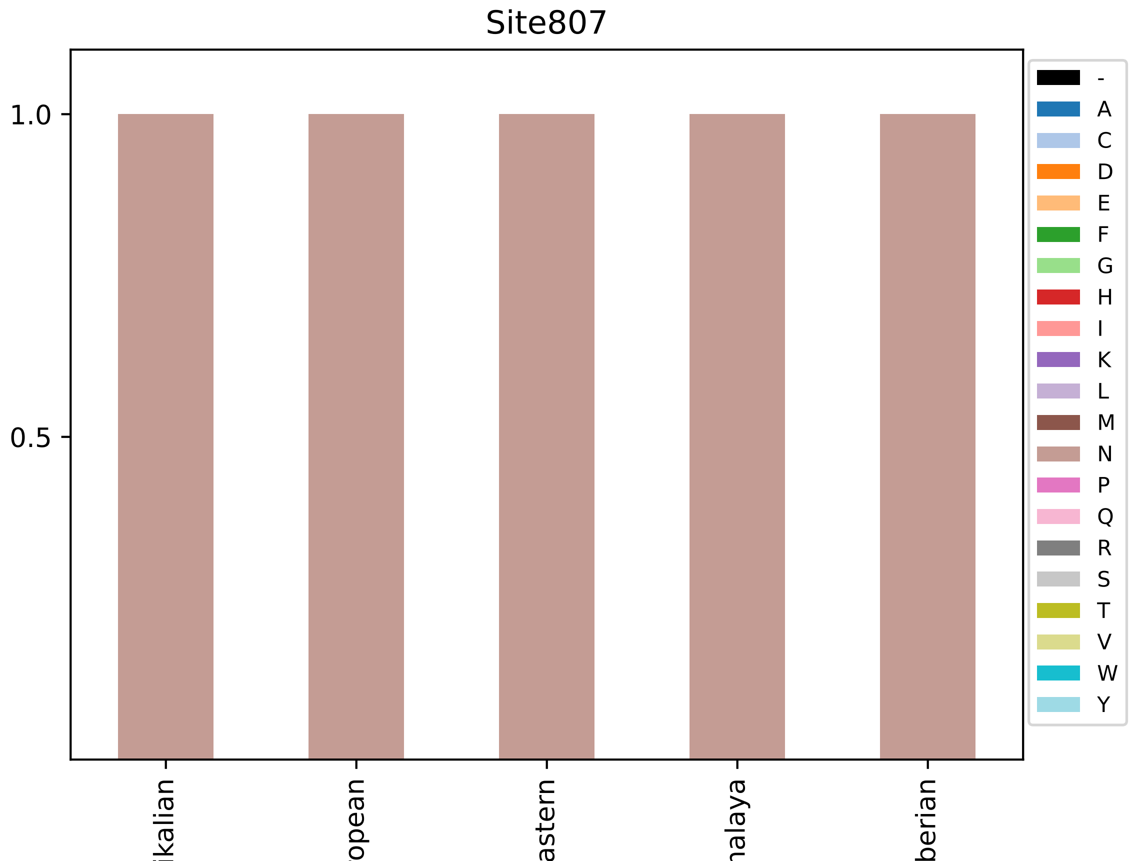

Site807
1.0
0.5
-
A
C
D
E
F
G
H
I
K
L
M
N
P
Q
R
S
T
V
W
Y
ikalian
opean
astern
nalaya
berian

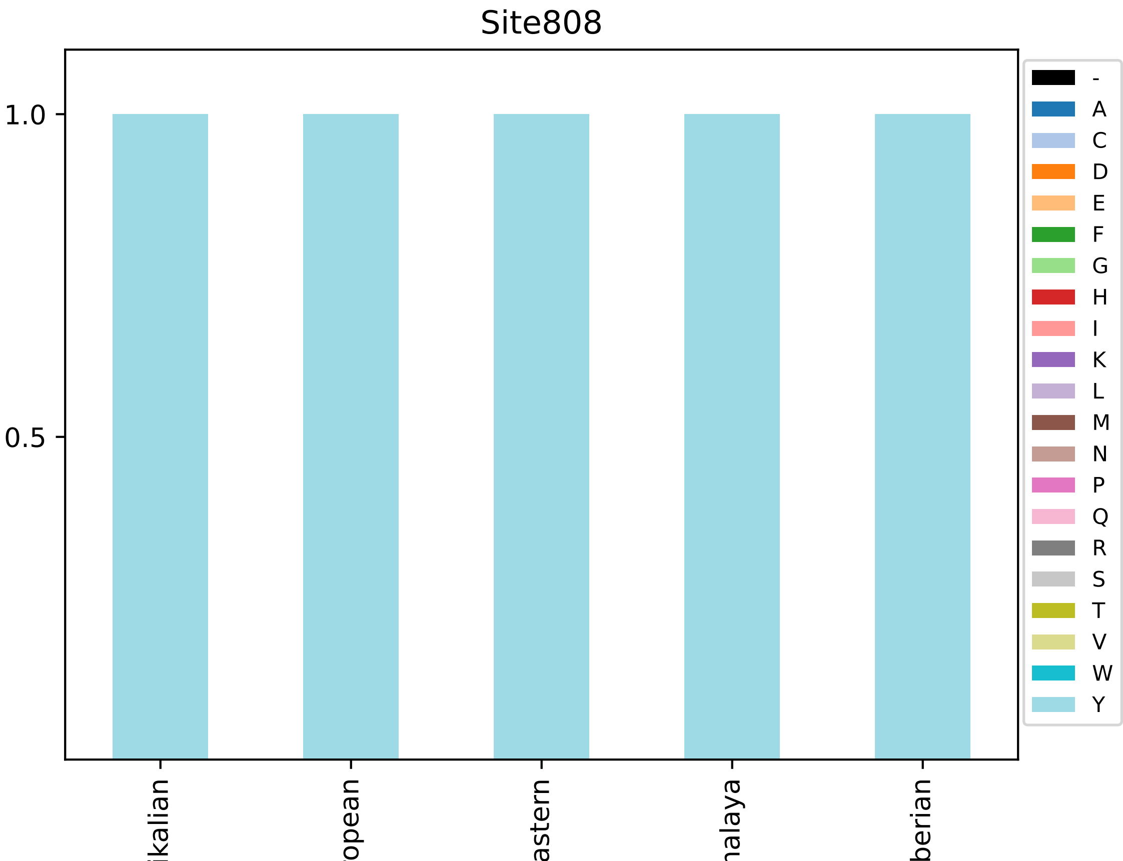

Site808
1.0
0.5
-
A
C
D
E
F
G
H
I
K
L
M
N
P
Q
R
S
T
V
W
Y
ikalian
opean
astern
nalaya
berian

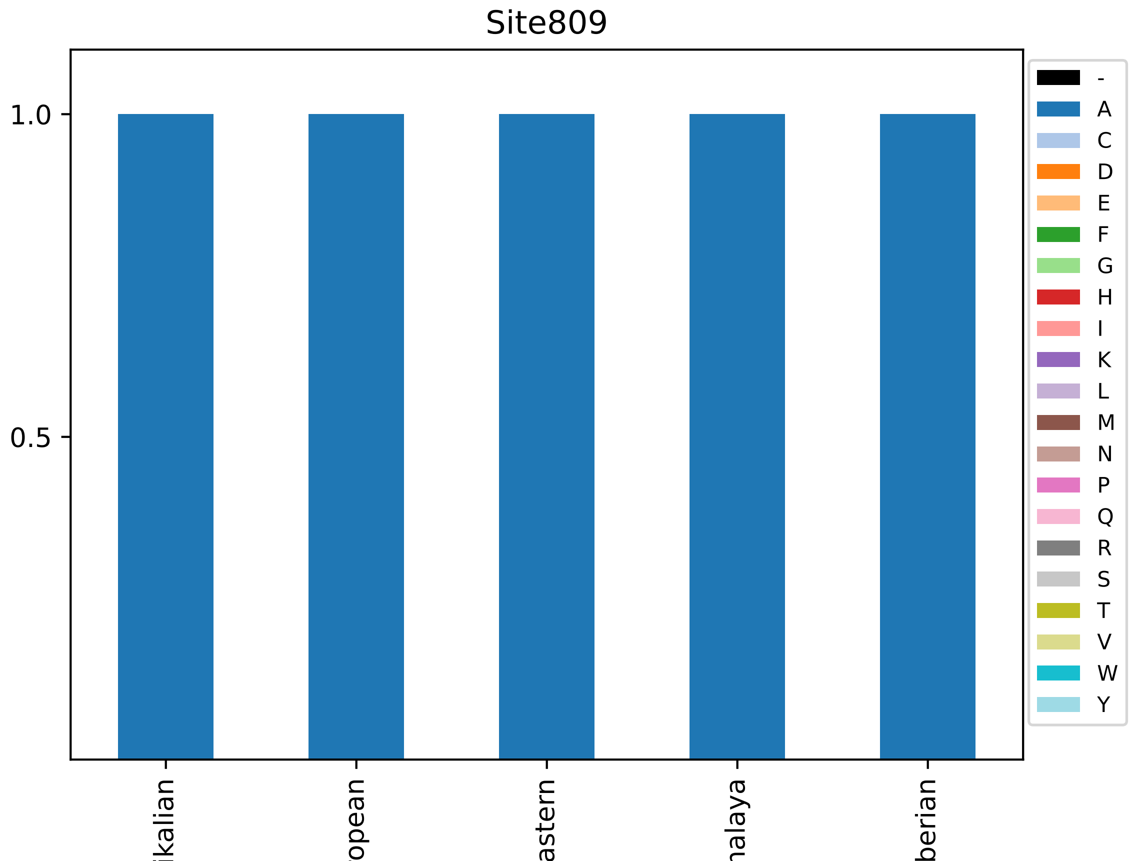
Site809
1.0
0.5
ikalian
opean
astern
nalaya
berian
-
A
C
D
E
F
G
H
I
K
L
M
N
P
Q
R
S
T
V
W
Y

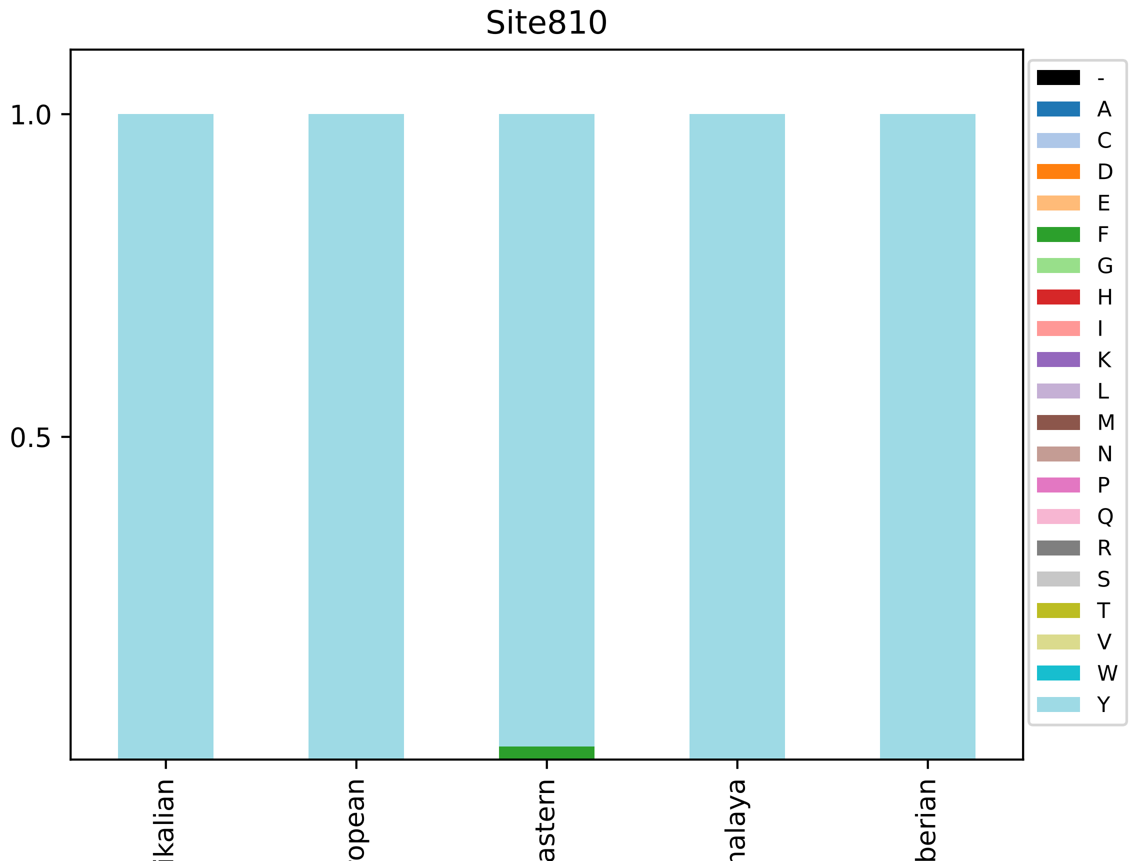

Site810
1.0
0.5
-
A
C
D
E
F
G
H
I
K
L
M
N
P
Q
R
S
T
V
W
Y
ikalian
opean
astern
nalaya
berian

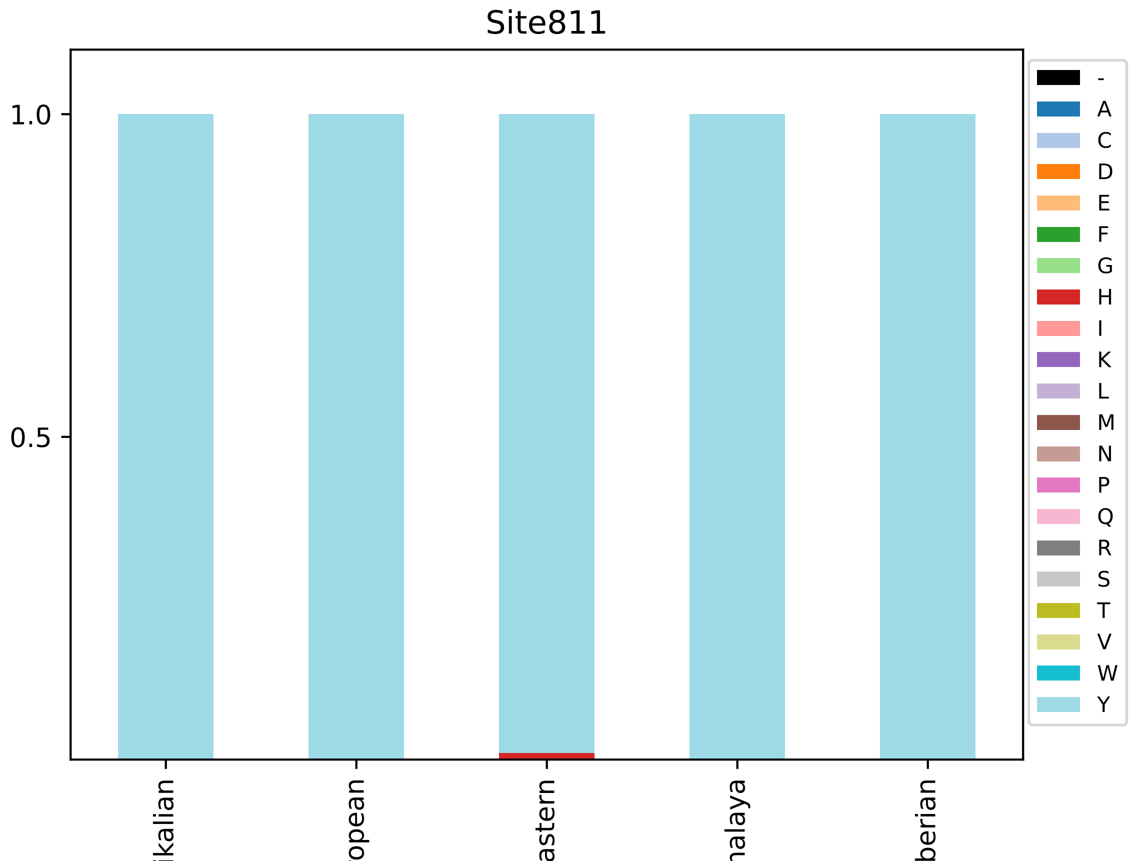

Site811
1.0
0.5
-
A
C
D
E
F
G
H
I
K
L
M
N
P
Q
R
S
T
V
W
Y
ikalian
opean
astern
nalaya
berian

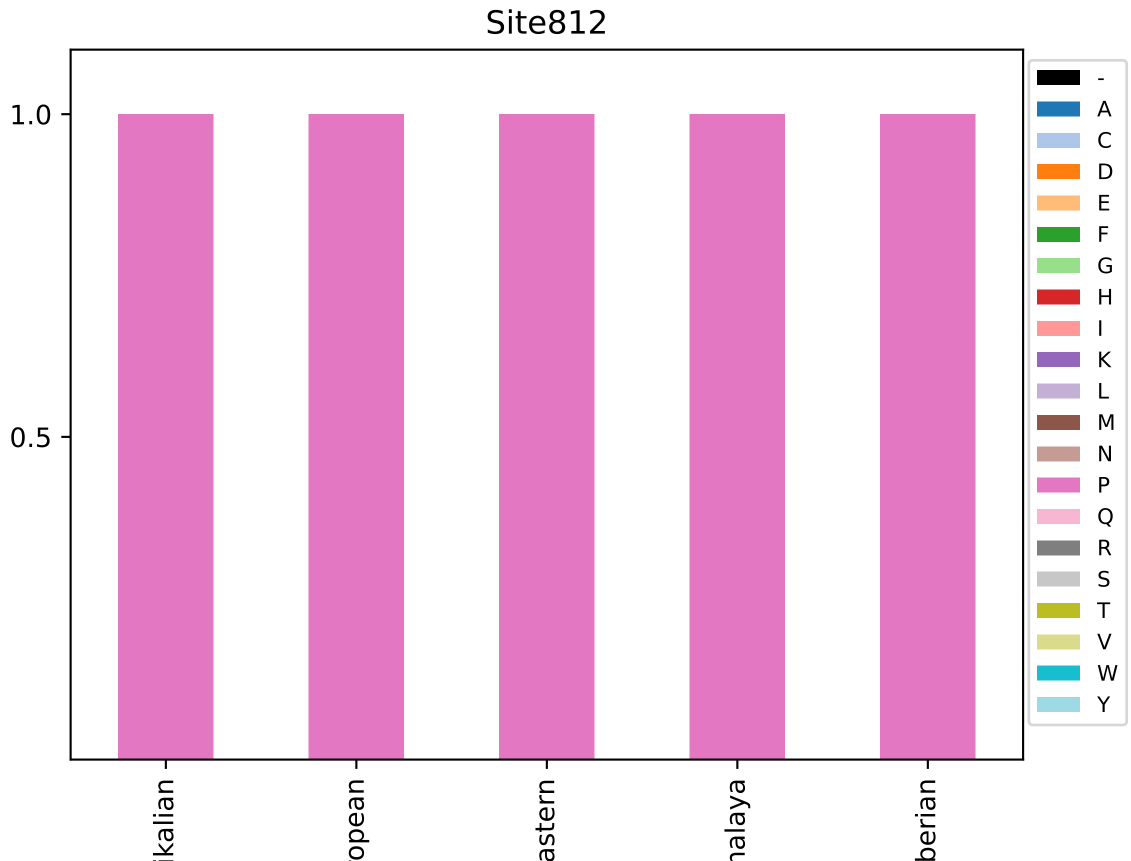

Site812
1.0
0.5
-
A
C
D
E
F
G
H
I
K
L
M
N
P
Q
R
S
T
V
W
Y
ikalian
opean
astern
nalaya
berian

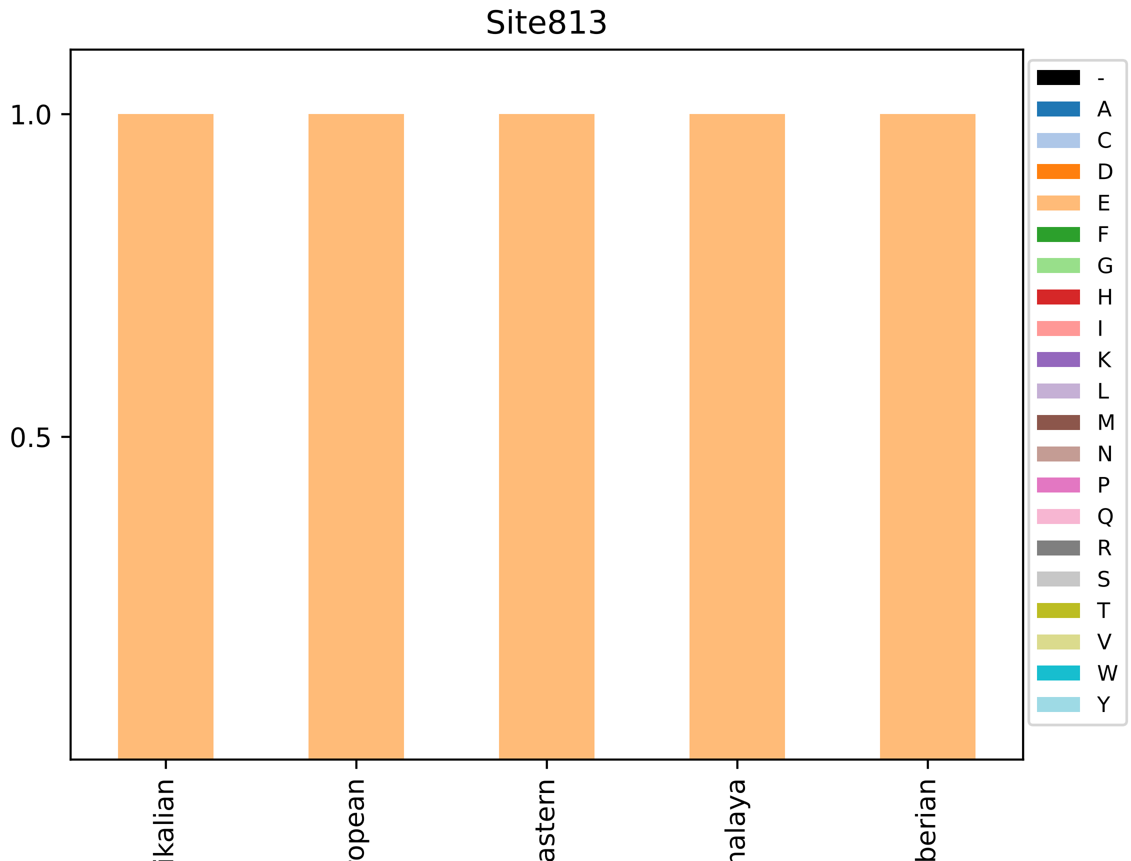

Site813
1.0
0.5
-
A
C
D
E
F
G
H
I
K
L
M
N
P
Q
R
S
T
V
W
Y
ikalian
opean
astern
nalaya
berian

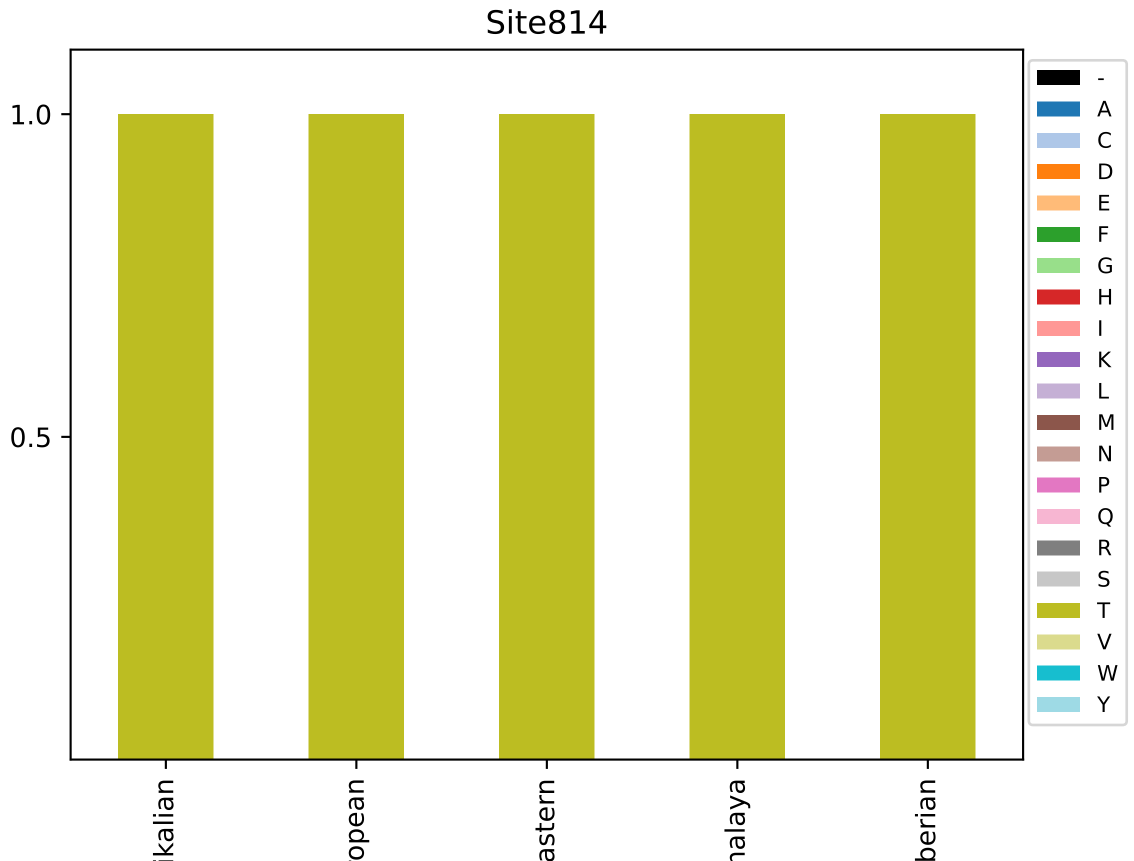
Site814
1.0
0.5
-
A
C
D
E
F
G
H
I
K
L
M
N
P
Q
R
S
T
V
W
Y
ikalian
opean
astern
nalaya
berian

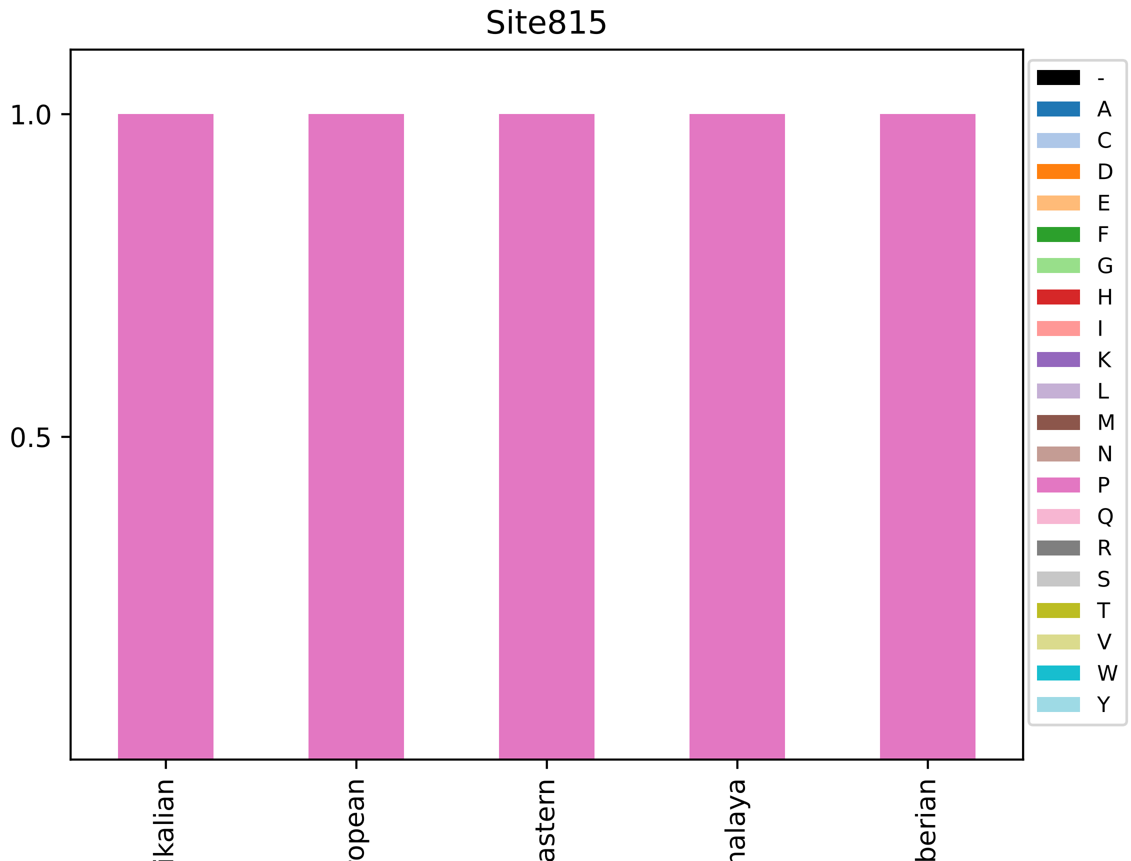
Site815
1.0
0.5
-
A
C
D
E
F
G
H
I
K
L
M
N
P
Q
R
S
T
V
W
Y
ikalian
opean
astern
nalaya
berian

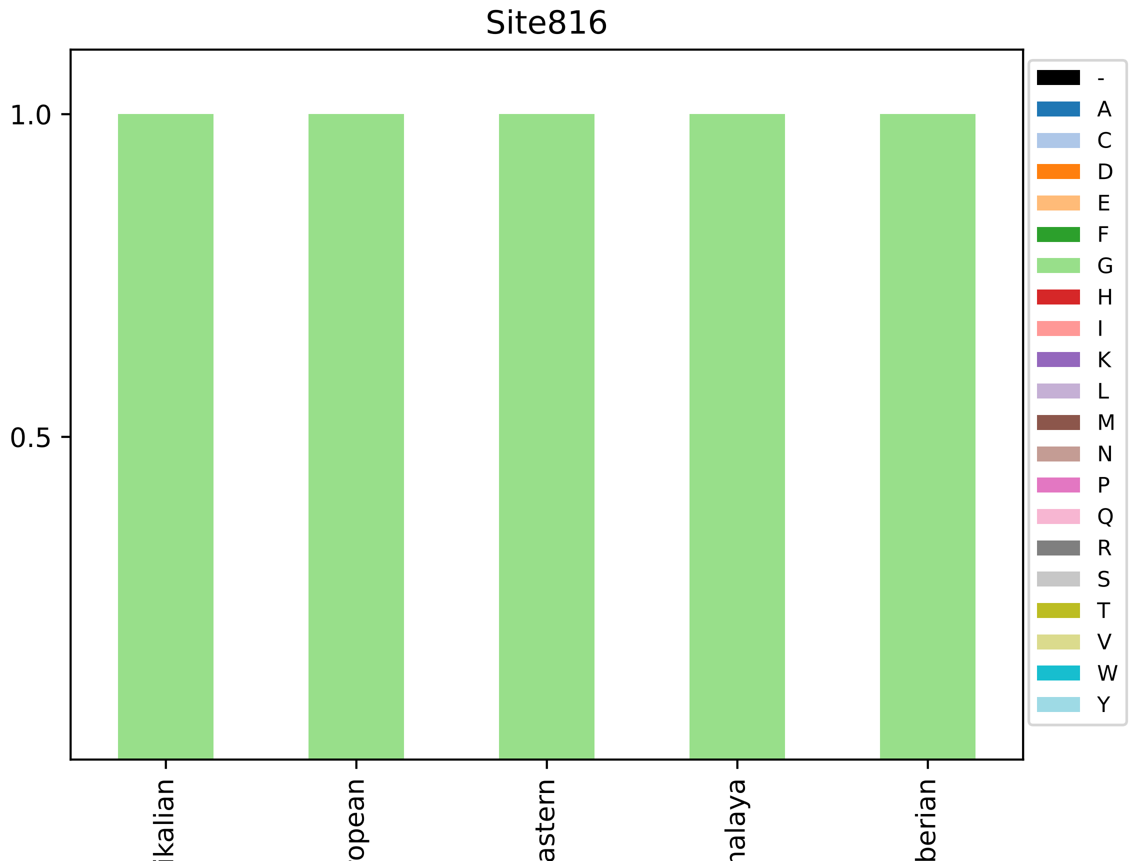
Site816
1.0
0.5
-
A
C
D
E
F
G
H
I
K
L
M
N
P
Q
R
S
T
V
W
Y
ikalian
opean
astern
nalaya
berian

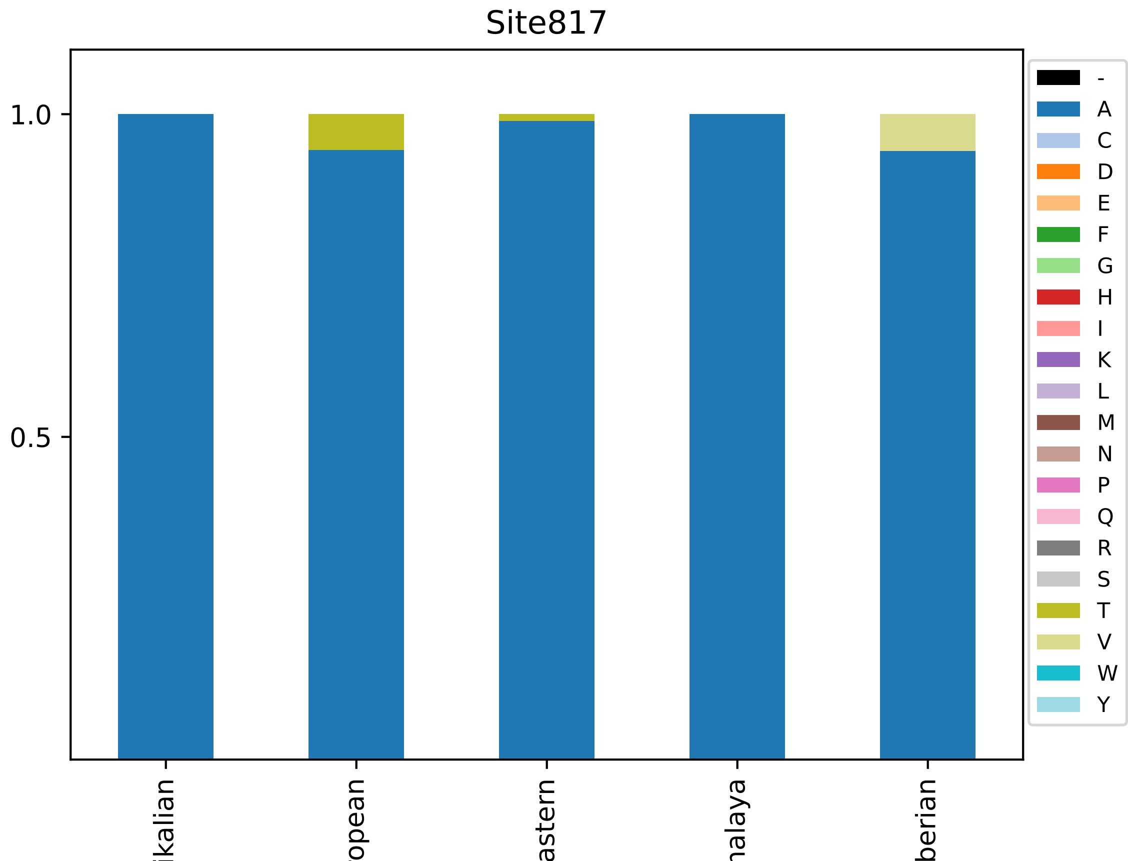

Site817
1.0
0.5
ikalian
opean
astern
nalaya
berian
-
A
C
D
E
F
G
H
I
K
L
M
N
P
Q
R
S
T
V
W
Y

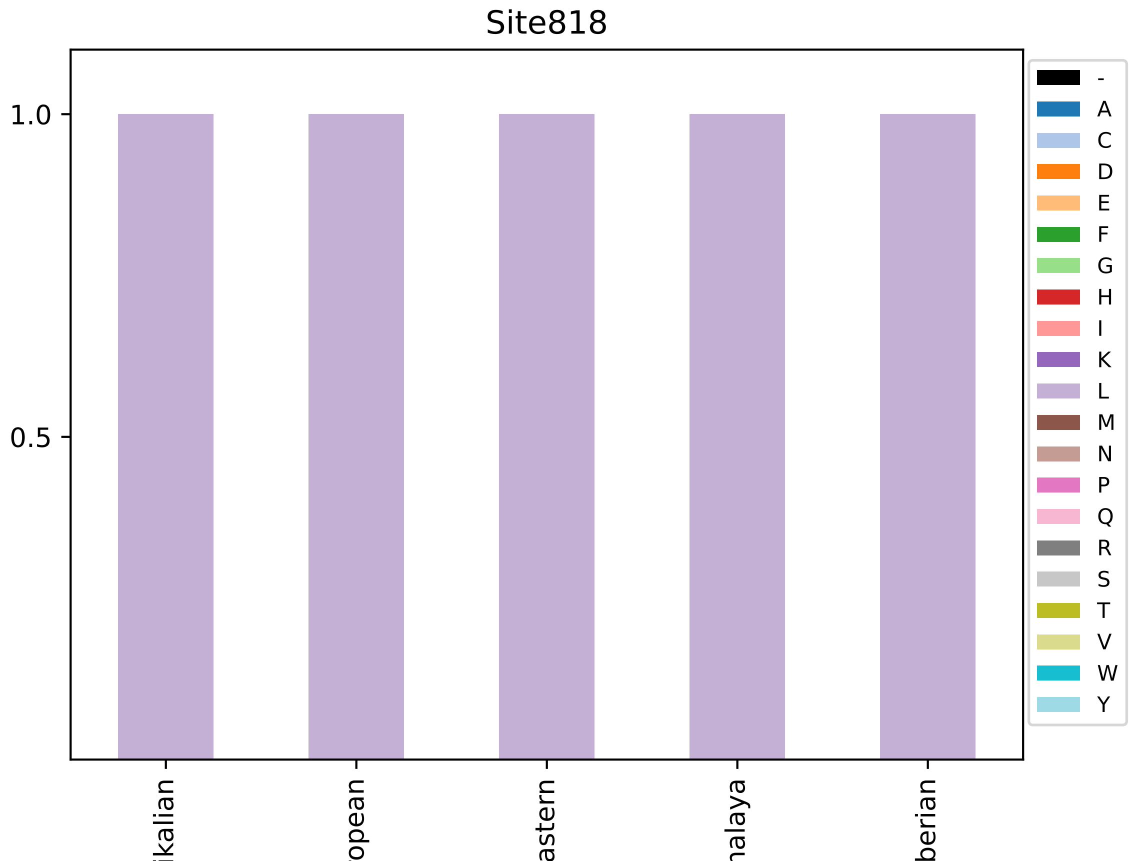
Site818
1.0
0.5
-
A
C
D
E
F
G
H
I
K
L
M
N
P
Q
R
S
T
V
W
Y
ikalian
opean
astern
nalaya
berian

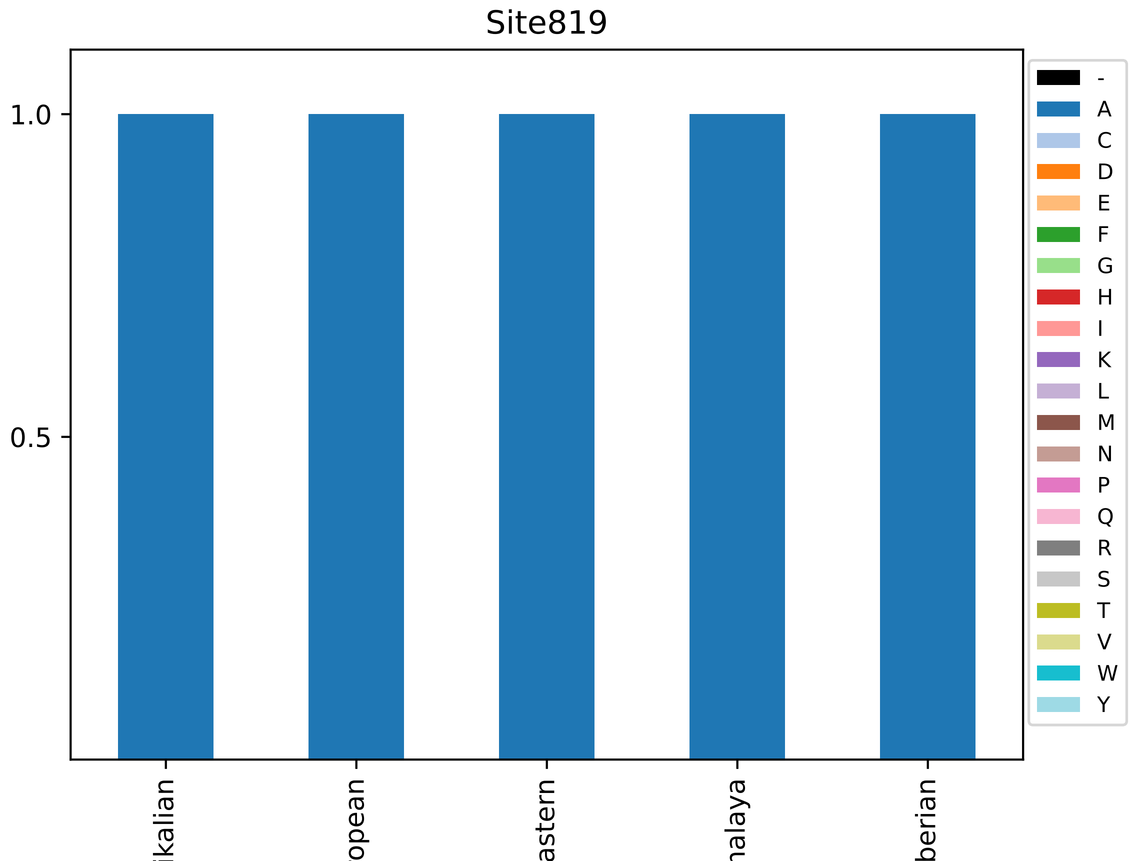

Site819
1.0
0.5
-
A
C
D
E
F
G
H
I
K
L
M
N
P
Q
R
S
T
V
W
Y

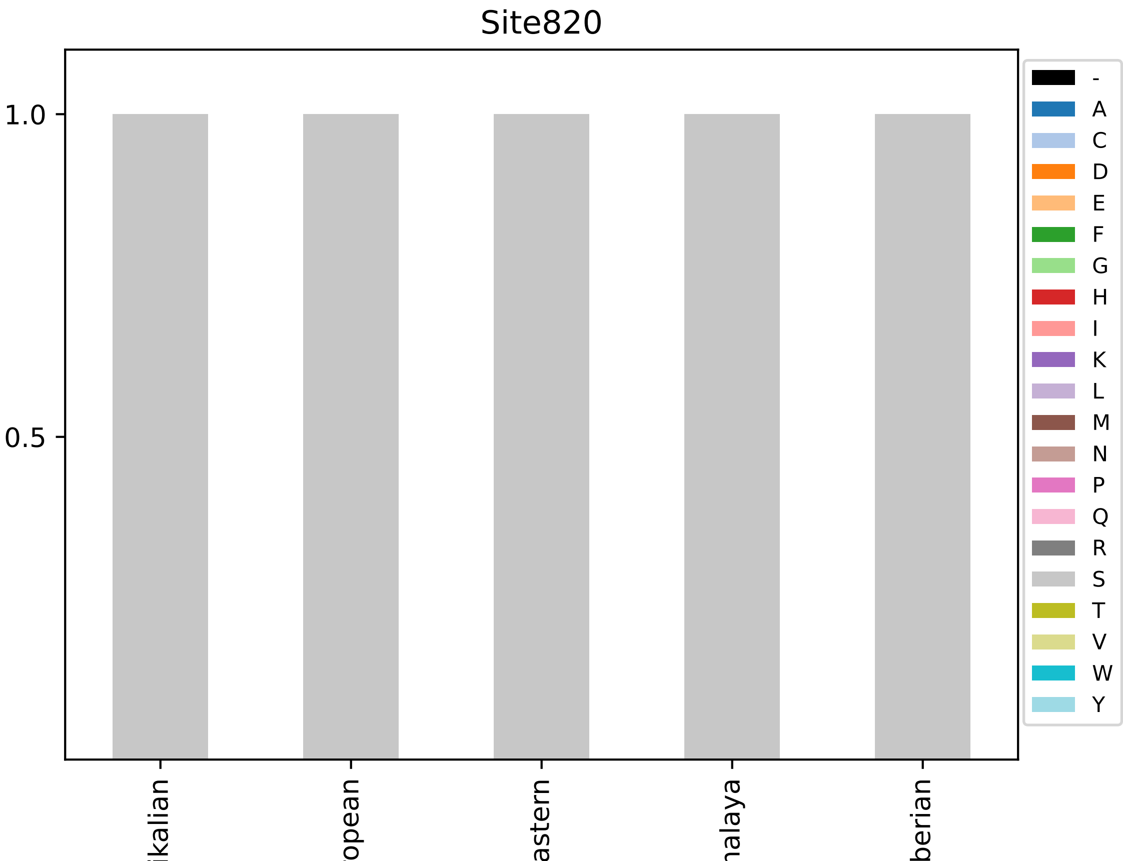

Site820
1.0
0.5
-
A
C
D
E
F
G
H
I
K
L
M
N
P
Q
R
S
T
V
W
Y
ikalian
opean
astern
nalaya
berian

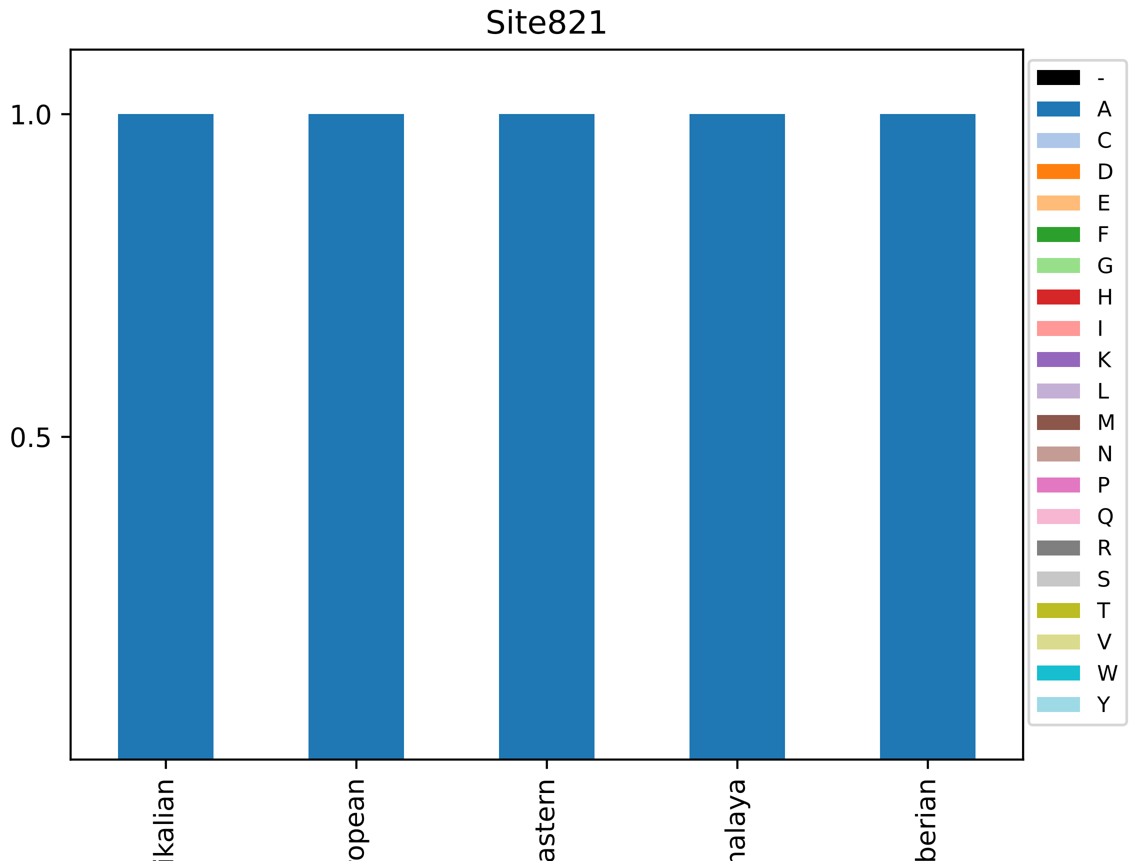
Site821
1.0
0.5
-
A
C
D
E
F
G
H
I
K
L
M
N
P
Q
R
S
T
V
W
Y
ikalian
opean
astern
nalaya
berian

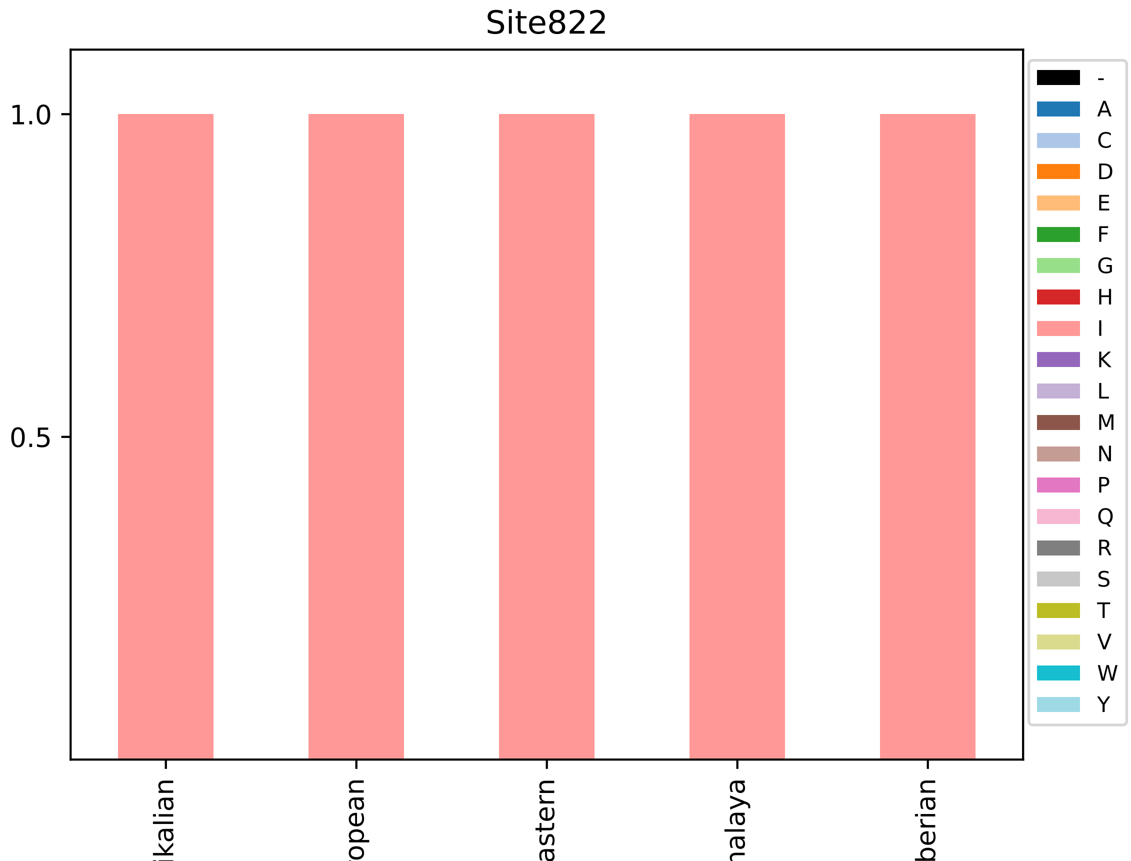
Site822
1.0
0.5
-
A
C
D
E
F
G
H
I
K
L
M
N
P
Q
R
S
T
V
W
Y
ikalian
opean
astern
nalaya
berian

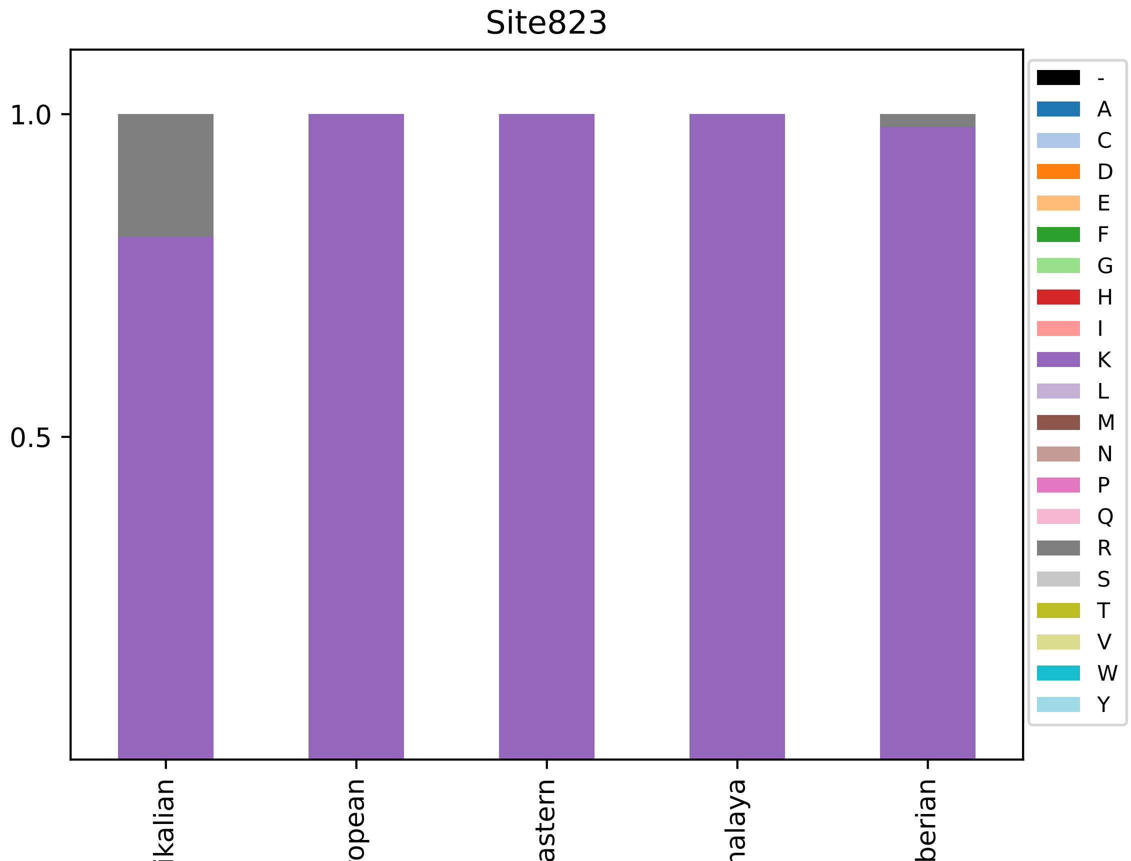
Site823
1.0
0.5
-
A
C
D
E
F
G
H
I
K
L
M
N
P
Q
R
S
T
V
W
Y
ikalian
opean
astern
nalaya
berian

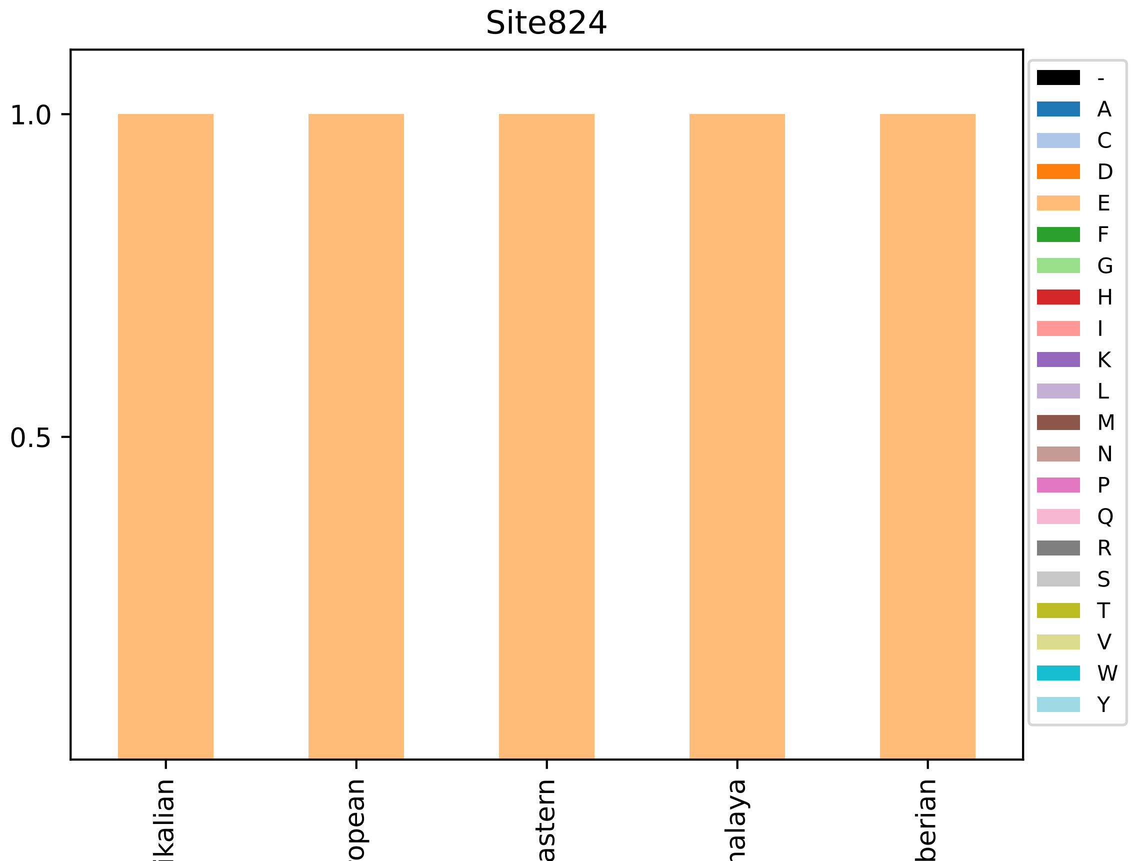
Site824
1.0
0.5
-
A
C
D
E
F
G
H
I
K
L
M
N
P
Q
R
S
T
V
W
Y
ikalian
opean
astern
nalaya
berian

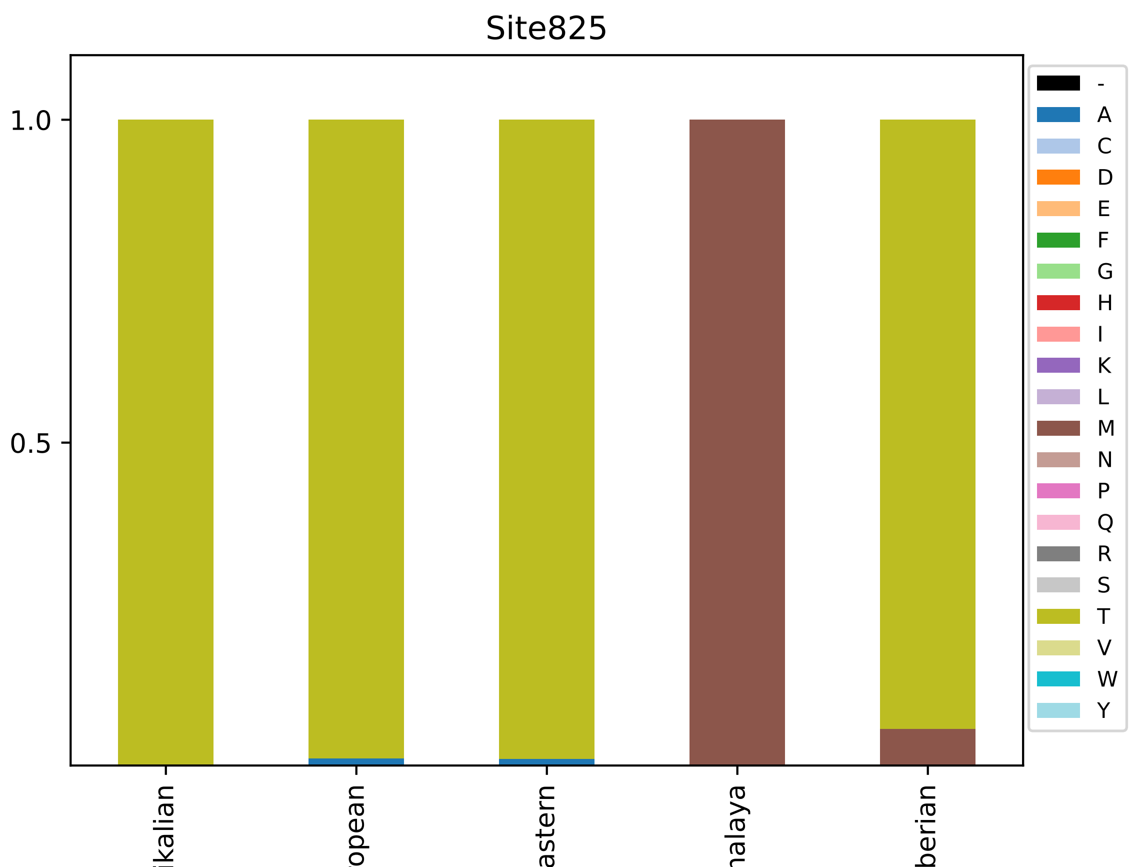
Site825
1.0
0.5
-
A
C
D
E
F
G
H
I
K
L
M
N
P
Q
R
S
T
V
W
Y
ikalian
opean
astern
nalaya
berian

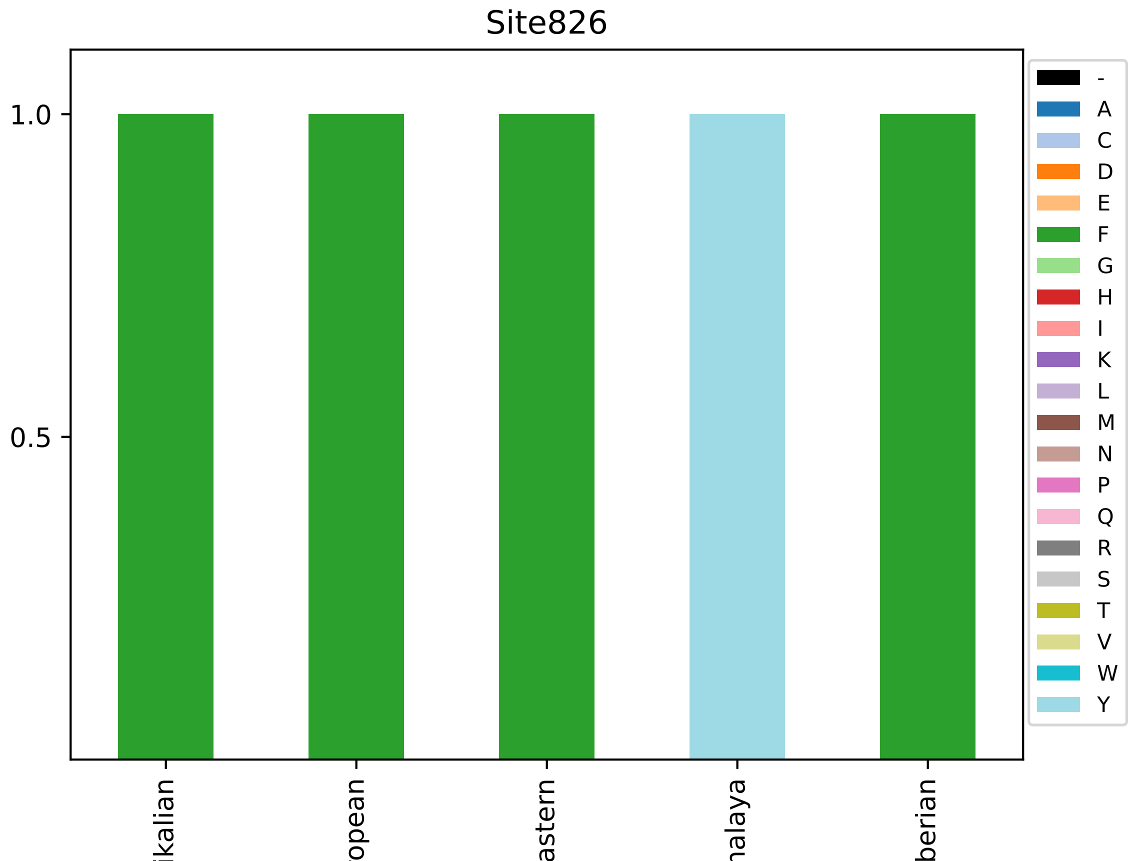
Site826
1.0
0.5
-
A
C
D
E
F
G
H
I
K
L
M
N
P
Q
R
S
T
V
W
Y
ikalian
ropean
astern
nalaya
berian

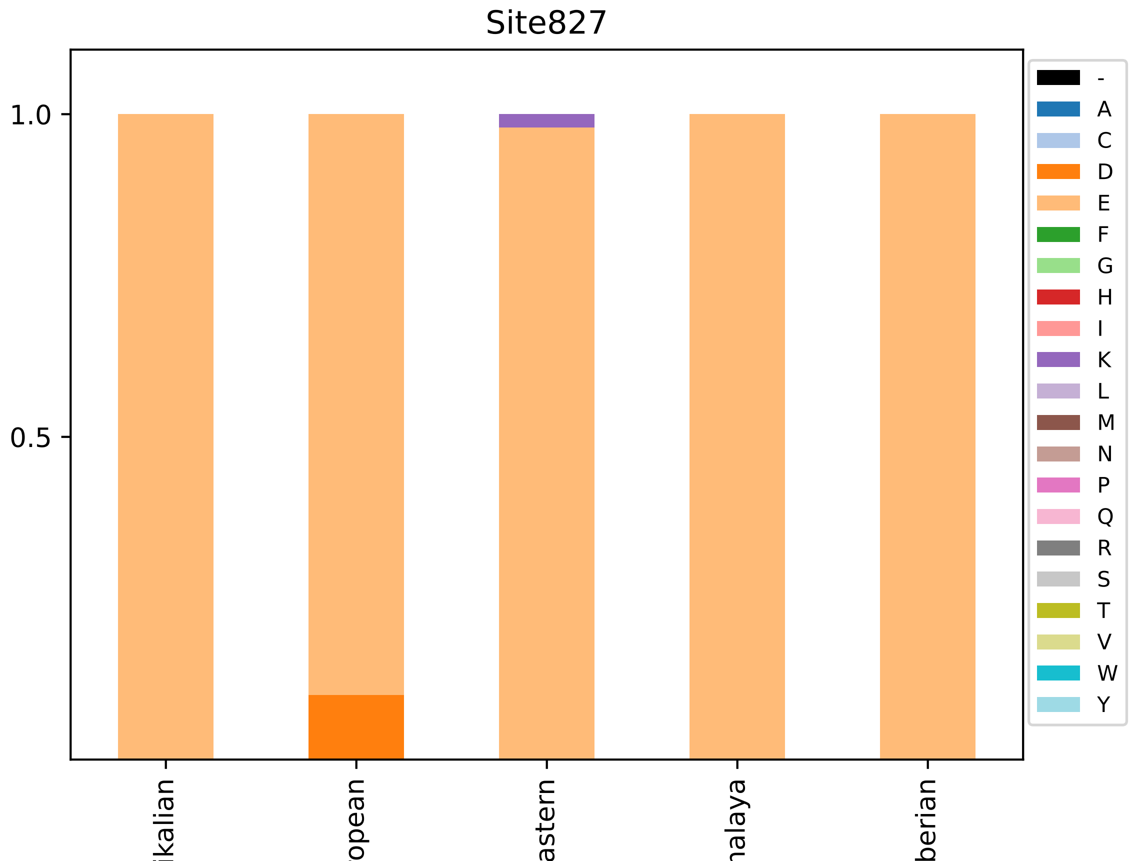
Site827
1.0
0.5
-
A
C
D
E
F
G
H
I
K
L
M
N
P
Q
R
S
T
V
W
Y
ikalian
opean
astern
nalaya
berian

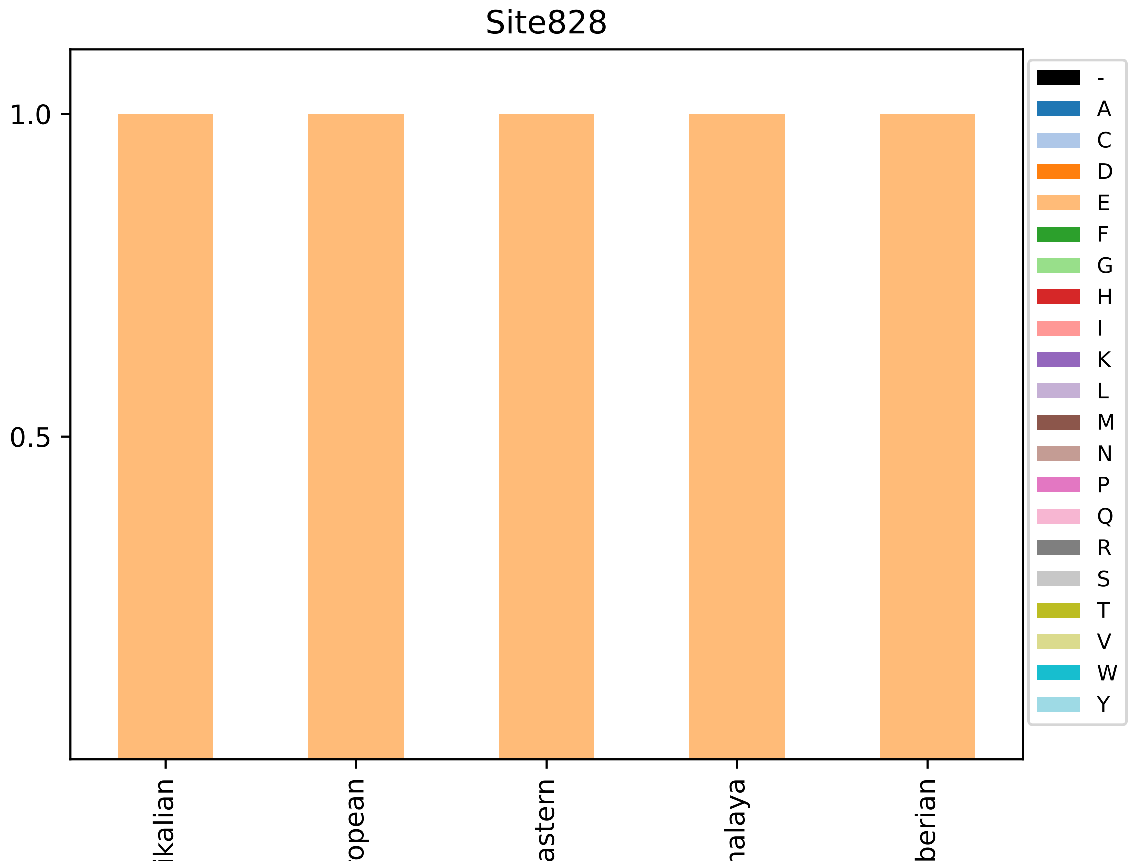

Site828
1.0
0.5
-
A
C
D
E
F
G
H
I
K
L
M
N
P
Q
R
S
T
V
W
Y
ikalian
opean
astern
nalaya
berian

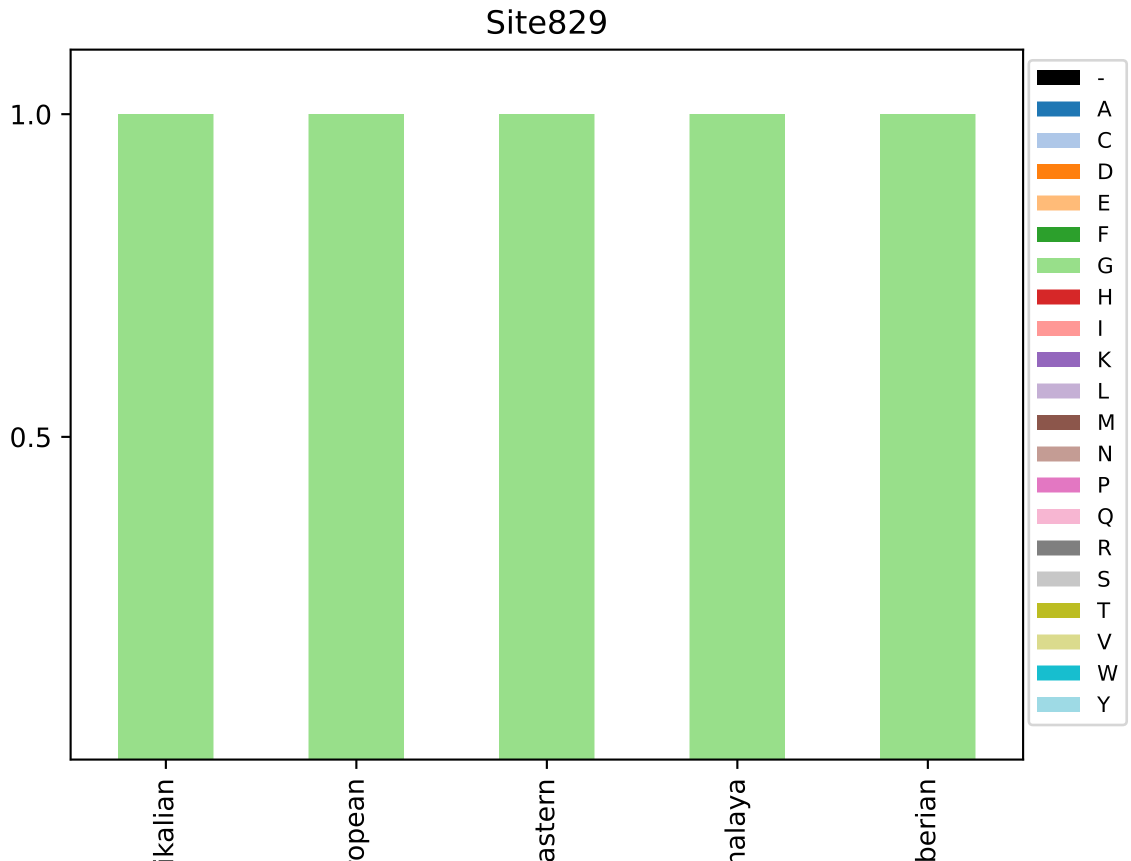
Site829
1.0
0.5
-
A
C
D
E
F
G
H
I
K
L
M
N
P
Q
R
S
T
V
W
Y
ikalian
opean
astern
nalaya
berian

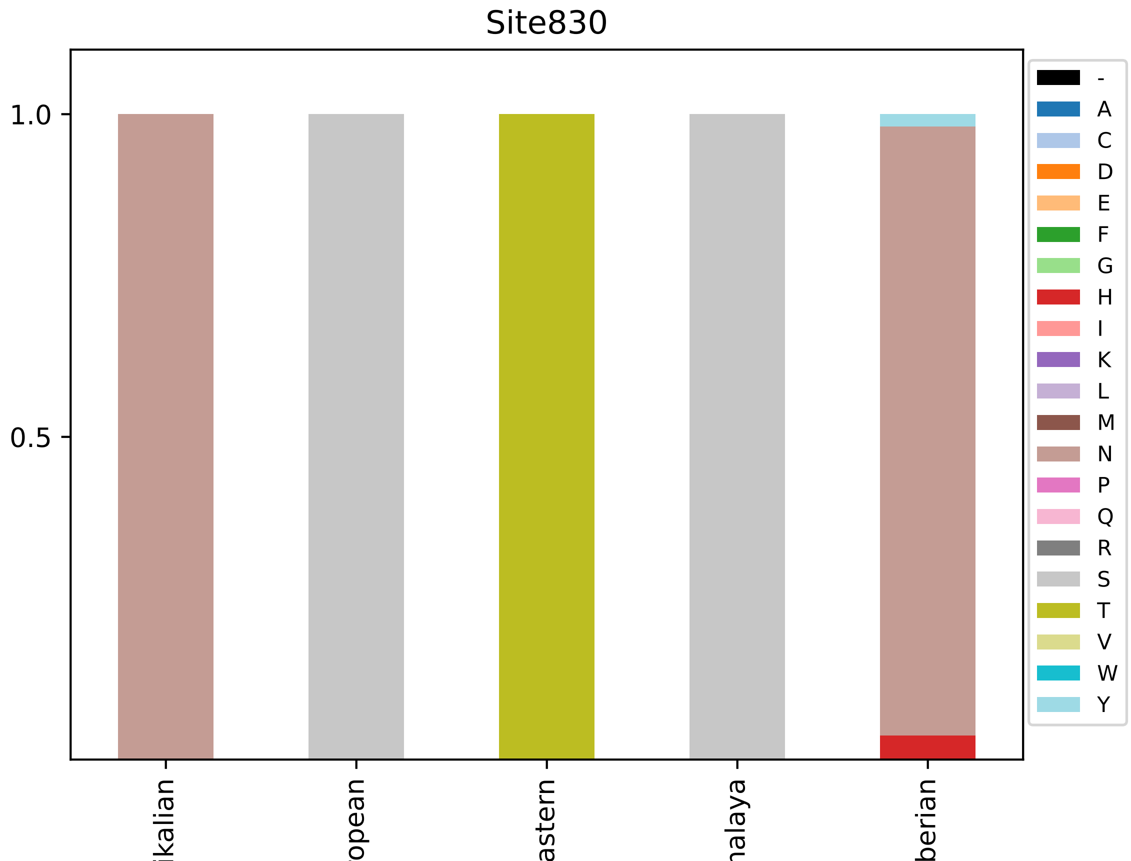

Site830
1.0
0.5
ikalian
opean
astern
nalaya
berian
-
A
C
D
E
F
G
H
I
K
L
M
N
P
Q
R
S
T
V
W
Y

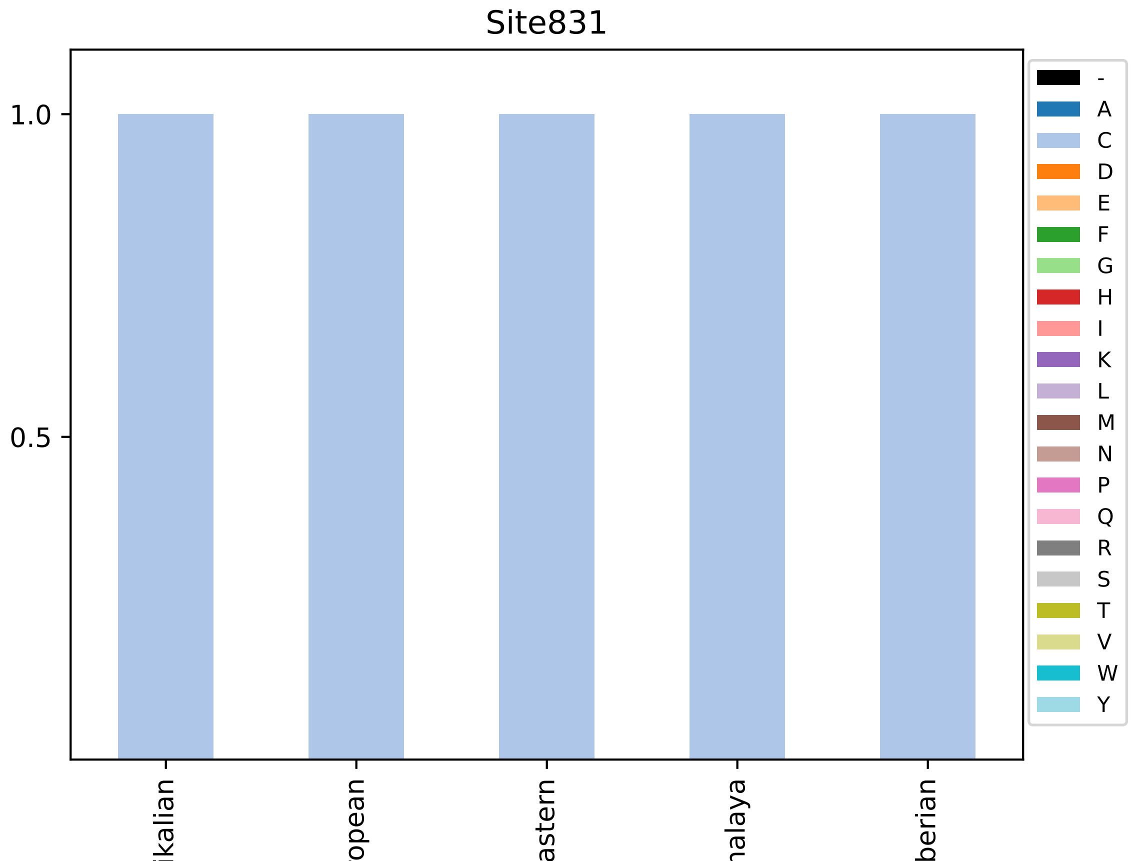
Site831
1.0
0.5
-
A
C
D
E
F
G
H
I
K
L
M
N
P
Q
R
S
T
V
W
Y
ikalian
opean
astern
nalaya
berian

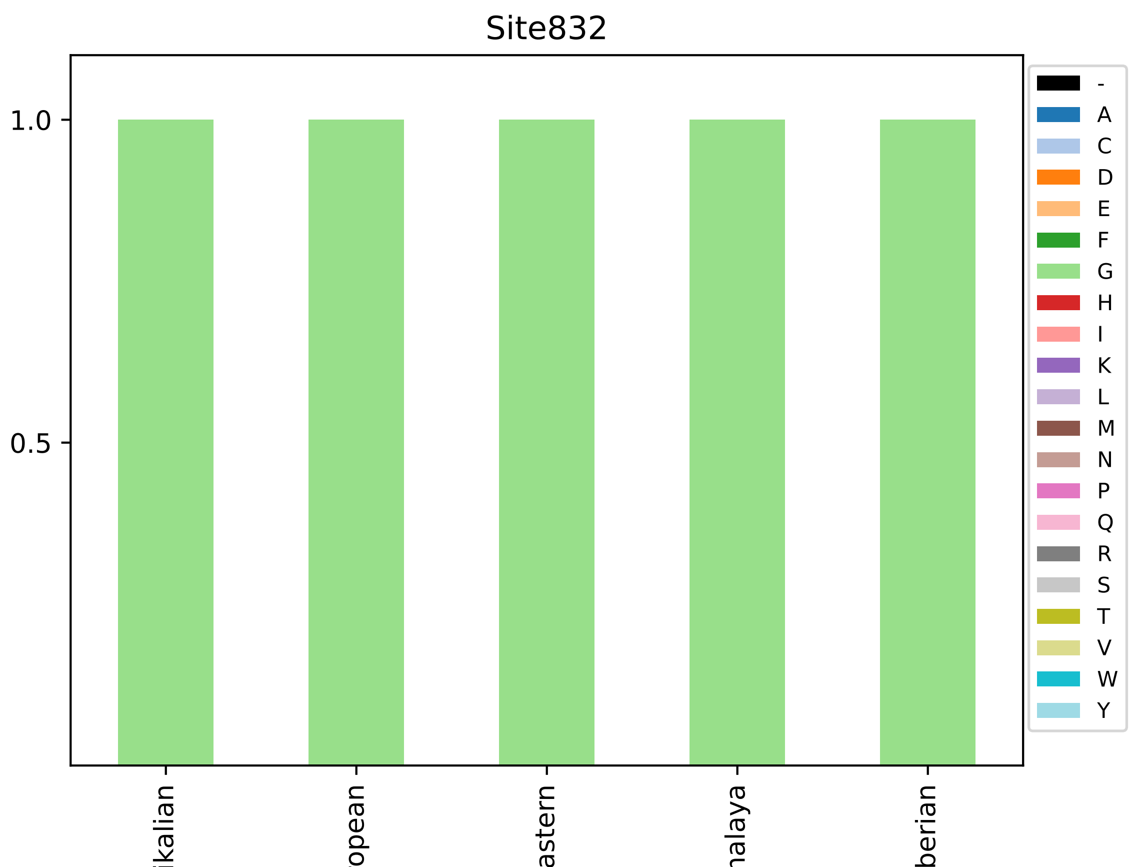

Site832
1.0
0.5
-
A
C
D
E
F
G
H
I
K
L
M
N
P
Q
R
S
T
V
W
Y
ikalian
opean
astern
nalaya
berian

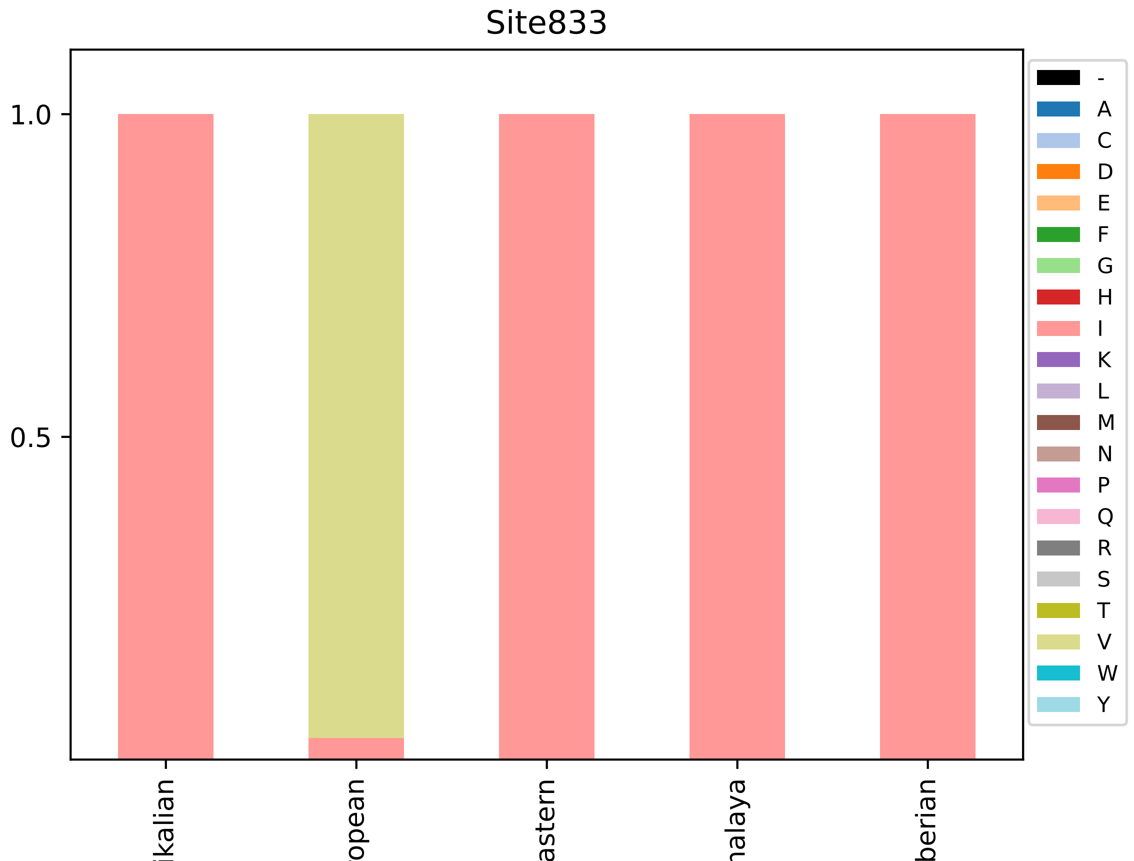
Site833
1.0
0.5
-
A
C
D
E
F
G
H
I
K
L
M
N
P
Q
R
S
T
V
W
Y
ikalian
opean
astern
nalaya
berian

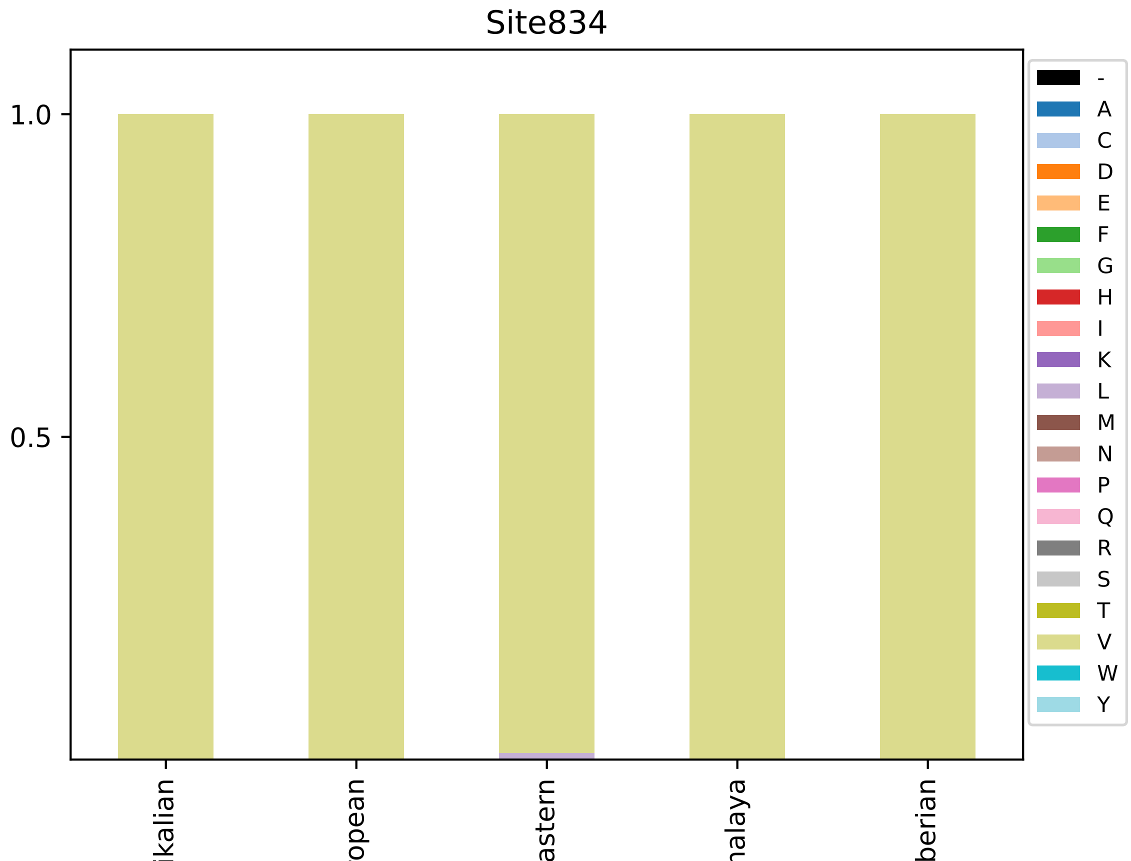
Site834
1.0
0.5
-
A
C
D
E
F
G
H
I
K
L
M
N
P
Q
R
S
T
V
W
Y
ikalian
opean
astern
nalaya
berian

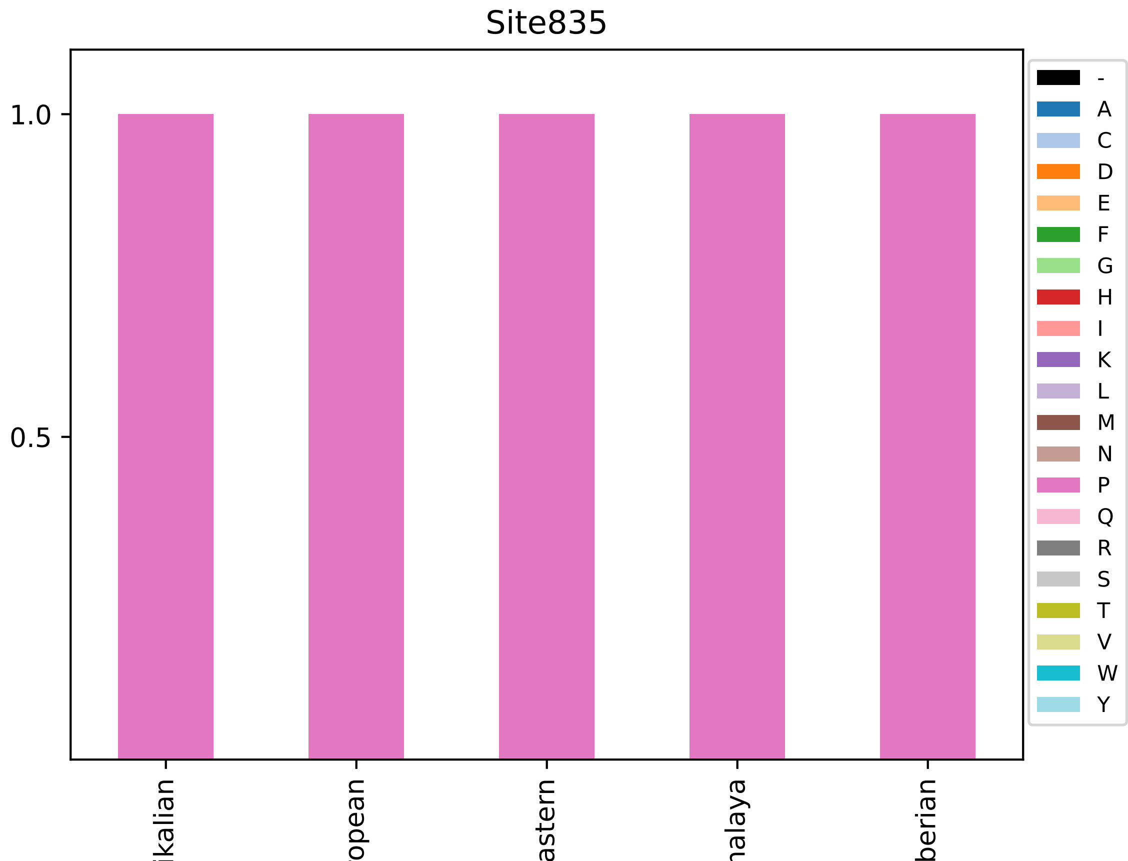

Site835
1.0
0.5
-
A
C
D
E
F
G
H
I
K
L
M
N
P
Q
R
S
T
V
W
Y

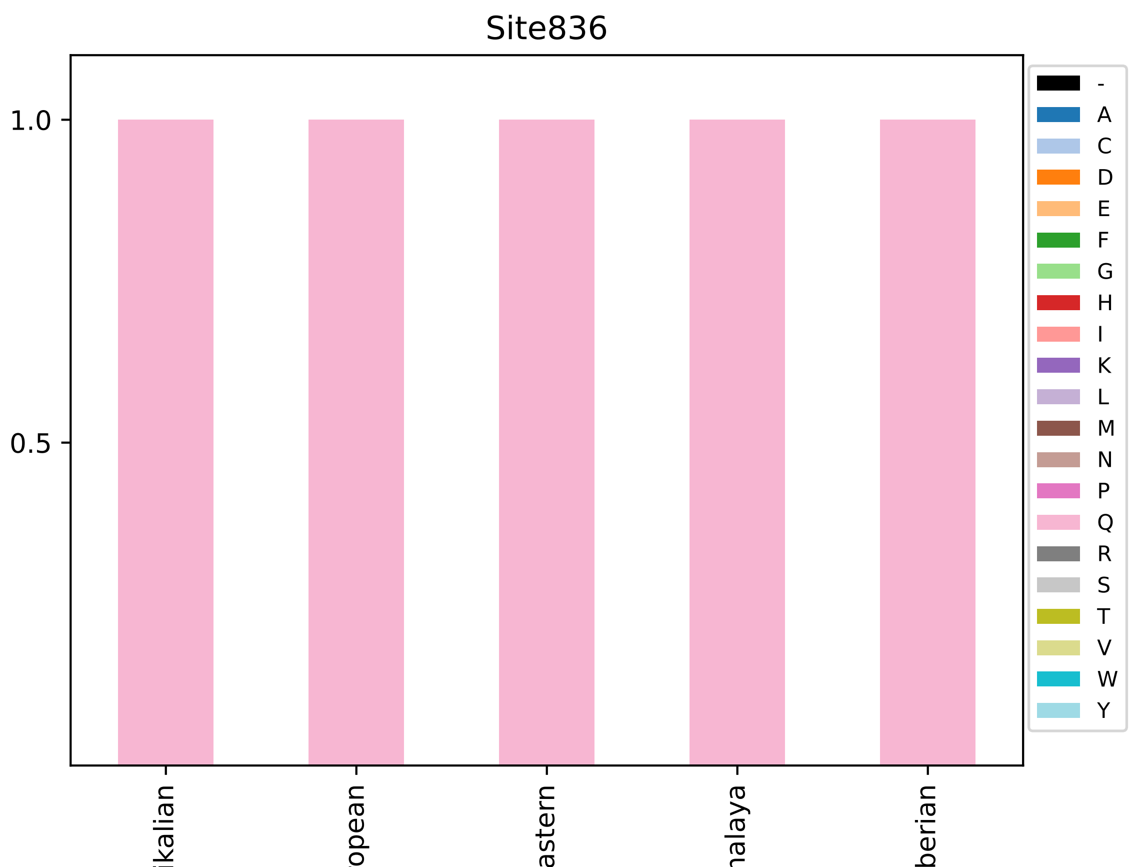
Site836
1.0
0.5
-
A
C
D
E
F
G
H
I
K
L
M
N
P
Q
R
S
T
V
W
Y
ikalian
opean
astern
nalaya
berian

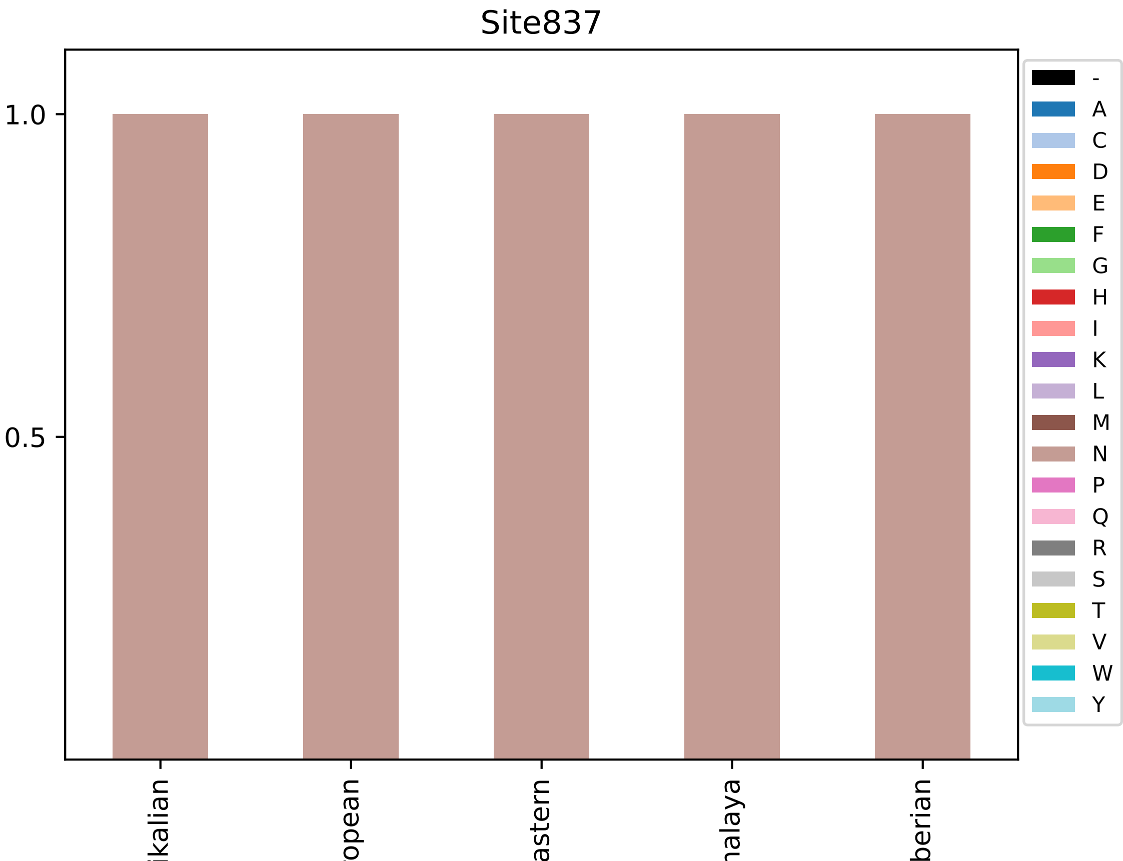
Site837
1.0
0.5
ikalian
opean
astern
nalaya
berian
-
A
C
D
E
F
G
H
I
K
L
M
N
P
Q
R
S
T
V
W
Y

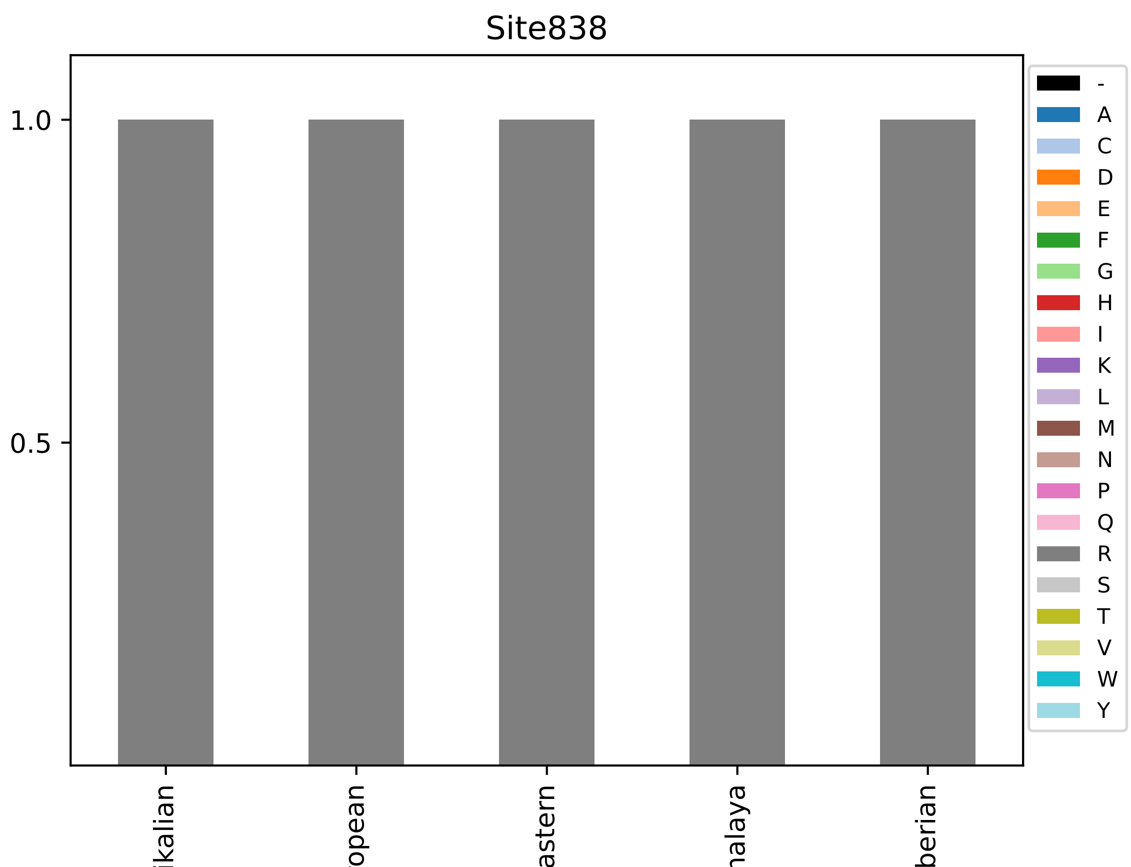

Site838
1.0
0.5
-
A
C
D
E
F
G
H
I
K
L
M
N
P
Q
R
S
T
V
W
Y

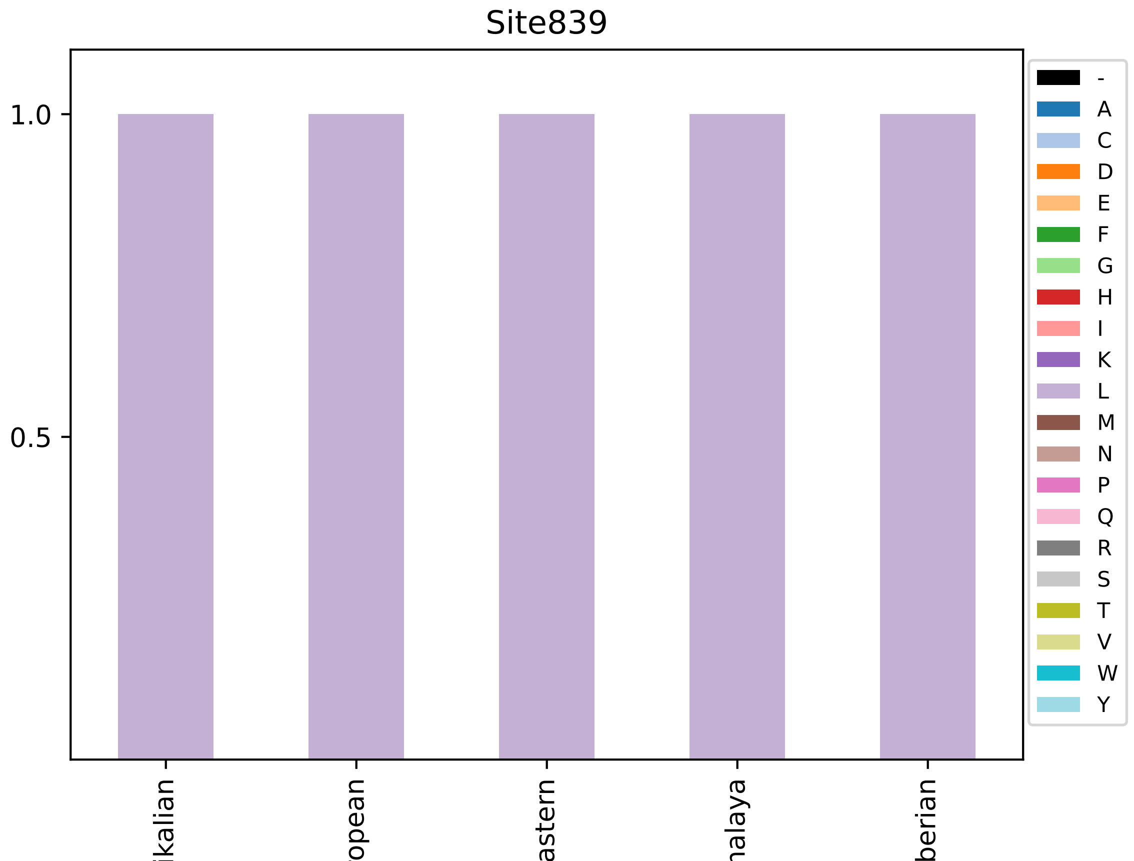
Site839
1.0
0.5
-
A
C
D
E
F
G
H
I
K
L
M
N
P
Q
R
S
T
V
W
Y
ikalian
opean
astern
nalaya
berian

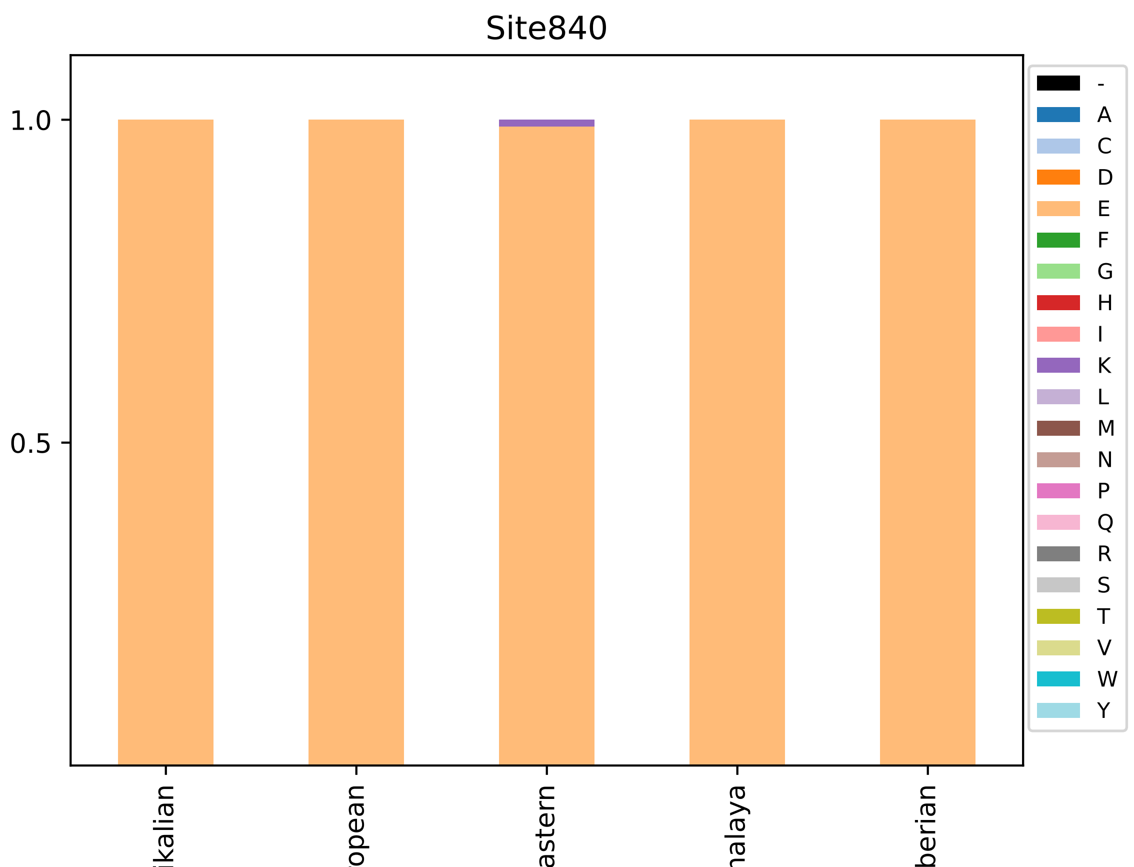
Site840
1.0
0.5
ikalian
opean
astern
nalaya
berian
-
A
C
D
E
F
G
H
I
K
L
M
N
P
Q
R
S
T
V
W
Y

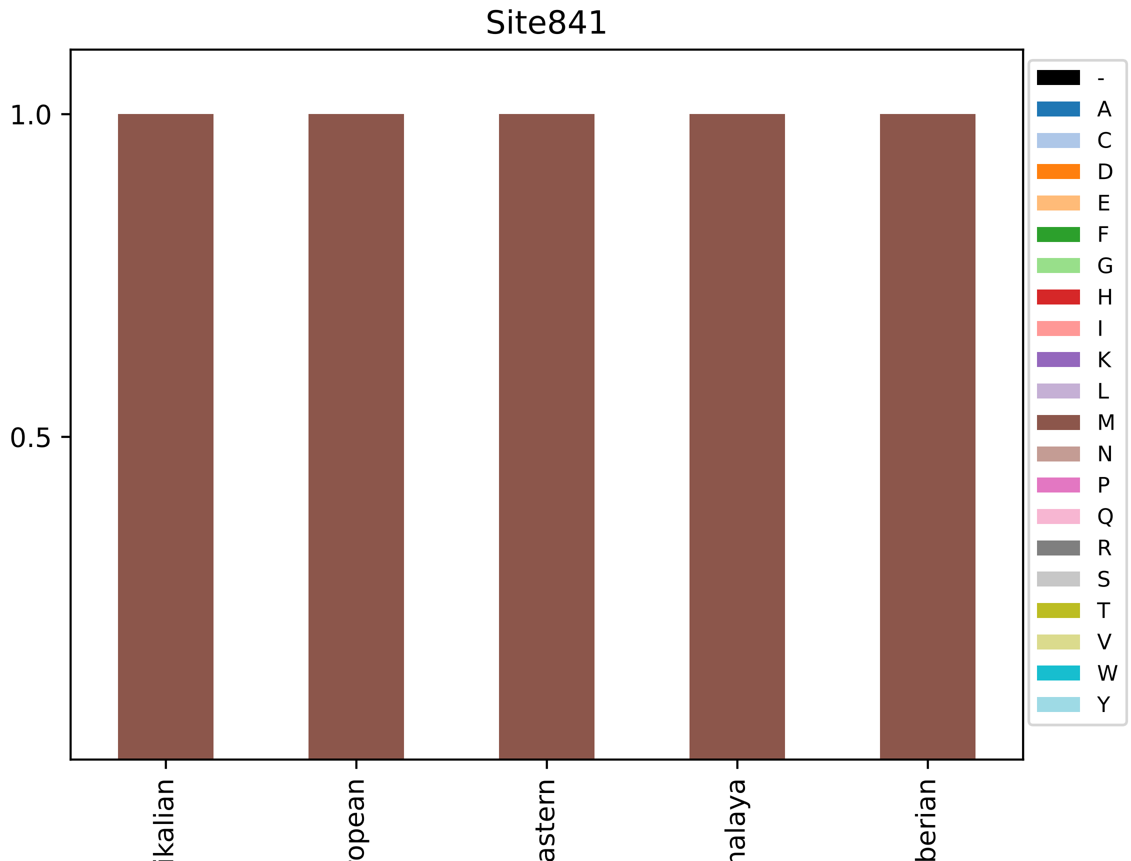
Site841
1.0
0.5
-
A
C
D
E
F
G
H
I
K
L
M
N
P
Q
R
S
T
V
W
Y
ikalian
opean
astern
nalaya
berian

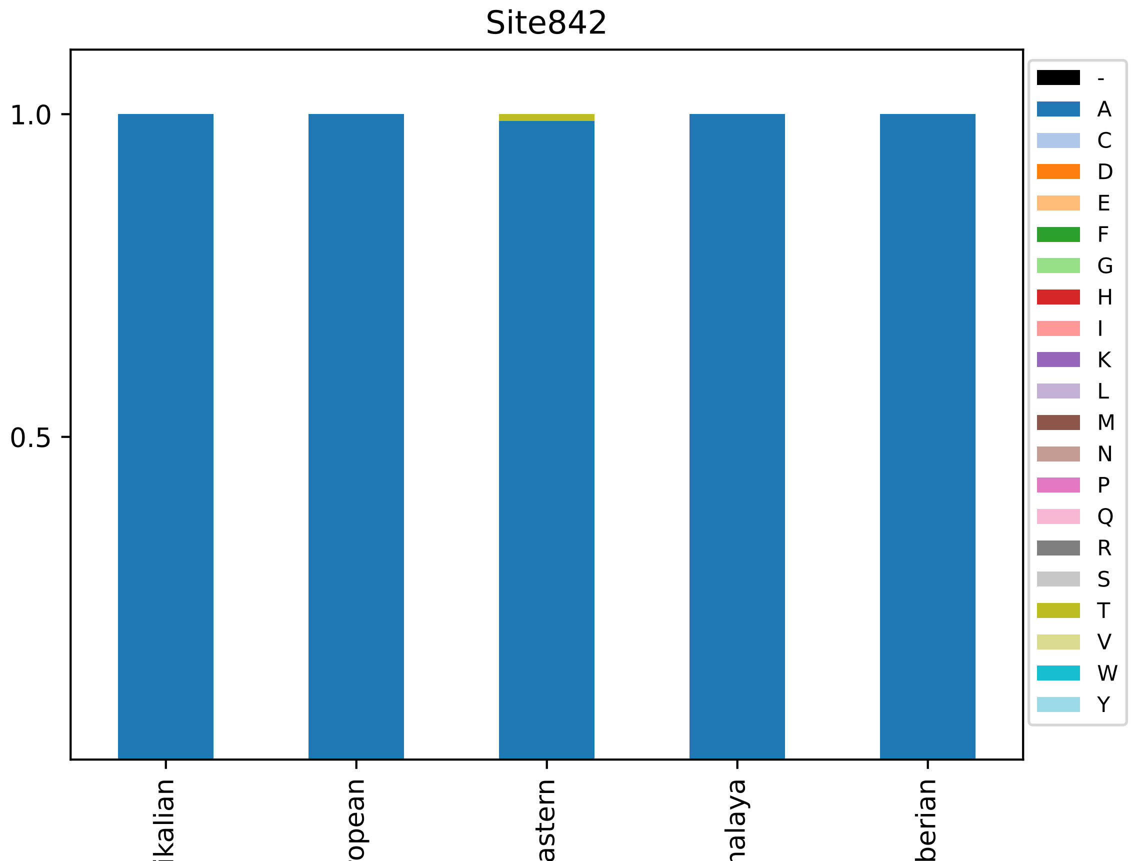
Site842
1.0
0.5
-
A
C
D
E
F
G
H
I
K
L
M
N
P
Q
R
S
T
V
W
Y
ikalian
opean
astern
nalaya
berian

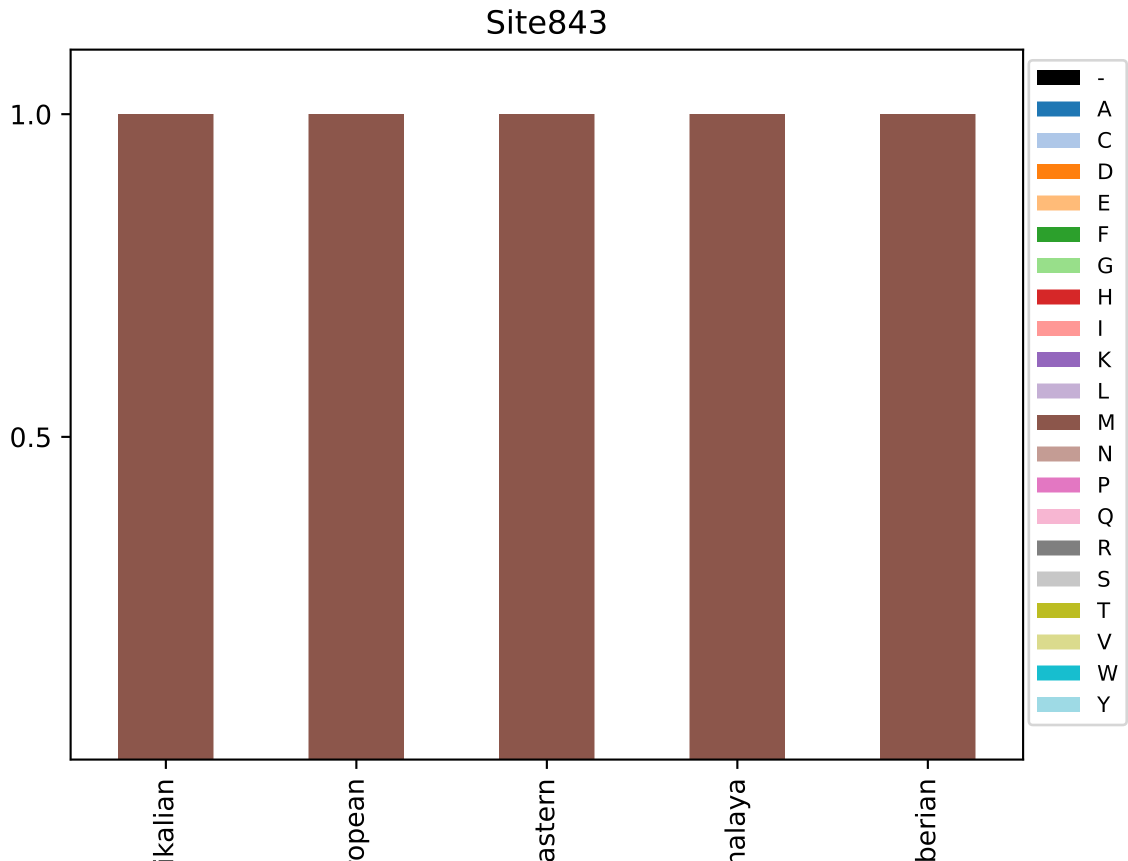

Site843
1.0
0.5
-
A
C
D
E
F
G
H
I
K
L
M
N
P
Q
R
S
T
V
W
Y
ikalian
opean
astern
nalaya
berian

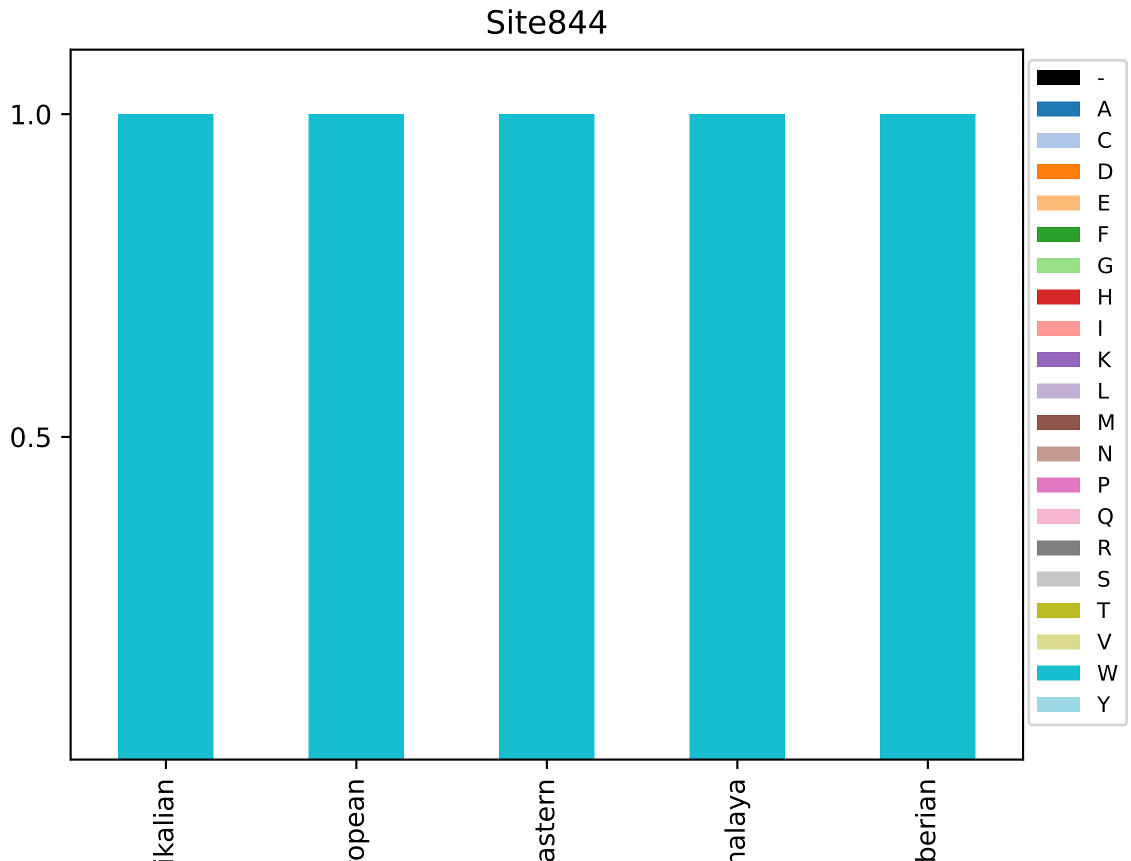

Site844
1.0
0.5
-
A
C
D
E
F
G
H
I
K
L
M
N
P
Q
R
S
T
V
W
Y
ikalian
opean
astern
nalaya
berian

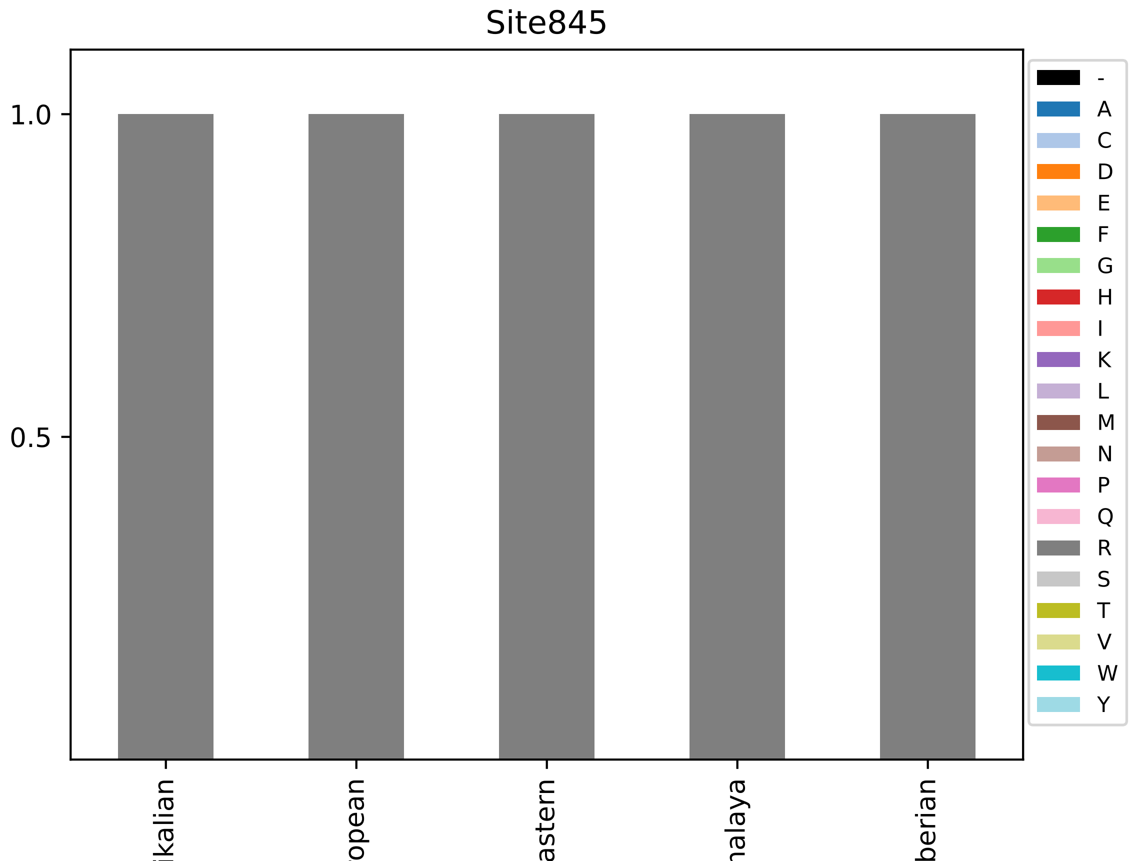

Site845
1.0
0.5
-
A
C
D
E
F
G
H
I
K
L
M
N
P
Q
R
S
T
V
W
Y
ikalian
opean
astern
nalaya
berian

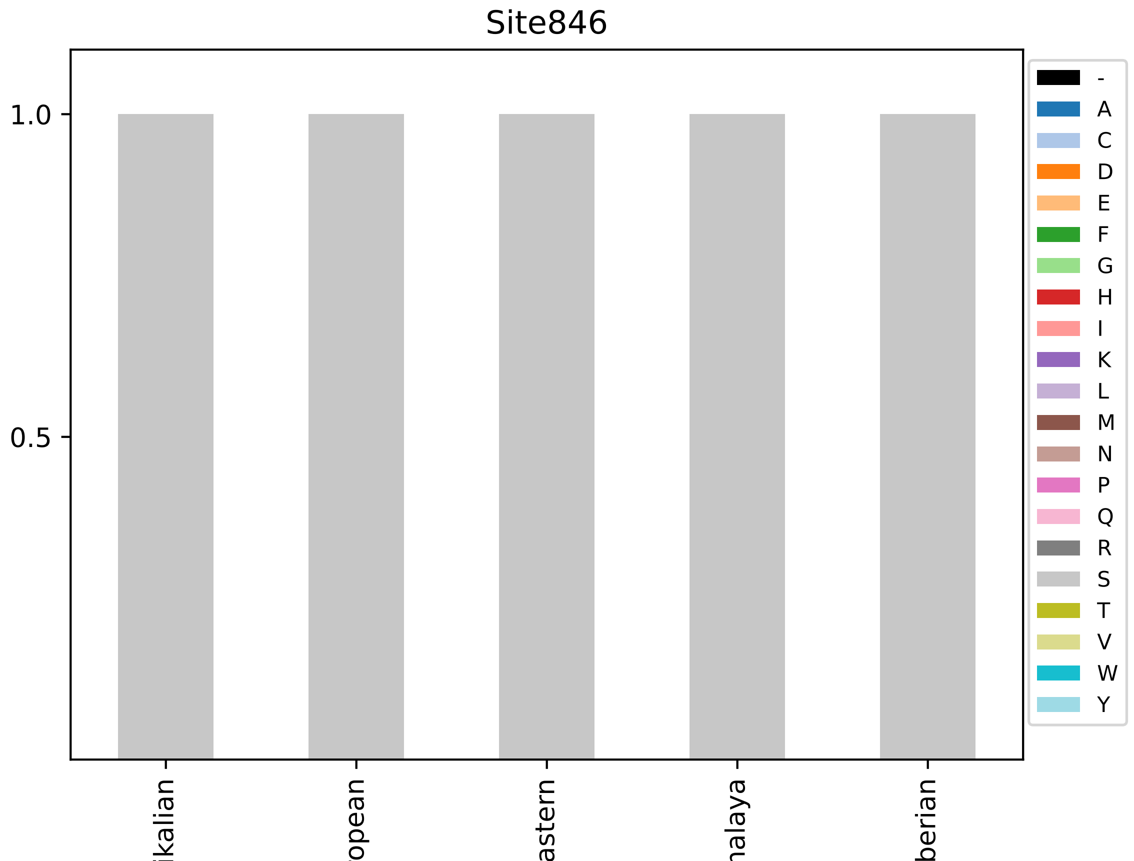

Site846
1.0
0.5
-
A
C
D
E
F
G
H
I
K
L
M
N
P
Q
R
S
T
V
W
Y
ikalian
opean
astern
nalaya
berian

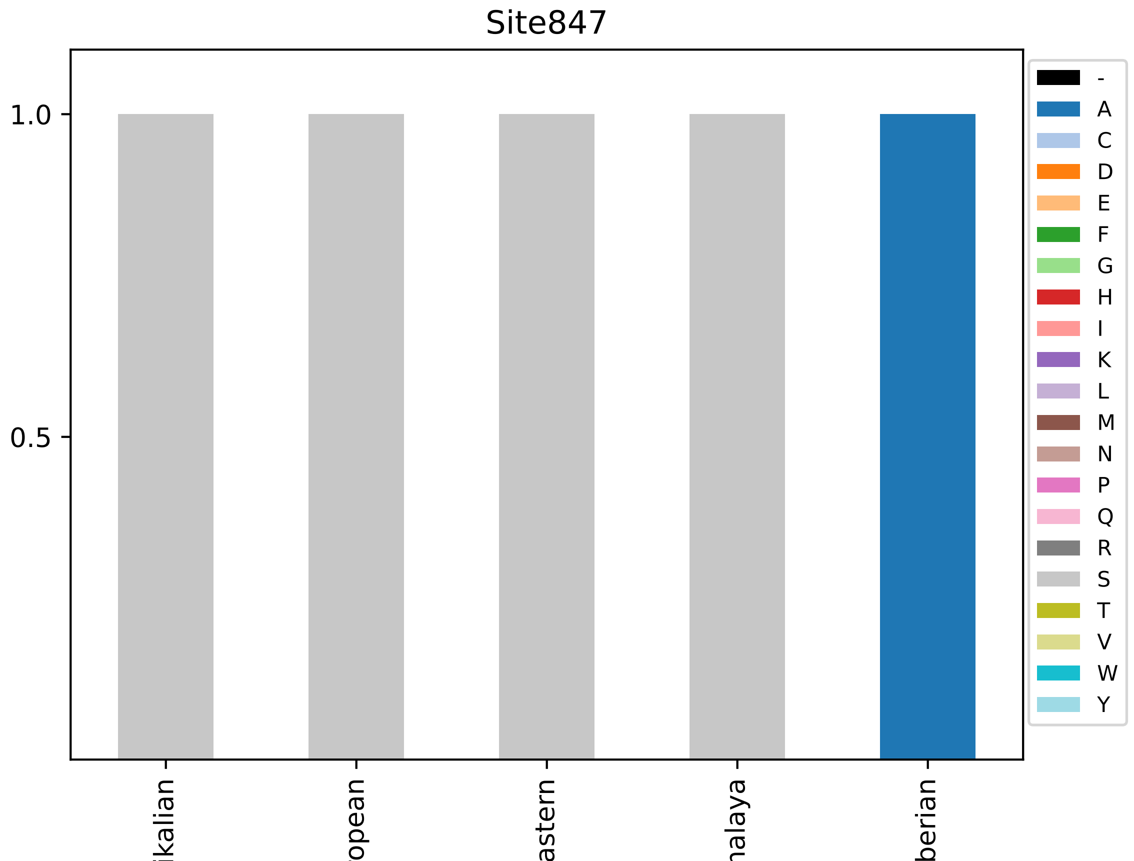
Site847
1.0
0.5
-
A
C
D
E
F
G
H
I
K
L
M
N
P
Q
R
S
T
V
W
Y
ikalian
opean
astern
nalaya
berian

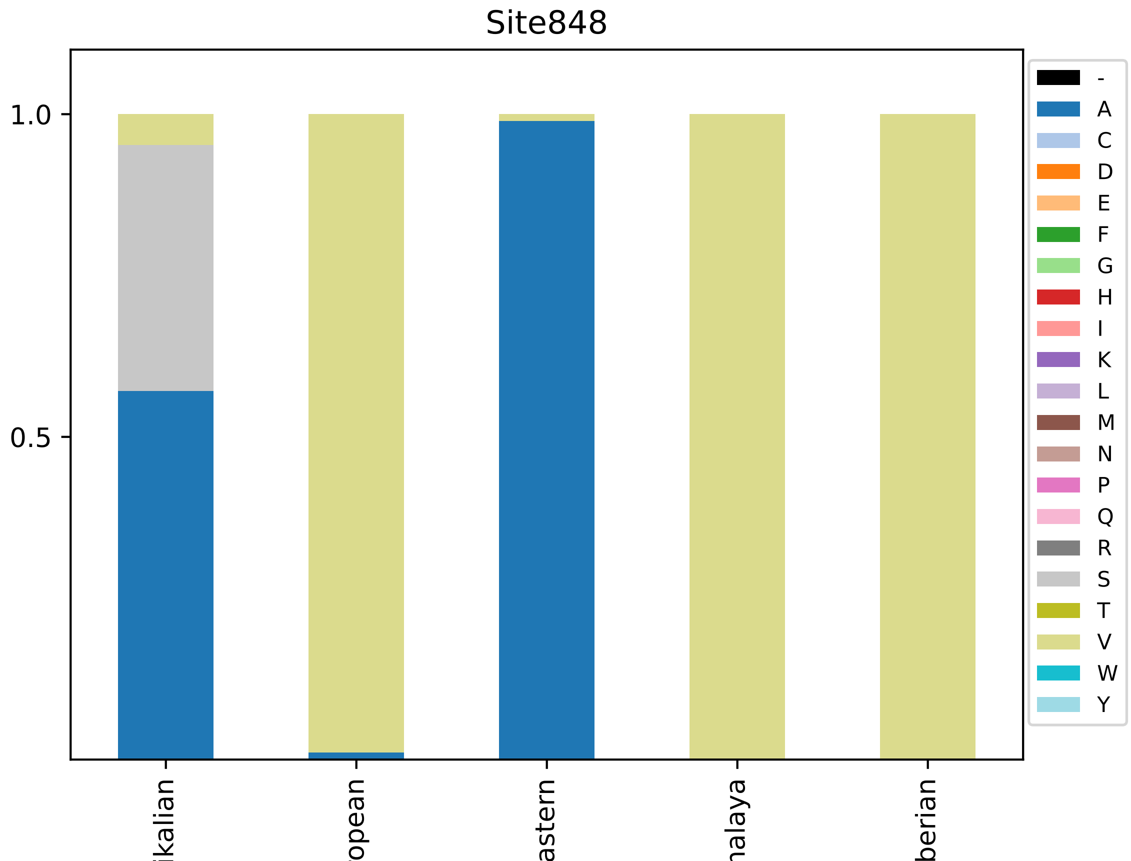
Site848
1.0
0.5
-
A
C
D
E
F
G
H
I
K
L
M
N
P
Q
R
S
T
V
W
Y
ikalian
opean
astern
nalaya
berian

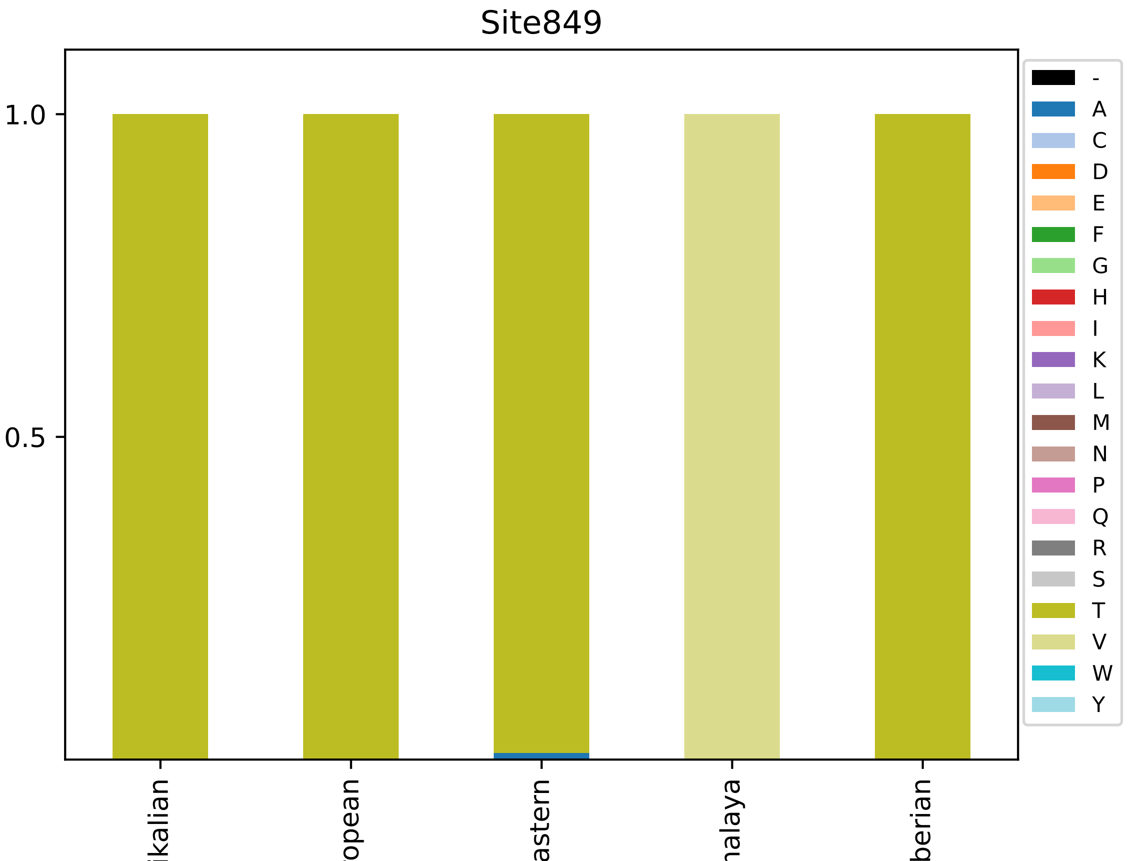

Site849
1.0
0.5
-
A
C
D
E
F
G
H
I
K
L
M
N
P
Q
R
S
T
V
W
Y
ikalian
opean
astern
nalaya
berian

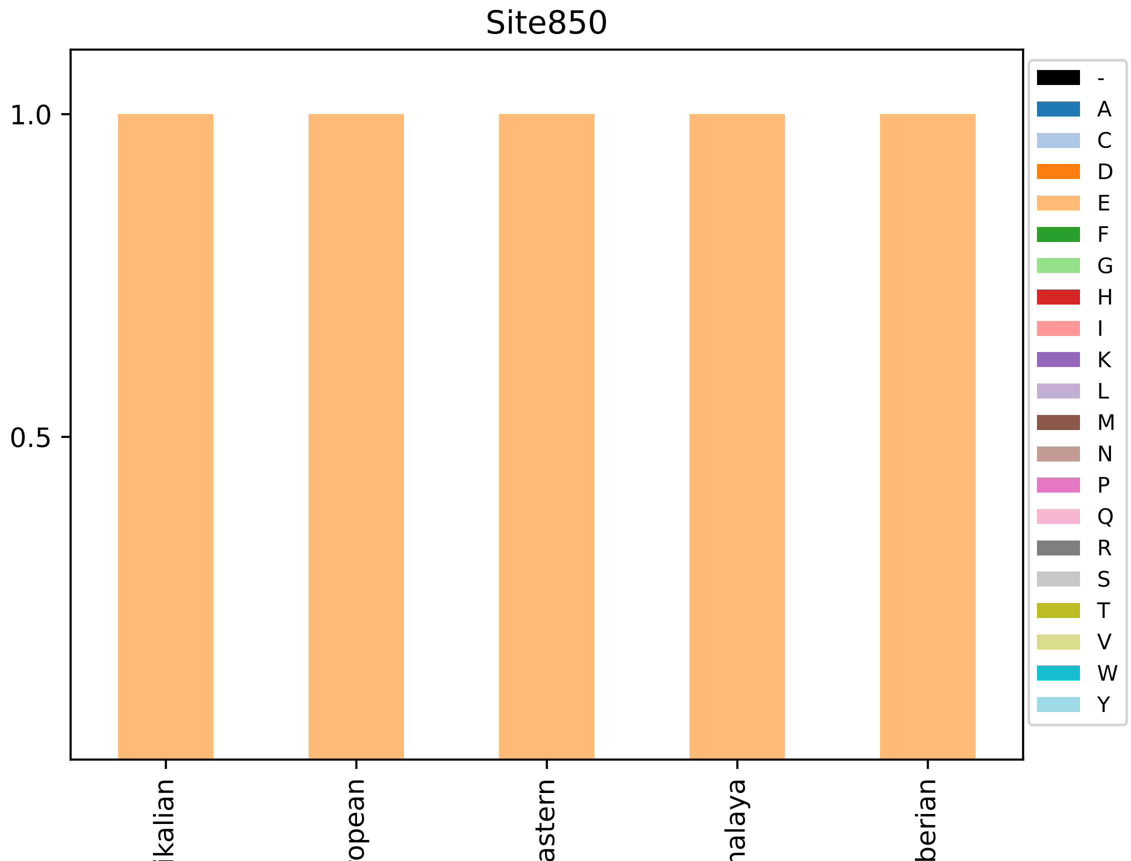

Site850
1.0
0.5
-
A
C
D
E
F
G
H
I
K
L
M
N
P
Q
R
S
T
V
W
Y
ikalian
opean
astern
nalaya
berian

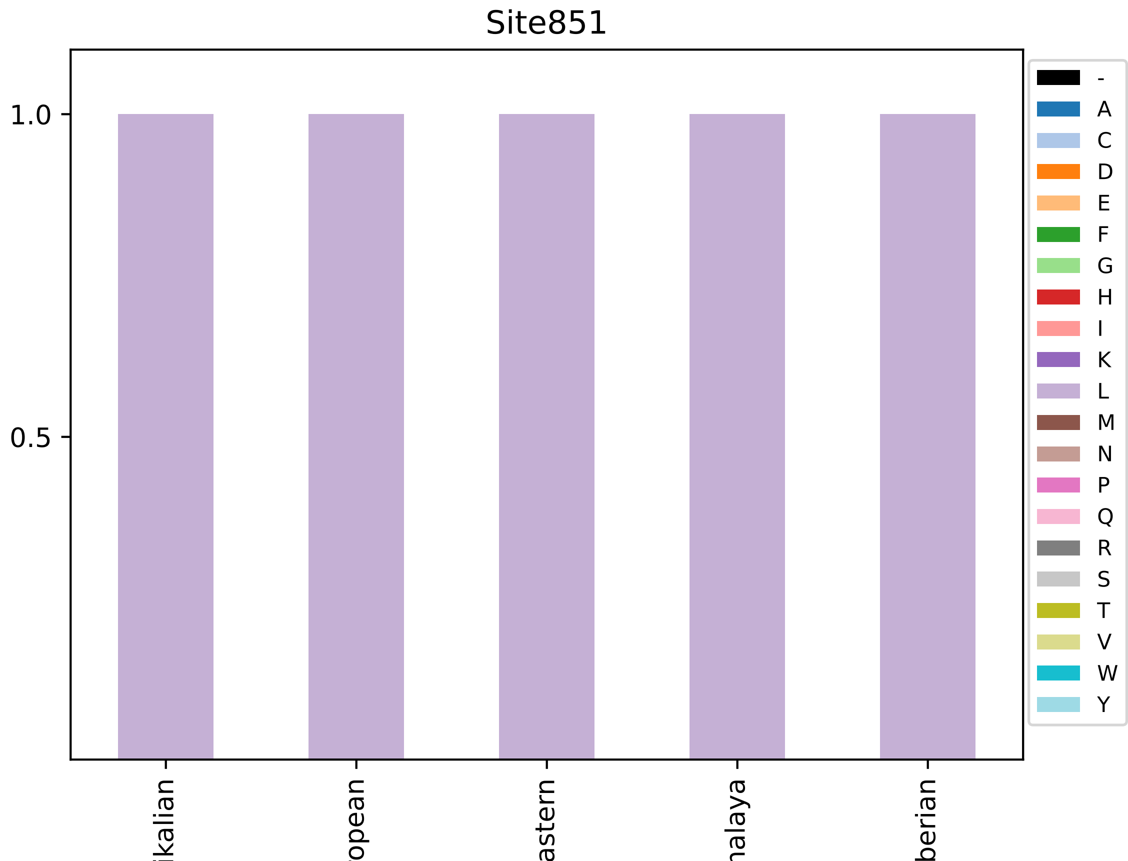

Site851
1.0
0.5
-
A
C
D
E
F
G
H
I
K
L
M
N
P
Q
R
S
T
V
W
Y
ikalian
opean
astern
nalaya
berian

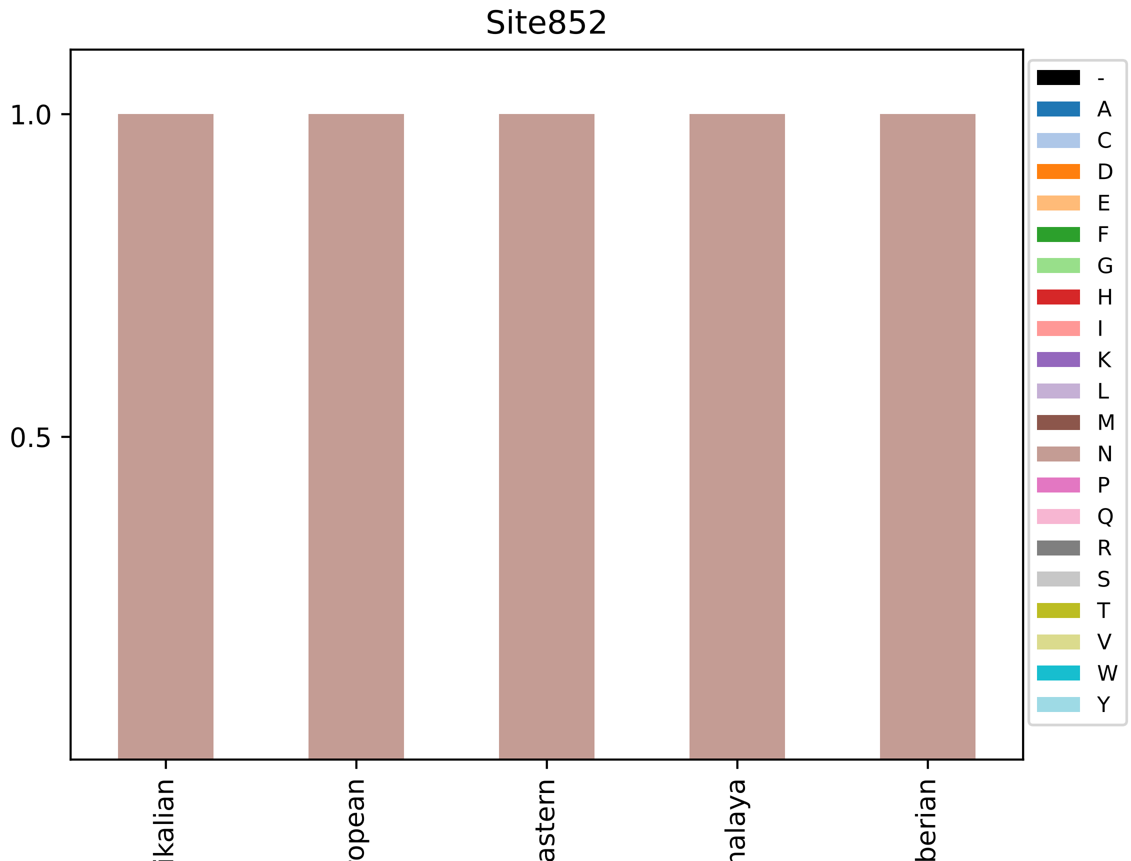

Site852
1.0
0.5
-
A
C
D
E
F
G
H
I
K
L
M
N
P
Q
R
S
T
V
W
Y
ikalian
opean
astern
nalaya
berian

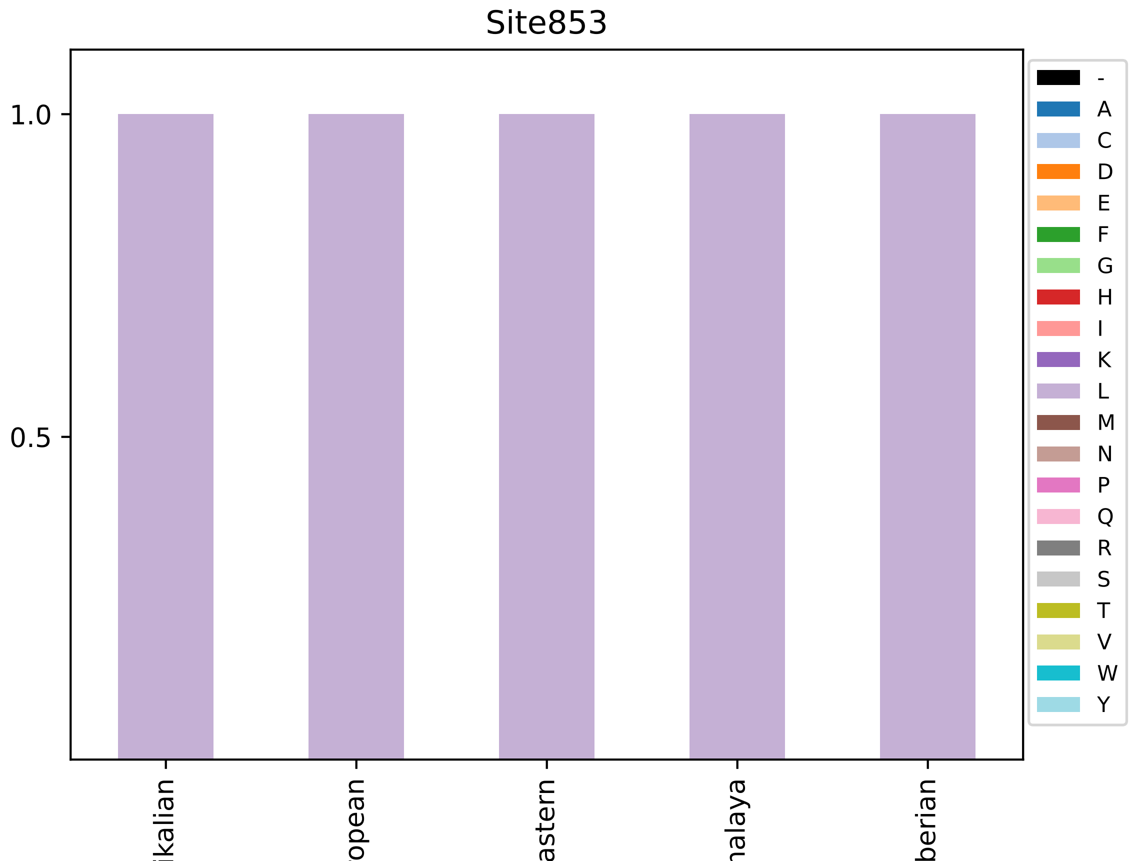

Site853
1.0
0.5
-
A
C
D
E
F
G
H
I
K
L
M
N
P
Q
R
S
T
V
W
Y
ikalian
opean
astern
halaya
berian

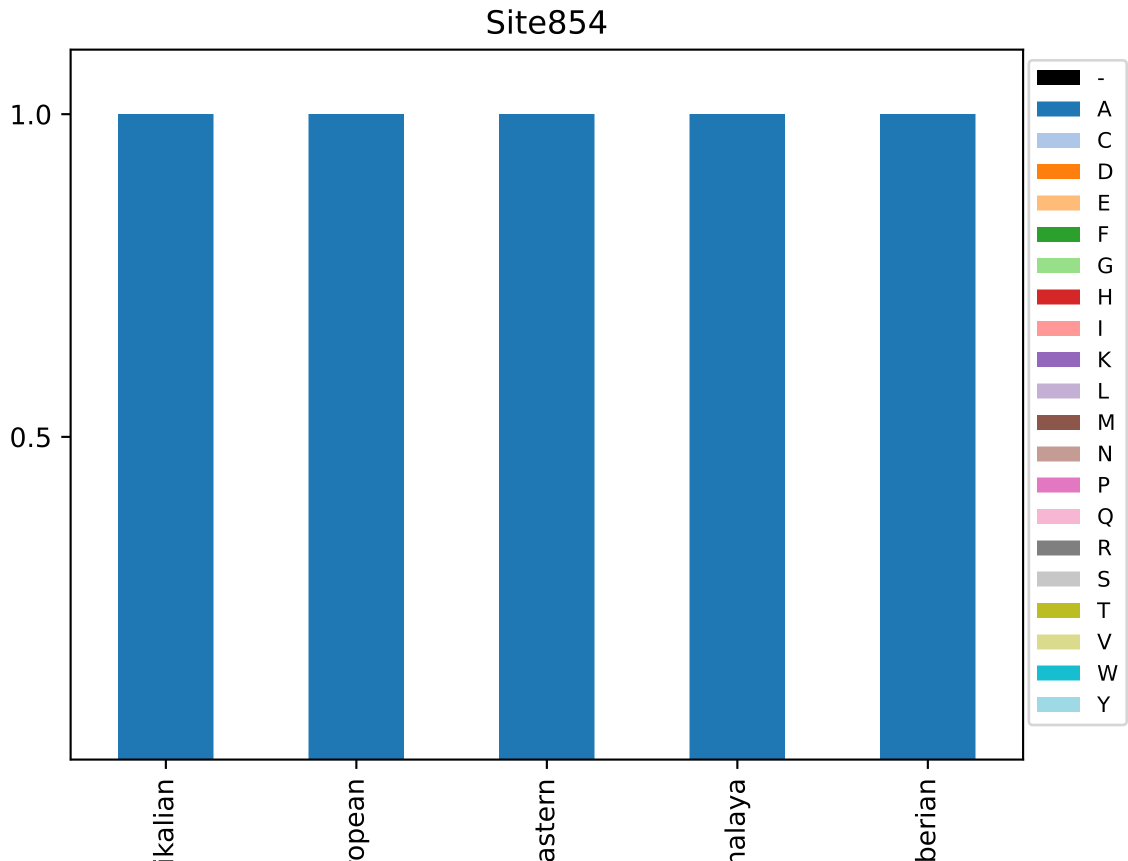

Site854
1.0
0.5
-
A
C
D
E
F
G
H
I
K
L
M
N
P
Q
R
S
T
V
W
Y

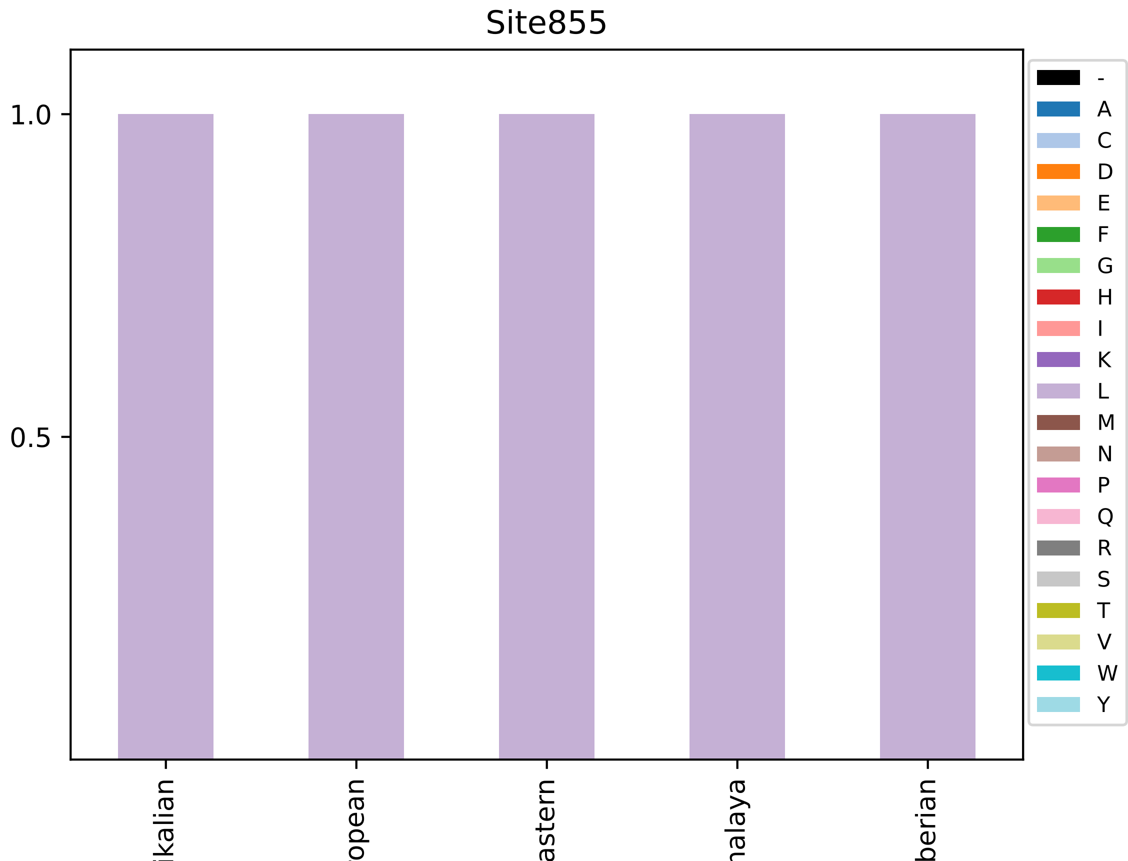

Site855
1.0
0.5
ikalian
opean
astern
nalaya
berian
-
A
C
D
E
F
G
H
I
K
L
M
N
P
Q
R
S
T
V
W
Y

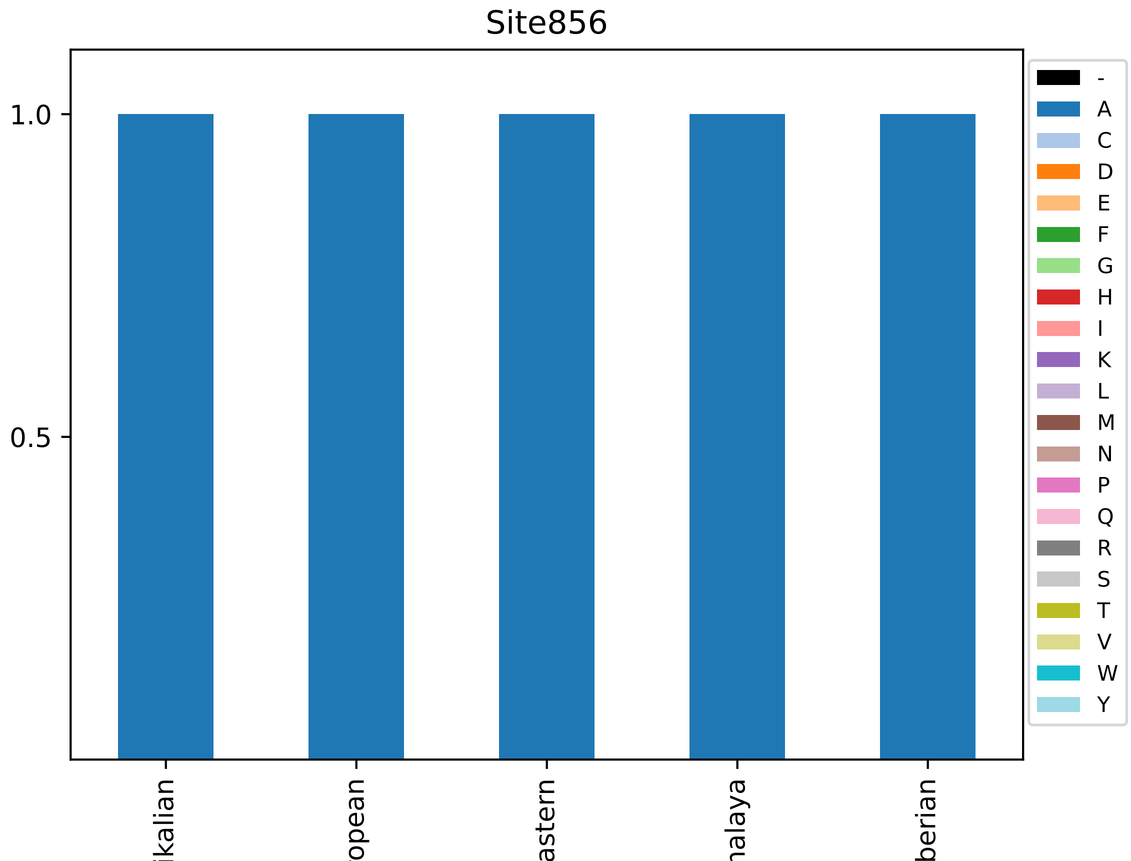
Site856
1.0
0.5
-
A
C
D
E
F
G
H
I
K
L
M
N
P
Q
R
S
T
V
W
Y
ikalian
opean
astern
nalaya
berian

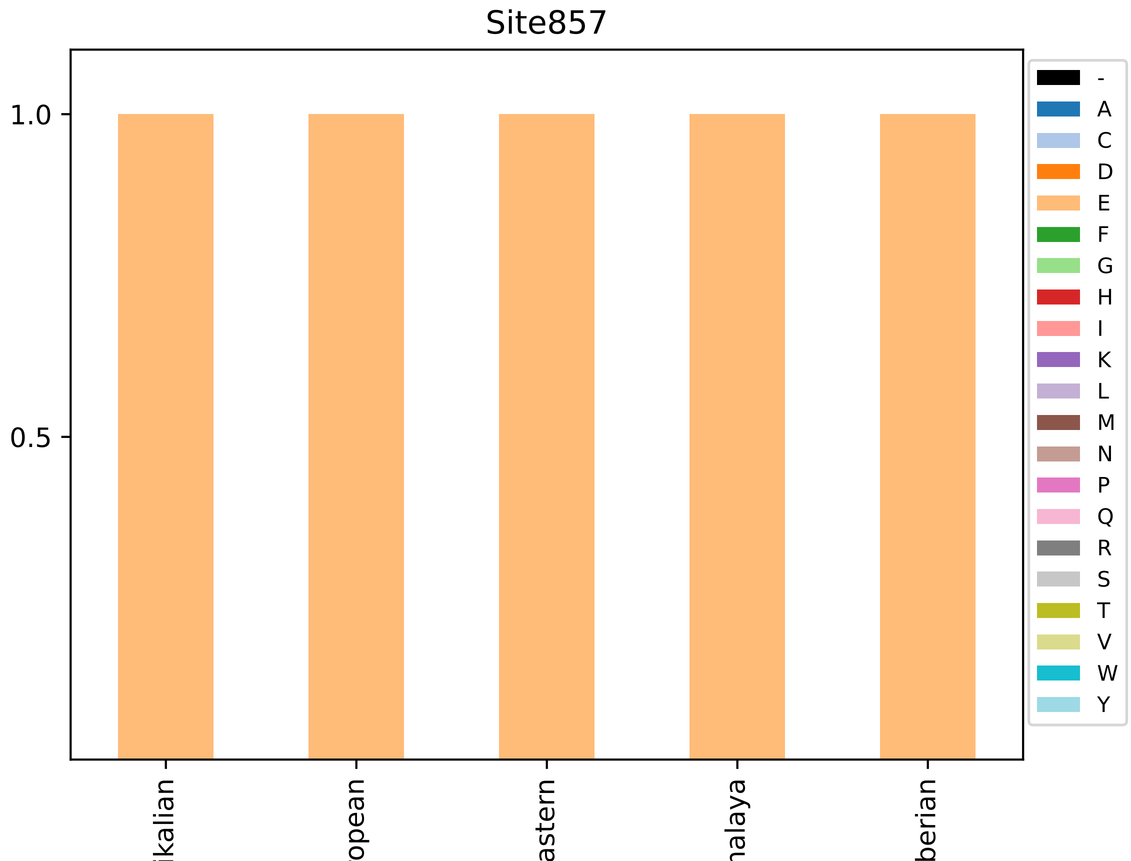

Site857
1.0
0.5
ikalian
opean
astern
nalaya
berian
-
A
C
D
E
F
G
H
I
K
L
M
N
P
Q
R
S
T
V
W
Y

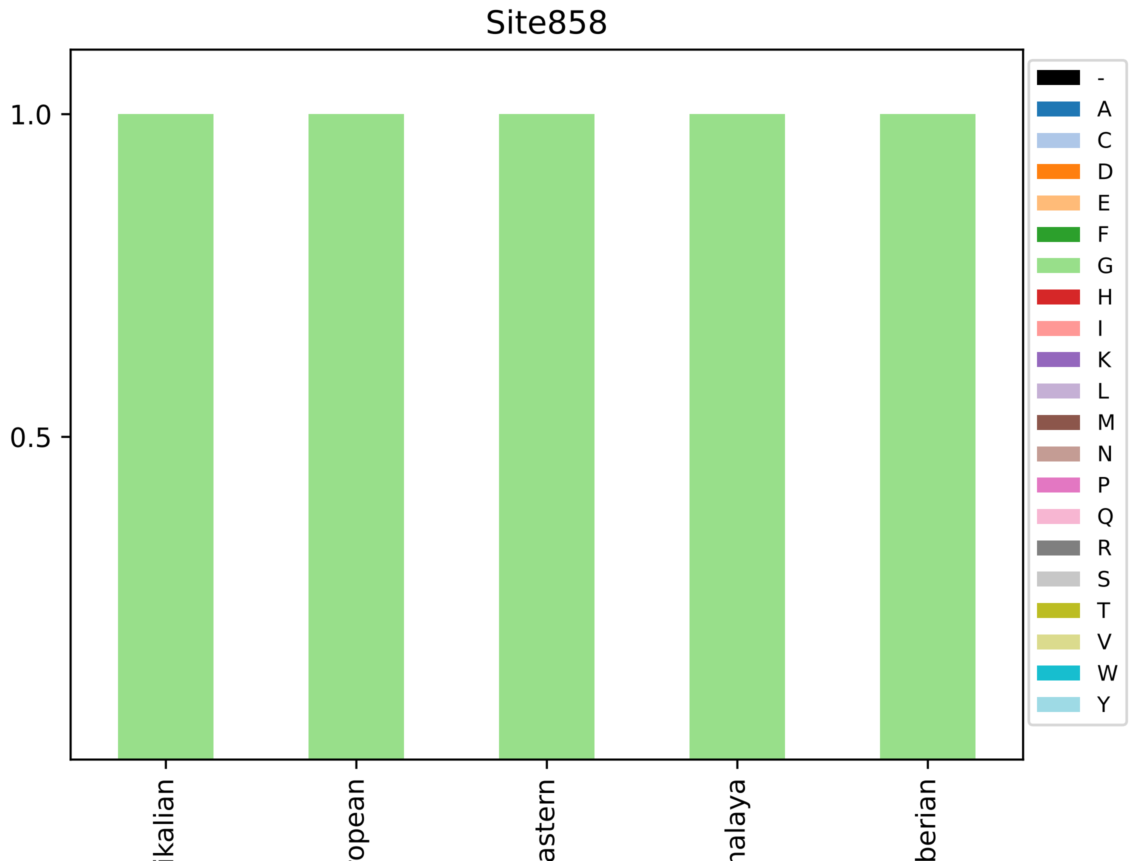
Site858
1.0
0.5
ikalian
opean
astern
nalaya
berian
-
A
C
D
E
F
G
H
I
K
L
M
N
P
Q
R
S
T
V
W
Y

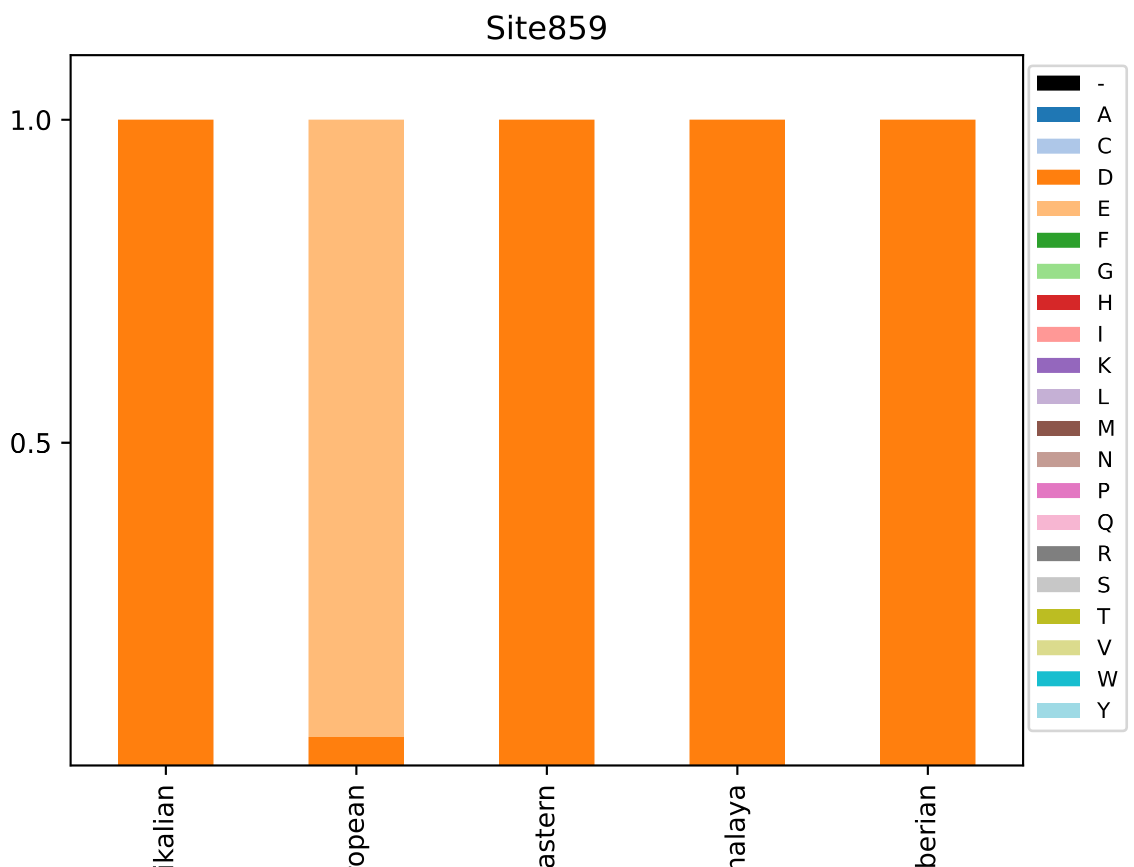

Site859
1.0
0.5
-
A
C
D
E
F
G
H
I
K
L
M
N
P
Q
R
S
T
V
W
Y
ikalian
opean
astern
nalaya
berian

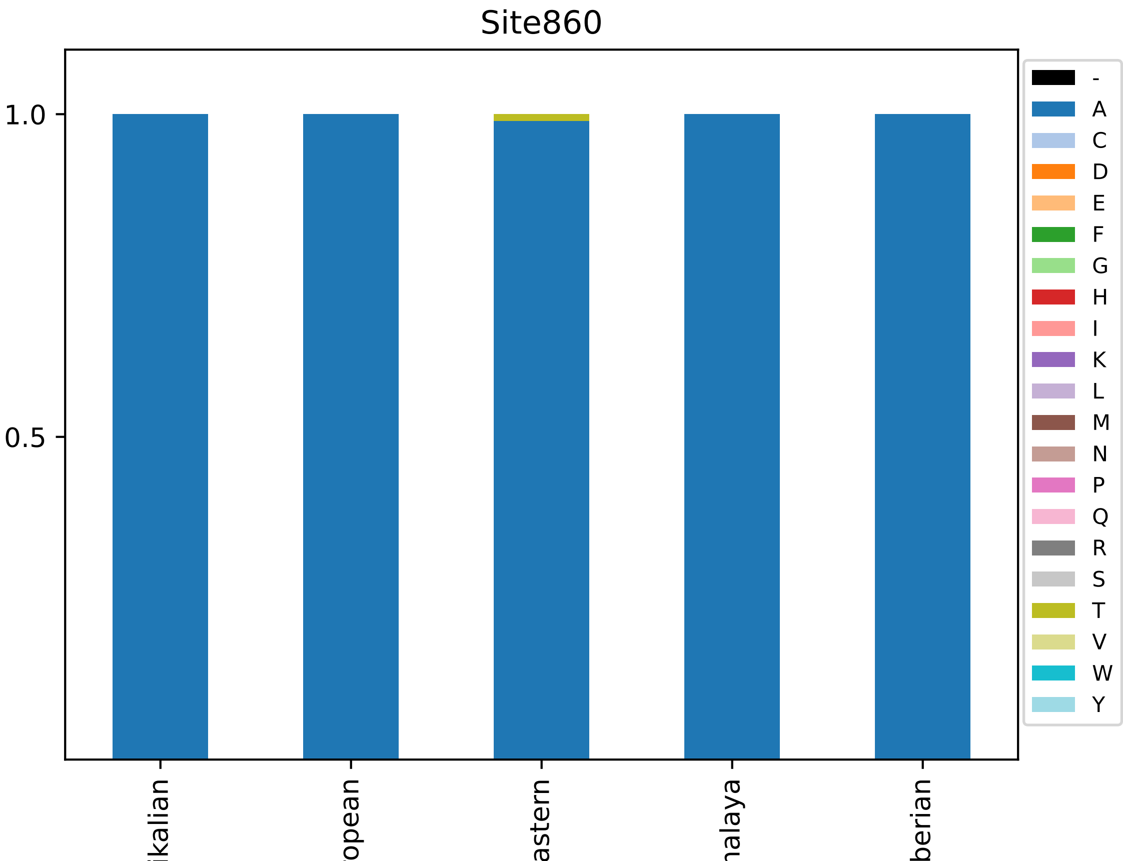

Site860
1.0
0.5
-
A
C
D
E
F
G
H
I
K
L
M
N
P
Q
R
S
T
V
W
Y
ikalian
opean
astern
nalaya
berian

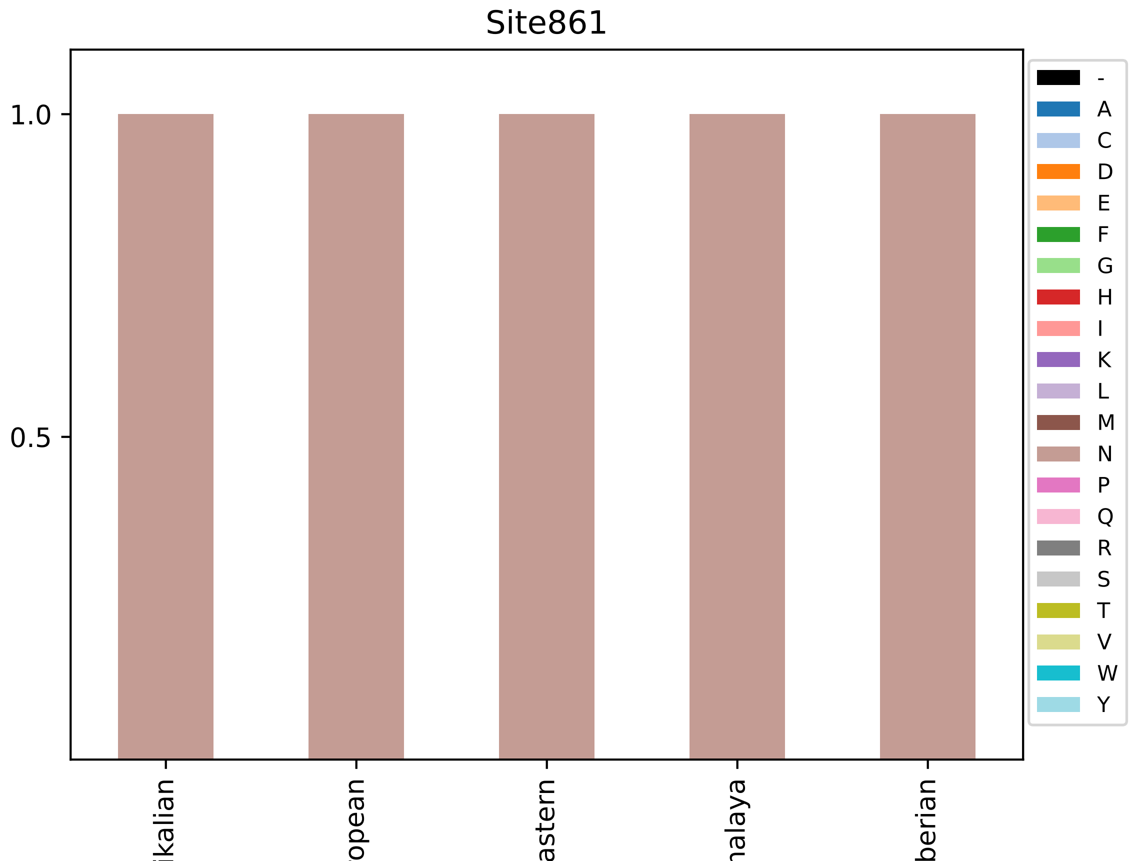
Site861
1.0
0.5
-
A
C
D
E
F
G
H
I
K
L
M
N
P
Q
R
S
T
V
W
Y
ikalian
opean
astern
nalaya
berian

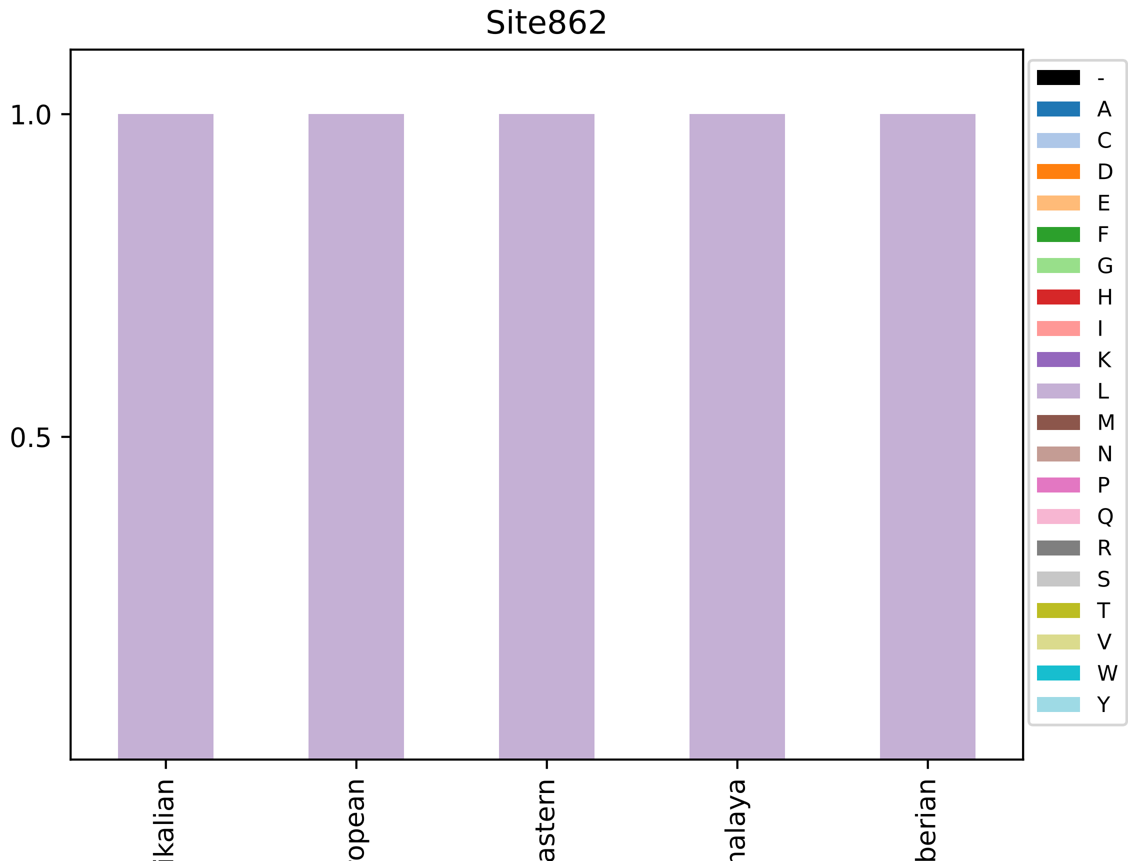

Site862
1.0
0.5
ikalian
opean
astern
nalaya
berian
-
A
C
D
E
F
G
H
I
K
L
M
N
P
Q
R
S
T
V
W
Y

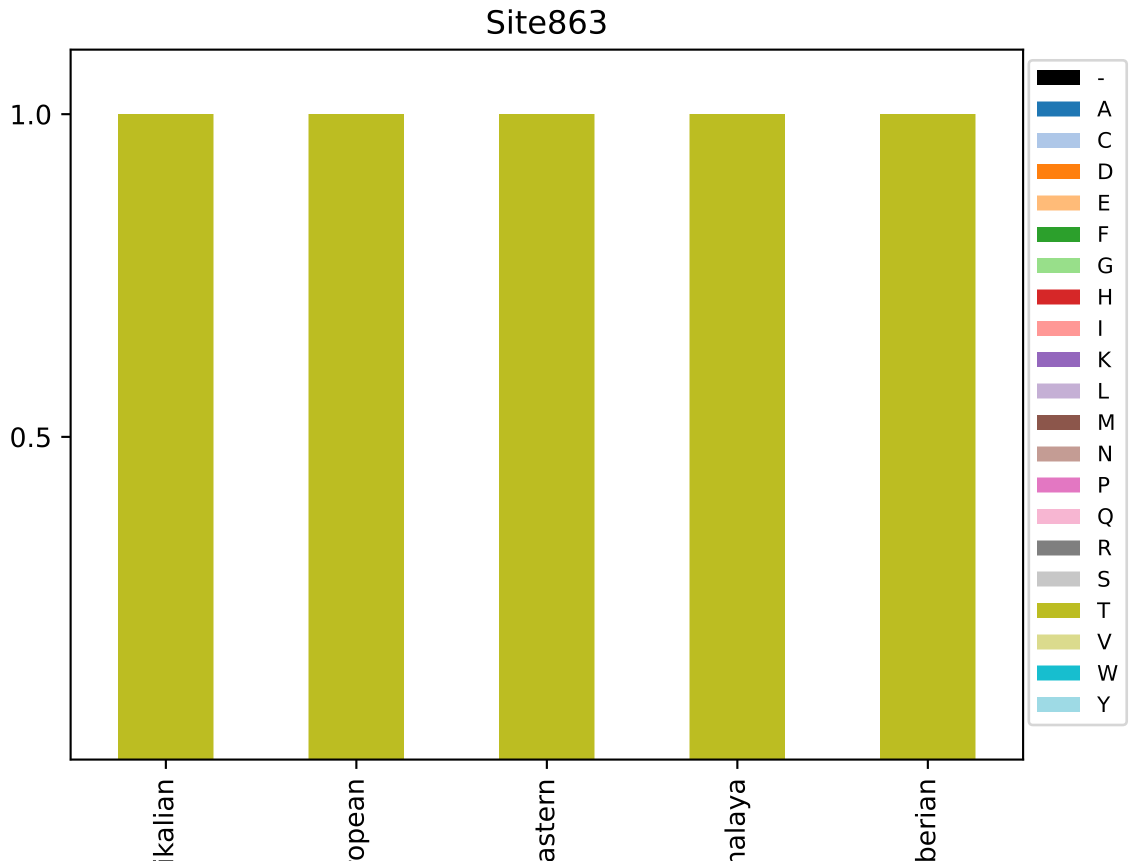
Site863
1.0
0.5
-
A
C
D
E
F
G
H
I
K
L
M
N
P
Q
R
S
T
V
W
Y
ikalian
opean
astern
nalaya
berian

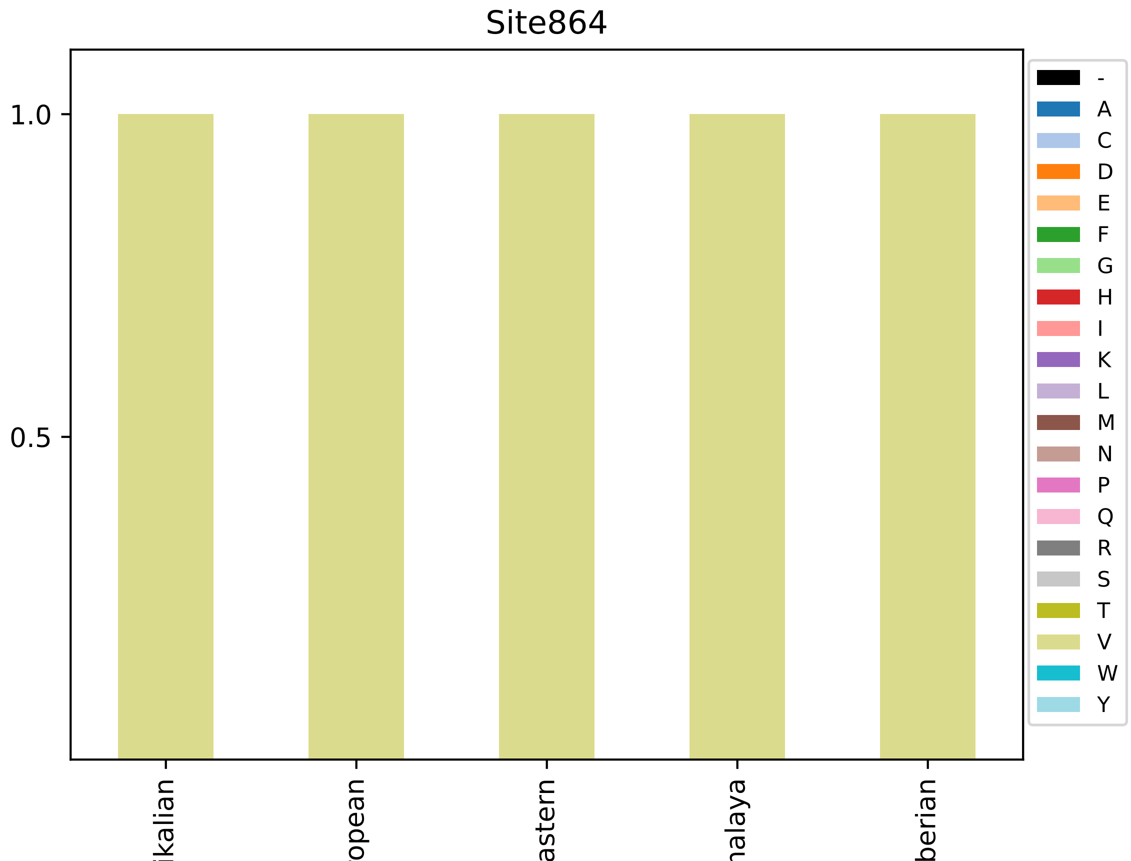
Site864
1.0
0.5
ikalian
opean
astern
nalaya
berian
-
A
C
D
E
F
G
H
I
K
L
M
N
P
Q
R
S
T
V
W
Y

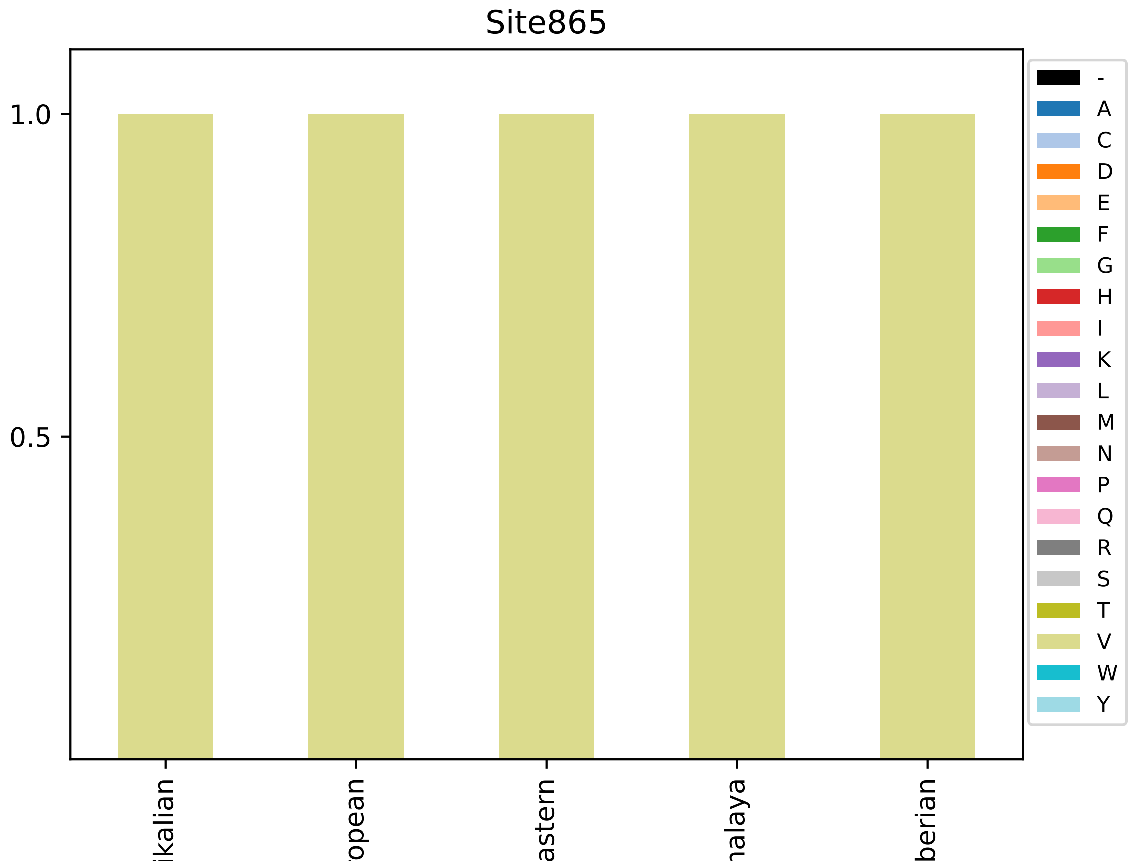
Site865
1.0
0.5
-
A
C
D
E
F
G
H
I
K
L
M
N
P
Q
R
S
T
V
W
Y
ikalian
opean
astern
nalaya
berian

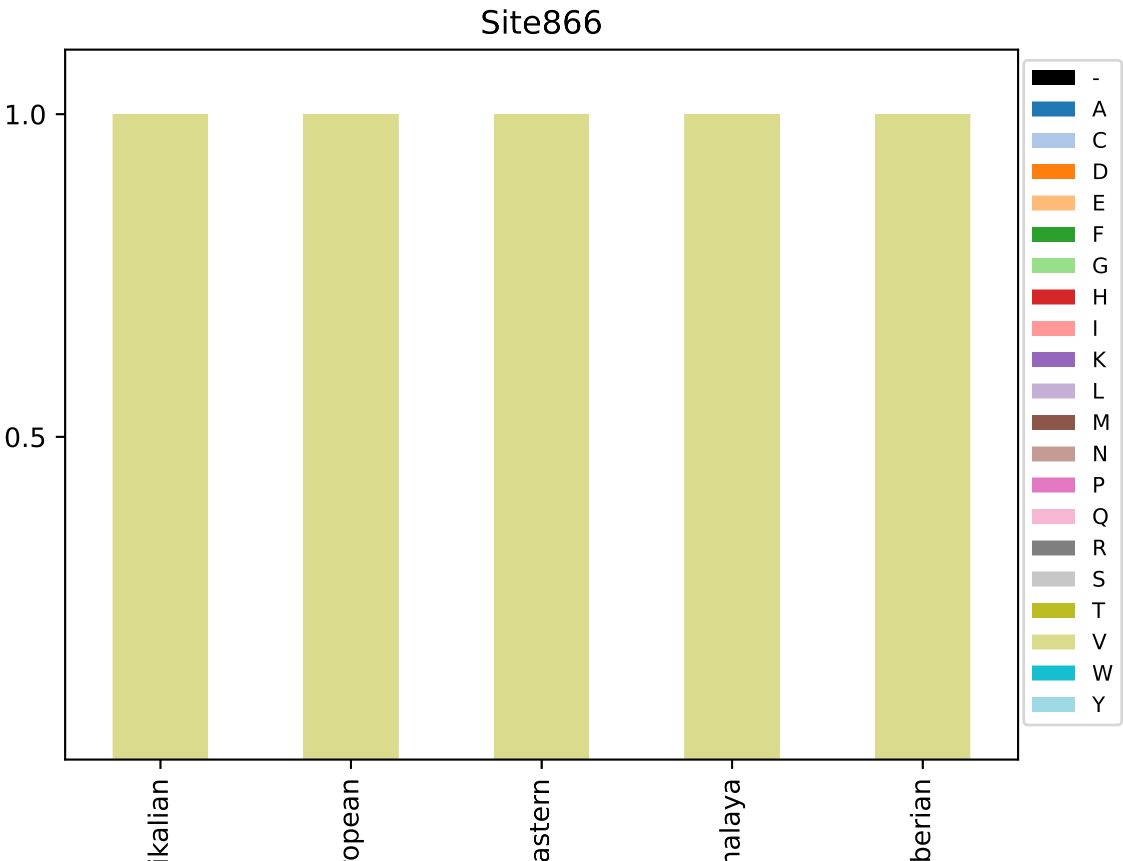
Site866
1.0
0.5
-
A
C
D
E
F
G
H
I
K
L
M
N
P
Q
R
S
T
V
W
Y
ikalian
opean
astern
nalaya
berian

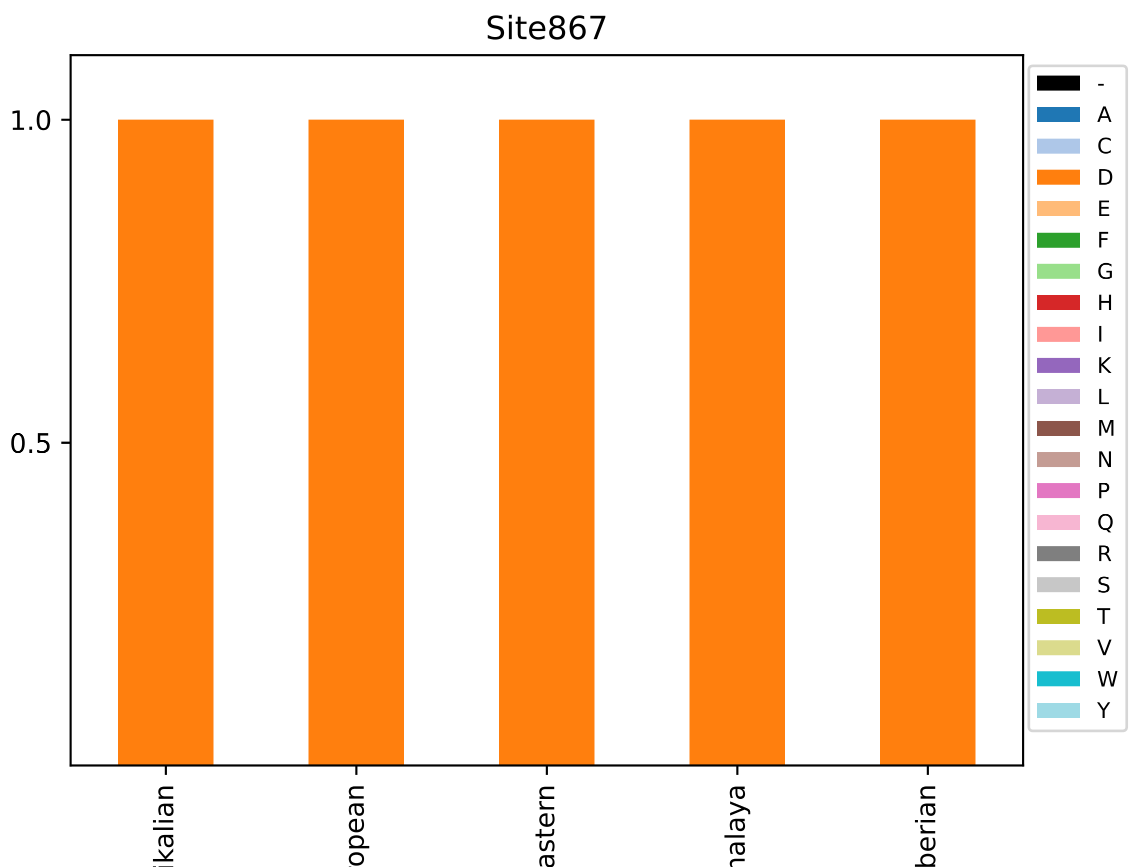
Site867
1.0
0.5
ikalian
opean
astern
nalaya
berian
-
A
C
D
E
F
G
H
I
K
L
M
N
P
Q
R
S
T
V
W
Y

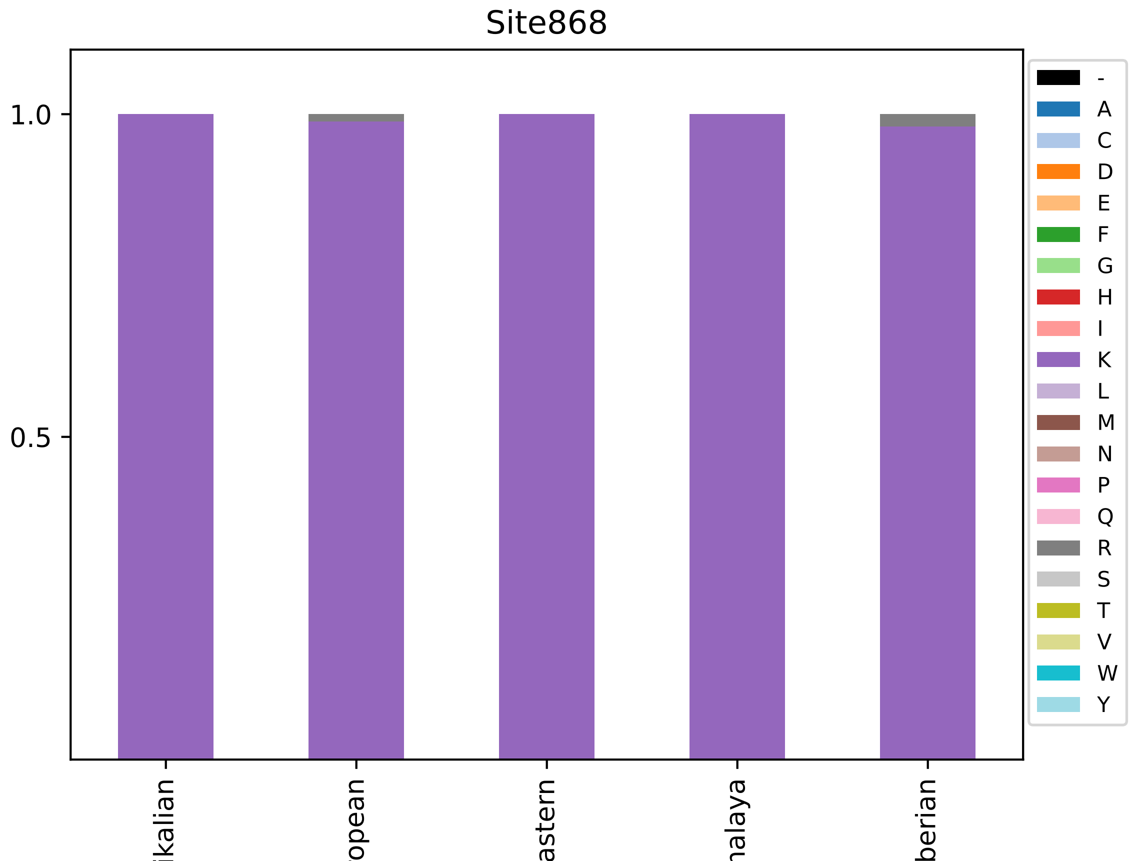

Site868
1.0
0.5
ikalian
opean
astern
nalaya
berian
-
A
C
D
E
F
G
H
I
K
L
M
N
P
Q
R
S
T
V
W
Y

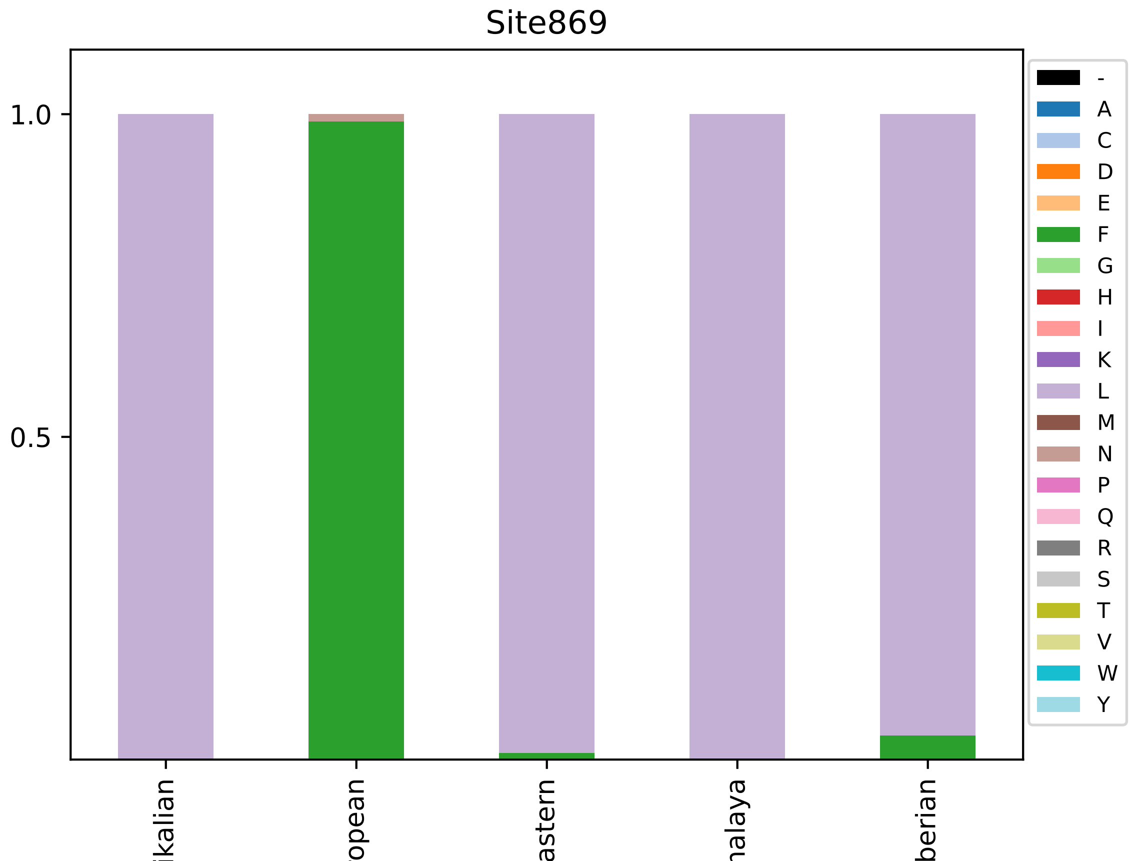

Site869
1.0
0.5
-
A
C
D
E
F
G
H
I
K
L
M
N
P
Q
R
S
T
V
W
Y
ikalian
opean
astern
nalaya
berian

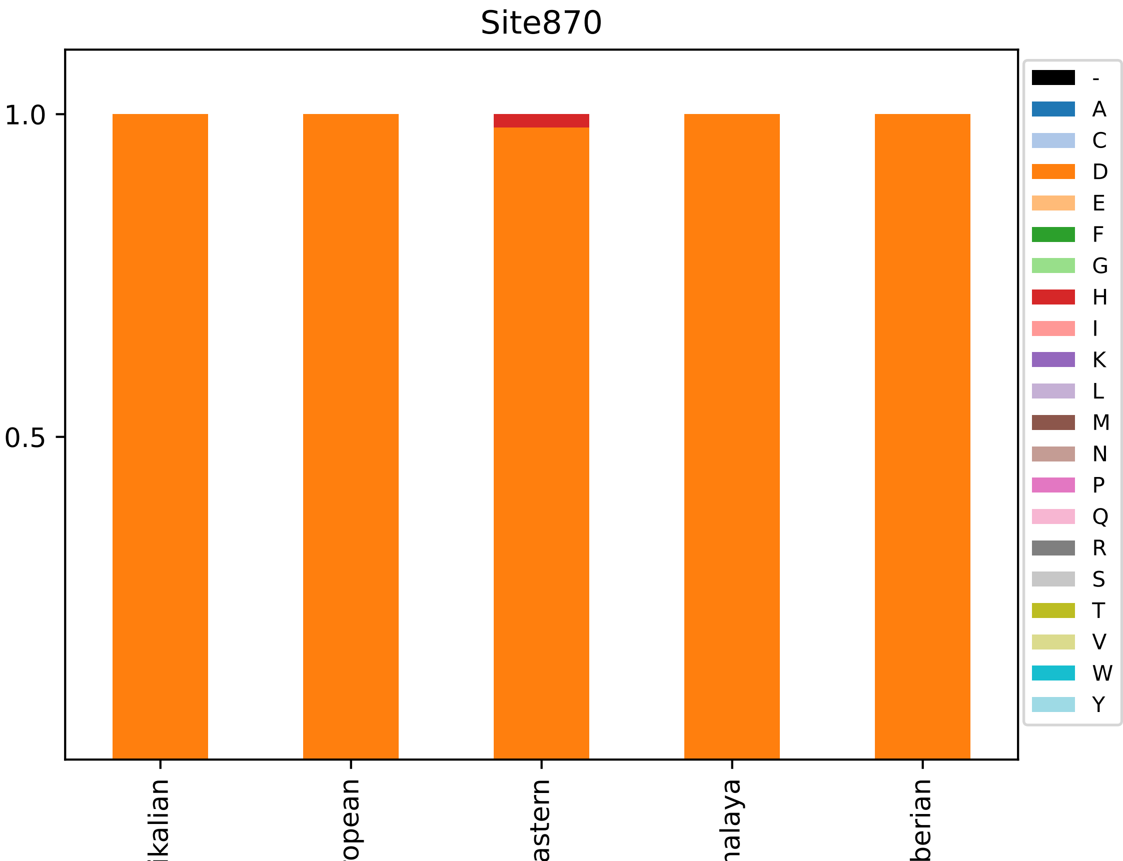
Site870
1.0
0.5
ikalian
opean
astern
nalaya
berian
-
A
C
D
E
F
G
H
I
K
L
M
N
P
Q
R
S
T
V
W
Y

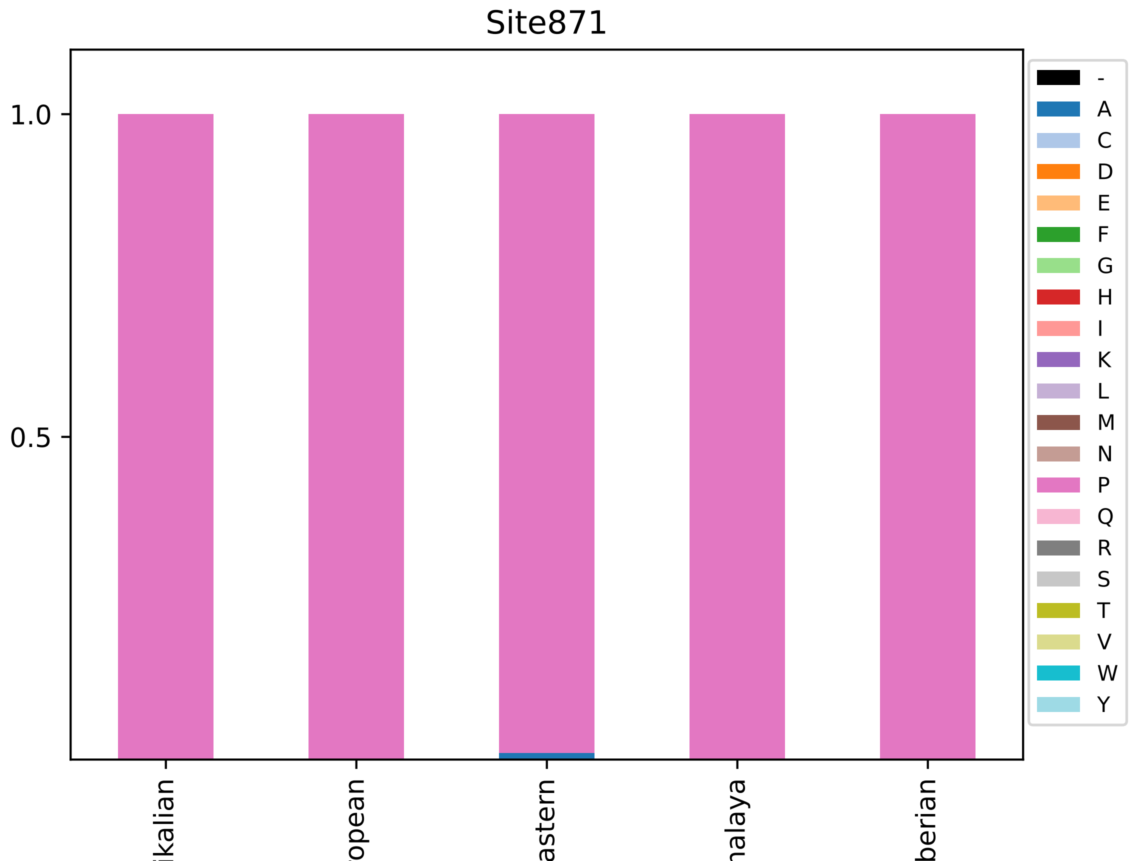

Site871
1.0
0.5
-
A
C
D
E
F
G
H
I
K
L
M
N
P
Q
R
S
T
V
W
Y
ikalian
opean
astern
nalaya
berian

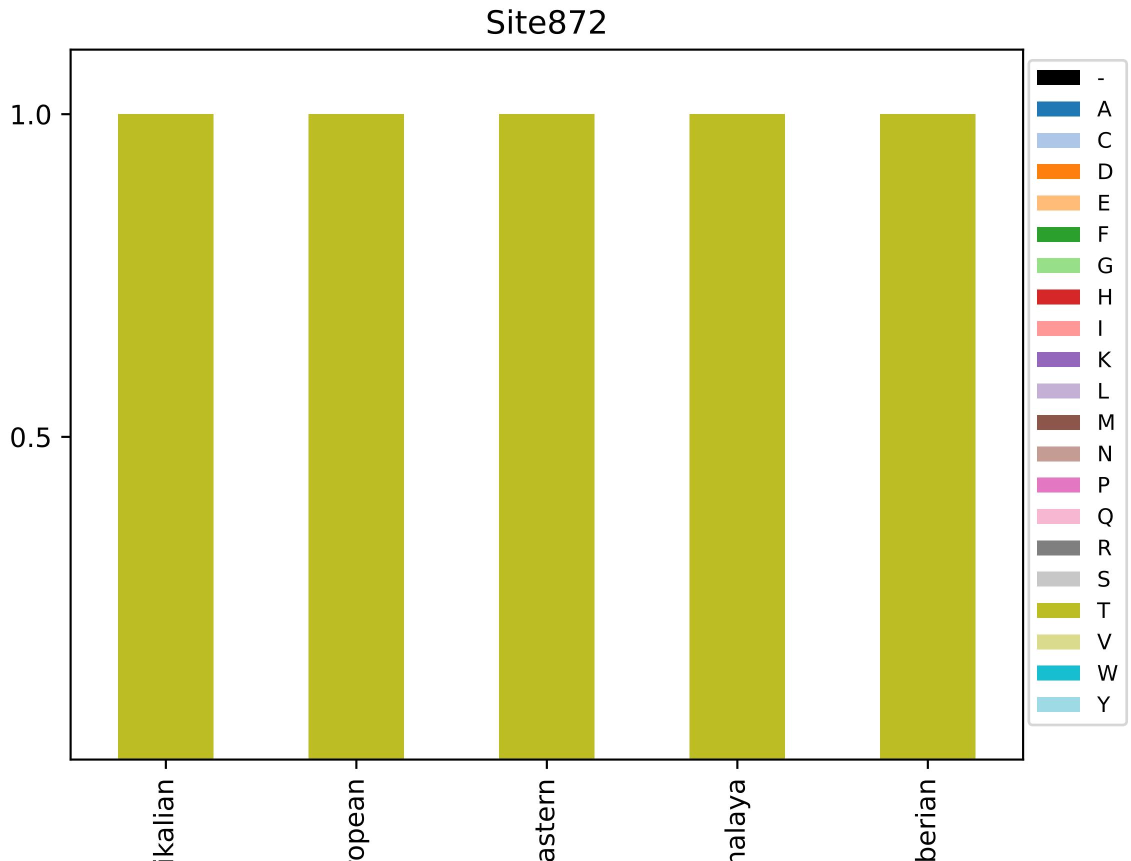

Site872
1.0
0.5
-
A
C
D
E
F
G
H
I
K
L
M
N
P
Q
R
S
T
V
W
Y
ikalian
opean
astern
nalaya
berian

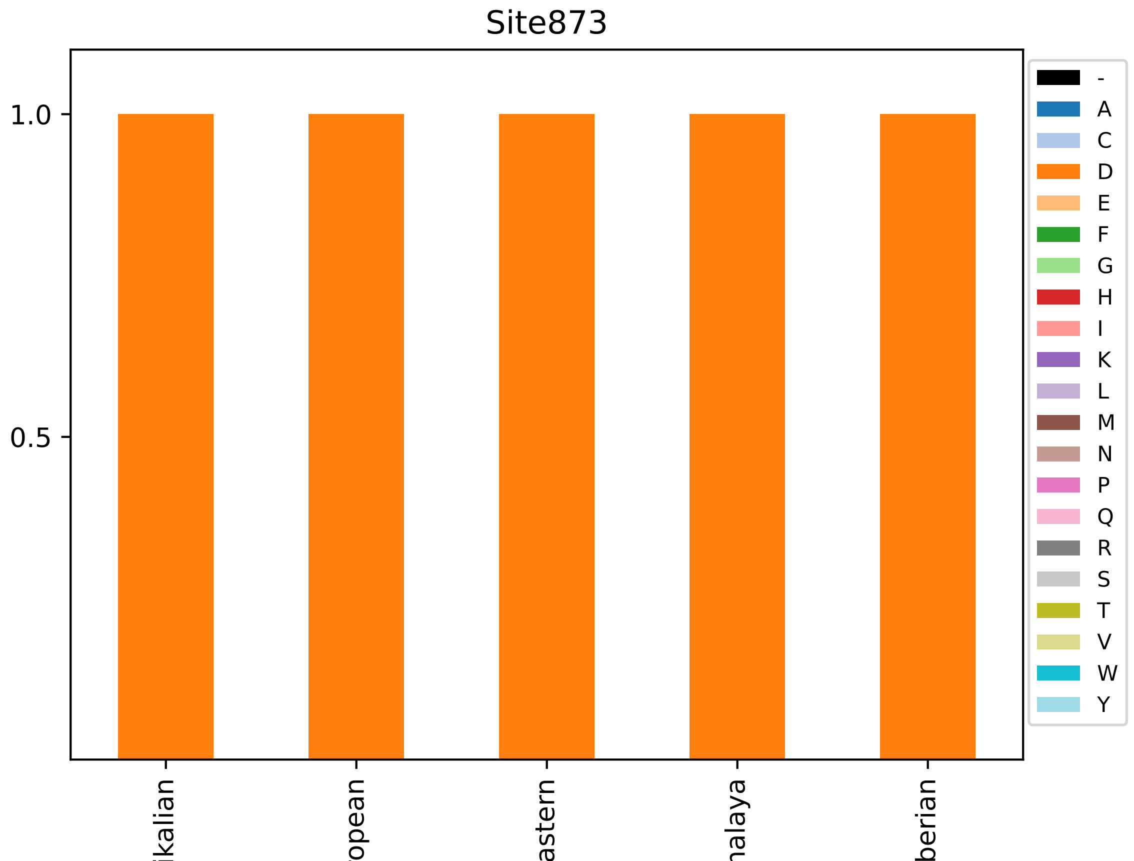

Site873
1.0
0.5
-
A
C
D
E
F
G
H
I
K
L
M
N
P
Q
R
S
T
V
W
Y
ikalian
opean
astern
nalaya
berian

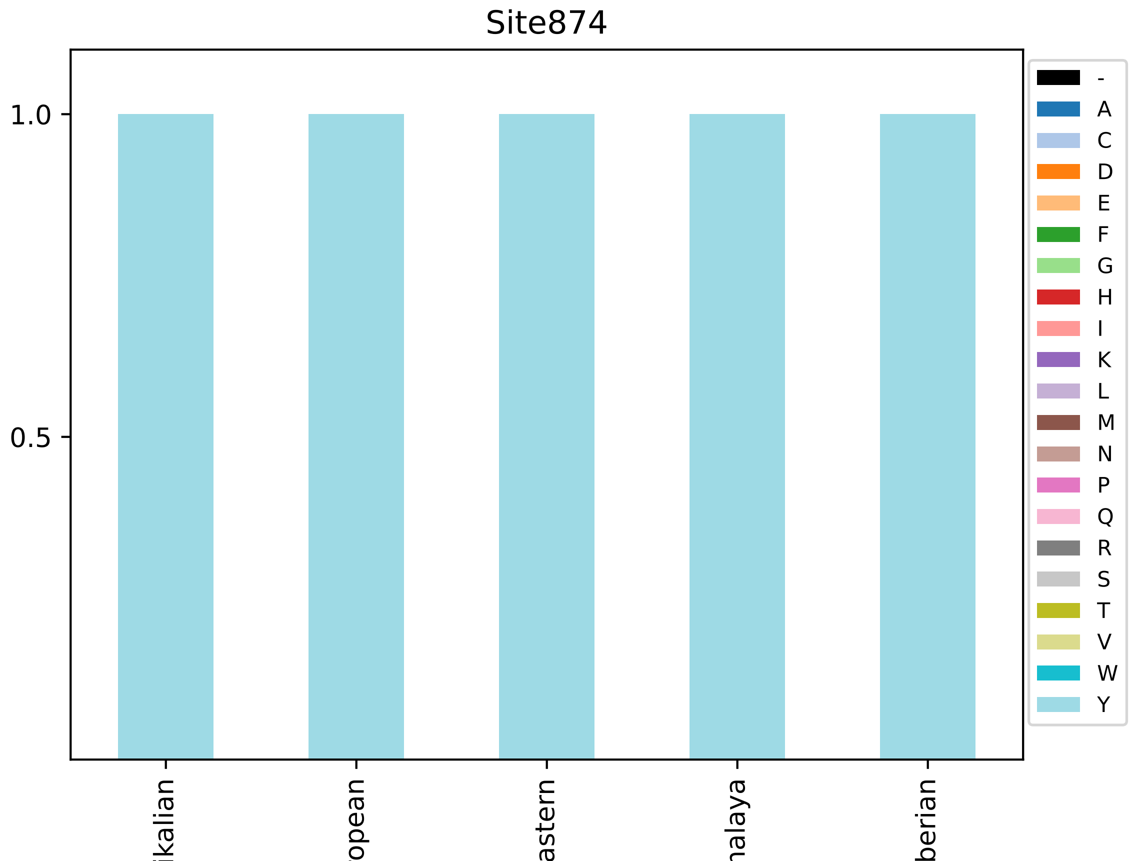
Site874
1.0
0.5
-
A
C
D
E
F
G
H
I
K
L
M
N
P
Q
R
S
T
V
W
Y
ikalian
opean
astern
nalaya
berian

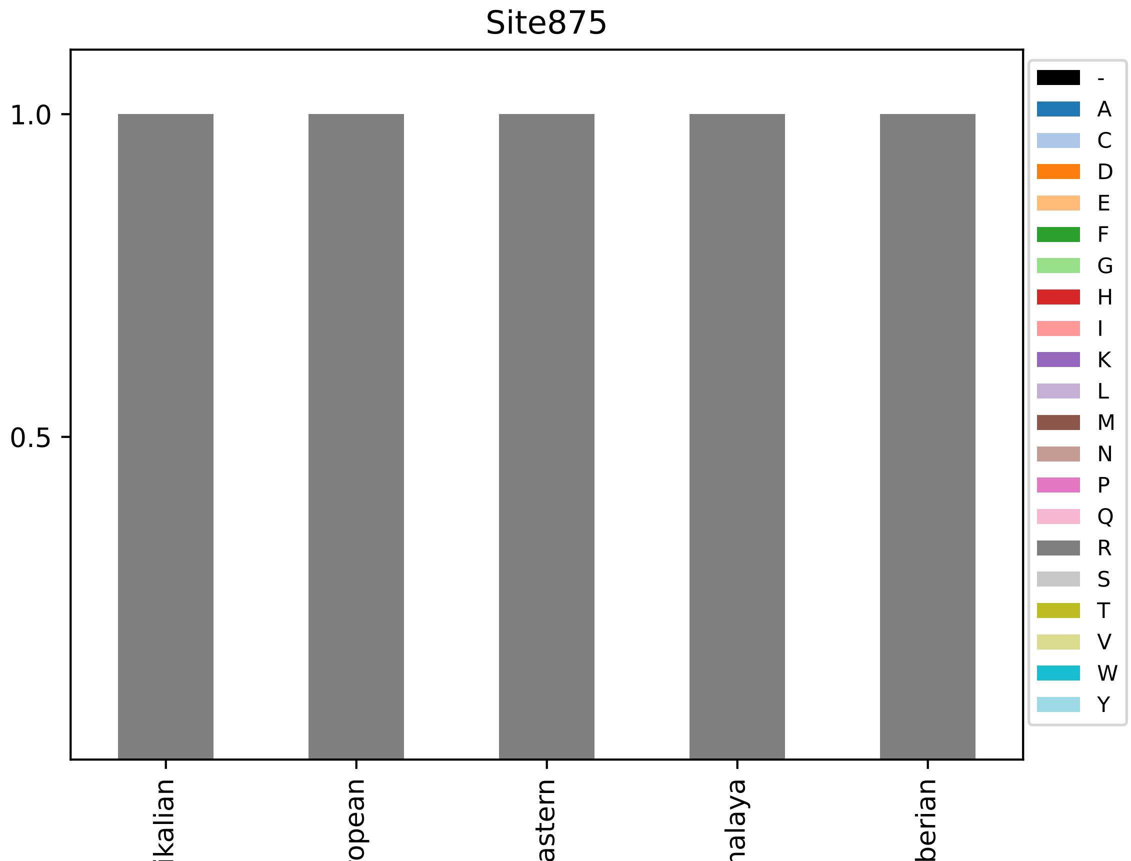
Site875
1.0
0.5
-
A
C
D
E
F
G
H
I
K
L
M
N
P
Q
R
S
T
V
W
Y
ikalian
ropean
astern
nalaya
berian

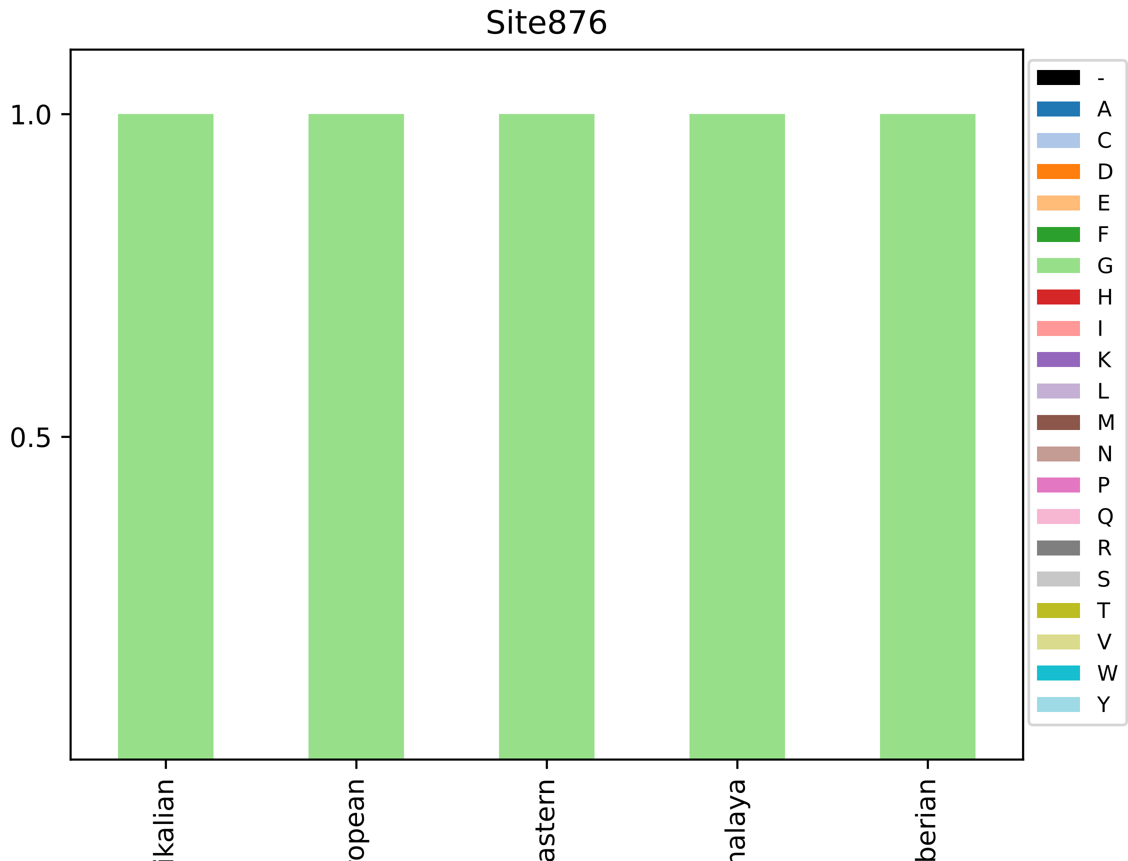

Site876
1.0
0.5
-
A
C
D
E
F
G
H
I
K
L
M
N
P
Q
R
S
T
V
W
Y

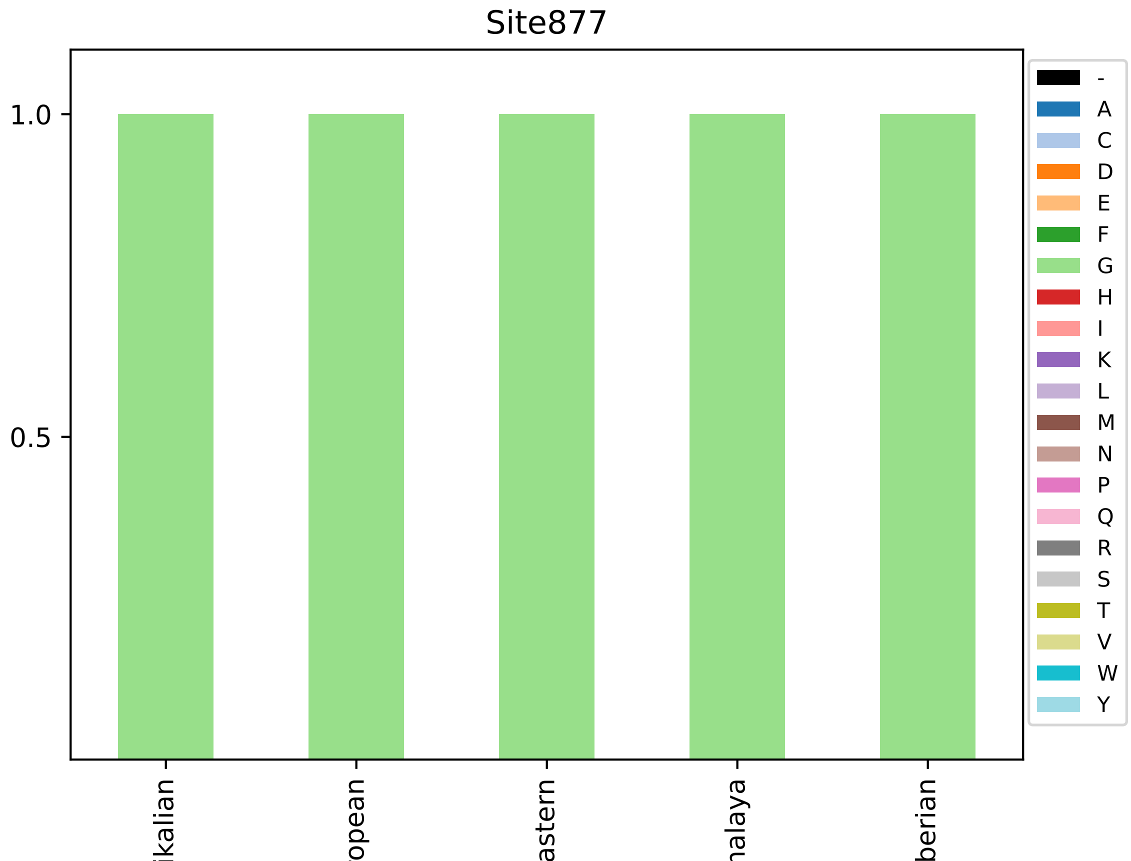
Site877
1.0
0.5
-
A
C
D
E
F
G
H
I
K
L
M
N
P
Q
R
S
T
V
W
Y
ikalian
opean
astern
nalaya
berian

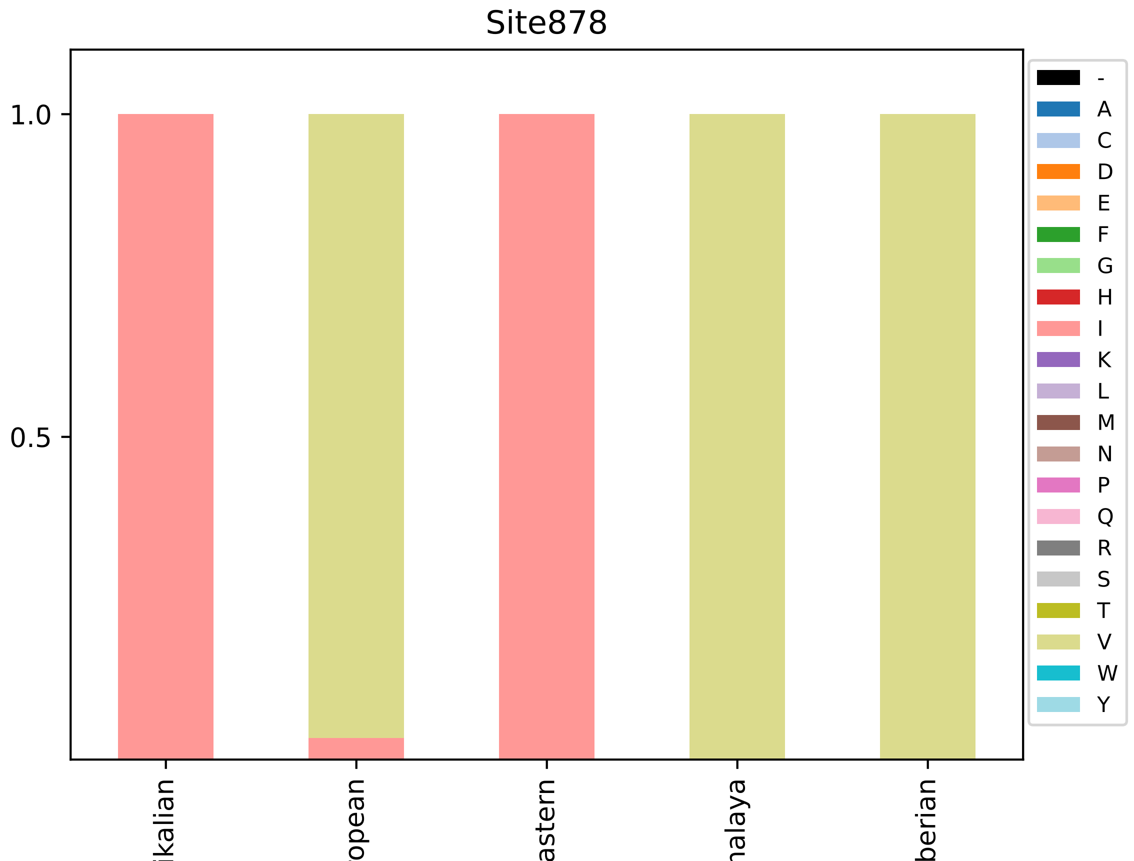
Site878
1.0
0.5
-
A
C
D
E
F
G
H
I
K
L
M
N
P
Q
R
S
T
V
W
Y
ikalian
opean
astern
nalaya
berian

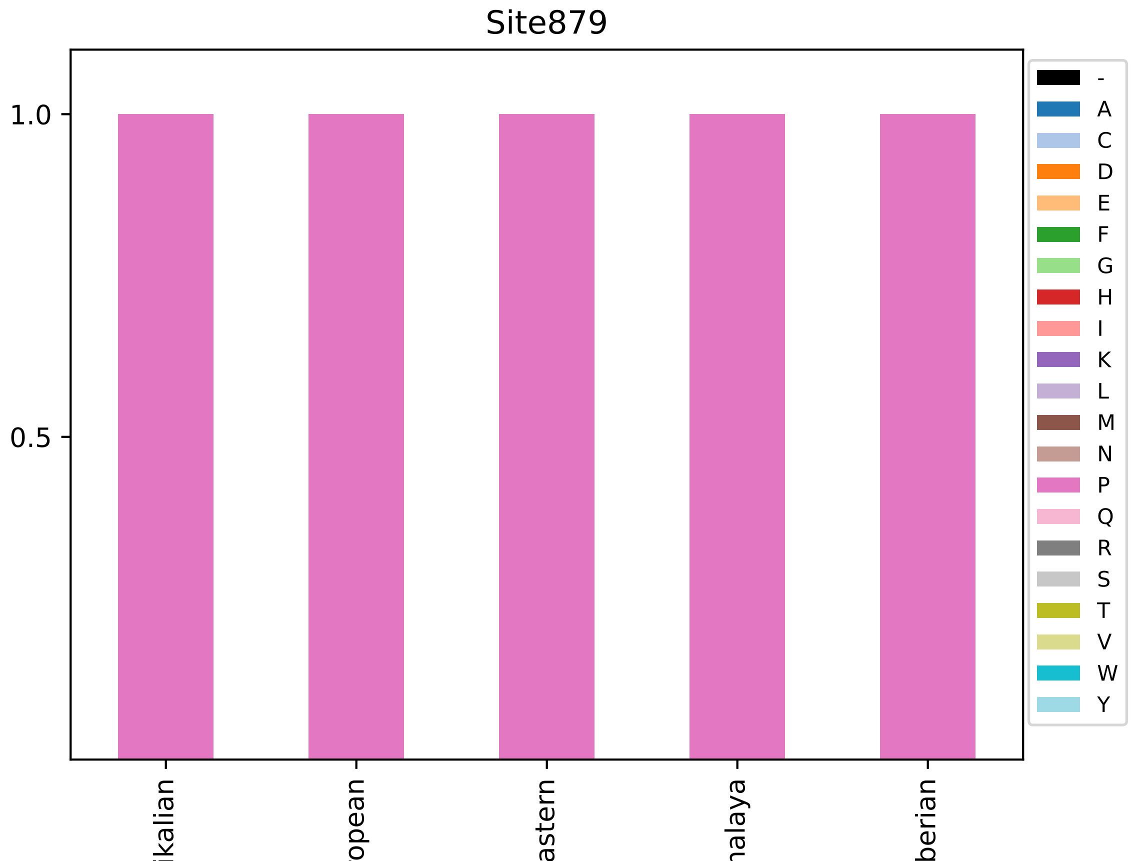

Site879
1.0
0.5
-
A
C
D
E
F
G
H
I
K
L
M
N
P
Q
R
S
T
V
W
Y
ikalian
opean
astern
nalaya
berian

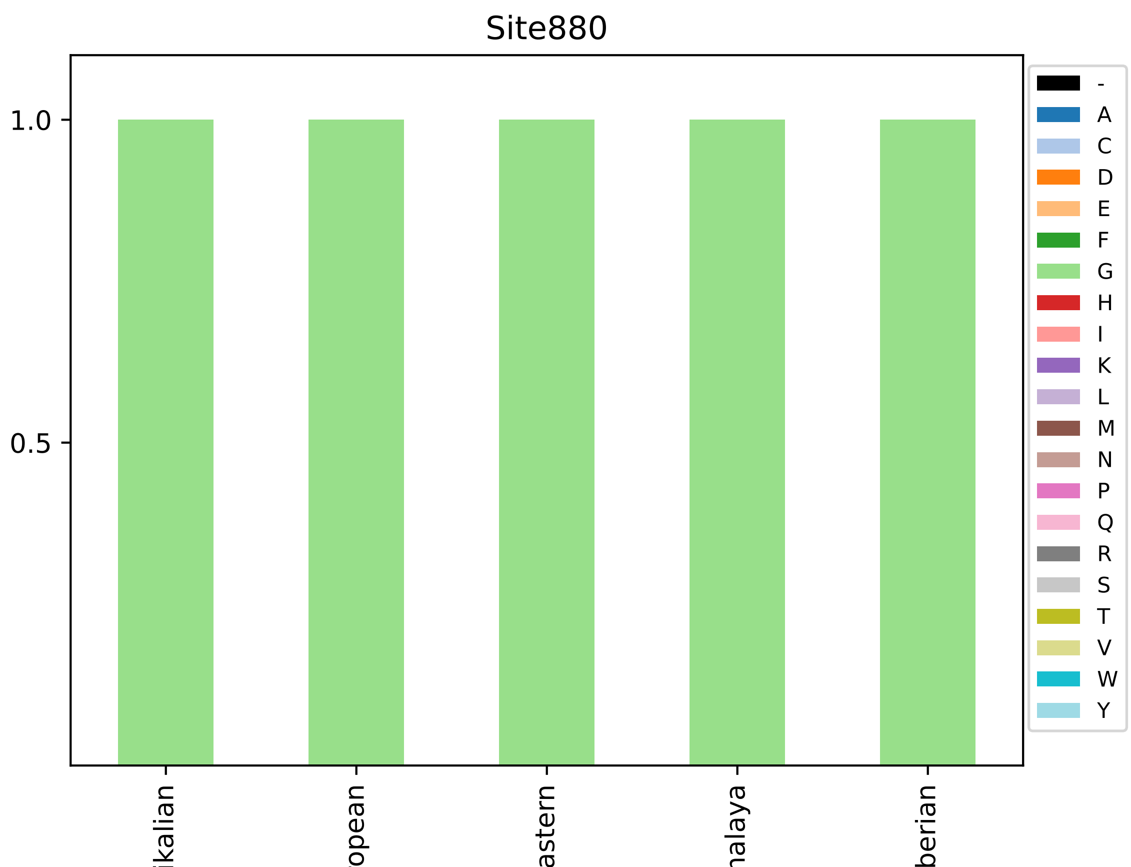

Site880
1.0
0.5
-
A
C
D
E
F
G
H
I
K
L
M
N
P
Q
R
S
T
V
W
Y
ikalian
opean
astern
nalaya
berian

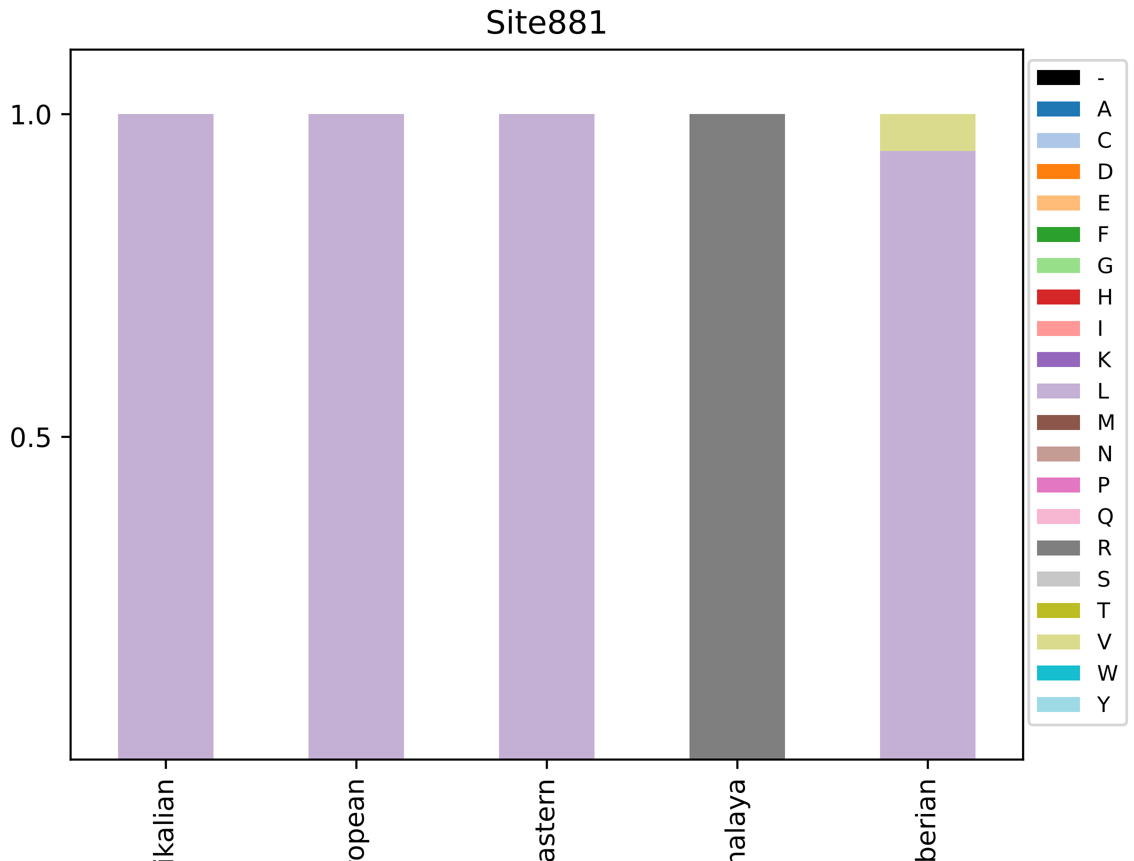
Site881
1.0
0.5
-
A
C
D
E
F
G
H
I
K
L
M
N
P
Q
R
S
T
V
W
Y
ikalian
opean
astern
nalaya
berian

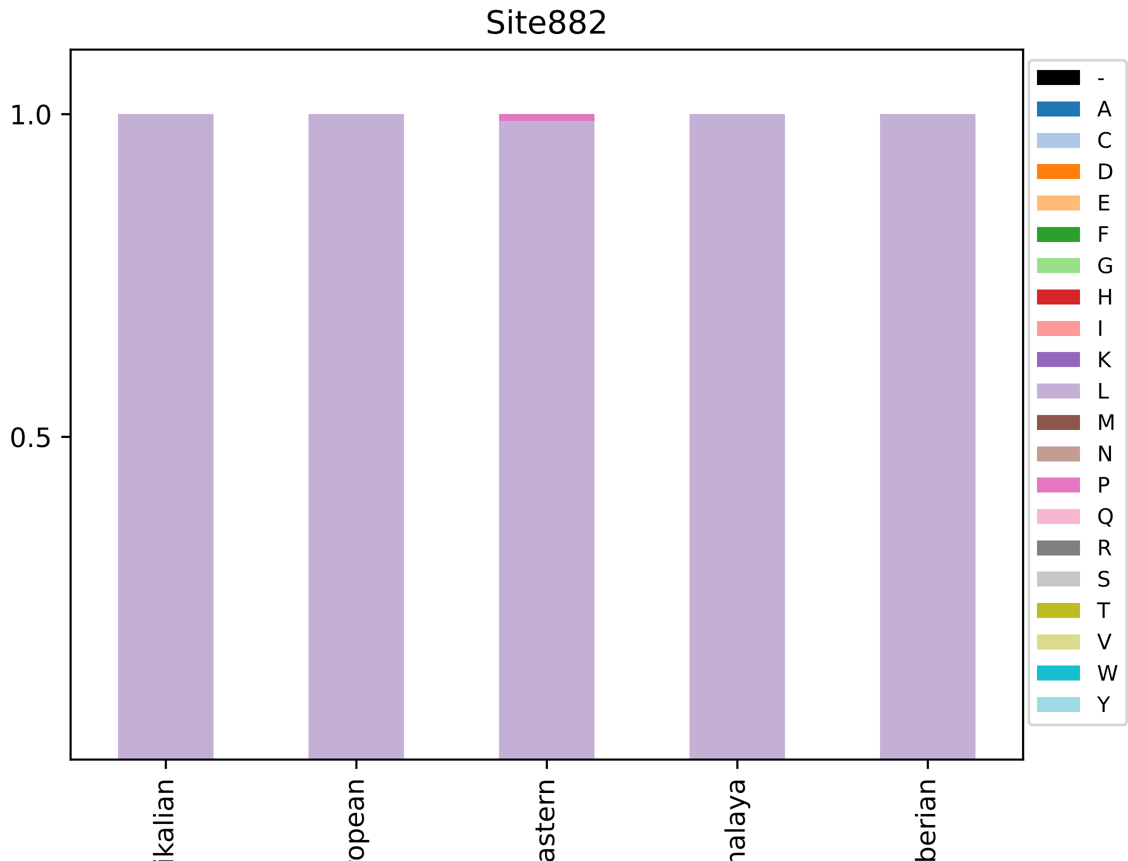

Site882
1.0
0.5
-
A
C
D
E
F
G
H
I
K
L
M
N
P
Q
R
S
T
V
W
Y
ikalian
opean
astern
nalaya
berian

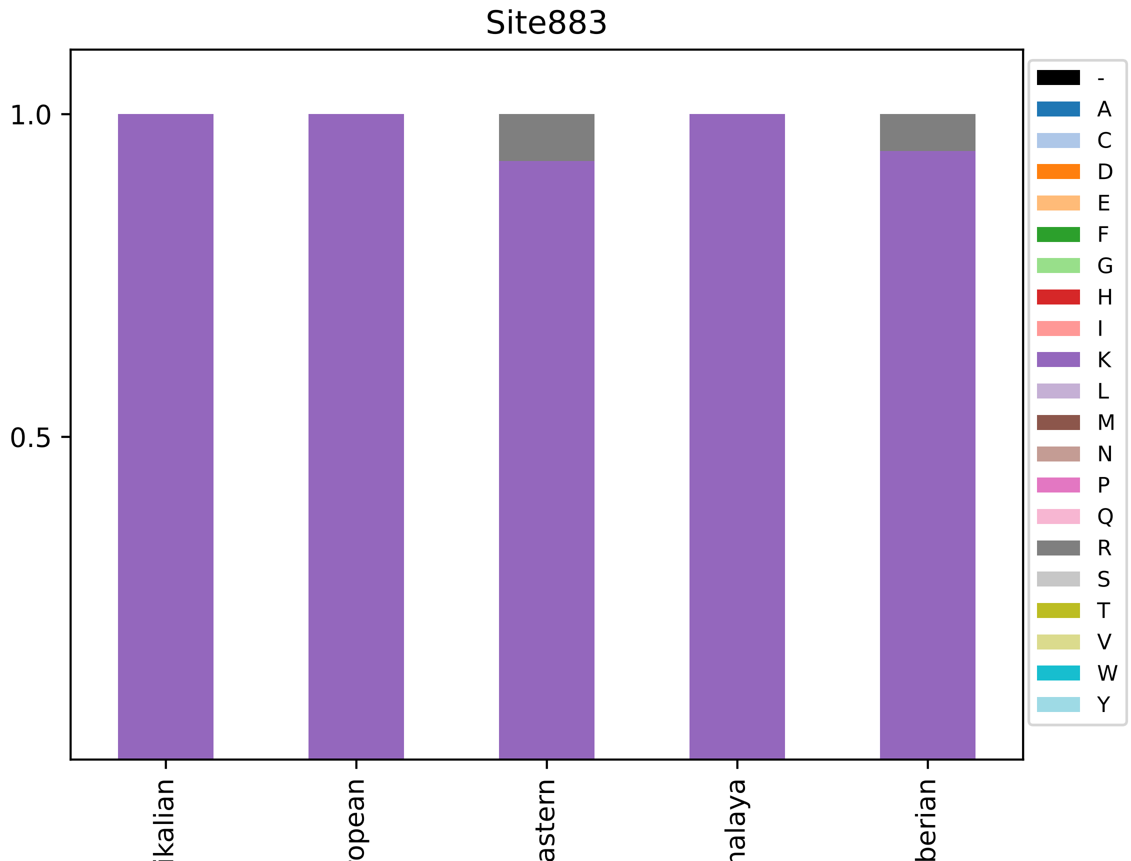
Site883
1.0
0.5
-
A
C
D
E
F
G
H
I
K
L
M
N
P
Q
R
S
T
V
W
Y
ikalian
opean
astern
nalaya
berian

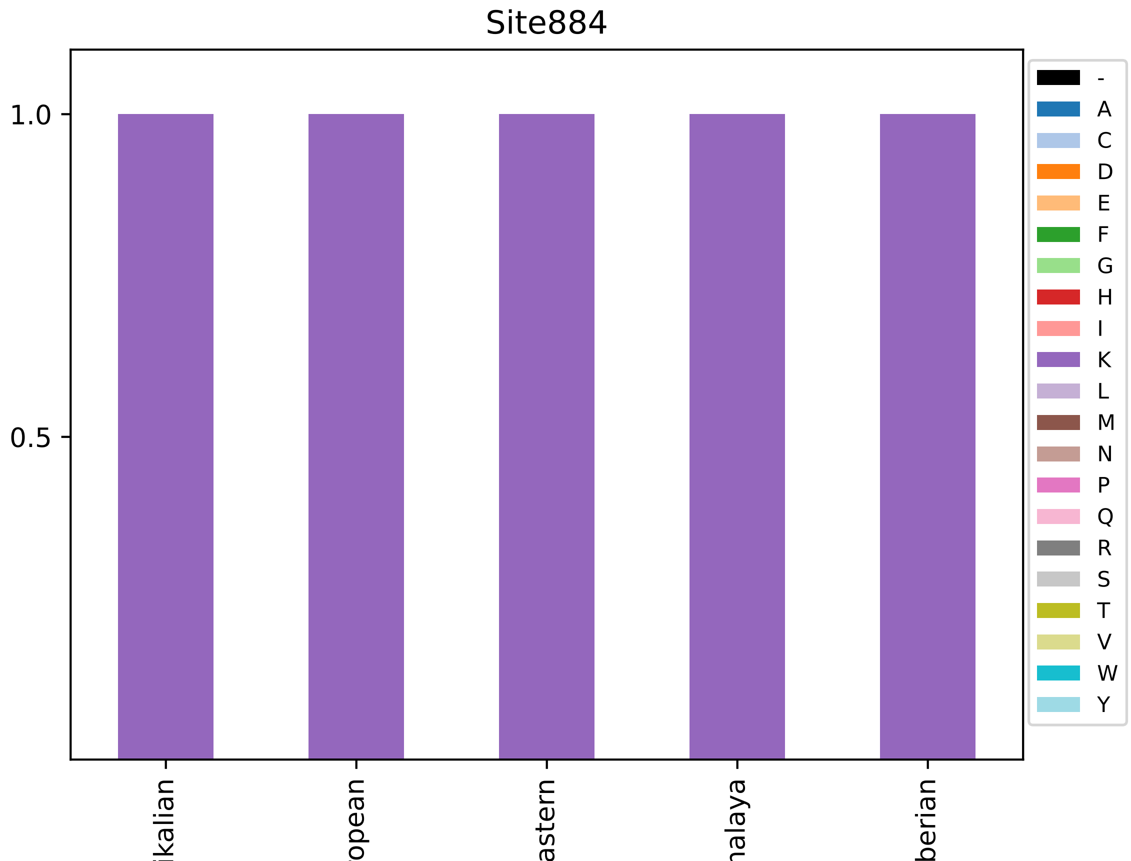
Site884
1.0
0.5
-
A
C
D
E
F
G
H
I
K
L
M
N
P
Q
R
S
T
V
W
Y
ikalian
opean
astern
nalaya
berian

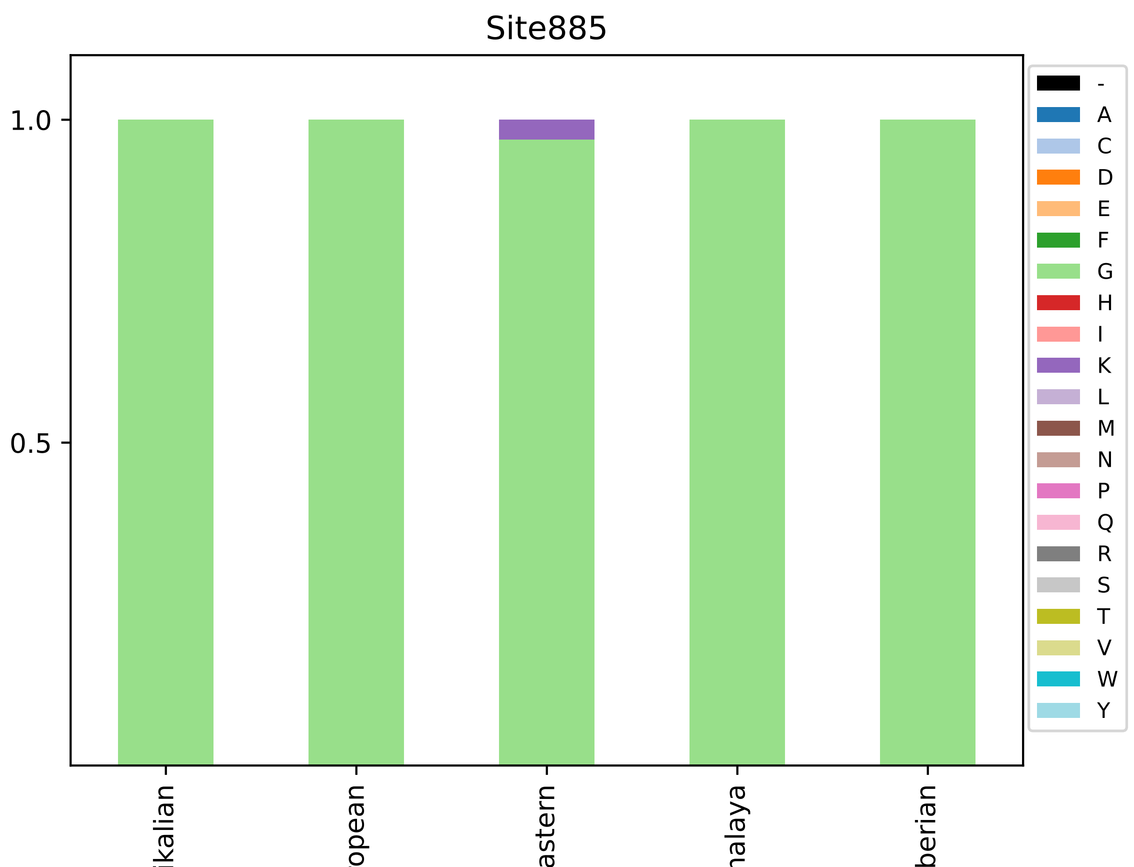
Site885
1.0
0.5
ikalian
opean
astern
nalaya
berian
-
A
C
D
E
F
G
H
I
K
L
M
N
P
Q
R
S
T
V
W
Y

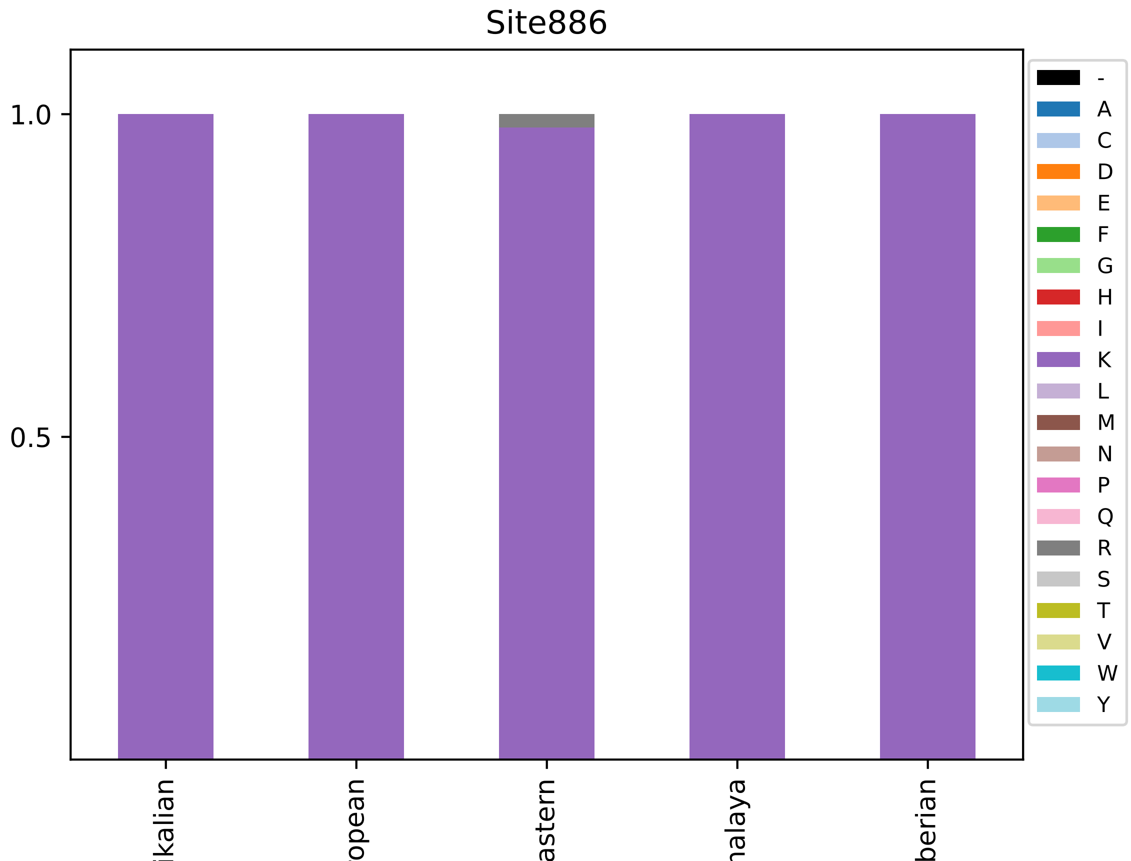
Site886
1.0
0.5
-
A
C
D
E
F
G
H
I
K
L
M
N
P
Q
R
S
T
V
W
Y

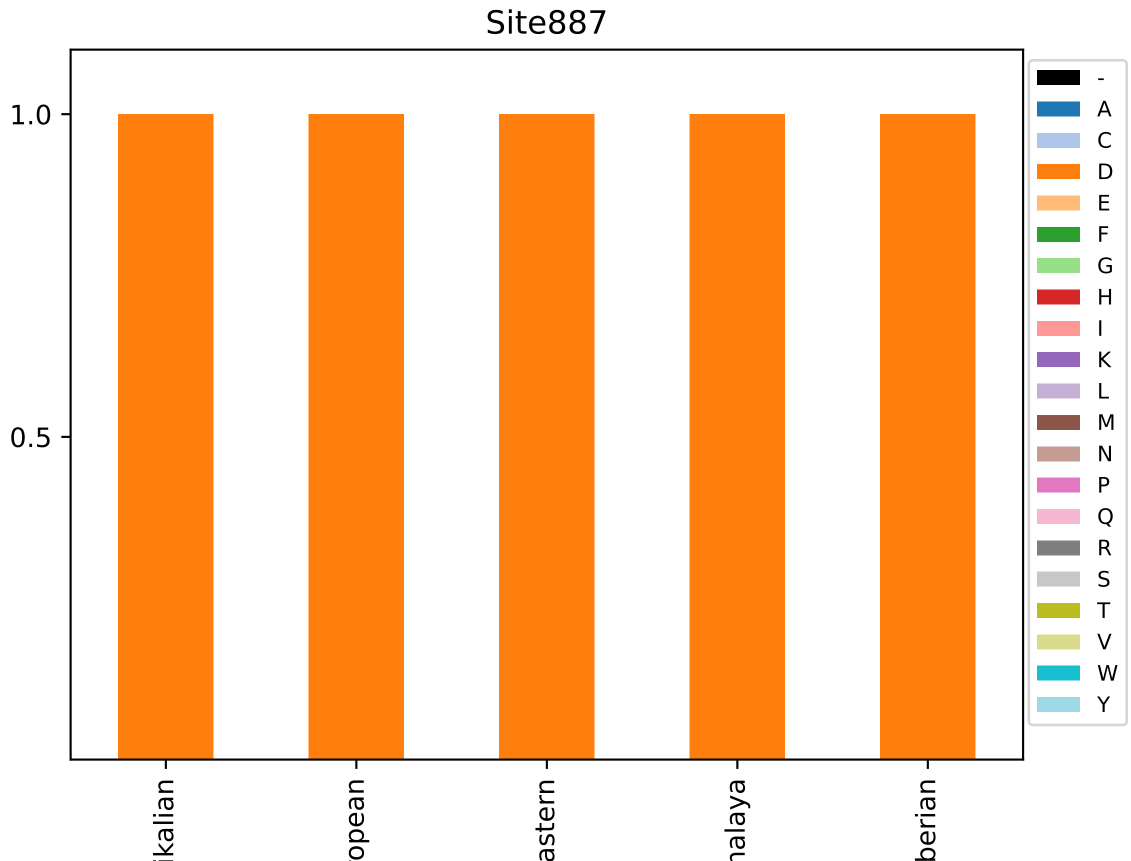
Site887
1.0
0.5
-
A
C
D
E
F
G
H
I
K
L
M
N
P
Q
R
S
T
V
W
Y

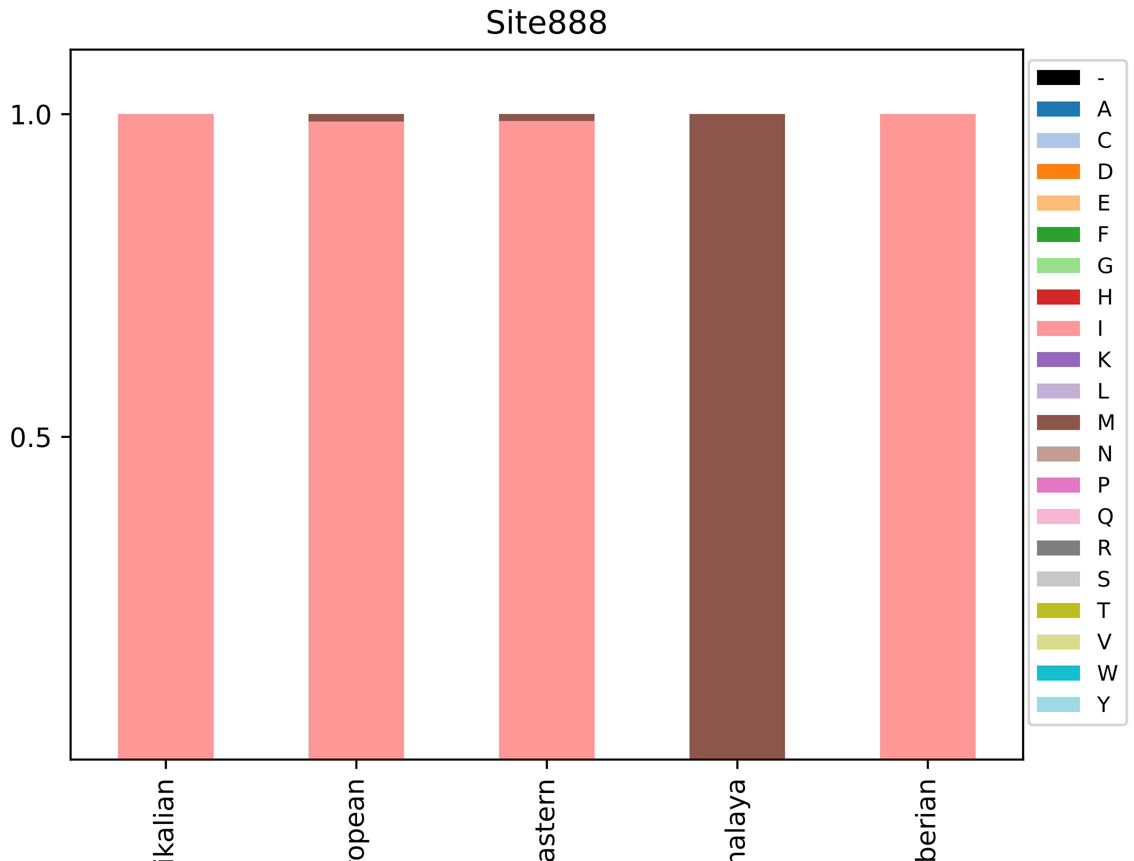
Site888
1.0
0.5
-
A
C
D
E
F
G
H
I
K
L
M
N
P
Q
R
S
T
V
W
Y
ikalian
opean
astern
nalaya
berian

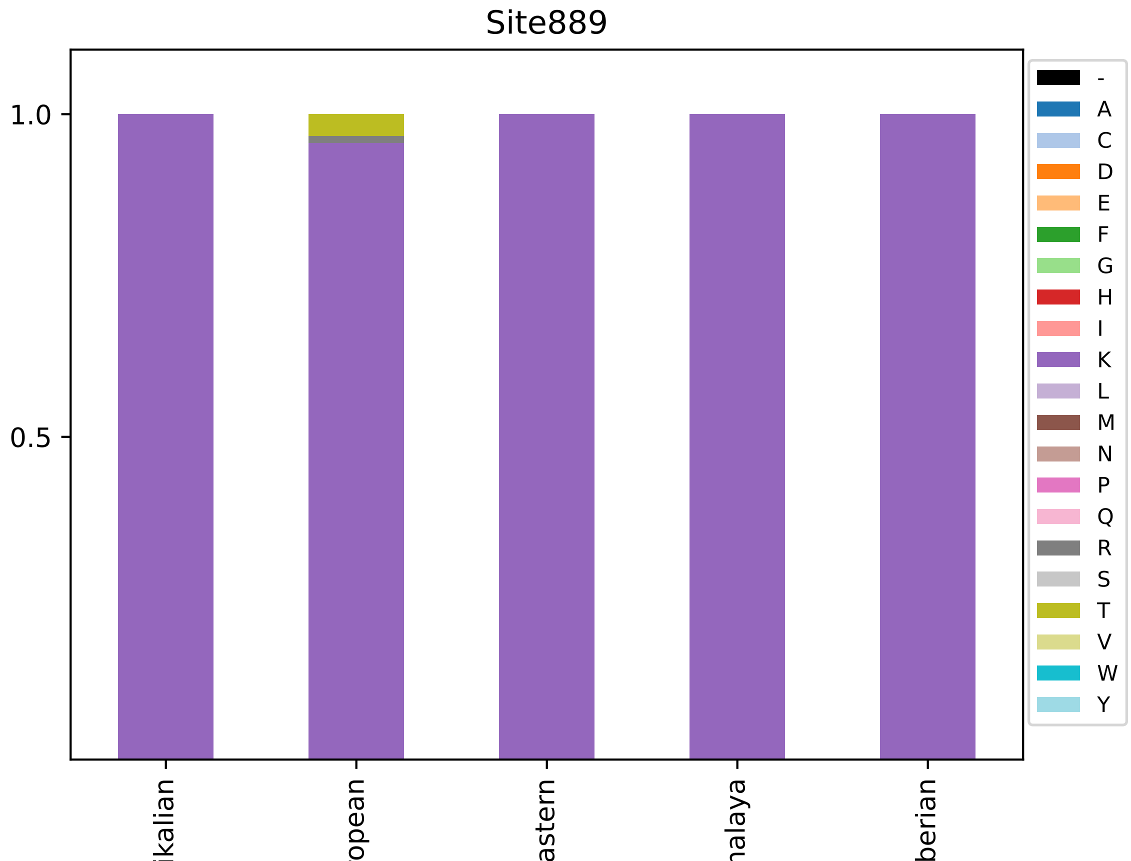
Site889
1.0
0.5
-
A
C
D
E
F
G
H
I
K
L
M
N
P
Q
R
S
T
V
W
Y

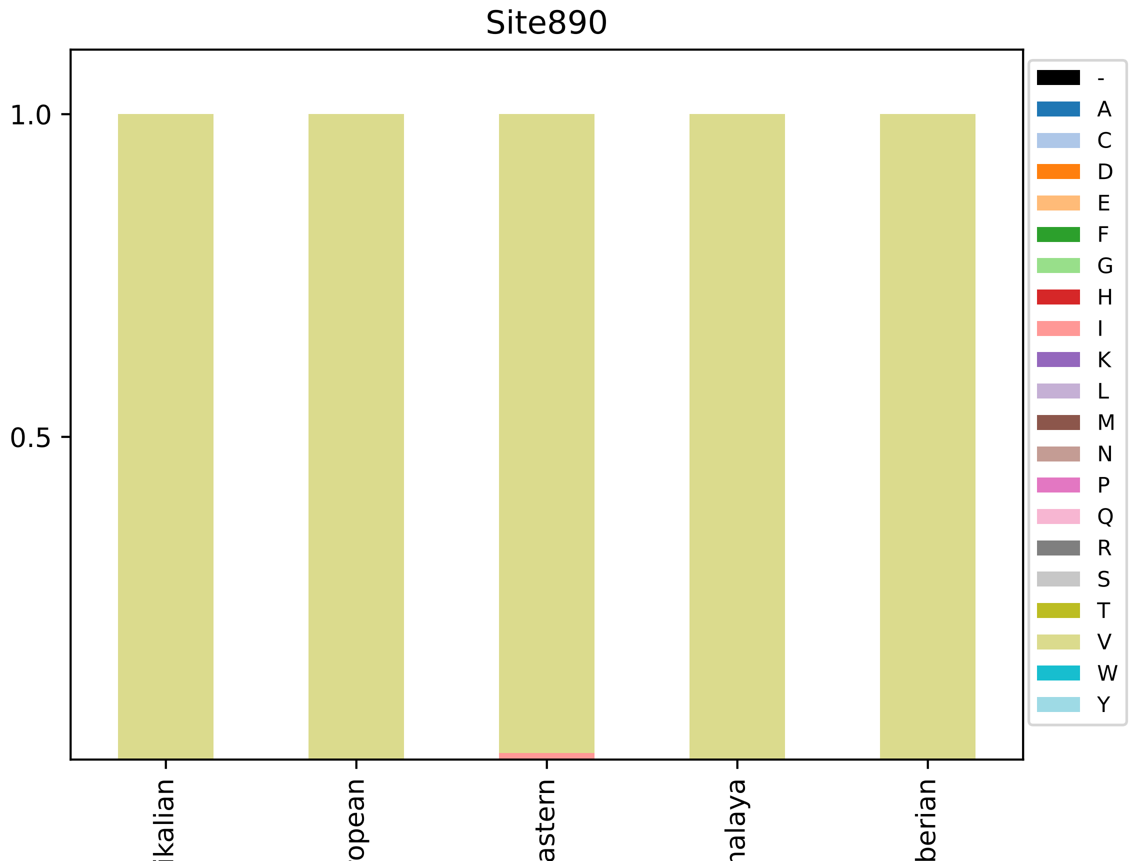

Site890
1.0
0.5
-
A
C
D
E
F
G
H
I
K
L
M
N
P
Q
R
S
T
V
W
Y
ikalian
opean
astern
nalaya
berian

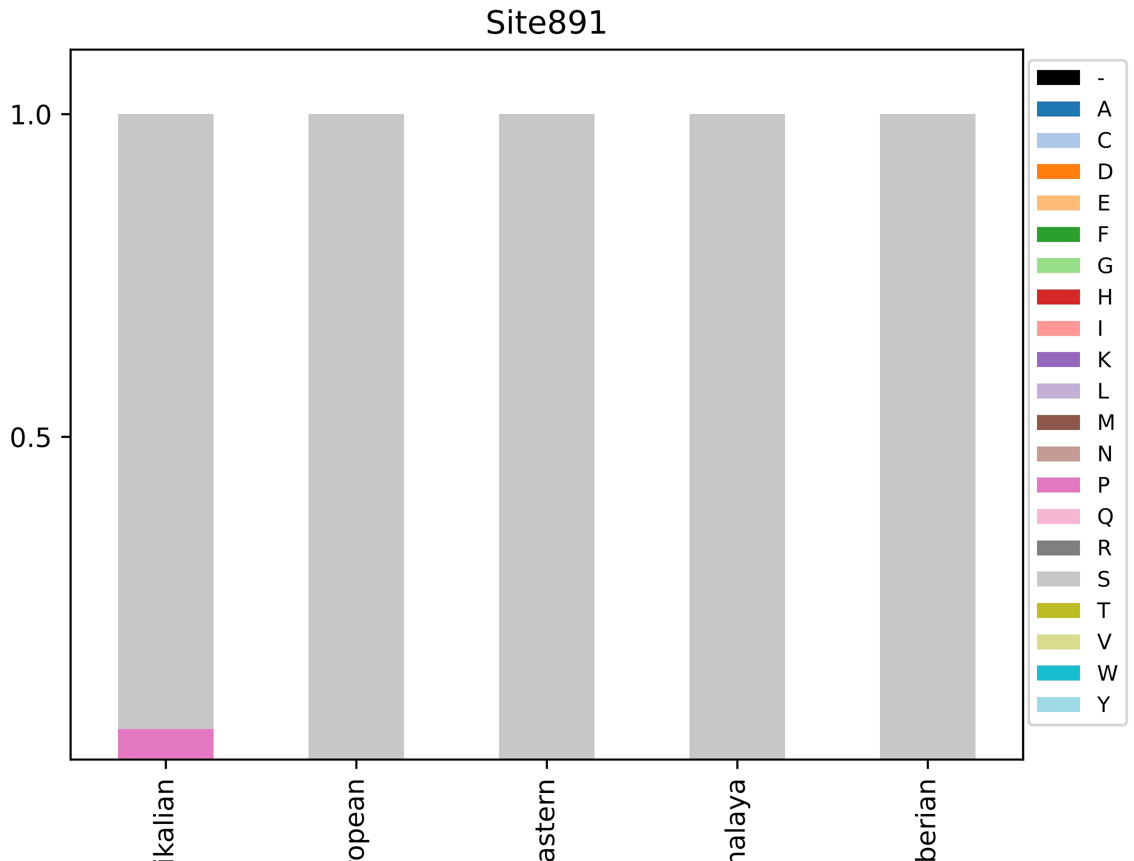
Site891
1.0
0.5
-
A
C
D
E
F
G
H
I
K
L
M
N
P
Q
R
S
T
V
W
Y
ikalian
opean
astern
nalaya
berian

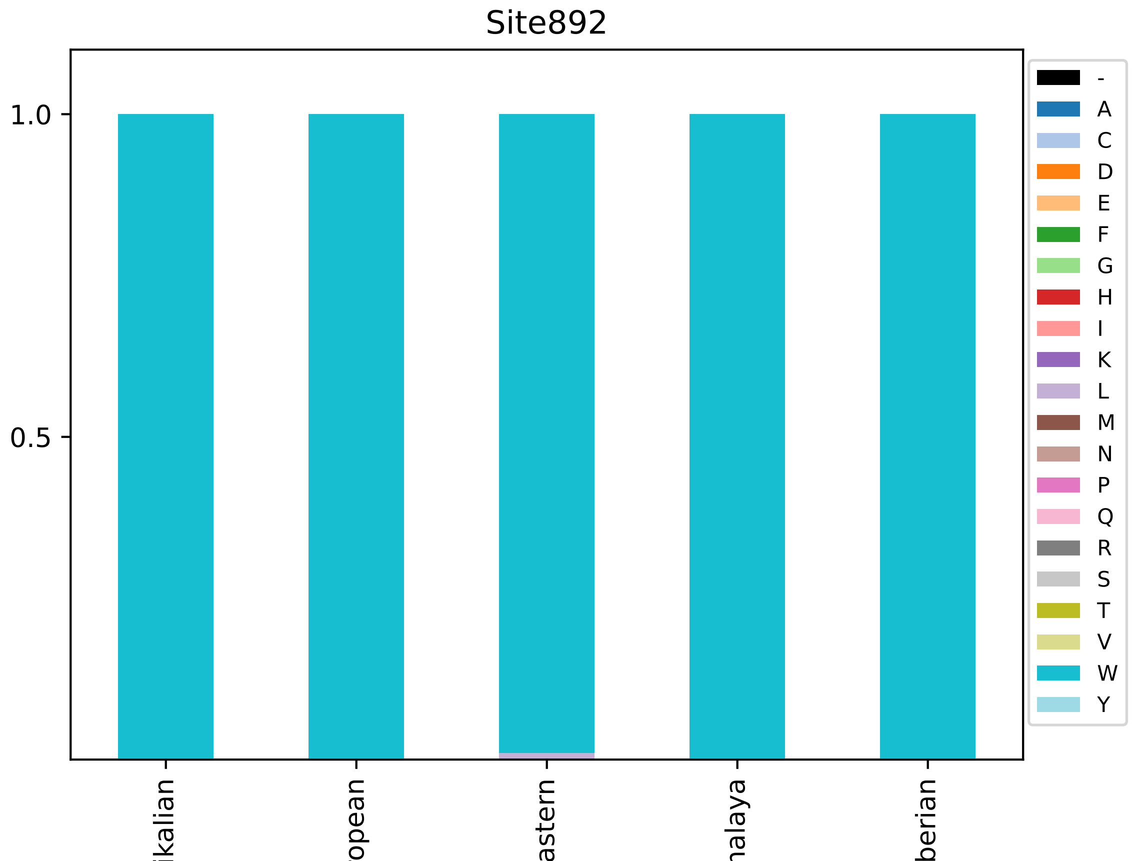
Site892
1.0
0.5
-
A
C
D
E
F
G
H
I
K
L
M
N
P
Q
R
S
T
V
W
Y
ikalian
opean
astern
nalaya
berian

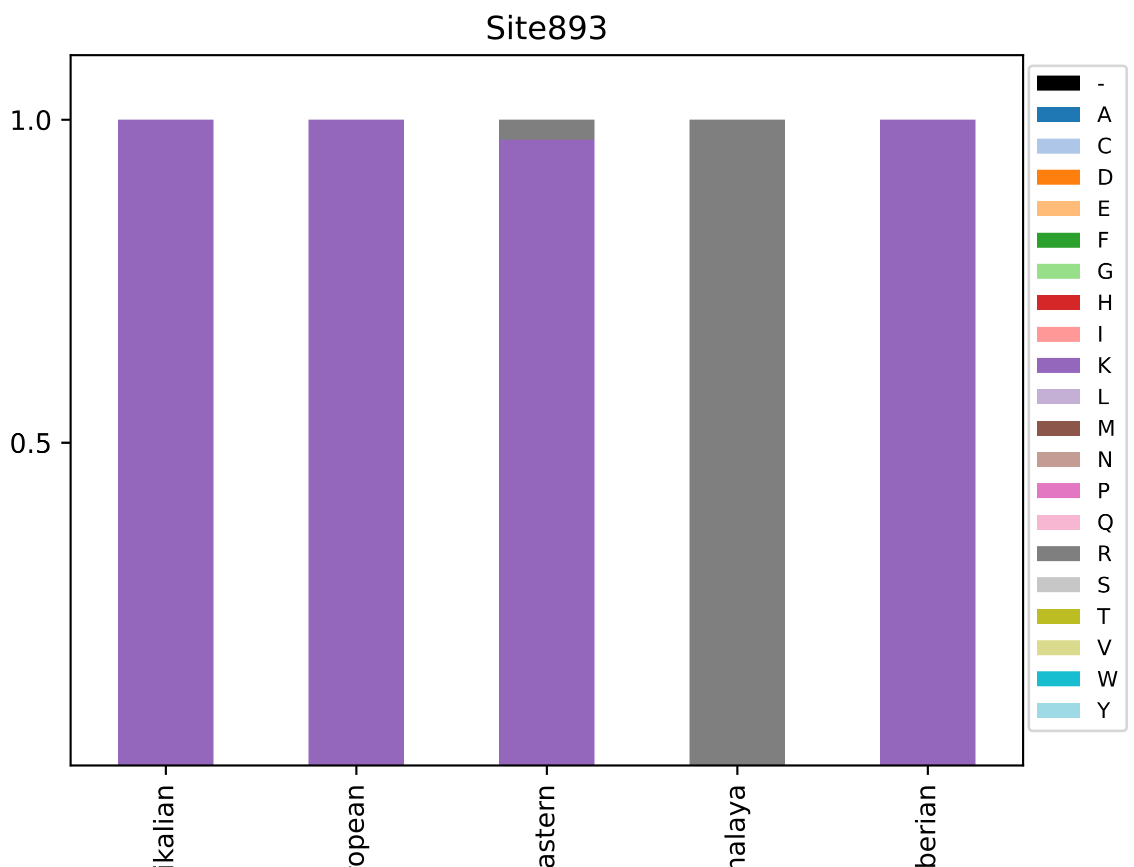
Site893
1.0
0.5
ikalian
opean
astern
nalaya
berian
-
A
C
D
E
F
G
H
I
K
L
M
N
P
Q
R
S
T
V
W
Y

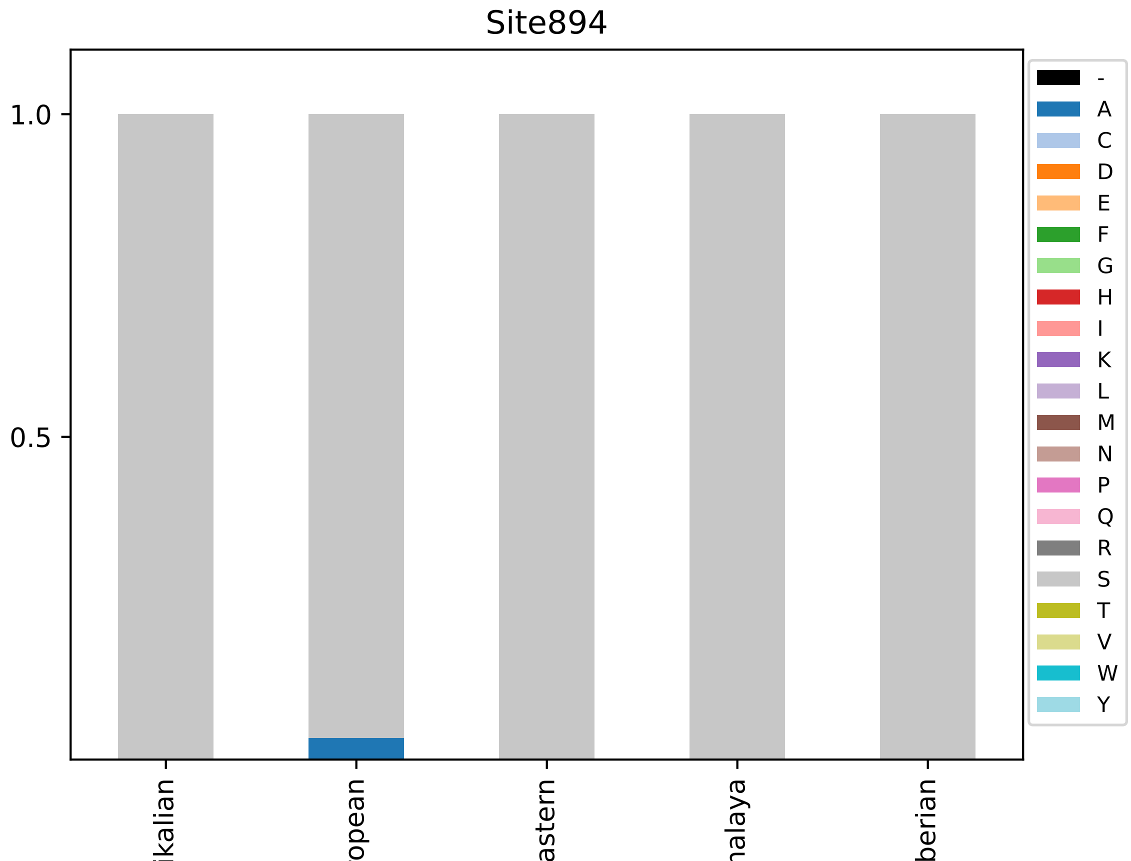
Site894
1.0
0.5
-
A
C
D
E
F
G
H
I
K
L
M
N
P
Q
R
S
T
V
W
Y
ikalian
opean
astern
nalaya
berian

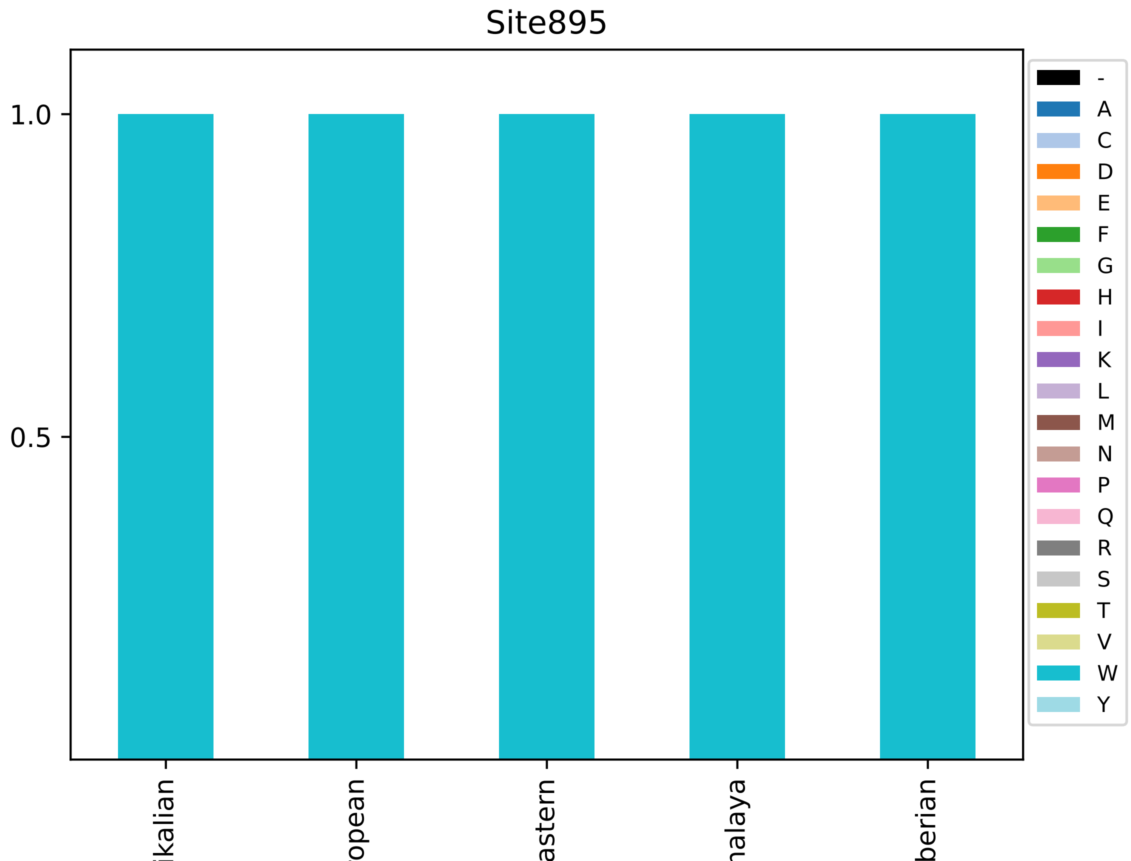

Site895
1.0
0.5
-
A
C
D
E
F
G
H
I
K
L
M
N
P
Q
R
S
T
V
W
Y
ikalian
opean
astern
nalaya
berian

Site896

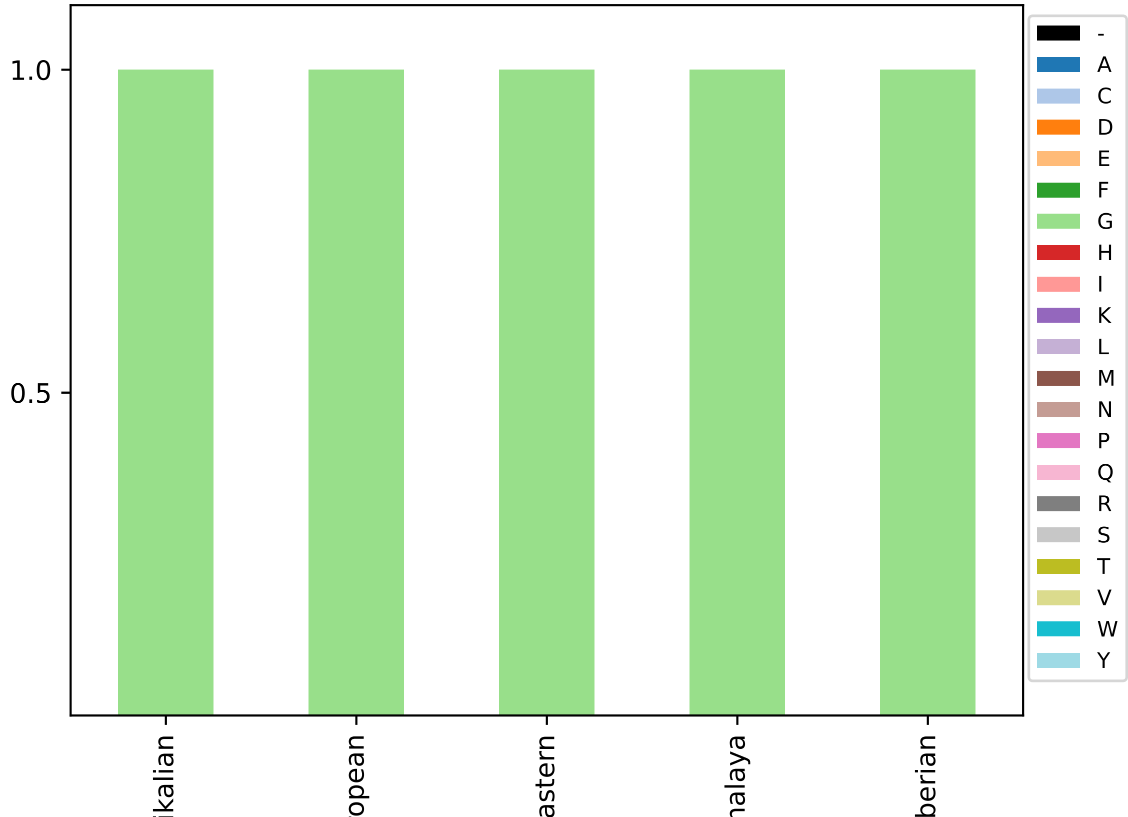

Site896
1.0
0.5
-
A
C
D
E
F
G
H
I
K
L
M
N
P
Q
R
S
T
V
W
Y
ikalian
opean
astern
nalaya
berian

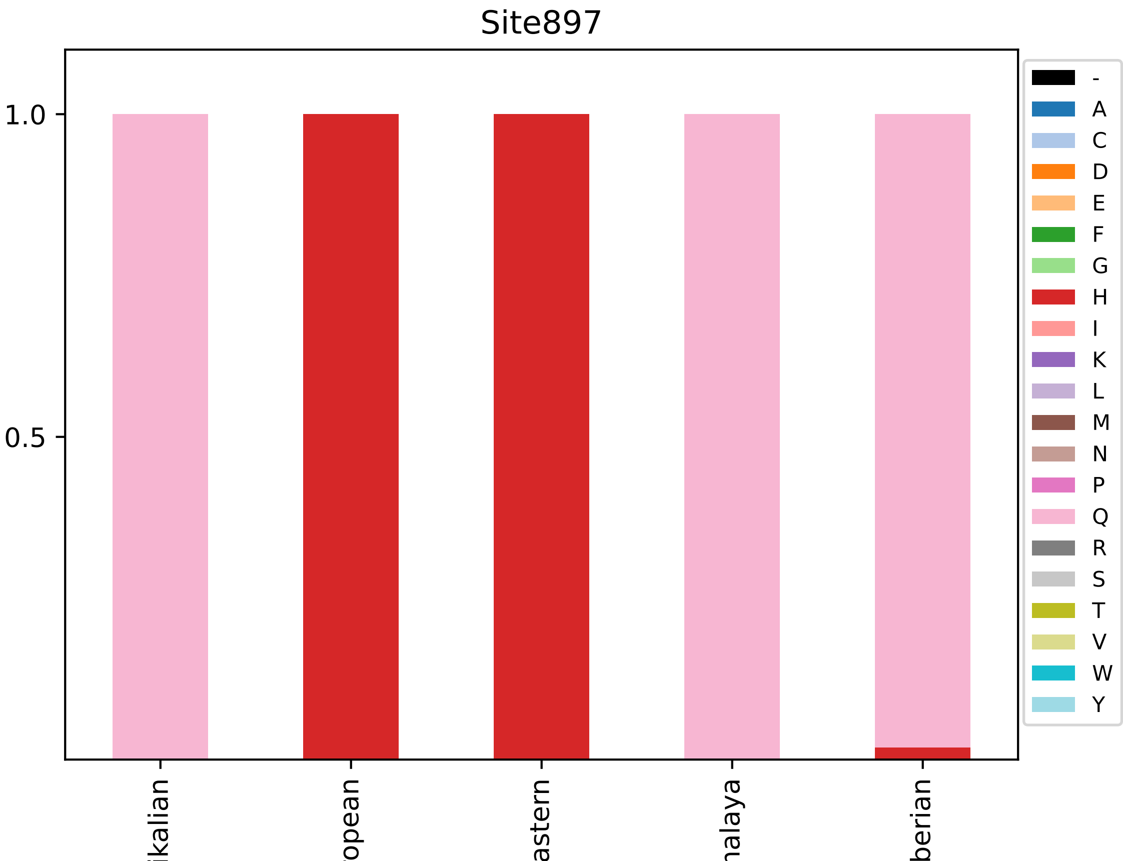

Site897
1.0
0.5
-
A
C
D
E
F
G
H
I
K
L
M
N
P
Q
R
S
T
V
W
Y
ikalian
opean
astern
nalaya
berian

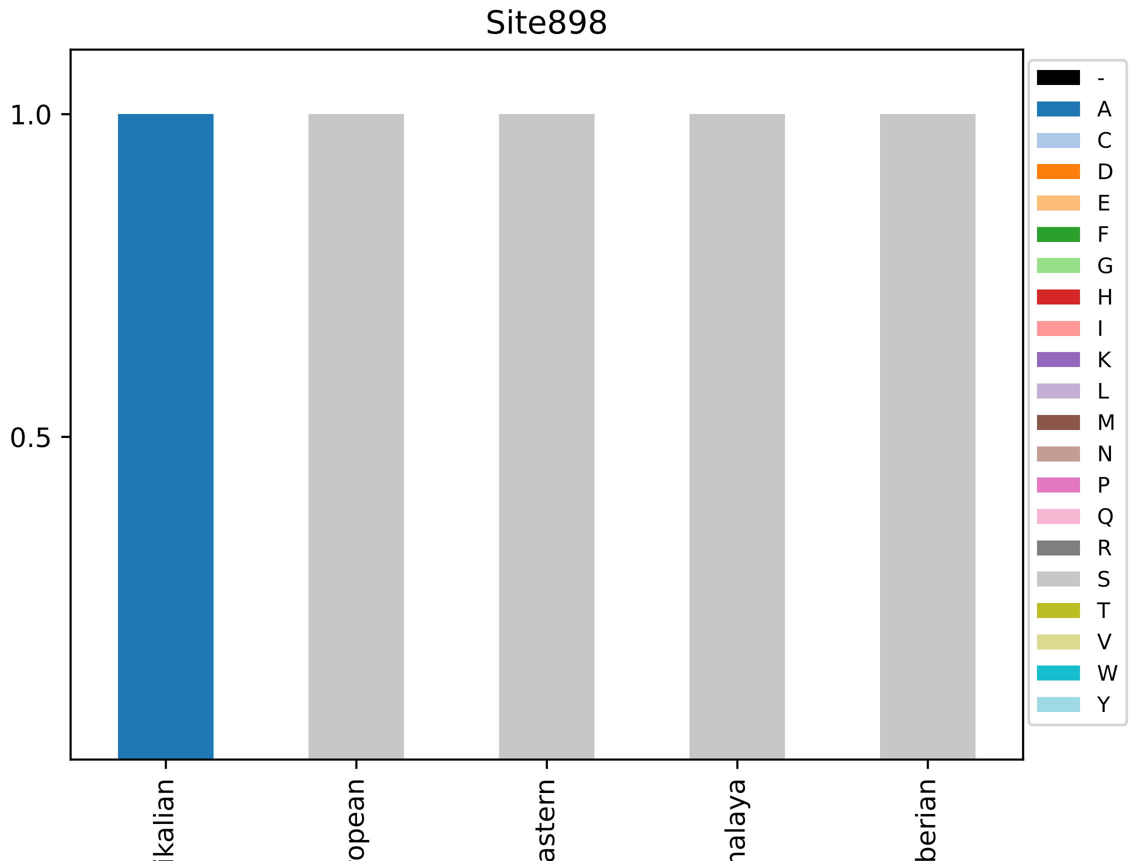

Site898
1.0
0.5
-
A
C
D
E
F
G
H
I
K
L
M
N
P
Q
R
S
T
V
W
Y
ikalian
opean
astern
nalaya
berian

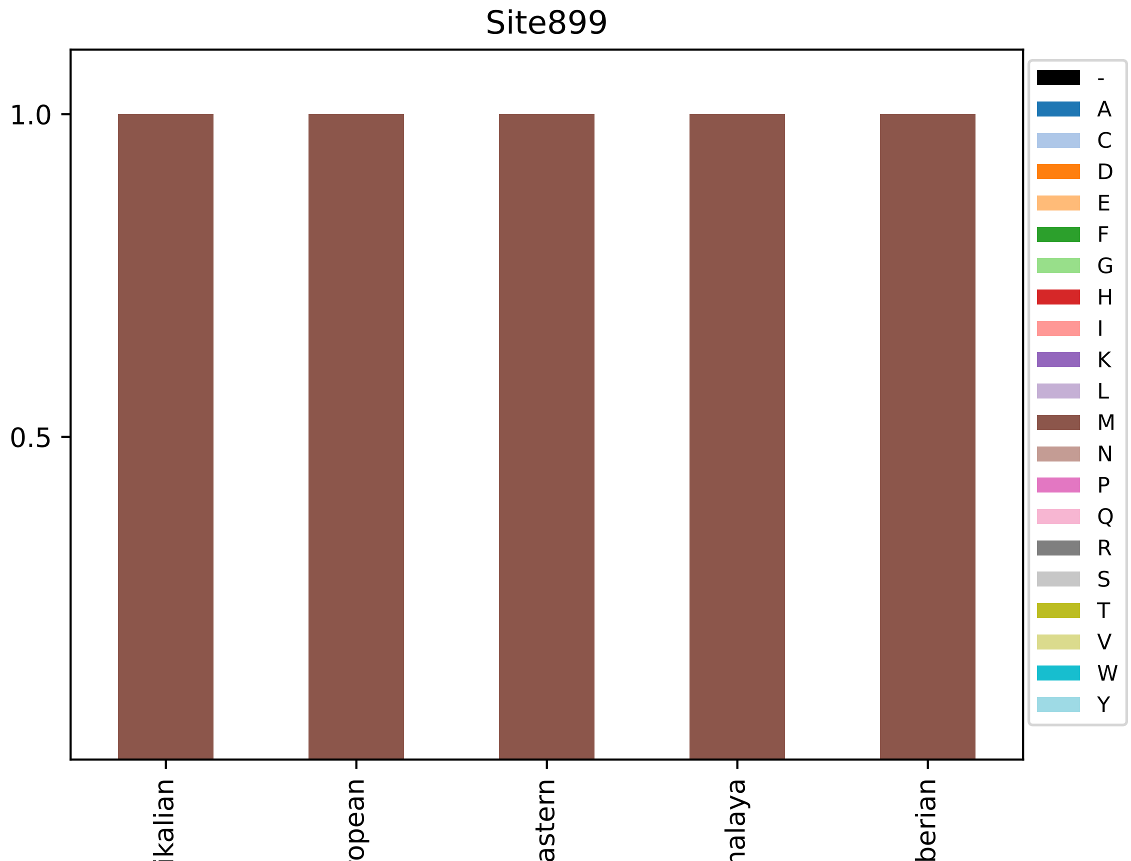
Site899
1.0
0.5
-
A
C
D
E
F
G
H
I
K
L
M
N
P
Q
R
S
T
V
W
Y
ikalian
opean
astern
nalaya
berian

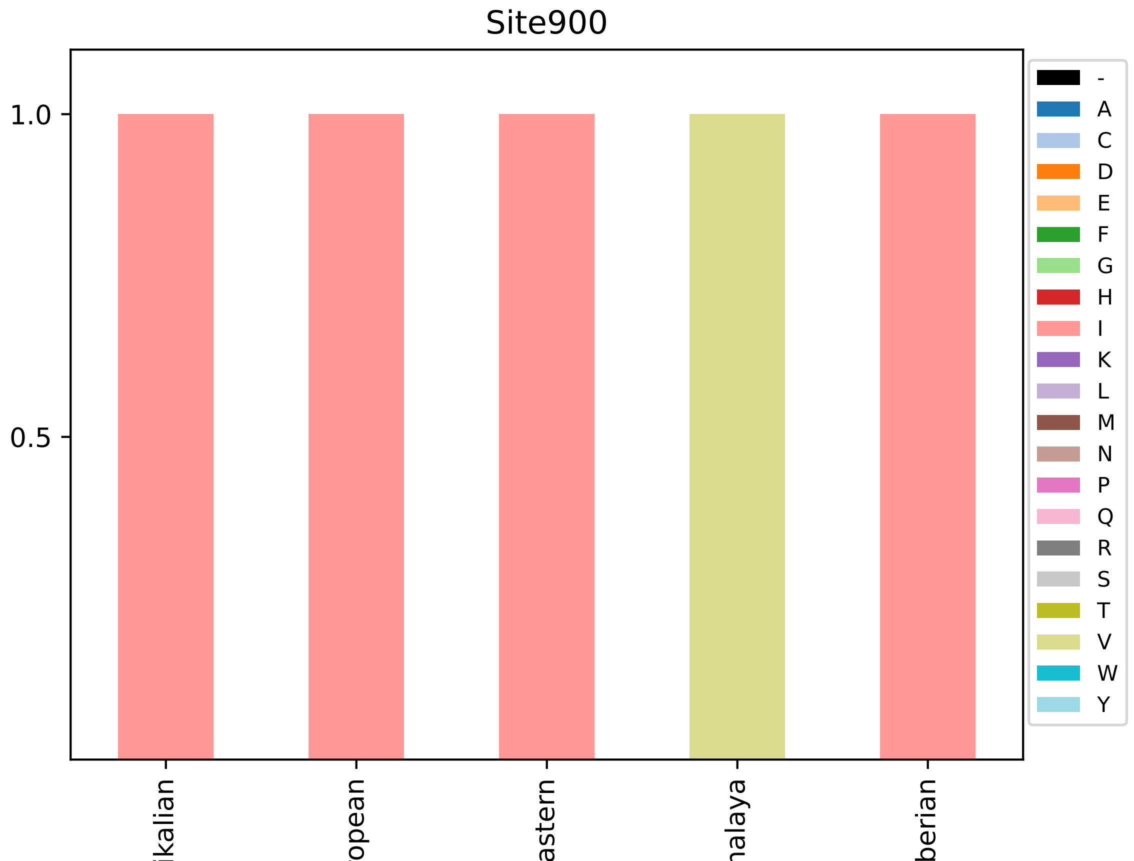
Site900
1.0
0.5
-
A
C
D
E
F
G
H
I
K
L
M
N
P
Q
R
S
T
V
W
Y
ikalian
opean
astern
nalaya
berian

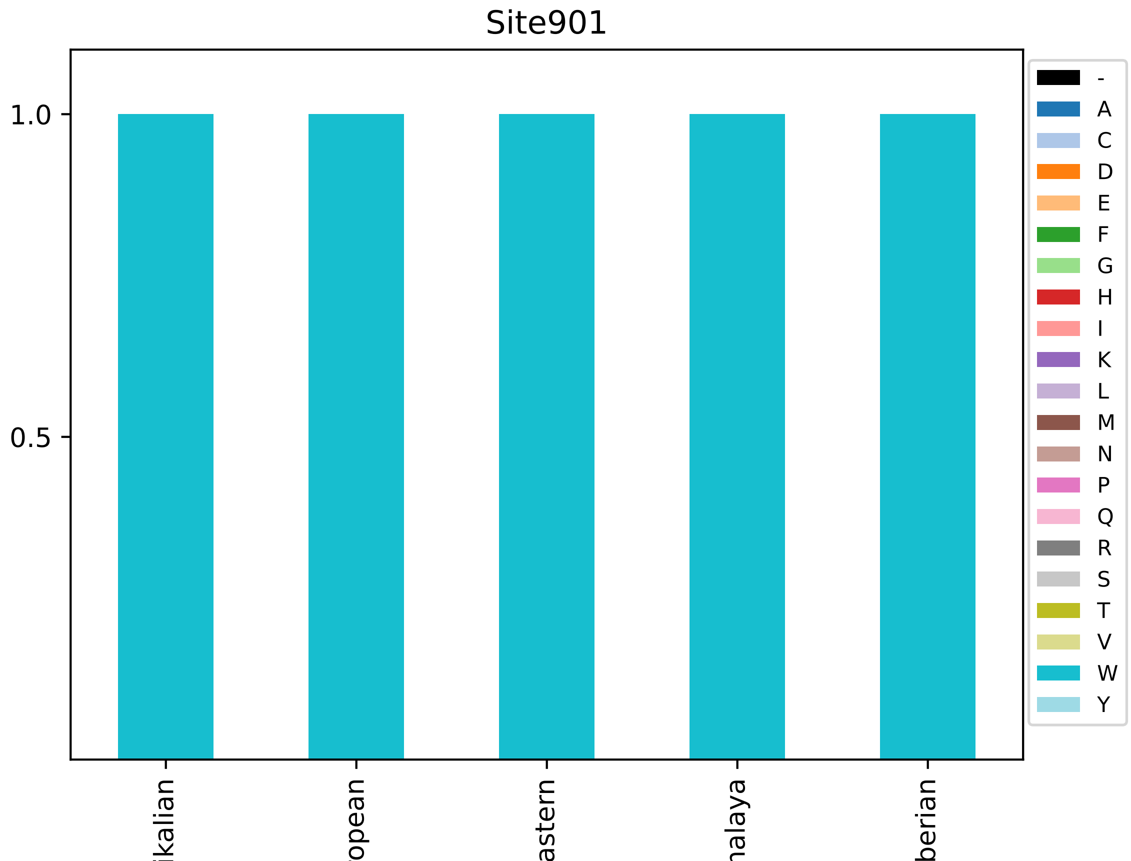

Site901
1.0
0.5
-
A
C
D
E
F
G
H
I
K
L
M
N
P
Q
R
S
T
V
W
Y
ikalian
opean
astern
nalaya
berian

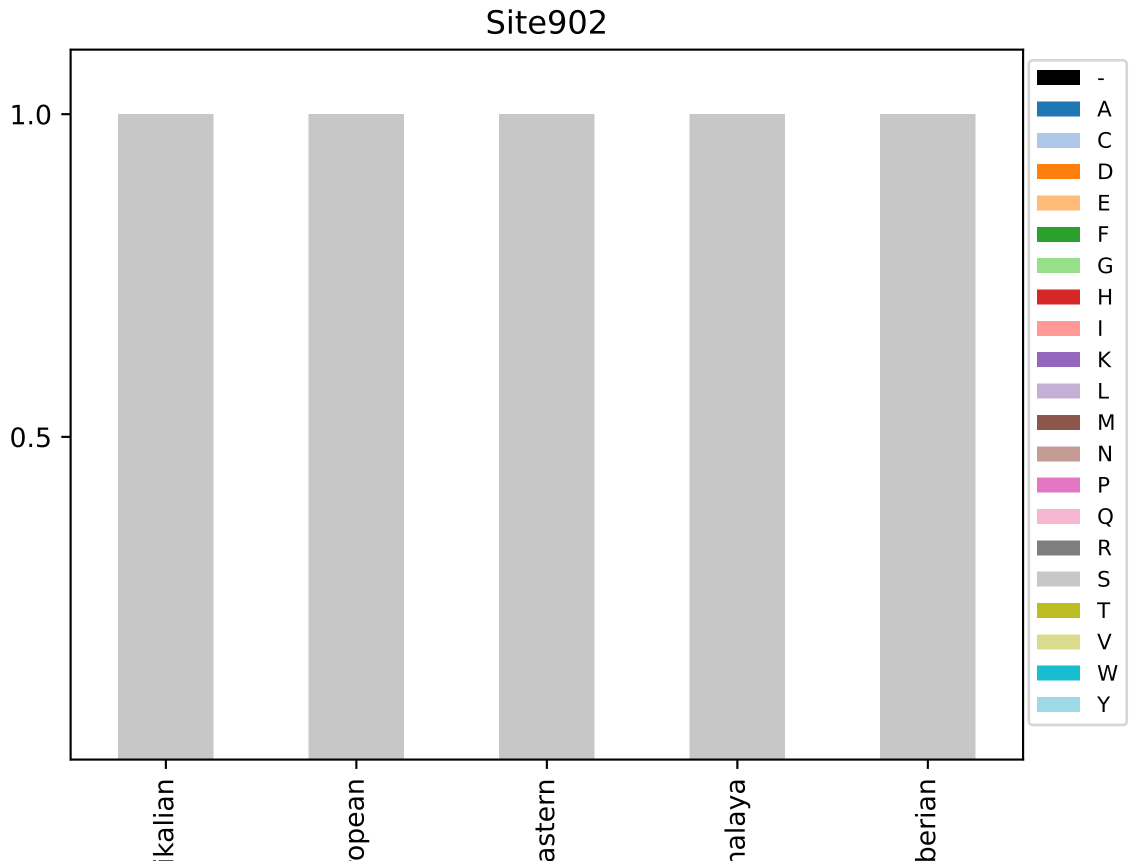

Site902
1.0
0.5
ikalian
opean
astern
nalaya
berian
-
A
C
D
E
F
G
H
I
K
L
M
N
P
Q
R
S
T
V
W
Y

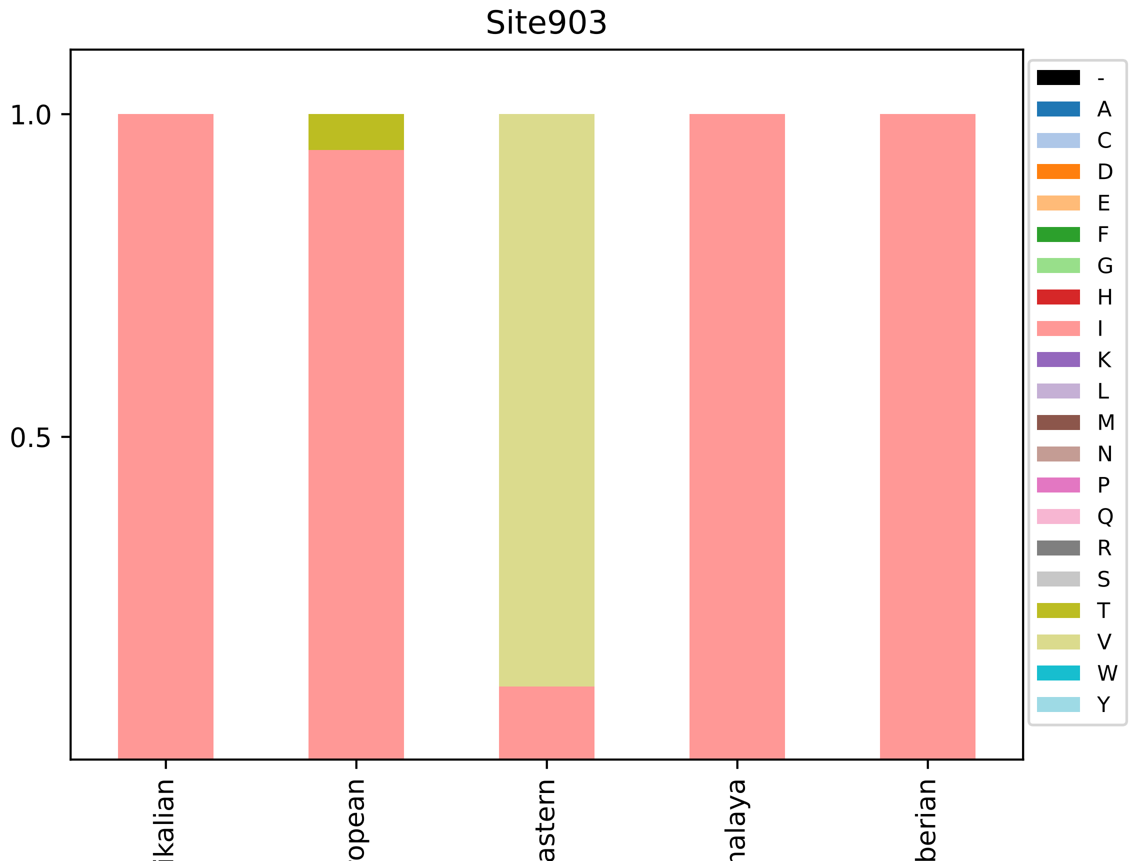

Site903
1.0
0.5
-
A
C
D
E
F
G
H
I
K
L
M
N
P
Q
R
S
T
V
W
Y
ikalian
opean
astern
nalaya
berian

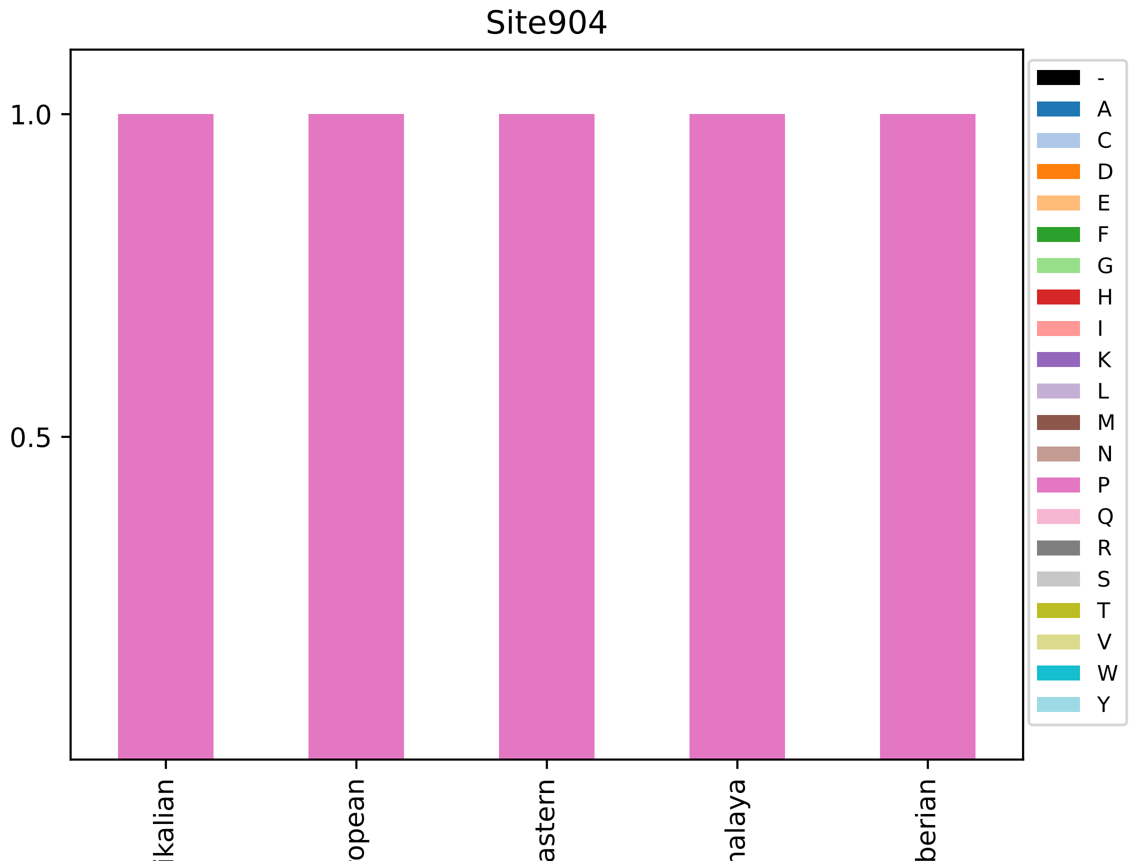

Site904
1.0
0.5
-
A
C
D
E
F
G
H
I
K
L
M
N
P
Q
R
S
T
V
W
Y

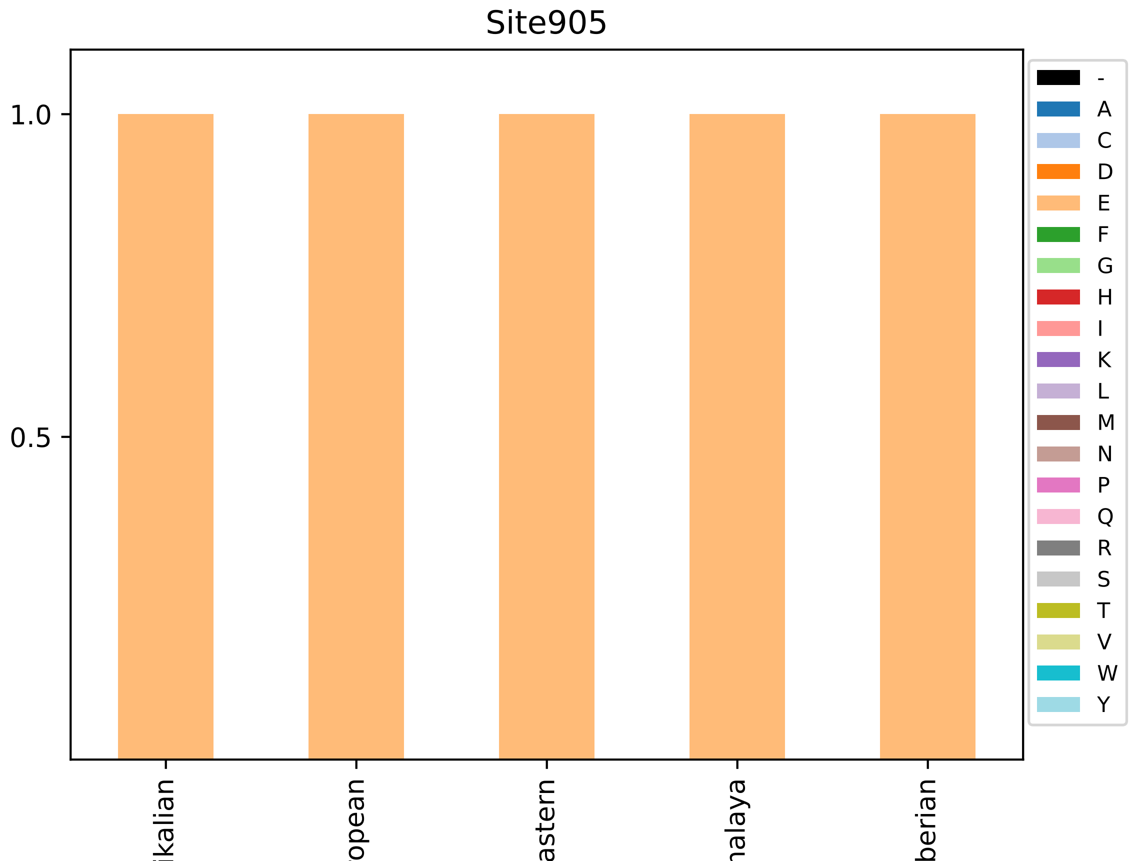

Site905
1.0
0.5
ikalian
ropean
astern
nalaya
berian
-
A
C
D
E
F
G
H
I
K
L
M
N
P
Q
R
S
T
V
W
Y

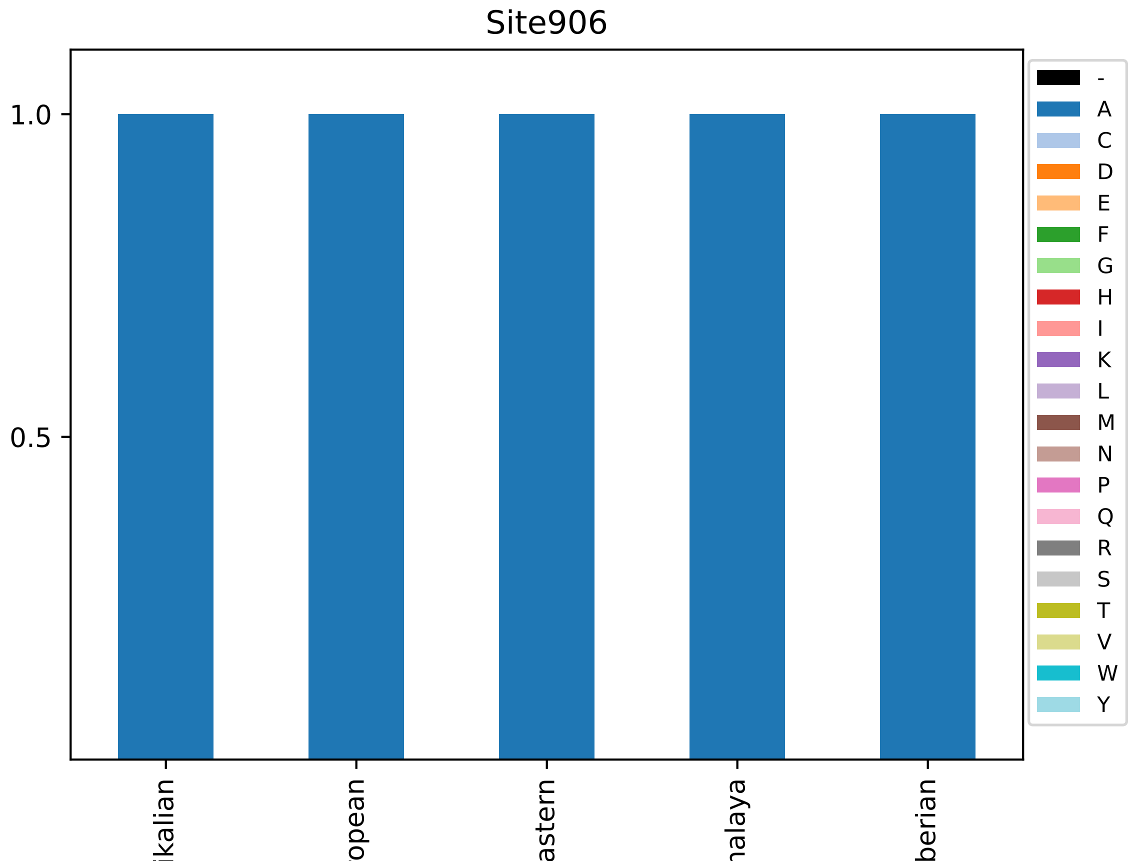
Site906
1.0
0.5
-
A
C
D
E
F
G
H
I
K
L
M
N
P
Q
R
S
T
V
W
Y
ikalian
opean
astern
nalaya
berian

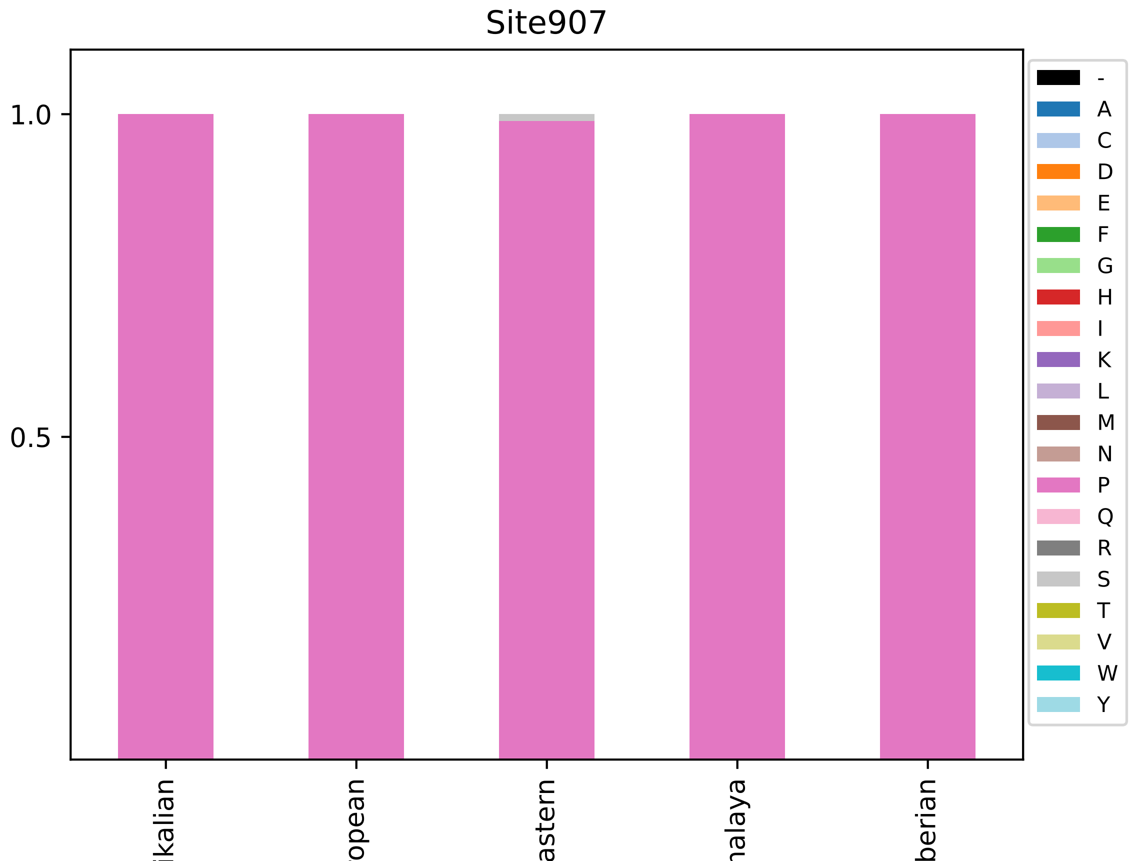
Site907
1.0
0.5
-
A
C
D
E
F
G
H
I
K
L
M
N
P
Q
R
S
T
V
W
Y

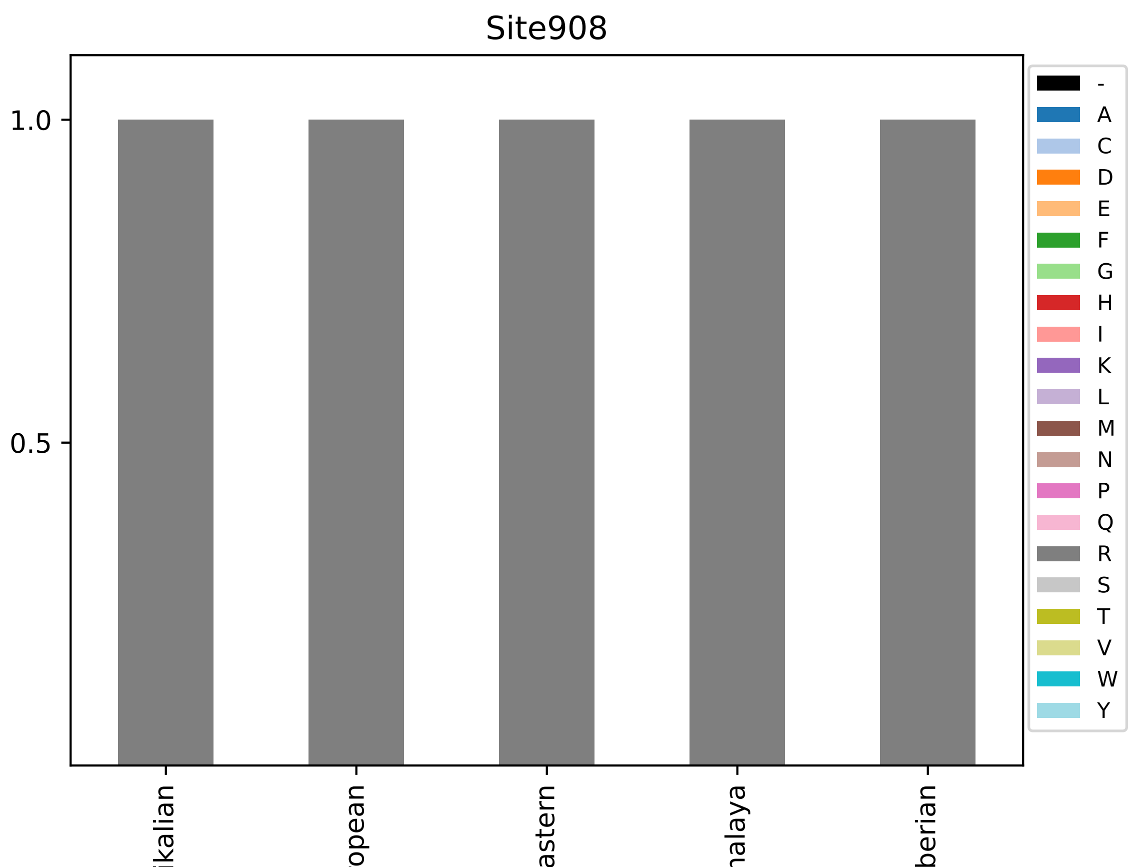

Site908
1.0
0.5
-
A
C
D
E
F
G
H
I
K
L
M
N
P
Q
R
S
T
V
W
Y

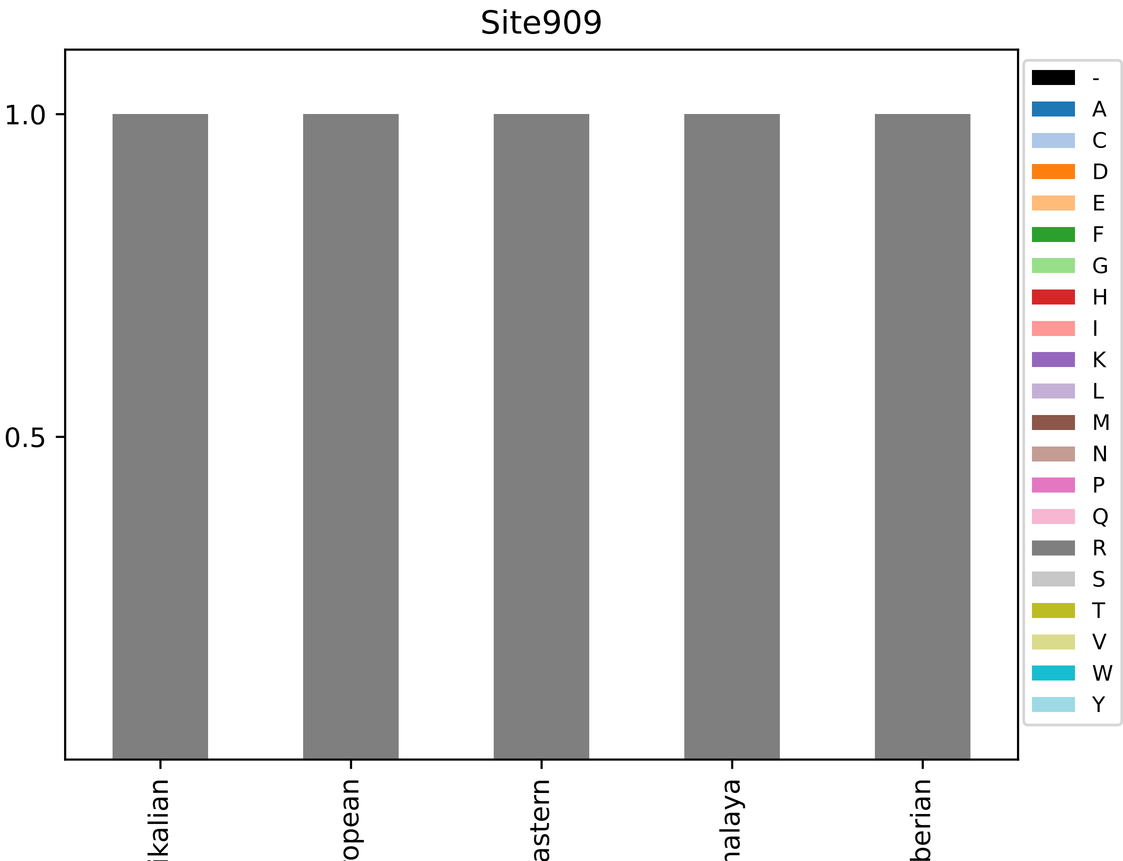

Site909
1.0
0.5
-
A
C
D
E
F
G
H
I
K
L
M
N
P
Q
R
S
T
V
W
Y

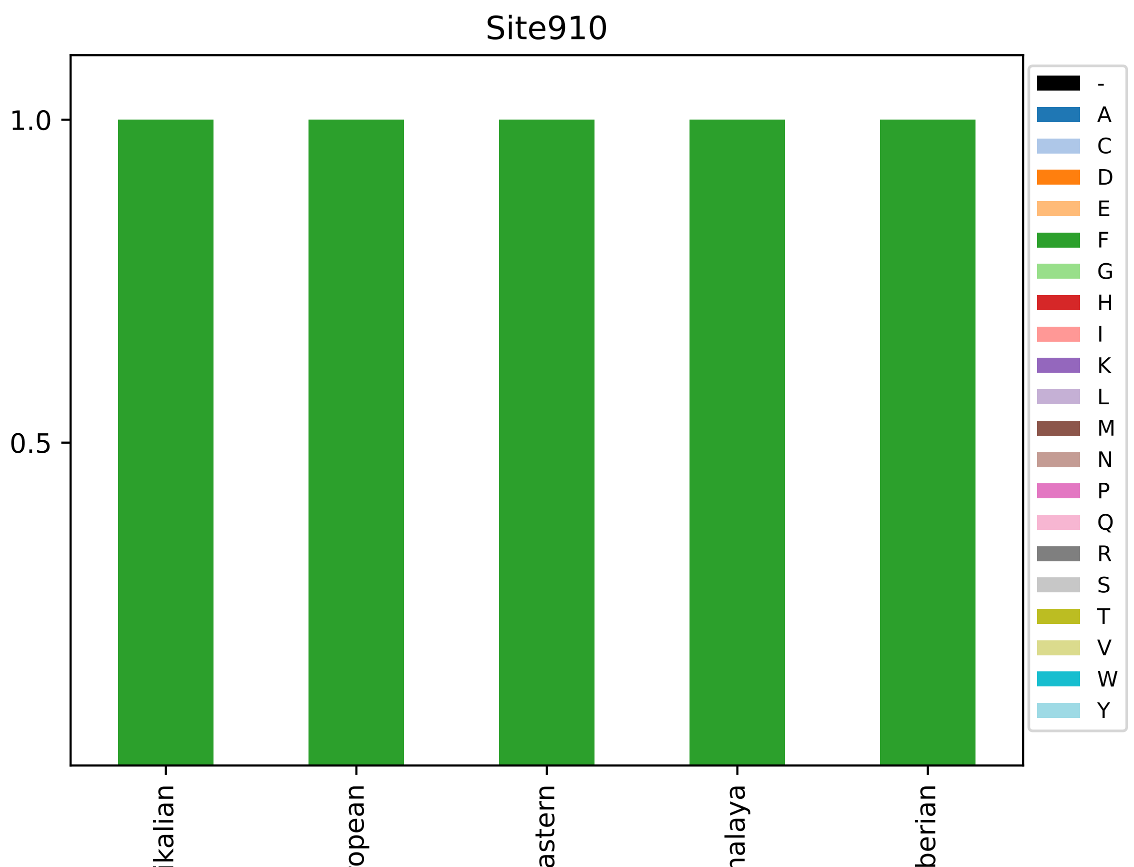
Site910
1.0
0.5
-
A
C
D
E
F
G
H
I
K
L
M
N
P
Q
R
S
T
V
W
Y
ikalian
ropean
astern
nalaya
berian

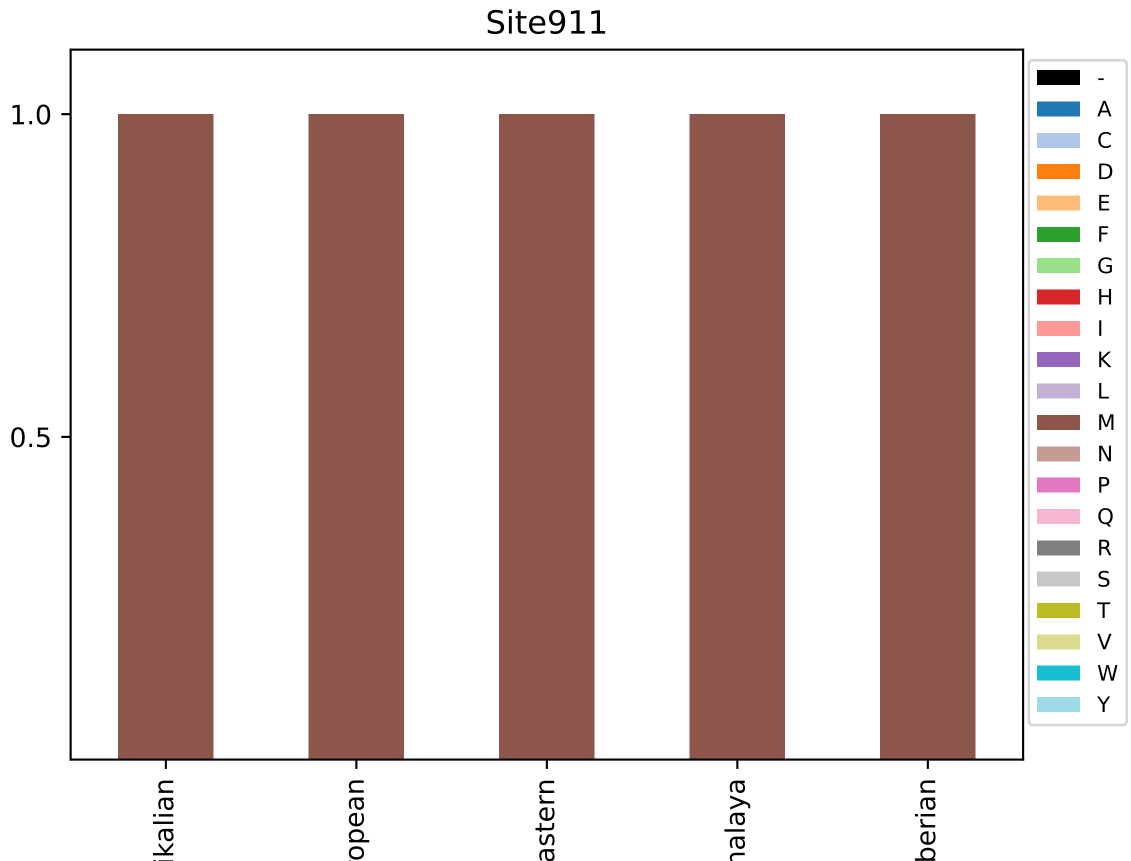
Site911
1.0
0.5
-
A
C
D
E
F
G
H
I
K
L
M
N
P
Q
R
S
T
V
W
Y
ikalian
opean
astern
nalaya
berian

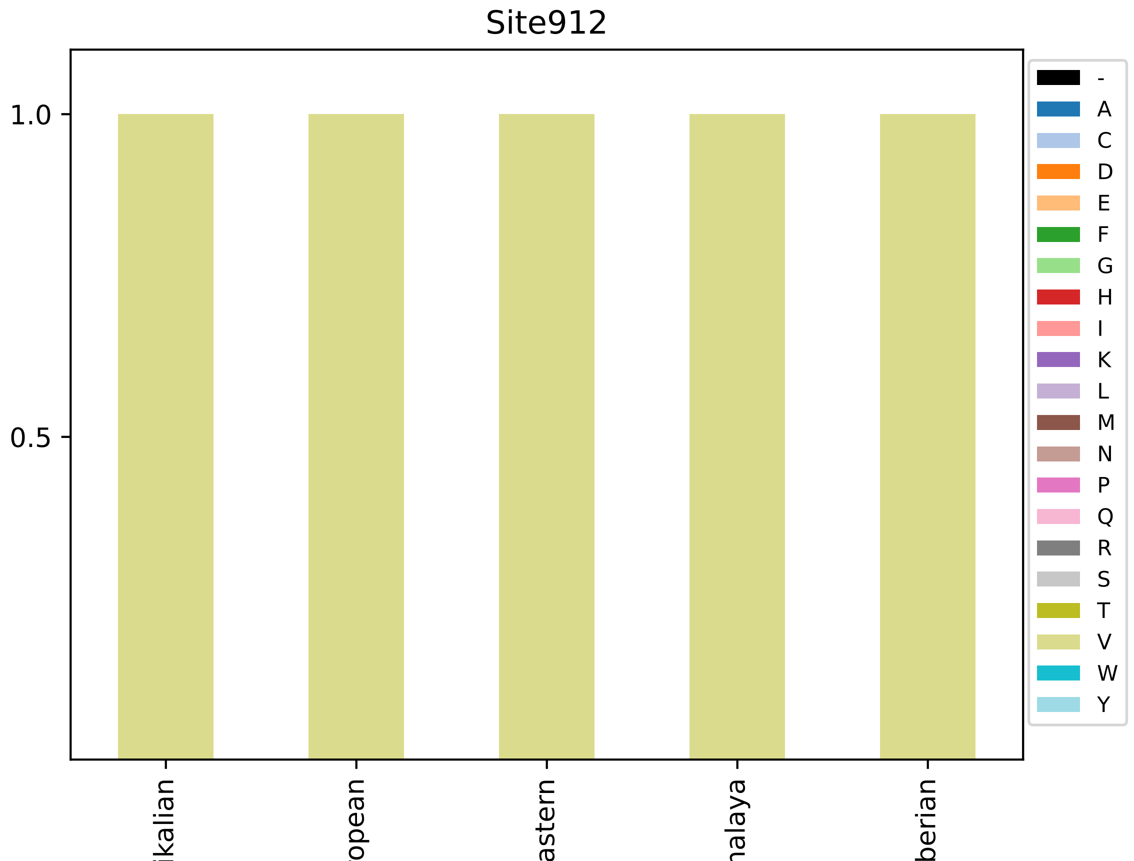

Site912
1.0
0.5
-
A
C
D
E
F
G
H
I
K
L
M
N
P
Q
R
S
T
V
W
Y
ikalian
opean
astern
nalaya
berian

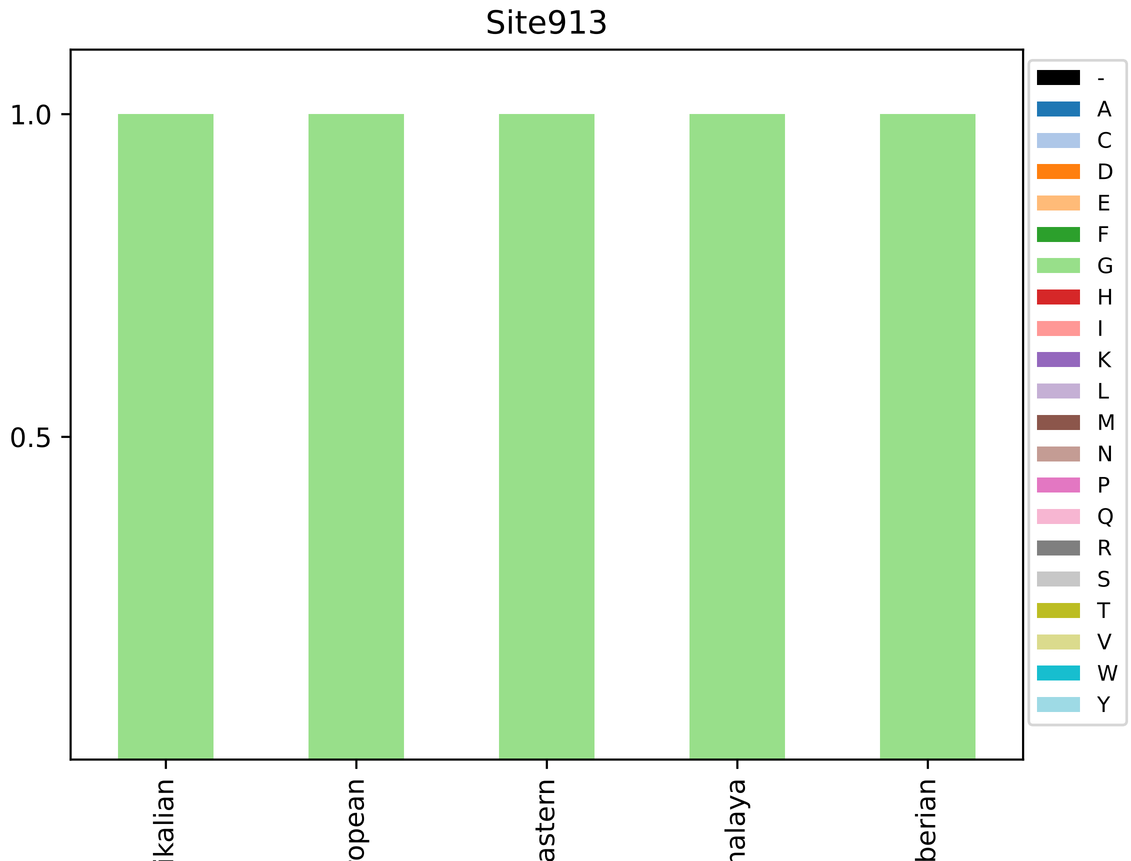

Site913
1.0
0.5
-
A
C
D
E
F
G
H
I
K
L
M
N
P
Q
R
S
T
V
W
Y
ikalian
opean
astern
nalaya
berian

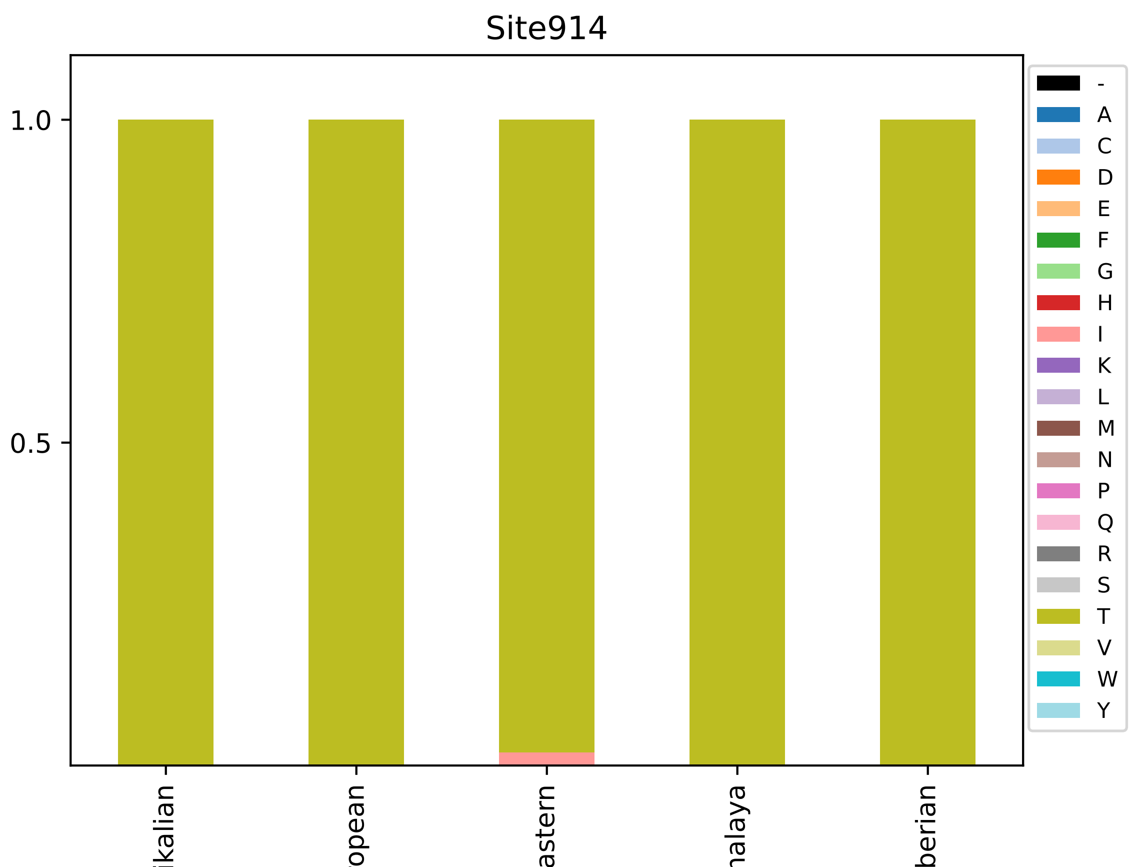
Site914
1.0
0.5
-
A
C
D
E
F
G
H
I
K
L
M
N
P
Q
R
S
T
V
W
Y
ikalian
opean
astern
nalaya
berian

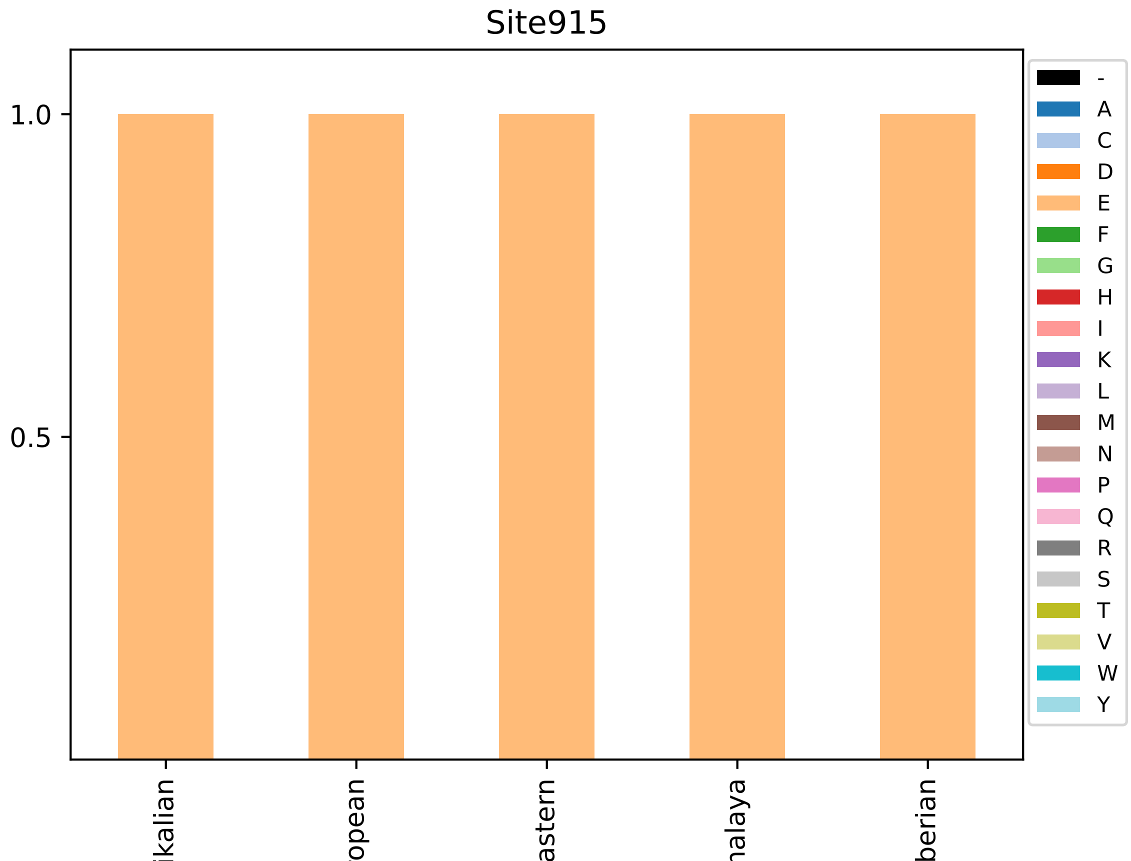

Site915
1.0
0.5
-
A
C
D
E
F
G
H
I
K
L
M
N
P
Q
R
S
T
V
W
Y

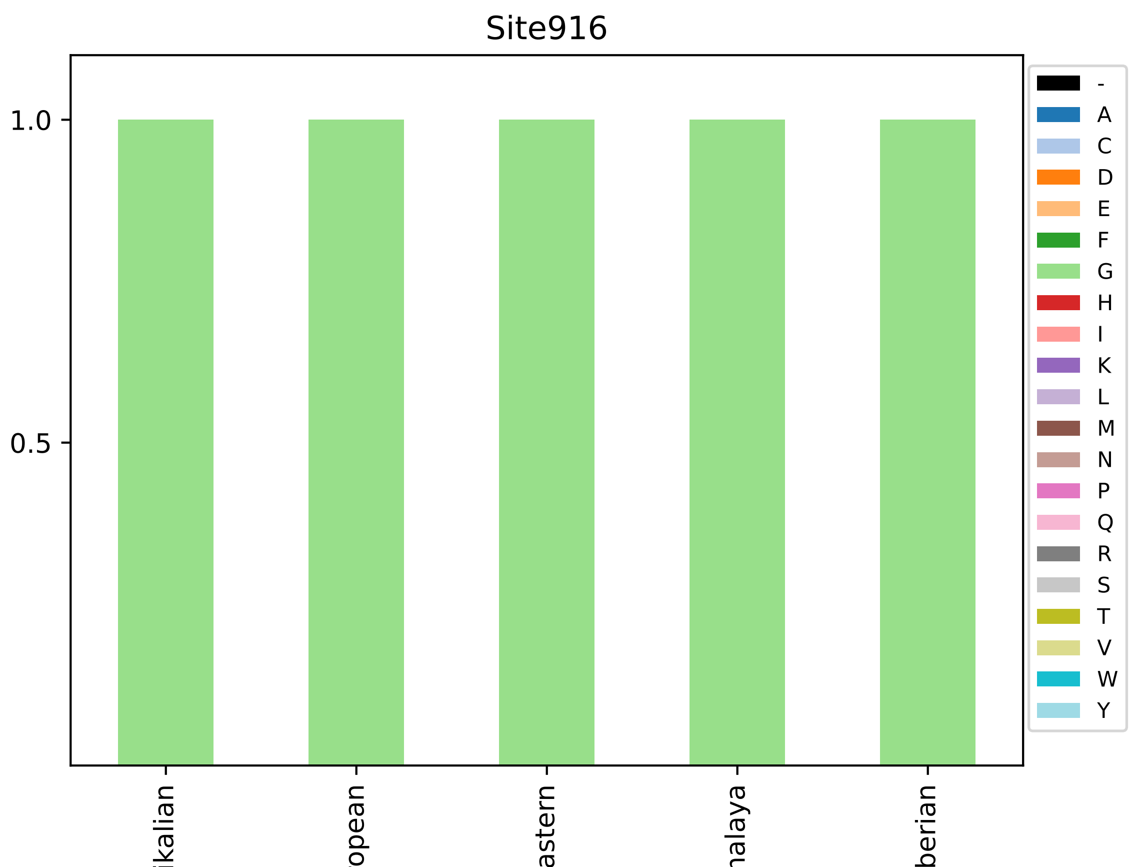

Site916
1.0
0.5
-
A
C
D
E
F
G
H
I
K
L
M
N
P
Q
R
S
T
V
W
Y
ikalian
opean
astern
nalaya
berian

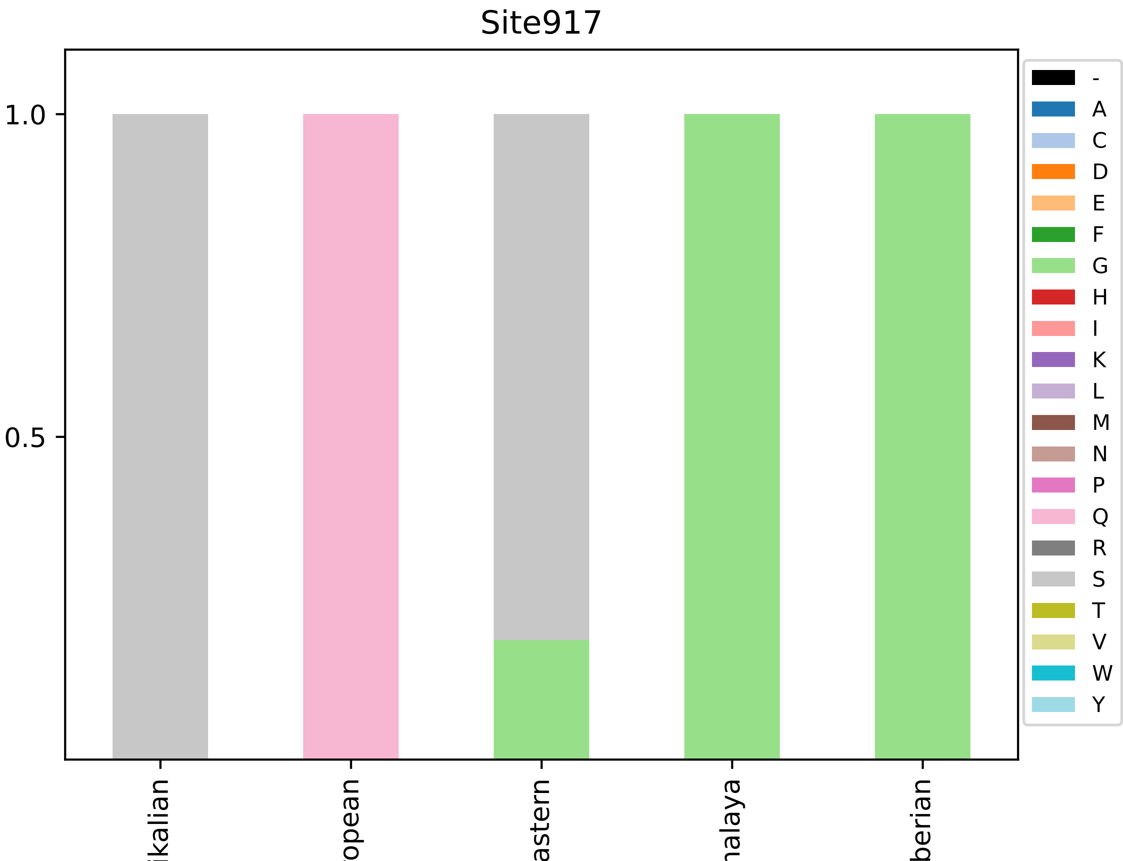
Site917
1.0
0.5
-
A
C
D
E
F
G
H
I
K
L
M
N
P
Q
R
S
T
V
W
Y
ikalian
opean
astern
nalaya
berian

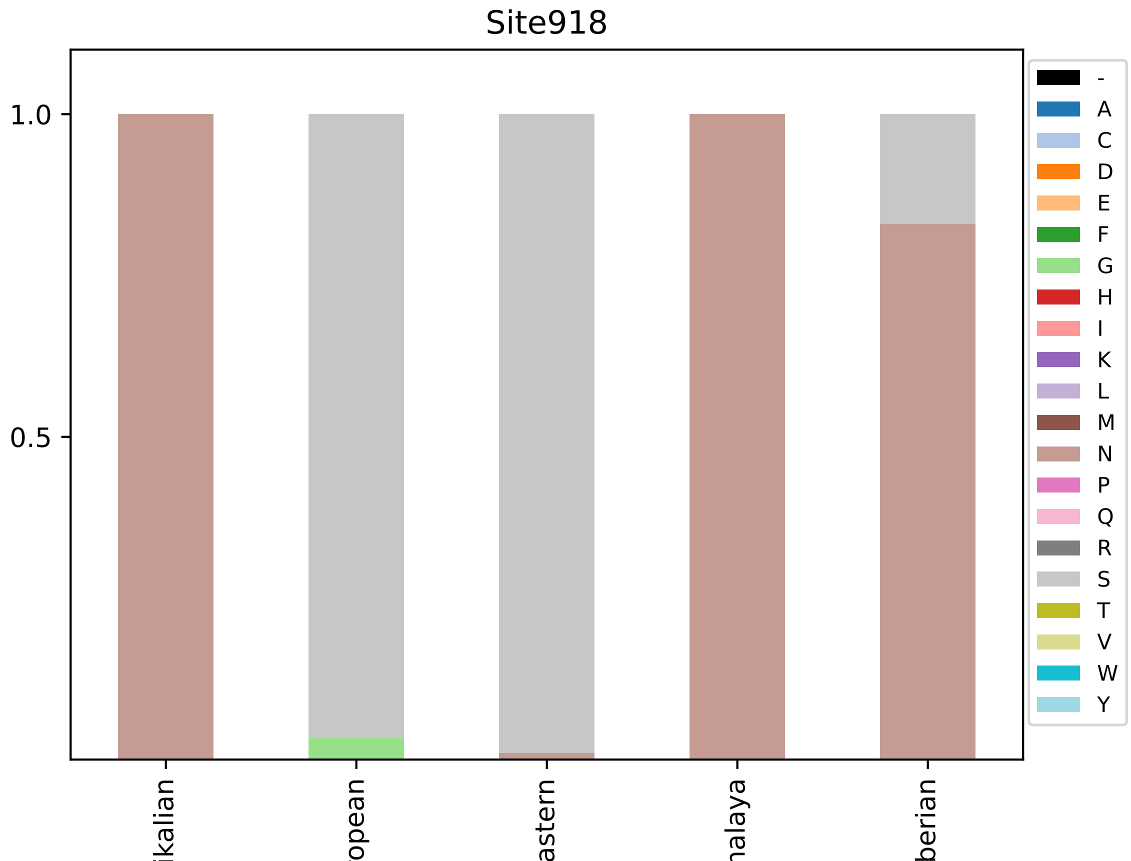

Site918
1.0
0.5
ikalian
ropean
astern
nalaya
berian
-
A
C
D
E
F
G
H
I
K
L
M
N
P
Q
R
S
T
V
W
Y

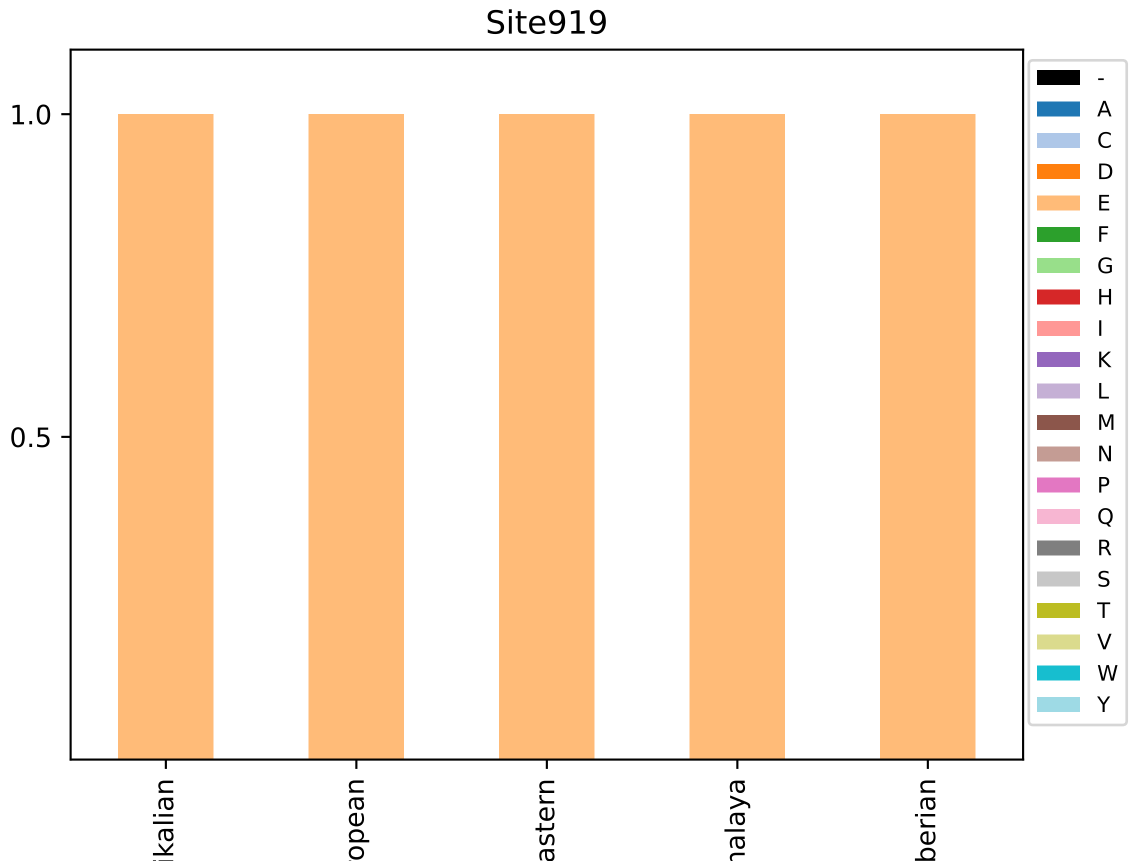
Site919
1.0
0.5
-
A
C
D
E
F
G
H
I
K
L
M
N
P
Q
R
S
T
V
W
Y
ikalian
opean
astern
nalaya
berian

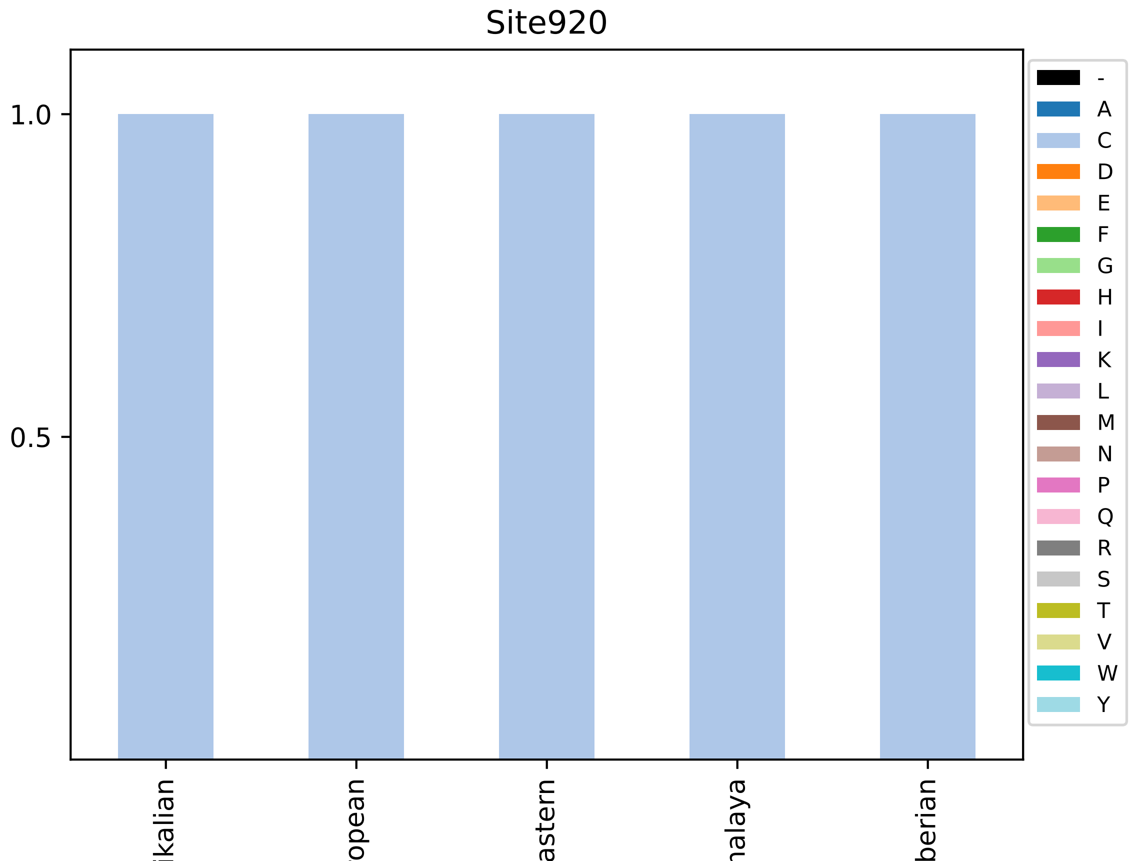
Site920
1.0
0.5
-
A
C
D
E
F
G
H
I
K
L
M
N
P
Q
R
S
T
V
W
Y
ikalian
opean
astern
nalaya
berian

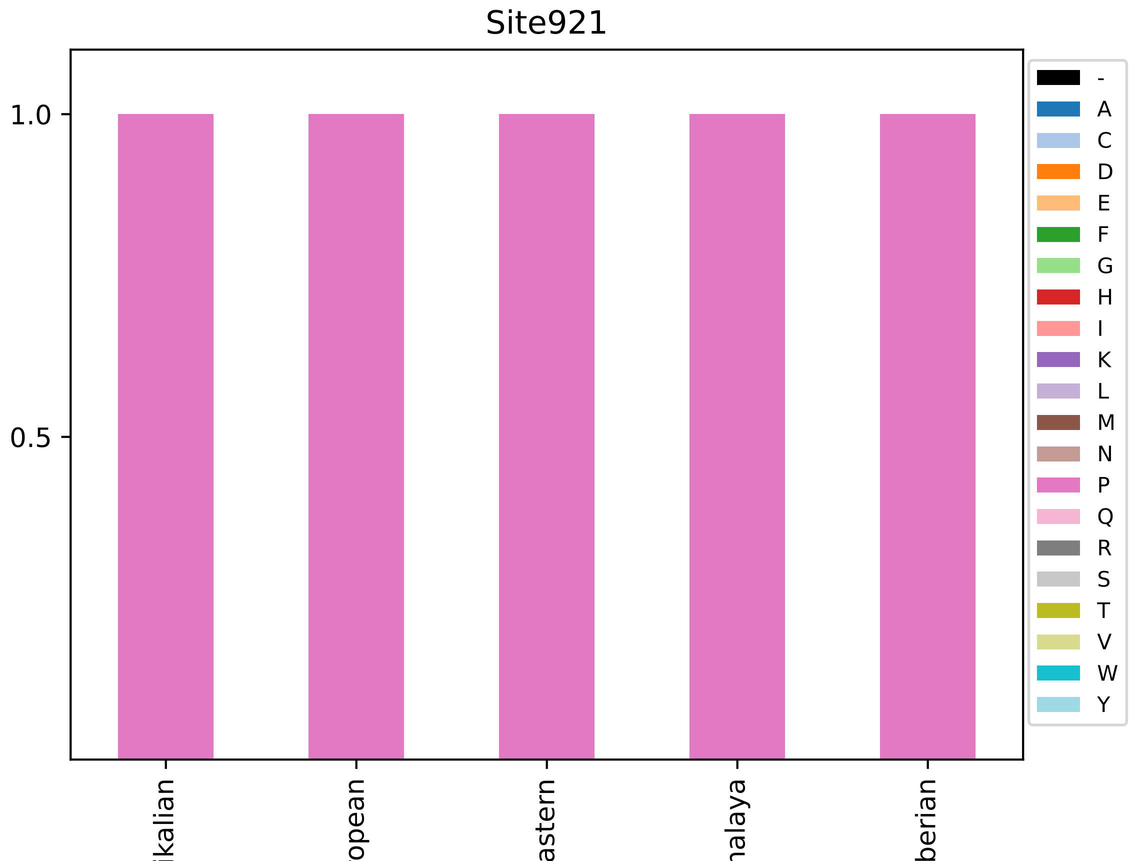

Site921
1.0
0.5
-
A
C
D
E
F
G
H
I
K
L
M
N
P
Q
R
S
T
V
W
Y
ikalian
opean
astern
nalaya
berian

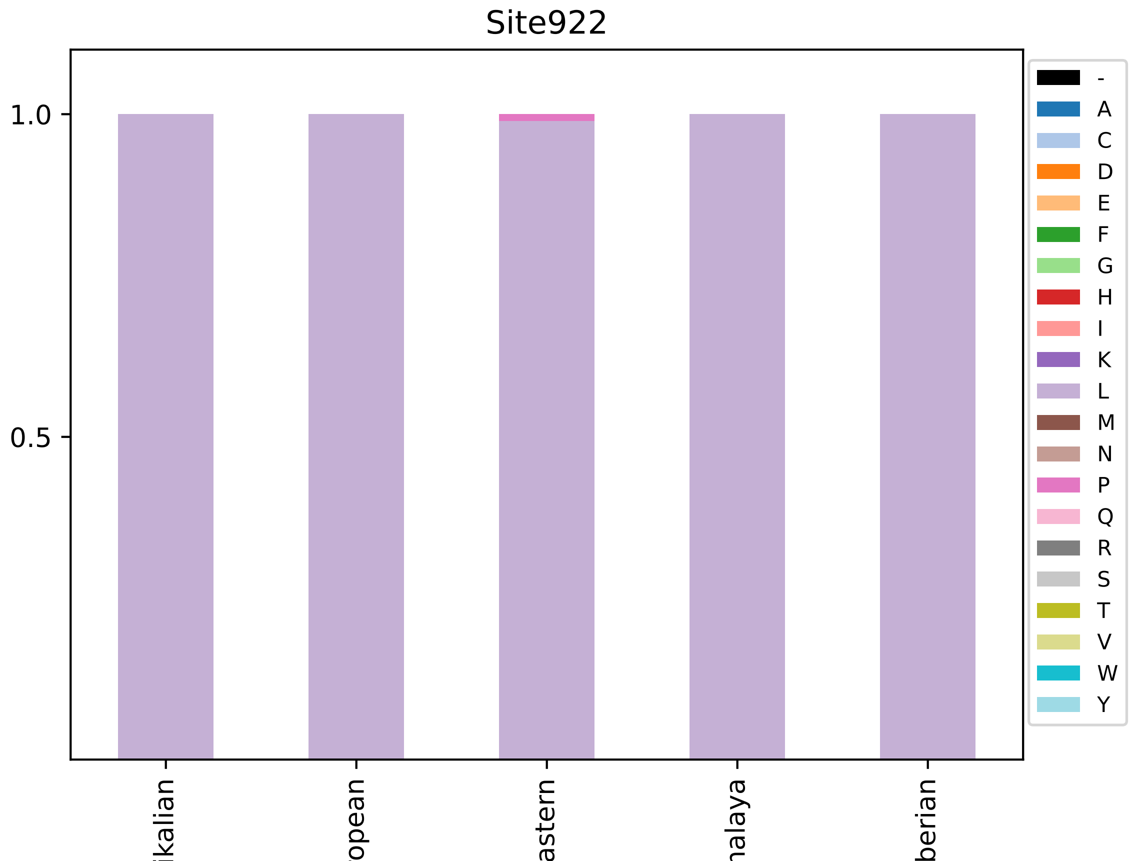
Site922
1.0
0.5
-
A
C
D
E
F
G
H
I
K
L
M
N
P
Q
R
S
T
V
W
Y
ikalian
opean
astern
nalaya
berian

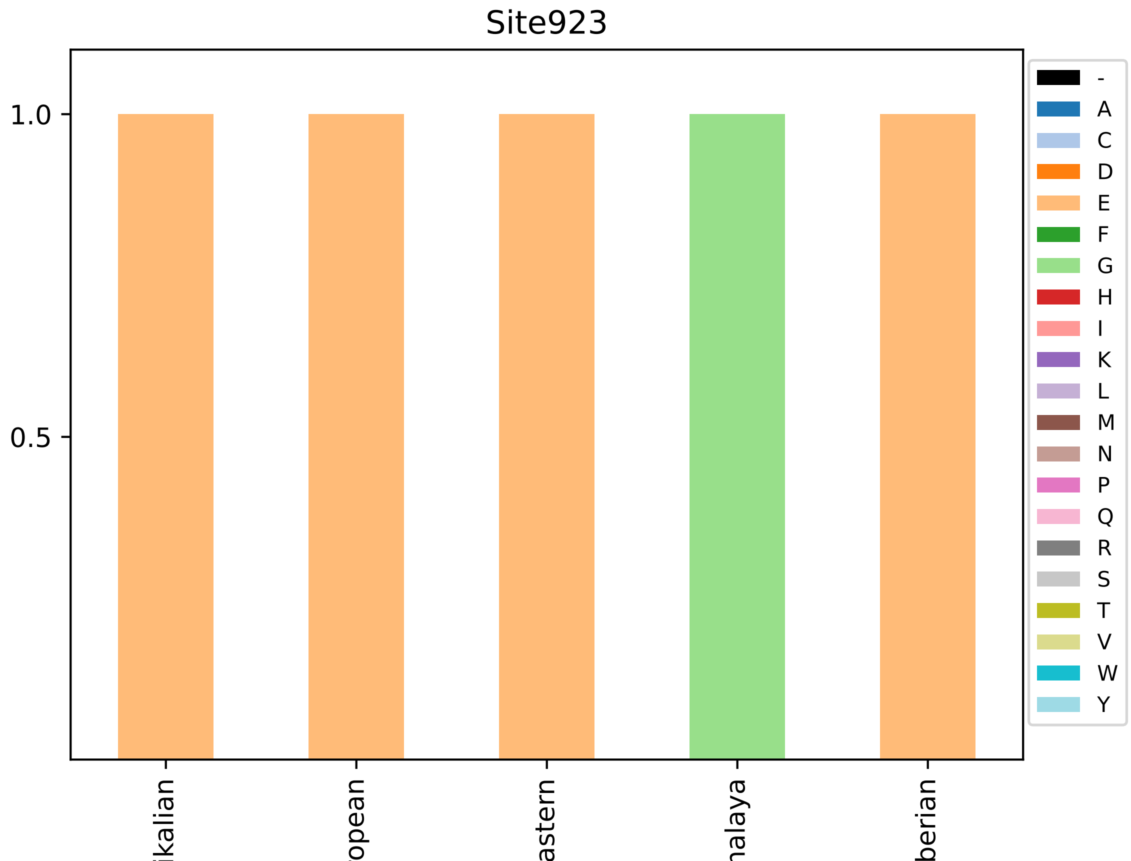
Site923
1.0
0.5
-
A
C
D
E
F
G
H
I
K
L
M
N
P
Q
R
S
T
V
W
Y
ikalian
opean
astern
nalaya
berian

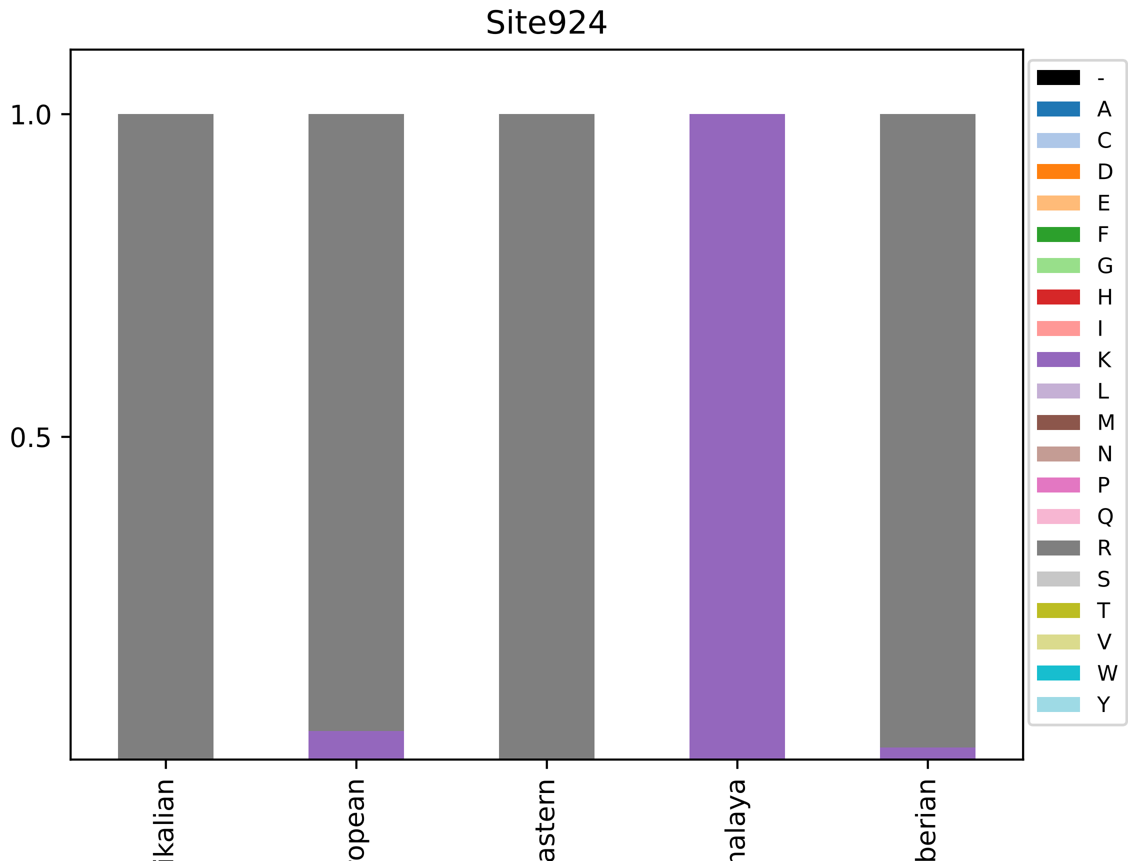

Site924
1.0
0.5
ikalian
opean
astern
nalaya
berian
-
A
C
D
E
F
G
H
I
K
L
M
N
P
Q
R
S
T
V
W
Y

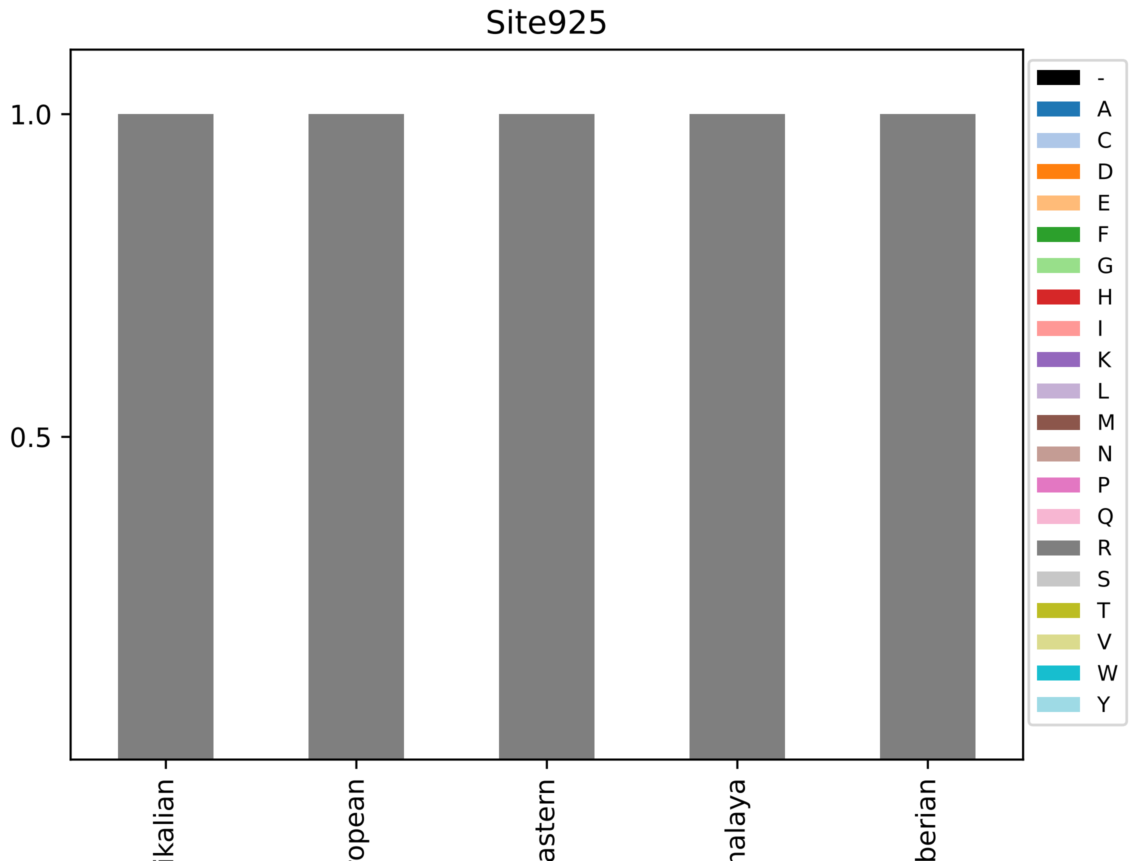
Site925
1.0
0.5
-
A
C
D
E
F
G
H
I
K
L
M
N
P
Q
R
S
T
V
W
Y
ikalian
opean
astern
nalaya
berian

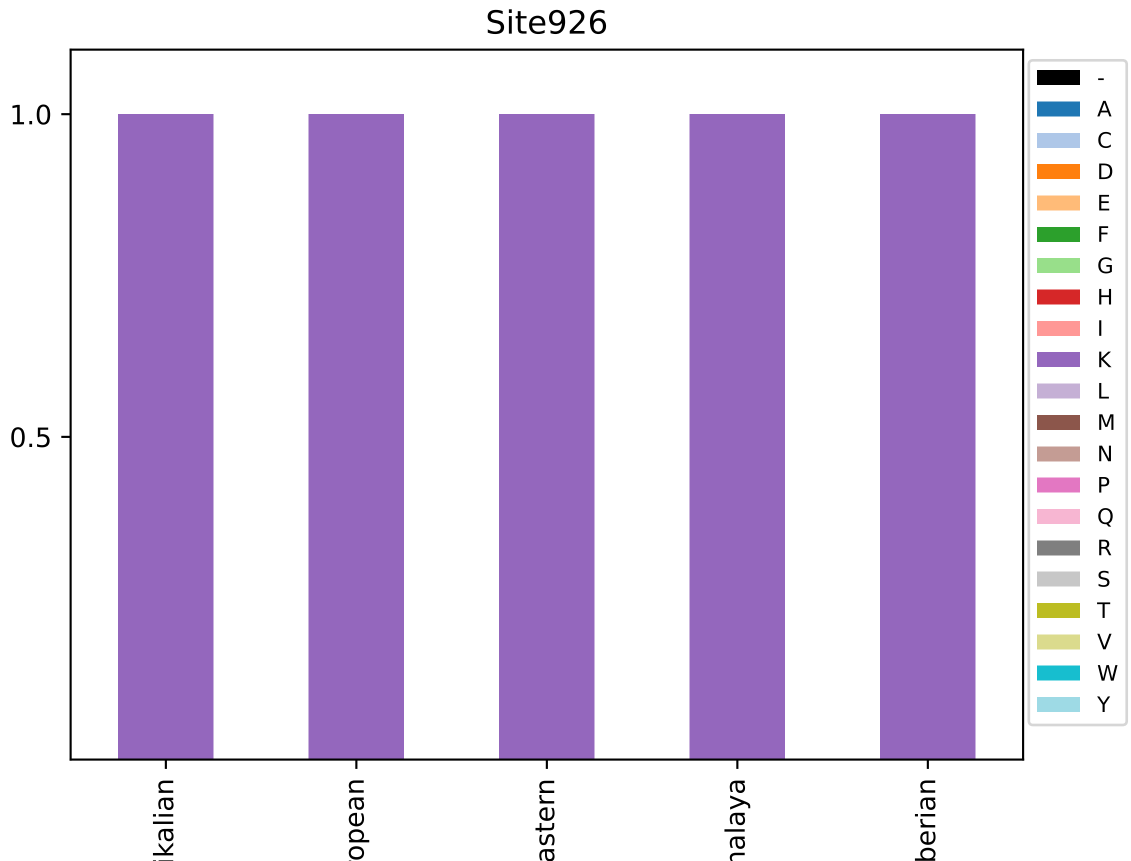

Site926
1.0
0.5
-
A
C
D
E
F
G
H
I
K
L
M
N
P
Q
R
S
T
V
W
Y
ikalian
opean
astern
nalaya
berian

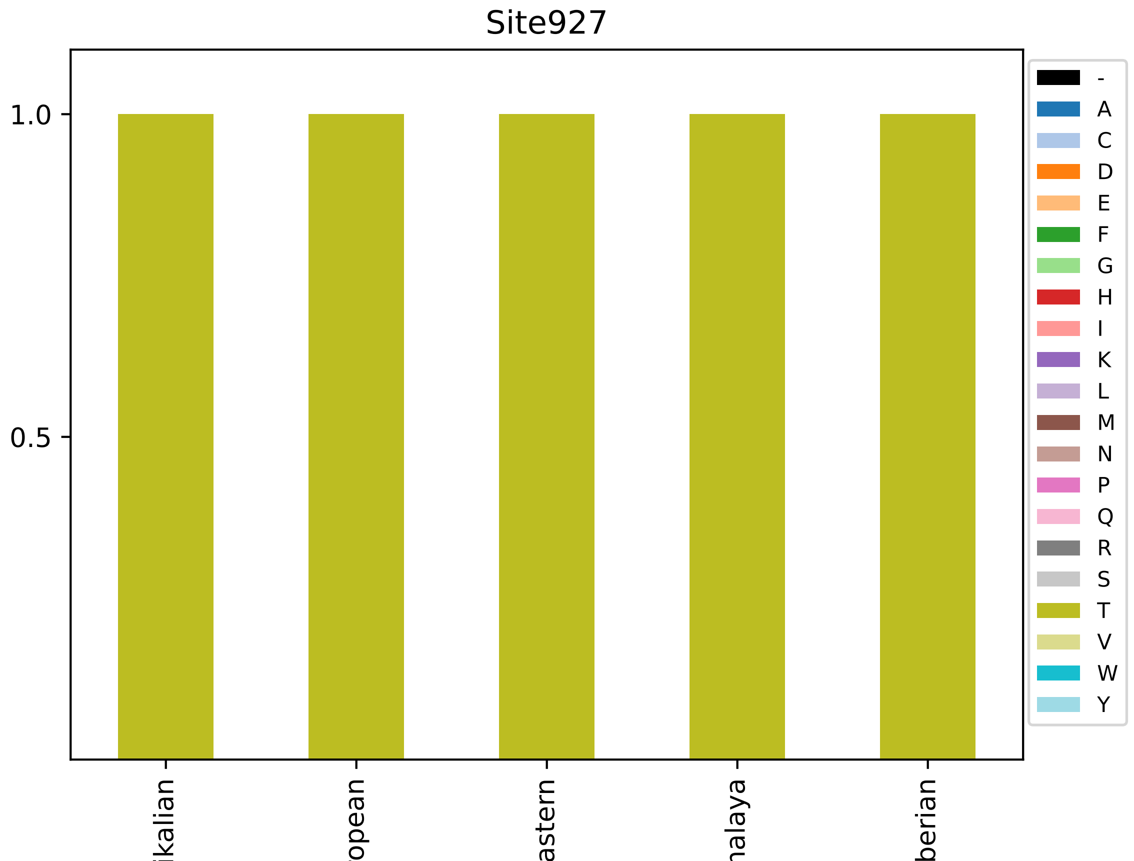

Site927
1.0
0.5
-
A
C
D
E
F
G
H
I
K
L
M
N
P
Q
R
S
T
V
W
Y
ikalian
opean
astern
nalaya
berian

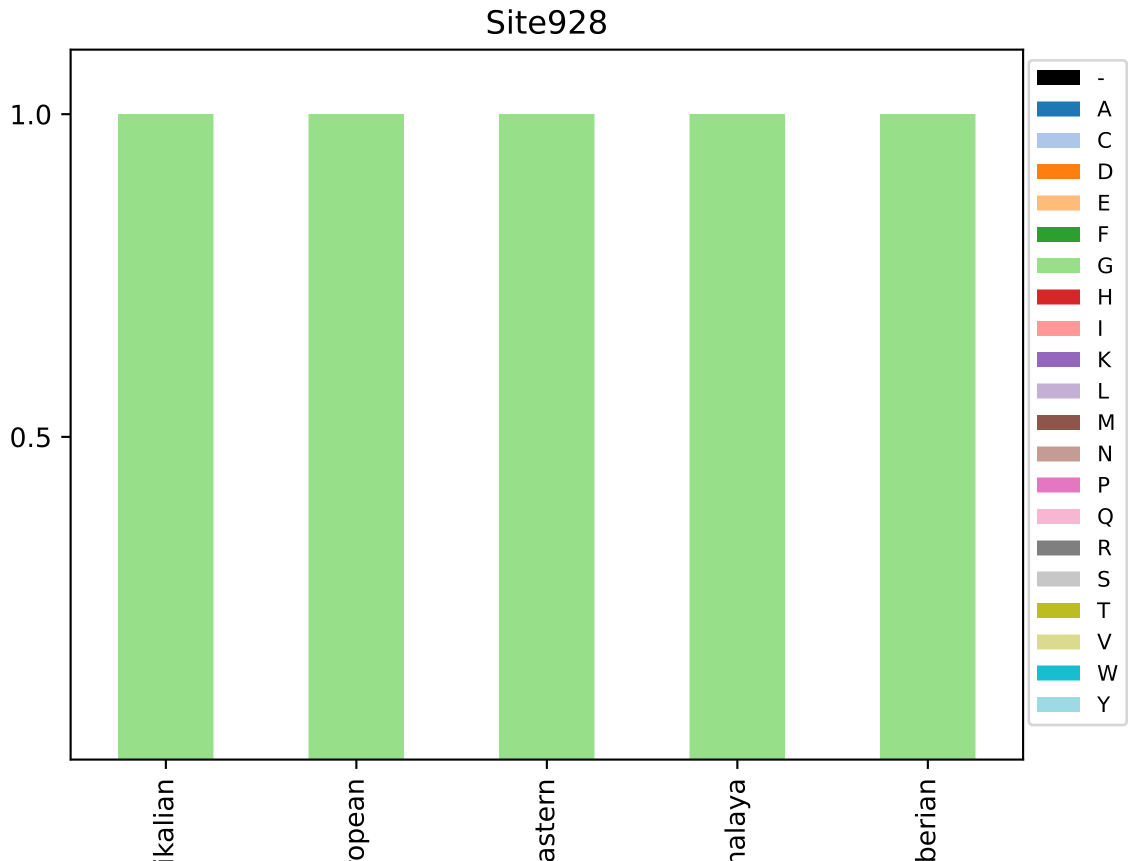

Site928
1.0
0.5
-
A
C
D
E
F
G
H
I
K
L
M
N
P
Q
R
S
T
V
W
Y

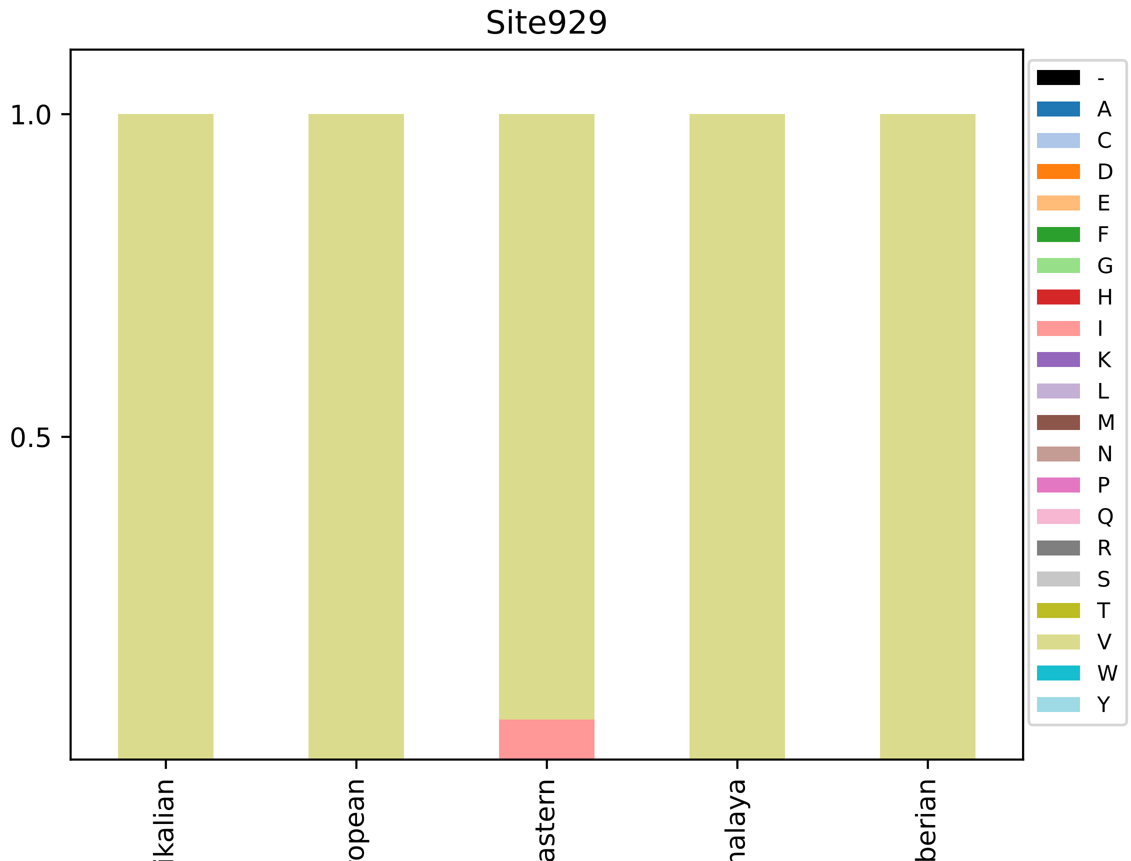

Site929
1.0
0.5
-
A
C
D
E
F
G
H
I
K
L
M
N
P
Q
R
S
T
V
W
Y
ikalian
opean
astern
nalaya
berian

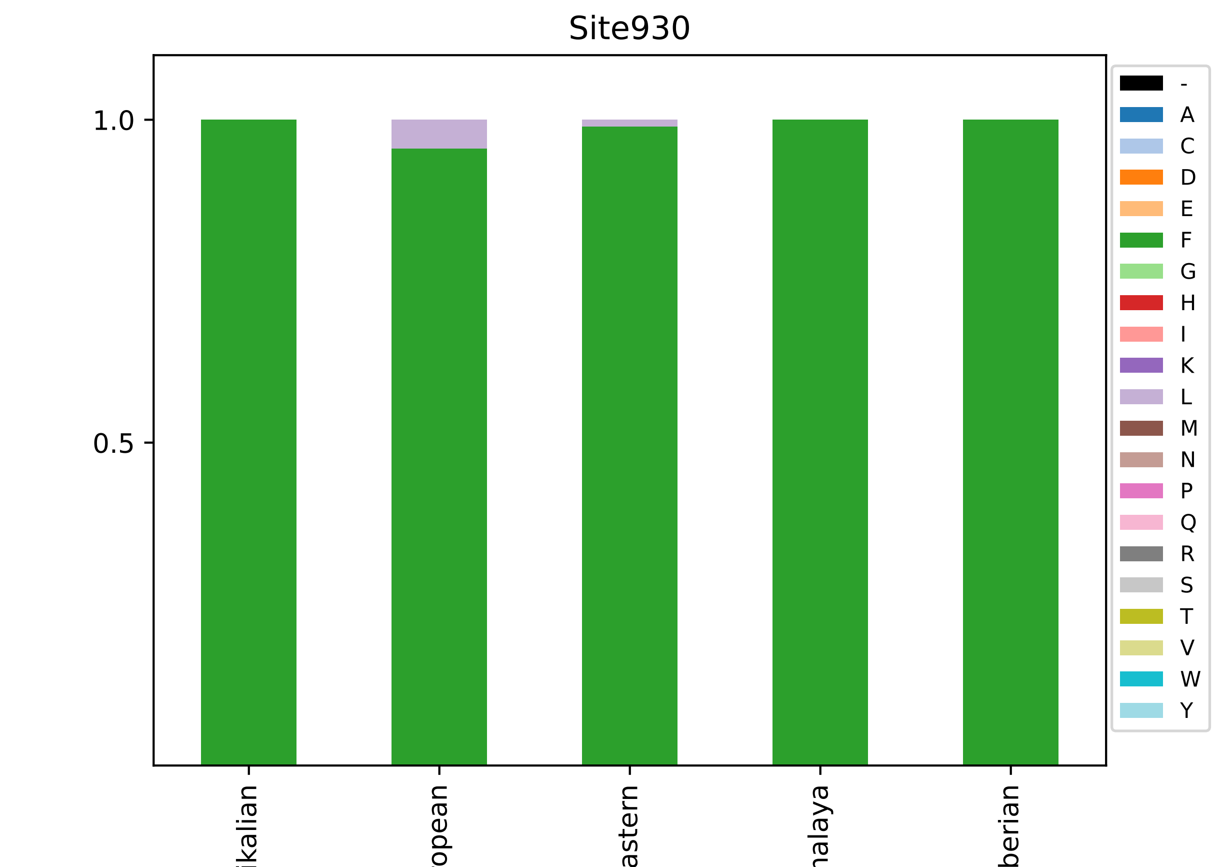
Site930
1.0
0.5
-
A
C
D
E
F
G
H
I
K
L
M
N
P
Q
R
S
T
V
W
Y
ikalian
opean
astern
nalaya
berian

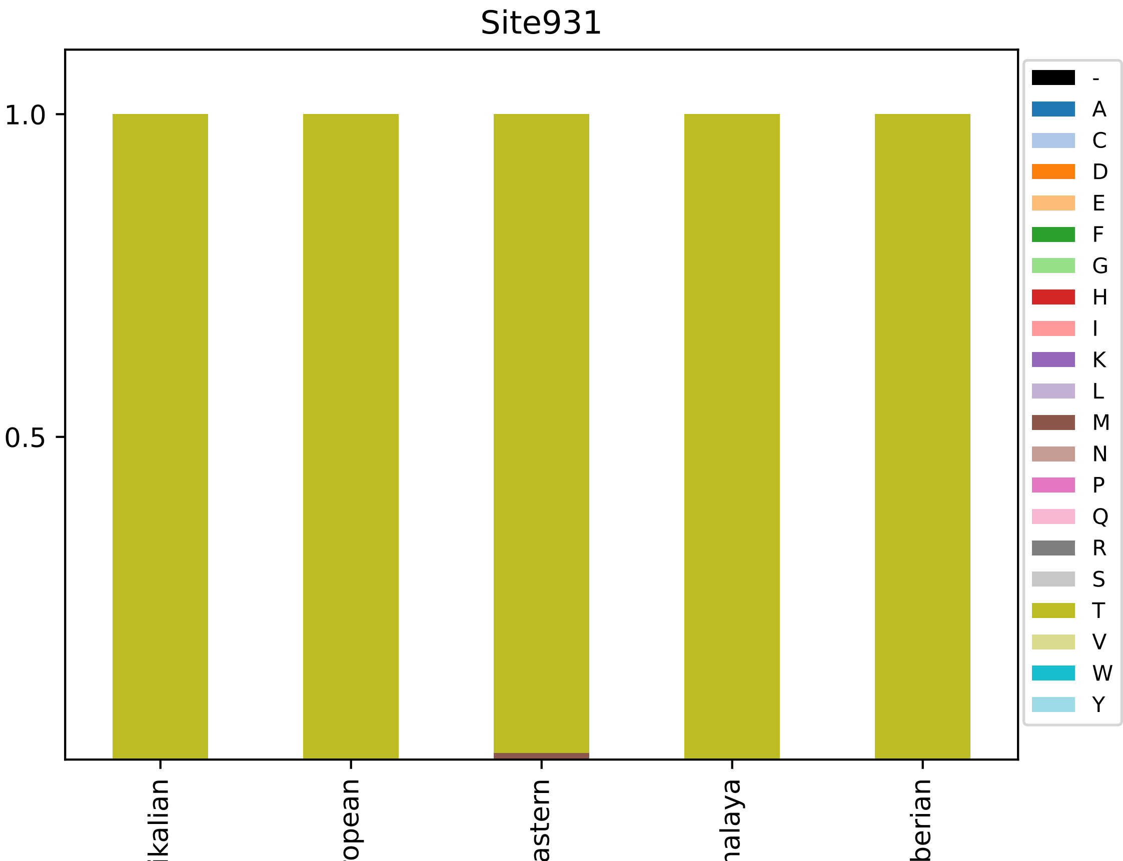

Site931
1.0
0.5
-
A
C
D
E
F
G
H
I
K
L
M
N
P
Q
R
S
T
V
W
Y

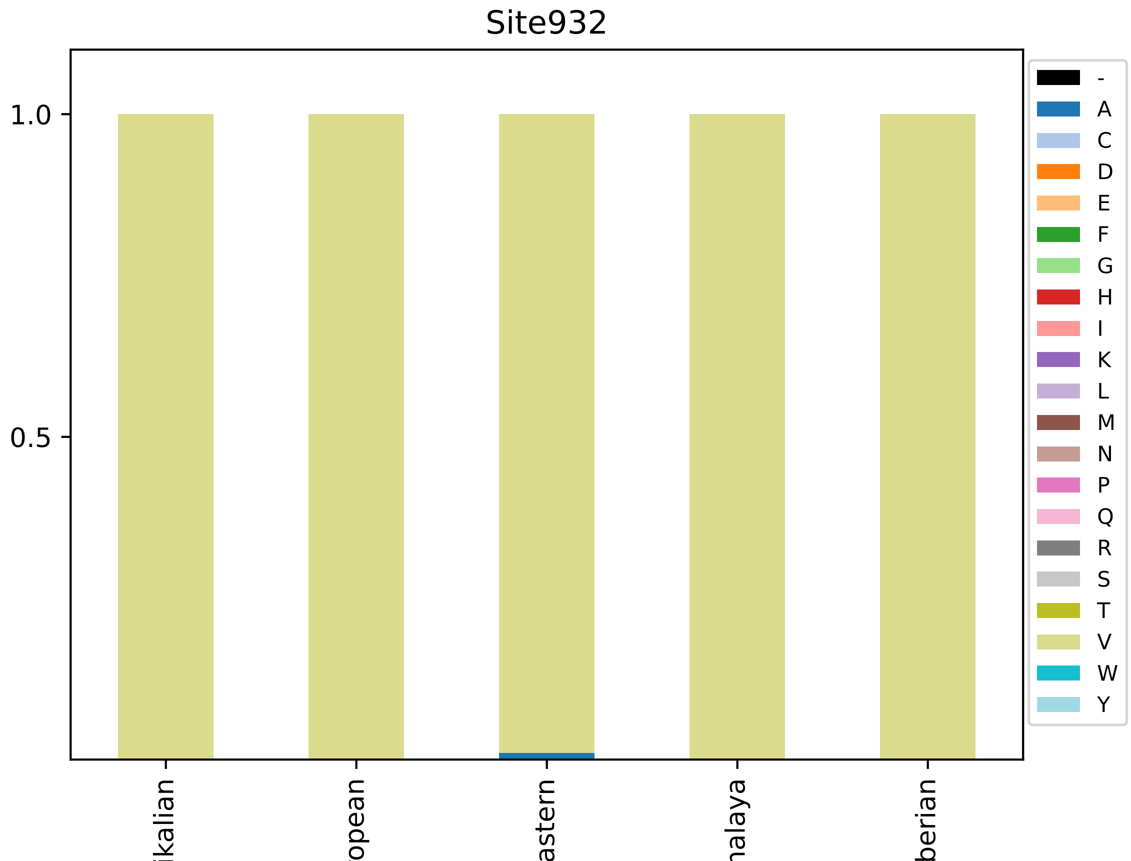

Site932
1.0
0.5
-
A
C
D
E
F
G
H
I
K
L
M
N
P
Q
R
S
T
V
W
Y
ikalian
opean
astern
nalaya
berian

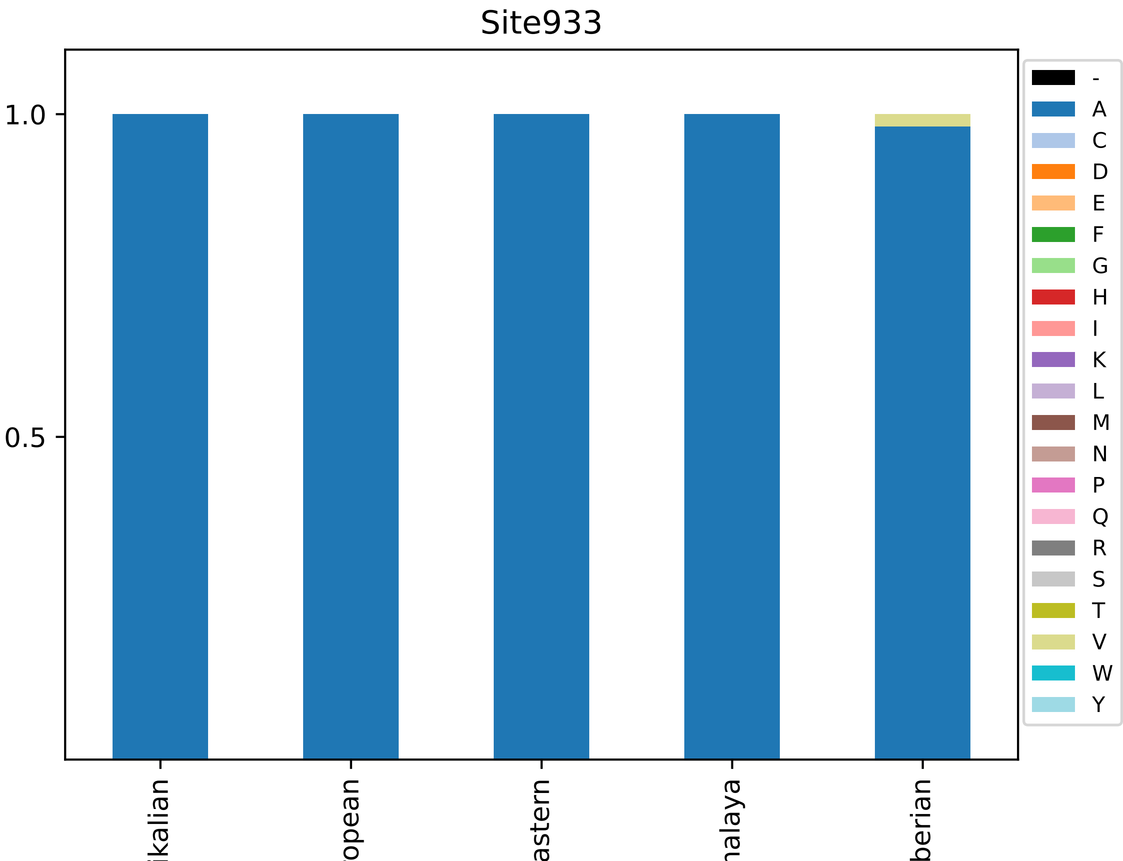

Site933
1.0
0.5
ikalian
opean
astern
nalaya
berian
-
A
C
D
E
F
G
H
I
K
L
M
N
P
Q
R
S
T
V
W
Y

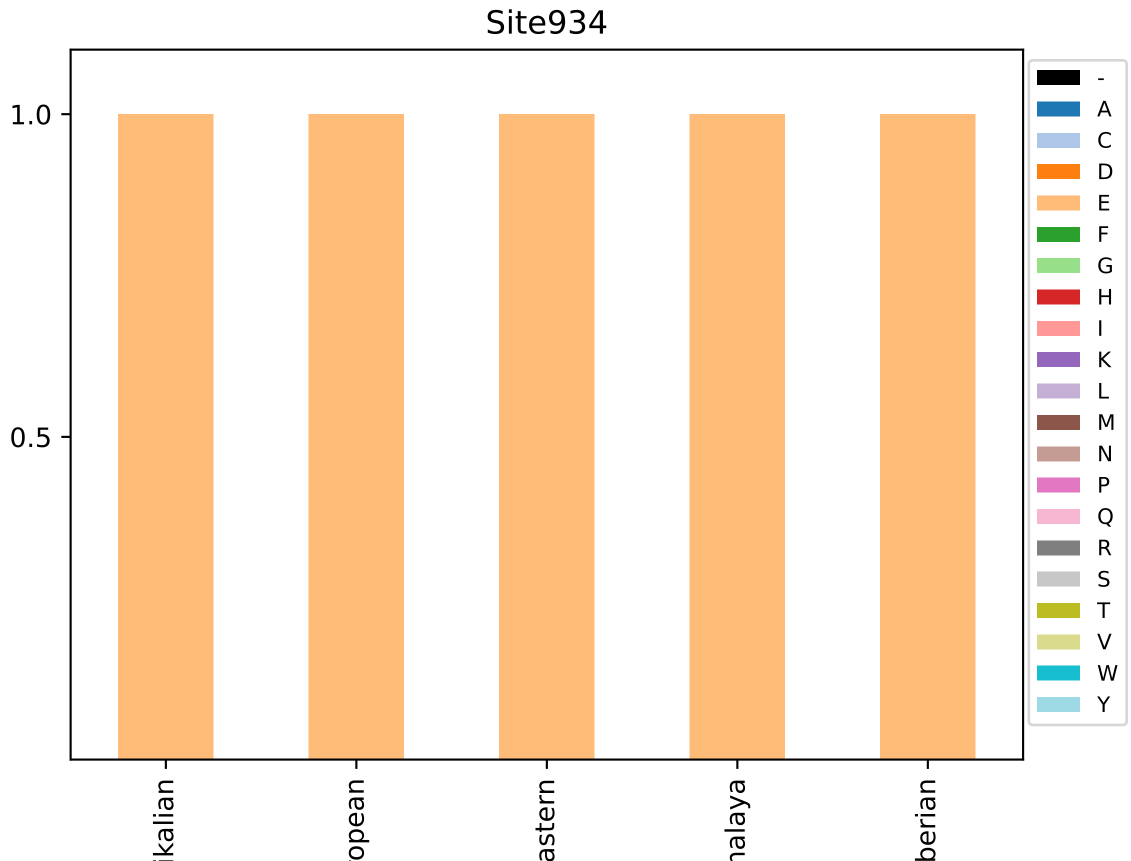

Site934
1.0
0.5
-
A
C
D
E
F
G
H
I
K
L
M
N
P
Q
R
S
T
V
W
Y
ikalian
opean
astern
nalaya
berian

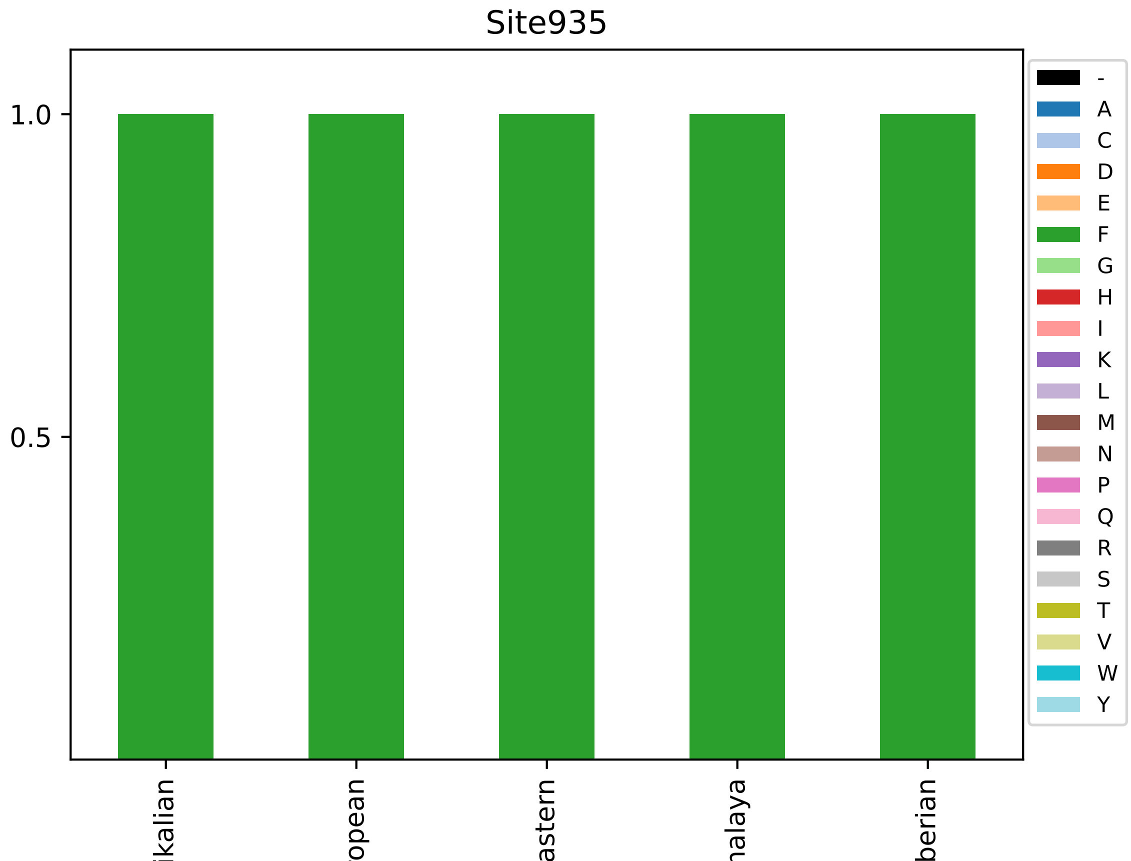

Site935
1.0
0.5
-
A
C
D
E
F
G
H
I
K
L
M
N
P
Q
R
S
T
V
W
Y

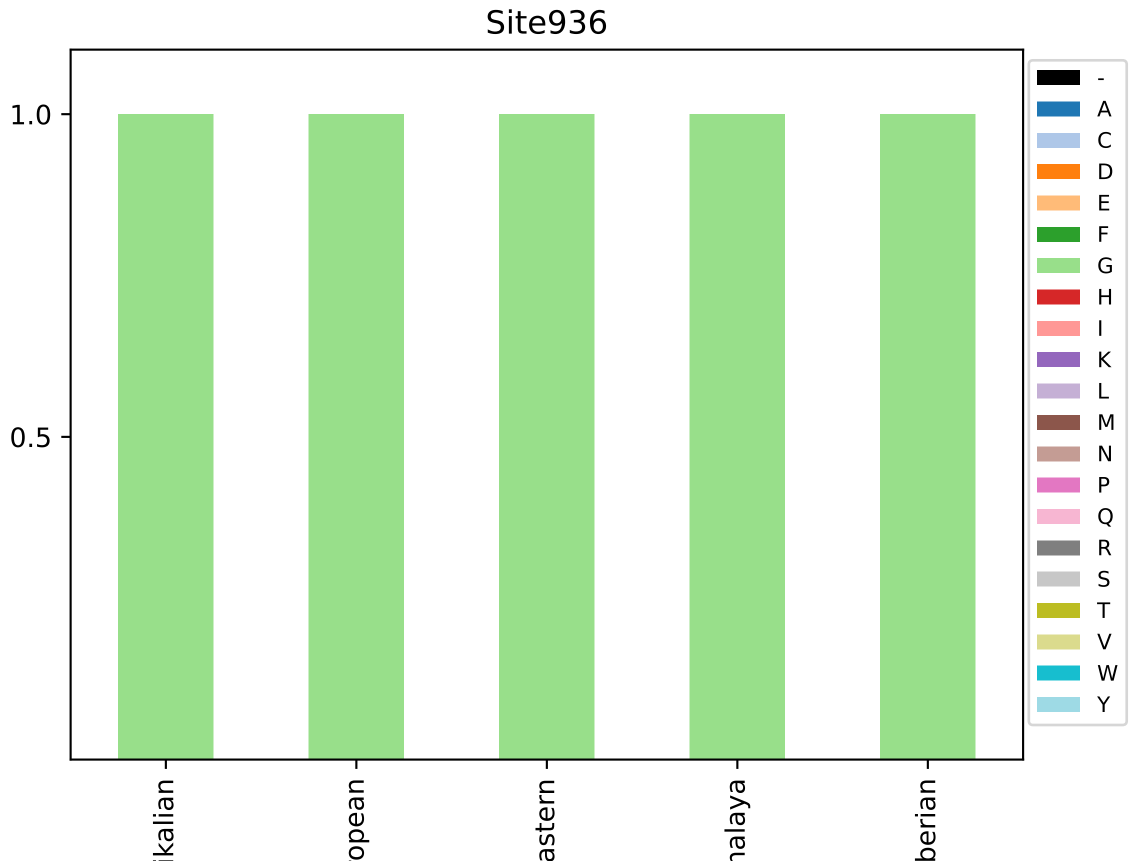
Site936
1.0
0.5
-
A
C
D
E
F
G
H
I
K
L
M
N
P
Q
R
S
T
V
W
Y
ikalian
opean
astern
nalaya
berian

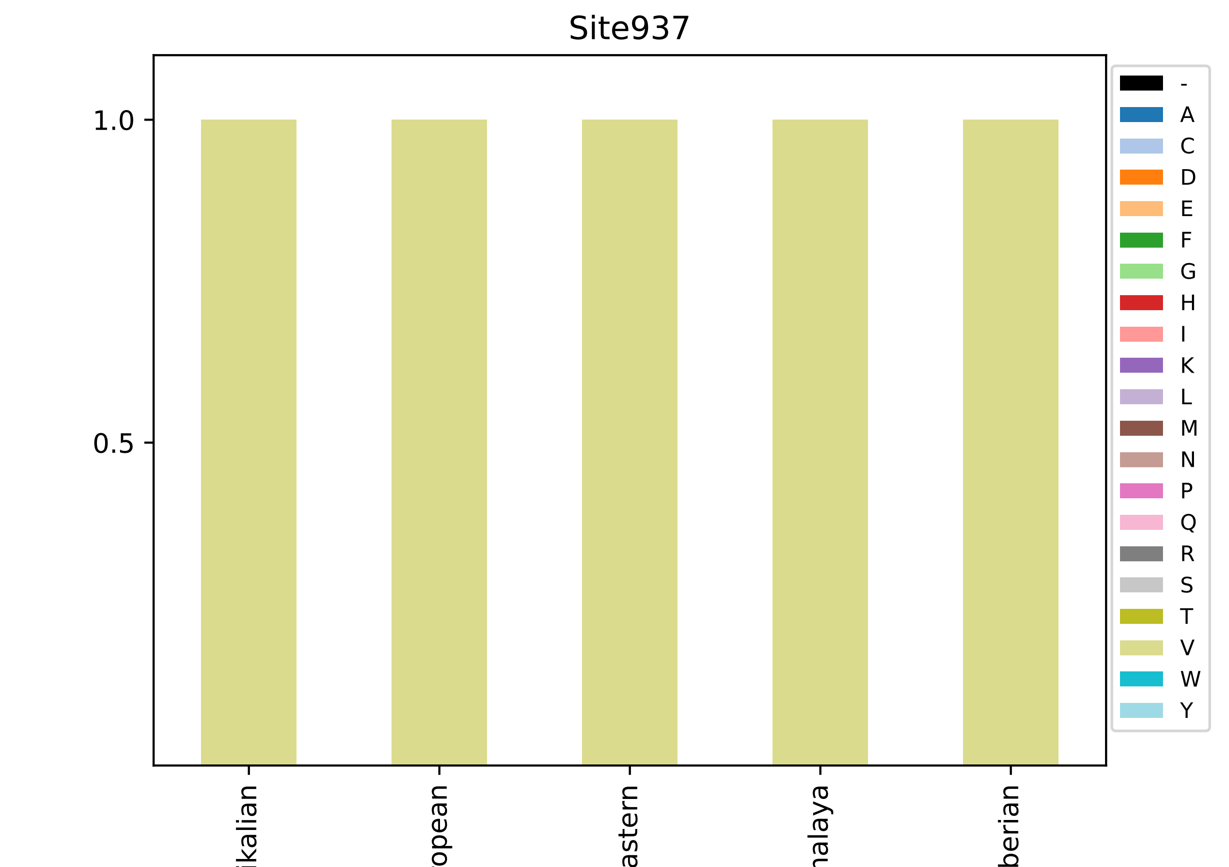
Site937
1.0
0.5
-
A
C
D
E
F
G
H
I
K
L
M
N
P
Q
R
S
T
V
W
Y
ikalian
opean
astern
nalaya
berian

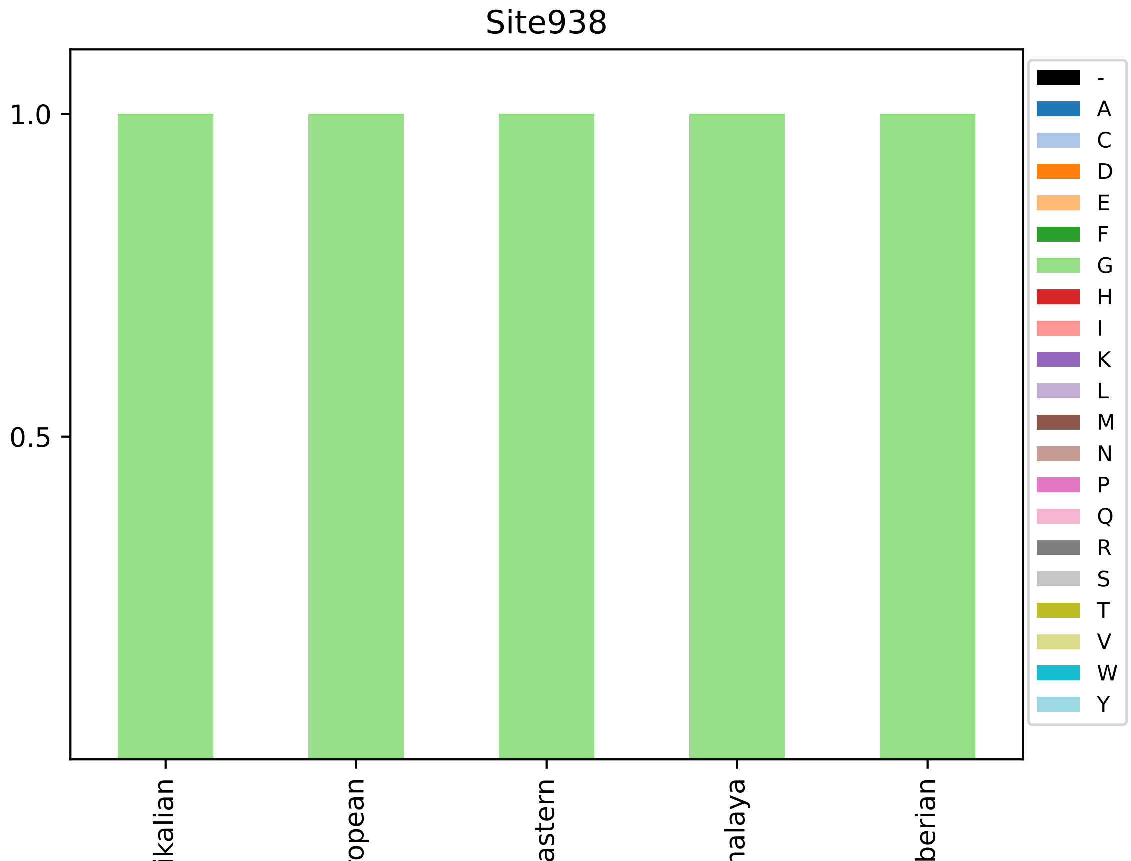
Site938
1.0
0.5
-
A
C
D
E
F
G
H
I
K
L
M
N
P
Q
R
S
T
V
W
Y
ikalian
opean
astern
nalaya
berian

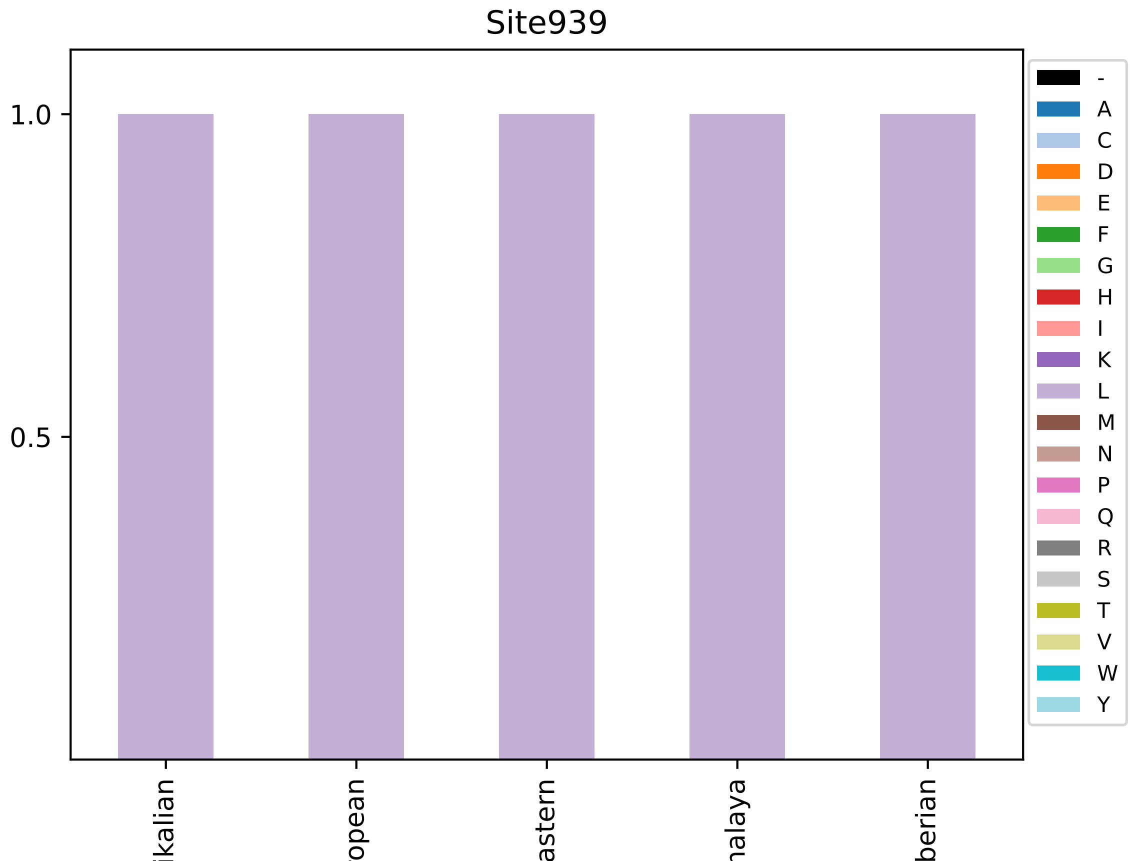
Site939
1.0
0.5
-
A
C
D
E
F
G
H
I
K
L
M
N
P
Q
R
S
T
V
W
Y
ikalian
opean
astern
nalaya
berian

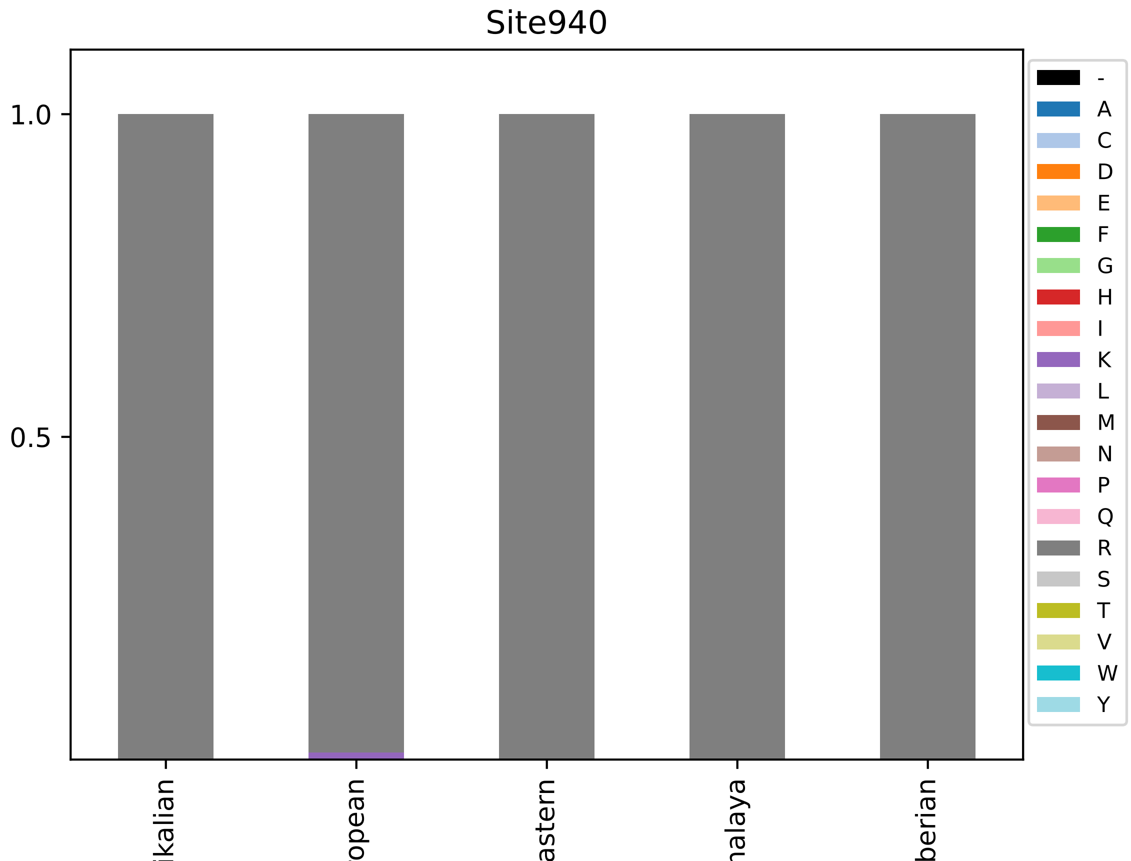

Site940
1.0
0.5
-
A
C
D
E
F
G
H
I
K
L
M
N
P
Q
R
S
T
V
W
Y
ikalian
opean
astern
nalaya
berian

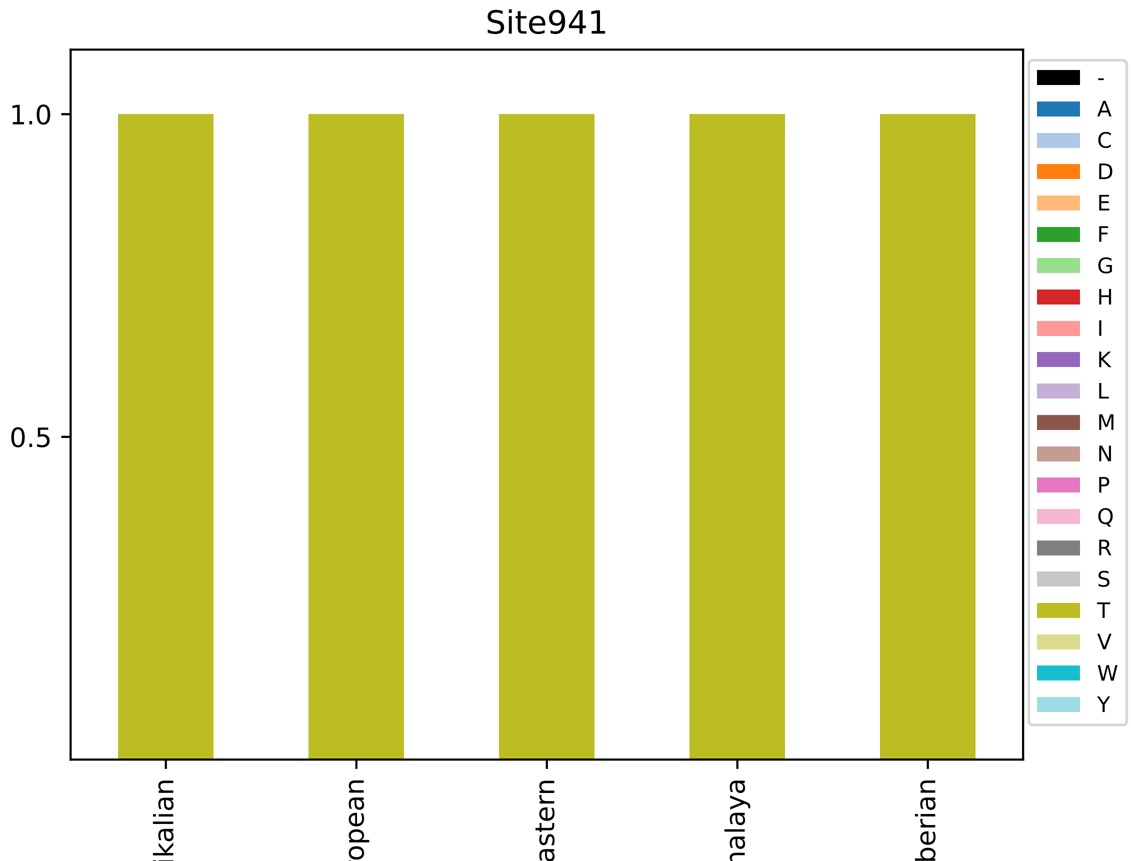

Site941
1.0
0.5
-
A
C
D
E
F
G
H
I
K
L
M
N
P
Q
R
S
T
V
W
Y
ikalian
opean
astern
nalaya
berian

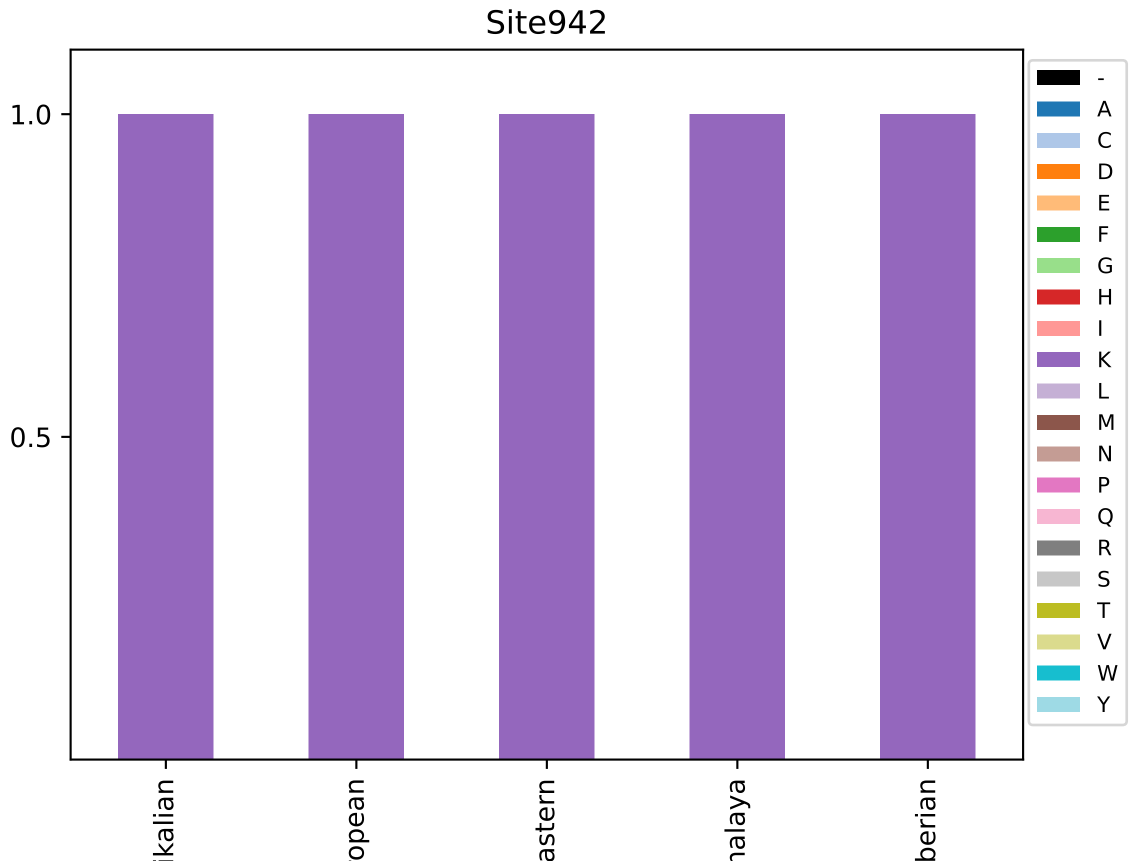

Site942
1.0
0.5
-
A
C
D
E
F
G
H
I
K
L
M
N
P
Q
R
S
T
V
W
Y
ikalian
opean
astern
nalaya
berian

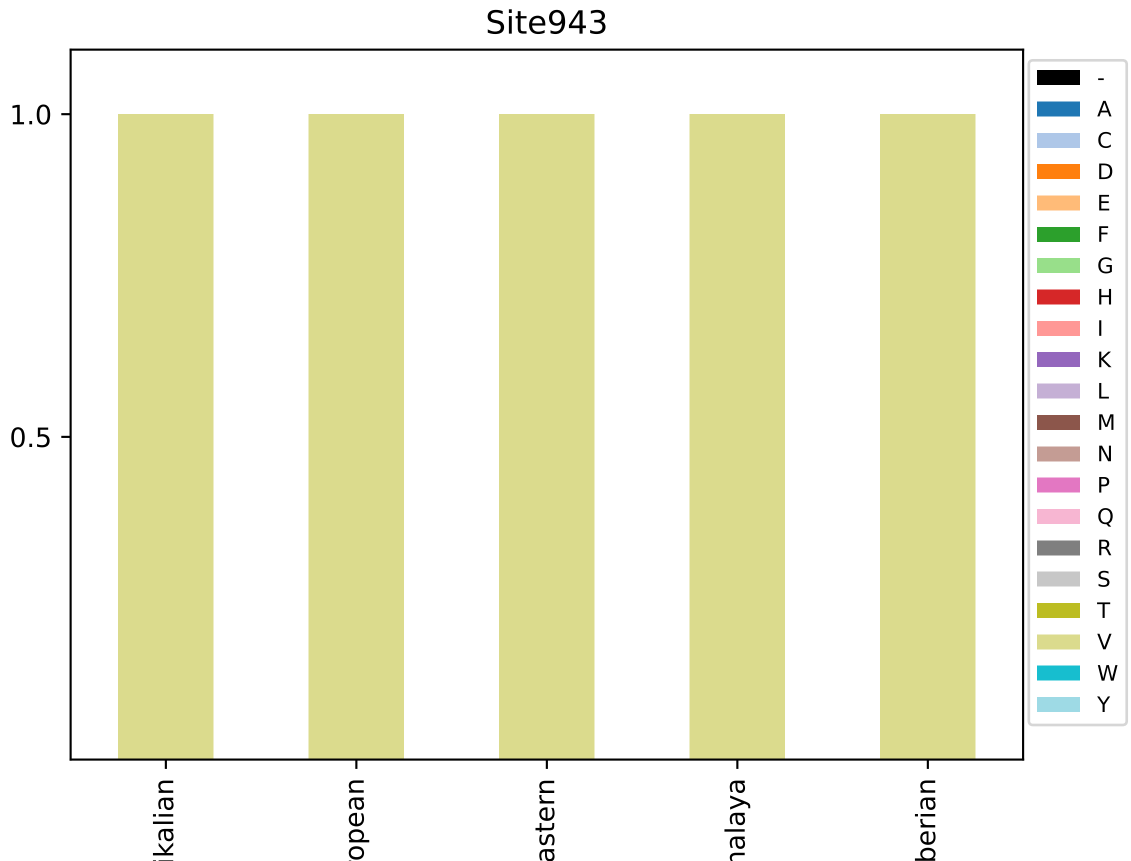
Site943
1.0
0.5
-
A
C
D
E
F
G
H
I
K
L
M
N
P
Q
R
S
T
V
W
Y
ikalian
opean
astern
nalaya
berian

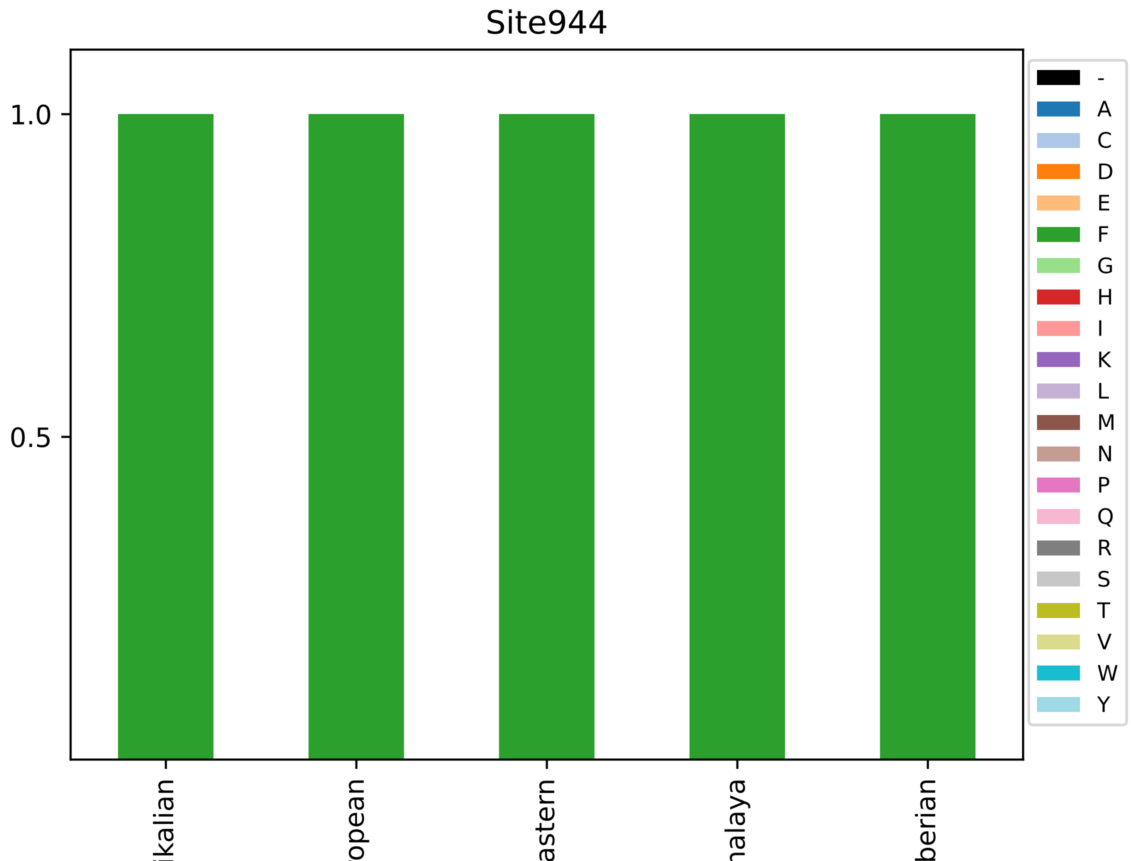

Site944
1.0
0.5
-
A
C
D
E
F
G
H
I
K
L
M
N
P
Q
R
S
T
V
W
Y

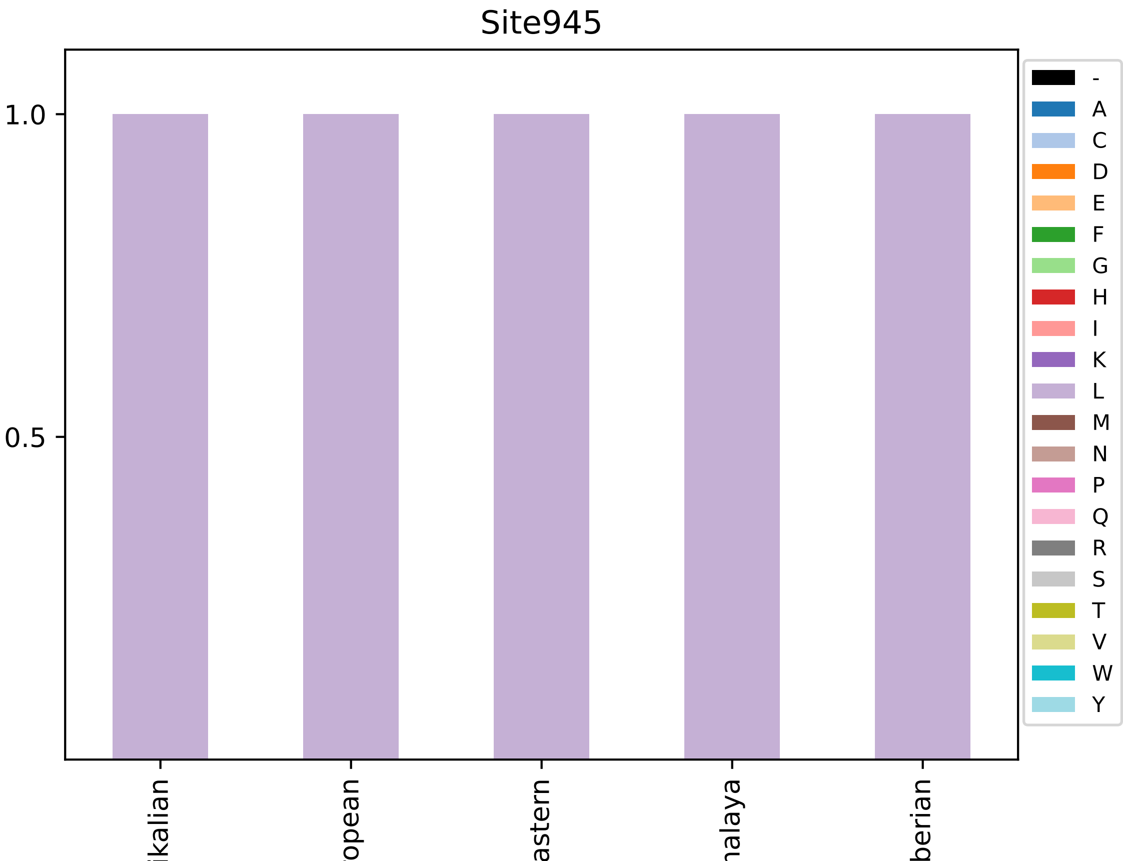
Site945
1.0
0.5
-
A
C
D
E
F
G
H
I
K
L
M
N
P
Q
R
S
T
V
W
Y

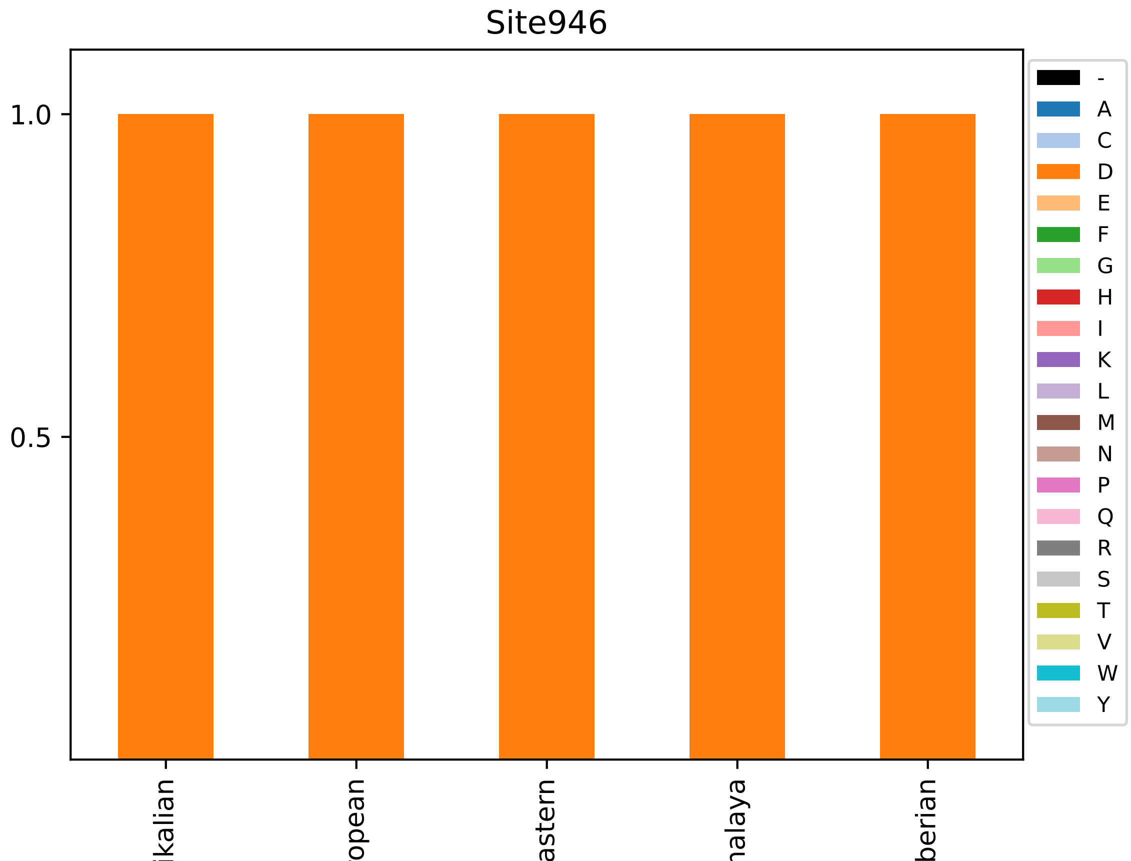
Site946
1.0
0.5
-
A
C
D
E
F
G
H
I
K
L
M
N
P
Q
R
S
T
V
W
Y
ikalian
opean
astern
nalaya
berian

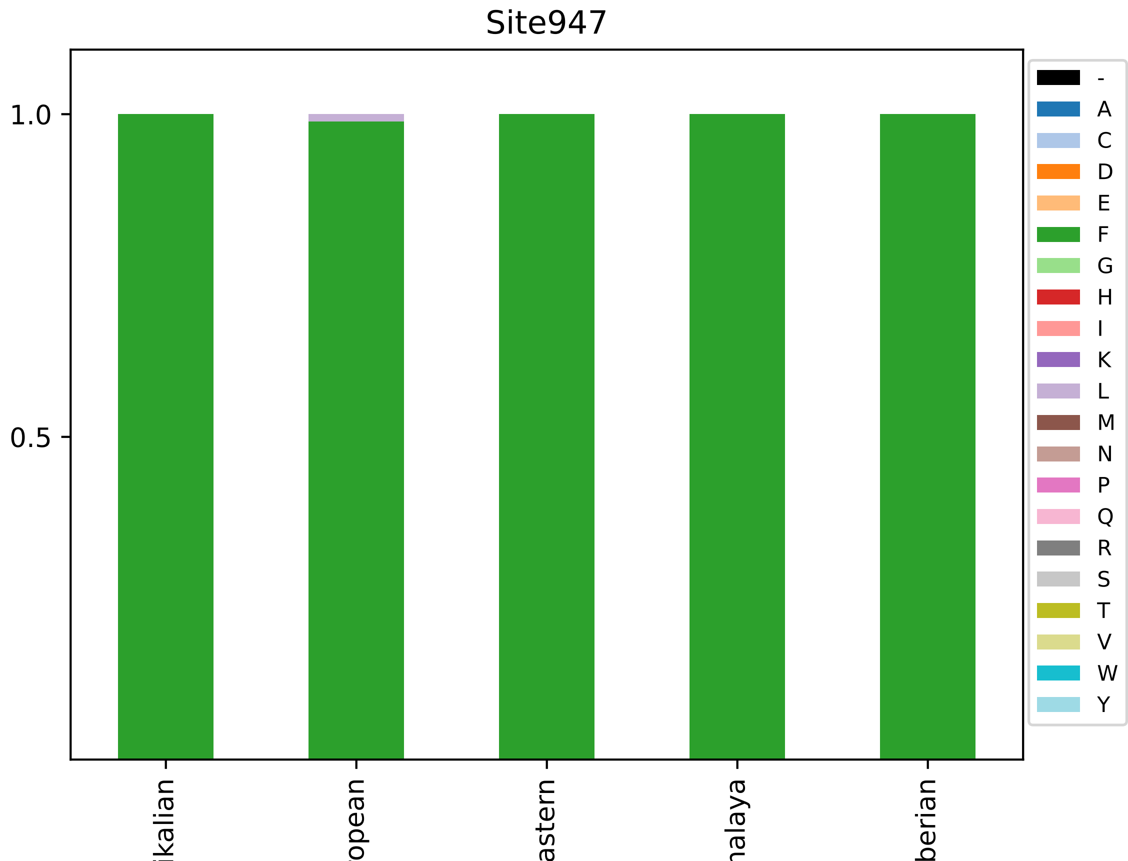

Site947
1.0
0.5
-
A
C
D
E
F
G
H
I
K
L
M
N
P
Q
R
S
T
V
W
Y
ikalian
opean
astern
nalaya
berian

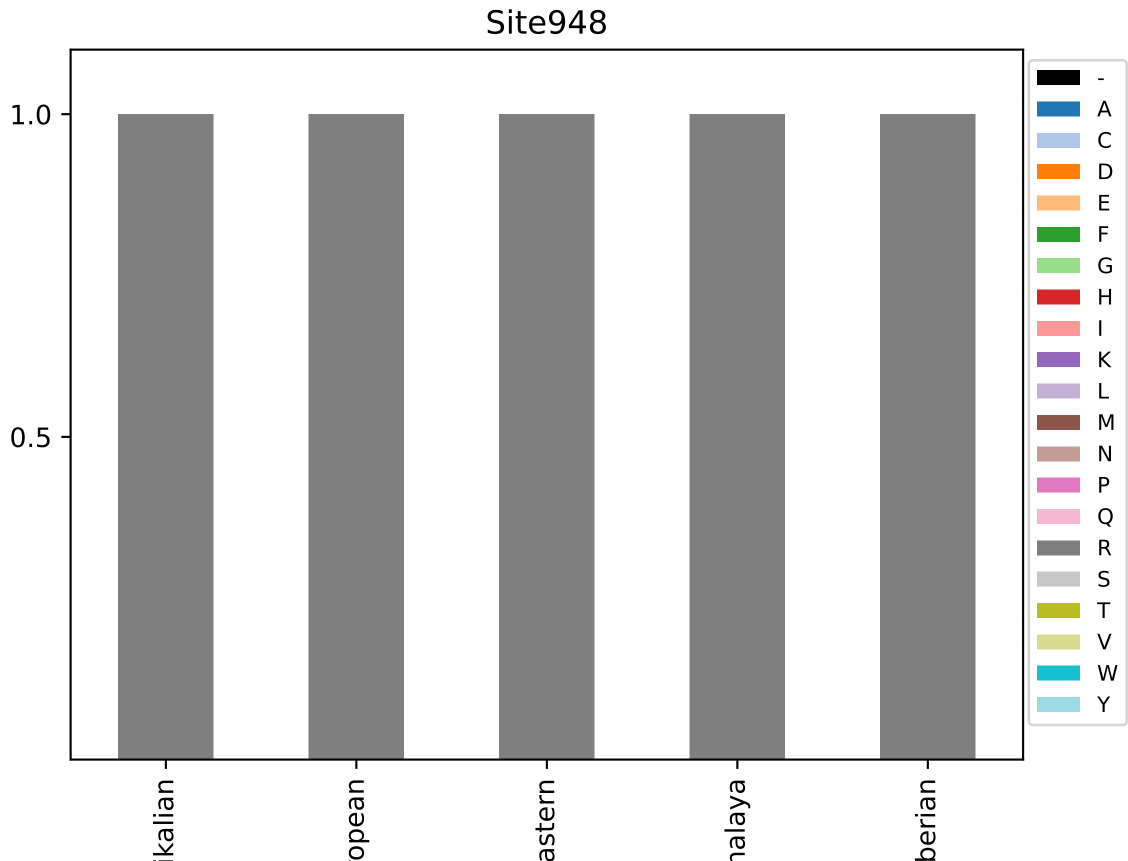

Site948
1.0
0.5
-
A
C
D
E
F
G
H
I
K
L
M
N
P
Q
R
S
T
V
W
Y
ikalian
opean
astern
nalaya
berian

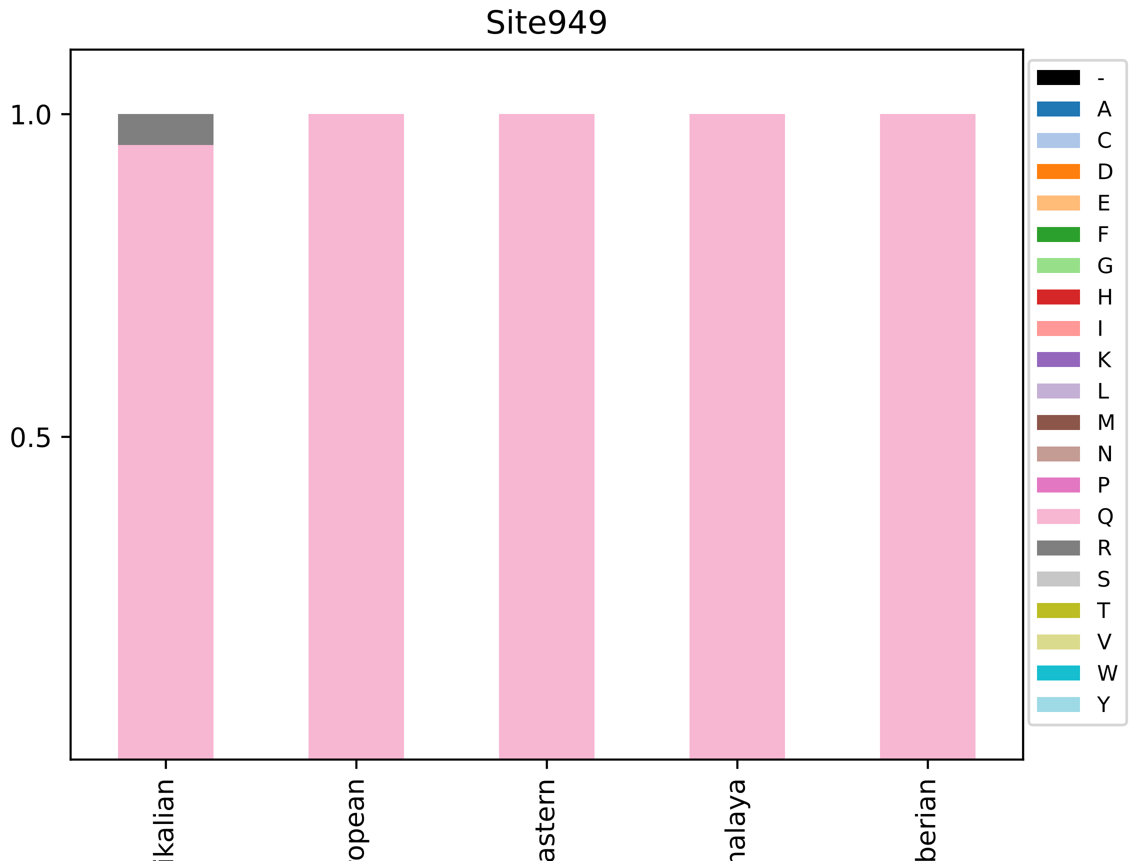
Site949
1.0
0.5
-
A
C
D
E
F
G
H
I
K
L
M
N
P
Q
R
S
T
V
W
Y
ikalian
opean
astern
nalaya
berian

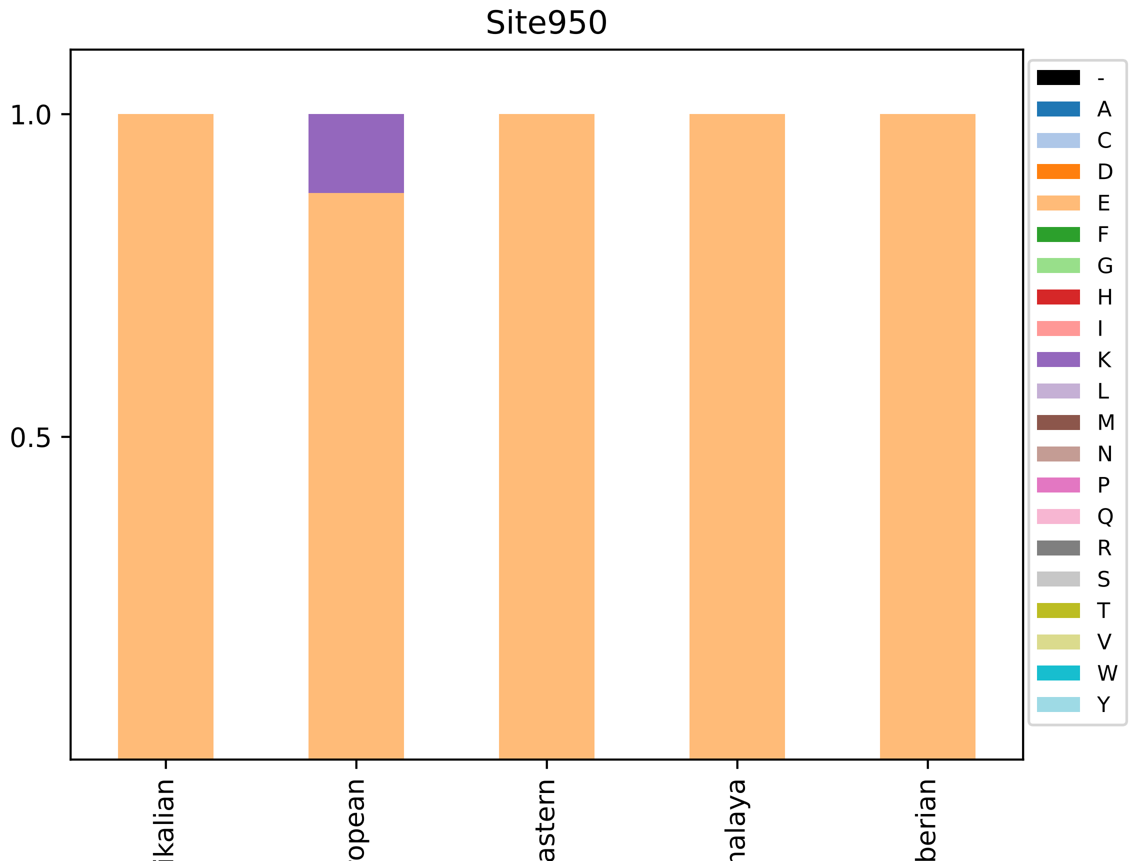
Site950
1.0
0.5
-
A
C
D
E
F
G
H
I
K
L
M
N
P
Q
R
S
T
V
W
Y
ikalian
ropean
astern
nalaya
berian

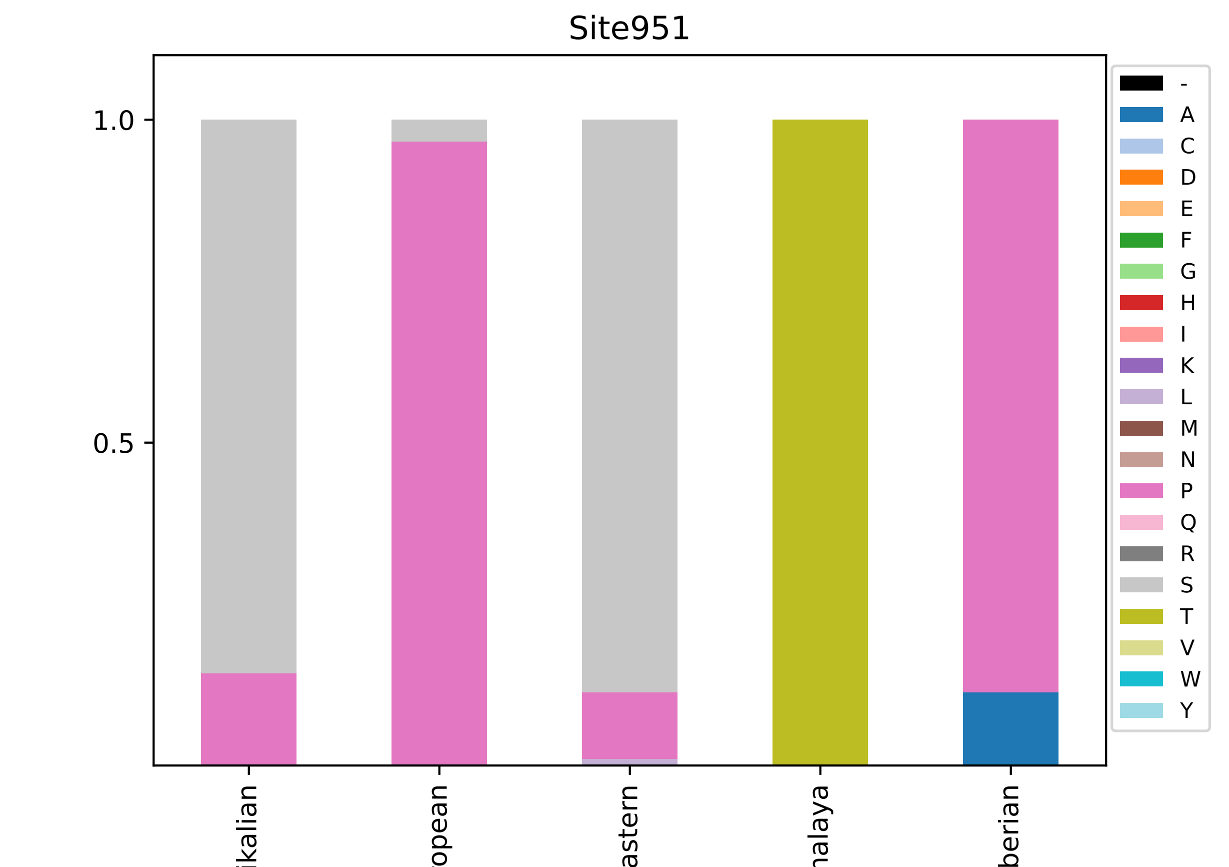
Site951
1.0
0.5
-
A
C
D
E
F
G
H
I
K
L
M
N
P
Q
R
S
T
V
W
Y
ikalian
opean
astern
nalaya
berian

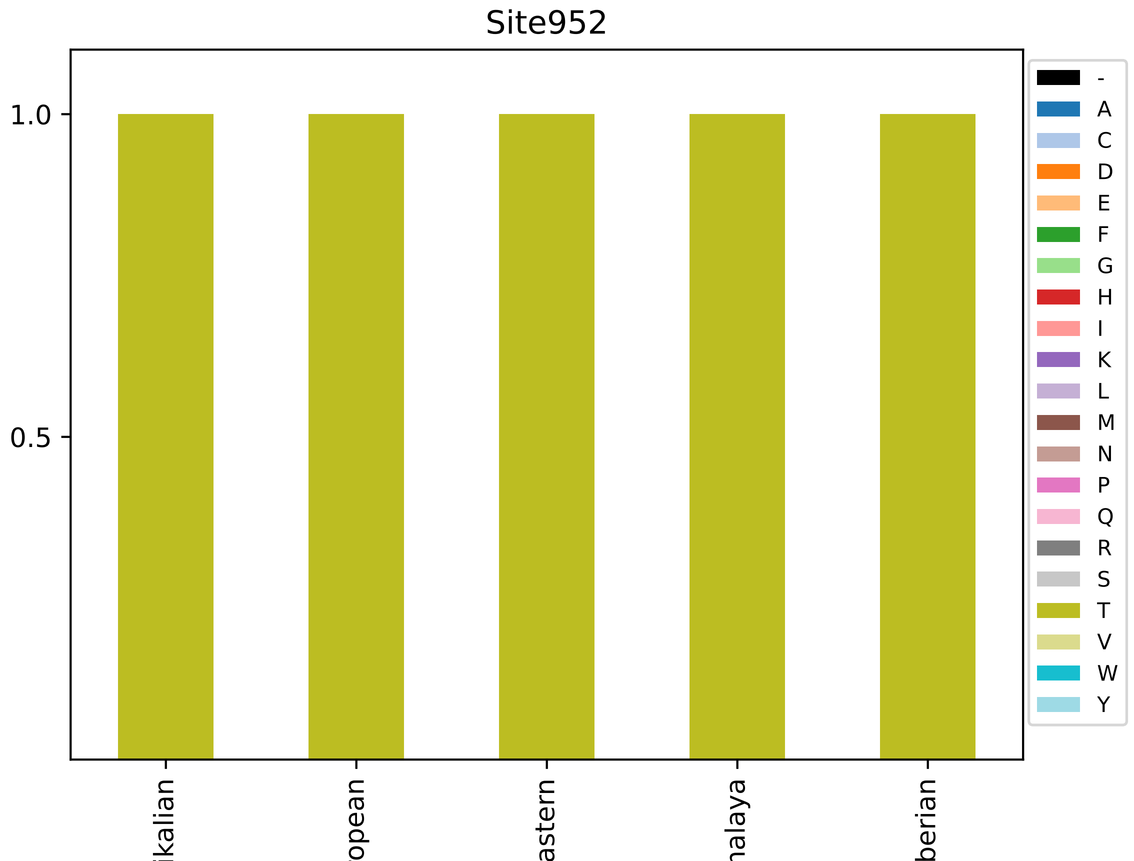

Site952
1.0
0.5
-
A
C
D
E
F
G
H
I
K
L
M
N
P
Q
R
S
T
V
W
Y
ikalian
opean
astern
nalaya
berian

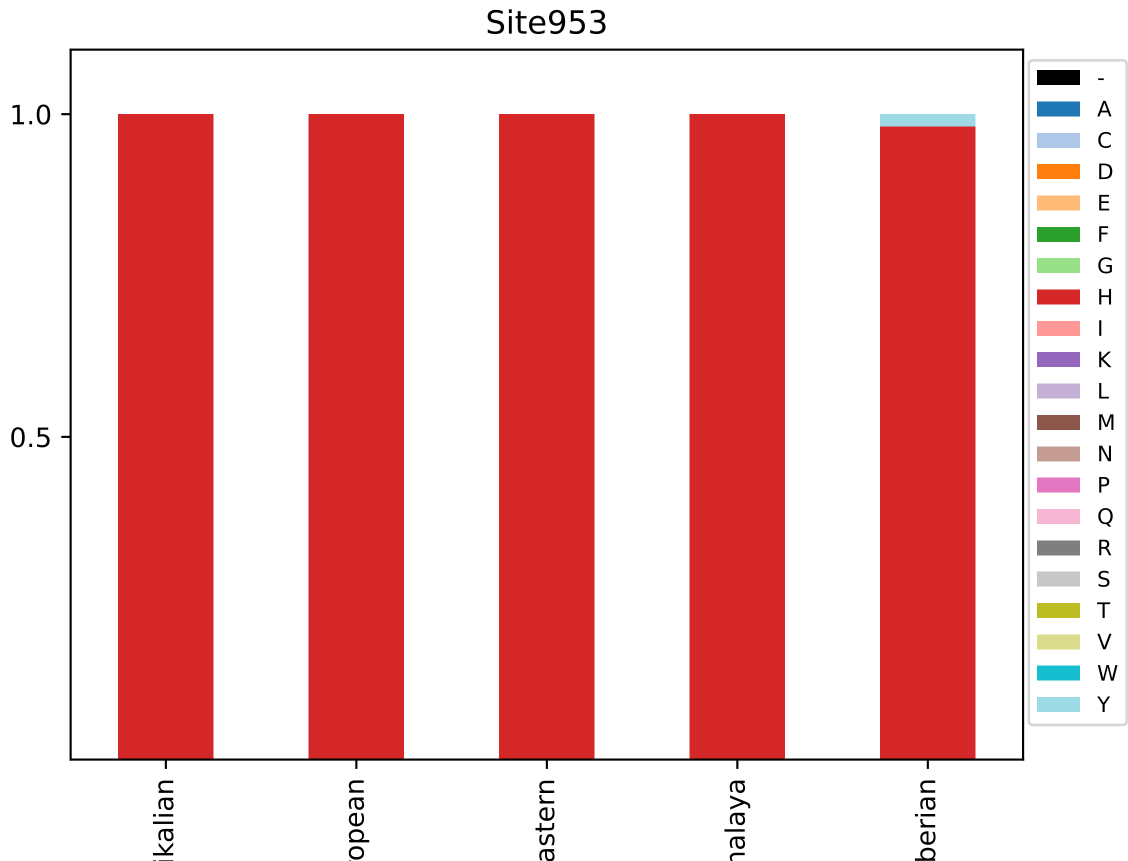

Site953
1.0
0.5
-
A
C
D
E
F
G
H
I
K
L
M
N
P
Q
R
S
T
V
W
Y
ikalian
opean
astern
nalaya
berian

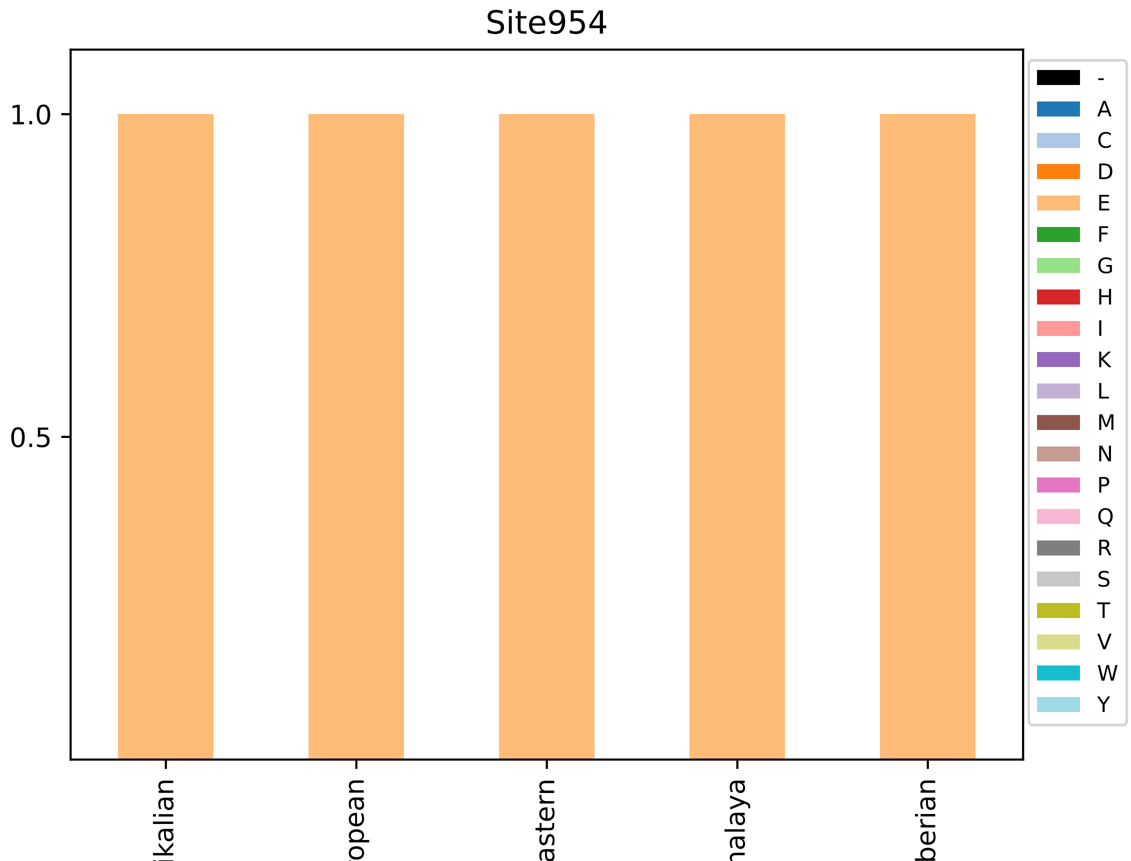
Site954
1.0
0.5
-
A
C
D
E
F
G
H
I
K
L
M
N
P
Q
R
S
T
V
W
Y
ikalian
opean
astern
nalaya
berian

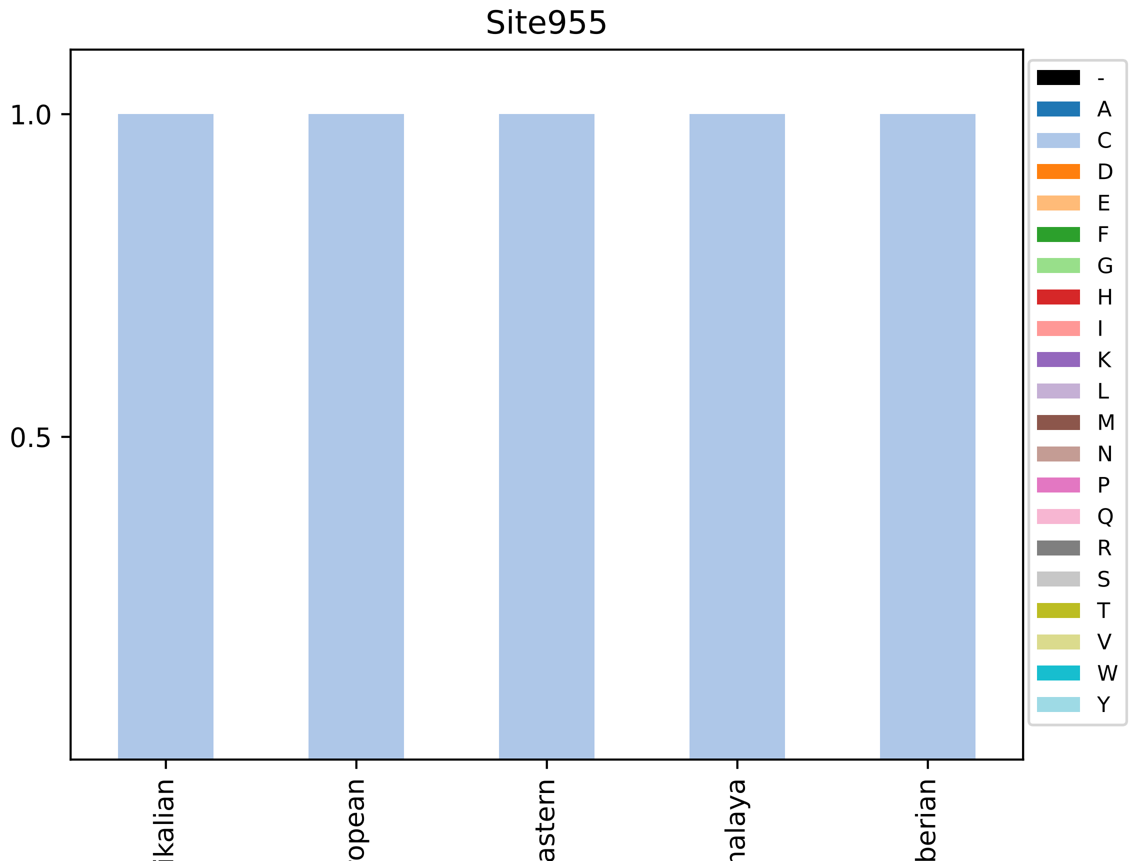

Site955
1.0
0.5
-
A
C
D
E
F
G
H
I
K
L
M
N
P
Q
R
S
T
V
W
Y
ikalian
opean
astern
nalaya
berian

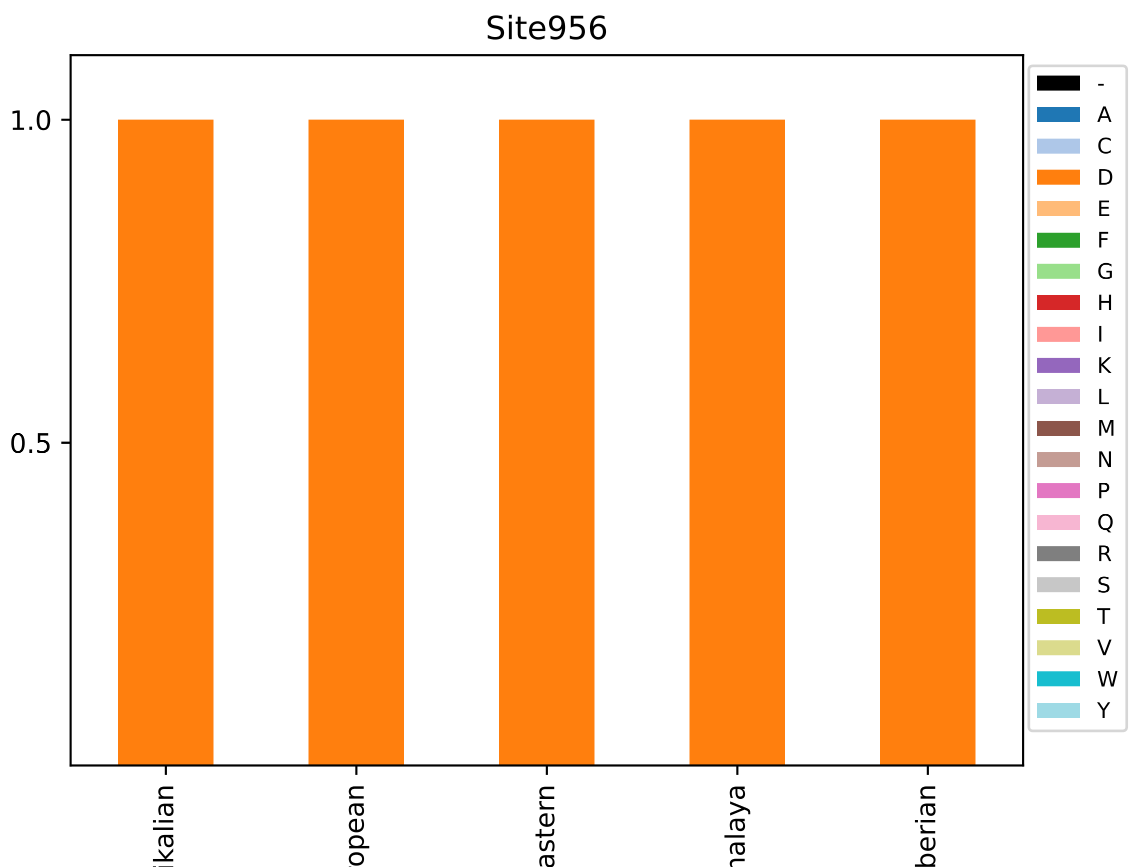
Site956
1.0
0.5
-
A
C
D
E
F
G
H
I
K
L
M
N
P
Q
R
S
T
V
W
Y
ikalian
opean
astern
nalaya
berian

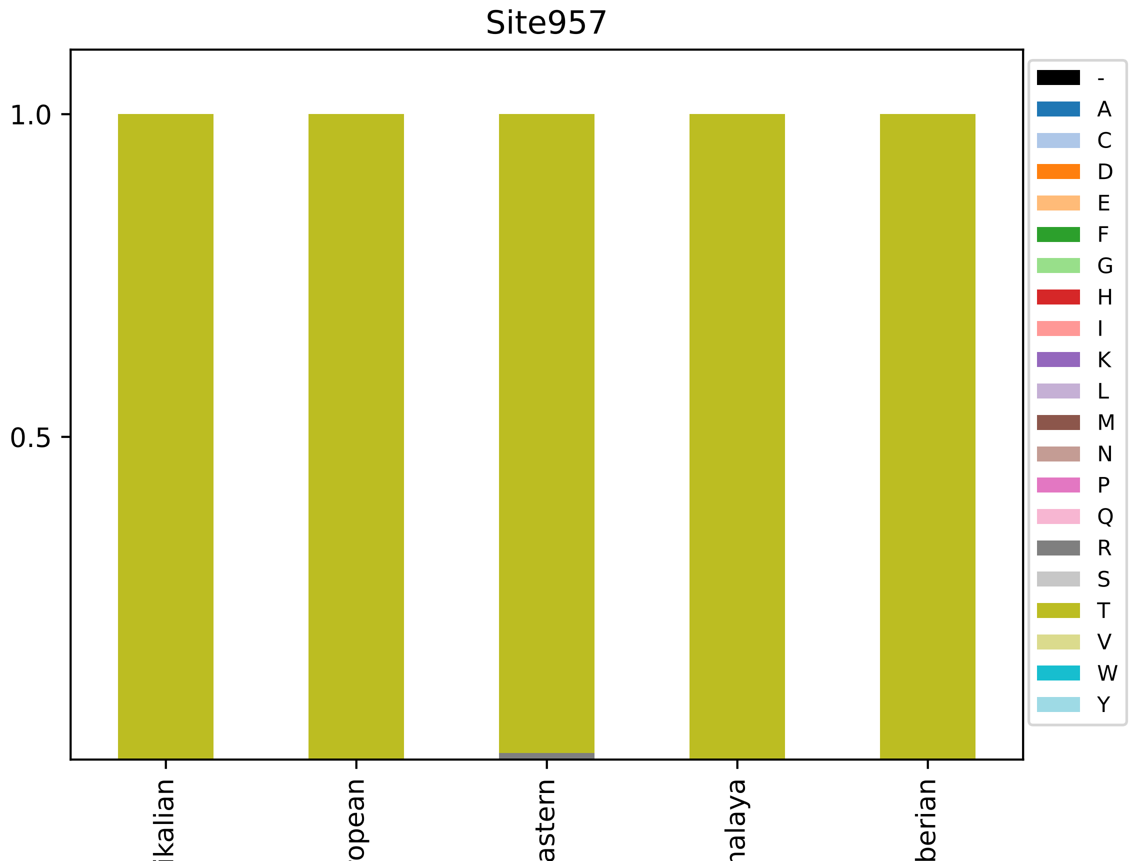

Site957
1.0
0.5
-
A
C
D
E
F
G
H
I
K
L
M
N
P
Q
R
S
T
V
W
Y
ikalian
opean
astern
nalaya
berian

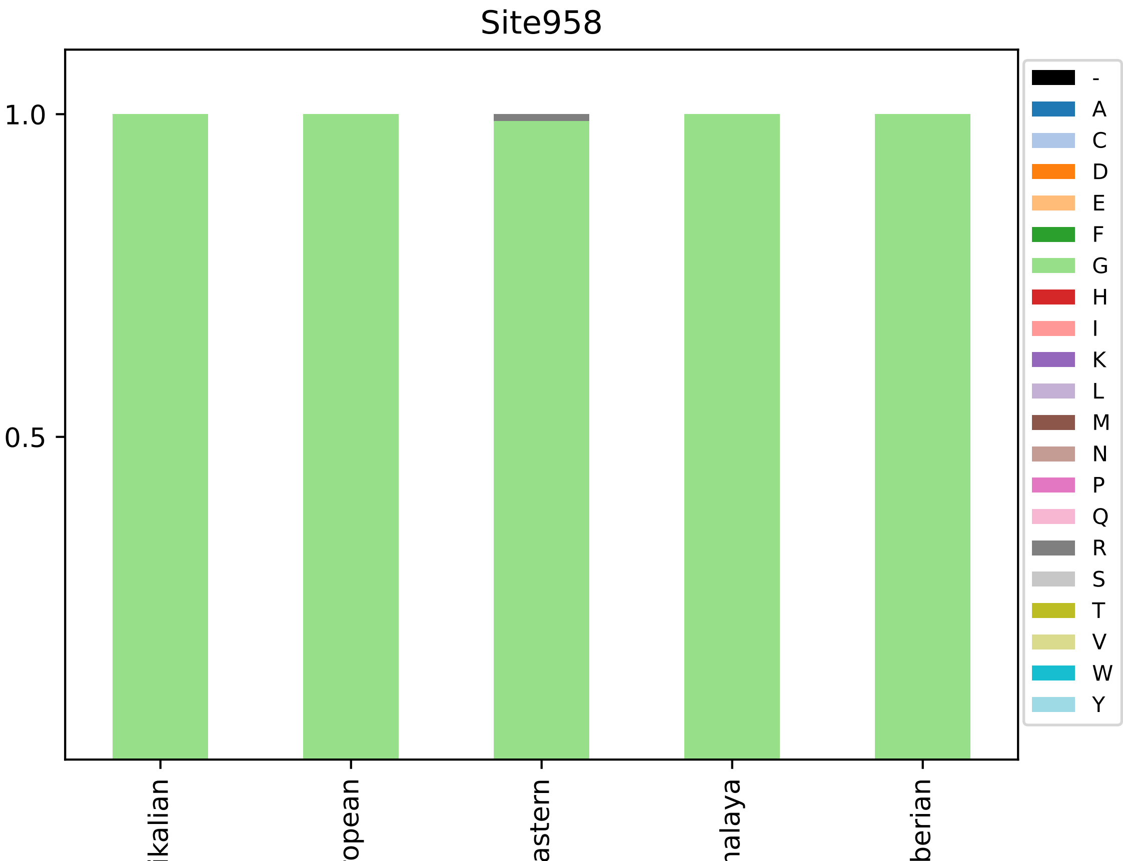
Site958
1.0
0.5
-
A
C
D
E
F
G
H
I
K
L
M
N
P
Q
R
S
T
V
W
Y
ikalian
opean
astern
nalaya
berian

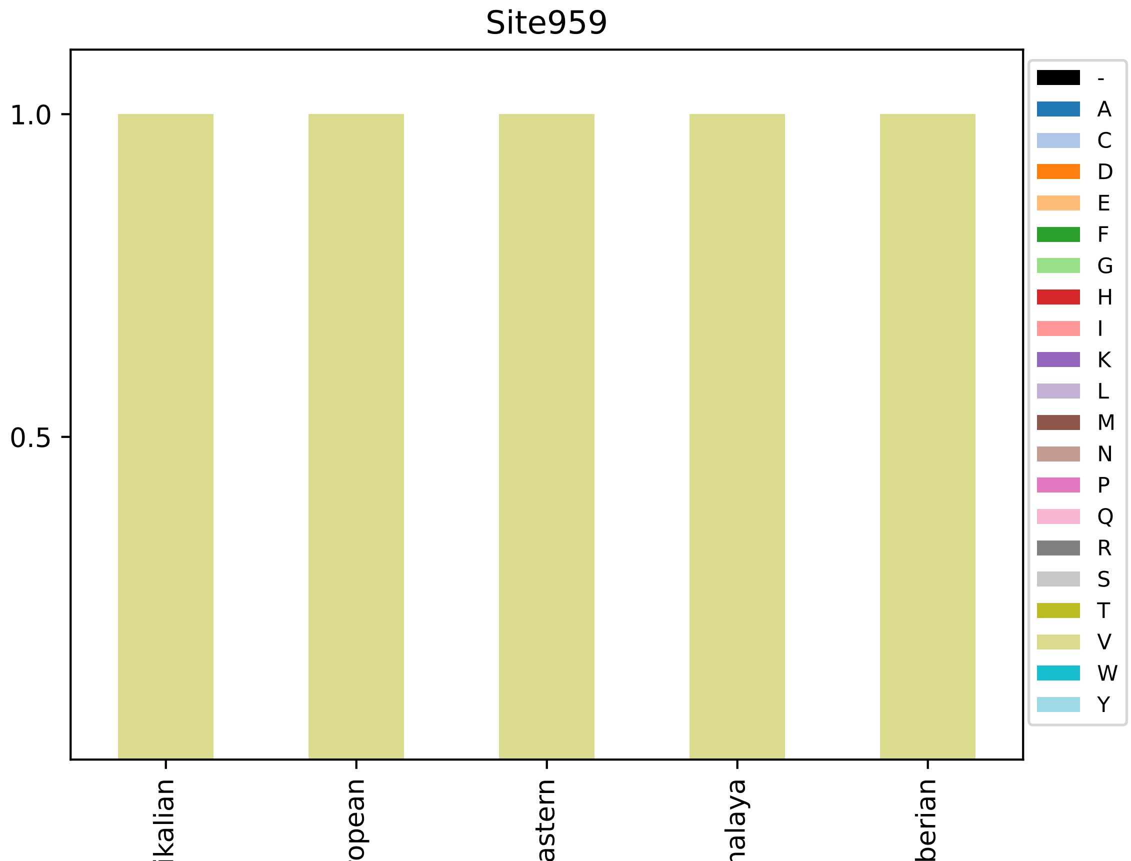
Site959
1.0
0.5
-
A
C
D
E
F
G
H
I
K
L
M
N
P
Q
R
S
T
V
W
Y

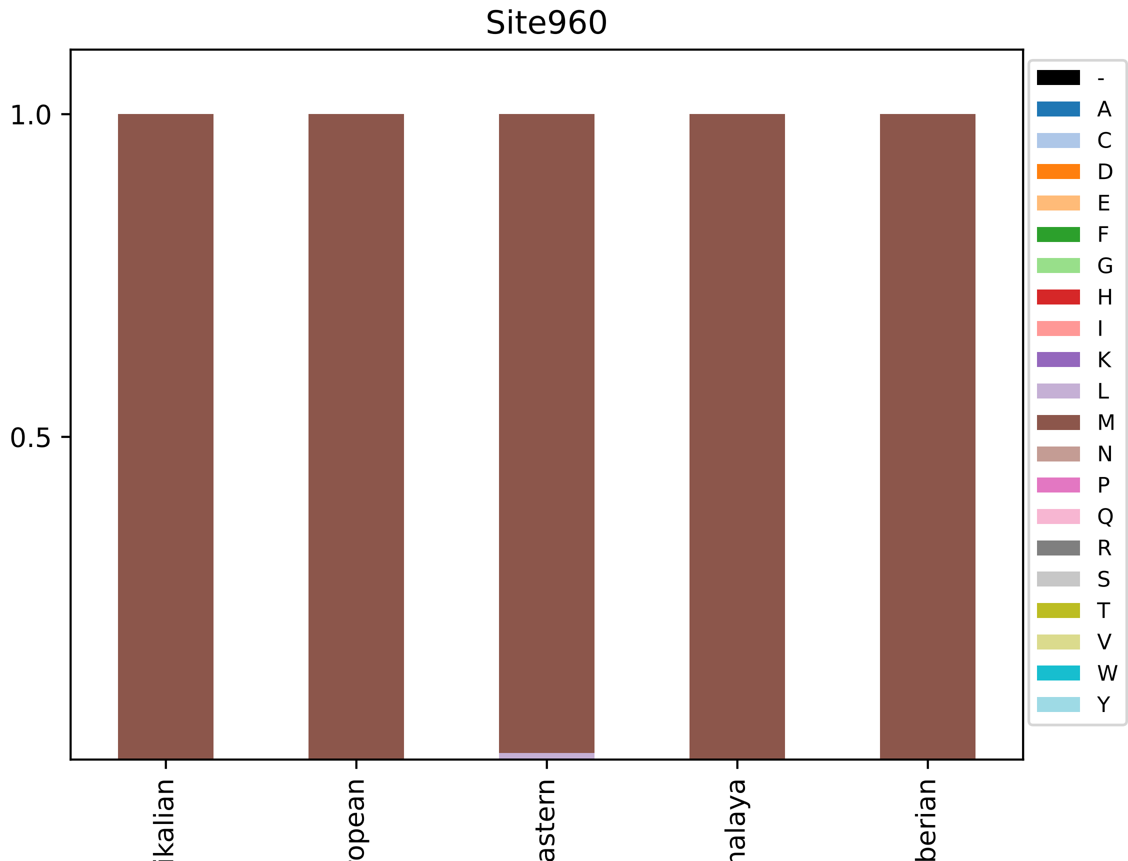
Site960
1.0
0.5
-
A
C
D
E
F
G
H
I
K
L
M
N
P
Q
R
S
T
V
W
Y
ikalian
opean
astern
nalaya
berian

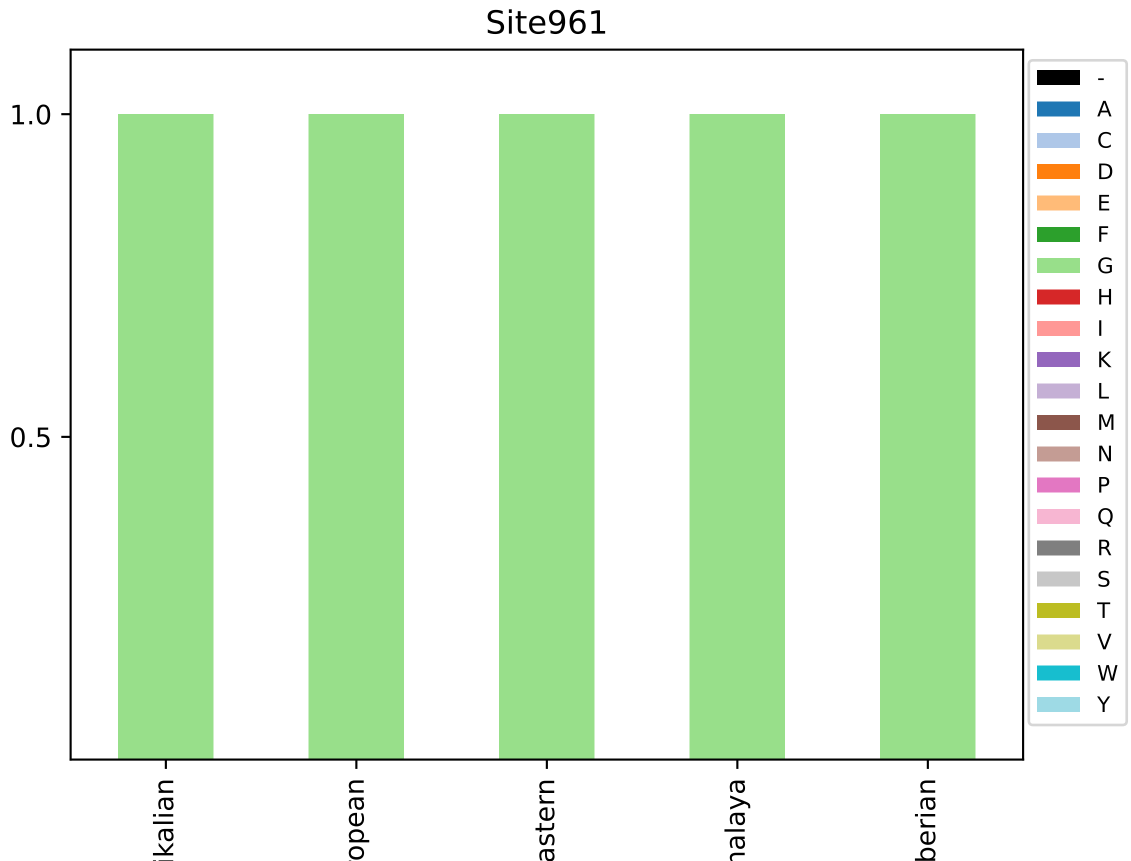

Site961
1.0
0.5
-
A
C
D
E
F
G
H
I
K
L
M
N
P
Q
R
S
T
V
W
Y
ikalian
opean
astern
nalaya
berian

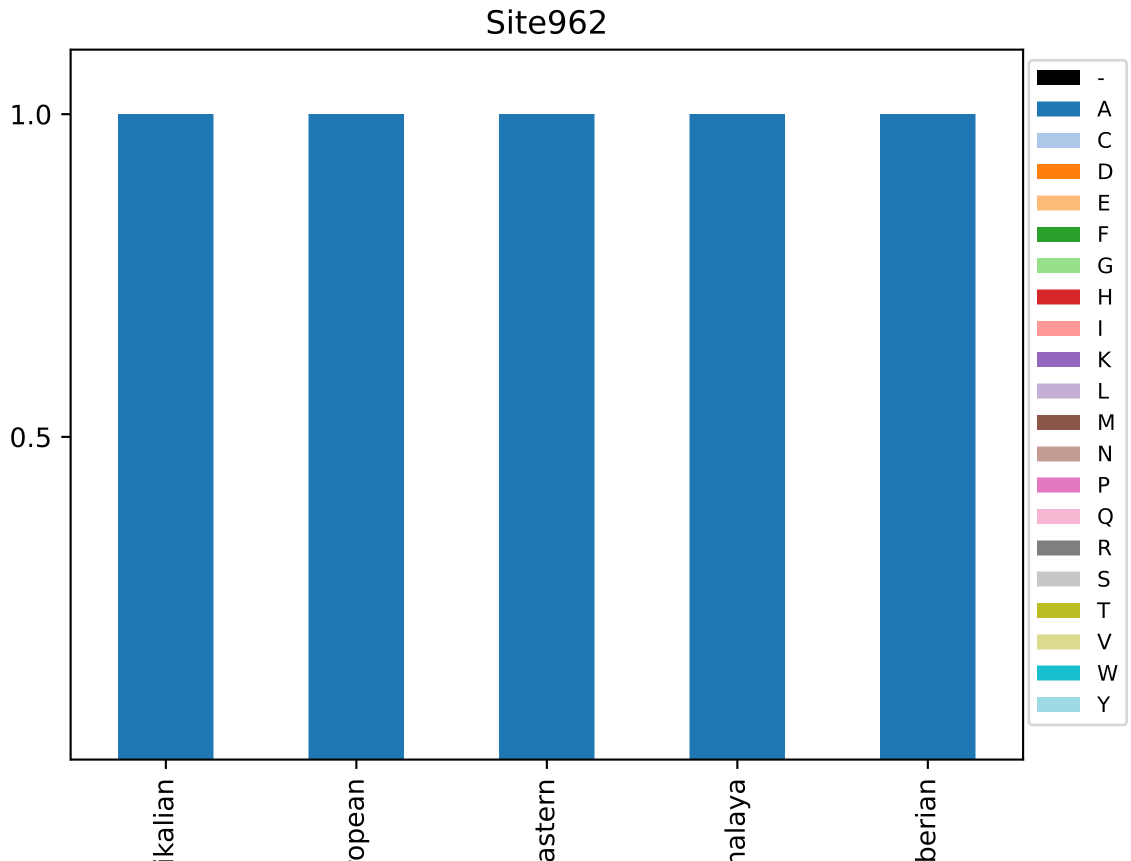
Site962
1.0
0.5
-
A
C
D
E
F
G
H
I
K
L
M
N
P
Q
R
S
T
V
W
Y
ikalian
opean
astern
nalaya
berian

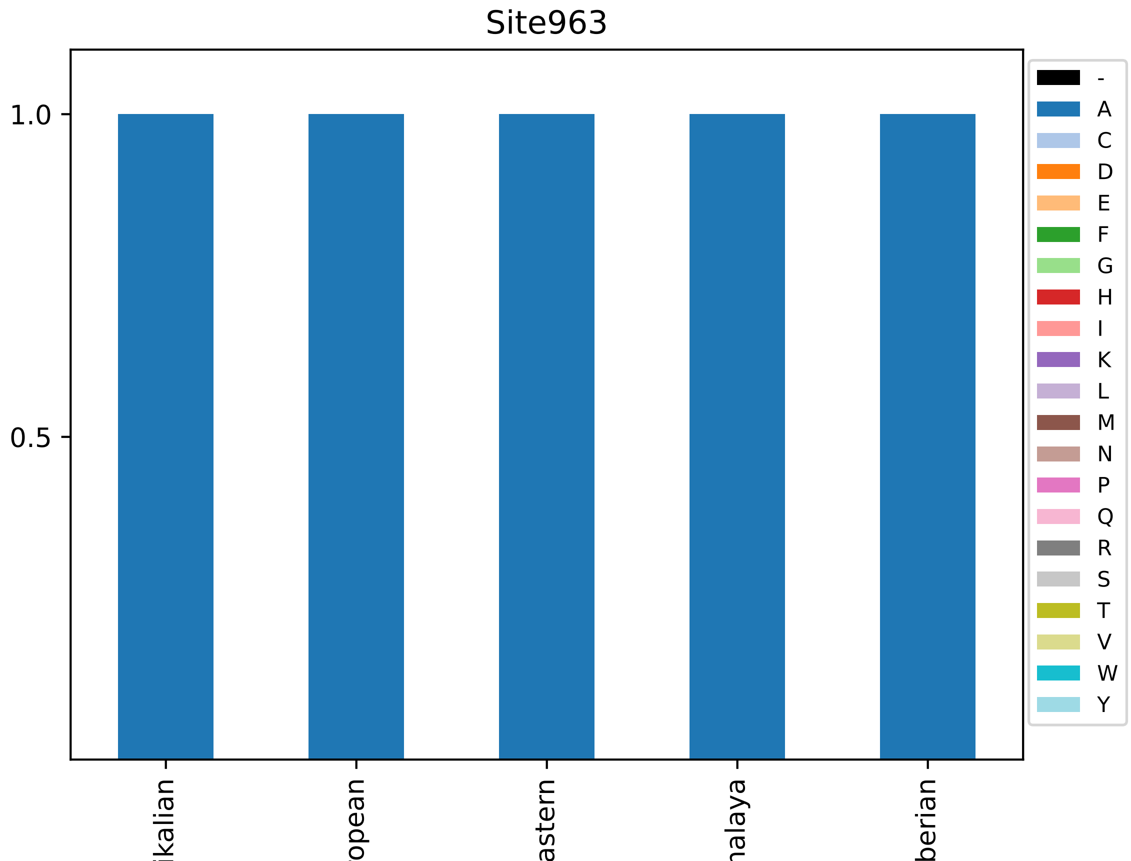

Site963
1.0
0.5
ikalian
opean
astern
nalaya
berian
-
A
C
D
E
F
G
H
I
K
L
M
N
P
Q
R
S
T
V
W
Y

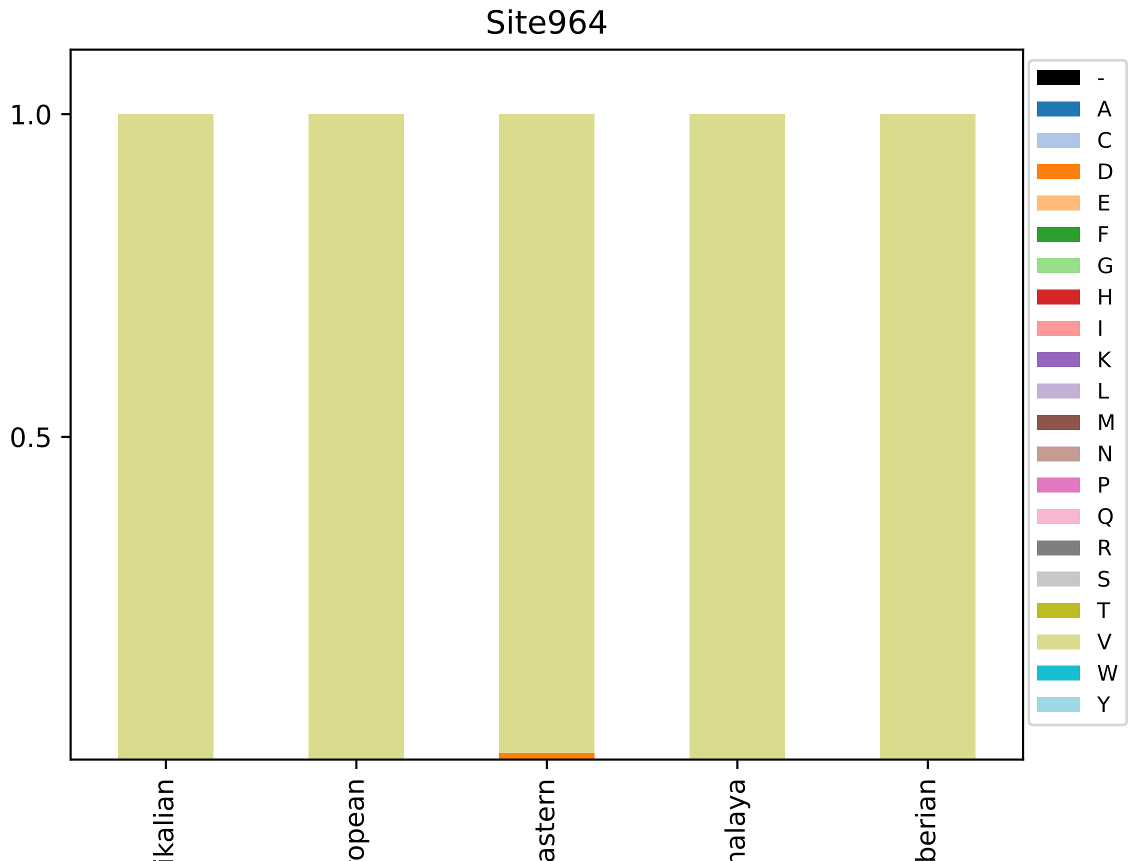

Site964
1.0
0.5
-
A
C
D
E
F
G
H
I
K
L
M
N
P
Q
R
S
T
V
W
Y
ikalian
opean
astern
nalaya
berian

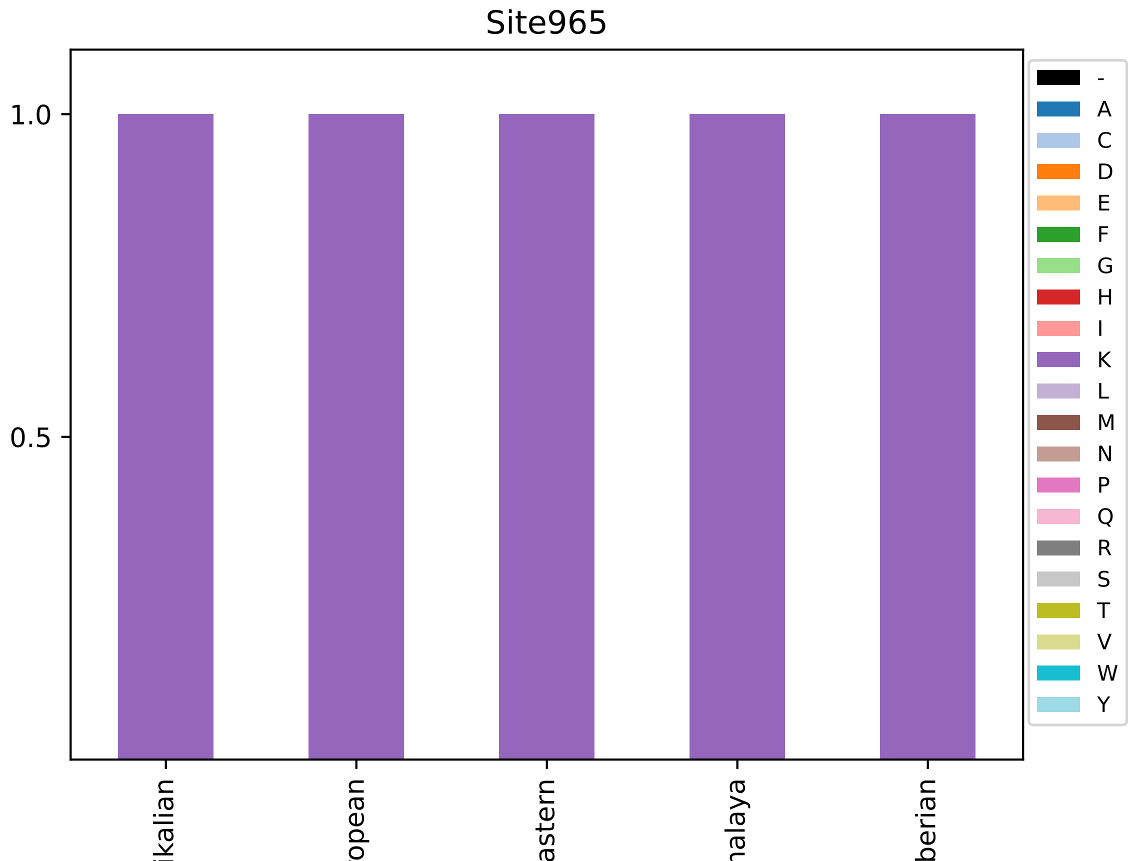

Site965
1.0
0.5
-
A
C
D
E
F
G
H
I
K
L
M
N
P
Q
R
S
T
V
W
Y
ikalian
ropean
astern
nalaya
berian

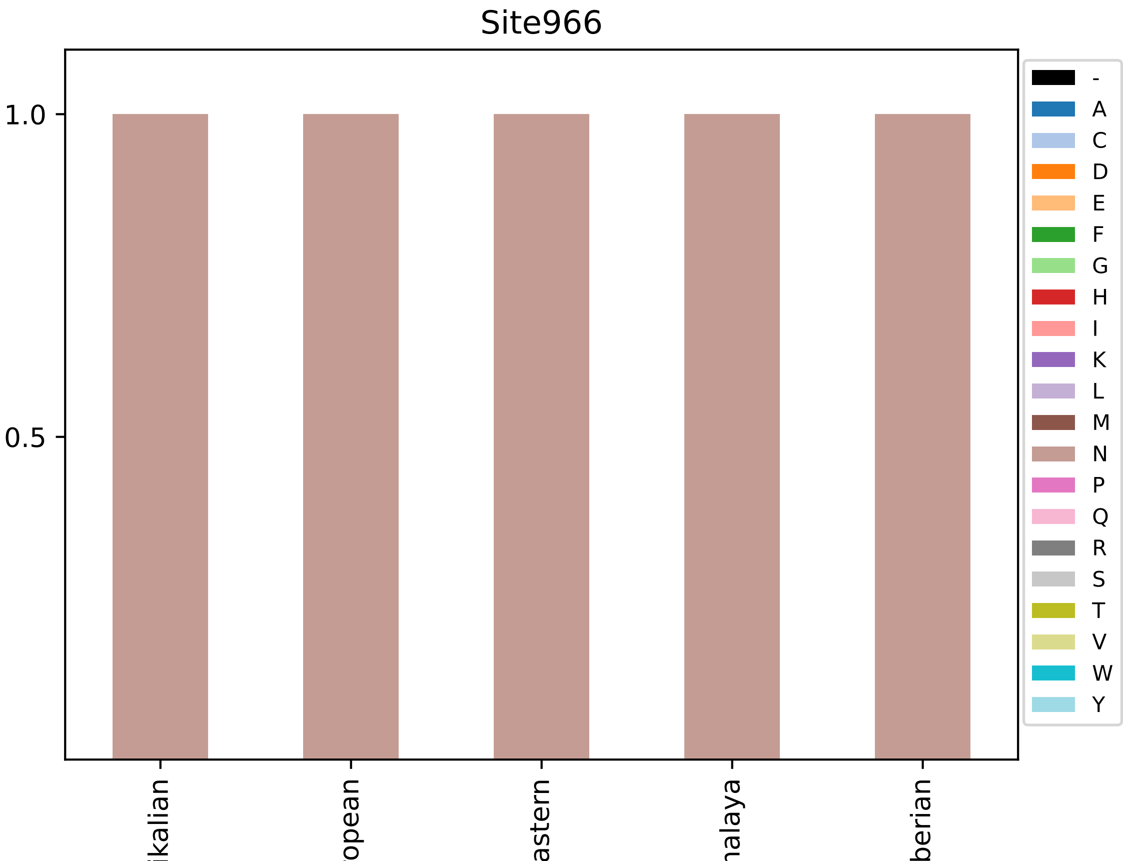

Site966
1.0
0.5
-
A
C
D
E
F
G
H
I
K
L
M
N
P
Q
R
S
T
V
W
Y
ikalian
opean
astern
nalaya
berian

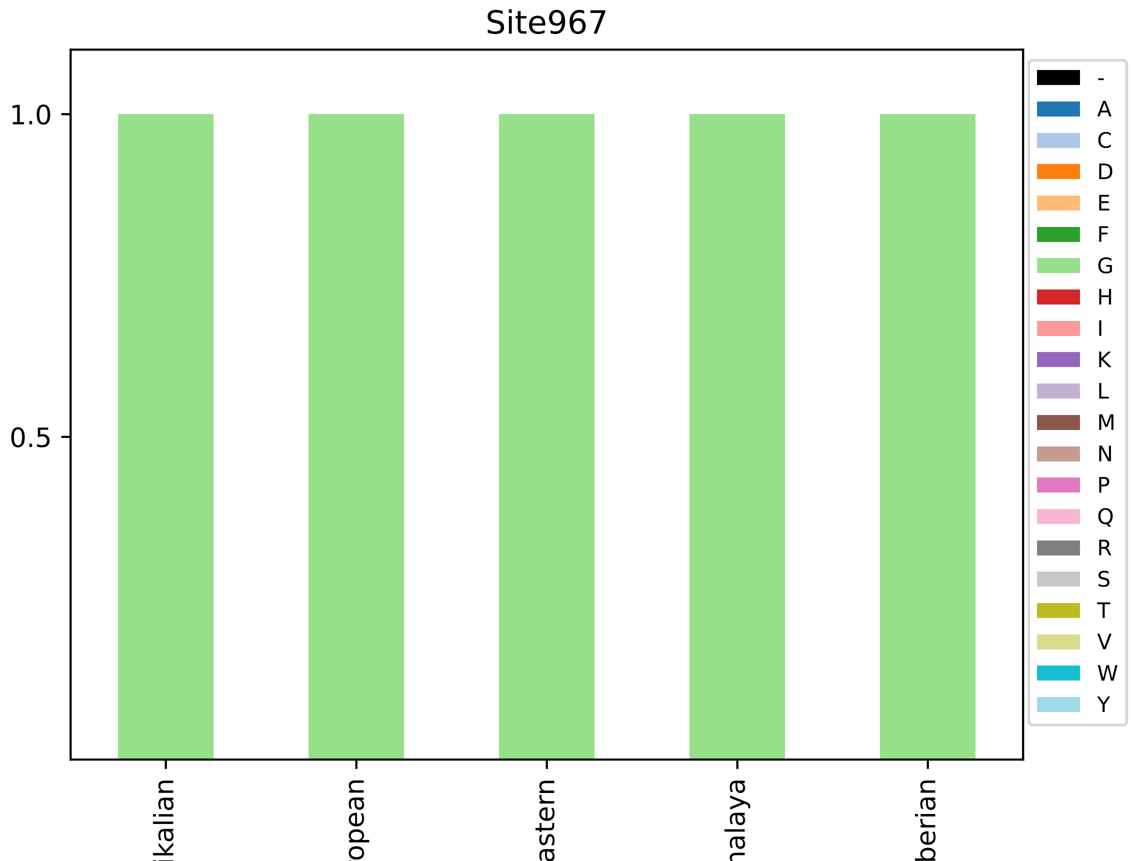
Site967
1.0
0.5
-
A
C
D
E
F
G
H
I
K
L
M
N
P
Q
R
S
T
V
W
Y
ikalian
opean
astern
nalaya
berian

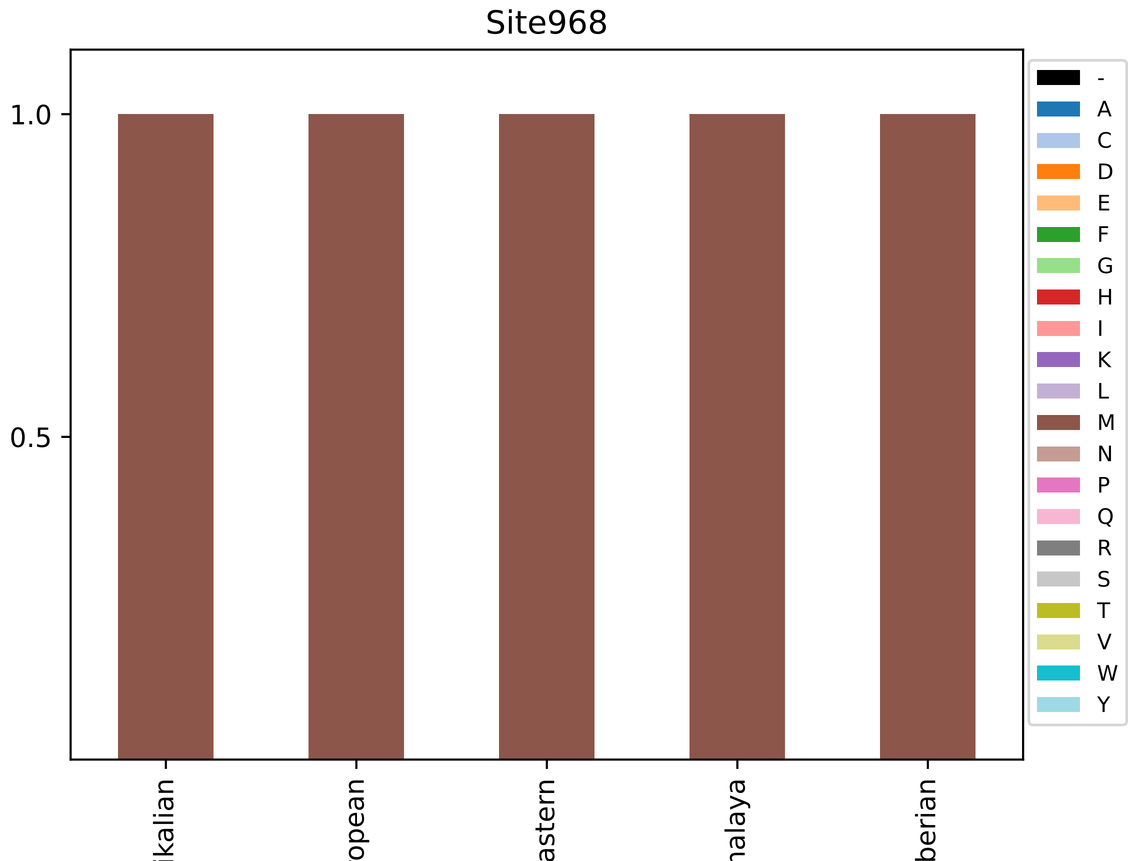
Site968
1.0
0.5
-
A
C
D
E
F
G
H
I
K
L
M
N
P
Q
R
S
T
V
W
Y
ikalian
opean
astern
nalaya
berian

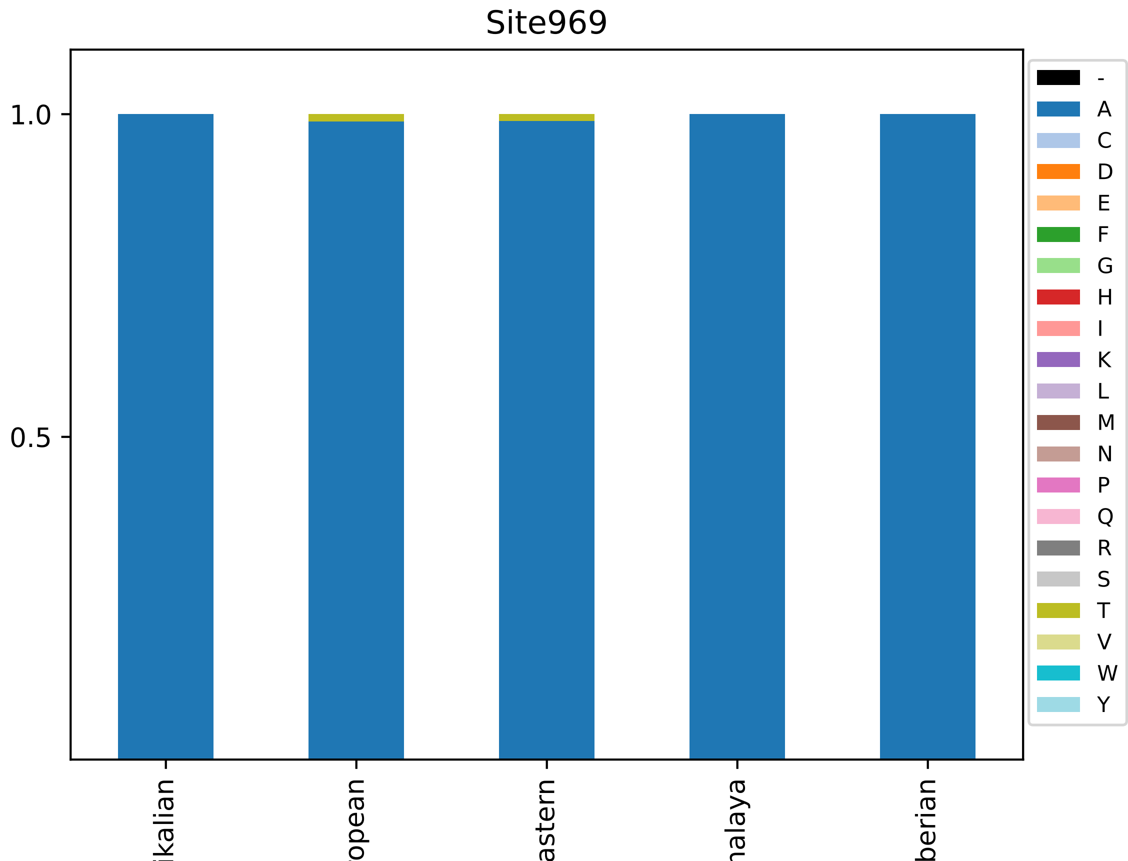

Site969
1.0
0.5
-
A
C
D
E
F
G
H
I
K
L
M
N
P
Q
R
S
T
V
W
Y
ikalian
opean
astern
nalaya
berian

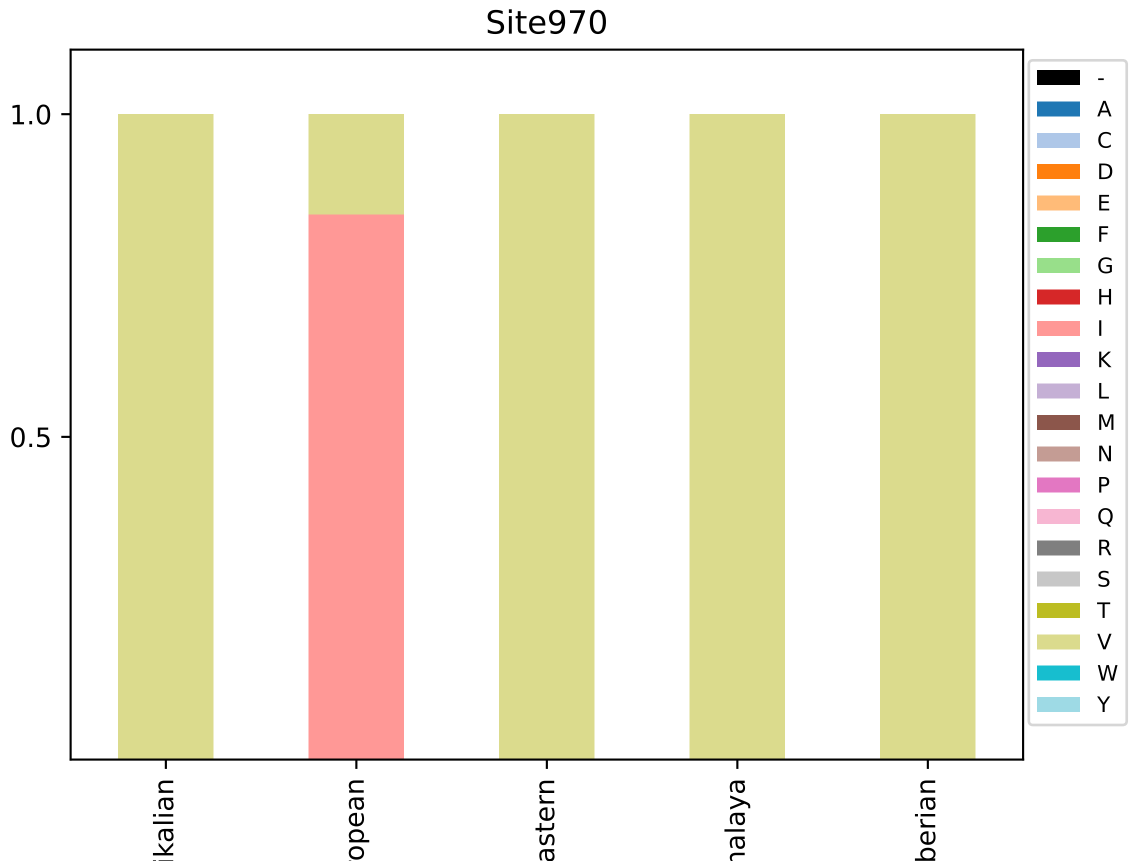

Site970
1.0
0.5
-
A
C
D
E
F
G
H
I
K
L
M
N
P
Q
R
S
T
V
W
Y
ikalian
opean
astern
nalaya
berian

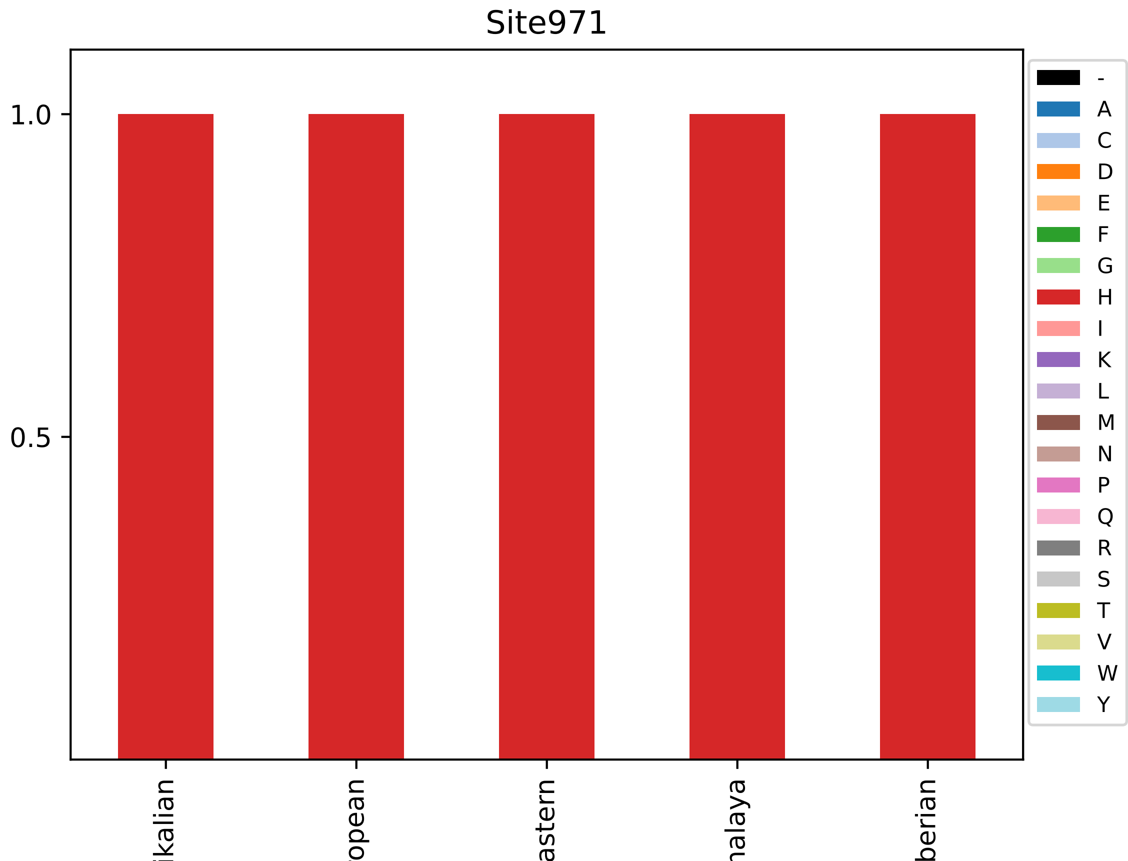
Site971
1.0
0.5
ikalian
opean
astern
nalaya
berian
-
A
C
D
E
F
G
H
I
K
L
M
N
P
Q
R
S
T
V
W
Y

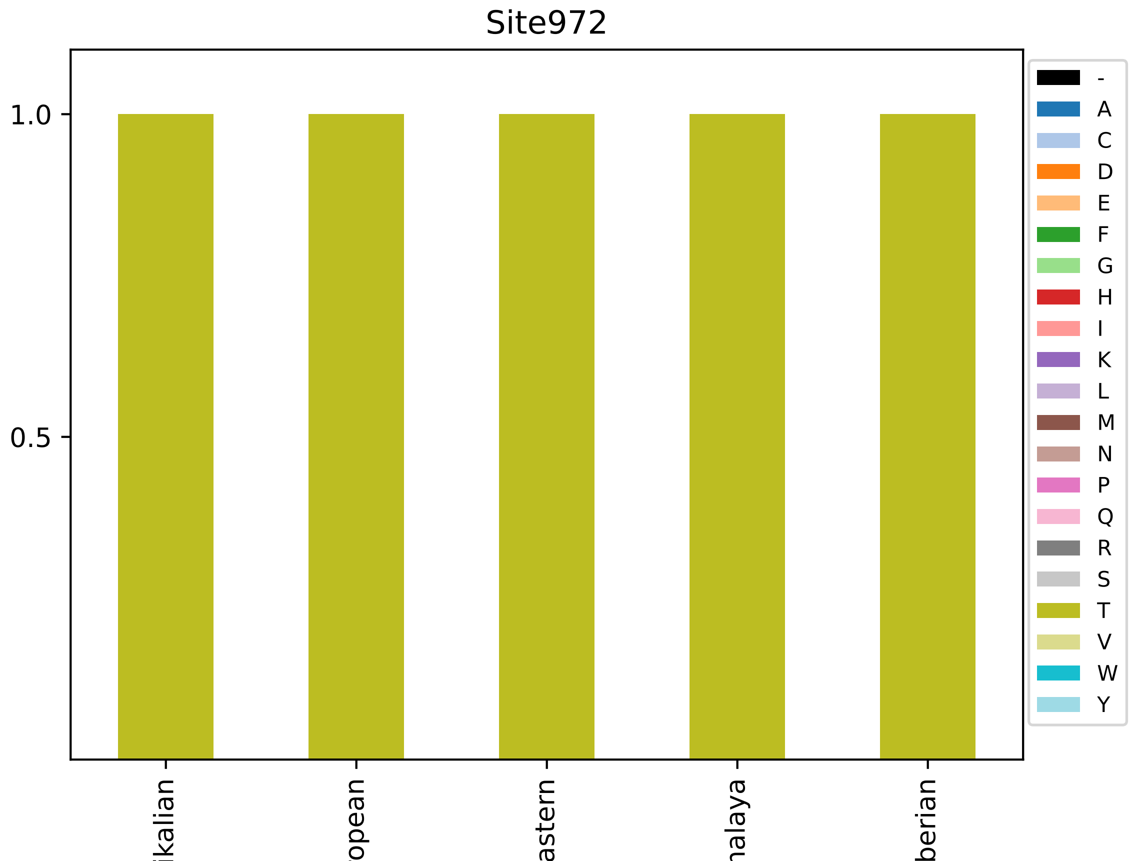

Site972
1.0
0.5
ikalian
opean
astern
nalaya
berian
-
A
C
D
E
F
G
H
I
K
L
M
N
P
Q
R
S
T
V
W
Y

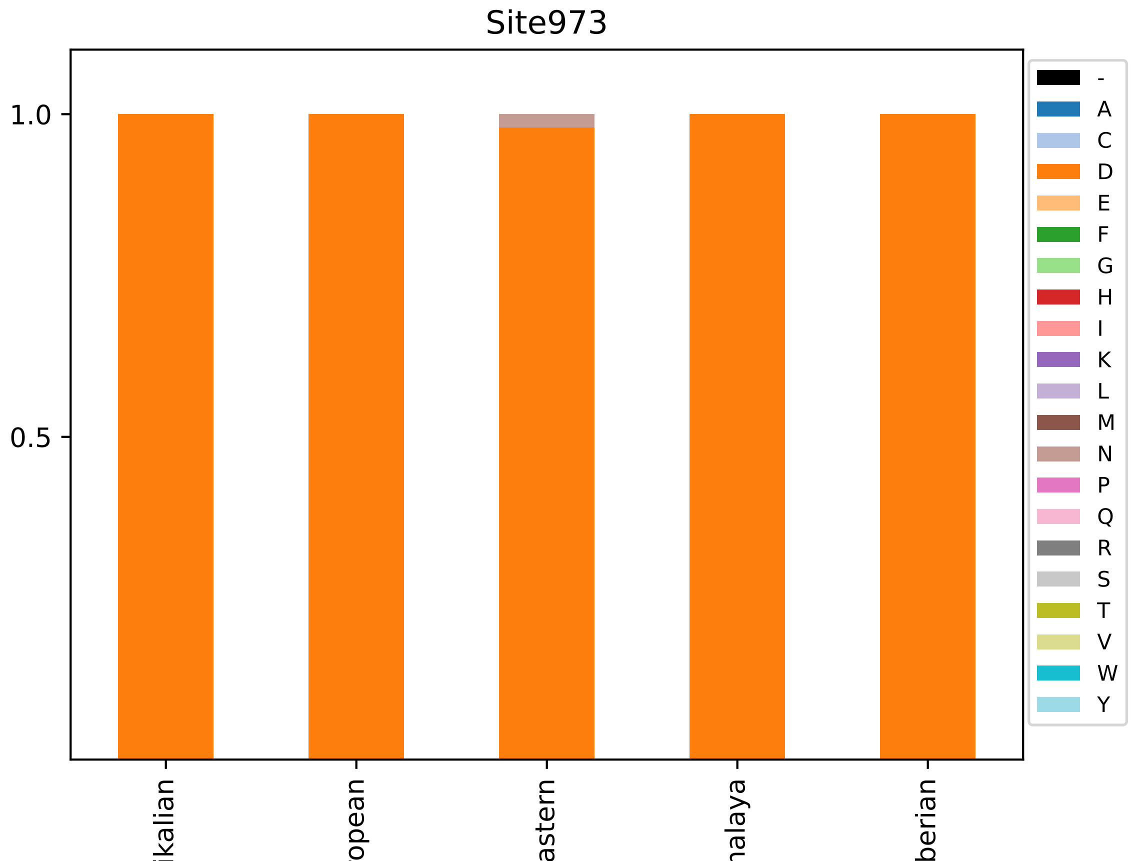
Site973
1.0
0.5
-
A
C
D
E
F
G
H
I
K
L
M
N
P
Q
R
S
T
V
W
Y

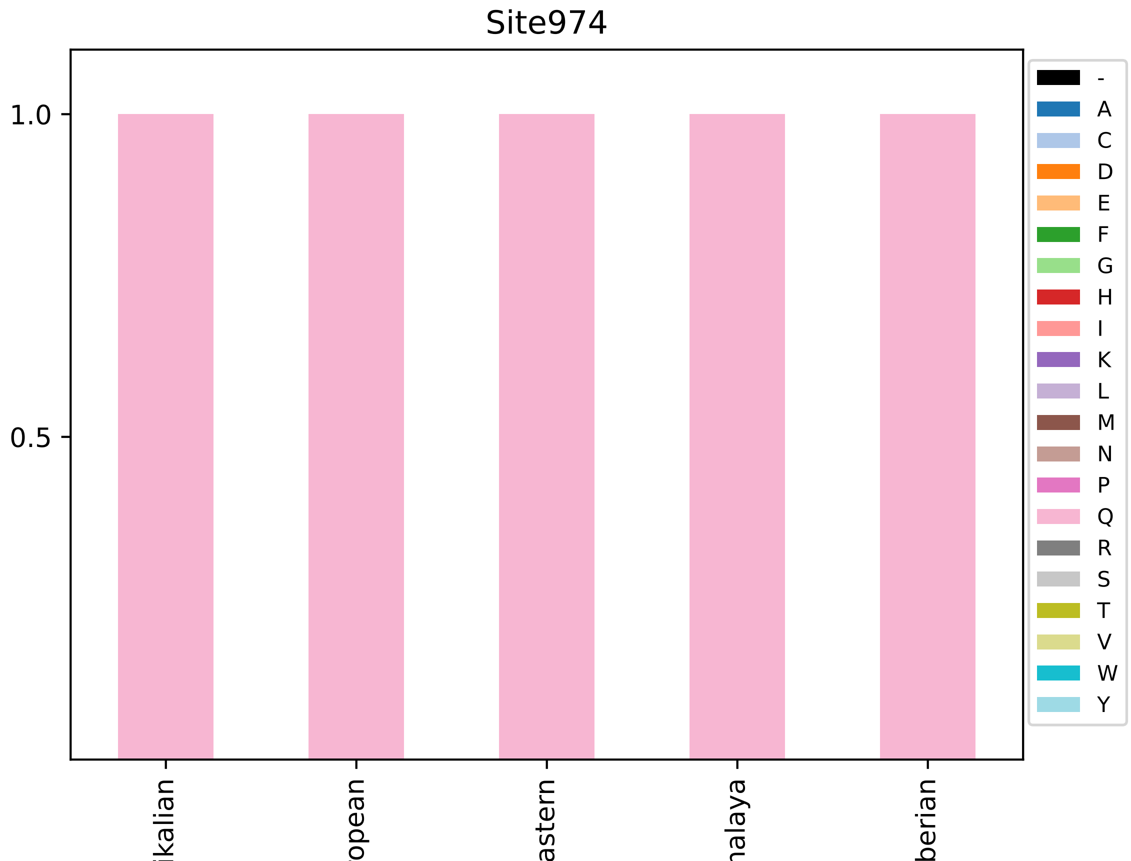
Site974
1.0
0.5
-
A
C
D
E
F
G
H
I
K
L
M
N
P
Q
R
S
T
V
W
Y
ikalian
opean
astern
nalaya
berian

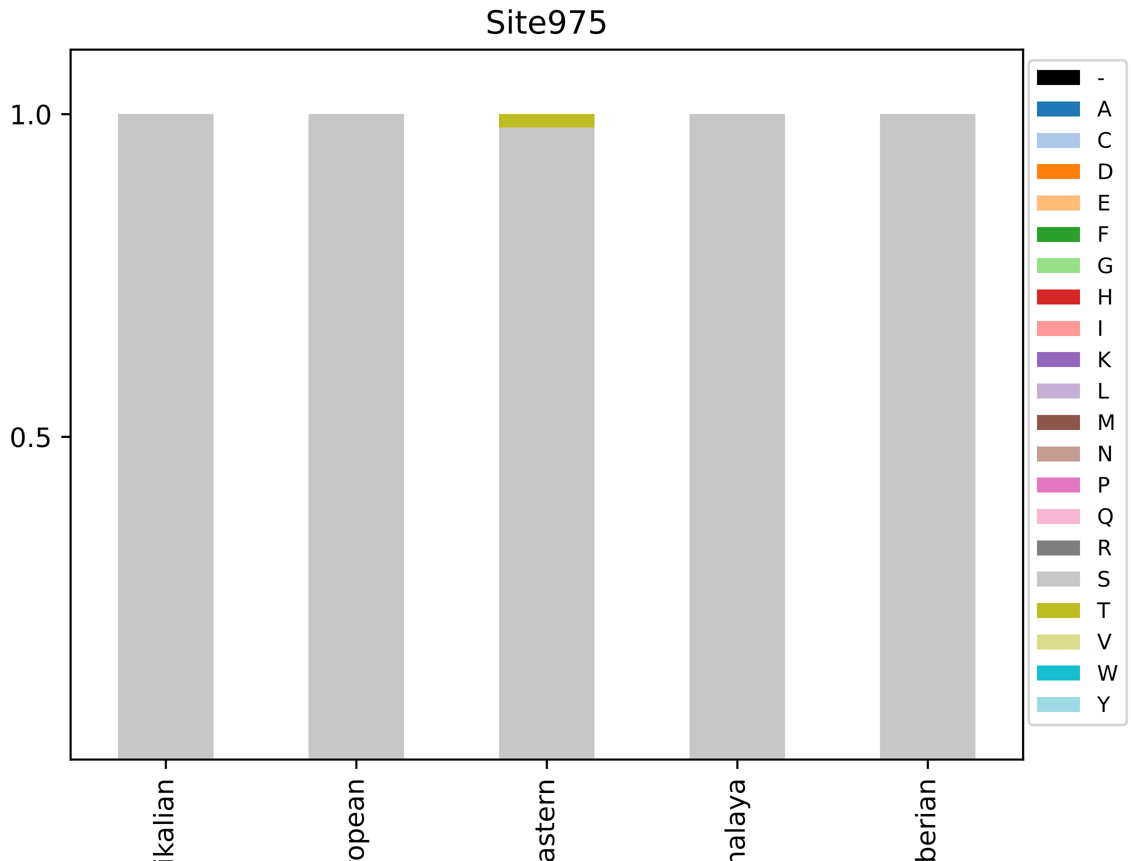

Site975
1.0
0.5
-
A
C
D
E
F
G
H
I
K
L
M
N
P
Q
R
S
T
V
W
Y
ikalian
opean
astern
halaya
berian

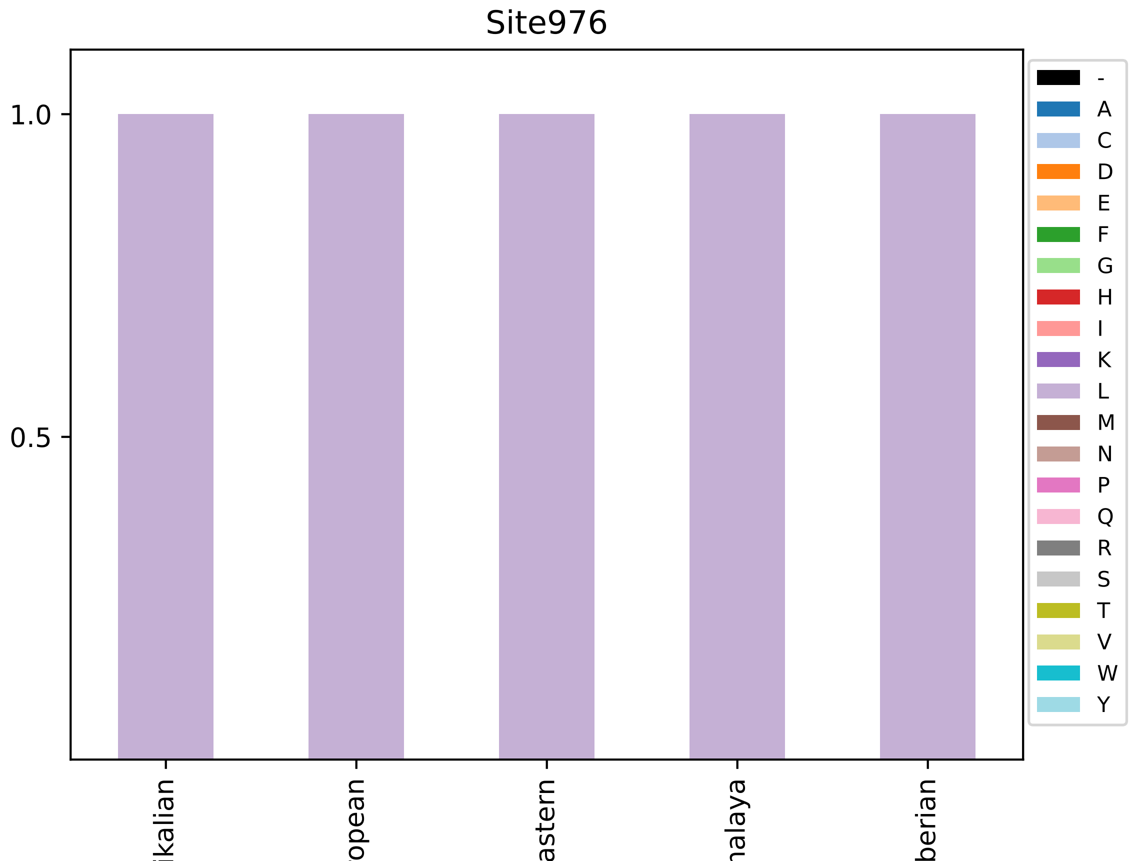

Site976
1.0
0.5
-
A
C
D
E
F
G
H
I
K
L
M
N
P
Q
R
S
T
V
W
Y

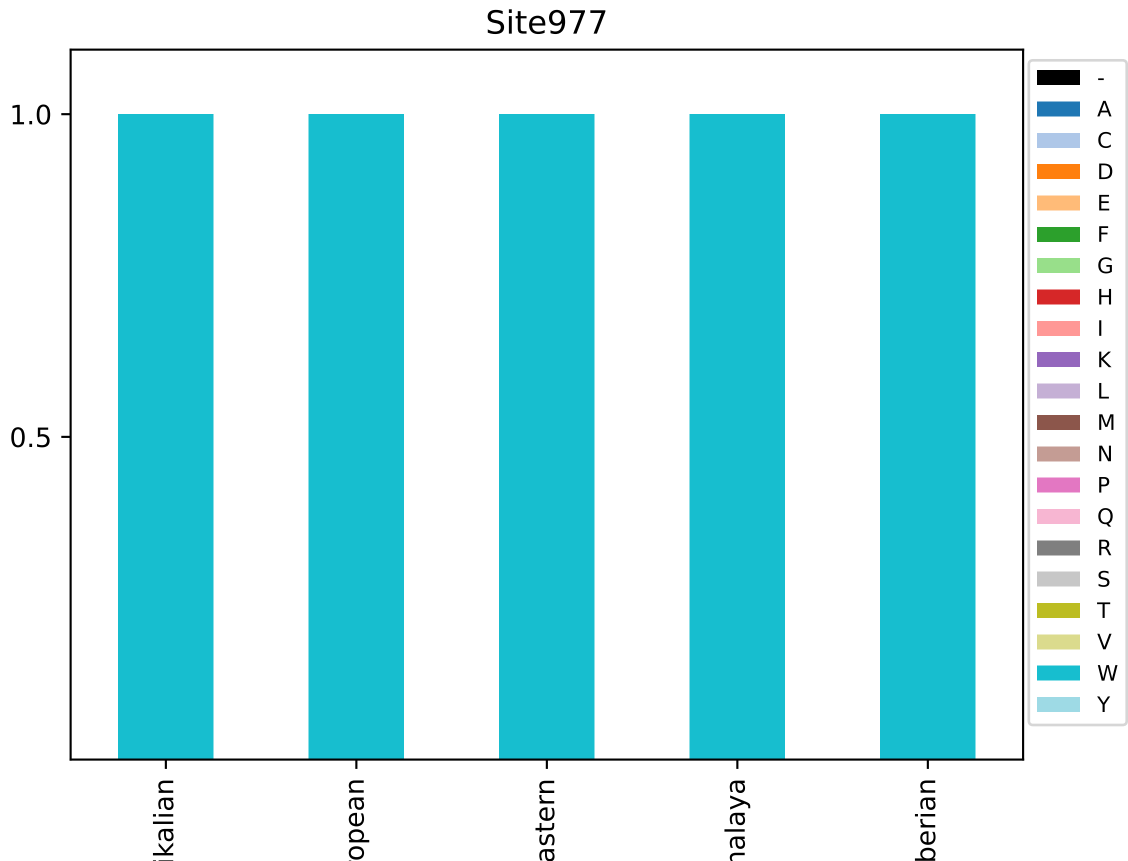
Site977
1.0
0.5
ikalian
opean
astern
nalaya
berian
-
A
C
D
E
F
G
H
I
K
L
M
N
P
Q
R
S
T
V
W
Y

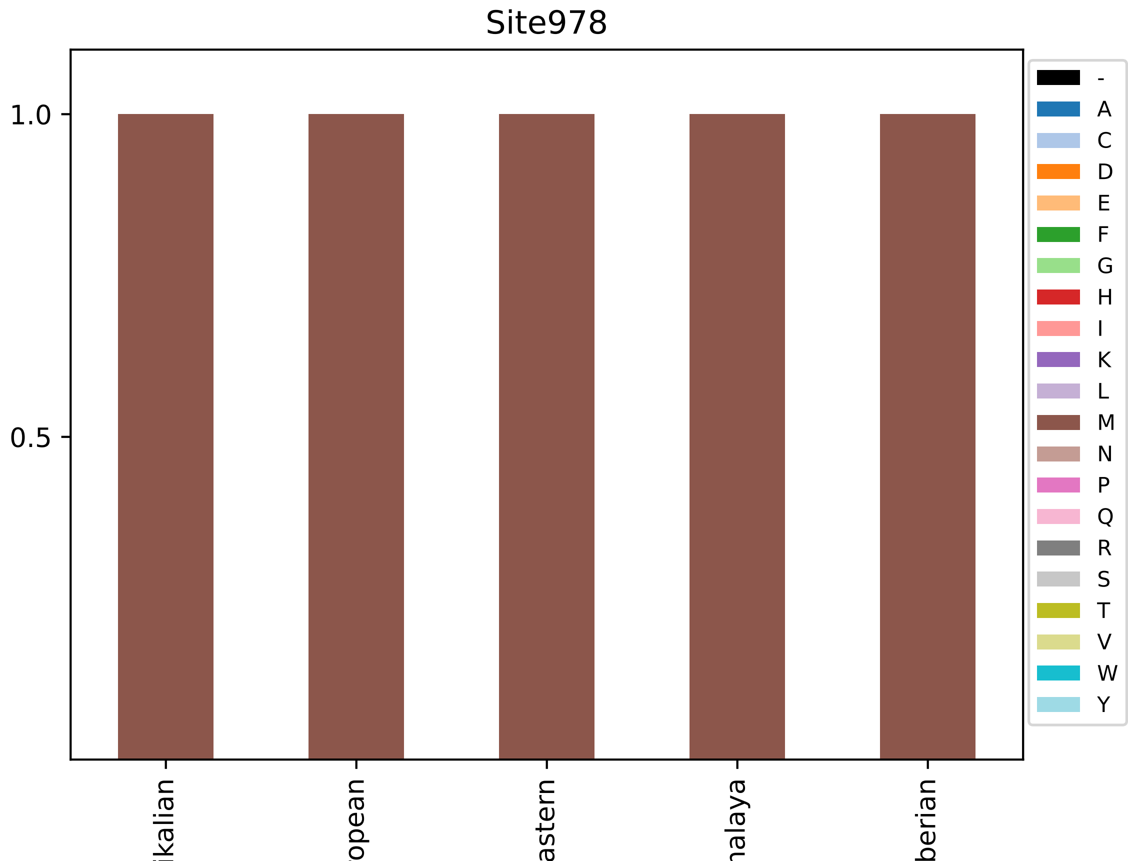
Site978
1.0
0.5
-
A
C
D
E
F
G
H
I
K
L
M
N
P
Q
R
S
T
V
W
Y
ikalian
opean
astern
nalaya
berian

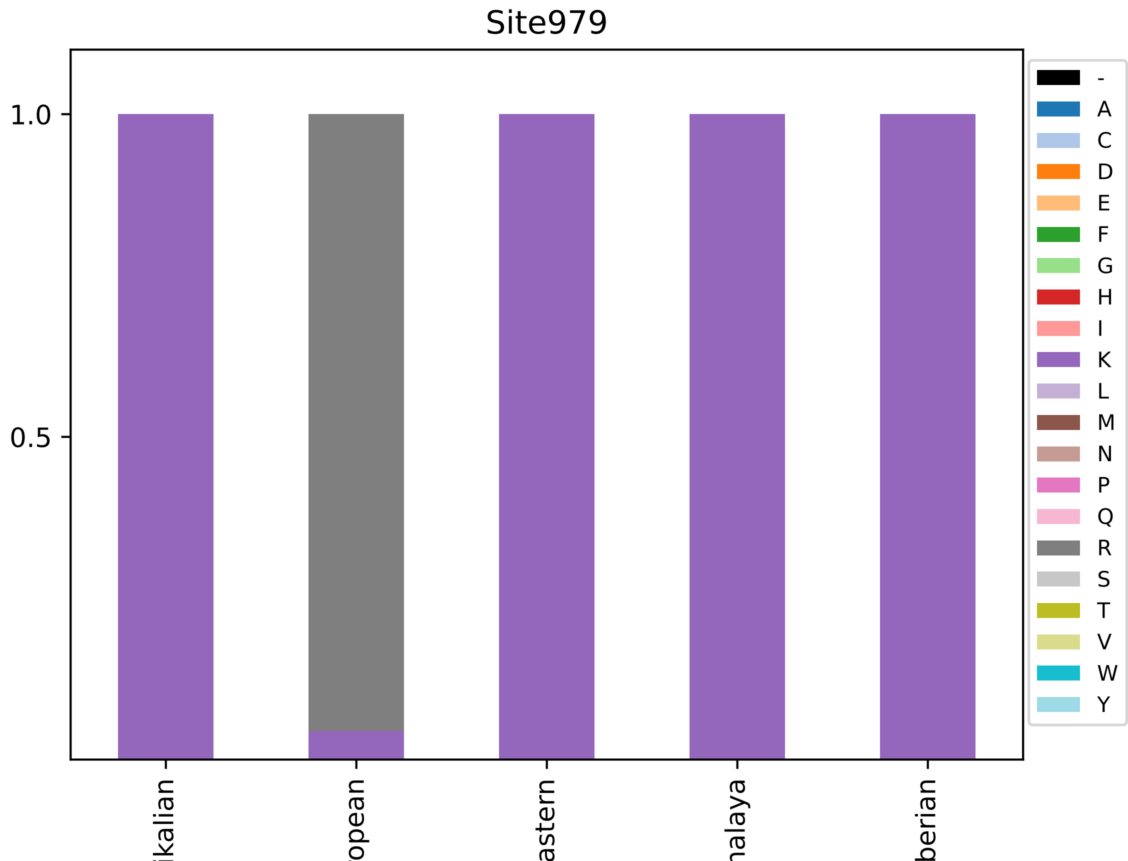
Site979
1.0
0.5
-
A
C
D
E
F
G
H
I
K
L
M
N
P
Q
R
S
T
V
W
Y

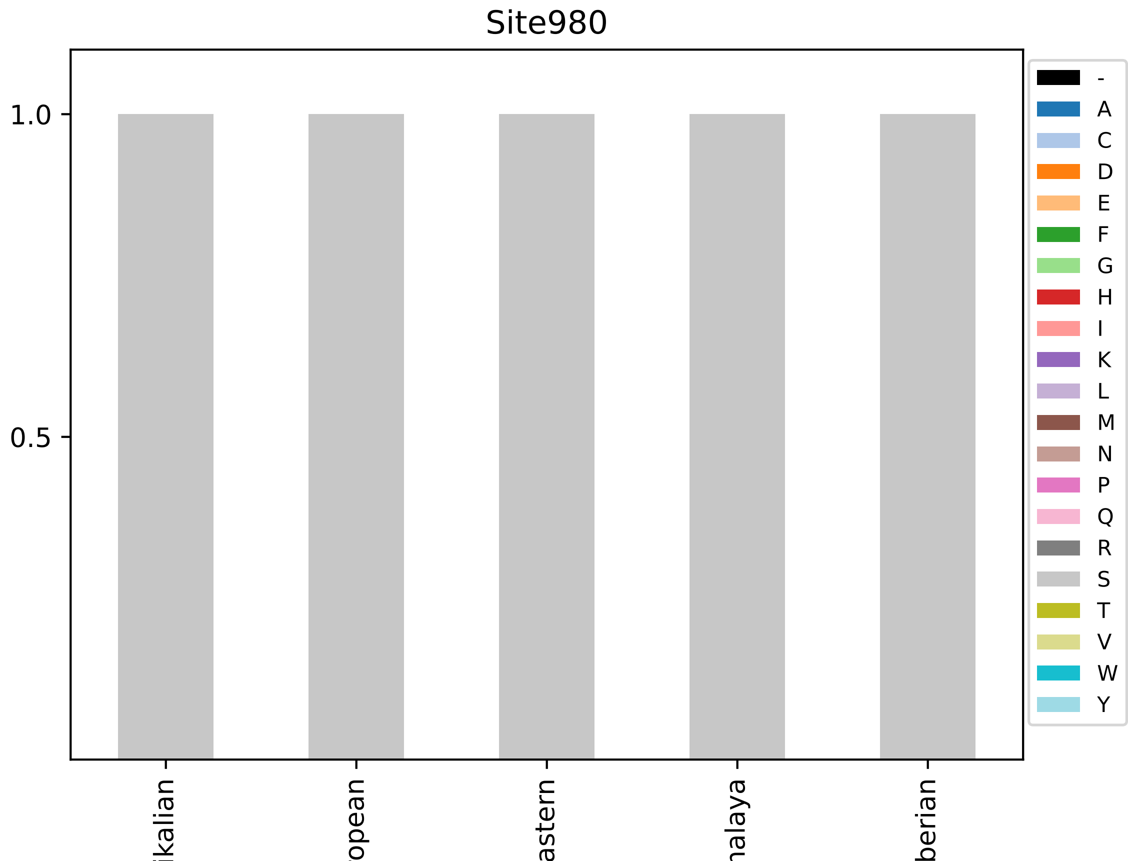

Site980
1.0
0.5
ikalian
opean
astern
nalaya
berian
-
A
C
D
E
F
G
H
I
K
L
M
N
P
Q
R
S
T
V
W
Y

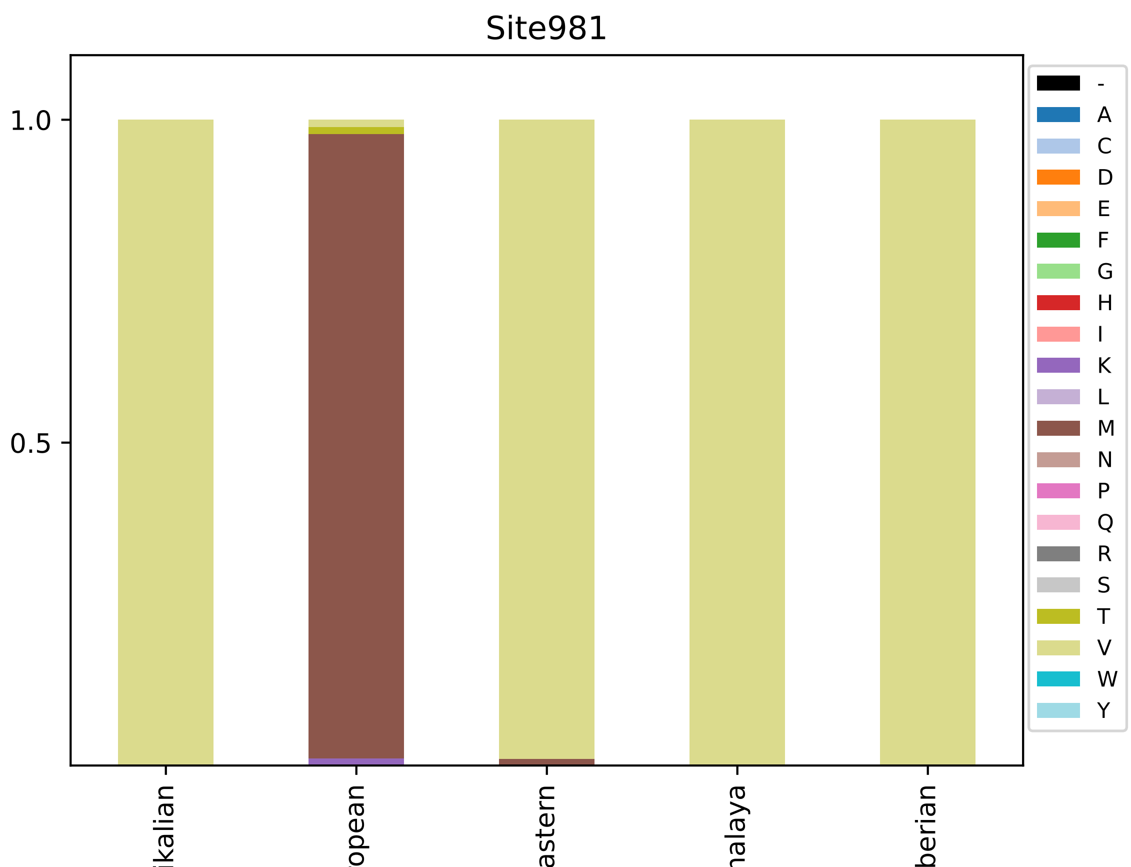
Site981
1.0
0.5
-
A
C
D
E
F
G
H
I
K
L
M
N
P
Q
R
S
T
V
W
Y
ikalian
opean
astern
nalaya
berian

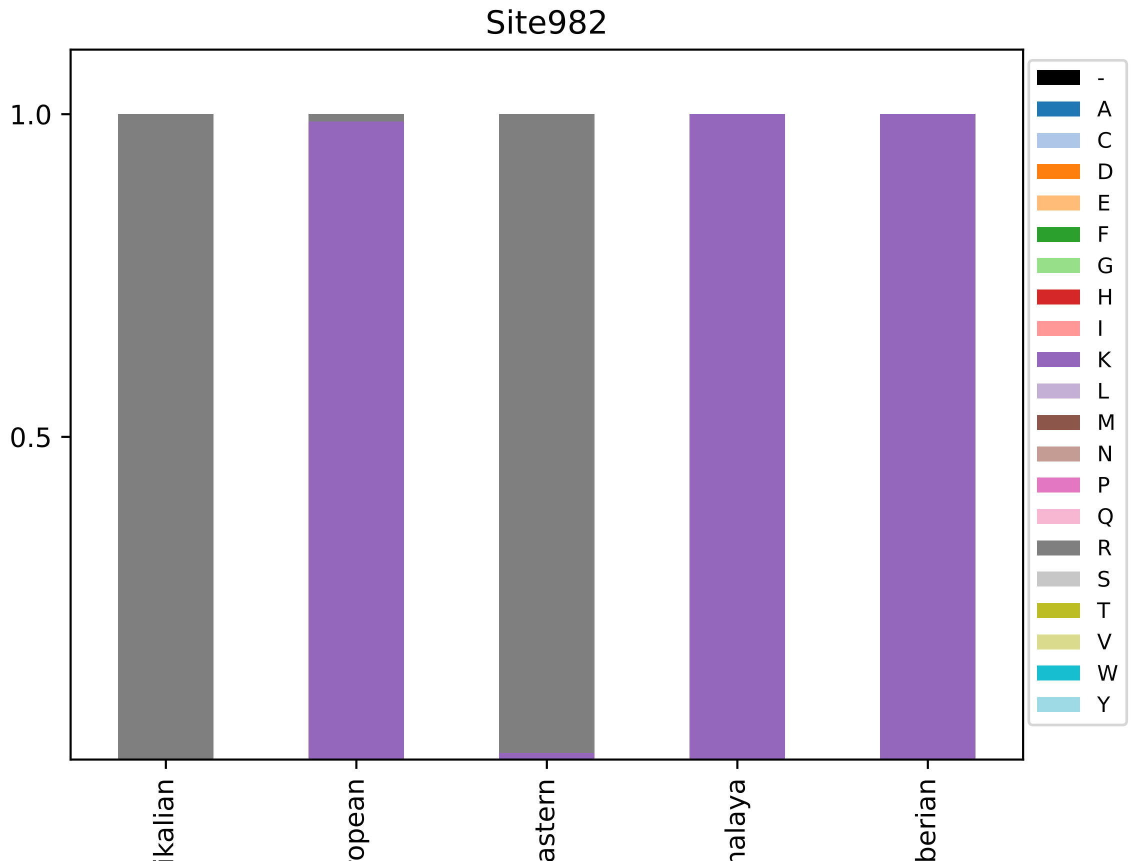

Site982
1.0
0.5
-
A
C
D
E
F
G
H
I
K
L
M
N
P
Q
R
S
T
V
W
Y
ikalian
opean
astern
nalaya
berian

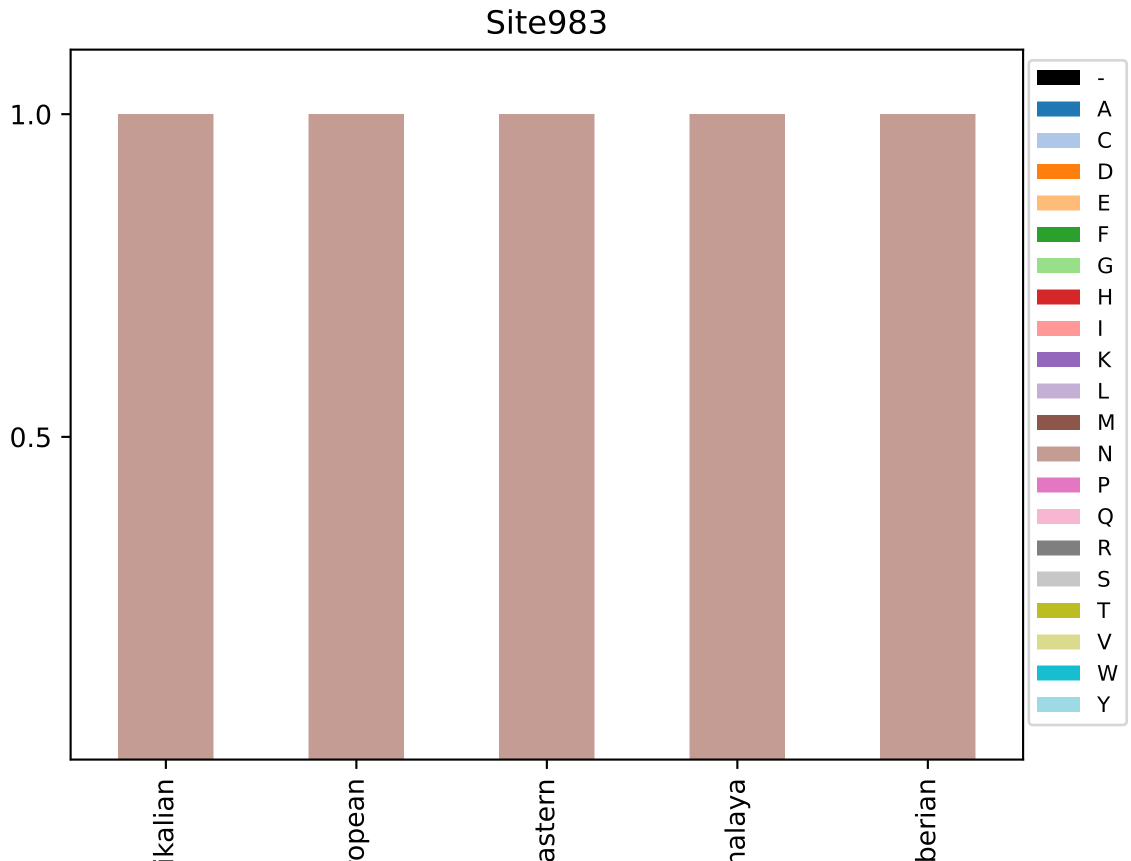

Site983
1.0
0.5
-
A
C
D
E
F
G
H
I
K
L
M
N
P
Q
R
S
T
V
W
Y
ikalian
opean
astern
nalaya
berian

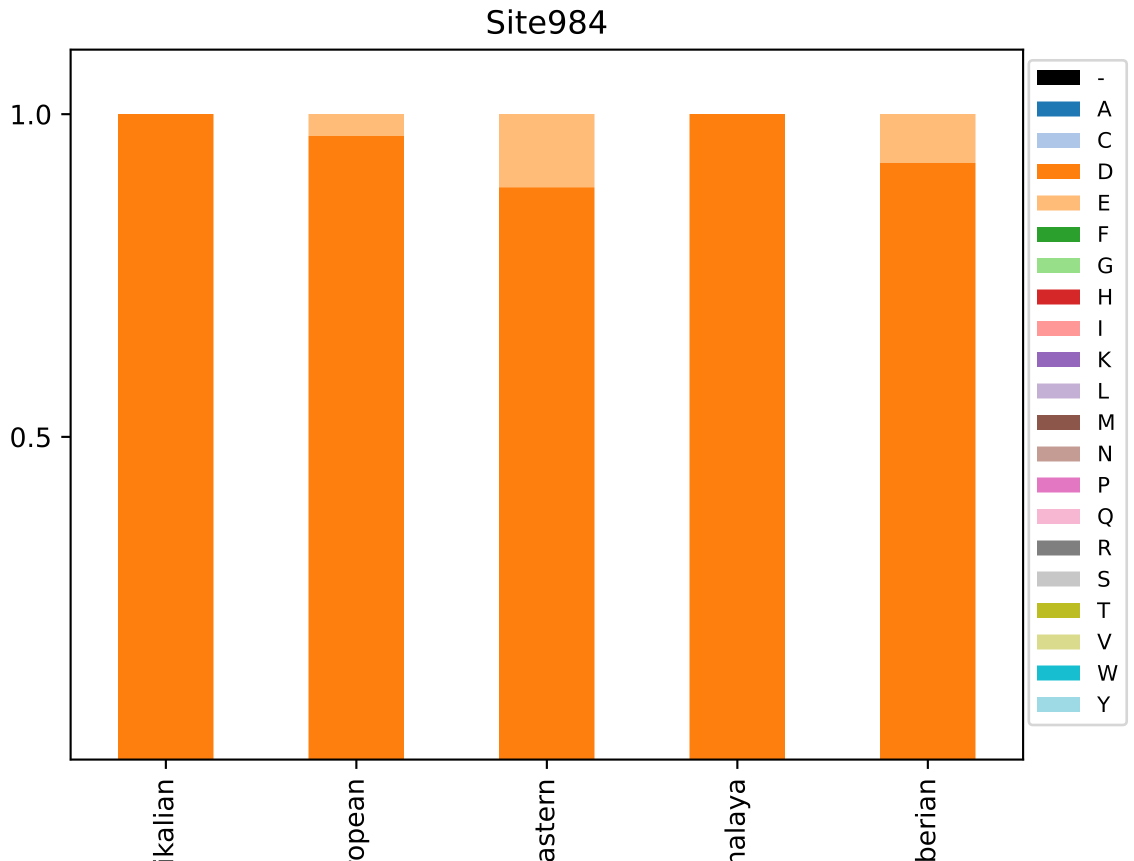

Site984
1.0
0.5
ikalian
opean
astern
nalaya
berian
-
A
C
D
E
F
G
H
I
K
L
M
N
P
Q
R
S
T
V
W
Y

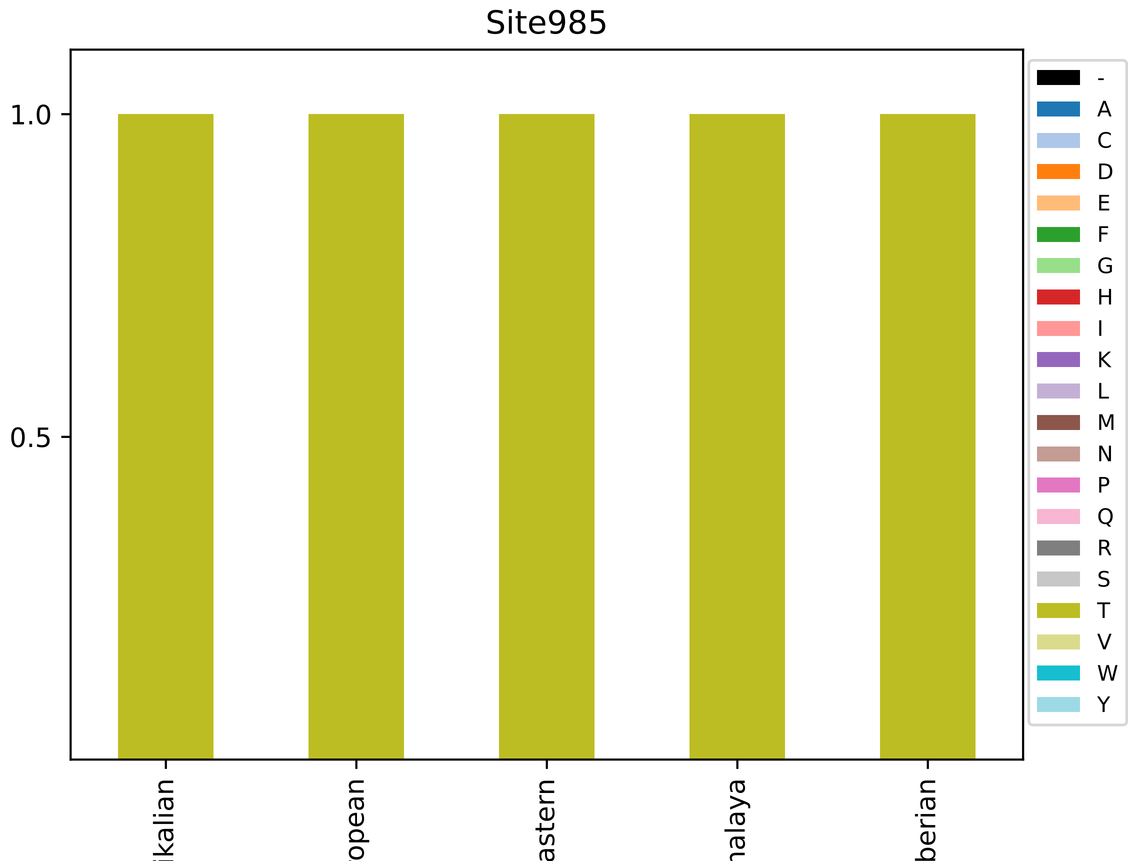

Site985
1.0
0.5
ikalian
opean
astern
nalaya
berian
-
A
C
D
E
F
G
H
I
K
L
M
N
P
Q
R
S
T
V
W
Y

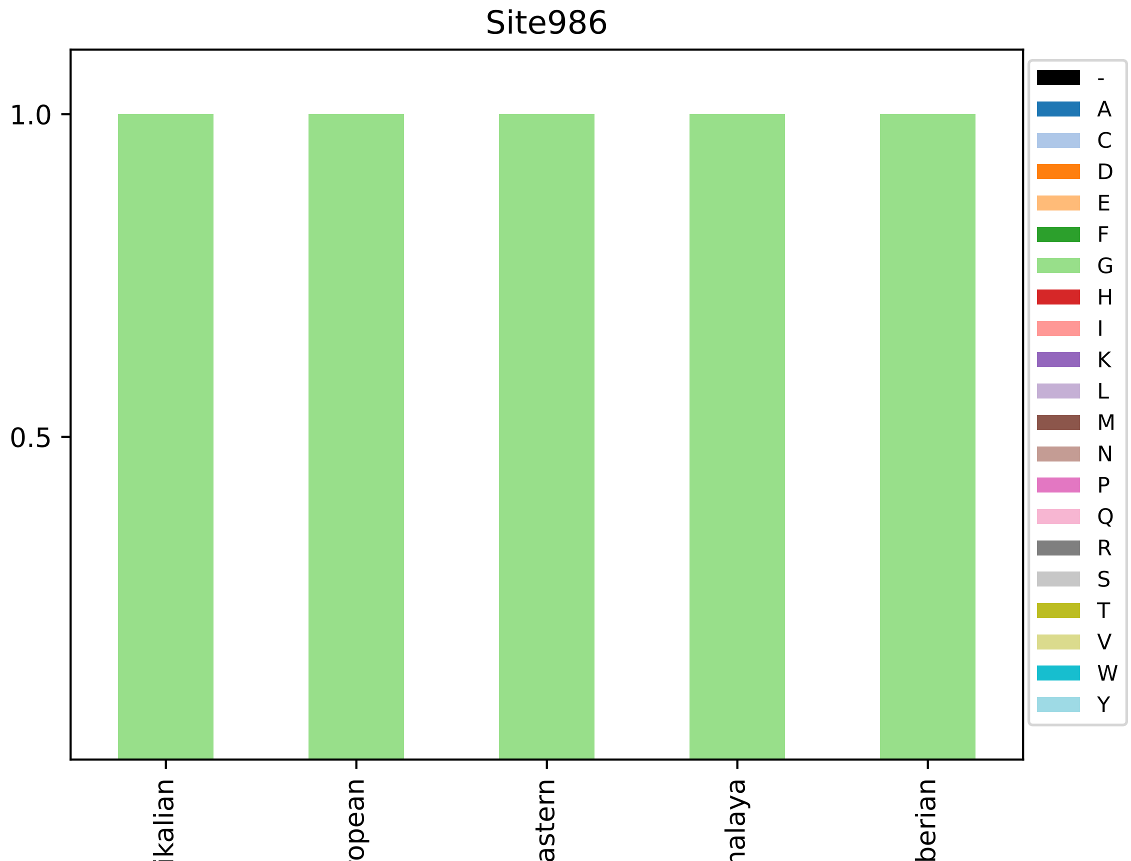
Site986
1.0
0.5
-
A
C
D
E
F
G
H
I
K
L
M
N
P
Q
R
S
T
V
W
Y
ikalian
opean
astern
nalaya
berian

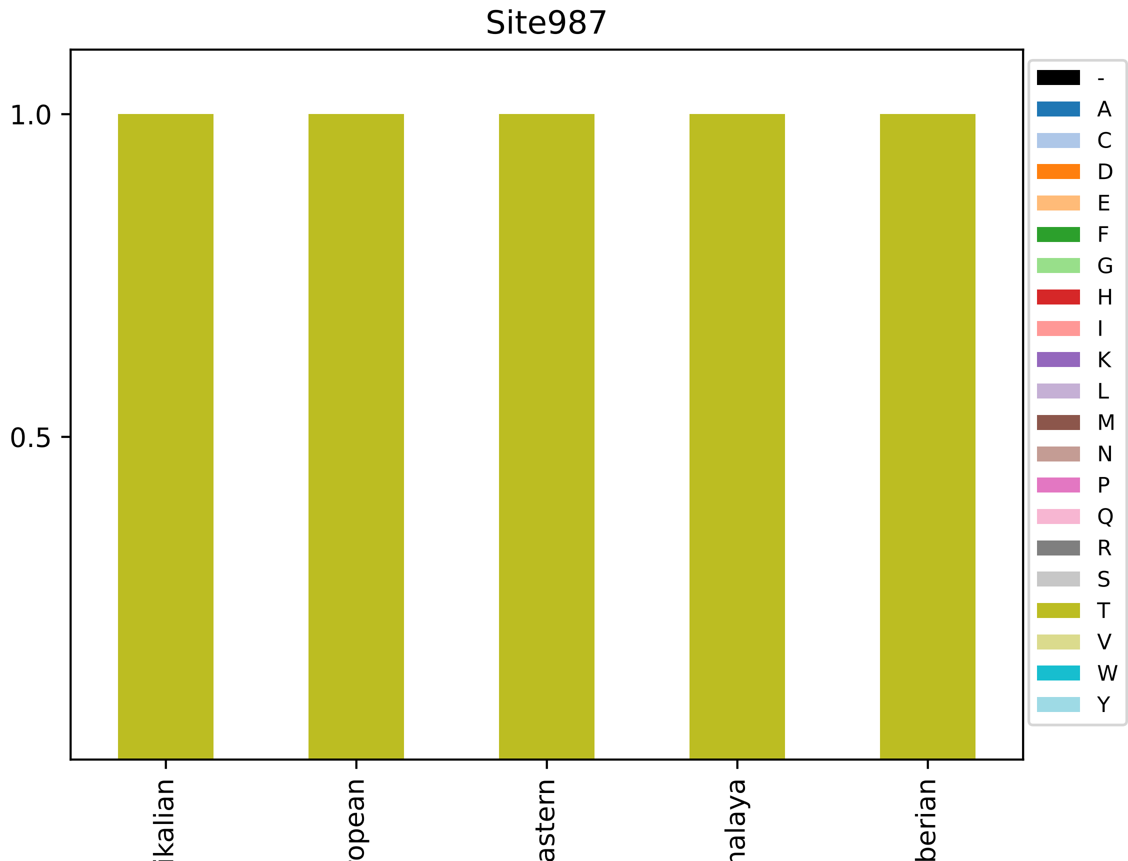

Site987
1.0
0.5
-
A
C
D
E
F
G
H
I
K
L
M
N
P
Q
R
S
T
V
W
Y

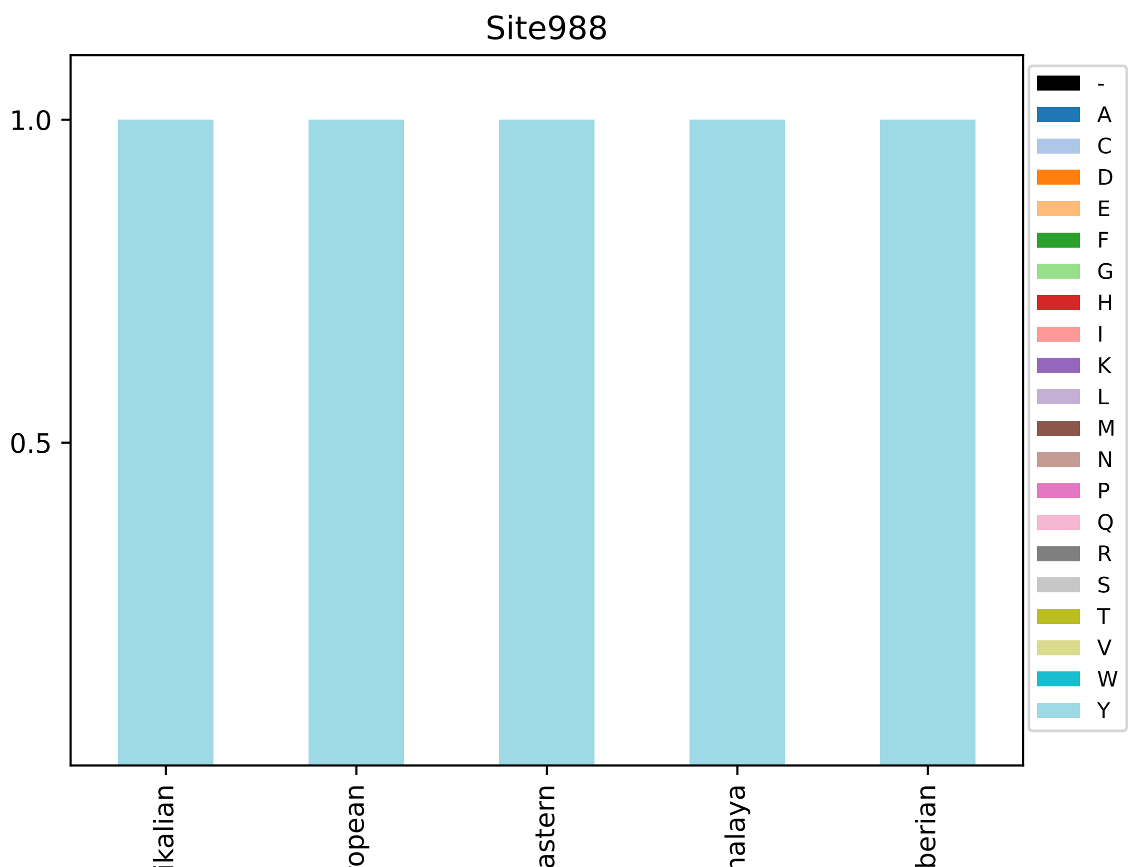
Site988
1.0
0.5
-
A
C
D
E
F
G
H
I
K
L
M
N
P
Q
R
S
T
V
W
Y
ikalian
opean
astern
nalaya
berian

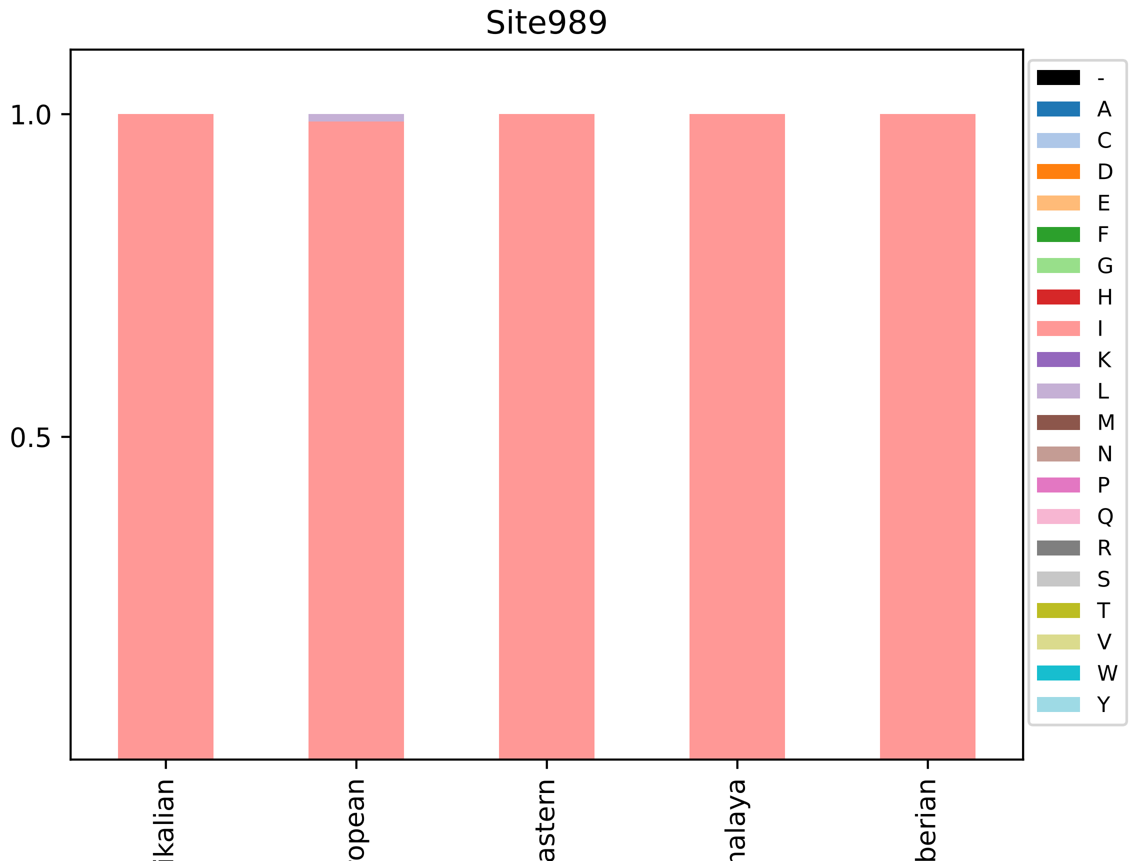
Site989
1.0
0.5
-
A
C
D
E
F
G
H
I
K
L
M
N
P
Q
R
S
T
V
W
Y

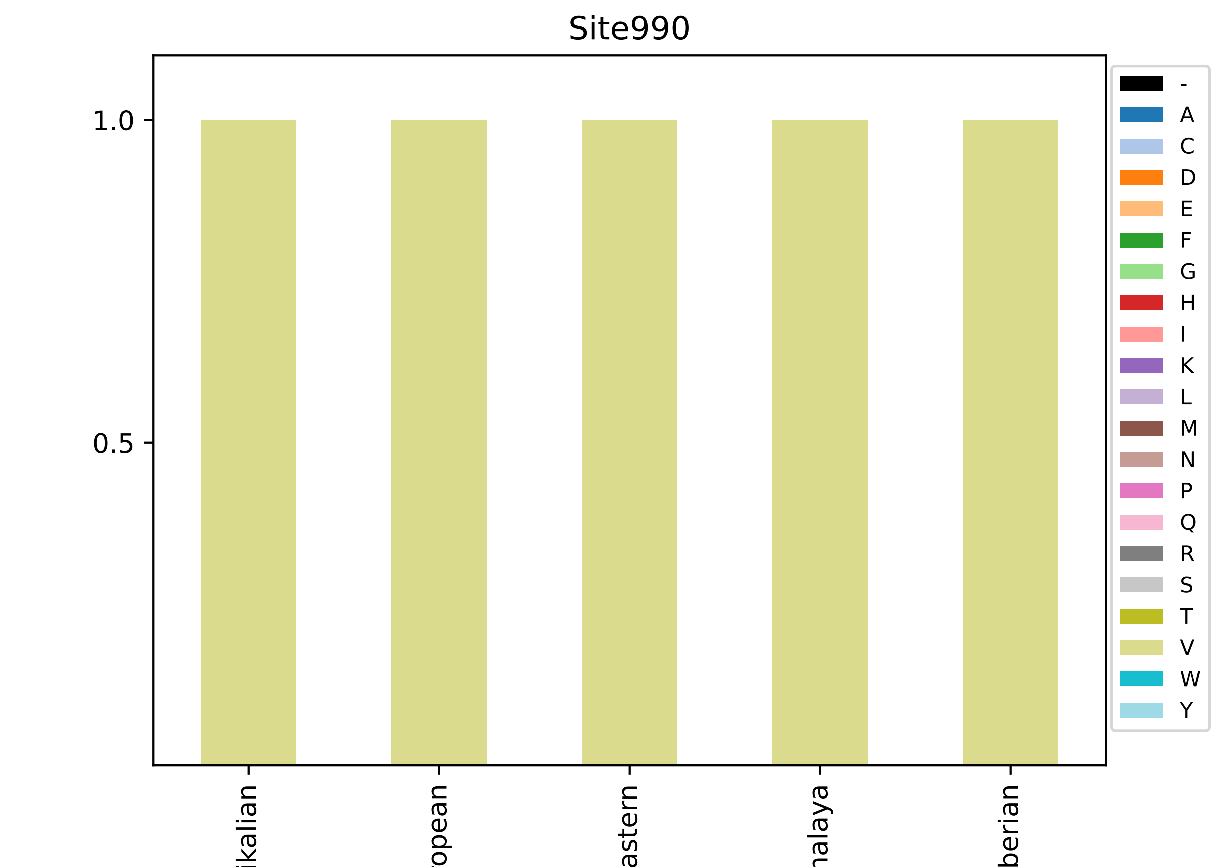
Site990
1.0
0.5
-
A
C
D
E
F
G
H
I
K
L
M
N
P
Q
R
S
T
V
W
Y
ikalian
opean
astern
nalaya
berian

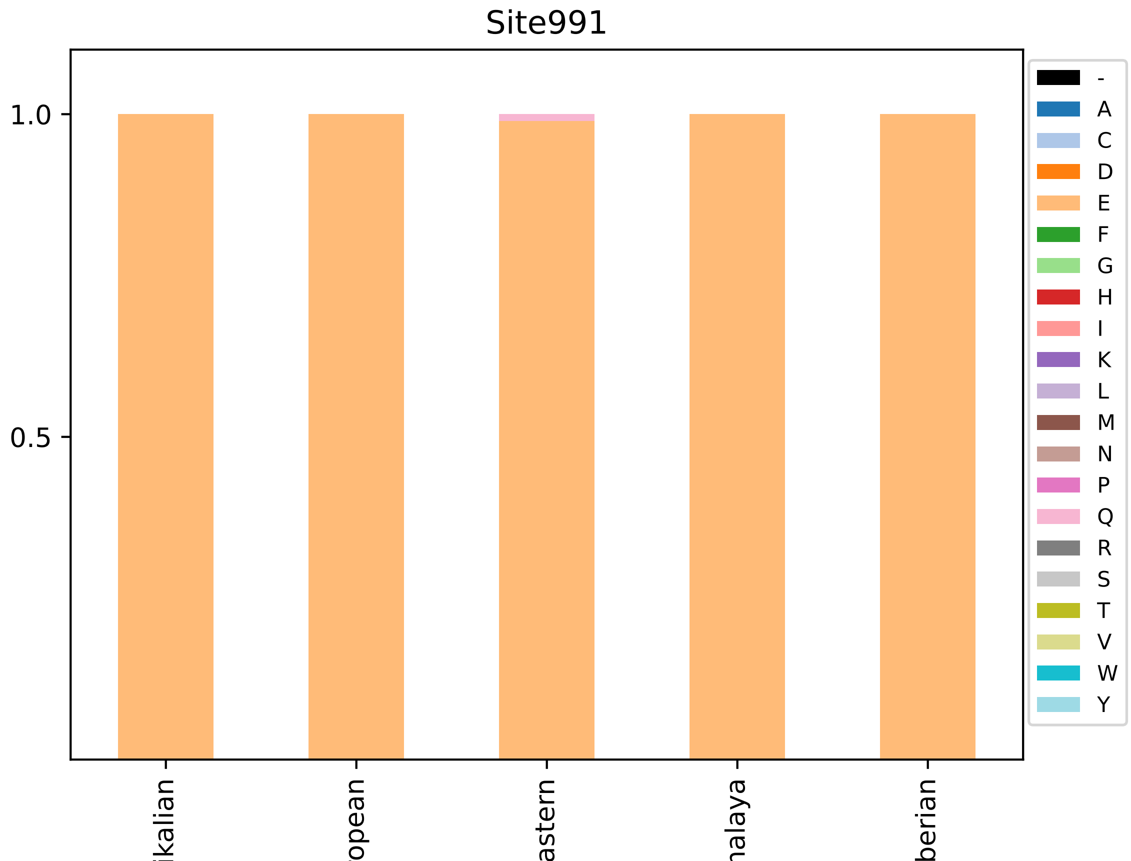
Site991
1.0
0.5
-
A
C
D
E
F
G
H
I
K
L
M
N
P
Q
R
S
T
V
W
Y
ikalian
opean
astern
nalaya
berian

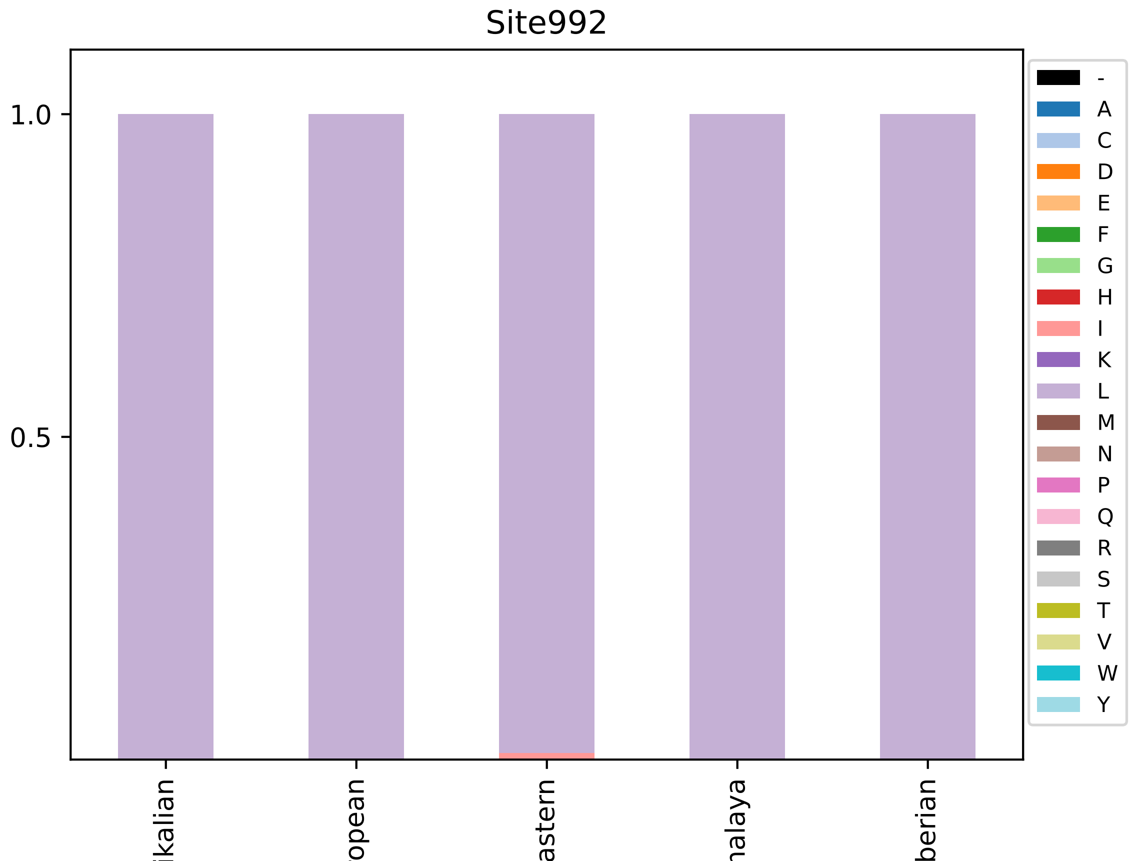
Site992
1.0
0.5
-
A
C
D
E
F
G
H
I
K
L
M
N
P
Q
R
S
T
V
W
Y
ikalian
opean
astern
nalaya
berian

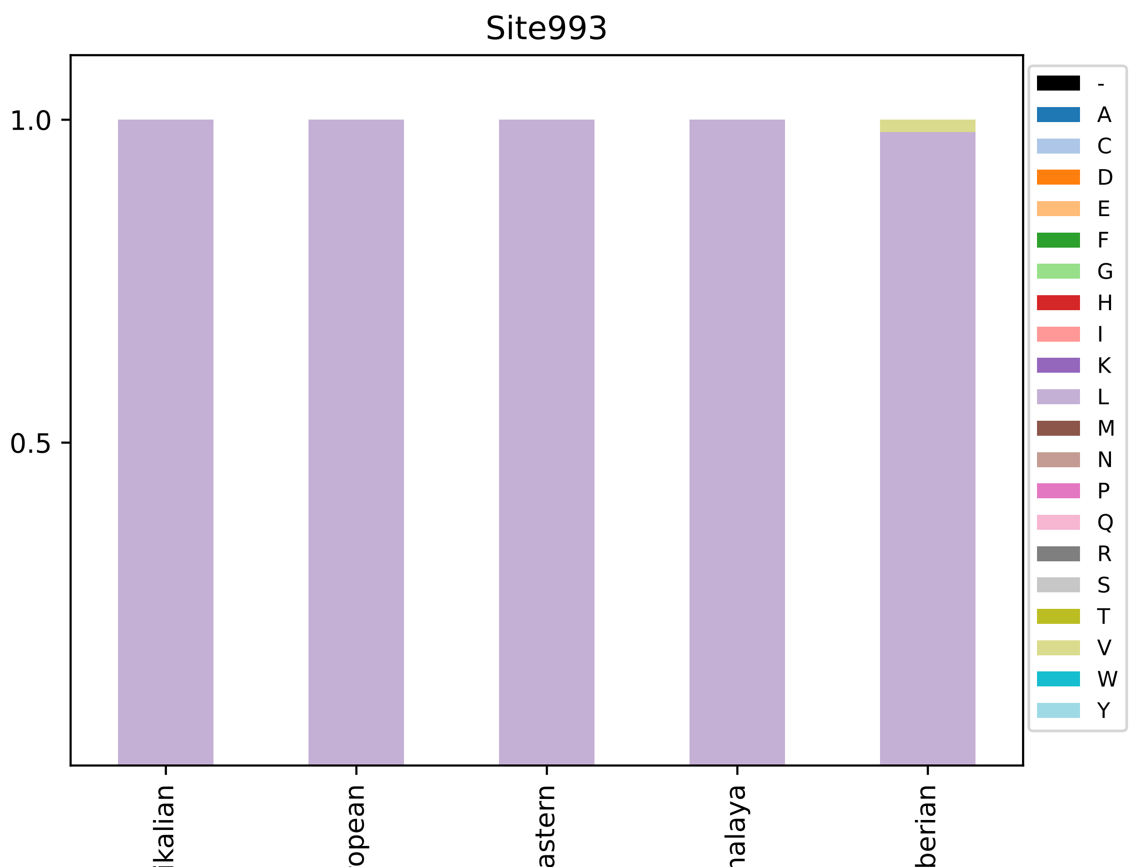

Site993
1.0
0.5
-
A
C
D
E
F
G
H
I
K
L
M
N
P
Q
R
S
T
V
W
Y
ikalian
opean
astern
nalaya
berian

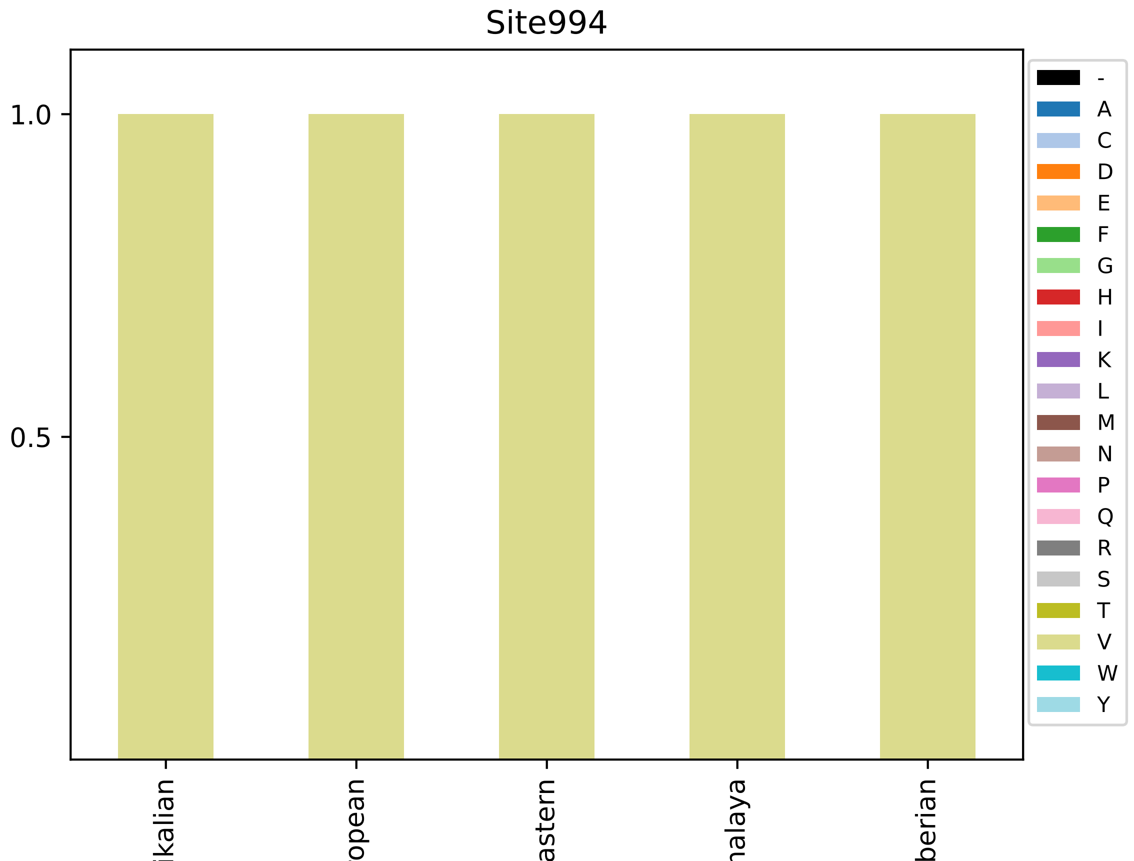

Site994
1.0
0.5
-
A
C
D
E
F
G
H
I
K
L
M
N
P
Q
R
S
T
V
W
Y
ikalian
opean
astern
nalaya
berian

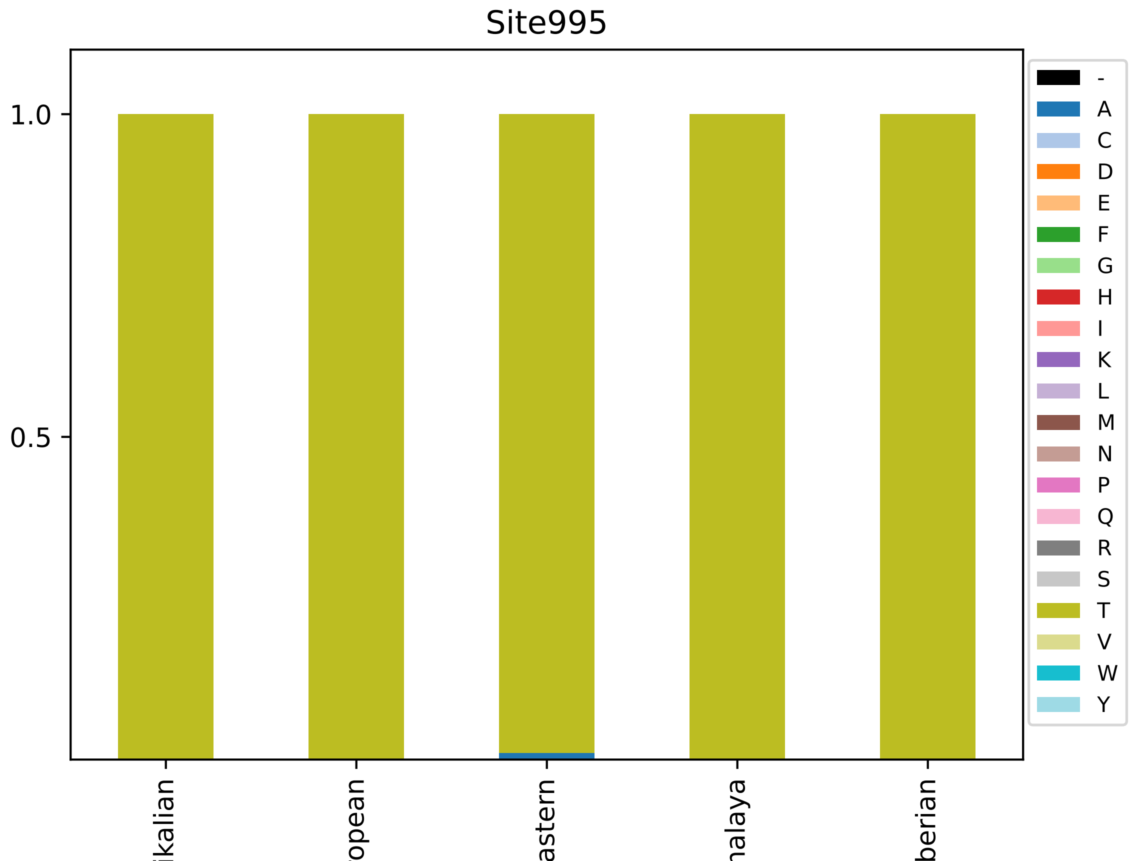

Site995
1.0
0.5
-
A
C
D
E
F
G
H
I
K
L
M
N
P
Q
R
S
T
V
W
Y
ikalian
opean
astern
nalaya
berian

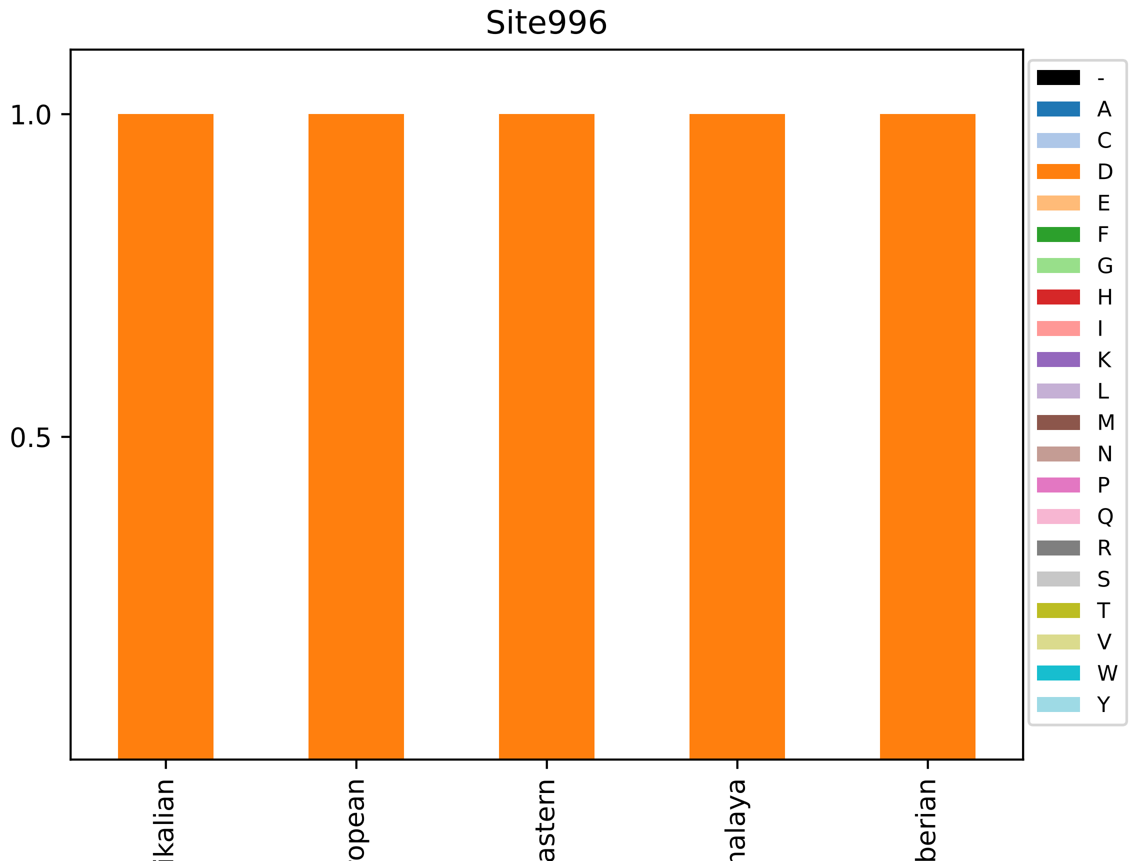
Site996
1.0
0.5
-
A
C
D
E
F
G
H
I
K
L
M
N
P
Q
R
S
T
V
W
Y
ikalian
opean
astern
nalaya
berian

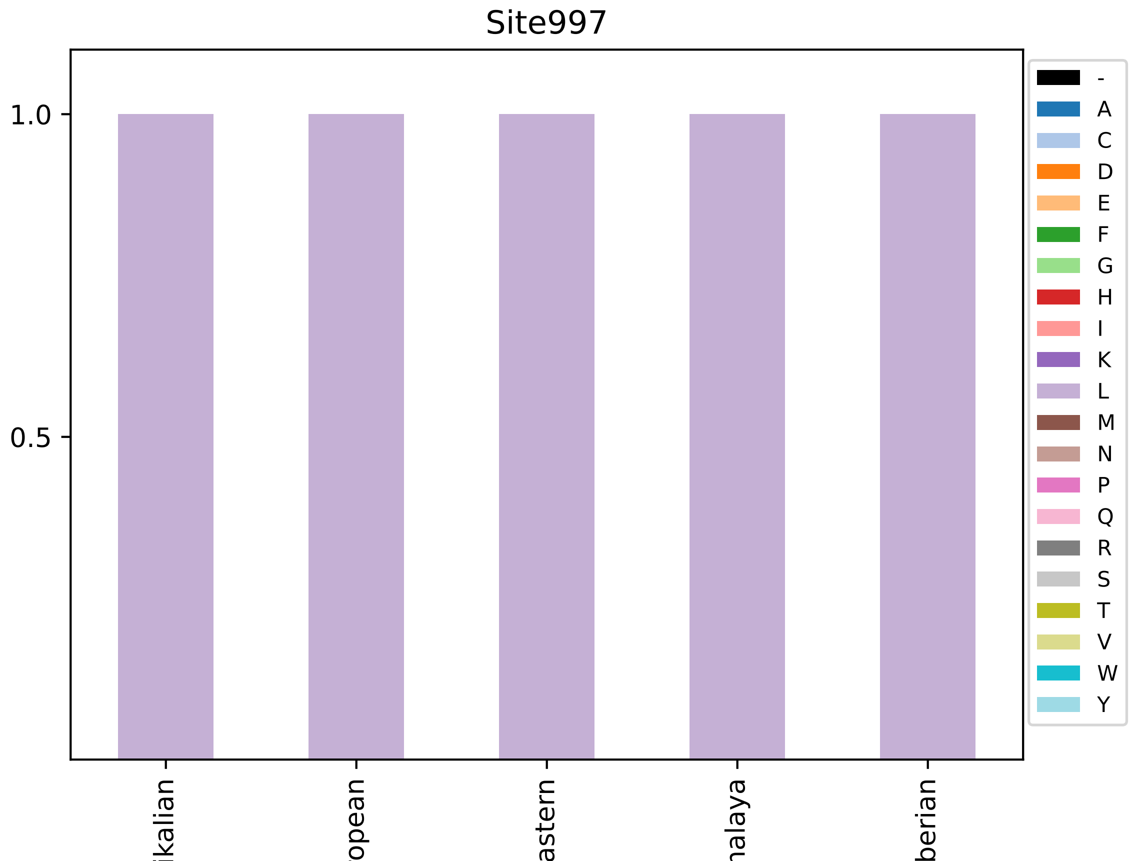

Site997
1.0
0.5
-
A
C
D
E
F
G
H
I
K
L
M
N
P
Q
R
S
T
V
W
Y
ikalian
opean
astern
nalaya
berian

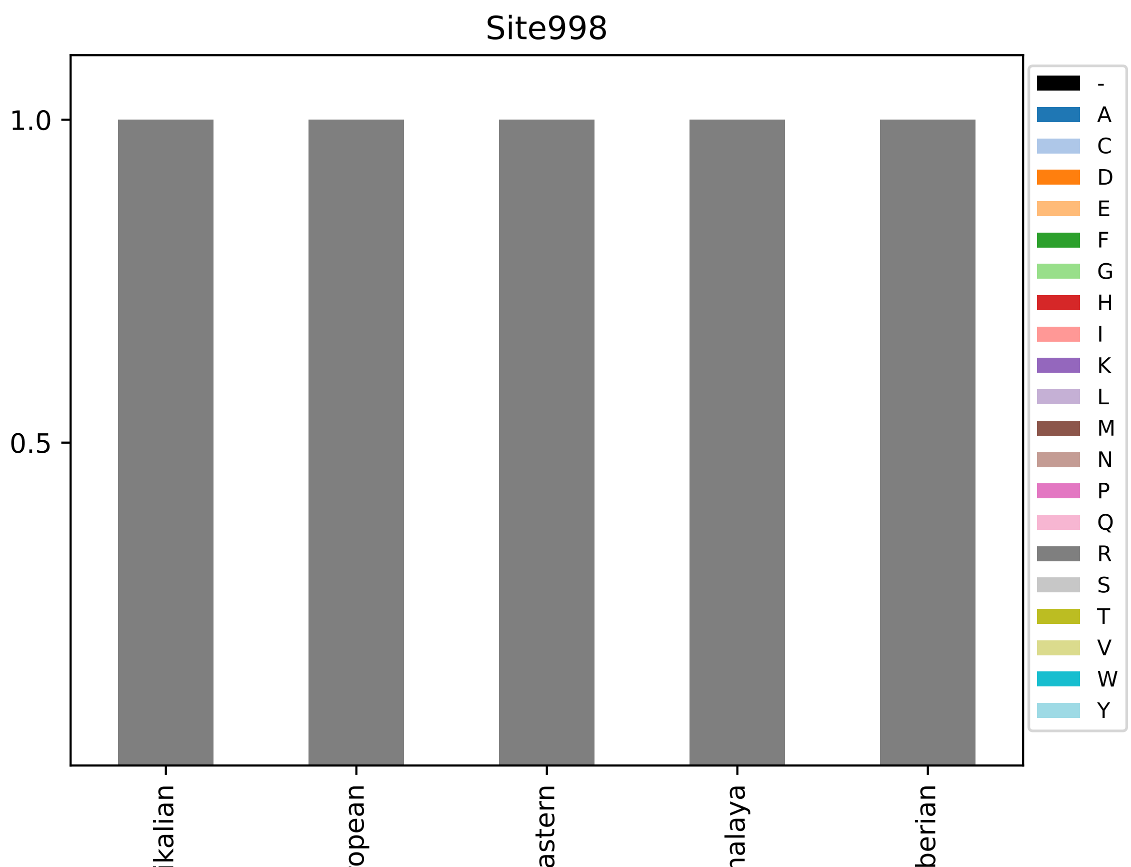
Site998
1.0
0.5
-
A
C
D
E
F
G
H
I
K
L
M
N
P
Q
R
S
T
V
W
Y
ikalian
opean
astern
nalaya
berian

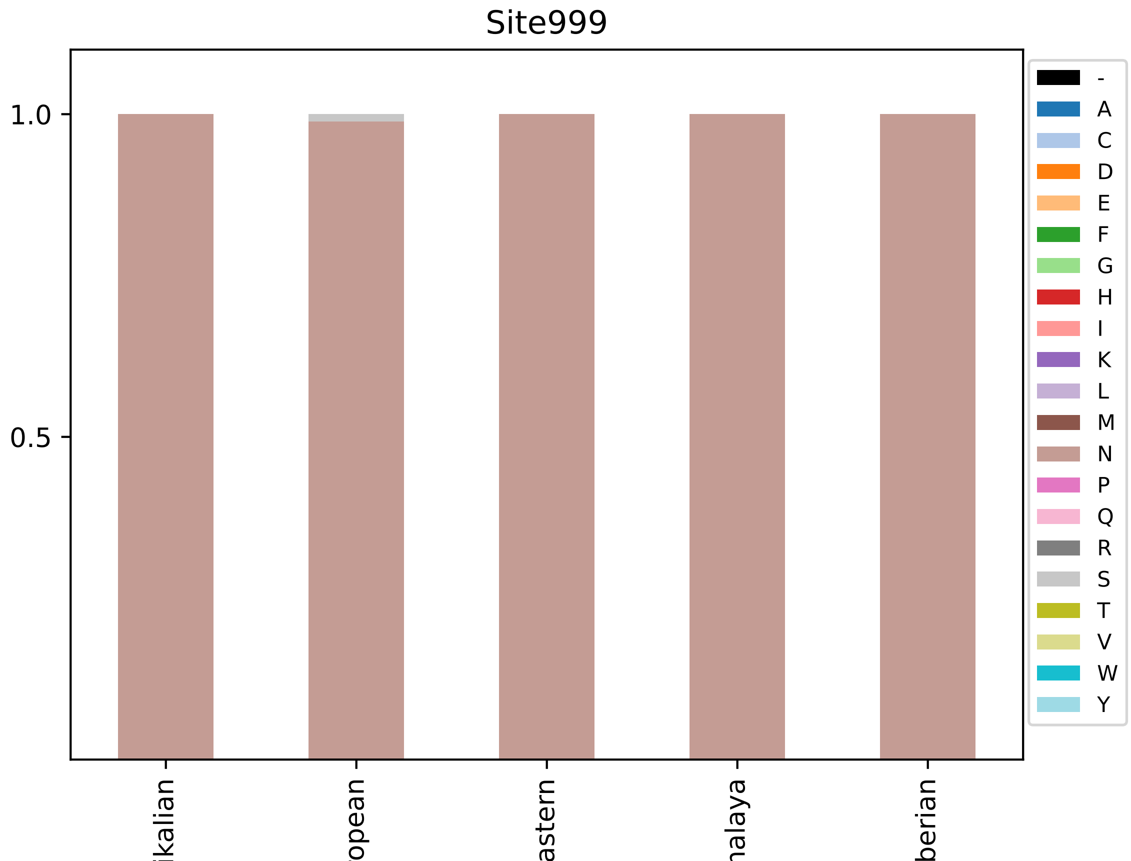
Site999
1.0
0.5
-
A
C
D
E
F
G
H
I
K
L
M
N
P
Q
R
S
T
V
W
Y

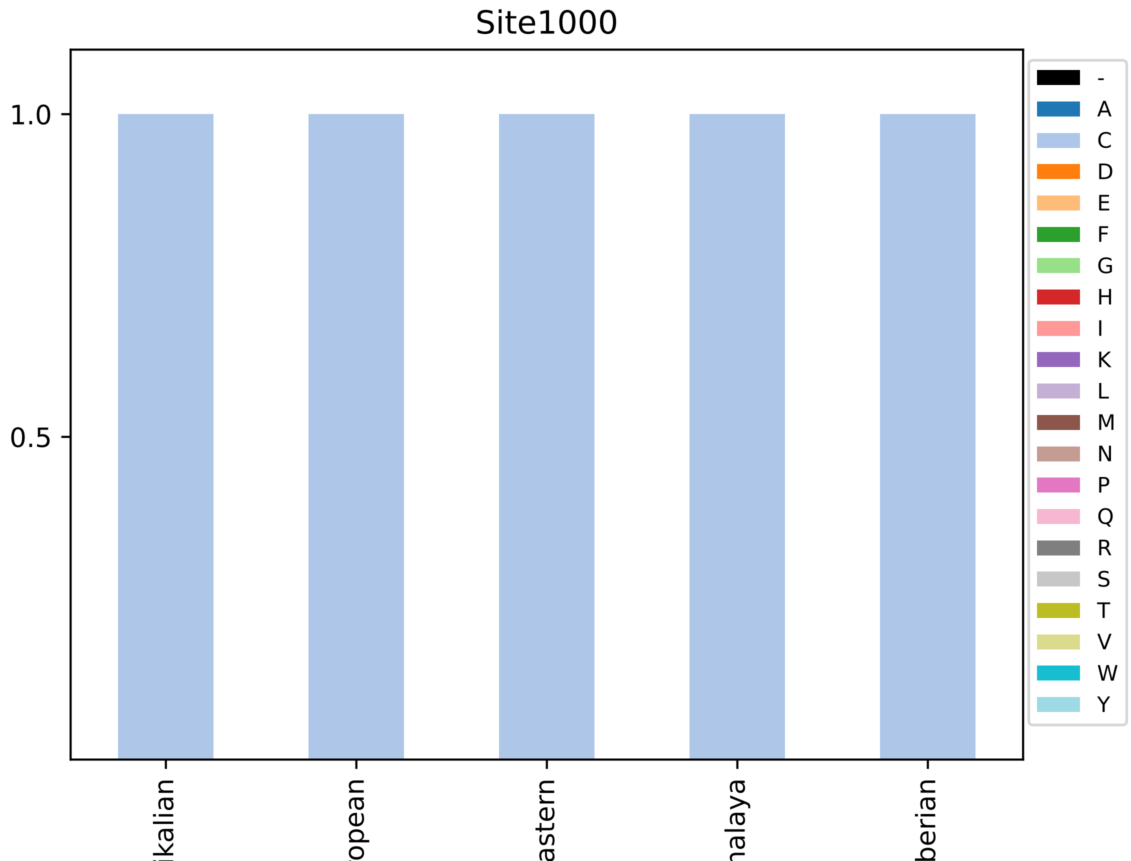
Site1000
1.0
0.5
-
A
C
D
E
F
G
H
I
K
L
M
N
P
Q
R
S
T
V
W
Y
ikalian
opean
astern
nalaya
berian

Site1001

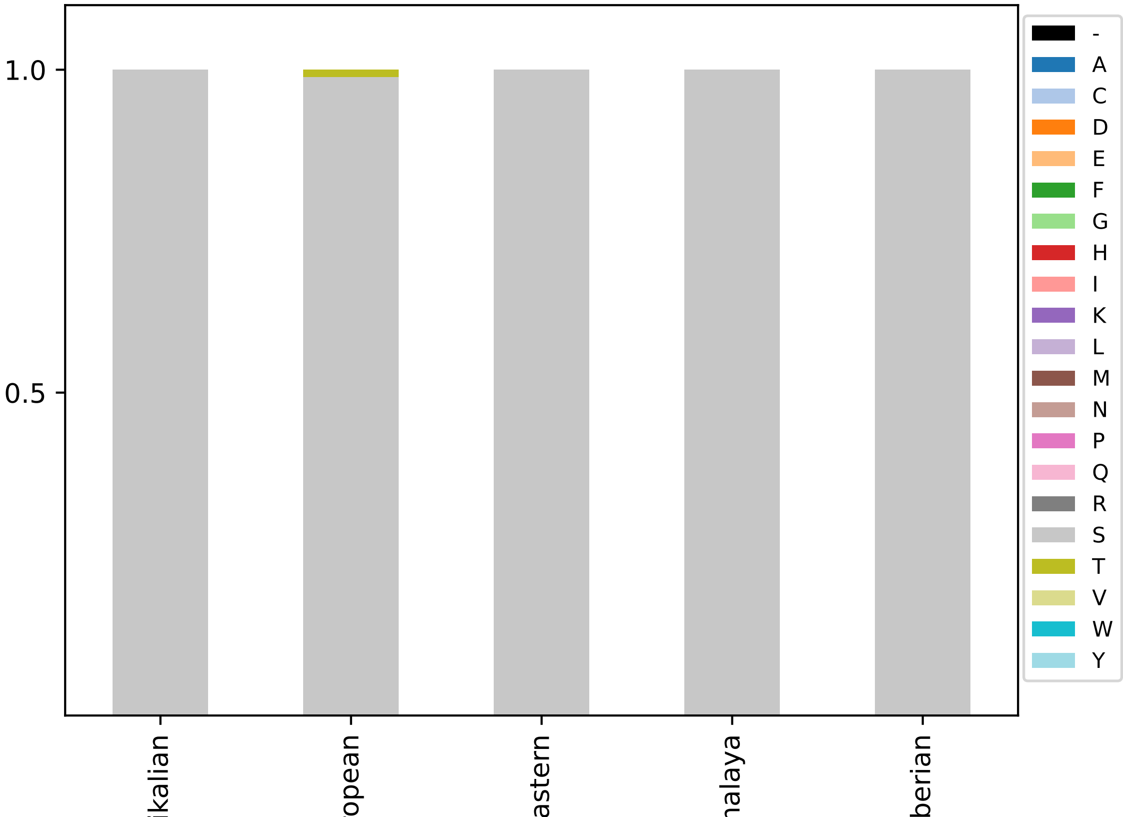

Site1001
1.0
0.5
ikalian
ropean
astern
nalaya
berian
-
A
C
D
E
F
G
H
I
K
L
M
N
P
Q
R
S
T
V
W
Y

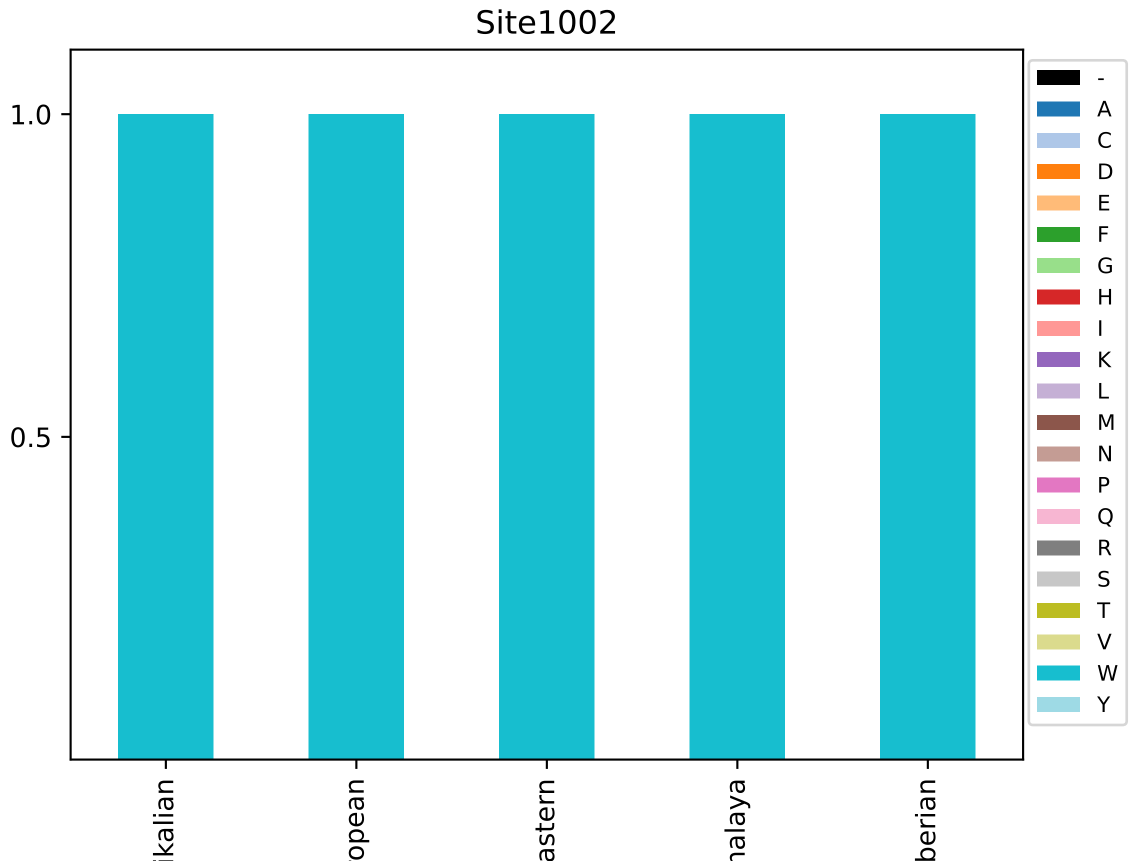

Site1002
1.0
0.5
-
A
C
D
E
F
G
H
I
K
L
M
N
P
Q
R
S
T
V
W
Y
ikalian
opean
astern
nalaya
berian

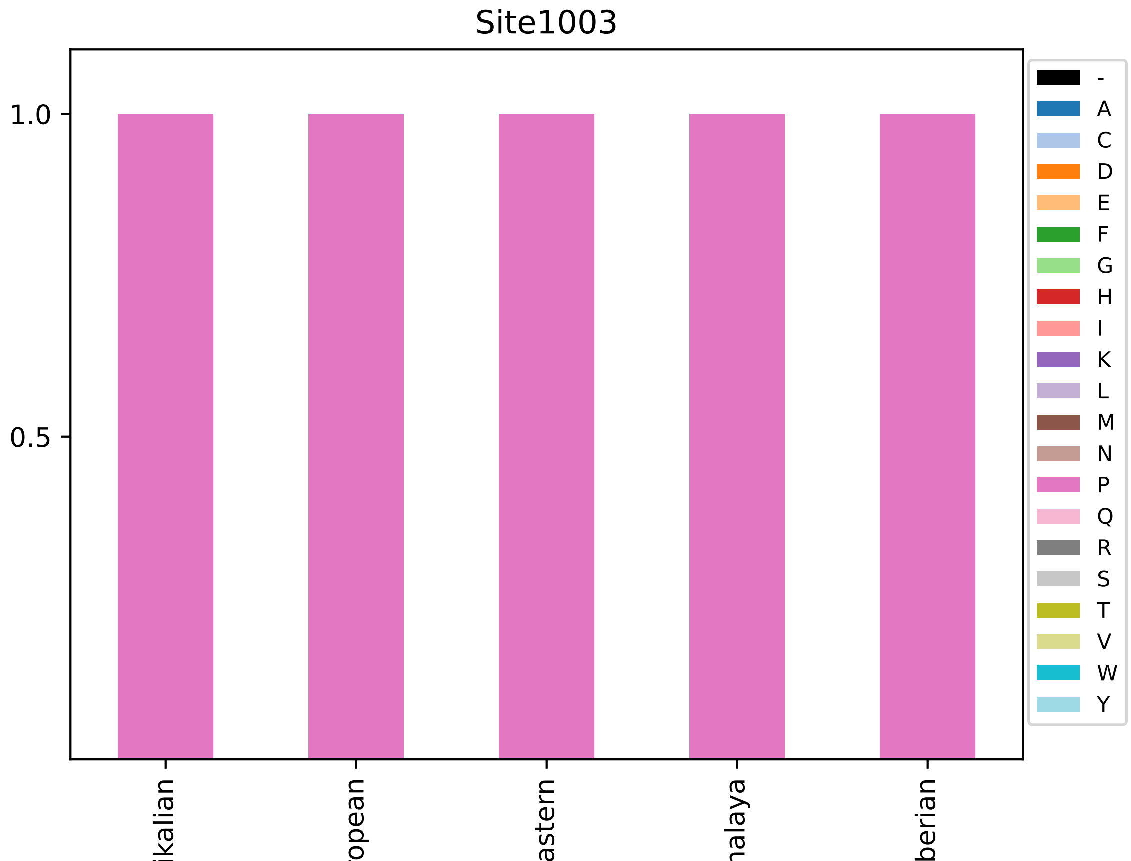
Site1003
1.0
0.5
-
A
C
D
E
F
G
H
I
K
L
M
N
P
Q
R
S
T
V
W
Y
ikalian
opean
astern
nalaya
berian

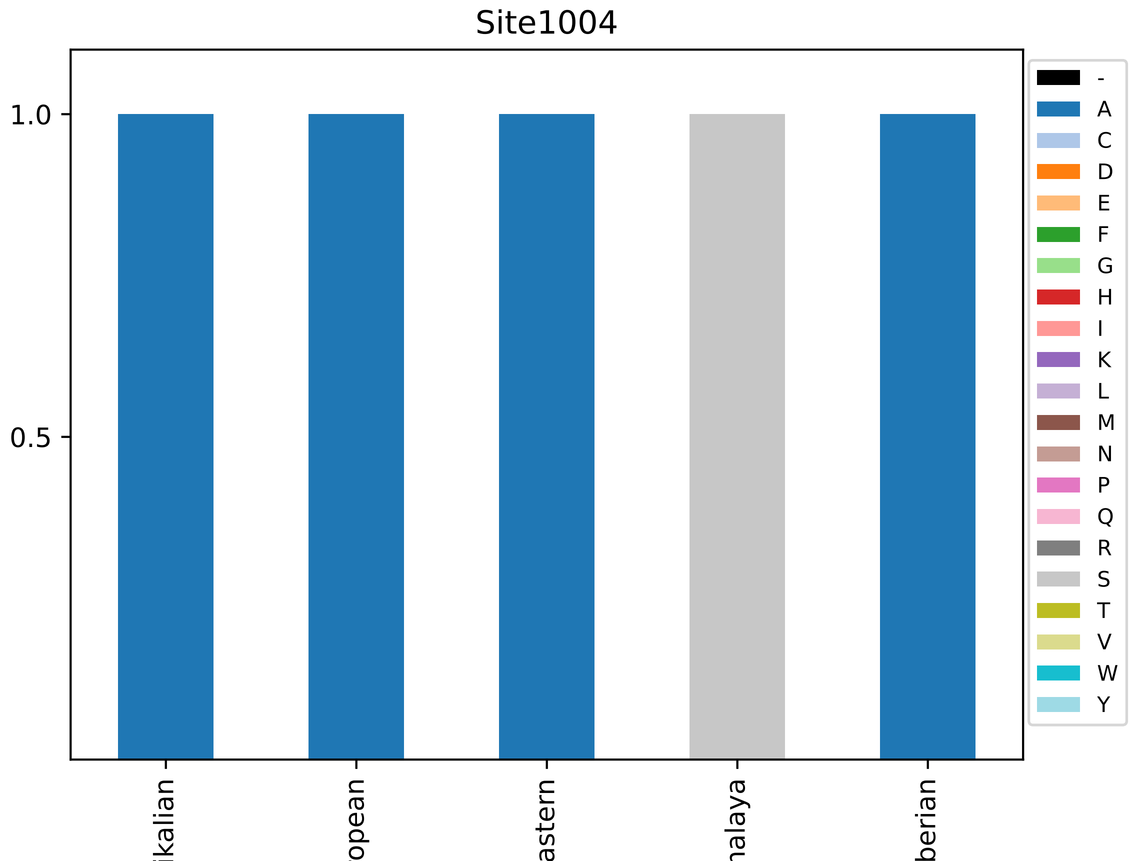
Site1004
1.0
0.5
-
A
C
D
E
F
G
H
I
K
L
M
N
P
Q
R
S
T
V
W
Y
ikalian
opean
astern
nalaya
berian

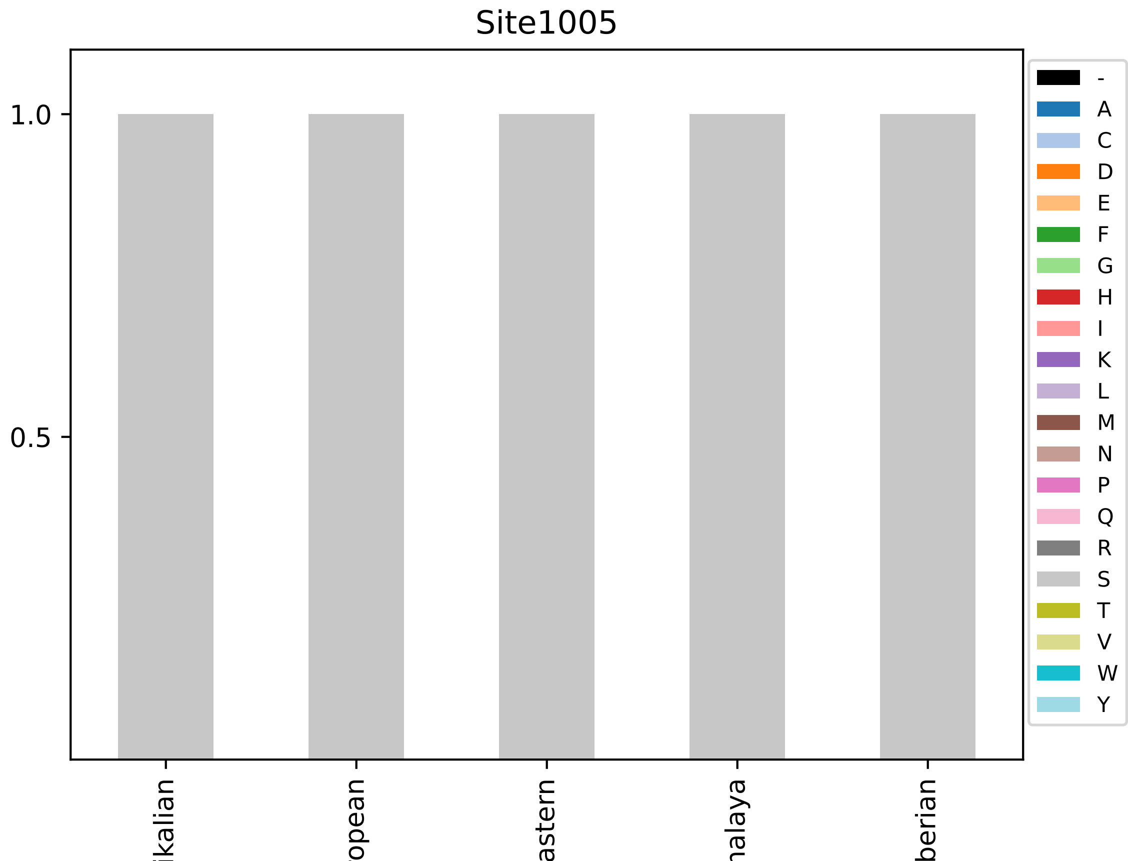

Site1005
1.0
0.5
ikalian
opean
astern
nalaya
berian
-
A
C
D
E
F
G
H
I
K
L
M
N
P
Q
R
S
T
V
W
Y

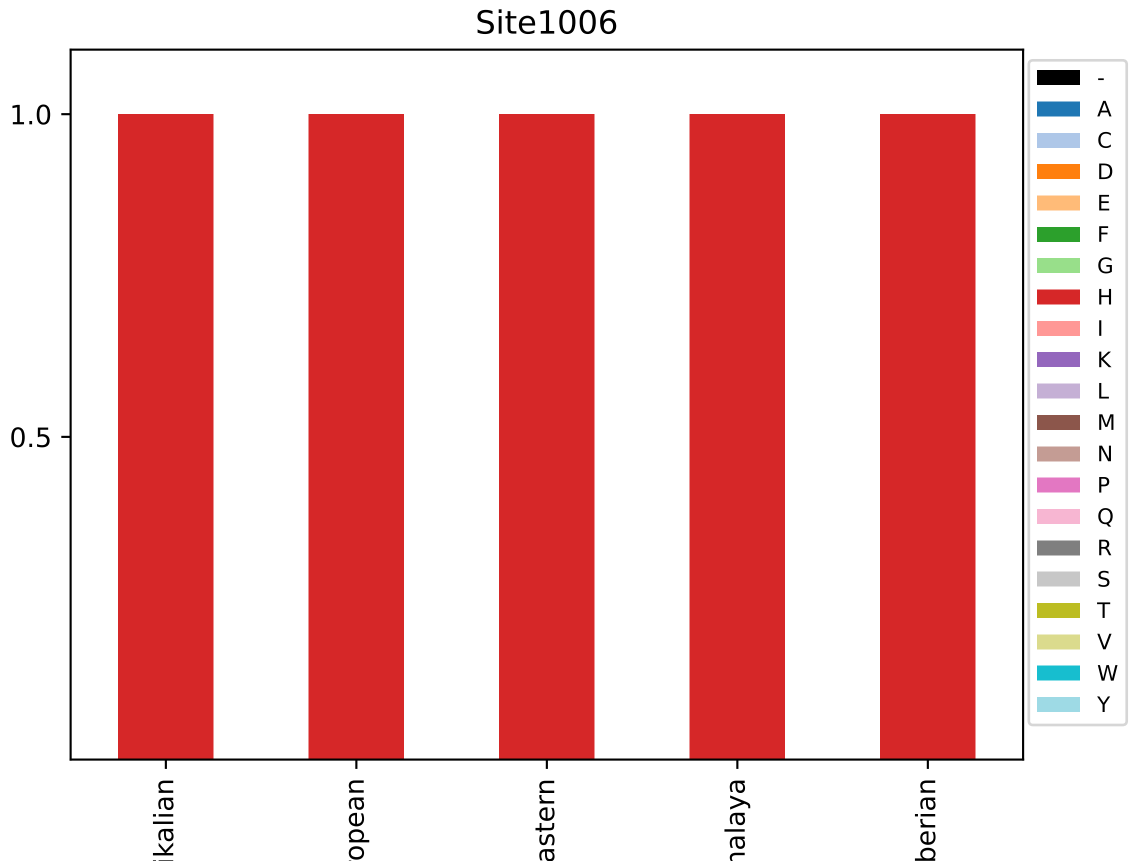

Site1006
1.0
0.5
-
A
C
D
E
F
G
H
I
K
L
M
N
P
Q
R
S
T
V
W
Y
ikalian
opean
astern
nalaya
berian

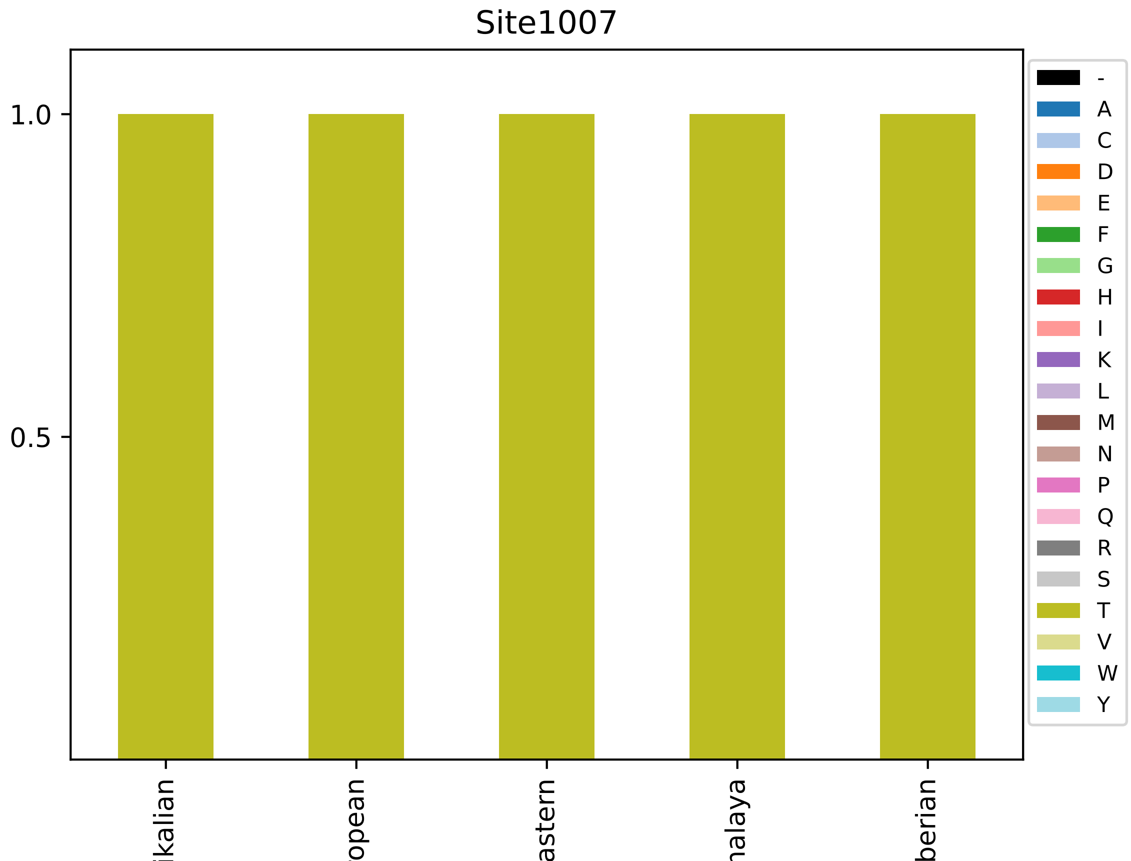
Site1007
1.0
0.5
-
A
C
D
E
F
G
H
I
K
L
M
N
P
Q
R
S
T
V
W
Y
ikalian
opean
astern
nalaya
berian

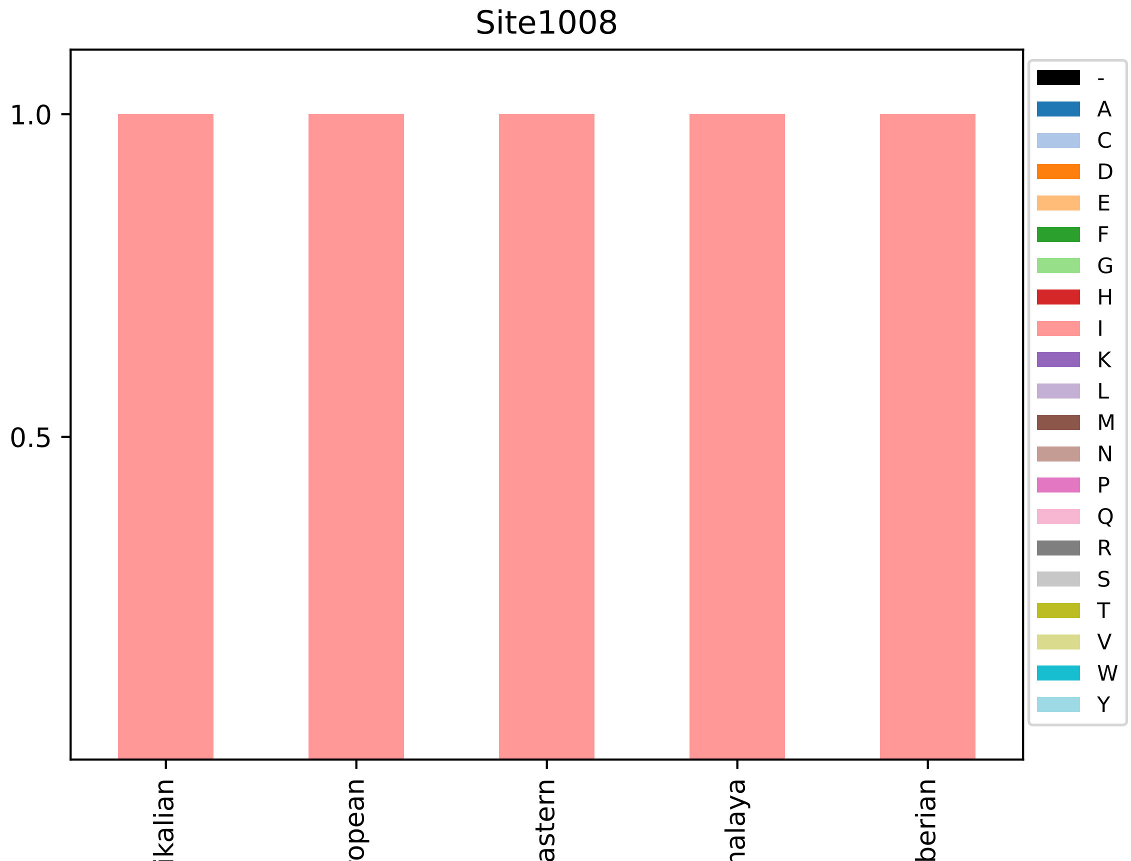

Site1008
1.0
0.5
-
A
C
D
E
F
G
H
I
K
L
M
N
P
Q
R
S
T
V
W
Y
ikalian
opean
astern
nalaya
berian

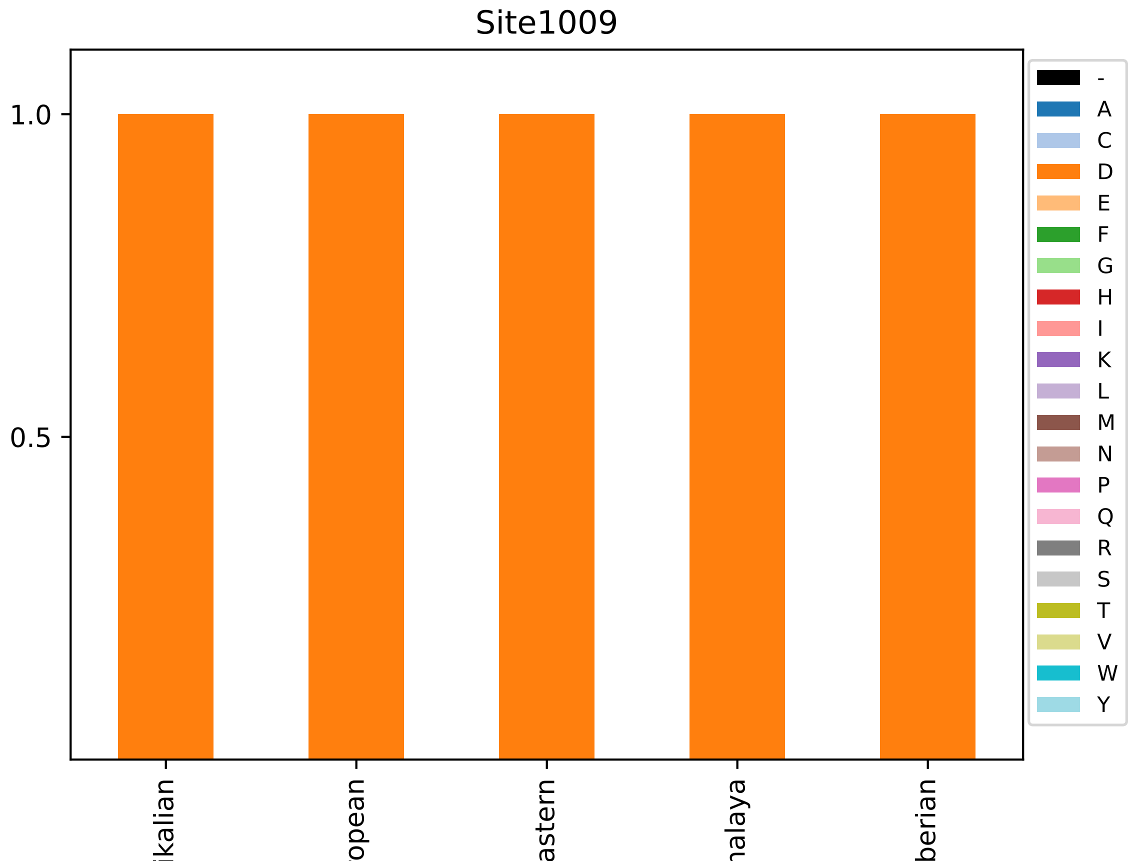

Site1009
1.0
0.5
ikalian
opean
astern
nalaya
berian
-
A
C
D
E
F
G
H
I
K
L
M
N
P
Q
R
S
T
V
W
Y

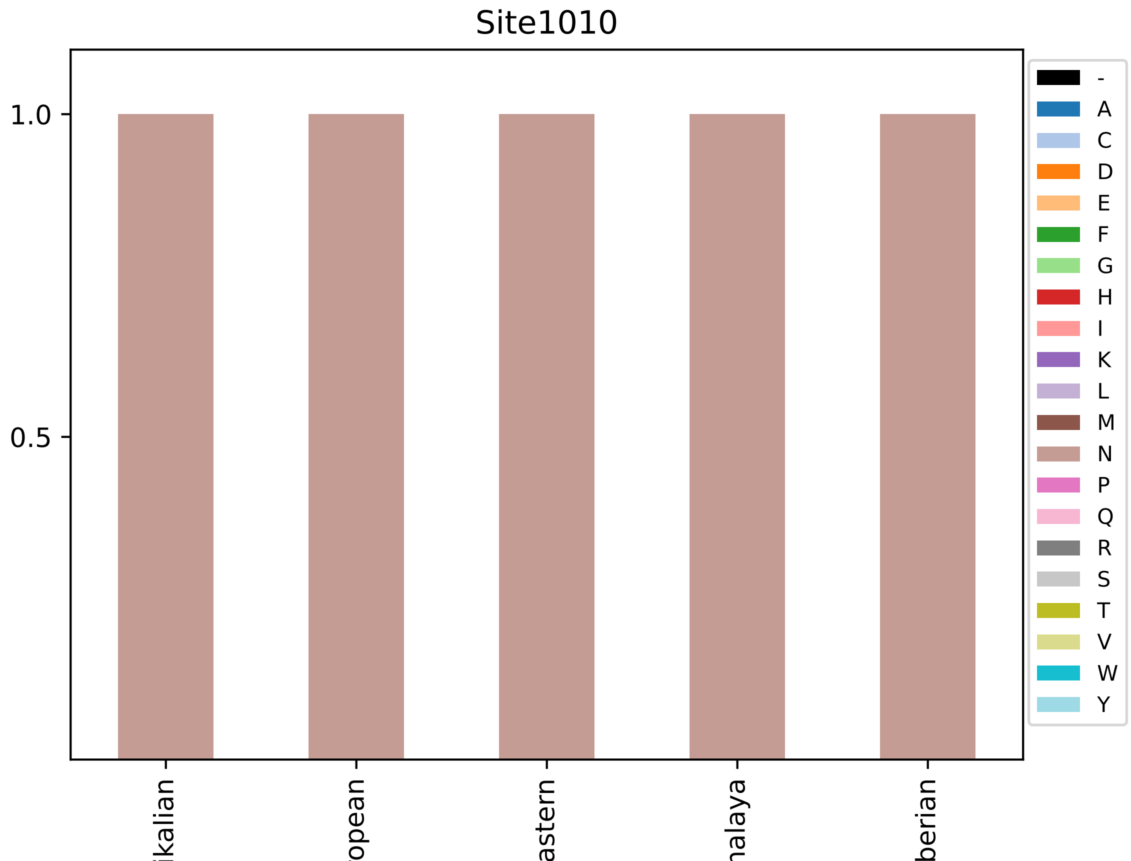
Site1010
1.0
0.5
-
A
C
D
E
F
G
H
I
K
L
M
N
P
Q
R
S
T
V
W
Y
ikalian
opean
astern
nalaya
berian

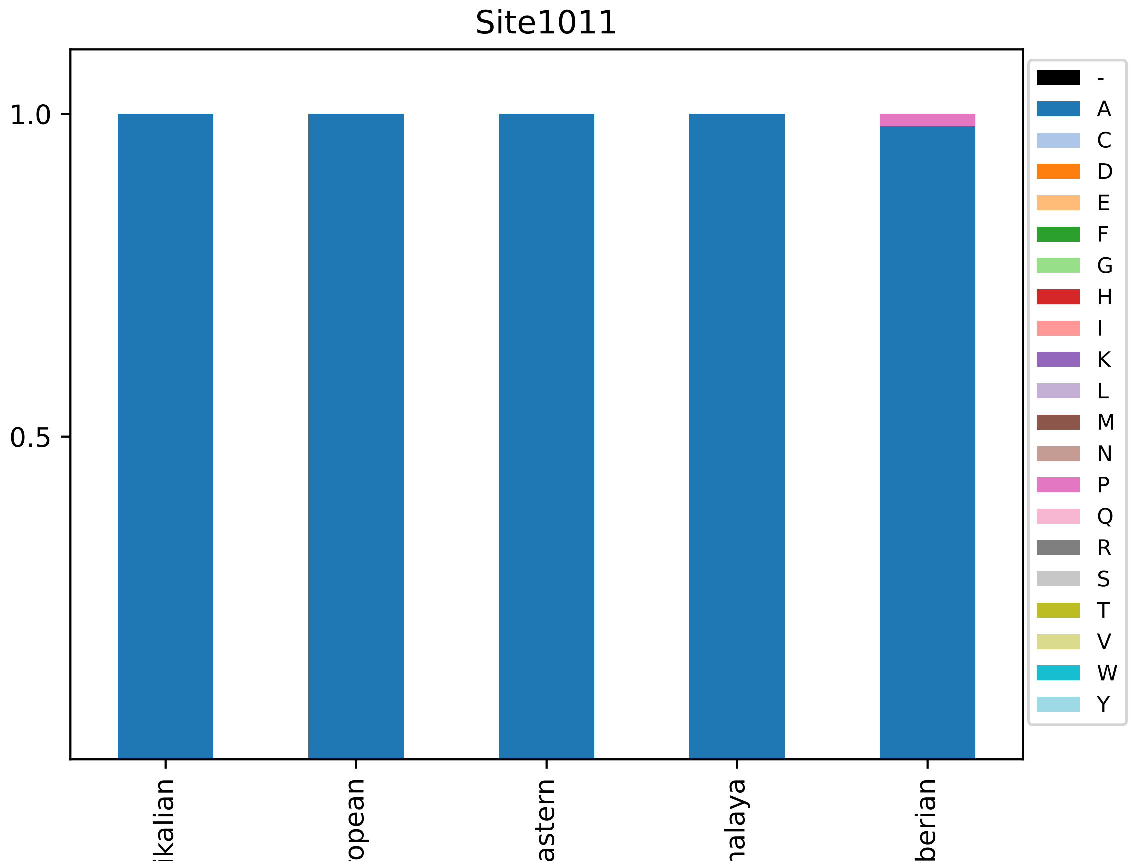
Site1011
1.0
0.5
-
A
C
D
E
F
G
H
I
K
L
M
N
P
Q
R
S
T
V
W
Y
ikalian
opean
astern
nalaya
berian

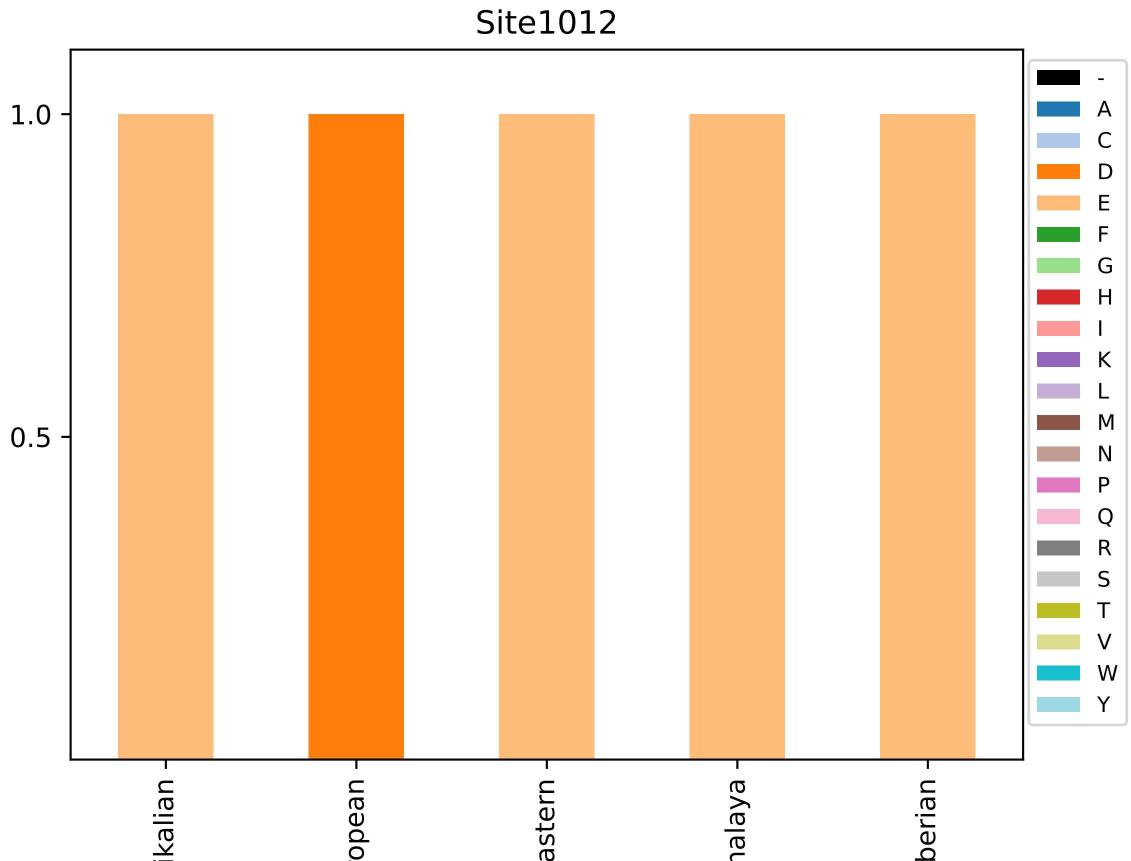

Site1012
1.0
0.5
-
A
C
D
E
F
G
H
I
K
L
M
N
P
Q
R
S
T
V
W
Y
ikalian
opean
astern
nalaya
berian

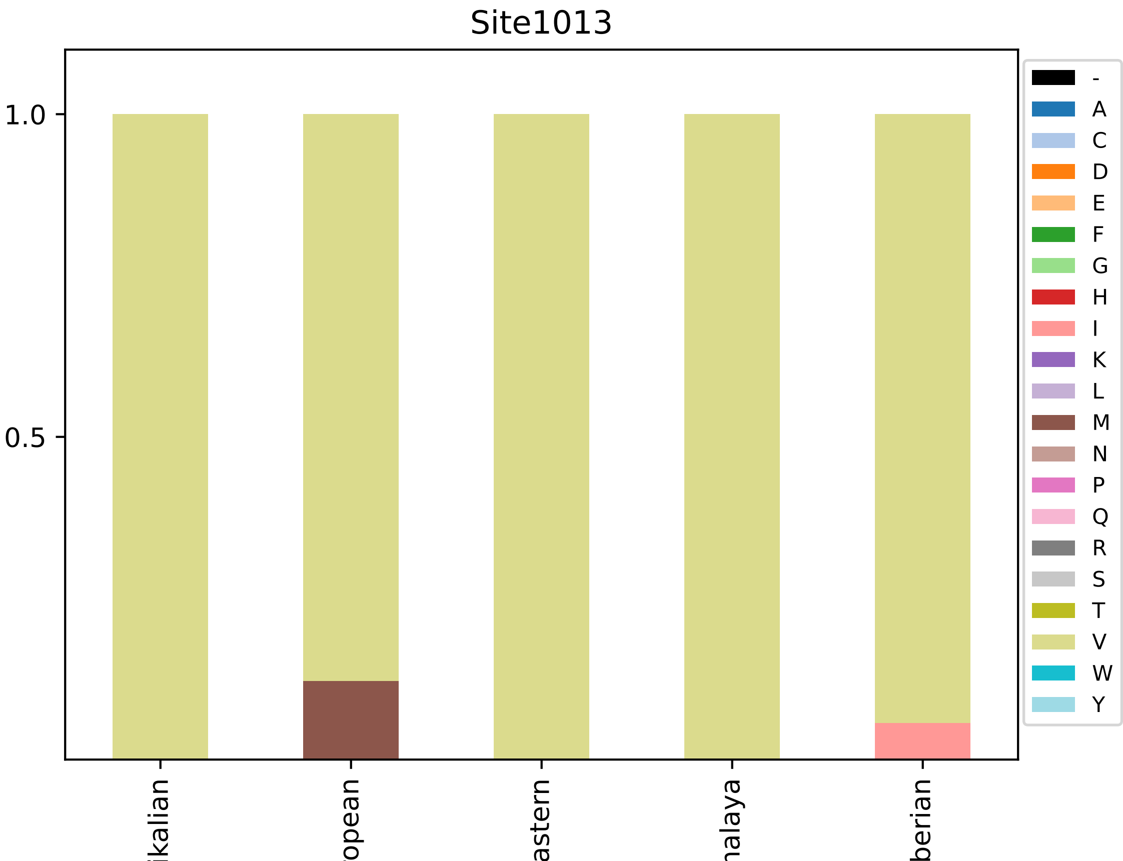

Site1013
1.0
0.5
-
A
C
D
E
F
G
H
I
K
L
M
N
P
Q
R
S
T
V
W
Y
ikalian
opean
astern
nalaya
berian

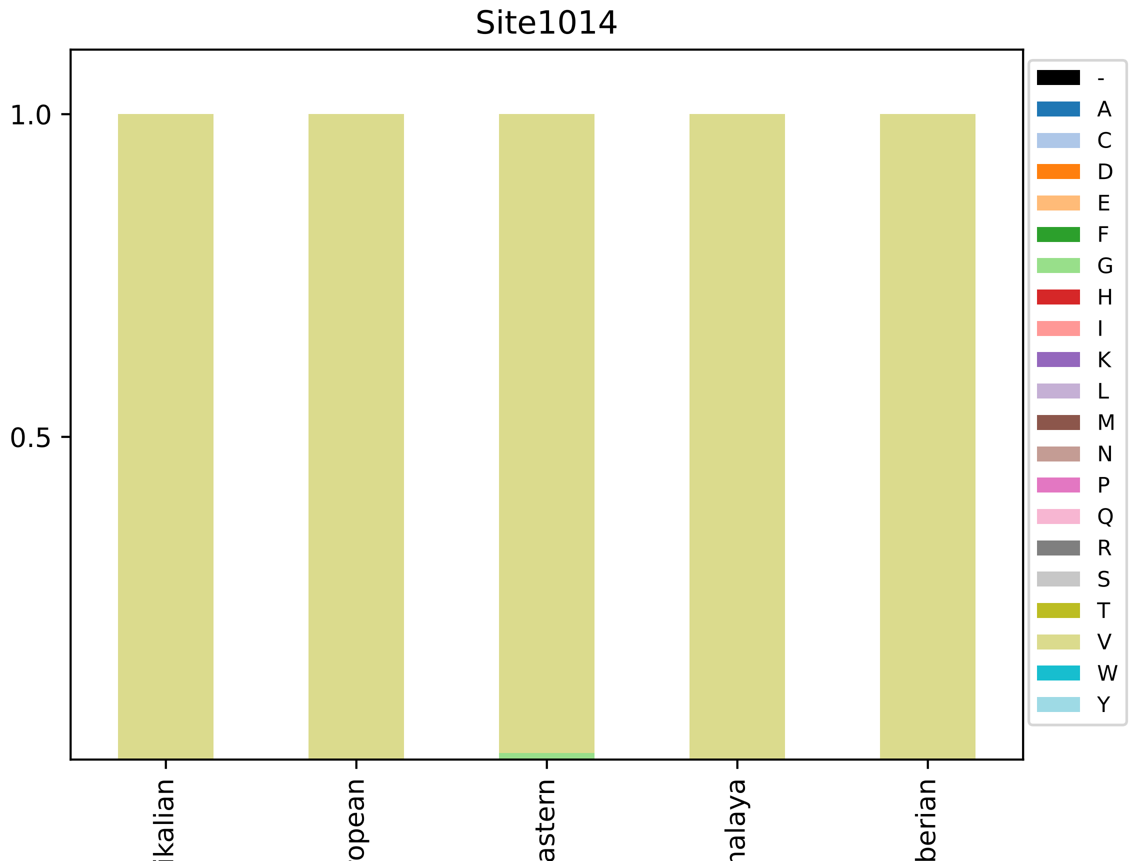

Site1014
1.0
0.5
-
A
C
D
E
F
G
H
I
K
L
M
N
P
Q
R
S
T
V
W
Y
ikalian
opean
astern
nalaya
berian

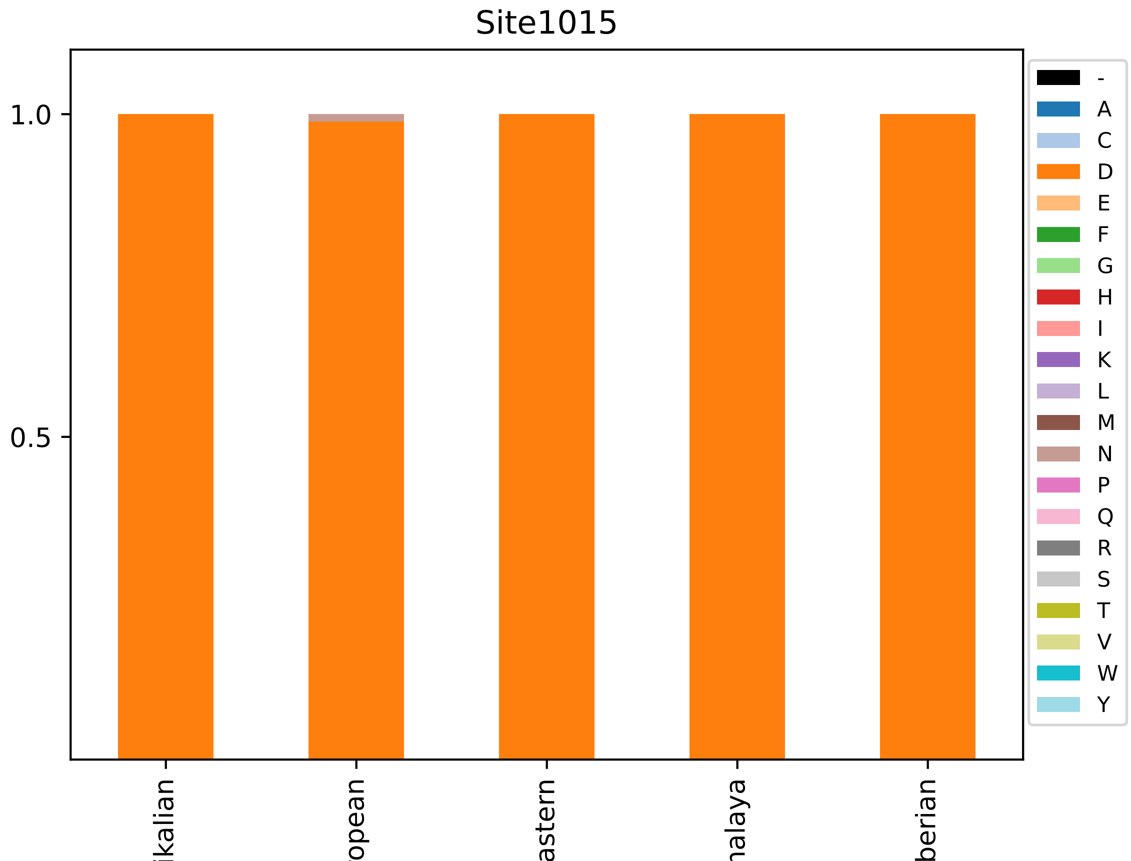
Site1015
1.0
0.5
-
A
C
D
E
F
G
H
I
K
L
M
N
P
Q
R
S
T
V
W
Y
ikalian
opean
astern
nalaya
berian

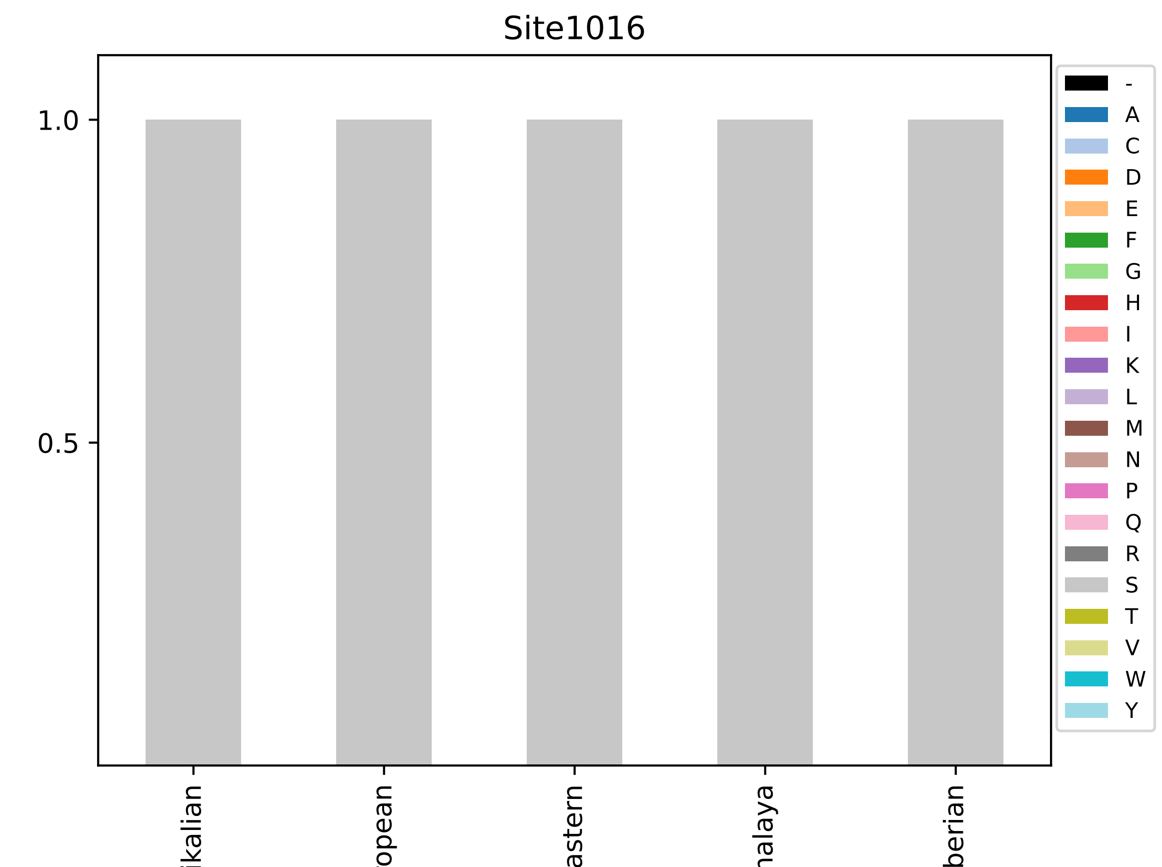
Site1016
1.0
0.5
-
A
C
D
E
F
G
H
I
K
L
M
N
P
Q
R
S
T
V
W
Y
ikalian
opean
astern
nalaya
berian

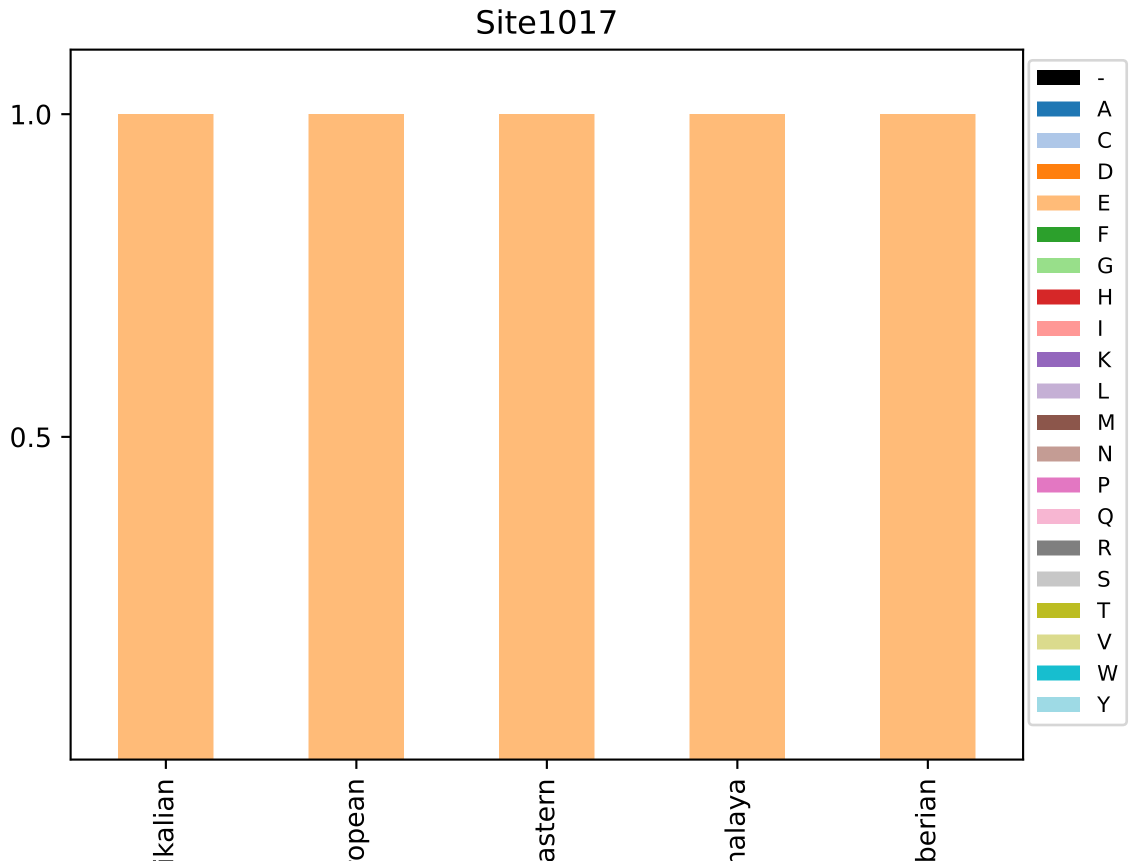
Site1017
1.0
0.5
-
A
C
D
E
F
G
H
I
K
L
M
N
P
Q
R
S
T
V
W
Y

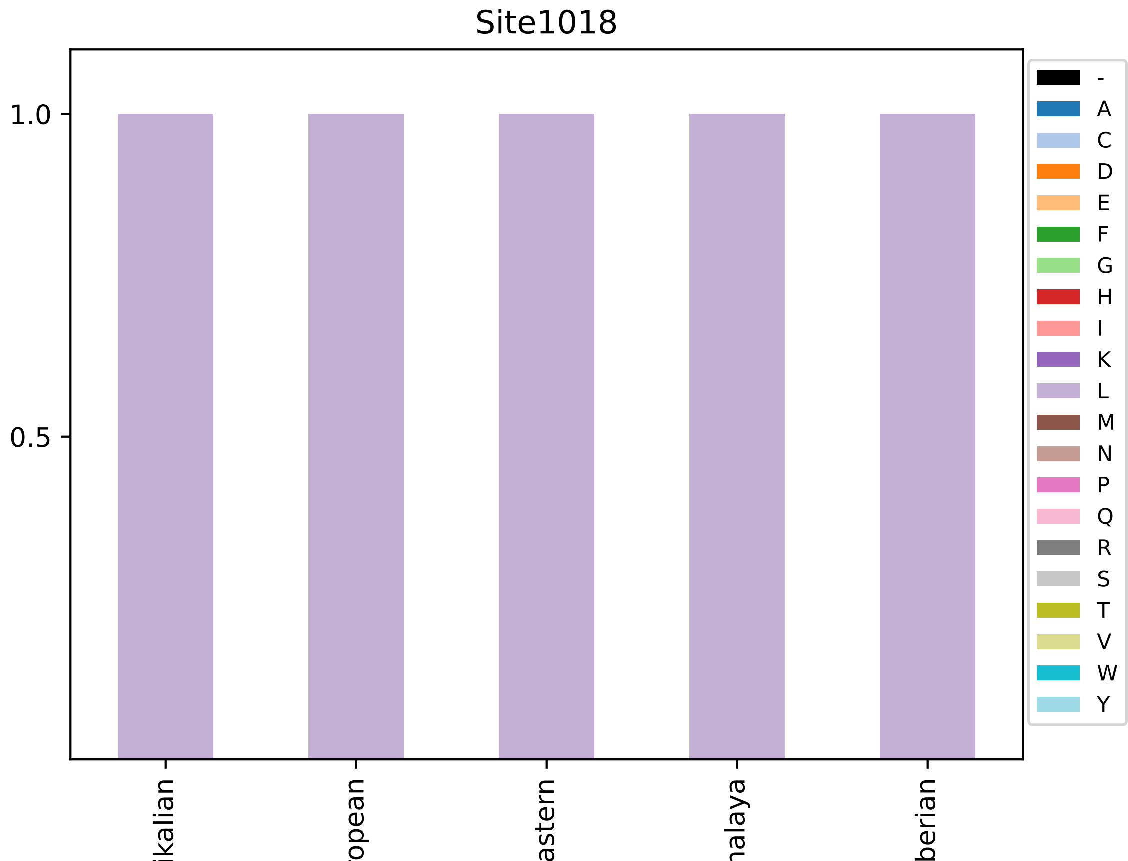
Site1018
1.0
0.5
-
A
C
D
E
F
G
H
I
K
L
M
N
P
Q
R
S
T
V
W
Y
ikalian
opean
astern
nalaya
berian

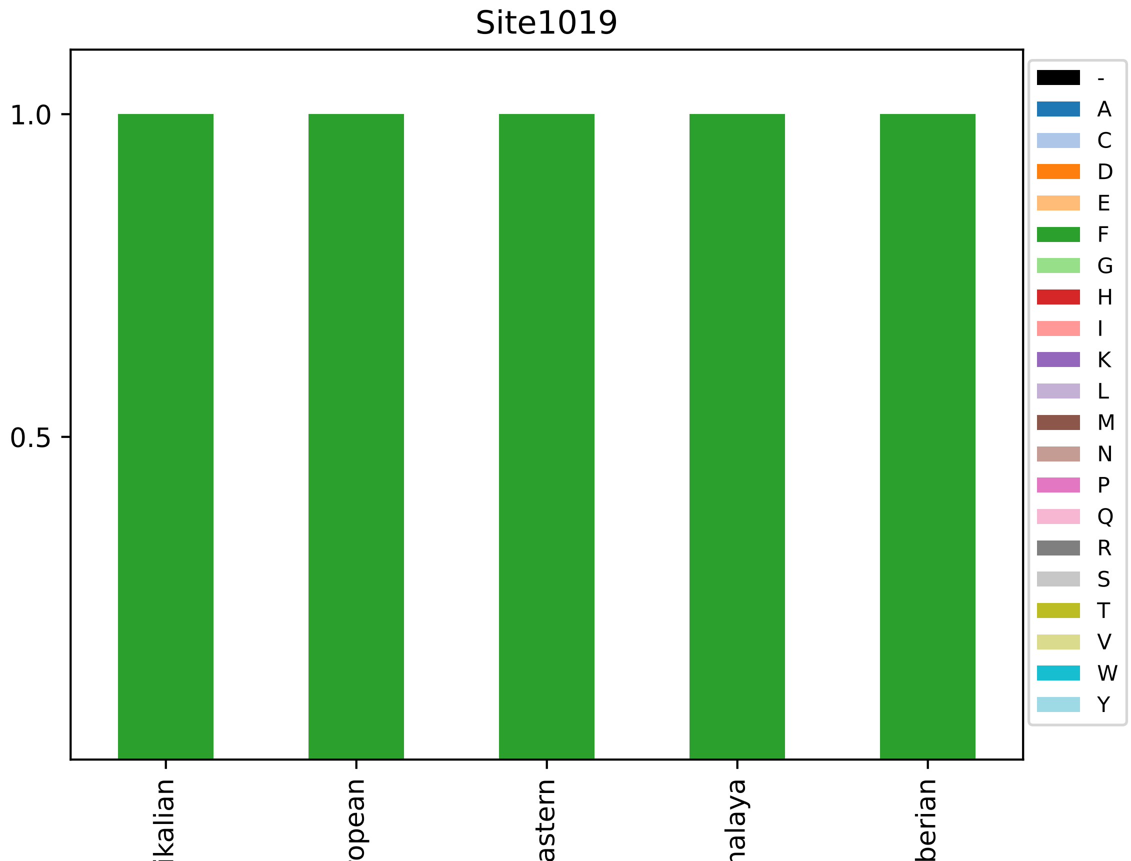

Site1019
1.0
0.5
-
A
C
D
E
F
G
H
I
K
L
M
N
P
Q
R
S
T
V
W
Y

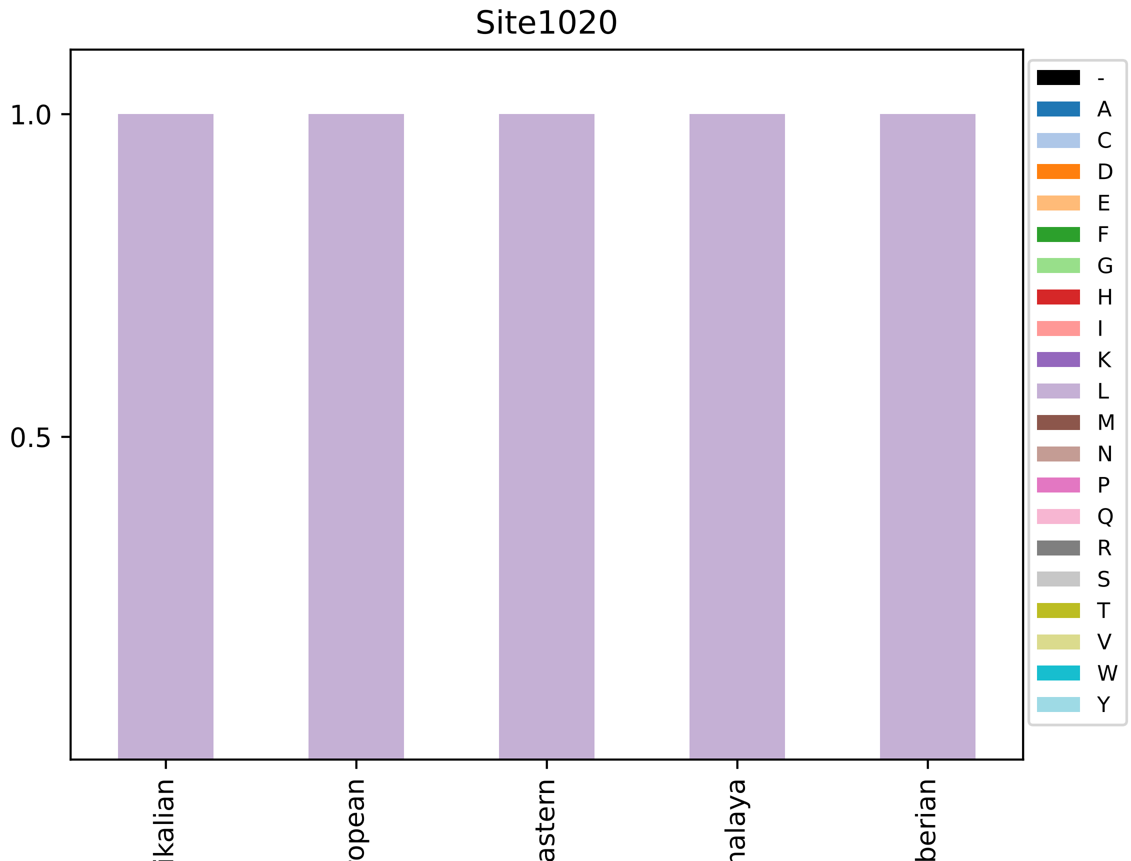

Site1020
1.0
0.5
-
A
C
D
E
F
G
H
I
K
L
M
N
P
Q
R
S
T
V
W
Y
ikalian
opean
astern
nalaya
berian

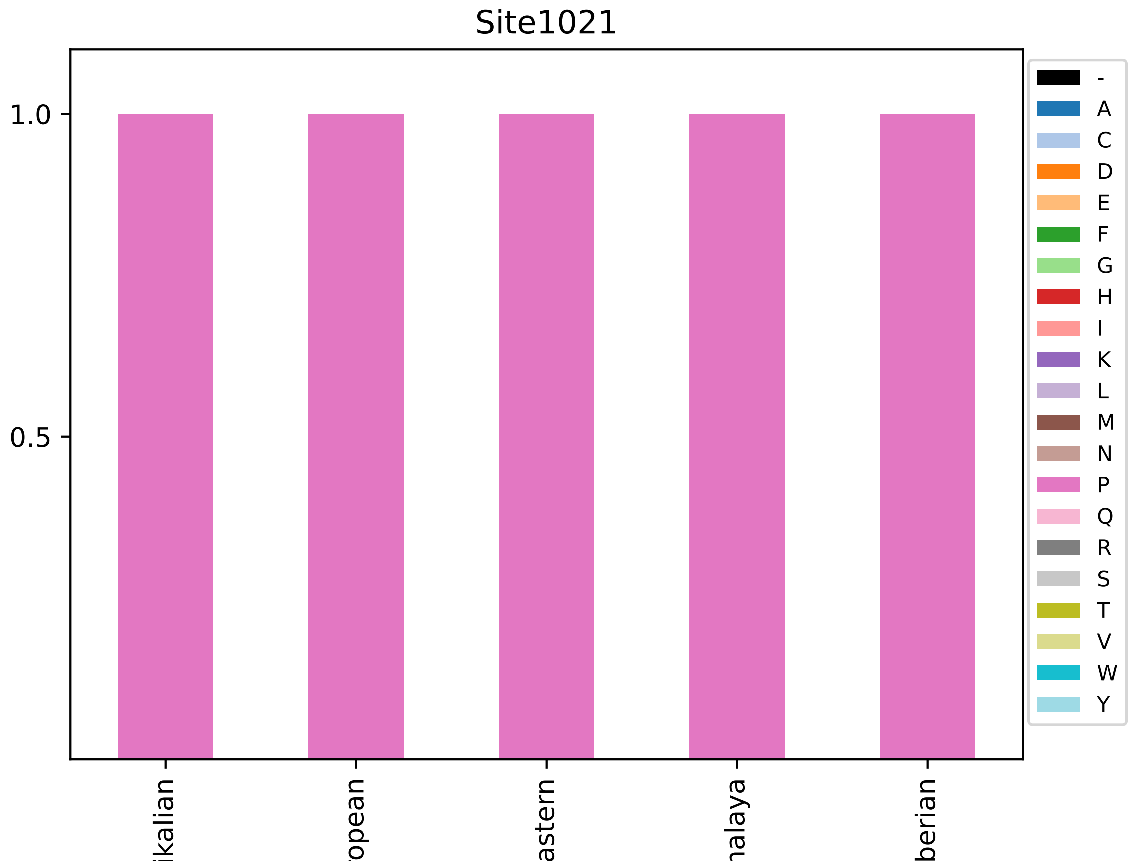
Site1021
1.0
0.5
ikalian
opean
astern
nalaya
berian
-
A
C
D
E
F
G
H
I
K
L
M
N
P
Q
R
S
T
V
W
Y

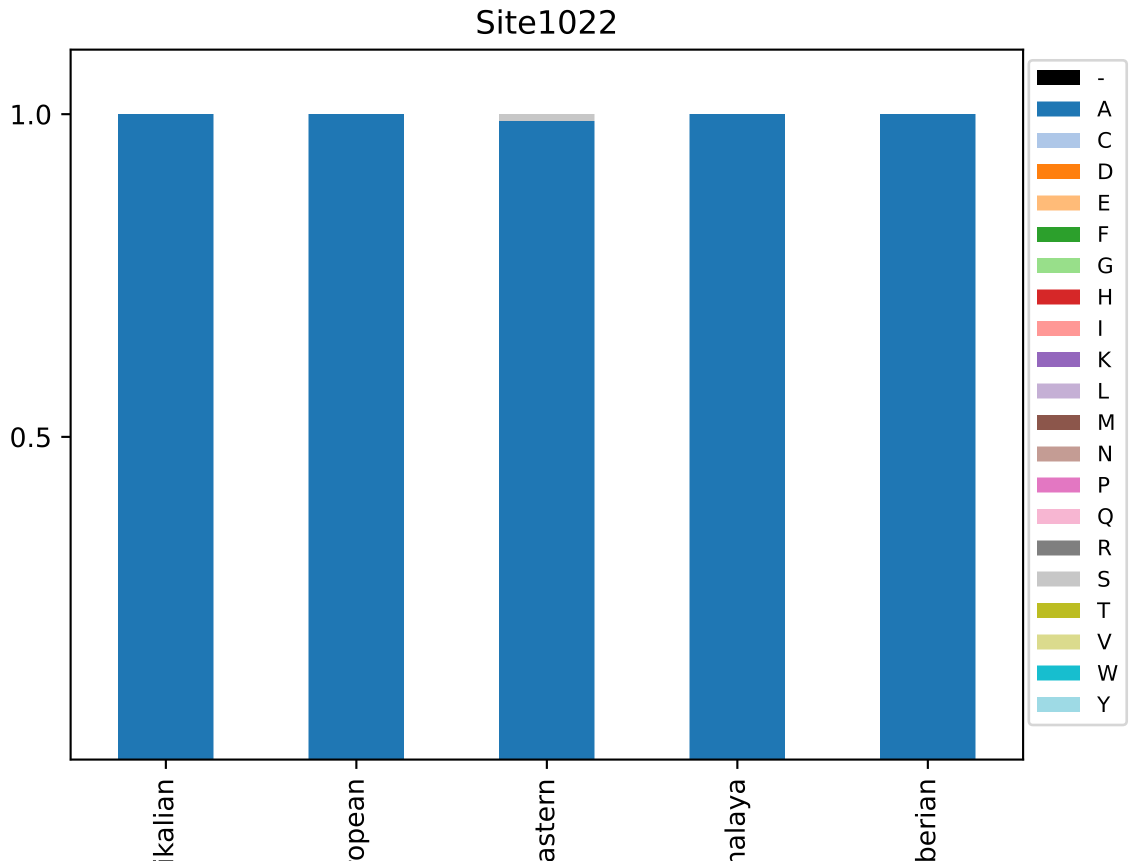
Site1022
1.0
0.5
ikalian
opean
astern
nalaya
berian
-
A
C
D
E
F
G
H
I
K
L
M
N
P
Q
R
S
T
V
W
Y

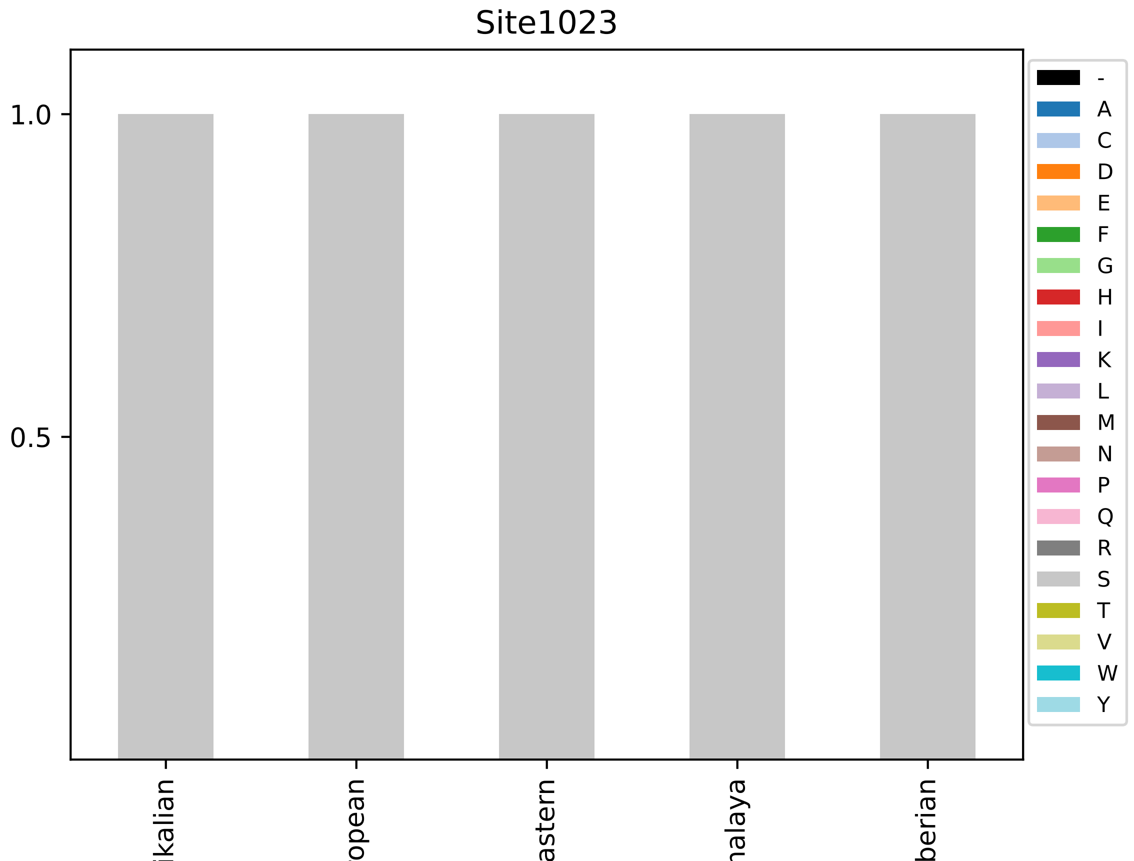

Site1023
1.0
0.5
-
A
C
D
E
F
G
H
I
K
L
M
N
P
Q
R
S
T
V
W
Y
ikalian
opean
astern
nalaya
berian

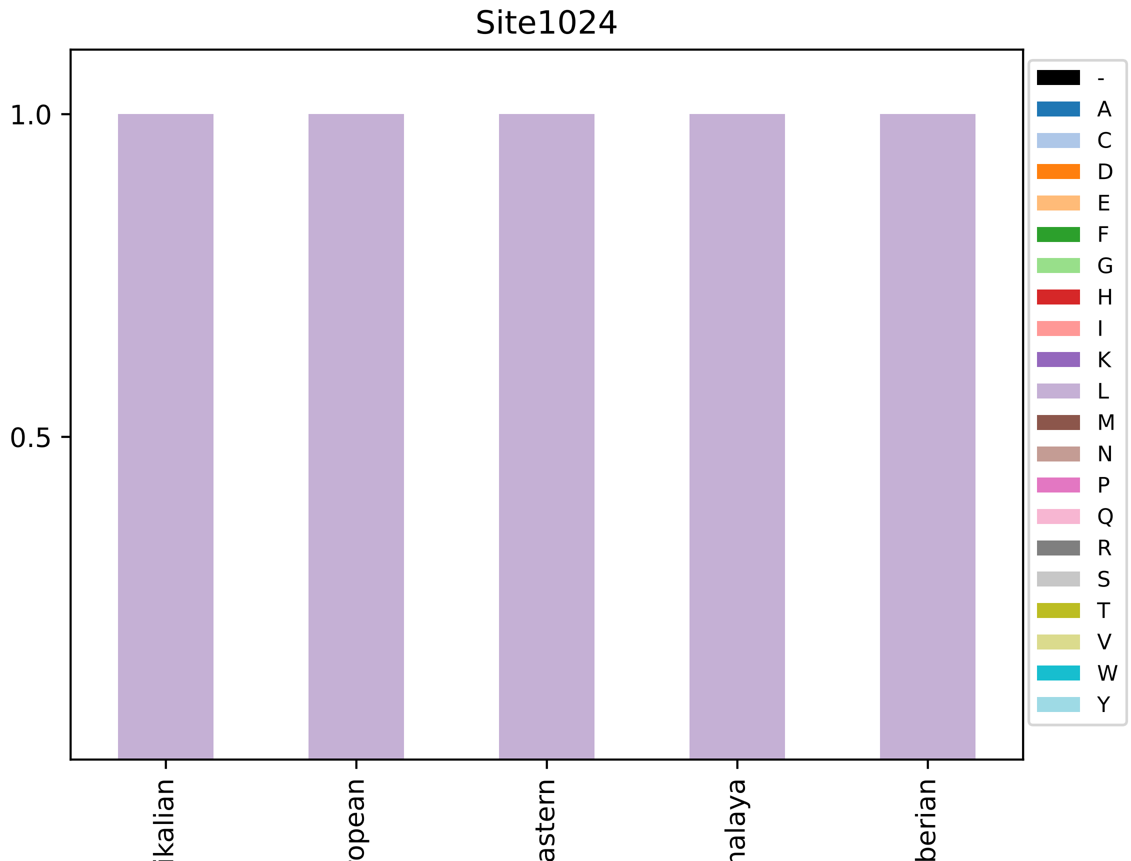
Site1024
1.0
0.5
-
A
C
D
E
F
G
H
I
K
L
M
N
P
Q
R
S
T
V
W
Y
ikalian
opean
astern
nalaya
berian

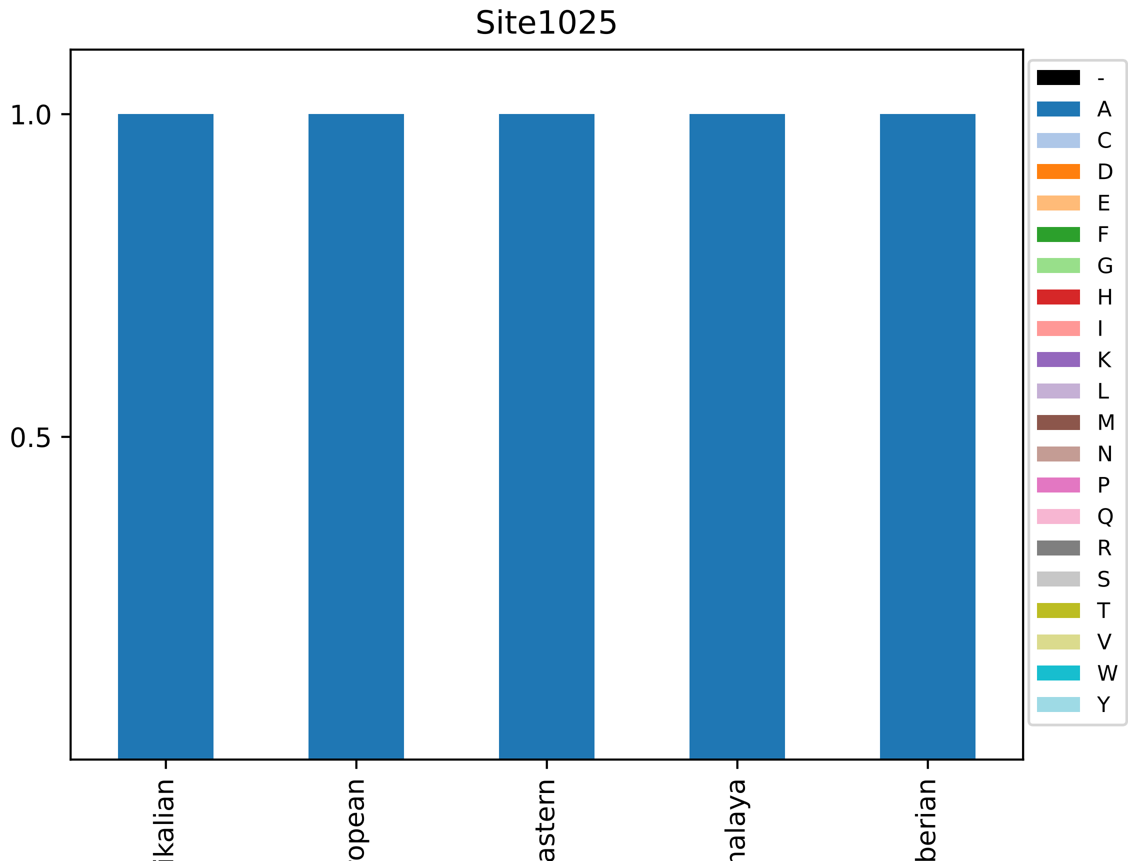

Site1025
1.0
0.5
ikalian
opean
astern
nalaya
berian
-
A
C
D
E
F
G
H
I
K
L
M
N
P
Q
R
S
T
V
W
Y

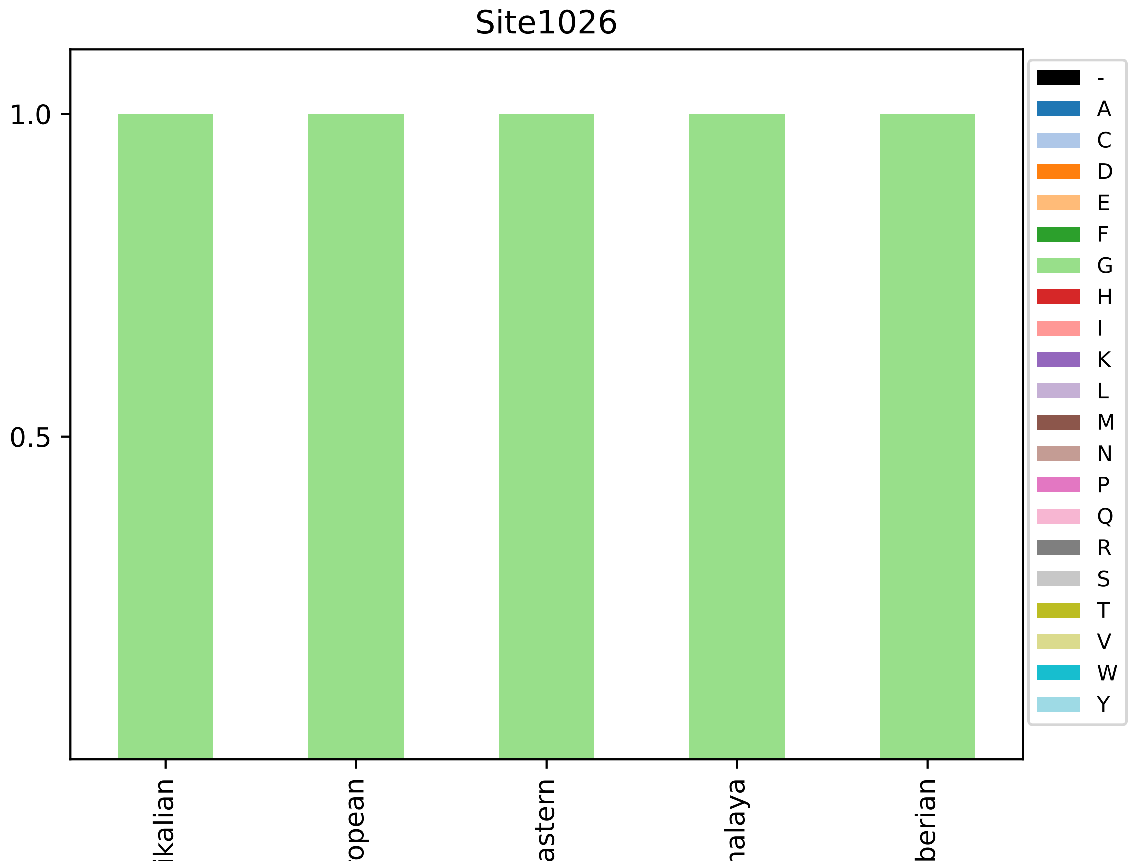

Site1026
1.0
0.5
-
A
C
D
E
F
G
H
I
K
L
M
N
P
Q
R
S
T
V
W
Y
ikalian
opean
astern
nalaya
berian

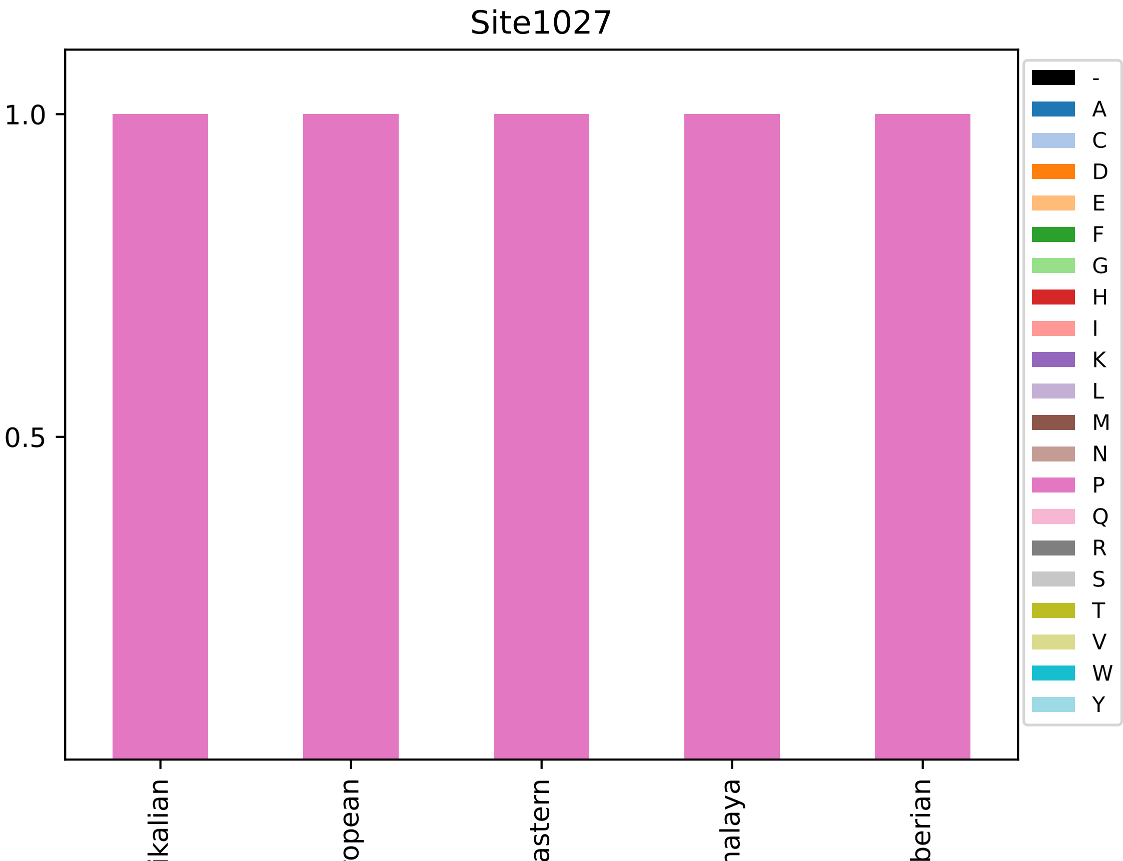

Site1027
1.0
0.5
-
A
C
D
E
F
G
H
I
K
L
M
N
P
Q
R
S
T
V
W
Y
ikalian
opean
astern
nalaya
berian

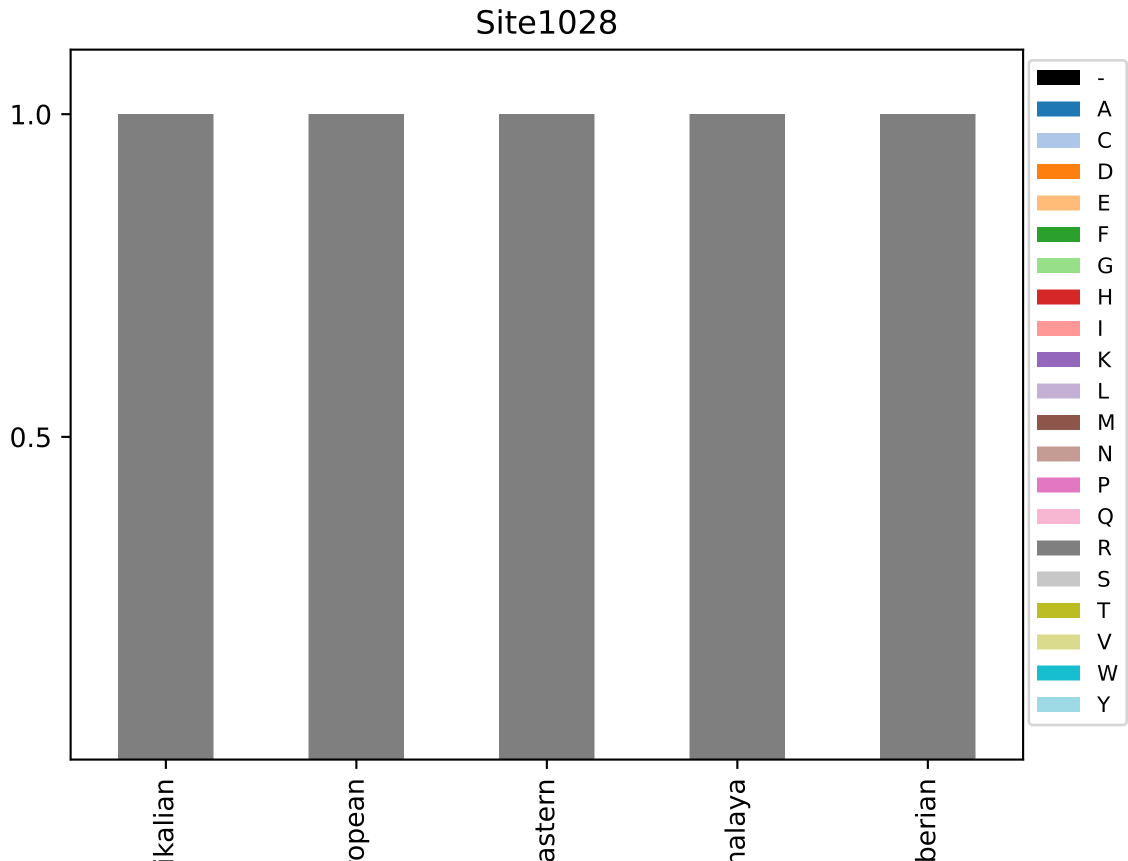
Site1028
1.0
0.5
-
A
C
D
E
F
G
H
I
K
L
M
N
P
Q
R
S
T
V
W
Y
ikalian
opean
astern
nalaya
berian

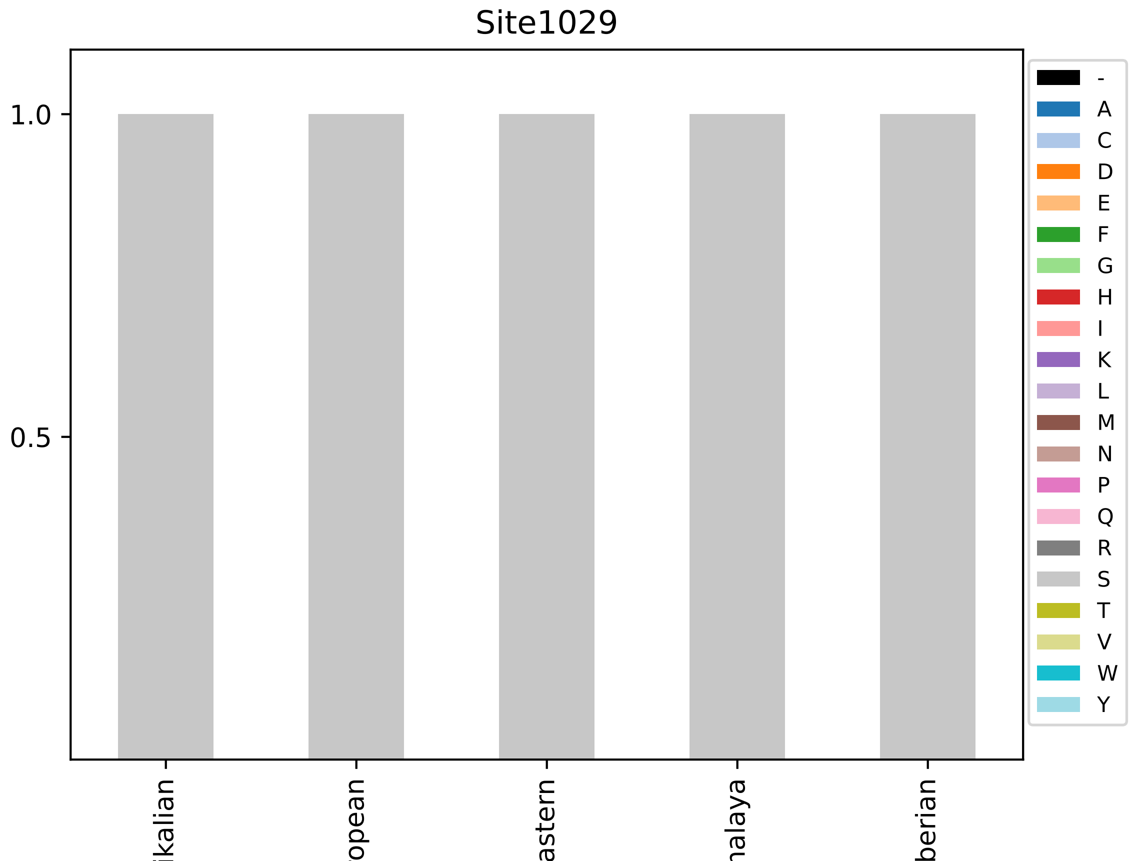
Site1029
1.0
0.5
ikalian
ropean
astern
nalaya
berian
-
A
C
D
E
F
G
H
I
K
L
M
N
P
Q
R
S
T
V
W
Y

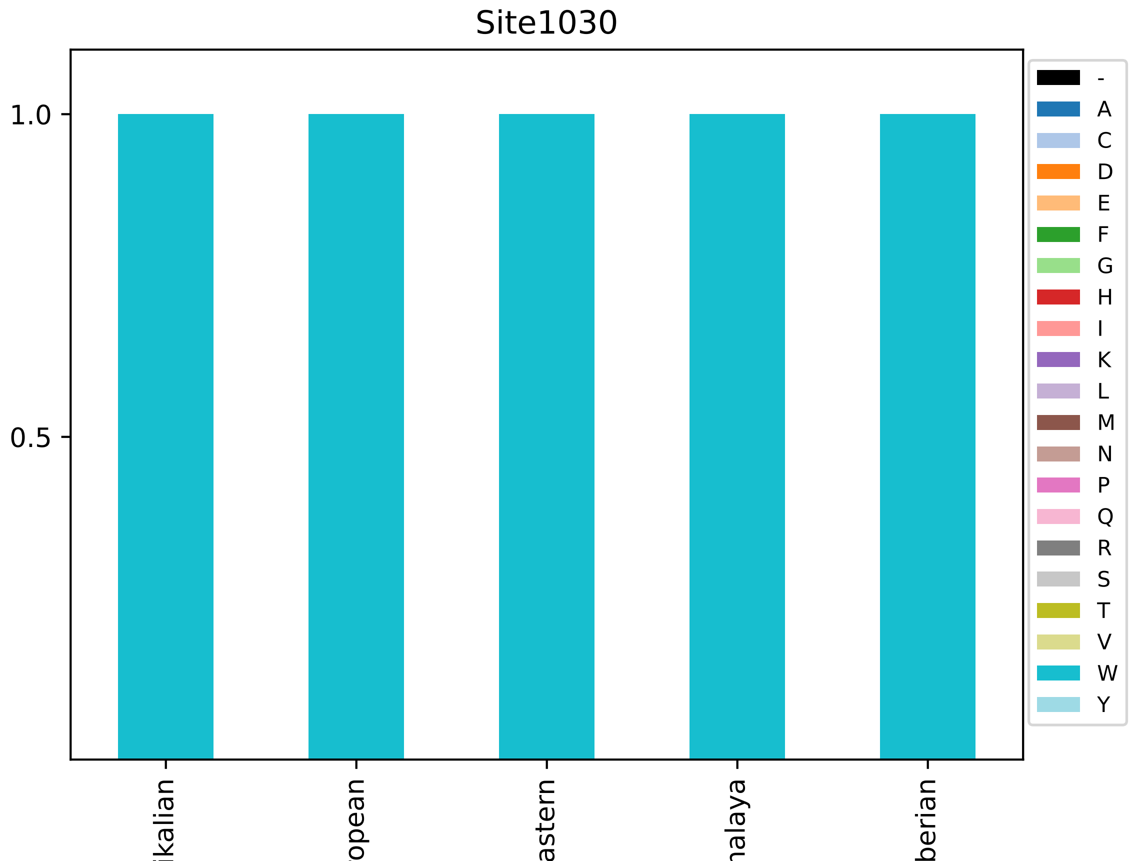
Site1030
1.0
0.5
-
A
C
D
E
F
G
H
I
K
L
M
N
P
Q
R
S
T
V
W
Y
ikalian
opean
astern
nalaya
berian

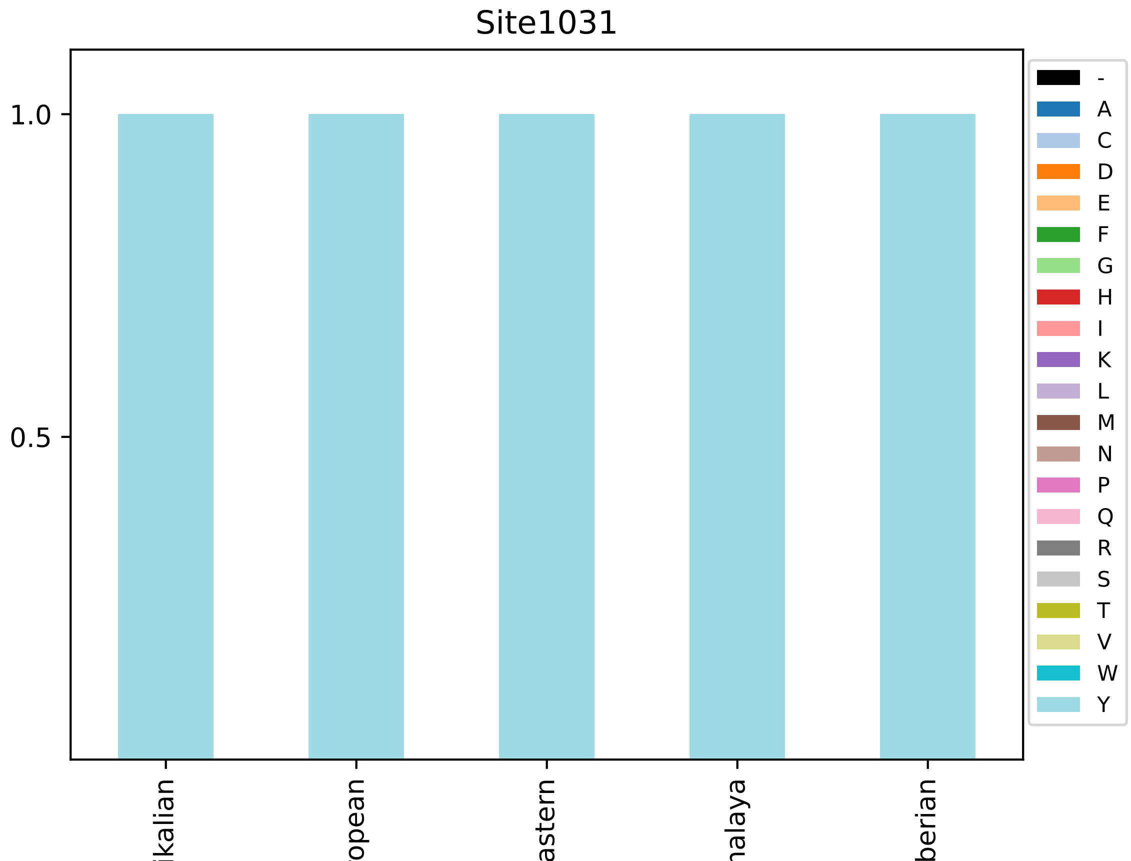
Site1031
1.0
0.5
-
A
C
D
E
F
G
H
I
K
L
M
N
P
Q
R
S
T
V
W
Y
ikalian
opean
astern
nalaya
berian

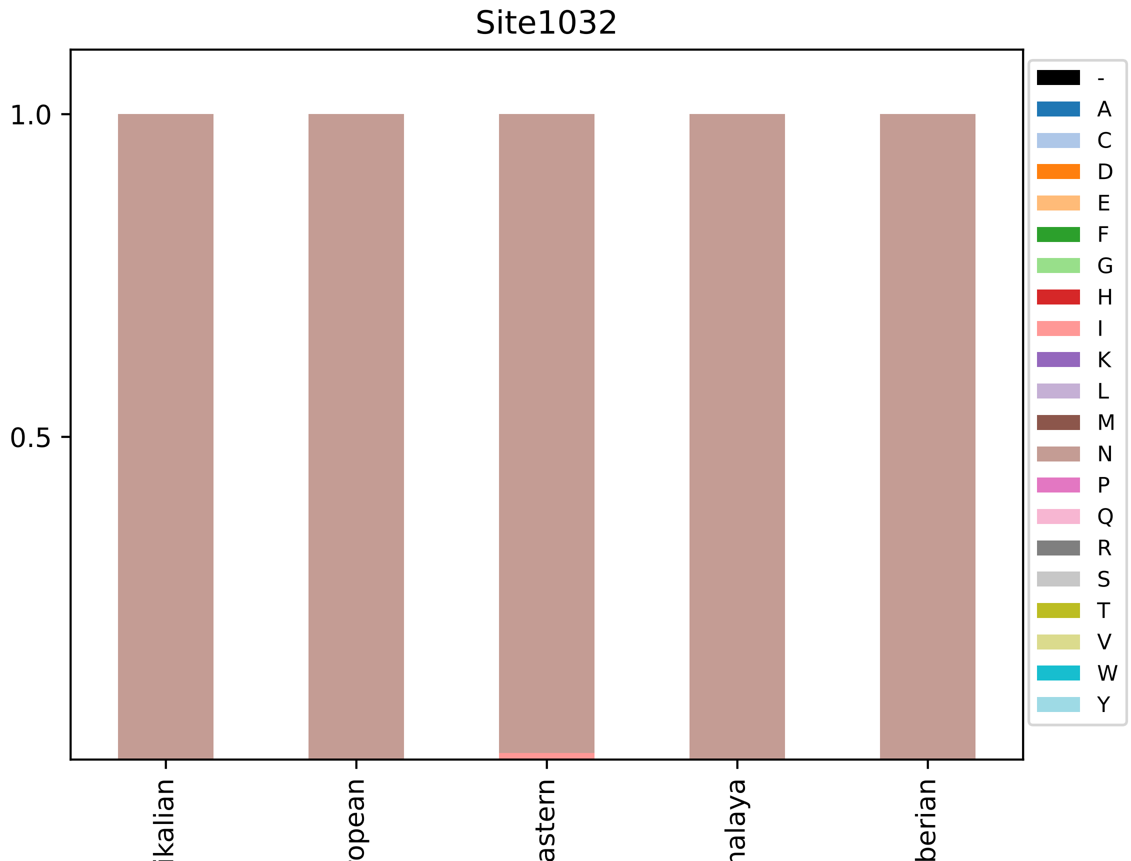

Site1032
1.0
0.5
-
A
C
D
E
F
G
H
I
K
L
M
N
P
Q
R
S
T
V
W
Y
ikalian
opean
astern
nalaya
berian

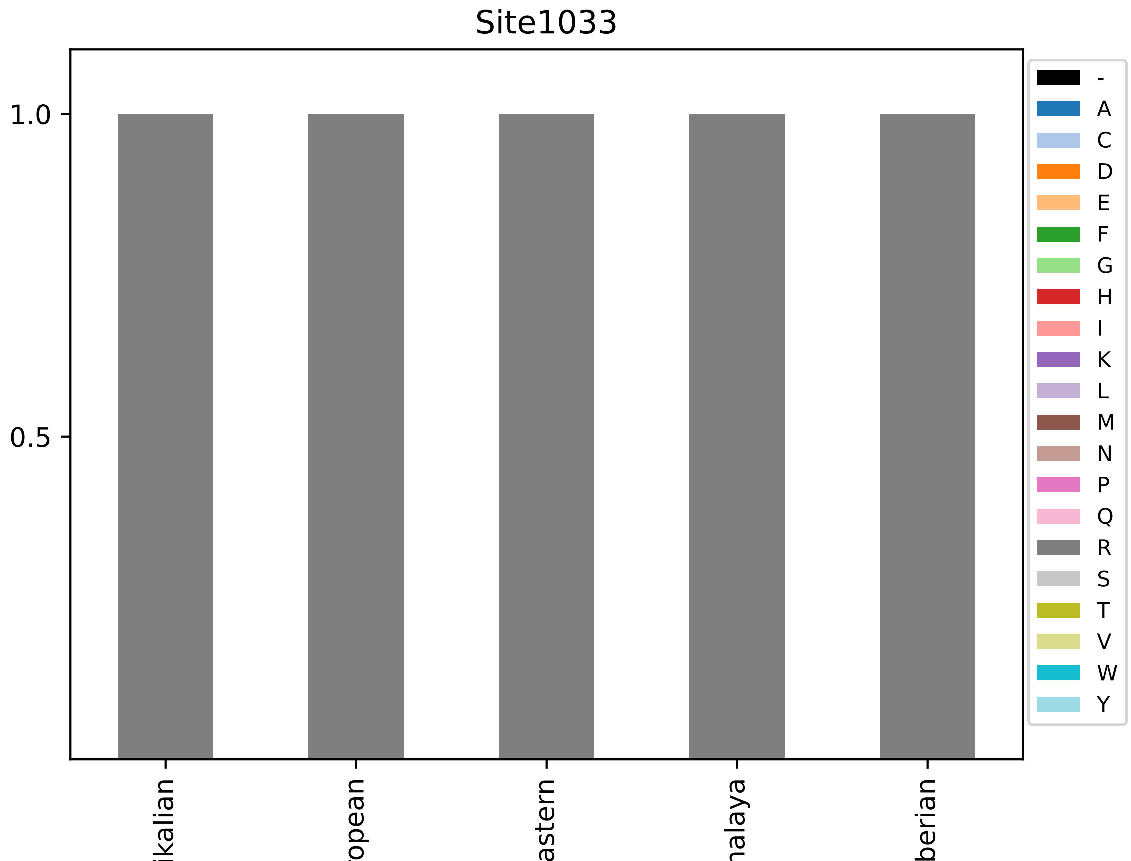
Site1033
1.0
0.5
ikalian
opean
astern
nalaya
berian
-
A
C
D
E
F
G
H
I
K
L
M
N
P
Q
R
S
T
V
W
Y

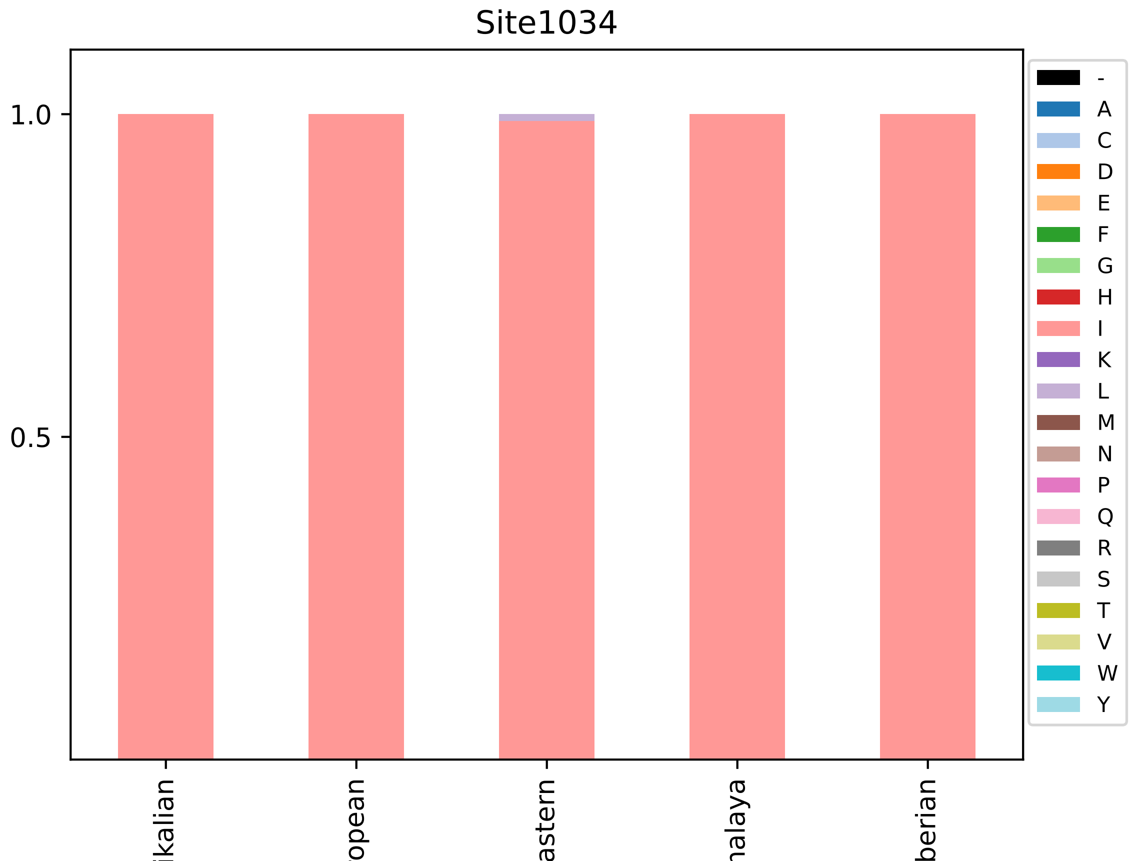
Site1034
1.0
0.5
-
A
C
D
E
F
G
H
I
K
L
M
N
P
Q
R
S
T
V
W
Y
ikalian
opean
astern
nalaya
berian

Site1035

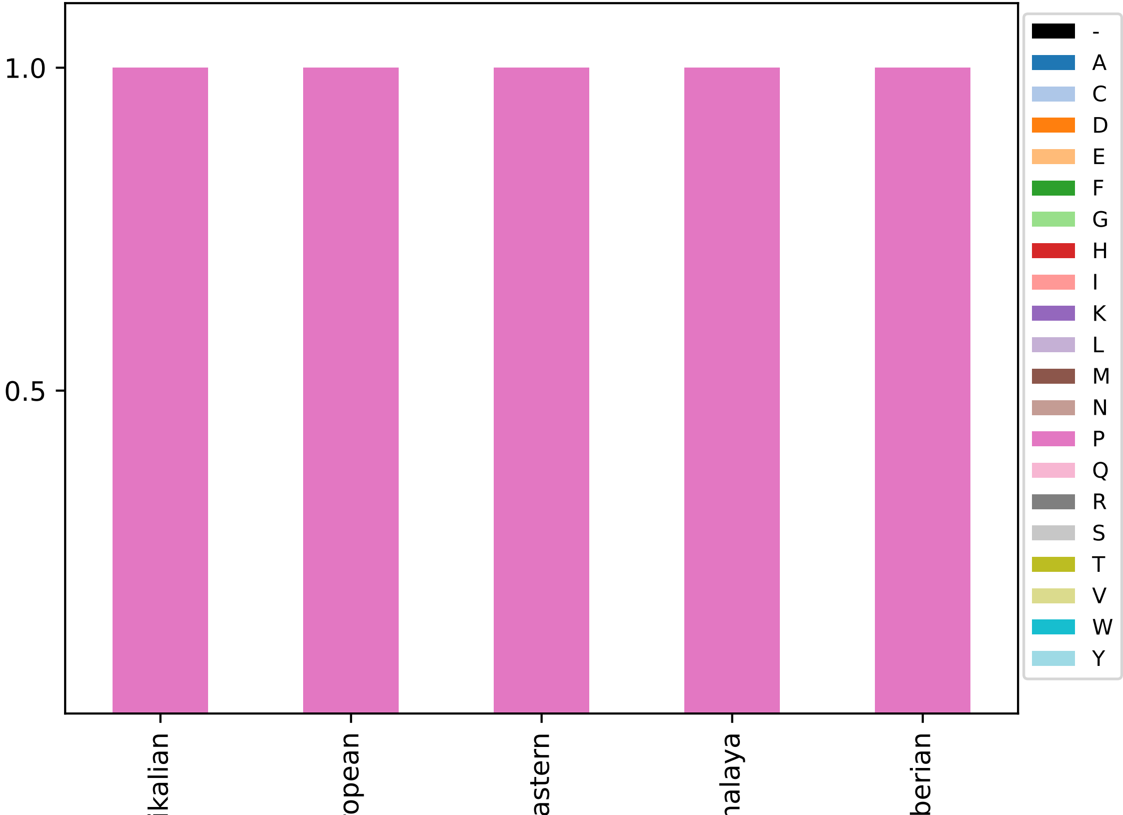

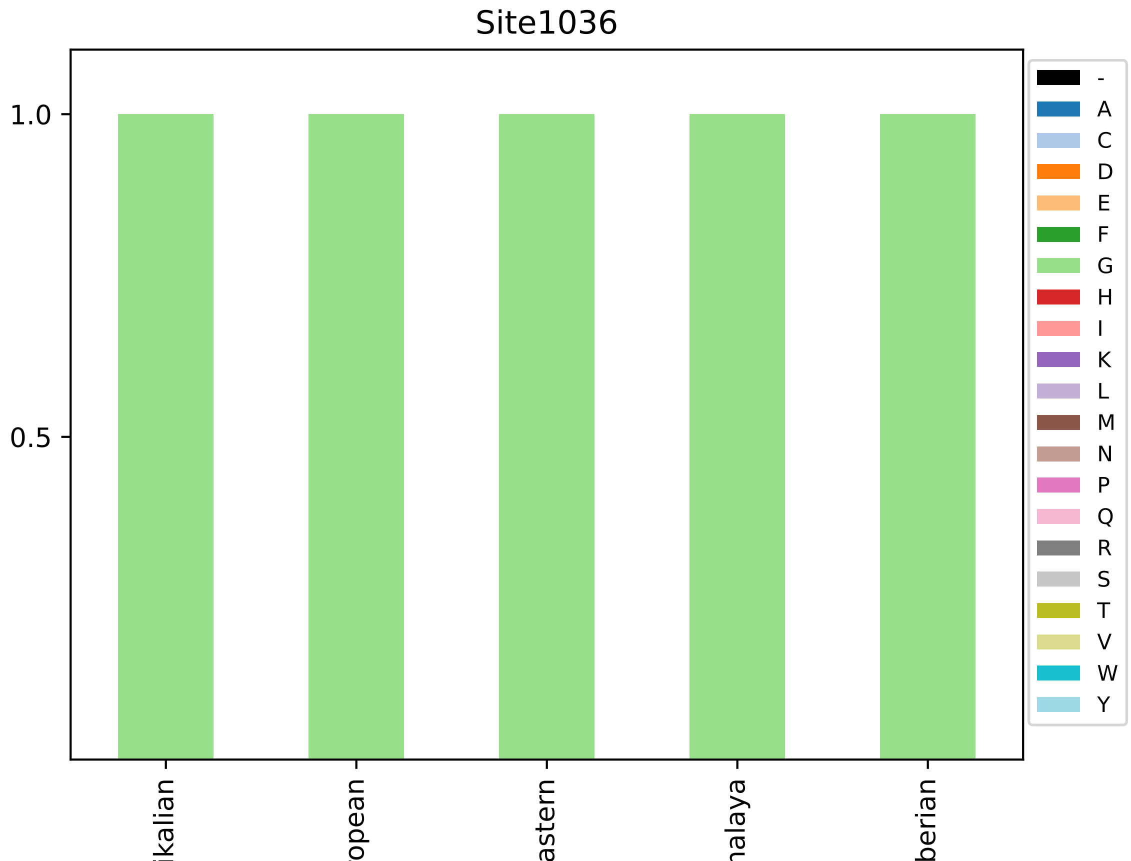

Site1036
1.0
0.5
-
A
C
D
E
F
G
H
I
K
L
M
N
P
Q
R
S
T
V
W
Y
ikalian
opean
astern
nalaya
berian

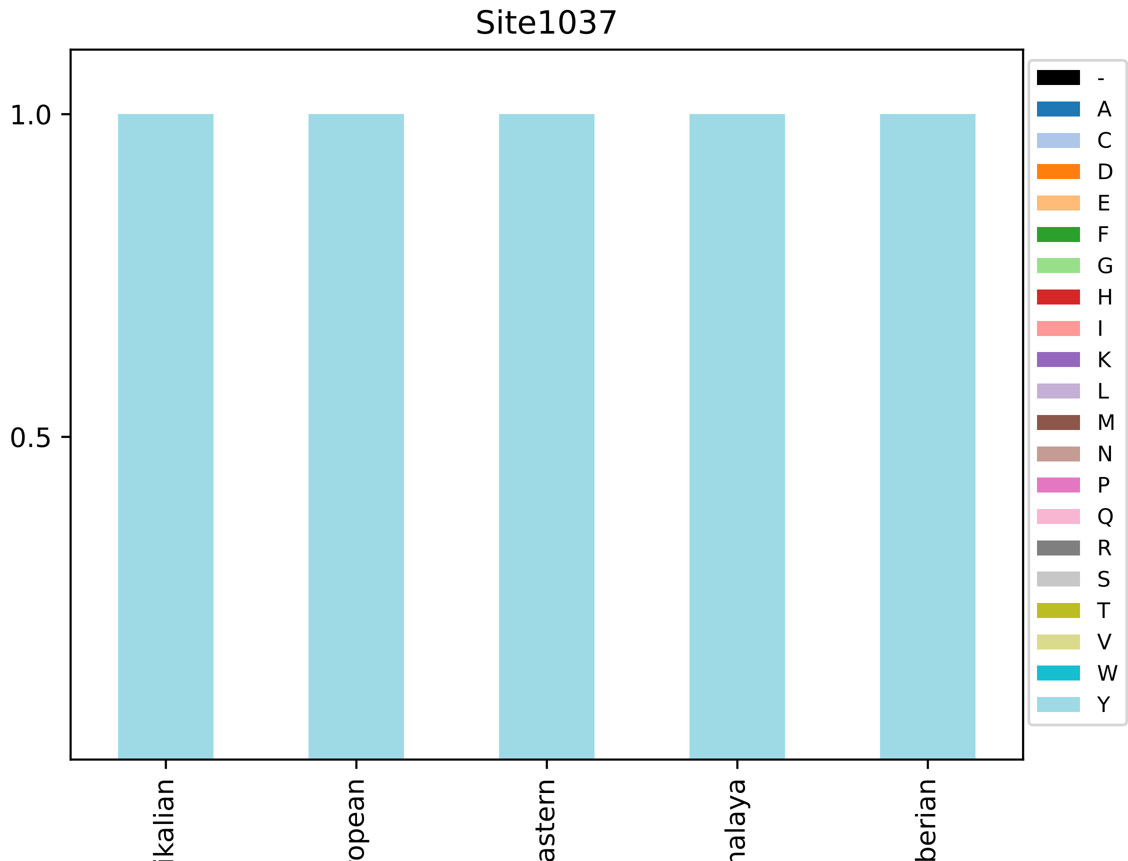
Site1037
1.0
0.5
-
A
C
D
E
F
G
H
I
K
L
M
N
P
Q
R
S
T
V
W
Y

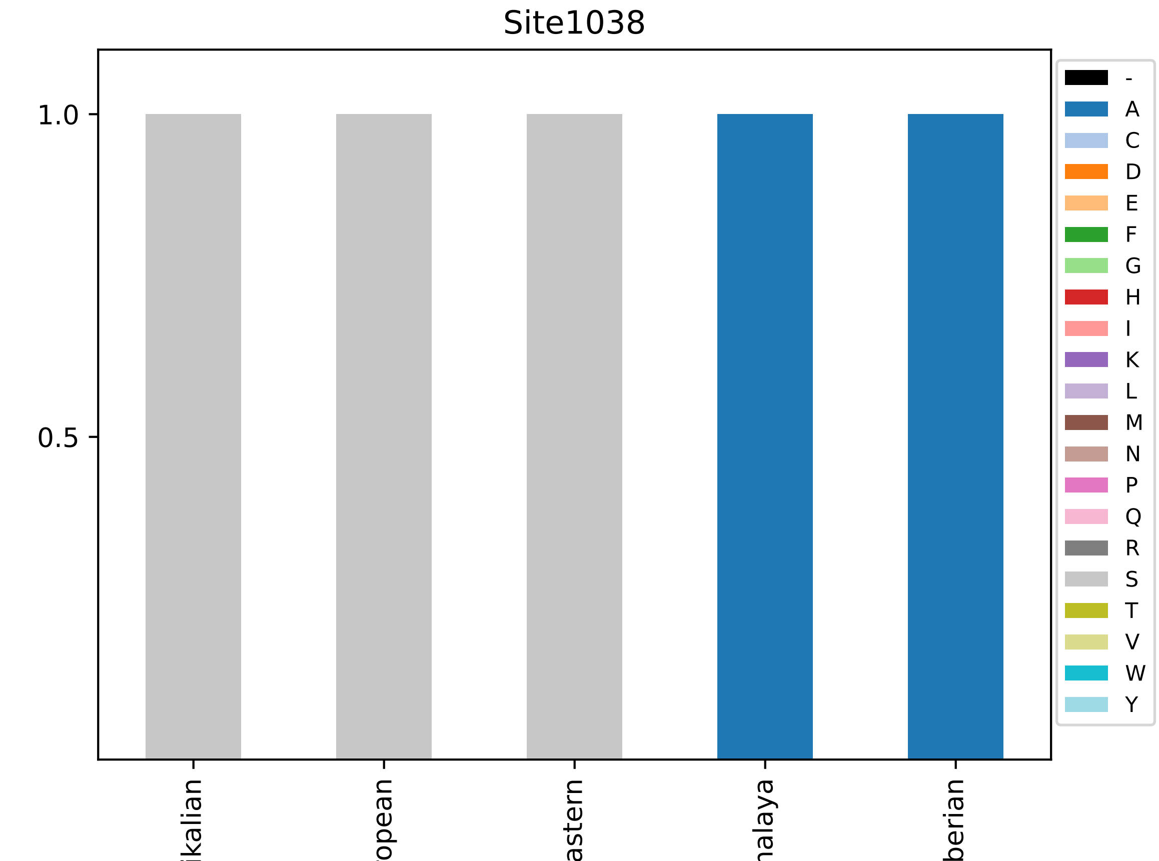
Site1038
1.0
0.5
-
A
C
D
E
F
G
H
I
K
L
M
N
P
Q
R
S
T
V
W
Y
ikalian
opean
astern
halaya
berian

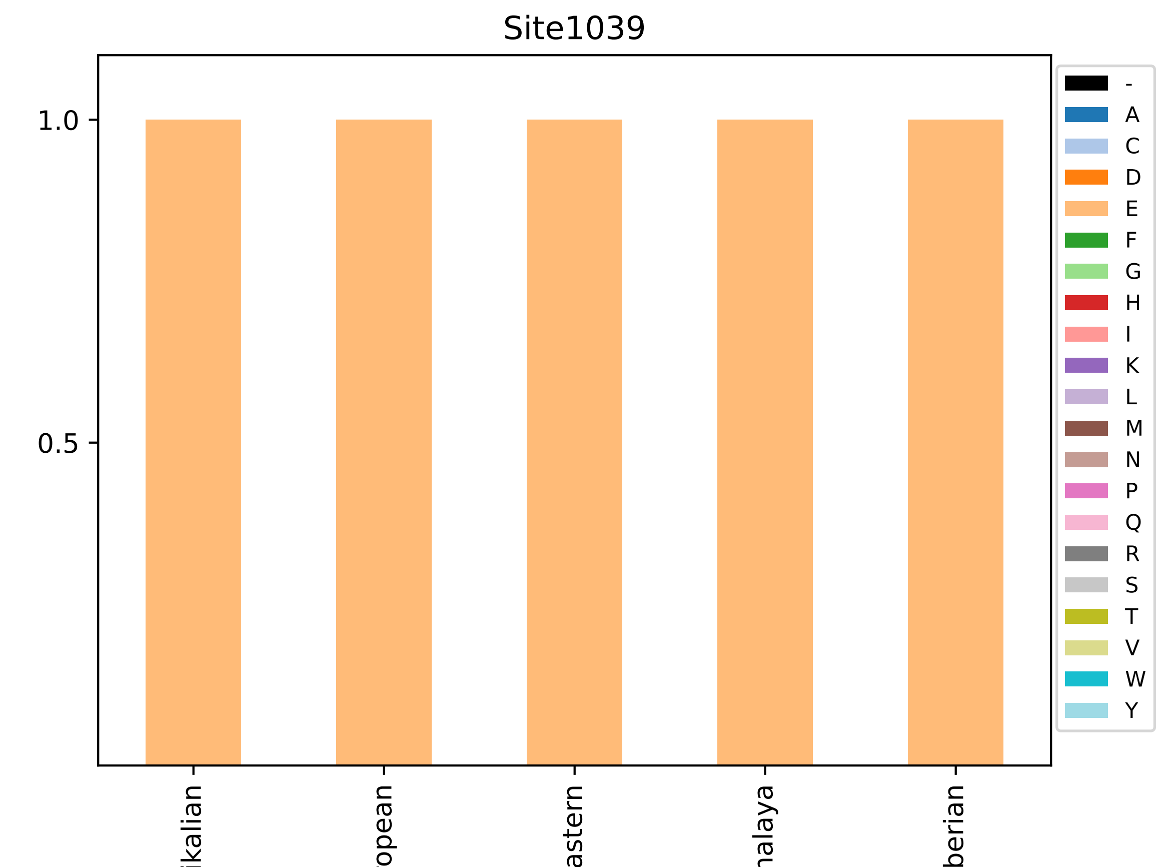

Site1039
1.0
0.5
-
A
C
D
E
F
G
H
I
K
L
M
N
P
Q
R
S
T
V
W
Y

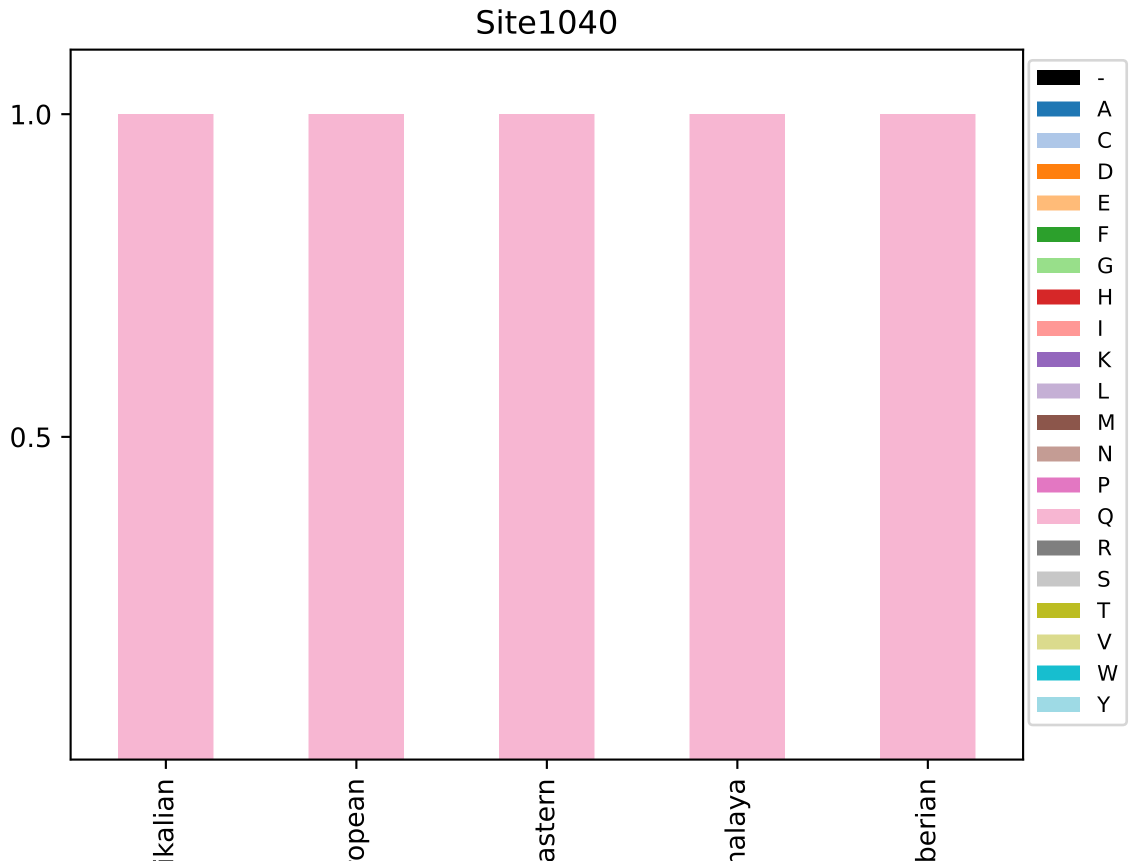
Site1040
1.0
0.5
ikalian
opean
astern
nalaya
berian
-
A
C
D
E
F
G
H
I
K
L
M
N
P
Q
R
S
T
V
W
Y

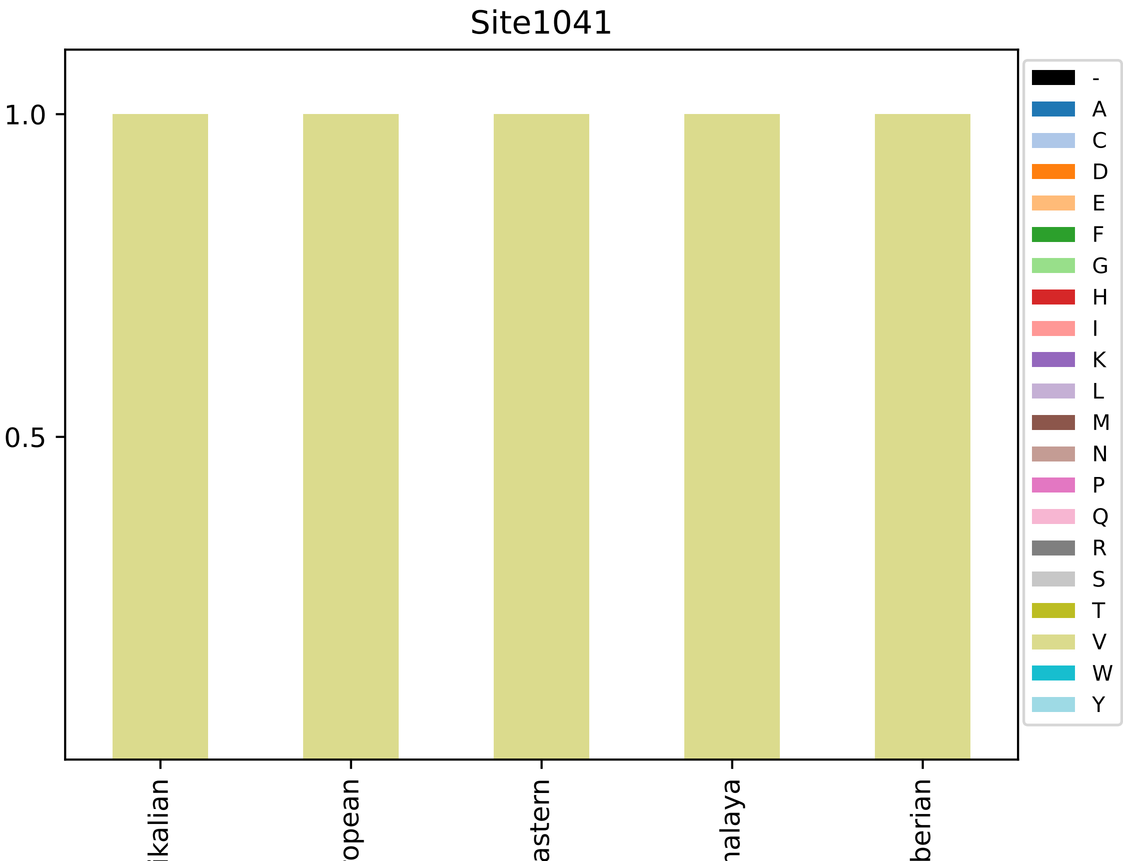

Site1041
1.0
0.5
-
A
C
D
E
F
G
H
I
K
L
M
N
P
Q
R
S
T
V
W
Y

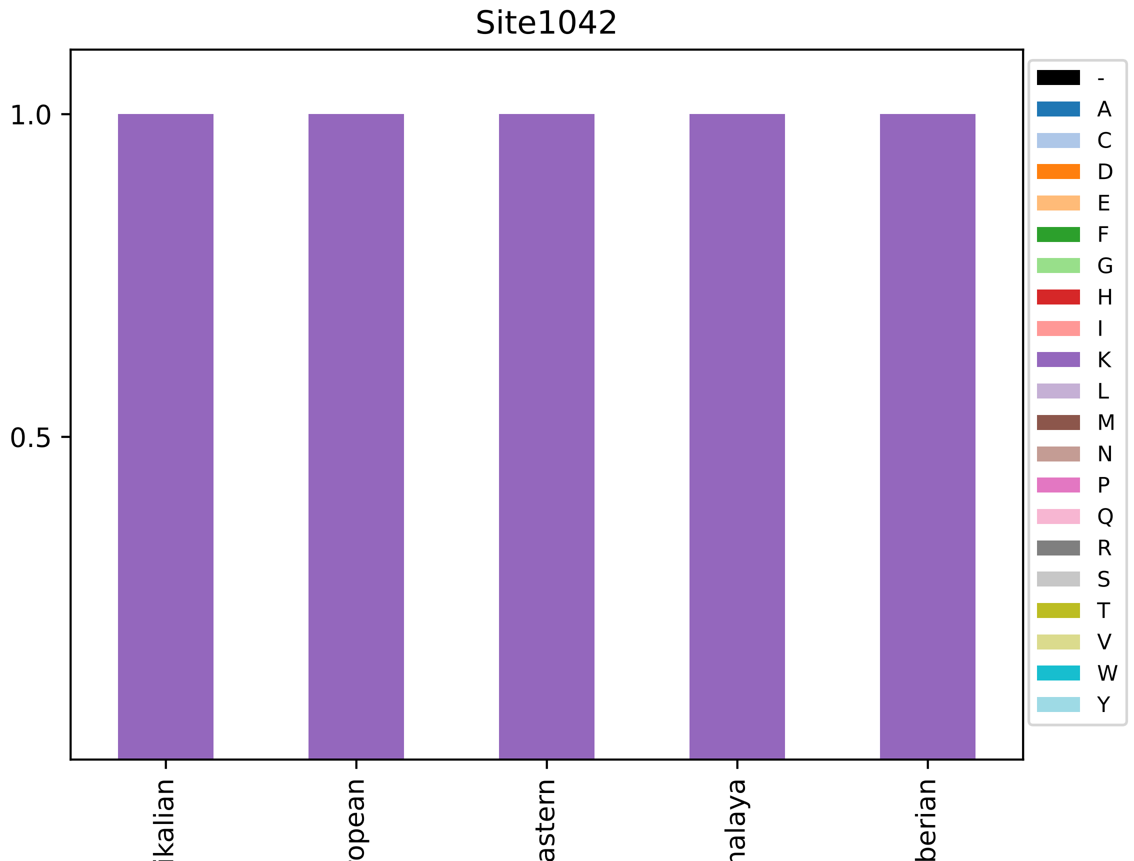
Site1042
1.0
0.5
-
A
C
D
E
F
G
H
I
K
L
M
N
P
Q
R
S
T
V
W
Y
ikalian
opean
astern
nalaya
berian

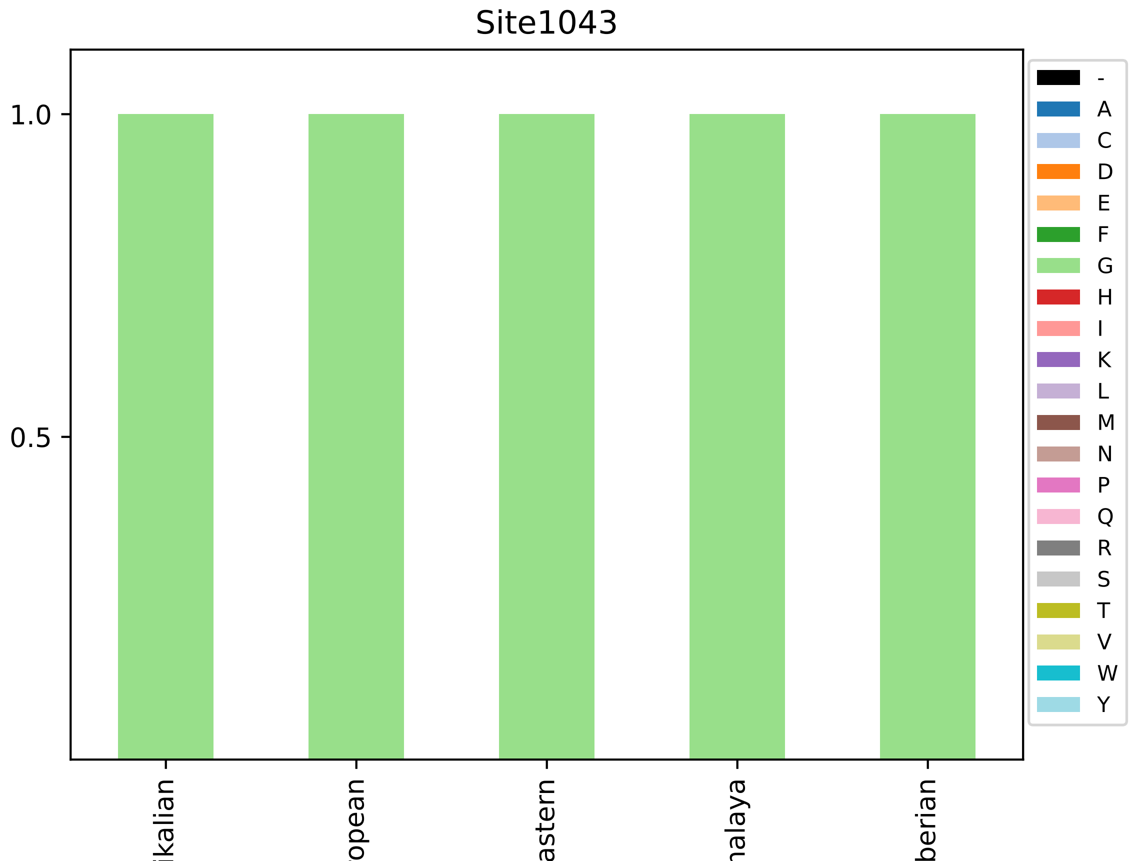
Site1043
1.0
0.5
ikalian
opean
astern
nalaya
berian
-
A
C
D
E
F
G
H
I
K
L
M
N
P
Q
R
S
T
V
W
Y

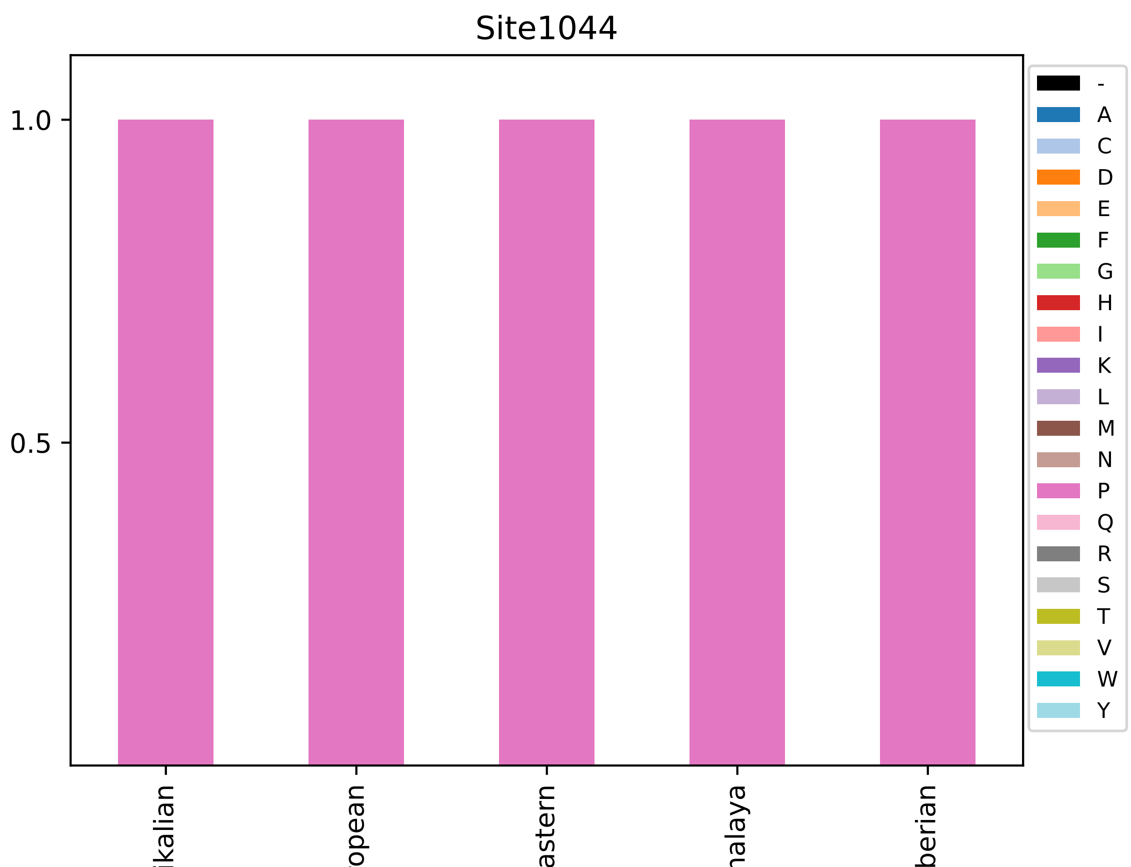

Site1044
1.0
0.5
-
A
C
D
E
F
G
H
I
K
L
M
N
P
Q
R
S
T
V
W
Y

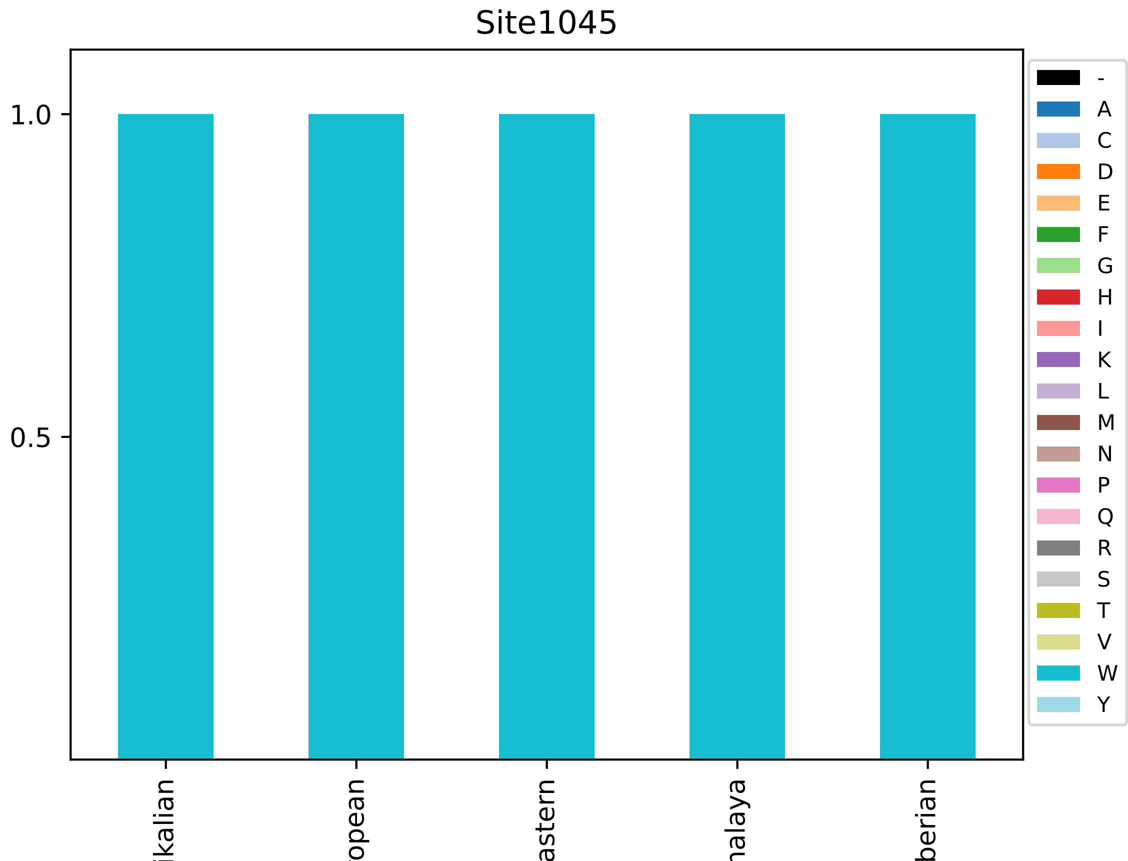

Site1045
1.0
0.5
-
A
C
D
E
F
G
H
I
K
L
M
N
P
Q
R
S
T
V
W
Y
ikalian
opean
astern
nalaya
berian

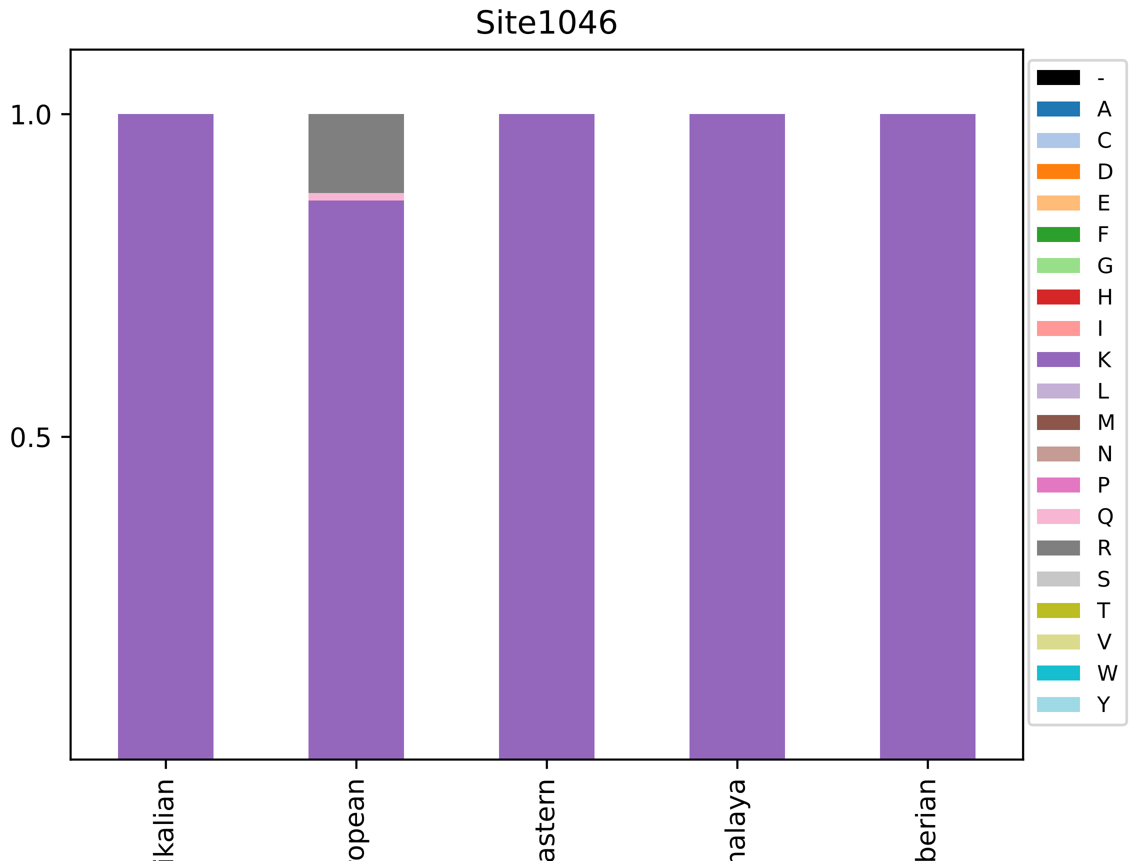
Site1046
1.0
0.5
-
A
C
D
E
F
G
H
I
K
L
M
N
P
Q
R
S
T
V
W
Y
ikalian
opean
astern
nalaya
berian

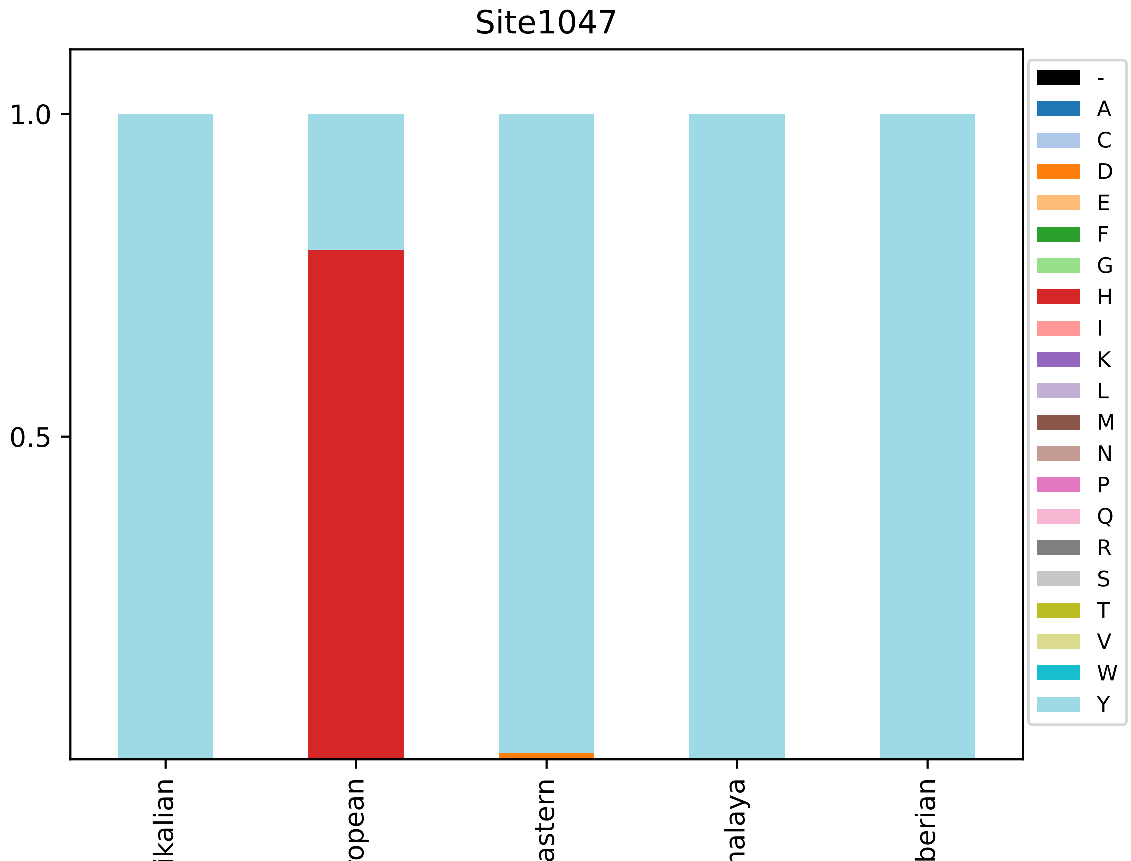
Site1047
1.0
0.5
-
A
C
D
E
F
G
H
I
K
L
M
N
P
Q
R
S
T
V
W
Y
ikalian
opean
astern
nalaya
berian

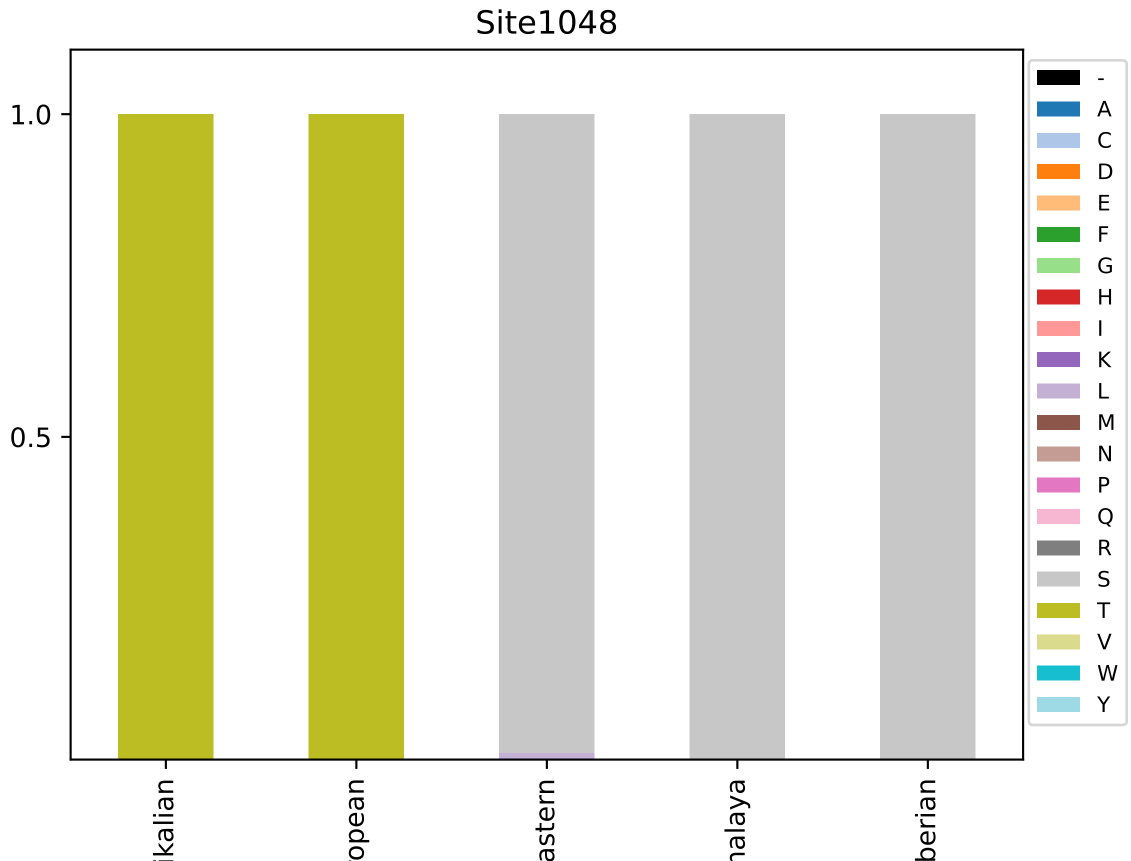
Site1048
1.0
0.5
-
A
C
D
E
F
G
H
I
K
L
M
N
P
Q
R
S
T
V
W
Y
ikalian
ropean
astern
nalaya
berian

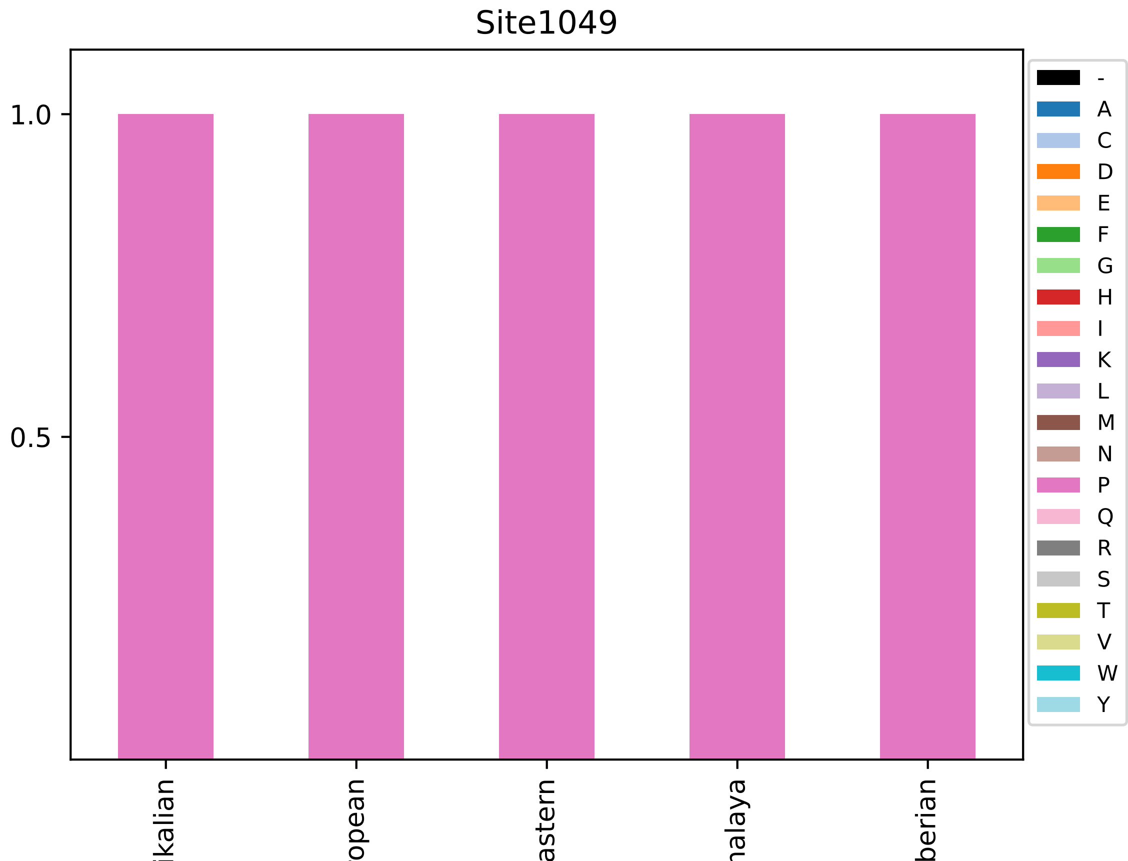

Site1049
1.0
0.5
-
A
C
D
E
F
G
H
I
K
L
M
N
P
Q
R
S
T
V
W
Y
ikalian
opean
astern
nalaya
berian

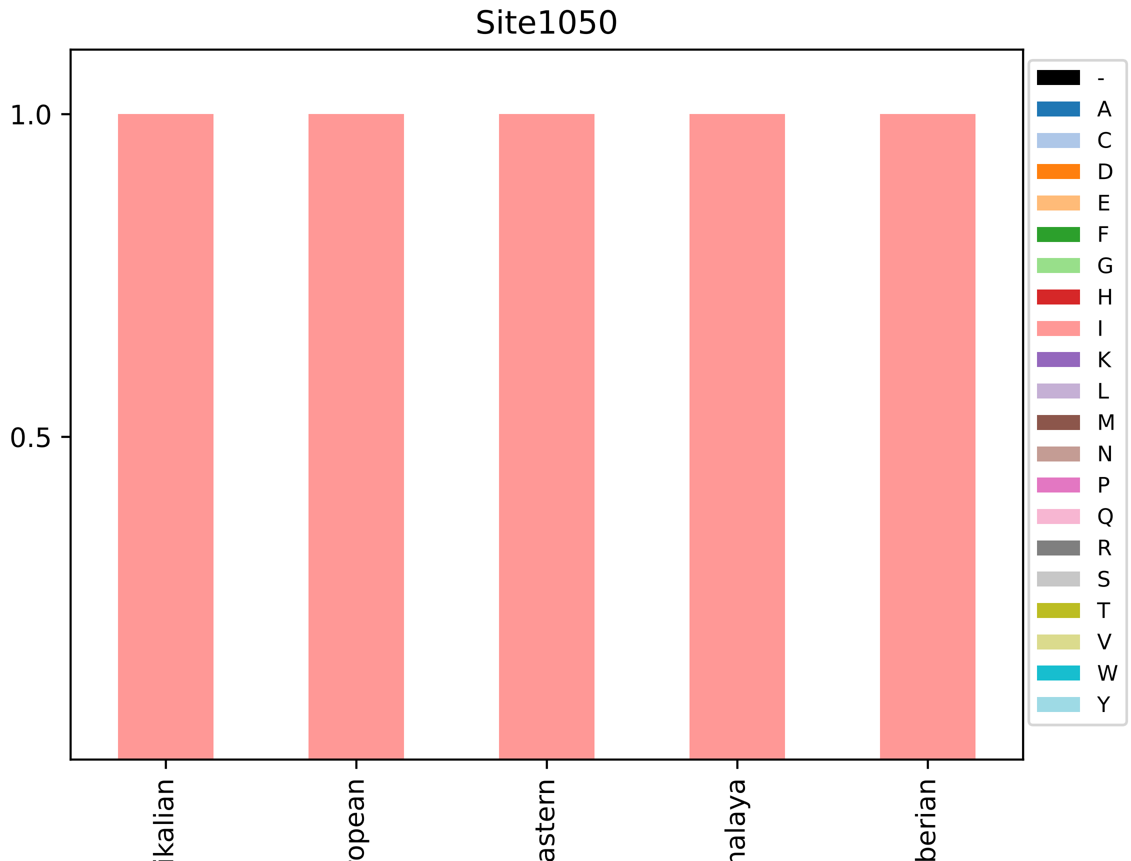

Site1050
1.0
0.5
ikalian
opean
astern
nalaya
berian
-
A
C
D
E
F
G
H
I
K
L
M
N
P
Q
R
S
T
V
W
Y

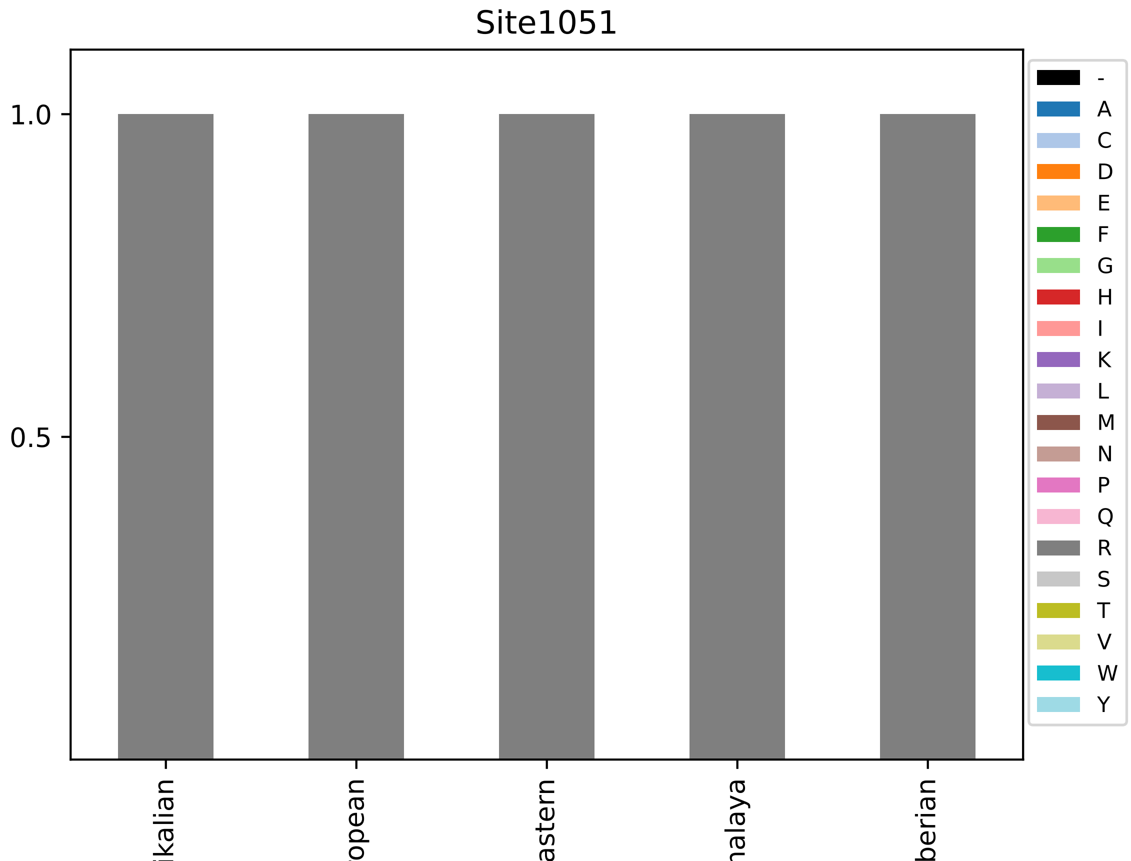

Site1051
1.0
0.5
ikalian
opean
astern
nalaya
berian
-
A
C
D
E
F
G
H
I
K
L
M
N
P
Q
R
S
T
V
W
Y

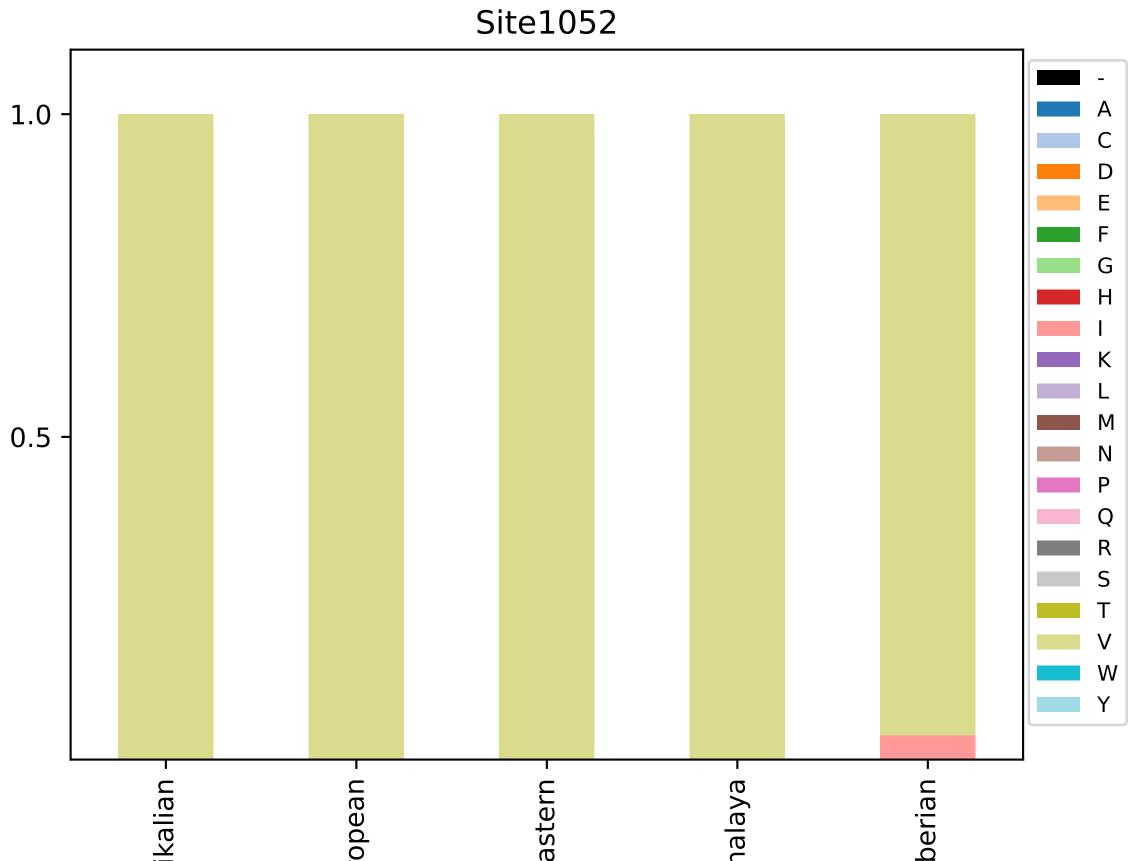
Site1052
1.0
0.5
ikalian
opean
astern
nalaya
berian
-
A
C
D
E
F
G
H
I
K
L
M
N
P
Q
R
S
T
V
W
Y

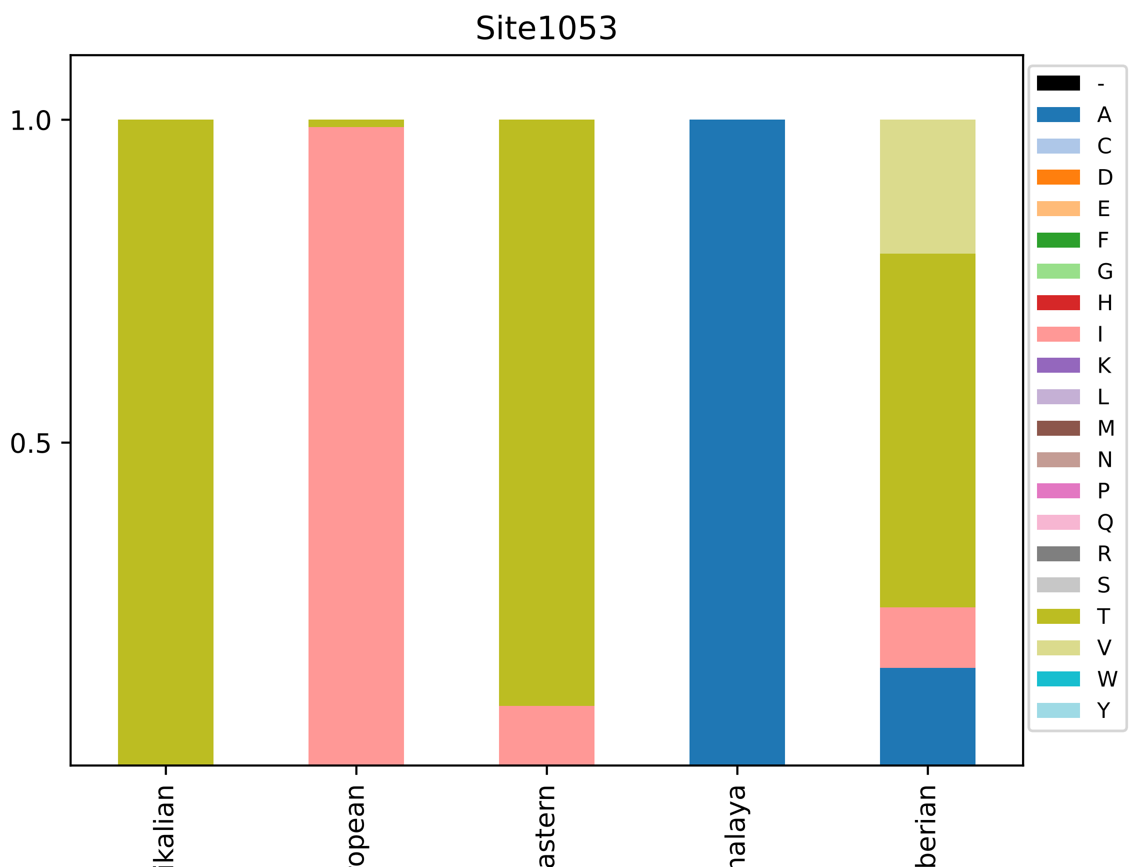
Site1053
1.0
0.5
-
A
C
D
E
F
G
H
I
K
L
M
N
P
Q
R
S
T
V
W
Y
ikalian
opean
astern
nalaya
berian

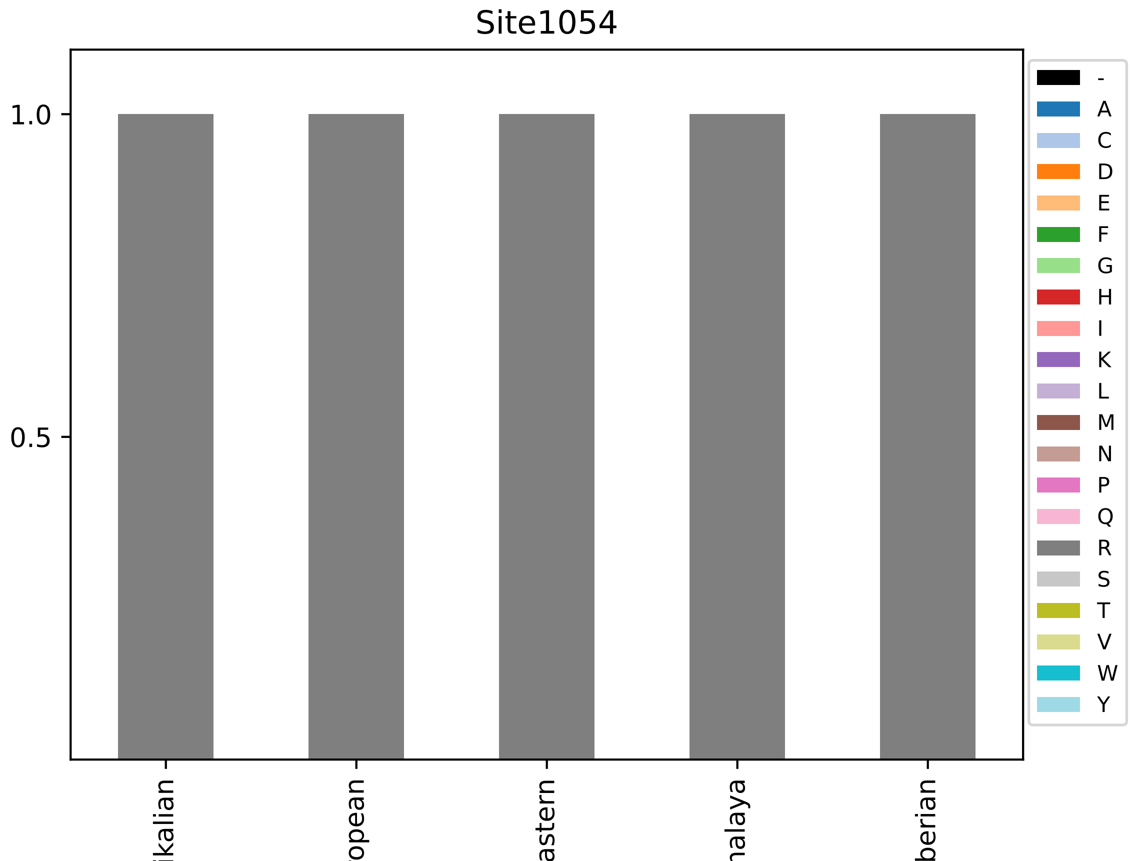
Site1054
1.0
0.5
-
A
C
D
E
F
G
H
I
K
L
M
N
P
Q
R
S
T
V
W
Y
ikalian
opean
astern
nalaya
berian

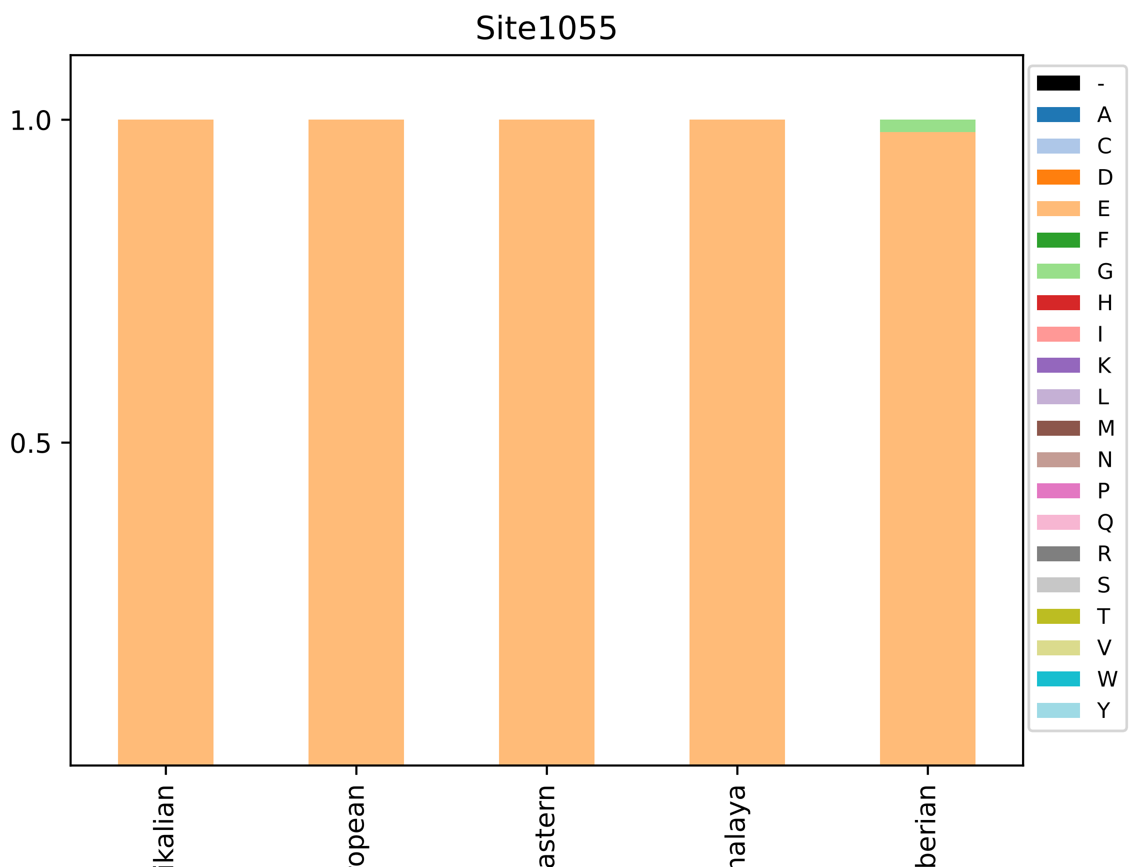
Site1055
1.0
0.5
-
A
C
D
E
F
G
H
I
K
L
M
N
P
Q
R
S
T
V
W
Y
ikalian
opean
astern
nalaya
berian

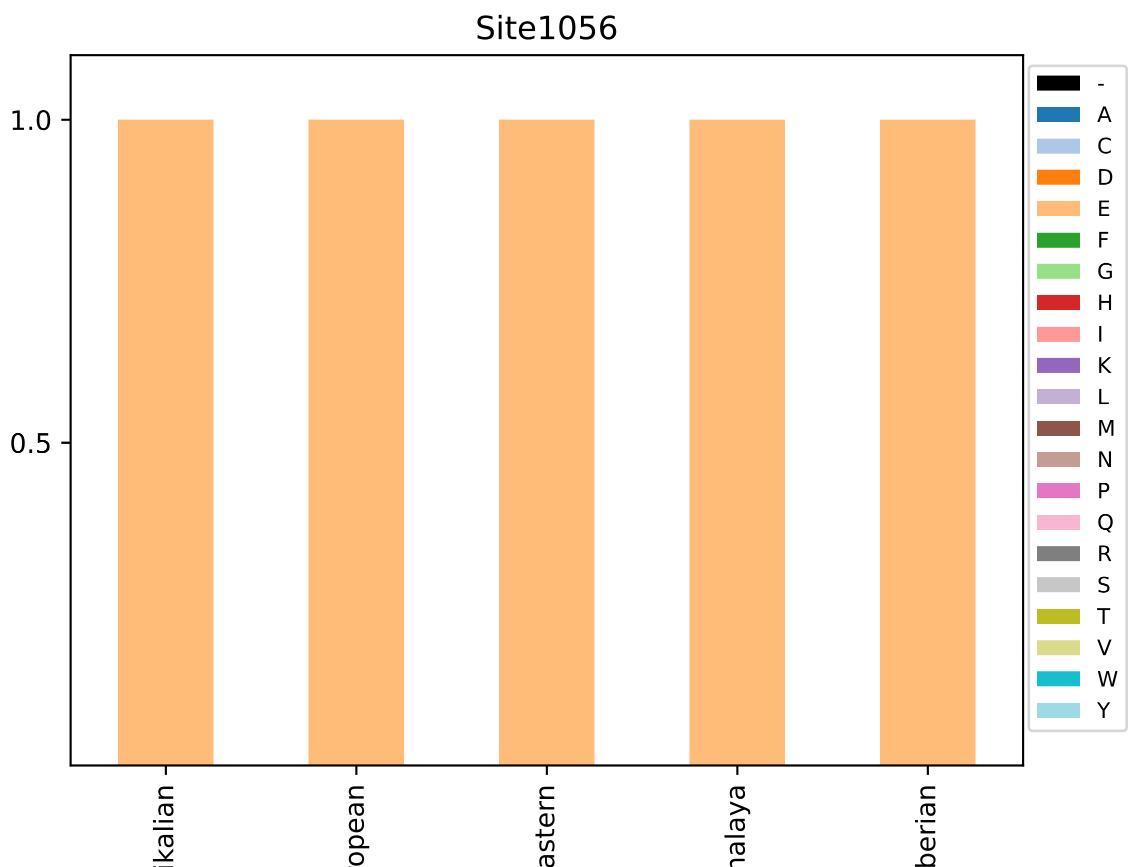
Site1056
1.0
0.5
-
A
C
D
E
F
G
H
I
K
L
M
N
P
Q
R
S
T
V
W
Y
ikalian
opean
astern
nalaya
berian

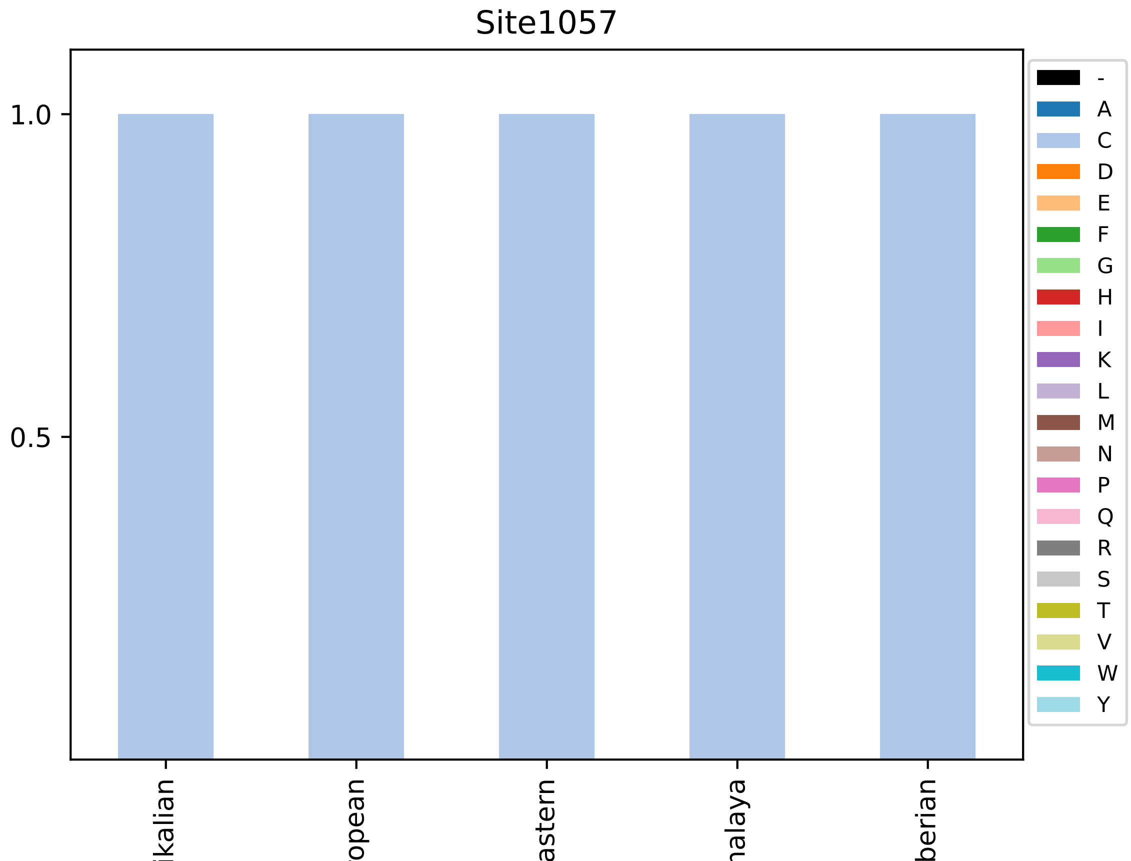
Site1057
1.0
0.5
-
A
C
D
E
F
G
H
I
K
L
M
N
P
Q
R
S
T
V
W
Y

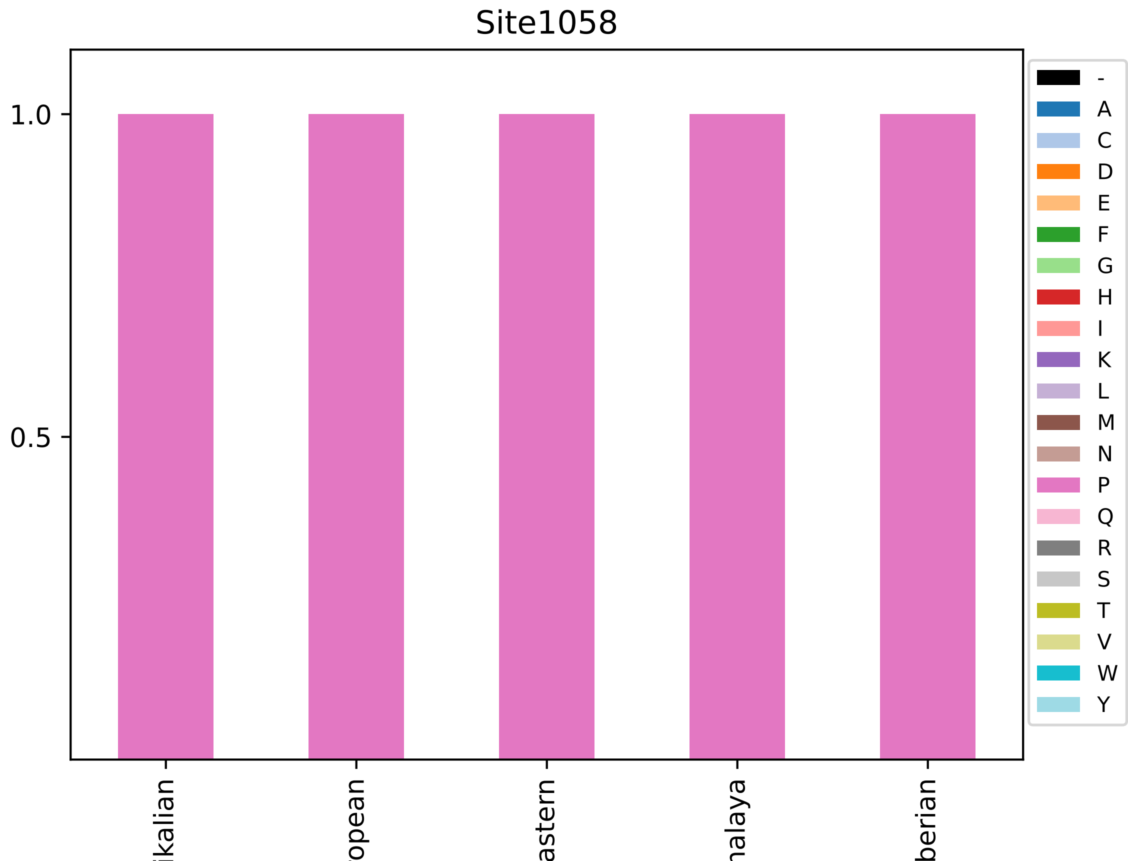
Site1058
1.0
0.5
-
A
C
D
E
F
G
H
I
K
L
M
N
P
Q
R
S
T
V
W
Y
ikalian
opean
astern
nalaya
berian

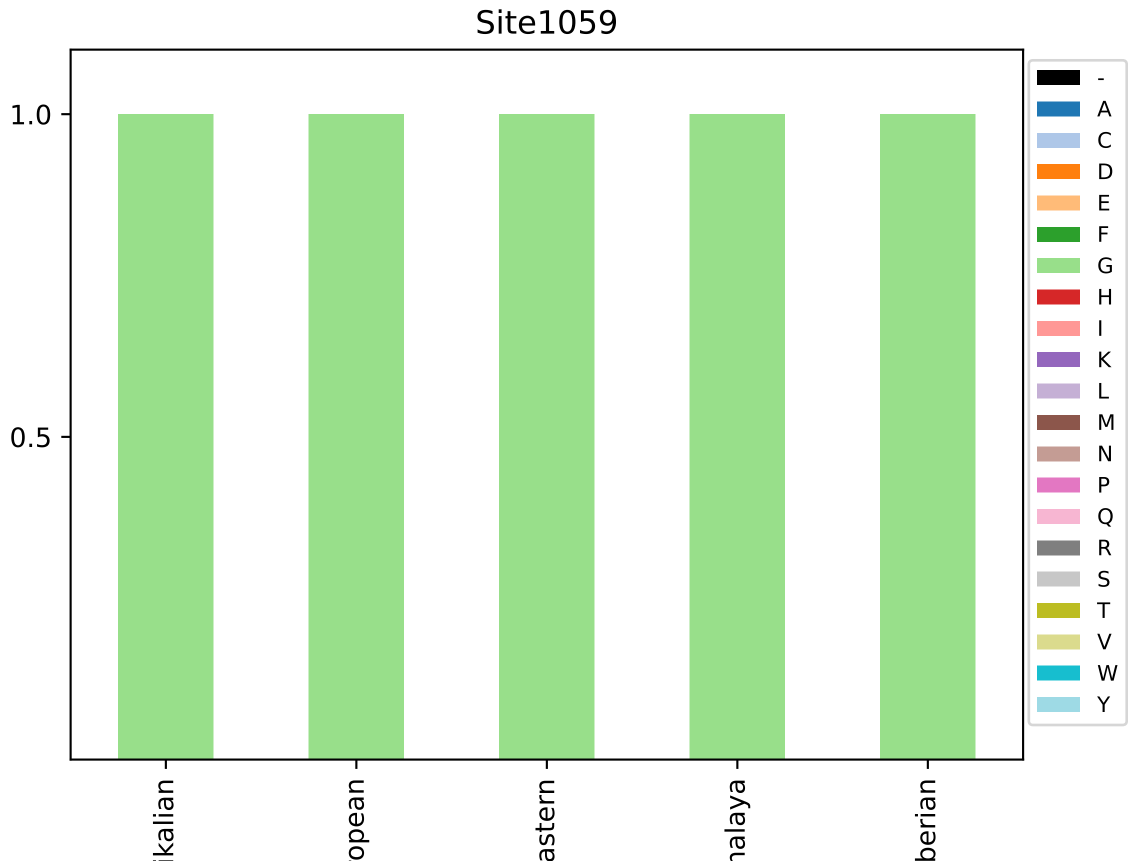
Site1059
1.0
0.5
-
A
C
D
E
F
G
H
I
K
L
M
N
P
Q
R
S
T
V
W
Y
ikalian
opean
astern
nalaya
berian

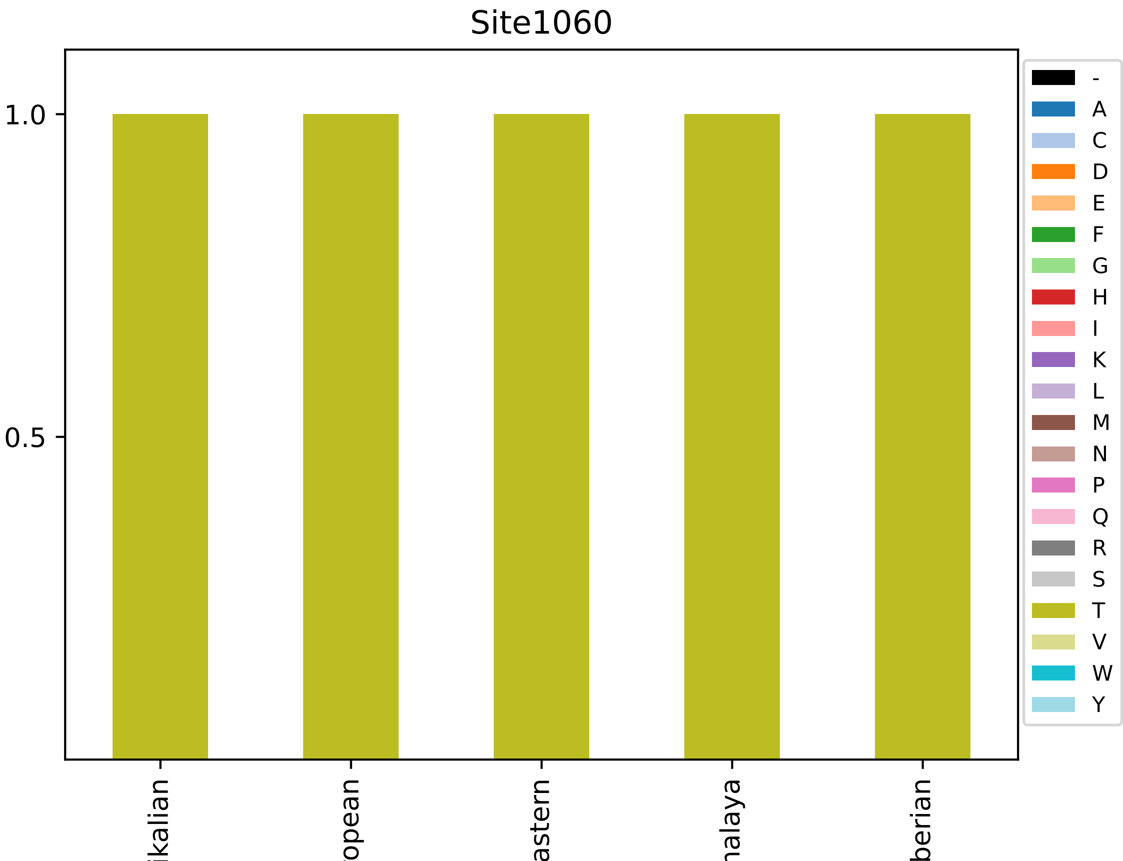

Site1060
1.0
0.5
-
A
C
D
E
F
G
H
I
K
L
M
N
P
Q
R
S
T
V
W
Y
ikalian
opean
astern
nalaya
berian

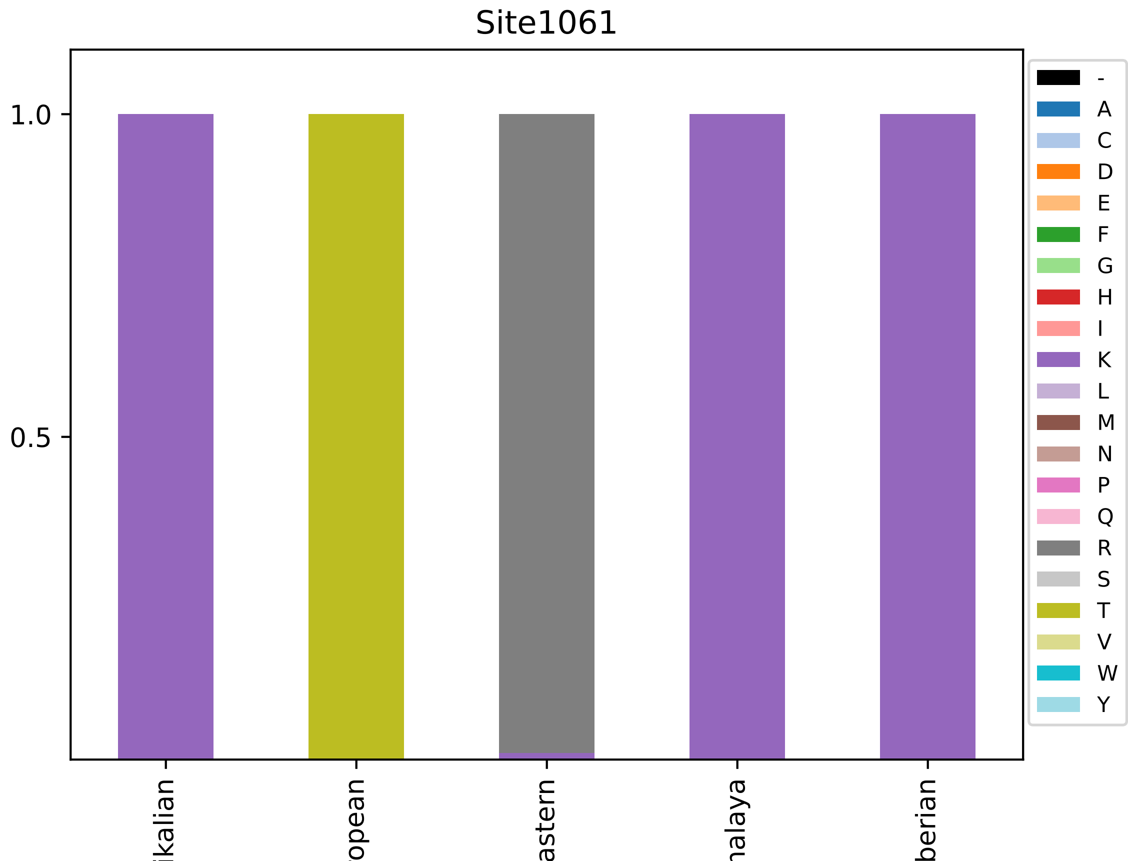
Site1061
1.0
0.5
-
A
C
D
E
F
G
H
I
K
L
M
N
P
Q
R
S
T
V
W
Y
ikalian
opean
astern
nalaya
berian

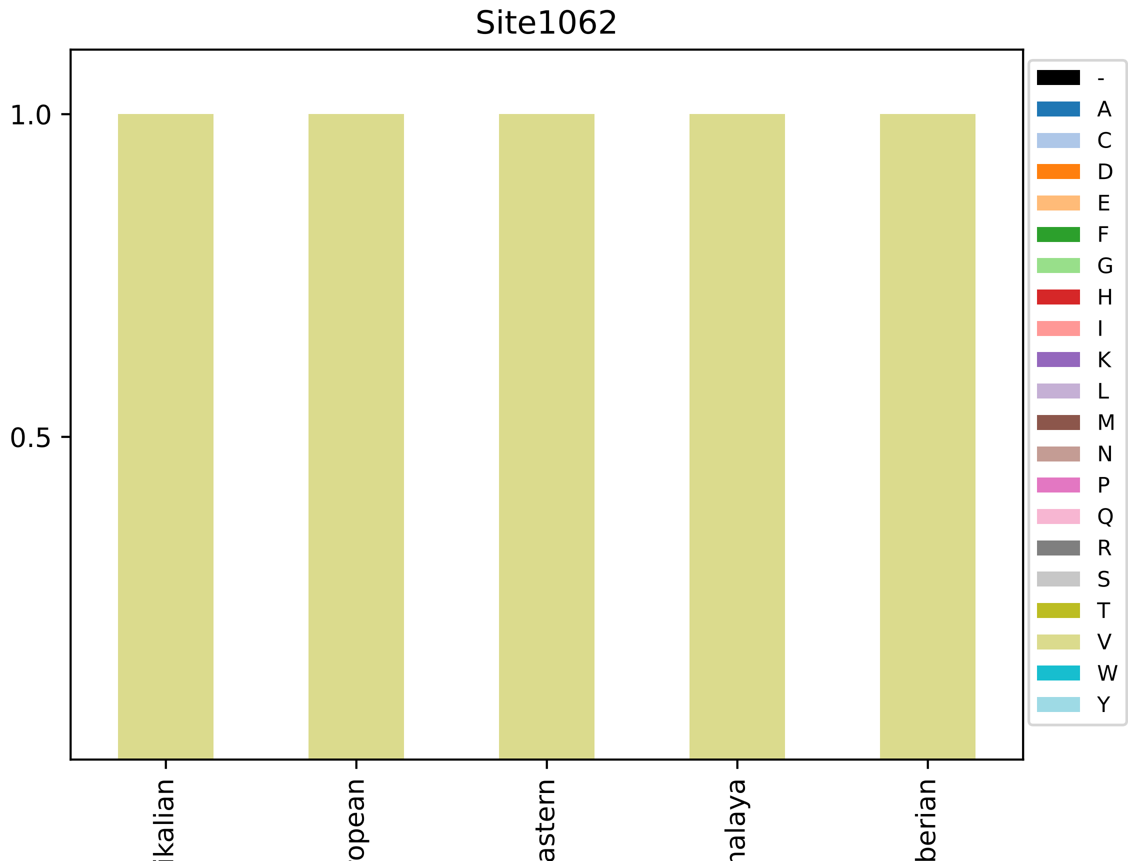
Site1062
1.0
0.5
-
A
C
D
E
F
G
H
I
K
L
M
N
P
Q
R
S
T
V
W
Y
ikalian
opean
astern
nalaya
berian

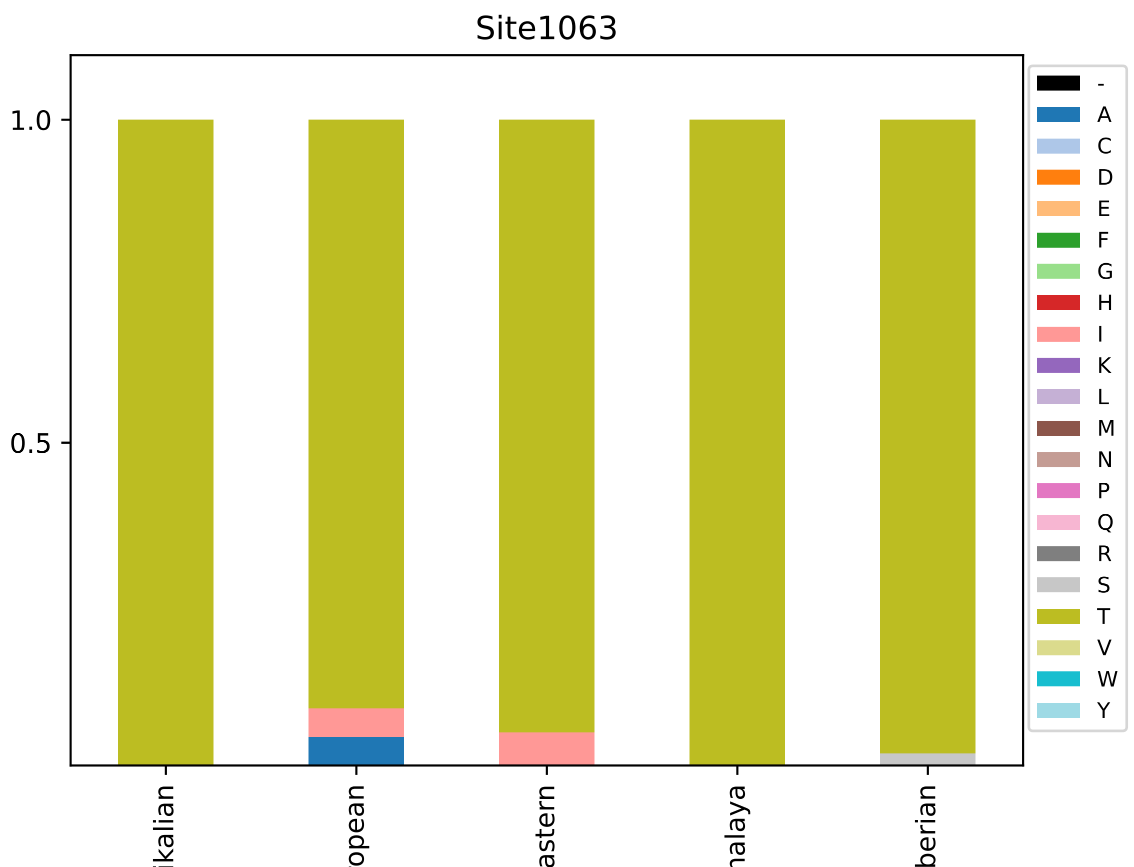

Site1063
1.0
0.5
ikalian
opean
astern
nalaya
berian
-
A
C
D
E
F
G
H
I
K
L
M
N
P
Q
R
S
T
V
W
Y

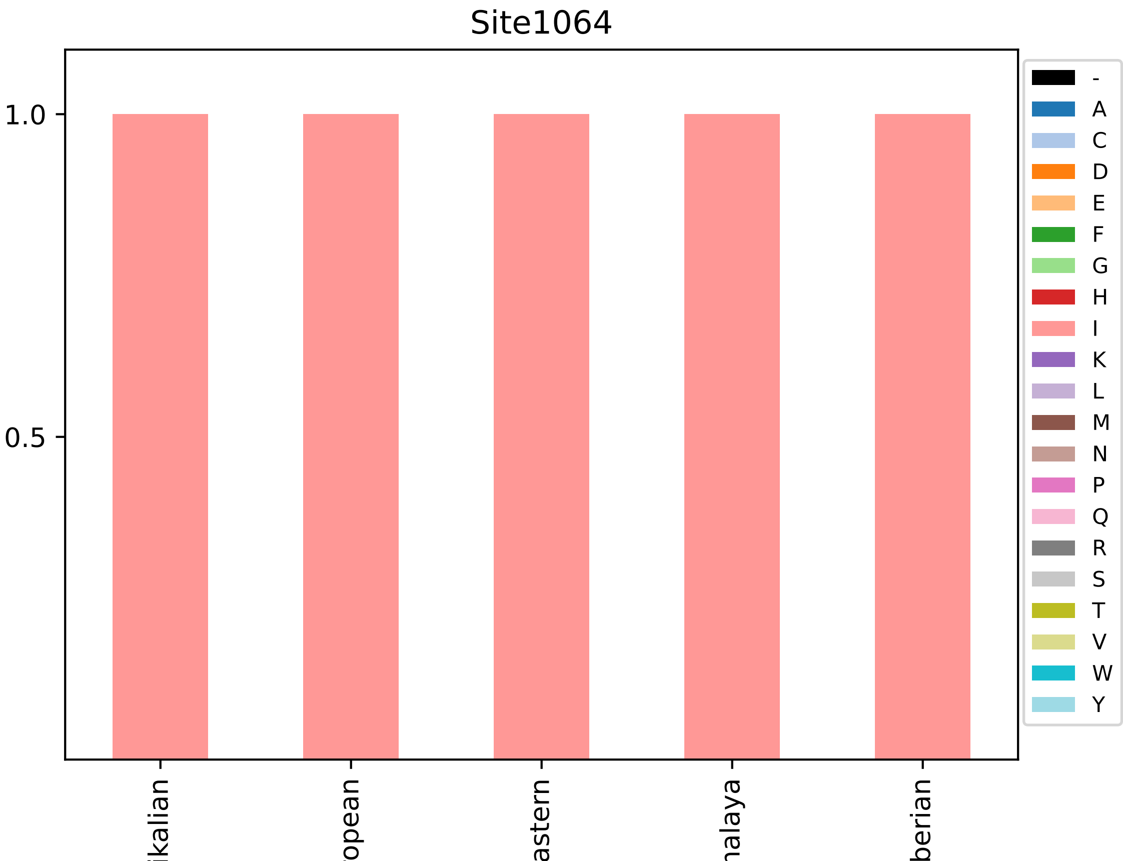
Site1064
1.0
0.5
ikalian
opean
astern
nalaya
berian
-
A
C
D
E
F
G
H
I
K
L
M
N
P
Q
R
S
T
V
W
Y

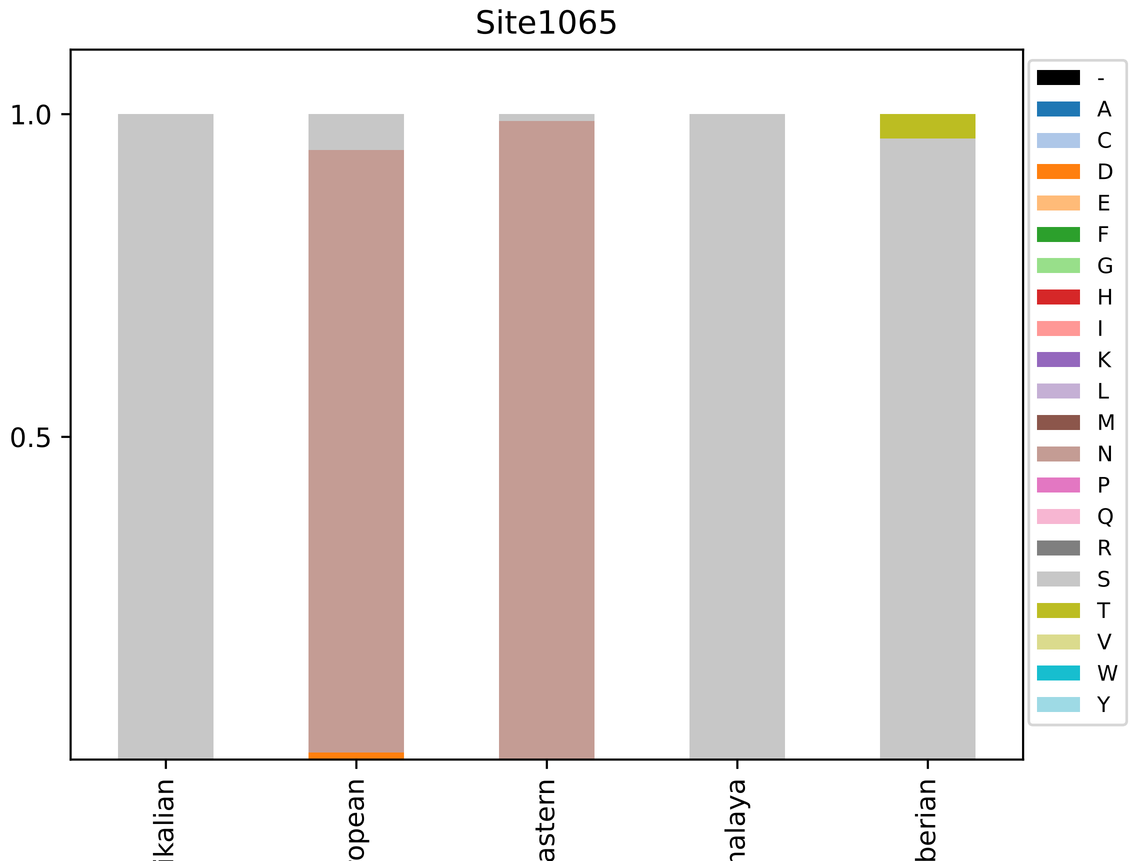

Site1065
1.0
0.5
-
A
C
D
E
F
G
H
I
K
L
M
N
P
Q
R
S
T
V
W
Y

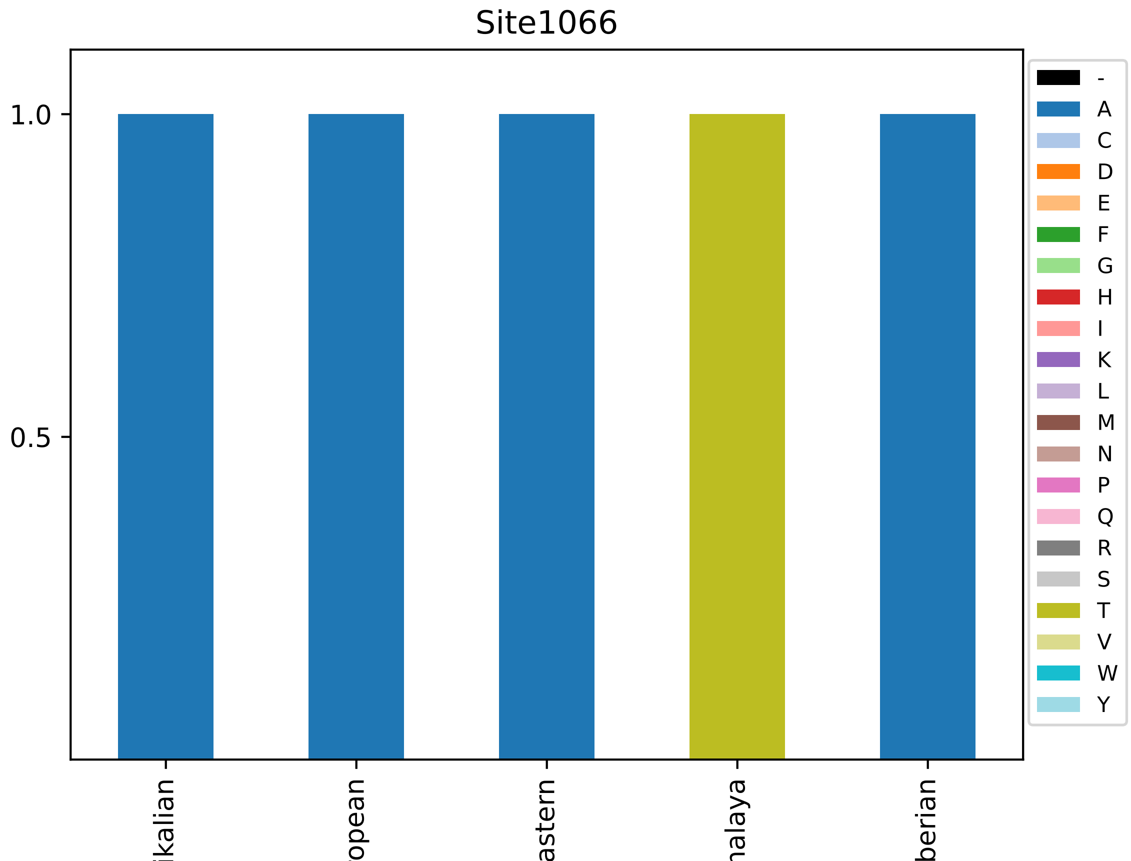
Site1066
1.0
0.5
-
A
C
D
E
F
G
H
I
K
L
M
N
P
Q
R
S
T
V
W
Y
ikalian
opean
astern
nalaya
berian

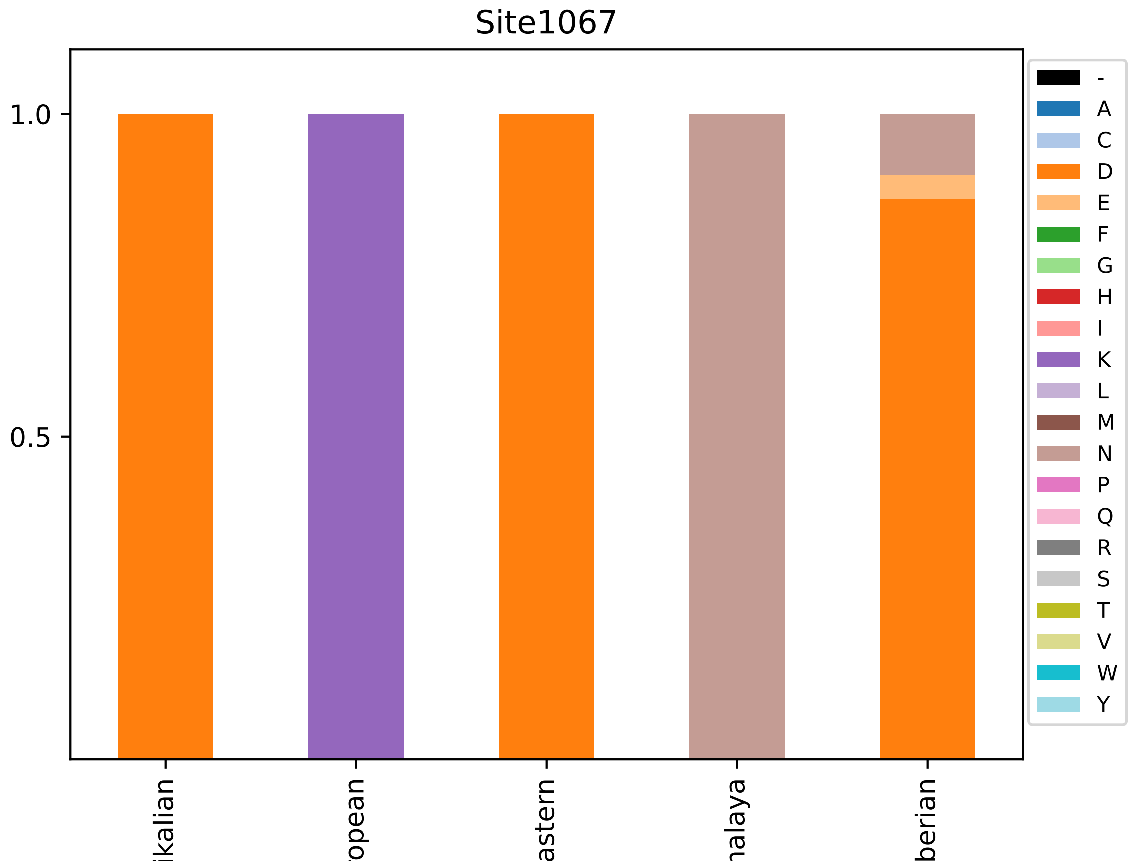
Site1067
1.0
0.5
-
A
C
D
E
F
G
H
I
K
L
M
N
P
Q
R
S
T
V
W
Y
ikalian
opean
astern
nalaya
berian

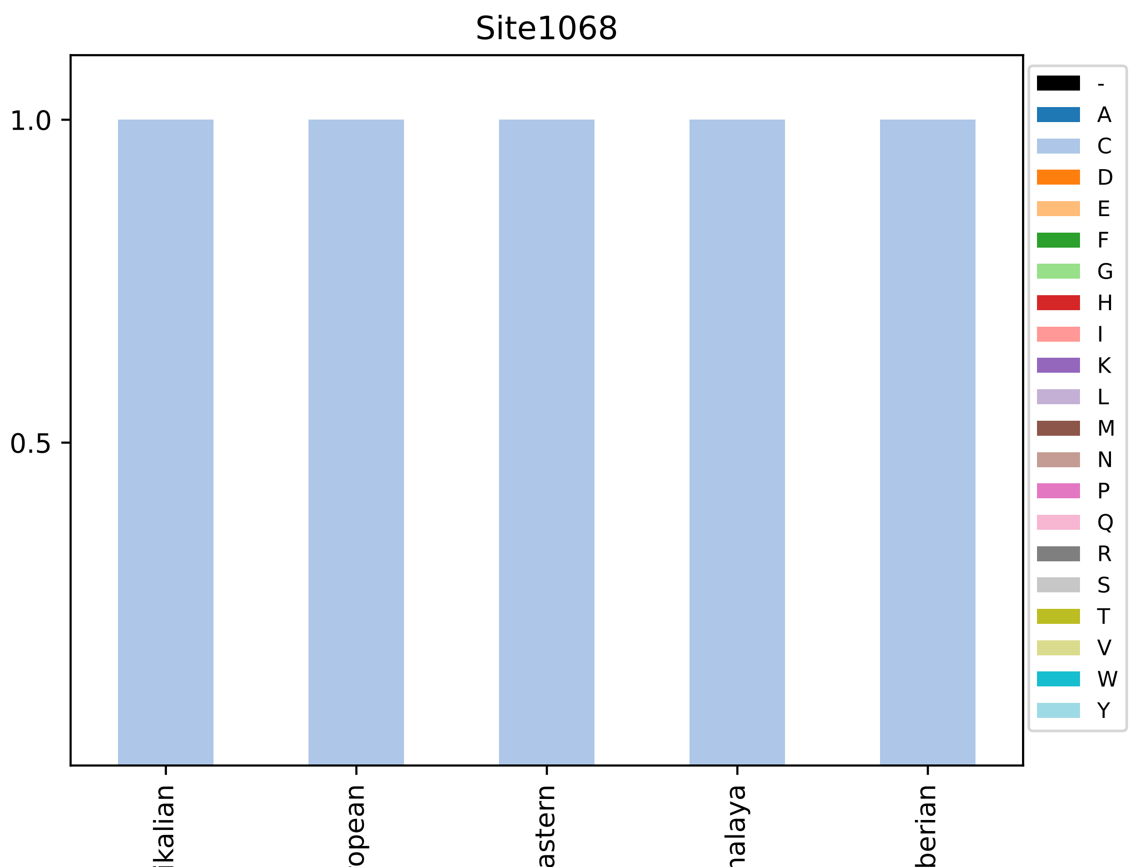
Site1068
1.0
0.5
-
A
C
D
E
F
G
H
I
K
L
M
N
P
Q
R
S
T
V
W
Y
ikalian
opean
astern
nalaya
berian

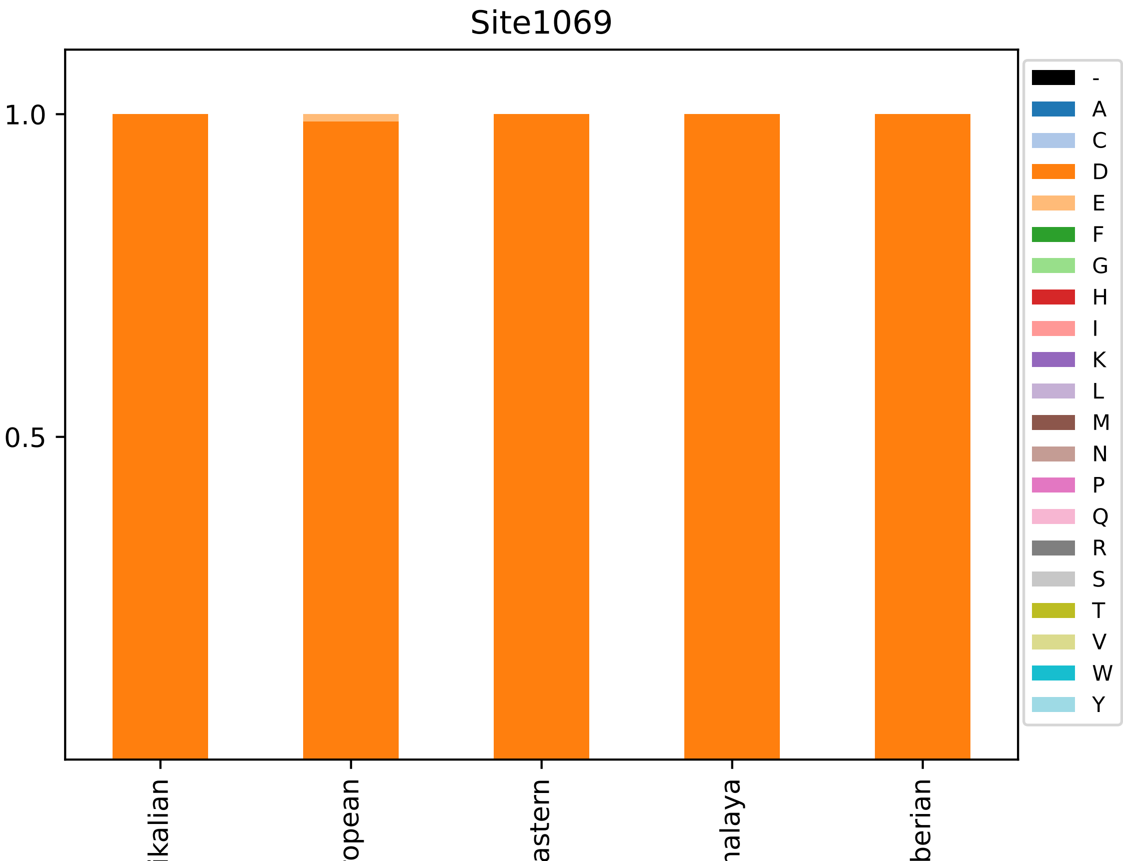
Site1069
1.0
0.5
-
A
C
D
E
F
G
H
I
K
L
M
N
P
Q
R
S
T
V
W
Y

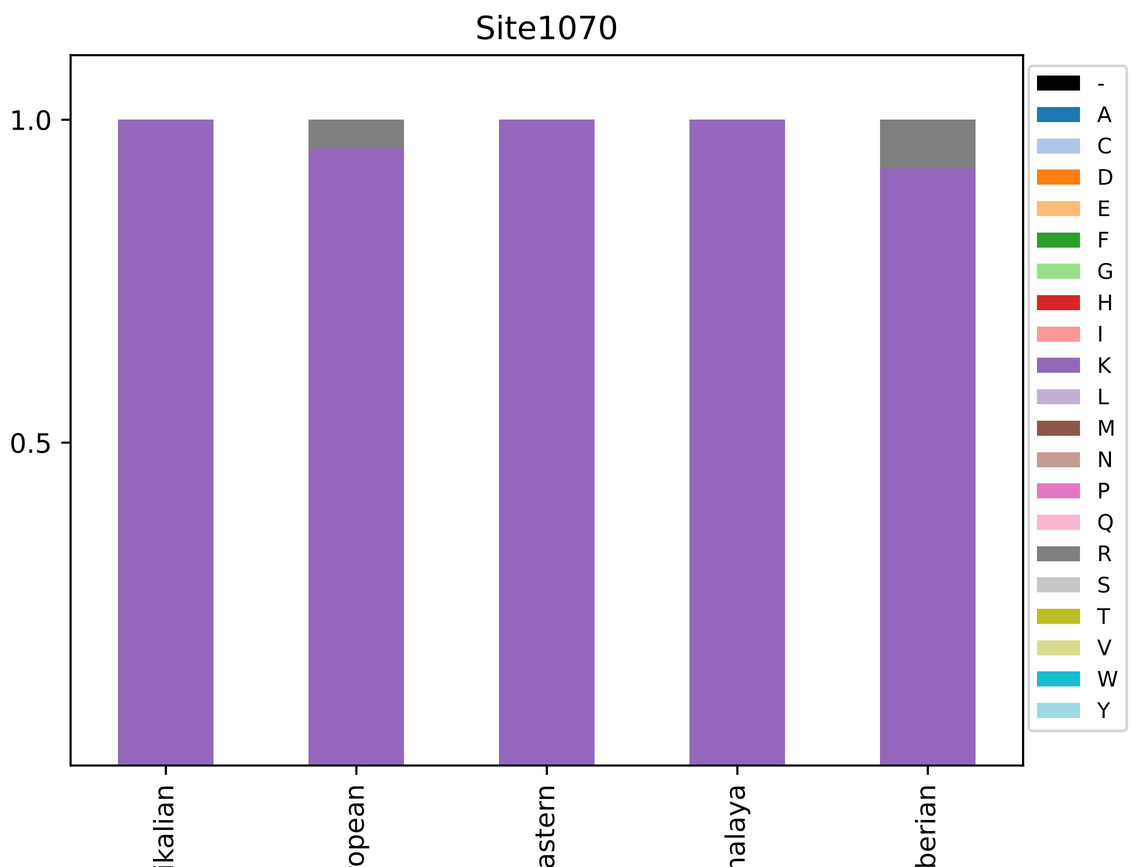
Site1070
1.0
0.5
ikalian
opean
astern
nalaya
berian
-
A
C
D
E
F
G
H
I
K
L
M
N
P
Q
R
S
T
V
W
Y

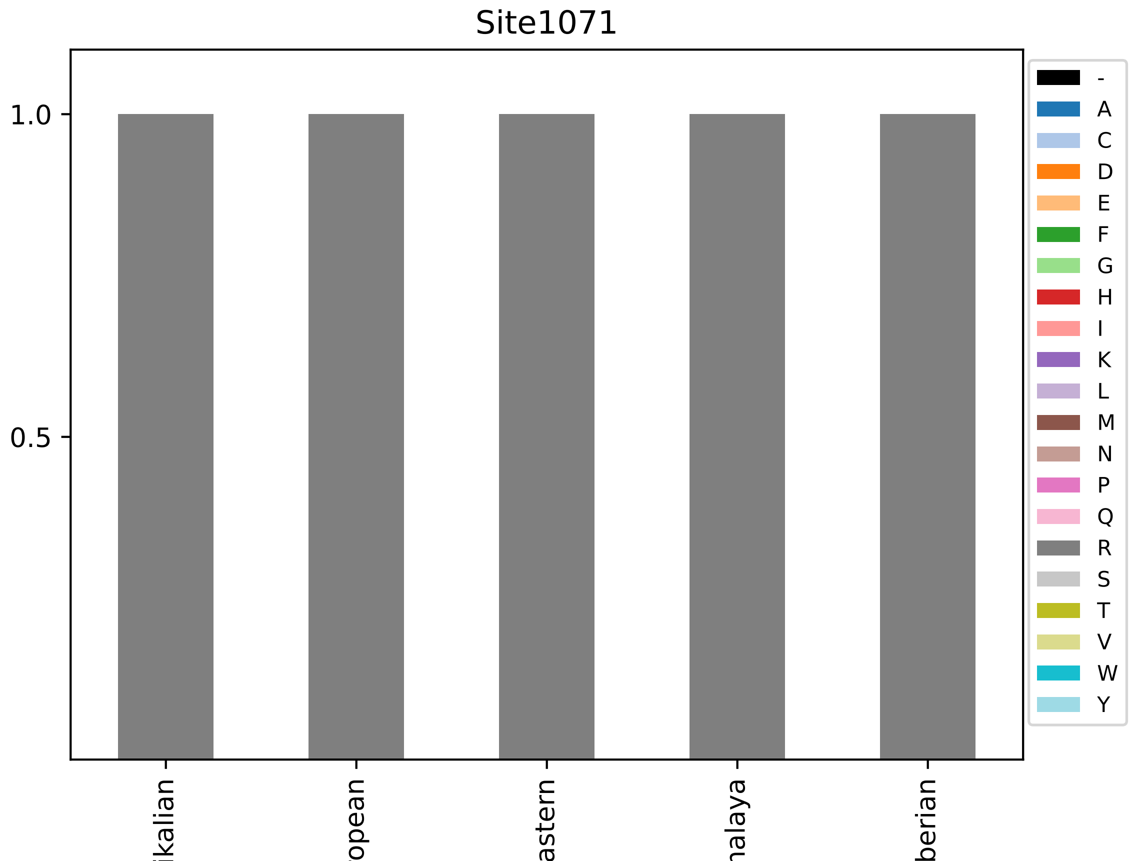
Site1071
1.0
0.5
-
A
C
D
E
F
G
H
I
K
L
M
N
P
Q
R
S
T
V
W
Y
ikalian
ropean
astern
nalaya
berian

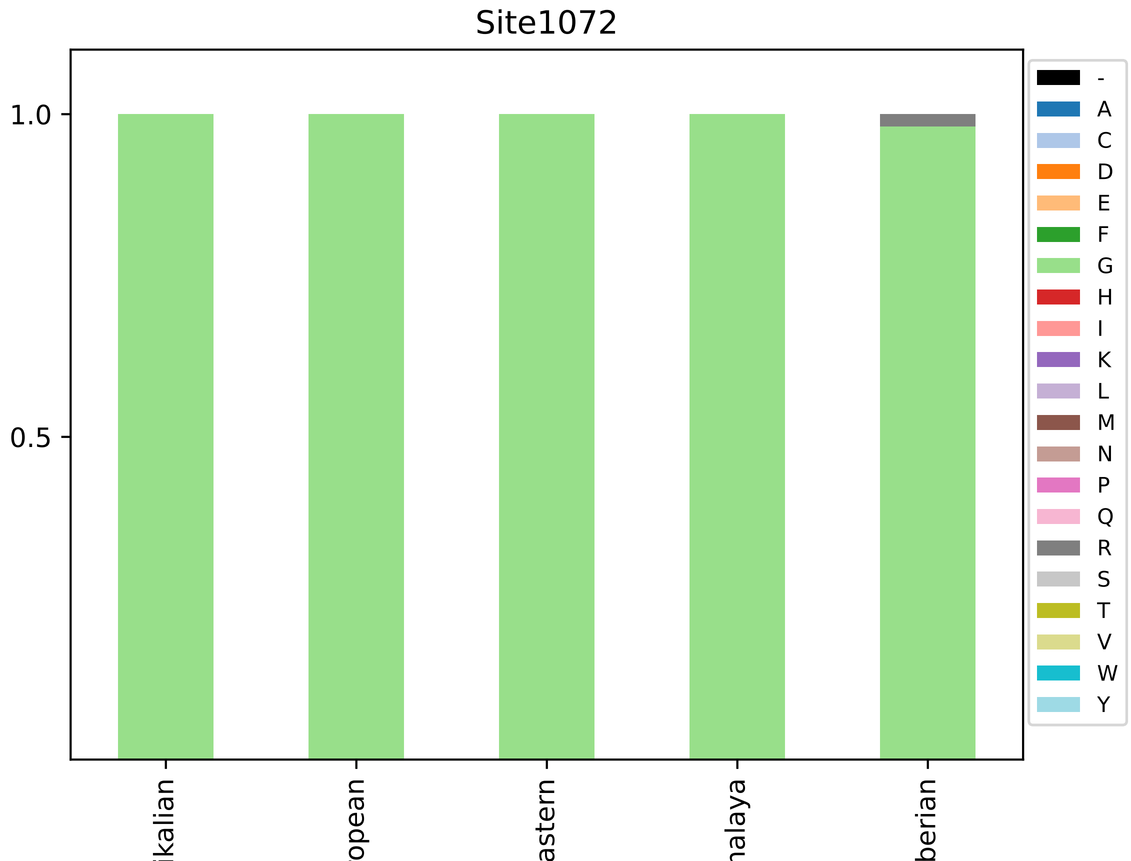
Site1072
1.0
0.5
-
A
C
D
E
F
G
H
I
K
L
M
N
P
Q
R
S
T
V
W
Y
ikalian
opean
astern
nalaya
berian

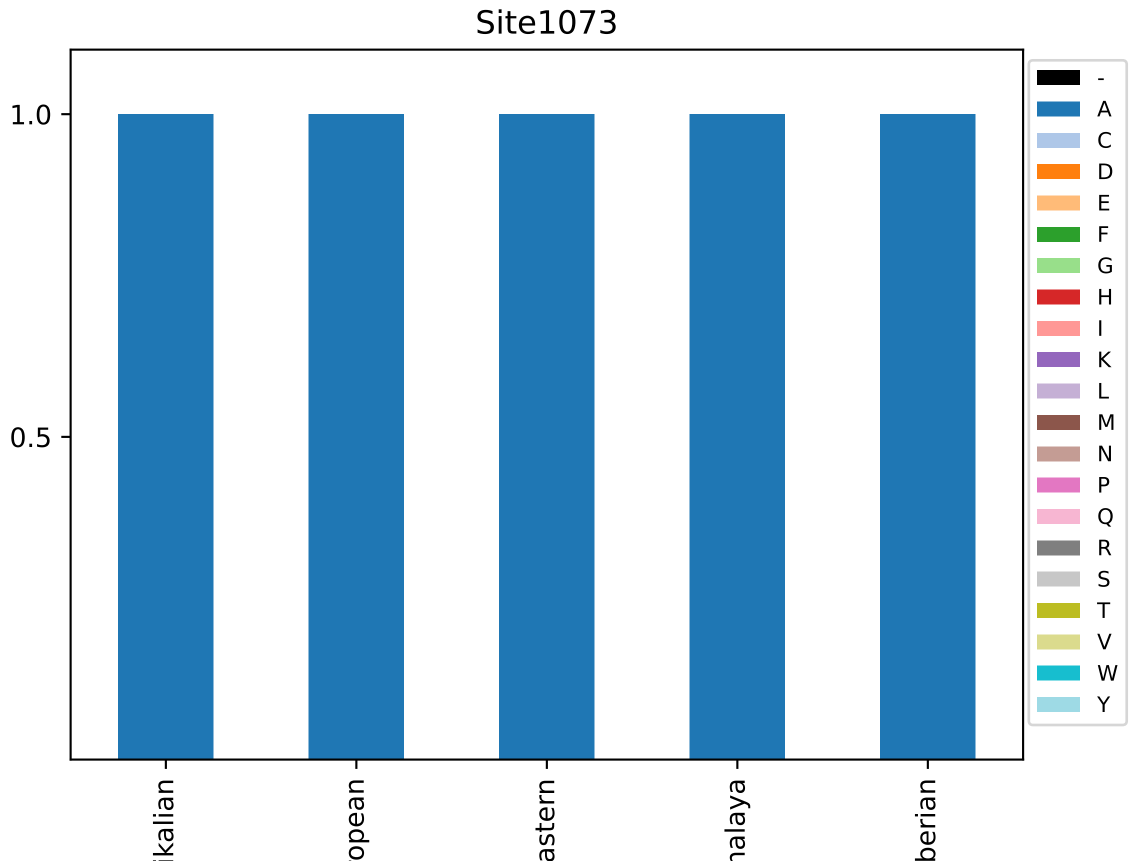
Site1073
1.0
0.5
-
A
C
D
E
F
G
H
I
K
L
M
N
P
Q
R
S
T
V
W
Y
ikalian
opean
astern
nalaya
berian

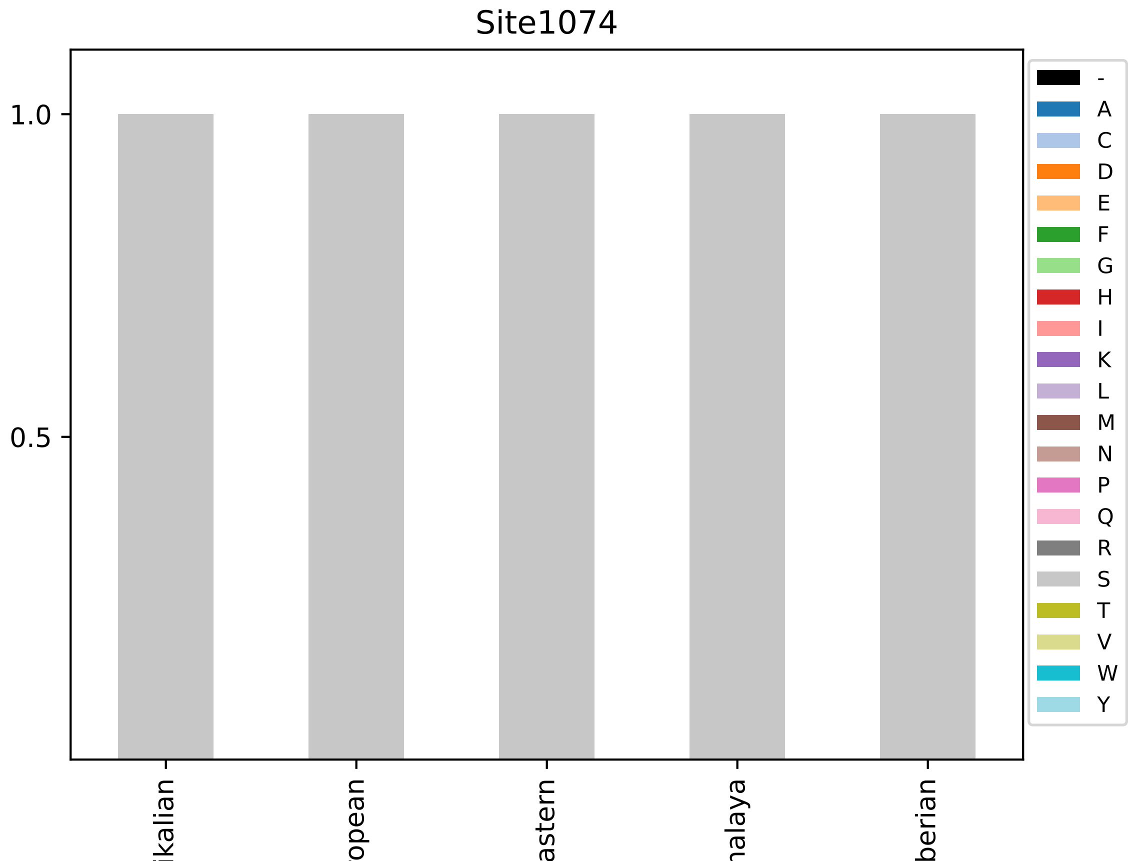
Site1074
1.0
0.5
ikalian
opean
astern
nalaya
berian
-
A
C
D
E
F
G
H
I
K
L
M
N
P
Q
R
S
T
V
W
Y

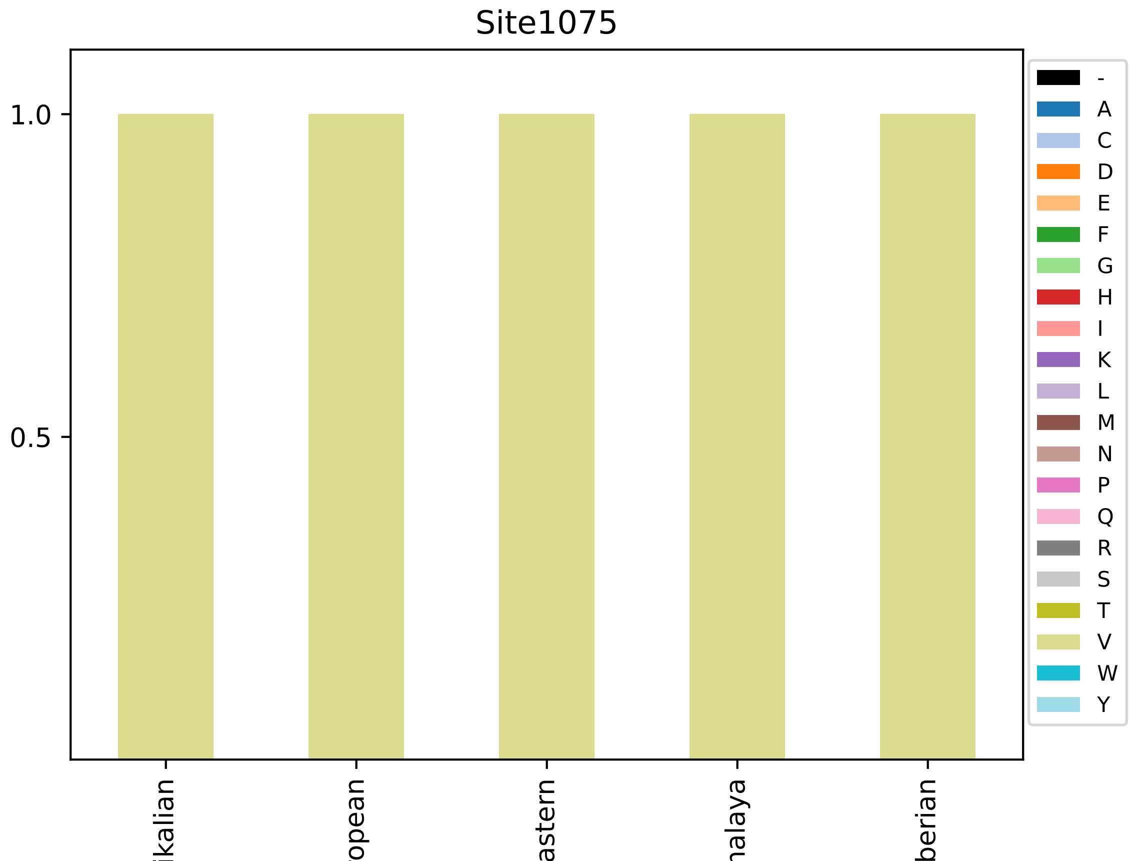

Site1075
1.0
0.5
-
A
C
D
E
F
G
H
I
K
L
M
N
P
Q
R
S
T
V
W
Y
ikalian
opean
astern
nalaya
berian

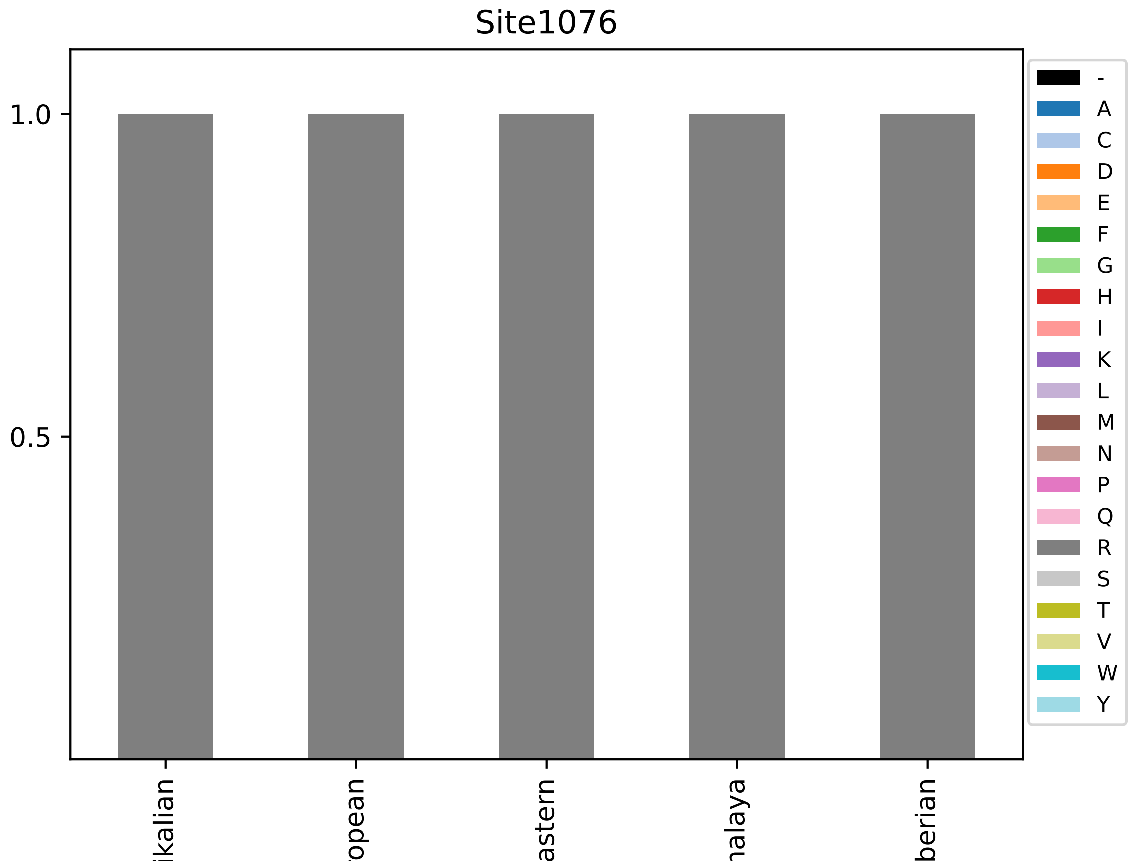
Site1076
1.0
0.5
ikalian
opean
astern
nalaya
berian
-
A
C
D
E
F
G
H
I
K
L
M
N
P
Q
R
S
T
V
W
Y

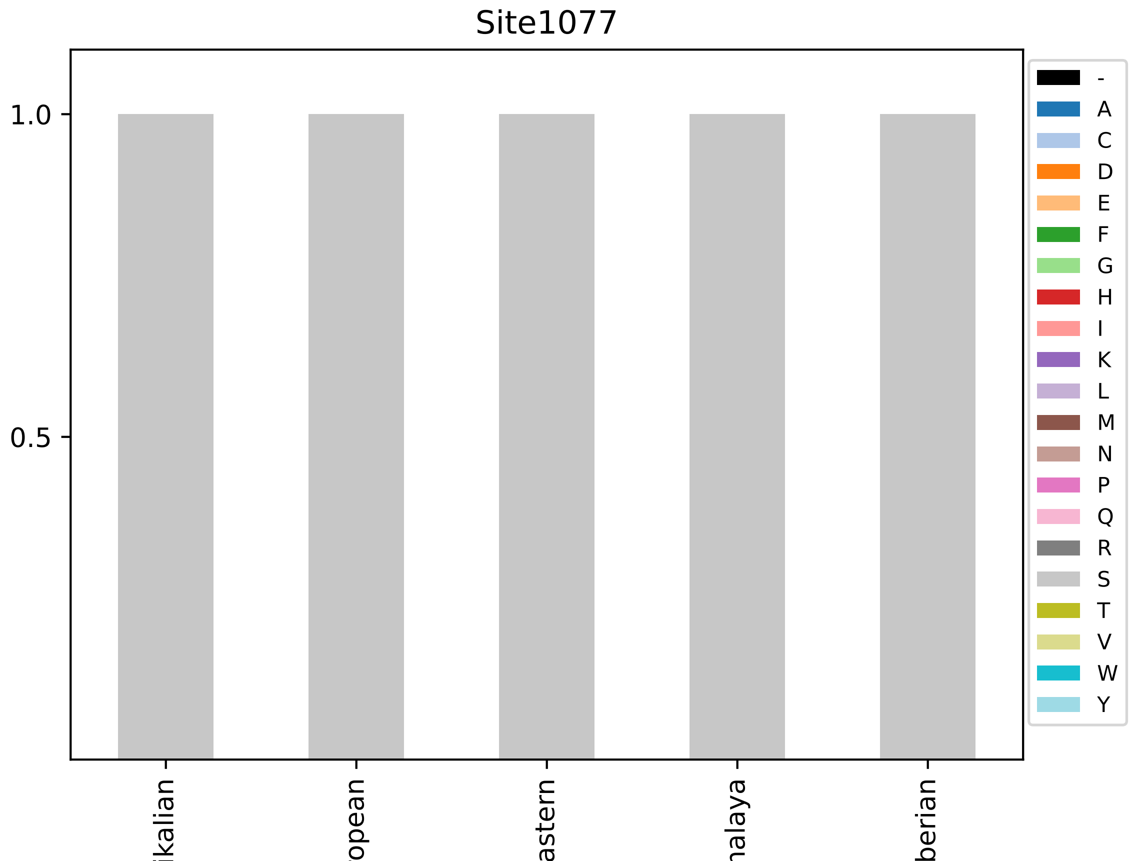

Site1077
1.0
0.5
-
A
C
D
E
F
G
H
I
K
L
M
N
P
Q
R
S
T
V
W
Y

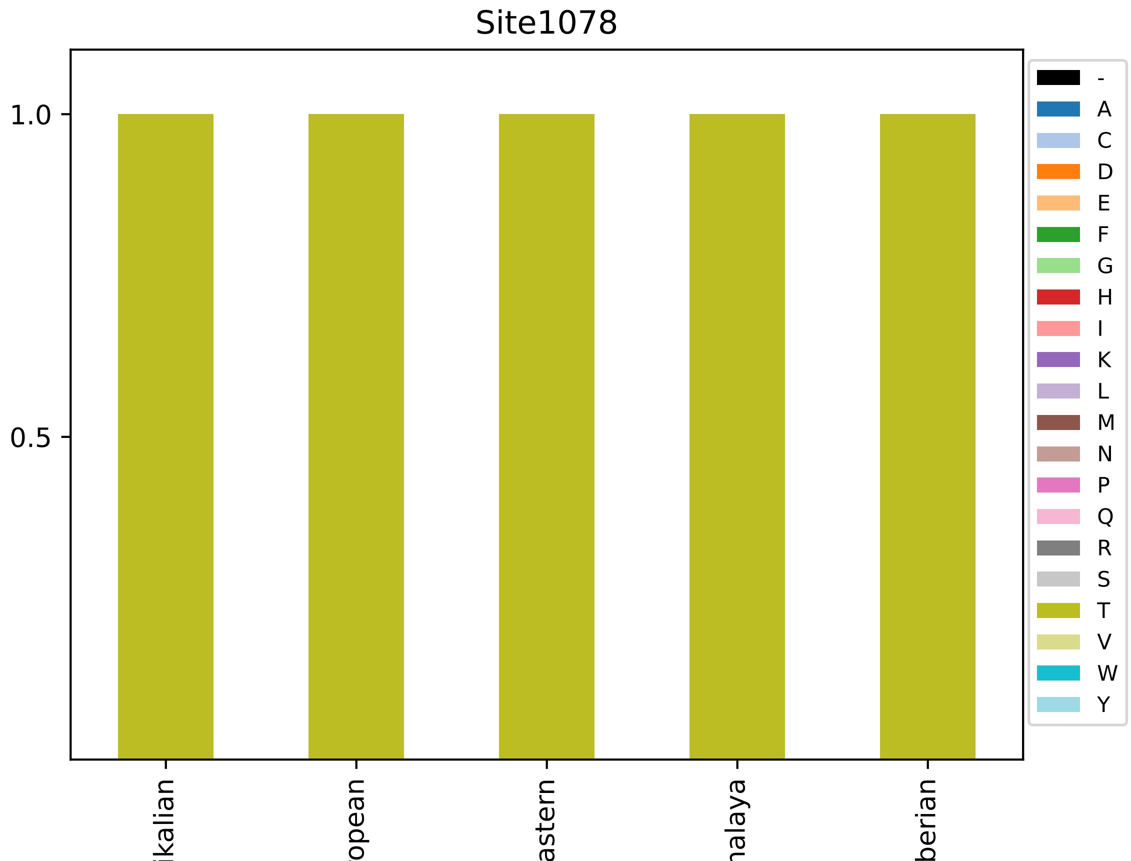
Site1078
1.0
0.5
-
A
C
D
E
F
G
H
I
K
L
M
N
P
Q
R
S
T
V
W
Y
ikalian
opean
astern
nalaya
berian

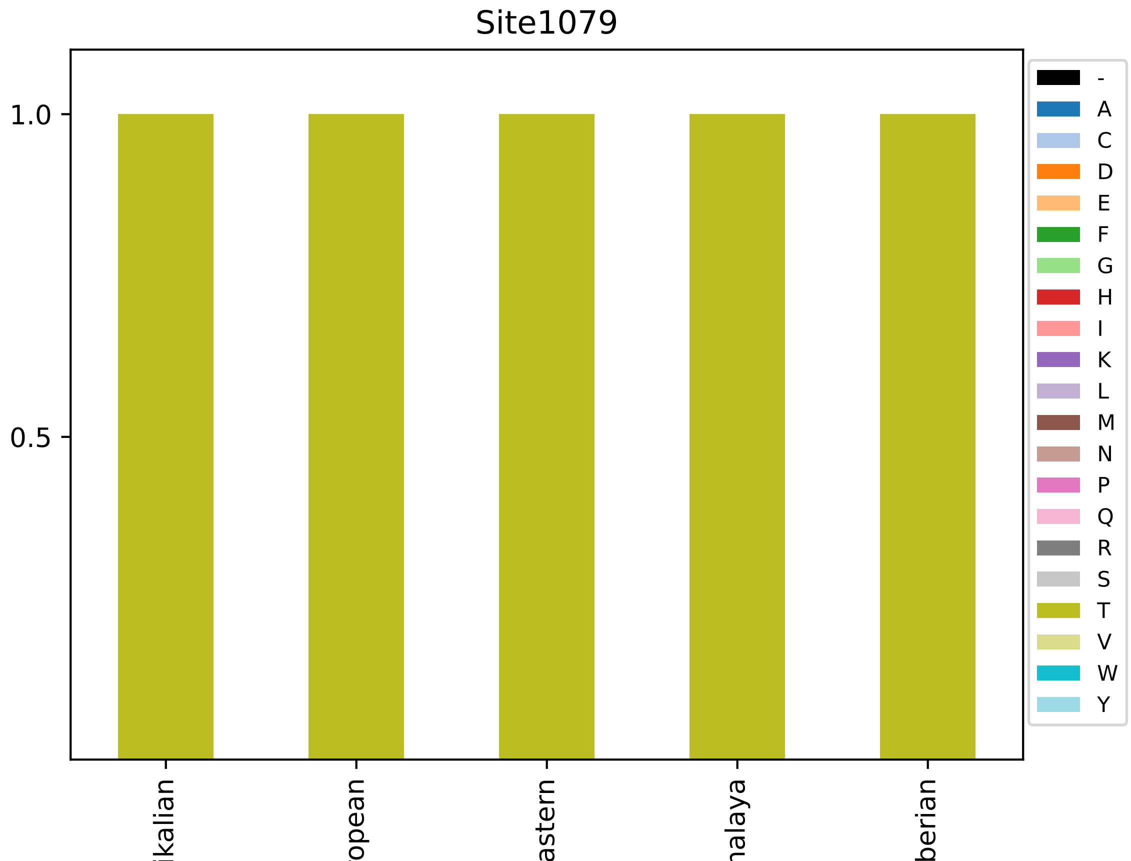
Site1079
1.0
0.5
-
A
C
D
E
F
G
H
I
K
L
M
N
P
Q
R
S
T
V
W
Y
ikalian
opean
astern
nalaya
berian

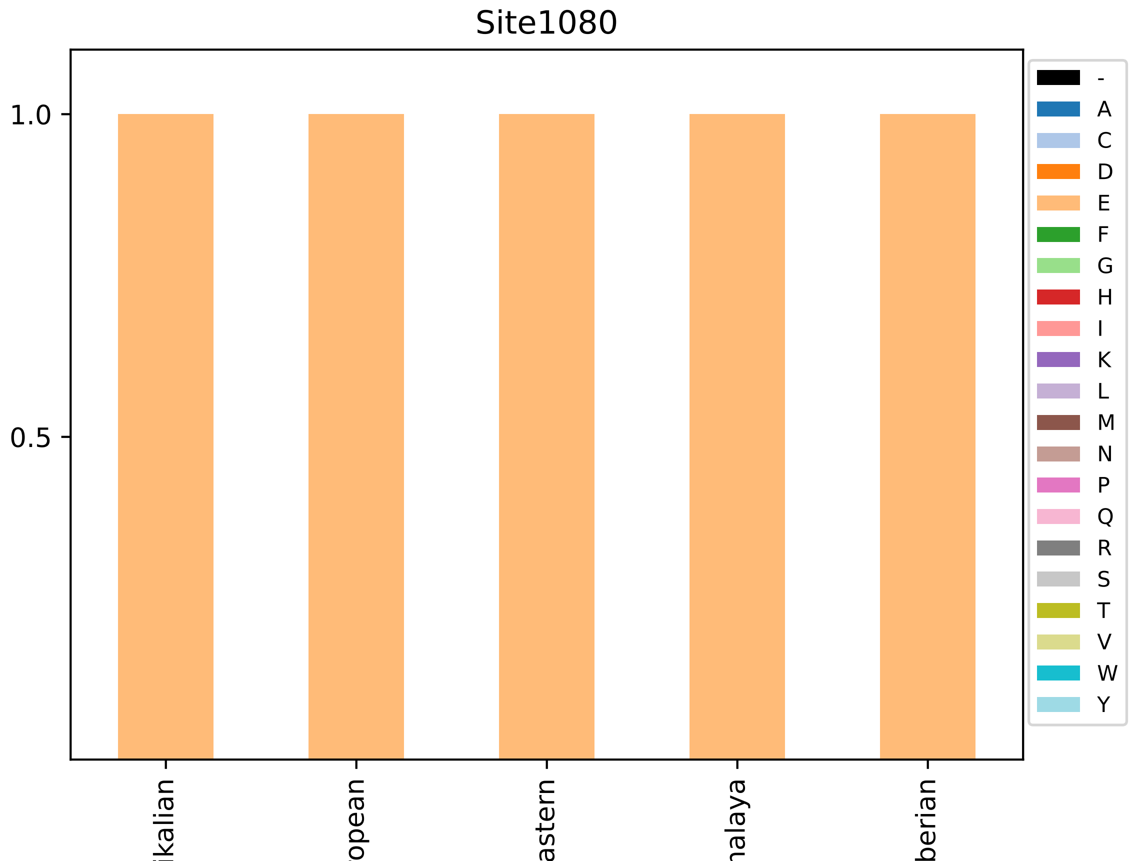

Site1080
1.0
0.5
ikalian
opean
astern
nalaya
berian
-
A
C
D
E
F
G
H
I
K
L
M
N
P
Q
R
S
T
V
W
Y

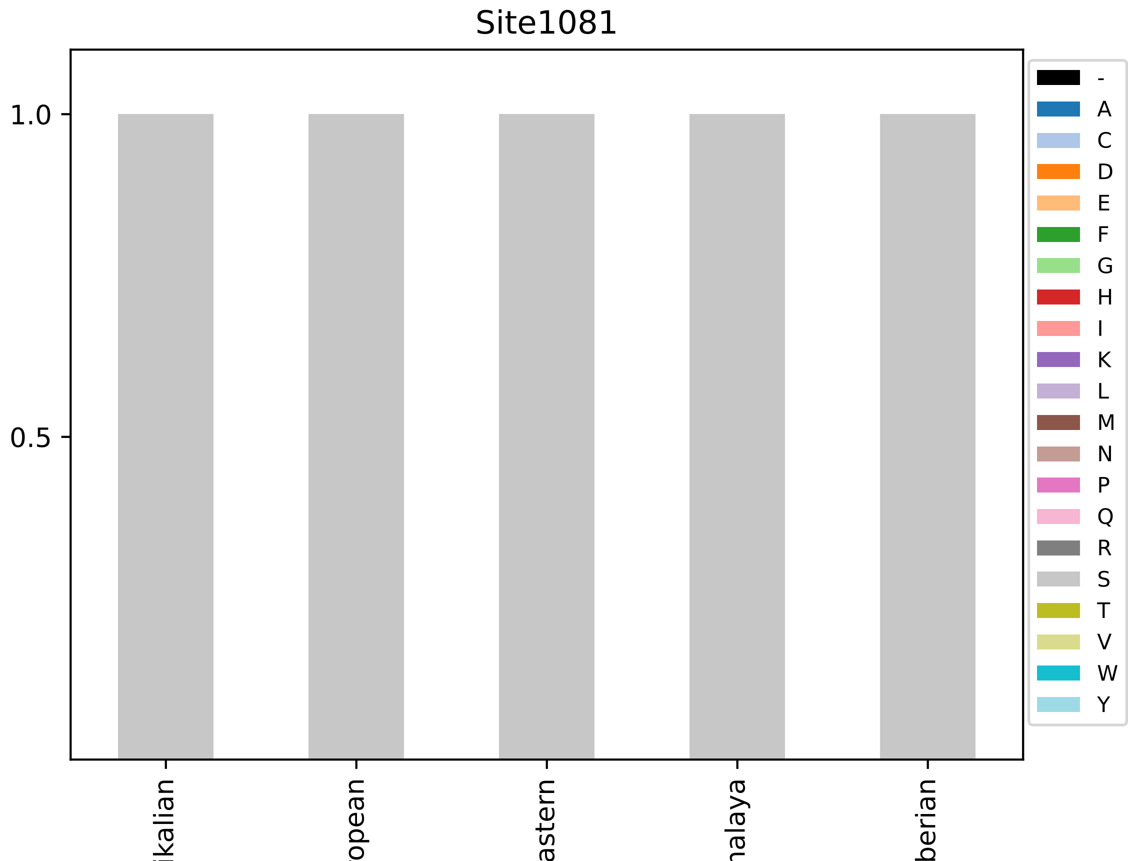

Site1081
1.0
0.5
-
A
C
D
E
F
G
H
I
K
L
M
N
P
Q
R
S
T
V
W
Y
ikalian
opean
astern
nalaya
berian

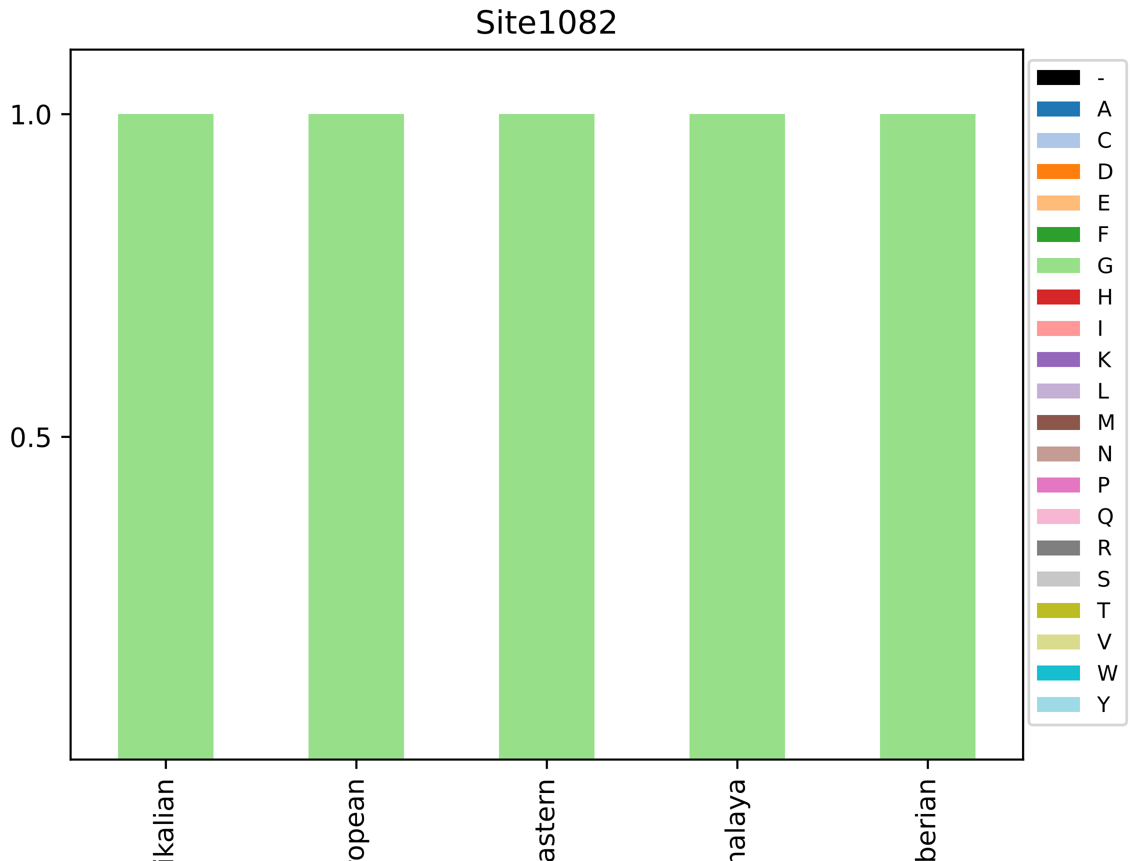

Site1082
1.0
0.5
-
A
C
D
E
F
G
H
I
K
L
M
N
P
Q
R
S
T
V
W
Y
ikalian
opean
astern
nalaya
berian

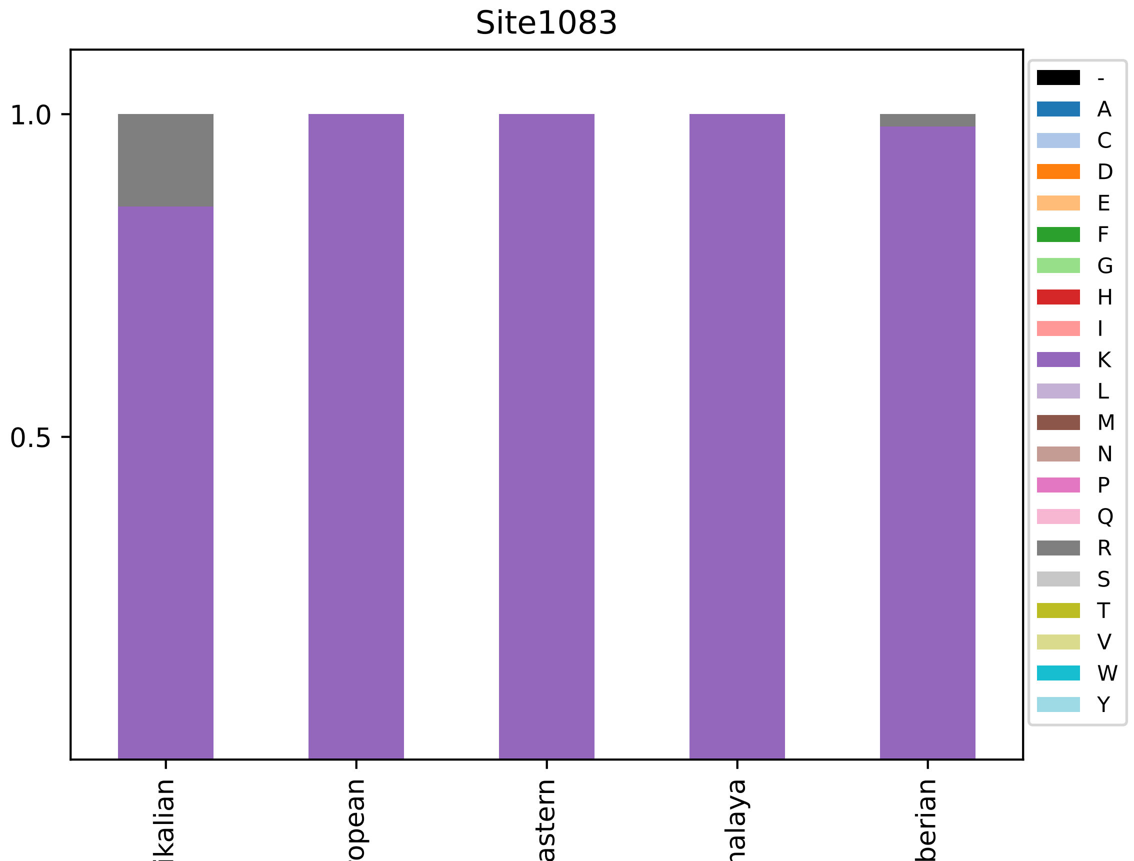

Site1083
1.0
0.5
-
A
C
D
E
F
G
H
I
K
L
M
N
P
Q
R
S
T
V
W
Y
ikalian
opean
astern
nalaya
berian

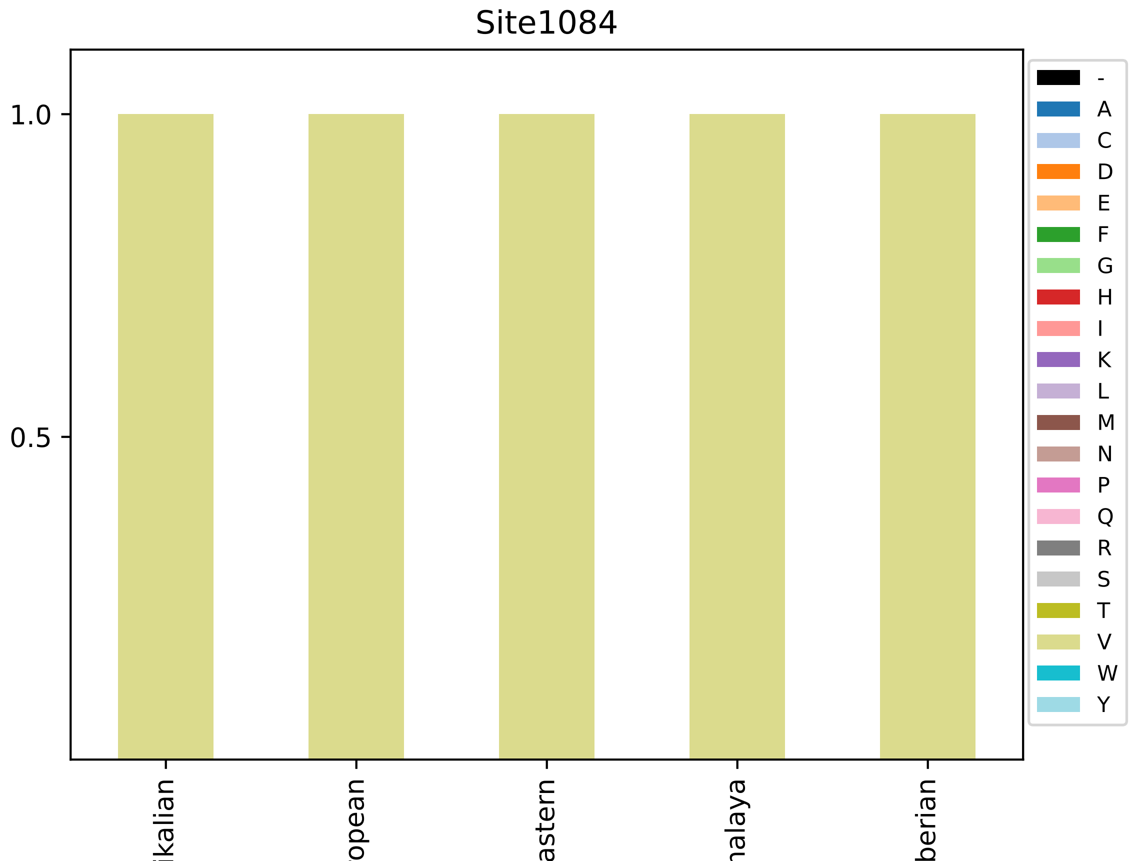
Site1084
1.0
0.5
-
A
C
D
E
F
G
H
I
K
L
M
N
P
Q
R
S
T
V
W
Y
ikalian
opean
astern
nalaya
berian

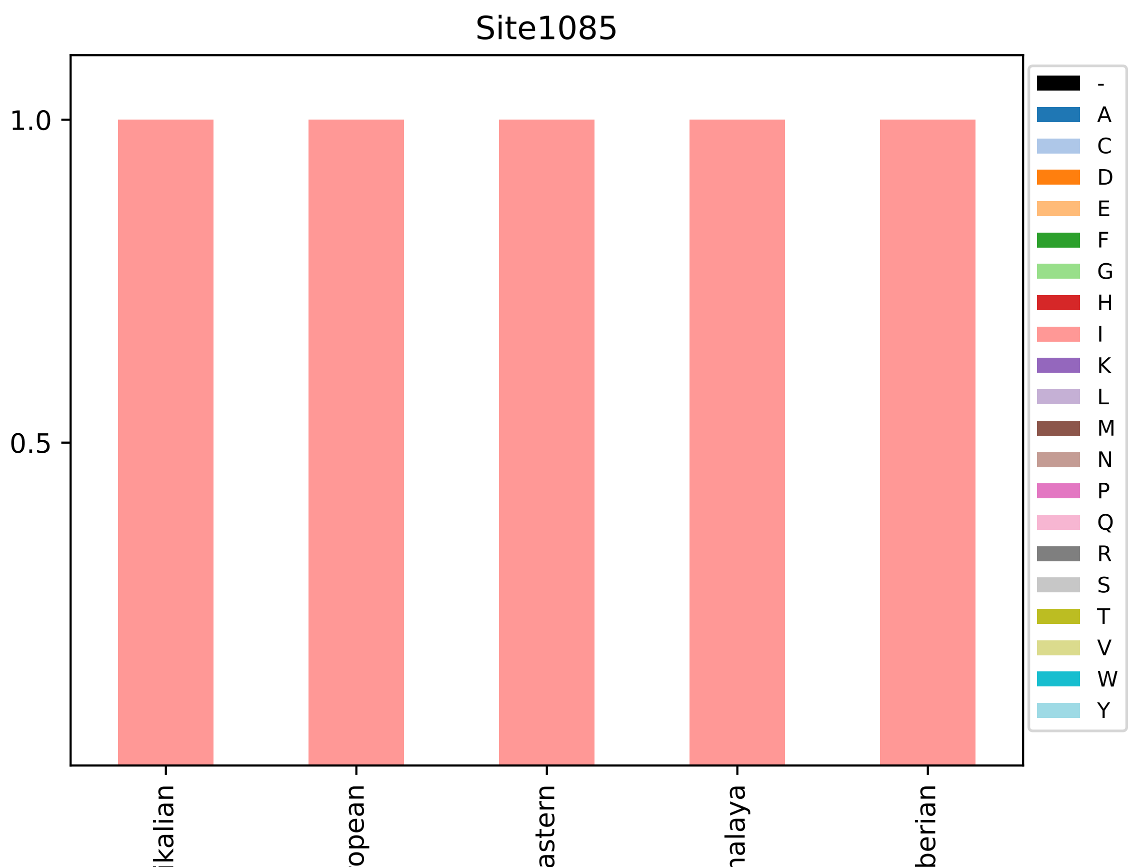

Site1085
1.0
0.5
-
A
C
D
E
F
G
H
I
K
L
M
N
P
Q
R
S
T
V
W
Y
ikalian
opean
astern
nalaya
berian

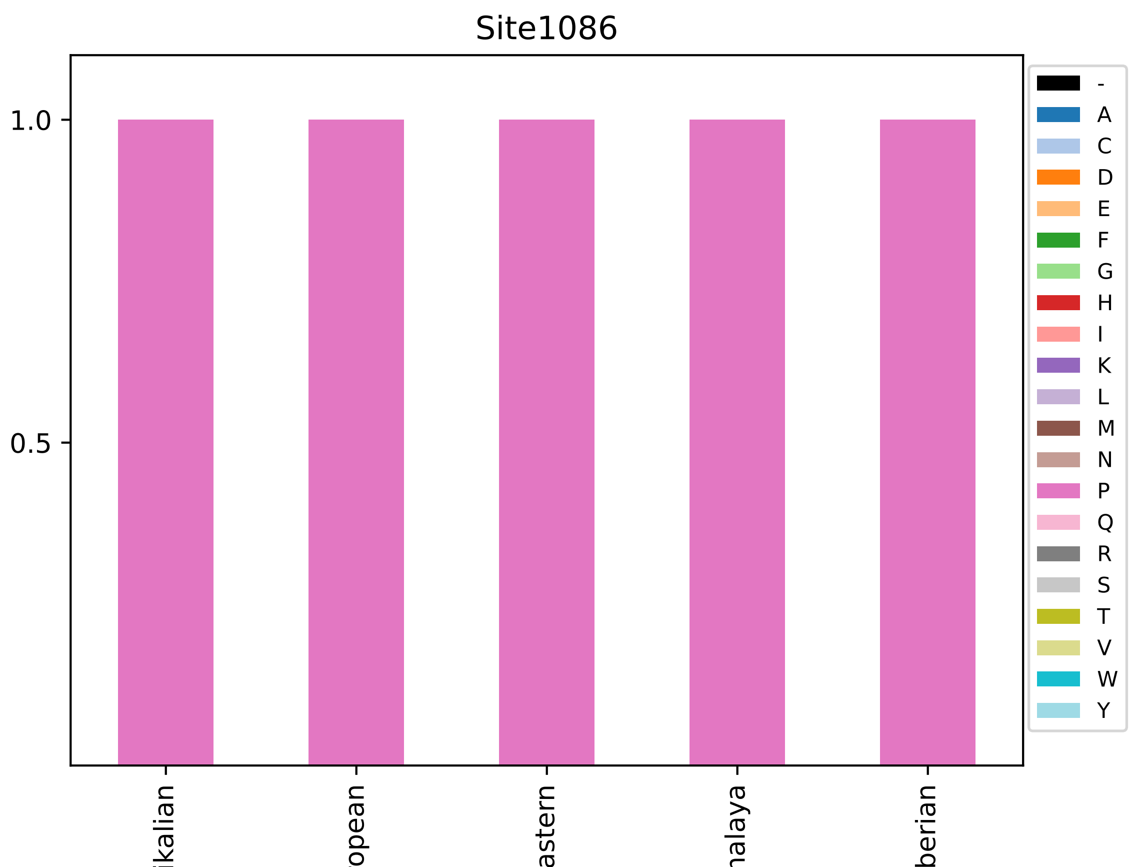

Site1086
1.0
0.5
ikalian
opean
astern
nalaya
berian
-
A
C
D
E
F
G
H
I
K
L
M
N
P
Q
R
S
T
V
W
Y

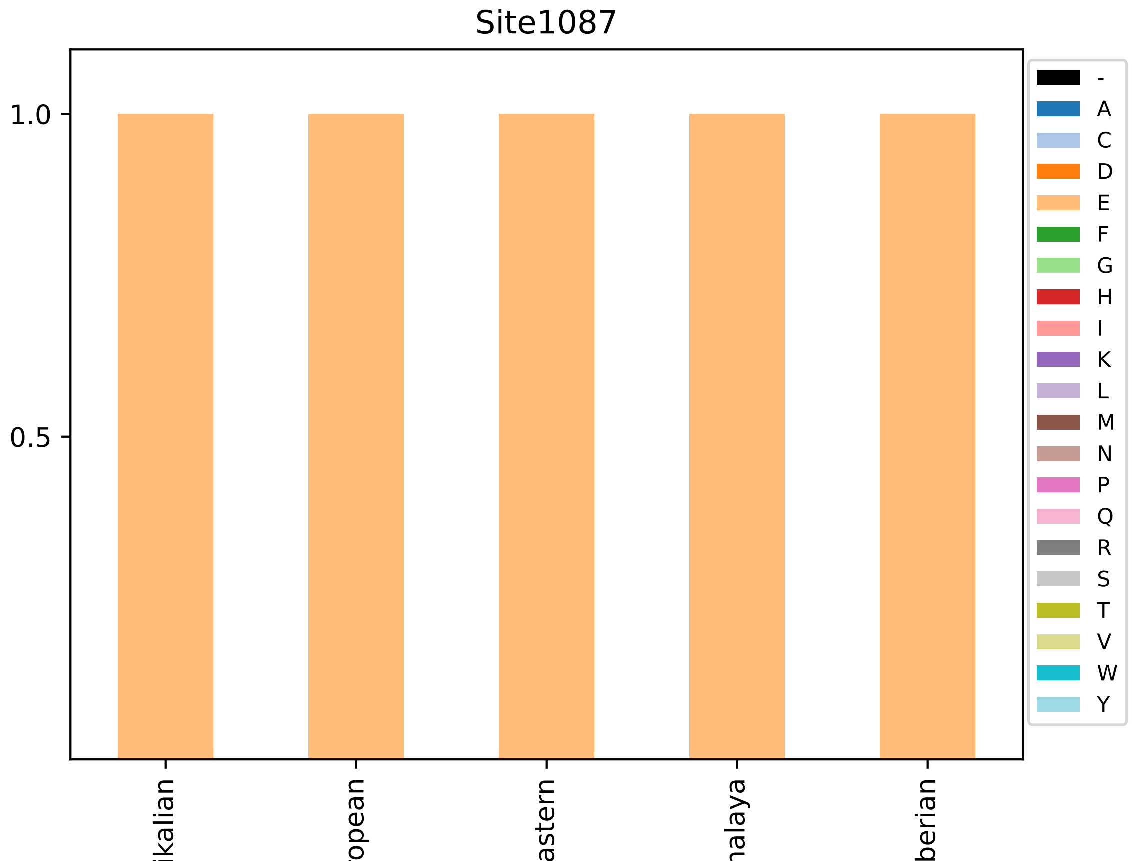

Site1087
1.0
0.5
-
A
C
D
E
F
G
H
I
K
L
M
N
P
Q
R
S
T
V
W
Y
ikalian
opean
astern
nalaya
berian

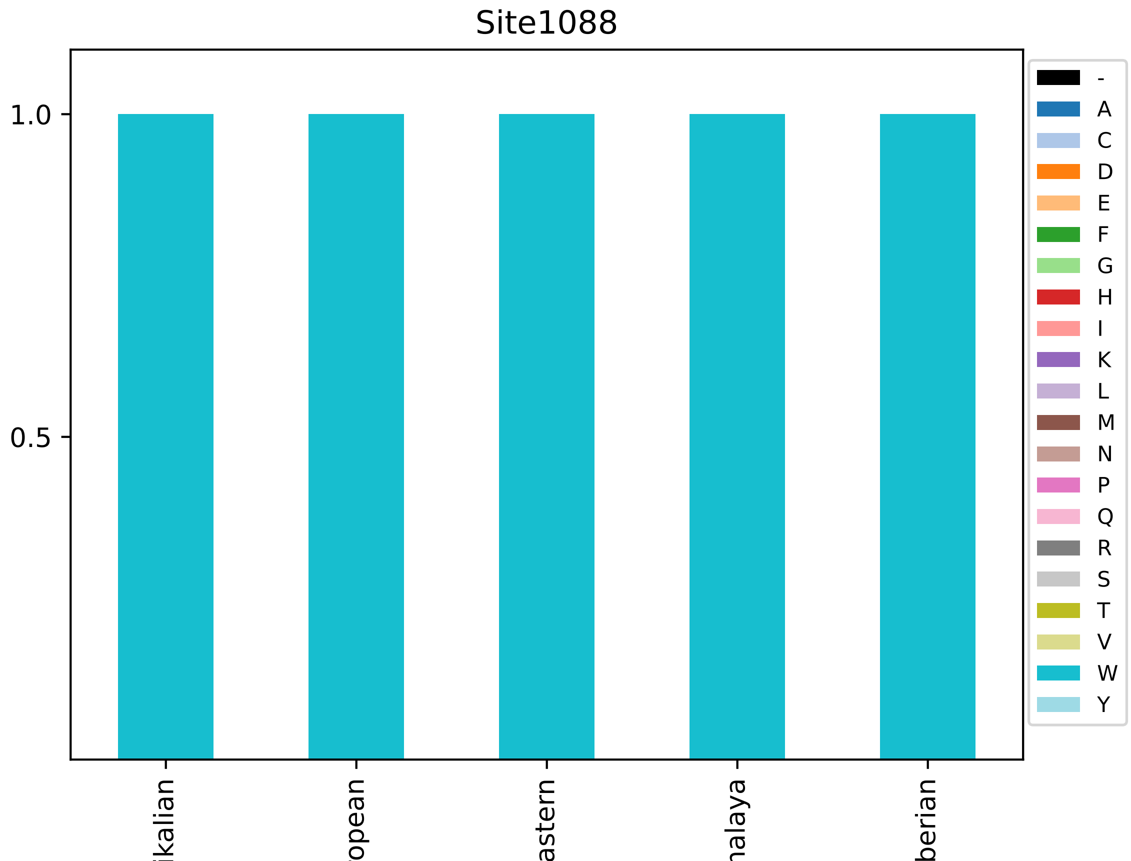
Site1088
1.0
0.5
-
A
C
D
E
F
G
H
I
K
L
M
N
P
Q
R
S
T
V
W
Y
ikalian
opean
astern
nalaya
berian

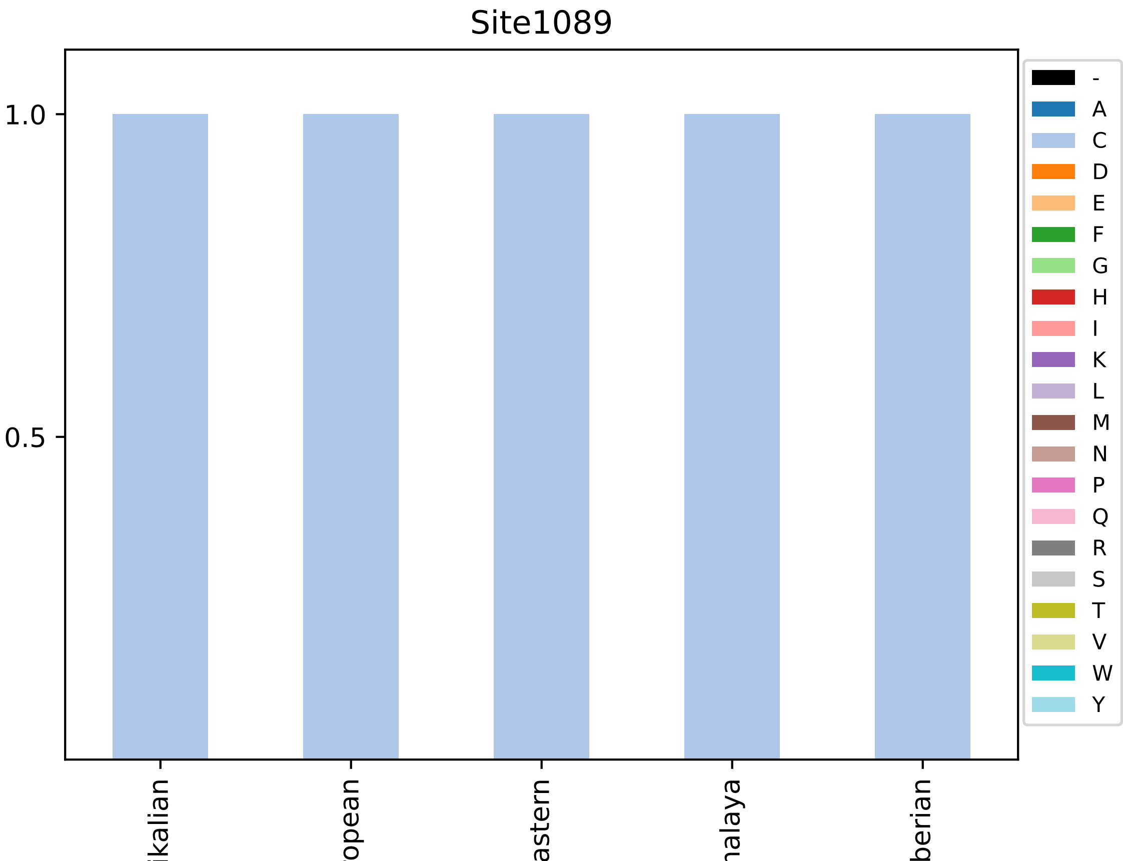
Site1089
1.0
0.5
ikalian
opean
astern
nalaya
berian
-
A
C
D
E
F
G
H
I
K
L
M
N
P
Q
R
S
T
V
W
Y

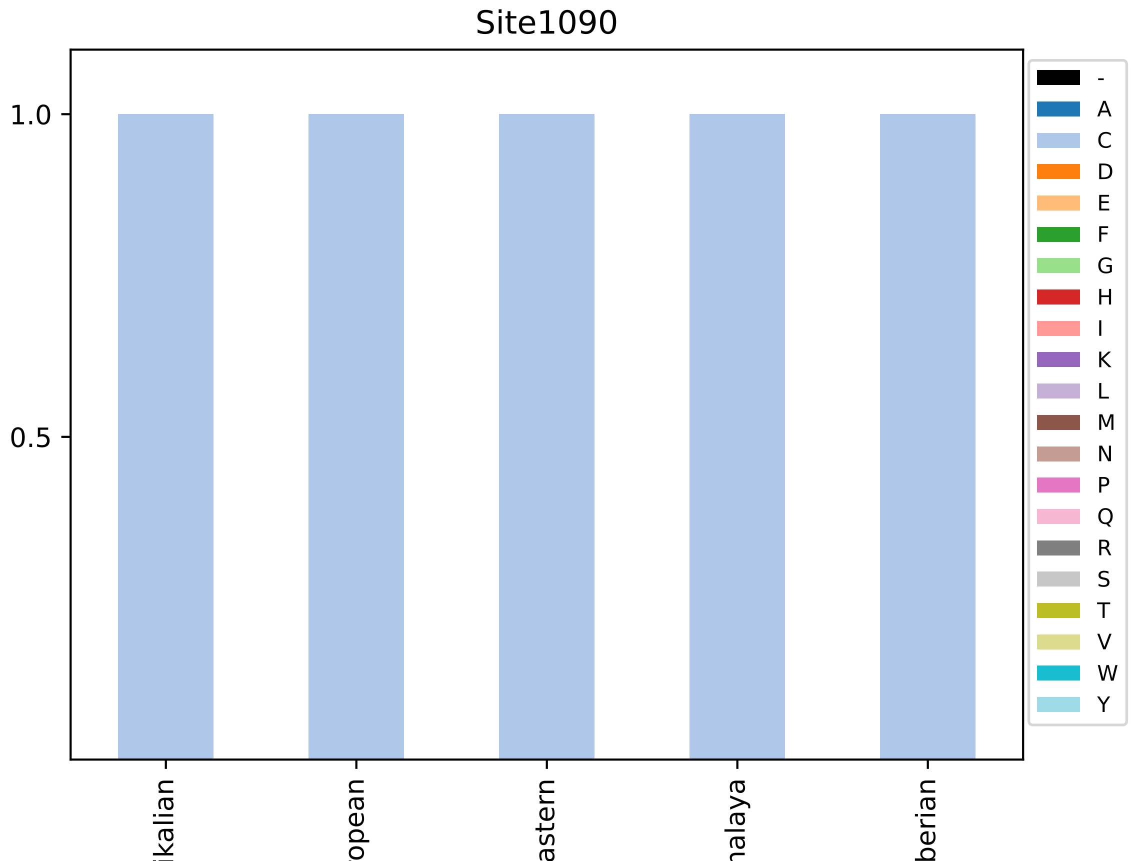
Site1090
1.0
0.5
-
A
C
D
E
F
G
H
I
K
L
M
N
P
Q
R
S
T
V
W
Y
ikalian
opean
astern
halaya
berian

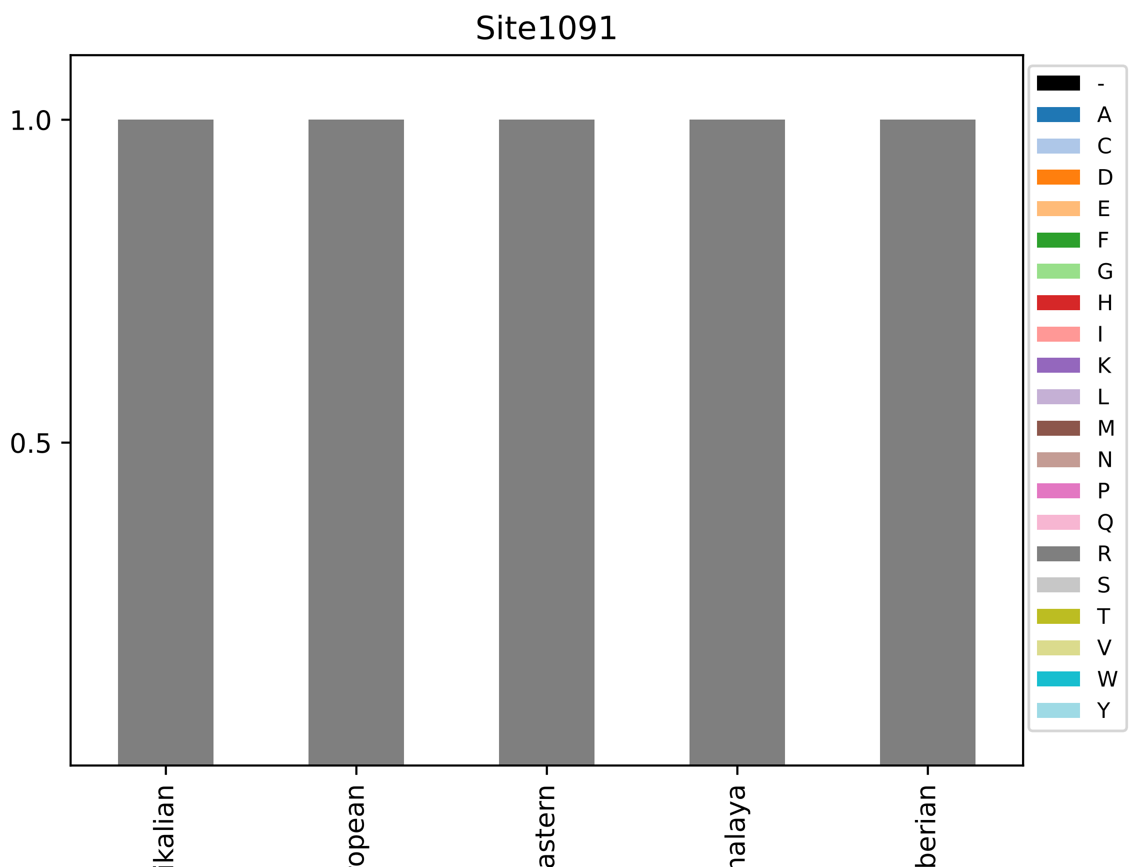
Site1091
1.0
0.5
-
A
C
D
E
F
G
H
I
K
L
M
N
P
Q
R
S
T
V
W
Y

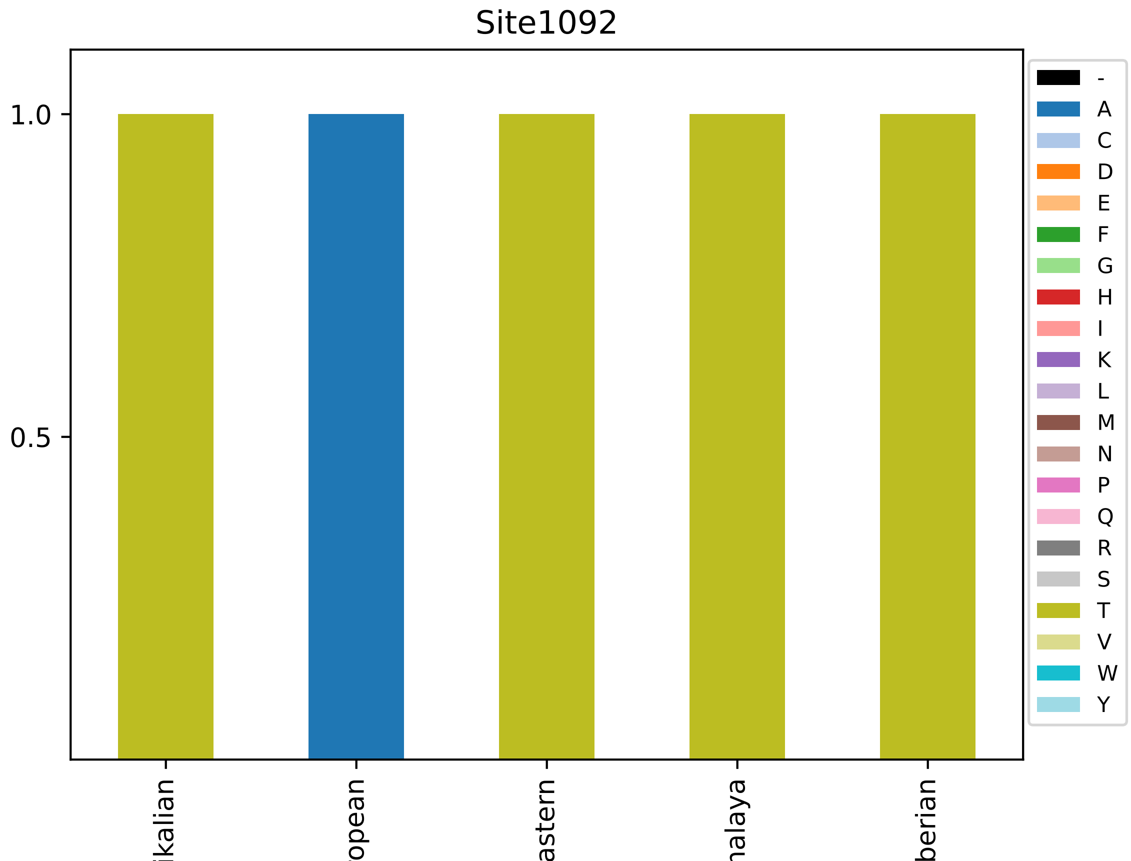

Site1092
1.0
0.5
ikalian
ropean
astern
nalaya
berian
-
A
C
D
E
F
G
H
I
K
L
M
N
P
Q
R
S
T
V
W
Y

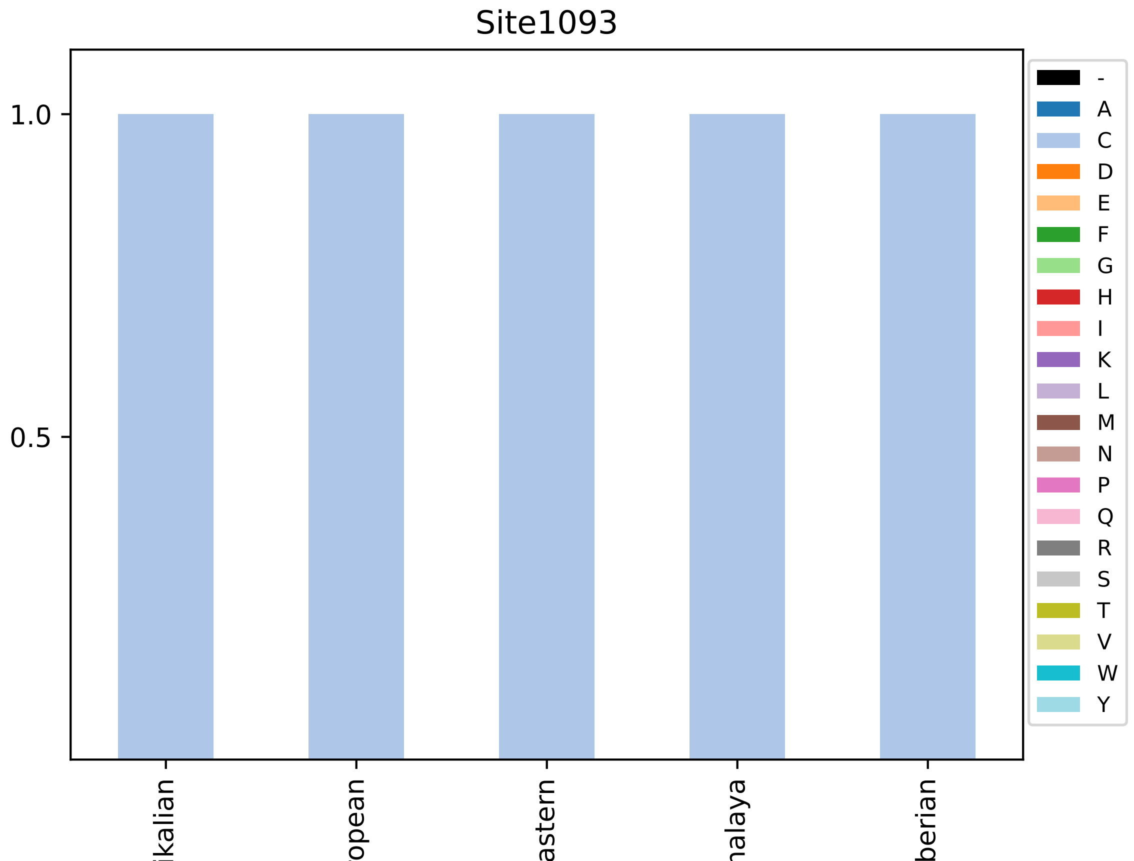
Site1093
1.0
0.5
ikalian
opean
astern
nalaya
berian
-
A
C
D
E
F
G
H
I
K
L
M
N
P
Q
R
S
T
V
W
Y

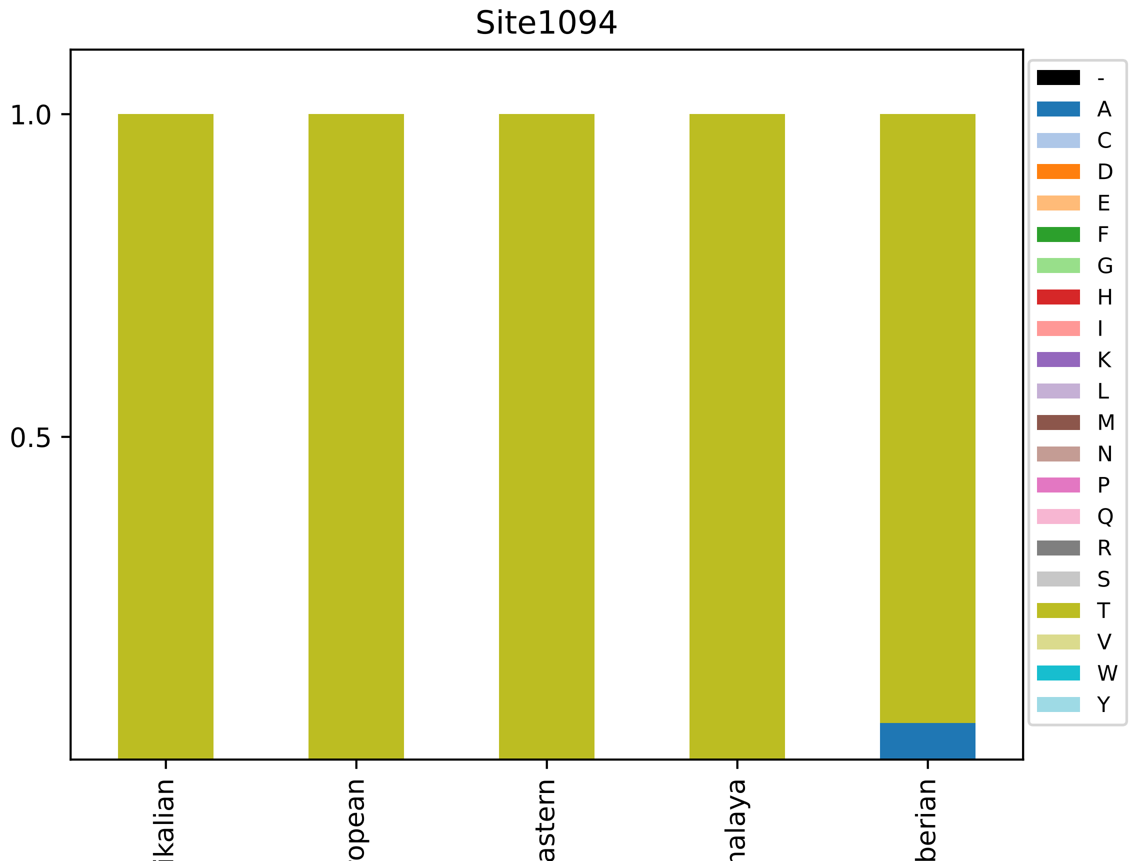
Site1094
1.0
0.5
ikalian
opean
astern
nalaya
berian
-
A
C
D
E
F
G
H
I
K
L
M
N
P
Q
R
S
T
V
W
Y

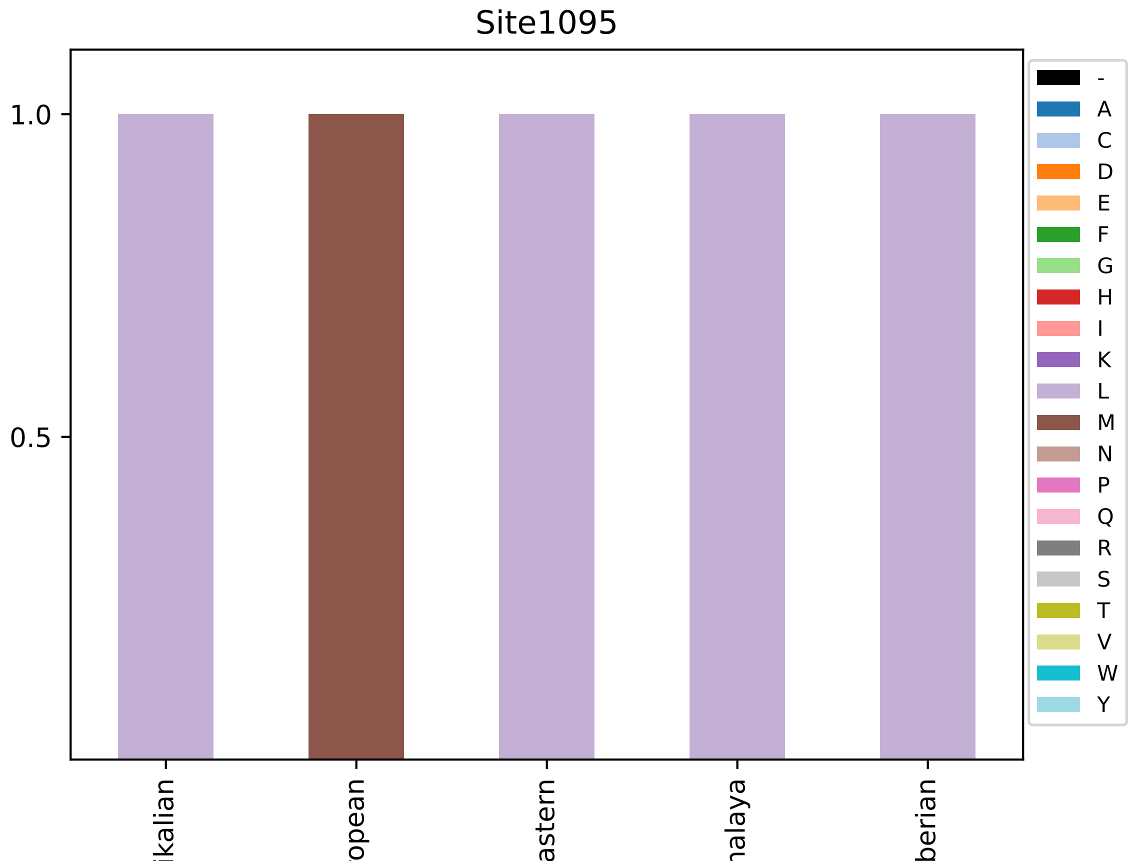
Site1095
1.0
0.5
ikalian
opean
astern
nalaya
berian
-
A
C
D
E
F
G
H
I
K
L
M
N
P
Q
R
S
T
V
W
Y

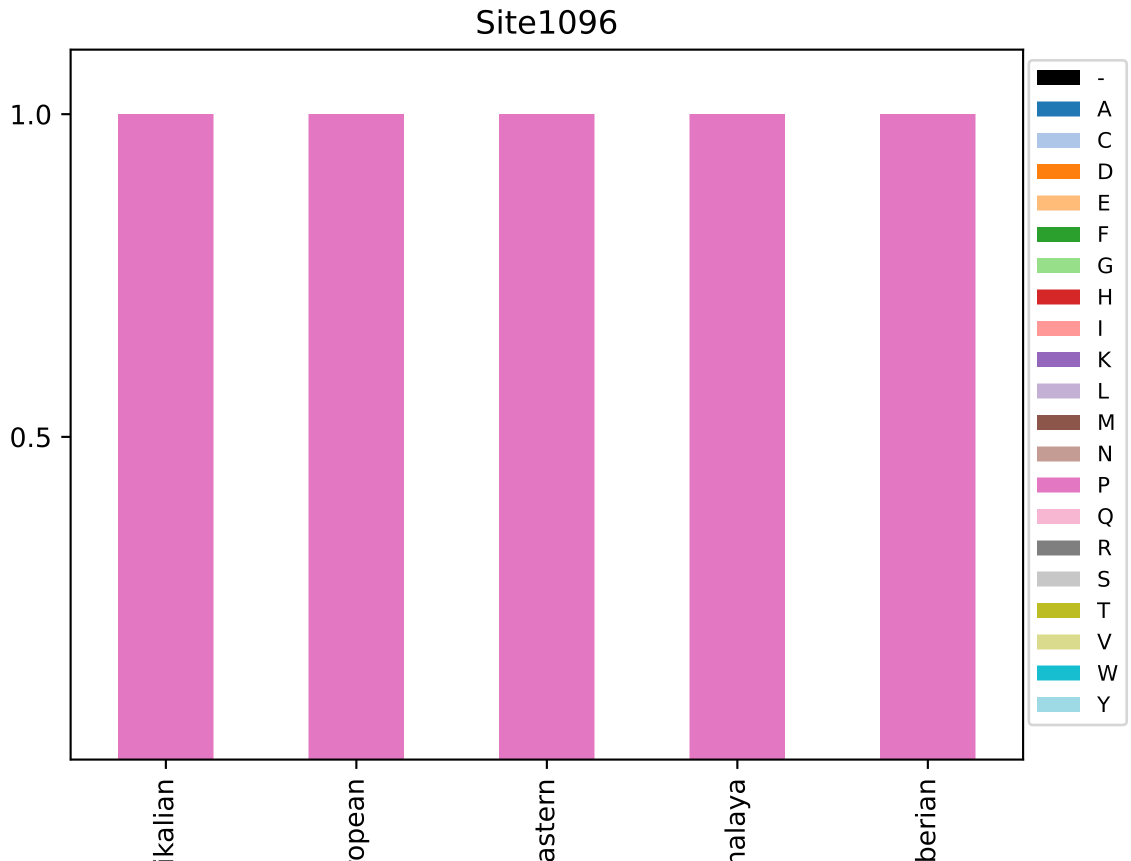
Site1096
1.0
0.5
-
A
C
D
E
F
G
H
I
K
L
M
N
P
Q
R
S
T
V
W
Y
ikalian
opean
astern
nalaya
berian

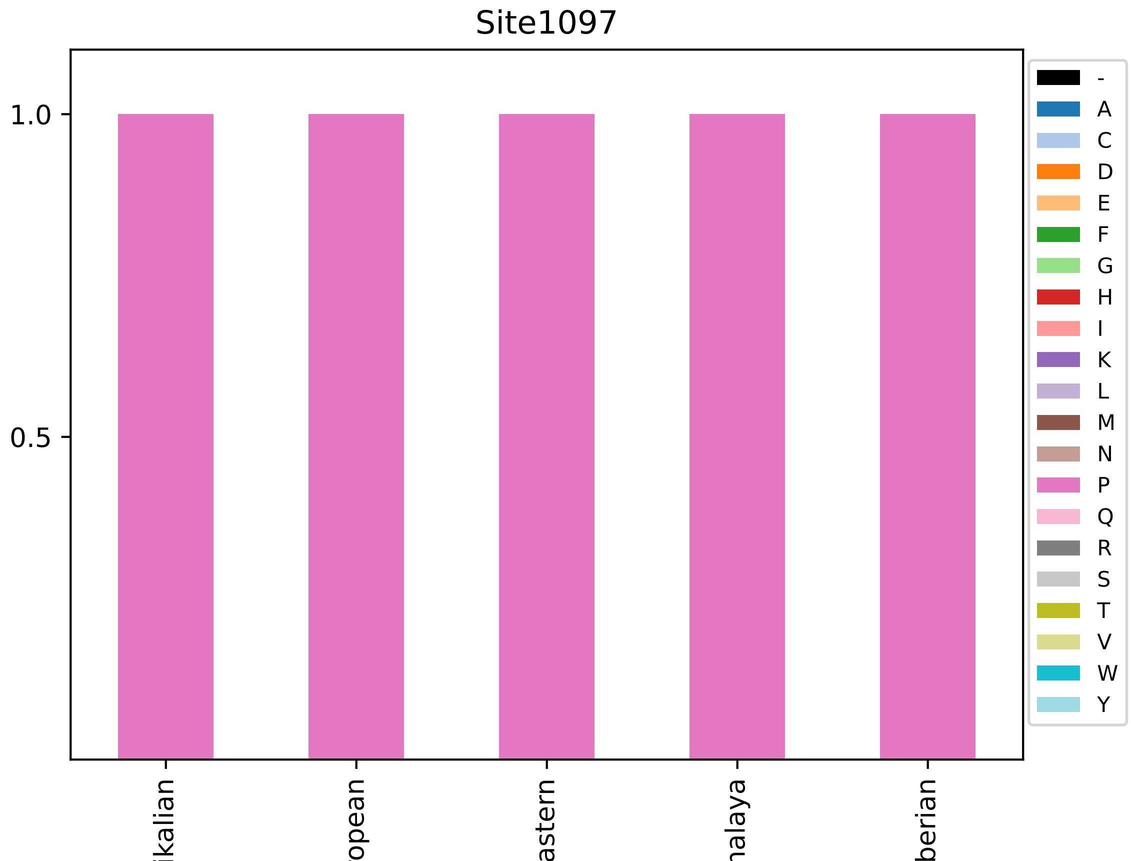

Site1097
1.0
0.5
-
A
C
D
E
F
G
H
I
K
L
M
N
P
Q
R
S
T
V
W
Y

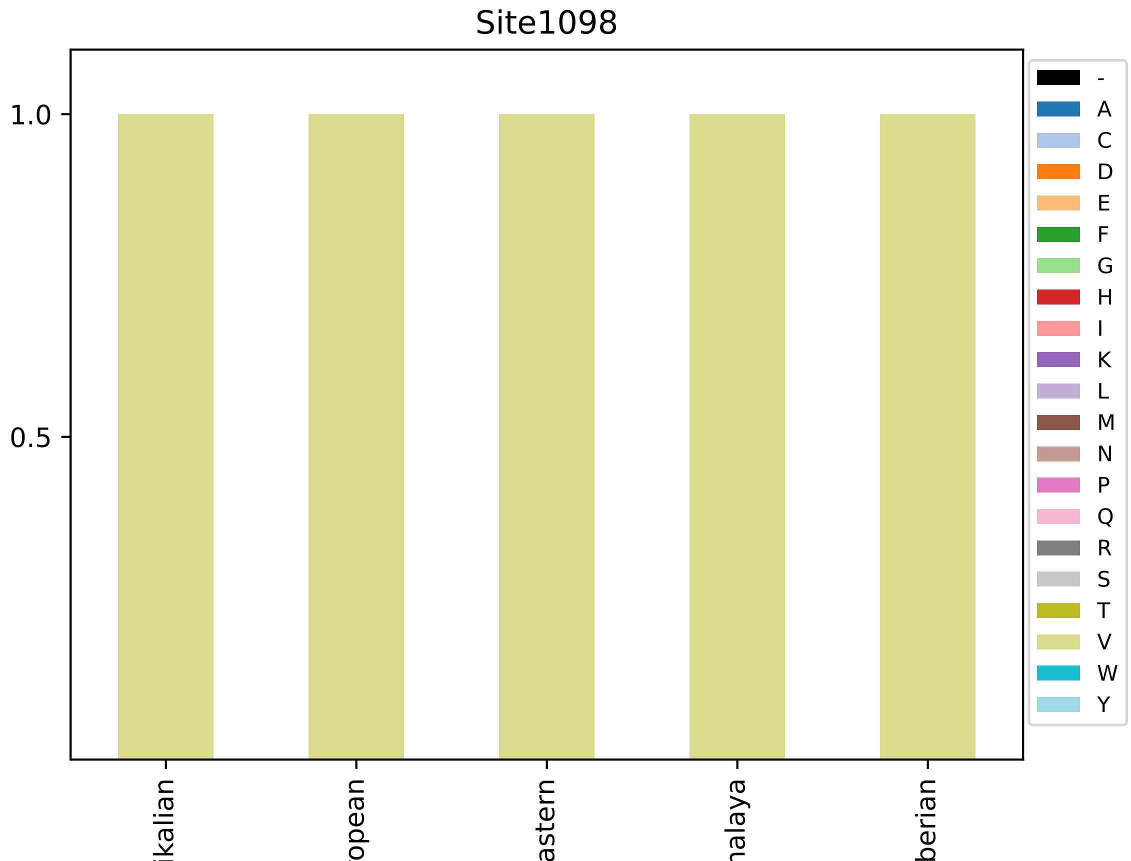

Site1098
1.0
0.5
-
A
C
D
E
F
G
H
I
K
L
M
N
P
Q
R
S
T
V
W
Y
ikalian
ropean
astern
nalaya
berian

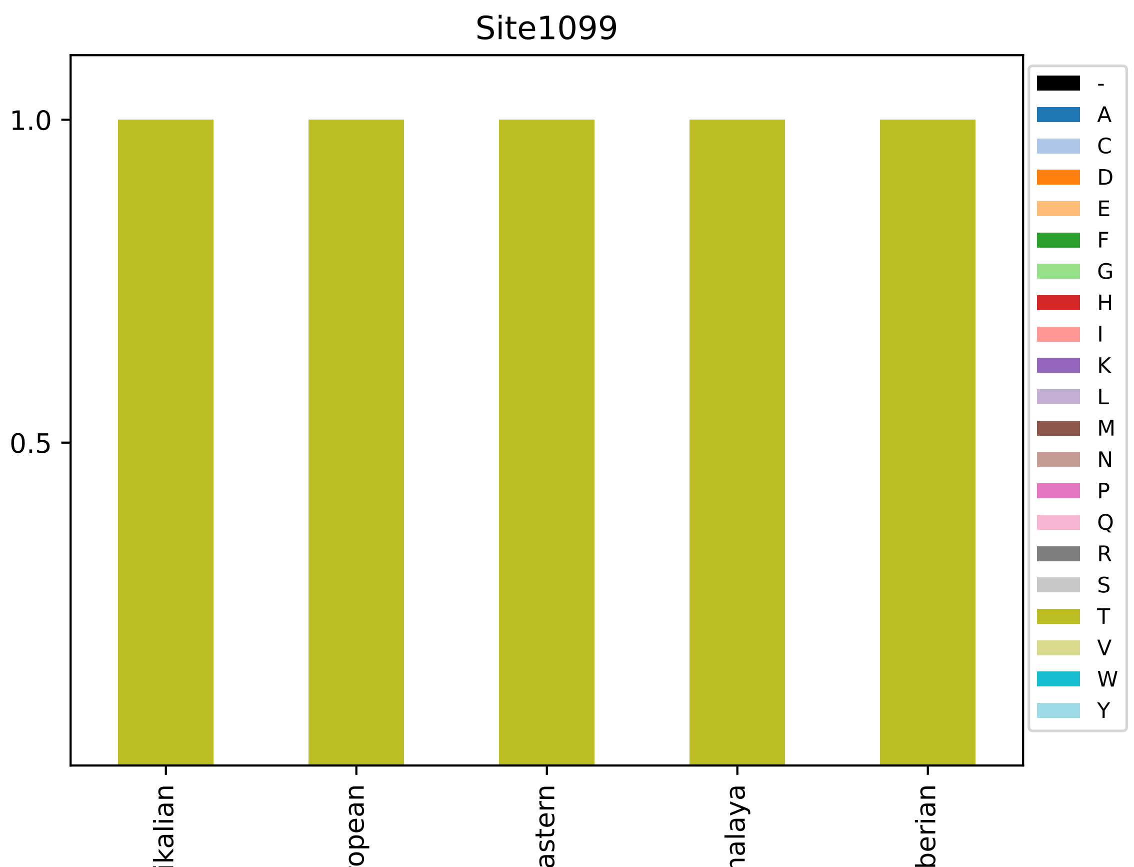
Site1099
1.0
0.5
-
A
C
D
E
F
G
H
I
K
L
M
N
P
Q
R
S
T
V
W
Y
ikalian
opean
astern
nalaya
berian

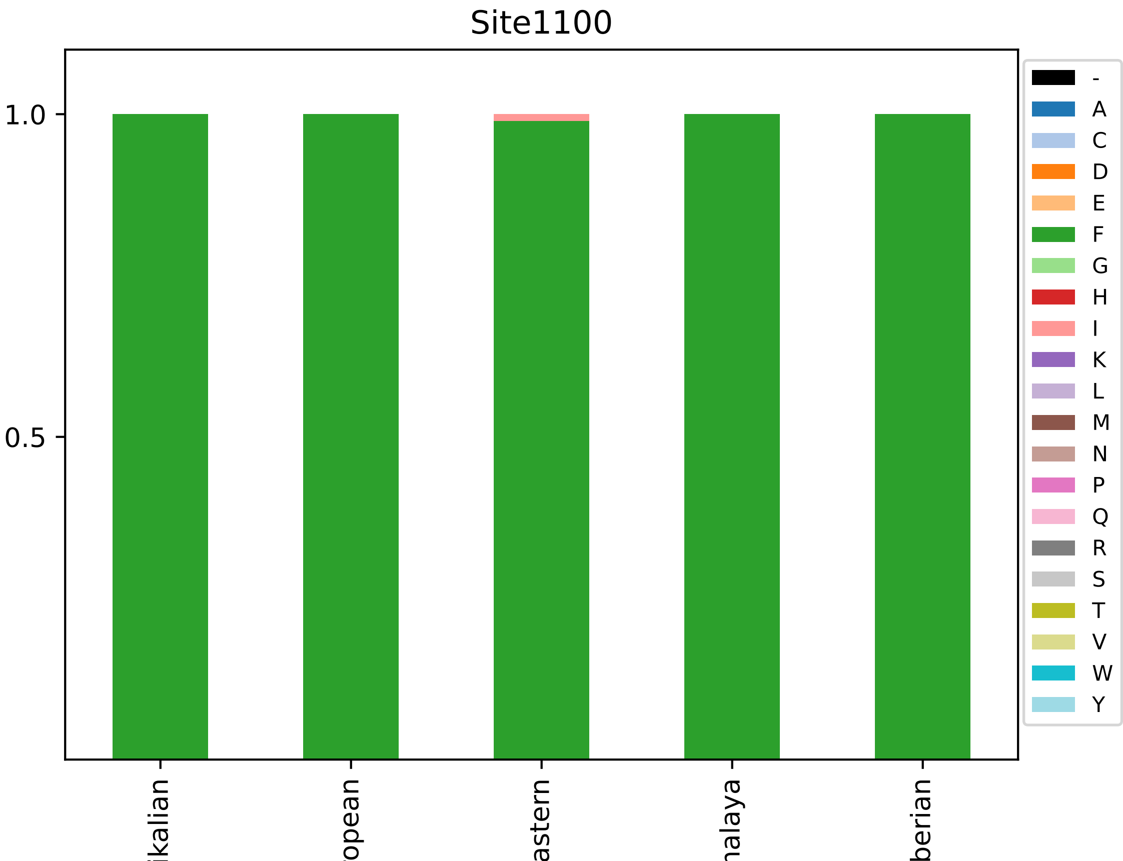
Site1100
1.0
0.5
-
A
C
D
E
F
G
H
I
K
L
M
N
P
Q
R
S
T
V
W
Y
ikalian
opean
astern
nalaya
berian

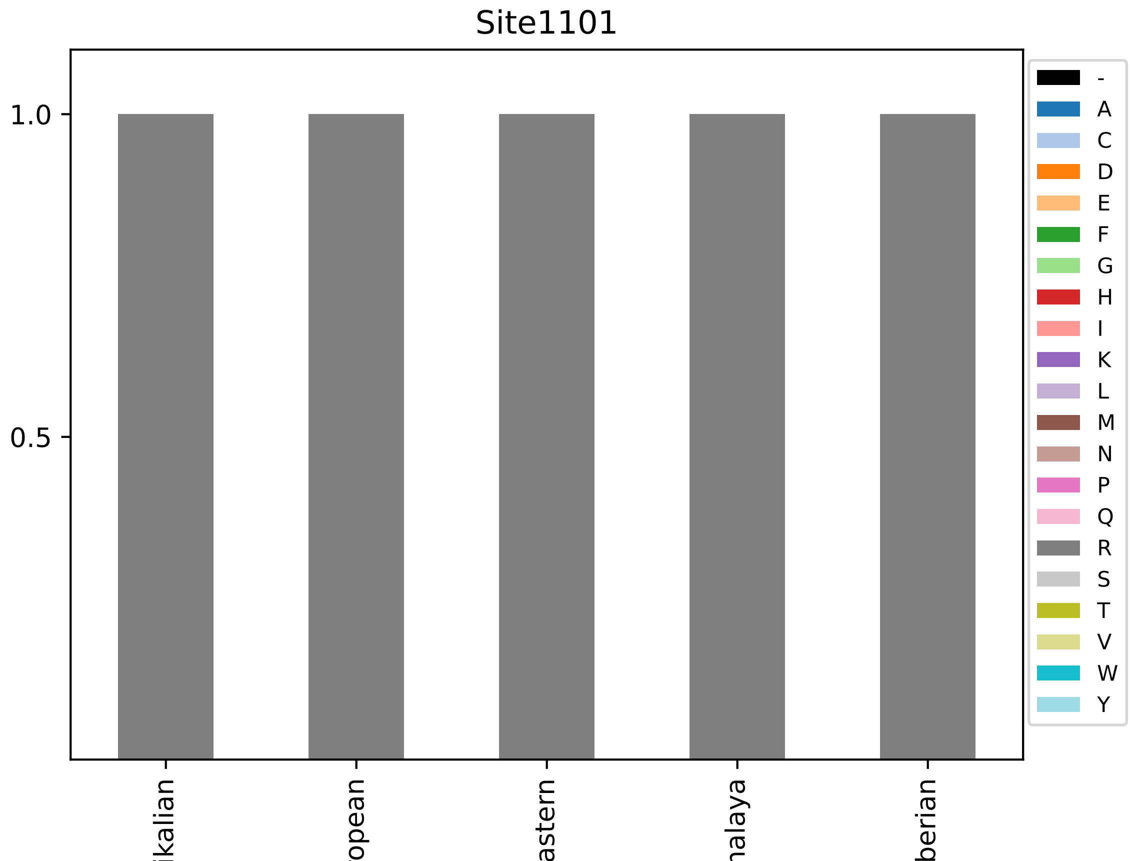
Site1101
1.0
0.5
-
A
C
D
E
F
G
H
I
K
L
M
N
P
Q
R
S
T
V
W
Y
ikalian
opean
astern
nalaya
berian

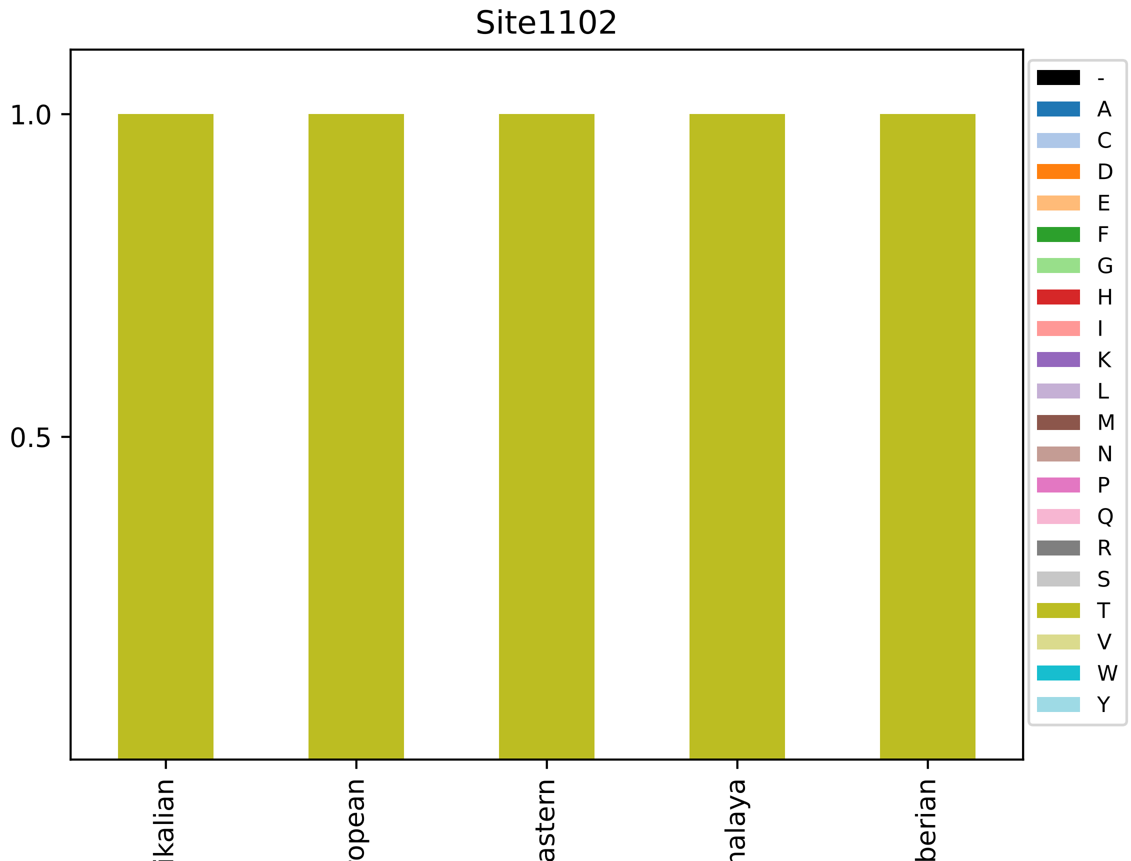
Site1102
1.0
0.5
ikalian
opean
astern
nalaya
berian
-
A
C
D
E
F
G
H
I
K
L
M
N
P
Q
R
S
T
V
W
Y

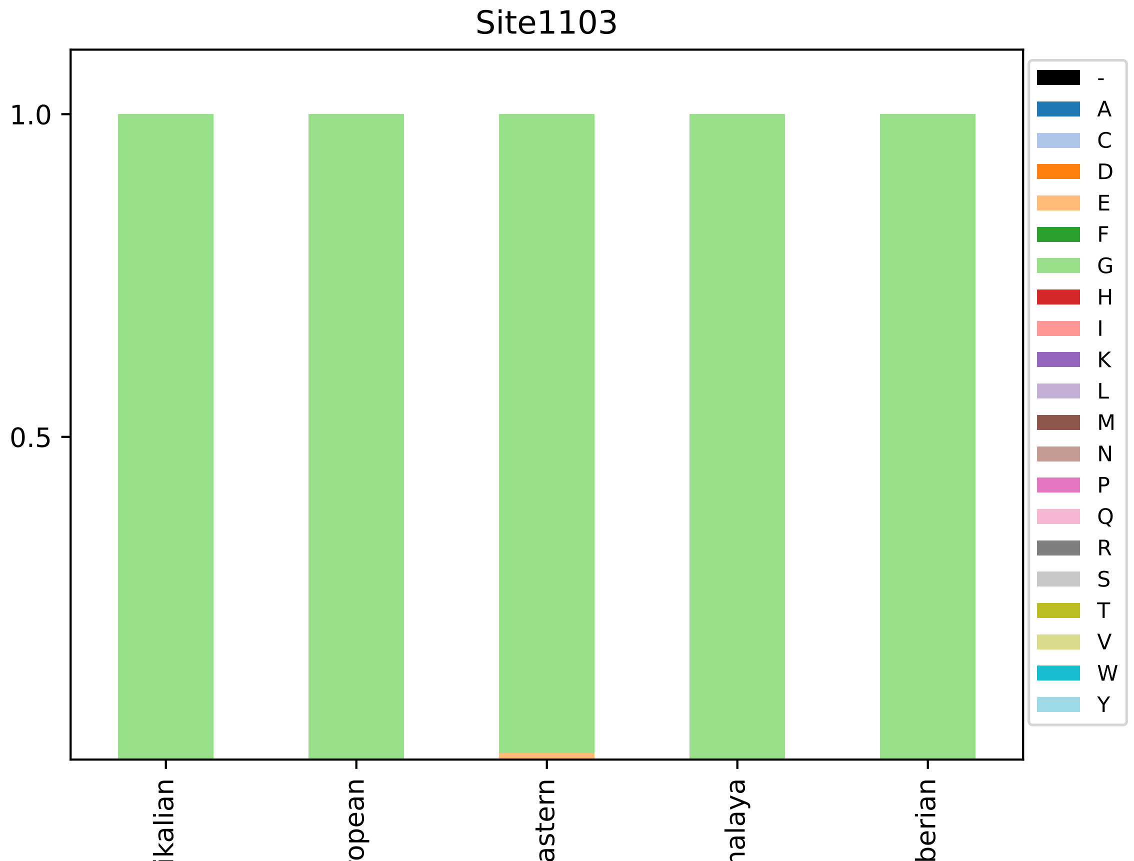
Site1103
1.0
0.5
-
A
C
D
E
F
G
H
I
K
L
M
N
P
Q
R
S
T
V
W
Y
ikalian
opean
astern
nalaya
berian

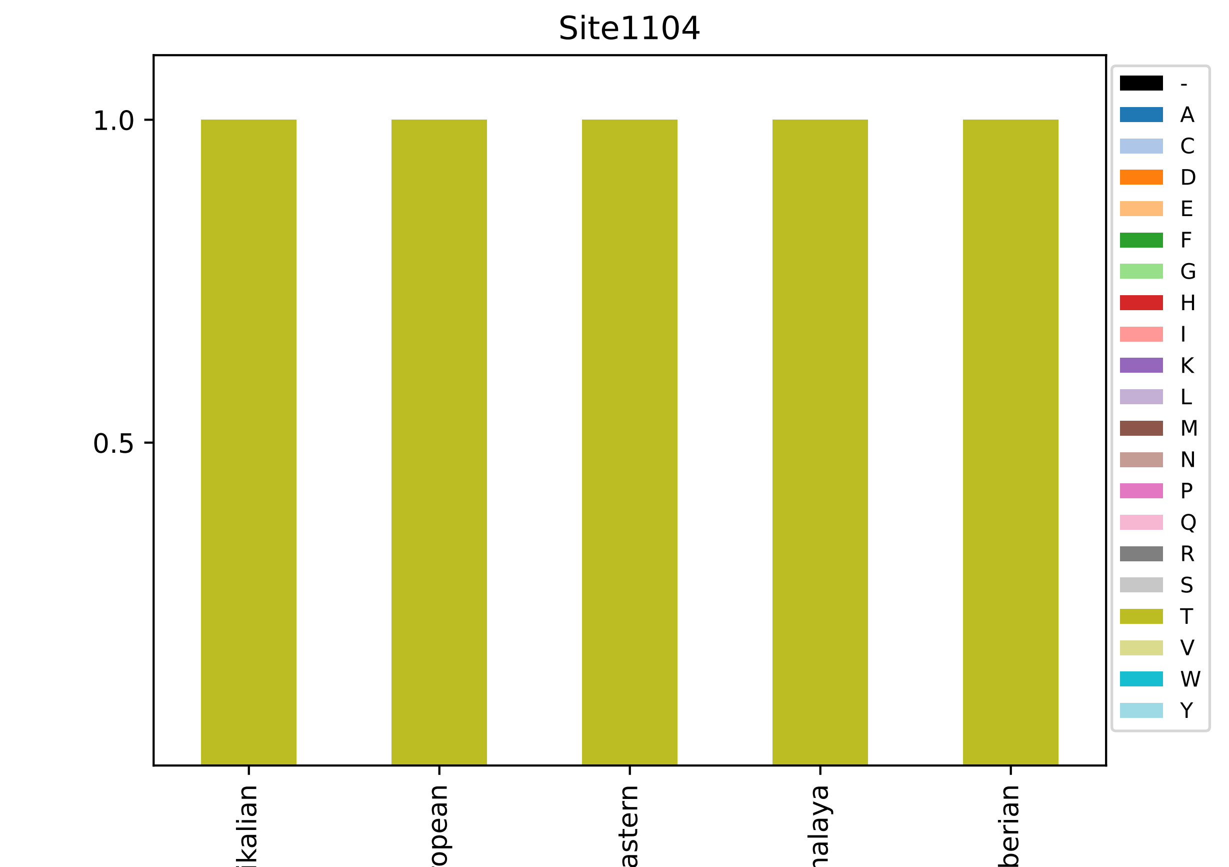
Site1104
1.0
0.5
-
A
C
D
E
F
G
H
I
K
L
M
N
P
Q
R
S
T
V
W
Y
ikalian
opean
astern
nalaya
berian

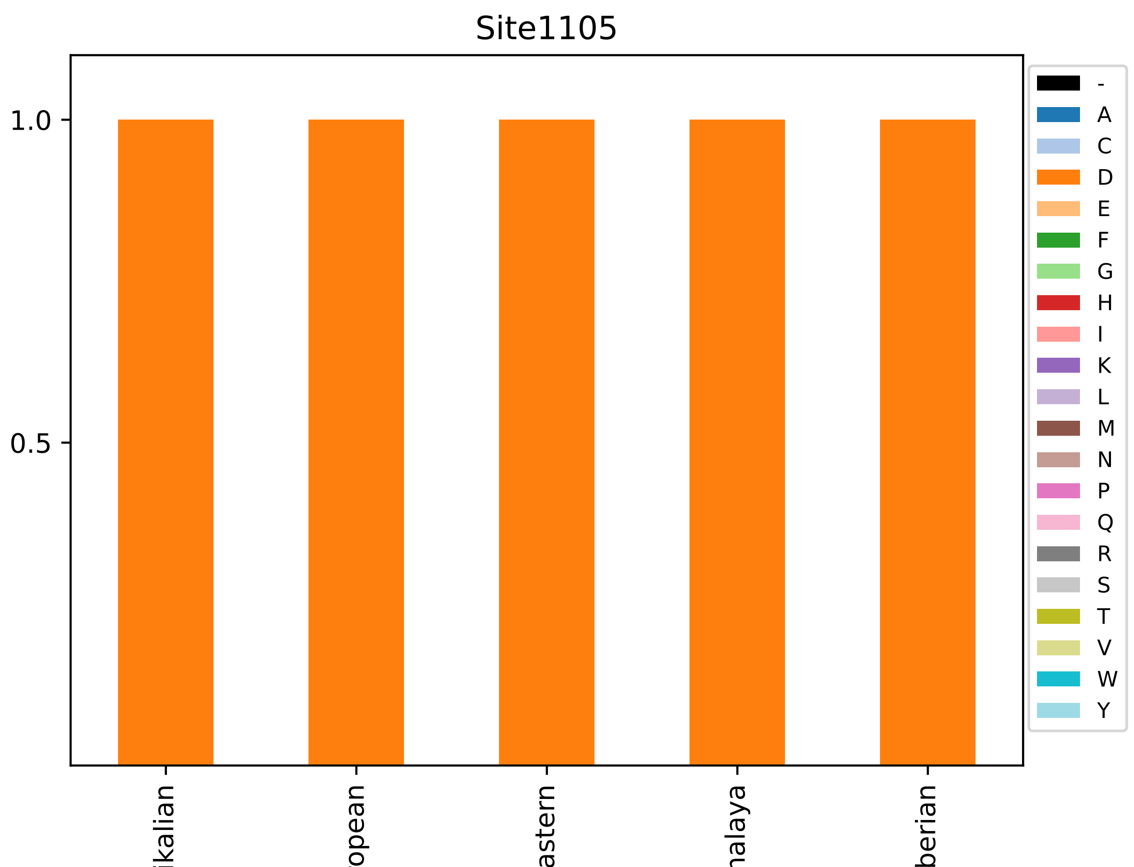
Site1105
1.0
0.5
-
A
C
D
E
F
G
H
I
K
L
M
N
P
Q
R
S
T
V
W
Y
ikalian
opean
astern
nalaya
berian

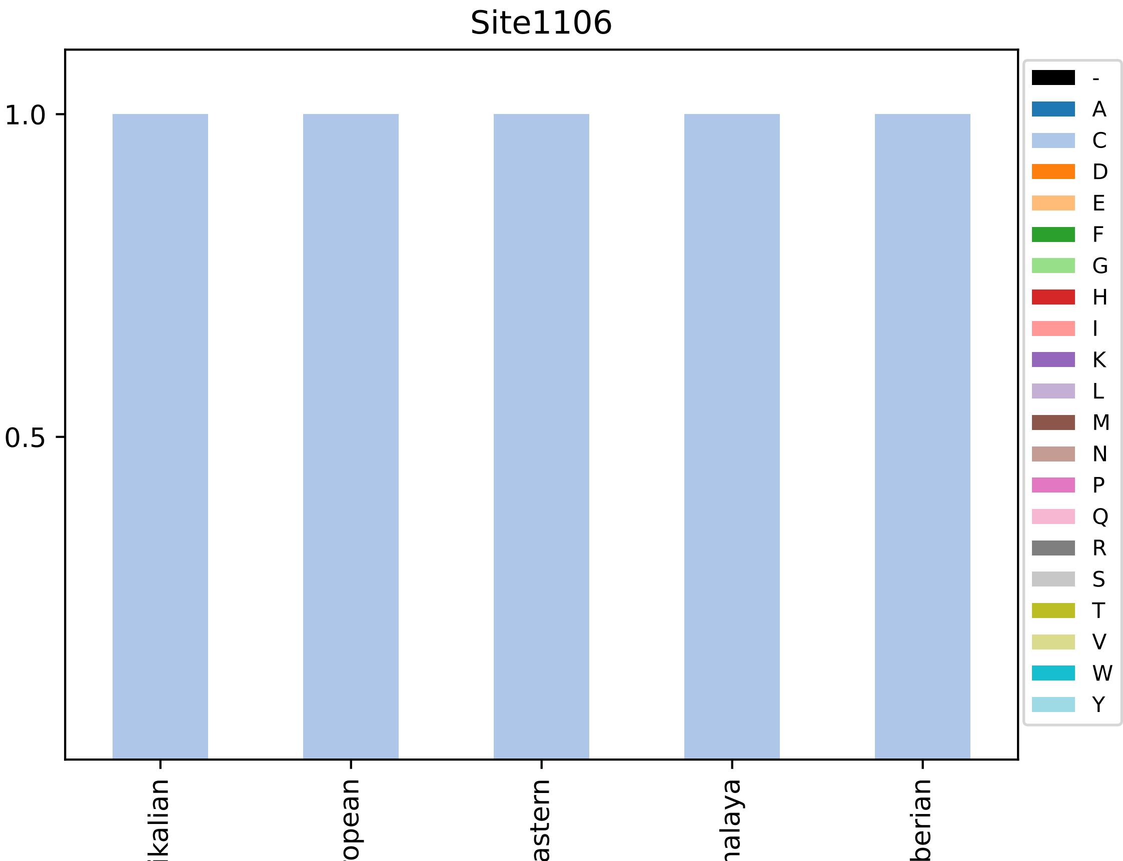

Site1106
1.0
0.5
-
A
C
D
E
F
G
H
I
K
L
M
N
P
Q
R
S
T
V
W
Y

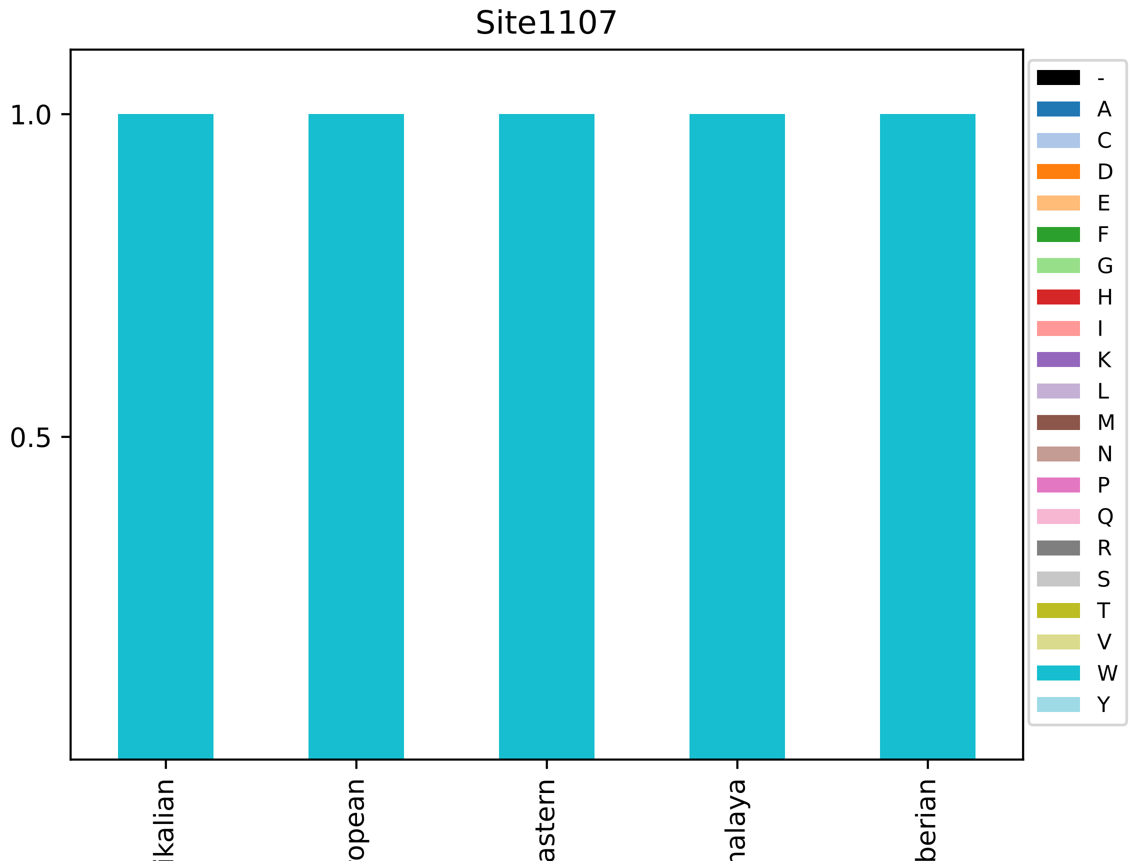

Site1107
1.0
0.5
-
A
C
D
E
F
G
H
I
K
L
M
N
P
Q
R
S
T
V
W
Y
ikalian
opean
astern
nalaya
berian

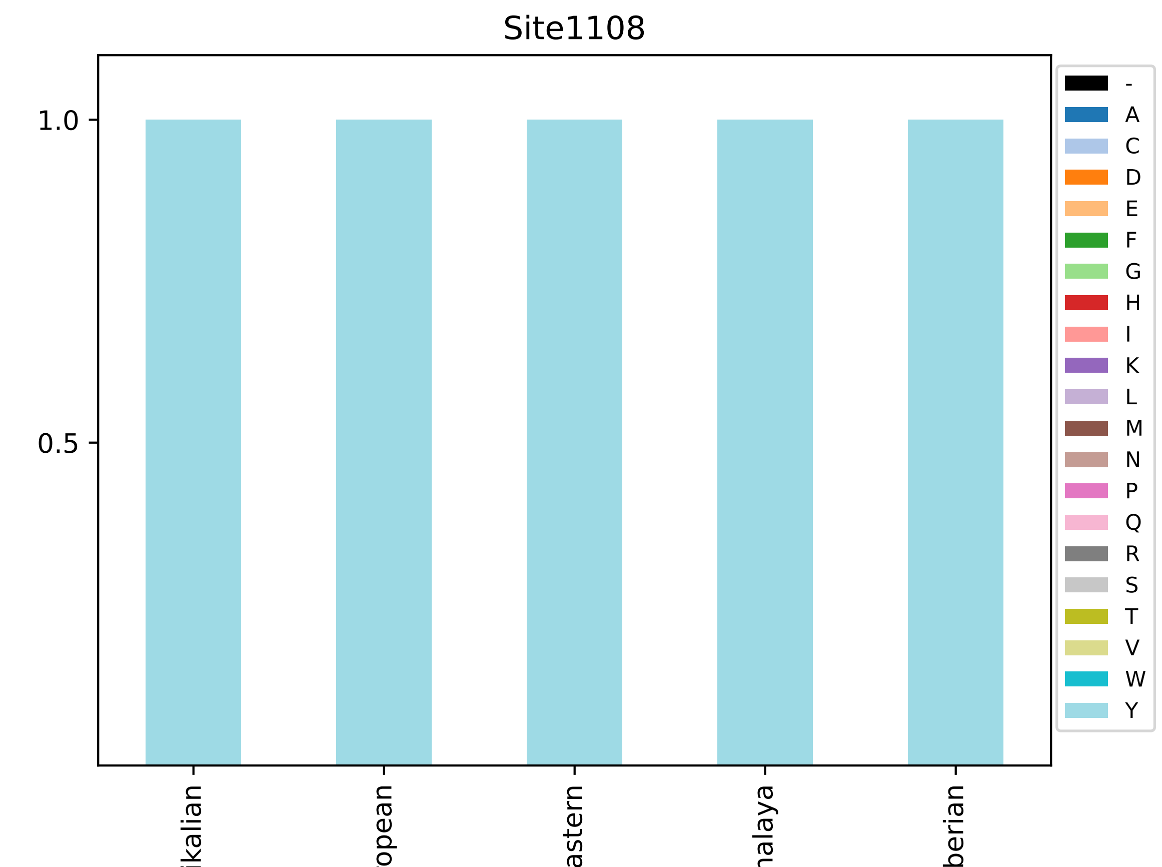

Site1108
1.0
0.5
ikalian
opean
astern
nalaya
berian
-
A
C
D
E
F
G
H
I
K
L
M
N
P
Q
R
S
T
V
W
Y

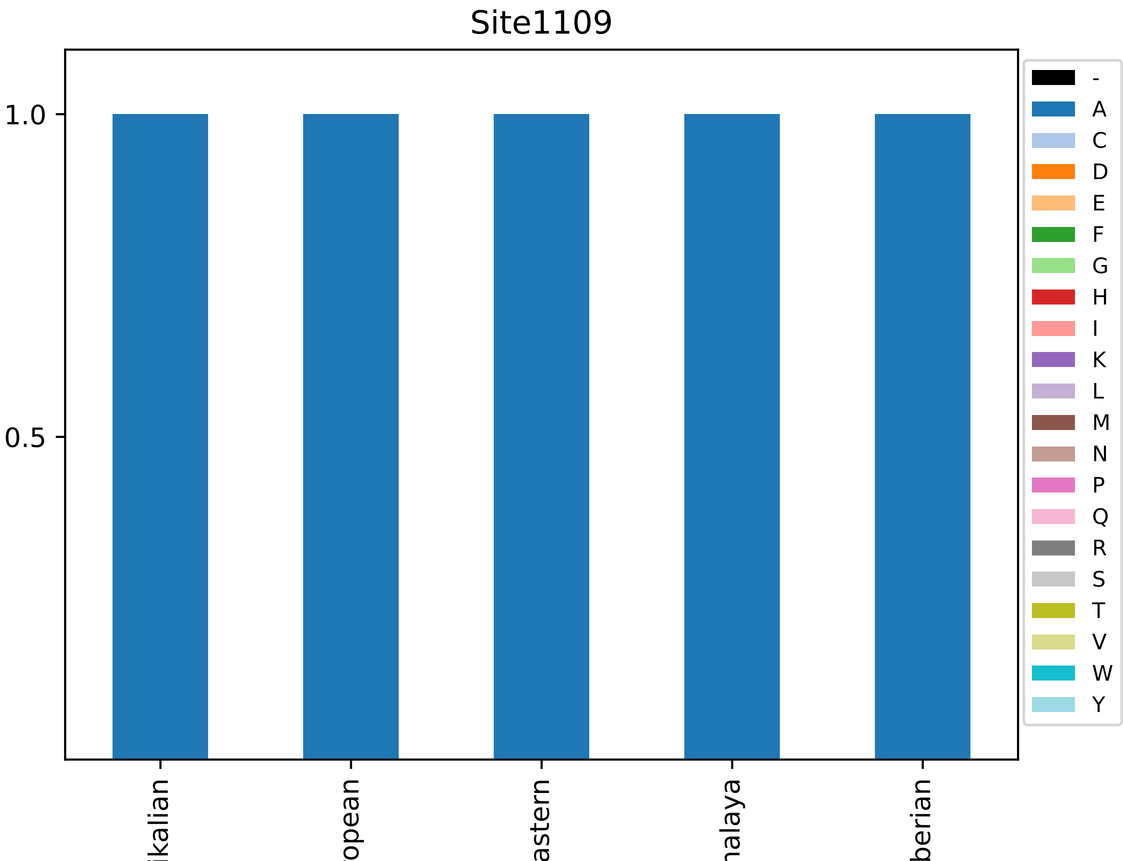
Site1109
1.0
0.5
ikalian
opean
astern
nalaya
berian
-
A
C
D
E
F
G
H
I
K
L
M
N
P
Q
R
S
T
V
W
Y

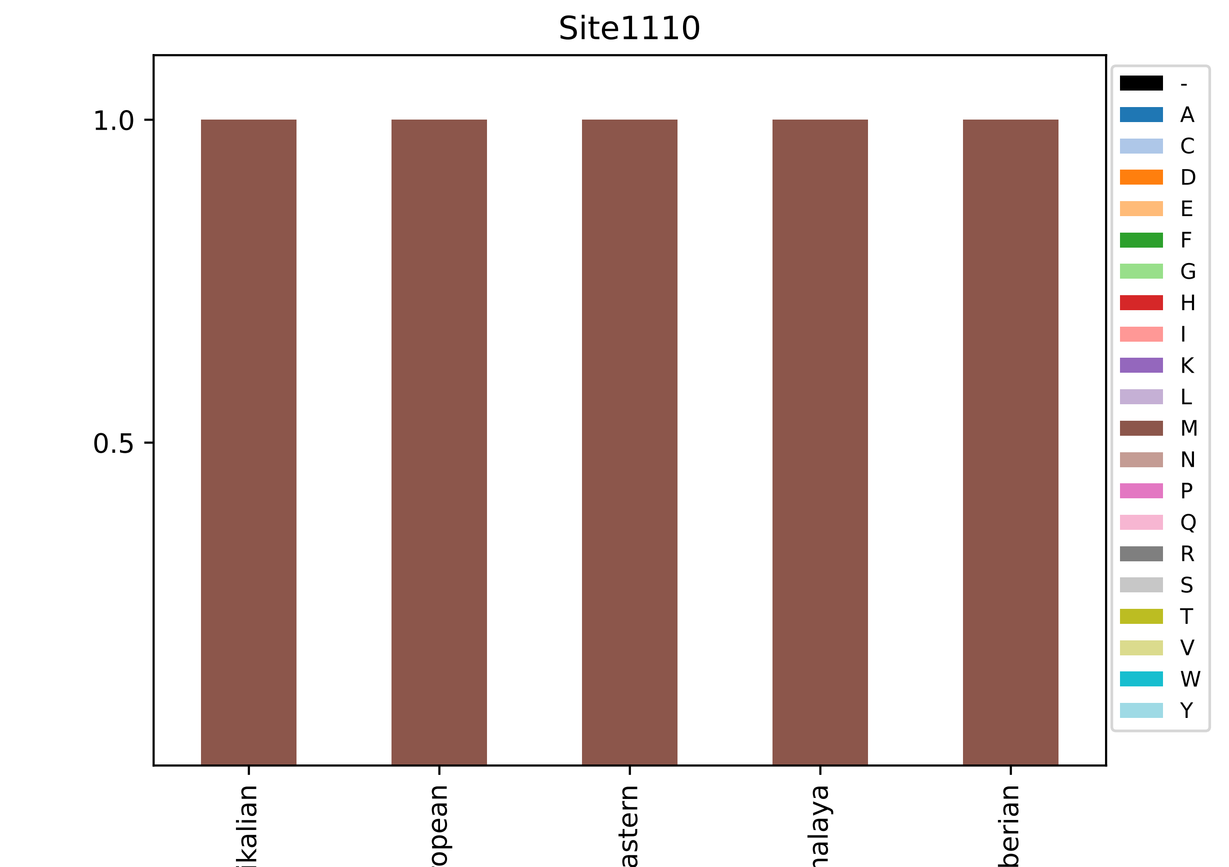
Site1110
1.0
0.5
-
A
C
D
E
F
G
H
I
K
L
M
N
P
Q
R
S
T
V
W
Y
ikalian
opean
astern
nalaya
berian

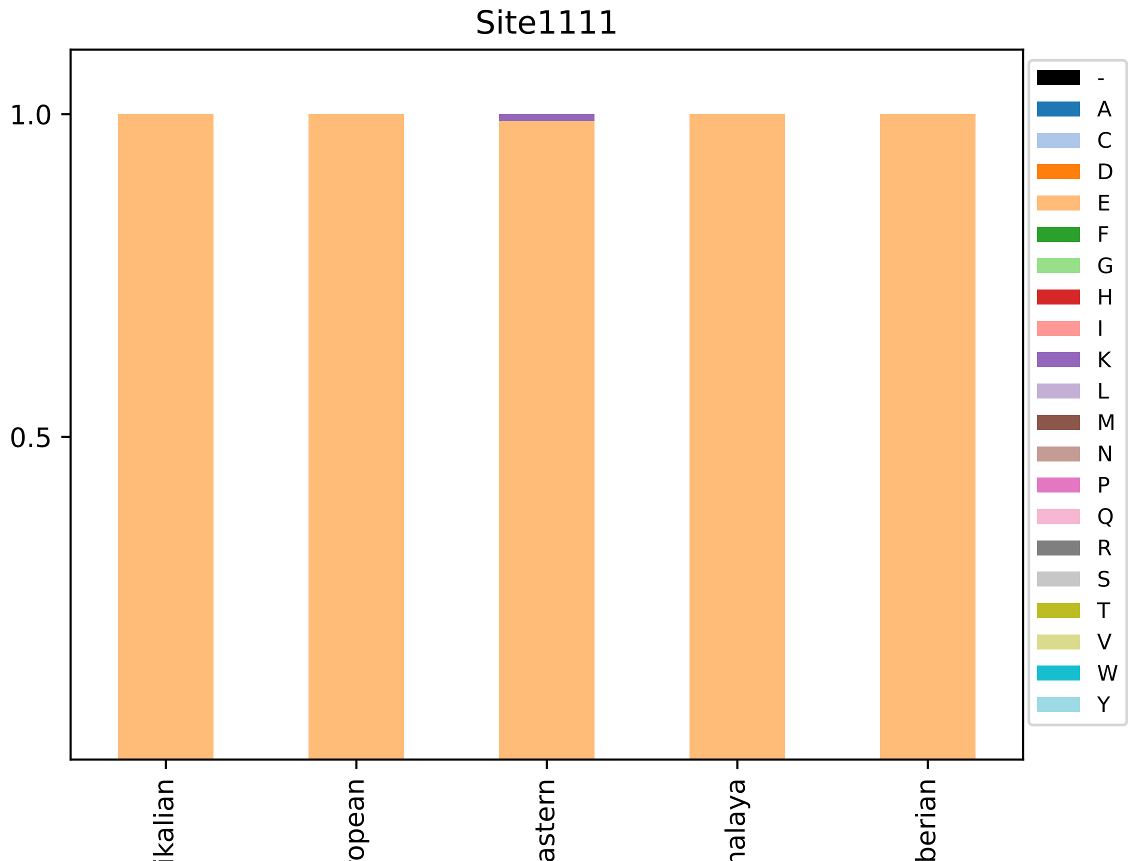

Site1111
1.0
0.5
-
A
C
D
E
F
G
H
I
K
L
M
N
P
Q
R
S
T
V
W
Y
ikalian
opean
astern
nalaya
berian

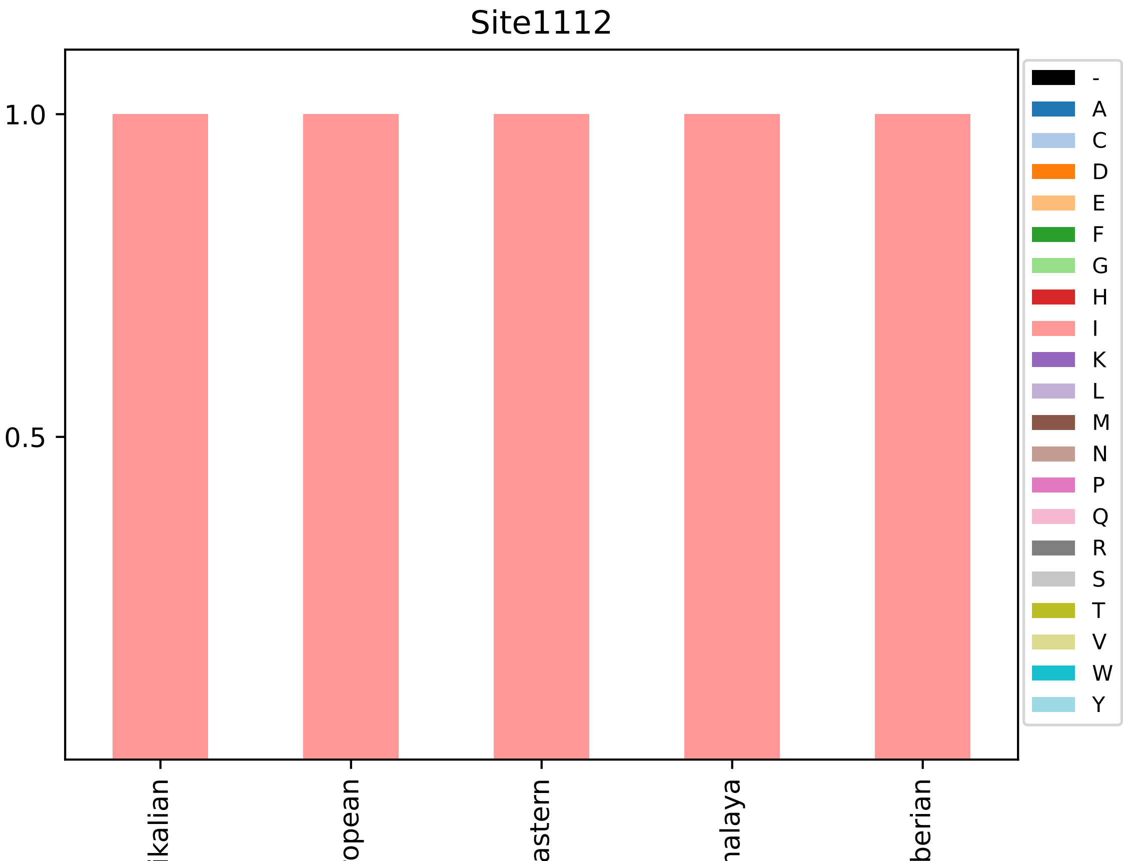

Site1112
1.0
0.5
-
A
C
D
E
F
G
H
I
K
L
M
N
P
Q
R
S
T
V
W
Y
ikalian
opean
astern
nalaya
berian

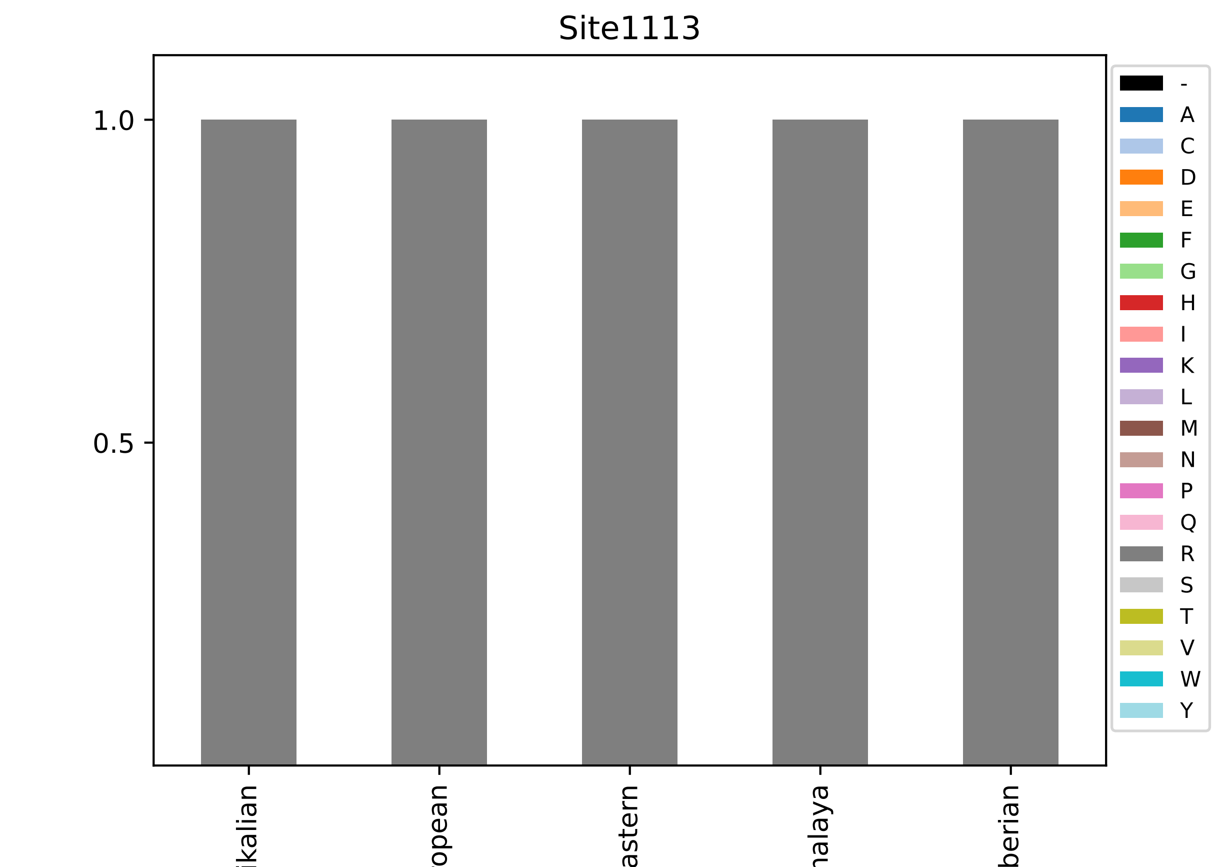

Site1113
1.0
0.5
-
A
C
D
E
F
G
H
I
K
L
M
N
P
Q
R
S
T
V
W
Y
ikalian
opean
astern
nalaya
berian

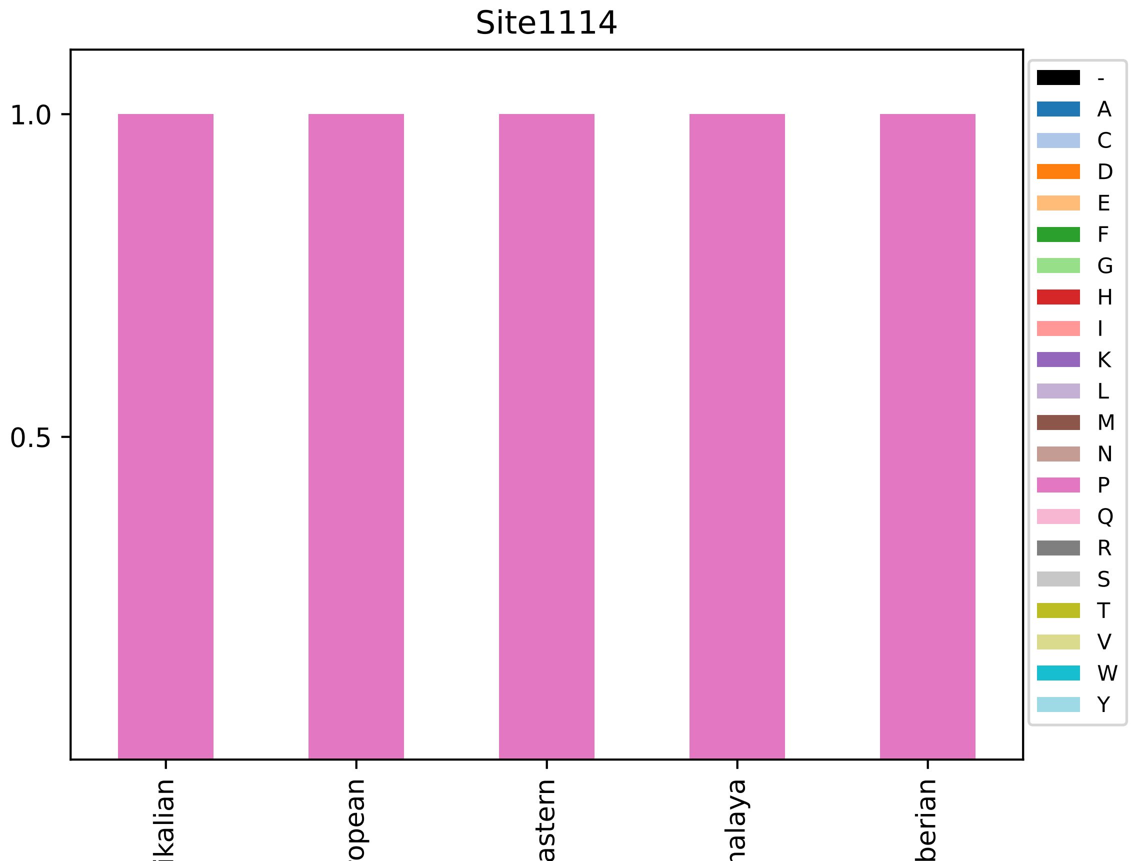
Site1114
1.0
0.5
-
A
C
D
E
F
G
H
I
K
L
M
N
P
Q
R
S
T
V
W
Y

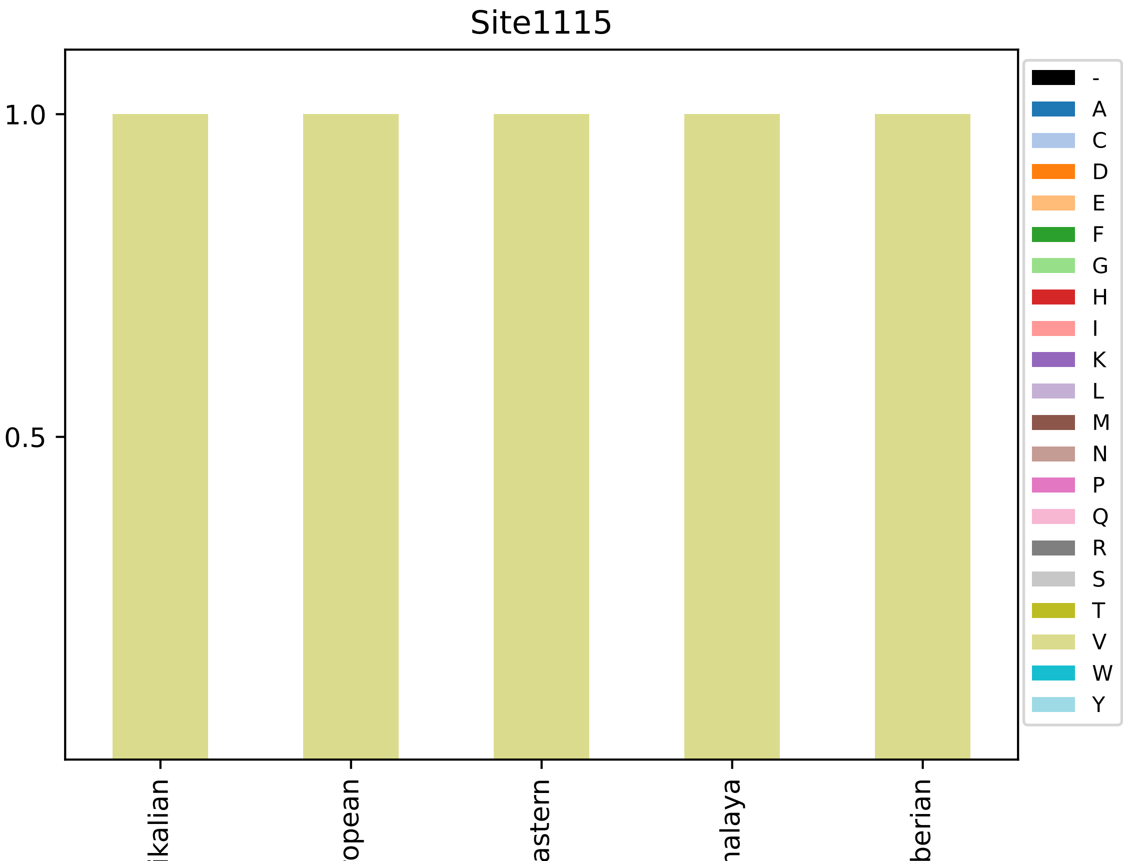
Site1115
1.0
0.5
ikalian
opean
astern
nalaya
berian
-
A
C
D
E
F
G
H
I
K
L
M
N
P
Q
R
S
T
V
W
Y

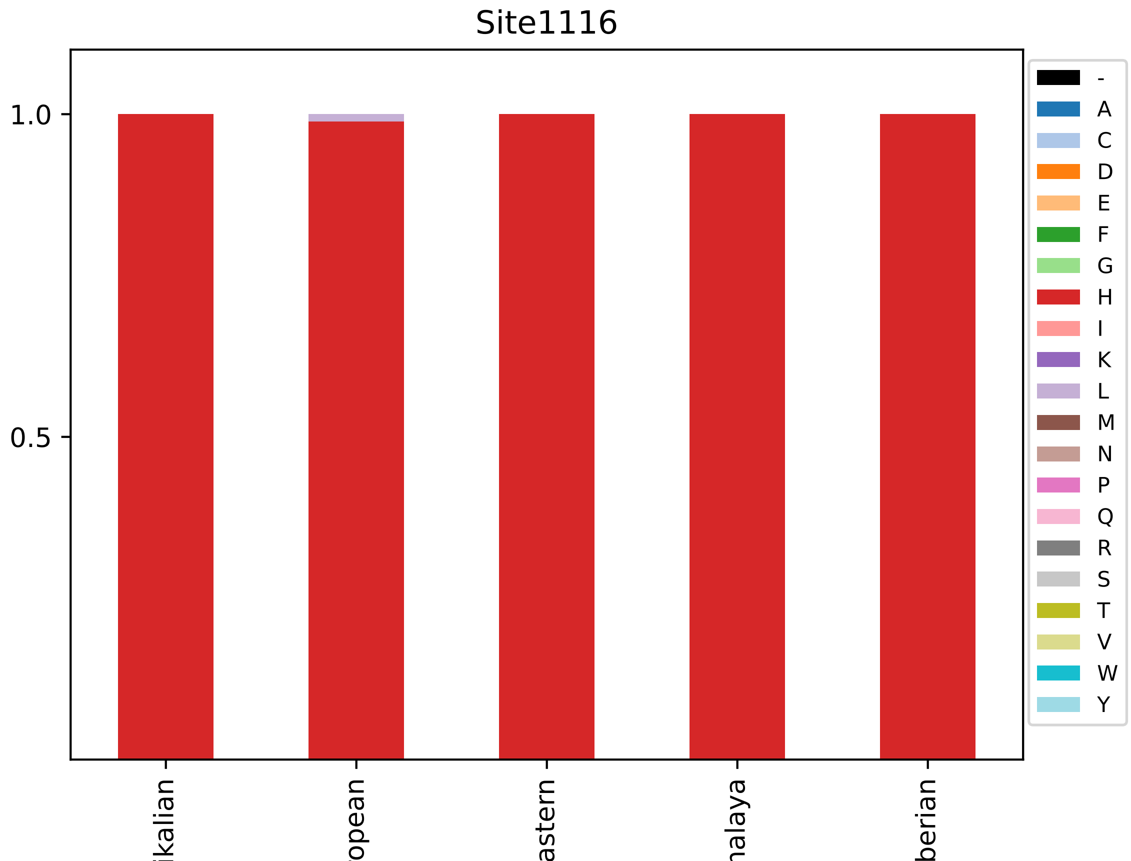

Site1116
1.0
0.5
-
A
C
D
E
F
G
H
I
K
L
M
N
P
Q
R
S
T
V
W
Y
ikalian
opean
astern
nalaya
berian

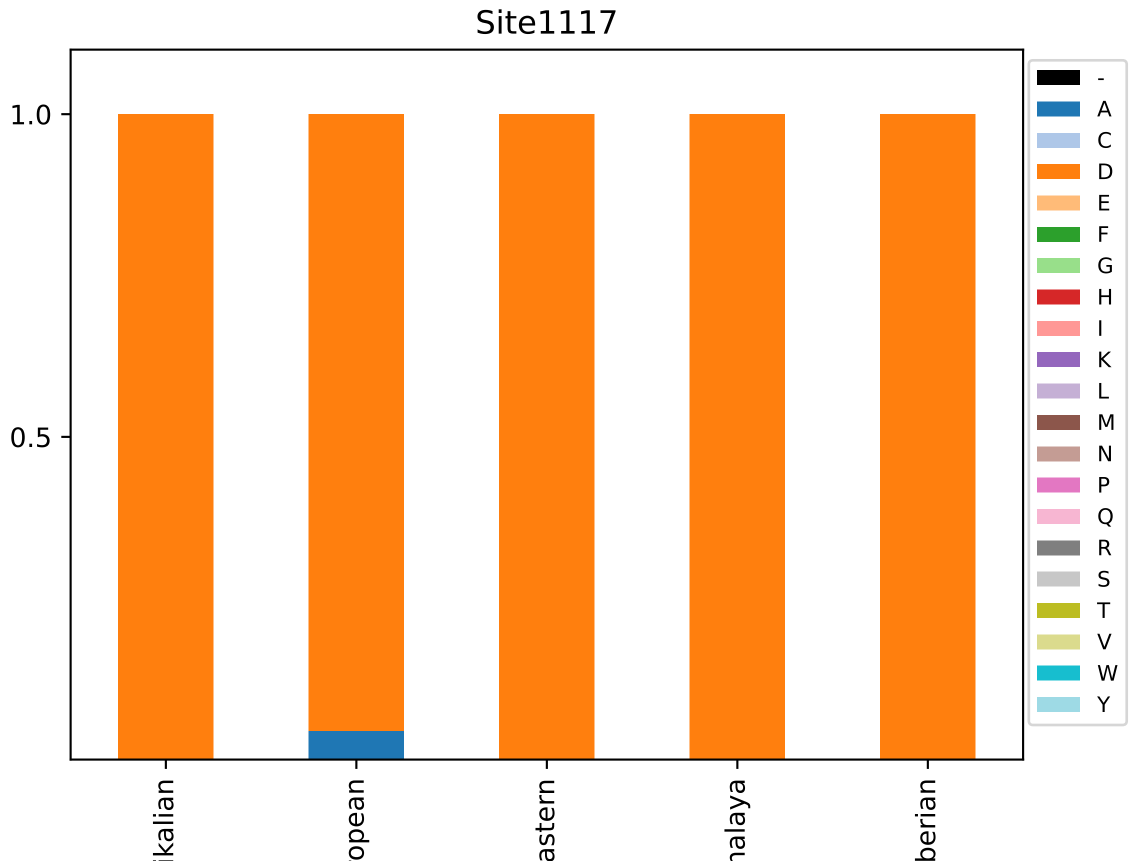
Site1117
1.0
0.5
-
A
C
D
E
F
G
H
I
K
L
M
N
P
Q
R
S
T
V
W
Y
ikalian
opean
astern
nalaya
berian

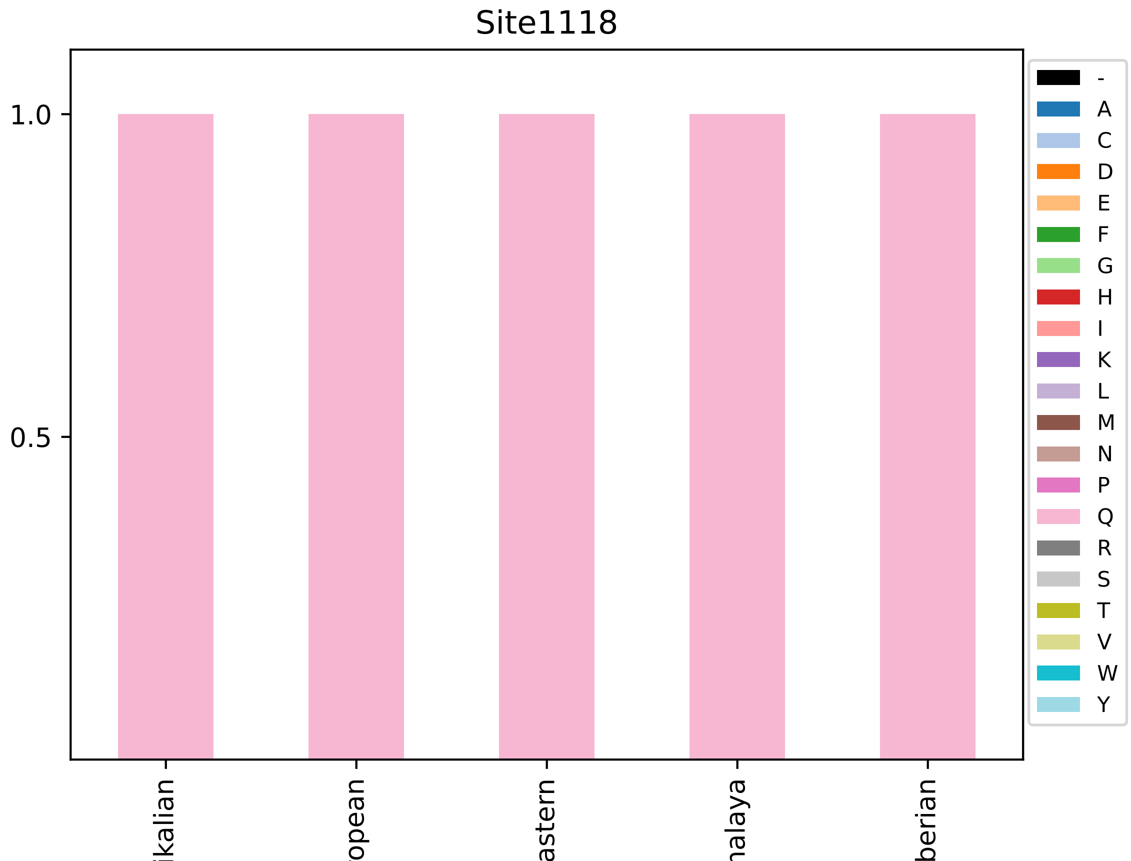

Site1118
1.0
0.5
-
A
C
D
E
F
G
H
I
K
L
M
N
P
Q
R
S
T
V
W
Y
ikalian
opean
astern
nalaya
berian

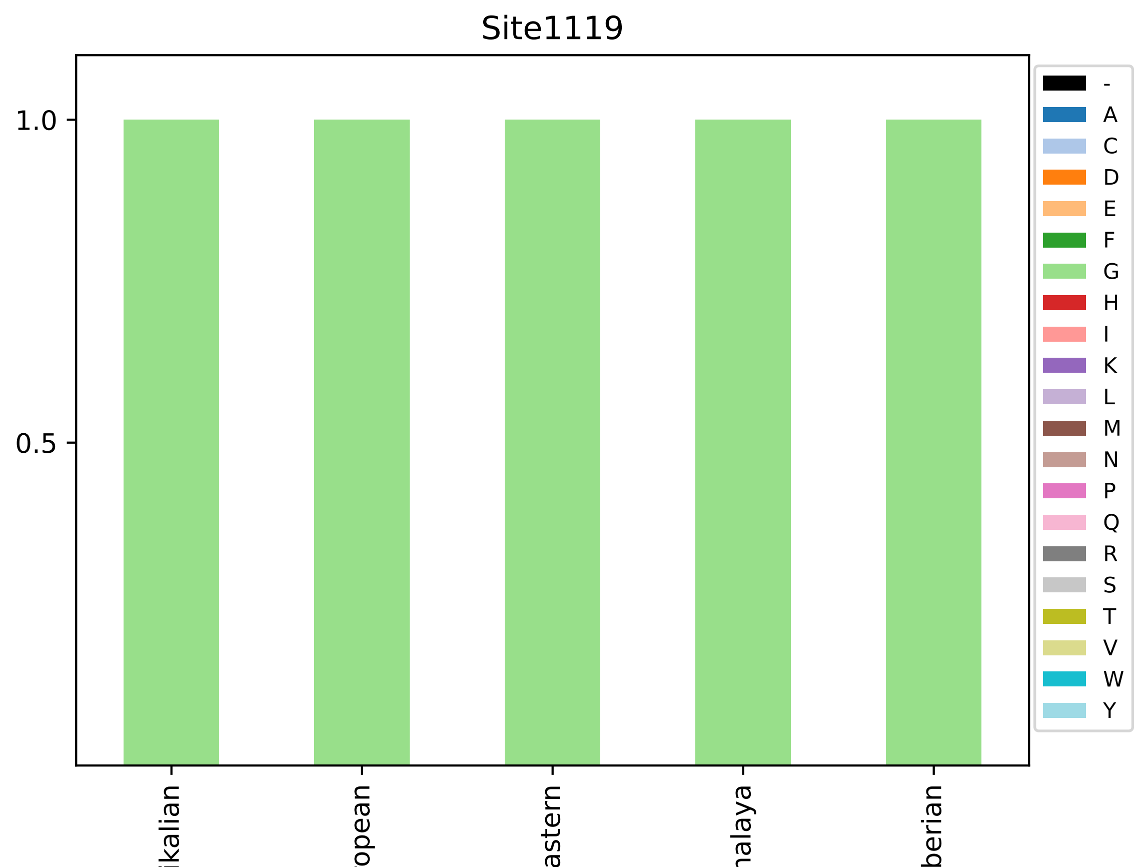

Site1119
1.0
0.5
ikalian
opean
astern
nalaya
berian
-
A
C
D
E
F
G
H
I
K
L
M
N
P
Q
R
S
T
V
W
Y

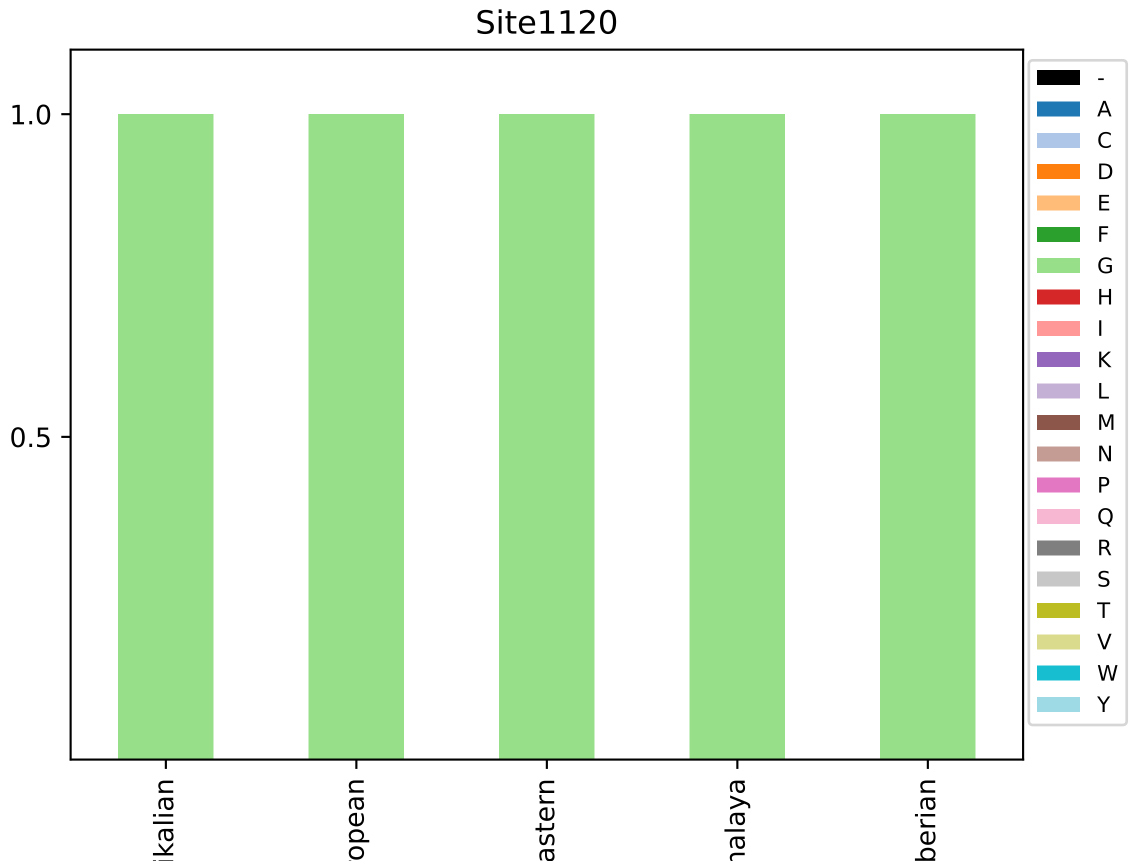
Site1120
1.0
0.5
-
A
C
D
E
F
G
H
I
K
L
M
N
P
Q
R
S
T
V
W
Y
ikalian
opean
astern
nalaya
berian

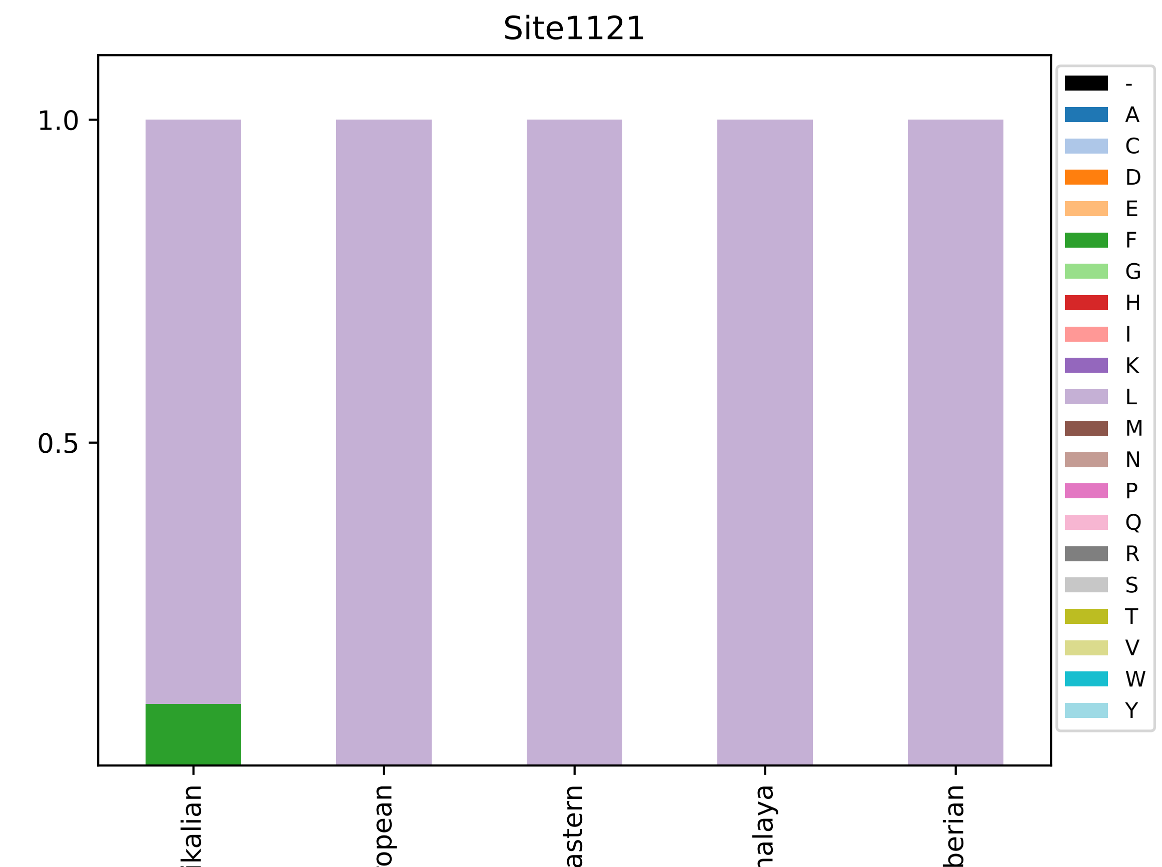
Site1121
1.0
0.5
-
A
C
D
E
F
G
H
I
K
L
M
N
P
Q
R
S
T
V
W
Y
ikalian
ropean
astern
nalaya
berian

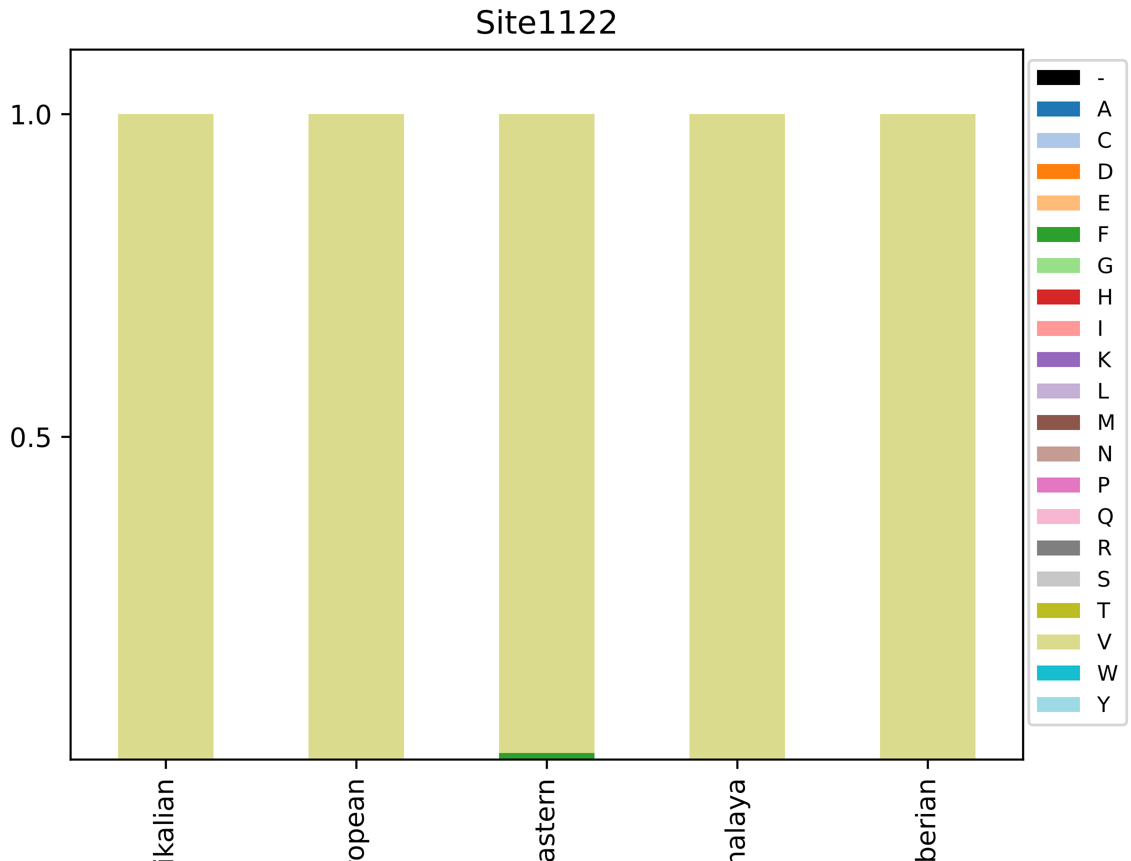
Site1122
1.0
0.5
ikalian
opean
astern
nalaya
berian
-
A
C
D
E
F
G
H
I
K
L
M
N
P
Q
R
S
T
V
W
Y

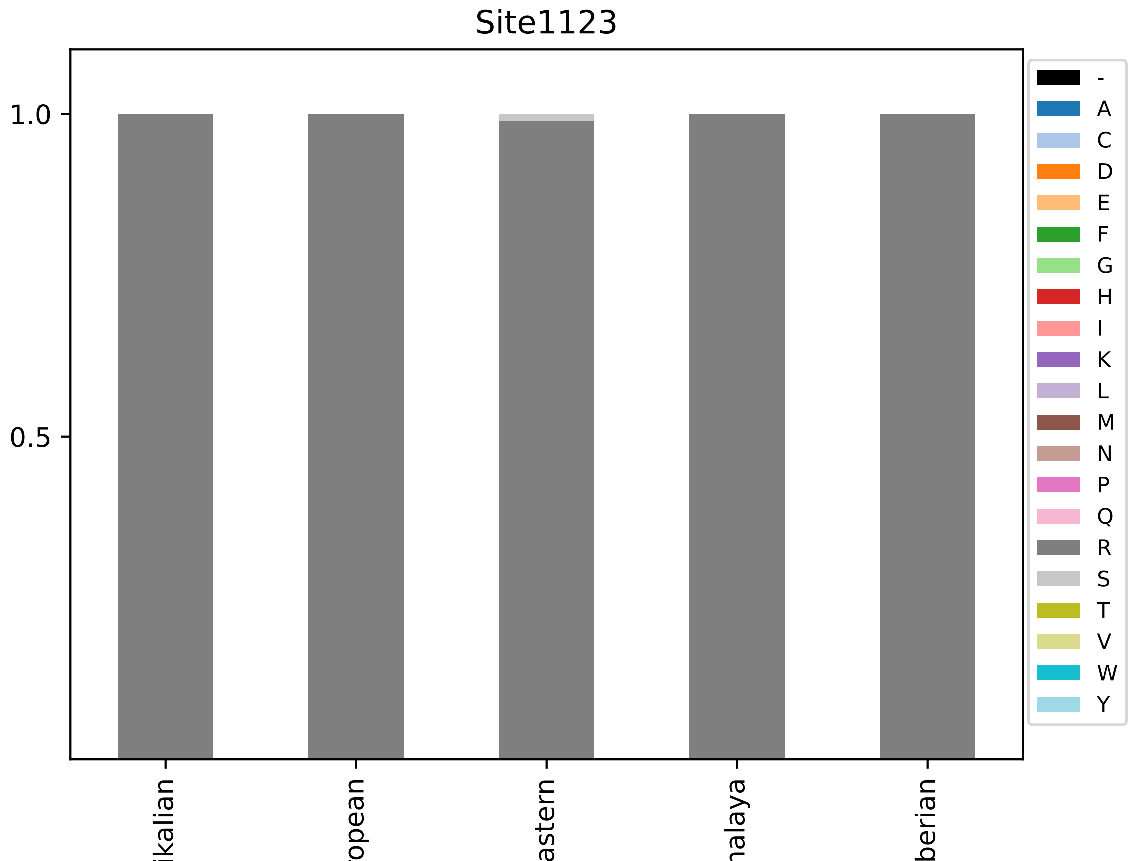

Site1123
1.0
0.5
-
A
C
D
E
F
G
H
I
K
L
M
N
P
Q
R
S
T
V
W
Y
ikalian
opean
astern
nalaya
berian

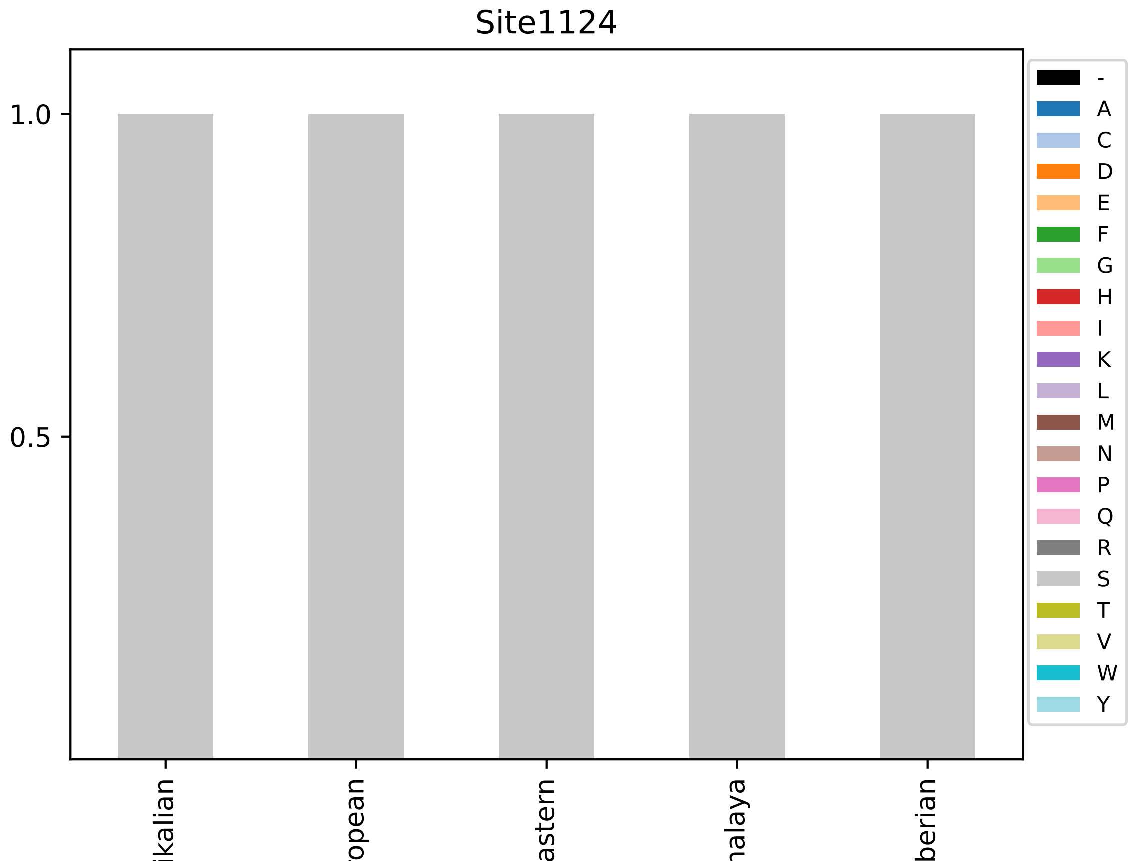
Site1124
1.0
0.5
-
A
C
D
E
F
G
H
I
K
L
M
N
P
Q
R
S
T
V
W
Y
ikalian
opean
astern
nalaya
berian

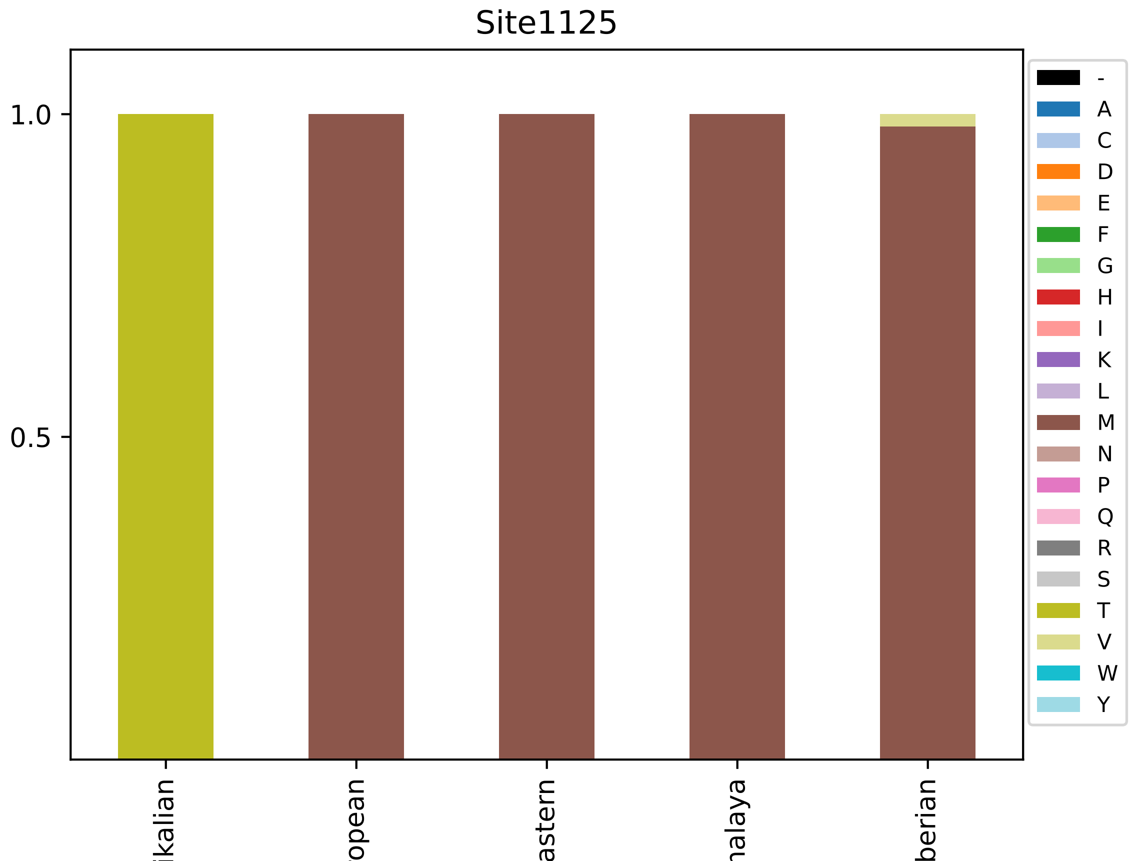
Site1125
1.0
0.5
ikalian
opean
astern
nalaya
berian
-
A
C
D
E
F
G
H
I
K
L
M
N
P
Q
R
S
T
V
W
Y

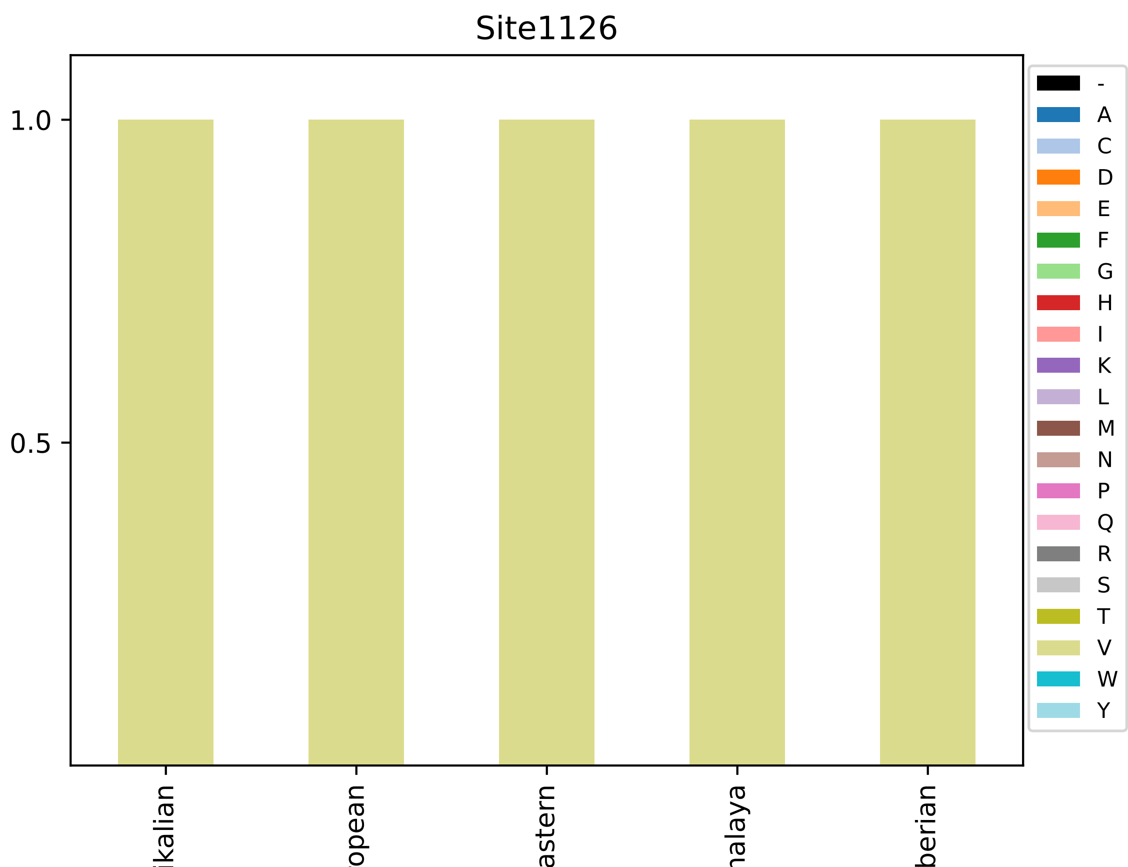
Site1126
1.0
0.5
ikalian
opean
astern
nalaya
berian
-
A
C
D
E
F
G
H
I
K
L
M
N
P
Q
R
S
T
V
W
Y

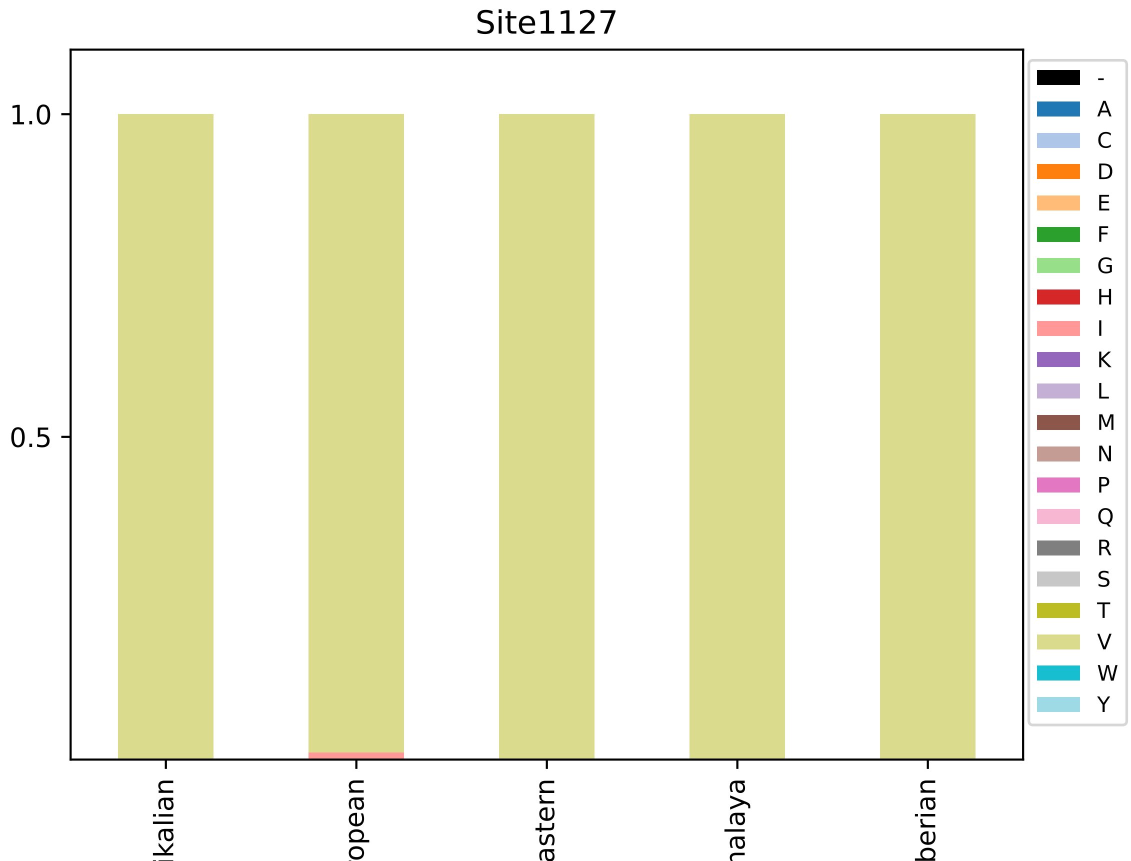
Site1127
1.0
0.5
-
A
C
D
E
F
G
H
I
K
L
M
N
P
Q
R
S
T
V
W
Y
ikalian
opean
astern
nalaya
berian

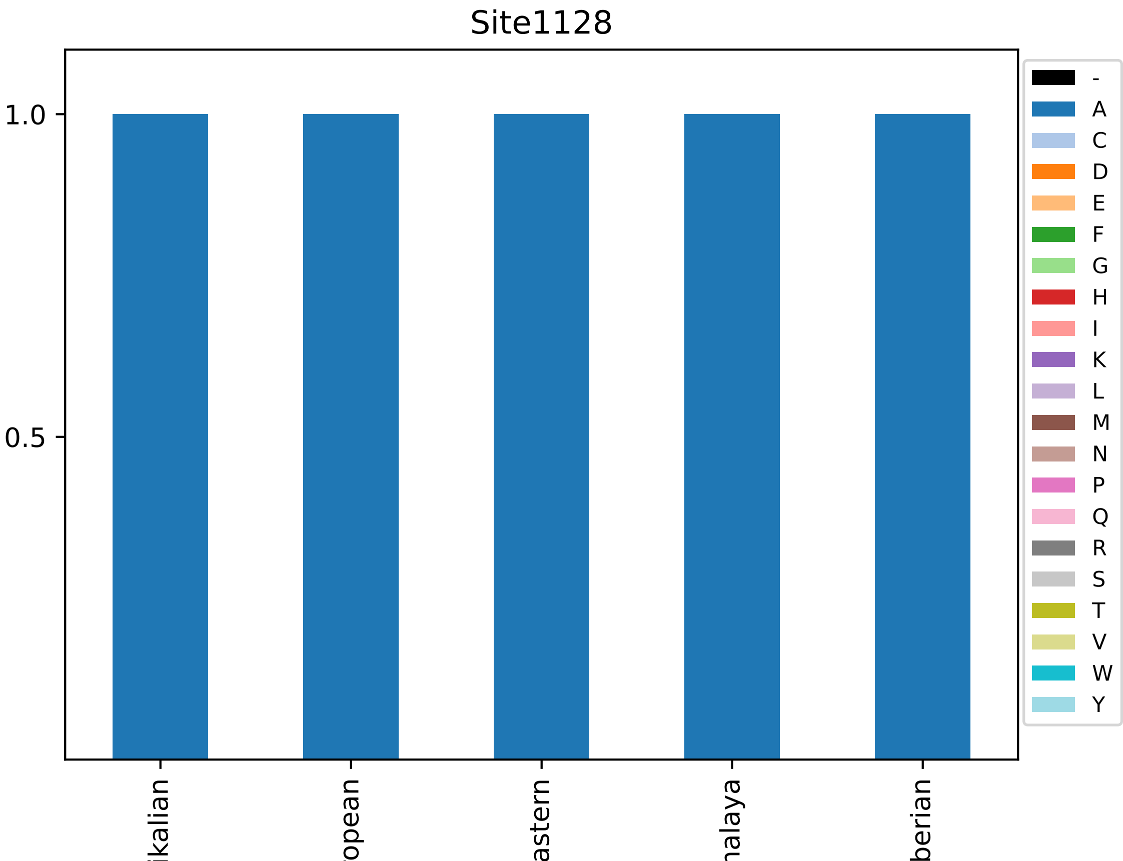
Site1128
1.0
0.5
ikalian
opean
astern
nalaya
berian
-
A
C
D
E
F
G
H
I
K
L
M
N
P
Q
R
S
T
V
W
Y

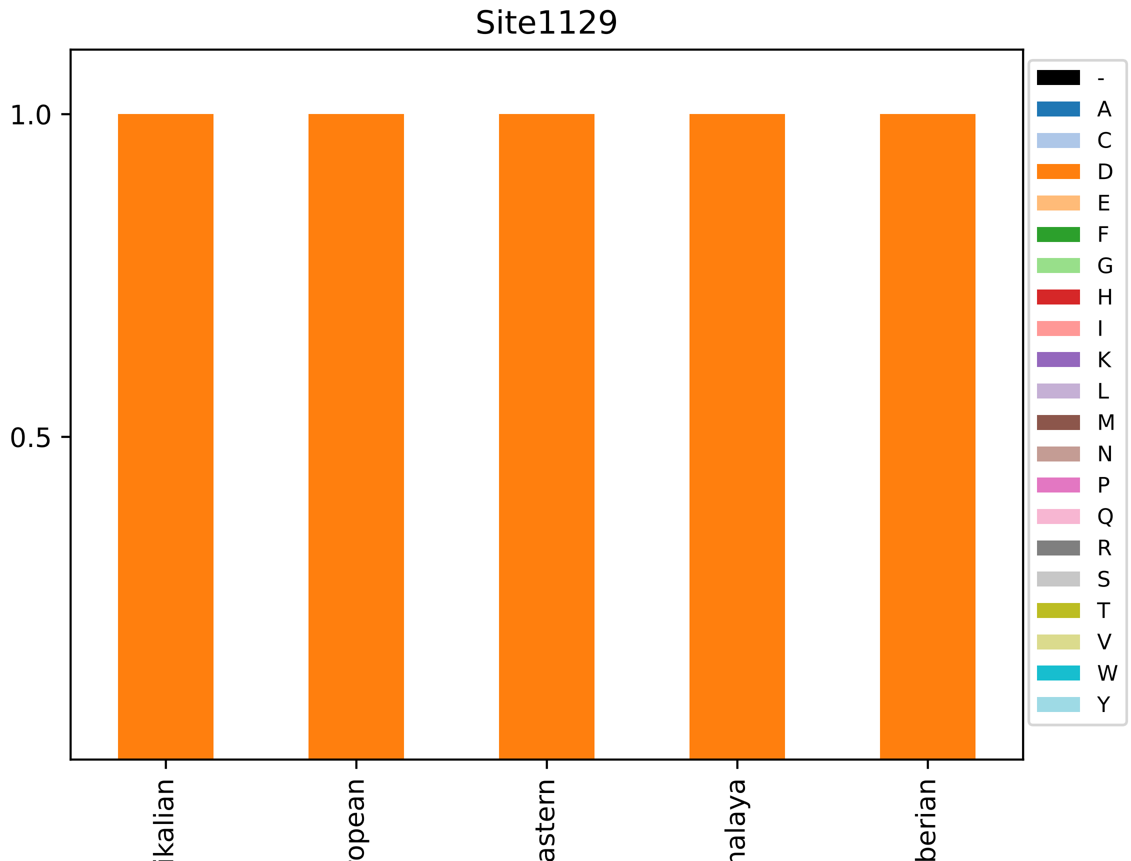
Site1129
1.0
0.5
ikalian
ropean
astern
nalaya
berian
-
A
C
D
E
F
G
H
I
K
L
M
N
P
Q
R
S
T
V
W
Y

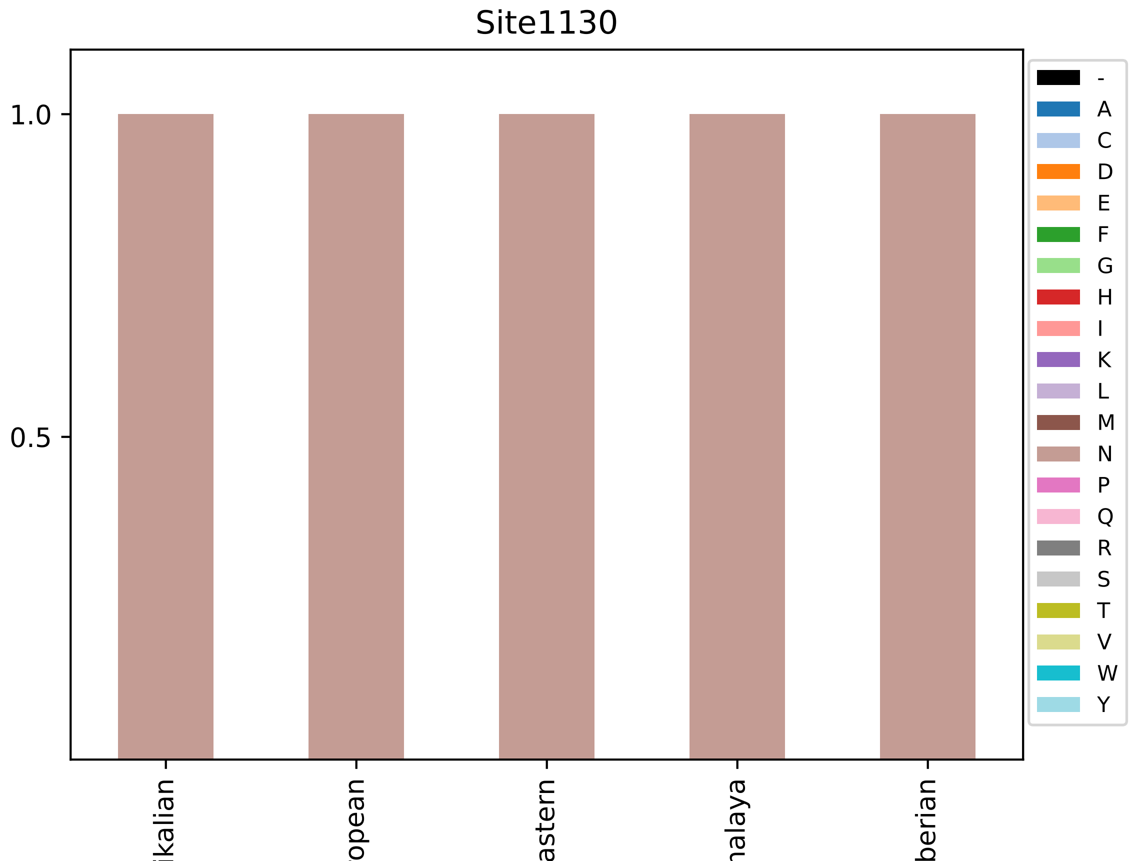
Site1130
1.0
0.5
-
A
C
D
E
F
G
H
I
K
L
M
N
P
Q
R
S
T
V
W
Y
ikalian
opean
astern
nalaya
berian

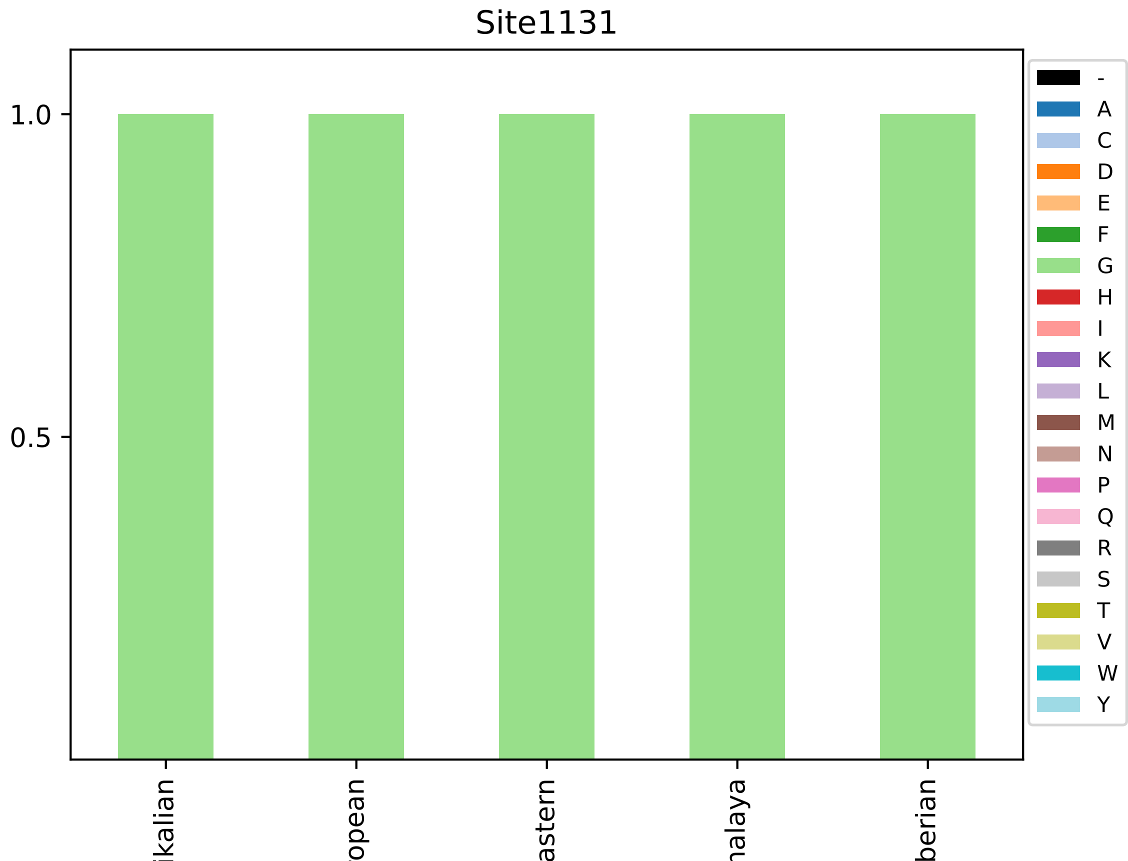

Site1131
1.0
0.5
-
A
C
D
E
F
G
H
I
K
L
M
N
P
Q
R
S
T
V
W
Y
ikalian
opean
astern
nalaya
berian

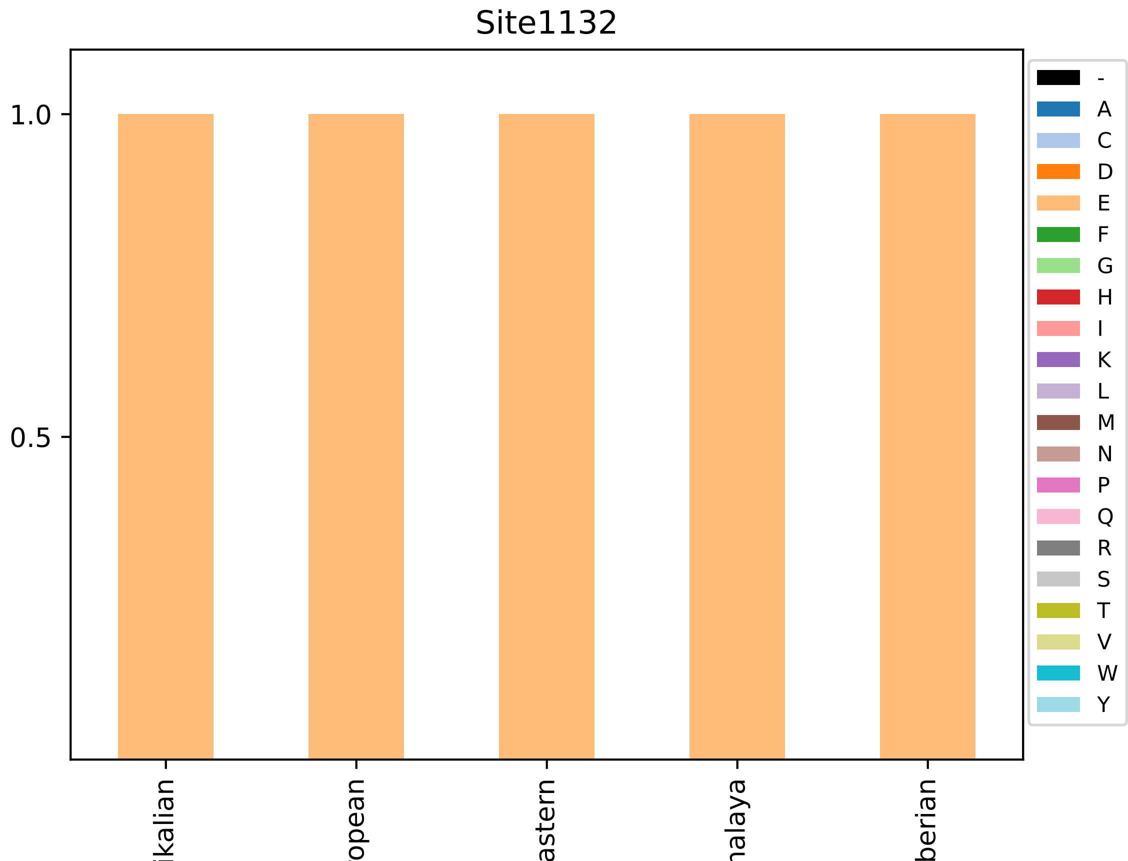

Site1132
1.0
0.5
ikalian
opean
astern
nalaya
berian
-
A
C
D
E
F
G
H
I
K
L
M
N
P
Q
R
S
T
V
W
Y

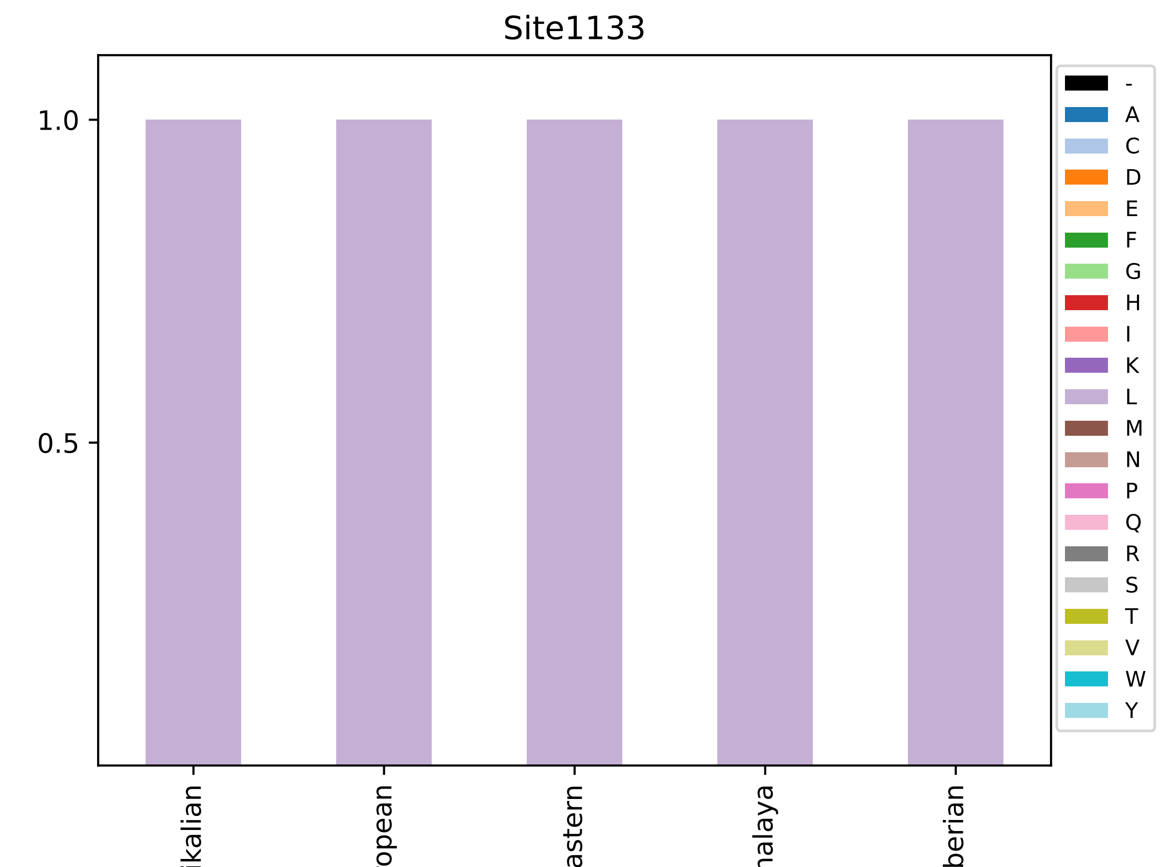

Site1133
1.0
0.5
ikalian
opean
astern
nalaya
berian
-
A
C
D
E
F
G
H
I
K
L
M
N
P
Q
R
S
T
V
W
Y

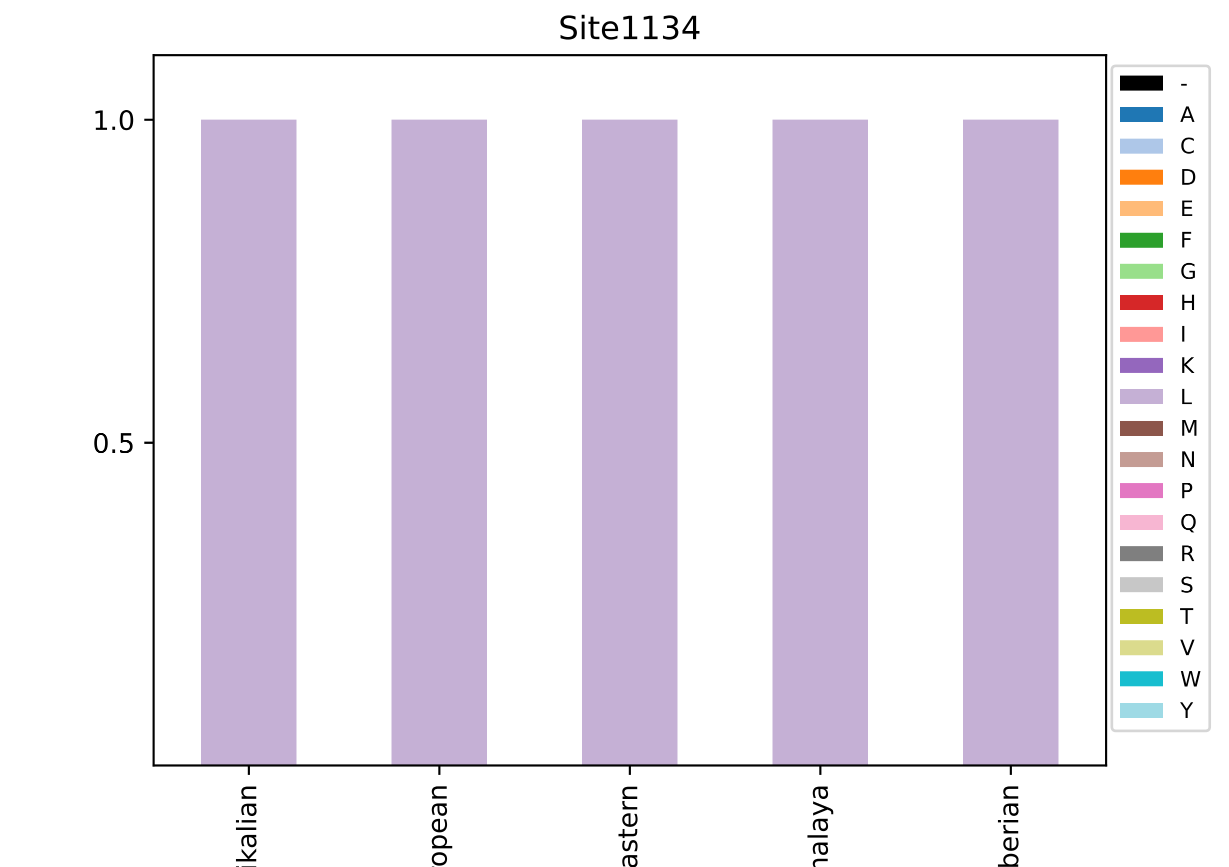
Site1134
1.0
0.5
-
A
C
D
E
F
G
H
I
K
L
M
N
P
Q
R
S
T
V
W
Y

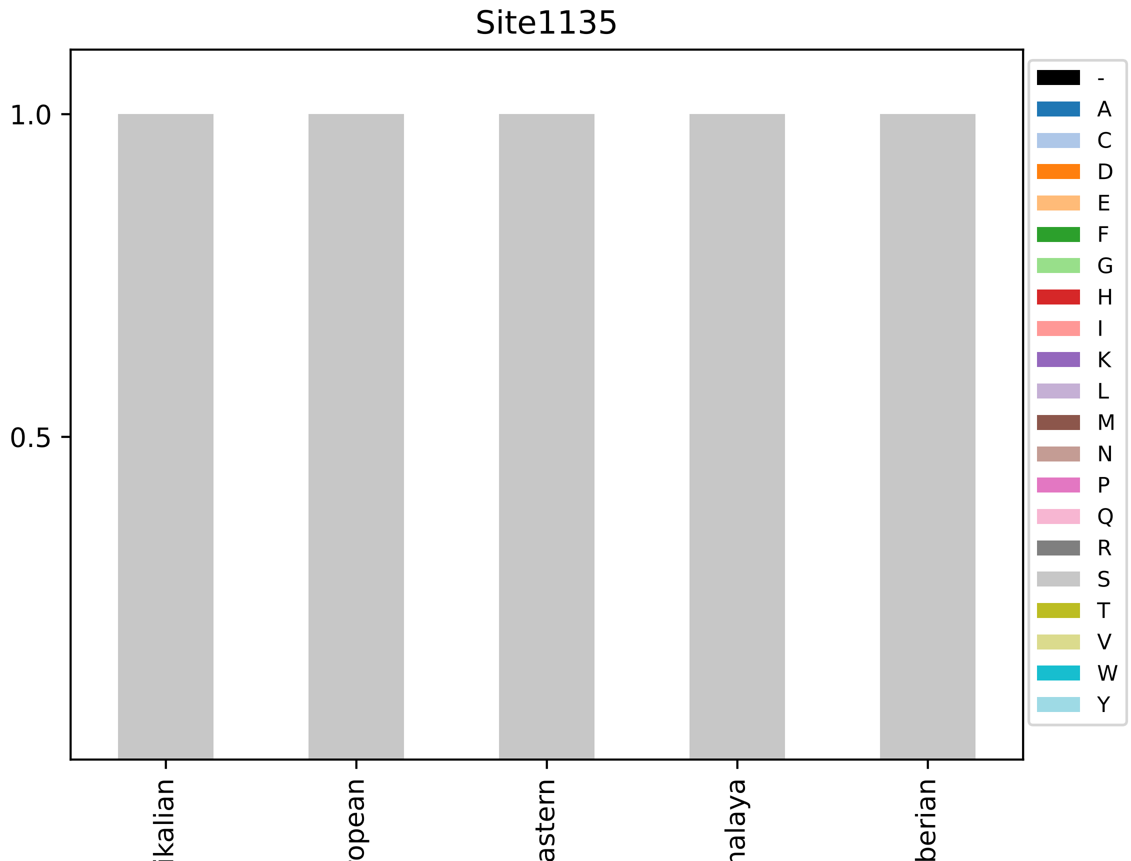

Site1135
1.0
0.5
-
A
C
D
E
F
G
H
I
K
L
M
N
P
Q
R
S
T
V
W
Y

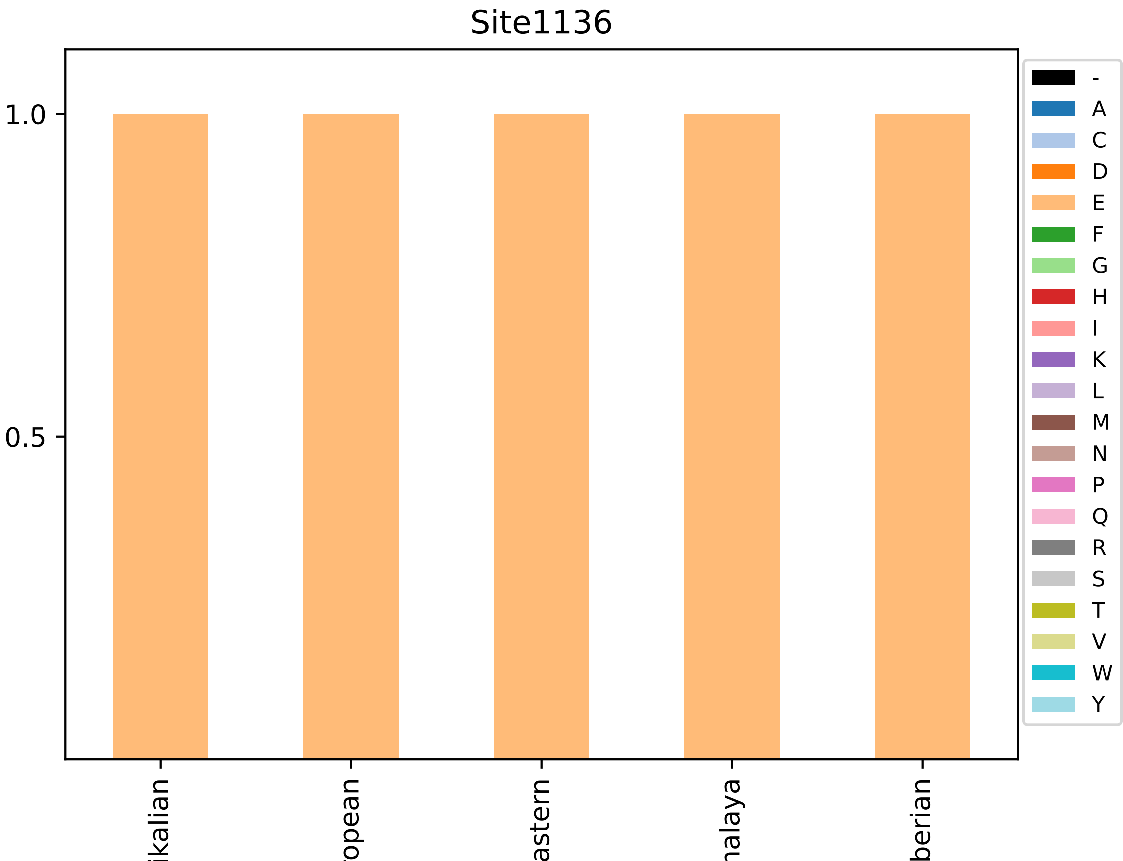

Site1136
1.0
0.5
-
A
C
D
E
F
G
H
I
K
L
M
N
P
Q
R
S
T
V
W
Y

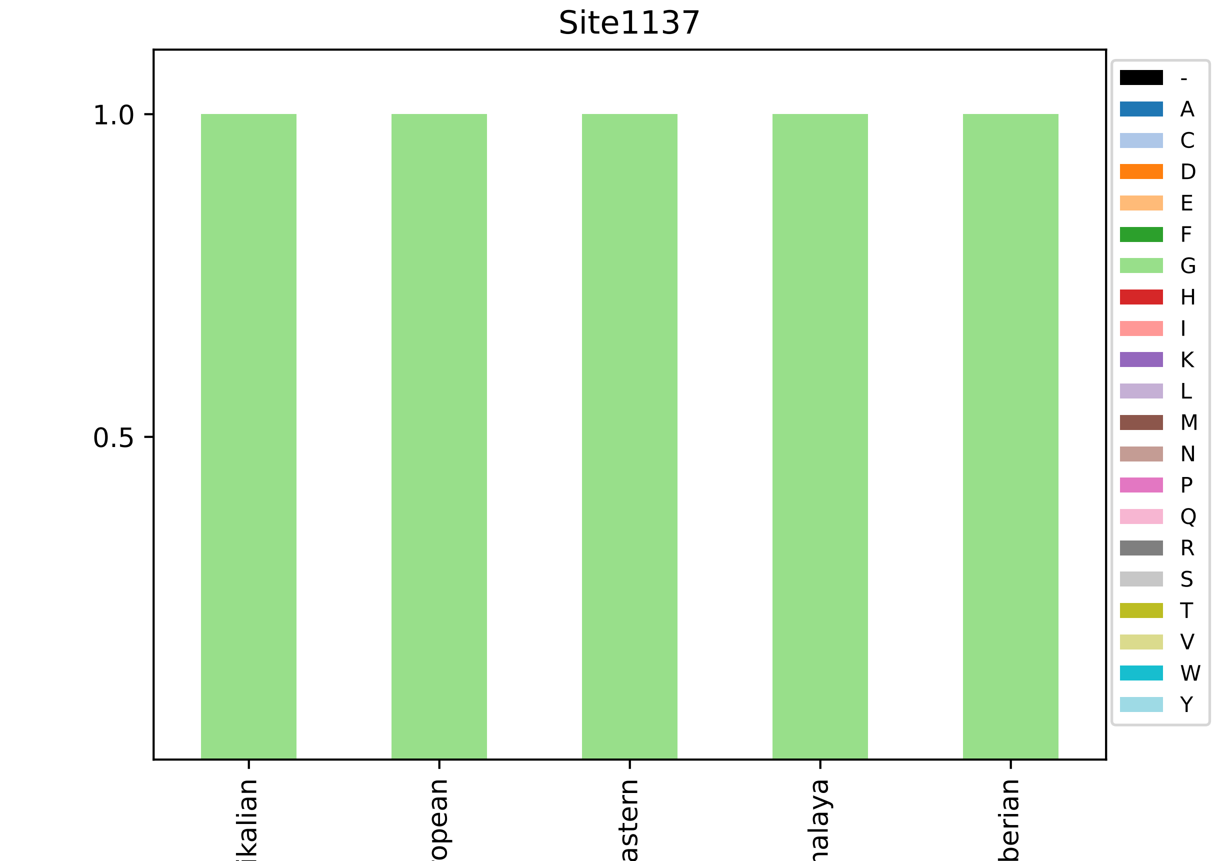

Site1137
1.0
0.5
-
A
C
D
E
F
G
H
I
K
L
M
N
P
Q
R
S
T
V
W
Y
ikalian
opean
astern
nalaya
berian

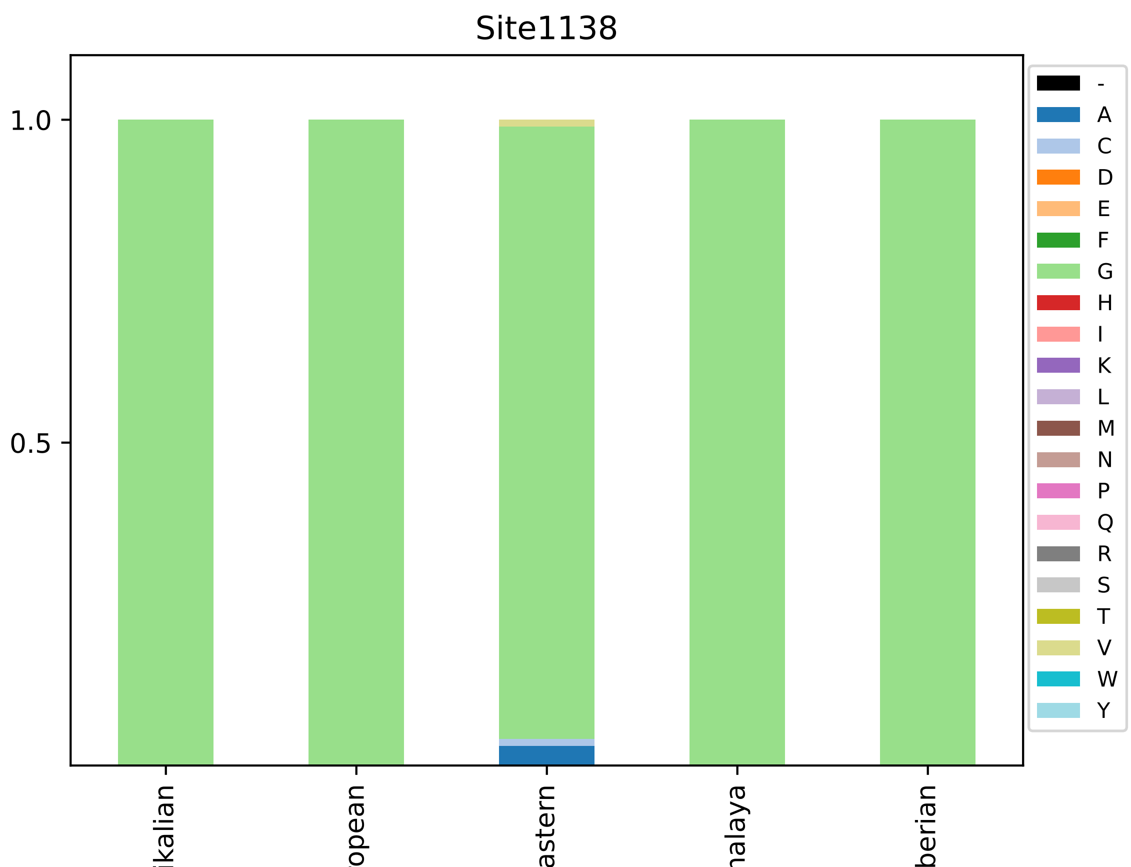

Site1138
1.0
0.5
ikalian
opean
astern
nalaya
berian
-
A
C
D
E
F
G
H
I
K
L
M
N
P
Q
R
S
T
V
W
Y

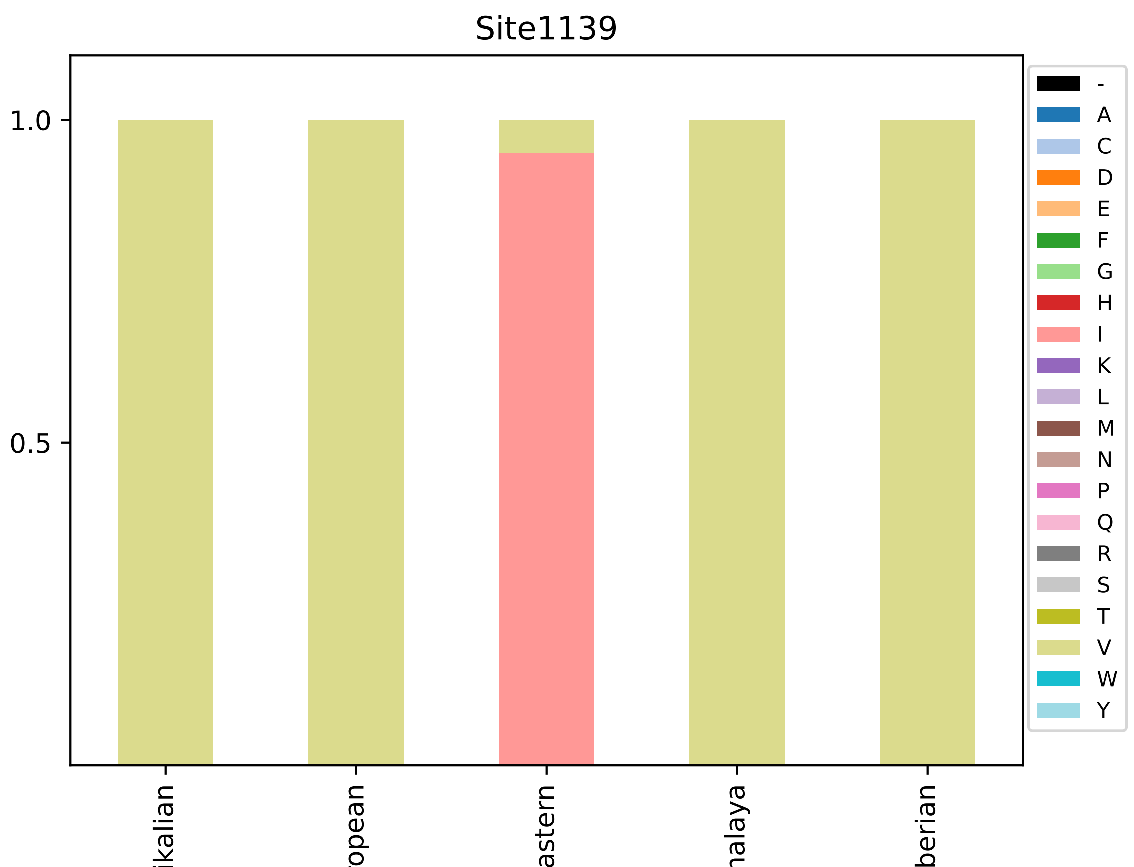
Site1139
1.0
0.5
-
A
C
D
E
F
G
H
I
K
L
M
N
P
Q
R
S
T
V
W
Y
ikalian
opean
astern
nalaya
berian

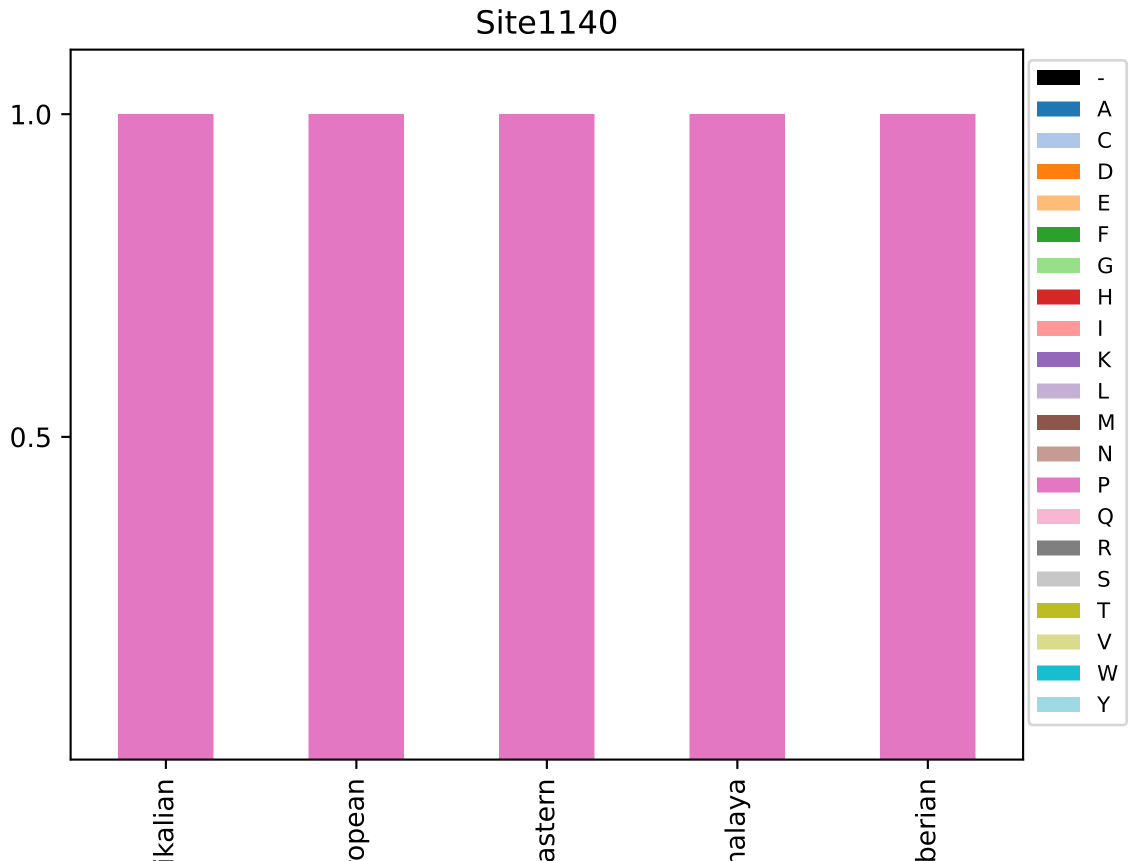
Site1140
1.0
0.5
-
A
C
D
E
F
G
H
I
K
L
M
N
P
Q
R
S
T
V
W
Y

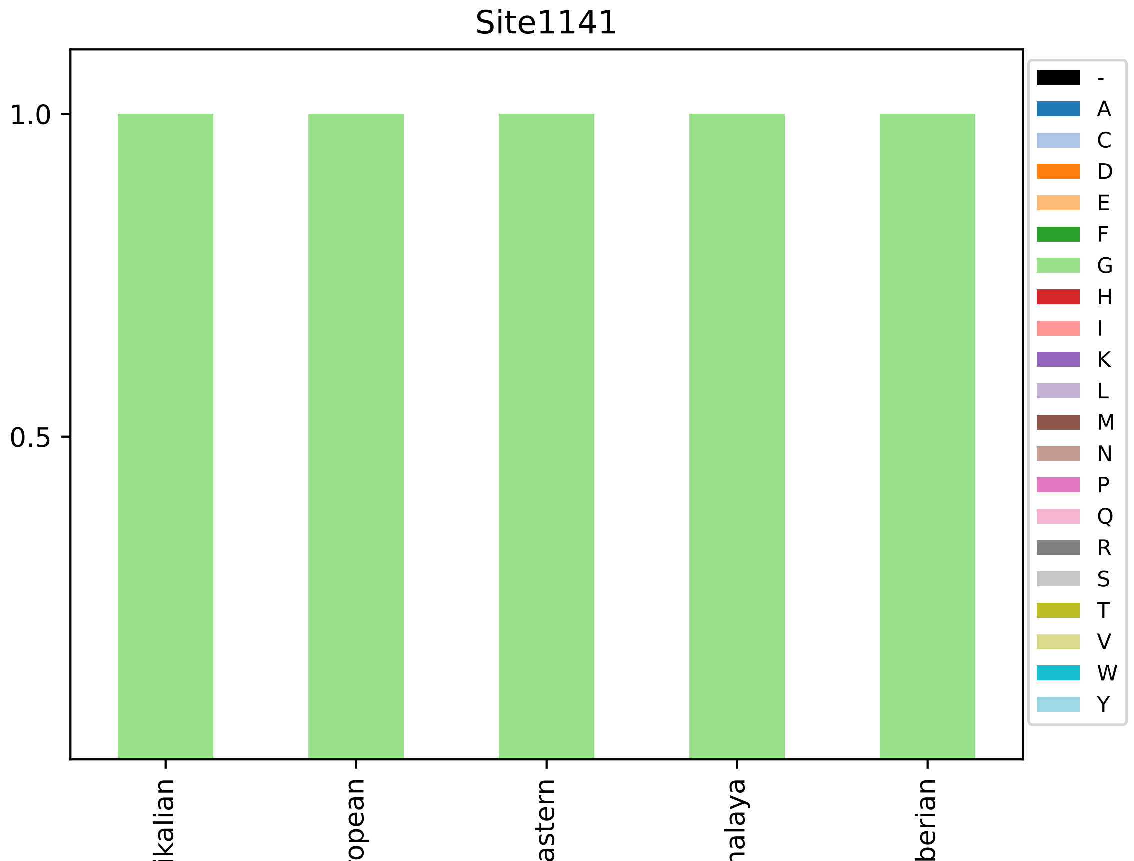
Site1141
1.0
0.5
-
A
C
D
E
F
G
H
I
K
L
M
N
P
Q
R
S
T
V
W
Y
ikalian
opean
astern
nalaya
berian

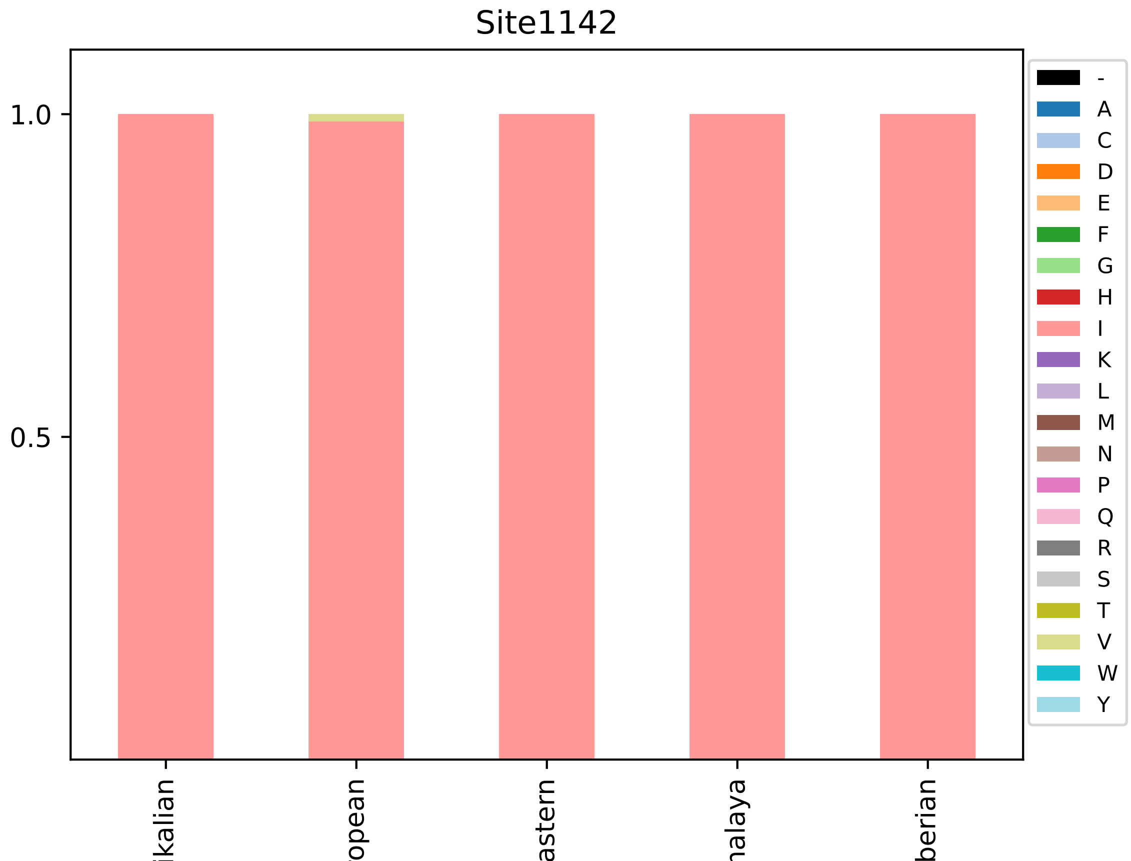
Site1142
1.0
0.5
-
A
C
D
E
F
G
H
I
K
L
M
N
P
Q
R
S
T
V
W
Y
ikalian
opean
astern
nalaya
berian

Site1143

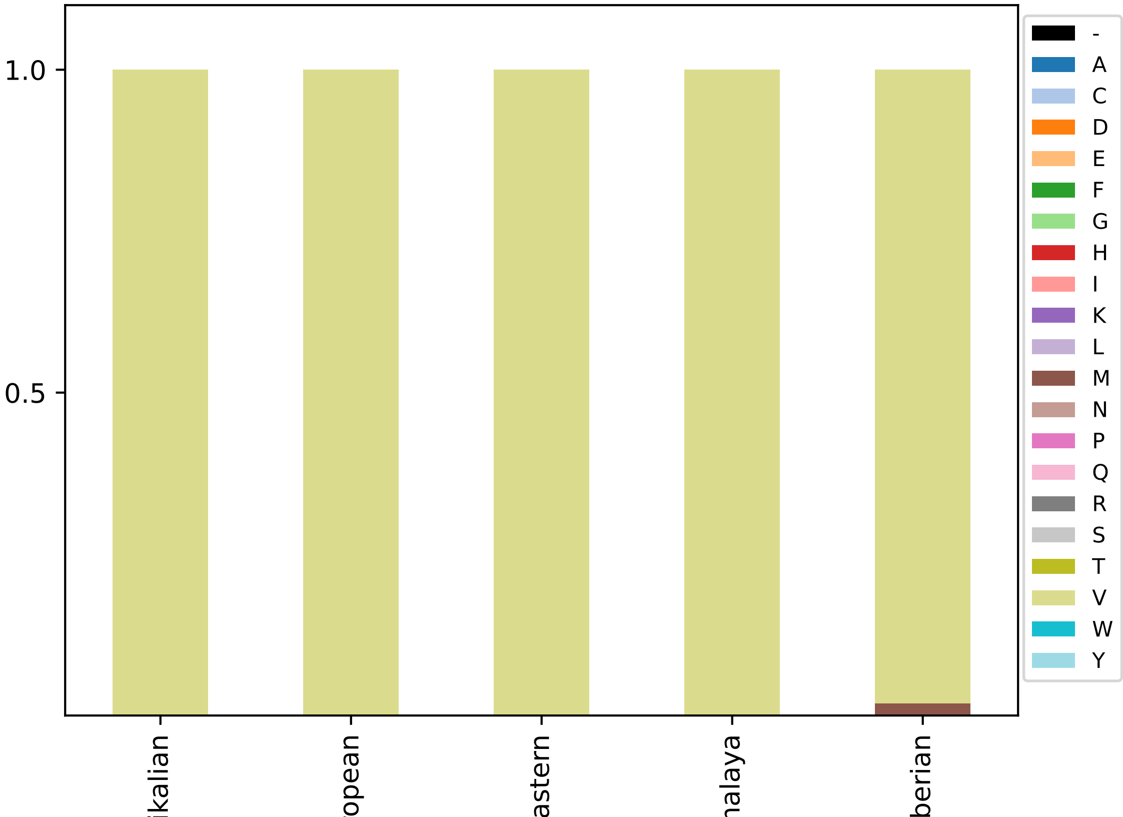
1.0
0.5
ikalian
opean
astern
nalaya
berian
-
A
C
D
E
F
G
H
I
K
L
M
N
P
Q
R
S
T
V
W
Y

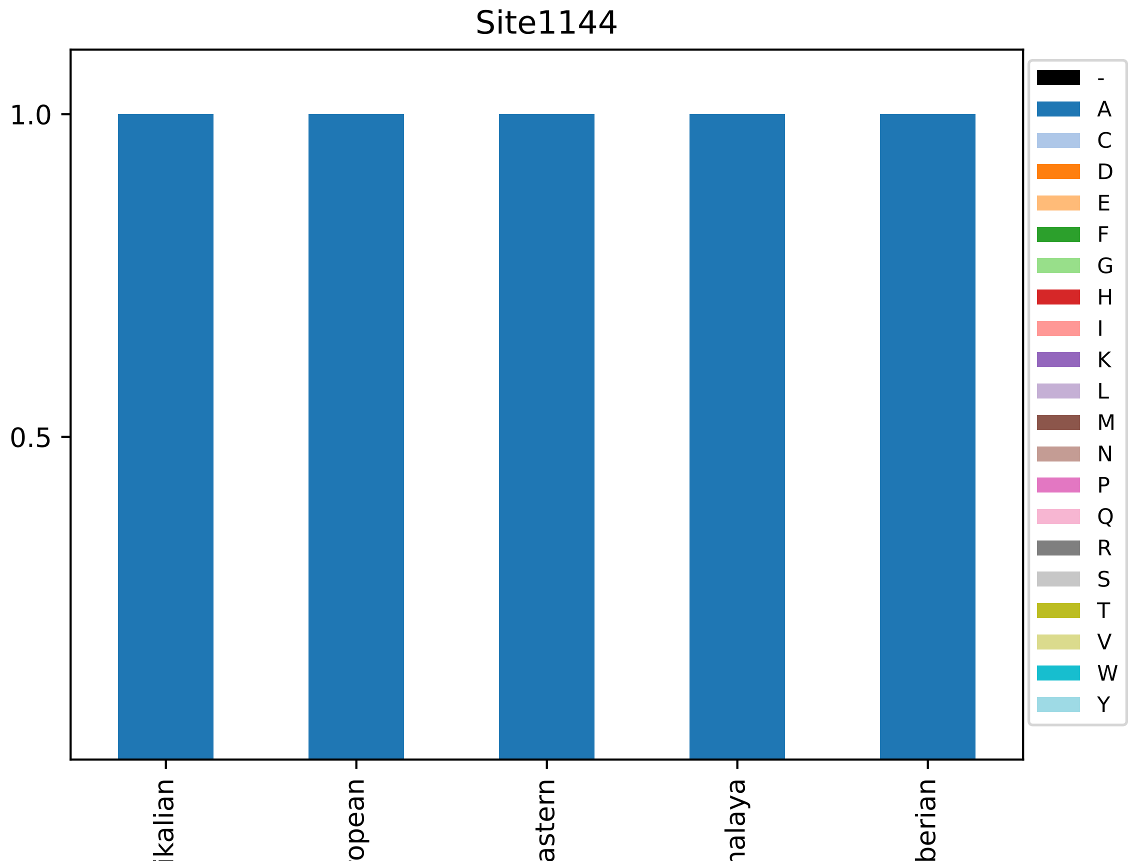
Site1144
1.0
0.5
-
A
C
D
E
F
G
H
I
K
L
M
N
P
Q
R
S
T
V
W
Y
ikalian
opean
astern
nalaya
berian

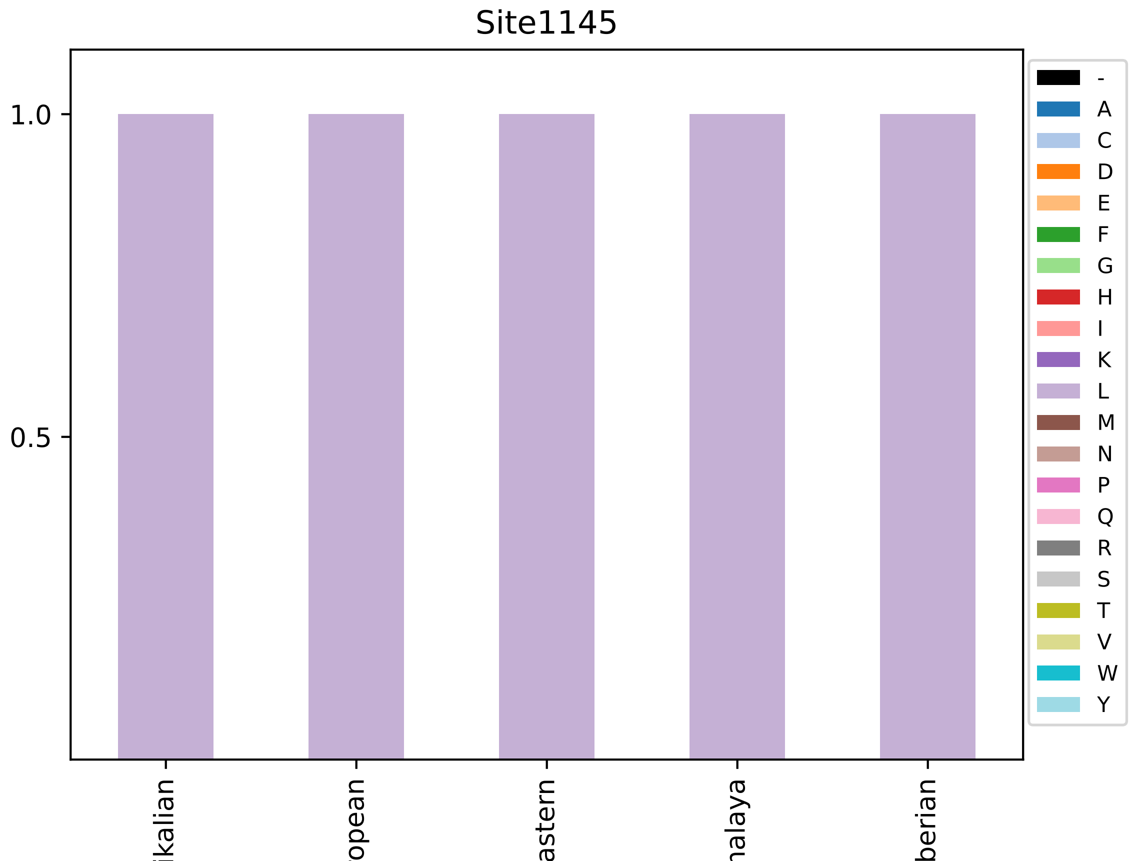
Site1145
1.0
0.5
-
A
C
D
E
F
G
H
I
K
L
M
N
P
Q
R
S
T
V
W
Y
ikalian
ropean
astern
nalaya
berian

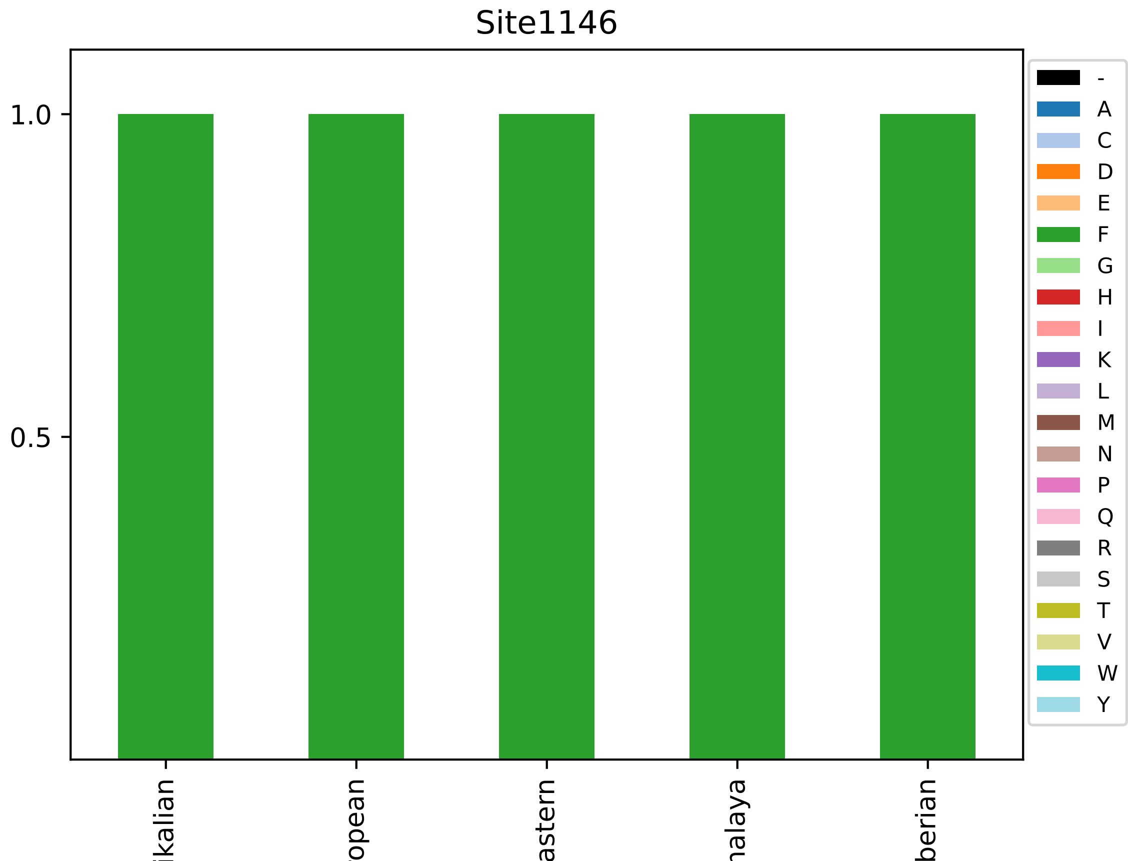
Site1146
1.0
0.5
-
A
C
D
E
F
G
H
I
K
L
M
N
P
Q
R
S
T
V
W
Y

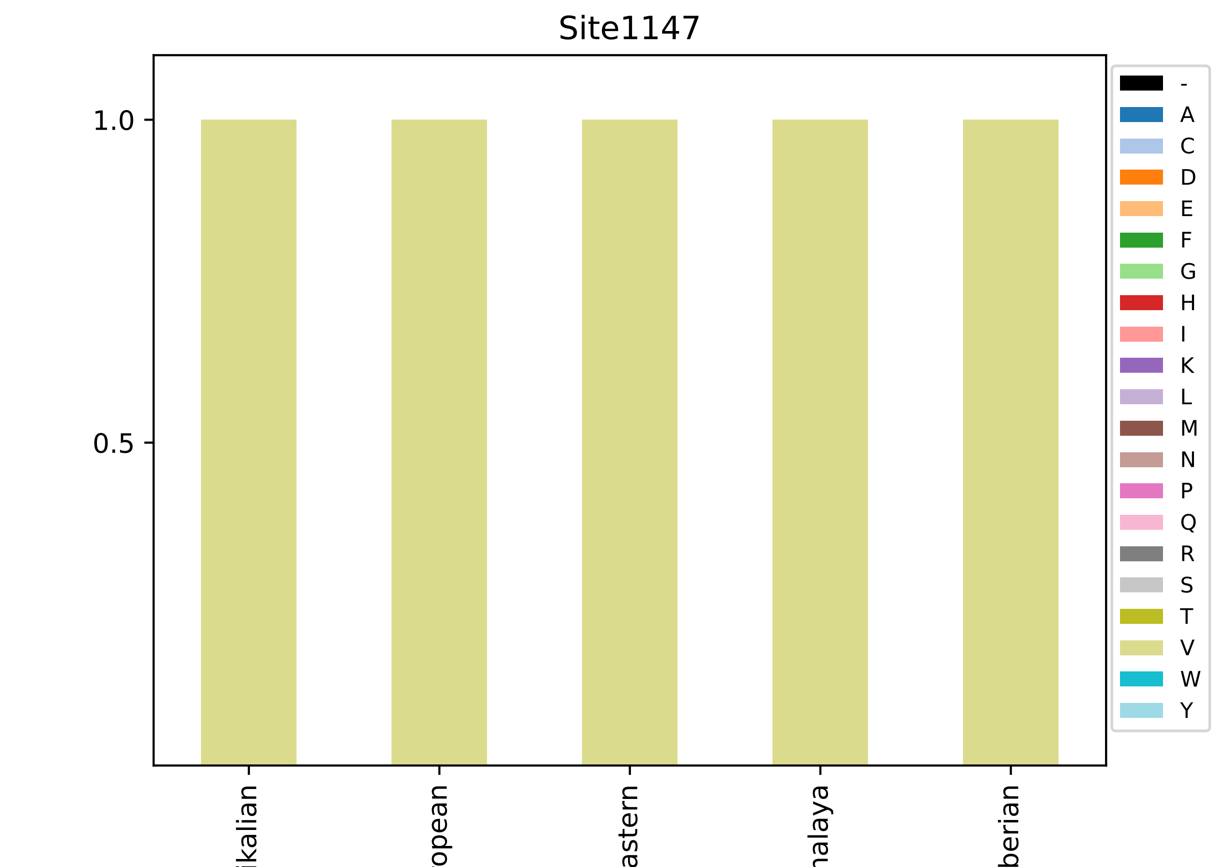

Site1147
1.0
0.5
ikalian
opean
astern
nalaya
berian
-
A
C
D
E
F
G
H
I
K
L
M
N
P
Q
R
S
T
V
W
Y

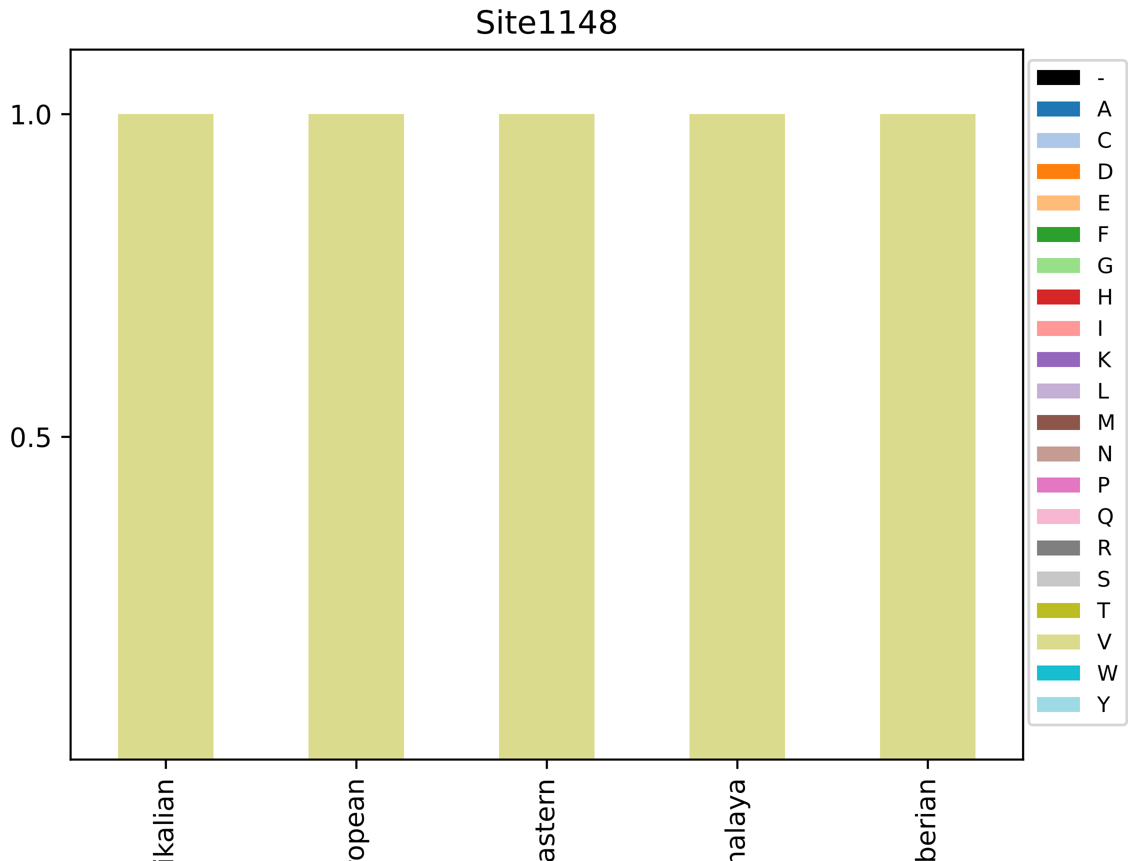
Site1148
1.0
0.5
-
A
C
D
E
F
G
H
I
K
L
M
N
P
Q
R
S
T
V
W
Y
ikalian
opean
astern
nalaya
berian

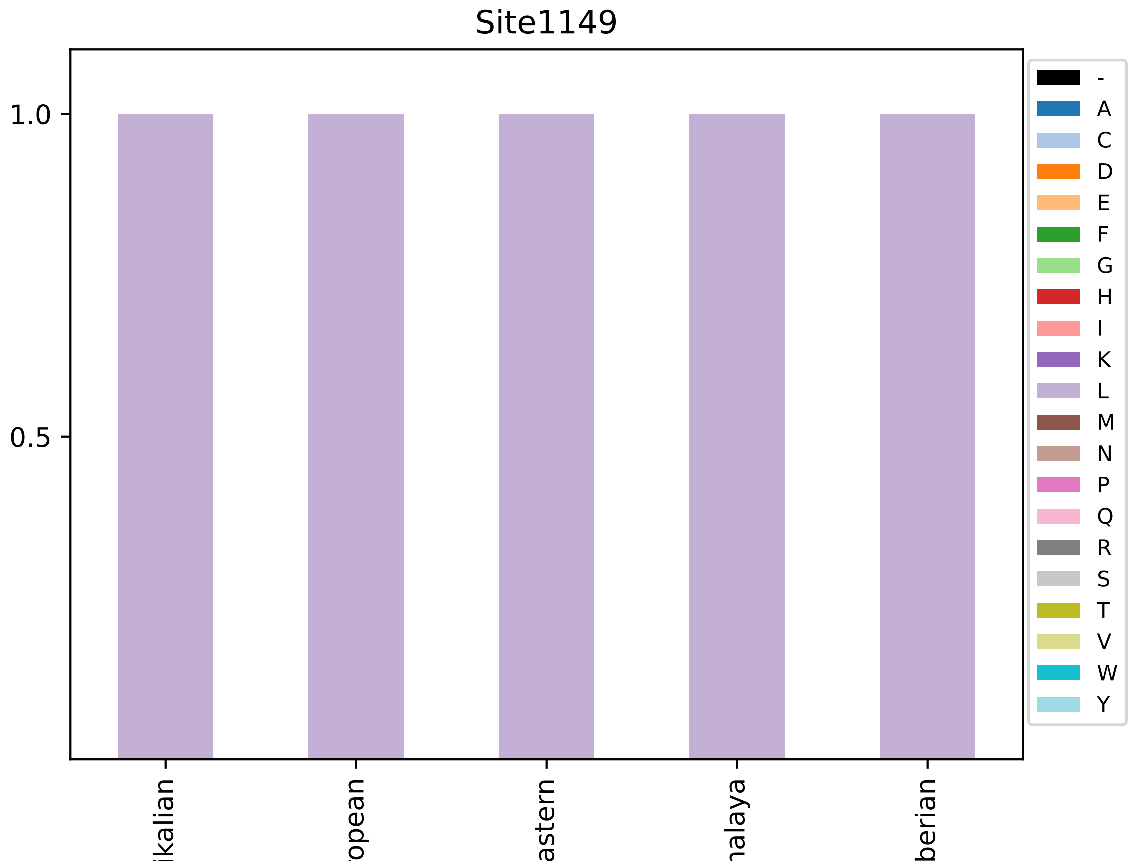
Site1149
1.0
0.5
ikalian
ropean
astern
nalaya
berian
-
A
C
D
E
F
G
H
I
K
L
M
N
P
Q
R
S
T
V
W
Y

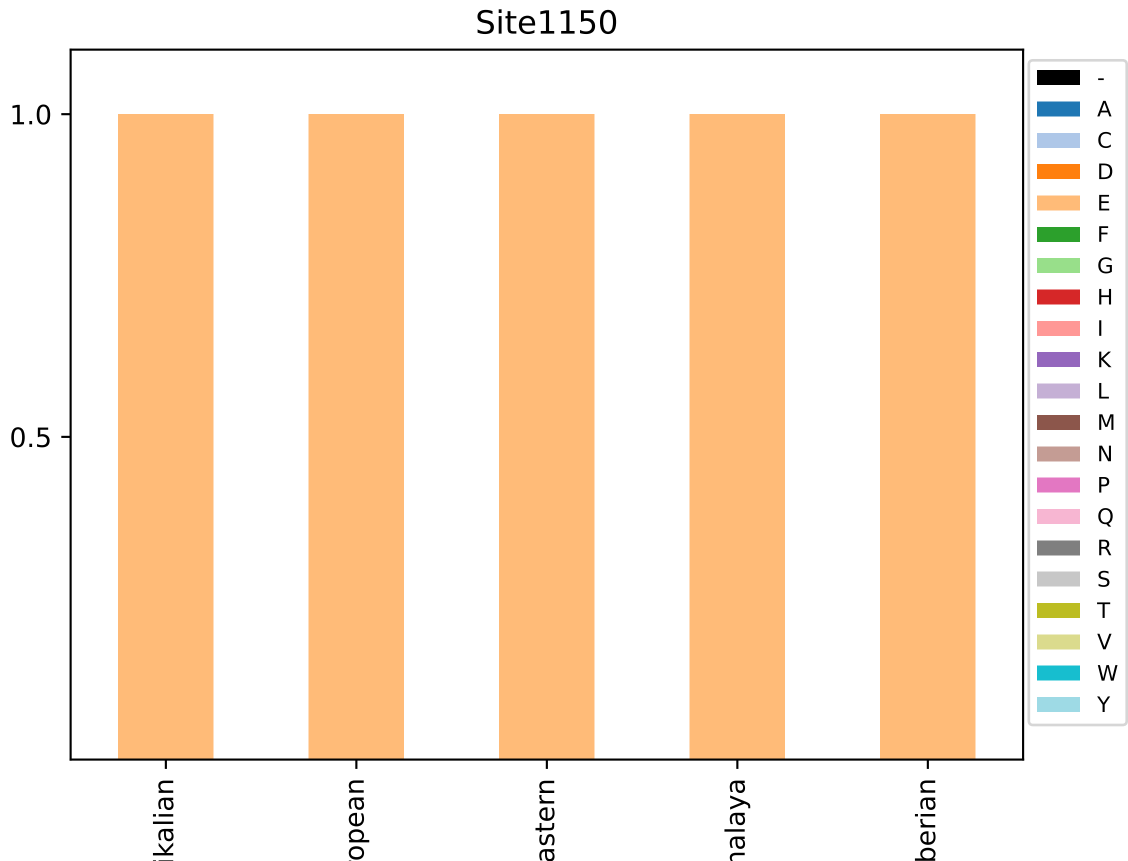

Site1150
1.0
0.5
-
A
C
D
E
F
G
H
I
K
L
M
N
P
Q
R
S
T
V
W
Y
ikalian
ropean
astern
nalaya
berian

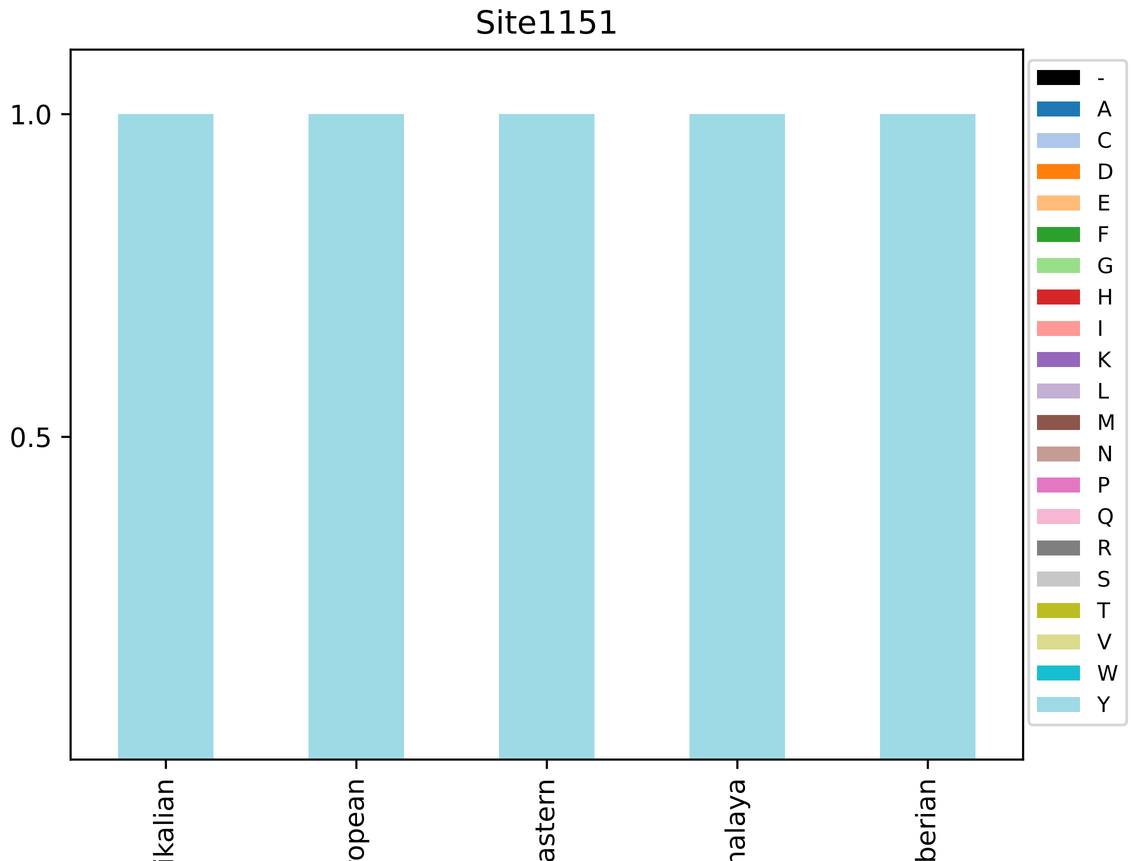

Site1151
1.0
0.5
-
A
C
D
E
F
G
H
I
K
L
M
N
P
Q
R
S
T
V
W
Y
ikalian
opean
astern
nalaya
berian

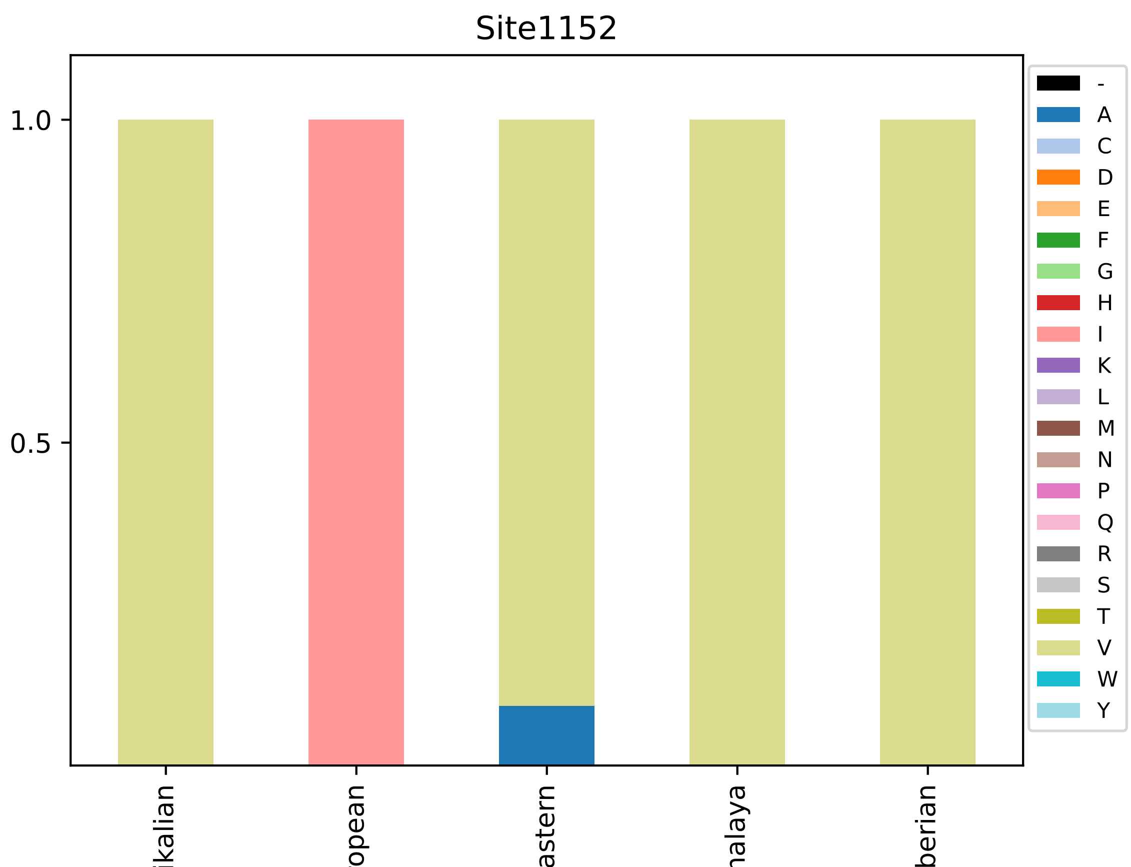

Site1152
1.0
0.5
-
A
C
D
E
F
G
H
I
K
L
M
N
P
Q
R
S
T
V
W
Y
ikalian
opean
astern
nalaya
berian

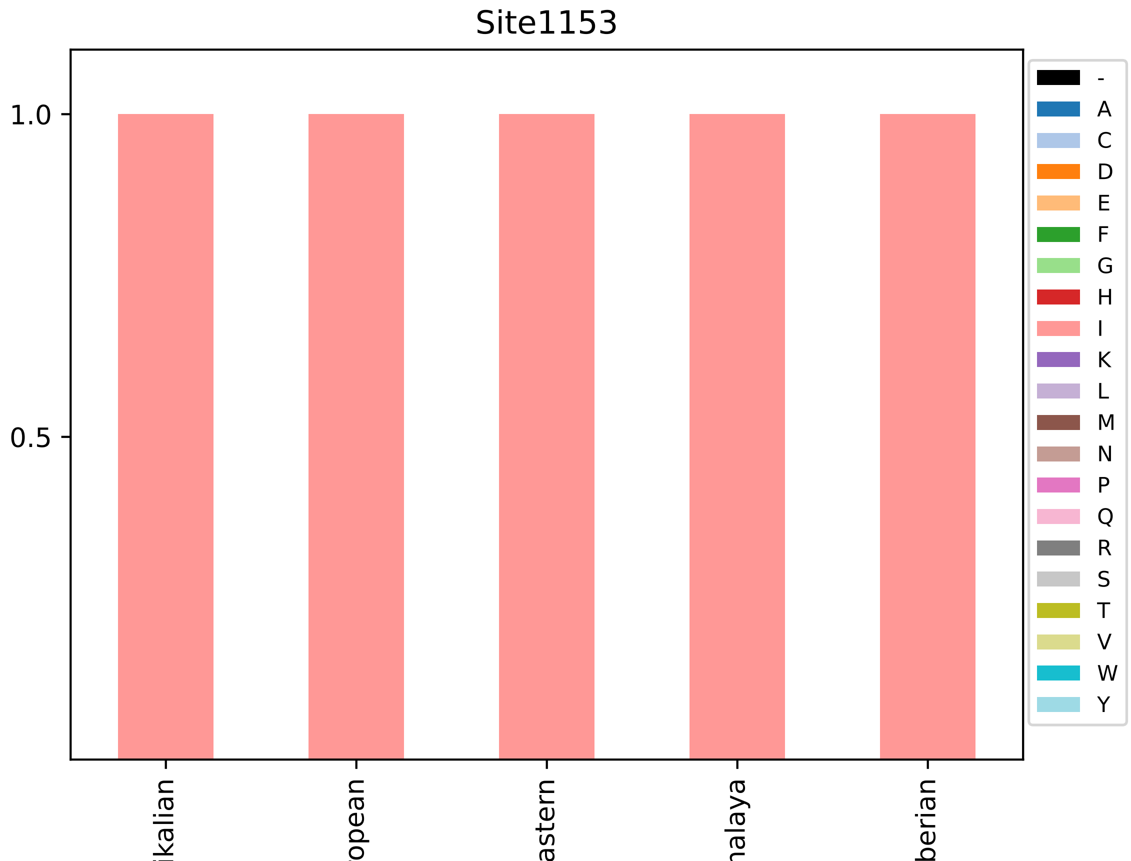
Site1153
1.0
0.5
ikalian
opean
astern
nalaya
berian
-
A
C
D
E
F
G
H
I
K
L
M
N
P
Q
R
S
T
V
W
Y

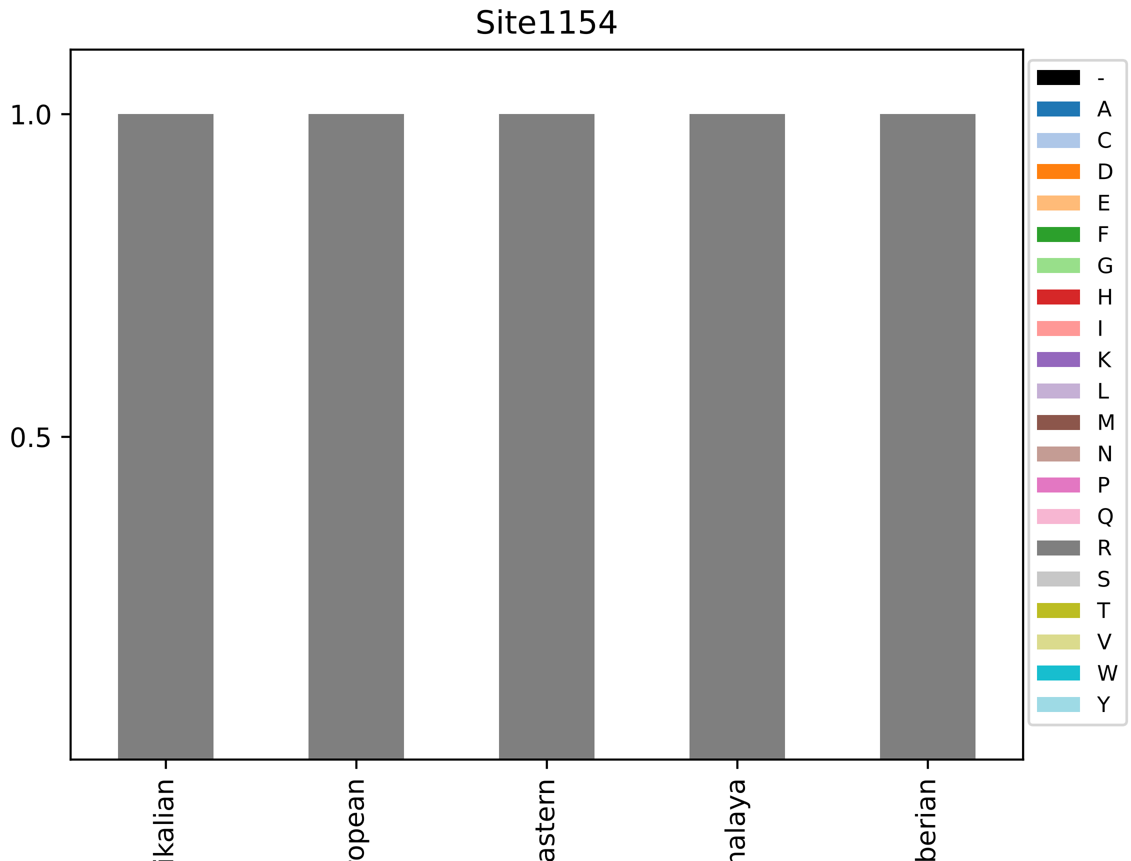
Site1154
1.0
0.5
ikalian
opean
astern
nalaya
berian
-
A
C
D
E
F
G
H
I
K
L
M
N
P
Q
R
S
T
V
W
Y

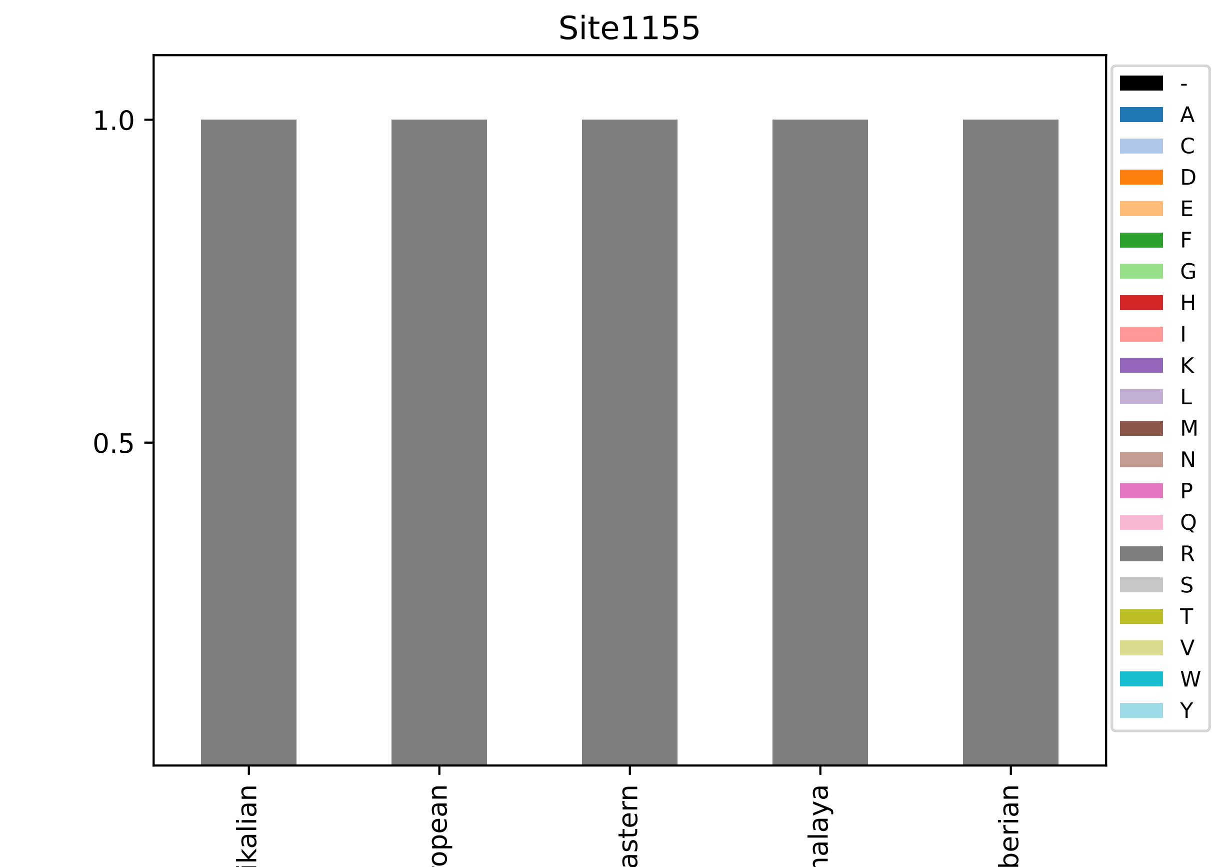

Site1155
1.0
0.5
-
A
C
D
E
F
G
H
I
K
L
M
N
P
Q
R
S
T
V
W
Y

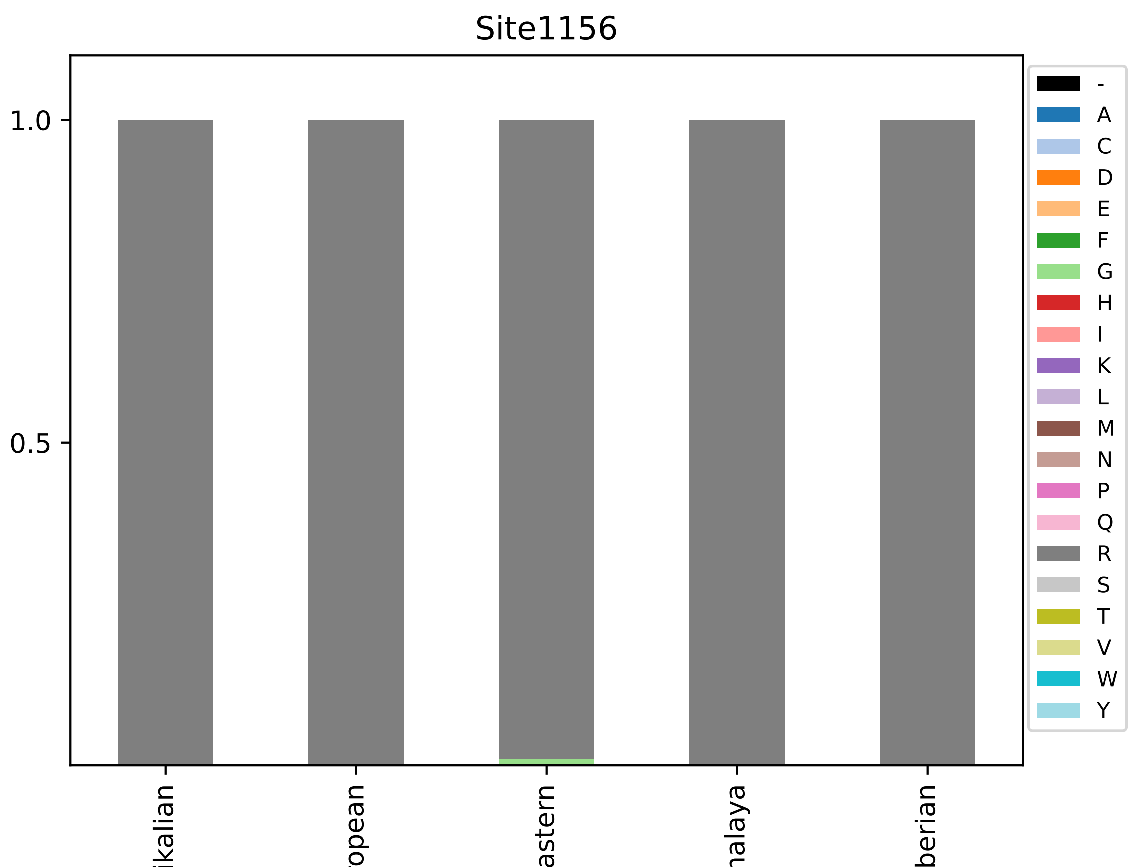

Site1156
1.0
0.5
-
A
C
D
E
F
G
H
I
K
L
M
N
P
Q
R
S
T
V
W
Y
ikalian
ropean
astern
nalaya
berian

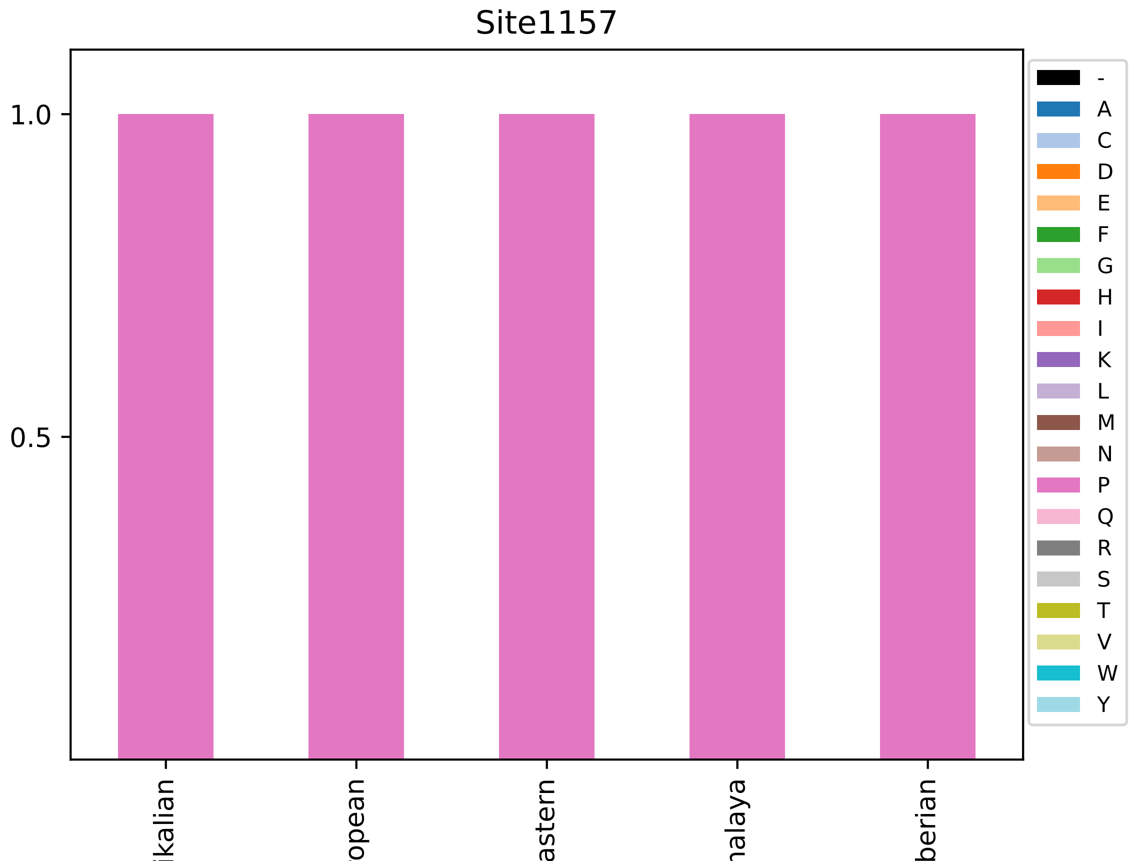

Site1157
1.0
0.5
-
A
C
D
E
F
G
H
I
K
L
M
N
P
Q
R
S
T
V
W
Y
ikalian
opean
astern
nalaya
berian

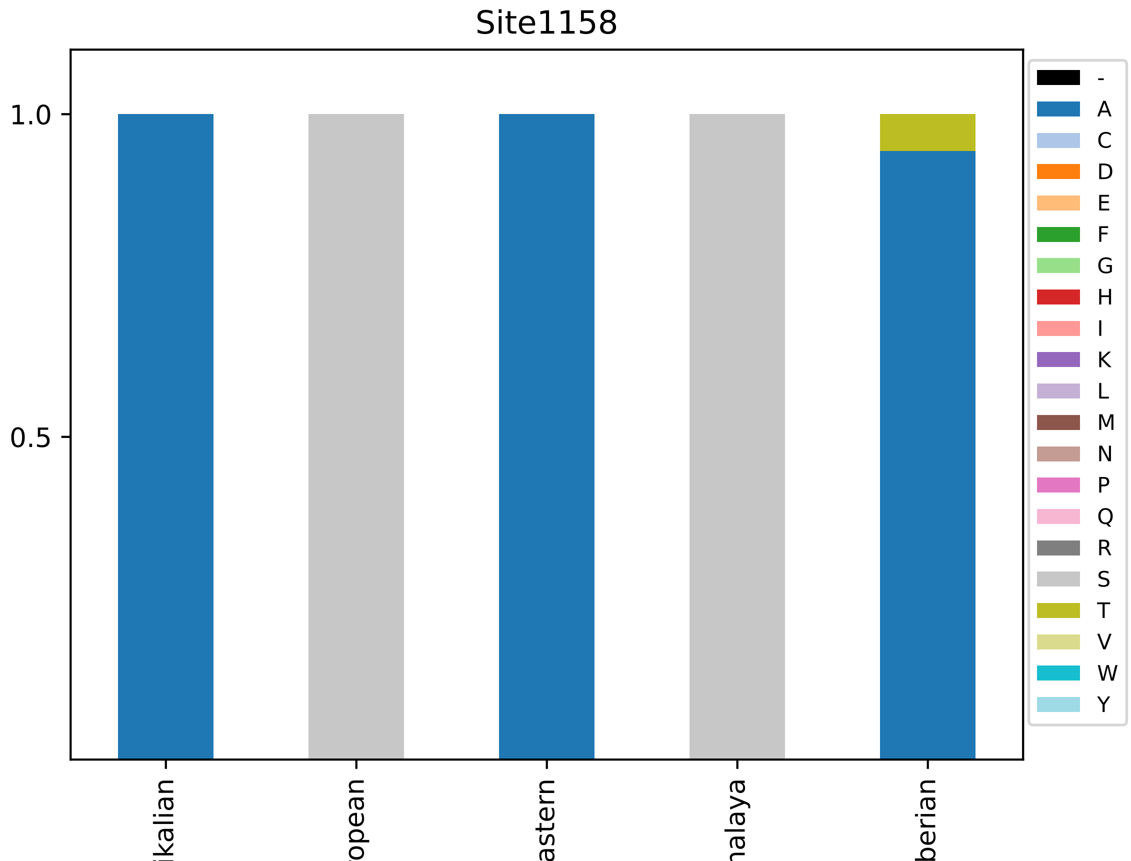

Site1158
1.0
0.5
-
A
C
D
E
F
G
H
I
K
L
M
N
P
Q
R
S
T
V
W
Y
ikalian
opean
astern
nalaya
berian

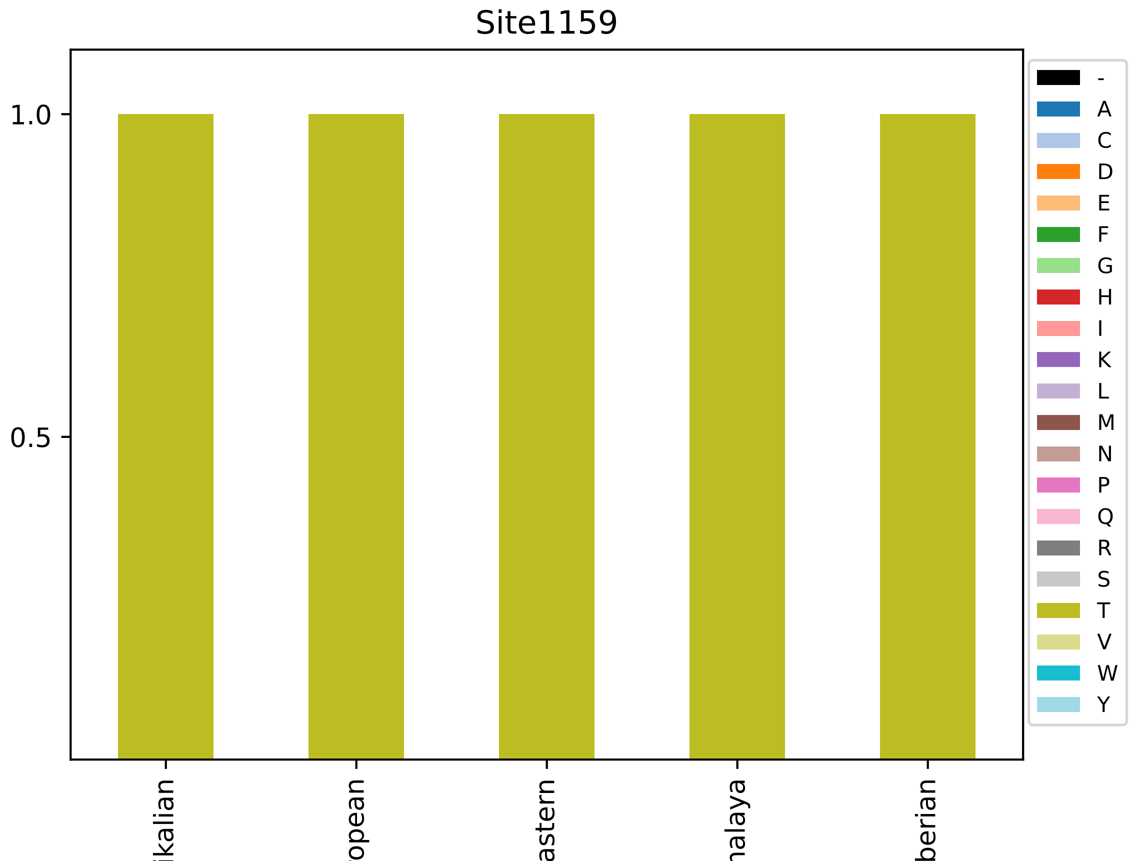
Site1159
1.0
0.5
-
A
C
D
E
F
G
H
I
K
L
M
N
P
Q
R
S
T
V
W
Y
ikalian
opean
astern
nalaya
berian

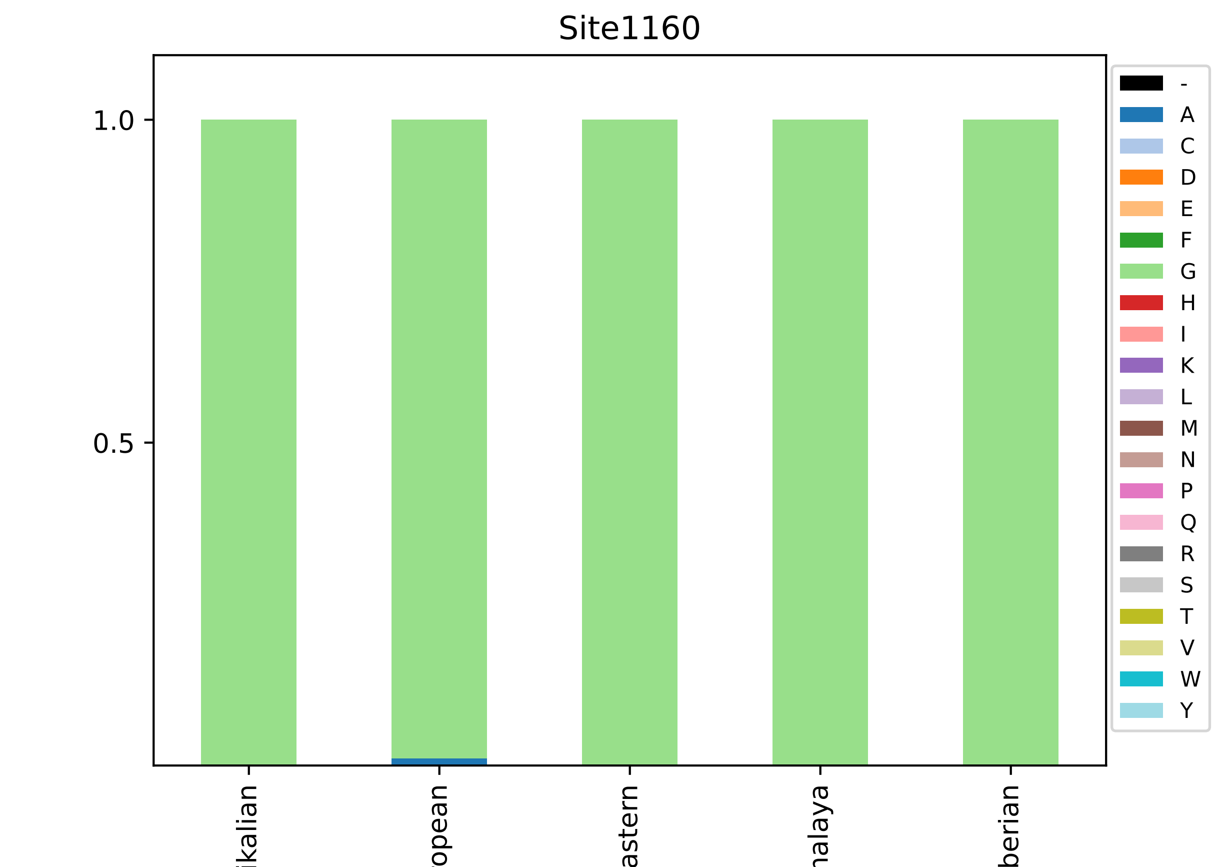

Site1160
1.0
0.5
-
A
C
D
E
F
G
H
I
K
L
M
N
P
Q
R
S
T
V
W
Y
ikalian
opean
astern
nalaya
berian

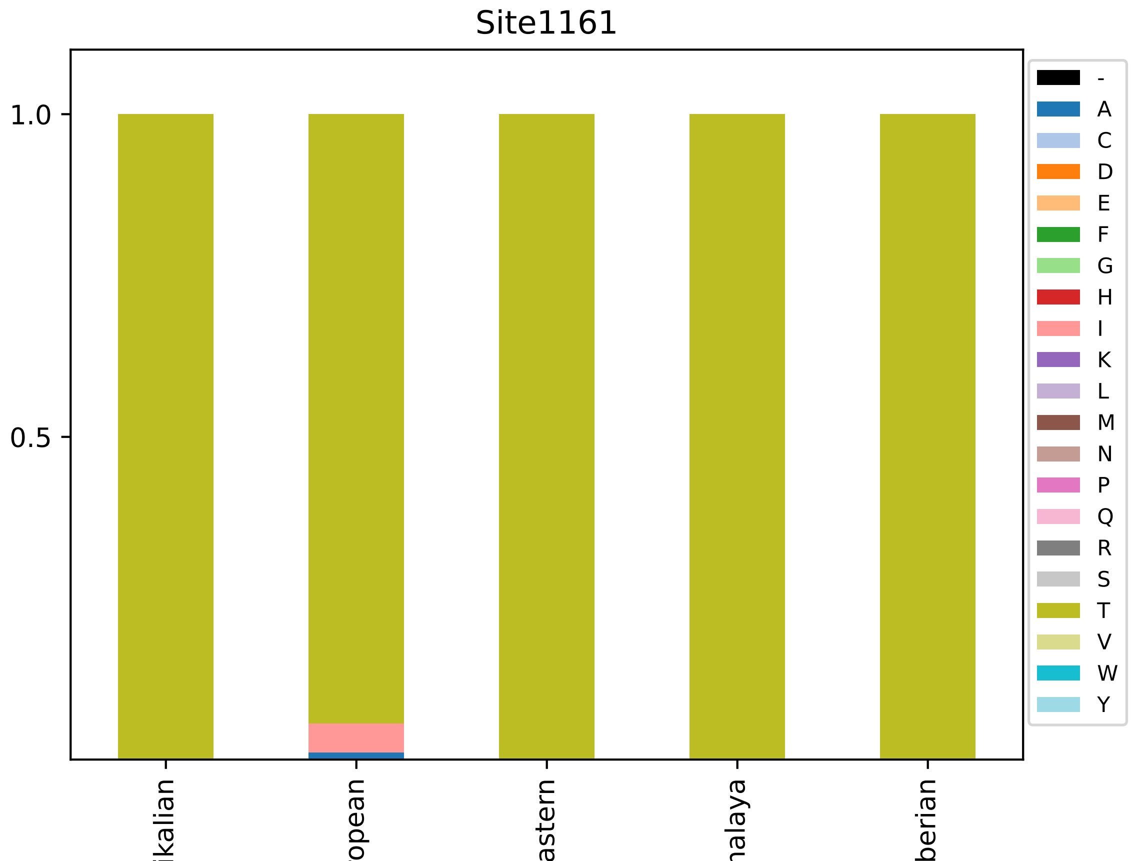
Site1161
1.0
0.5
-
A
C
D
E
F
G
H
I
K
L
M
N
P
Q
R
S
T
V
W
Y
ikalian
opean
astern
nalaya
berian

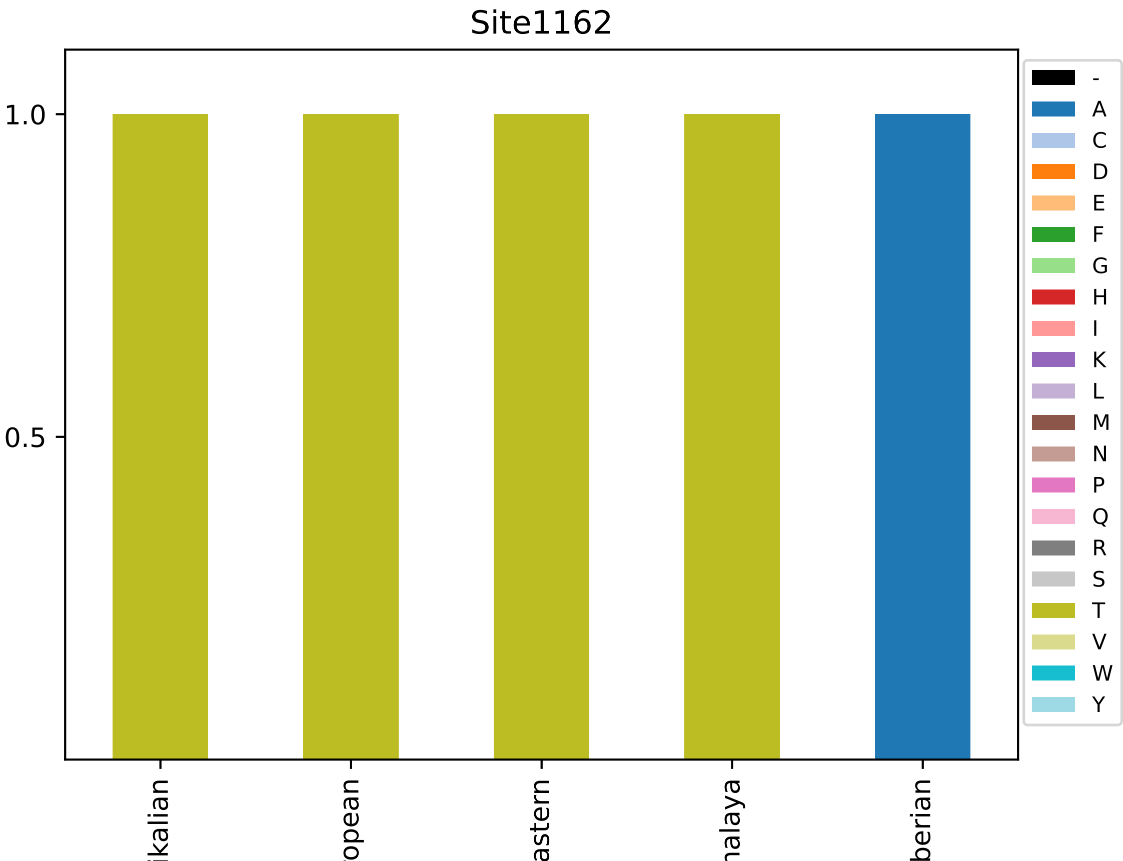
Site1162
1.0
0.5
-
A
C
D
E
F
G
H
I
K
L
M
N
P
Q
R
S
T
V
W
Y
ikalian
opean
astern
nalaya
berian

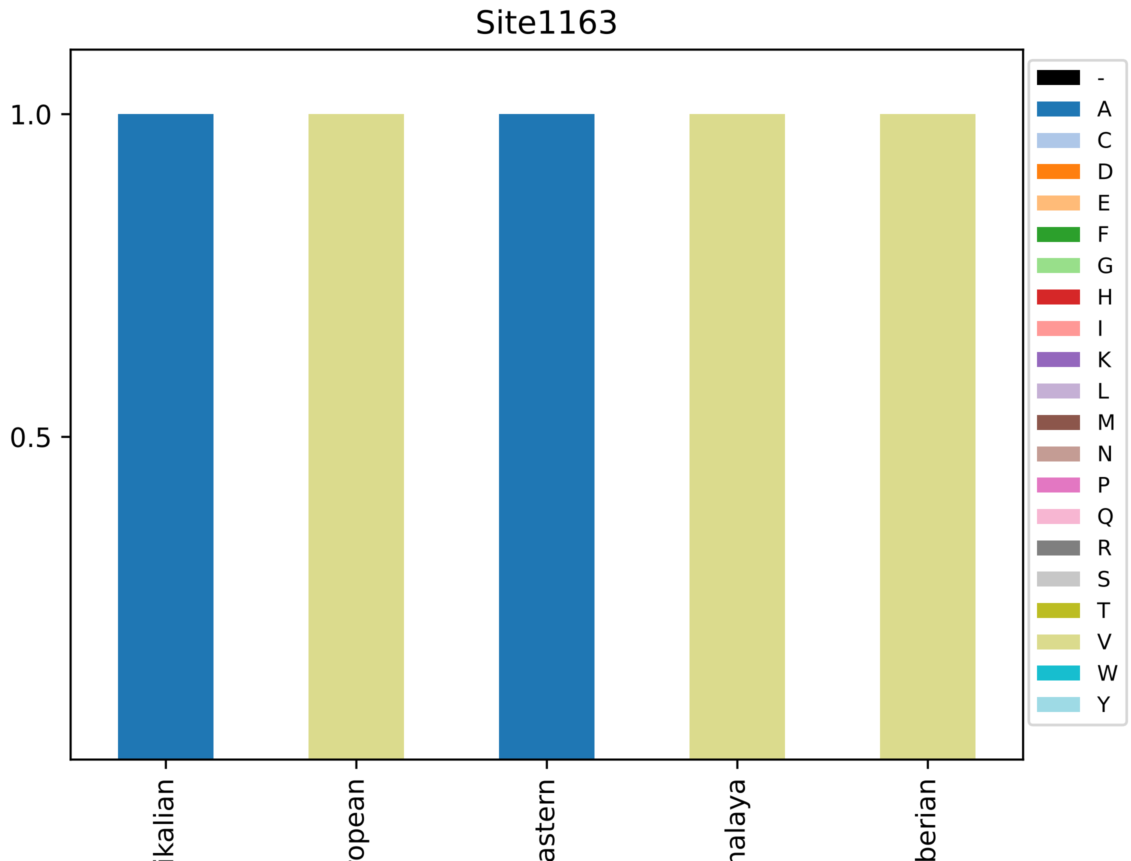
Site1163
1.0
0.5
-
A
C
D
E
F
G
H
I
K
L
M
N
P
Q
R
S
T
V
W
Y
ikalian
opean
astern
nalaya
berian

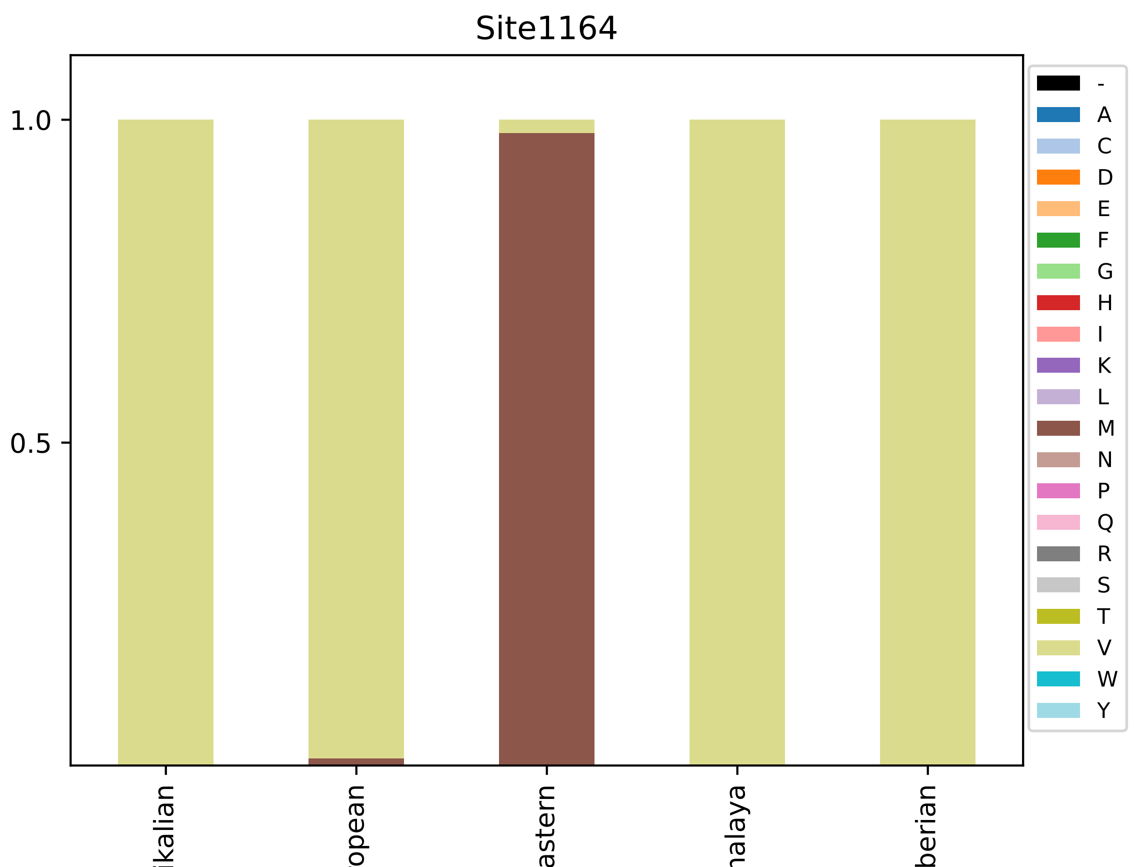
Site1164
1.0
0.5
ikalian
opean
astern
nalaya
berian
-
A
C
D
E
F
G
H
I
K
L
M
N
P
Q
R
S
T
V
W
Y

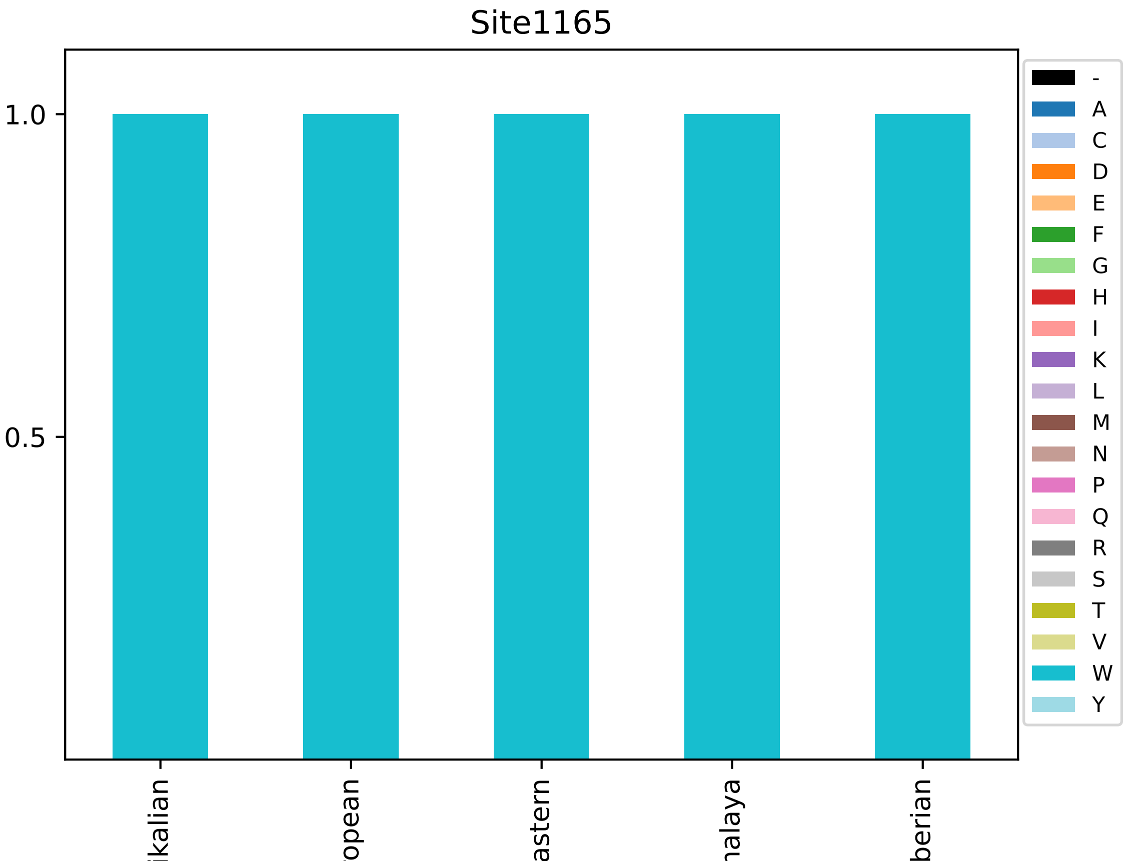
Site1165
1.0
0.5
-
A
C
D
E
F
G
H
I
K
L
M
N
P
Q
R
S
T
V
W
Y
ikalian
opean
astern
nalaya
berian

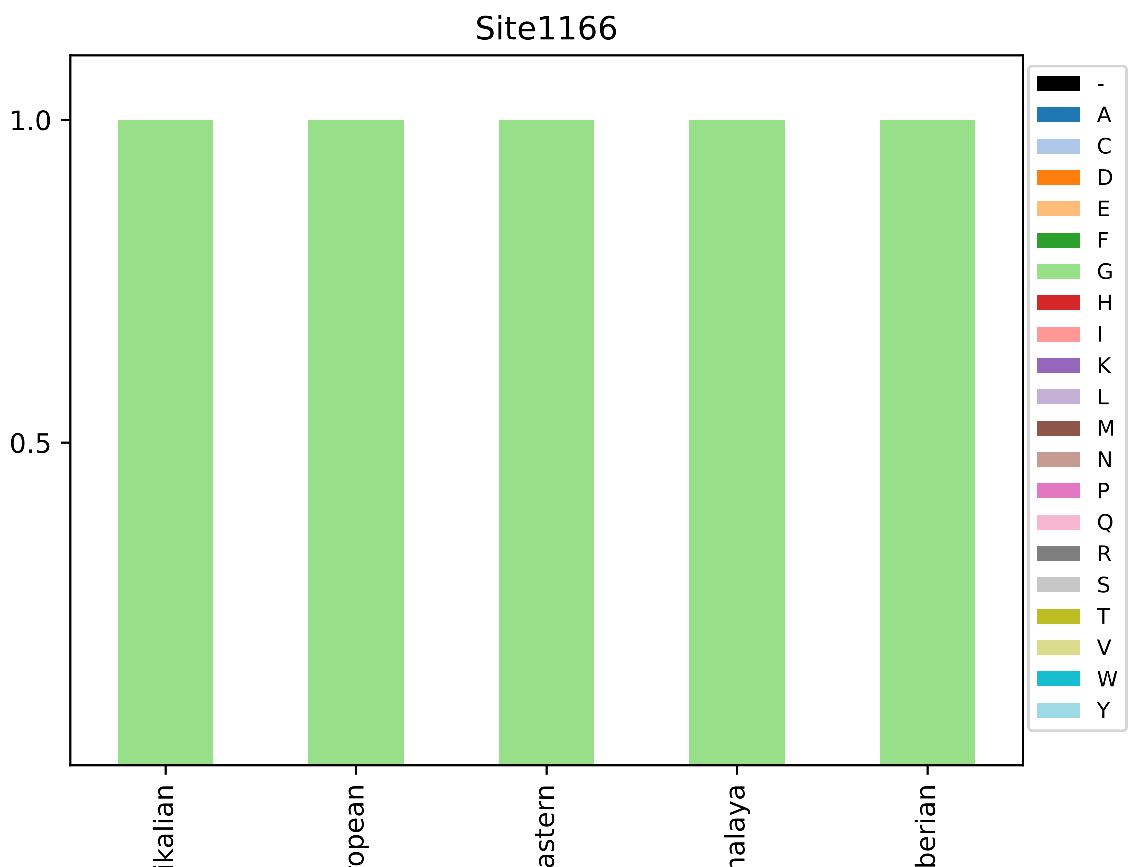

Site1166
1.0
0.5
ikalian
opean
astern
nalaya
berian
-
A
C
D
E
F
G
H
I
K
L
M
N
P
Q
R
S
T
V
W
Y

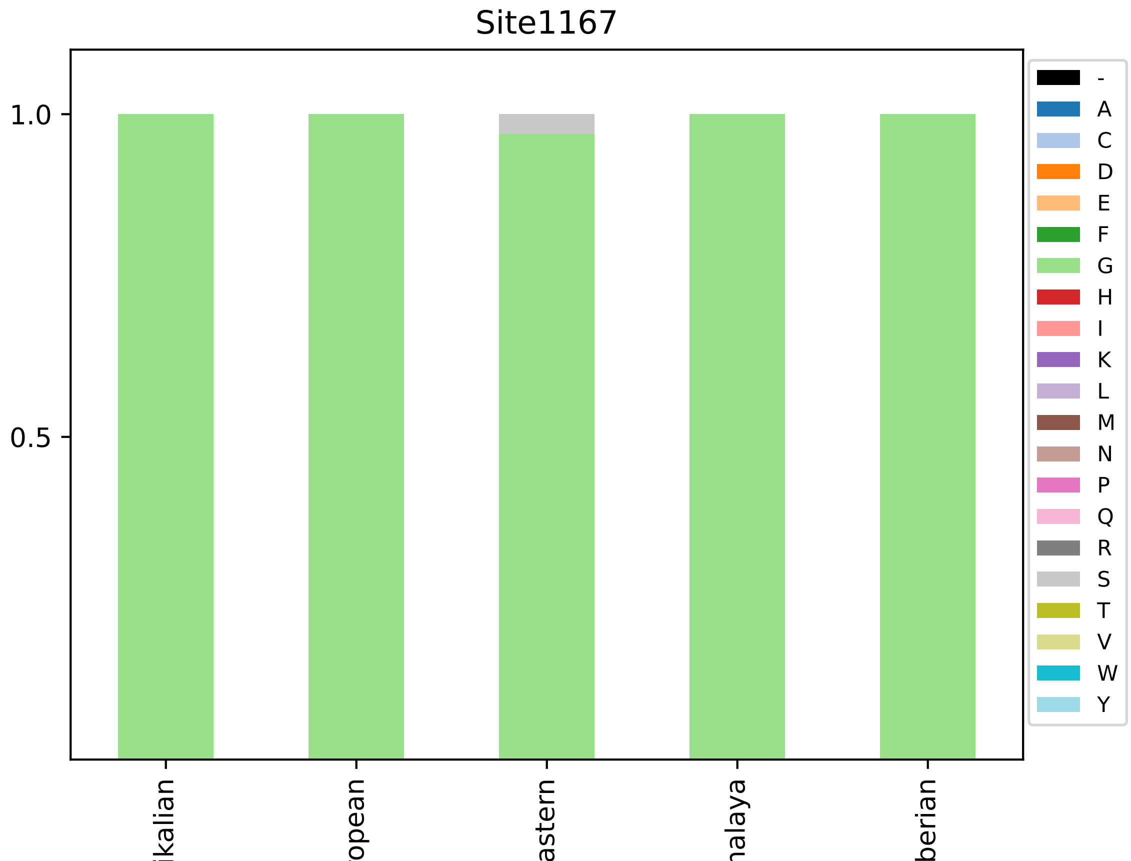
Site1167
1.0
0.5
ikalian
opean
astern
nalaya
berian
-
A
C
D
E
F
G
H
I
K
L
M
N
P
Q
R
S
T
V
W
Y

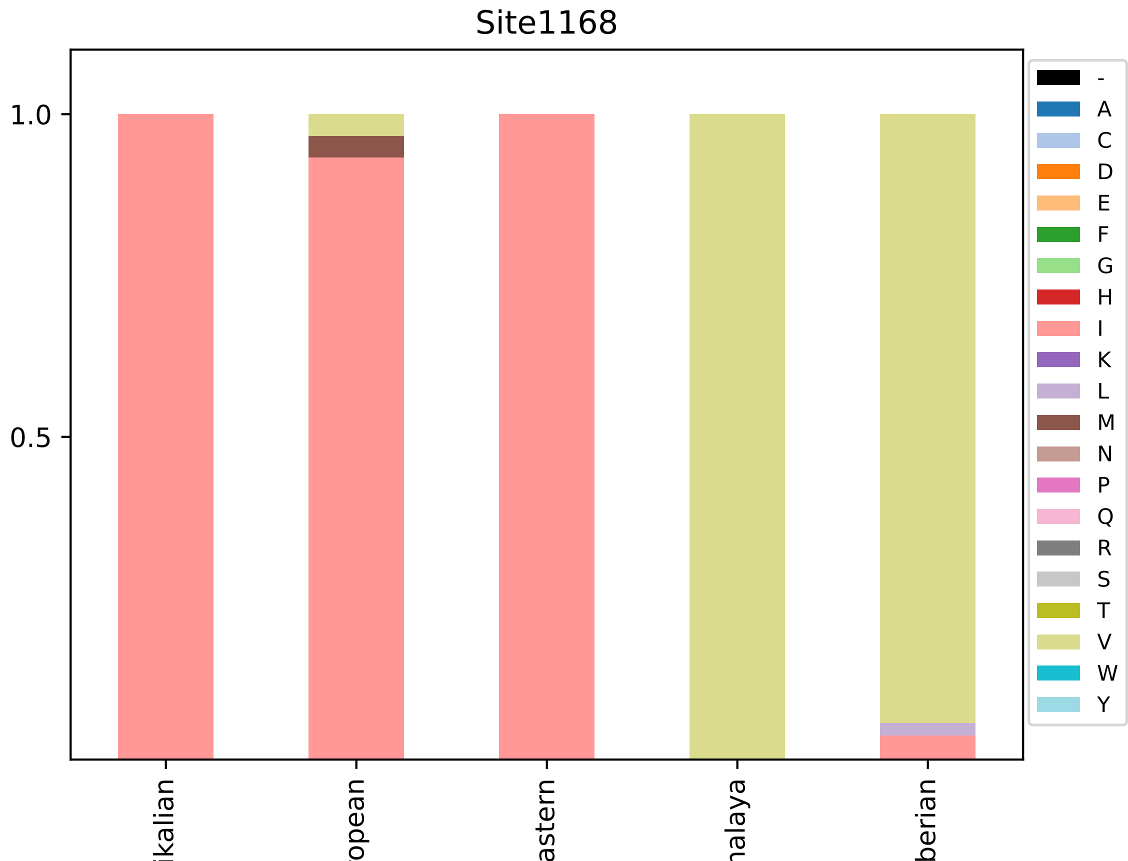
Site1168
1.0
0.5
-
A
C
D
E
F
G
H
I
K
L
M
N
P
Q
R
S
T
V
W
Y
ikalian
ropean
astern
nalaya
berian

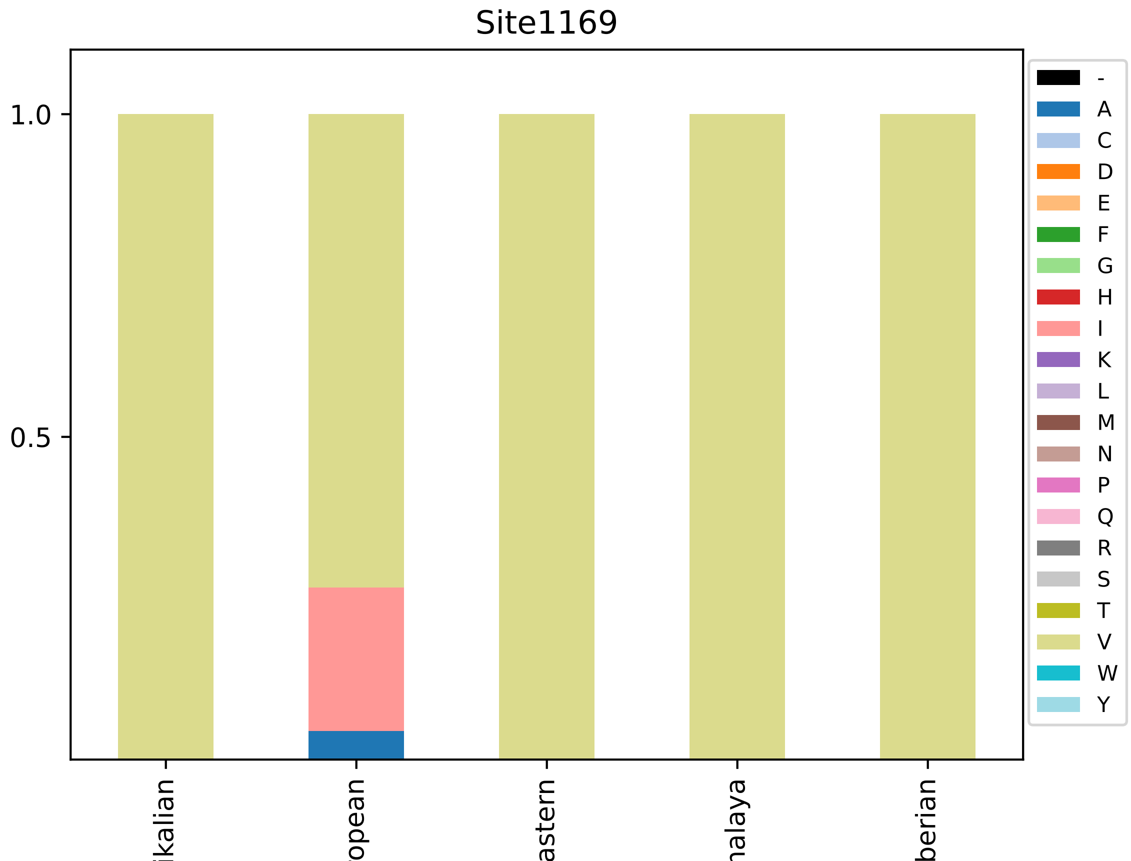

Site1169
1.0
0.5
-
A
C
D
E
F
G
H
I
K
L
M
N
P
Q
R
S
T
V
W
Y
ikalian
opean
astern
nalaya
berian

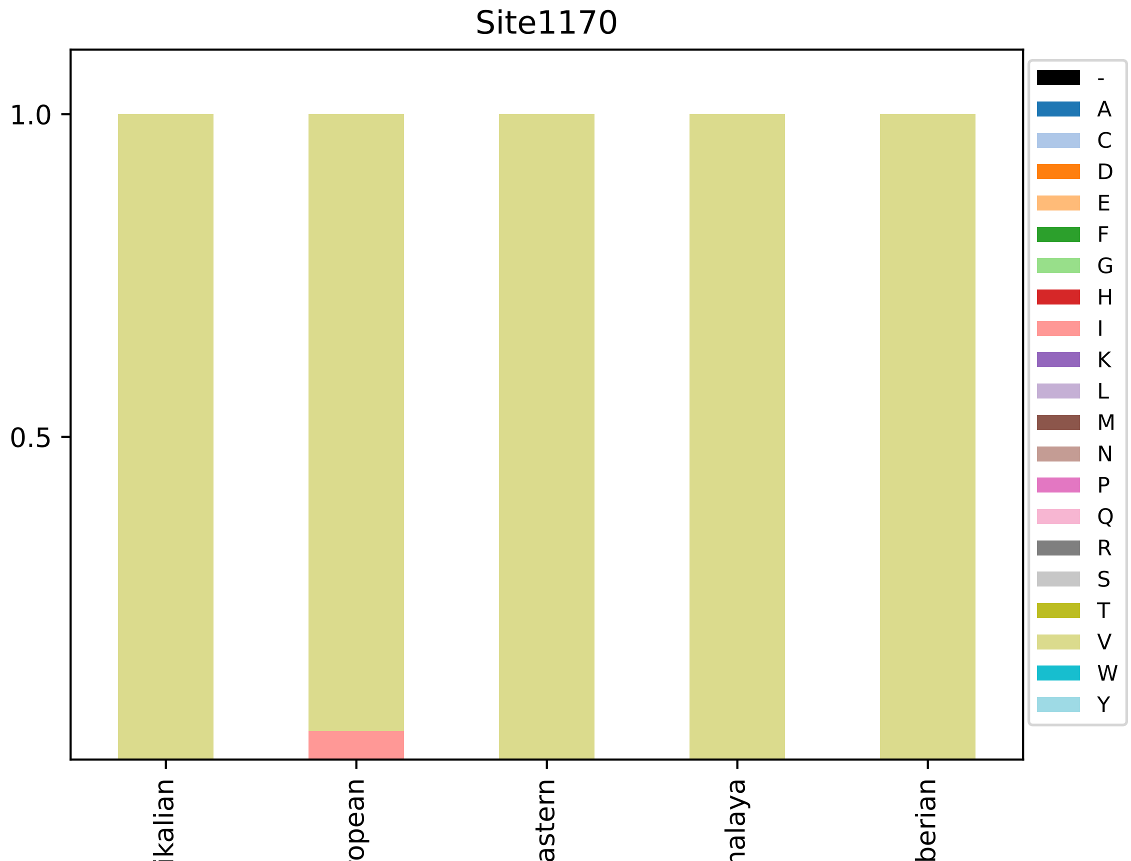

Site1170
1.0
0.5
-
A
C
D
E
F
G
H
I
K
L
M
N
P
Q
R
S
T
V
W
Y
ikalian
opean
astern
nalaya
berian

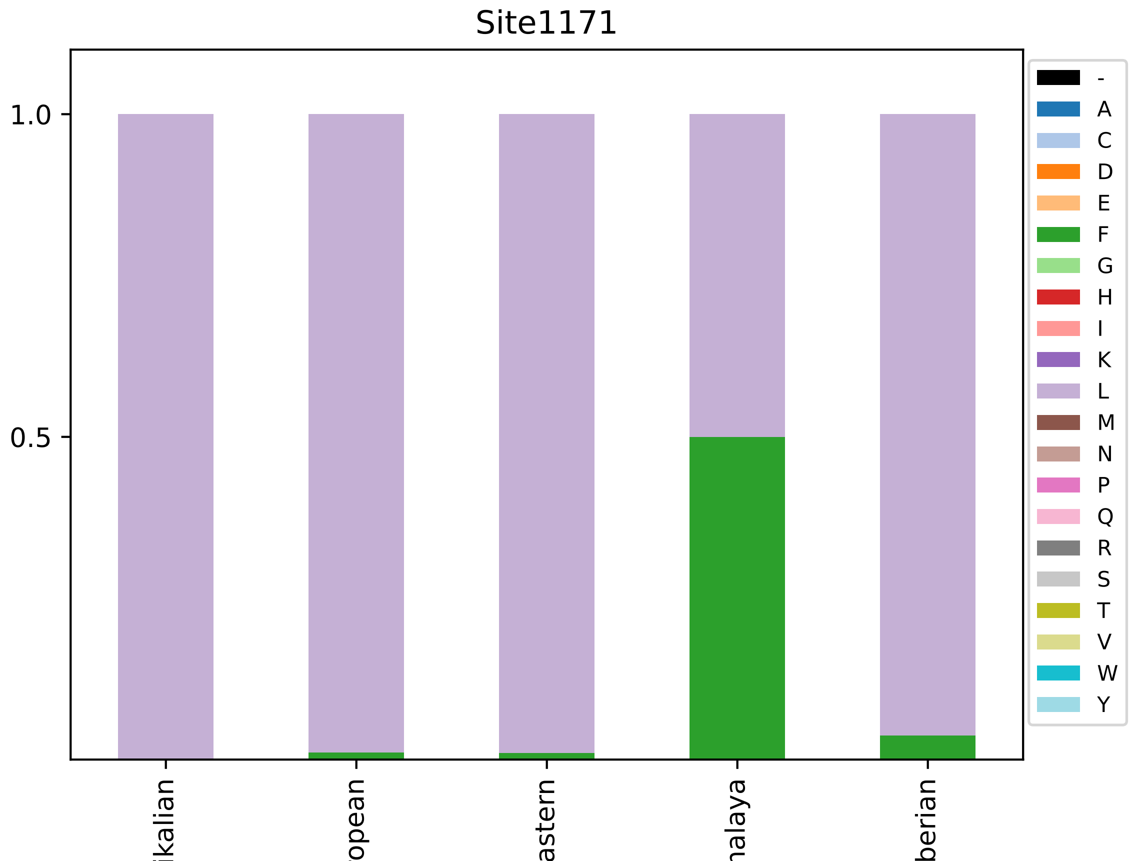
Site1171
1.0
0.5
-
A
C
D
E
F
G
H
I
K
L
M
N
P
Q
R
S
T
V
W
Y
ikalian
opean
astern
nalaya
berian

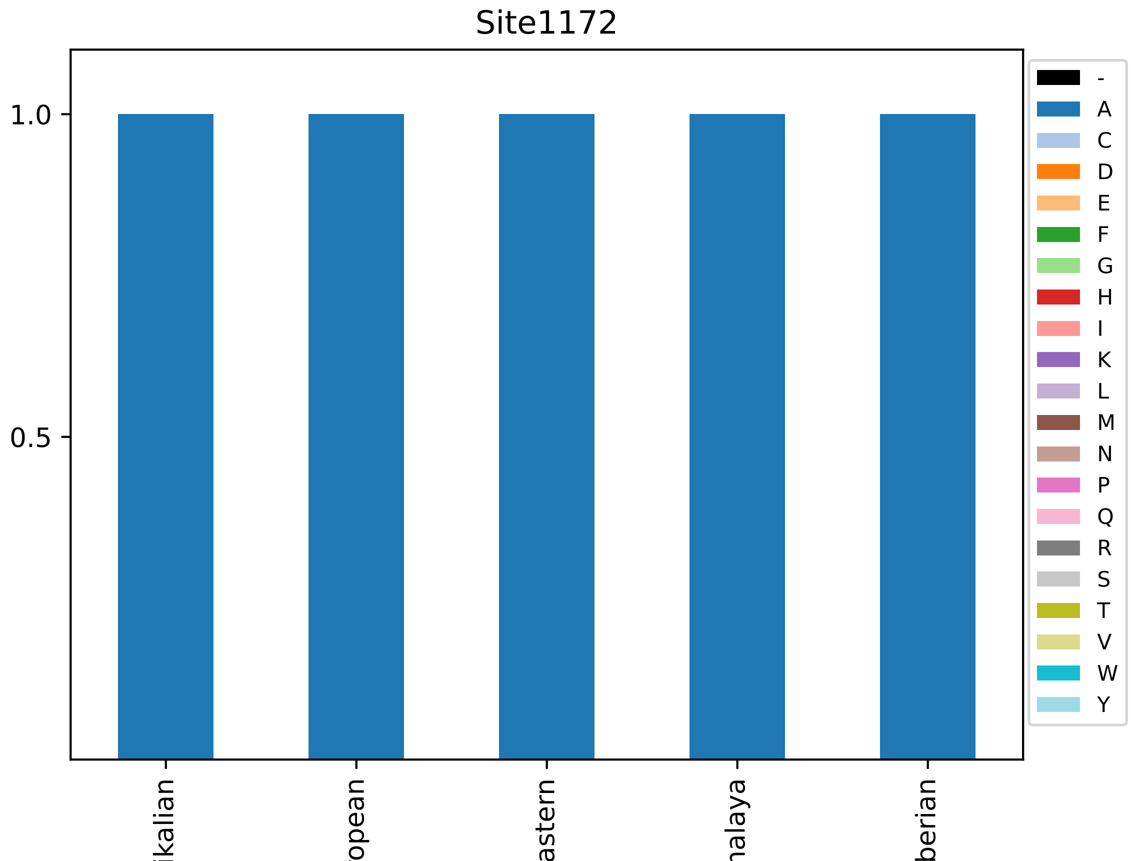

Site1172
1.0
0.5
-
A
C
D
E
F
G
H
I
K
L
M
N
P
Q
R
S
T
V
W
Y
ikalian
opean
astern
nalaya
berian

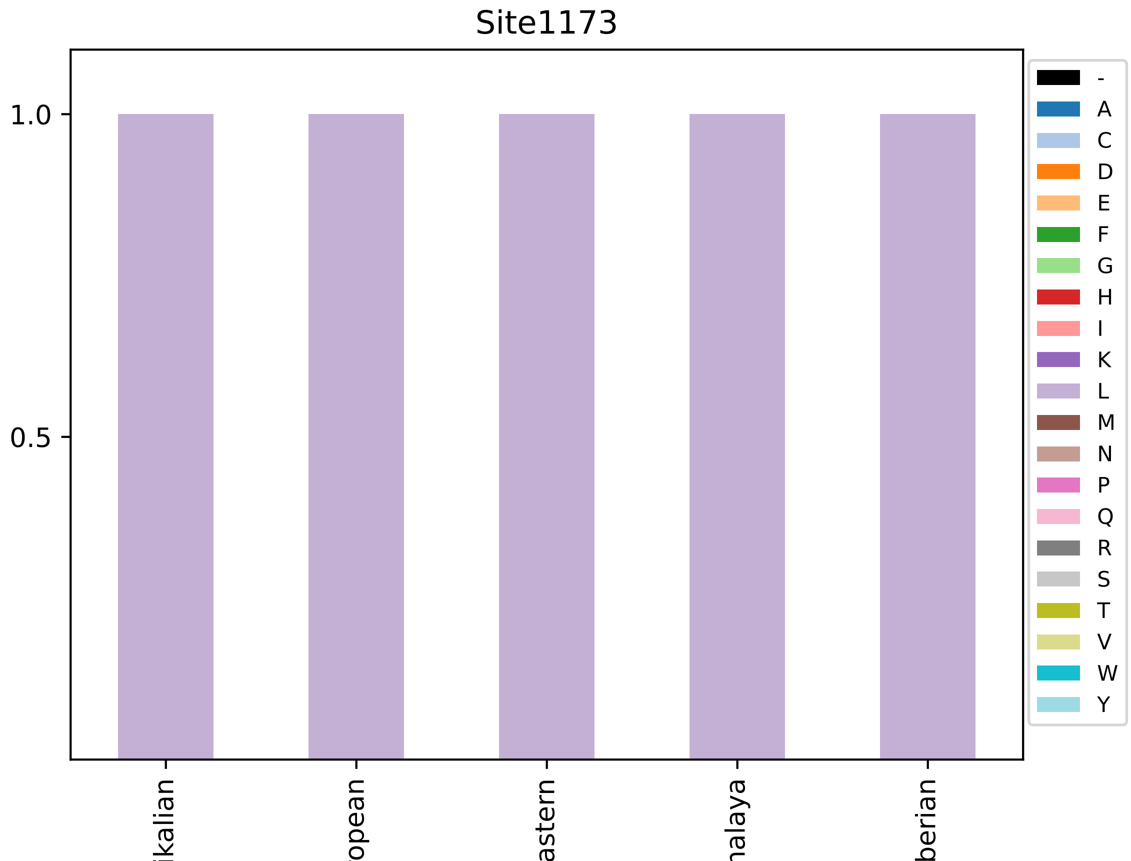

Site1173
1.0
0.5
ikalian
opean
astern
nalaya
berian
-
A
C
D
E
F
G
H
I
K
L
M
N
P
Q
R
S
T
V
W
Y

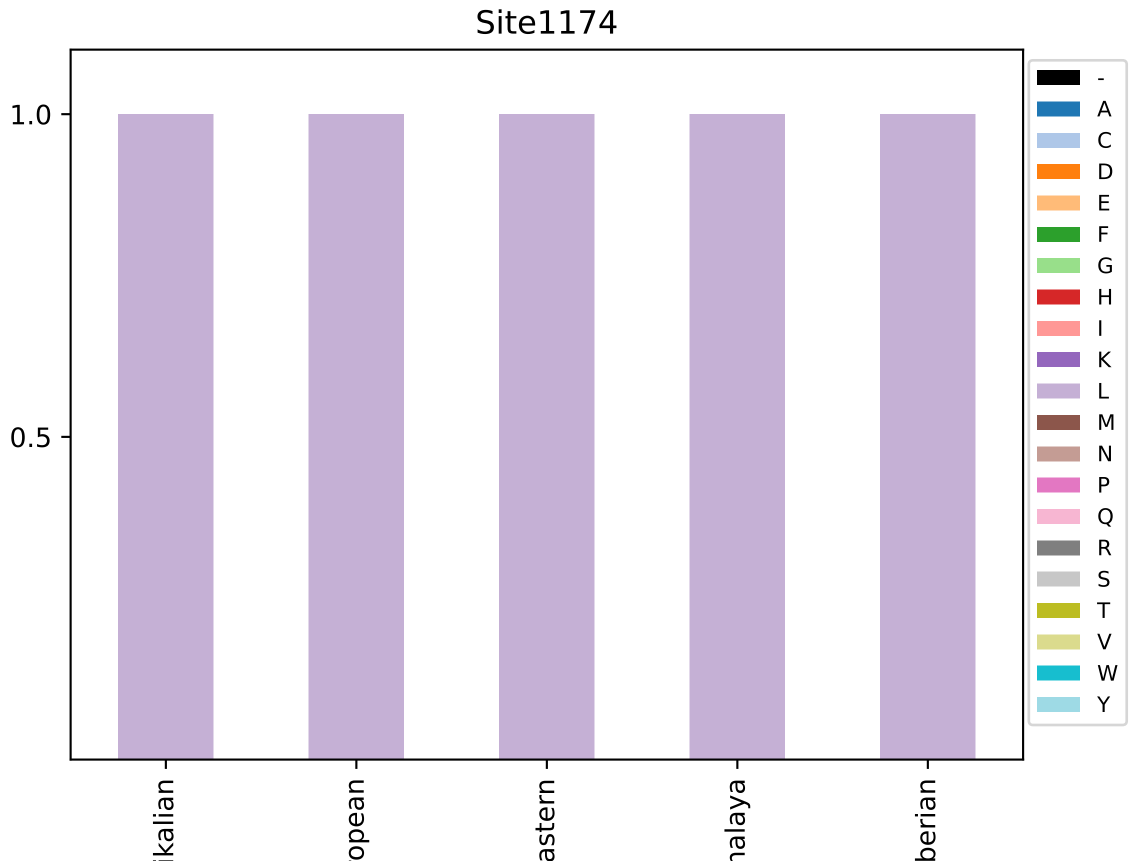

Site1174
1.0
0.5
-
A
C
D
E
F
G
H
I
K
L
M
N
P
Q
R
S
T
V
W
Y

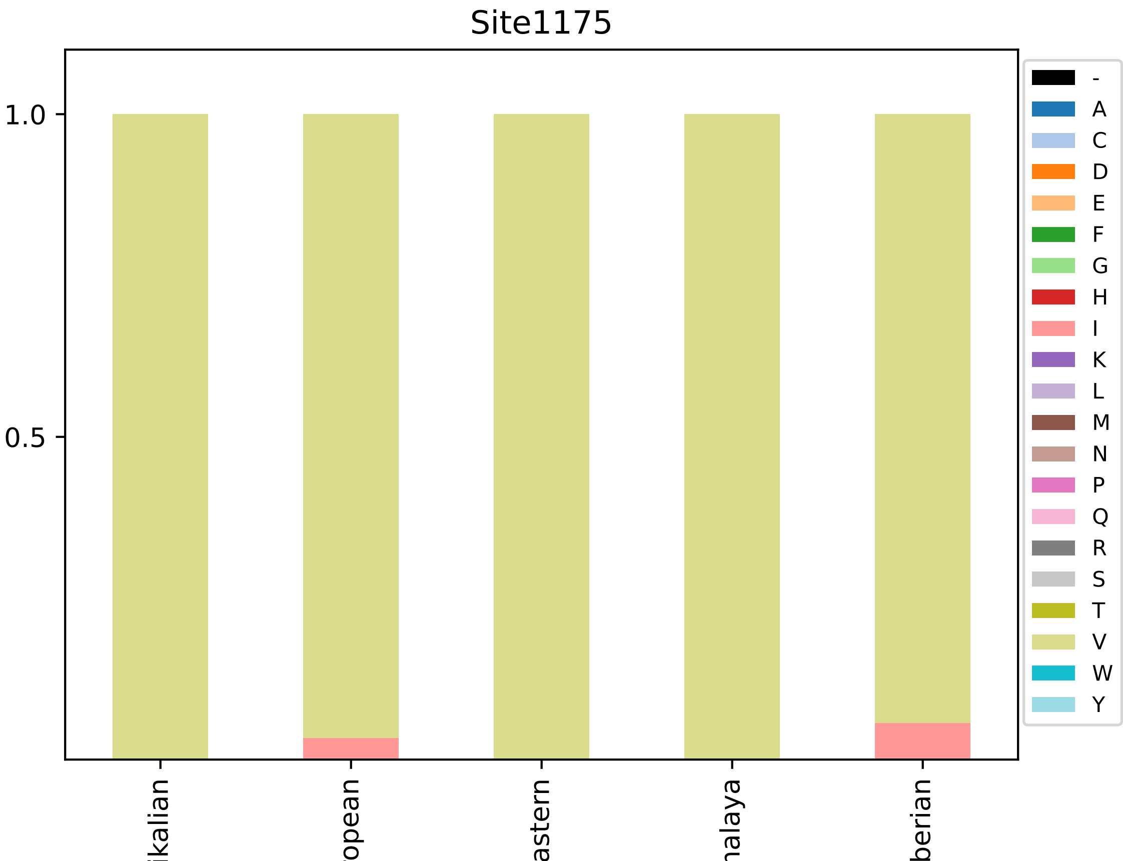
Site1175
1.0
0.5
-
A
C
D
E
F
G
H
I
K
L
M
N
P
Q
R
S
T
V
W
Y
ikalian
opean
astern
nalaya
berian

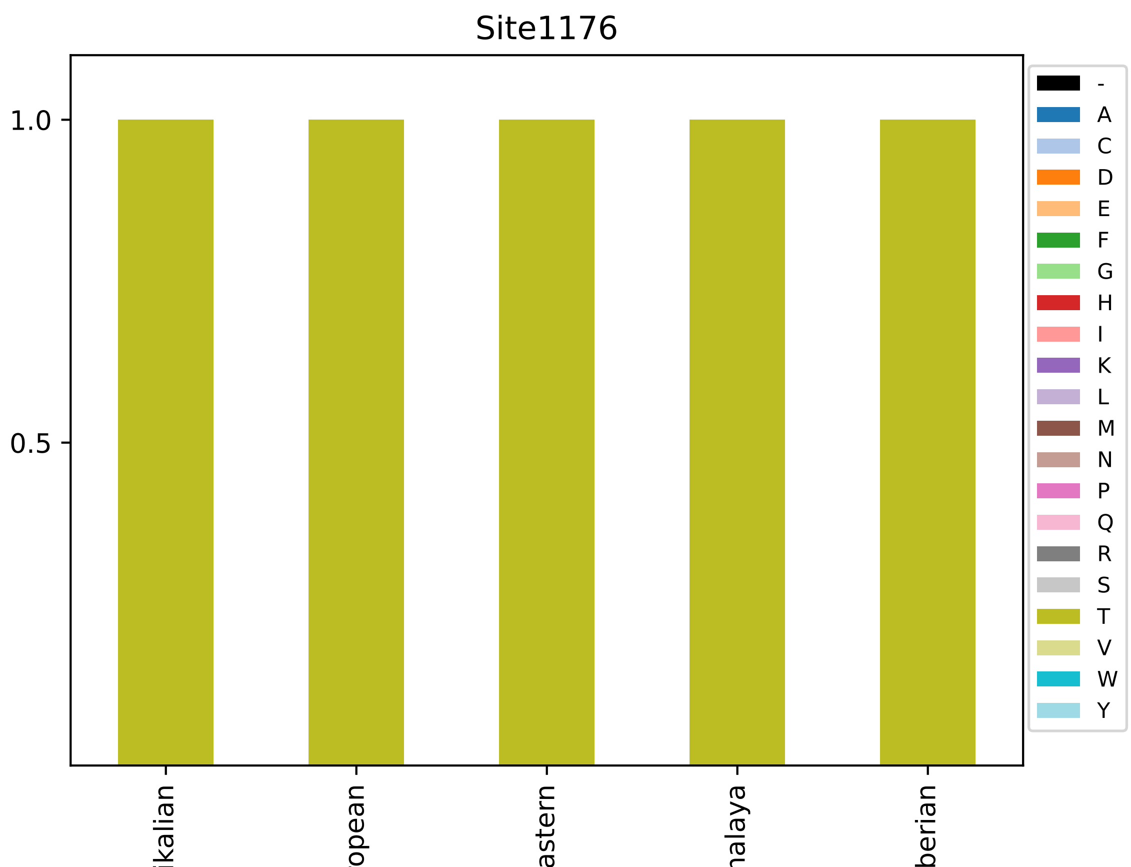
Site1176
1.0
0.5
-
A
C
D
E
F
G
H
I
K
L
M
N
P
Q
R
S
T
V
W
Y
ikalian
opean
astern
nalaya
berian

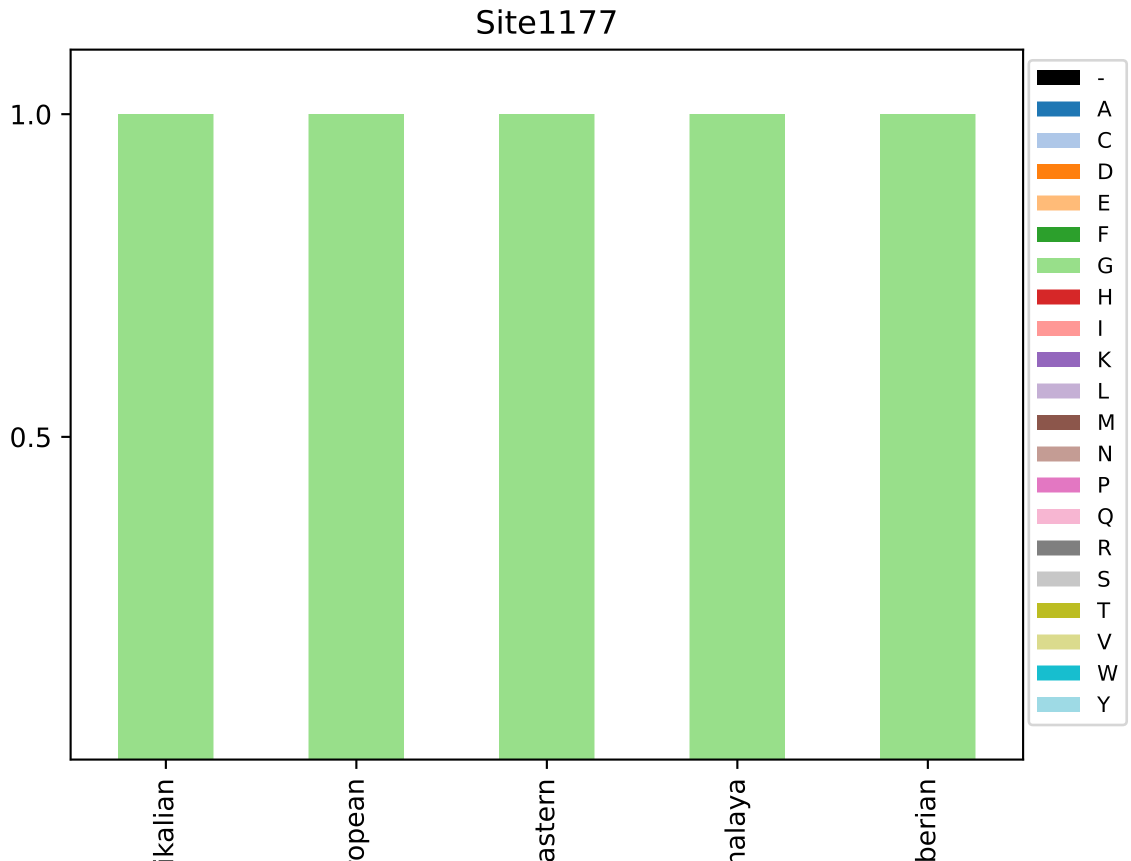

Site1177
1.0
0.5
-
A
C
D
E
F
G
H
I
K
L
M
N
P
Q
R
S
T
V
W
Y
ikalian
opean
astern
nalaya
berian

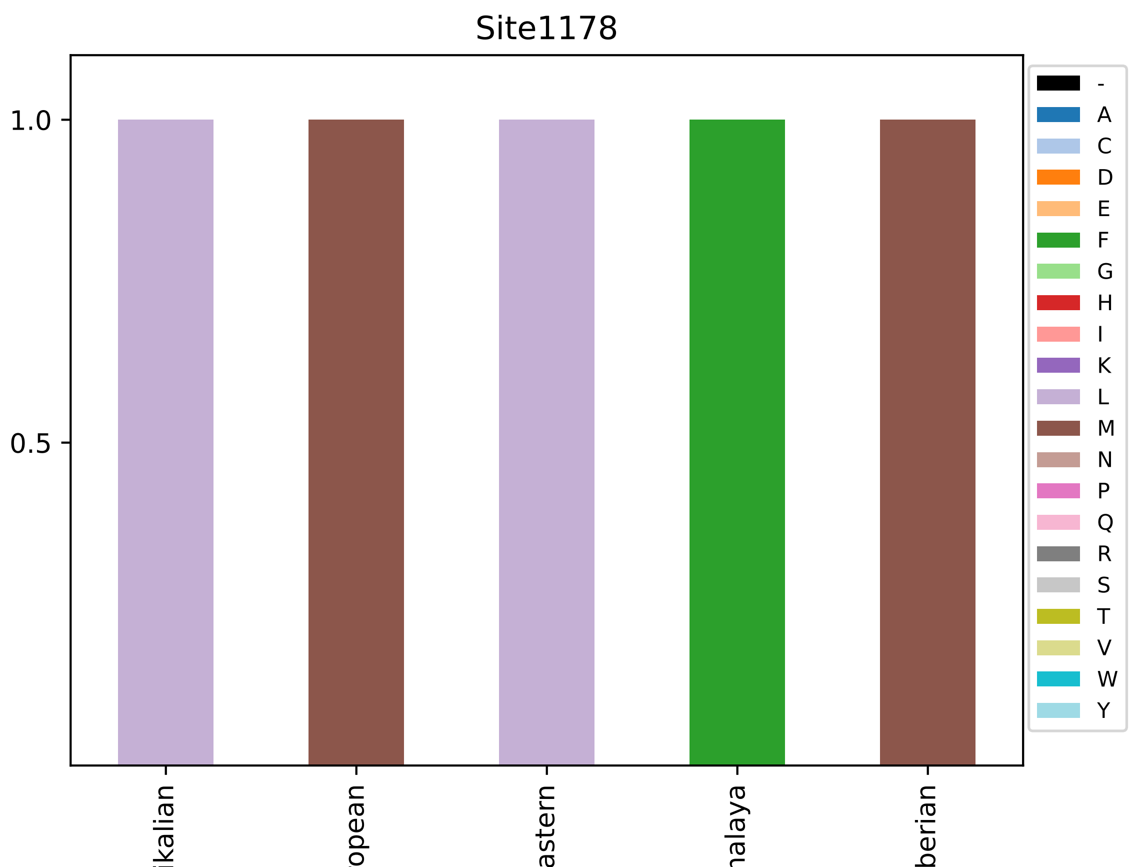

Site1178
1.0
0.5
-
A
C
D
E
F
G
H
I
K
L
M
N
P
Q
R
S
T
V
W
Y
ikalian
opean
astern
nalaya
berian

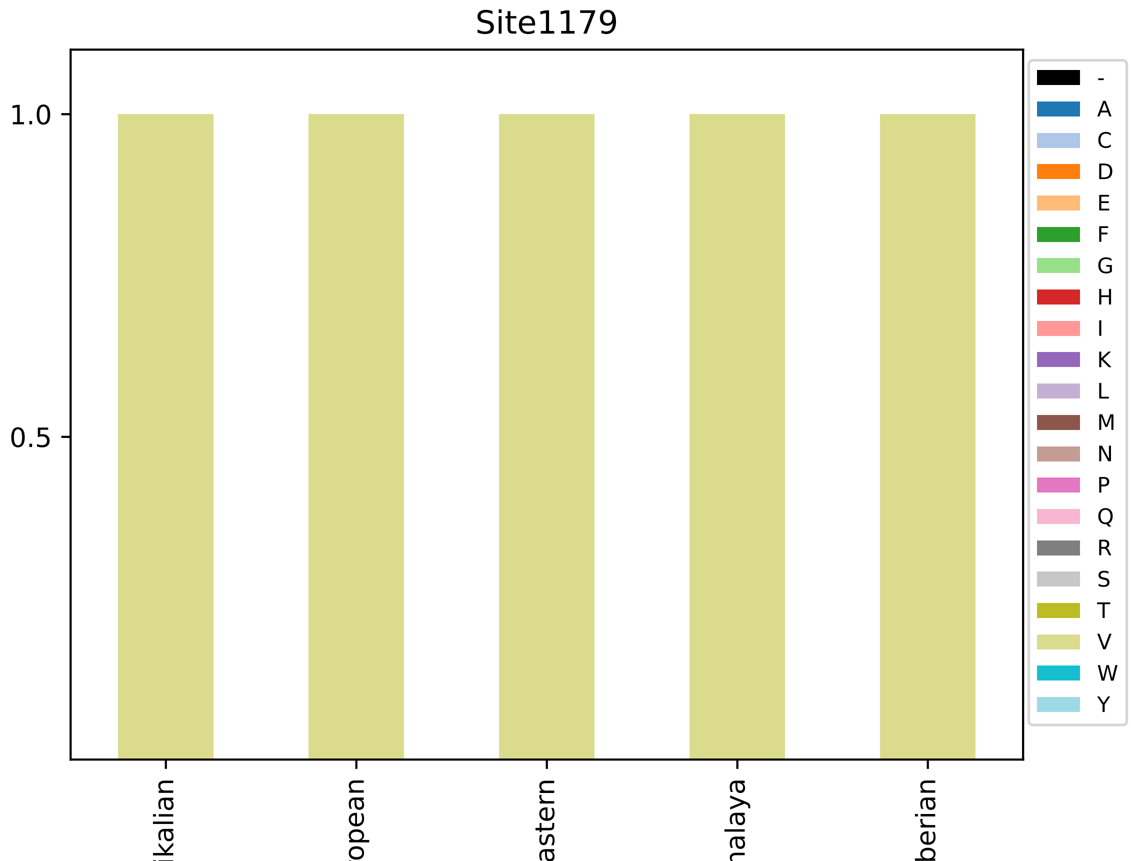
Site1179
1.0
0.5
-
A
C
D
E
F
G
H
I
K
L
M
N
P
Q
R
S
T
V
W
Y
ikalian
opean
astern
nalaya
berian

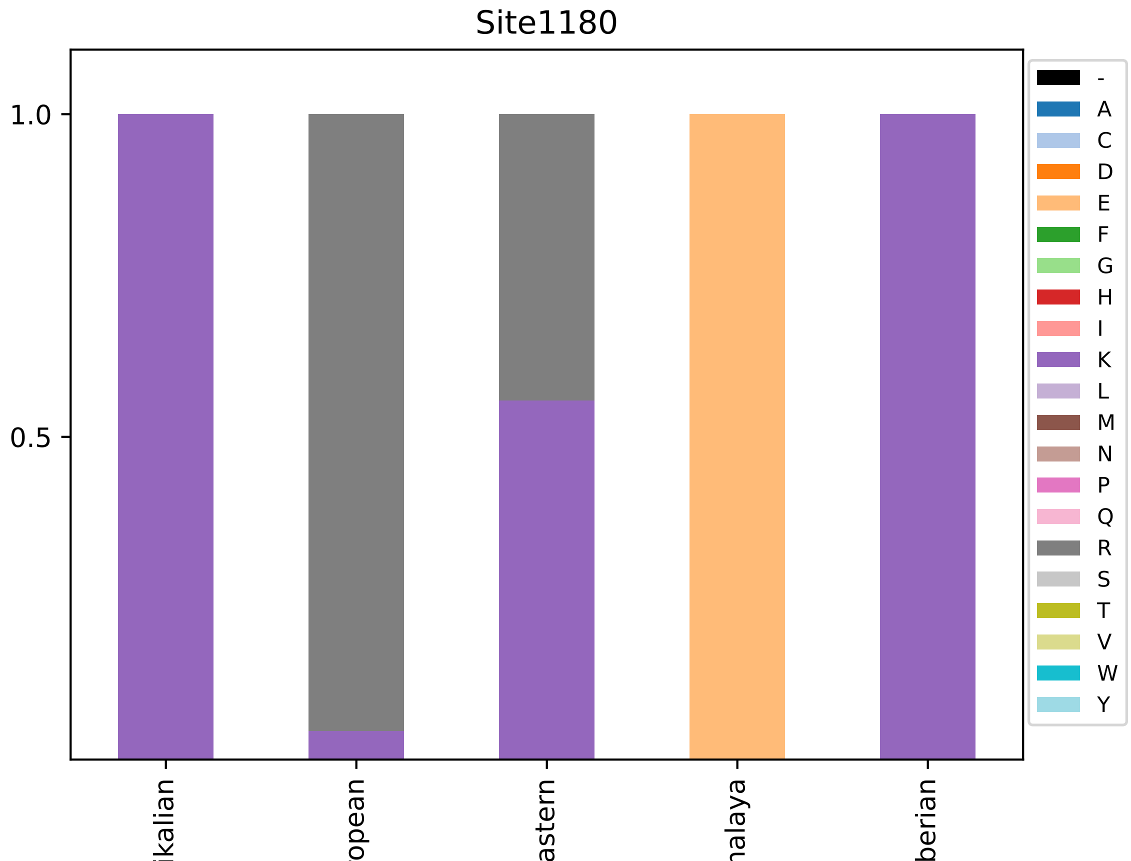

Site1180
1.0
0.5
ikalian
opean
astern
nalaya
berian
-
A
C
D
E
F
G
H
I
K
L
M
N
P
Q
R
S
T
V
W
Y

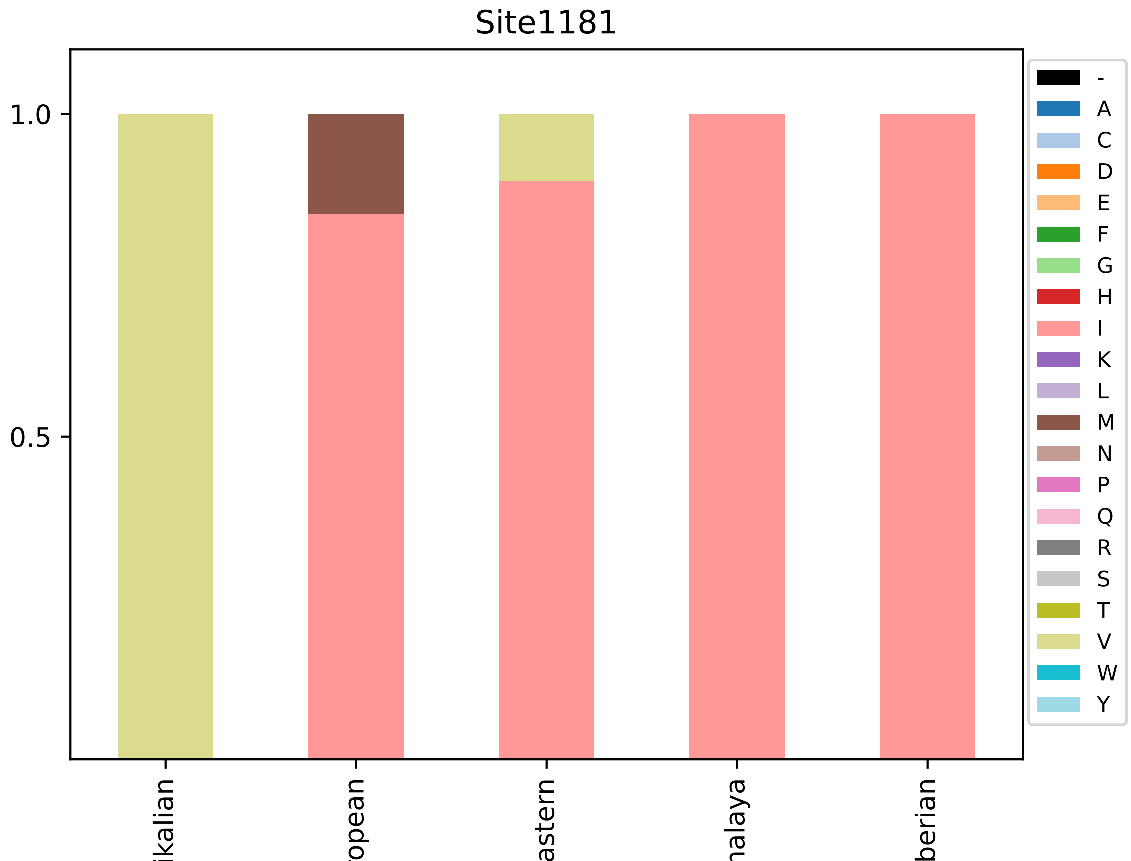
Site1181
1.0
0.5
-
A
C
D
E
F
G
H
I
K
L
M
N
P
Q
R
S
T
V
W
Y
ikalian
opean
astern
nalaya
berian

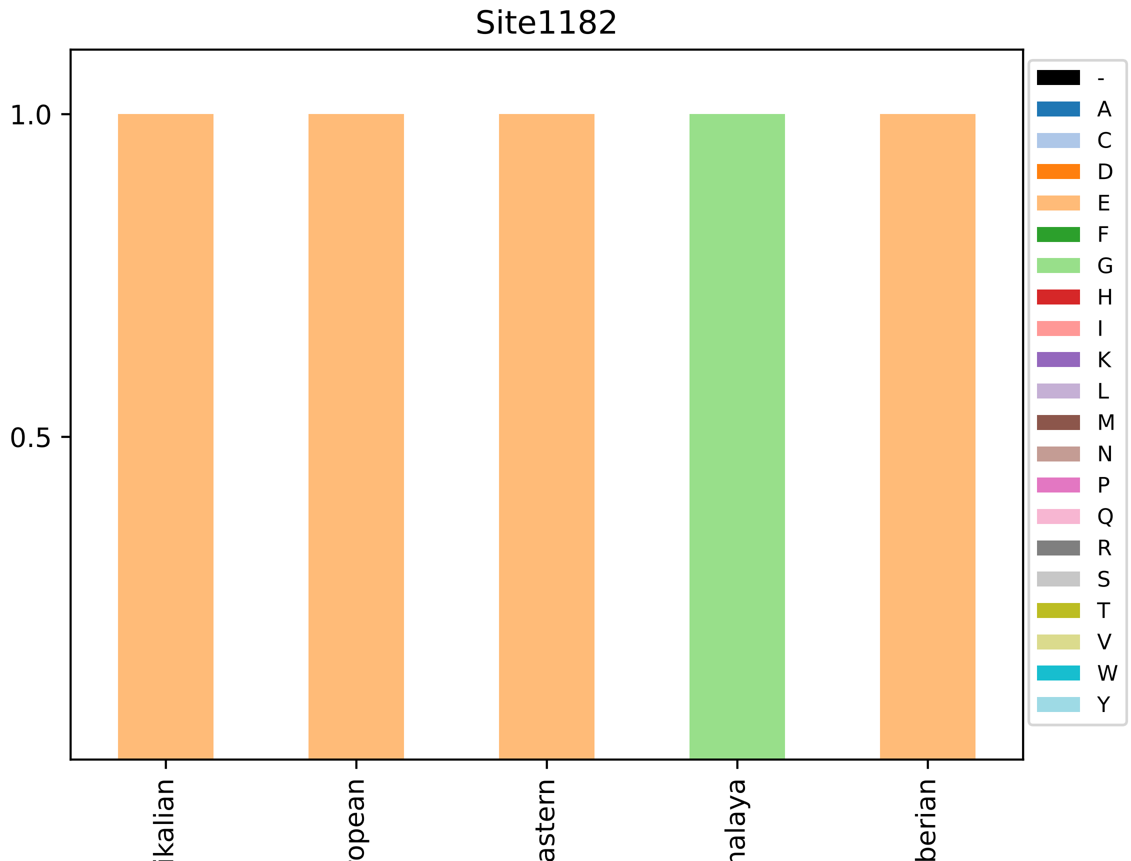

Site1182
1.0
0.5
ikalian
opean
astern
nalaya
berian
-
A
C
D
E
F
G
H
I
K
L
M
N
P
Q
R
S
T
V
W
Y

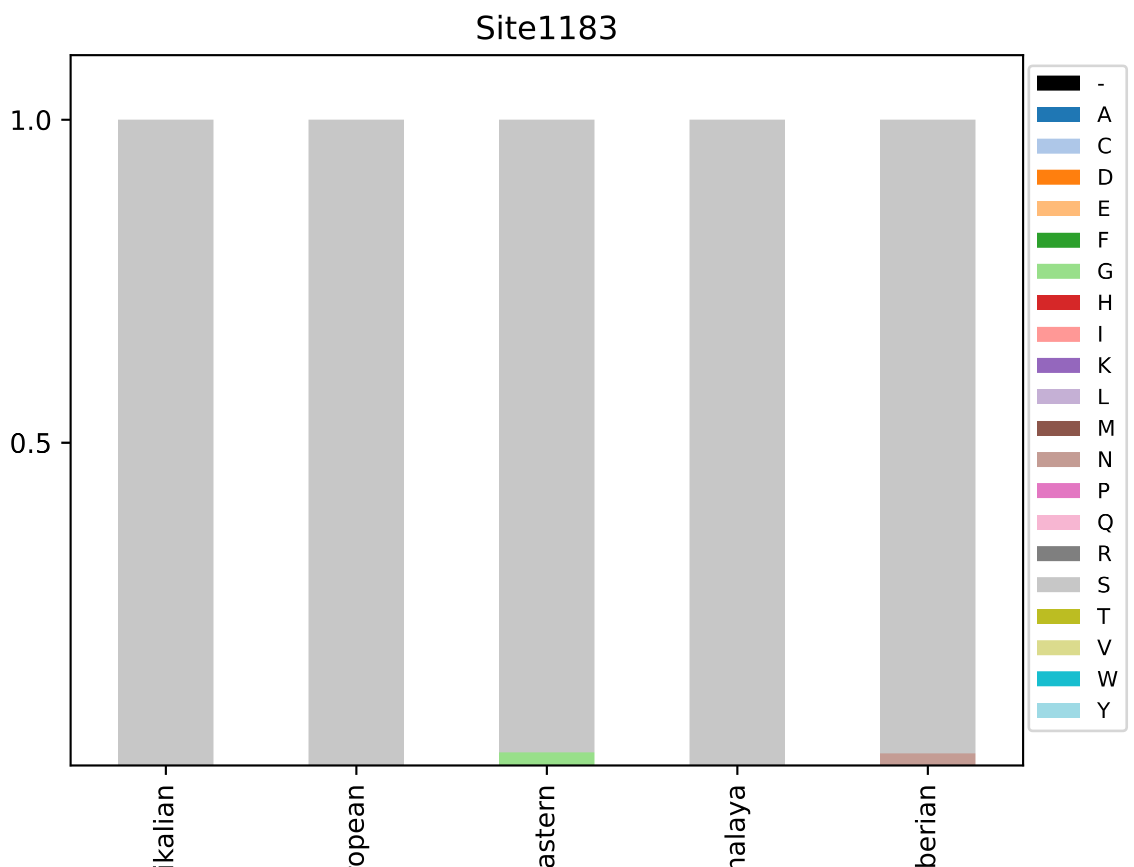

Site1183
1.0
0.5
-
A
C
D
E
F
G
H
I
K
L
M
N
P
Q
R
S
T
V
W
Y
ikalian
opean
astern
nalaya
berian

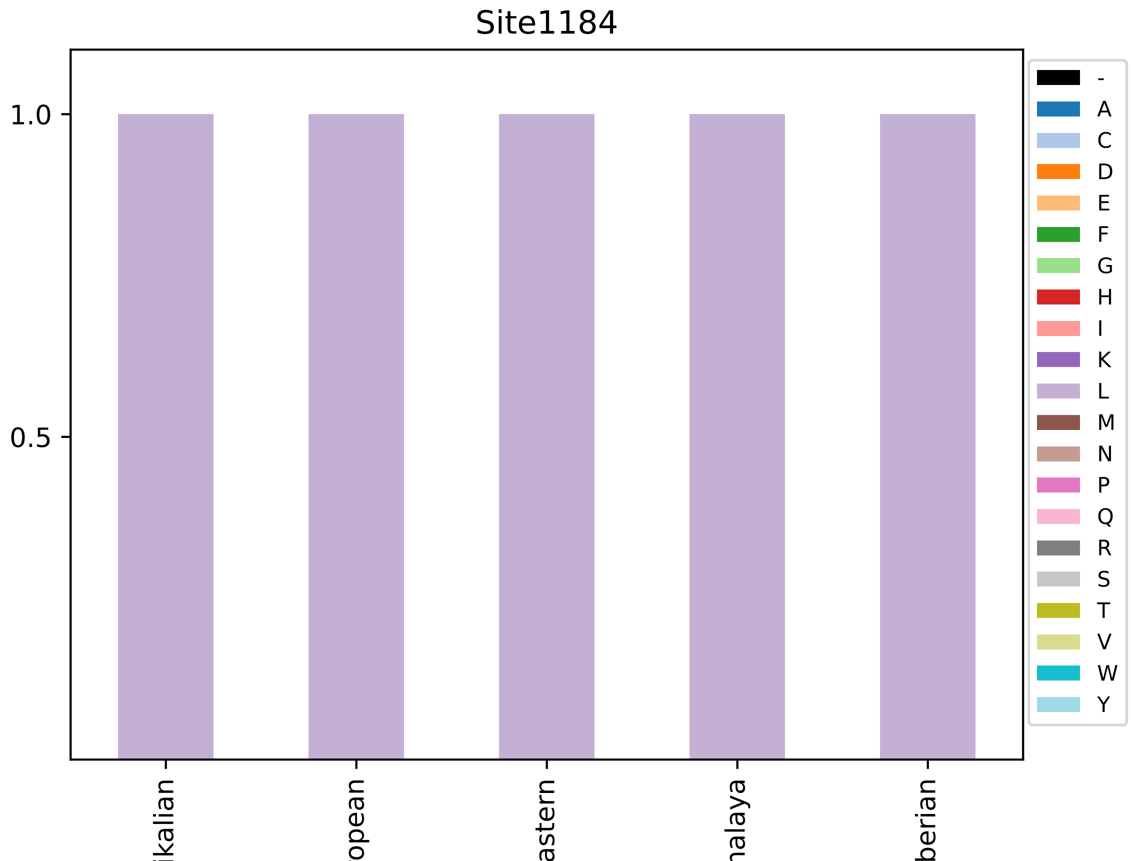

Site1184
1.0
0.5
-
A
C
D
E
F
G
H
I
K
L
M
N
P
Q
R
S
T
V
W
Y
ikalian
opean
astern
nalaya
berian

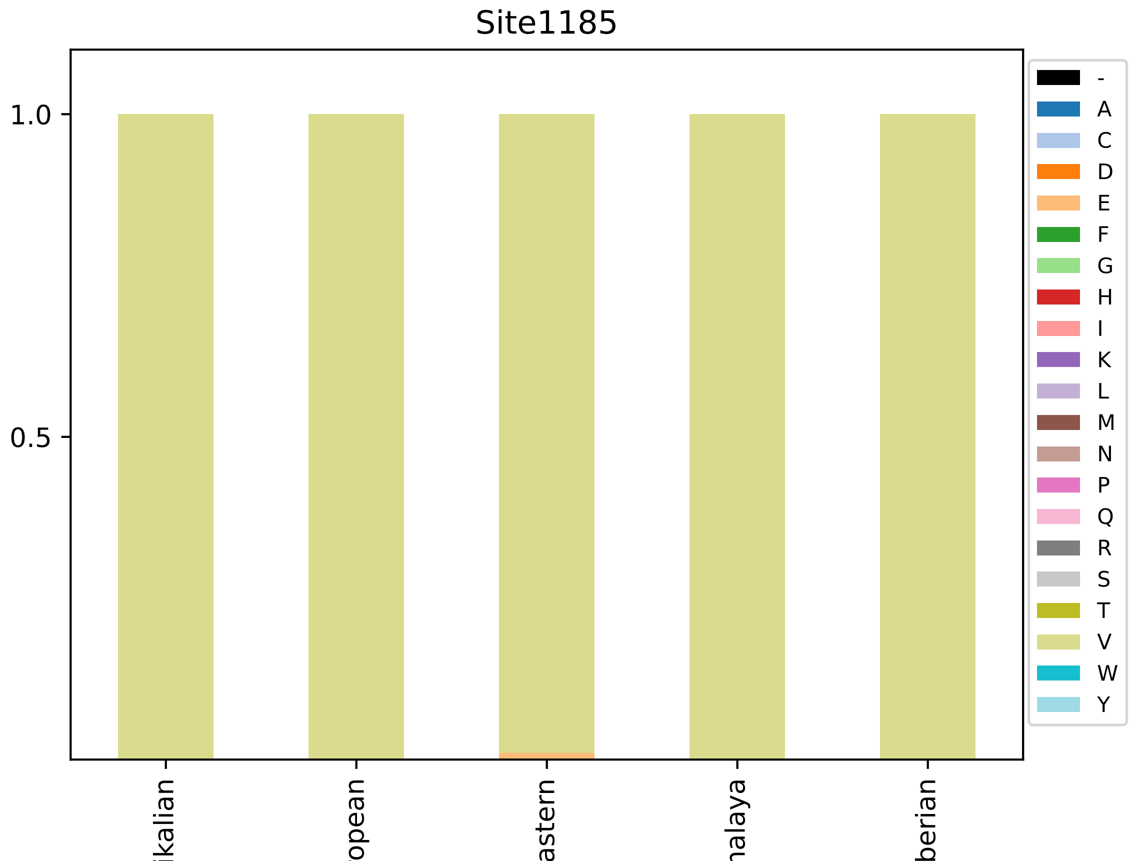
Site1185
1.0
0.5
-
A
C
D
E
F
G
H
I
K
L
M
N
P
Q
R
S
T
V
W
Y
ikalian
opean
astern
nalaya
berian

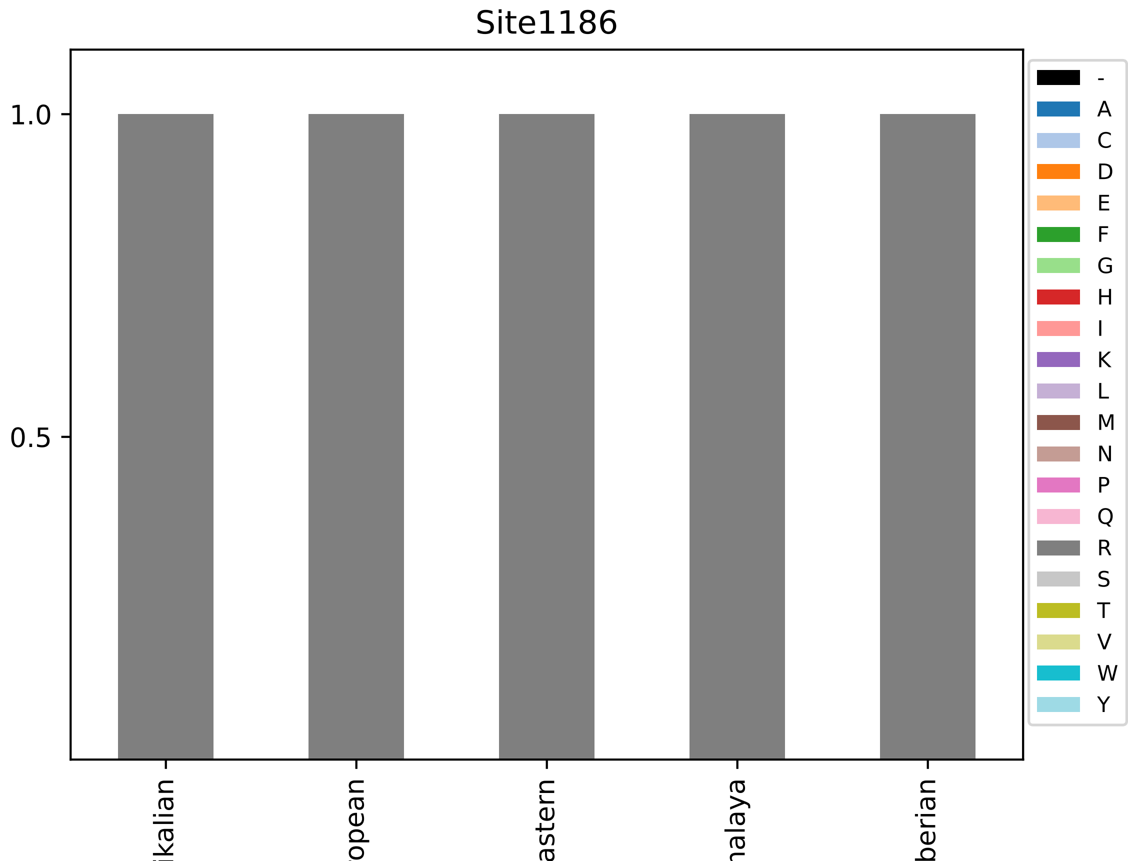

Site1186
1.0
0.5
-
A
C
D
E
F
G
H
I
K
L
M
N
P
Q
R
S
T
V
W
Y

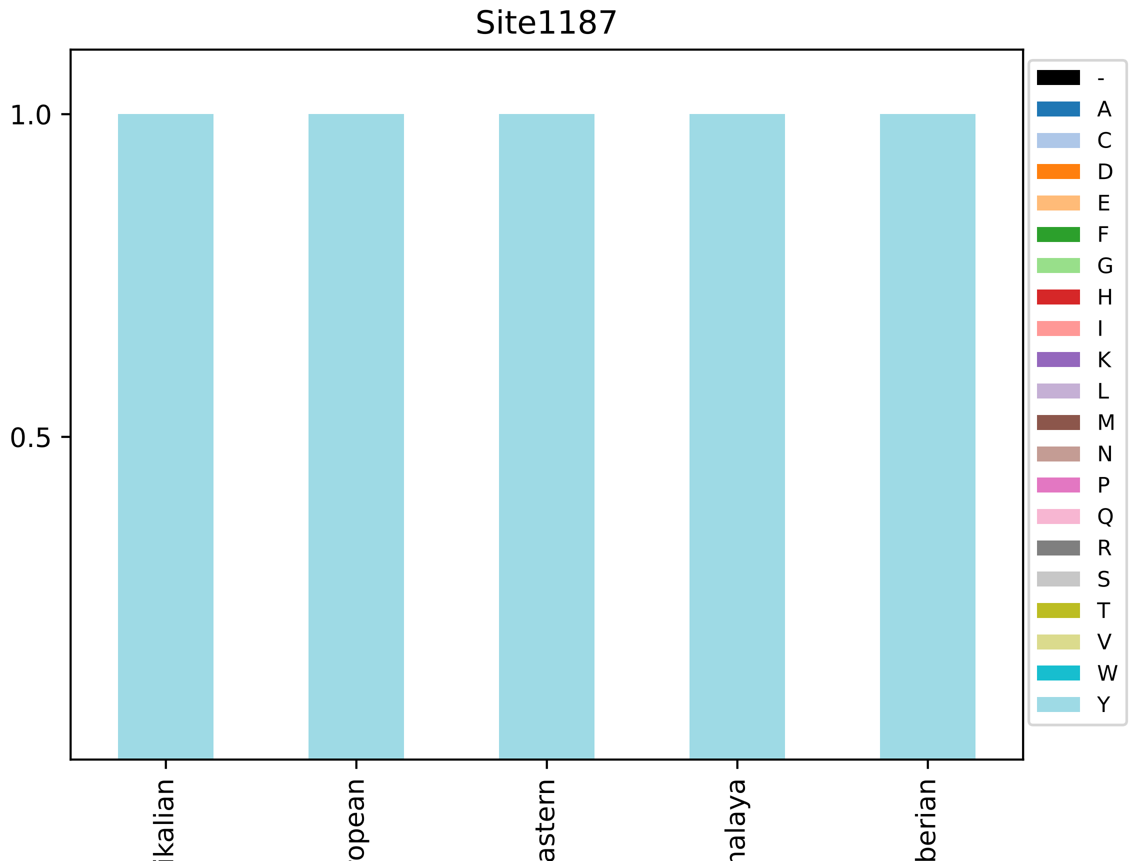
Site1187
1.0
0.5
-
A
C
D
E
F
G
H
I
K
L
M
N
P
Q
R
S
T
V
W
Y
ikalian
opean
astern
nalaya
berian

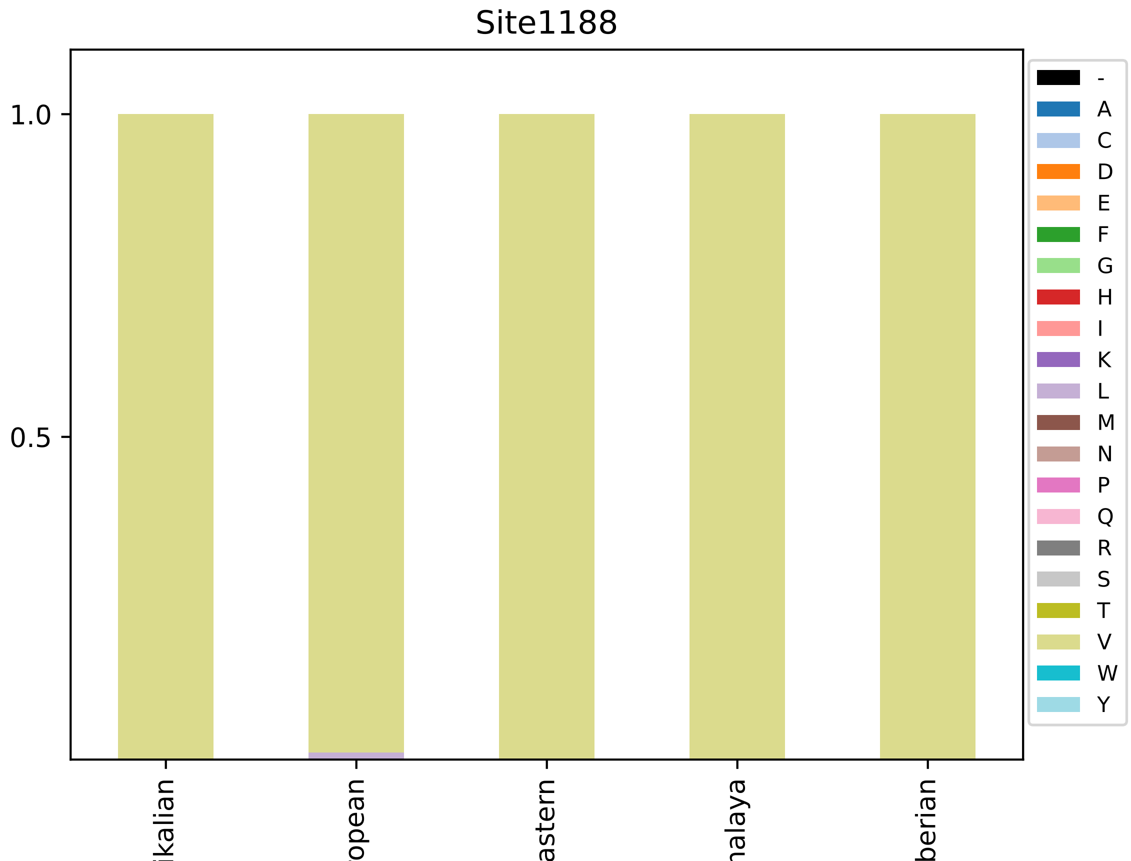
Site1188
1.0
0.5
-
A
C
D
E
F
G
H
I
K
L
M
N
P
Q
R
S
T
V
W
Y
ikalian
opean
astern
nalaya
berian

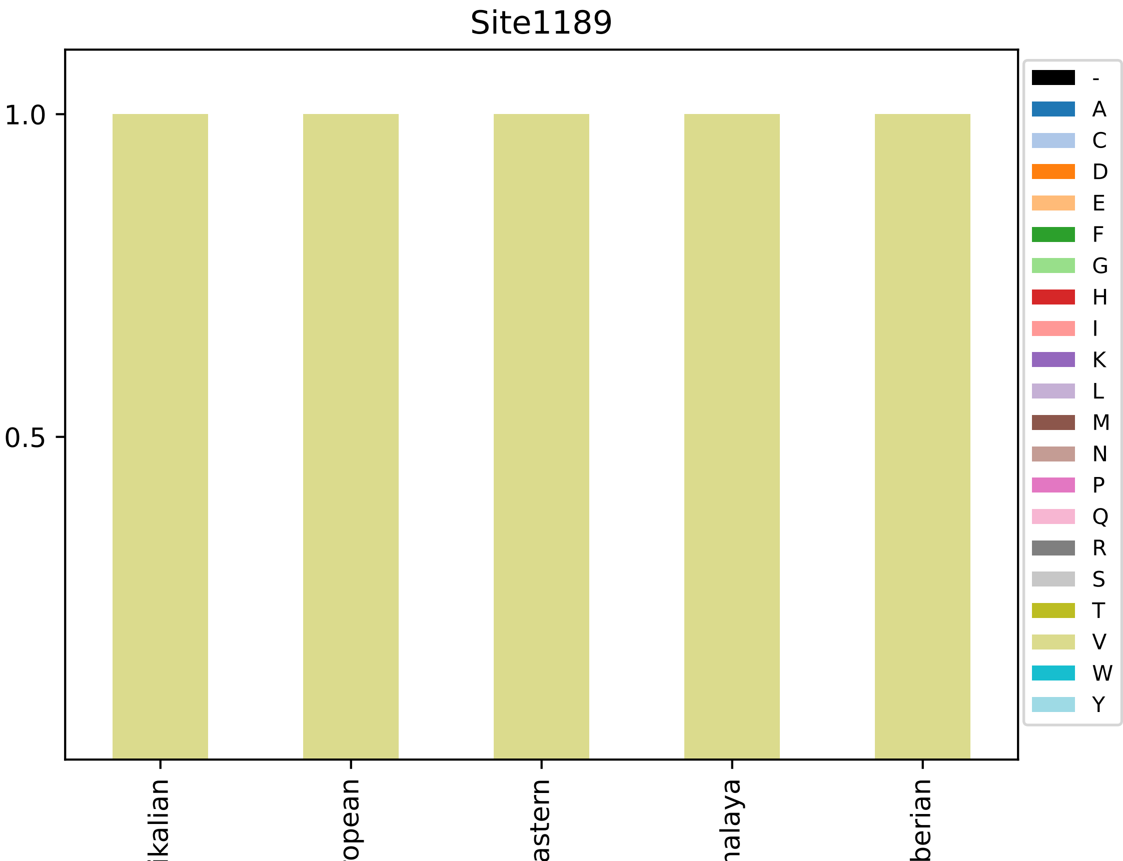

Site1189
1.0
0.5
-
A
C
D
E
F
G
H
I
K
L
M
N
P
Q
R
S
T
V
W
Y
ikalian
opean
astern
nalaya
berian

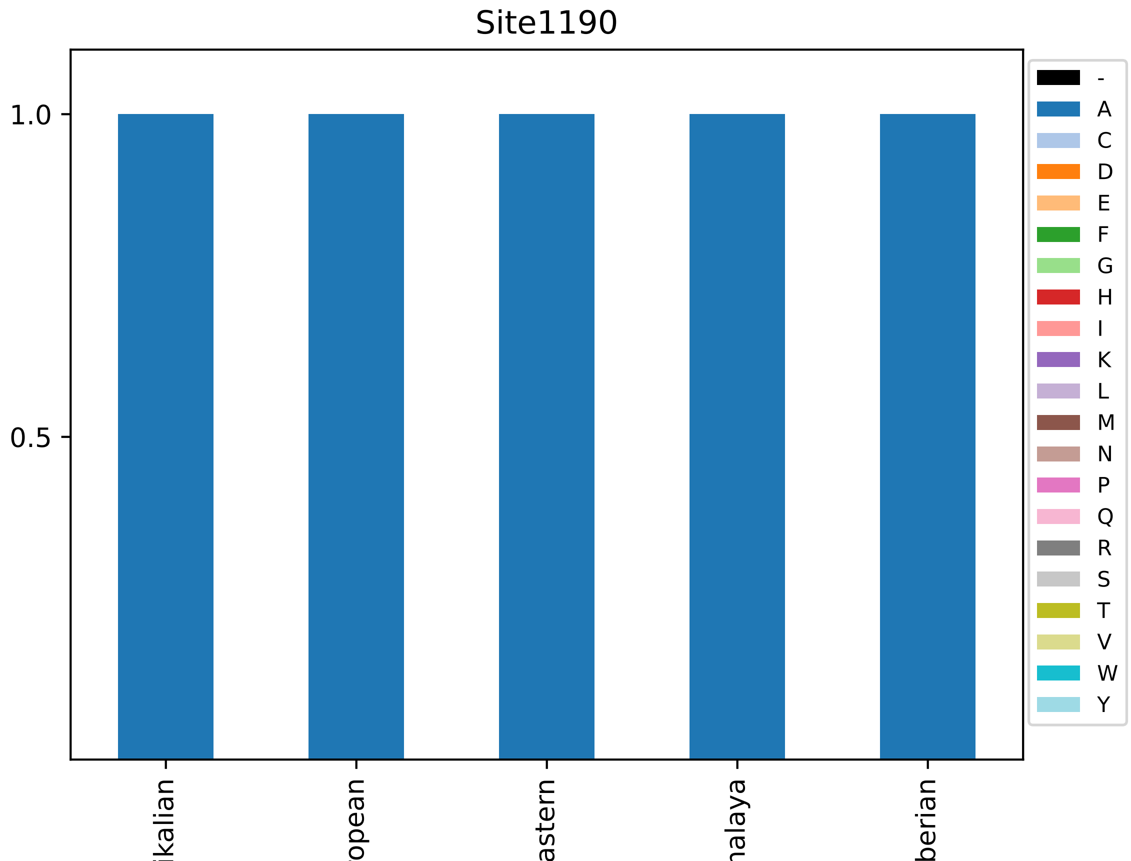
Site1190
1.0
0.5
-
A
C
D
E
F
G
H
I
K
L
M
N
P
Q
R
S
T
V
W
Y
ikalian
opean
astern
nalaya
berian

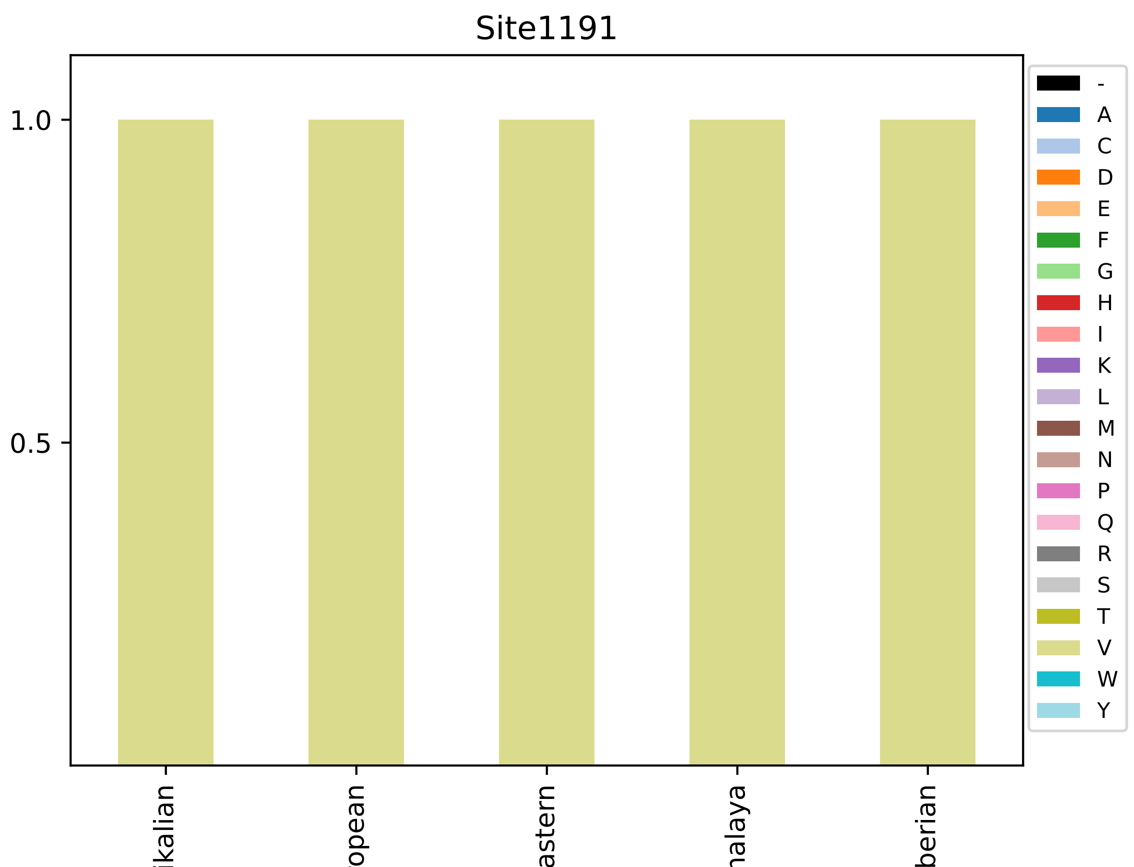
Site1191
1.0
0.5
-
A
C
D
E
F
G
H
I
K
L
M
N
P
Q
R
S
T
V
W
Y
ikalian
ropean
astern
nalaya
berian

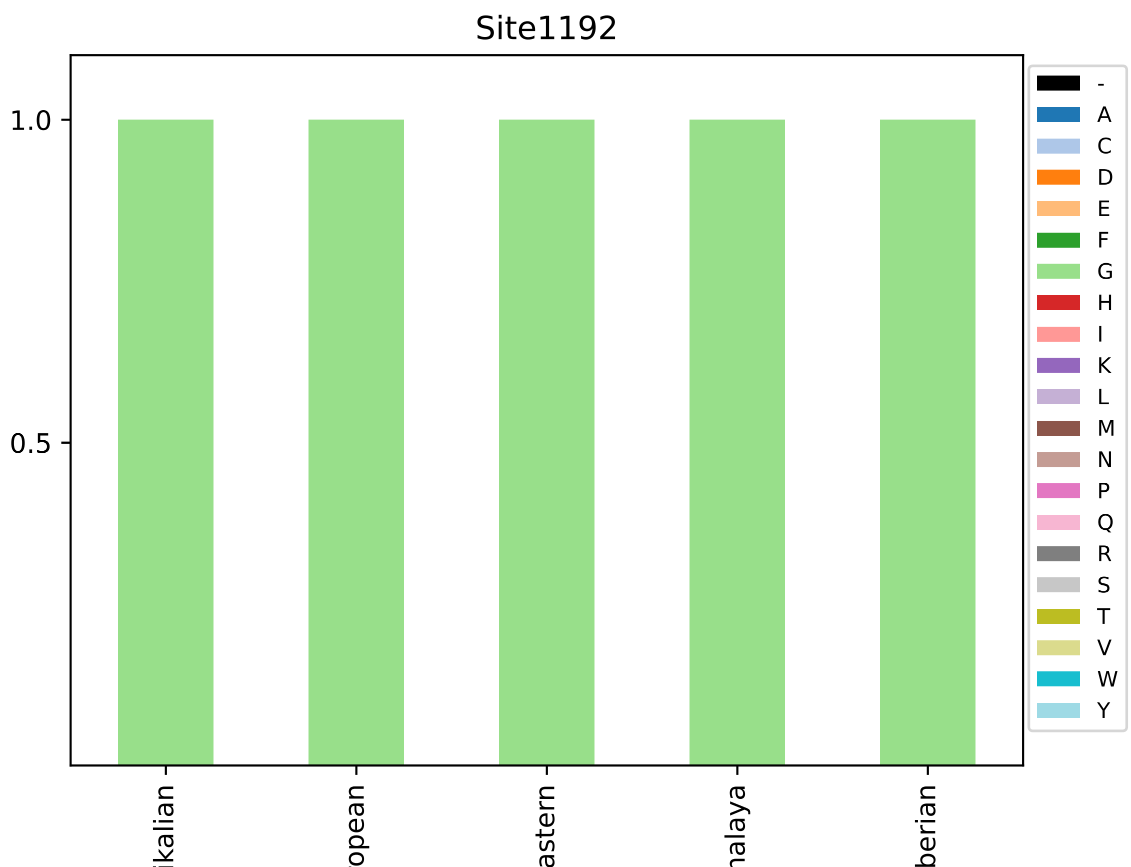
Site1192
1.0
0.5
-
A
C
D
E
F
G
H
I
K
L
M
N
P
Q
R
S
T
V
W
Y
ikalian
opean
astern
nalaya
berian

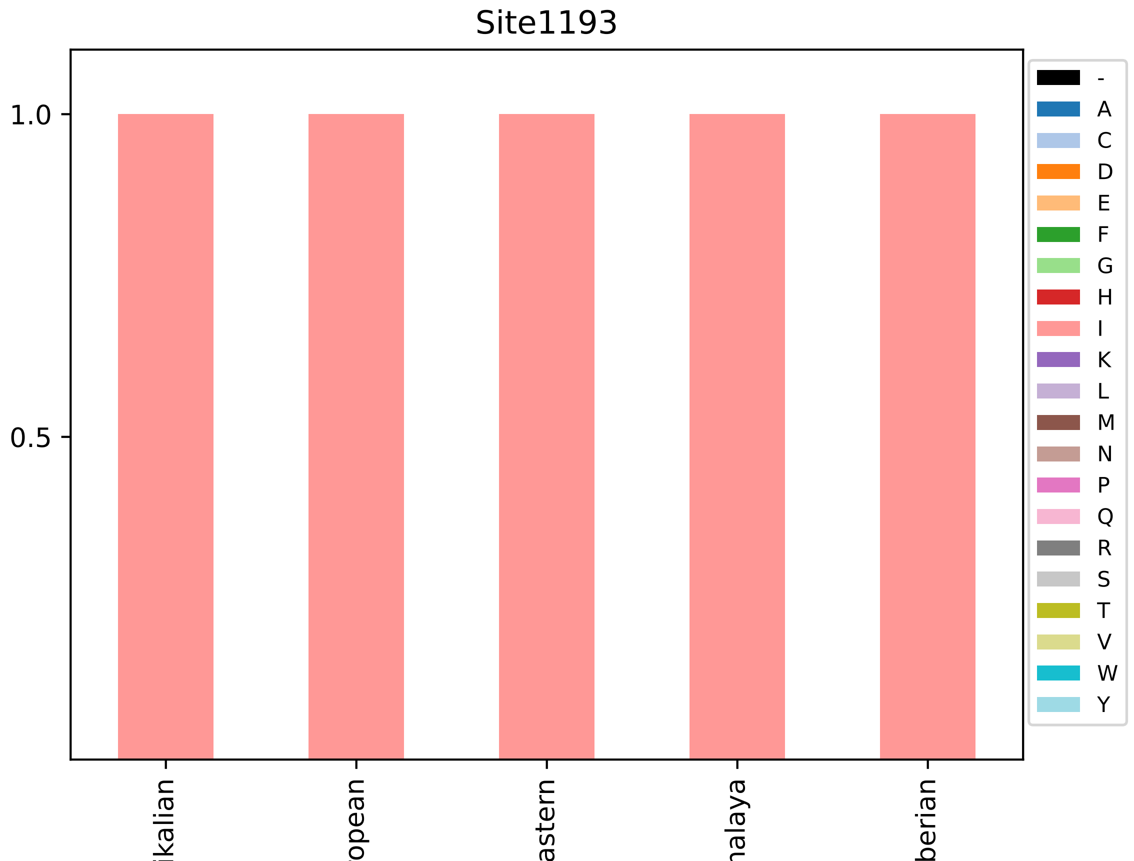
Site1193
1.0
0.5
-
A
C
D
E
F
G
H
I
K
L
M
N
P
Q
R
S
T
V
W
Y
ikalian
opean
astern
nalaya
berian

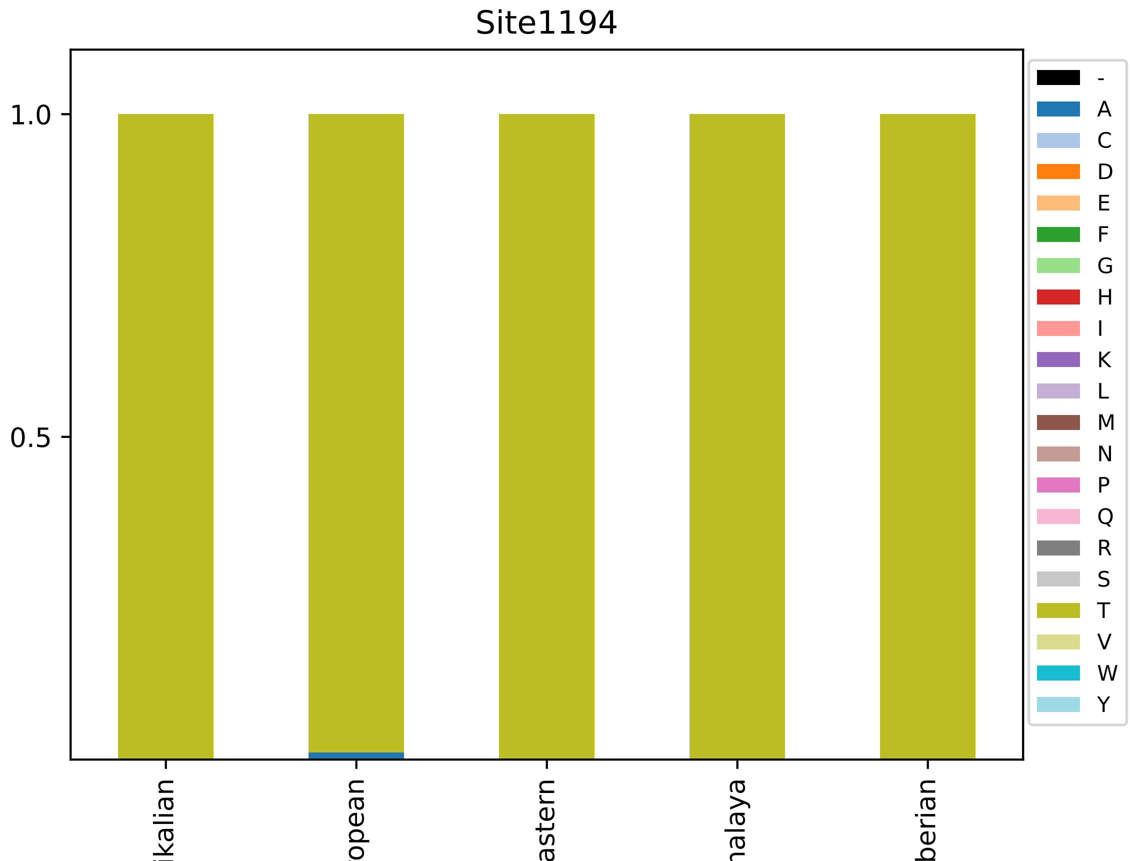

Site1194
1.0
0.5
-
A
C
D
E
F
G
H
I
K
L
M
N
P
Q
R
S
T
V
W
Y
ikalian
opean
astern
nalaya
berian

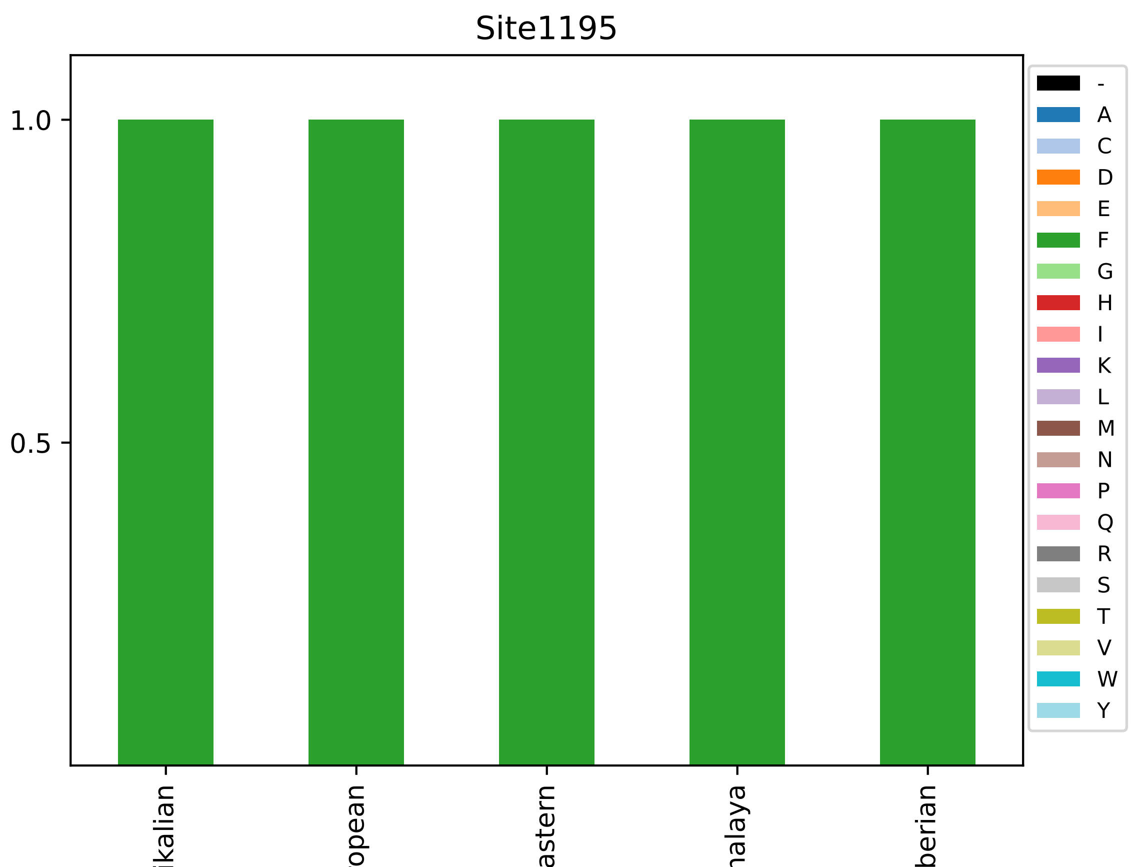
Site1195
1.0
0.5
-
A
C
D
E
F
G
H
I
K
L
M
N
P
Q
R
S
T
V
W
Y
ikalian
opean
astern
nalaya
berian

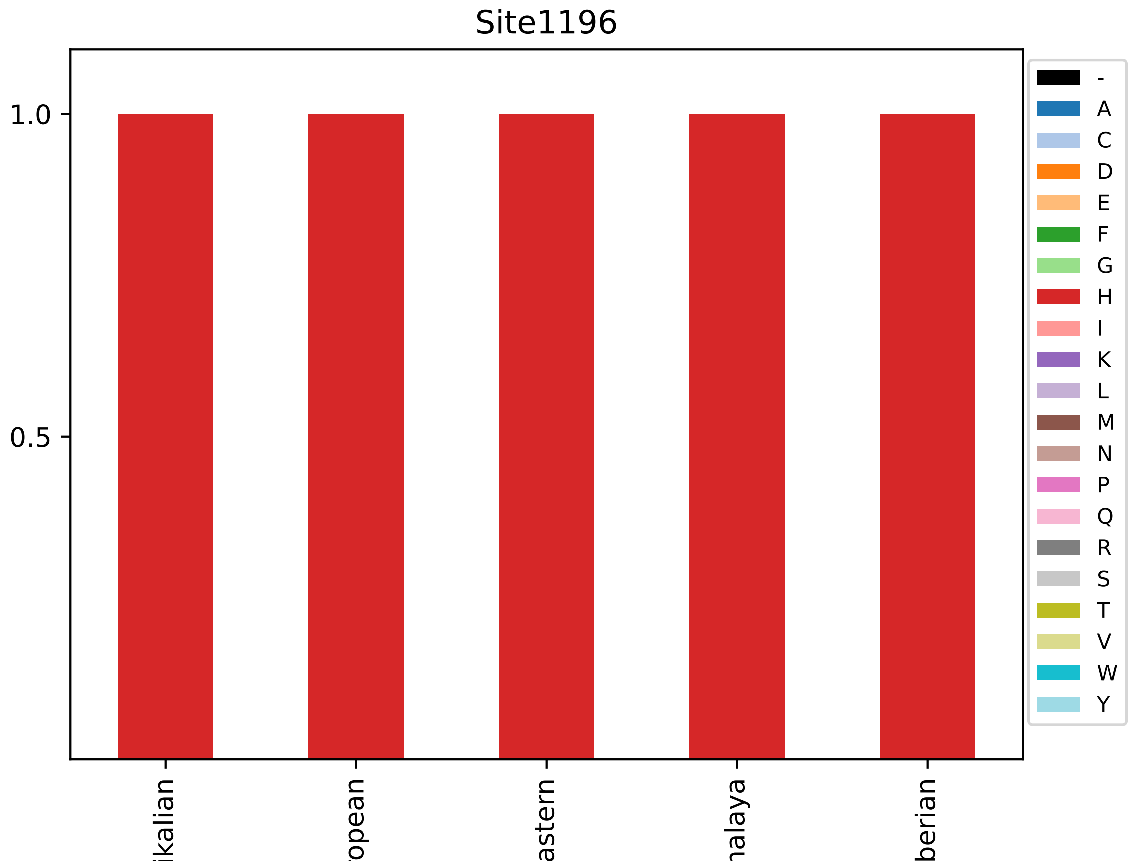
Site1196
1.0
0.5
-
A
C
D
E
F
G
H
I
K
L
M
N
P
Q
R
S
T
V
W
Y
ikalian
opean
astern
nalaya
berian

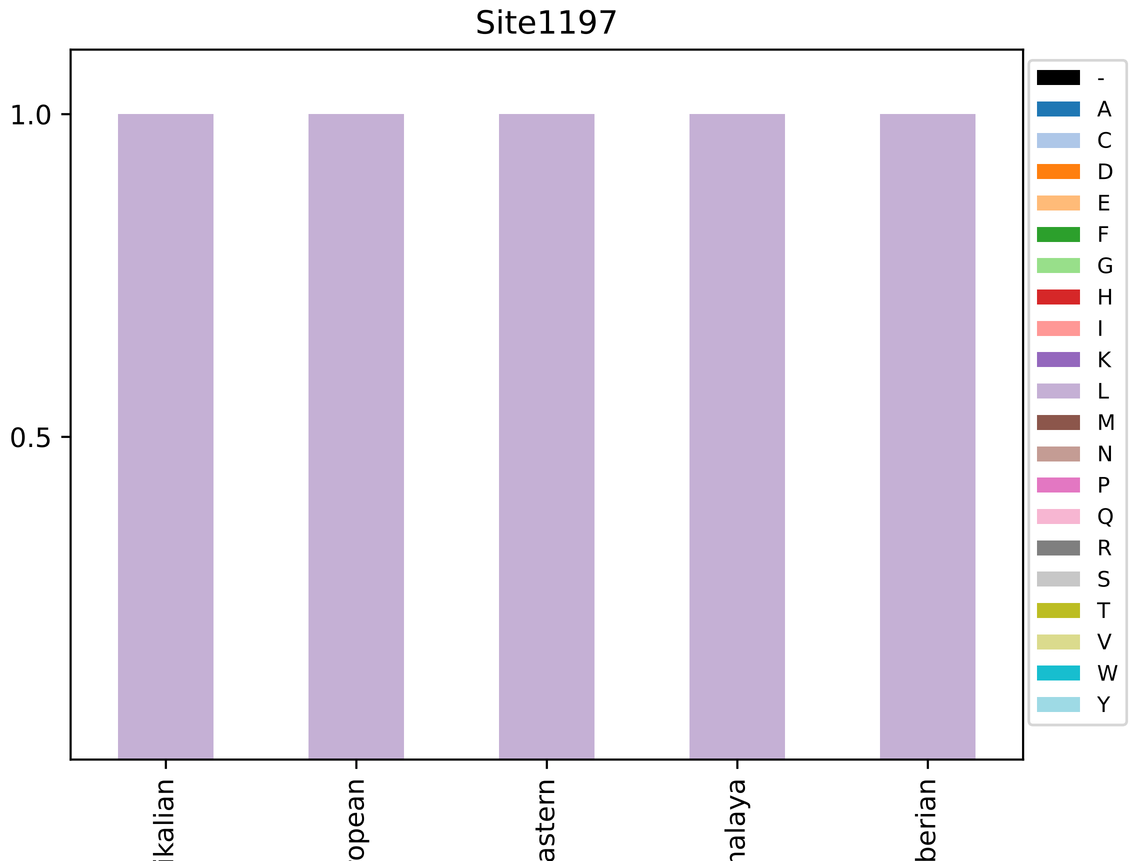
Site1197
1.0
0.5
-
A
C
D
E
F
G
H
I
K
L
M
N
P
Q
R
S
T
V
W
Y
ikalian
opean
astern
nalaya
berian

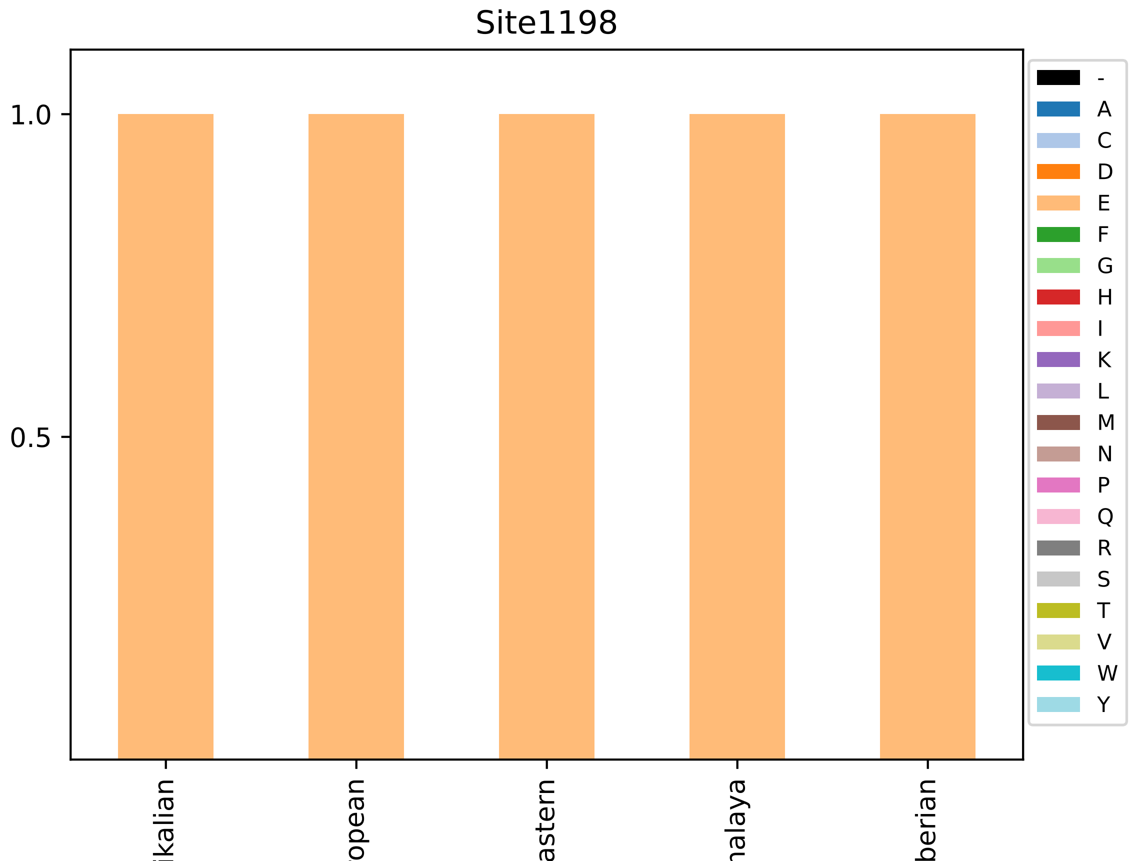
Site1198
1.0
0.5
ikalian
ropean
astern
nalaya
berian
-
A
C
D
E
F
G
H
I
K
L
M
N
P
Q
R
S
T
V
W
Y

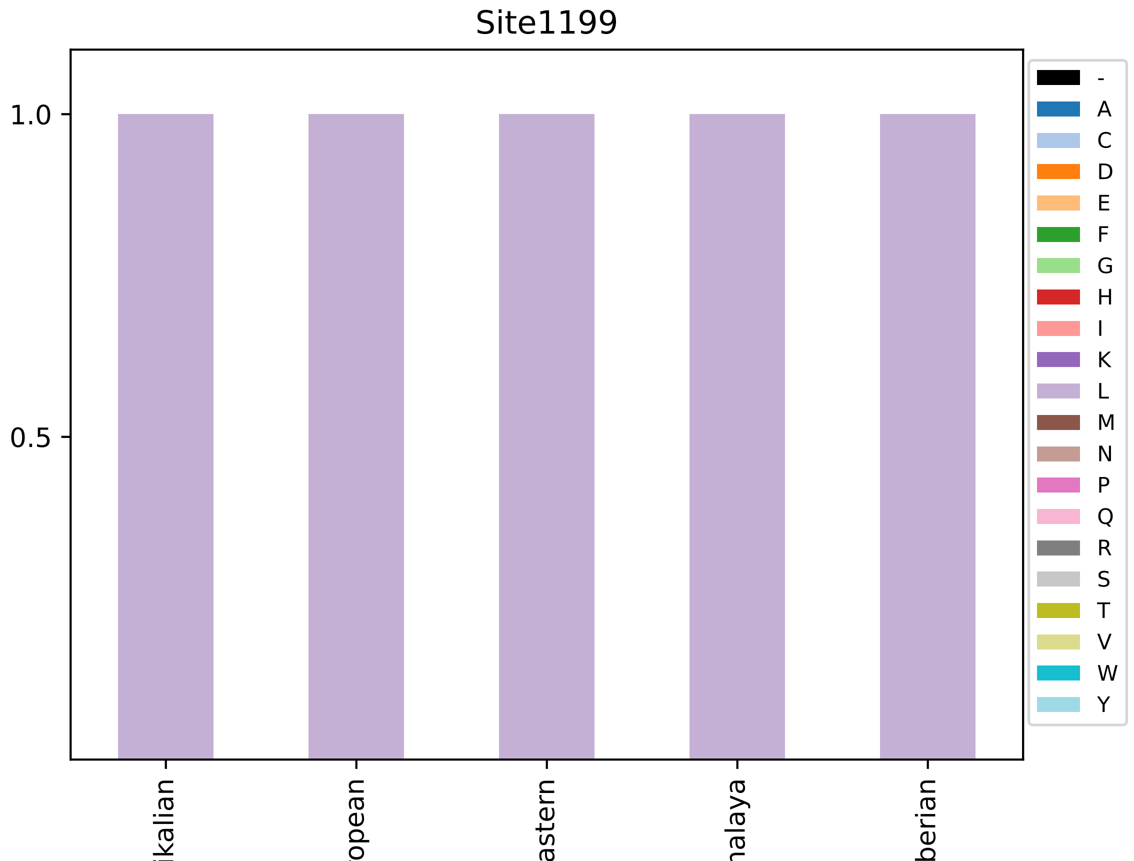
Site1199
1.0
0.5
-
A
C
D
E
F
G
H
I
K
L
M
N
P
Q
R
S
T
V
W
Y
ikalian
opean
astern
nalaya
berian

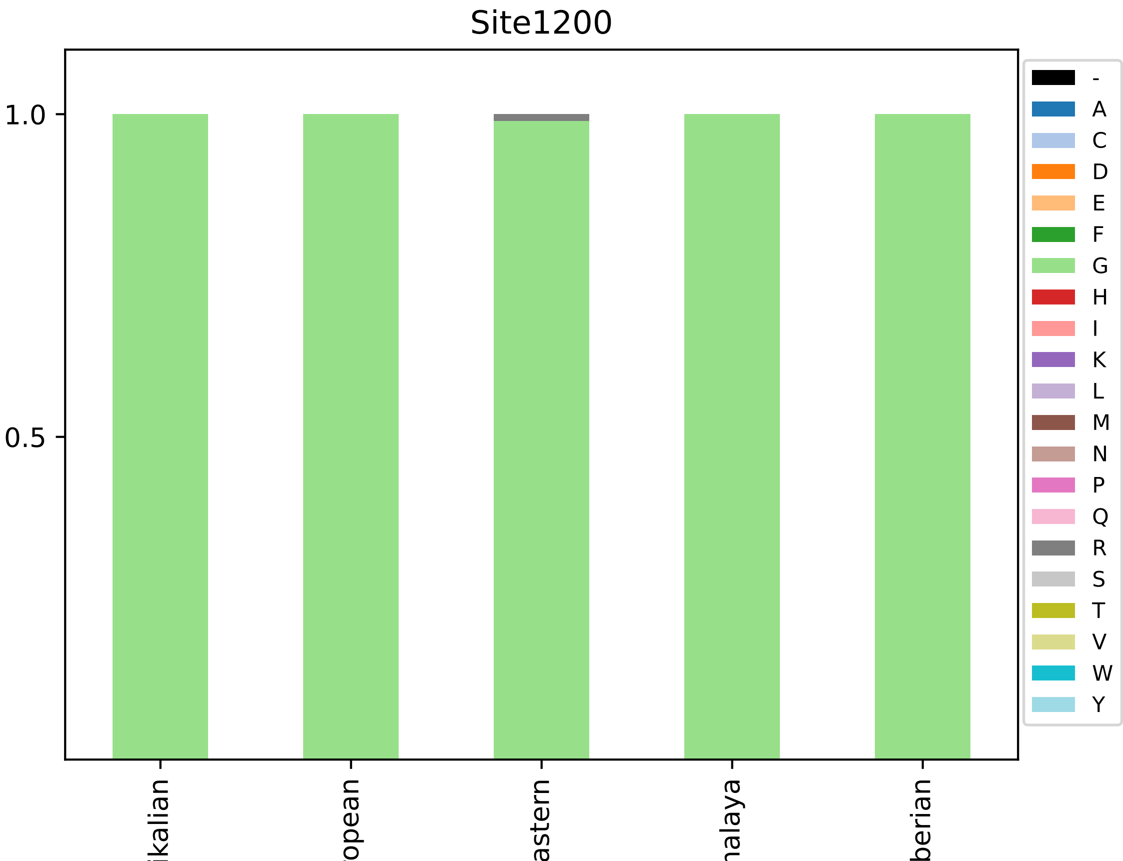

Site1200
1.0
0.5
-
A
C
D
E
F
G
H
I
K
L
M
N
P
Q
R
S
T
V
W
Y
ikalian
opean
astern
nalaya
berian

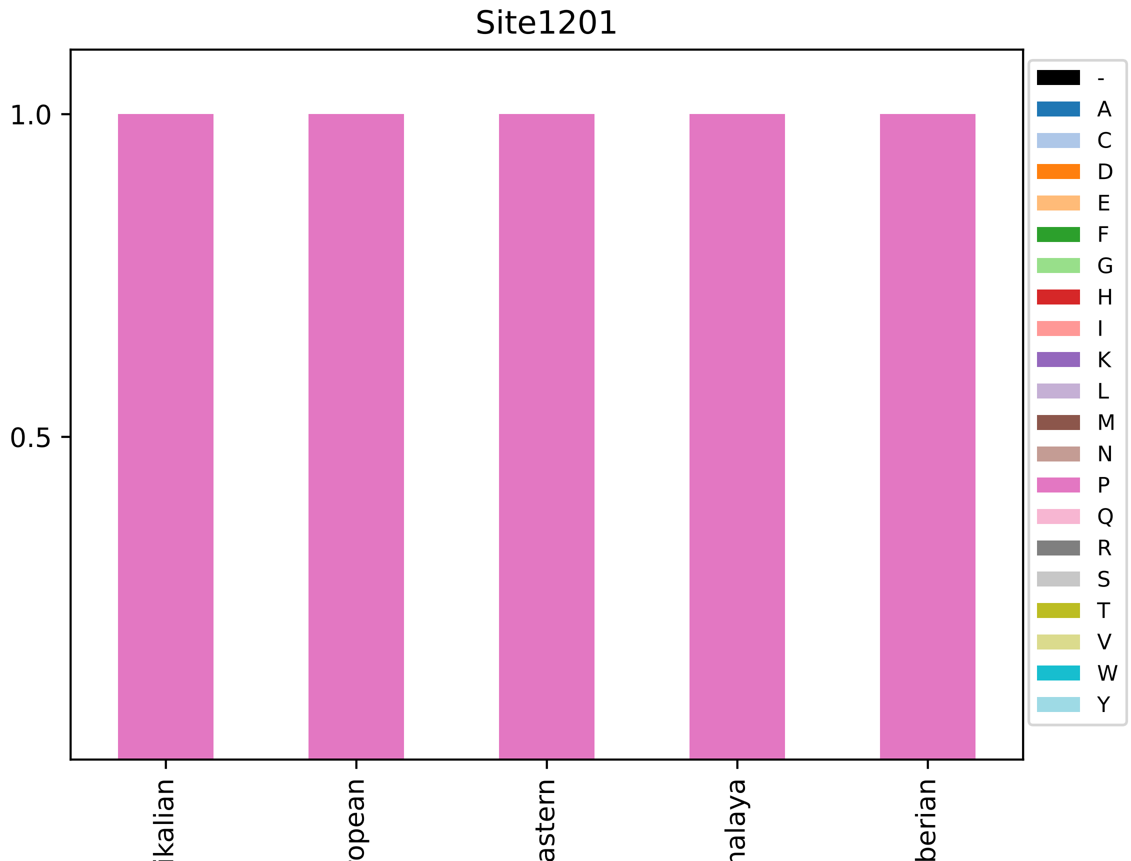
Site1201
1.0
0.5
-
A
C
D
E
F
G
H
I
K
L
M
N
P
Q
R
S
T
V
W
Y
ikalian
opean
astern
nalaya
berian

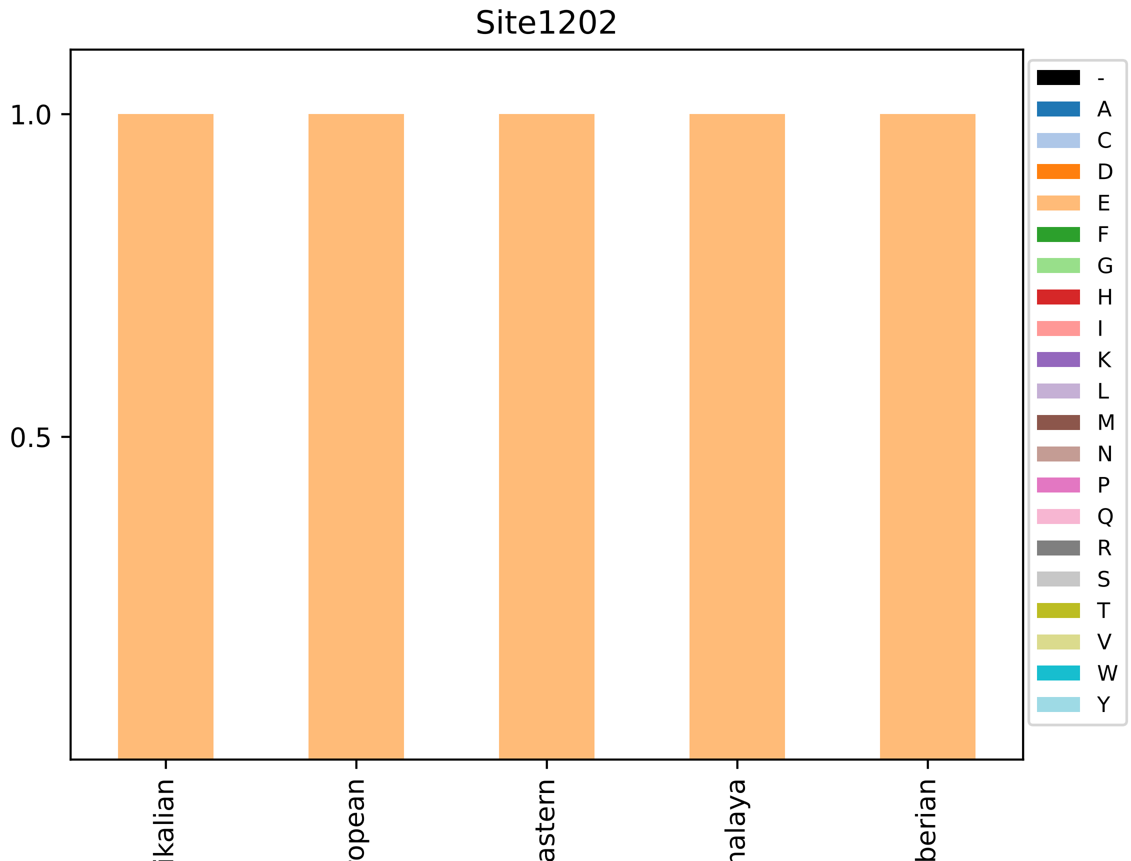

Site1202
1.0
0.5
-
A
C
D
E
F
G
H
I
K
L
M
N
P
Q
R
S
T
V
W
Y
ikalian
opean
astern
nalaya
berian

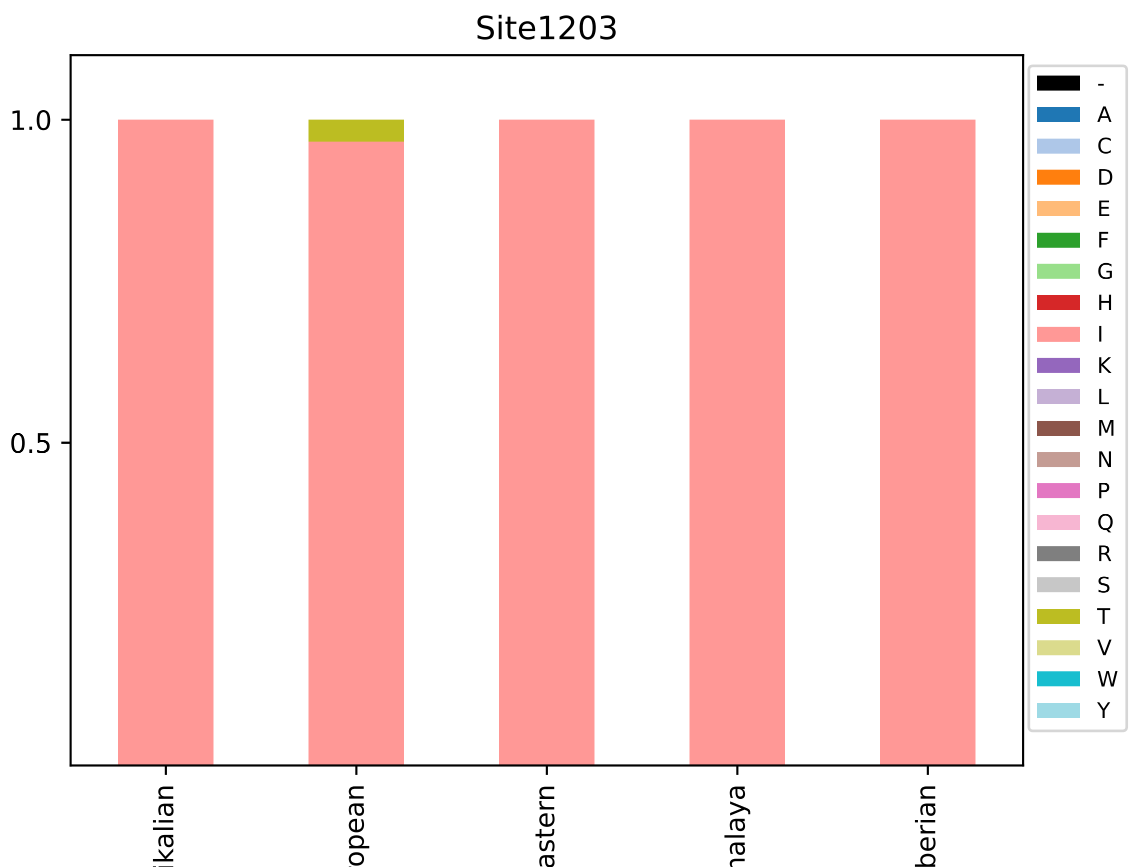
Site1203
1.0
0.5
ikalian
opean
astern
nalaya
berian
-
A
C
D
E
F
G
H
I
K
L
M
N
P
Q
R
S
T
V
W
Y

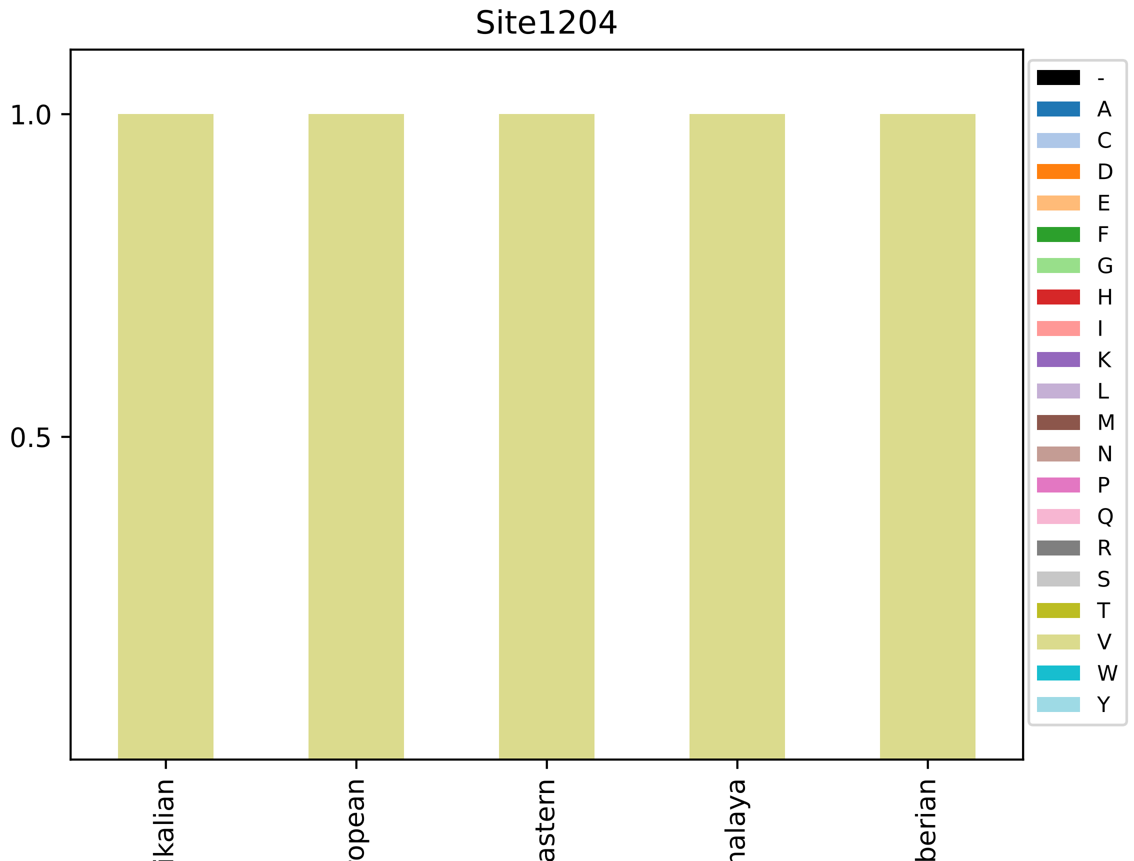
Site1204
1.0
0.5
-
A
C
D
E
F
G
H
I
K
L
M
N
P
Q
R
S
T
V
W
Y
ikalian
opean
astern
nalaya
berian

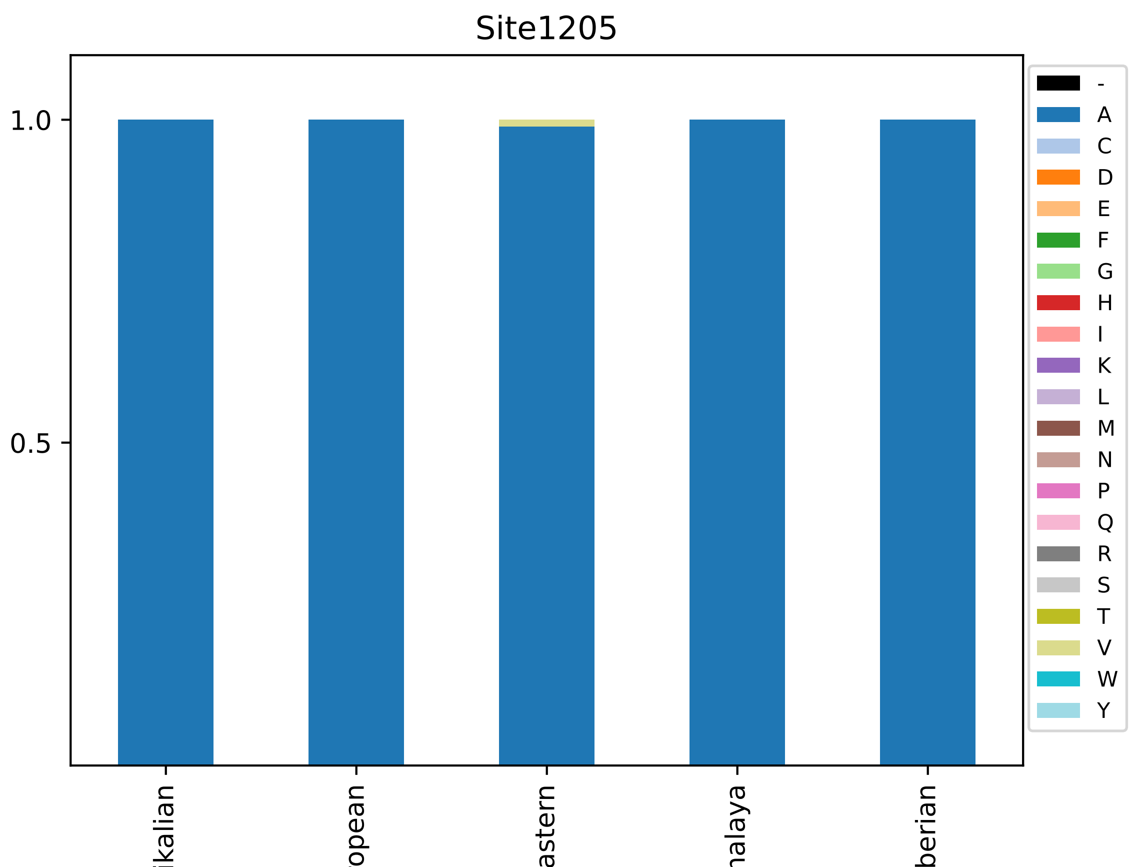

Site1205
1.0
0.5
ikalian
opean
astern
nalaya
berian
-
A
C
D
E
F
G
H
I
K
L
M
N
P
Q
R
S
T
V
W
Y

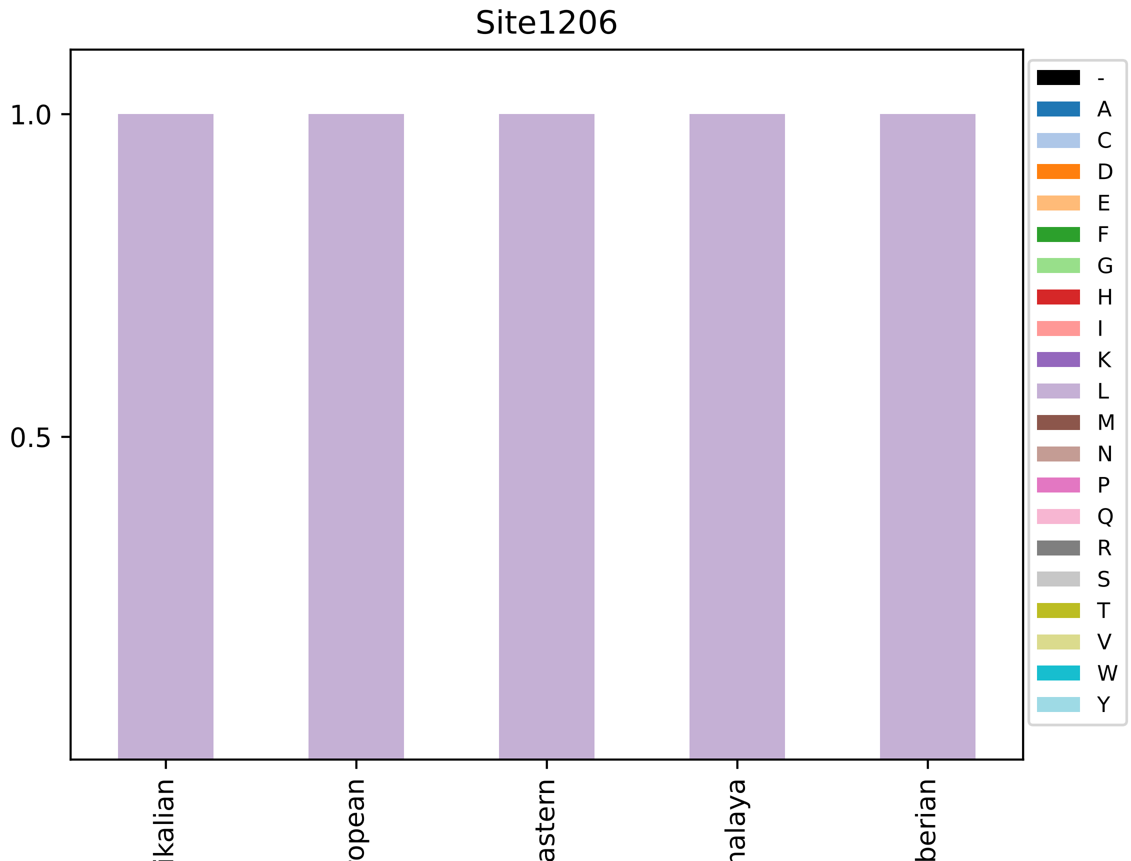

Site1206
1.0
0.5
ikalian
ropean
astern
nalaya
berian
-
A
C
D
E
F
G
H
I
K
L
M
N
P
Q
R
S
T
V
W
Y

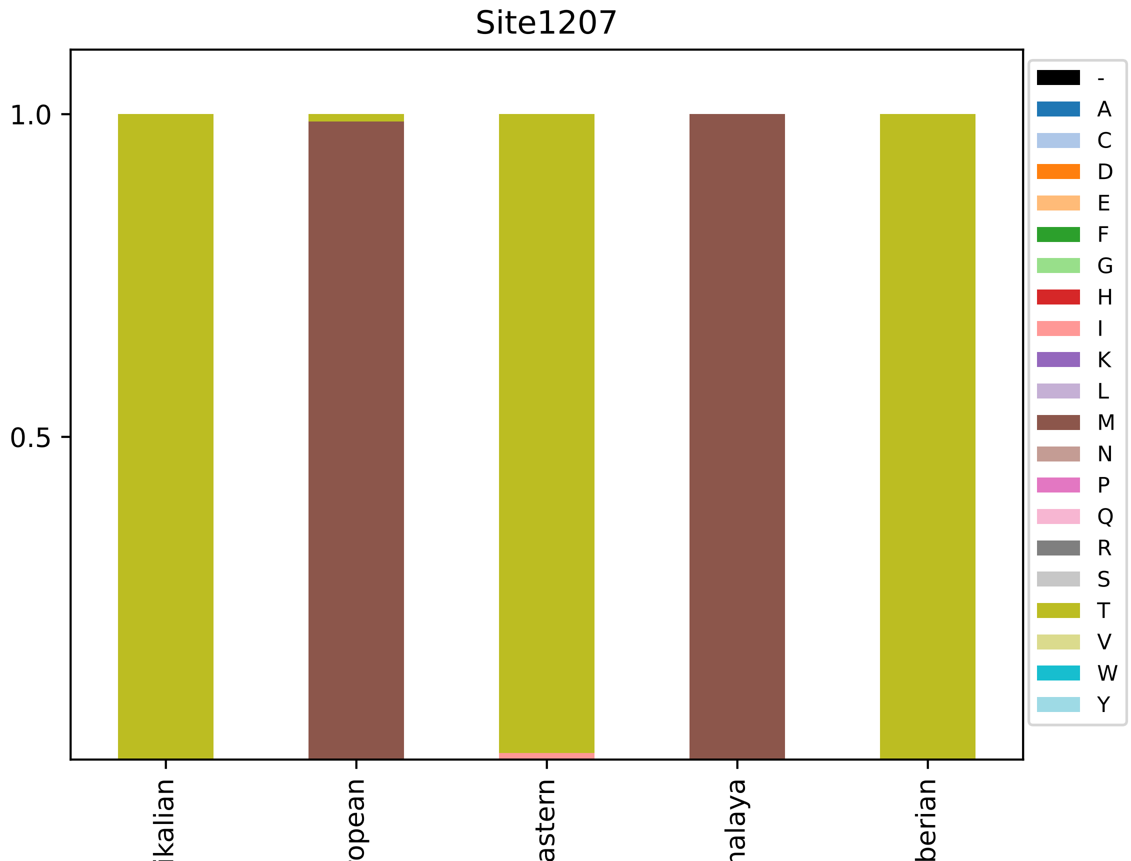

Site1207
1.0
0.5
ikalian
opean
astern
nalaya
berian
-
A
C
D
E
F
G
H
I
K
L
M
N
P
Q
R
S
T
V
W
Y

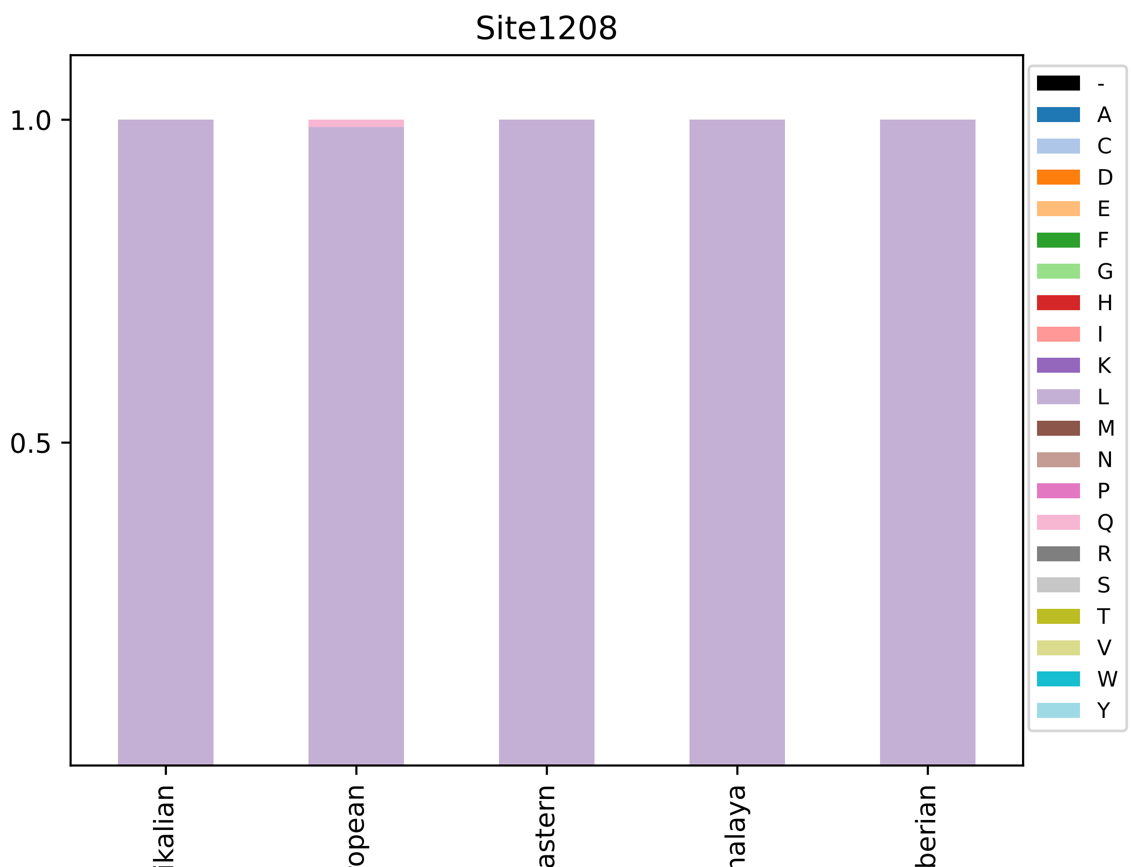

Site1208
1.0
0.5
-
A
C
D
E
F
G
H
I
K
L
M
N
P
Q
R
S
T
V
W
Y
ikalian
opean
astern
nalaya
berian

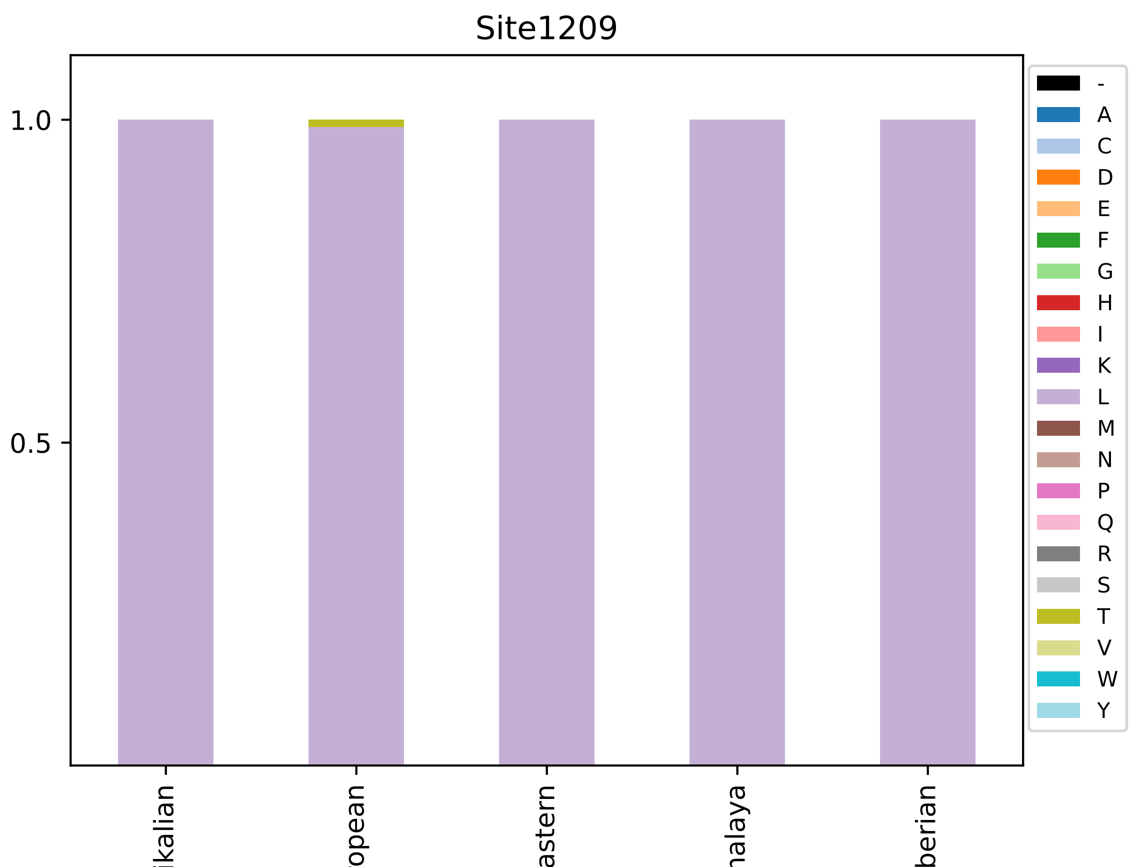
Site1209
1.0
0.5
-
A
C
D
E
F
G
H
I
K
L
M
N
P
Q
R
S
T
V
W
Y
ikalian
opean
astern
halaya
berian

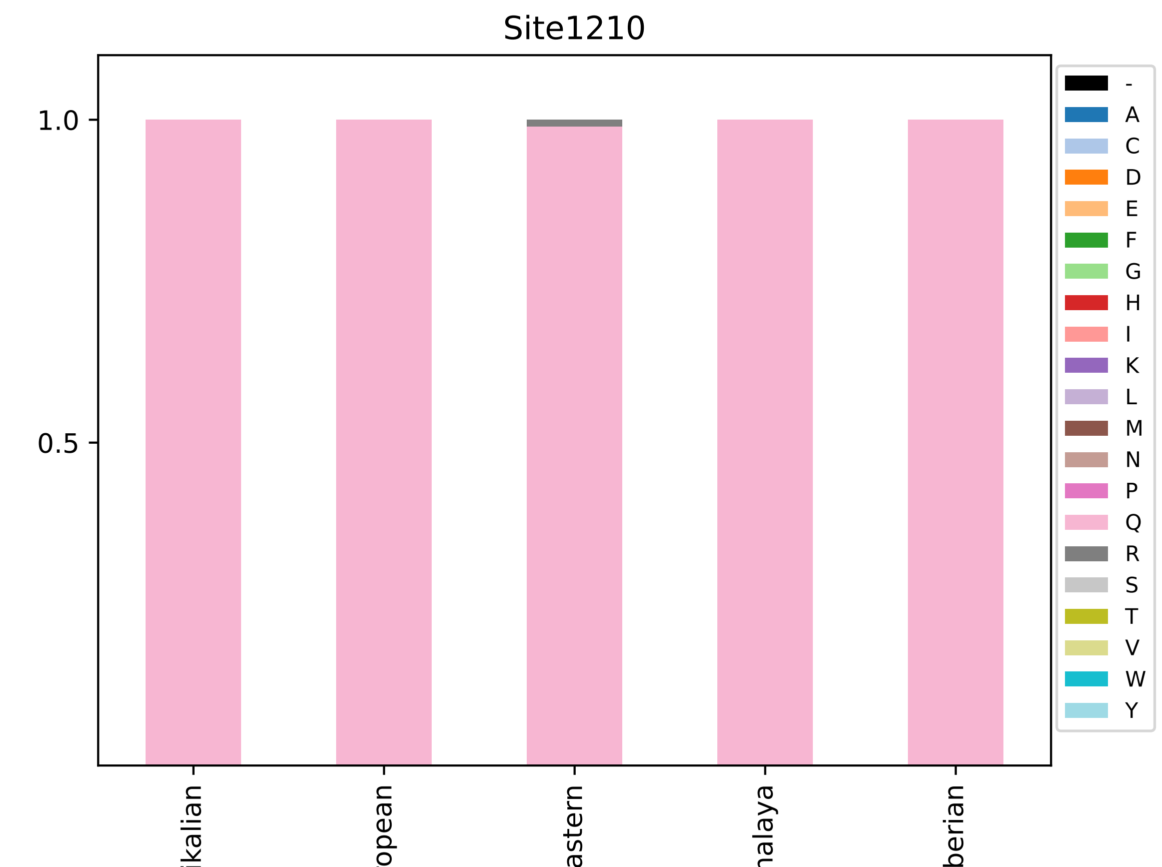
Site1210
1.0
0.5
-
A
C
D
E
F
G
H
I
K
L
M
N
P
Q
R
S
T
V
W
Y
ikalian
opean
astern
nalaya
berian

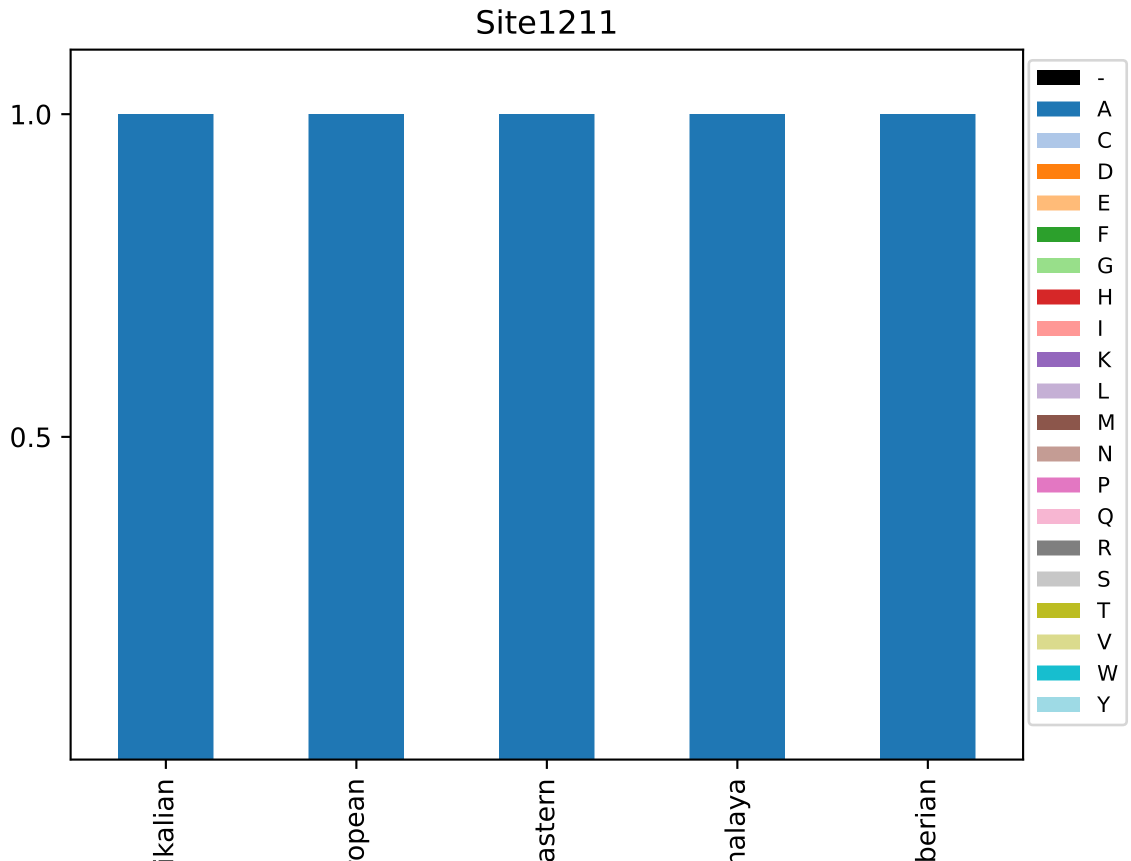

Site1211
1.0
0.5
-
A
C
D
E
F
G
H
I
K
L
M
N
P
Q
R
S
T
V
W
Y
ikalian
opean
astern
nalaya
berian

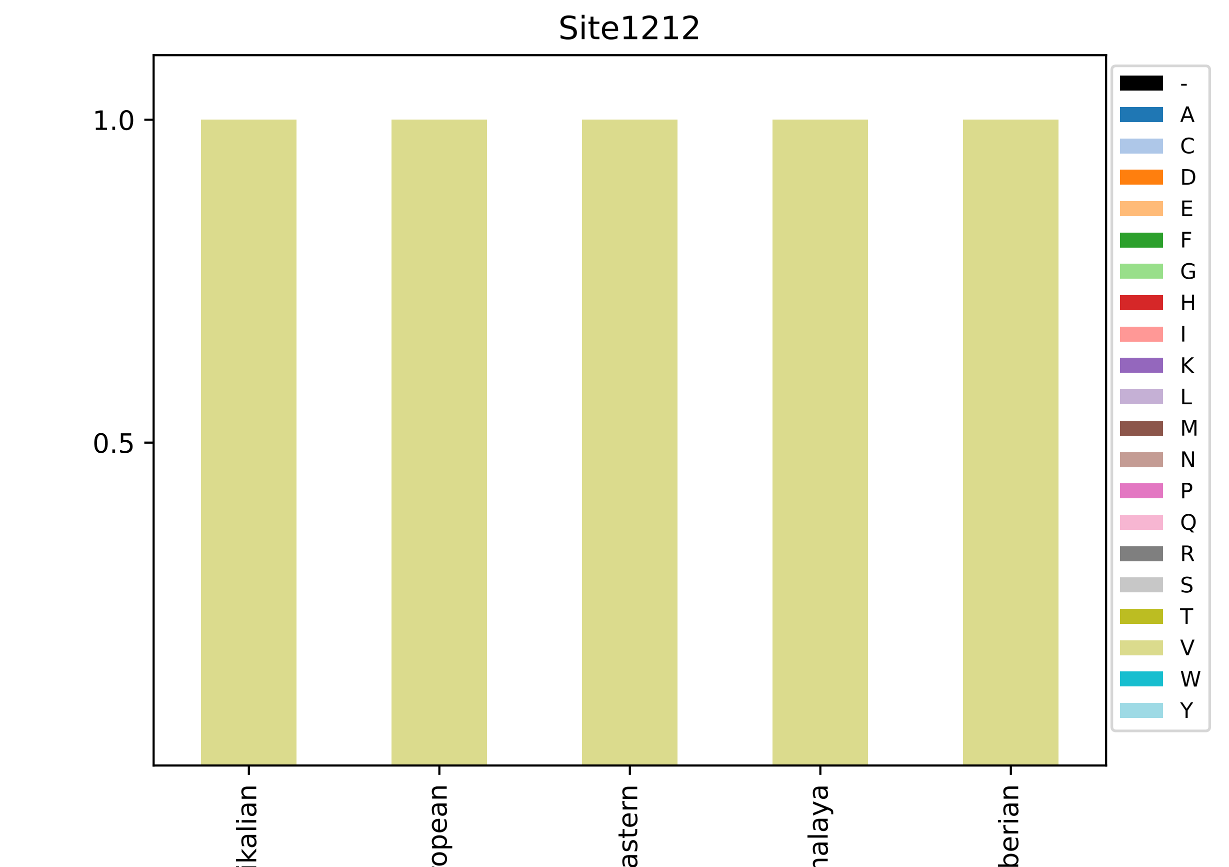

Site1212
1.0
0.5
ikalian
opean
astern
nalaya
berian
-
A
C
D
E
F
G
H
I
K
L
M
N
P
Q
R
S
T
V
W
Y

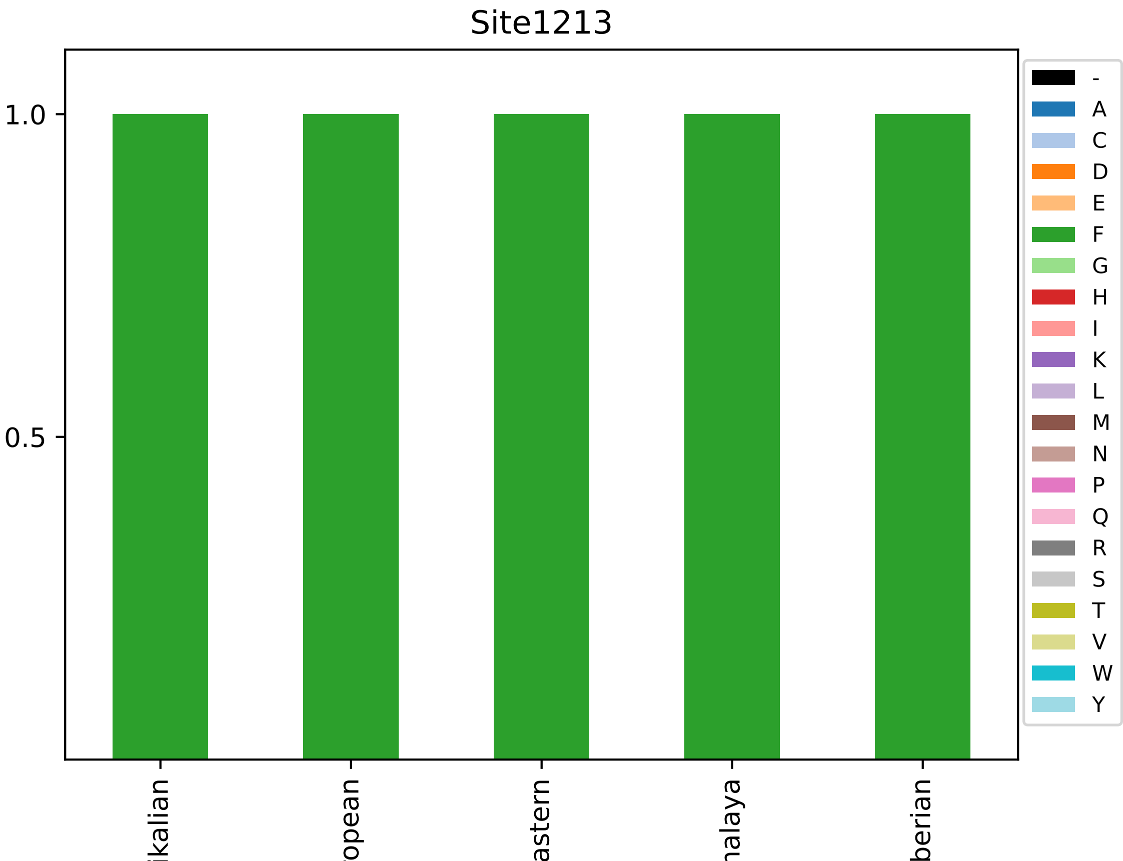

Site1213
1.0
0.5
ikalian
opean
astern
nalaya
berian
-
A
C
D
E
F
G
H
I
K
L
M
N
P
Q
R
S
T
V
W
Y

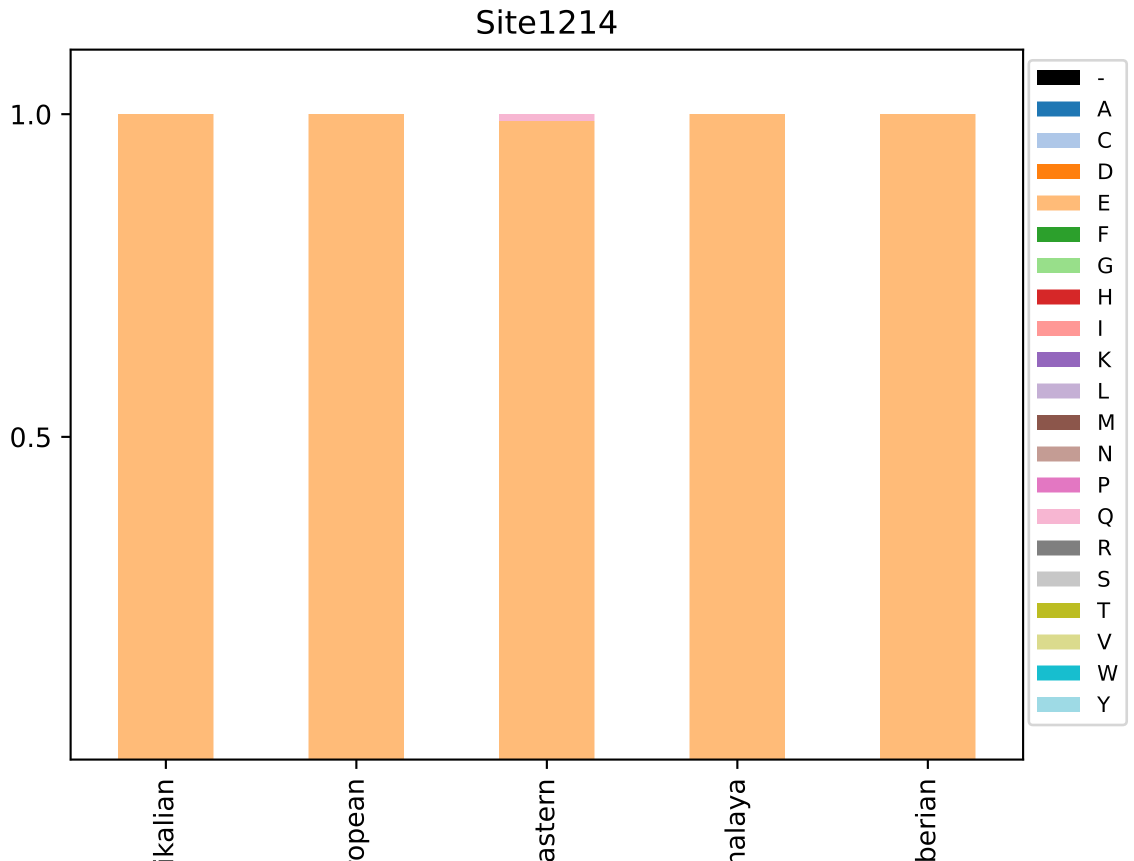
Site1214
1.0
0.5
ikalian
opean
astern
nalaya
berian
-
A
C
D
E
F
G
H
I
K
L
M
N
P
Q
R
S
T
V
W
Y

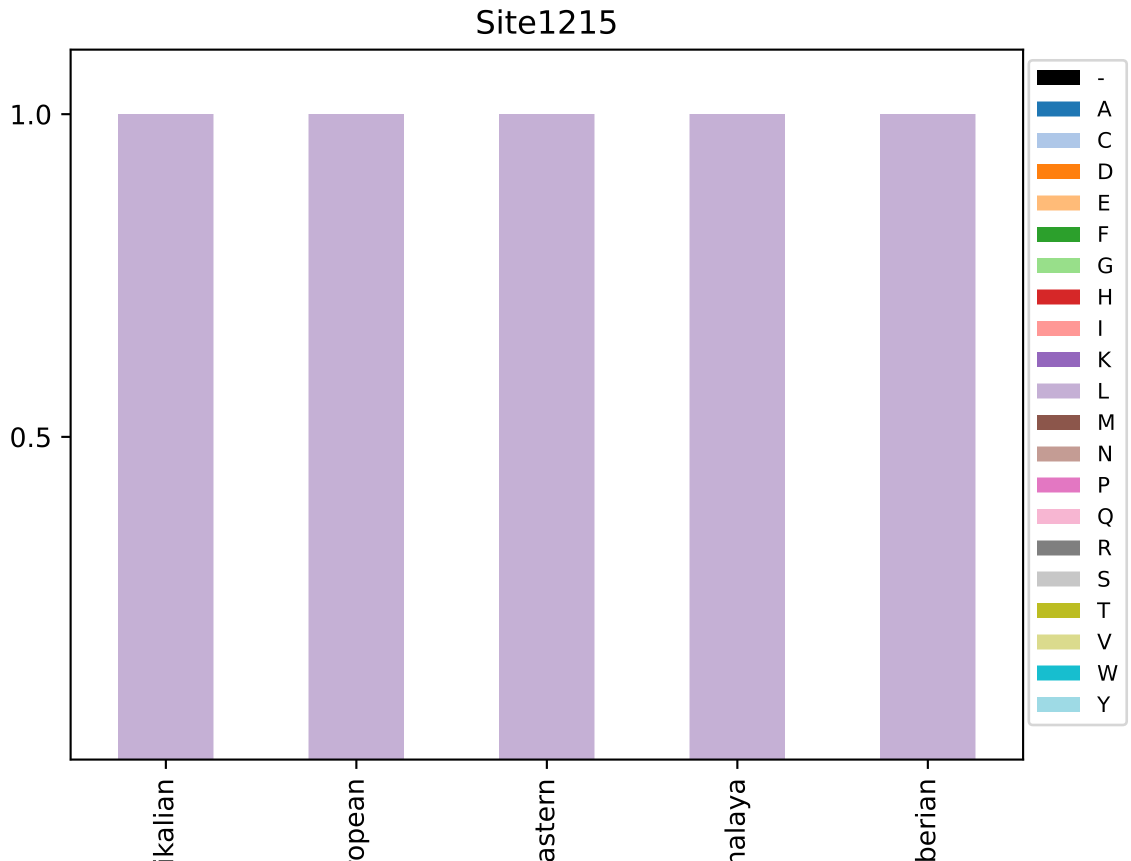

Site1215
1.0
0.5
-
A
C
D
E
F
G
H
I
K
L
M
N
P
Q
R
S
T
V
W
Y
ikalian
opean
astern
nalaya
berian

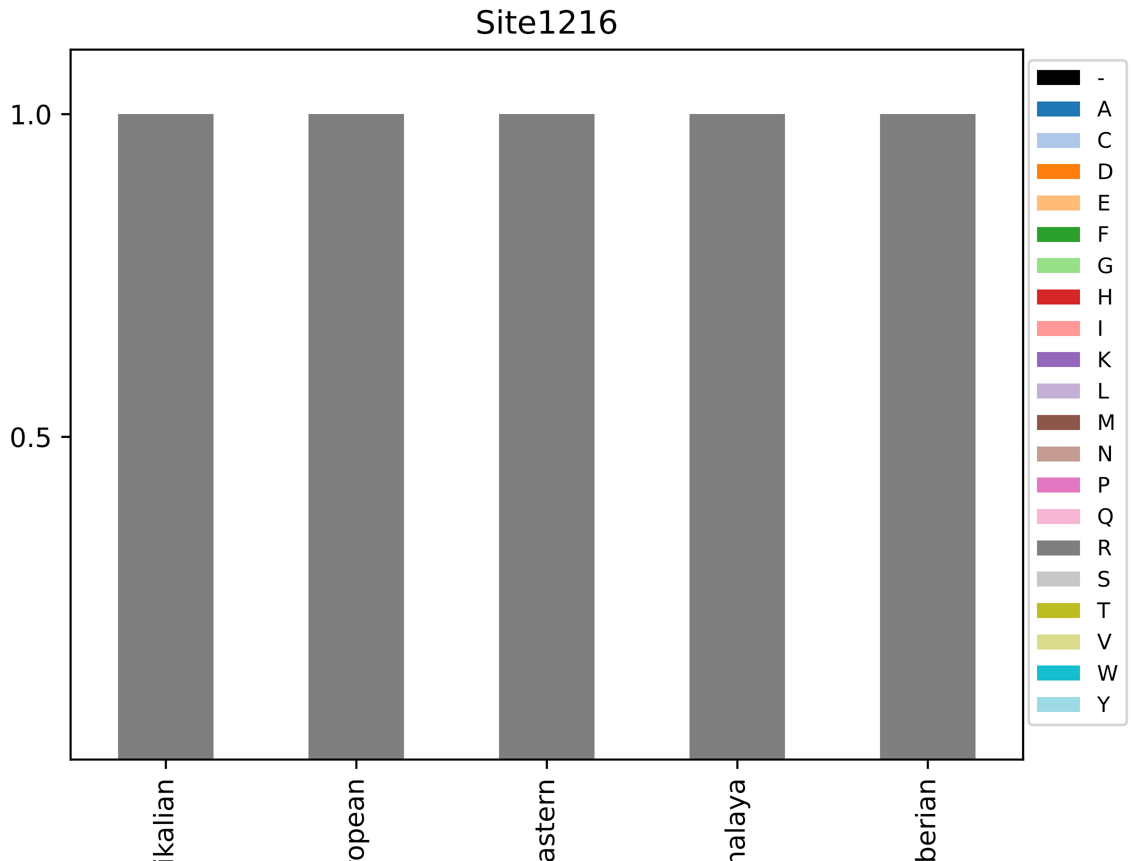
Site1216
1.0
0.5
-
A
C
D
E
F
G
H
I
K
L
M
N
P
Q
R
S
T
V
W
Y
ikalian
opean
astern
nalaya
berian

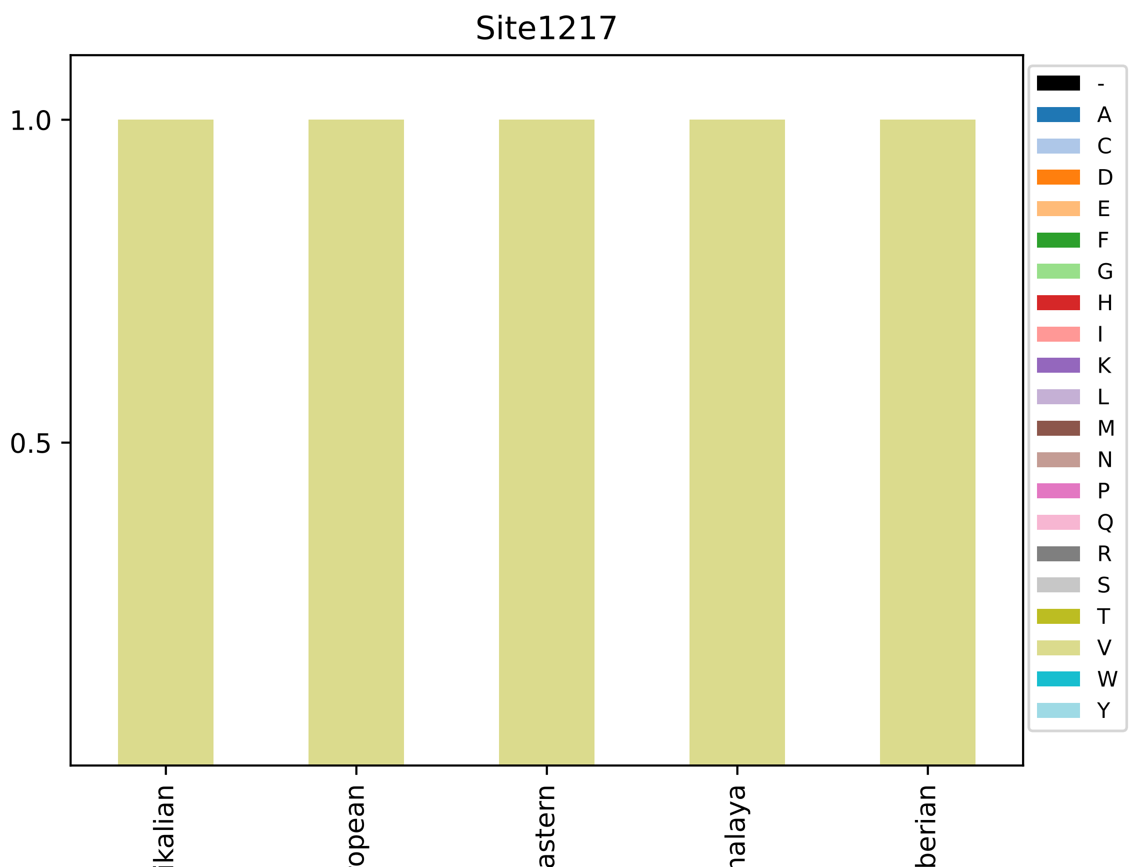
Site1217
1.0
0.5
-
A
C
D
E
F
G
H
I
K
L
M
N
P
Q
R
S
T
V
W
Y
ikalian
opean
astern
nalaya
berian

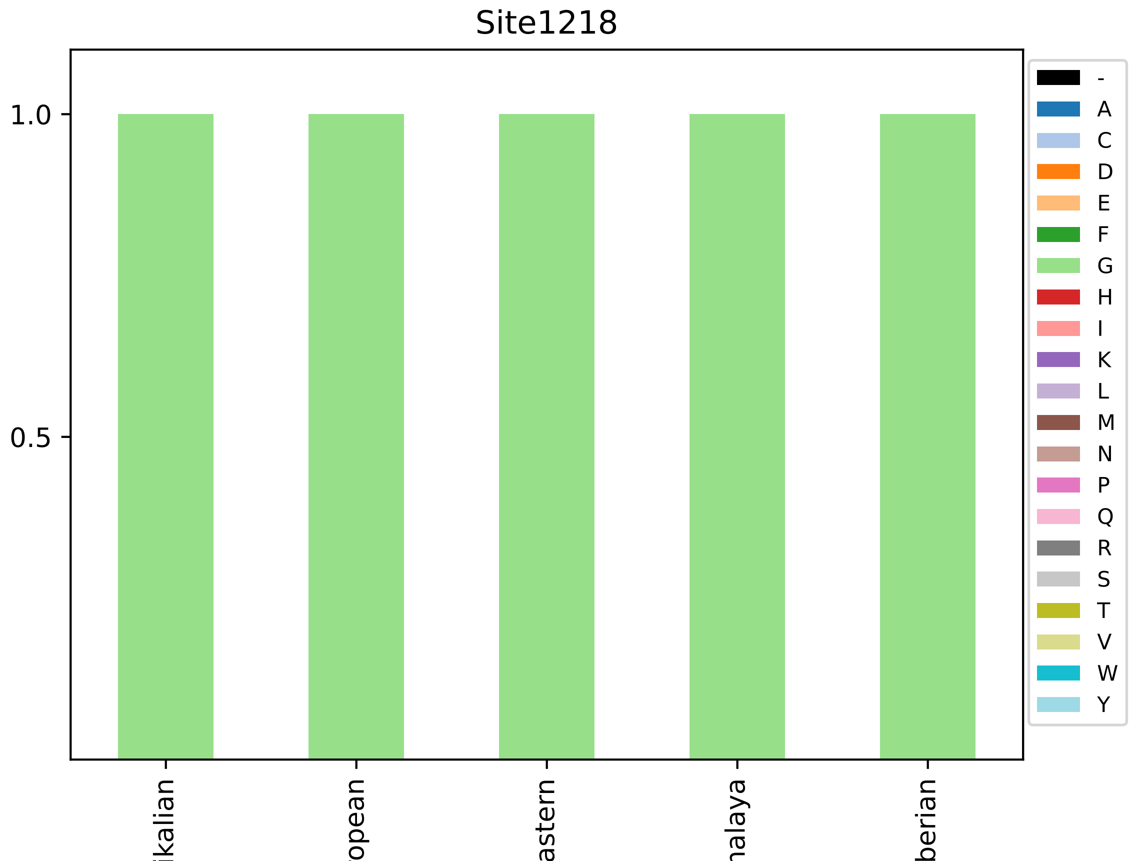
Site1218
1.0
0.5
ikalian
opean
astern
nalaya
berian
-
A
C
D
E
F
G
H
I
K
L
M
N
P
Q
R
S
T
V
W
Y

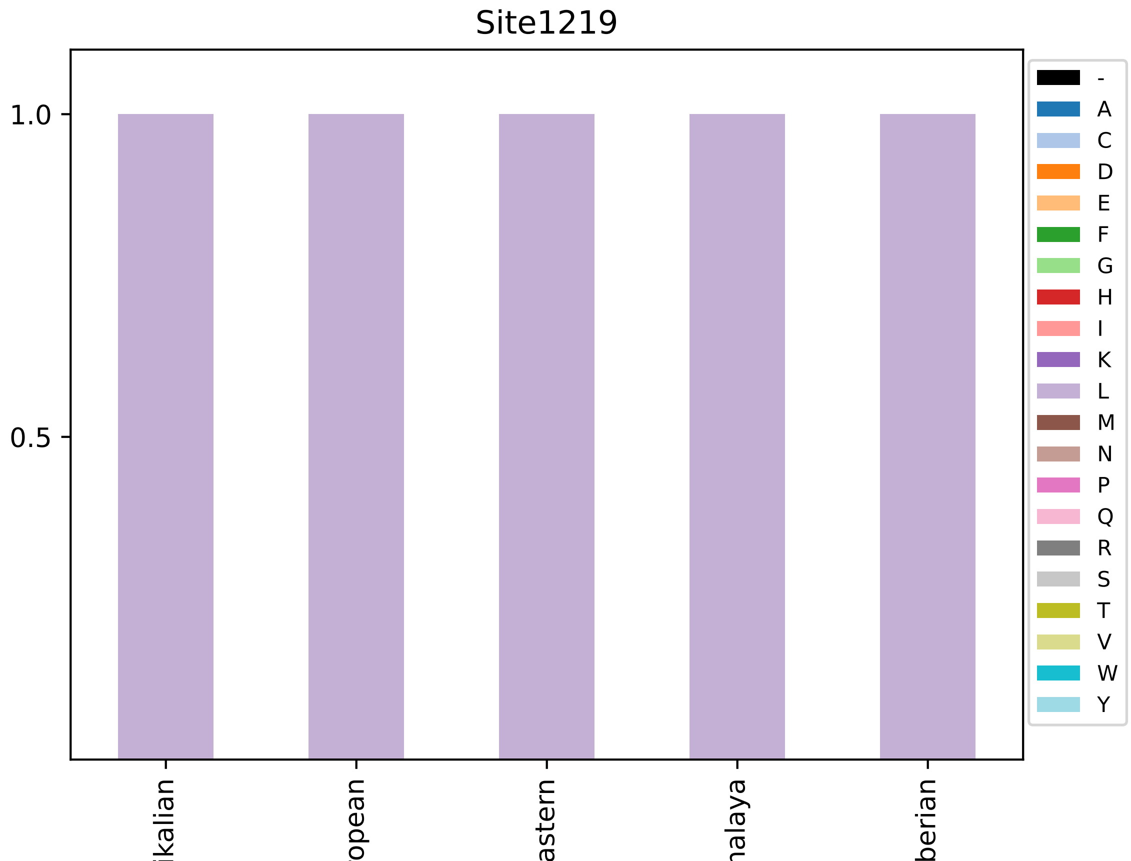
Site1219
1.0
0.5
-
A
C
D
E
F
G
H
I
K
L
M
N
P
Q
R
S
T
V
W
Y
ikalian
opean
astern
nalaya
berian

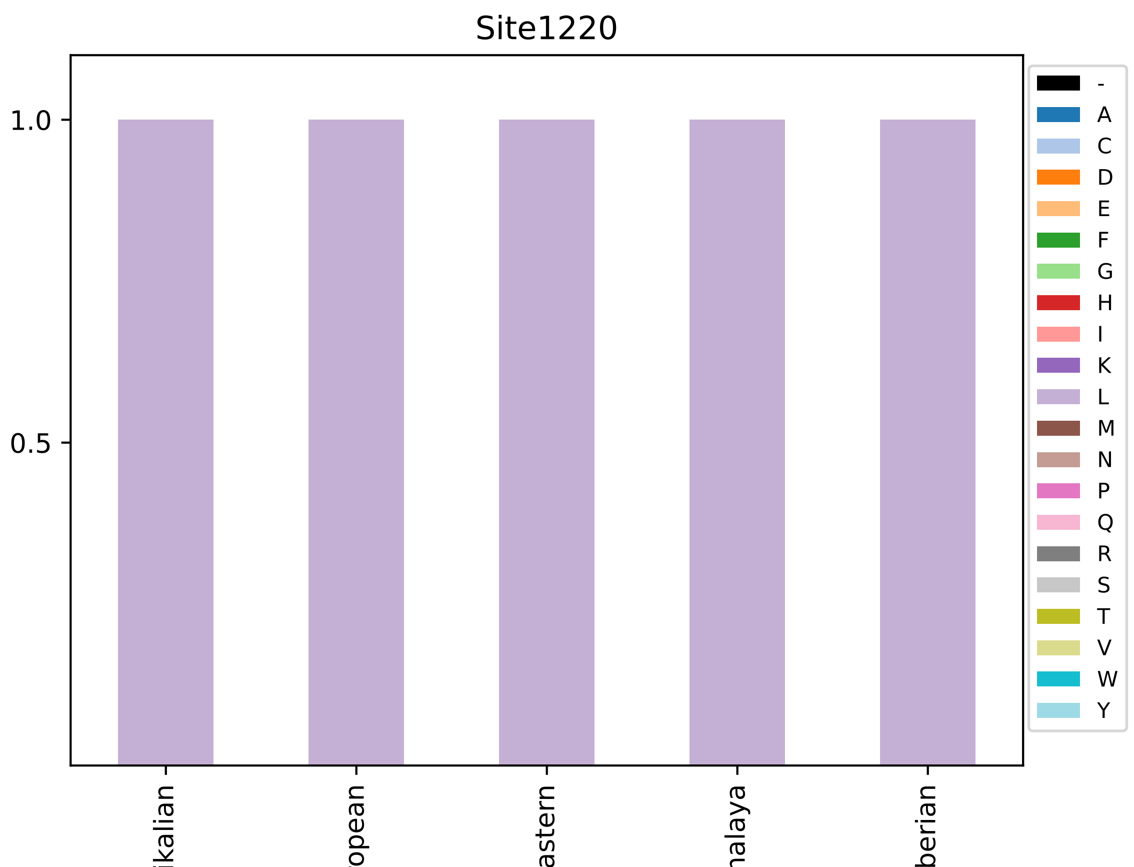

Site1220
1.0
0.5
ikalian
opean
astern
alaya
berian
-
A
C
D
E
F
G
H
I
K
L
M
N
P
Q
R
S
T
V
W
Y

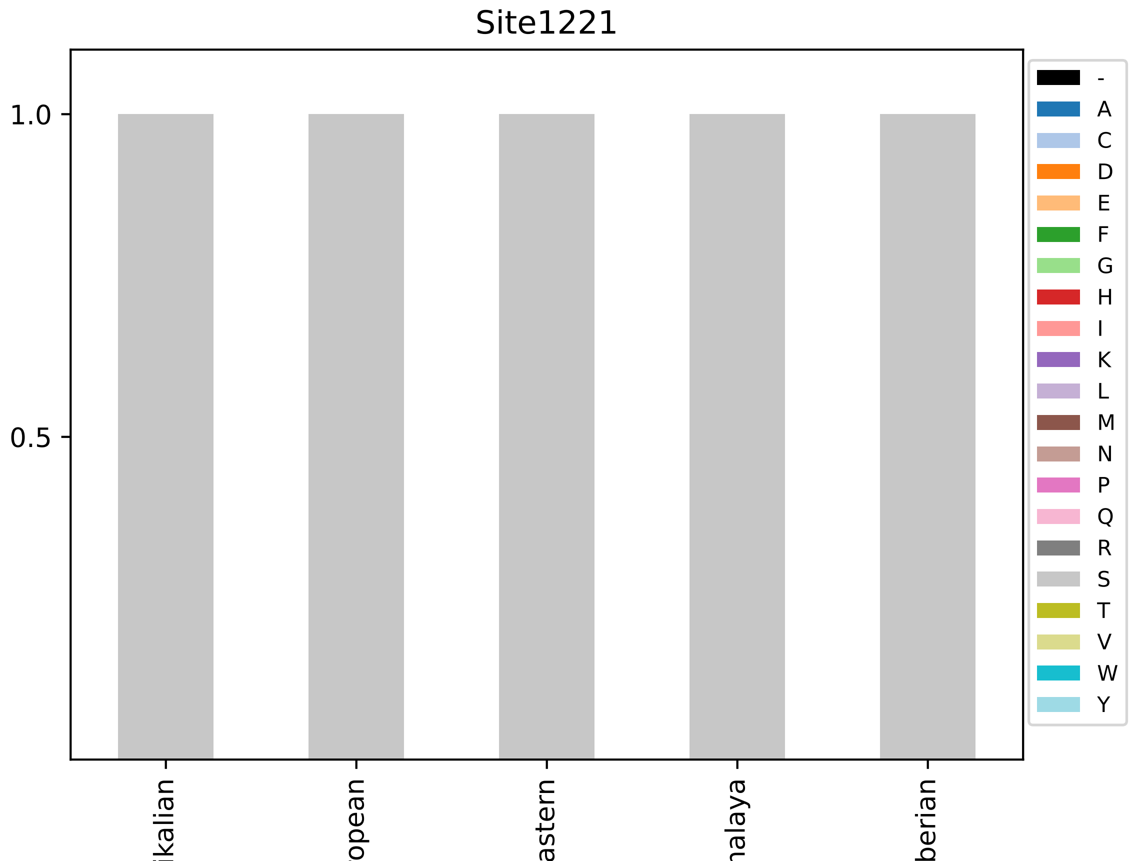
Site1221
1.0
0.5
-
A
C
D
E
F
G
H
I
K
L
M
N
P
Q
R
S
T
V
W
Y
ikalian
opean
astern
nalaya
berian

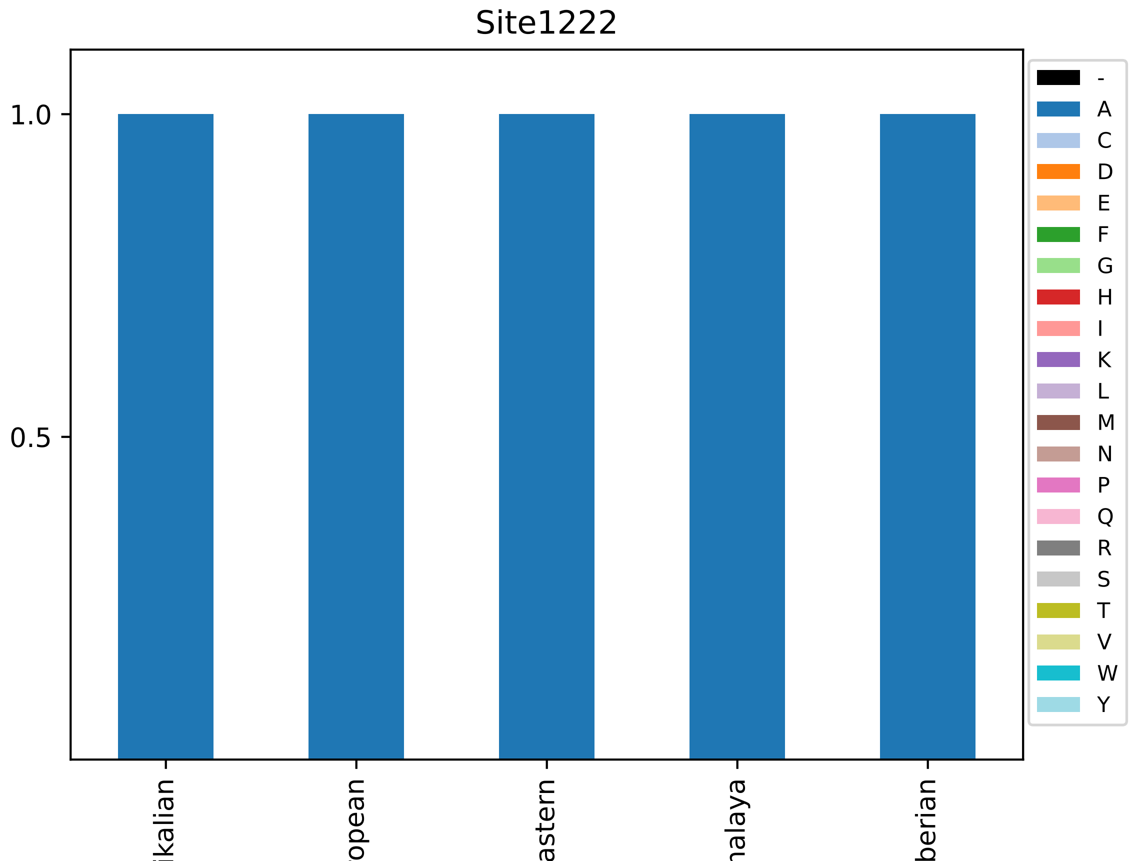

Site1222
1.0
0.5
-
A
C
D
E
F
G
H
I
K
L
M
N
P
Q
R
S
T
V
W
Y
ikalian
opean
astern
halaya
berian

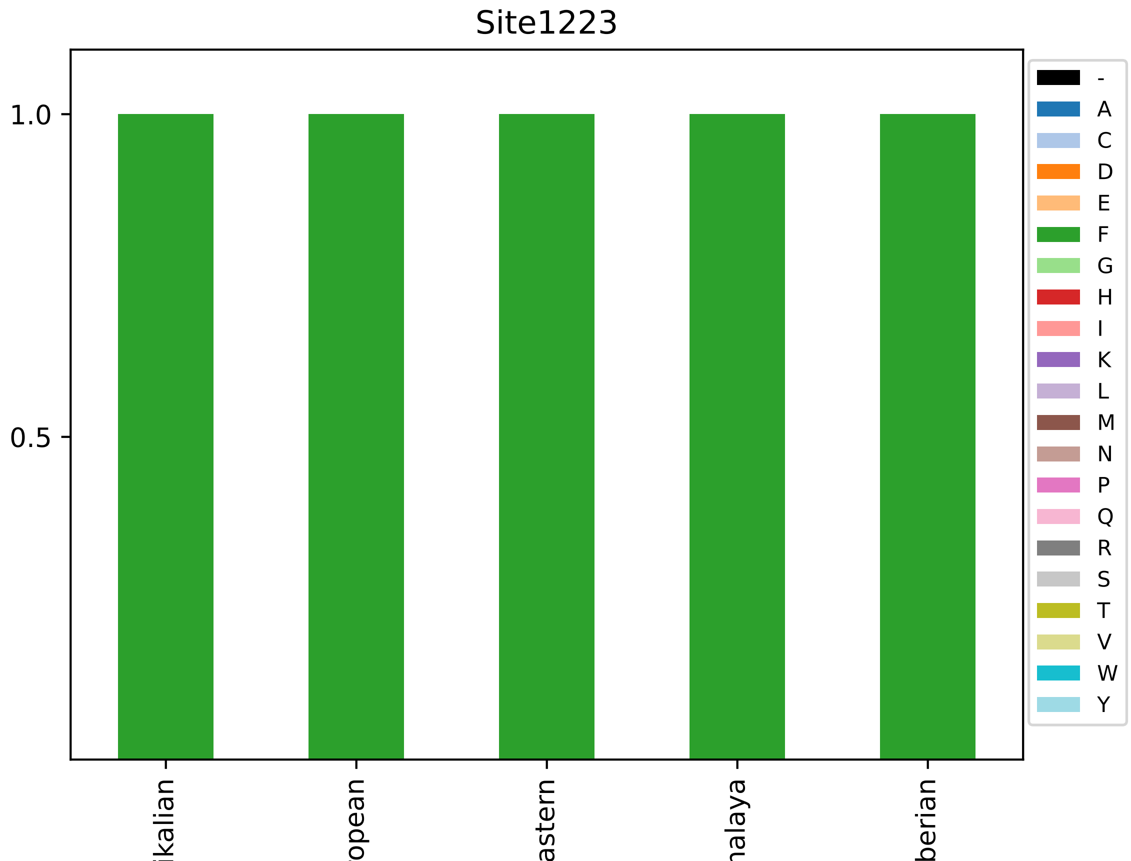

Site1223
1.0
0.5
ikalian
opean
astern
nalaya
berian
-
A
C
D
E
F
G
H
I
K
L
M
N
P
Q
R
S
T
V
W
Y

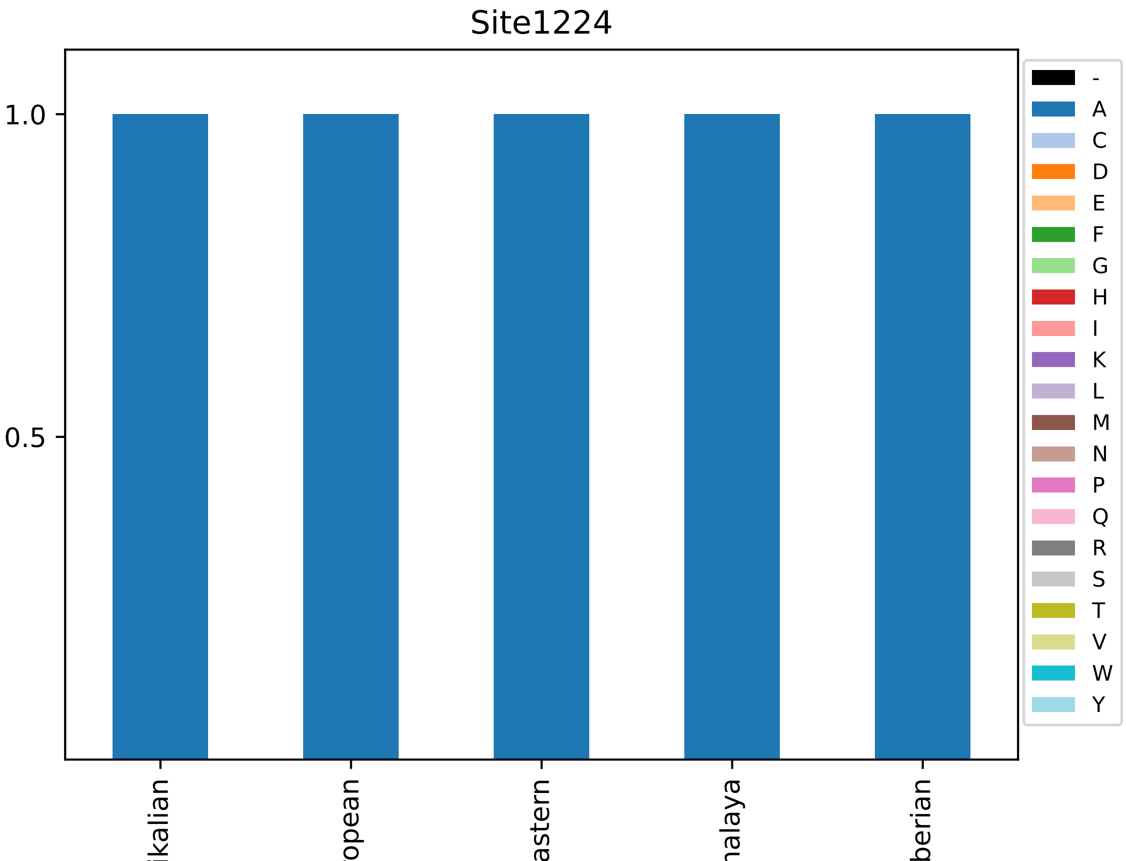
Site1224
1.0
0.5
-
A
C
D
E
F
G
H
I
K
L
M
N
P
Q
R
S
T
V
W
Y
ikalian
opean
astern
nalaya
berian

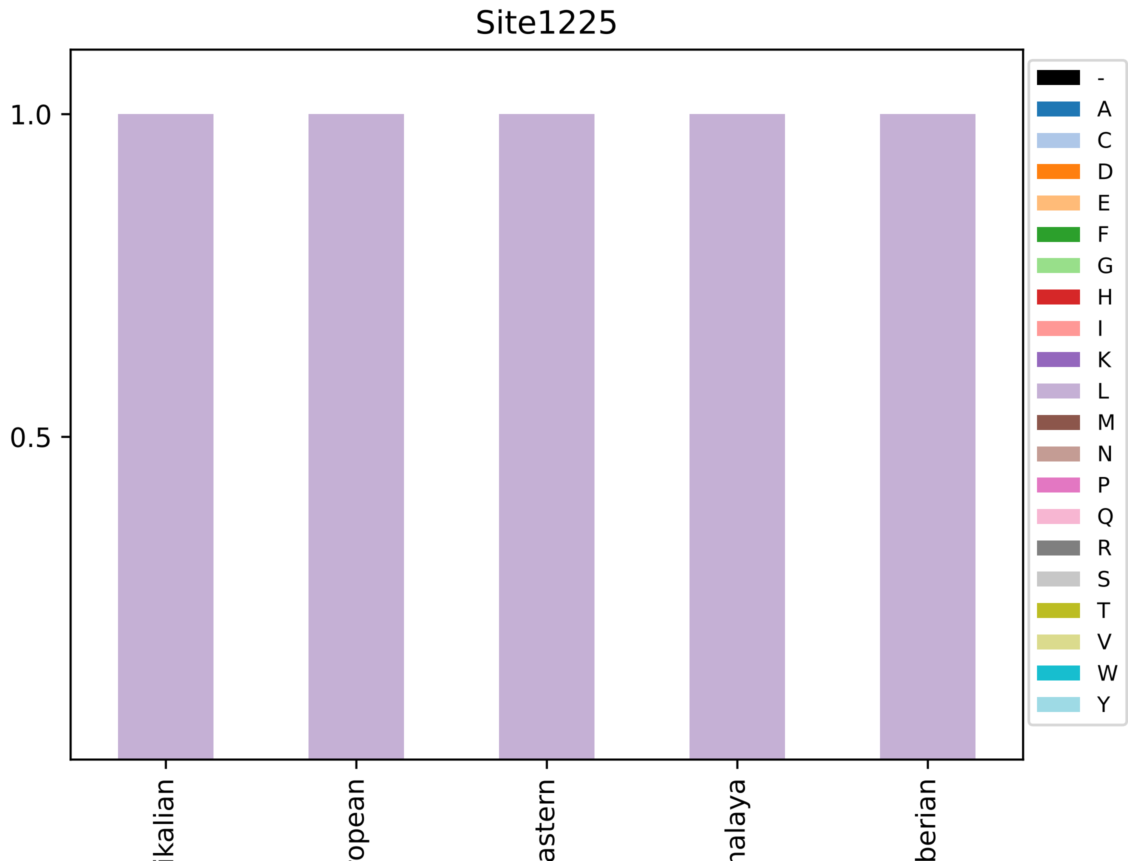

Site1225
1.0
0.5
ikalian
opean
astern
nalaya
berian
-
A
C
D
E
F
G
H
I
K
L
M
N
P
Q
R
S
T
V
W
Y

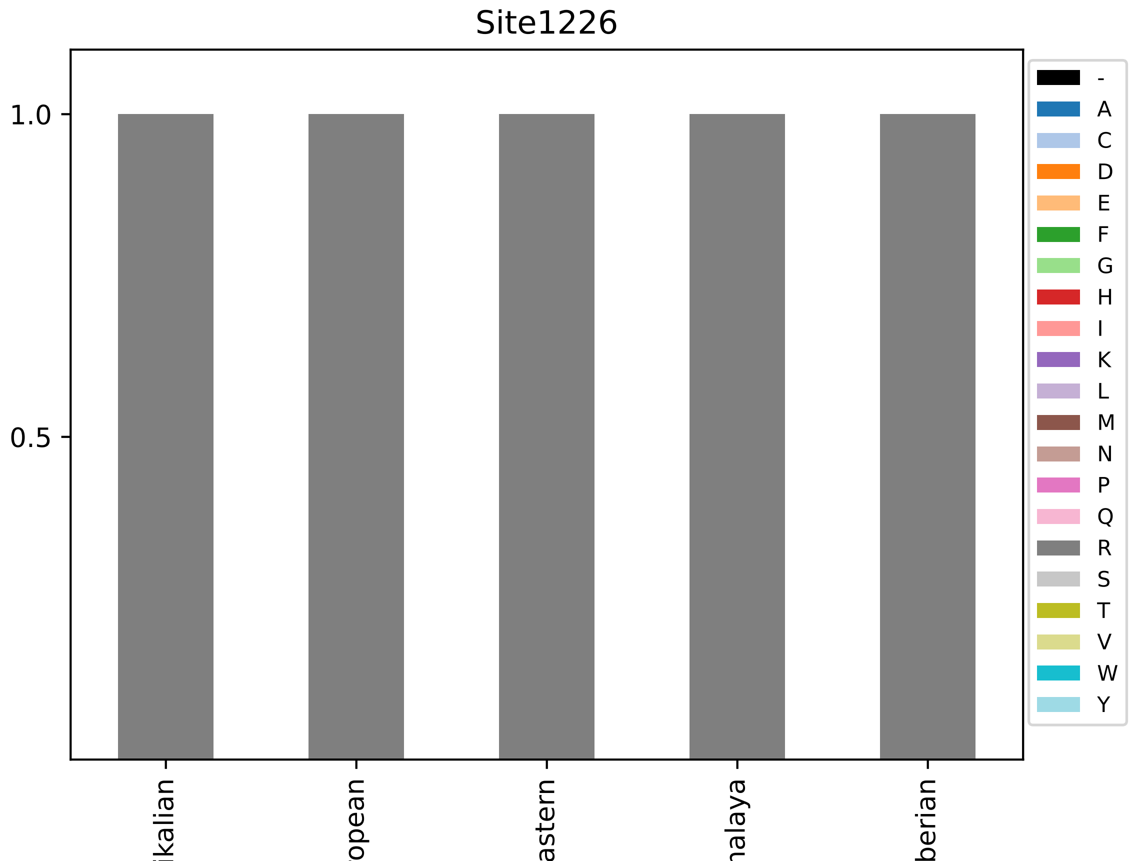
Site1226
1.0
0.5
-
A
C
D
E
F
G
H
I
K
L
M
N
P
Q
R
S
T
V
W
Y
ikalian
opean
astern
nalaya
berian

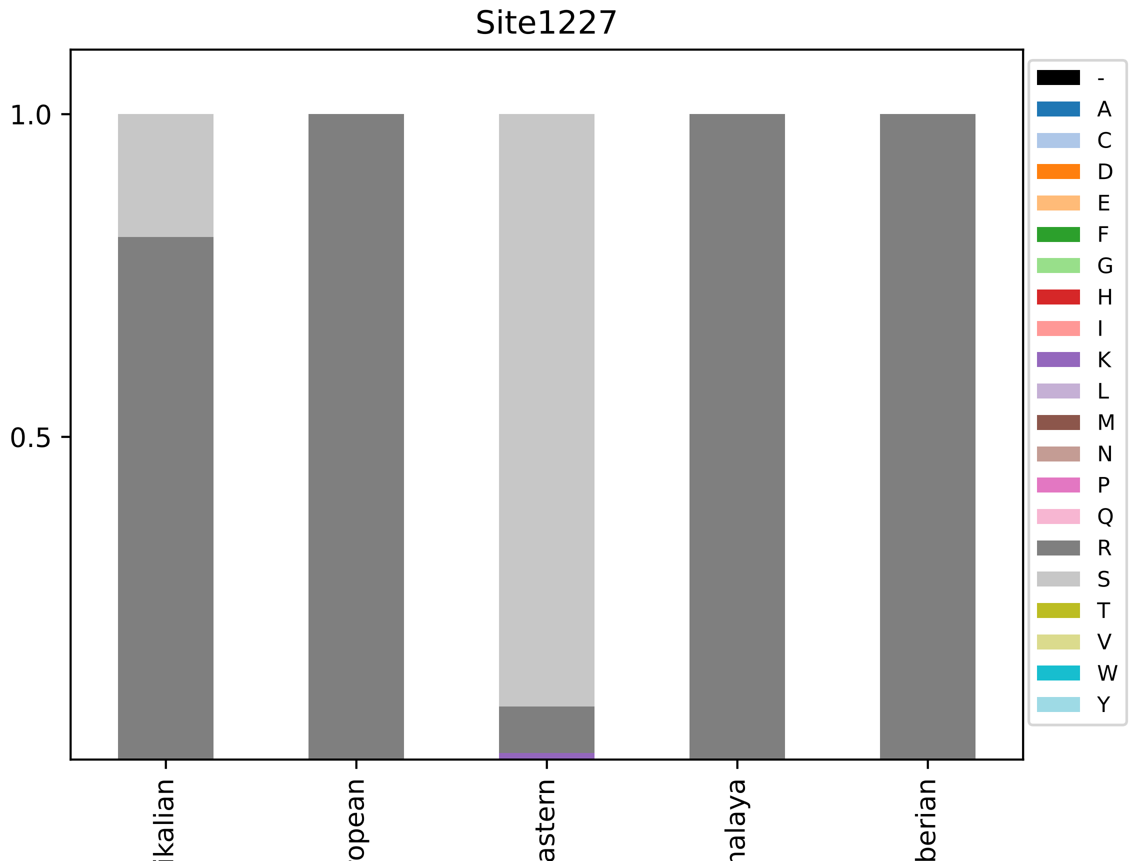

Site1227
1.0
0.5
-
A
C
D
E
F
G
H
I
K
L
M
N
P
Q
R
S
T
V
W
Y
ikalian
opean
astern
nalaya
berian

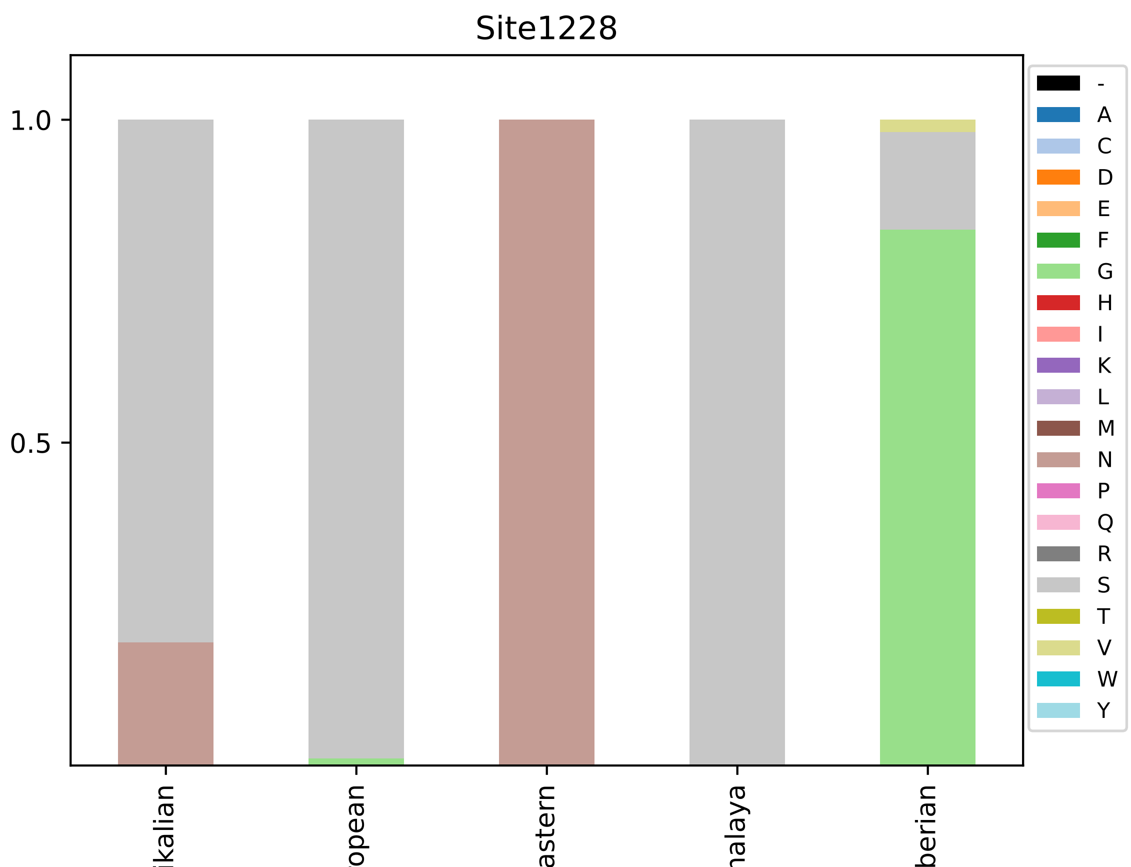
Site1228
1.0
0.5
-
A
C
D
E
F
G
H
I
K
L
M
N
P
Q
R
S
T
V
W
Y
ikalian
opean
astern
nalaya
berian

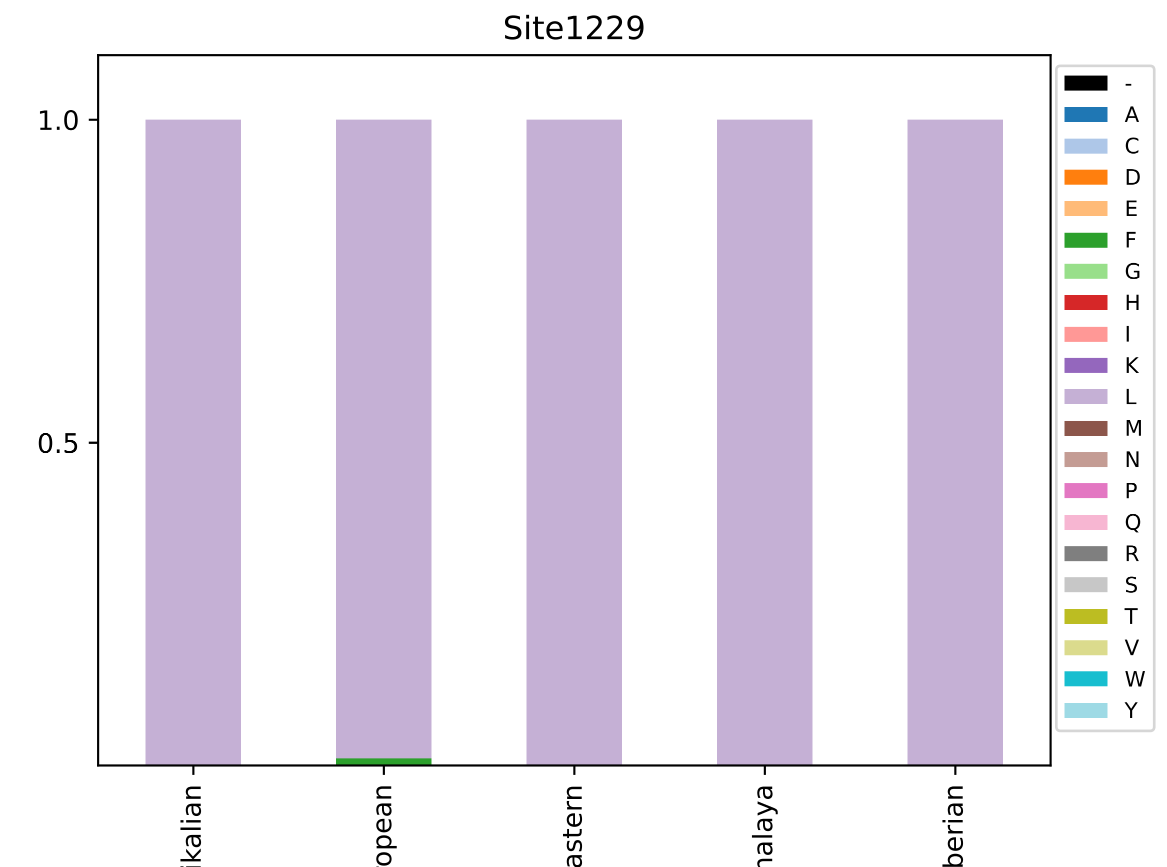

Site1229
1.0
0.5
-
A
C
D
E
F
G
H
I
K
L
M
N
P
Q
R
S
T
V
W
Y
ikalian
opean
astern
nalaya
berian

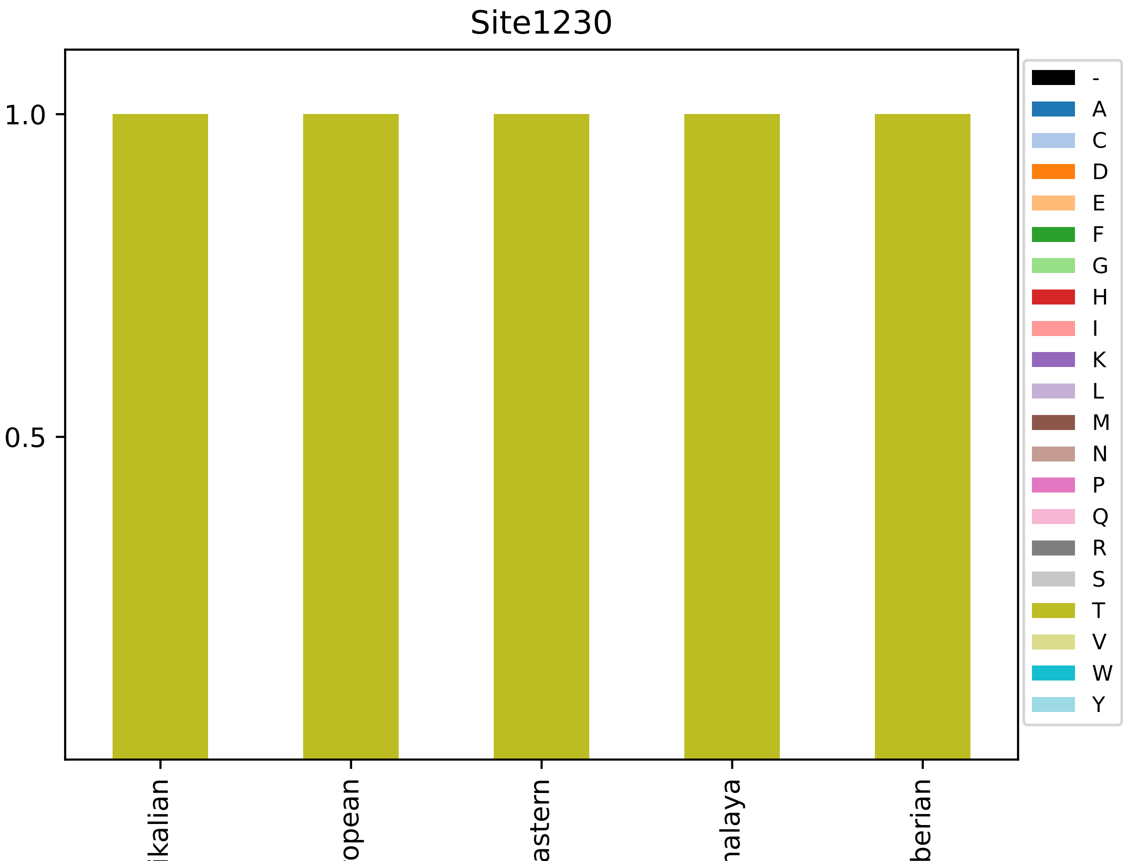
Site1230
1.0
0.5
-
A
C
D
E
F
G
H
I
K
L
M
N
P
Q
R
S
T
V
W
Y

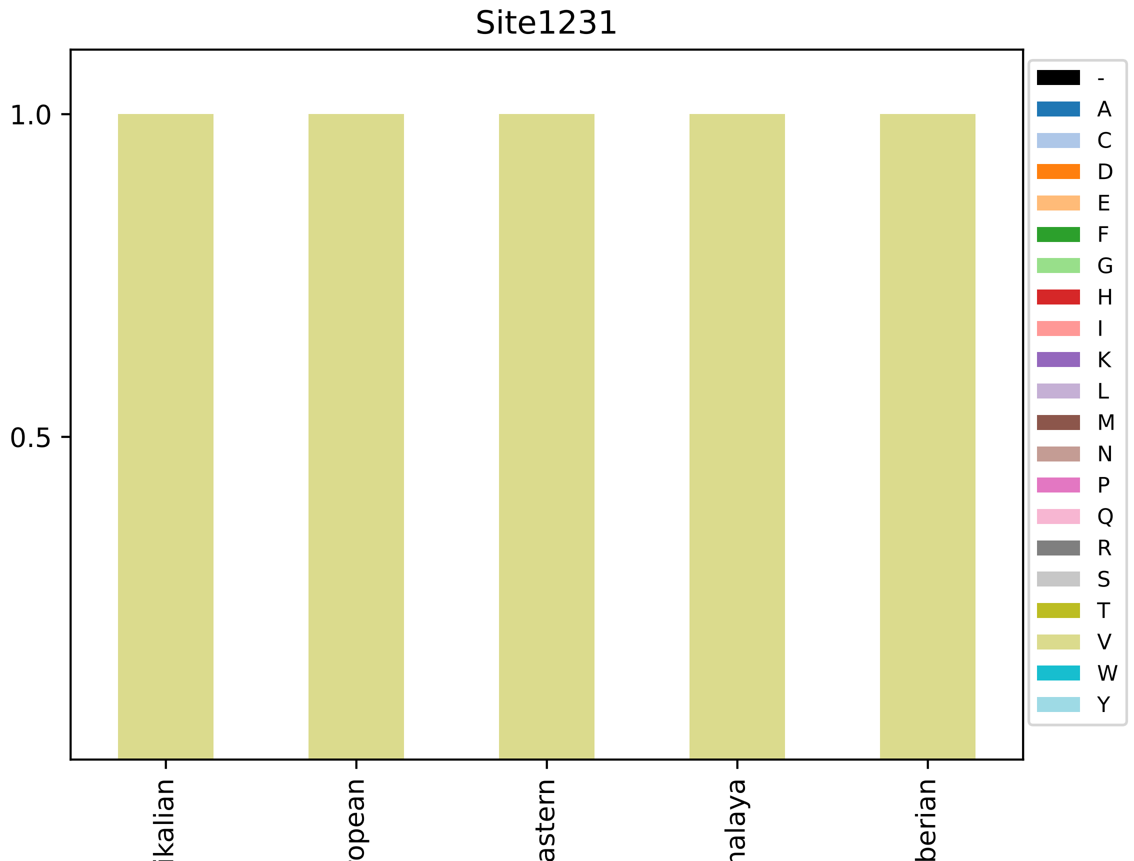
Site1231
1.0
0.5
-
A
C
D
E
F
G
H
I
K
L
M
N
P
Q
R
S
T
V
W
Y
ikalian
opean
astern
nalaya
berian

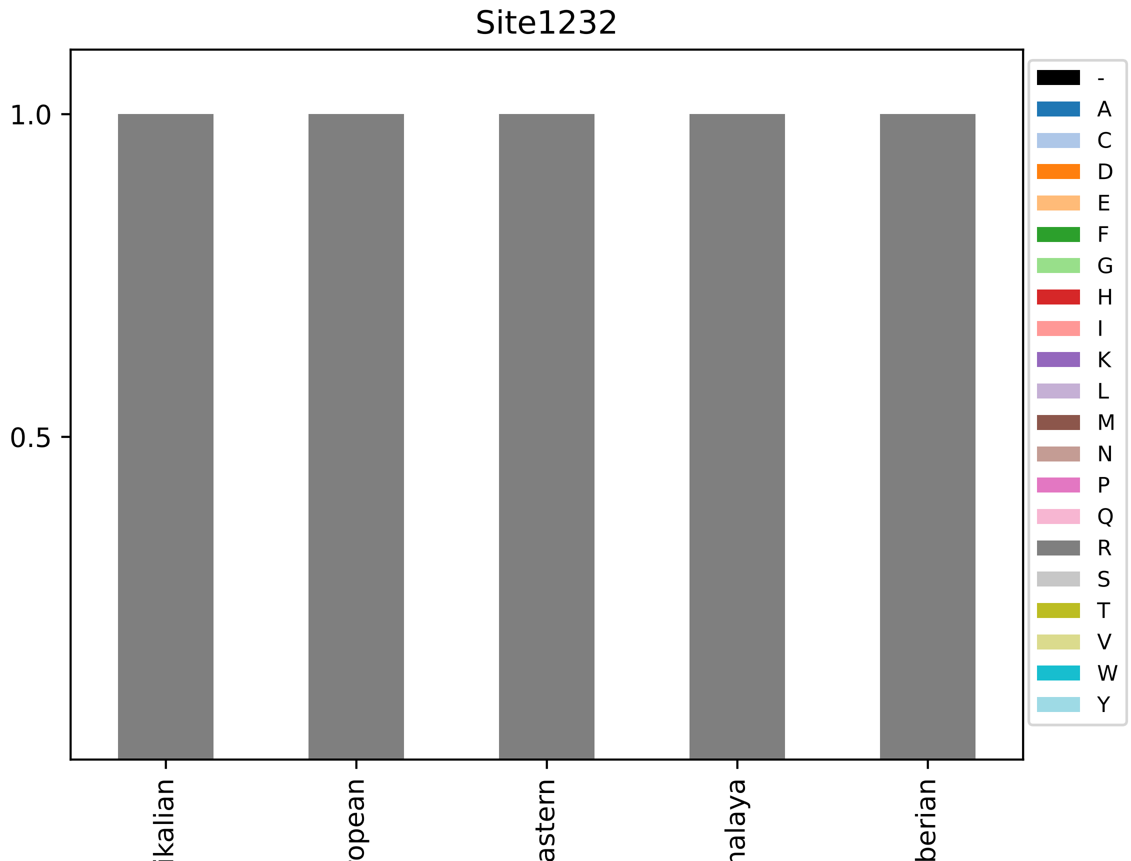
Site1232
1.0
0.5
-
A
C
D
E
F
G
H
I
K
L
M
N
P
Q
R
S
T
V
W
Y
ikalian
opean
astern
nalaya
berian

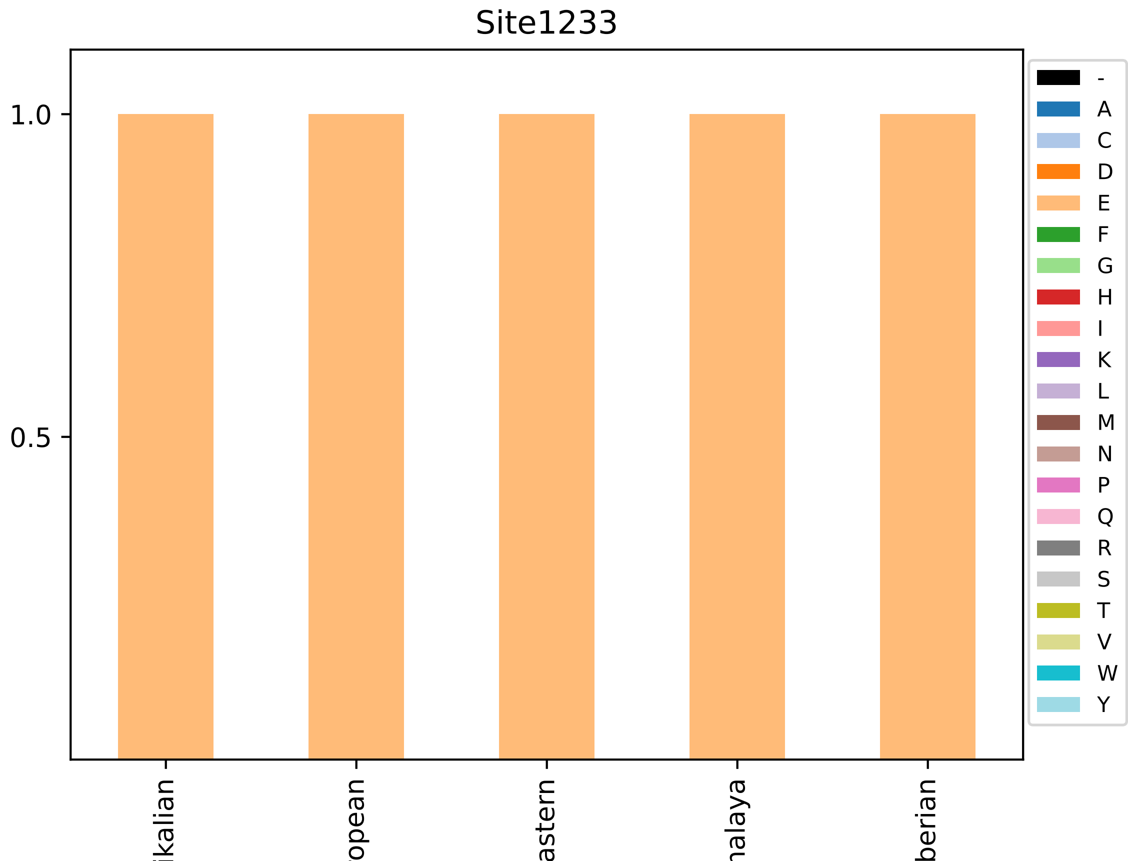

Site1233
1.0
0.5
-
A
C
D
E
F
G
H
I
K
L
M
N
P
Q
R
S
T
V
W
Y
ikalian
opean
astern
nalaya
berian

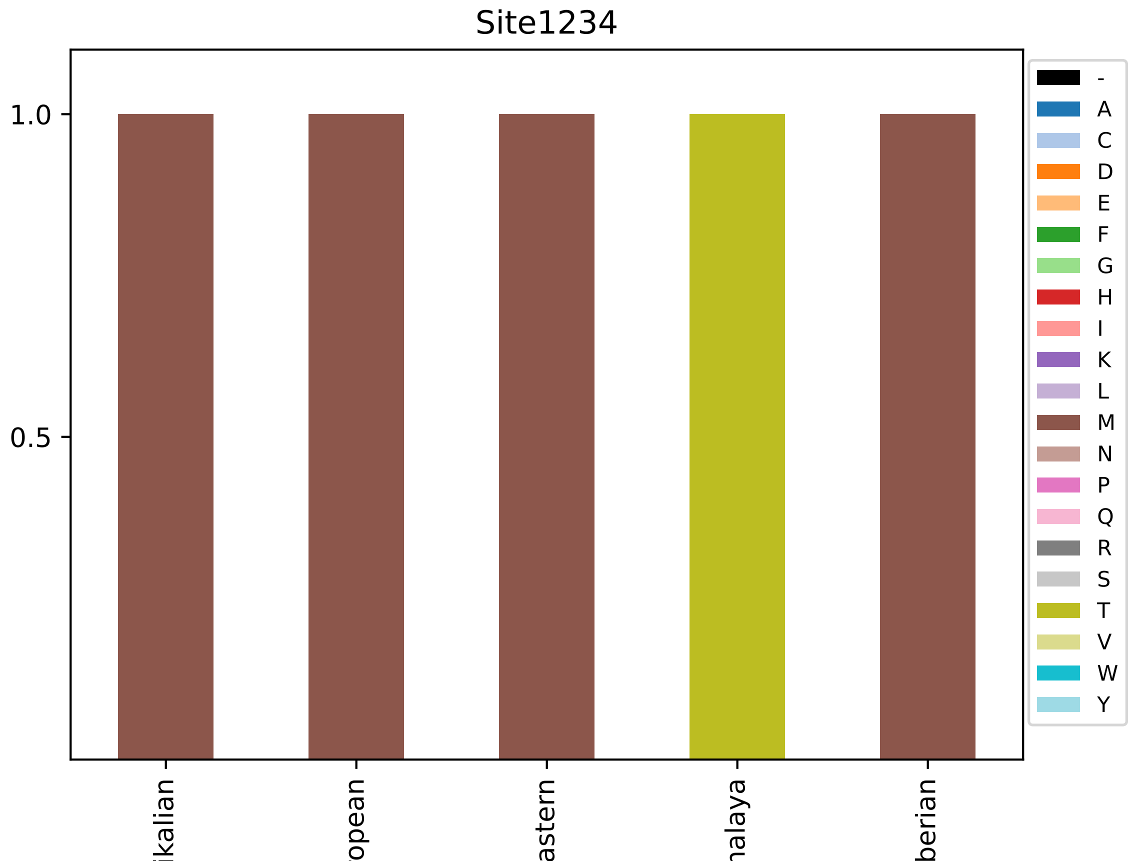

Site1234
1.0
0.5
-
A
C
D
E
F
G
H
I
K
L
M
N
P
Q
R
S
T
V
W
Y
ikalian
opean
astern
nalaya
berian

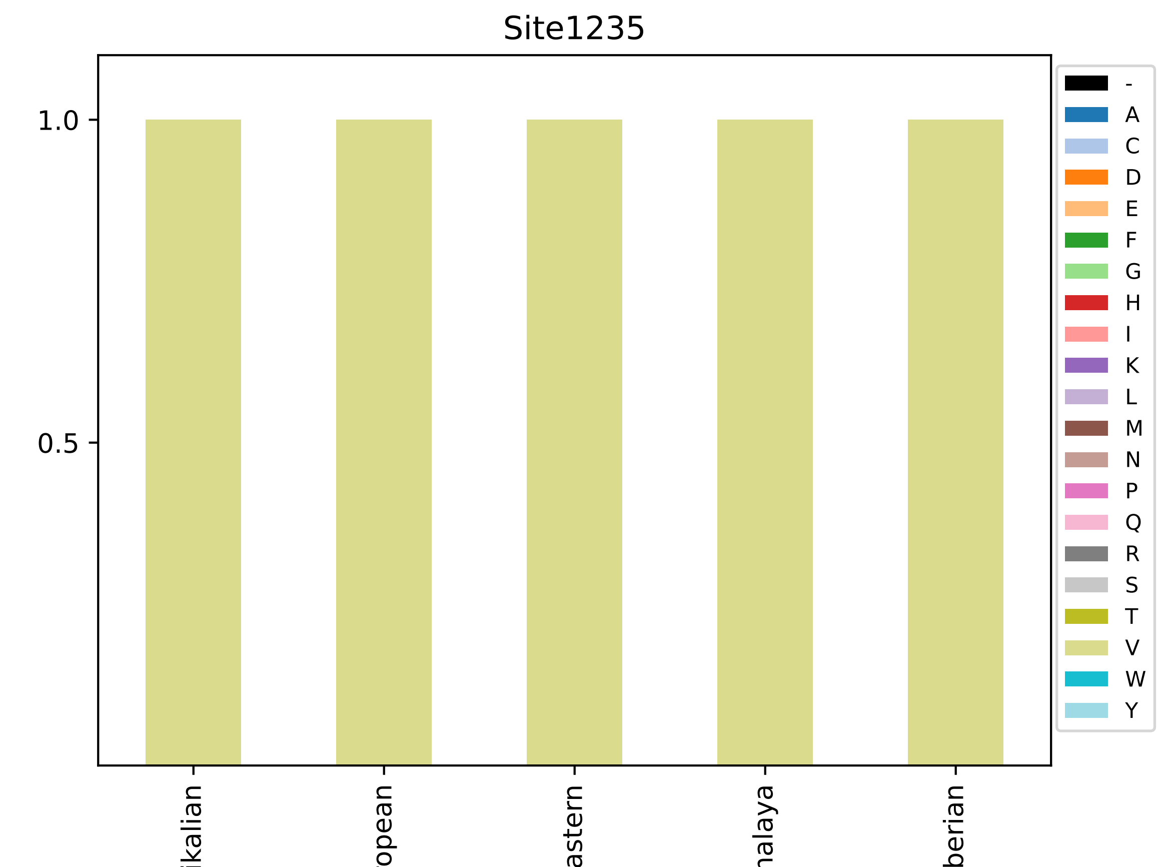
Site1235
1.0
0.5
-
A
C
D
E
F
G
H
I
K
L
M
N
P
Q
R
S
T
V
W
Y
ikalian
opean
astern
nalaya
berian

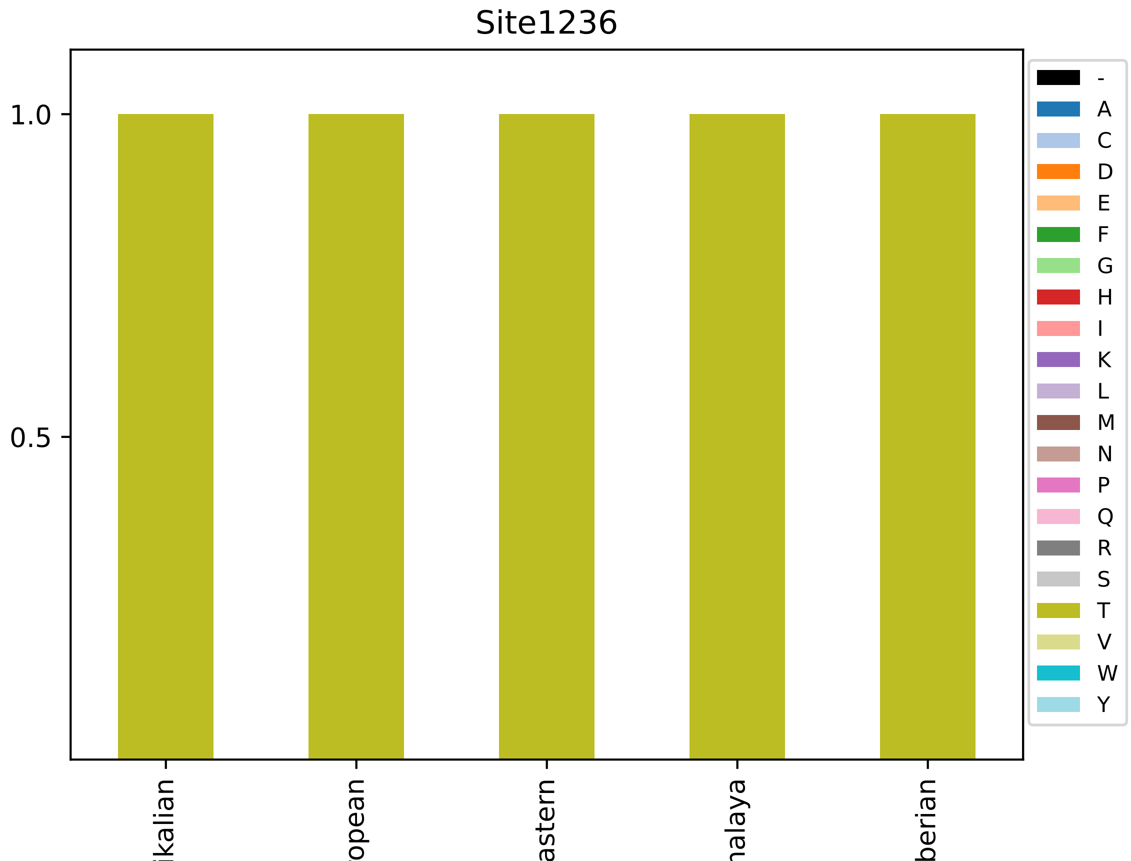

Site1236
1.0
0.5
-
A
C
D
E
F
G
H
I
K
L
M
N
P
Q
R
S
T
V
W
Y
ikalian
opean
astern
nalaya
berian

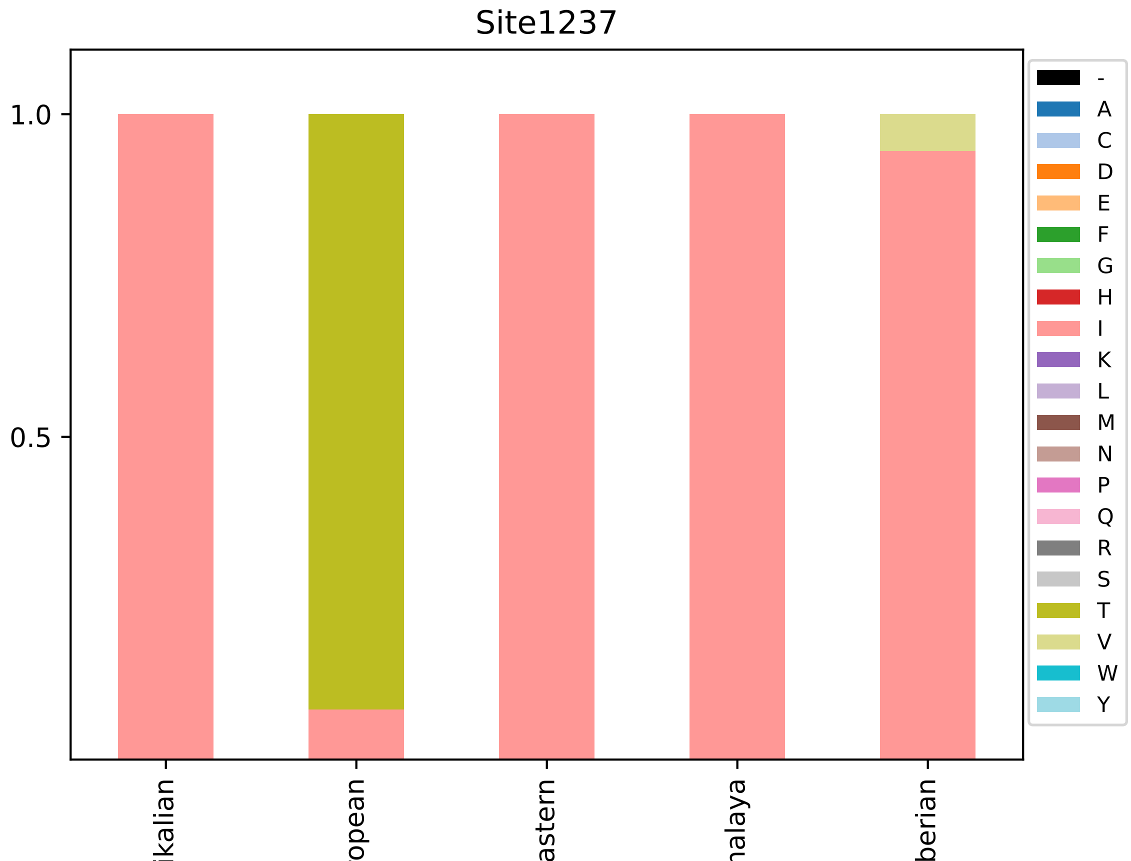

Site1237
1.0
0.5
-
A
C
D
E
F
G
H
I
K
L
M
N
P
Q
R
S
T
V
W
Y
ikalian
ropean
astern
nalaya
berian

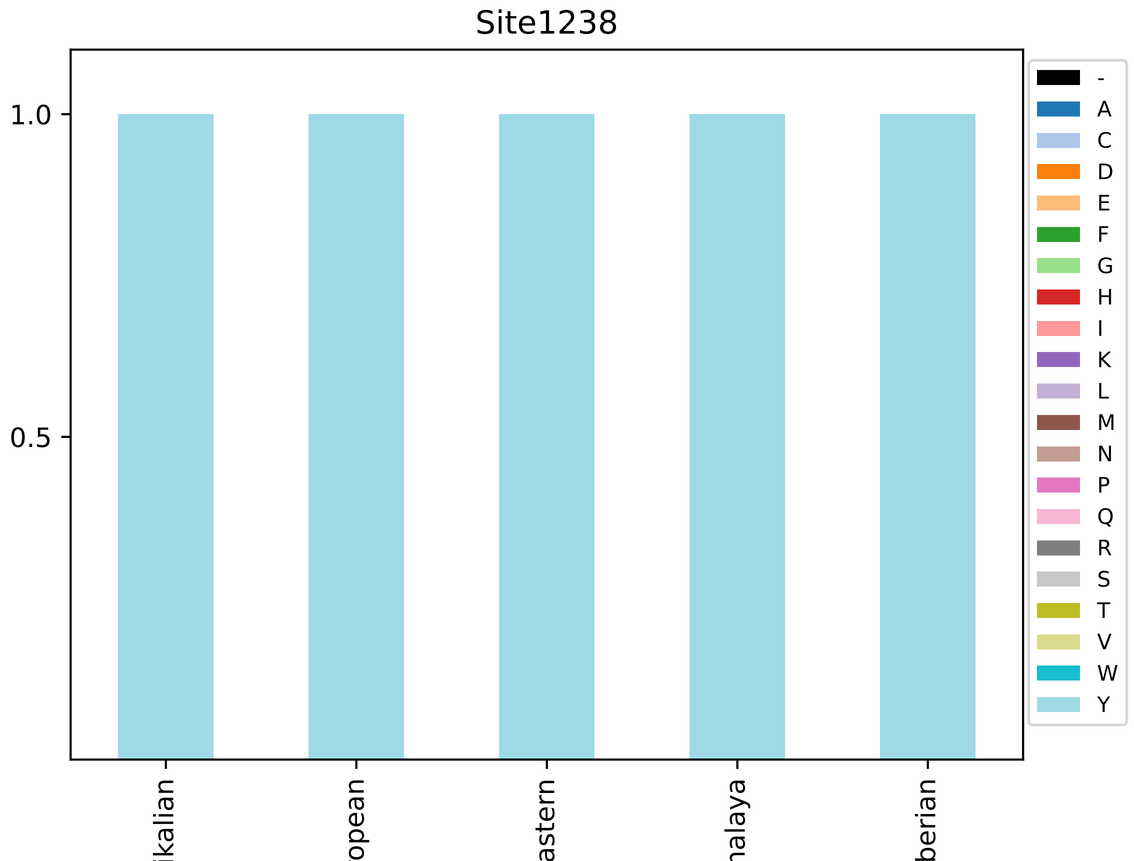
Site1238
1.0
0.5
-
A
C
D
E
F
G
H
I
K
L
M
N
P
Q
R
S
T
V
W
Y

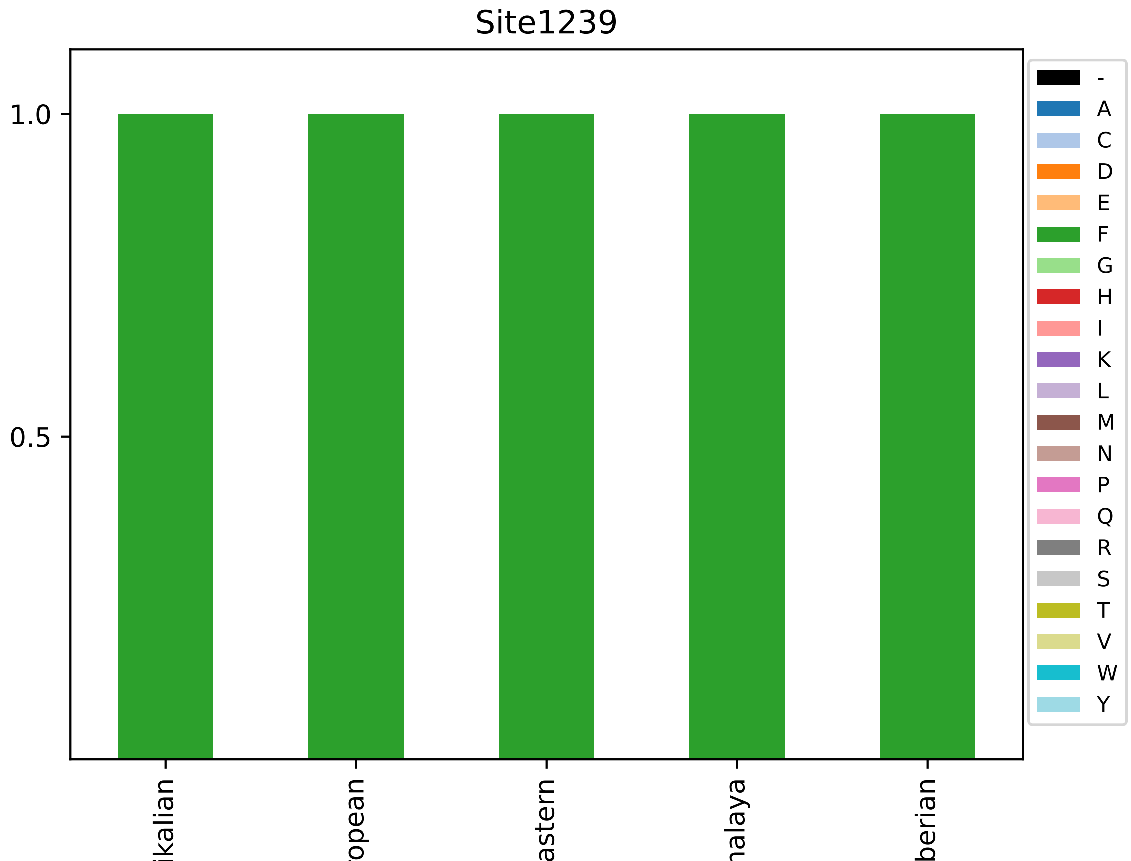

Site1239
1.0
0.5
-
A
C
D
E
F
G
H
I
K
L
M
N
P
Q
R
S
T
V
W
Y
ikalian
opean
astern
nalaya
berian

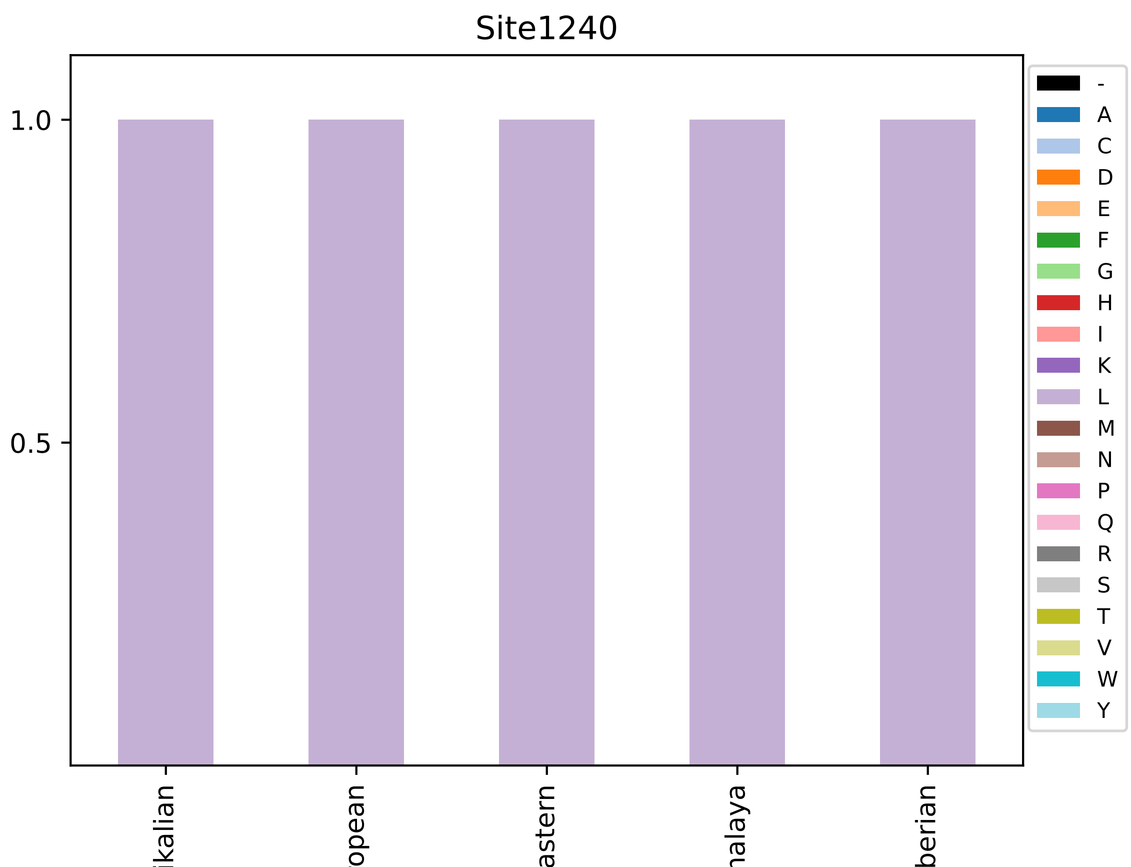
Site1240
1.0
0.5
-
A
C
D
E
F
G
H
I
K
L
M
N
P
Q
R
S
T
V
W
Y
ikalian
opean
astern
nalaya
berian

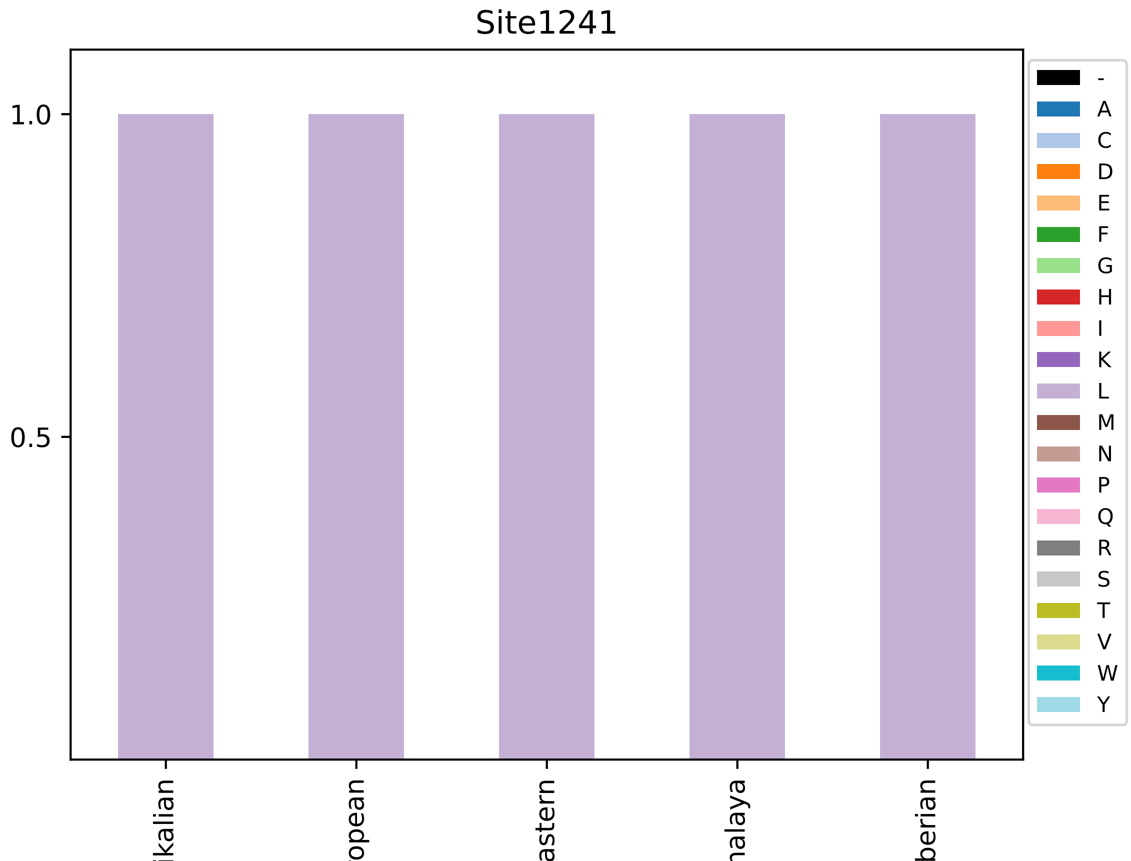
Site1241
1.0
0.5
-
A
C
D
E
F
G
H
I
K
L
M
N
P
Q
R
S
T
V
W
Y
ikalian
opean
astern
nalaya
berian

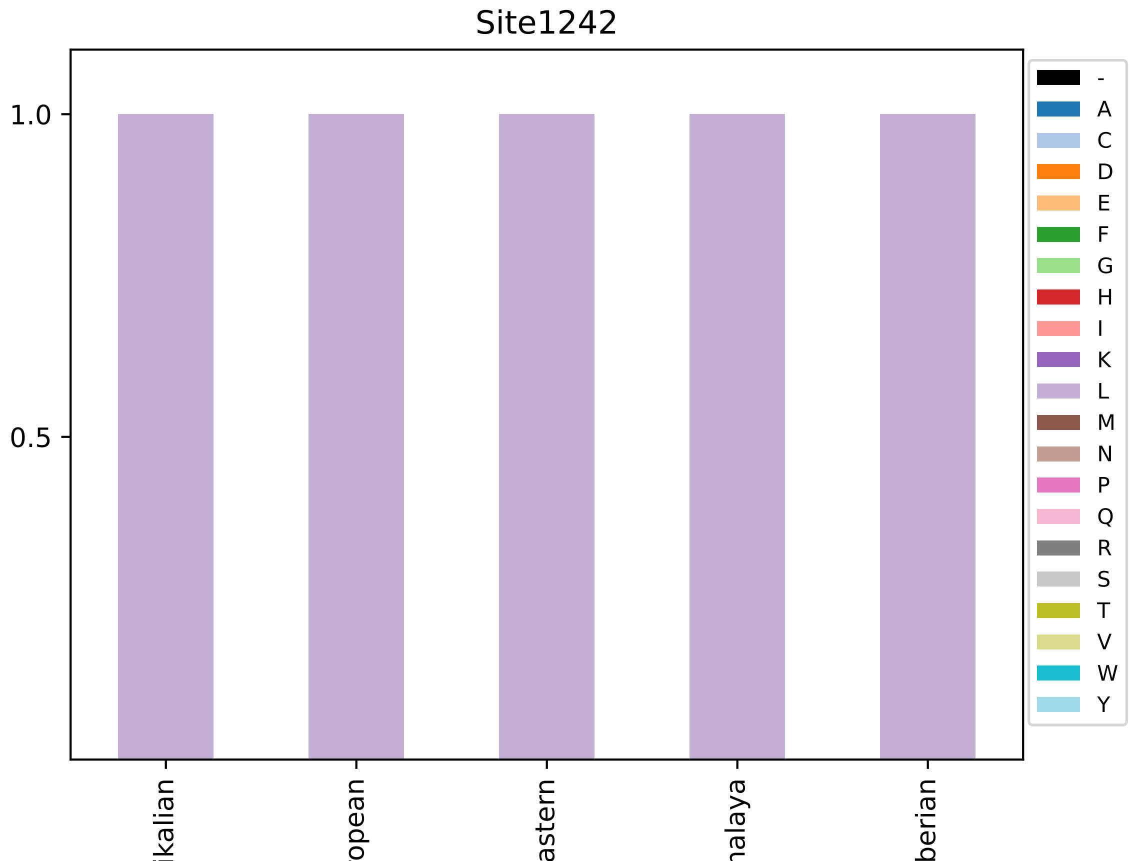
Site1242
1.0
0.5
-
A
C
D
E
F
G
H
I
K
L
M
N
P
Q
R
S
T
V
W
Y
ikalian
opean
astern
nalaya
berian

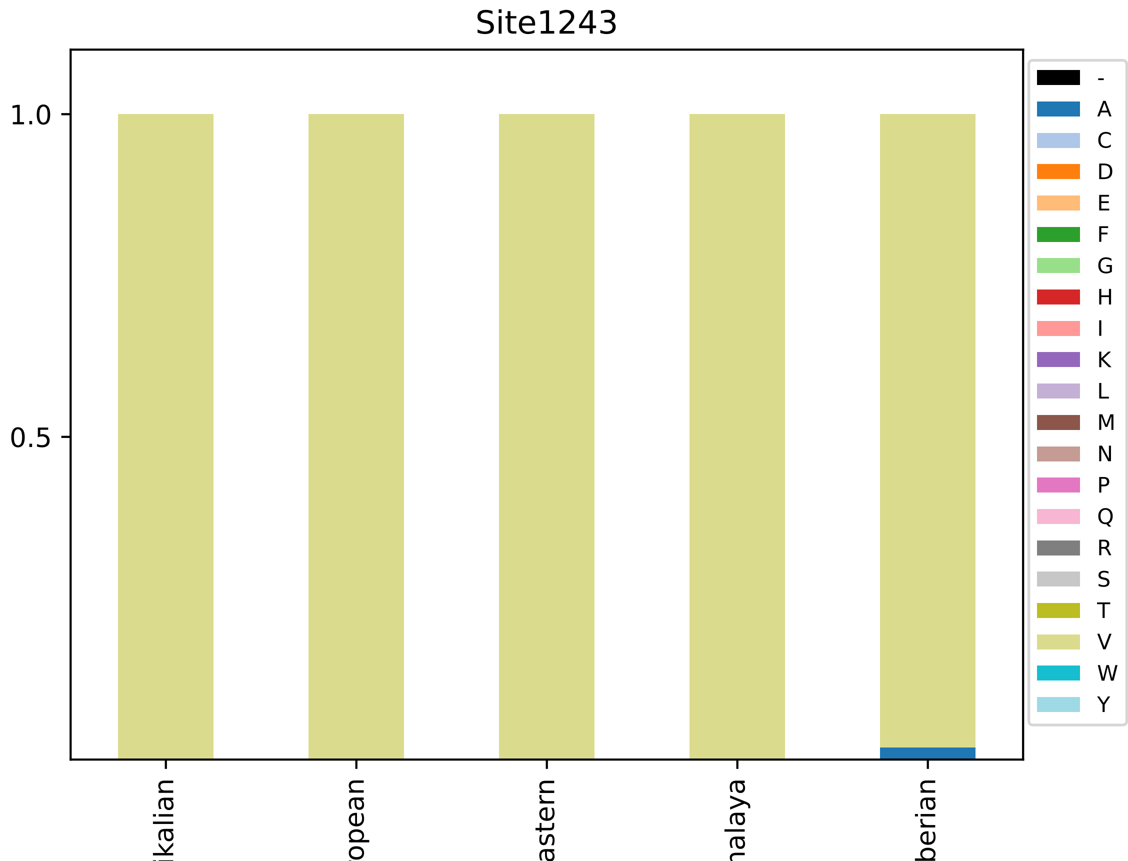
Site1243
1.0
0.5
-
A
C
D
E
F
G
H
I
K
L
M
N
P
Q
R
S
T
V
W
Y
ikalian
opean
astern
nalaya
berian

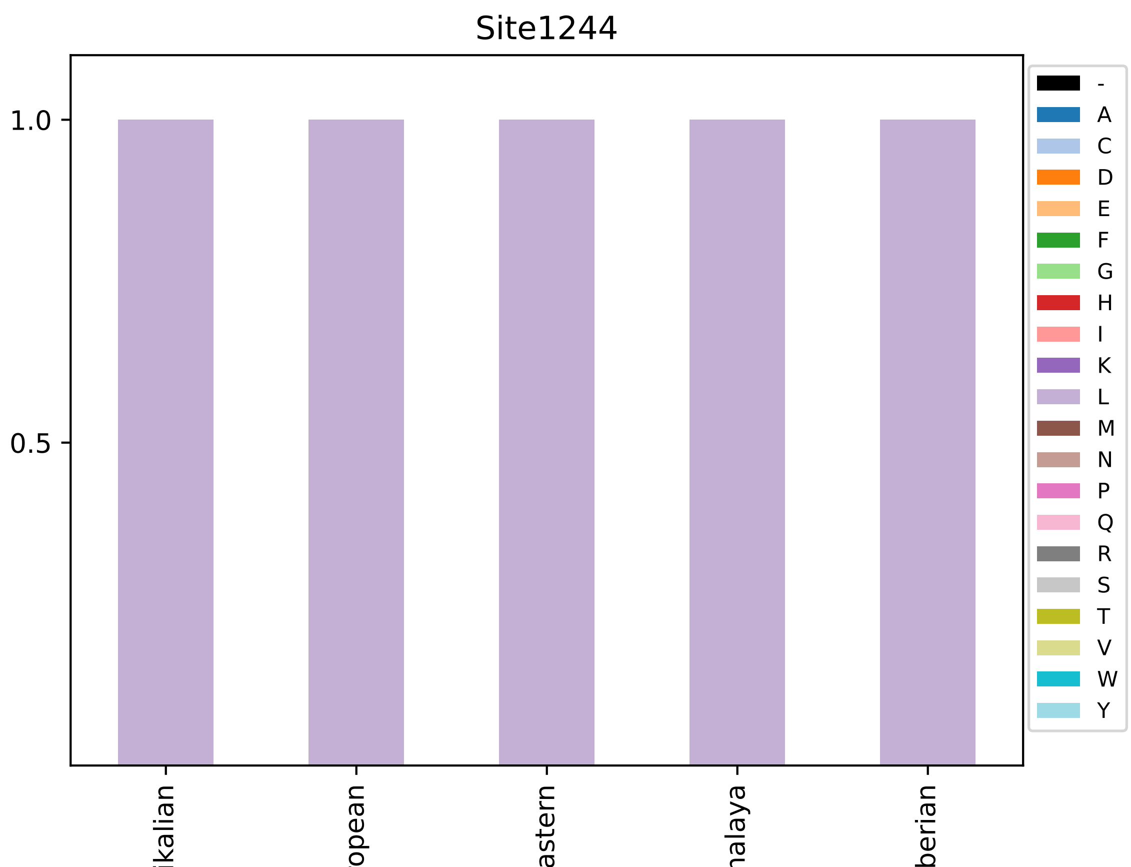

Site1244
1.0
0.5
-
A
C
D
E
F
G
H
I
K
L
M
N
P
Q
R
S
T
V
W
Y
ikalian
opean
astern
nalaya
berian

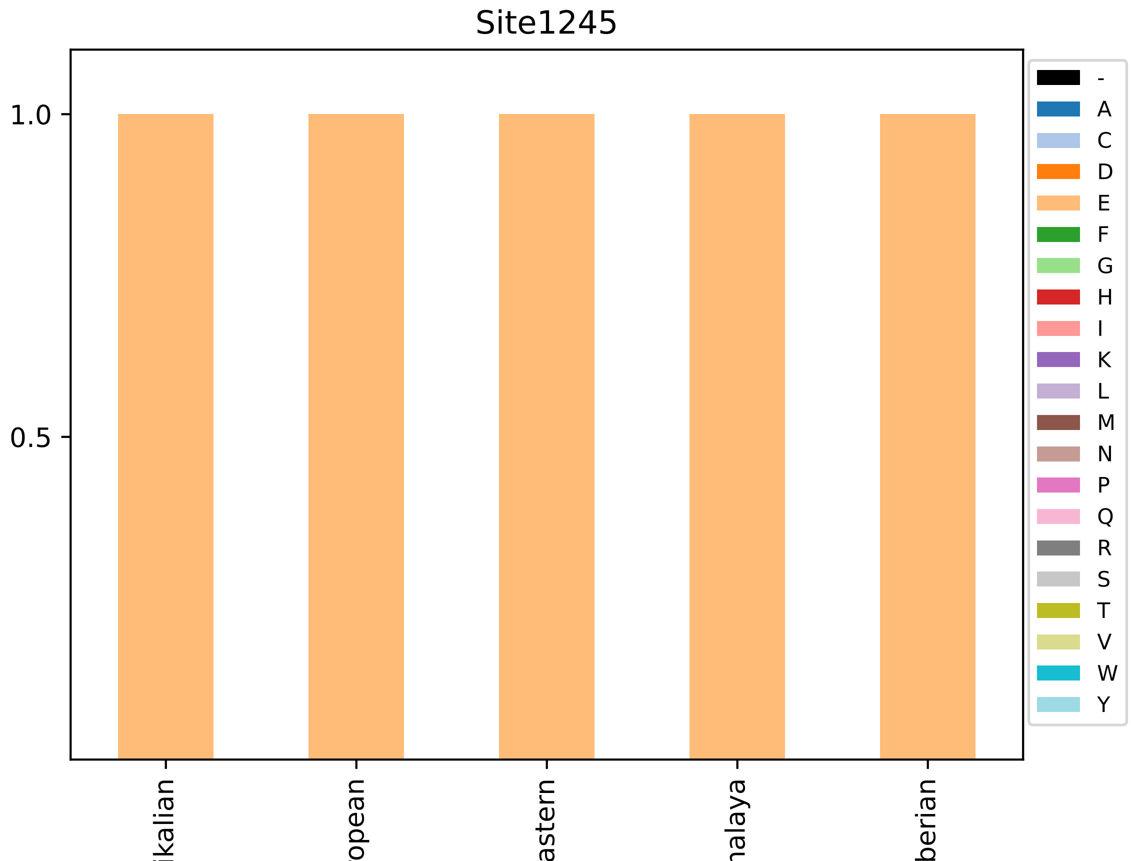
Site1245
1.0
0.5
-
A
C
D
E
F
G
H
I
K
L
M
N
P
Q
R
S
T
V
W
Y
ikalian
opean
astern
halaya
berian

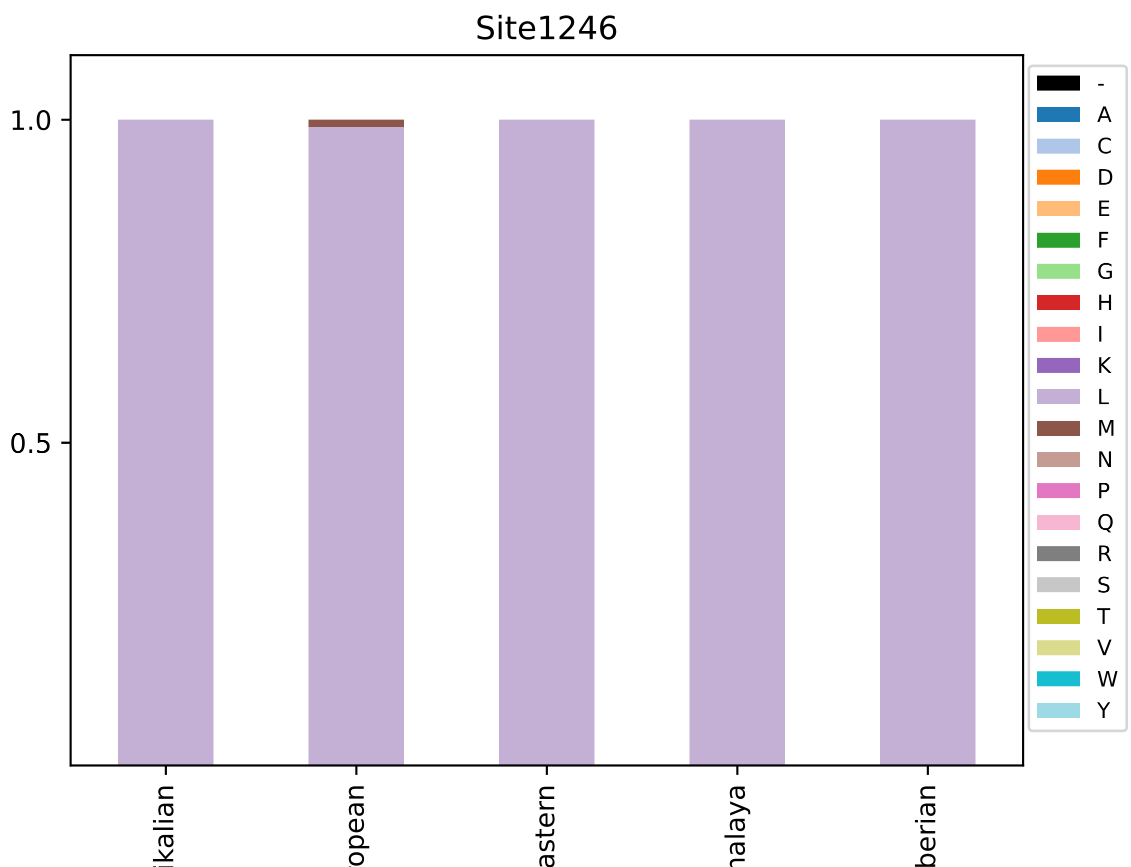
Site1246
1.0
0.5
-
A
C
D
E
F
G
H
I
K
L
M
N
P
Q
R
S
T
V
W
Y
ikalian
opean
astern
nalaya
berian

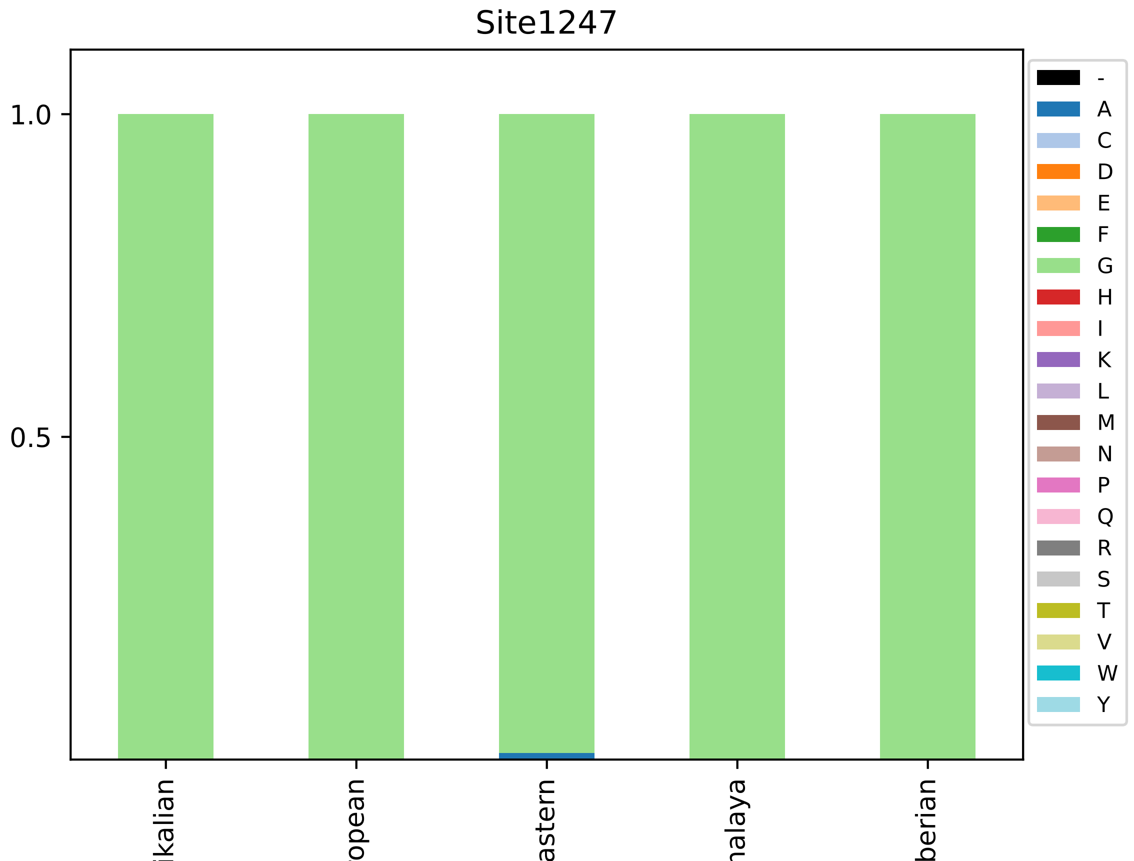

Site1247
1.0
0.5
-
A
C
D
E
F
G
H
I
K
L
M
N
P
Q
R
S
T
V
W
Y
ikalian
opean
astern
nalaya
berian

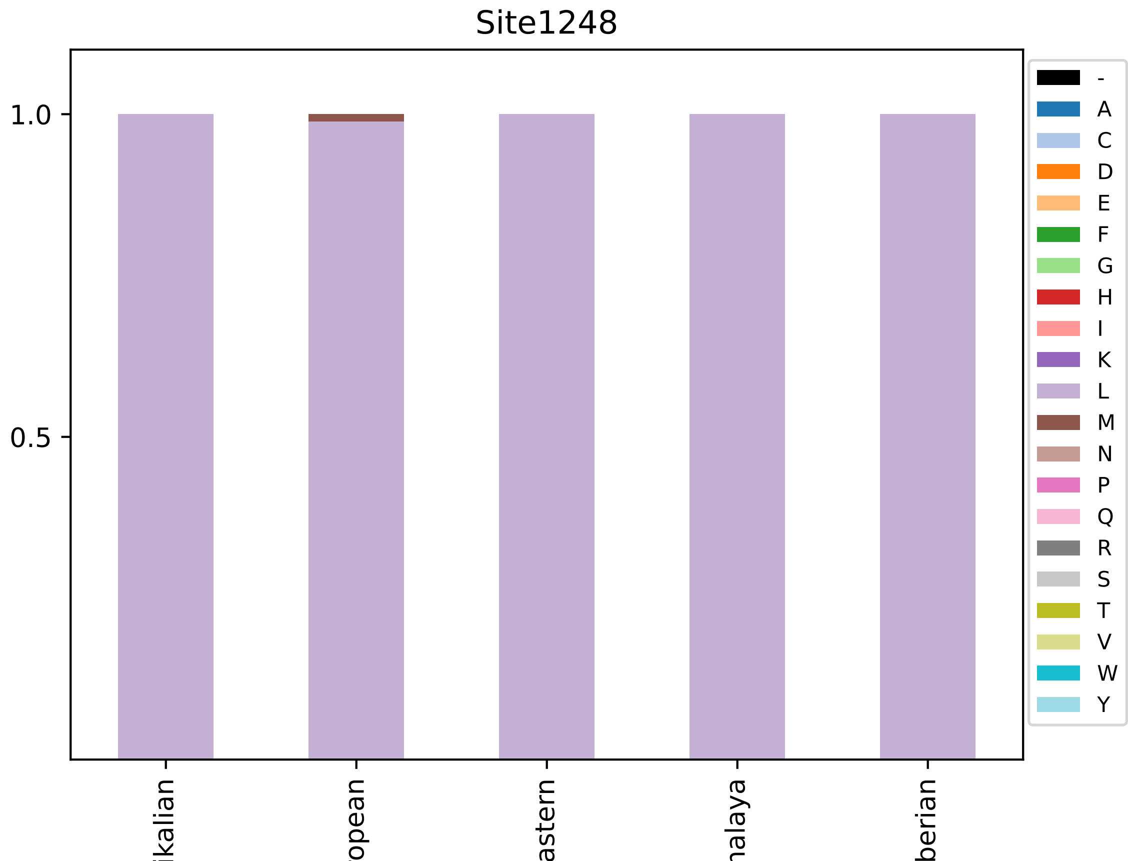
Site1248
1.0
0.5
-
A
C
D
E
F
G
H
I
K
L
M
N
P
Q
R
S
T
V
W
Y
ikalian
opean
astern
nalaya
berian

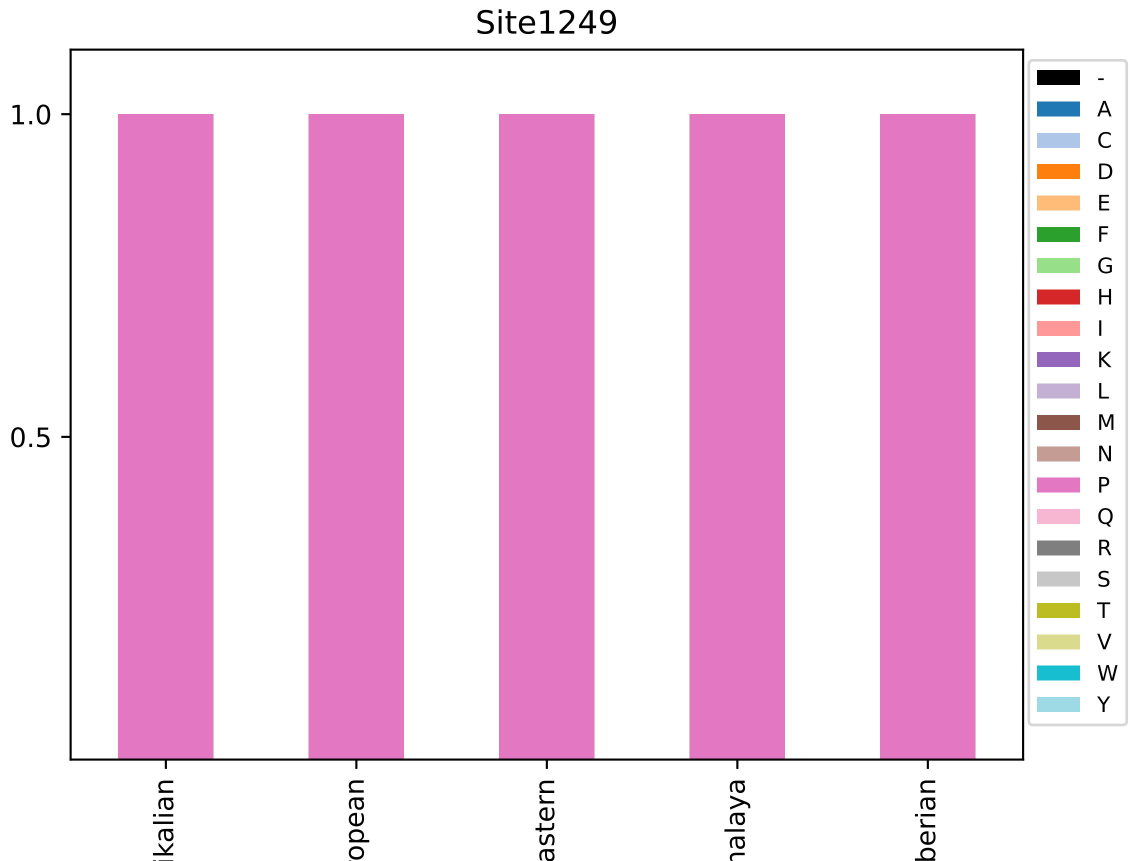

Site1249
1.0
0.5
ikalian
opean
astern
nalaya
berian
-
A
C
D
E
F
G
H
I
K
L
M
N
P
Q
R
S
T
V
W
Y

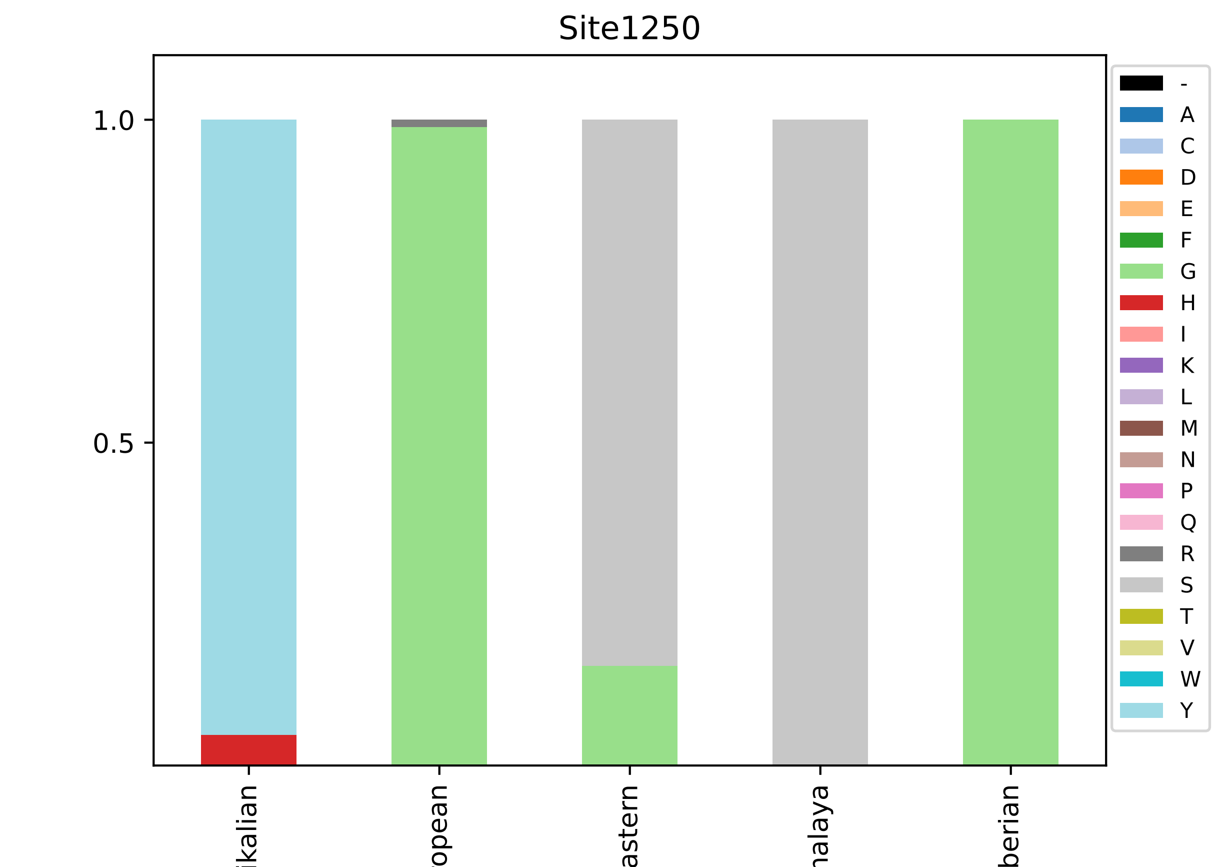
Site1250
1.0
0.5
-
A
C
D
E
F
G
H
I
K
L
M
N
P
Q
R
S
T
V
W
Y
ikalian
opean
astern
nalaya
berian

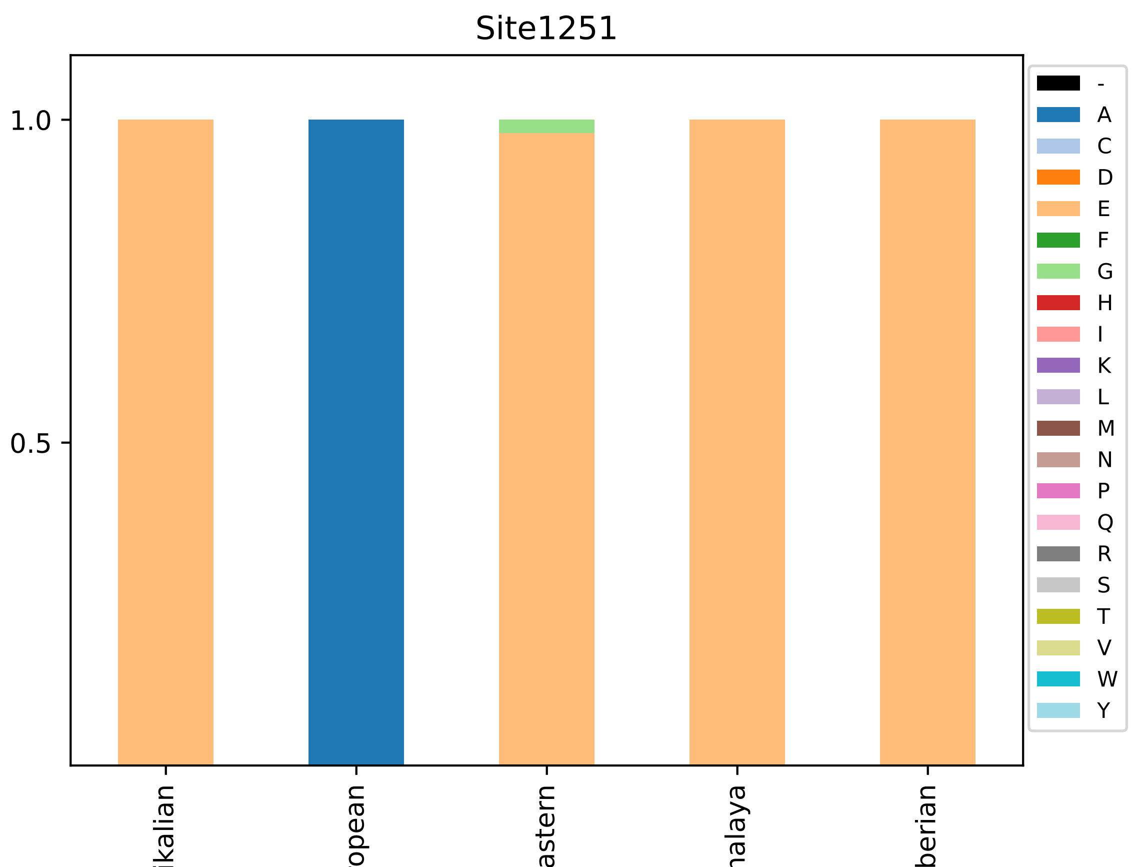

Site1251
1.0
0.5
-
A
C
D
E
F
G
H
I
K
L
M
N
P
Q
R
S
T
V
W
Y
ikalian
opean
astern
nalaya
berian

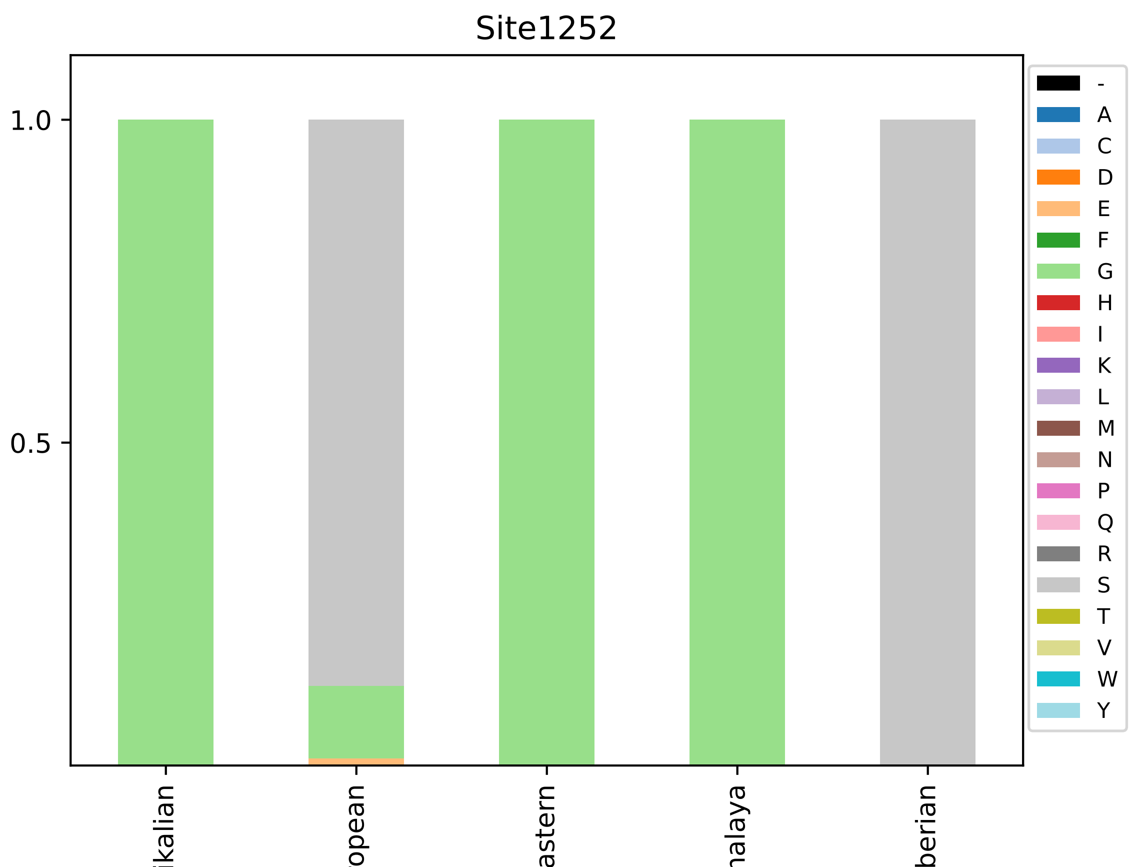

Site1252
1.0
0.5
-
A
C
D
E
F
G
H
I
K
L
M
N
P
Q
R
S
T
V
W
Y
ikalian
opean
astern
nalaya
berian

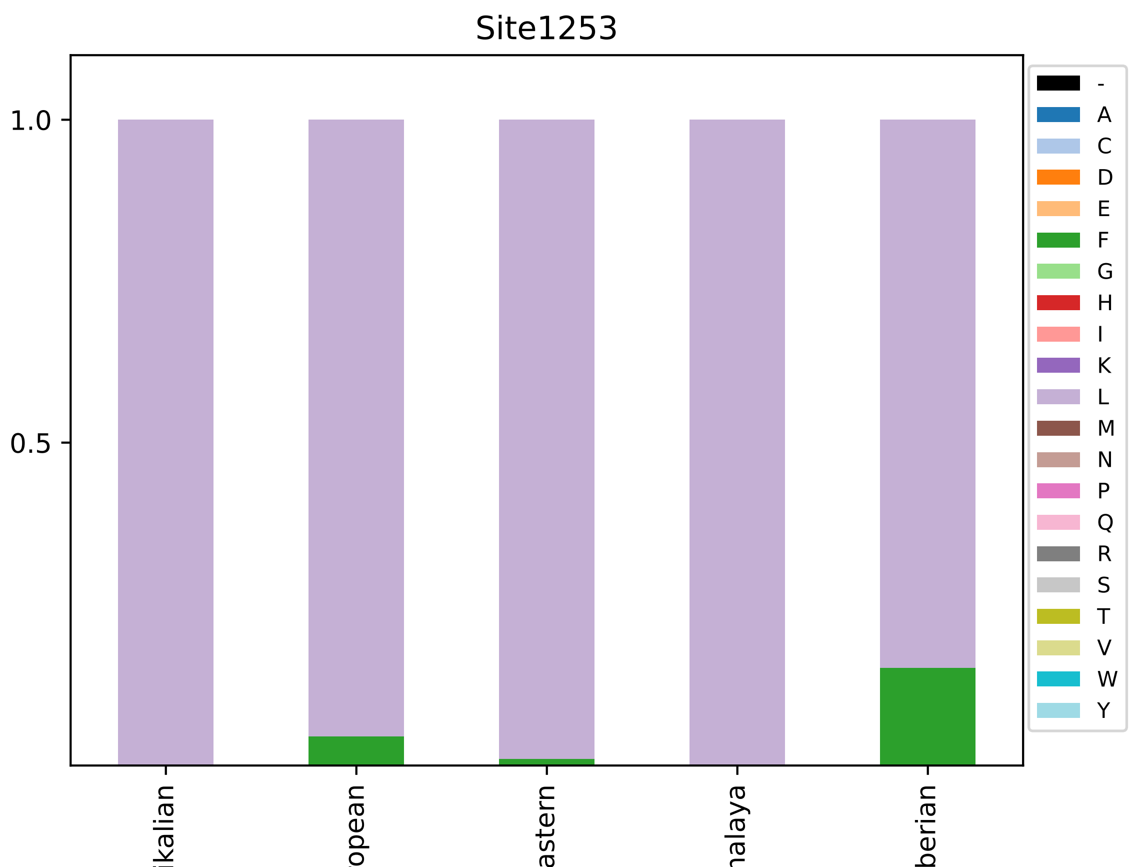

Site1253
1.0
0.5
ikalian
opean
astern
nalaya
berian
-
A
C
D
E
F
G
H
I
K
L
M
N
P
Q
R
S
T
V
W
Y

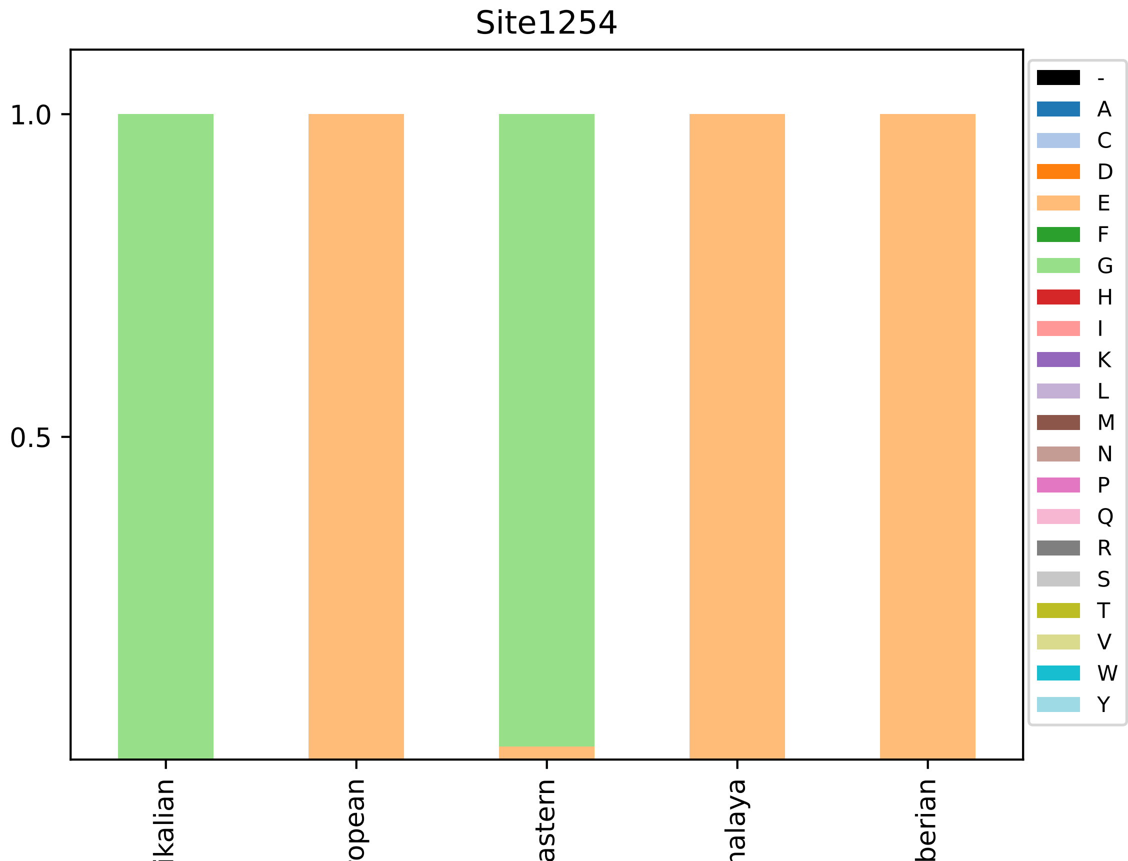
Site1254
1.0
0.5
-
A
C
D
E
F
G
H
I
K
L
M
N
P
Q
R
S
T
V
W
Y
ikalian
opean
astern
nalaya
berian

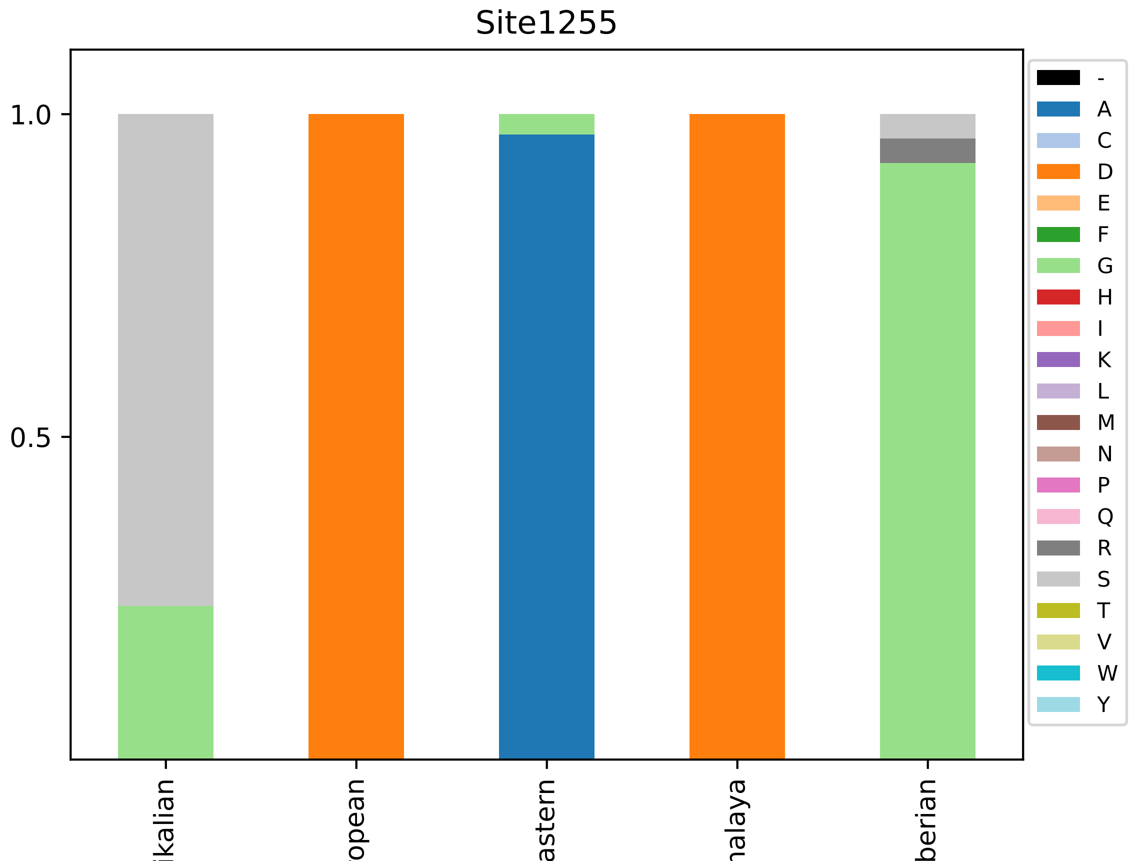
Site1255
1.0
0.5
-
A
C
D
E
F
G
H
I
K
L
M
N
P
Q
R
S
T
V
W
Y
ikalian
opean
astern
nalaya
berian

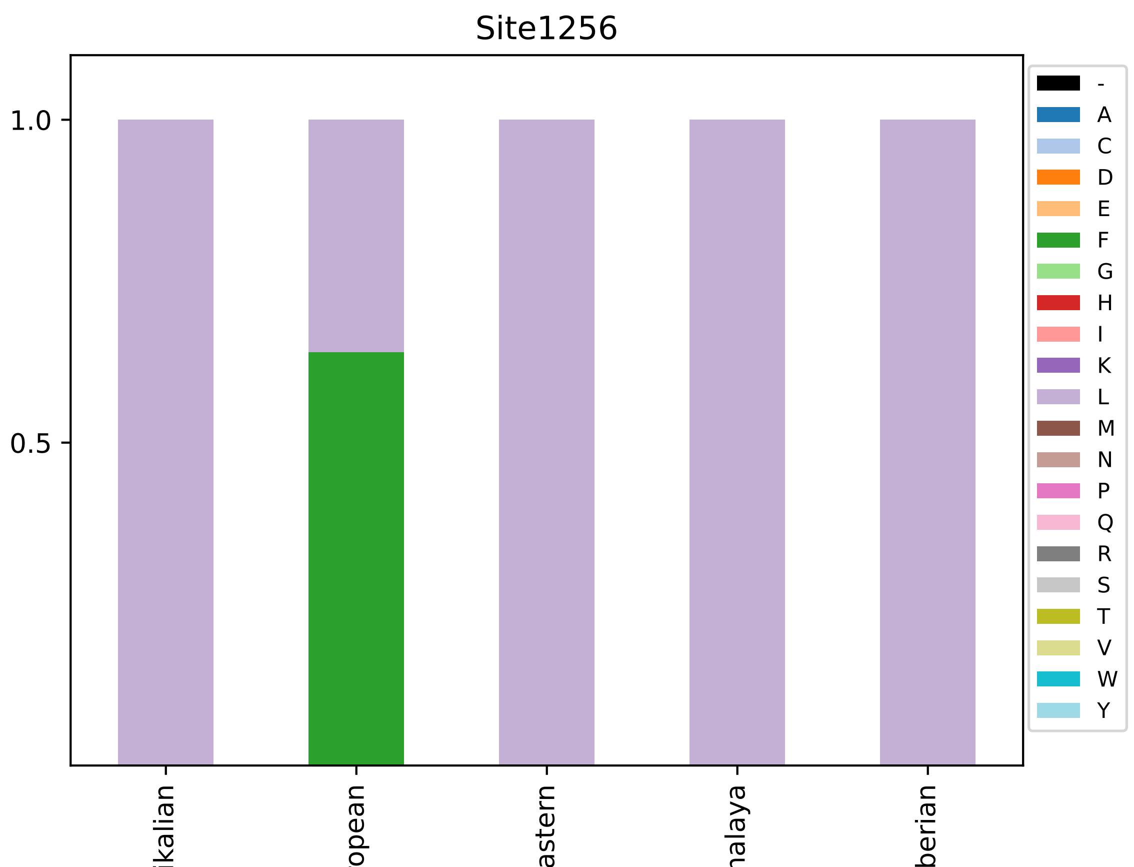
Site1256
1.0
0.5
-
A
C
D
E
F
G
H
I
K
L
M
N
P
Q
R
S
T
V
W
Y
ikalian
opean
astern
halaya
berian

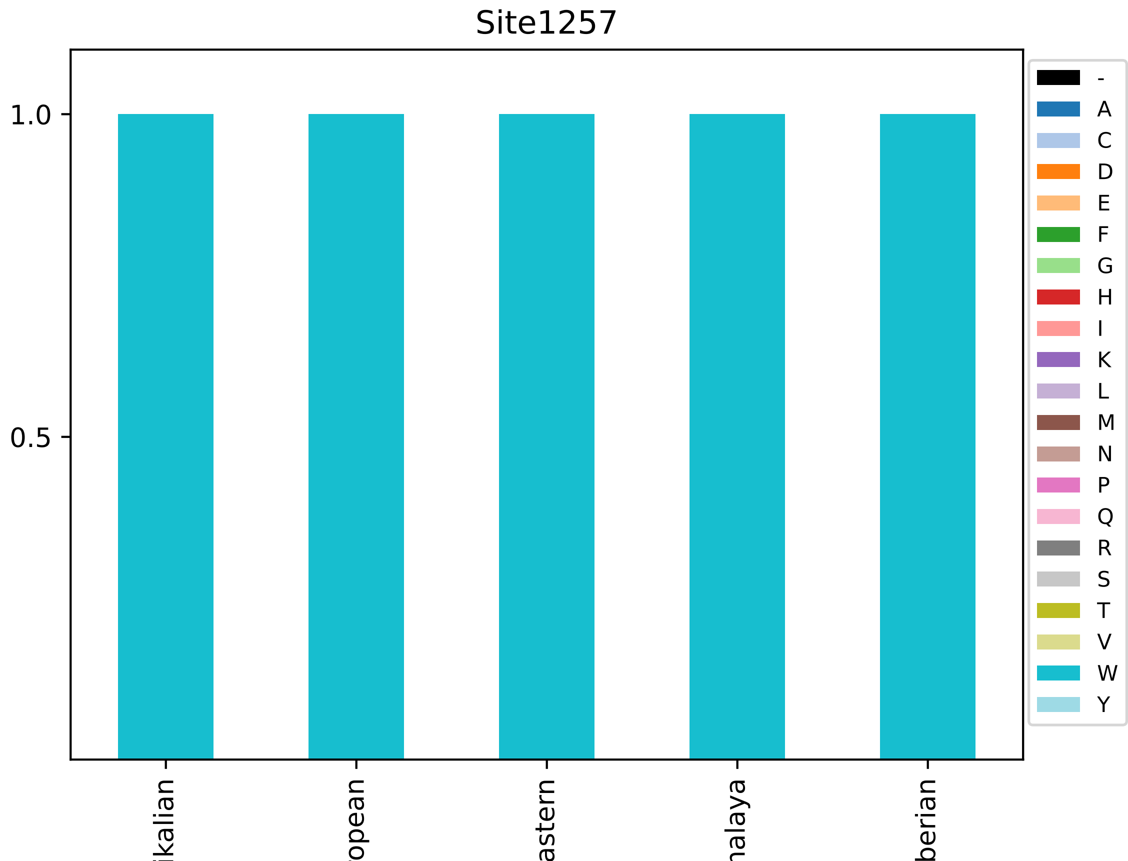

Site1257
1.0
0.5
-
A
C
D
E
F
G
H
I
K
L
M
N
P
Q
R
S
T
V
W
Y
ikalian
opean
astern
nalaya
berian

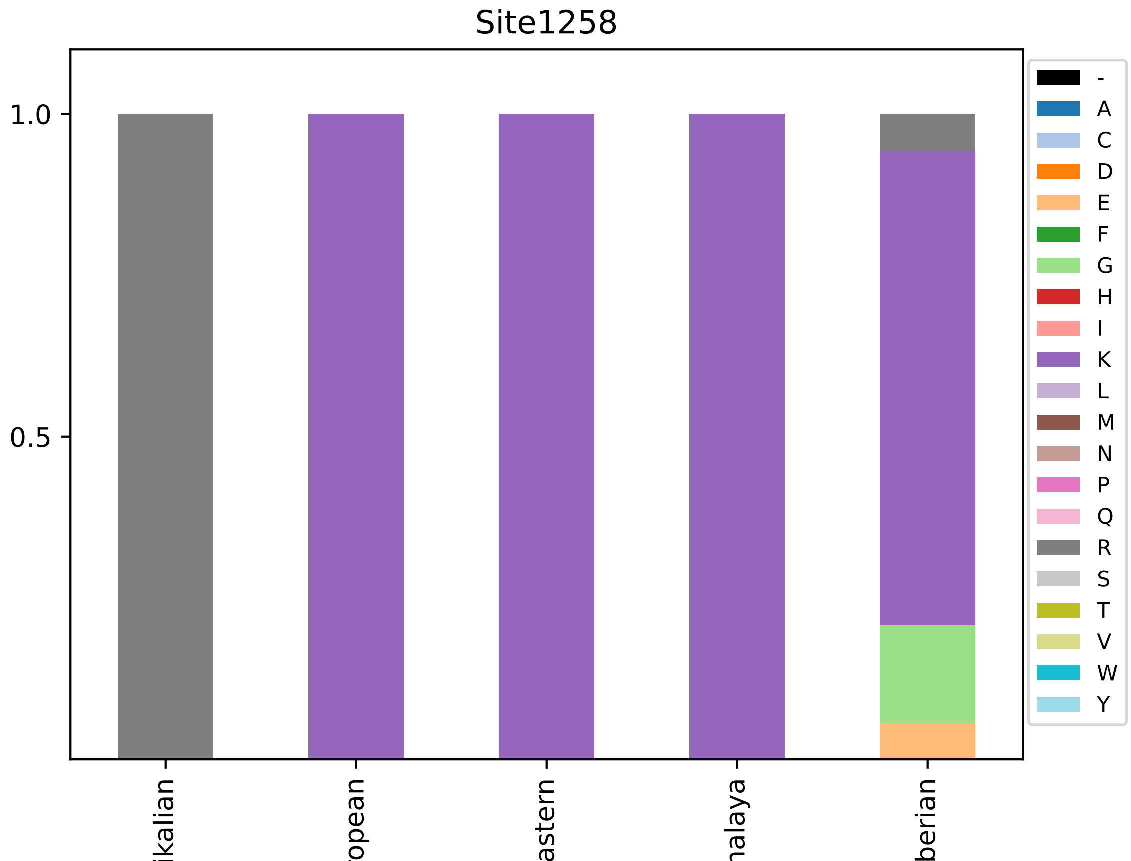

Site1258
1.0
0.5
-
A
C
D
E
F
G
H
I
K
L
M
N
P
Q
R
S
T
V
W
Y
ikalian
opean
astern
nalaya
berian

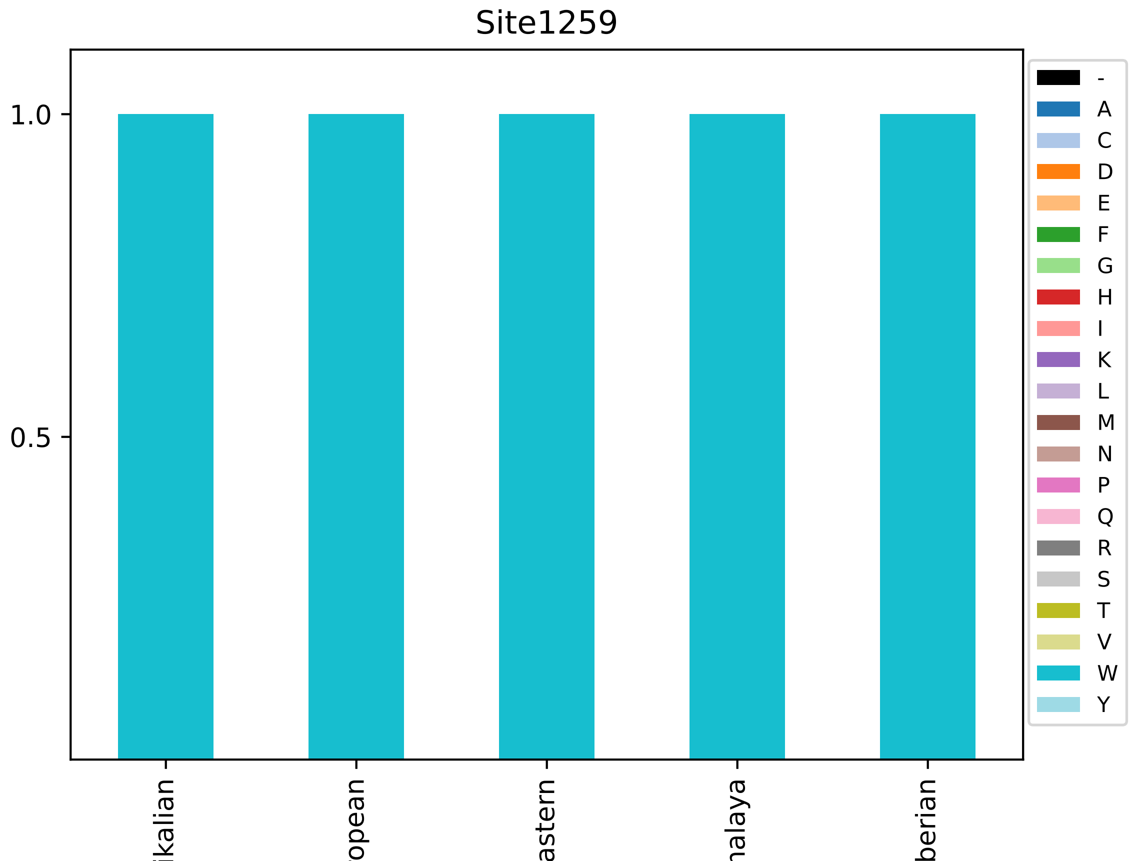

Site1259
1.0
0.5
-
A
C
D
E
F
G
H
I
K
L
M
N
P
Q
R
S
T
V
W
Y

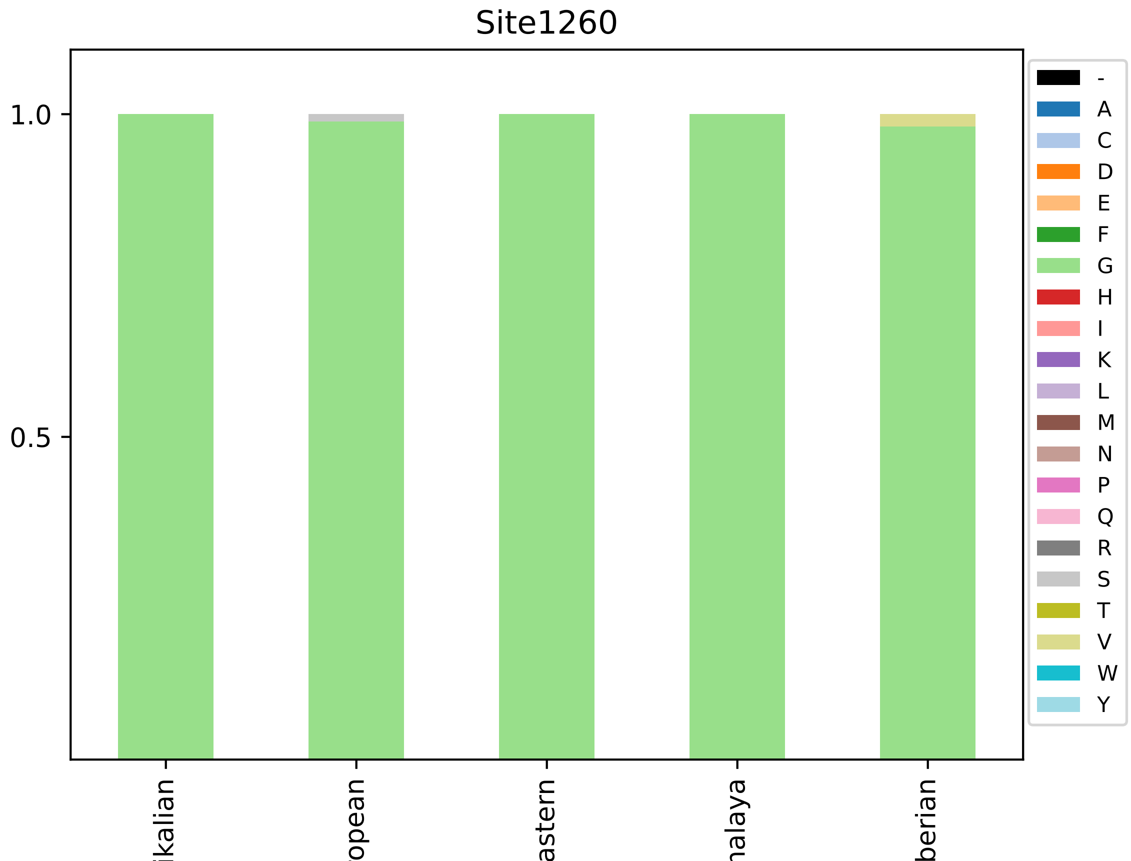
Site1260
1.0
0.5
-
A
C
D
E
F
G
H
I
K
L
M
N
P
Q
R
S
T
V
W
Y
ikalian
opean
astern
nalaya
berian

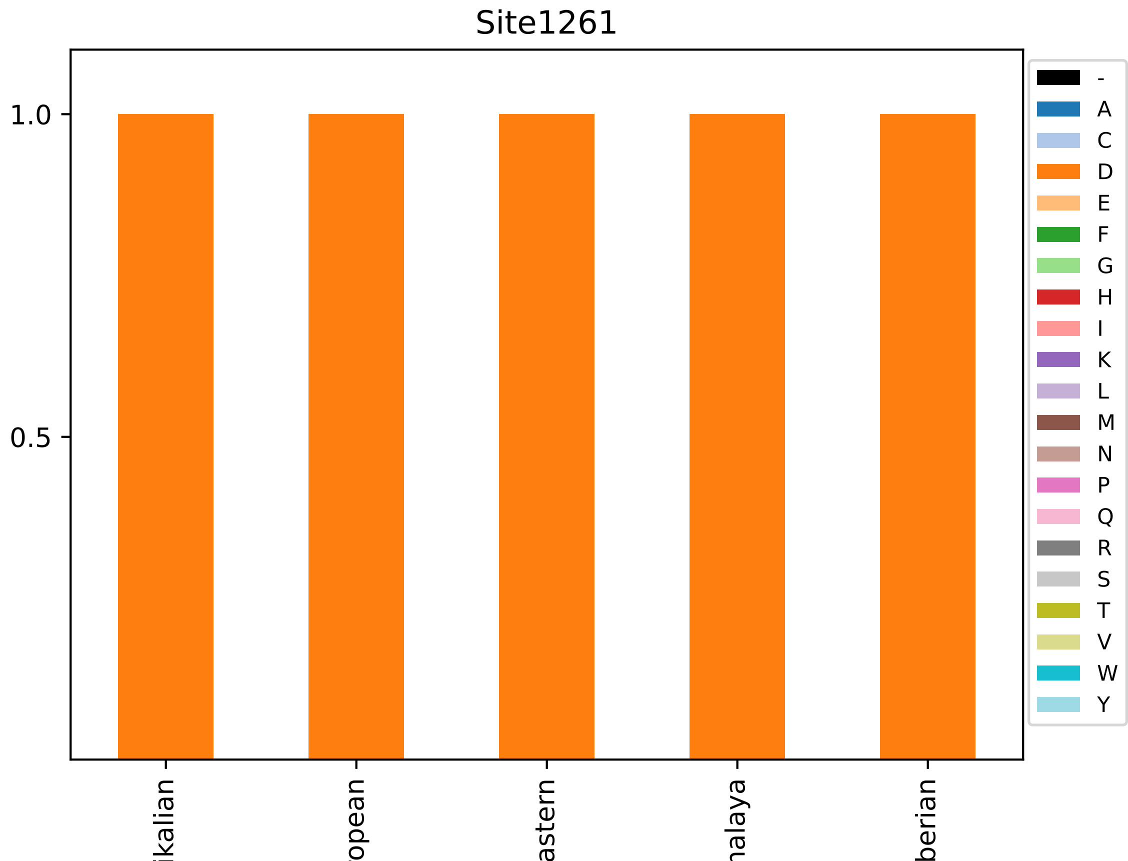

Site1261
1.0
0.5
-
A
C
D
E
F
G
H
I
K
L
M
N
P
Q
R
S
T
V
W
Y
ikalian
opean
astern
nalaya
berian

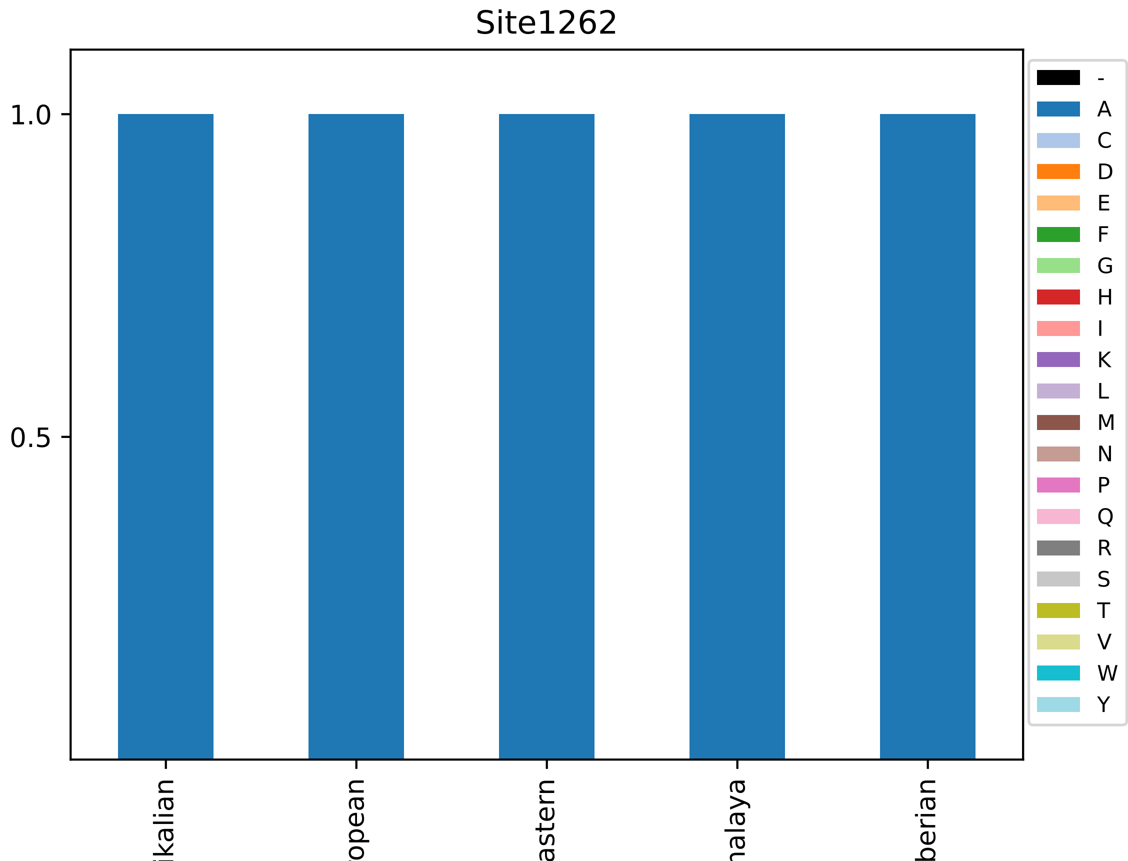

Site1262
1.0
0.5
-
A
C
D
E
F
G
H
I
K
L
M
N
P
Q
R
S
T
V
W
Y

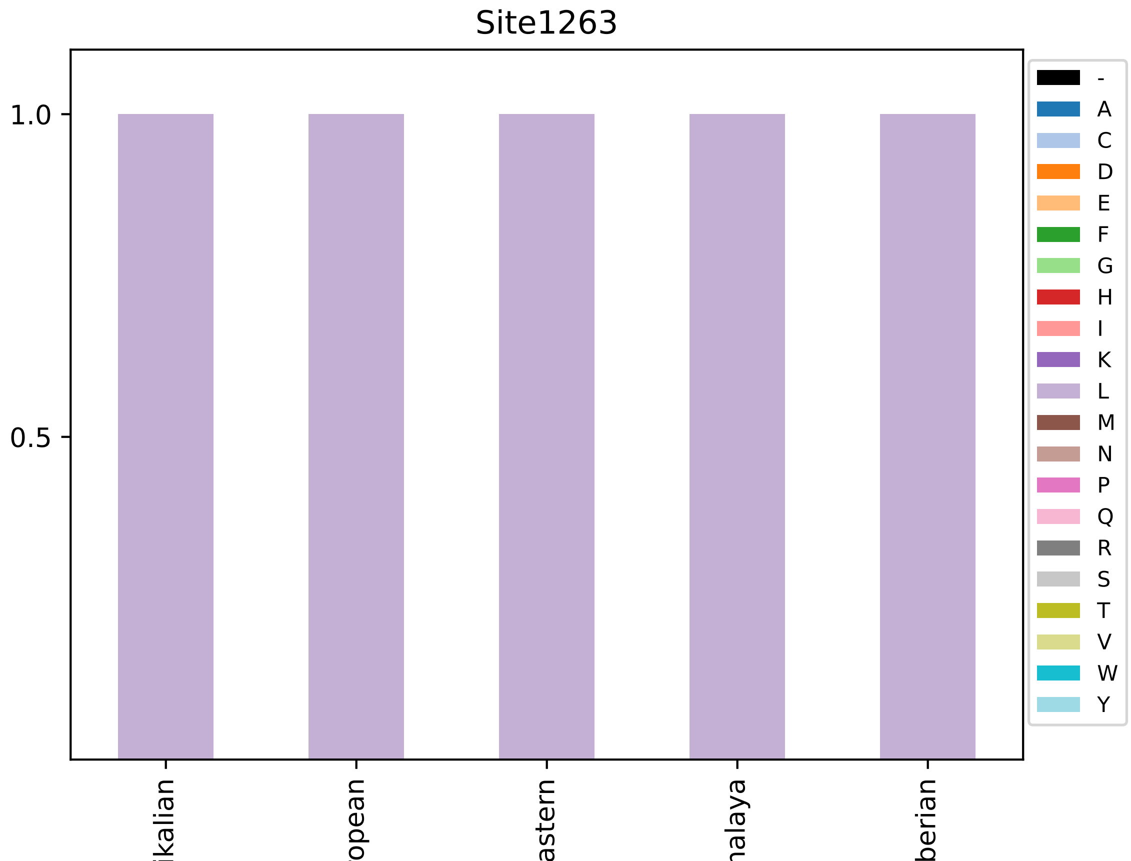

Site1263
1.0
0.5
ikalian
opean
astern
nalaya
berian
-
A
C
D
E
F
G
H
I
K
L
M
N
P
Q
R
S
T
V
W
Y

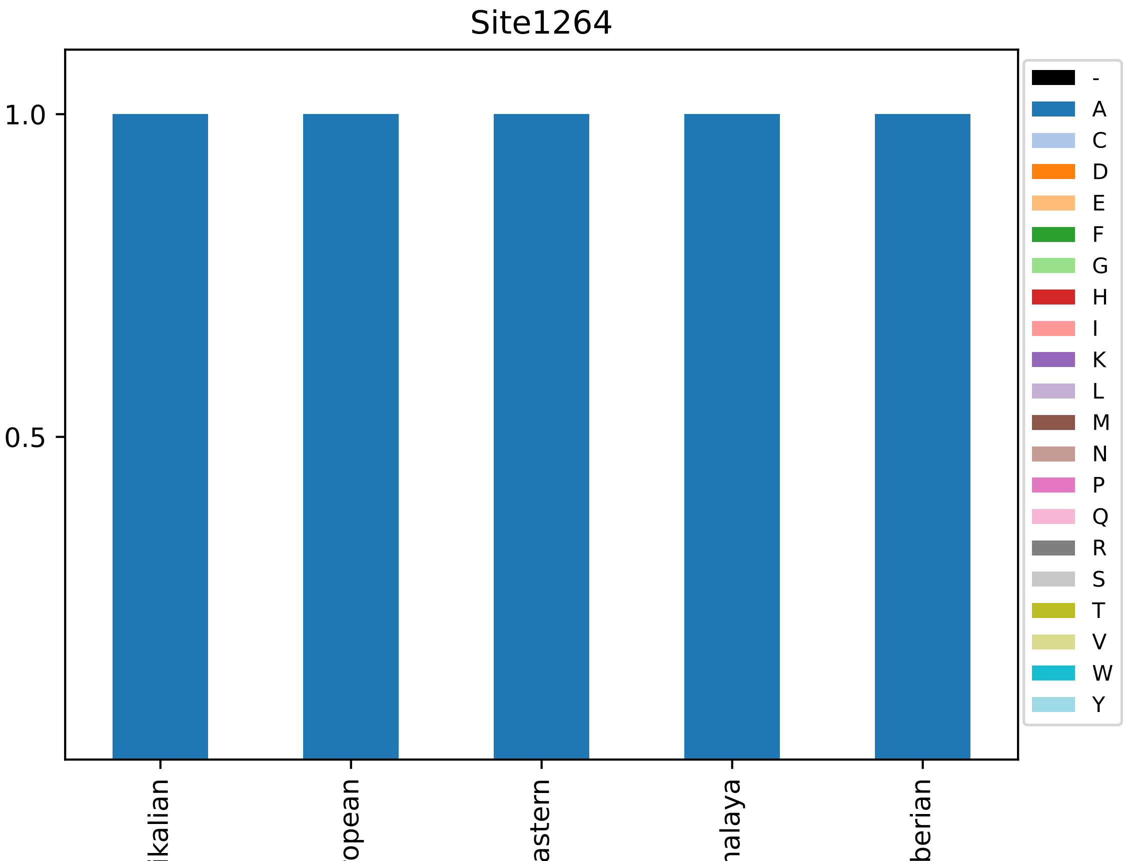

Site1264
1.0
0.5
-
A
C
D
E
F
G
H
I
K
L
M
N
P
Q
R
S
T
V
W
Y
ikalian
opean
astern
nalaya
berian

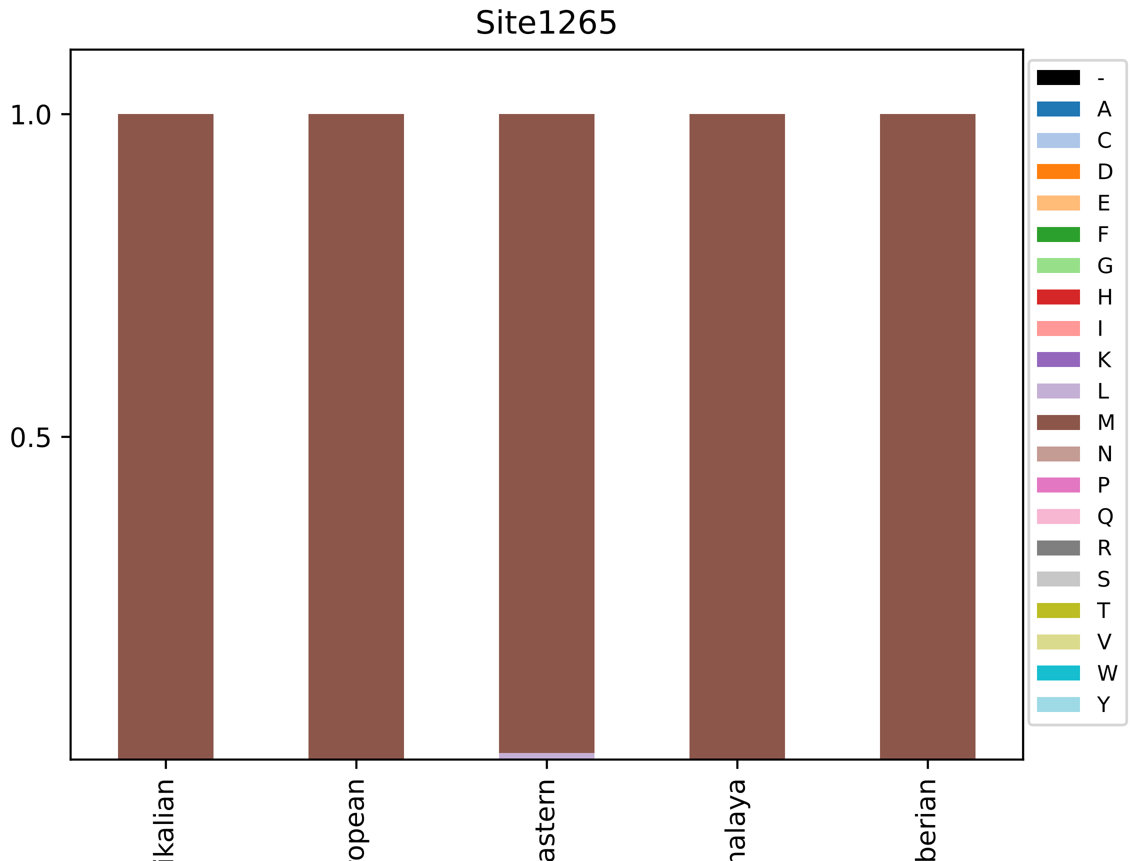
Site1265
1.0
0.5
-
A
C
D
E
F
G
H
I
K
L
M
N
P
Q
R
S
T
V
W
Y
ikalian
opean
astern
alaya
berian

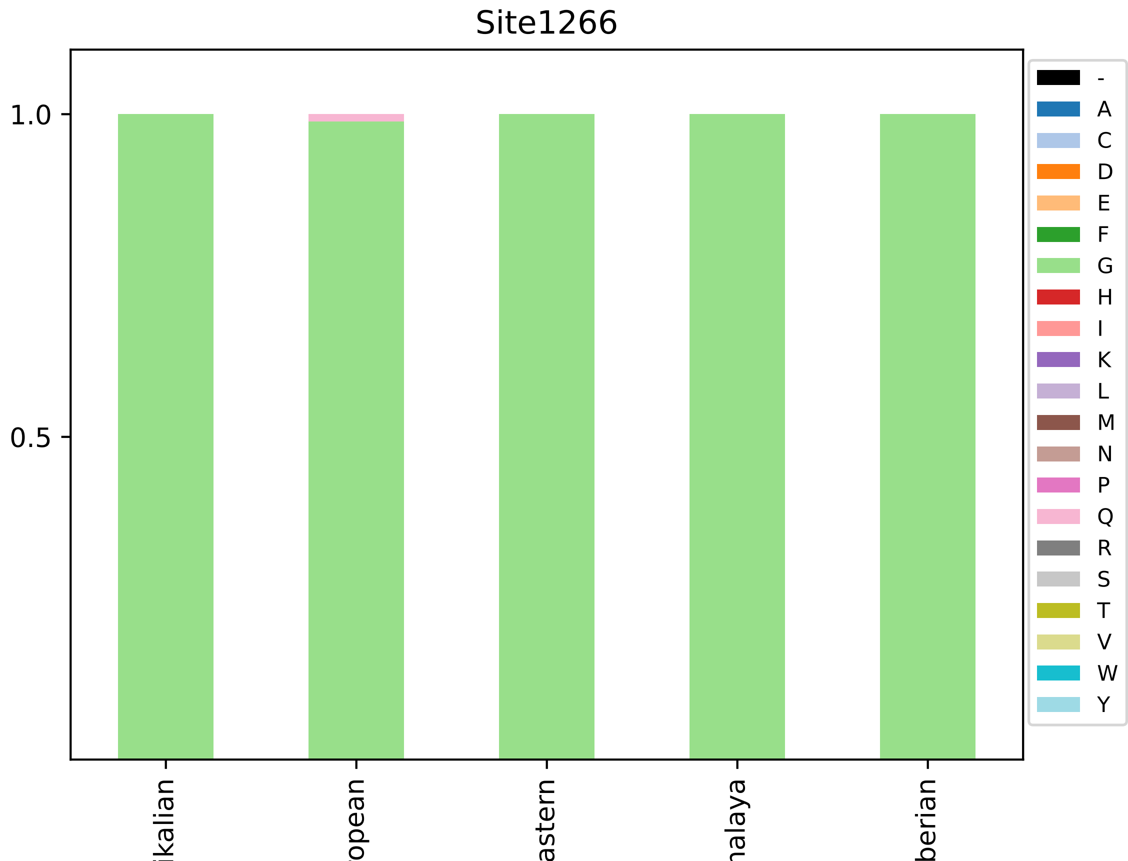
Site1266
1.0
0.5
ikalian
ropean
astern
nalaya
berian
-
A
C
D
E
F
G
H
I
K
L
M
N
P
Q
R
S
T
V
W
Y

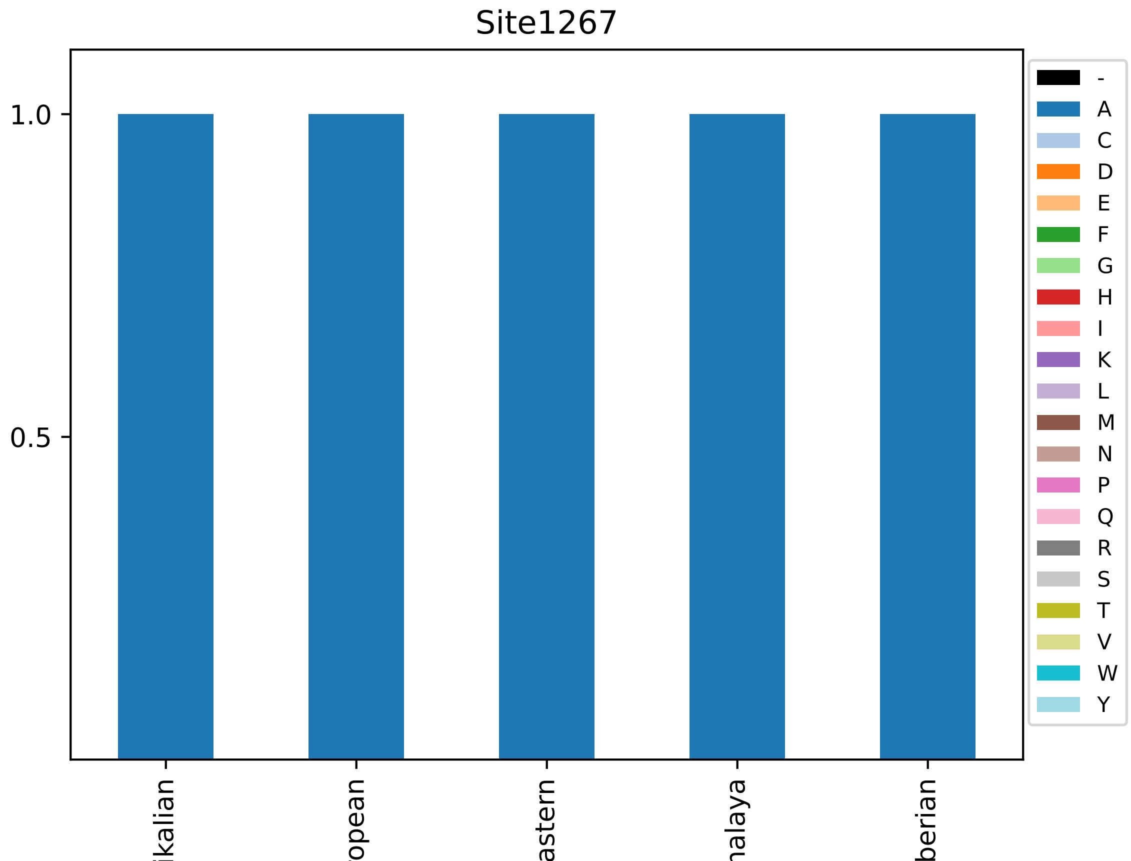

Site1267
1.0
0.5
-
A
C
D
E
F
G
H
I
K
L
M
N
P
Q
R
S
T
V
W
Y
ikalian
opean
astern
nalaya
berian

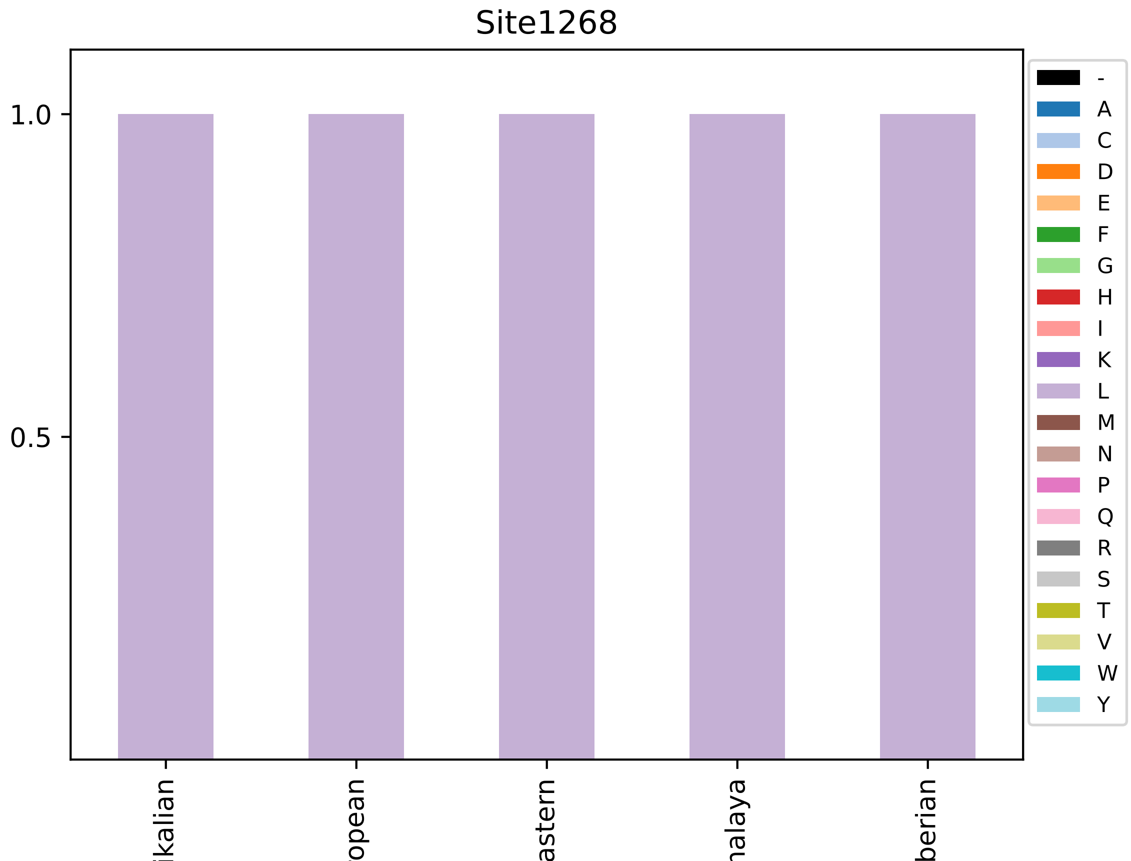
Site1268
1.0
0.5
-
A
C
D
E
F
G
H
I
K
L
M
N
P
Q
R
S
T
V
W
Y
ikalian
opean
astern
nalaya
berian

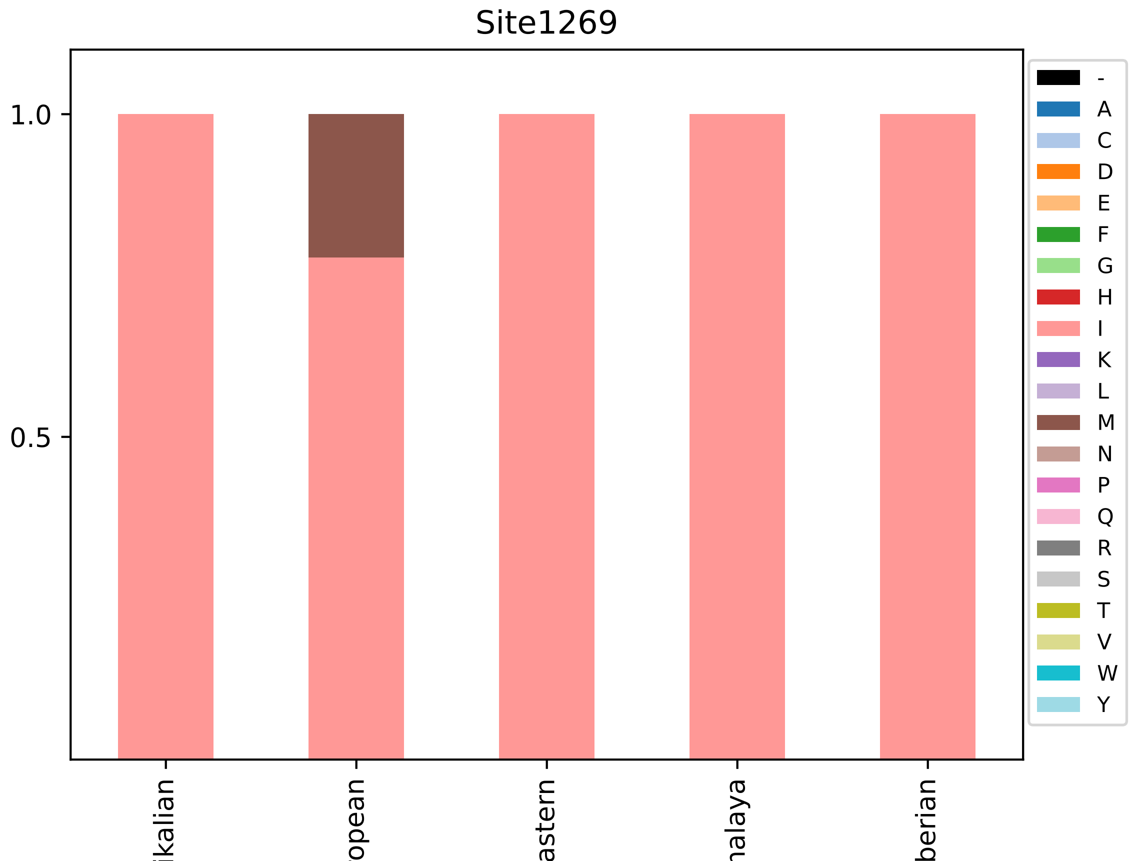
Site1269
1.0
0.5
ikalian
opean
astern
nalaya
berian
-
A
C
D
E
F
G
H
I
K
L
M
N
P
Q
R
S
T
V
W
Y

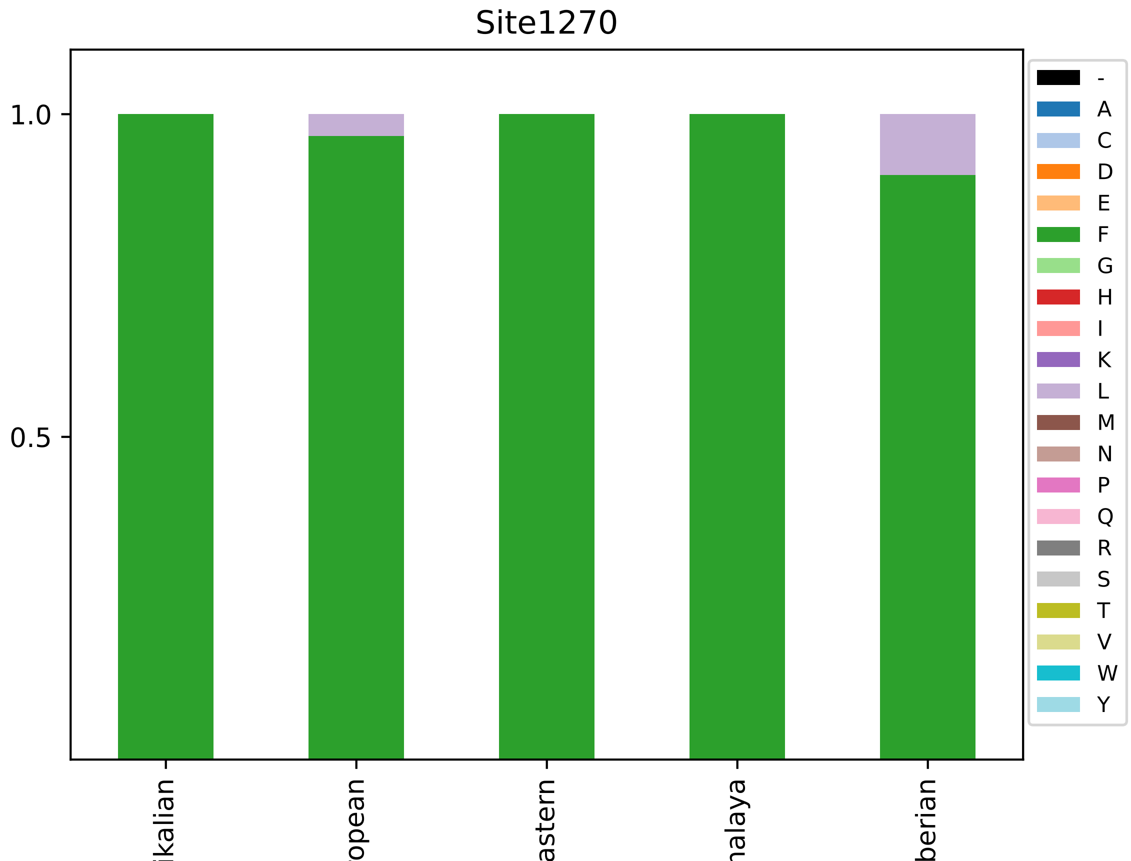

Site1270
1.0
0.5
-
A
C
D
E
F
G
H
I
K
L
M
N
P
Q
R
S
T
V
W
Y
ikalian
opean
astern
nalaya
berian

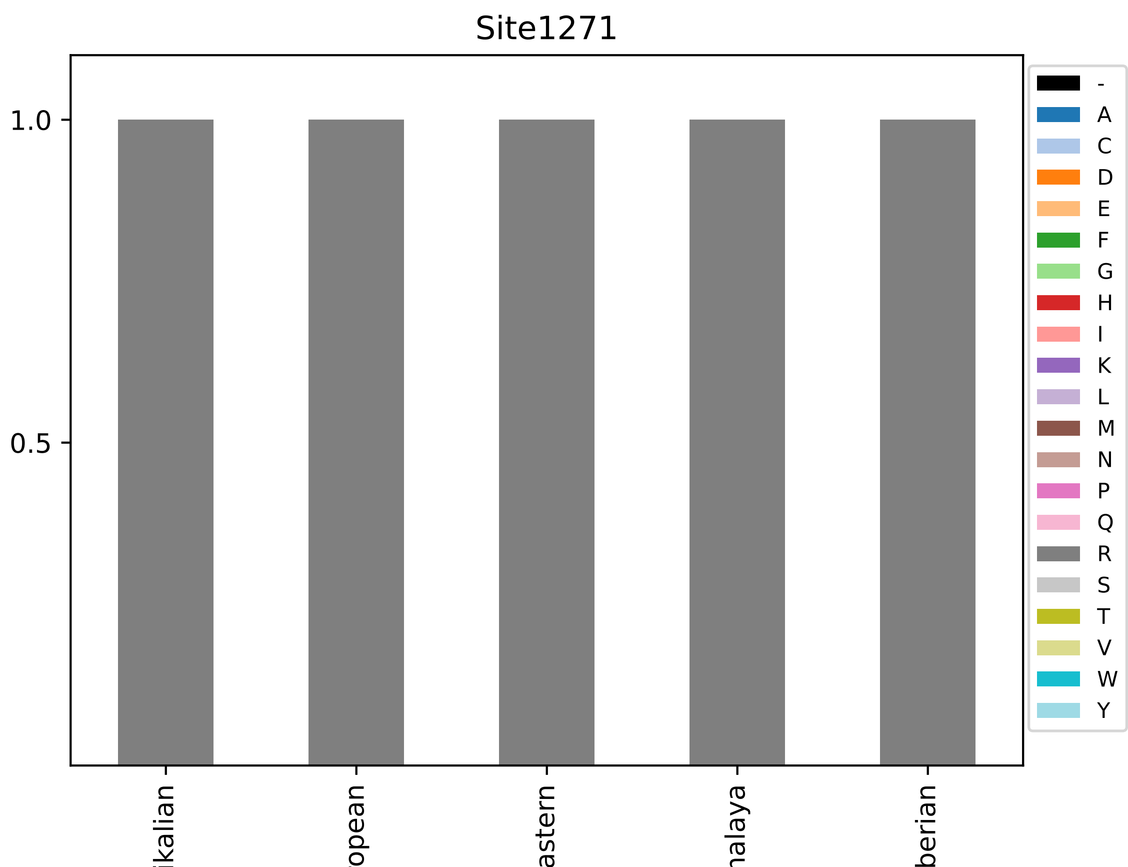
Site1271
1.0
0.5
-
A
C
D
E
F
G
H
I
K
L
M
N
P
Q
R
S
T
V
W
Y
ikalian
opean
astern
nalaya
berian

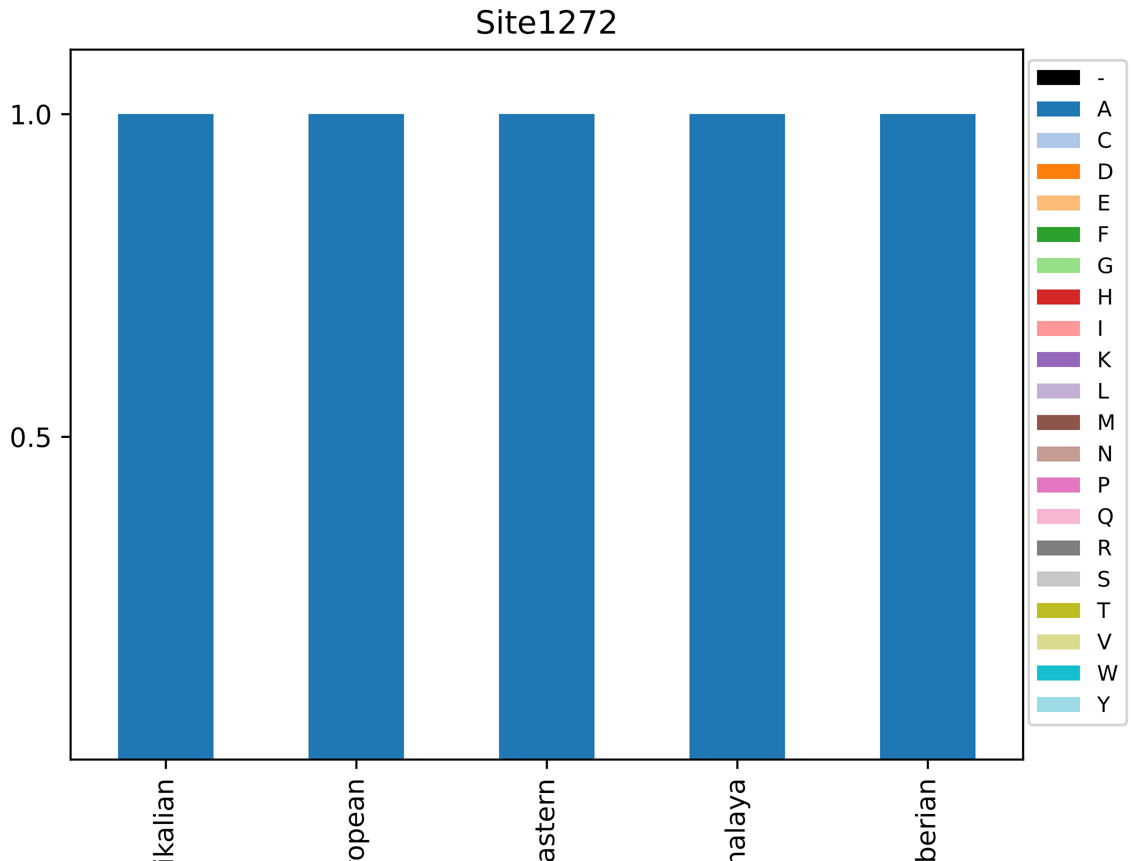
Site1272
1.0
0.5
-
A
C
D
E
F
G
H
I
K
L
M
N
P
Q
R
S
T
V
W
Y
ikalian
opean
astern
nalaya
berian

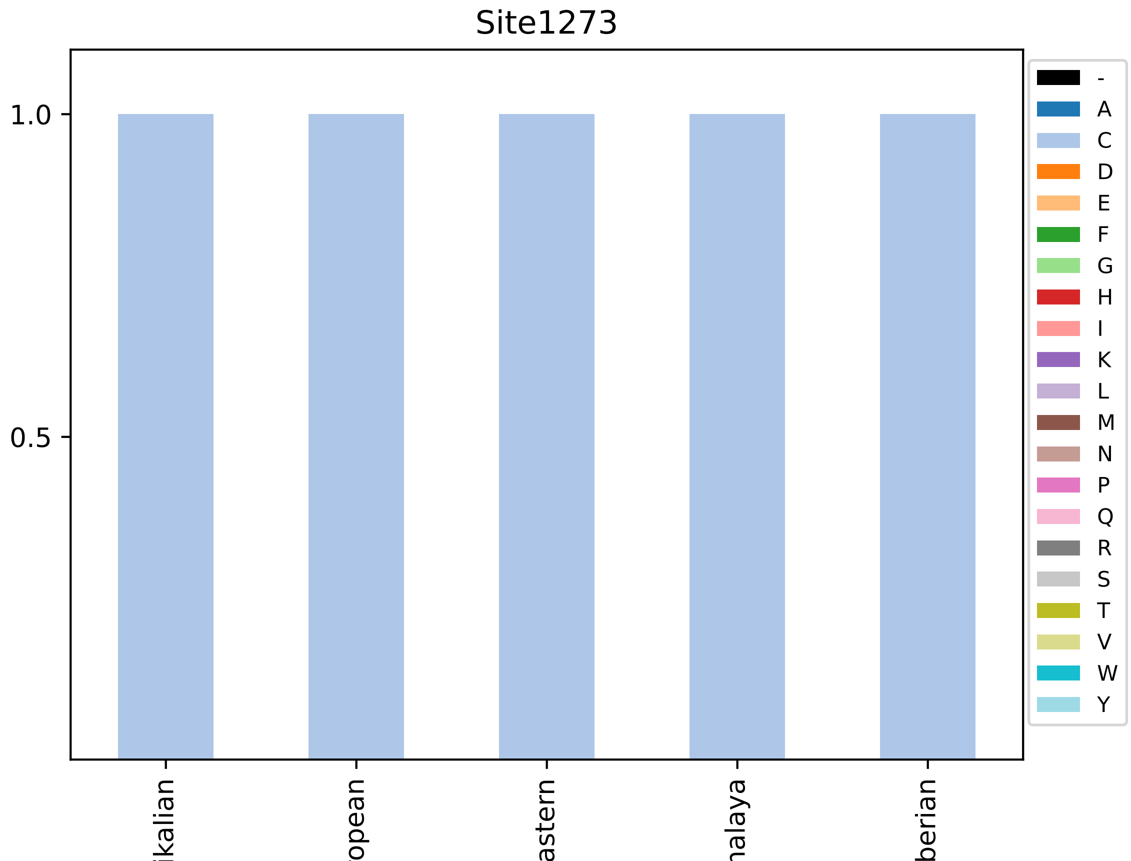
Site1273
1.0
0.5
ikalian
opean
astern
nalaya
berian
-
A
C
D
E
F
G
H
I
K
L
M
N
P
Q
R
S
T
V
W
Y

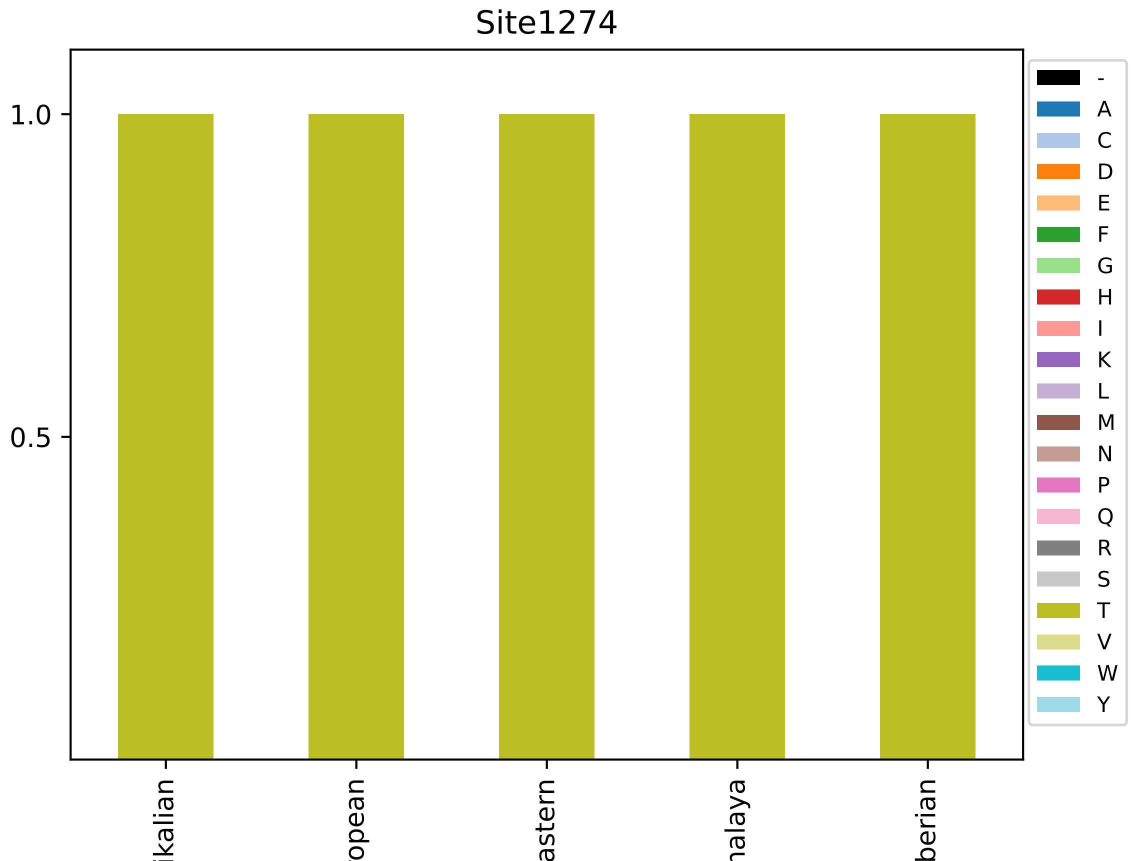

Site1274
1.0
0.5
-
A
C
D
E
F
G
H
I
K
L
M
N
P
Q
R
S
T
V
W
Y
ikalian
opean
astern
nalaya
berian

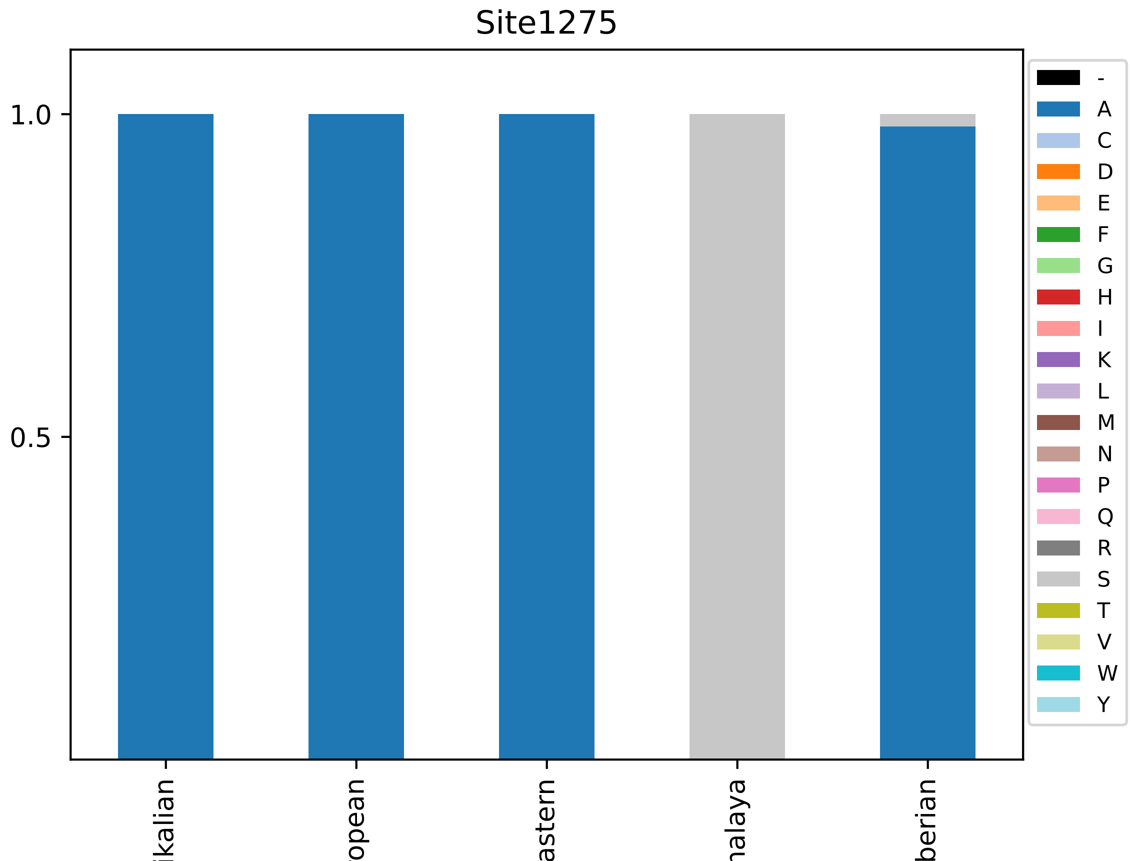
Site1275
1.0
0.5
ikalian
opean
astern
nalaya
berian
-
A
C
D
E
F
G
H
I
K
L
M
N
P
Q
R
S
T
V
W
Y

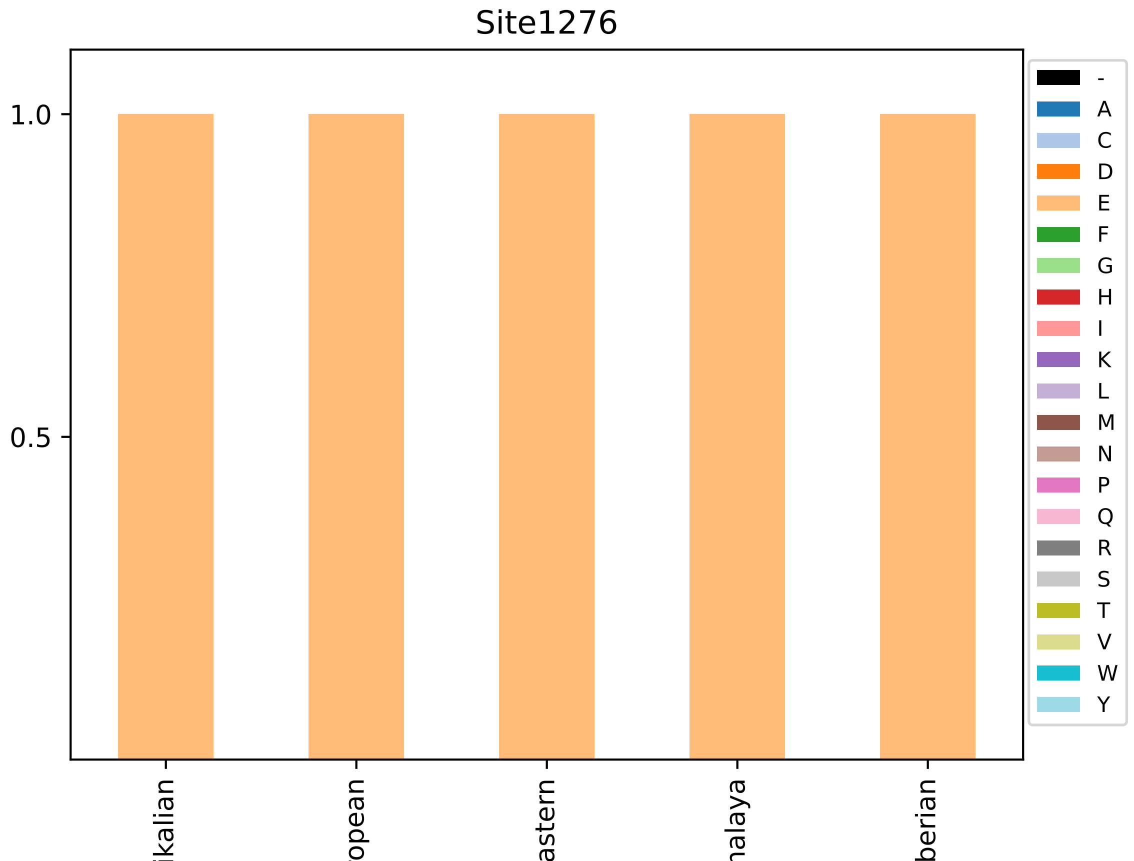
Site1276
1.0
0.5
-
A
C
D
E
F
G
H
I
K
L
M
N
P
Q
R
S
T
V
W
Y
ikalian
opean
astern
nalaya
berian

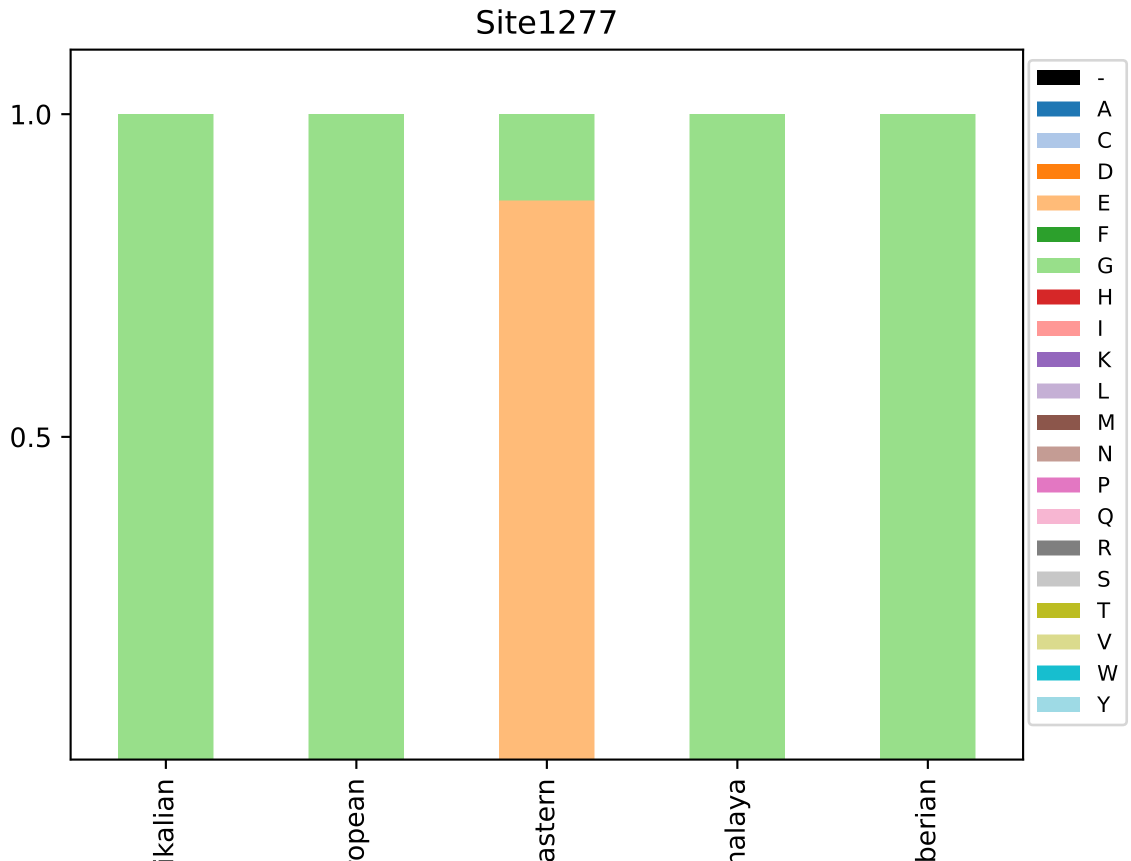
Site1277
1.0
0.5
ikalian
opean
astern
nalaya
berian
-
A
C
D
E
F
G
H
I
K
L
M
N
P
Q
R
S
T
V
W
Y

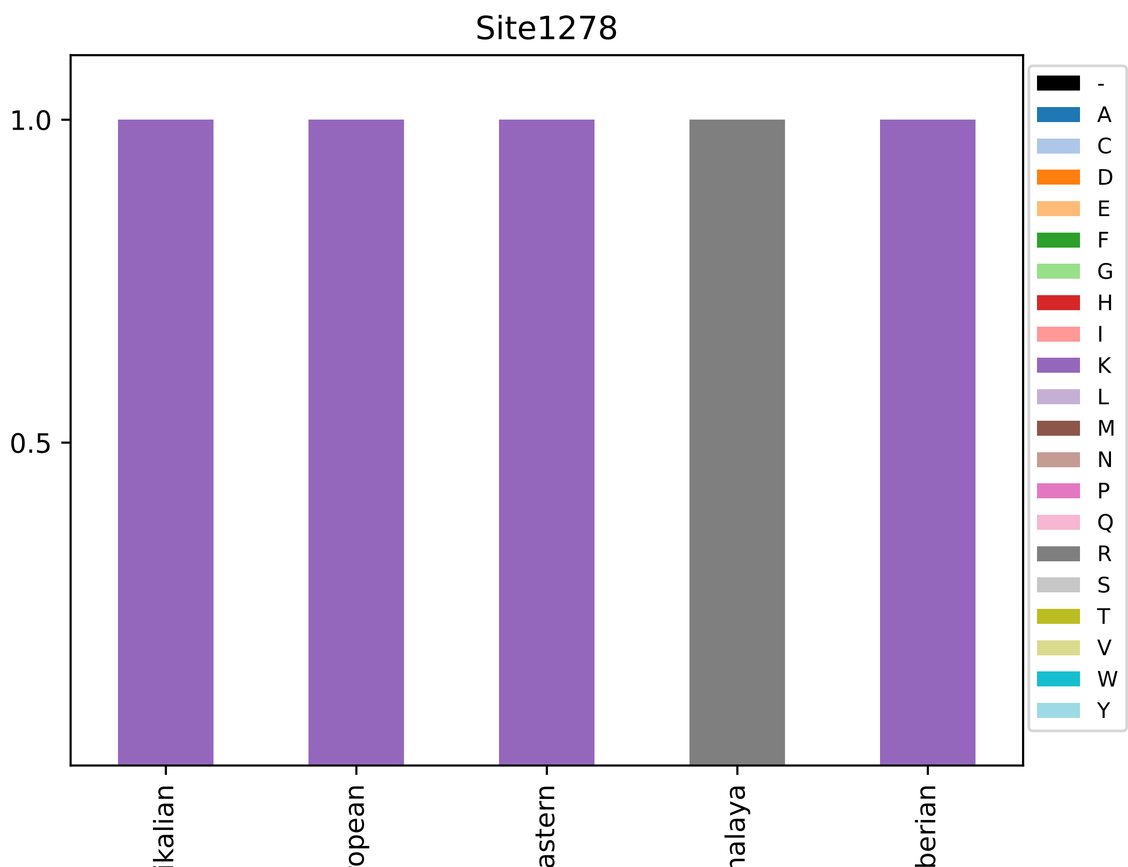
Site1278
1.0
0.5
-
A
C
D
E
F
G
H
I
K
L
M
N
P
Q
R
S
T
V
W
Y
ikalian
opean
astern
nalaya
berian

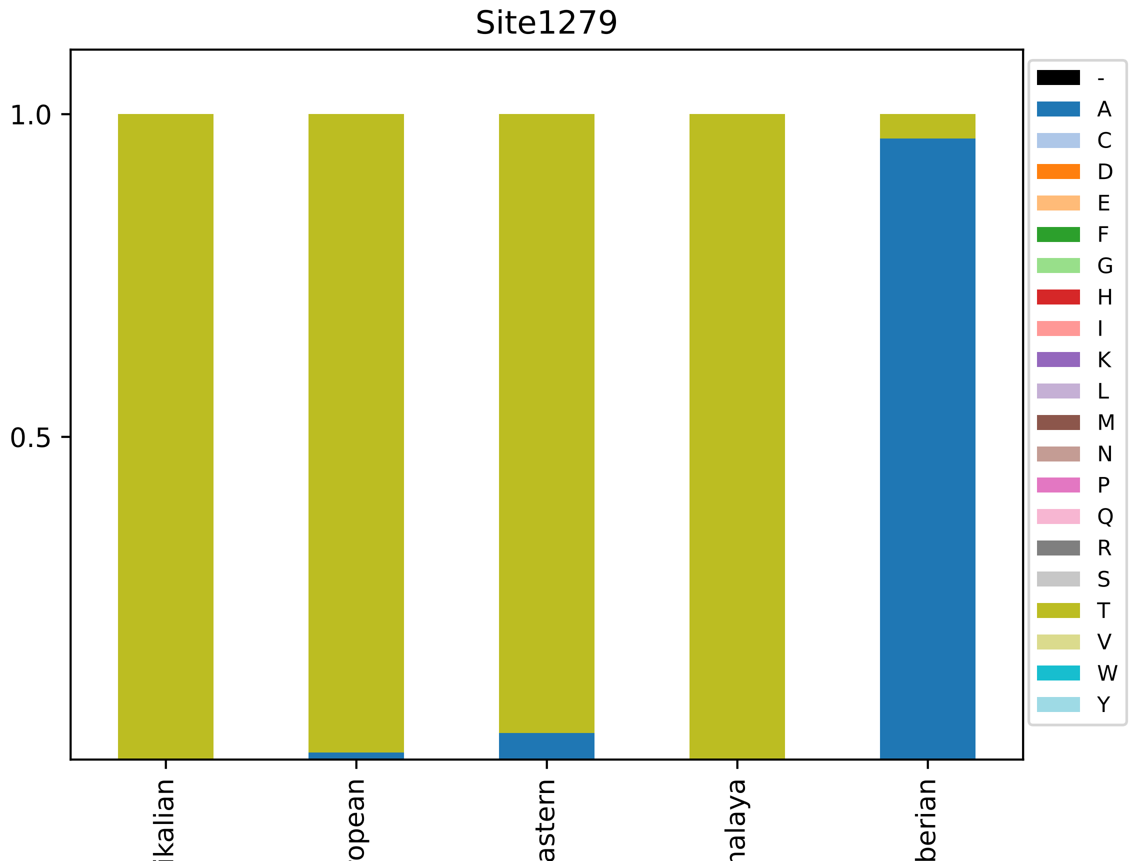
Site1279
1.0
0.5
-
A
C
D
E
F
G
H
I
K
L
M
N
P
Q
R
S
T
V
W
Y
ikalian
opean
astern
nalaya
berian

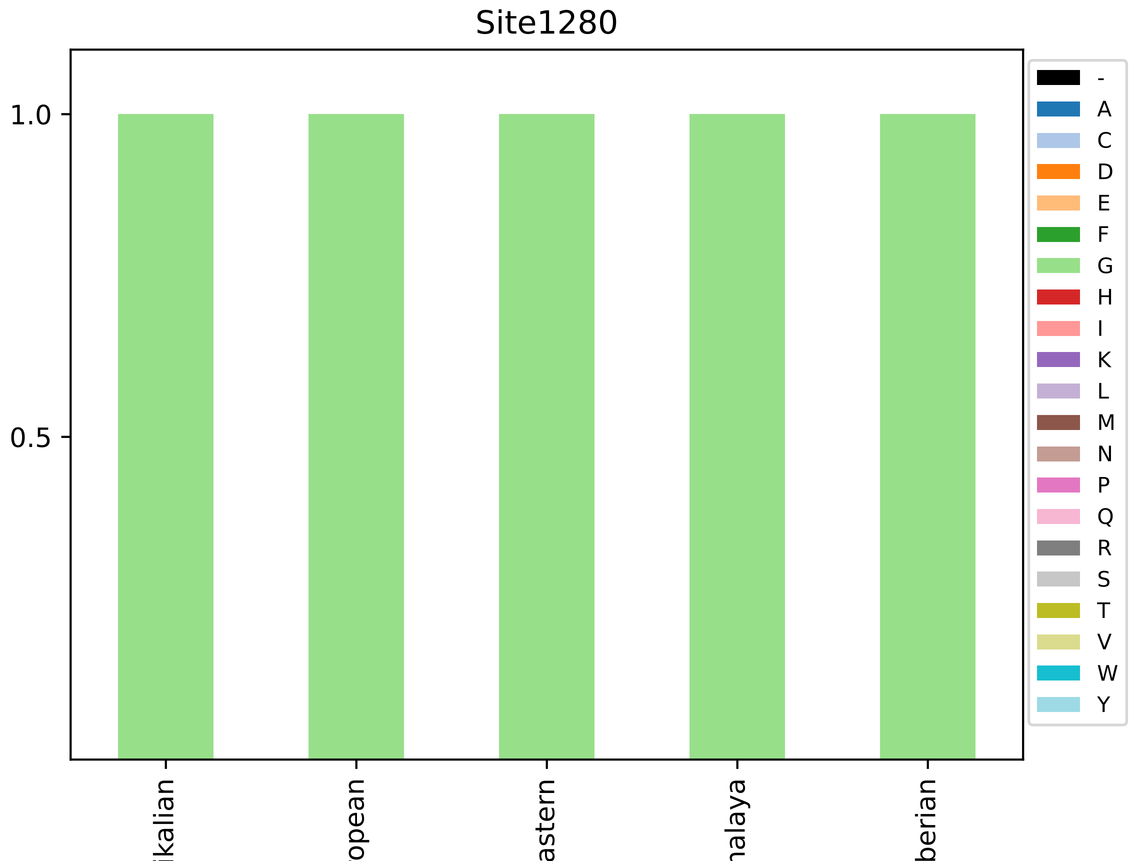

Site1280
1.0
0.5
-
A
C
D
E
F
G
H
I
K
L
M
N
P
Q
R
S
T
V
W
Y
ikalian
opean
astern
nalaya
berian

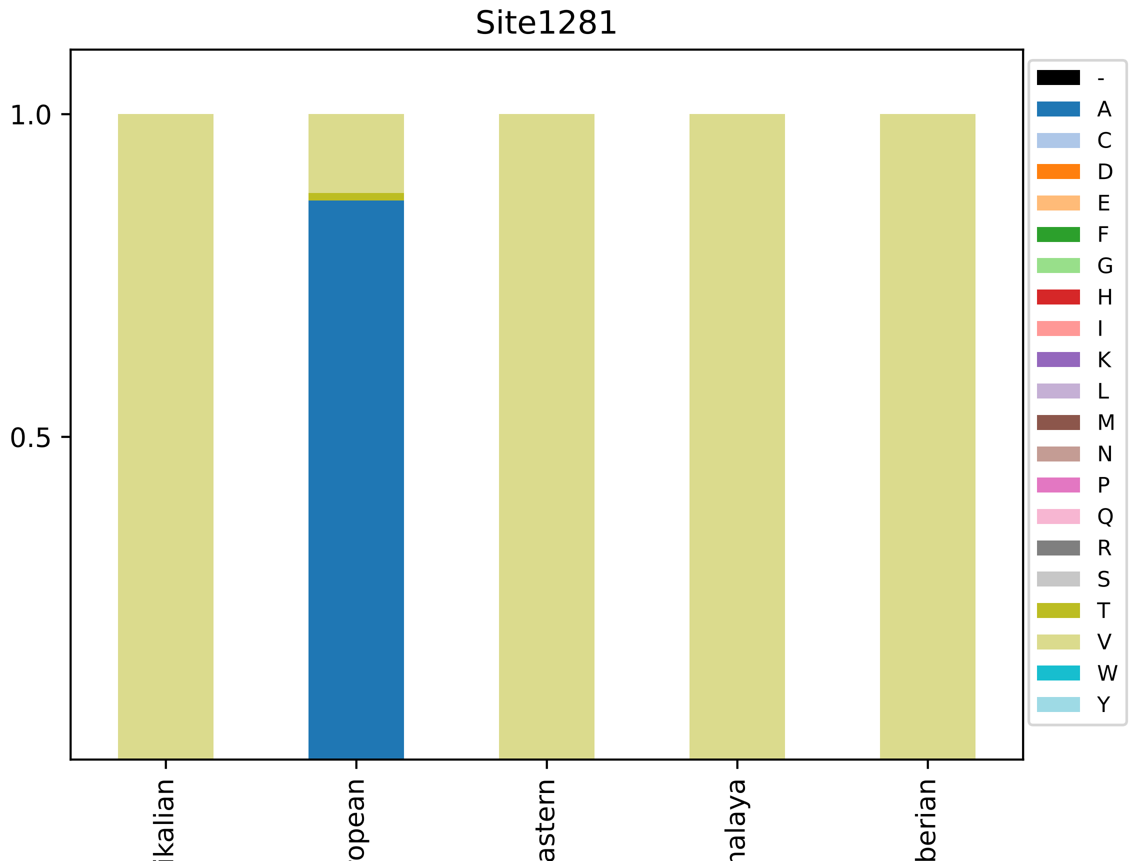

Site1281
1.0
0.5
-
A
C
D
E
F
G
H
I
K
L
M
N
P
Q
R
S
T
V
W
Y
ikalian
opean
astern
nalaya
berian

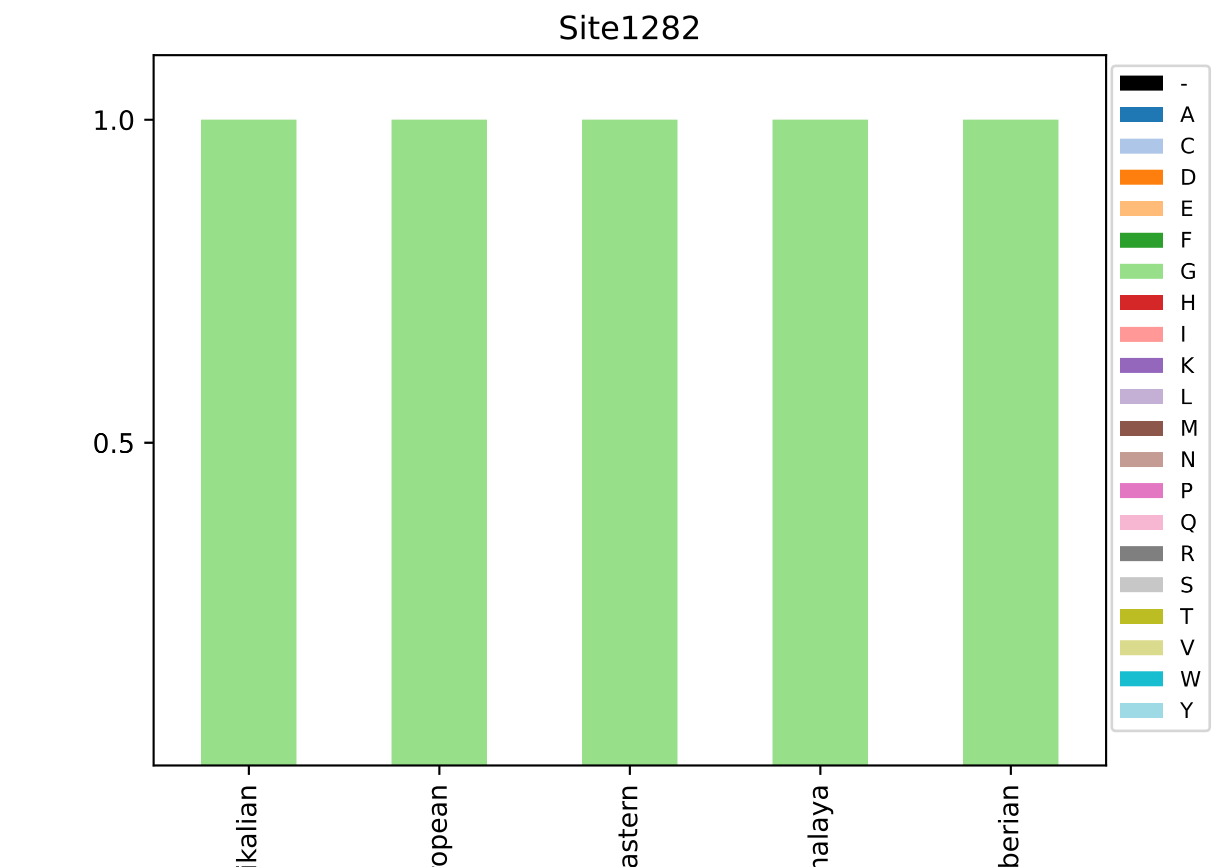
Site1282
1.0
0.5
-
A
C
D
E
F
G
H
I
K
L
M
N
P
Q
R
S
T
V
W
Y
ikalian
opean
astern
nalaya
berian

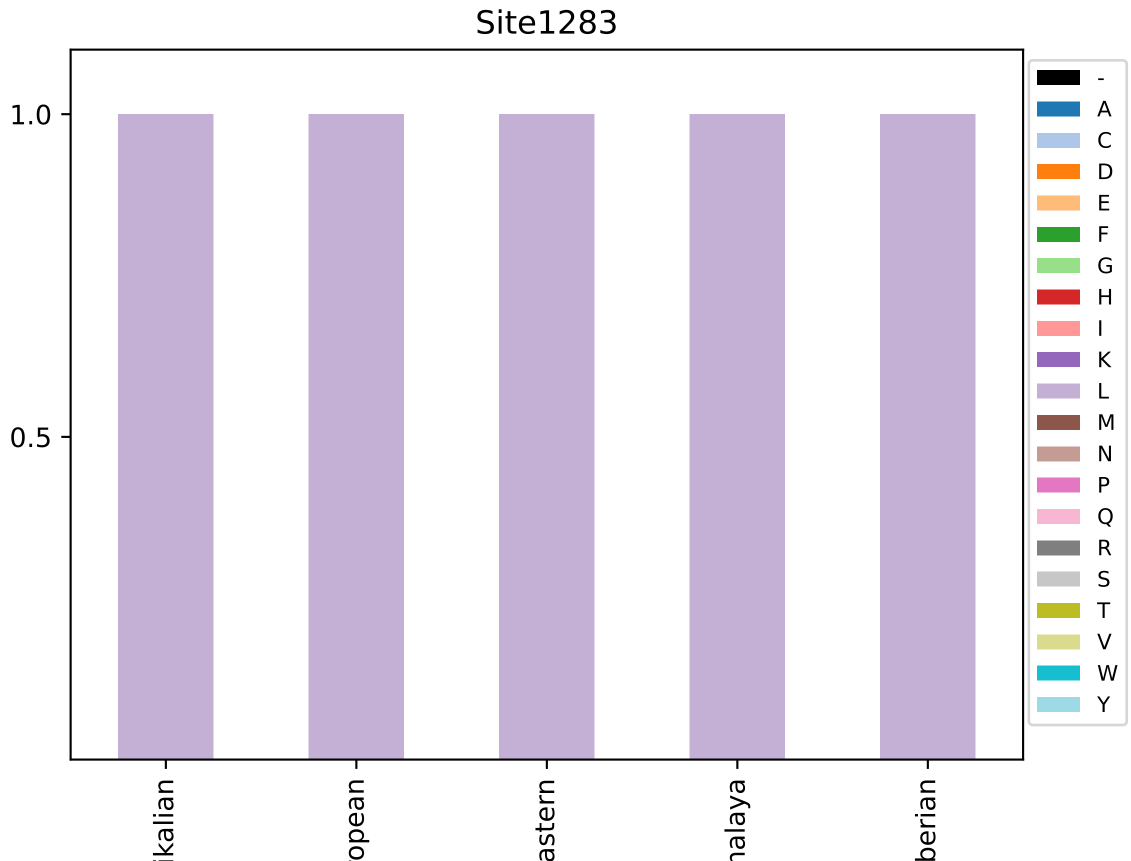
Site1283
1.0
0.5
-
A
C
D
E
F
G
H
I
K
L
M
N
P
Q
R
S
T
V
W
Y
ikalian
opean
astern
nalaya
berian

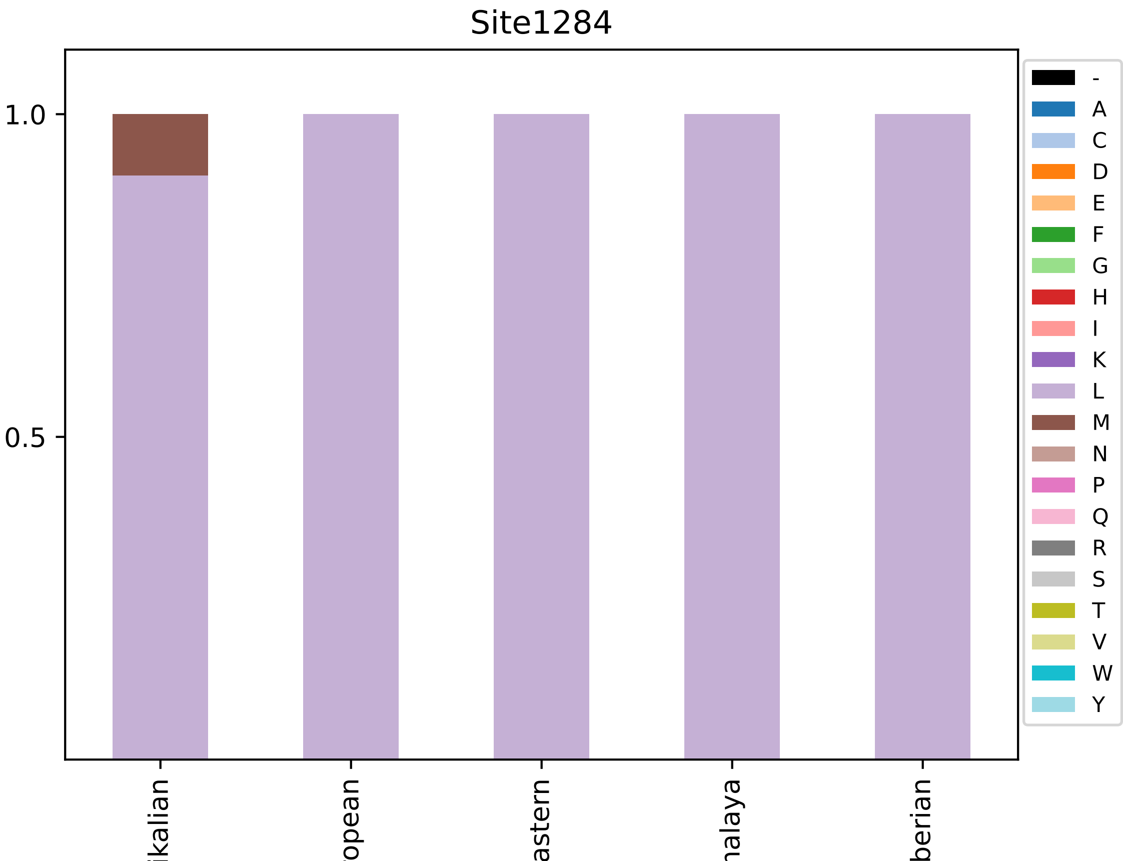

Site1284
1.0
0.5
ikalian
opean
astern
nalaya
berian
-
A
C
D
E
F
G
H
I
K
L
M
N
P
Q
R
S
T
V
W
Y

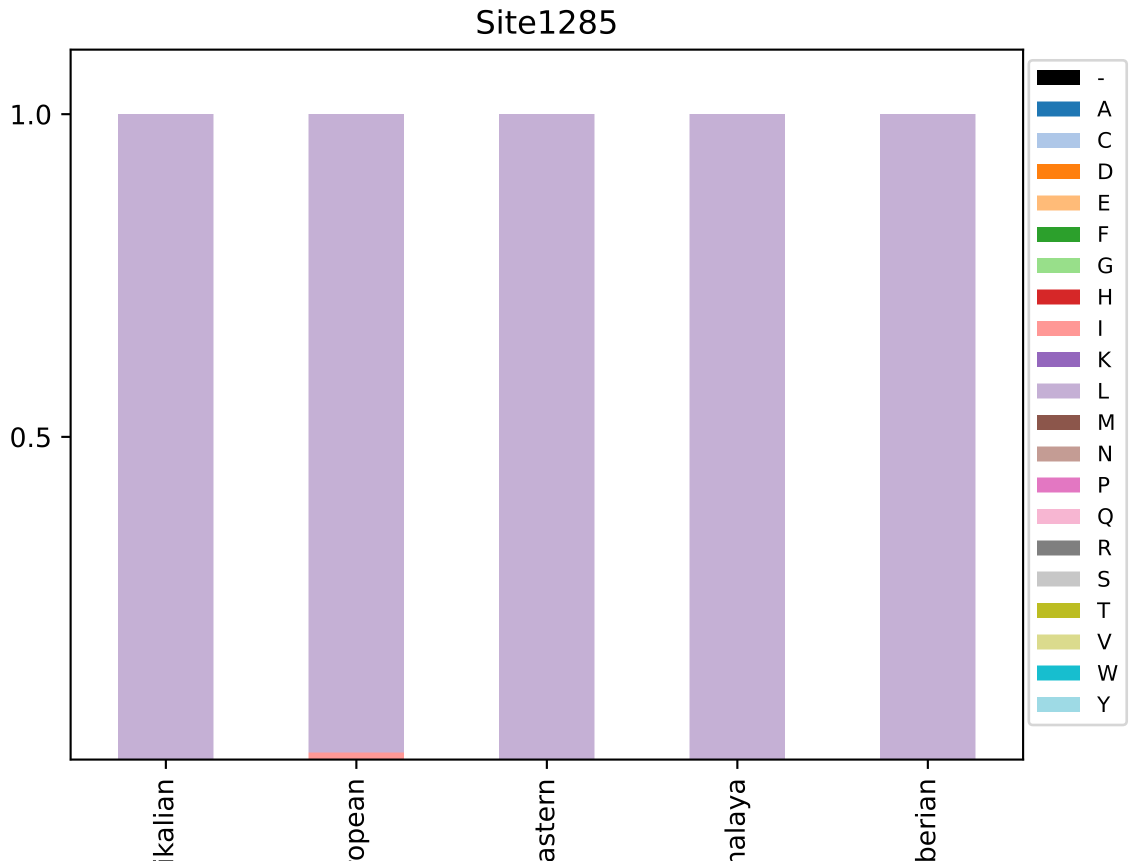
Site1285
1.0
0.5
-
A
C
D
E
F
G
H
I
K
L
M
N
P
Q
R
S
T
V
W
Y
ikalian
opean
astern
nalaya
berian

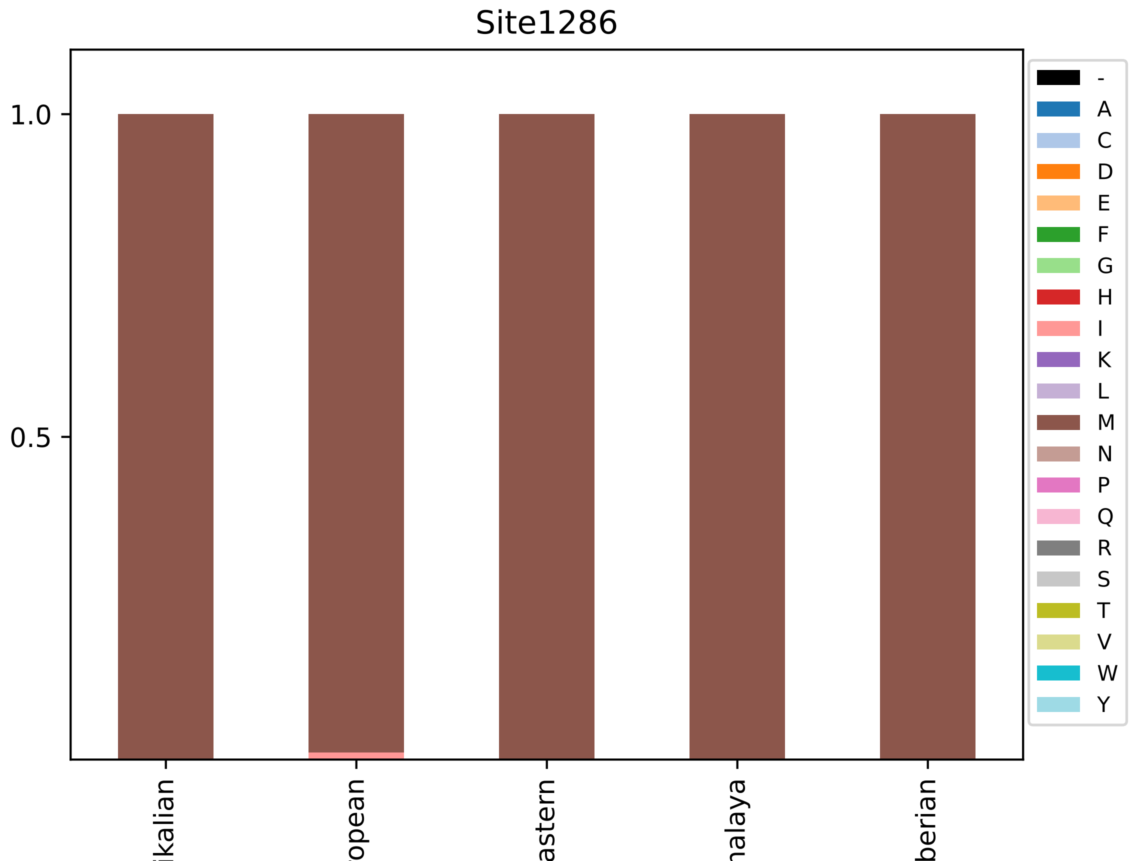
Site1286
1.0
0.5
-
A
C
D
E
F
G
H
I
K
L
M
N
P
Q
R
S
T
V
W
Y
ikalian
opean
astern
nalaya
berian

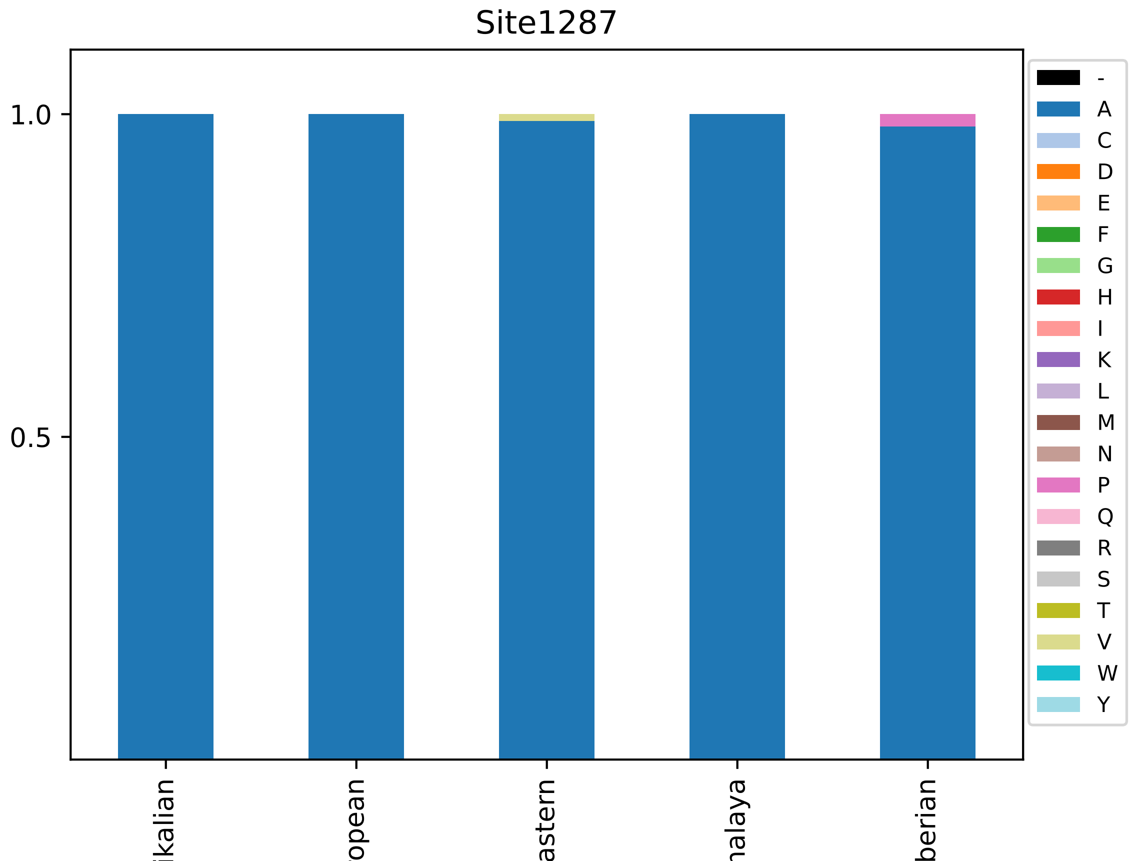

Site1287
1.0
0.5
-
A
C
D
E
F
G
H
I
K
L
M
N
P
Q
R
S
T
V
W
Y
ikalian
opean
astern
nalaya
berian

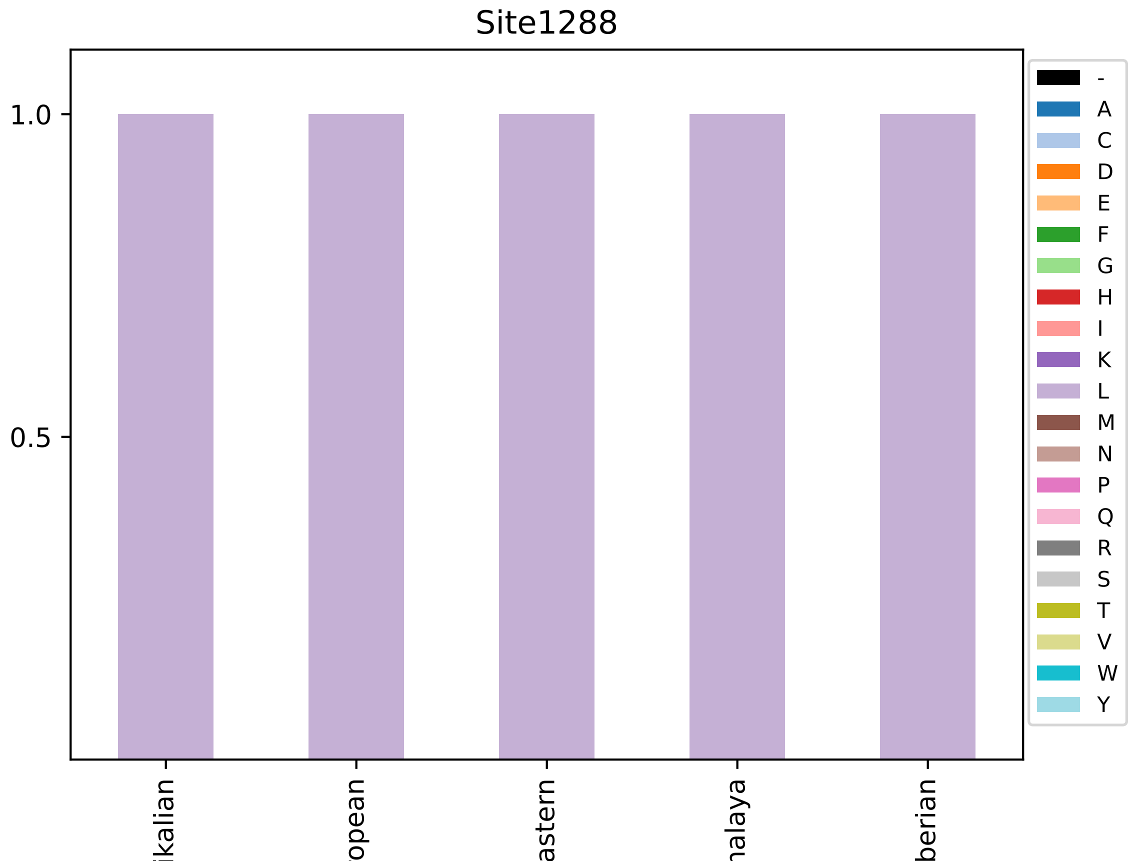

Site1288
1.0
0.5
-
A
C
D
E
F
G
H
I
K
L
M
N
P
Q
R
S
T
V
W
Y
ikalian
opean
astern
nalaya
berian

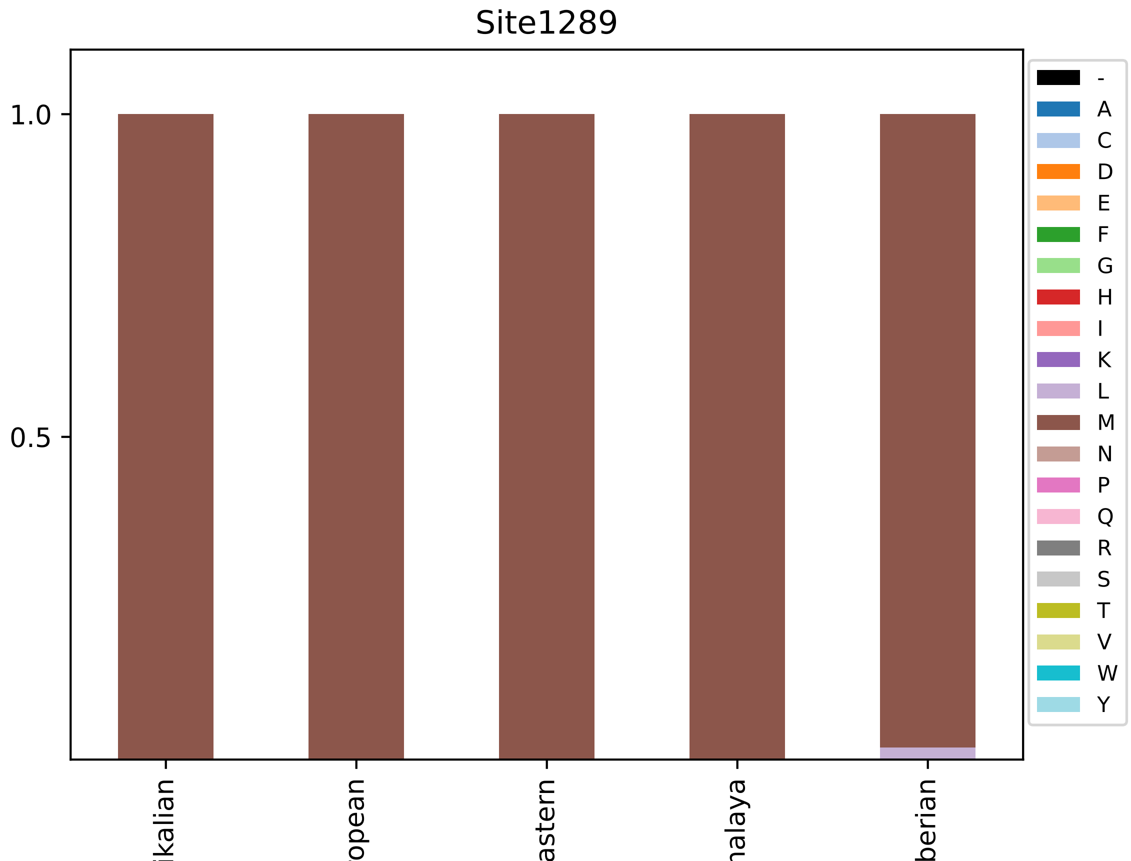

Site1289
1.0
0.5
ikalian
opean
astern
nalaya
berian
-
A
C
D
E
F
G
H
I
K
L
M
N
P
Q
R
S
T
V
W
Y

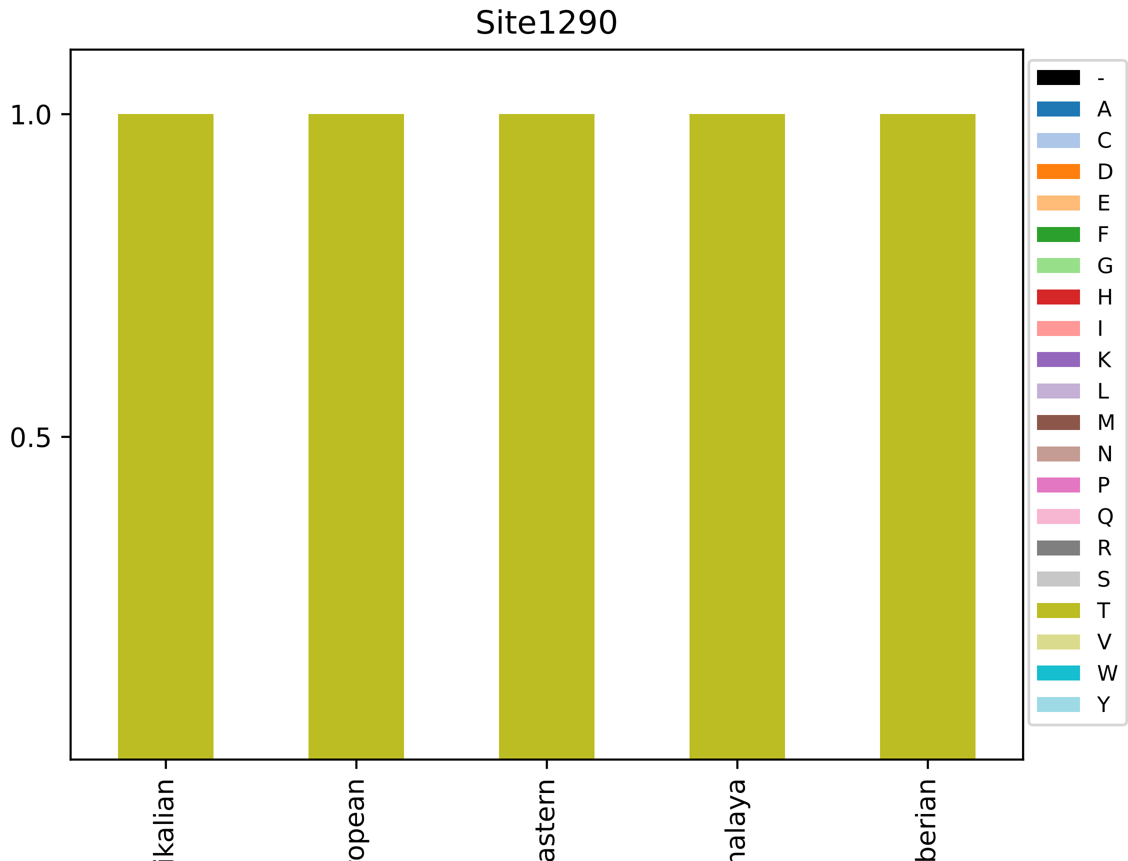

Site1290
1.0
0.5
-
A
C
D
E
F
G
H
I
K
L
M
N
P
Q
R
S
T
V
W
Y
ikalian
opean
astern
nalaya
berian

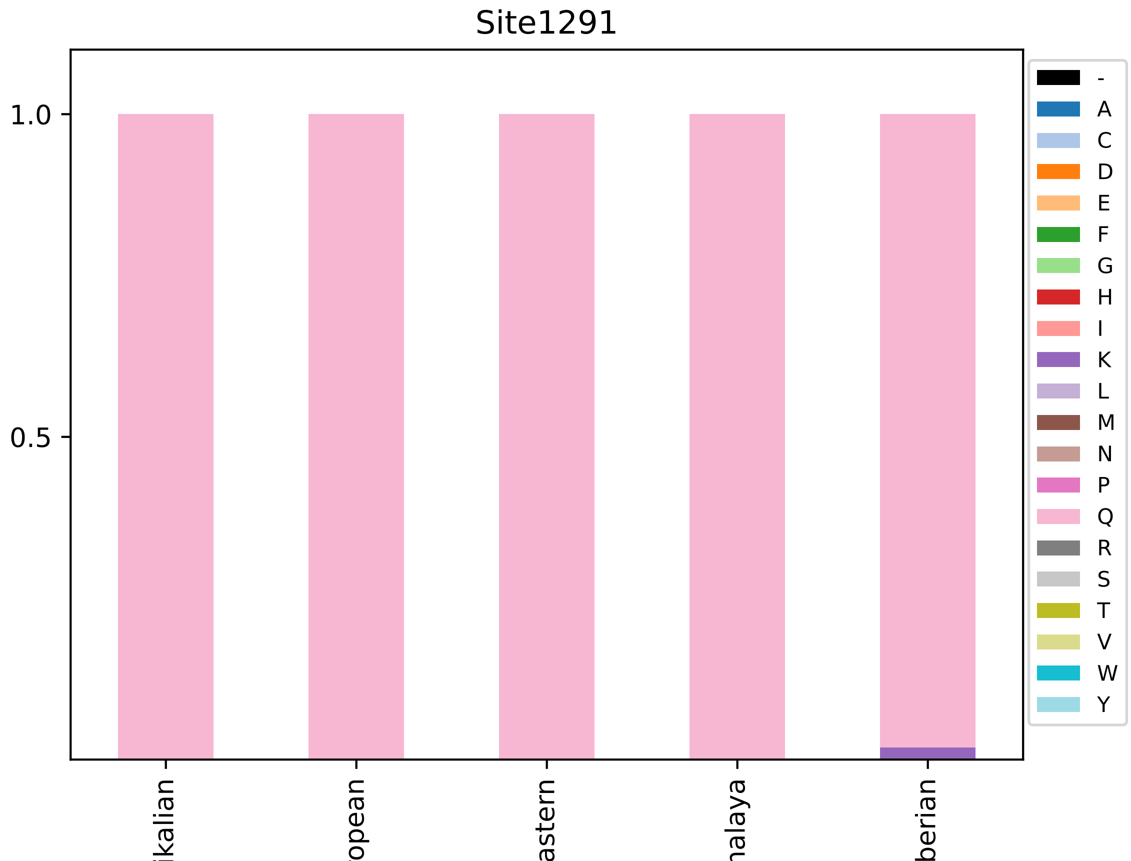
Site1291
1.0
0.5
-
A
C
D
E
F
G
H
I
K
L
M
N
P
Q
R
S
T
V
W
Y
ikalian
opean
astern
nalaya
berian

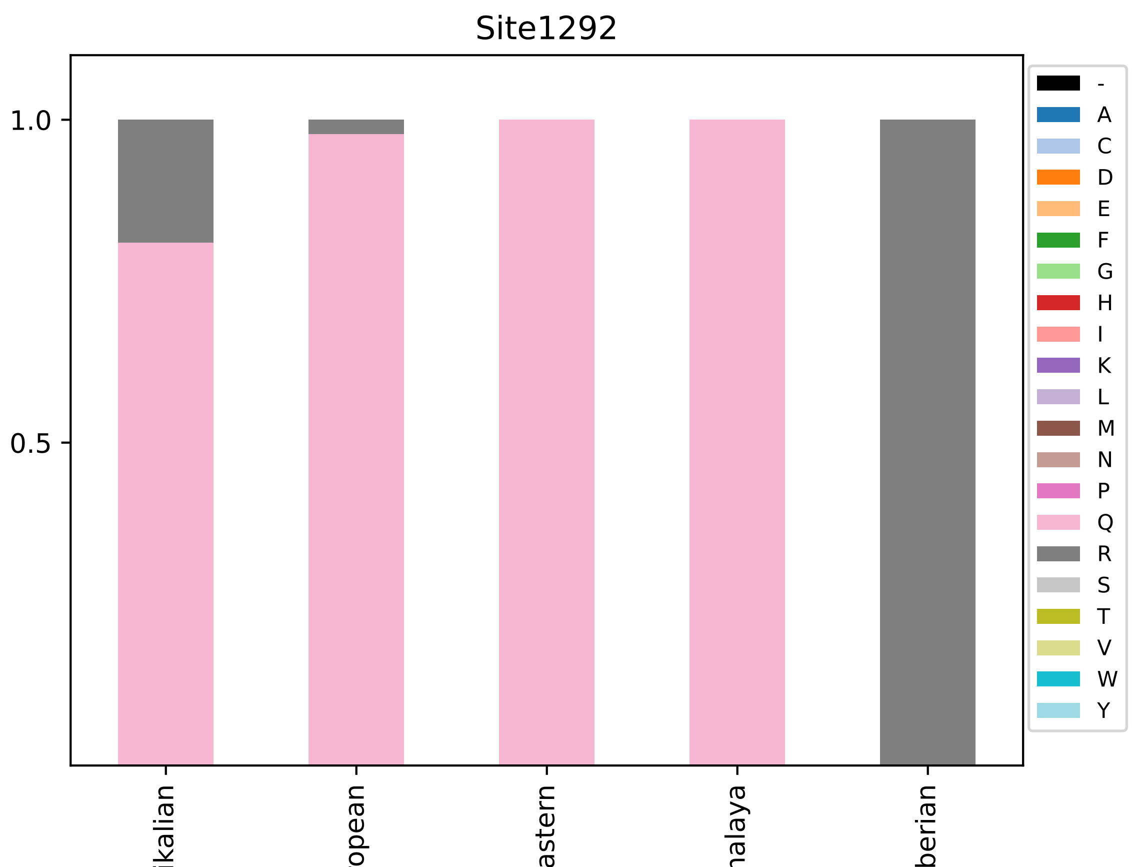

Site1292
1.0
0.5
ikalian
opean
astern
nalaya
berian
-
A
C
D
E
F
G
H
I
K
L
M
N
P
Q
R
S
T
V
W
Y

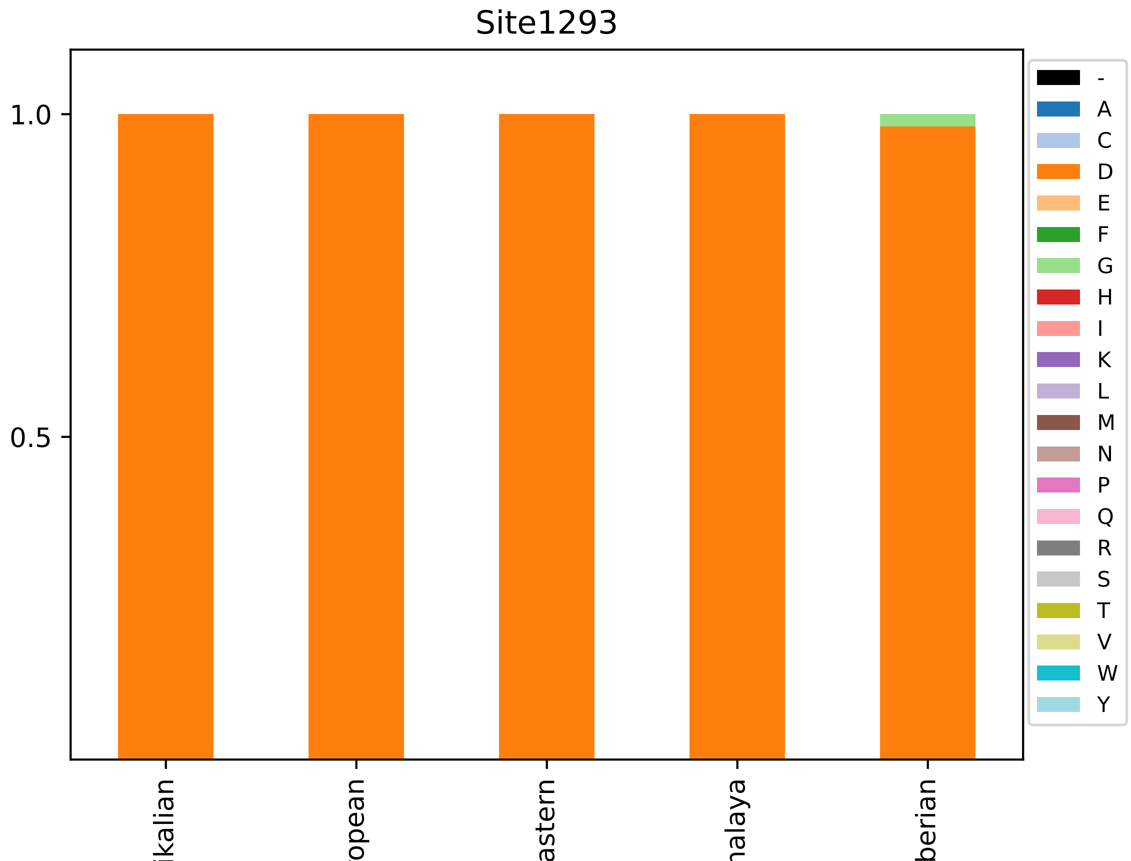
Site1293
1.0
0.5
-
A
C
D
E
F
G
H
I
K
L
M
N
P
Q
R
S
T
V
W
Y
ikalian
opean
astern
nalaya
berian

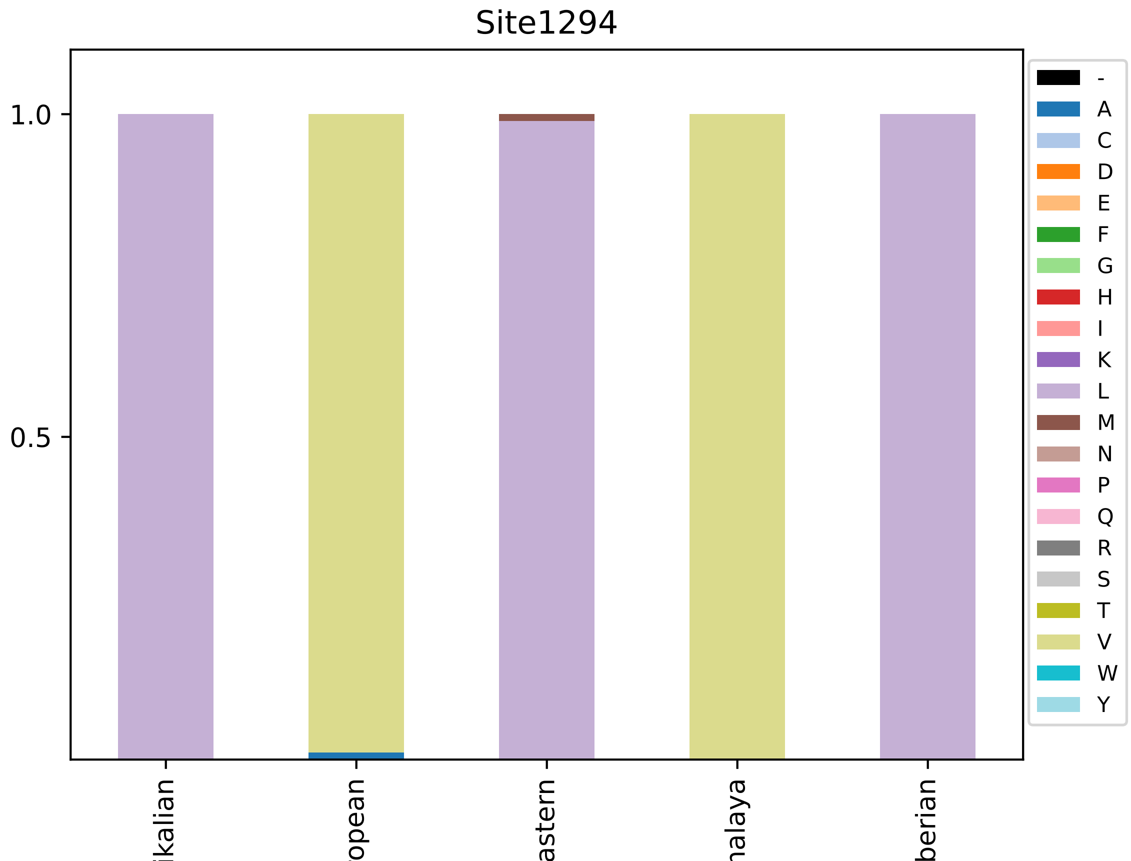
Site1294
1.0
0.5
-
A
C
D
E
F
G
H
I
K
L
M
N
P
Q
R
S
T
V
W
Y
ikalian
opean
astern
nalaya
berian

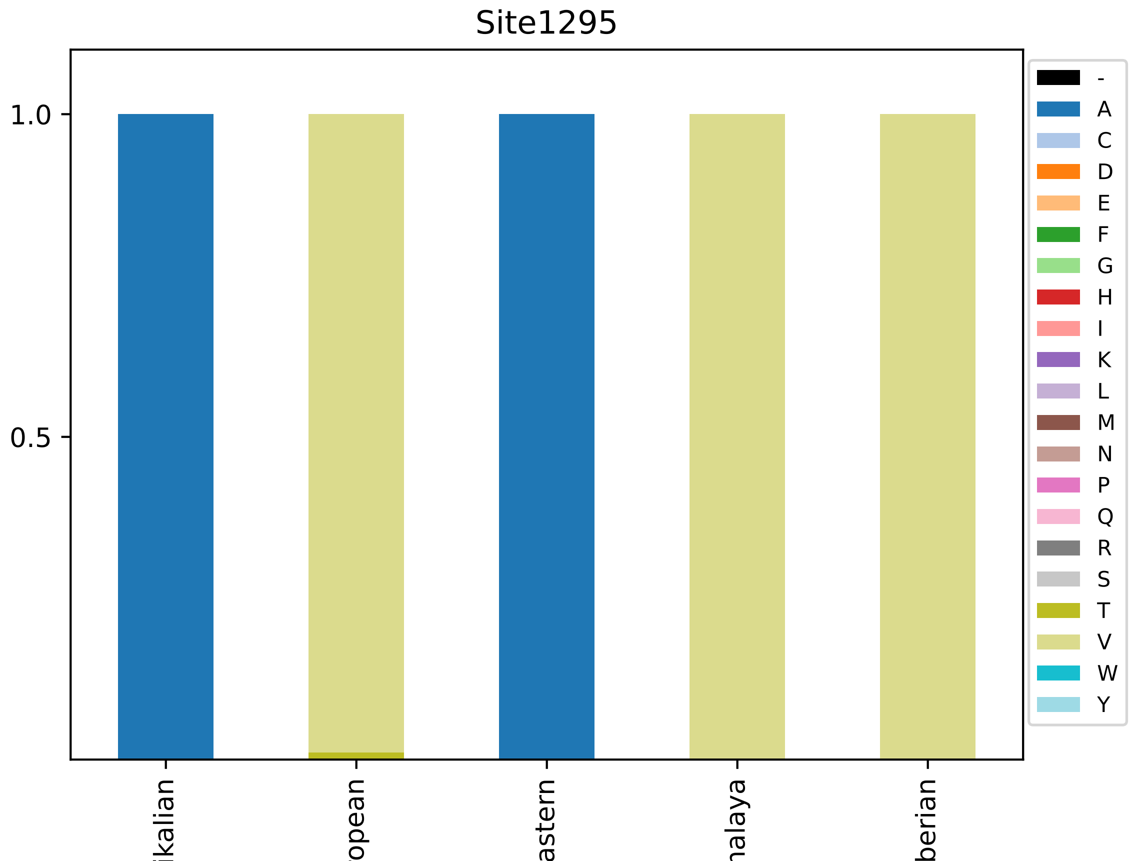
Site1295
1.0
0.5
ikalian
opean
astern
nalaya
berian
-
A
C
D
E
F
G
H
I
K
L
M
N
P
Q
R
S
T
V
W
Y

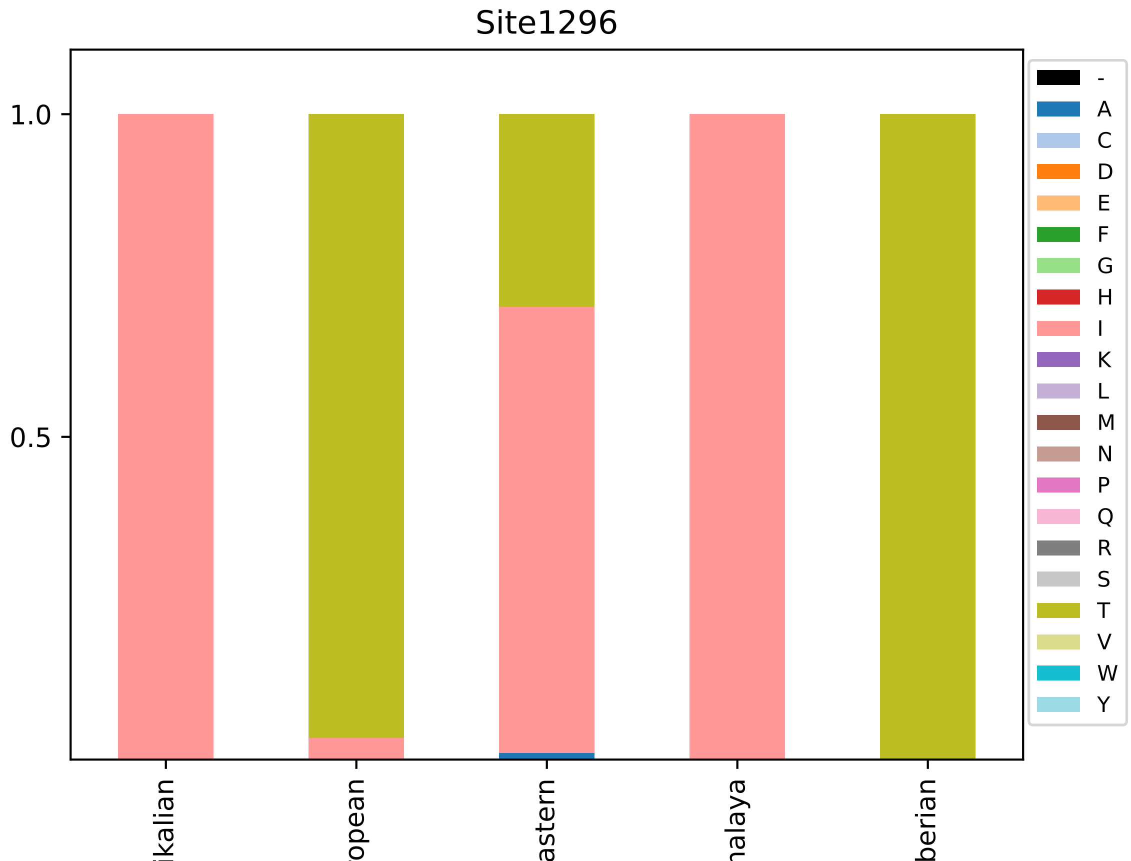
Site1296
1.0
0.5
-
A
C
D
E
F
G
H
I
K
L
M
N
P
Q
R
S
T
V
W
Y
ikalian
opean
astern
nalaya
berian

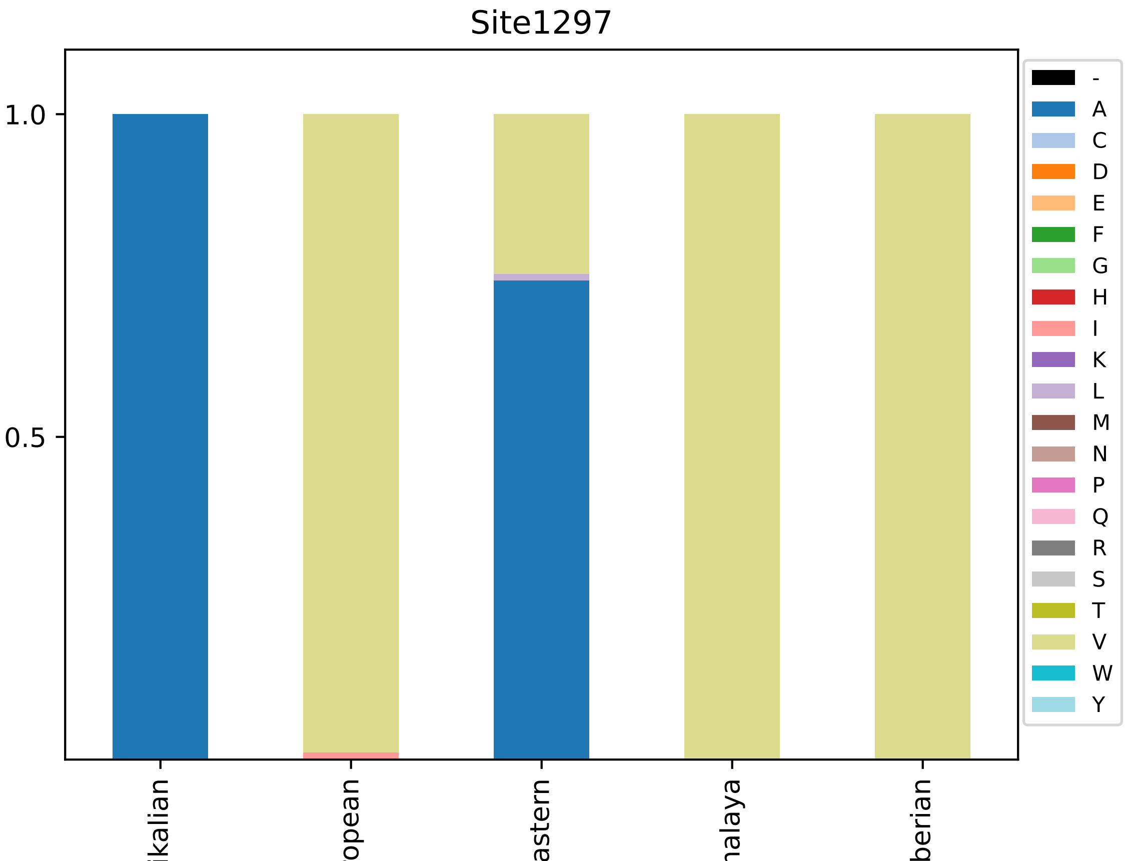

Site1297
1.0
0.5
-
A
C
D
E
F
G
H
I
K
L
M
N
P
Q
R
S
T
V
W
Y
ikalian
opean
astern
nalaya
berian

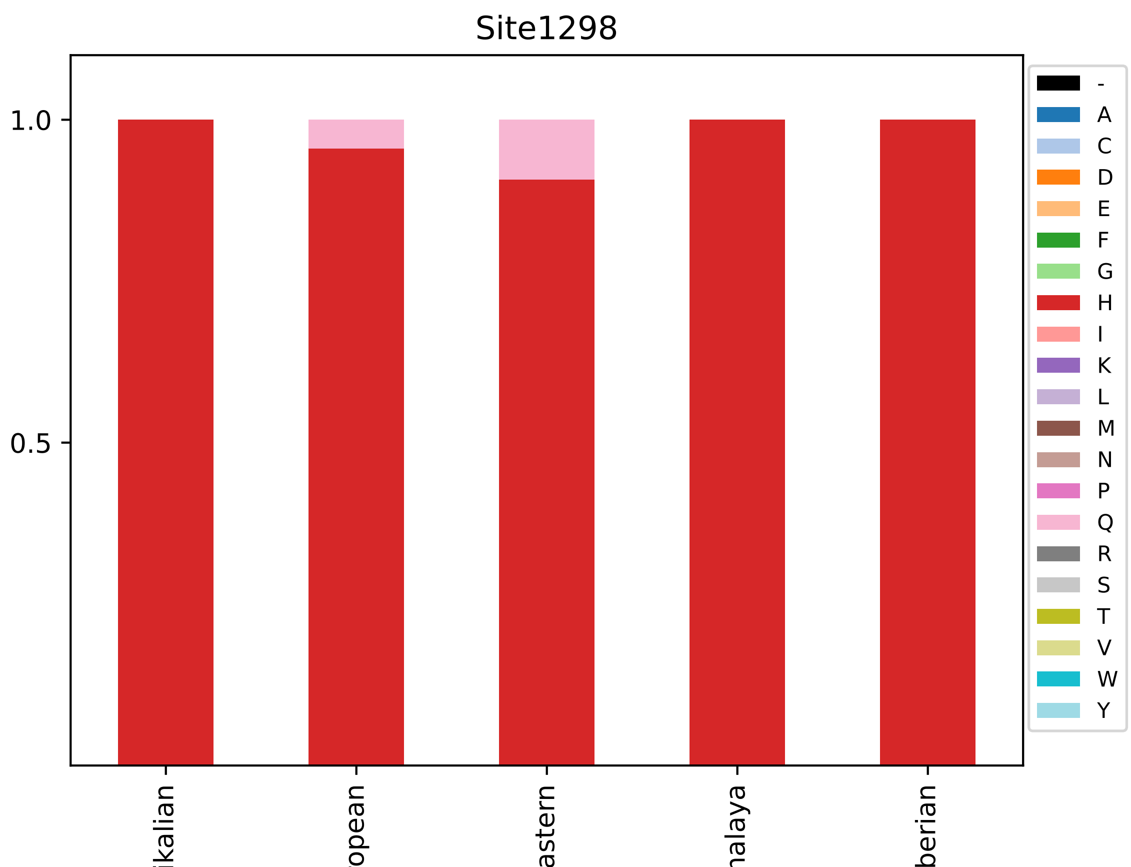
Site1298
1.0
0.5
-
A
C
D
E
F
G
H
I
K
L
M
N
P
Q
R
S
T
V
W
Y
ikalian
opean
astern
nalaya
berian

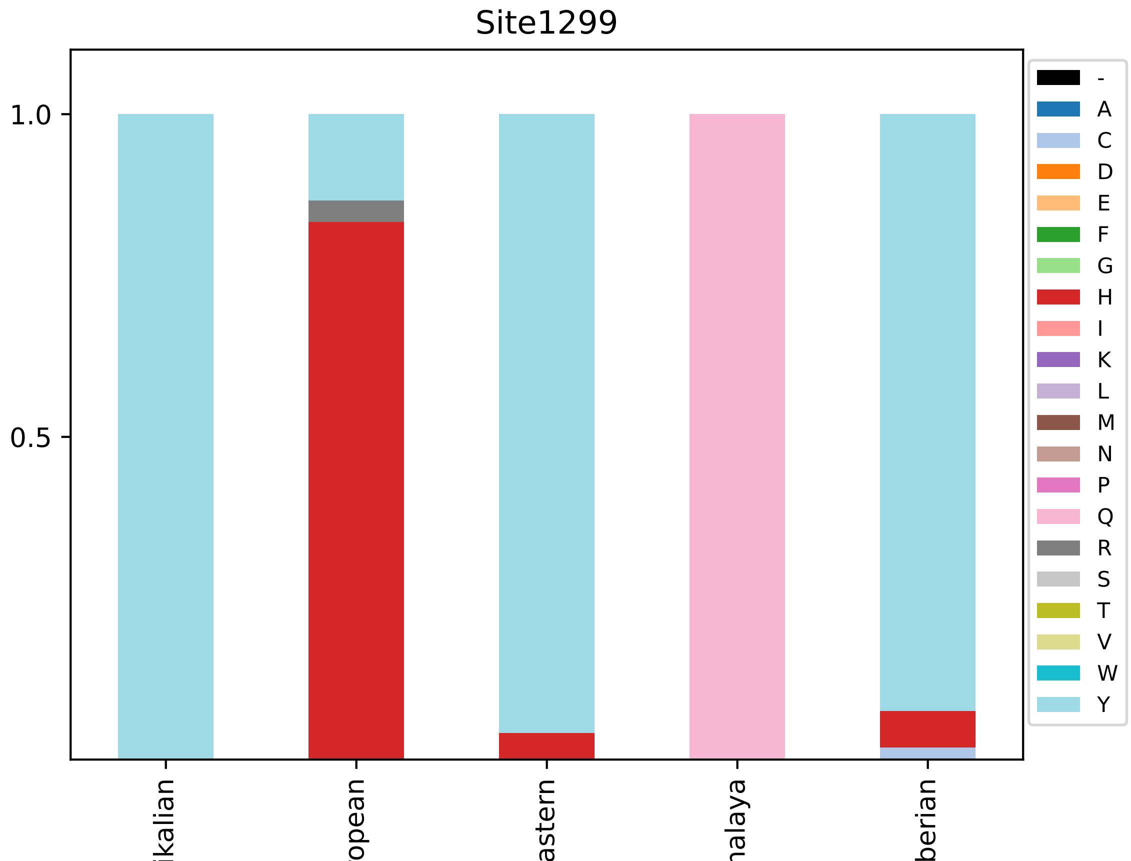

Site1299
1.0
0.5
-
A
C
D
E
F
G
H
I
K
L
M
N
P
Q
R
S
T
V
W
Y
ikalian
opean
astern
nalaya
berian

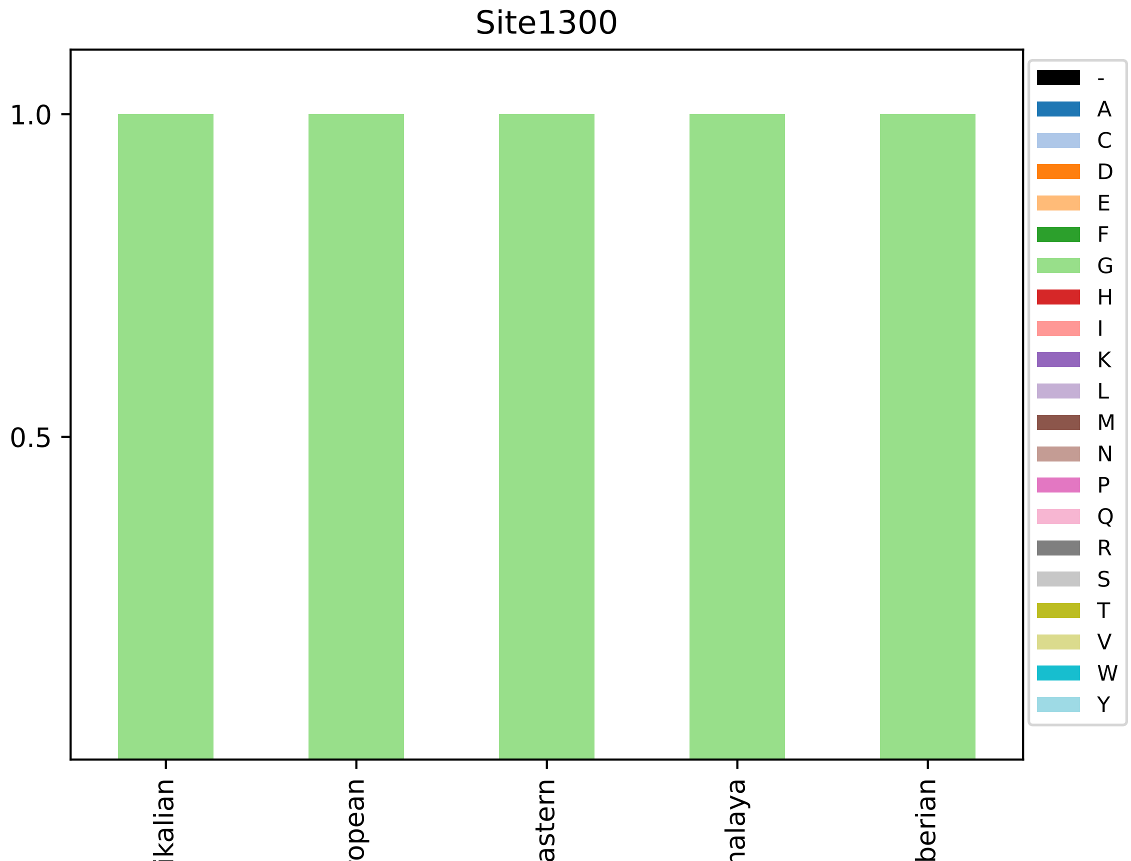

Site1300
1.0
0.5
-
A
C
D
E
F
G
H
I
K
L
M
N
P
Q
R
S
T
V
W
Y
ikalian
opean
astern
nalaya
berian

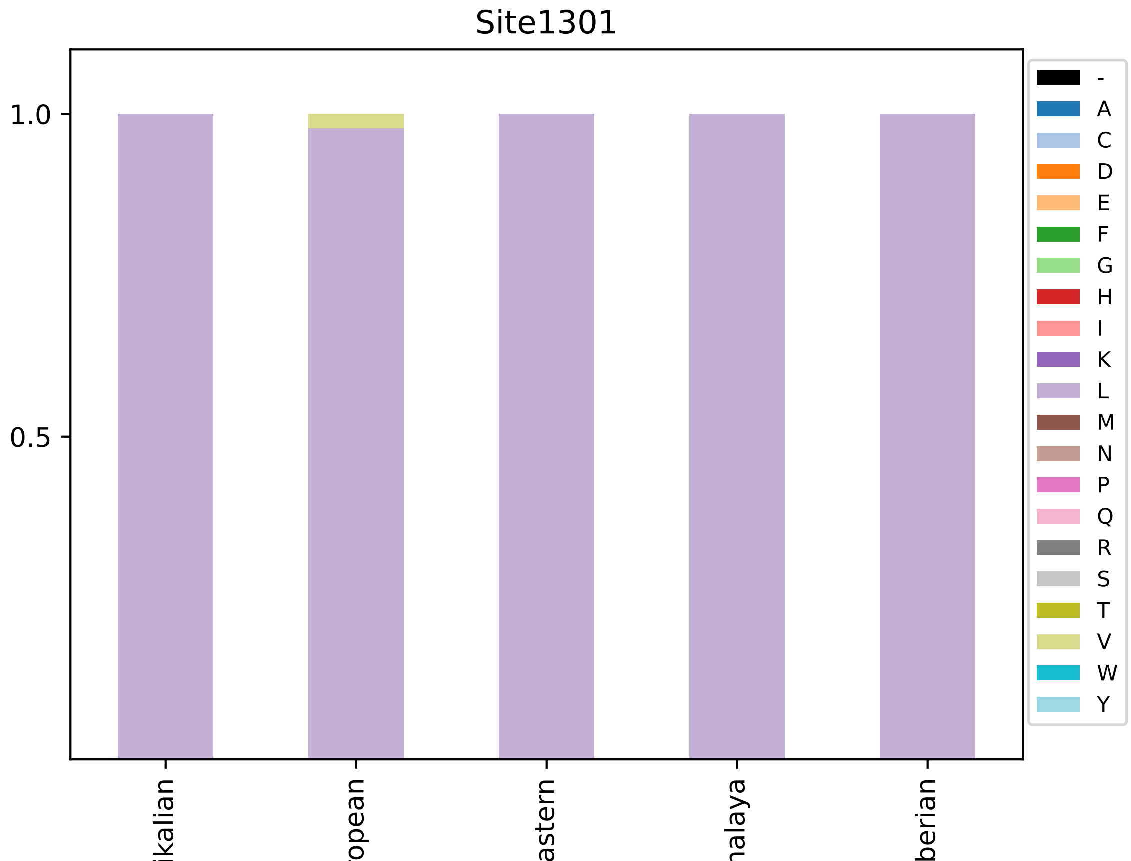
Site1301
1.0
0.5
-
A
C
D
E
F
G
H
I
K
L
M
N
P
Q
R
S
T
V
W
Y
ikalian
opean
astern
nalaya
berian

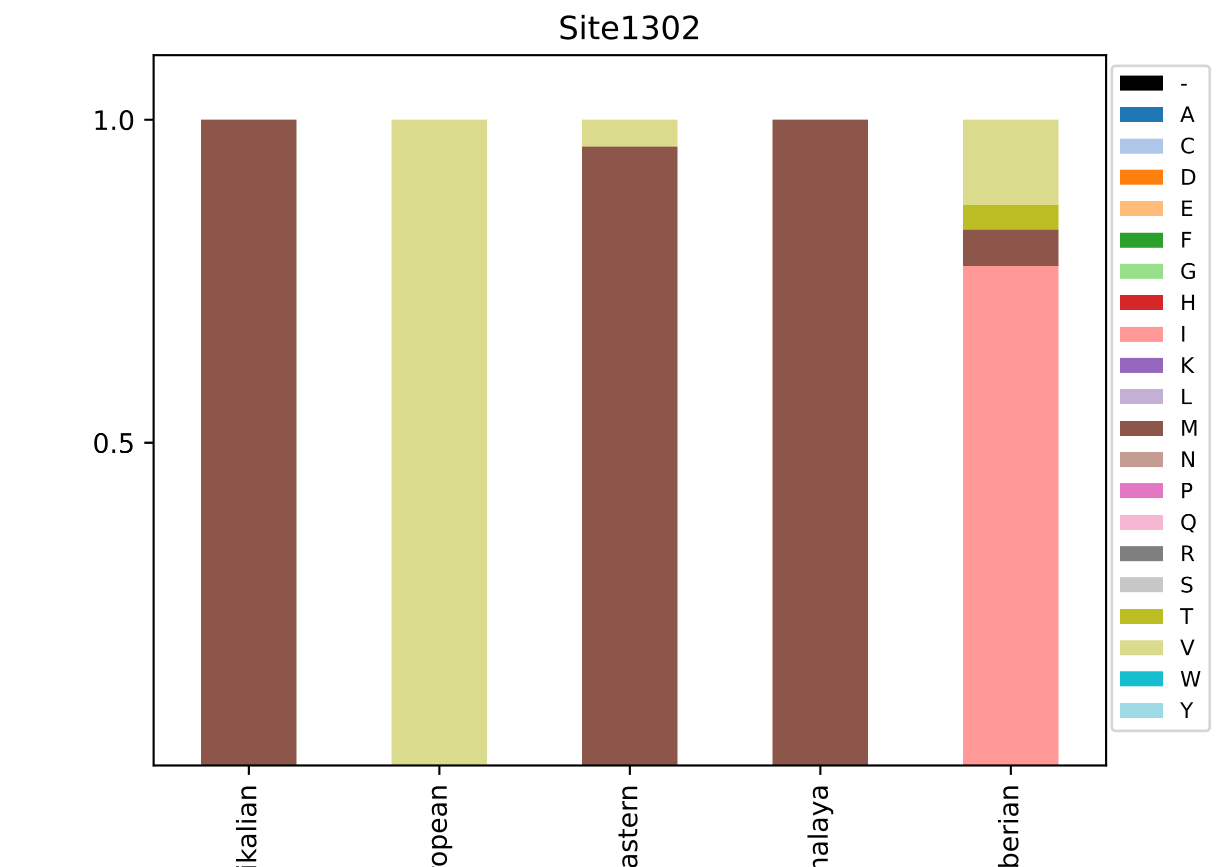

Site1302
1.0
0.5
ikalian
opean
astern
nalaya
berian
-
A
C
D
E
F
G
H
I
K
L
M
N
P
Q
R
S
T
V
W
Y

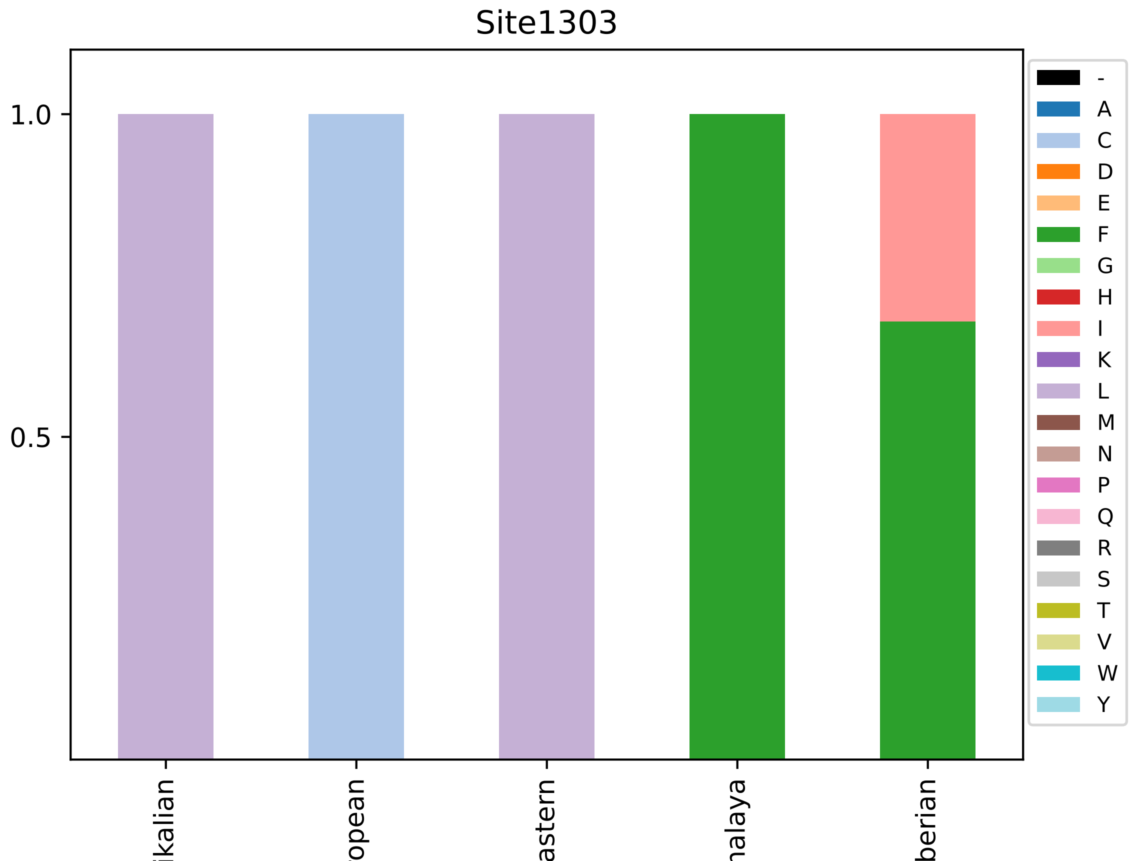
Site1303
1.0
0.5
-
A
C
D
E
F
G
H
I
K
L
M
N
P
Q
R
S
T
V
W
Y
ikalian
opean
astern
nalaya
berian

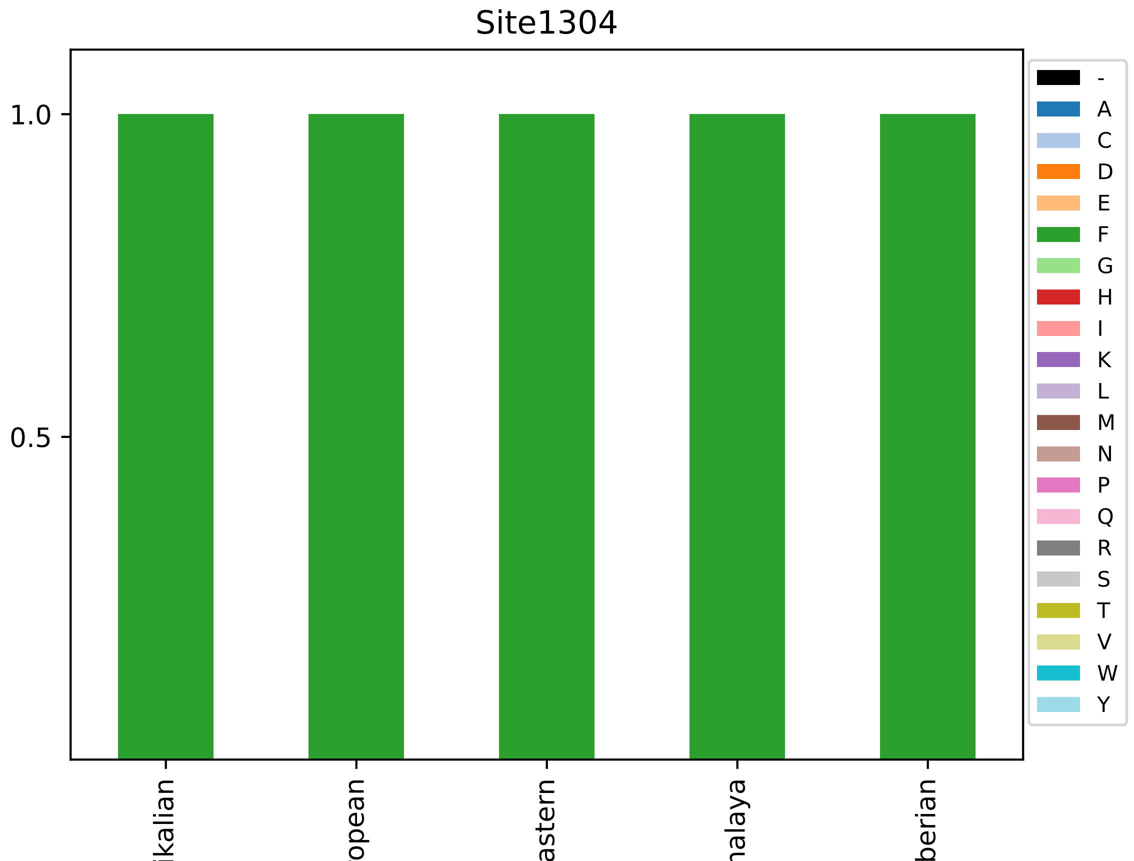
Site1304
1.0
0.5
-
A
C
D
E
F
G
H
I
K
L
M
N
P
Q
R
S
T
V
W
Y
ikalian
opean
astern
nalaya
berian

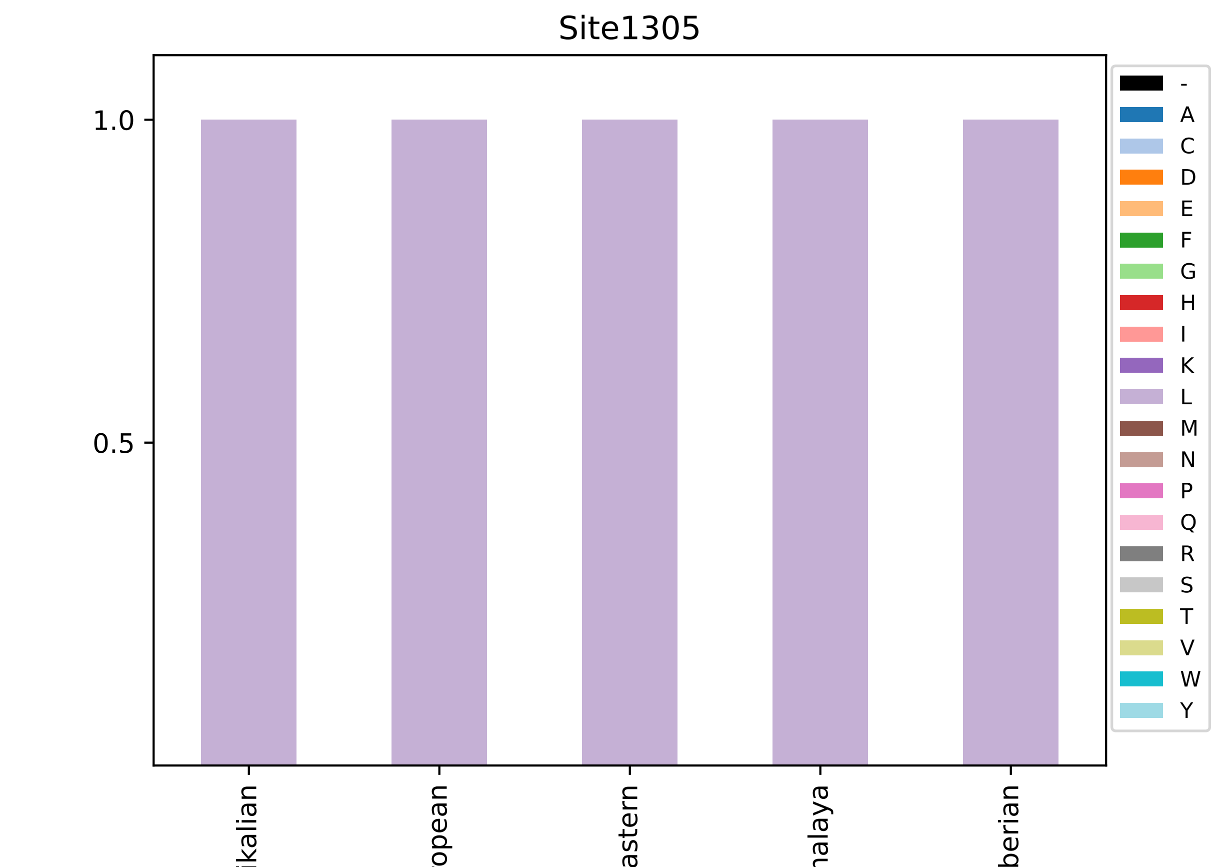

Site1305
1.0
0.5
-
A
C
D
E
F
G
H
I
K
L
M
N
P
Q
R
S
T
V
W
Y
ikalian
opean
astern
nalaya
berian

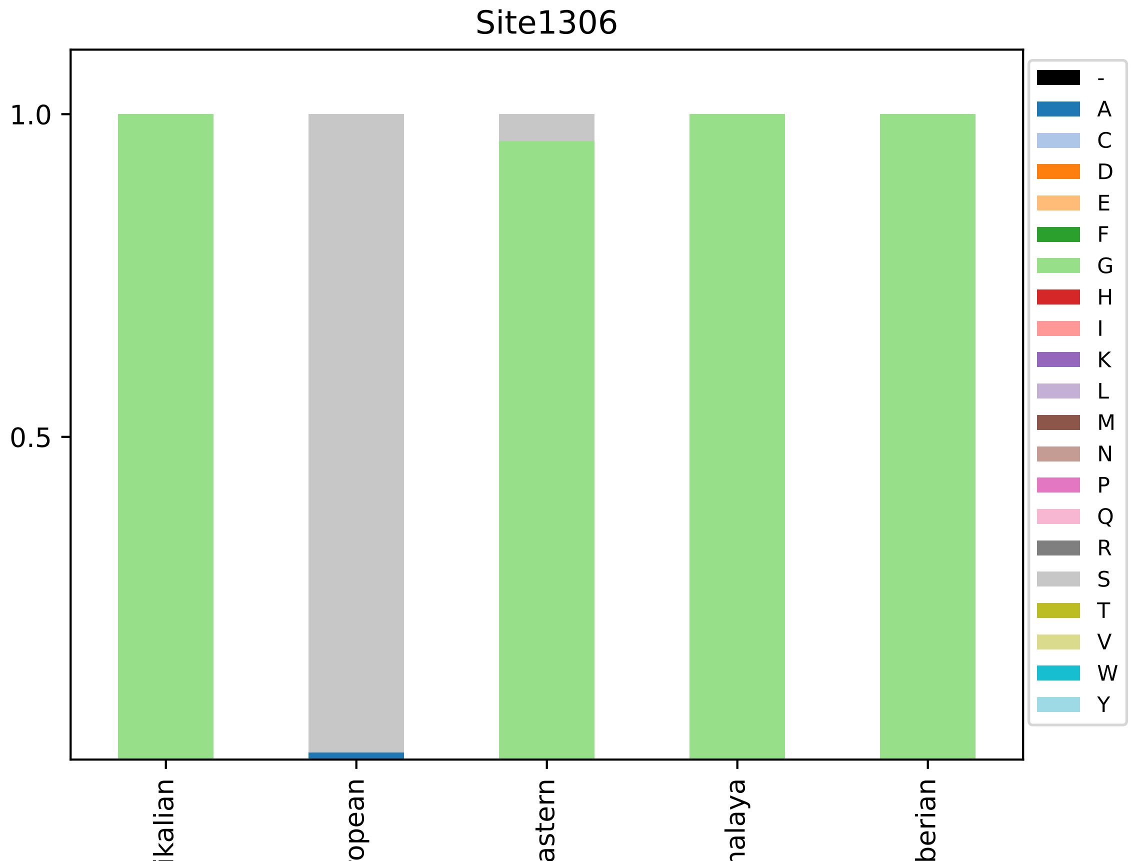

Site1306
1.0
0.5
-
A
C
D
E
F
G
H
I
K
L
M
N
P
Q
R
S
T
V
W
Y
ikalian
opean
astern
nalaya
berian

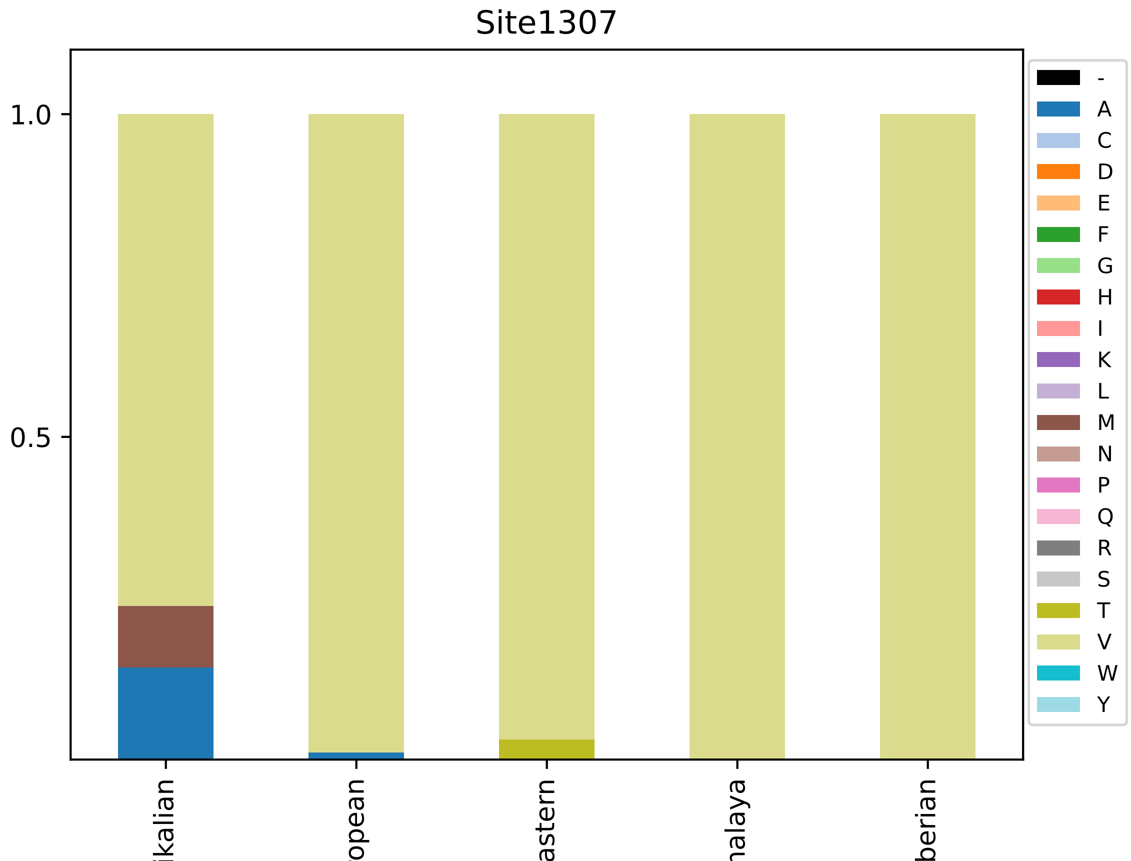

Site1307
1.0
0.5
-
A
C
D
E
F
G
H
I
K
L
M
N
P
Q
R
S
T
V
W
Y
ikalian
opean
astern
nalaya
berian

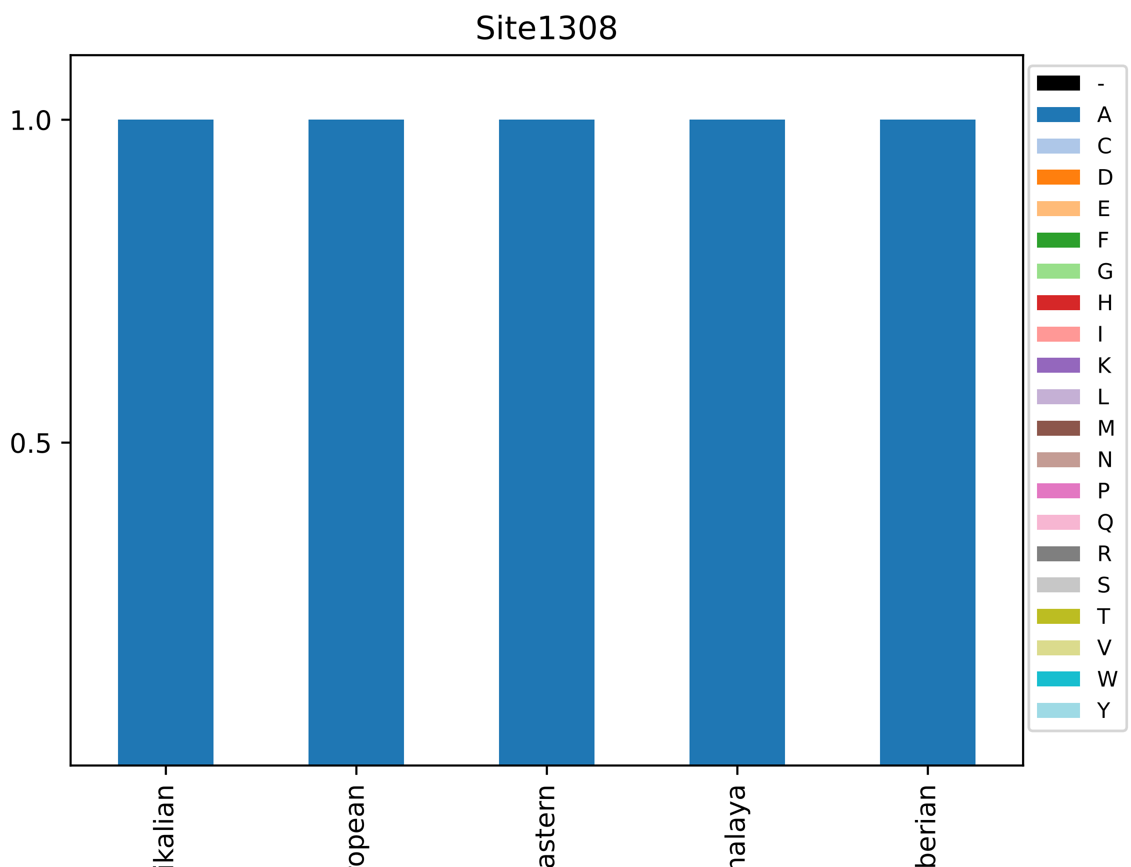

Site1308
1.0
0.5
-
A
C
D
E
F
G
H
I
K
L
M
N
P
Q
R
S
T
V
W
Y
ikalian
opean
astern
nalaya
berian

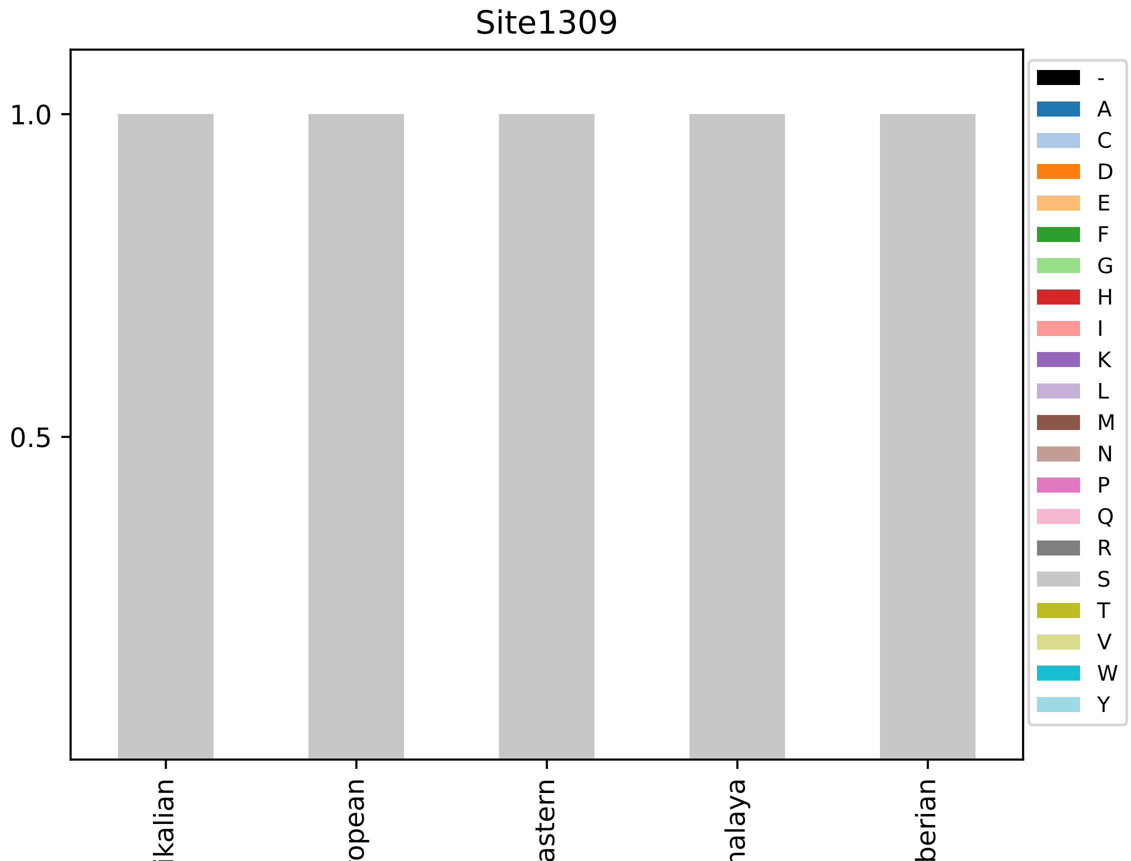

Site1309
1.0
0.5
-
A
C
D
E
F
G
H
I
K
L
M
N
P
Q
R
S
T
V
W
Y
ikalian
opean
astern
nalaya
berian

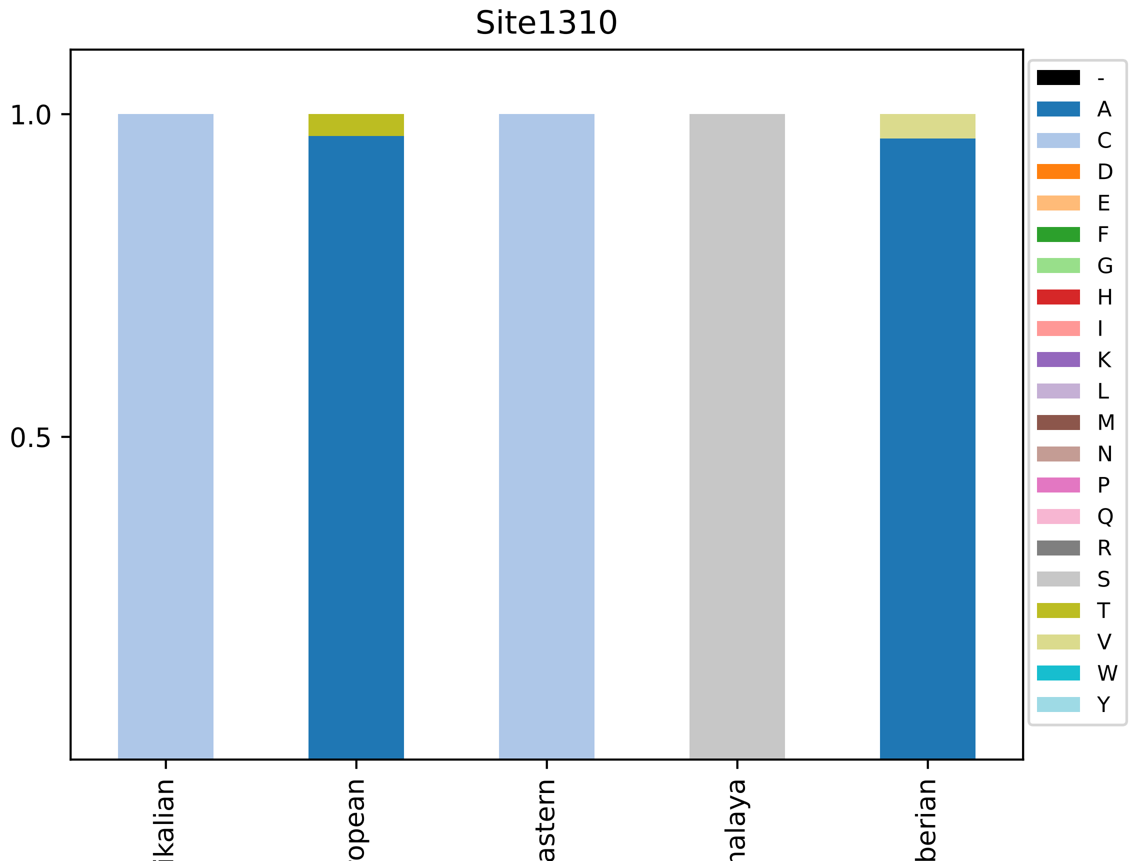

Site1310
1.0
0.5
ikalian
opean
astern
halaya
berian
-
A
C
D
E
F
G
H
I
K
L
M
N
P
Q
R
S
T
V
W
Y

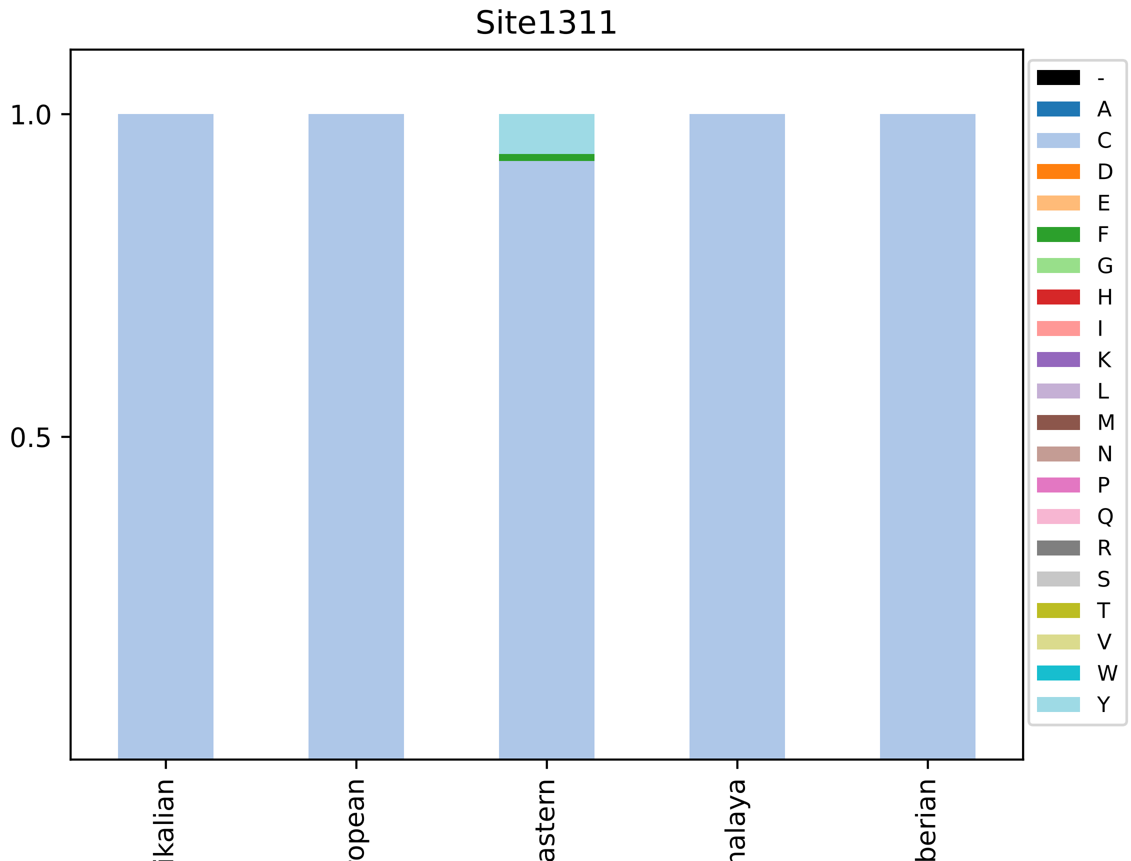

Site1311
1.0
0.5
-
A
C
D
E
F
G
H
I
K
L
M
N
P
Q
R
S
T
V
W
Y
ikalian
opean
astern
nalaya
berian

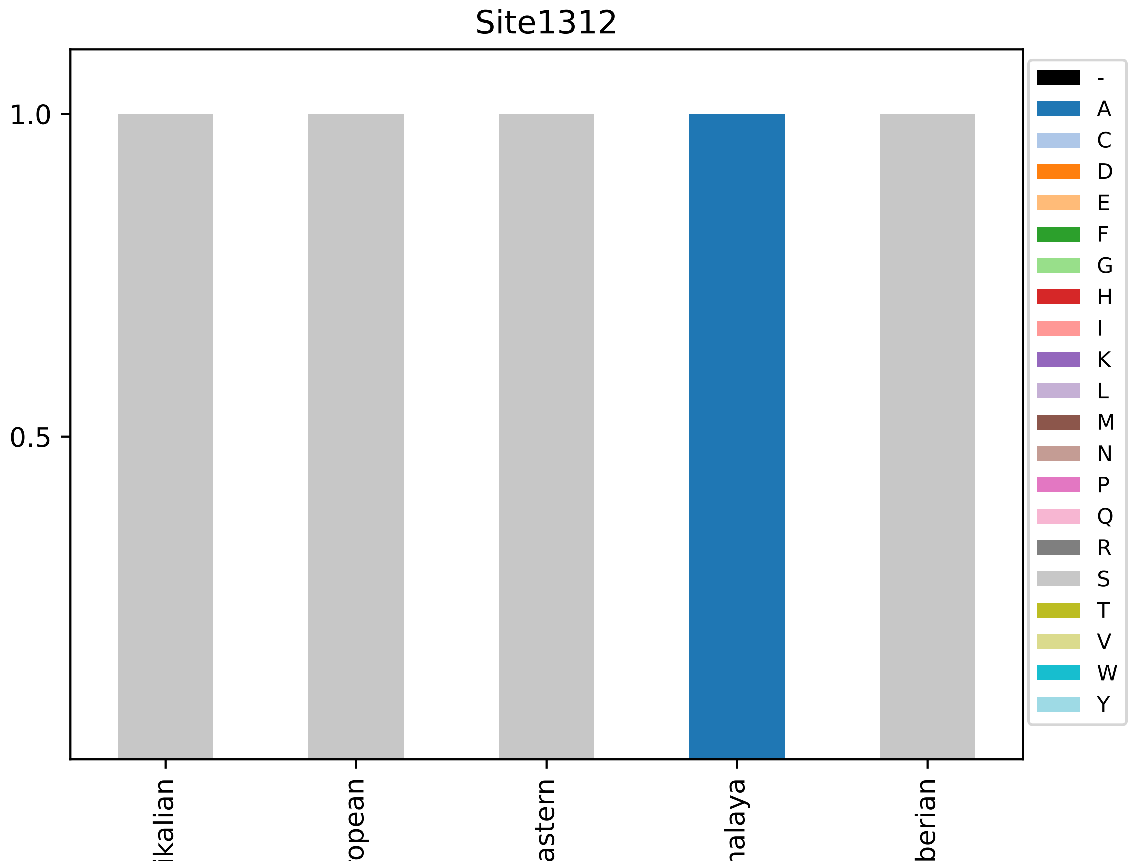

Site1312
1.0
0.5
-
A
C
D
E
F
G
H
I
K
L
M
N
P
Q
R
S
T
V
W
Y
ikalian
opean
astern
nalaya
berian

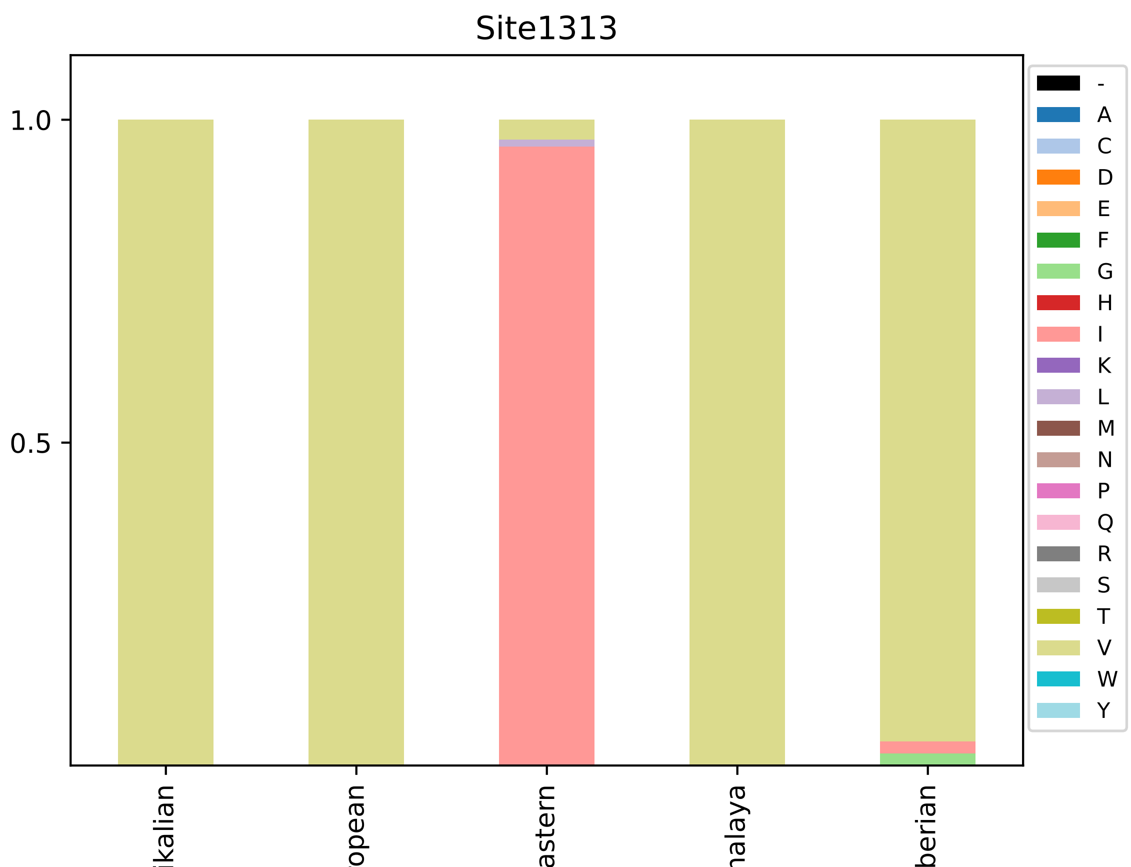
Site1313
1.0
0.5
ikalian
opean
astern
nalaya
berian
-
A
C
D
E
F
G
H
I
K
L
M
N
P
Q
R
S
T
V
W
Y

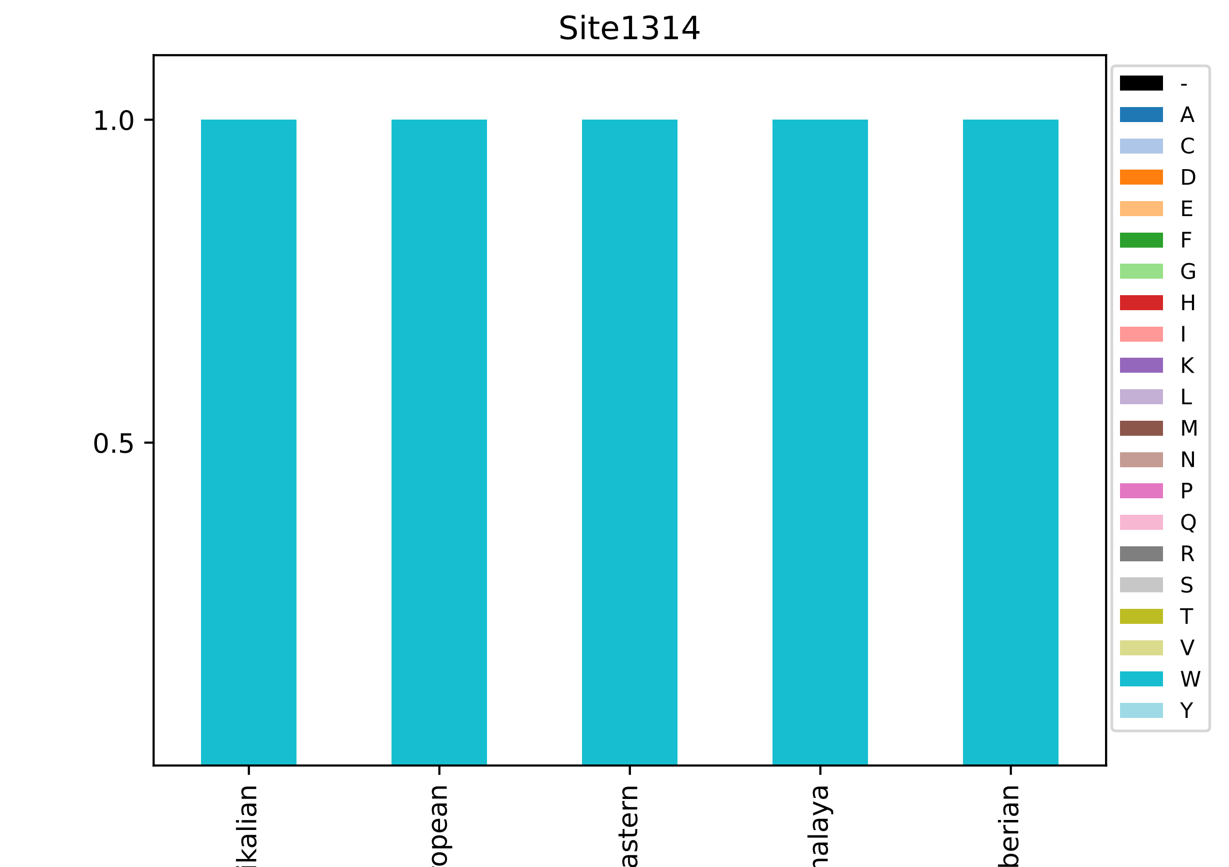

Site1314
1.0
0.5
-
A
C
D
E
F
G
H
I
K
L
M
N
P
Q
R
S
T
V
W
Y
ikalian
opean
astern
nalaya
berian

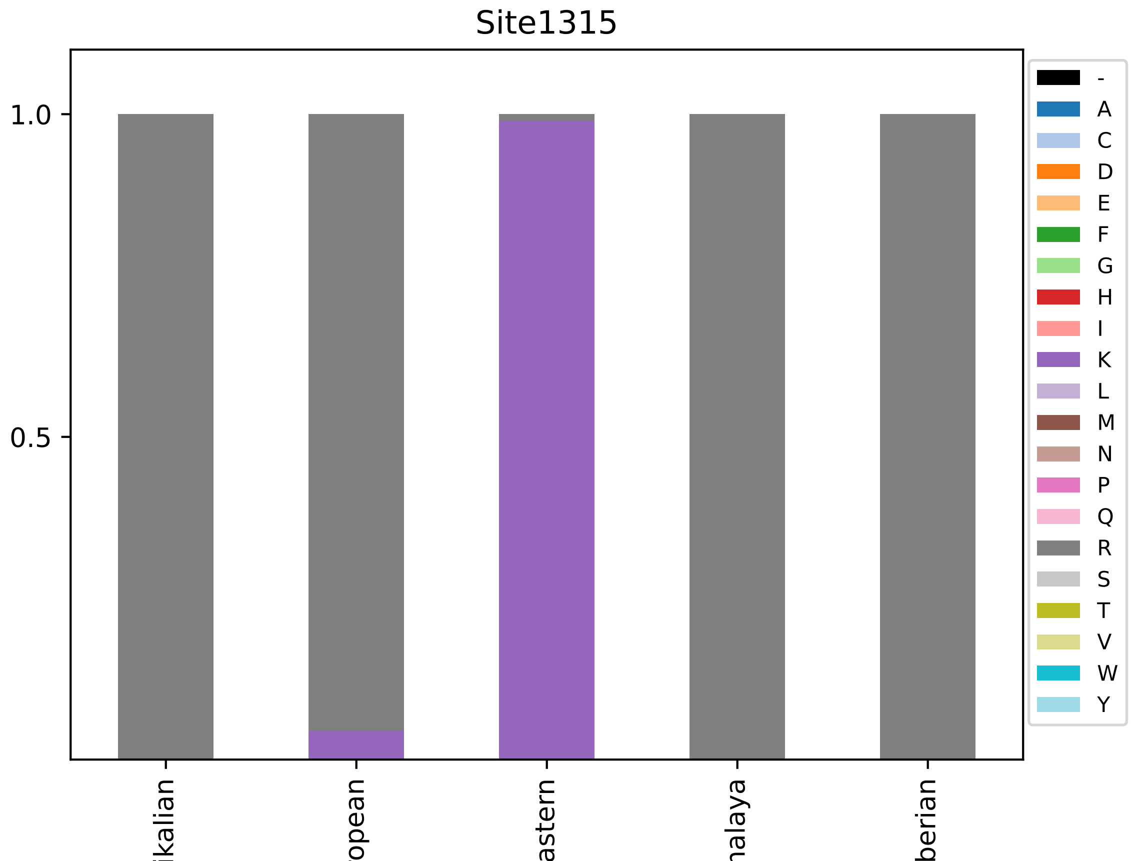

Site1315
1.0
0.5
-
A
C
D
E
F
G
H
I
K
L
M
N
P
Q
R
S
T
V
W
Y
ikalian
opean
astern
nalaya
berian

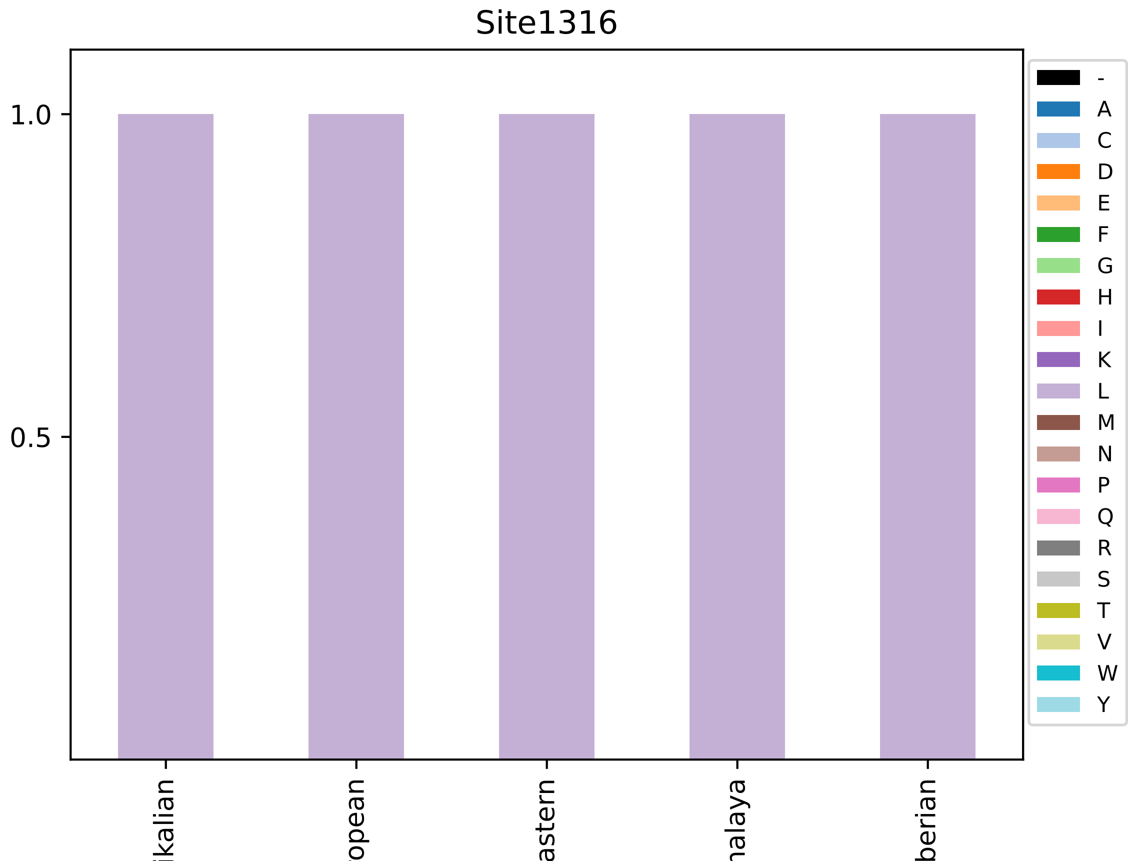
Site1316
1.0
0.5
-
A
C
D
E
F
G
H
I
K
L
M
N
P
Q
R
S
T
V
W
Y

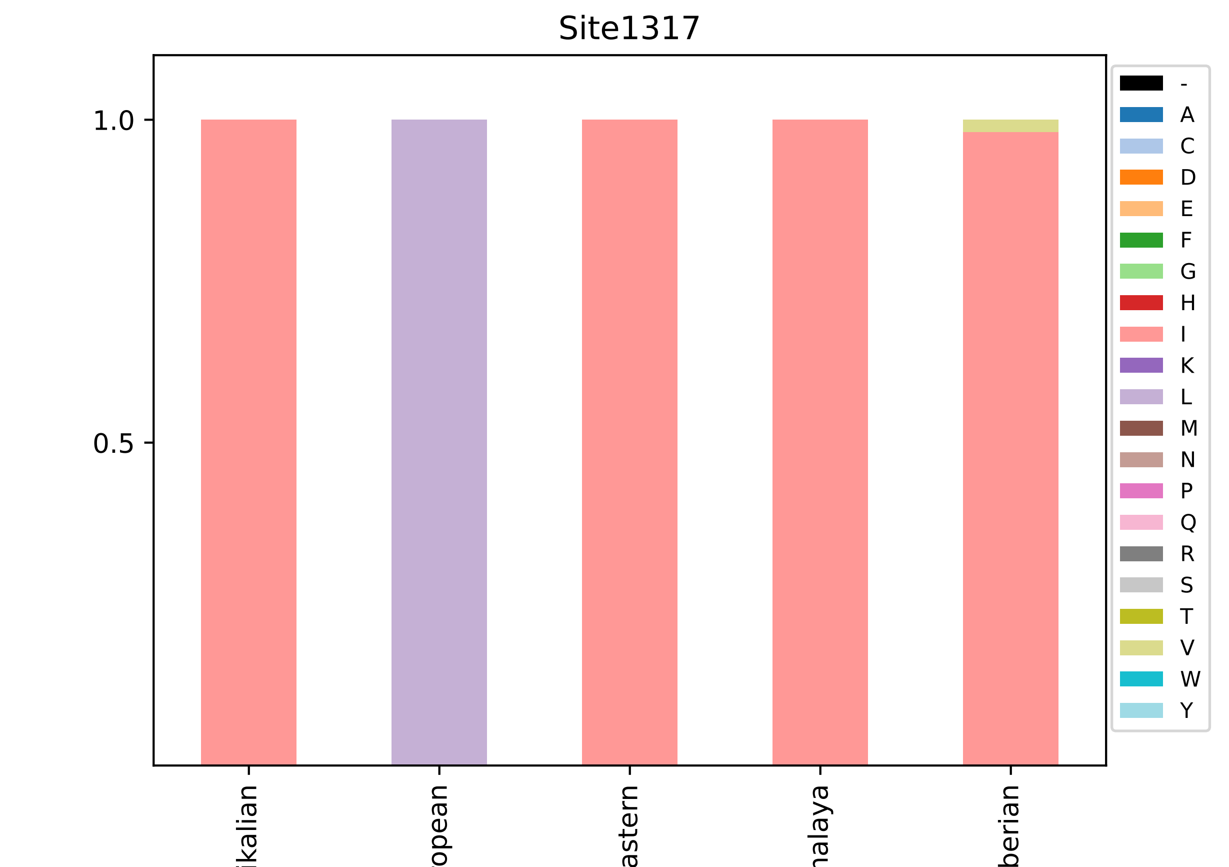

Site1317
1.0
0.5
-
A
C
D
E
F
G
H
I
K
L
M
N
P
Q
R
S
T
V
W
Y
ikalian
opean
astern
nalaya
berian

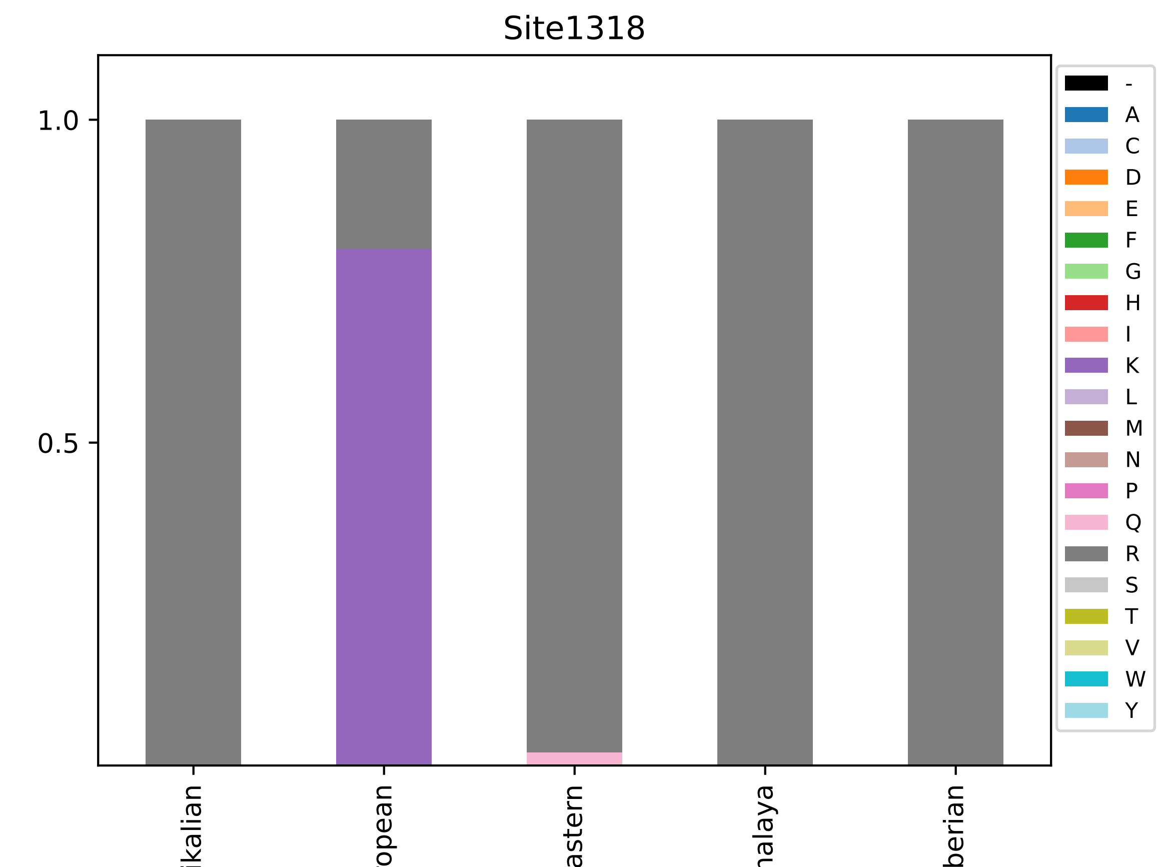

Site1318
1.0
0.5
-
A
C
D
E
F
G
H
I
K
L
M
N
P
Q
R
S
T
V
W
Y
ikalian
opean
astern
nalaya
berian

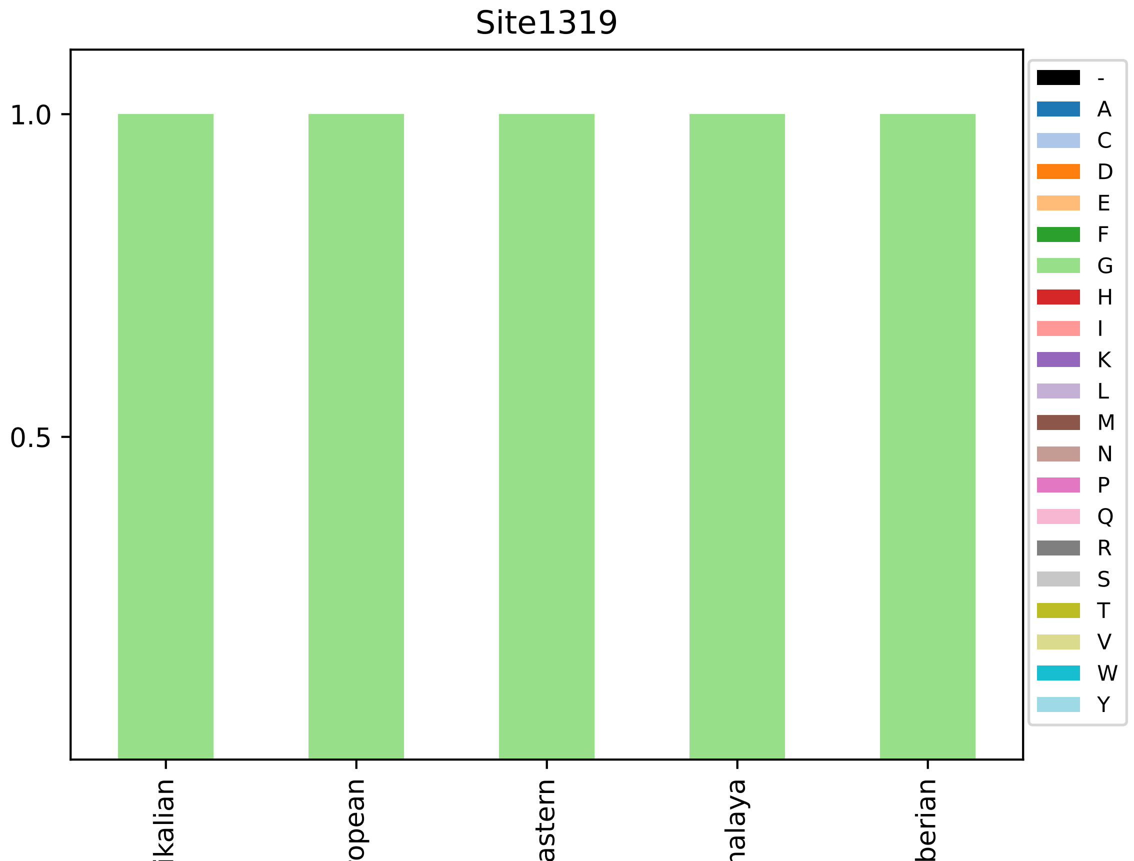
Site1319
1.0
0.5
-
A
C
D
E
F
G
H
I
K
L
M
N
P
Q
R
S
T
V
W
Y
ikalian
opean
astern
nalaya
berian

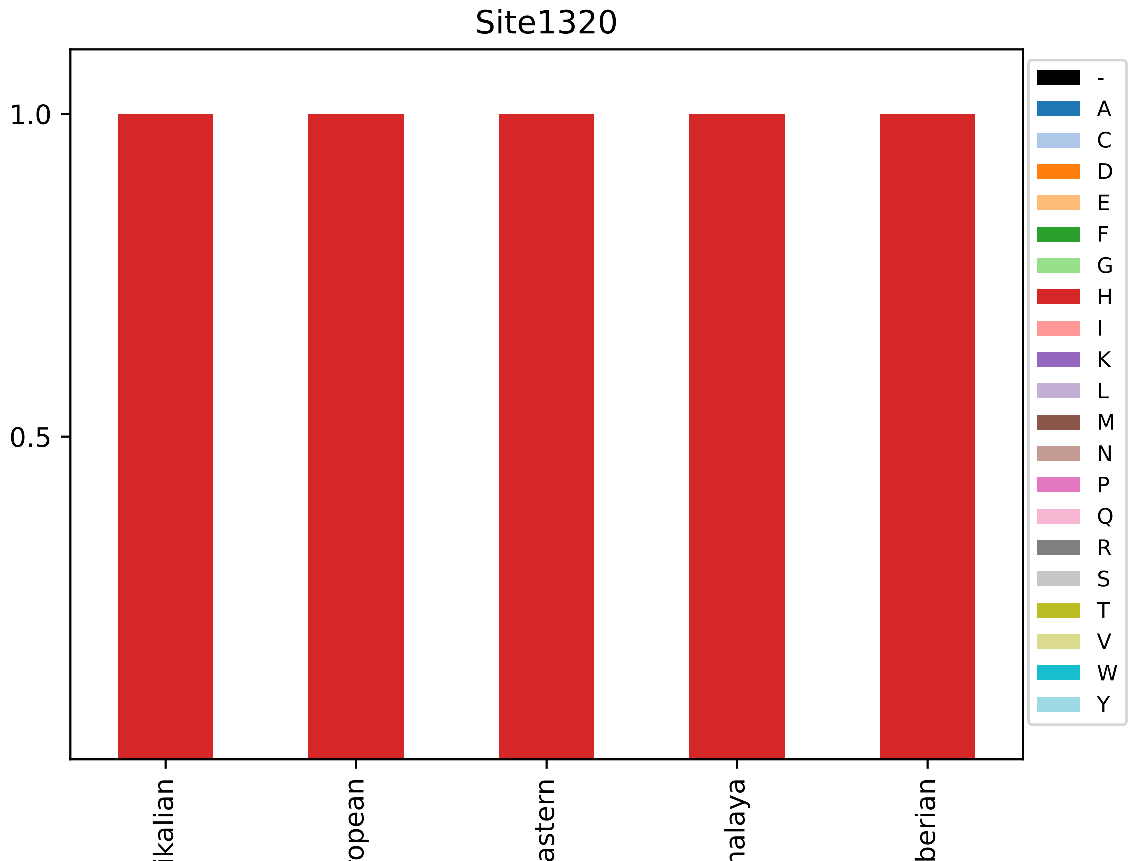

Site1320
1.0
0.5
-
A
C
D
E
F
G
H
I
K
L
M
N
P
Q
R
S
T
V
W
Y
ikalian
opean
astern
nalaya
berian

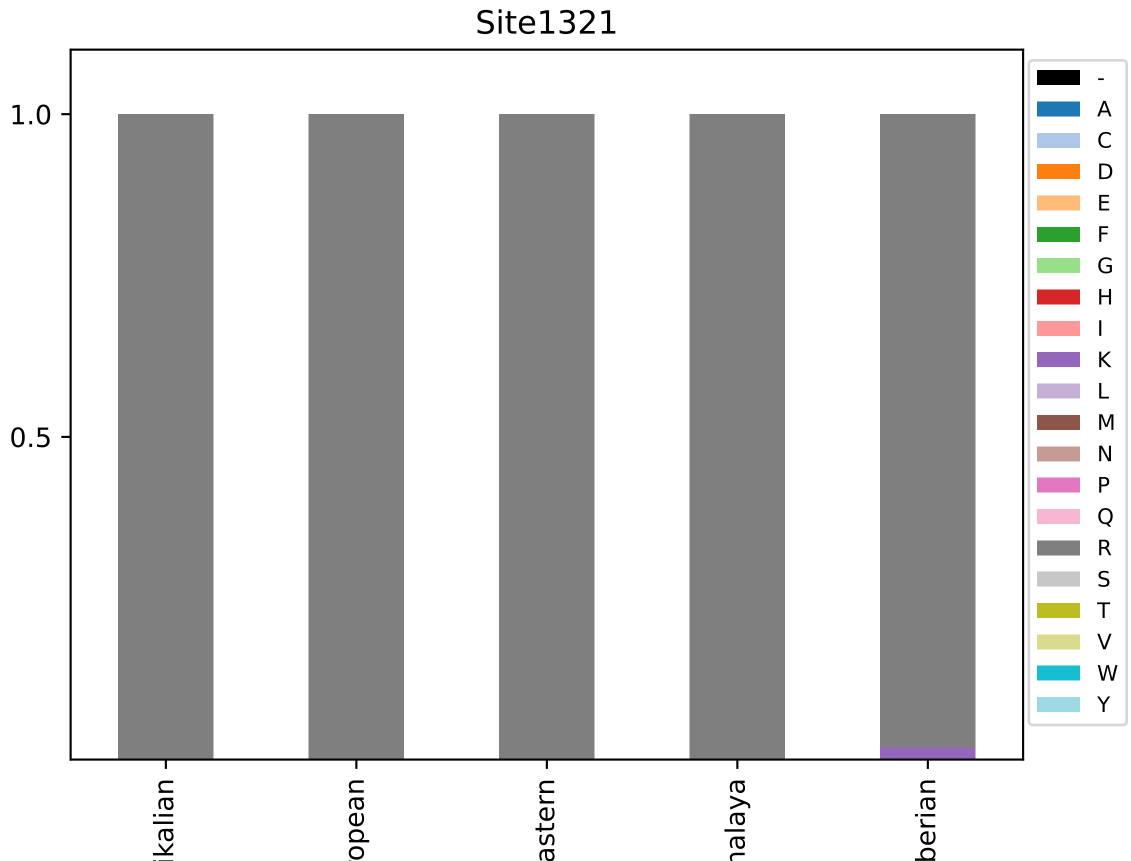
Site1321
1.0
0.5
-
A
C
D
E
F
G
H
I
K
L
M
N
P
Q
R
S
T
V
W
Y

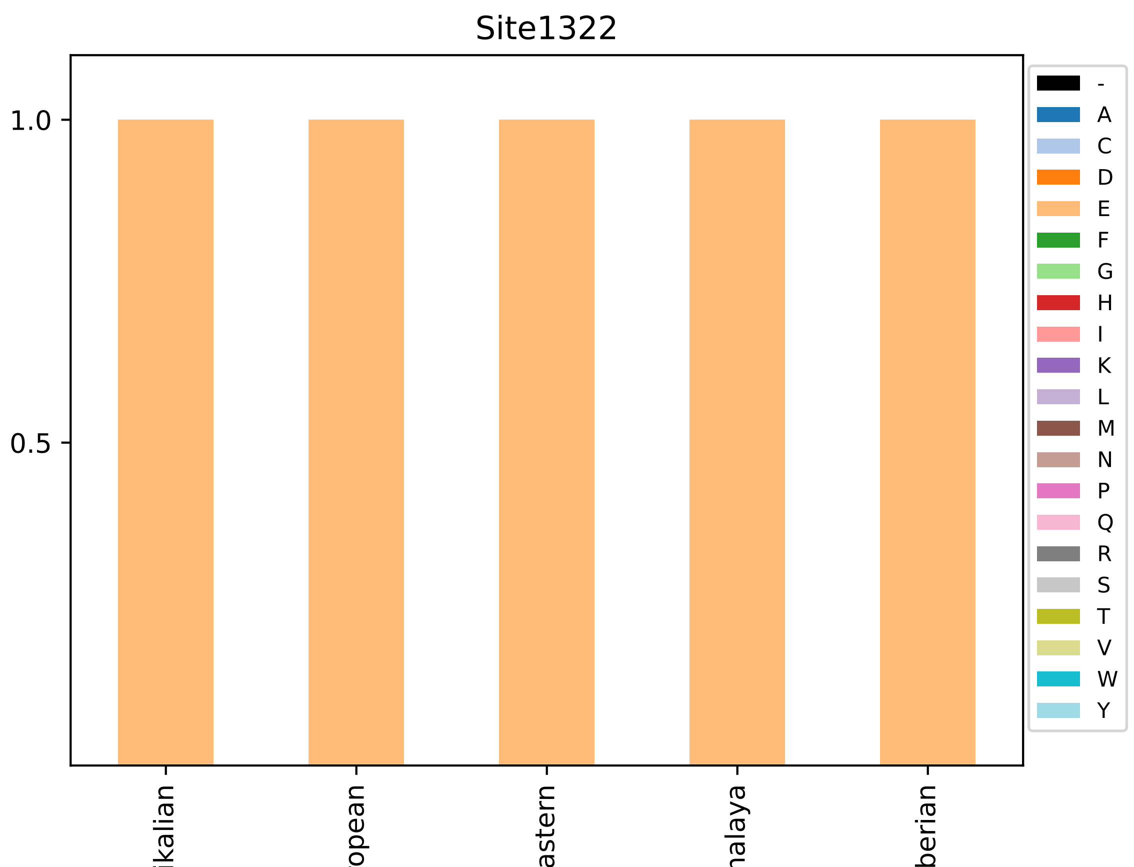

Site1322
1.0
0.5
-
A
C
D
E
F
G
H
I
K
L
M
N
P
Q
R
S
T
V
W
Y
ikalian
opean
astern
halaya
berian

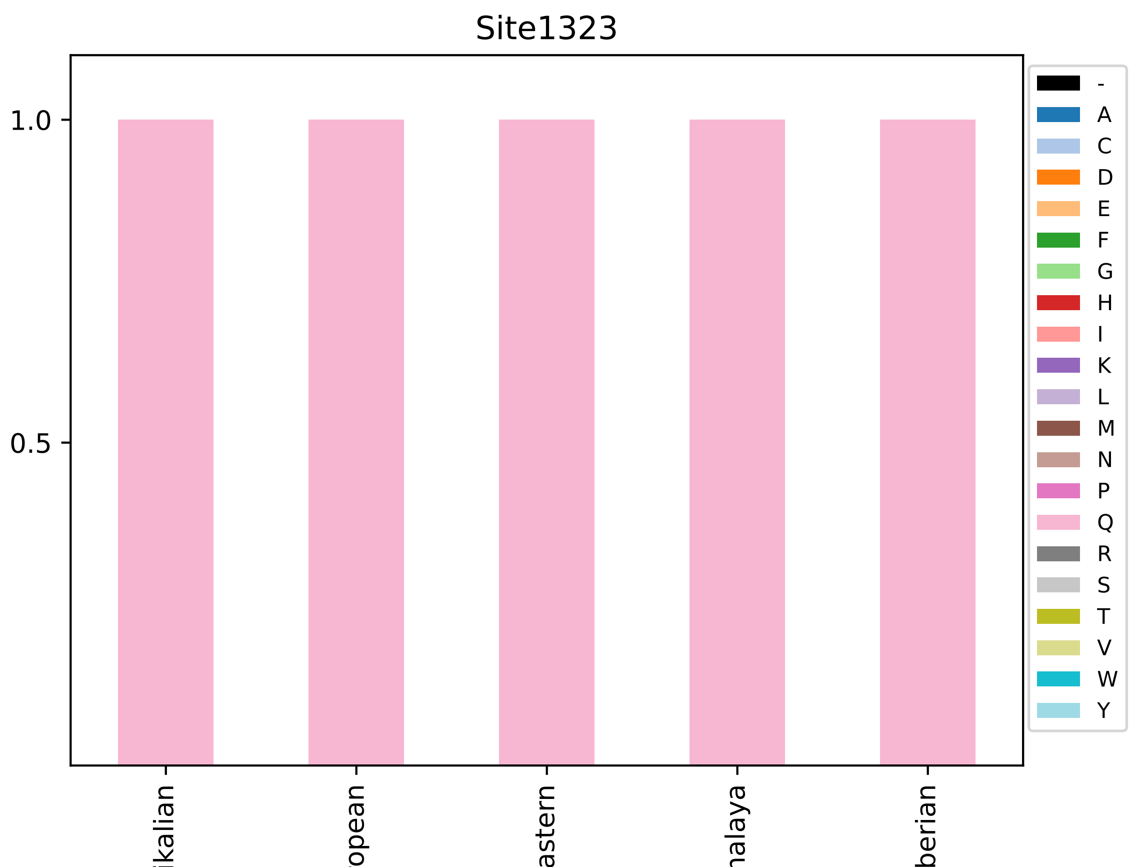

Site1323
1.0
0.5
-
A
C
D
E
F
G
H
I
K
L
M
N
P
Q
R
S
T
V
W
Y
ikalian
opean
astern
nalaya
berian

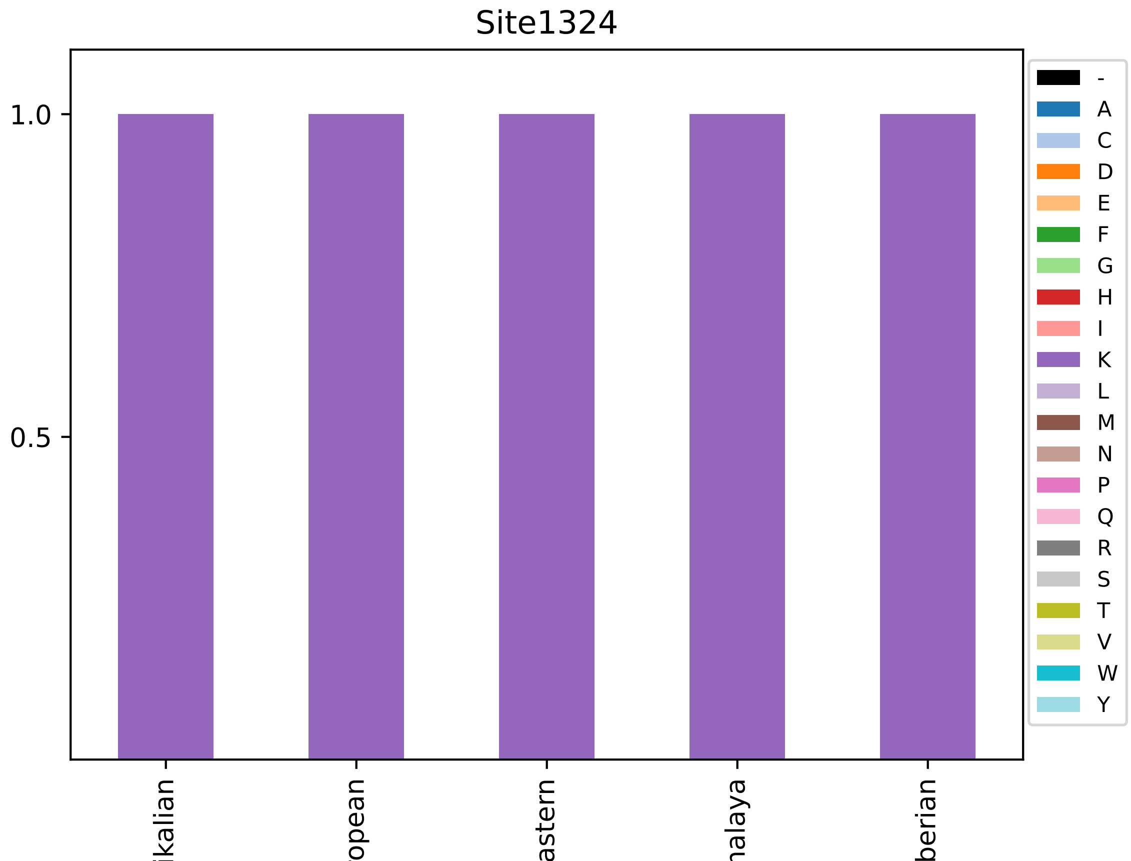

Site1324
1.0
0.5
-
A
C
D
E
F
G
H
I
K
L
M
N
P
Q
R
S
T
V
W
Y
ikalian
opean
astern
nalaya
berian

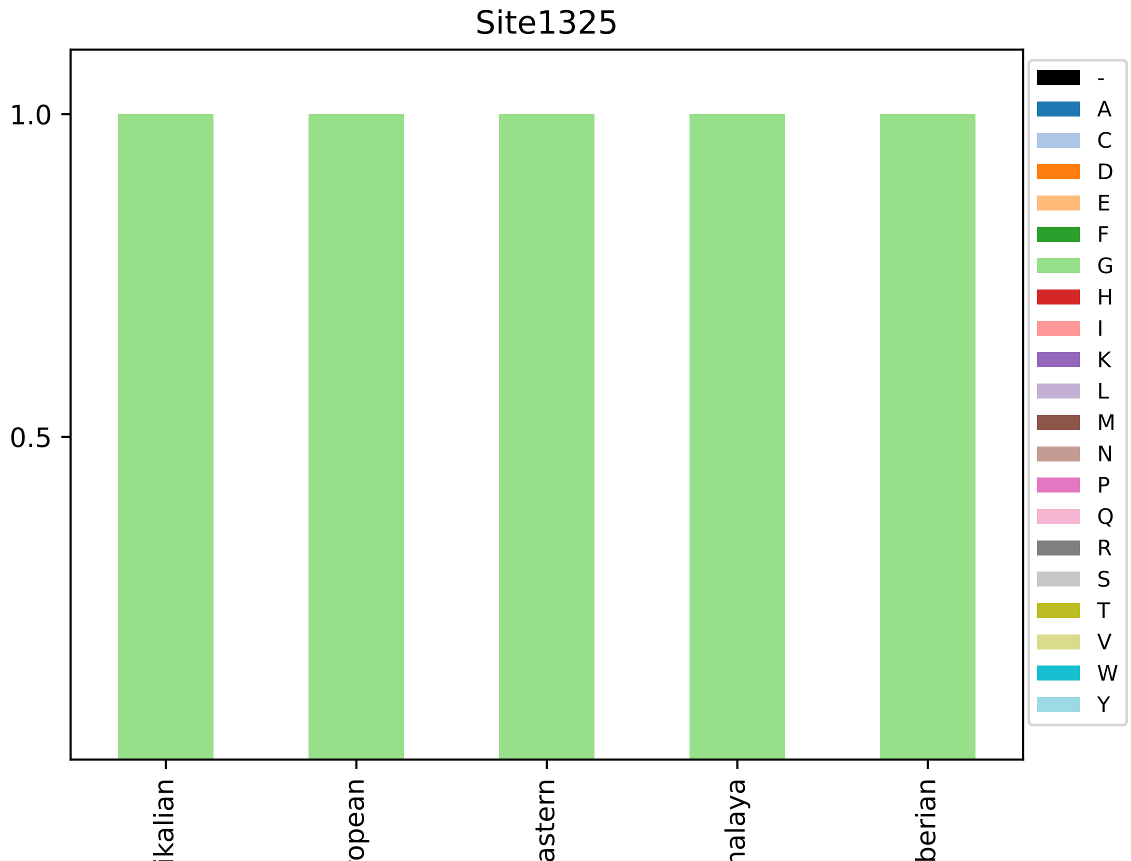

Site1325
1.0
0.5
-
A
C
D
E
F
G
H
I
K
L
M
N
P
Q
R
S
T
V
W
Y
ikalian
opean
astern
nalaya
berian

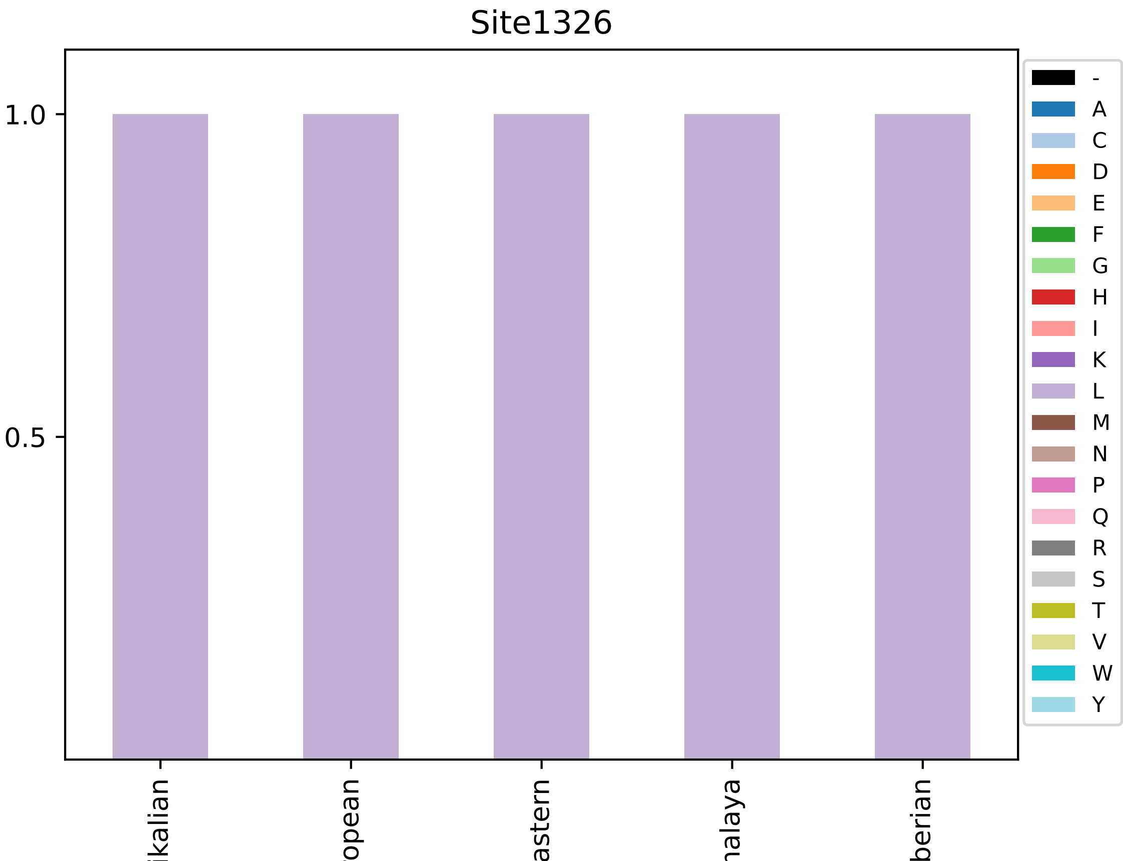

Site1326
1.0
0.5
-
A
C
D
E
F
G
H
I
K
L
M
N
P
Q
R
S
T
V
W
Y
ikalian
opean
astern
nalaya
berian

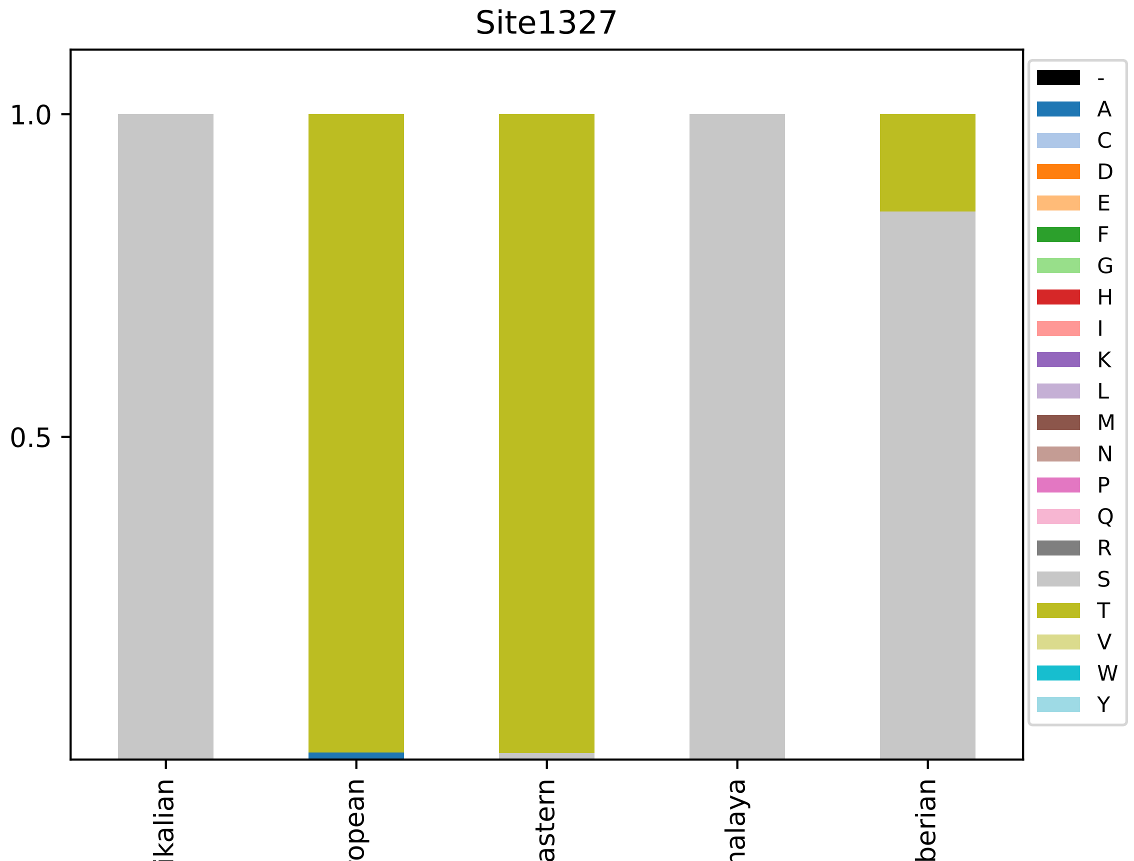

Site1327
1.0
0.5
ikalian
opean
astern
nalaya
berian
-
A
C
D
E
F
G
H
I
K
L
M
N
P
Q
R
S
T
V
W
Y

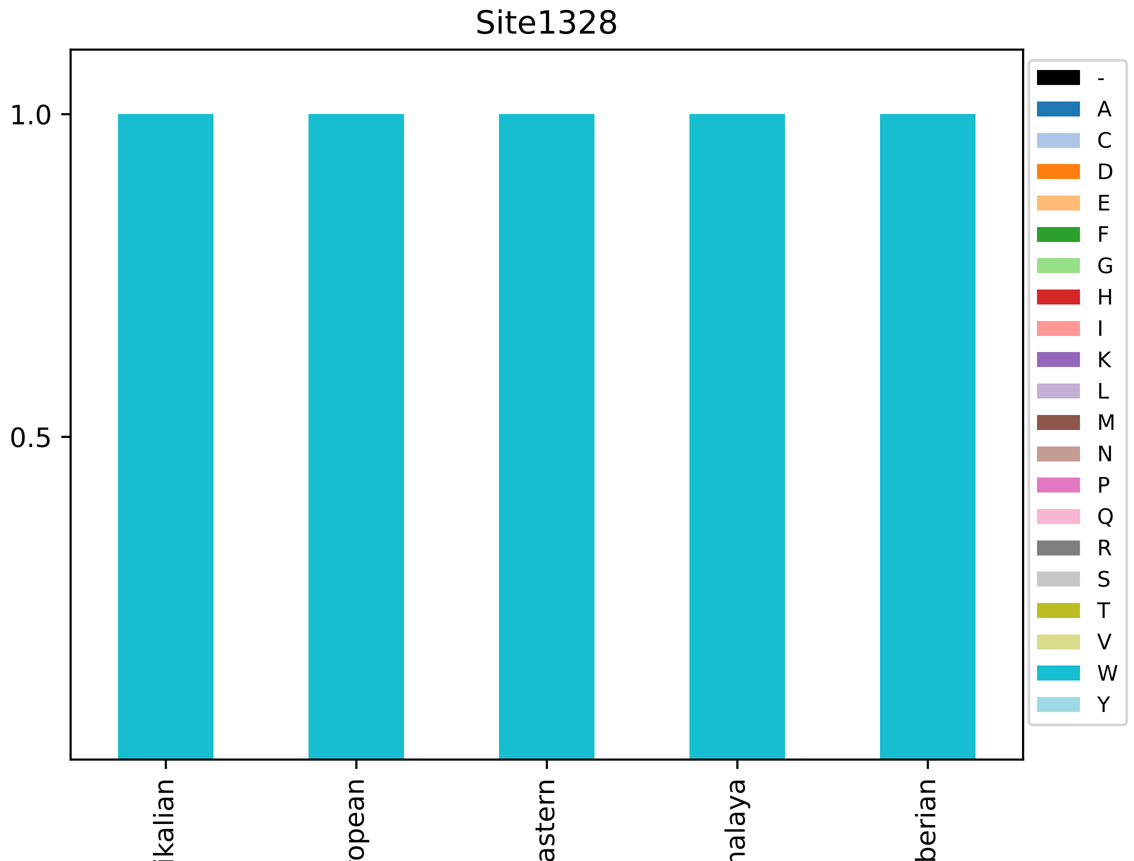
Site1328
1.0
0.5
ikalian
opean
astern
nalaya
berian
-
A
C
D
E
F
G
H
I
K
L
M
N
P
Q
R
S
T
V
W
Y

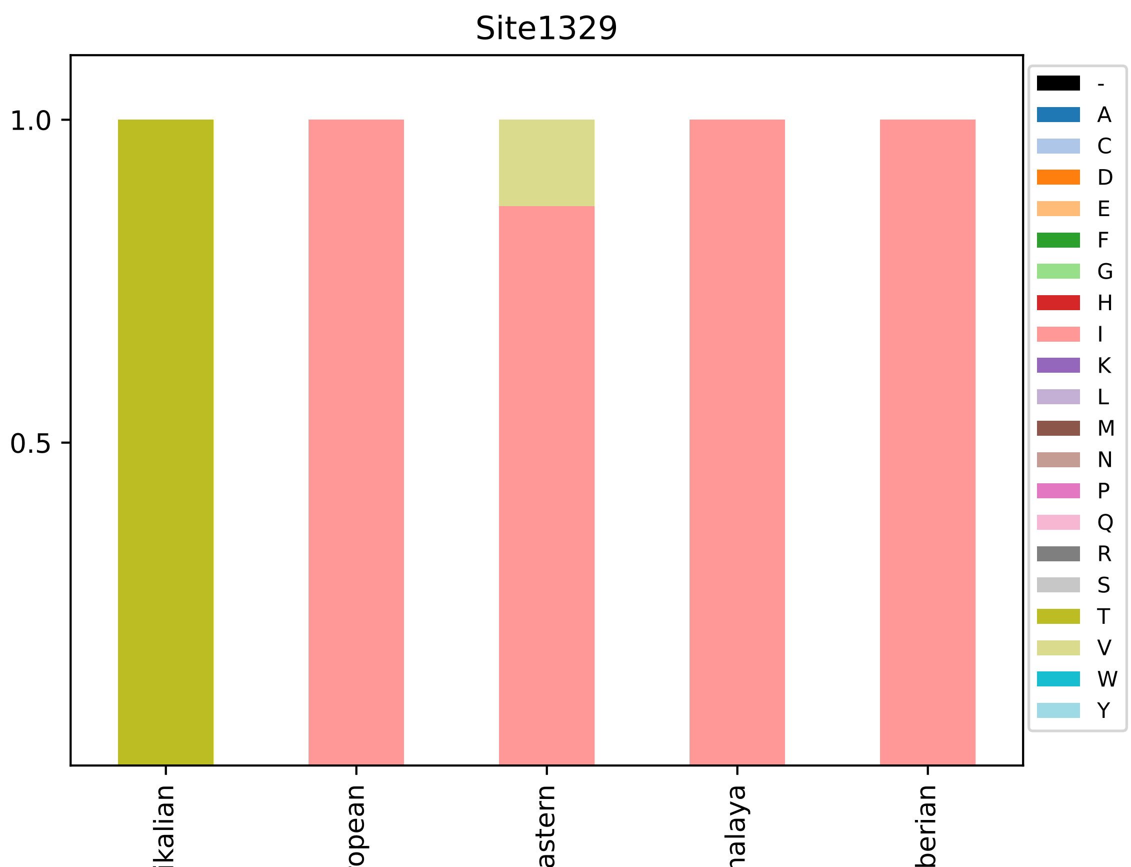
Site1329
1.0
0.5
-
A
C
D
E
F
G
H
I
K
L
M
N
P
Q
R
S
T
V
W
Y
ikalian
opean
astern
nalaya
berian

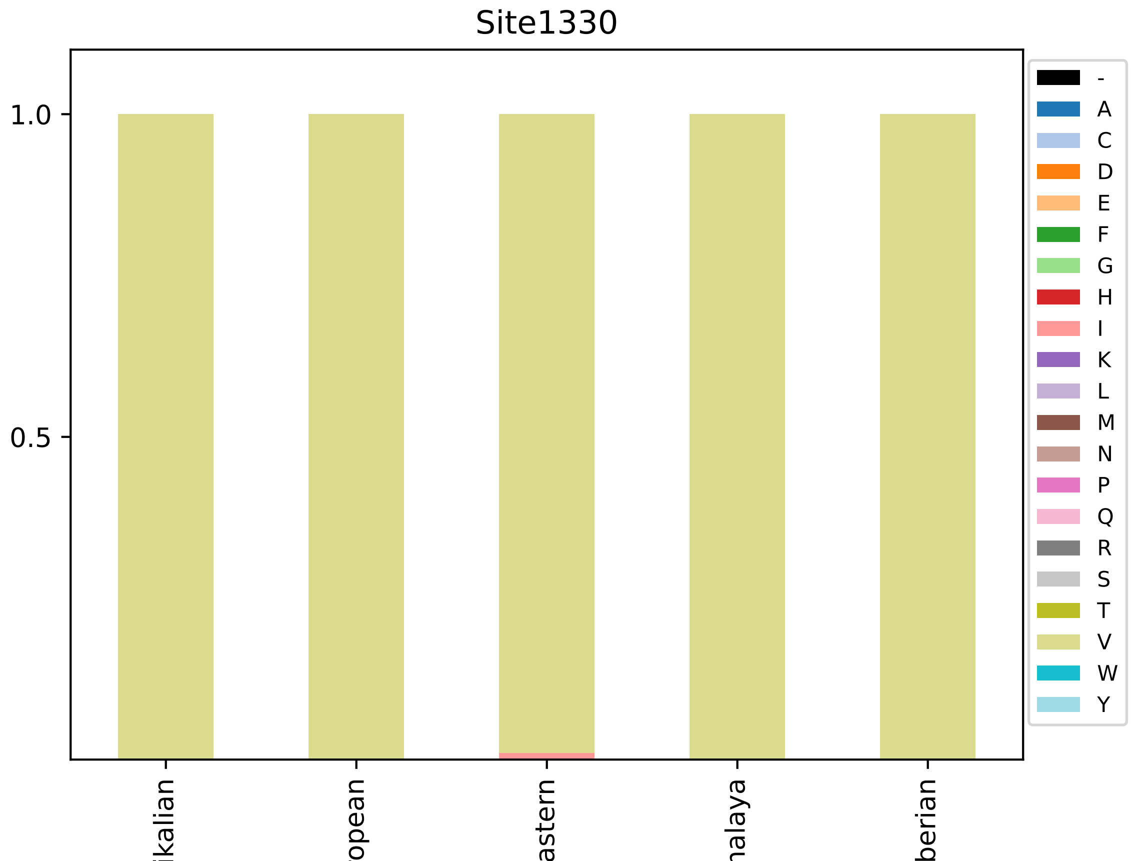
Site1330
1.0
0.5
-
A
C
D
E
F
G
H
I
K
L
M
N
P
Q
R
S
T
V
W
Y
ikalian
opean
astern
nalaya
berian

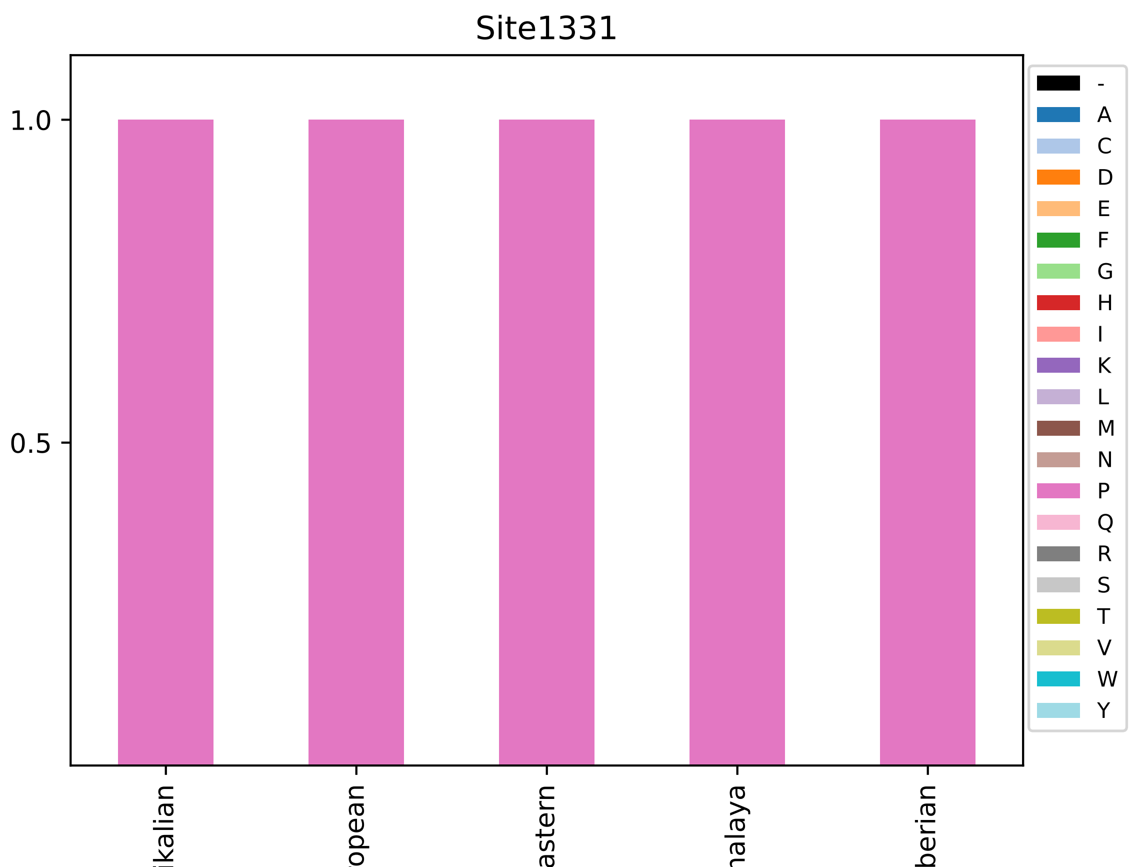

Site1331
1.0
0.5
-
A
C
D
E
F
G
H
I
K
L
M
N
P
Q
R
S
T
V
W
Y
ikalian
opean
astern
nalaya
berian

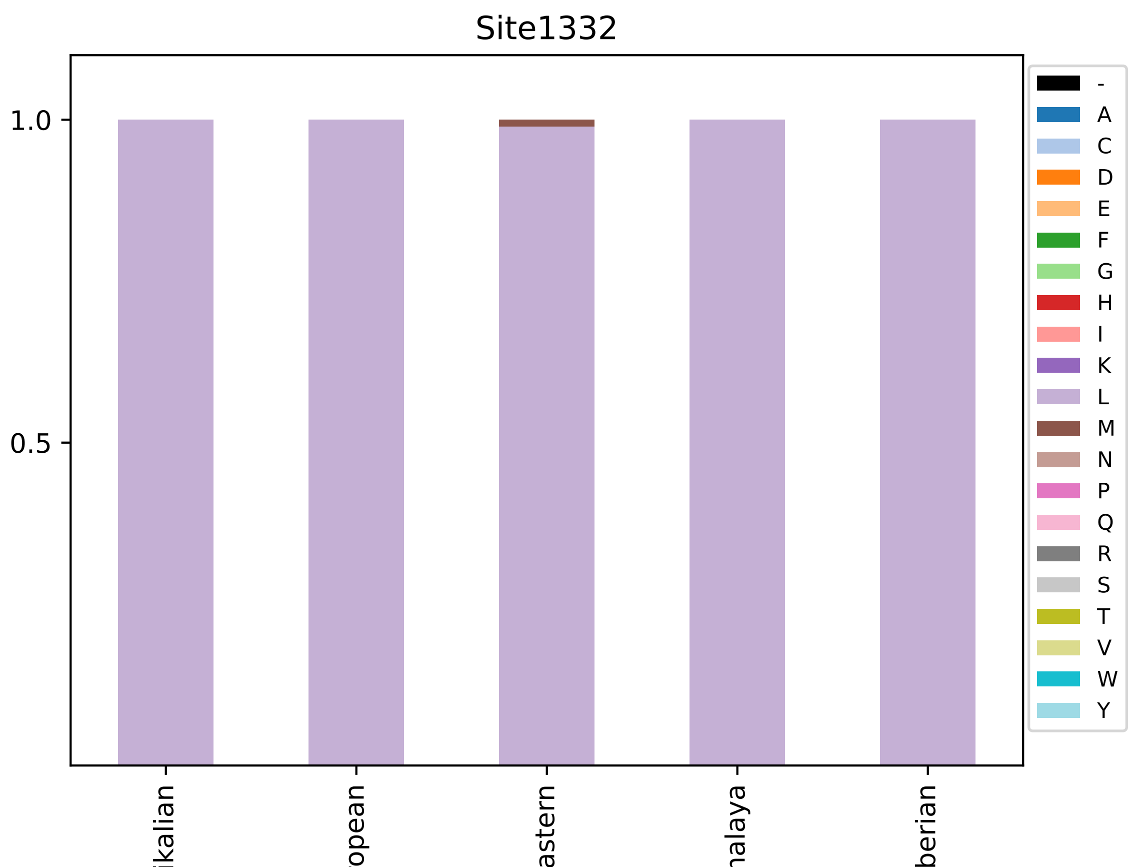
Site1332
1.0
0.5
-
A
C
D
E
F
G
H
I
K
L
M
N
P
Q
R
S
T
V
W
Y
ikalian
opean
astern
nalaya
berian

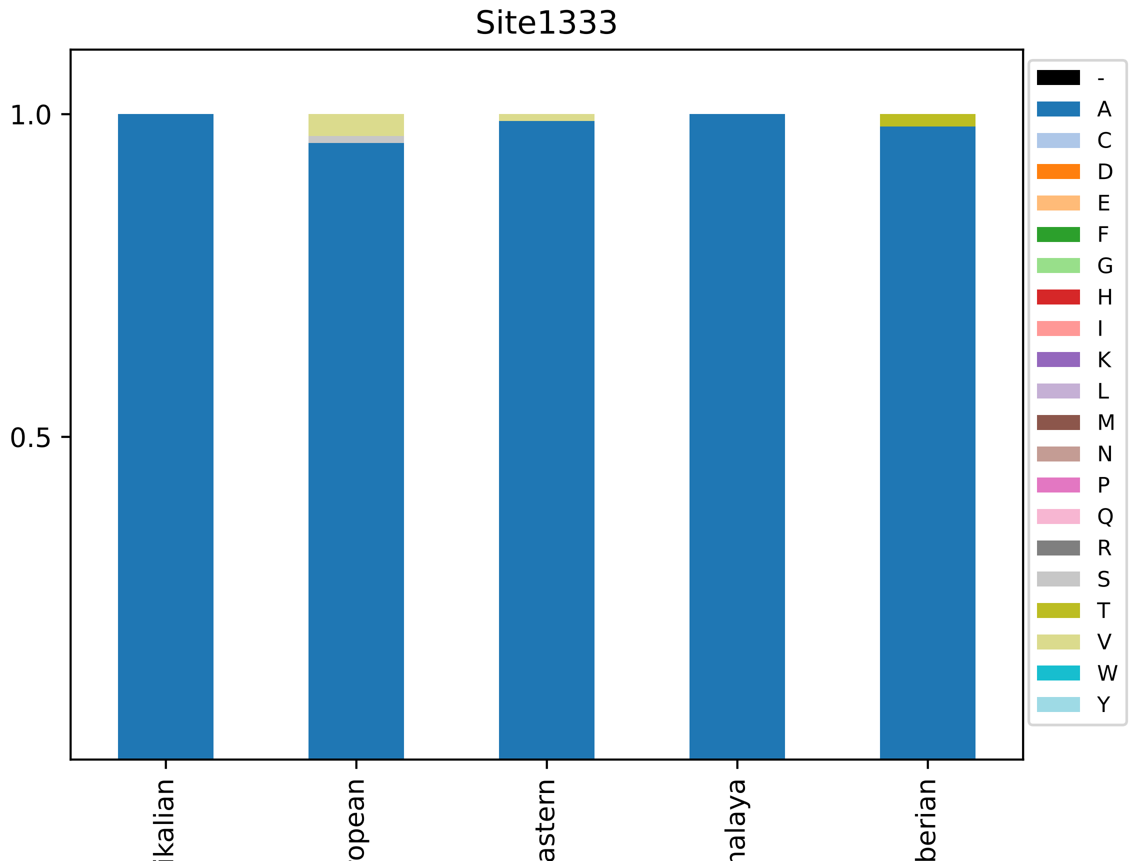

Site1333
1.0
0.5
-
A
C
D
E
F
G
H
I
K
L
M
N
P
Q
R
S
T
V
W
Y
ikalian
opean
astern
nalaya
berian

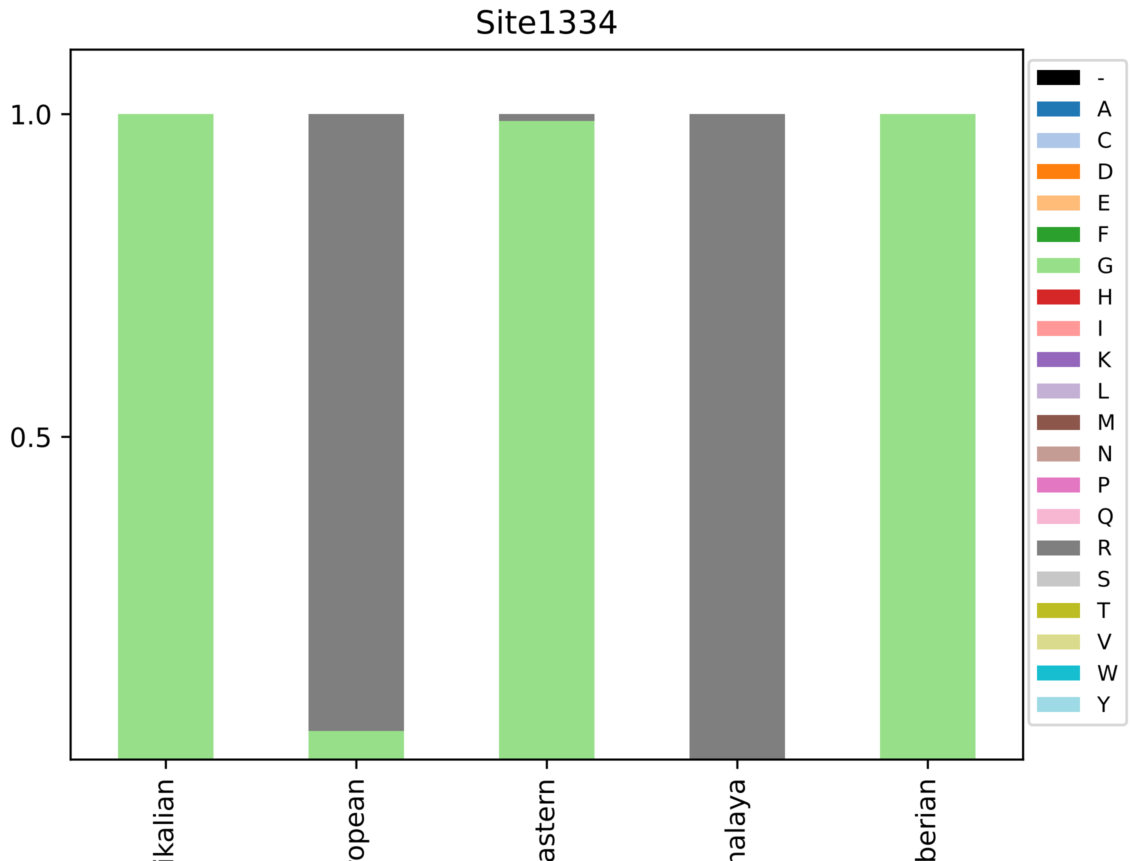

Site1334
1.0
0.5
ikalian
opean
astern
nalaya
berian
-
A
C
D
E
F
G
H
I
K
L
M
N
P
Q
R
S
T
V
W
Y

Site1335

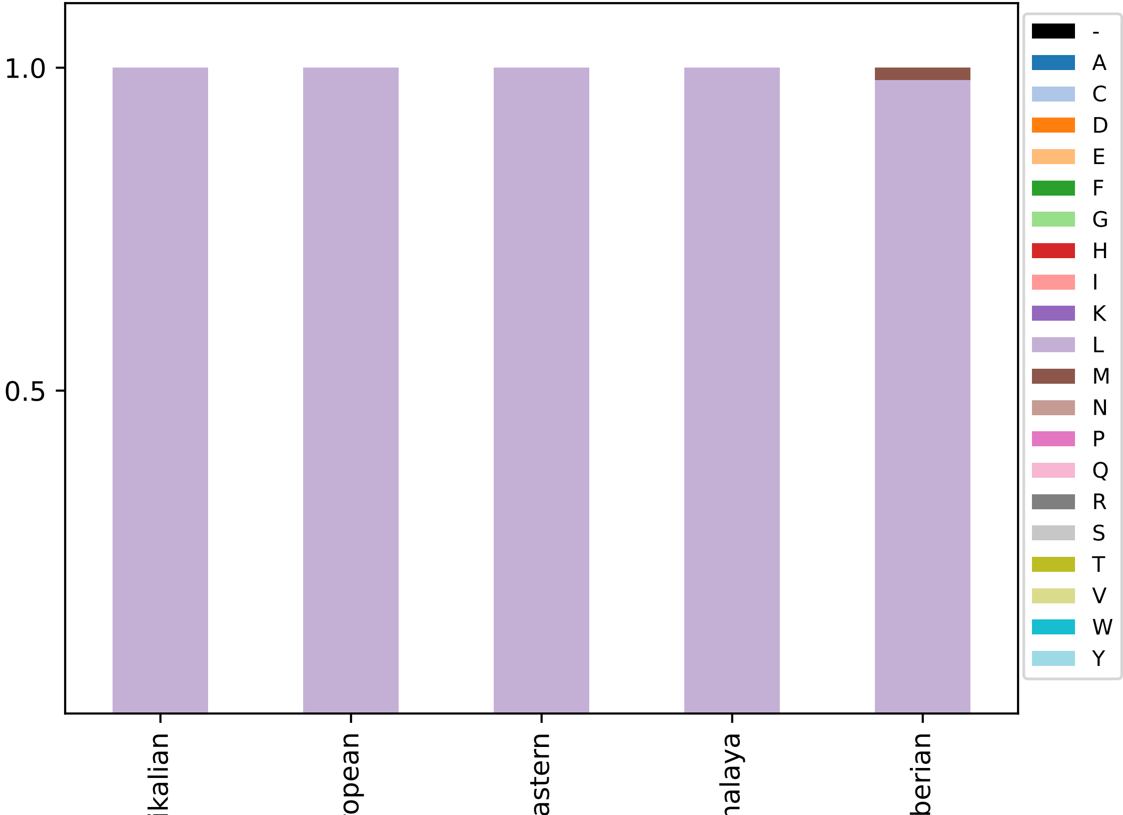

1.0
0.5
ikalian
opean
astern
nalaya
berian
-
A
C
D
E
F
G
H
I
K
L
M
N
P
Q
R
S
T
V
W
Y

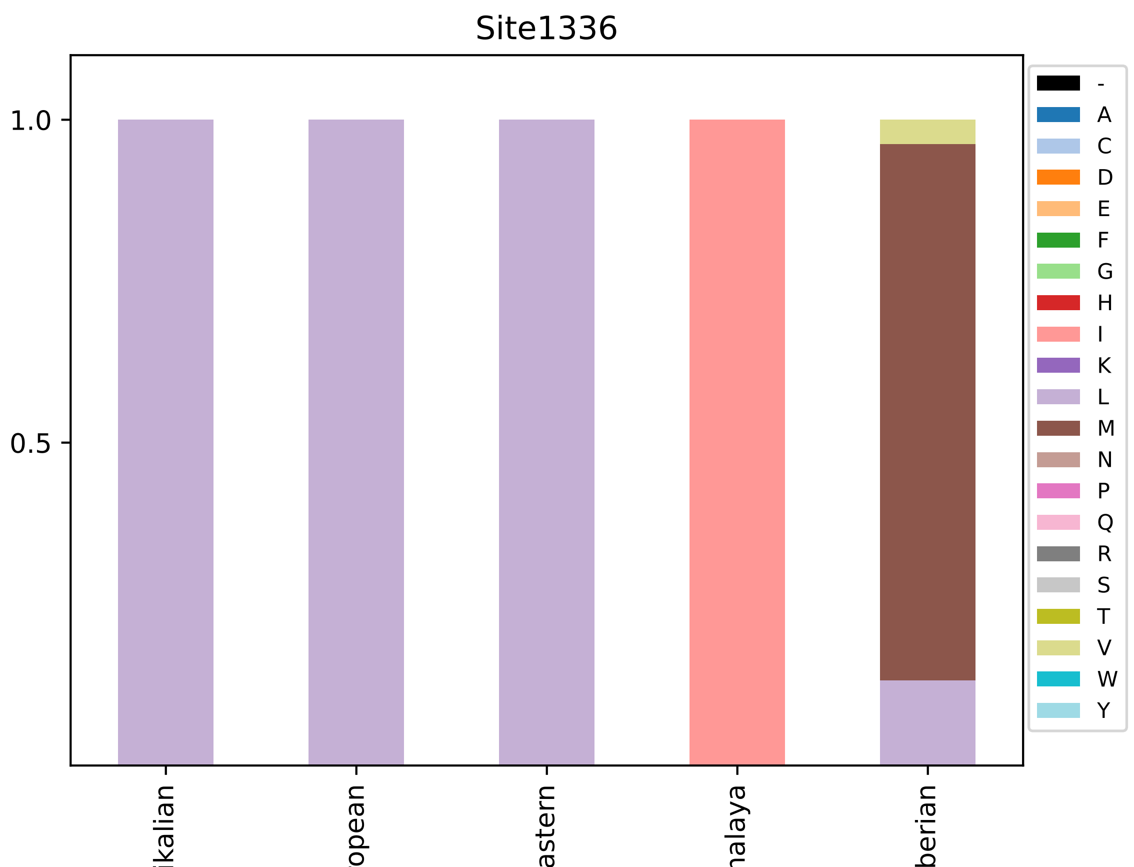
Site1336
1.0
0.5
ikalian
opean
astern
nalaya
berian
-
A
C
D
E
F
G
H
I
K
L
M
N
P
Q
R
S
T
V
W
Y

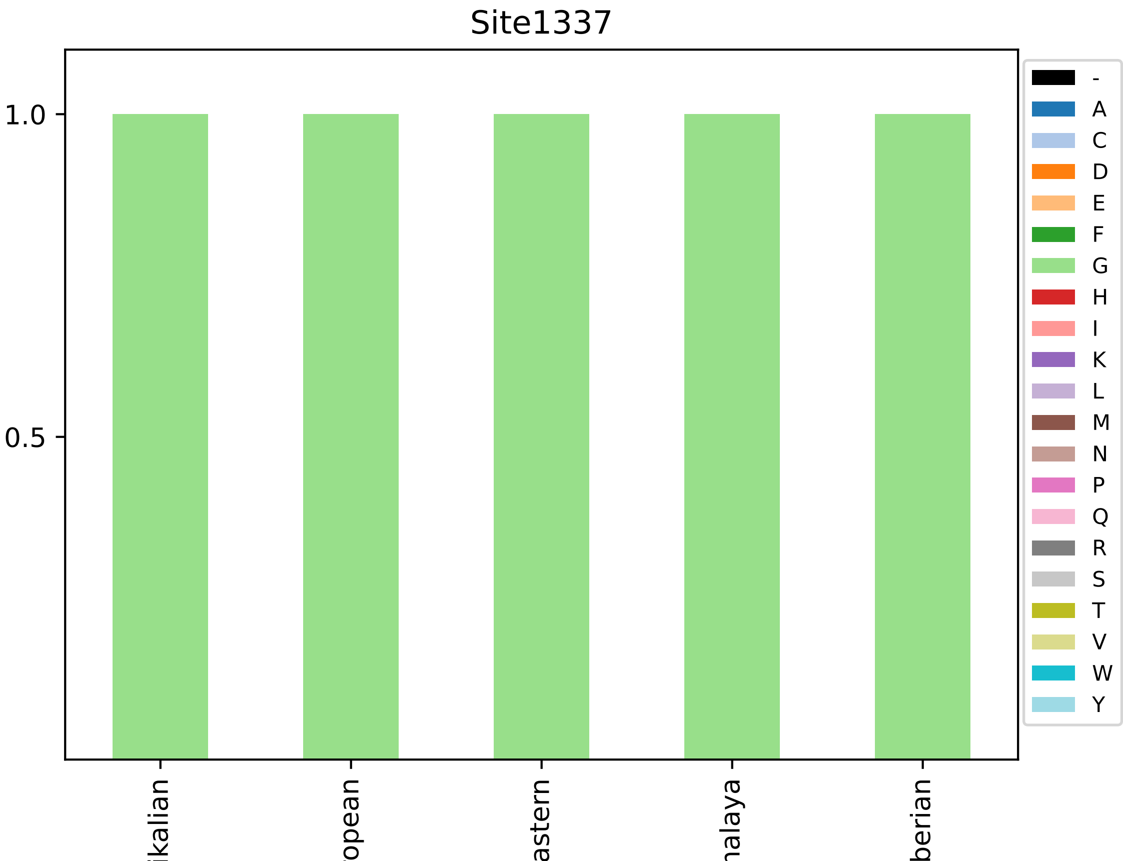

Site1337
1.0
0.5
-
A
C
D
E
F
G
H
I
K
L
M
N
P
Q
R
S
T
V
W
Y
ikalian
opean
astern
nalaya
berian

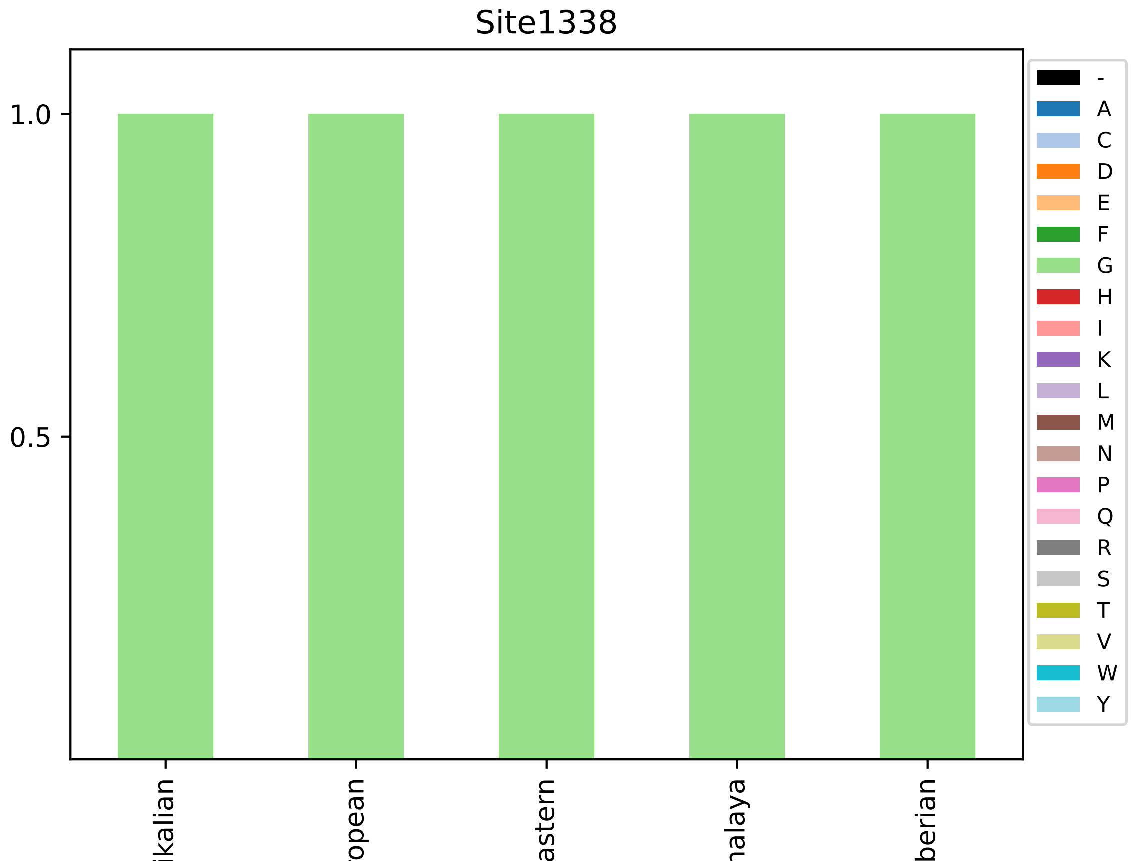
Site1338
1.0
0.5
-
A
C
D
E
F
G
H
I
K
L
M
N
P
Q
R
S
T
V
W
Y
ikalian
opean
astern
nalaya
berian

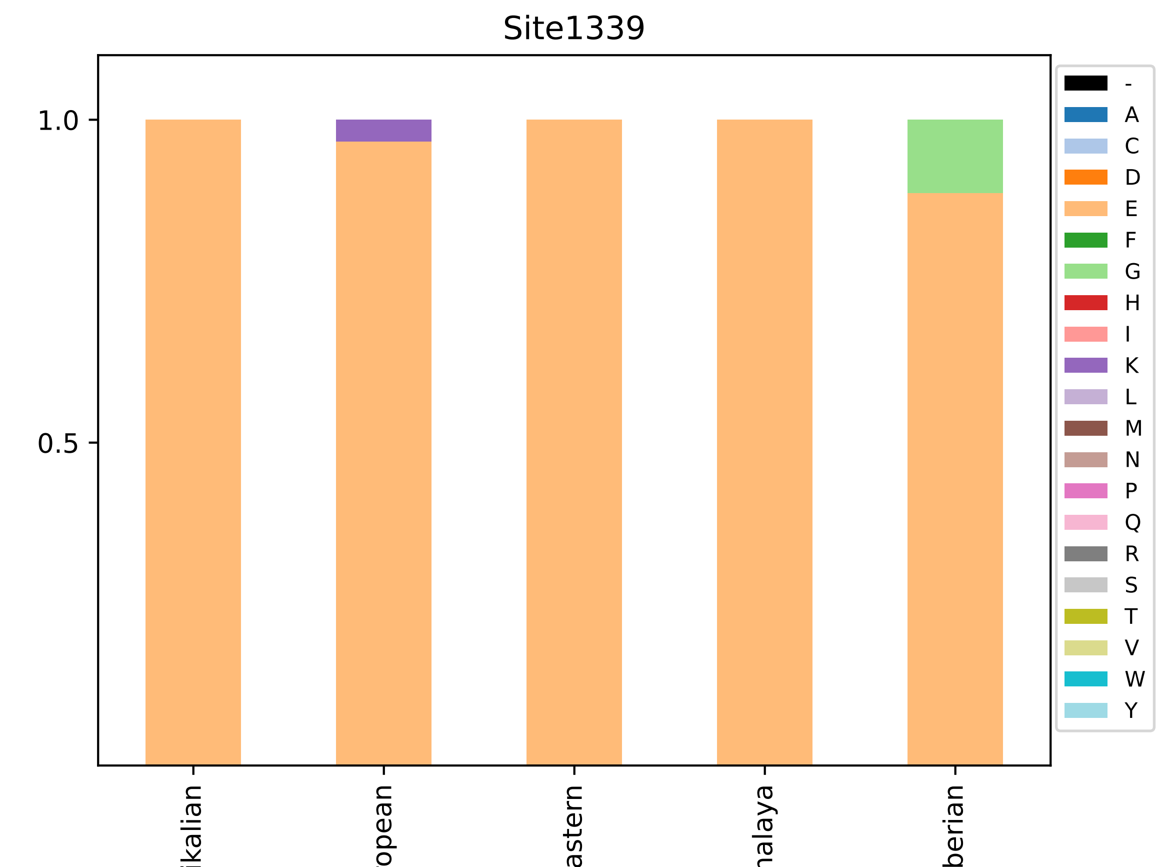
Site1339
1.0
0.5
-
A
C
D
E
F
G
H
I
K
L
M
N
P
Q
R
S
T
V
W
Y
ikalian
opean
astern
nalaya
berian

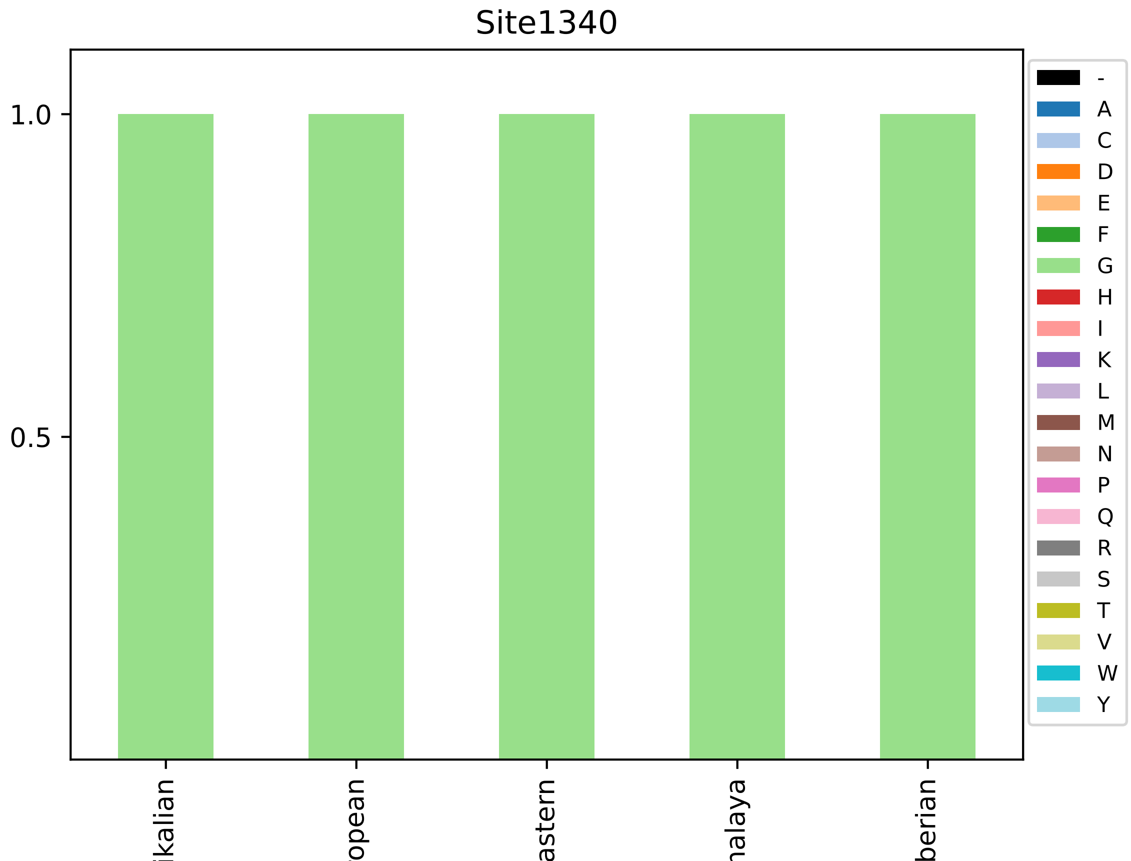

Site1340
1.0
0.5
-
A
C
D
E
F
G
H
I
K
L
M
N
P
Q
R
S
T
V
W
Y
ikalian
opean
astern
nalaya
berian

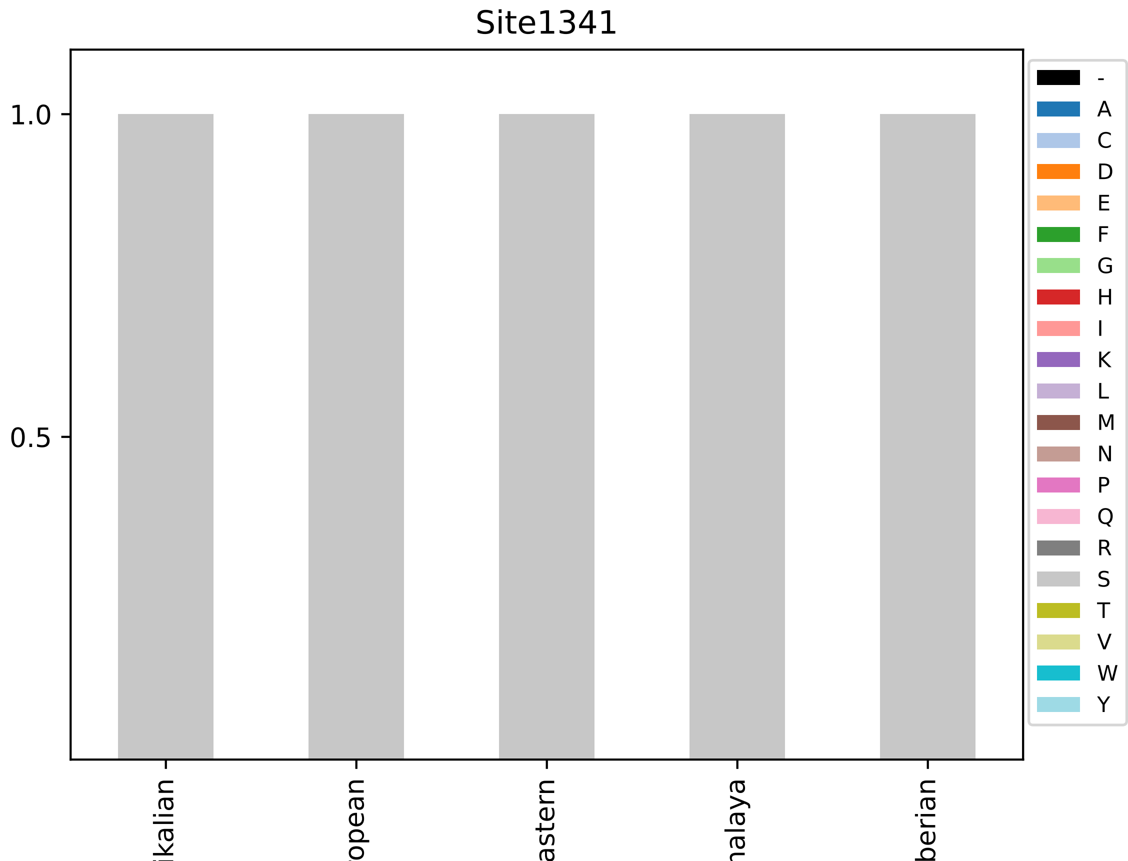
Site1341
1.0
0.5
-
A
C
D
E
F
G
H
I
K
L
M
N
P
Q
R
S
T
V
W
Y
ikalian
opean
astern
nalaya
berian

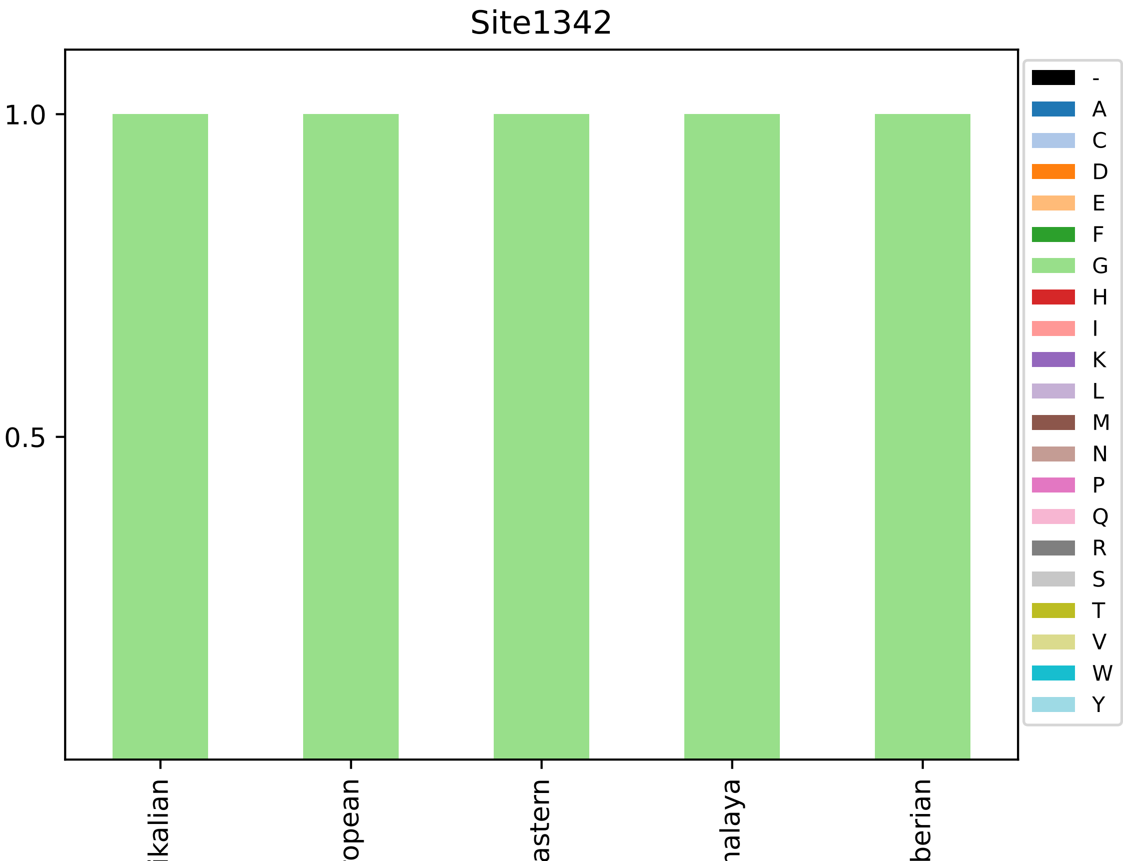

Site1342
1.0
0.5
-
A
C
D
E
F
G
H
I
K
L
M
N
P
Q
R
S
T
V
W
Y

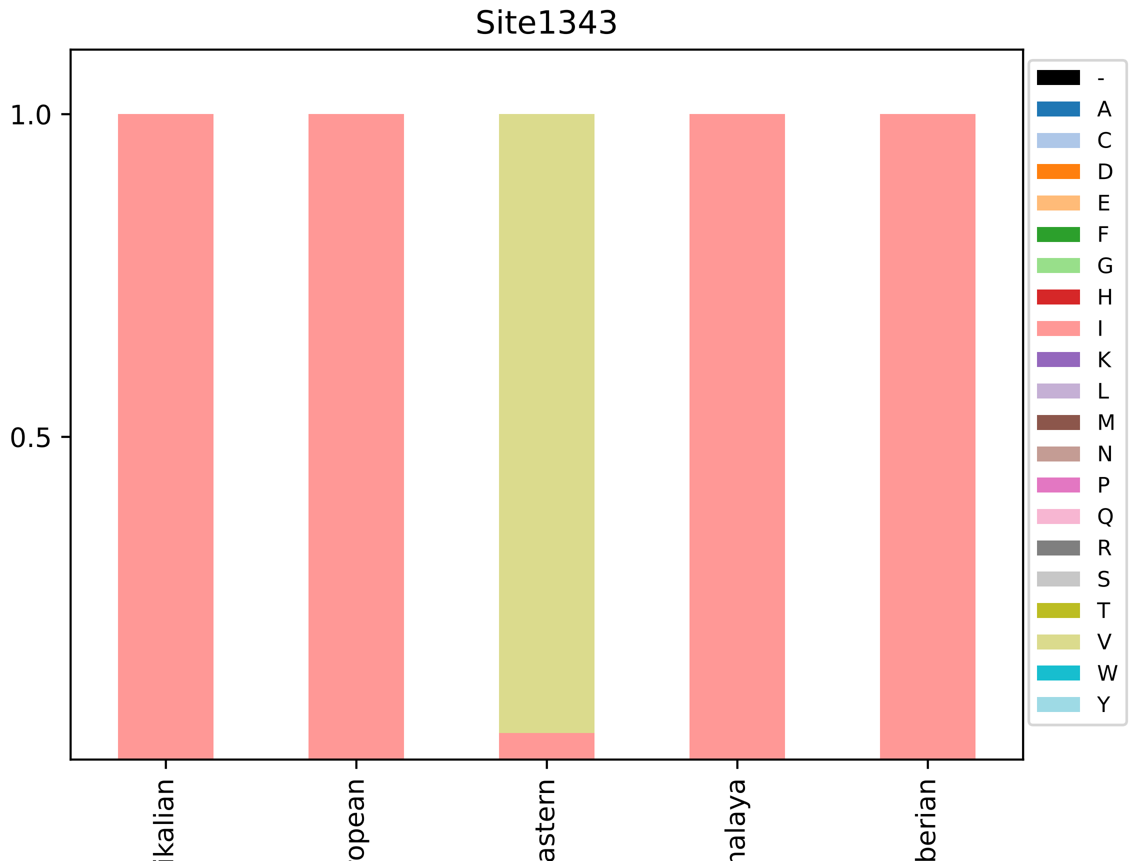
Site1343
1.0
0.5
-
A
C
D
E
F
G
H
I
K
L
M
N
P
Q
R
S
T
V
W
Y

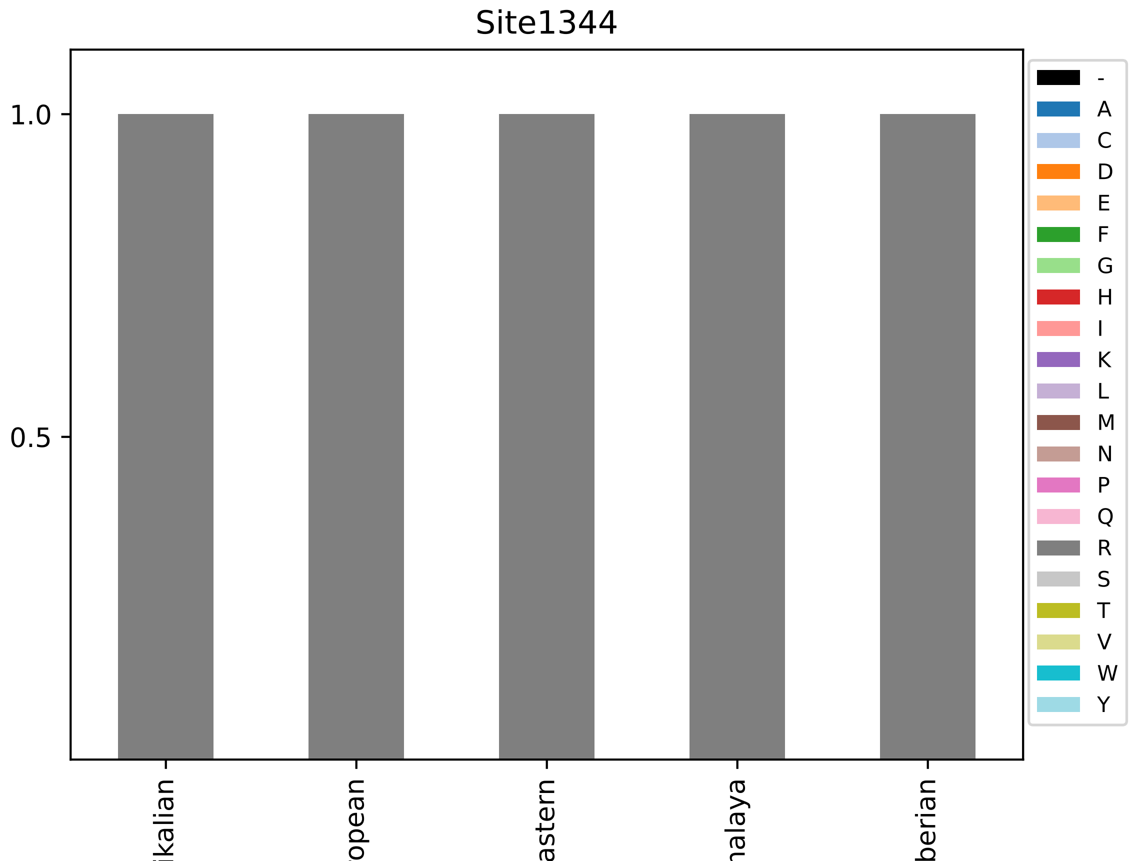

Site1344
1.0
0.5
-
A
C
D
E
F
G
H
I
K
L
M
N
P
Q
R
S
T
V
W
Y
ikalian
opean
astern
nalaya
berian

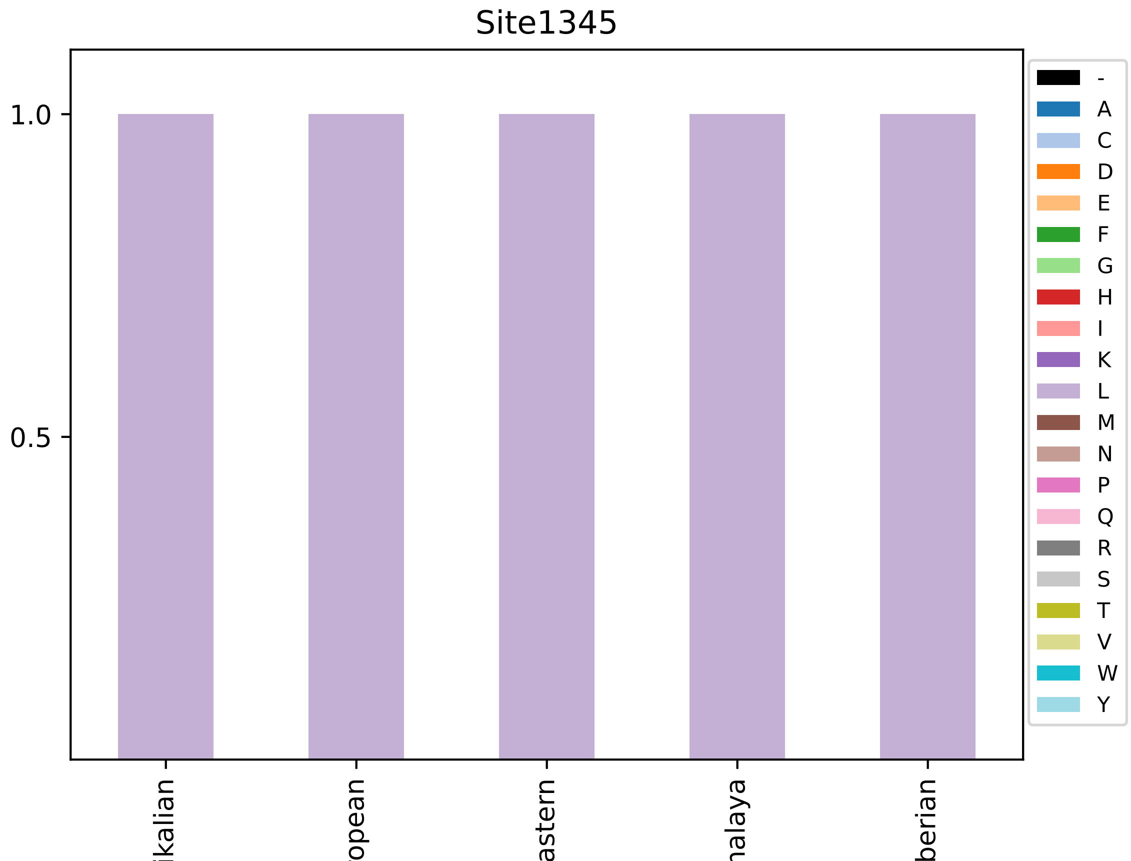

Site1345
1.0
0.5
-
A
C
D
E
F
G
H
I
K
L
M
N
P
Q
R
S
T
V
W
Y
ikalian
ropean
astern
nalaya
berian

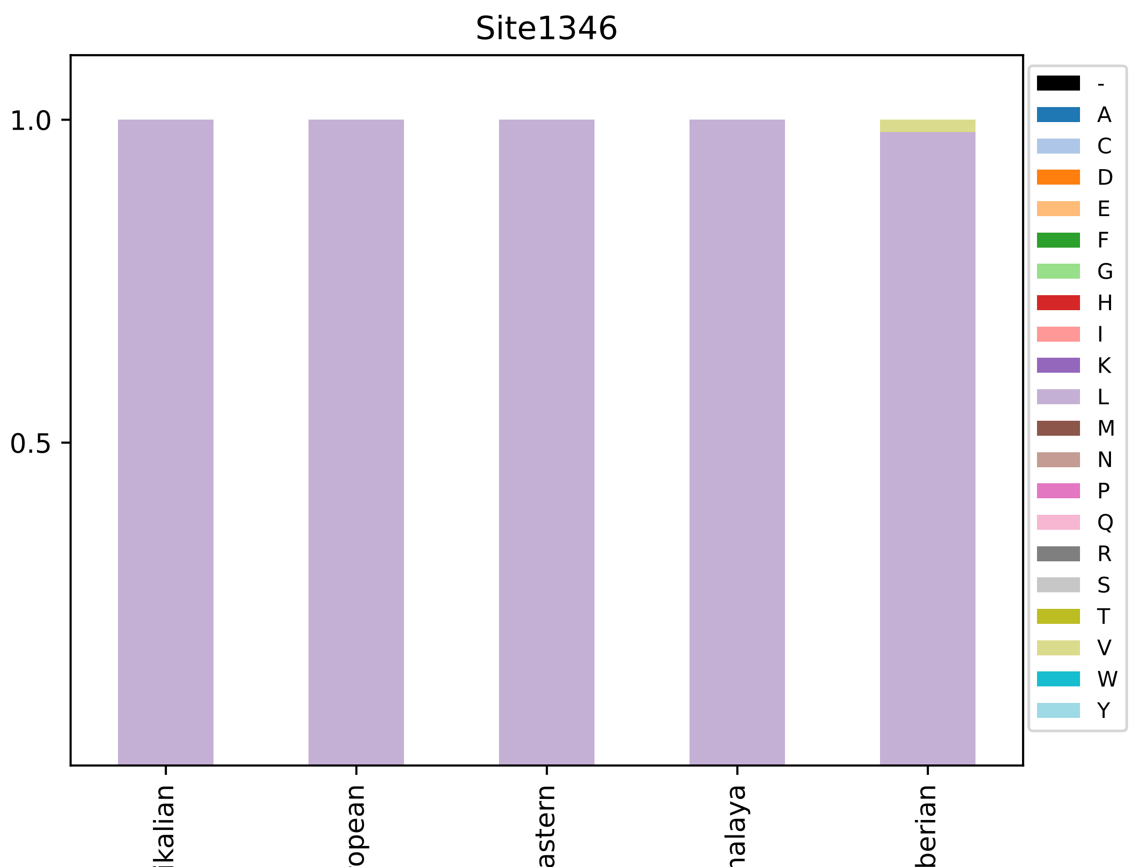

Site1346
1.0
0.5
-
A
C
D
E
F
G
H
I
K
L
M
N
P
Q
R
S
T
V
W
Y
ikalian
opean
astern
nalaya
berian

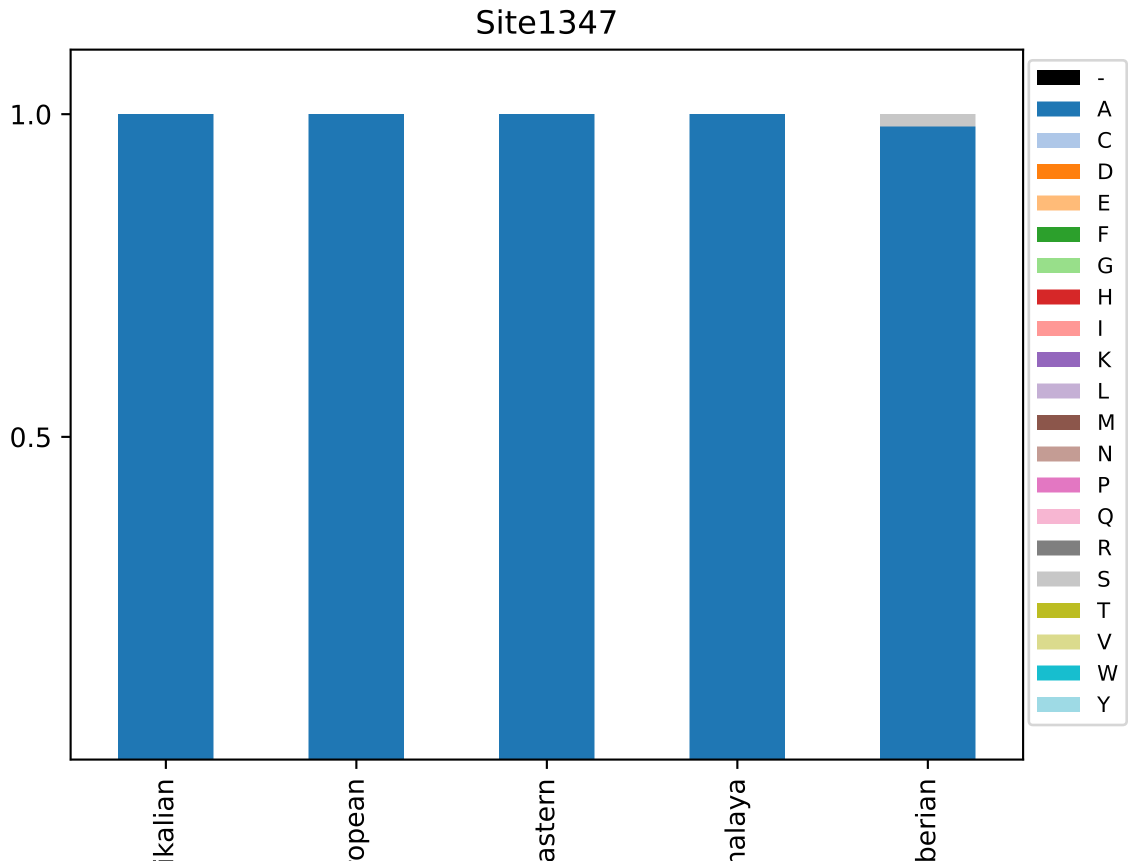
Site1347
1.0
0.5
ikalian
opean
astern
nalaya
berian
-
A
C
D
E
F
G
H
I
K
L
M
N
P
Q
R
S
T
V
W
Y

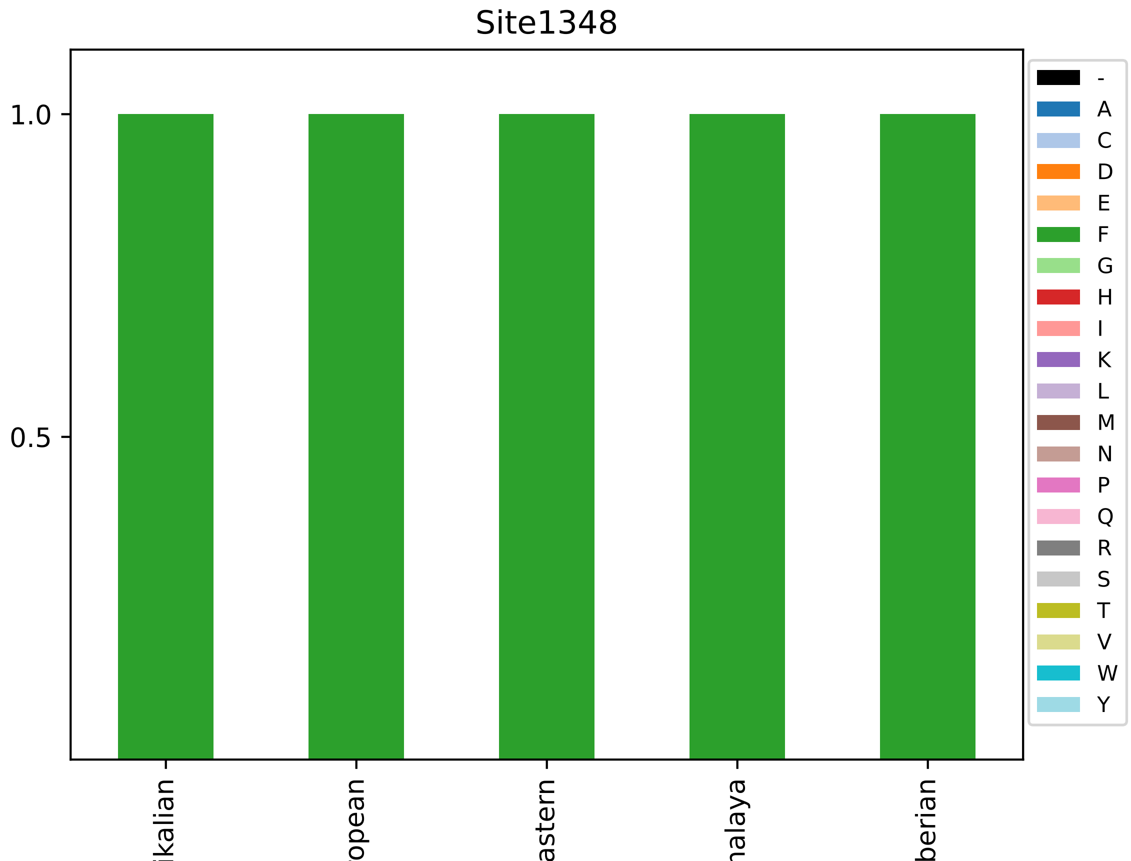

Site1348
1.0
0.5
ikalian
opean
astern
nalaya
berian
-
A
C
D
E
F
G
H
I
K
L
M
N
P
Q
R
S
T
V
W
Y

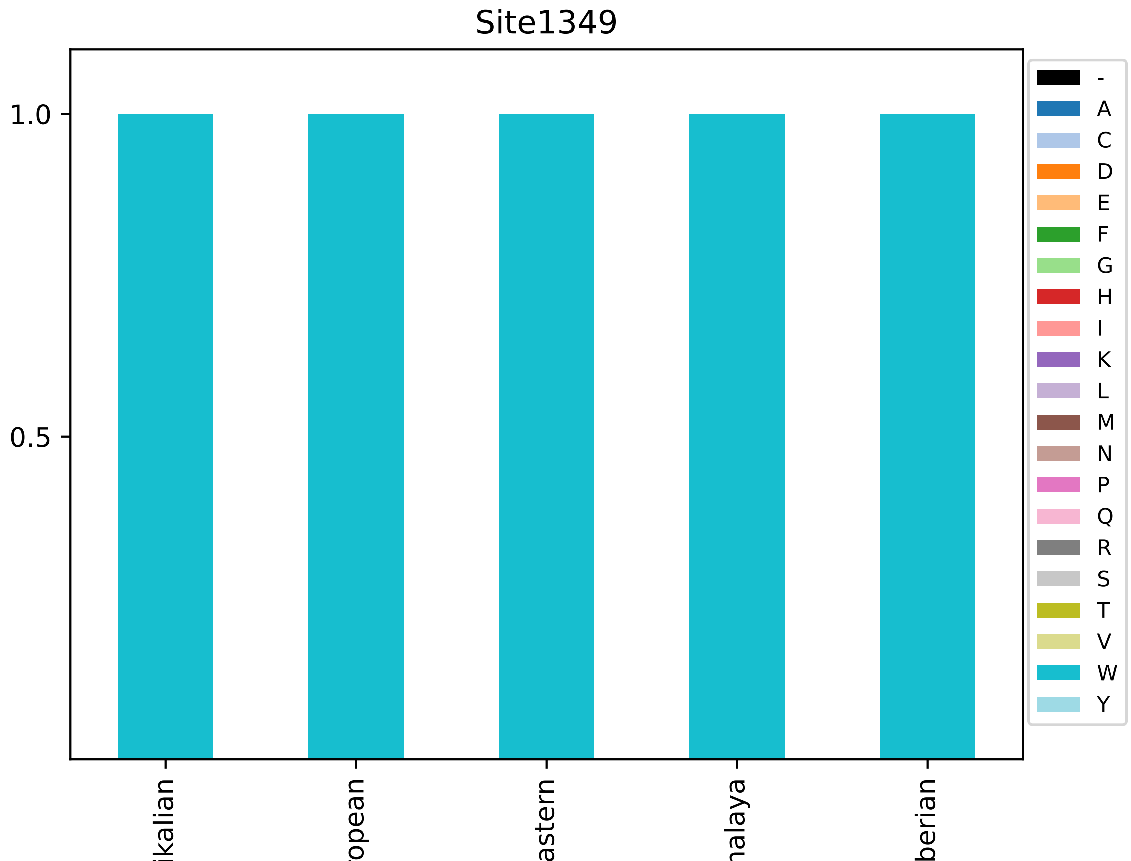
Site1349
1.0
0.5
-
A
C
D
E
F
G
H
I
K
L
M
N
P
Q
R
S
T
V
W
Y
ikalian
opean
astern
nalaya
berian

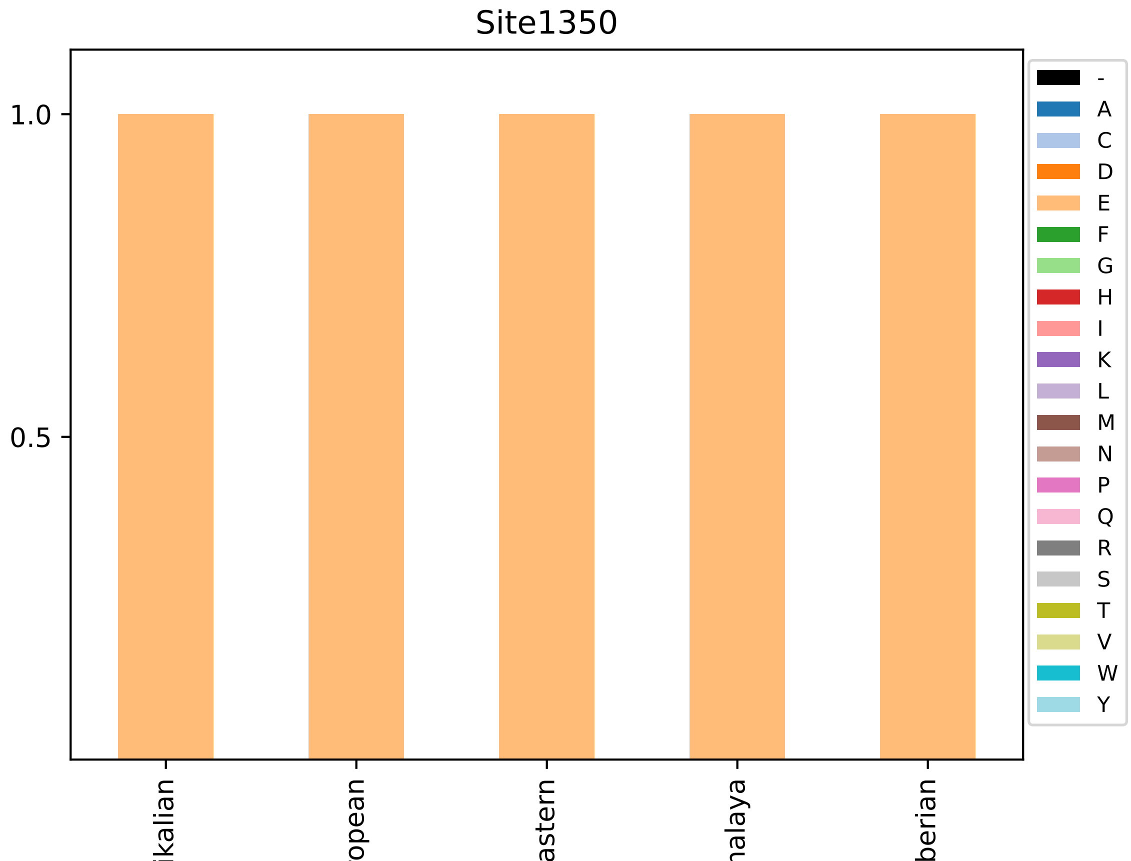

Site1350
1.0
0.5
-
A
C
D
E
F
G
H
I
K
L
M
N
P
Q
R
S
T
V
W
Y

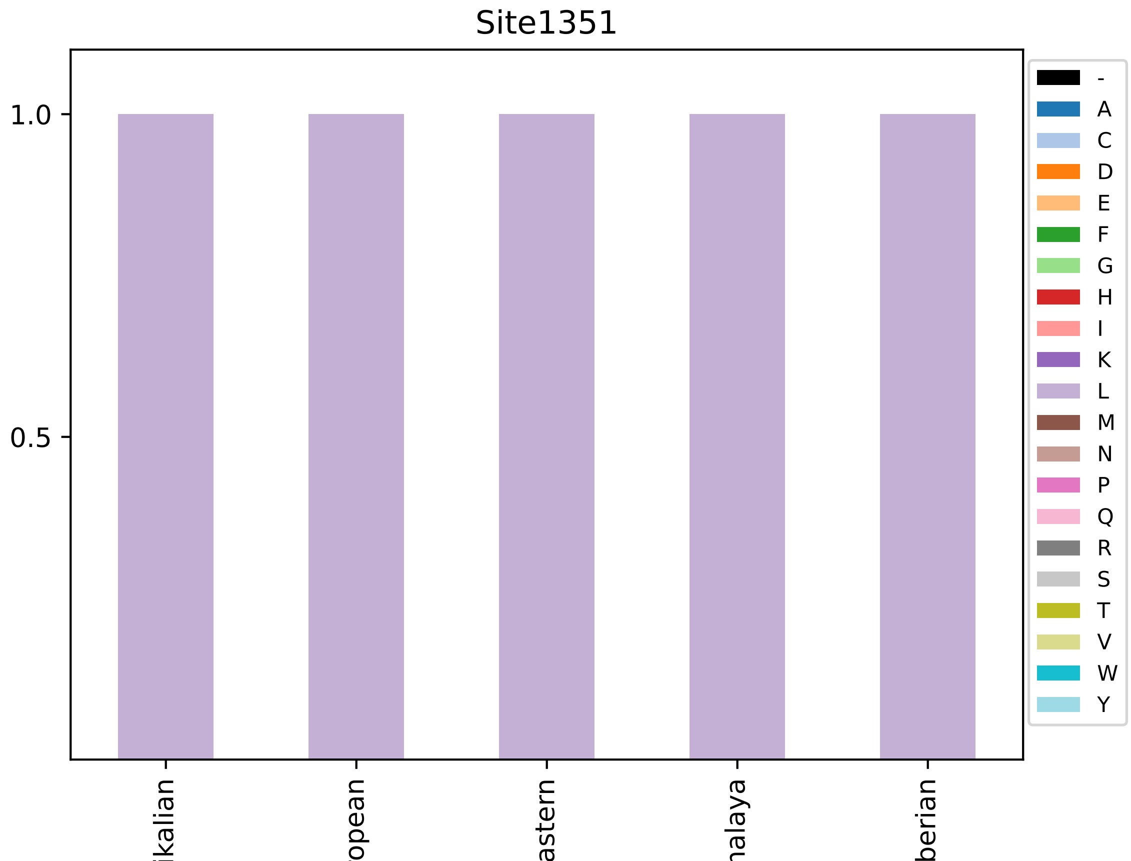
Site1351
1.0
0.5
-
A
C
D
E
F
G
H
I
K
L
M
N
P
Q
R
S
T
V
W
Y
ikalian
opean
astern
nalaya
berian

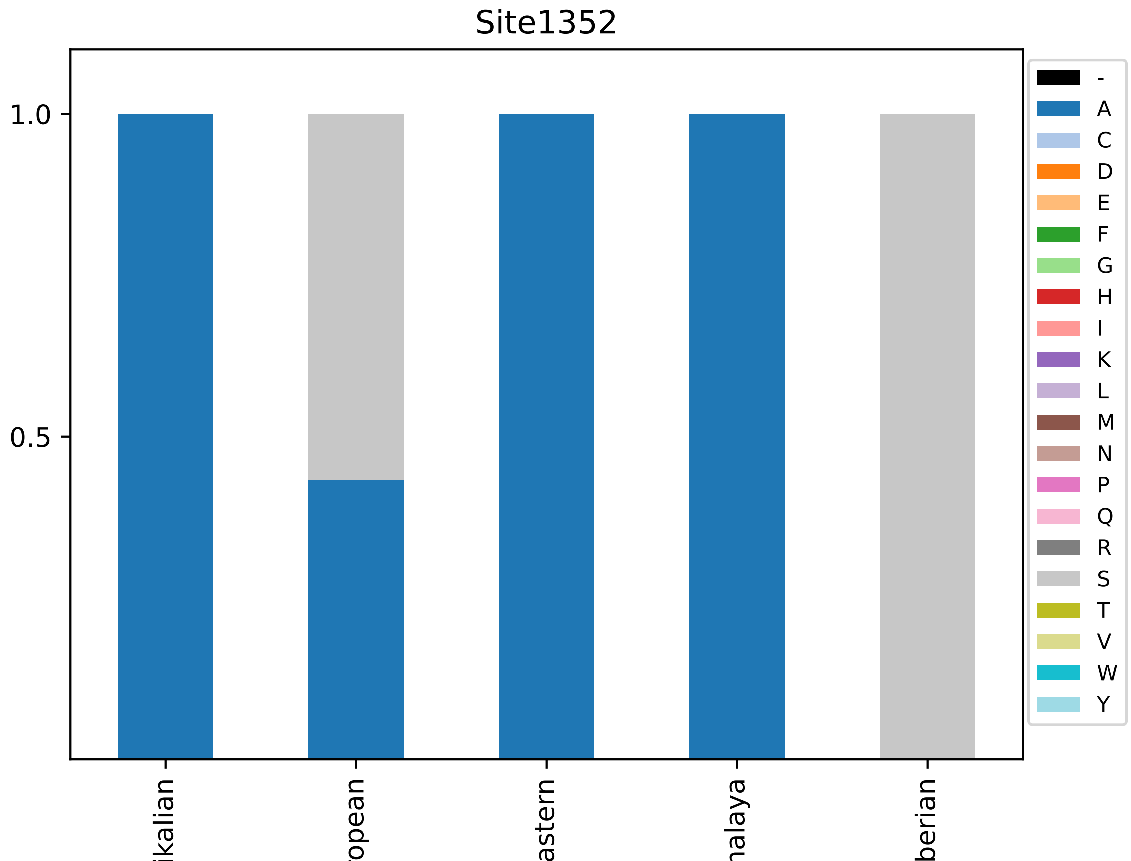
Site1352
1.0
0.5
-
A
C
D
E
F
G
H
I
K
L
M
N
P
Q
R
S
T
V
W
Y
ikalian
opean
astern
nalaya
berian

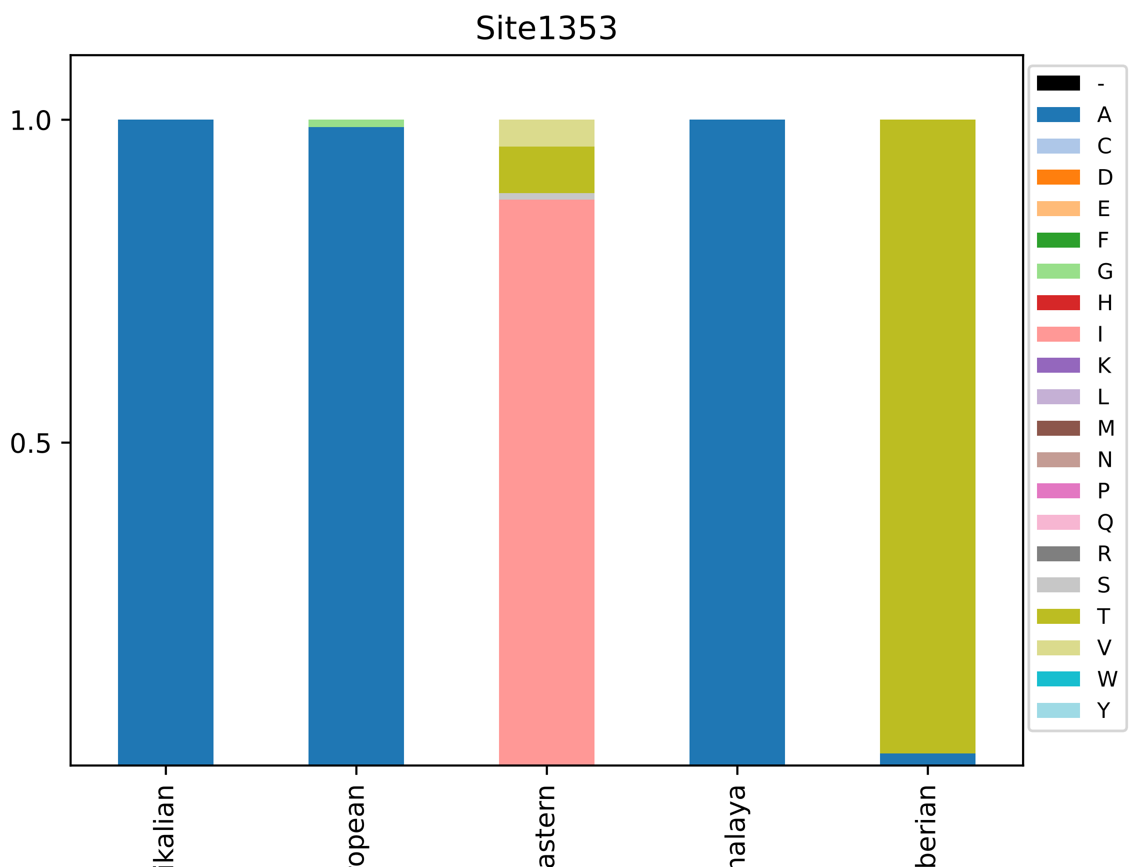

Site1353
1.0
0.5
-
A
C
D
E
F
G
H
I
K
L
M
N
P
Q
R
S
T
V
W
Y
ikalian
opean
astern
nalaya
berian

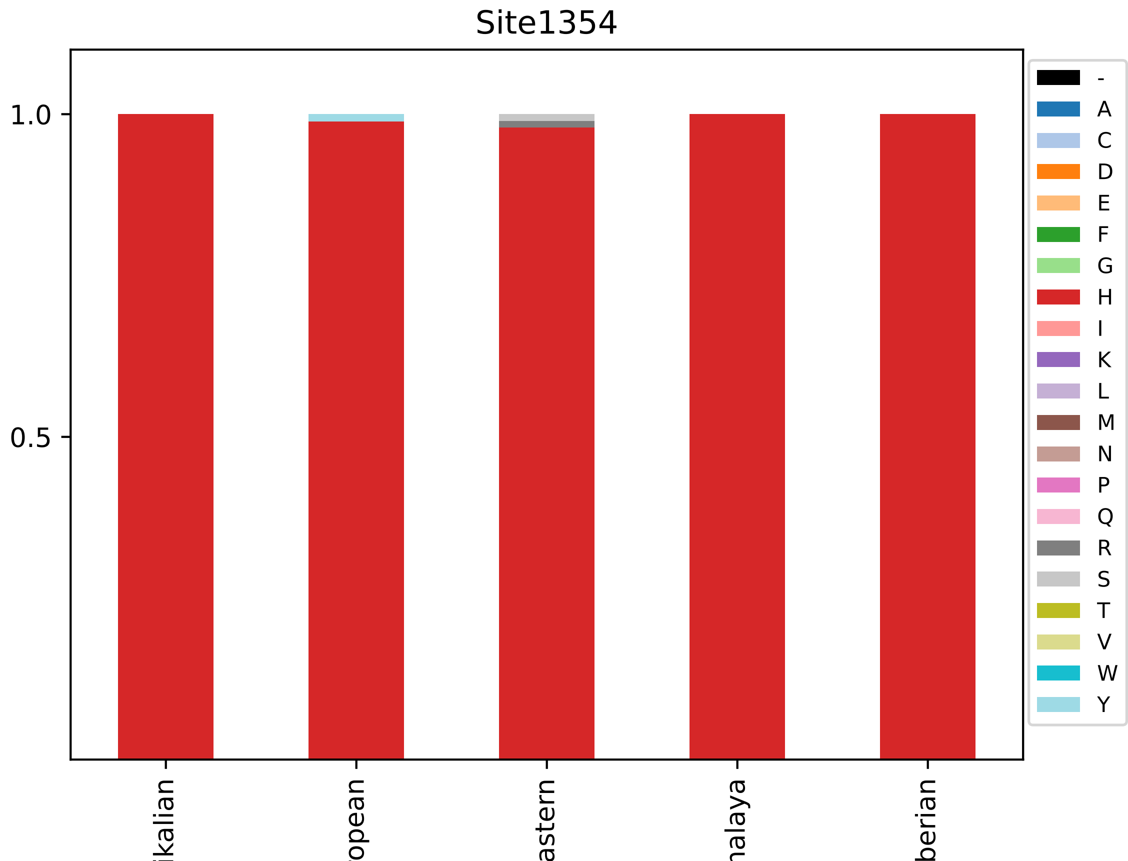
Site1354
1.0
0.5
ikalian
opean
astern
nalaya
berian
-
A
C
D
E
F
G
H
I
K
L
M
N
P
Q
R
S
T
V
W
Y

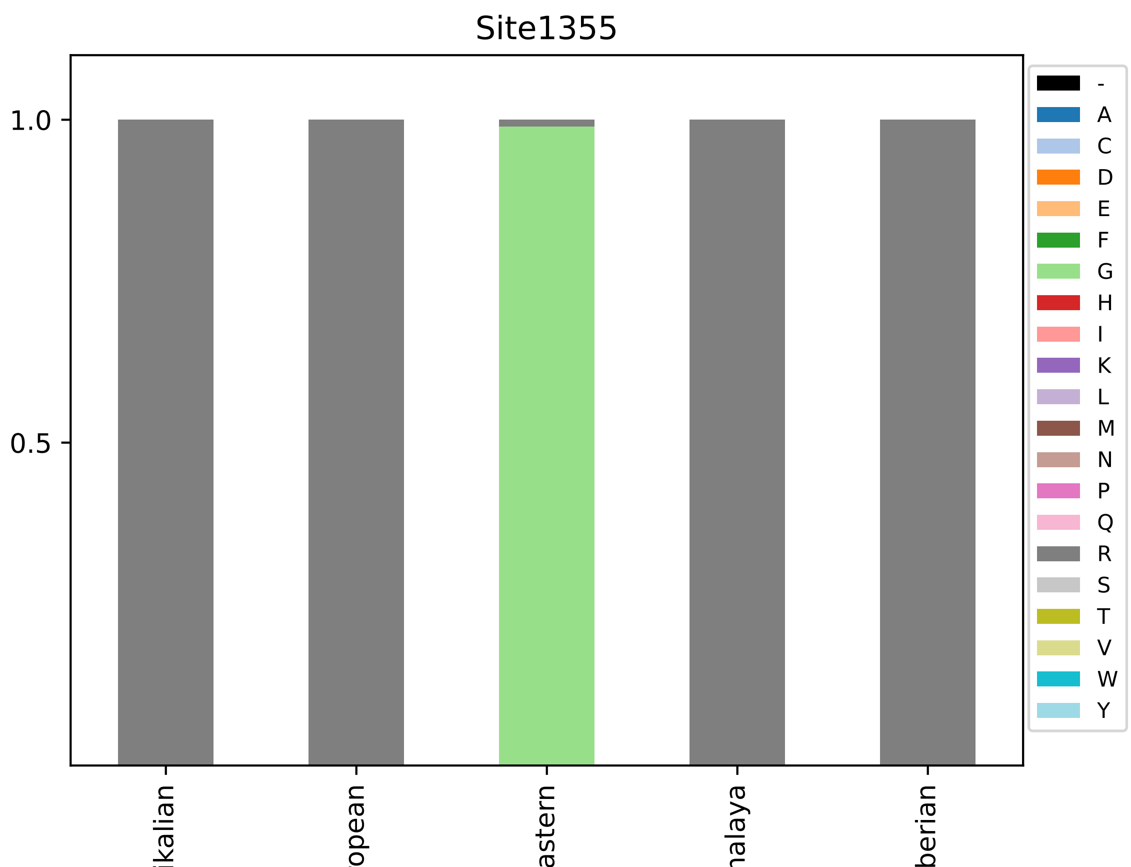
Site1355
1.0
0.5
-
A
C
D
E
F
G
H
I
K
L
M
N
P
Q
R
S
T
V
W
Y
ikalian
opean
astern
nalaya
berian

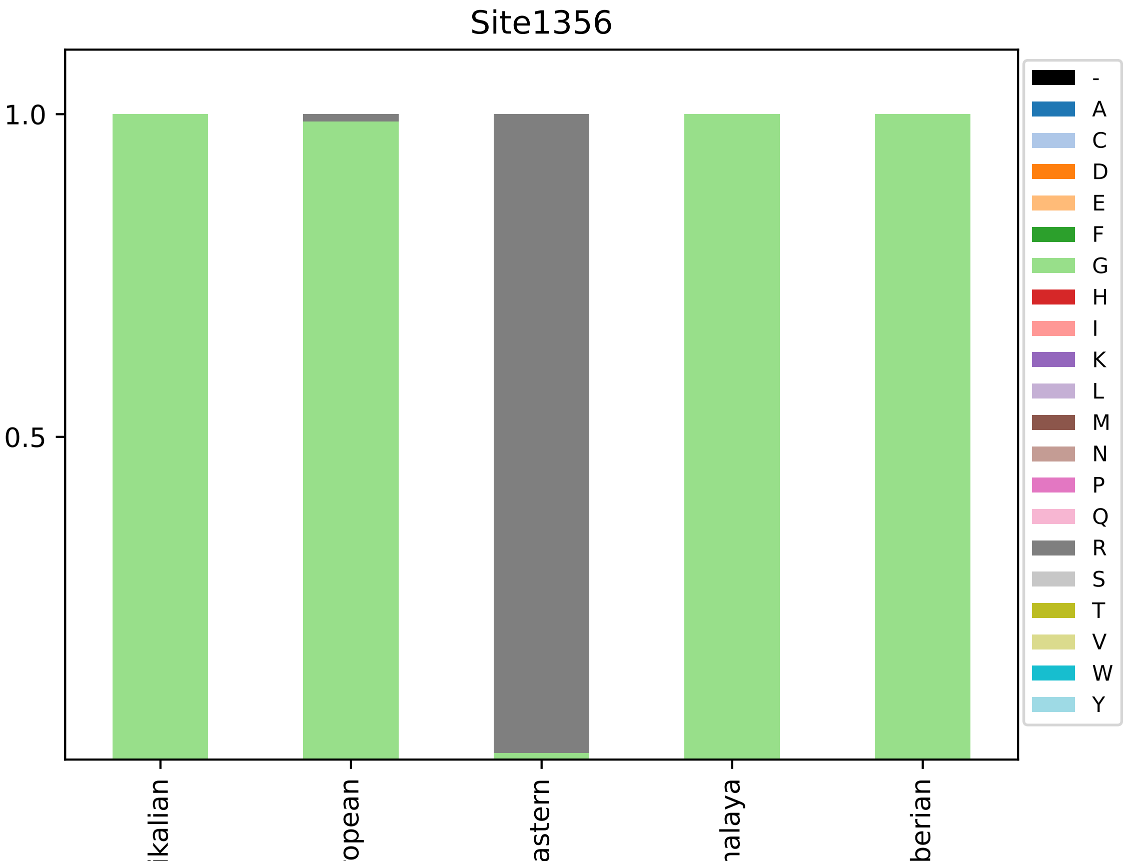
Site1356
1.0
0.5
ikalian
opean
astern
nalaya
berian
-
A
C
D
E
F
G
H
I
K
L
M
N
P
Q
R
S
T
V
W
Y

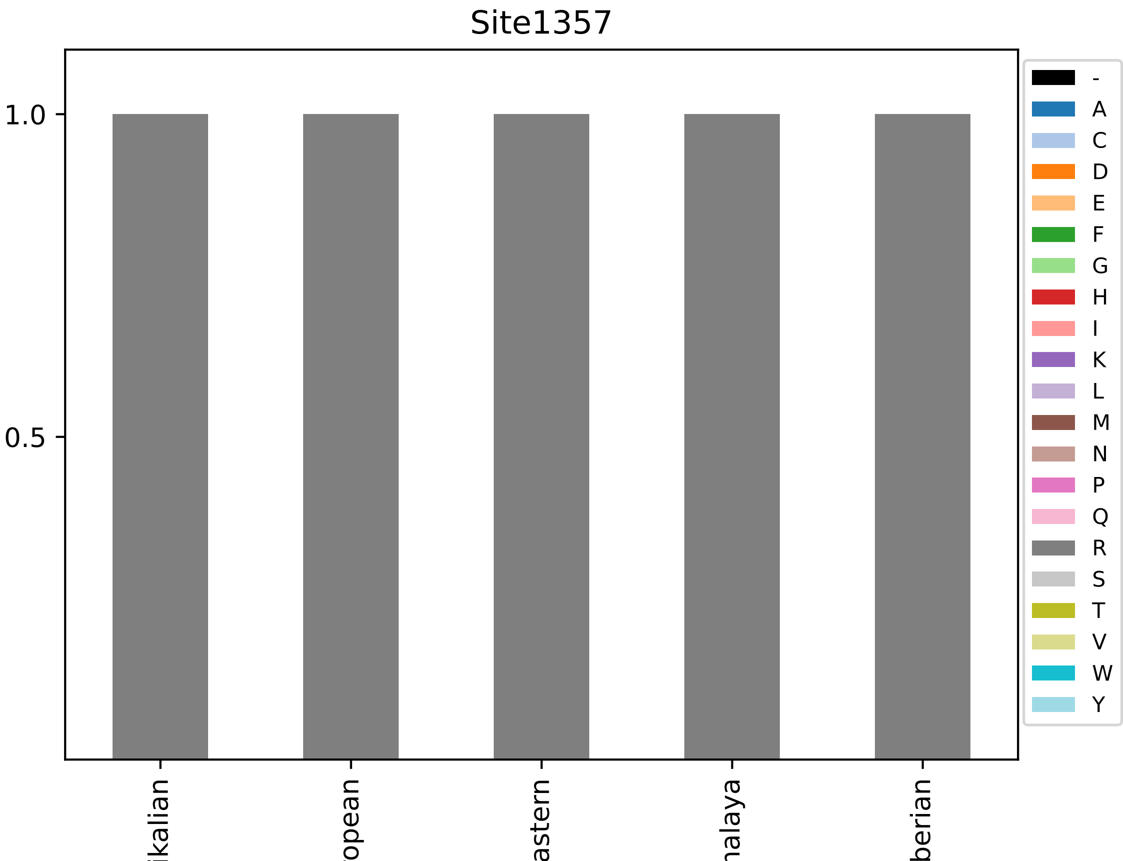

Site1357
1.0
0.5
-
A
C
D
E
F
G
H
I
K
L
M
N
P
Q
R
S
T
V
W
Y

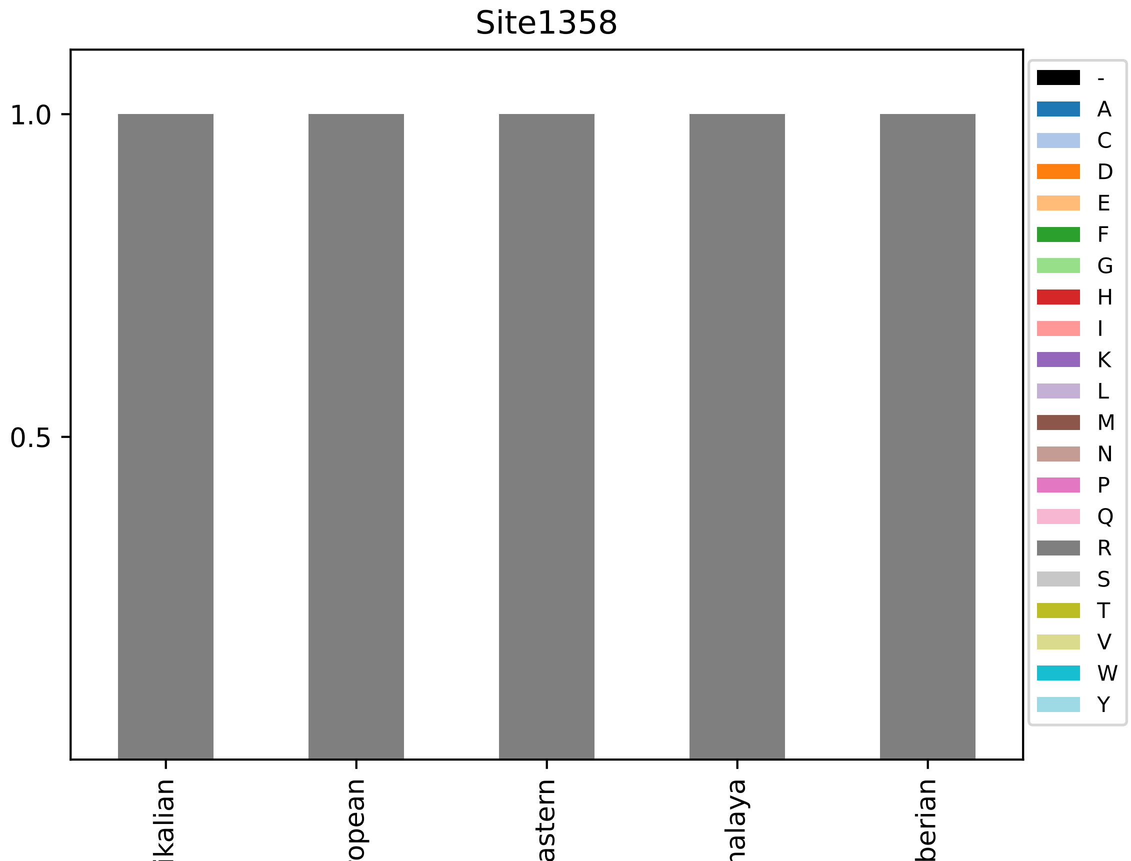

Site1358
1.0
0.5
-
A
C
D
E
F
G
H
I
K
L
M
N
P
Q
R
S
T
V
W
Y
ikalian
opean
astern
nalaya
berian

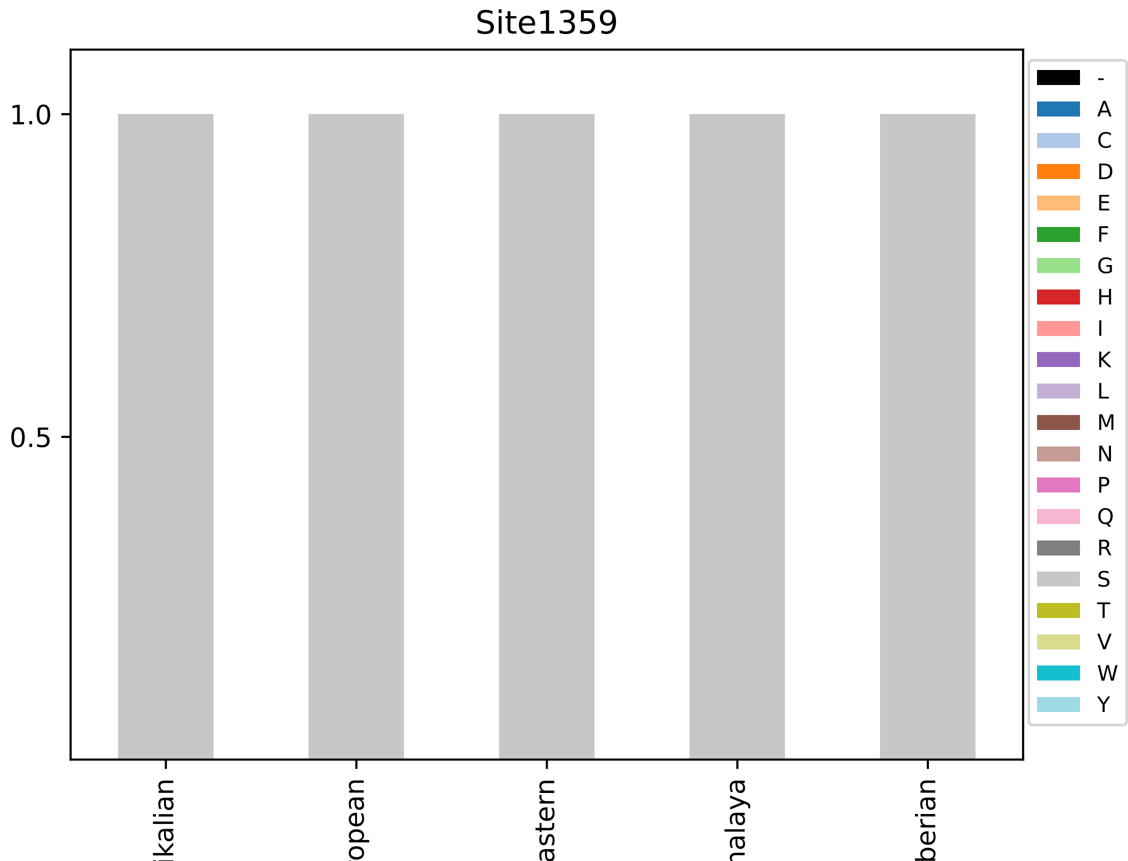

Site1359
1.0
0.5
ikalian
opean
astern
nalaya
berian
-
A
C
D
E
F
G
H
I
K
L
M
N
P
Q
R
S
T
V
W
Y

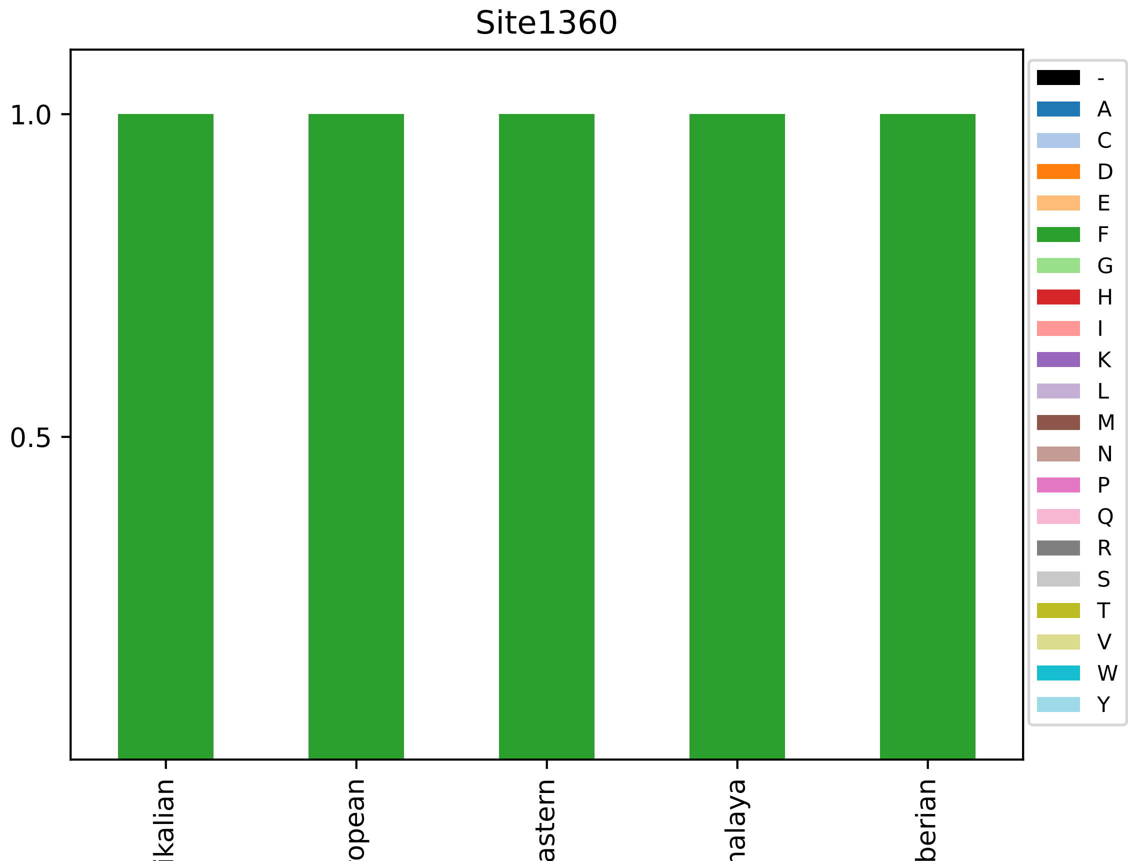

Site1360
1.0
0.5
-
A
C
D
E
F
G
H
I
K
L
M
N
P
Q
R
S
T
V
W
Y
ikalian
opean
astern
nalaya
berian

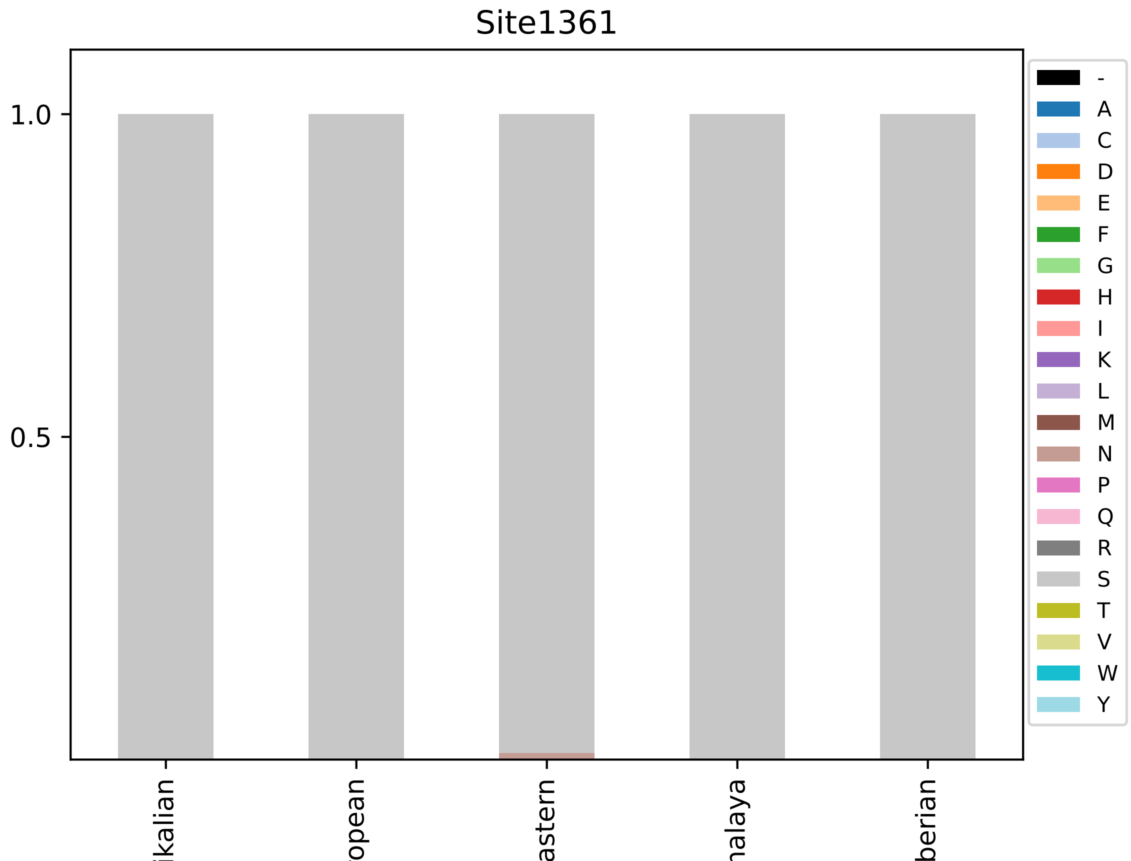
Site1361
1.0
0.5
ikalian
ropean
astern
nalaya
berian
-
A
C
D
E
F
G
H
I
K
L
M
N
P
Q
R
S
T
V
W
Y

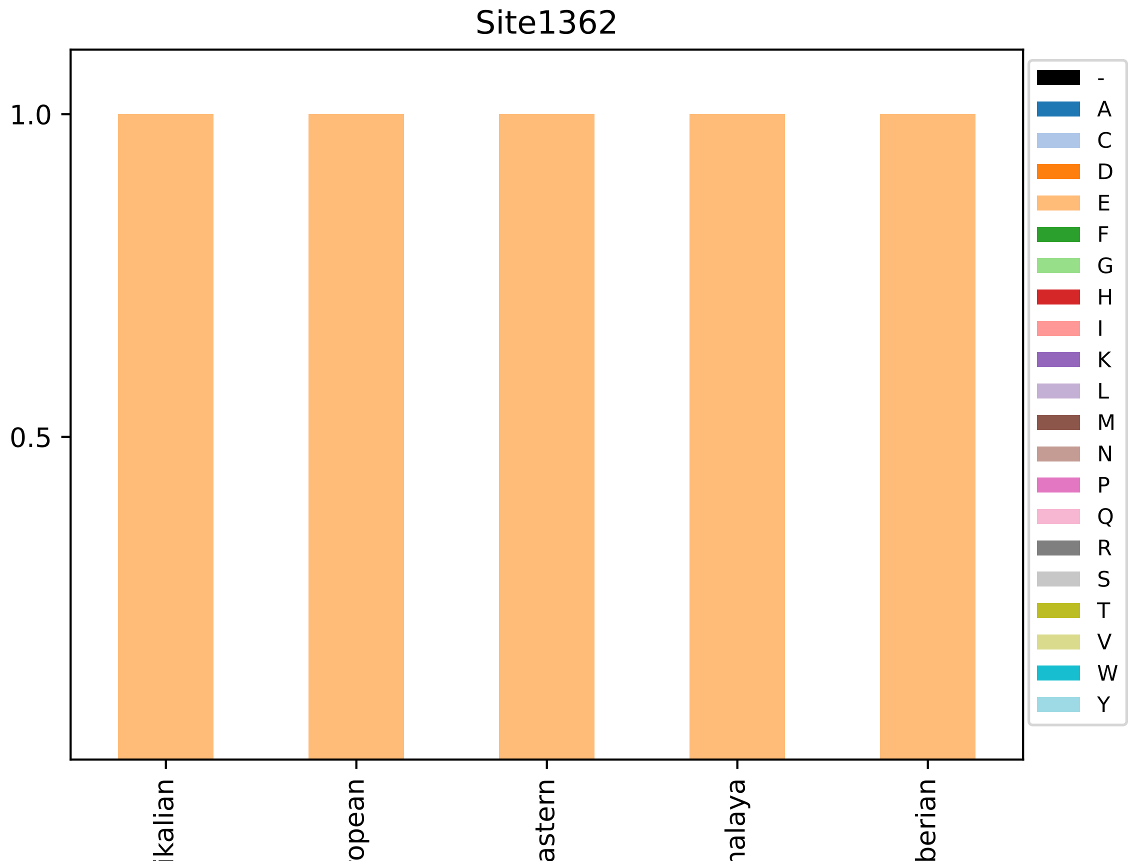

Site1362
1.0
0.5
-
A
C
D
E
F
G
H
I
K
L
M
N
P
Q
R
S
T
V
W
Y
ikalian
opean
astern
nalaya
berian

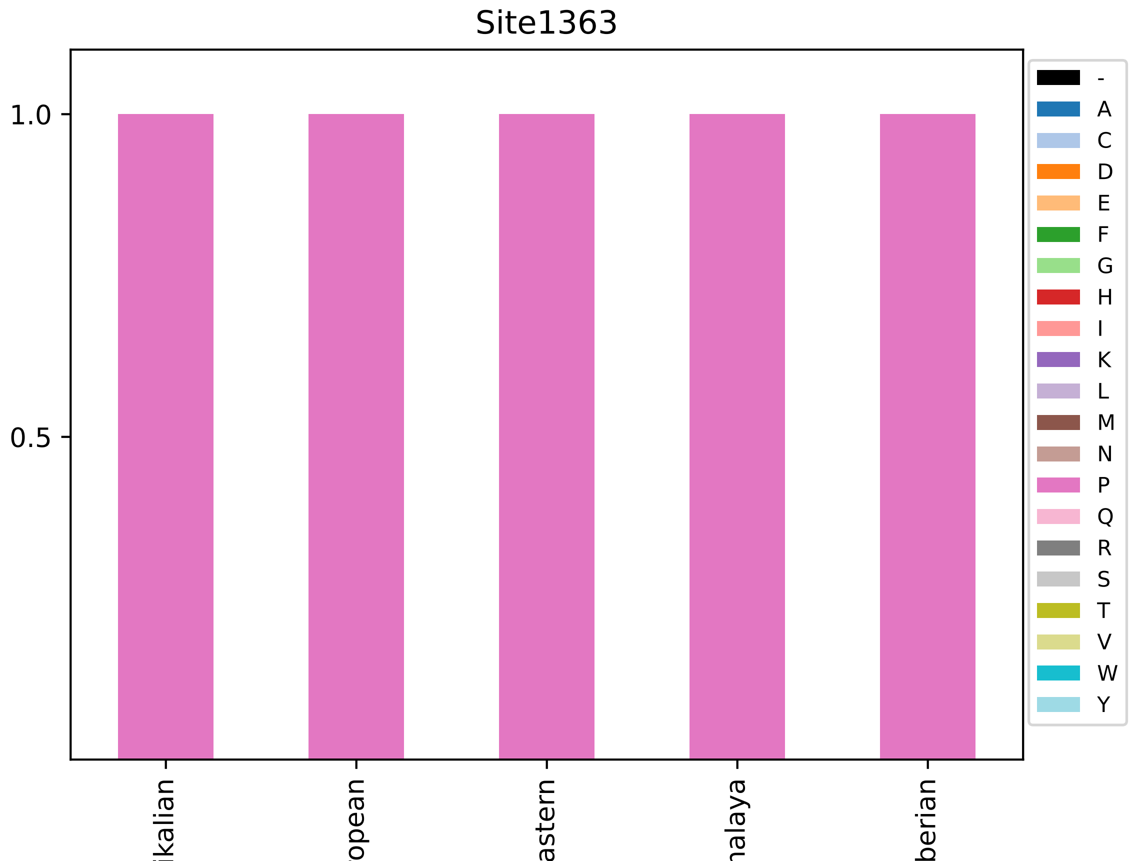
Site1363
1.0
0.5
-
A
C
D
E
F
G
H
I
K
L
M
N
P
Q
R
S
T
V
W
Y
ikalian
opean
astern
alaya
berian

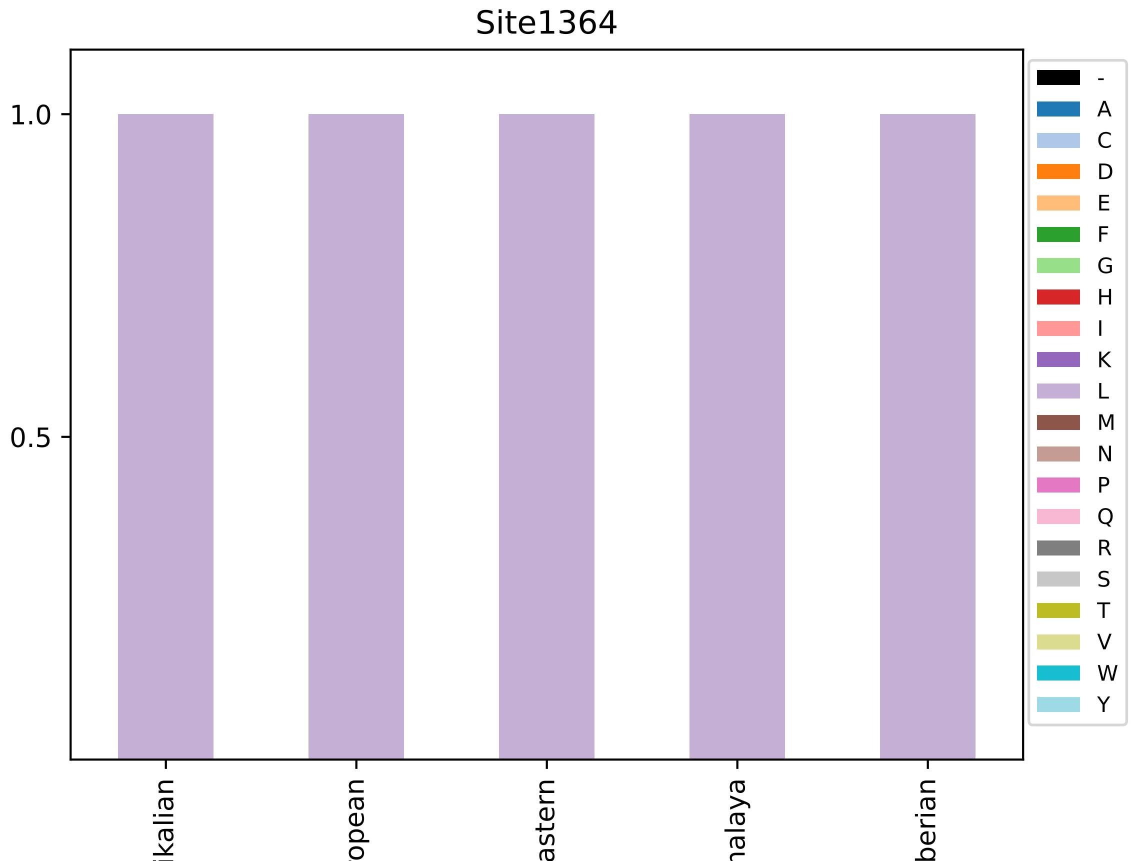

Site1364
1.0
0.5
ikalian
opean
astern
nalaya
berian
-
A
C
D
E
F
G
H
I
K
L
M
N
P
Q
R
S
T
V
W
Y

Site1365

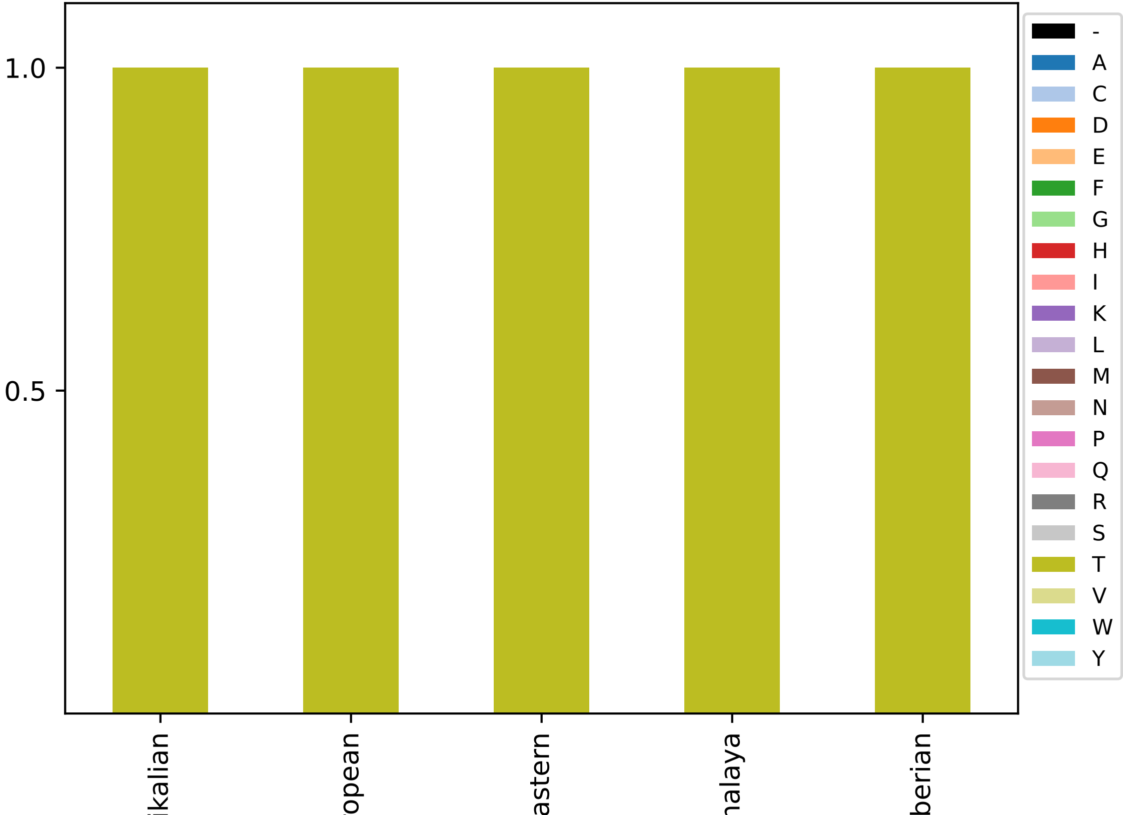

1.0
0.5
ikalian
opean
astern
nalaya
berian
-
A
C
D
E
F
G
H
I
K
L
M
N
P
Q
R
S
T
V
W
Y

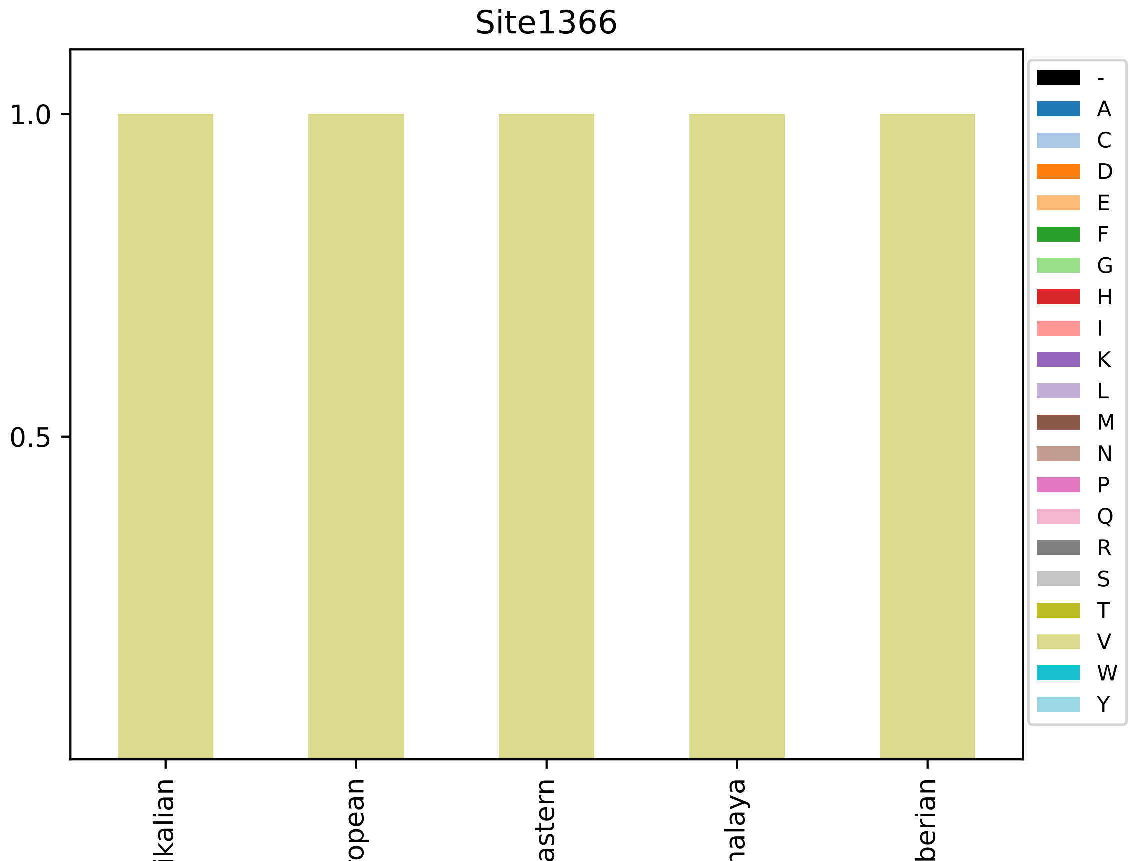
Site1366
1.0
0.5
-
A
C
D
E
F
G
H
I
K
L
M
N
P
Q
R
S
T
V
W
Y
ikalian
opean
astern
halaya
berian

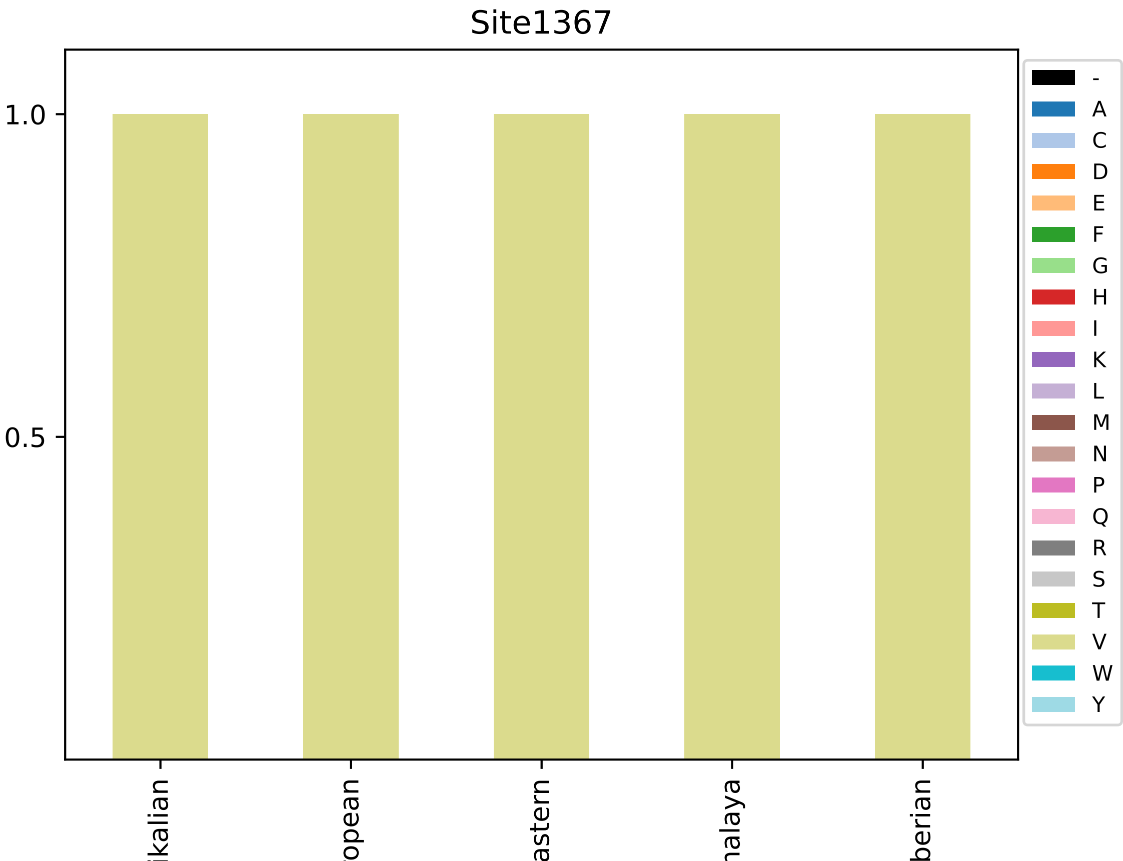

Site1367
1.0
0.5
-
A
C
D
E
F
G
H
I
K
L
M
N
P
Q
R
S
T
V
W
Y
ikalian
opean
astern
nalaya
berian

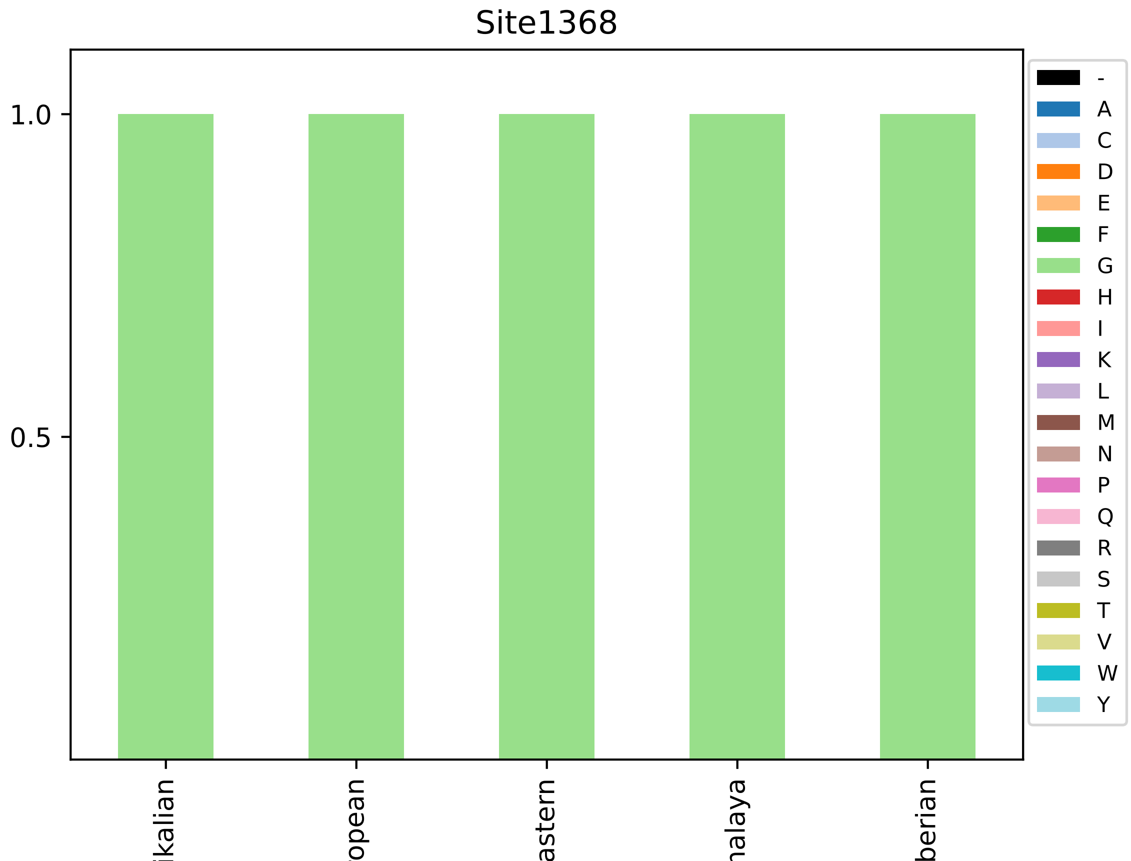
Site1368
1.0
0.5
-
A
C
D
E
F
G
H
I
K
L
M
N
P
Q
R
S
T
V
W
Y
ikalian
opean
astern
nalaya
berian

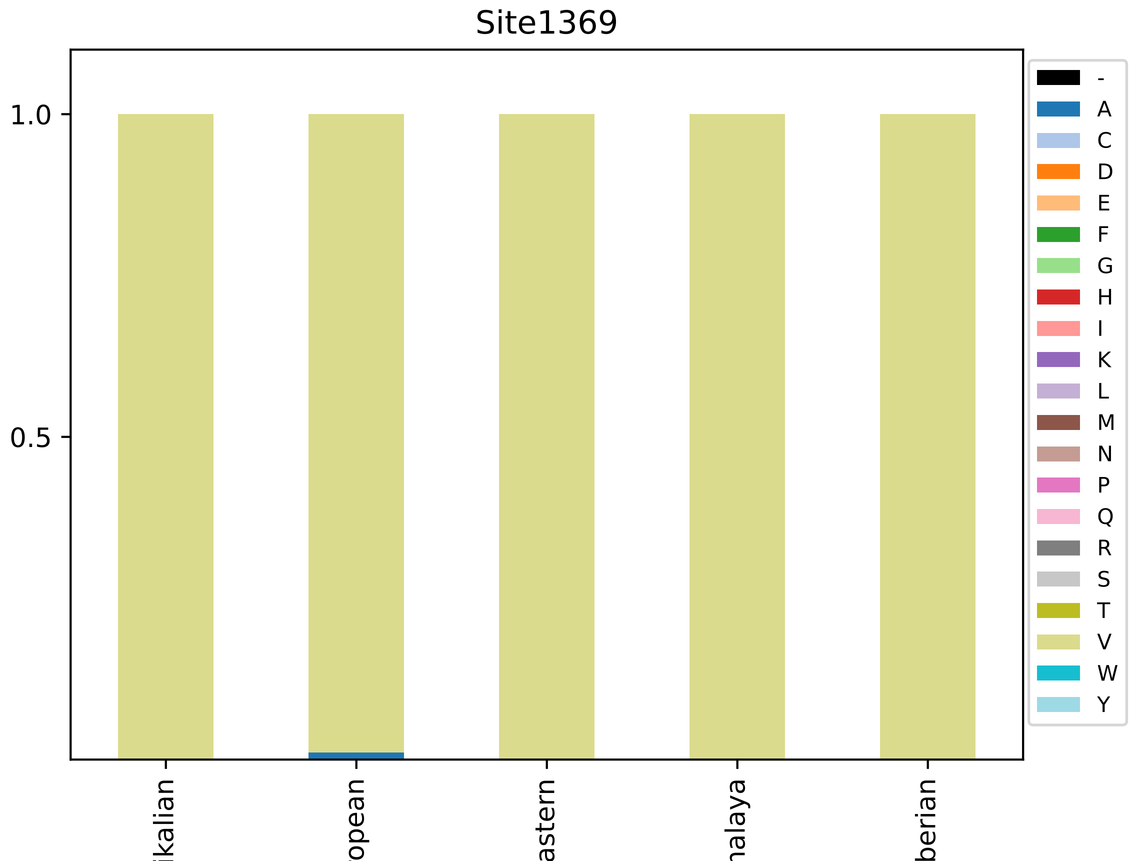

Site1369
1.0
0.5
-
A
C
D
E
F
G
H
I
K
L
M
N
P
Q
R
S
T
V
W
Y
ikalian
opean
astern
nalaya
berian

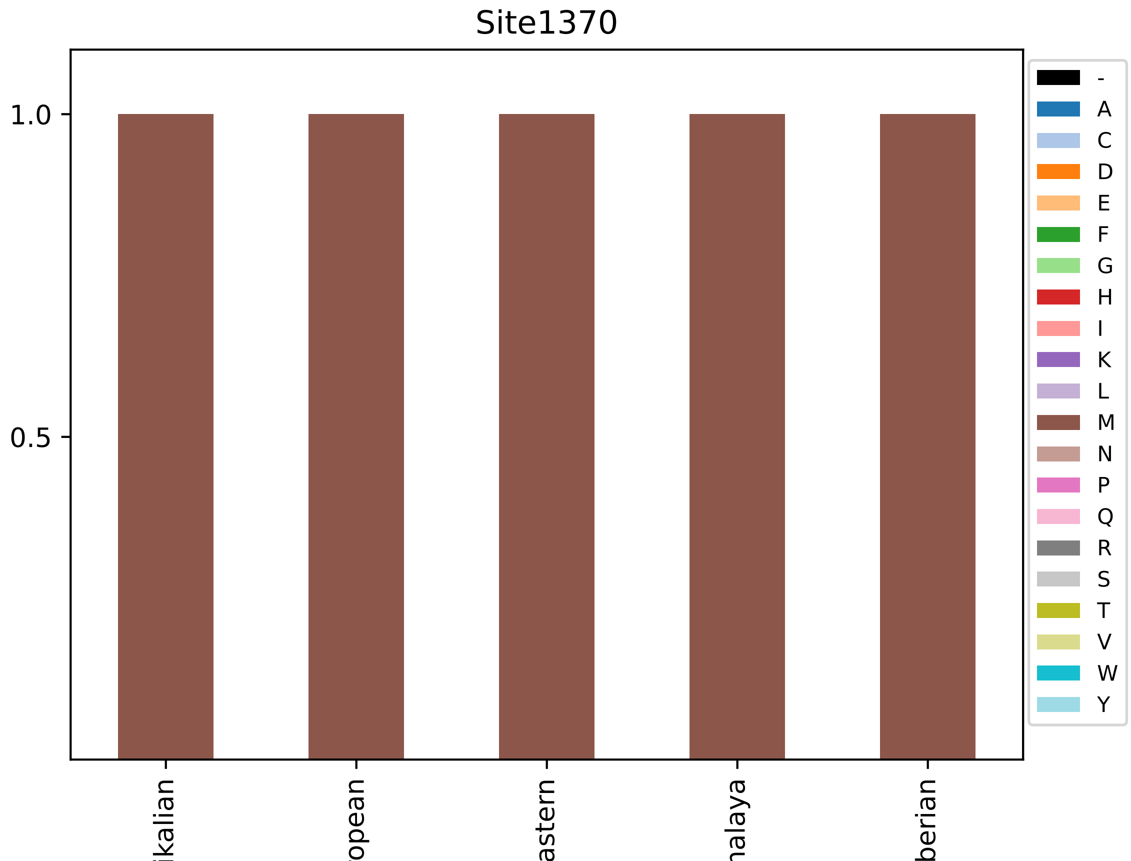

Site1370
1.0
0.5
ikalian
opean
astern
nalaya
berian
-
A
C
D
E
F
G
H
I
K
L
M
N
P
Q
R
S
T
V
W
Y

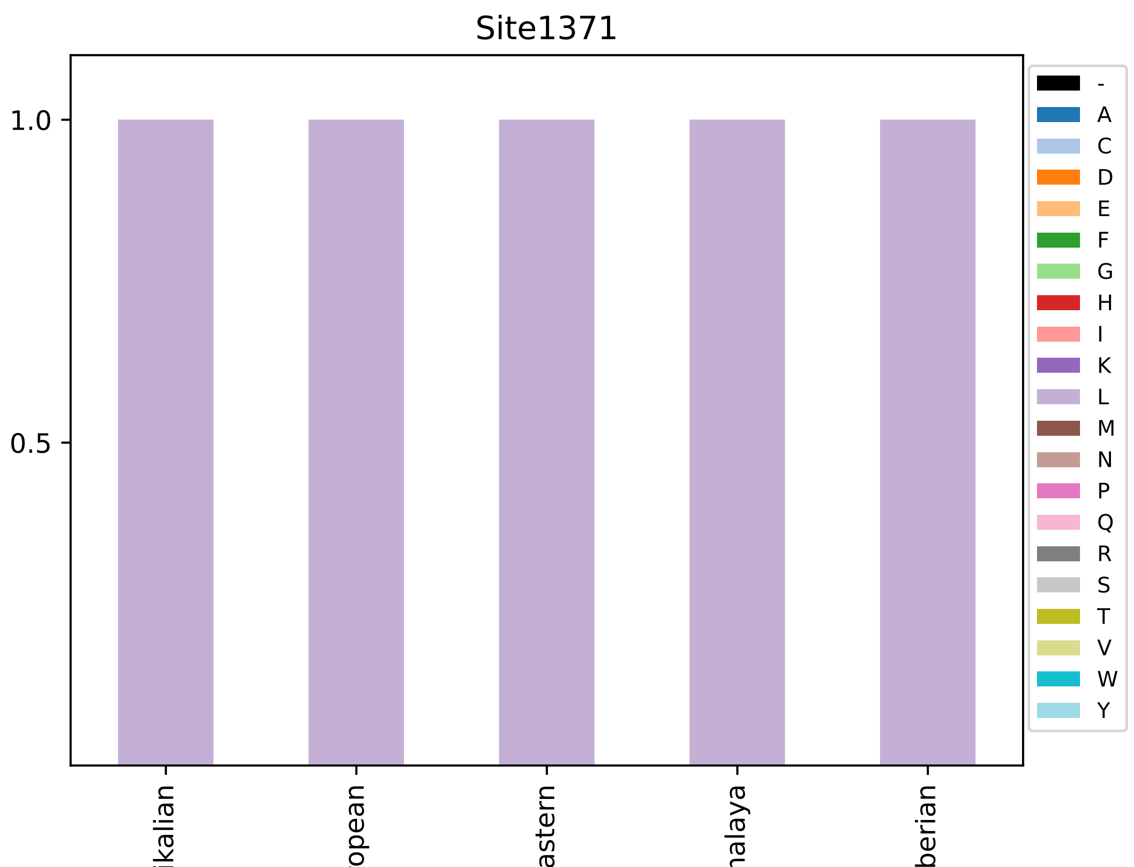
Site1371
1.0
0.5
-
A
C
D
E
F
G
H
I
K
L
M
N
P
Q
R
S
T
V
W
Y
ikalian
opean
astern
nalaya
berian

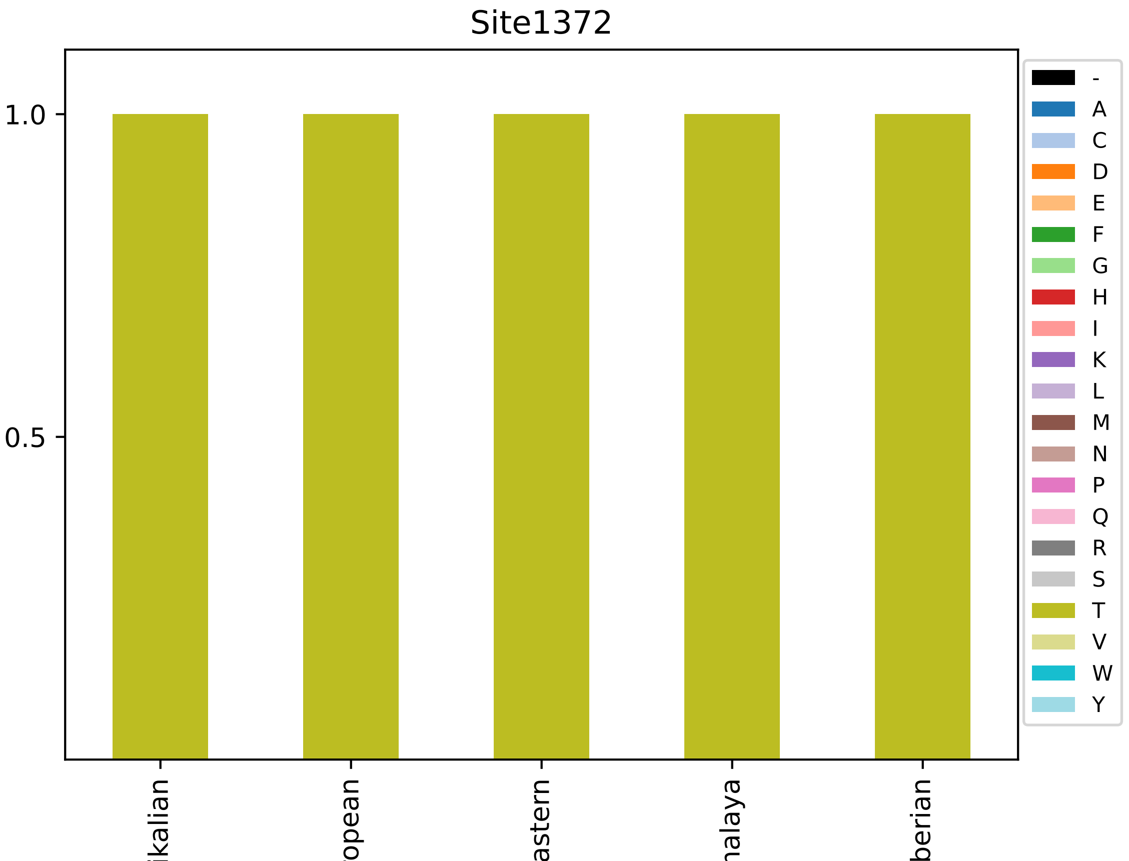
Site1372
1.0
0.5
-
A
C
D
E
F
G
H
I
K
L
M
N
P
Q
R
S
T
V
W
Y
ikalian
opean
astern
nalaya
berian

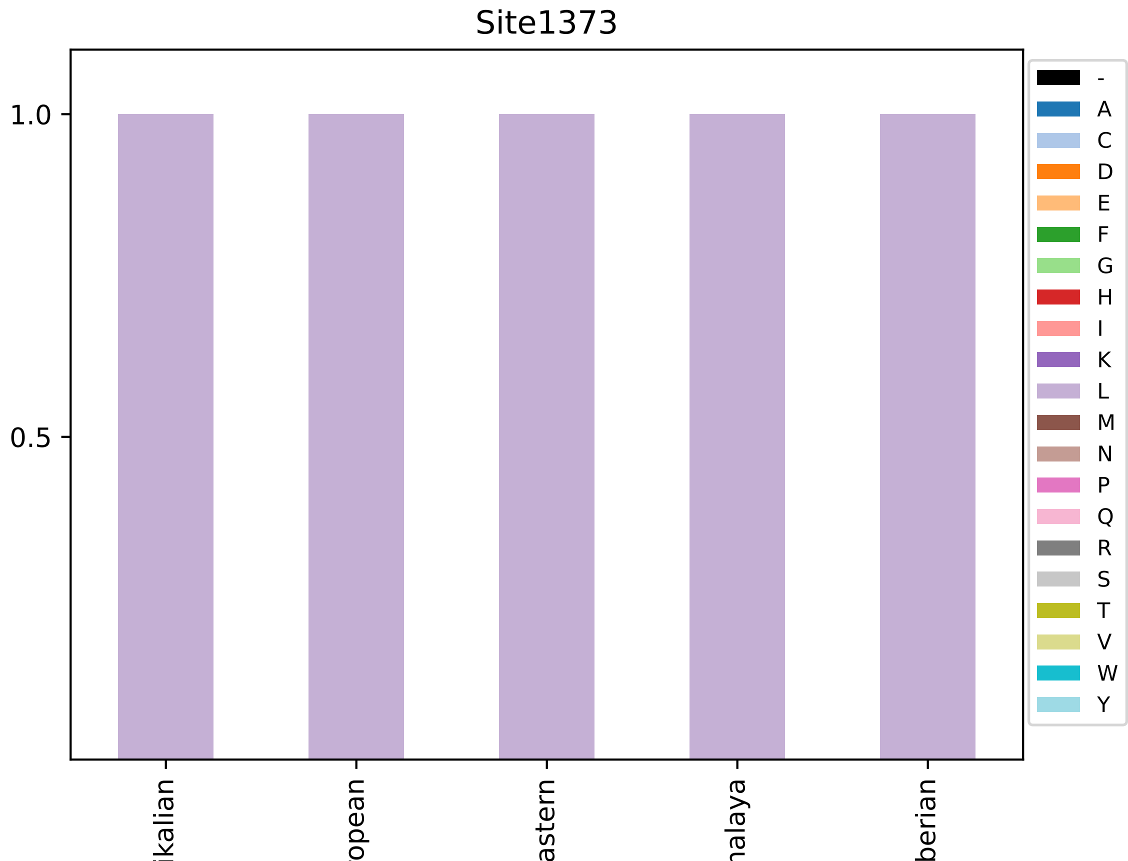

Site1373
1.0
0.5
ikalian
opean
astern
nalaya
berian
-
A
C
D
E
F
G
H
I
K
L
M
N
P
Q
R
S
T
V
W
Y

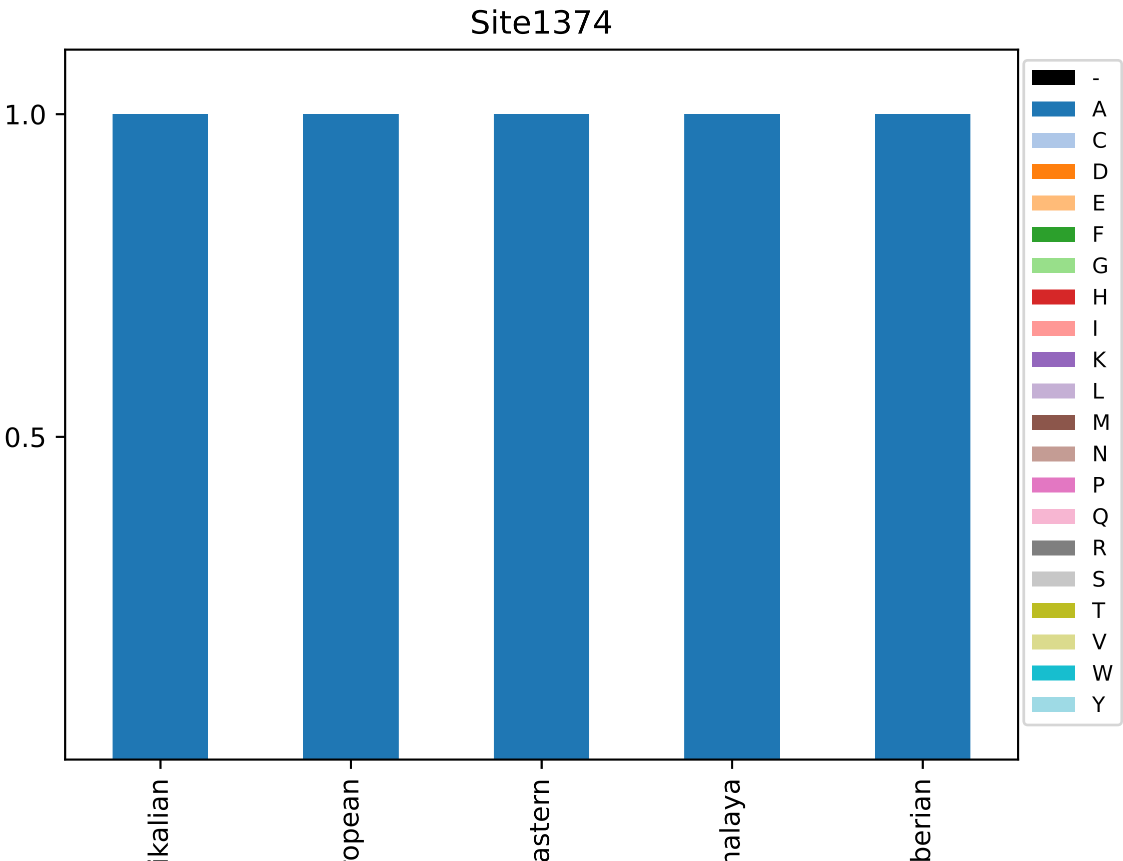
Site1374
1.0
0.5
-
A
C
D
E
F
G
H
I
K
L
M
N
P
Q
R
S
T
V
W
Y
ikalian
opean
astern
nalaya
berian

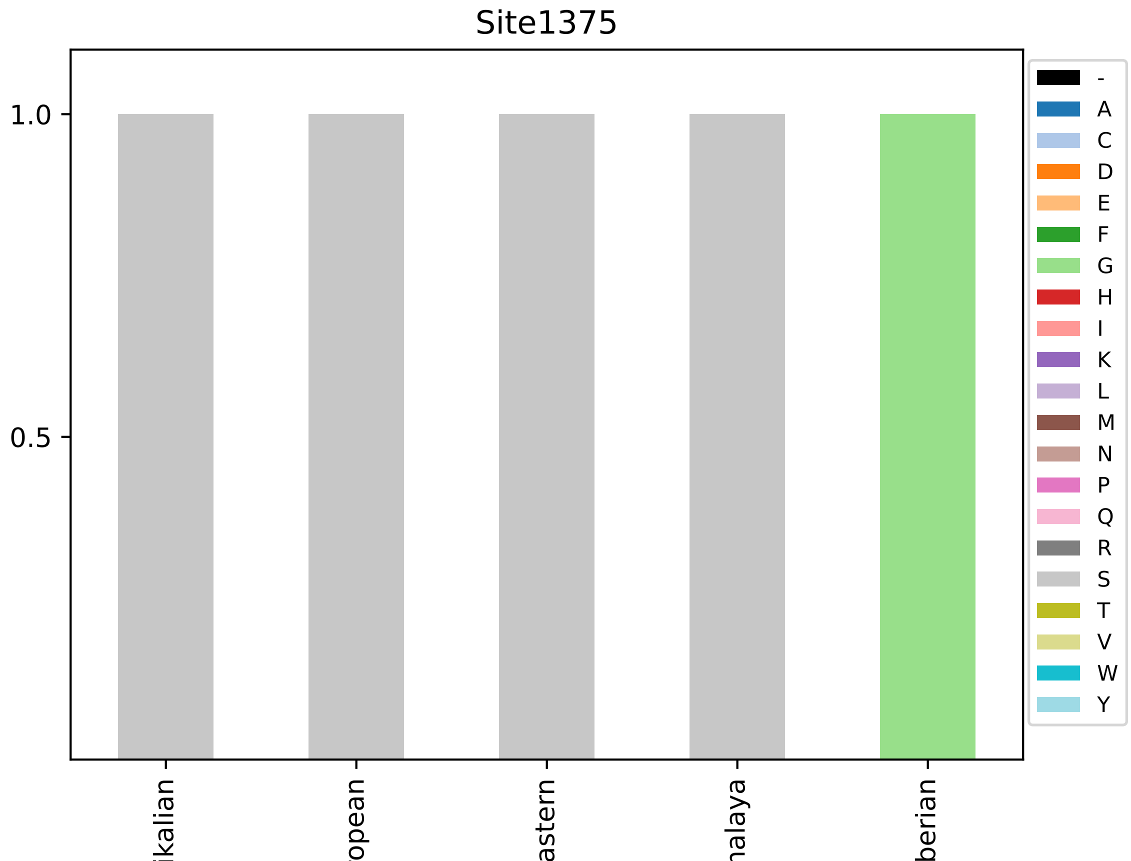
Site1375
1.0
0.5
-
A
C
D
E
F
G
H
I
K
L
M
N
P
Q
R
S
T
V
W
Y
ikalian
ropean
astern
nalaya
berian

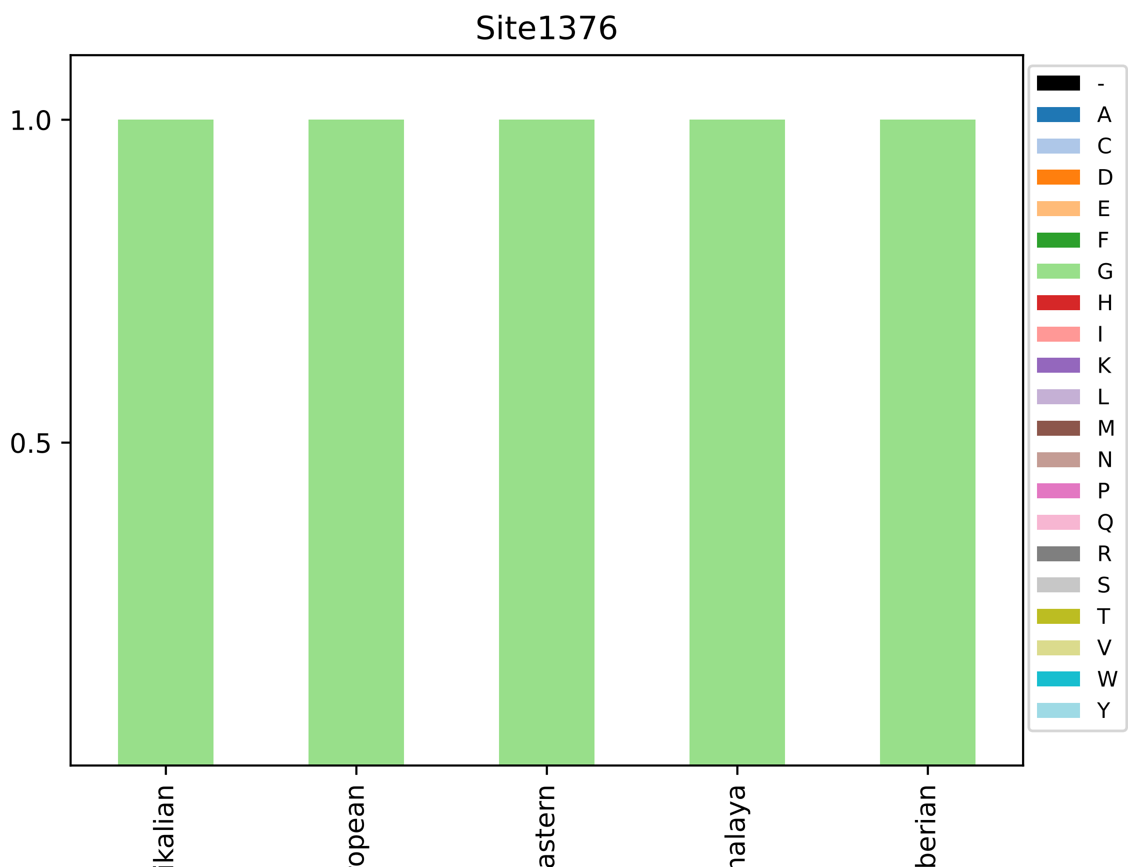

Site1376
1.0
0.5
ikalian
opean
astern
nalaya
berian
-
A
C
D
E
F
G
H
I
K
L
M
N
P
Q
R
S
T
V
W
Y

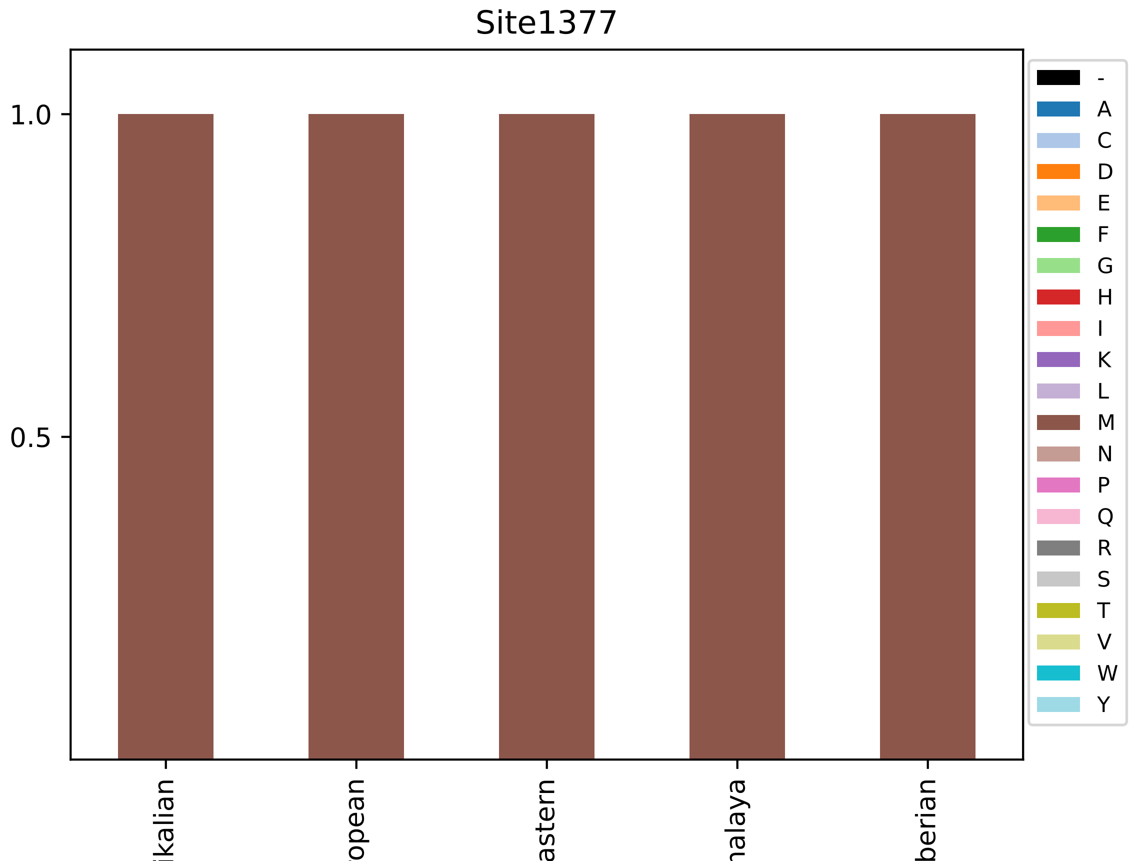
Site1377
1.0
0.5
-
A
C
D
E
F
G
H
I
K
L
M
N
P
Q
R
S
T
V
W
Y
ikalian
opean
astern
nalaya
berian

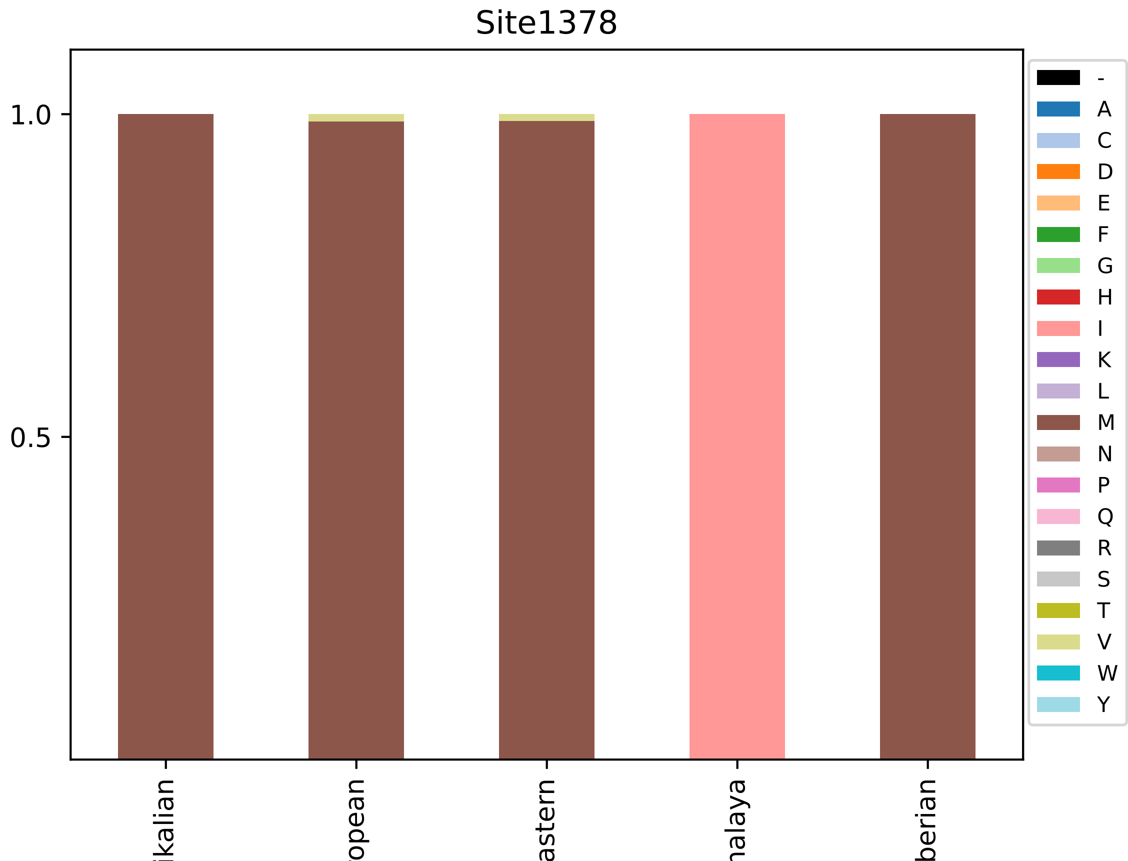
Site1378
1.0
0.5
-
A
C
D
E
F
G
H
I
K
L
M
N
P
Q
R
S
T
V
W
Y
ikalian
opean
astern
nalaya
berian

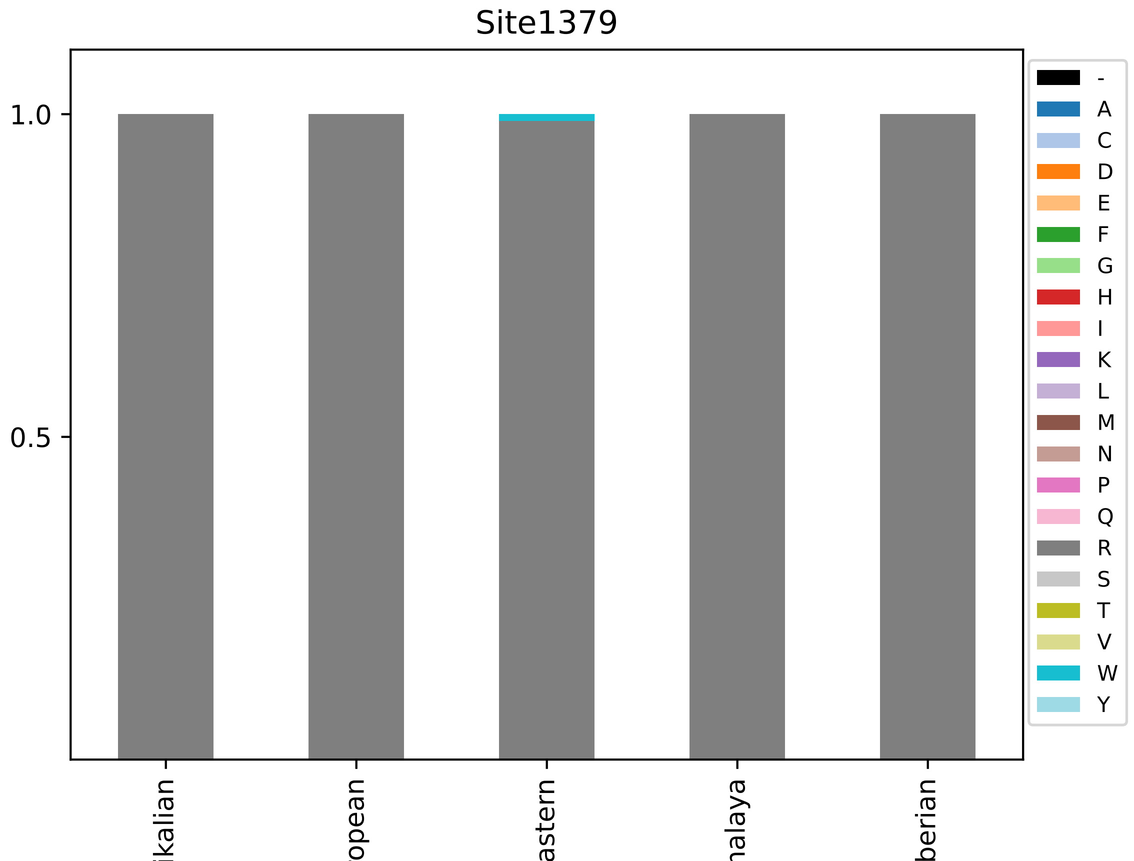
Site1379
1.0
0.5
-
A
C
D
E
F
G
H
I
K
L
M
N
P
Q
R
S
T
V
W
Y

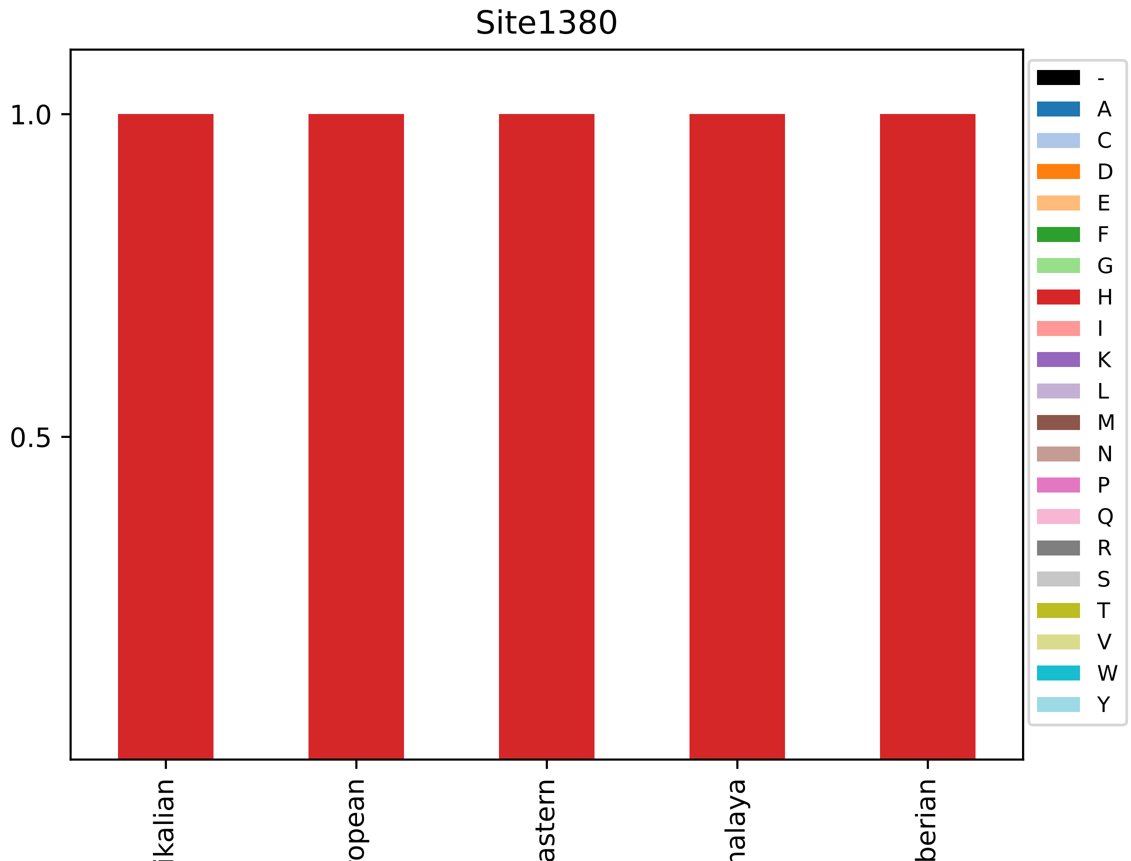
Site1380
1.0
0.5
-
A
C
D
E
F
G
H
I
K
L
M
N
P
Q
R
S
T
V
W
Y
ikalian
opean
astern
nalaya
berian

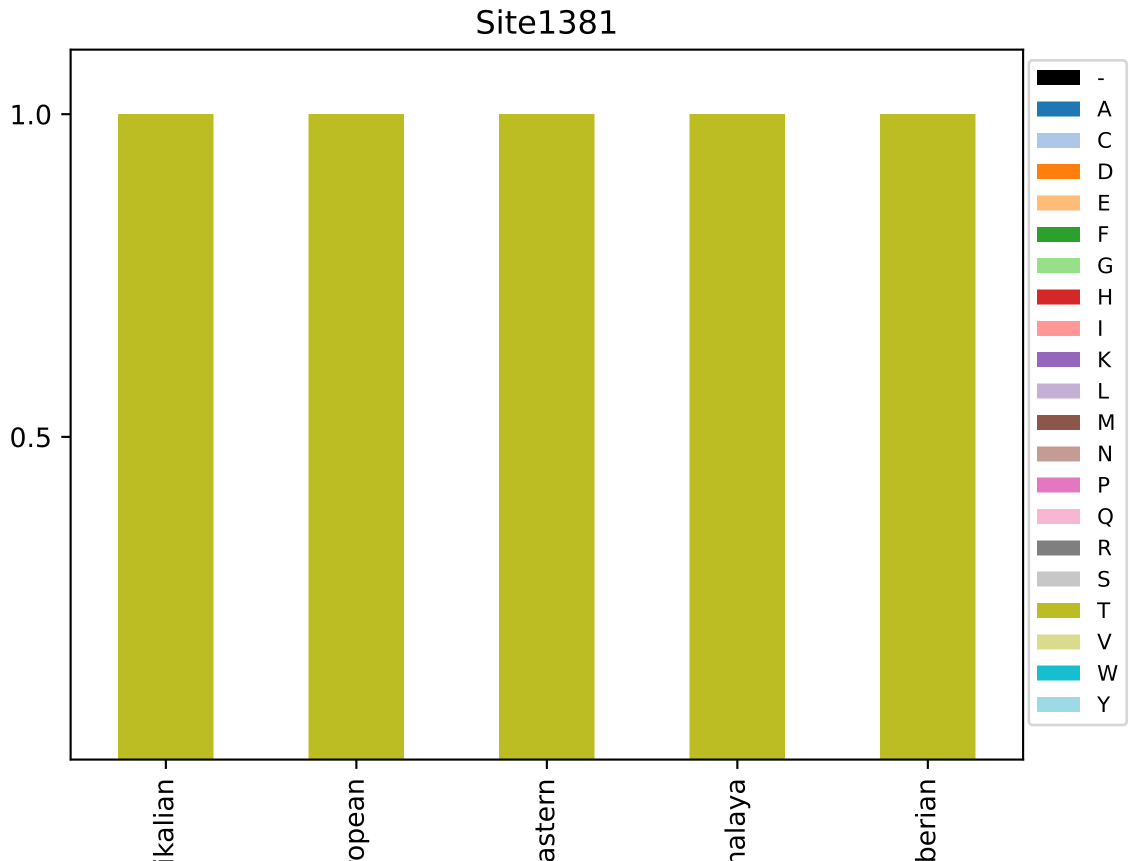
Site1381
1.0
0.5
-
A
C
D
E
F
G
H
I
K
L
M
N
P
Q
R
S
T
V
W
Y
ikalian
opean
astern
nalaya
berian

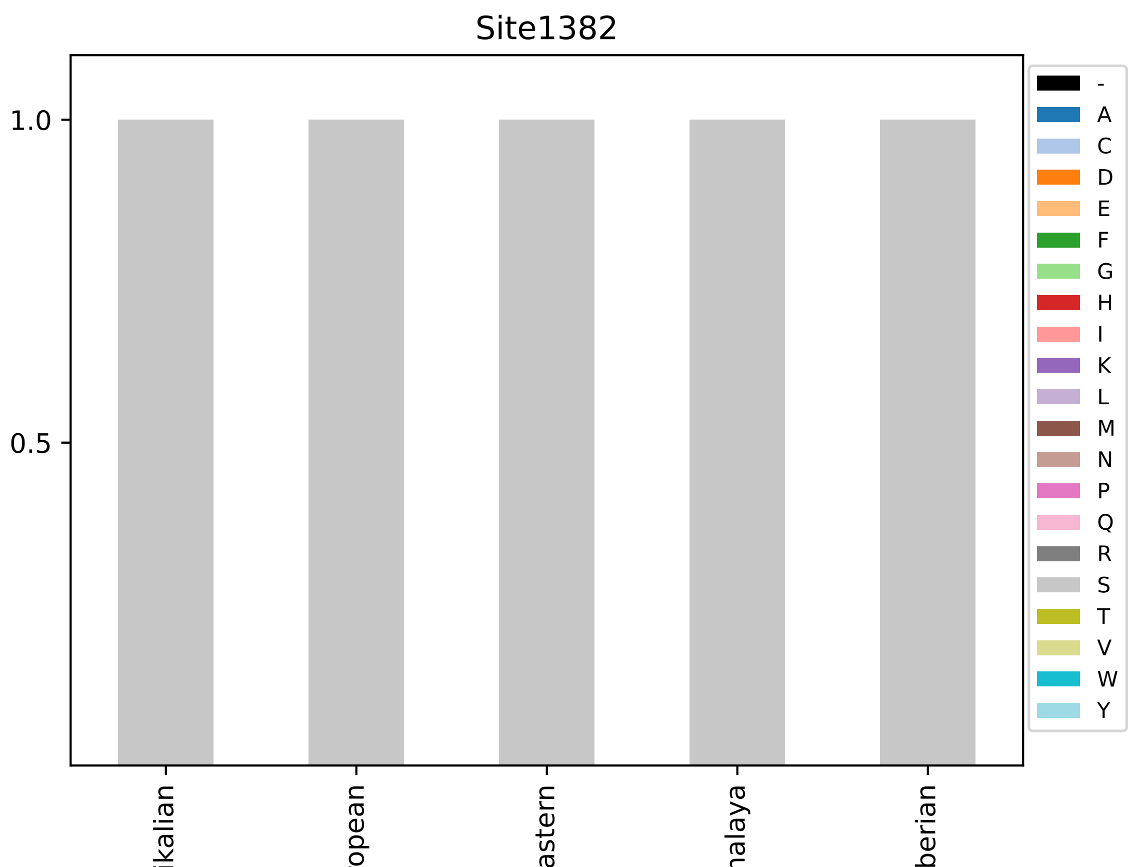
Site1382
1.0
0.5
-
A
C
D
E
F
G
H
I
K
L
M
N
P
Q
R
S
T
V
W
Y
ikalian
opean
astern
nalaya
berian

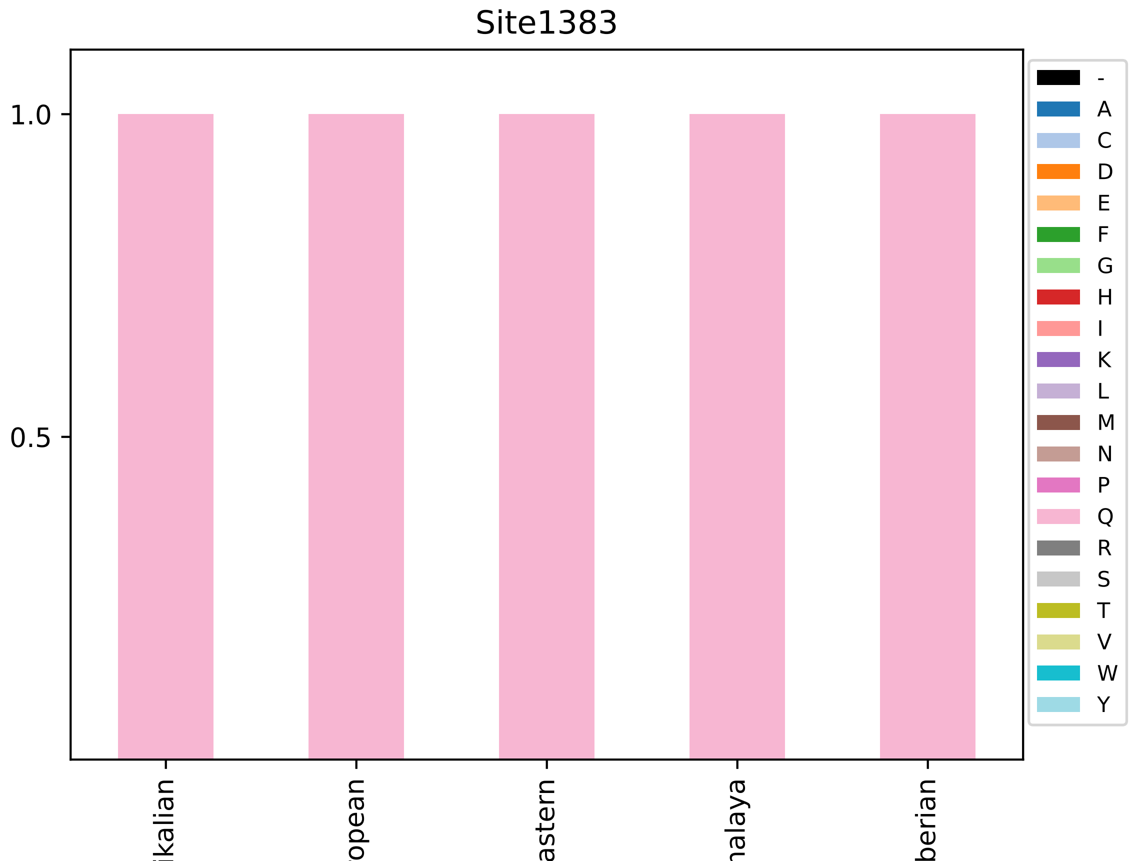
Site1383
1.0
0.5
-
A
C
D
E
F
G
H
I
K
L
M
N
P
Q
R
S
T
V
W
Y
ikalian
opean
astern
nalaya
berian

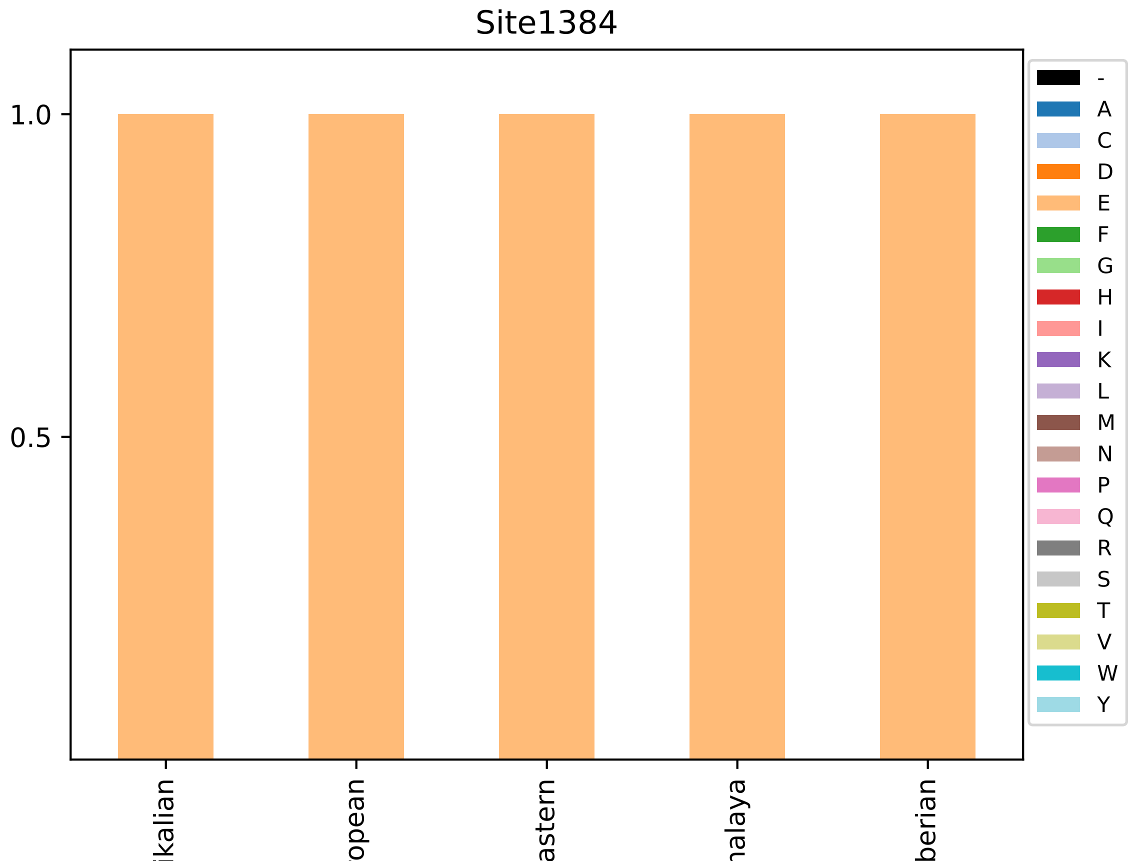

Site1384
1.0
0.5
-
A
C
D
E
F
G
H
I
K
L
M
N
P
Q
R
S
T
V
W
Y
ikalian
opean
astern
nalaya
berian

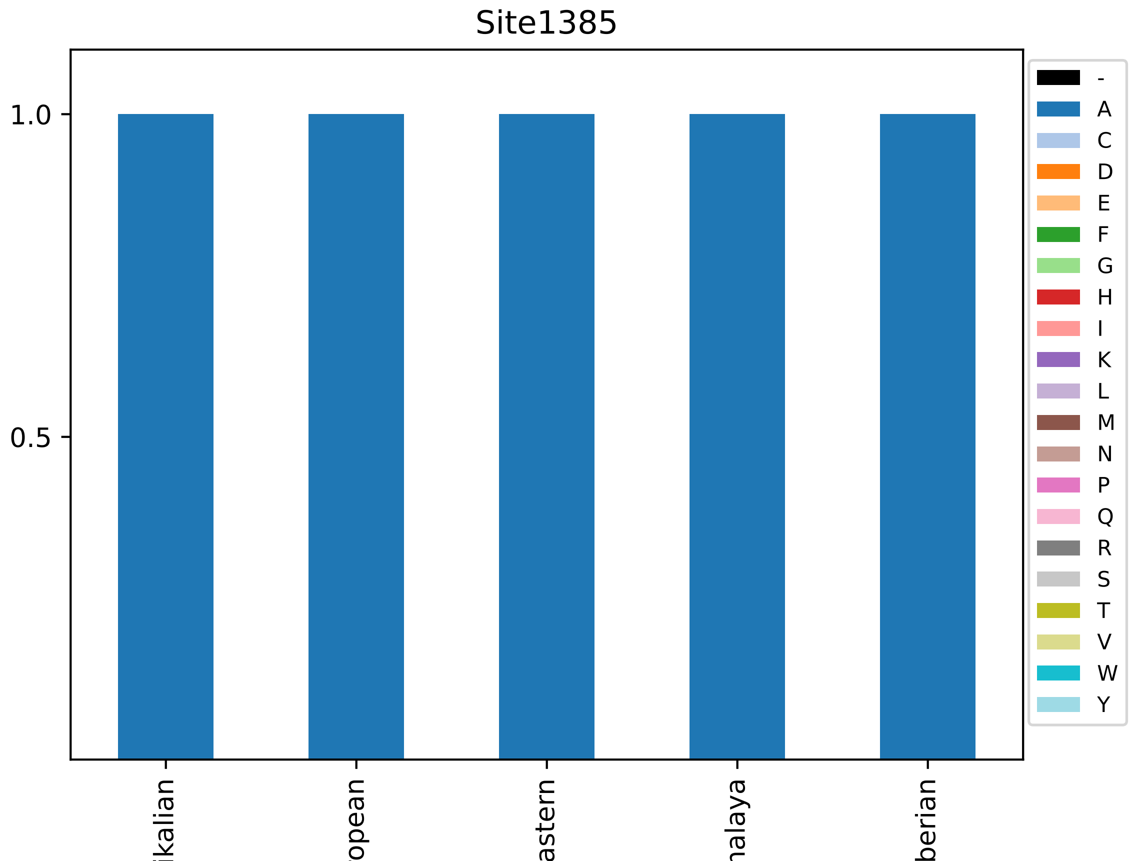
Site1385
1.0
0.5
-
A
C
D
E
F
G
H
I
K
L
M
N
P
Q
R
S
T
V
W
Y
ikalian
opean
astern
nalaya
berian

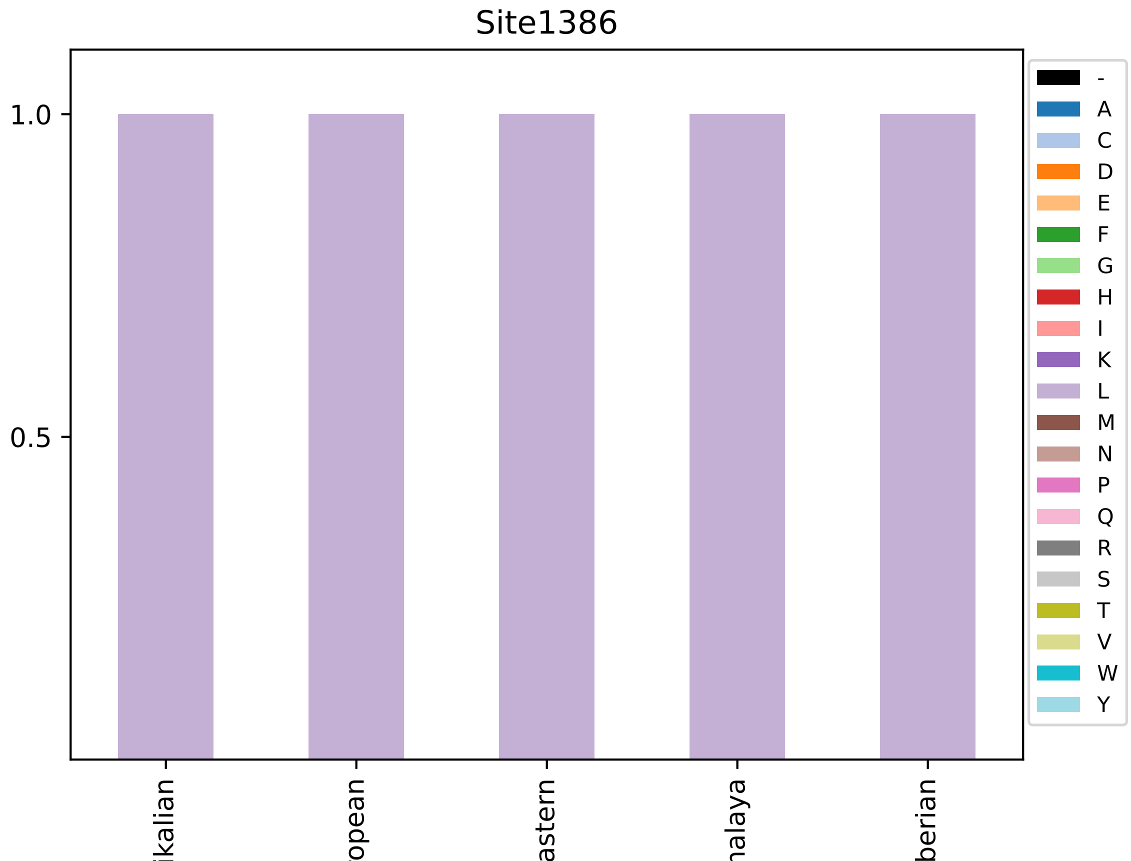
Site1386
1.0
0.5
-
A
C
D
E
F
G
H
I
K
L
M
N
P
Q
R
S
T
V
W
Y
ikalian
opean
astern
nalaya
berian

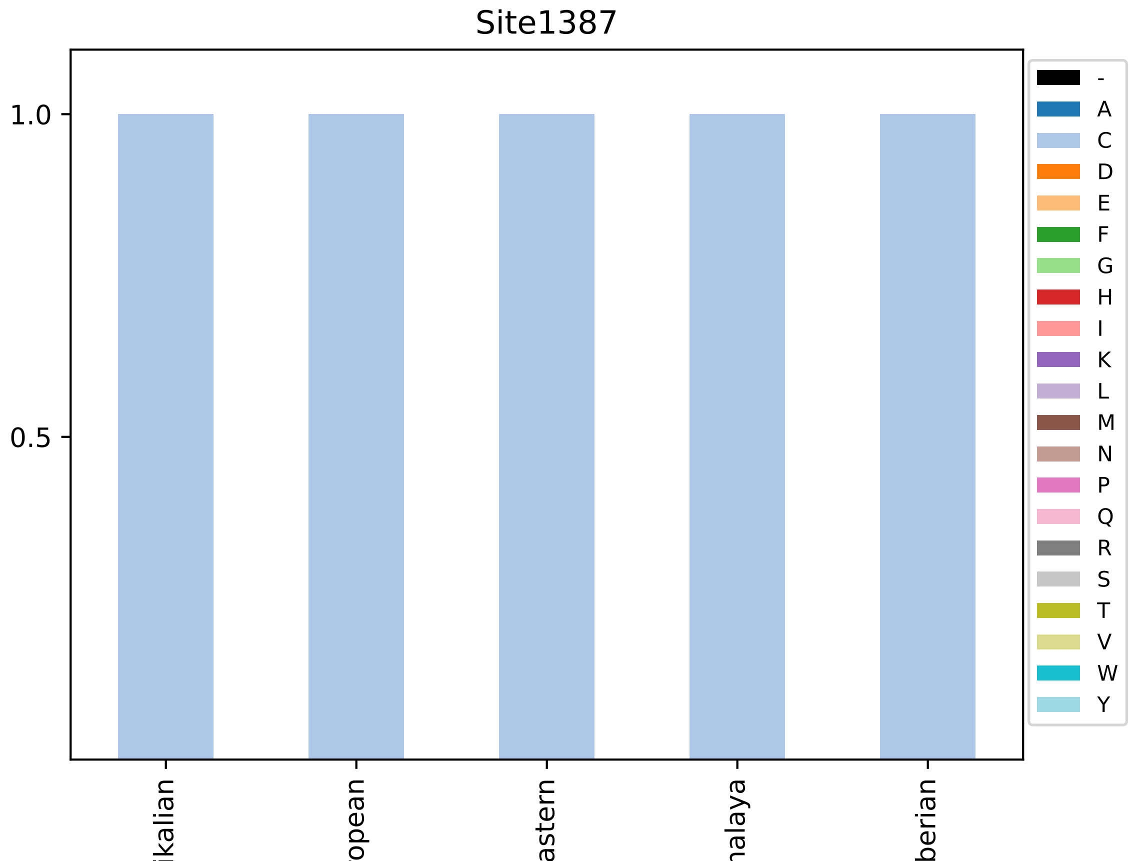
Site1387
1.0
0.5
-
A
C
D
E
F
G
H
I
K
L
M
N
P
Q
R
S
T
V
W
Y
ikalian
opean
astern
nalaya
berian

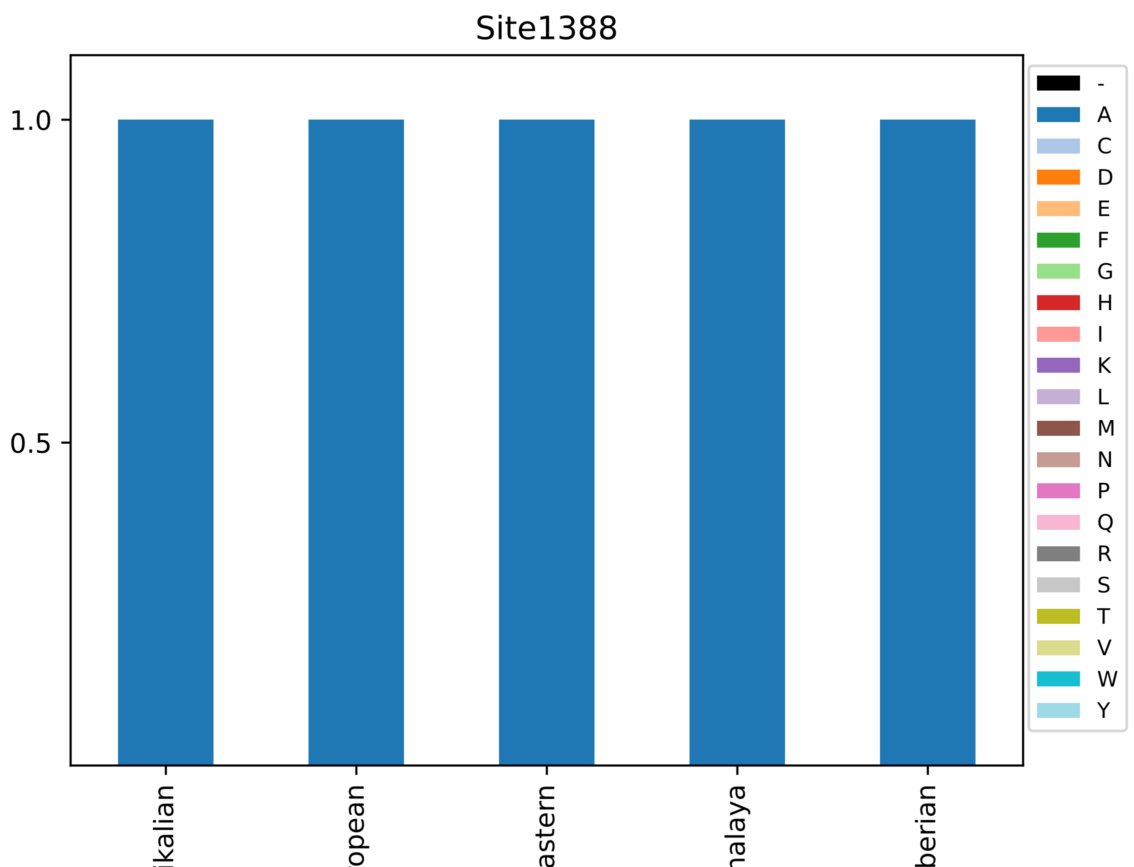
Site1388
1.0
0.5
ikalian
opean
astern
nalaya
berian
-
A
C
D
E
F
G
H
I
K
L
M
N
P
Q
R
S
T
V
W
Y

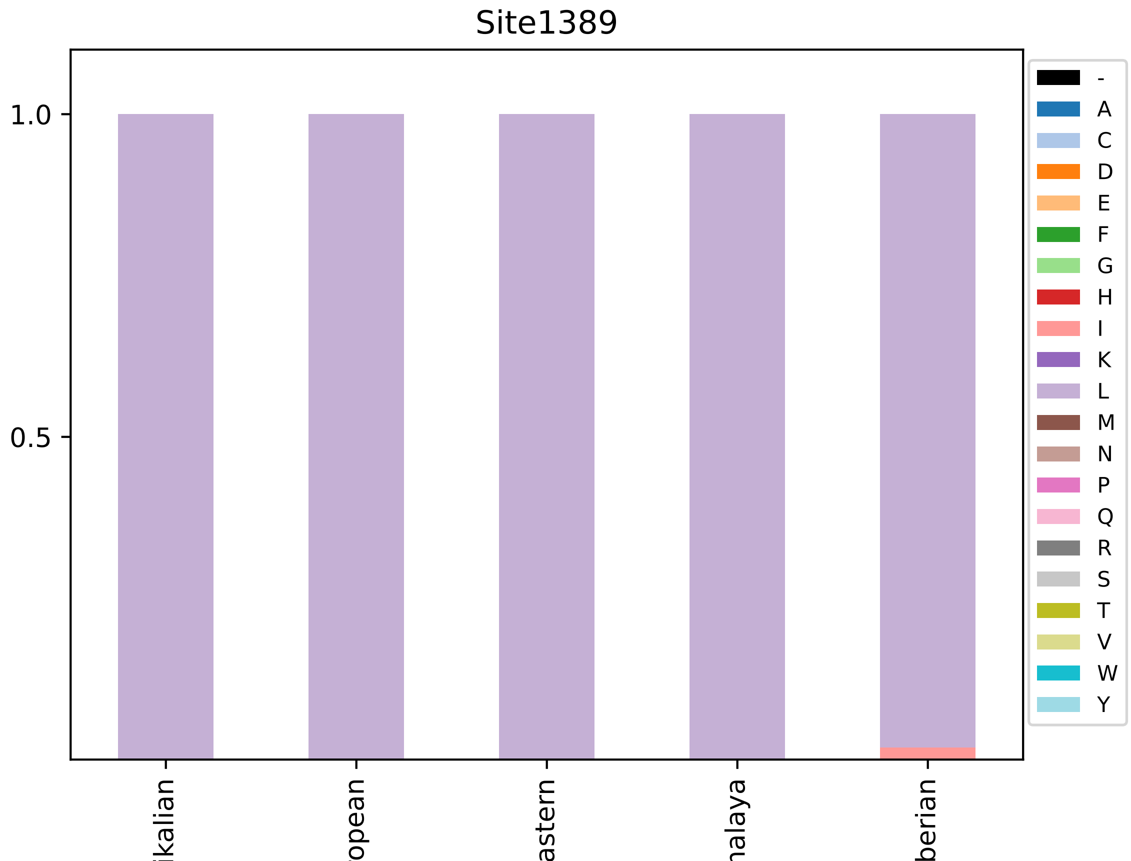

Site1389
1.0
0.5
-
A
C
D
E
F
G
H
I
K
L
M
N
P
Q
R
S
T
V
W
Y
ikalian
opean
astern
nalaya
berian

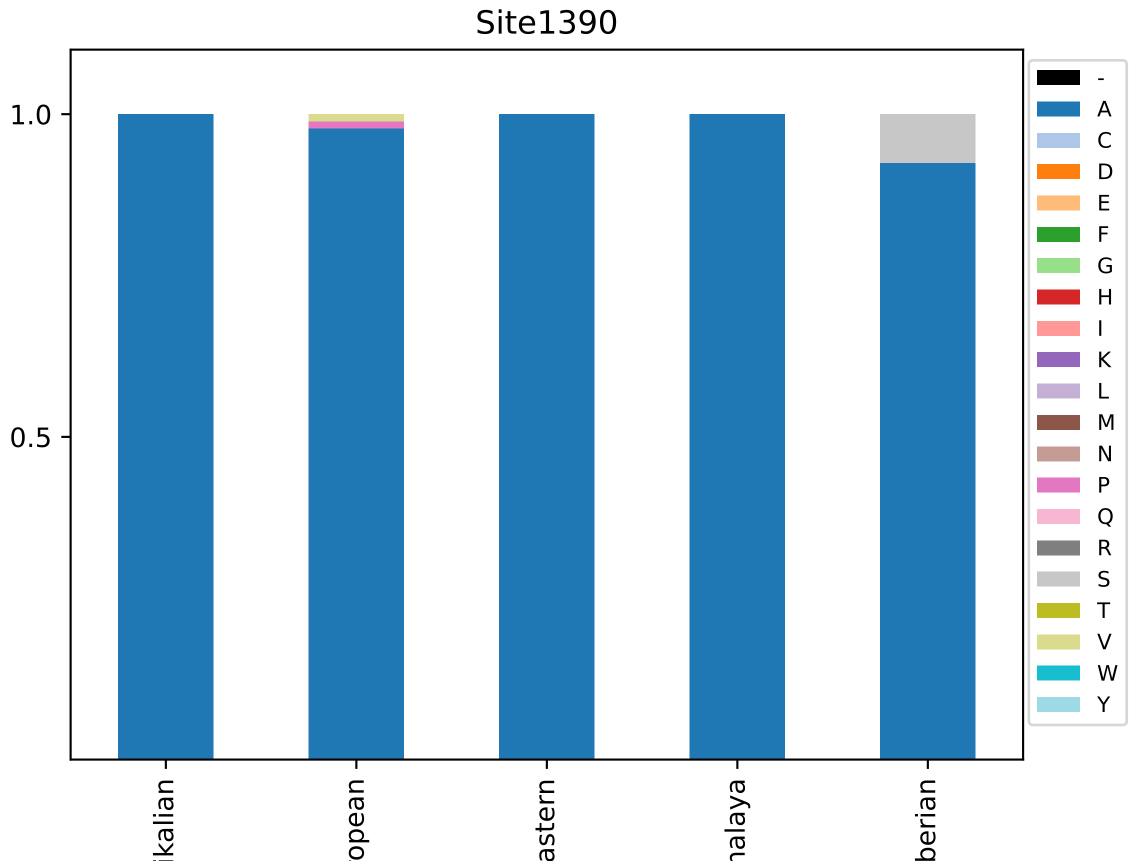
Site1390
1.0
0.5
-
A
C
D
E
F
G
H
I
K
L
M
N
P
Q
R
S
T
V
W
Y
ikalian
opean
astern
nalaya
berian

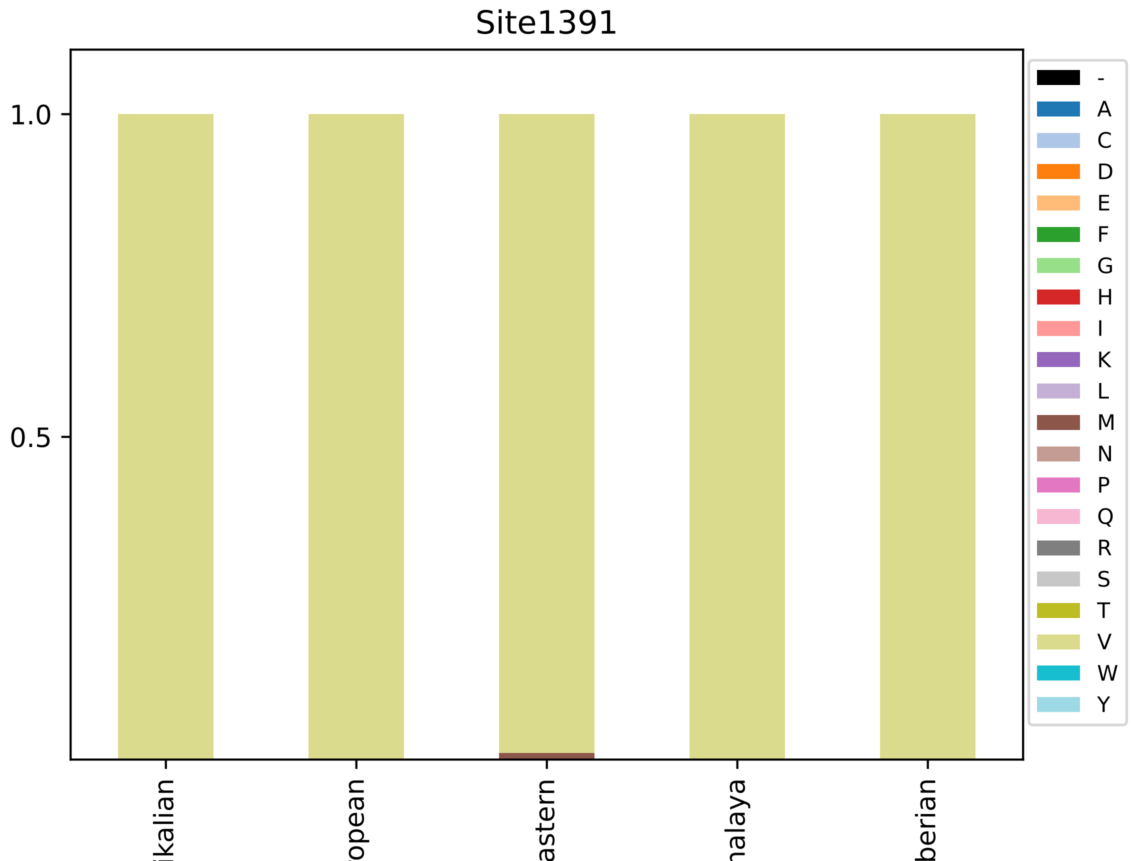
Site1391
1.0
0.5
-
A
C
D
E
F
G
H
I
K
L
M
N
P
Q
R
S
T
V
W
Y
ikalian
opean
astern
nalaya
berian

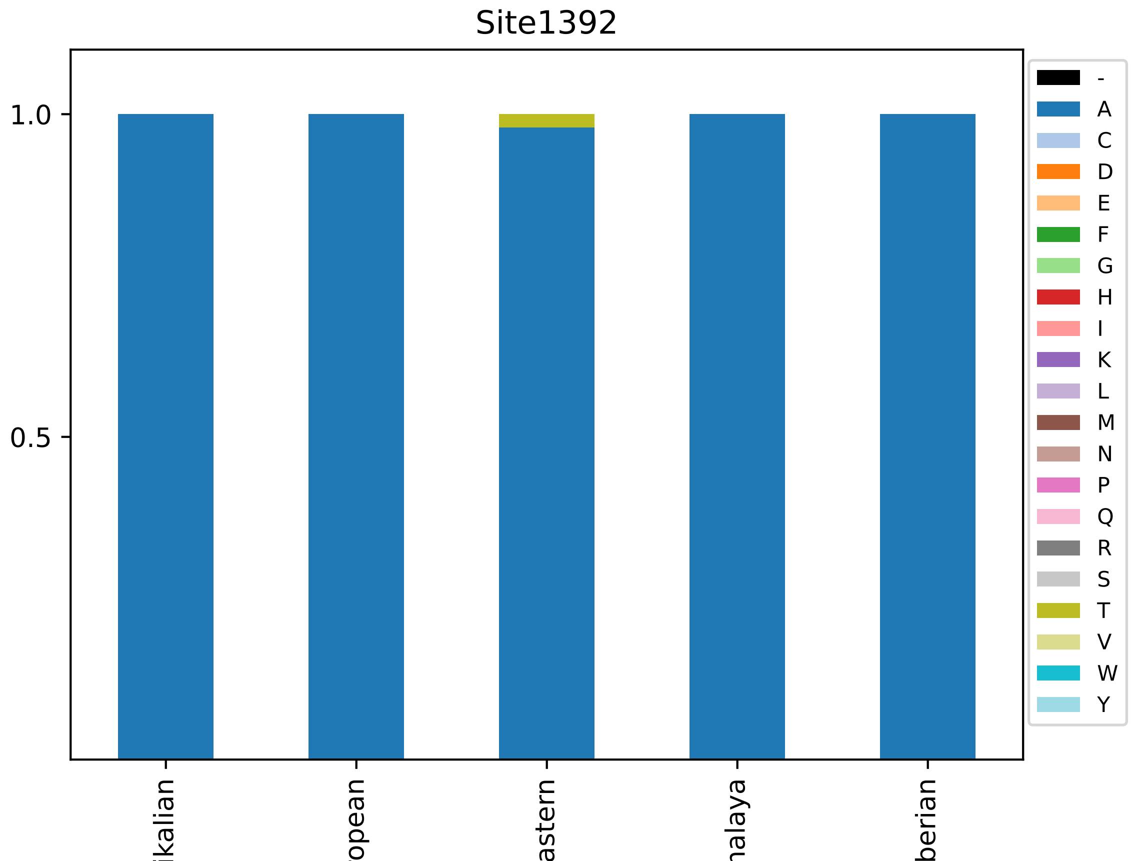

Site1392
1.0
0.5
-
A
C
D
E
F
G
H
I
K
L
M
N
P
Q
R
S
T
V
W
Y
ikalian
opean
astern
nalaya
berian

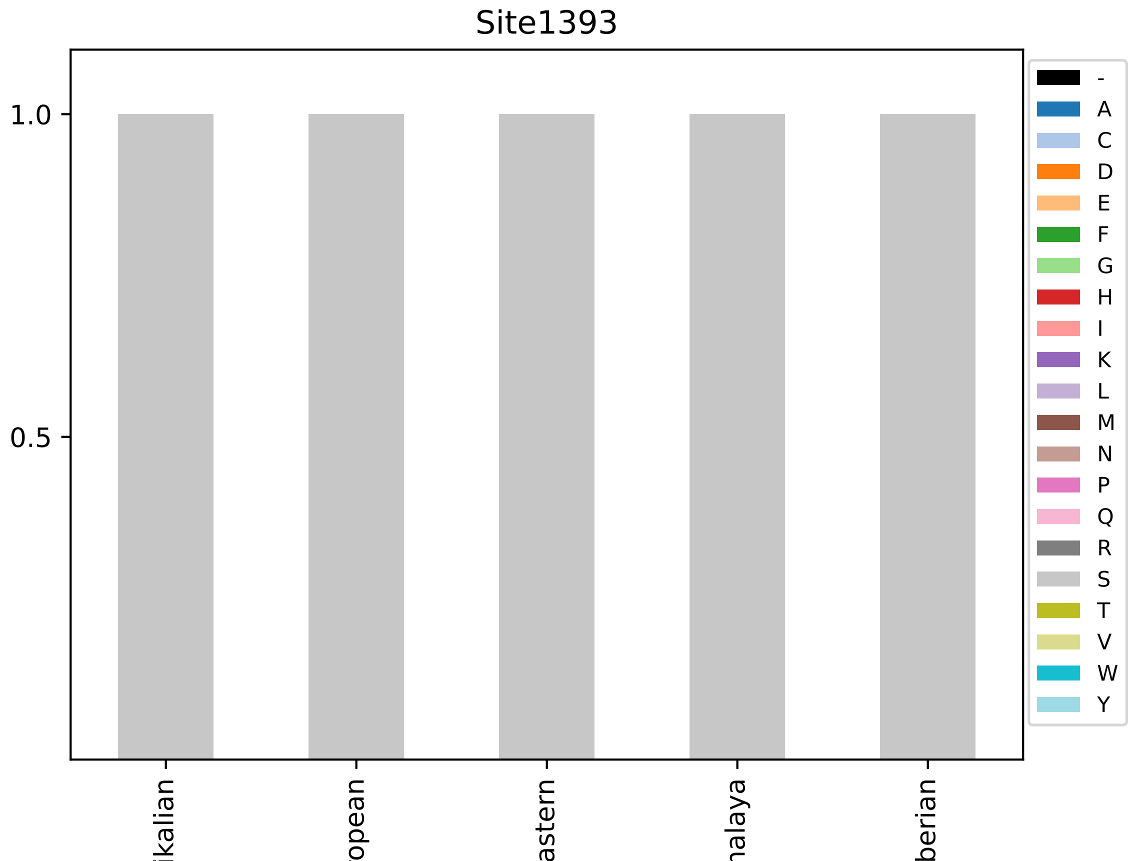
Site1393
1.0
0.5
-
A
C
D
E
F
G
H
I
K
L
M
N
P
Q
R
S
T
V
W
Y
ikalian
opean
astern
nalaya
berian

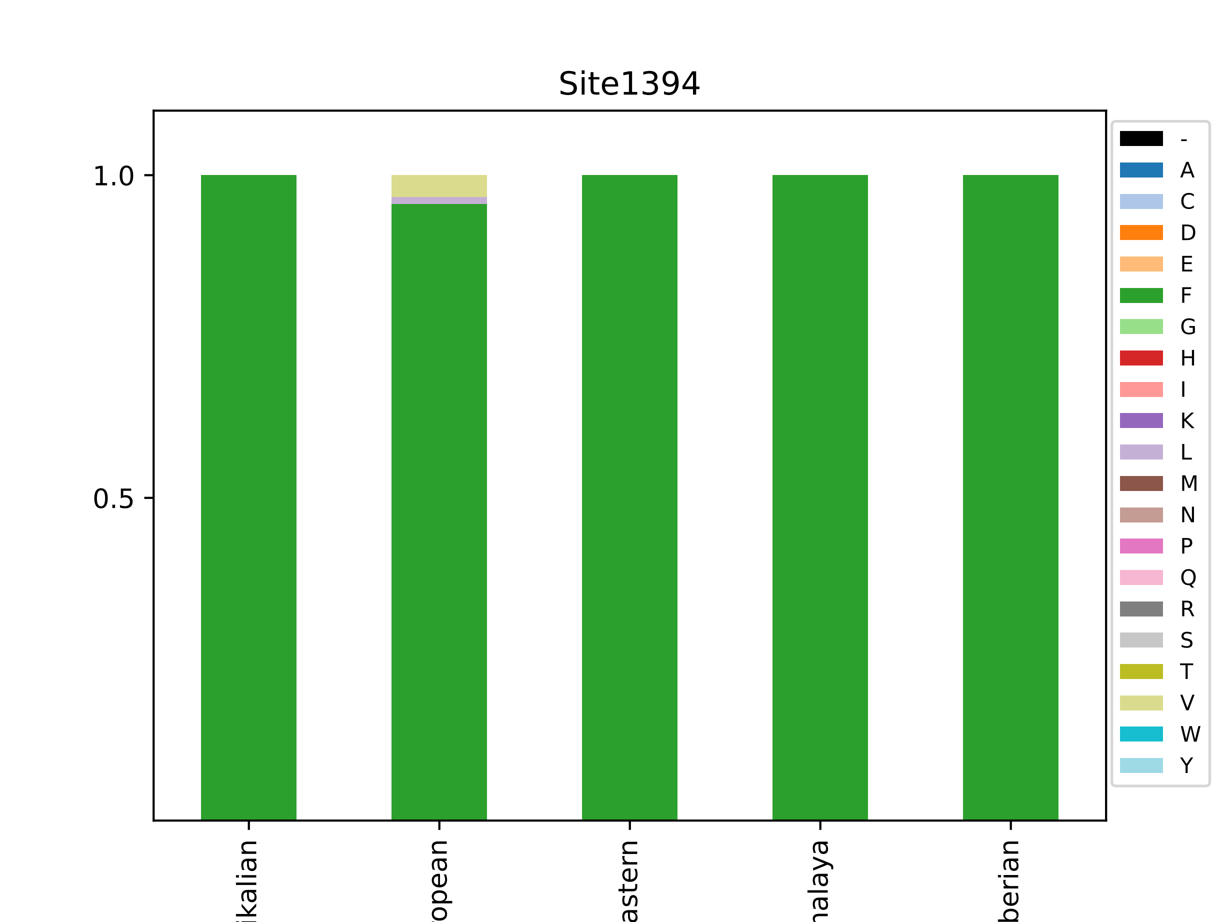

Site1394
1.0
0.5
-
A
C
D
E
F
G
H
I
K
L
M
N
P
Q
R
S
T
V
W
Y
ikalian
opean
astern
nalaya
berian

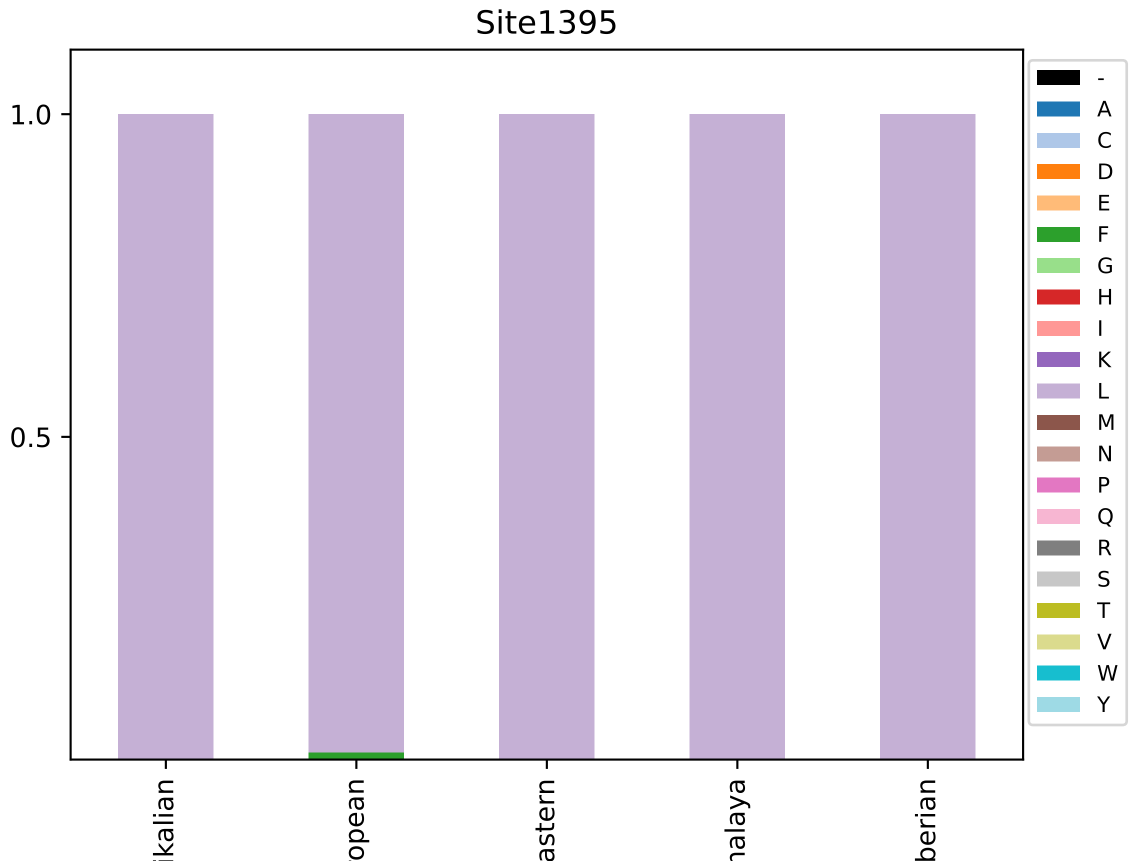

Site1395
1.0
0.5
-
A
C
D
E
F
G
H
I
K
L
M
N
P
Q
R
S
T
V
W
Y
ikalian
opean
astern
nalaya
berian

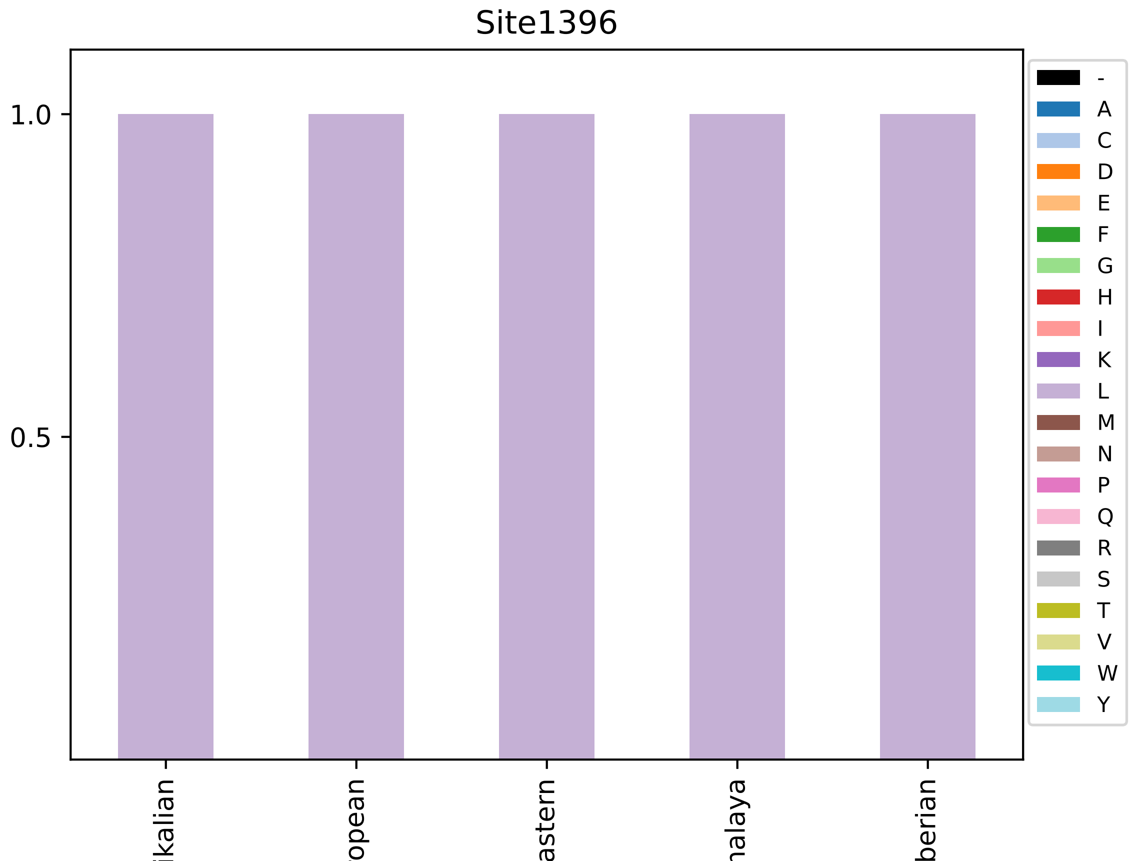
Site1396
1.0
0.5
-
A
C
D
E
F
G
H
I
K
L
M
N
P
Q
R
S
T
V
W
Y
ikalian
opean
astern
nalaya
berian

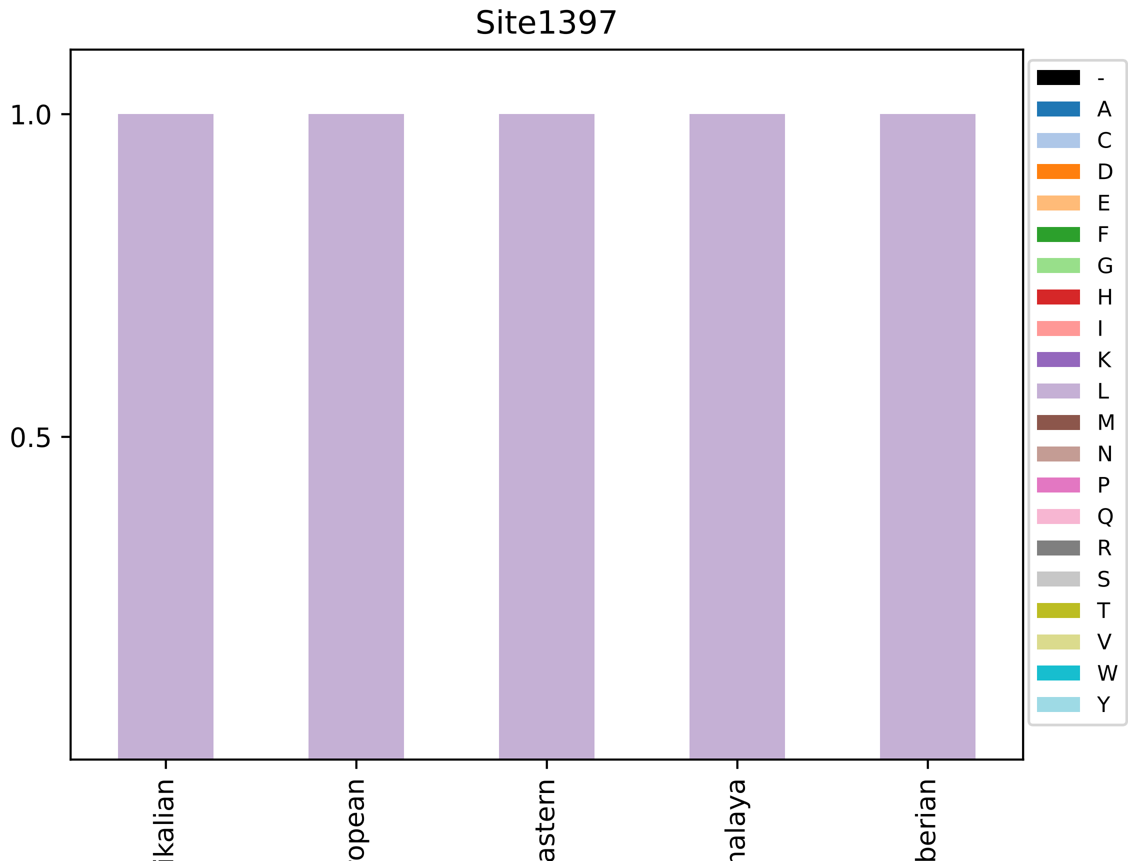

Site1397
1.0
0.5
-
A
C
D
E
F
G
H
I
K
L
M
N
P
Q
R
S
T
V
W
Y
ikalian
opean
astern
nalaya
berian

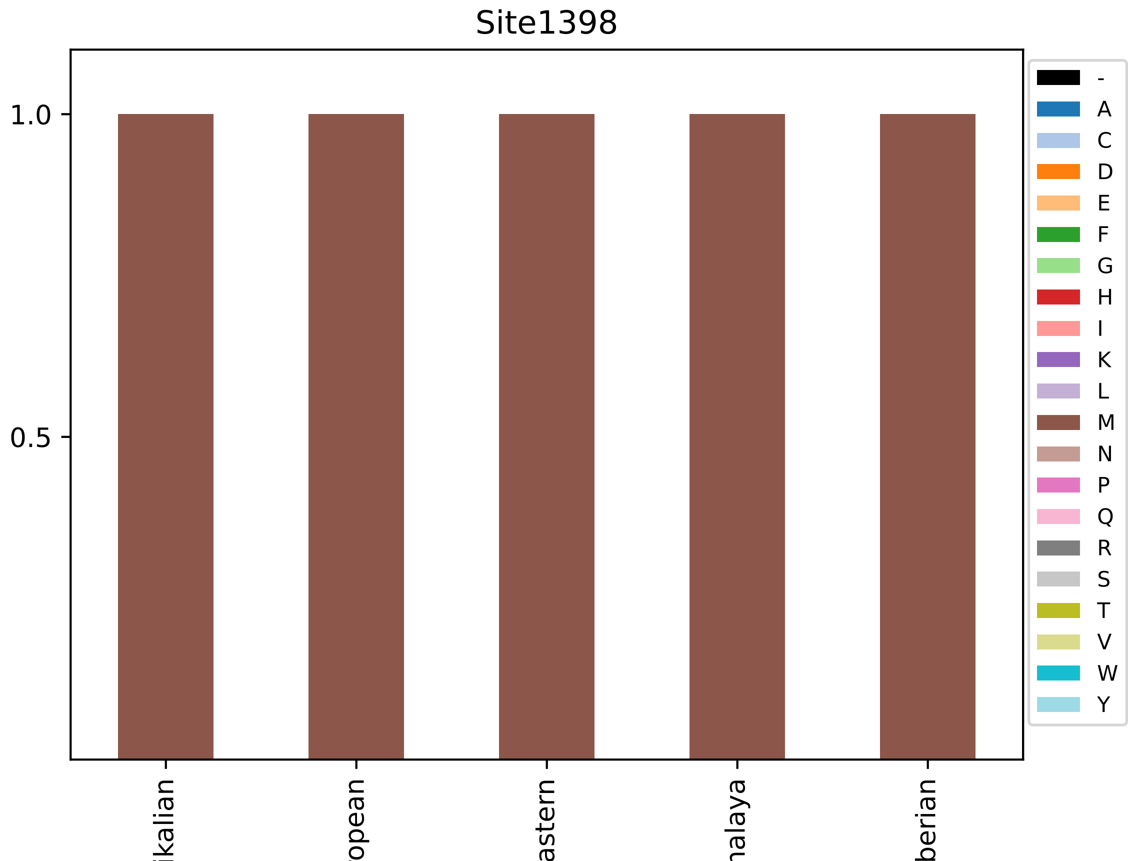
Site1398
1.0
0.5
-
A
C
D
E
F
G
H
I
K
L
M
N
P
Q
R
S
T
V
W
Y
ikalian
ropean
astern
nalaya
berian

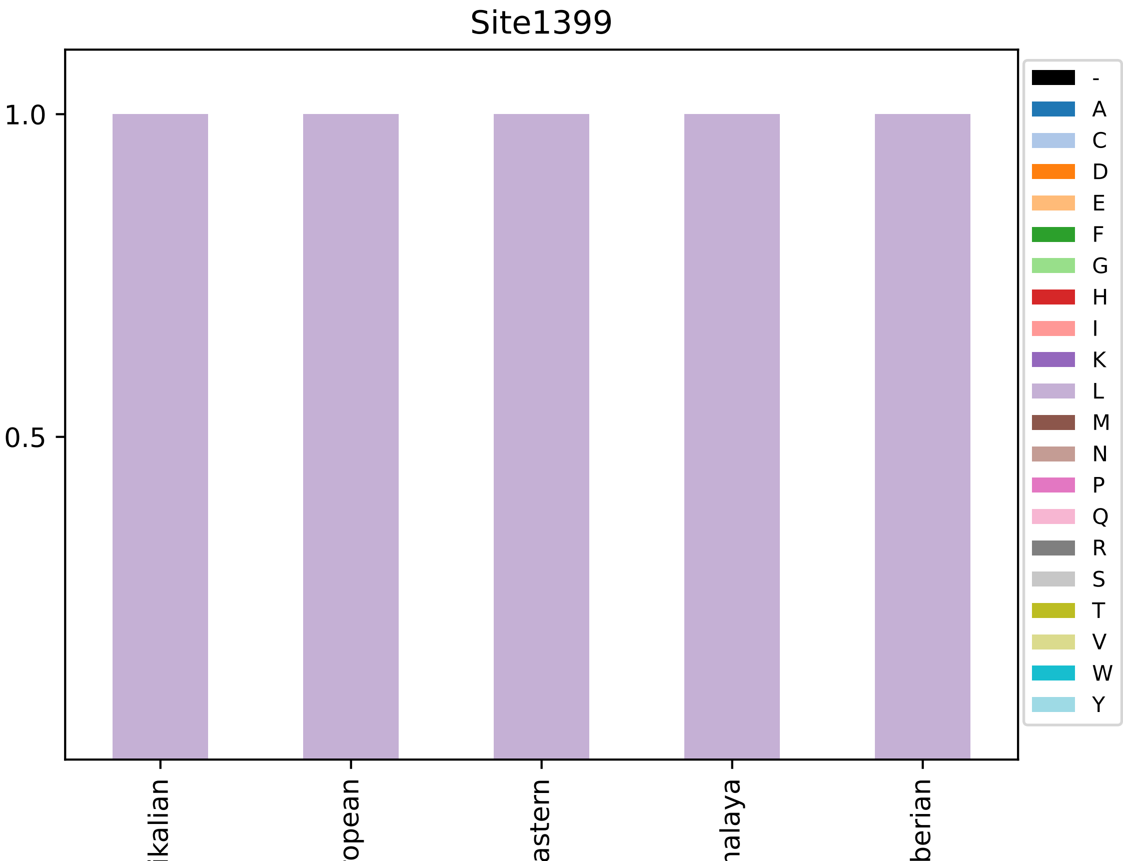

Site1399
1.0
0.5
-
A
C
D
E
F
G
H
I
K
L
M
N
P
Q
R
S
T
V
W
Y
ikalian
opean
astern
nalaya
berian

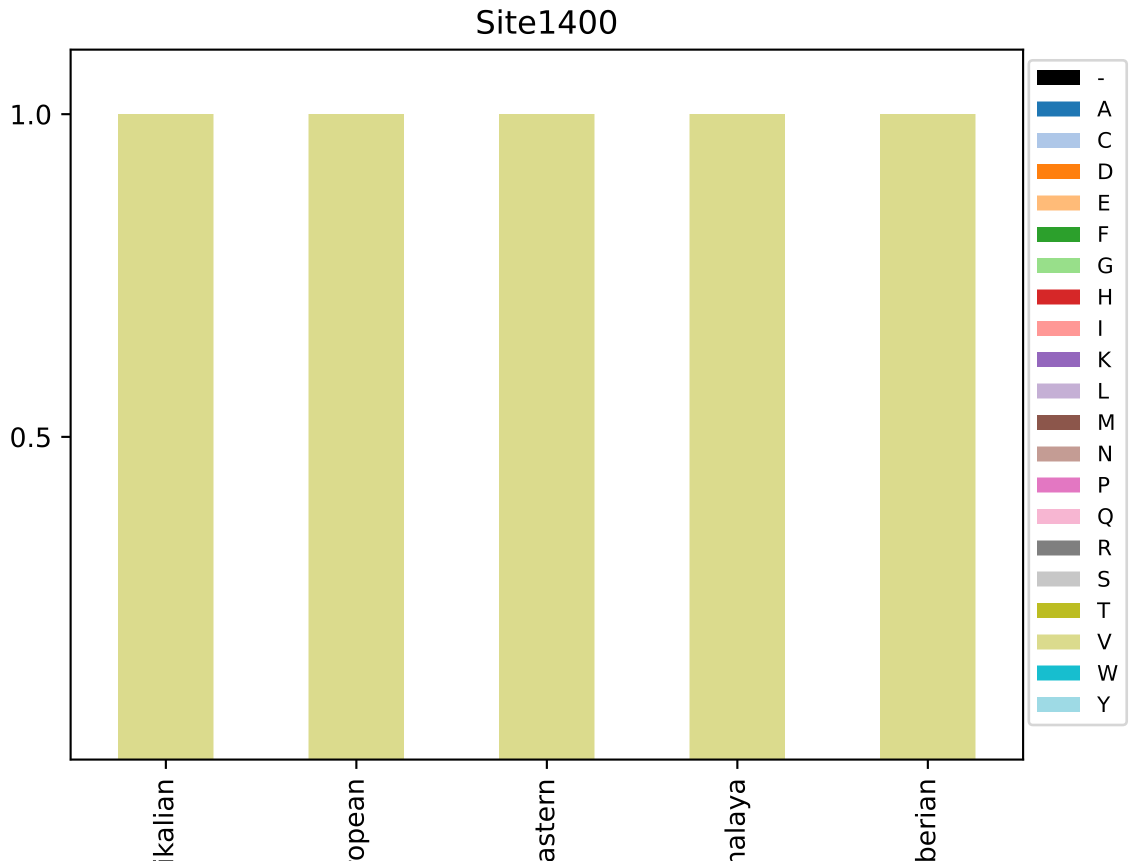

Site1400
1.0
0.5
-
A
C
D
E
F
G
H
I
K
L
M
N
P
Q
R
S
T
V
W
Y
ikalian
opean
astern
nalaya
berian

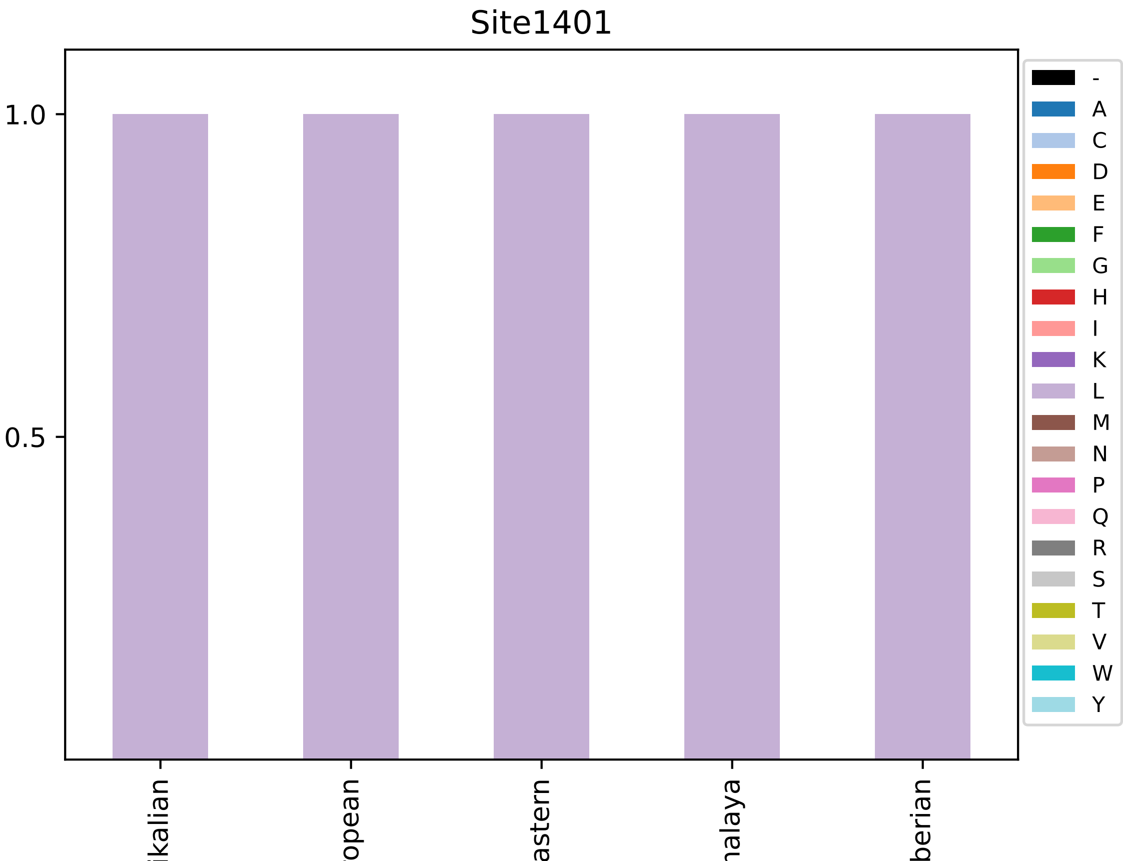

Site1401
1.0
0.5
-
A
C
D
E
F
G
H
I
K
L
M
N
P
Q
R
S
T
V
W
Y
ikalian
opean
astern
nalaya
berian

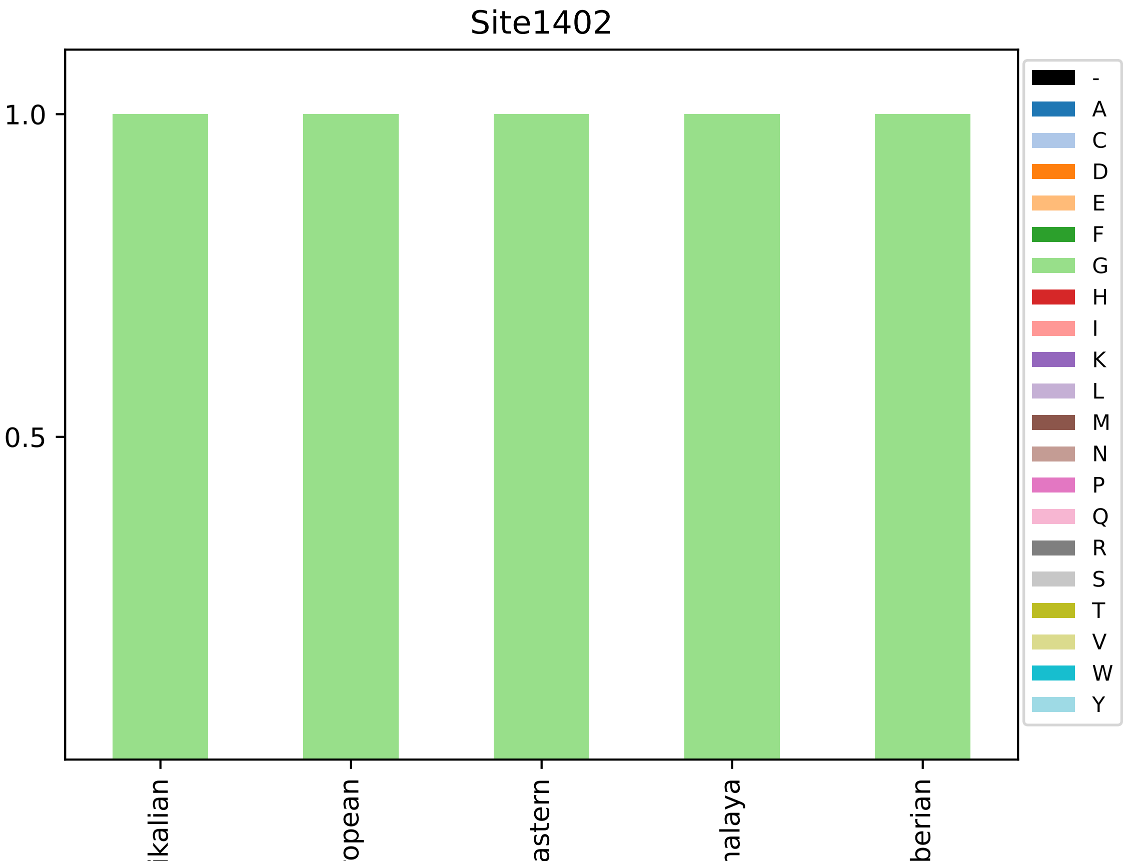

Site1402
1.0
0.5
-
A
C
D
E
F
G
H
I
K
L
M
N
P
Q
R
S
T
V
W
Y
ikalian
opean
astern
nalaya
berian

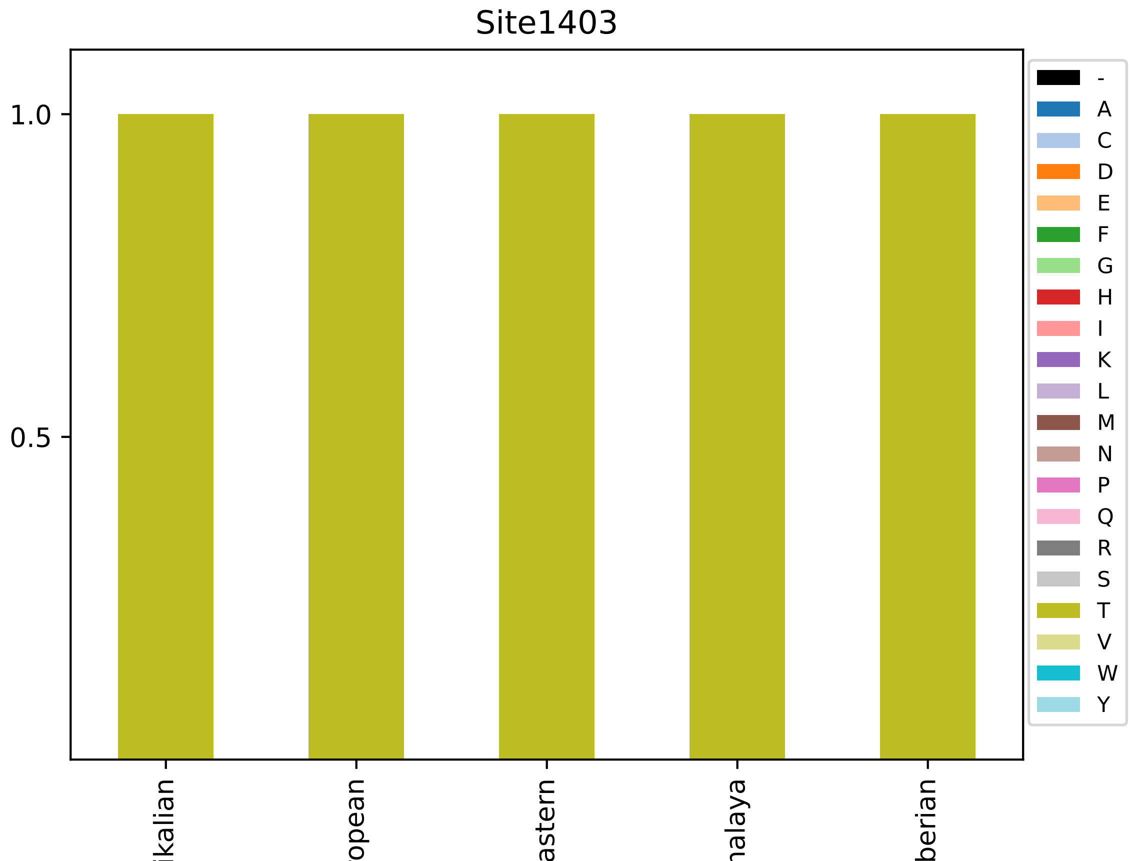
Site1403
1.0
0.5
-
A
C
D
E
F
G
H
I
K
L
M
N
P
Q
R
S
T
V
W
Y
ikalian
opean
astern
nalaya
berian

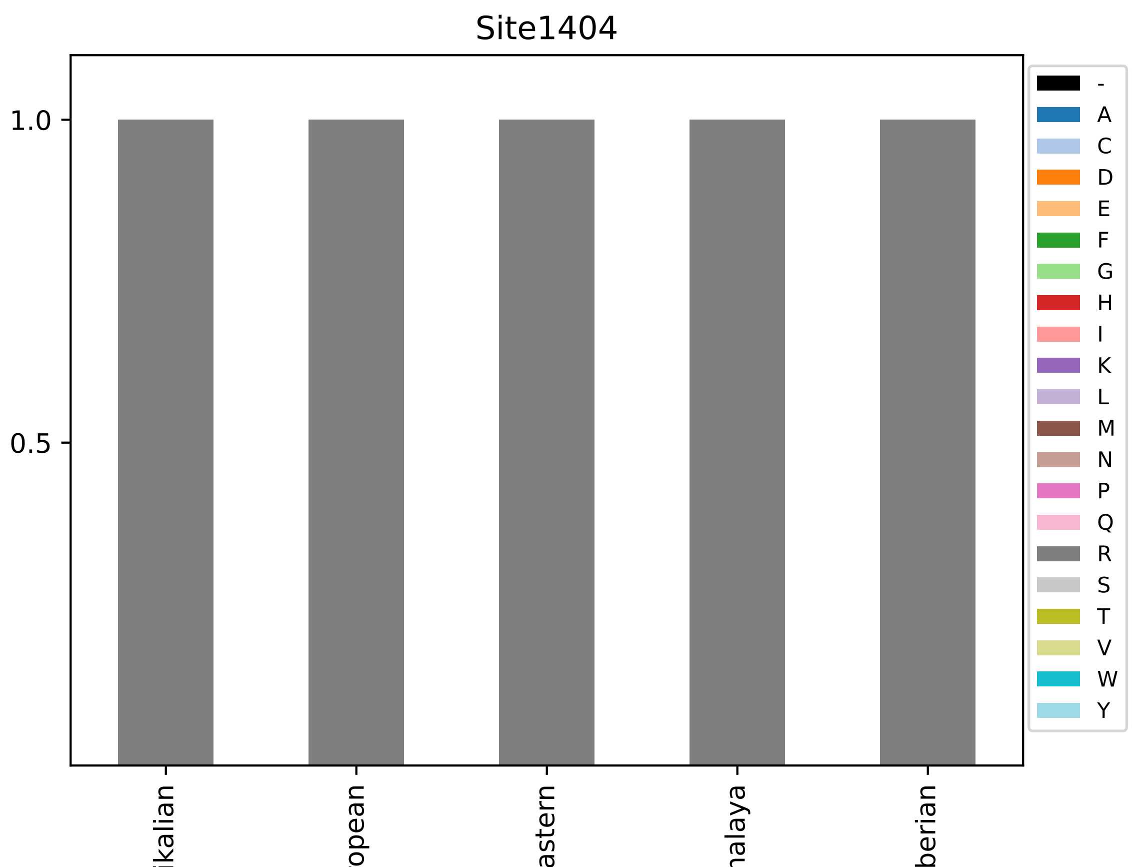
Site1404
1.0
0.5
-
A
C
D
E
F
G
H
I
K
L
M
N
P
Q
R
S
T
V
W
Y
ikalian
ropean
astern
nalaya
berian

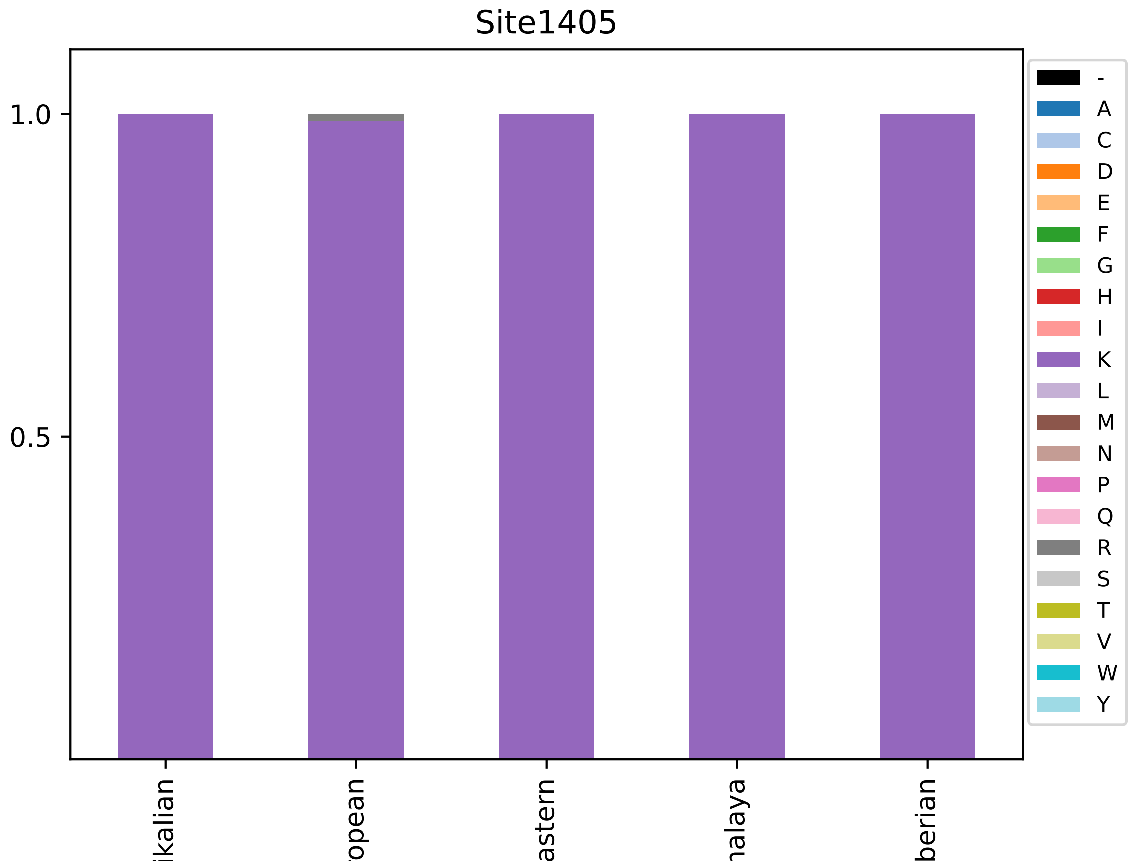
Site1405
1.0
0.5
ikalian
opean
astern
nalaya
berian
-
A
C
D
E
F
G
H
I
K
L
M
N
P
Q
R
S
T
V
W
Y

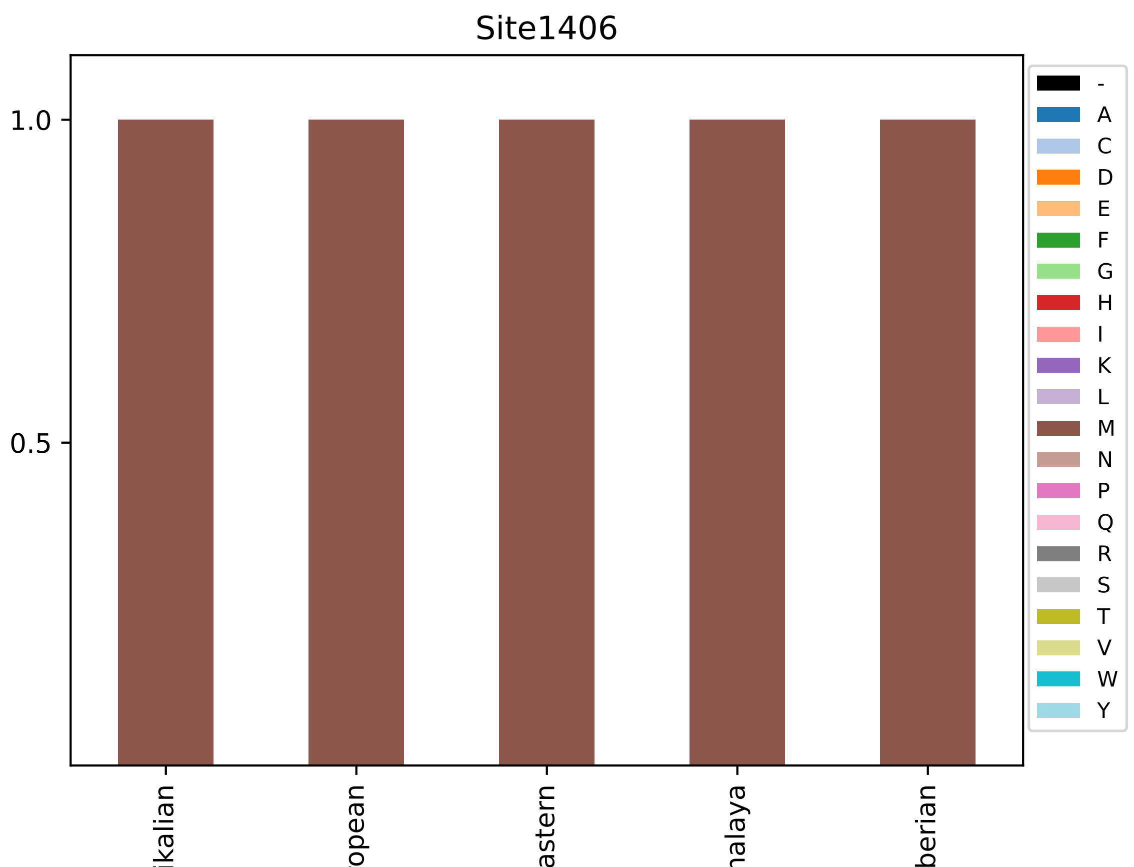
Site1406
1.0
0.5
ikalian
opean
astern
alaya
berian
-
A
C
D
E
F
G
H
I
K
L
M
N
P
Q
R
S
T
V
W
Y

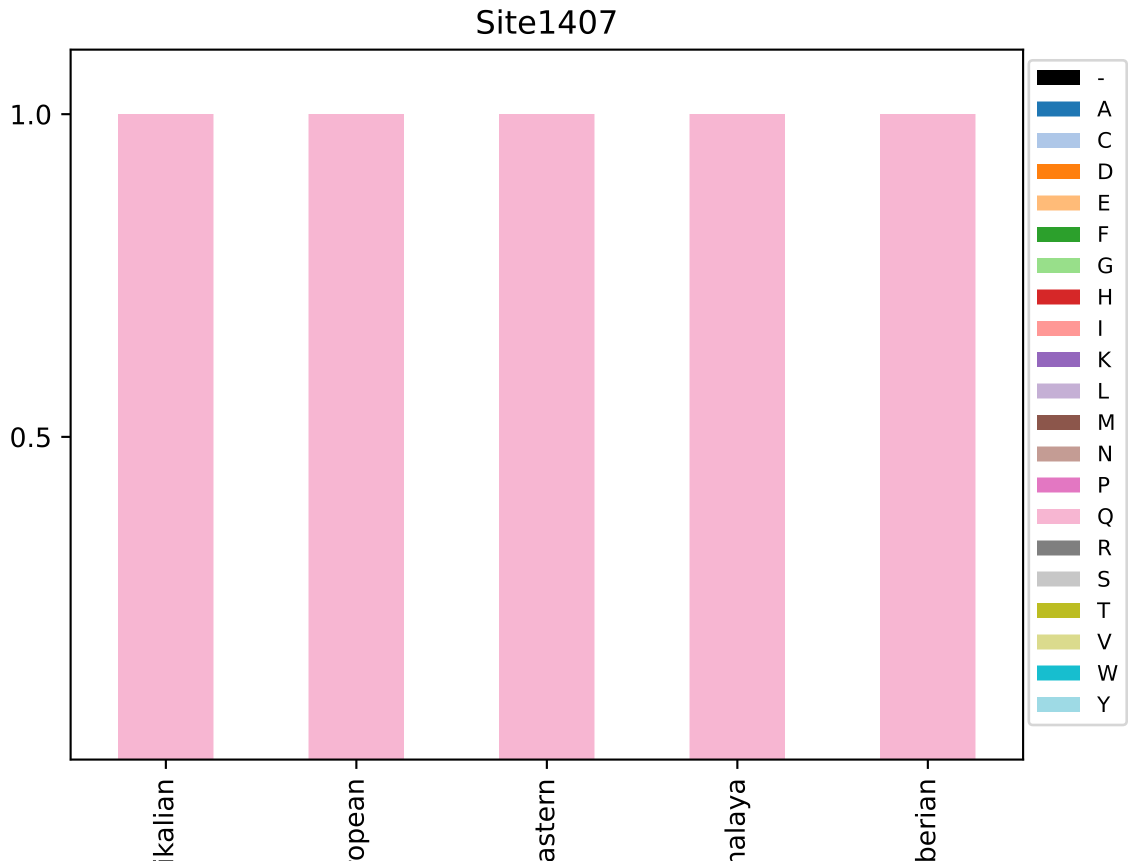
Site1407
1.0
0.5
ikalian
ropean
astern
nalaya
berian
-
A
C
D
E
F
G
H
I
K
L
M
N
P
Q
R
S
T
V
W
Y

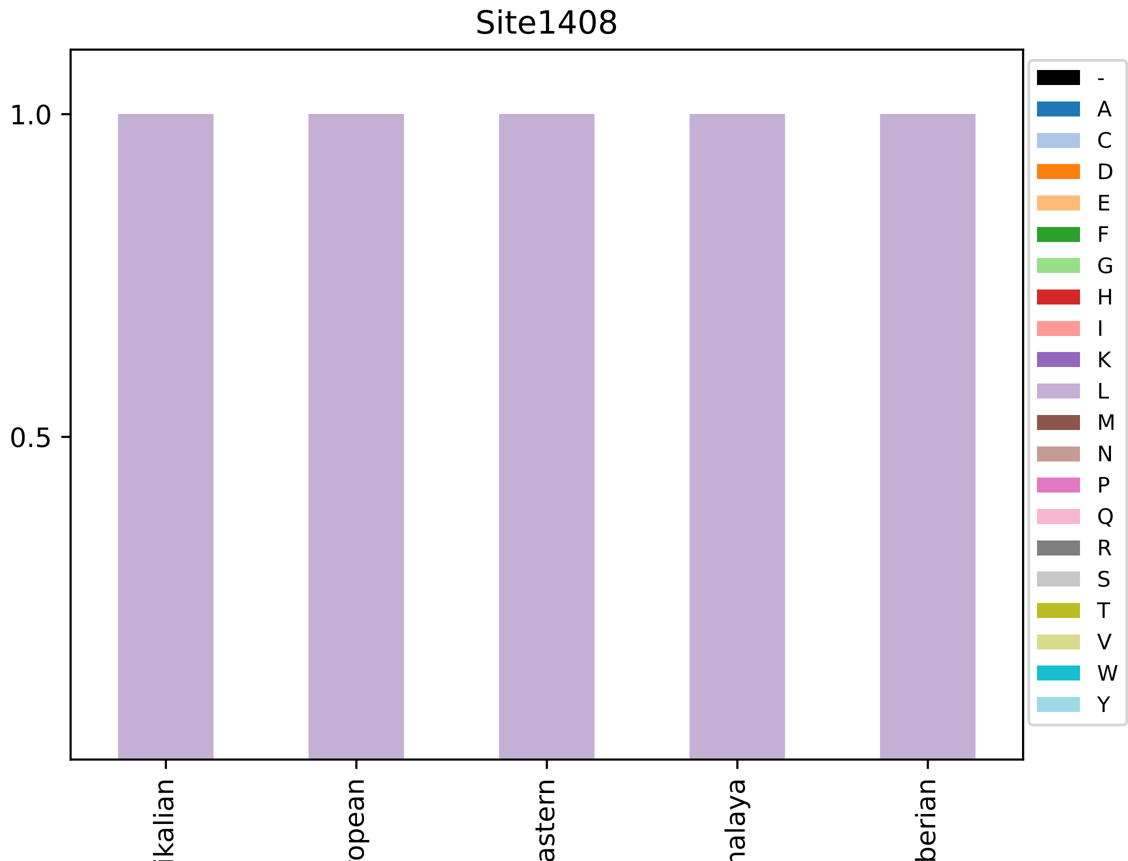

Site1408
1.0
0.5
ikalian
ropean
astern
nalaya
berian
-
A
C
D
E
F
G
H
I
K
L
M
N
P
Q
R
S
T
V
W
Y

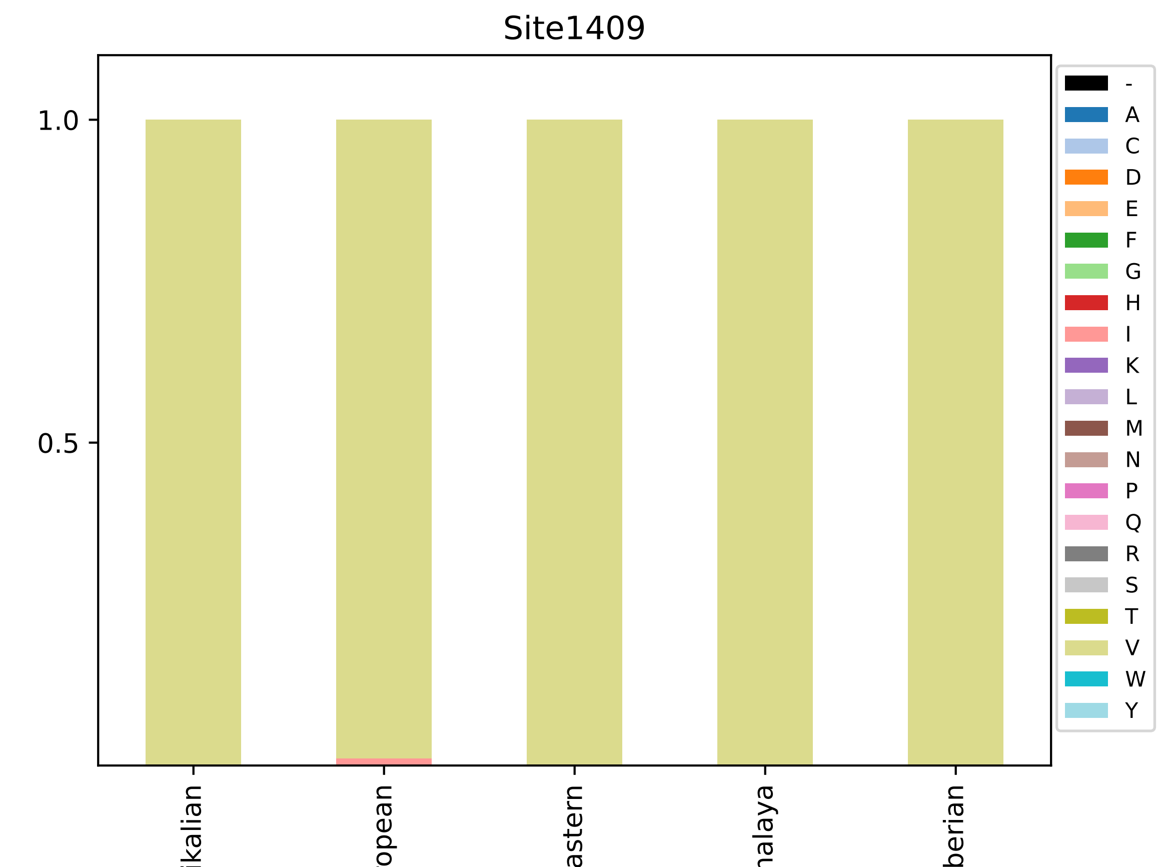
Site1409
1.0
0.5
-
A
C
D
E
F
G
H
I
K
L
M
N
P
Q
R
S
T
V
W
Y
ikalian
ropean
astern
nalaya
berian

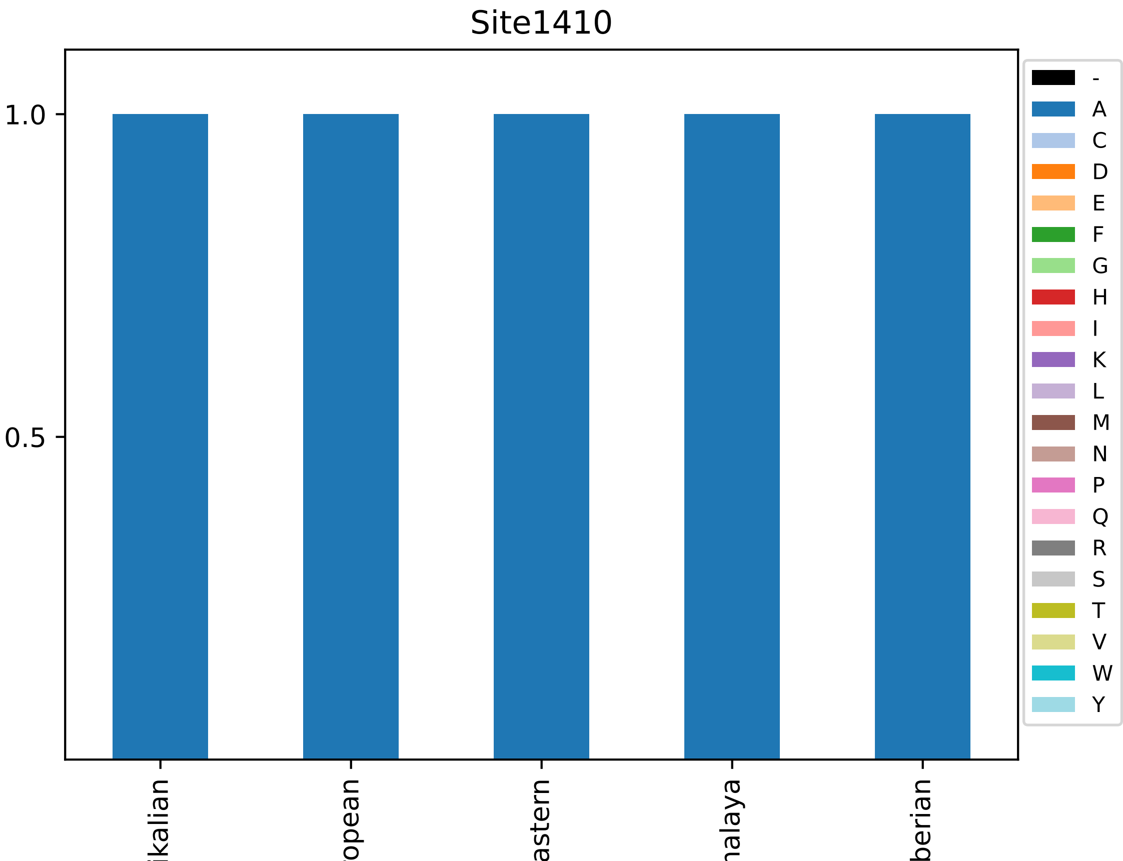
Site1410
1.0
0.5
-
A
C
D
E
F
G
H
I
K
L
M
N
P
Q
R
S
T
V
W
Y
ikalian
opean
astern
nalaya
berian

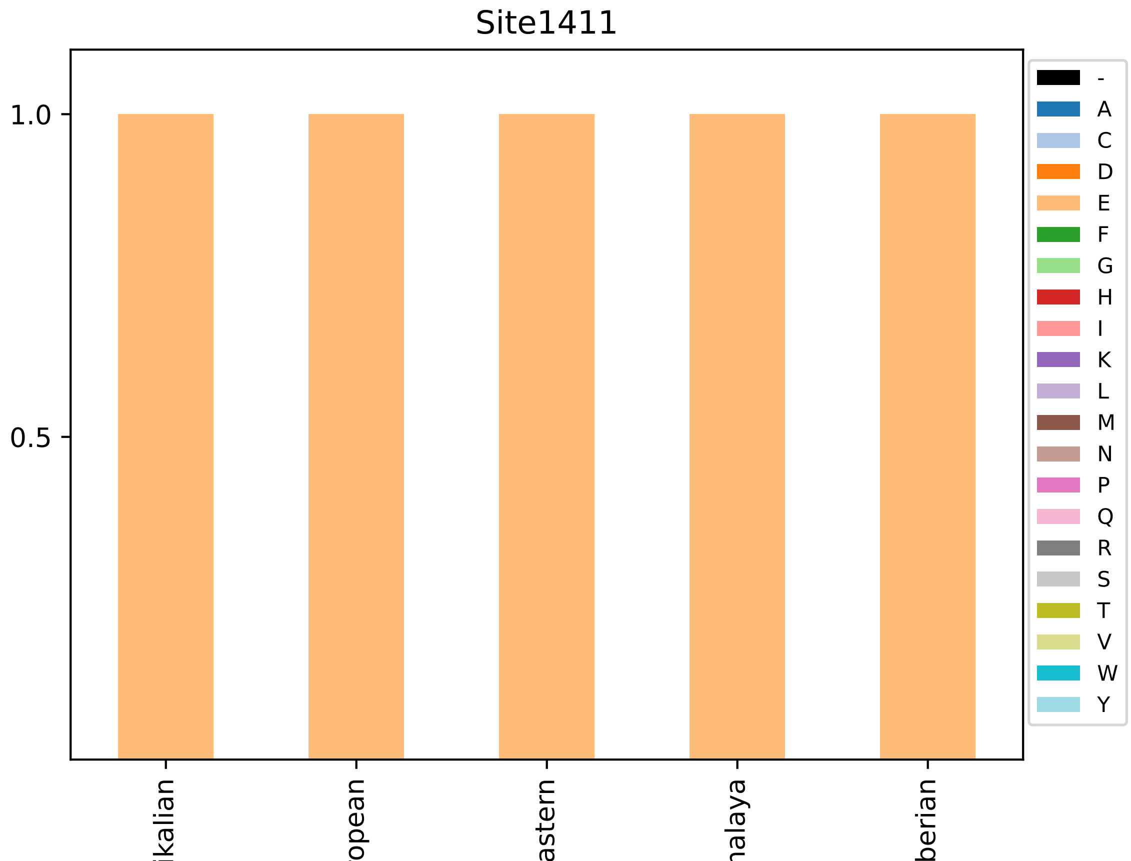

Site1411
1.0
0.5
-
A
C
D
E
F
G
H
I
K
L
M
N
P
Q
R
S
T
V
W
Y
ikalian
opean
astern
nalaya
berian

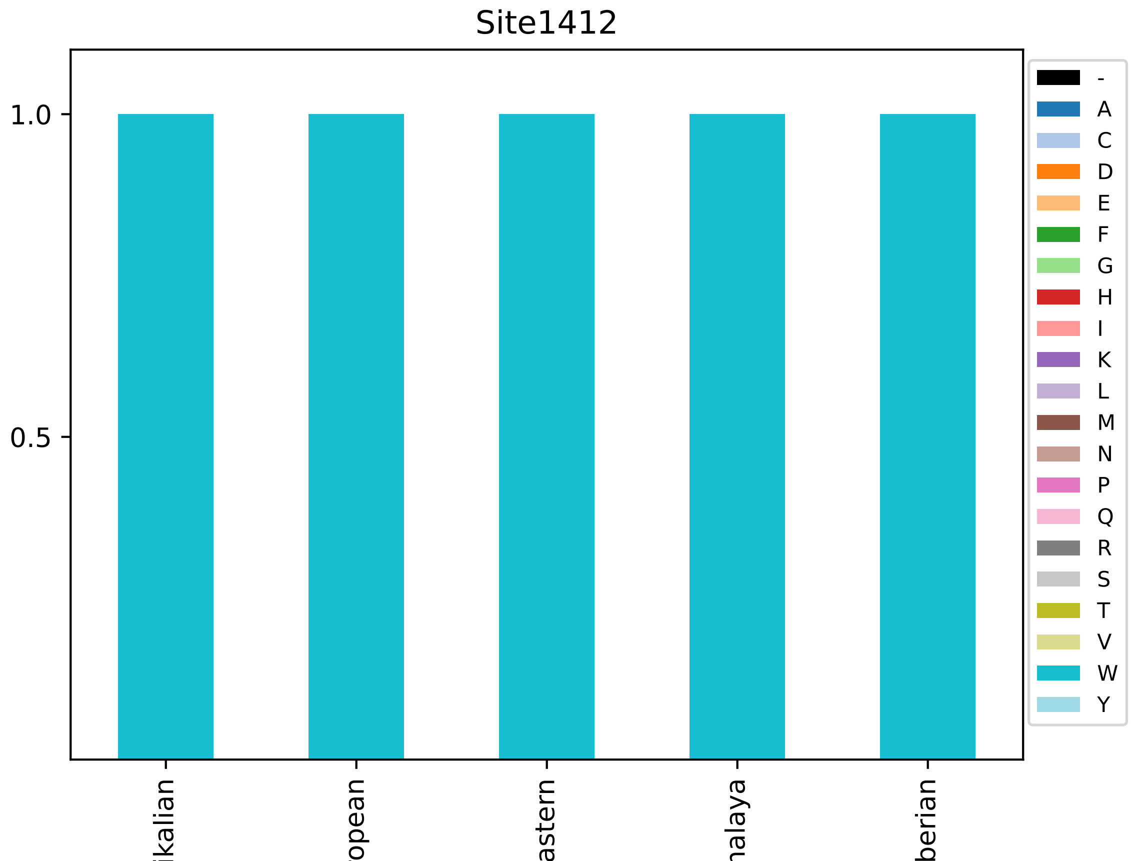
Site1412
1.0
0.5
ikalian
opean
astern
nalaya
berian
-
A
C
D
E
F
G
H
I
K
L
M
N
P
Q
R
S
T
V
W
Y

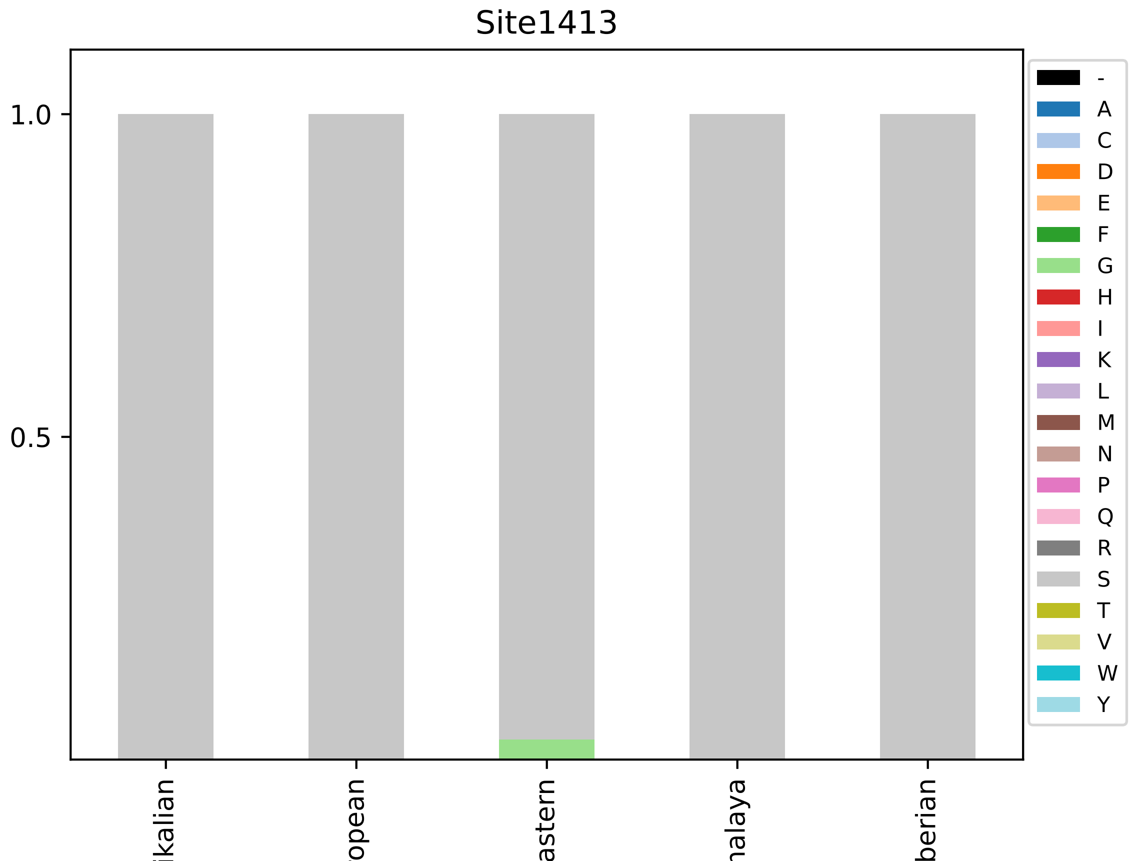

Site1413
1.0
0.5
ikalian
opean
astern
nalaya
berian
-
A
C
D
E
F
G
H
I
K
L
M
N
P
Q
R
S
T
V
W
Y

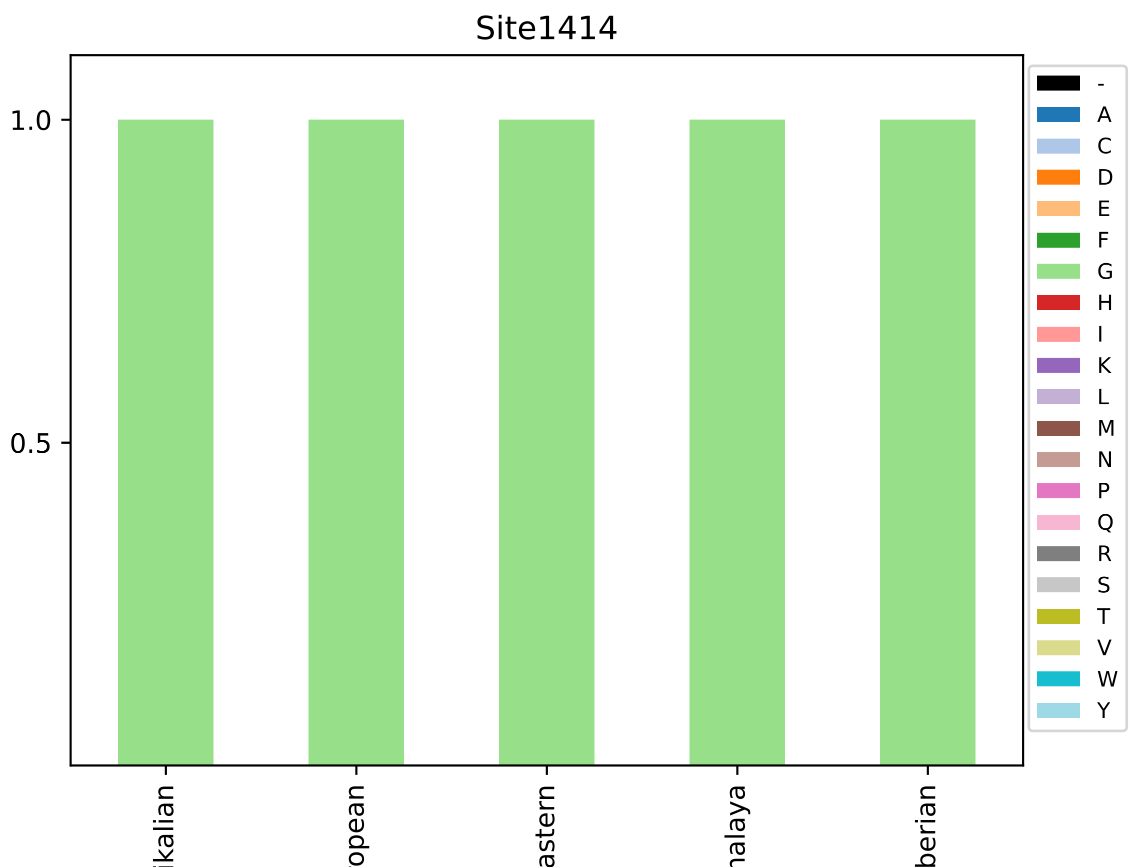

Site1414
1.0
0.5
-
A
C
D
E
F
G
H
I
K
L
M
N
P
Q
R
S
T
V
W
Y
ikalian
opean
astern
nalaya
berian

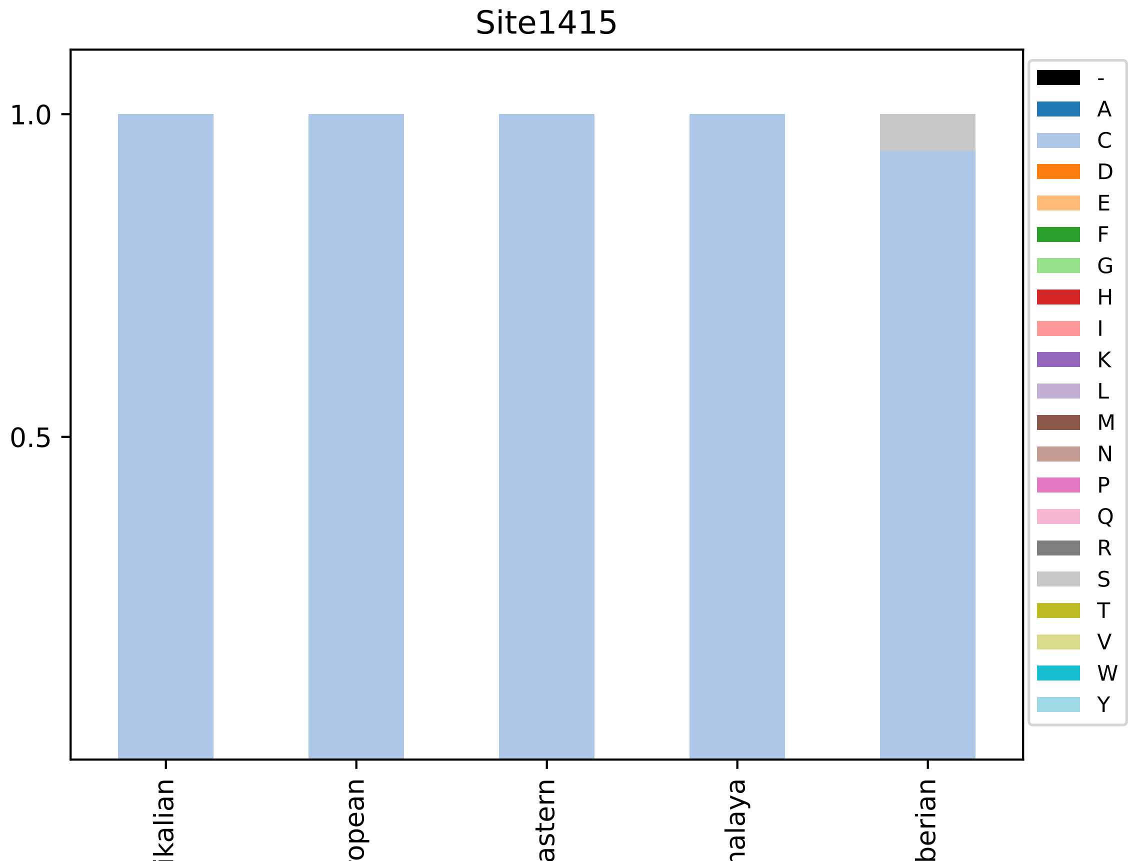

Site1415
1.0
0.5
ikalian
opean
astern
nalaya
berian
-
A
C
D
E
F
G
H
I
K
L
M
N
P
Q
R
S
T
V
W
Y

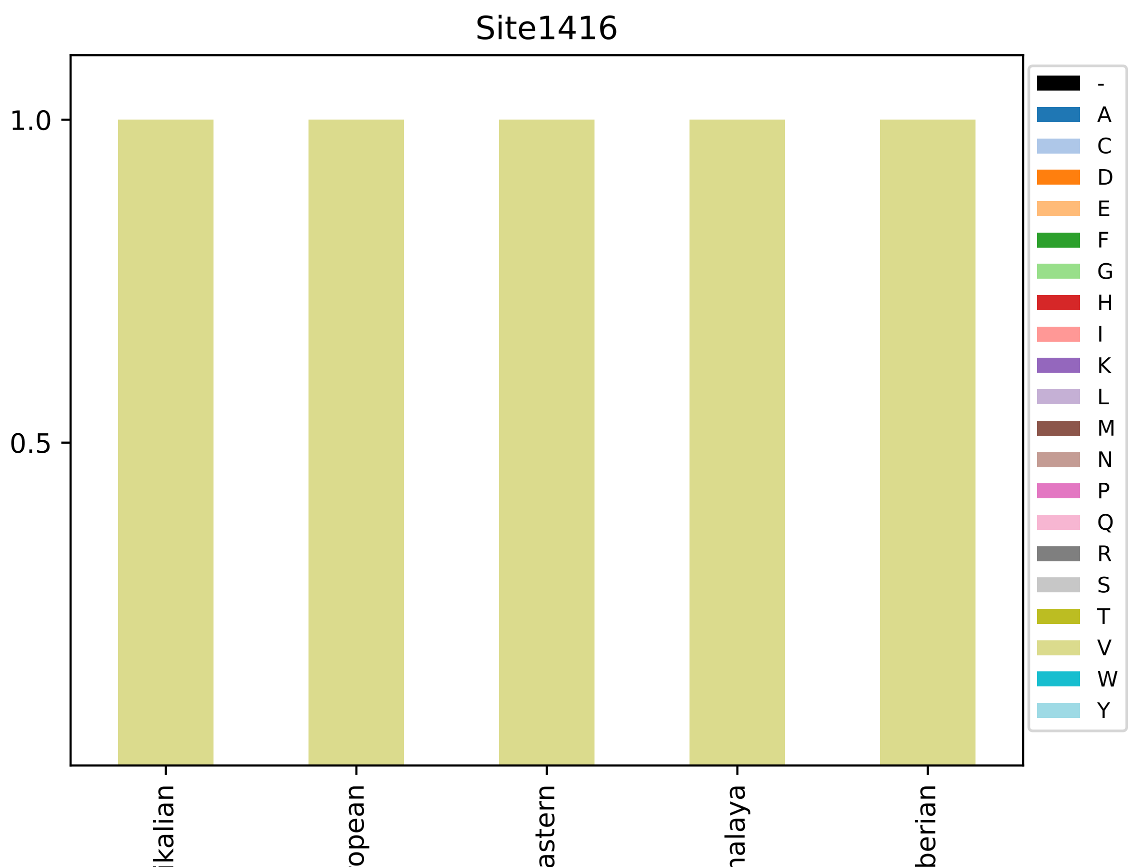

Site1416
1.0
0.5
-
A
C
D
E
F
G
H
I
K
L
M
N
P
Q
R
S
T
V
W
Y
ikalian
ropean
astern
nalaya
berian

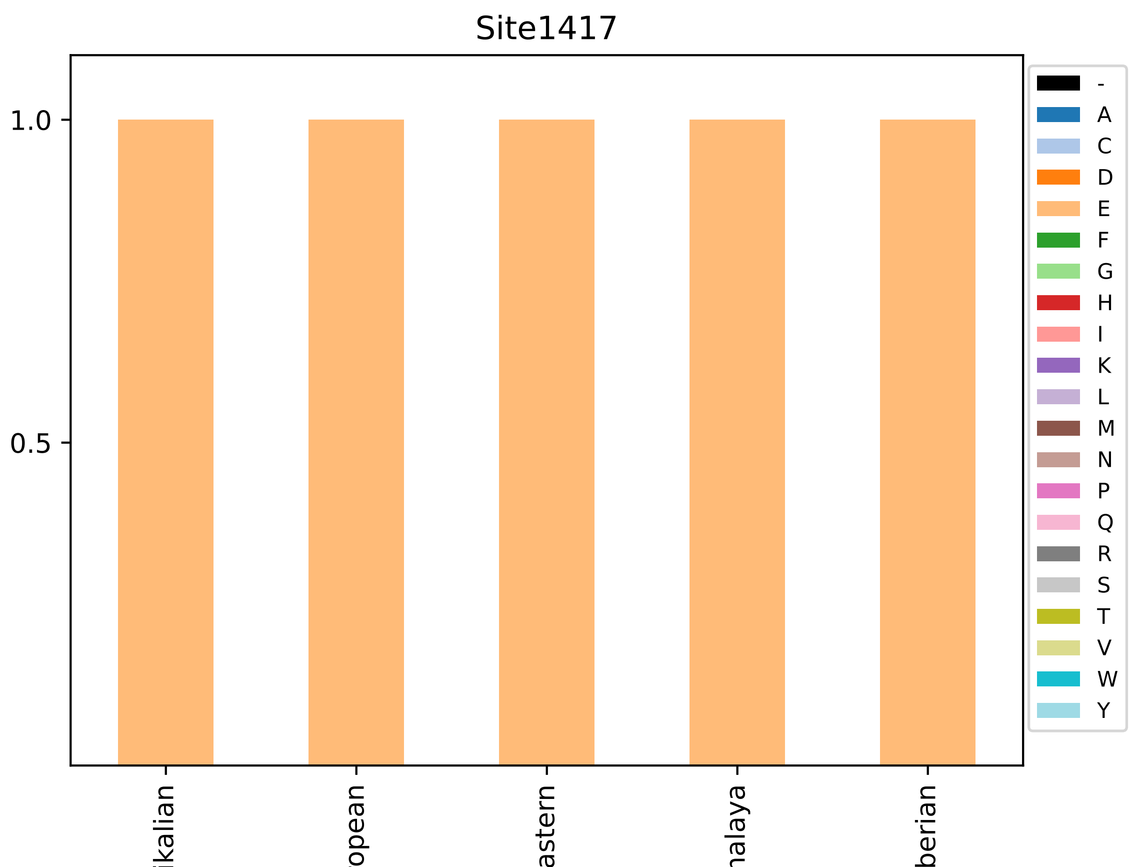
Site1417
1.0
0.5
-
A
C
D
E
F
G
H
I
K
L
M
N
P
Q
R
S
T
V
W
Y
ikalian
opean
astern
nalaya
berian

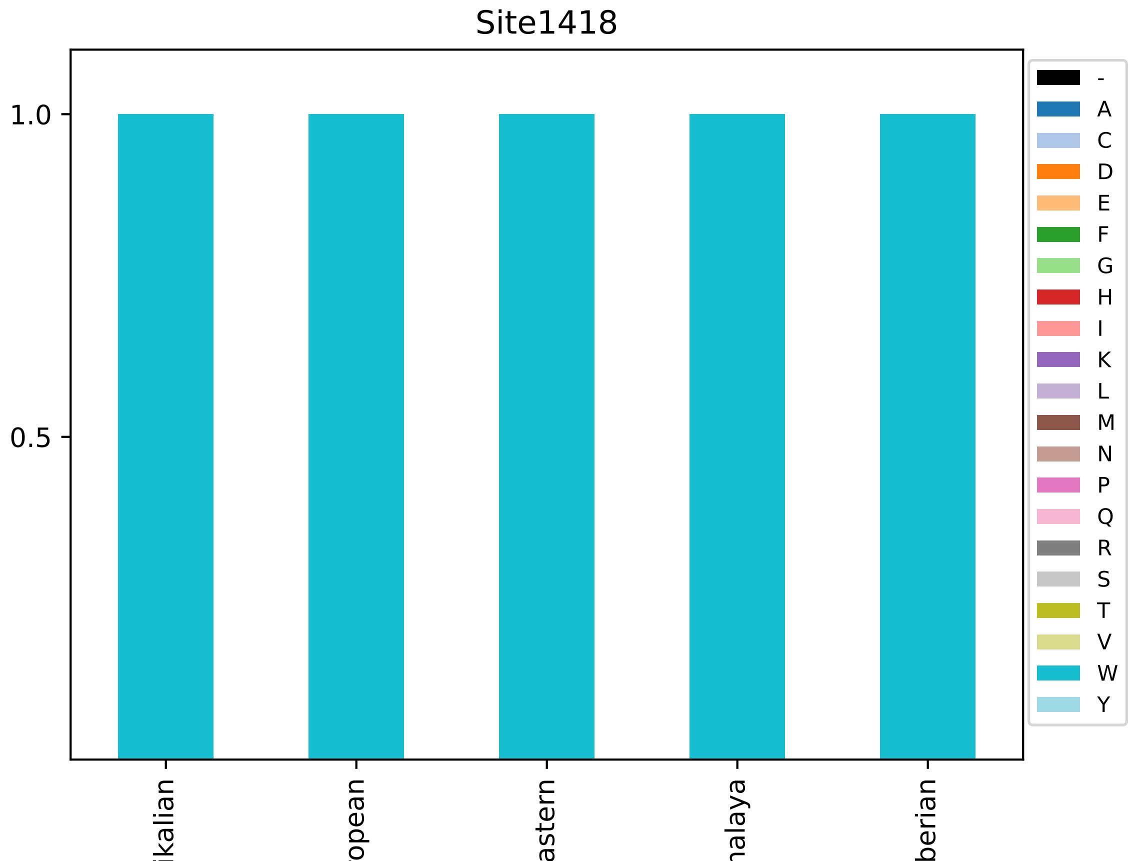

Site1418
1.0
0.5
ikalian
opean
astern
nalaya
berian
-
A
C
D
E
F
G
H
I
K
L
M
N
P
Q
R
S
T
V
W
Y

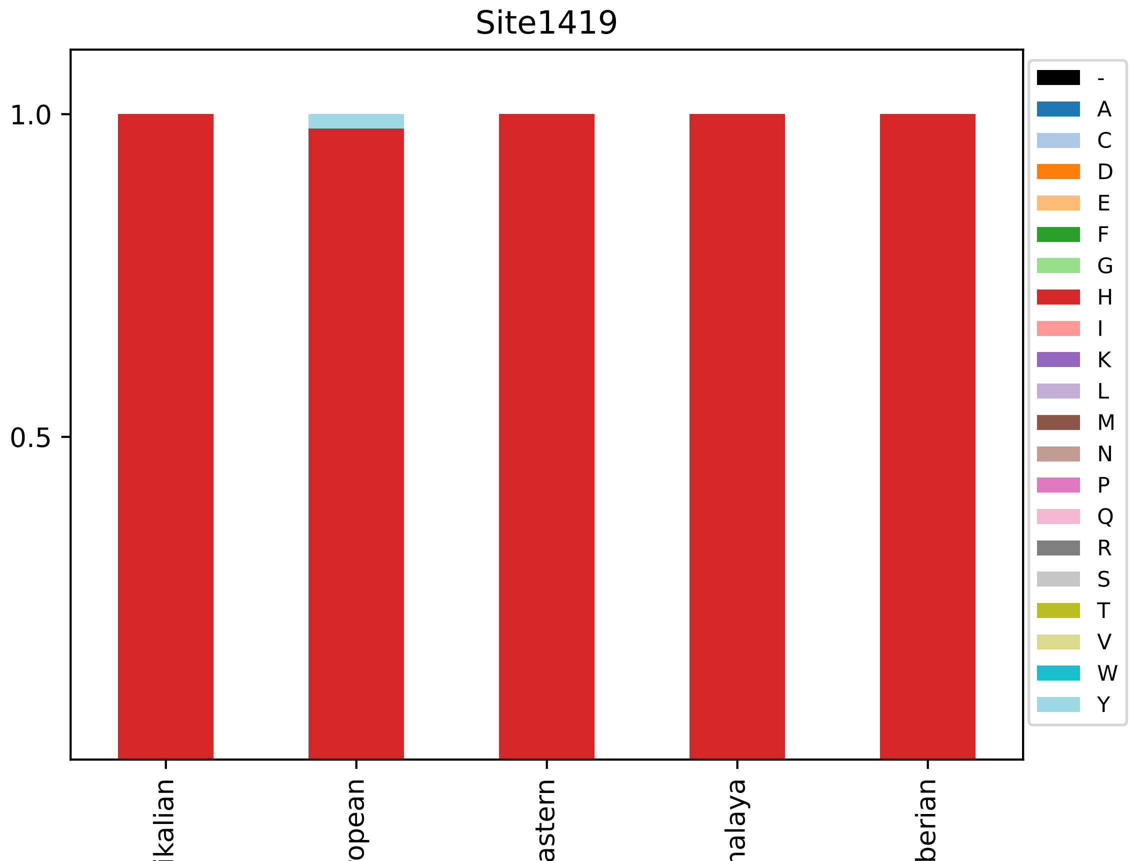
Site1419
1.0
0.5
-
A
C
D
E
F
G
H
I
K
L
M
N
P
Q
R
S
T
V
W
Y
ikalian
opean
astern
nalaya
berian

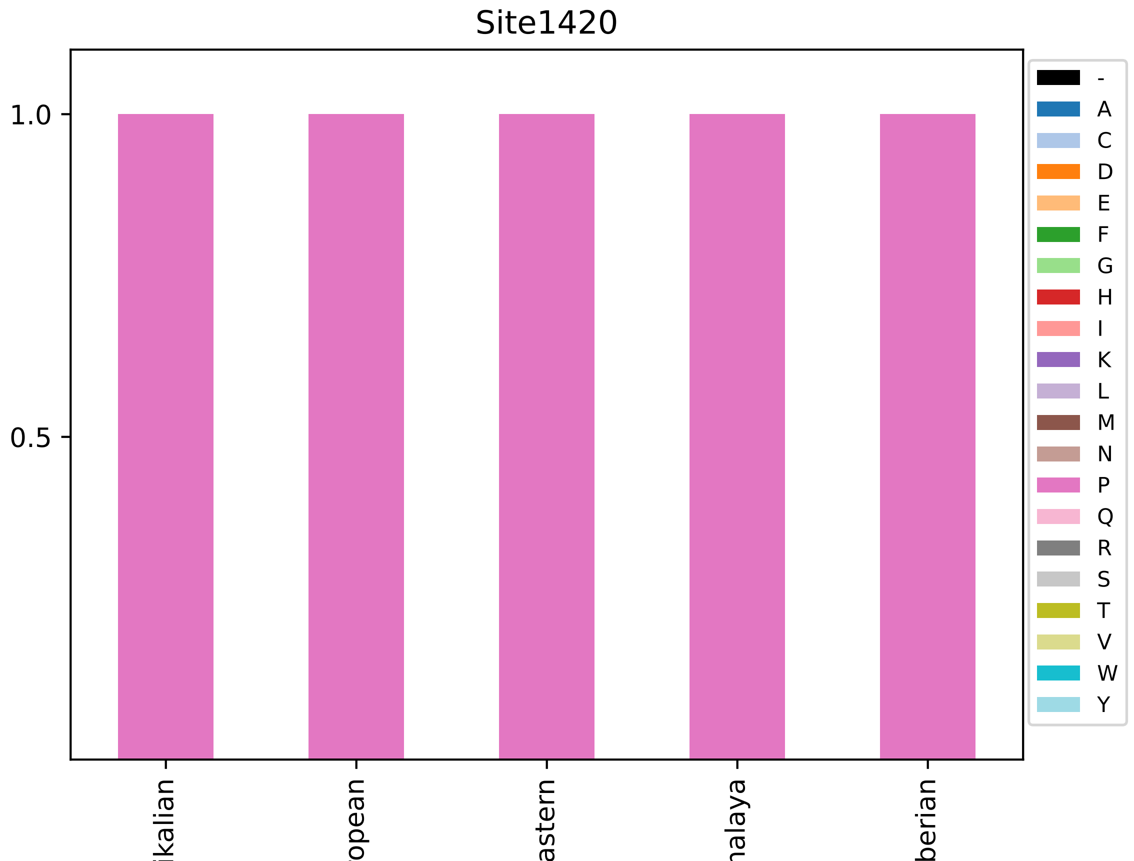

Site1420
1.0
0.5
ikalian
opean
astern
nalaya
berian
-
A
C
D
E
F
G
H
I
K
L
M
N
P
Q
R
S
T
V
W
Y

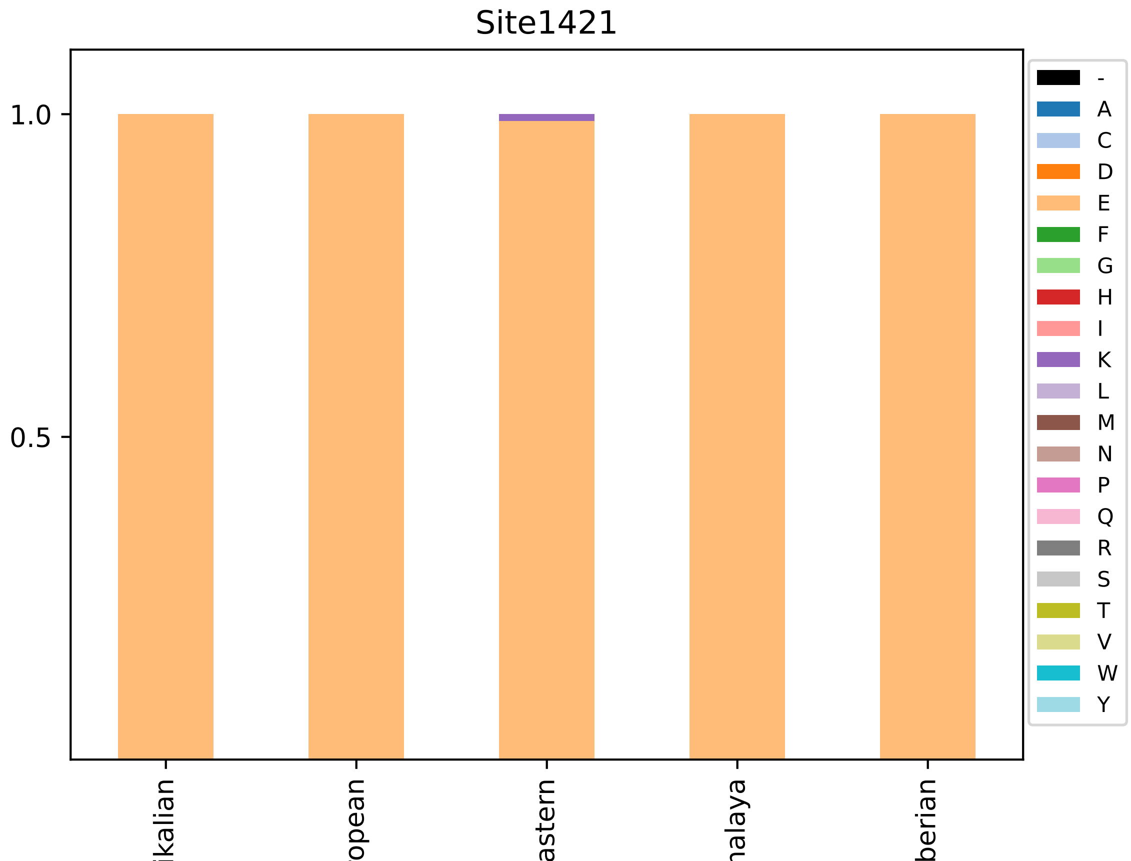
Site1421
1.0
0.5
-
A
C
D
E
F
G
H
I
K
L
M
N
P
Q
R
S
T
V
W
Y
ikalian
opean
astern
nalaya
berian

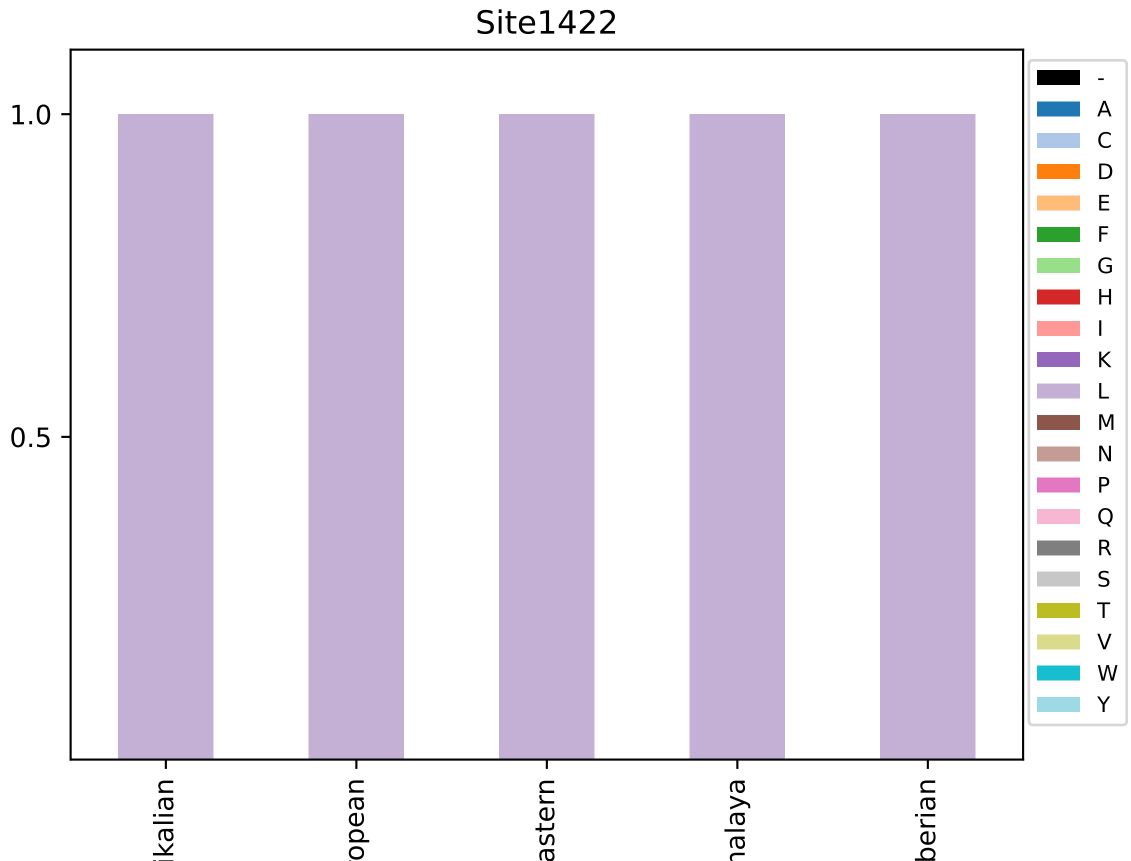

Site1422
1.0
0.5
-
A
C
D
E
F
G
H
I
K
L
M
N
P
Q
R
S
T
V
W
Y
ikalian
opean
astern
nalaya
berian

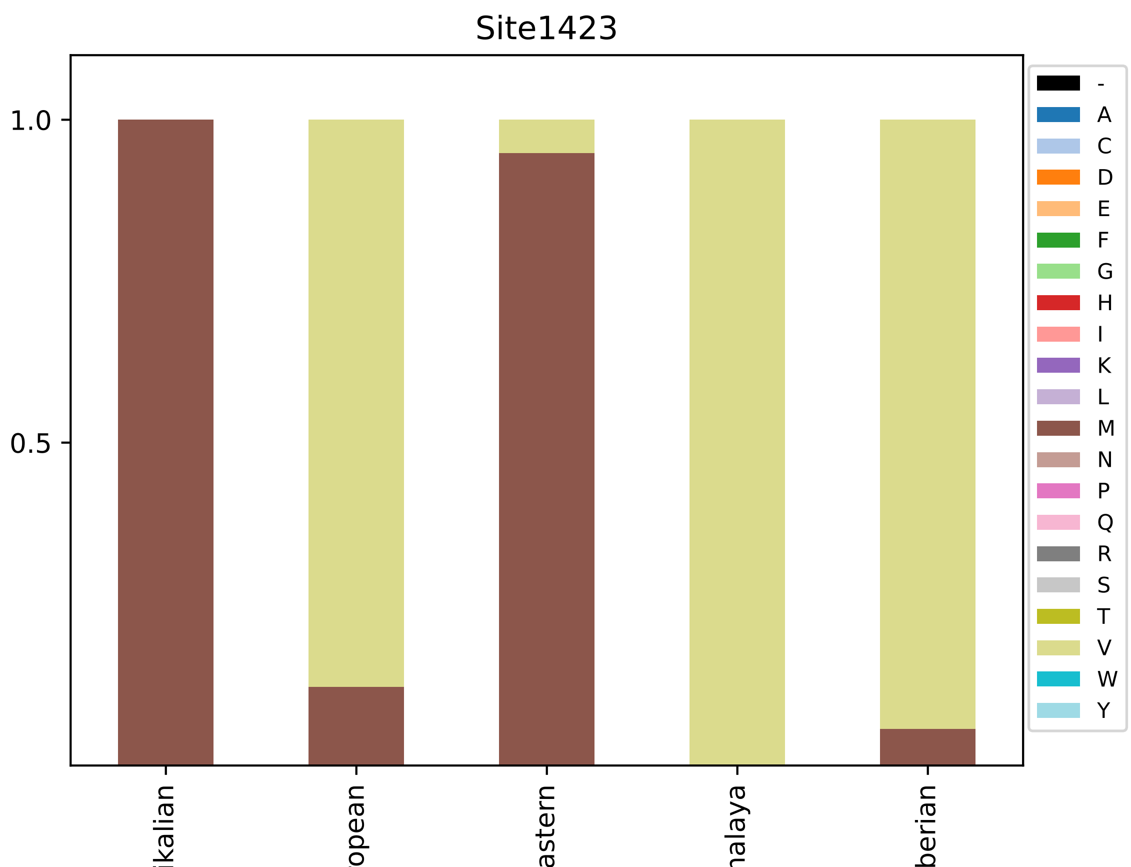
Site1423
1.0
0.5
-
A
C
D
E
F
G
H
I
K
L
M
N
P
Q
R
S
T
V
W
Y
ikalian
opean
astern
nalaya
berian

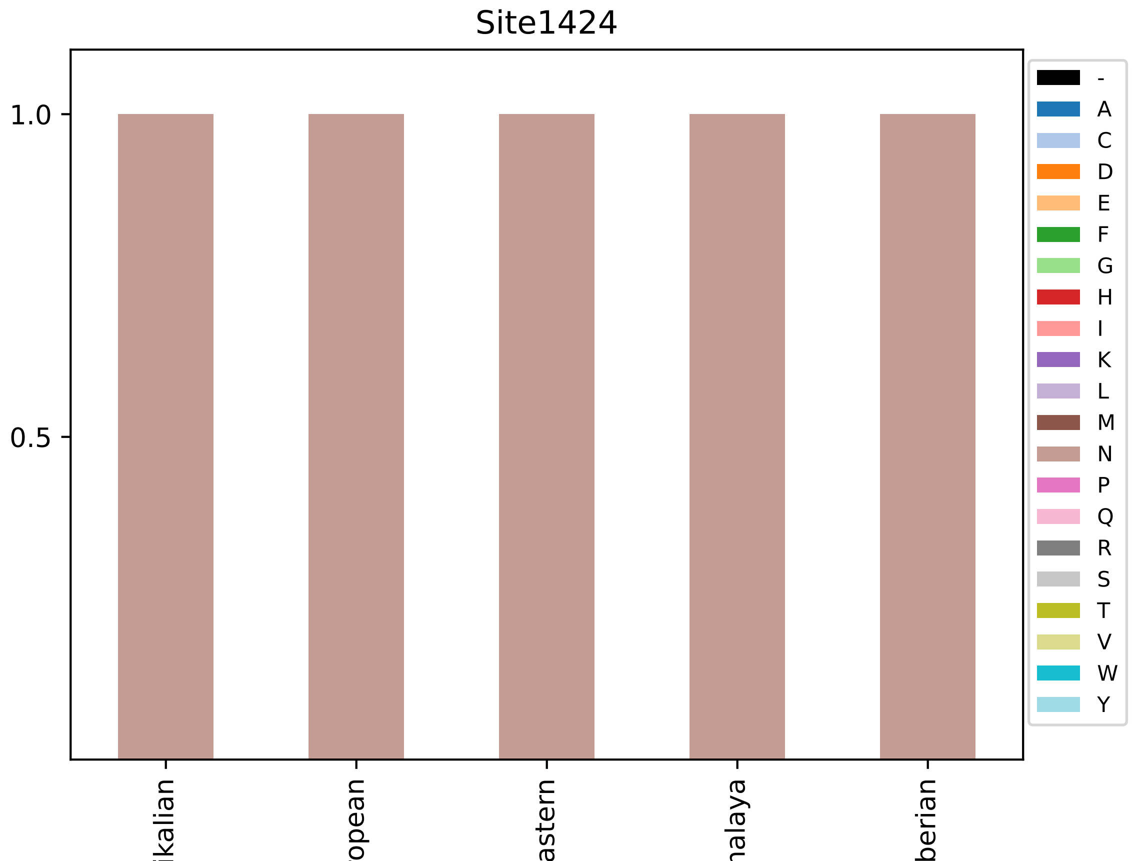
Site1424
1.0
0.5
ikalian
opean
astern
nalaya
berian
-
A
C
D
E
F
G
H
I
K
L
M
N
P
Q
R
S
T
V
W
Y

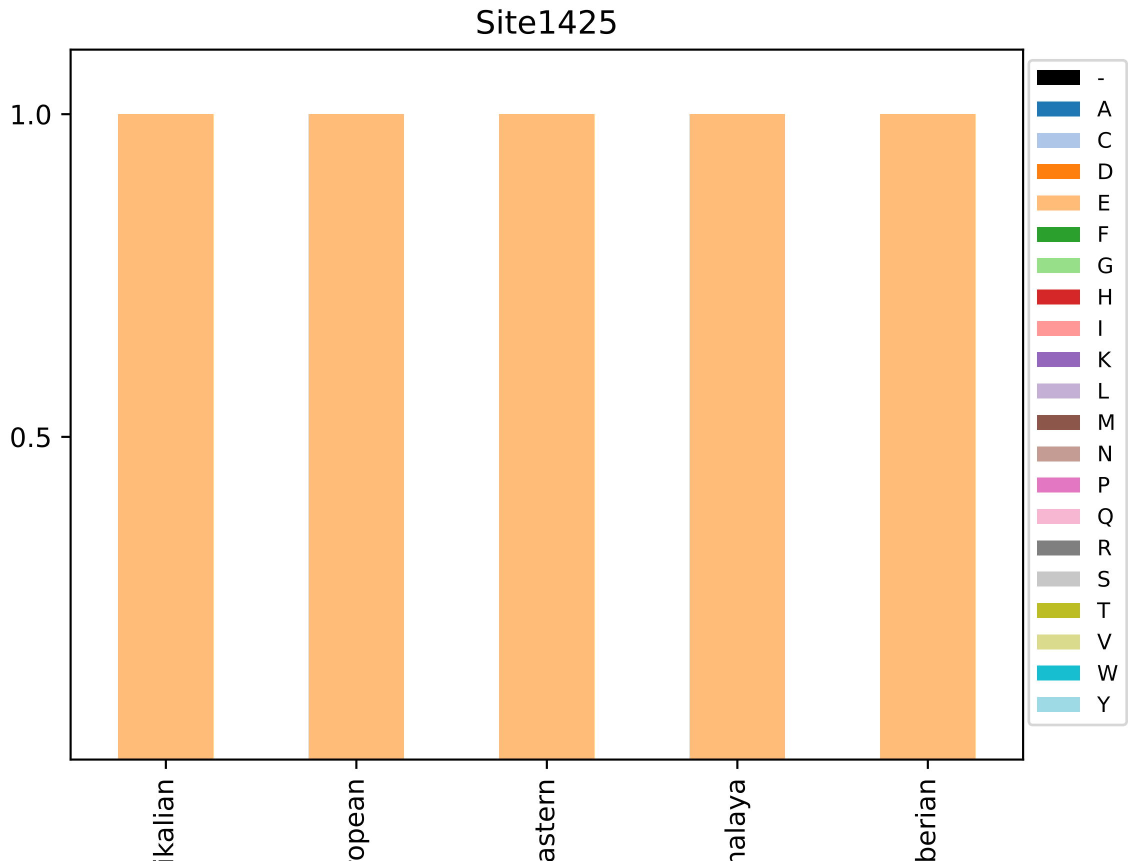
Site1425
1.0
0.5
-
A
C
D
E
F
G
H
I
K
L
M
N
P
Q
R
S
T
V
W
Y
ikalian
opean
astern
nalaya
berian

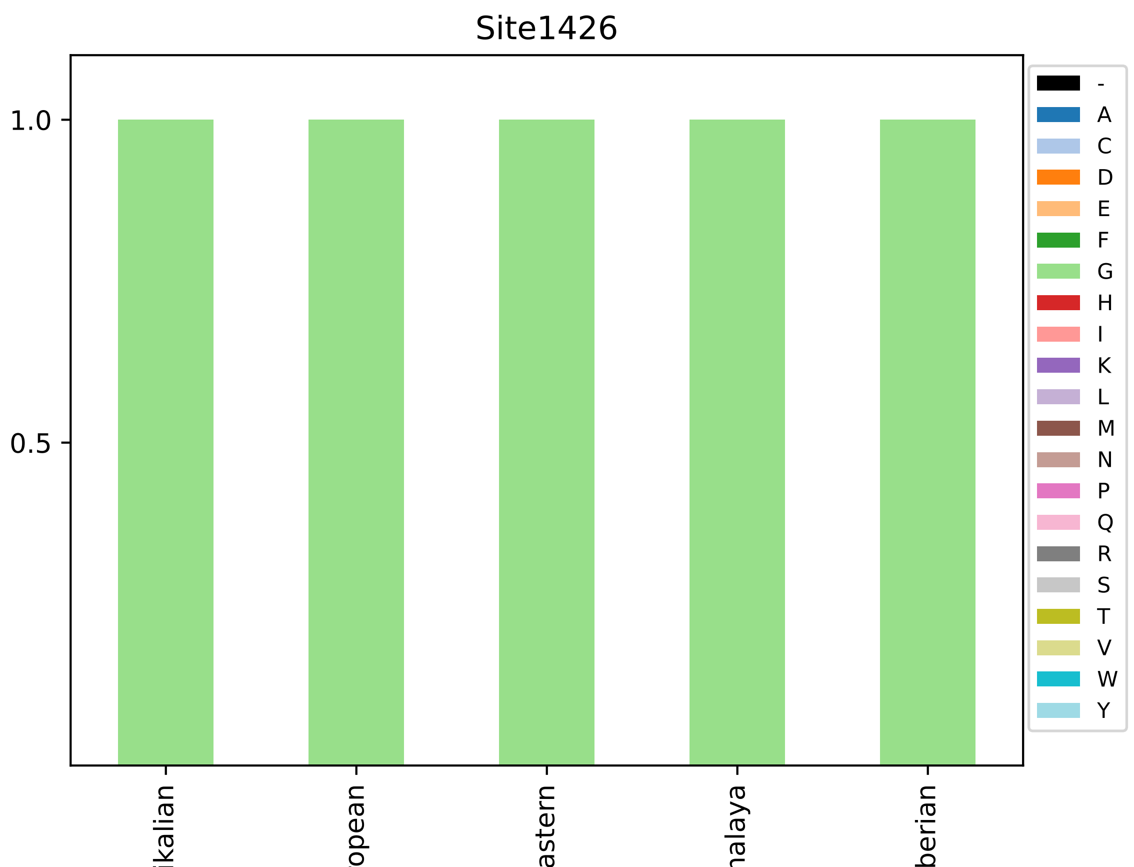
Site1426
1.0
0.5
-
A
C
D
E
F
G
H
I
K
L
M
N
P
Q
R
S
T
V
W
Y
ikalian
opean
astern
nalaya
berian

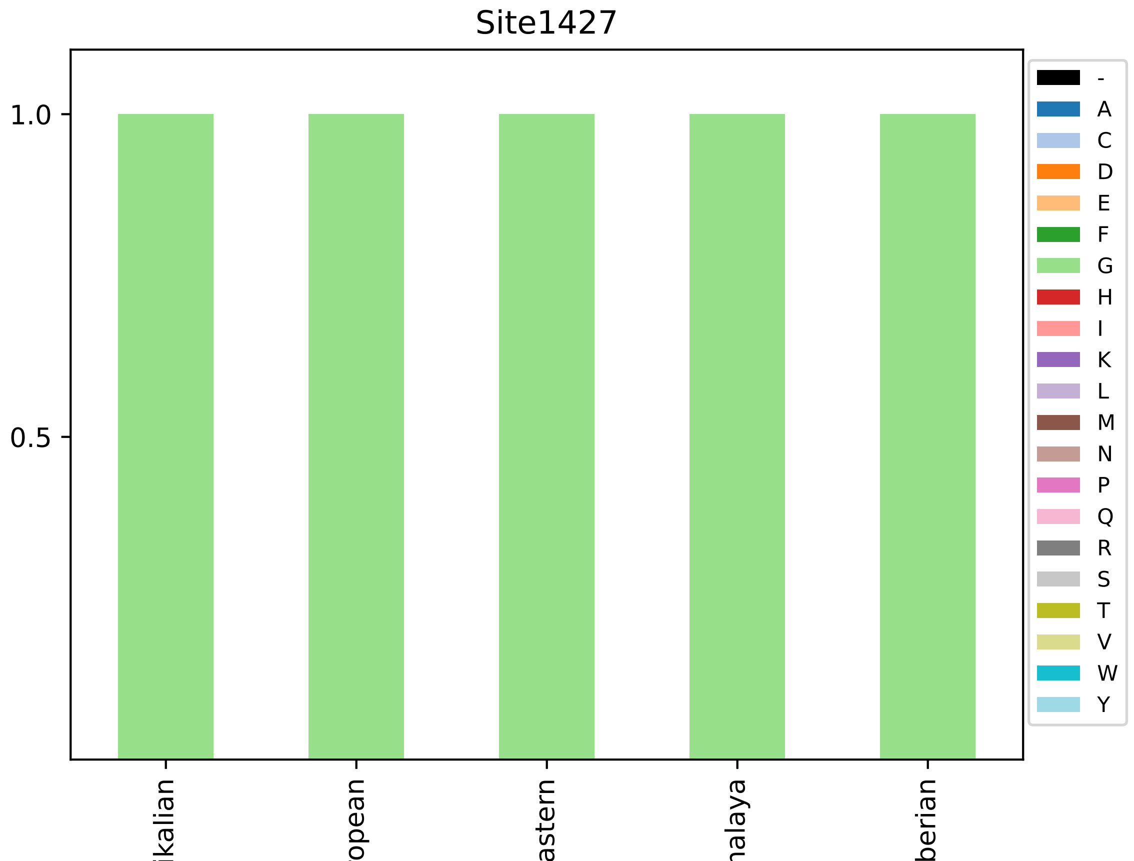
Site1427
1.0
0.5
-
A
C
D
E
F
G
H
I
K
L
M
N
P
Q
R
S
T
V
W
Y
ikalian
ropean
astern
nalaya
berian

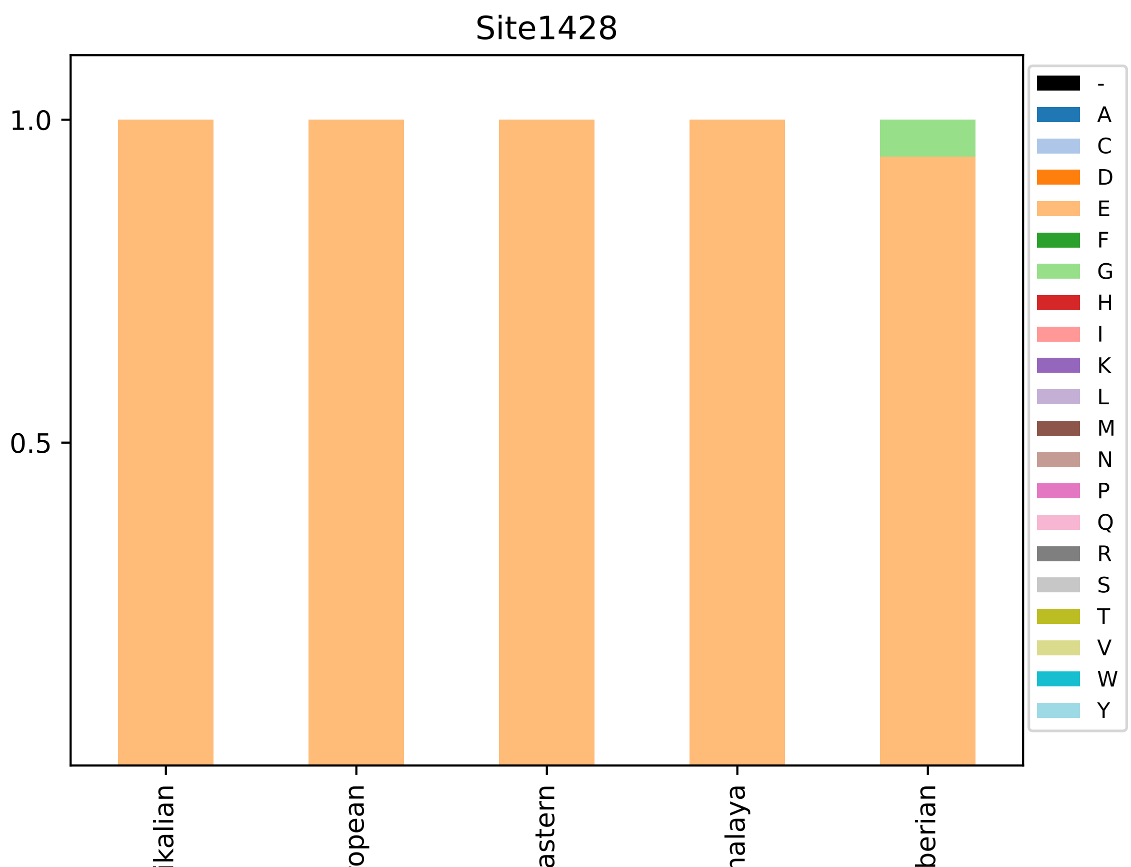

Site1428
1.0
0.5
-
A
C
D
E
F
G
H
I
K
L
M
N
P
Q
R
S
T
V
W
Y
ikalian
opean
astern
nalaya
berian

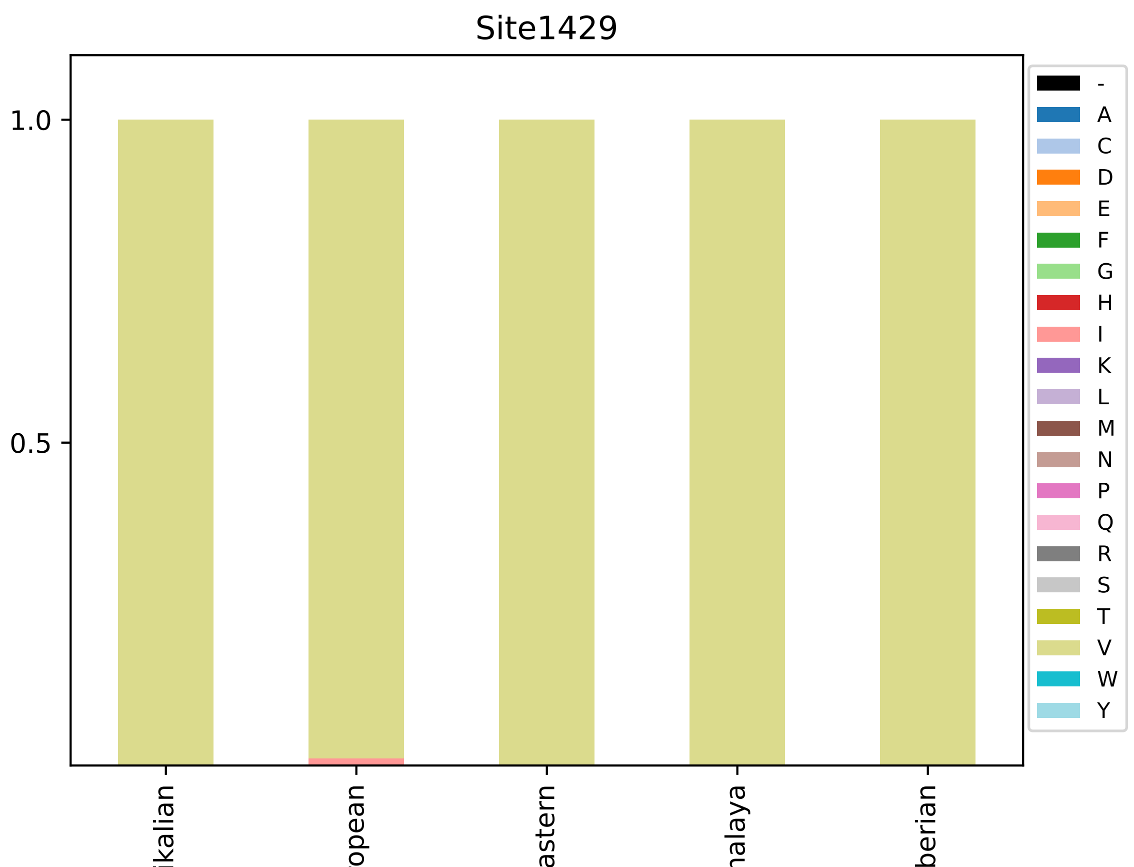

Site1429
1.0
0.5
-
A
C
D
E
F
G
H
I
K
L
M
N
P
Q
R
S
T
V
W
Y
ikalian
opean
astern
nalaya
berian

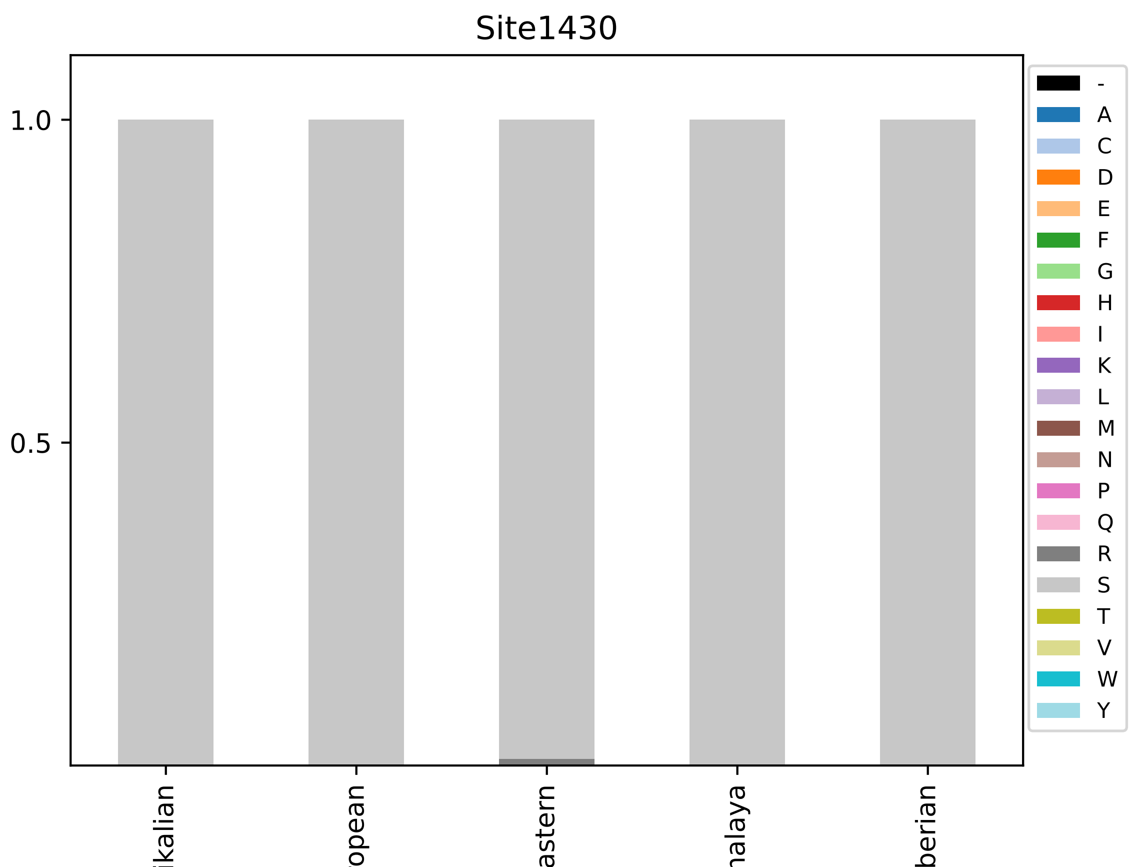
Site1430
1.0
0.5
-
A
C
D
E
F
G
H
I
K
L
M
N
P
Q
R
S
T
V
W
Y
ikalian
opean
astern
nalaya
berian

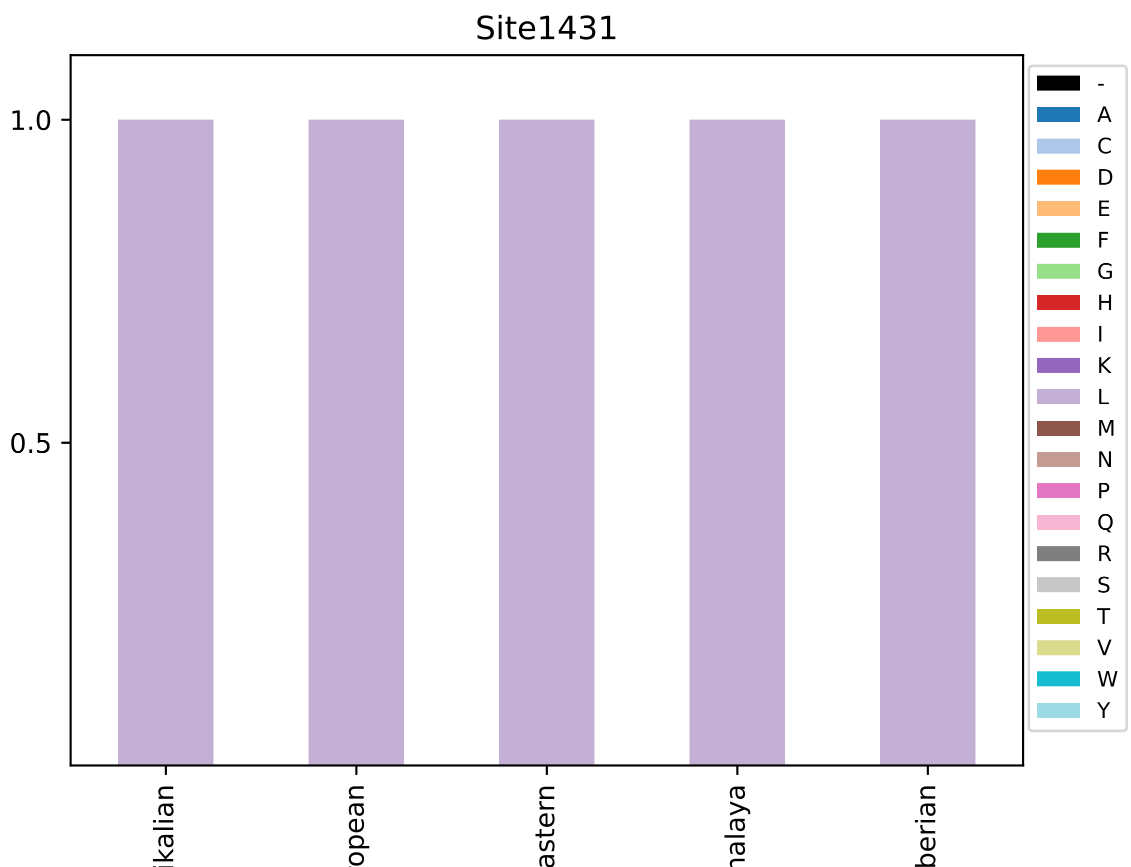
Site1431
1.0
0.5
-
A
C
D
E
F
G
H
I
K
L
M
N
P
Q
R
S
T
V
W
Y
ikalian
opean
astern
halaya
berian

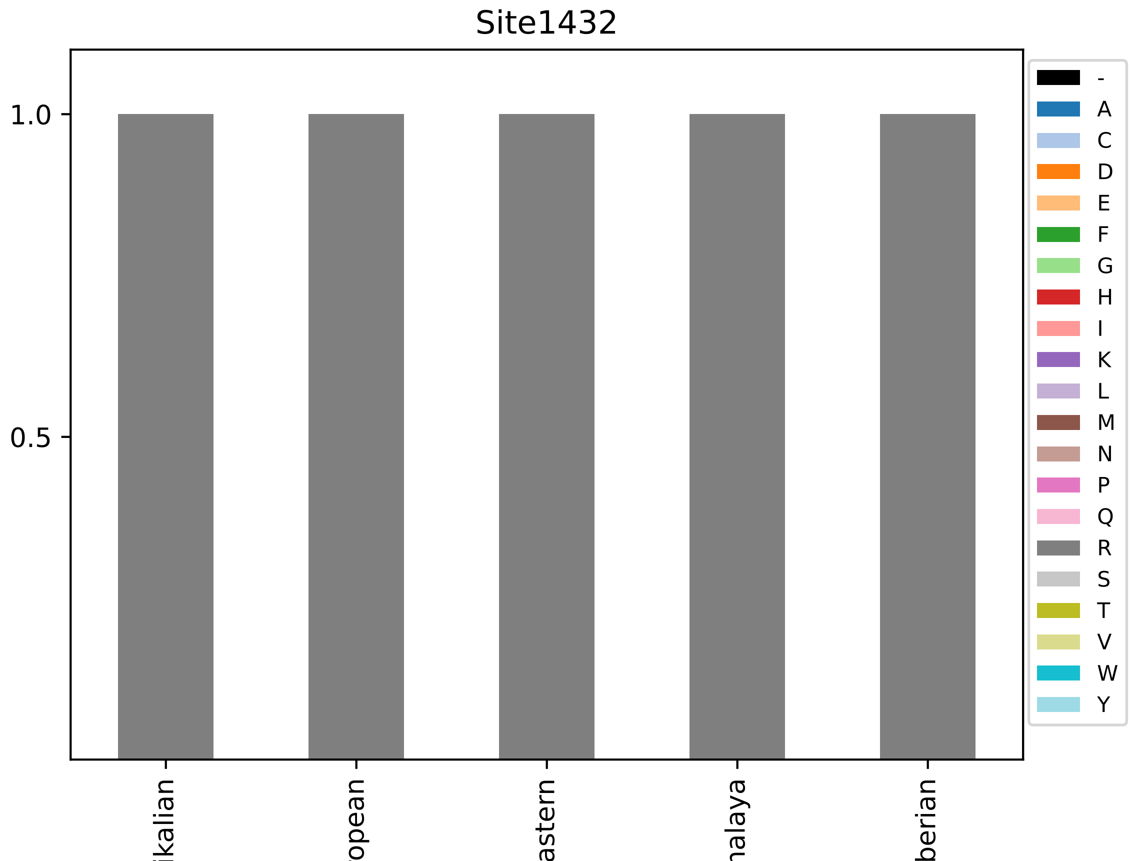
Site1432
1.0
0.5
ikalian
opean
astern
nalaya
berian
-
A
C
D
E
F
G
H
I
K
L
M
N
P
Q
R
S
T
V
W
Y

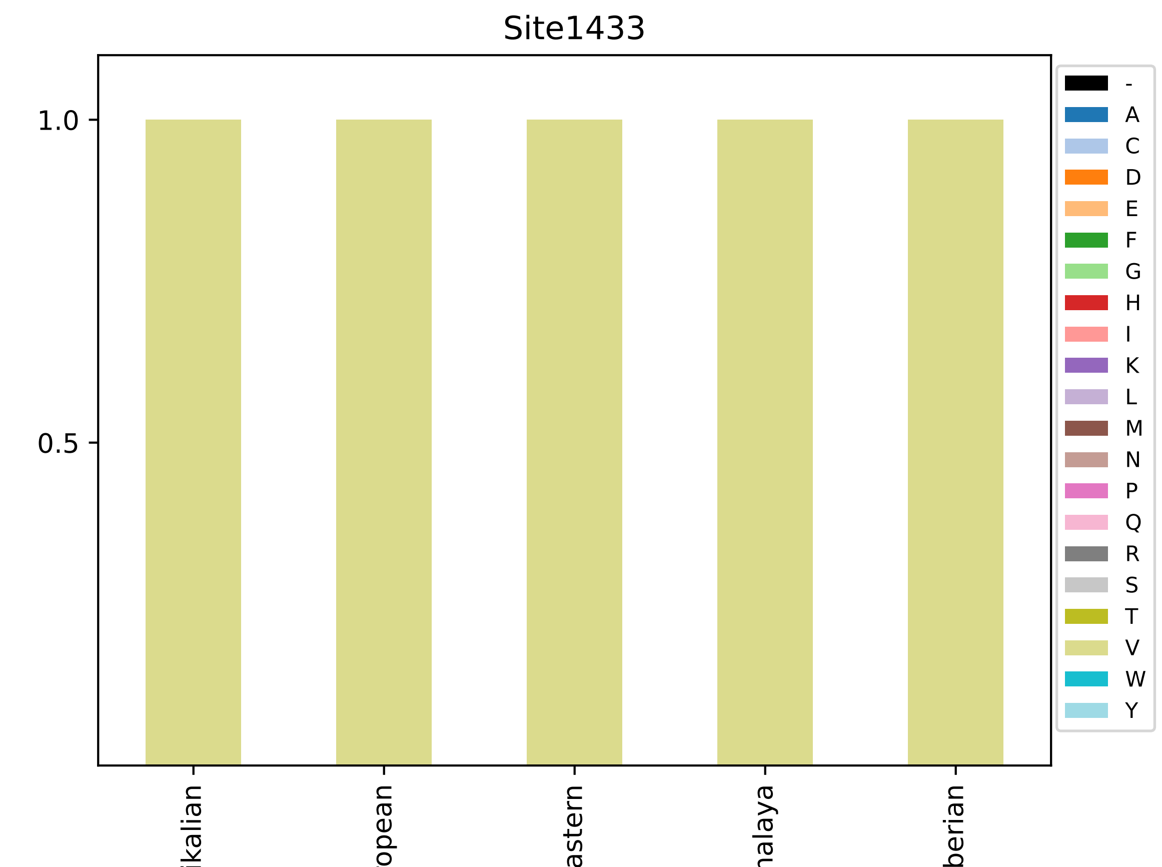

Site1433
1.0
0.5
-
A
C
D
E
F
G
H
I
K
L
M
N
P
Q
R
S
T
V
W
Y

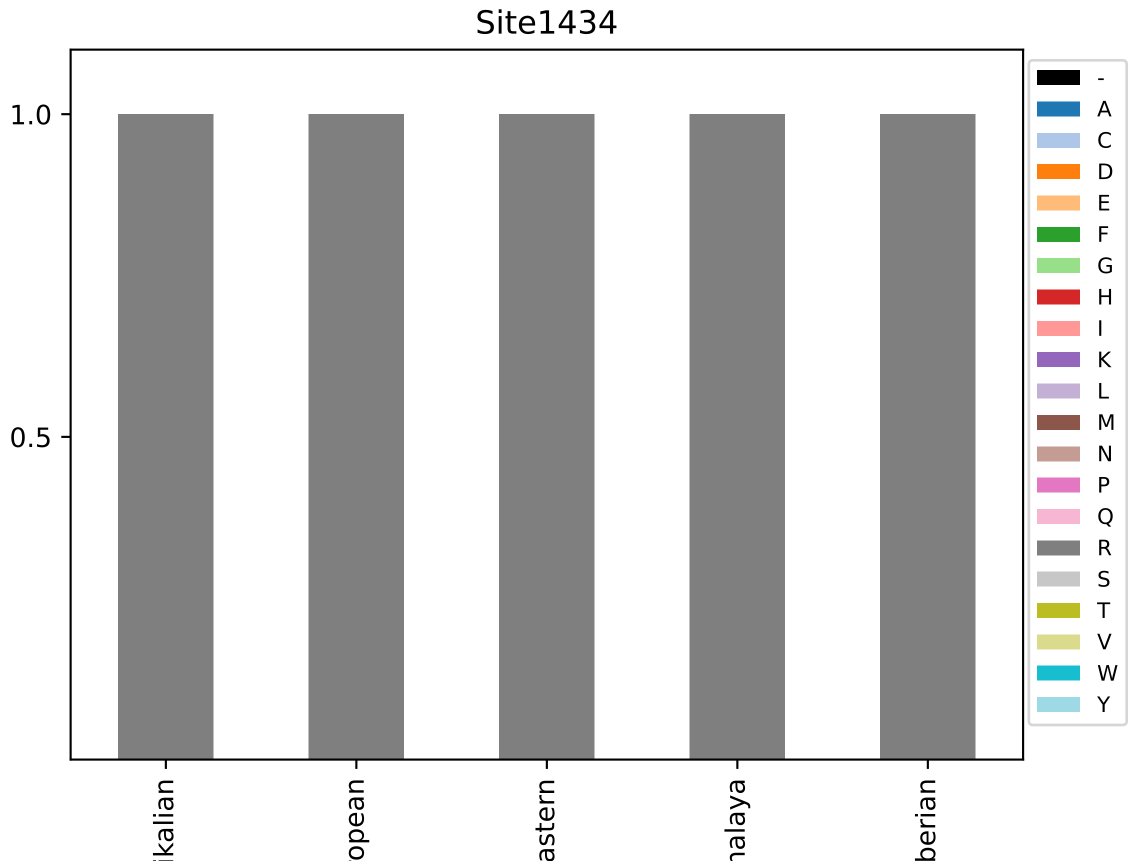
Site1434
1.0
0.5
-
A
C
D
E
F
G
H
I
K
L
M
N
P
Q
R
S
T
V
W
Y
ikalian
opean
astern
nalaya
berian

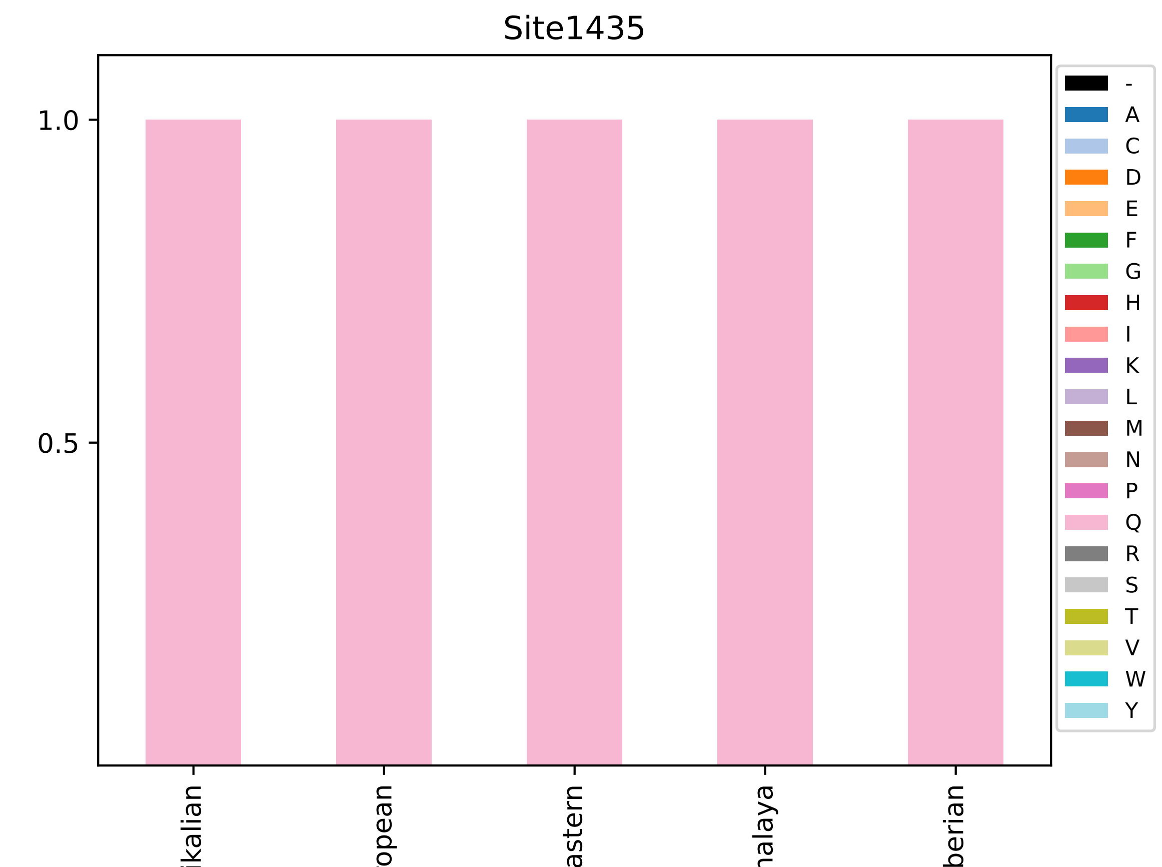
Site1435
1.0
0.5
-
A
C
D
E
F
G
H
I
K
L
M
N
P
Q
R
S
T
V
W
Y

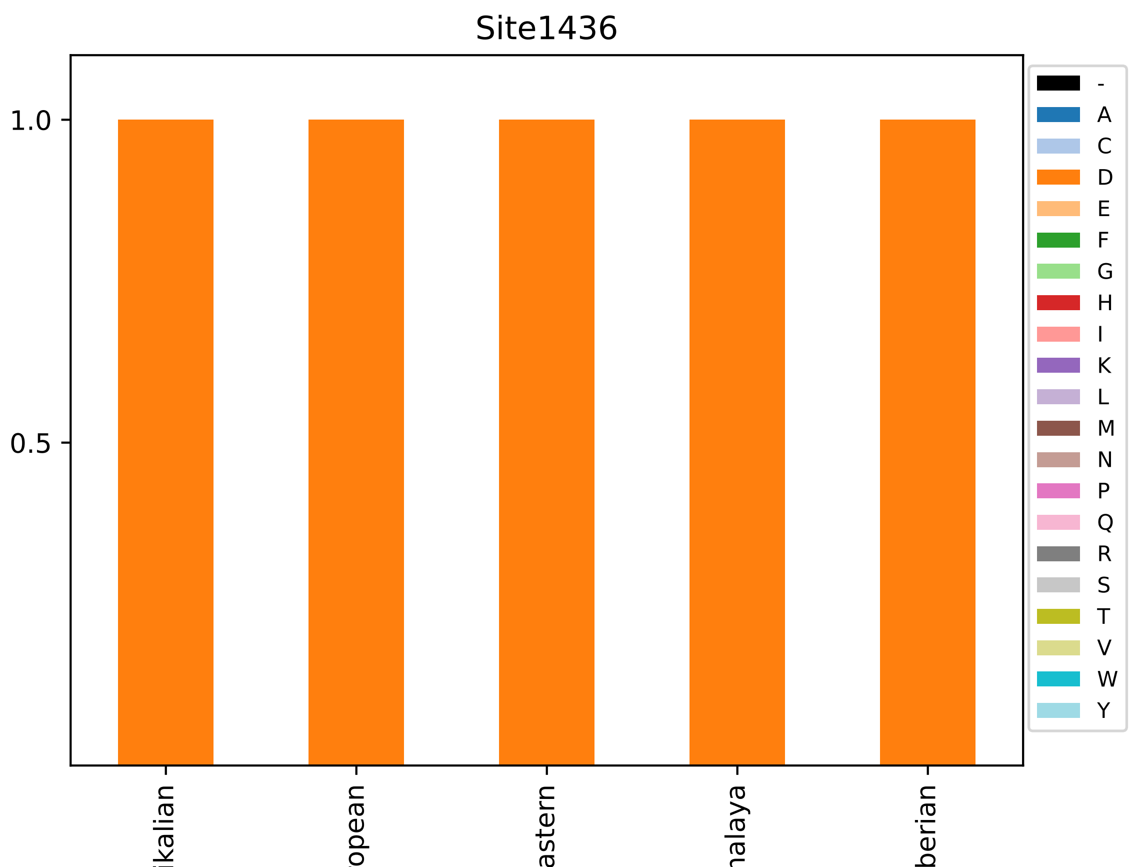
Site1436
1.0
0.5
-
A
C
D
E
F
G
H
I
K
L
M
N
P
Q
R
S
T
V
W
Y
ikalian
opean
astern
nalaya
berian

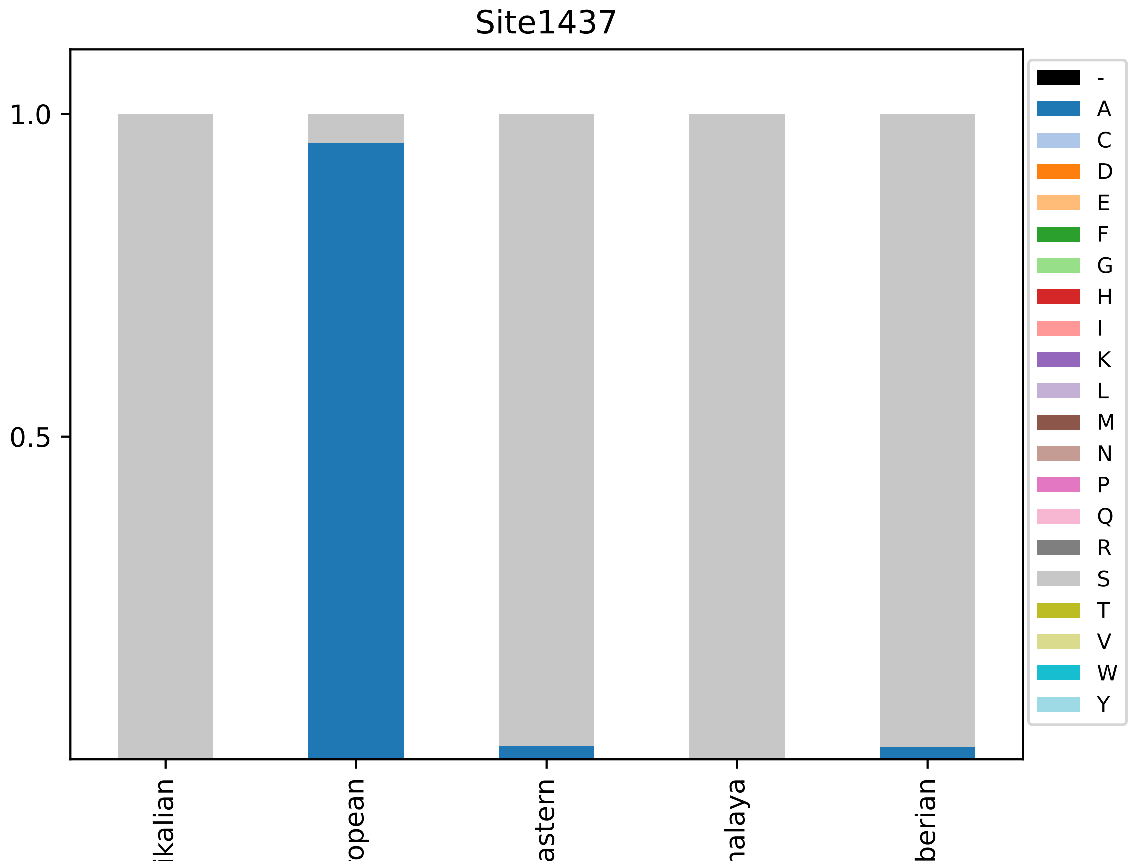

Site1437
1.0
0.5
-
A
C
D
E
F
G
H
I
K
L
M
N
P
Q
R
S
T
V
W
Y
ikalian
opean
astern
nalaya
berian

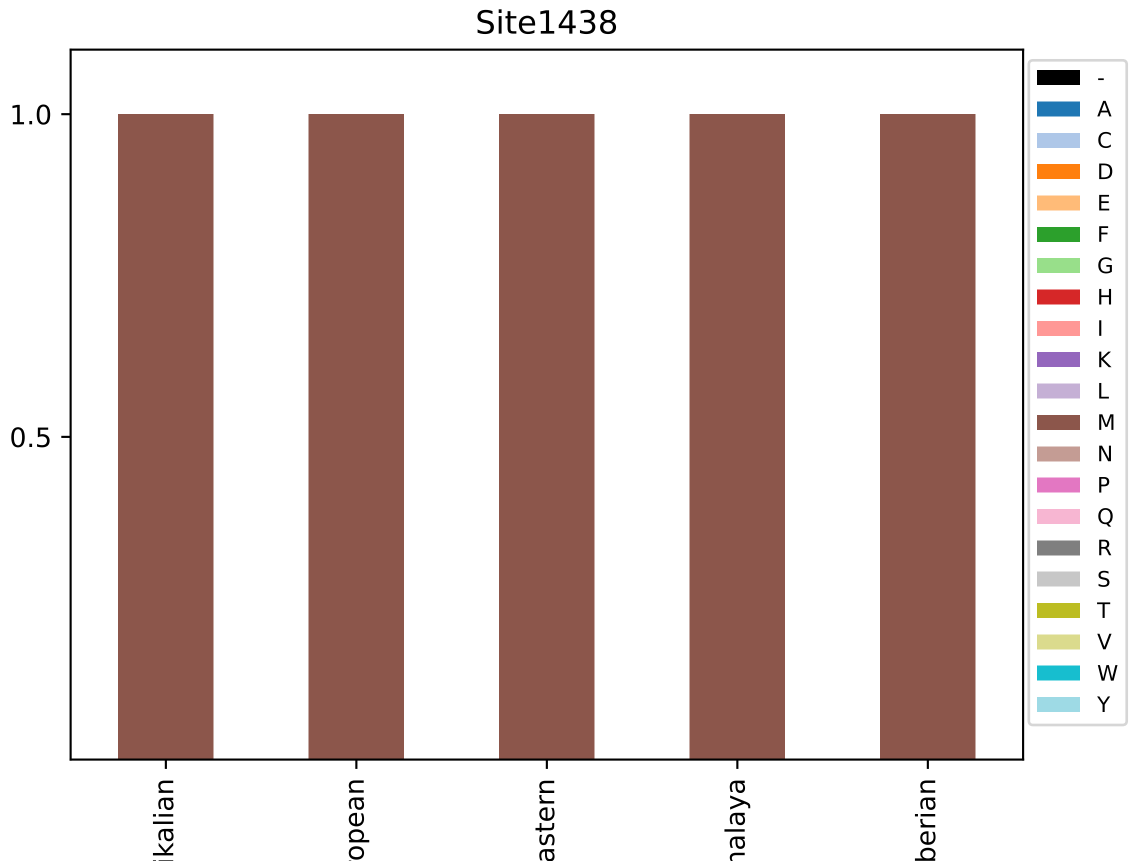

Site1438
1.0
0.5
ikalian
opean
astern
nalaya
berian
-
A
C
D
E
F
G
H
I
K
L
M
N
P
Q
R
S
T
V
W
Y

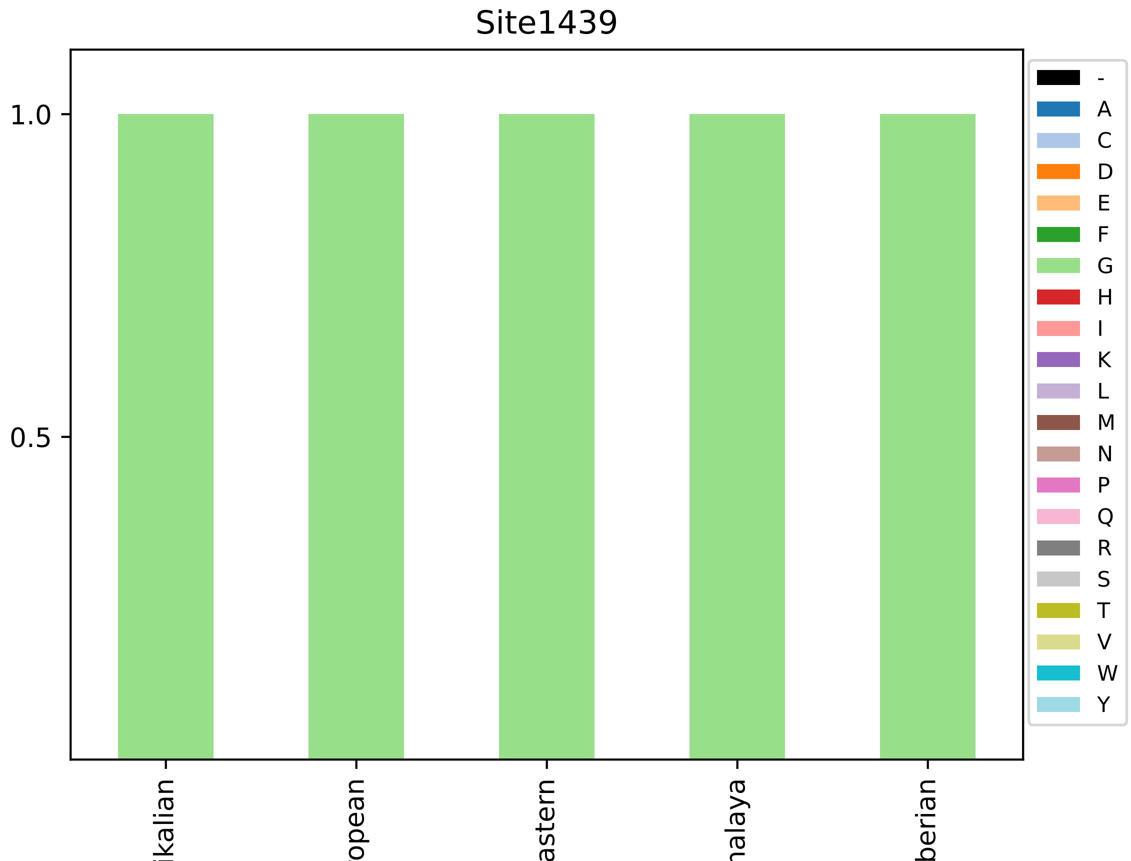
Site1439
1.0
0.5
ikalian
opean
astern
nalaya
berian
-
A
C
D
E
F
G
H
I
K
L
M
N
P
Q
R
S
T
V
W
Y

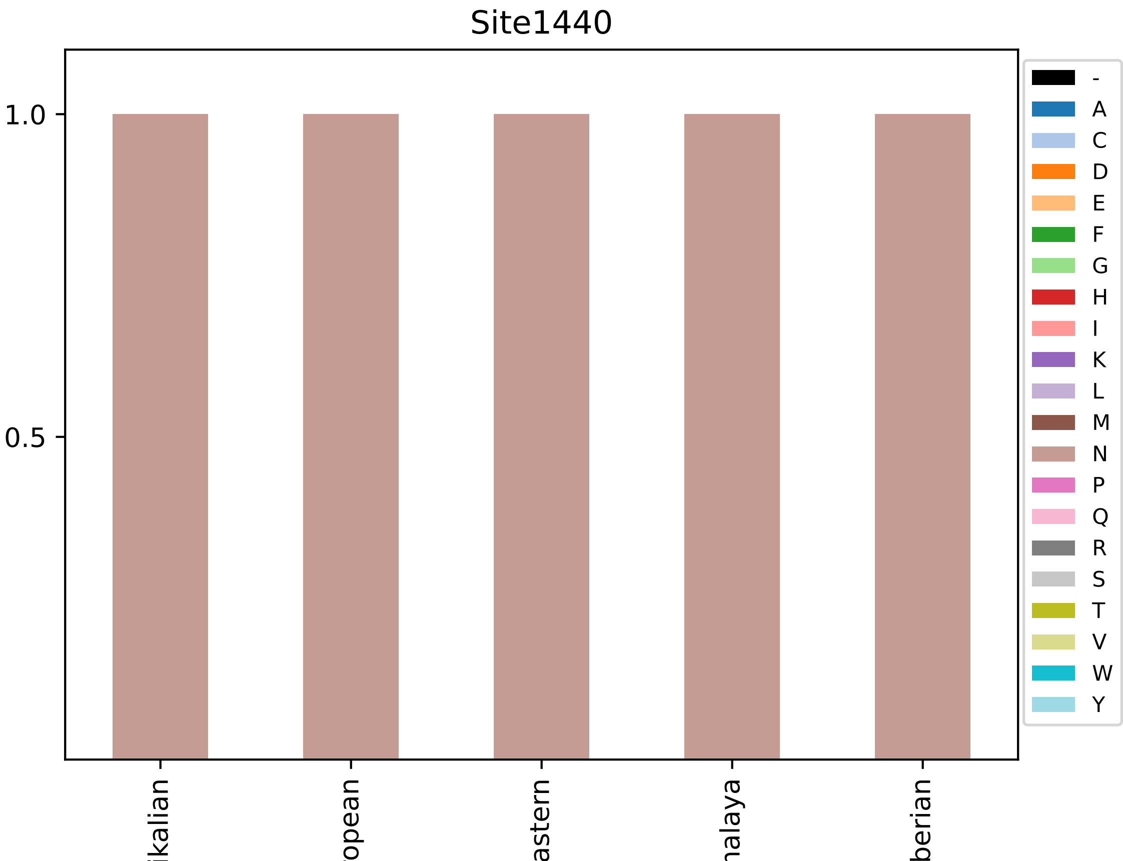
Site1440
1.0
0.5
-
A
C
D
E
F
G
H
I
K
L
M
N
P
Q
R
S
T
V
W
Y
ikalian
opean
astern
nalaya
berian

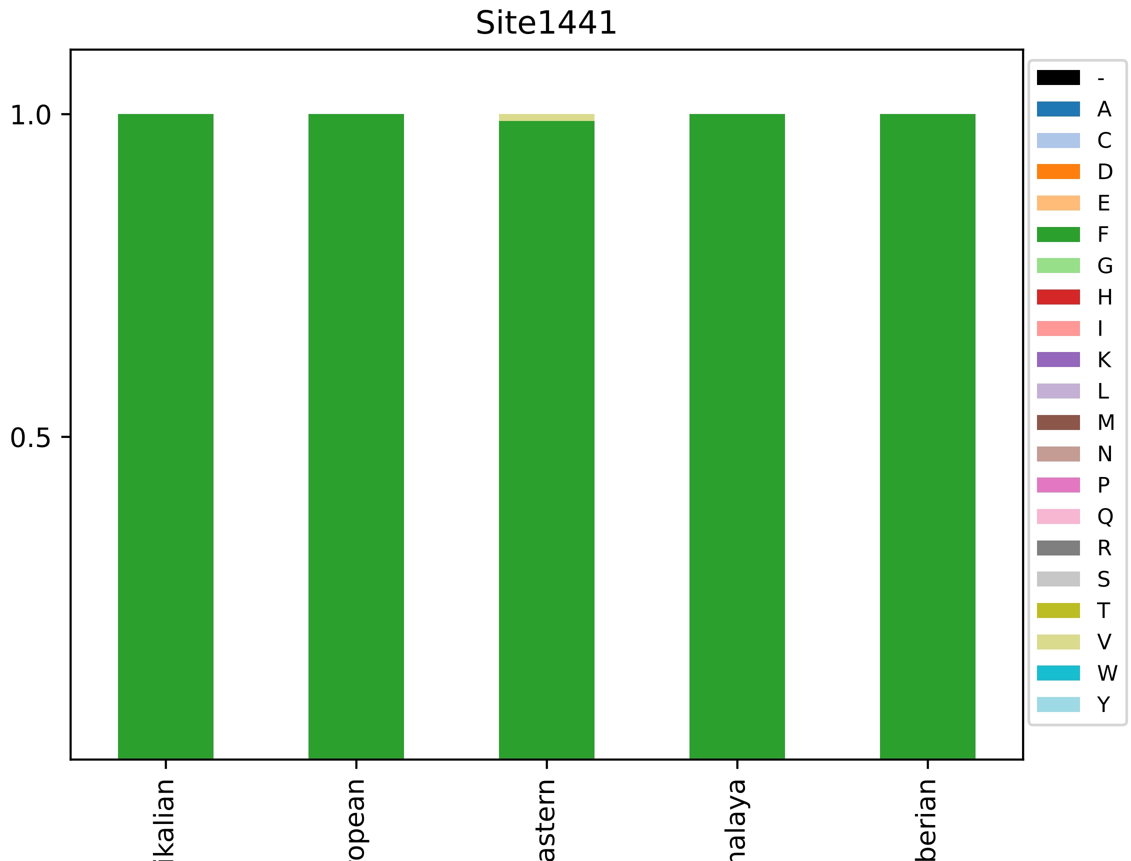
Site1441
1.0
0.5
ikalian
opean
astern
nalaya
berian
-
A
C
D
E
F
G
H
I
K
L
M
N
P
Q
R
S
T
V
W
Y

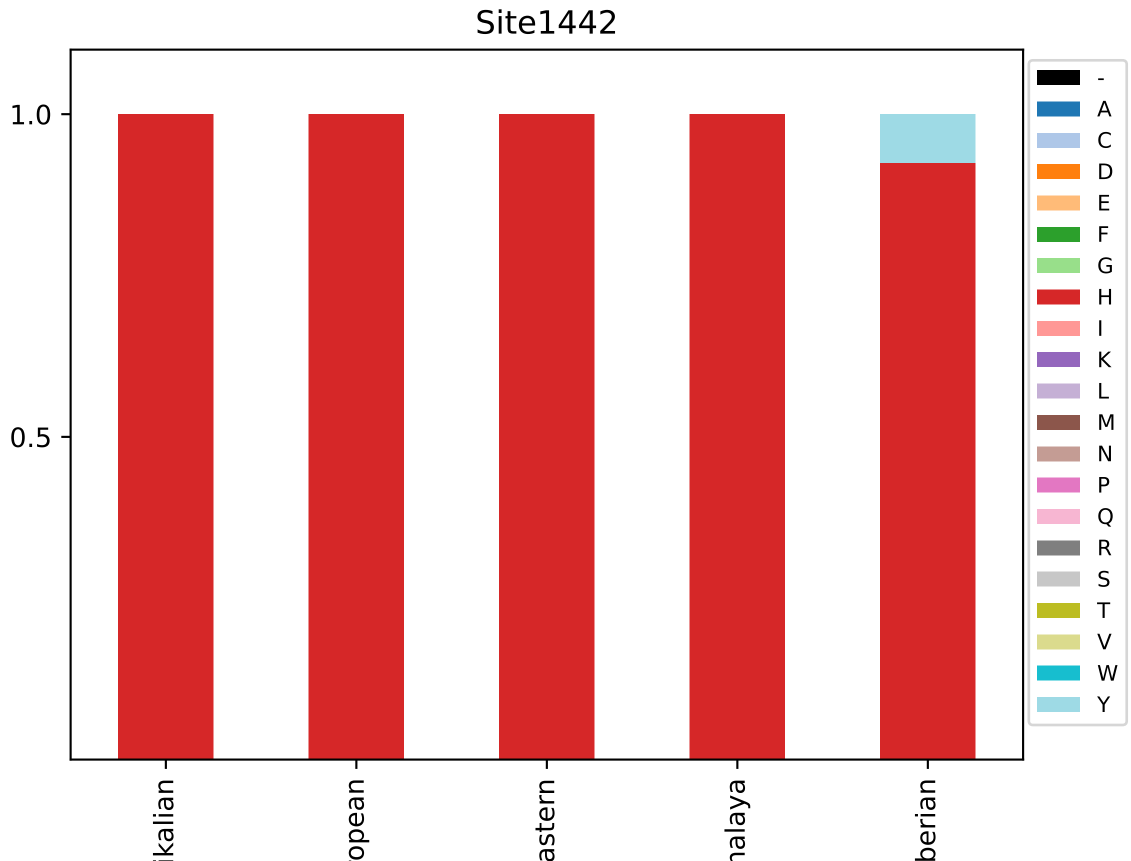
Site1442
1.0
0.5
-
A
C
D
E
F
G
H
I
K
L
M
N
P
Q
R
S
T
V
W
Y
ikalian
opean
astern
nalaya
berian

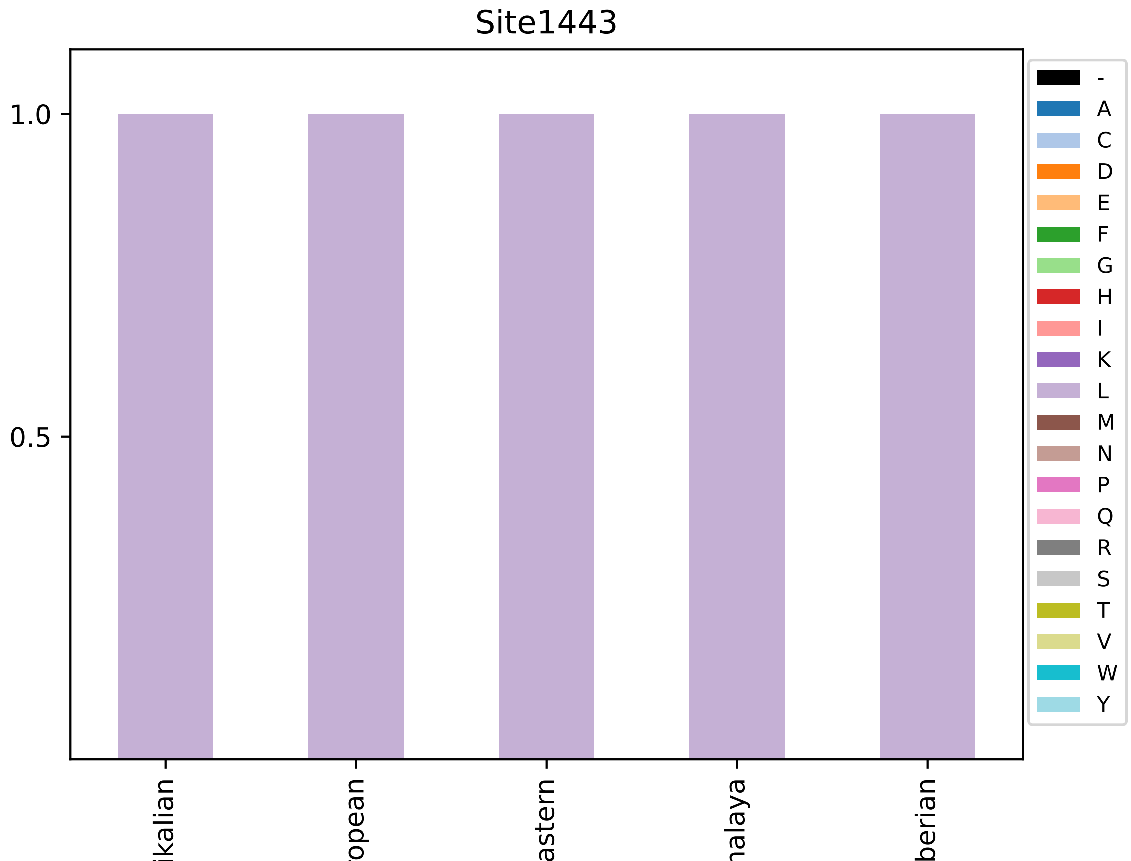
Site1443
1.0
0.5
-
A
C
D
E
F
G
H
I
K
L
M
N
P
Q
R
S
T
V
W
Y
ikalian
ropean
astern
nalaya
berian

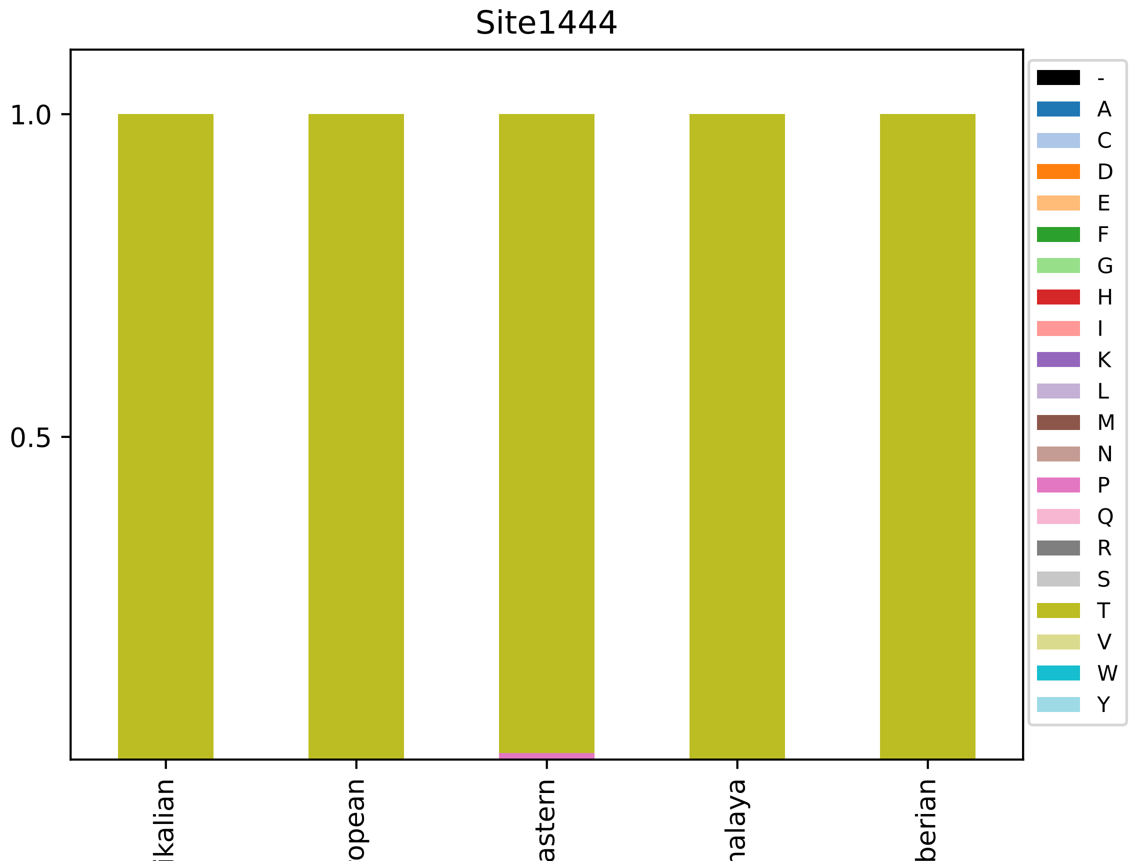
Site1444
1.0
0.5
-
A
C
D
E
F
G
H
I
K
L
M
N
P
Q
R
S
T
V
W
Y
ikalian
opean
astern
nalaya
berian

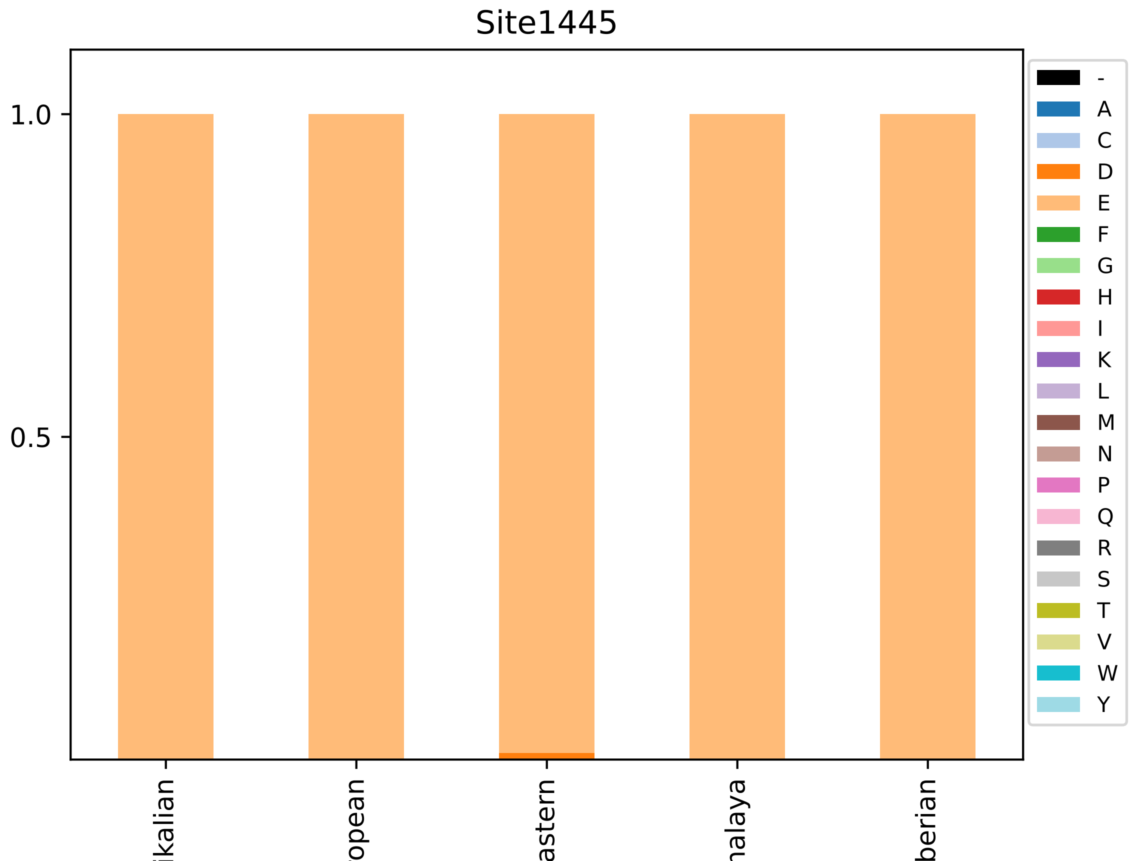

Site1445
1.0
0.5
ikalian
opean
astern
halaya
berian
-
A
C
D
E
F
G
H
I
K
L
M
N
P
Q
R
S
T
V
W
Y

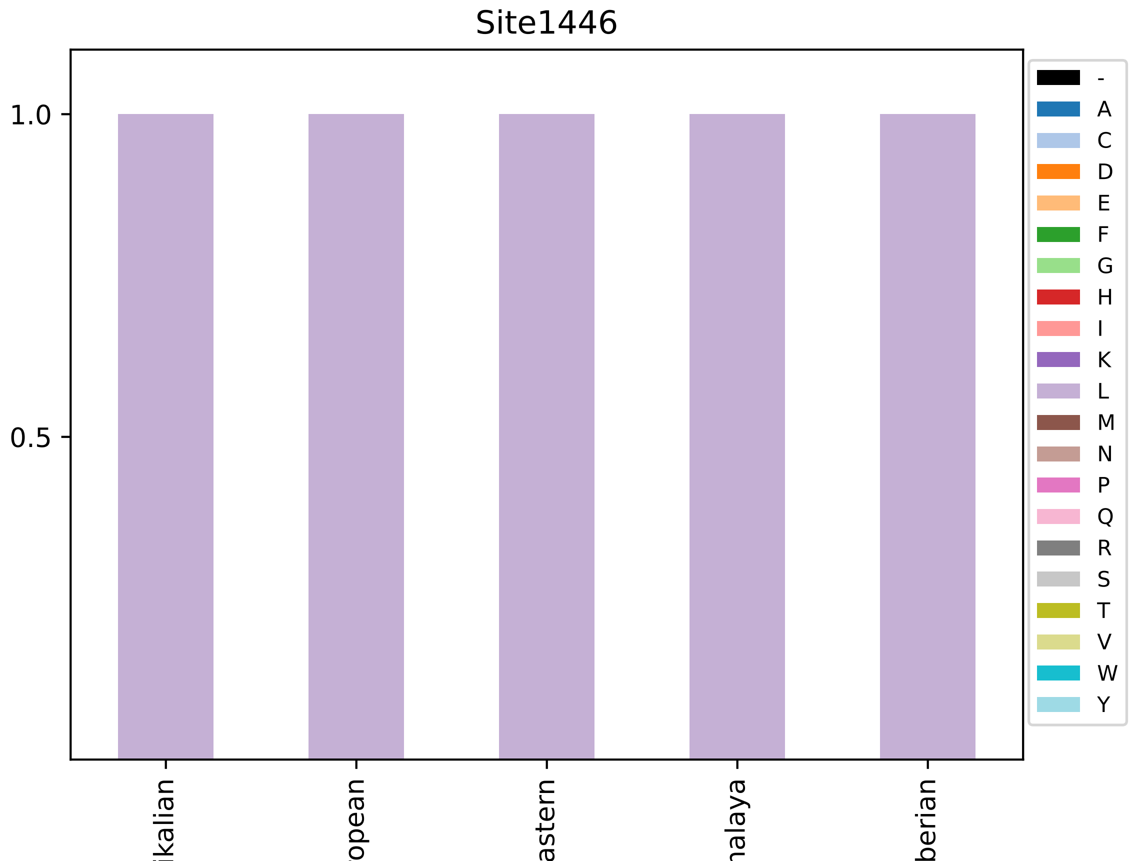

Site1446
1.0
0.5
ikalian
opean
astern
nalaya
berian
-
A
C
D
E
F
G
H
I
K
L
M
N
P
Q
R
S
T
V
W
Y

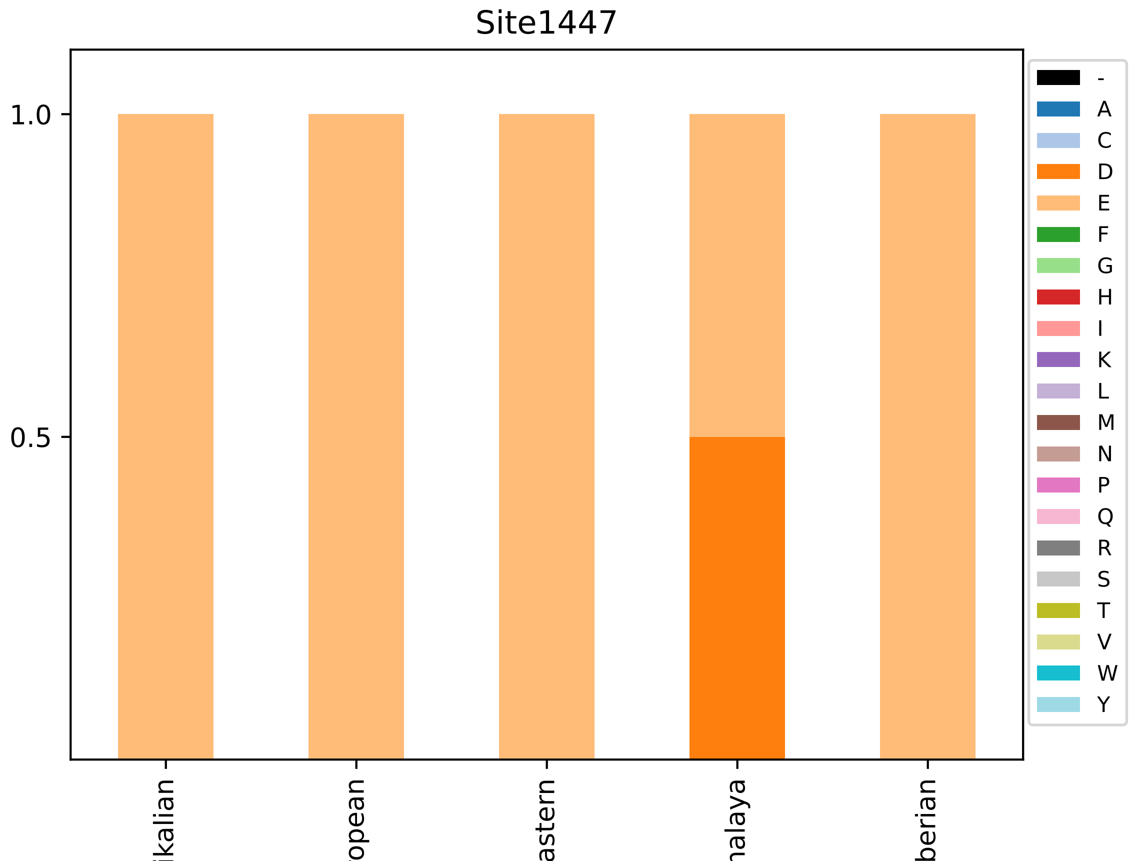

Site1447
1.0
0.5
-
A
C
D
E
F
G
H
I
K
L
M
N
P
Q
R
S
T
V
W
Y
ikalian
opean
astern
nalaya
berian

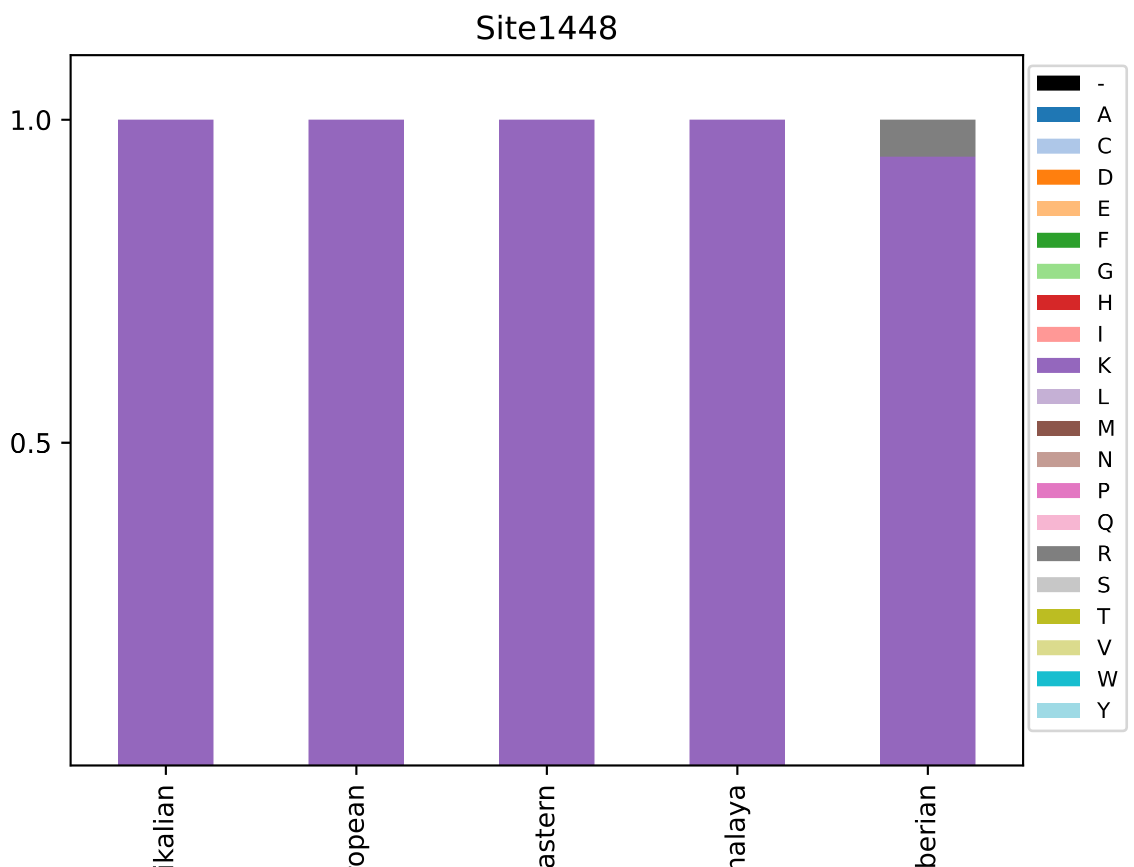

Site1448
1.0
0.5
-
A
C
D
E
F
G
H
I
K
L
M
N
P
Q
R
S
T
V
W
Y
ikalian
opean
astern
nalaya
berian

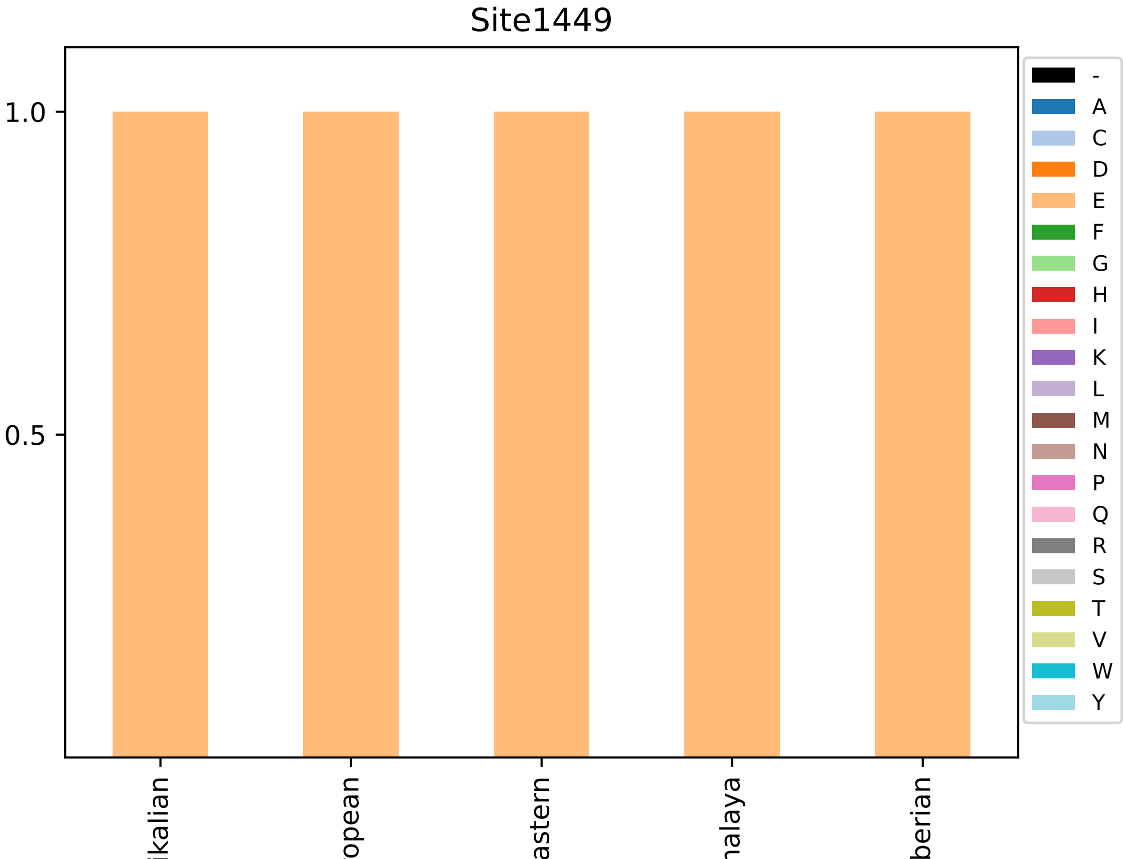

Site1449
1.0
0.5
-
A
C
D
E
F
G
H
I
K
L
M
N
P
Q
R
S
T
V
W
Y
ikalian
opean
astern
nalaya
berian

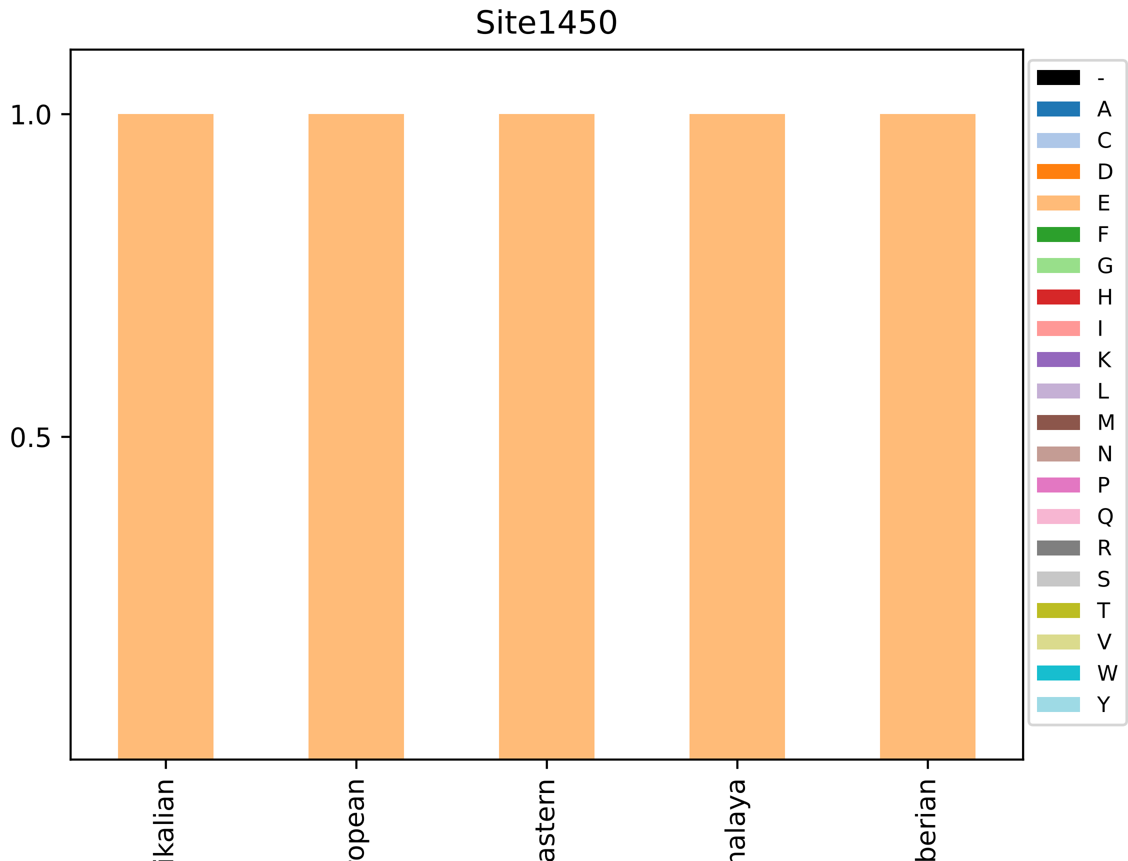

Site1450
1.0
0.5
ikalian
ropean
astern
nalaya
berian
-
A
C
D
E
F
G
H
I
K
L
M
N
P
Q
R
S
T
V
W
Y

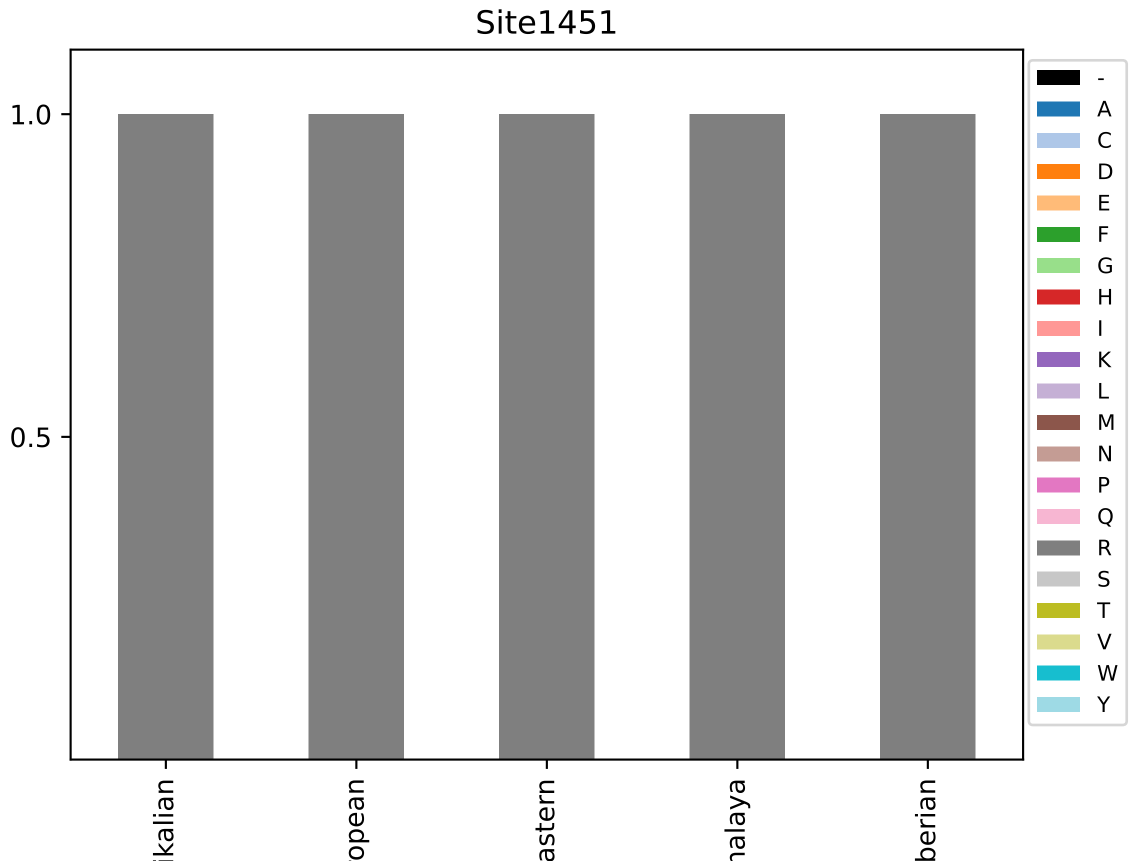

Site1451
1.0
0.5
-
A
C
D
E
F
G
H
I
K
L
M
N
P
Q
R
S
T
V
W
Y
ikalian
opean
astern
nalaya
berian

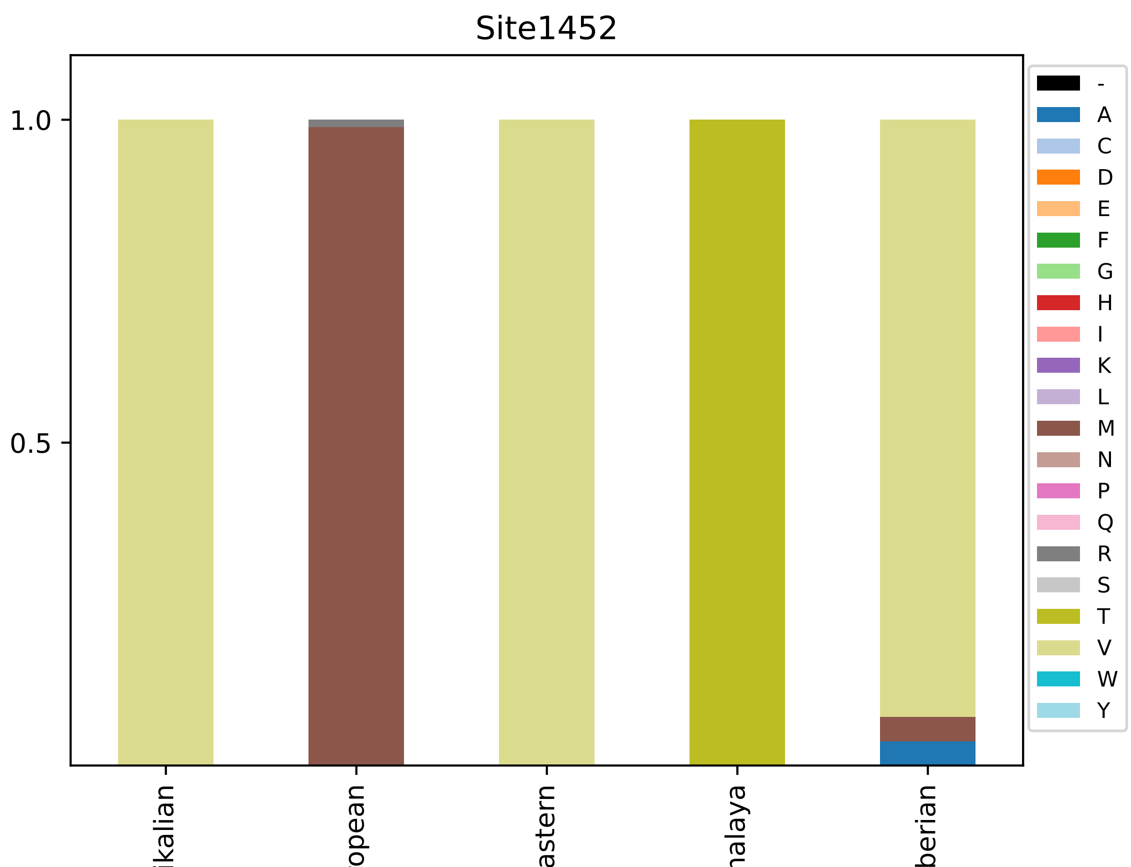
Site1452
1.0
0.5
-
A
C
D
E
F
G
H
I
K
L
M
N
P
Q
R
S
T
V
W
Y
ikalian
opean
astern
nalaya
berian

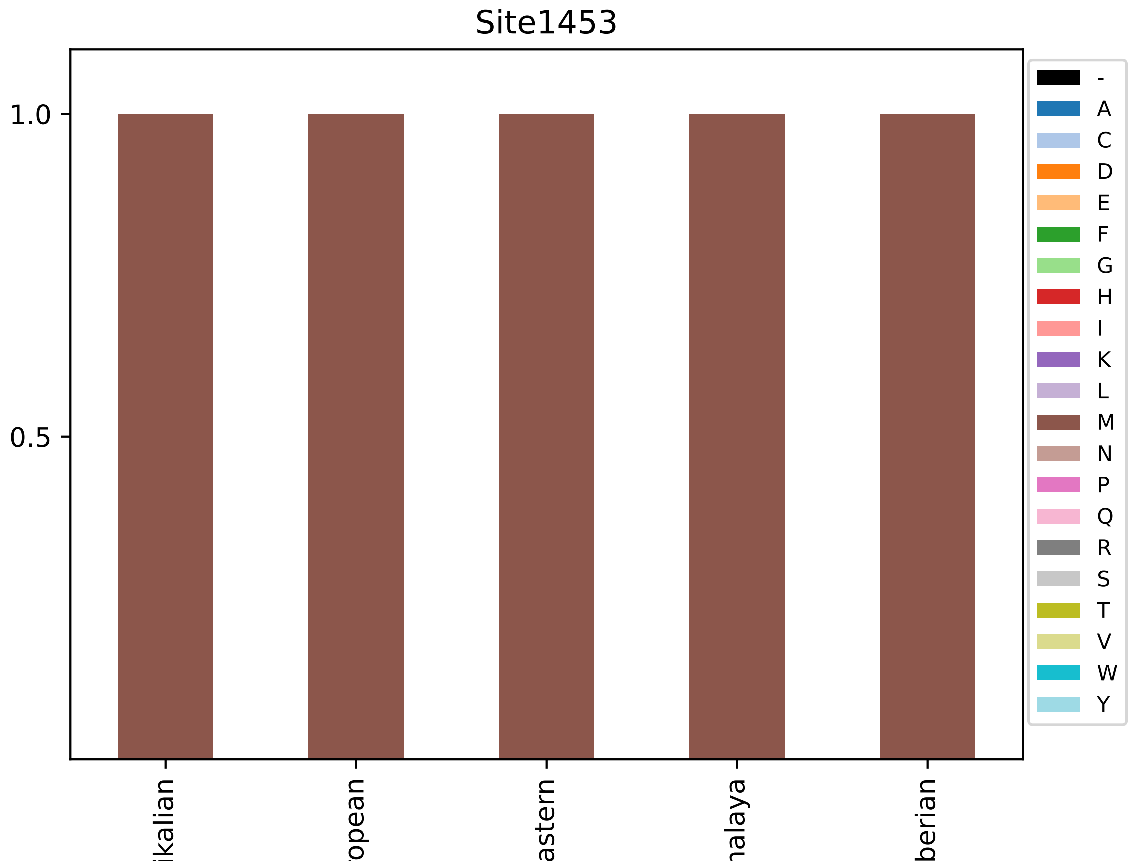

Site1453
1.0
0.5
-
A
C
D
E
F
G
H
I
K
L
M
N
P
Q
R
S
T
V
W
Y
ikalian
opean
astern
nalaya
berian

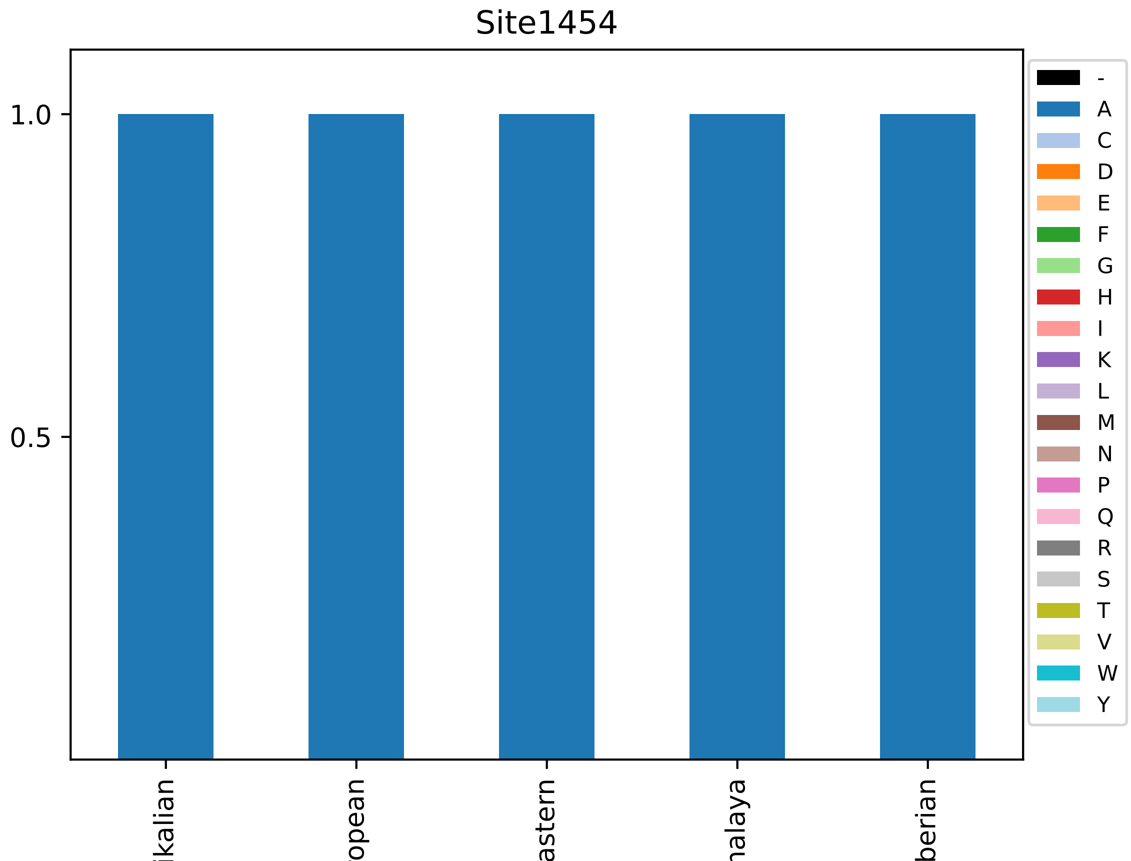
Site1454
1.0
0.5
ikalian
opean
astern
nalaya
berian
-
A
C
D
E
F
G
H
I
K
L
M
N
P
Q
R
S
T
V
W
Y

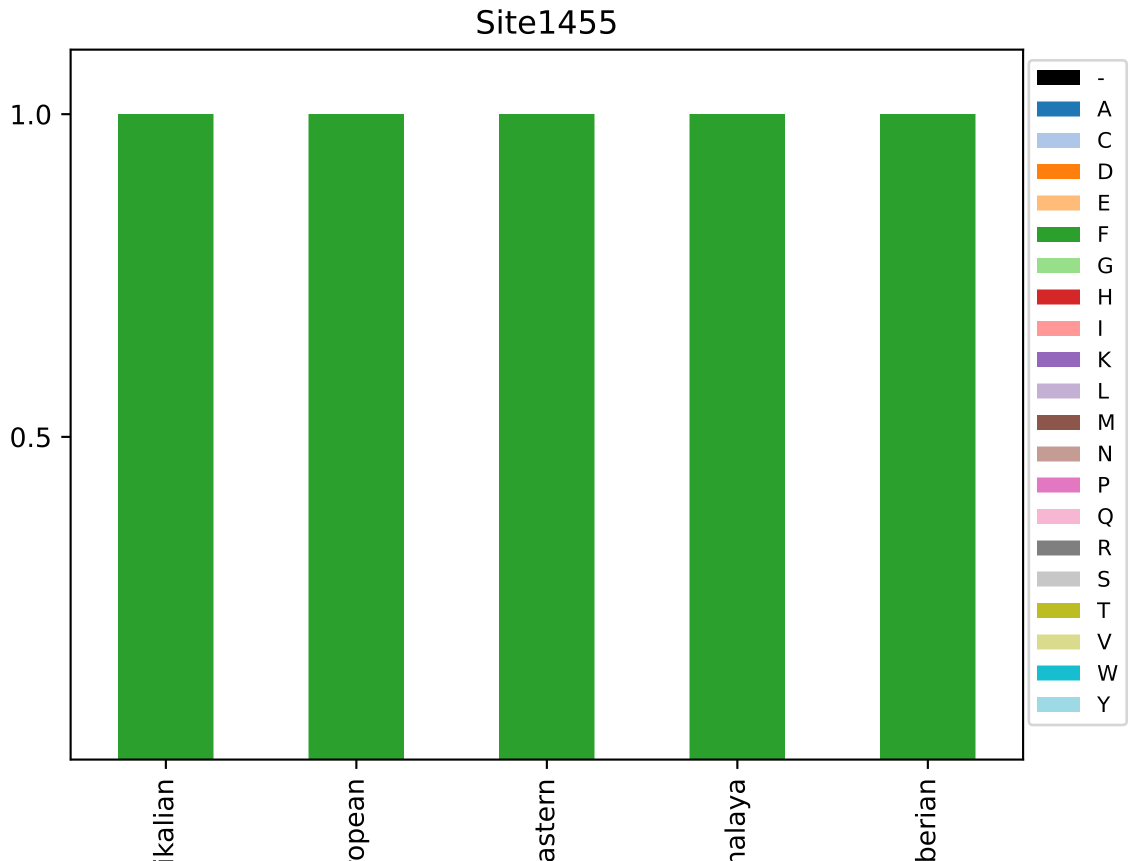
Site1455
1.0
0.5
-
A
C
D
E
F
G
H
I
K
L
M
N
P
Q
R
S
T
V
W
Y

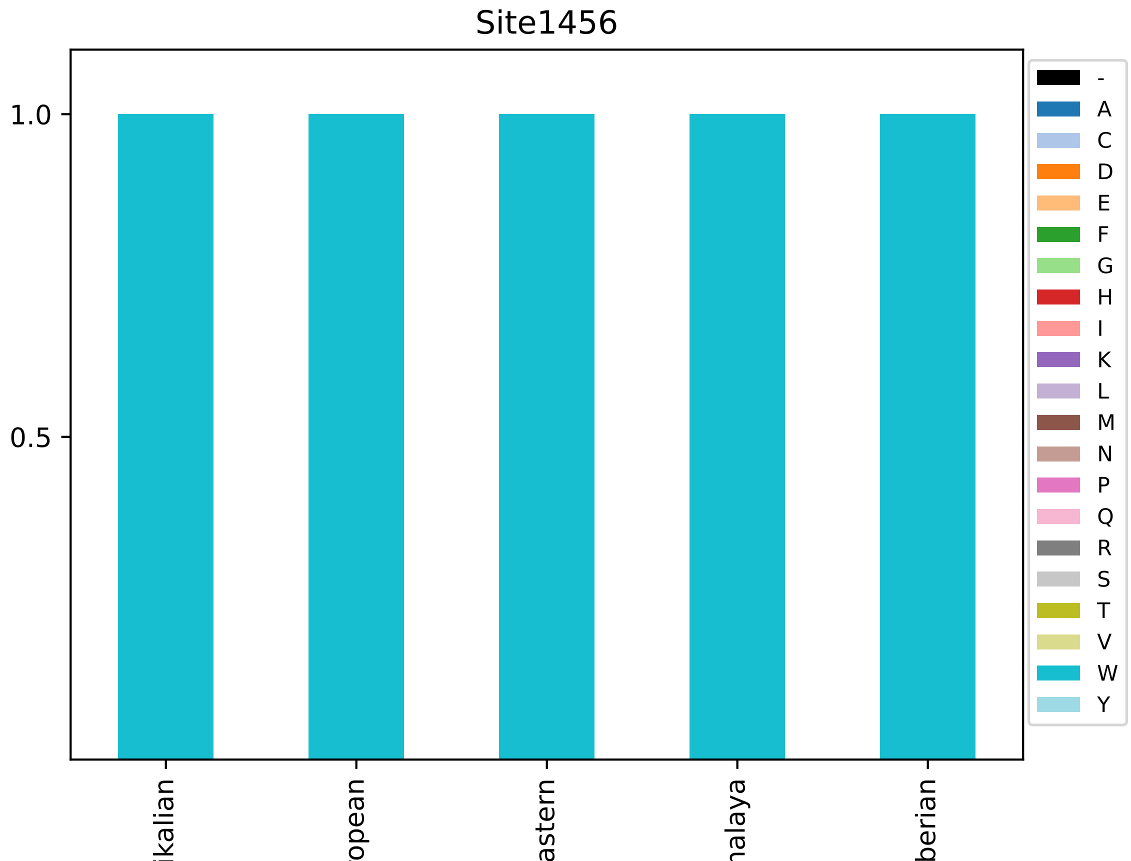

Site1456
1.0
0.5
ikalian
opean
astern
halaya
berian
-
A
C
D
E
F
G
H
I
K
L
M
N
P
Q
R
S
T
V
W
Y

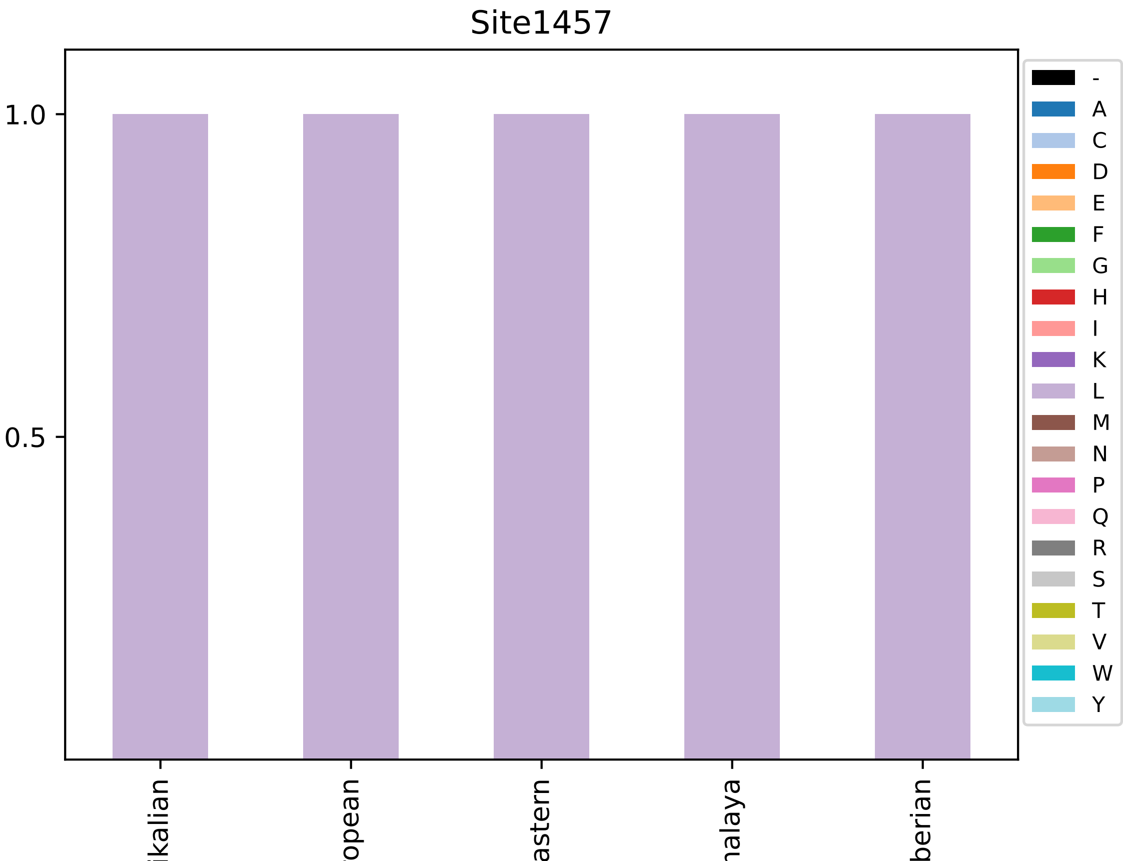
Site1457
1.0
0.5
-
A
C
D
E
F
G
H
I
K
L
M
N
P
Q
R
S
T
V
W
Y
ikalian
opean
astern
nalaya
berian

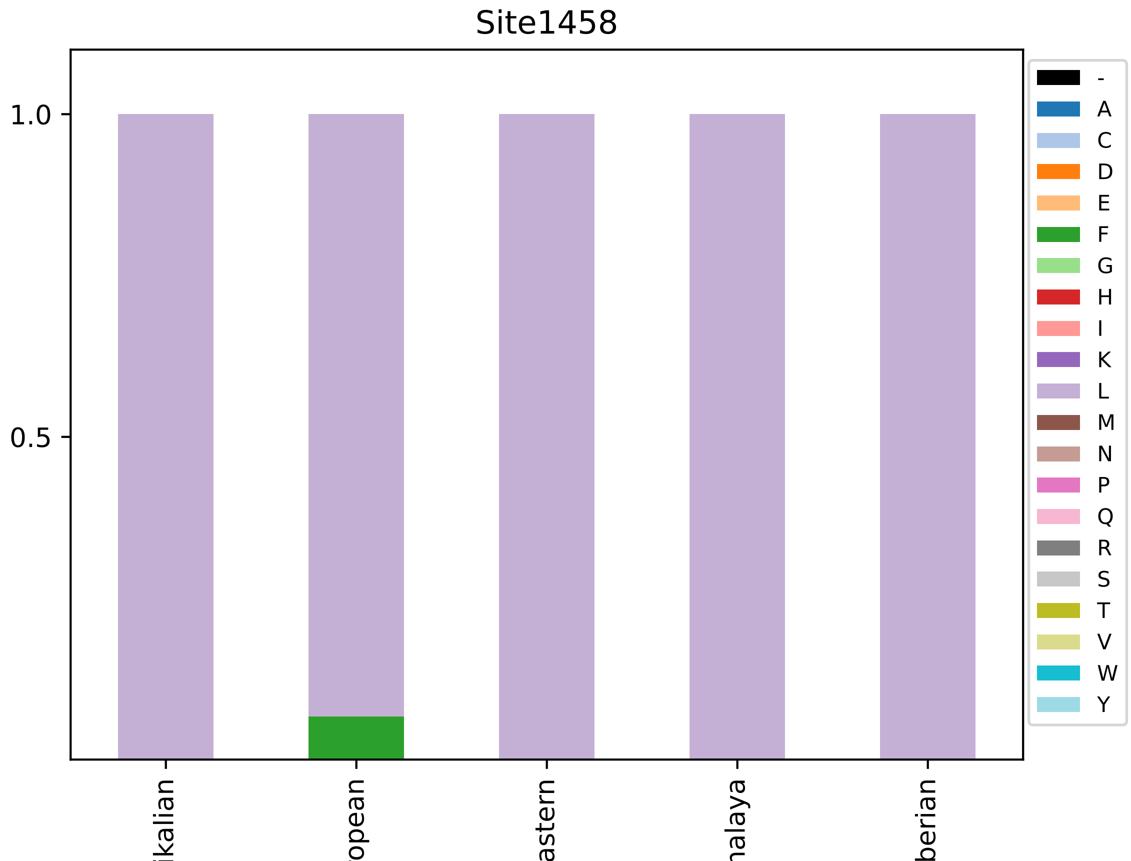

Site1458
1.0
0.5
-
A
C
D
E
F
G
H
I
K
L
M
N
P
Q
R
S
T
V
W
Y
ikalian
opean
astern
nalaya
berian

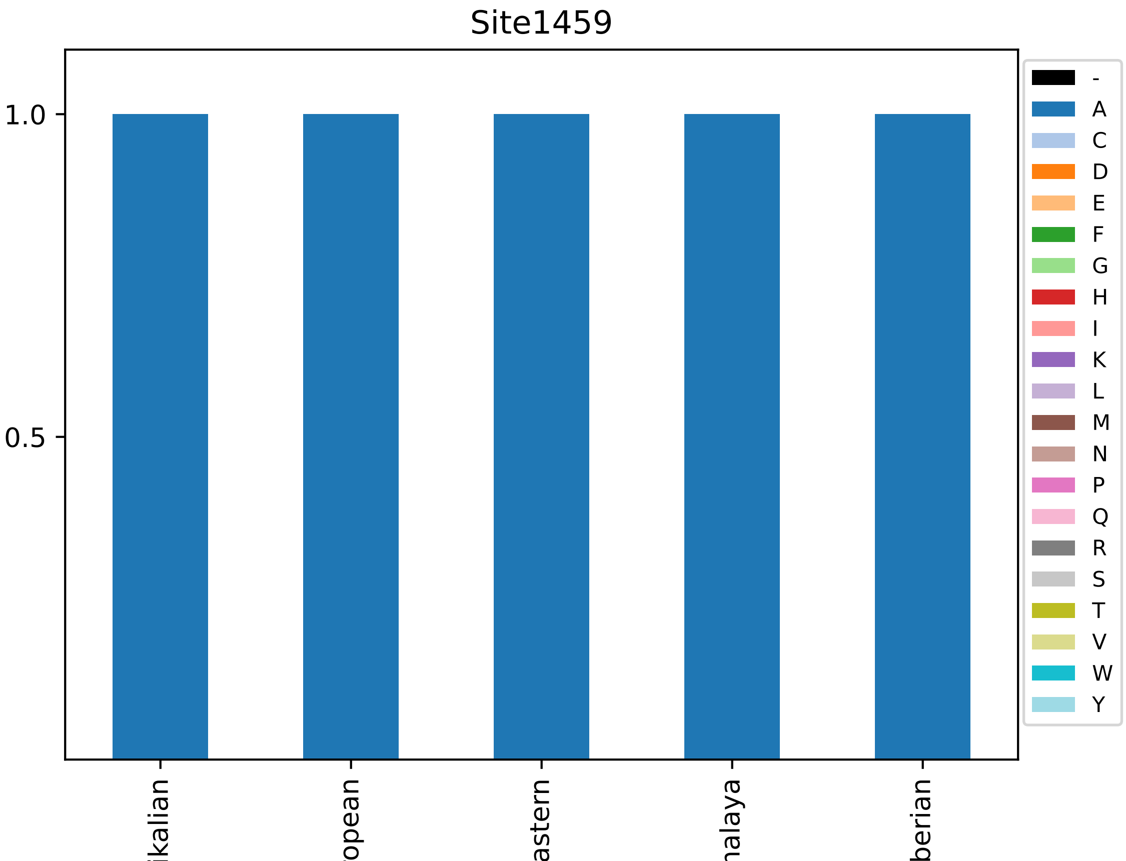
Site1459
1.0
0.5
-
A
C
D
E
F
G
H
I
K
L
M
N
P
Q
R
S
T
V
W
Y
ikalian
opean
astern
nalaya
berian

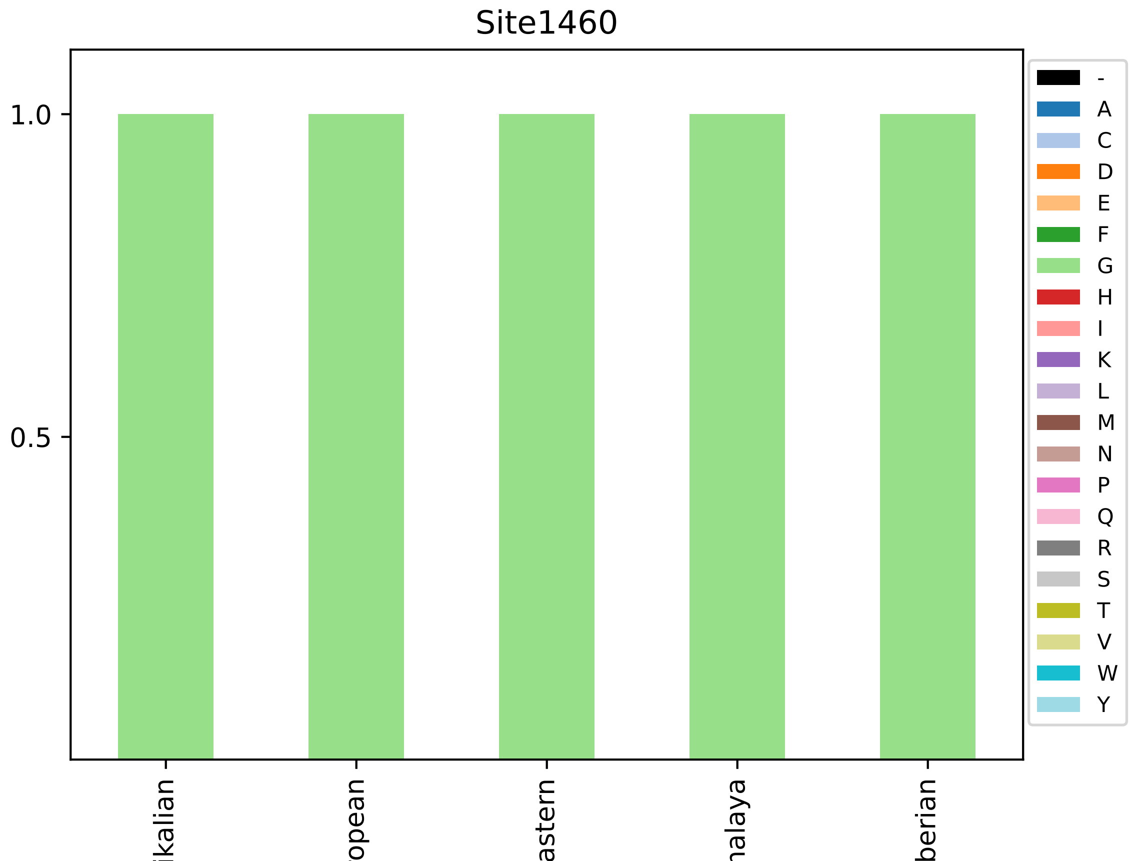

Site1460
1.0
0.5
-
A
C
D
E
F
G
H
I
K
L
M
N
P
Q
R
S
T
V
W
Y
ikalian
opean
astern
nalaya
berian

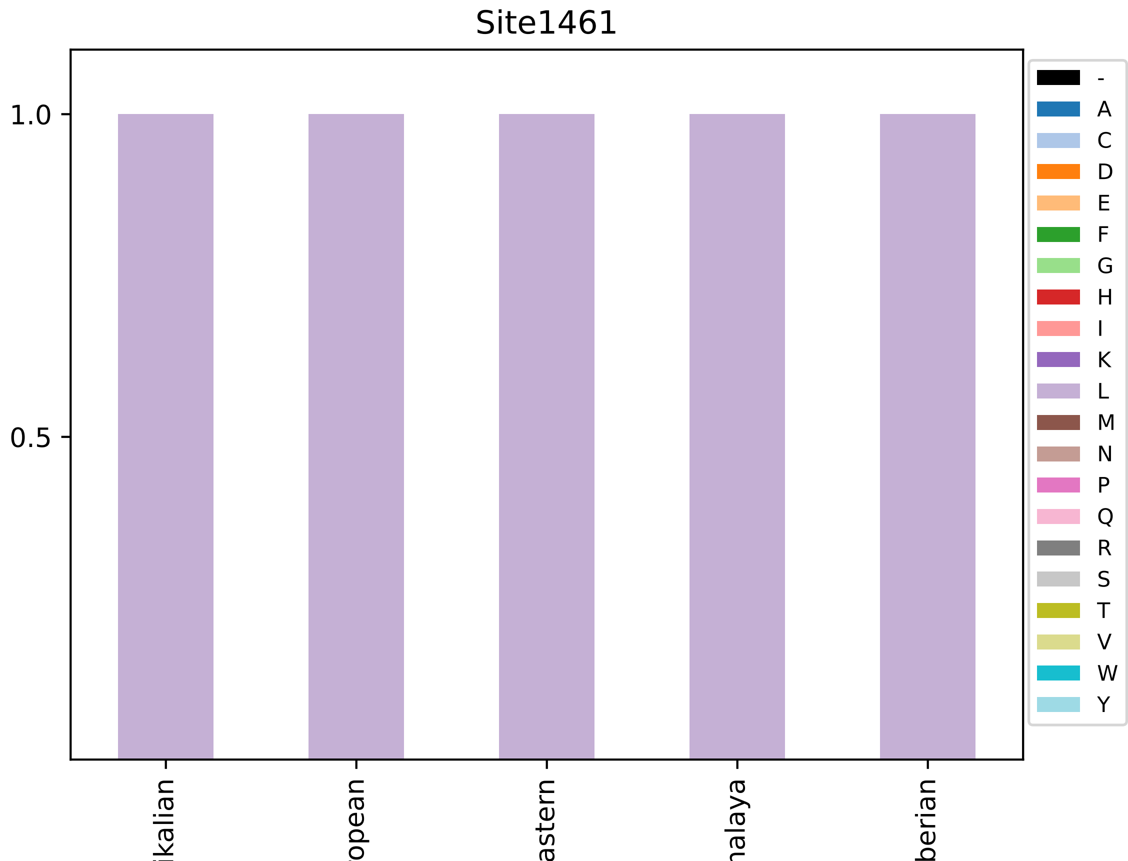

Site1461
1.0
0.5
-
A
C
D
E
F
G
H
I
K
L
M
N
P
Q
R
S
T
V
W
Y
ikalian
opean
astern
nalaya
berian

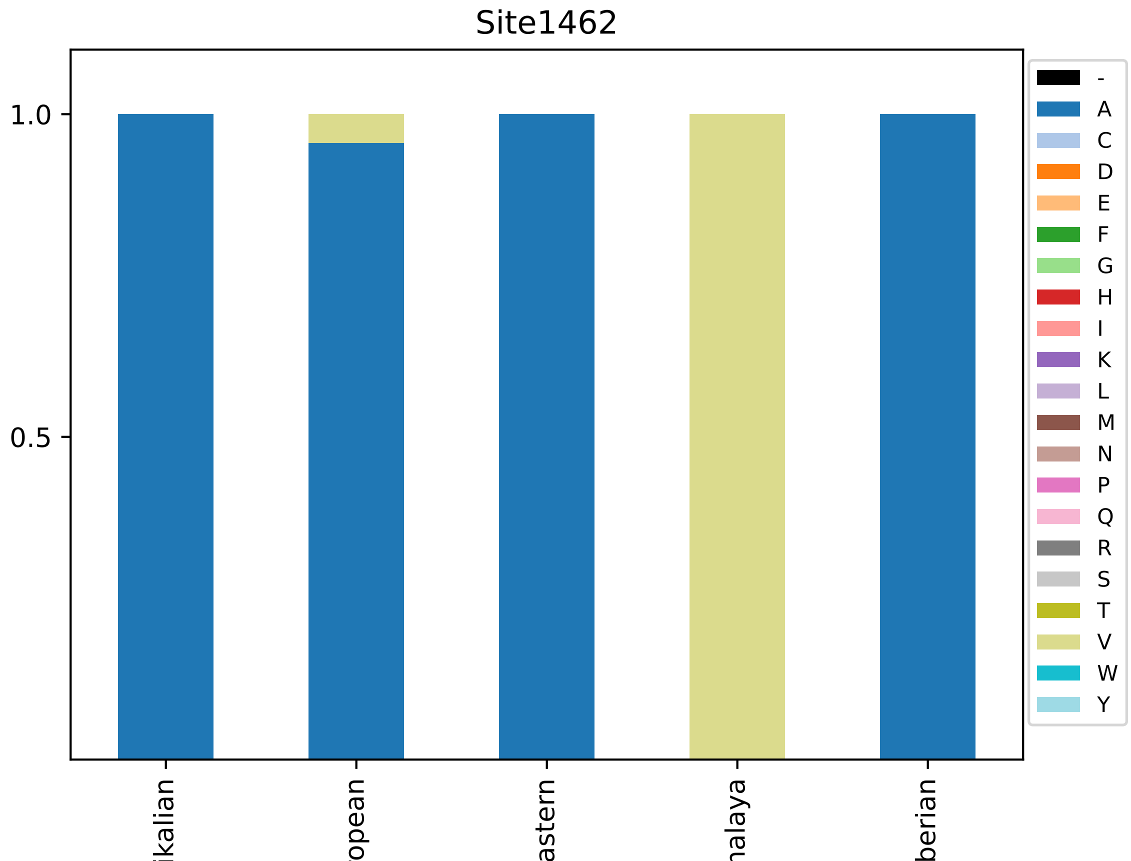

Site1462
1.0
0.5
-
A
C
D
E
F
G
H
I
K
L
M
N
P
Q
R
S
T
V
W
Y
ikalian
opean
astern
nalaya
berian

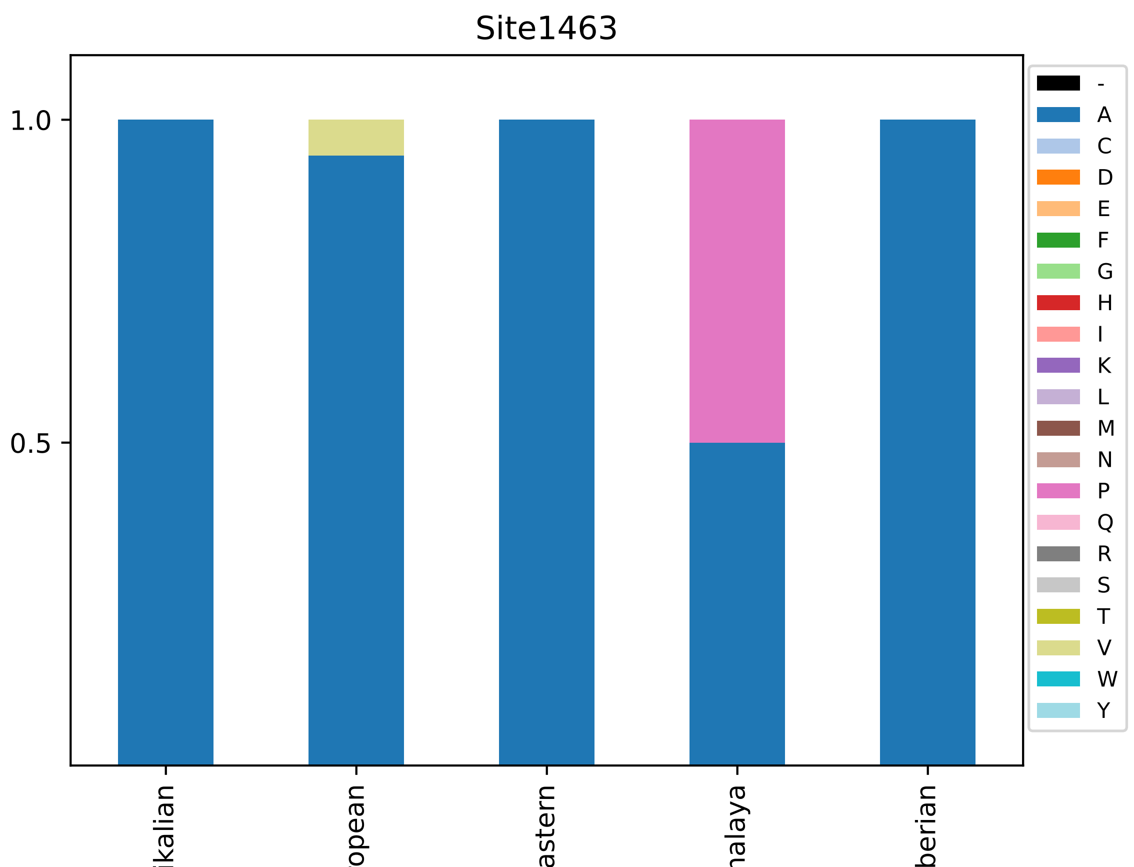
Site1463
1.0
0.5
-
A
C
D
E
F
G
H
I
K
L
M
N
P
Q
R
S
T
V
W
Y
ikalian
opean
astern
nalaya
berian

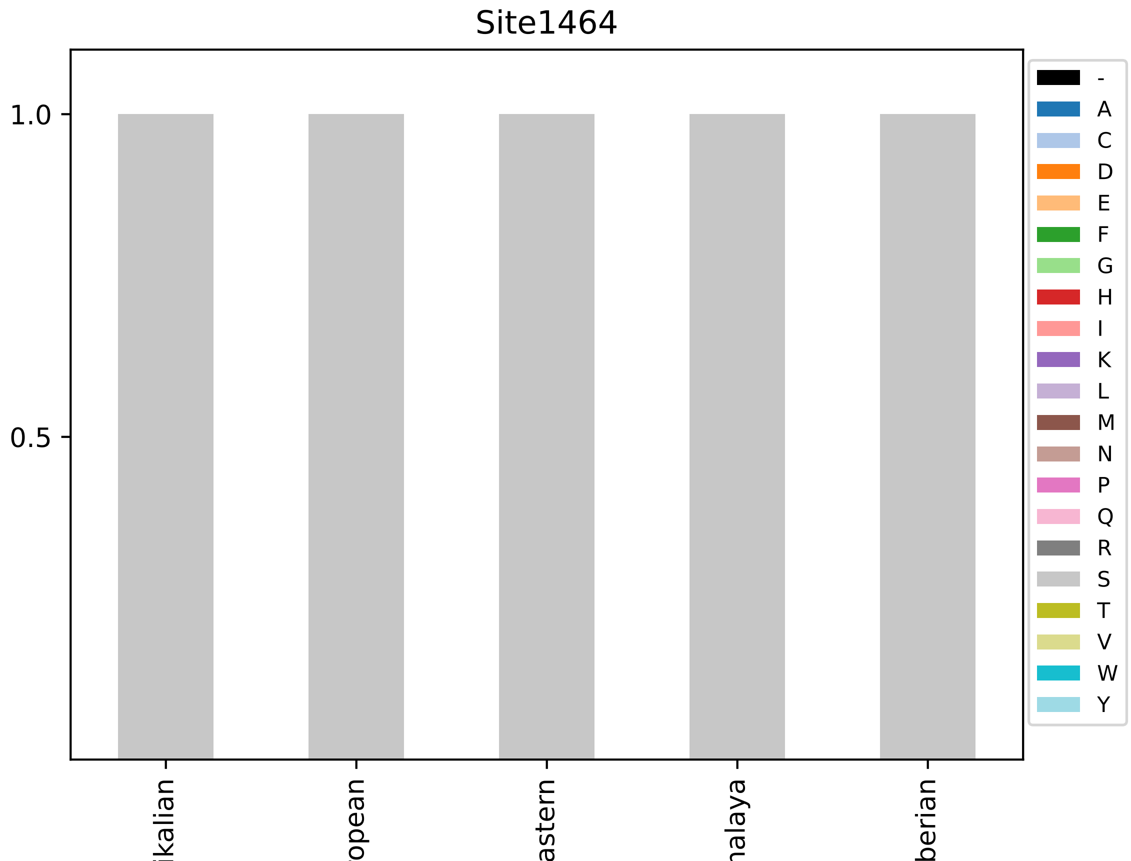
Site1464
1.0
0.5
-
A
C
D
E
F
G
H
I
K
L
M
N
P
Q
R
S
T
V
W
Y
ikalian
opean
astern
nalaya
berian

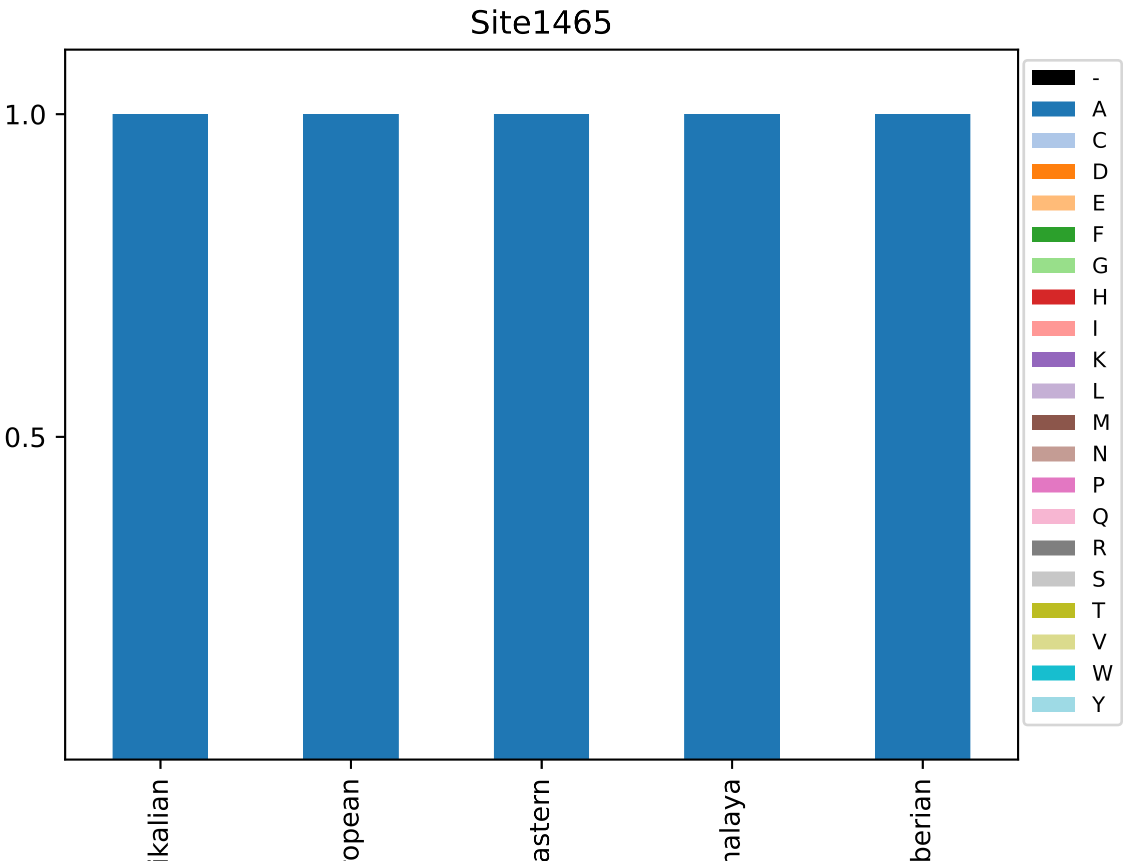
Site1465
1.0
0.5
-
A
C
D
E
F
G
H
I
K
L
M
N
P
Q
R
S
T
V
W
Y
ikalian
opean
astern
nalaya
berian

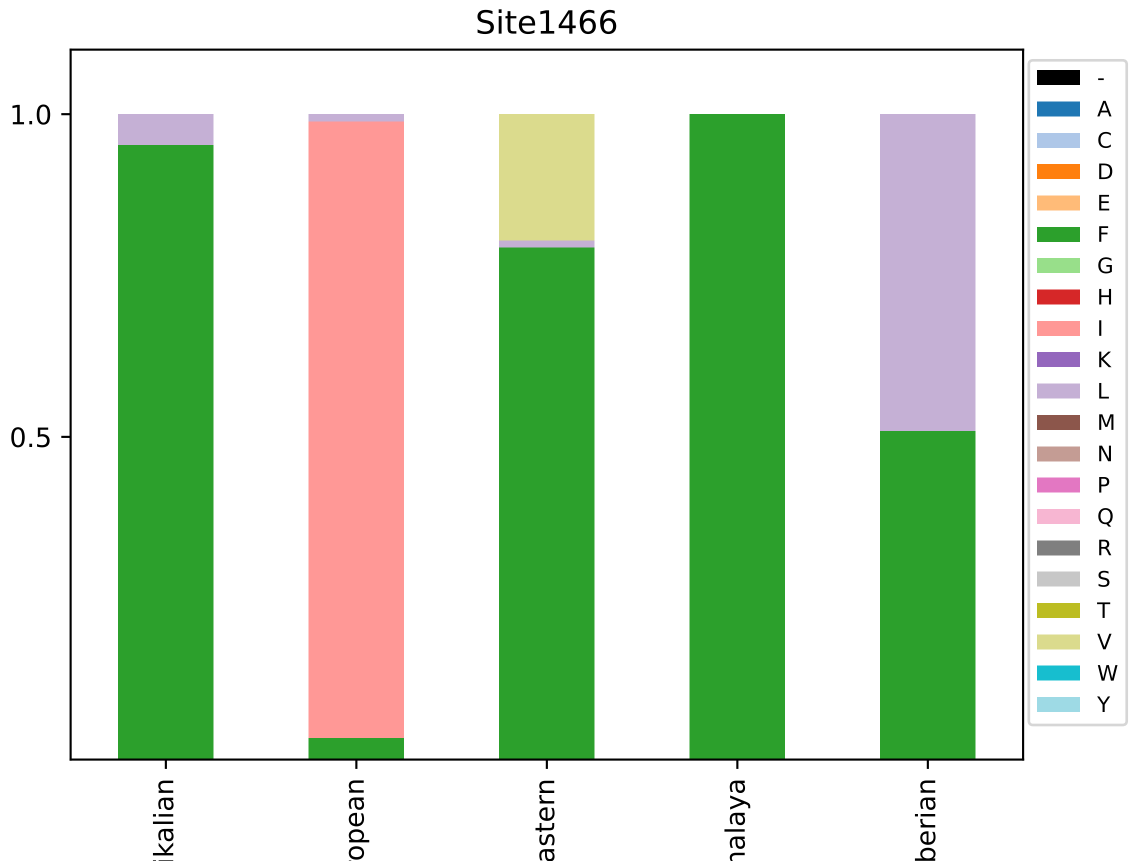
Site1466
1.0
0.5
-
A
C
D
E
F
G
H
I
K
L
M
N
P
Q
R
S
T
V
W
Y
ikalian
opean
astern
nalaya
berian

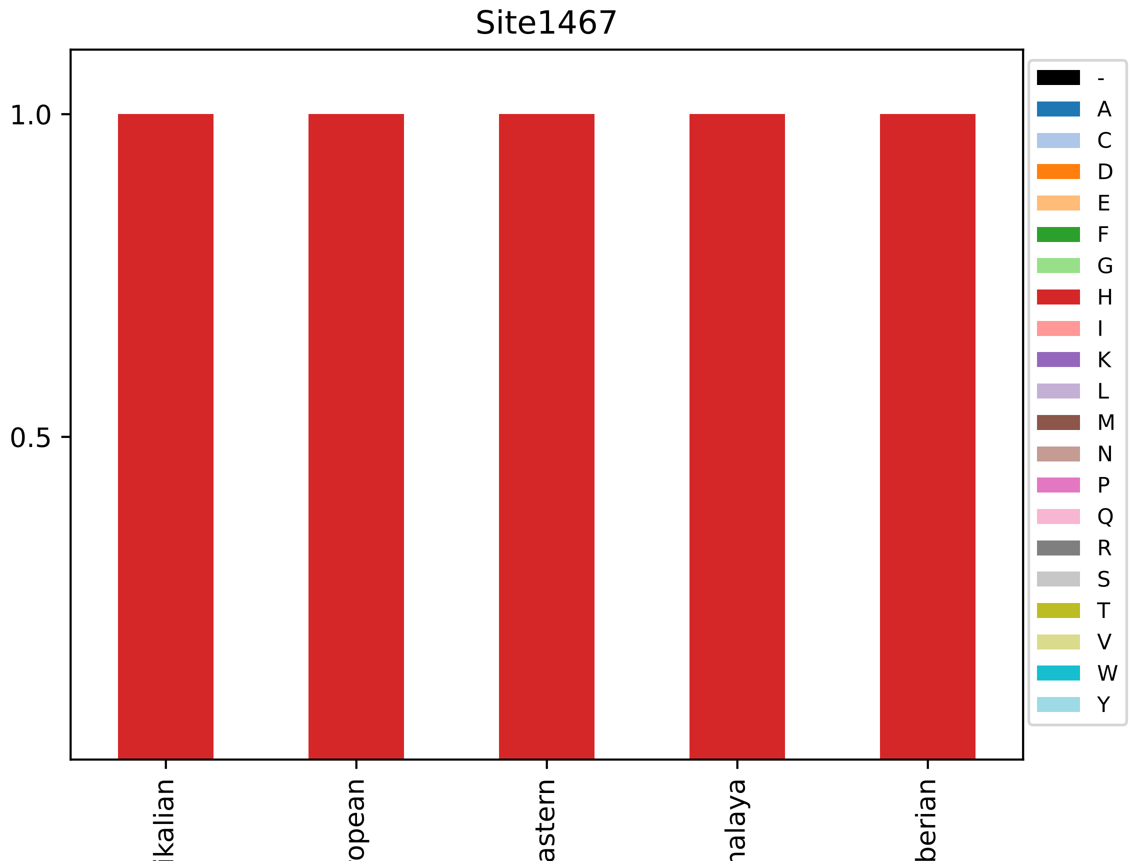
Site1467
1.0
0.5
-
A
C
D
E
F
G
H
I
K
L
M
N
P
Q
R
S
T
V
W
Y

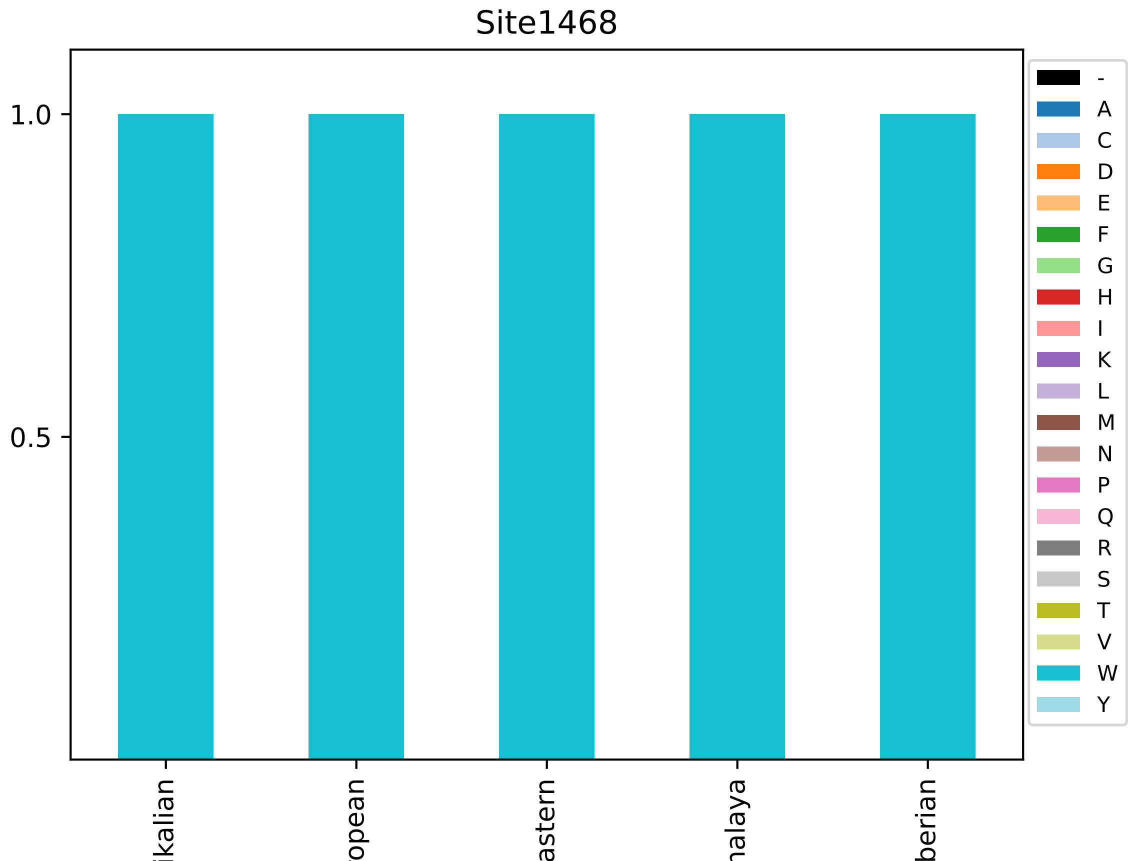
Site1468
1.0
0.5
ikalian
opean
astern
nalaya
berian
-
A
C
D
E
F
G
H
I
K
L
M
N
P
Q
R
S
T
V
W
Y

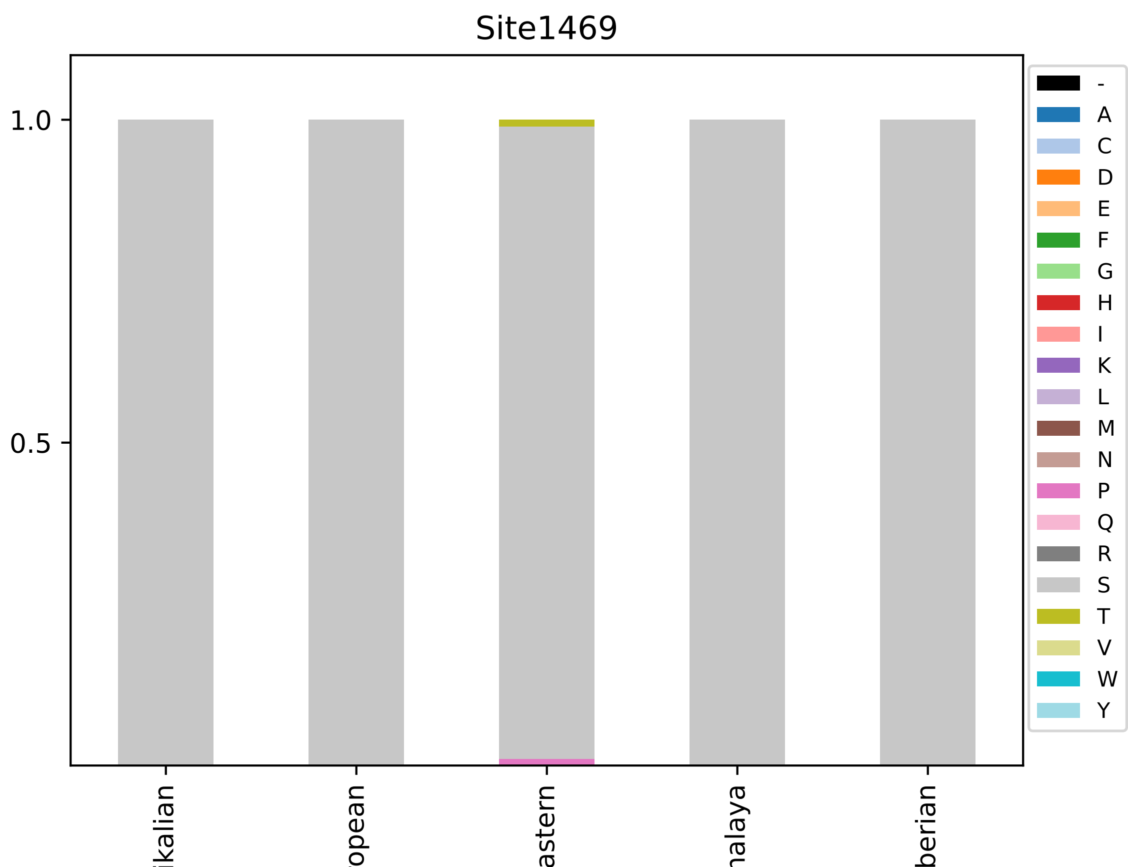

Site1469
1.0
0.5
ikalian
opean
astern
nalaya
berian
-
A
C
D
E
F
G
H
I
K
L
M
N
P
Q
R
S
T
V
W
Y

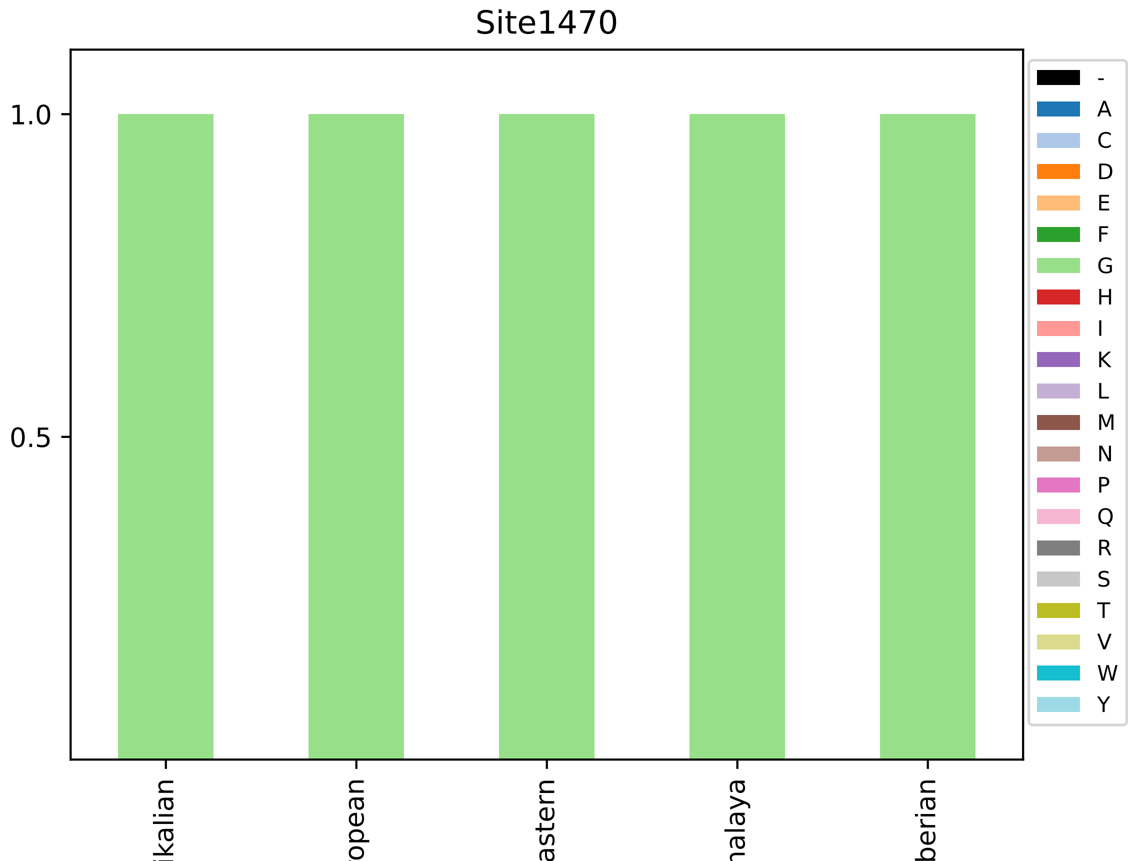

Site1470
1.0
0.5
-
A
C
D
E
F
G
H
I
K
L
M
N
P
Q
R
S
T
V
W
Y
ikalian
opean
astern
nalaya
berian

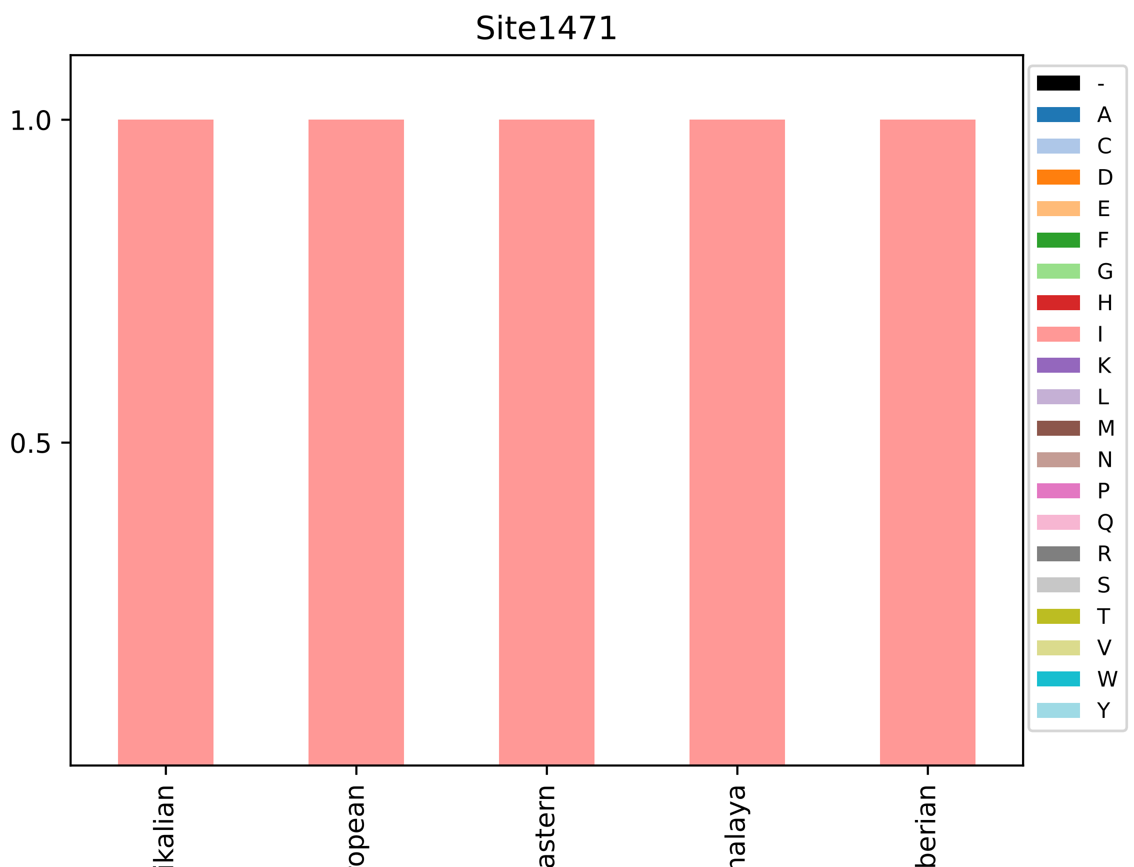
Site1471
1.0
0.5
-
A
C
D
E
F
G
H
I
K
L
M
N
P
Q
R
S
T
V
W
Y
ikalian
ropean
astern
nalaya
berian

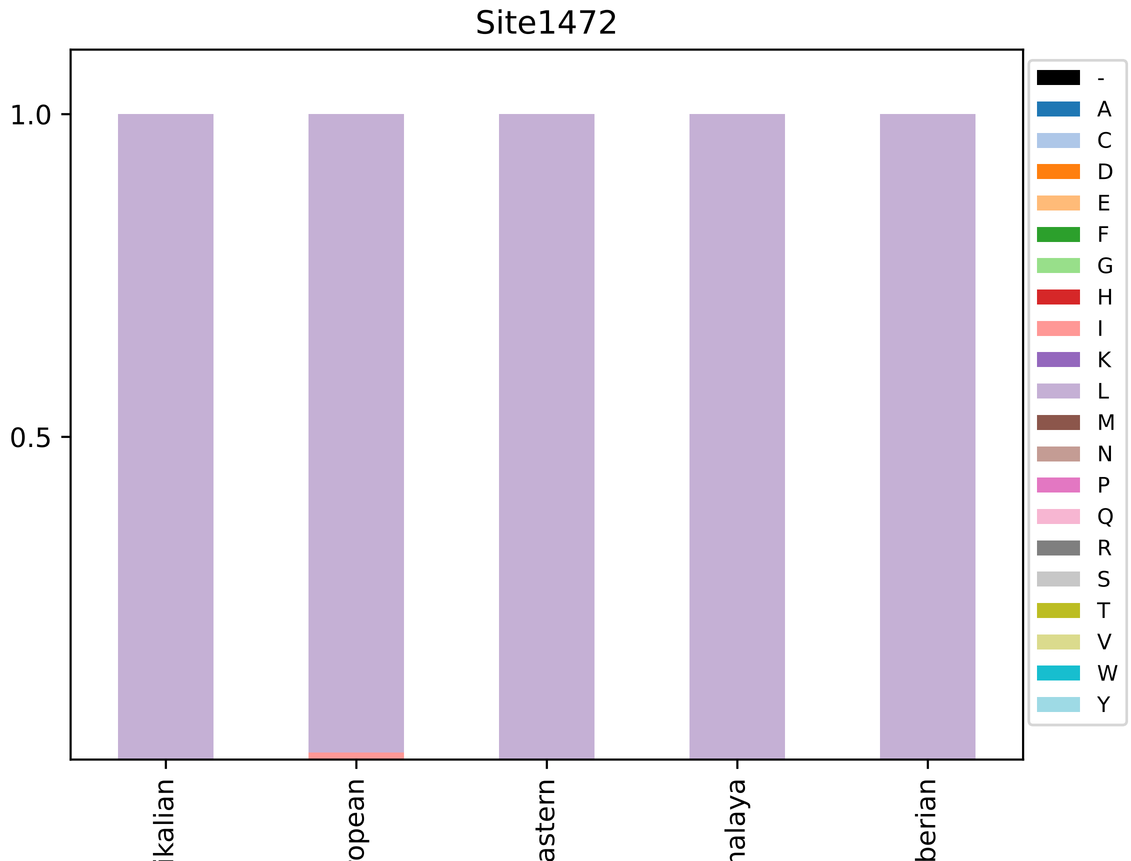

Site1472
1.0
0.5
ikalian
opean
astern
nalaya
berian
-
A
C
D
E
F
G
H
I
K
L
M
N
P
Q
R
S
T
V
W
Y

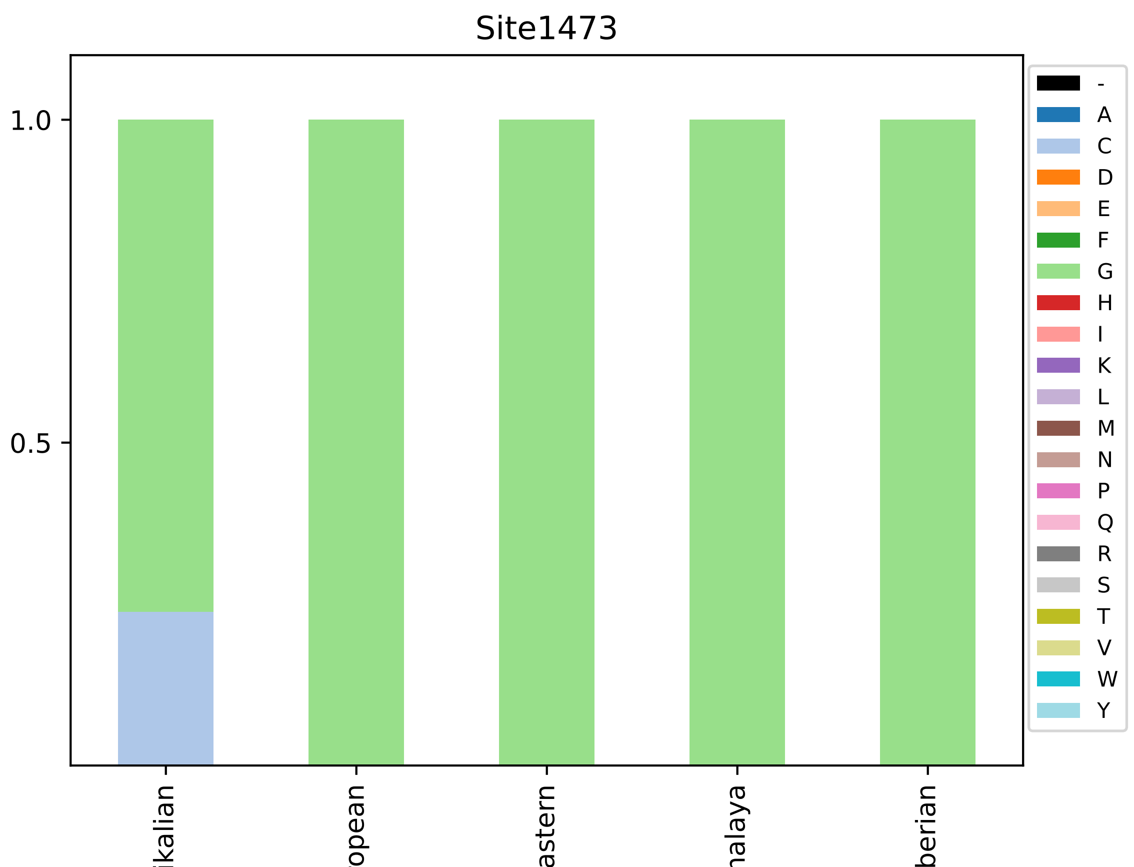
Site1473
1.0
0.5
-
A
C
D
E
F
G
H
I
K
L
M
N
P
Q
R
S
T
V
W
Y
ikalian
opean
astern
nalaya
berian

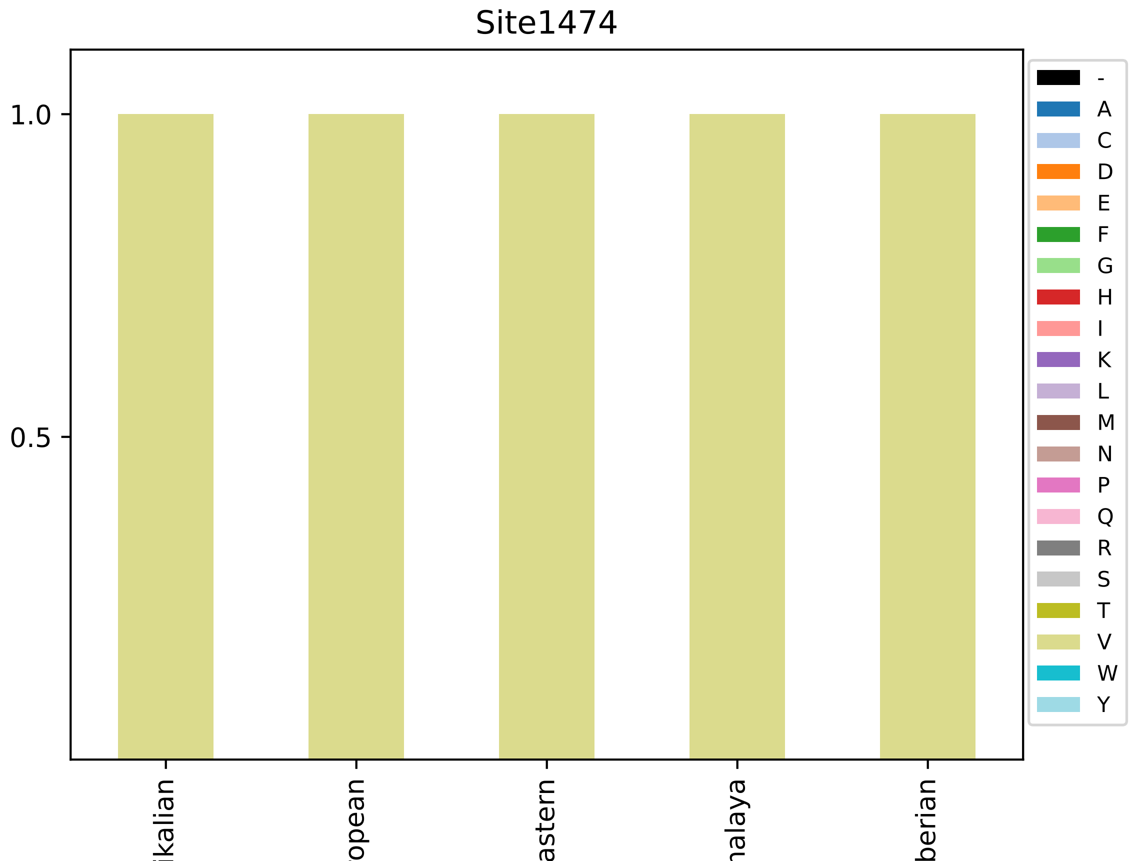
Site1474
1.0
0.5
-
A
C
D
E
F
G
H
I
K
L
M
N
P
Q
R
S
T
V
W
Y
ikalian
opean
astern
nalaya
berian

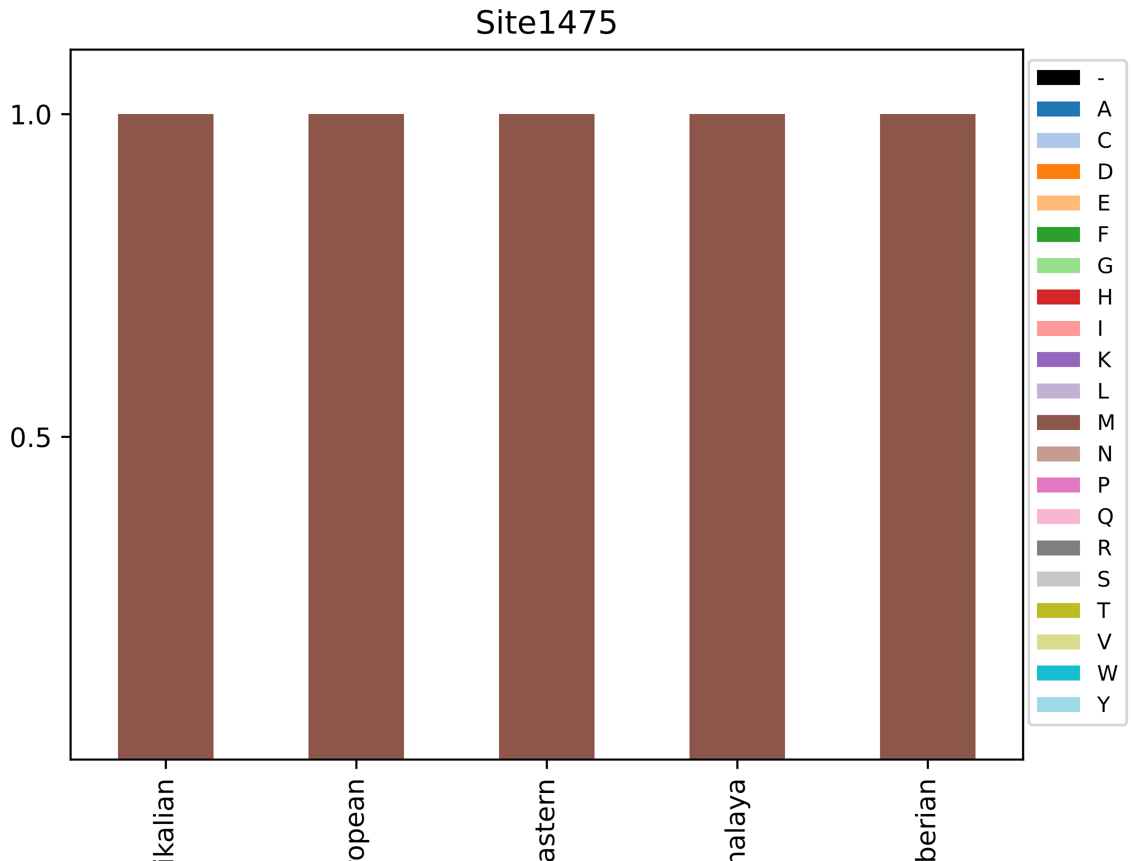

Site1475
1.0
0.5
-
A
C
D
E
F
G
H
I
K
L
M
N
P
Q
R
S
T
V
W
Y
ikalian
opean
astern
nalaya
berian

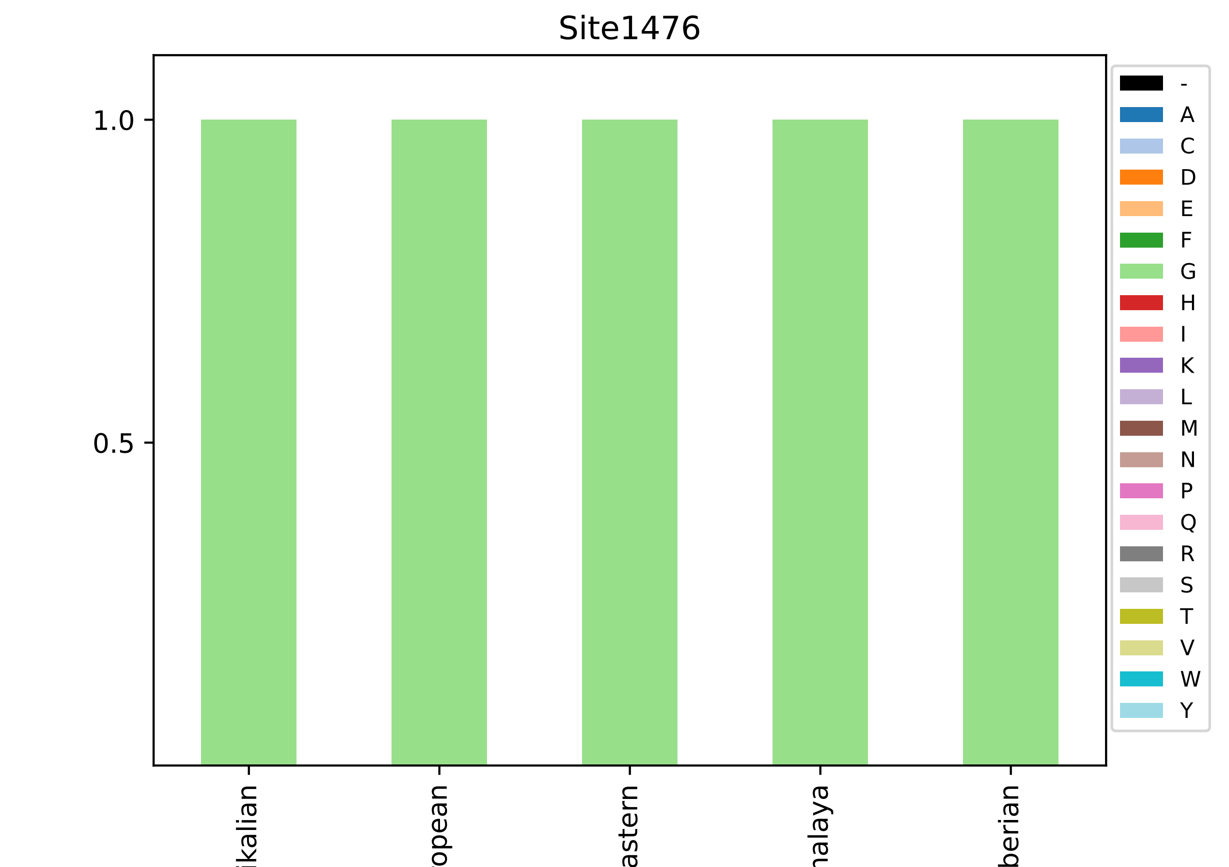
Site1476
1.0
0.5
-
A
C
D
E
F
G
H
I
K
L
M
N
P
Q
R
S
T
V
W
Y
ikalian
opean
astern
nalaya
berian

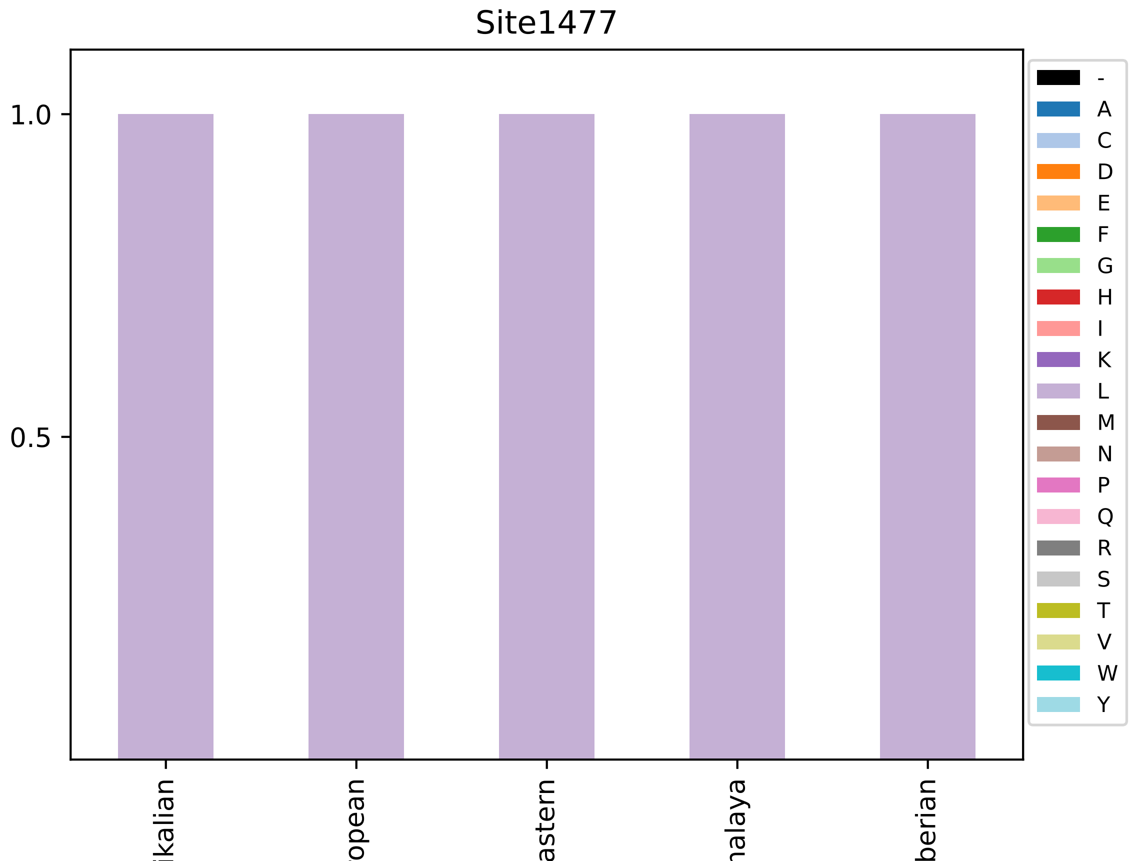
Site1477
1.0
0.5
-
A
C
D
E
F
G
H
I
K
L
M
N
P
Q
R
S
T
V
W
Y

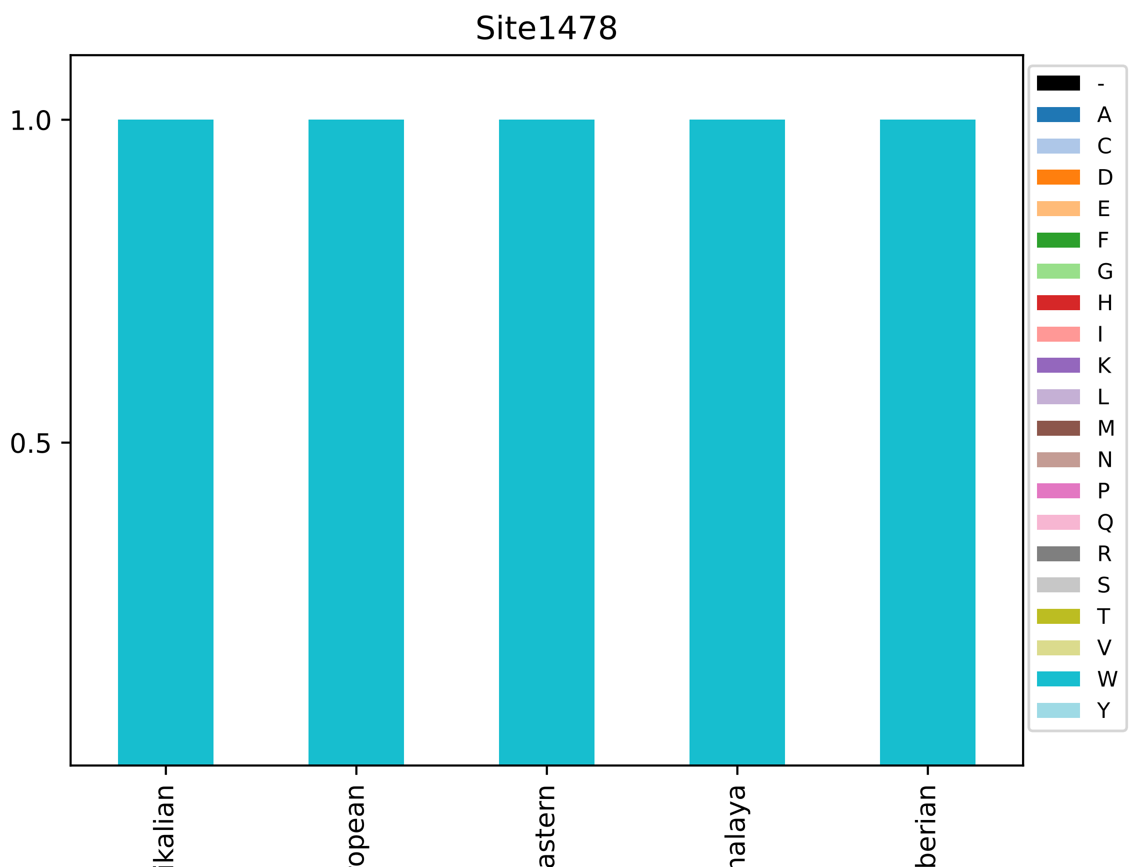

Site1478
1.0
0.5
-
A
C
D
E
F
G
H
I
K
L
M
N
P
Q
R
S
T
V
W
Y
ikalian
opean
astern
nalaya
berian

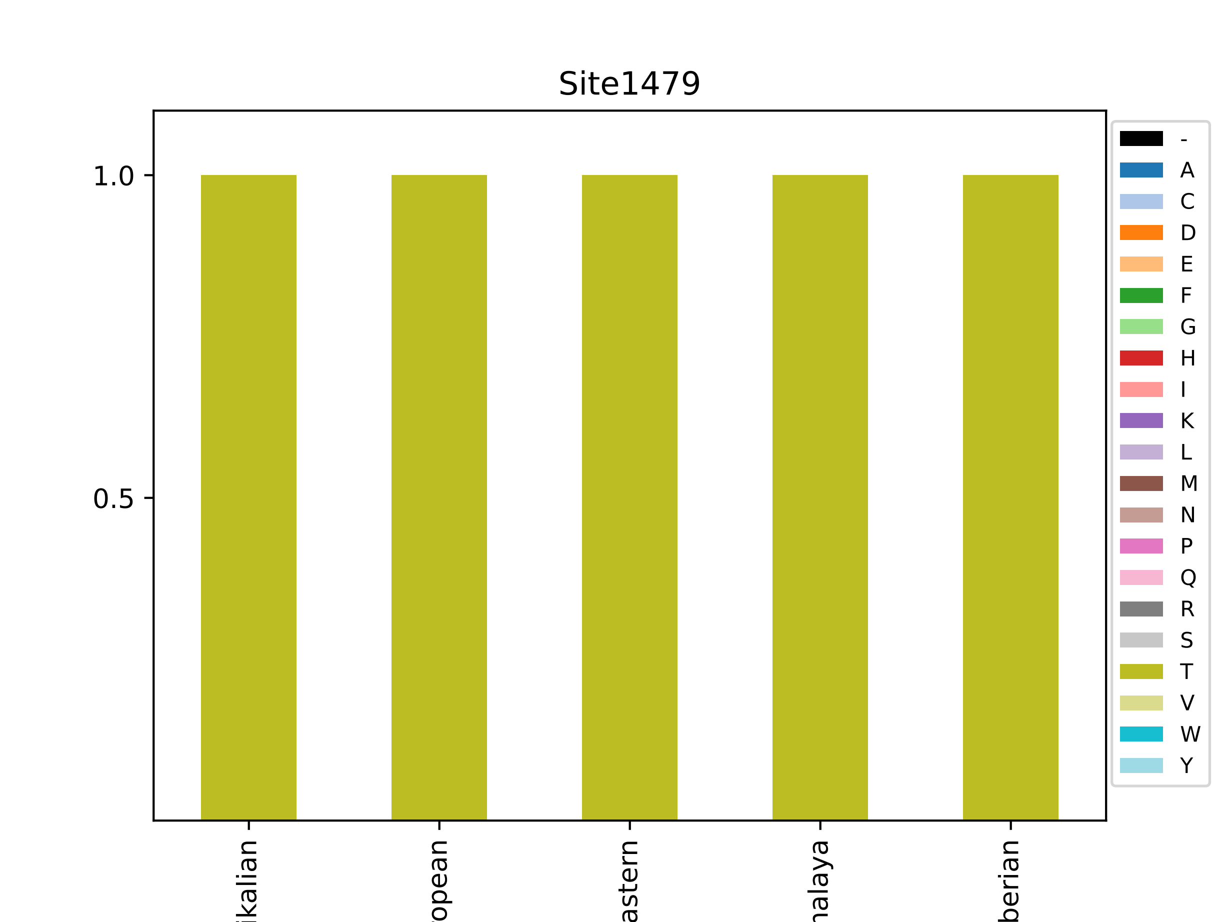

Site1479
1.0
0.5
-
A
C
D
E
F
G
H
I
K
L
M
N
P
Q
R
S
T
V
W
Y
ikalian
opean
astern
nalaya
berian

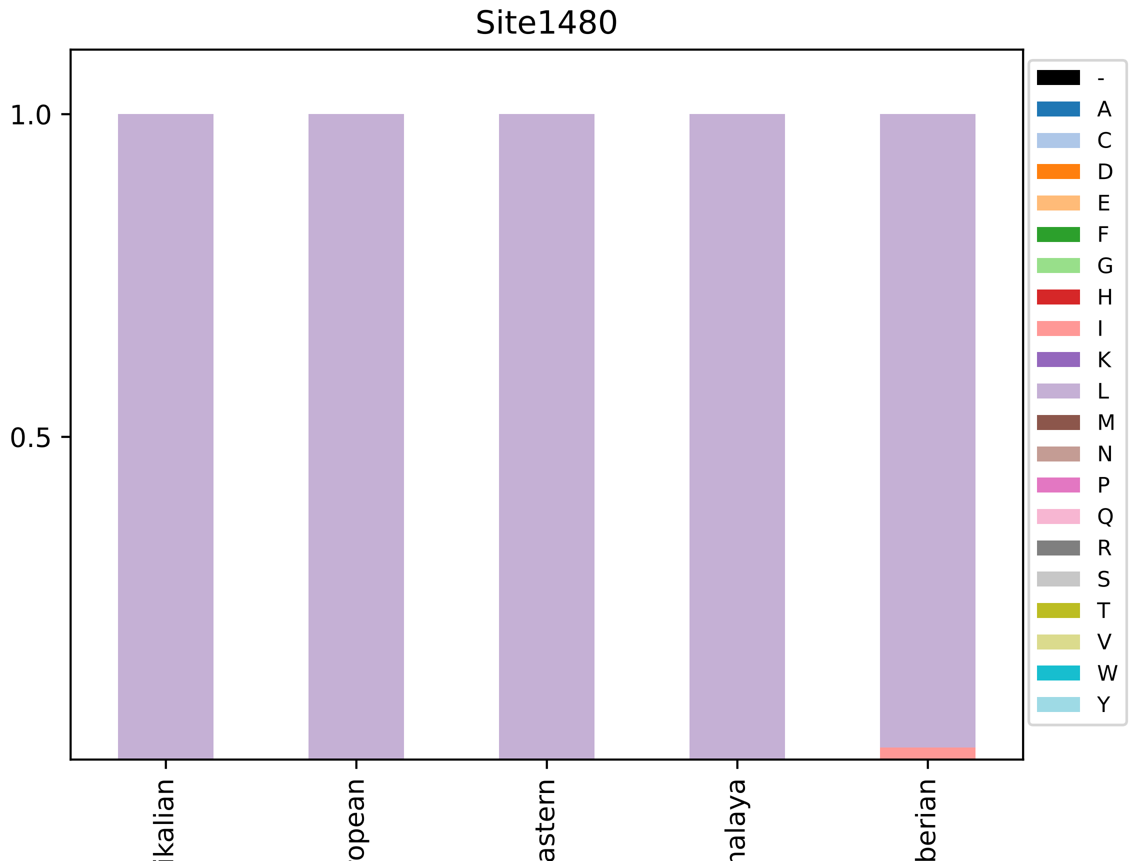

Site1480
1.0
0.5
-
A
C
D
E
F
G
H
I
K
L
M
N
P
Q
R
S
T
V
W
Y
ikalian
opean
astern
nalaya
berian

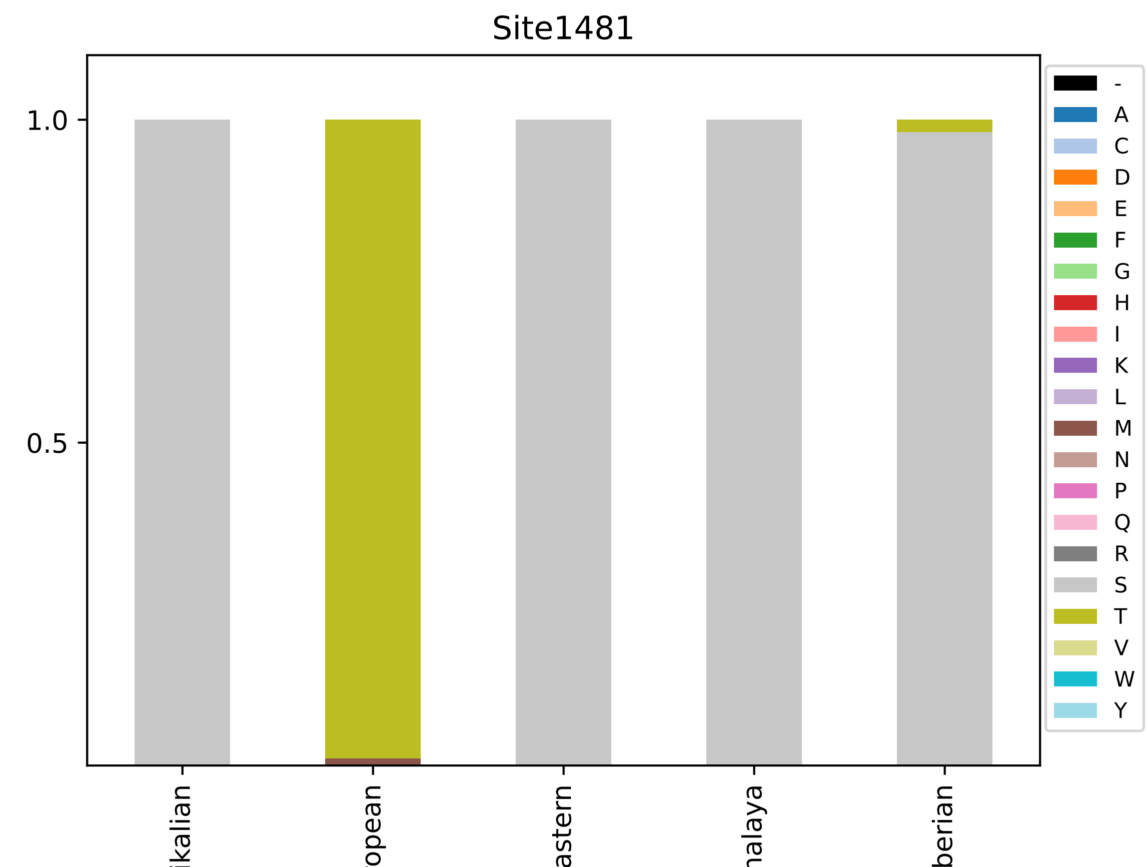

Site1481
1.0
0.5
-
A
C
D
E
F
G
H
I
K
L
M
N
P
Q
R
S
T
V
W
Y

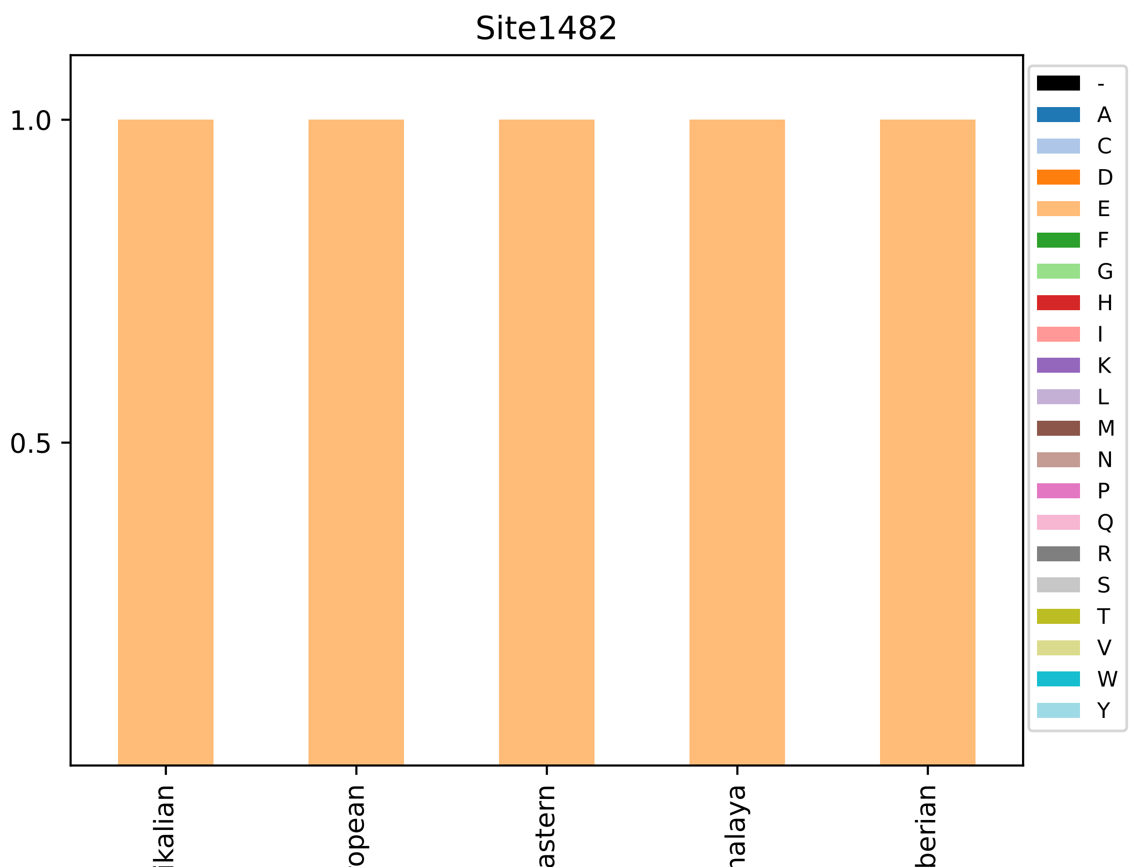
Site1482
1.0
0.5
-
A
C
D
E
F
G
H
I
K
L
M
N
P
Q
R
S
T
V
W
Y
ikalian
ropean
astern
nalaya
berian

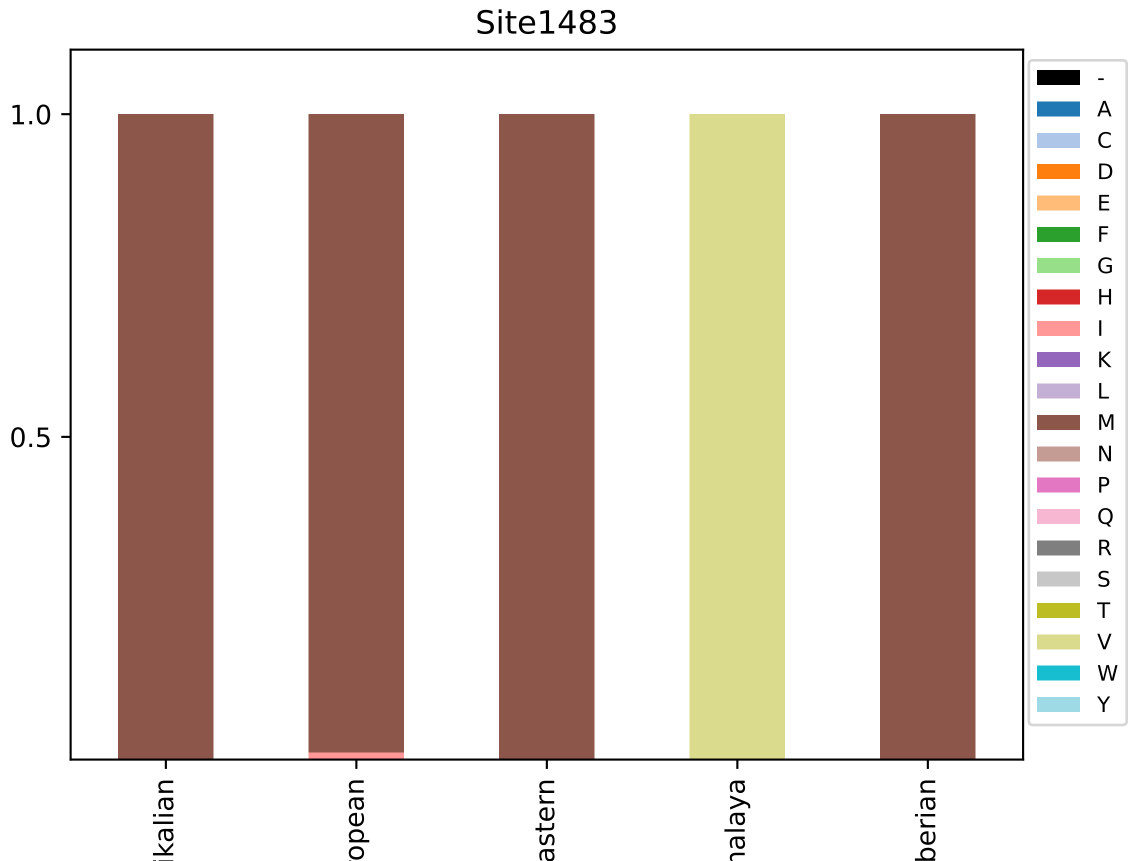
Site1483
1.0
0.5
-
A
C
D
E
F
G
H
I
K
L
M
N
P
Q
R
S
T
V
W
Y
ikalian
opean
astern
nalaya
berian

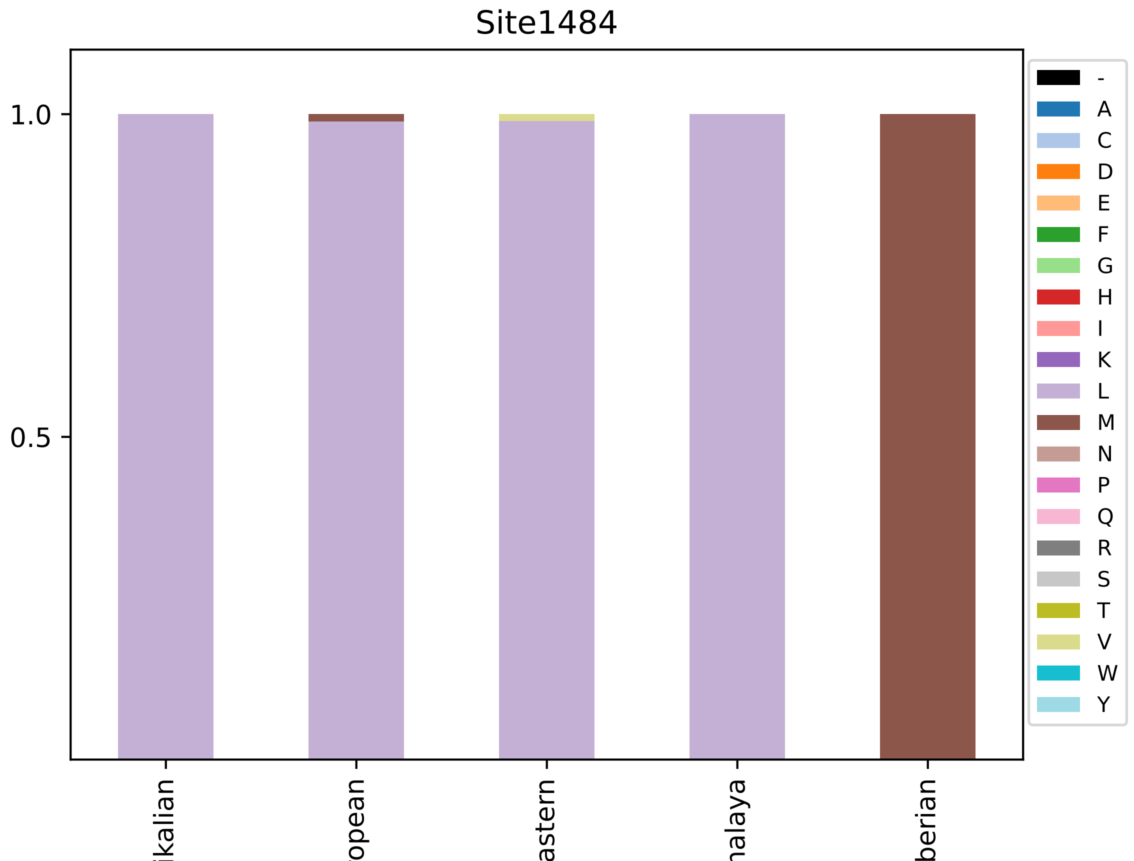
Site1484
1.0
0.5
-
A
C
D
E
F
G
H
I
K
L
M
N
P
Q
R
S
T
V
W
Y
ikalian
opean
astern
nalaya
berian

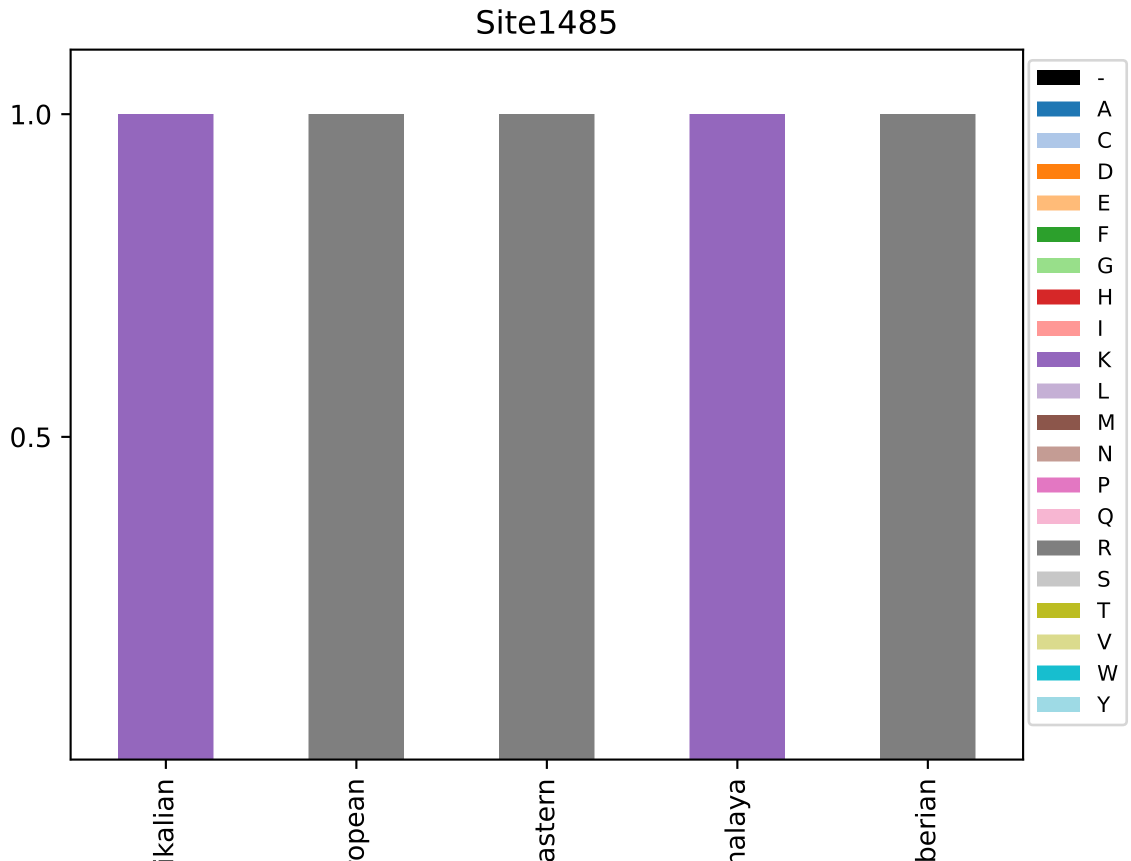
Site1485
1.0
0.5
-
A
C
D
E
F
G
H
I
K
L
M
N
P
Q
R
S
T
V
W
Y
ikalian
opean
astern
nalaya
berian

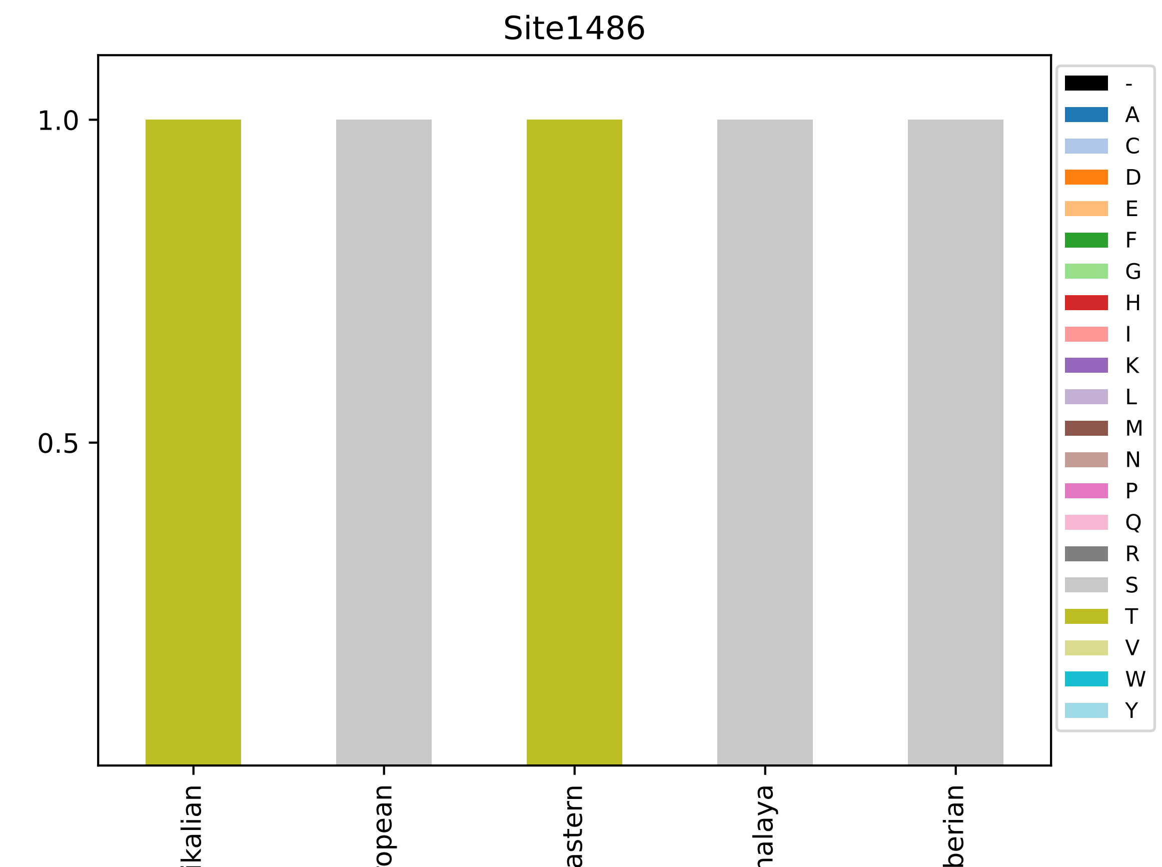
Site1486
1.0
0.5
ikalian
opean
astern
nalaya
berian
-
A
C
D
E
F
G
H
I
K
L
M
N
P
Q
R
S
T
V
W
Y

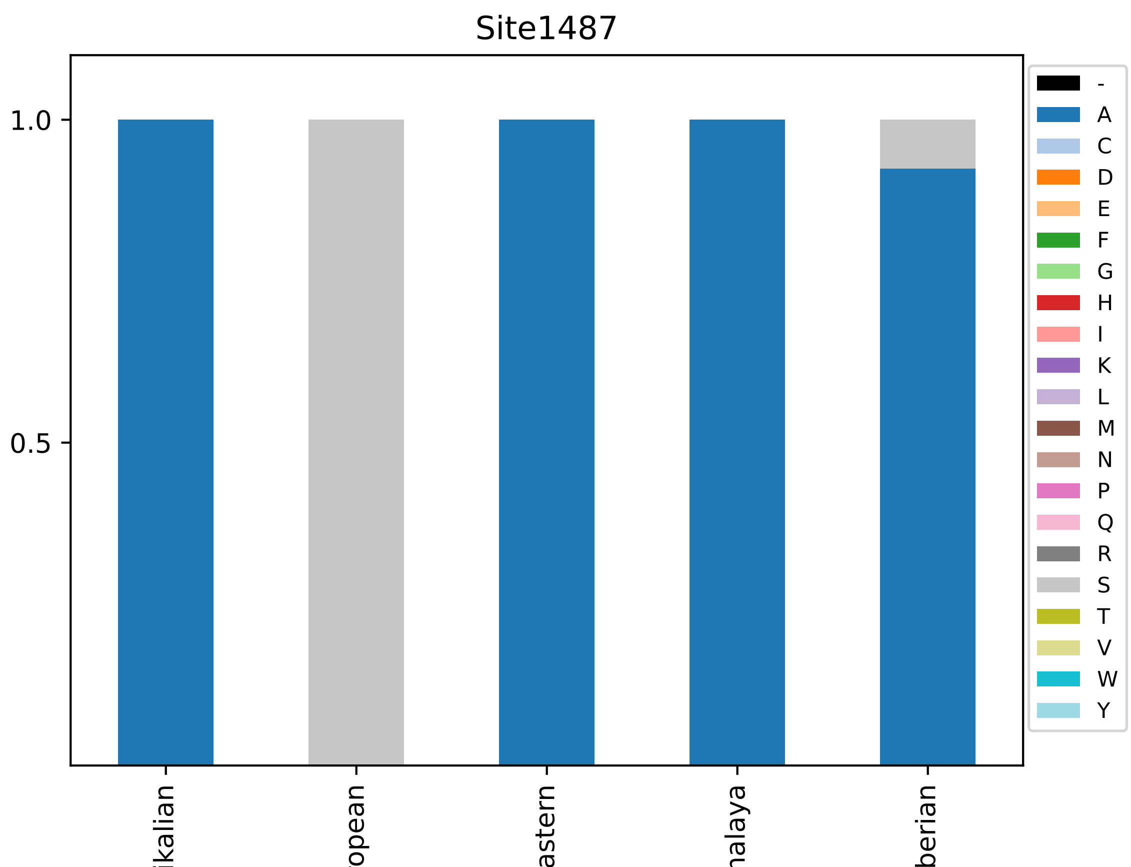

Site1487
1.0
0.5
-
A
C
D
E
F
G
H
I
K
L
M
N
P
Q
R
S
T
V
W
Y
ikalian
opean
astern
nalaya
berian

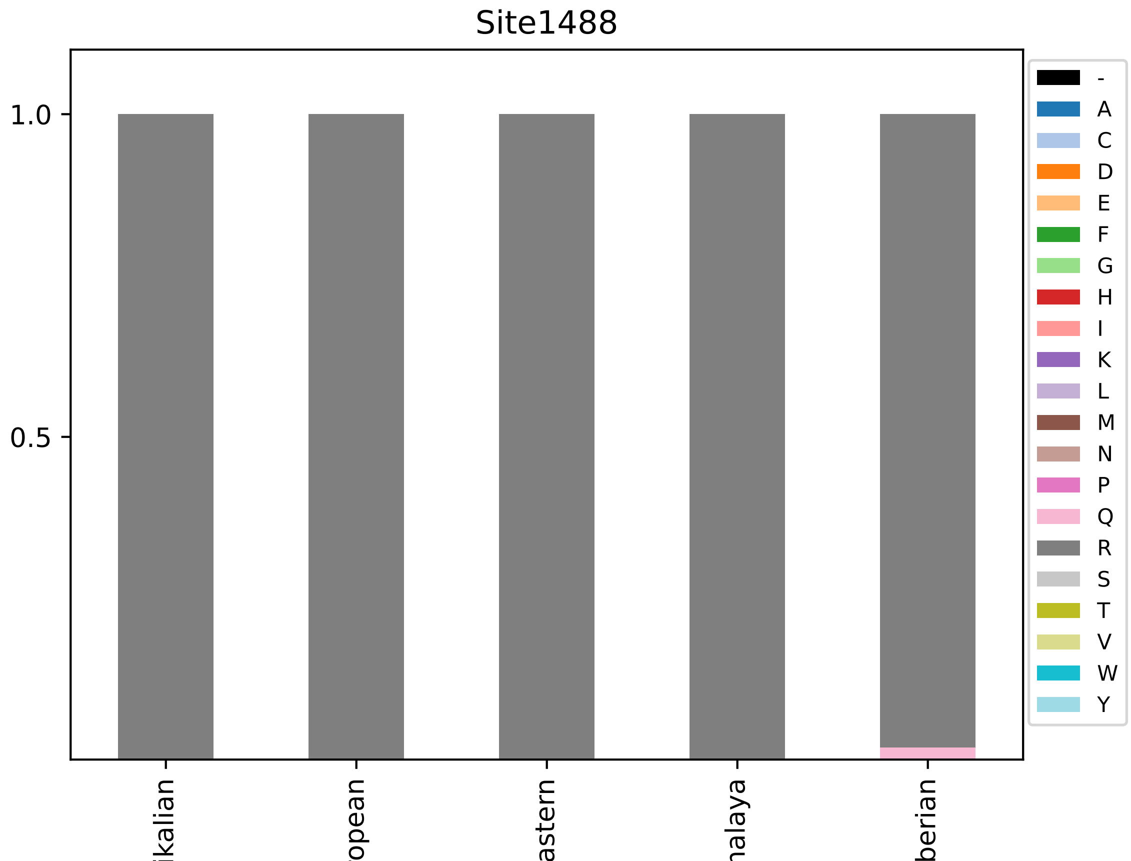

Site1488
1.0
0.5
-
A
C
D
E
F
G
H
I
K
L
M
N
P
Q
R
S
T
V
W
Y
ikalian
opean
astern
nalaya
berian

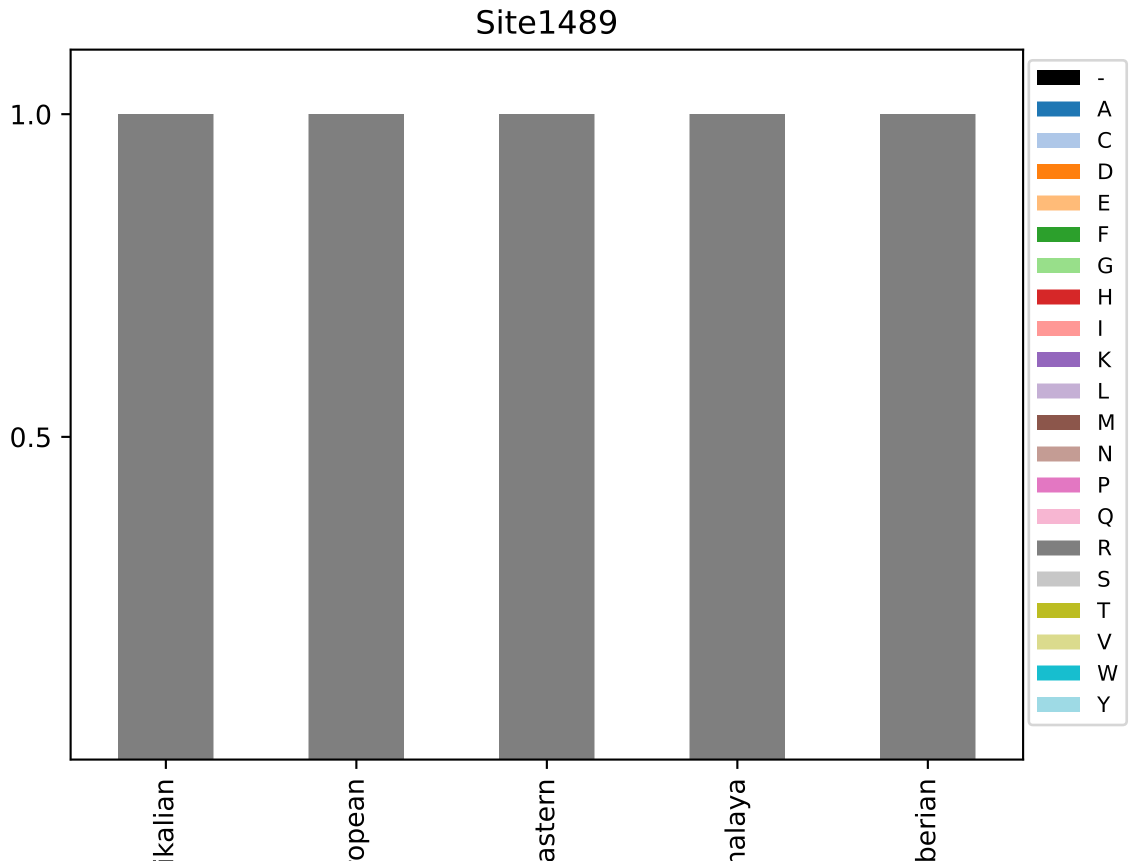
Site1489
1.0
0.5
-
A
C
D
E
F
G
H
I
K
L
M
N
P
Q
R
S
T
V
W
Y

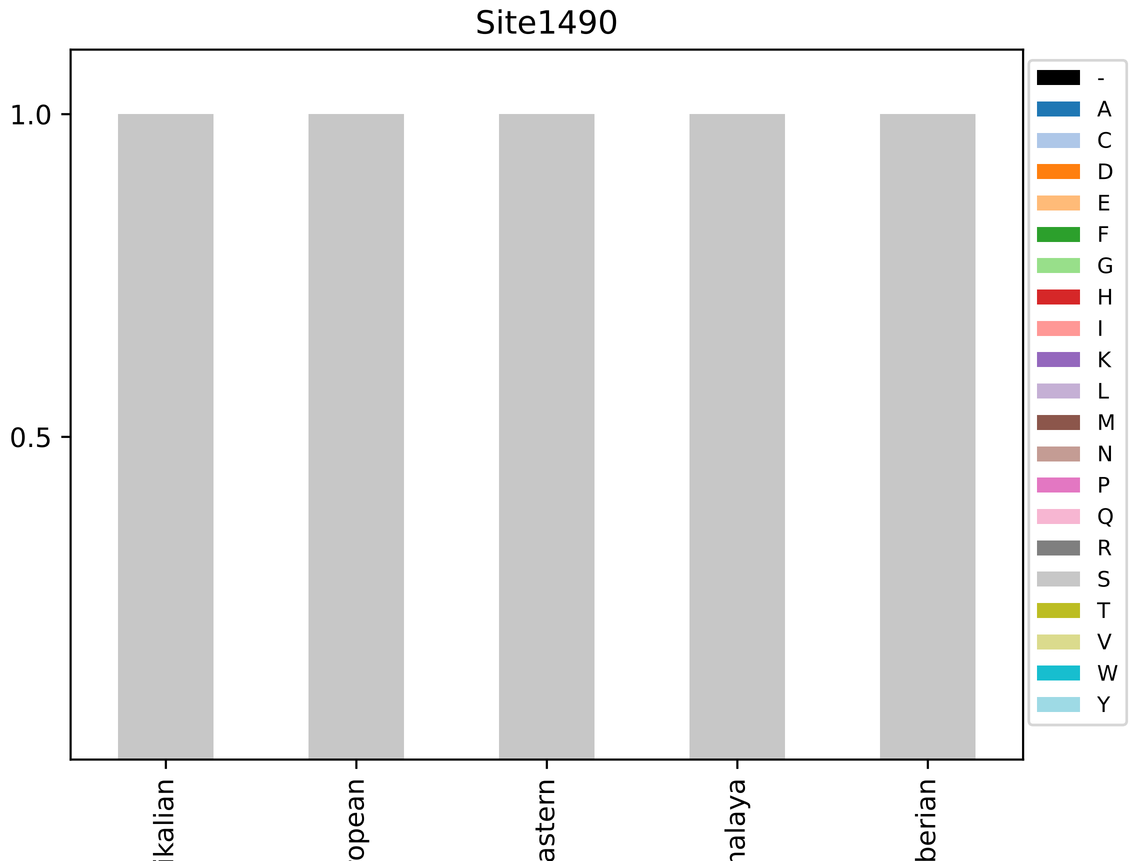
Site1490
1.0
0.5
-
A
C
D
E
F
G
H
I
K
L
M
N
P
Q
R
S
T
V
W
Y
ikalian
opean
astern
nalaya
berian

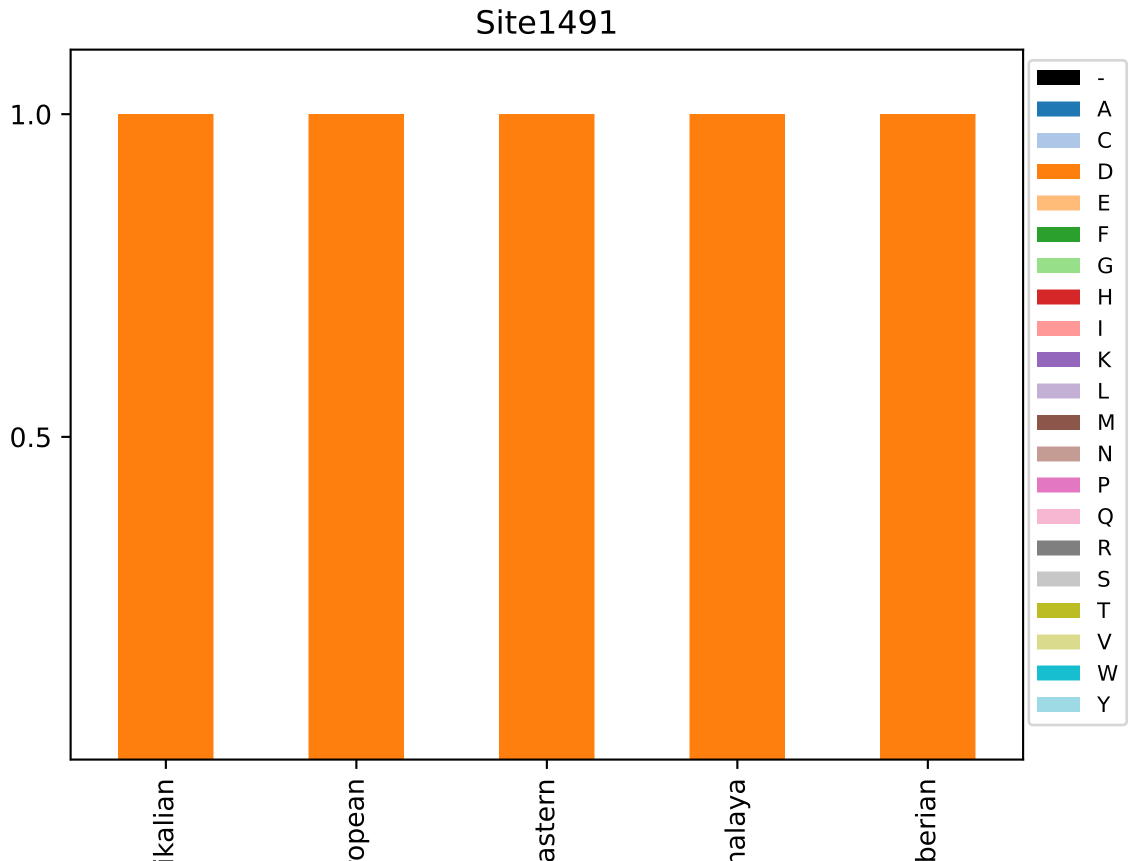
Site1491
1.0
0.5
-
A
C
D
E
F
G
H
I
K
L
M
N
P
Q
R
S
T
V
W
Y

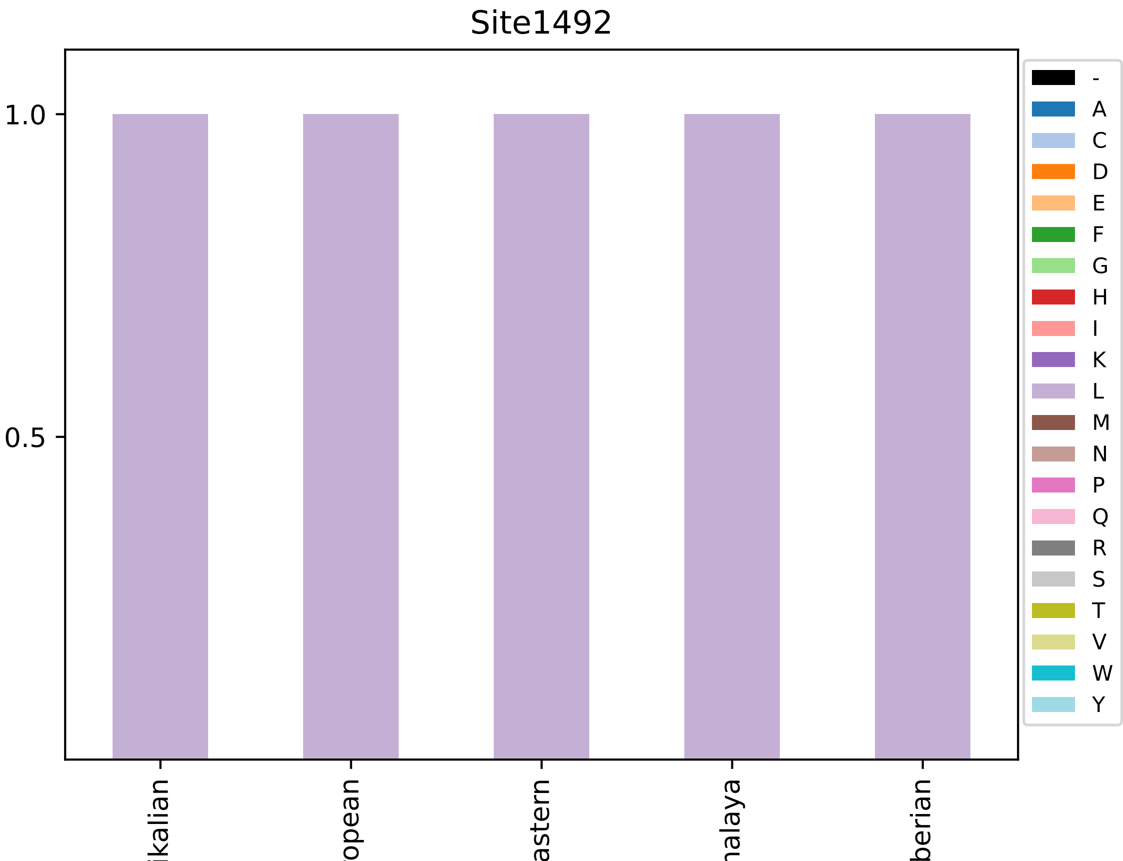
Site1492
1.0
0.5
ikalian
opean
astern
nalaya
berian
-
A
C
D
E
F
G
H
I
K
L
M
N
P
Q
R
S
T
V
W
Y

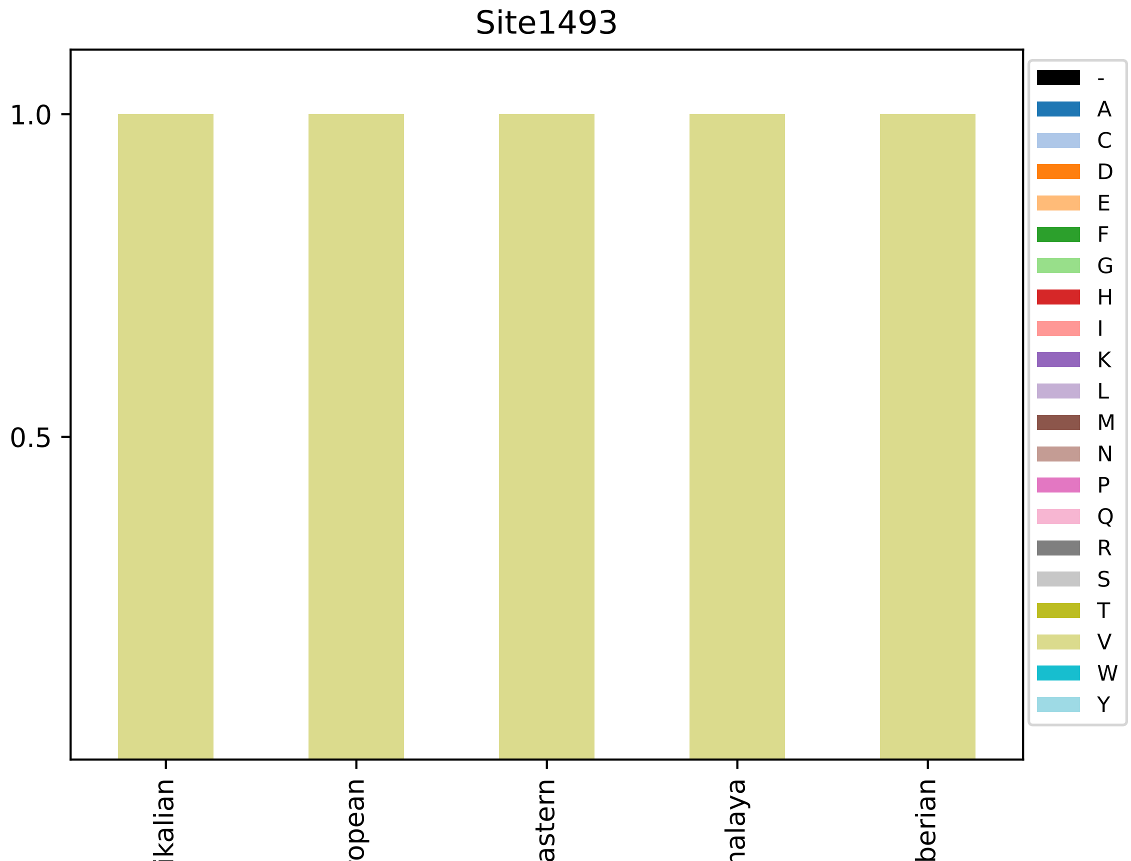

Site1493
1.0
0.5
-
A
C
D
E
F
G
H
I
K
L
M
N
P
Q
R
S
T
V
W
Y
ikalian
opean
astern
alaya
berian

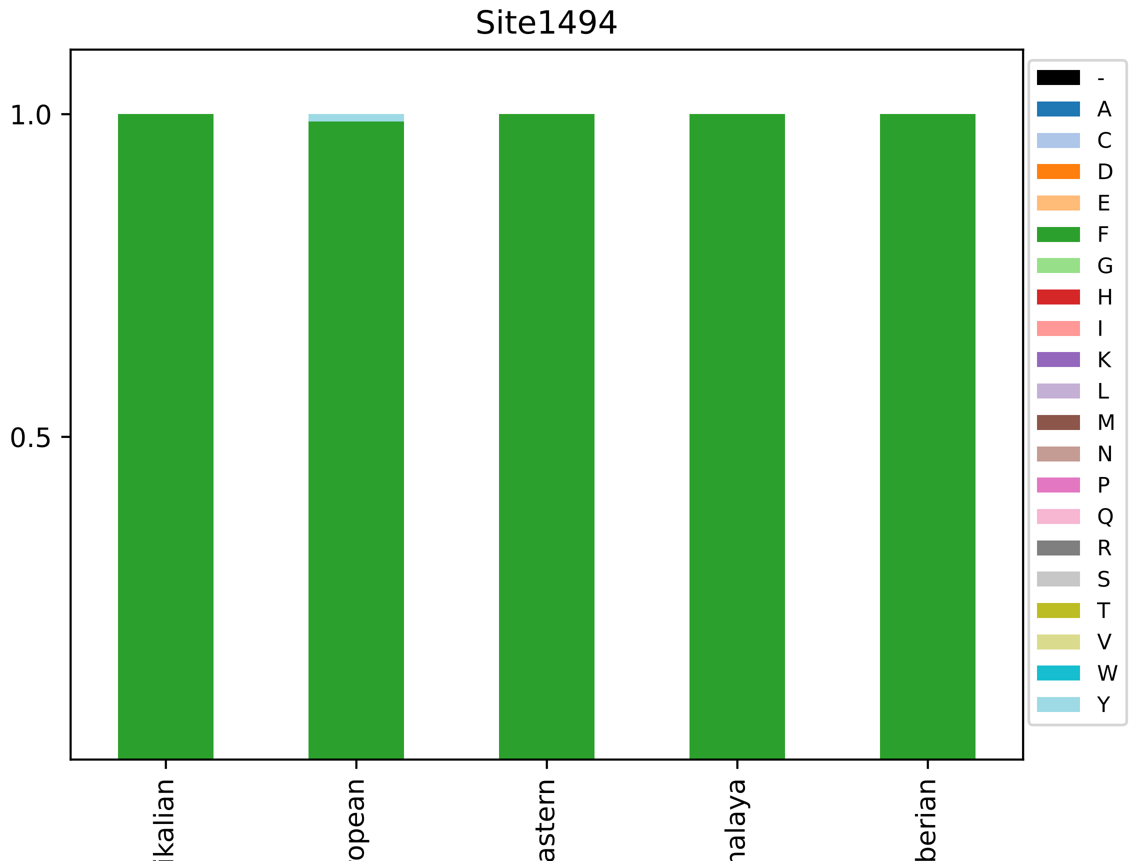

Site1494
1.0
0.5
ikalian
opean
astern
nalaya
berian
-
A
C
D
E
F
G
H
I
K
L
M
N
P
Q
R
S
T
V
W
Y

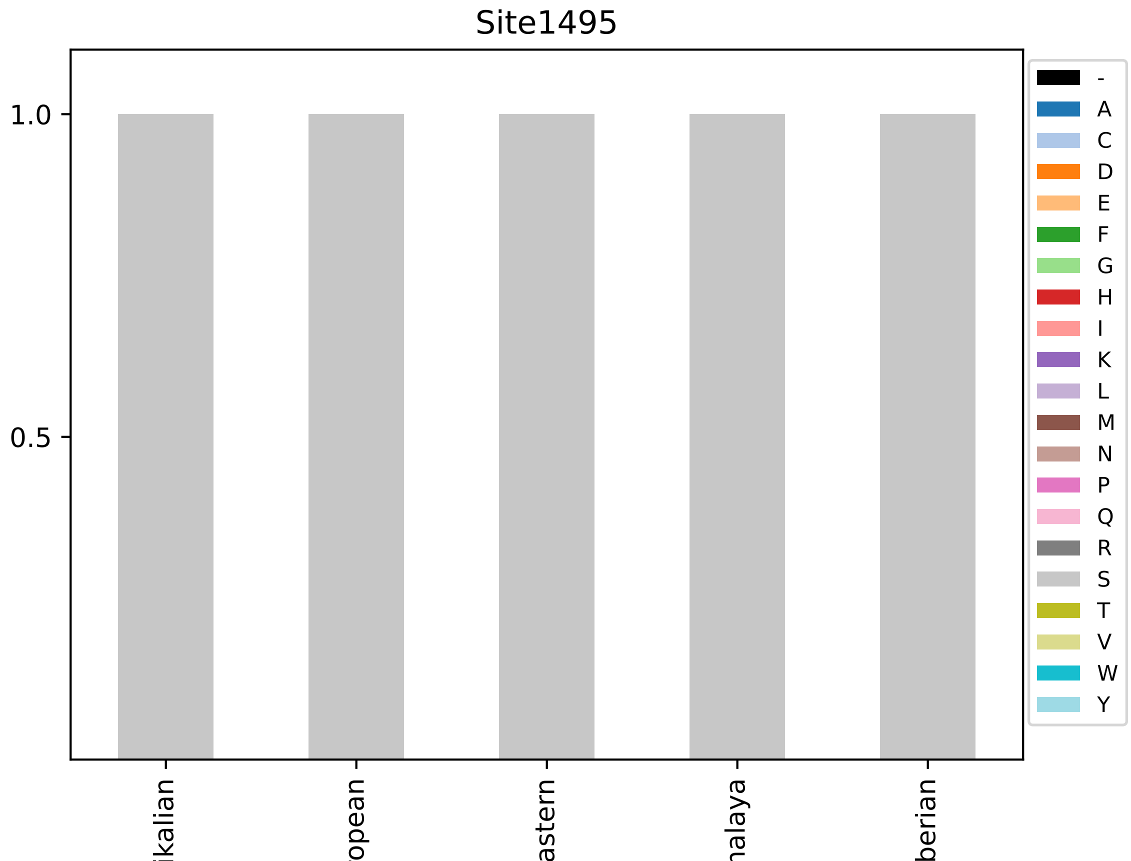
Site1495
1.0
0.5
-
A
C
D
E
F
G
H
I
K
L
M
N
P
Q
R
S
T
V
W
Y
ikalian
opean
astern
nalaya
berian

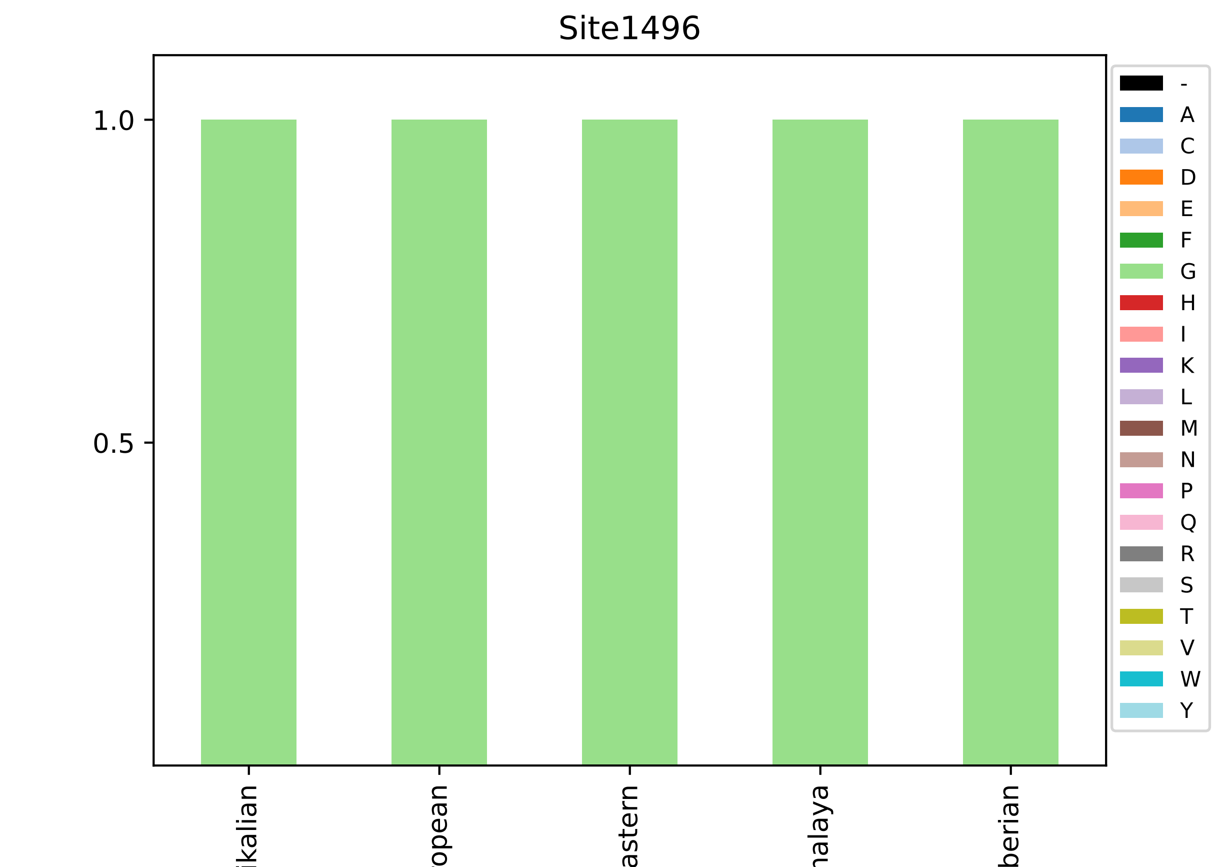
Site1496
1.0
0.5
-
A
C
D
E
F
G
H
I
K
L
M
N
P
Q
R
S
T
V
W
Y
ikalian
opean
astern
nalaya
berian

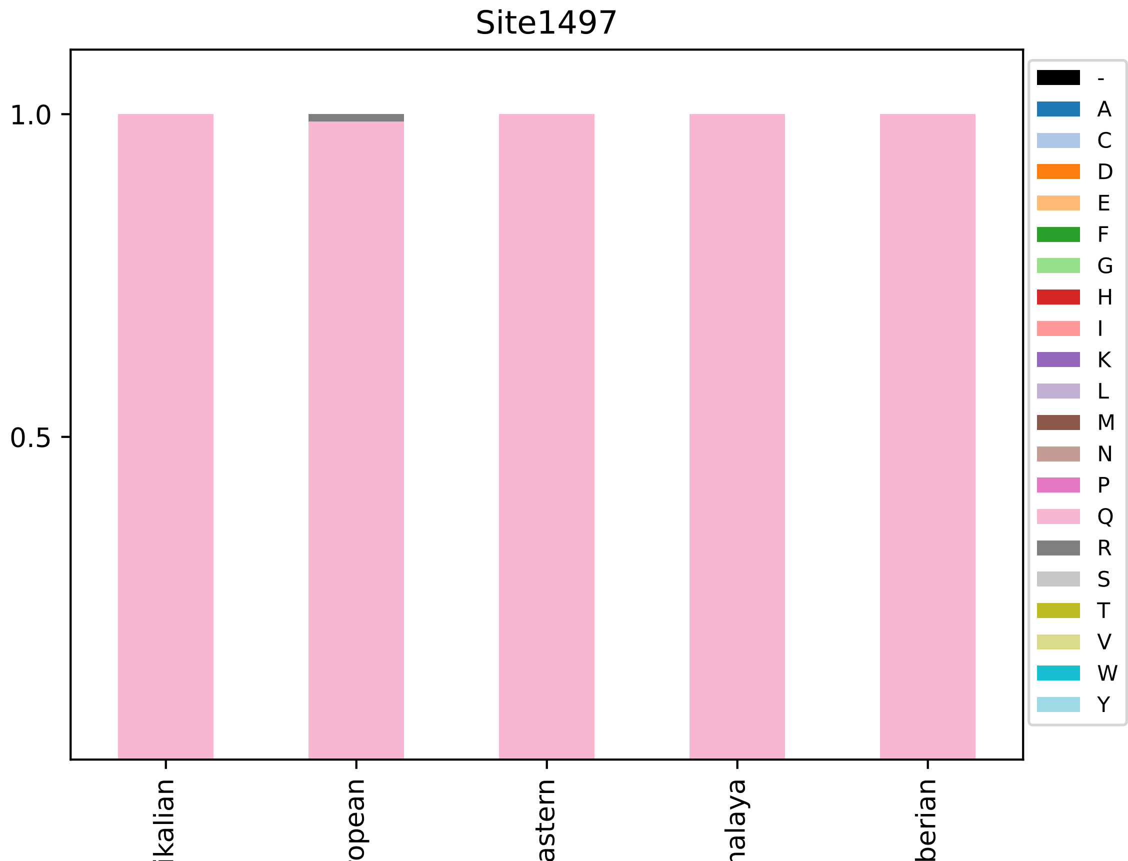
Site1497
1.0
0.5
-
A
C
D
E
F
G
H
I
K
L
M
N
P
Q
R
S
T
V
W
Y
ikalian
opean
astern
nalaya
berian

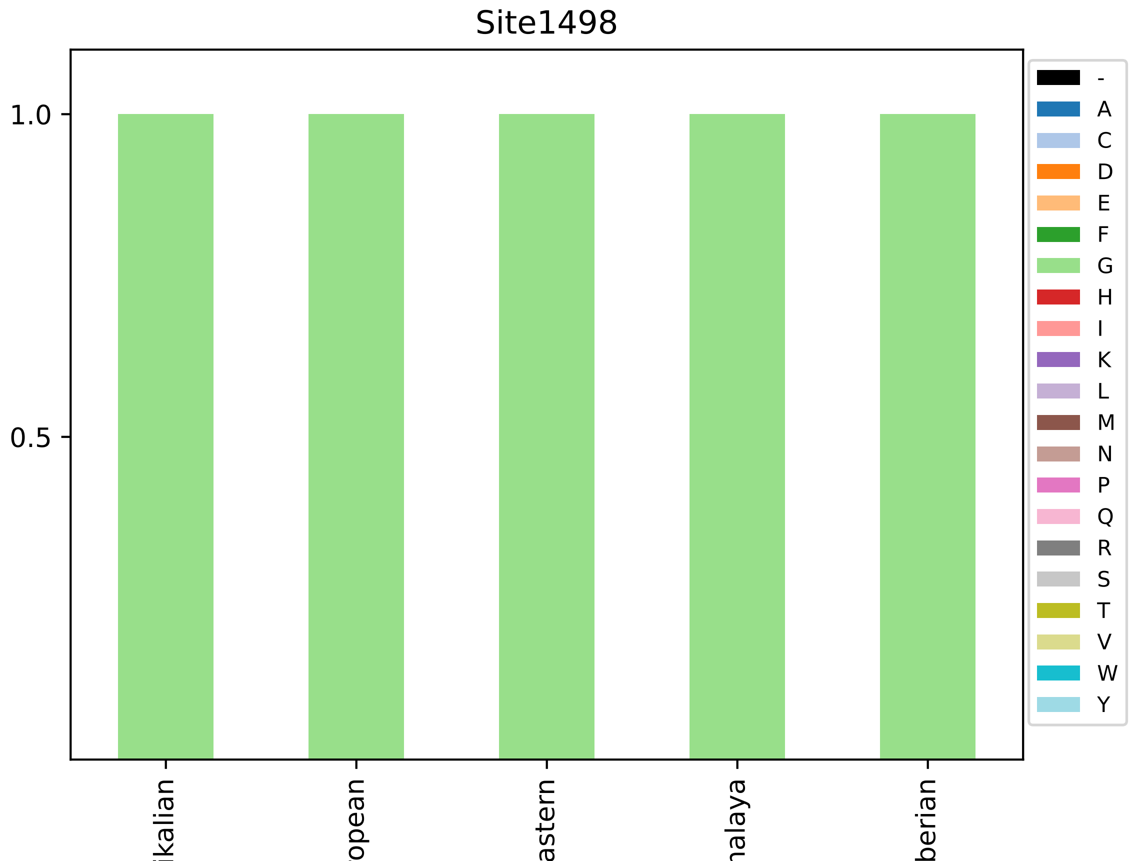
Site1498
1.0
0.5
-
A
C
D
E
F
G
H
I
K
L
M
N
P
Q
R
S
T
V
W
Y
ikalian
opean
astern
nalaya
berian

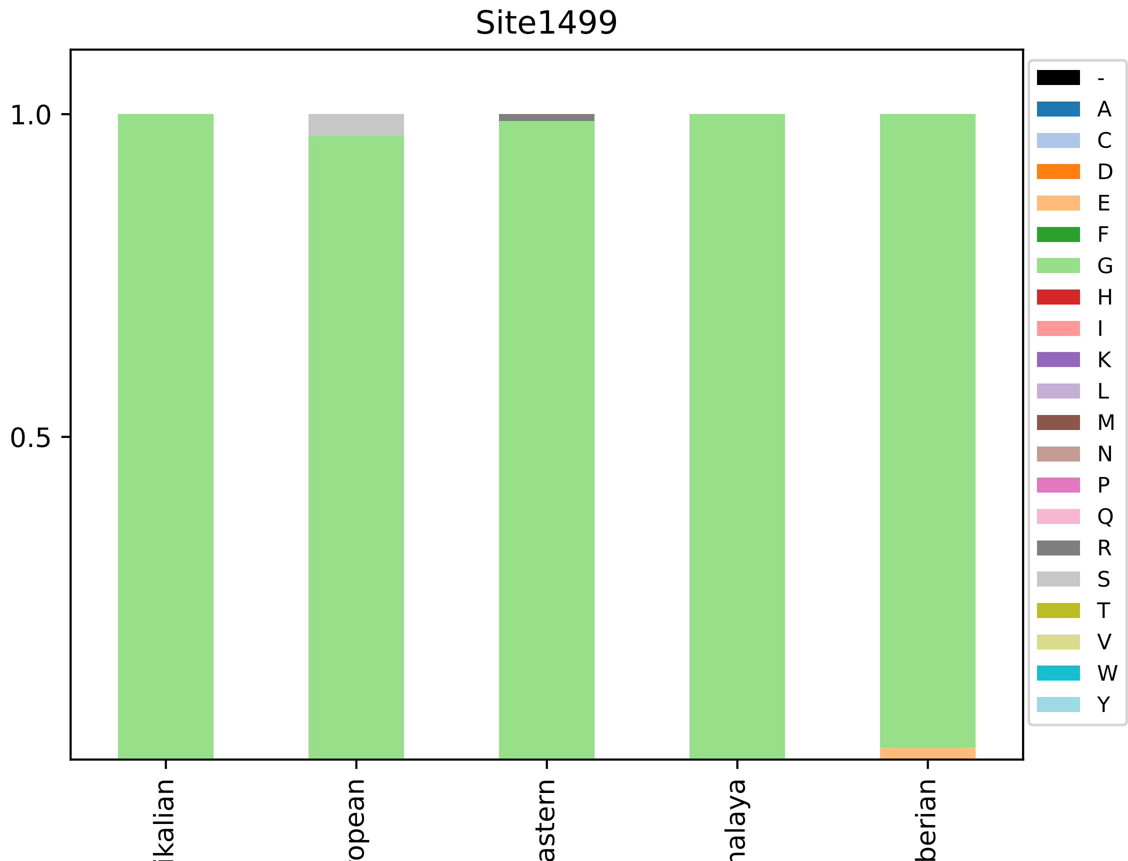

Site1499
1.0
0.5
-
A
C
D
E
F
G
H
I
K
L
M
N
P
Q
R
S
T
V
W
Y
ikalian
opean
astern
nalaya
berian

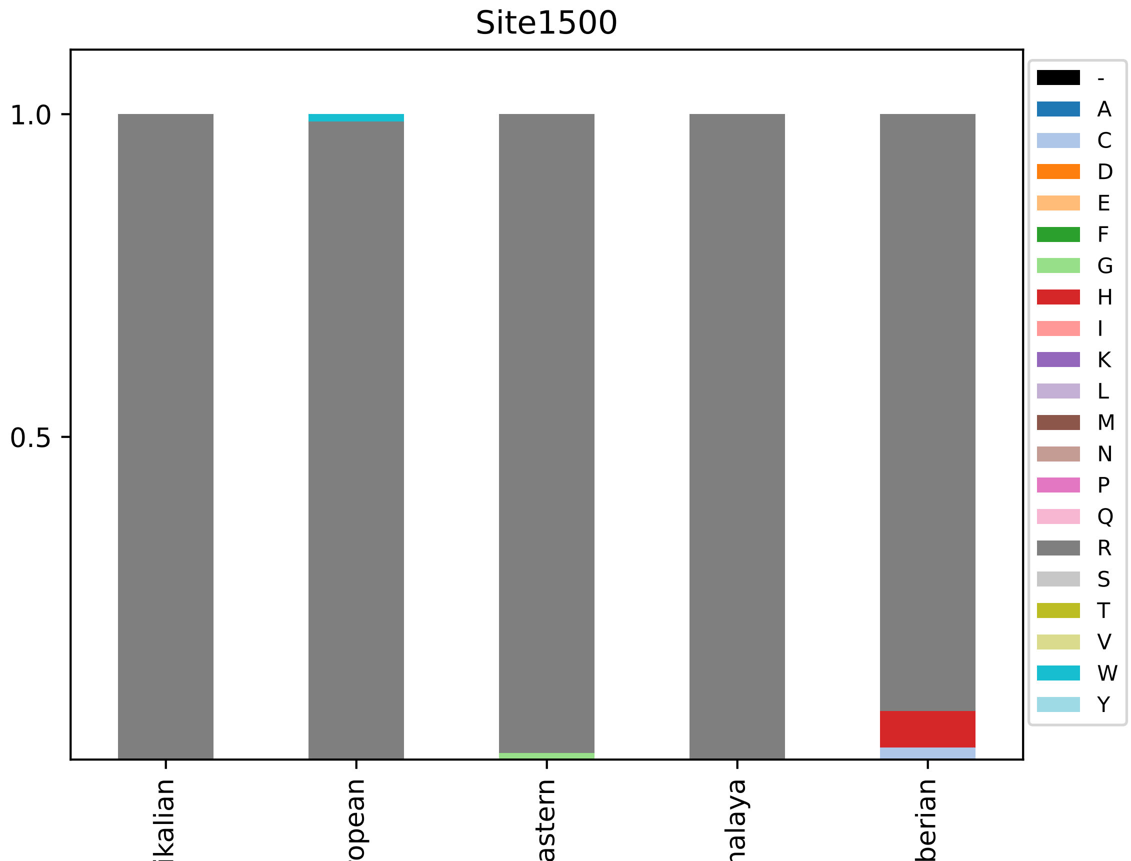

Site1500
1.0
0.5
ikalian
opean
astern
nalaya
berian
-
A
C
D
E
F
G
H
I
K
L
M
N
P
Q
R
S
T
V
W
Y

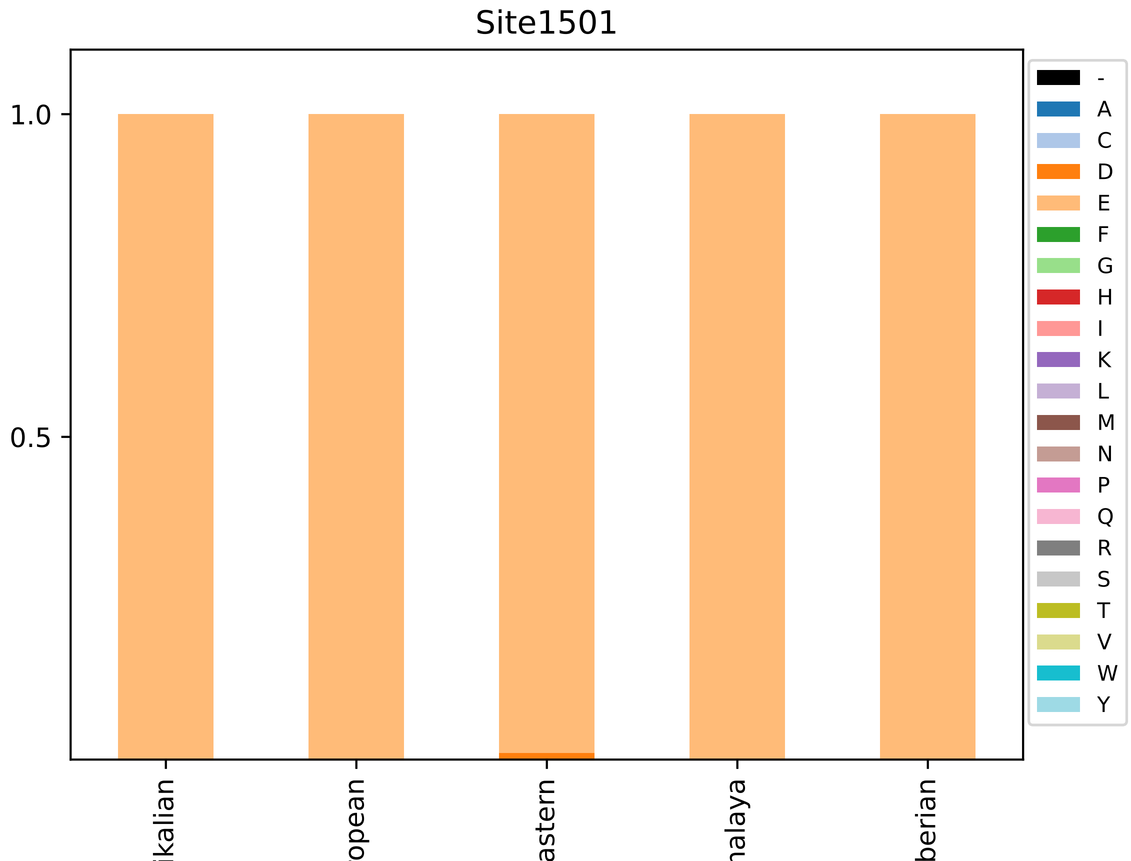
Site1501
1.0
0.5
-
A
C
D
E
F
G
H
I
K
L
M
N
P
Q
R
S
T
V
W
Y
ikalian
opean
astern
nalaya
berian

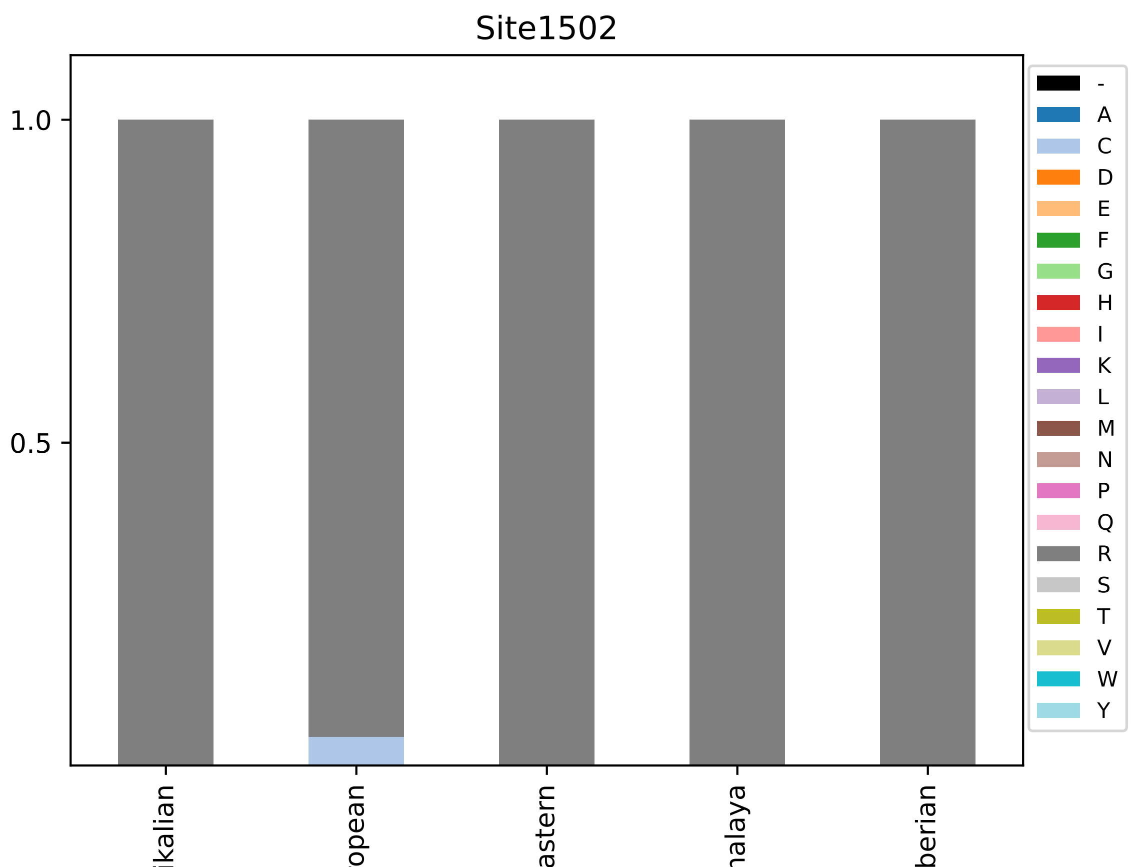
Site1502
1.0
0.5
-
A
C
D
E
F
G
H
I
K
L
M
N
P
Q
R
S
T
V
W
Y
ikalian
opean
astern
nalaya
berian

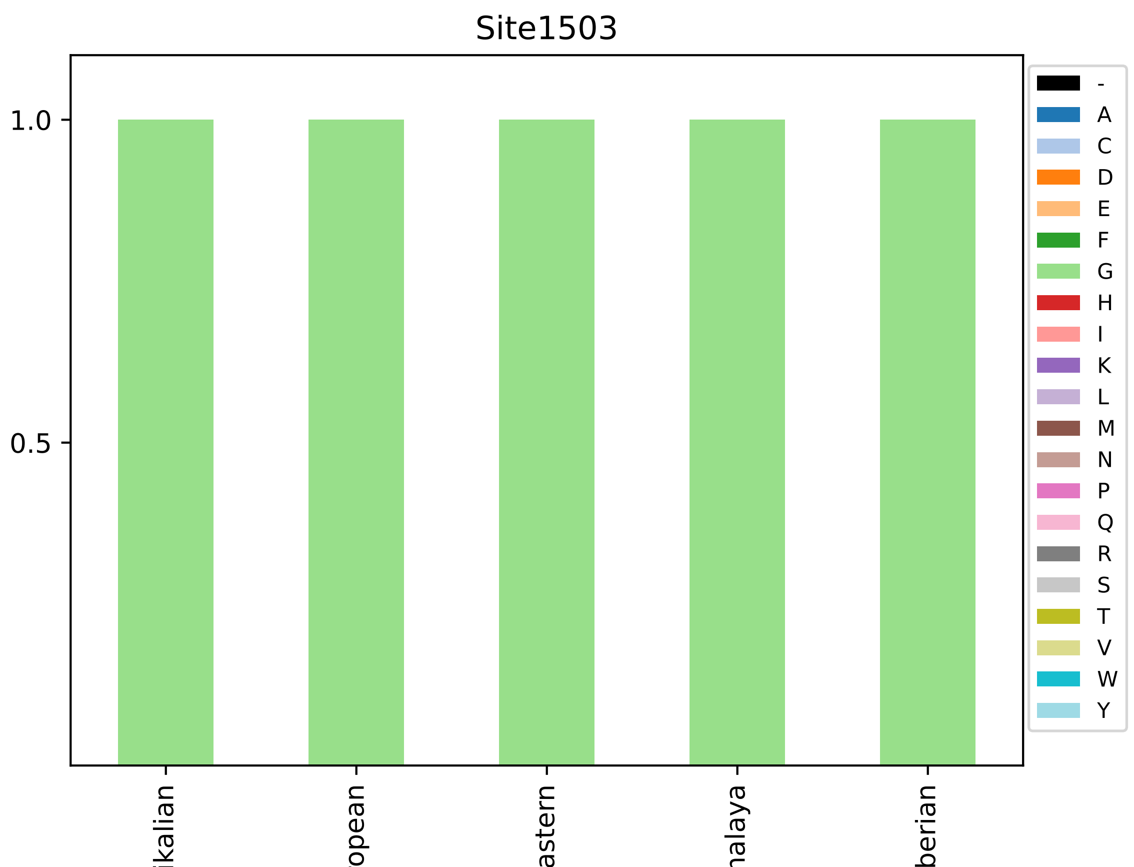

Site1503
1.0
0.5
-
A
C
D
E
F
G
H
I
K
L
M
N
P
Q
R
S
T
V
W
Y
ikalian
opean
astern
nalaya
berian

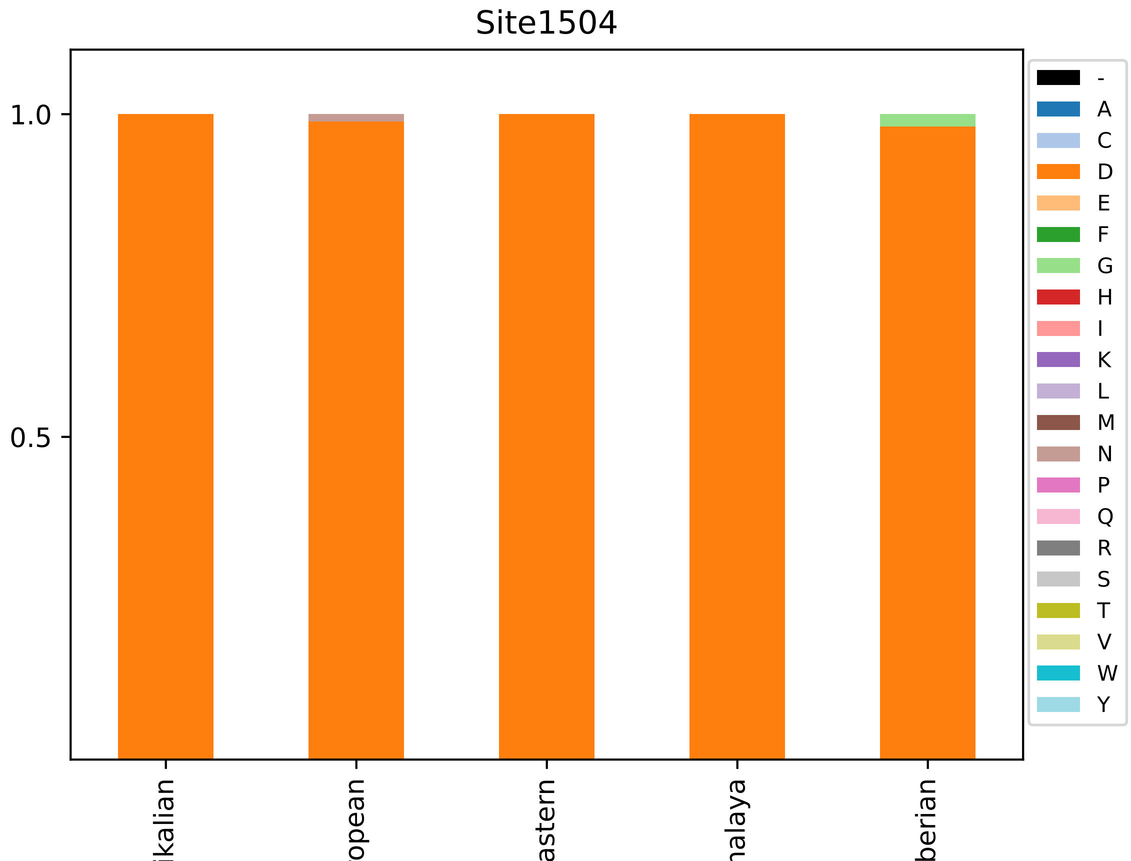

Site1504
1.0
0.5
ikalian
opean
astern
nalaya
berian
-
A
C
D
E
F
G
H
I
K
L
M
N
P
Q
R
S
T
V
W
Y

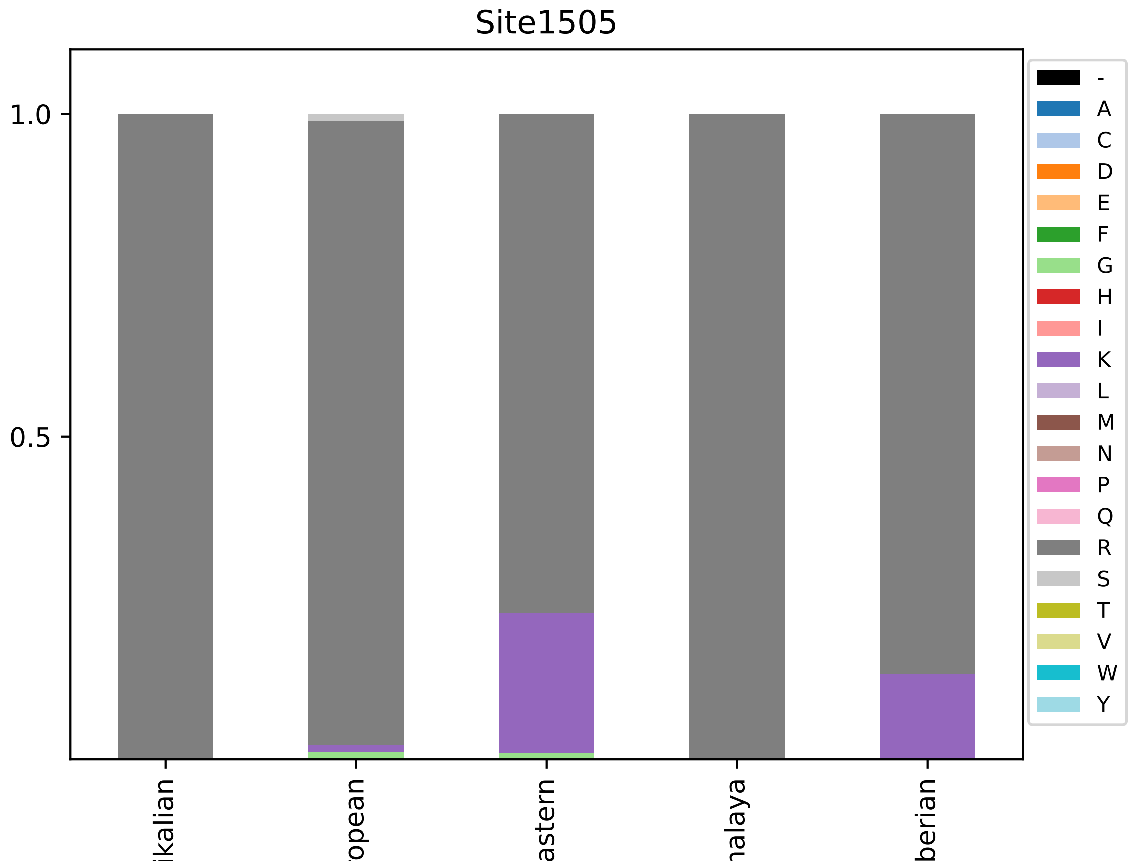
Site1505
1.0
0.5
-
A
C
D
E
F
G
H
I
K
L
M
N
P
Q
R
S
T
V
W
Y
ikalian
opean
astern
nalaya
berian

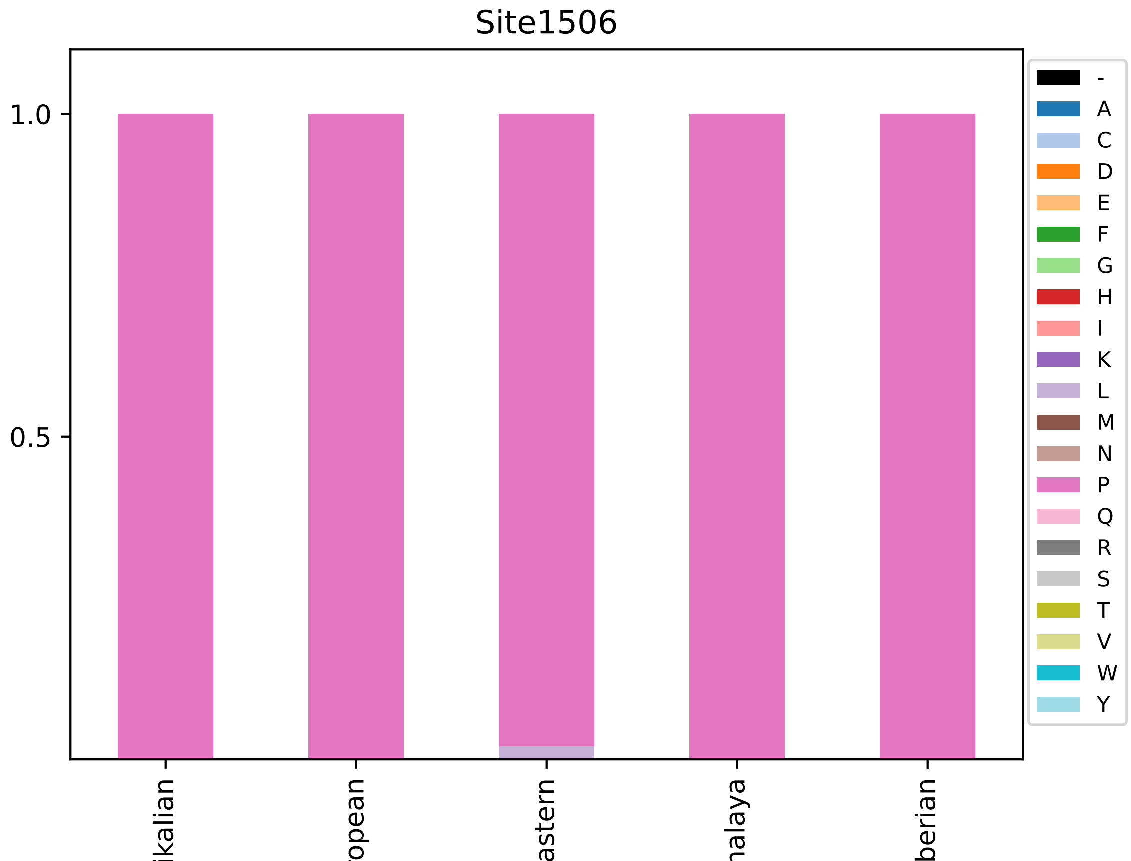

Site1506
1.0
0.5
-
A
C
D
E
F
G
H
I
K
L
M
N
P
Q
R
S
T
V
W
Y

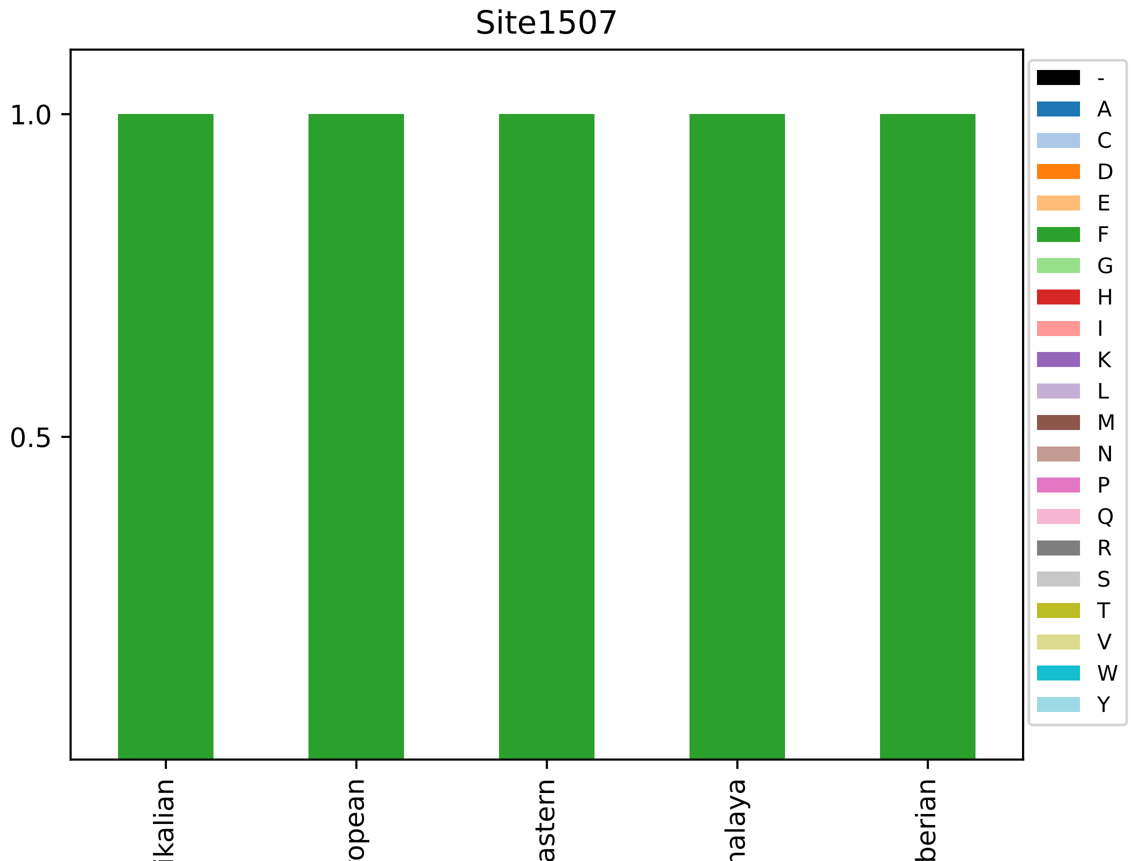
Site1507
1.0
0.5
-
A
C
D
E
F
G
H
I
K
L
M
N
P
Q
R
S
T
V
W
Y
ikalian
opean
astern
nalaya
berian

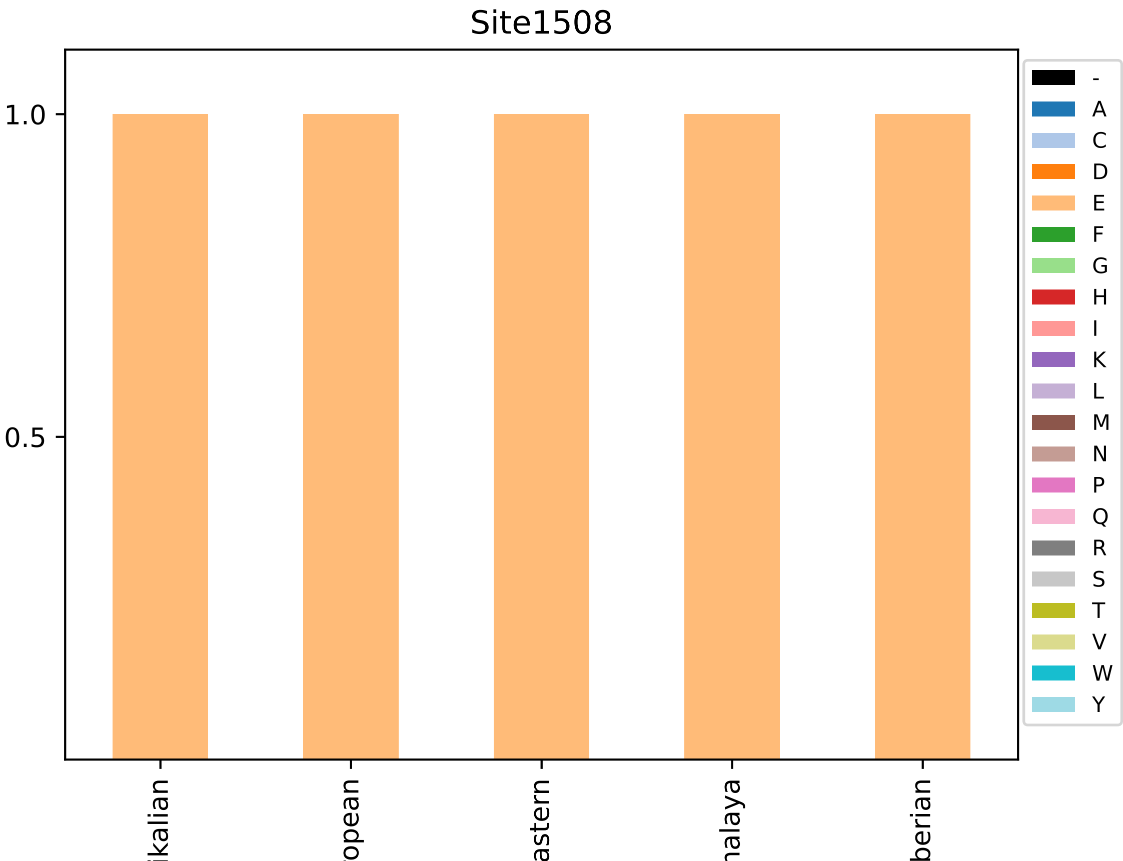

Site1508
1.0
0.5
-
A
C
D
E
F
G
H
I
K
L
M
N
P
Q
R
S
T
V
W
Y

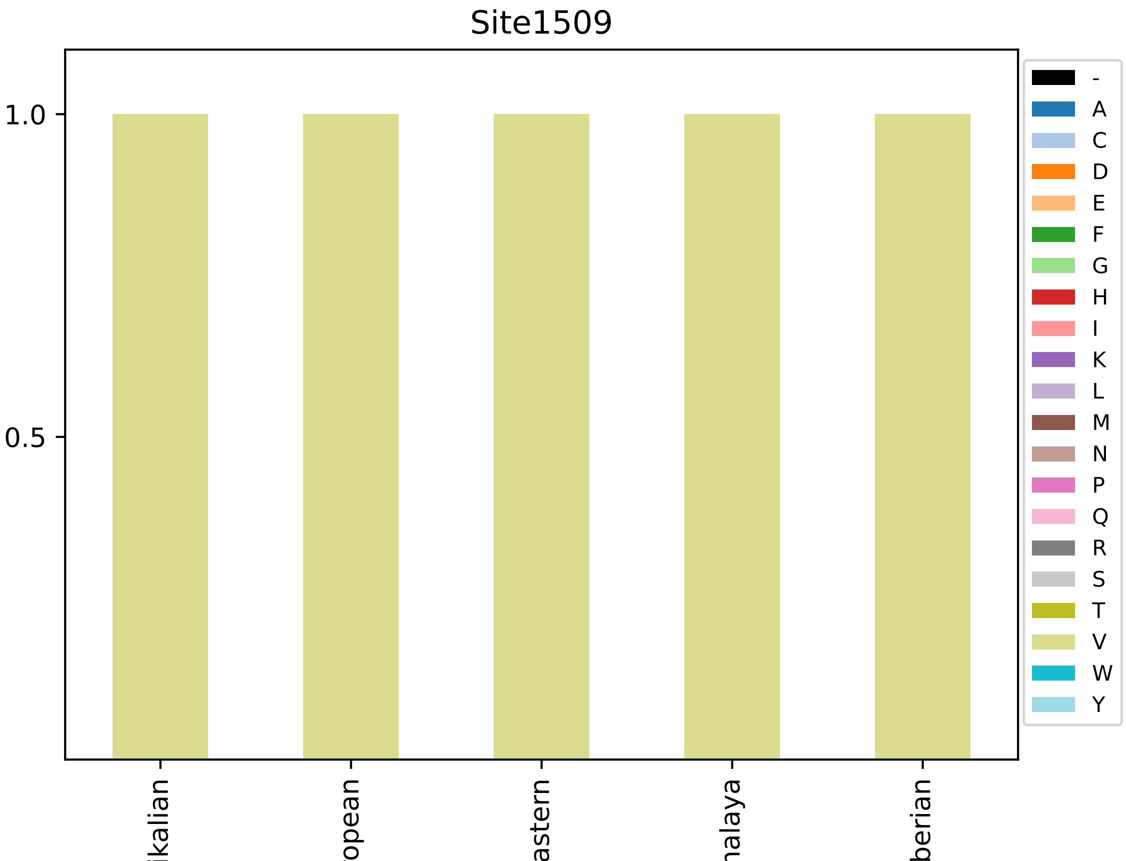
Site1509
1.0
0.5
-
A
C
D
E
F
G
H
I
K
L
M
N
P
Q
R
S
T
V
W
Y
ikalian
opean
astern
nalaya
berian

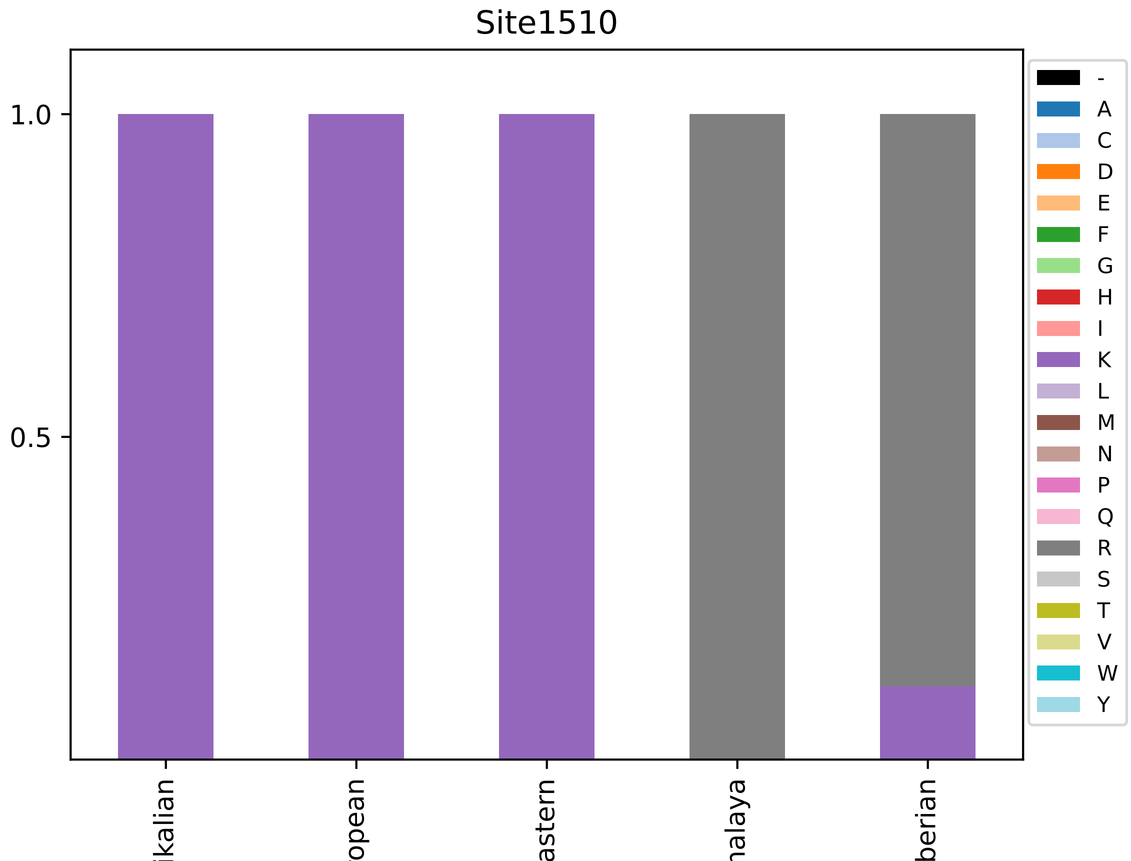
Site1510
1.0
0.5
ikalian
opean
astern
nalaya
berian
-
A
C
D
E
F
G
H
I
K
L
M
N
P
Q
R
S
T
V
W
Y

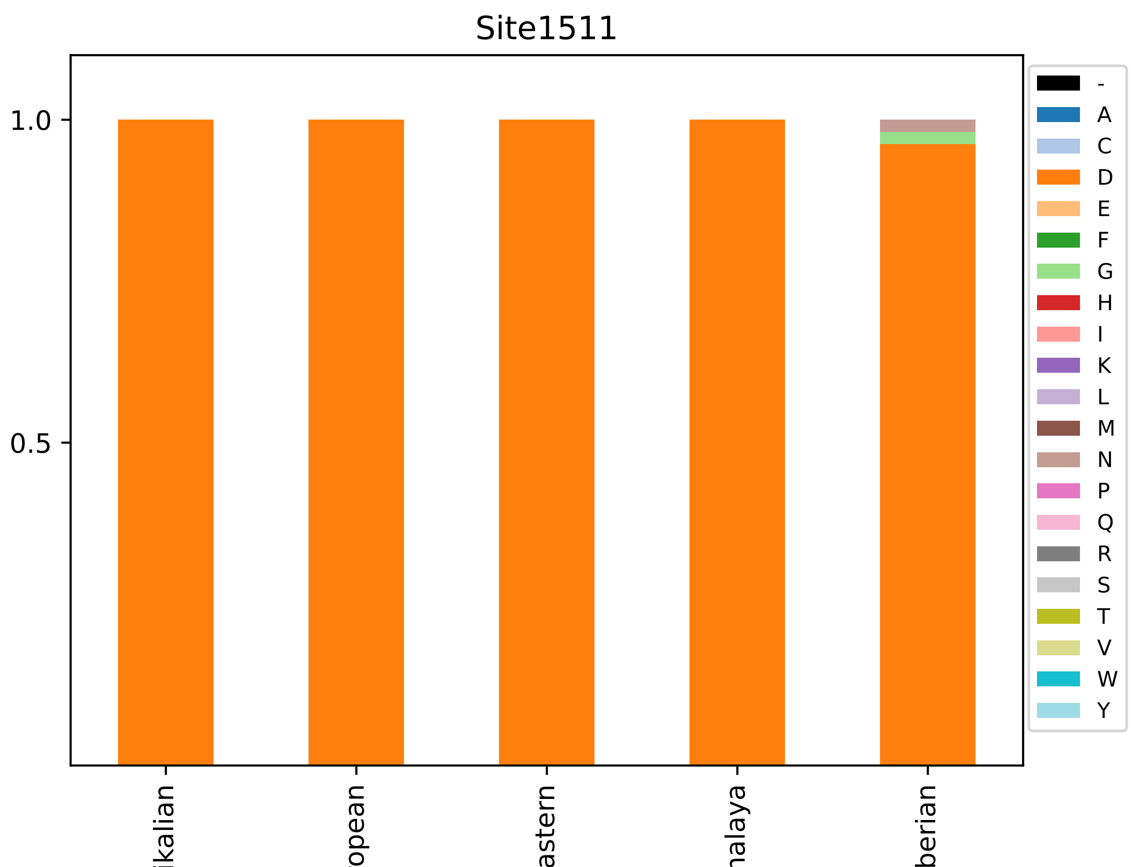
Site1511
1.0
0.5
-
A
C
D
E
F
G
H
I
K
L
M
N
P
Q
R
S
T
V
W
Y
ikalian
opean
astern
nalaya
berian

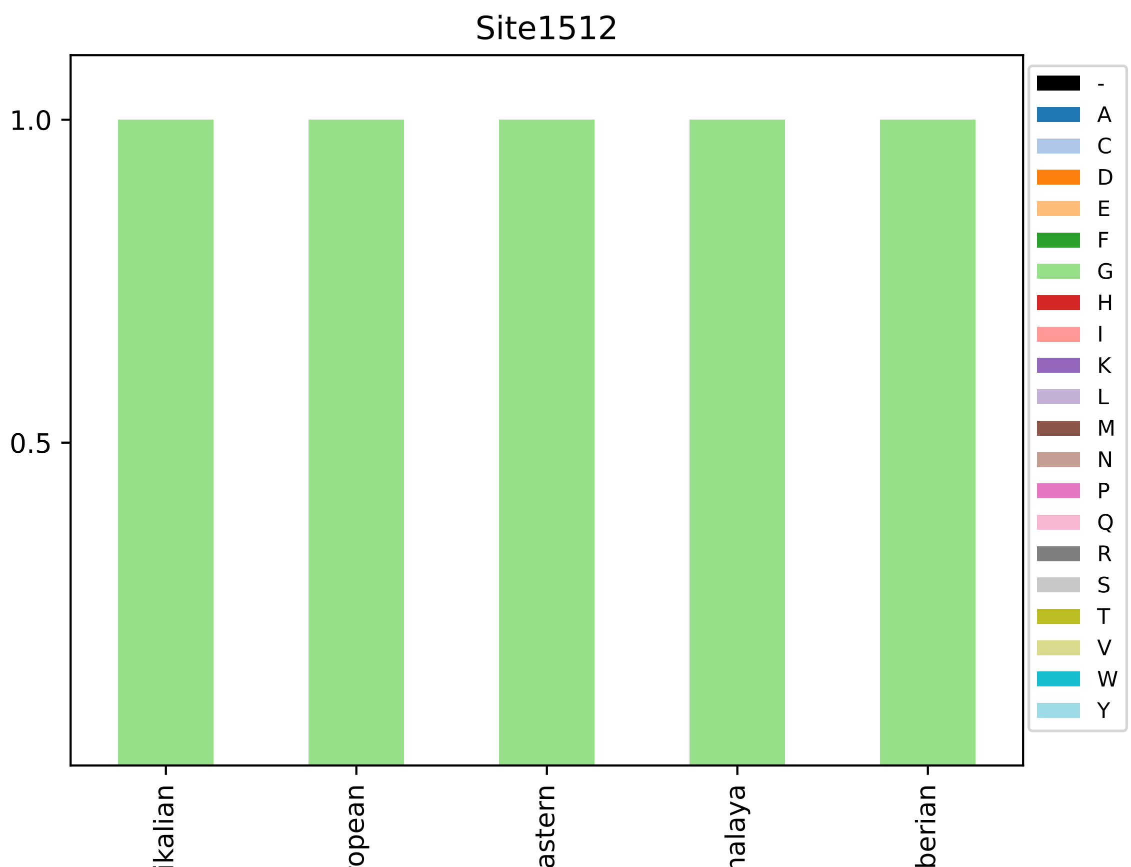
Site1512
1.0
0.5
-
A
C
D
E
F
G
H
I
K
L
M
N
P
Q
R
S
T
V
W
Y

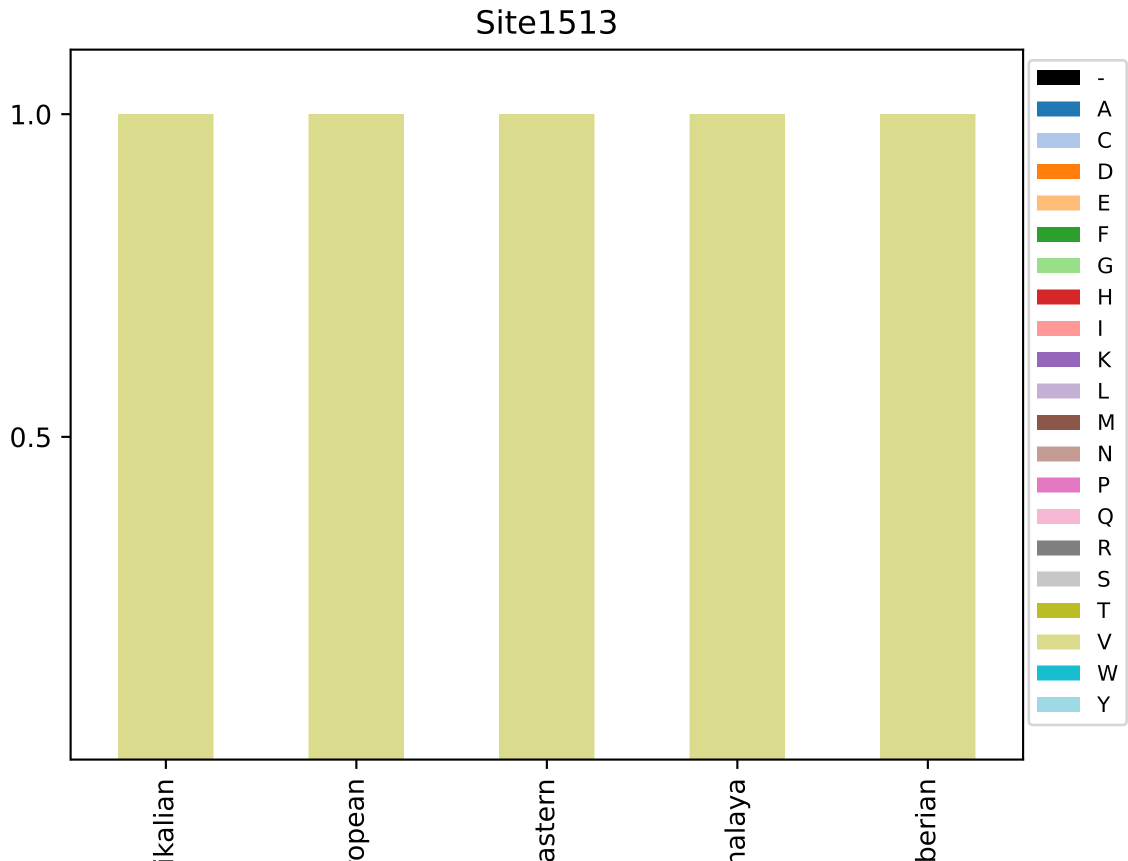
Site1513
1.0
0.5
-
A
C
D
E
F
G
H
I
K
L
M
N
P
Q
R
S
T
V
W
Y
ikalian
opean
astern
nalaya
berian

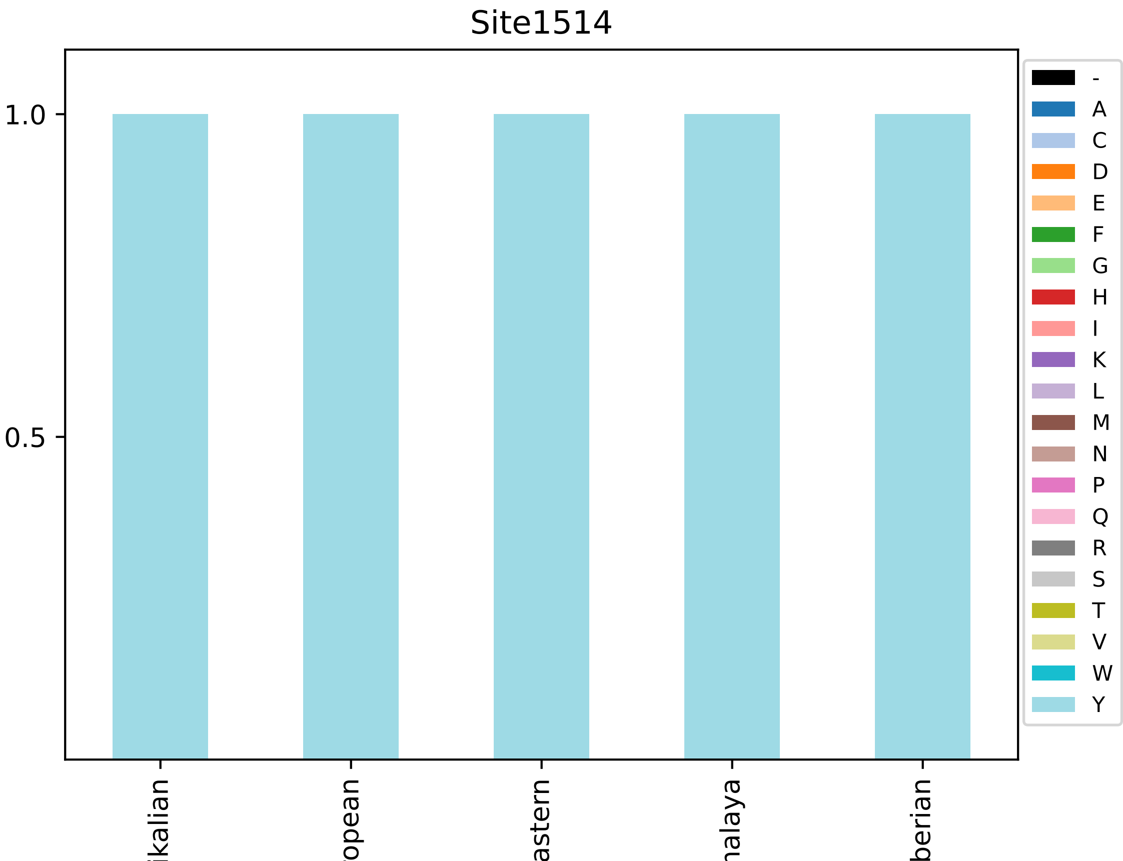
Site1514
1.0
0.5
-
A
C
D
E
F
G
H
I
K
L
M
N
P
Q
R
S
T
V
W
Y
ikalian
opean
astern
nalaya
berian

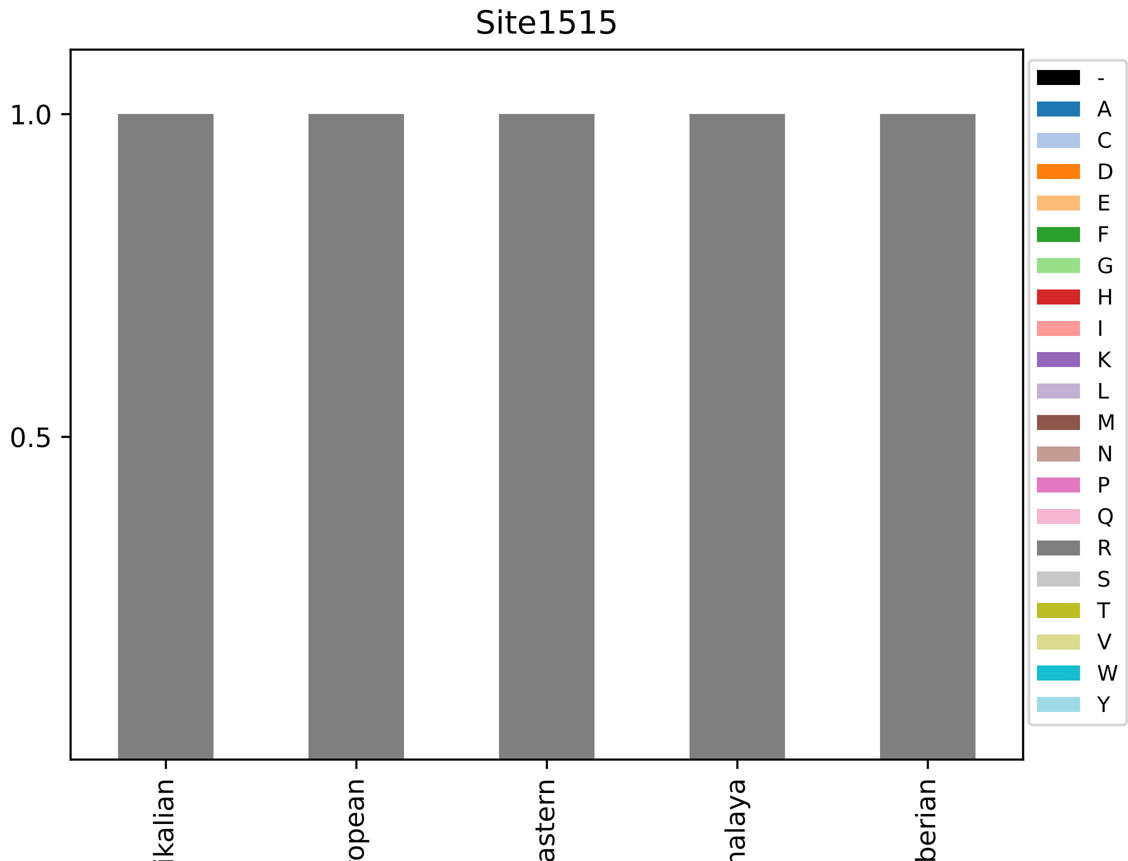
Site1515
1.0
0.5
-
A
C
D
E
F
G
H
I
K
L
M
N
P
Q
R
S
T
V
W
Y
ikalian
opean
astern
nalaya
berian

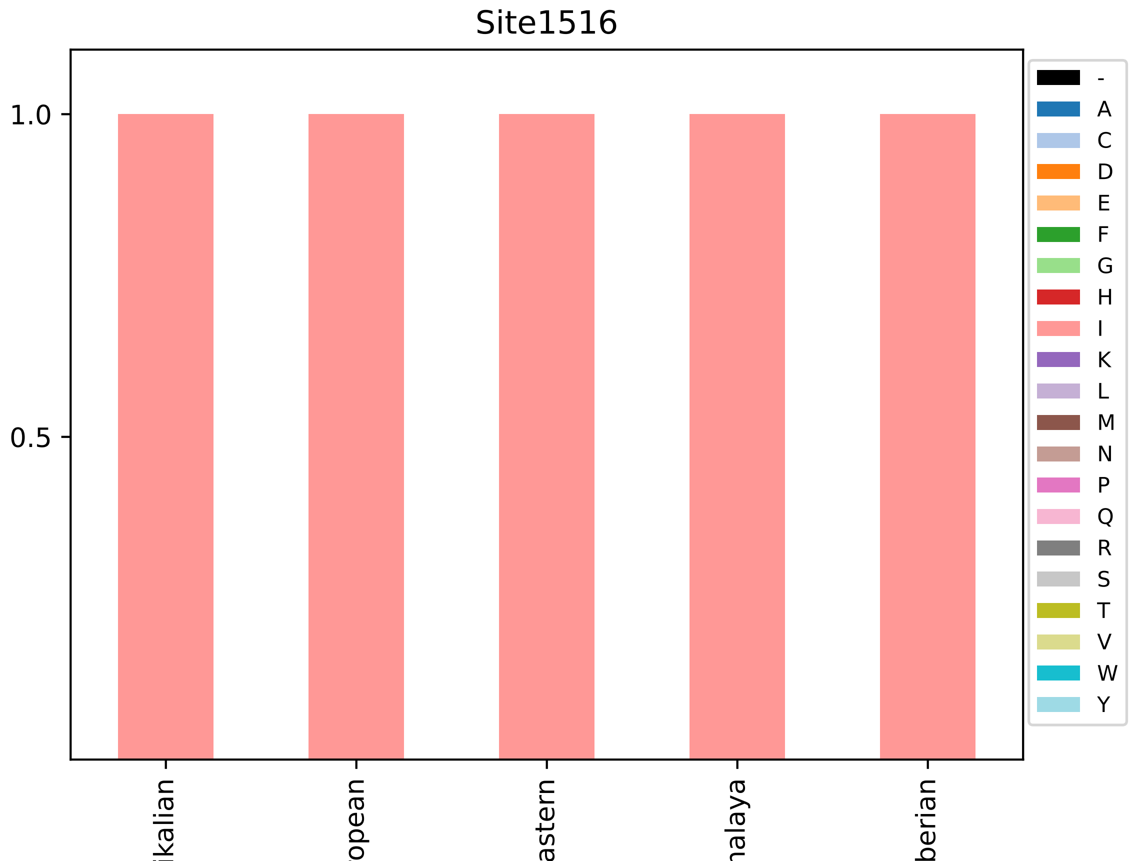
Site1516
1.0
0.5
-
A
C
D
E
F
G
H
I
K
L
M
N
P
Q
R
S
T
V
W
Y
ikalian
opean
astern
nalaya
berian

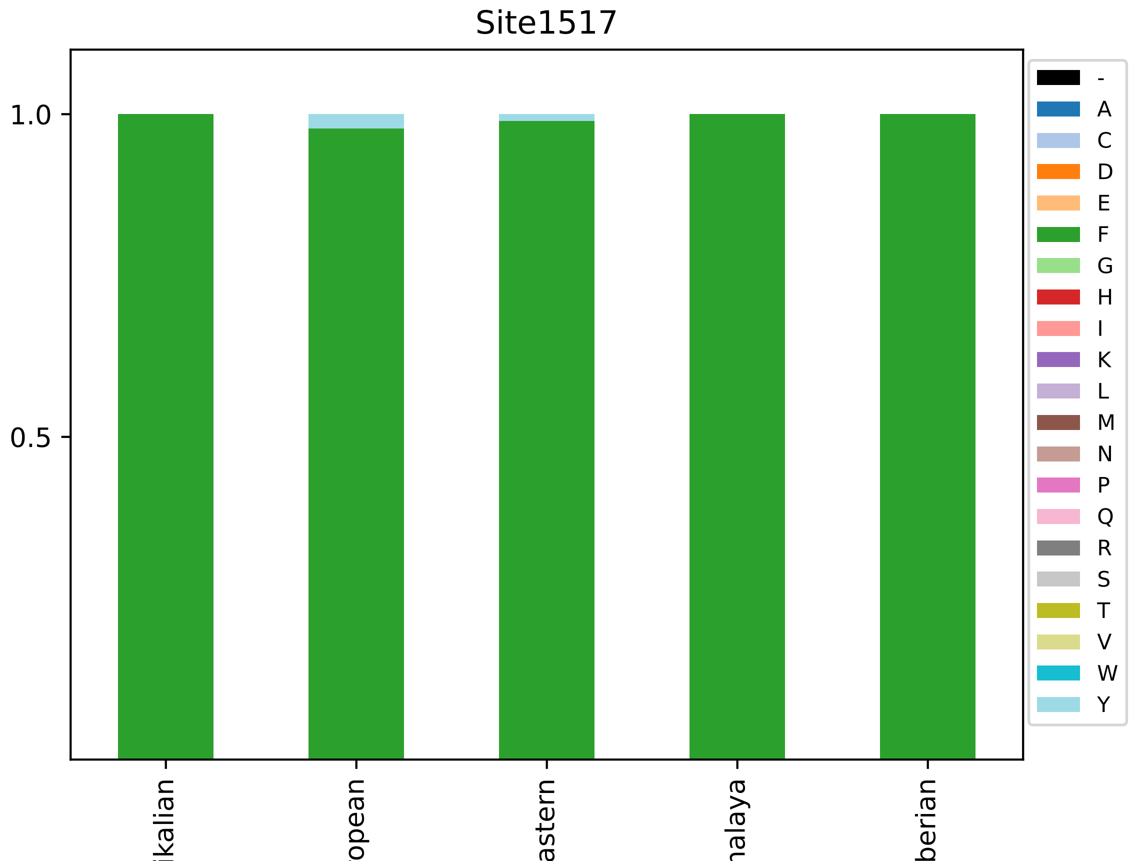
Site1517
1.0
0.5
-
A
C
D
E
F
G
H
I
K
L
M
N
P
Q
R
S
T
V
W
Y
ikalian
opean
astern
nalaya
berian

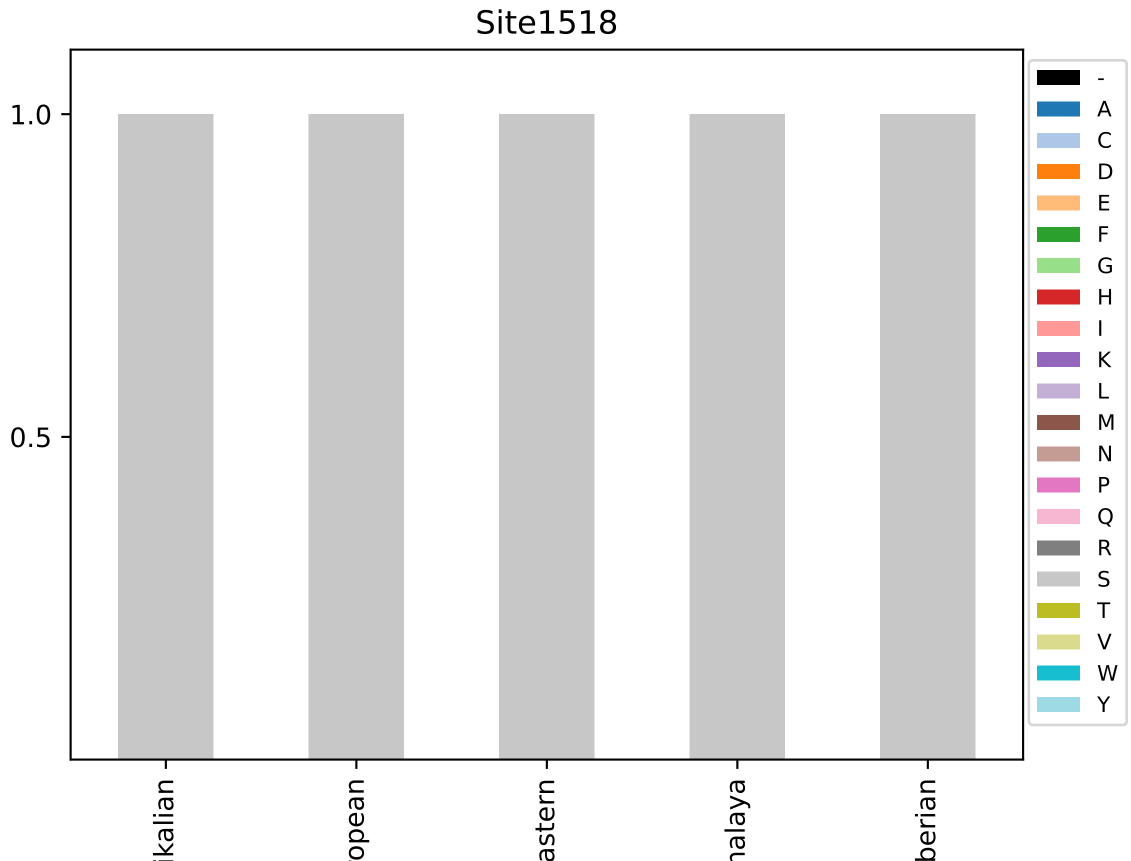
Site1518
1.0
0.5
-
A
C
D
E
F
G
H
I
K
L
M
N
P
Q
R
S
T
V
W
Y
ikalian
opean
astern
nalaya
berian

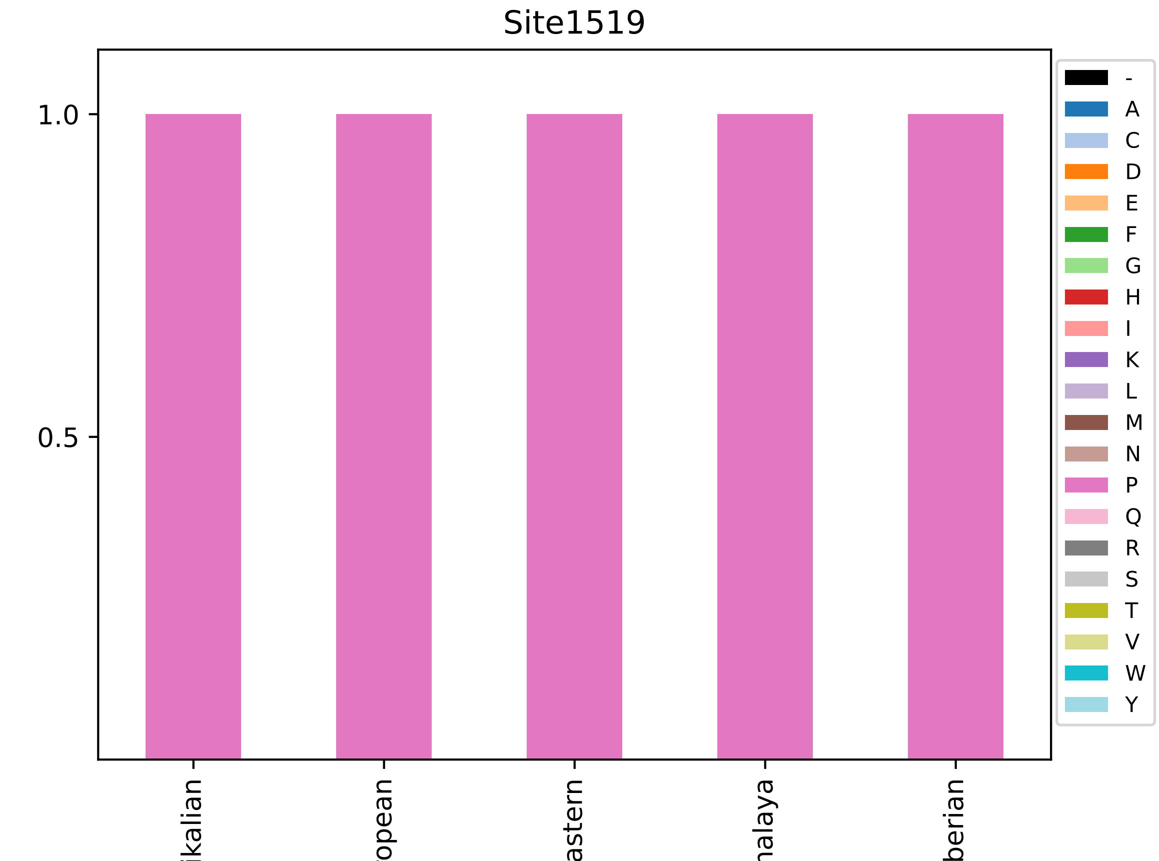
Site1519
1.0
0.5
-
A
C
D
E
F
G
H
I
K
L
M
N
P
Q
R
S
T
V
W
Y
ikalian
opean
astern
nalaya
berian

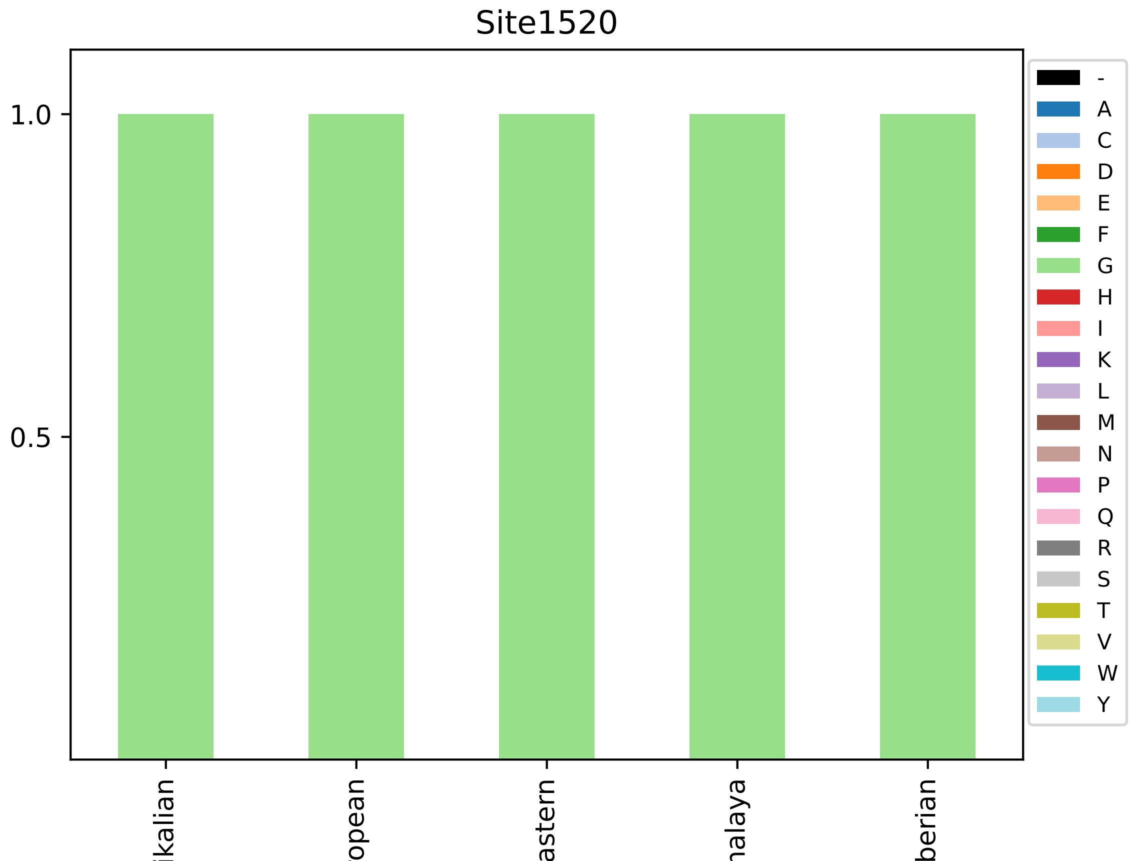
Site1520
1.0
0.5
-
A
C
D
E
F
G
H
I
K
L
M
N
P
Q
R
S
T
V
W
Y
ikalian
opean
astern
nalaya
berian

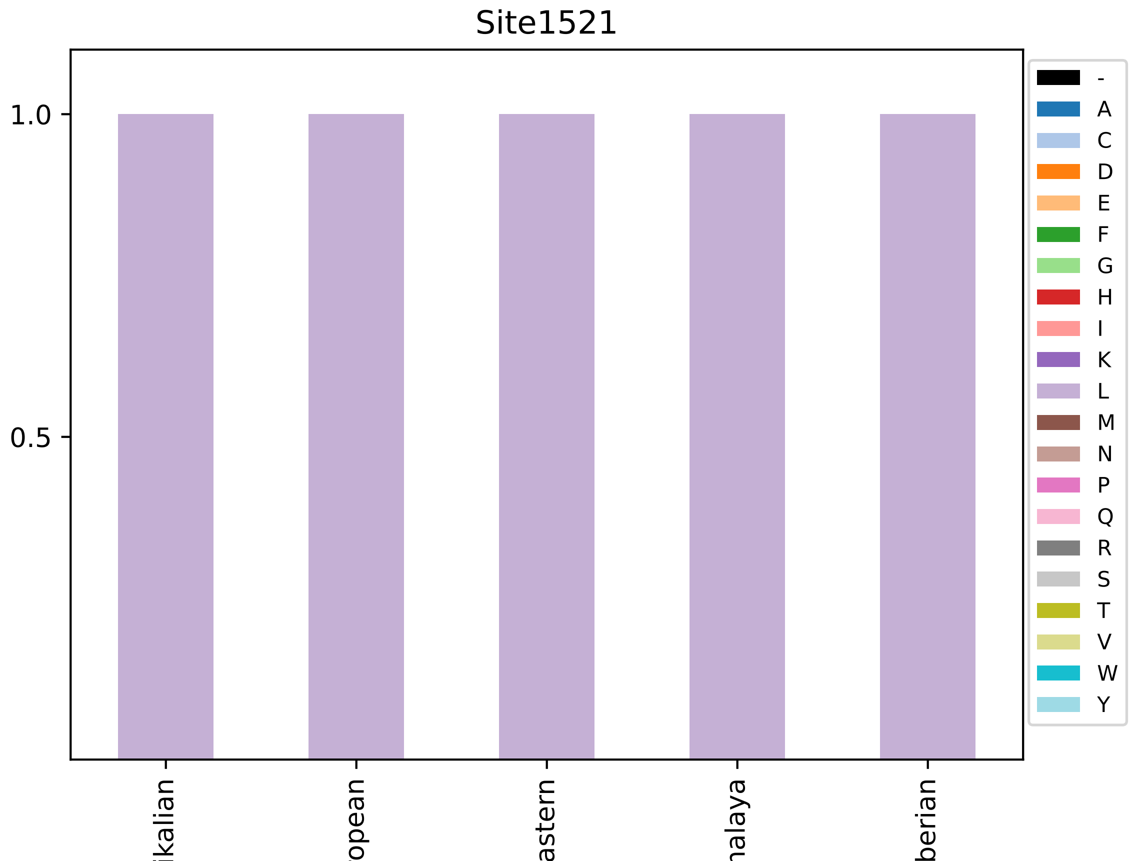
Site1521
1.0
0.5
-
A
C
D
E
F
G
H
I
K
L
M
N
P
Q
R
S
T
V
W
Y
ikalian
opean
astern
nalaya
berian

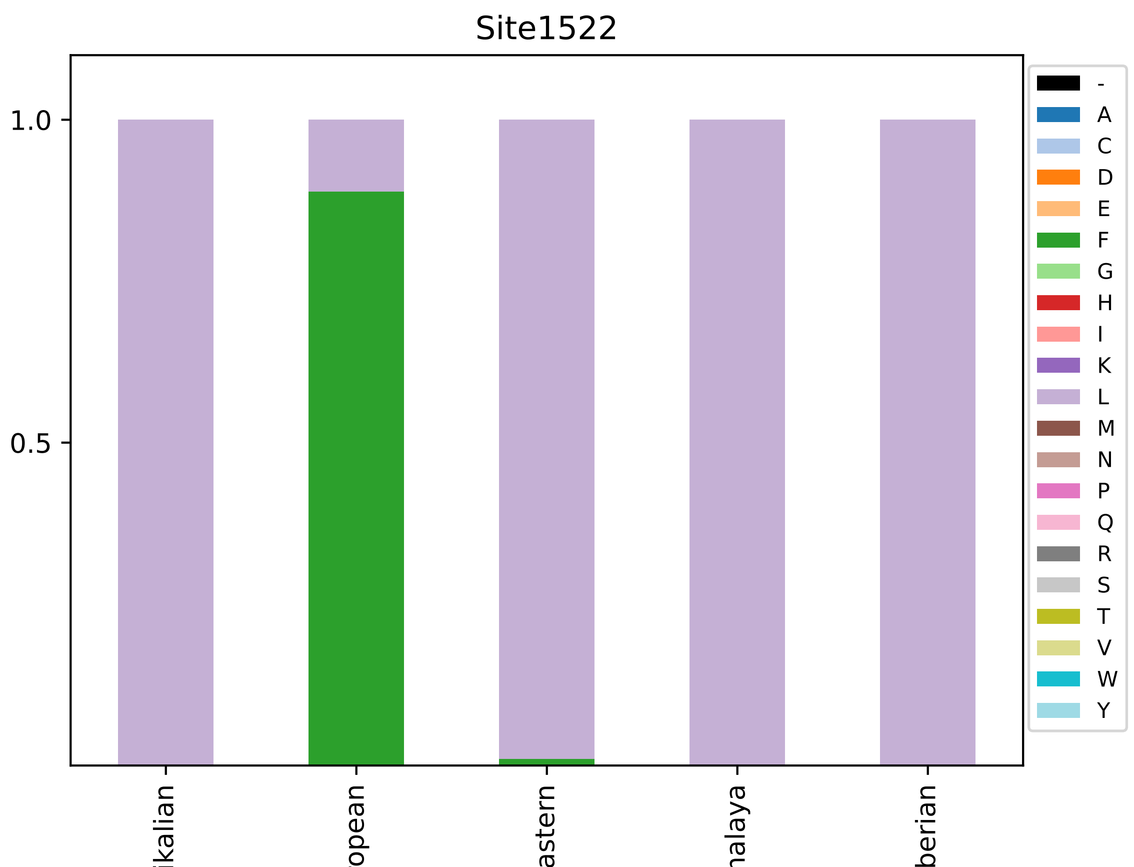

Site1522
1.0
0.5
ikalian
ropean
astern
nalaya
berian
-
A
C
D
E
F
G
H
I
K
L
M
N
P
Q
R
S
T
V
W
Y

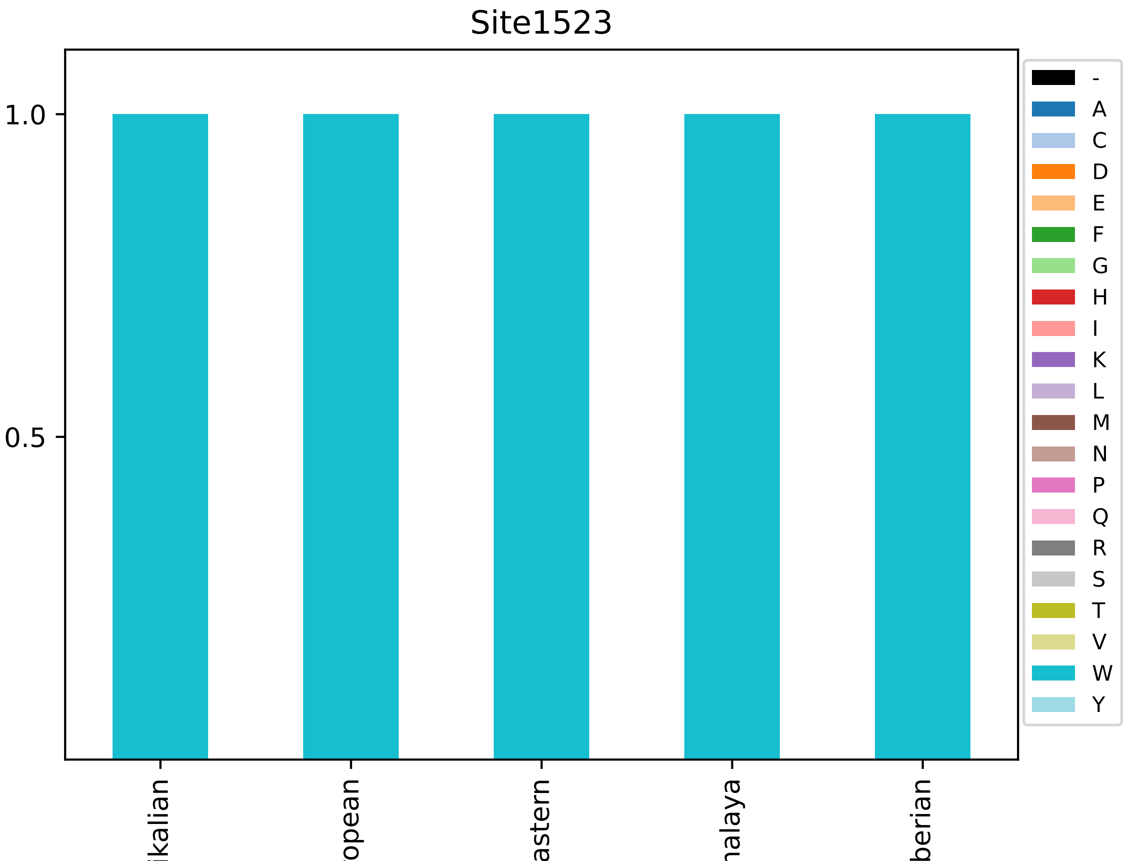

Site1523
1.0
0.5
-
A
C
D
E
F
G
H
I
K
L
M
N
P
Q
R
S
T
V
W
Y
ikalian
opean
astern
halaya
berian

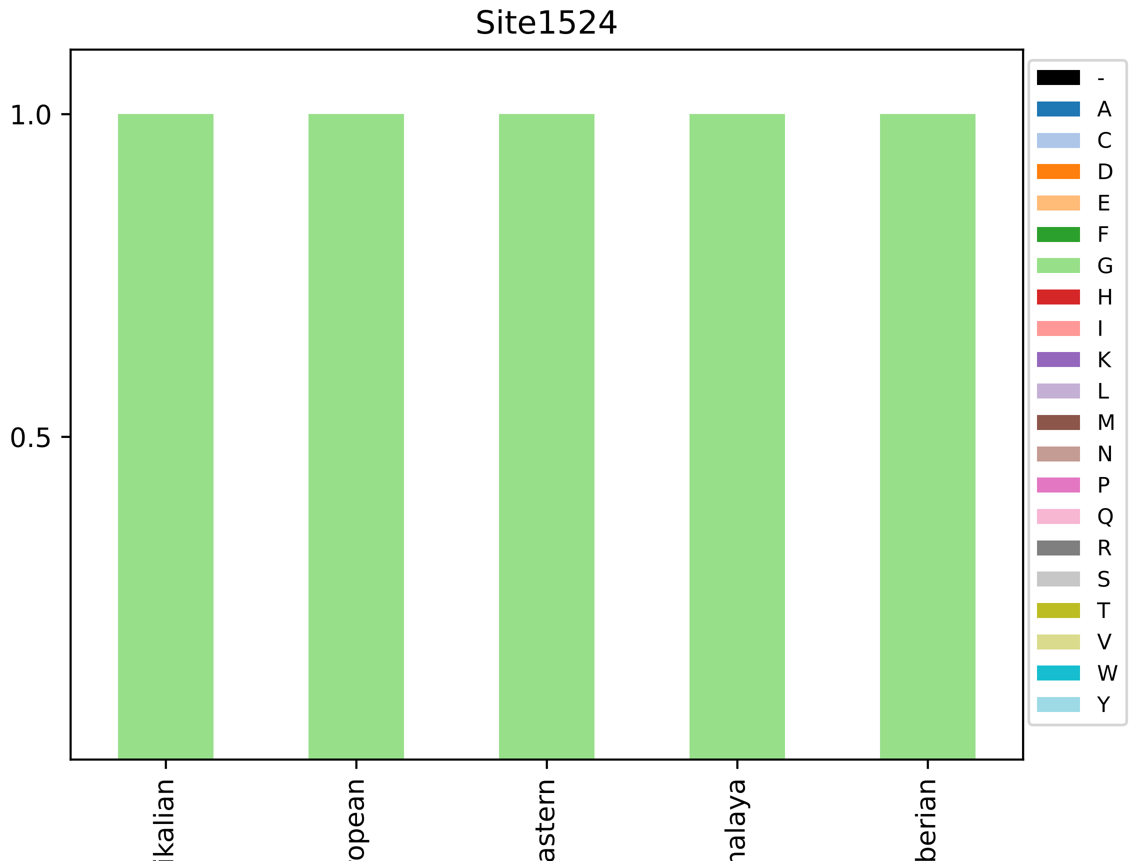
Site1524
1.0
0.5
-
A
C
D
E
F
G
H
I
K
L
M
N
P
Q
R
S
T
V
W
Y
ikalian
opean
astern
nalaya
berian

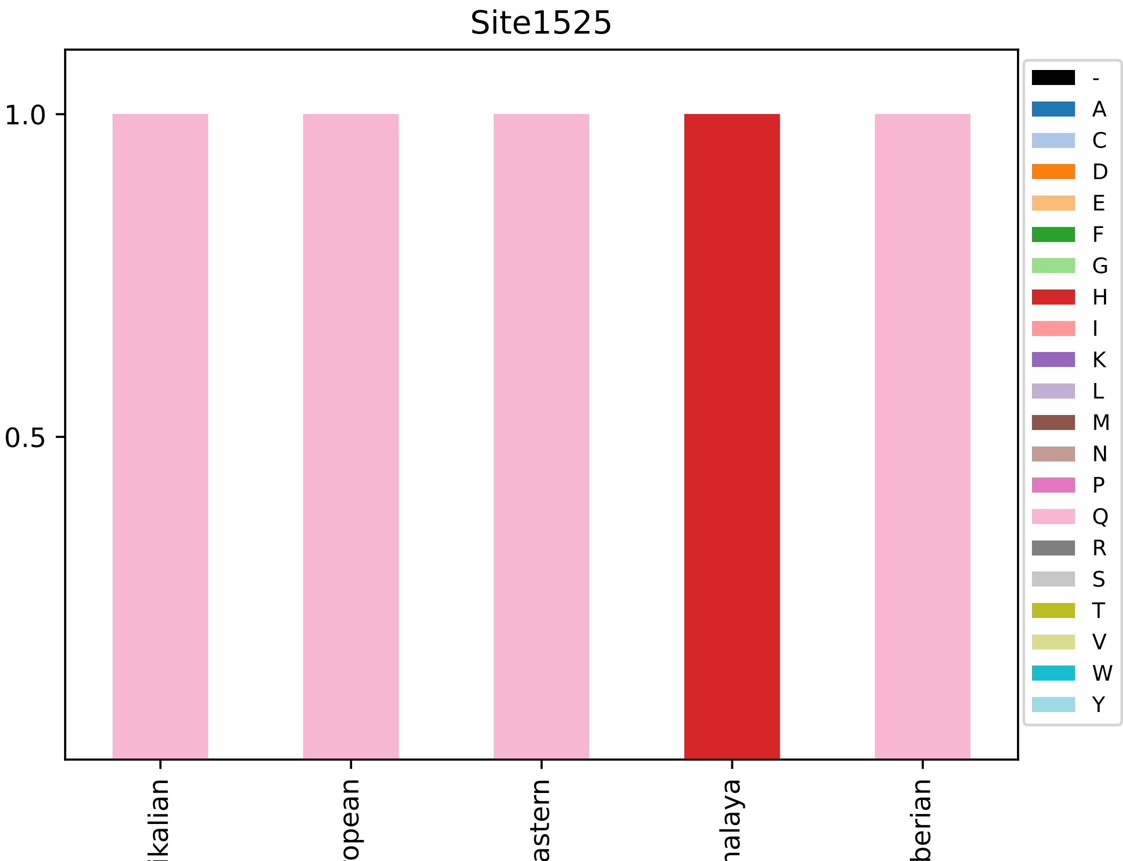

Site1525
1.0
0.5
-
A
C
D
E
F
G
H
I
K
L
M
N
P
Q
R
S
T
V
W
Y

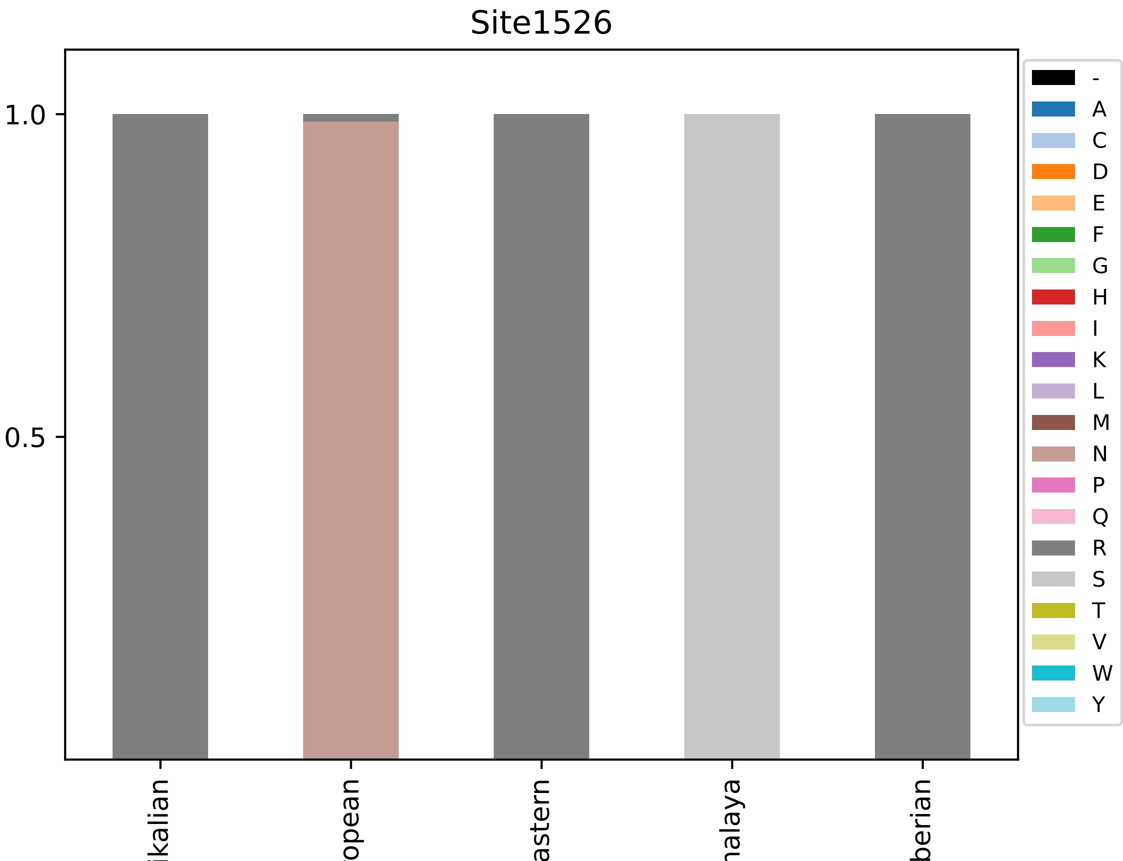
Site1526
1.0
0.5
-
A
C
D
E
F
G
H
I
K
L
M
N
P
Q
R
S
T
V
W
Y
ikalian
opean
astern
nalaya
berian

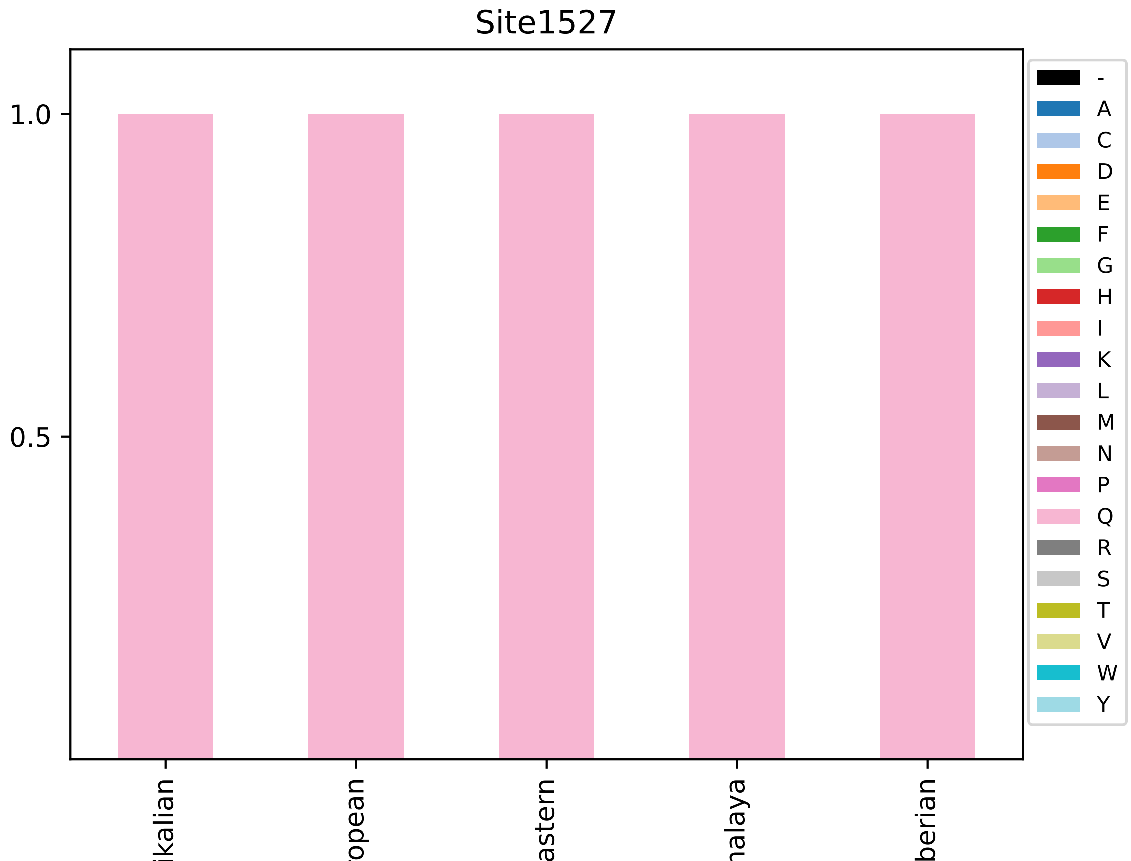
Site1527
1.0
0.5
ikalian
ropean
astern
nalaya
berian
-
A
C
D
E
F
G
H
I
K
L
M
N
P
Q
R
S
T
V
W
Y

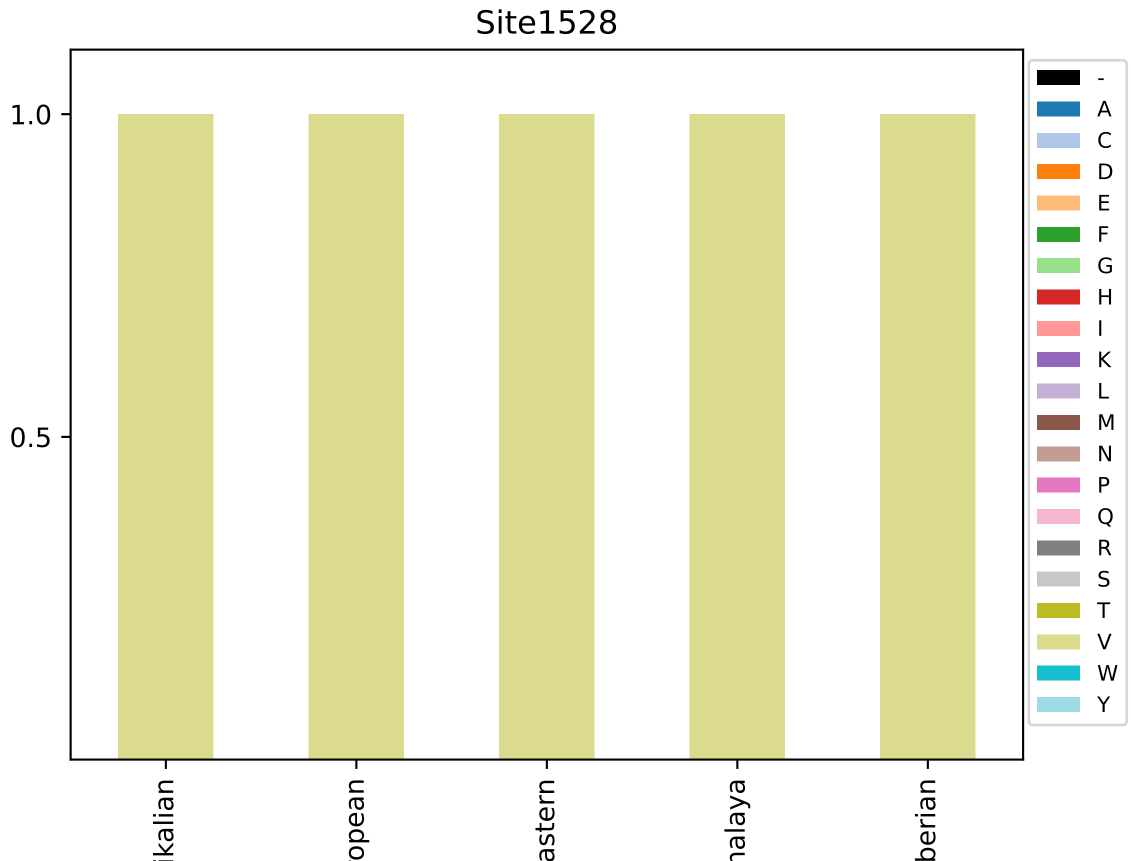

Site1528
1.0
0.5
-
A
C
D
E
F
G
H
I
K
L
M
N
P
Q
R
S
T
V
W
Y
ikalian
opean
astern
nalaya
berian

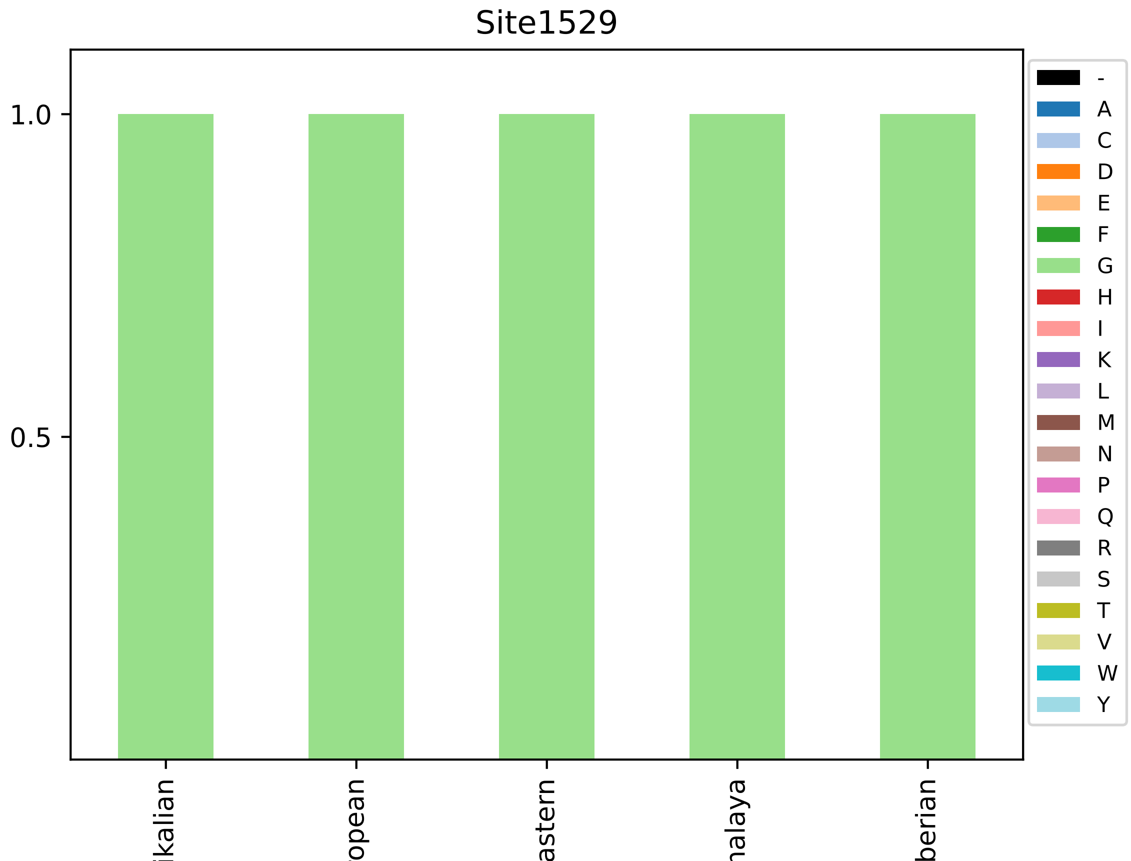

Site1529
1.0
0.5
ikalian
opean
astern
nalaya
berian
-
A
C
D
E
F
G
H
I
K
L
M
N
P
Q
R
S
T
V
W
Y

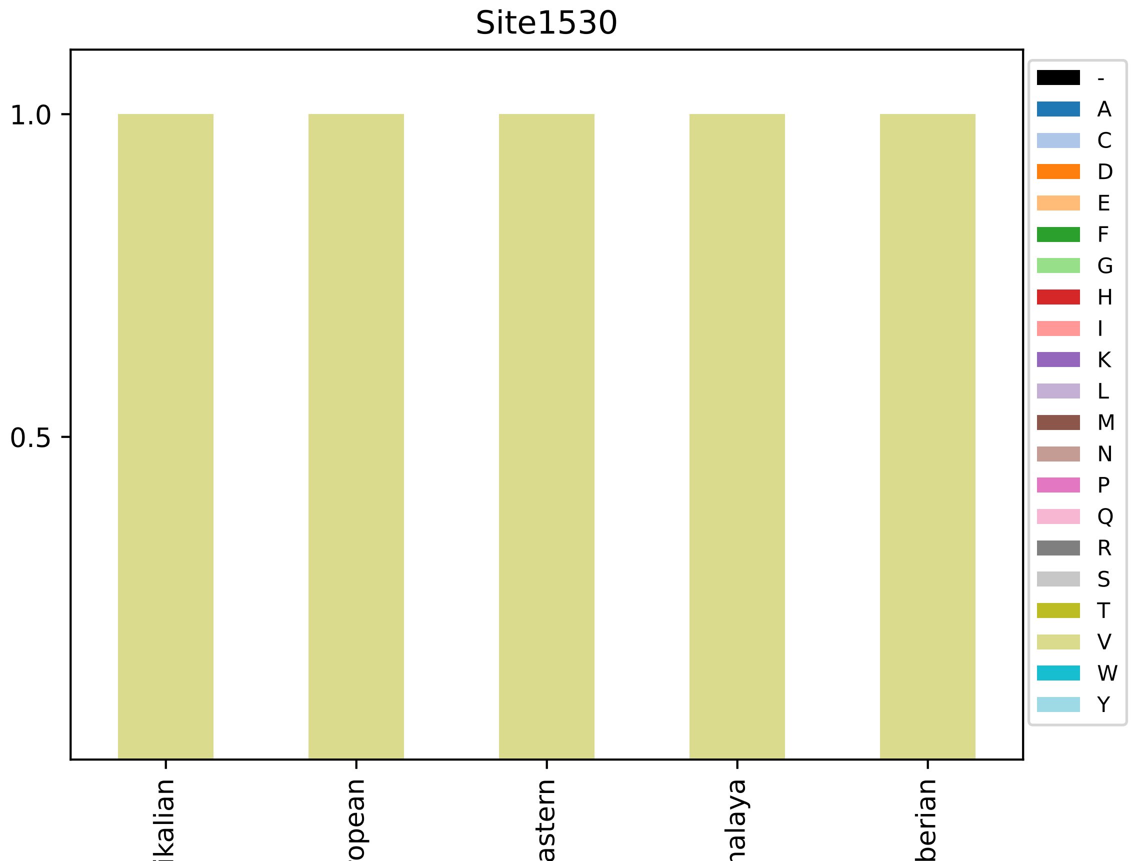

Site1530
1.0
0.5
-
A
C
D
E
F
G
H
I
K
L
M
N
P
Q
R
S
T
V
W
Y
ikalian
opean
astern
nalaya
berian

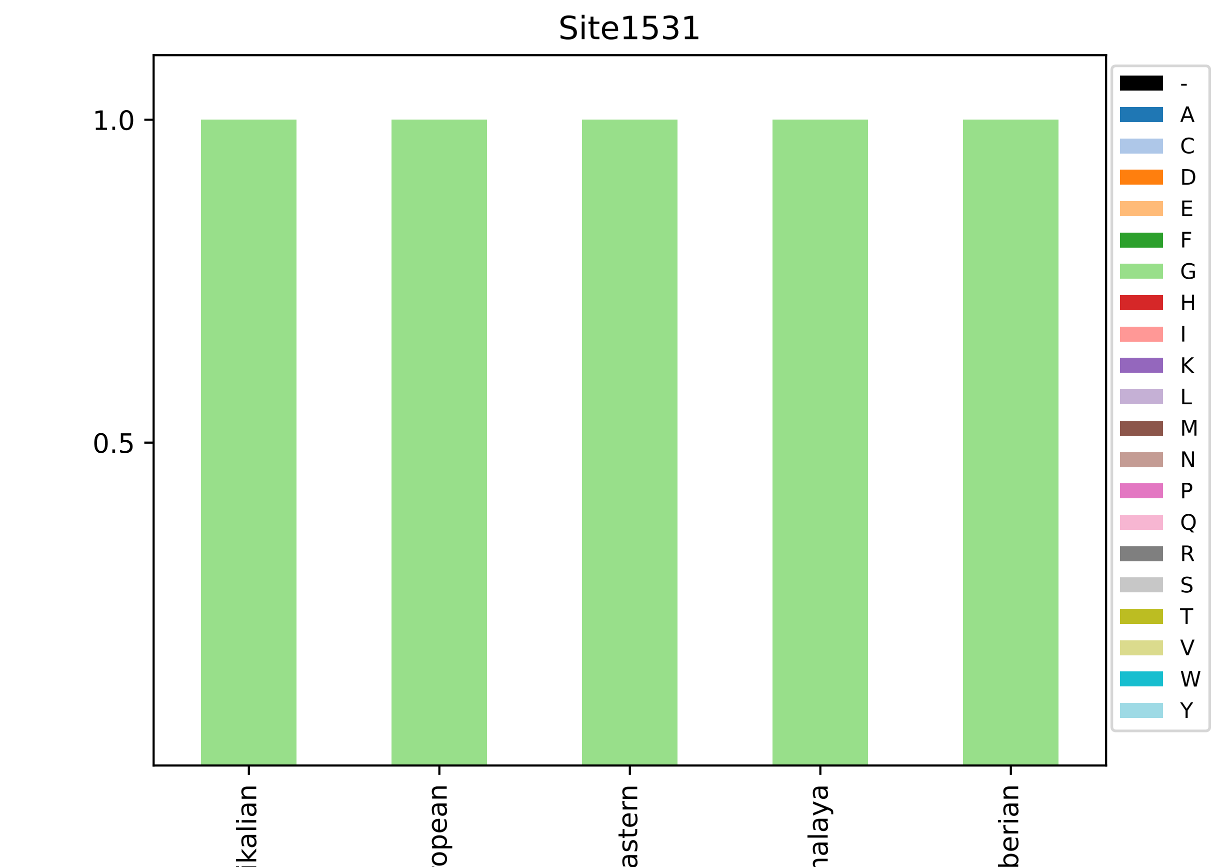
Site1531
1.0
0.5
-
A
C
D
E
F
G
H
I
K
L
M
N
P
Q
R
S
T
V
W
Y

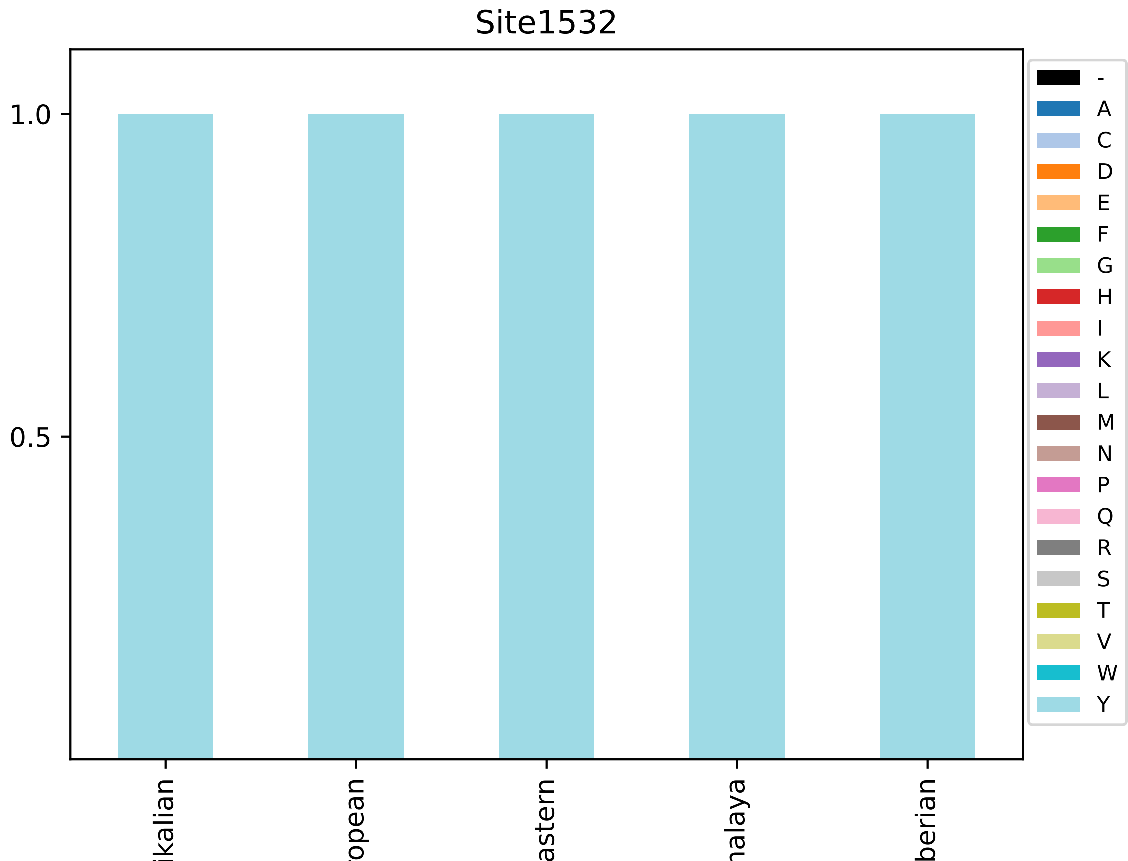
Site1532
1.0
0.5
-
A
C
D
E
F
G
H
I
K
L
M
N
P
Q
R
S
T
V
W
Y
ikalian
opean
astern
nalaya
berian

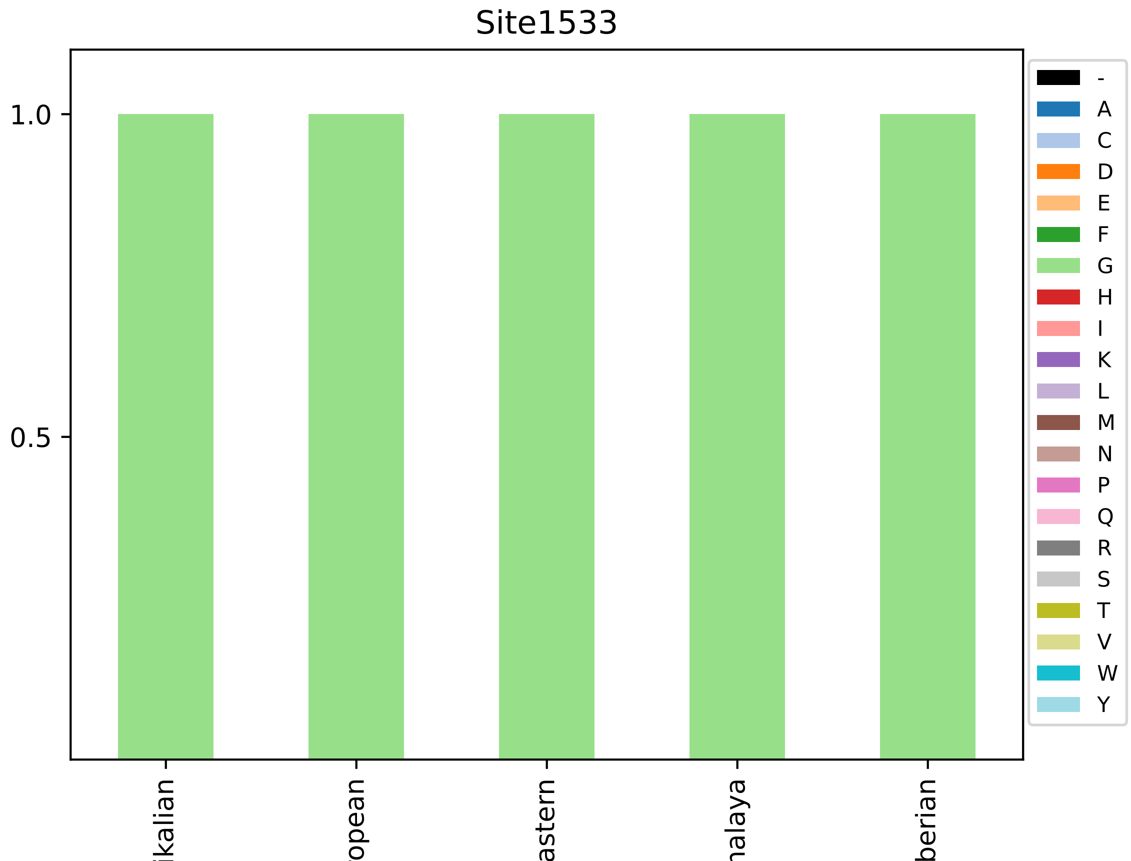

Site1533
1.0
0.5
-
A
C
D
E
F
G
H
I
K
L
M
N
P
Q
R
S
T
V
W
Y
ikalian
opean
astern
nalaya
berian

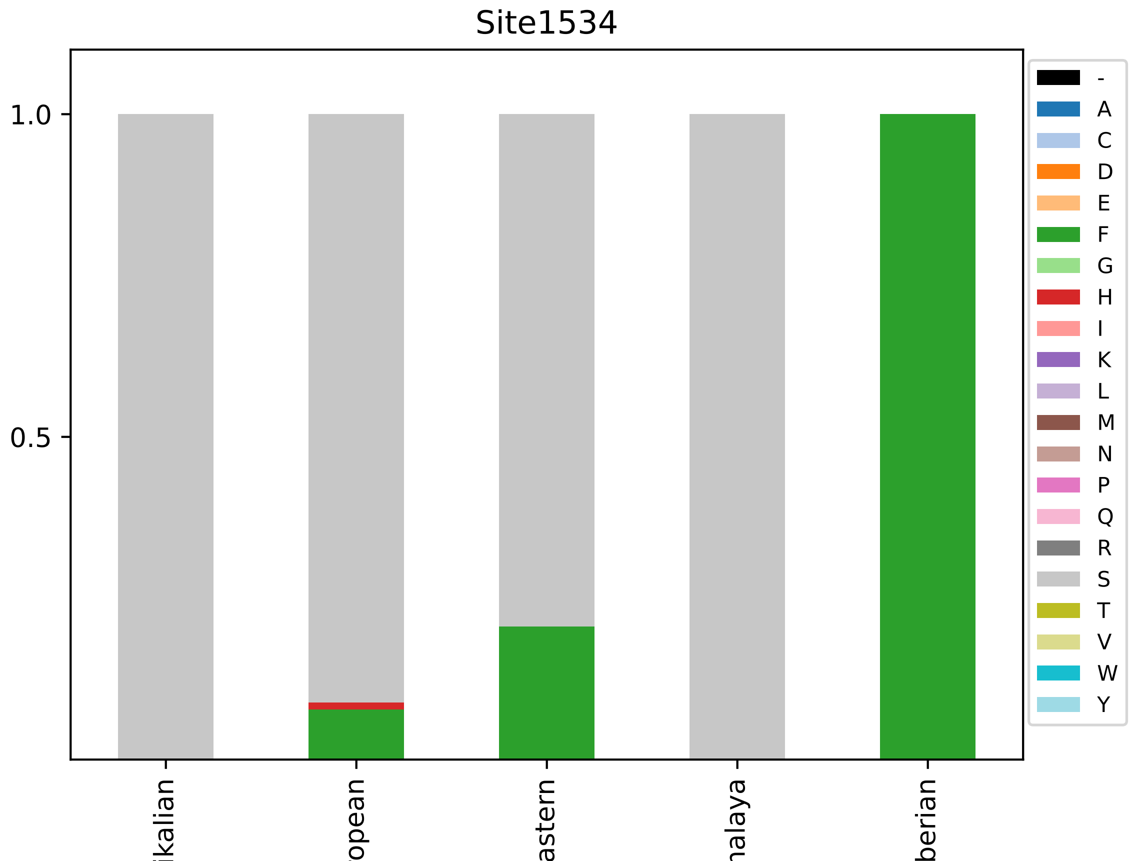

Site1534
1.0
0.5
-
A
C
D
E
F
G
H
I
K
L
M
N
P
Q
R
S
T
V
W
Y
ikalian
opean
astern
nalaya
berian

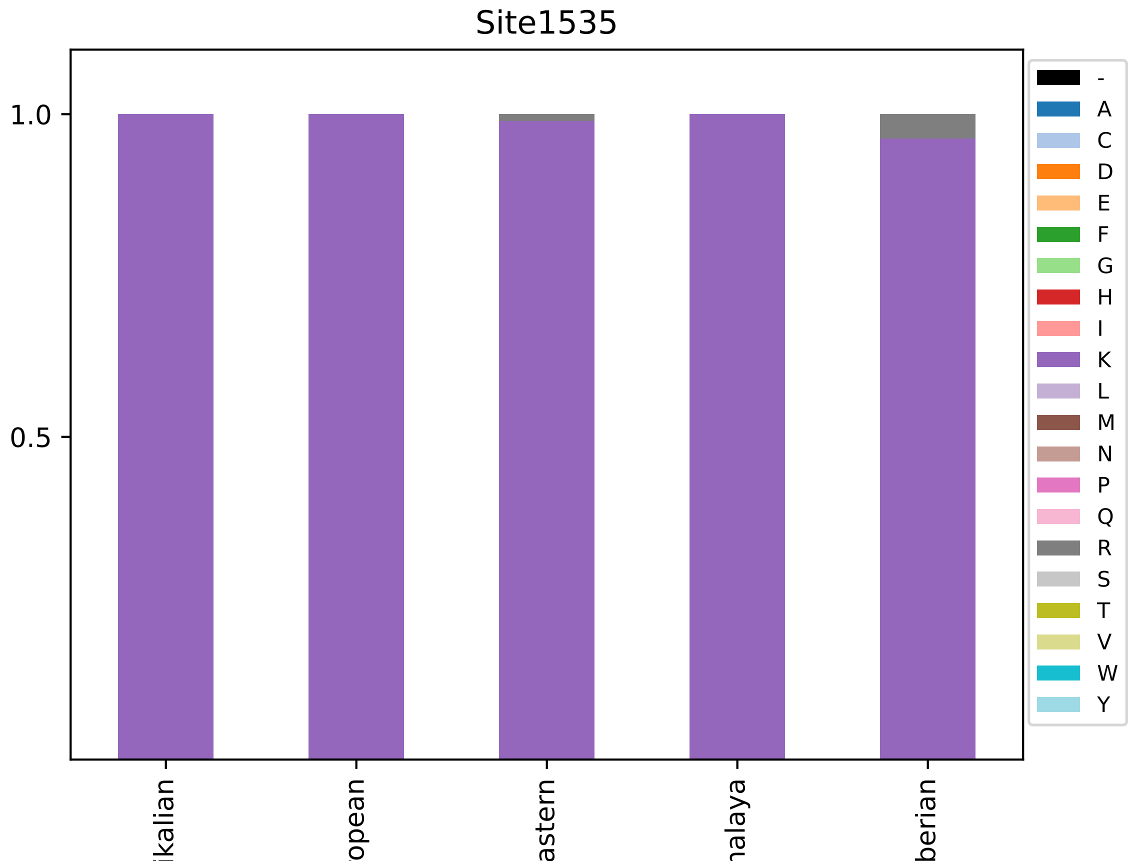
Site1535
1.0
0.5
-
A
C
D
E
F
G
H
I
K
L
M
N
P
Q
R
S
T
V
W
Y
ikalian
opean
astern
nalaya
berian

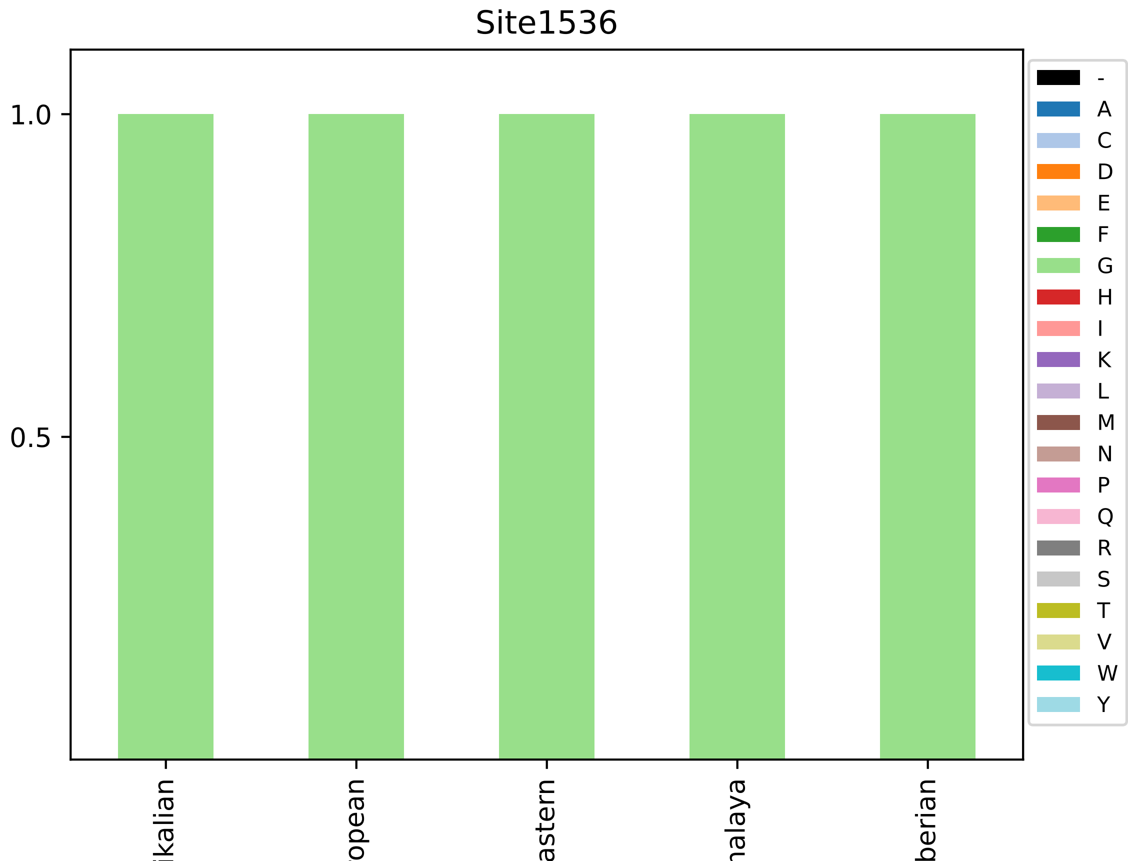

Site1536
1.0
0.5
-
A
C
D
E
F
G
H
I
K
L
M
N
P
Q
R
S
T
V
W
Y
ikalian
ropean
astern
nalaya
berian

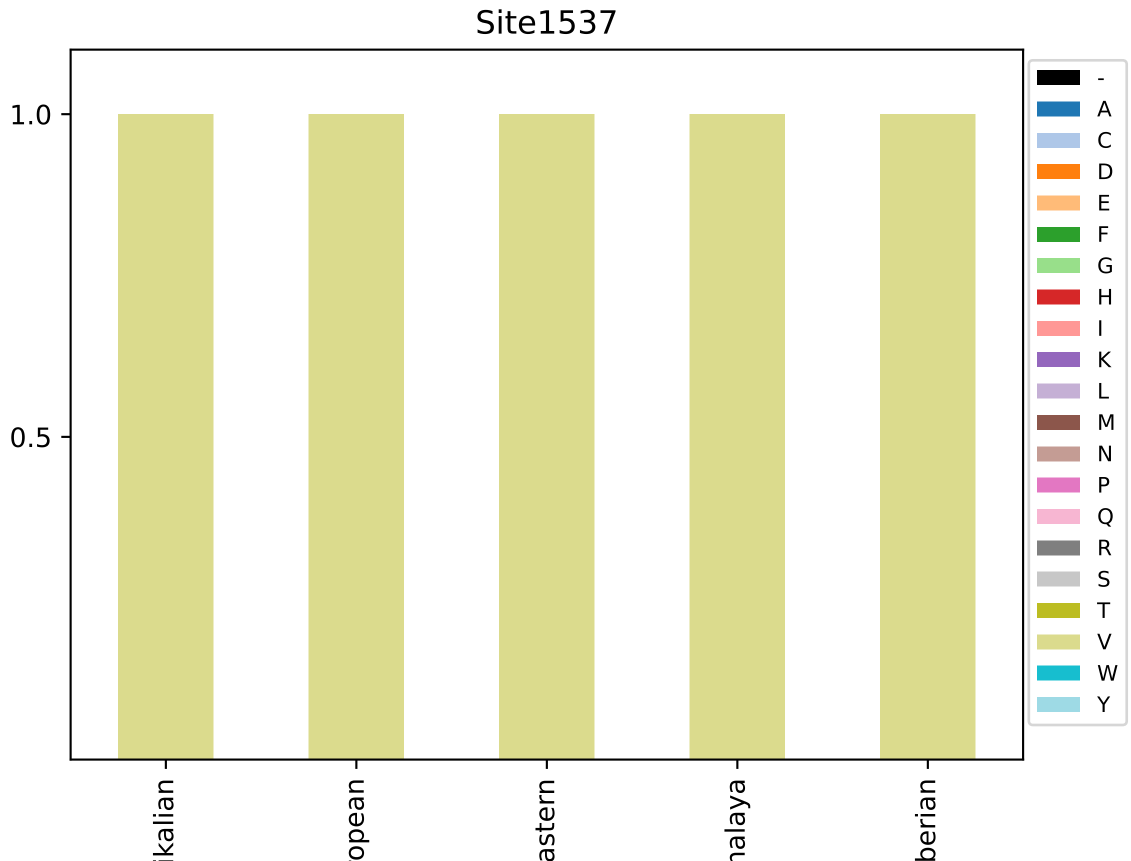

Site1537
1.0
0.5
-
A
C
D
E
F
G
H
I
K
L
M
N
P
Q
R
S
T
V
W
Y
ikalian
opean
astern
nalaya
berian

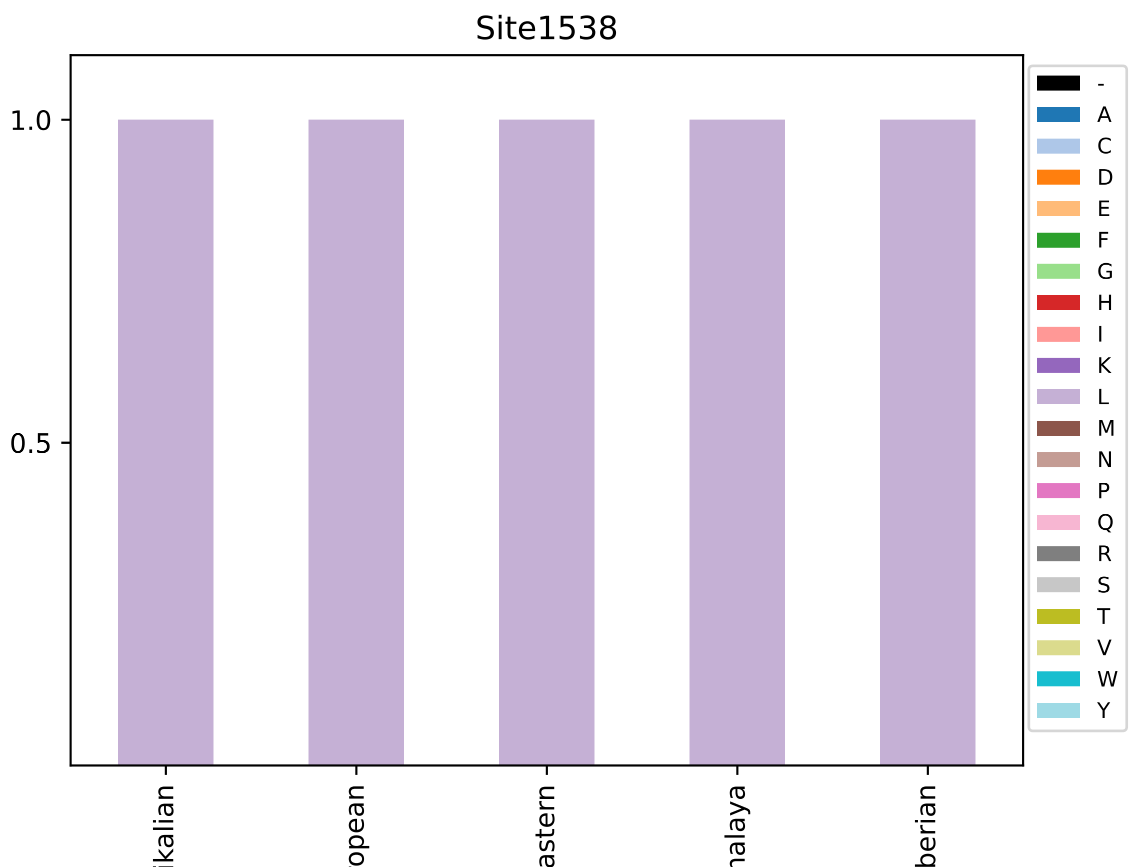
Site1538
1.0
0.5
-
A
C
D
E
F
G
H
I
K
L
M
N
P
Q
R
S
T
V
W
Y
ikalian
opean
astern
nalaya
berian

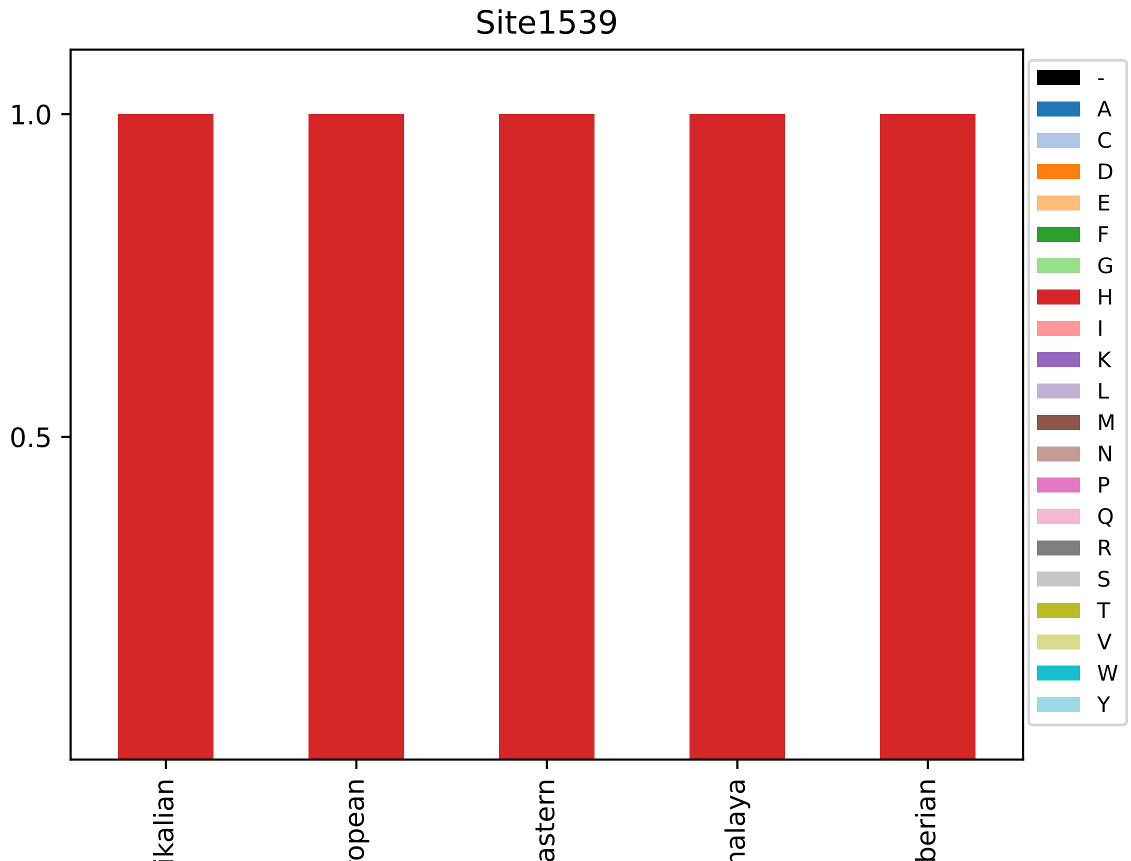
Site1539
1.0
0.5
-
A
C
D
E
F
G
H
I
K
L
M
N
P
Q
R
S
T
V
W
Y
ikalian
opean
astern
nalaya
berian

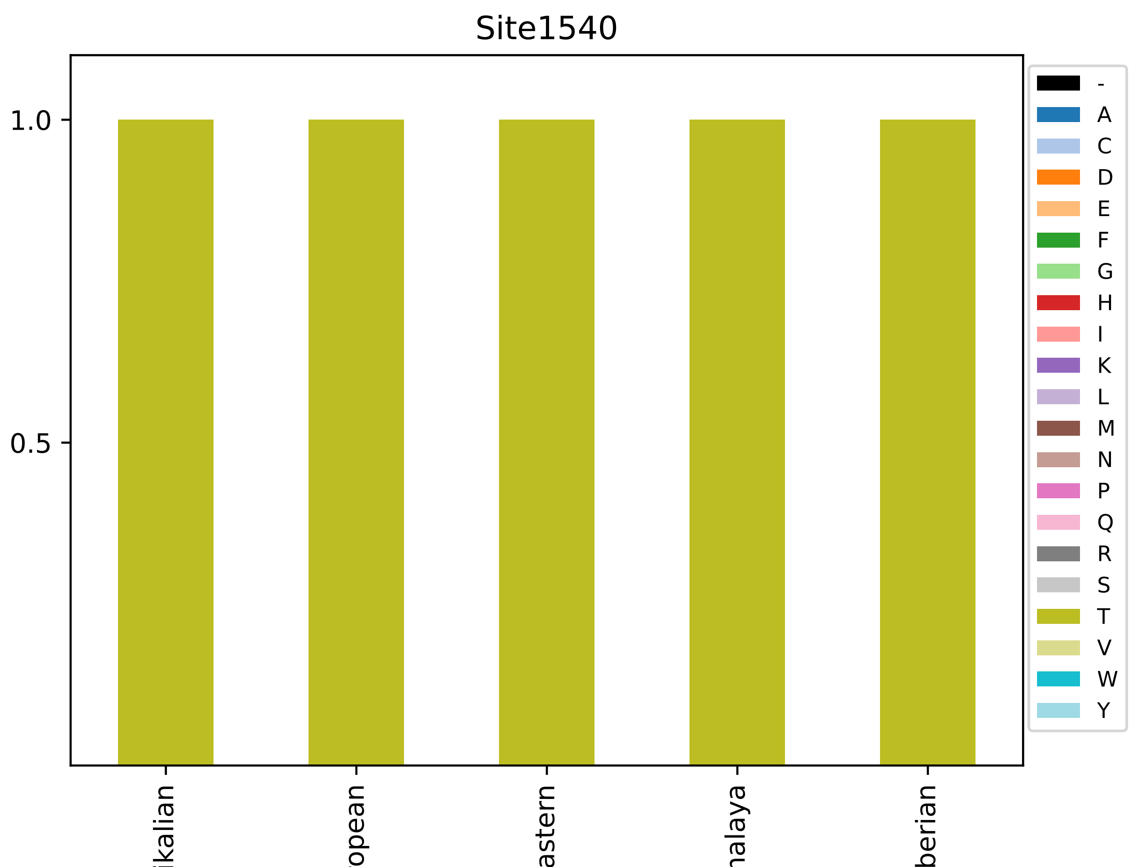

Site1540
1.0
0.5
-
A
C
D
E
F
G
H
I
K
L
M
N
P
Q
R
S
T
V
W
Y
ikalian
opean
astern
nalaya
berian

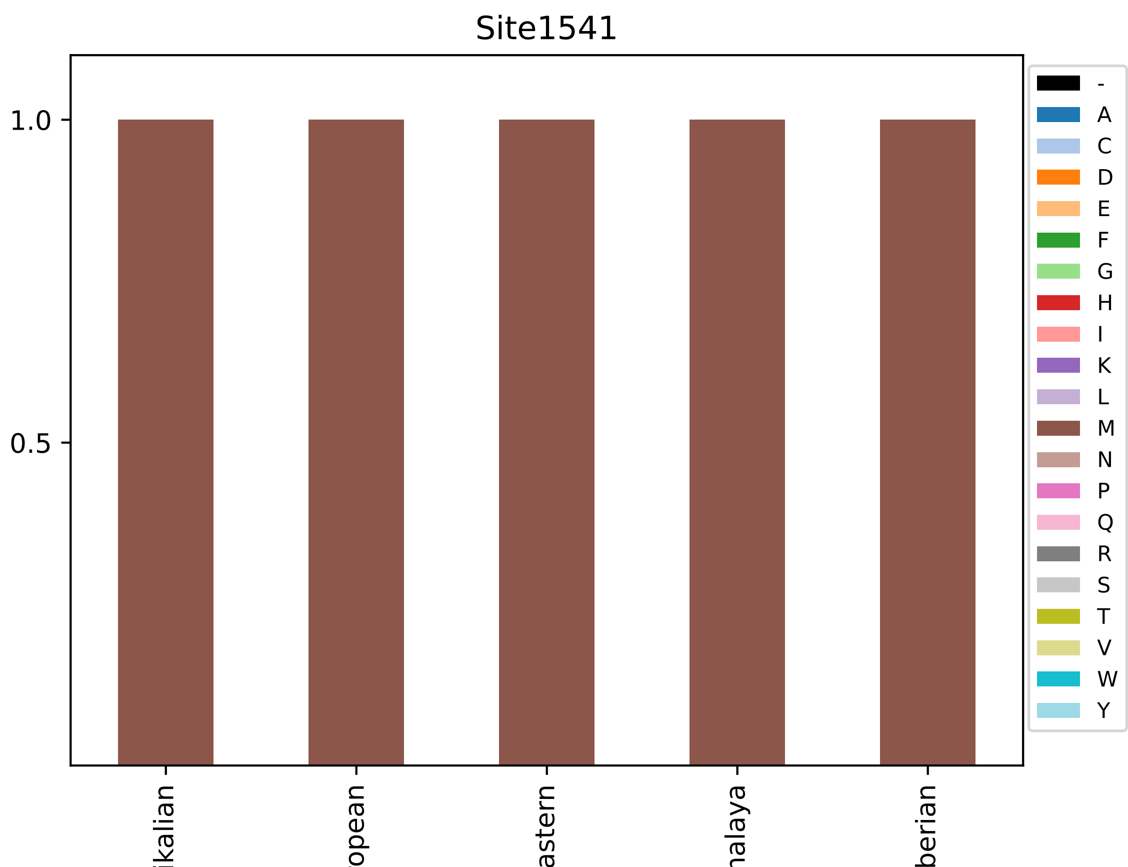

Site1541
1.0
0.5
ikalian
opean
astern
nalaya
berian
-
A
C
D
E
F
G
H
I
K
L
M
N
P
Q
R
S
T
V
W
Y

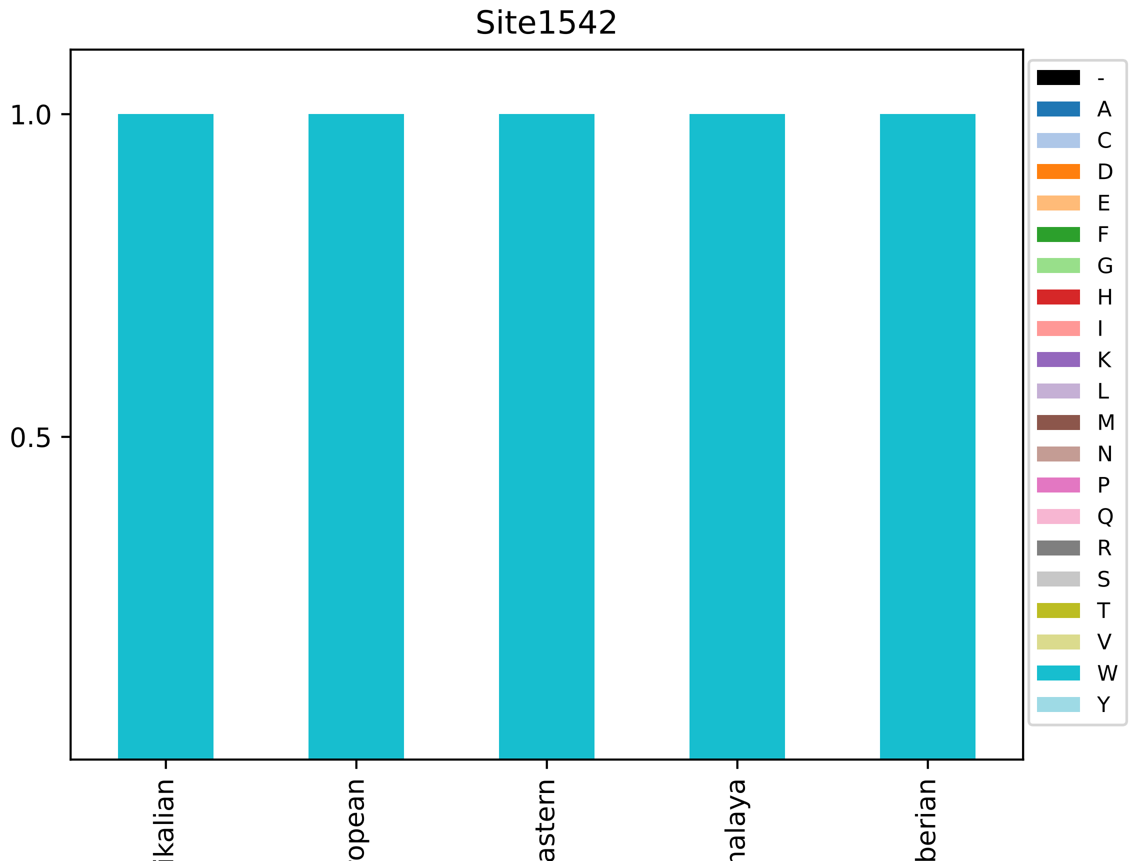
Site1542
1.0
0.5
ikalian
opean
astern
nalaya
berian
-
A
C
D
E
F
G
H
I
K
L
M
N
P
Q
R
S
T
V
W
Y

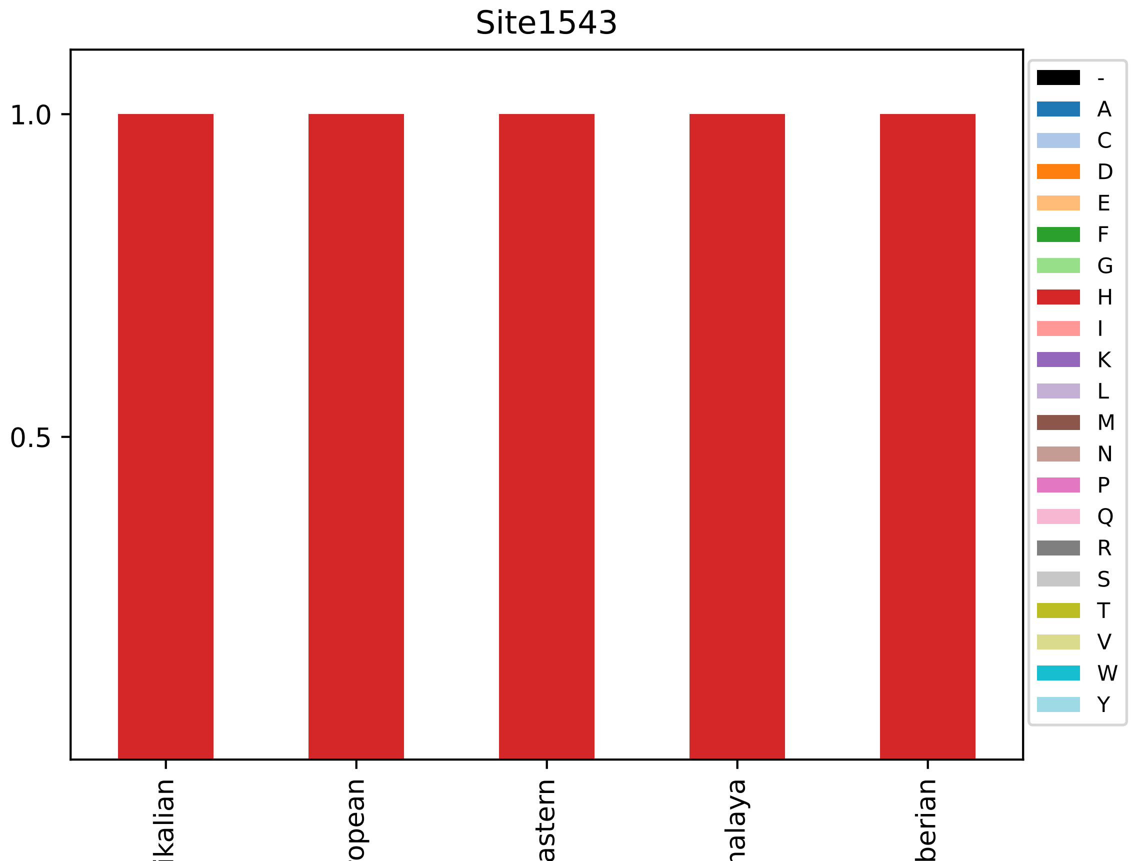

Site1543
1.0
0.5
-
A
C
D
E
F
G
H
I
K
L
M
N
P
Q
R
S
T
V
W
Y
ikalian
opean
astern
nalaya
berian

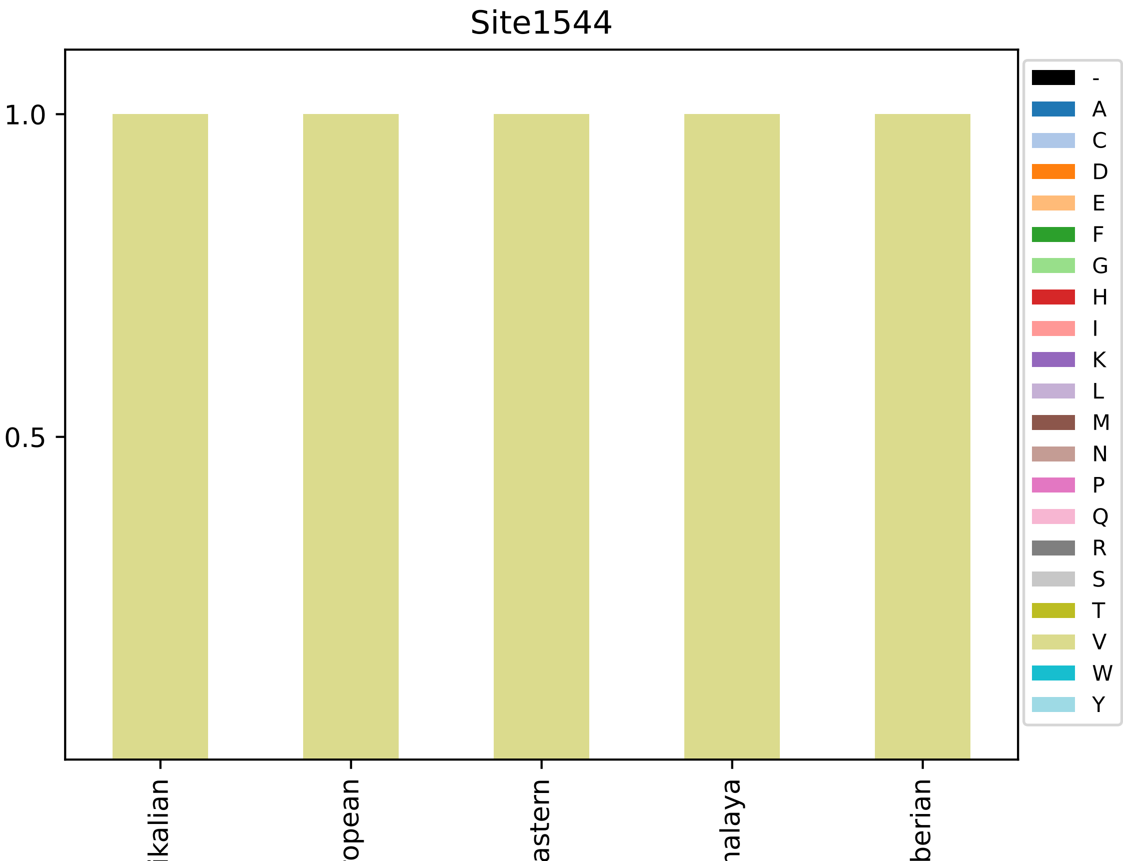
Site1544
1.0
0.5
-
A
C
D
E
F
G
H
I
K
L
M
N
P
Q
R
S
T
V
W
Y
ikalian
opean
astern
nalaya
berian

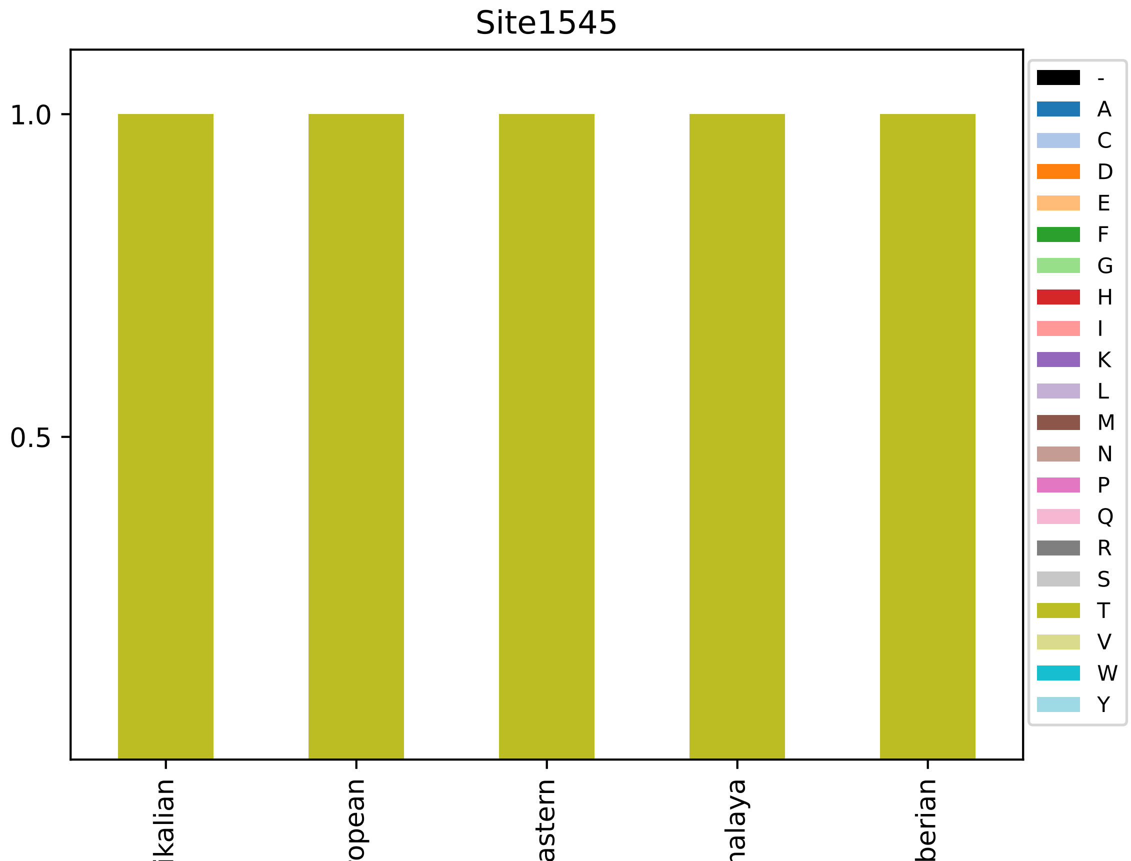

Site1545
1.0
0.5
-
A
C
D
E
F
G
H
I
K
L
M
N
P
Q
R
S
T
V
W
Y
ikalian
opean
astern
nalaya
berian

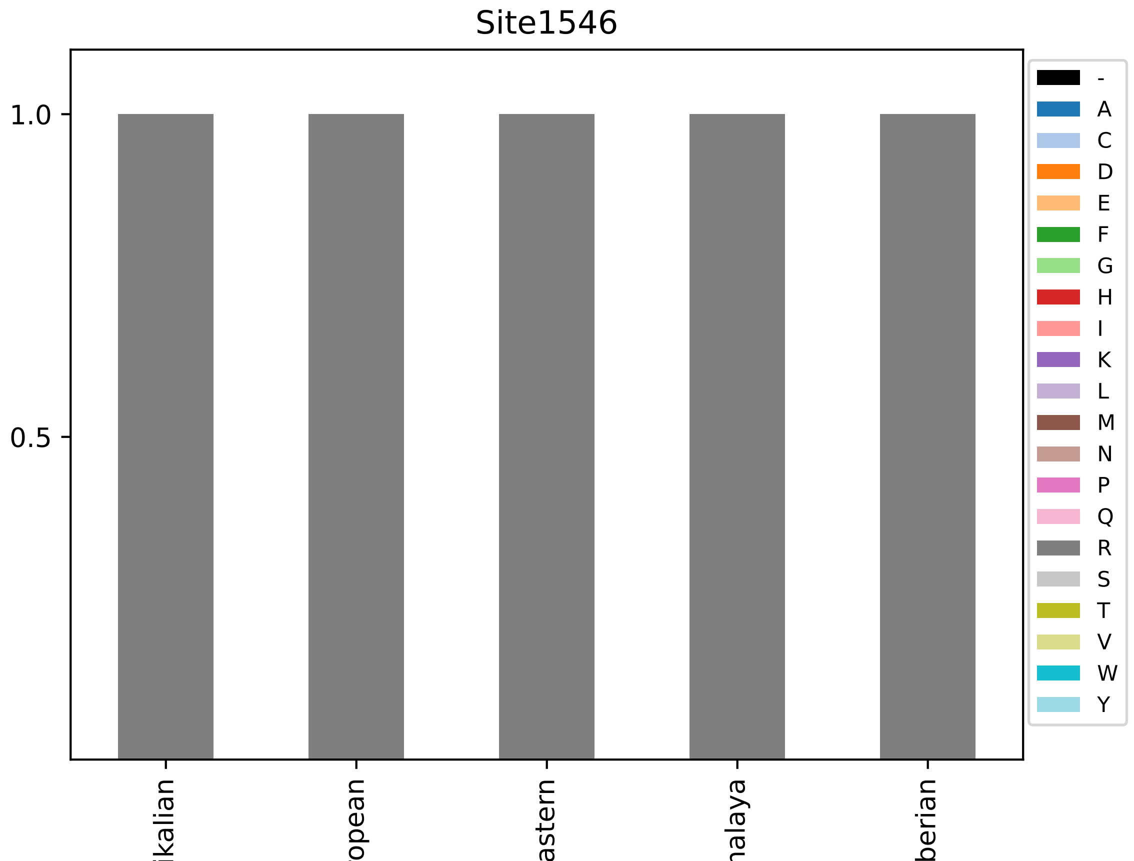

Site1546
1.0
0.5
-
A
C
D
E
F
G
H
I
K
L
M
N
P
Q
R
S
T
V
W
Y
ikalian
opean
astern
nalaya
berian

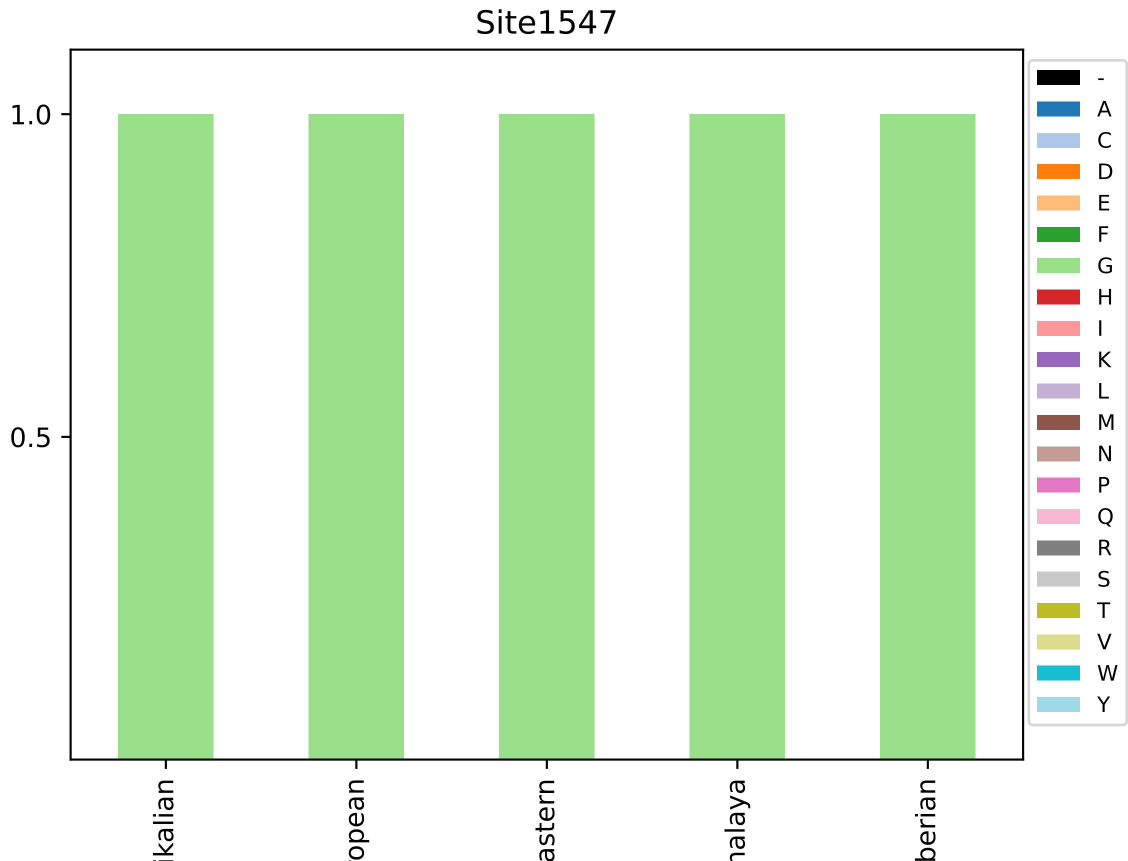

Site1547
1.0
0.5
-
A
C
D
E
F
G
H
I
K
L
M
N
P
Q
R
S
T
V
W
Y
ikalian
opean
astern
nalaya
berian

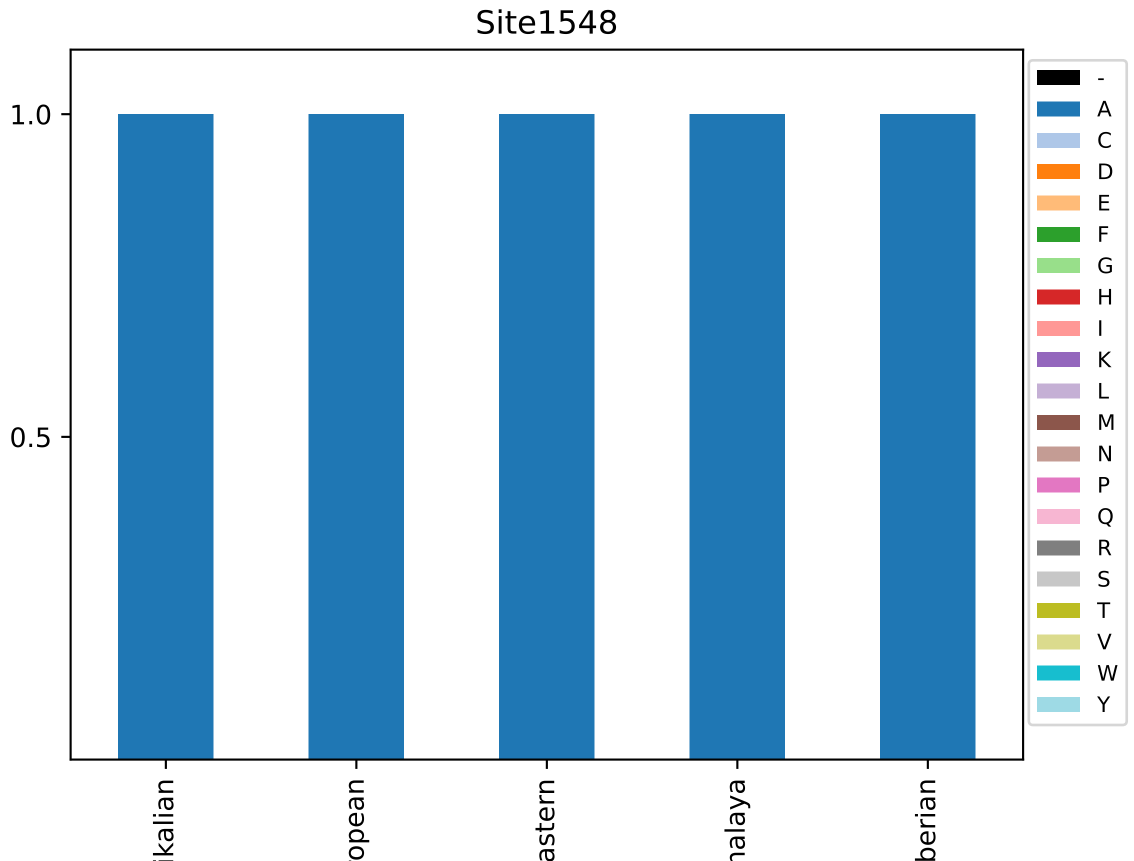

Site1548
1.0
0.5
ikalian
opean
astern
nalaya
berian
-
A
C
D
E
F
G
H
I
K
L
M
N
P
Q
R
S
T
V
W
Y

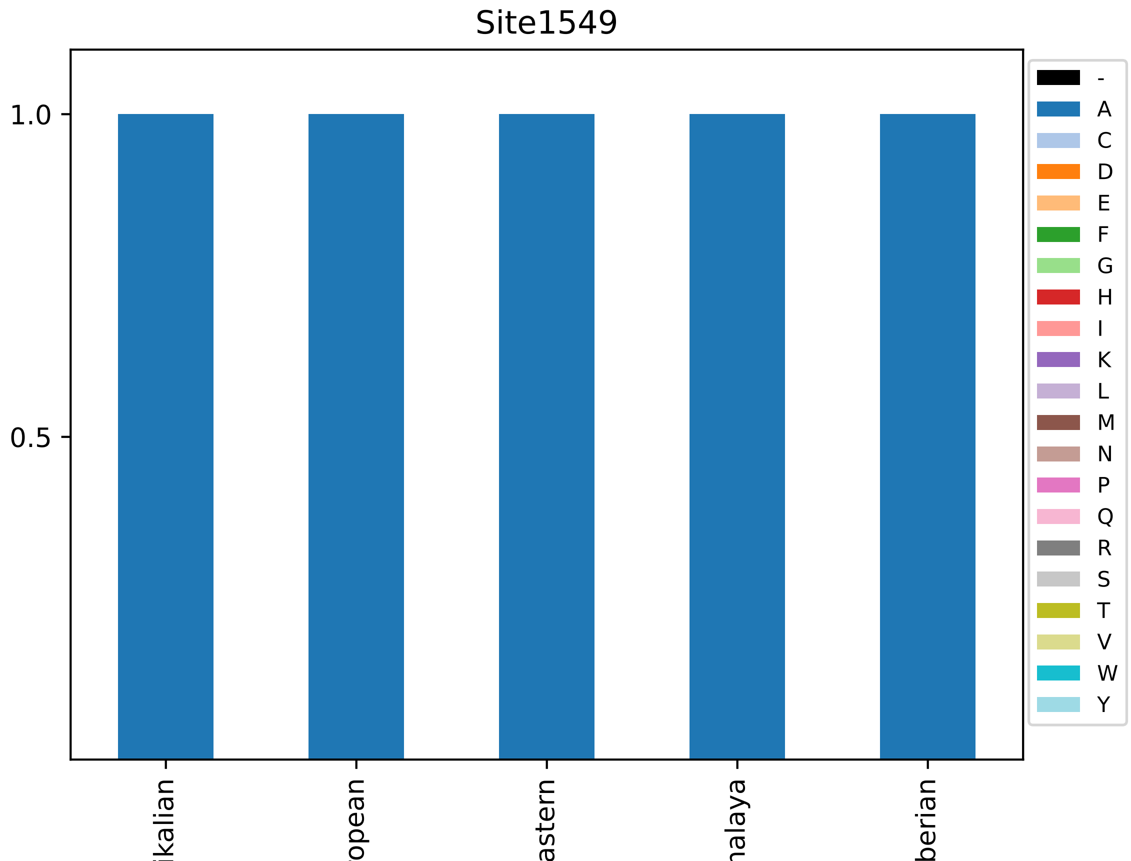

Site1549
1.0
0.5
-
A
C
D
E
F
G
H
I
K
L
M
N
P
Q
R
S
T
V
W
Y
ikalian
opean
astern
nalaya
berian

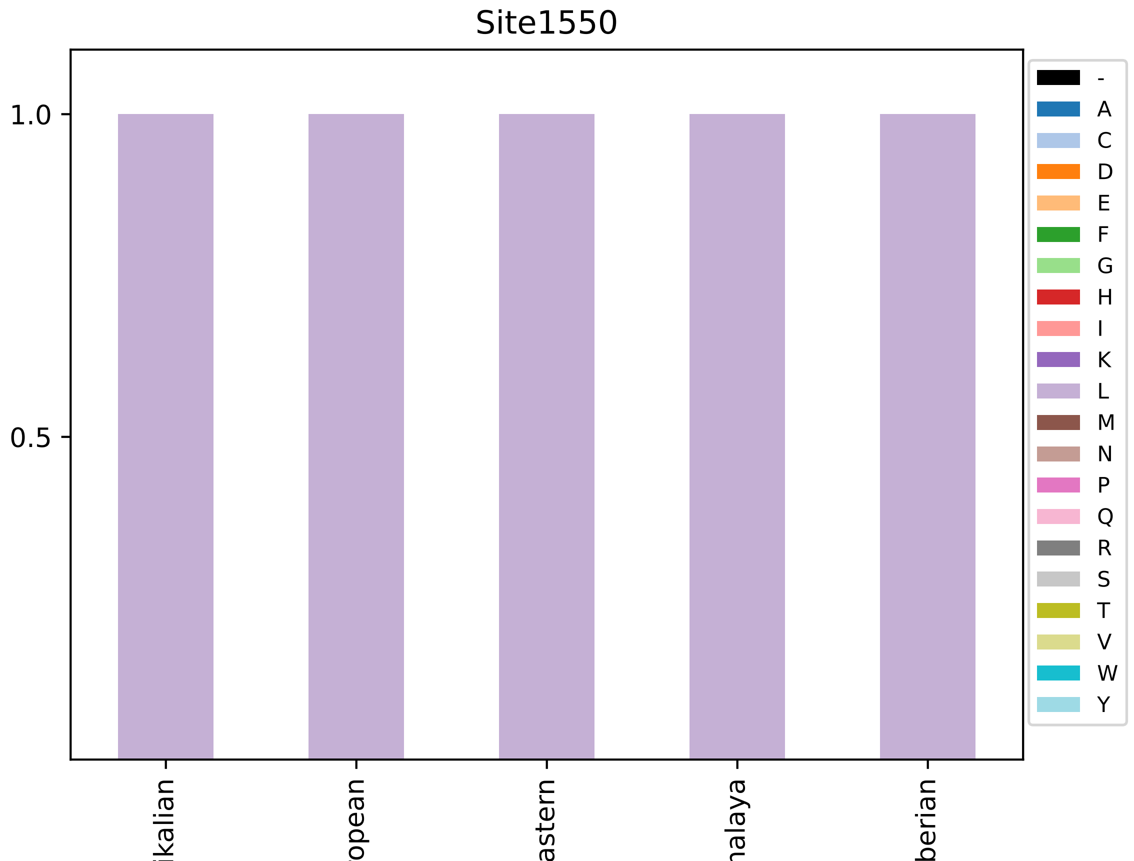
Site1550
1.0
0.5
ikalian
ropean
astern
nalaya
berian
-
A
C
D
E
F
G
H
I
K
L
M
N
P
Q
R
S
T
V
W
Y

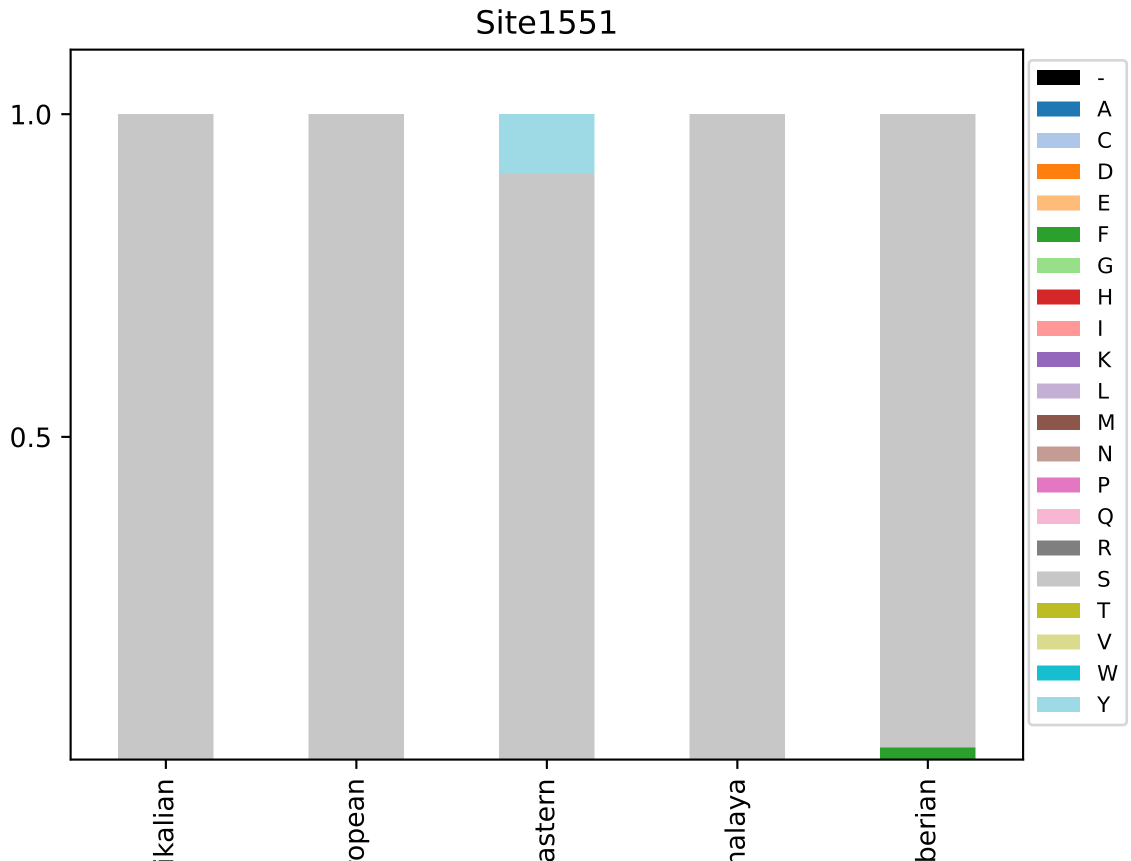
Site1551
1.0
0.5
-
A
C
D
E
F
G
H
I
K
L
M
N
P
Q
R
S
T
V
W
Y
ikalian
opean
astern
nalaya
berian

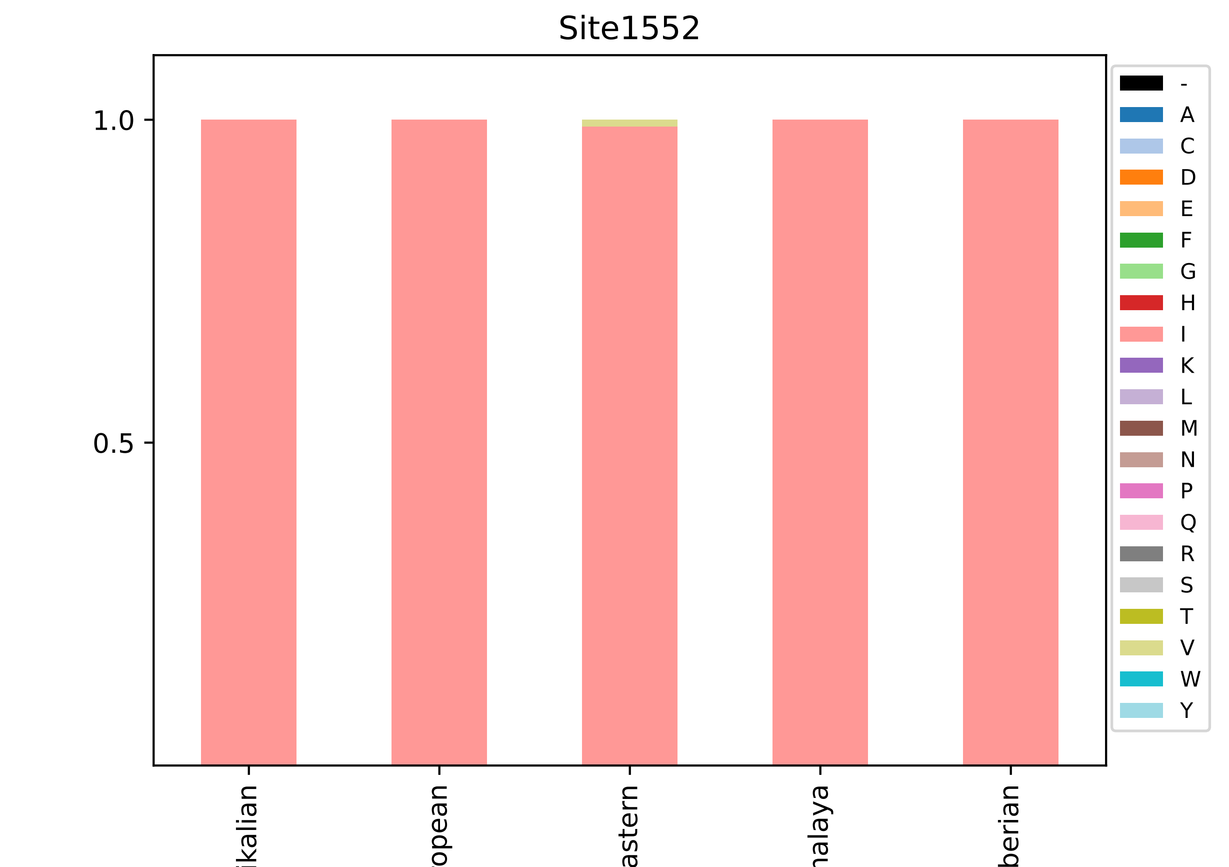
Site1552
1.0
0.5
ikalian
opean
astern
nalaya
berian
-
A
C
D
E
F
G
H
I
K
L
M
N
P
Q
R
S
T
V
W
Y

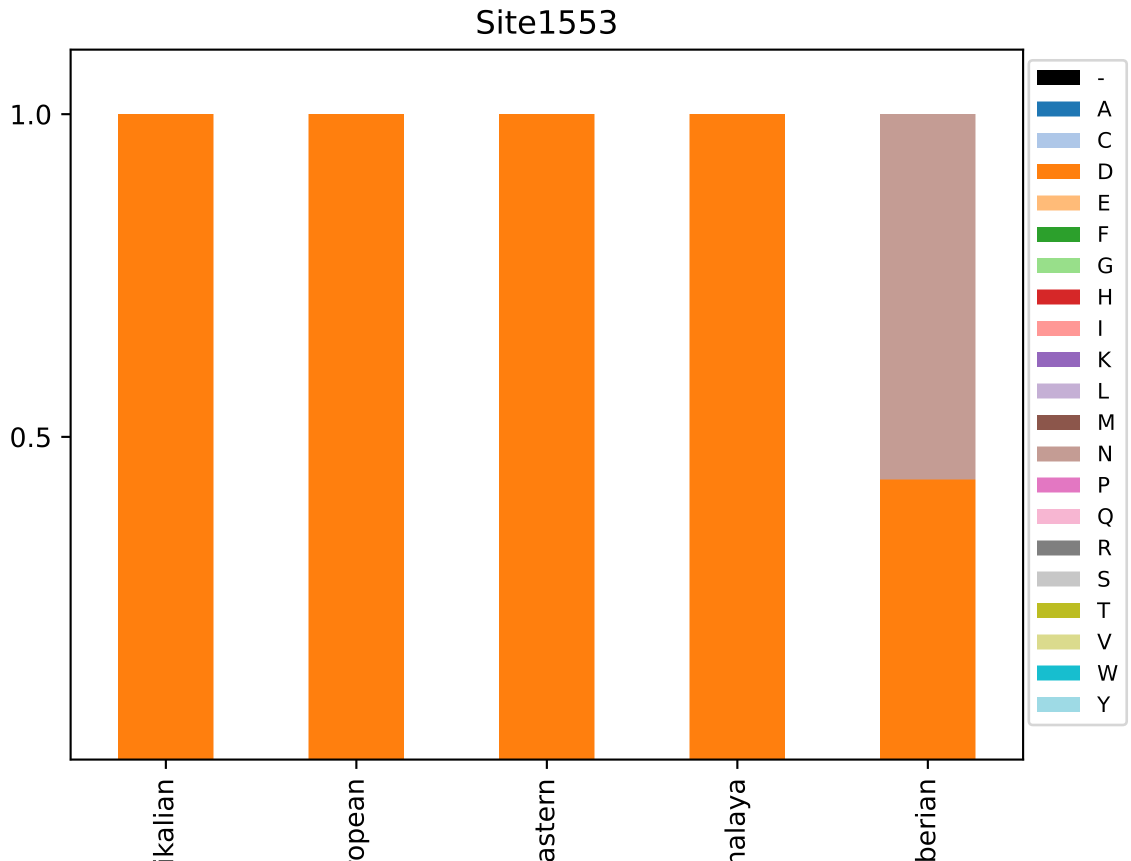
Site1553
1.0
0.5
-
A
C
D
E
F
G
H
I
K
L
M
N
P
Q
R
S
T
V
W
Y
ikalian
ropean
astern
nalaya
berian

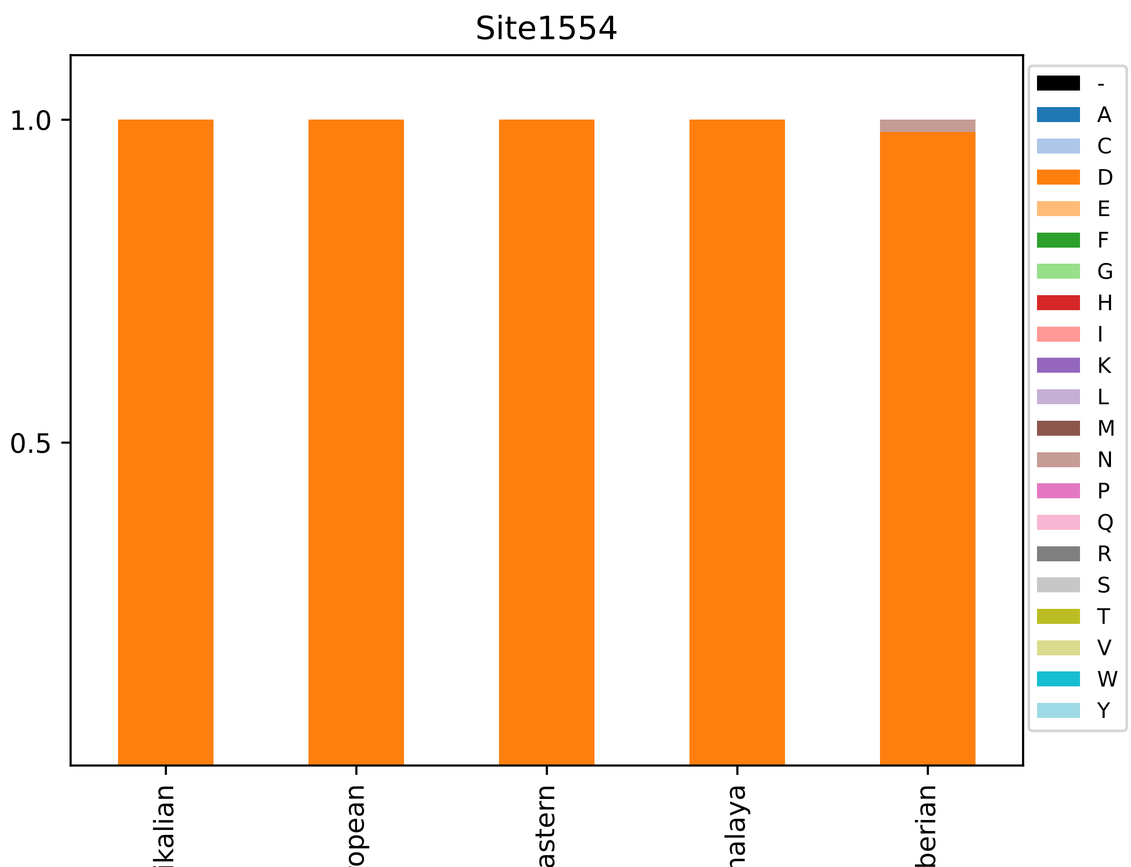

Site1554
1.0
0.5
-
A
C
D
E
F
G
H
I
K
L
M
N
P
Q
R
S
T
V
W
Y
ikalian
opean
astern
nalaya
berian

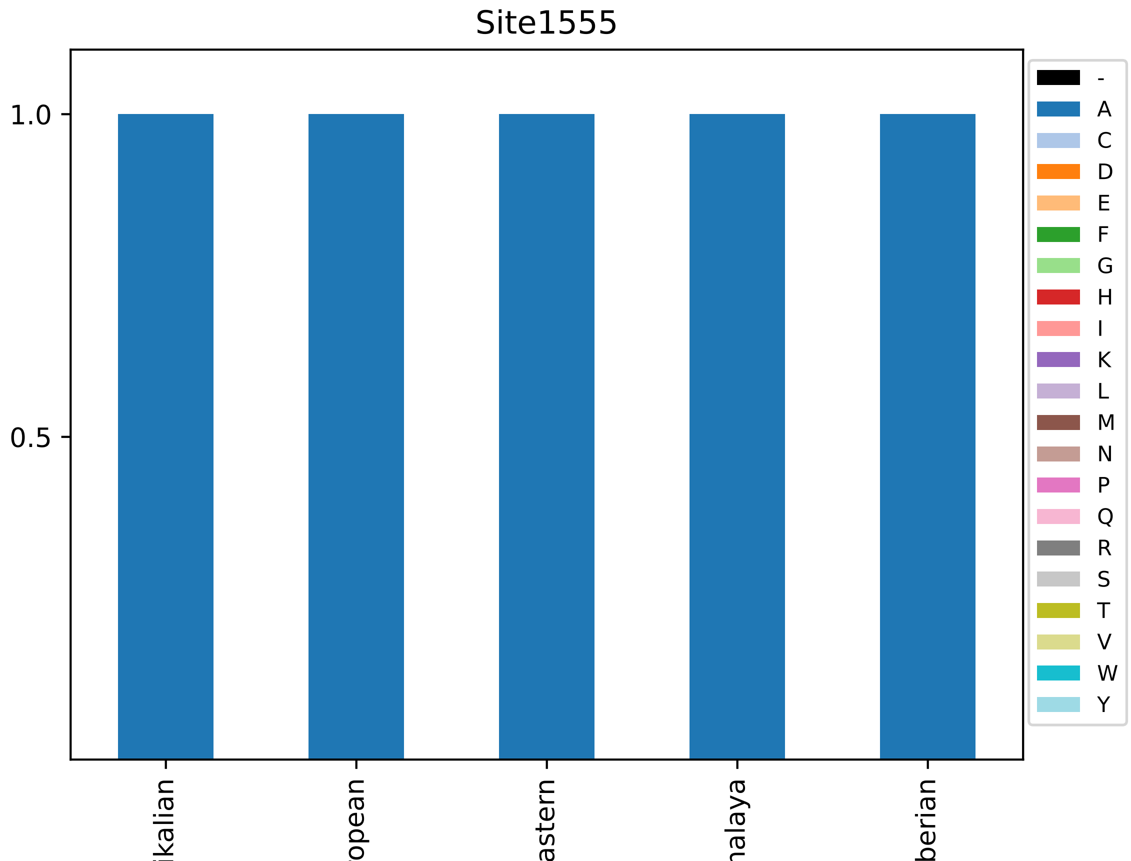
Site1555
1.0
0.5
-
A
C
D
E
F
G
H
I
K
L
M
N
P
Q
R
S
T
V
W
Y

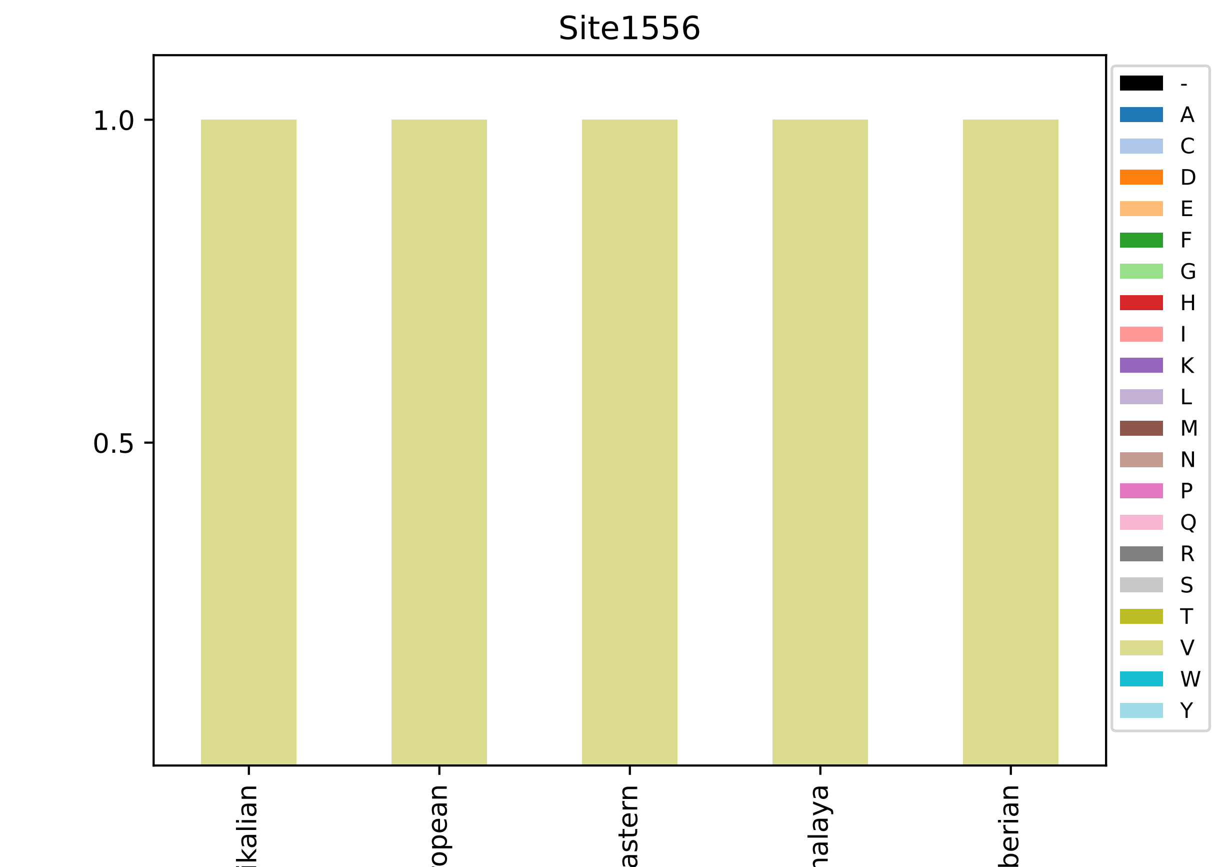

Site1556
1.0
0.5
-
A
C
D
E
F
G
H
I
K
L
M
N
P
Q
R
S
T
V
W
Y

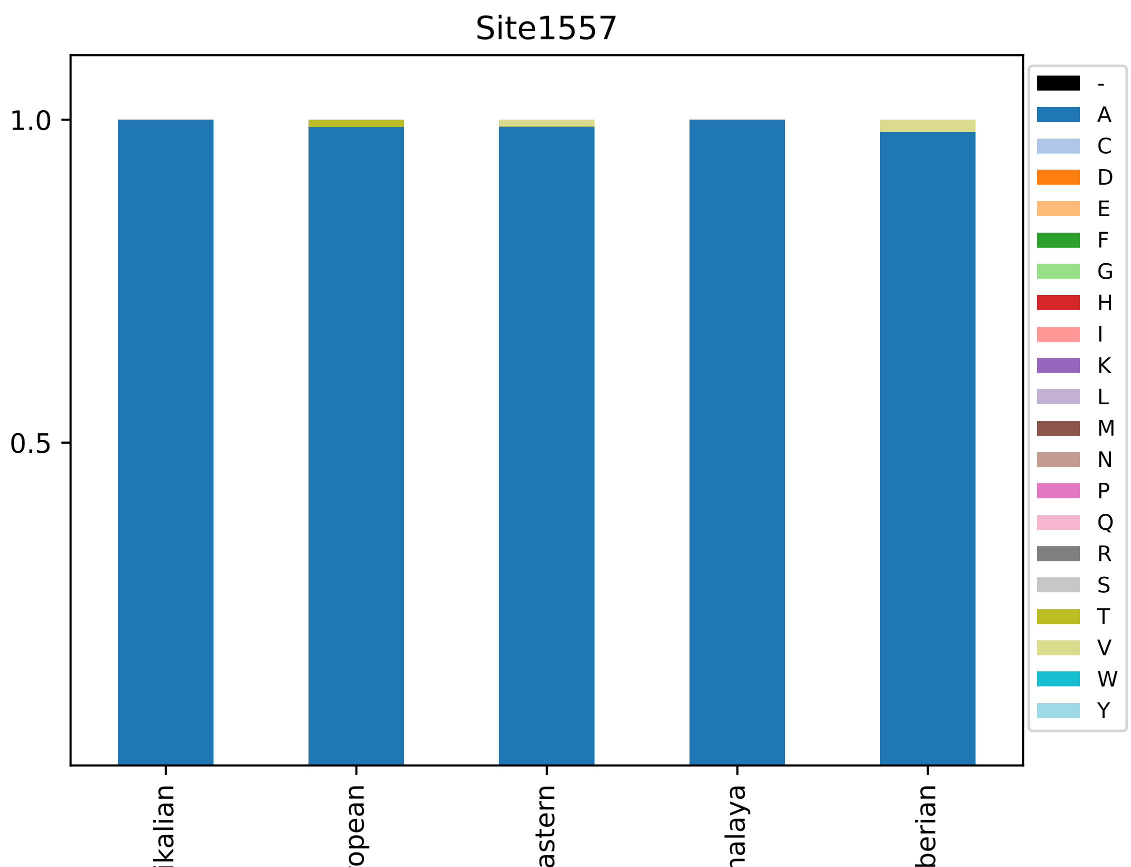

Site1557
1.0
0.5
ikalian
opean
astern
nalaya
berian
-
A
C
D
E
F
G
H
I
K
L
M
N
P
Q
R
S
T
V
W
Y

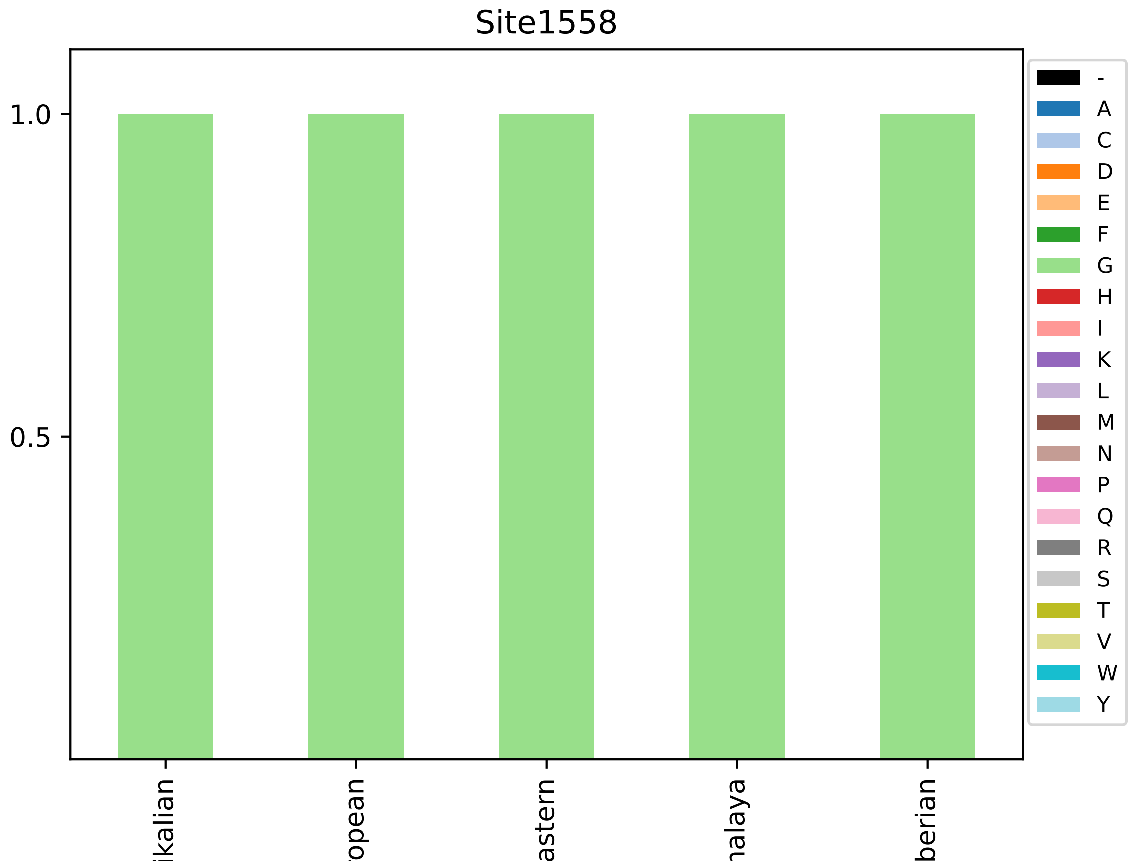
Site1558
1.0
0.5
-
A
C
D
E
F
G
H
I
K
L
M
N
P
Q
R
S
T
V
W
Y
ikalian
opean
astern
nalaya
berian

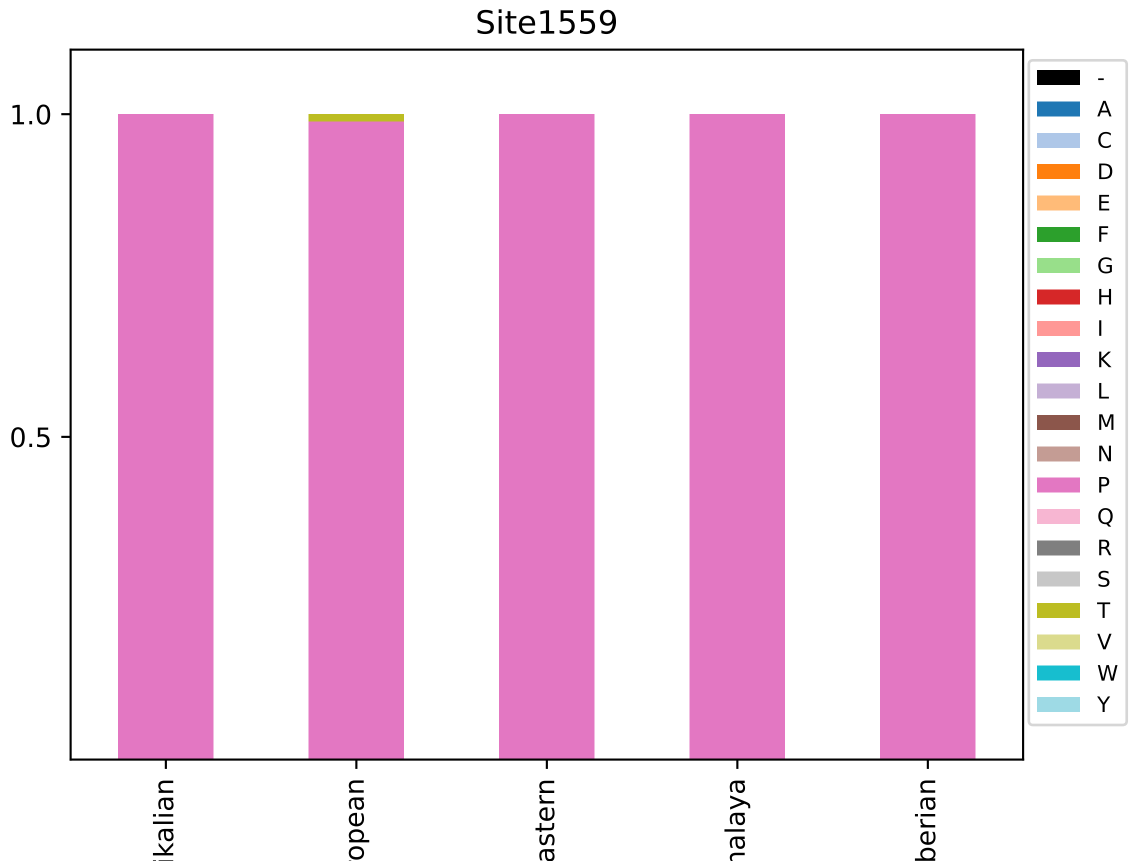

Site1559
1.0
0.5
ikalian
opean
astern
nalaya
berian
-
A
C
D
E
F
G
H
I
K
L
M
N
P
Q
R
S
T
V
W
Y

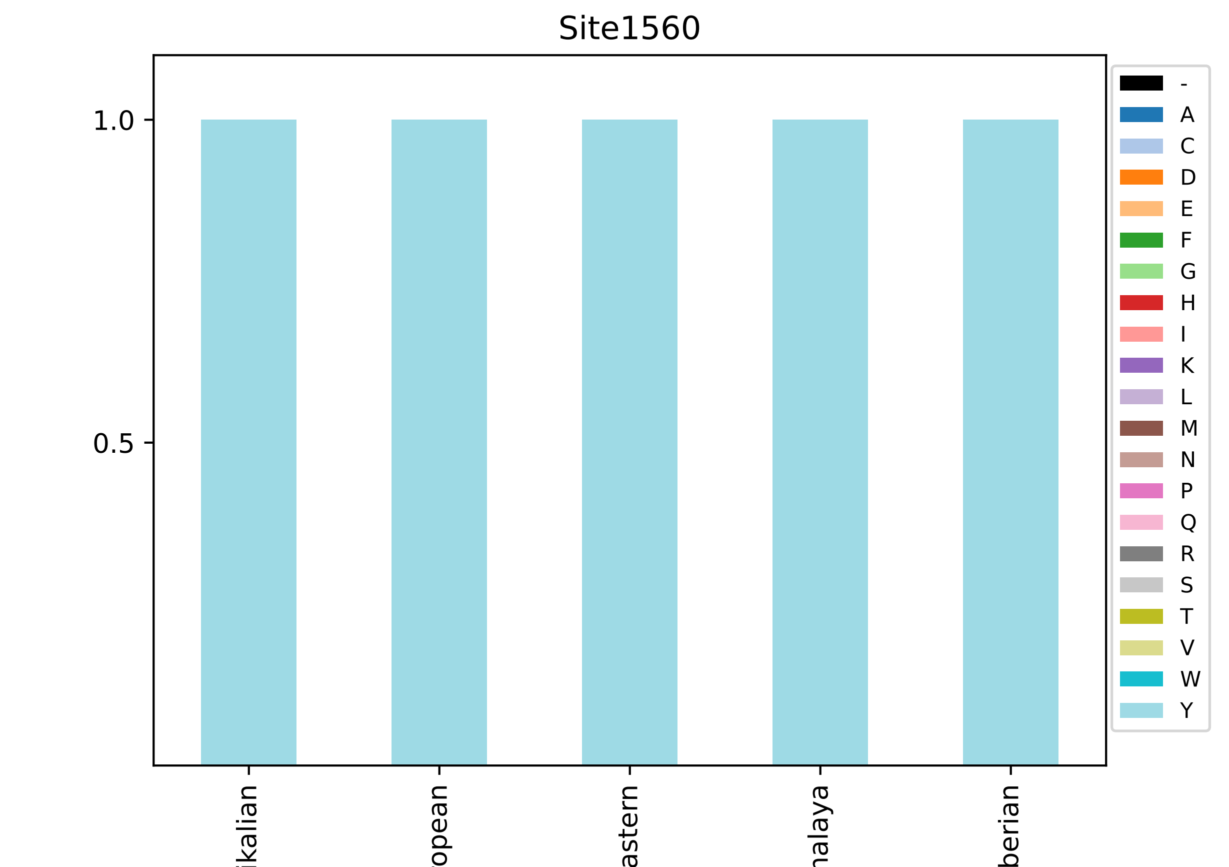
Site1560
1.0
0.5
-
A
C
D
E
F
G
H
I
K
L
M
N
P
Q
R
S
T
V
W
Y
ikalian
opean
astern
nalaya
berian

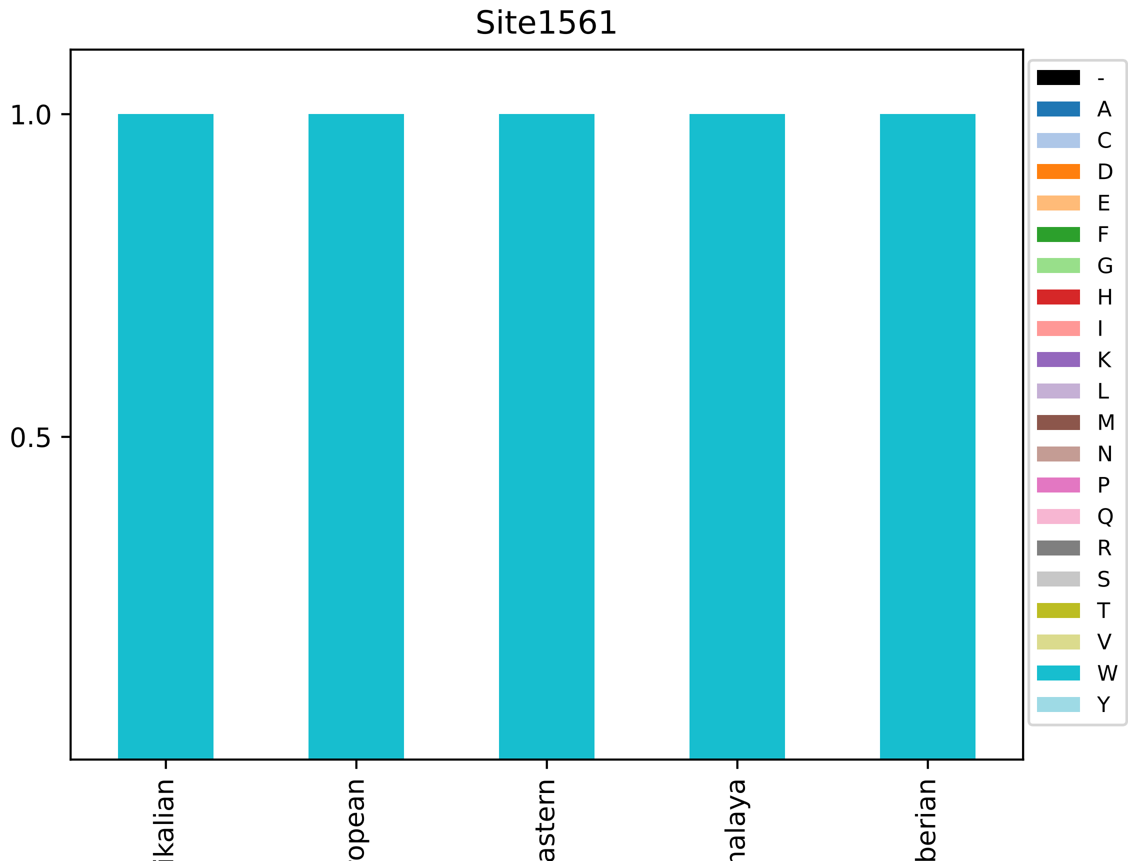

Site1561
1.0
0.5
-
A
C
D
E
F
G
H
I
K
L
M
N
P
Q
R
S
T
V
W
Y
ikalian
opean
astern
nalaya
berian

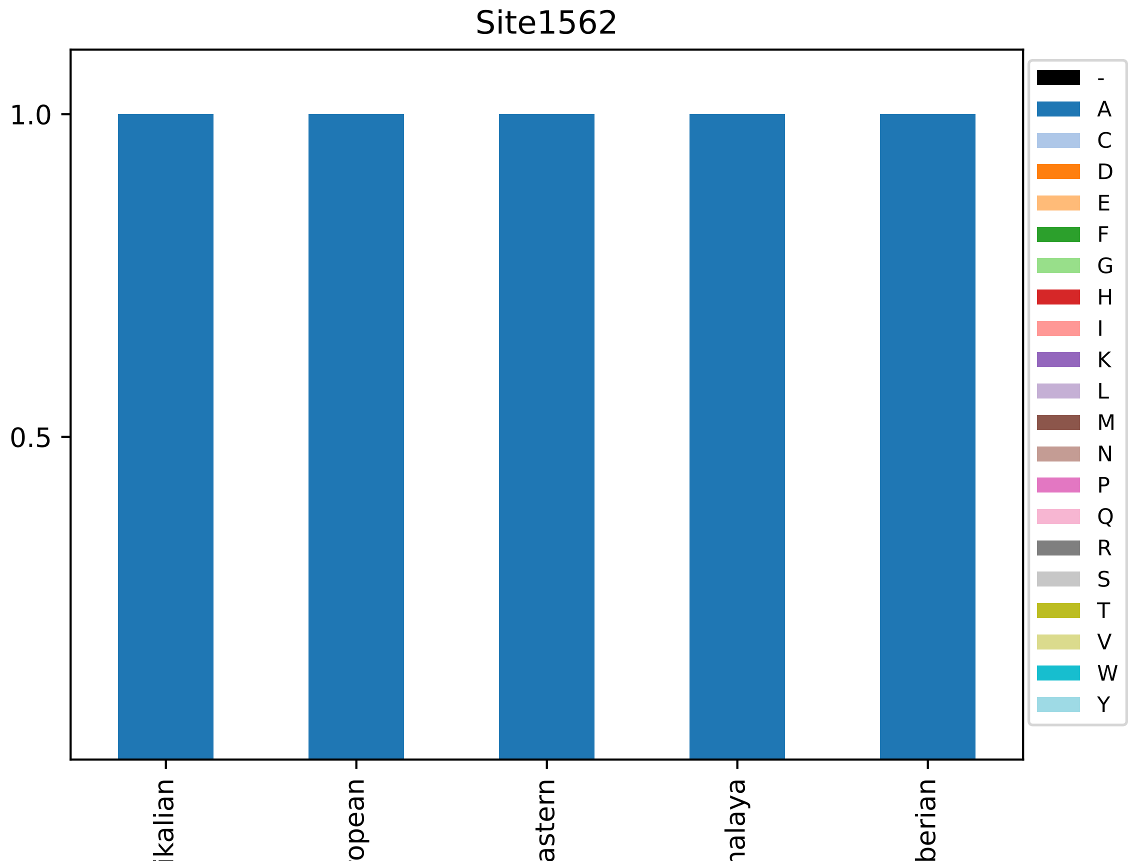

Site1562
1.0
0.5
-
A
C
D
E
F
G
H
I
K
L
M
N
P
Q
R
S
T
V
W
Y
ikalian
opean
astern
nalaya
berian

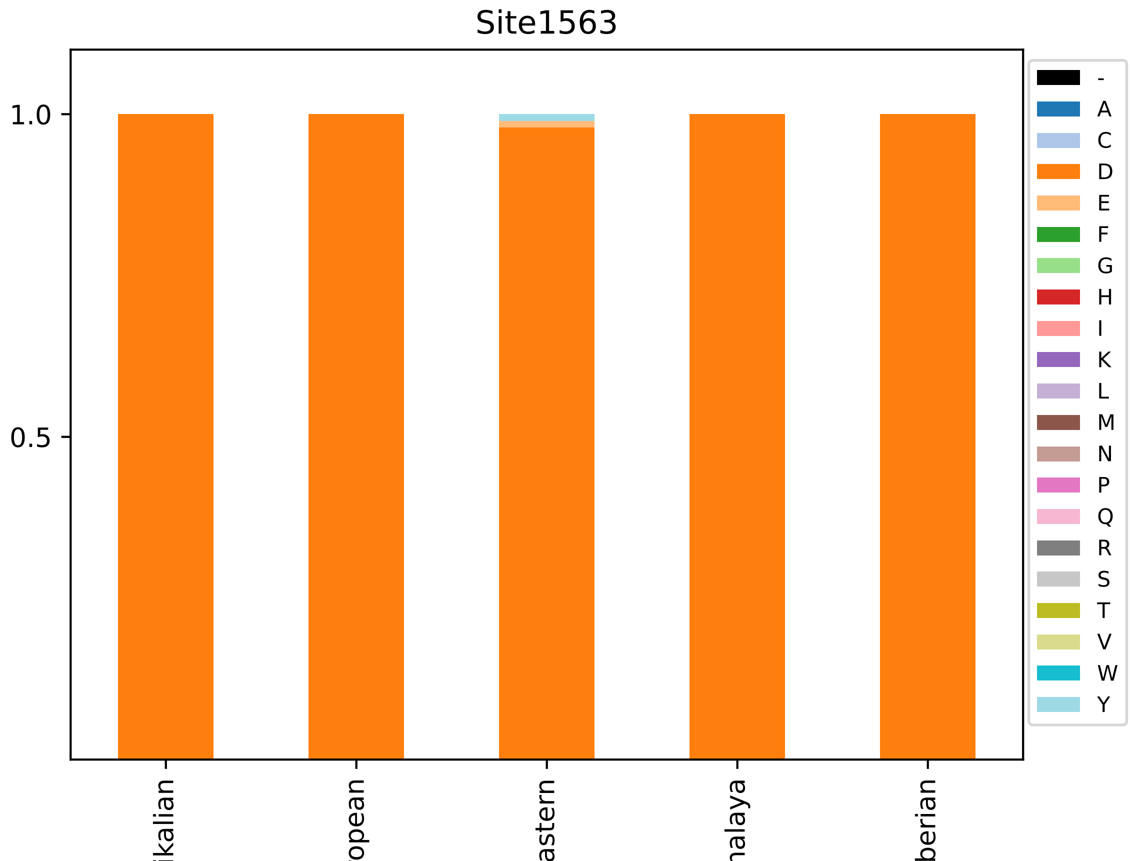

Site1563
1.0
0.5
-
A
C
D
E
F
G
H
I
K
L
M
N
P
Q
R
S
T
V
W
Y
ikalian
opean
astern
nalaya
berian

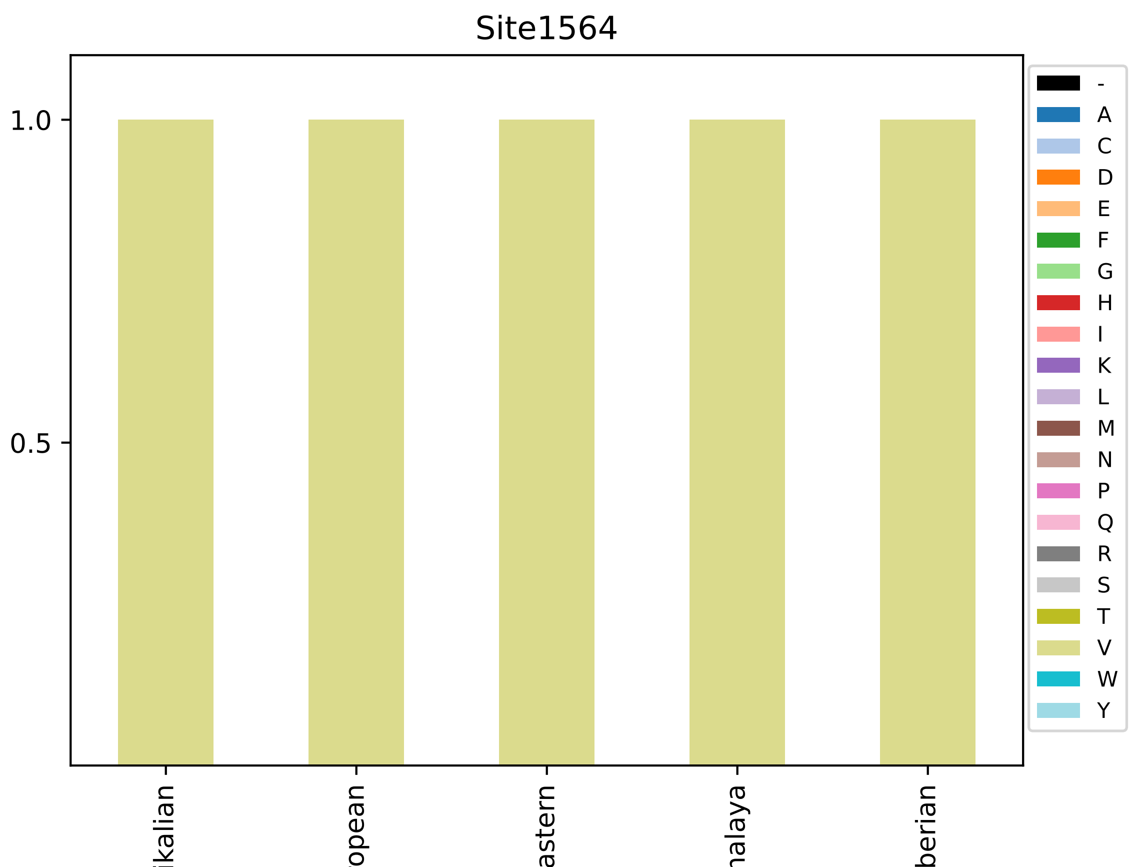

Site1564
1.0
0.5
-
A
C
D
E
F
G
H
I
K
L
M
N
P
Q
R
S
T
V
W
Y
ikalian
opean
astern
nalaya
berian

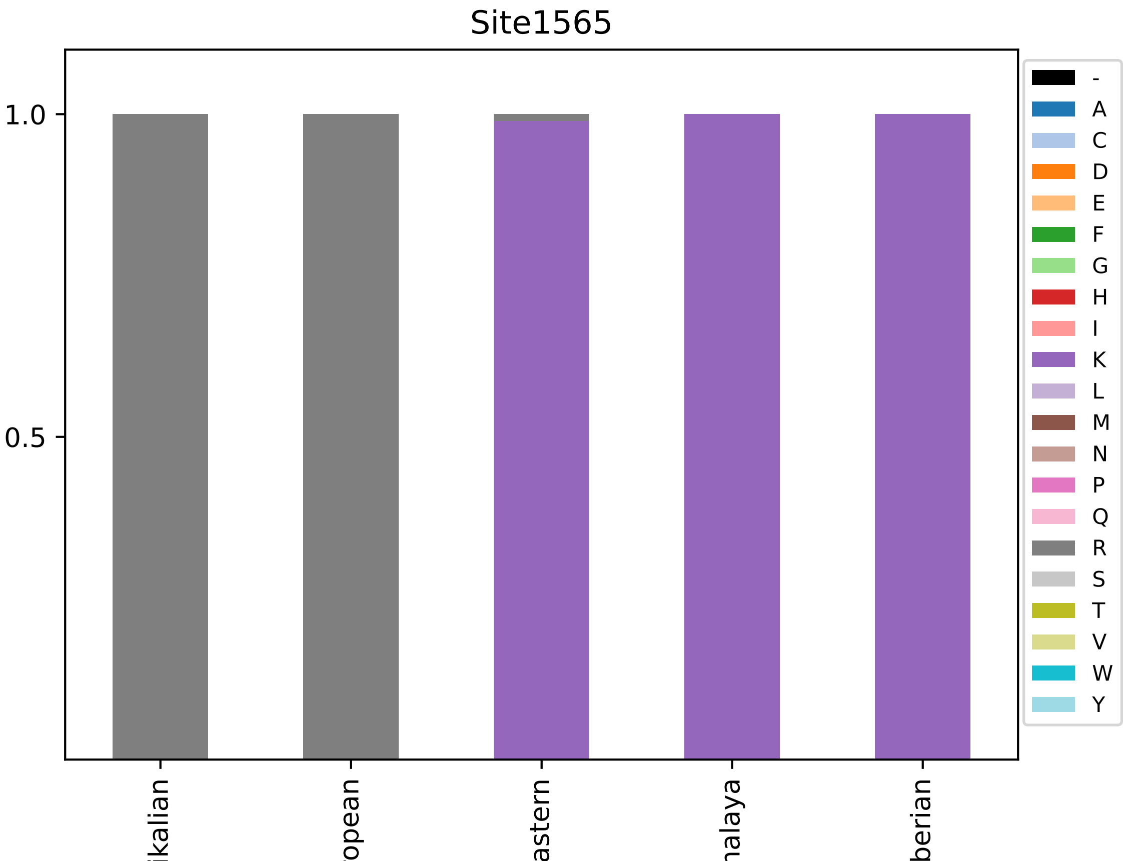
Site1565
1.0
0.5
-
A
C
D
E
F
G
H
I
K
L
M
N
P
Q
R
S
T
V
W
Y
ikalian
opean
astern
nalaya
berian

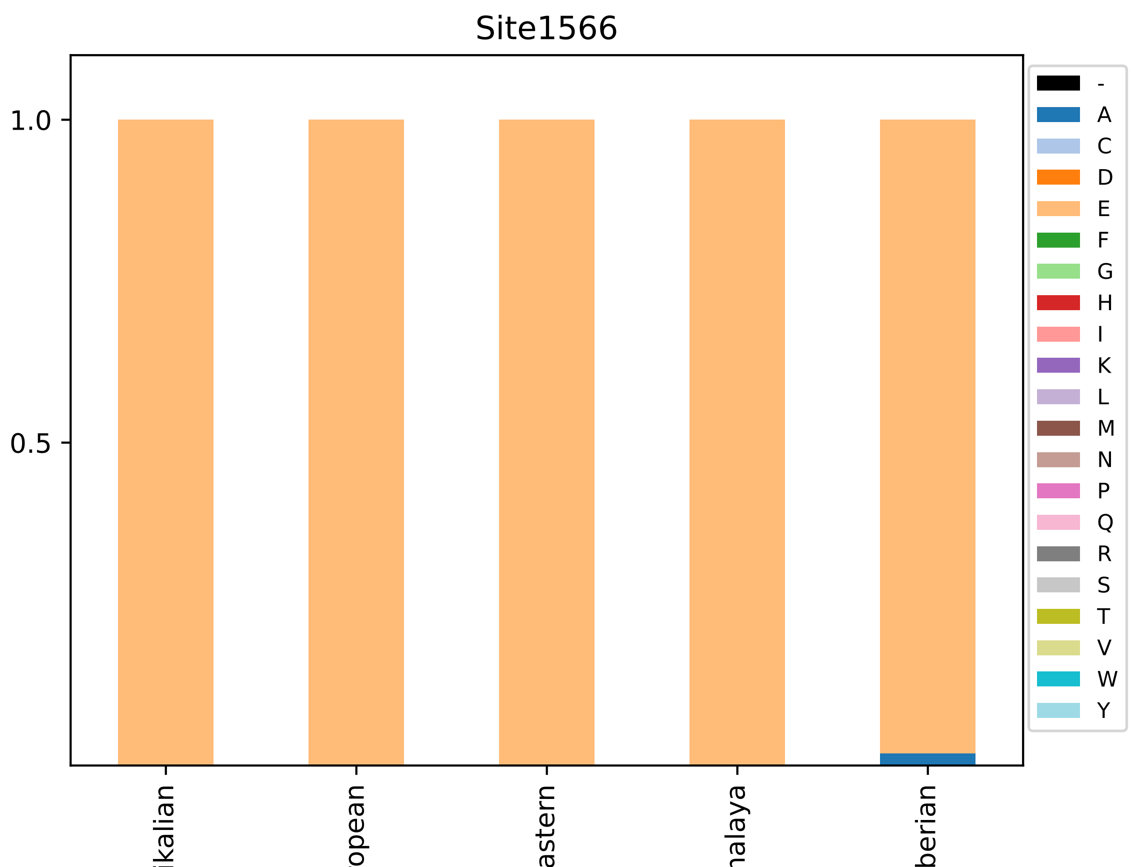
Site1566
1.0
0.5
-
A
C
D
E
F
G
H
I
K
L
M
N
P
Q
R
S
T
V
W
Y
ikalian
opean
astern
nalaya
berian

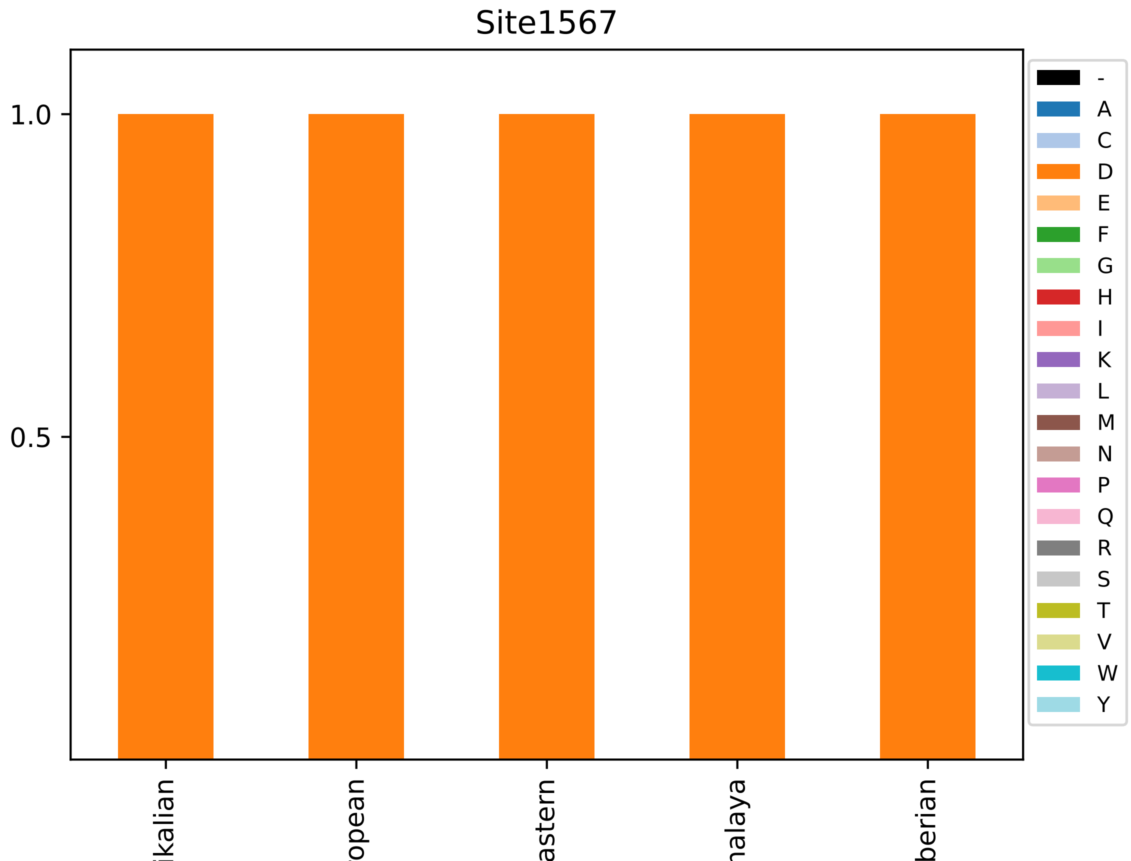
Site1567
1.0
0.5
-
A
C
D
E
F
G
H
I
K
L
M
N
P
Q
R
S
T
V
W
Y
ikalian
opean
astern
nalaya
berian

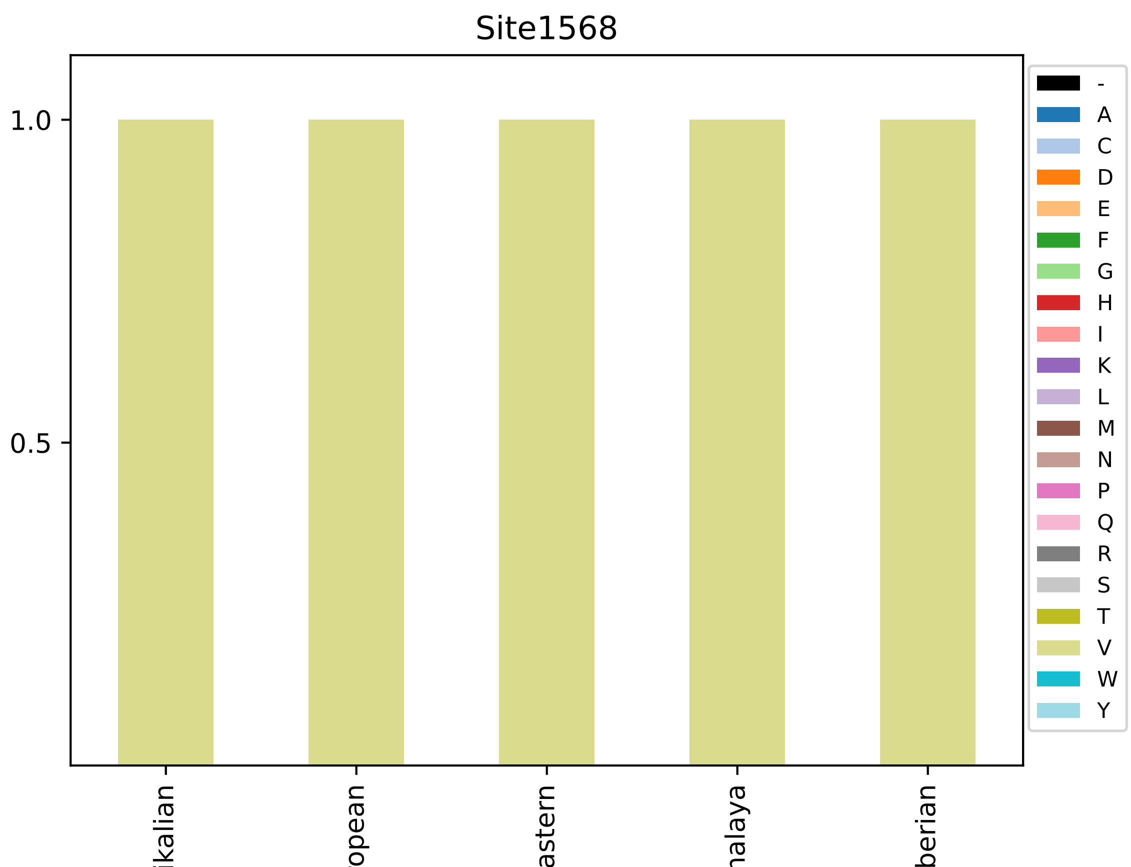
Site1568
1.0
0.5
ikalian
opean
astern
nalaya
berian
-
A
C
D
E
F
G
H
I
K
L
M
N
P
Q
R
S
T
V
W
Y

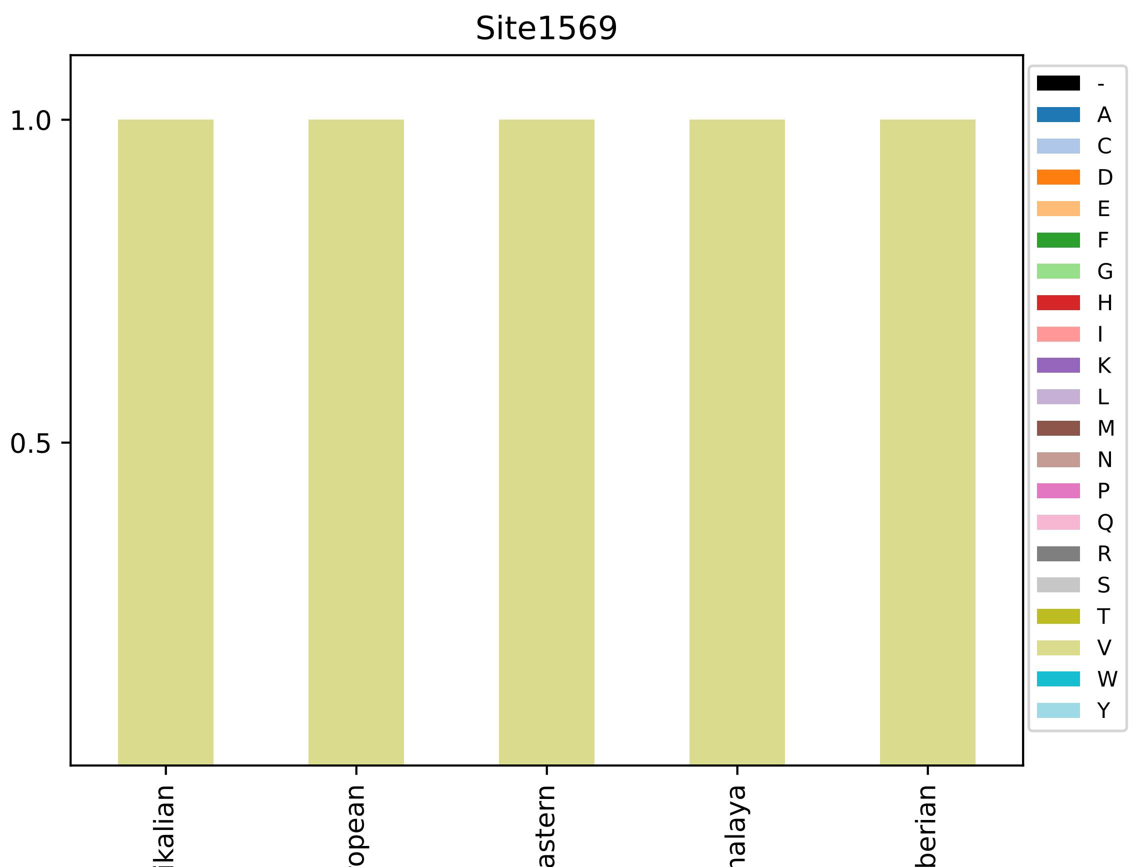
Site1569
1.0
0.5
ikalian
opean
astern
nalaya
berian
-
A
C
D
E
F
G
H
I
K
L
M
N
P
Q
R
S
T
V
W
Y

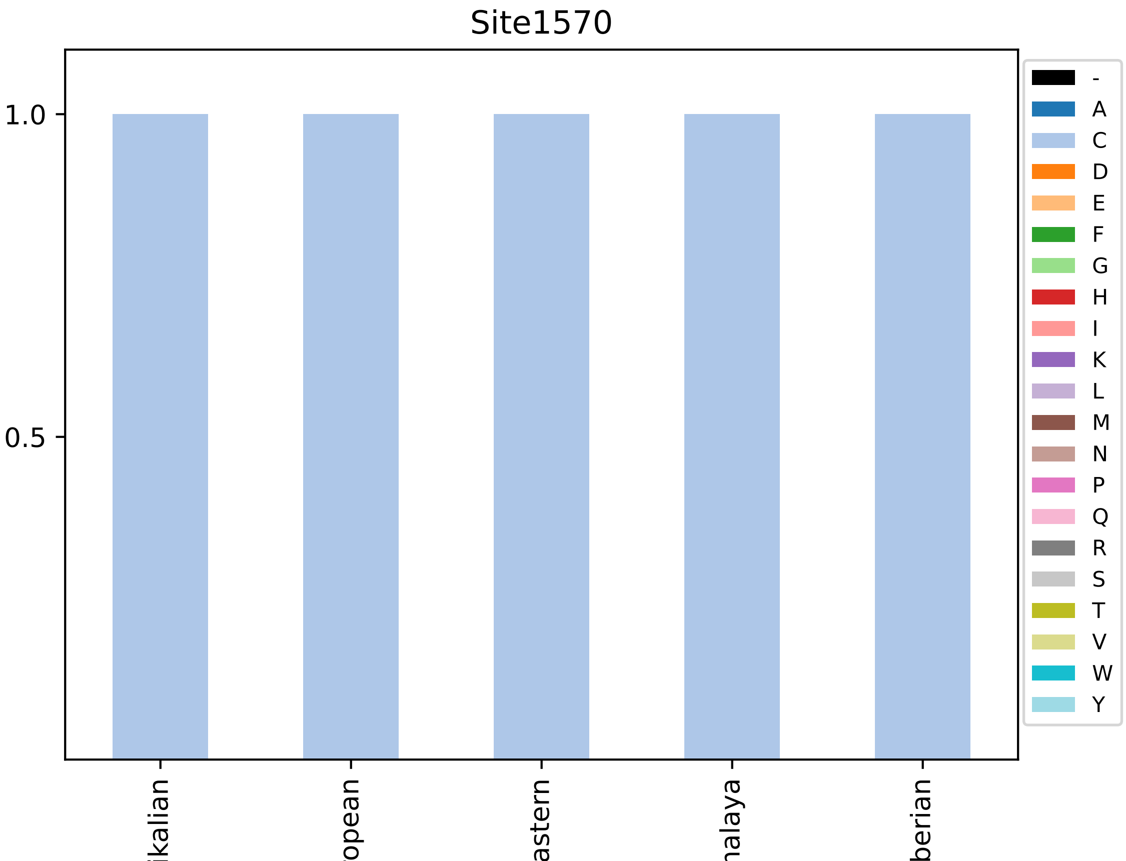
Site1570
1.0
0.5
-
A
C
D
E
F
G
H
I
K
L
M
N
P
Q
R
S
T
V
W
Y
ikalian
opean
astern
nalaya
berian

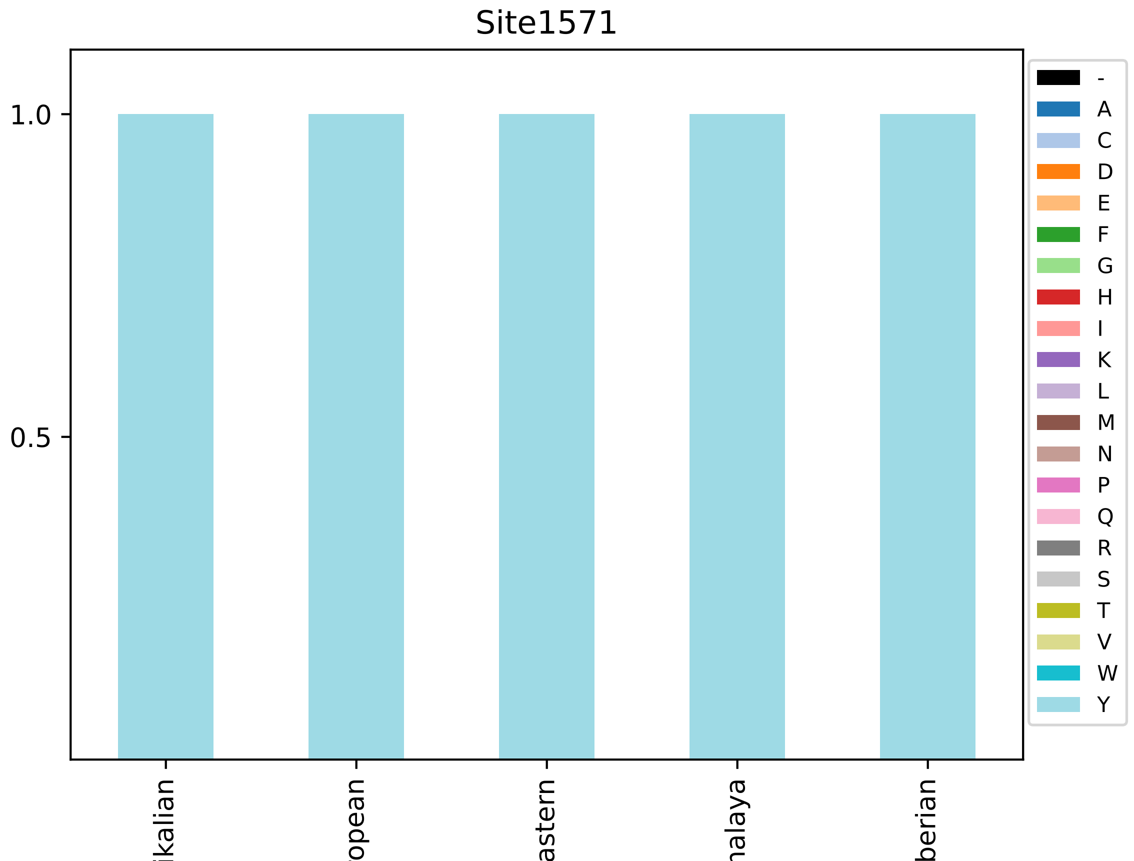

Site1571
1.0
0.5
-
A
C
D
E
F
G
H
I
K
L
M
N
P
Q
R
S
T
V
W
Y
ikalian
opean
astern
nalaya
berian

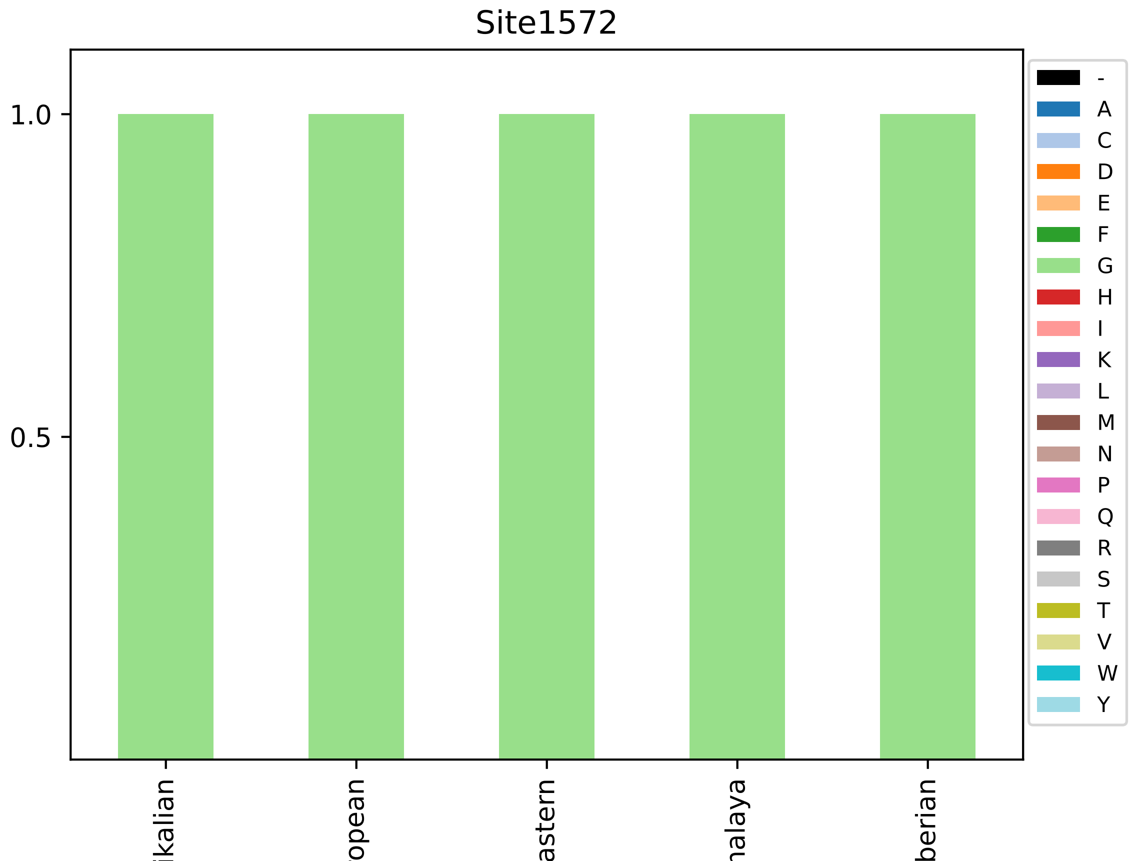

Site1572
1.0
0.5
-
A
C
D
E
F
G
H
I
K
L
M
N
P
Q
R
S
T
V
W
Y
ikalian
opean
astern
nalaya
berian

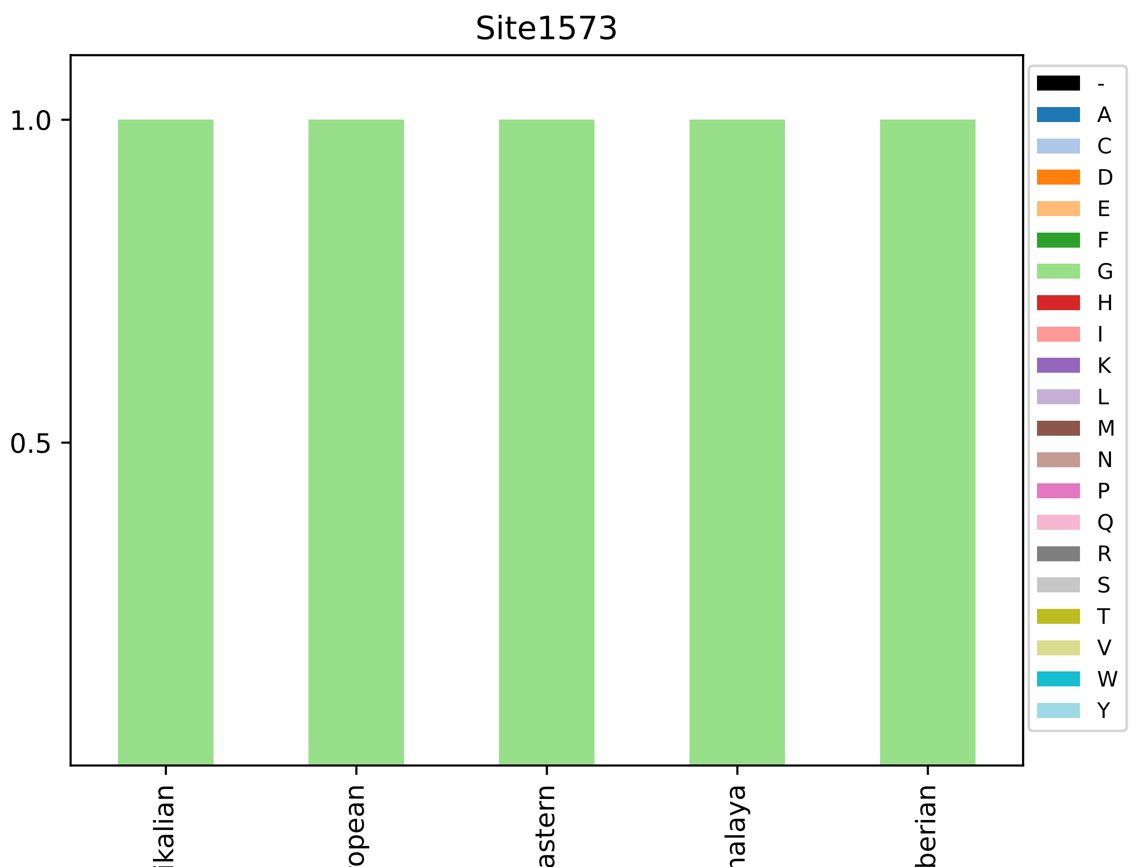

Site1573
1.0
0.5
-
A
C
D
E
F
G
H
I
K
L
M
N
P
Q
R
S
T
V
W
Y
ikalian
opean
astern
nalaya
berian

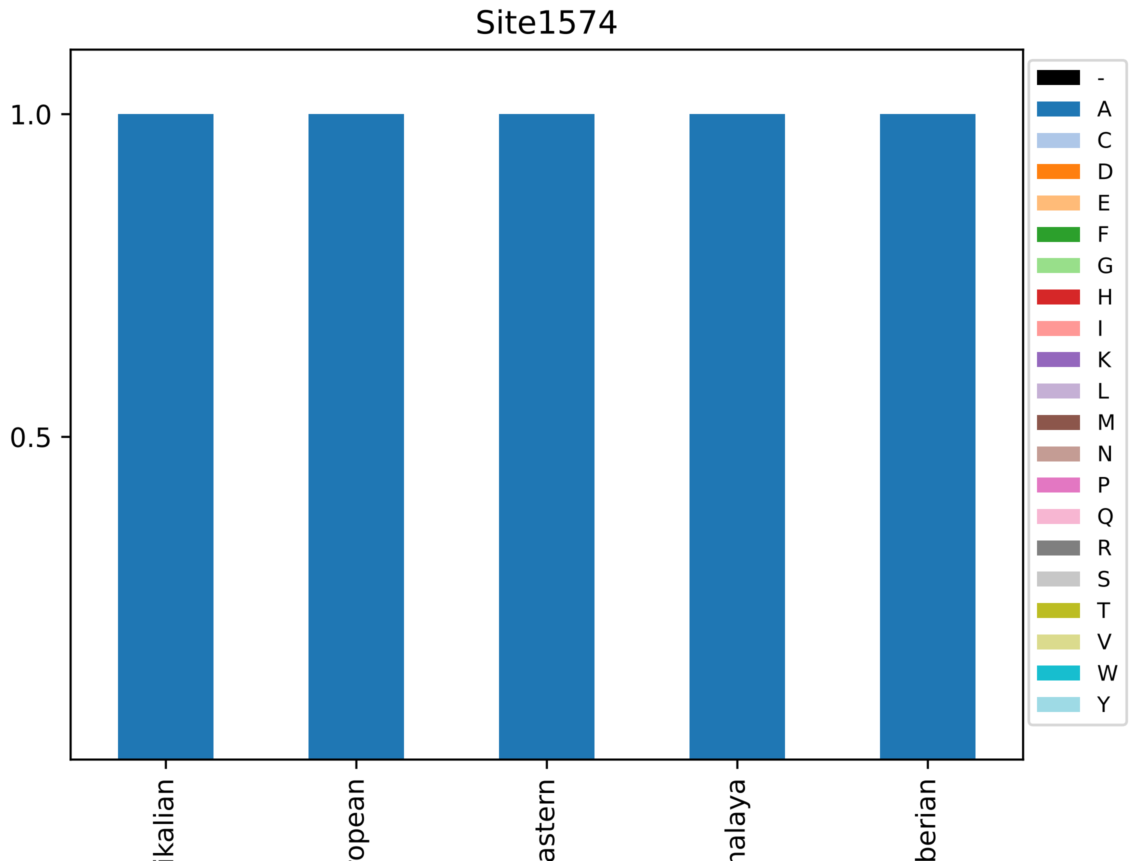
Site1574
1.0
0.5
ikalian
opean
astern
nalaya
berian
-
A
C
D
E
F
G
H
I
K
L
M
N
P
Q
R
S
T
V
W
Y

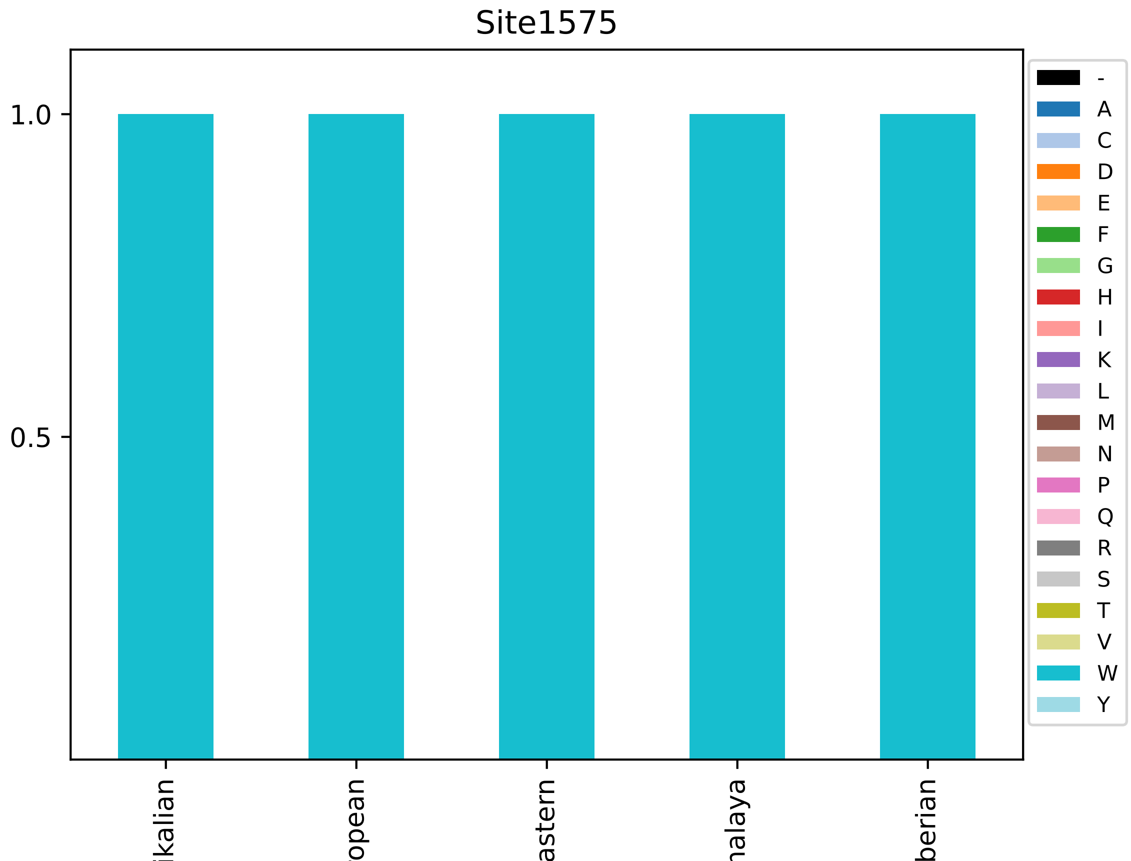
Site1575
1.0
0.5
-
A
C
D
E
F
G
H
I
K
L
M
N
P
Q
R
S
T
V
W
Y
ikalian
opean
astern
nalaya
berian

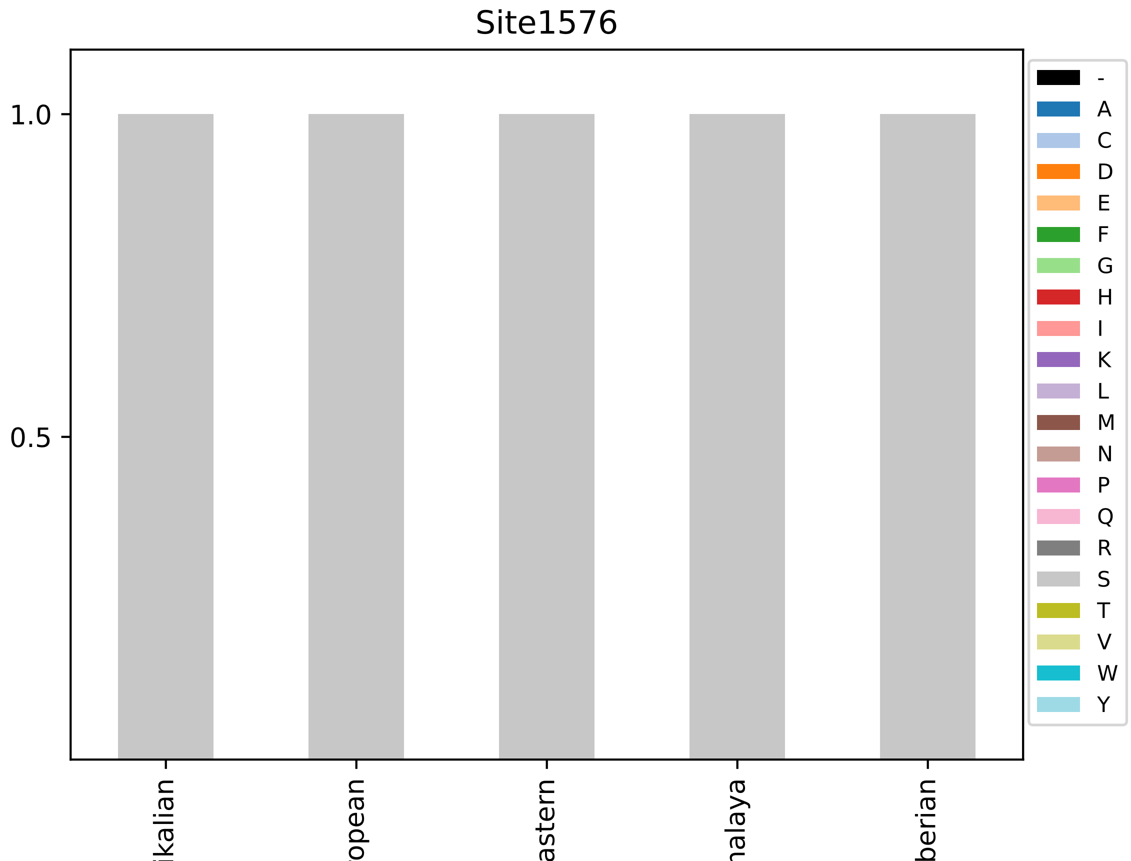

Site1576
1.0
0.5
ikalian
opean
astern
nalaya
berian
-
A
C
D
E
F
G
H
I
K
L
M
N
P
Q
R
S
T
V
W
Y

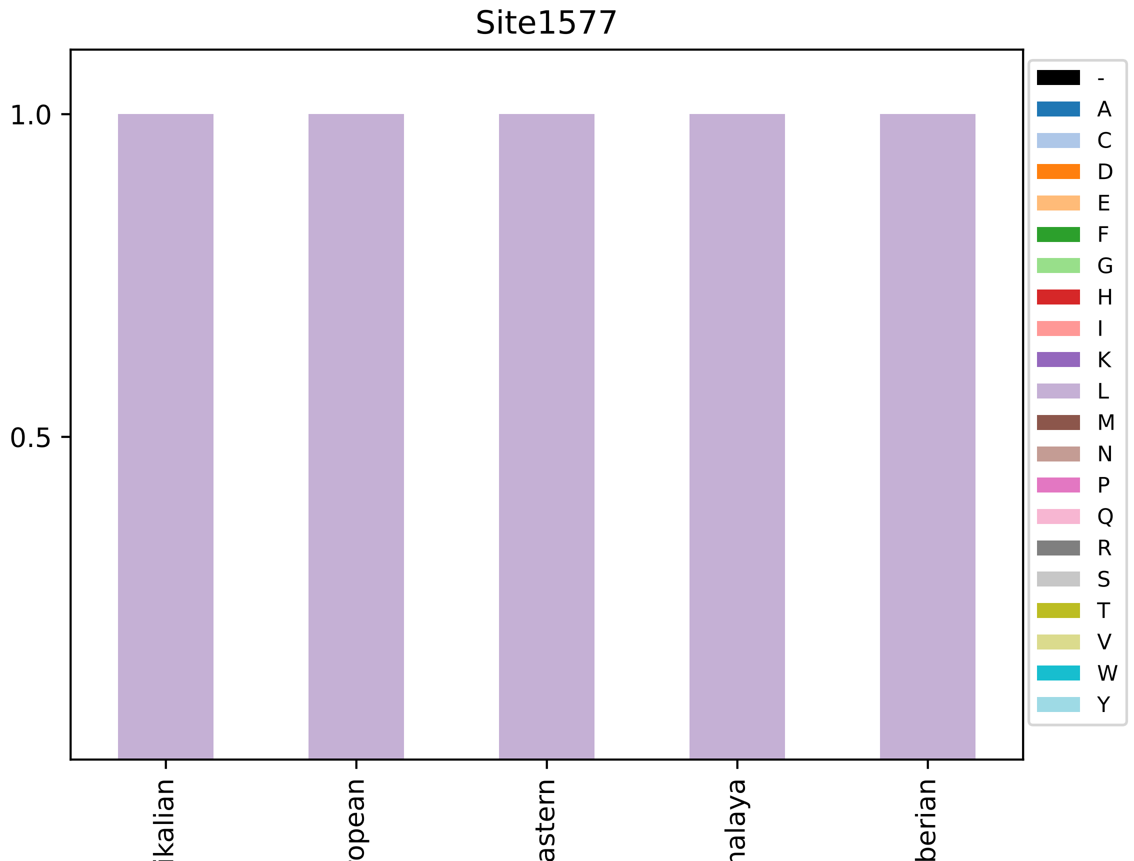
Site1577
1.0
0.5
-
A
C
D
E
F
G
H
I
K
L
M
N
P
Q
R
S
T
V
W
Y
ikalian
opean
astern
nalaya
berian

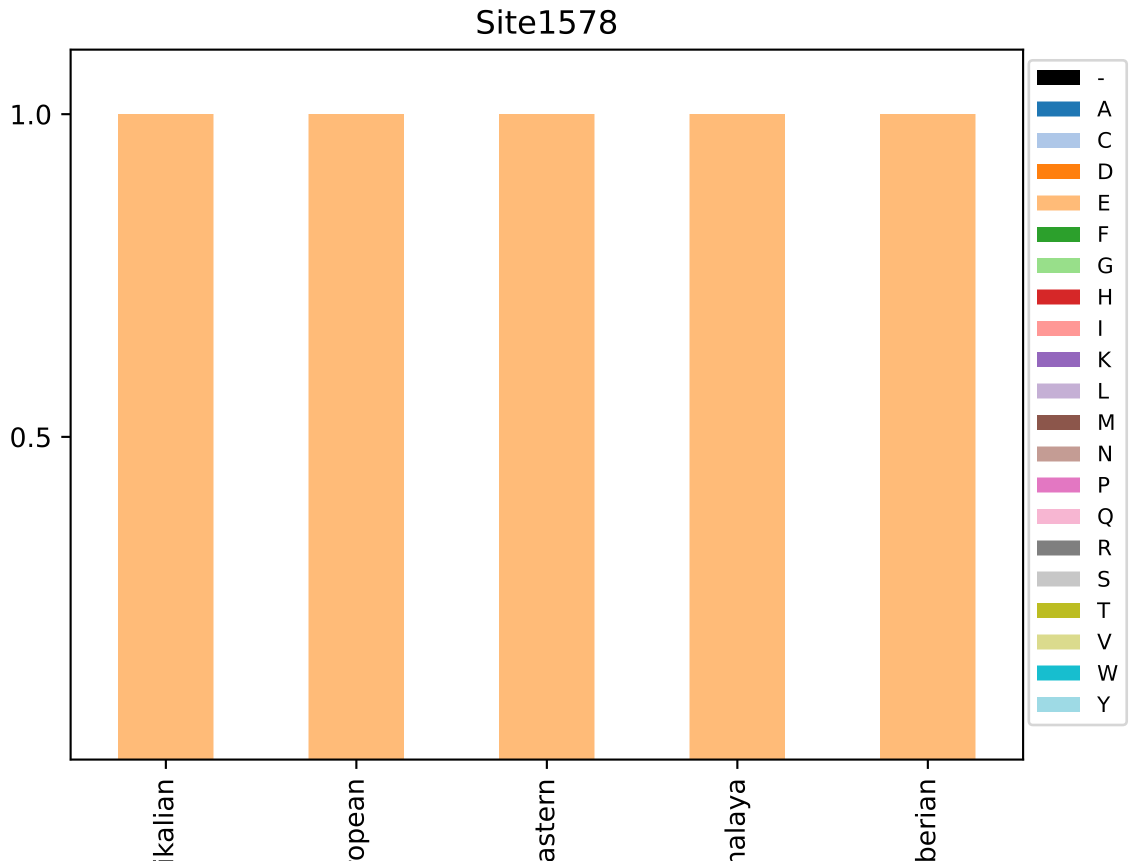

Site1578
1.0
0.5
ikalian
ropean
astern
nalaya
berian
-
A
C
D
E
F
G
H
I
K
L
M
N
P
Q
R
S
T
V
W
Y

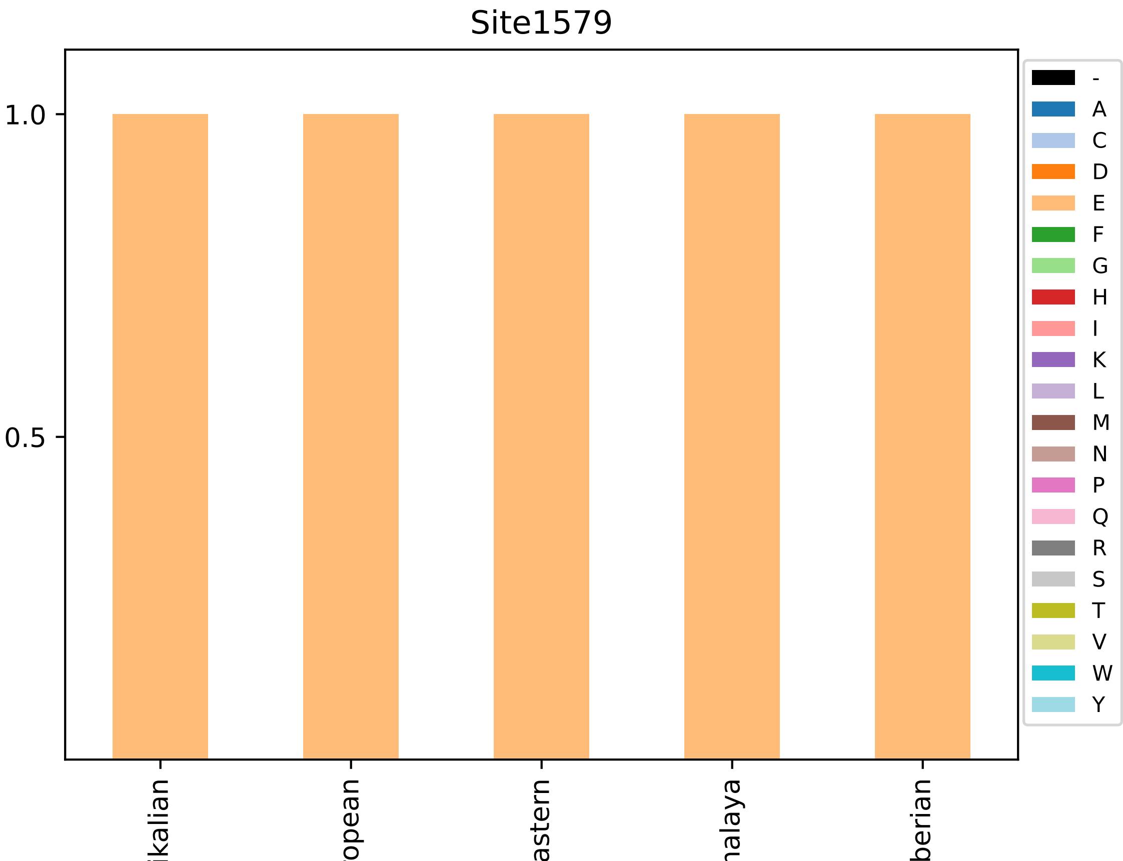

Site1579
1.0
0.5
-
A
C
D
E
F
G
H
I
K
L
M
N
P
Q
R
S
T
V
W
Y
ikalian
opean
astern
nalaya
berian

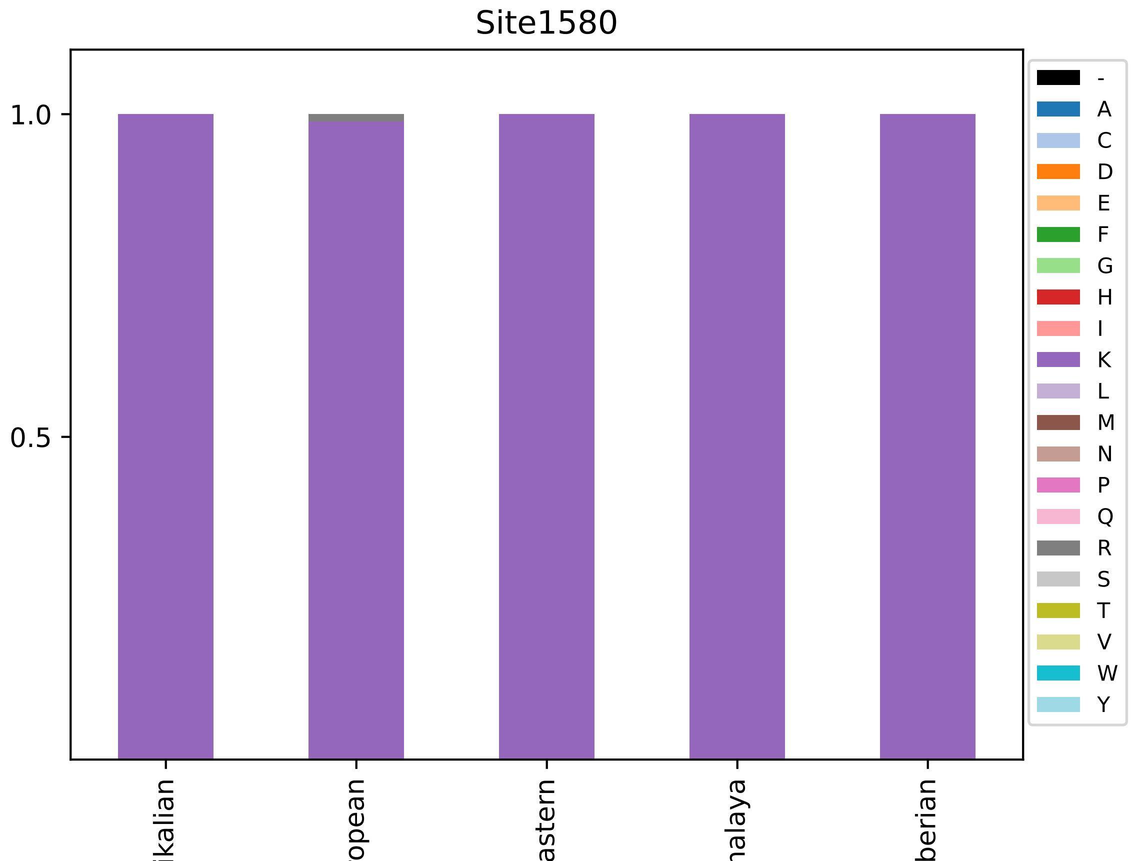

Site1580
1.0
0.5
ikalian
opean
astern
nalaya
berian
-
A
C
D
E
F
G
H
I
K
L
M
N
P
Q
R
S
T
V
W
Y

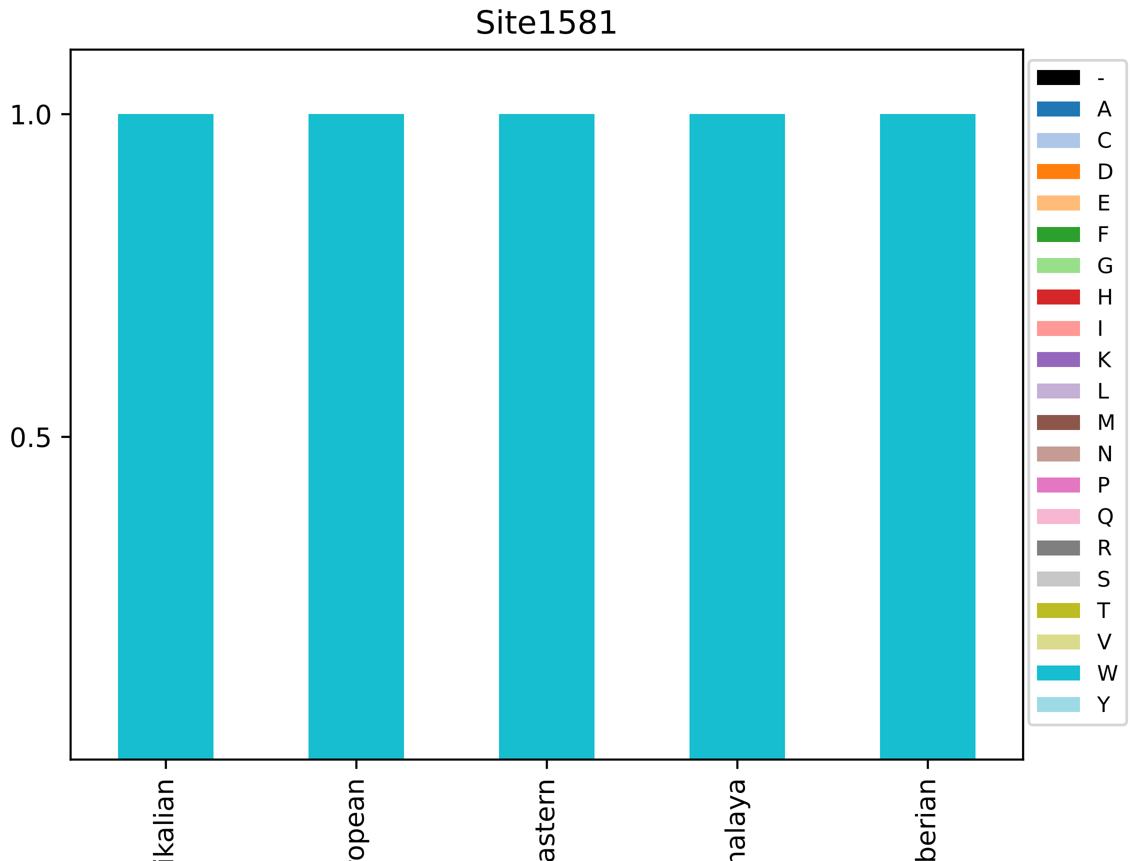
Site1581
1.0
0.5
-
A
C
D
E
F
G
H
I
K
L
M
N
P
Q
R
S
T
V
W
Y
ikalian
opean
astern
nalaya
berian

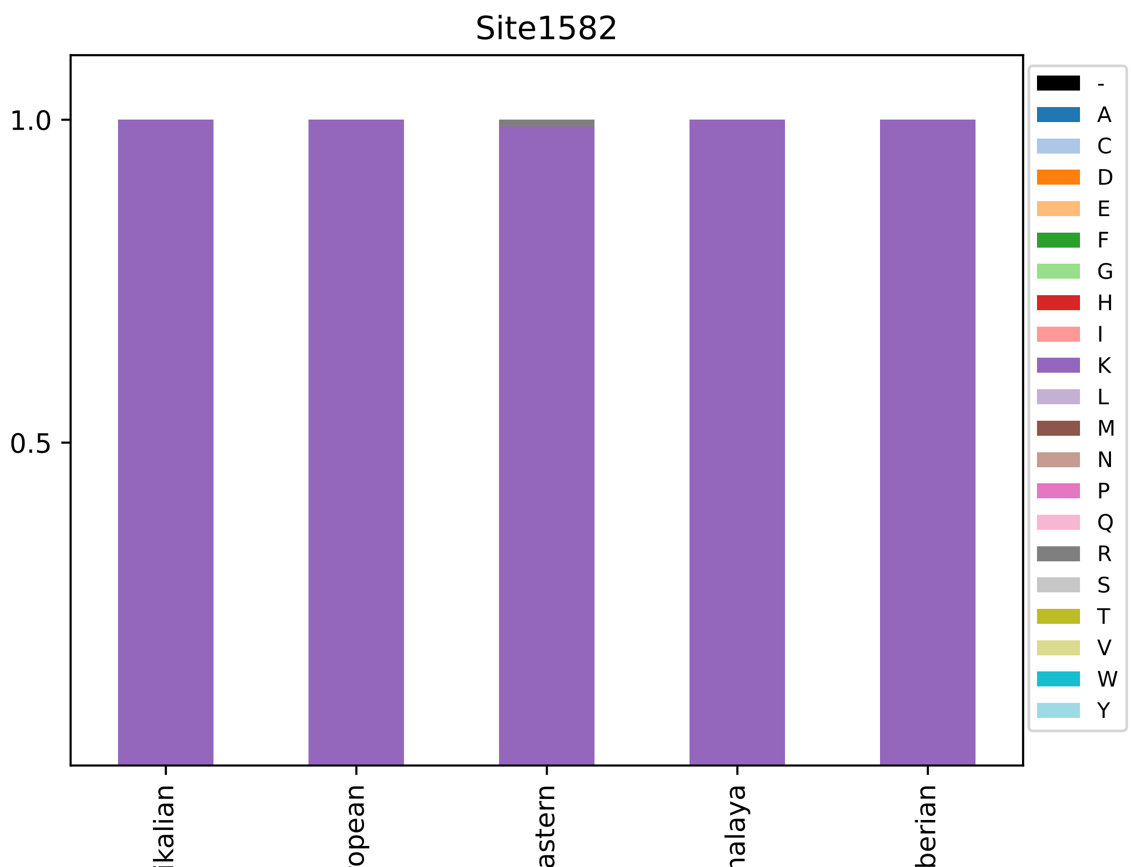

Site1582
1.0
0.5
-
A
C
D
E
F
G
H
I
K
L
M
N
P
Q
R
S
T
V
W
Y
ikalian
opean
astern
nalaya
berian

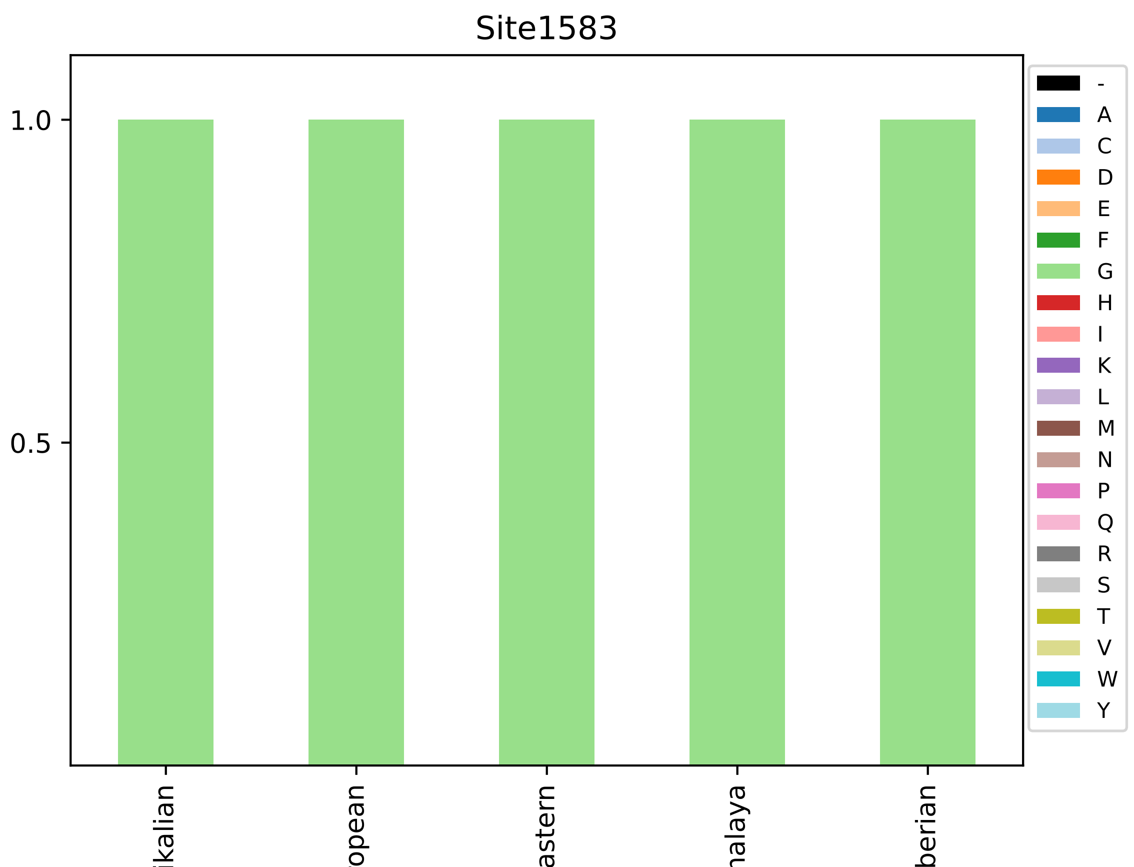

Site1583
1.0
0.5
-
A
C
D
E
F
G
H
I
K
L
M
N
P
Q
R
S
T
V
W
Y
ikalian
opean
astern
nalaya
berian

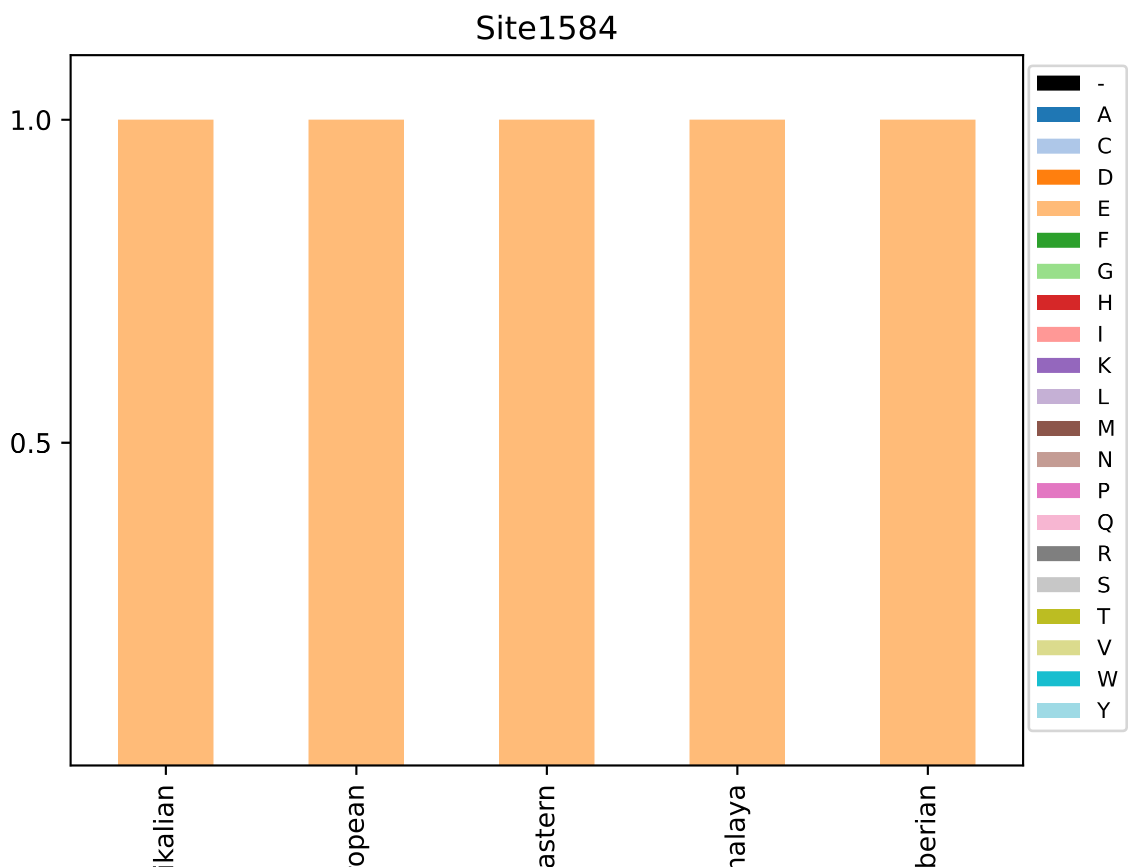

Site1584
1.0
0.5
-
A
C
D
E
F
G
H
I
K
L
M
N
P
Q
R
S
T
V
W
Y
ikalian
opean
astern
nalaya
berian

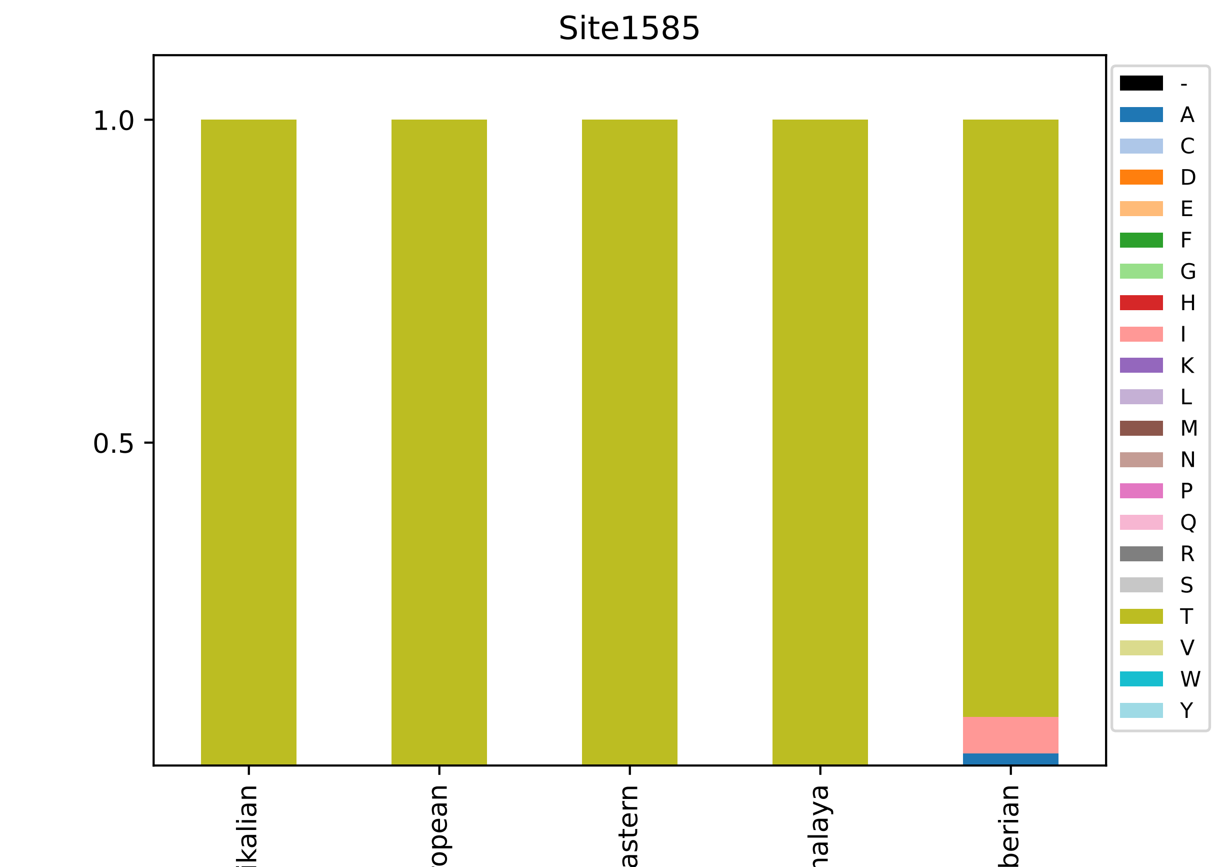
Site1585
1.0
0.5
-
A
C
D
E
F
G
H
I
K
L
M
N
P
Q
R
S
T
V
W
Y
ikalian
opean
astern
nalaya
berian

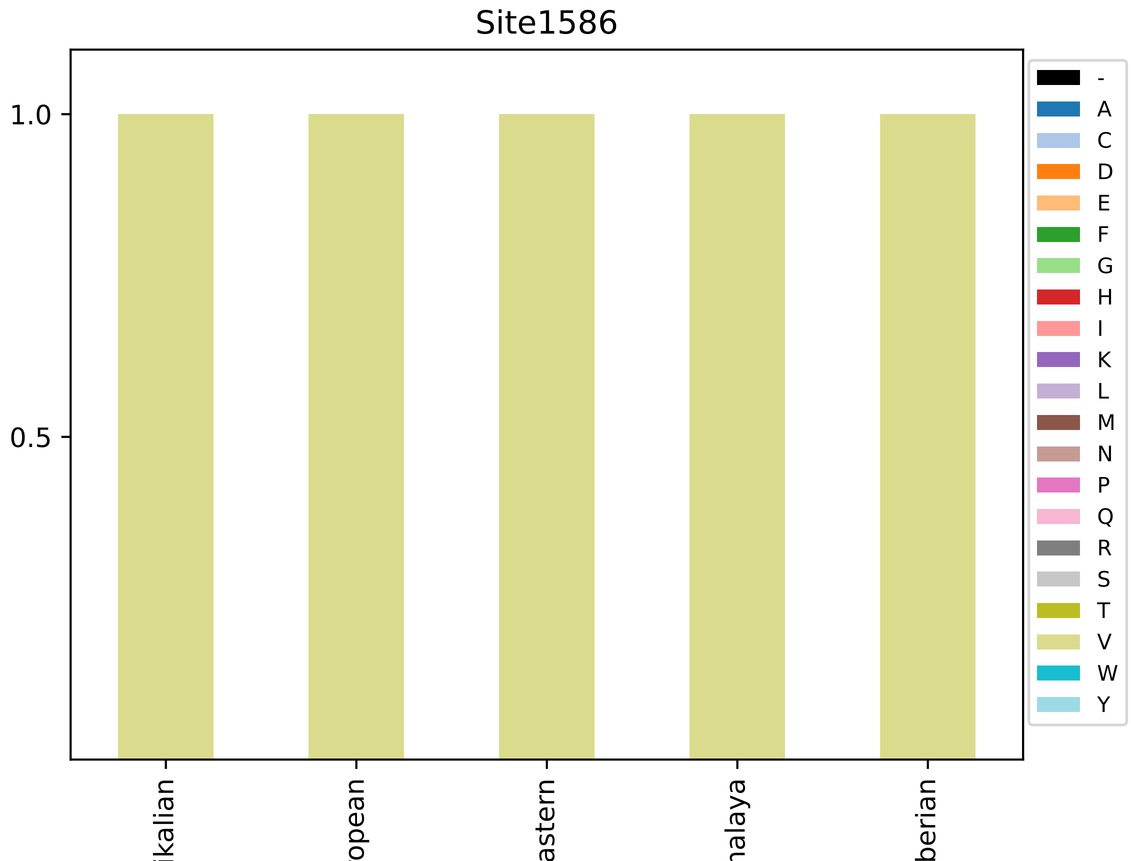
Site1586
1.0
0.5
-
A
C
D
E
F
G
H
I
K
L
M
N
P
Q
R
S
T
V
W
Y
ikalian
opean
astern
nalaya
berian

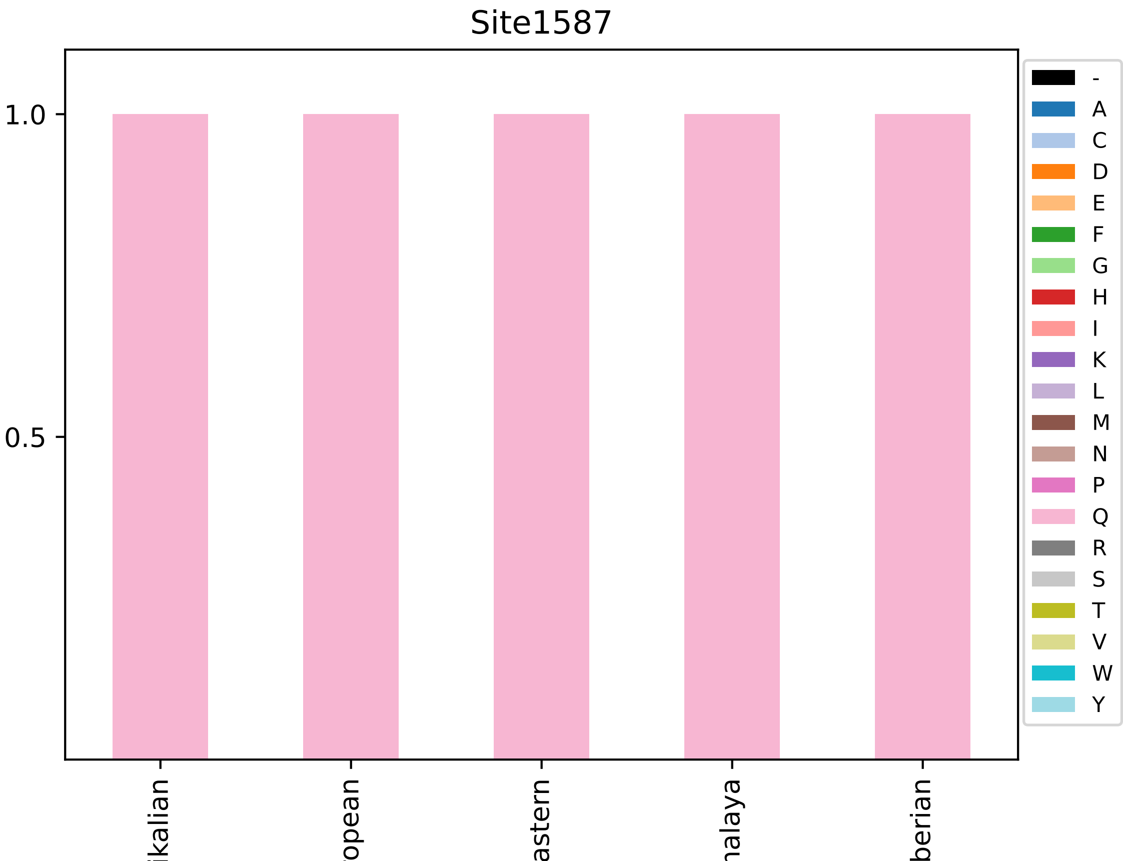
Site1587
1.0
0.5
-
A
C
D
E
F
G
H
I
K
L
M
N
P
Q
R
S
T
V
W
Y
ikalian
opean
astern
nalaya
berian

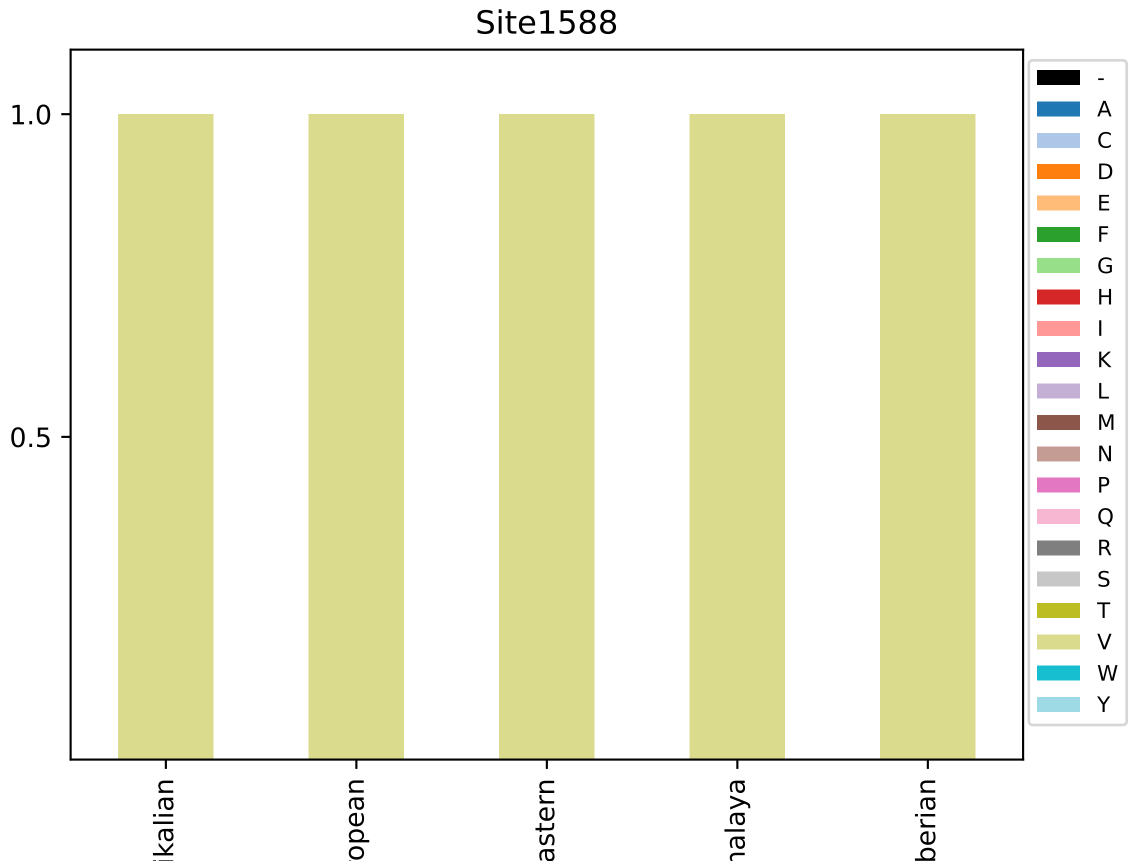
Site1588
1.0
0.5
-
A
C
D
E
F
G
H
I
K
L
M
N
P
Q
R
S
T
V
W
Y
ikalian
opean
astern
nalaya
berian

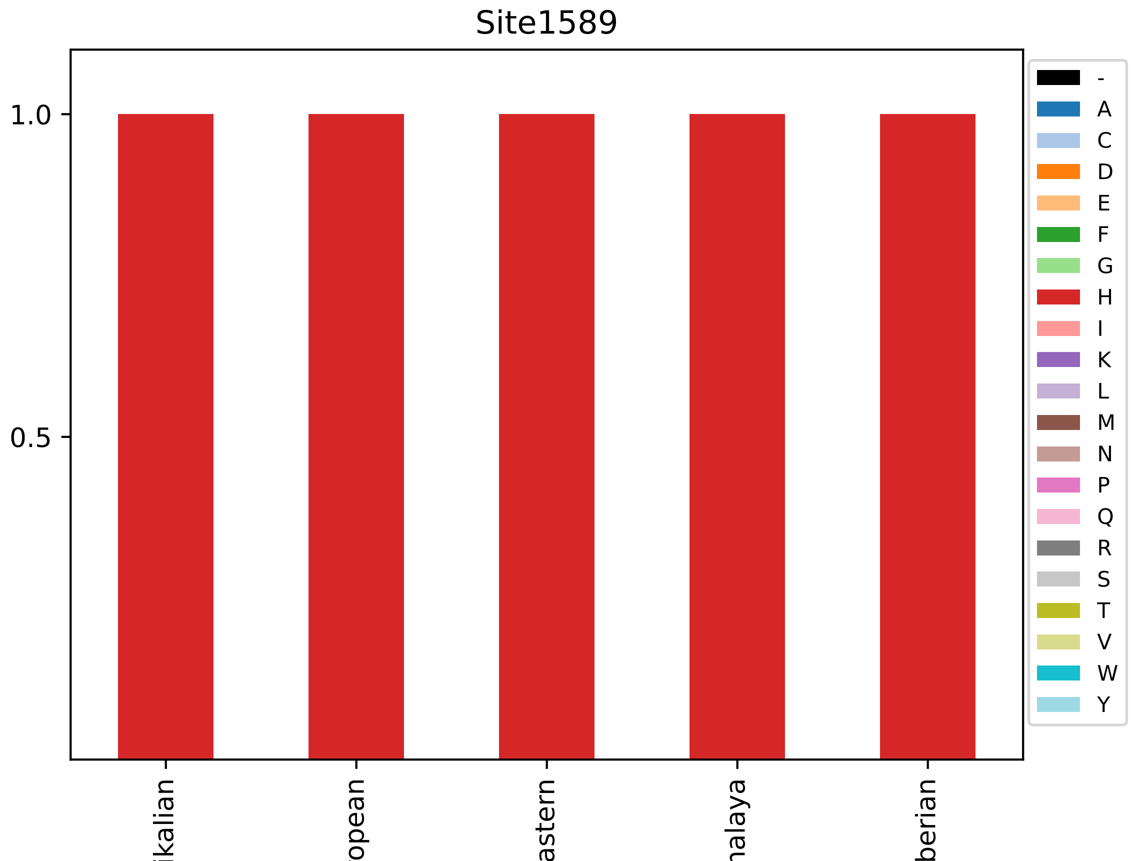
Site1589
1.0
0.5
-
A
C
D
E
F
G
H
I
K
L
M
N
P
Q
R
S
T
V
W
Y
ikalian
opean
astern
nalaya
berian

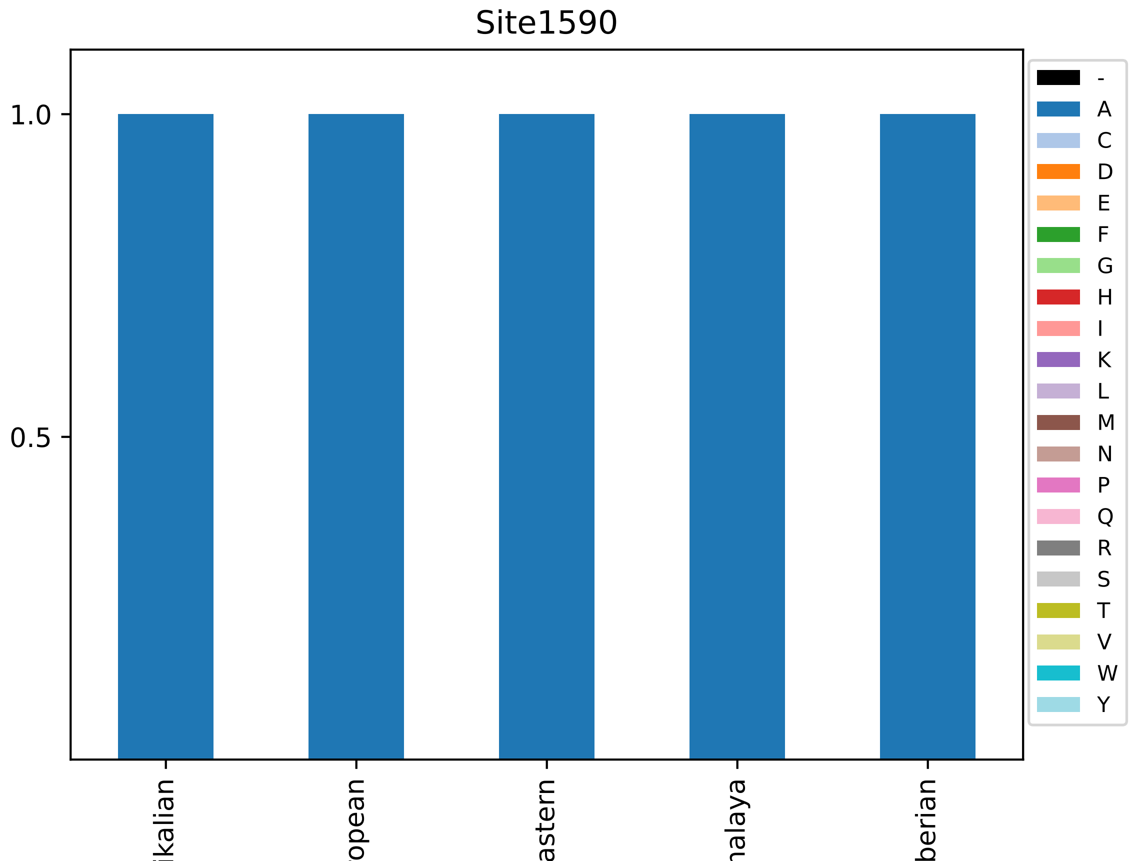

Site1590
1.0
0.5
ikalian
opean
astern
nalaya
berian
-
A
C
D
E
F
G
H
I
K
L
M
N
P
Q
R
S
T
V
W
Y

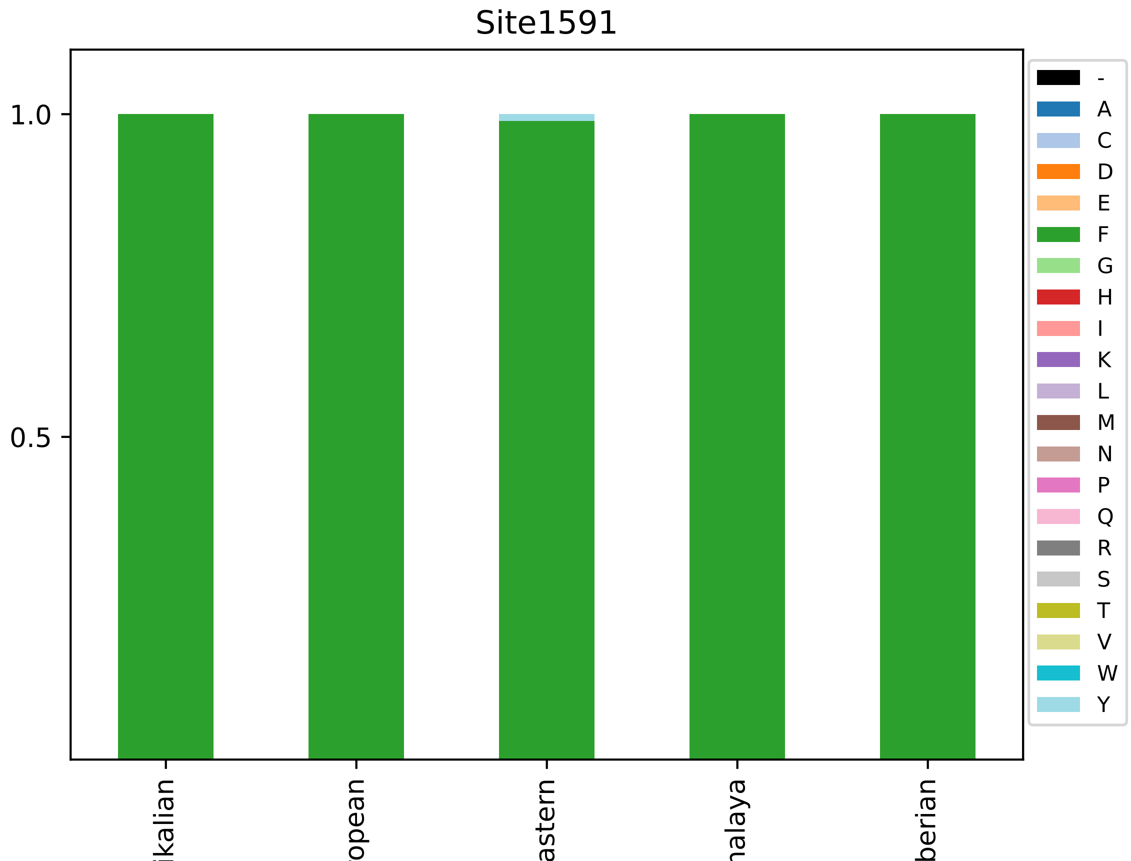

Site1591
1.0
0.5
-
A
C
D
E
F
G
H
I
K
L
M
N
P
Q
R
S
T
V
W
Y
ikalian
opean
astern
nalaya
berian

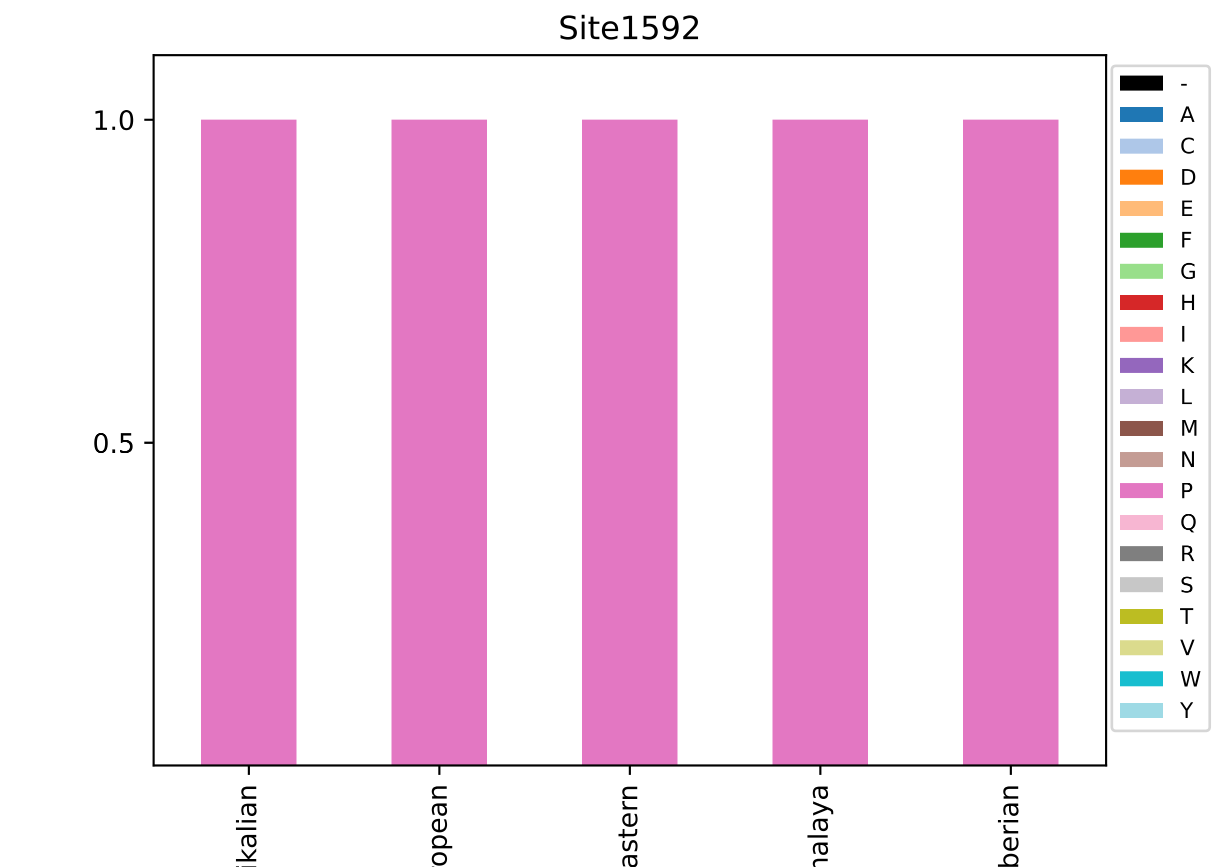

Site1592
1.0
0.5
ikalian
opean
astern
nalaya
berian
-
A
C
D
E
F
G
H
I
K
L
M
N
P
Q
R
S
T
V
W
Y

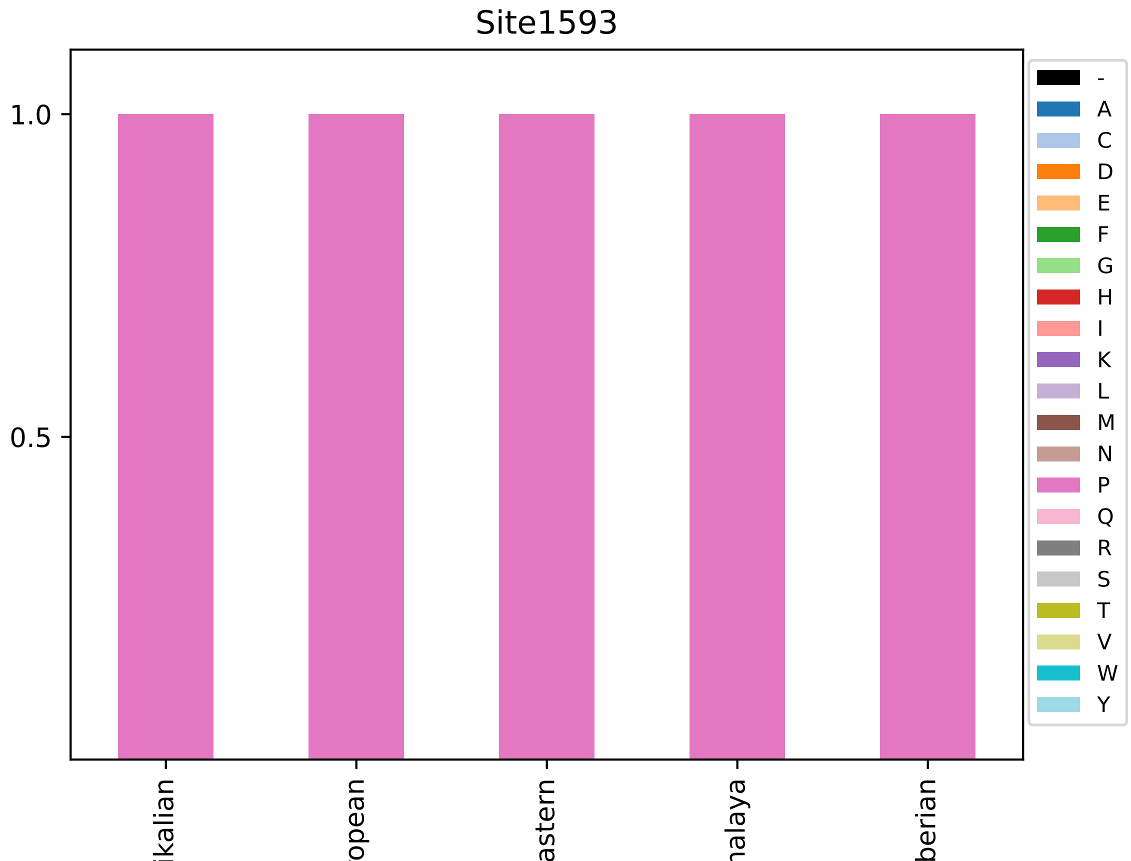

Site1593
1.0
0.5
-
A
C
D
E
F
G
H
I
K
L
M
N
P
Q
R
S
T
V
W
Y
ikalian
opean
astern
nalaya
berian

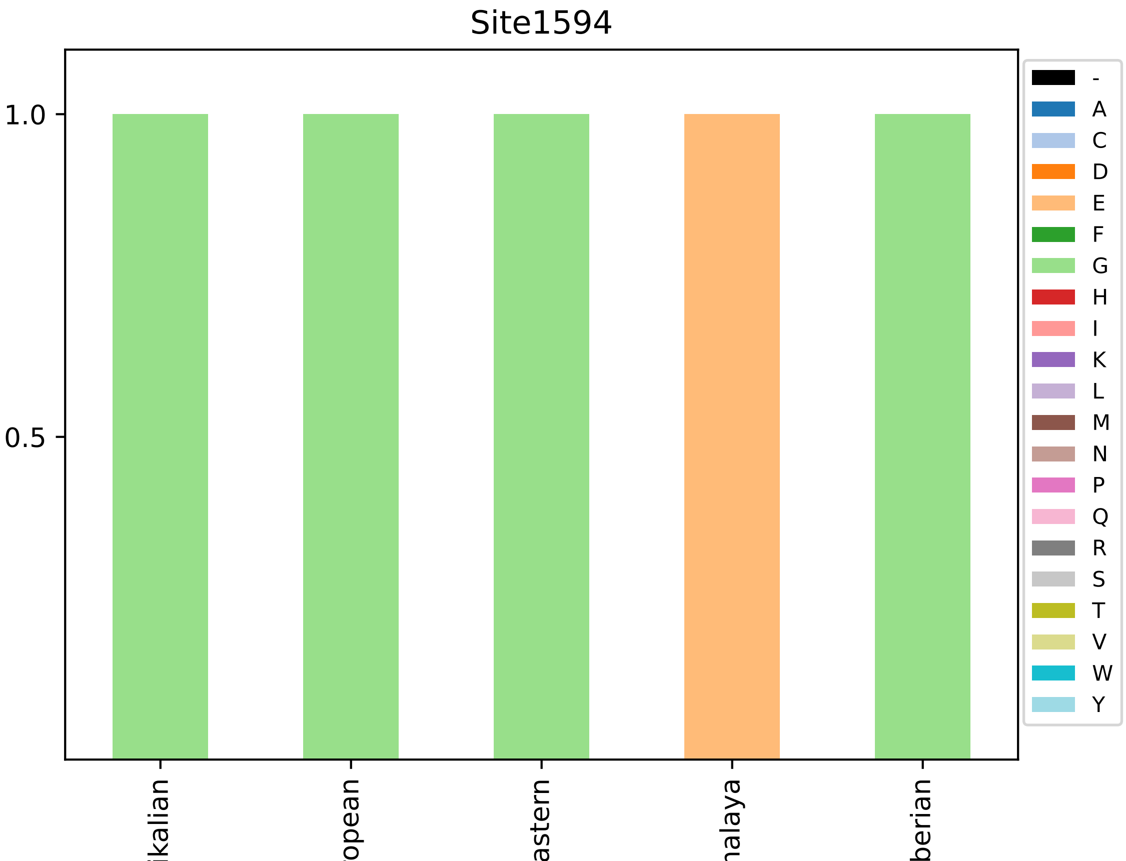

Site1594
1.0
0.5
-
A
C
D
E
F
G
H
I
K
L
M
N
P
Q
R
S
T
V
W
Y
ikalian
opean
astern
nalaya
berian

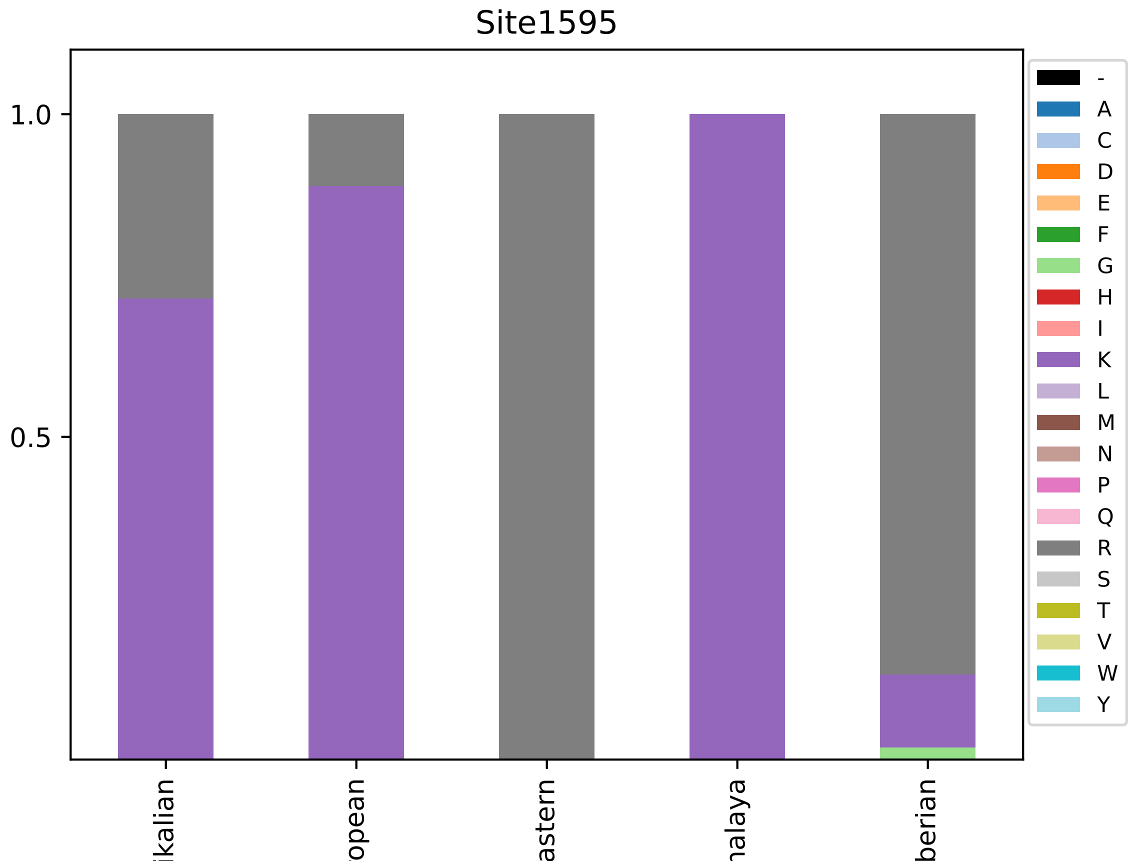

Site1595
1.0
0.5
-
A
C
D
E
F
G
H
I
K
L
M
N
P
Q
R
S
T
V
W
Y
ikalian
ropean
astern
nalaya
berian

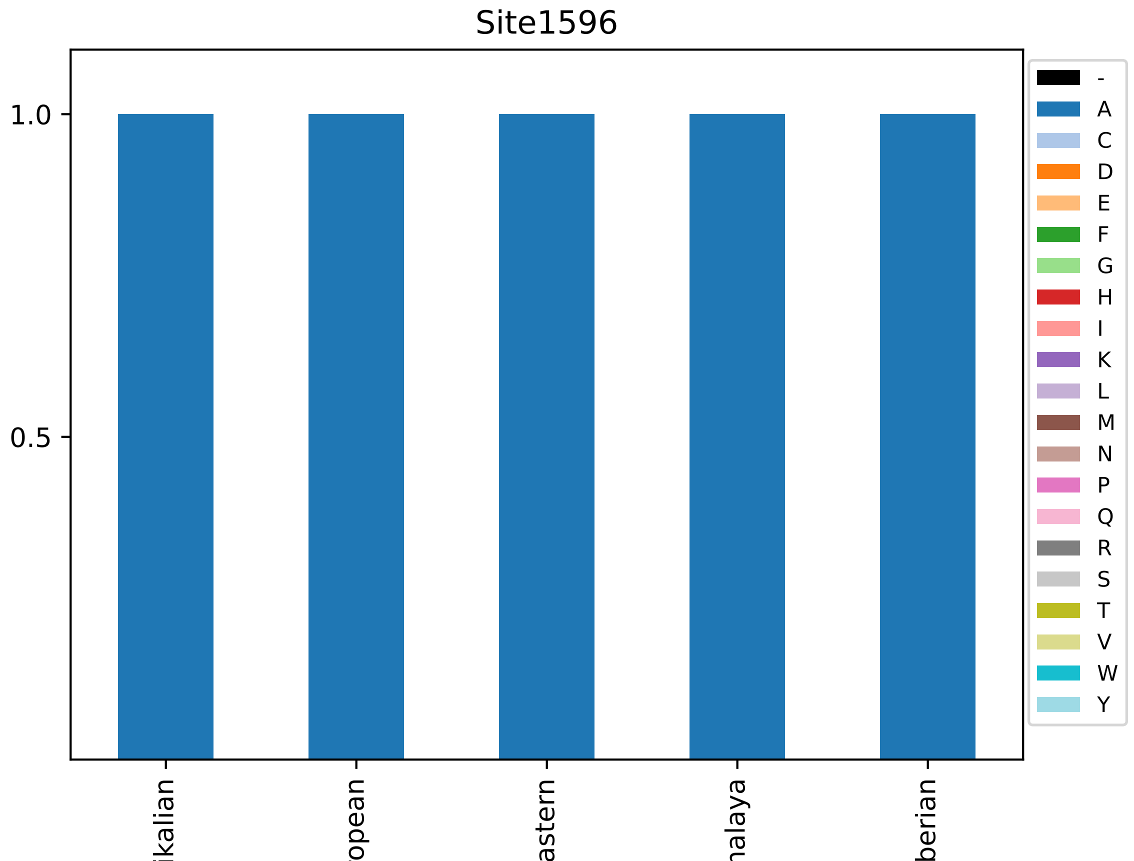
Site1596
1.0
0.5
ikalian
opean
astern
nalaya
berian
-
A
C
D
E
F
G
H
I
K
L
M
N
P
Q
R
S
T
V
W
Y

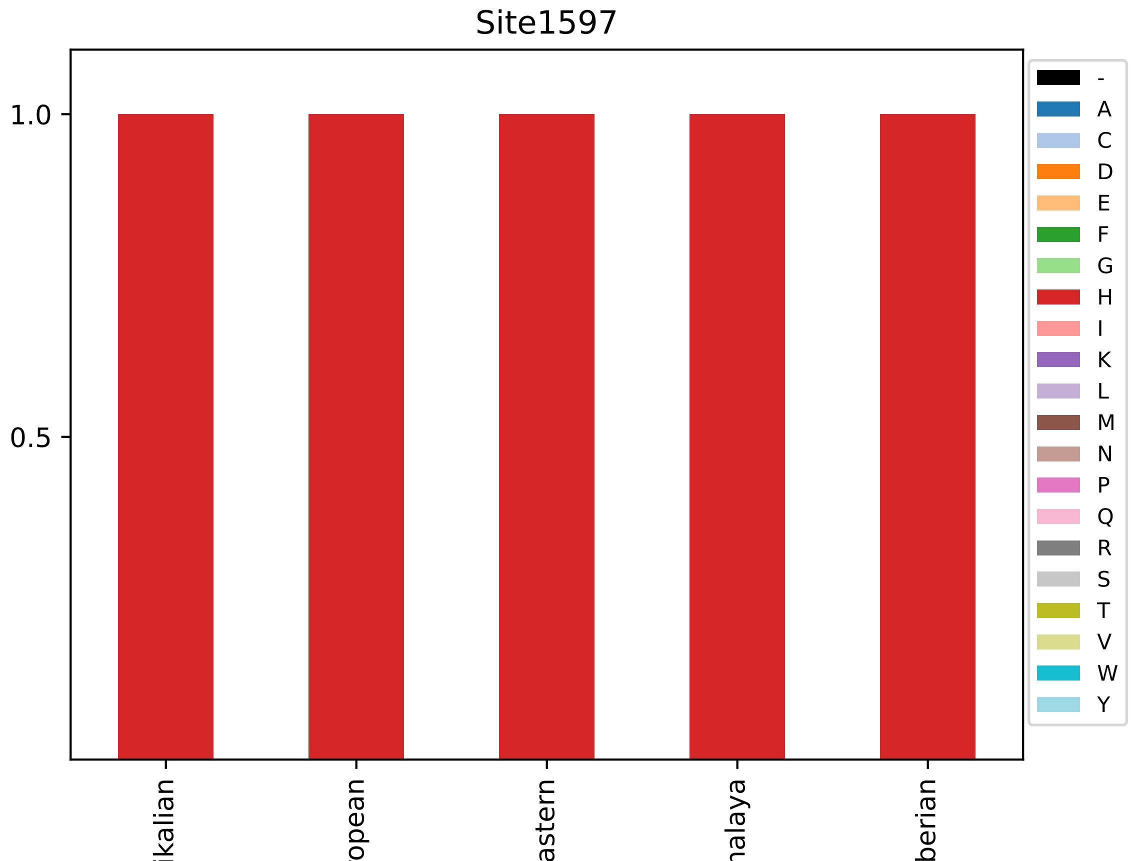
Site1597
1.0
0.5
ikalian
opean
astern
nalaya
berian
-
A
C
D
E
F
G
H
I
K
L
M
N
P
Q
R
S
T
V
W
Y

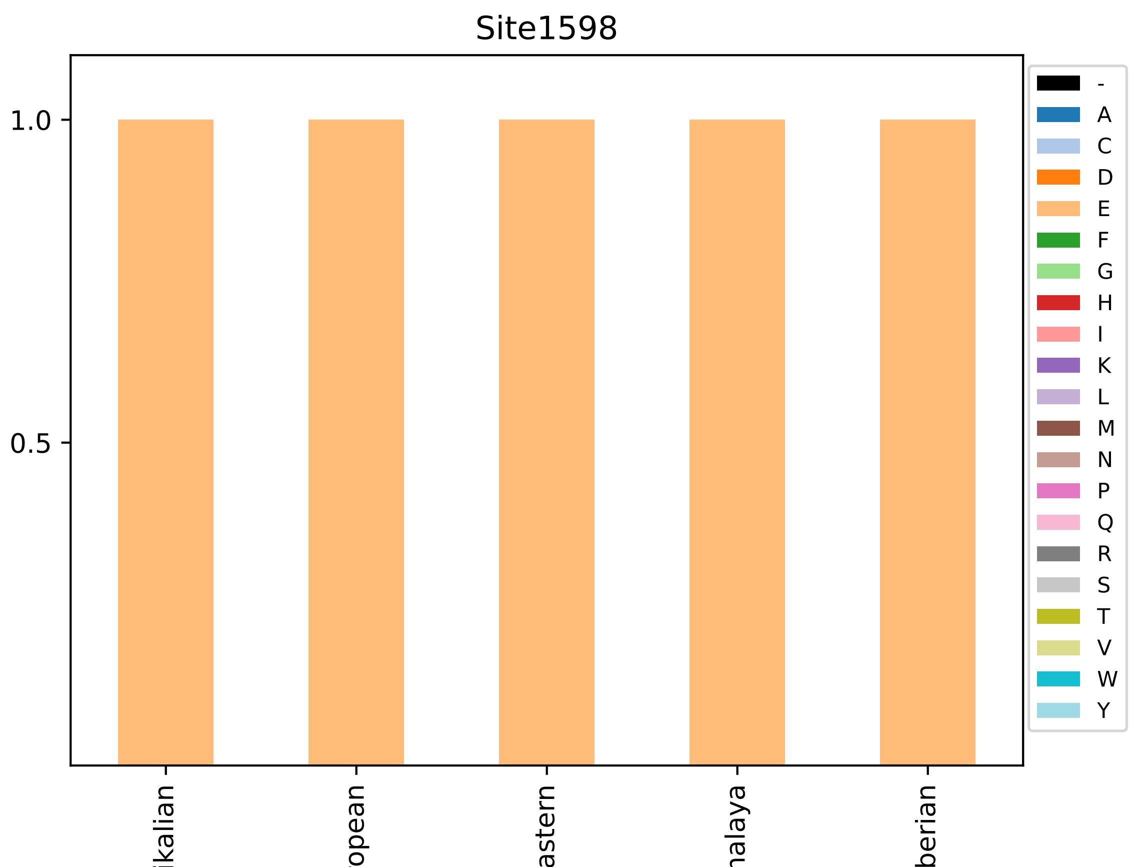

Site1598
1.0
0.5
ikalian
opean
astern
nalaya
berian
-
A
C
D
E
F
G
H
I
K
L
M
N
P
Q
R
S
T
V
W
Y

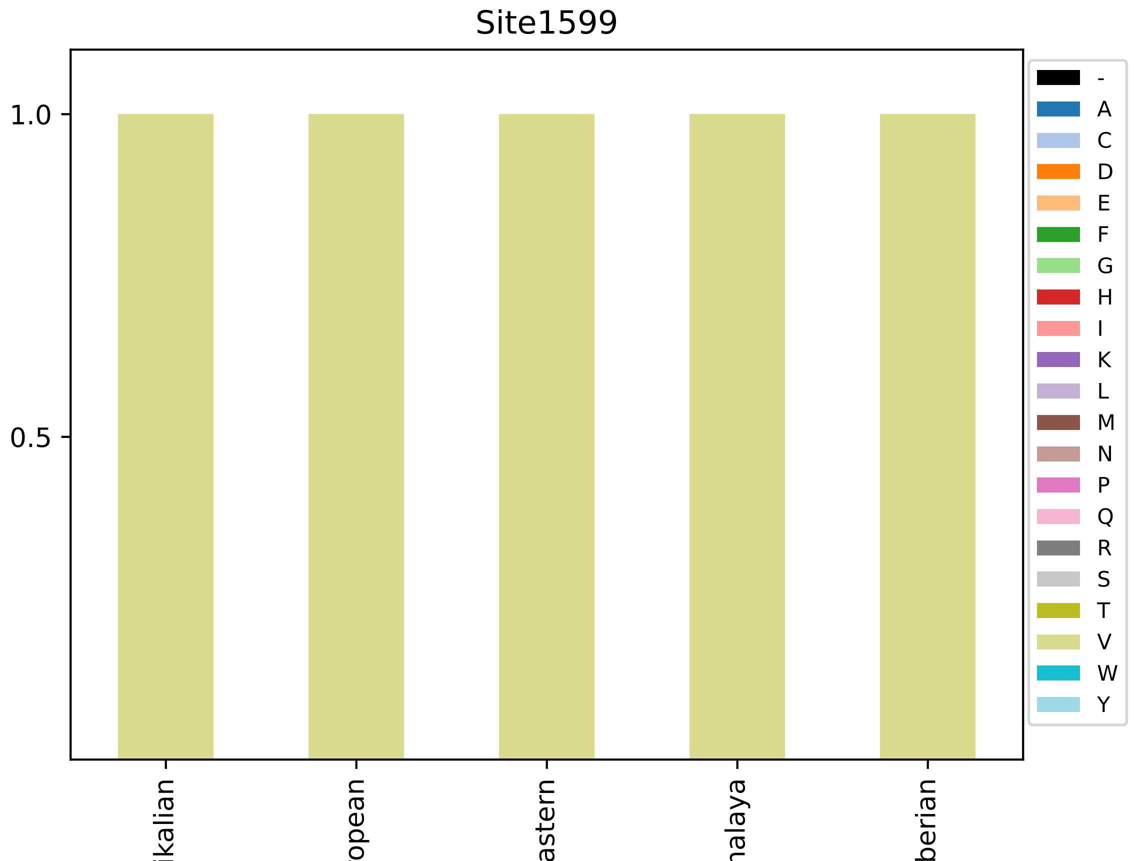

Site1599
1.0
0.5
ikalian
opean
astern
nalaya
berian
-
A
C
D
E
F
G
H
I
K
L
M
N
P
Q
R
S
T
V
W
Y

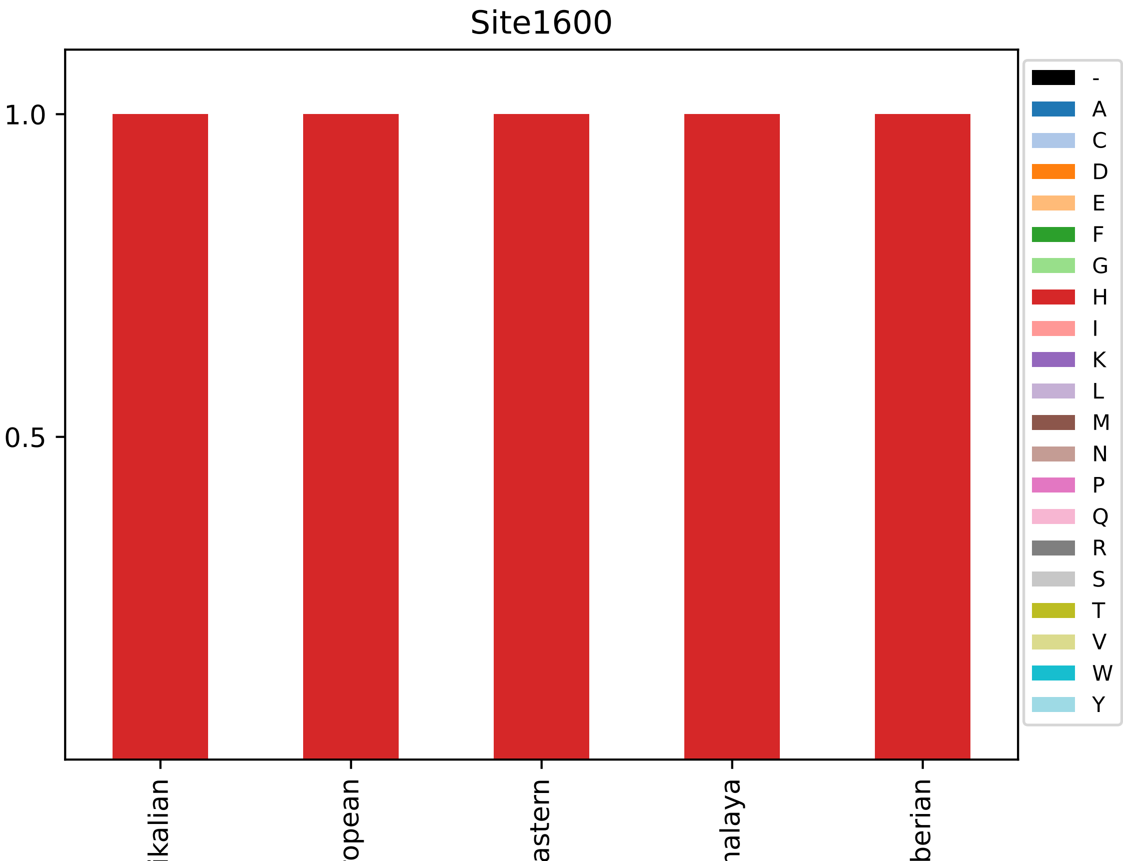
Site1600
1.0
0.5
-
A
C
D
E
F
G
H
I
K
L
M
N
P
Q
R
S
T
V
W
Y
ikalian
opean
astern
nalaya
berian

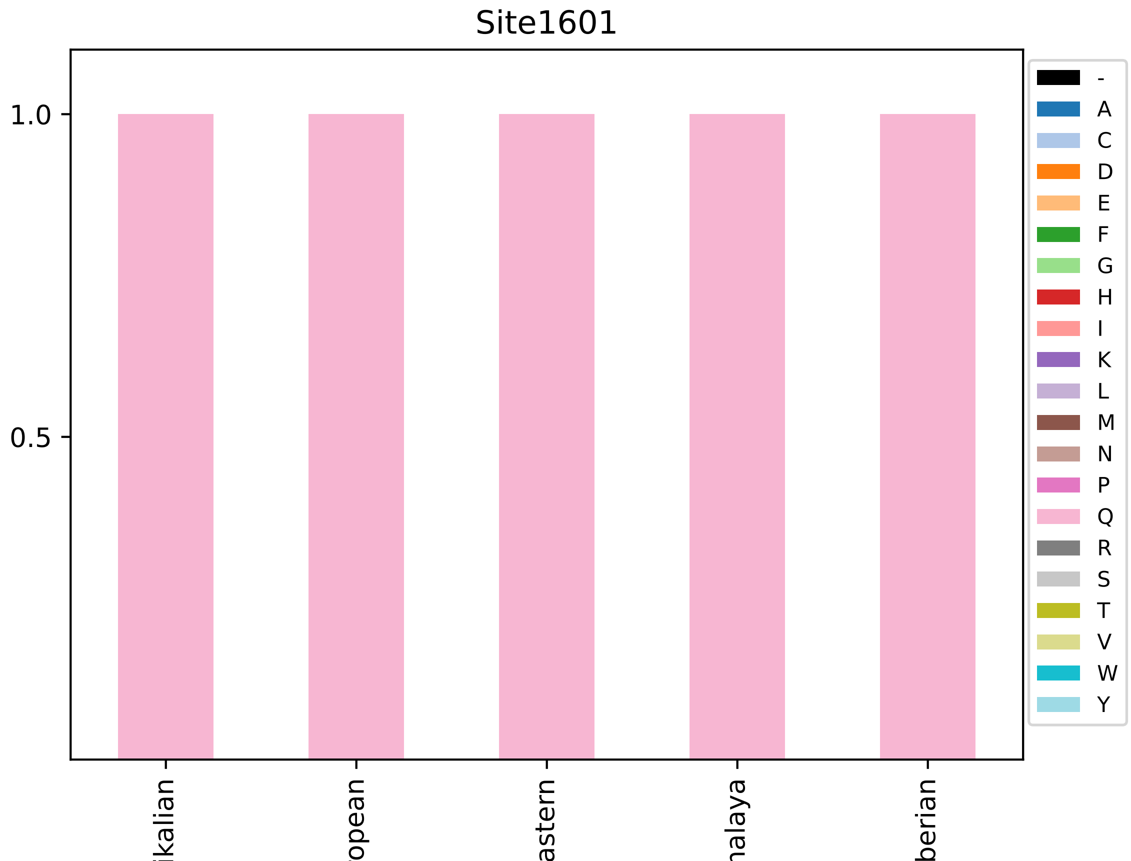
Site1601
1.0
0.5
ikalian
opean
astern
nalaya
berian
-
A
C
D
E
F
G
H
I
K
L
M
N
P
Q
R
S
T
V
W
Y

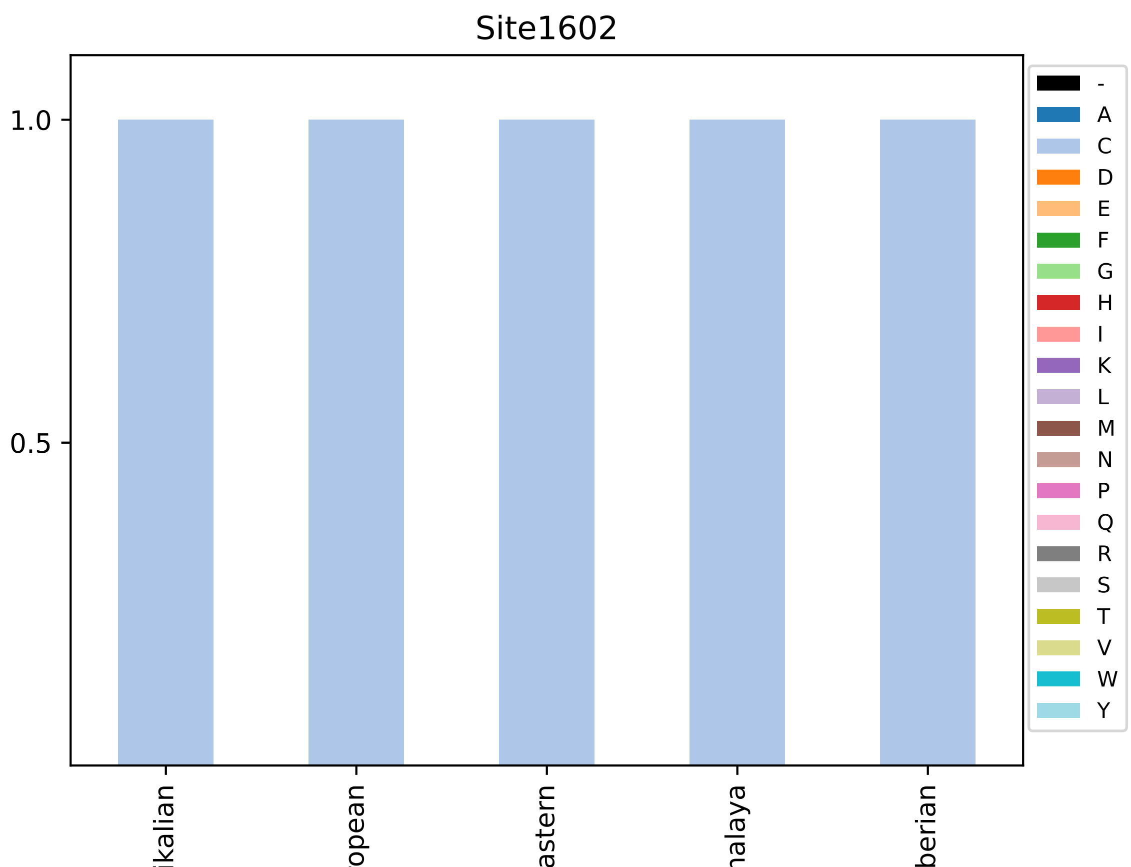
Site1602
1.0
0.5
-
A
C
D
E
F
G
H
I
K
L
M
N
P
Q
R
S
T
V
W
Y
ikalian
opean
astern
nalaya
berian

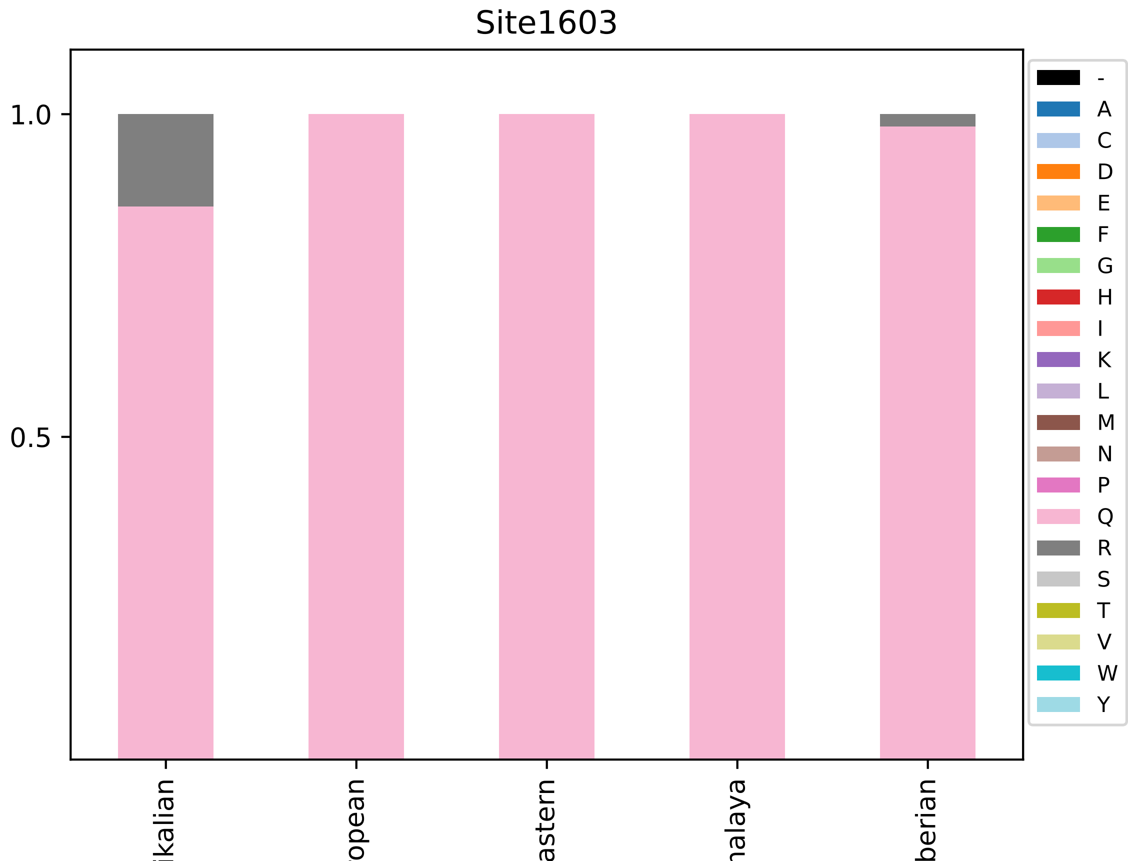
Site1603
1.0
0.5
-
A
C
D
E
F
G
H
I
K
L
M
N
P
Q
R
S
T
V
W
Y
ikalian
opean
astern
nalaya
berian

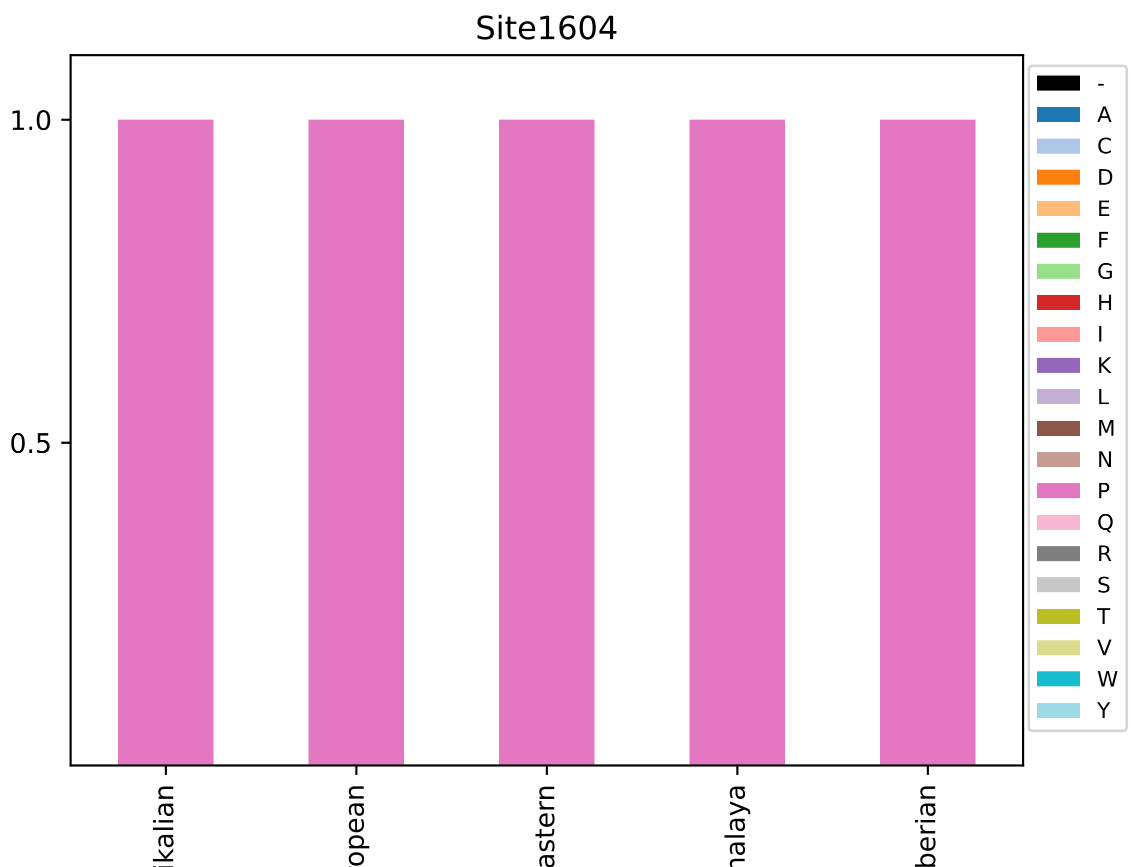
Site1604
1.0
0.5
-
A
C
D
E
F
G
H
I
K
L
M
N
P
Q
R
S
T
V
W
Y
ikalian
opean
astern
nalaya
berian

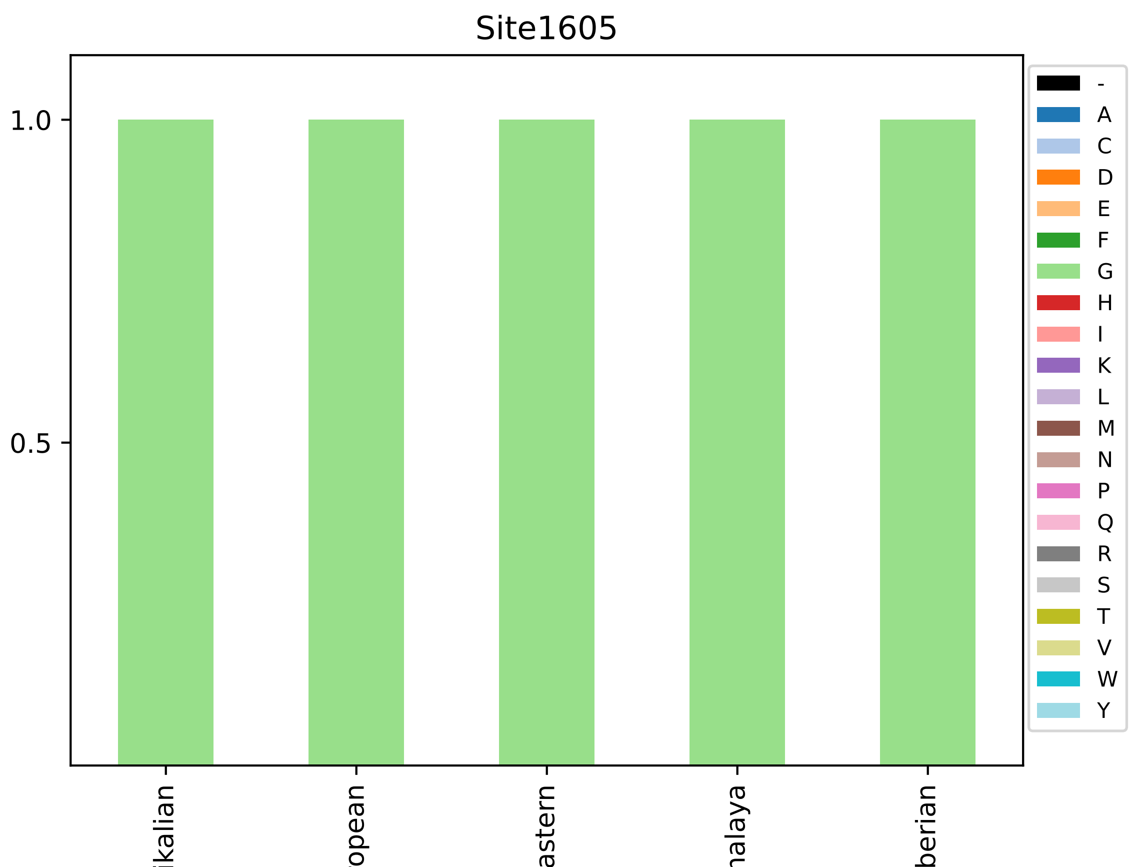
Site1605
1.0
0.5
ikalian
opean
astern
nalaya
berian
-
A
C
D
E
F
G
H
I
K
L
M
N
P
Q
R
S
T
V
W
Y

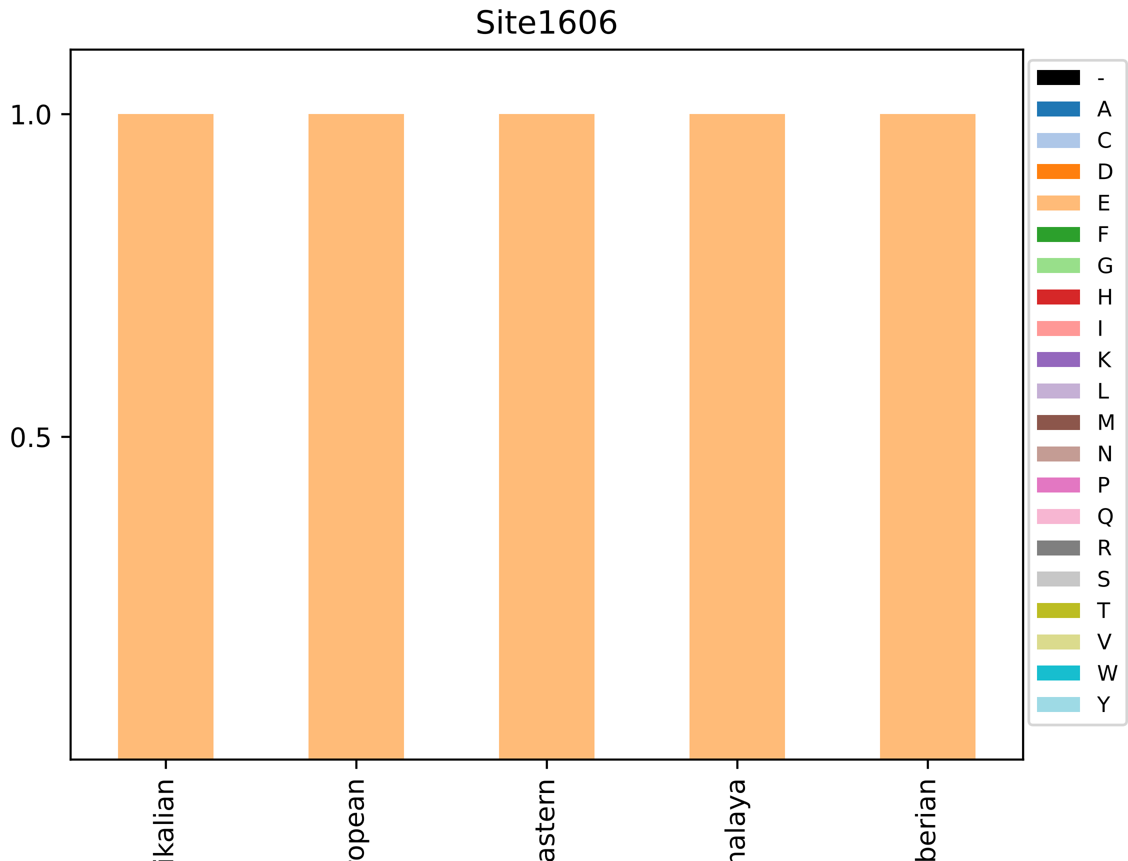

Site1606
1.0
0.5
-
A
C
D
E
F
G
H
I
K
L
M
N
P
Q
R
S
T
V
W
Y
ikalian
opean
astern
nalaya
berian

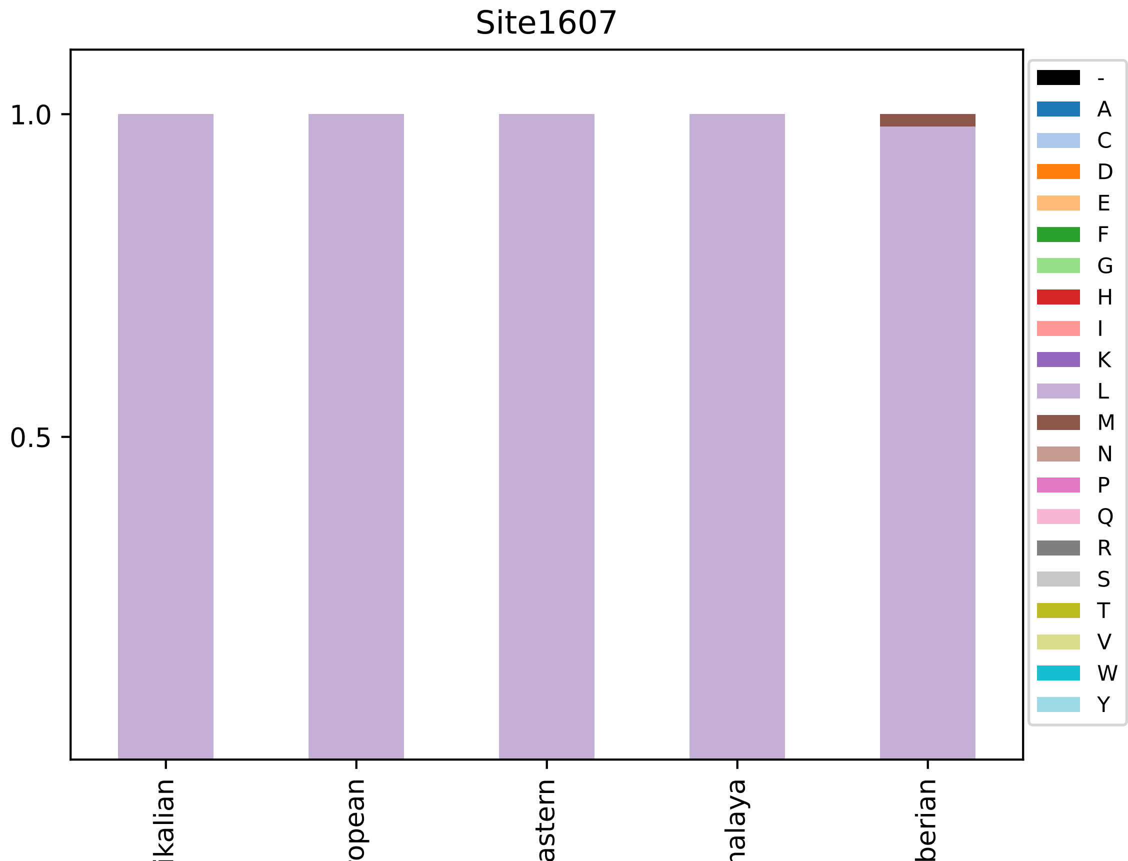

Site1607
1.0
0.5
ikalian
opean
astern
nalaya
berian
-
A
C
D
E
F
G
H
I
K
L
M
N
P
Q
R
S
T
V
W
Y

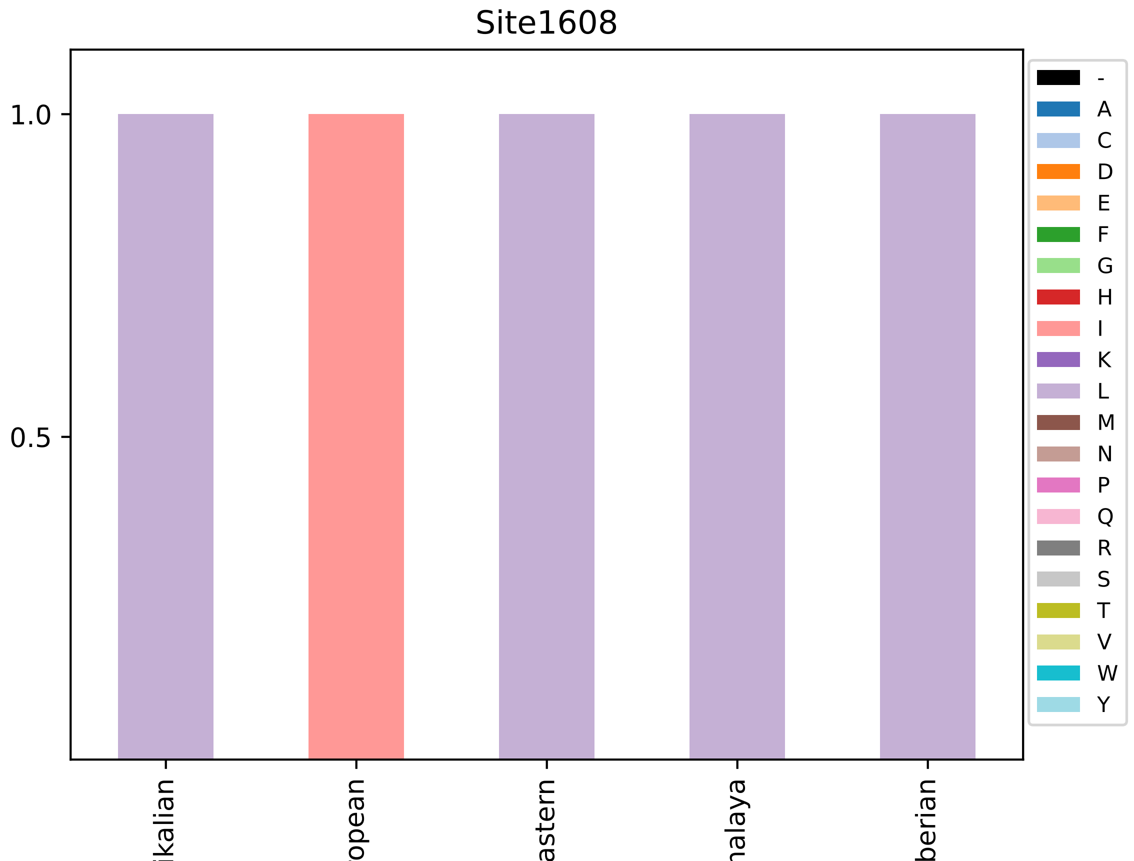

Site1608
1.0
0.5
-
A
C
D
E
F
G
H
I
K
L
M
N
P
Q
R
S
T
V
W
Y
ikalian
opean
astern
nalaya
berian

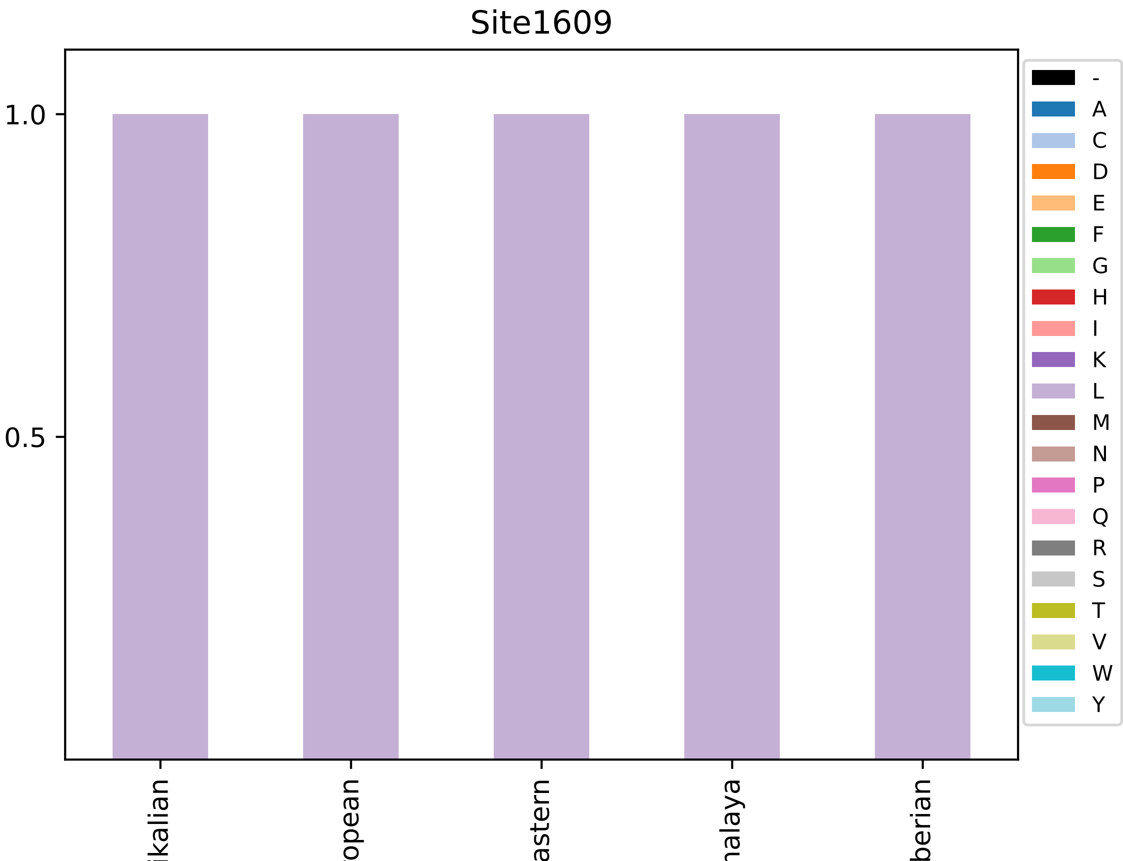

Site1609
1.0
0.5
-
A
C
D
E
F
G
H
I
K
L
M
N
P
Q
R
S
T
V
W
Y
ikalian
opean
astern
nalaya
berian

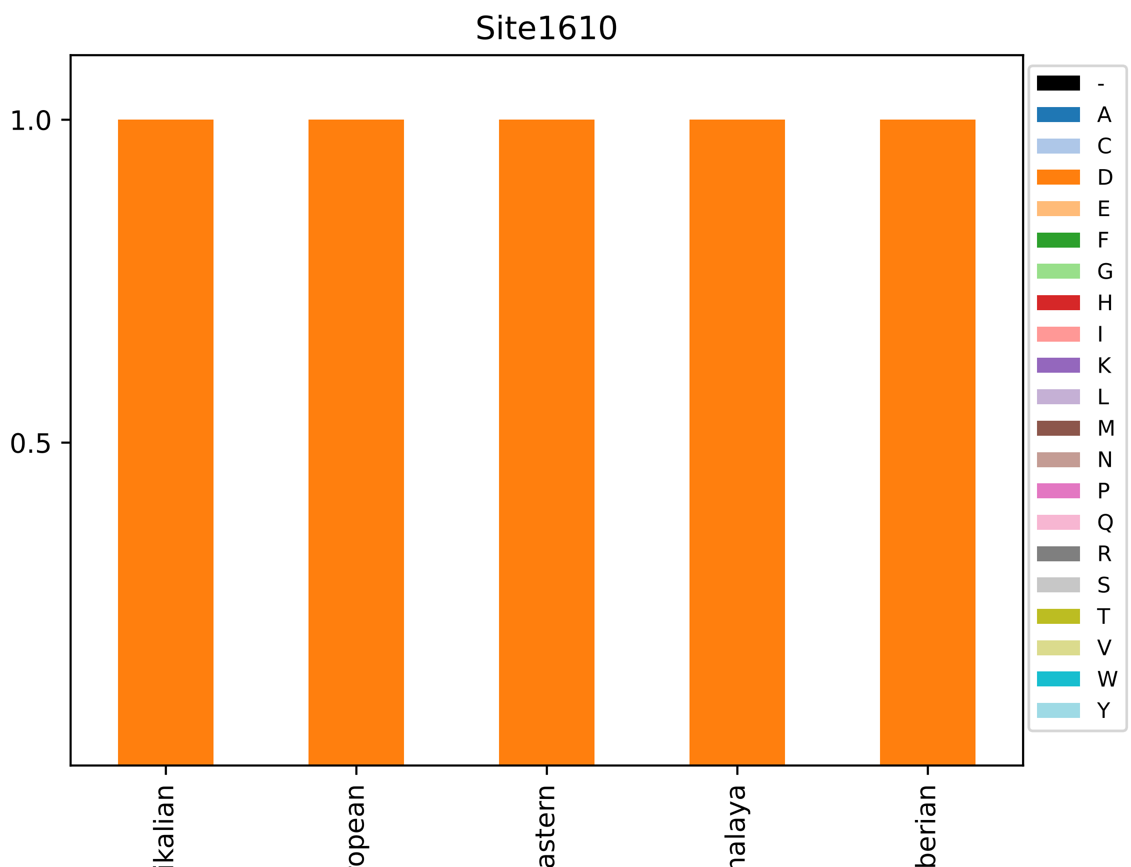

Site1610
1.0
0.5
-
A
C
D
E
F
G
H
I
K
L
M
N
P
Q
R
S
T
V
W
Y
ikalian
opean
astern
nalaya
berian

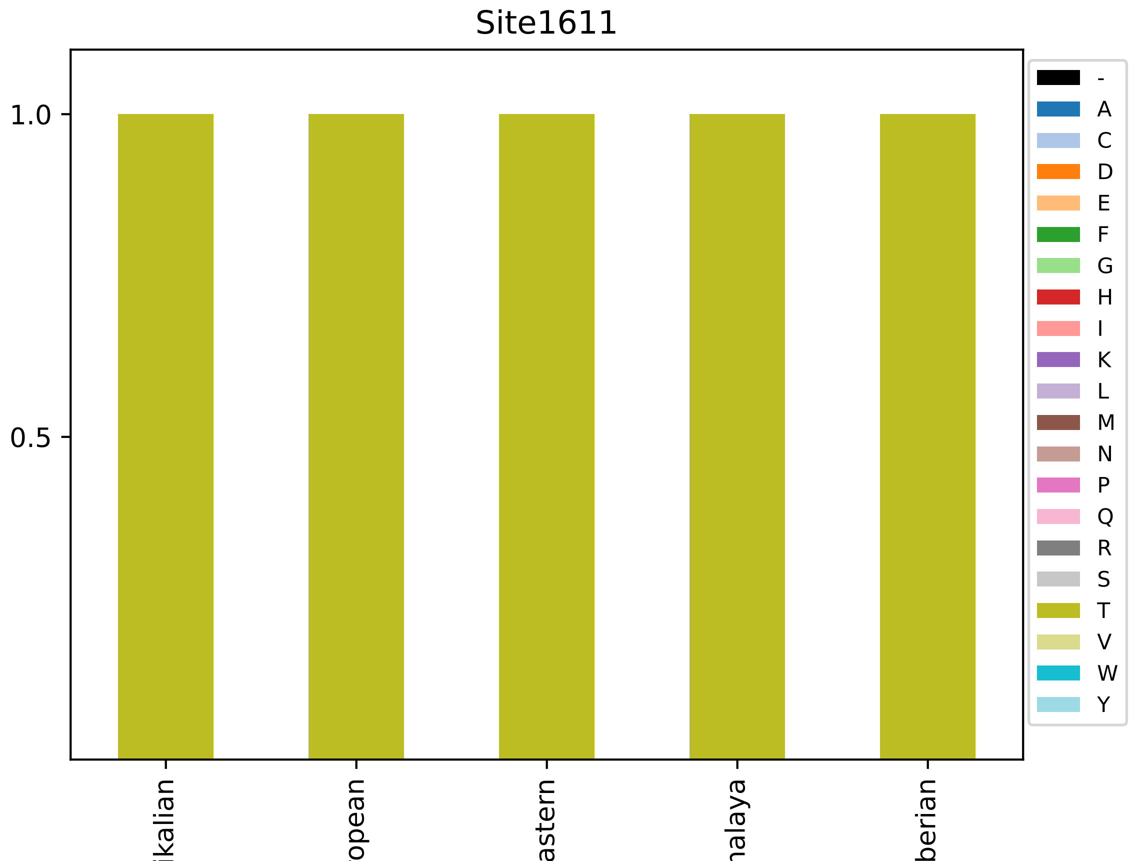

Site1611
1.0
0.5
-
A
C
D
E
F
G
H
I
K
L
M
N
P
Q
R
S
T
V
W
Y
ikalian
ropean
astern
nalaya
berian

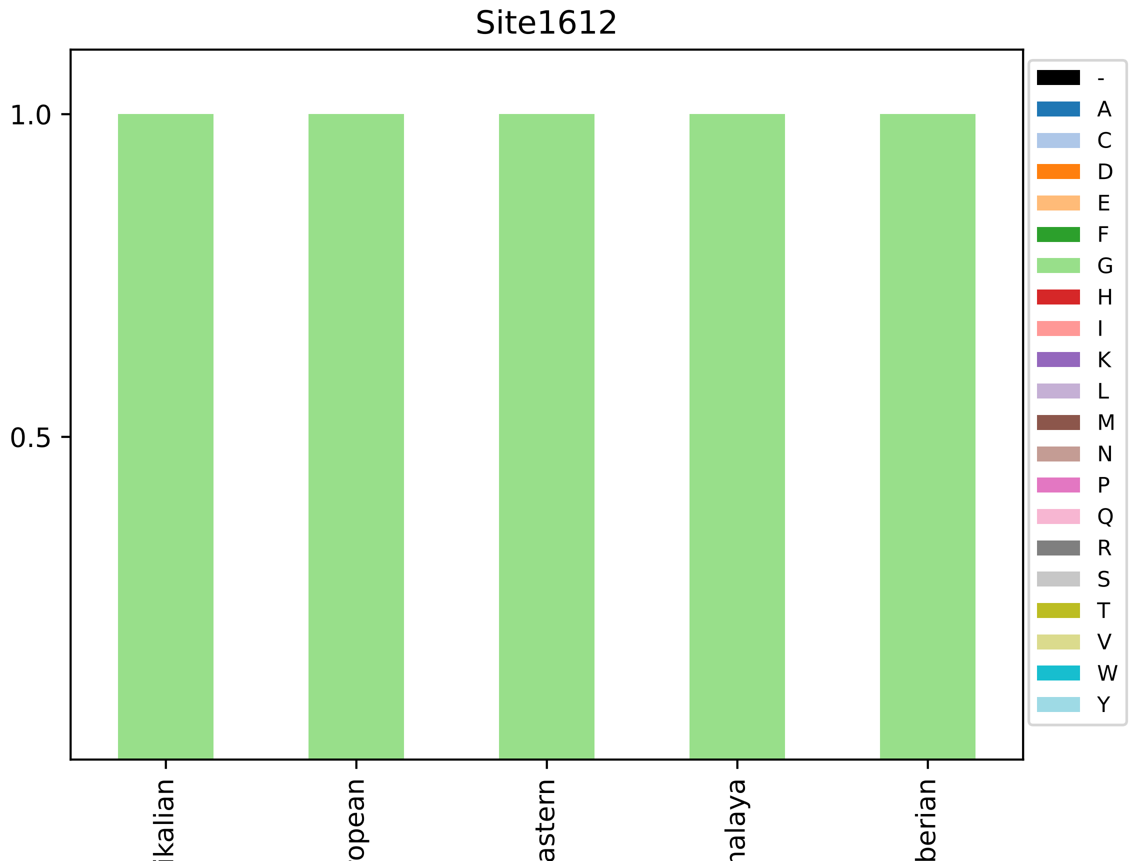
Site1612
1.0
0.5
-
A
C
D
E
F
G
H
I
K
L
M
N
P
Q
R
S
T
V
W
Y
ikalian
opean
astern
nalaya
berian

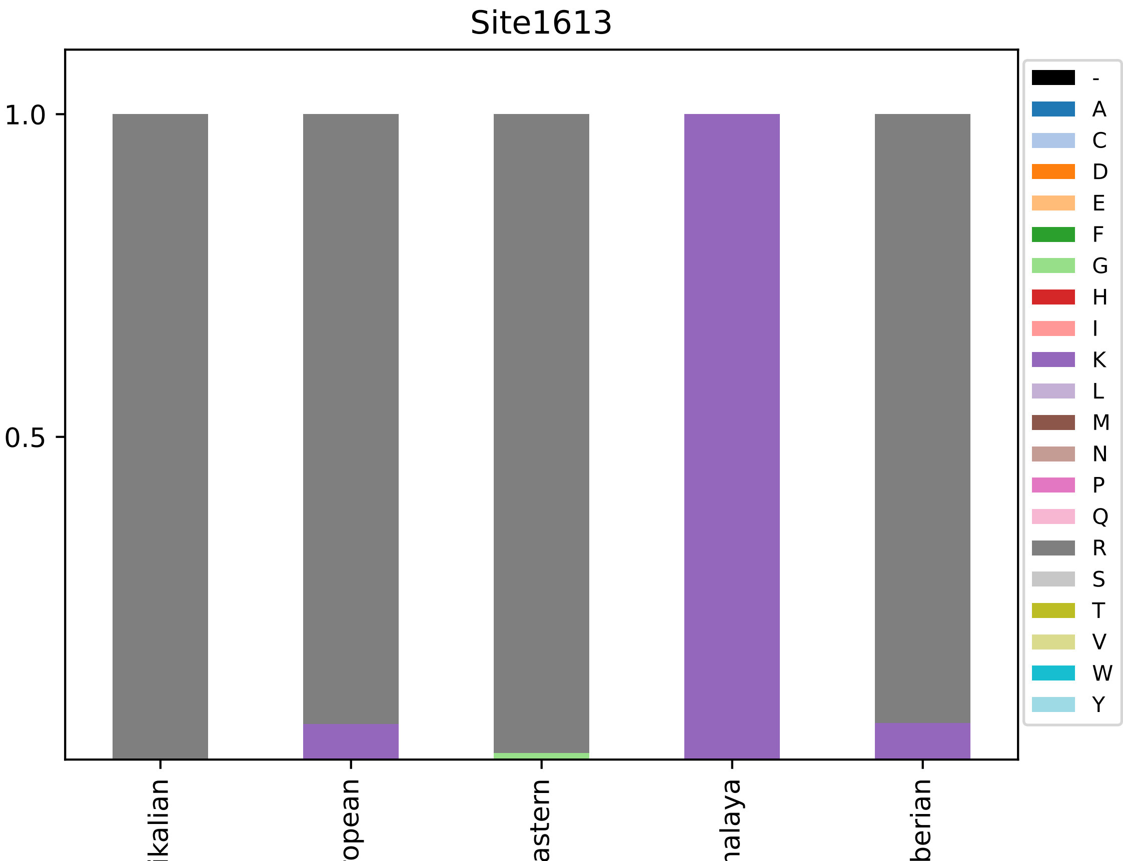
Site1613
1.0
0.5
-
A
C
D
E
F
G
H
I
K
L
M
N
P
Q
R
S
T
V
W
Y

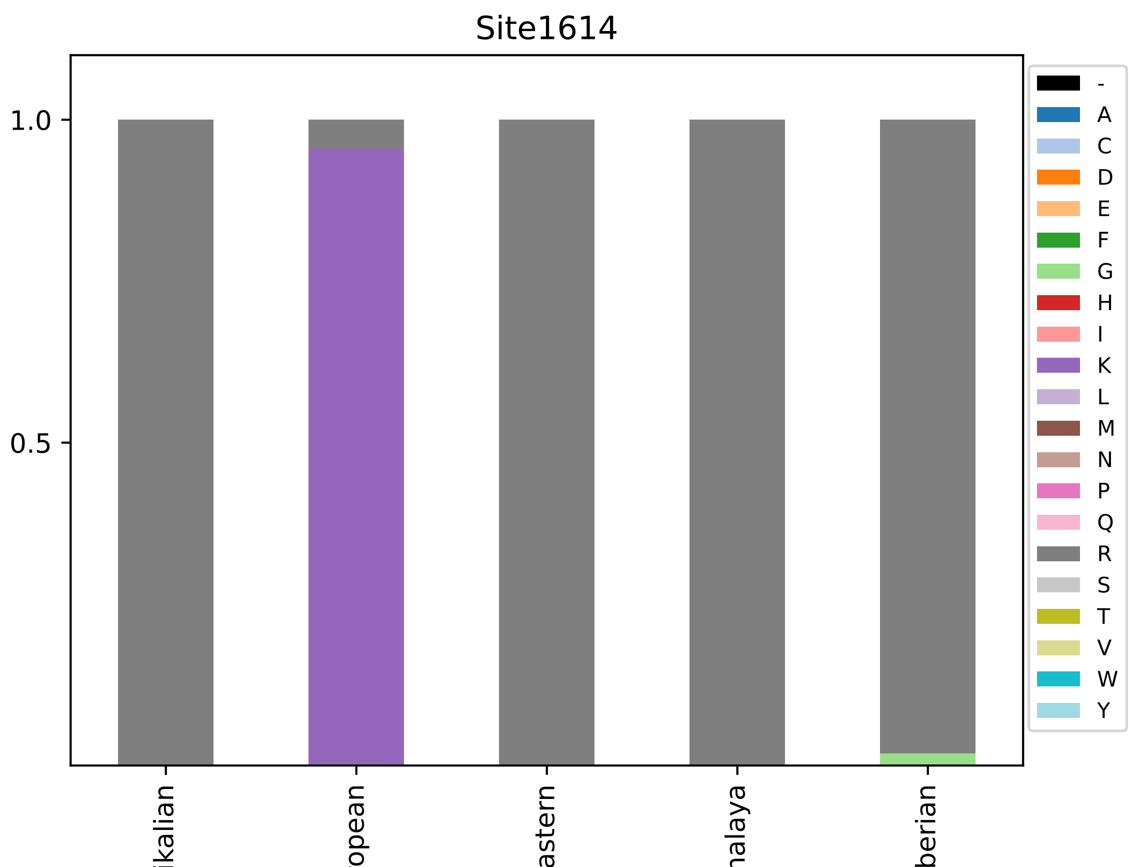

Site1614
1.0
0.5
ikalian
opean
astern
nalaya
berian
-
A
C
D
E
F
G
H
I
K
L
M
N
P
Q
R
S
T
V
W
Y

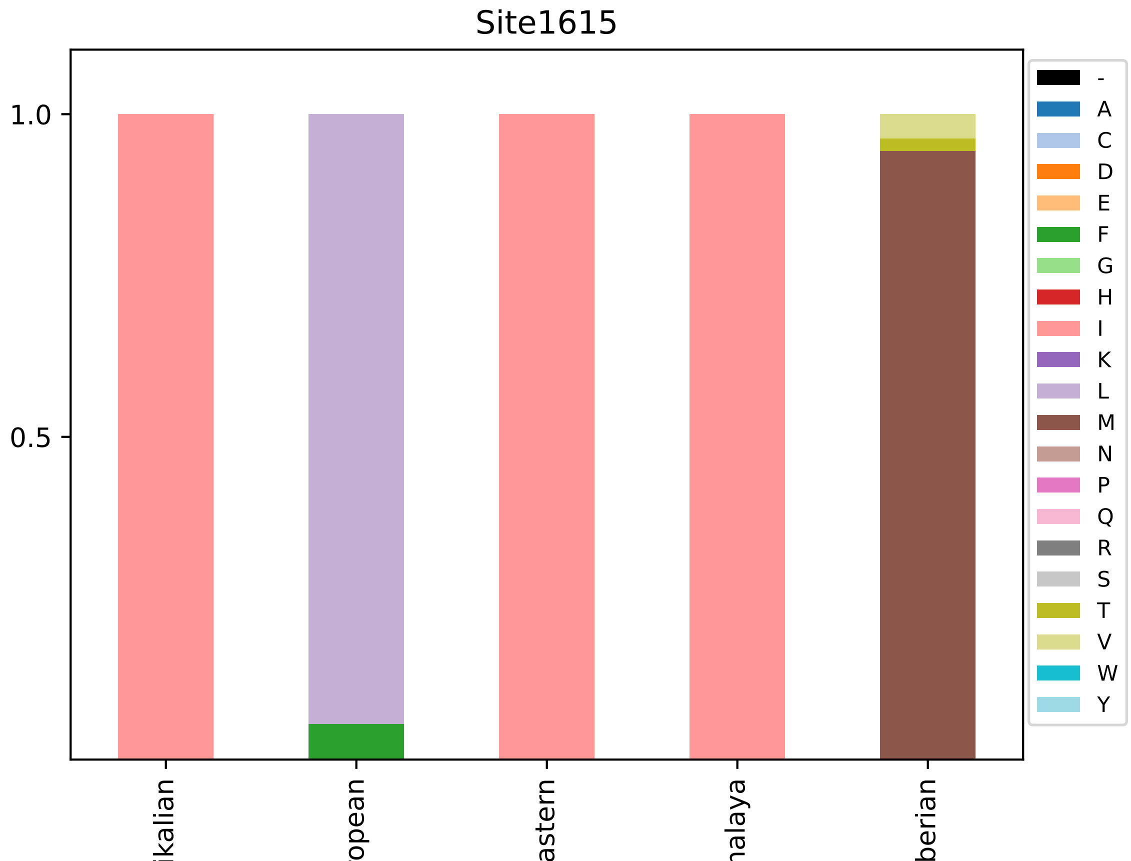
Site1615
1.0
0.5
-
A
C
D
E
F
G
H
I
K
L
M
N
P
Q
R
S
T
V
W
Y
ikalian
opean
astern
nalaya
berian

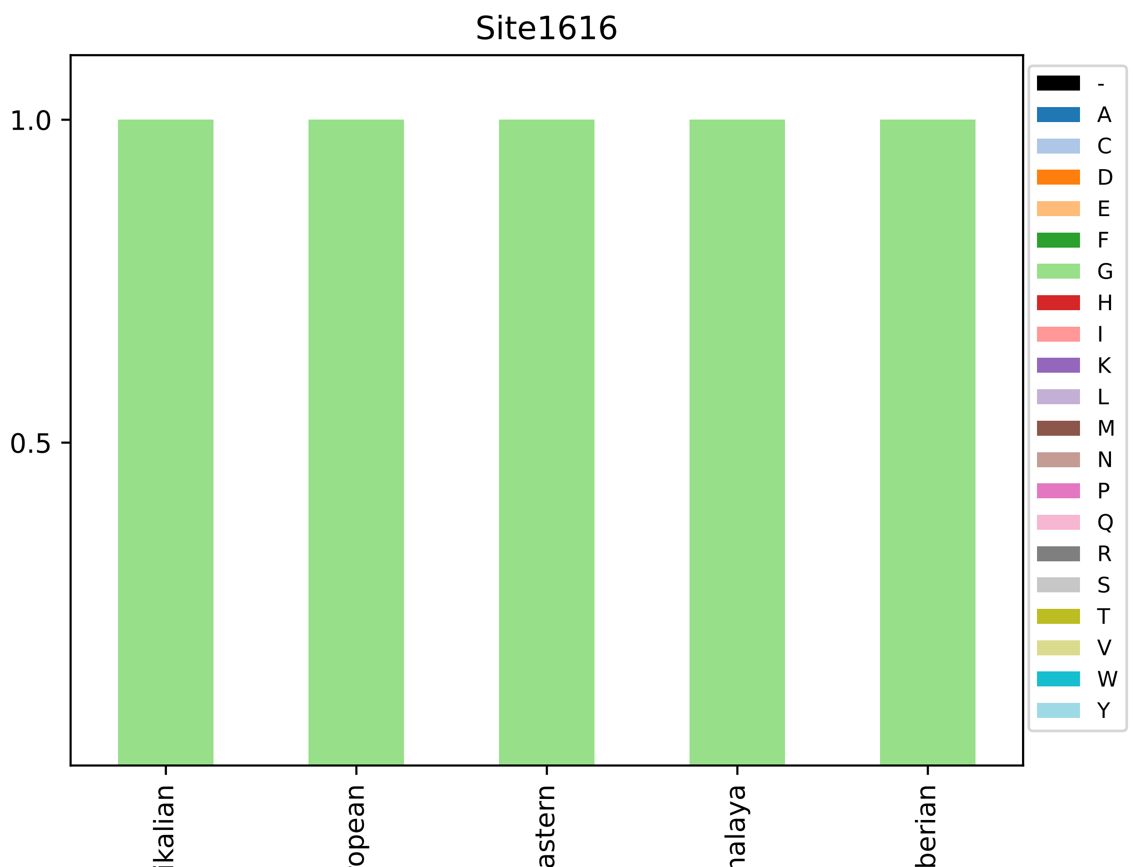
Site1616
1.0
0.5
-
A
C
D
E
F
G
H
I
K
L
M
N
P
Q
R
S
T
V
W
Y
ikalian
opean
astern
nalaya
berian

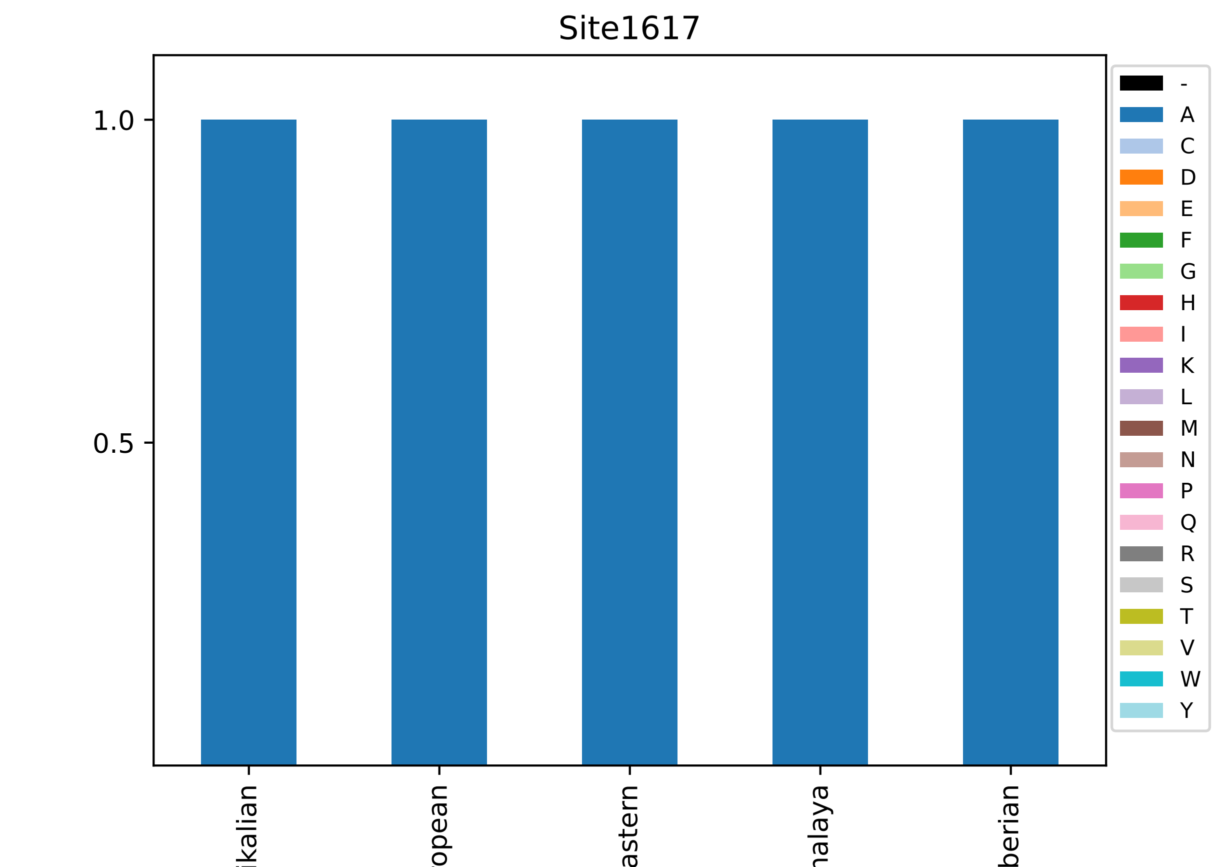

Site1617
1.0
0.5
-
A
C
D
E
F
G
H
I
K
L
M
N
P
Q
R
S
T
V
W
Y
ikalian
ropean
astern
nalaya
berian

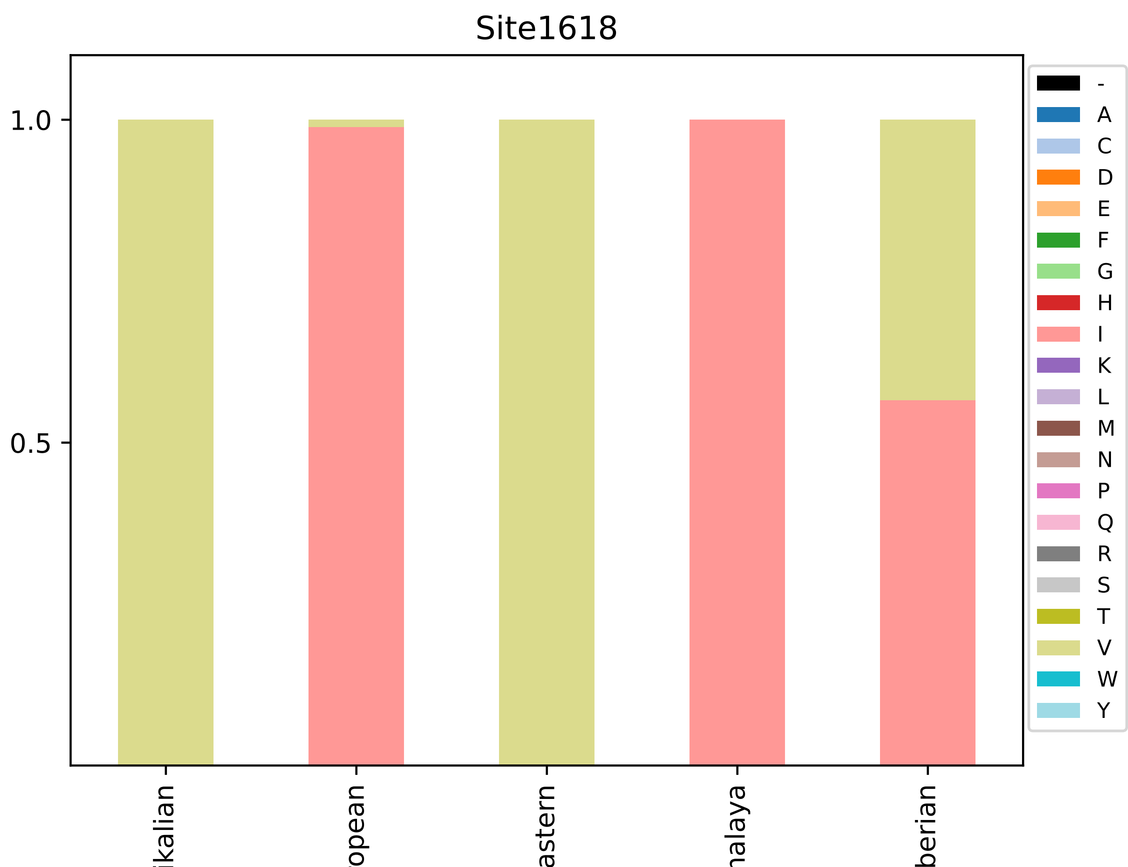
Site1618
1.0
0.5
ikalian
opean
astern
nalaya
berian
-
A
C
D
E
F
G
H
I
K
L
M
N
P
Q
R
S
T
V
W
Y

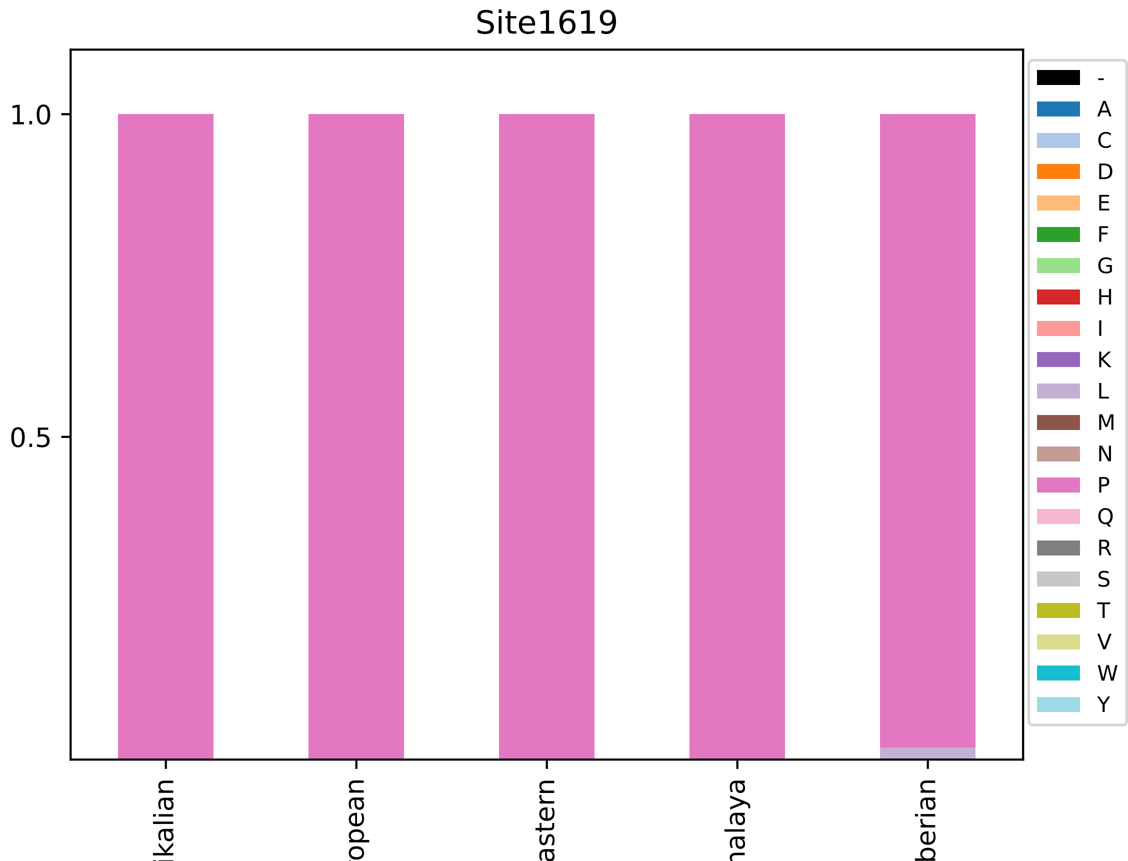
Site1619
1.0
0.5
-
A
C
D
E
F
G
H
I
K
L
M
N
P
Q
R
S
T
V
W
Y
ikalian
opean
astern
nalaya
berian

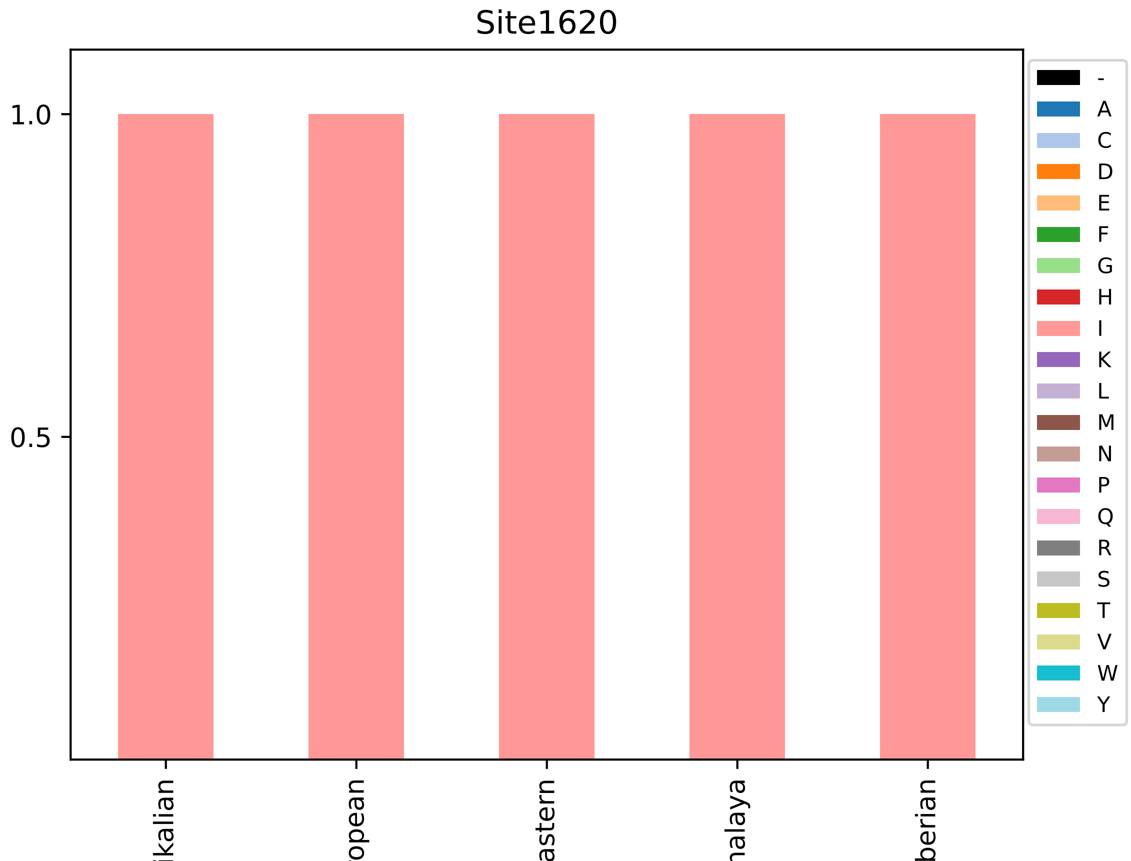
Site1620
1.0
0.5
-
A
C
D
E
F
G
H
I
K
L
M
N
P
Q
R
S
T
V
W
Y
ikalian
opean
astern
nalaya
berian

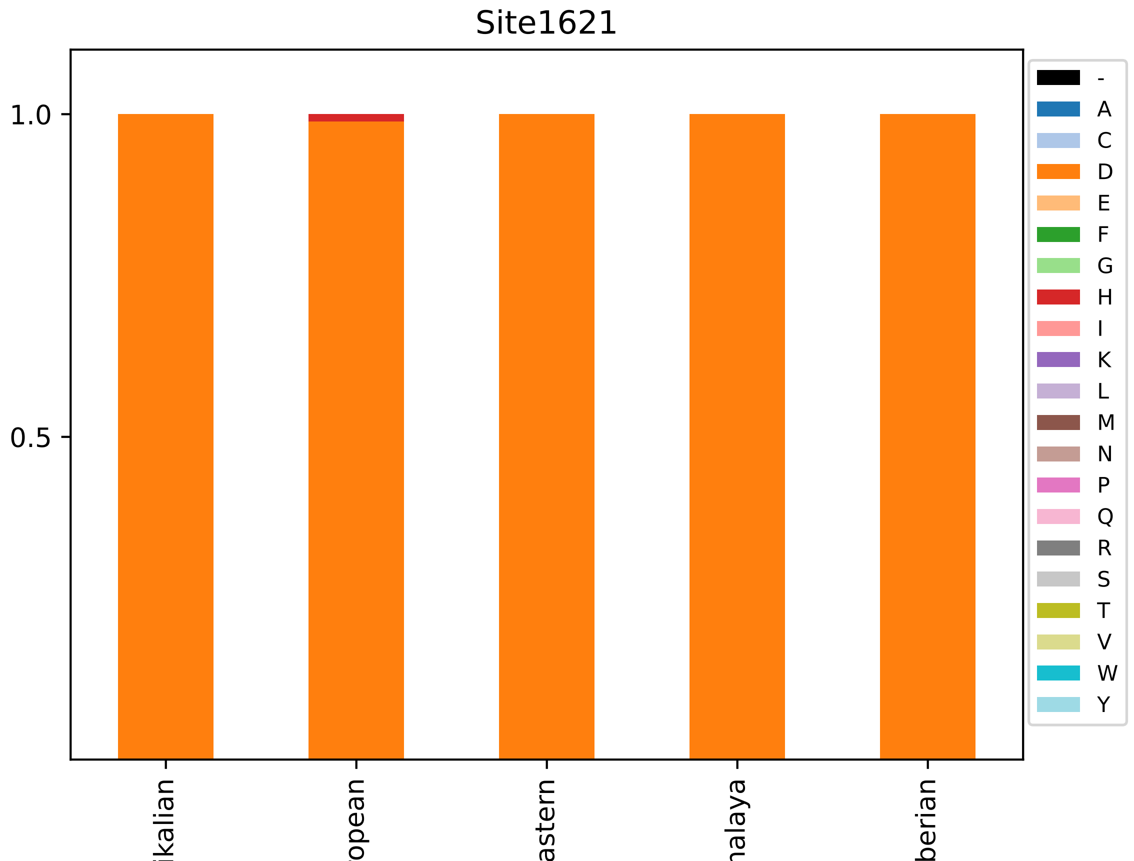
Site1621
1.0
0.5
-
A
C
D
E
F
G
H
I
K
L
M
N
P
Q
R
S
T
V
W
Y
ikalian
opean
astern
nalaya
berian

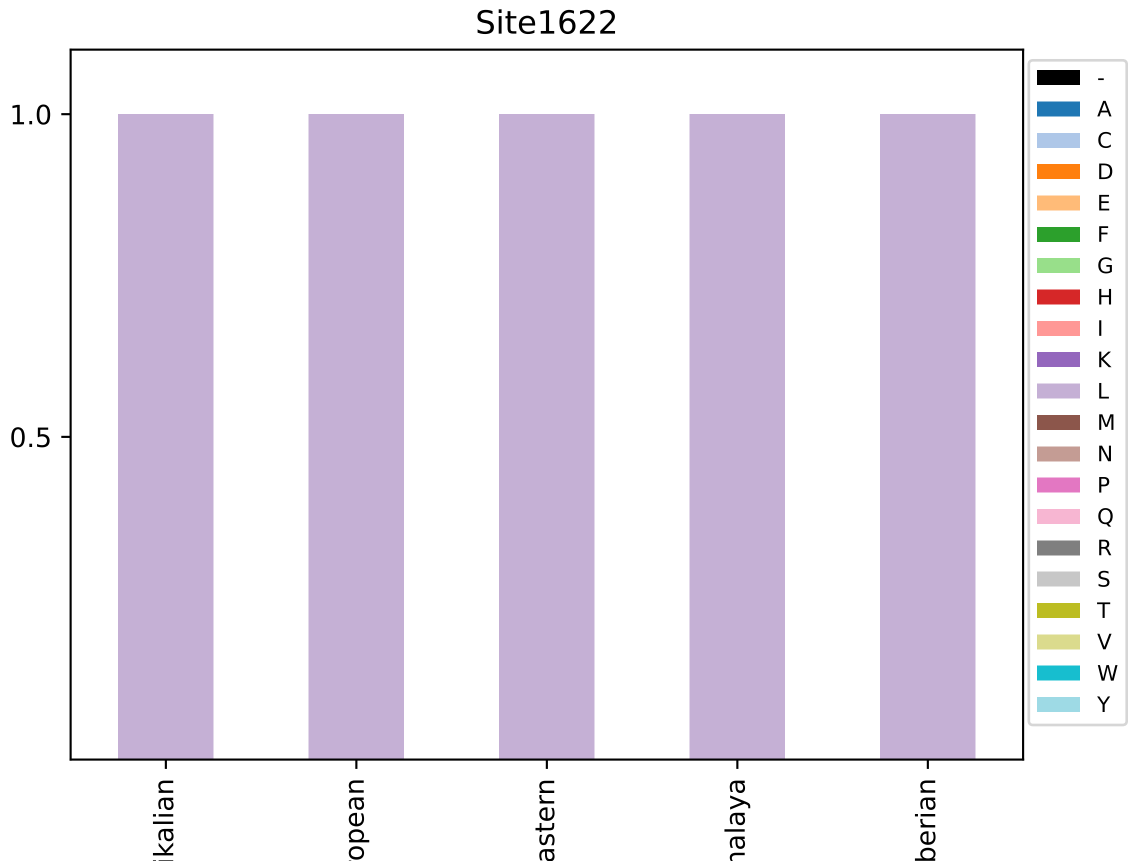
Site1622
1.0
0.5
-
A
C
D
E
F
G
H
I
K
L
M
N
P
Q
R
S
T
V
W
Y
ikalian
opean
astern
nalaya
berian

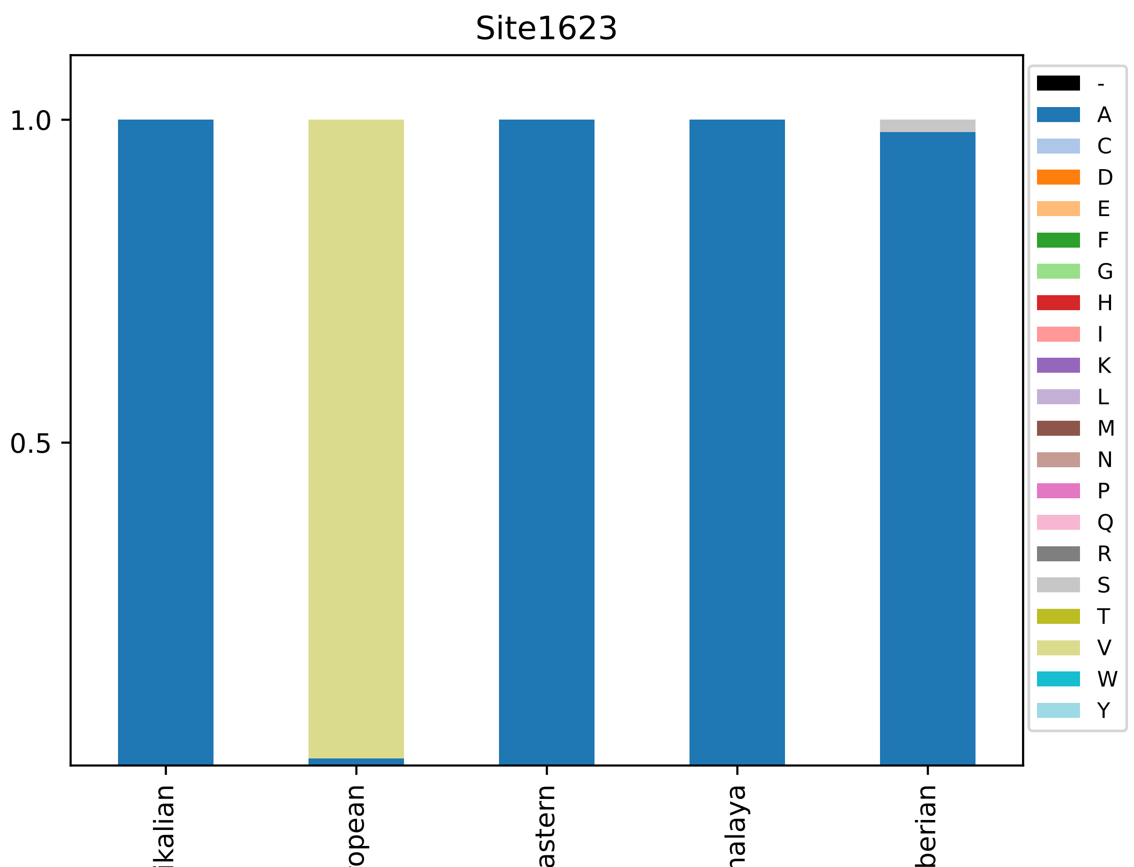

Site1623
1.0
0.5
-
A
C
D
E
F
G
H
I
K
L
M
N
P
Q
R
S
T
V
W
Y
ikalian
opean
astern
halaya
berian

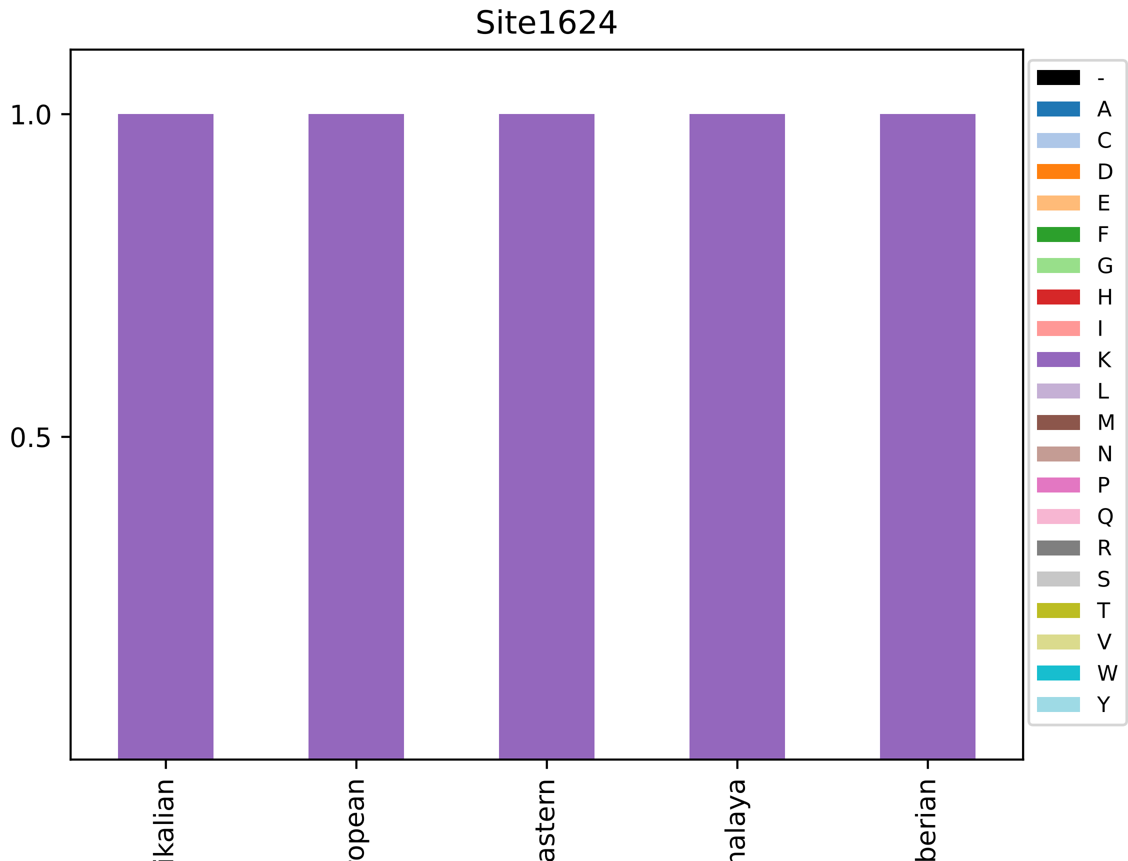

Site1624
1.0
0.5
-
A
C
D
E
F
G
H
I
K
L
M
N
P
Q
R
S
T
V
W
Y
ikalian
opean
astern
nalaya
berian

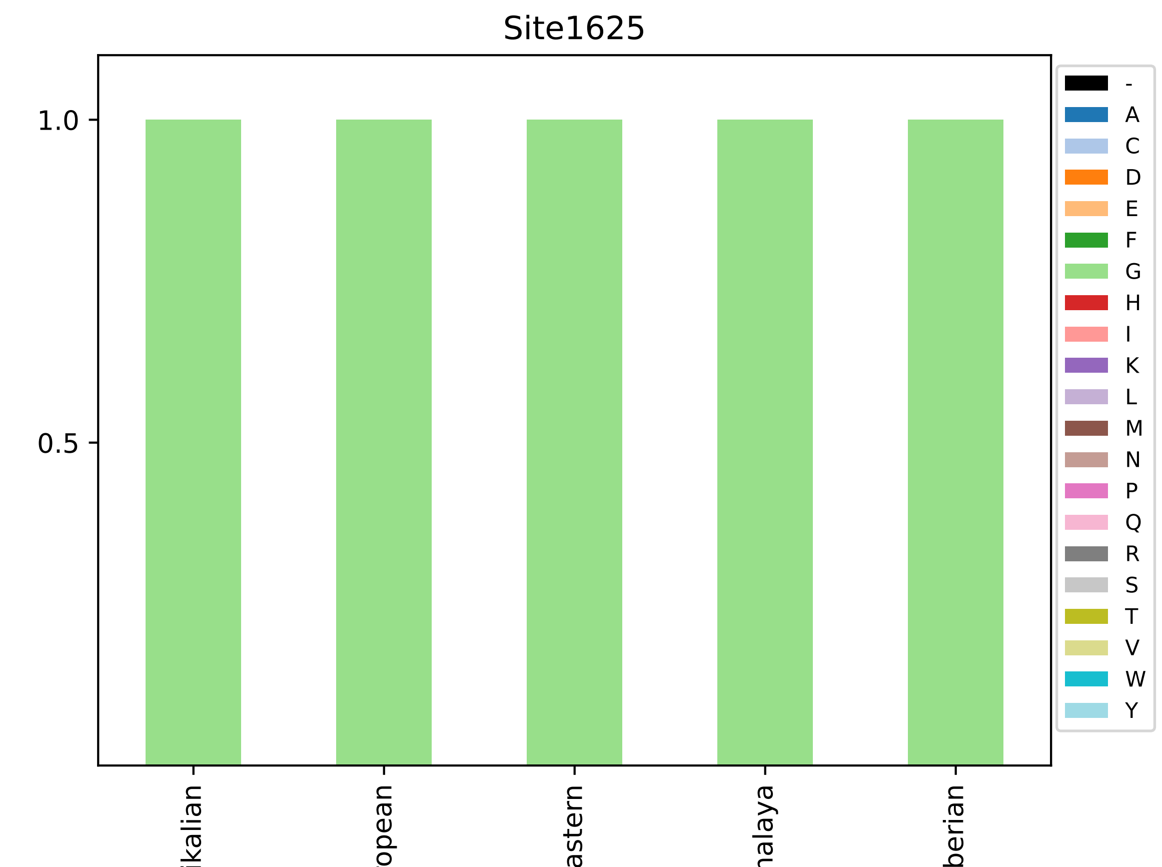
Site1625
1.0
0.5
ikalian
opean
astern
nalaya
berian
-
A
C
D
E
F
G
H
I
K
L
M
N
P
Q
R
S
T
V
W
Y

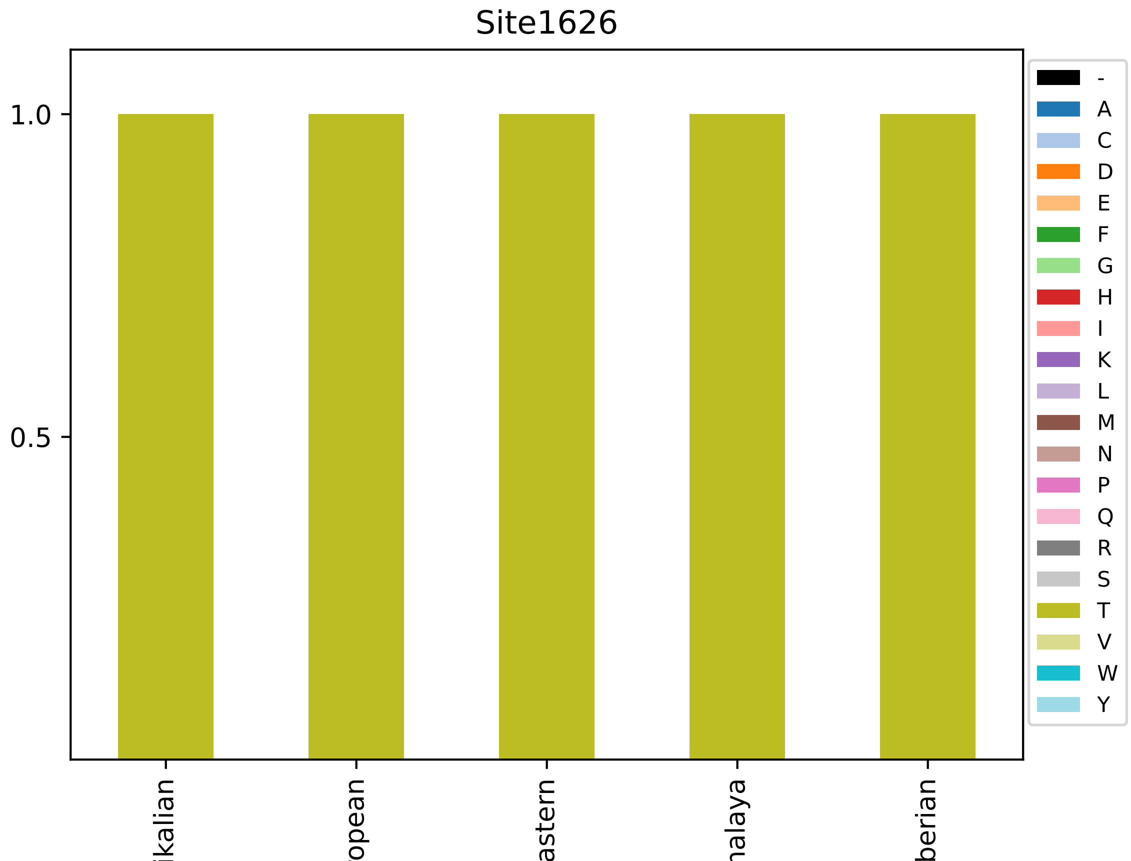
Site1626
1.0
0.5
-
A
C
D
E
F
G
H
I
K
L
M
N
P
Q
R
S
T
V
W
Y
ikalian
opean
astern
nalaya
berian

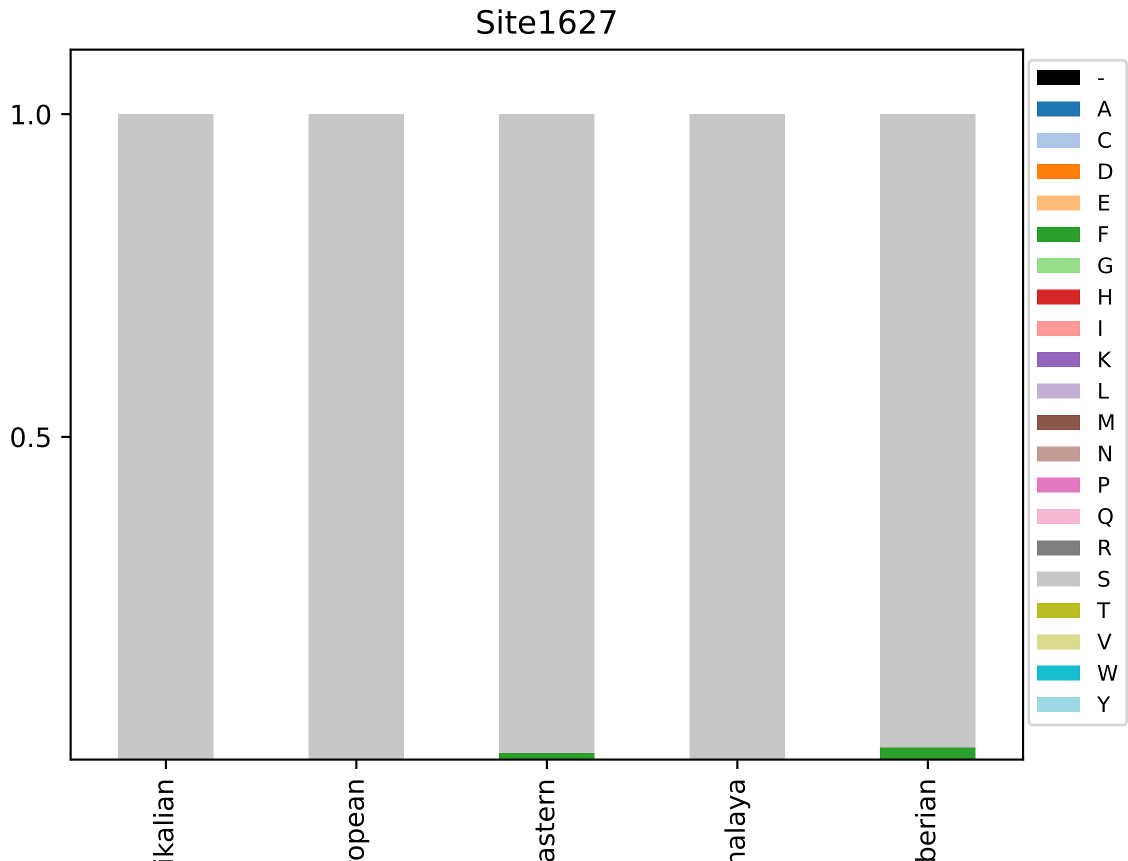
Site1627
1.0
0.5
ikalian
ropean
astern
nalaya
berian
-
A
C
D
E
F
G
H
I
K
L
M
N
P
Q
R
S
T
V
W
Y

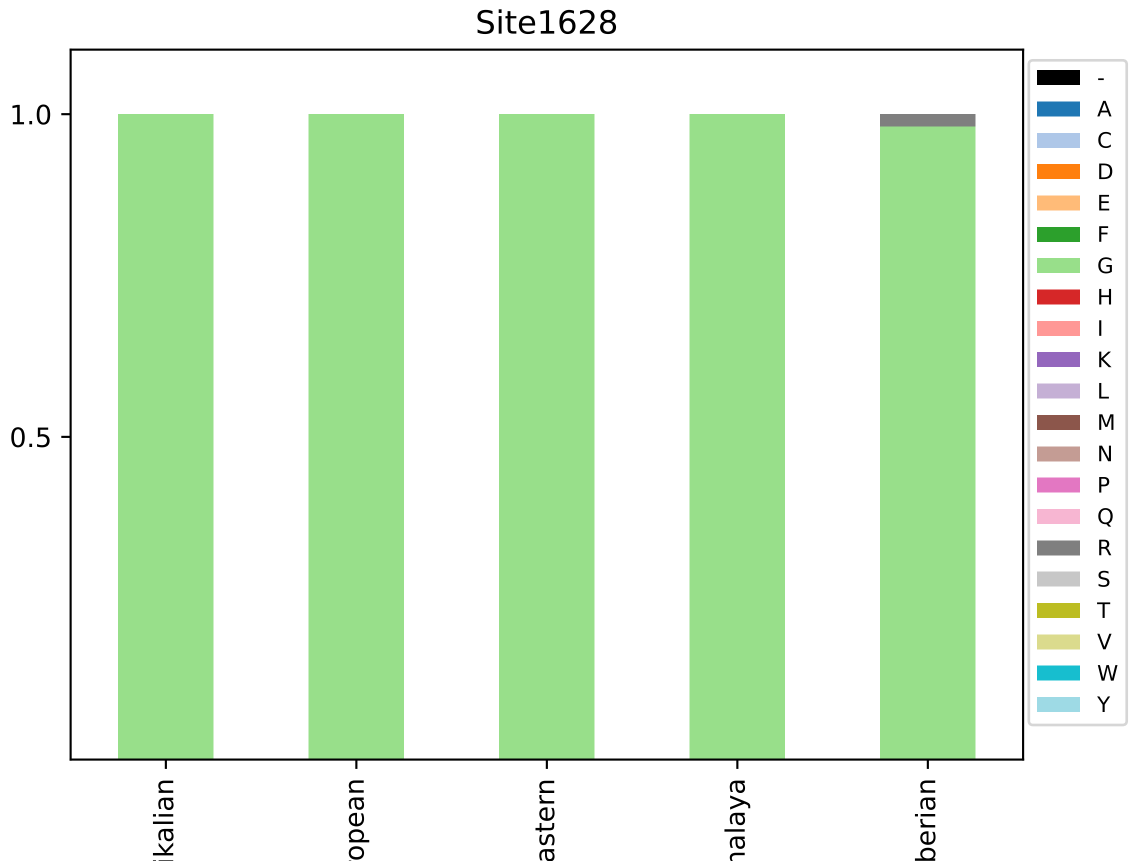
Site1628
1.0
0.5
-
A
C
D
E
F
G
H
I
K
L
M
N
P
Q
R
S
T
V
W
Y
ikalian
opean
astern
nalaya
berian

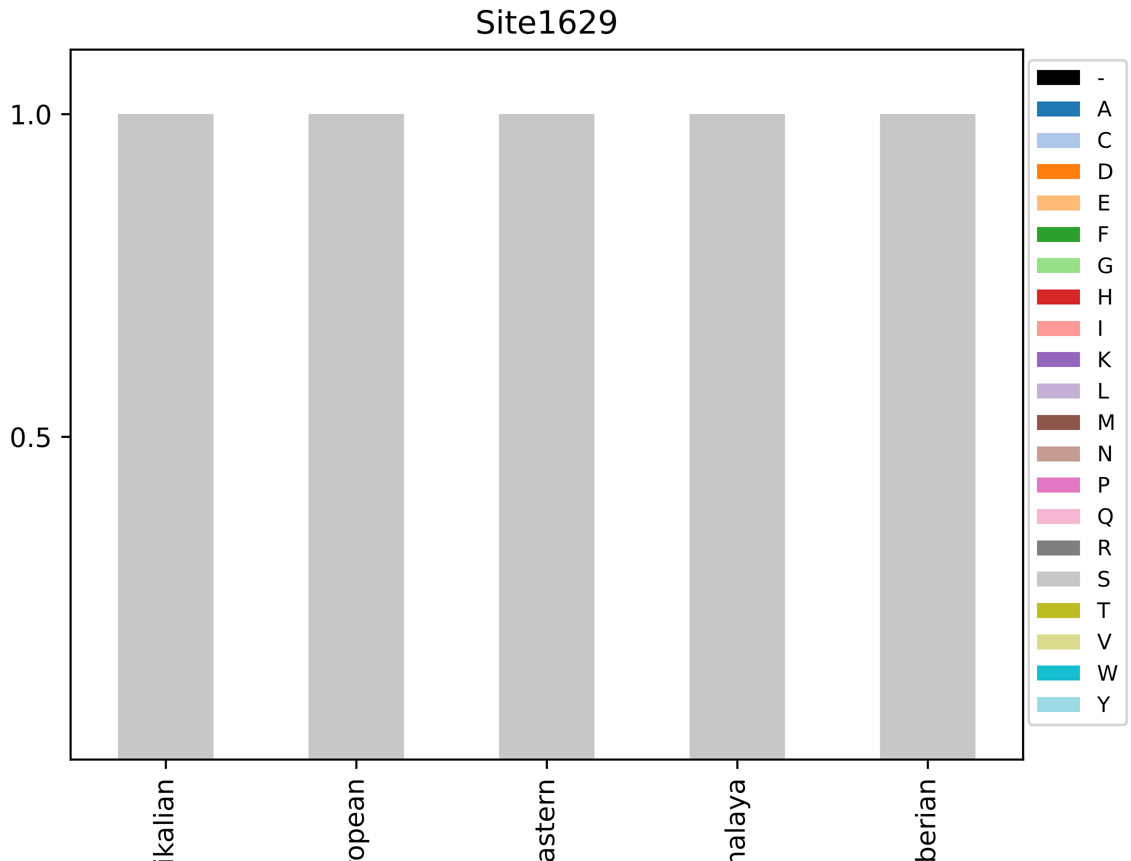
Site1629
1.0
0.5
-
A
C
D
E
F
G
H
I
K
L
M
N
P
Q
R
S
T
V
W
Y
ikalian
opean
astern
nalaya
berian

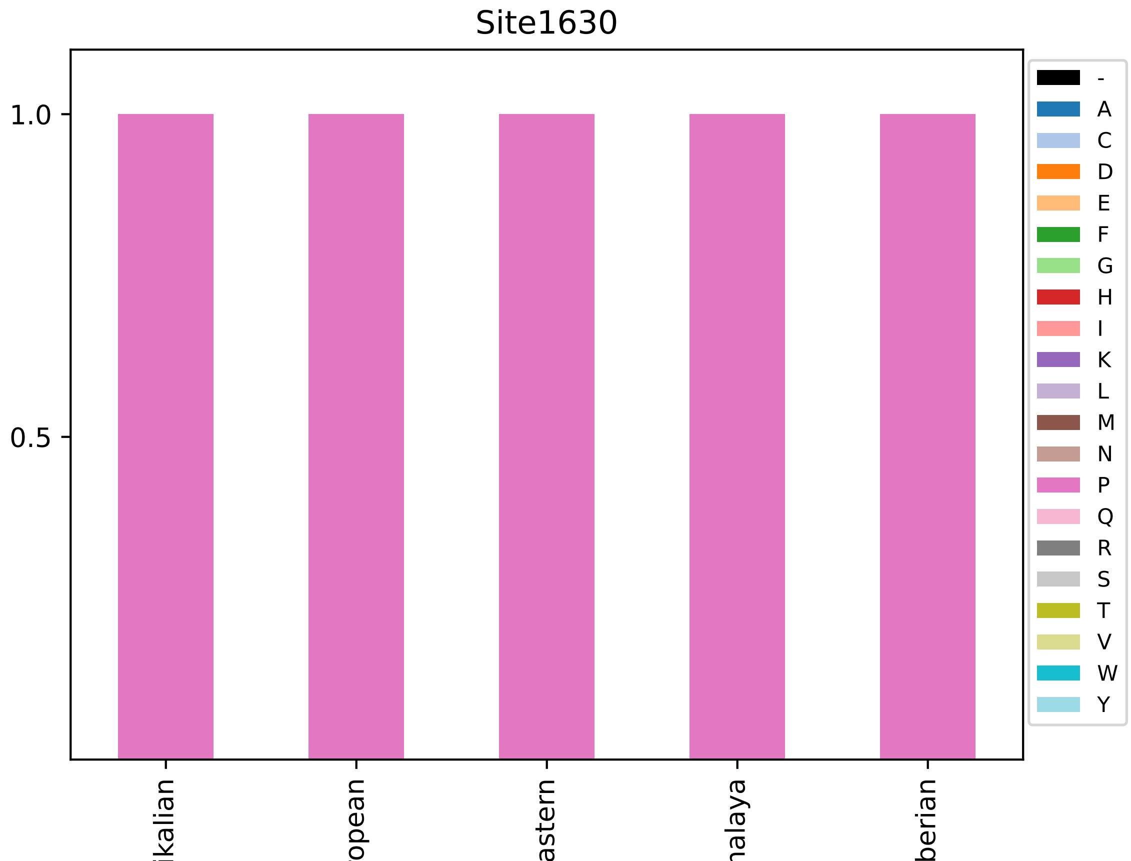

Site1630
1.0
0.5
-
A
C
D
E
F
G
H
I
K
L
M
N
P
Q
R
S
T
V
W
Y
ikalian
opean
astern
nalaya
berian

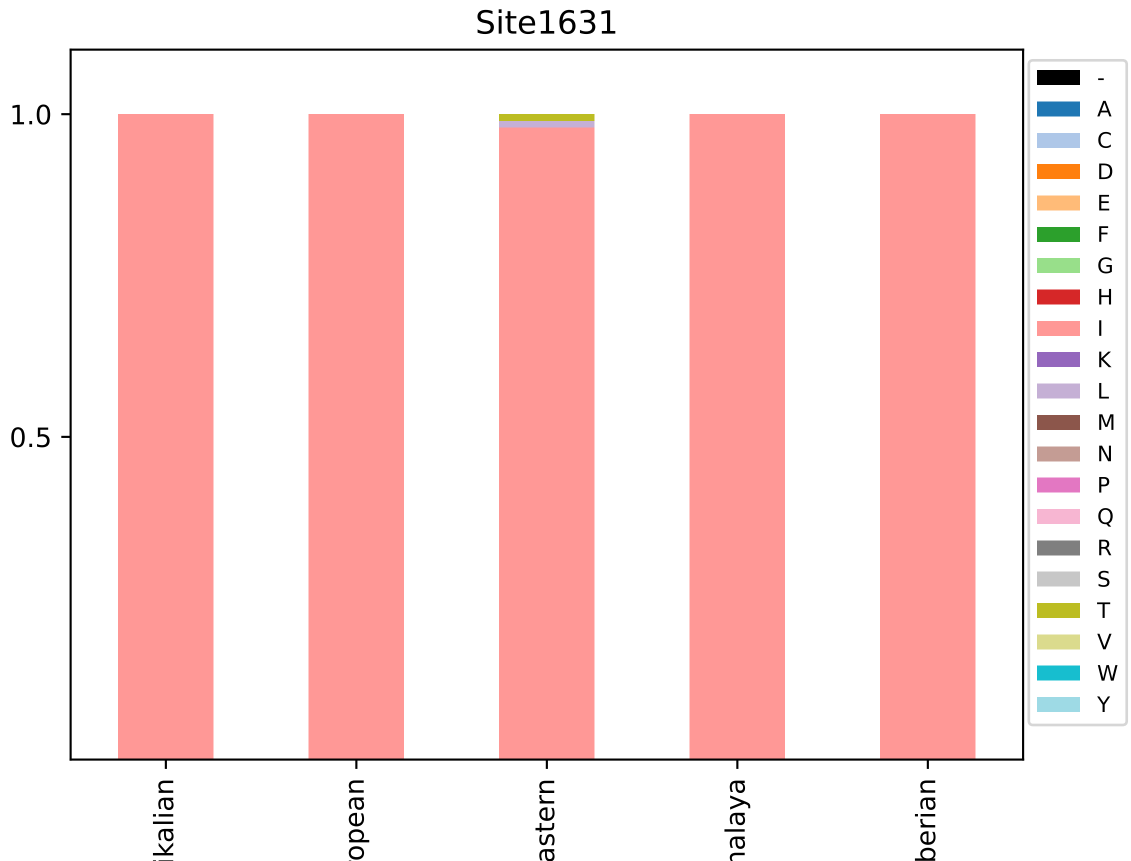

Site1631
1.0
0.5
-
A
C
D
E
F
G
H
I
K
L
M
N
P
Q
R
S
T
V
W
Y
ikalian
opean
astern
nalaya
berian

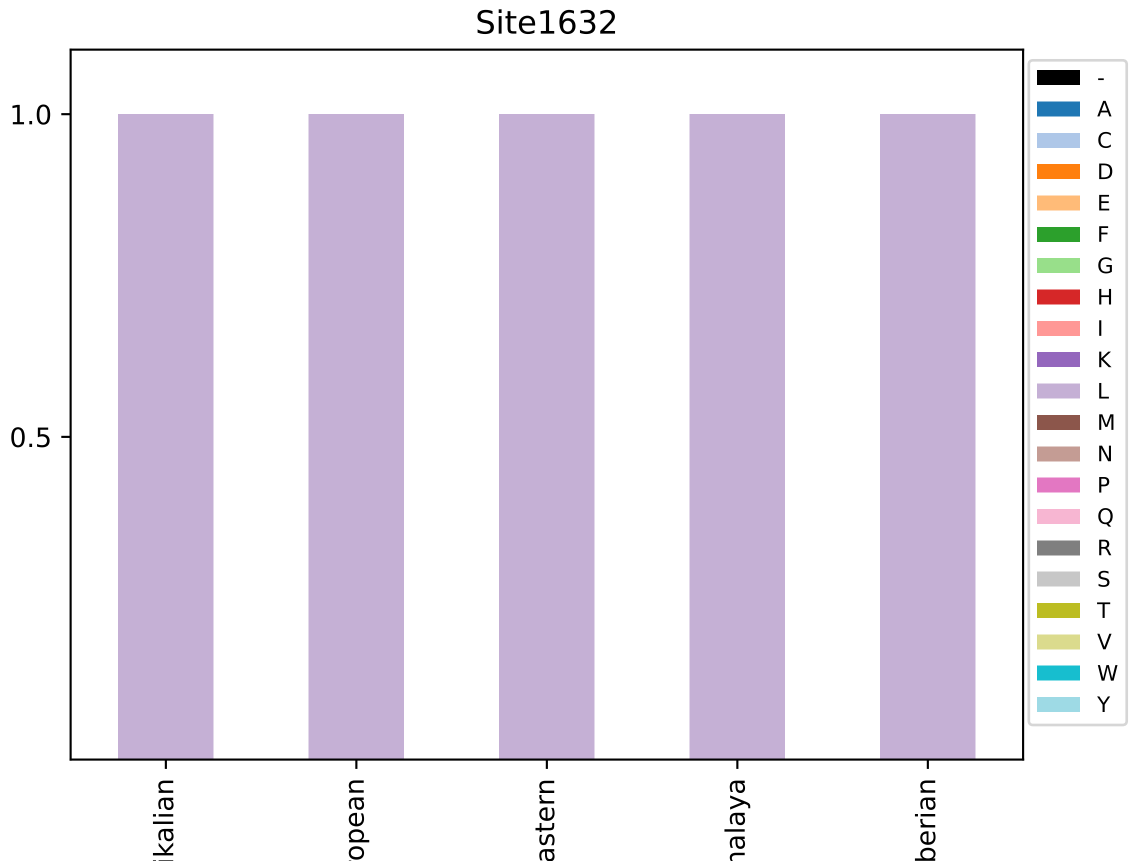
Site1632
1.0
0.5
-
A
C
D
E
F
G
H
I
K
L
M
N
P
Q
R
S
T
V
W
Y
ikalian
opean
astern
nalaya
berian

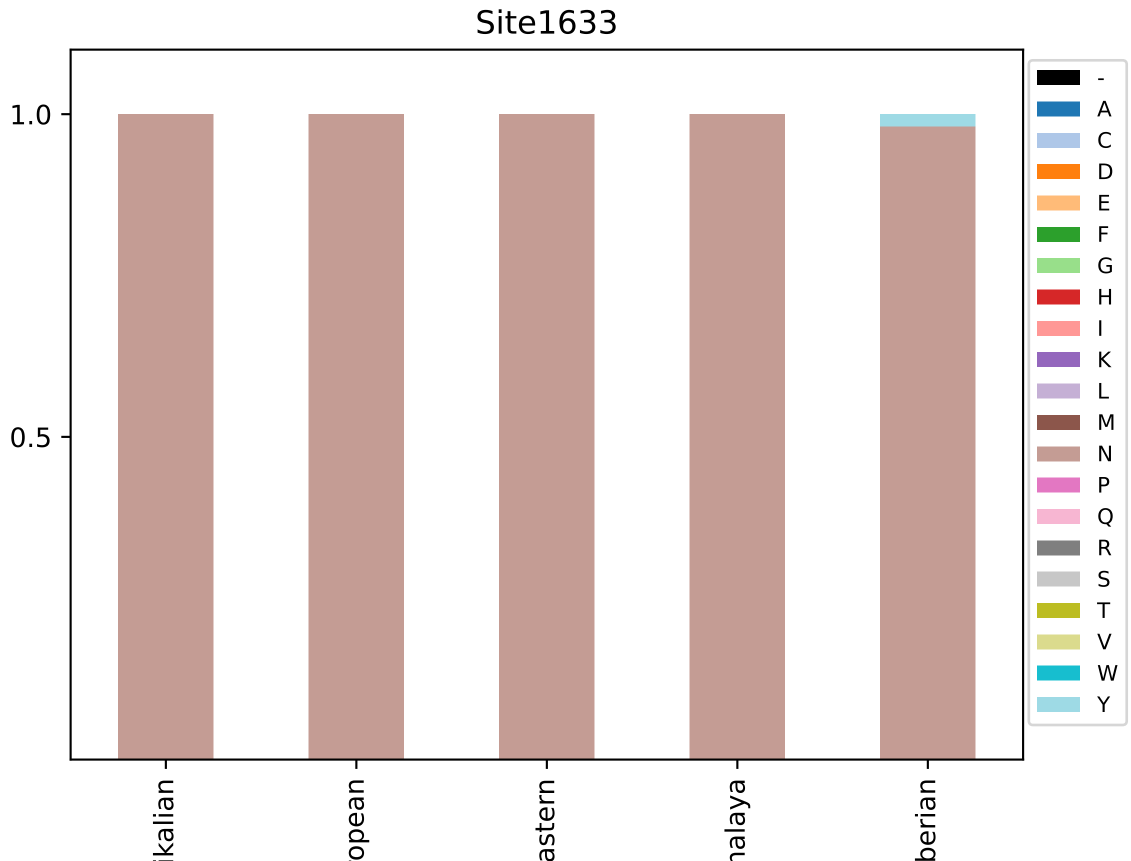
Site1633
1.0
0.5
-
A
C
D
E
F
G
H
I
K
L
M
N
P
Q
R
S
T
V
W
Y
ikalian
opean
astern
nalaya
berian

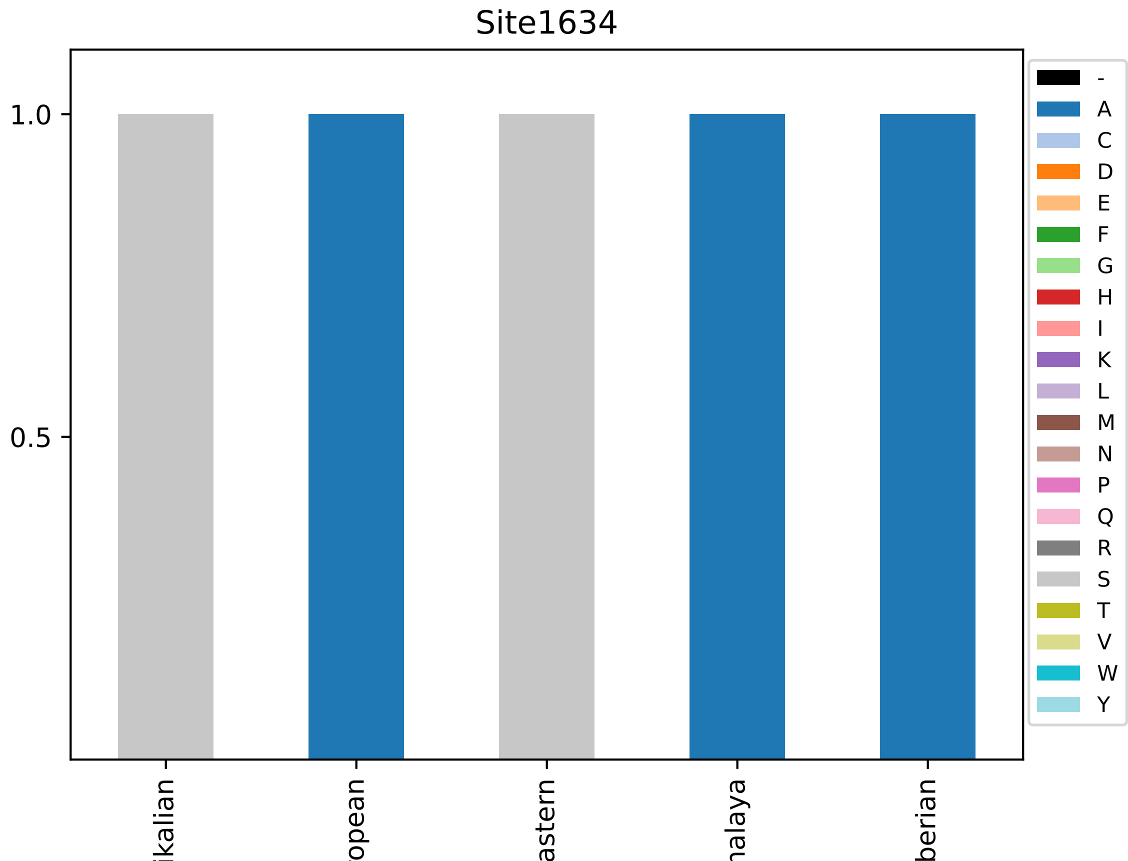

Site1634
1.0
0.5
-
A
C
D
E
F
G
H
I
K
L
M
N
P
Q
R
S
T
V
W
Y
ikalian
opean
astern
nalaya
berian

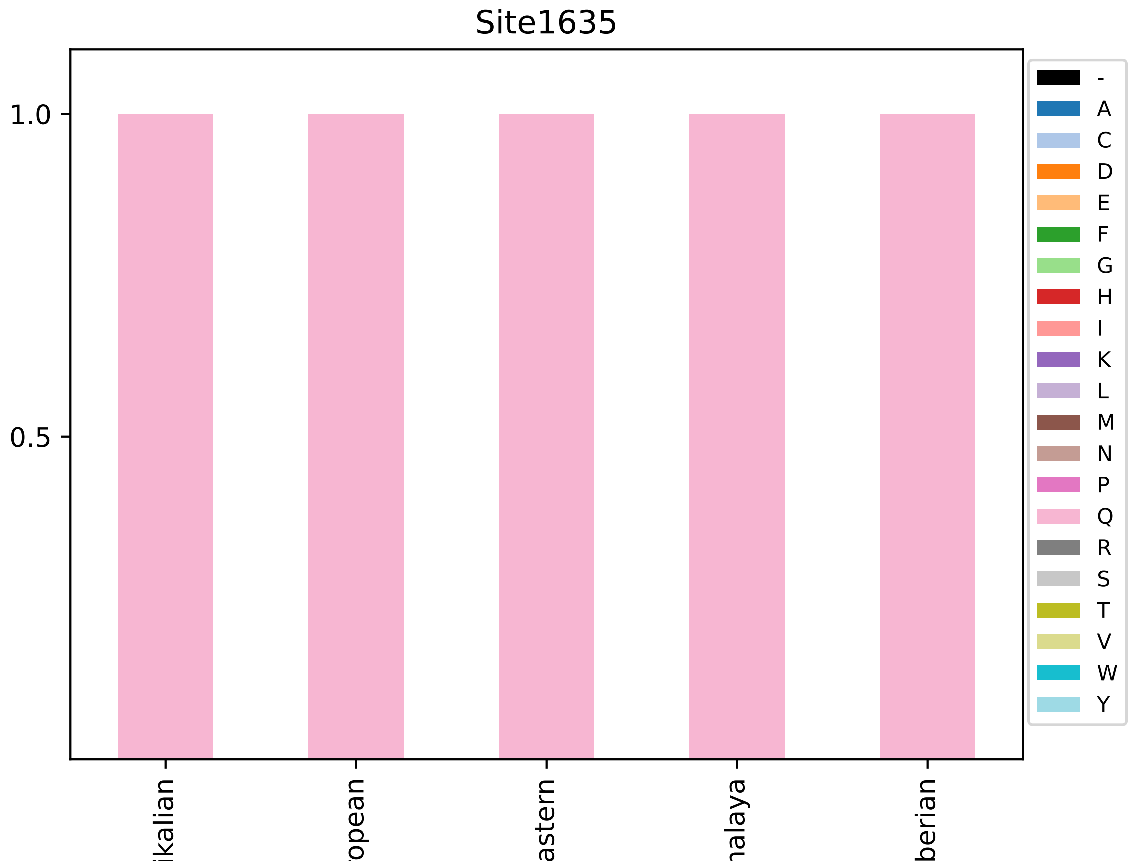
Site1635
1.0
0.5
ikalian
opean
astern
nalaya
berian
-
A
C
D
E
F
G
H
I
K
L
M
N
P
Q
R
S
T
V
W
Y

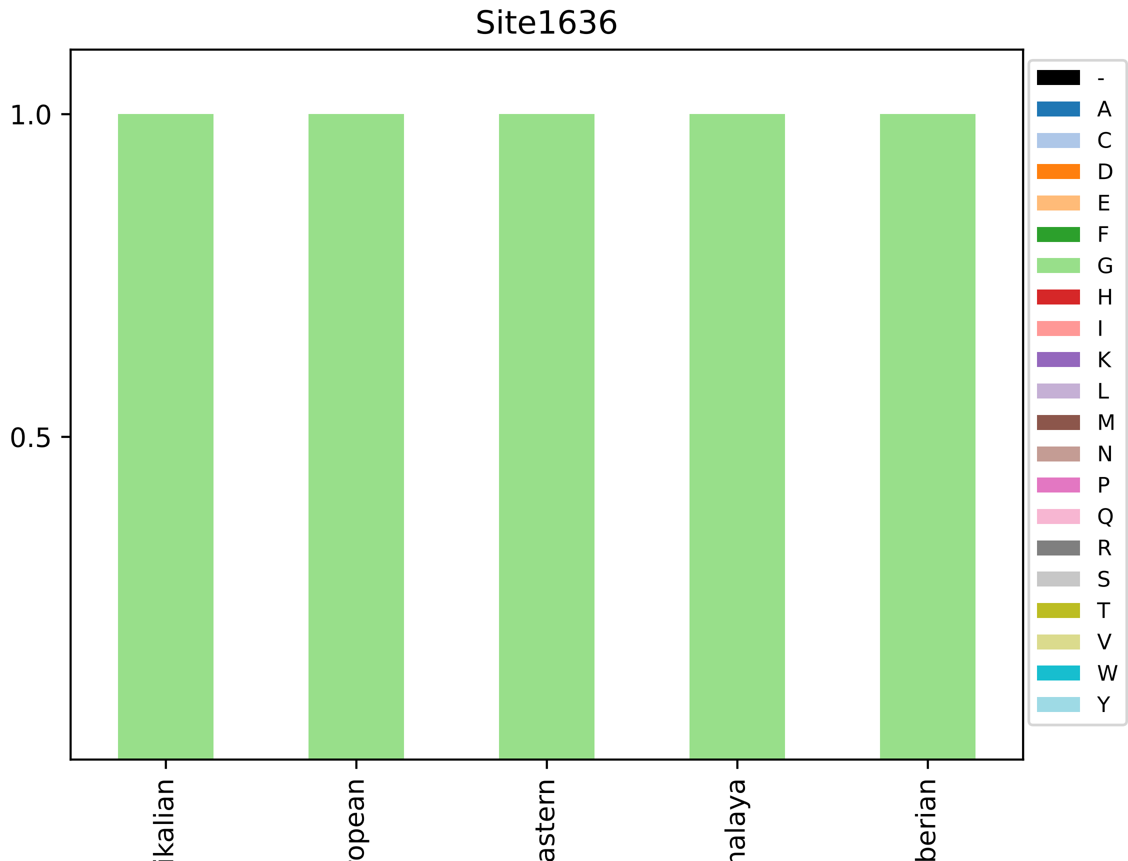

Site1636
1.0
0.5
-
A
C
D
E
F
G
H
I
K
L
M
N
P
Q
R
S
T
V
W
Y
ikalian
opean
astern
nalaya
berian

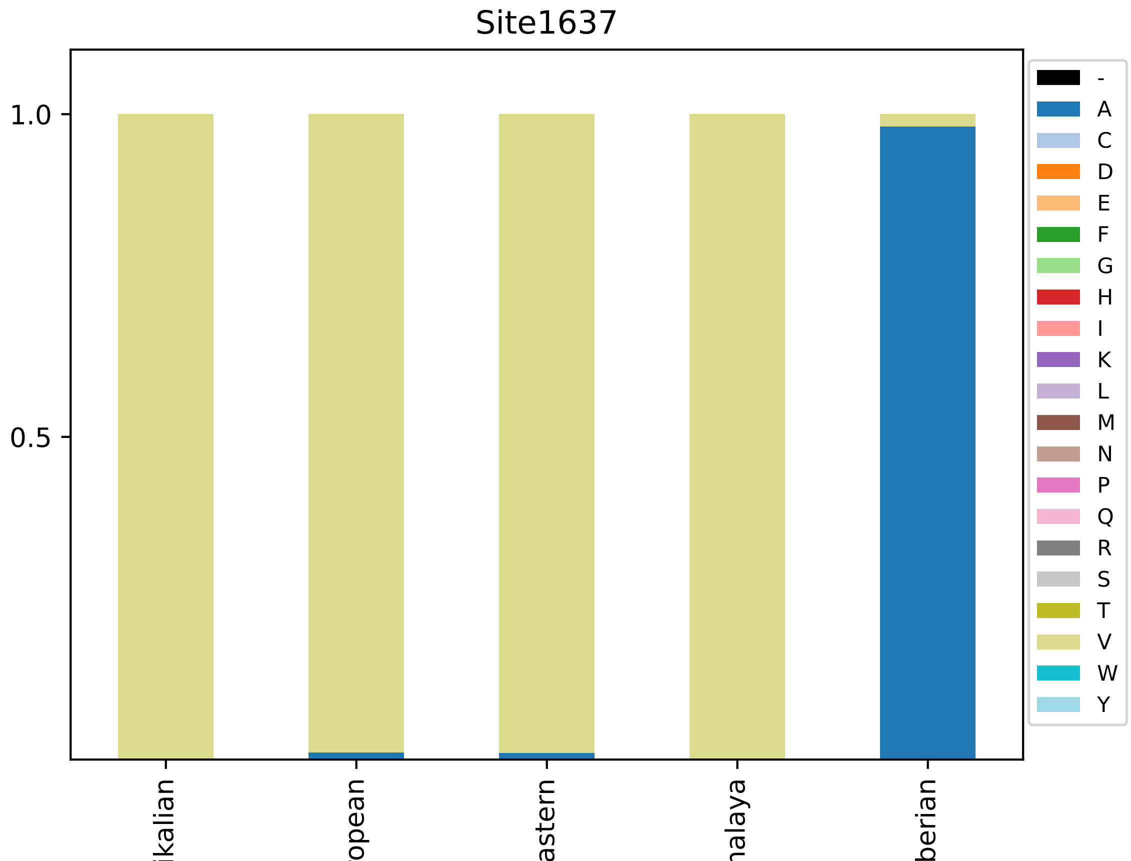

Site1637
1.0
0.5
-
A
C
D
E
F
G
H
I
K
L
M
N
P
Q
R
S
T
V
W
Y
ikalian
opean
astern
nalaya
berian

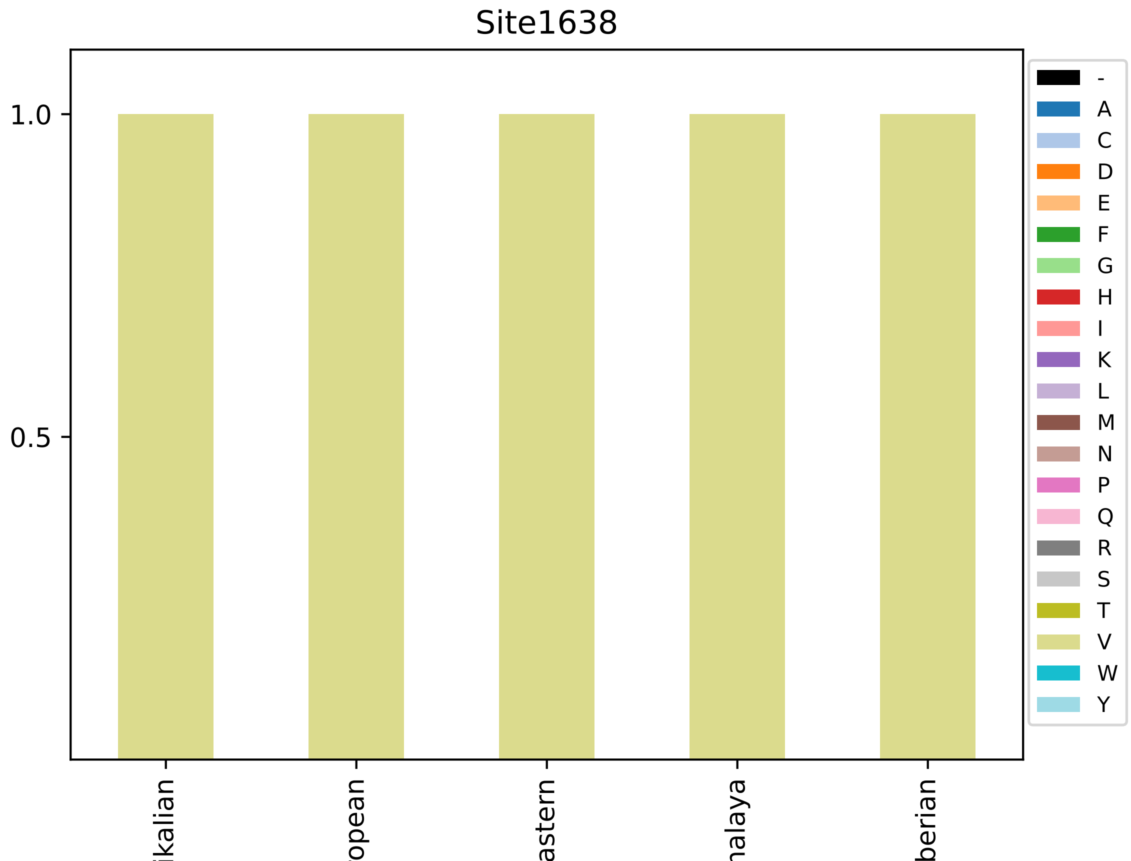

Site1638
1.0
0.5
-
A
C
D
E
F
G
H
I
K
L
M
N
P
Q
R
S
T
V
W
Y
ikalian
opean
astern
nalaya
berian

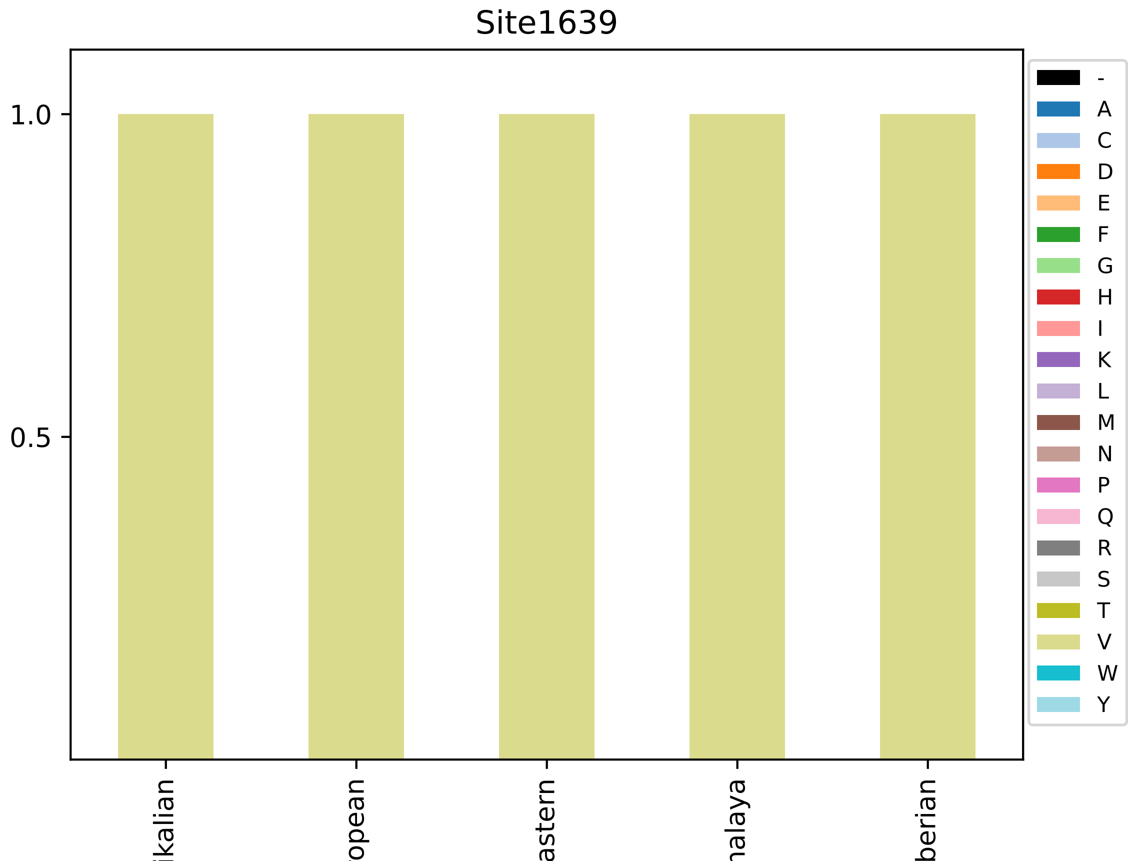

Site1639
1.0
0.5
ikalian
ropean
astern
nalaya
berian
-
A
C
D
E
F
G
H
I
K
L
M
N
P
Q
R
S
T
V
W
Y

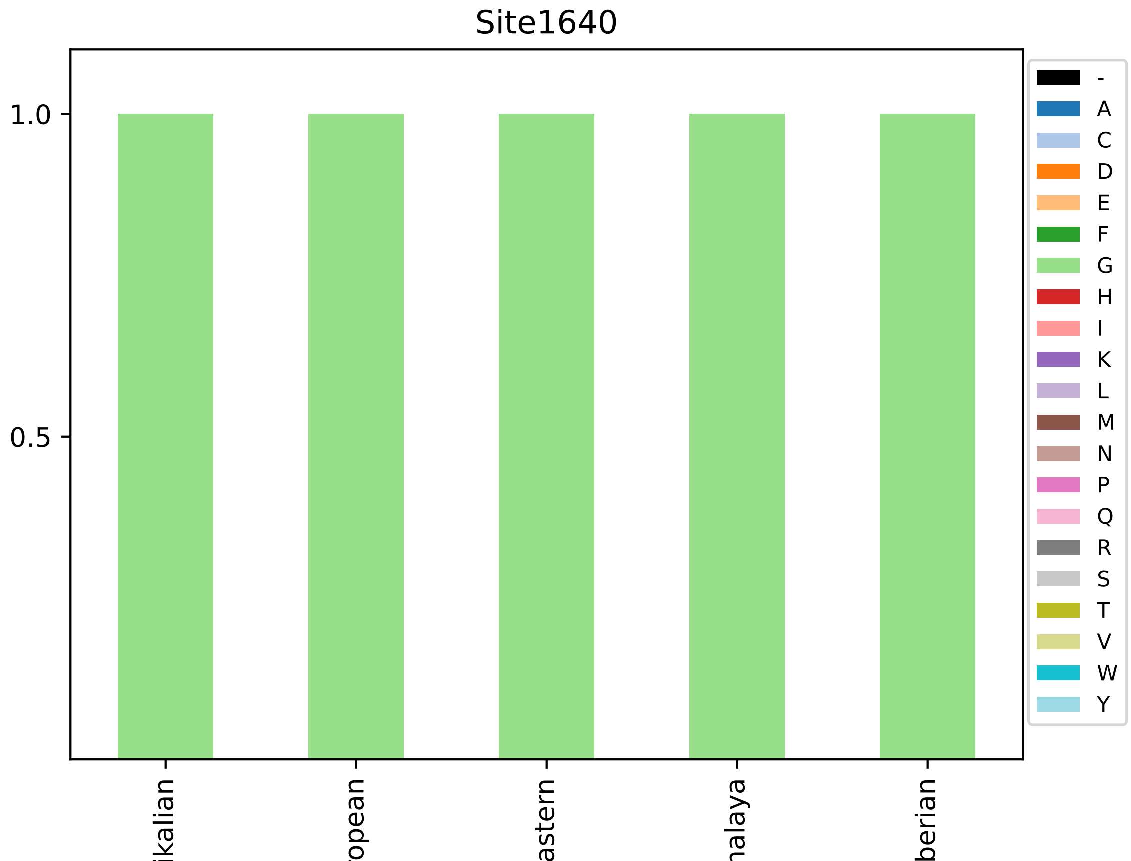

Site1640
1.0
0.5
-
A
C
D
E
F
G
H
I
K
L
M
N
P
Q
R
S
T
V
W
Y
ikalian
opean
astern
halaya
berian

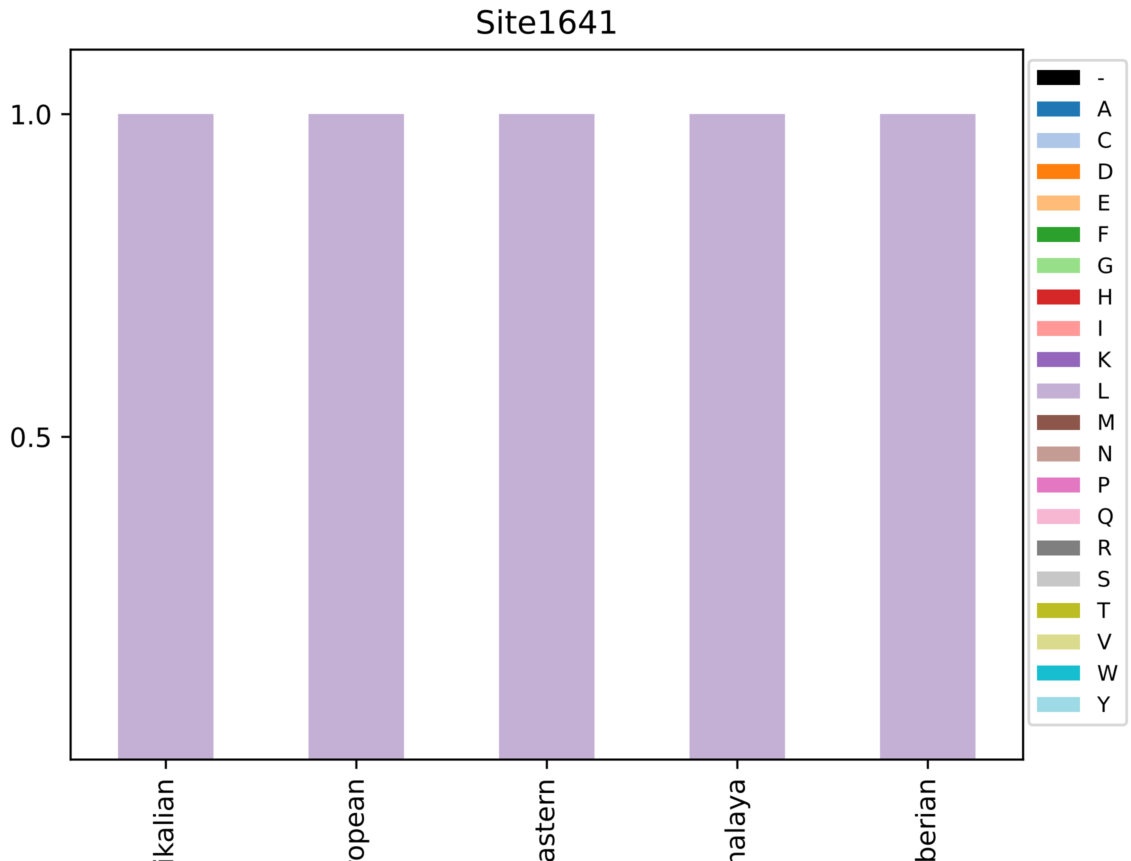

Site1641
1.0
0.5
-
A
C
D
E
F
G
H
I
K
L
M
N
P
Q
R
S
T
V
W
Y
ikalian
opean
astern
nalaya
berian

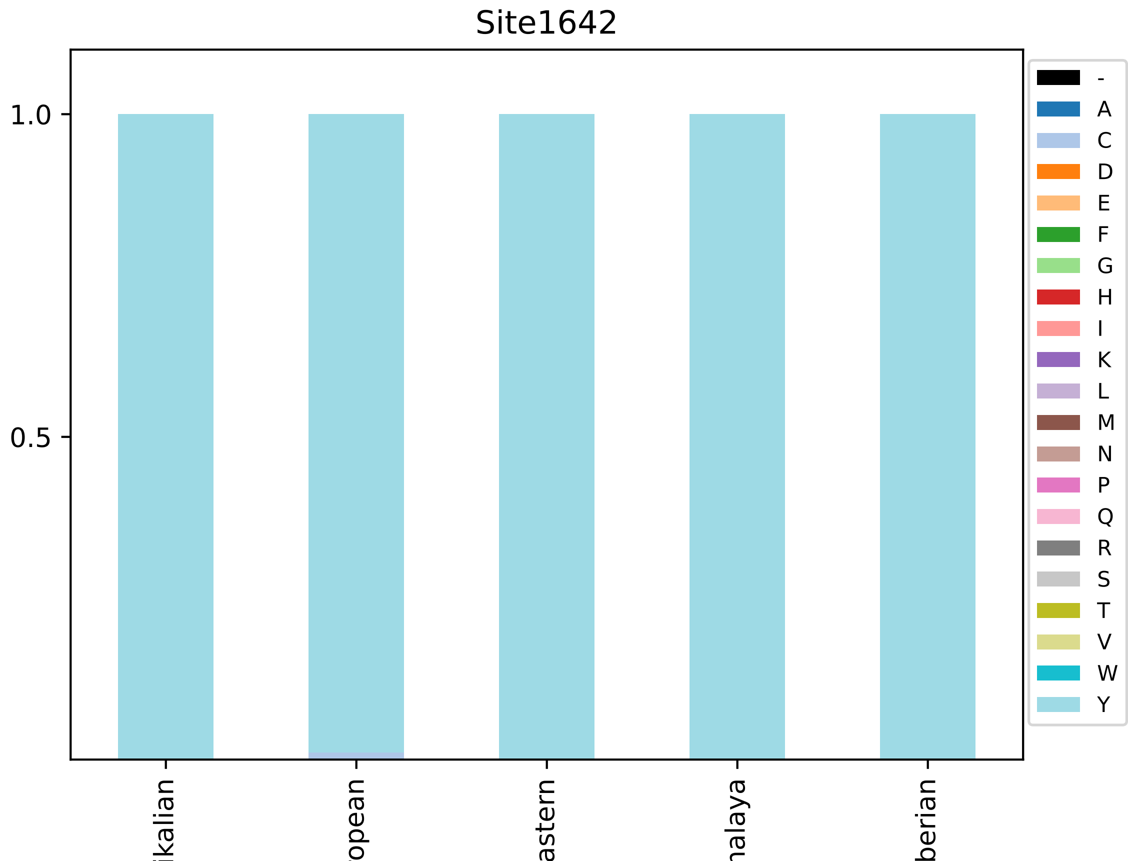
Site1642
1.0
0.5
-
A
C
D
E
F
G
H
I
K
L
M
N
P
Q
R
S
T
V
W
Y

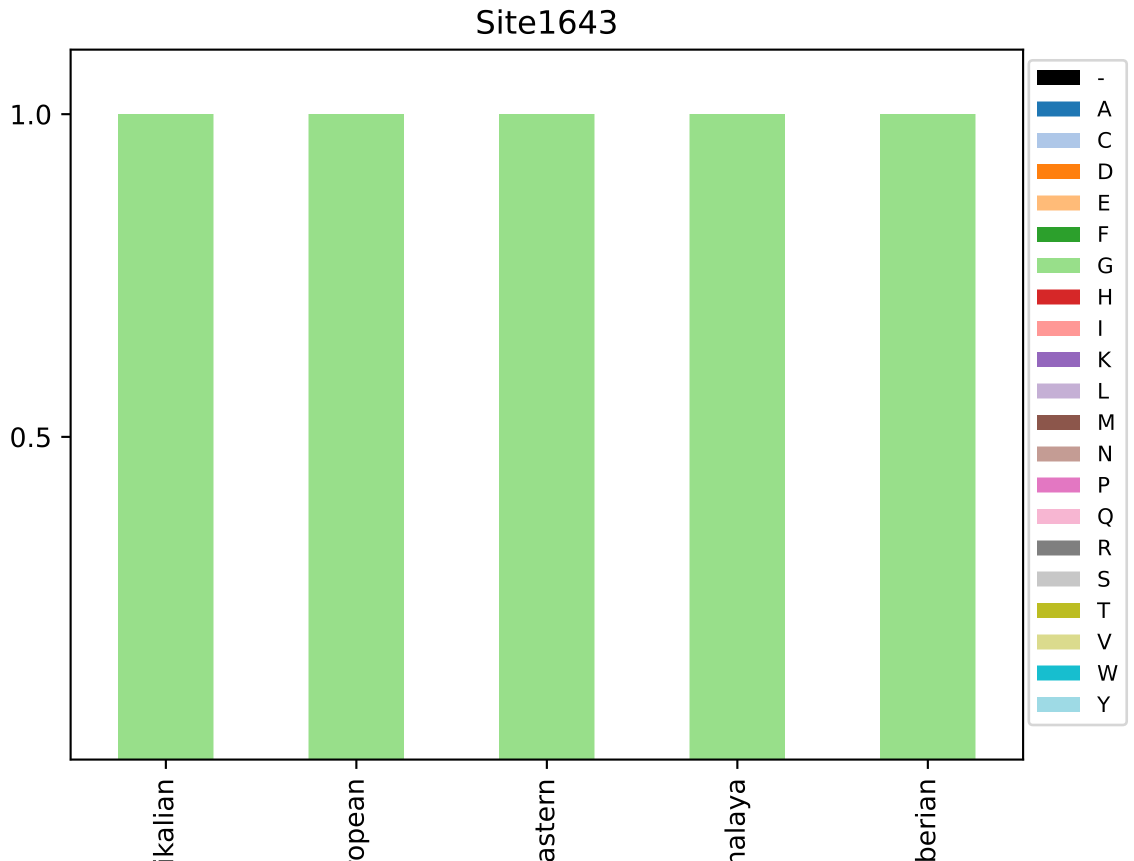

Site1643
1.0
0.5
-
A
C
D
E
F
G
H
I
K
L
M
N
P
Q
R
S
T
V
W
Y
ikalian
opean
astern
nalaya
berian

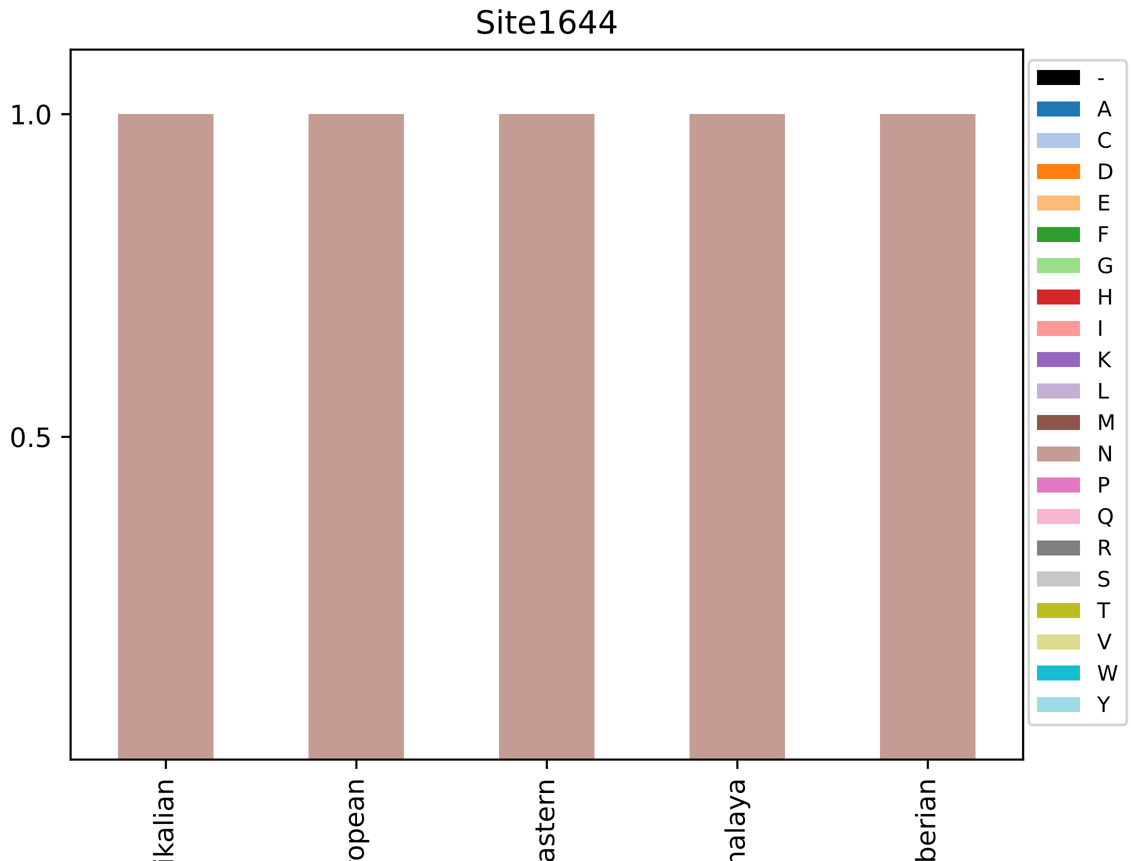

Site1644
1.0
0.5
-
A
C
D
E
F
G
H
I
K
L
M
N
P
Q
R
S
T
V
W
Y
ikalian
opean
astern
nalaya
berian

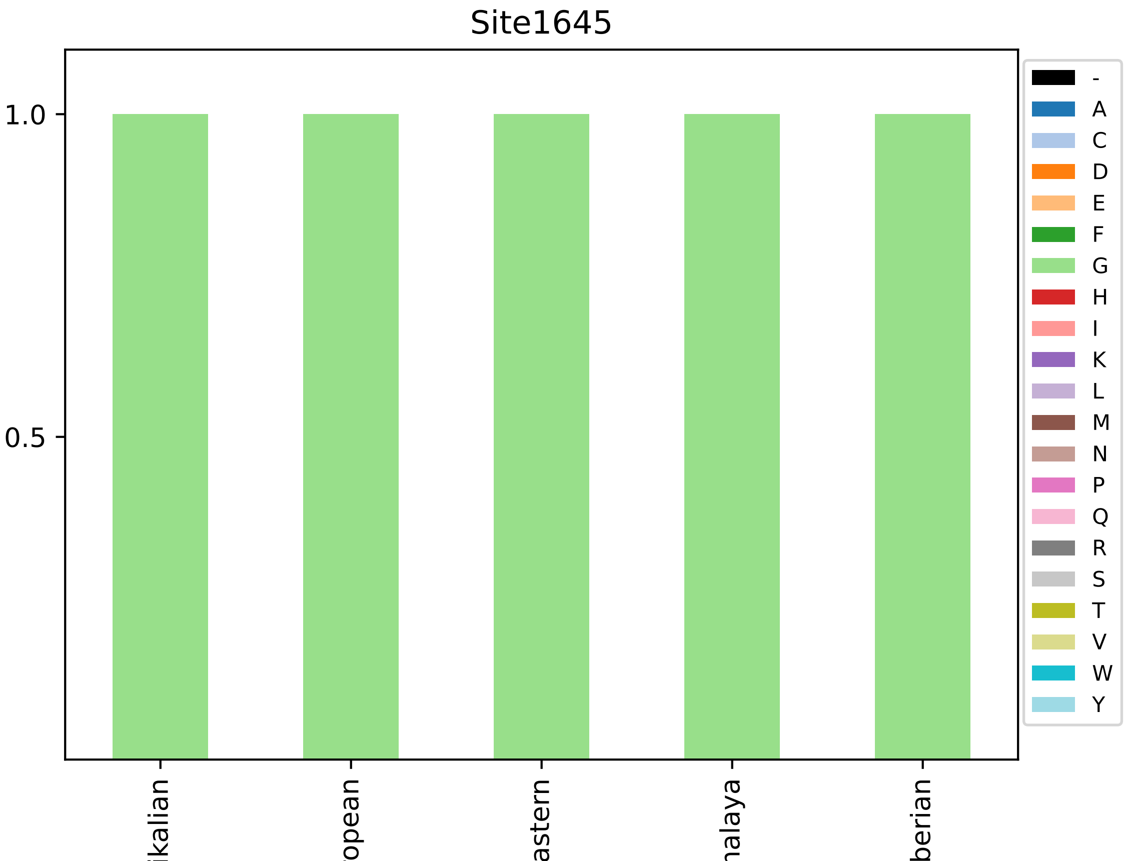

Site1645
1.0
0.5
-
A
C
D
E
F
G
H
I
K
L
M
N
P
Q
R
S
T
V
W
Y
ikalian
opean
astern
nalaya
berian

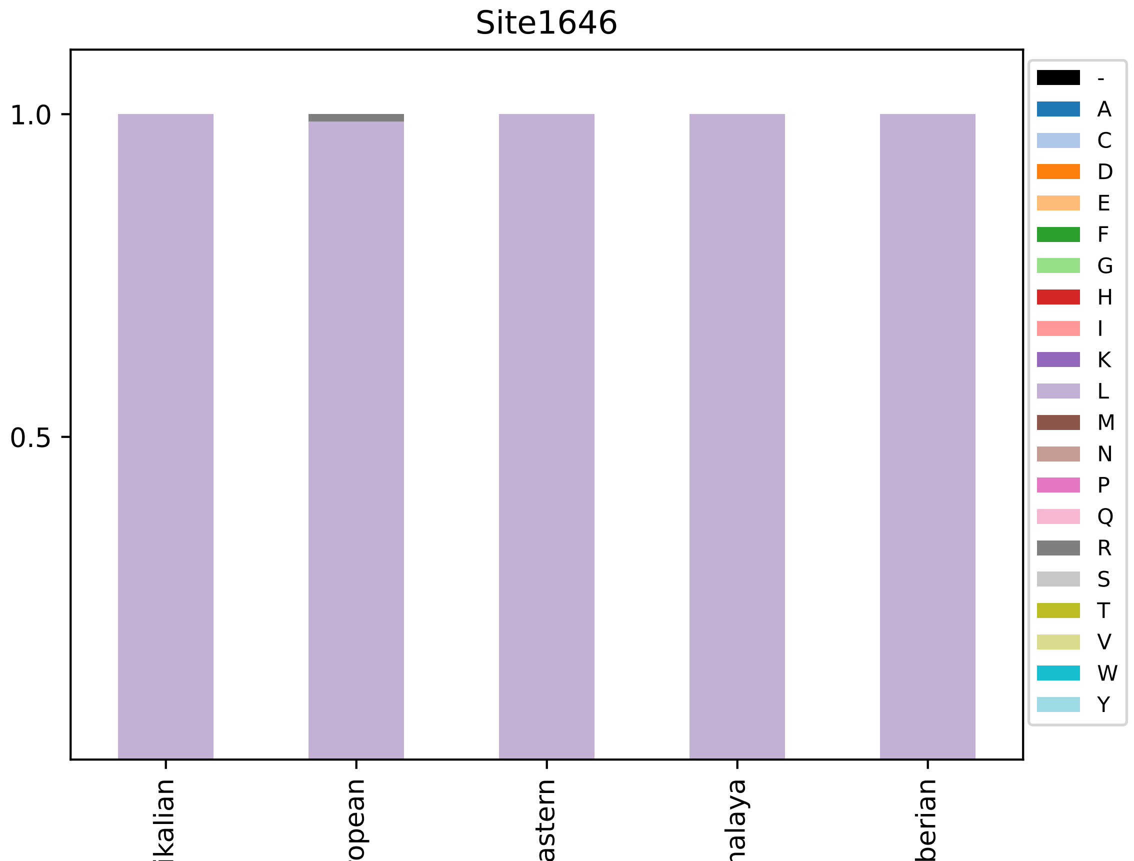

Site1646
1.0
0.5
-
A
C
D
E
F
G
H
I
K
L
M
N
P
Q
R
S
T
V
W
Y
ikalian
opean
astern
nalaya
berian

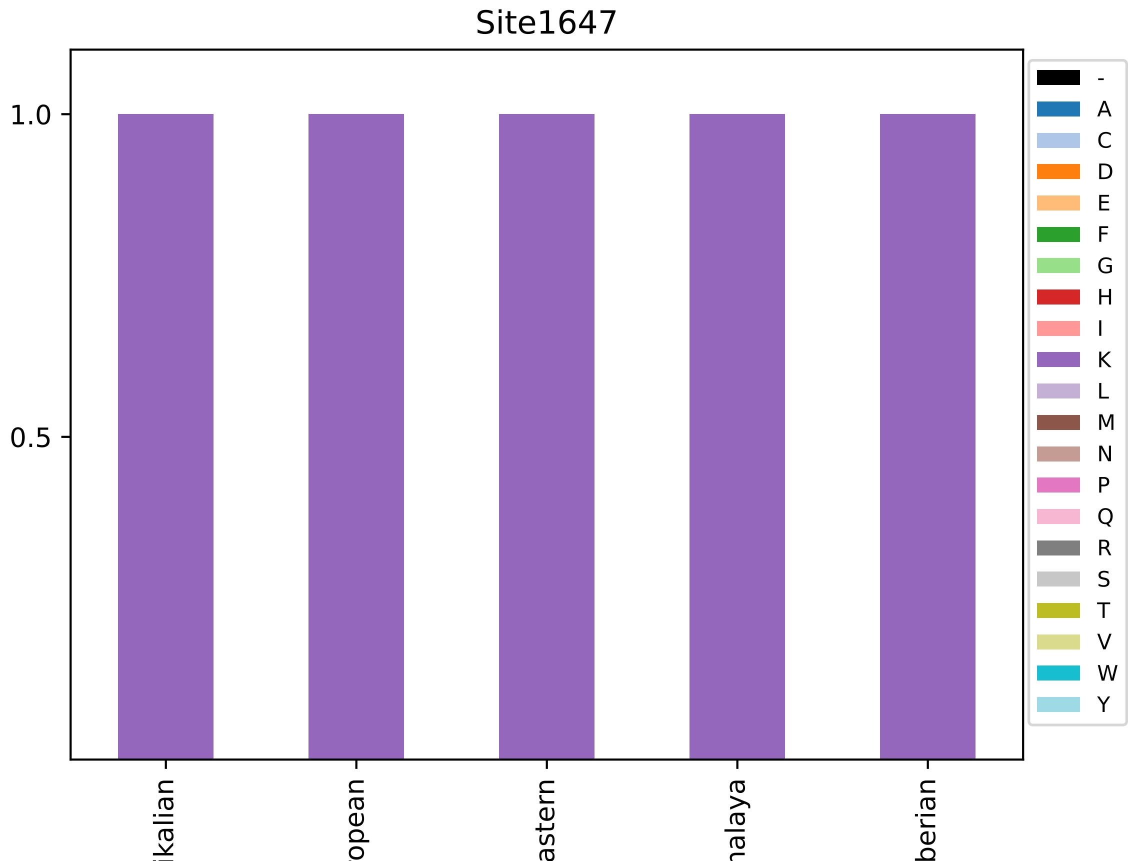
Site1647
1.0
0.5
-
A
C
D
E
F
G
H
I
K
L
M
N
P
Q
R
S
T
V
W
Y

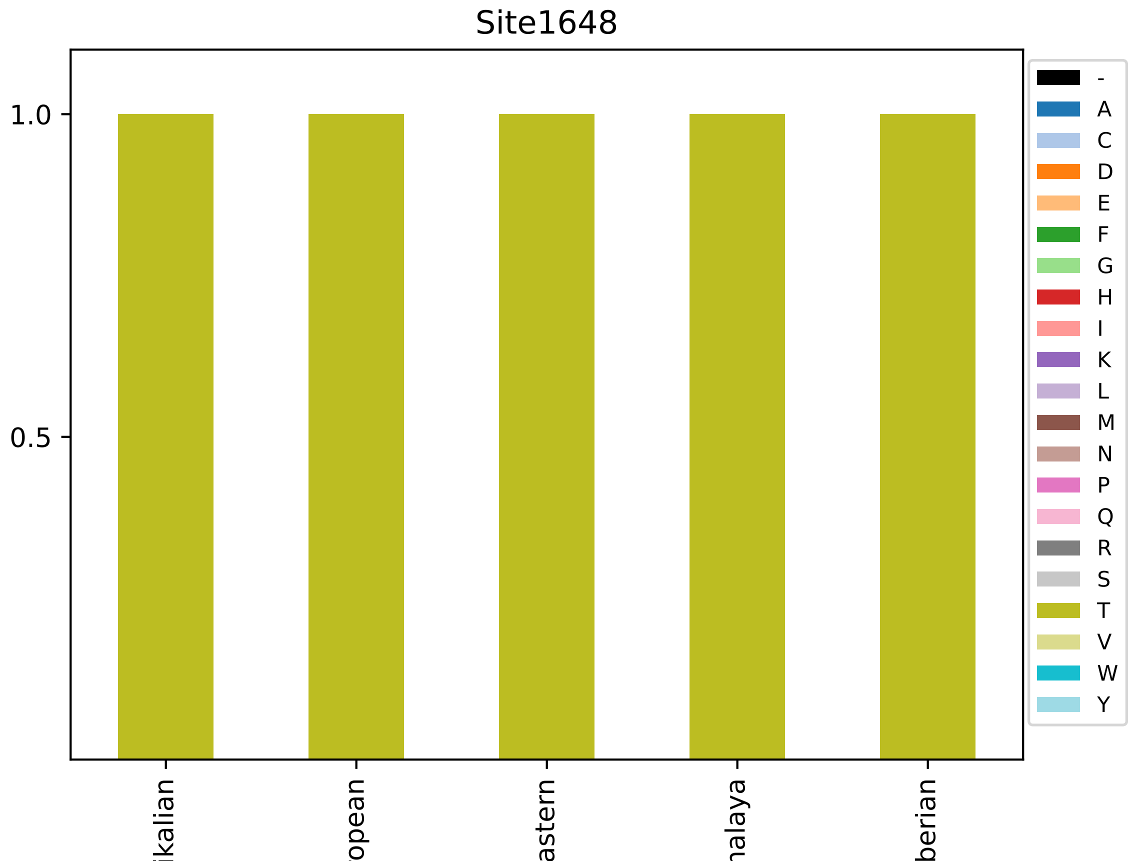

Site1648
1.0
0.5
ikalian
opean
astern
nalaya
berian
-
A
C
D
E
F
G
H
I
K
L
M
N
P
Q
R
S
T
V
W
Y

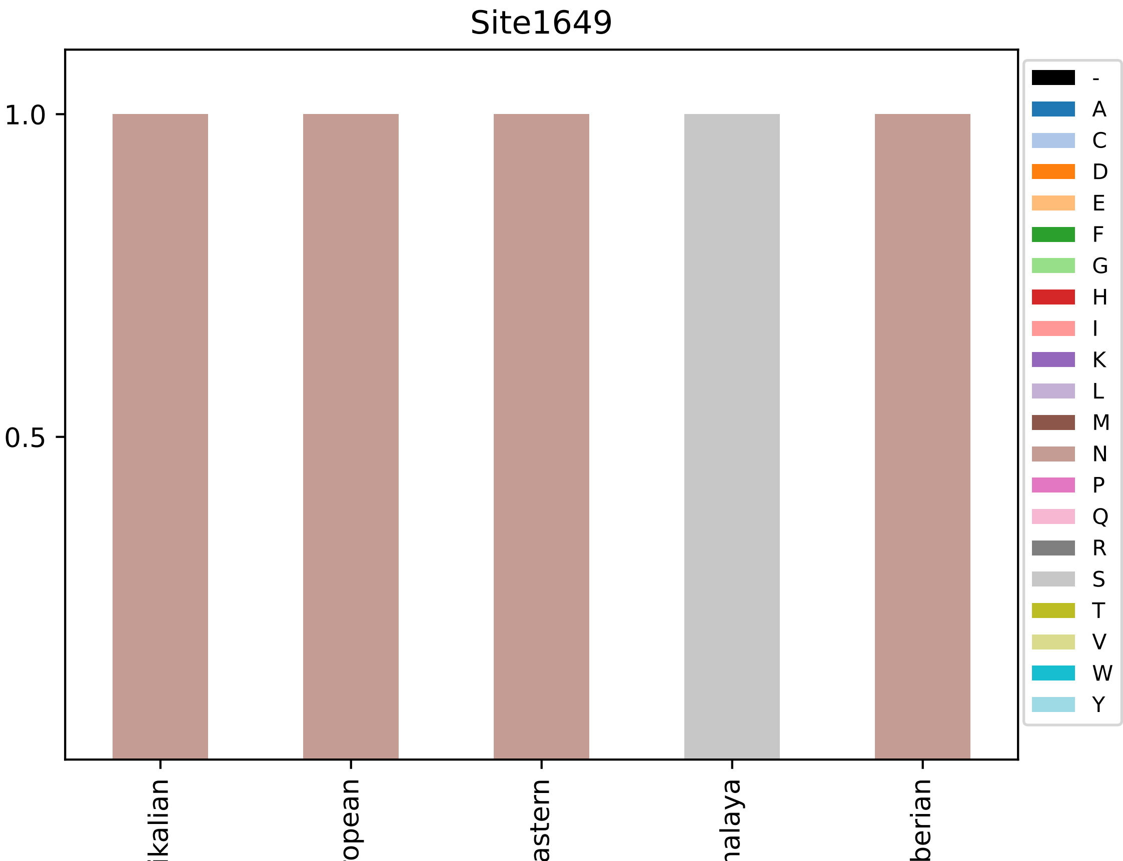

Site1649
1.0
0.5
-
A
C
D
E
F
G
H
I
K
L
M
N
P
Q
R
S
T
V
W
Y

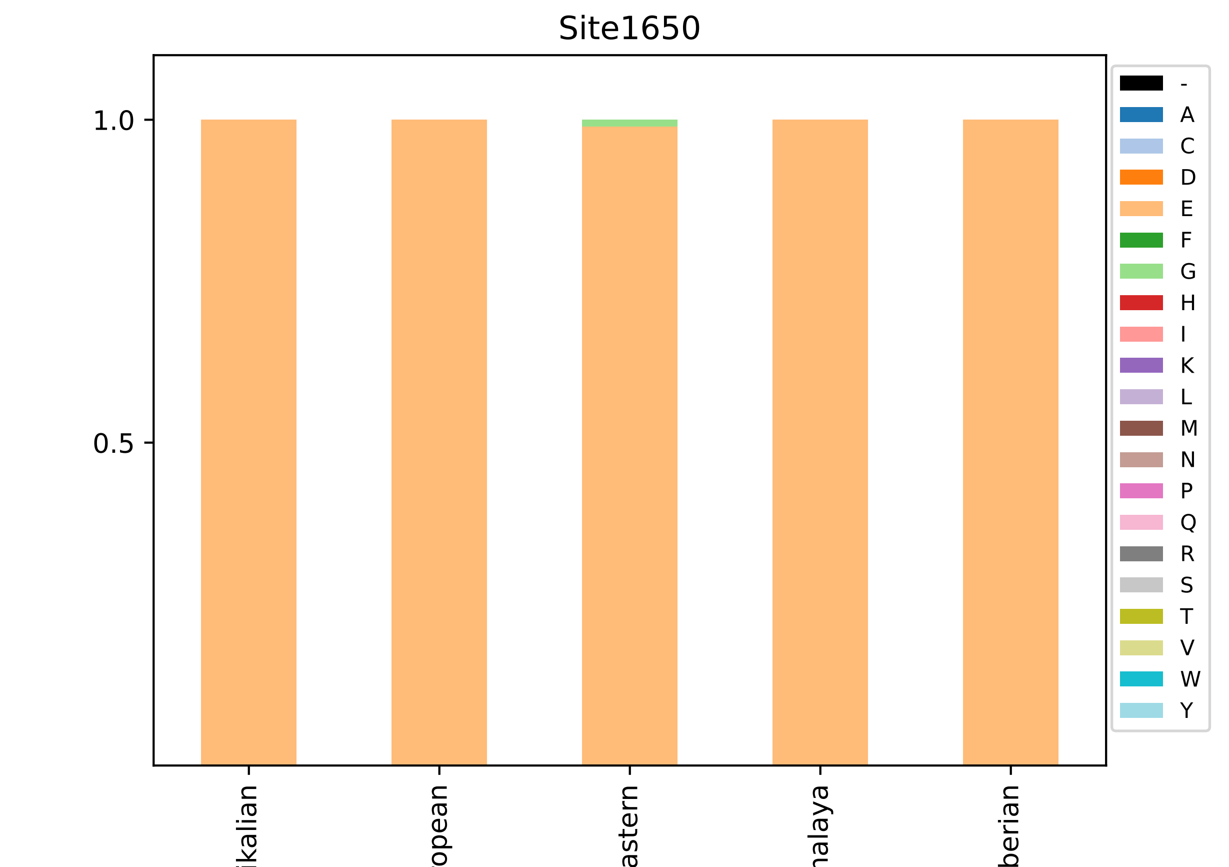

Site1650
1.0
0.5
-
A
C
D
E
F
G
H
I
K
L
M
N
P
Q
R
S
T
V
W
Y
ikalian
opean
astern
nalaya
berian

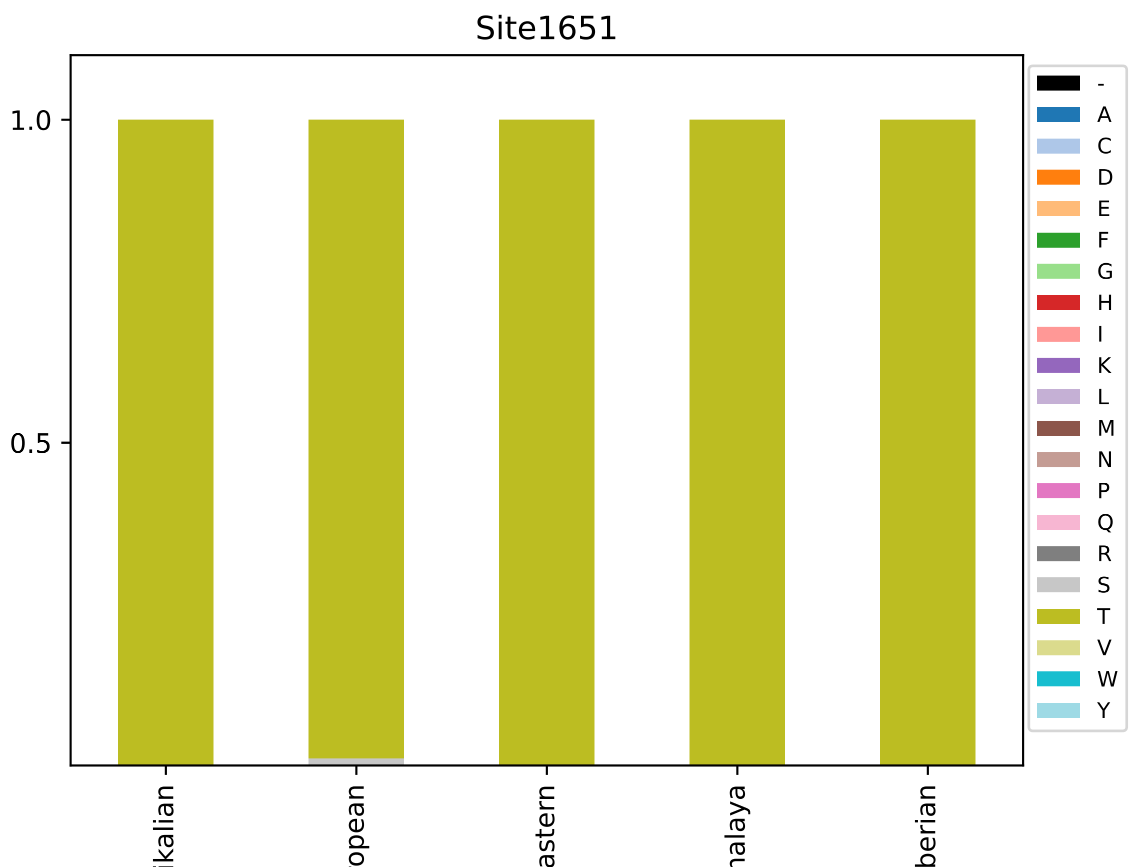

Site1651
1.0
0.5
-
A
C
D
E
F
G
H
I
K
L
M
N
P
Q
R
S
T
V
W
Y
ikalian
opean
astern
nalaya
berian

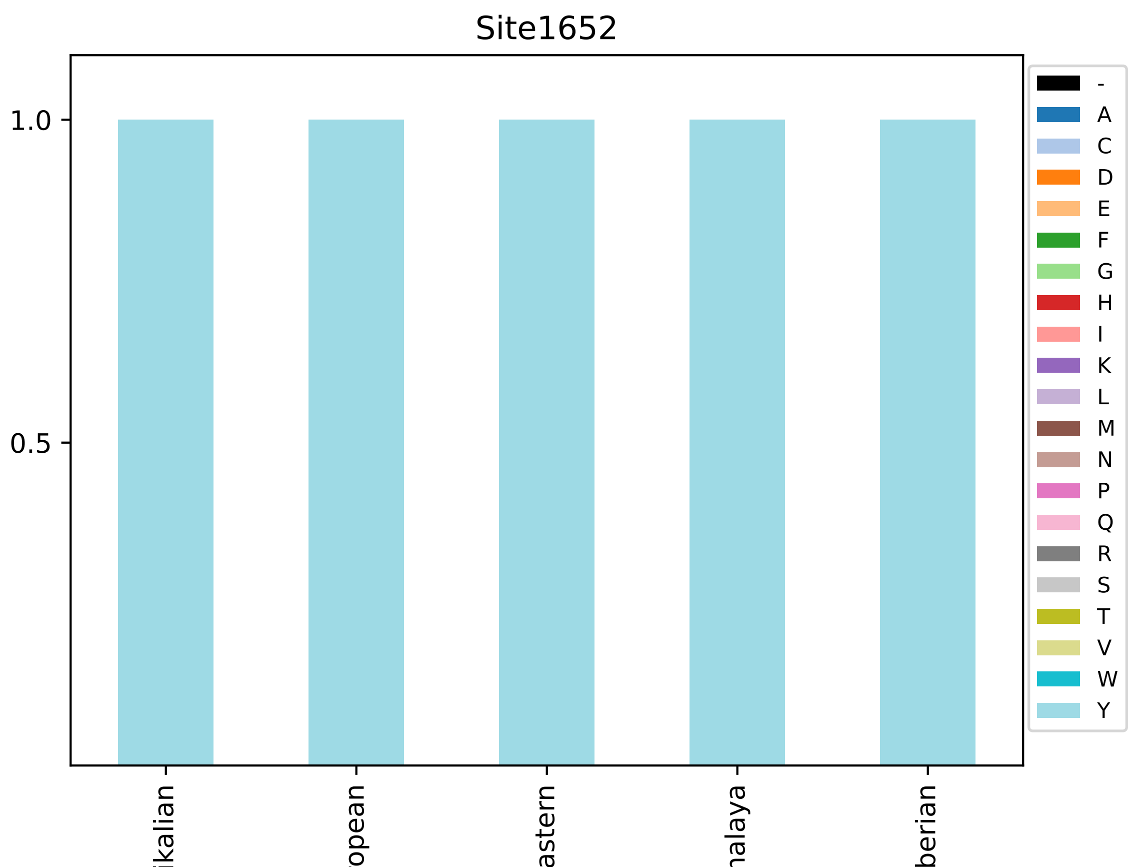

Site1652
1.0
0.5
-
A
C
D
E
F
G
H
I
K
L
M
N
P
Q
R
S
T
V
W
Y
ikalian
opean
astern
nalaya
berian

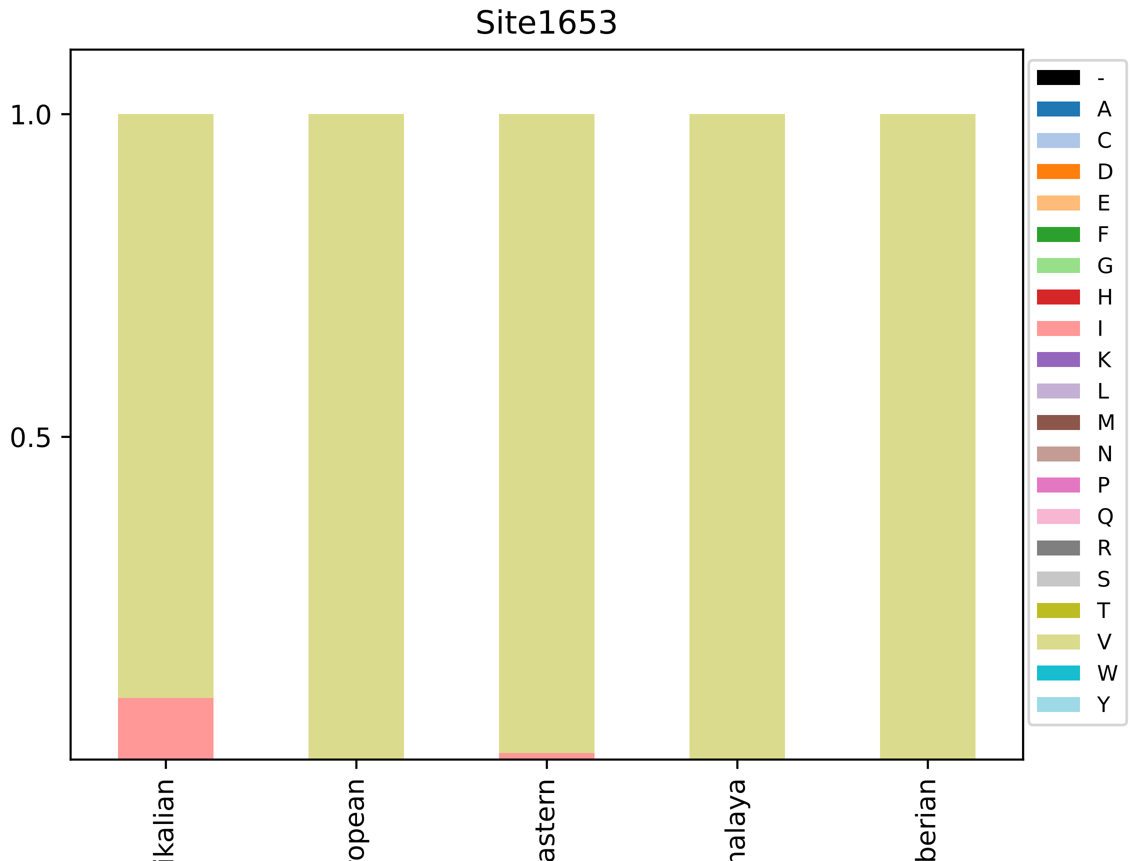

Site1653
1.0
0.5
-
A
C
D
E
F
G
H
I
K
L
M
N
P
Q
R
S
T
V
W
Y
ikalian
opean
astern
nalaya
berian

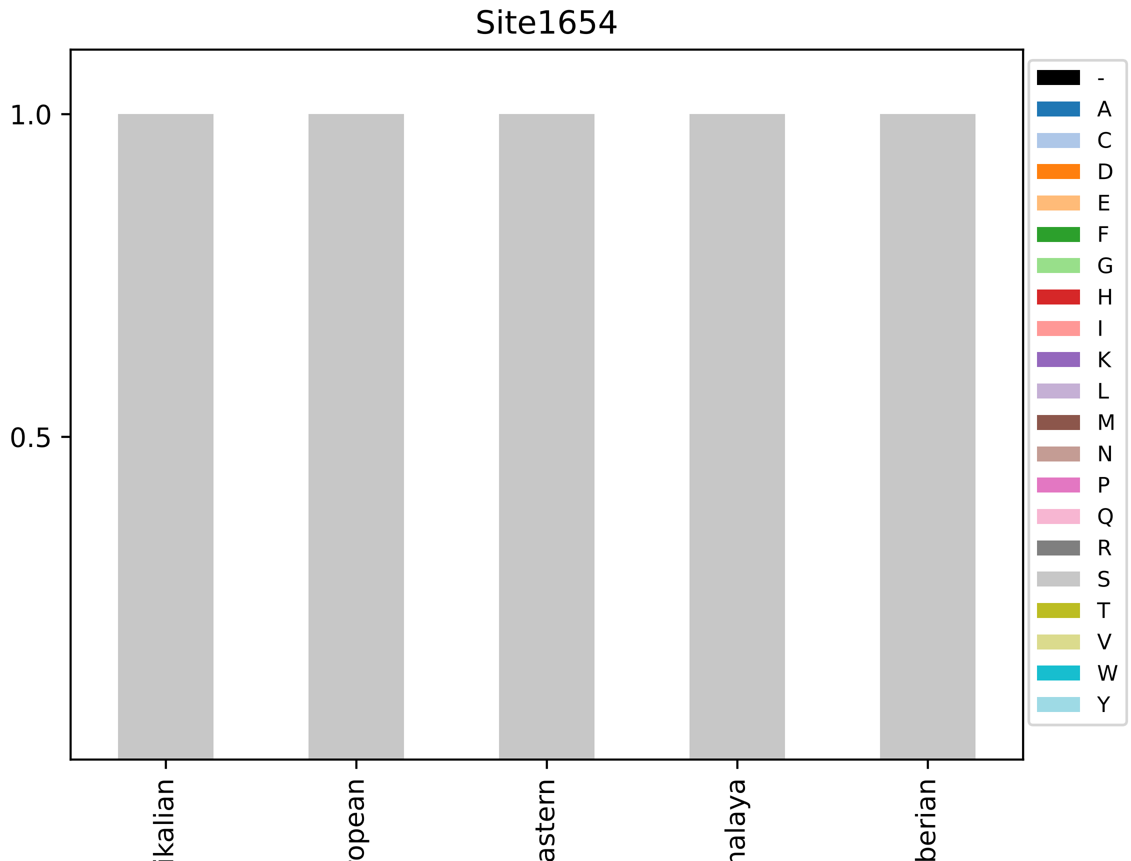
Site1654
1.0
0.5
-
A
C
D
E
F
G
H
I
K
L
M
N
P
Q
R
S
T
V
W
Y
ikalian
opean
astern
nalaya
berian

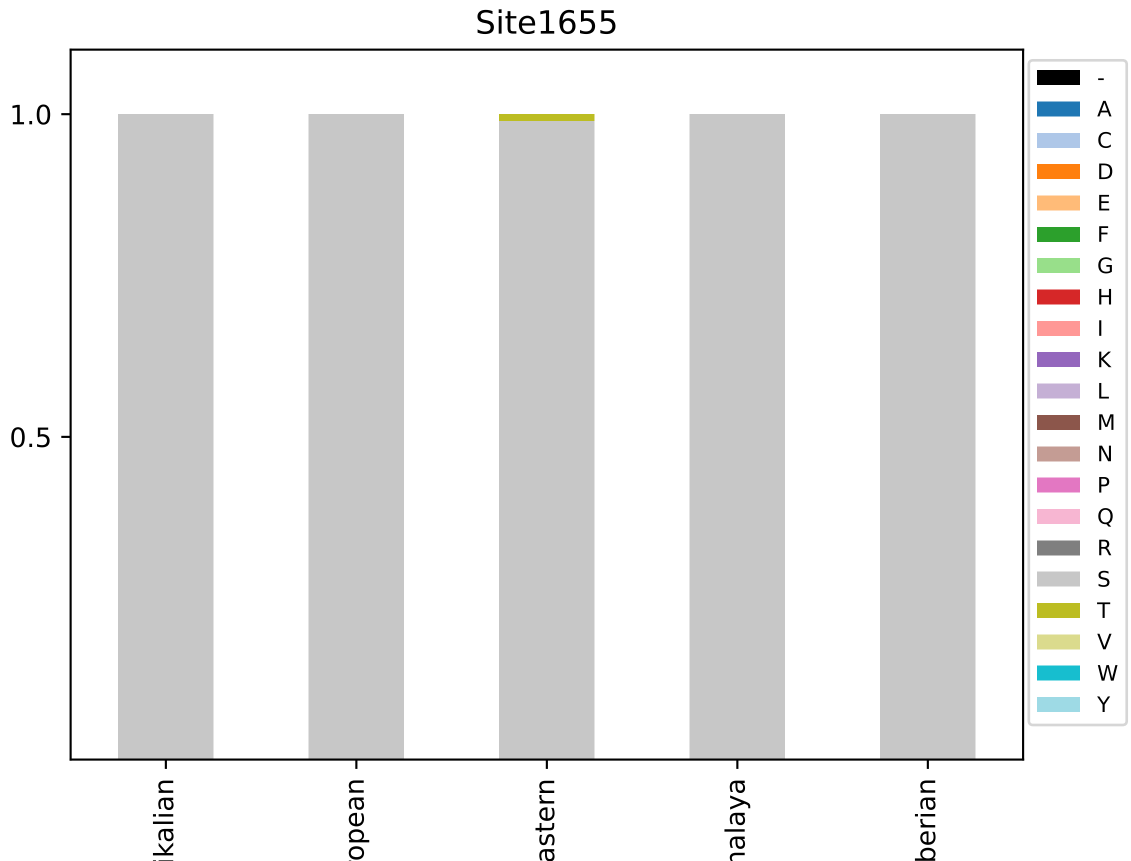

Site1655
1.0
0.5
ikalian
opean
astern
nalaya
berian
-
A
C
D
E
F
G
H
I
K
L
M
N
P
Q
R
S
T
V
W
Y

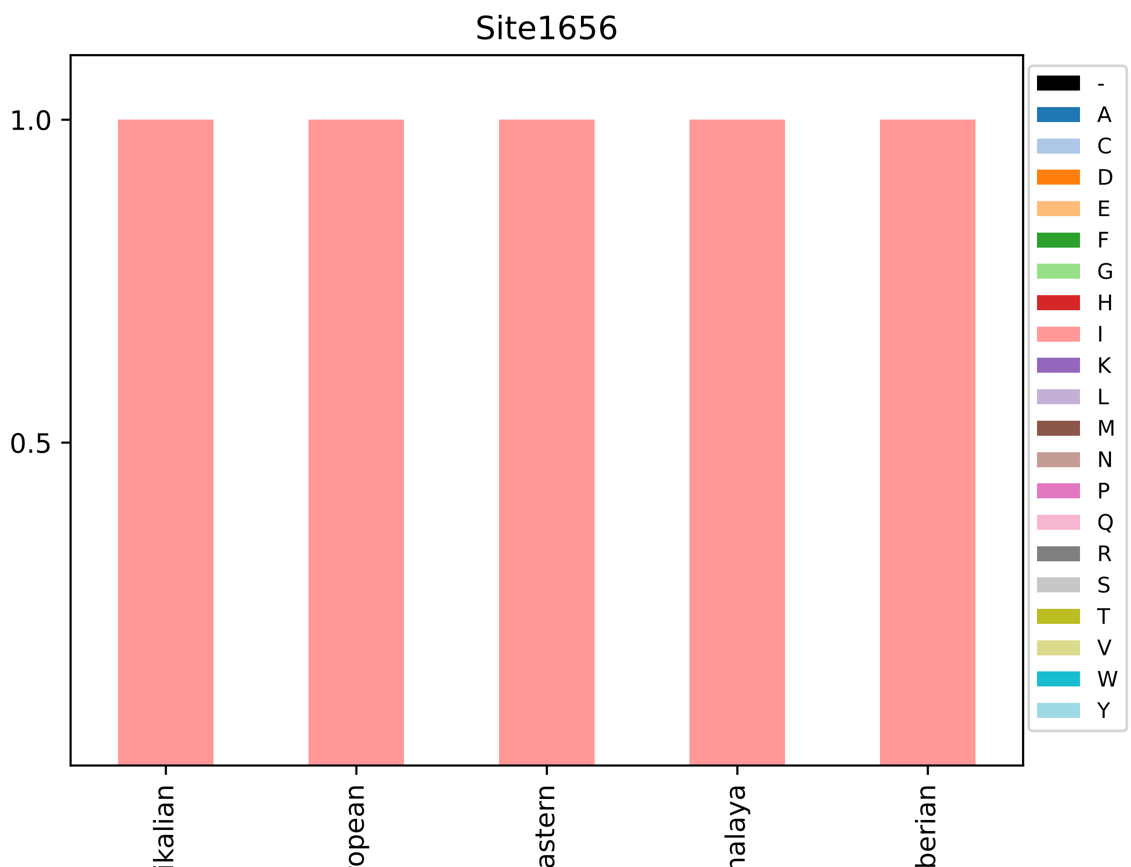

Site1656
1.0
0.5
-
A
C
D
E
F
G
H
I
K
L
M
N
P
Q
R
S
T
V
W
Y
ikalian
opean
astern
nalaya
berian

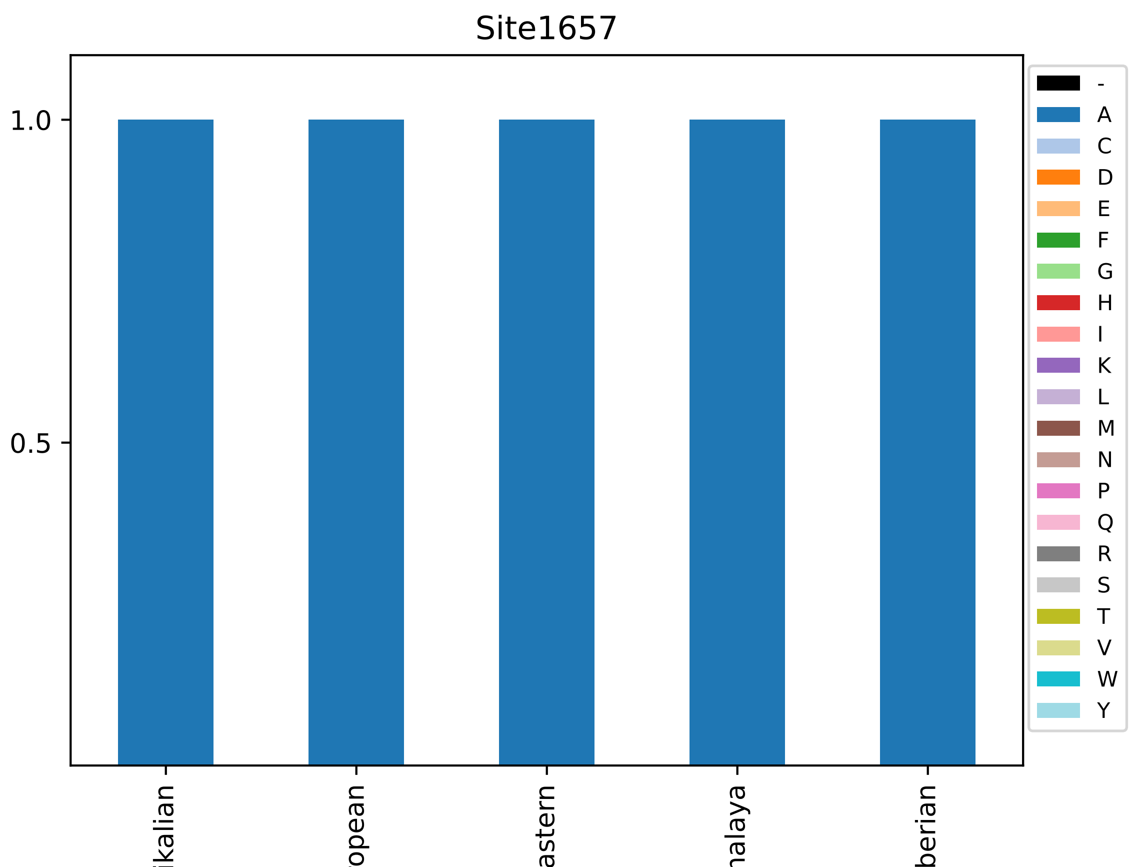

Site1657
1.0
0.5
-
A
C
D
E
F
G
H
I
K
L
M
N
P
Q
R
S
T
V
W
Y
ikalian
opean
astern
nalaya
berian

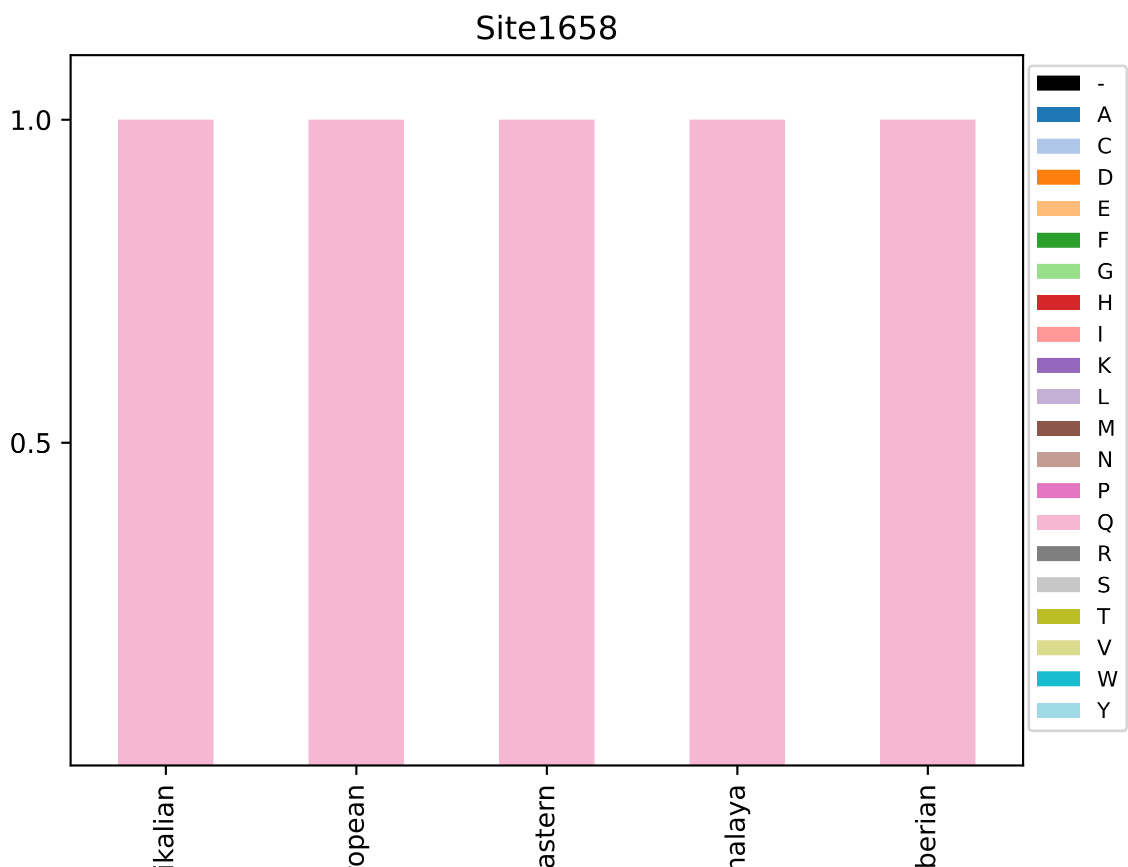

Site1658
1.0
0.5
-
A
C
D
E
F
G
H
I
K
L
M
N
P
Q
R
S
T
V
W
Y
ikalian
opean
astern
nalaya
berian

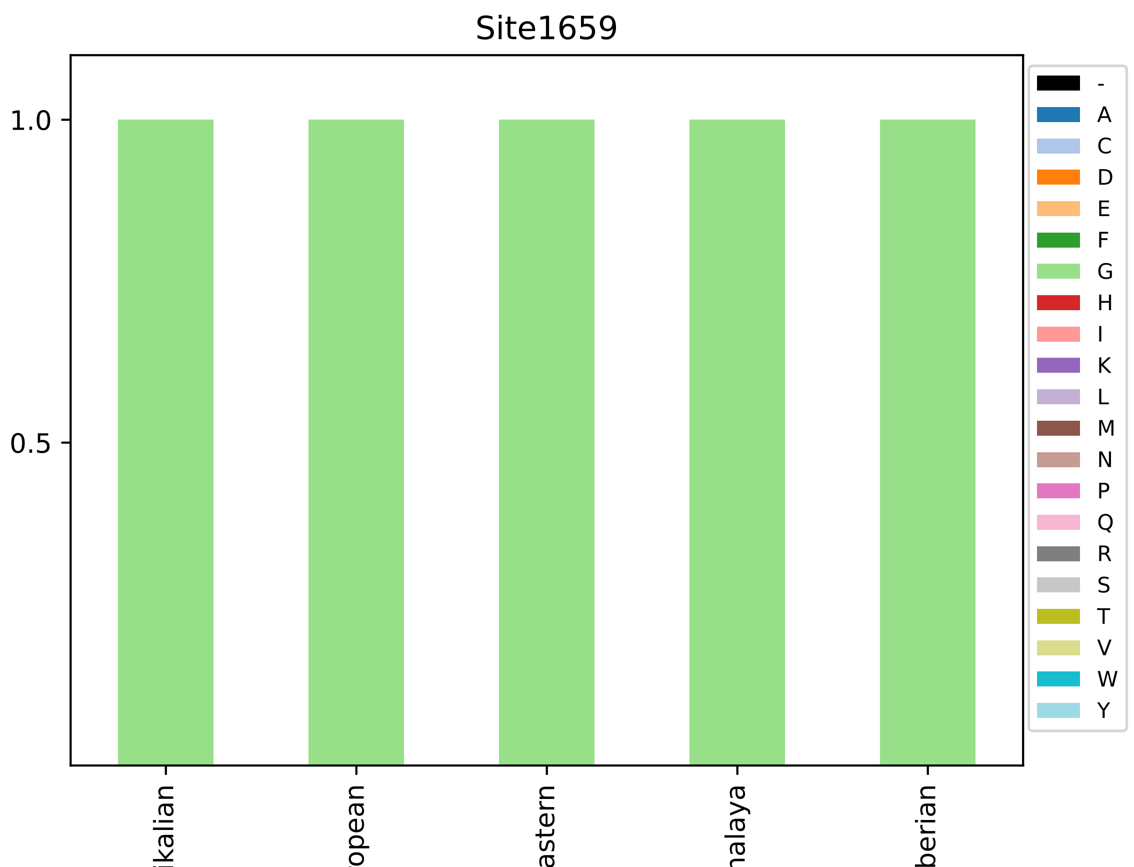

Site1659
1.0
0.5
-
A
C
D
E
F
G
H
I
K
L
M
N
P
Q
R
S
T
V
W
Y
ikalian
opean
astern
nalaya
berian

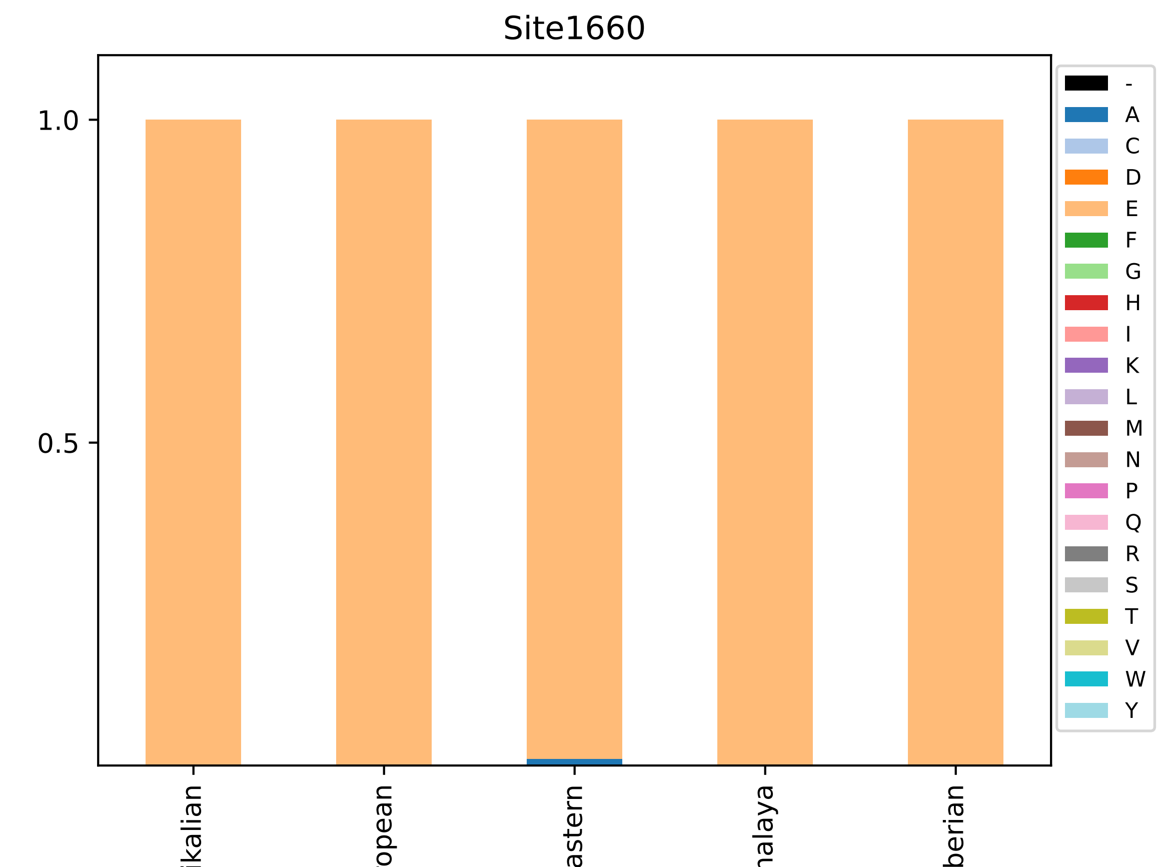

Site1660
1.0
0.5
-
A
C
D
E
F
G
H
I
K
L
M
N
P
Q
R
S
T
V
W
Y
ikalian
opean
astern
nalaya
berian

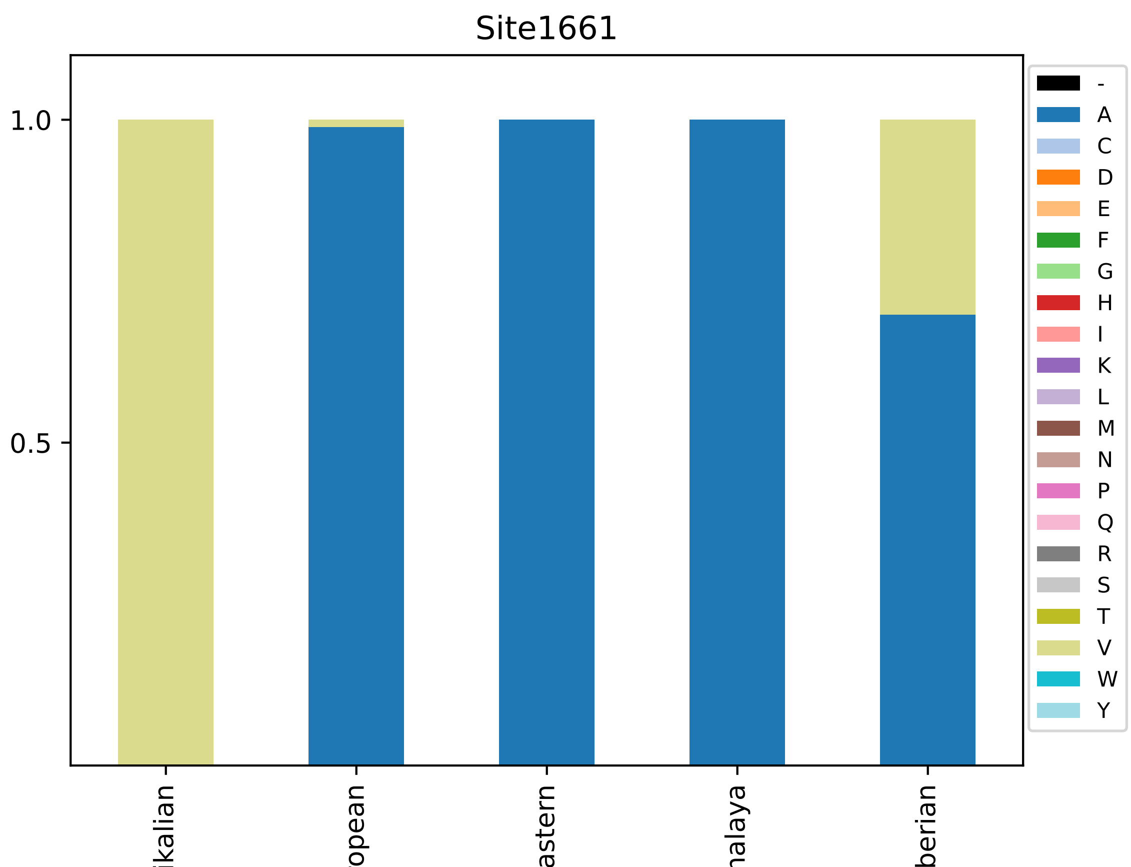

Site1661
1.0
0.5
-
A
C
D
E
F
G
H
I
K
L
M
N
P
Q
R
S
T
V
W
Y
ikalian
opean
astern
nalaya
berian

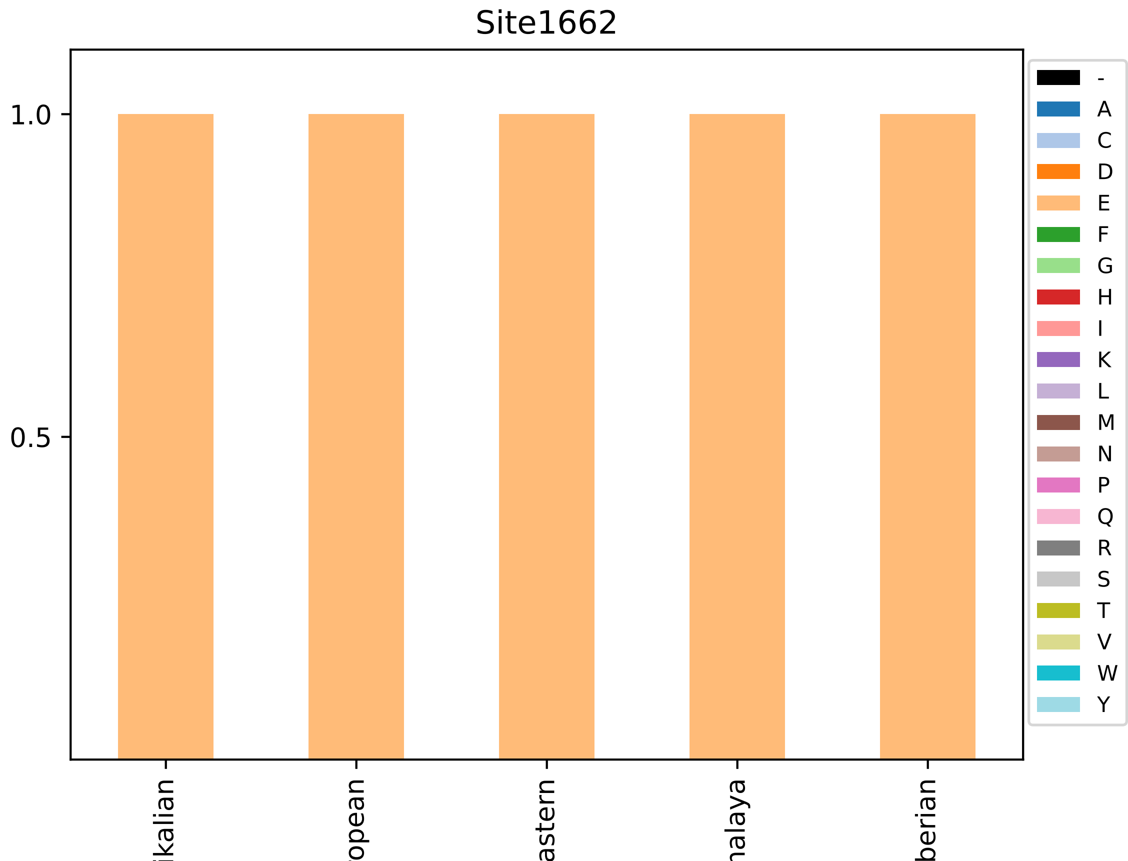
Site1662
1.0
0.5
-
A
C
D
E
F
G
H
I
K
L
M
N
P
Q
R
S
T
V
W
Y
ikalian
opean
astern
nalaya
berian

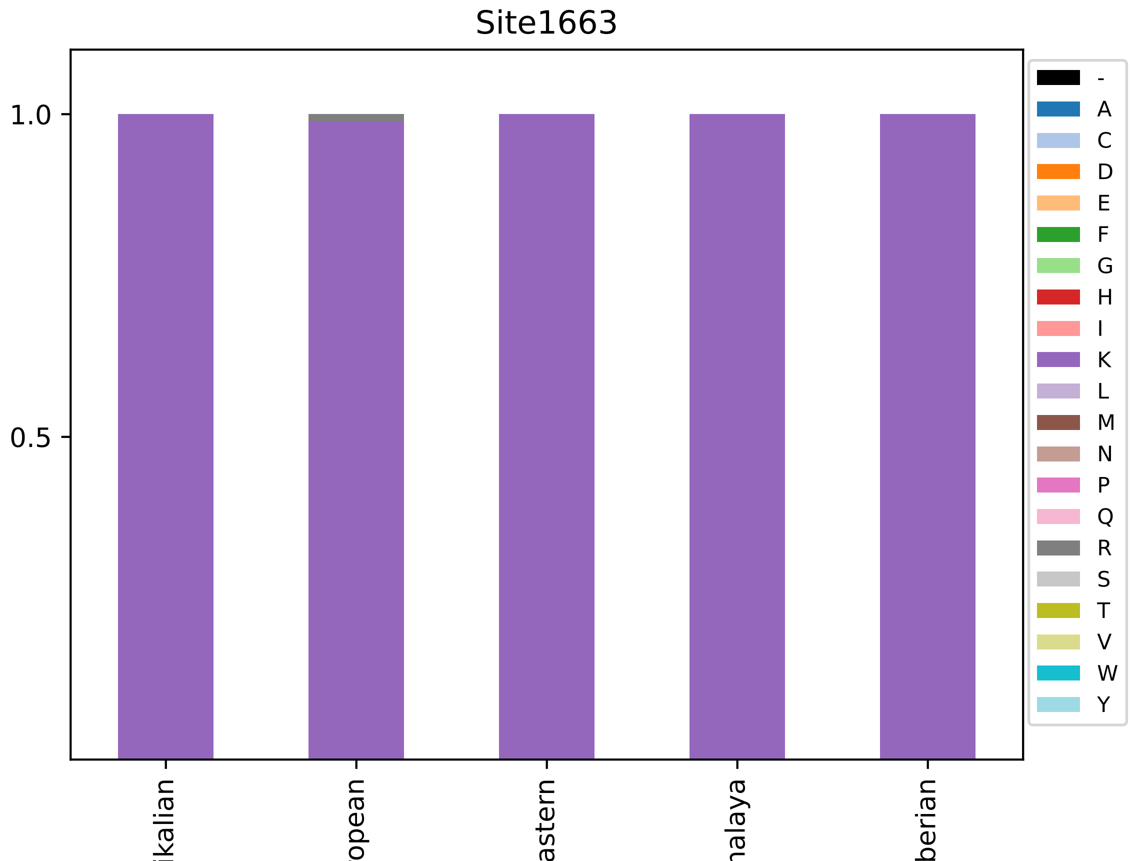

Site1663
1.0
0.5
-
A
C
D
E
F
G
H
I
K
L
M
N
P
Q
R
S
T
V
W
Y
ikalian
opean
astern
nalaya
berian

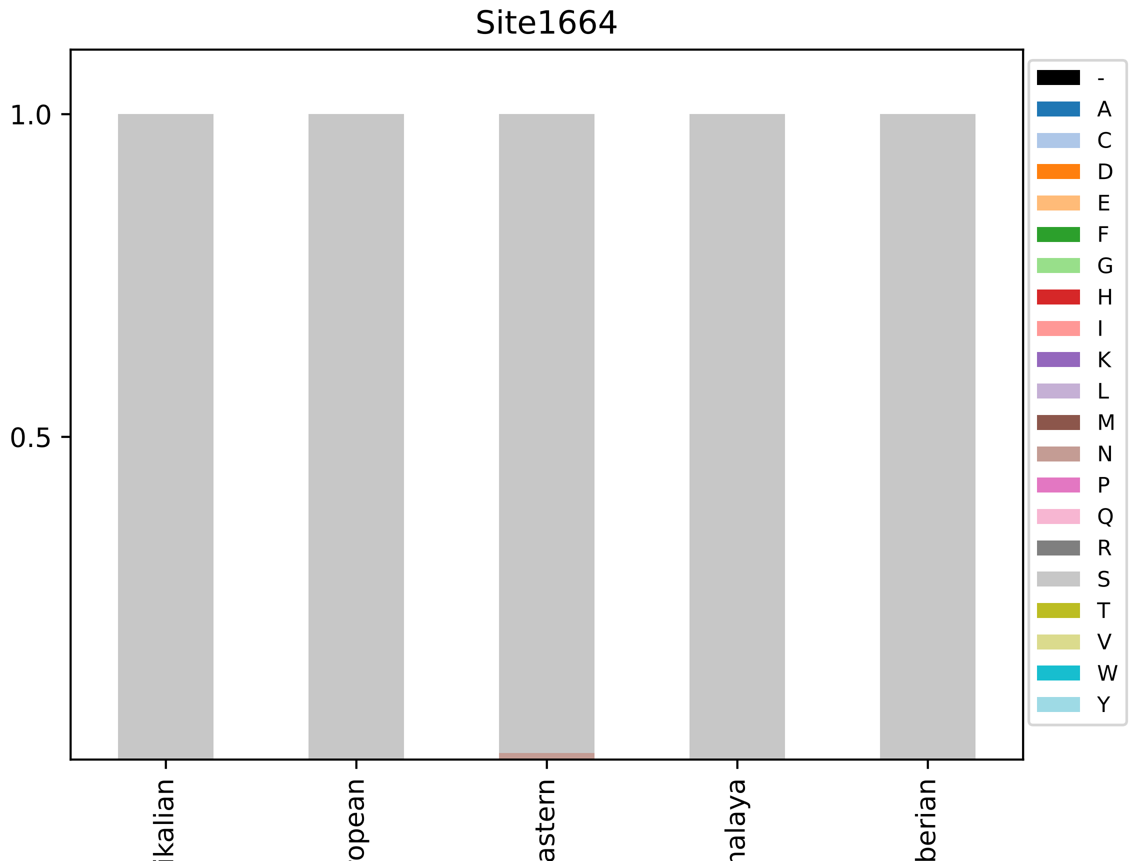
Site1664
1.0
0.5
-
A
C
D
E
F
G
H
I
K
L
M
N
P
Q
R
S
T
V
W
Y
ikalian
opean
astern
nalaya
berian

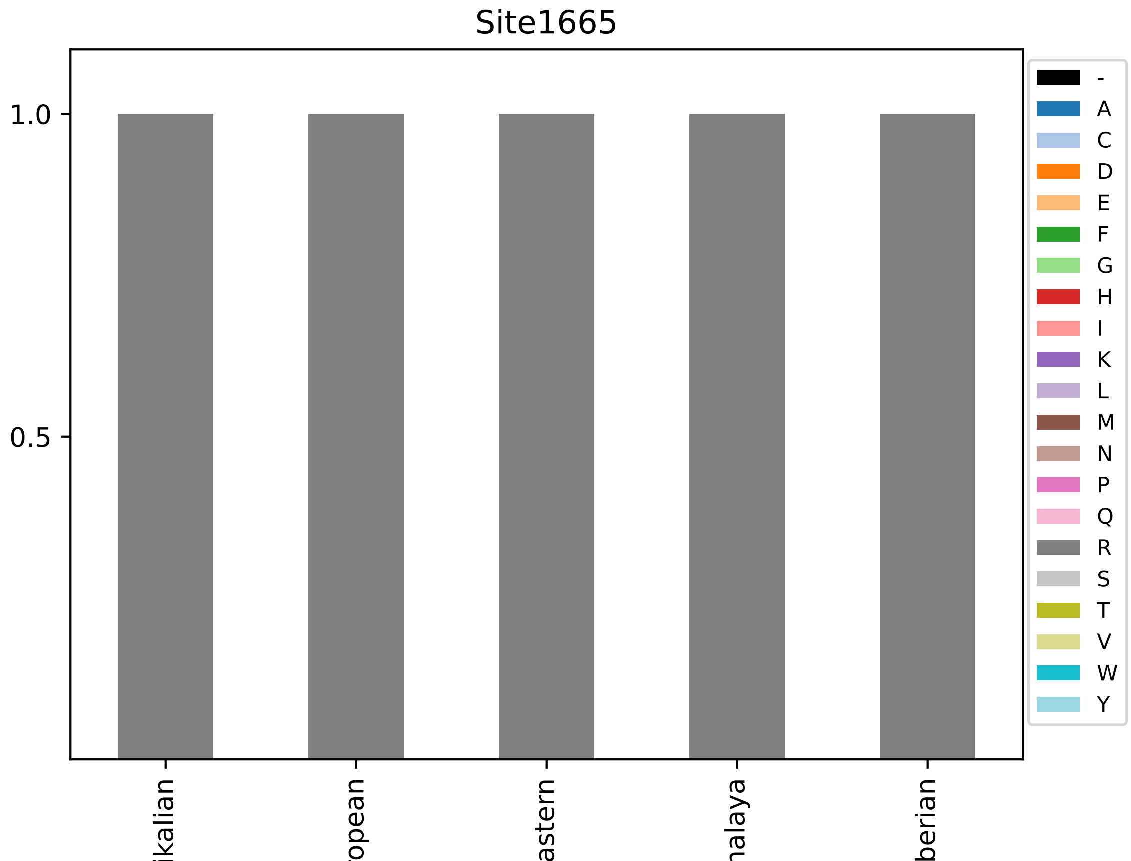

Site1665
1.0
0.5
ikalian
opean
astern
nalaya
berian
-
A
C
D
E
F
G
H
I
K
L
M
N
P
Q
R
S
T
V
W
Y

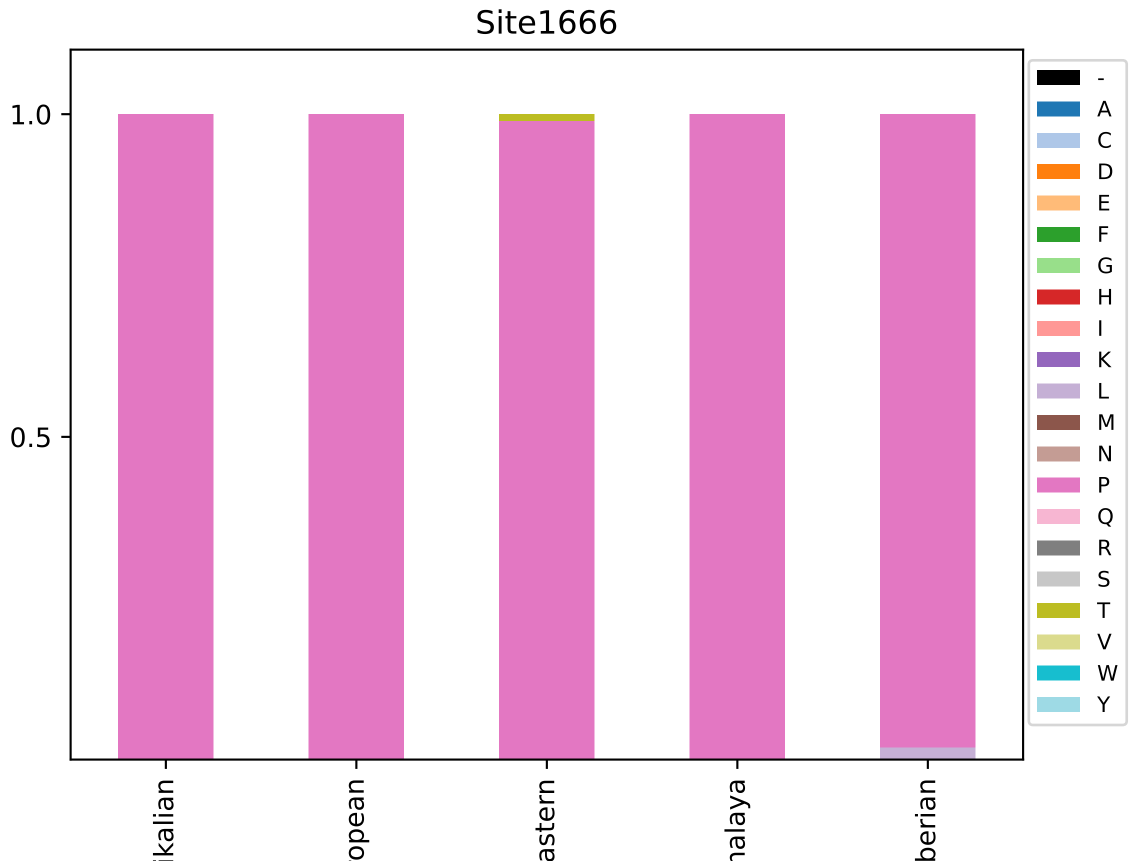
Site1666
1.0
0.5
-
A
C
D
E
F
G
H
I
K
L
M
N
P
Q
R
S
T
V
W
Y
ikalian
opean
astern
nalaya
berian

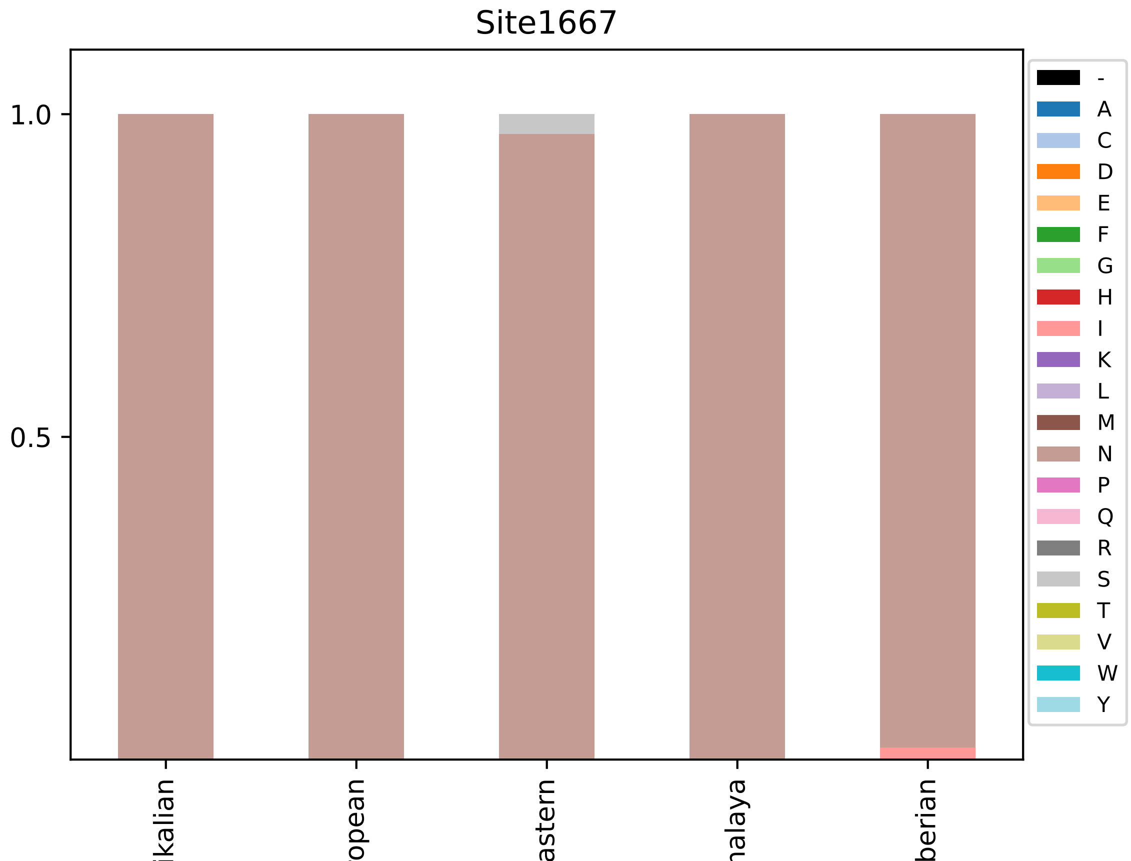
Site1667
1.0
0.5
-
A
C
D
E
F
G
H
I
K
L
M
N
P
Q
R
S
T
V
W
Y
ikalian
opean
astern
nalaya
berian

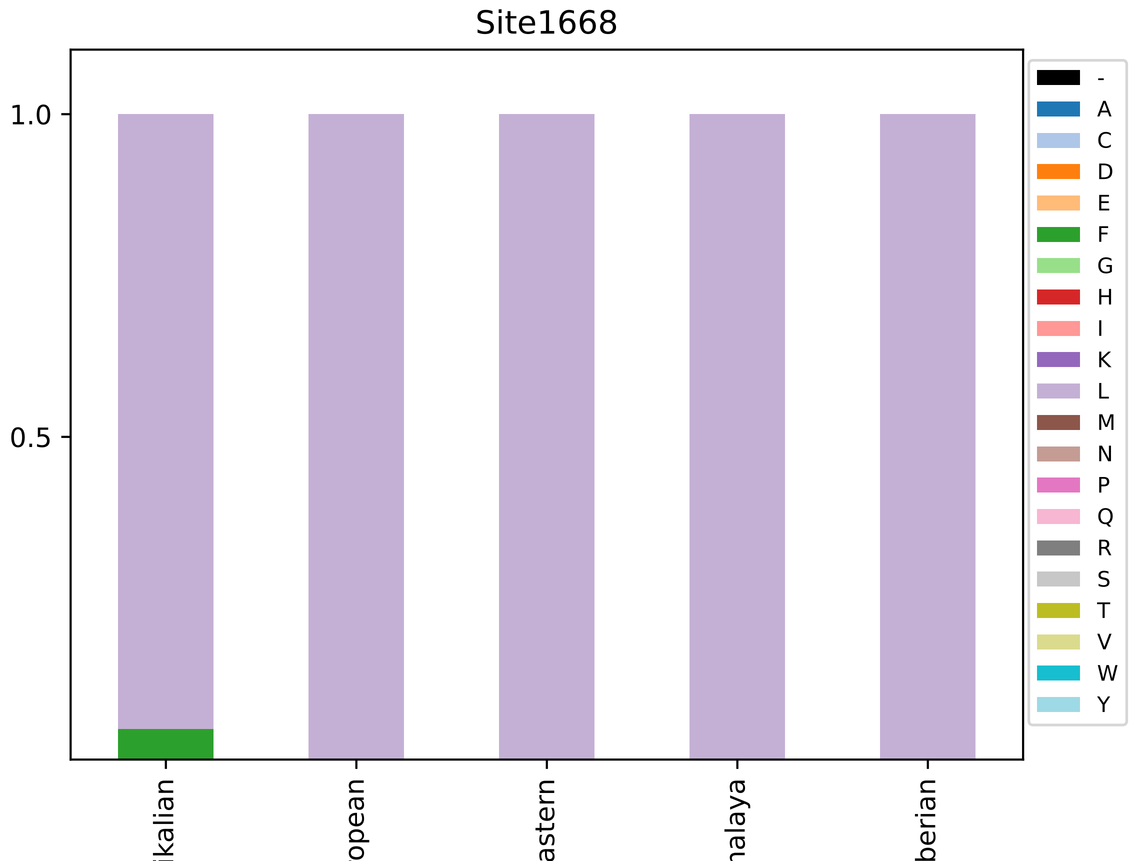
Site1668
1.0
0.5
-
A
C
D
E
F
G
H
I
K
L
M
N
P
Q
R
S
T
V
W
Y
ikalian
opean
astern
nalaya
berian

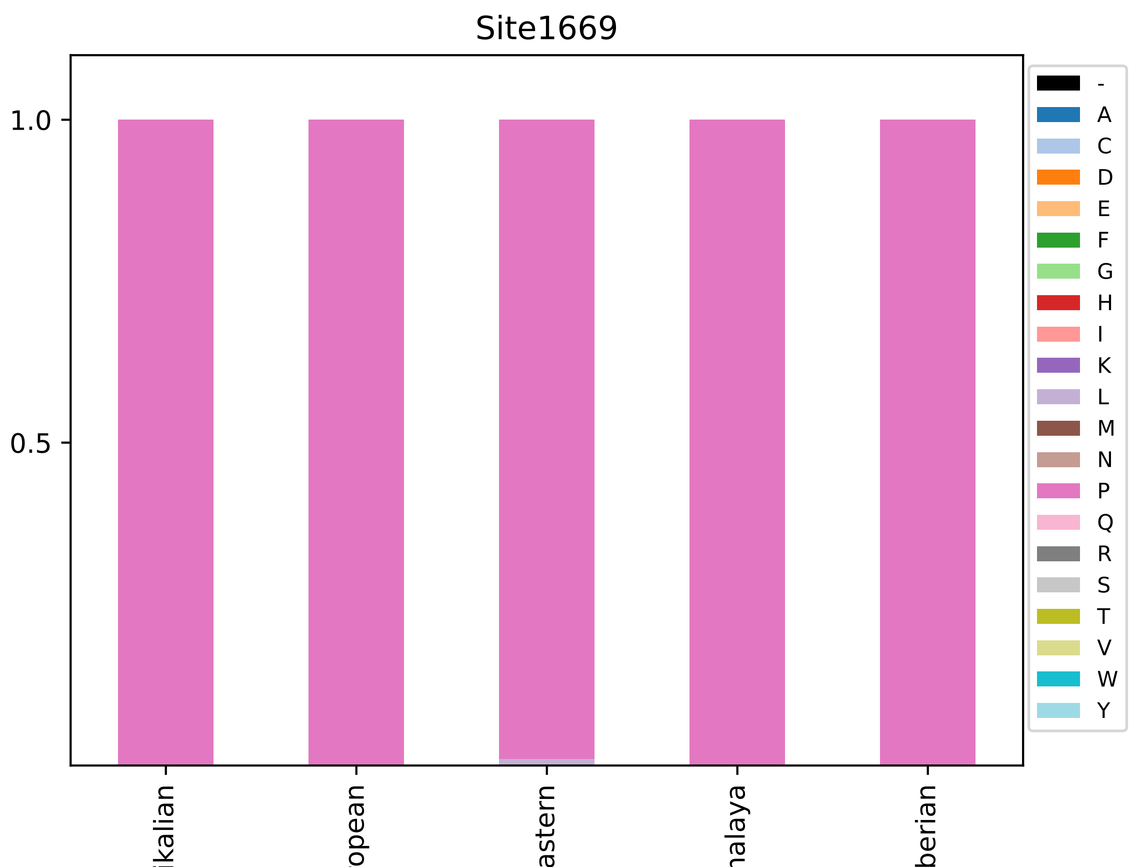
Site1669
1.0
0.5
-
A
C
D
E
F
G
H
I
K
L
M
N
P
Q
R
S
T
V
W
Y
ikalian
opean
astern
nalaya
berian

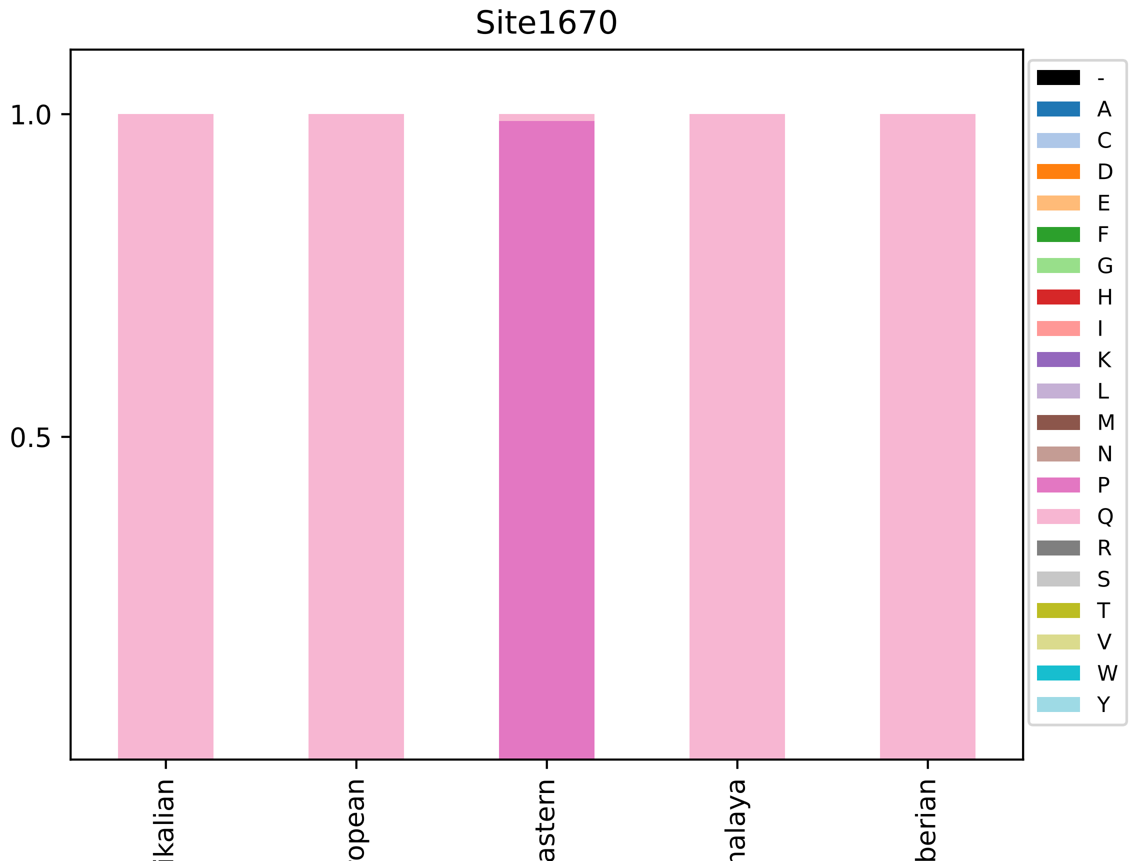

Site1670
1.0
0.5
-
A
C
D
E
F
G
H
I
K
L
M
N
P
Q
R
S
T
V
W
Y
ikalian
opean
astern
nalaya
berian

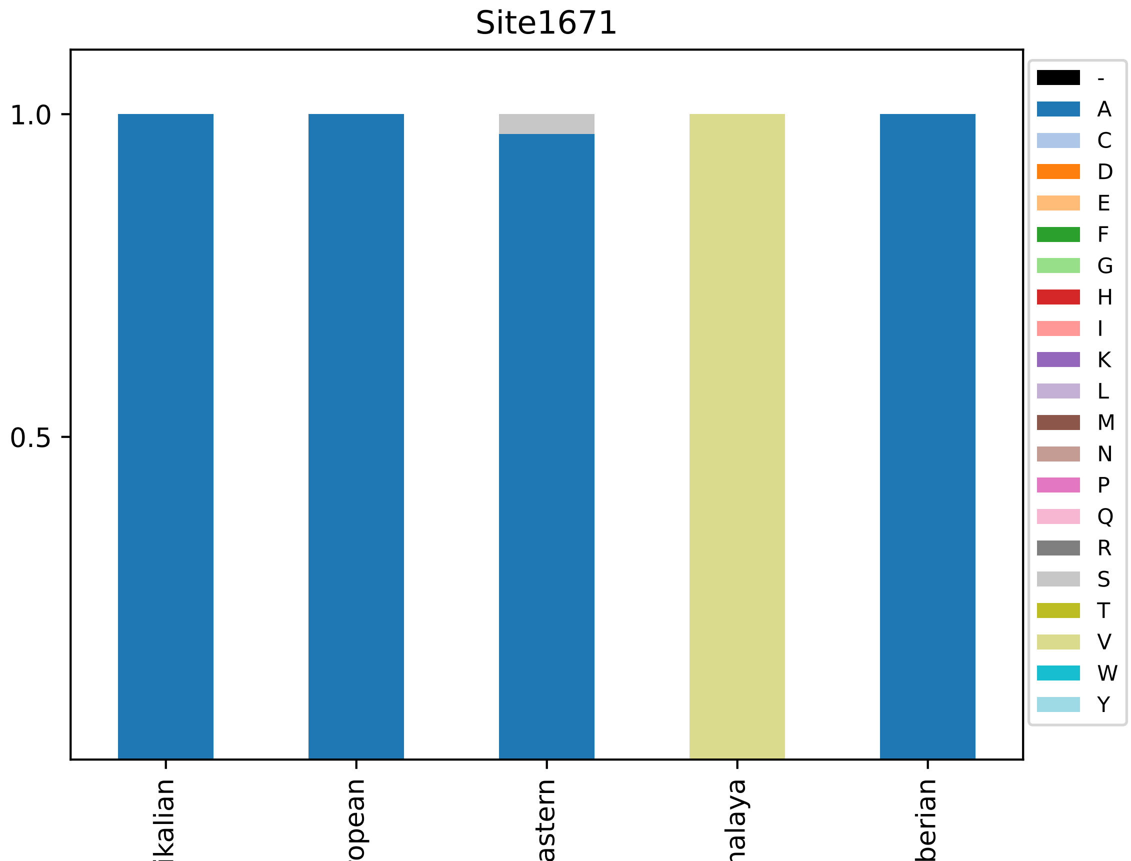

Site1671
1.0
0.5
-
A
C
D
E
F
G
H
I
K
L
M
N
P
Q
R
S
T
V
W
Y
ikalian
opean
astern
nalaya
berian

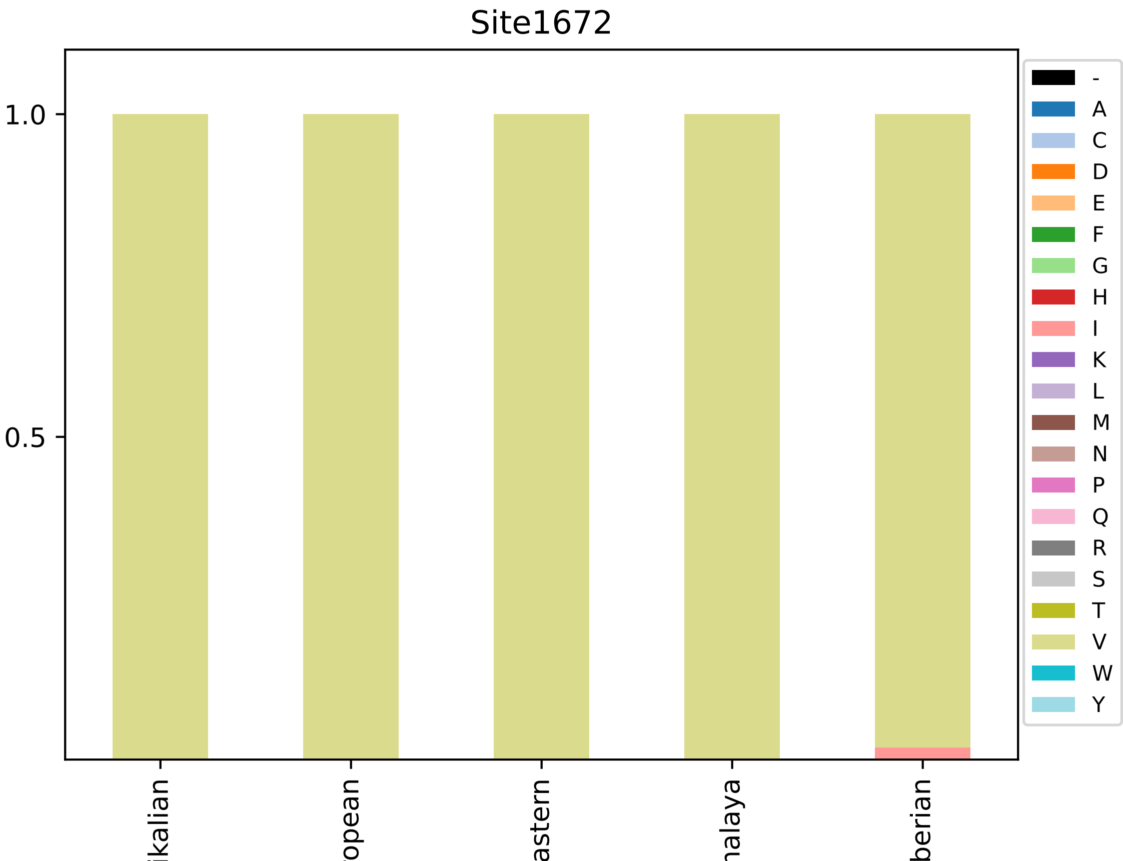
Site1672
1.0
0.5
-
A
C
D
E
F
G
H
I
K
L
M
N
P
Q
R
S
T
V
W
Y
ikalian
opean
astern
nalaya
berian

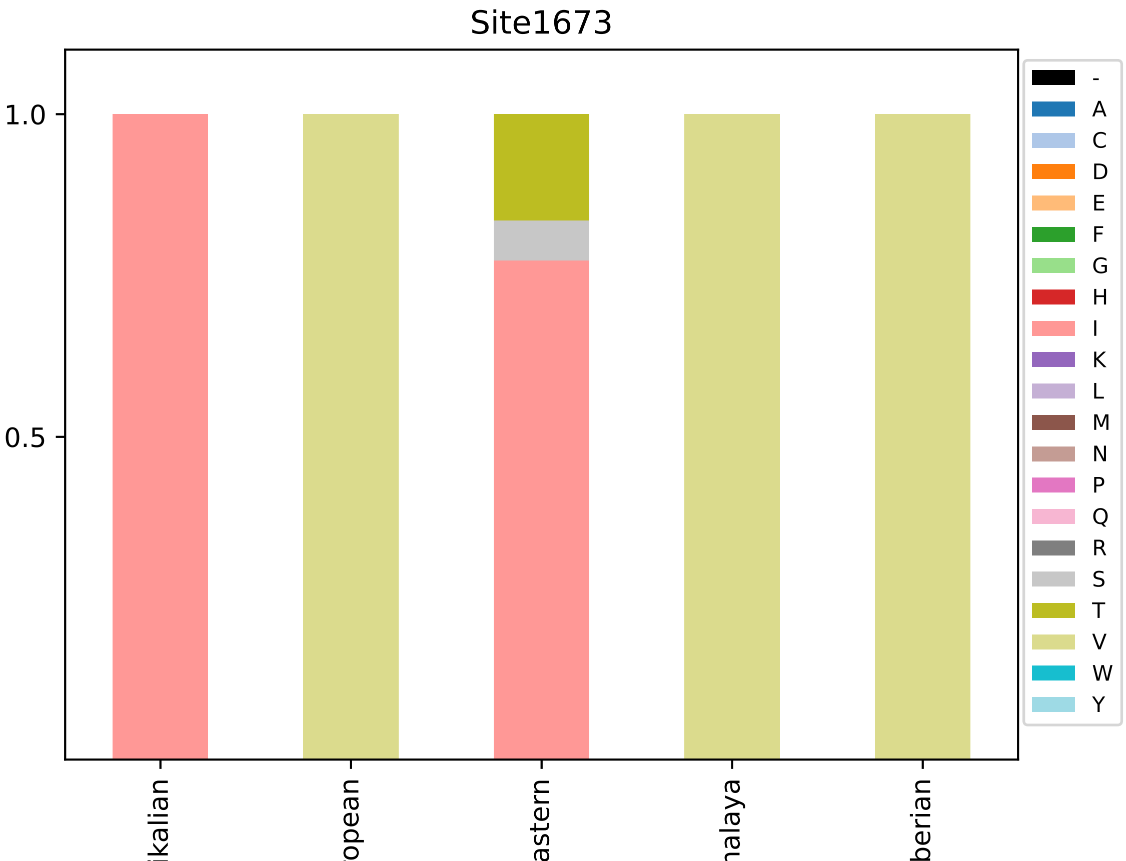

Site1673
1.0
0.5
-
A
C
D
E
F
G
H
I
K
L
M
N
P
Q
R
S
T
V
W
Y
ikalian
opean
astern
nalaya
berian

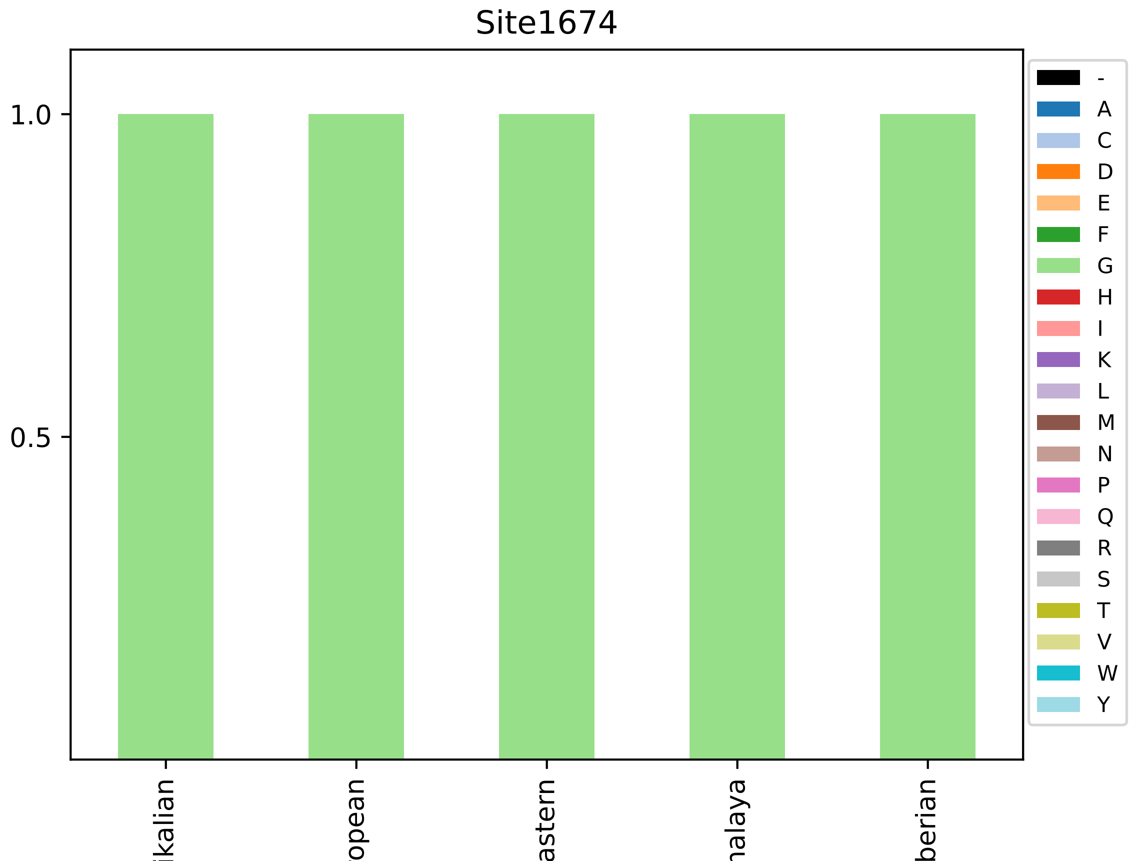

Site1674
1.0
0.5
ikalian
opean
astern
nalaya
berian
-
A
C
D
E
F
G
H
I
K
L
M
N
P
Q
R
S
T
V
W
Y

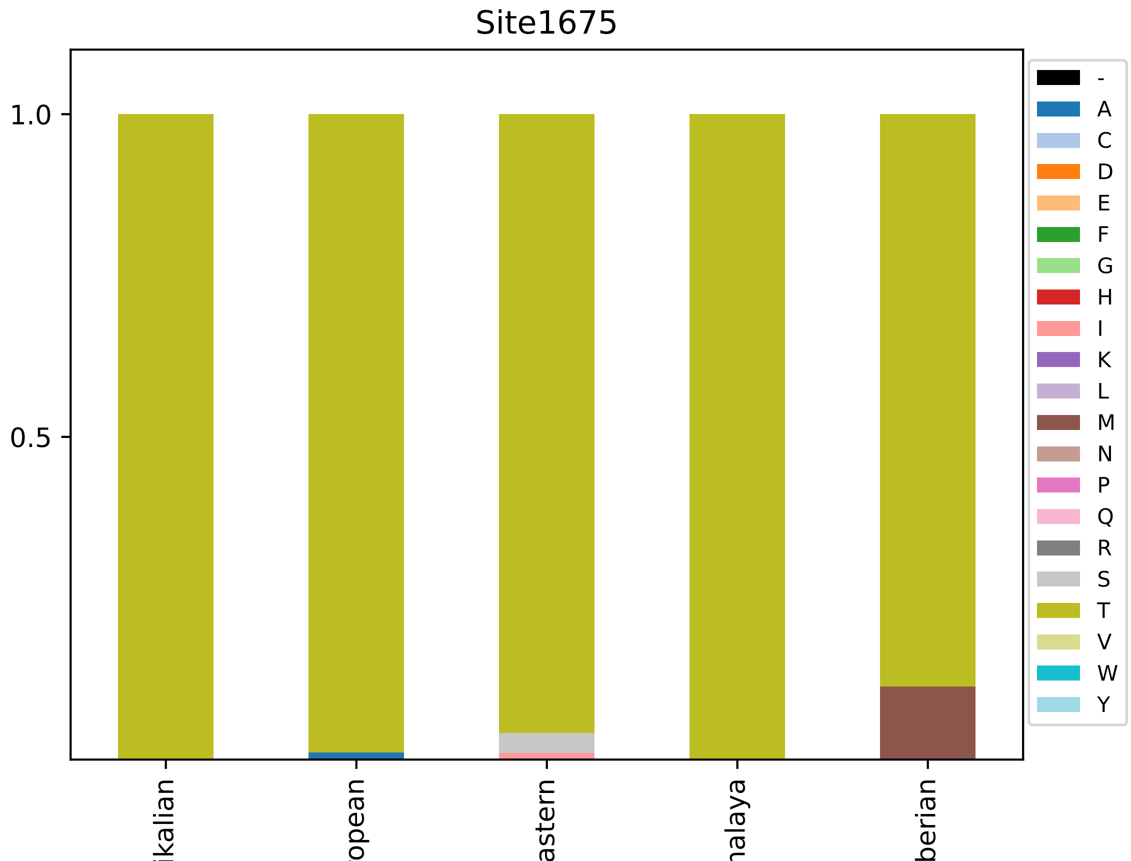

Site1675
1.0
0.5
-
A
C
D
E
F
G
H
I
K
L
M
N
P
Q
R
S
T
V
W
Y

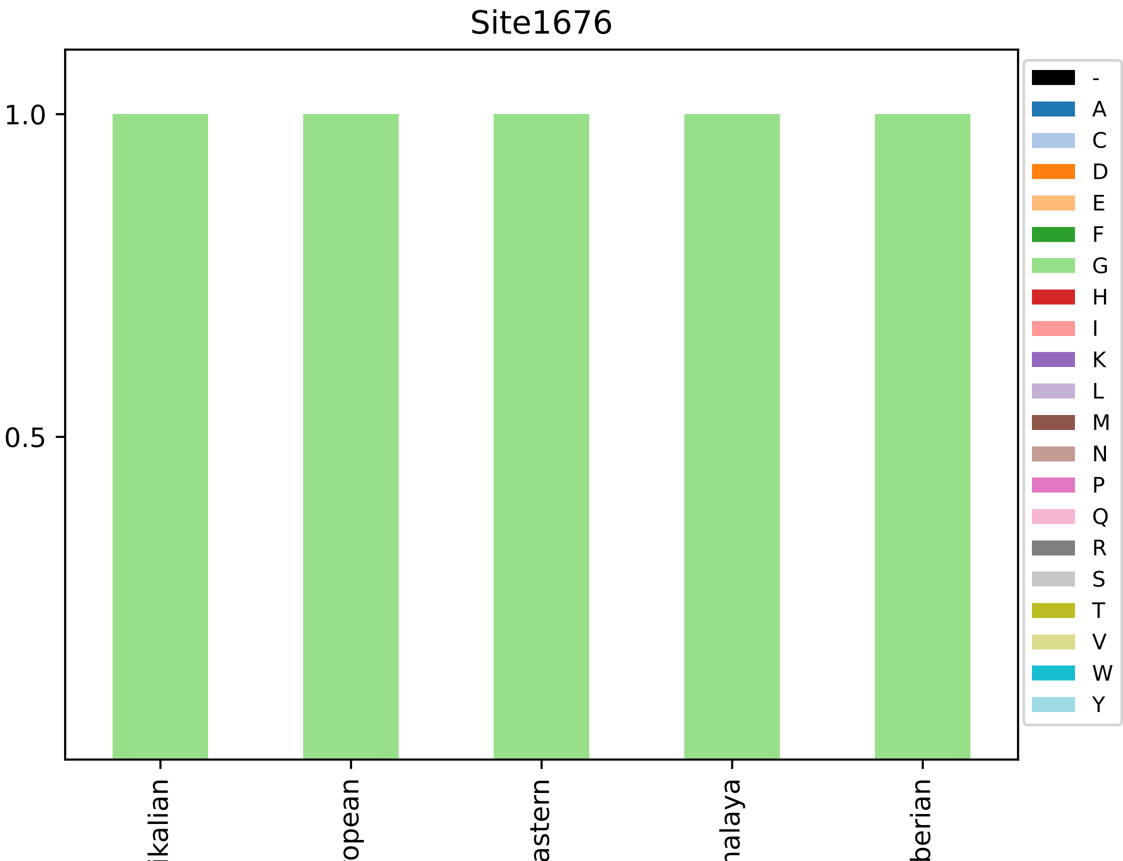

Site1676
1.0
0.5
-
A
C
D
E
F
G
H
I
K
L
M
N
P
Q
R
S
T
V
W
Y
ikalian
opean
astern
nalaya
berian

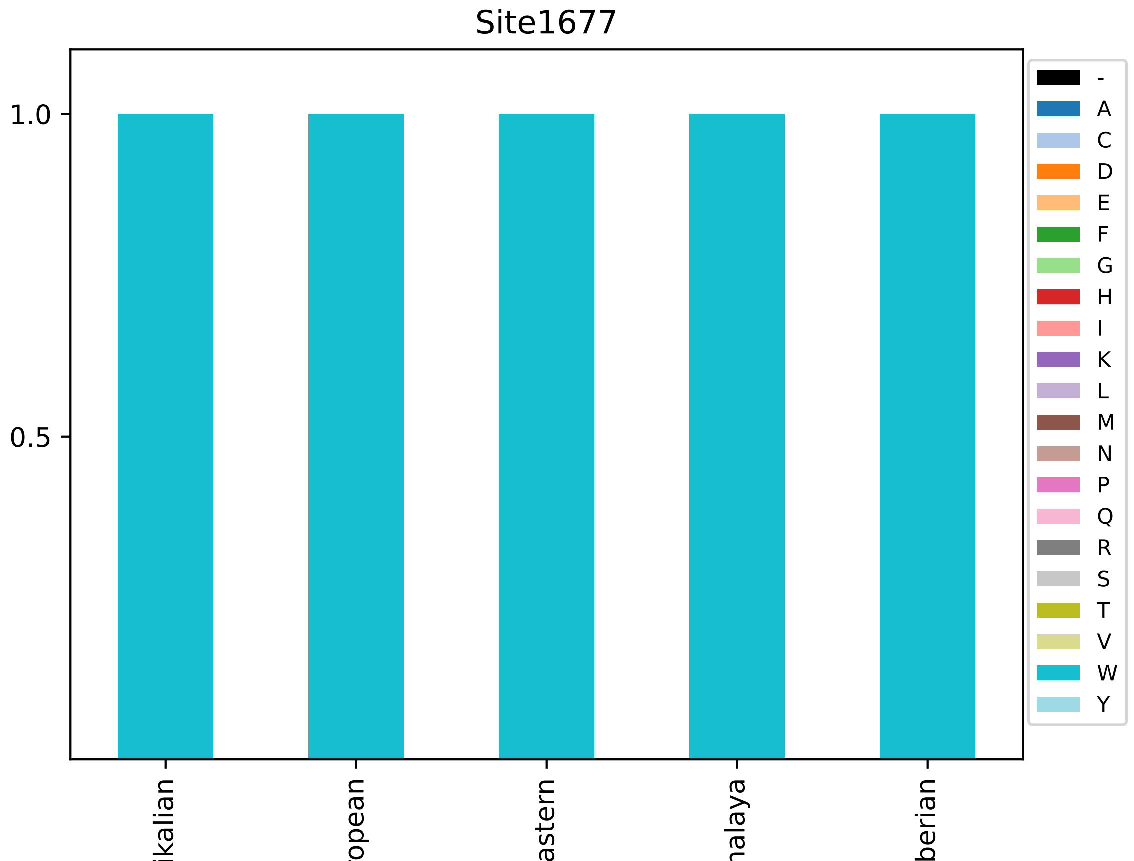

Site1677
1.0
0.5
-
A
C
D
E
F
G
H
I
K
L
M
N
P
Q
R
S
T
V
W
Y
ikalian
ropean
astern
nalaya
berian

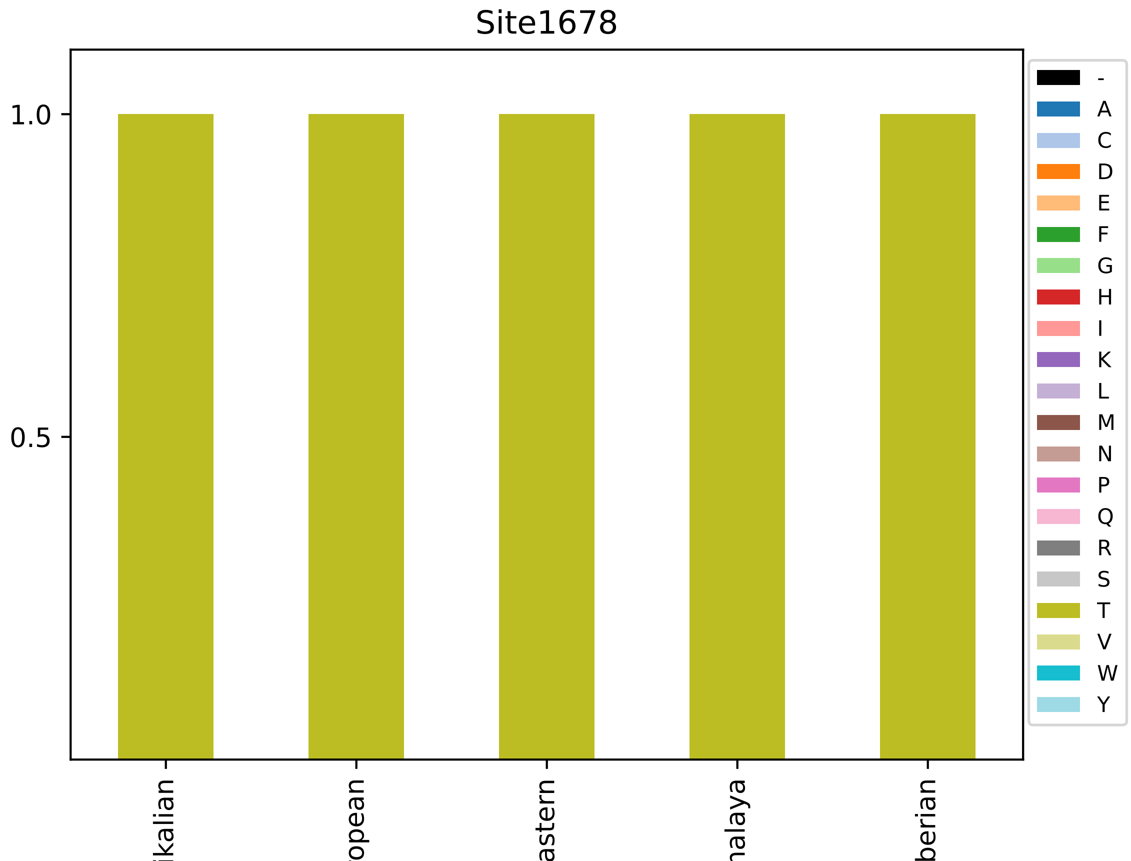

Site1678
1.0
0.5
-
A
C
D
E
F
G
H
I
K
L
M
N
P
Q
R
S
T
V
W
Y
ikalian
opean
astern
nalaya
berian

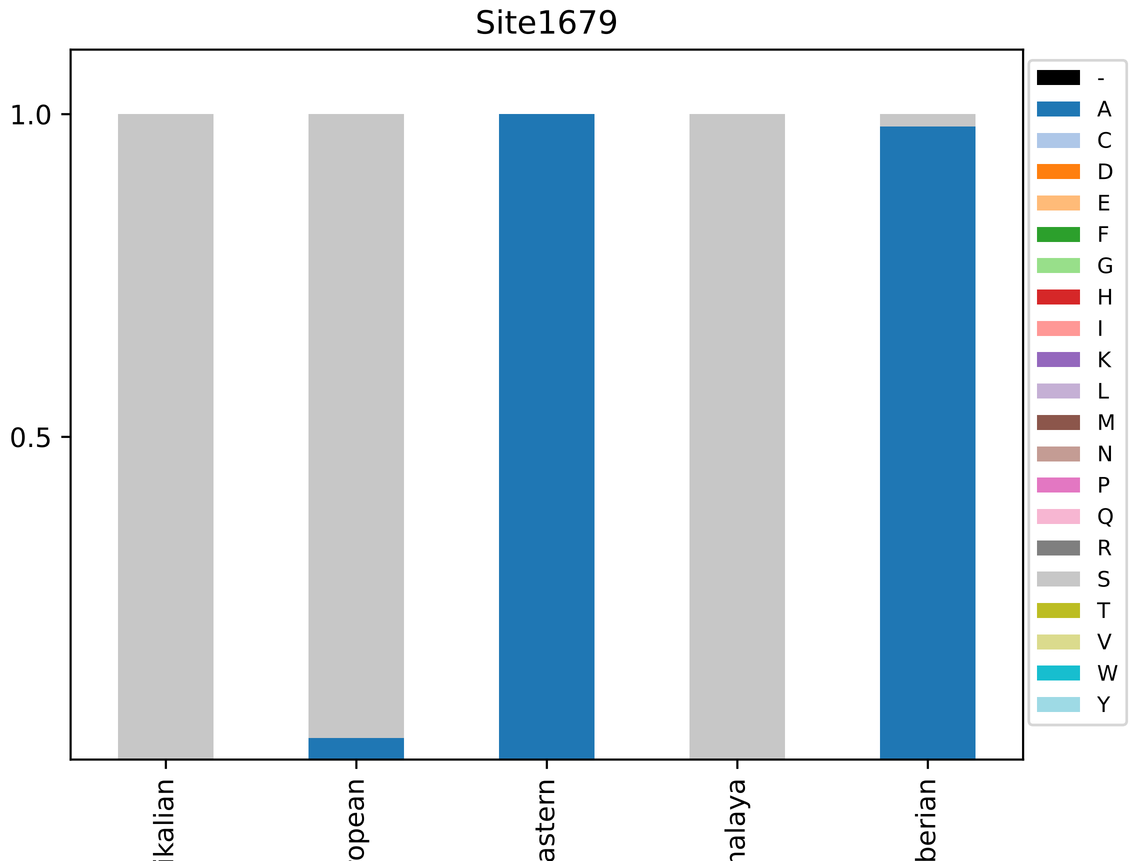

Site1679
1.0
0.5
ikalian
opean
astern
nalaya
berian
-
A
C
D
E
F
G
H
I
K
L
M
N
P
Q
R
S
T
V
W
Y

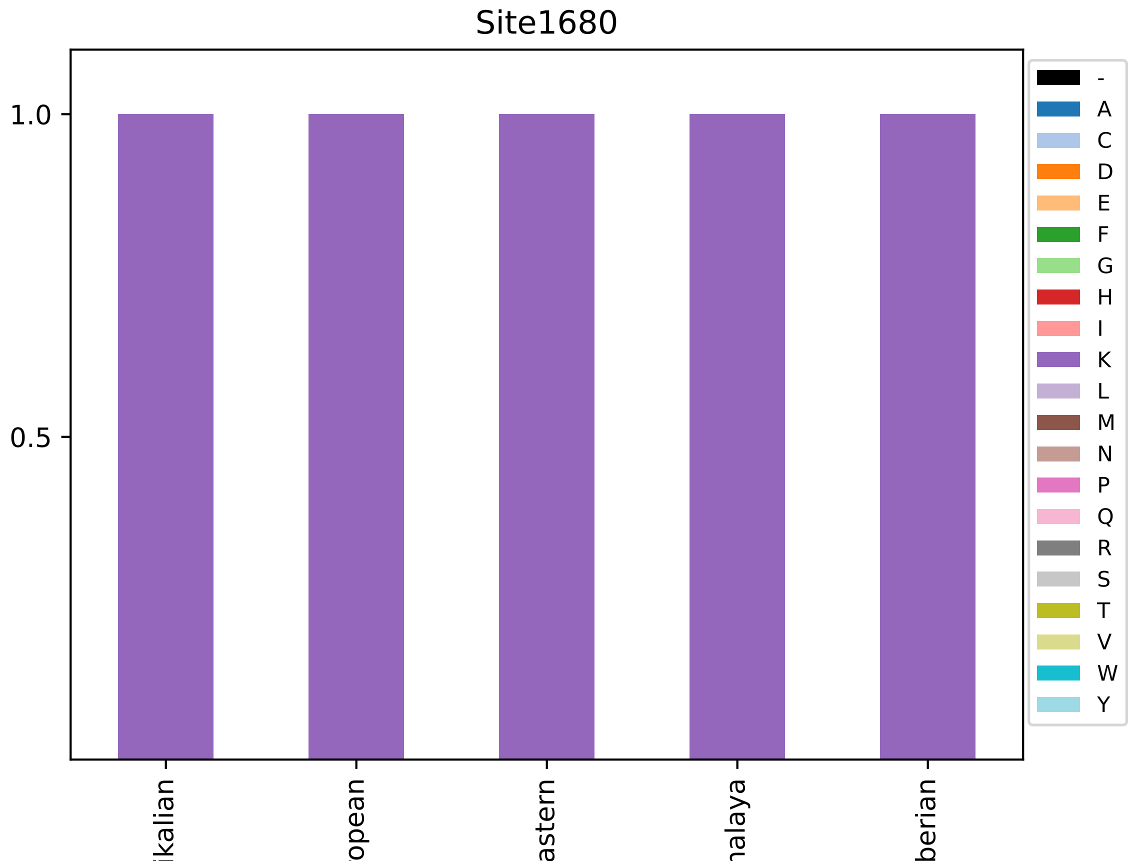

Site1680
1.0
0.5
-
A
C
D
E
F
G
H
I
K
L
M
N
P
Q
R
S
T
V
W
Y
ikalian
opean
astern
nalaya
berian

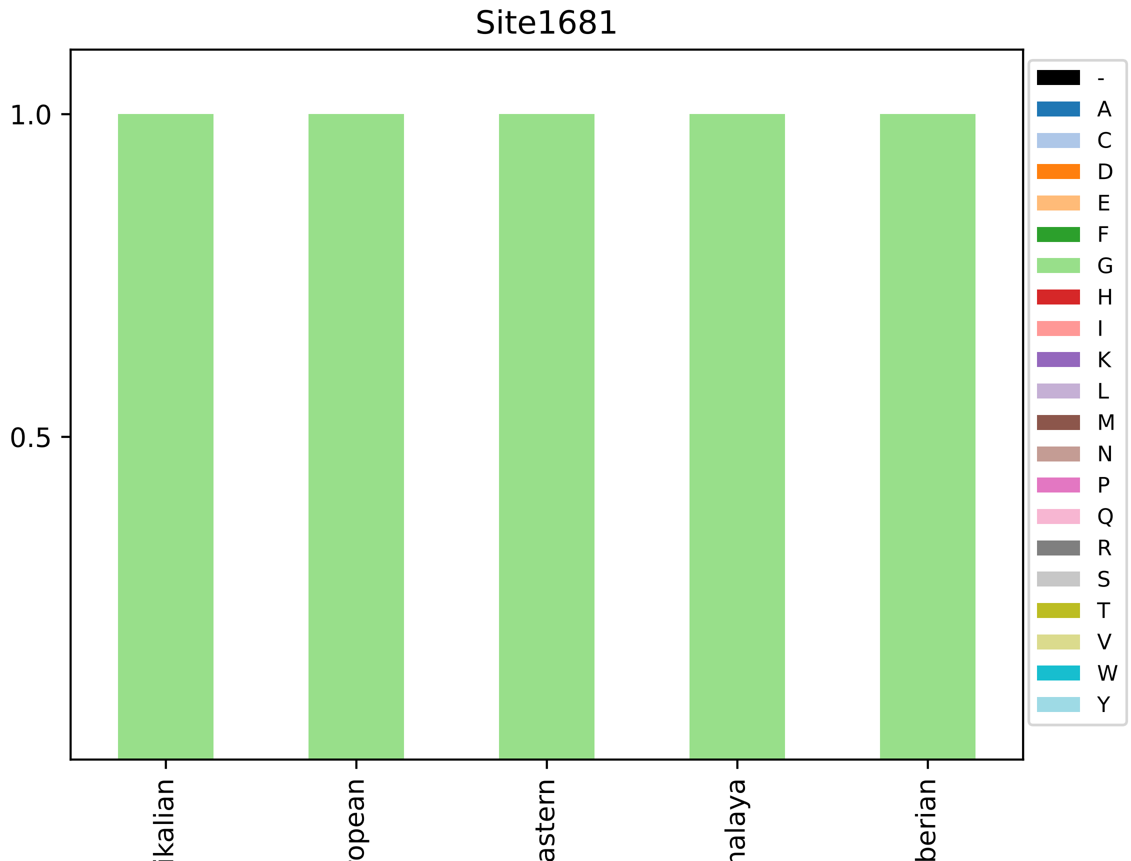
Site1681
1.0
0.5
ikalian
opean
astern
nalaya
berian
-
A
C
D
E
F
G
H
I
K
L
M
N
P
Q
R
S
T
V
W
Y

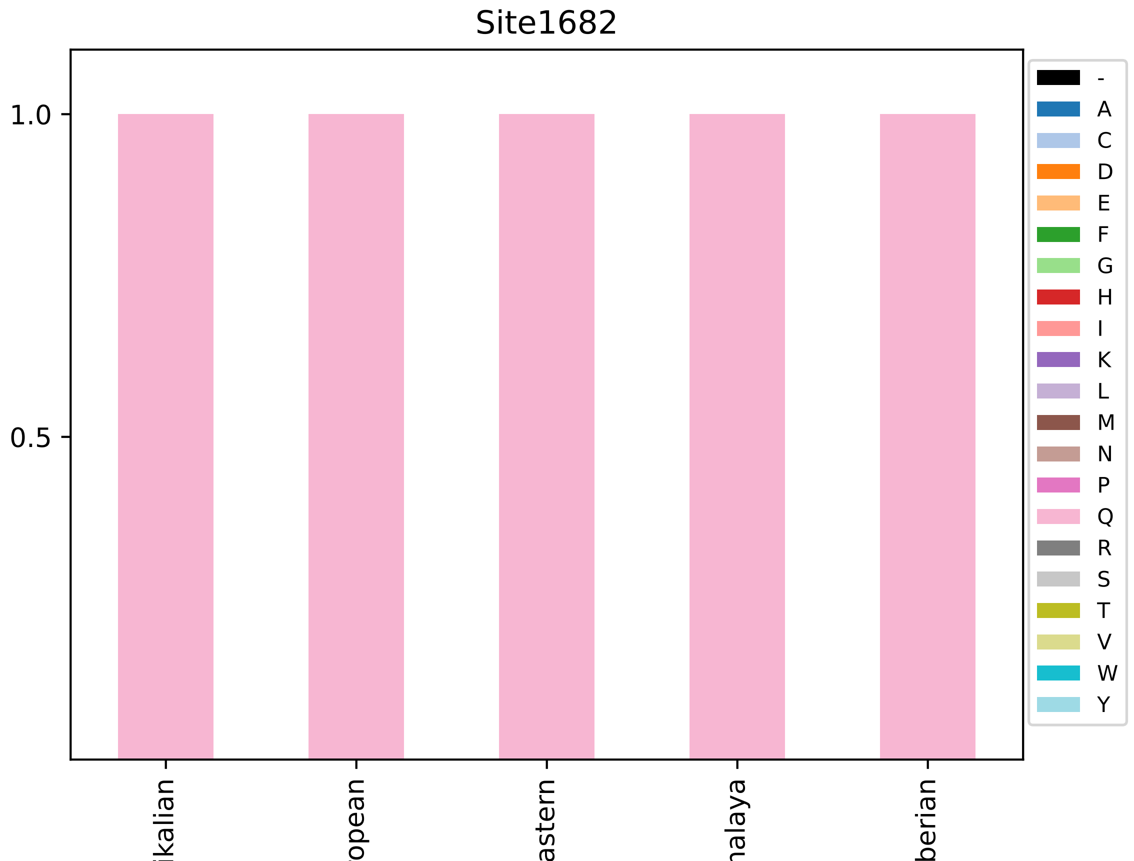
Site1682
1.0
0.5
-
A
C
D
E
F
G
H
I
K
L
M
N
P
Q
R
S
T
V
W
Y
ikalian
opean
astern
nalaya
berian

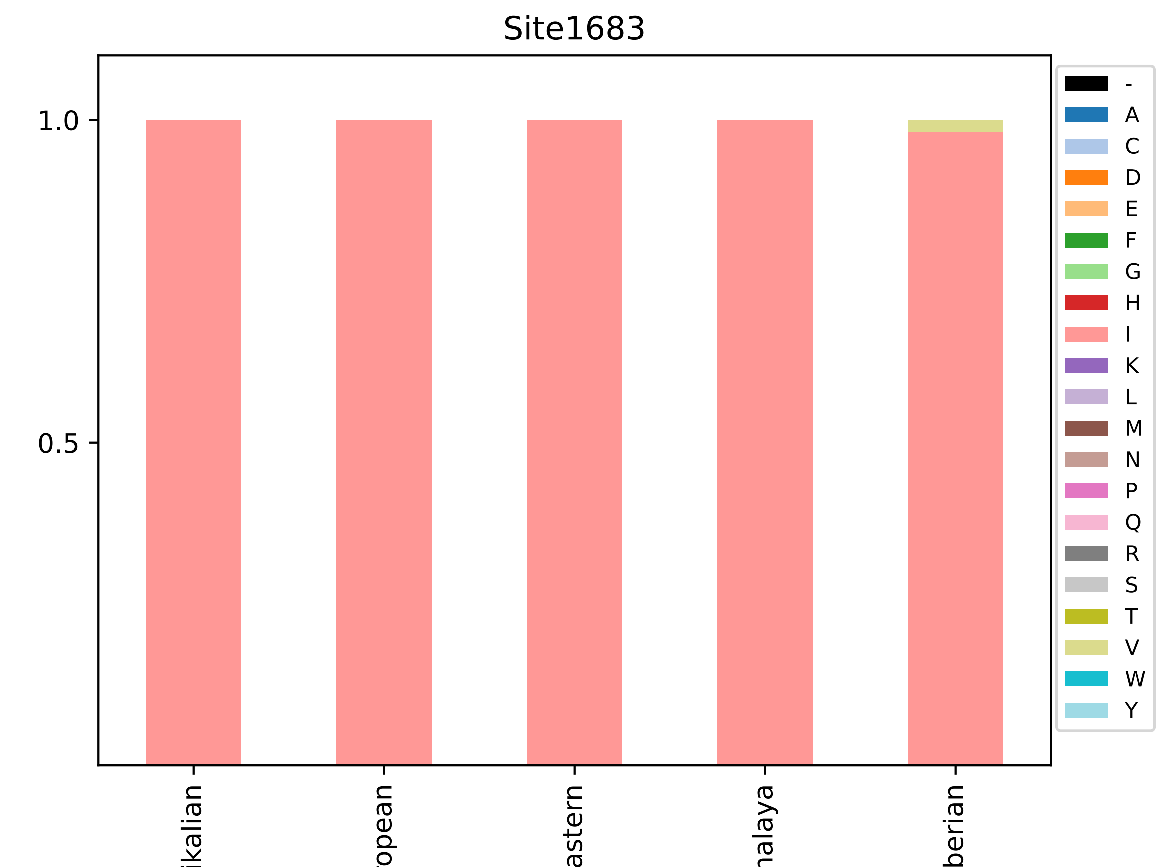

Site1683
1.0
0.5
-
A
C
D
E
F
G
H
I
K
L
M
N
P
Q
R
S
T
V
W
Y
ikalian
opean
astern
nalaya
berian

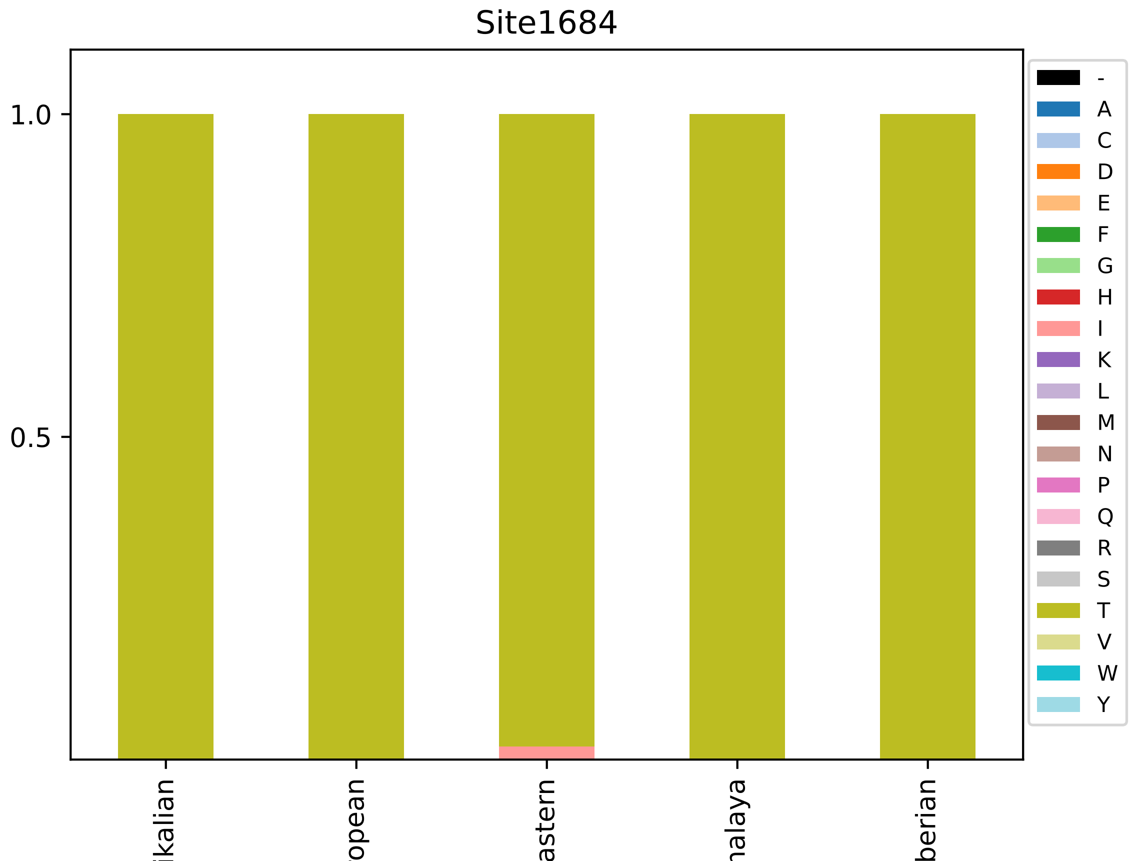

Site1684
1.0
0.5
-
A
C
D
E
F
G
H
I
K
L
M
N
P
Q
R
S
T
V
W
Y
ikalian
opean
astern
nalaya
berian

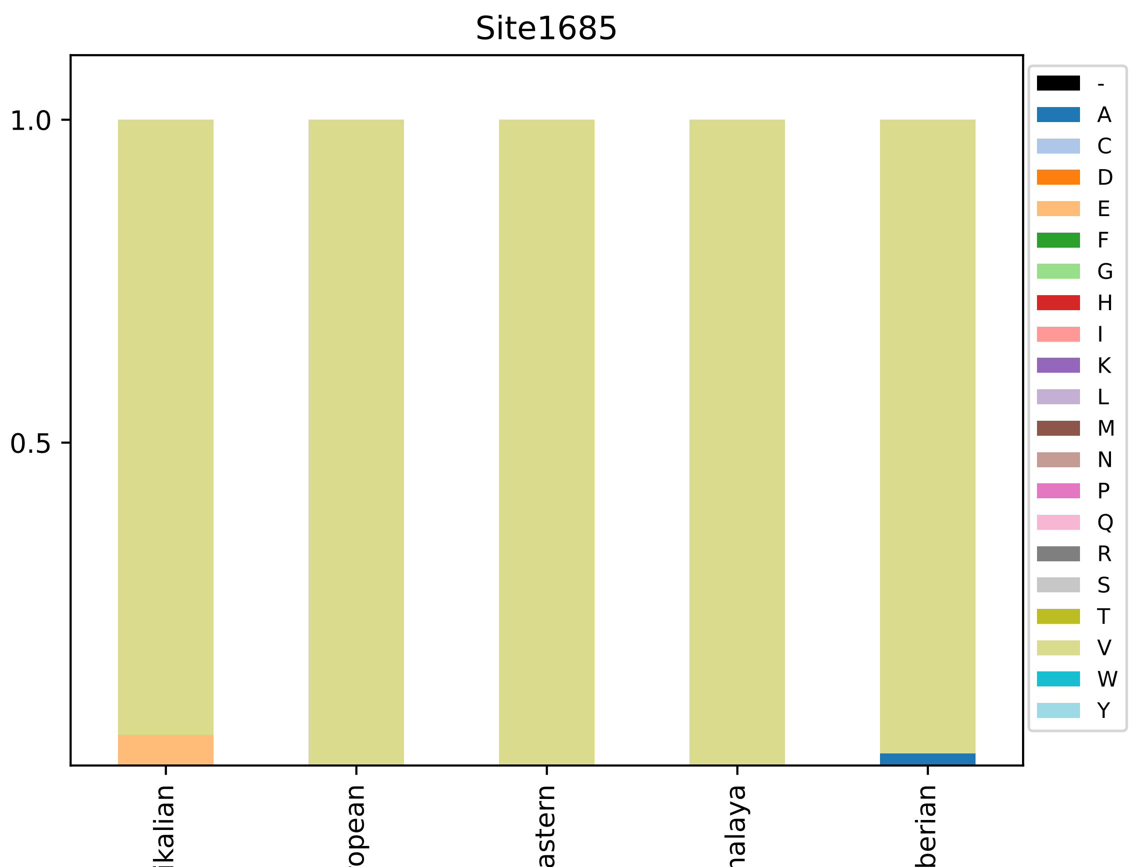
Site1685
1.0
0.5
-
A
C
D
E
F
G
H
I
K
L
M
N
P
Q
R
S
T
V
W
Y
ikalian
opean
astern
nalaya
berian

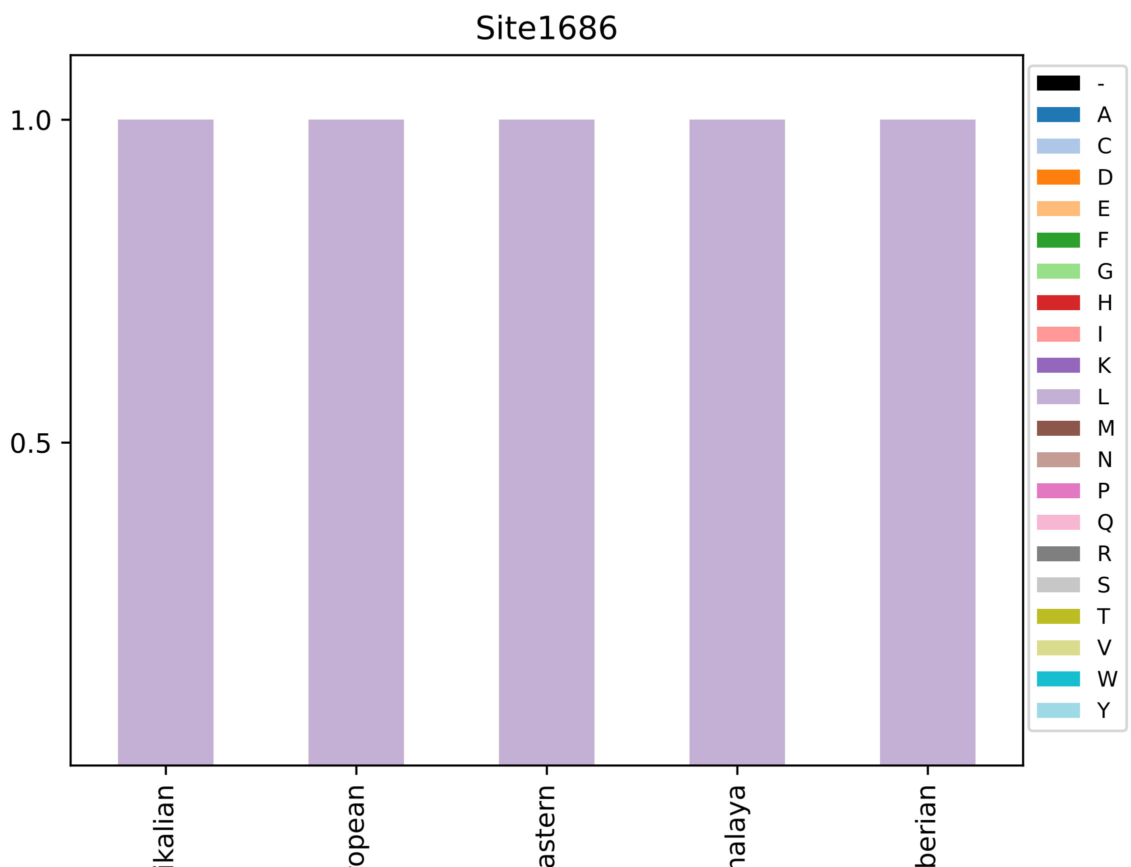
Site1686
1.0
0.5
-
A
C
D
E
F
G
H
I
K
L
M
N
P
Q
R
S
T
V
W
Y
ikalian
opean
astern
nalaya
berian

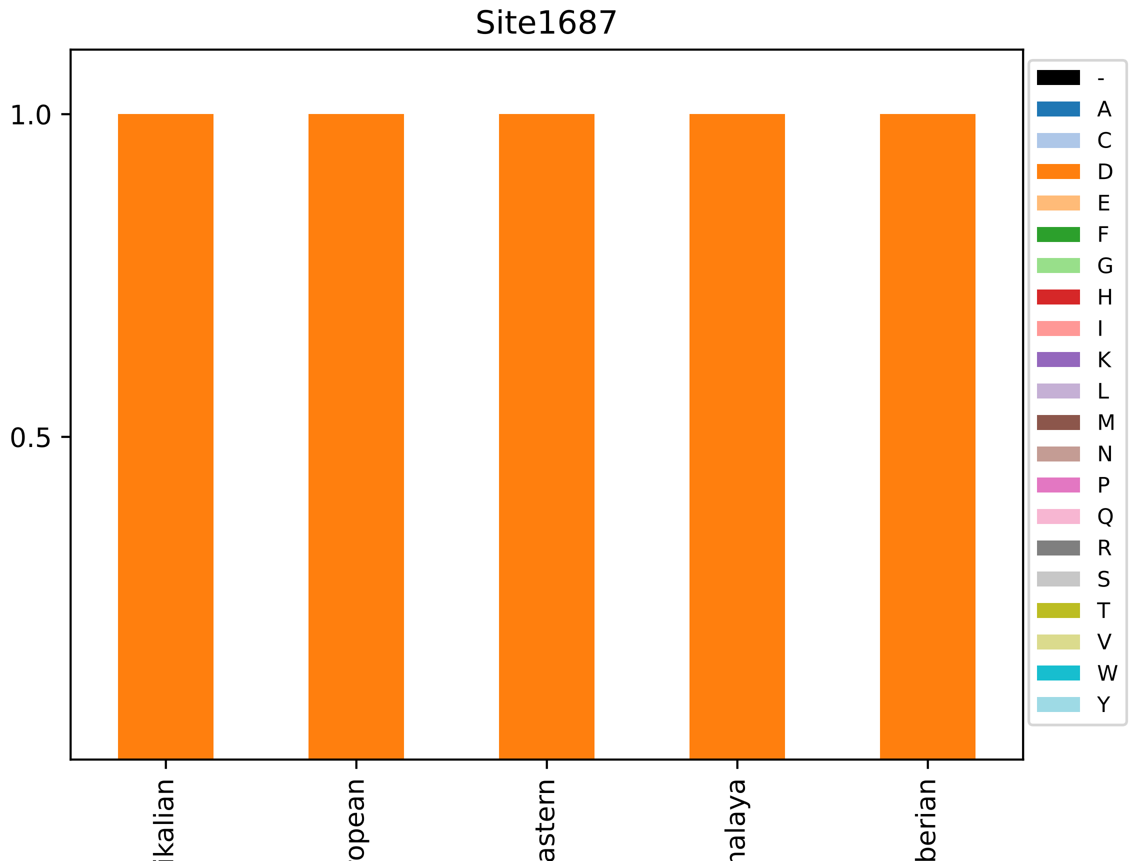
Site1687
1.0
0.5
-
A
C
D
E
F
G
H
I
K
L
M
N
P
Q
R
S
T
V
W
Y
ikalian
opean
astern
nalaya
berian

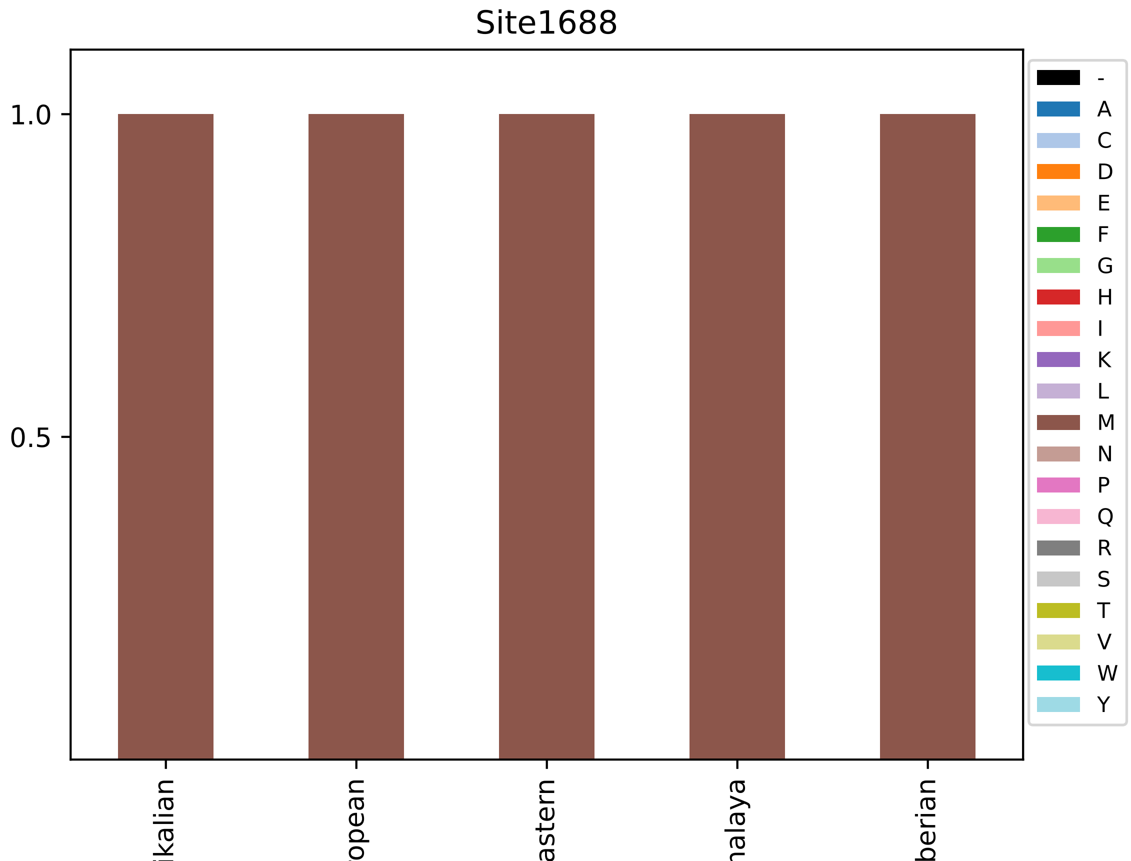
Site1688
1.0
0.5
ikalian
opean
astern
nalaya
berian
-
A
C
D
E
F
G
H
I
K
L
M
N
P
Q
R
S
T
V
W
Y

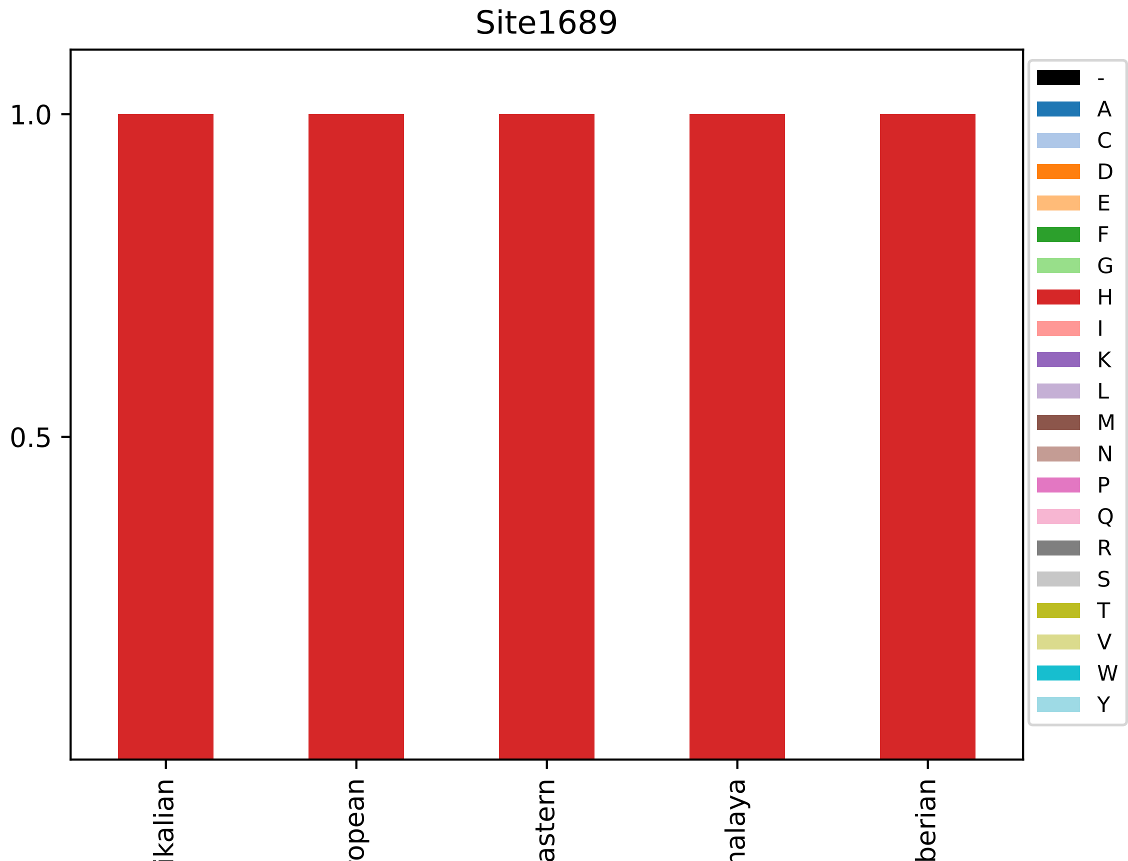
Site1689
1.0
0.5
-
A
C
D
E
F
G
H
I
K
L
M
N
P
Q
R
S
T
V
W
Y
ikalian
opean
astern
nalaya
berian

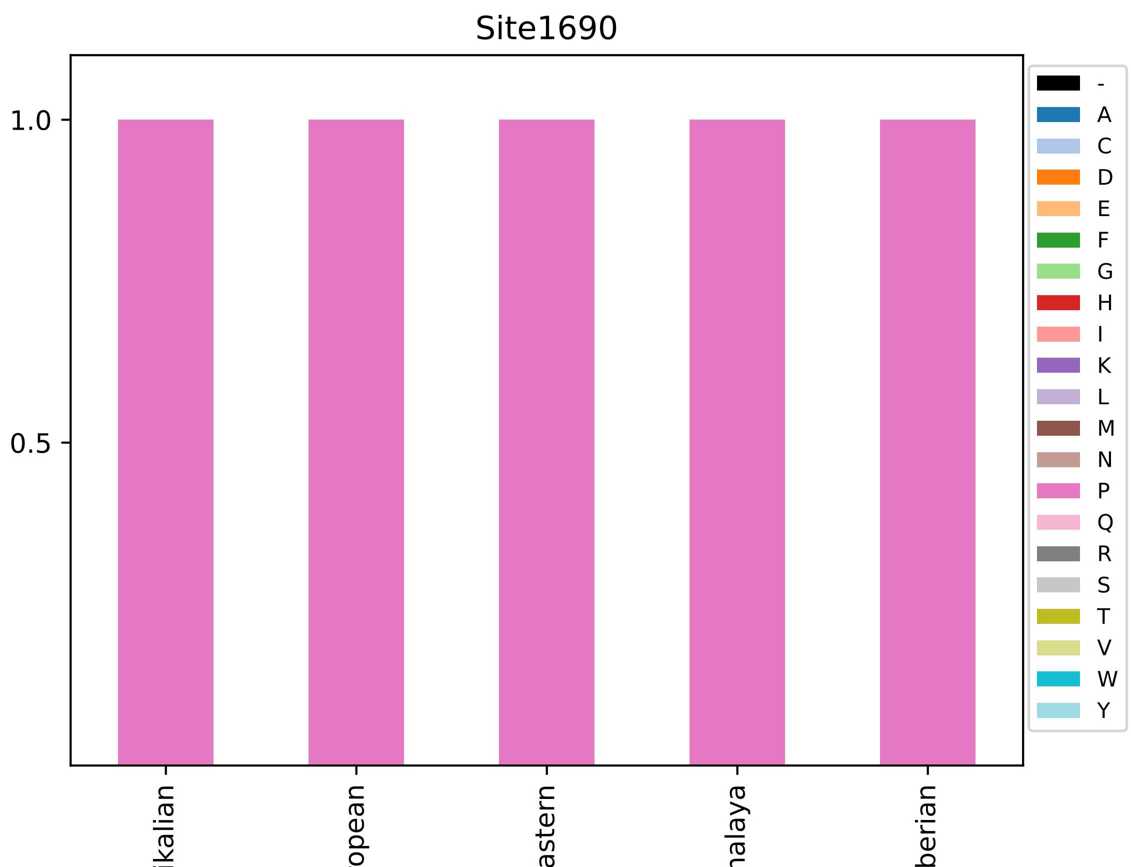
Site1690
1.0
0.5
-
A
C
D
E
F
G
H
I
K
L
M
N
P
Q
R
S
T
V
W
Y
ikalian
opean
astern
nalaya
berian

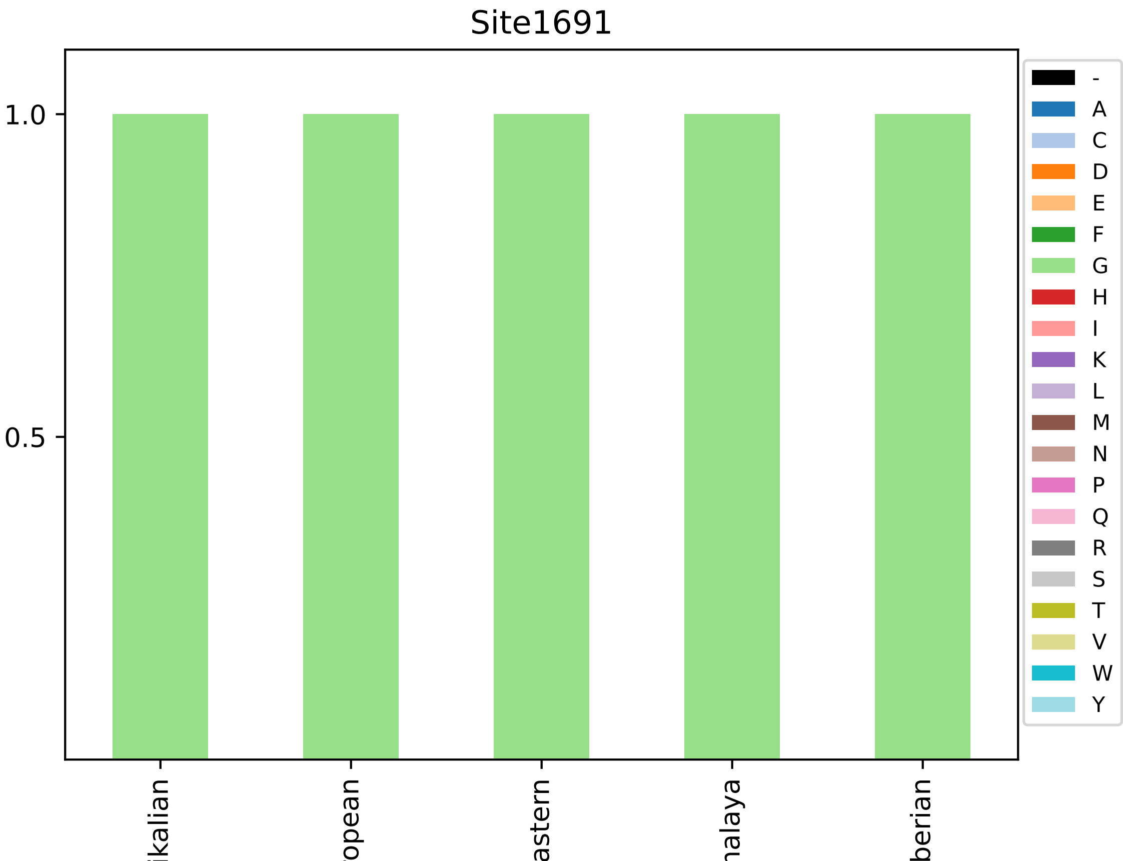
Site1691
1.0
0.5
-
A
C
D
E
F
G
H
I
K
L
M
N
P
Q
R
S
T
V
W
Y
ikalian
ropean
astern
nalaya
berian

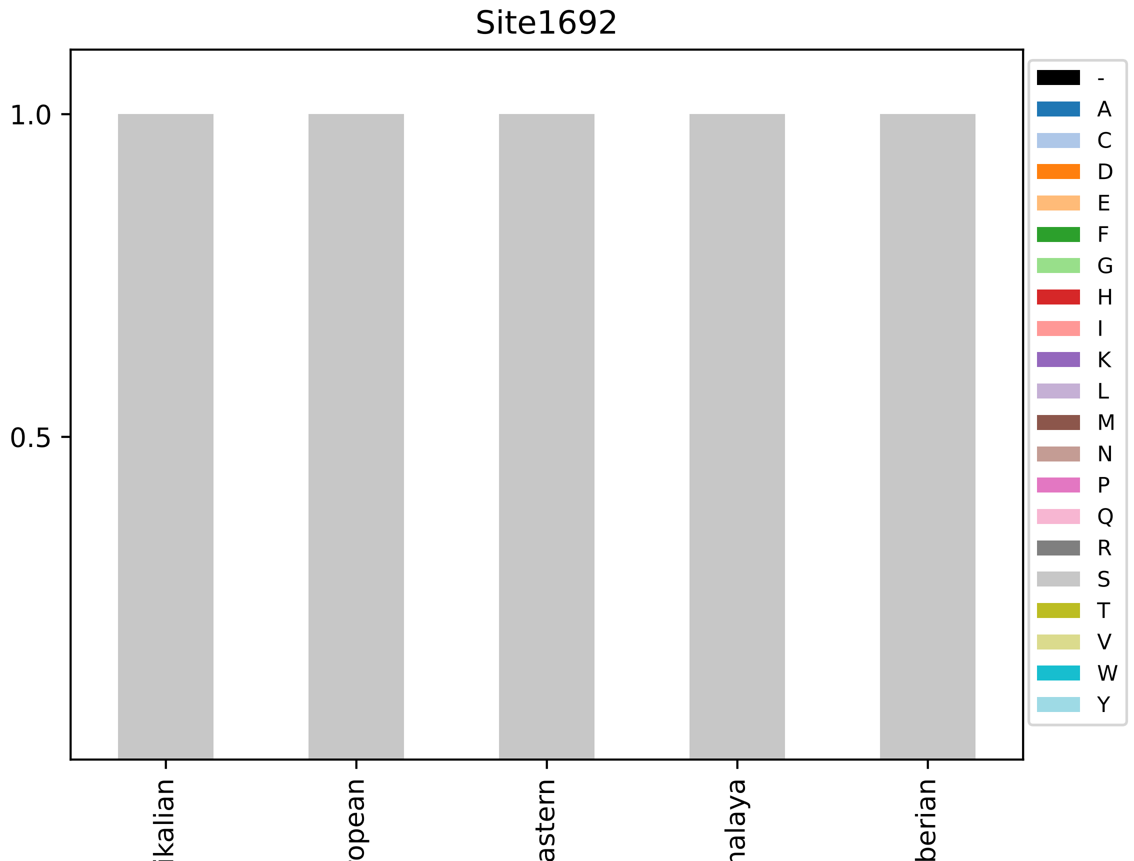

Site1692
1.0
0.5
-
A
C
D
E
F
G
H
I
K
L
M
N
P
Q
R
S
T
V
W
Y
ikalian
opean
astern
nalaya
berian

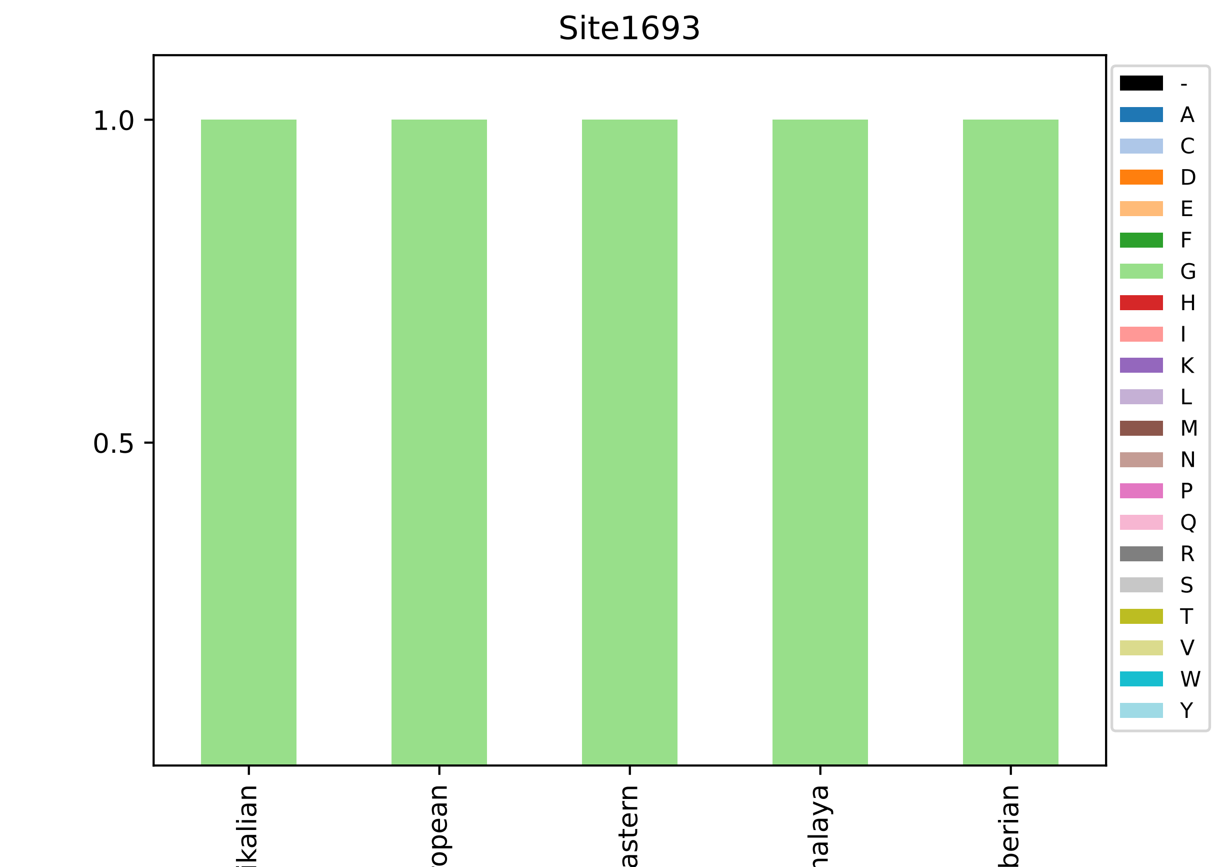

Site1693
1.0
0.5
-
A
C
D
E
F
G
H
I
K
L
M
N
P
Q
R
S
T
V
W
Y
ikalian
ropean
astern
nalaya
berian

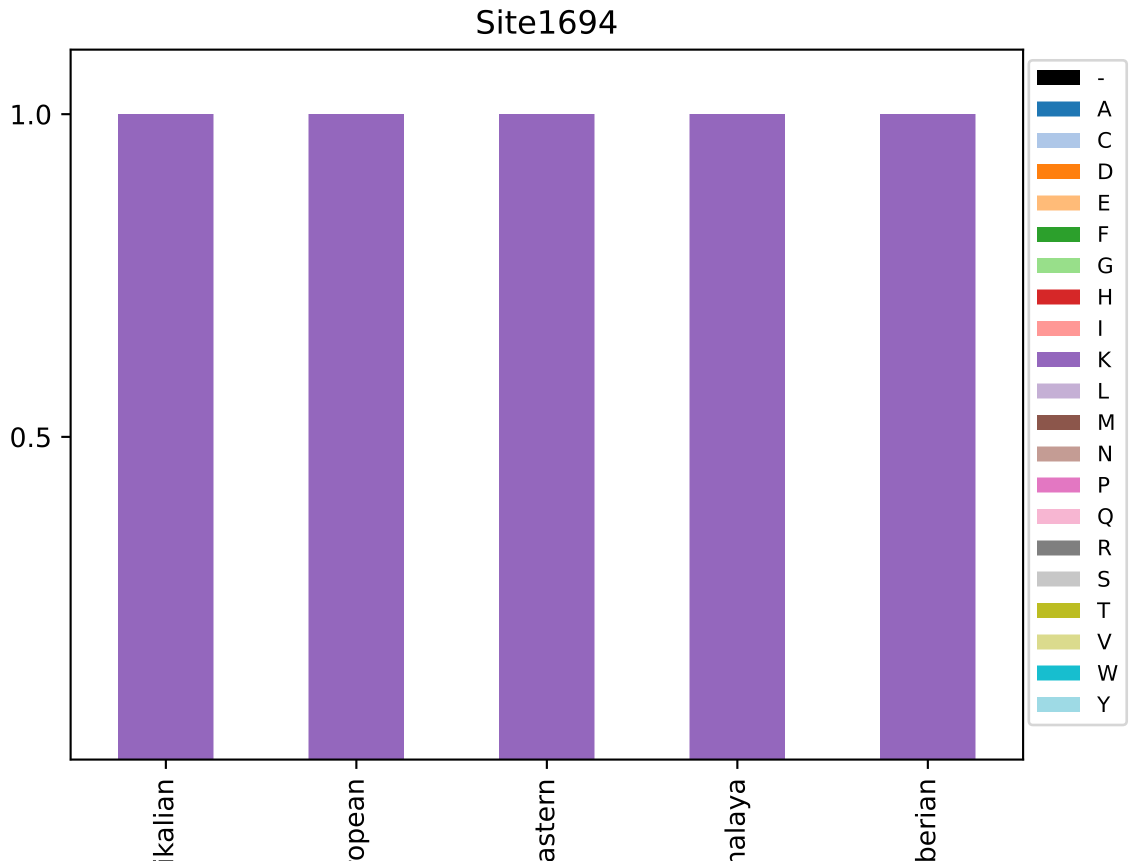
Site1694
1.0
0.5
-
A
C
D
E
F
G
H
I
K
L
M
N
P
Q
R
S
T
V
W
Y
ikalian
opean
astern
nalaya
berian

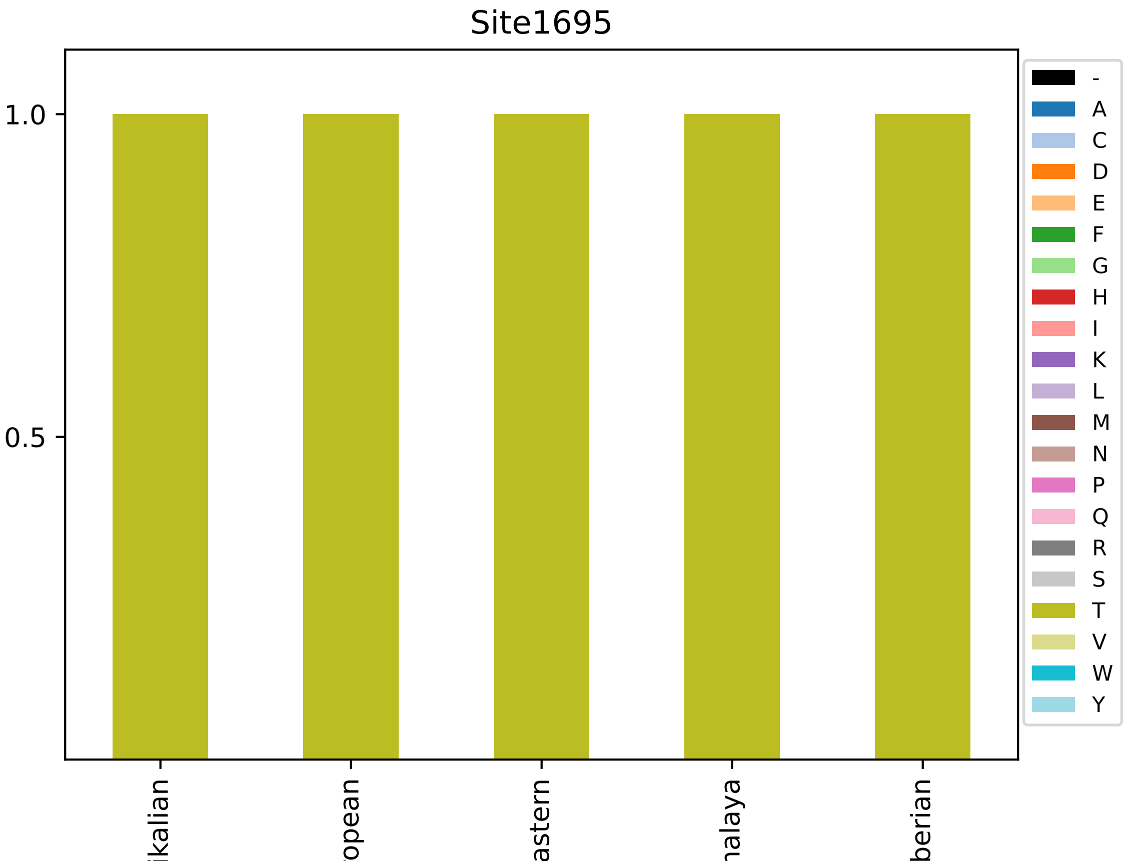

Site1695
1.0
0.5
ikalian
opean
astern
nalaya
berian
-
A
C
D
E
F
G
H
I
K
L
M
N
P
Q
R
S
T
V
W
Y

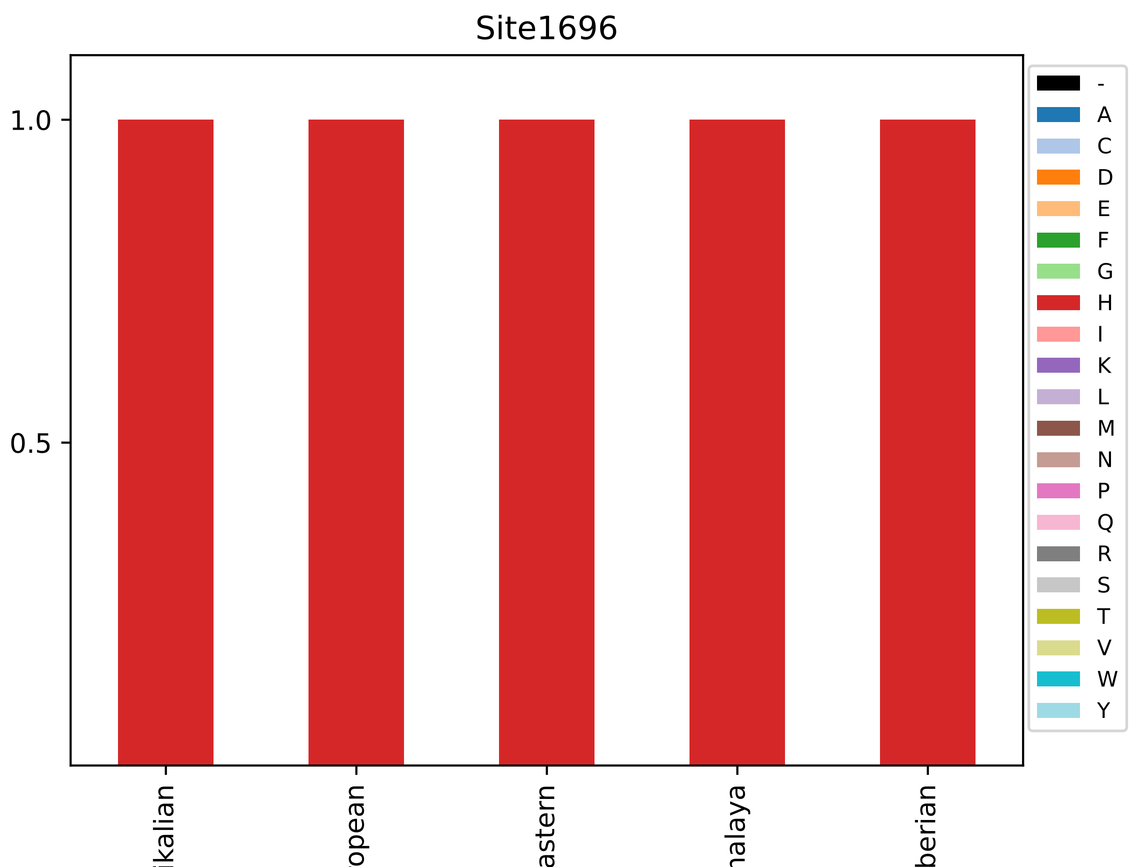
Site1696
1.0
0.5
ikalian
opean
astern
nalaya
berian
-
A
C
D
E
F
G
H
I
K
L
M
N
P
Q
R
S
T
V
W
Y

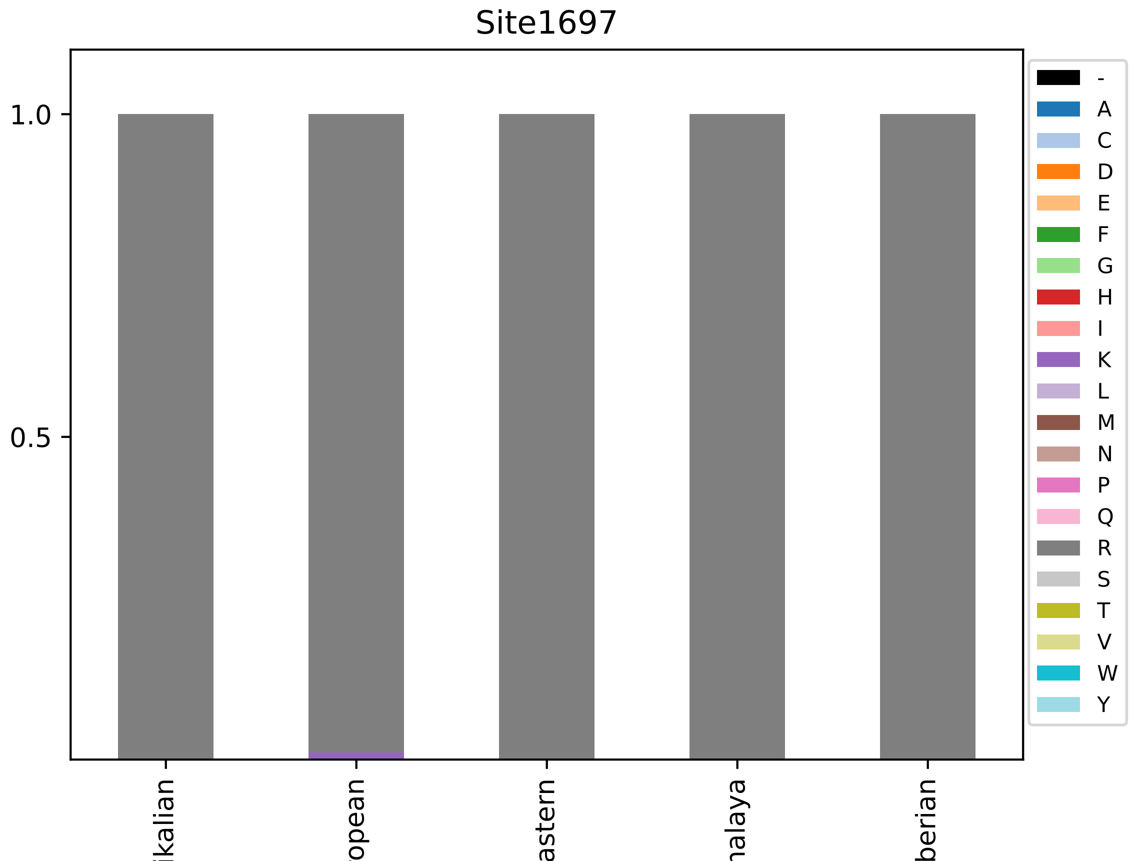

Site1697
1.0
0.5
-
A
C
D
E
F
G
H
I
K
L
M
N
P
Q
R
S
T
V
W
Y
ikalian
opean
astern
nalaya
berian

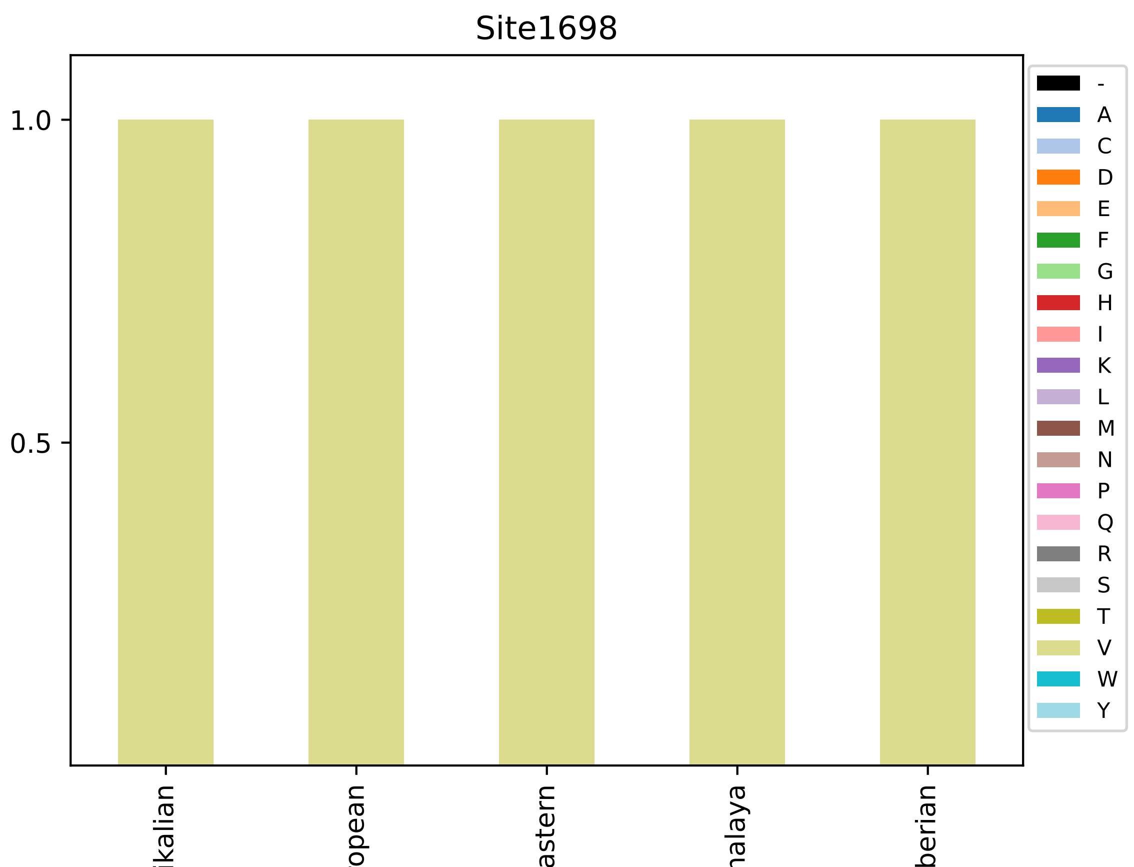
Site1698
1.0
0.5
-
A
C
D
E
F
G
H
I
K
L
M
N
P
Q
R
S
T
V
W
Y

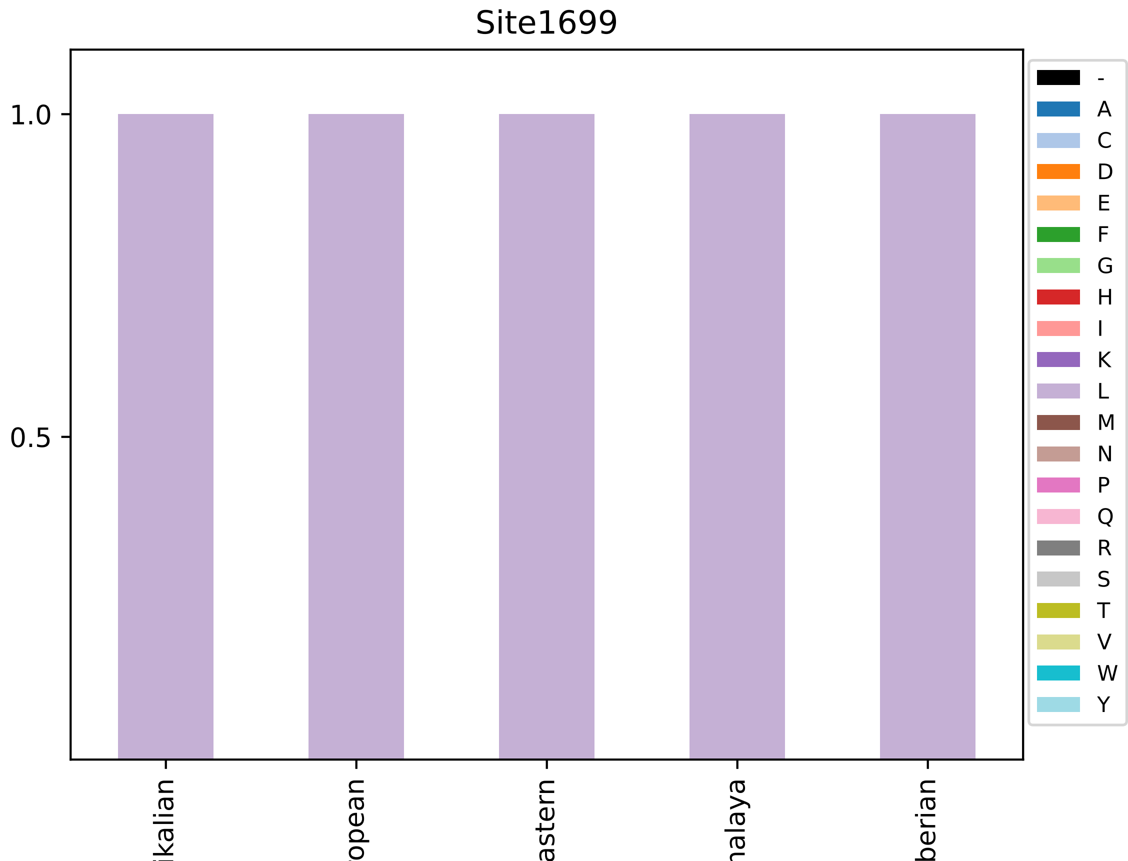

Site1699
1.0
0.5
-
A
C
D
E
F
G
H
I
K
L
M
N
P
Q
R
S
T
V
W
Y
ikalian
opean
astern
nalaya
berian

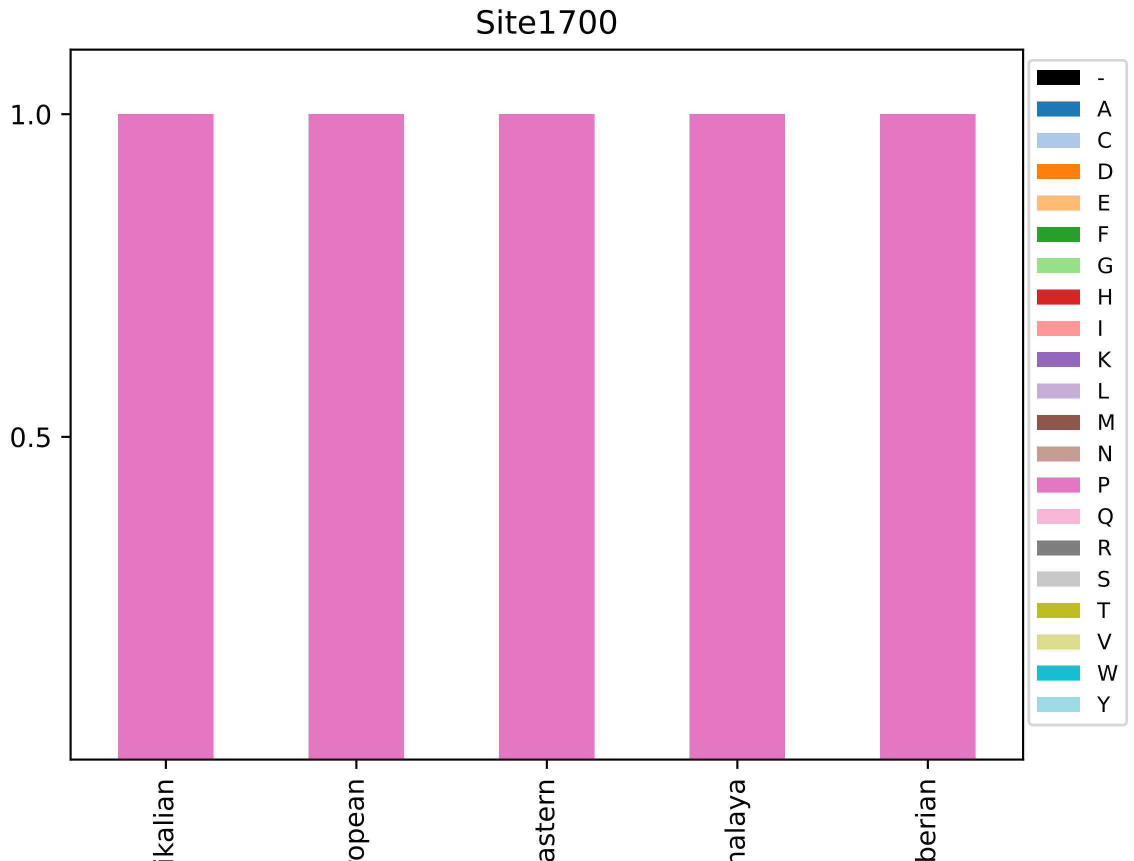

Site1700
1.0
0.5
-
A
C
D
E
F
G
H
I
K
L
M
N
P
Q
R
S
T
V
W
Y
ikalian
opean
astern
nalaya
berian

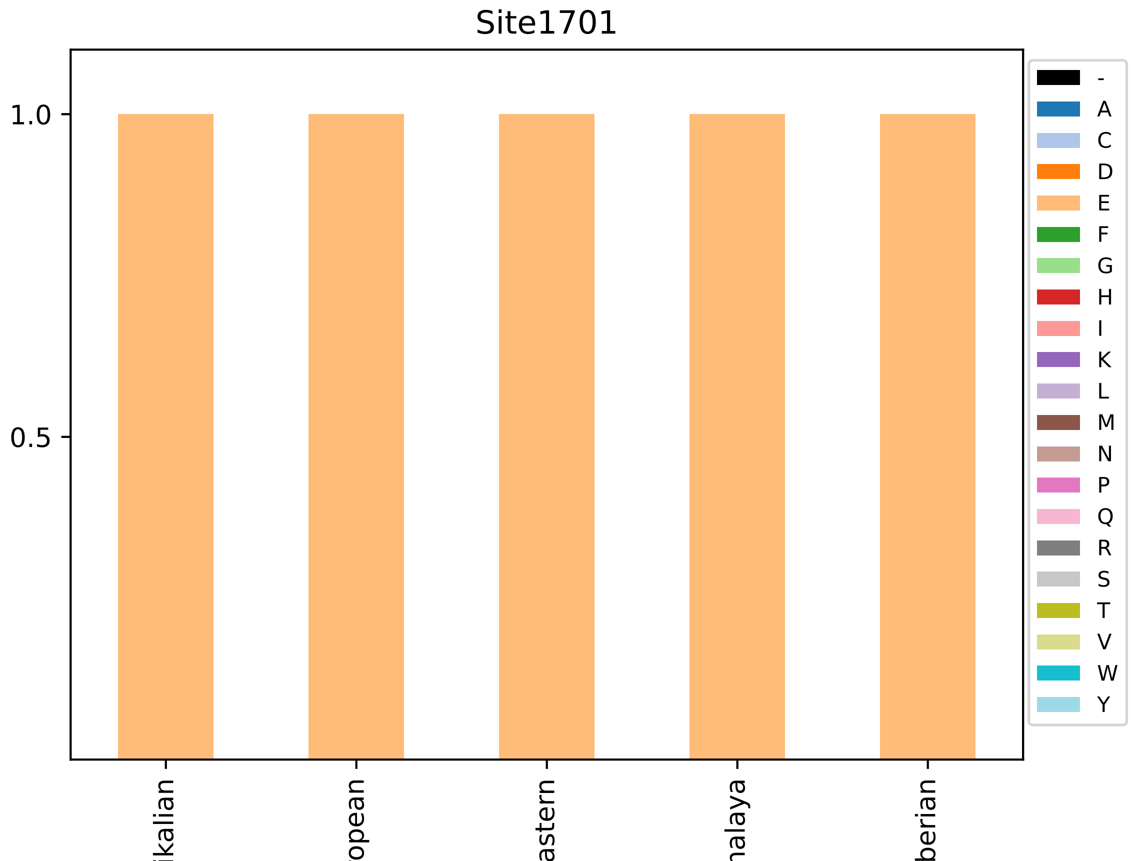
Site1701
1.0
0.5
ikalian
opean
astern
nalaya
berian
-
A
C
D
E
F
G
H
I
K
L
M
N
P
Q
R
S
T
V
W
Y

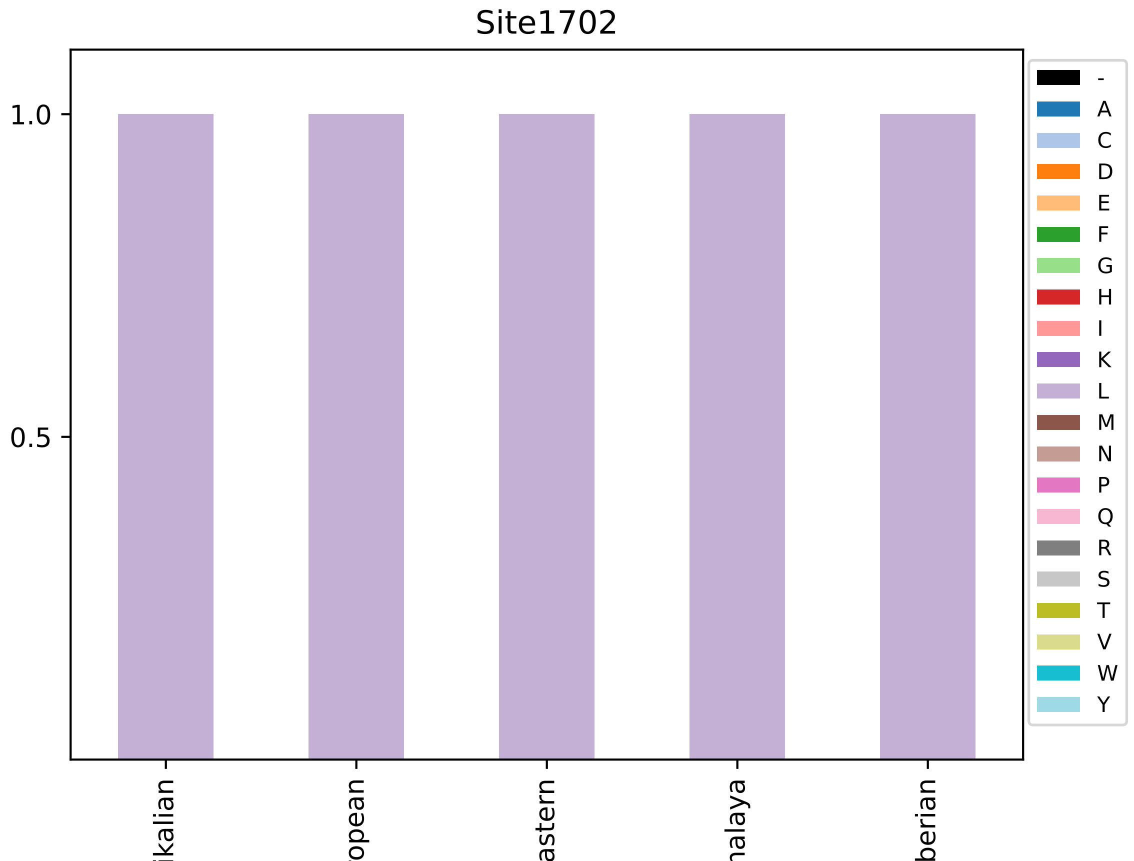
Site1702
1.0
0.5
-
A
C
D
E
F
G
H
I
K
L
M
N
P
Q
R
S
T
V
W
Y
ikalian
opean
astern
nalaya
berian

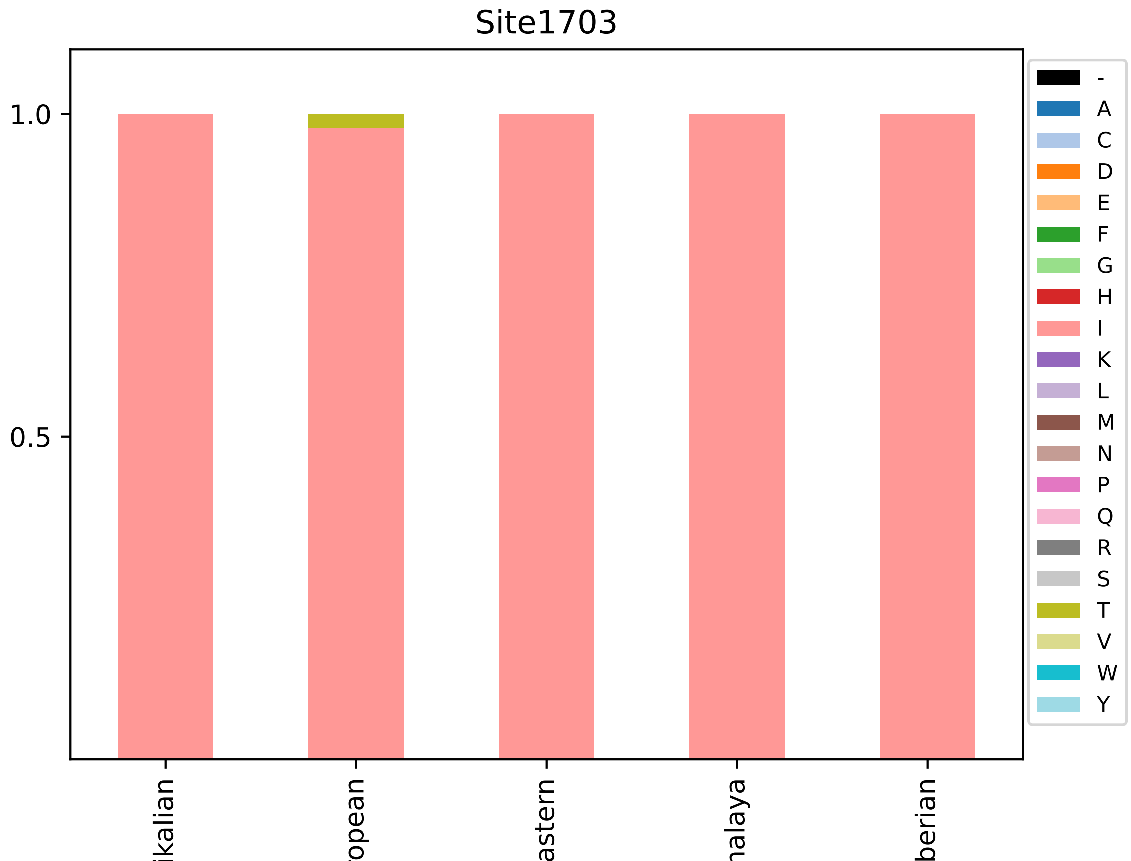
Site1703
1.0
0.5
-
A
C
D
E
F
G
H
I
K
L
M
N
P
Q
R
S
T
V
W
Y
ikalian
opean
astern
nalaya
berian

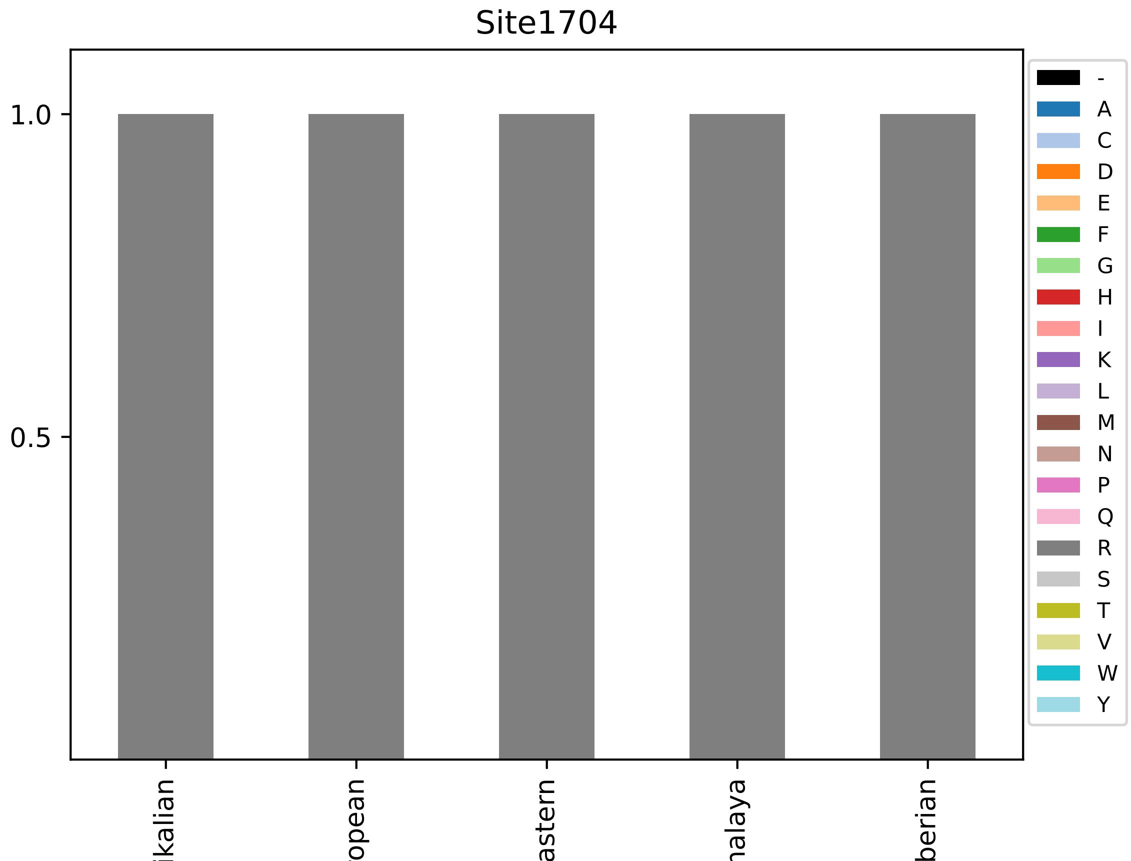
Site1704
1.0
0.5
-
A
C
D
E
F
G
H
I
K
L
M
N
P
Q
R
S
T
V
W
Y
ikalian
opean
astern
nalaya
berian

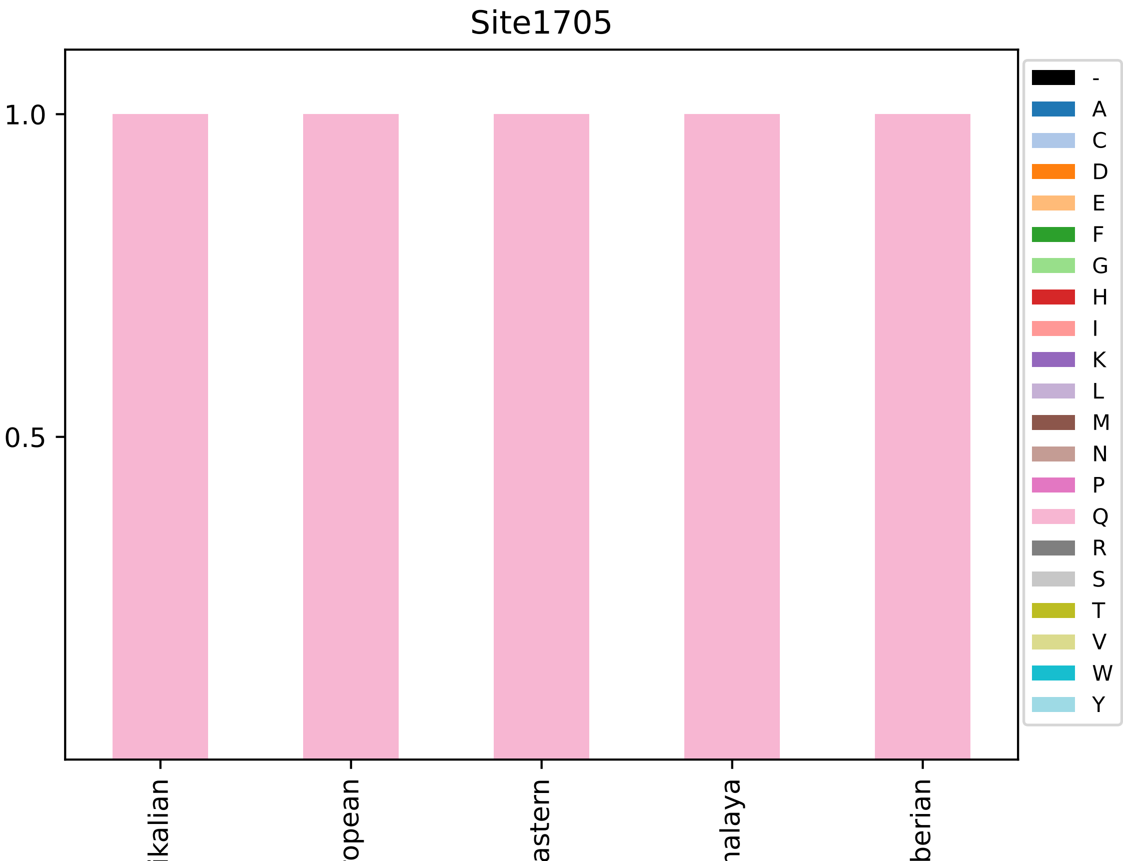

Site1705
1.0
0.5
-
A
C
D
E
F
G
H
I
K
L
M
N
P
Q
R
S
T
V
W
Y

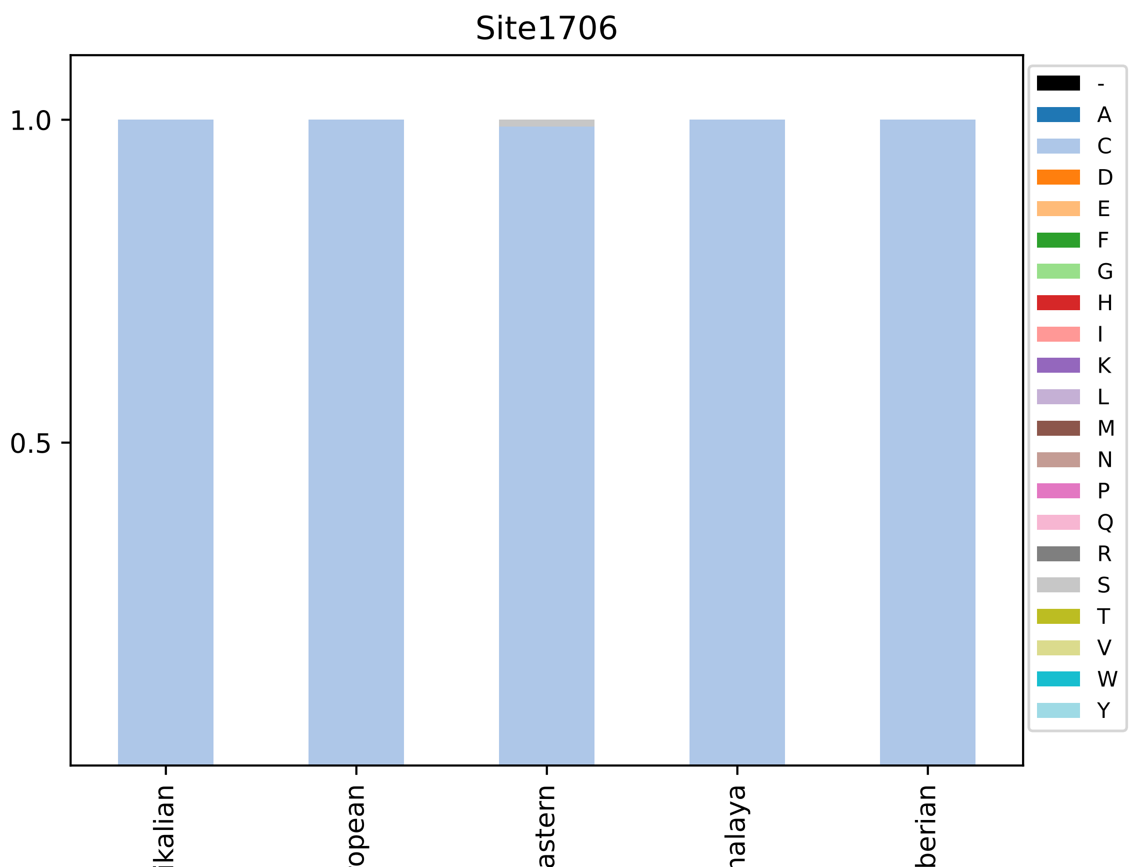

Site1706
1.0
0.5
-
A
C
D
E
F
G
H
I
K
L
M
N
P
Q
R
S
T
V
W
Y
ikalian
opean
astern
nalaya
berian

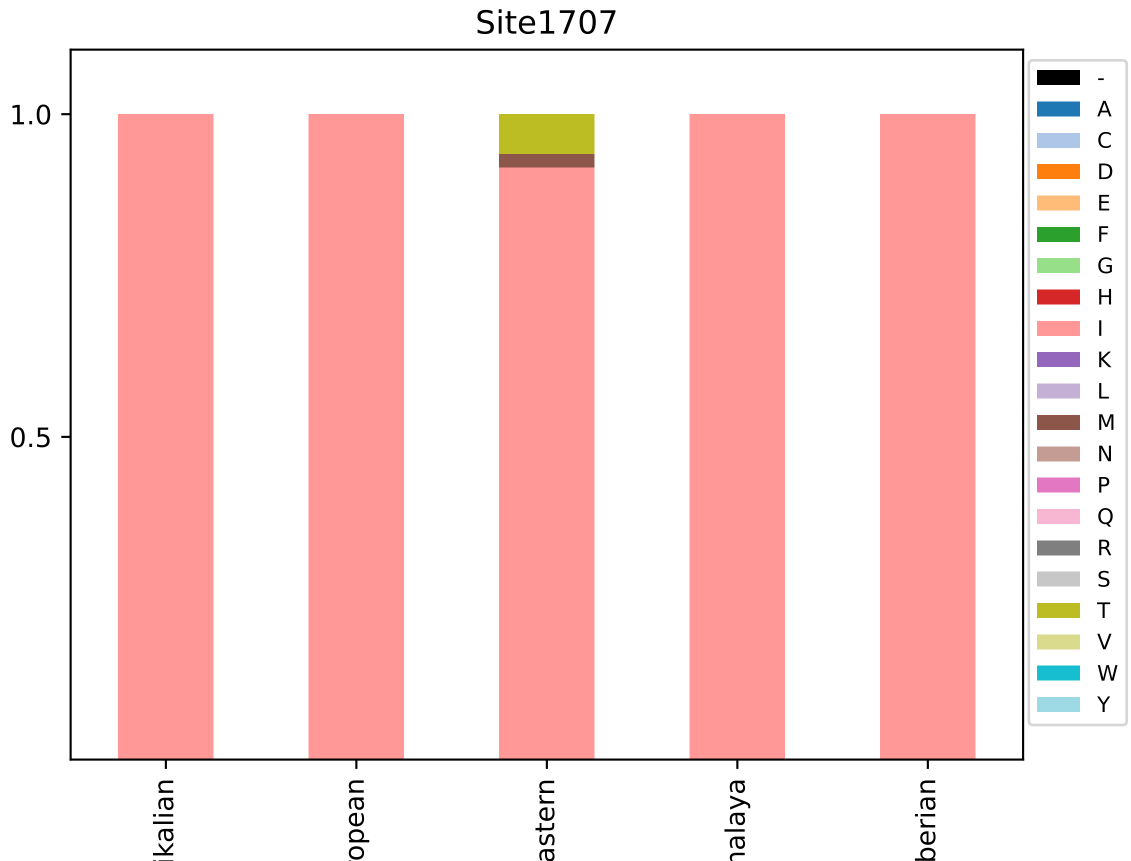

Site1707
1.0
0.5
-
A
C
D
E
F
G
H
I
K
L
M
N
P
Q
R
S
T
V
W
Y
ikalian
opean
astern
nalaya
berian

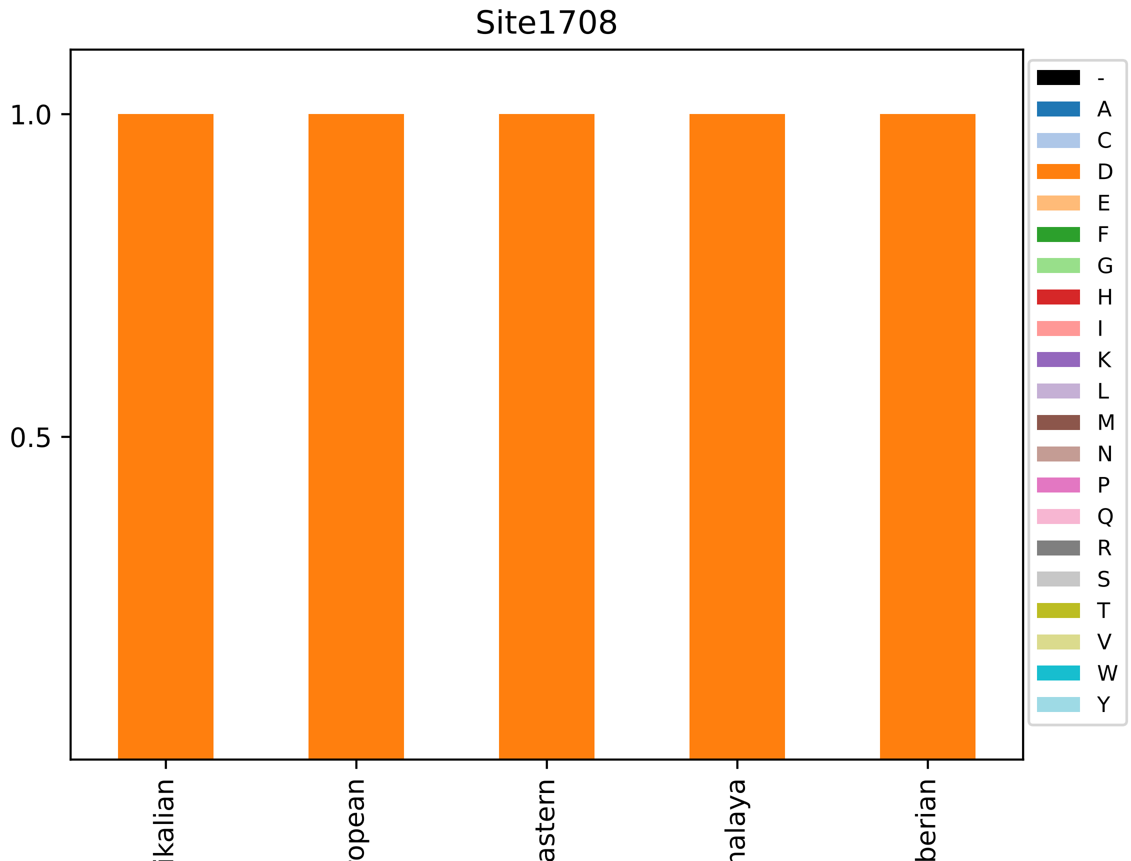

Site1708
1.0
0.5
ikalian
opean
astern
nalaya
berian
-
A
C
D
E
F
G
H
I
K
L
M
N
P
Q
R
S
T
V
W
Y

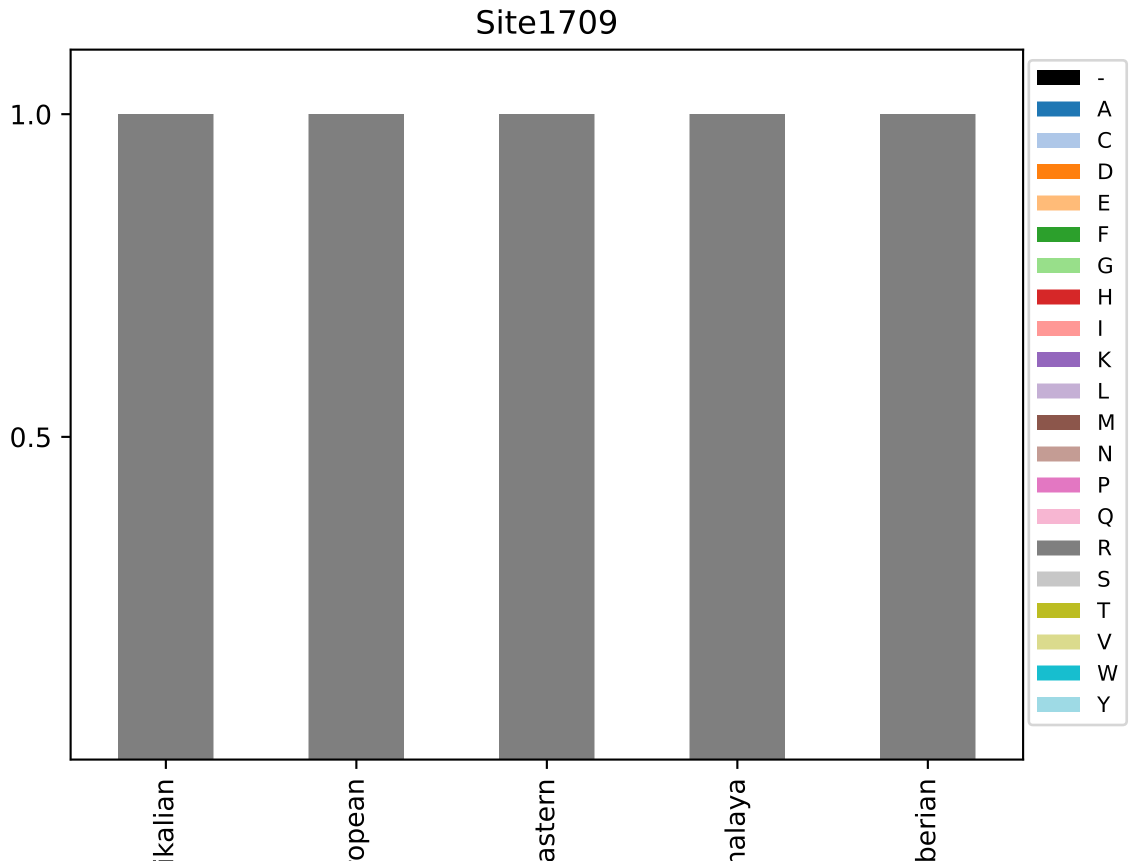
Site1709
1.0
0.5
-
A
C
D
E
F
G
H
I
K
L
M
N
P
Q
R
S
T
V
W
Y

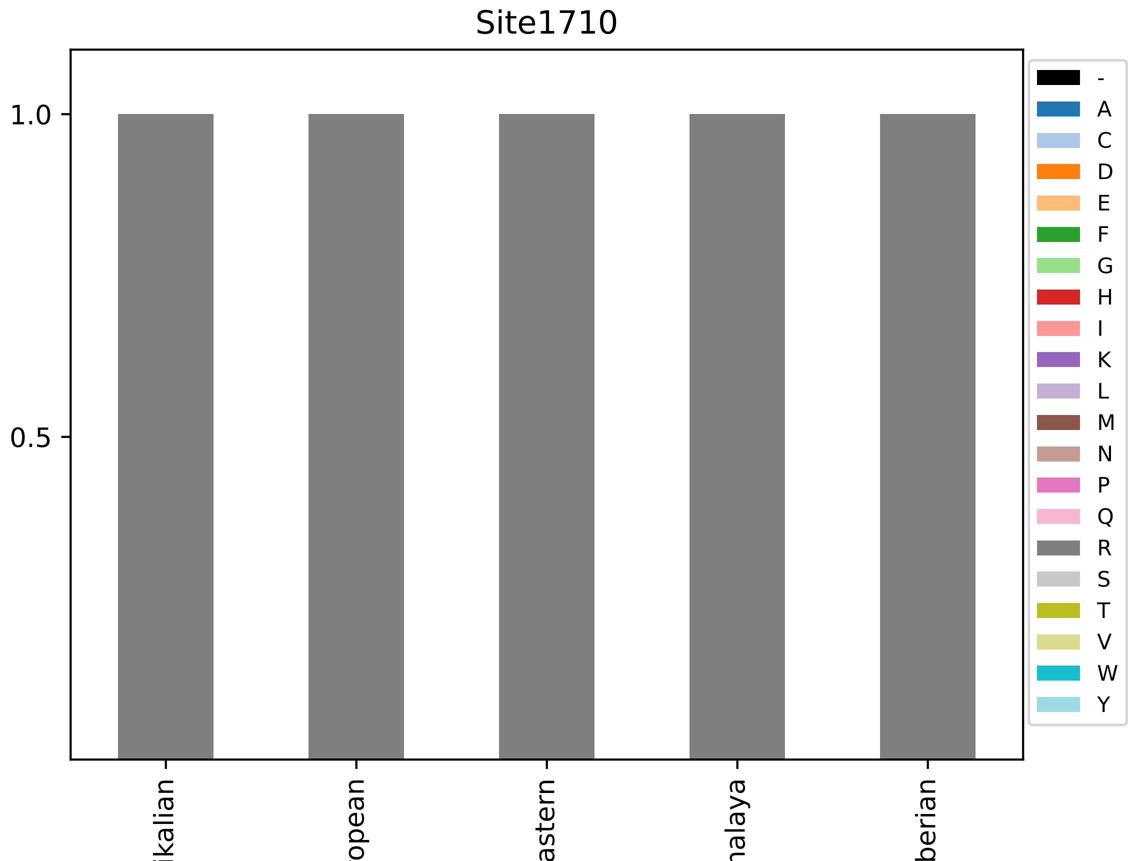

Site1710
1.0
0.5
-
A
C
D
E
F
G
H
I
K
L
M
N
P
Q
R
S
T
V
W
Y
ikalian
opean
astern
nalaya
berian

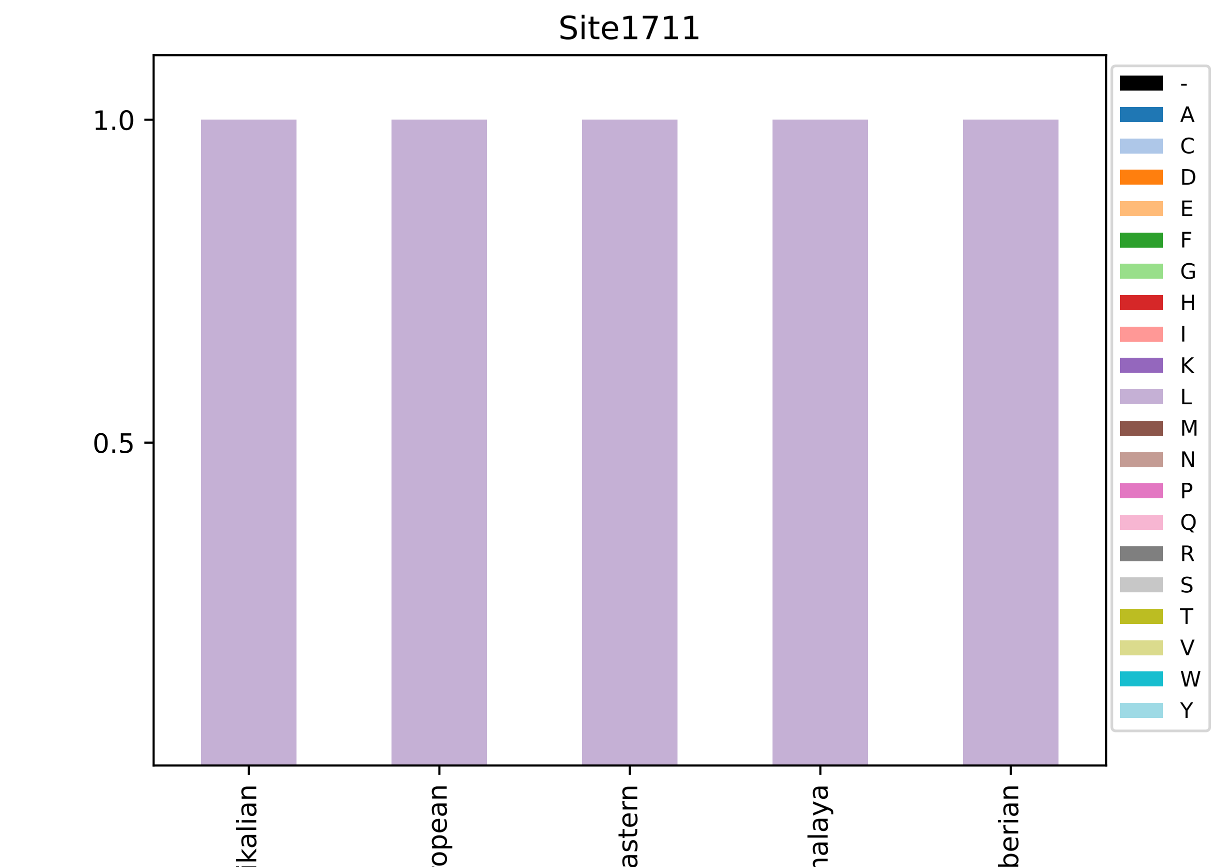

Site1711
1.0
0.5
ikalian
opean
astern
nalaya
berian
-
A
C
D
E
F
G
H
I
K
L
M
N
P
Q
R
S
T
V
W
Y

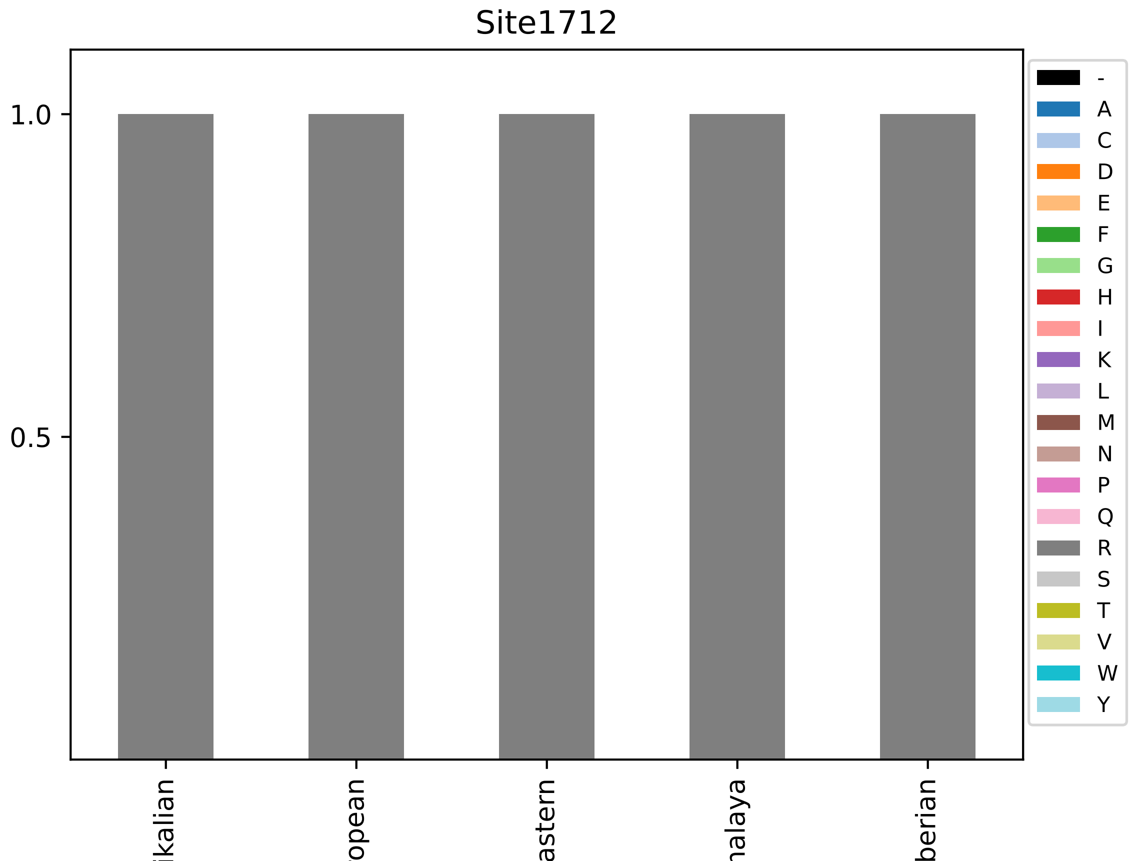
Site1712
1.0
0.5
ikalian
opean
astern
nalaya
berian
-
A
C
D
E
F
G
H
I
K
L
M
N
P
Q
R
S
T
V
W
Y

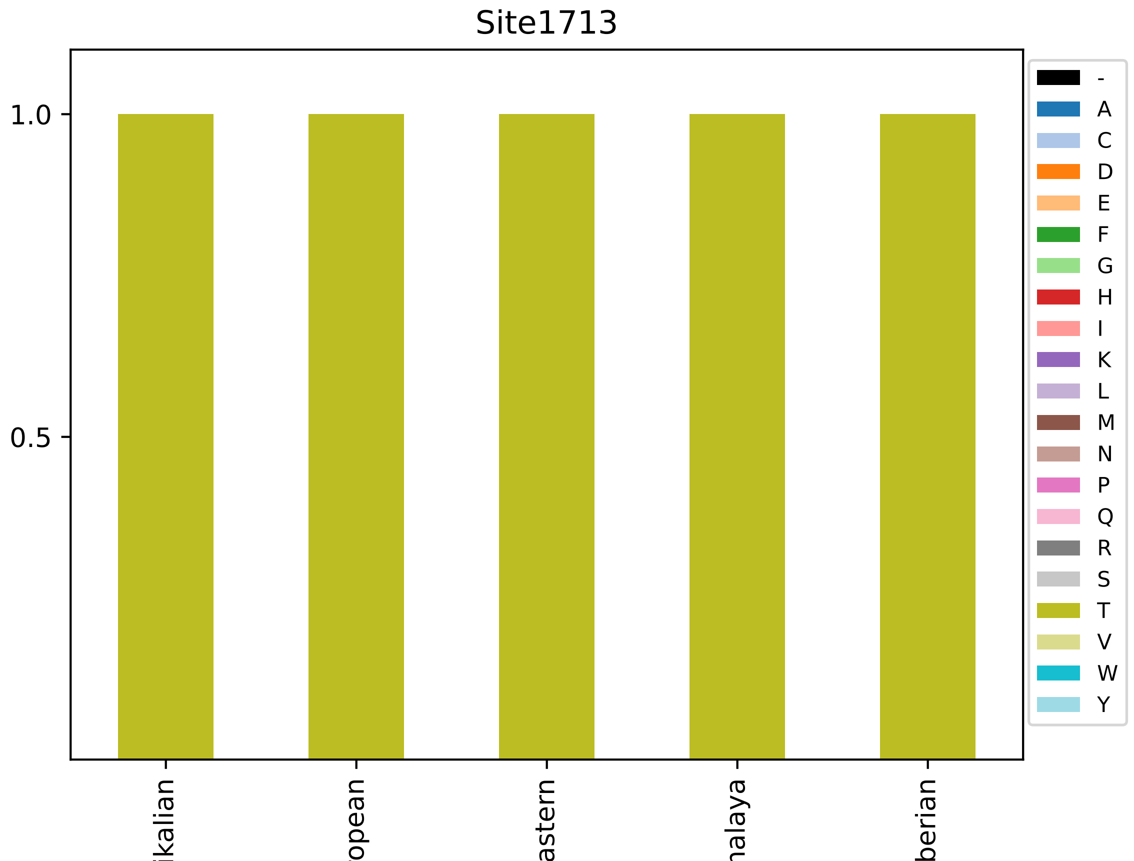

Site1713
1.0
0.5
ikalian
opean
astern
nalaya
berian
-
A
C
D
E
F
G
H
I
K
L
M
N
P
Q
R
S
T
V
W
Y

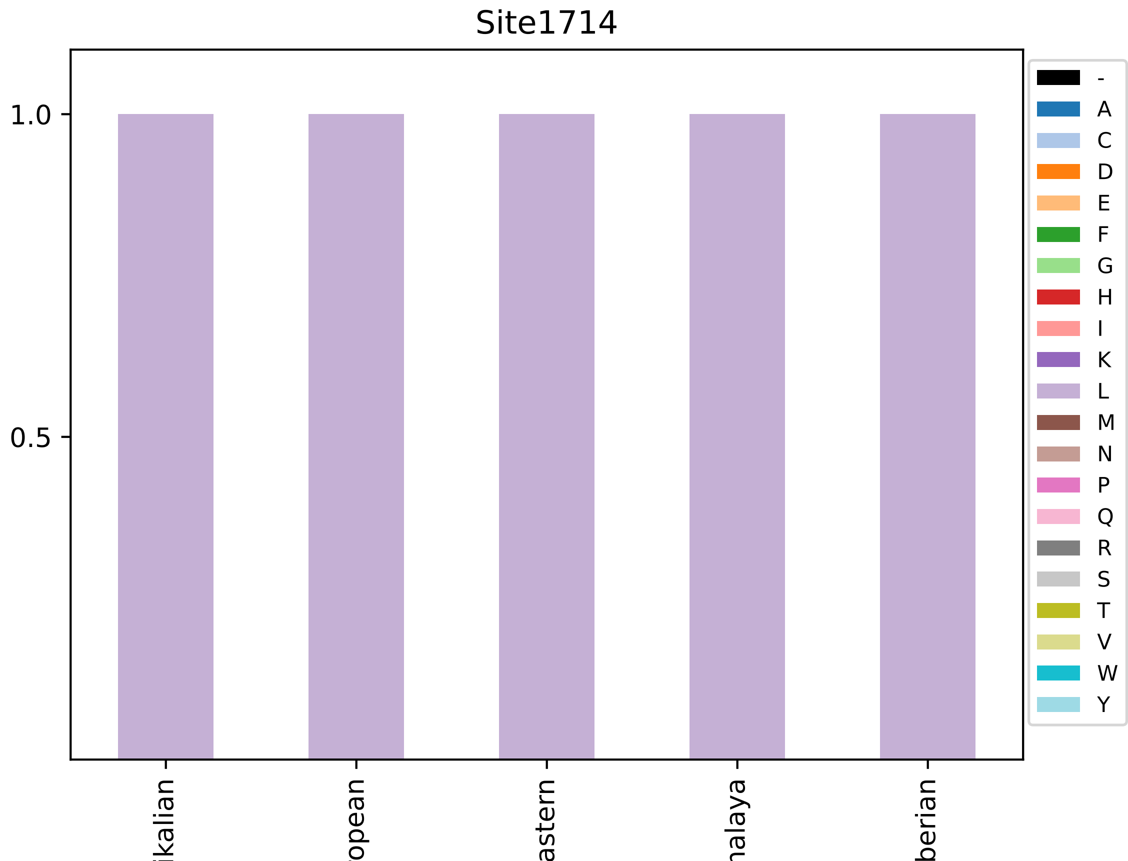

Site1714
1.0
0.5
-
A
C
D
E
F
G
H
I
K
L
M
N
P
Q
R
S
T
V
W
Y
ikalian
ropean
astern
nalaya
berian

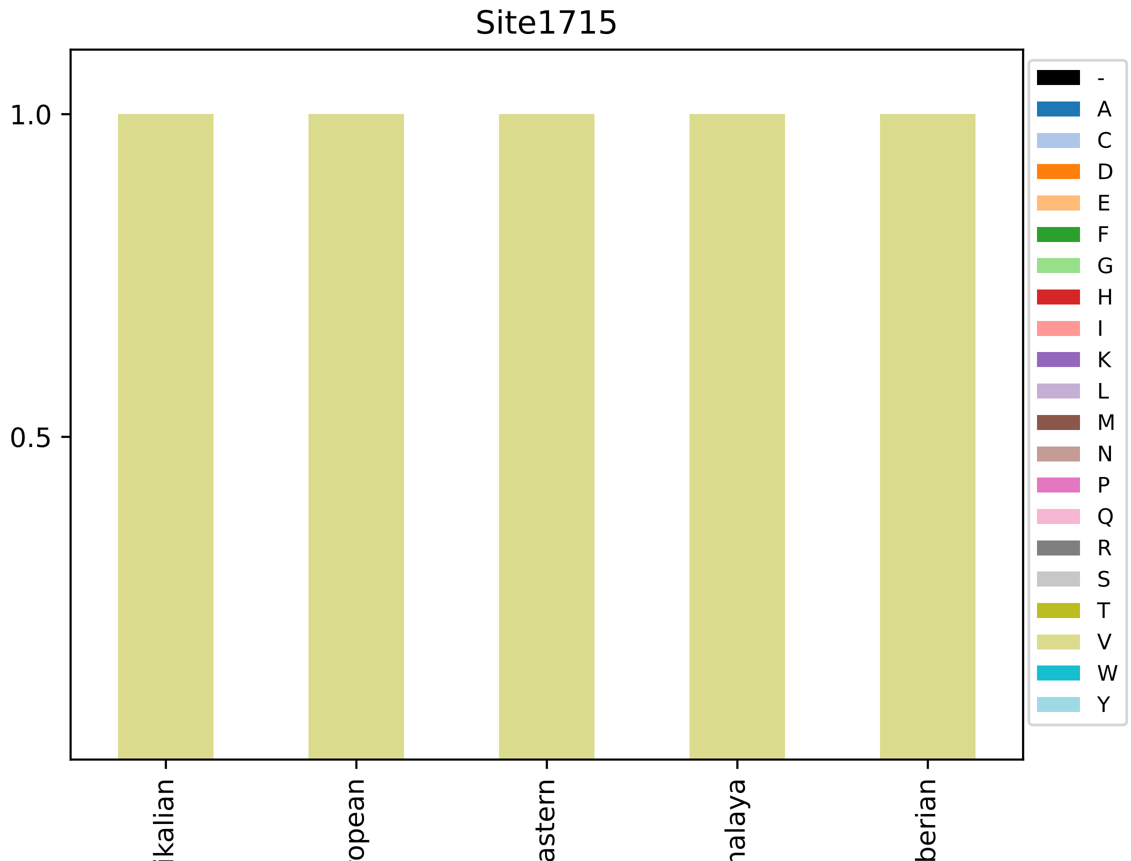

Site1715
1.0
0.5
ikalian
opean
astern
nalaya
berian
-
A
C
D
E
F
G
H
I
K
L
M
N
P
Q
R
S
T
V
W
Y

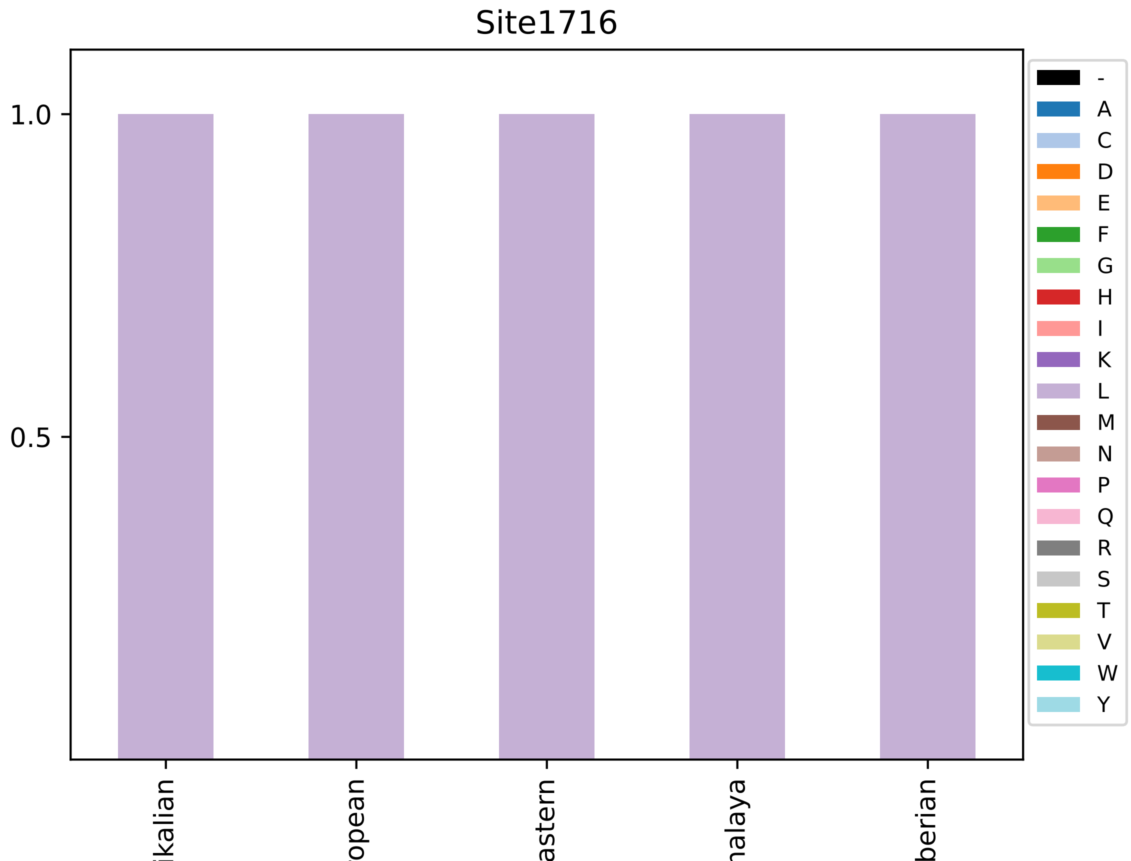

Site1716
1.0
0.5
ikalian
opean
astern
nalaya
berian
-
A
C
D
E
F
G
H
I
K
L
M
N
P
Q
R
S
T
V
W
Y

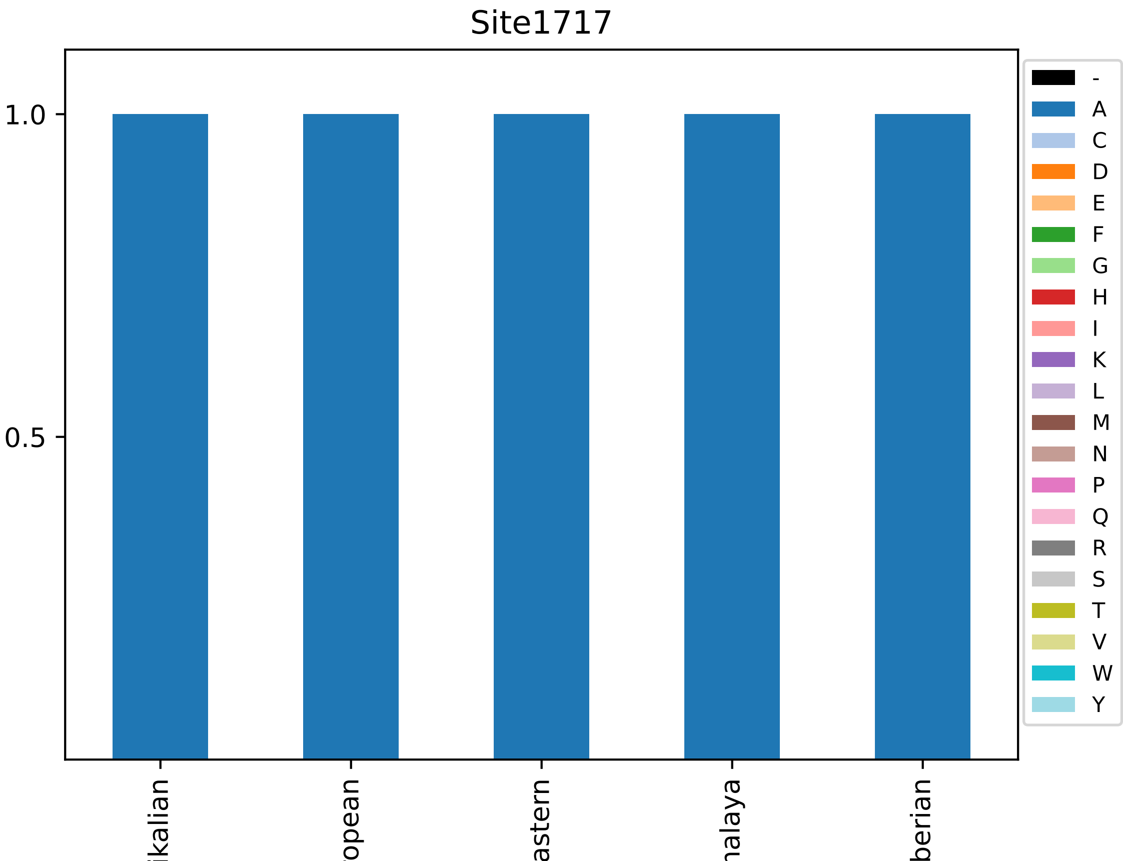
Site1717
1.0
0.5
-
A
C
D
E
F
G
H
I
K
L
M
N
P
Q
R
S
T
V
W
Y
ikalian
opean
astern
nalaya
berian

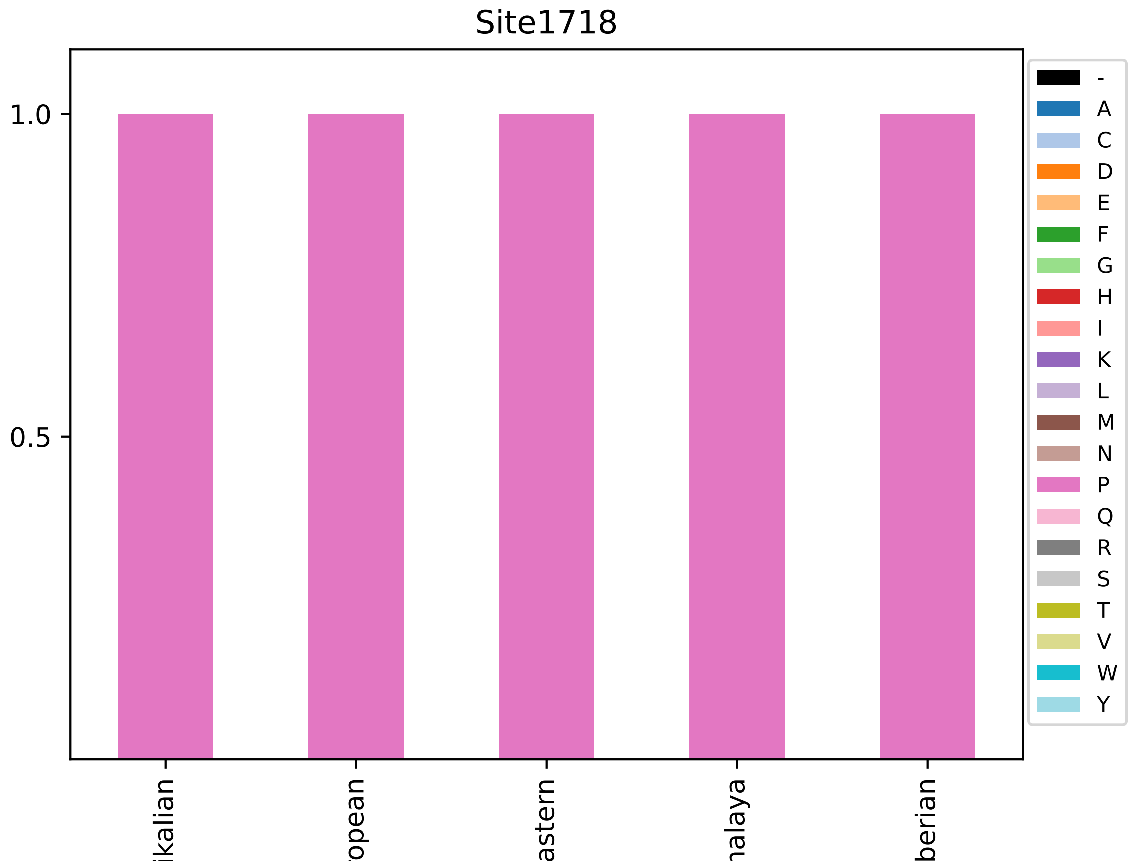

Site1718
1.0
0.5
-
A
C
D
E
F
G
H
I
K
L
M
N
P
Q
R
S
T
V
W
Y
ikalian
opean
astern
nalaya
berian

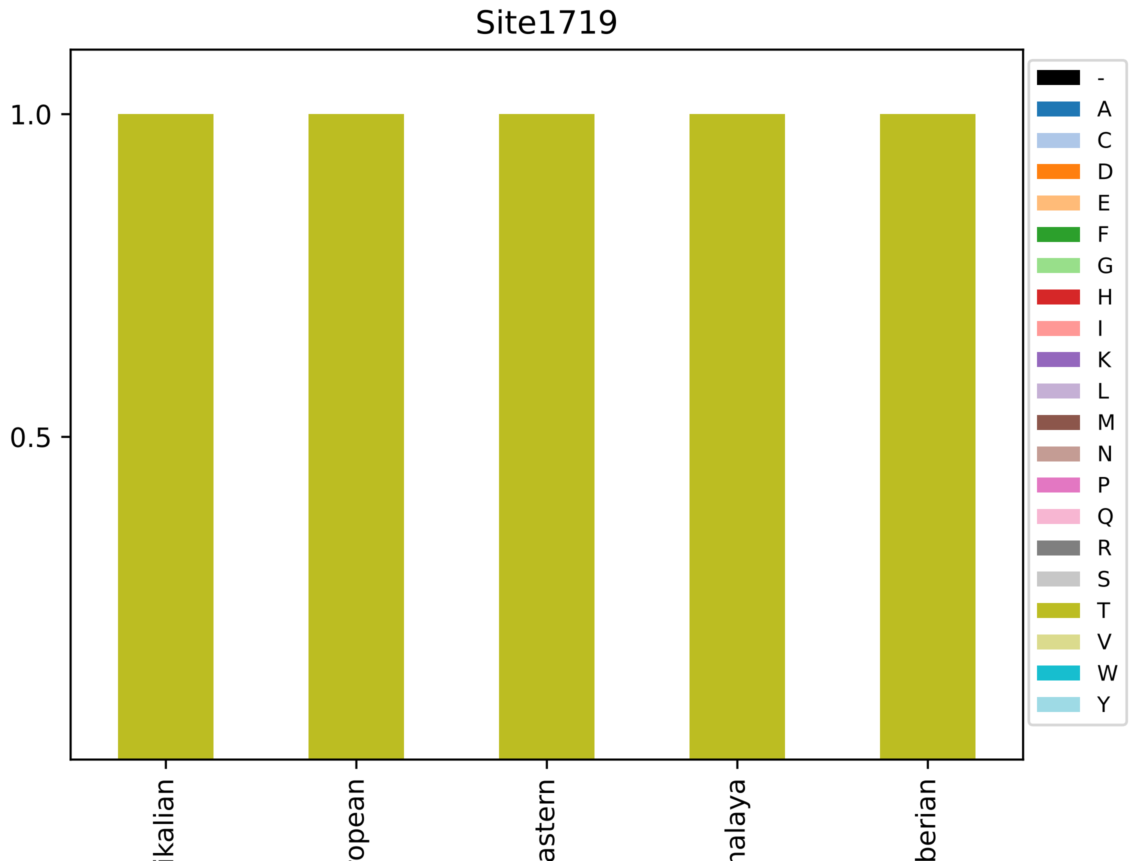

Site1719
1.0
0.5
ikalian
ropean
astern
nalaya
berian
-
A
C
D
E
F
G
H
I
K
L
M
N
P
Q
R
S
T
V
W
Y

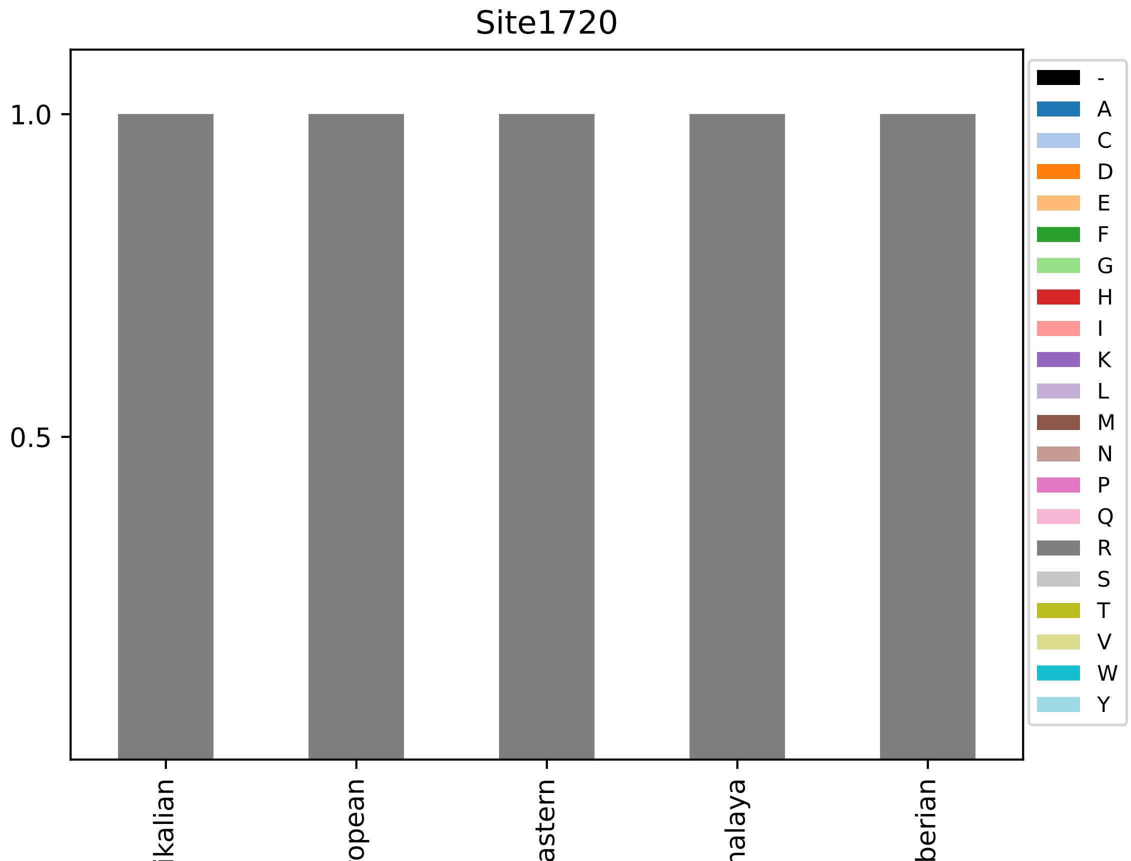

Site1720
1.0
0.5
-
A
C
D
E
F
G
H
I
K
L
M
N
P
Q
R
S
T
V
W
Y
ikalian
opean
astern
nalaya
berian

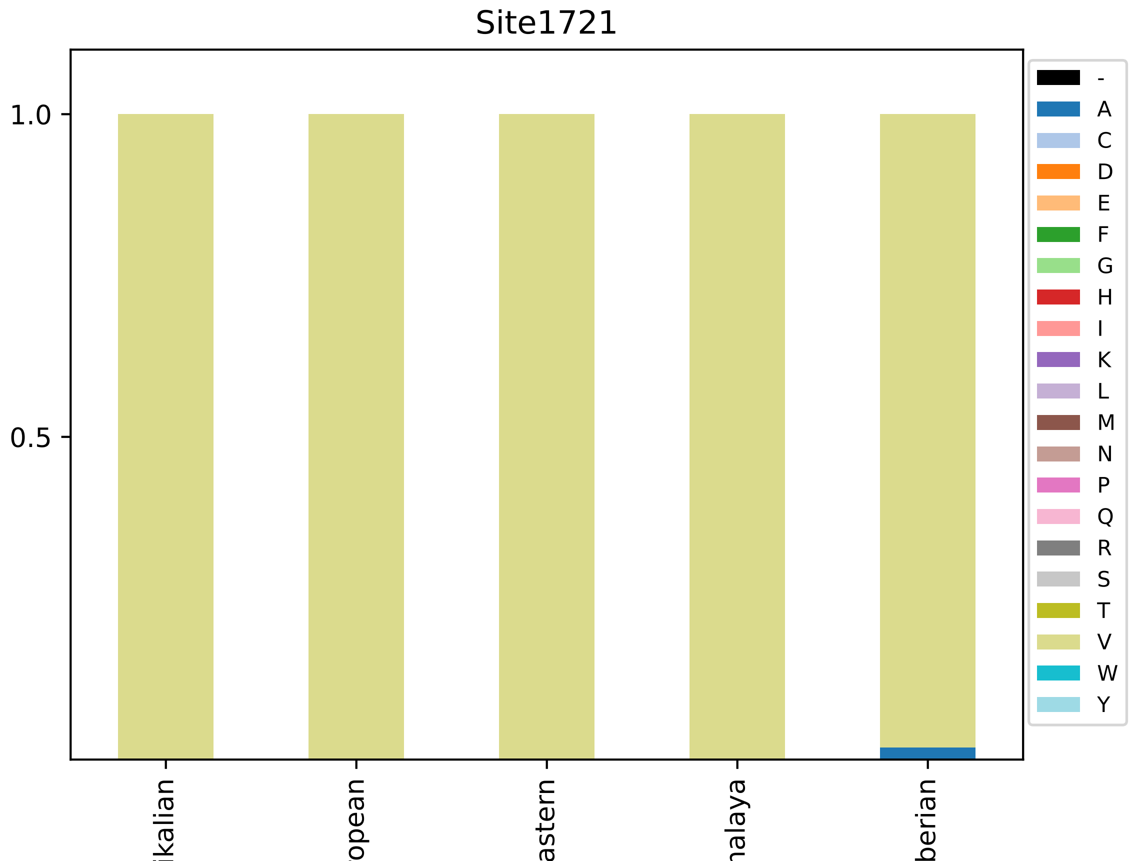

Site1721
1.0
0.5
-
A
C
D
E
F
G
H
I
K
L
M
N
P
Q
R
S
T
V
W
Y

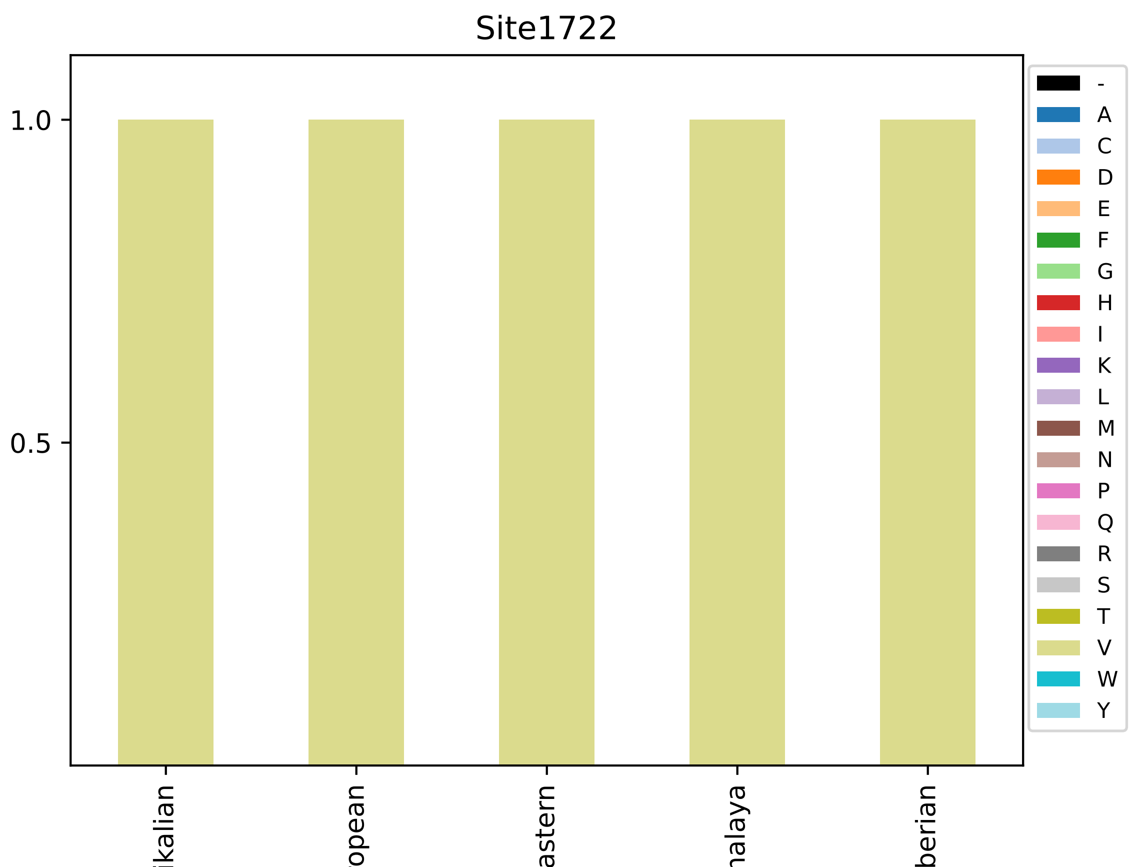
Site1722
1.0
0.5
-
A
C
D
E
F
G
H
I
K
L
M
N
P
Q
R
S
T
V
W
Y
ikalian
opean
astern
nalaya
berian

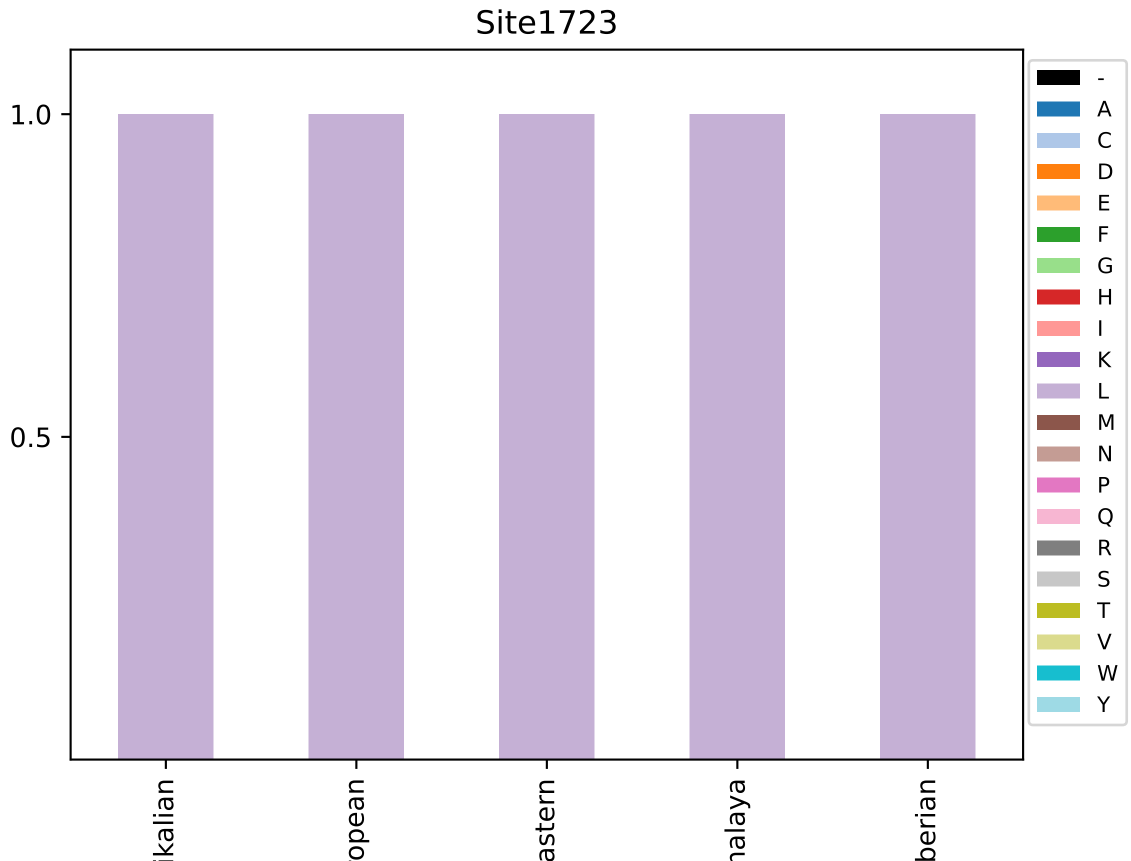

Site1723
1.0
0.5
-
A
C
D
E
F
G
H
I
K
L
M
N
P
Q
R
S
T
V
W
Y
ikalian
opean
astern
nalaya
berian

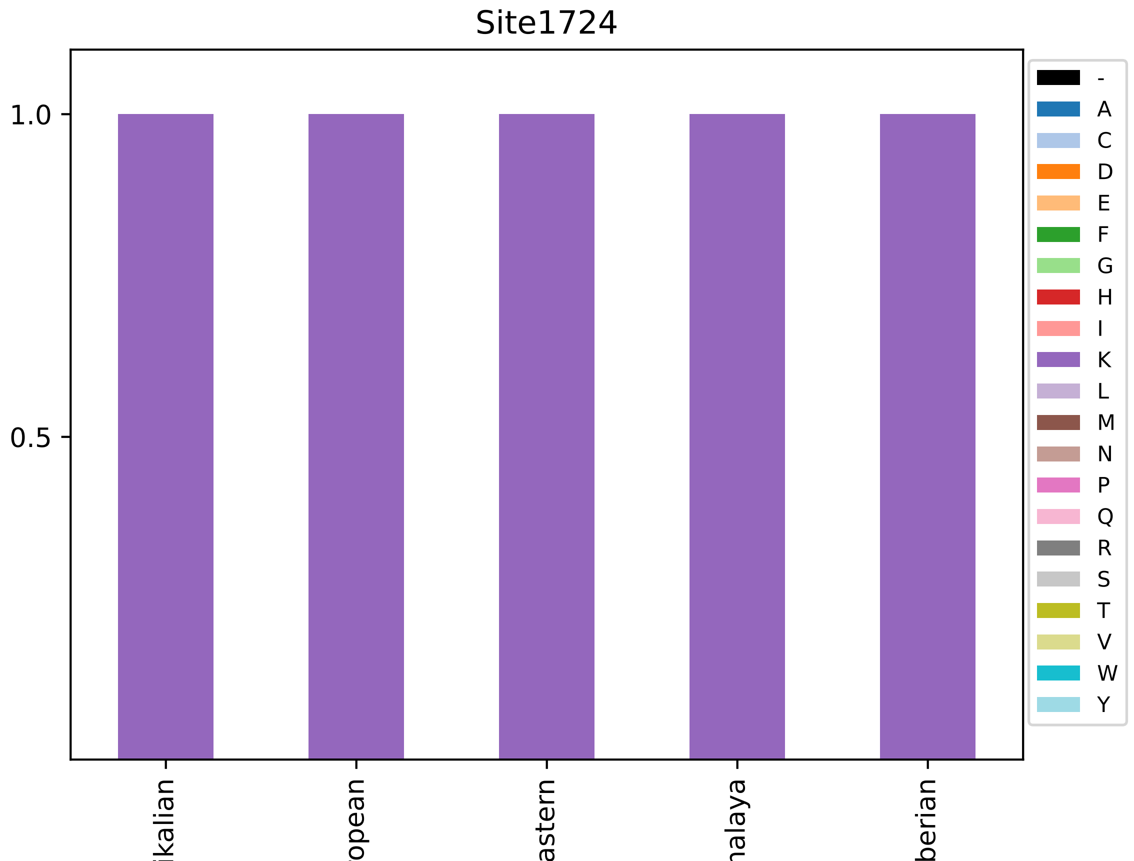
Site1724
1.0
0.5
-
A
C
D
E
F
G
H
I
K
L
M
N
P
Q
R
S
T
V
W
Y
ikalian
opean
astern
nalaya
berian

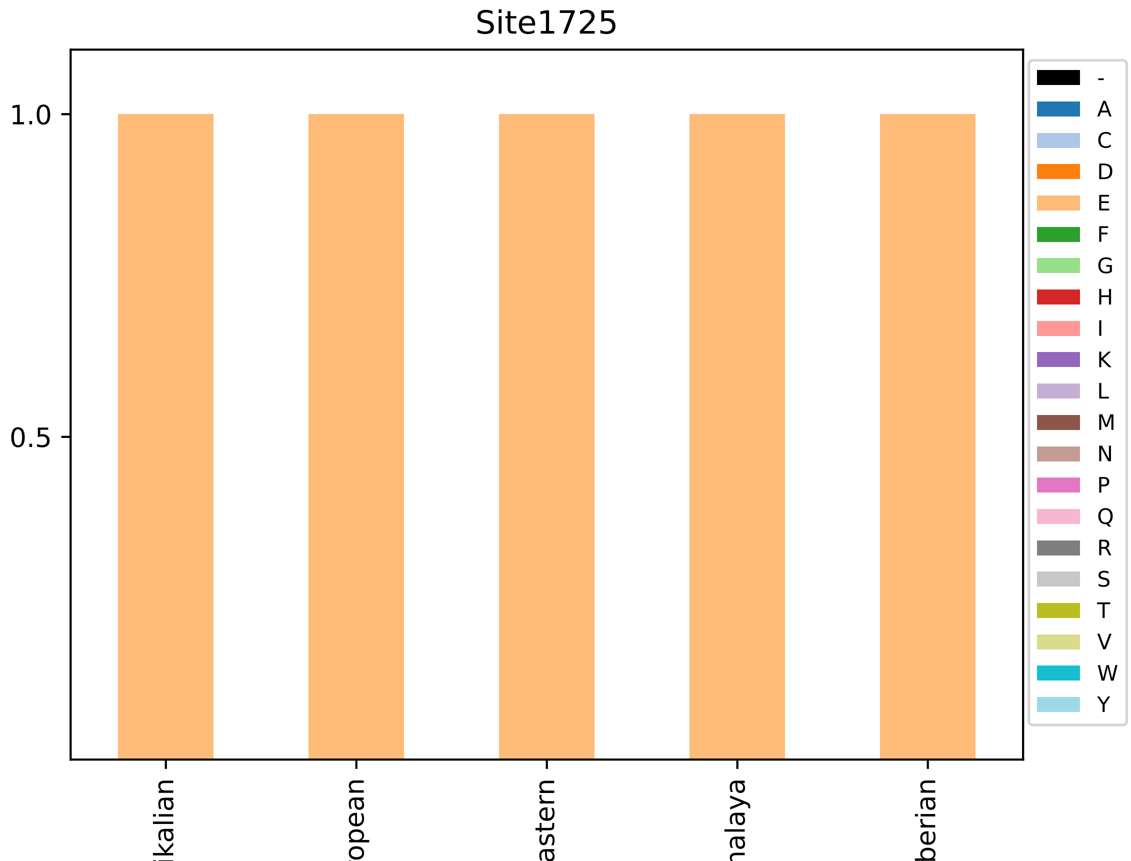
Site1725
1.0
0.5
ikalian
opean
astern
nalaya
berian
-
A
C
D
E
F
G
H
I
K
L
M
N
P
Q
R
S
T
V
W
Y

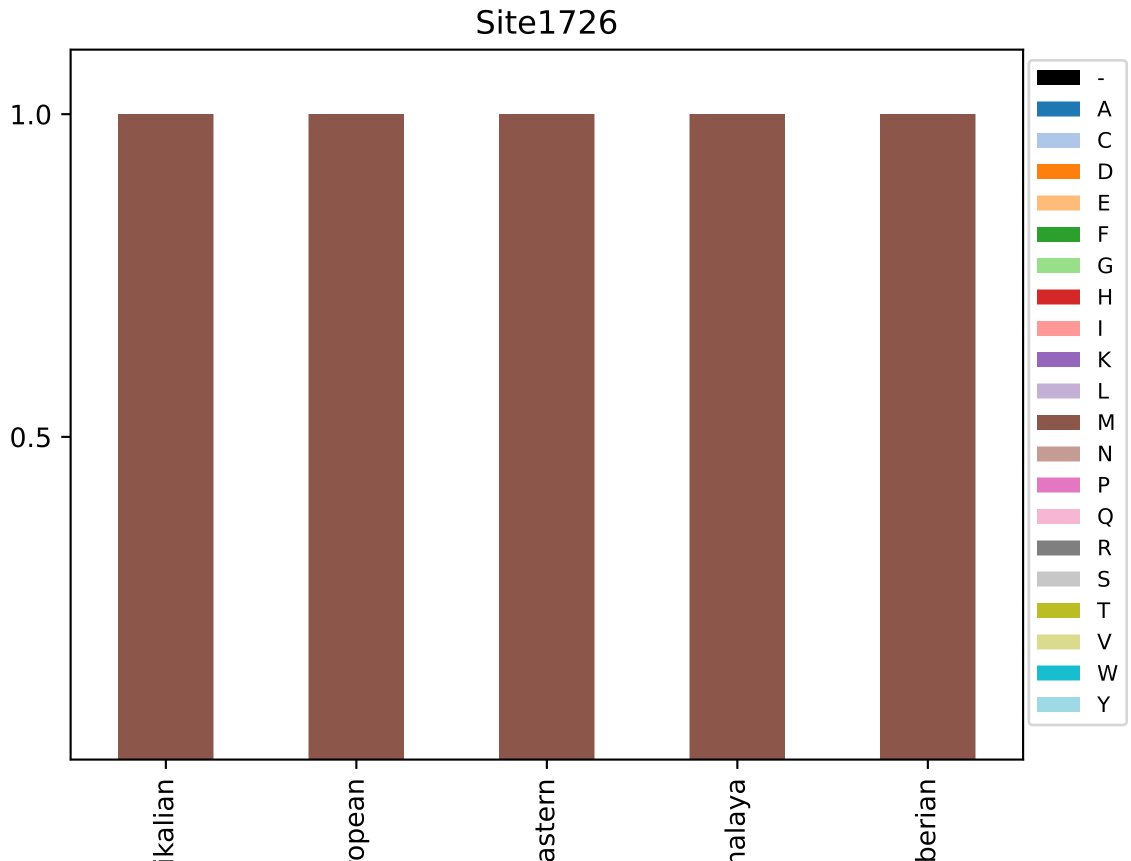
Site1726
1.0
0.5
-
A
C
D
E
F
G
H
I
K
L
M
N
P
Q
R
S
T
V
W
Y
ikalian
opean
astern
nalaya
berian

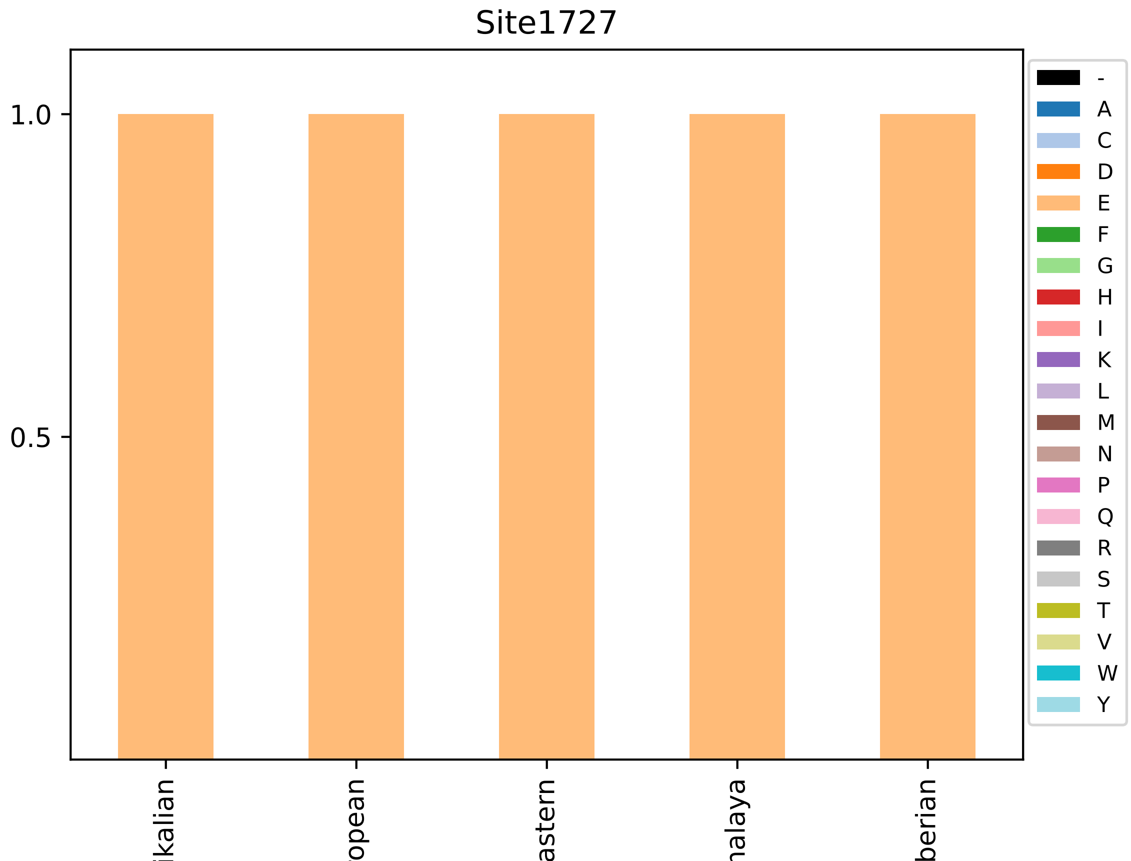

Site1727
1.0
0.5
-
A
C
D
E
F
G
H
I
K
L
M
N
P
Q
R
S
T
V
W
Y
ikalian
opean
astern
nalaya
berian

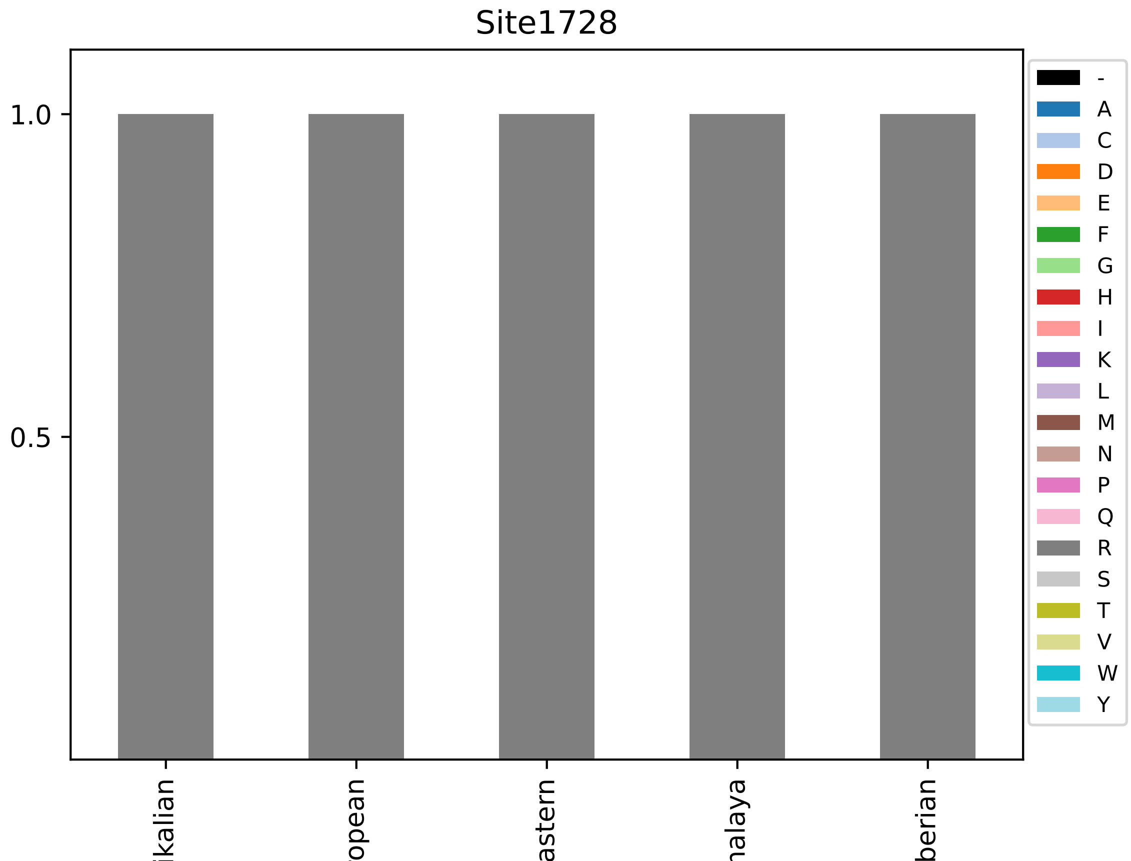
Site1728
1.0
0.5
-
A
C
D
E
F
G
H
I
K
L
M
N
P
Q
R
S
T
V
W
Y
ikalian
opean
astern
nalaya
berian

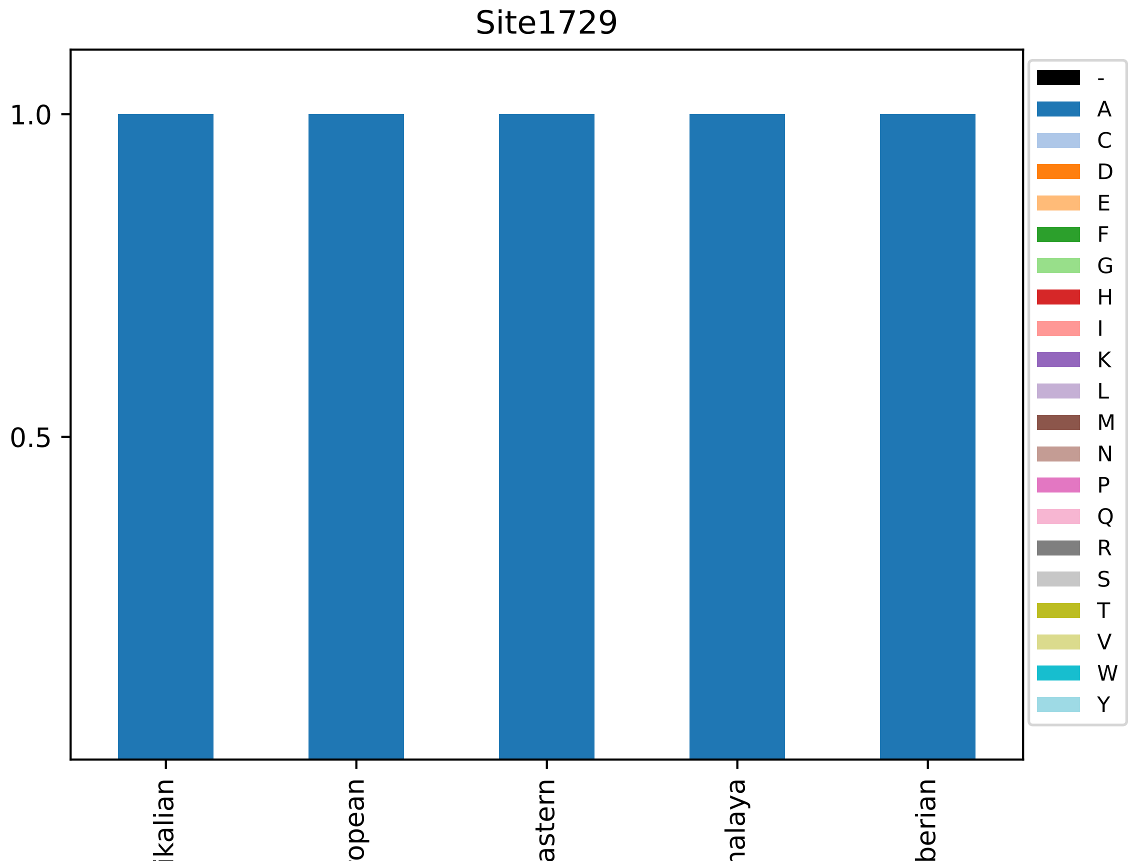
Site1729
1.0
0.5
-
A
C
D
E
F
G
H
I
K
L
M
N
P
Q
R
S
T
V
W
Y
ikalian
opean
astern
nalaya
berian

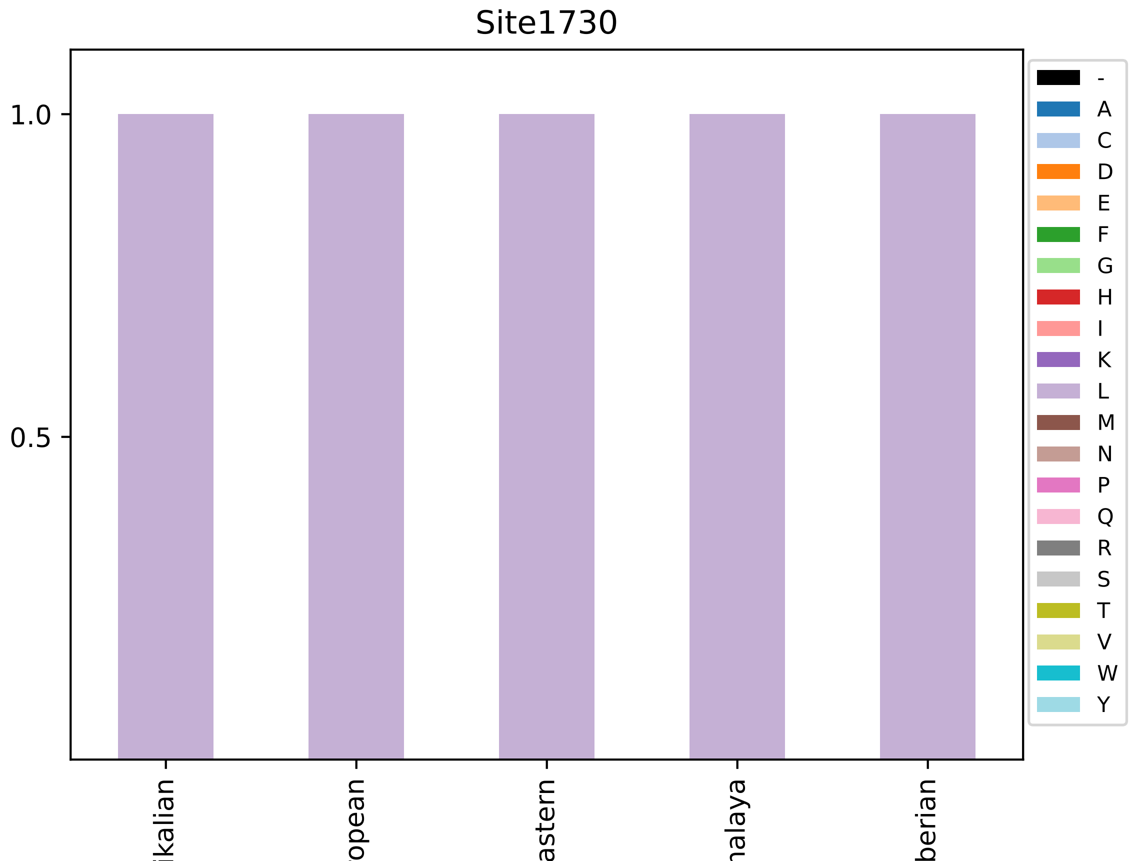

Site1730
1.0
0.5
-
A
C
D
E
F
G
H
I
K
L
M
N
P
Q
R
S
T
V
W
Y
ikalian
opean
astern
nalaya
berian

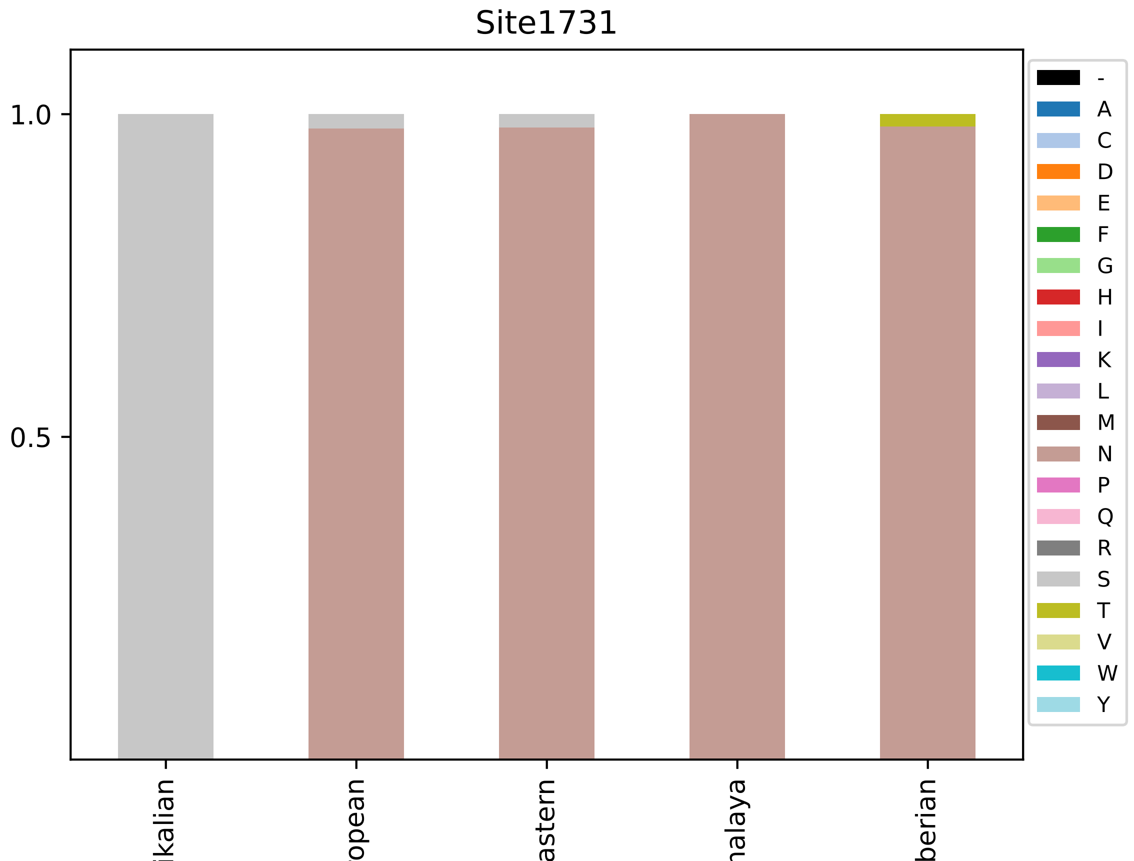

Site1731
1.0
0.5
-
A
C
D
E
F
G
H
I
K
L
M
N
P
Q
R
S
T
V
W
Y
ikalian
opean
astern
nalaya
berian

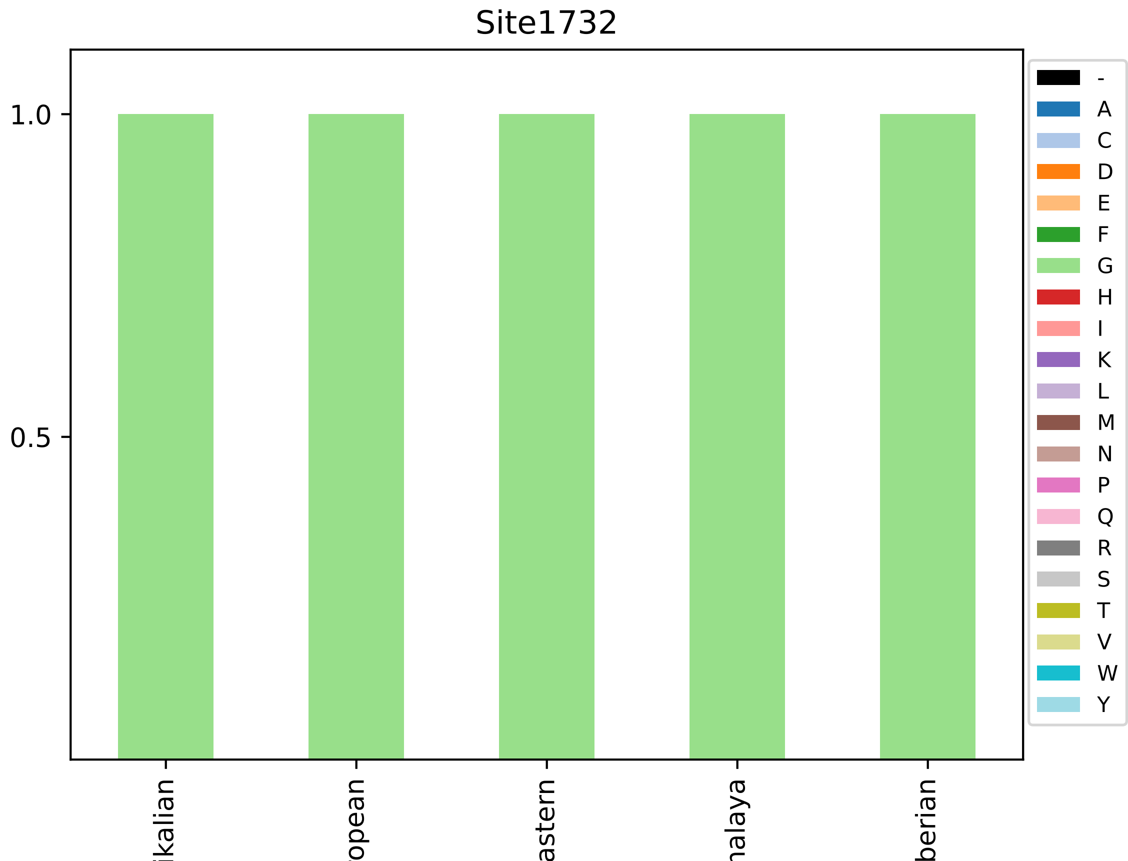

Site1732
1.0
0.5
-
A
C
D
E
F
G
H
I
K
L
M
N
P
Q
R
S
T
V
W
Y
ikalian
opean
astern
nalaya
berian

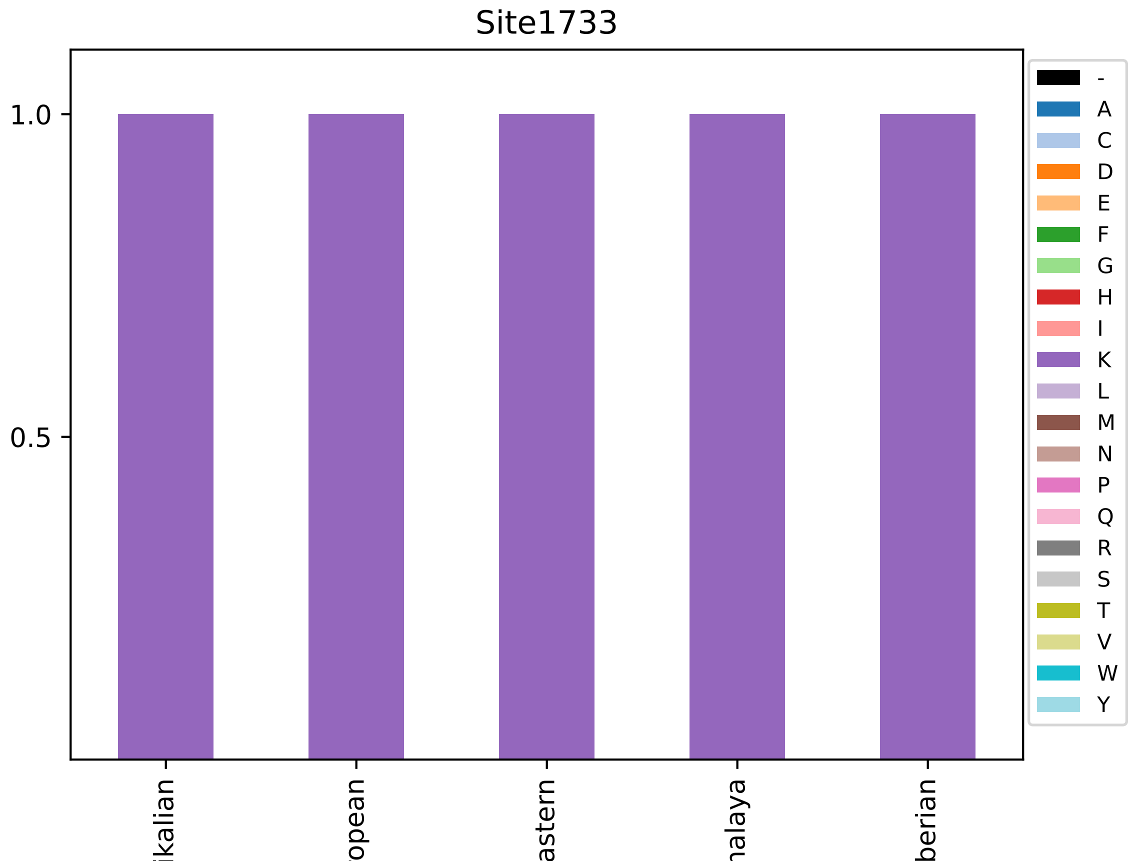
Site1733
1.0
0.5
ikalian
opean
astern
nalaya
berian
-
A
C
D
E
F
G
H
I
K
L
M
N
P
Q
R
S
T
V
W
Y

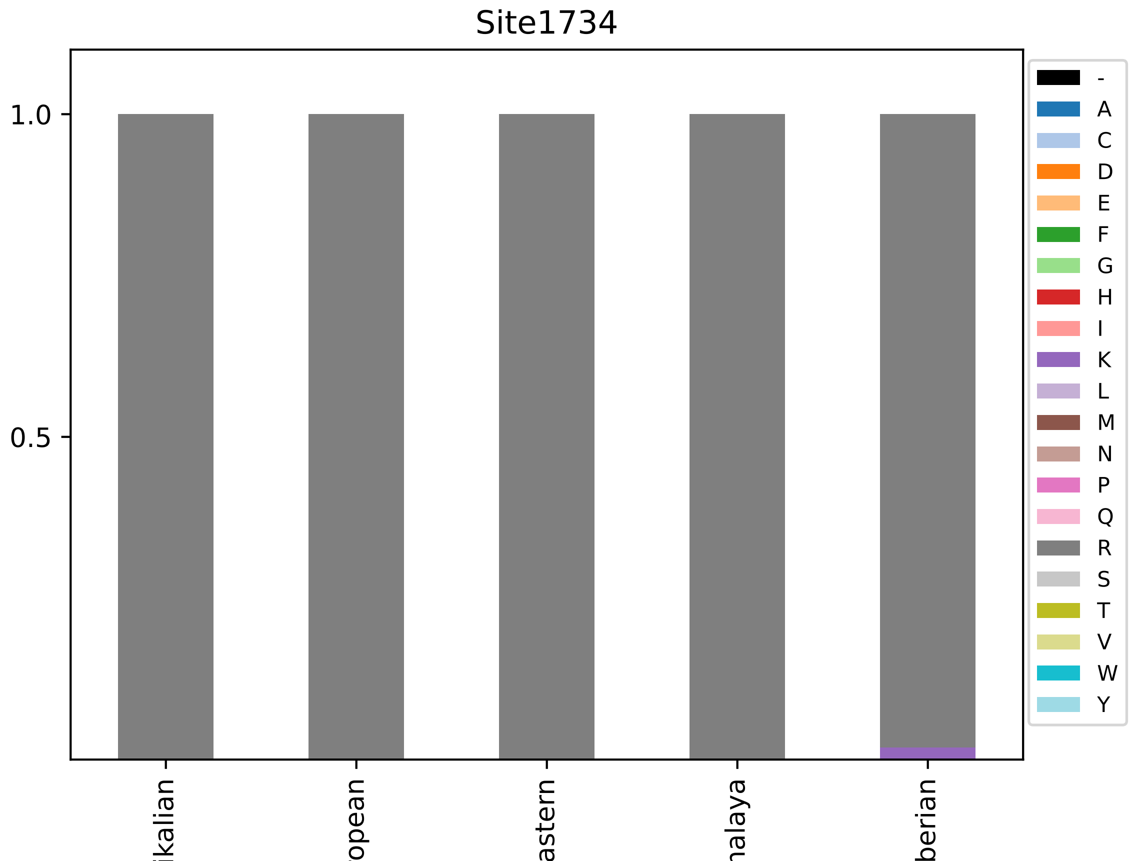
Site1734
1.0
0.5
ikalian
opean
astern
nalaya
berian
-
A
C
D
E
F
G
H
I
K
L
M
N
P
Q
R
S
T
V
W
Y

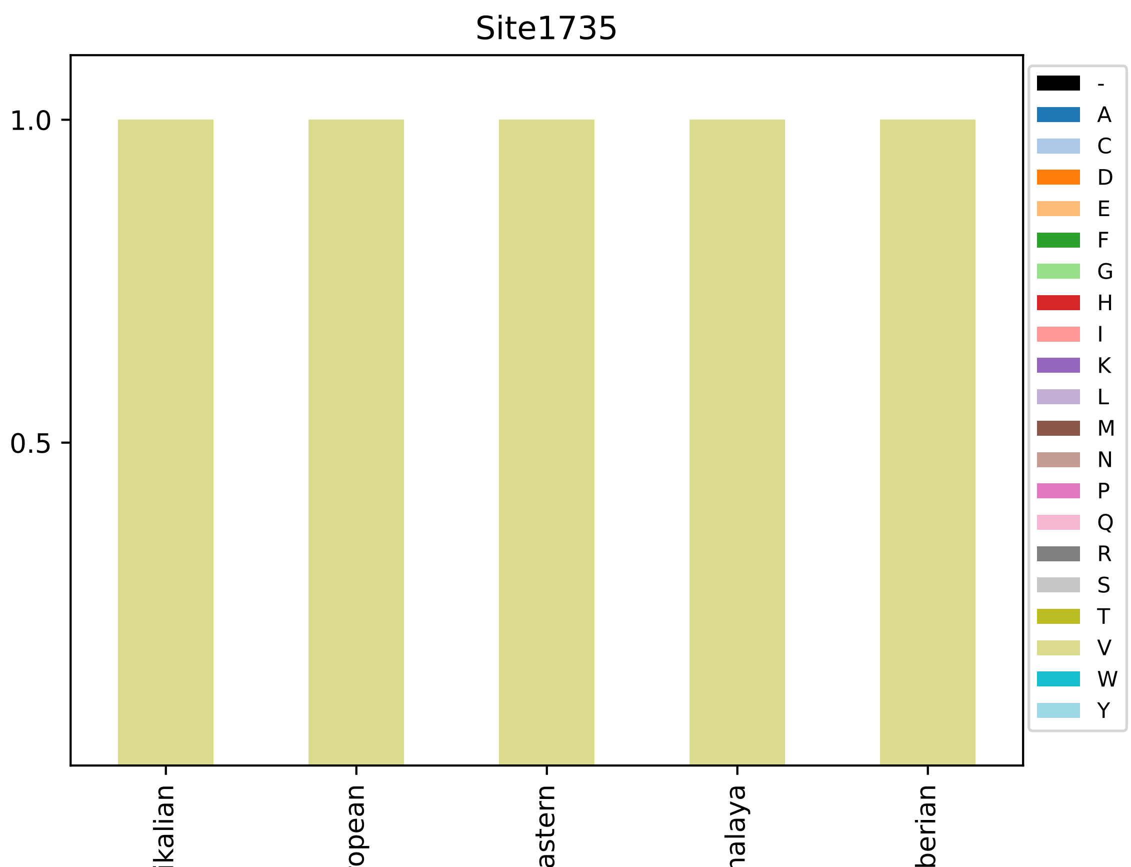
Site1735
1.0
0.5
-
A
C
D
E
F
G
H
I
K
L
M
N
P
Q
R
S
T
V
W
Y
ikalian
opean
astern
nalaya
berian

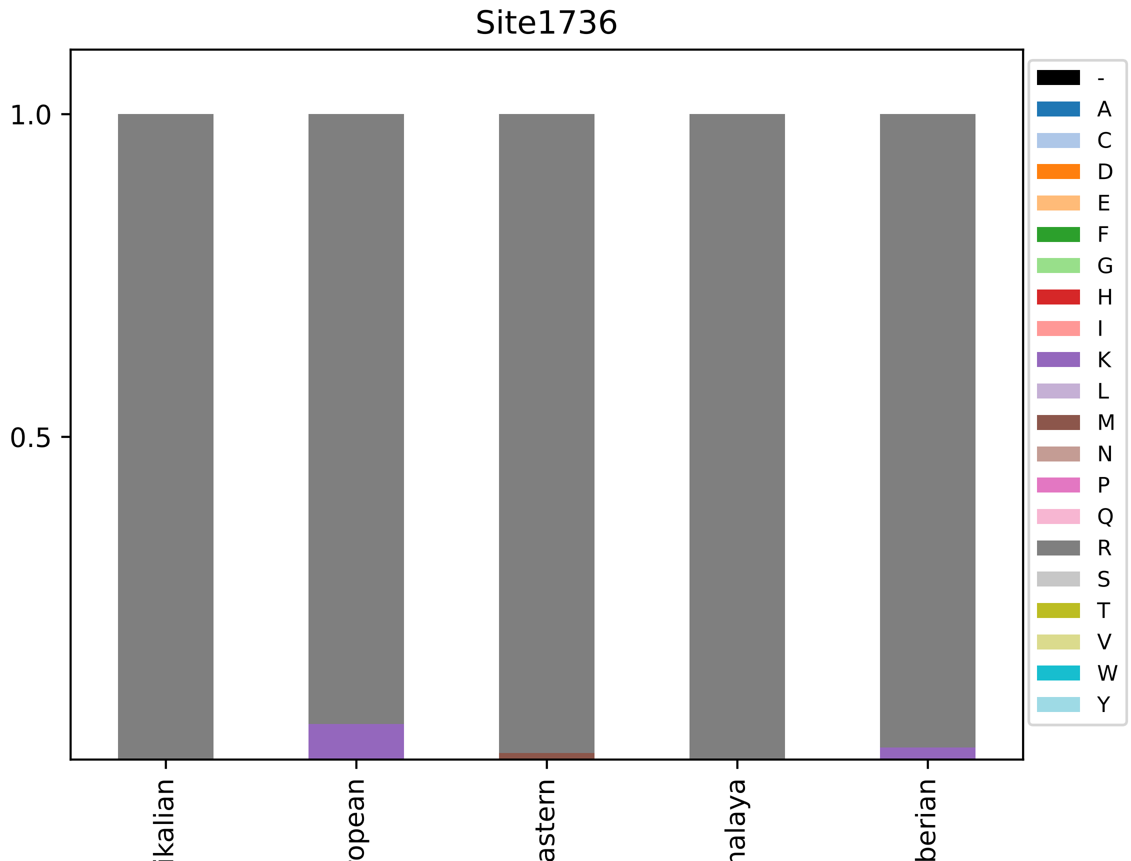
Site1736
1.0
0.5
-
A
C
D
E
F
G
H
I
K
L
M
N
P
Q
R
S
T
V
W
Y
ikalian
opean
astern
nalaya
berian

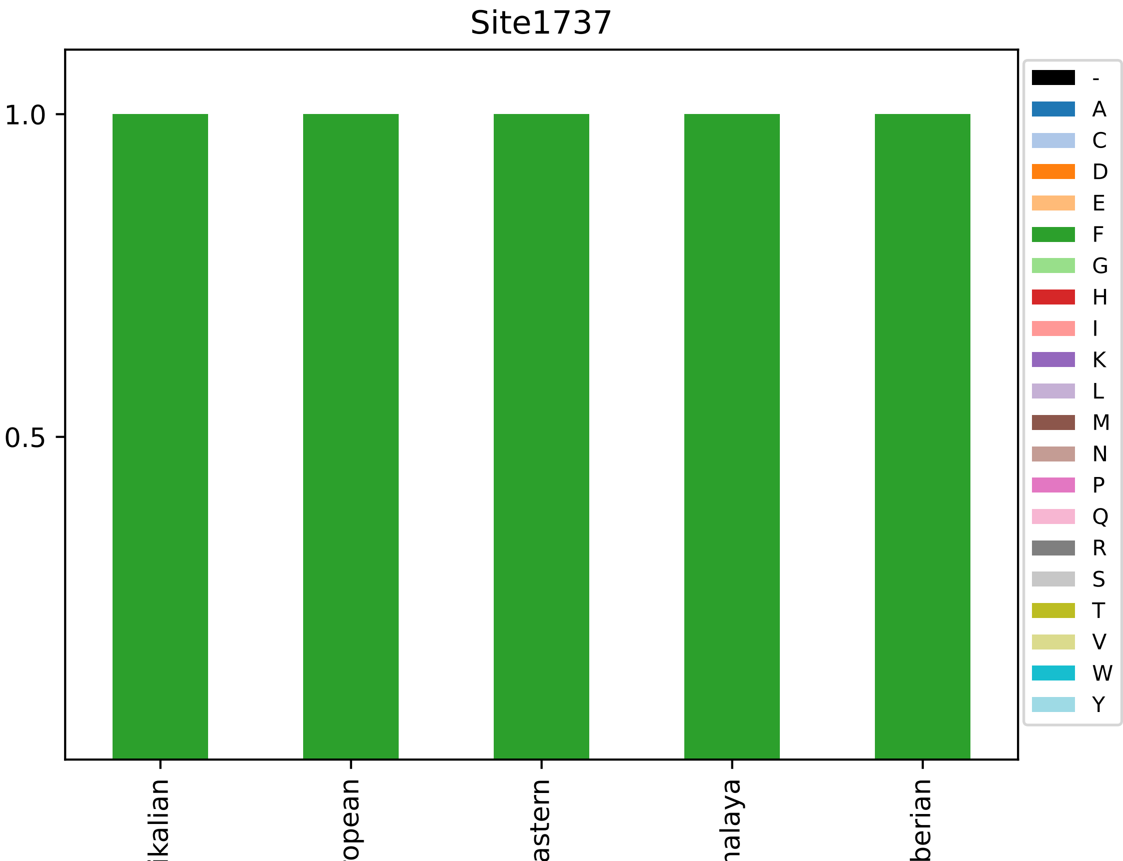

Site1737
1.0
0.5
-
A
C
D
E
F
G
H
I
K
L
M
N
P
Q
R
S
T
V
W
Y
ikalian
opean
astern
nalaya
berian

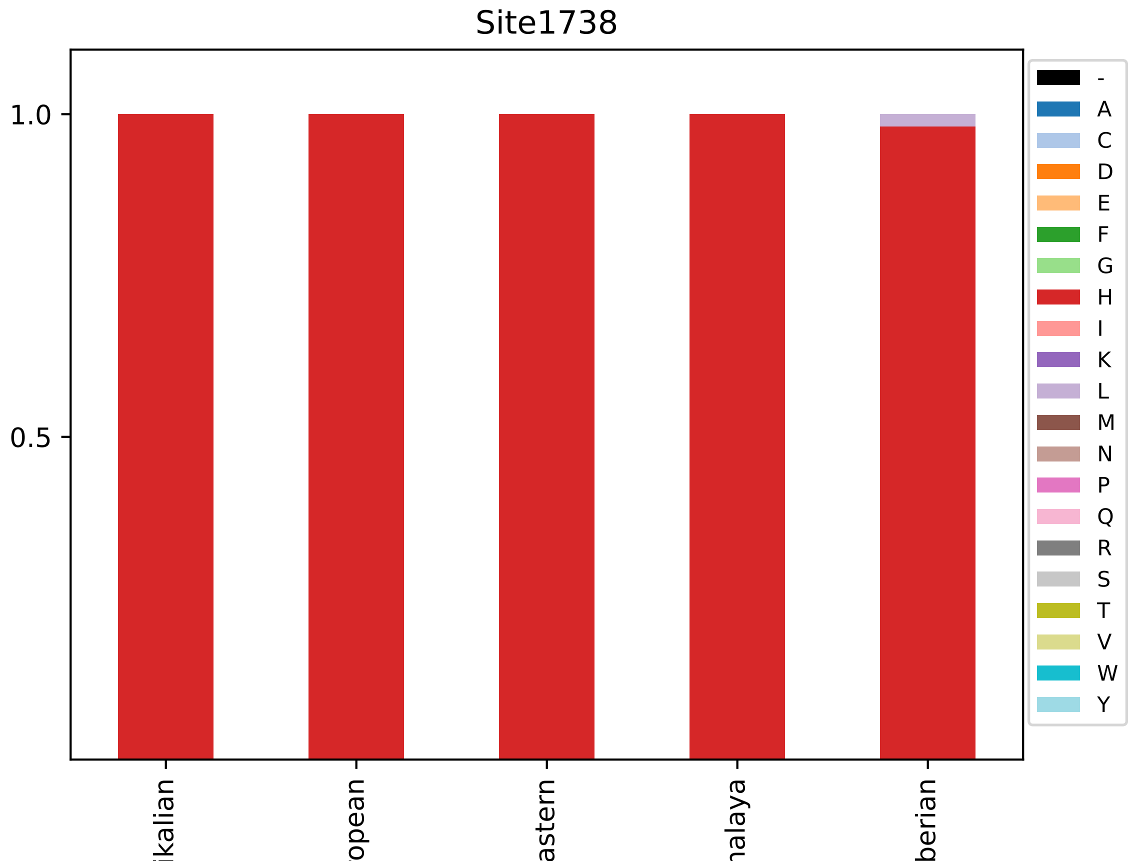
Site1738
1.0
0.5
-
A
C
D
E
F
G
H
I
K
L
M
N
P
Q
R
S
T
V
W
Y
ikalian
opean
astern
nalaya
berian

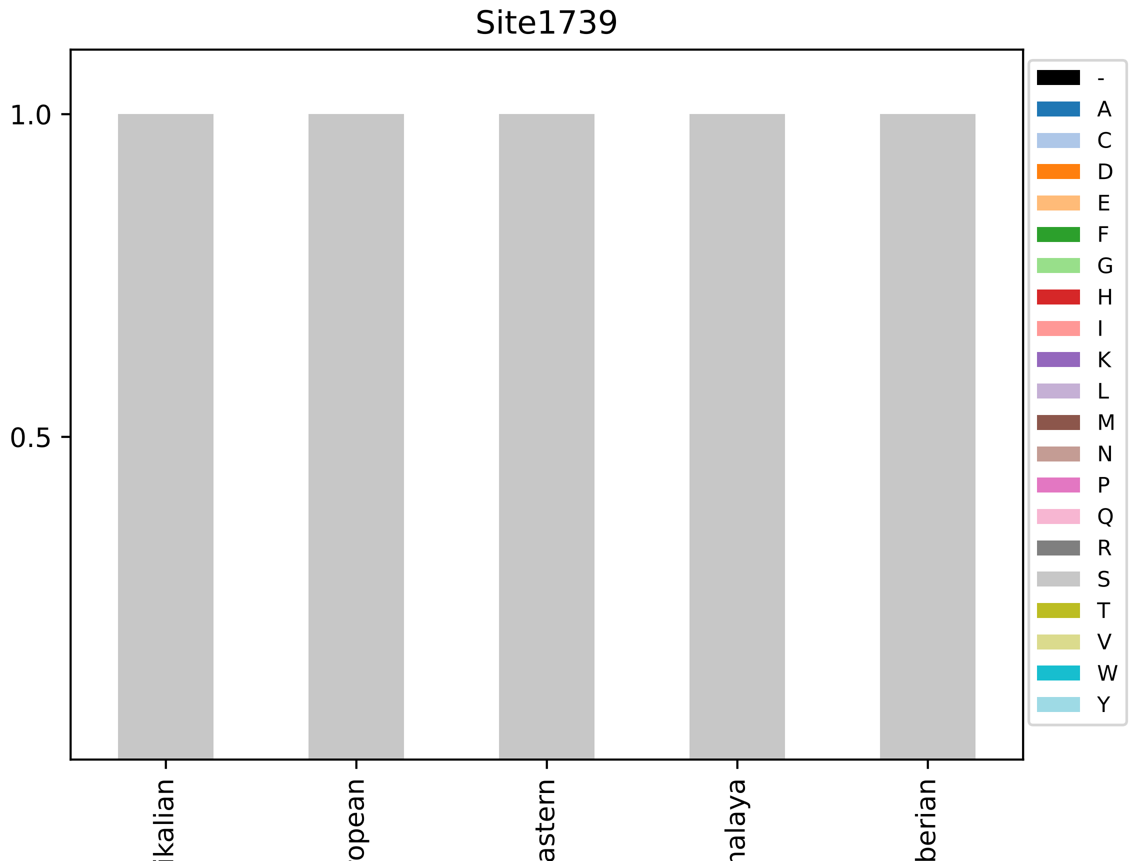

Site1739
1.0
0.5
-
A
C
D
E
F
G
H
I
K
L
M
N
P
Q
R
S
T
V
W
Y
ikalian
opean
astern
nalaya
berian

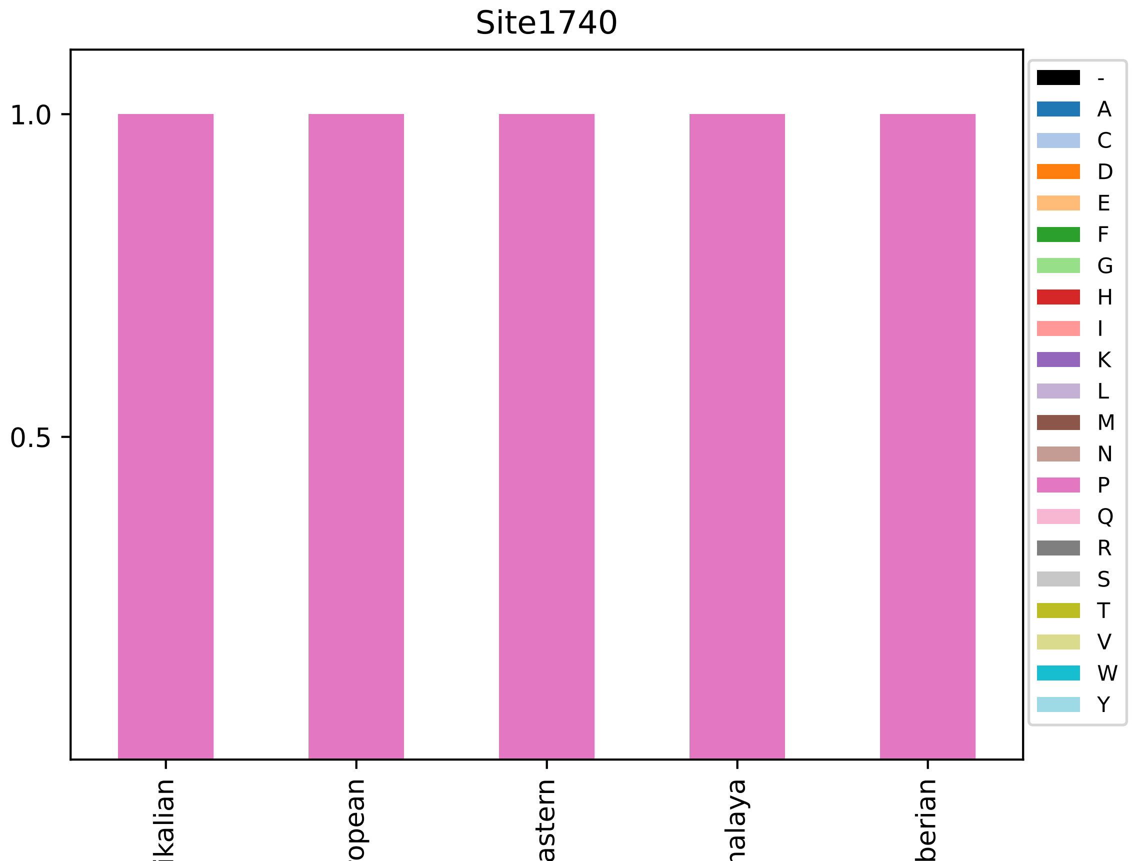
Site1740
1.0
0.5
-
A
C
D
E
F
G
H
I
K
L
M
N
P
Q
R
S
T
V
W
Y

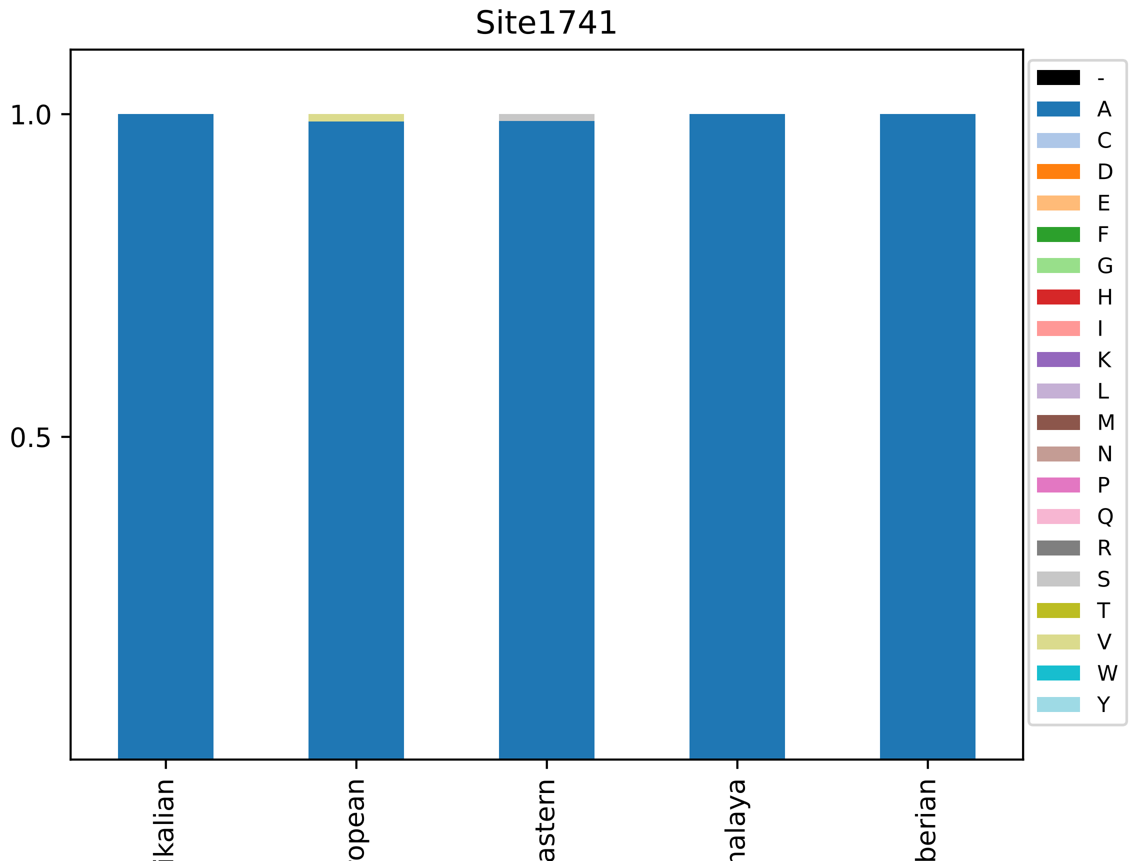

Site1741
1.0
0.5
ikalian
opean
astern
nalaya
berian
-
A
C
D
E
F
G
H
I
K
L
M
N
P
Q
R
S
T
V
W
Y

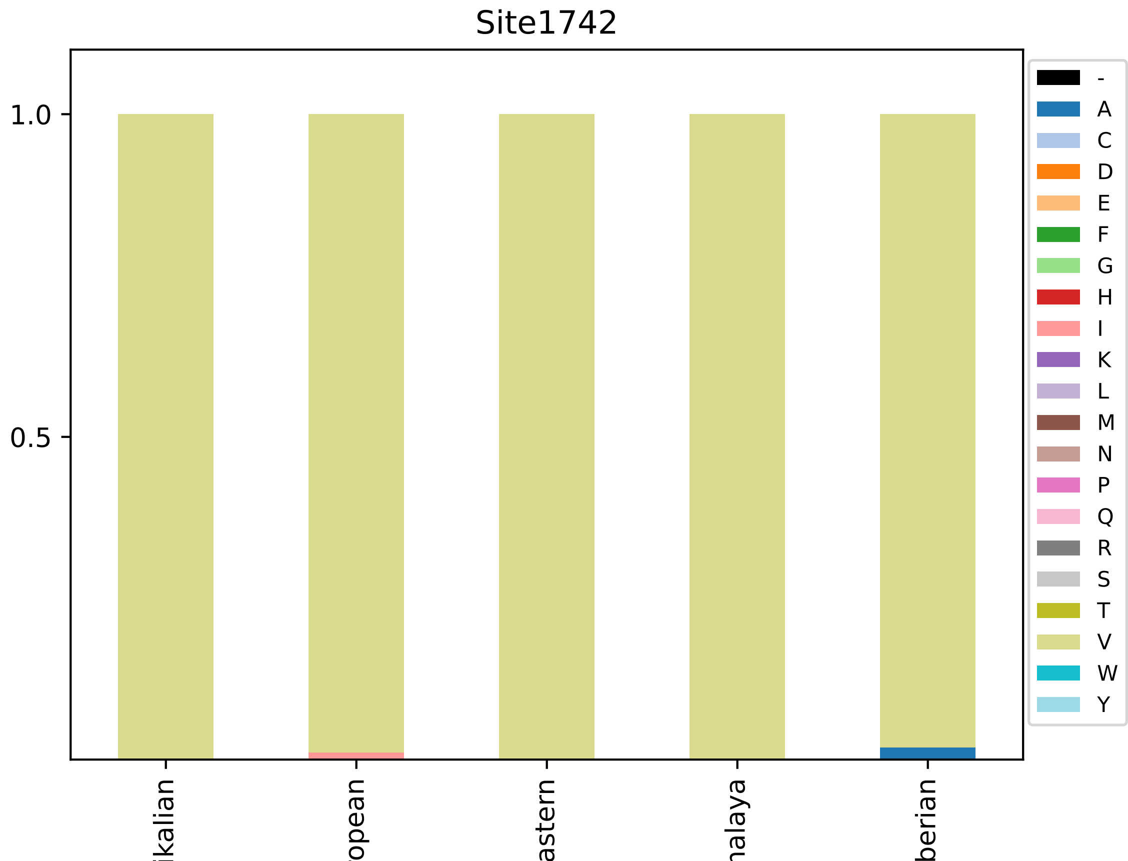

Site1742
1.0
0.5
-
A
C
D
E
F
G
H
I
K
L
M
N
P
Q
R
S
T
V
W
Y
ikalian
opean
astern
nalaya
berian

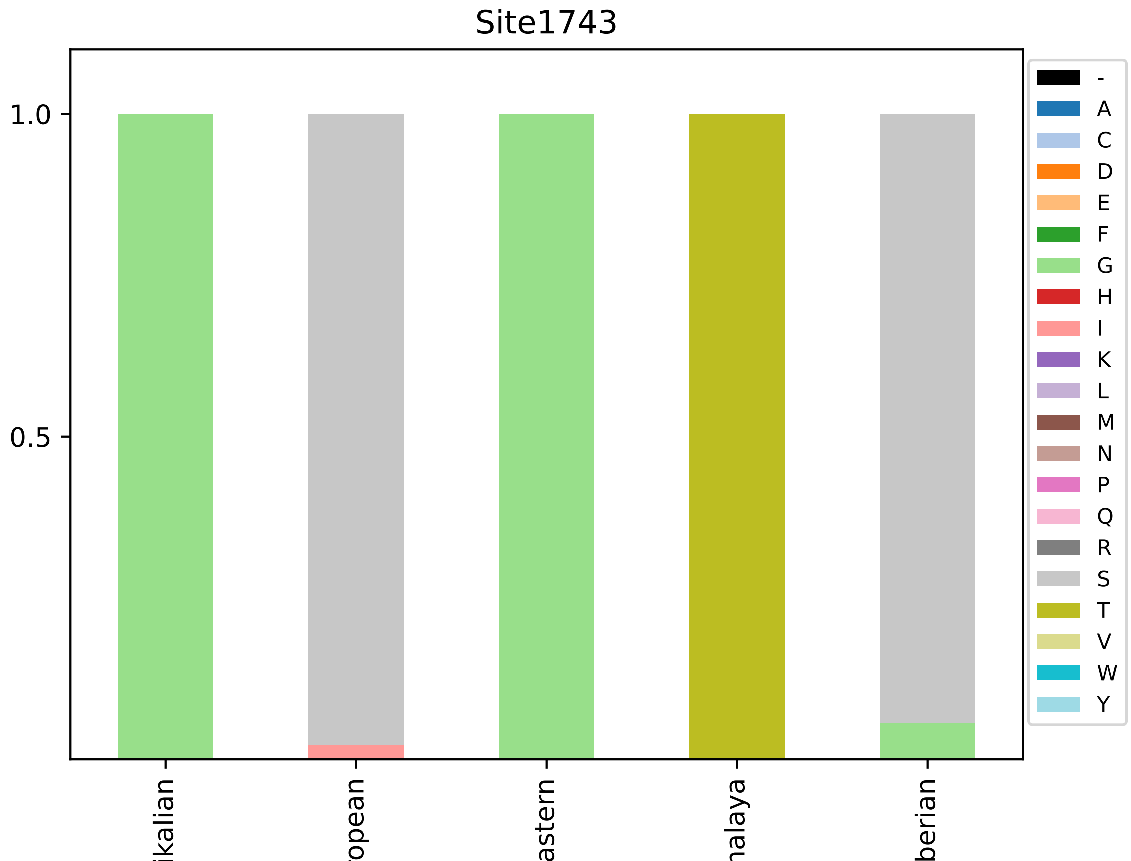
Site1743
1.0
0.5
ikalian
opean
astern
nalaya
berian
-
A
C
D
E
F
G
H
I
K
L
M
N
P
Q
R
S
T
V
W
Y

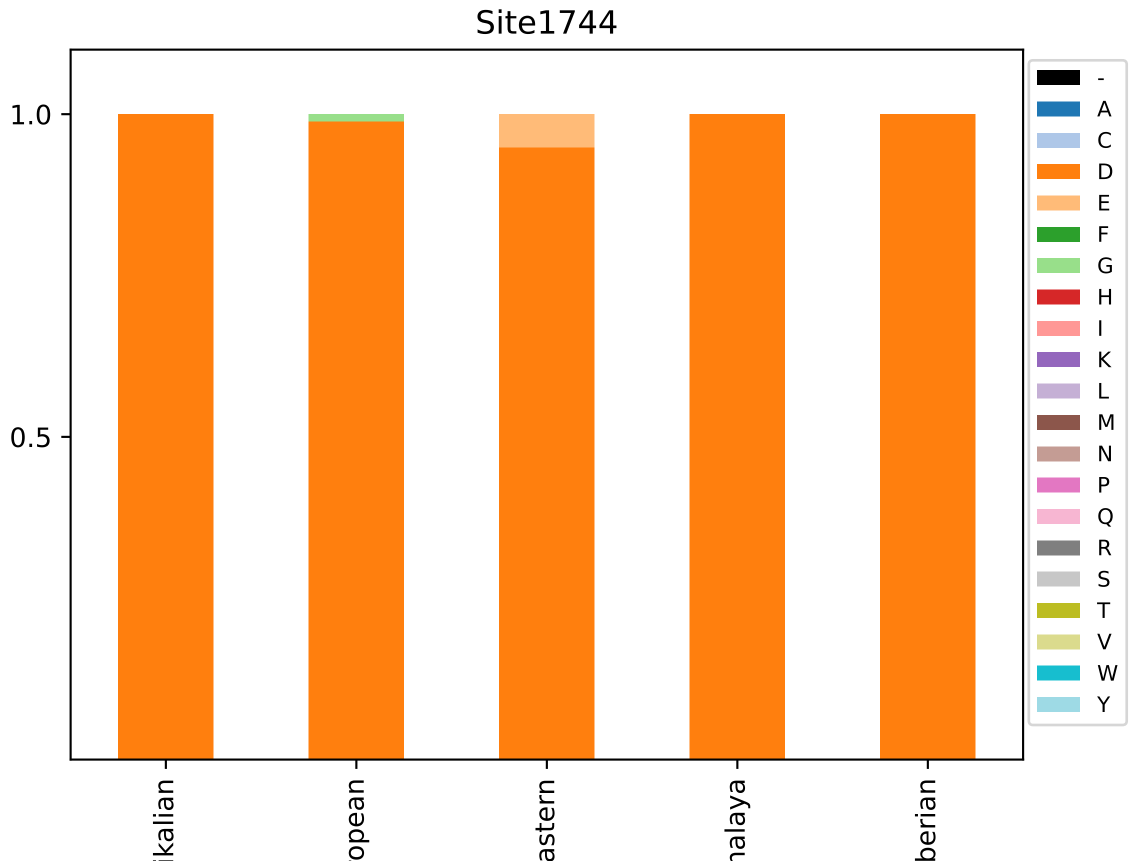

Site1744
1.0
0.5
-
A
C
D
E
F
G
H
I
K
L
M
N
P
Q
R
S
T
V
W
Y
ikalian
opean
astern
nalaya
berian

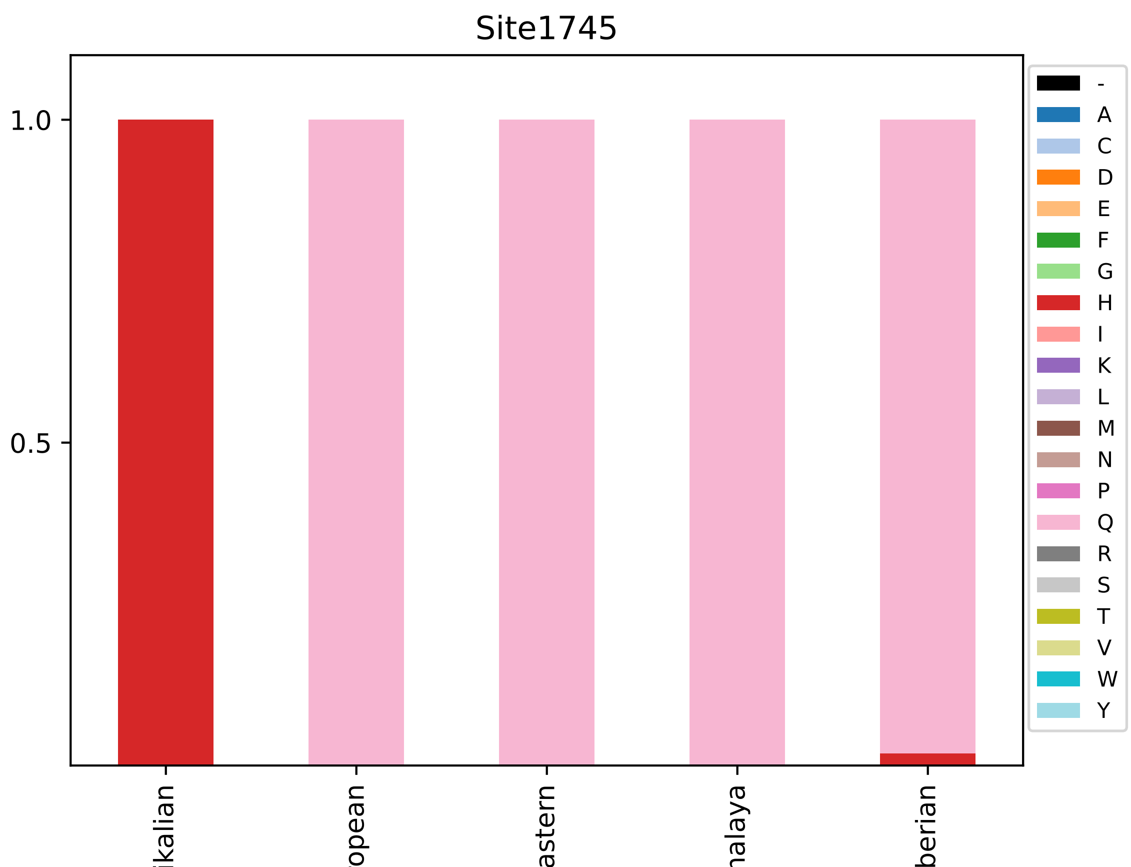

Site1745
1.0
0.5
-
A
C
D
E
F
G
H
I
K
L
M
N
P
Q
R
S
T
V
W
Y
ikalian
opean
astern
nalaya
berian

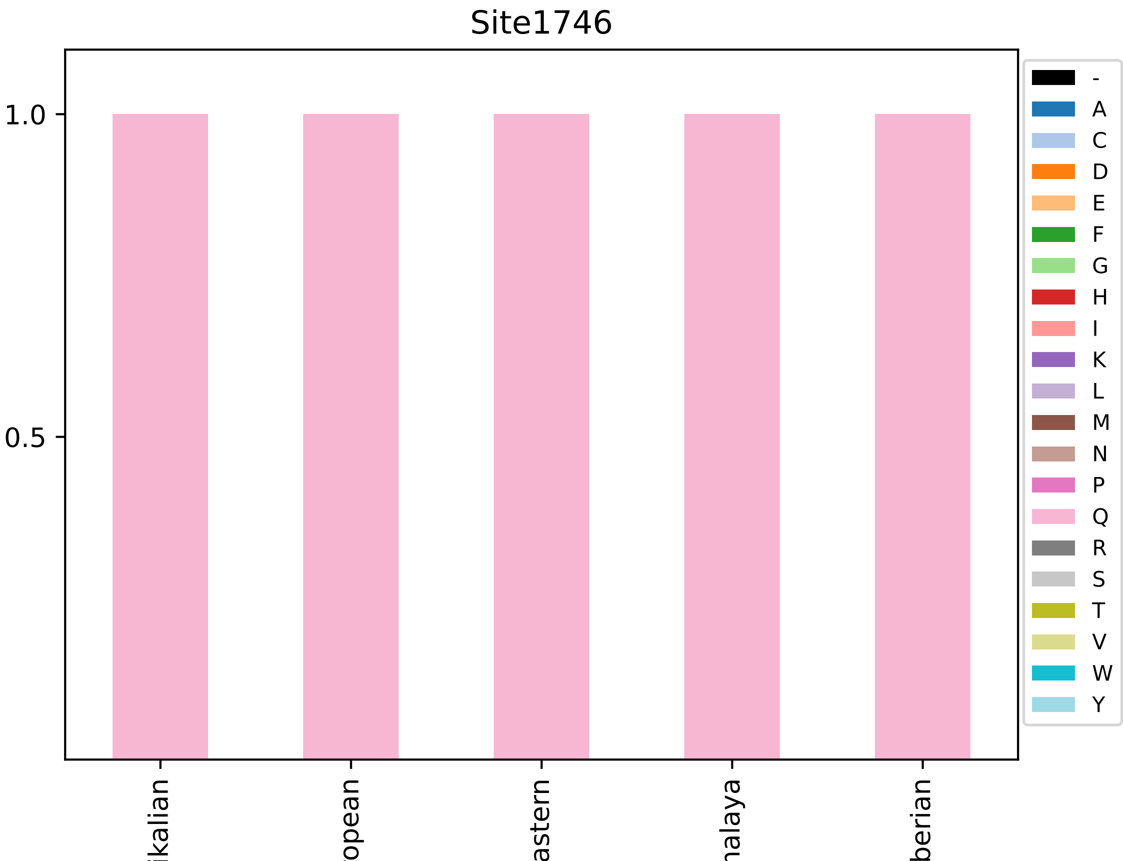

Site1746
1.0
0.5
-
A
C
D
E
F
G
H
I
K
L
M
N
P
Q
R
S
T
V
W
Y
ikalian
ropean
astern
nalaya
berian

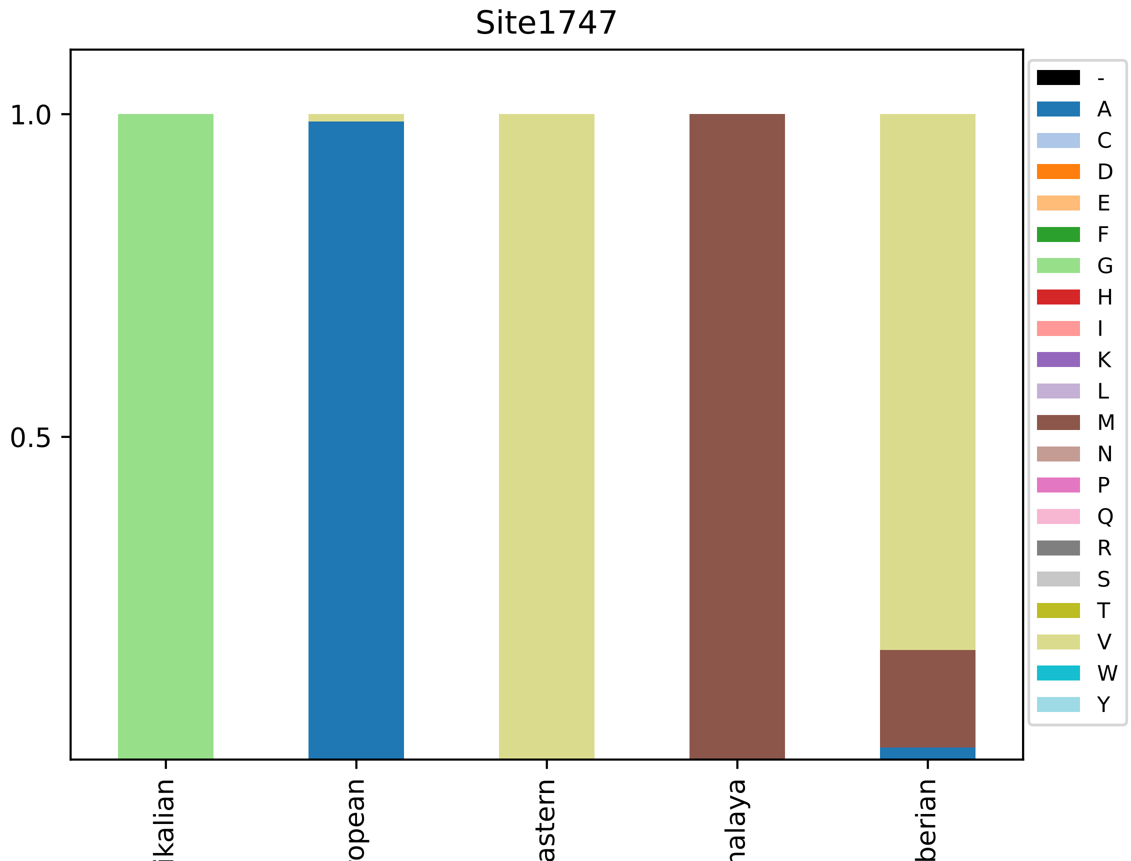

Site1747
1.0
0.5
-
A
C
D
E
F
G
H
I
K
L
M
N
P
Q
R
S
T
V
W
Y
ikalian
opean
astern
nalaya
berian

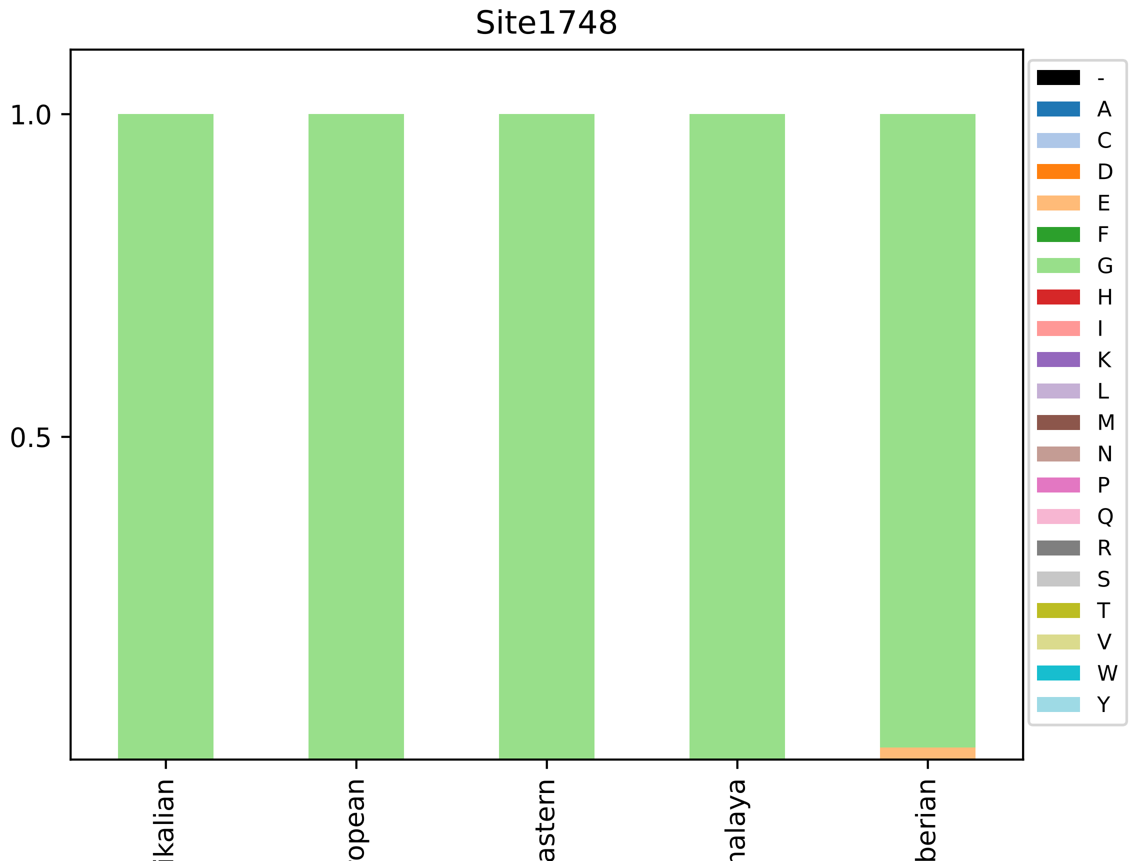

Site1748
1.0
0.5
-
A
C
D
E
F
G
H
I
K
L
M
N
P
Q
R
S
T
V
W
Y
ikalian
opean
astern
nalaya
berian

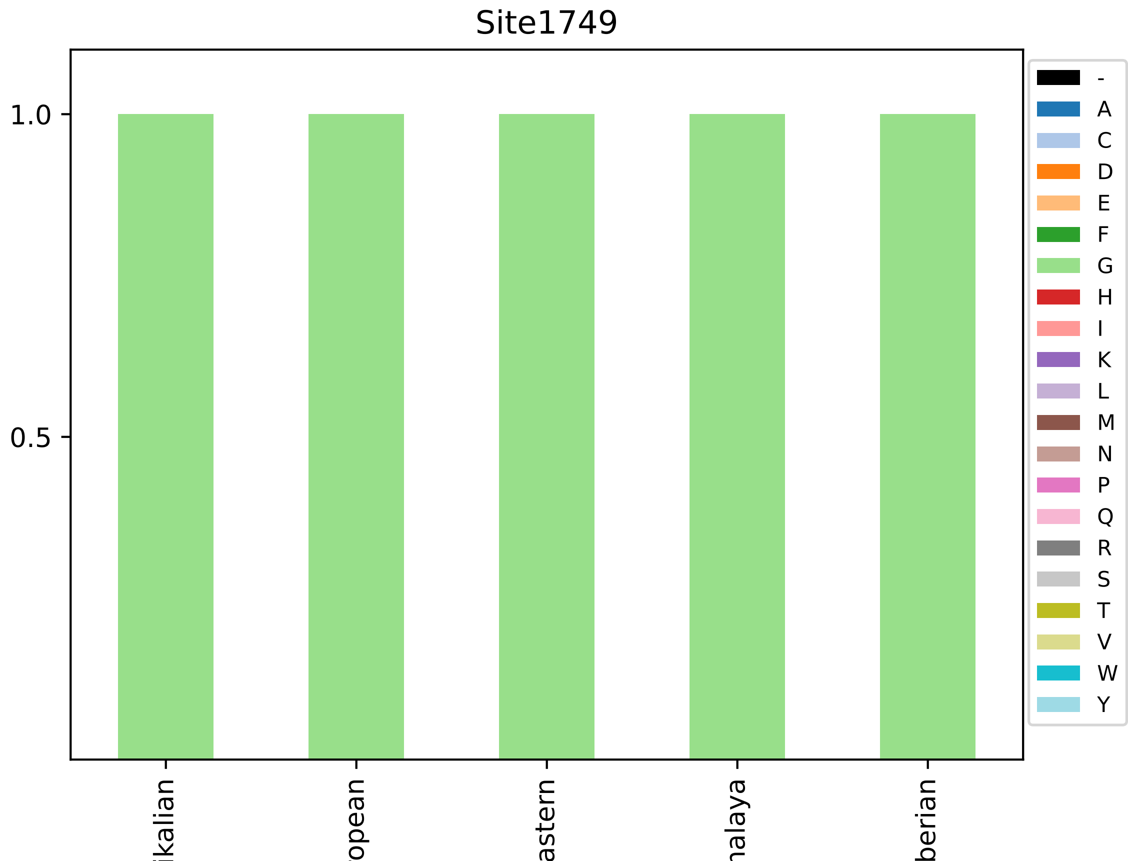
Site1749
1.0
0.5
-
A
C
D
E
F
G
H
I
K
L
M
N
P
Q
R
S
T
V
W
Y
ikalian
opean
astern
nalaya
berian

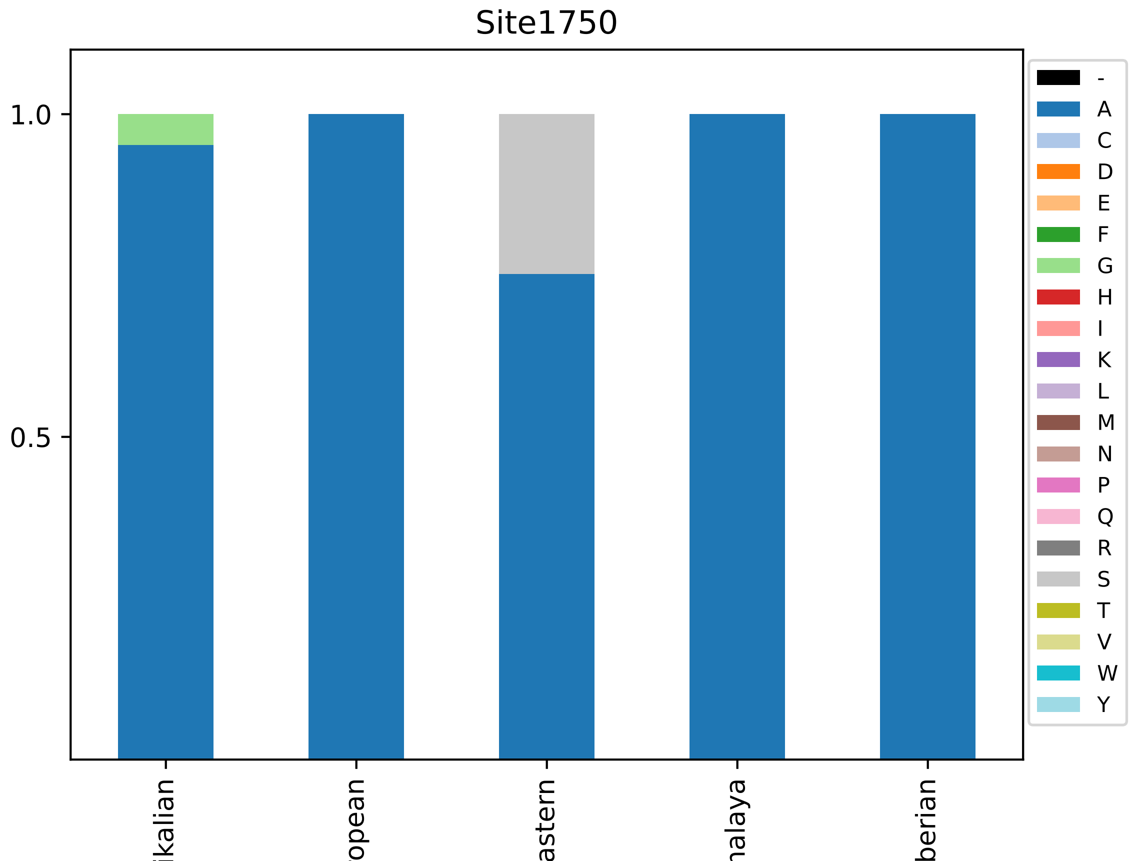
Site1750
1.0
0.5
-
A
C
D
E
F
G
H
I
K
L
M
N
P
Q
R
S
T
V
W
Y
ikalian
opean
astern
nalaya
berian

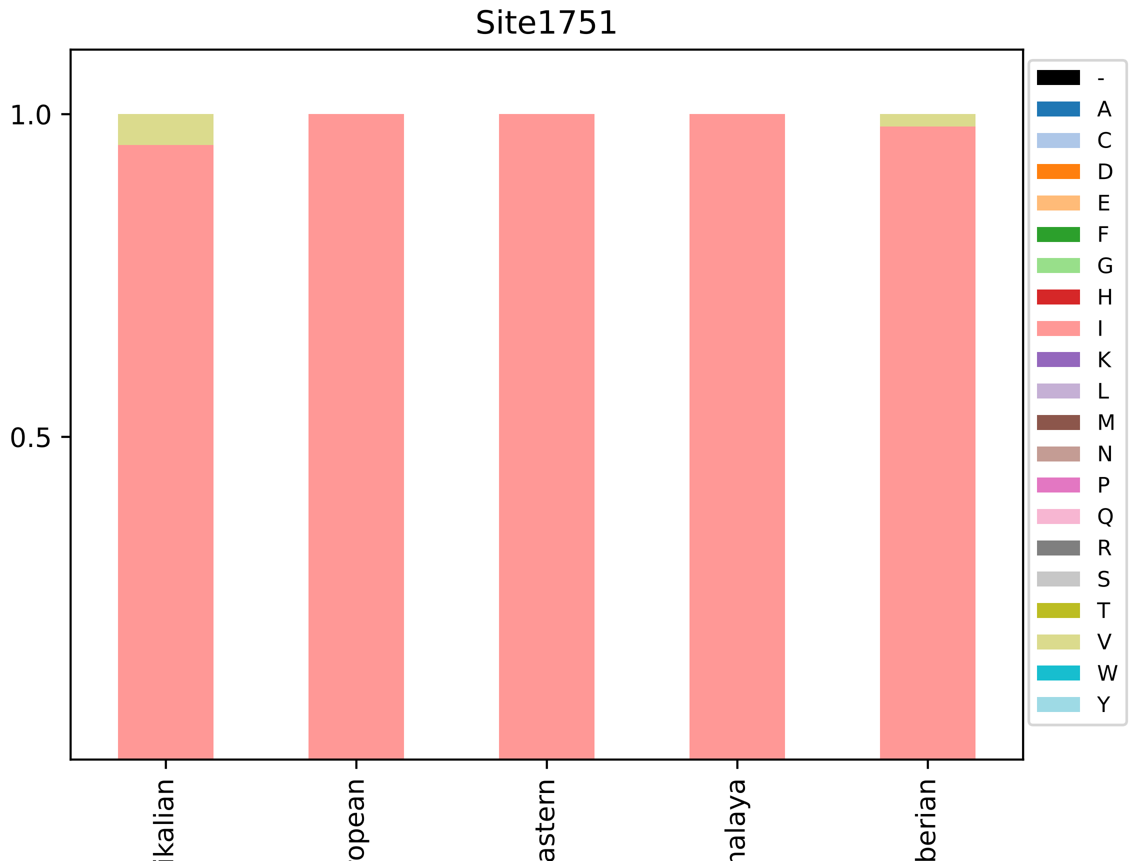
Site1751
1.0
0.5
-
A
C
D
E
F
G
H
I
K
L
M
N
P
Q
R
S
T
V
W
Y

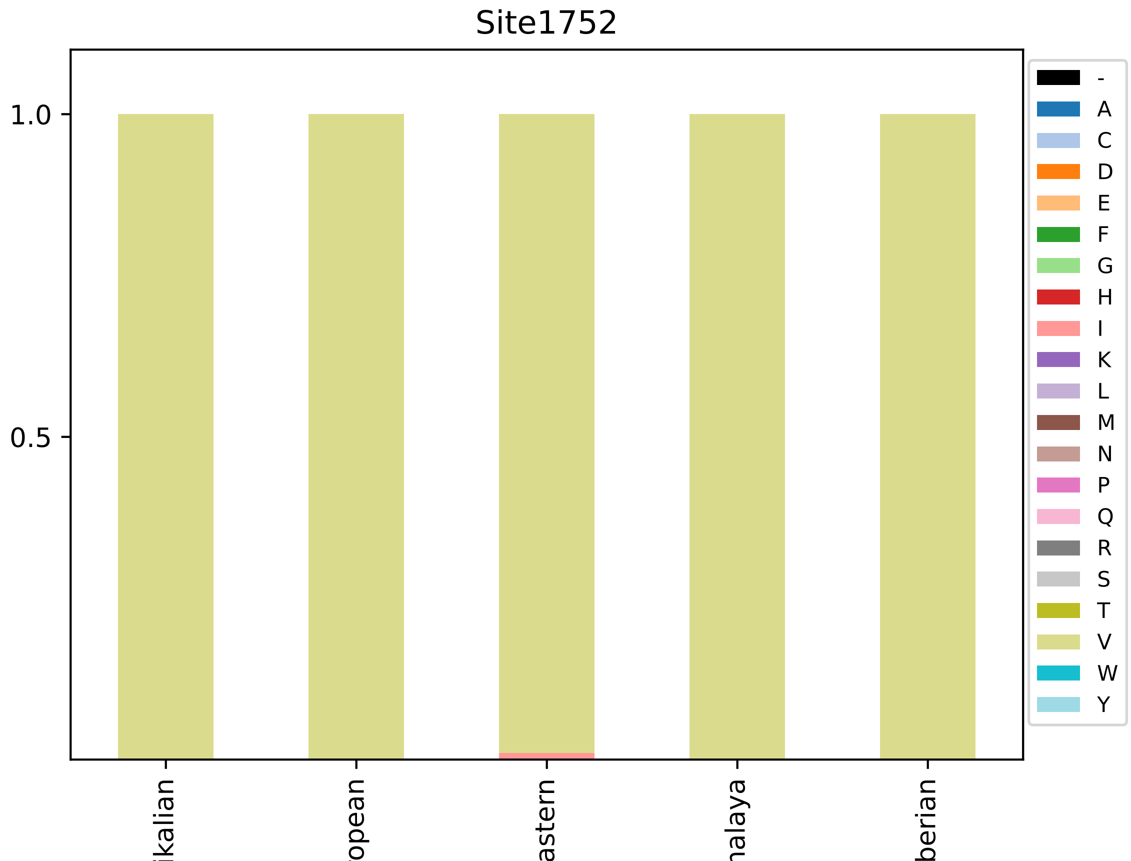

Site1752
1.0
0.5
-
A
C
D
E
F
G
H
I
K
L
M
N
P
Q
R
S
T
V
W
Y
ikalian
opean
astern
nalaya
berian

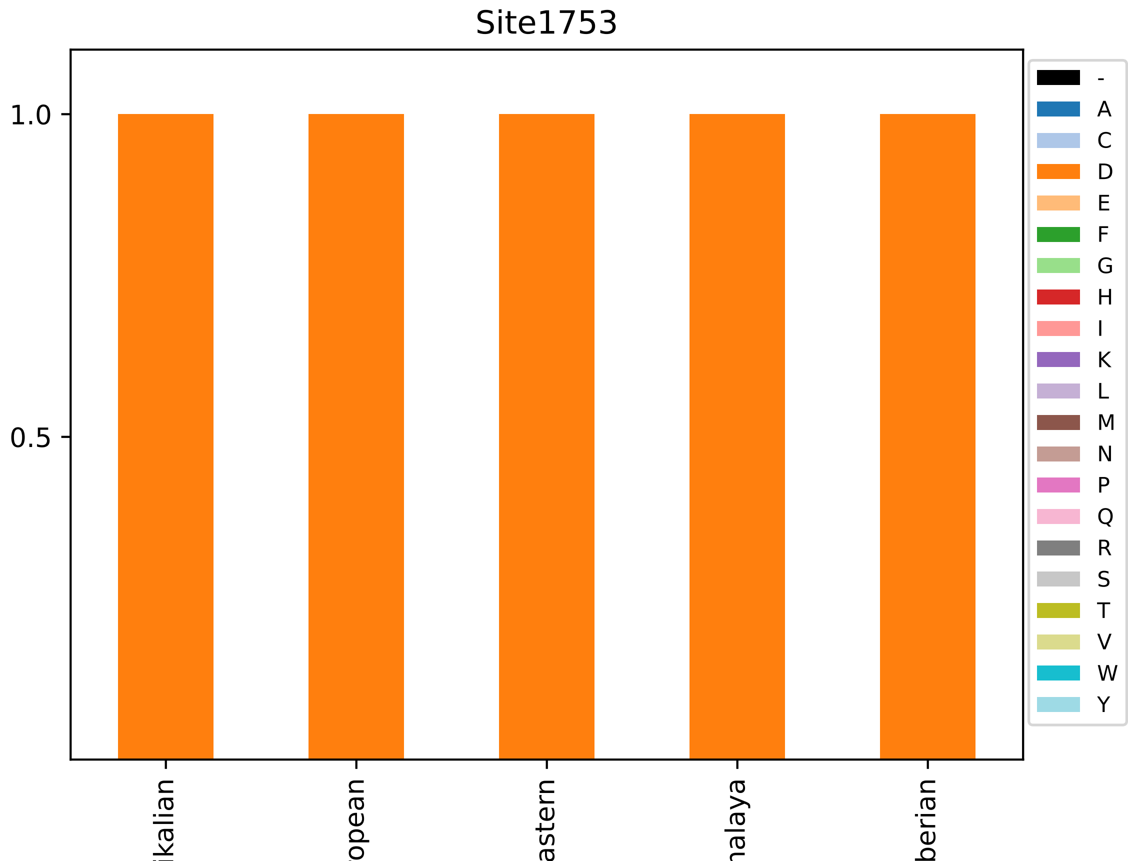

Site1753
1.0
0.5
-
A
C
D
E
F
G
H
I
K
L
M
N
P
Q
R
S
T
V
W
Y
ikalian
opean
astern
nalaya
berian

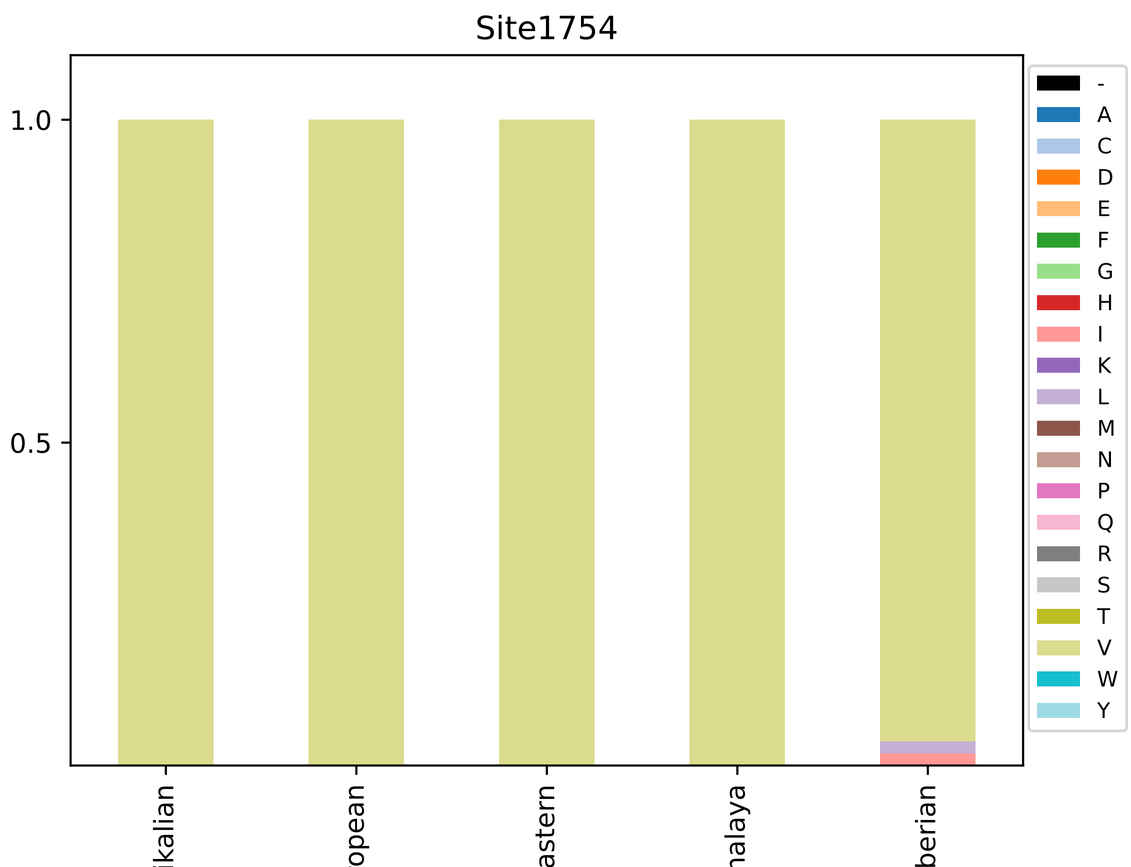
Site1754
1.0
0.5
ikalian
ropean
astern
nalaya
berian
-
A
C
D
E
F
G
H
I
K
L
M
N
P
Q
R
S
T
V
W
Y

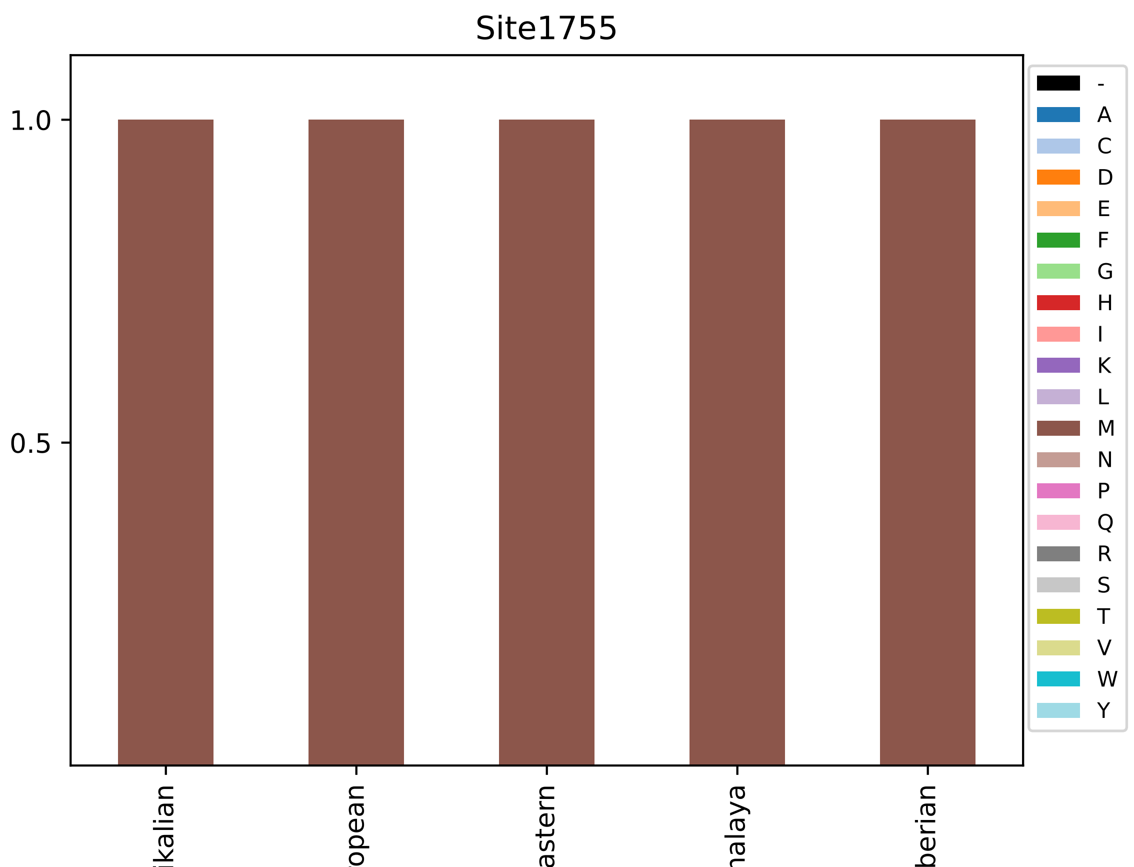

Site1755
1.0
0.5
-
A
C
D
E
F
G
H
I
K
L
M
N
P
Q
R
S
T
V
W
Y
ikalian
opean
astern
nalaya
berian

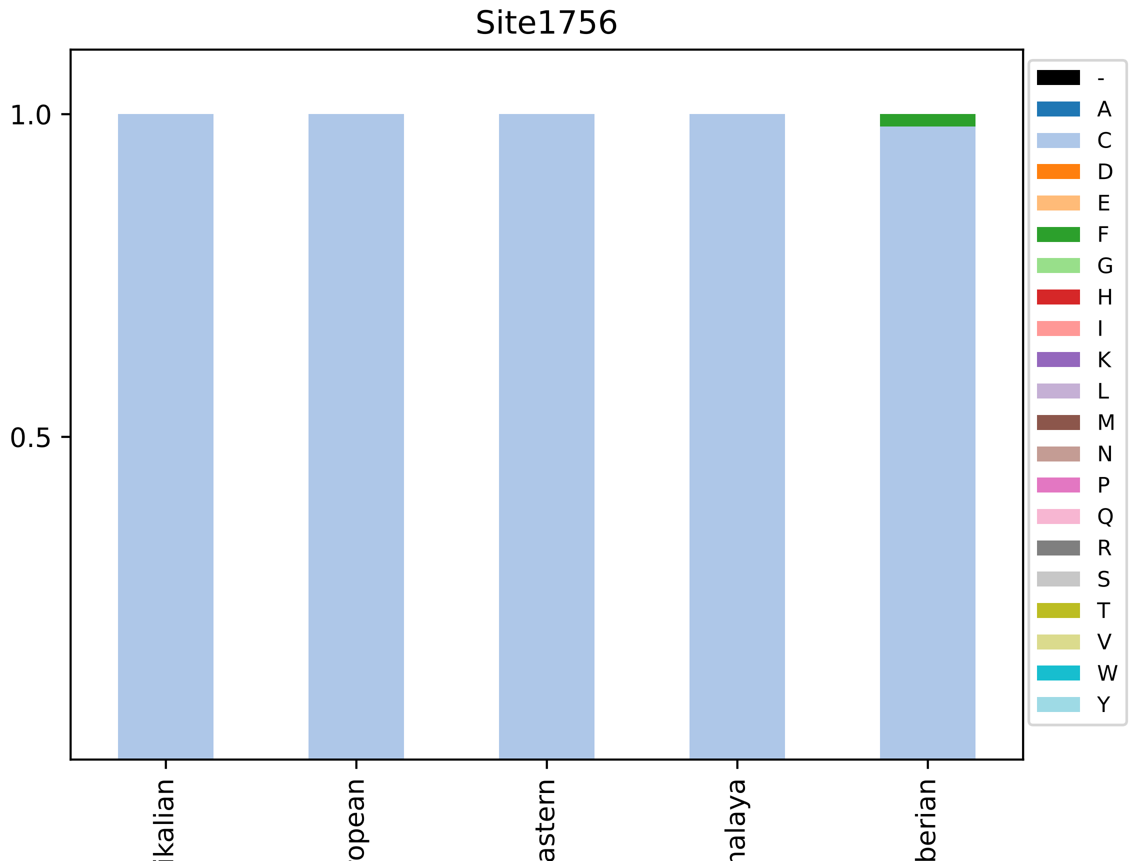
Site1756
1.0
0.5
-
A
C
D
E
F
G
H
I
K
L
M
N
P
Q
R
S
T
V
W
Y
ikalian
opean
astern
nalaya
berian

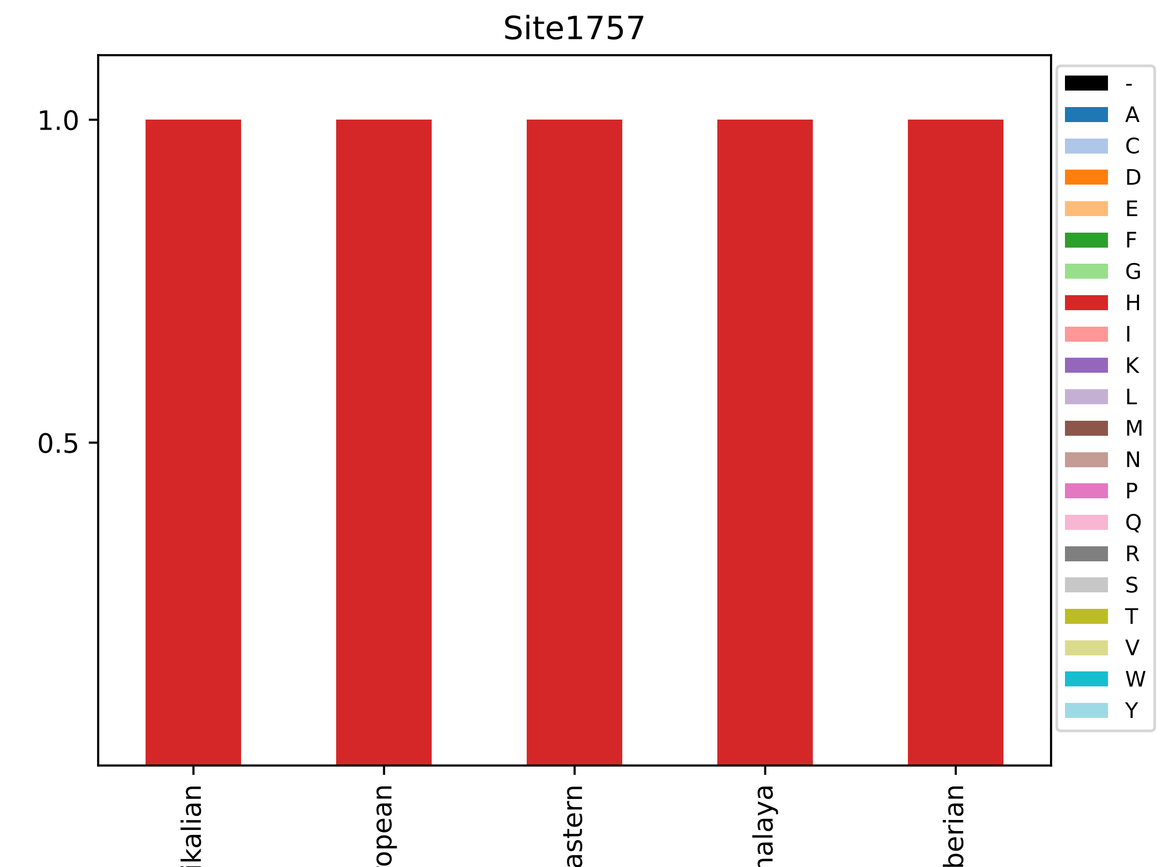

Site1757
1.0
0.5
-
A
C
D
E
F
G
H
I
K
L
M
N
P
Q
R
S
T
V
W
Y
ikalian
opean
astern
nalaya
berian

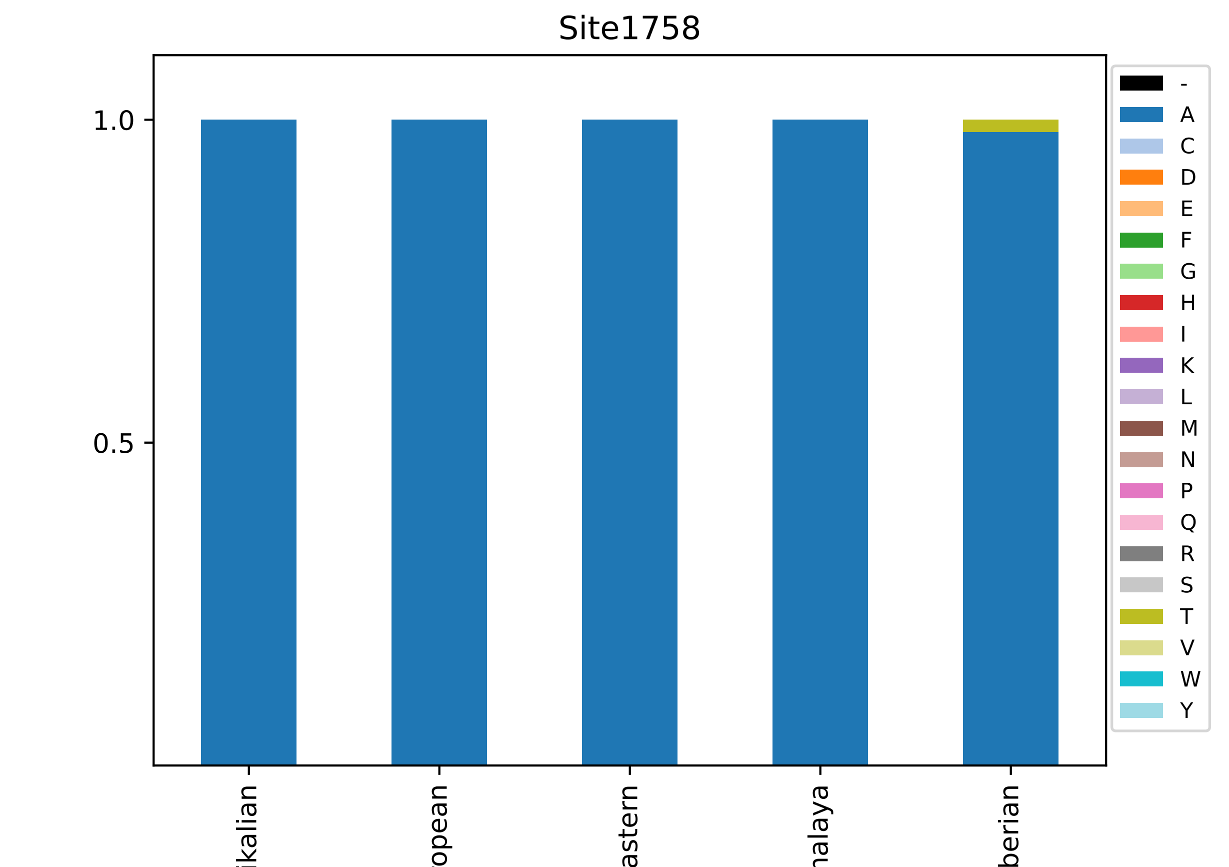
Site1758
1.0
0.5
-
A
C
D
E
F
G
H
I
K
L
M
N
P
Q
R
S
T
V
W
Y
ikalian
opean
astern
nalaya
berian

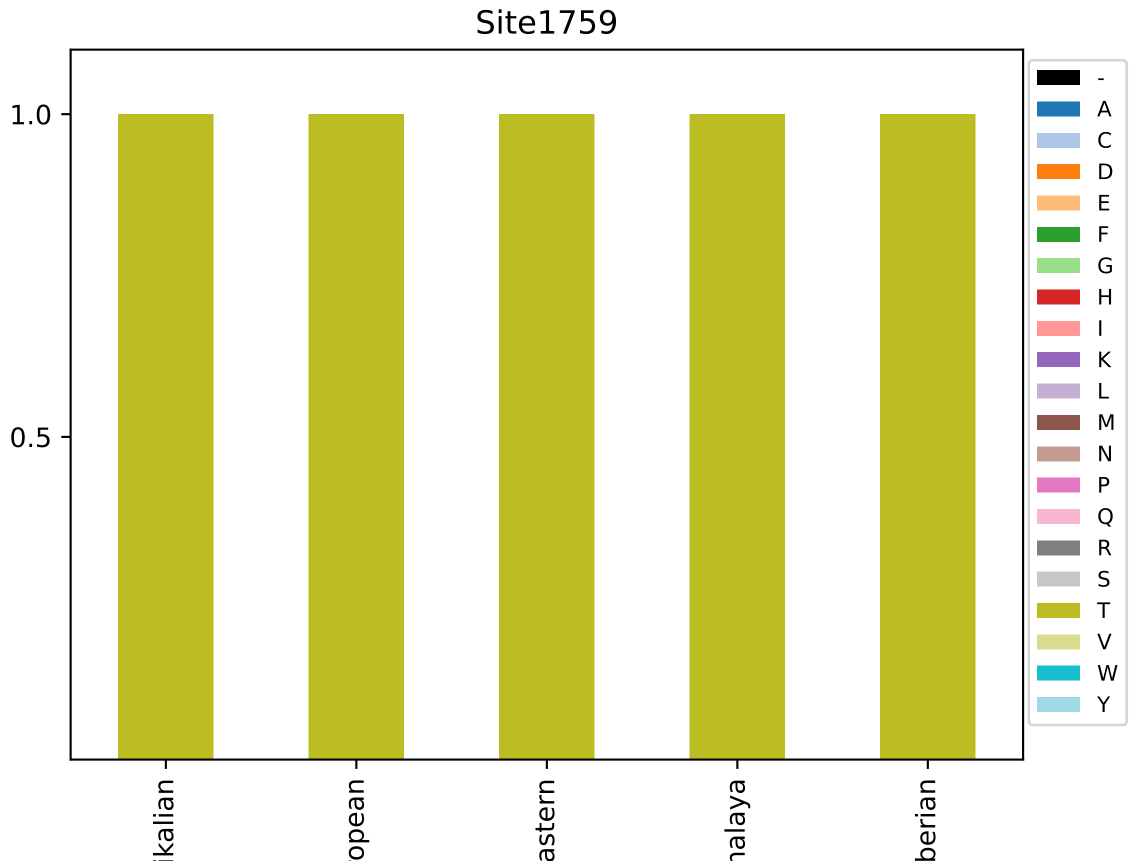

Site1759
1.0
0.5
-
A
C
D
E
F
G
H
I
K
L
M
N
P
Q
R
S
T
V
W
Y
ikalian
opean
astern
nalaya
berian

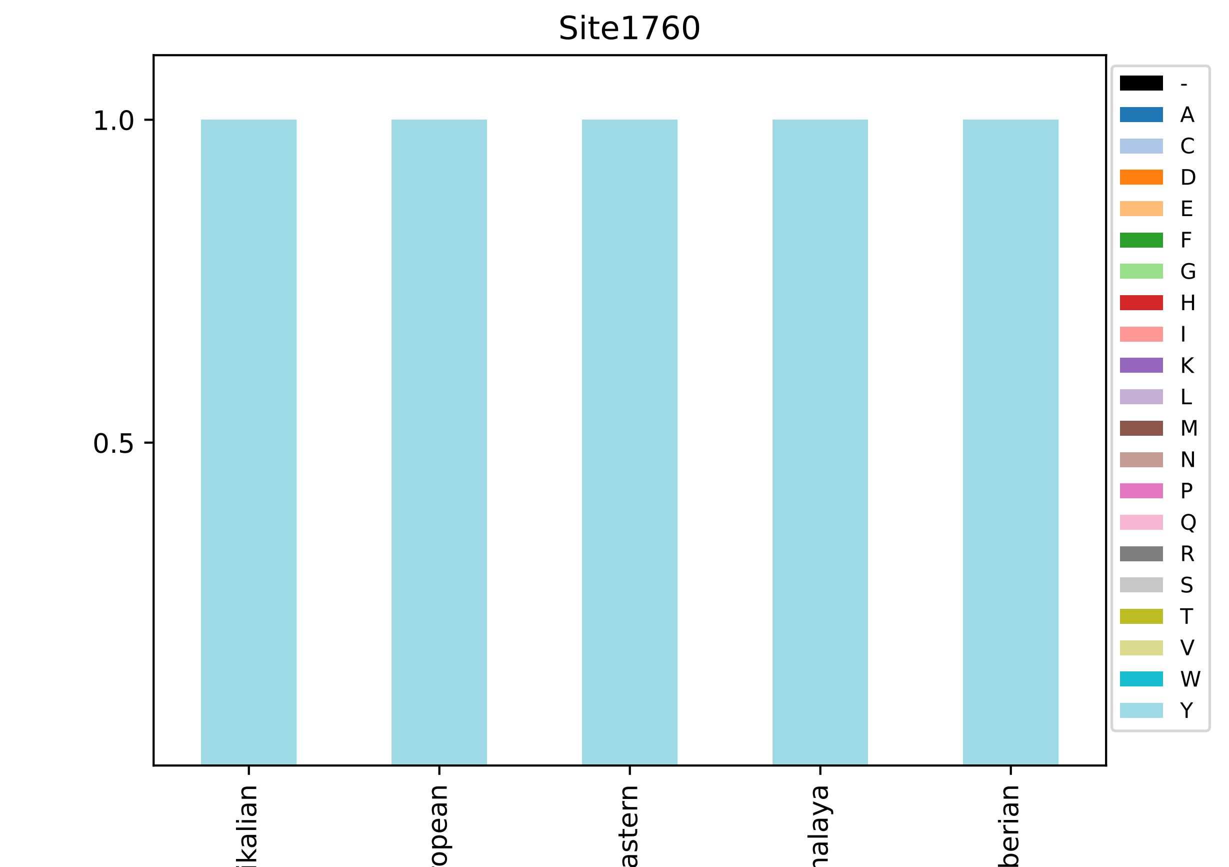

Site1760
1.0
0.5
-
A
C
D
E
F
G
H
I
K
L
M
N
P
Q
R
S
T
V
W
Y
ikalian
opean
astern
nalaya
berian

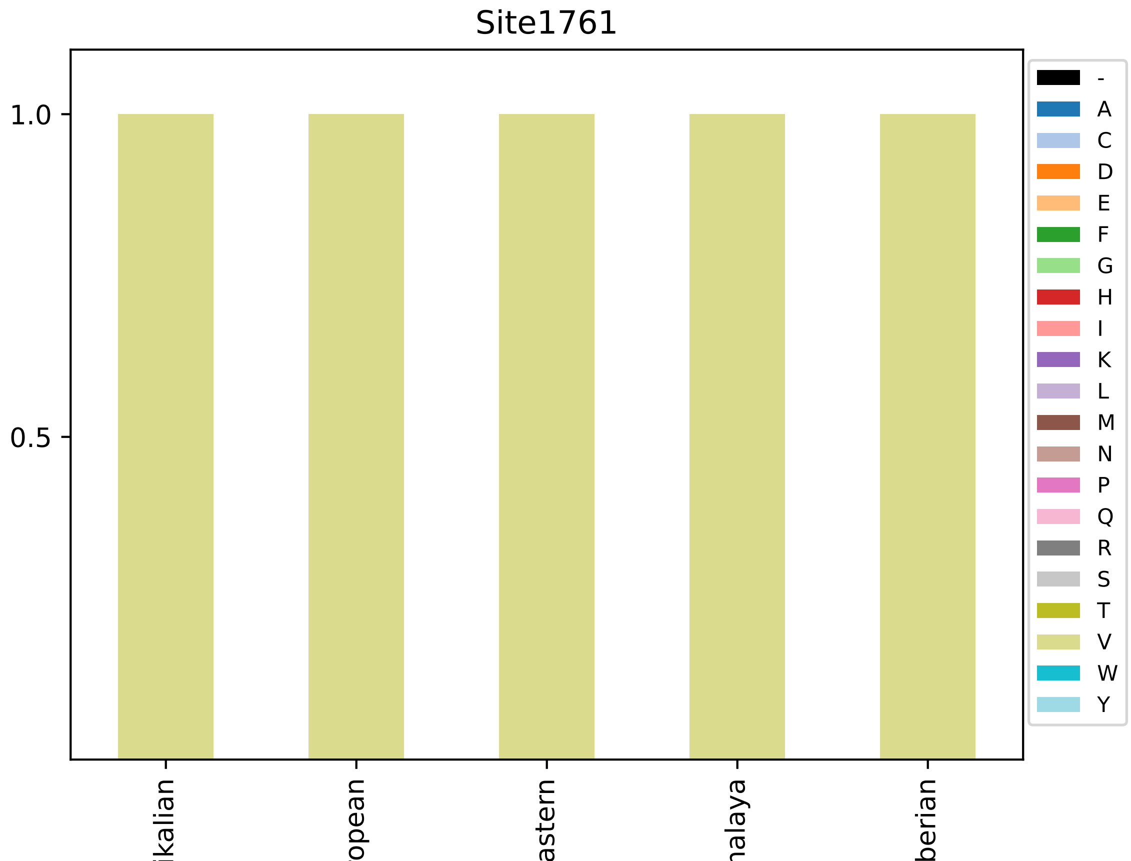
Site1761
1.0
0.5
-
A
C
D
E
F
G
H
I
K
L
M
N
P
Q
R
S
T
V
W
Y

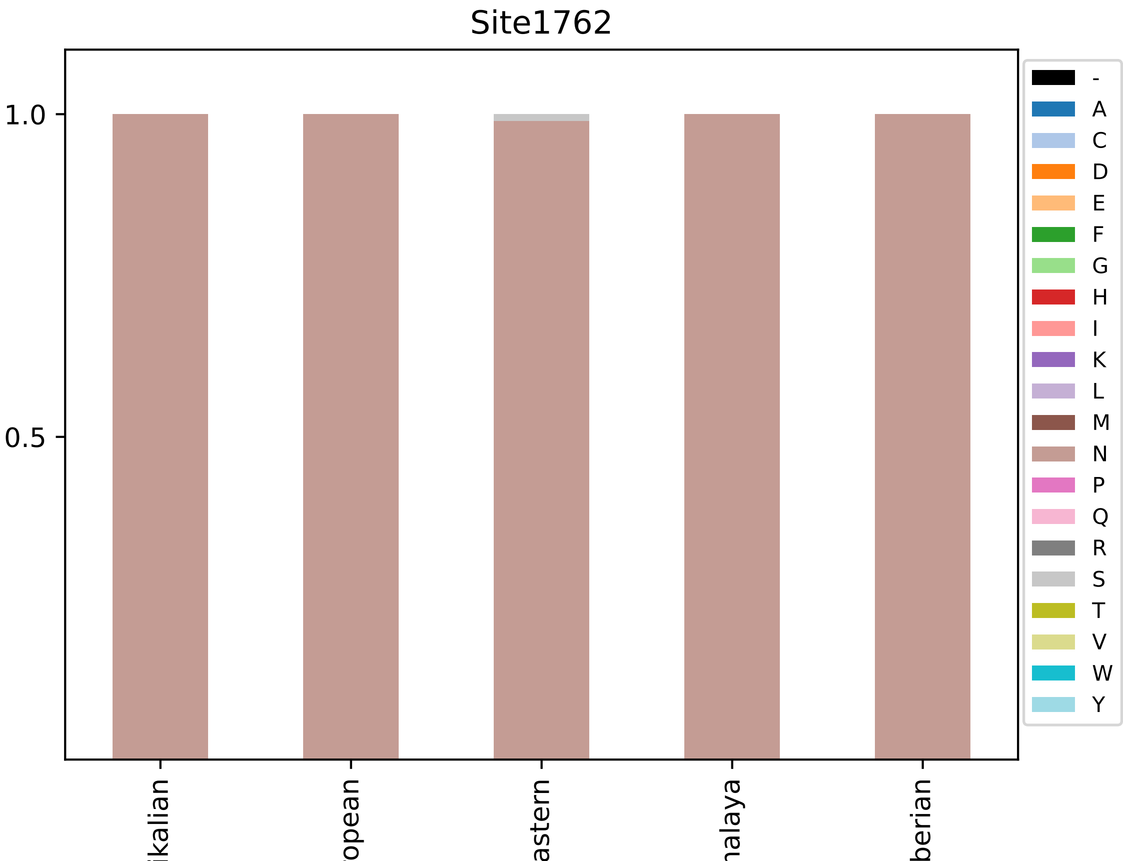

Site1762
1.0
0.5
-
A
C
D
E
F
G
H
I
K
L
M
N
P
Q
R
S
T
V
W
Y
ikalian
opean
astern
nalaya
berian

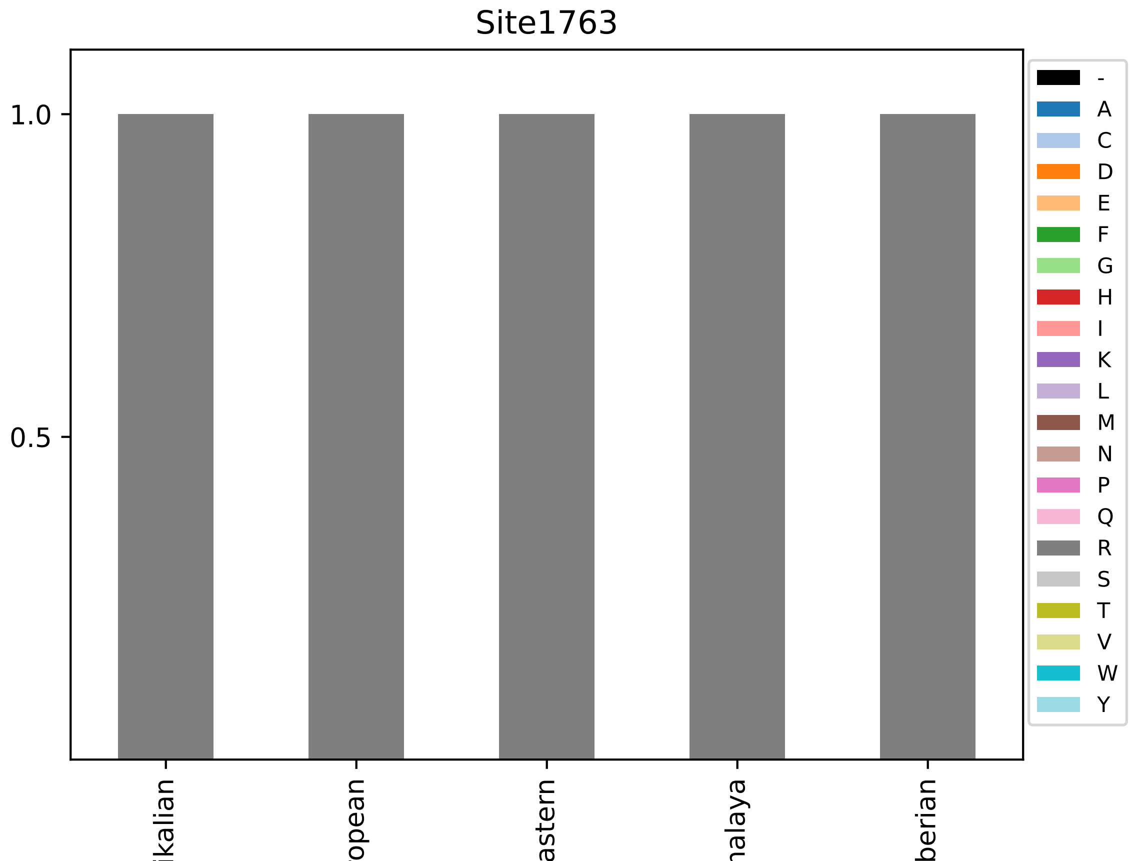

Site1763
1.0
0.5
ikalian
opean
astern
nalaya
berian
-
A
C
D
E
F
G
H
I
K
L
M
N
P
Q
R
S
T
V
W
Y

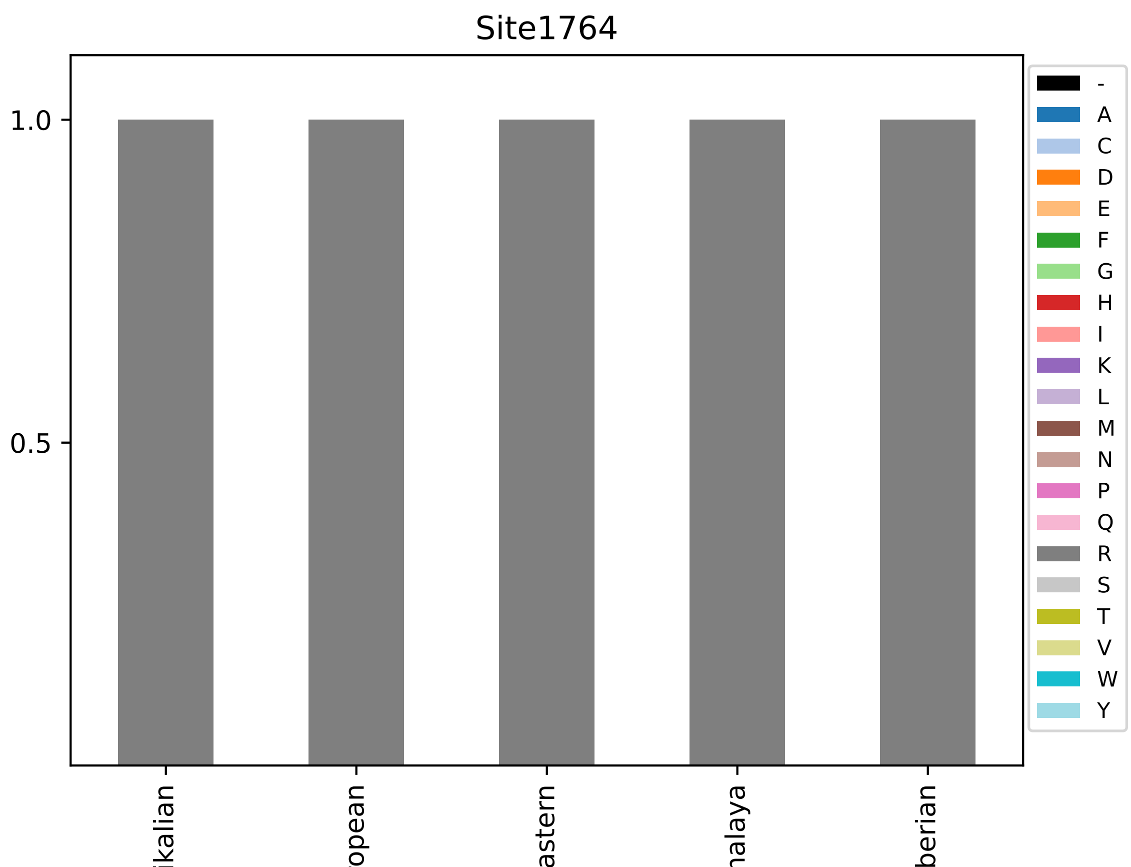

Site1764
1.0
0.5
-
A
C
D
E
F
G
H
I
K
L
M
N
P
Q
R
S
T
V
W
Y
ikalian
opean
astern
halaya
berian

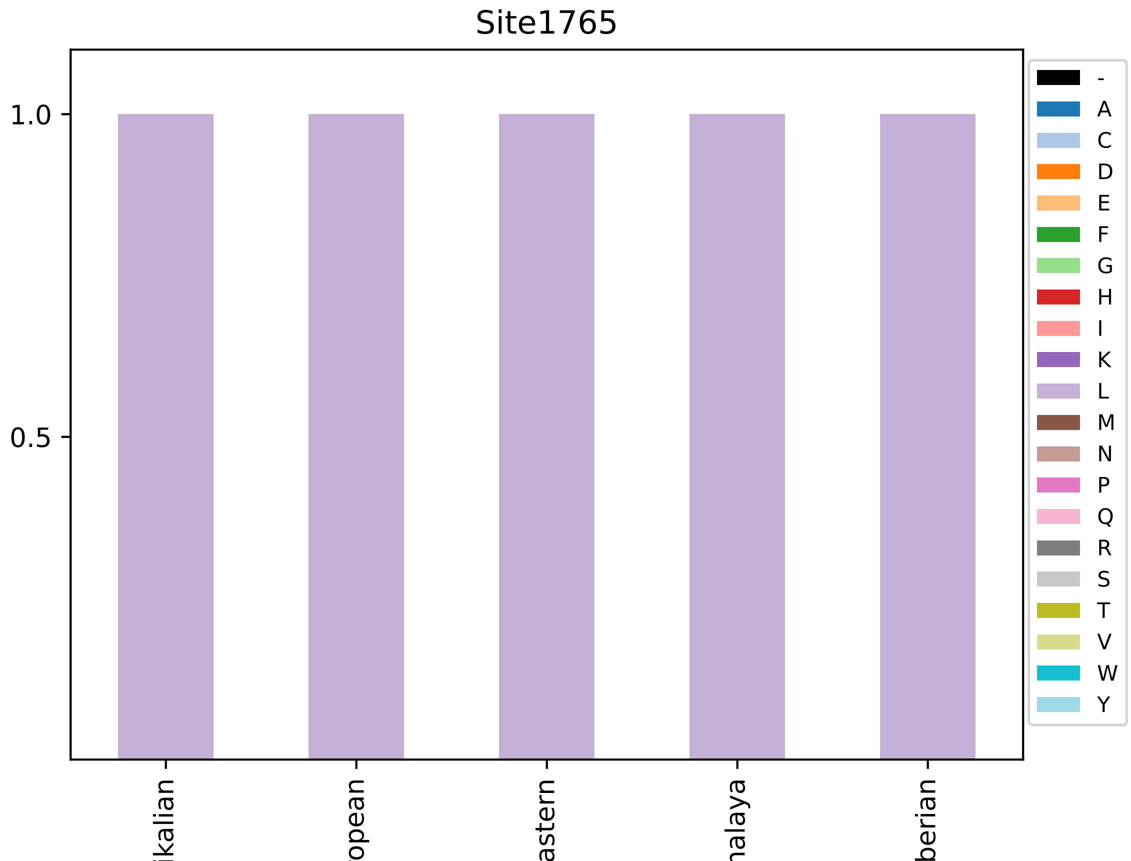

Site1765
1.0
0.5
-
A
C
D
E
F
G
H
I
K
L
M
N
P
Q
R
S
T
V
W
Y
ikalian
opean
astern
nalaya
berian

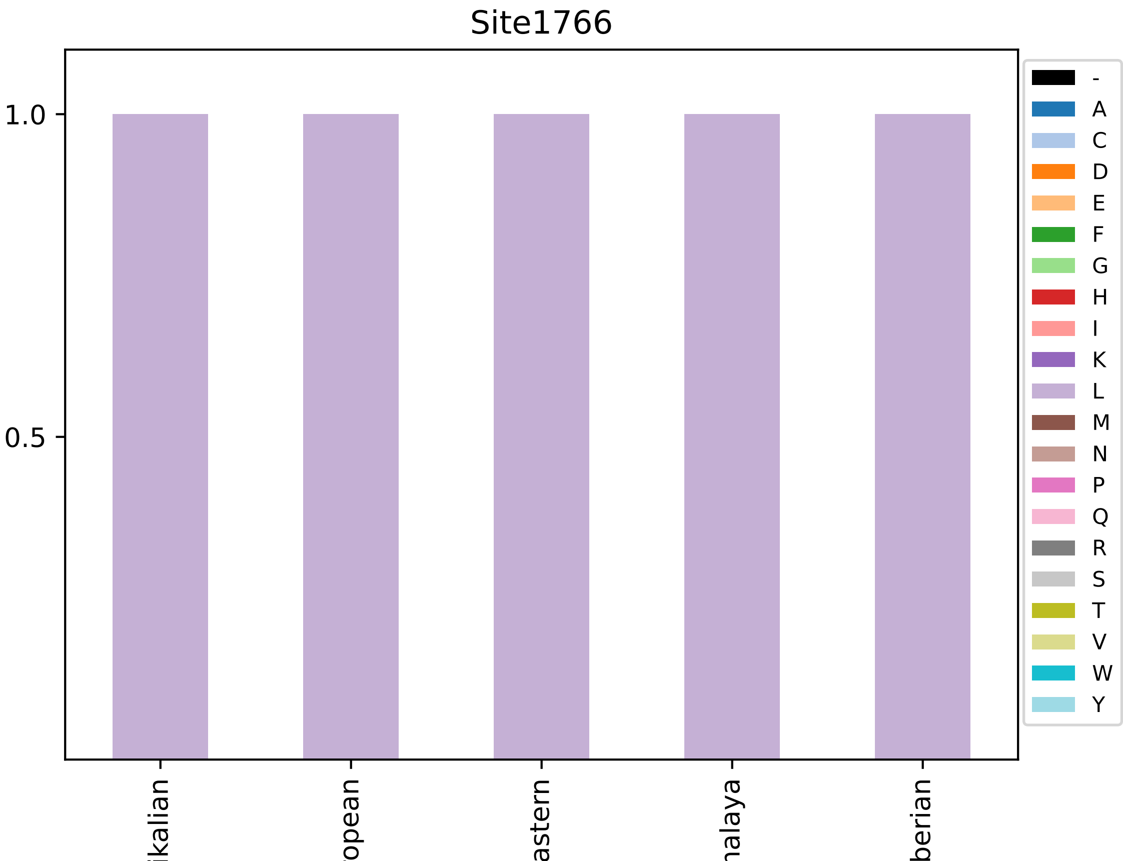
Site1766
1.0
0.5
-
A
C
D
E
F
G
H
I
K
L
M
N
P
Q
R
S
T
V
W
Y
ikalian
opean
astern
nalaya
berian

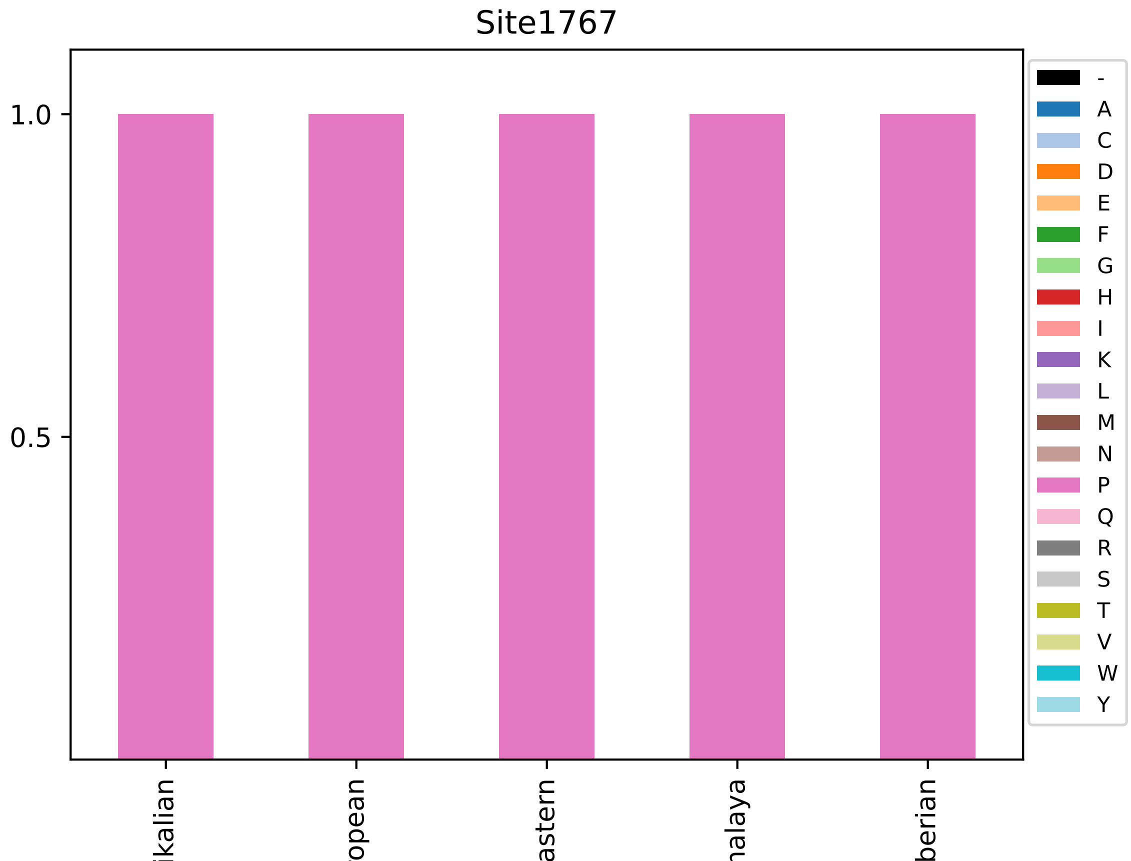
Site1767
1.0
0.5
-
A
C
D
E
F
G
H
I
K
L
M
N
P
Q
R
S
T
V
W
Y
ikalian
opean
astern
nalaya
berian

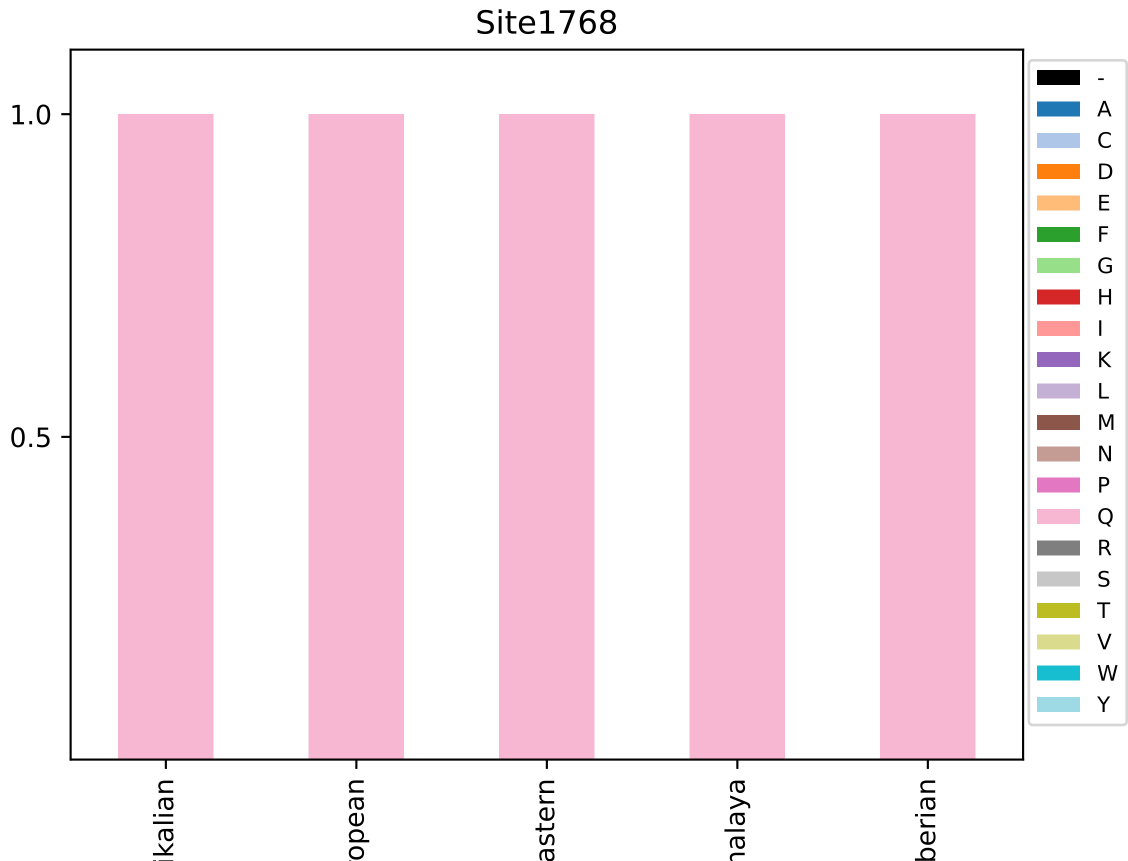
Site1768
1.0
0.5
-
A
C
D
E
F
G
H
I
K
L
M
N
P
Q
R
S
T
V
W
Y
ikalian
opean
astern
nalaya
berian

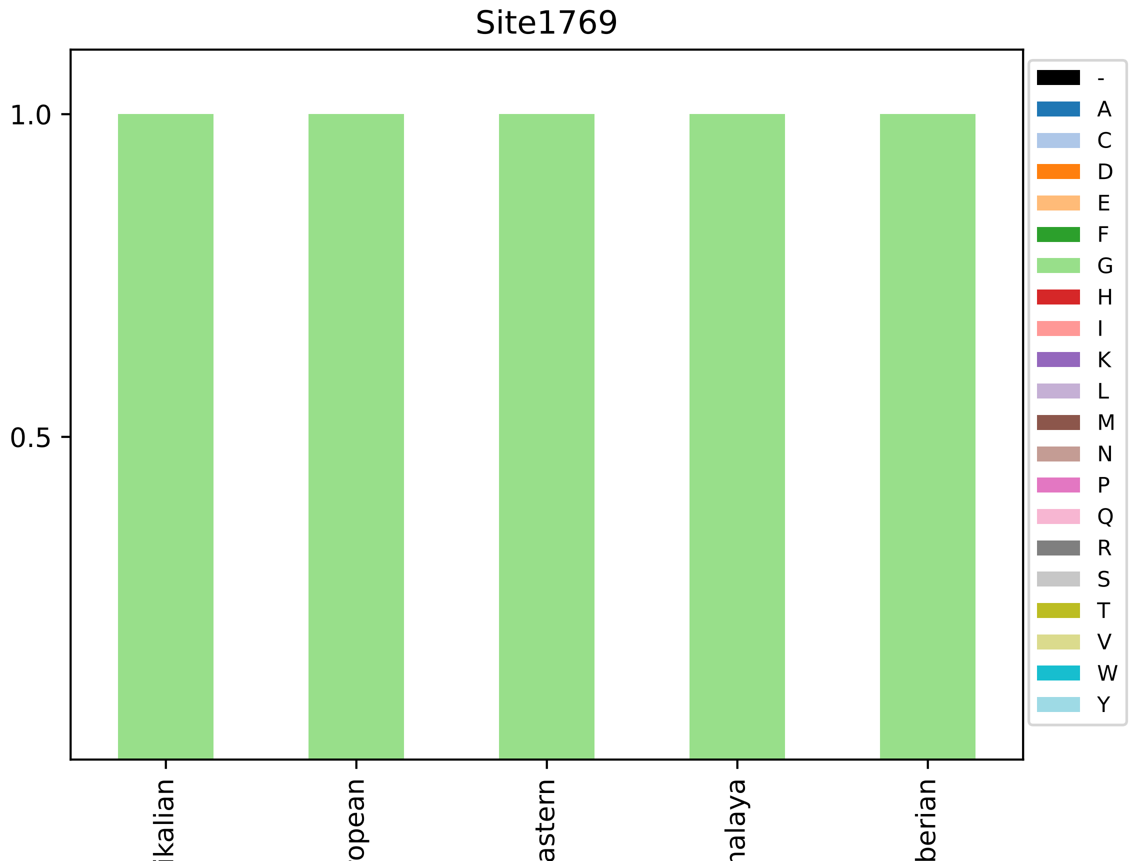

Site1769
1.0
0.5
-
A
C
D
E
F
G
H
I
K
L
M
N
P
Q
R
S
T
V
W
Y
ikalian
opean
astern
nalaya
berian

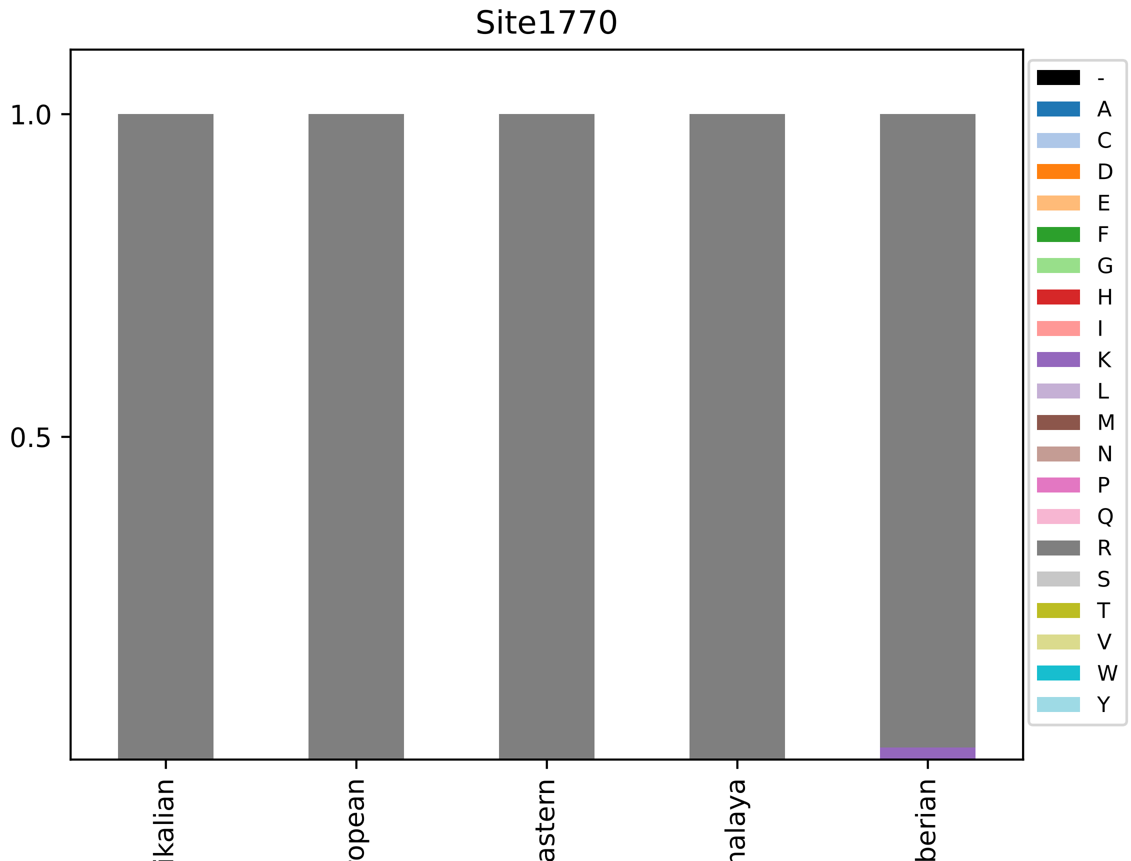
Site1770
1.0
0.5
ikalian
opean
astern
nalaya
berian
-
A
C
D
E
F
G
H
I
K
L
M
N
P
Q
R
S
T
V
W
Y

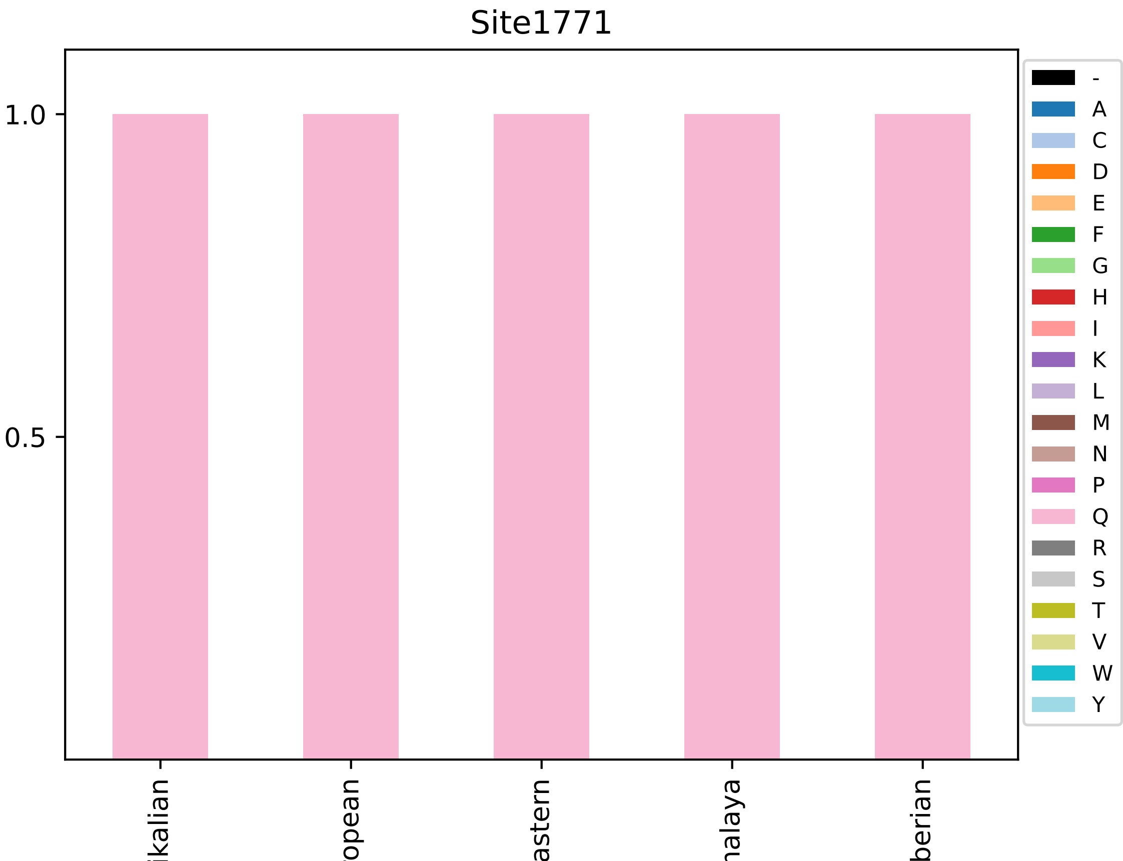

Site1771
1.0
0.5
-
A
C
D
E
F
G
H
I
K
L
M
N
P
Q
R
S
T
V
W
Y
ikalian
opean
astern
nalaya
berian

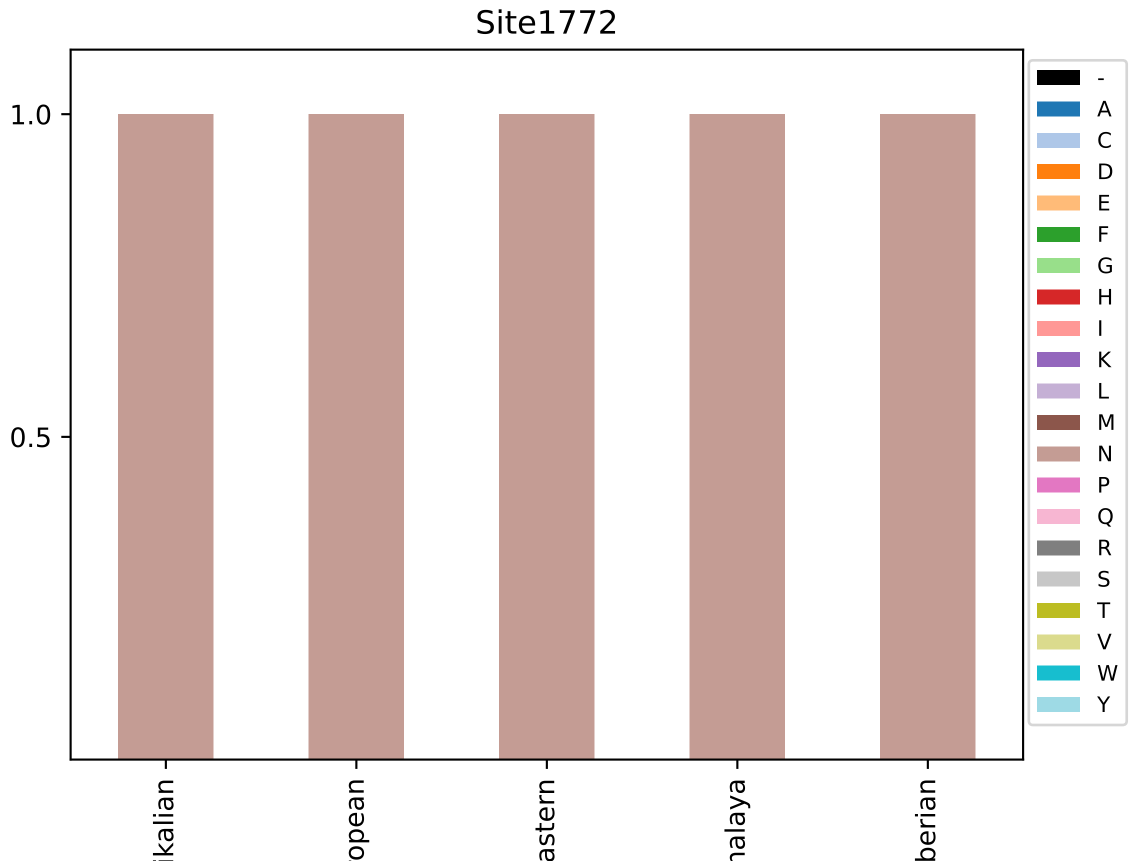

Site1772
1.0
0.5
-
A
C
D
E
F
G
H
I
K
L
M
N
P
Q
R
S
T
V
W
Y
ikalian
opean
astern
nalaya
berian

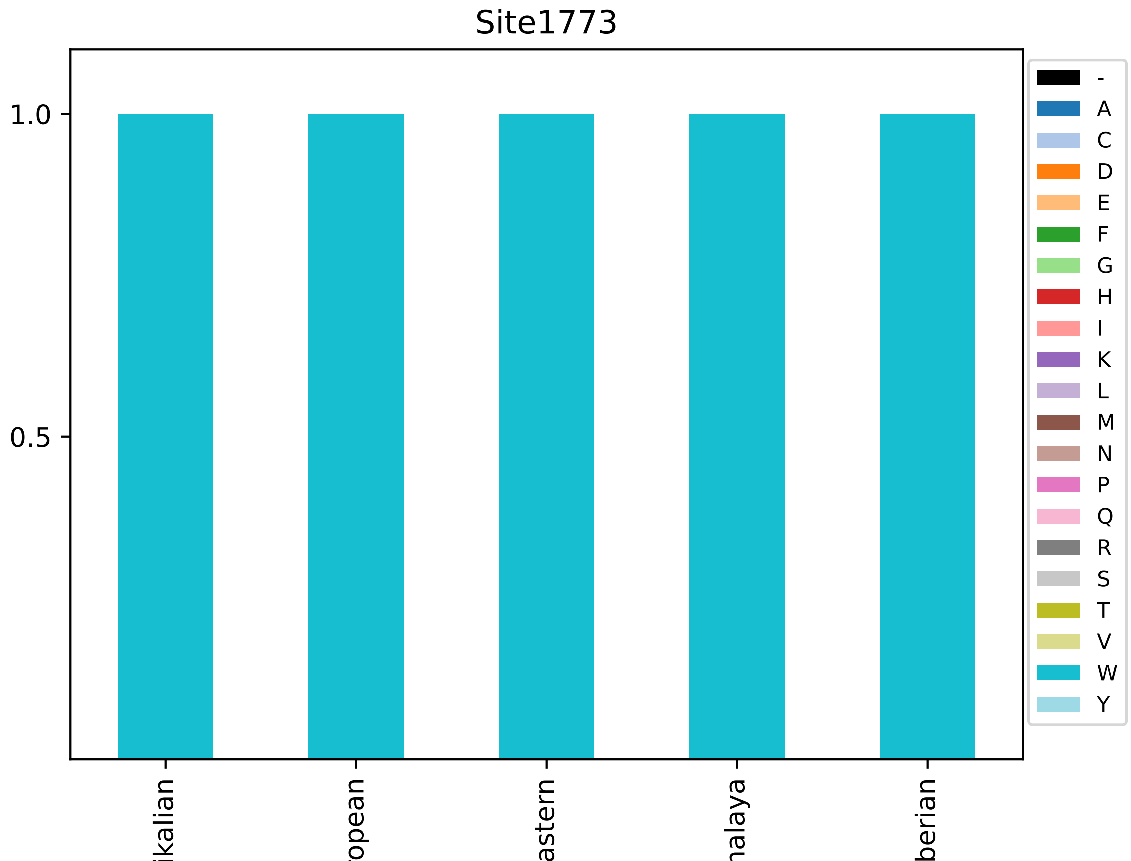

Site1773
1.0
0.5
-
A
C
D
E
F
G
H
I
K
L
M
N
P
Q
R
S
T
V
W
Y
ikalian
opean
astern
nalaya
berian

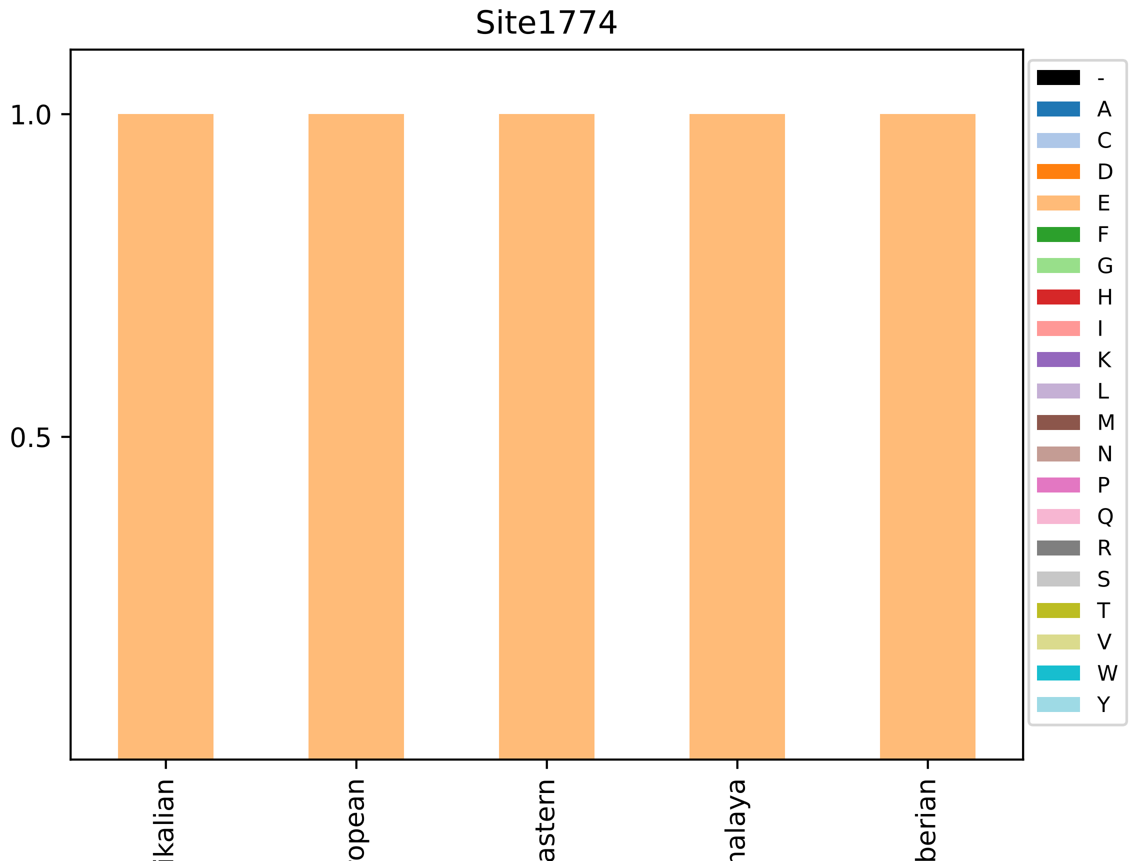
Site1774
1.0
0.5
-
A
C
D
E
F
G
H
I
K
L
M
N
P
Q
R
S
T
V
W
Y
ikalian
opean
astern
nalaya
berian

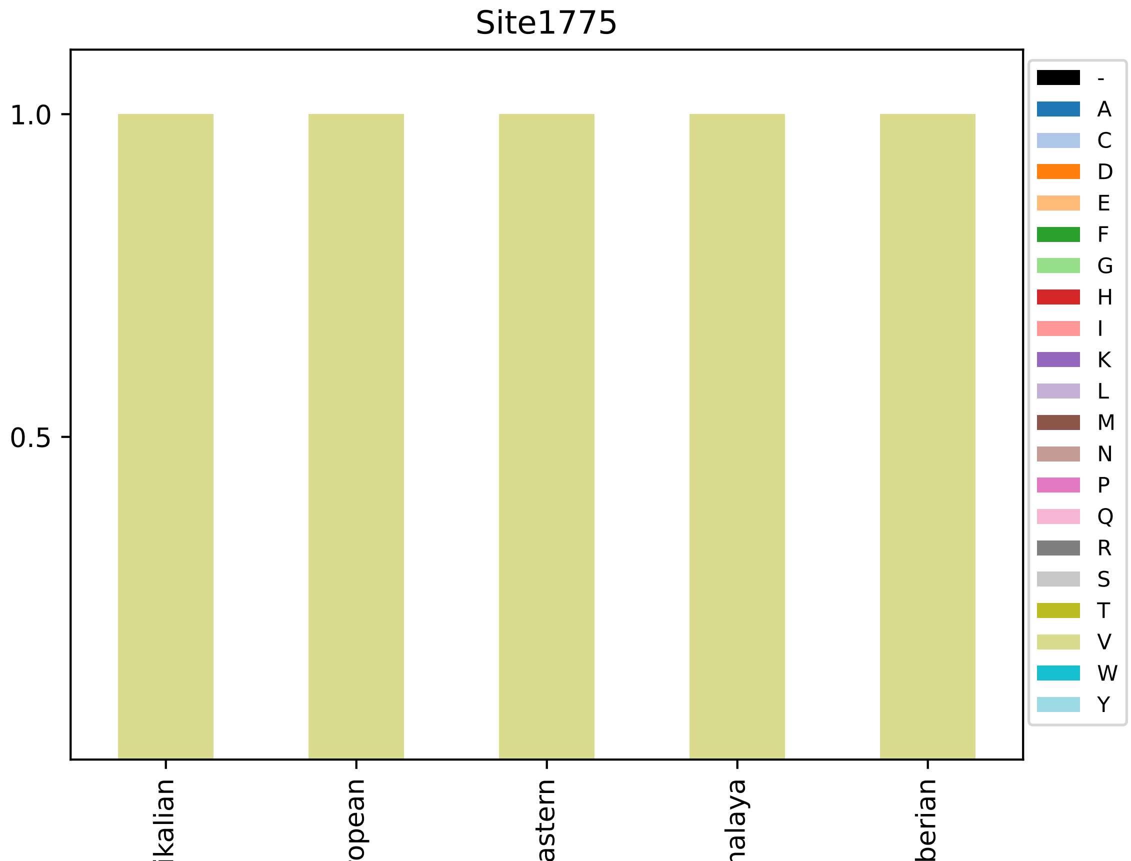

Site1775
1.0
0.5
-
A
C
D
E
F
G
H
I
K
L
M
N
P
Q
R
S
T
V
W
Y
ikalian
opean
astern
nalaya
berian

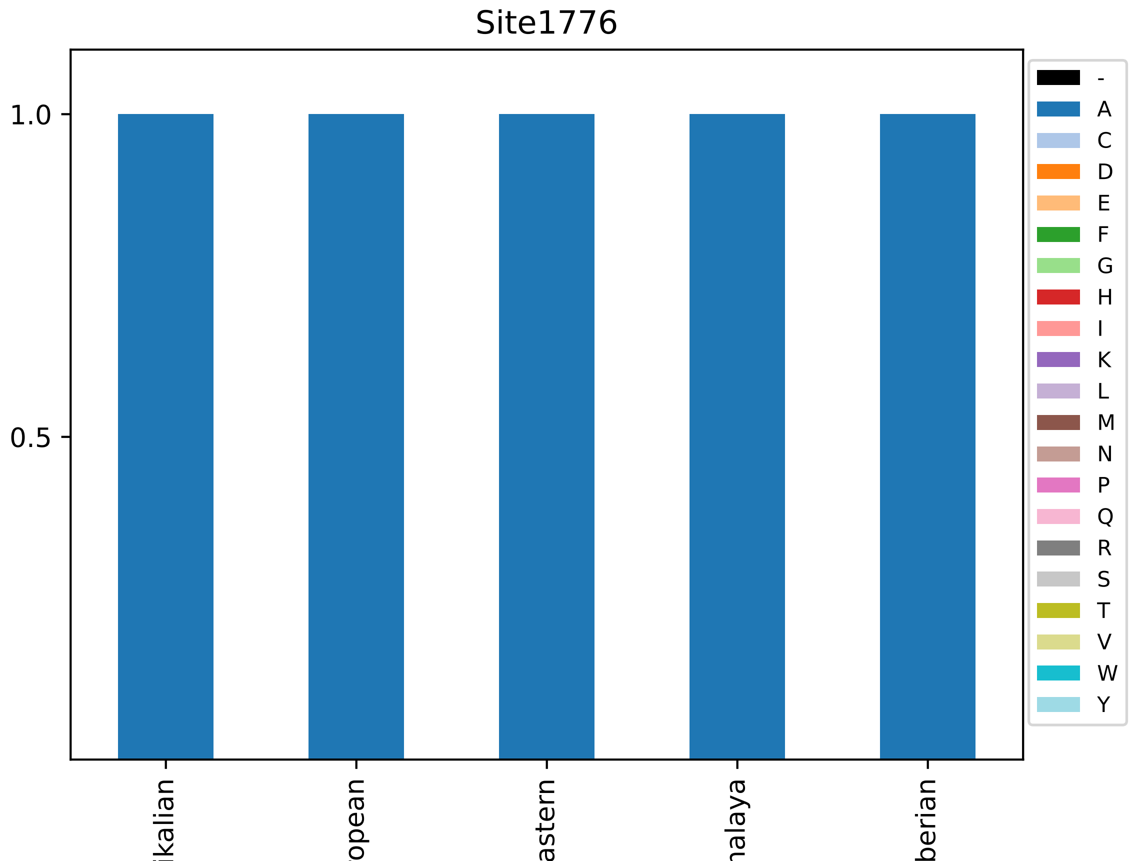

Site1776
1.0
0.5
-
A
C
D
E
F
G
H
I
K
L
M
N
P
Q
R
S
T
V
W
Y
ikalian
ropean
astern
nalaya
berian

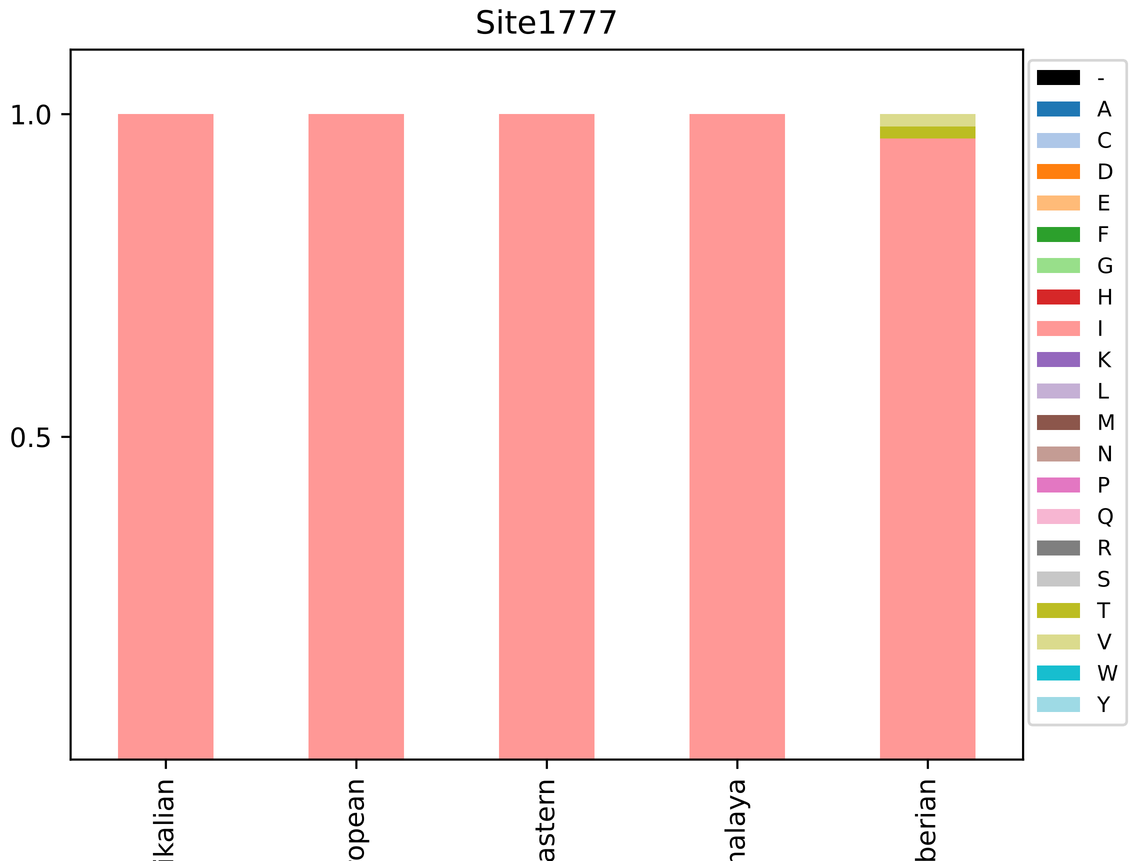
Site1777
1.0
0.5
-
A
C
D
E
F
G
H
I
K
L
M
N
P
Q
R
S
T
V
W
Y
ikalian
opean
astern
nalaya
berian

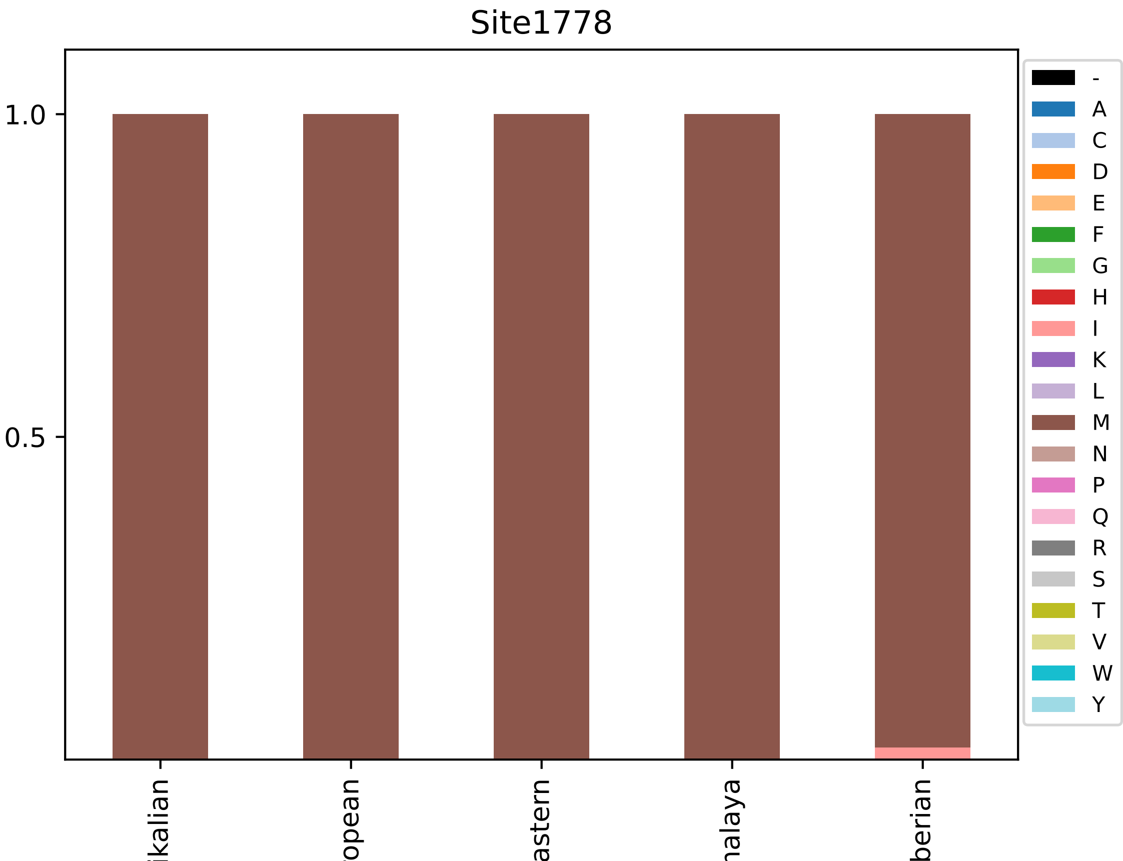

Site1778
1.0
0.5
ikalian
opean
astern
nalaya
berian
-
A
C
D
E
F
G
H
I
K
L
M
N
P
Q
R
S
T
V
W
Y

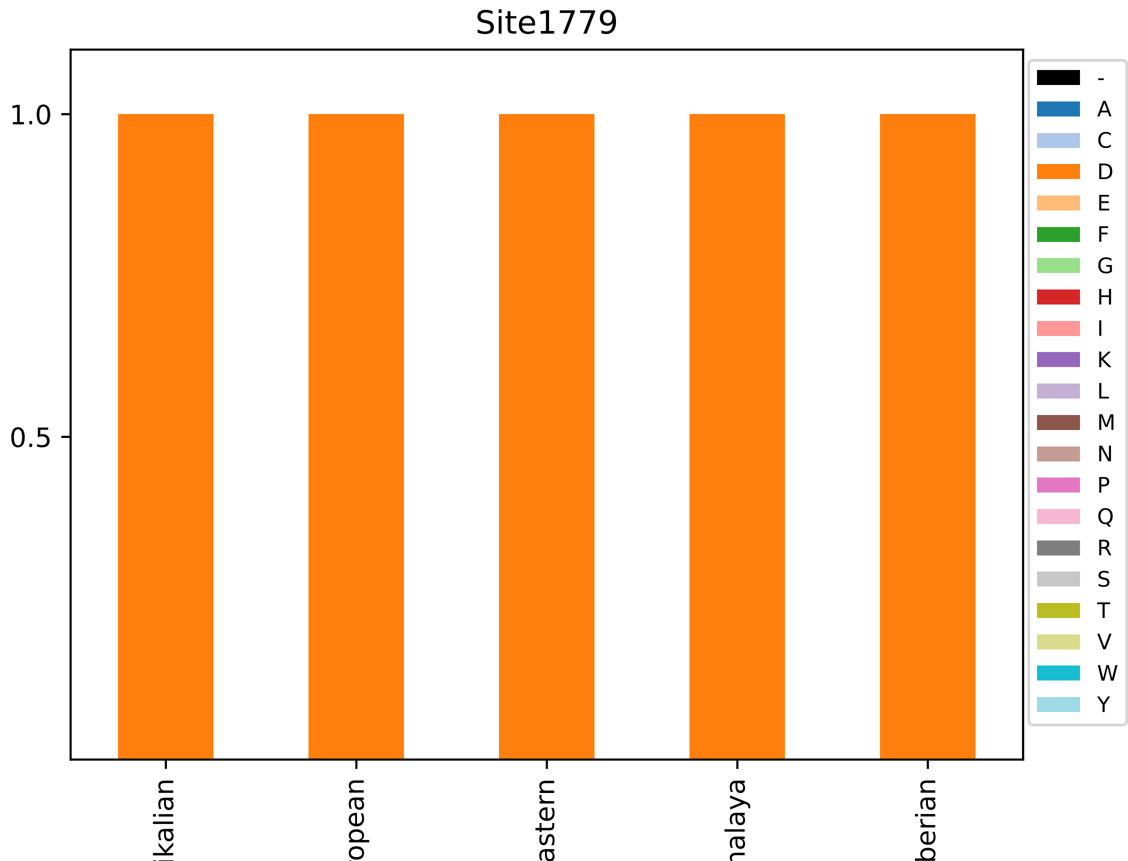
Site1779
1.0
0.5
-
A
C
D
E
F
G
H
I
K
L
M
N
P
Q
R
S
T
V
W
Y
ikalian
opean
astern
nalaya
berian

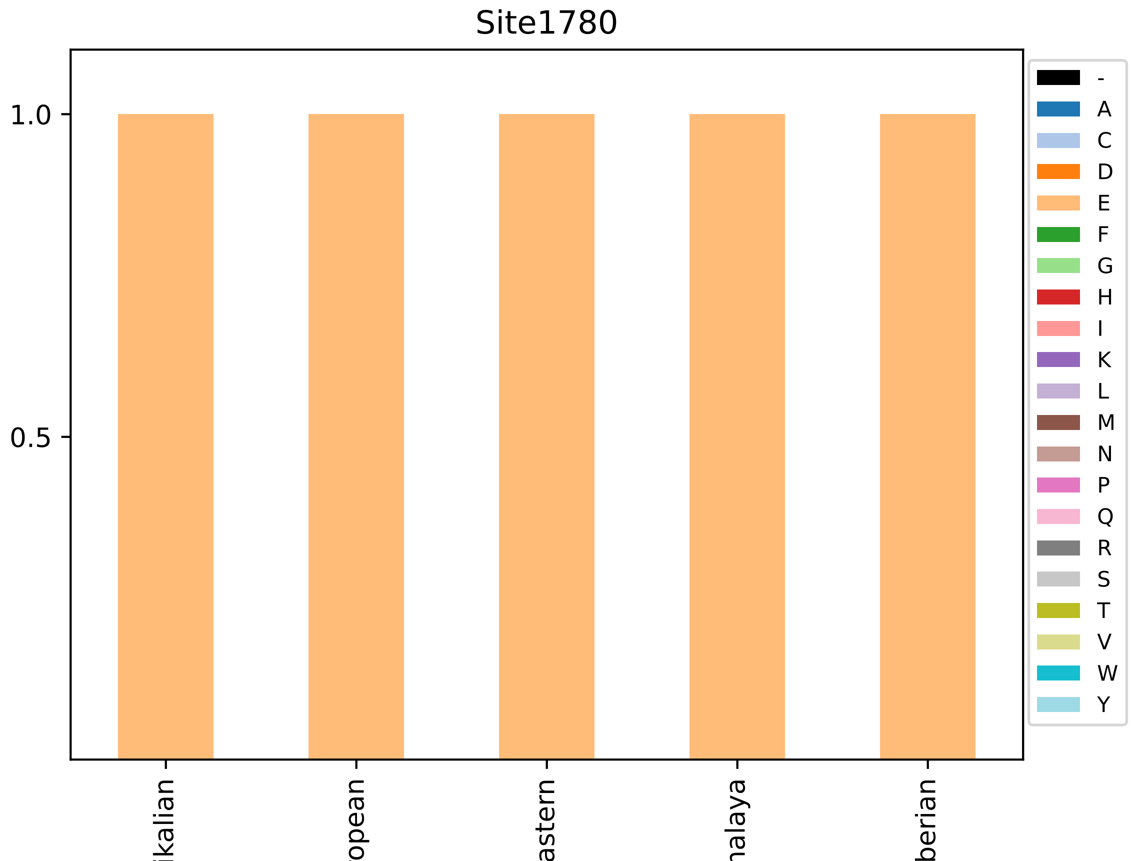

Site1780
1.0
0.5
-
A
C
D
E
F
G
H
I
K
L
M
N
P
Q
R
S
T
V
W
Y
ikalian
opean
astern
nalaya
berian

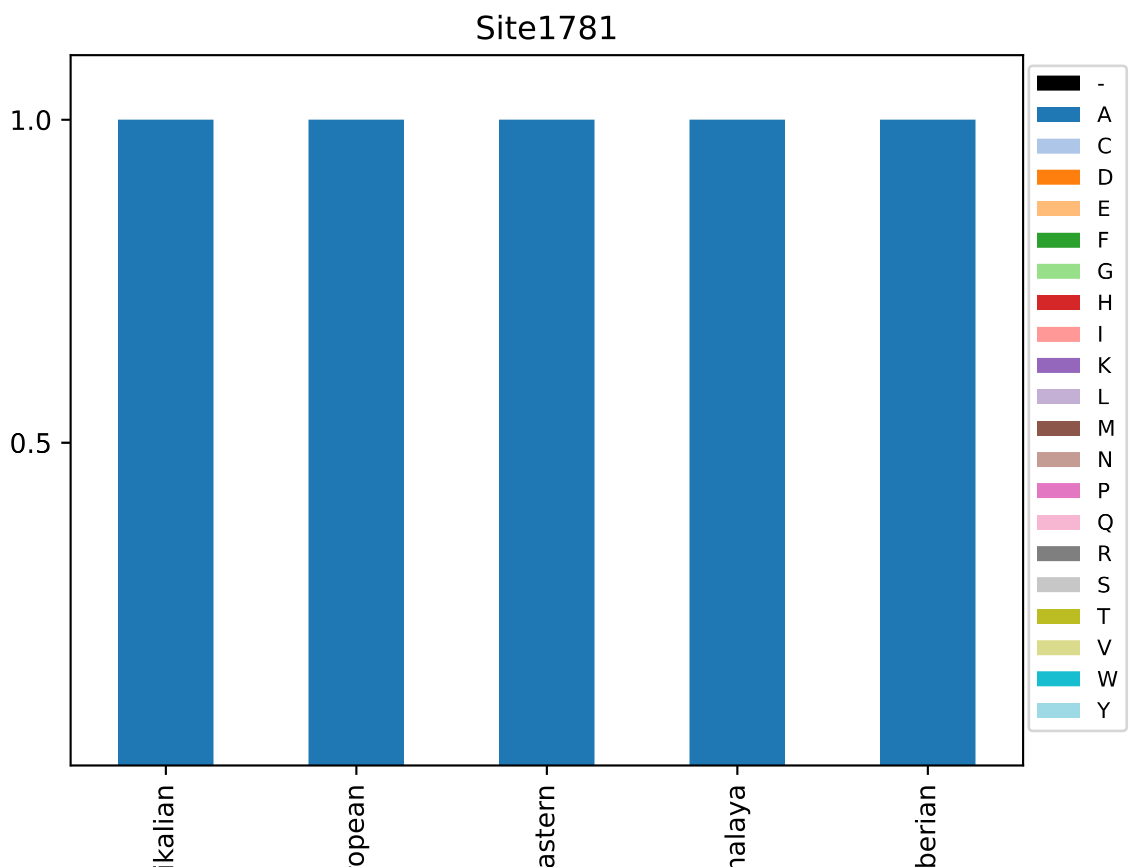
Site1781
1.0
0.5
-
A
C
D
E
F
G
H
I
K
L
M
N
P
Q
R
S
T
V
W
Y
ikalian
opean
astern
nalaya
berian

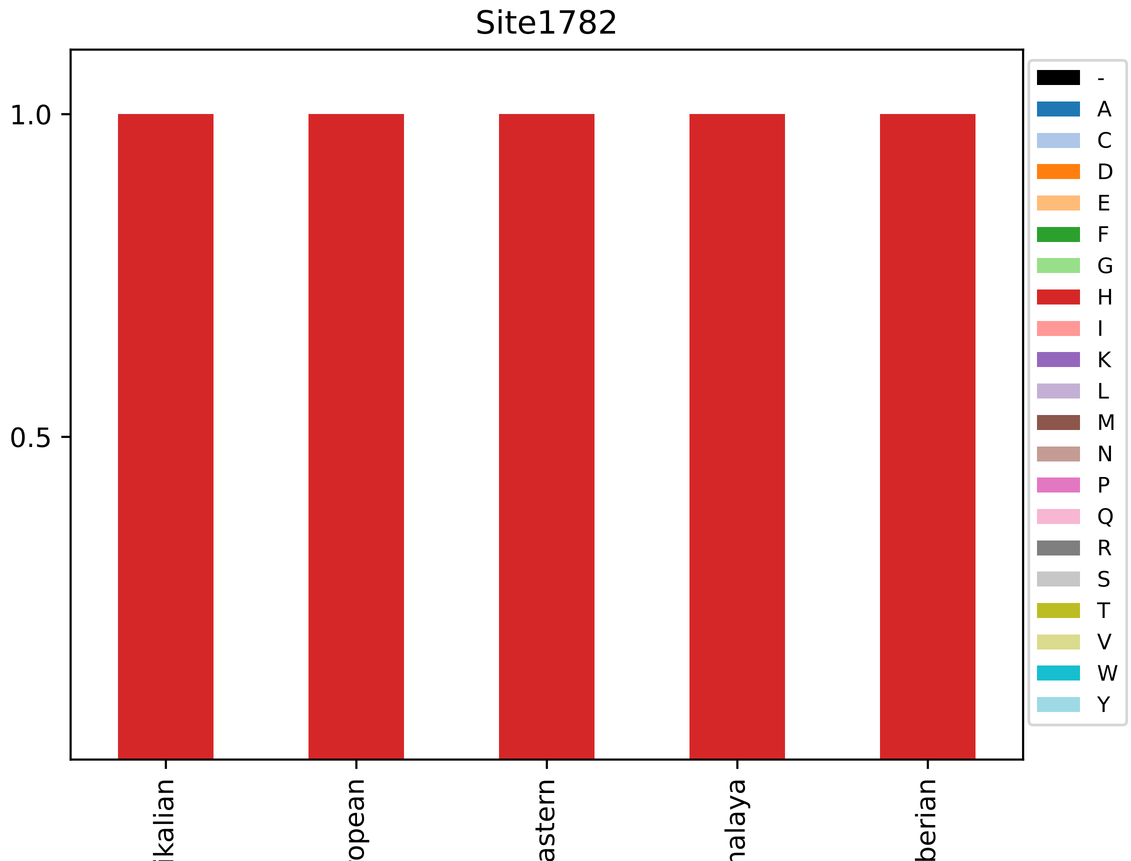

Site1782
1.0
0.5
-
A
C
D
E
F
G
H
I
K
L
M
N
P
Q
R
S
T
V
W
Y
ikalian
ropean
astern
nalaya
berian

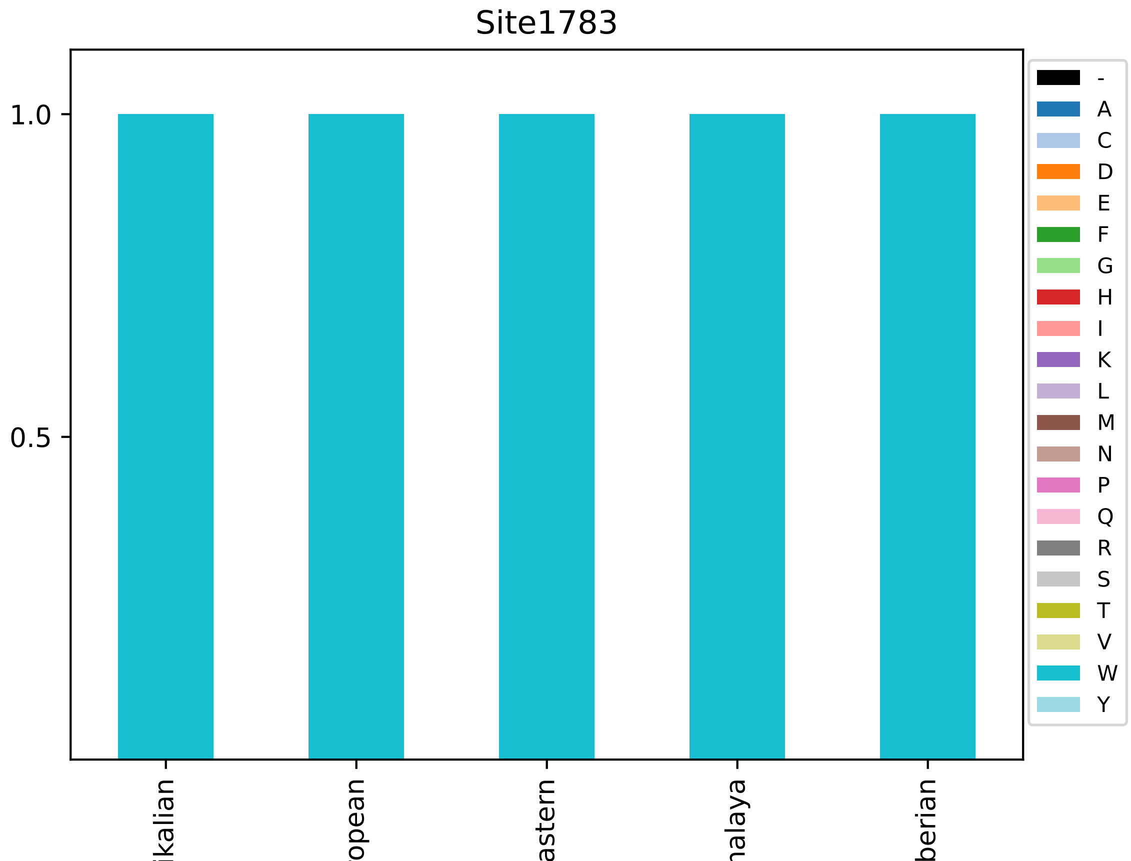
Site1783
1.0
0.5
-
A
C
D
E
F
G
H
I
K
L
M
N
P
Q
R
S
T
V
W
Y
ikalian
opean
astern
nalaya
berian

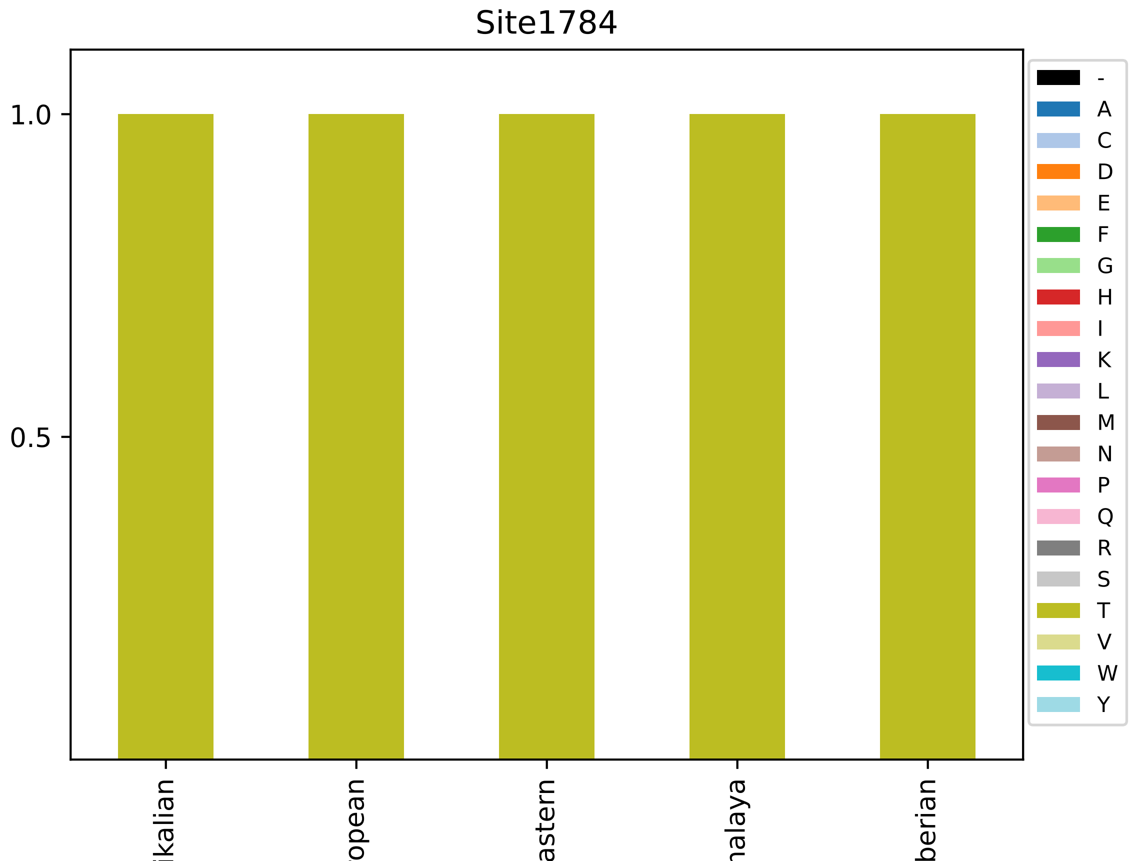

Site1784
1.0
0.5
-
A
C
D
E
F
G
H
I
K
L
M
N
P
Q
R
S
T
V
W
Y

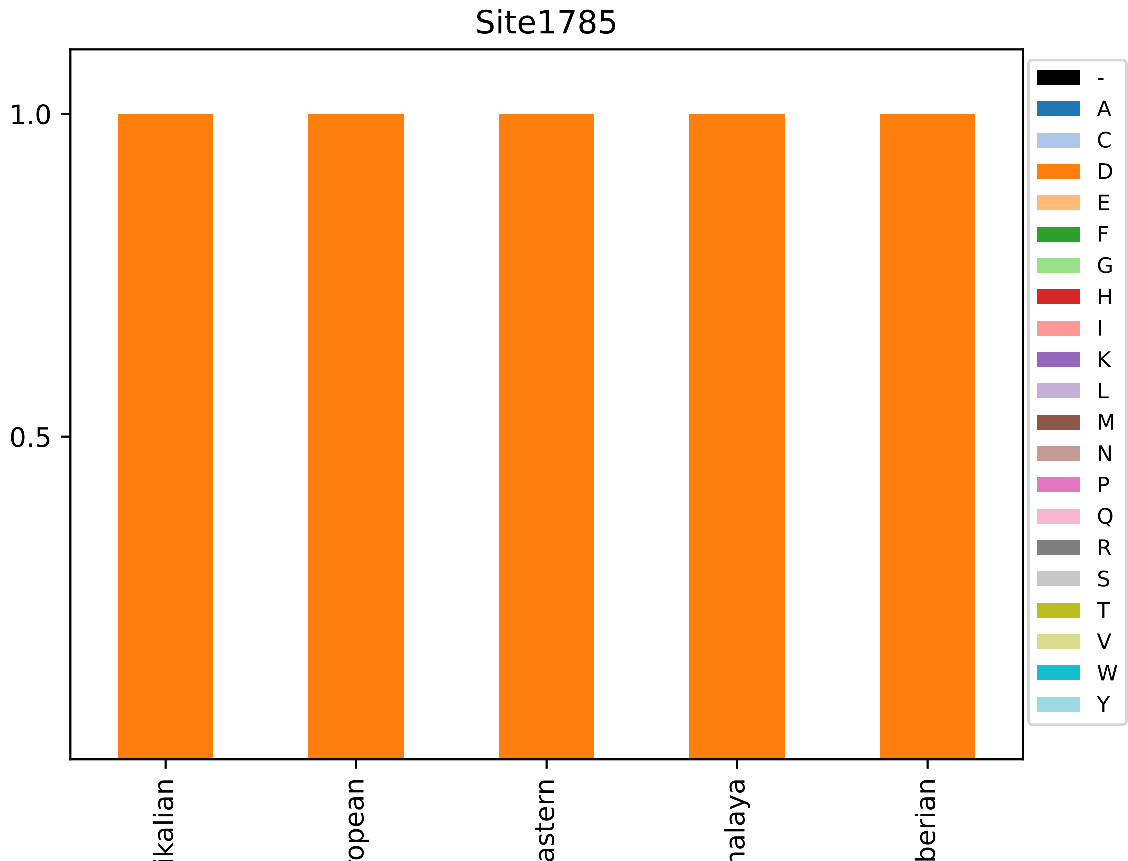

Site1785
1.0
0.5
ikalian
opean
astern
alaya
berian
-
A
C
D
E
F
G
H
I
K
L
M
N
P
Q
R
S
T
V
W
Y

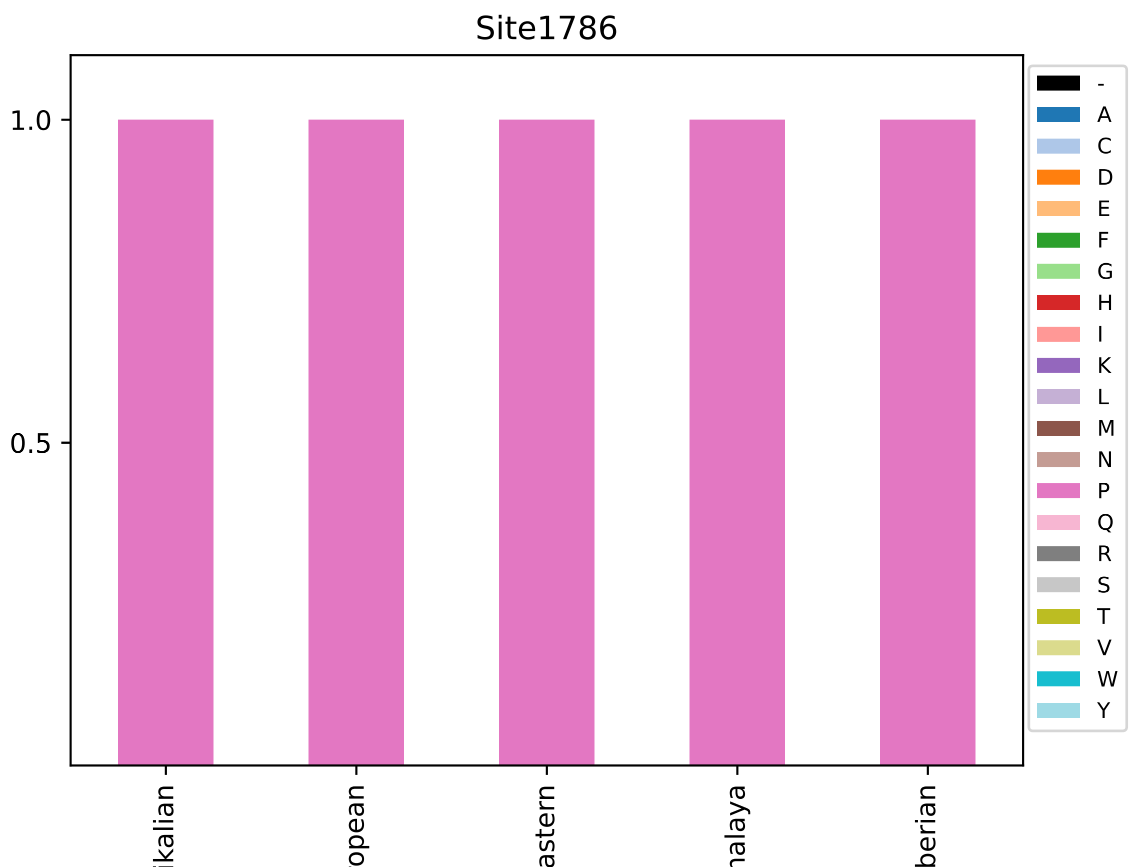
Site1786
1.0
0.5
-
A
C
D
E
F
G
H
I
K
L
M
N
P
Q
R
S
T
V
W
Y
ikalian
opean
astern
nalaya
berian

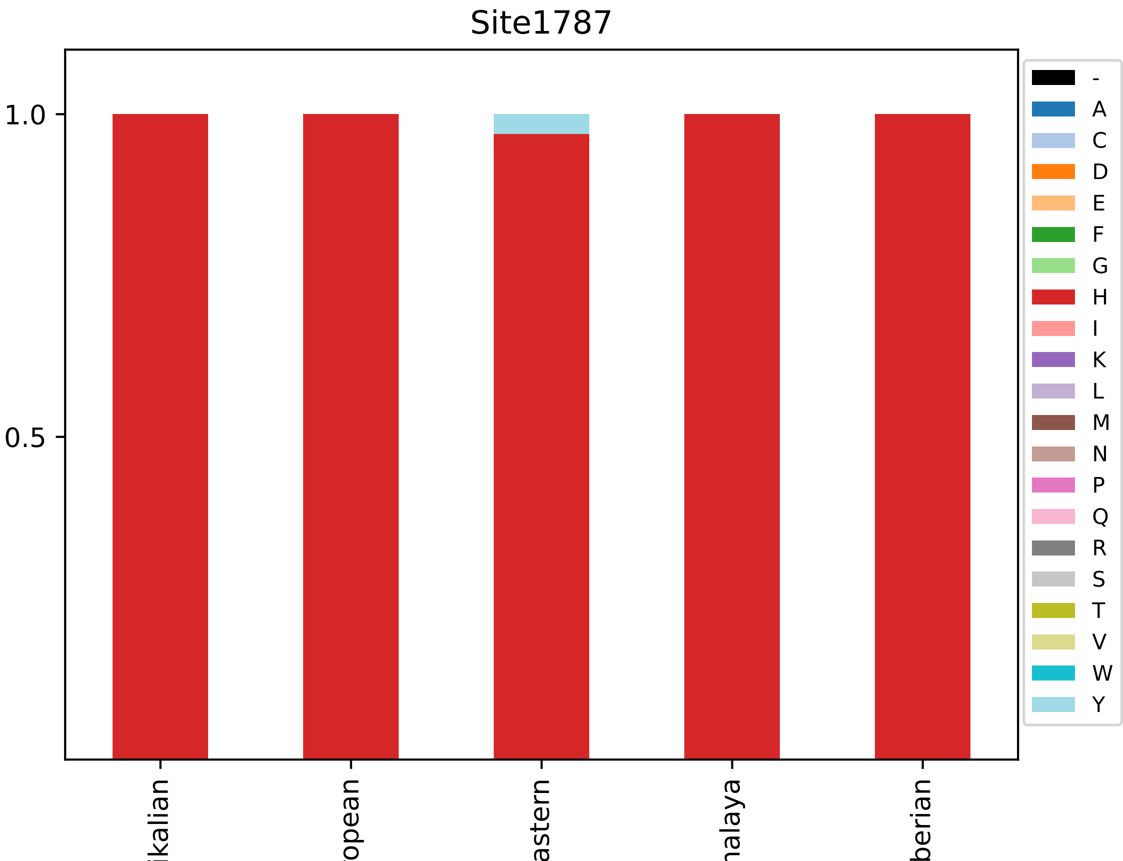

Site1787
1.0
0.5
ikalian
opean
astern
nalaya
berian
-
A
C
D
E
F
G
H
I
K
L
M
N
P
Q
R
S
T
V
W
Y

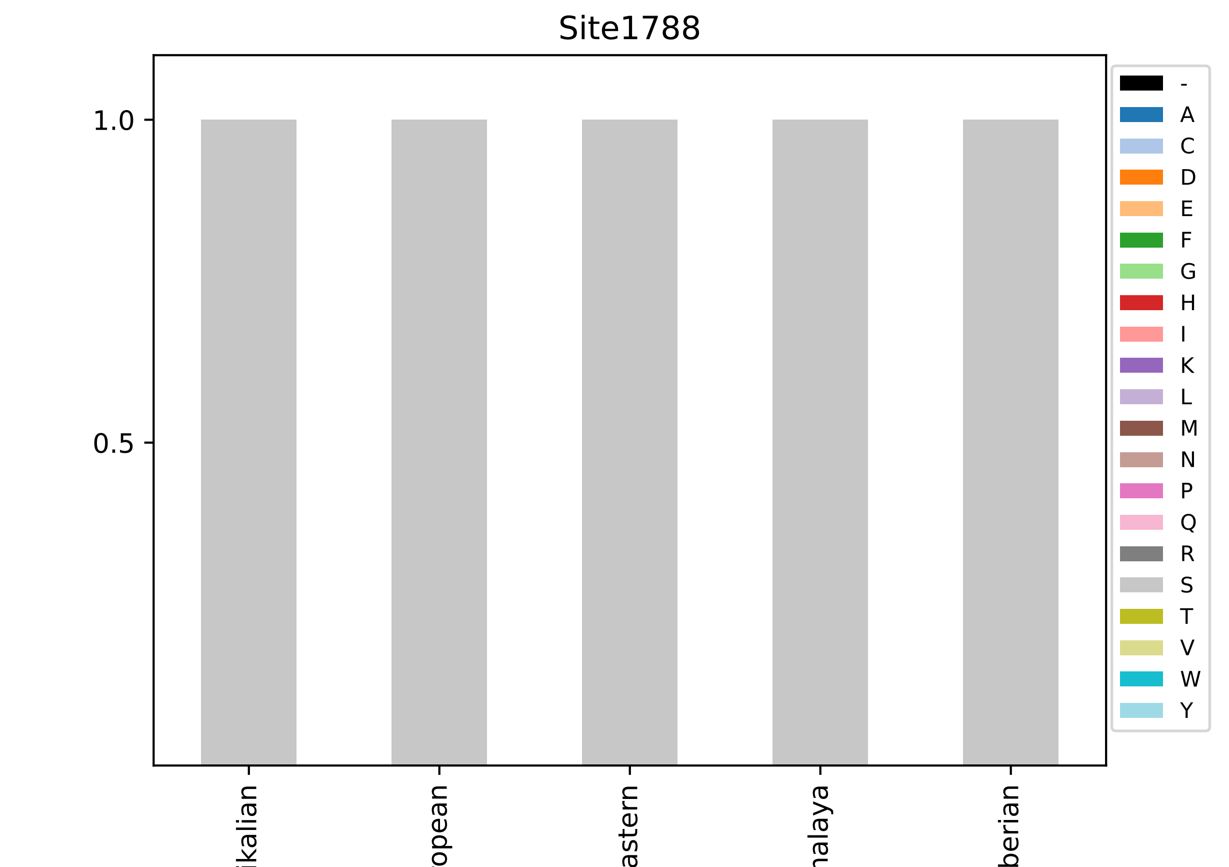

Site1788
1.0
0.5
-
A
C
D
E
F
G
H
I
K
L
M
N
P
Q
R
S
T
V
W
Y
ikalian
opean
astern
nalaya
berian

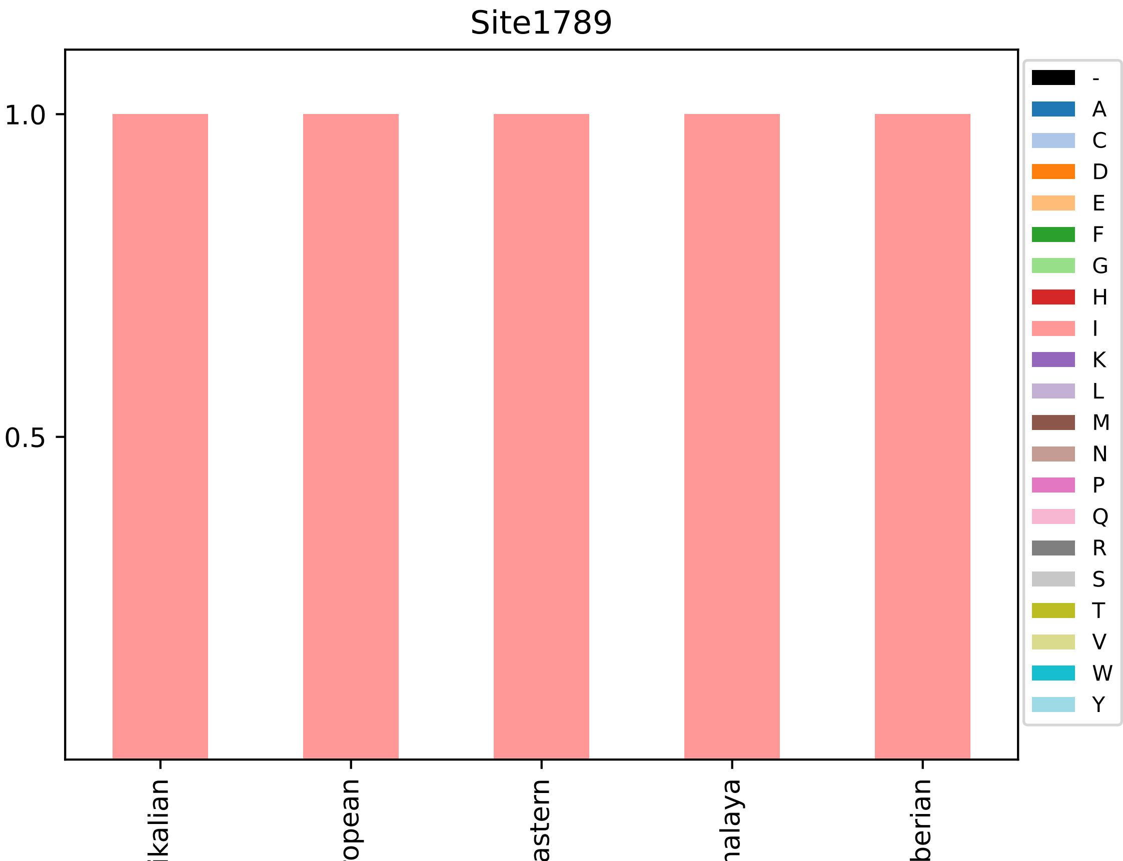

Site1789
1.0
0.5
-
A
C
D
E
F
G
H
I
K
L
M
N
P
Q
R
S
T
V
W
Y
ikalian
opean
astern
nalaya
berian

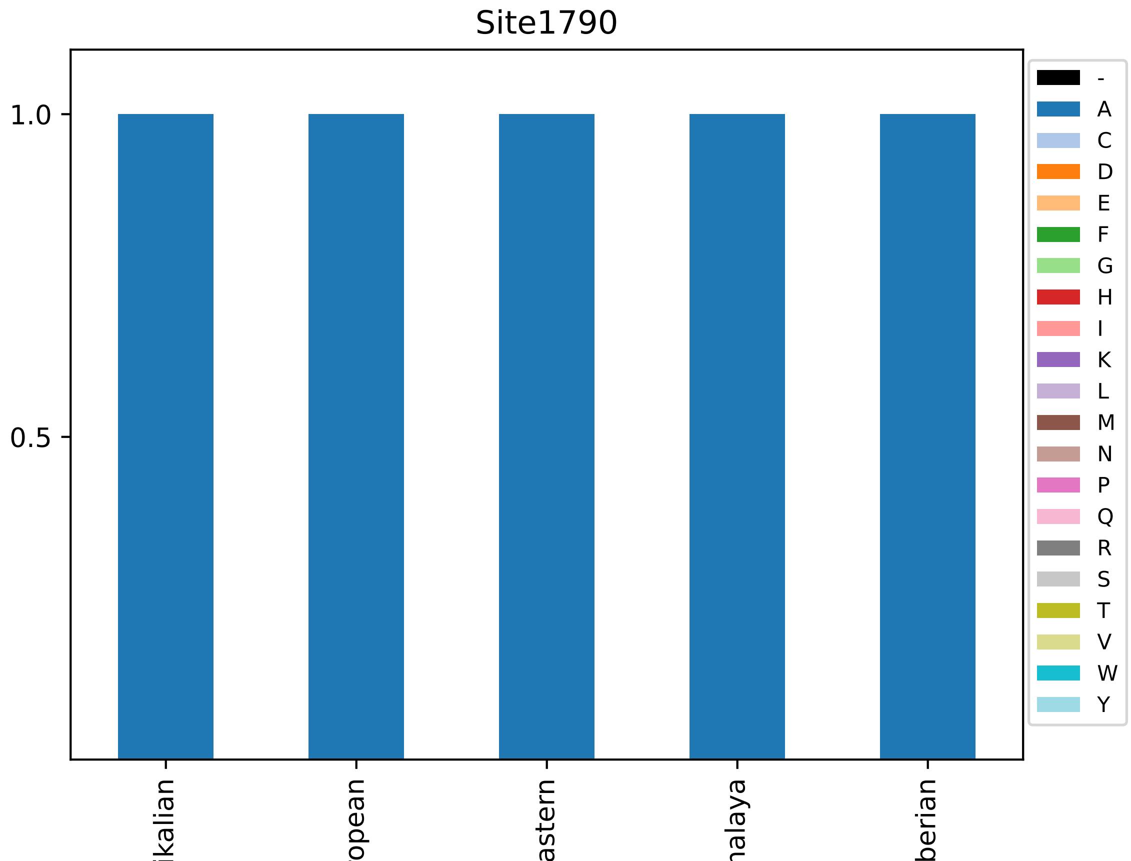

Site1790
1.0
0.5
-
A
C
D
E
F
G
H
I
K
L
M
N
P
Q
R
S
T
V
W
Y
ikalian
opean
astern
nalaya
berian

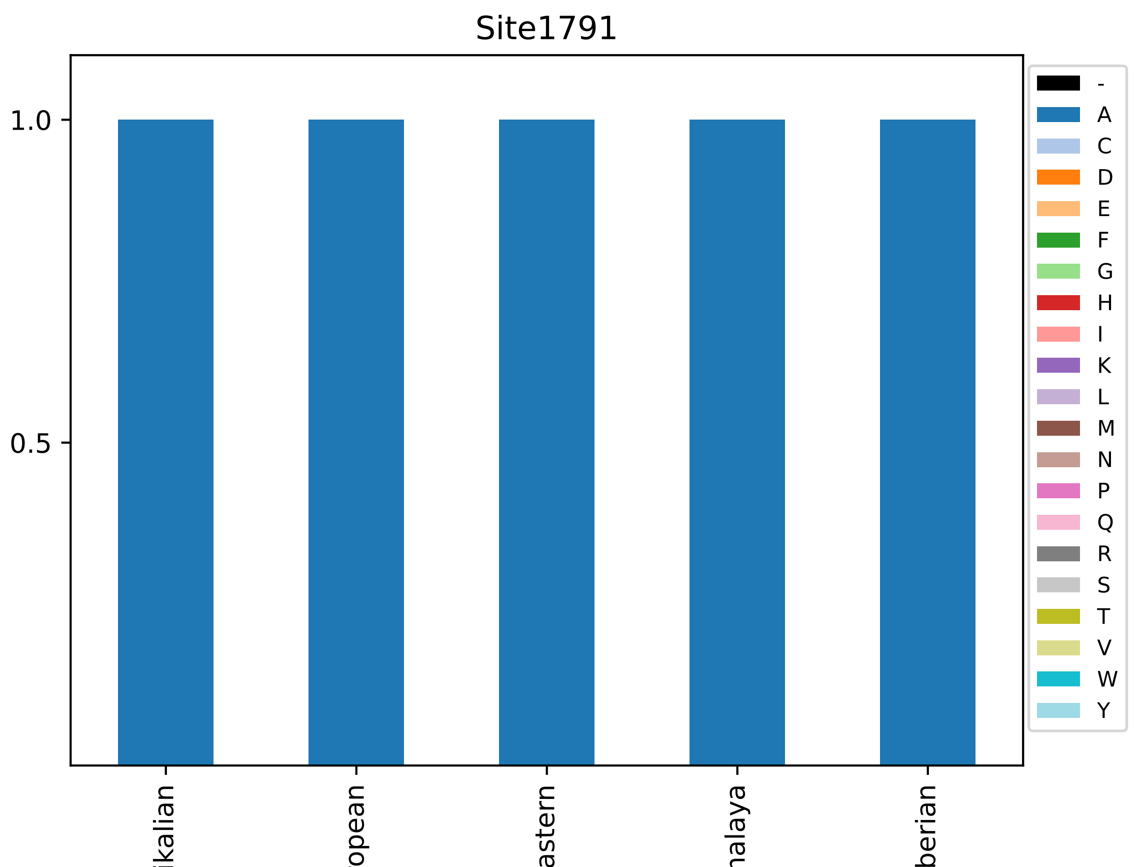

Site1791
1.0
0.5
-
A
C
D
E
F
G
H
I
K
L
M
N
P
Q
R
S
T
V
W
Y
ikalian
opean
astern
nalaya
berian

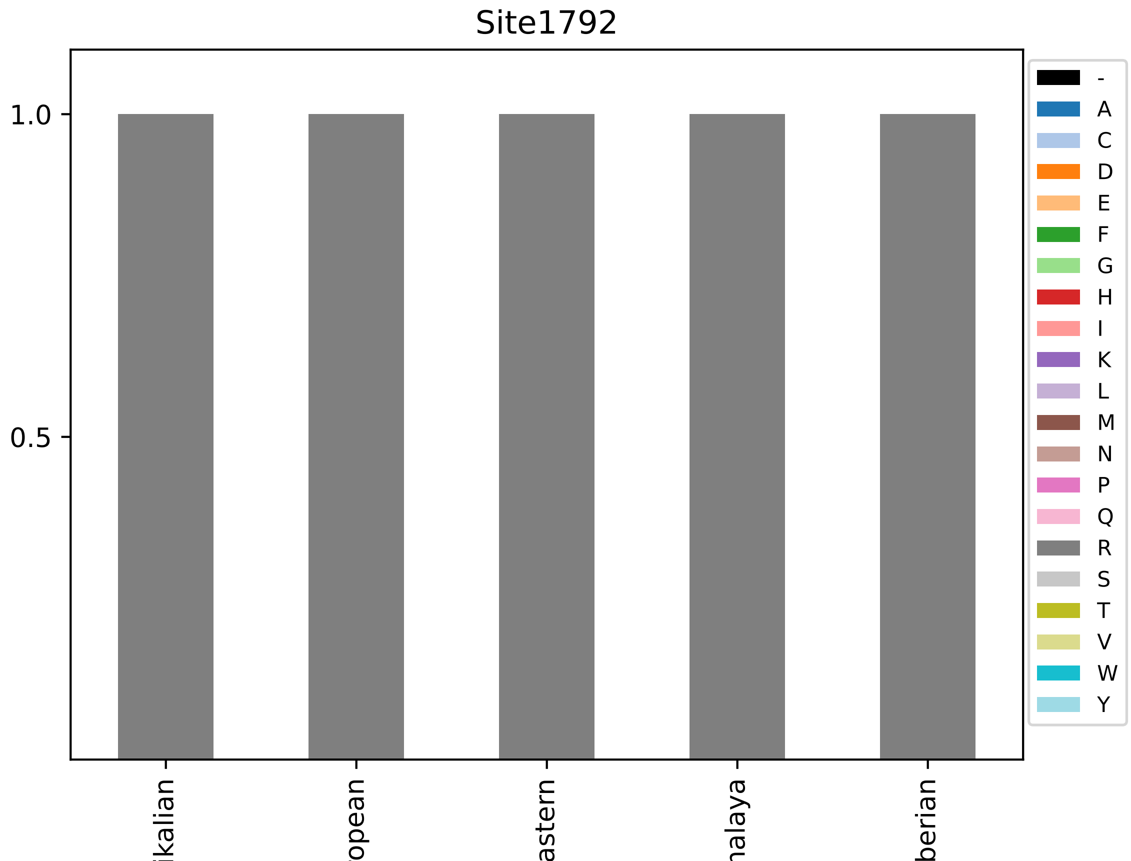

Site1792
1.0
0.5
-
A
C
D
E
F
G
H
I
K
L
M
N
P
Q
R
S
T
V
W
Y
ikalian
opean
astern
nalaya
berian

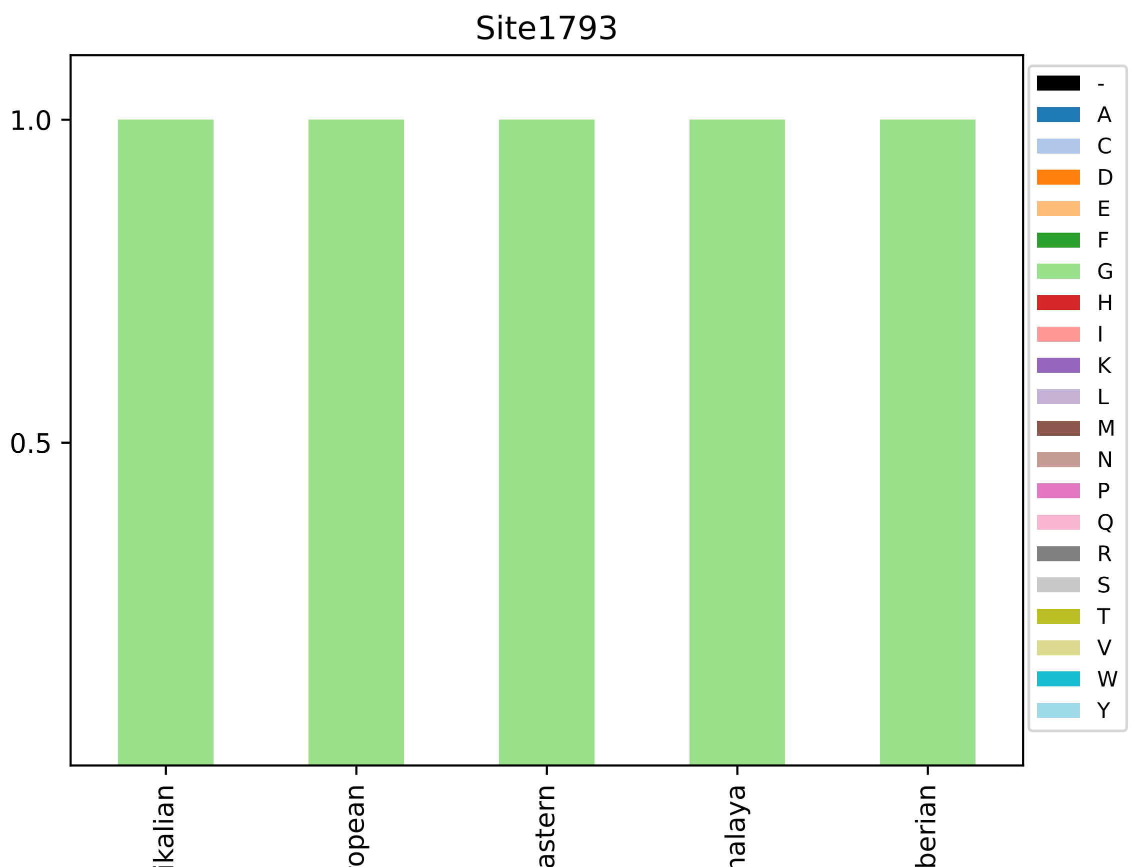
Site1793
1.0
0.5
-
A
C
D
E
F
G
H
I
K
L
M
N
P
Q
R
S
T
V
W
Y
ikalian
opean
astern
nalaya
berian

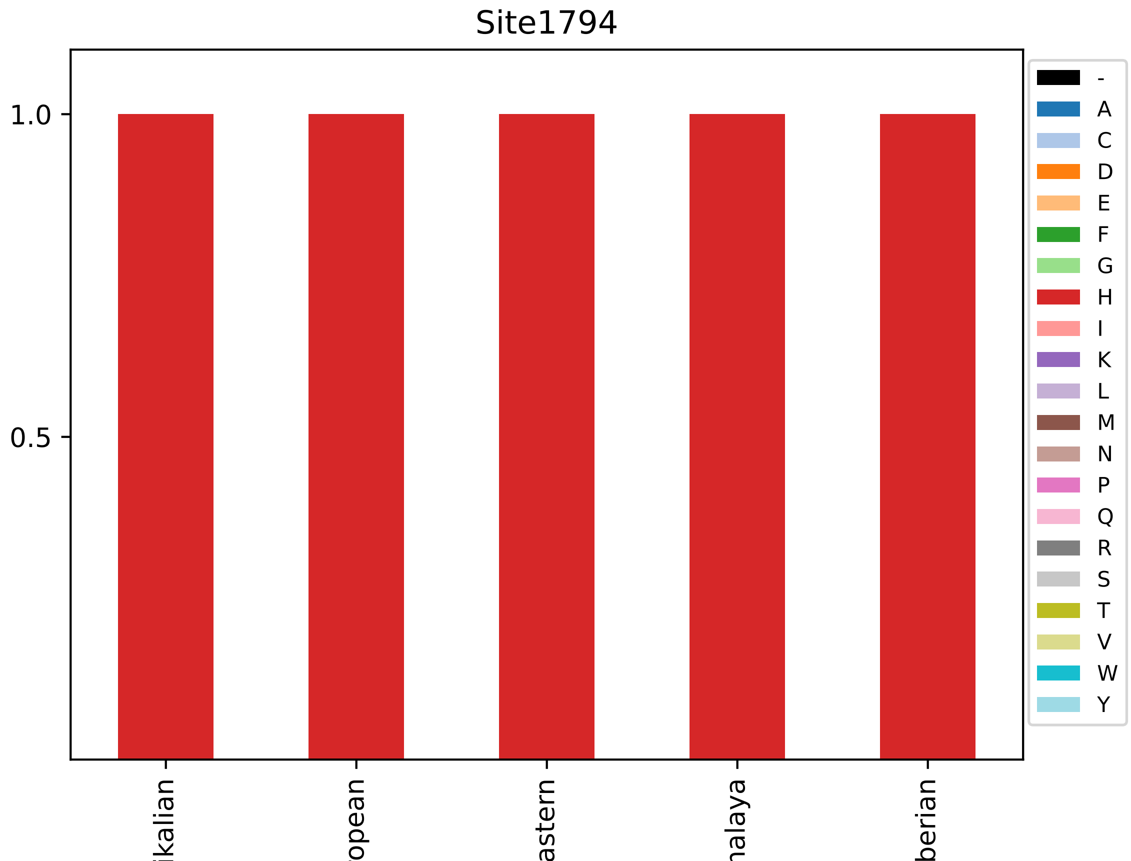
Site1794
1.0
0.5
-
A
C
D
E
F
G
H
I
K
L
M
N
P
Q
R
S
T
V
W
Y
ikalian
opean
astern
nalaya
berian

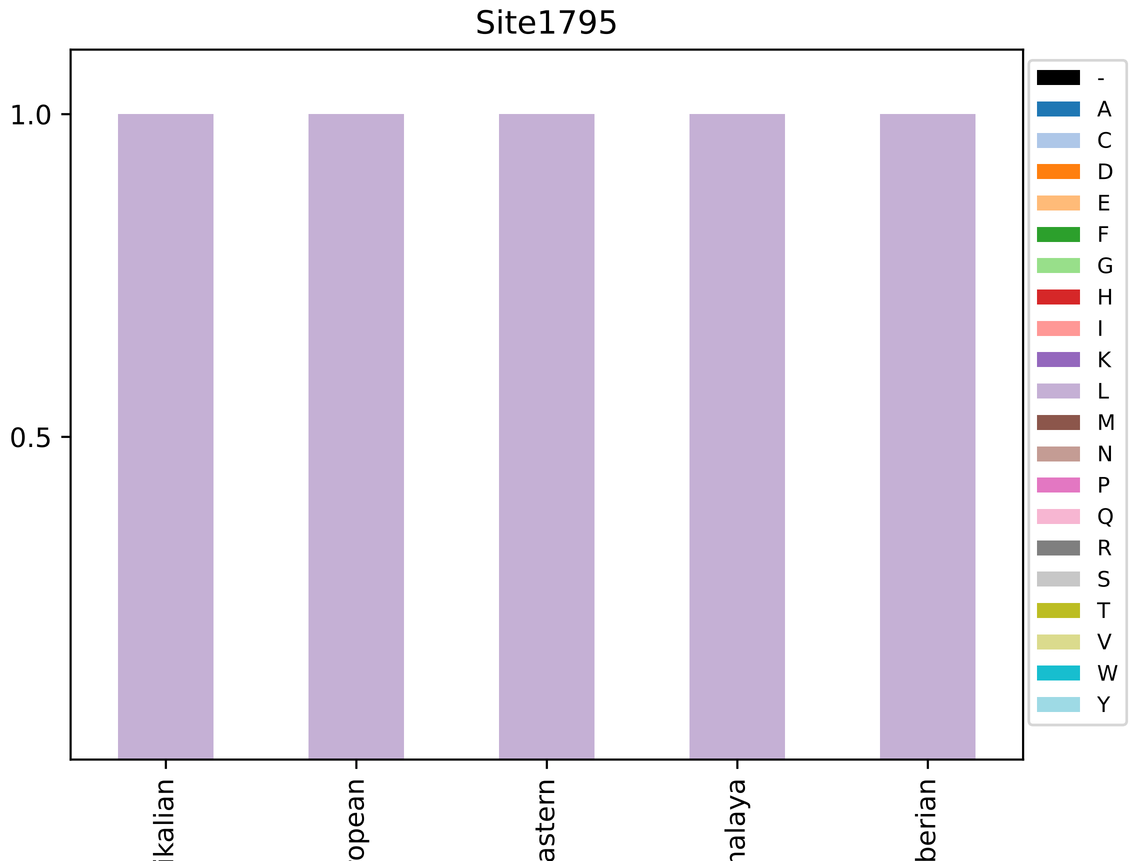

Site1795
1.0
0.5
-
A
C
D
E
F
G
H
I
K
L
M
N
P
Q
R
S
T
V
W
Y
ikalian
opean
astern
nalaya
berian

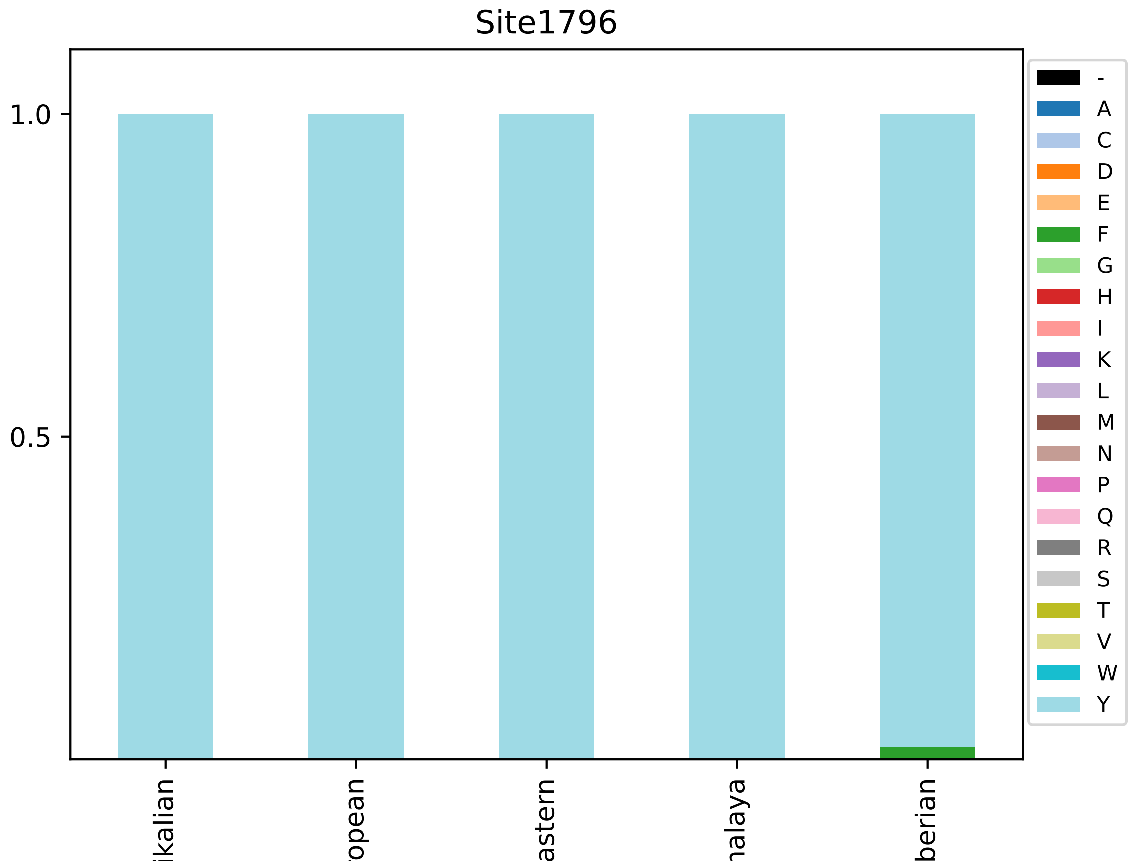

Site1796
1.0
0.5
-
A
C
D
E
F
G
H
I
K
L
M
N
P
Q
R
S
T
V
W
Y
ikalian
opean
astern
nalaya
berian

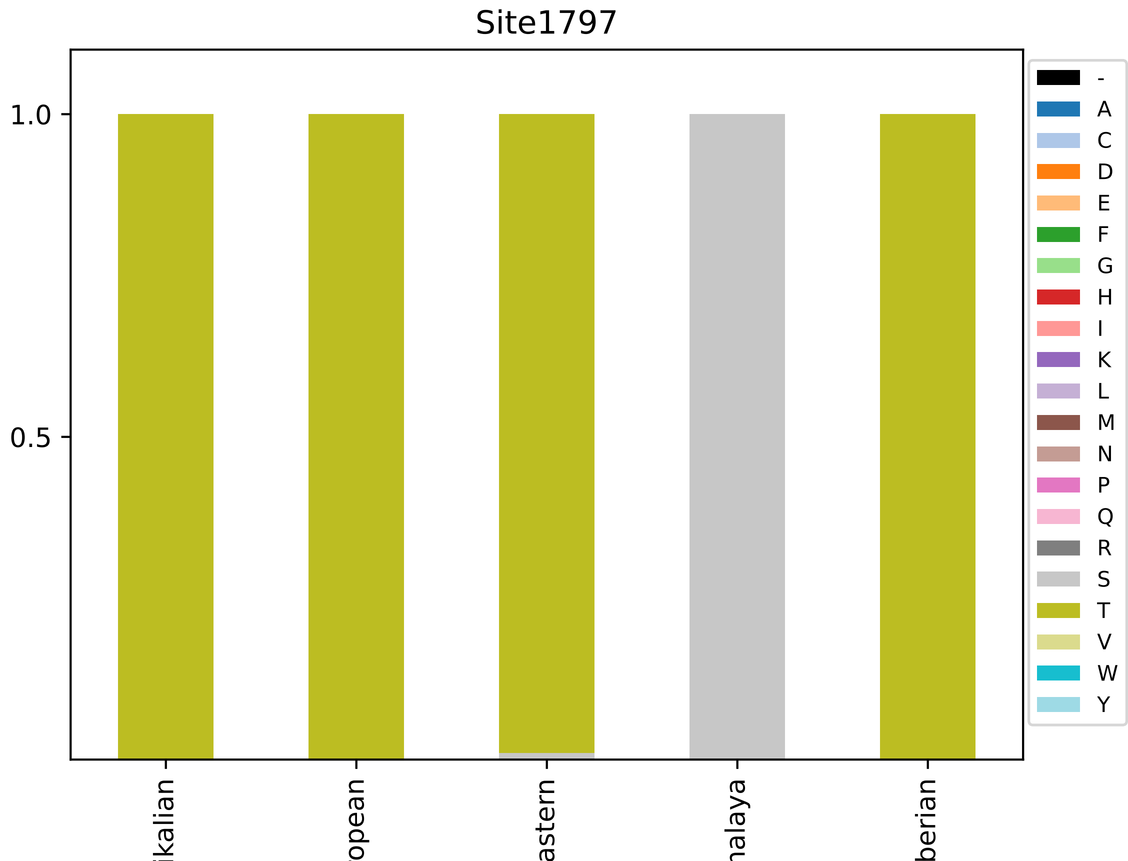

Site1797
1.0
0.5
ikalian
opean
astern
nalaya
berian
-
A
C
D
E
F
G
H
I
K
L
M
N
P
Q
R
S
T
V
W
Y

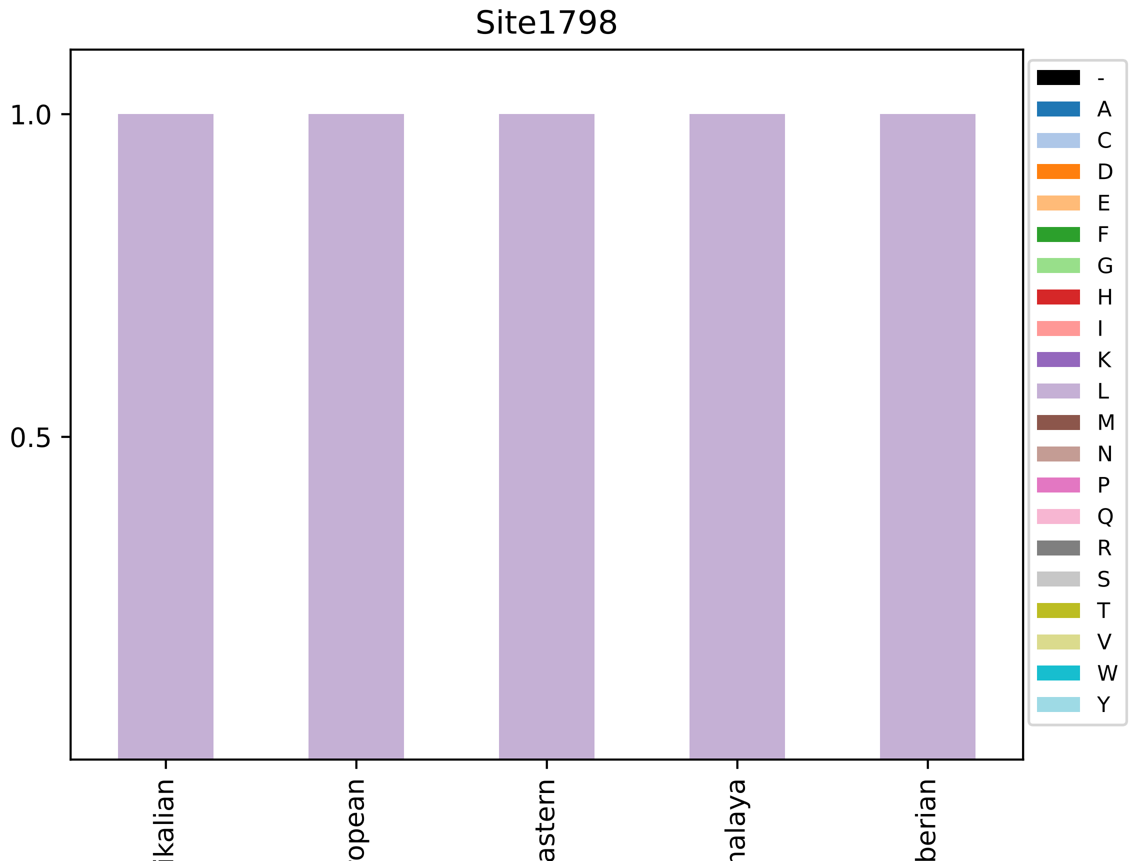
Site1798
1.0
0.5
-
A
C
D
E
F
G
H
I
K
L
M
N
P
Q
R
S
T
V
W
Y
ikalian
opean
astern
nalaya
berian

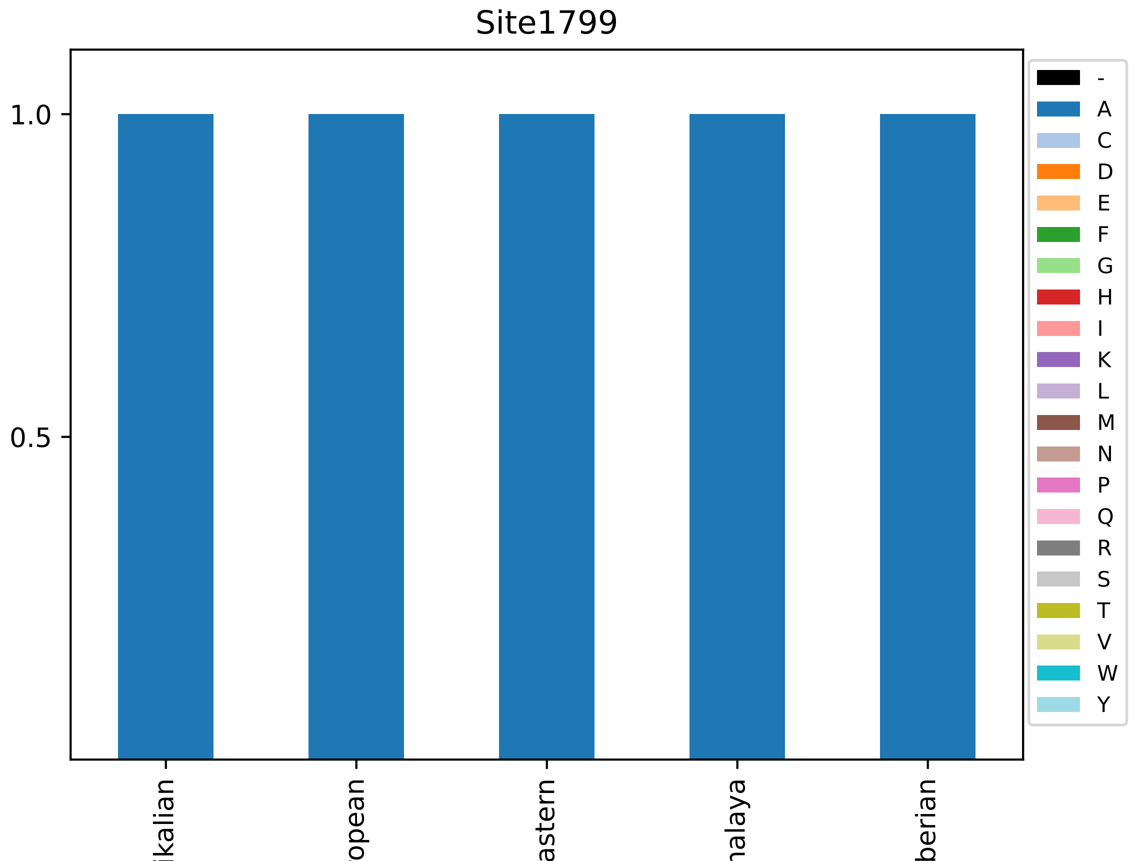
Site1799
1.0
0.5
-
A
C
D
E
F
G
H
I
K
L
M
N
P
Q
R
S
T
V
W
Y
ikalian
opean
astern
nalaya
berian

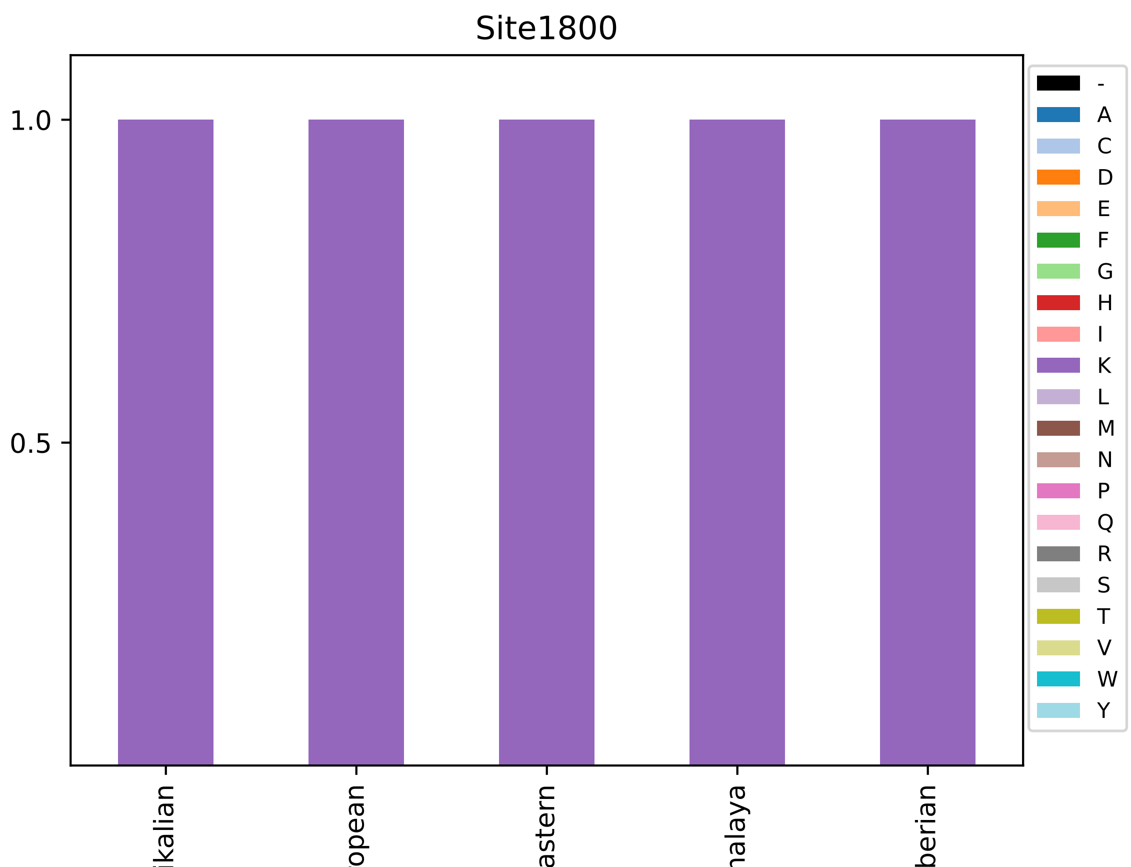

Site1800
1.0
0.5
-
A
C
D
E
F
G
H
I
K
L
M
N
P
Q
R
S
T
V
W
Y
ikalian
opean
astern
nalaya
berian

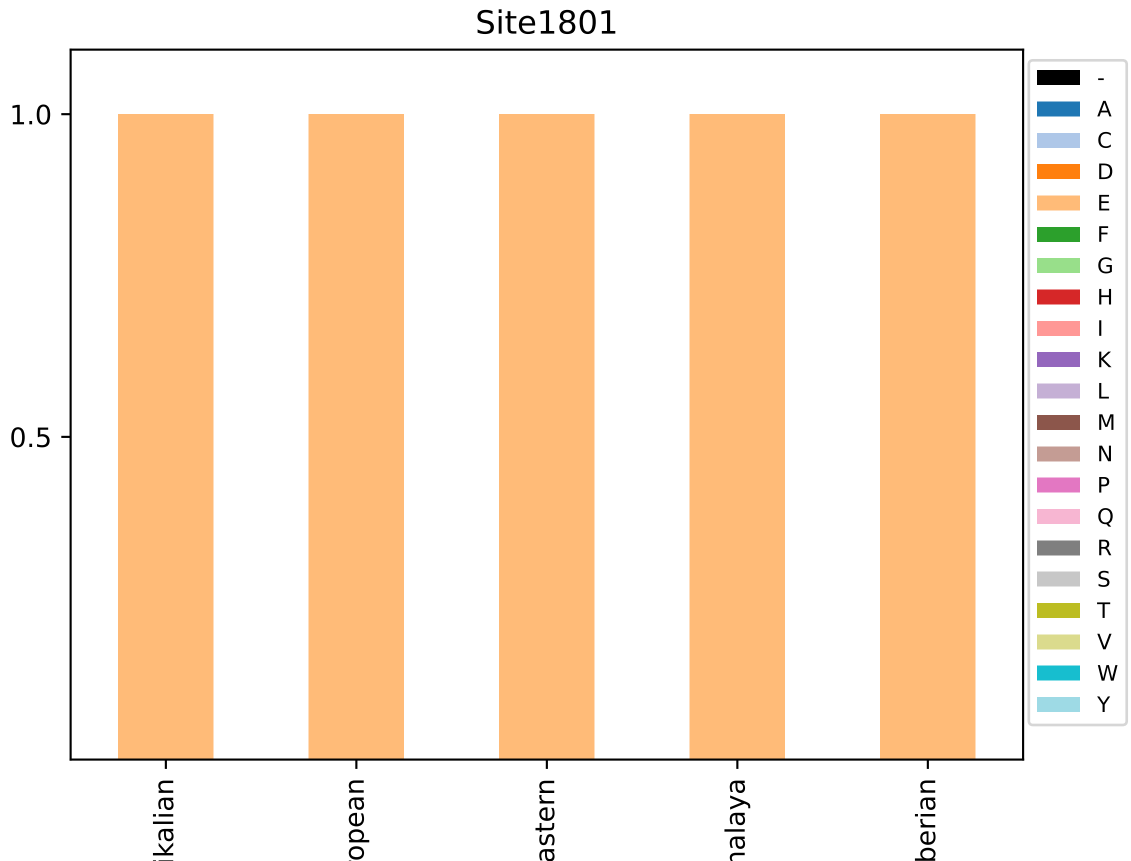

Site1801
1.0
0.5
-
A
C
D
E
F
G
H
I
K
L
M
N
P
Q
R
S
T
V
W
Y

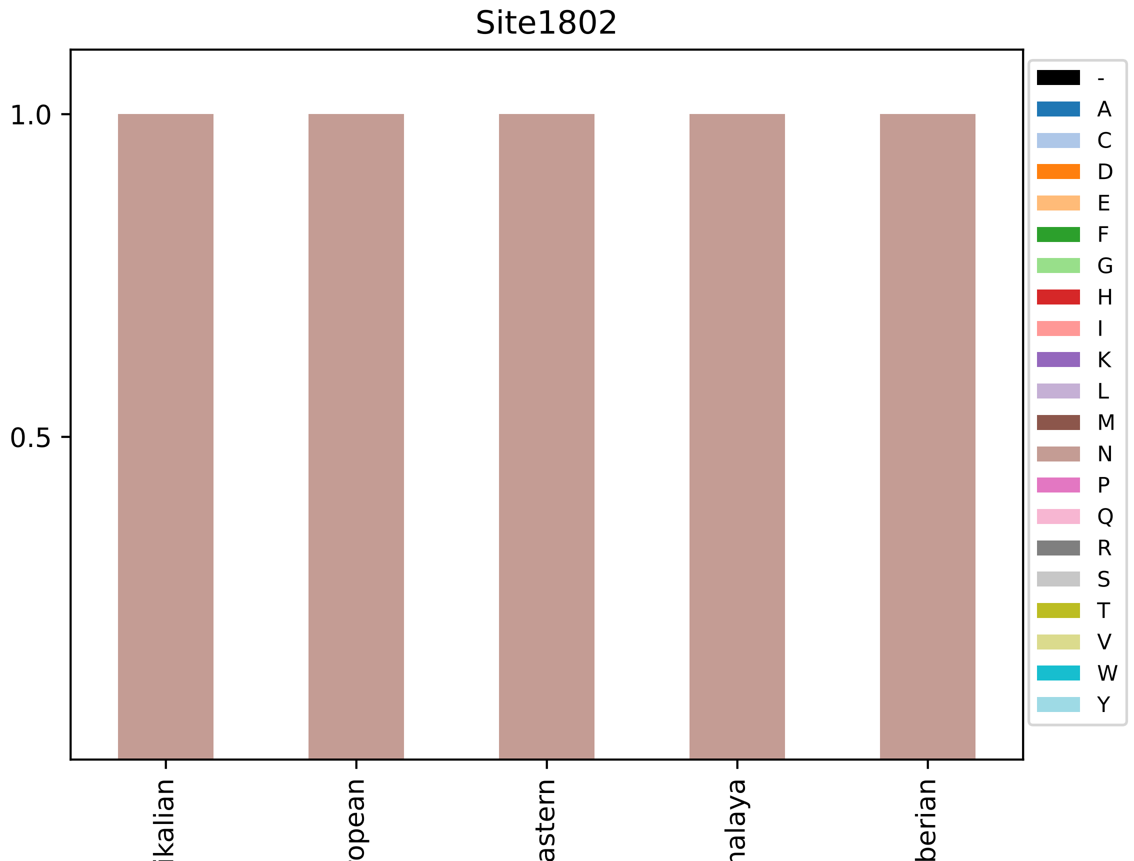

Site1802
1.0
0.5
-
A
C
D
E
F
G
H
I
K
L
M
N
P
Q
R
S
T
V
W
Y
ikalian
opean
astern
halaya
berian

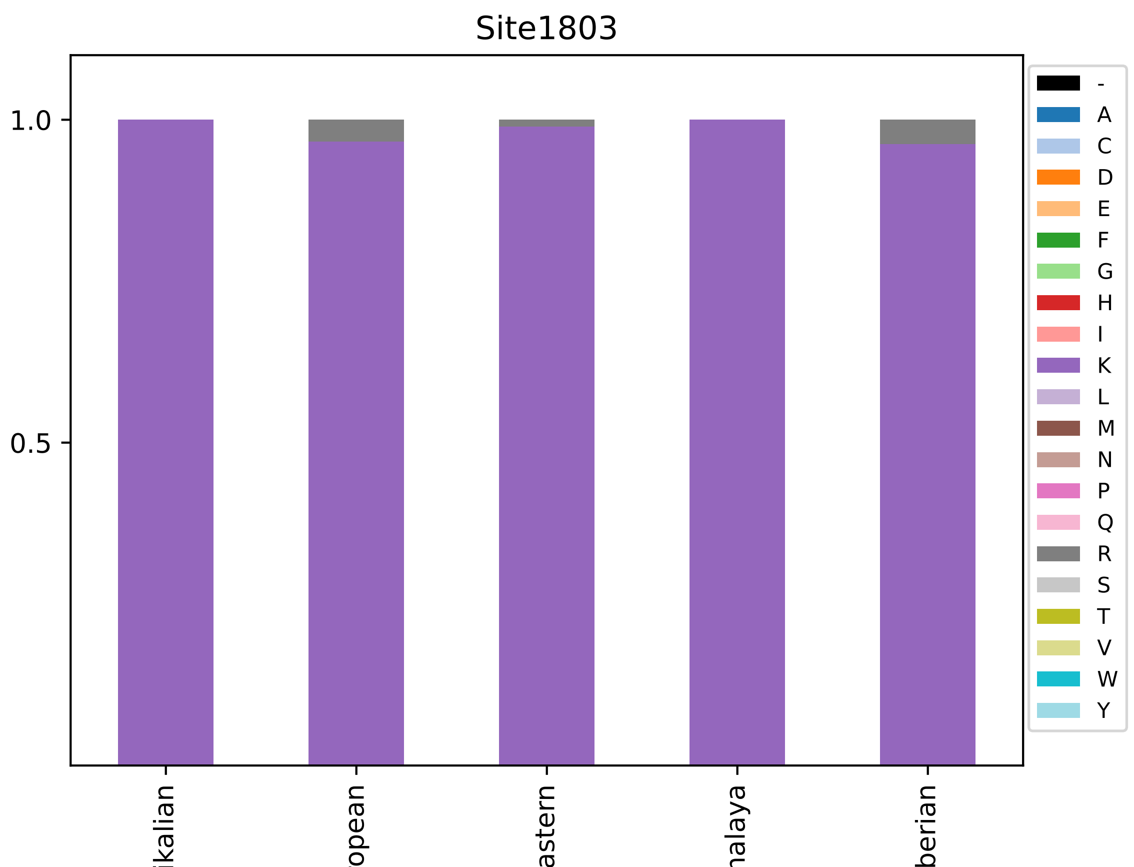

Site1803
1.0
0.5
-
A
C
D
E
F
G
H
I
K
L
M
N
P
Q
R
S
T
V
W
Y

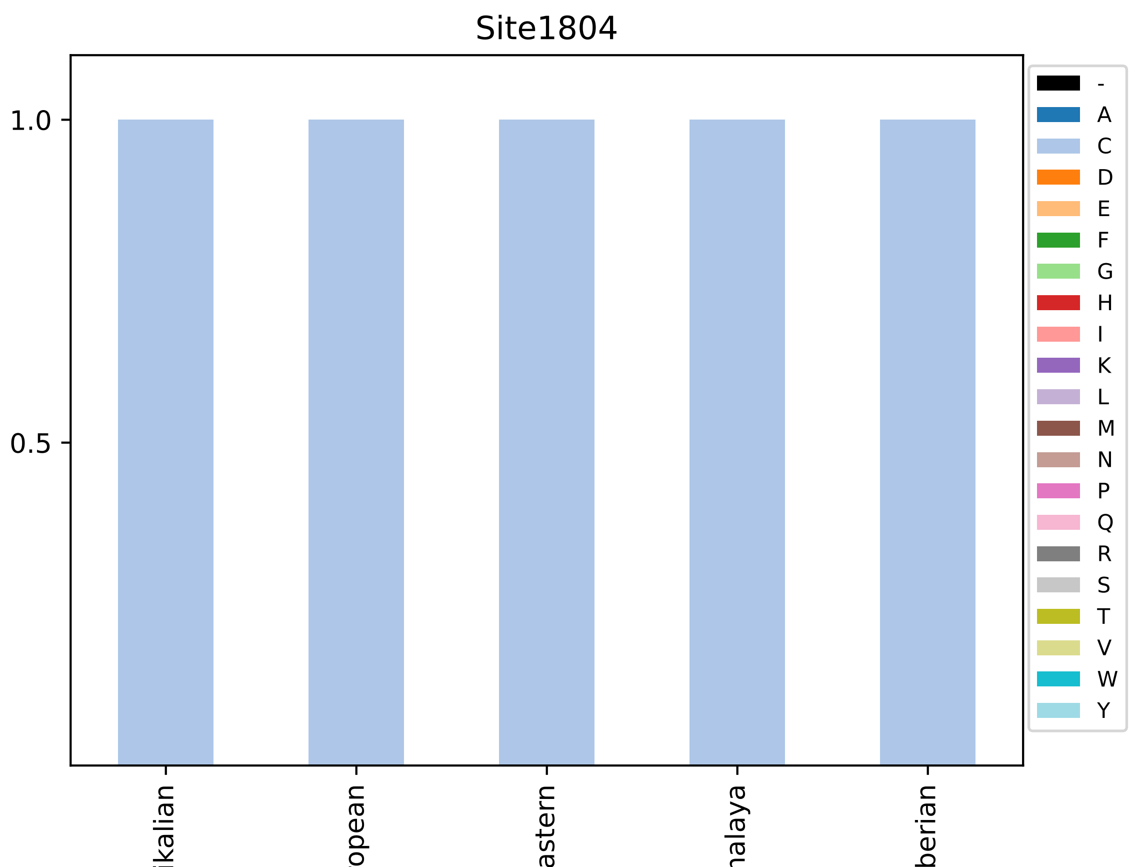
Site1804
1.0
0.5
-
A
C
D
E
F
G
H
I
K
L
M
N
P
Q
R
S
T
V
W
Y
ikalian
opean
astern
nalaya
berian

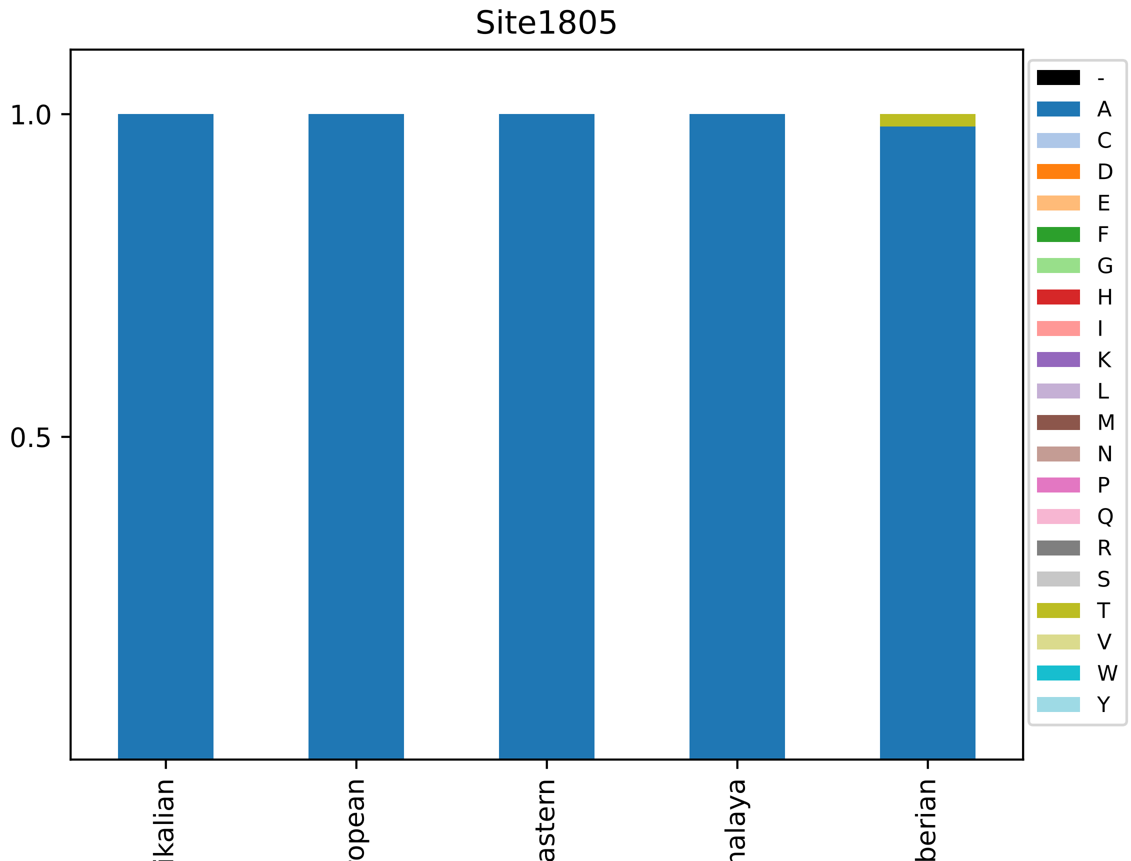
Site1805
1.0
0.5
-
A
C
D
E
F
G
H
I
K
L
M
N
P
Q
R
S
T
V
W
Y

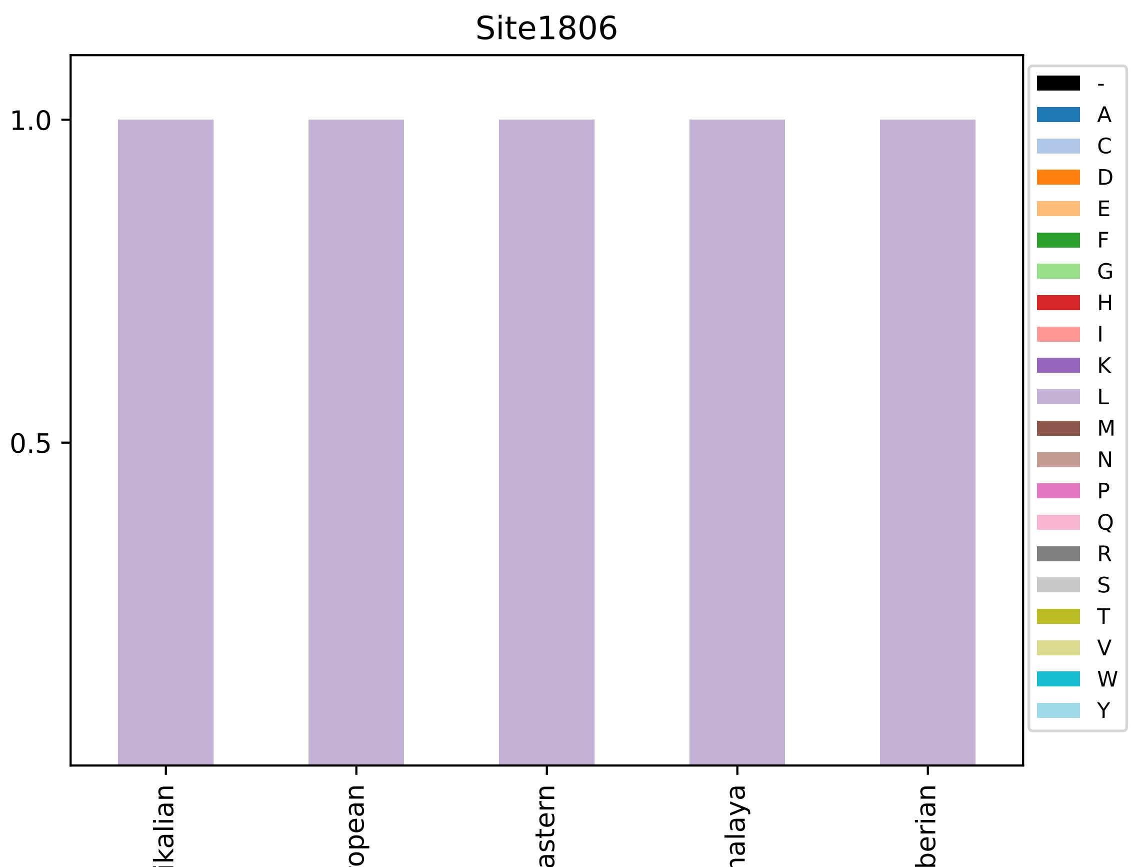

Site1806
1.0
0.5
-
A
C
D
E
F
G
H
I
K
L
M
N
P
Q
R
S
T
V
W
Y

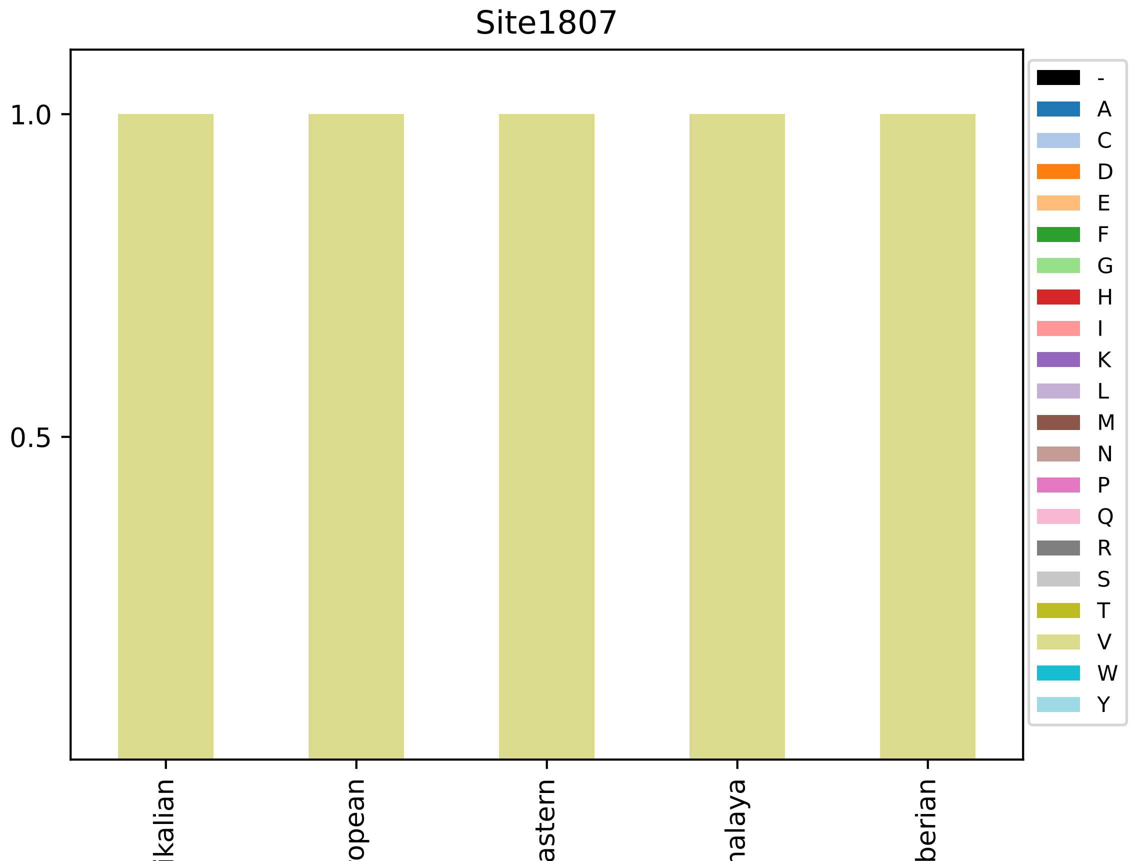
Site1807
1.0
0.5
-
A
C
D
E
F
G
H
I
K
L
M
N
P
Q
R
S
T
V
W
Y

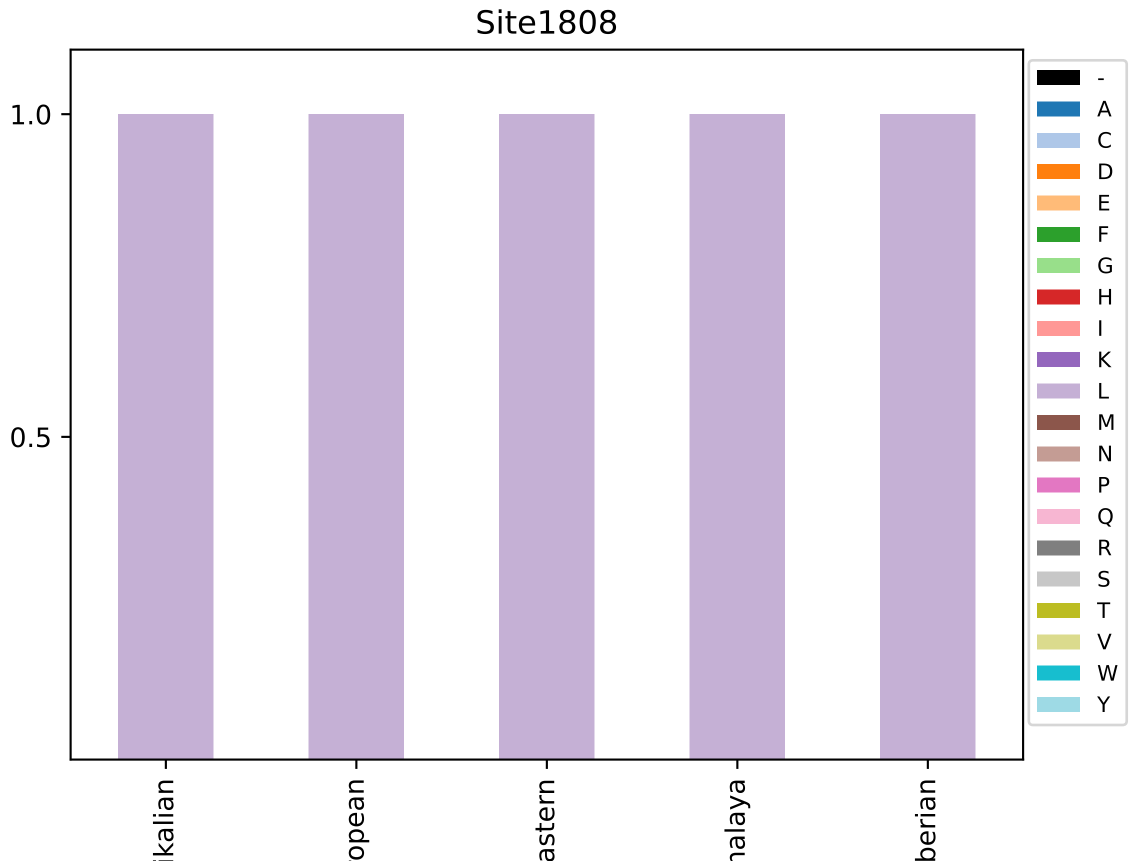

Site1808
1.0
0.5
ikalian
opean
astern
nalaya
berian
-
A
C
D
E
F
G
H
I
K
L
M
N
P
Q
R
S
T
V
W
Y

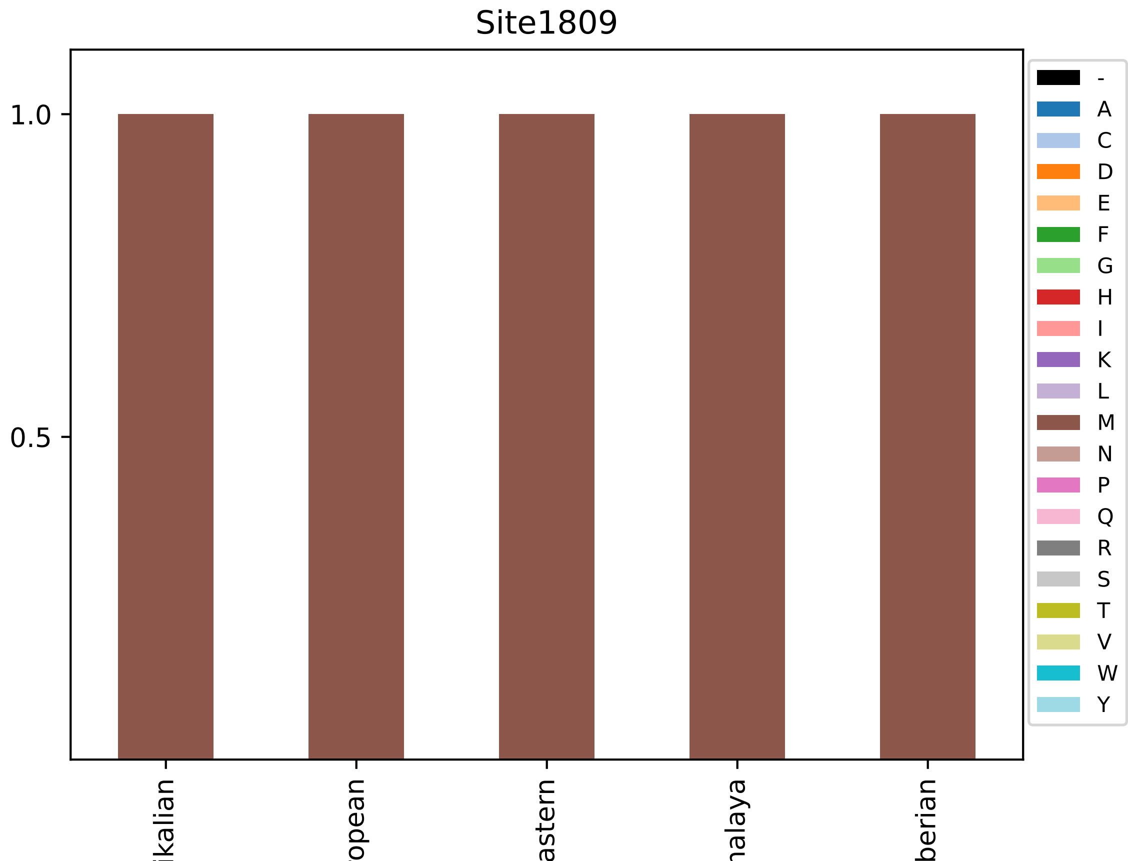
Site1809
1.0
0.5
-
A
C
D
E
F
G
H
I
K
L
M
N
P
Q
R
S
T
V
W
Y
ikalian
opean
astern
nalaya
berian

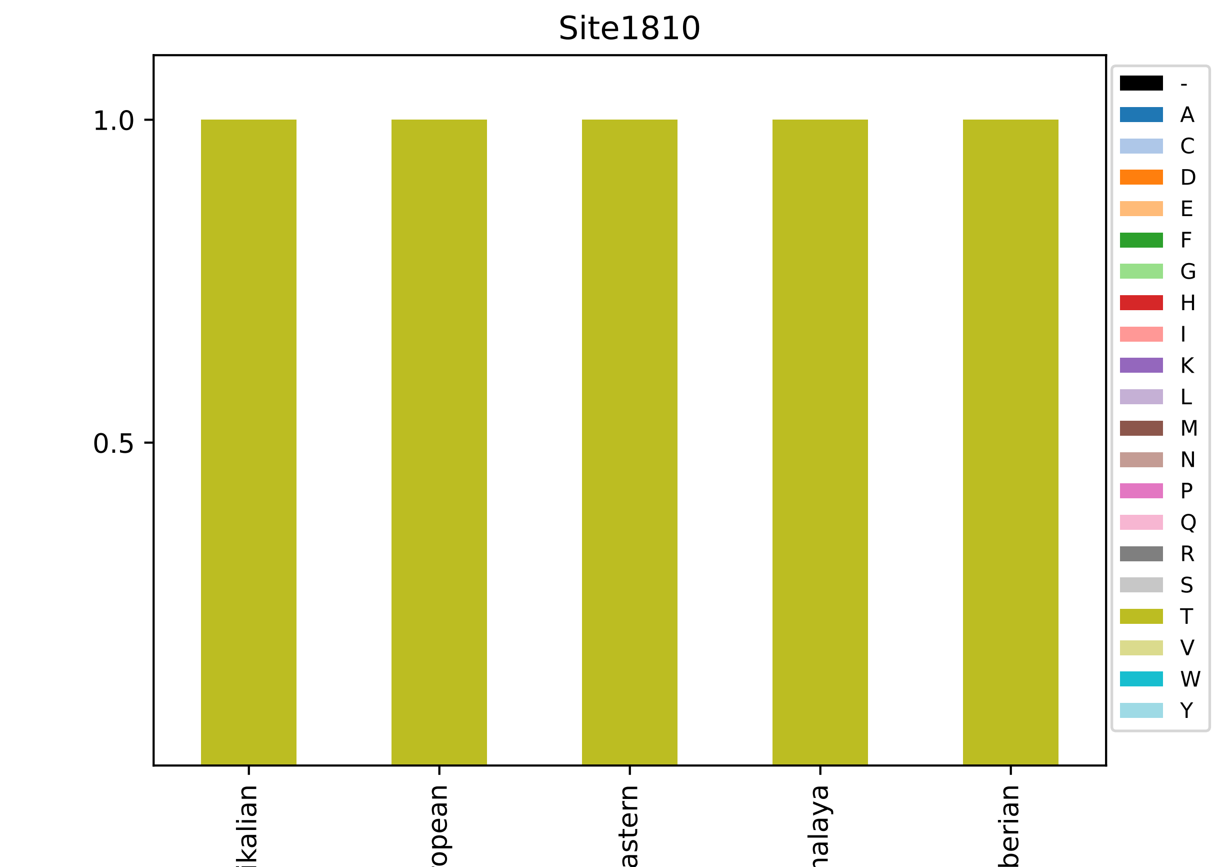

Site1810
1.0
0.5
ikalian
opean
astern
nalaya
berian
-
A
C
D
E
F
G
H
I
K
L
M
N
P
Q
R
S
T
V
W
Y

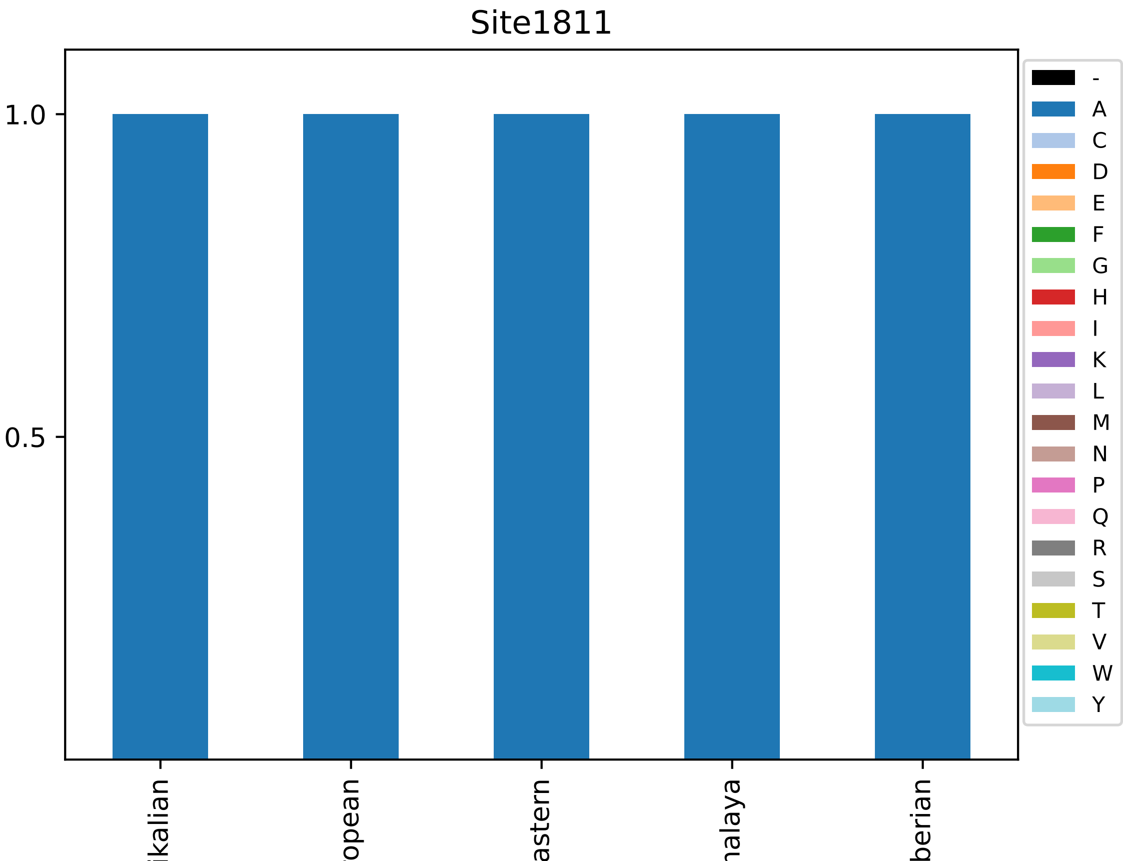

Site1811
1.0
0.5
-
A
C
D
E
F
G
H
I
K
L
M
N
P
Q
R
S
T
V
W
Y
ikalian
opean
astern
nalaya
berian

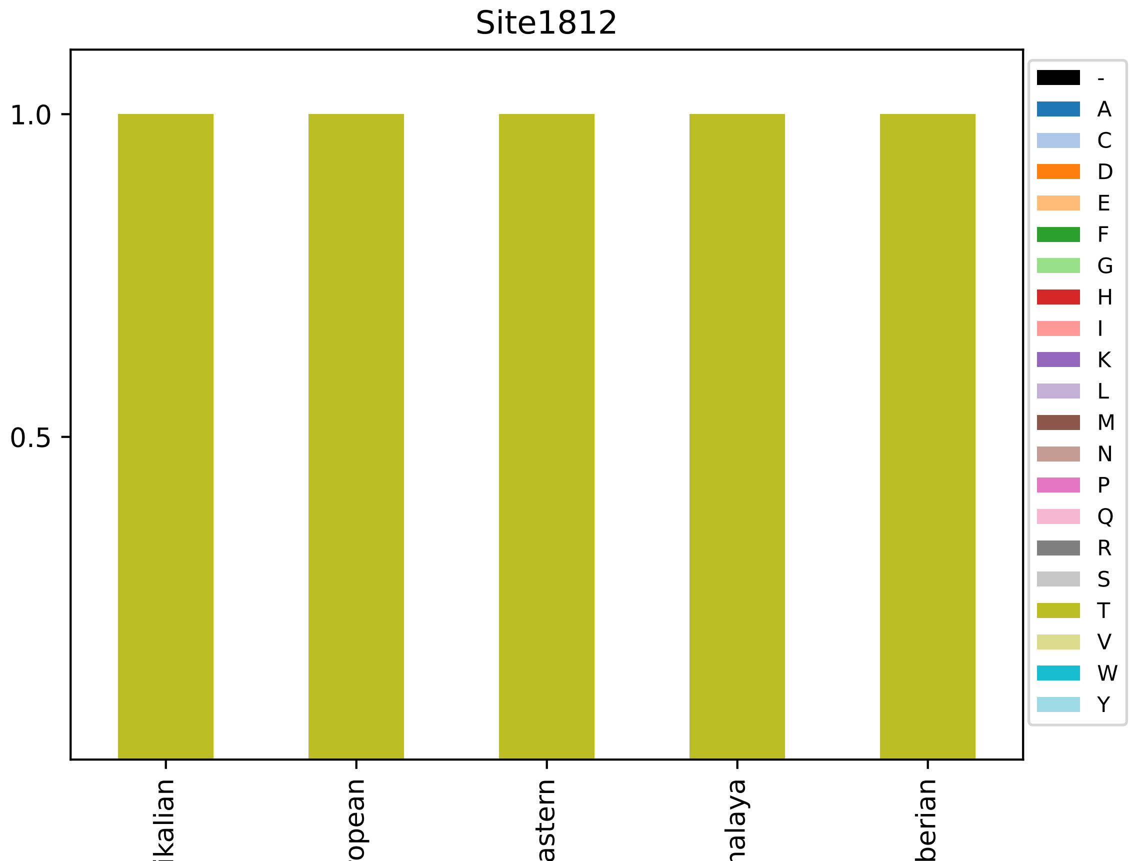
Site1812
1.0
0.5
-
A
C
D
E
F
G
H
I
K
L
M
N
P
Q
R
S
T
V
W
Y
ikalian
opean
astern
nalaya
berian

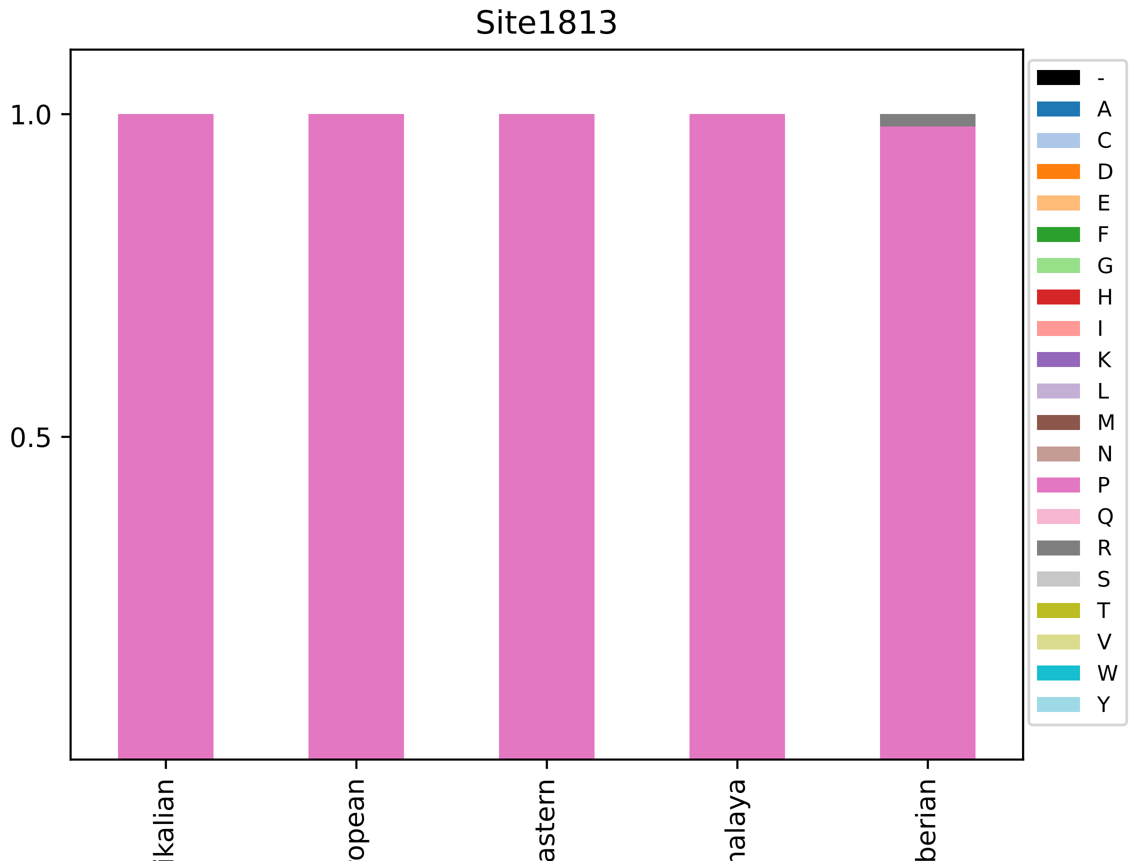

Site1813
1.0
0.5
ikalian
opean
astern
nalaya
berian
-
A
C
D
E
F
G
H
I
K
L
M
N
P
Q
R
S
T
V
W
Y

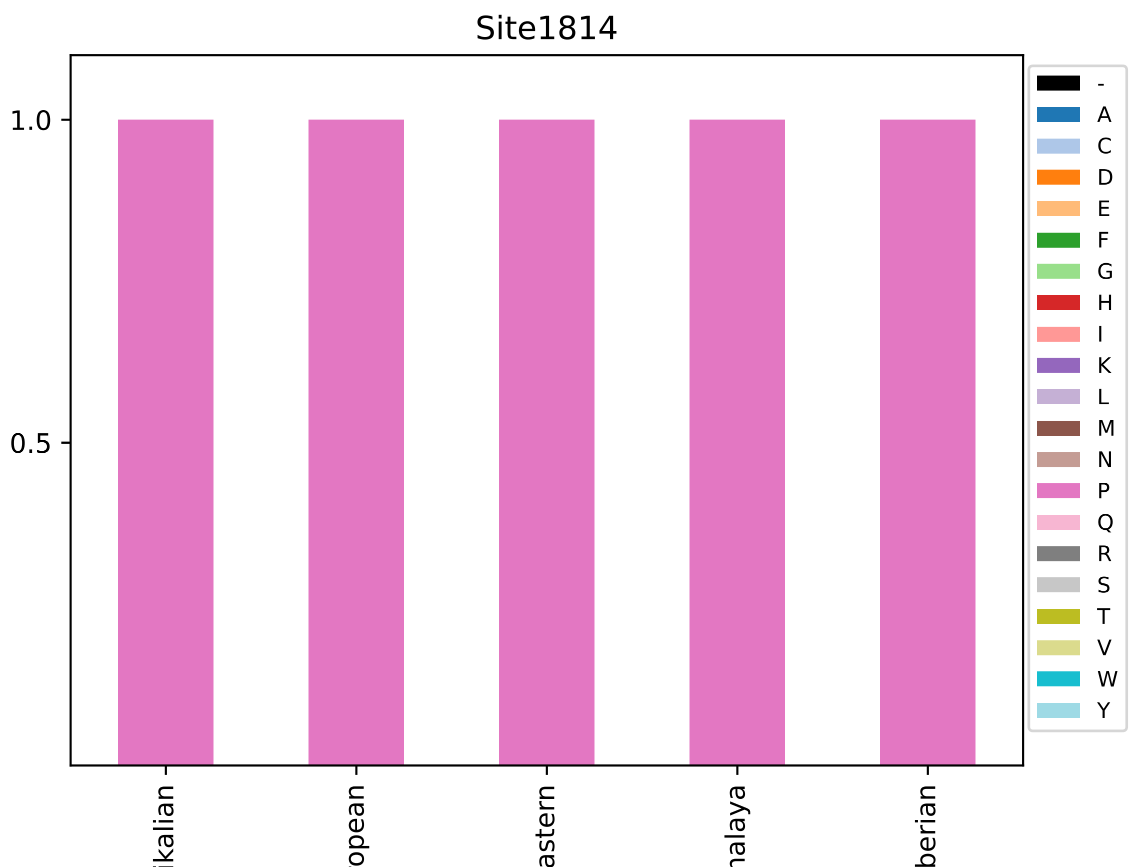

Site1814
1.0
0.5
-
A
C
D
E
F
G
H
I
K
L
M
N
P
Q
R
S
T
V
W
Y
ikalian
opean
astern
nalaya
berian

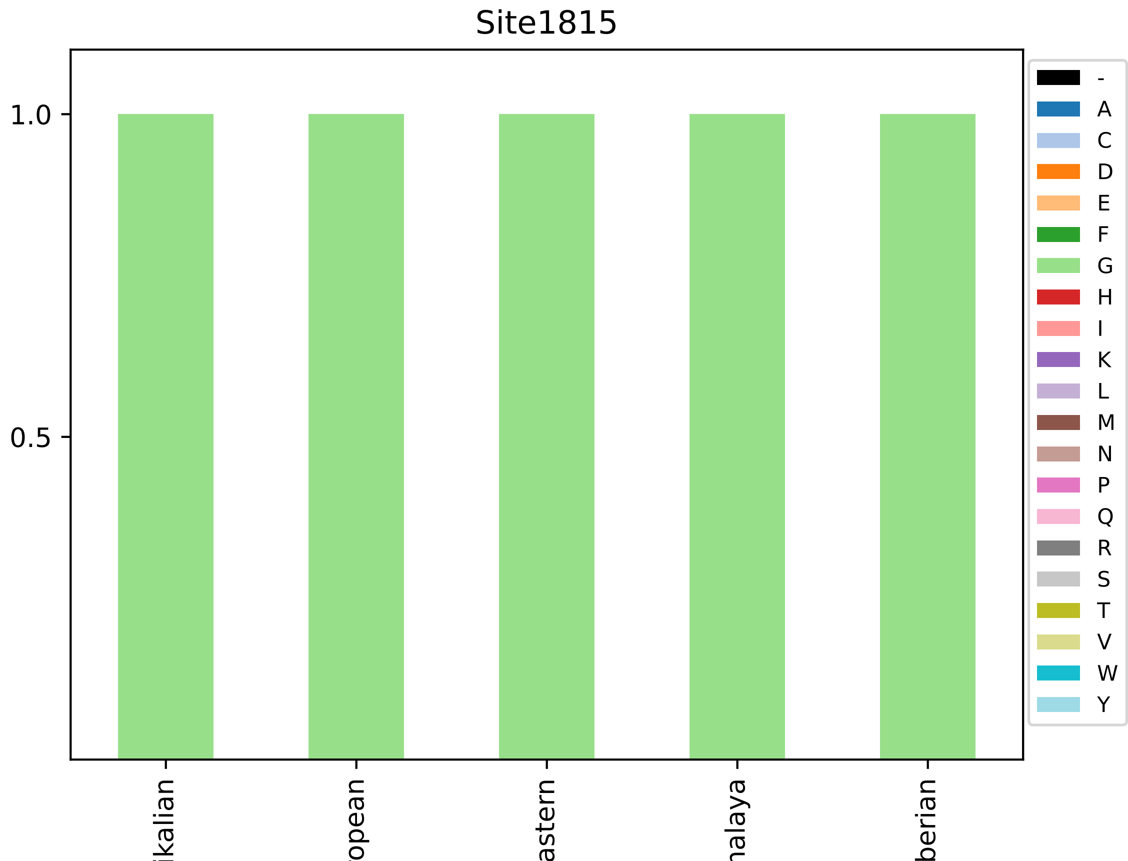

Site1815
1.0
0.5
-
A
C
D
E
F
G
H
I
K
L
M
N
P
Q
R
S
T
V
W
Y
ikalian
opean
astern
nalaya
berian

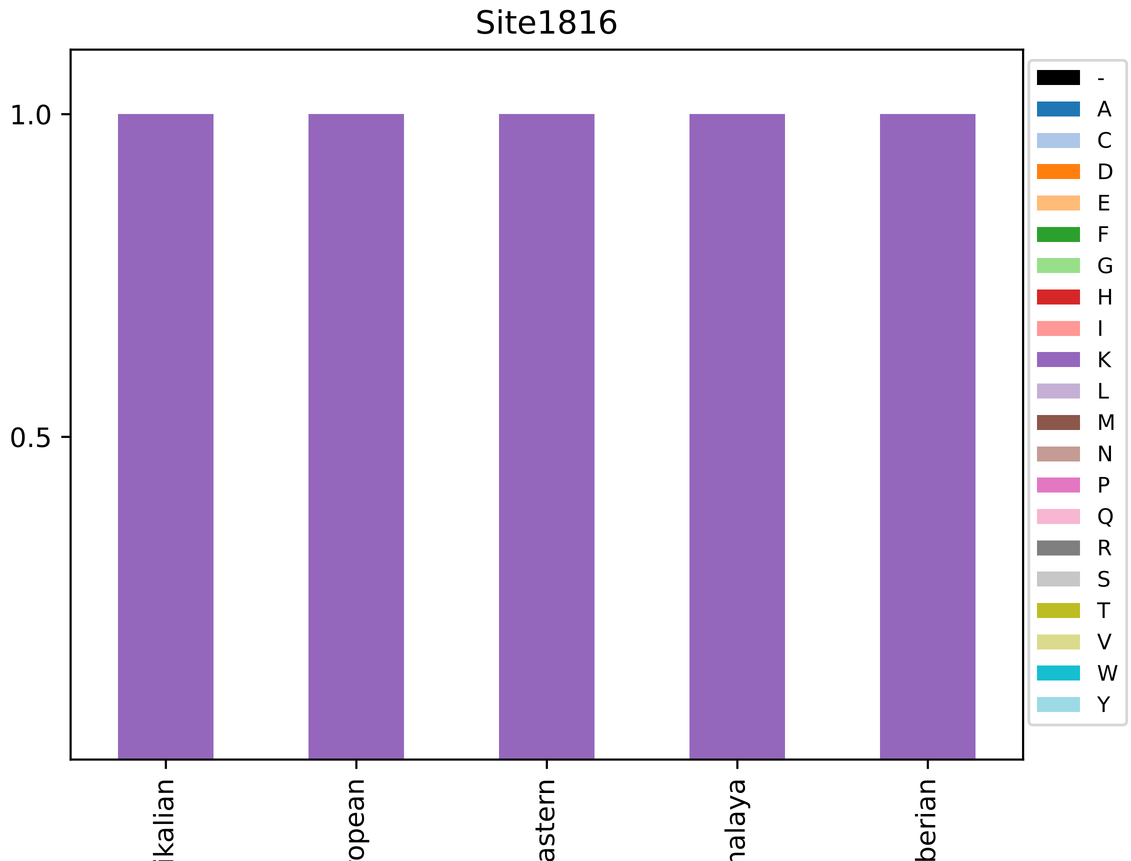
Site1816
1.0
0.5
-
A
C
D
E
F
G
H
I
K
L
M
N
P
Q
R
S
T
V
W
Y
ikalian
opean
astern
nalaya
berian

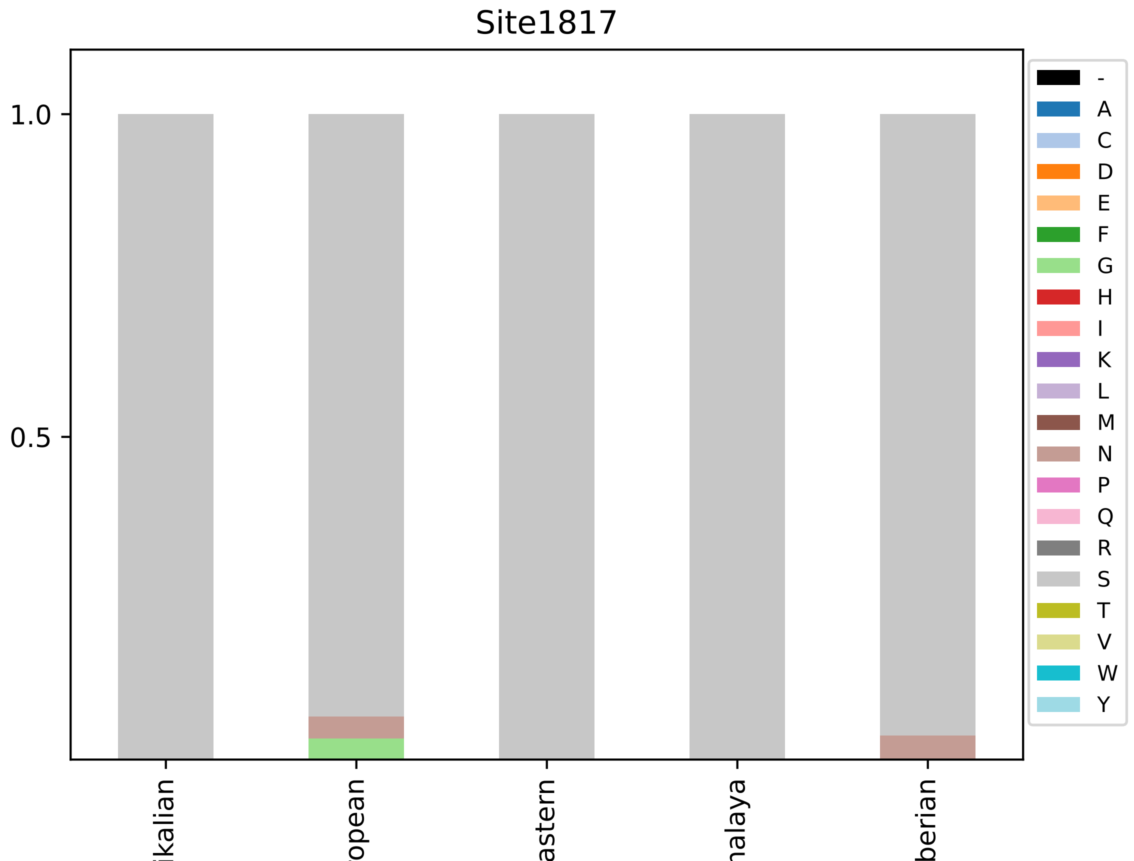

Site1817
1.0
0.5
-
A
C
D
E
F
G
H
I
K
L
M
N
P
Q
R
S
T
V
W
Y
ikalian
opean
astern
nalaya
berian

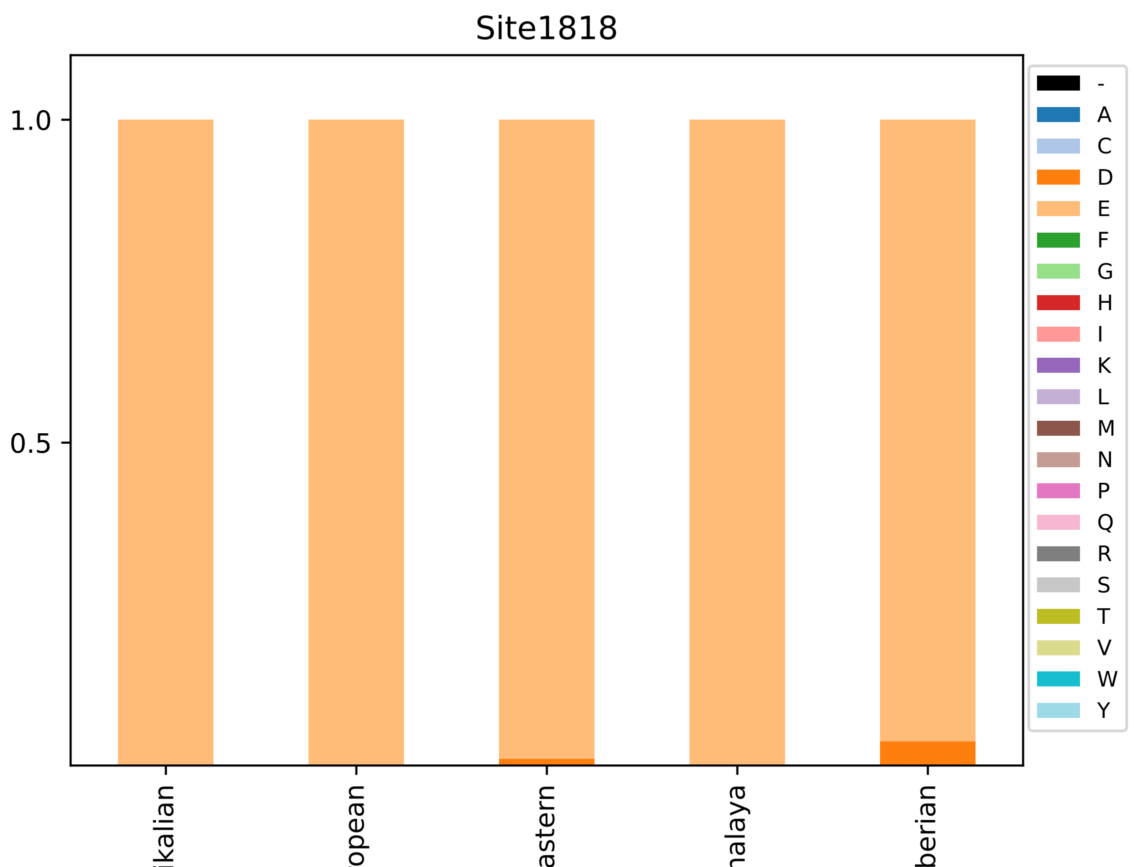

Site1818
1.0
0.5
-
A
C
D
E
F
G
H
I
K
L
M
N
P
Q
R
S
T
V
W
Y
ikalian
opean
astern
nalaya
berian

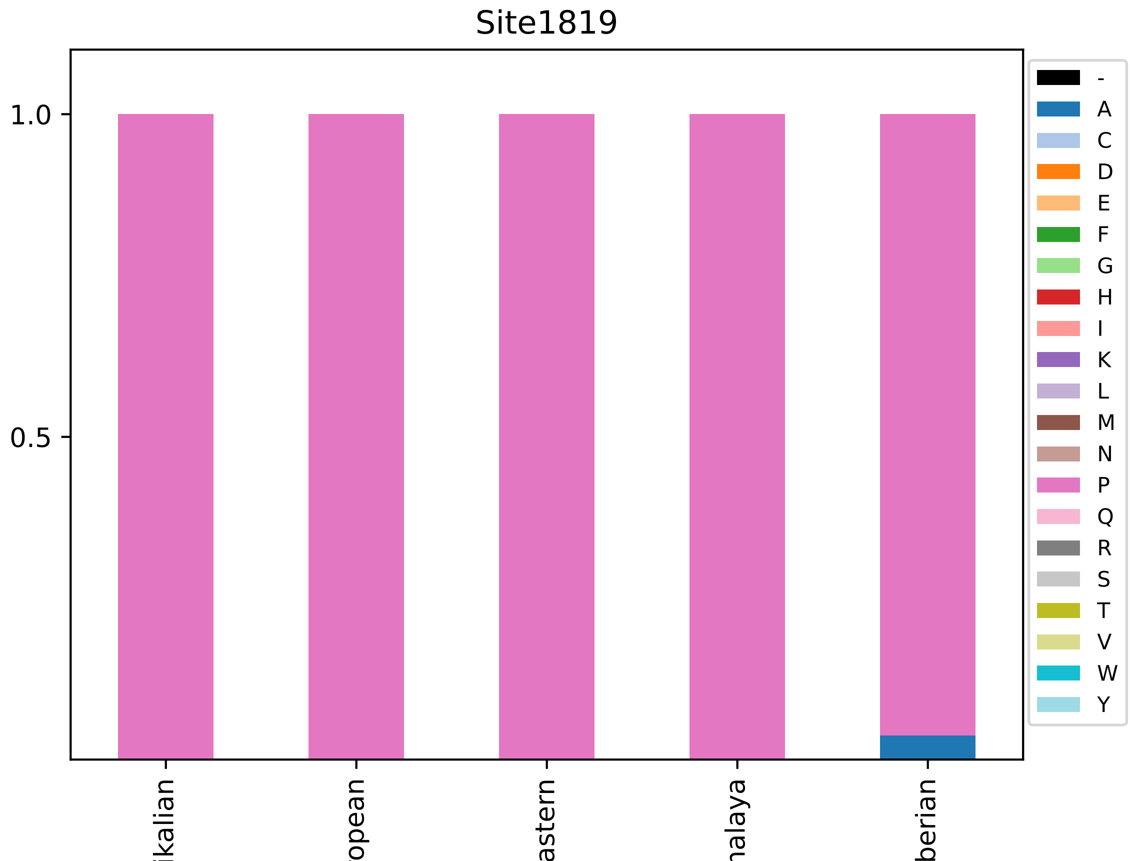

Site1819
1.0
0.5
-
A
C
D
E
F
G
H
I
K
L
M
N
P
Q
R
S
T
V
W
Y
ikalian
opean
astern
nalaya
berian

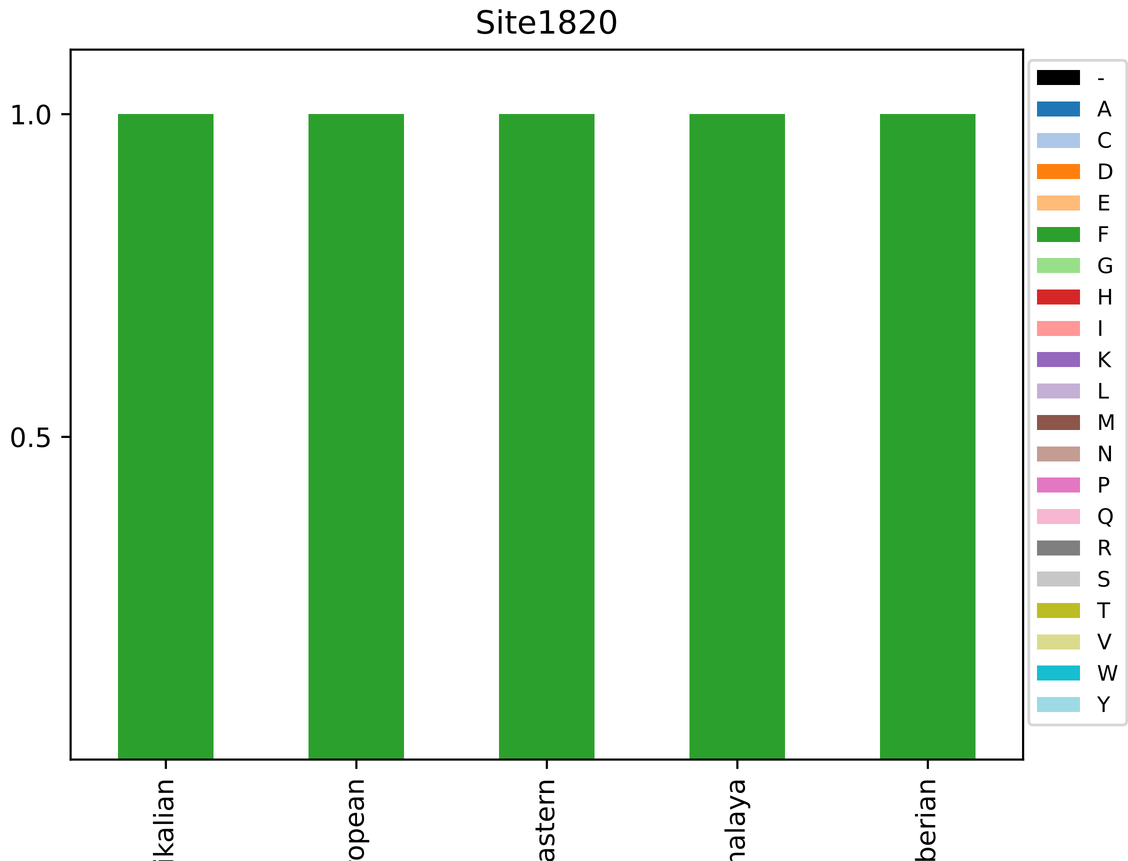

Site1820
1.0
0.5
ikalian
opean
astern
nalaya
berian
-
A
C
D
E
F
G
H
I
K
L
M
N
P
Q
R
S
T
V
W
Y

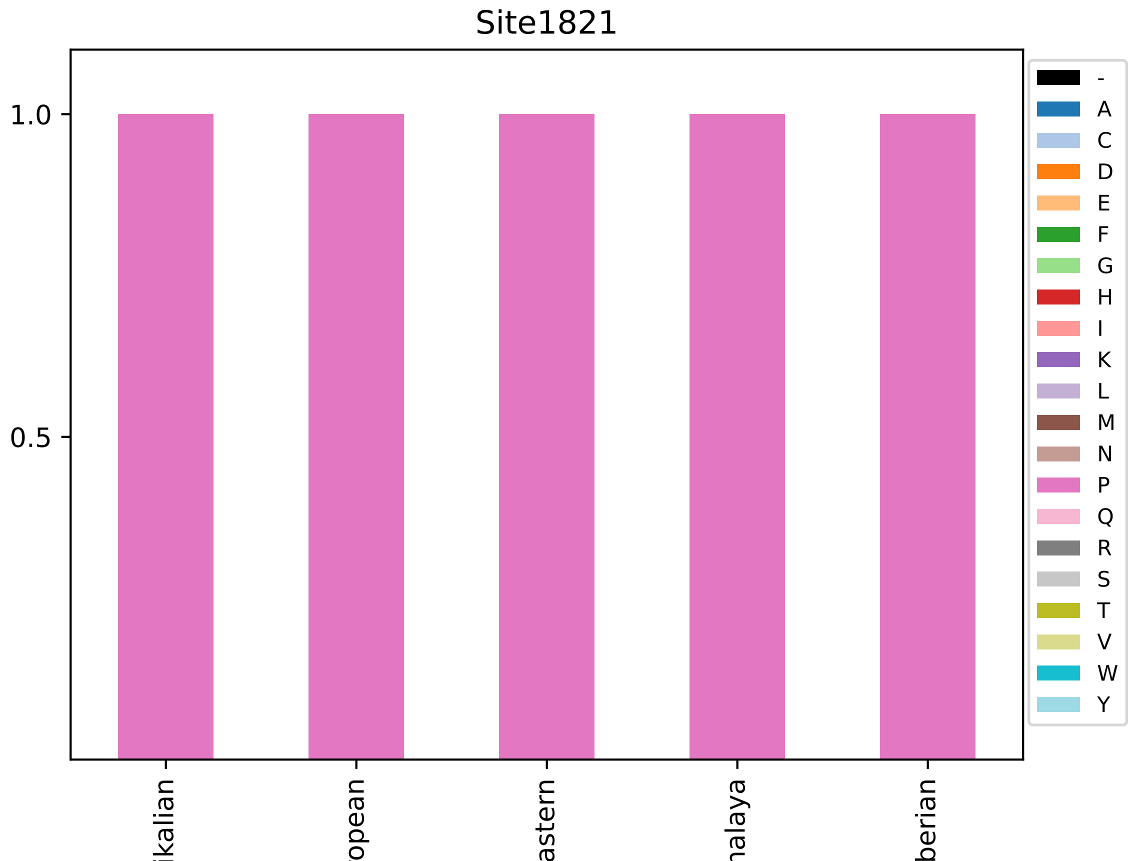

Site1821
1.0
0.5
-
A
C
D
E
F
G
H
I
K
L
M
N
P
Q
R
S
T
V
W
Y
ikalian
opean
astern
nalaya
berian

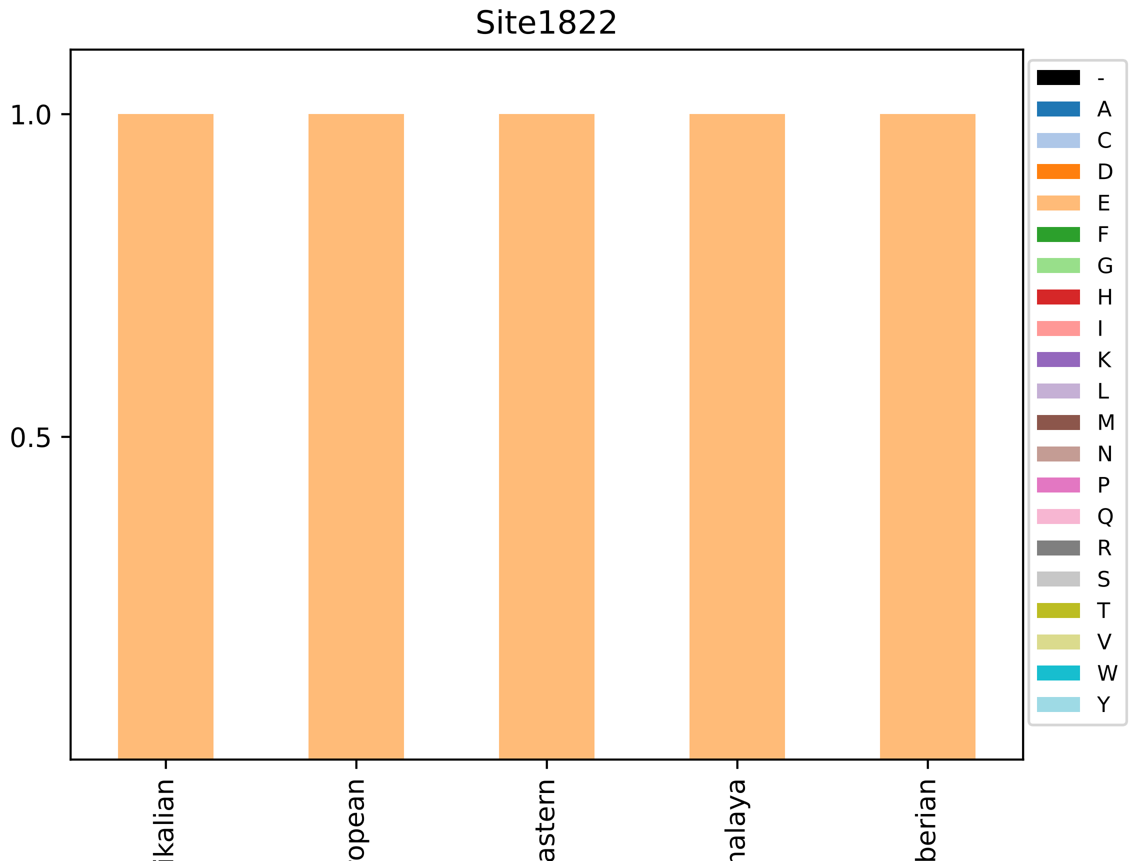

Site1822
1.0
0.5
-
A
C
D
E
F
G
H
I
K
L
M
N
P
Q
R
S
T
V
W
Y
ikalian
opean
astern
nalaya
berian

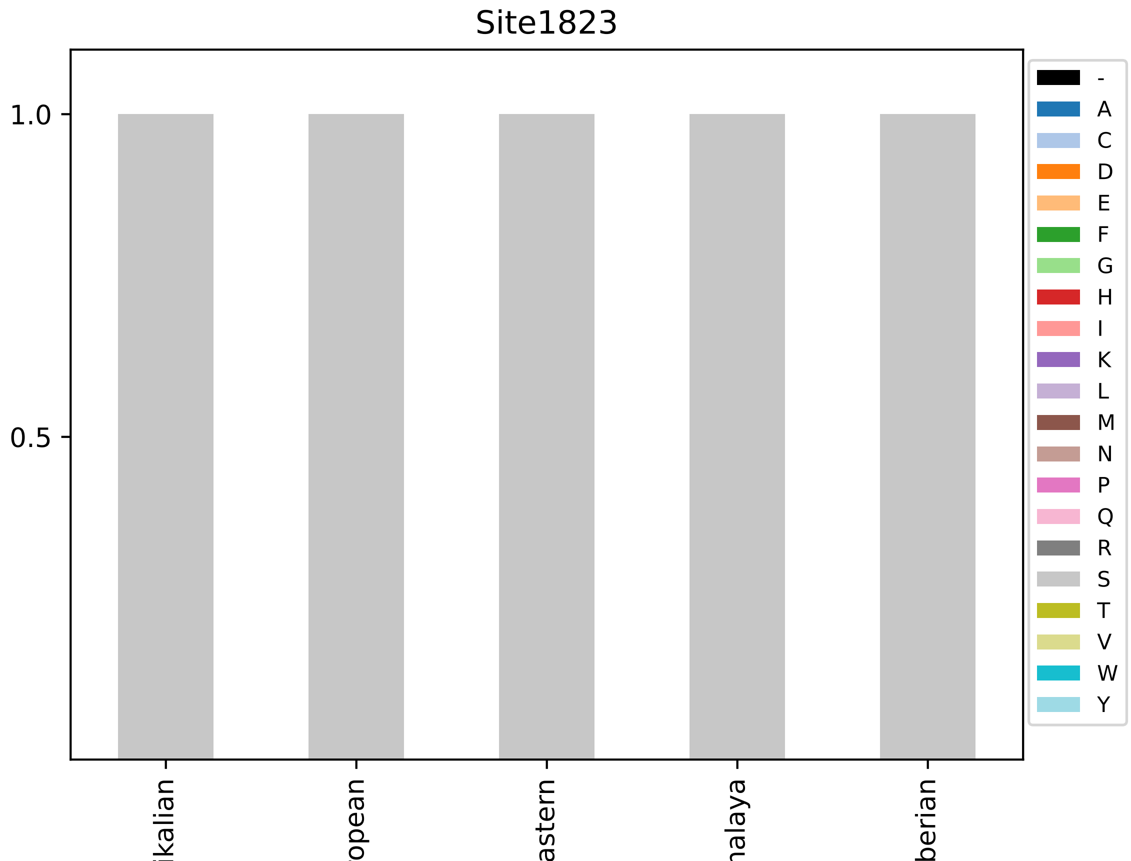

Site1823
1.0
0.5
-
A
C
D
E
F
G
H
I
K
L
M
N
P
Q
R
S
T
V
W
Y
ikalian
opean
astern
nalaya
berian

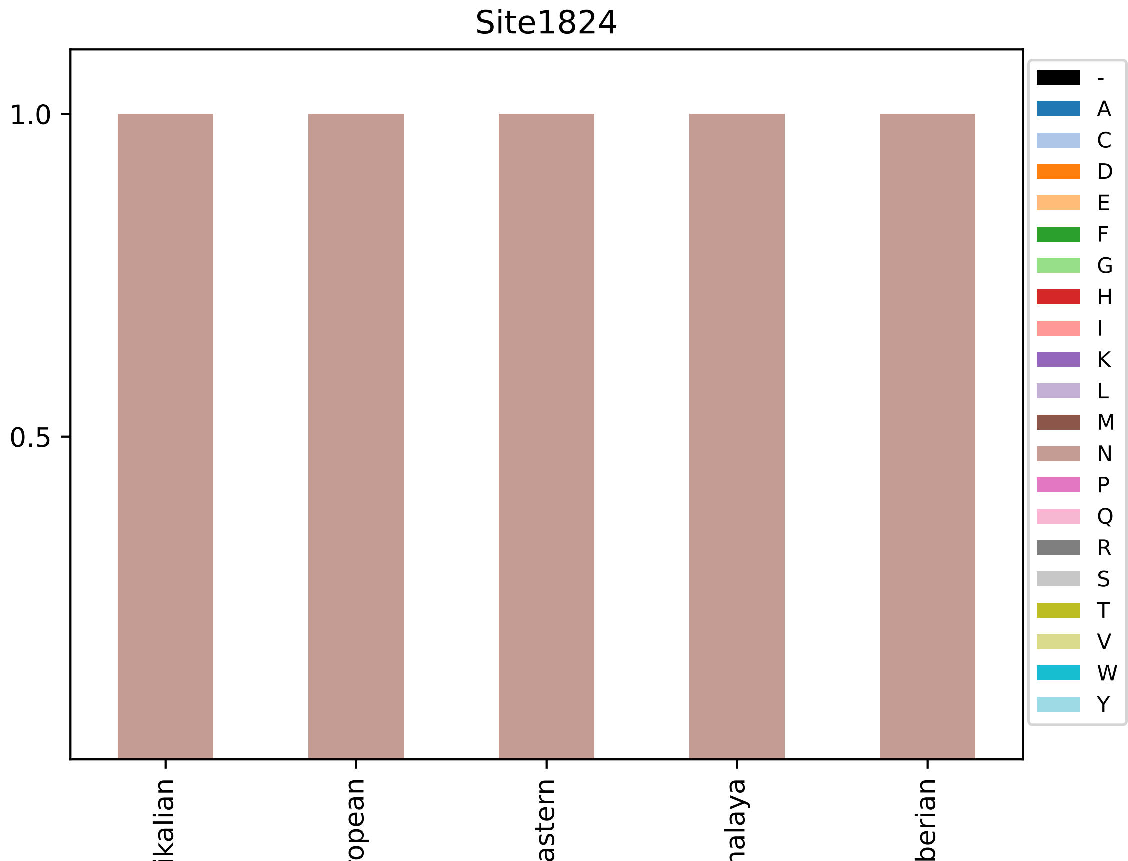

Site1824
1.0
0.5
-
A
C
D
E
F
G
H
I
K
L
M
N
P
Q
R
S
T
V
W
Y
ikalian
opean
astern
nalaya
berian

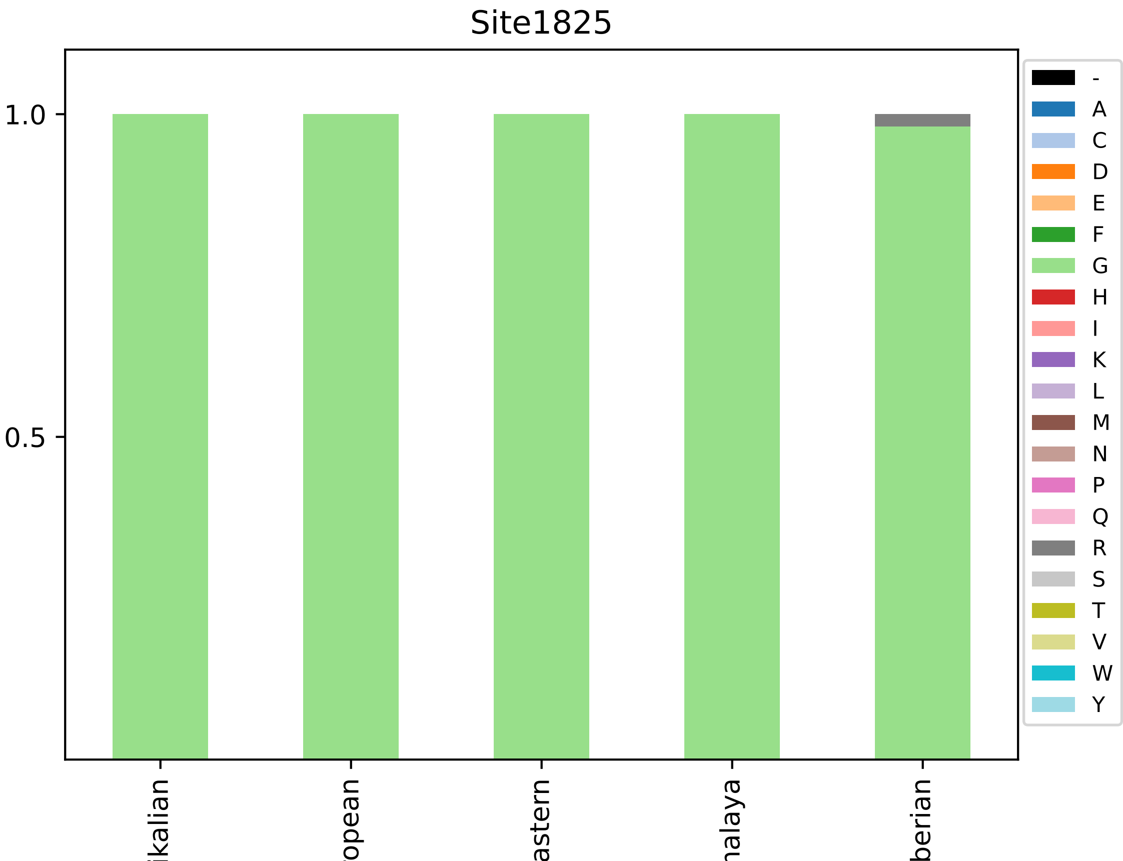
Site1825
1.0
0.5
-
A
C
D
E
F
G
H
I
K
L
M
N
P
Q
R
S
T
V
W
Y
ikalian
opean
astern
nalaya
berian

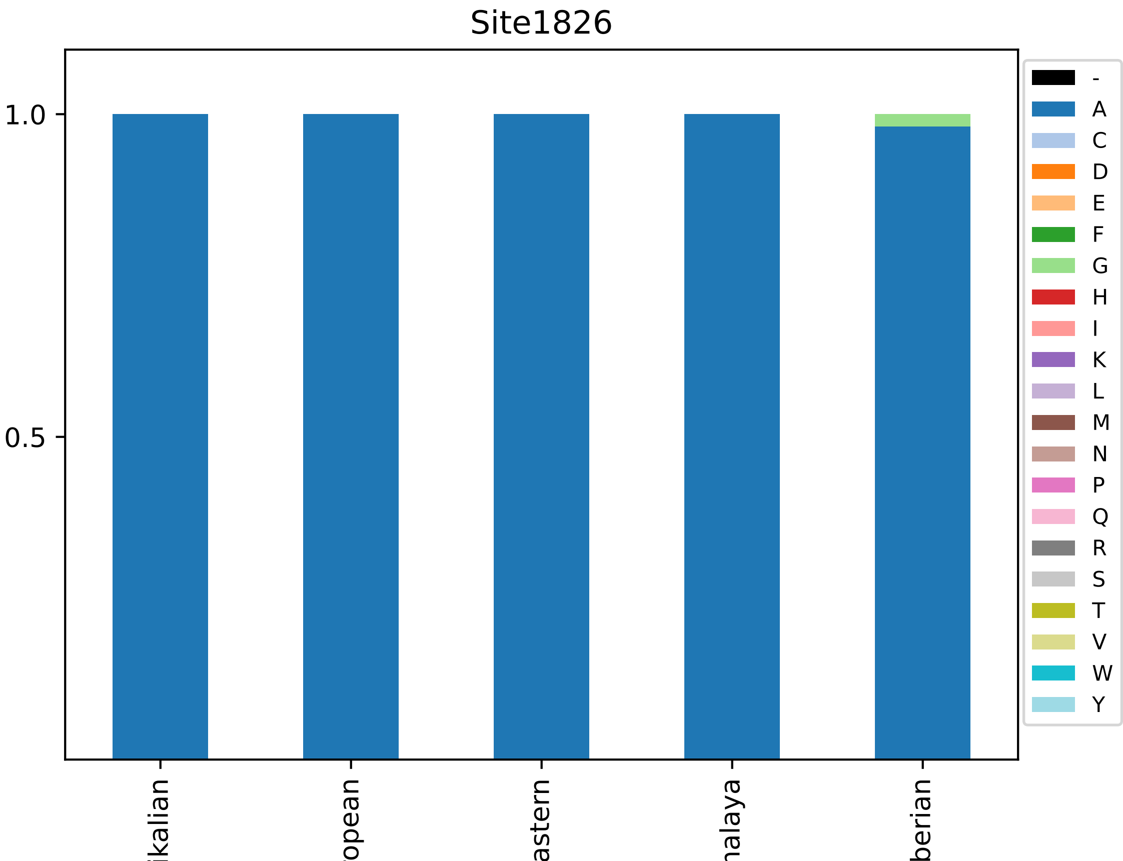

Site1826
1.0
0.5
ikalian
opean
astern
nalaya
berian
-
A
C
D
E
F
G
H
I
K
L
M
N
P
Q
R
S
T
V
W
Y

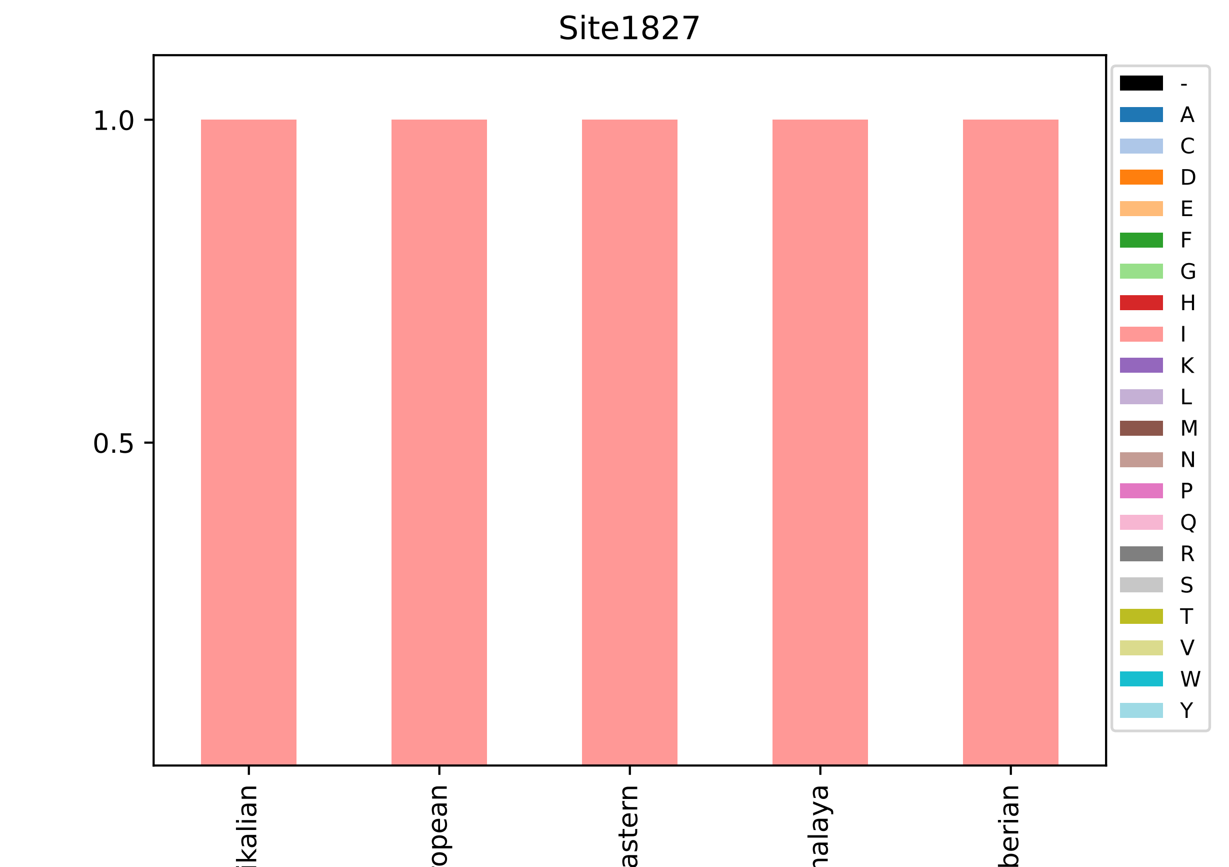

Site1827
1.0
0.5
-
A
C
D
E
F
G
H
I
K
L
M
N
P
Q
R
S
T
V
W
Y
ikalian
opean
astern
nalaya
berian

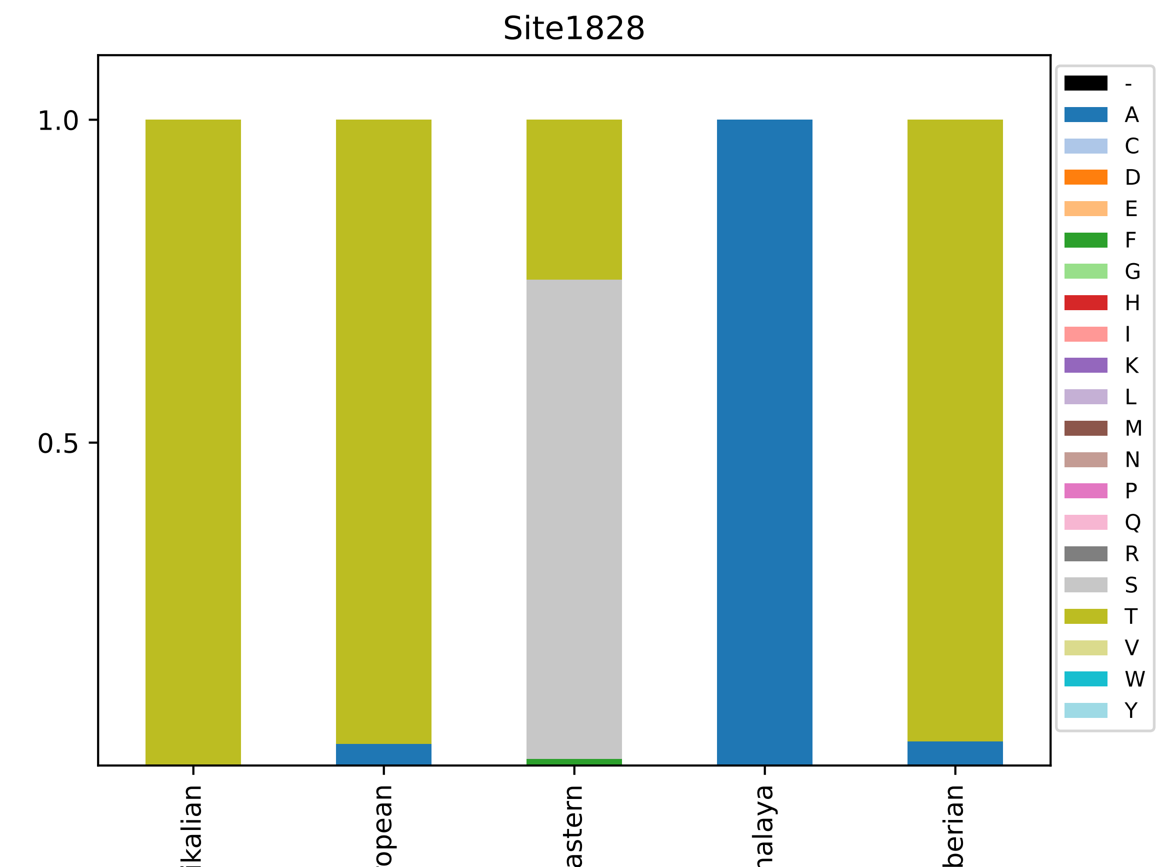
Site1828
1.0
0.5
ikalian
opean
astern
nalaya
berian
-
A
C
D
E
F
G
H
I
K
L
M
N
P
Q
R
S
T
V
W
Y

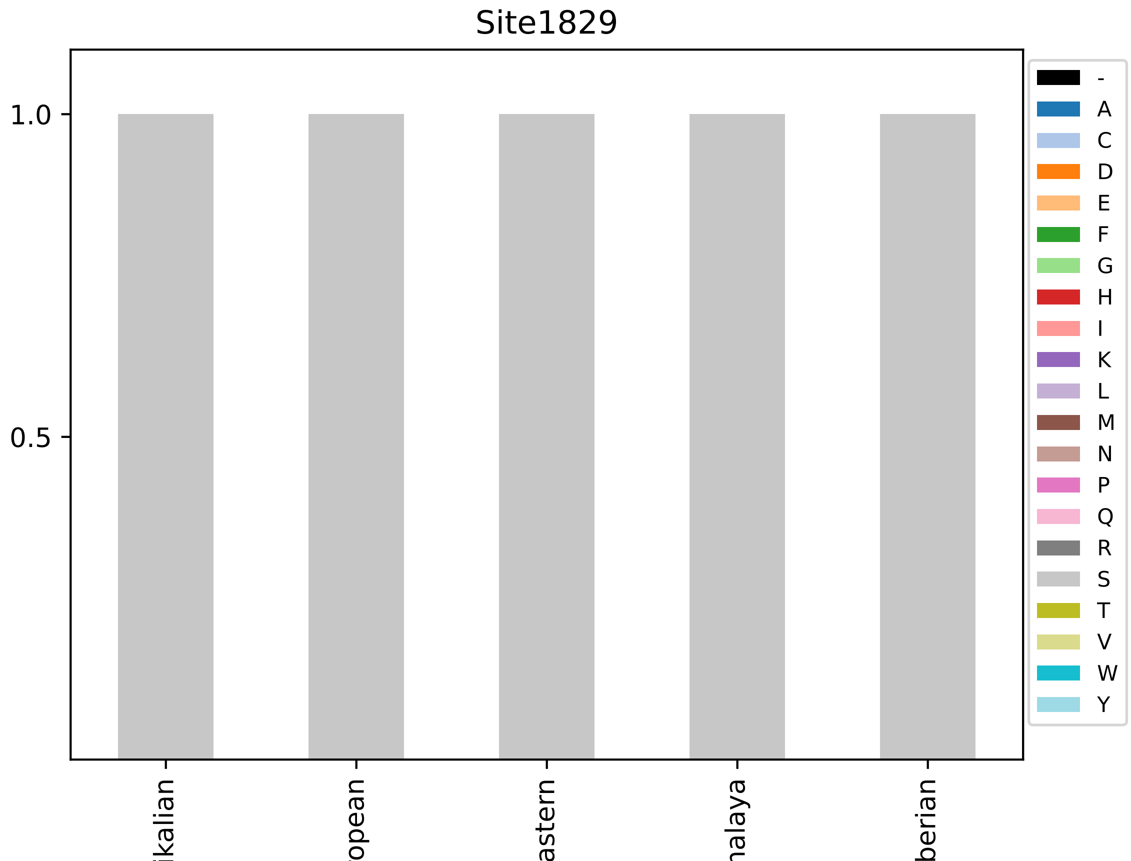

Site1829
1.0
0.5
-
A
C
D
E
F
G
H
I
K
L
M
N
P
Q
R
S
T
V
W
Y
ikalian
opean
astern
halaya
berian

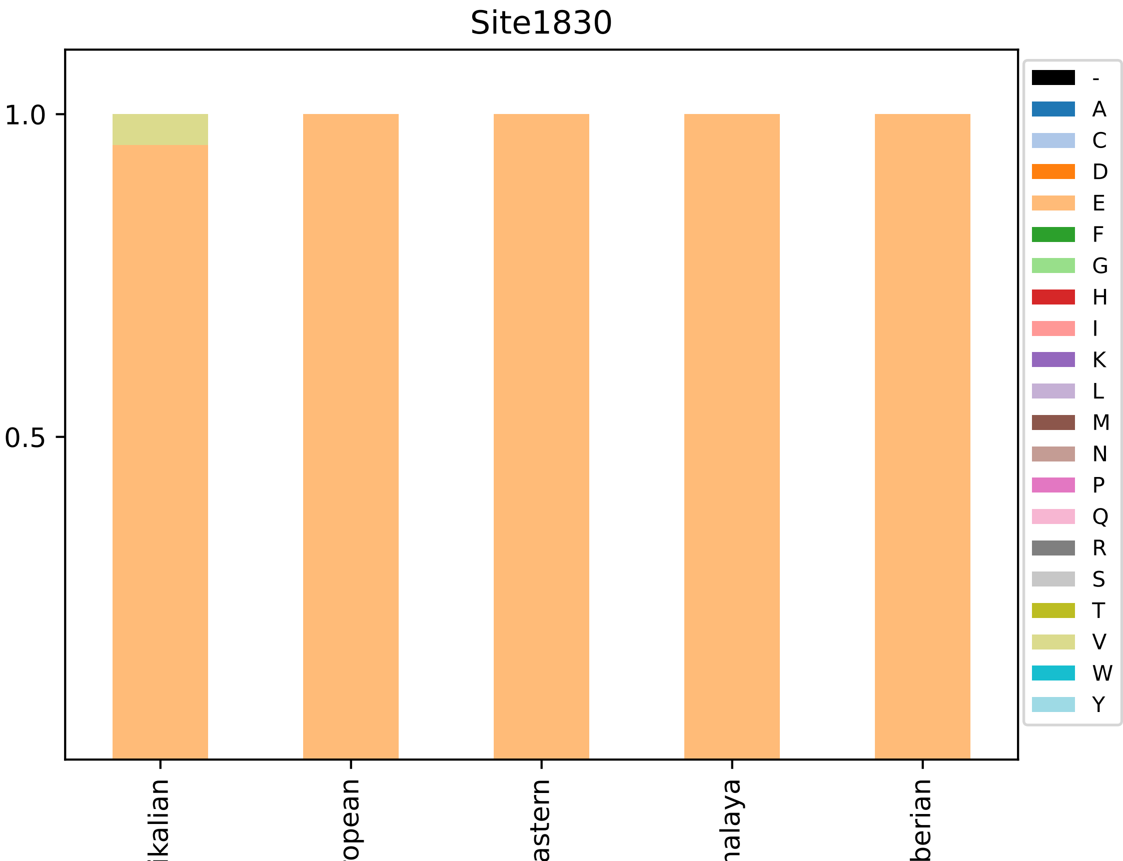

Site1830
1.0
0.5
ikalian
ropean
astern
nalaya
berian
-
A
C
D
E
F
G
H
I
K
L
M
N
P
Q
R
S
T
V
W
Y

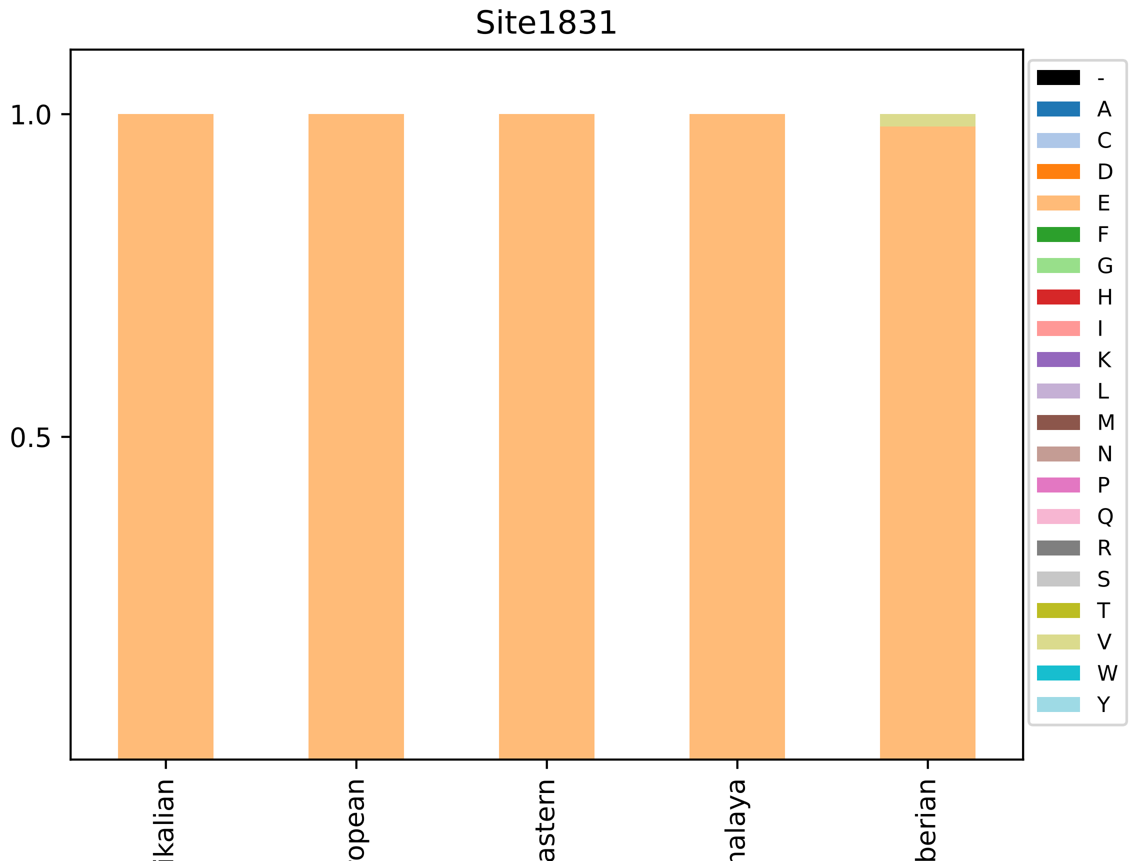
Site1831
1.0
0.5
ikalian
ropean
astern
halaya
berian
-
A
C
D
E
F
G
H
I
K
L
M
N
P
Q
R
S
T
V
W
Y

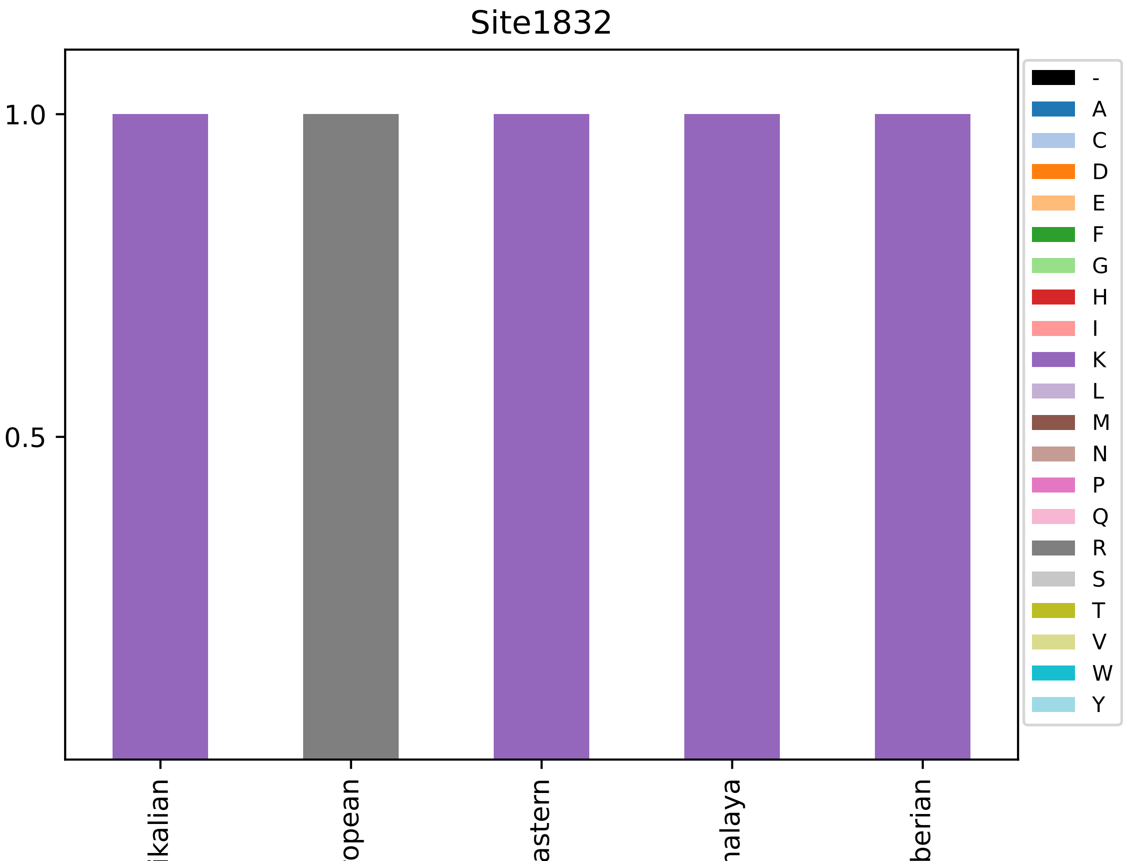
Site1832
1.0
0.5
-
A
C
D
E
F
G
H
I
K
L
M
N
P
Q
R
S
T
V
W
Y

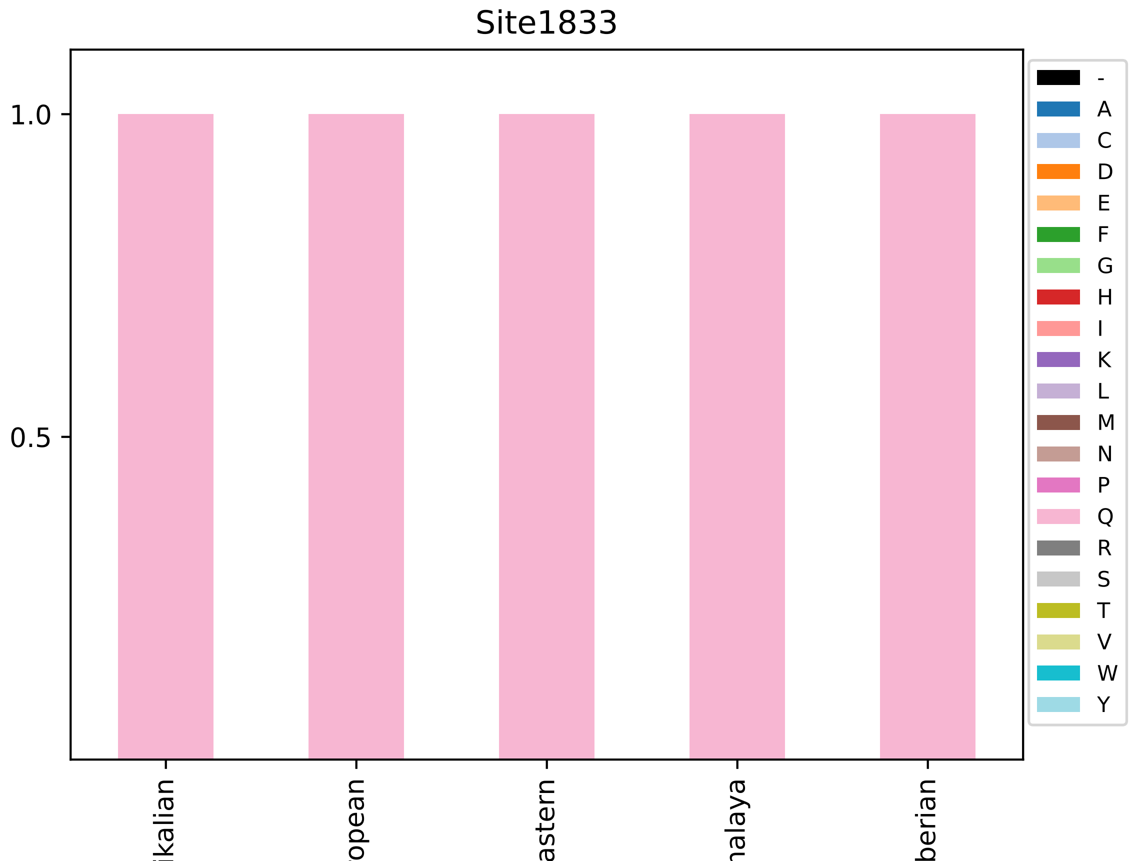
Site1833
1.0
0.5
-
A
C
D
E
F
G
H
I
K
L
M
N
P
Q
R
S
T
V
W
Y
ikalian
opean
astern
nalaya
berian

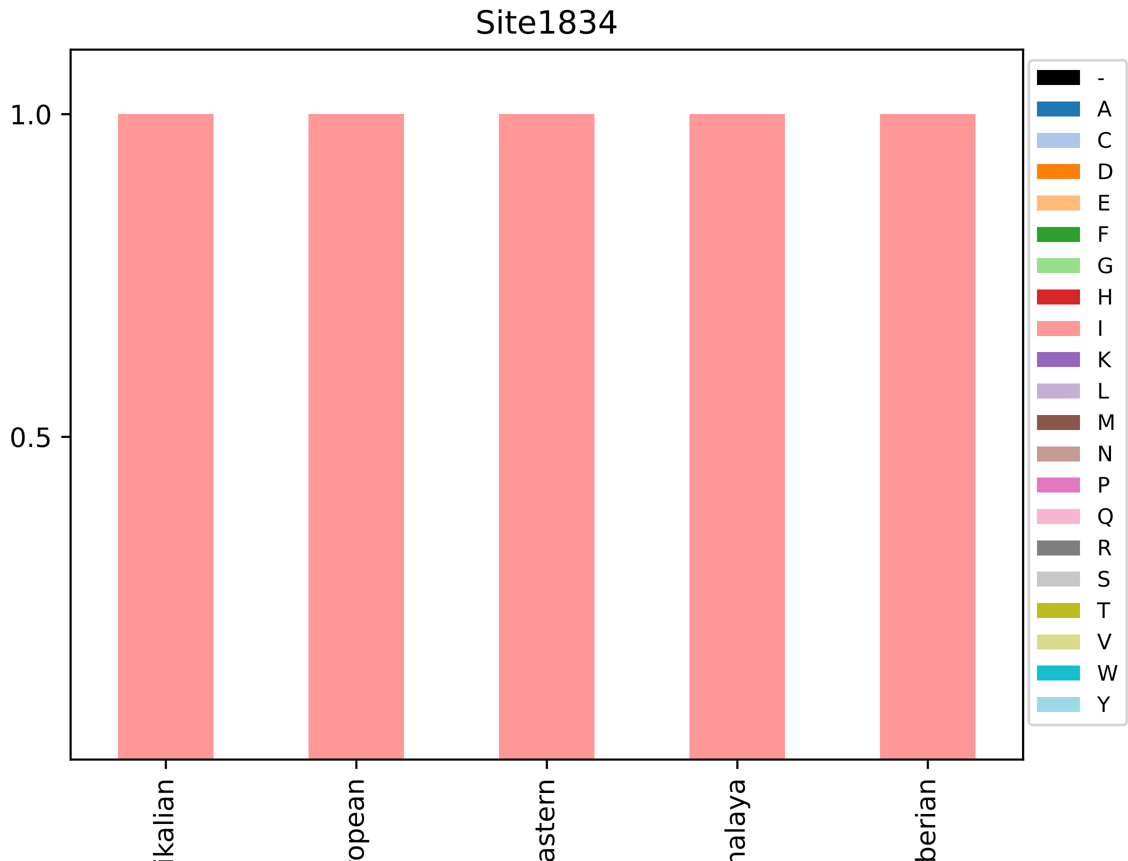
Site1834
1.0
0.5
-
A
C
D
E
F
G
H
I
K
L
M
N
P
Q
R
S
T
V
W
Y
ikalian
ropean
astern
nalaya
berian

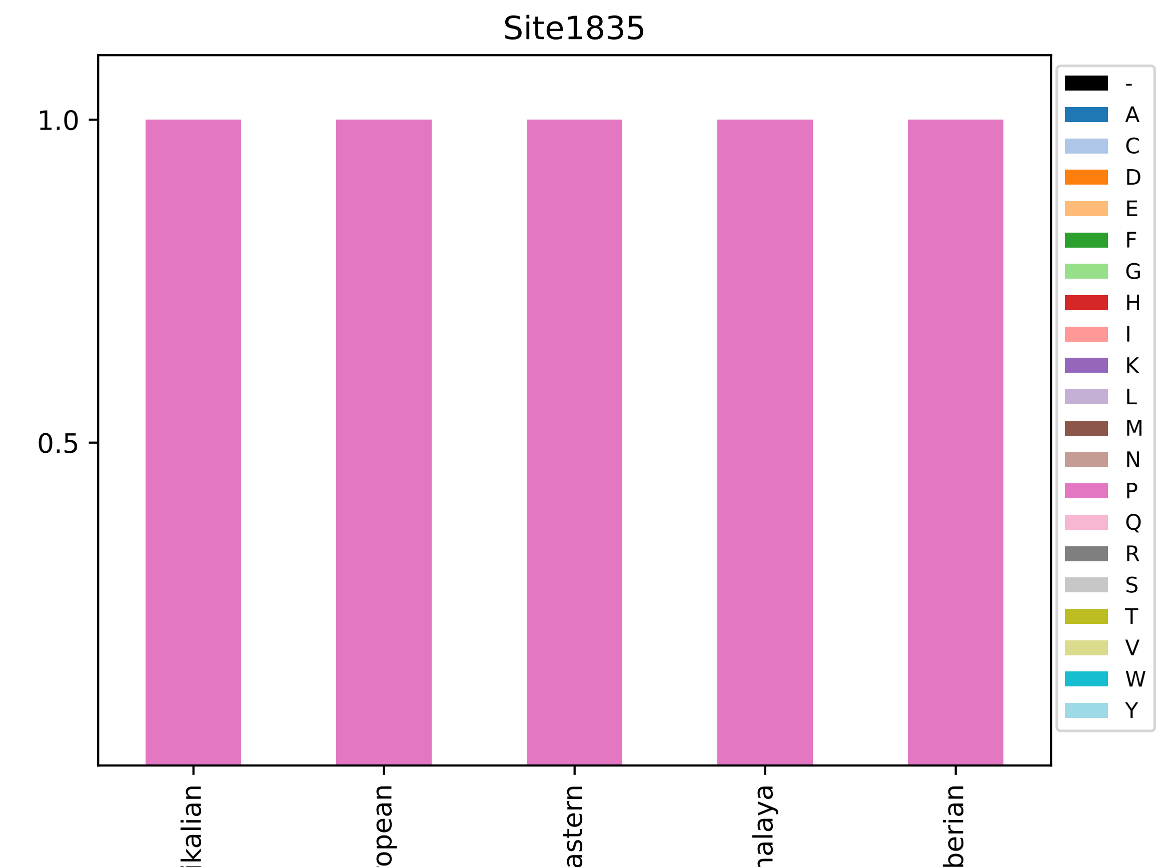
Site1835
1.0
0.5
-
A
C
D
E
F
G
H
I
K
L
M
N
P
Q
R
S
T
V
W
Y
ikalian
opean
astern
nalaya
berian

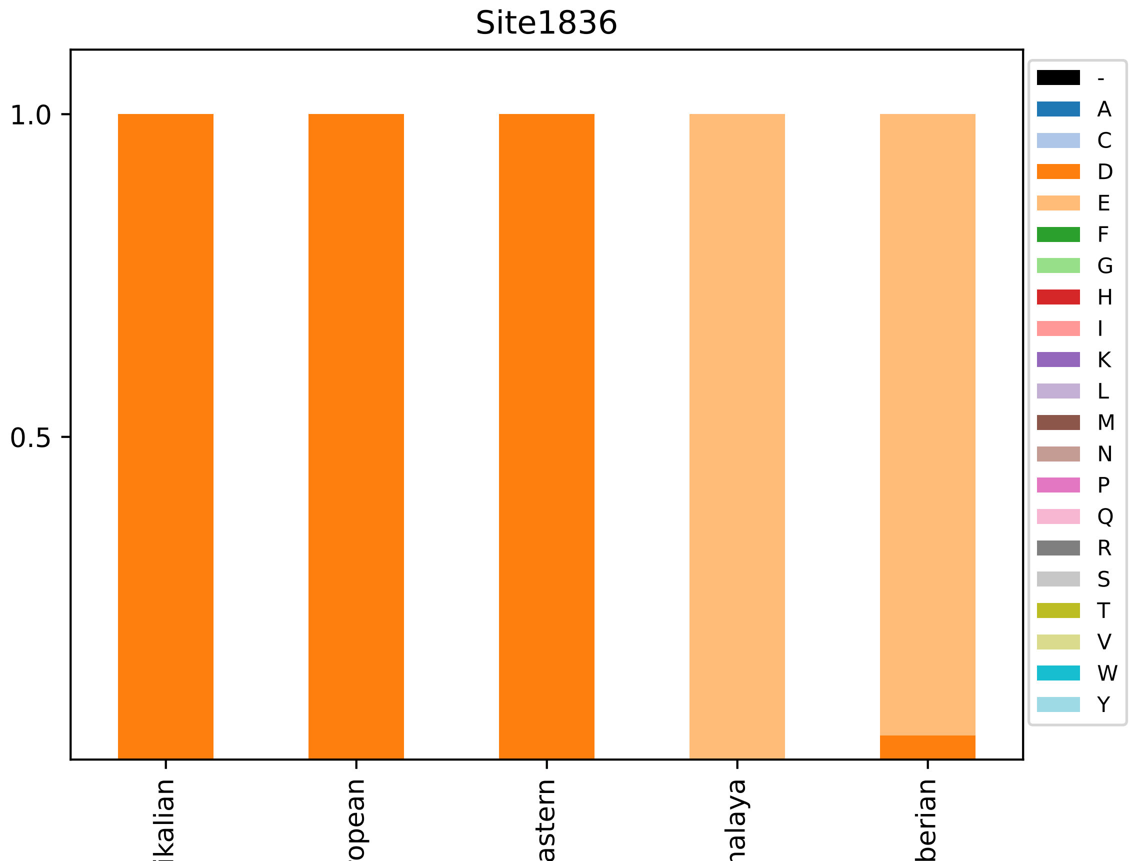
Site1836
1.0
0.5
-
A
C
D
E
F
G
H
I
K
L
M
N
P
Q
R
S
T
V
W
Y
ikalian
opean
astern
nalaya
berian

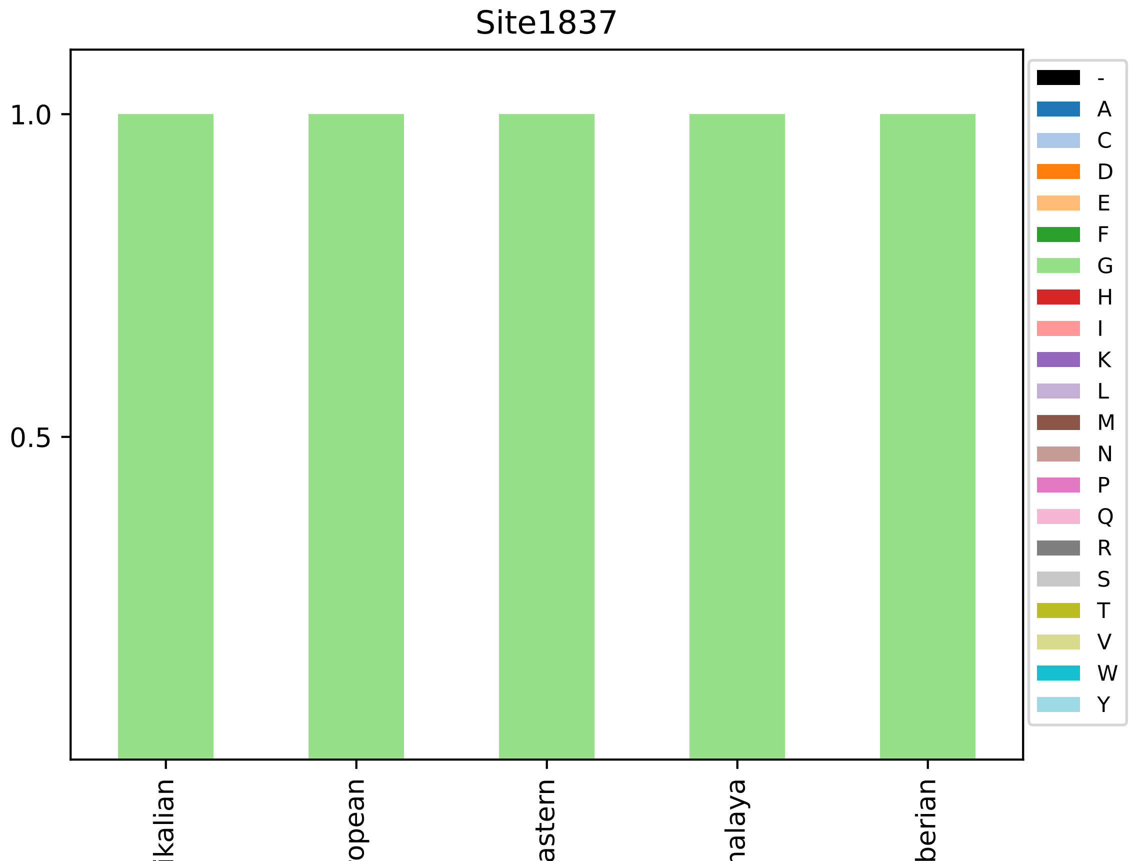
Site1837
1.0
0.5
-
A
C
D
E
F
G
H
I
K
L
M
N
P
Q
R
S
T
V
W
Y
ikalian
opean
astern
nalaya
berian

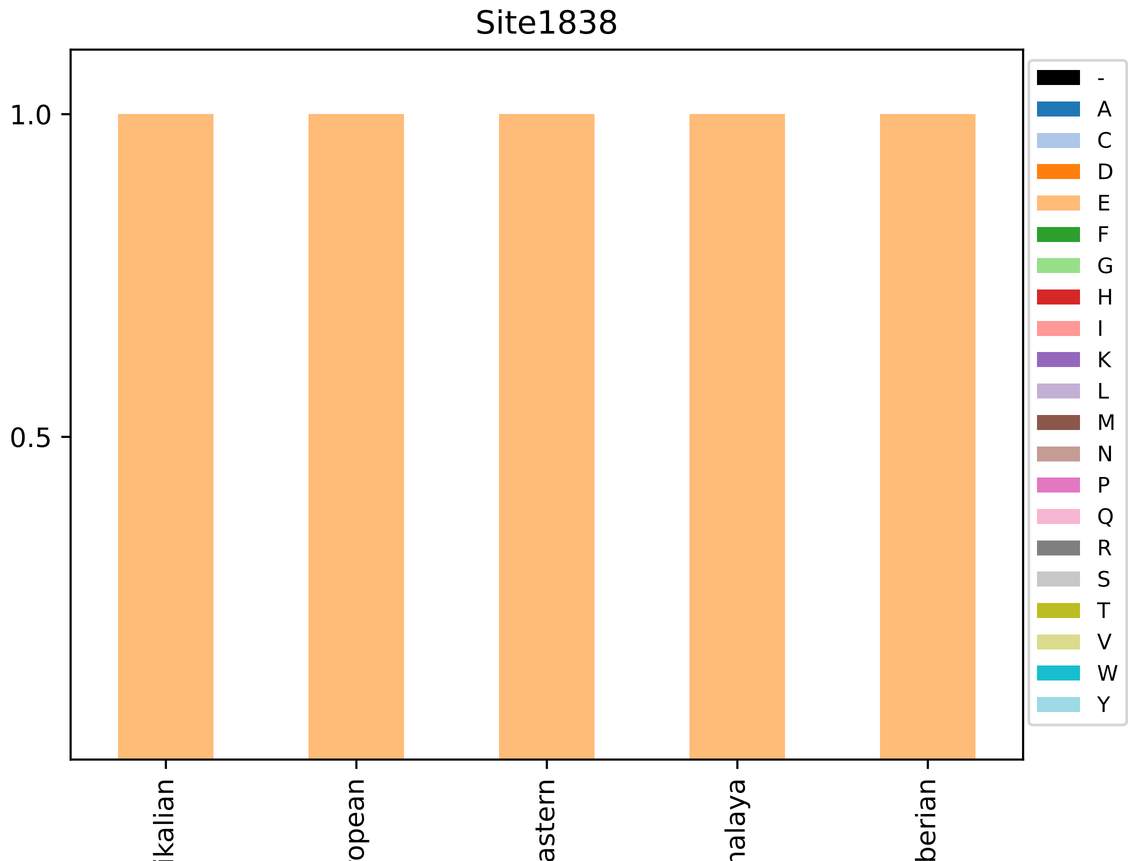

Site1838
1.0
0.5
-
A
C
D
E
F
G
H
I
K
L
M
N
P
Q
R
S
T
V
W
Y
ikalian
opean
astern
nalaya
berian

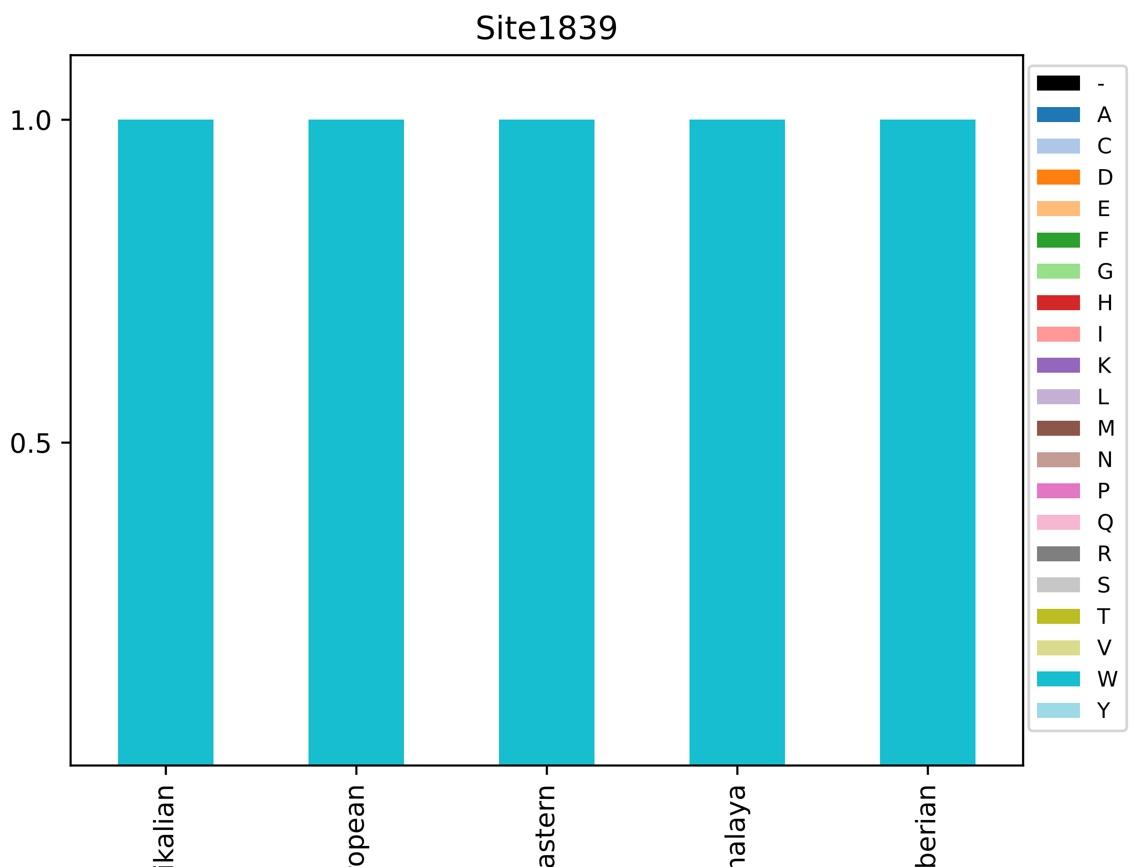
Site1839
1.0
0.5
-
A
C
D
E
F
G
H
I
K
L
M
N
P
Q
R
S
T
V
W
Y
ikalian
opean
astern
nalaya
berian

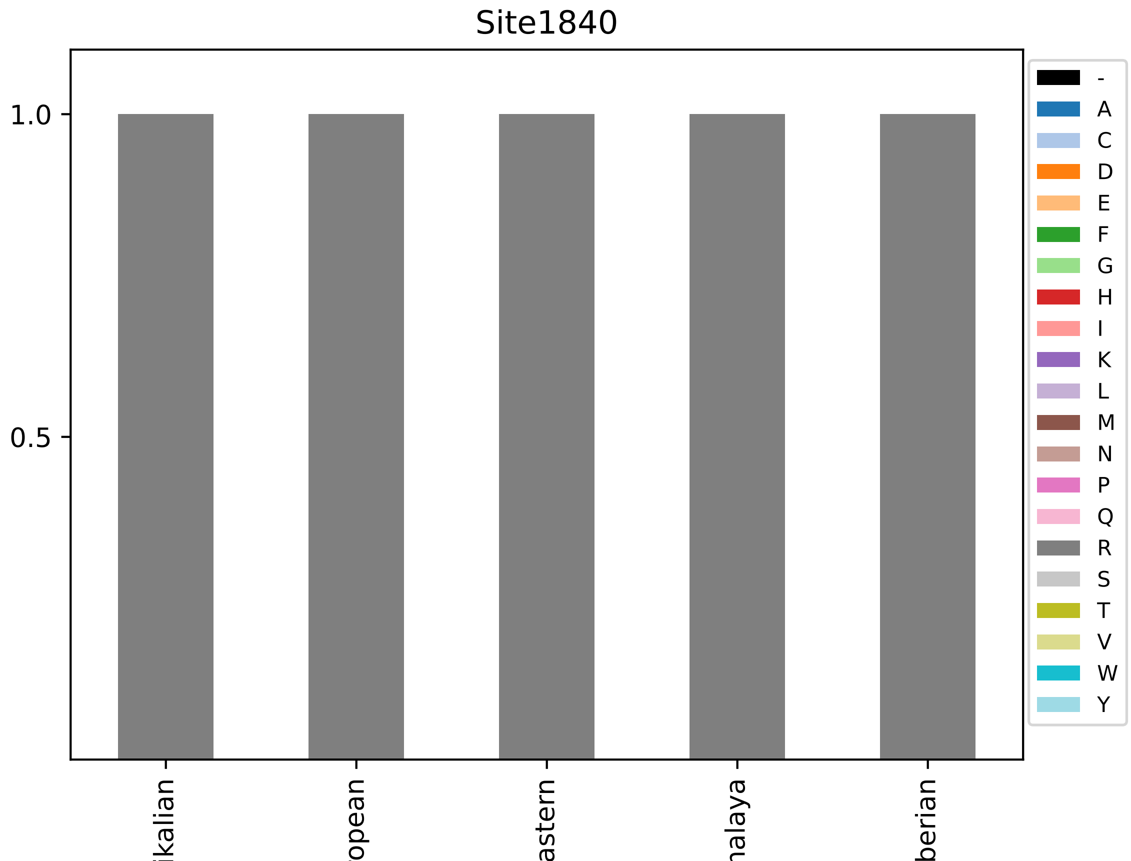
Site1840
1.0
0.5
-
A
C
D
E
F
G
H
I
K
L
M
N
P
Q
R
S
T
V
W
Y
ikalian
ropean
astern
nalaya
berian

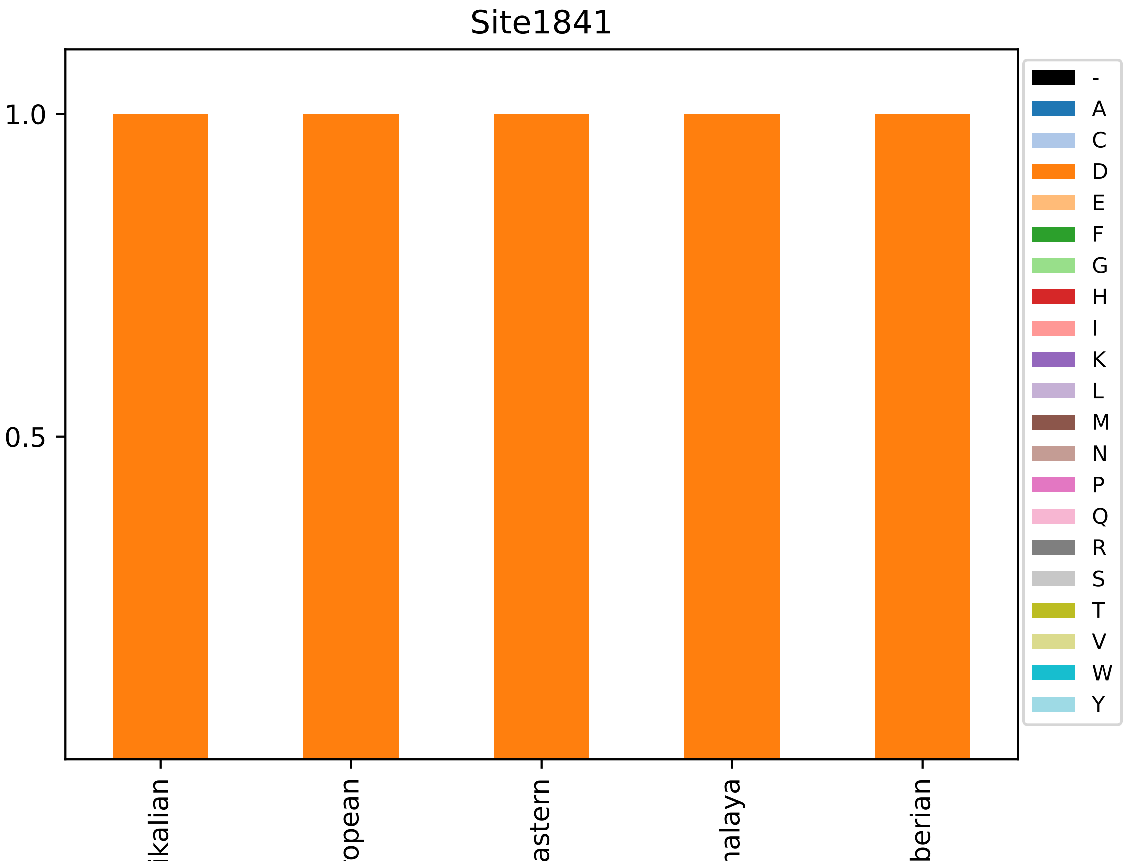

Site1841
1.0
0.5
ikalian
opean
astern
nalaya
berian
-
A
C
D
E
F
G
H
I
K
L
M
N
P
Q
R
S
T
V
W
Y

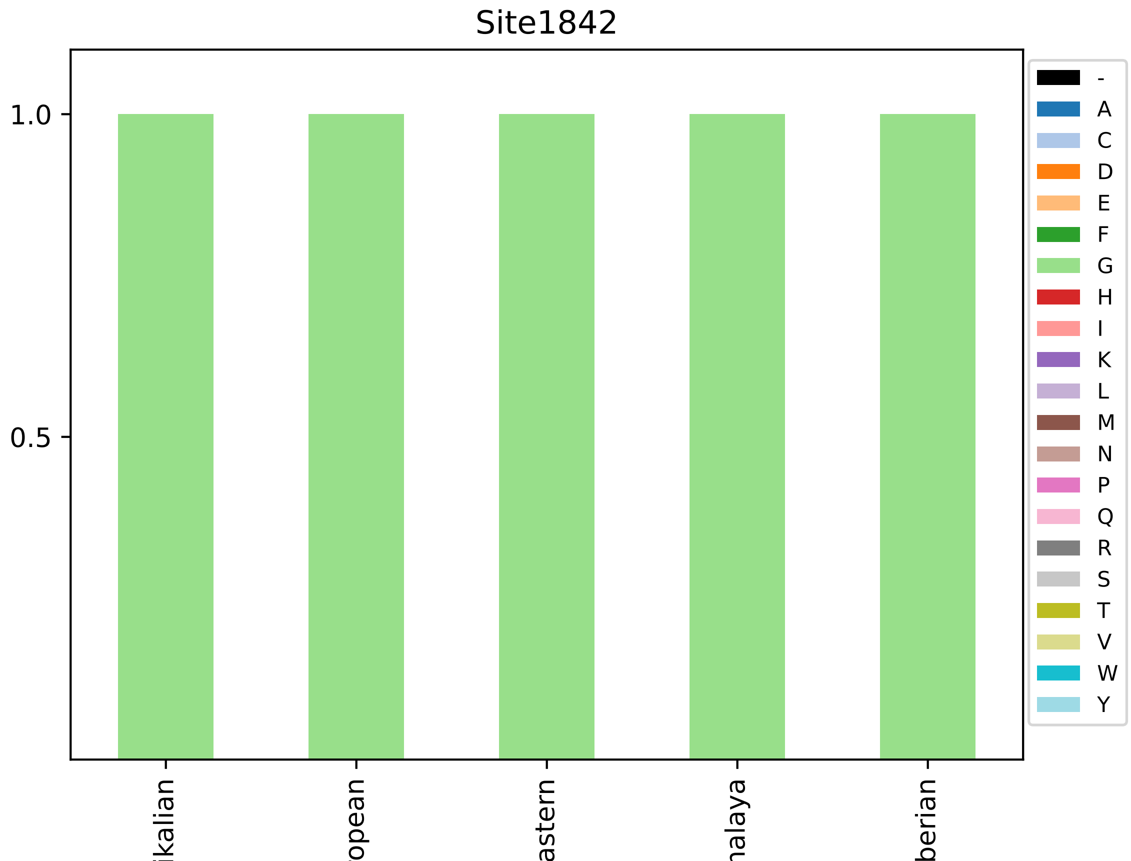
Site1842
1.0
0.5
ikalian
opean
astern
nalaya
berian
-
A
C
D
E
F
G
H
I
K
L
M
N
P
Q
R
S
T
V
W
Y

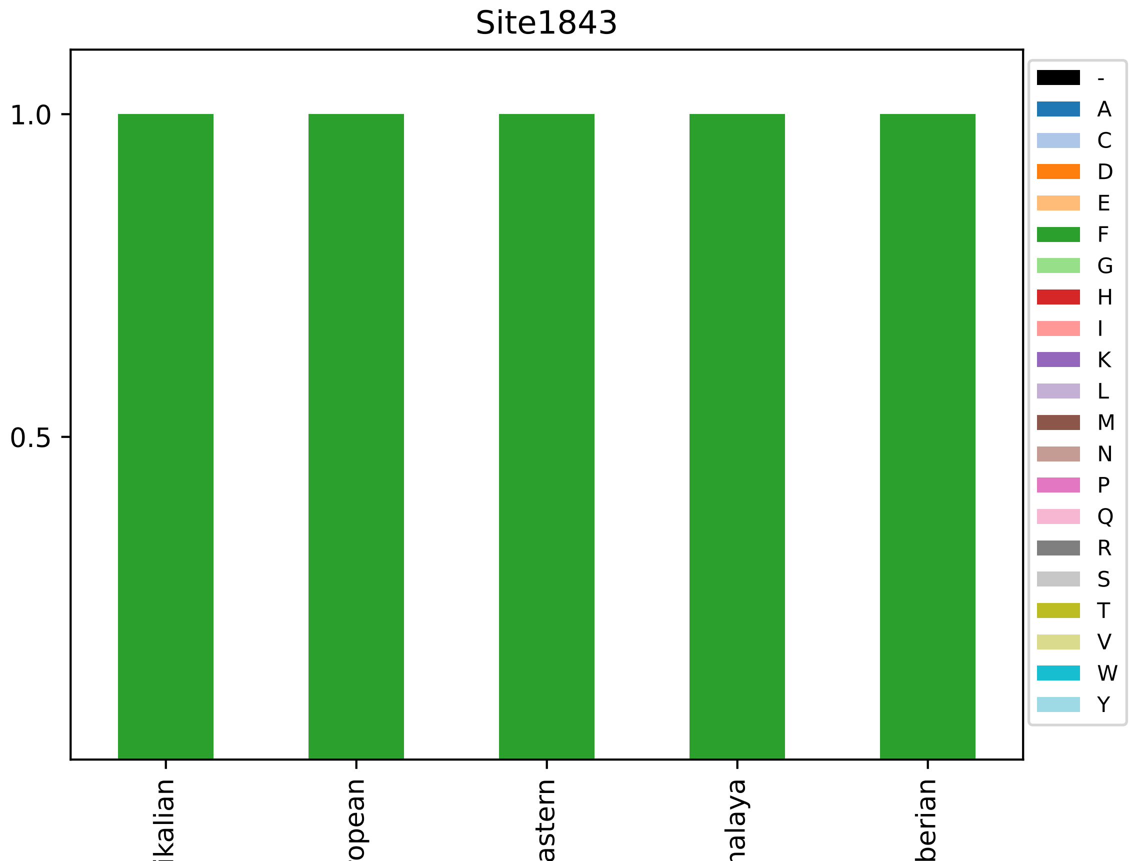

Site1843
1.0
0.5
-
A
C
D
E
F
G
H
I
K
L
M
N
P
Q
R
S
T
V
W
Y

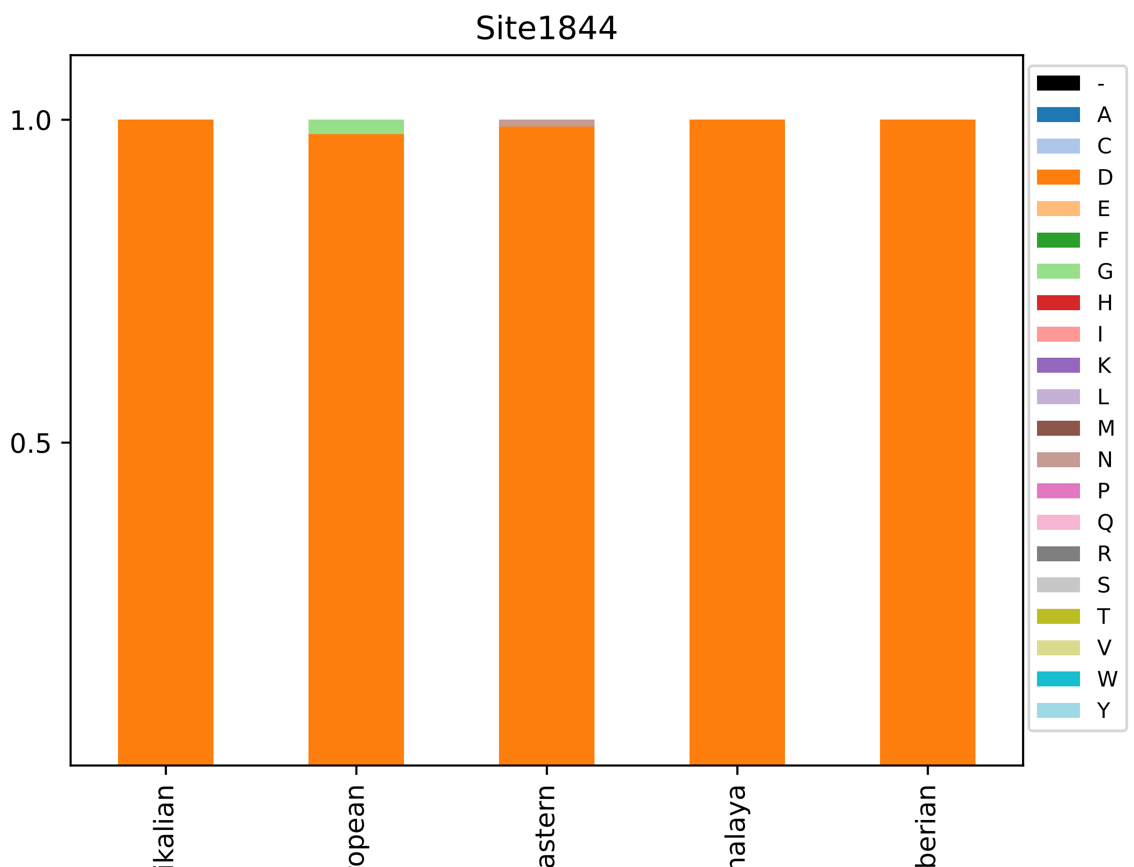

Site1844
1.0
0.5
-
A
C
D
E
F
G
H
I
K
L
M
N
P
Q
R
S
T
V
W
Y
ikalian
opean
astern
nalaya
berian

Site1845

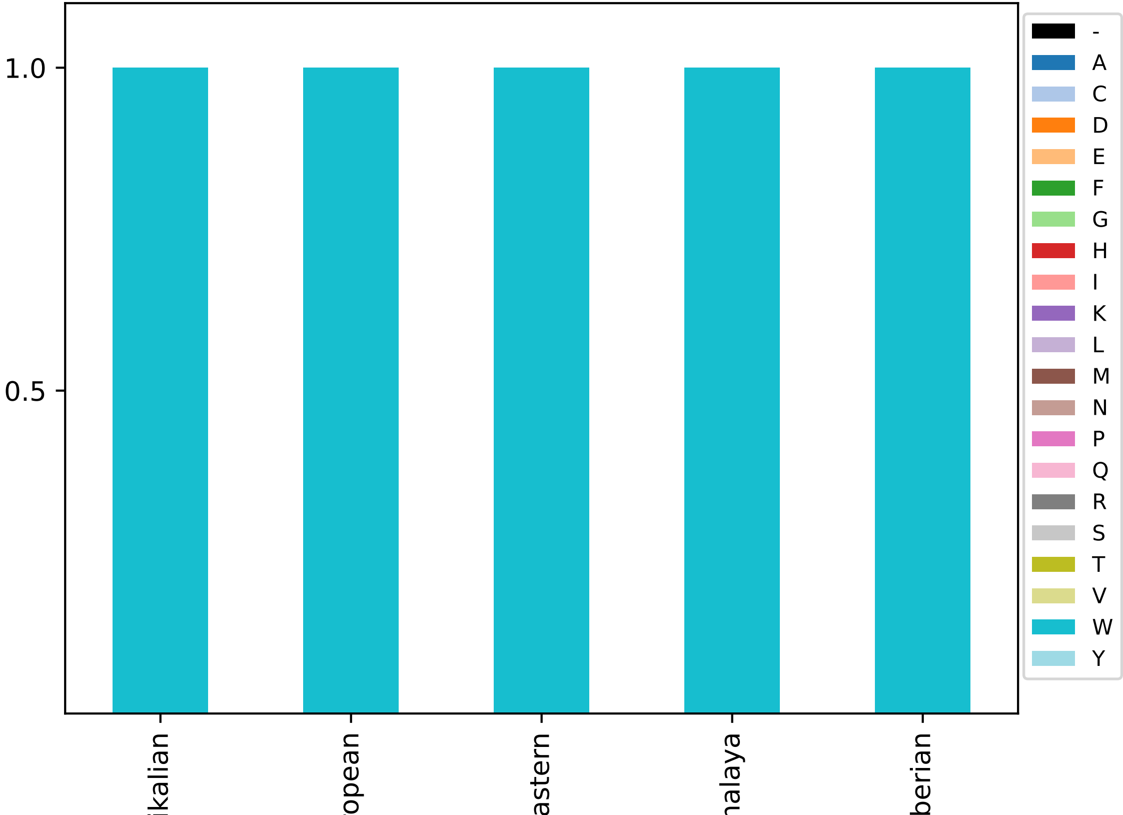

1.0
0.5
-
A
C
D
E
F
G
H
I
K
L
M
N
P
Q
R
S
T
V
W
Y
ikalian
opean
astern
nalaya
berian

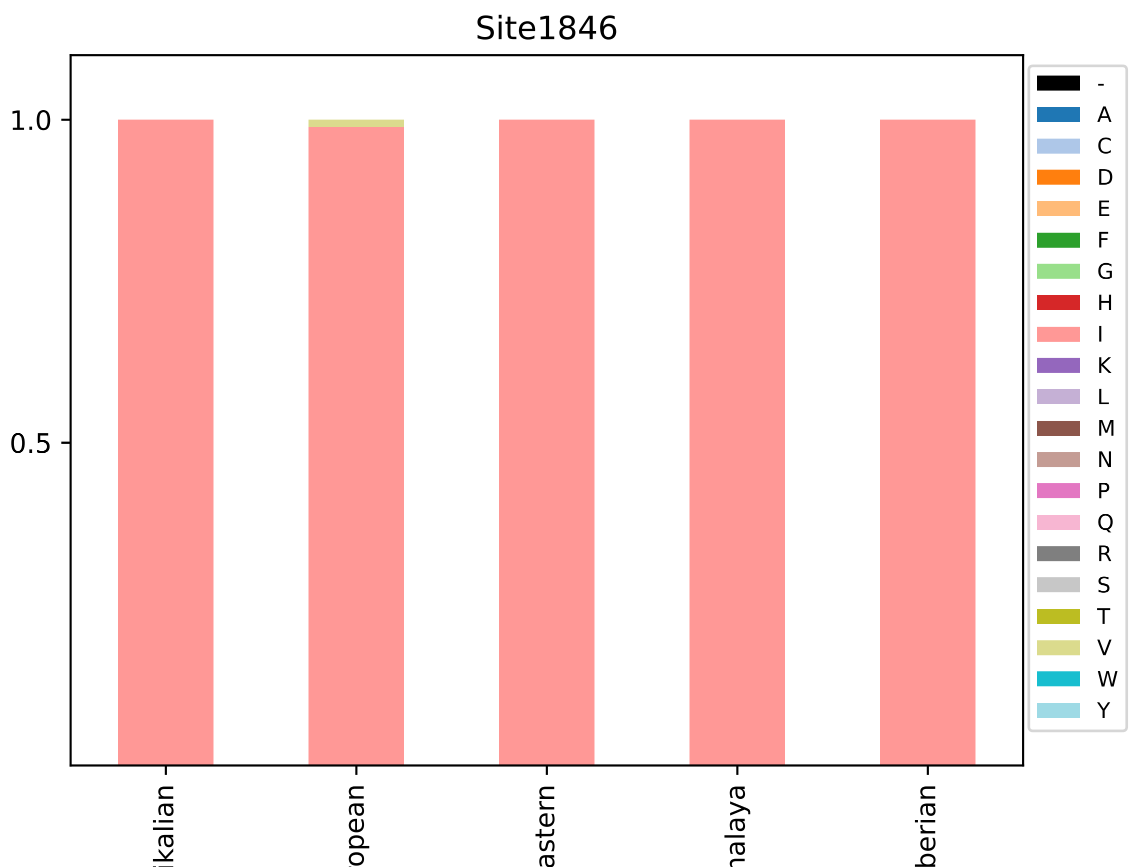

Site1846
1.0
0.5
-
A
C
D
E
F
G
H
I
K
L
M
N
P
Q
R
S
T
V
W
Y
ikalian
opean
astern
nalaya
berian

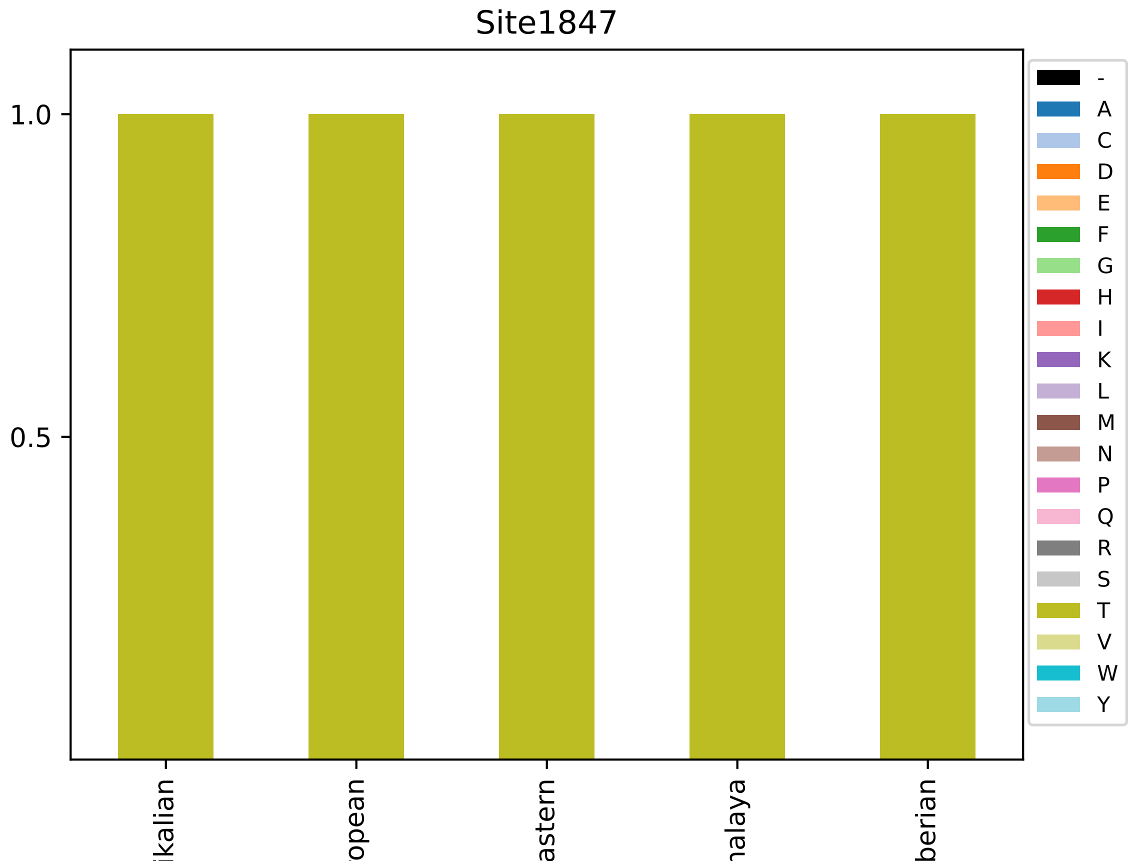
Site1847
1.0
0.5
-
A
C
D
E
F
G
H
I
K
L
M
N
P
Q
R
S
T
V
W
Y
ikalian
opean
astern
nalaya
berian

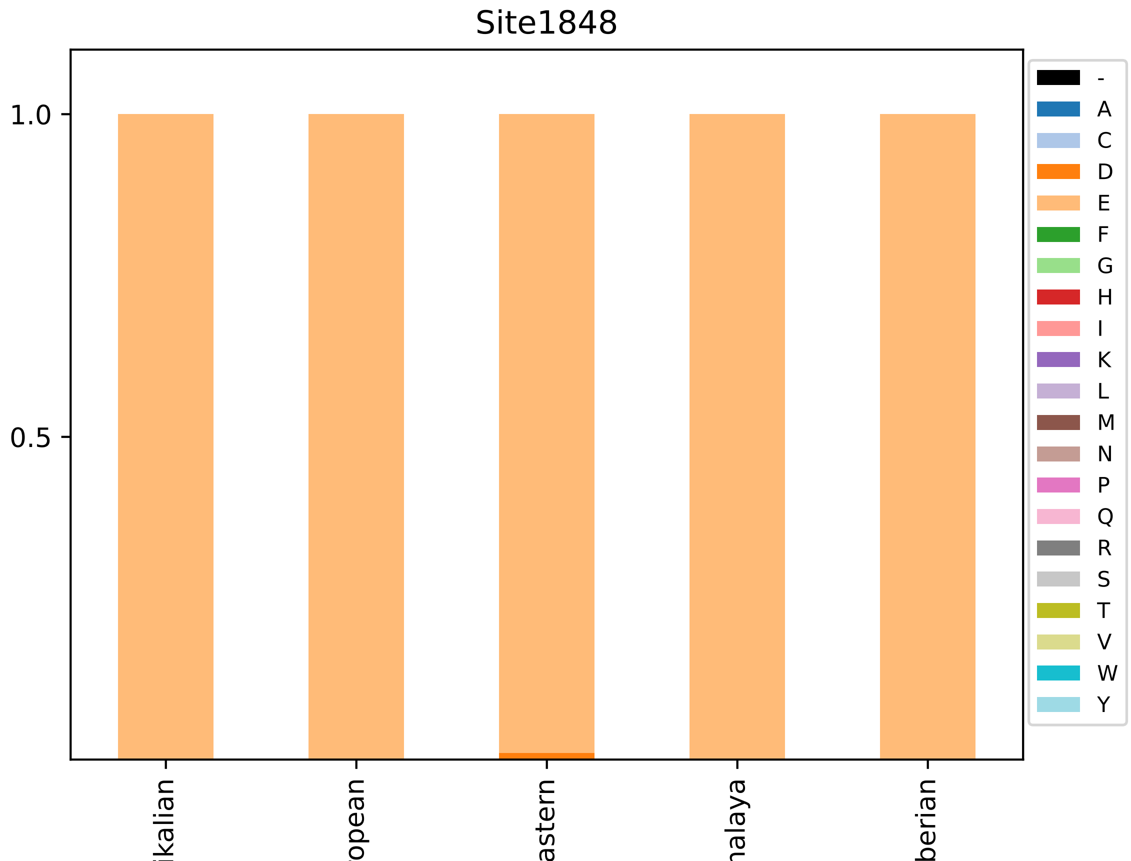
Site1848
1.0
0.5
ikalian
opean
astern
nalaya
berian
-
A
C
D
E
F
G
H
I
K
L
M
N
P
Q
R
S
T
V
W
Y

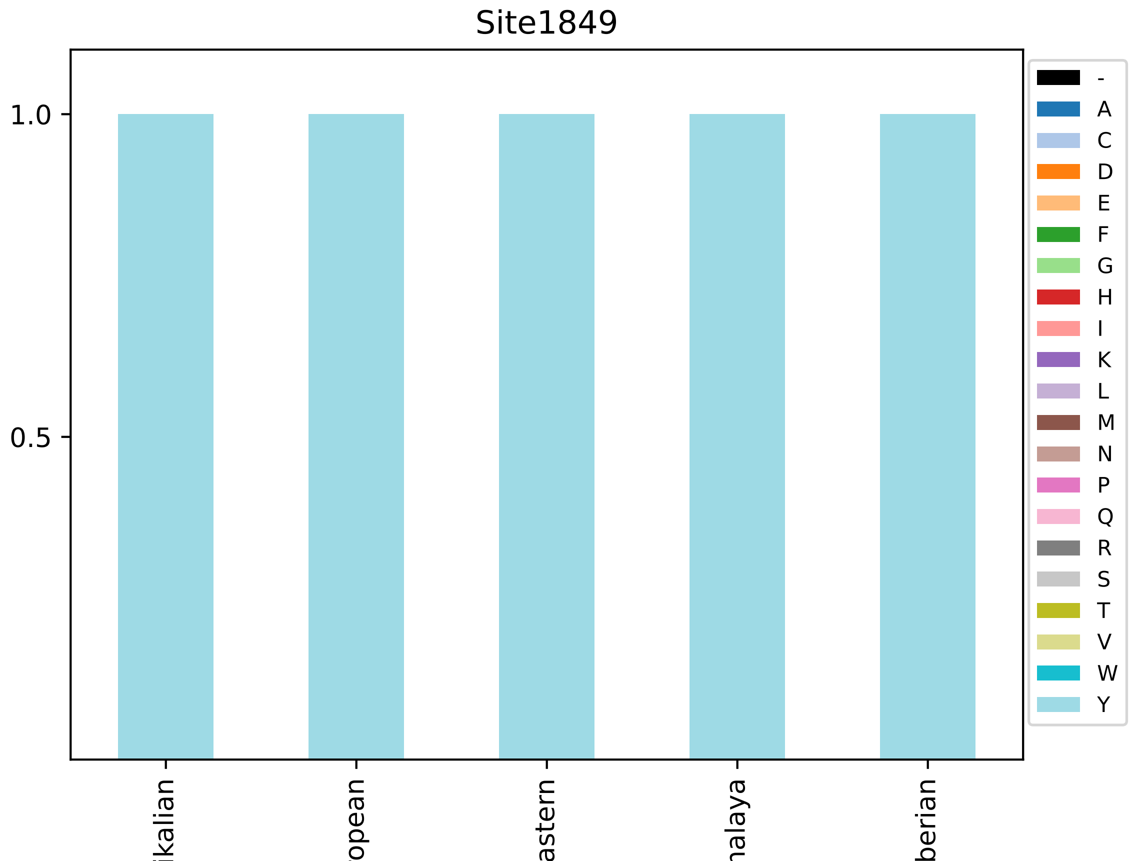
Site1849
1.0
0.5
-
A
C
D
E
F
G
H
I
K
L
M
N
P
Q
R
S
T
V
W
Y
ikalian
opean
astern
nalaya
berian

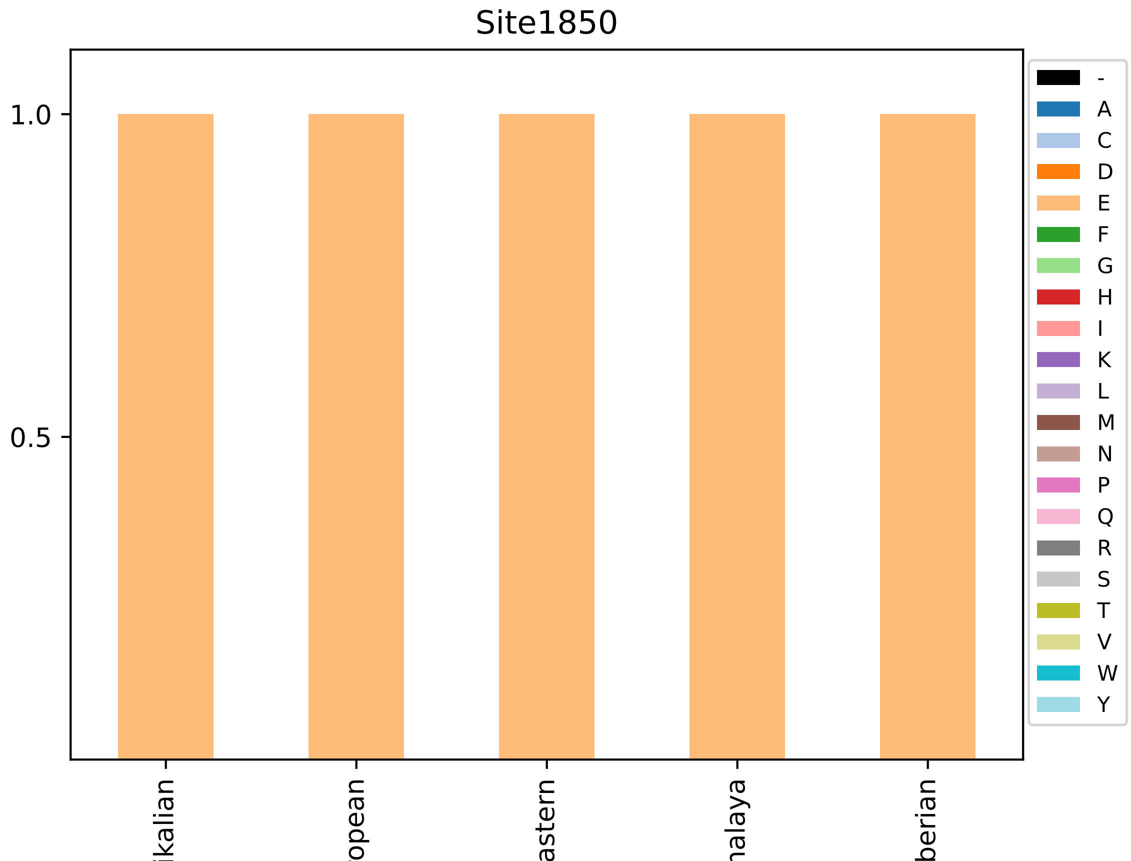
Site1850
1.0
0.5
-
A
C
D
E
F
G
H
I
K
L
M
N
P
Q
R
S
T
V
W
Y
ikalian
opean
astern
nalaya
berian

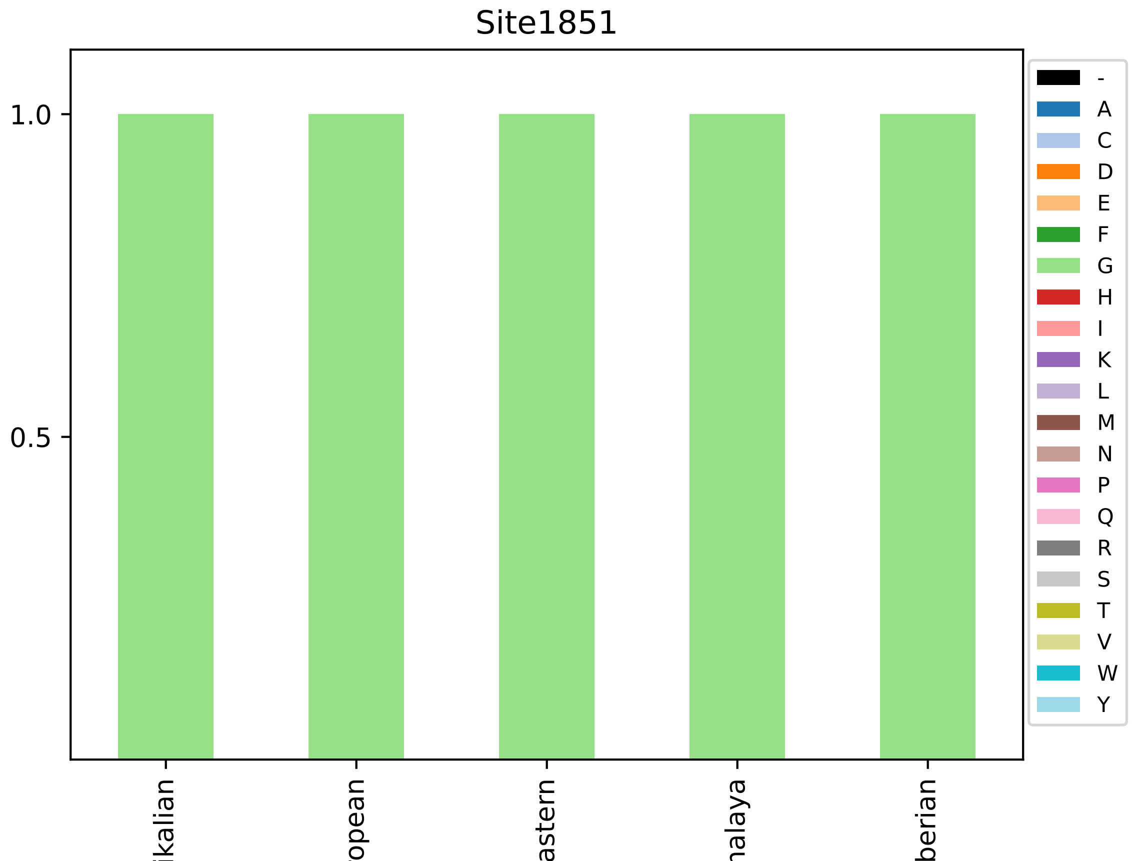
Site1851
1.0
0.5
ikalian
opean
astern
halaya
berian
-
A
C
D
E
F
G
H
I
K
L
M
N
P
Q
R
S
T
V
W
Y

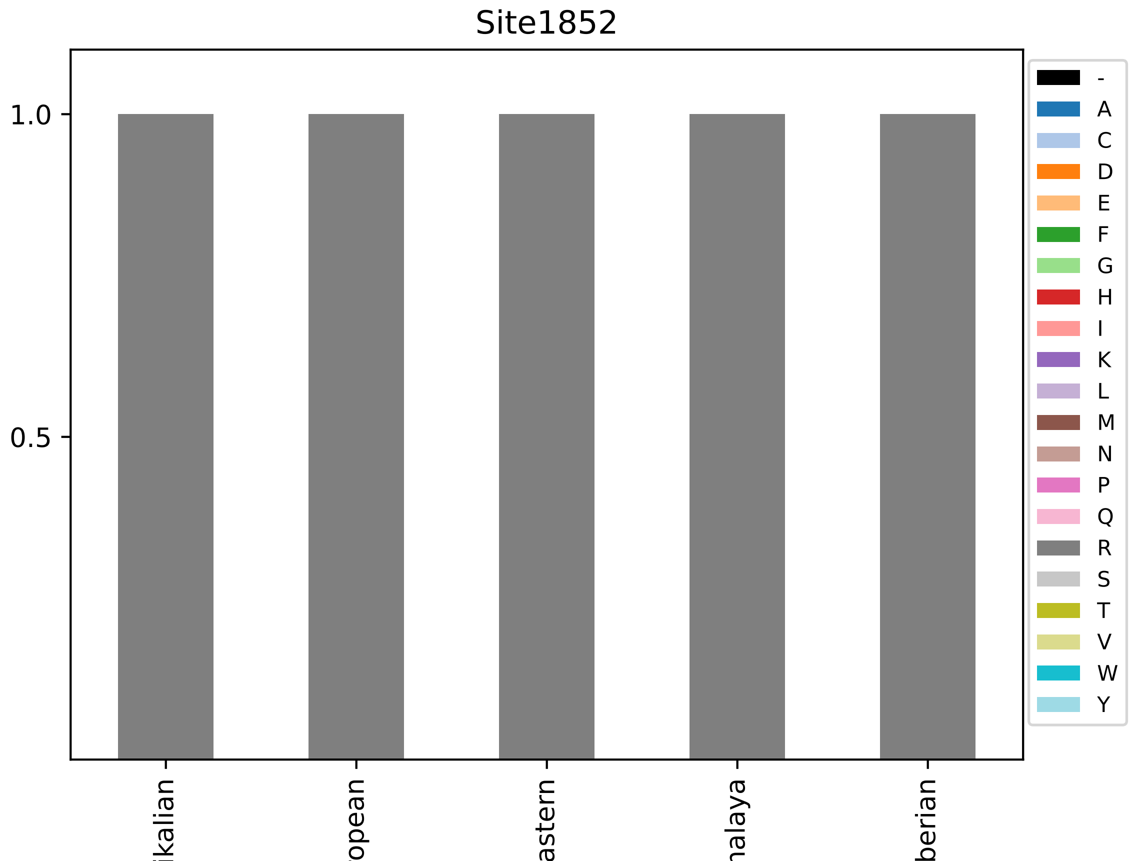
Site1852
1.0
0.5
-
A
C
D
E
F
G
H
I
K
L
M
N
P
Q
R
S
T
V
W
Y
ikalian
opean
astern
nalaya
berian

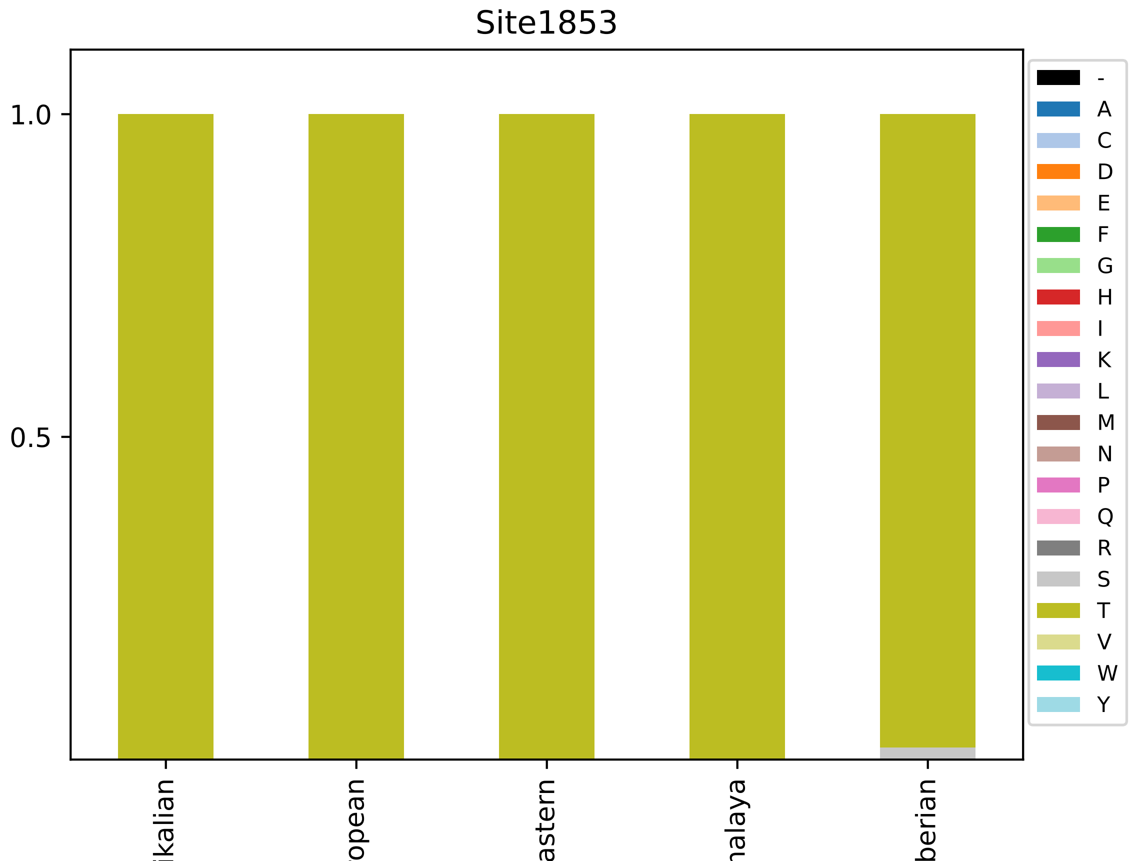
Site1853
1.0
0.5
-
A
C
D
E
F
G
H
I
K
L
M
N
P
Q
R
S
T
V
W
Y
ikalian
opean
astern
nalaya
berian

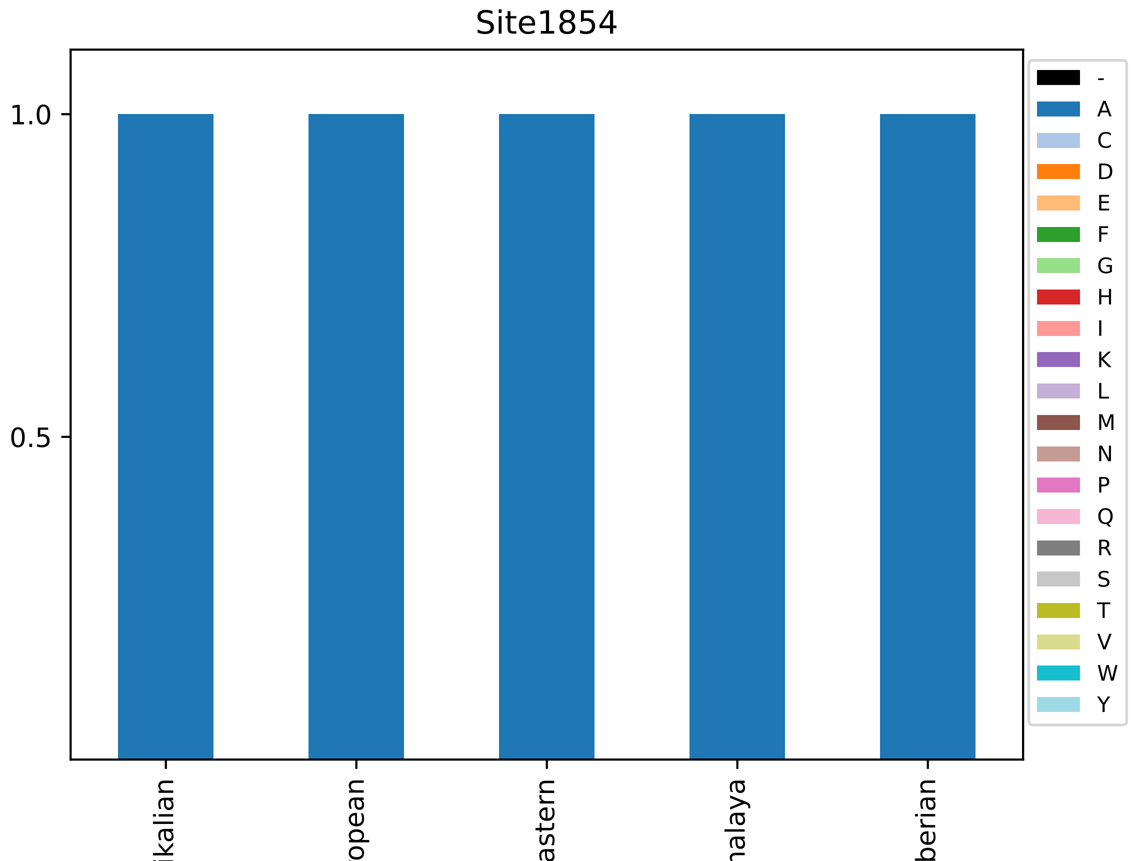

Site1854
1.0
0.5
-
A
C
D
E
F
G
H
I
K
L
M
N
P
Q
R
S
T
V
W
Y
ikalian
opean
astern
nalaya
berian

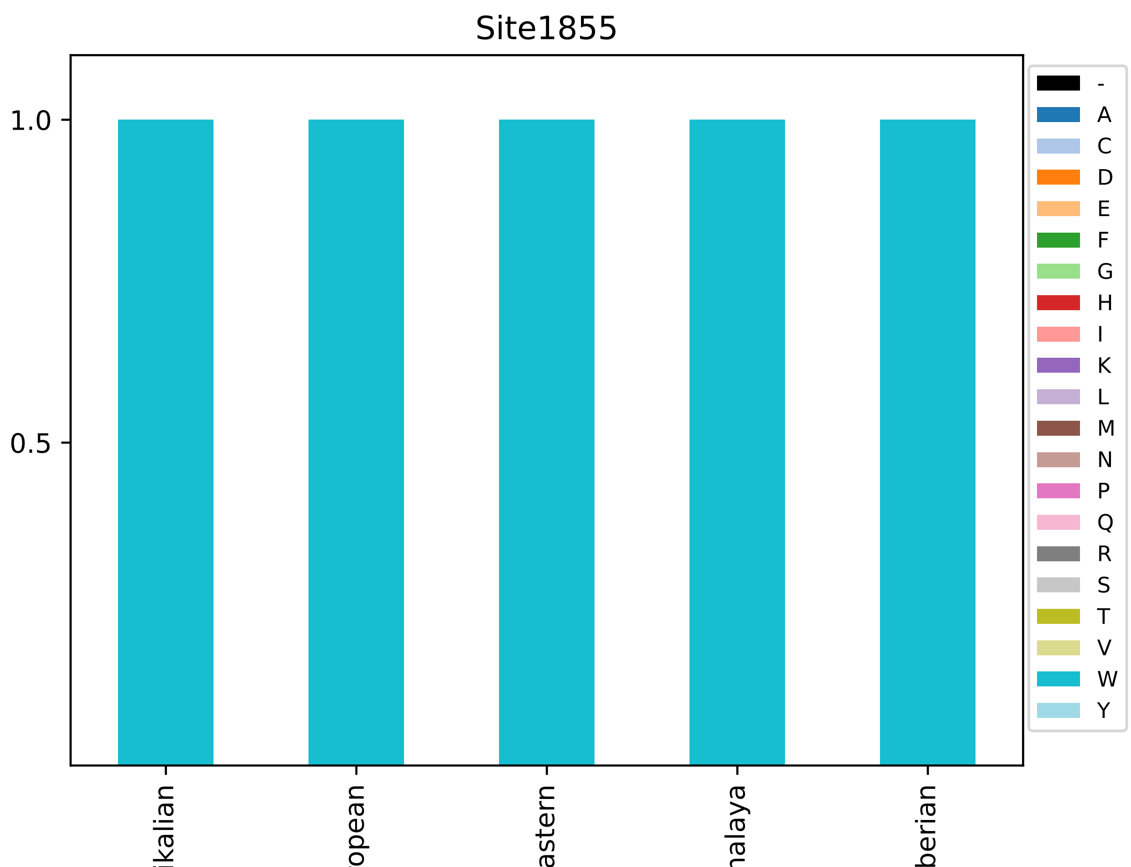

Site1855
1.0
0.5
-
A
C
D
E
F
G
H
I
K
L
M
N
P
Q
R
S
T
V
W
Y
ikalian
opean
astern
nalaya
berian

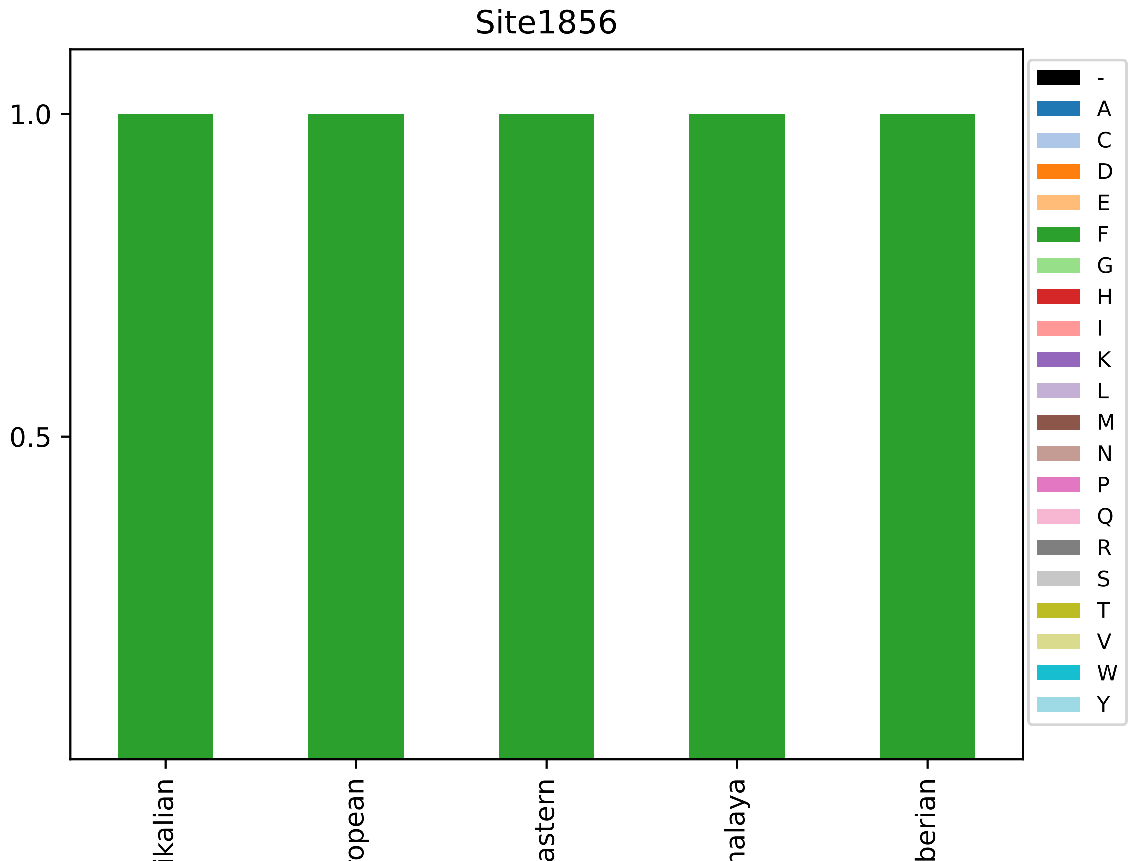
Site1856
1.0
0.5
-
A
C
D
E
F
G
H
I
K
L
M
N
P
Q
R
S
T
V
W
Y
ikalian
ropean
astern
nalaya
berian

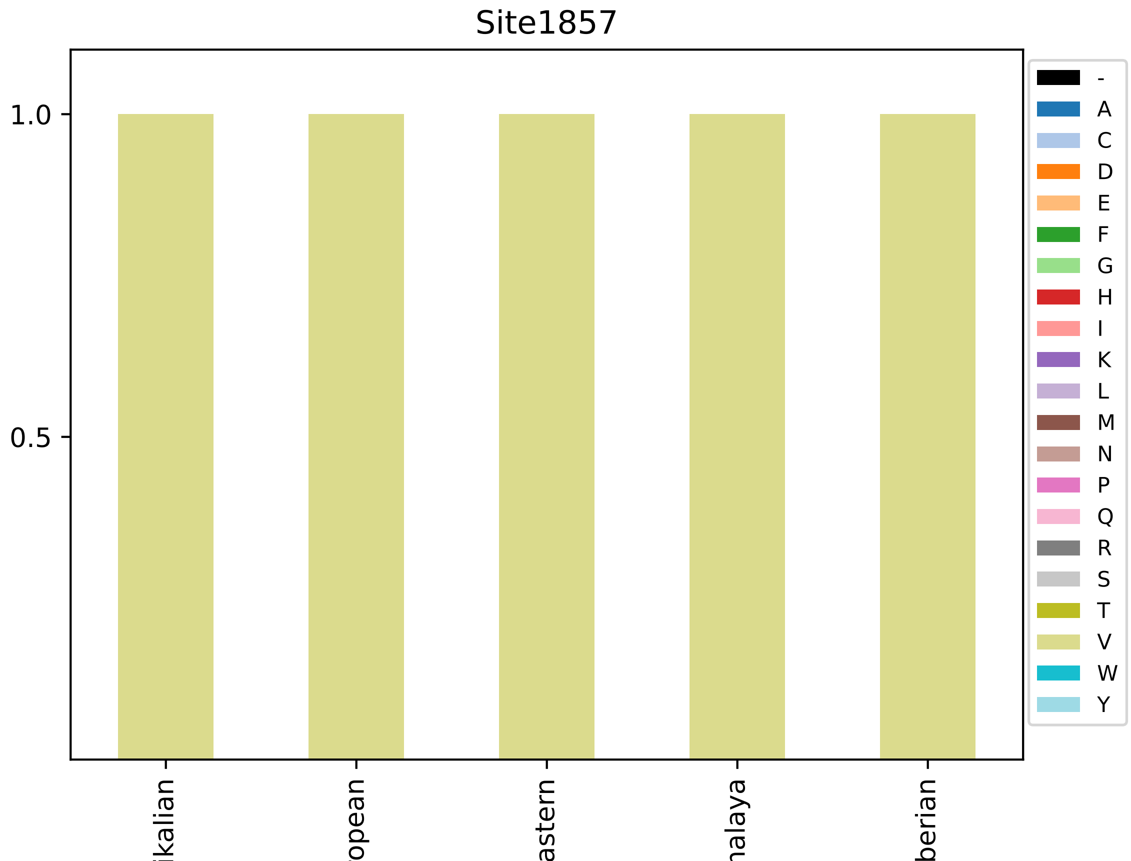

Site1857
1.0
0.5
-
A
C
D
E
F
G
H
I
K
L
M
N
P
Q
R
S
T
V
W
Y
ikalian
opean
astern
nalaya
berian

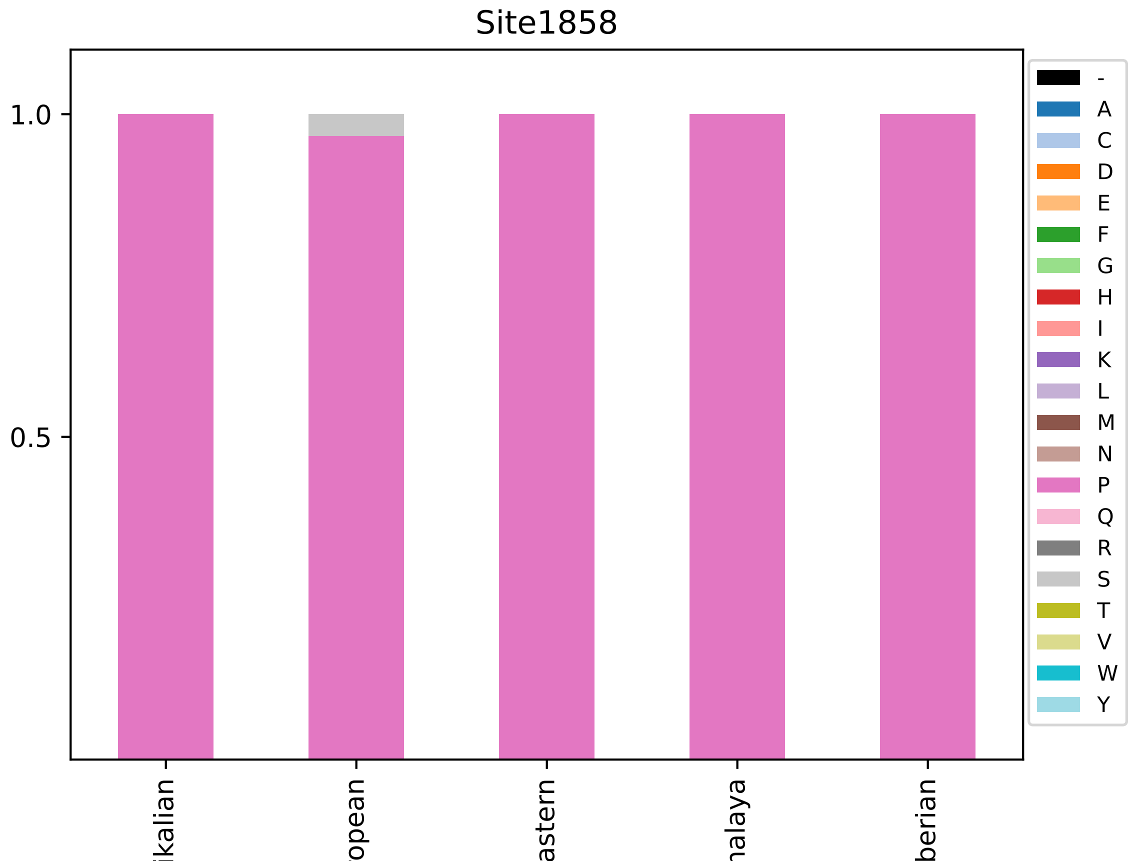

Site1858
1.0
0.5
-
A
C
D
E
F
G
H
I
K
L
M
N
P
Q
R
S
T
V
W
Y
ikalian
opean
astern
nalaya
berian

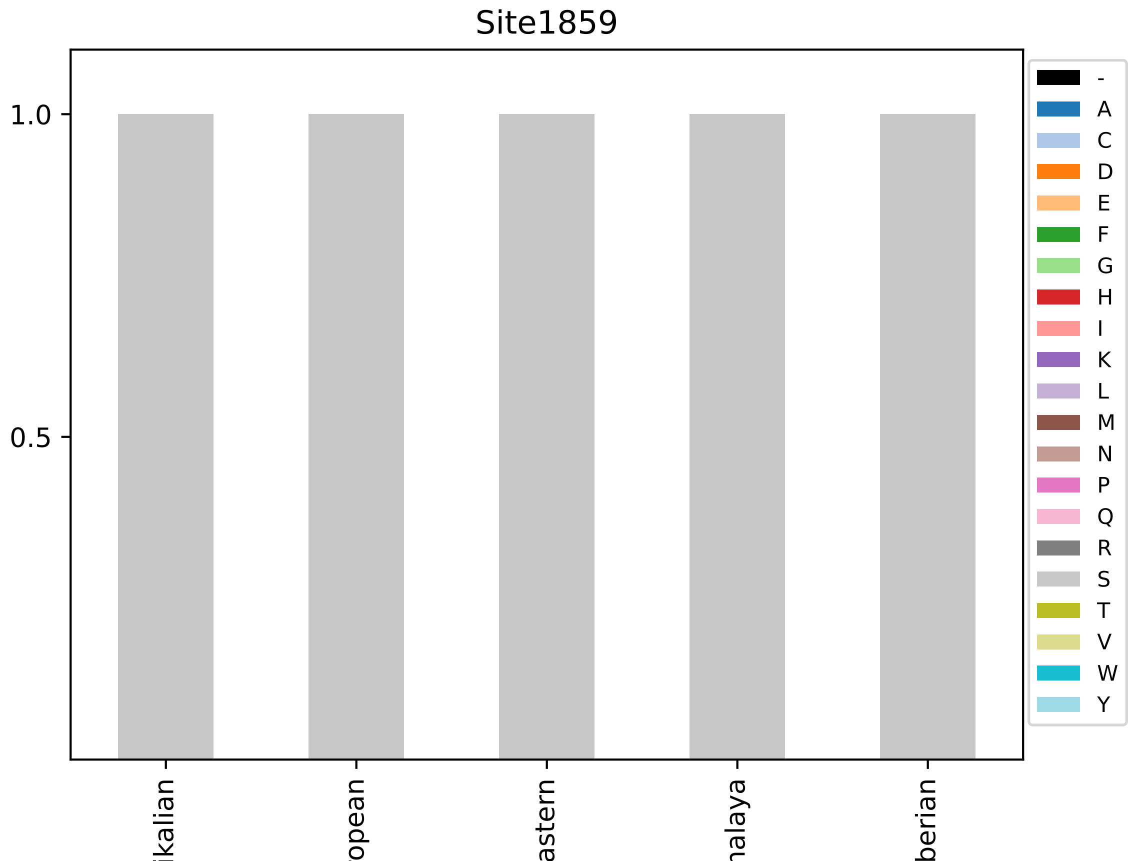
Site1859
1.0
0.5
-
A
C
D
E
F
G
H
I
K
L
M
N
P
Q
R
S
T
V
W
Y

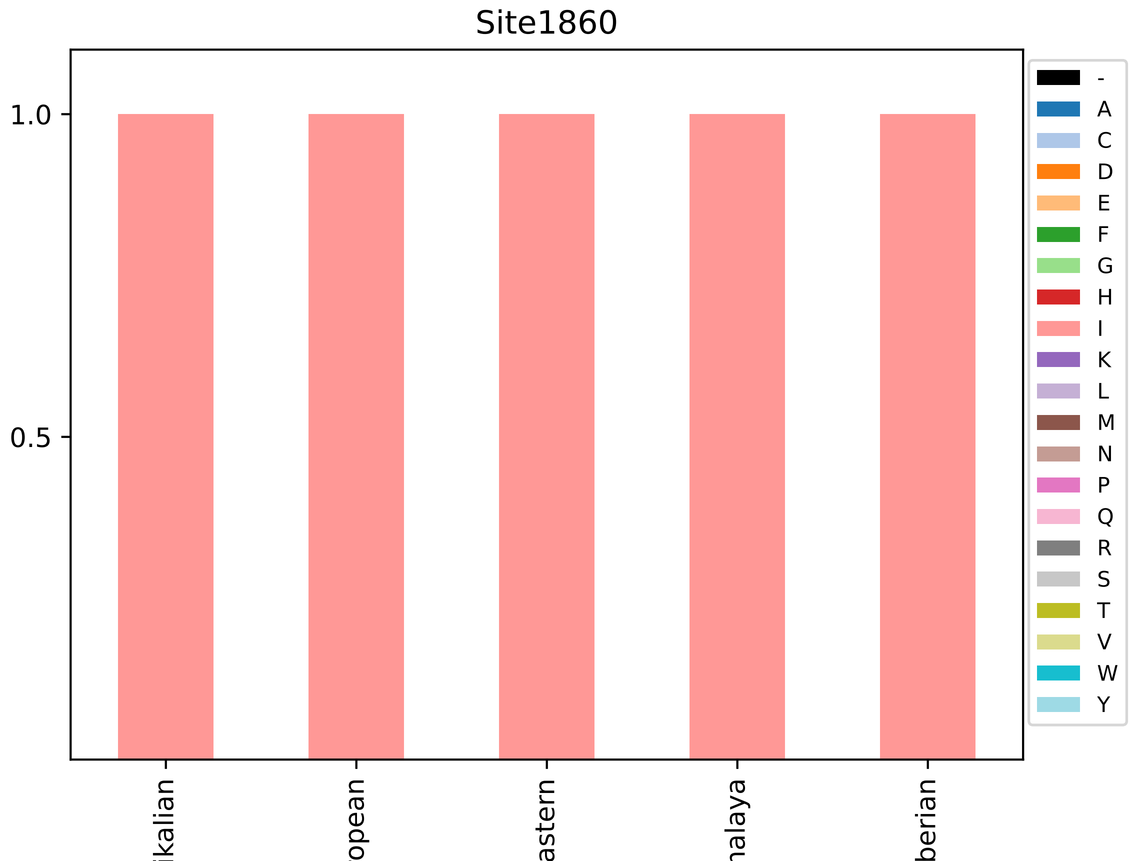
Site1860
1.0
0.5
-
A
C
D
E
F
G
H
I
K
L
M
N
P
Q
R
S
T
V
W
Y
ikalian
opean
astern
nalaya
berian

Site1861

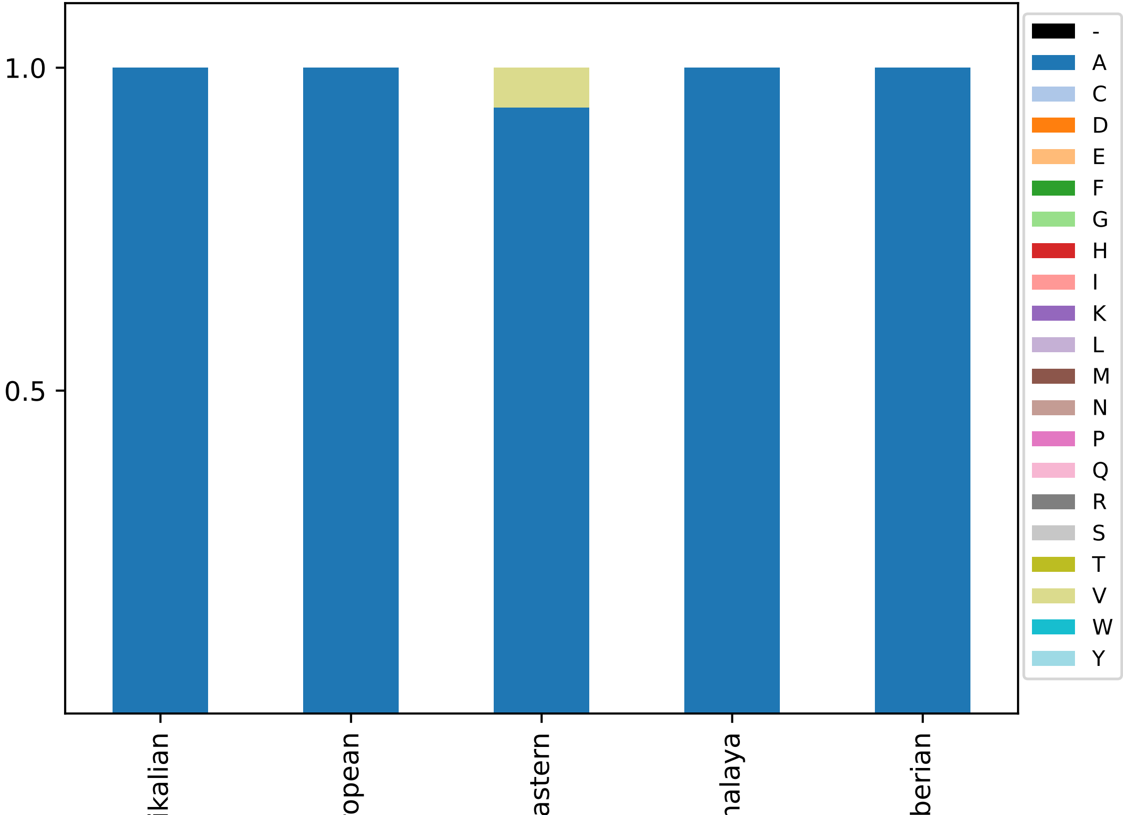
1.0
0.5
-
A
C
D
E
F
G
H
I
K
L
M
N
P
Q
R
S
T
V
W
Y
ikalian
opean
astern
nalaya
berian

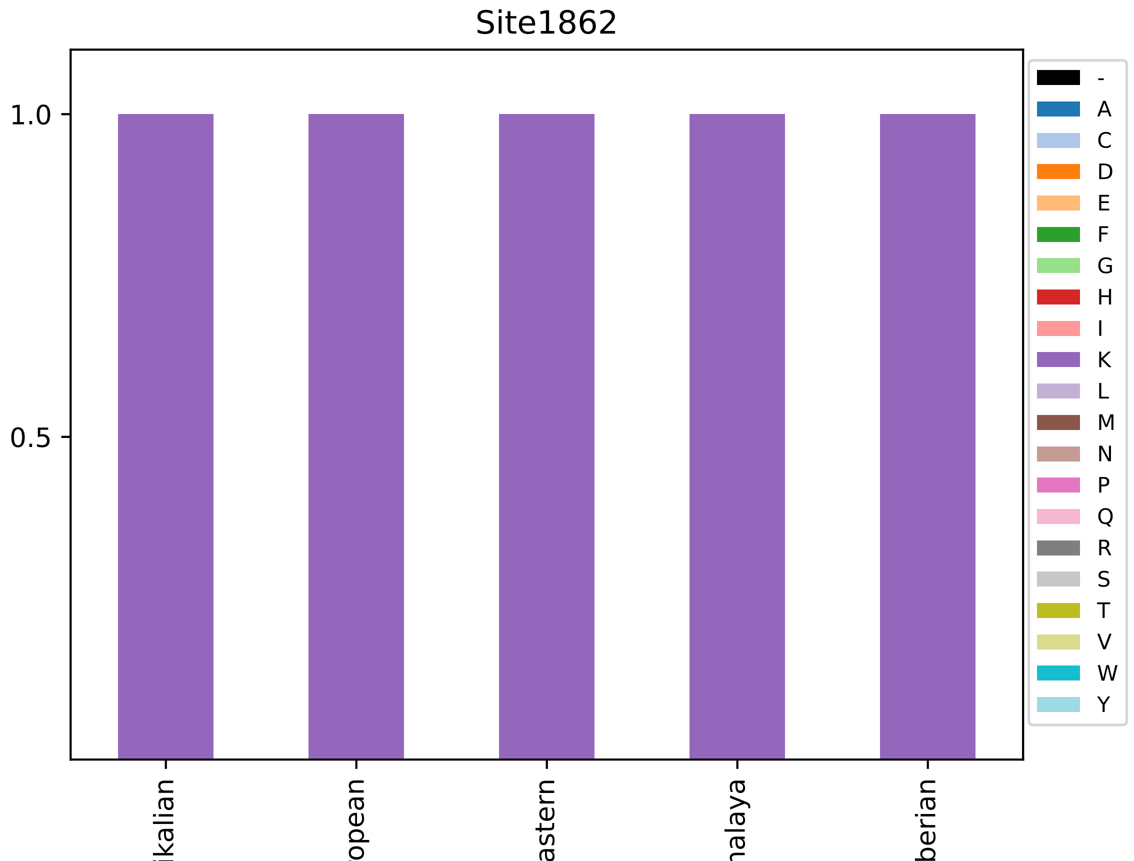

Site1862
1.0
0.5
-
A
C
D
E
F
G
H
I
K
L
M
N
P
Q
R
S
T
V
W
Y
ikalian
opean
astern
nalaya
berian

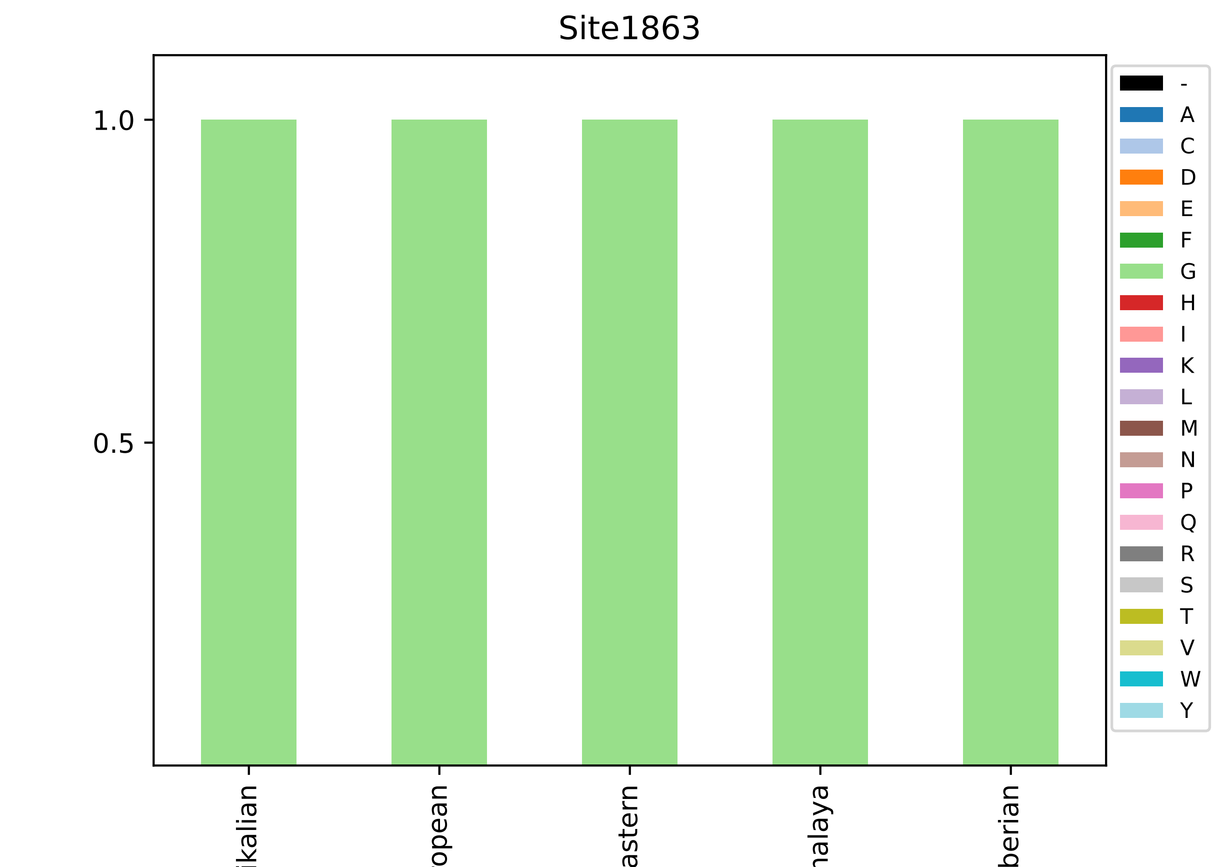

Site1863
1.0
0.5
-
A
C
D
E
F
G
H
I
K
L
M
N
P
Q
R
S
T
V
W
Y
ikalian
opean
astern
nalaya
berian

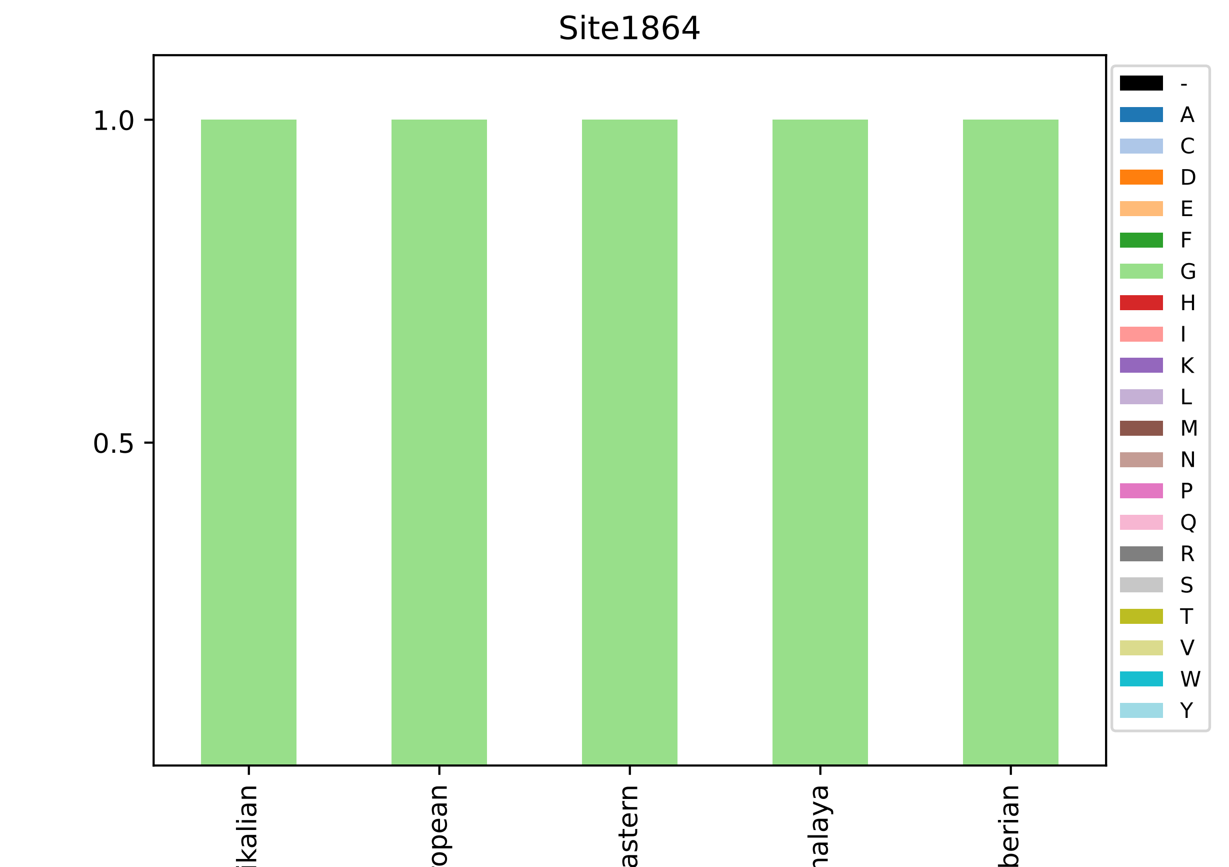
Site1864
1.0
0.5
ikalian
opean
astern
nalaya
berian
-
A
C
D
E
F
G
H
I
K
L
M
N
P
Q
R
S
T
V
W
Y

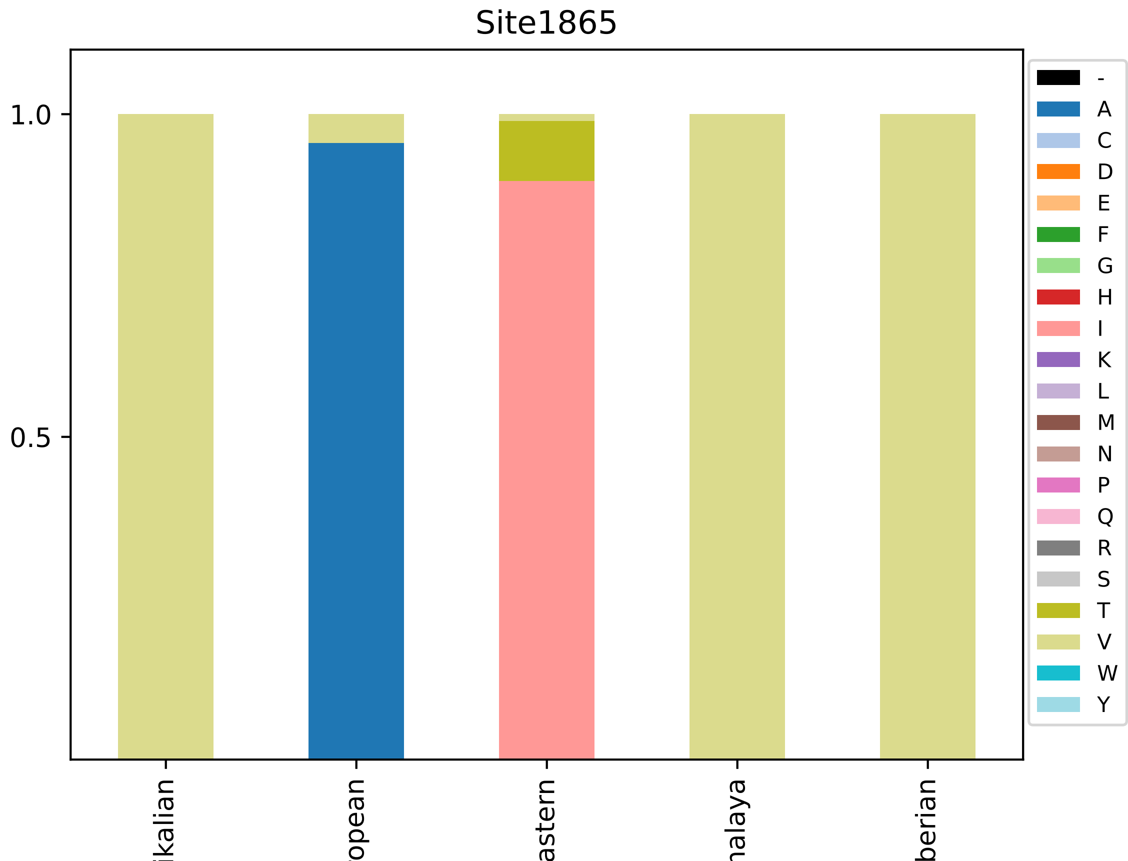
Site1865
1.0
0.5
-
A
C
D
E
F
G
H
I
K
L
M
N
P
Q
R
S
T
V
W
Y
ikalian
opean
astern
nalaya
berian

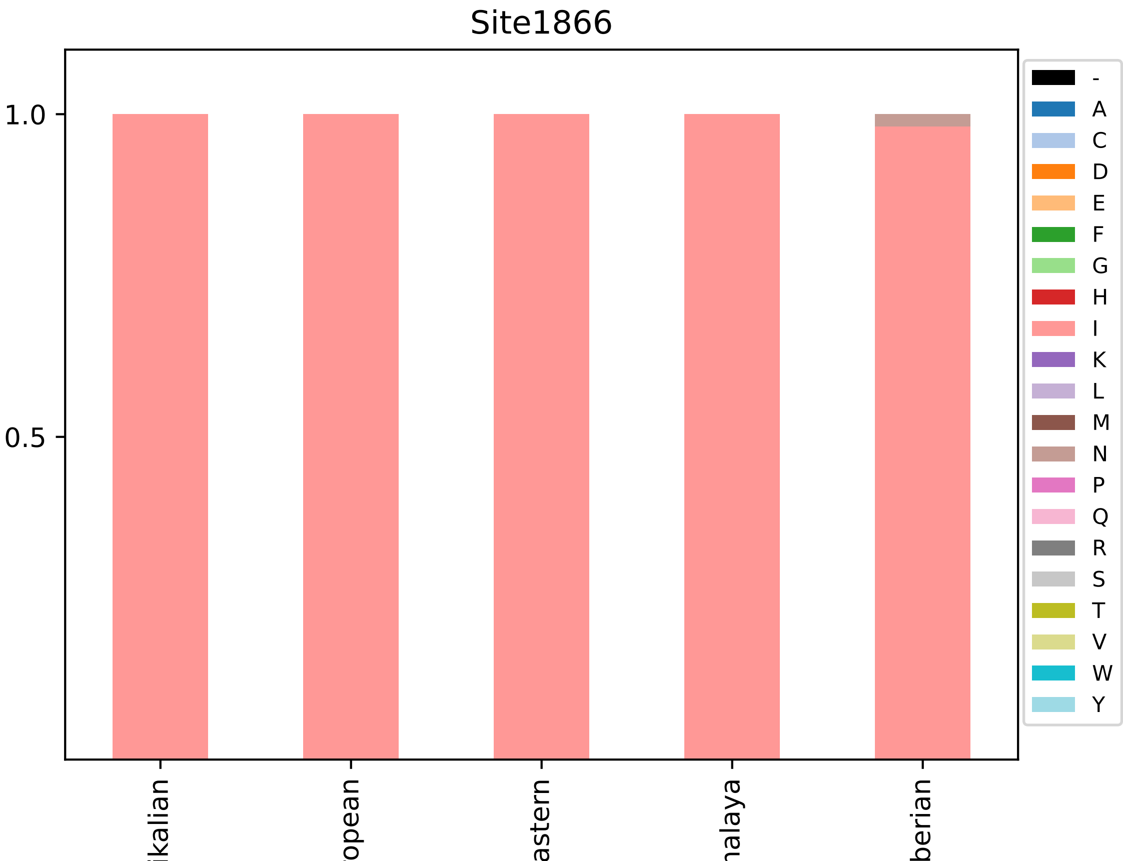
Site1866
1.0
0.5
ikalian
opean
astern
nalaya
berian
-
A
C
D
E
F
G
H
I
K
L
M
N
P
Q
R
S
T
V
W
Y

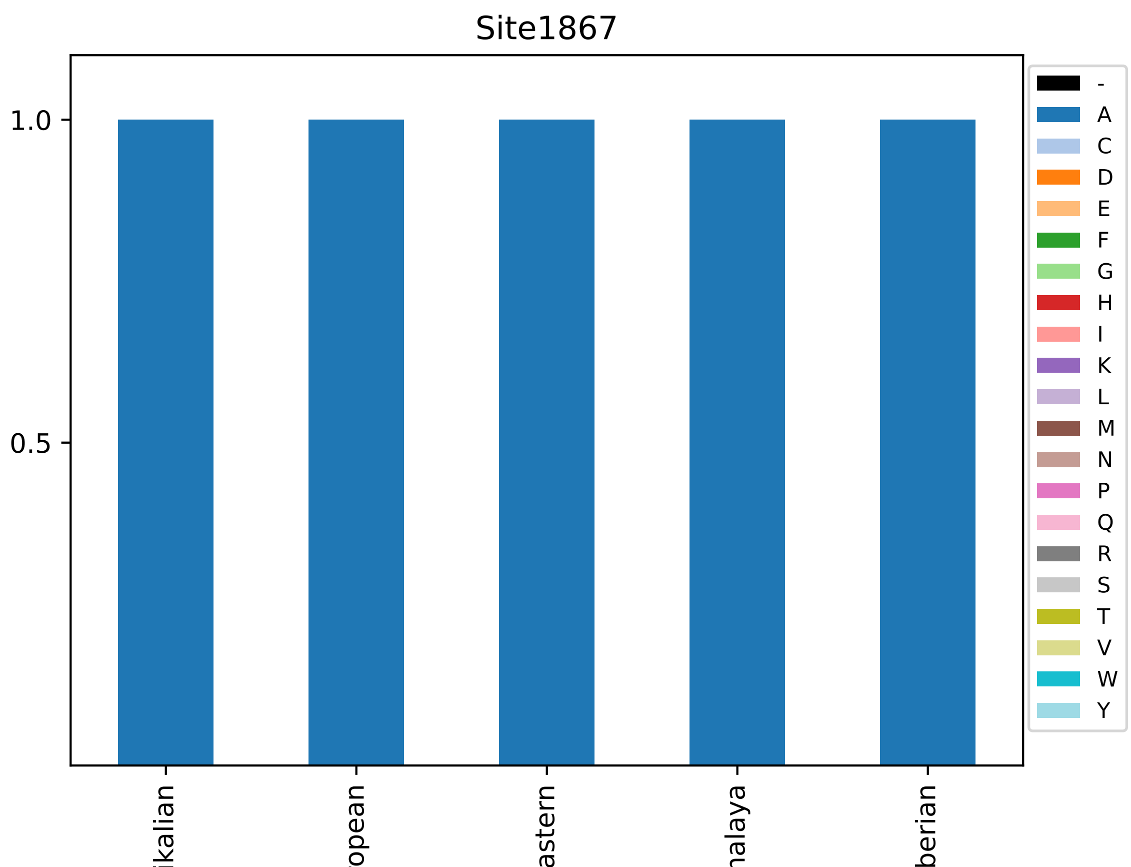

Site1867
1.0
0.5
-
A
C
D
E
F
G
H
I
K
L
M
N
P
Q
R
S
T
V
W
Y
ikalian
opean
astern
nalaya
berian

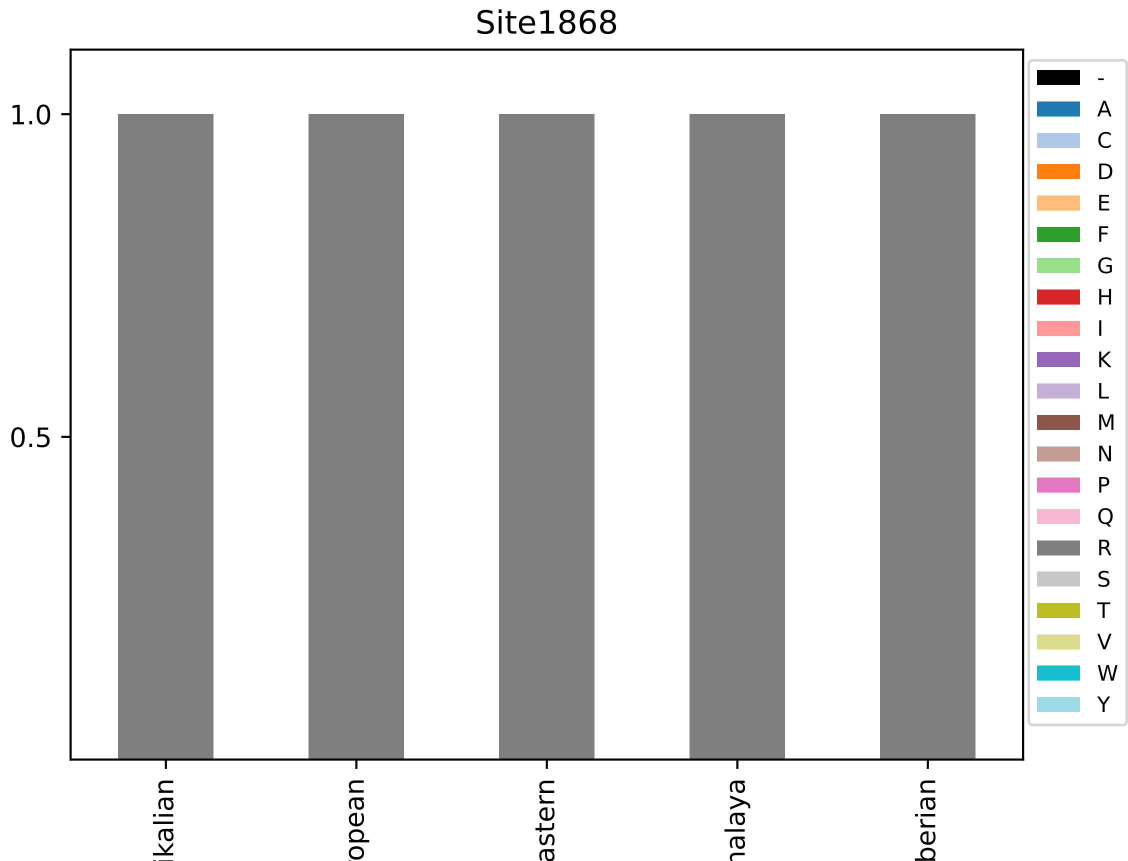

Site1868
1.0
0.5
-
A
C
D
E
F
G
H
I
K
L
M
N
P
Q
R
S
T
V
W
Y
ikalian
opean
astern
nalaya
berian

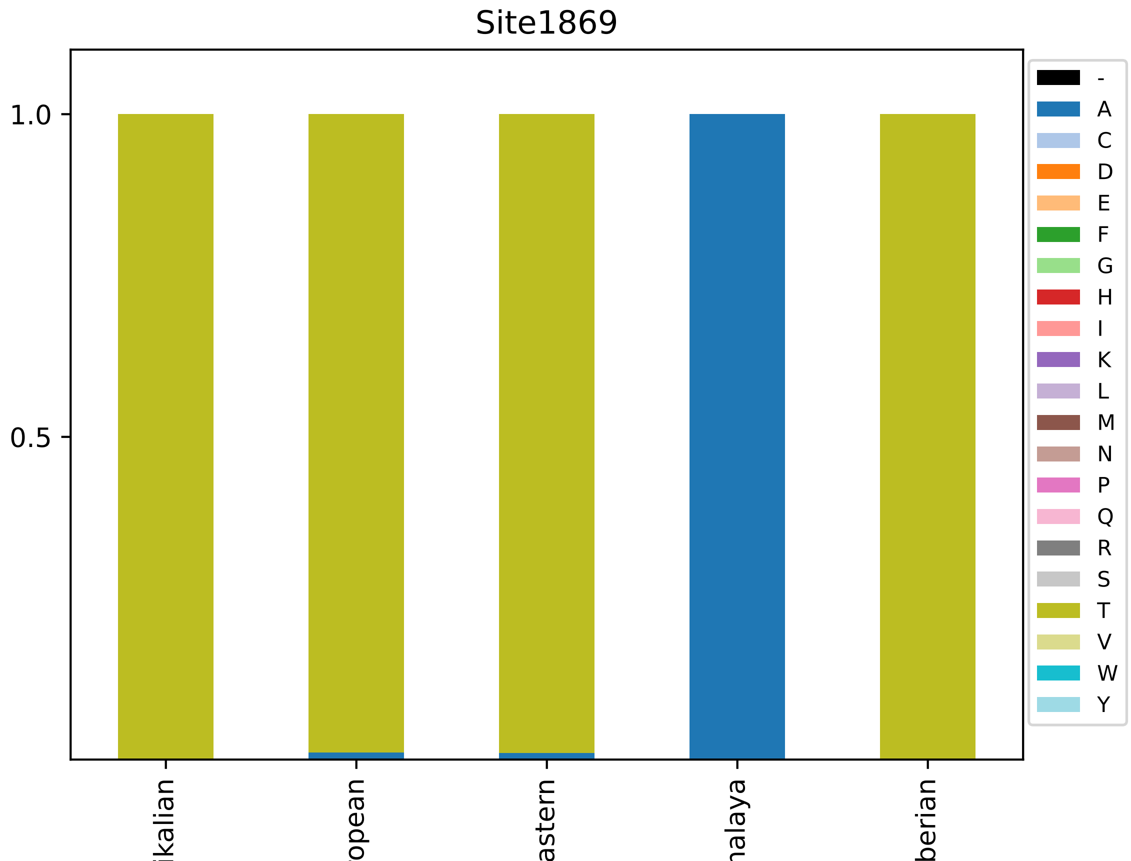
Site1869
1.0
0.5
-
A
C
D
E
F
G
H
I
K
L
M
N
P
Q
R
S
T
V
W
Y
ikalian
opean
astern
nalaya
berian

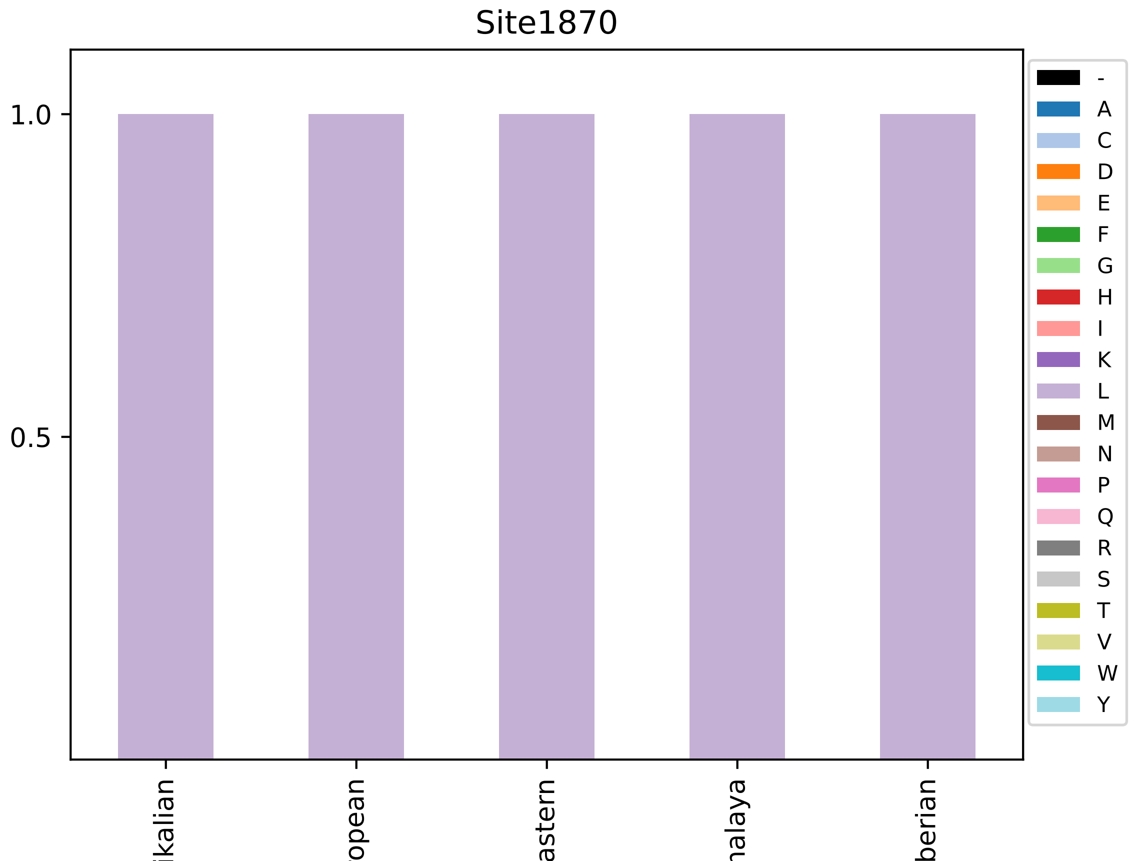
Site1870
1.0
0.5
-
A
C
D
E
F
G
H
I
K
L
M
N
P
Q
R
S
T
V
W
Y
ikalian
opean
astern
nalaya
berian

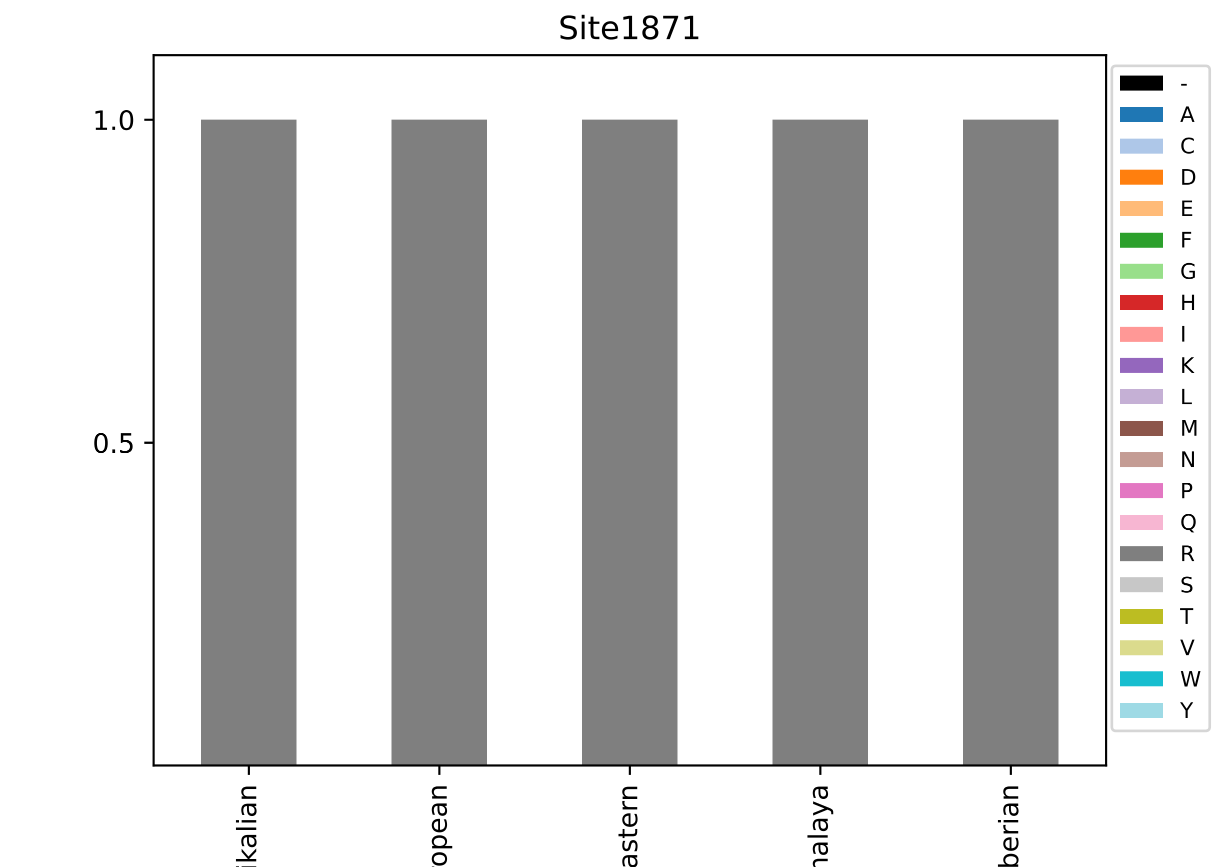
Site1871
1.0
0.5
-
A
C
D
E
F
G
H
I
K
L
M
N
P
Q
R
S
T
V
W
Y
ikalian
opean
astern
nalaya
berian

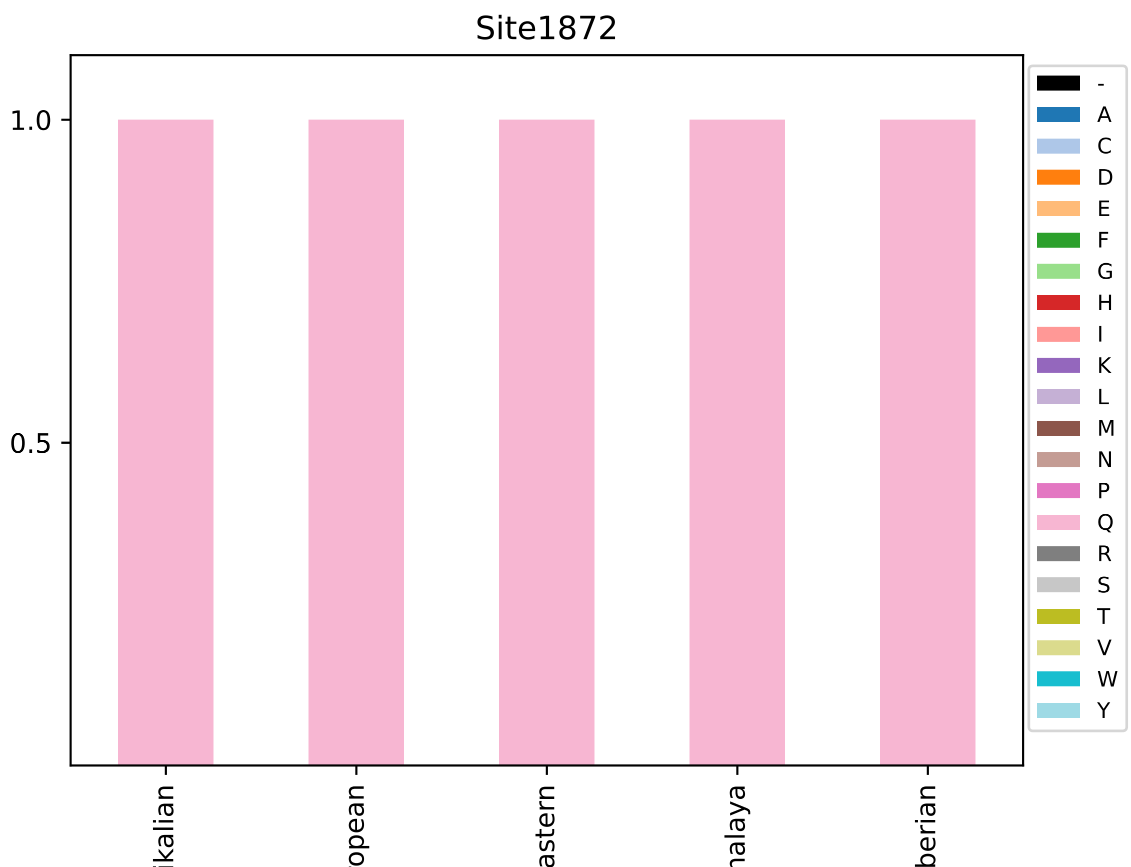
Site1872
1.0
0.5
ikalian
opean
astern
nalaya
berian
-
A
C
D
E
F
G
H
I
K
L
M
N
P
Q
R
S
T
V
W
Y

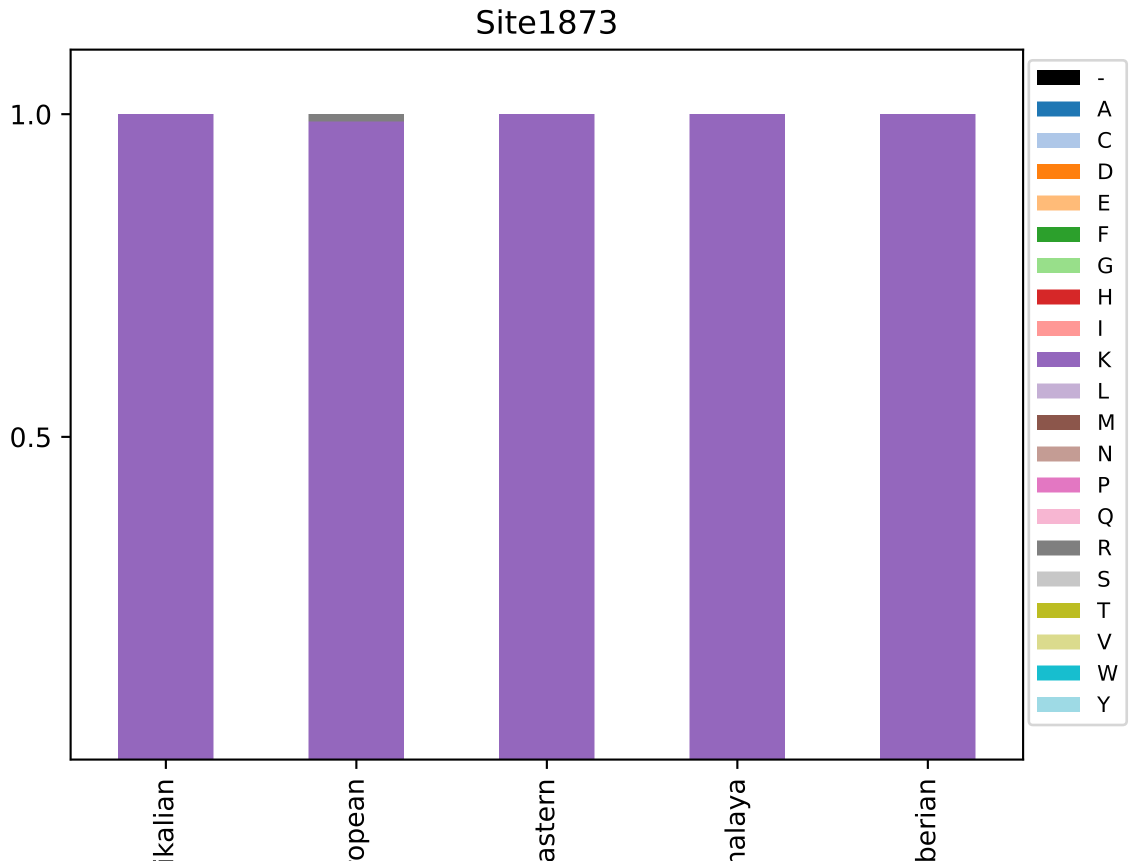
Site1873
1.0
0.5
ikalian
opean
astern
nalaya
berian
-
A
C
D
E
F
G
H
I
K
L
M
N
P
Q
R
S
T
V
W
Y

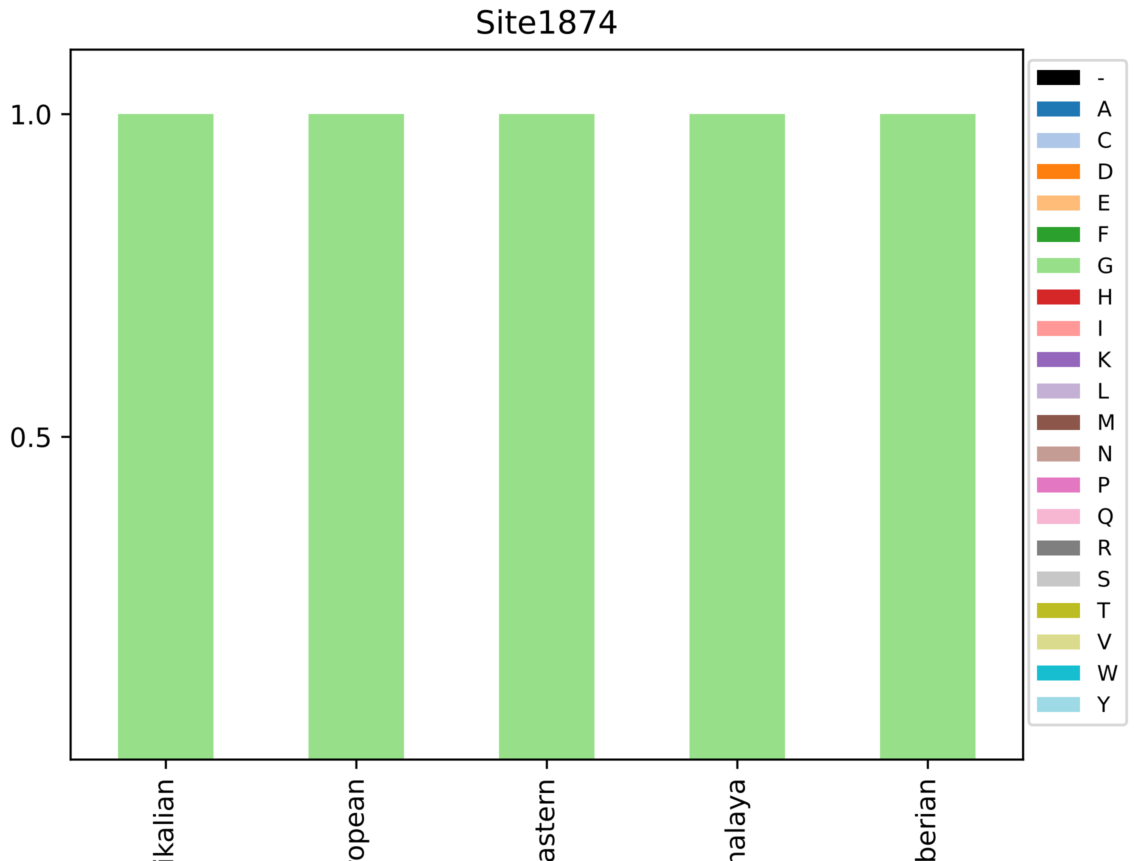
Site1874
1.0
0.5
-
A
C
D
E
F
G
H
I
K
L
M
N
P
Q
R
S
T
V
W
Y
ikalian
opean
astern
nalaya
berian

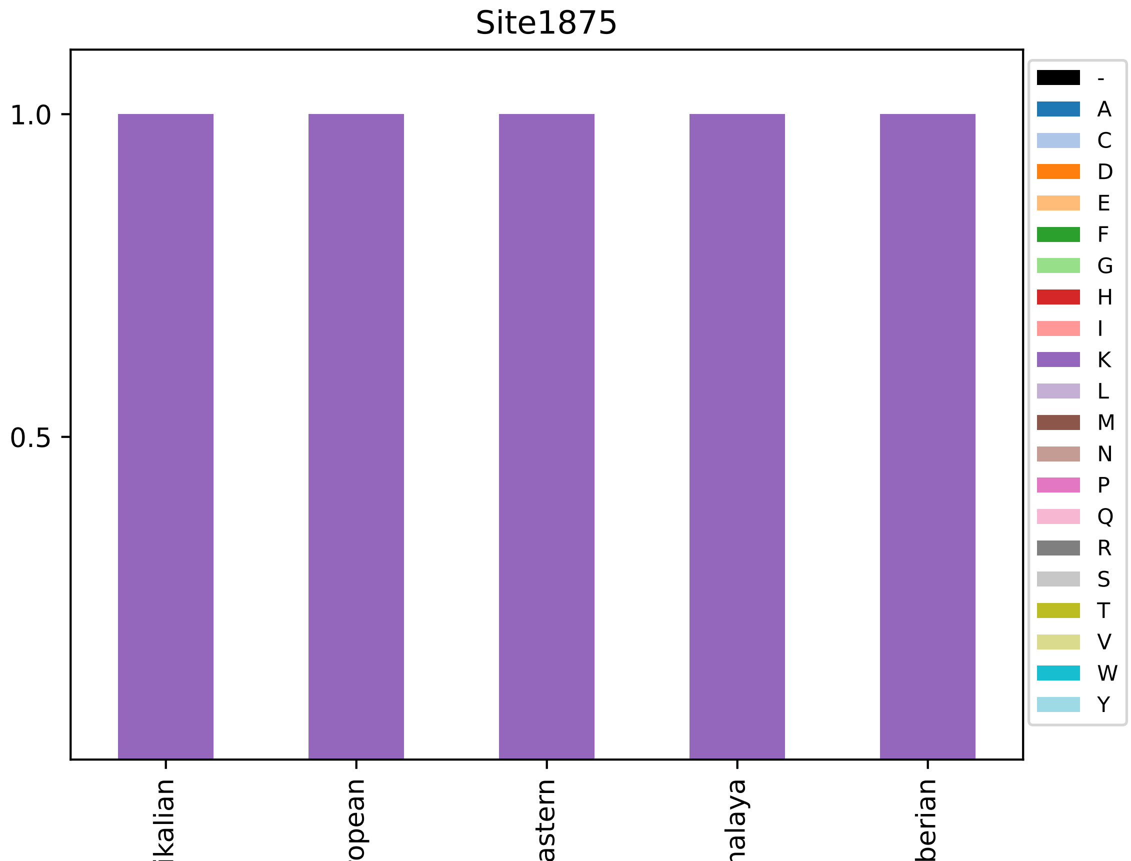

Site1875
1.0
0.5
-
A
C
D
E
F
G
H
I
K
L
M
N
P
Q
R
S
T
V
W
Y
ikalian
opean
astern
nalaya
berian

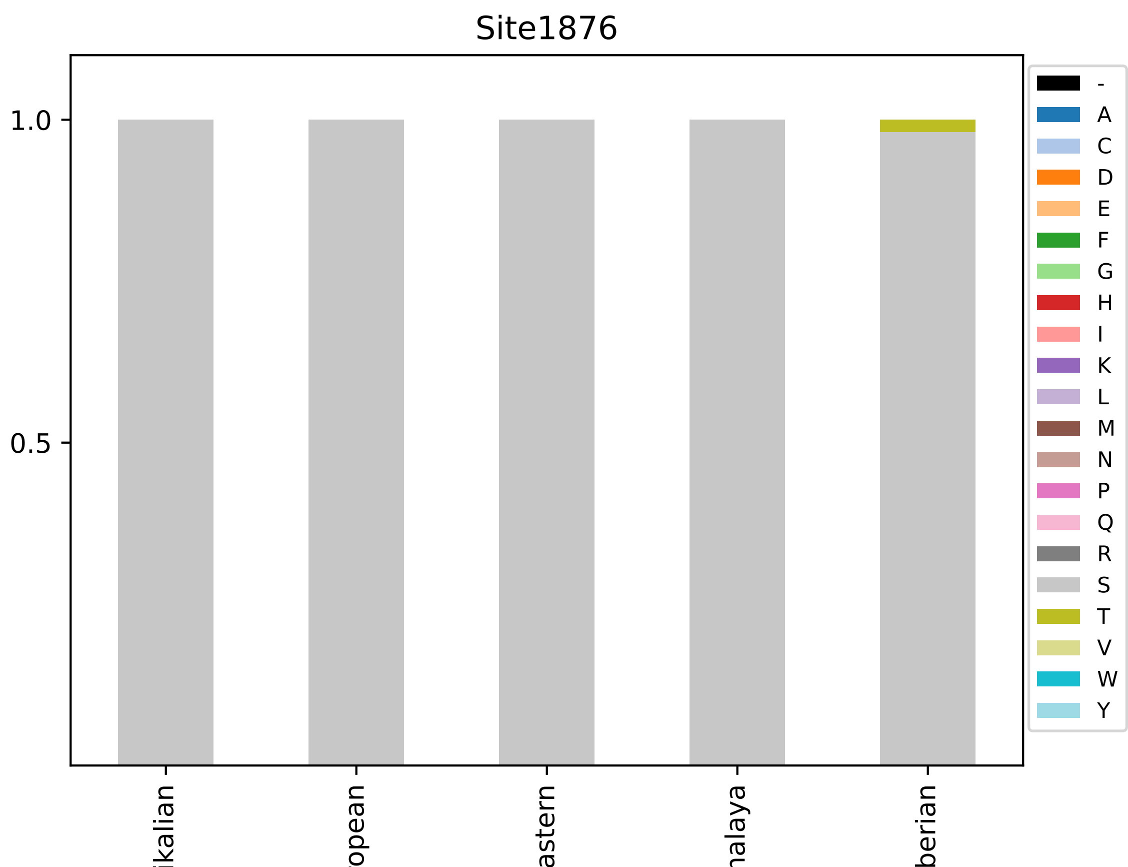

Site1876
1.0
0.5
ikalian
opean
astern
nalaya
berian
-
A
C
D
E
F
G
H
I
K
L
M
N
P
Q
R
S
T
V
W
Y

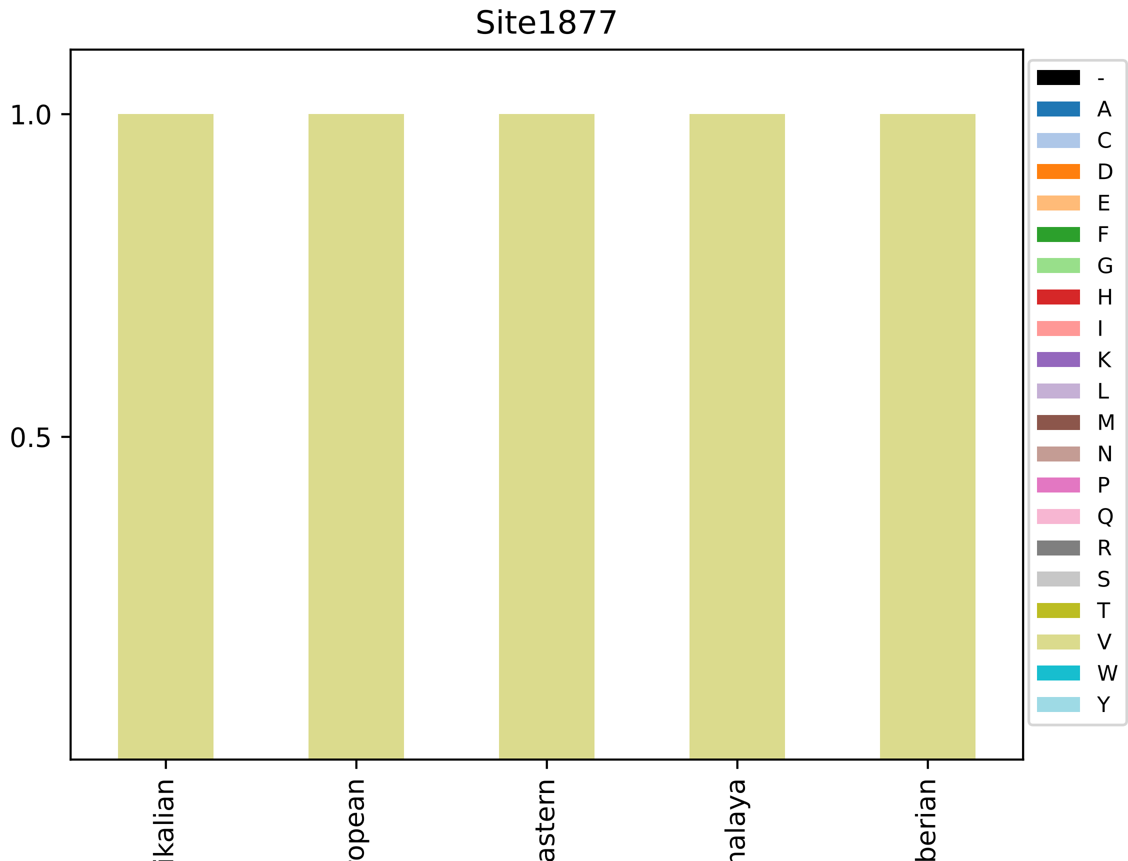

Site1877
1.0
0.5
-
A
C
D
E
F
G
H
I
K
L
M
N
P
Q
R
S
T
V
W
Y
ikalian
opean
astern
nalaya
berian

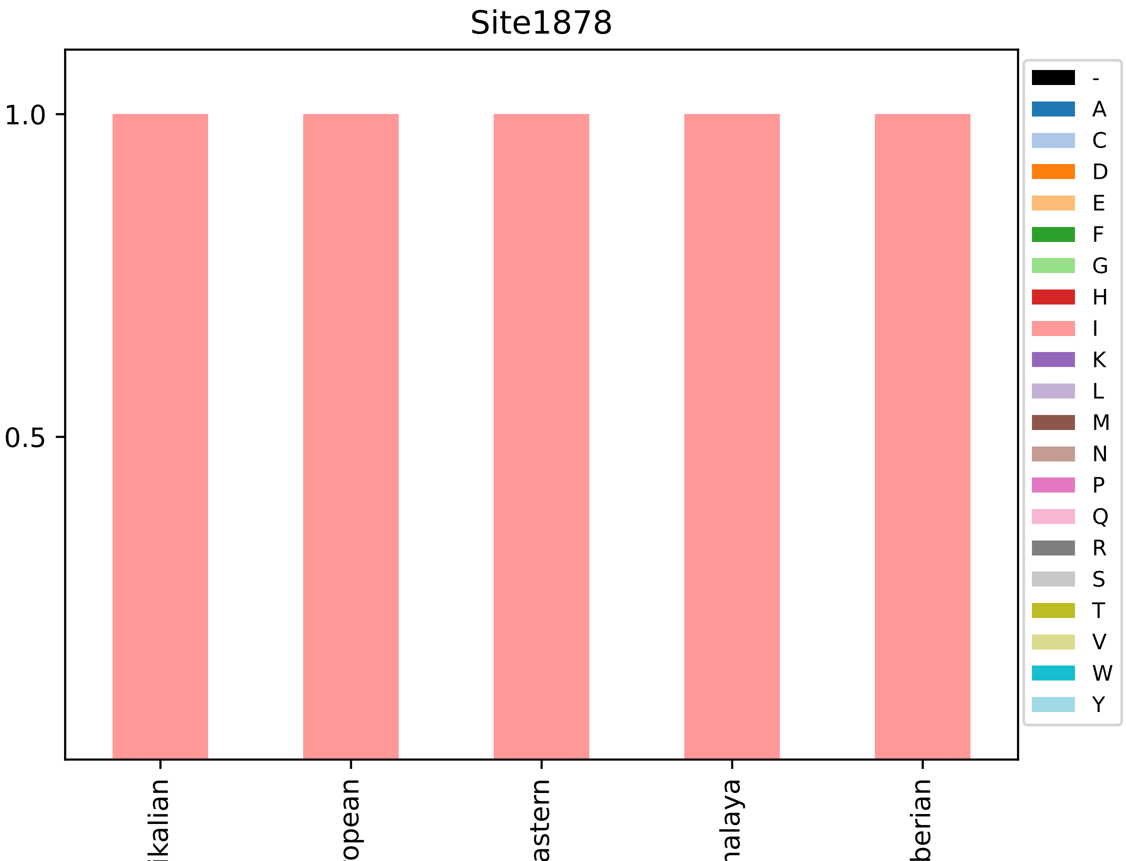

Site1878
1.0
0.5
ikalian
opean
astern
nalaya
berian
-
A
C
D
E
F
G
H
I
K
L
M
N
P
Q
R
S
T
V
W
Y

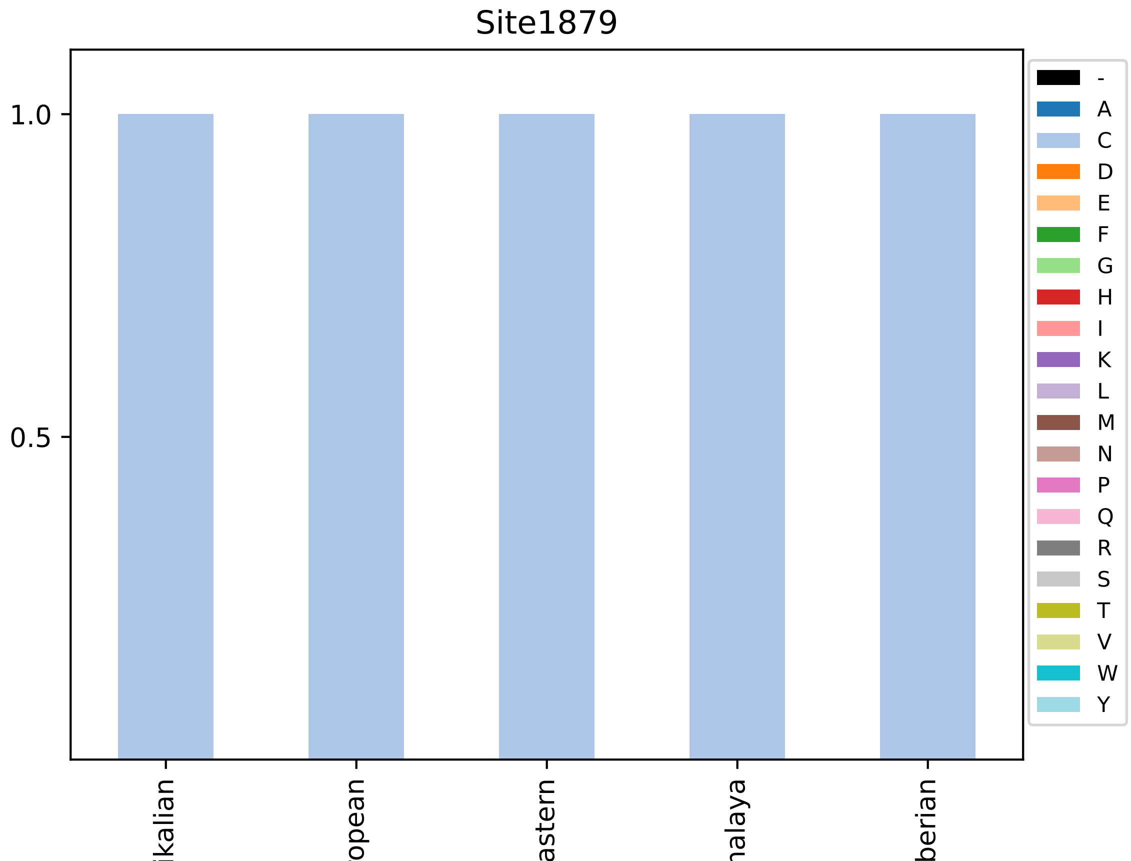

Site1879
1.0
0.5
ikalian
ropean
astern
nalaya
berian
-
A
C
D
E
F
G
H
I
K
L
M
N
P
Q
R
S
T
V
W
Y

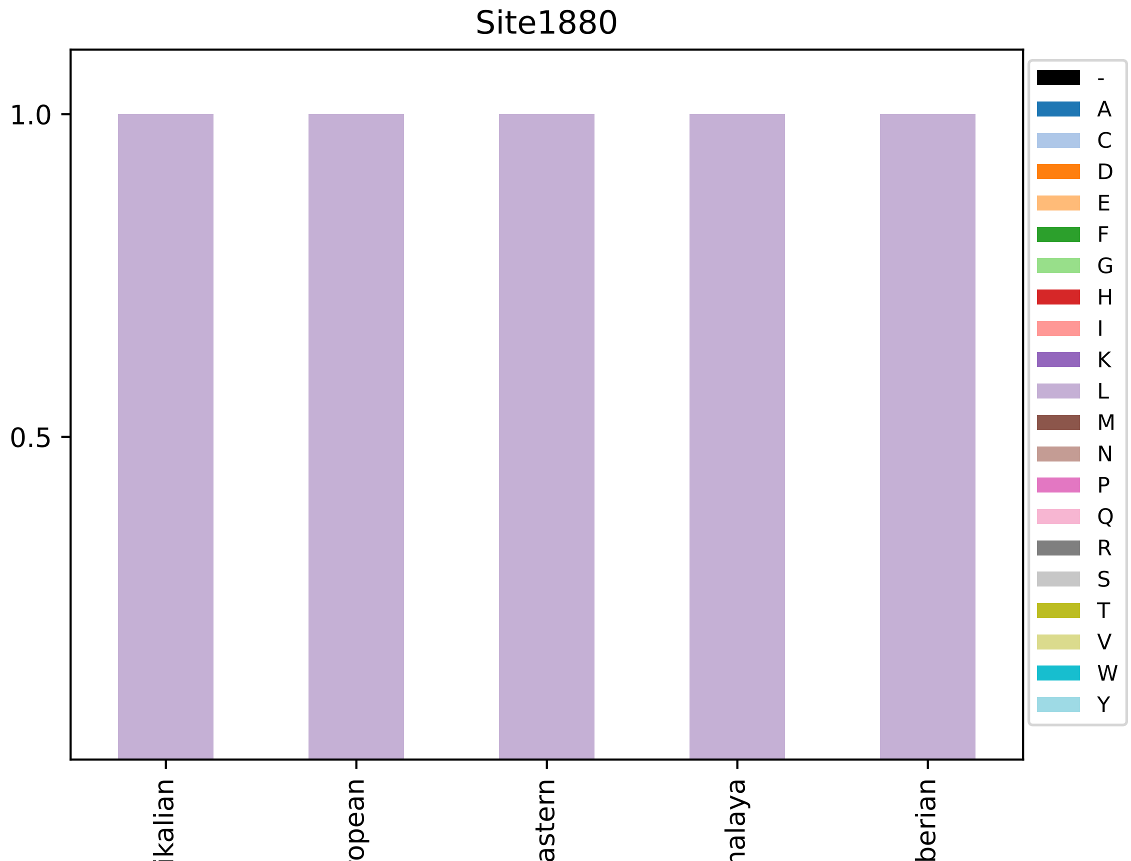
Site1880
1.0
0.5
-
A
C
D
E
F
G
H
I
K
L
M
N
P
Q
R
S
T
V
W
Y
ikalian
opean
astern
nalaya
berian

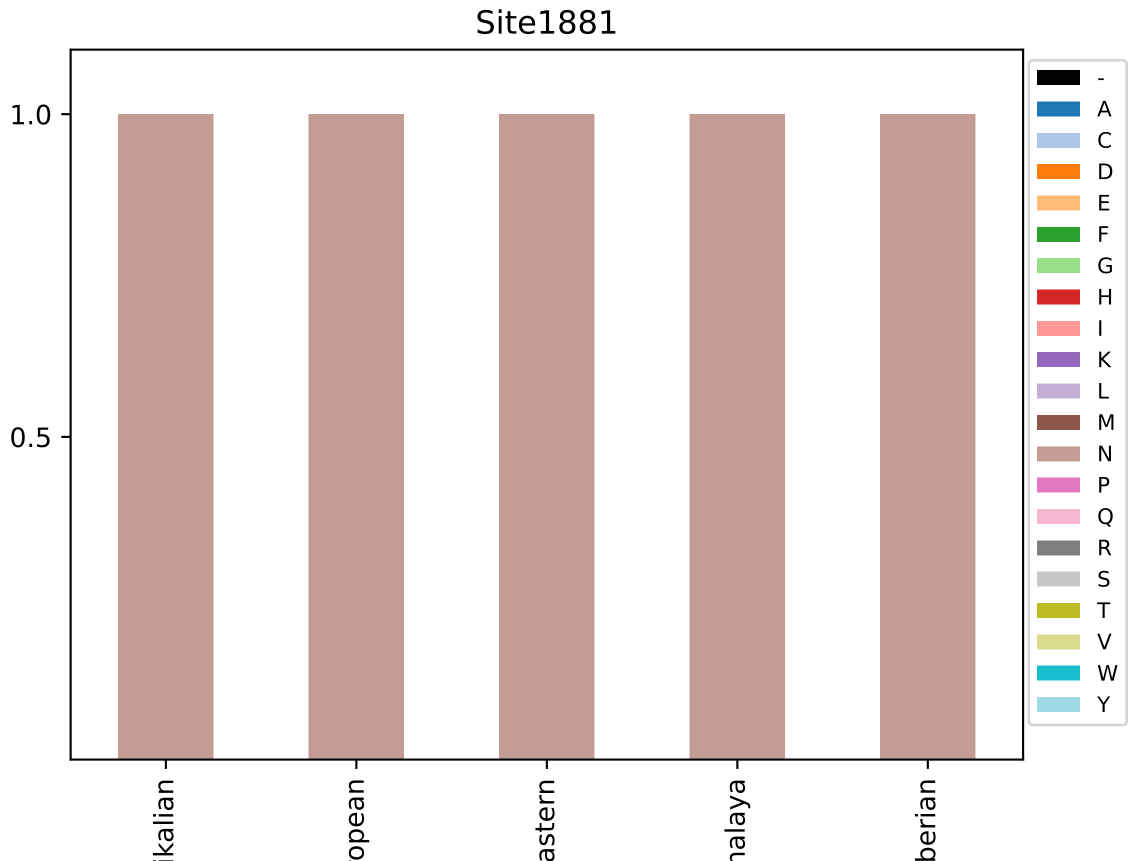
Site1881
1.0
0.5
-
A
C
D
E
F
G
H
I
K
L
M
N
P
Q
R
S
T
V
W
Y

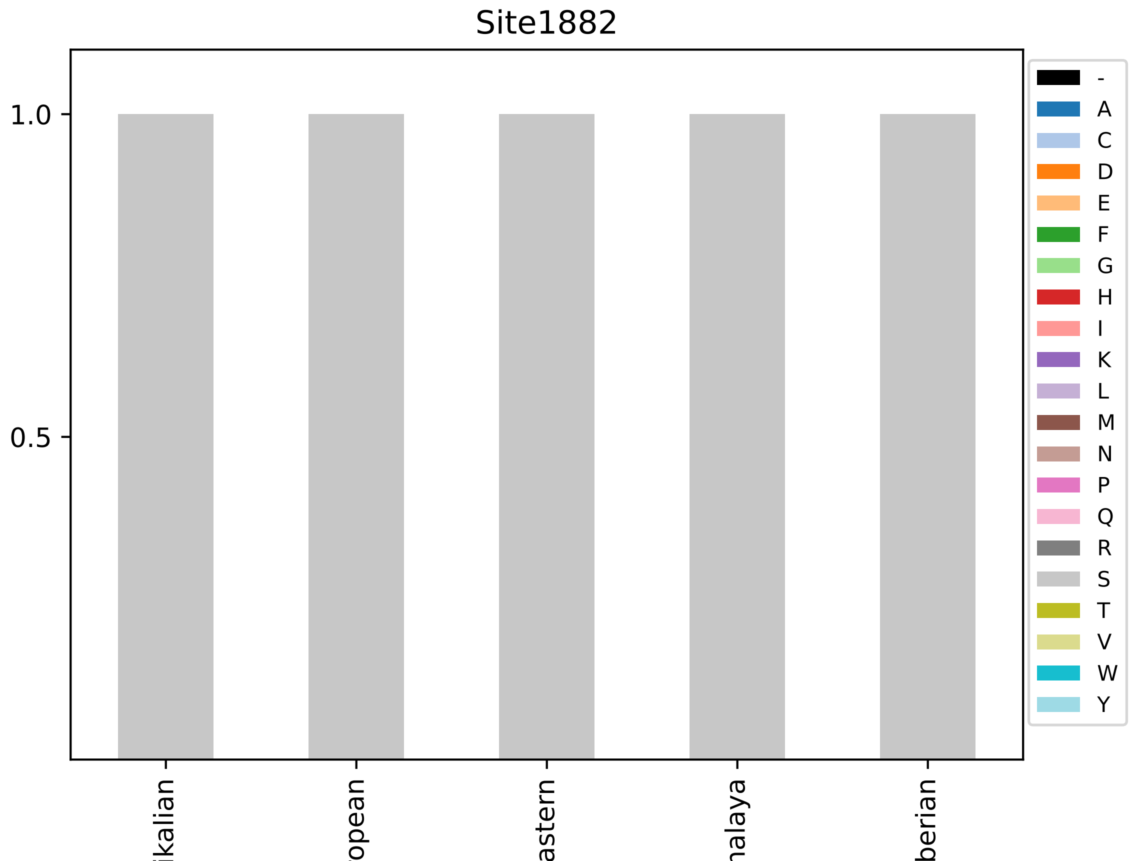
Site1882
1.0
0.5
-
A
C
D
E
F
G
H
I
K
L
M
N
P
Q
R
S
T
V
W
Y
ikalian
opean
astern
nalaya
berian

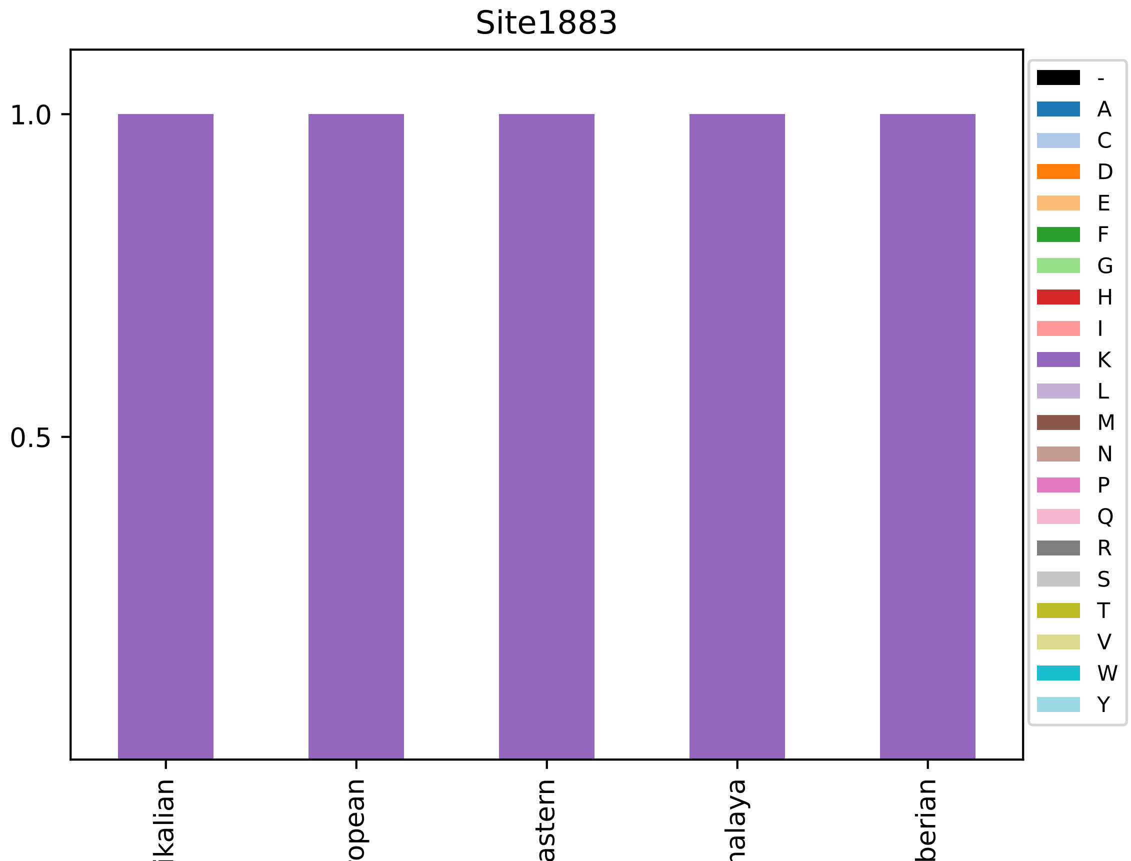
Site1883
1.0
0.5
-
A
C
D
E
F
G
H
I
K
L
M
N
P
Q
R
S
T
V
W
Y
ikalian
opean
astern
nalaya
berian

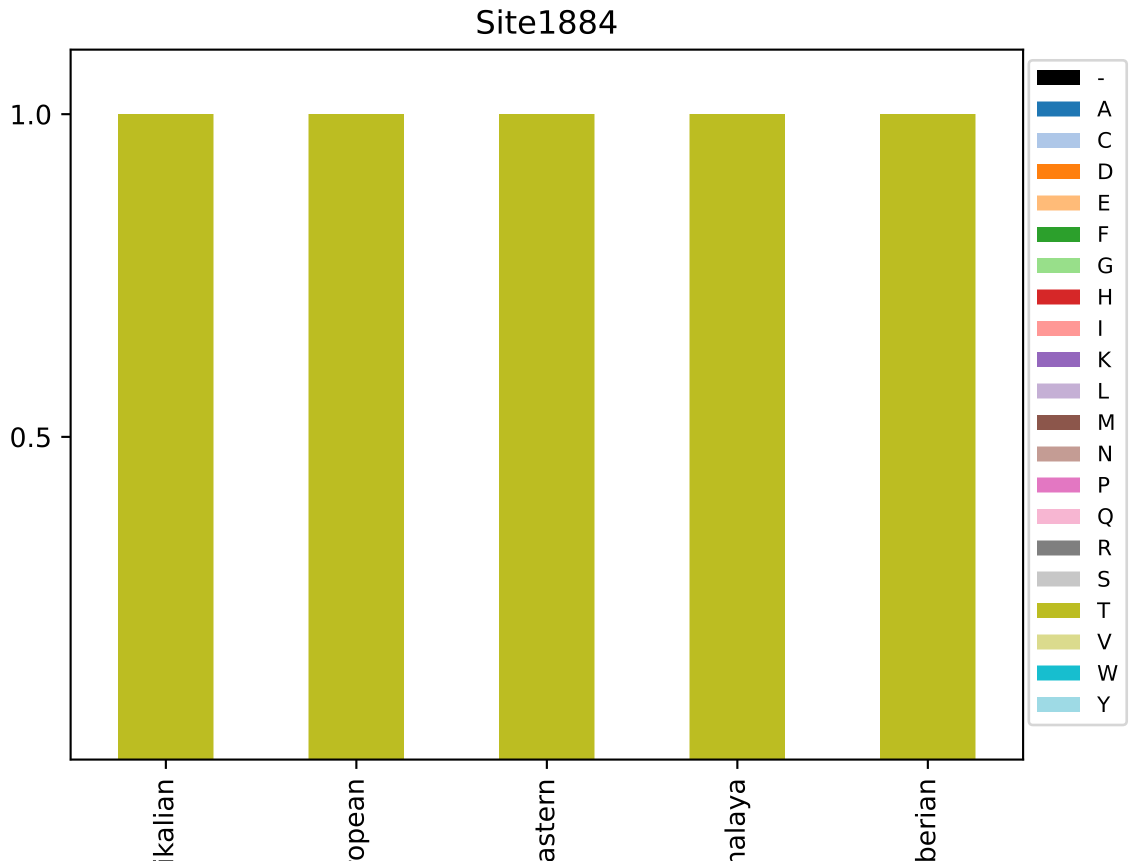
Site1884
1.0
0.5
-
A
C
D
E
F
G
H
I
K
L
M
N
P
Q
R
S
T
V
W
Y
ikalian
opean
astern
nalaya
berian

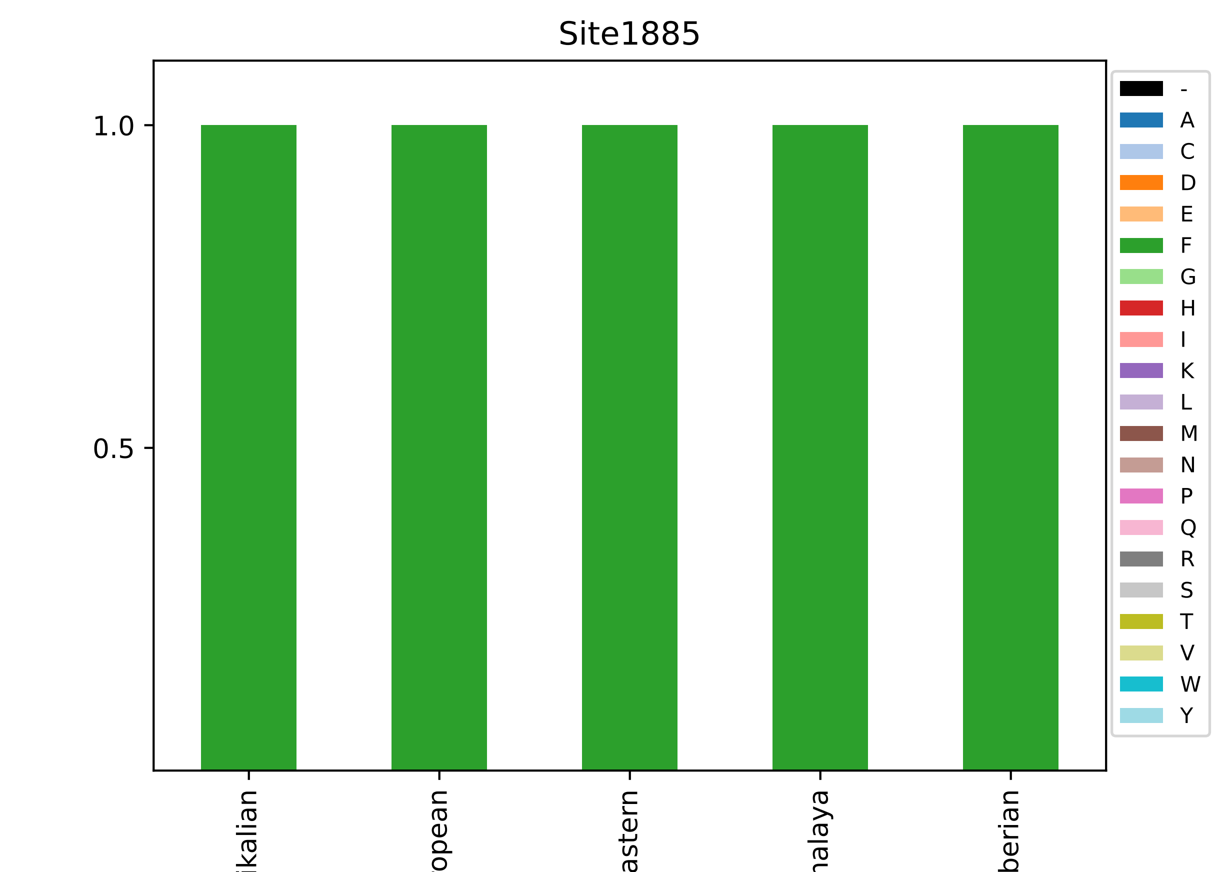
Site1885
1.0
0.5
-
A
C
D
E
F
G
H
I
K
L
M
N
P
Q
R
S
T
V
W
Y
ikalian
opean
astern
nalaya
berian

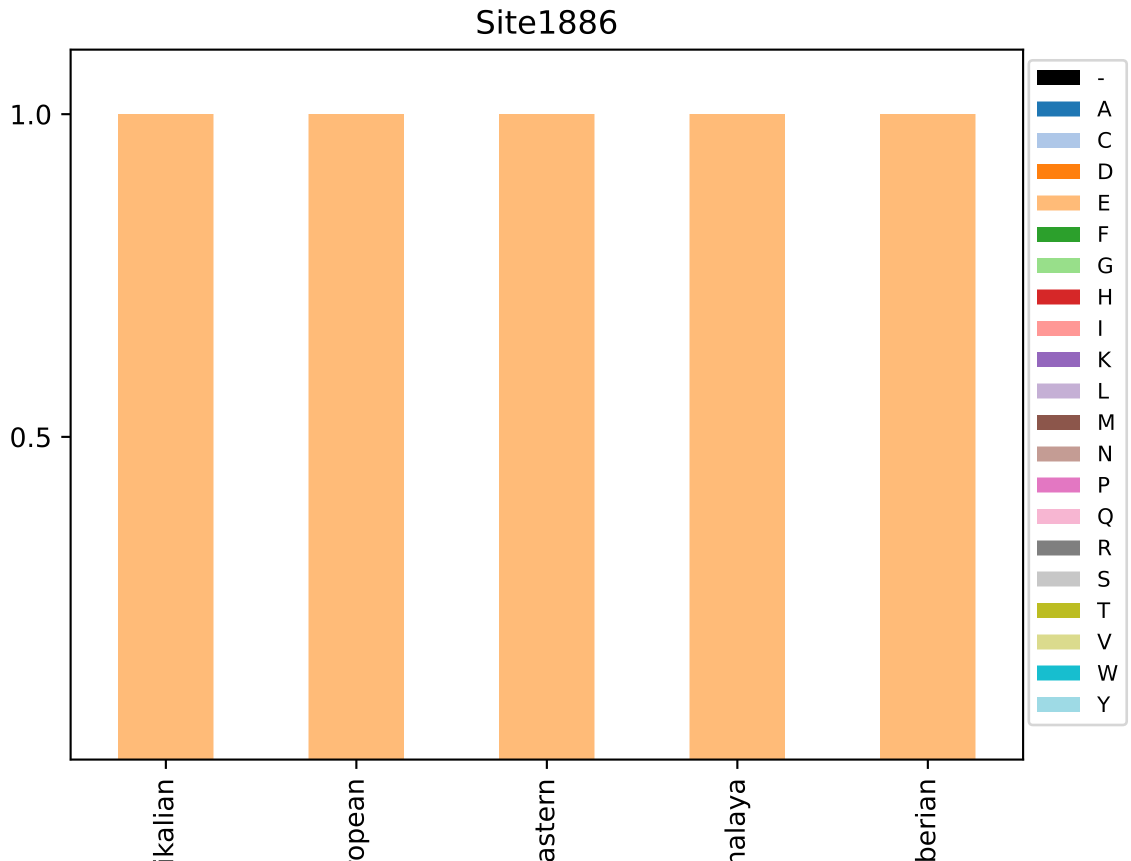
Site1886
1.0
0.5
-
A
C
D
E
F
G
H
I
K
L
M
N
P
Q
R
S
T
V
W
Y
ikalian
opean
astern
nalaya
berian

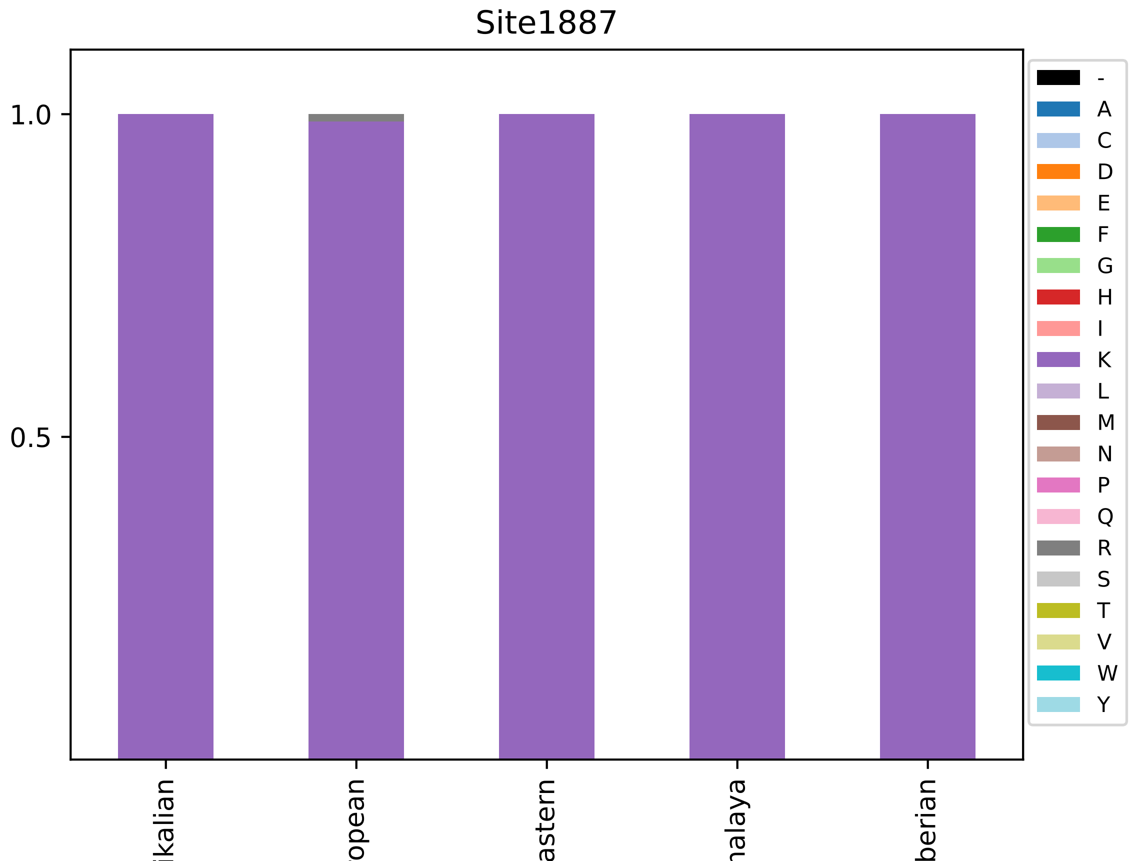
Site1887
1.0
0.5
ikalian
opean
astern
nalaya
berian
-
A
C
D
E
F
G
H
I
K
L
M
N
P
Q
R
S
T
V
W
Y

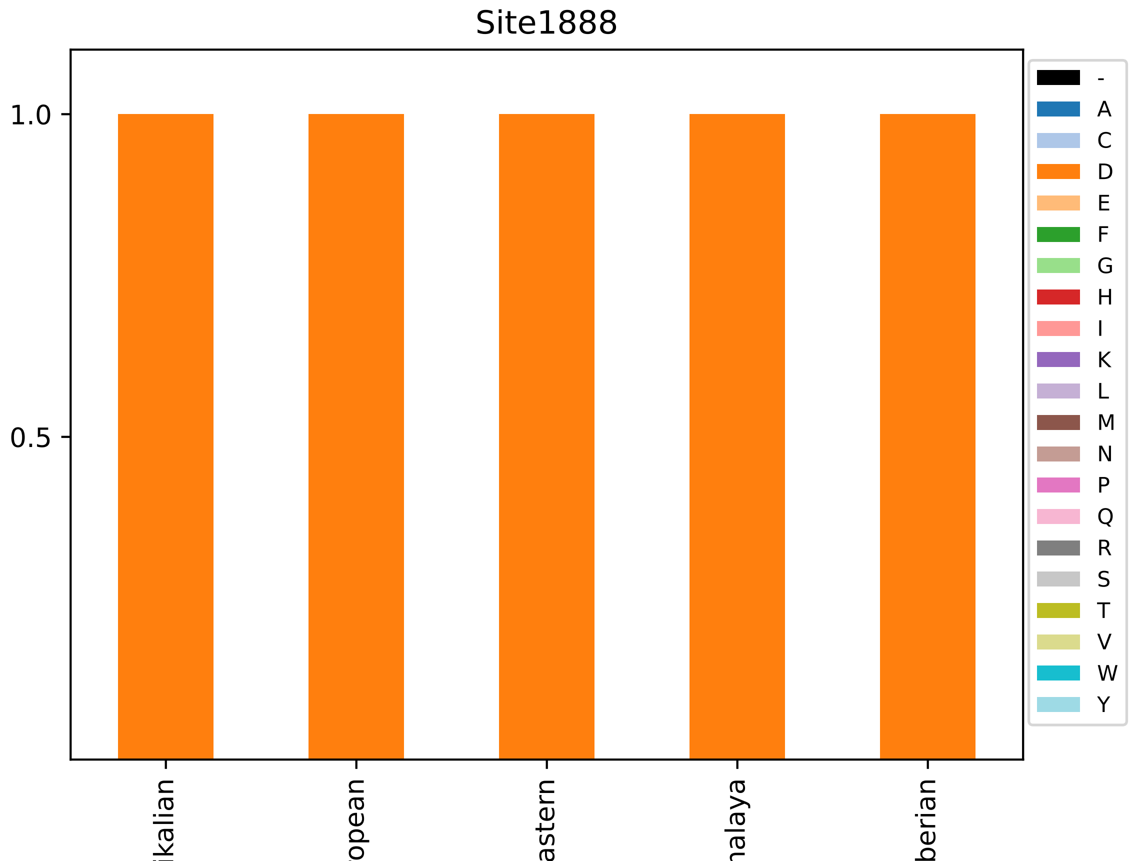

Site1888
1.0
0.5
-
A
C
D
E
F
G
H
I
K
L
M
N
P
Q
R
S
T
V
W
Y
ikalian
opean
astern
nalaya
berian

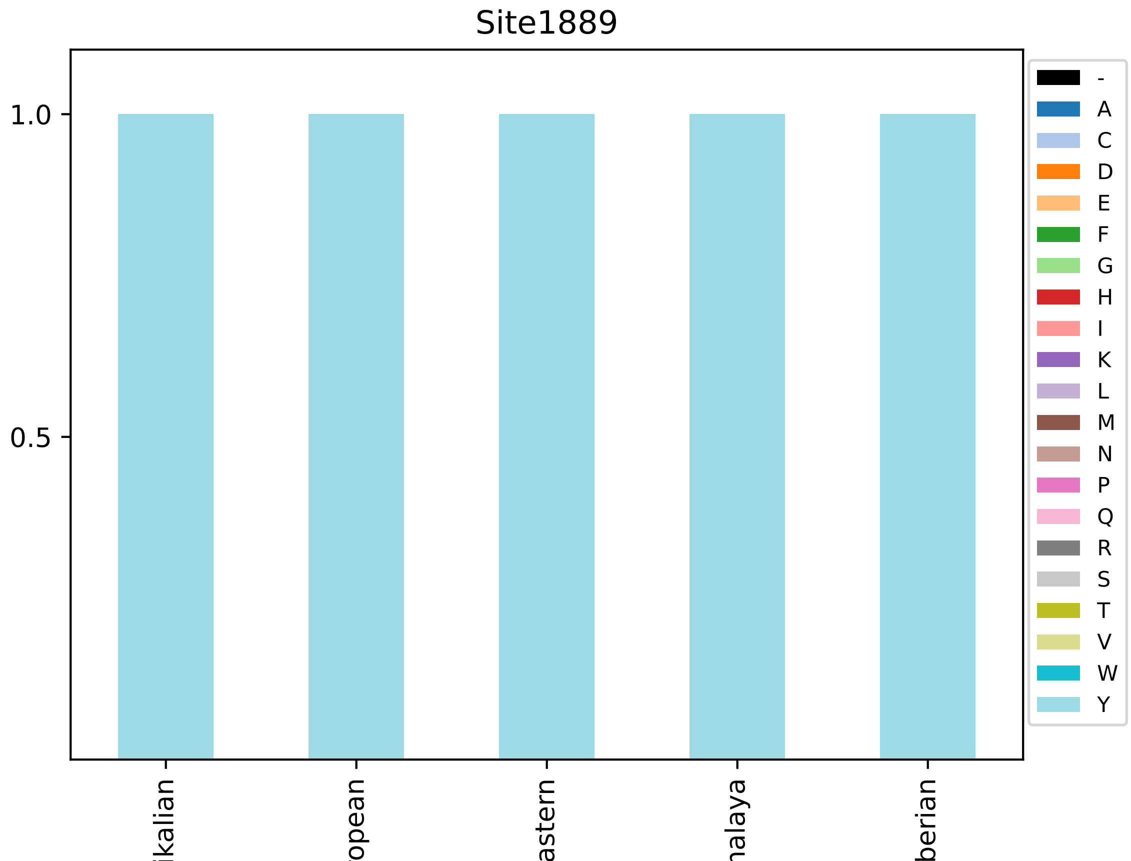
Site1889
1.0
0.5
-
A
C
D
E
F
G
H
I
K
L
M
N
P
Q
R
S
T
V
W
Y
ikalian
opean
astern
nalaya
berian

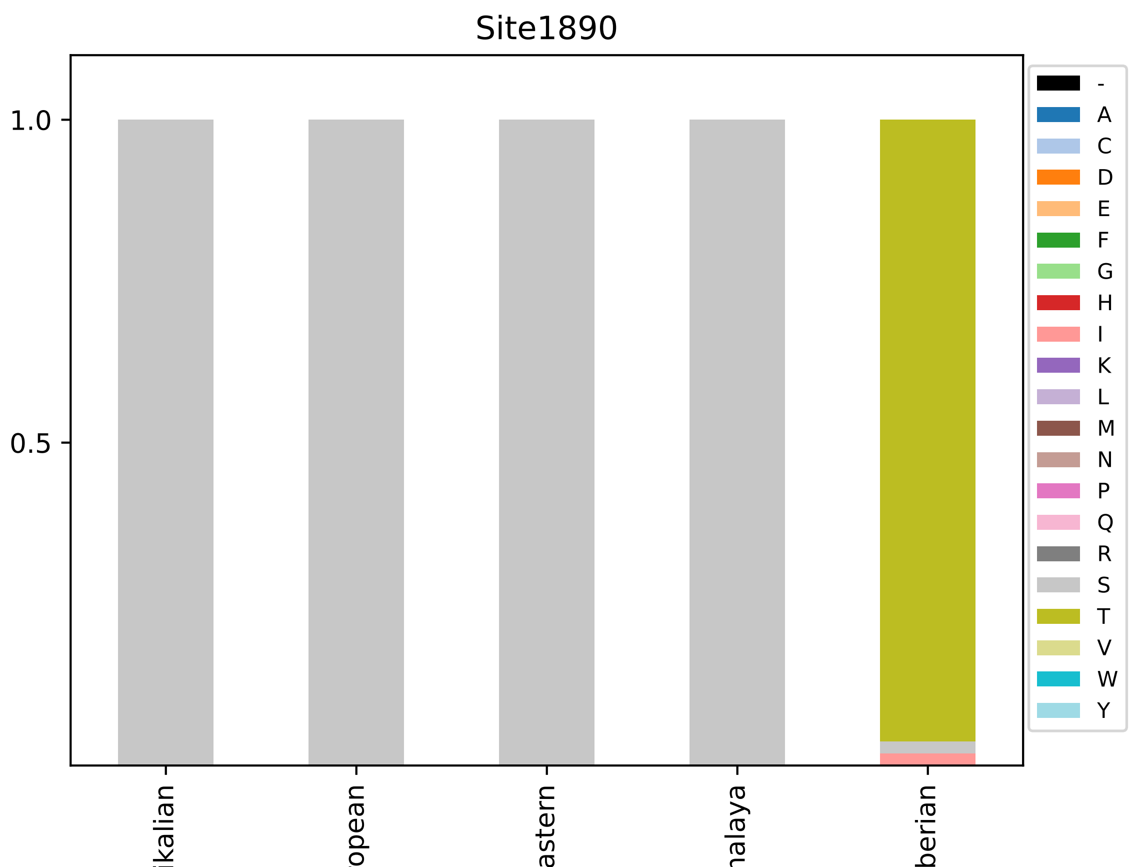

Site1890
1.0
0.5
-
A
C
D
E
F
G
H
I
K
L
M
N
P
Q
R
S
T
V
W
Y
ikalian
opean
astern
nalaya
berian

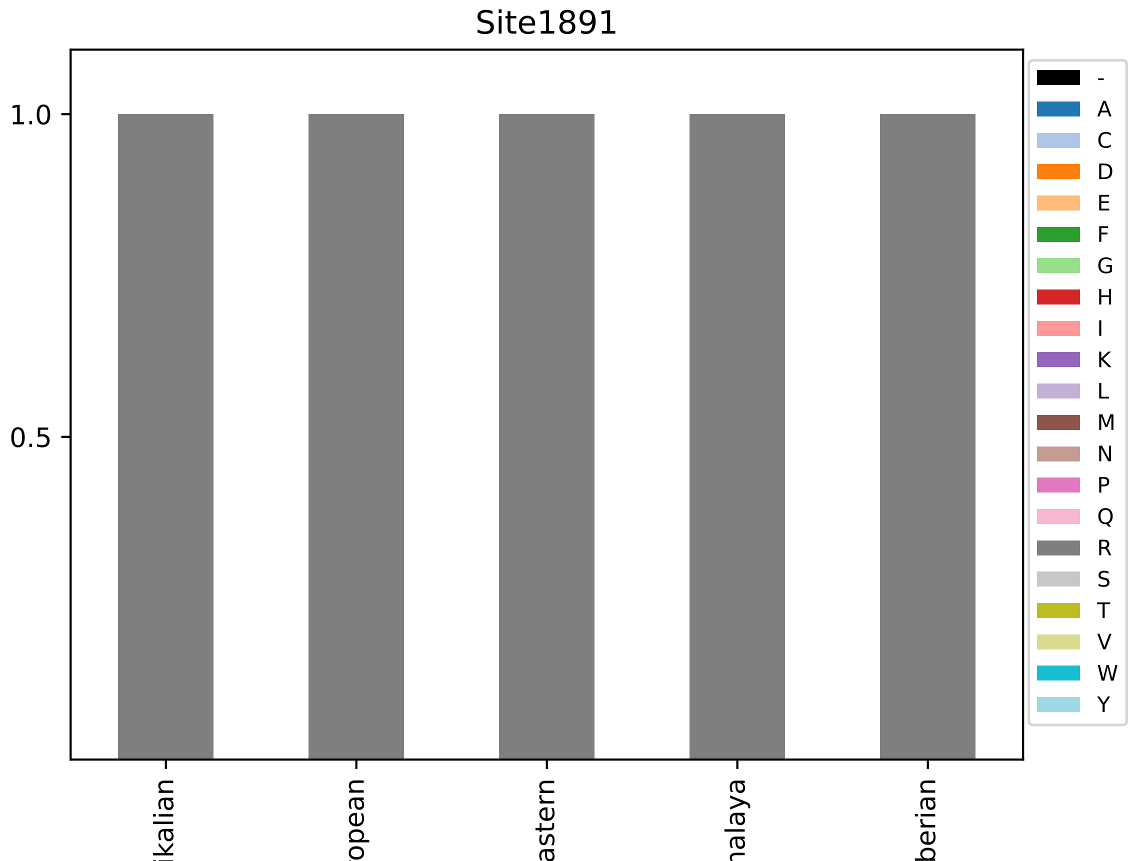
Site1891
1.0
0.5
-
A
C
D
E
F
G
H
I
K
L
M
N
P
Q
R
S
T
V
W
Y
ikalian
opean
astern
nalaya
berian

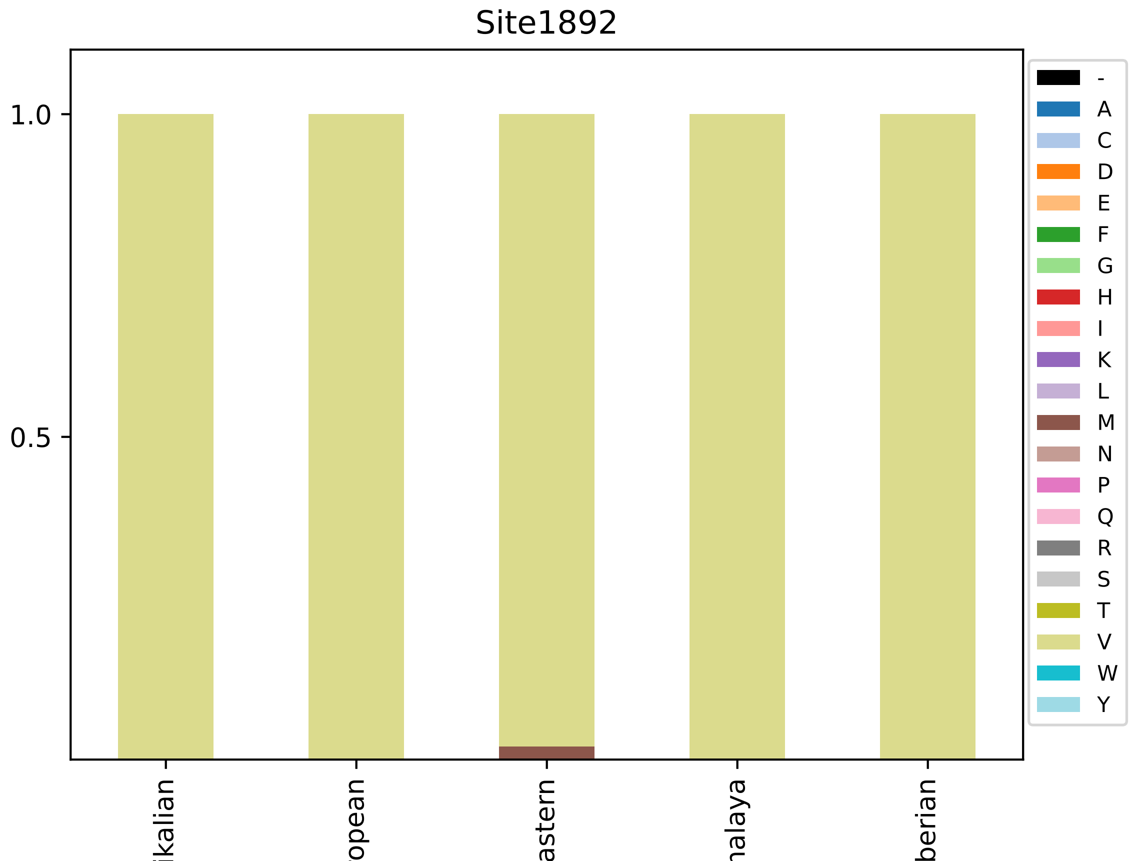

Site1892
1.0
0.5
-
A
C
D
E
F
G
H
I
K
L
M
N
P
Q
R
S
T
V
W
Y
ikalian
opean
astern
nalaya
berian

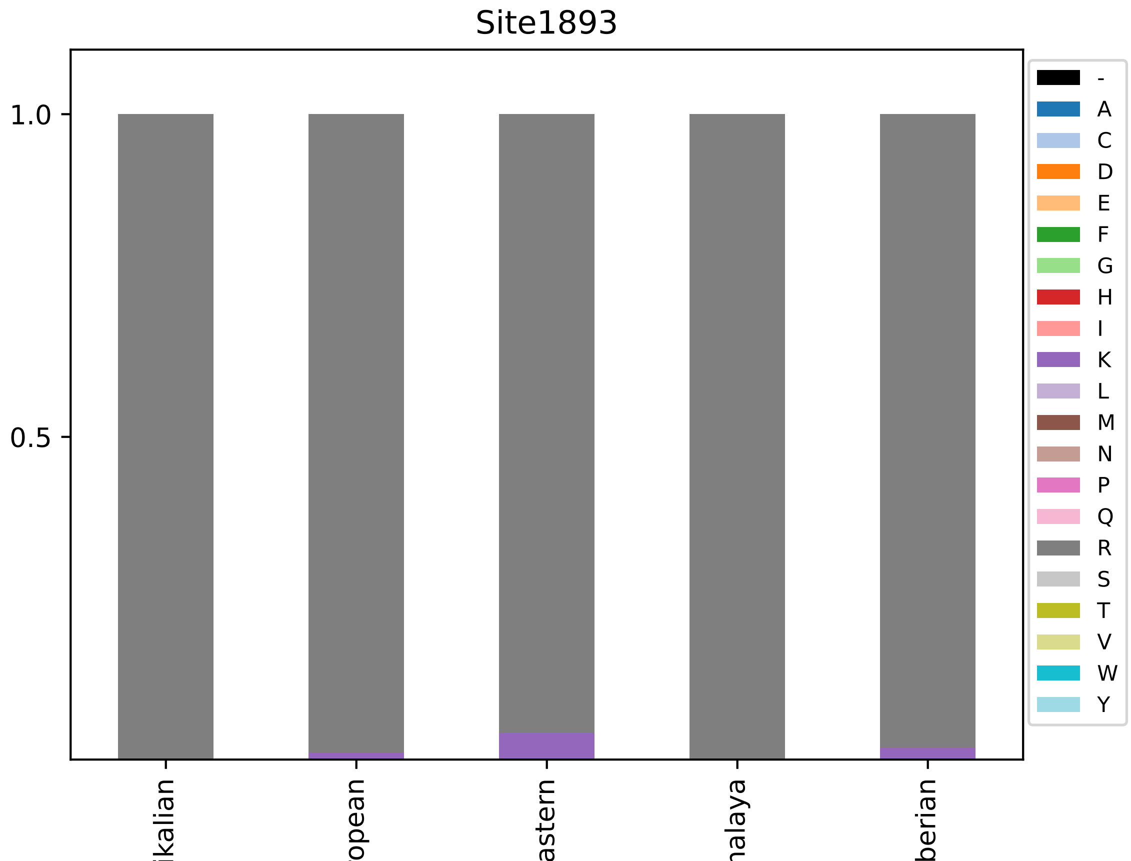

Site1893
1.0
0.5
-
A
C
D
E
F
G
H
I
K
L
M
N
P
Q
R
S
T
V
W
Y
ikalian
opean
astern
nalaya
berian

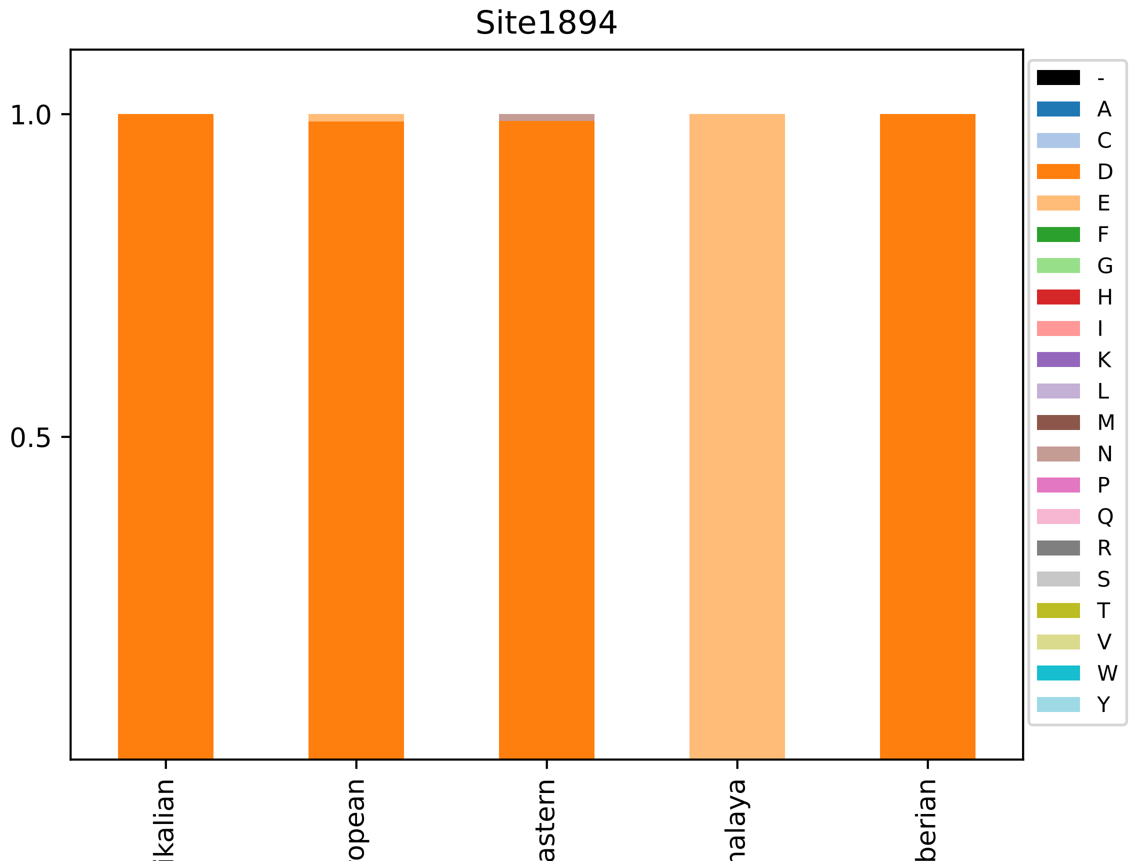

Site1894
1.0
0.5
ikalian
opean
astern
nalaya
berian
-
A
C
D
E
F
G
H
I
K
L
M
N
P
Q
R
S
T
V
W
Y

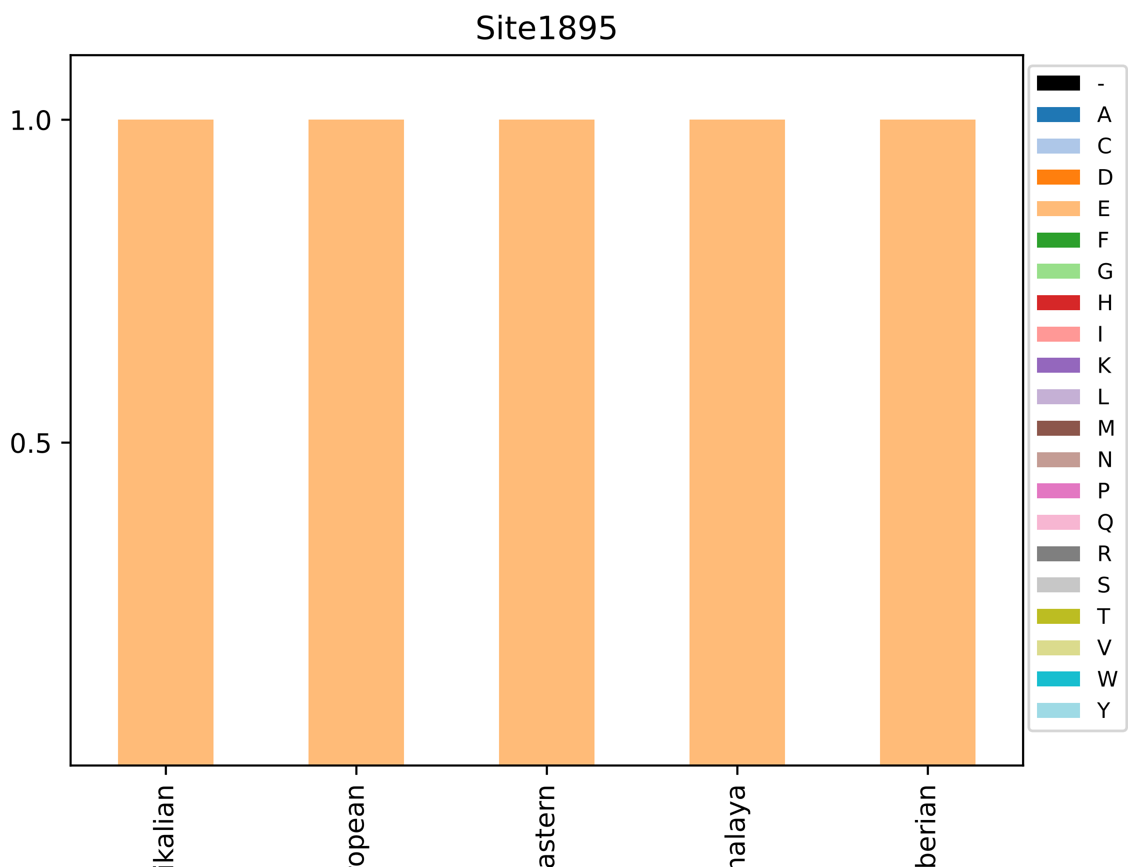
Site1895
1.0
0.5
-
A
C
D
E
F
G
H
I
K
L
M
N
P
Q
R
S
T
V
W
Y

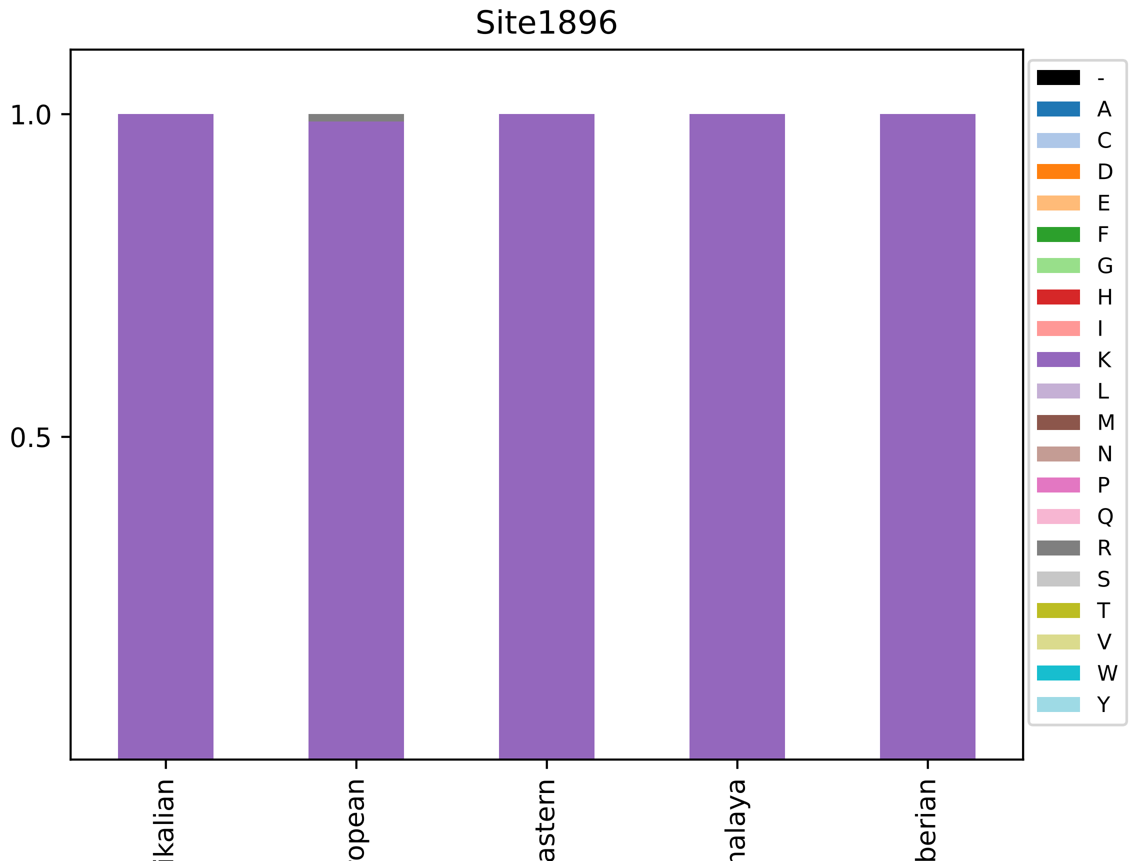
Site1896
1.0
0.5
-
A
C
D
E
F
G
H
I
K
L
M
N
P
Q
R
S
T
V
W
Y
ikalian
opean
astern
nalaya
berian

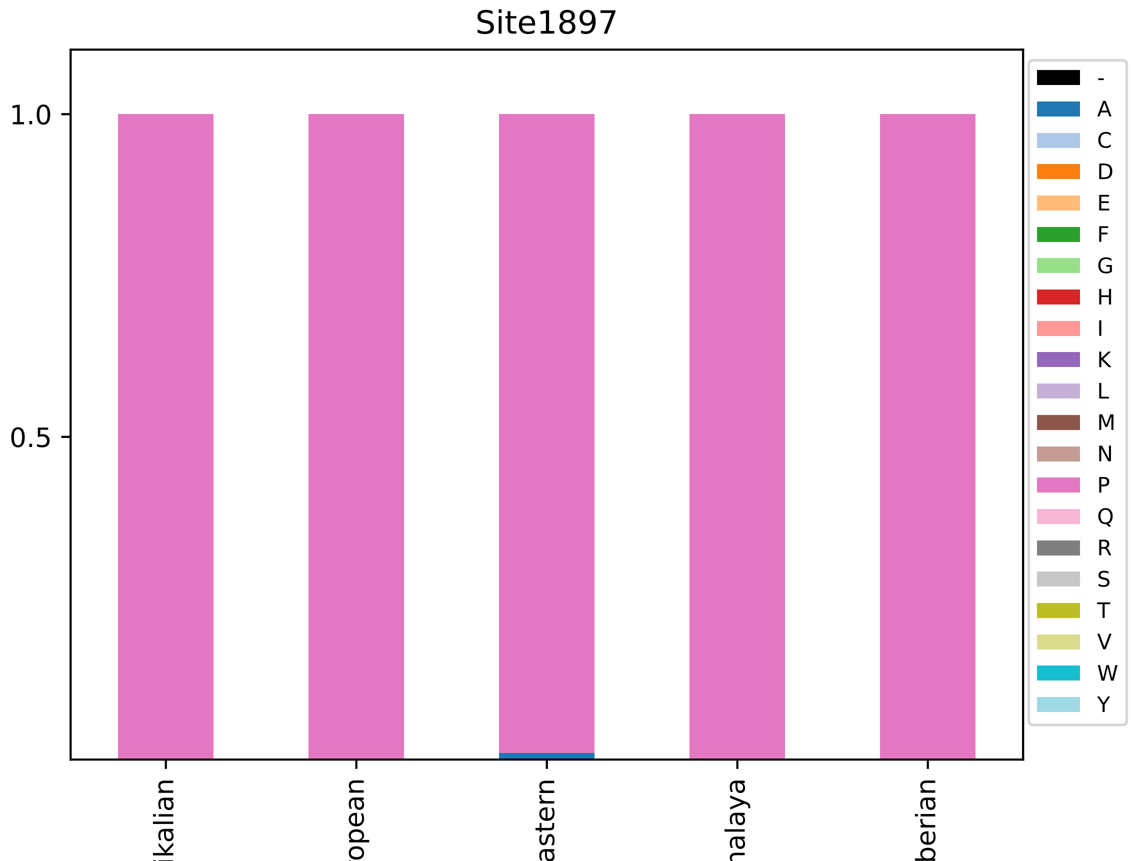
Site1897
1.0
0.5
-
A
C
D
E
F
G
H
I
K
L
M
N
P
Q
R
S
T
V
W
Y
ikalian
opean
astern
nalaya
berian

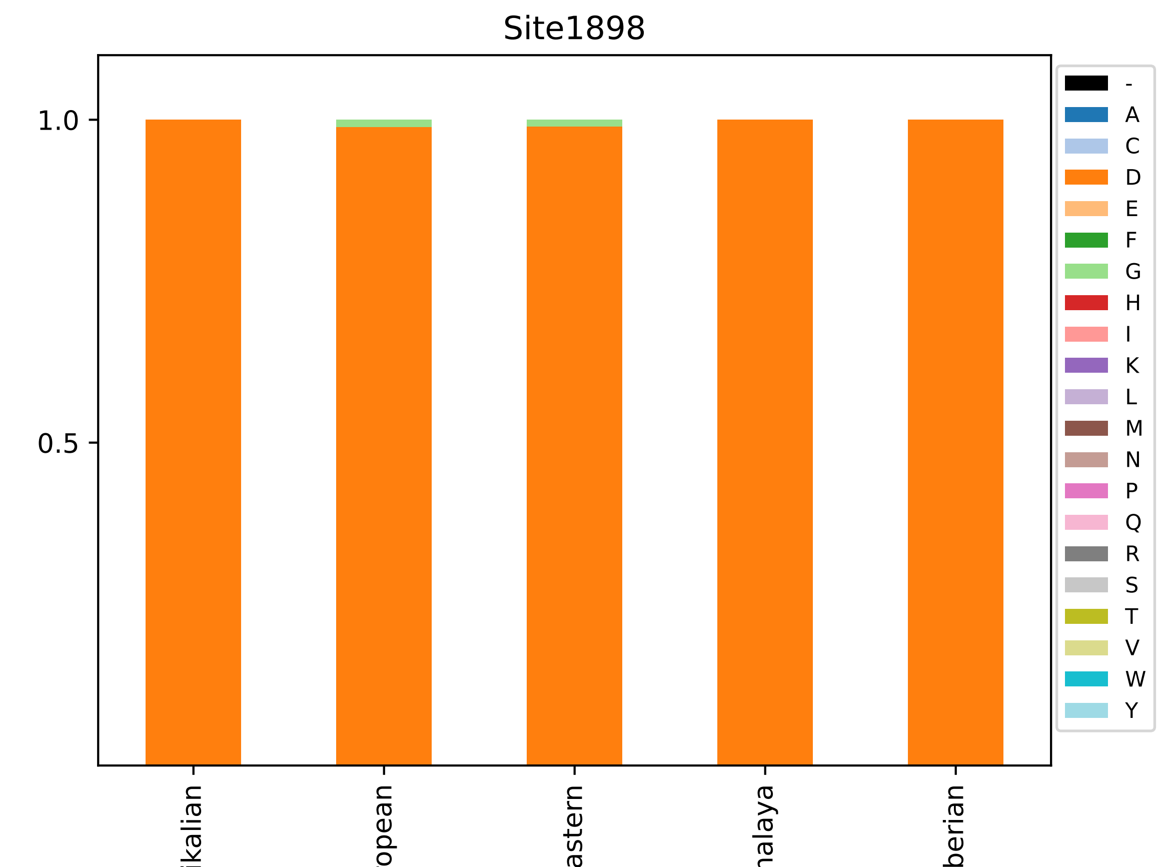
Site1898
1.0
0.5
-
A
C
D
E
F
G
H
I
K
L
M
N
P
Q
R
S
T
V
W
Y
ikalian
opean
astern
nalaya
berian

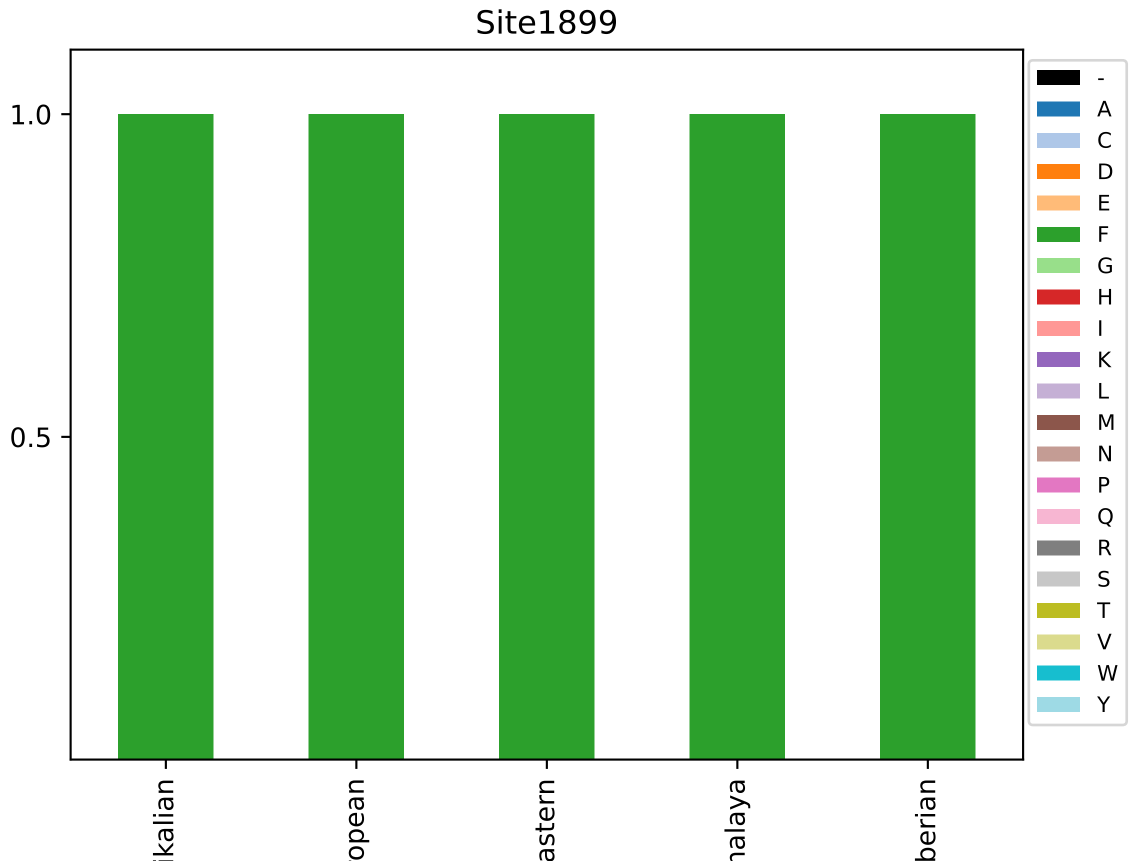

Site1899
1.0
0.5
-
A
C
D
E
F
G
H
I
K
L
M
N
P
Q
R
S
T
V
W
Y
ikalian
ropean
astern
nalaya
berian

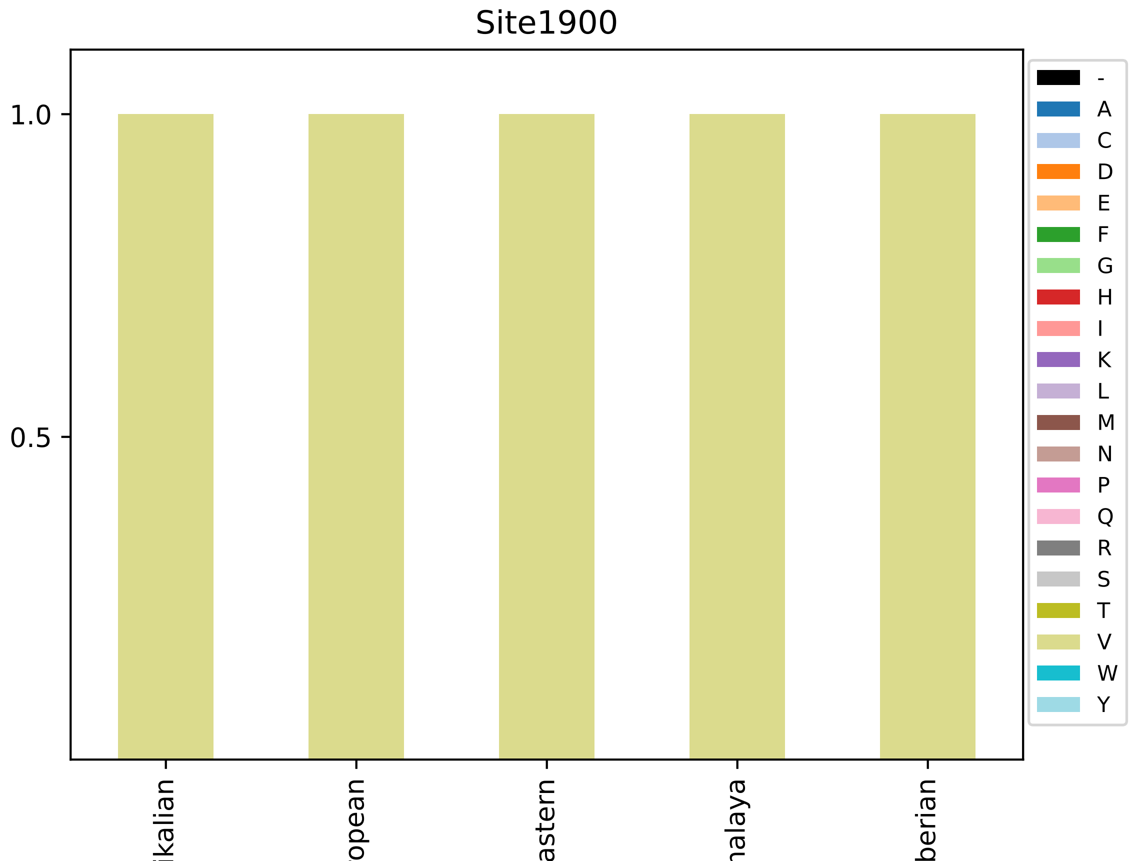

Site1900
1.0
0.5
-
A
C
D
E
F
G
H
I
K
L
M
N
P
Q
R
S
T
V
W
Y
ikalian
opean
astern
nalaya
berian

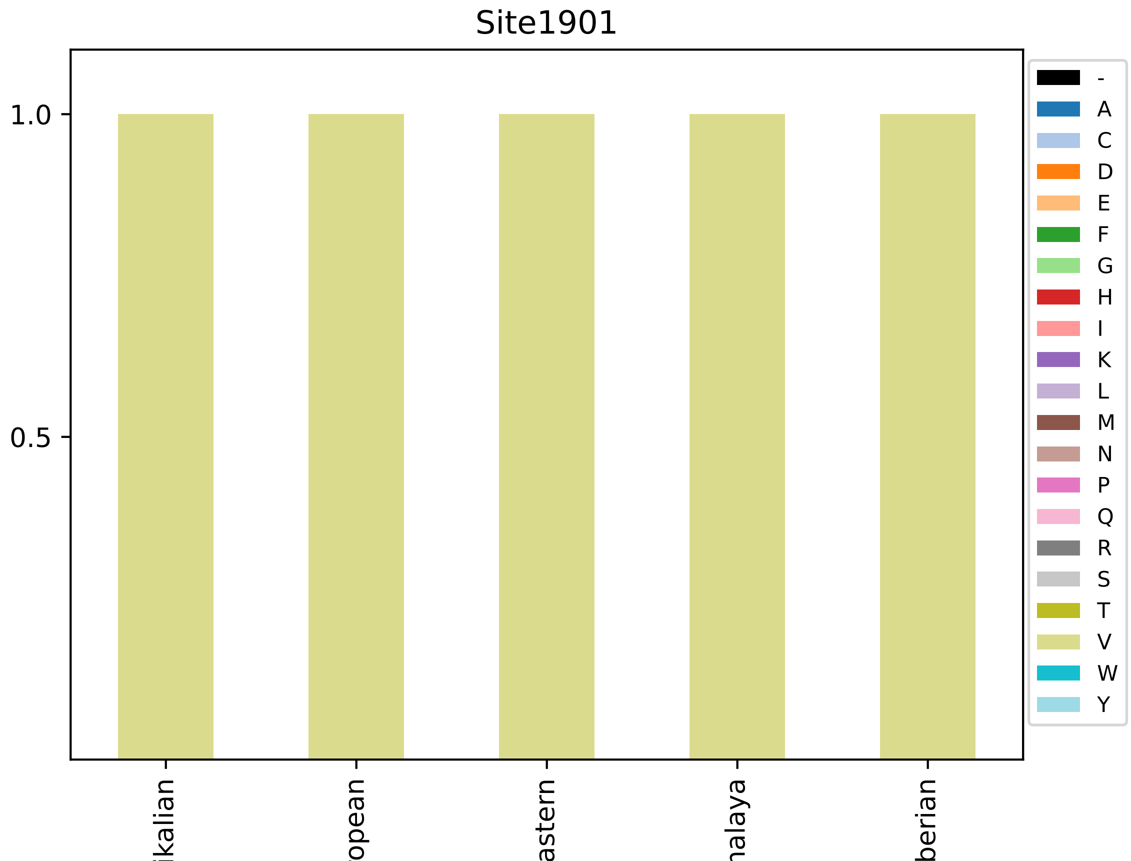

Site1901
1.0
0.5
-
A
C
D
E
F
G
H
I
K
L
M
N
P
Q
R
S
T
V
W
Y
ikalian
opean
astern
nalaya
berian

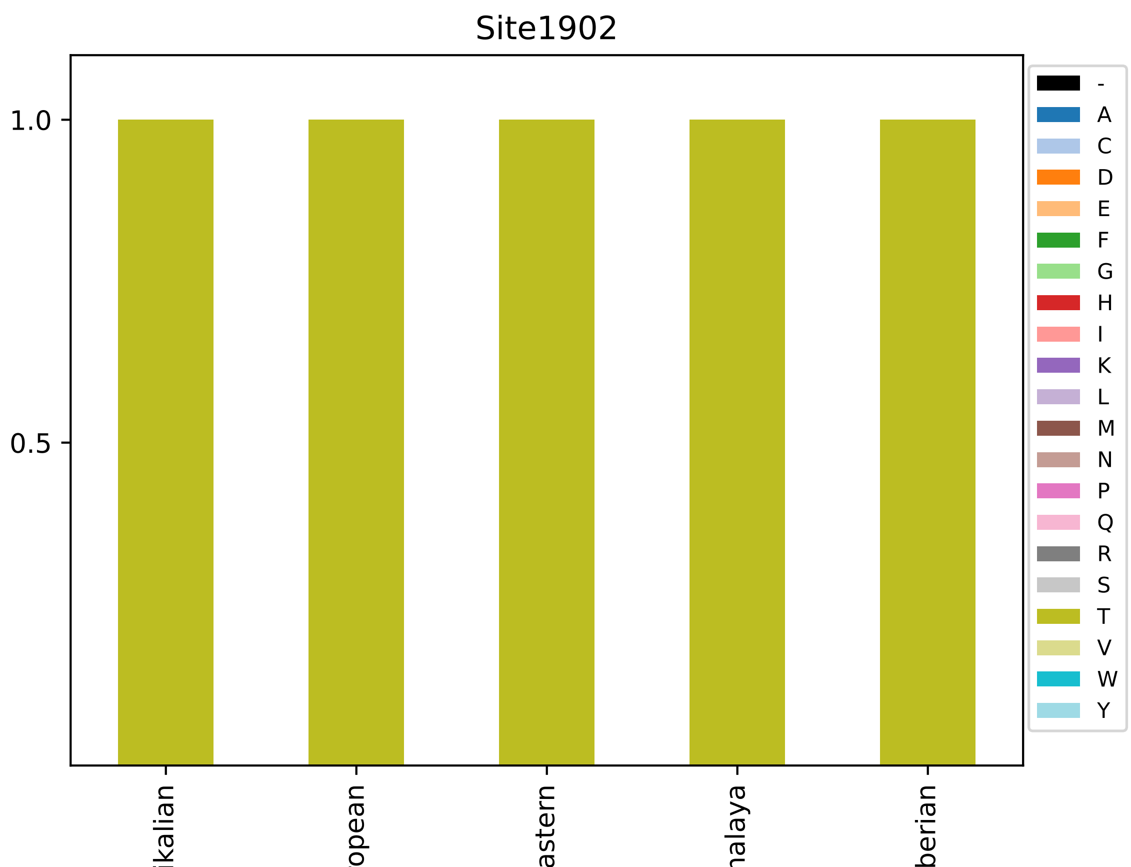
Site1902
1.0
0.5
-
A
C
D
E
F
G
H
I
K
L
M
N
P
Q
R
S
T
V
W
Y
ikalian
opean
astern
nalaya
berian

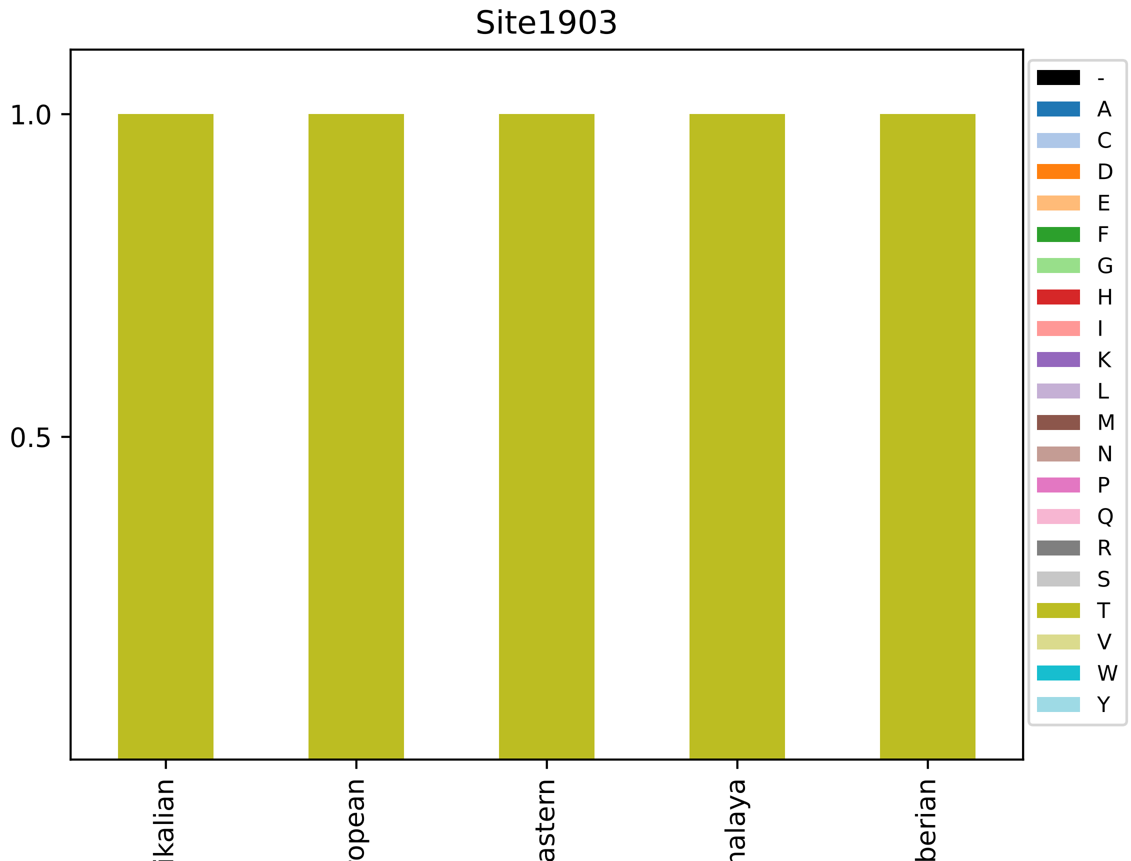
Site1903
1.0
0.5
-
A
C
D
E
F
G
H
I
K
L
M
N
P
Q
R
S
T
V
W
Y
ikalian
opean
astern
nalaya
berian

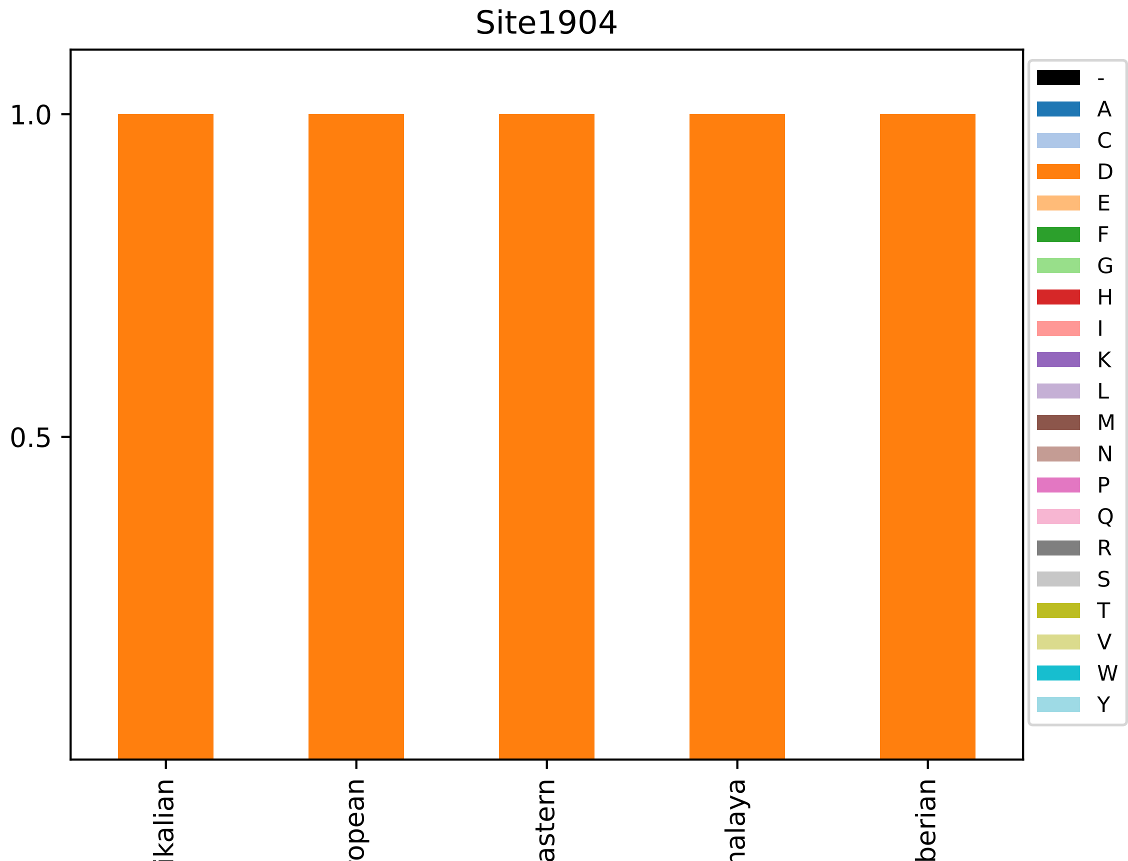

Site1904
1.0
0.5
-
A
C
D
E
F
G
H
I
K
L
M
N
P
Q
R
S
T
V
W
Y
ikalian
opean
astern
nalaya
berian

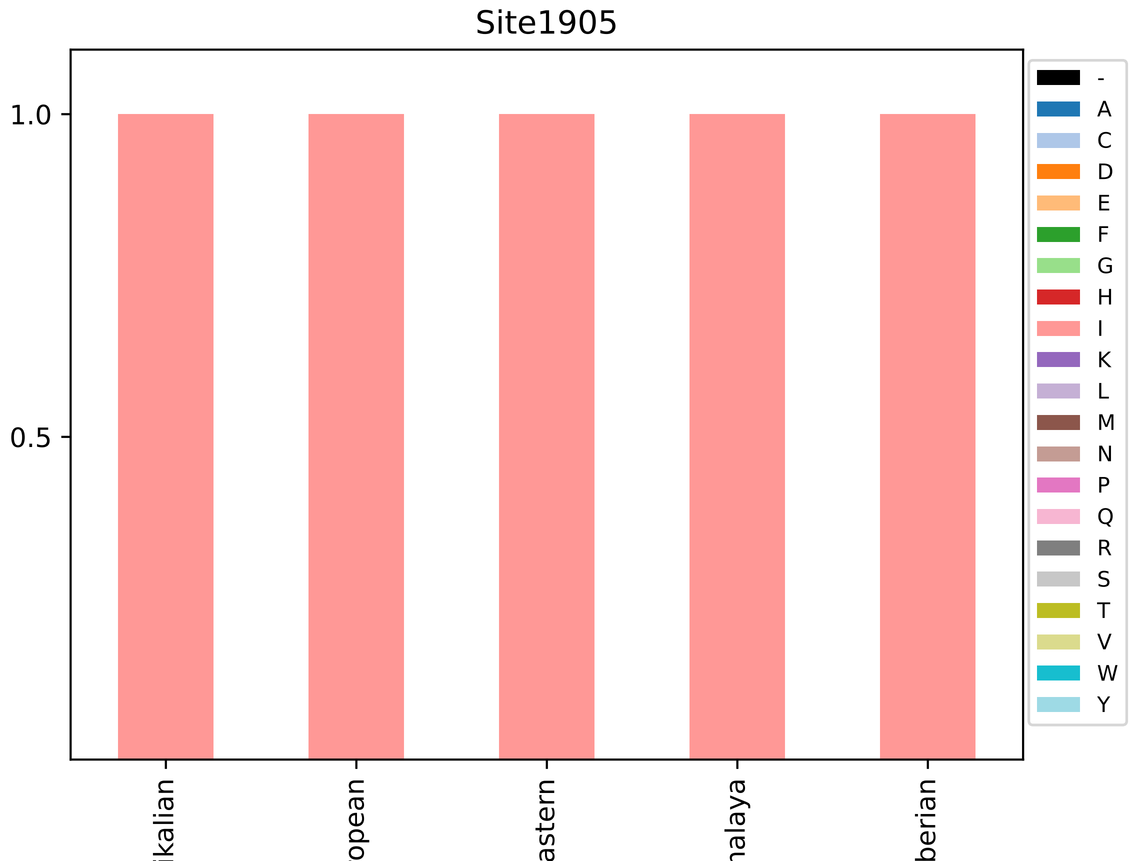

Site1905
1.0
0.5
-
A
C
D
E
F
G
H
I
K
L
M
N
P
Q
R
S
T
V
W
Y
ikalian
opean
astern
nalaya
berian

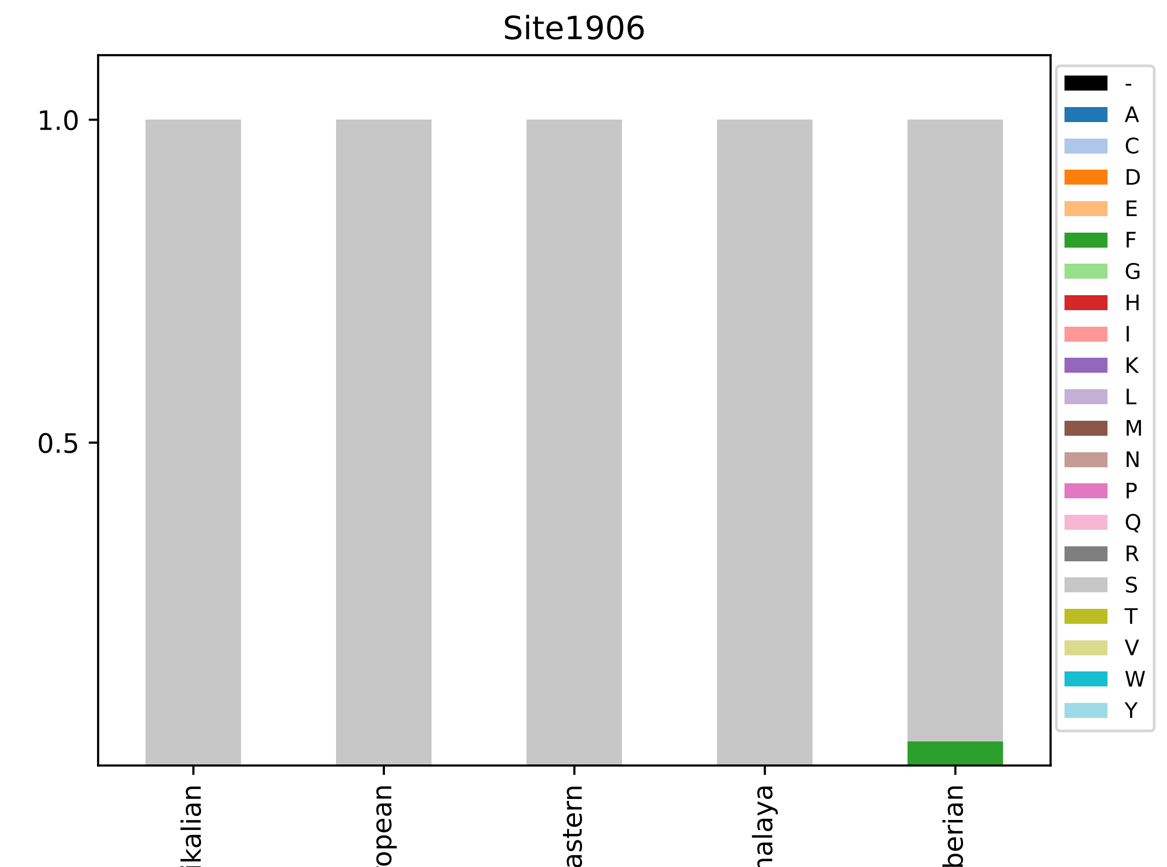

Site1906
1.0
0.5
-
A
C
D
E
F
G
H
I
K
L
M
N
P
Q
R
S
T
V
W
Y
ikalian
opean
astern
nalaya
berian

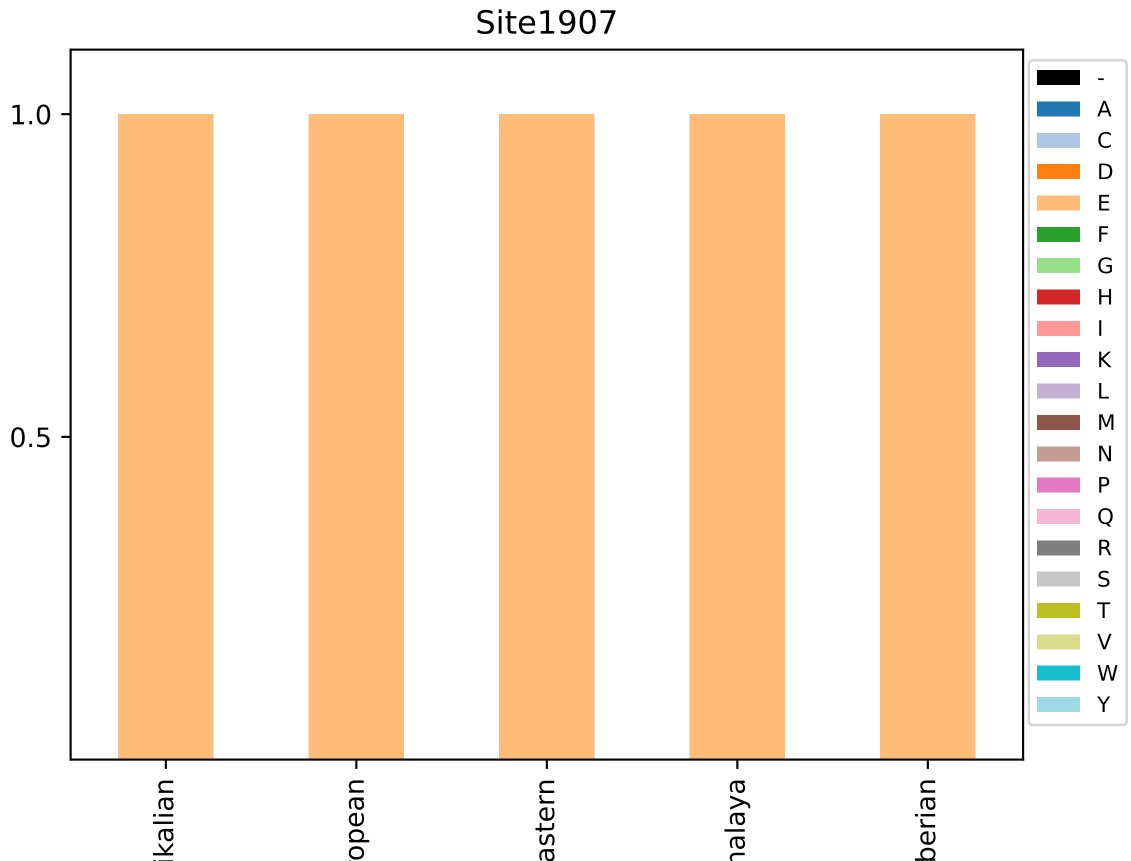

Site1907
1.0
0.5
-
A
C
D
E
F
G
H
I
K
L
M
N
P
Q
R
S
T
V
W
Y
ikalian
opean
astern
nalaya
berian

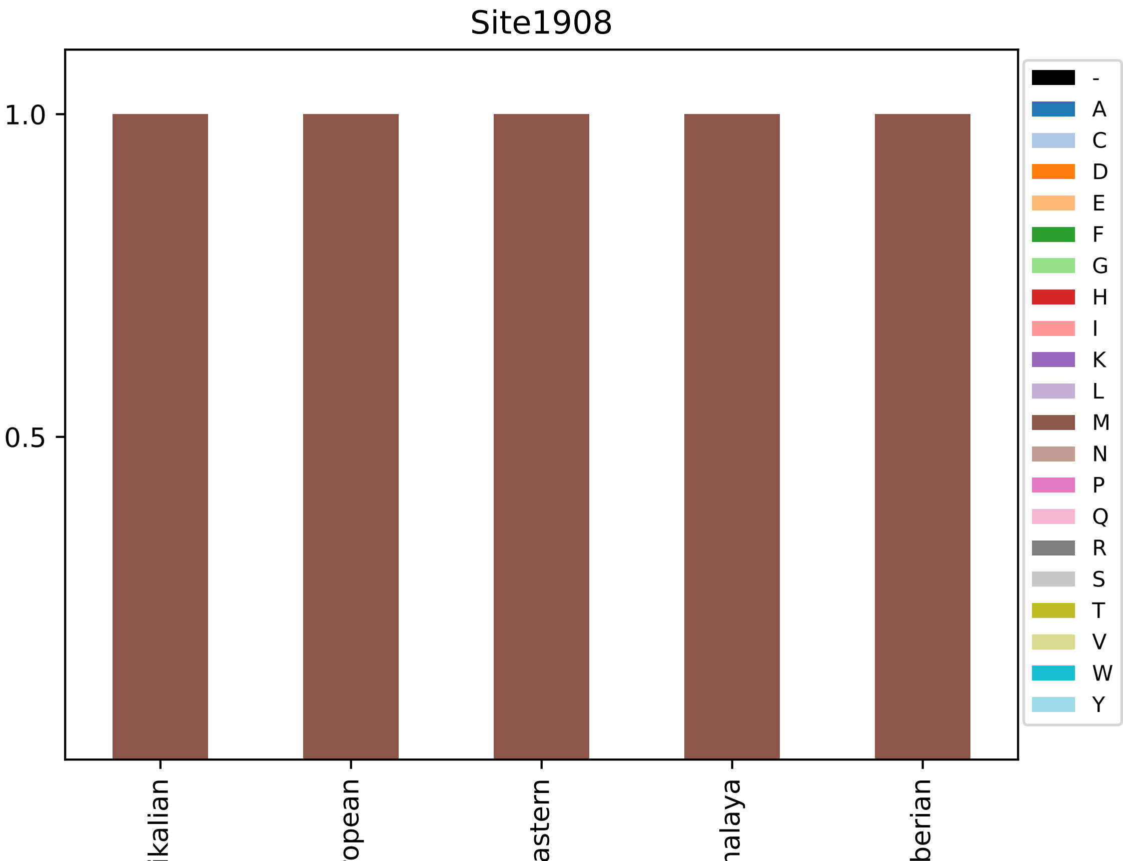
Site1908
1.0
0.5
-
A
C
D
E
F
G
H
I
K
L
M
N
P
Q
R
S
T
V
W
Y
ikalian
opean
astern
nalaya
berian

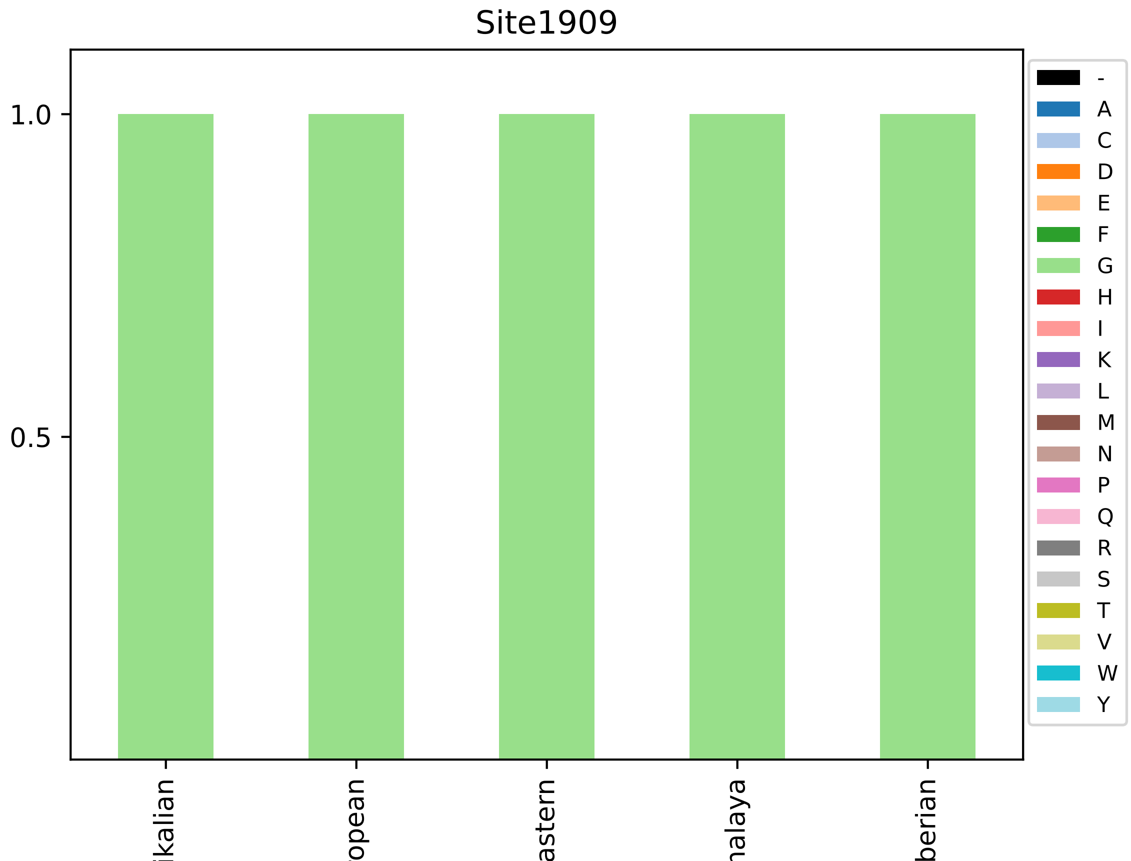
Site1909
1.0
0.5
-
A
C
D
E
F
G
H
I
K
L
M
N
P
Q
R
S
T
V
W
Y

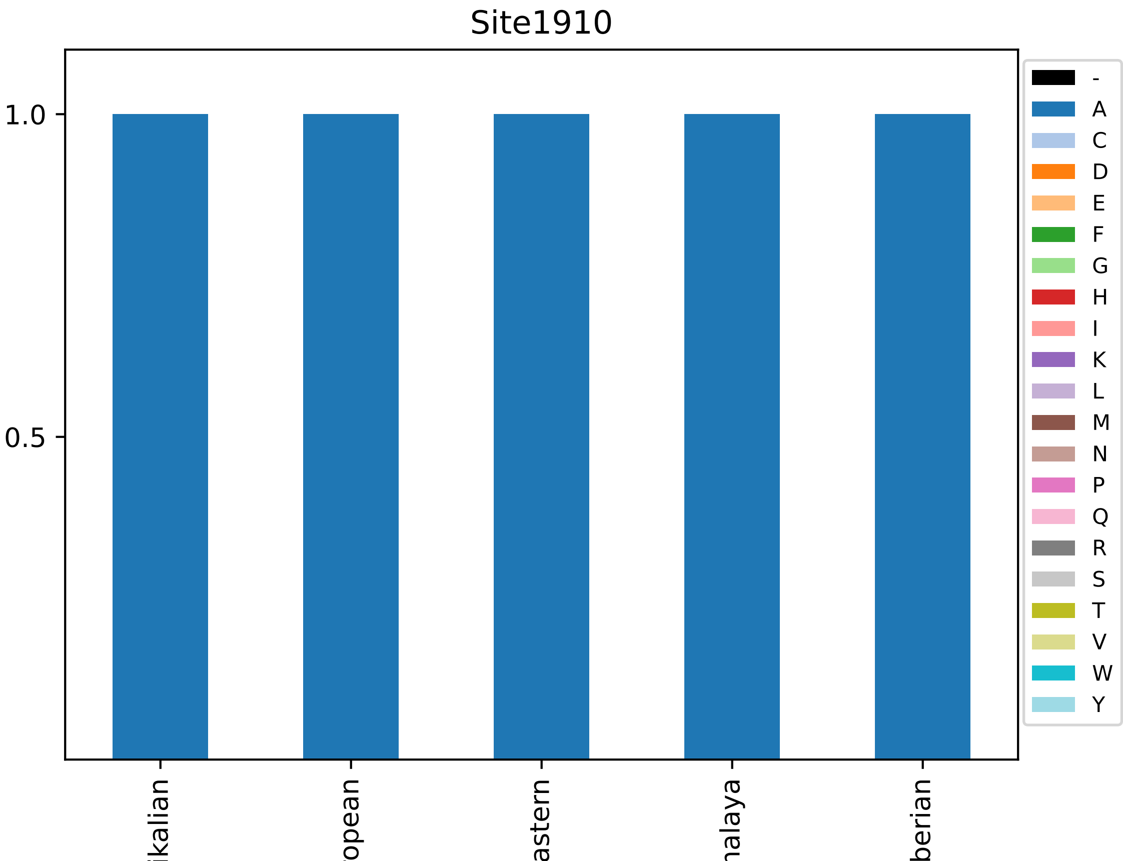
Site1910
1.0
0.5
-
A
C
D
E
F
G
H
I
K
L
M
N
P
Q
R
S
T
V
W
Y

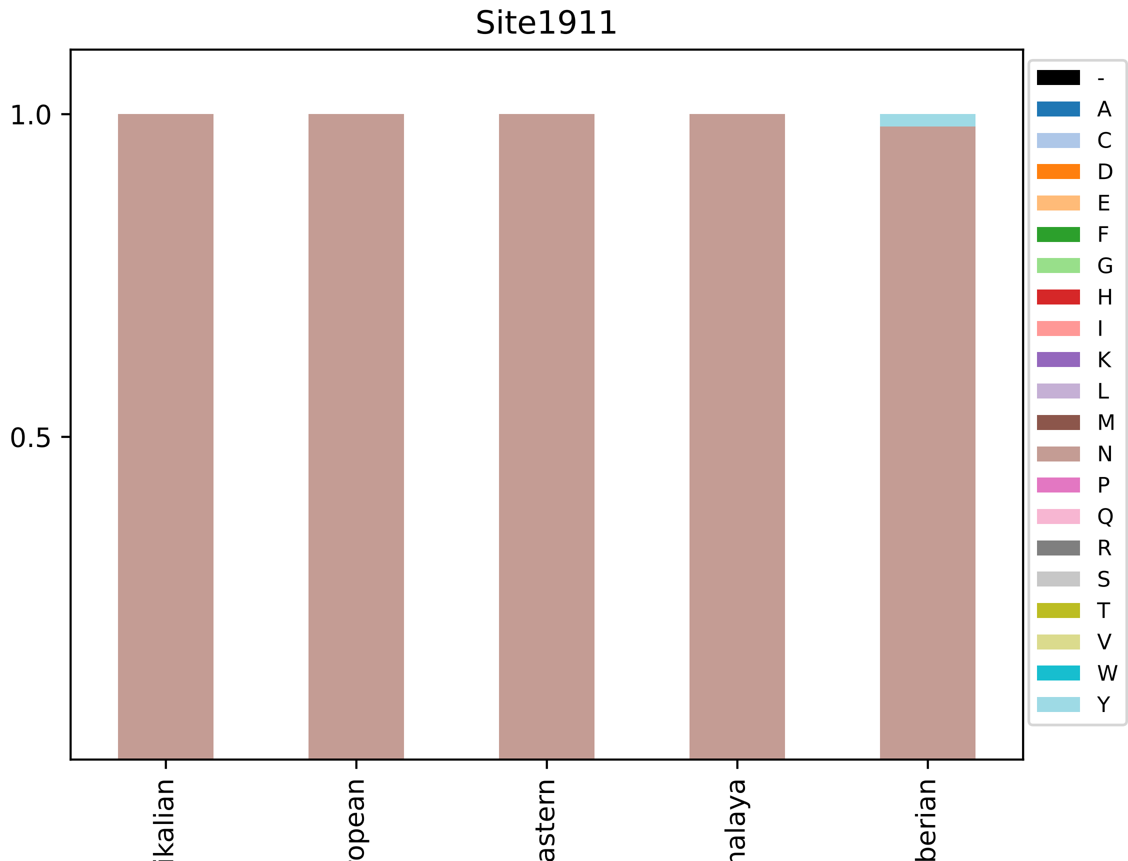
Site1911
1.0
0.5
-
A
C
D
E
F
G
H
I
K
L
M
N
P
Q
R
S
T
V
W
Y
ikalian
opean
astern
nalaya
berian

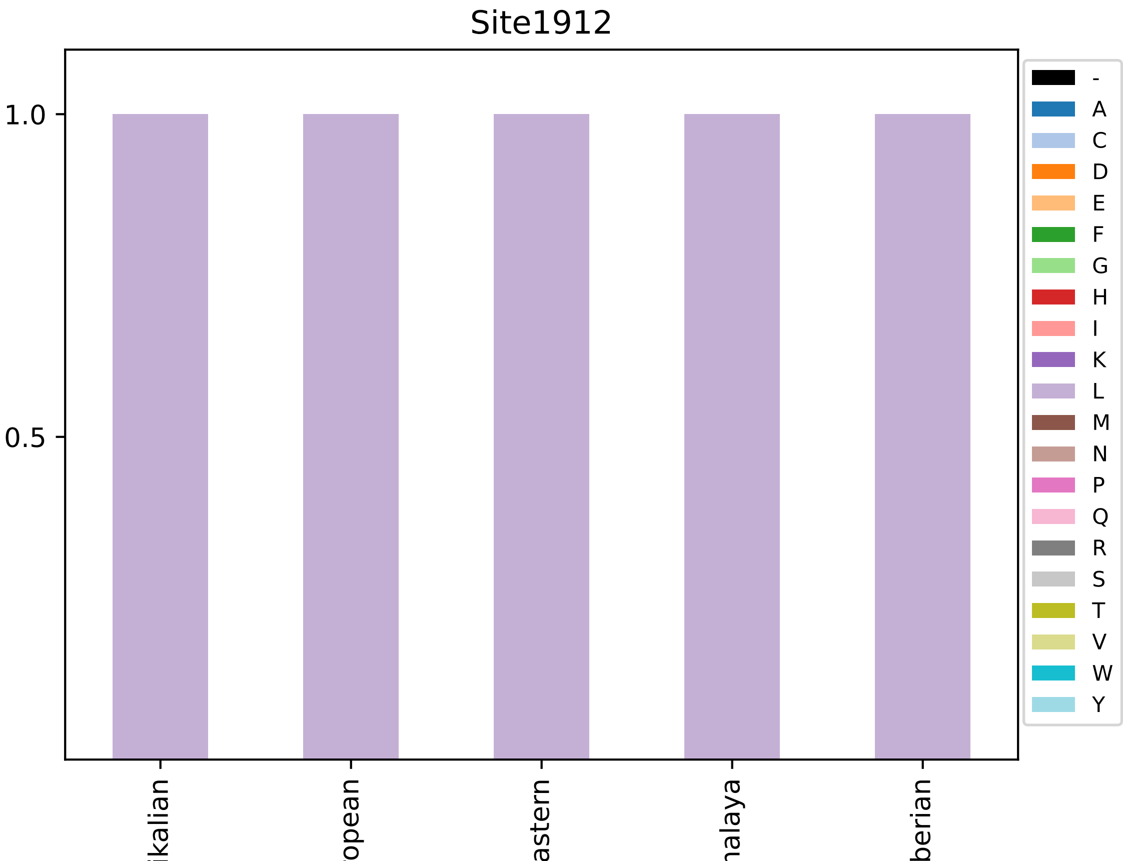
Site1912
1.0
0.5
ikalian
opean
astern
nalaya
berian
-
A
C
D
E
F
G
H
I
K
L
M
N
P
Q
R
S
T
V
W
Y

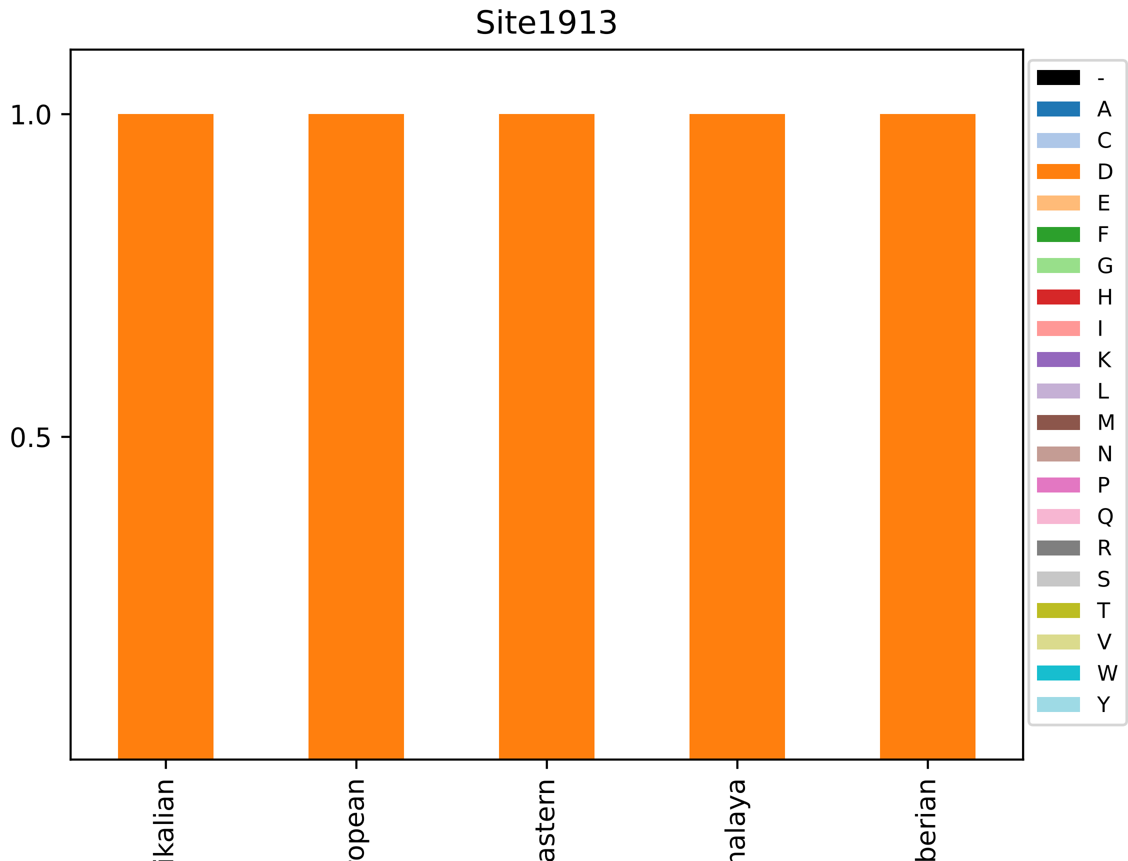

Site1913
1.0
0.5
-
A
C
D
E
F
G
H
I
K
L
M
N
P
Q
R
S
T
V
W
Y

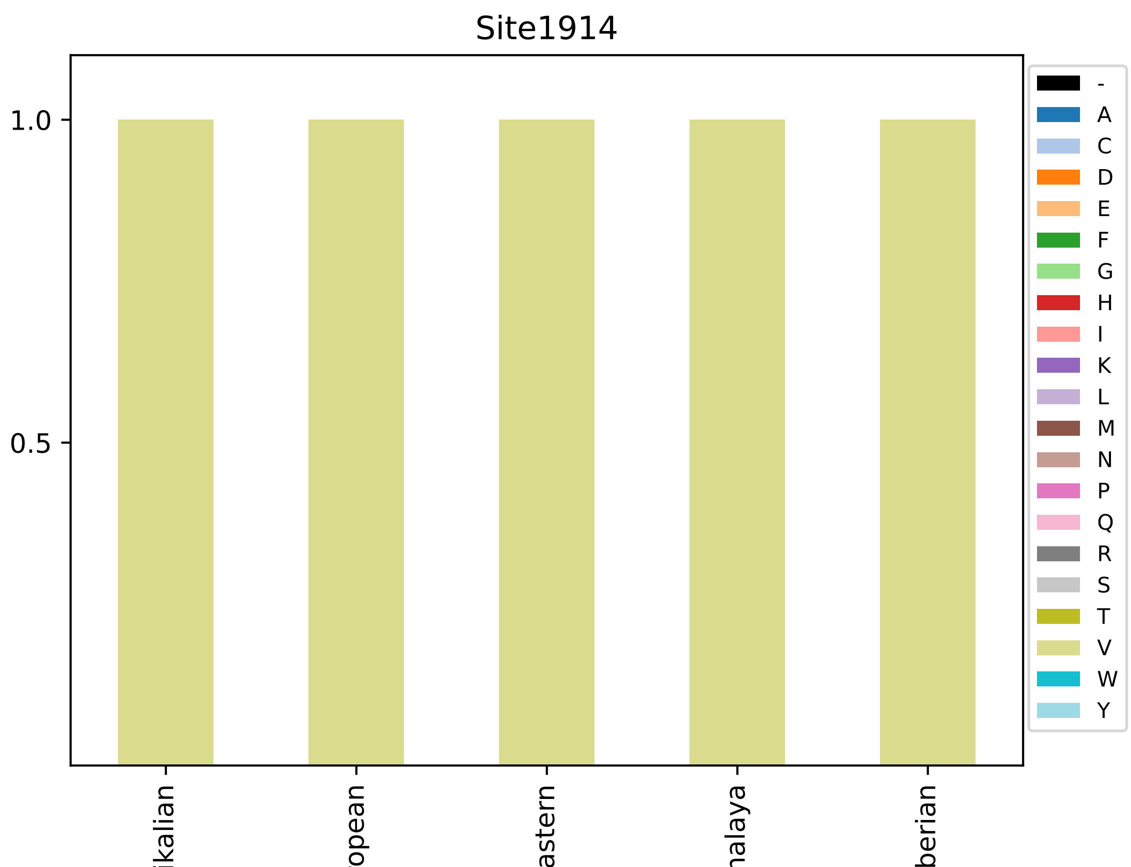

Site1914
1.0
0.5
-
A
C
D
E
F
G
H
I
K
L
M
N
P
Q
R
S
T
V
W
Y
ikalian
opean
astern
nalaya
berian

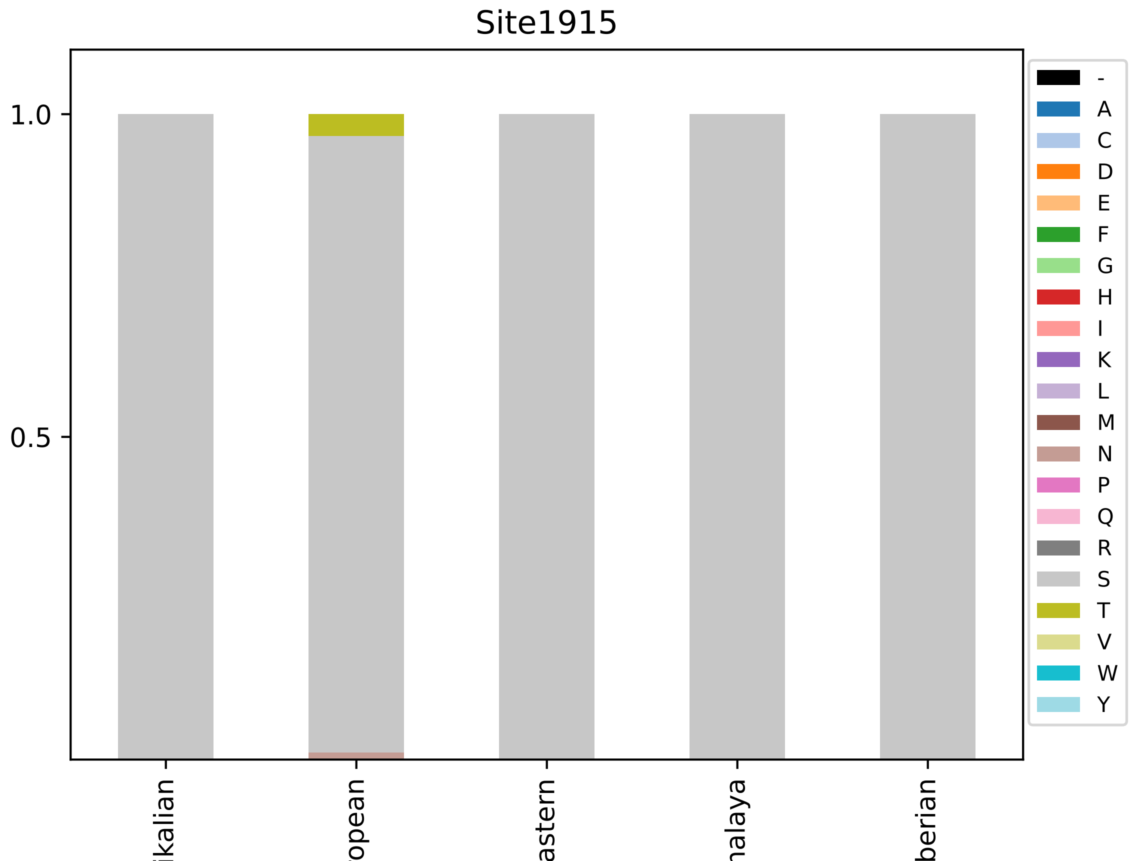

Site1915
1.0
0.5
-
A
C
D
E
F
G
H
I
K
L
M
N
P
Q
R
S
T
V
W
Y
ikalian
opean
astern
nalaya
berian

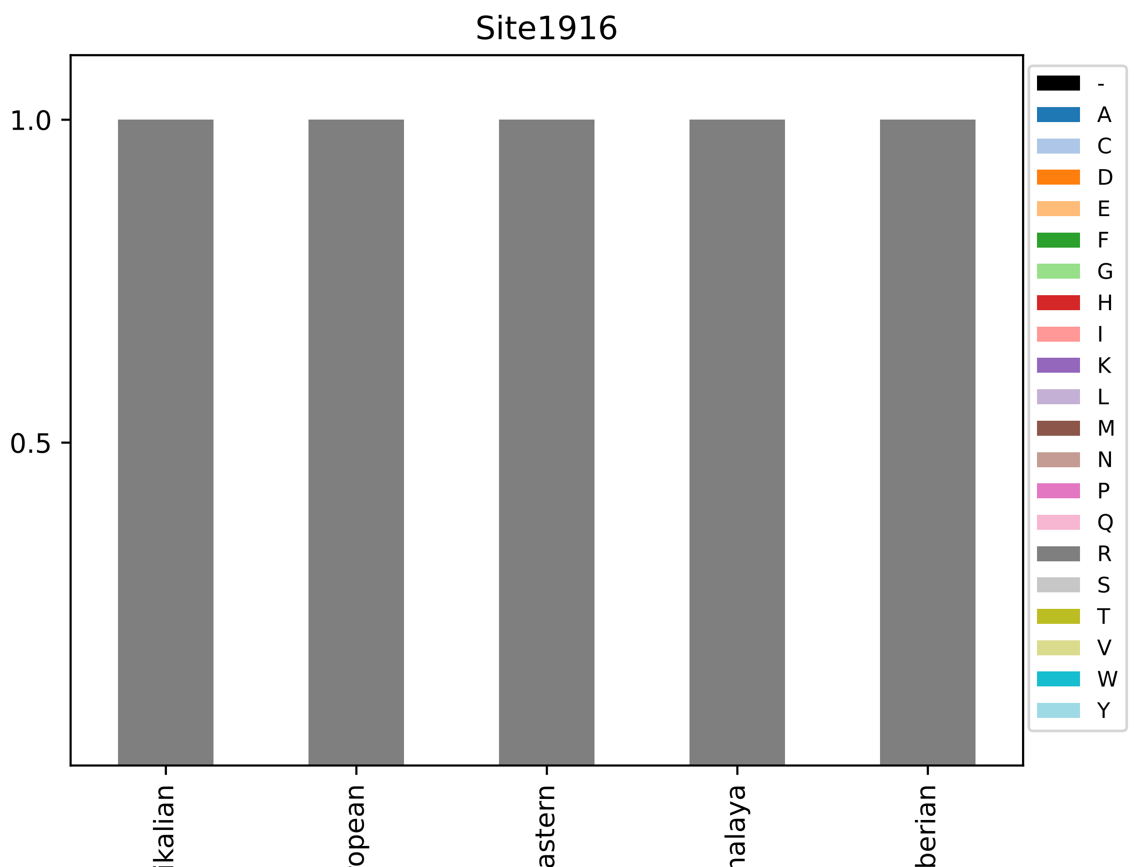
Site1916
1.0
0.5
-
A
C
D
E
F
G
H
I
K
L
M
N
P
Q
R
S
T
V
W
Y
ikalian
opean
astern
nalaya
berian

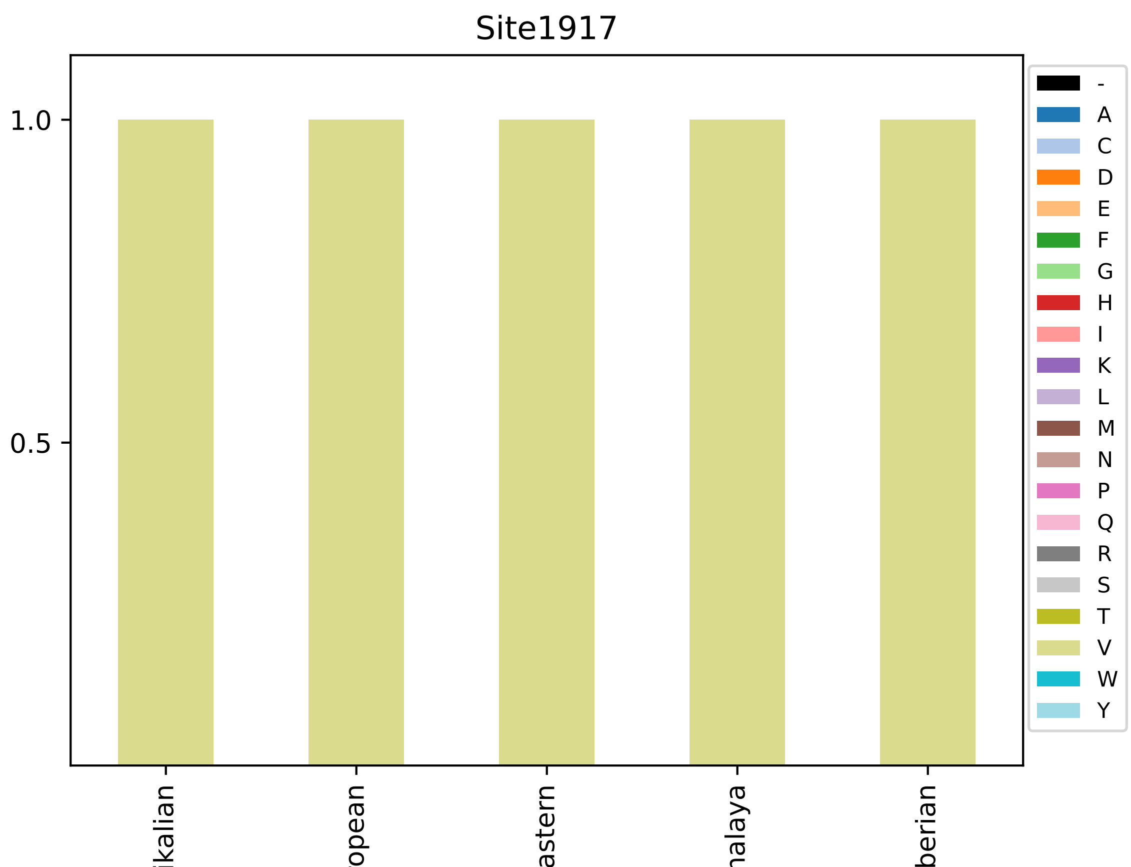
Site1917
1.0
0.5
-
A
C
D
E
F
G
H
I
K
L
M
N
P
Q
R
S
T
V
W
Y
ikalian
opean
astern
nalaya
berian

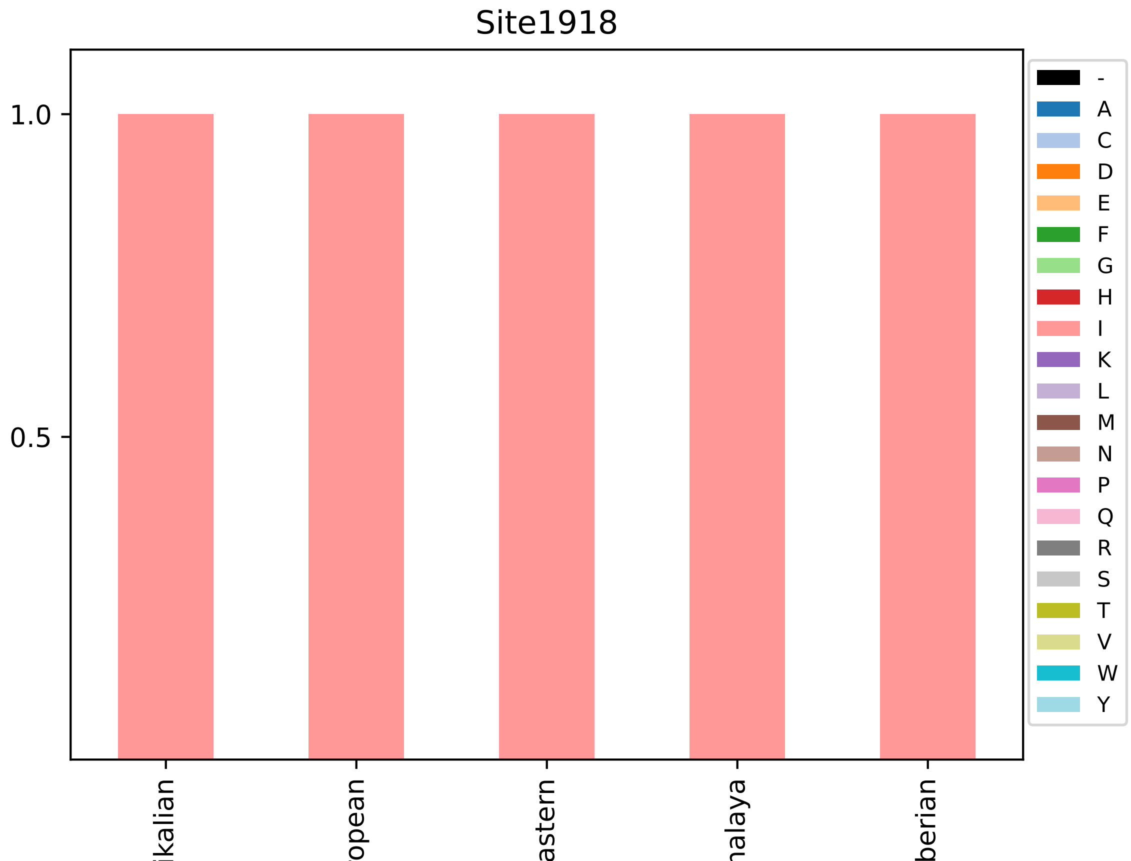
Site1918
1.0
0.5
ikalian
opean
astern
nalaya
berian
-
A
C
D
E
F
G
H
I
K
L
M
N
P
Q
R
S
T
V
W
Y

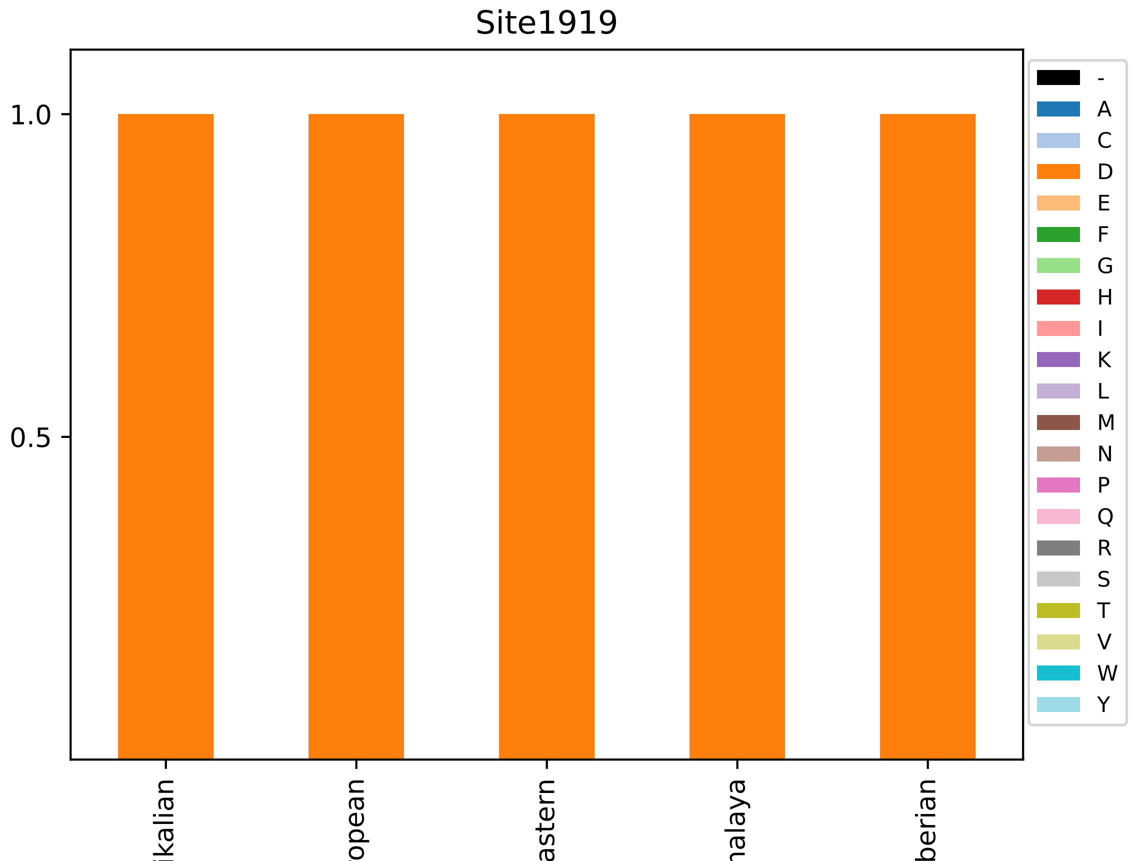
Site1919
1.0
0.5
-
A
C
D
E
F
G
H
I
K
L
M
N
P
Q
R
S
T
V
W
Y

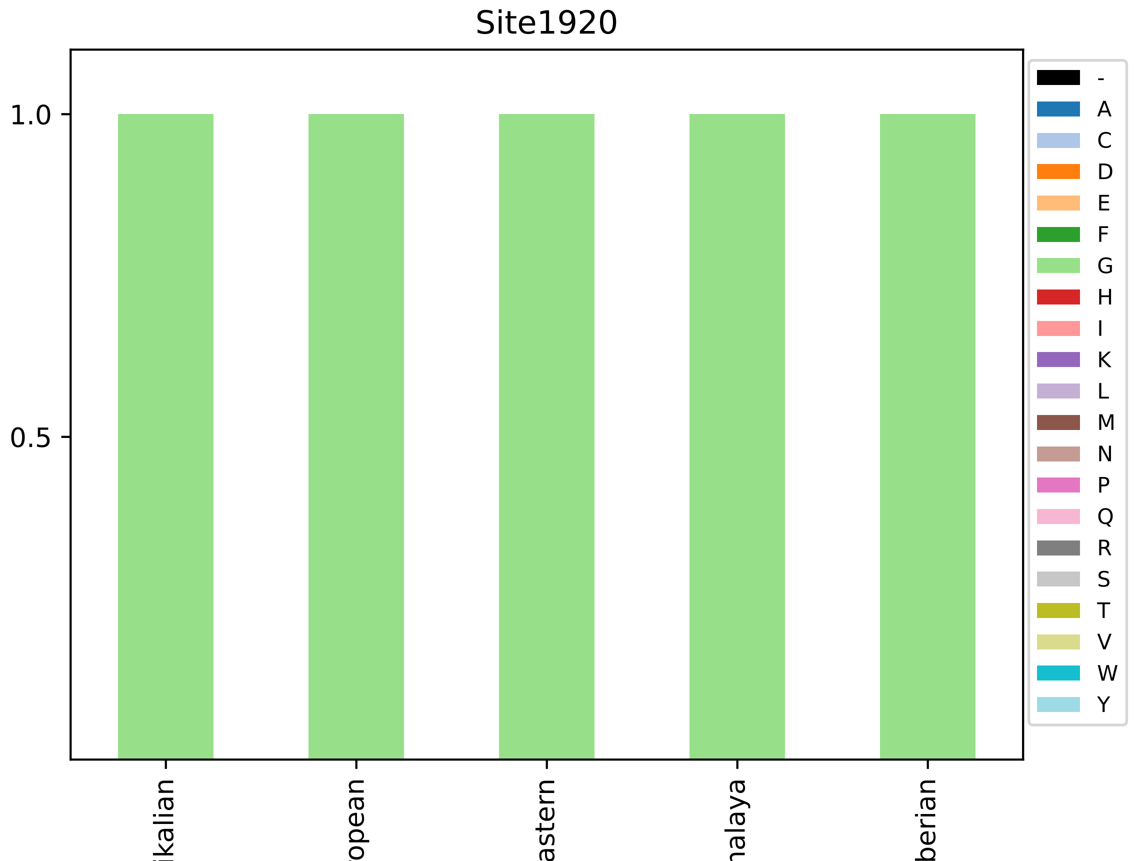

Site1920
1.0
0.5
-
A
C
D
E
F
G
H
I
K
L
M
N
P
Q
R
S
T
V
W
Y
ikalian
opean
astern
nalaya
berian

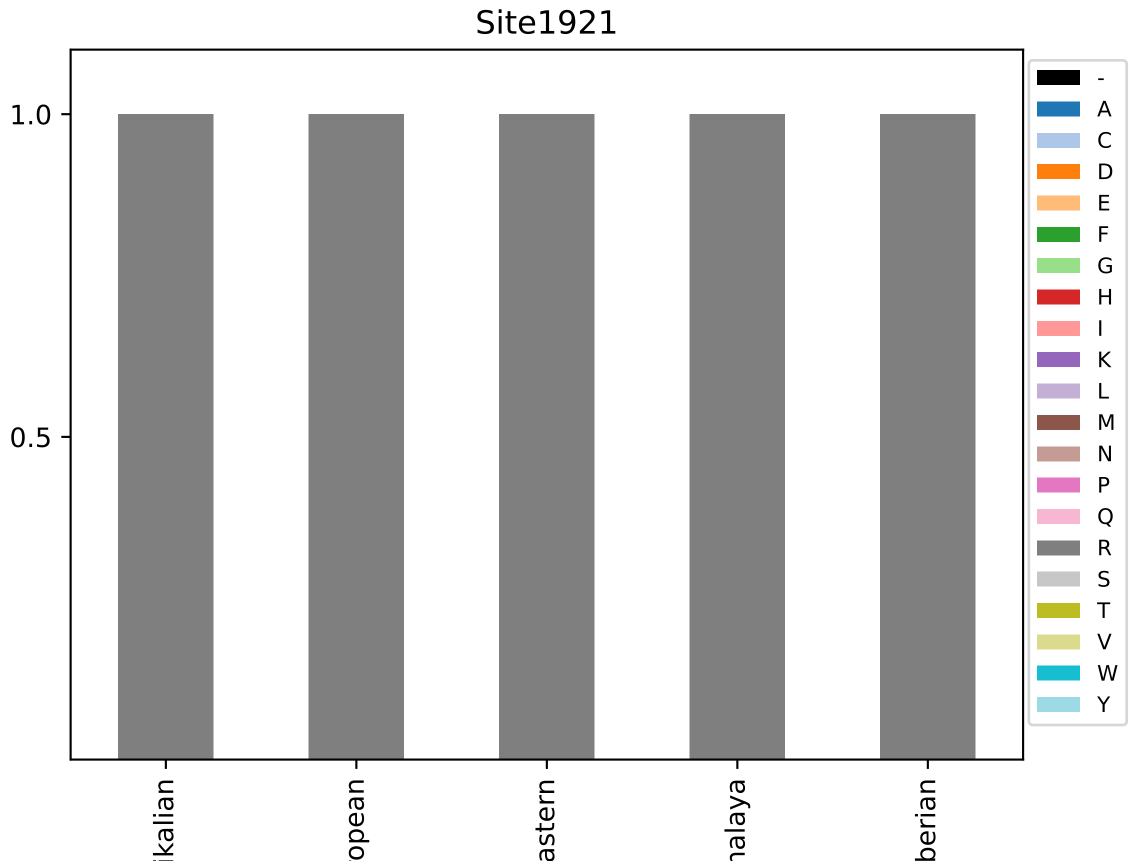
Site1921
1.0
0.5
-
A
C
D
E
F
G
H
I
K
L
M
N
P
Q
R
S
T
V
W
Y
ikalian
opean
astern
nalaya
berian

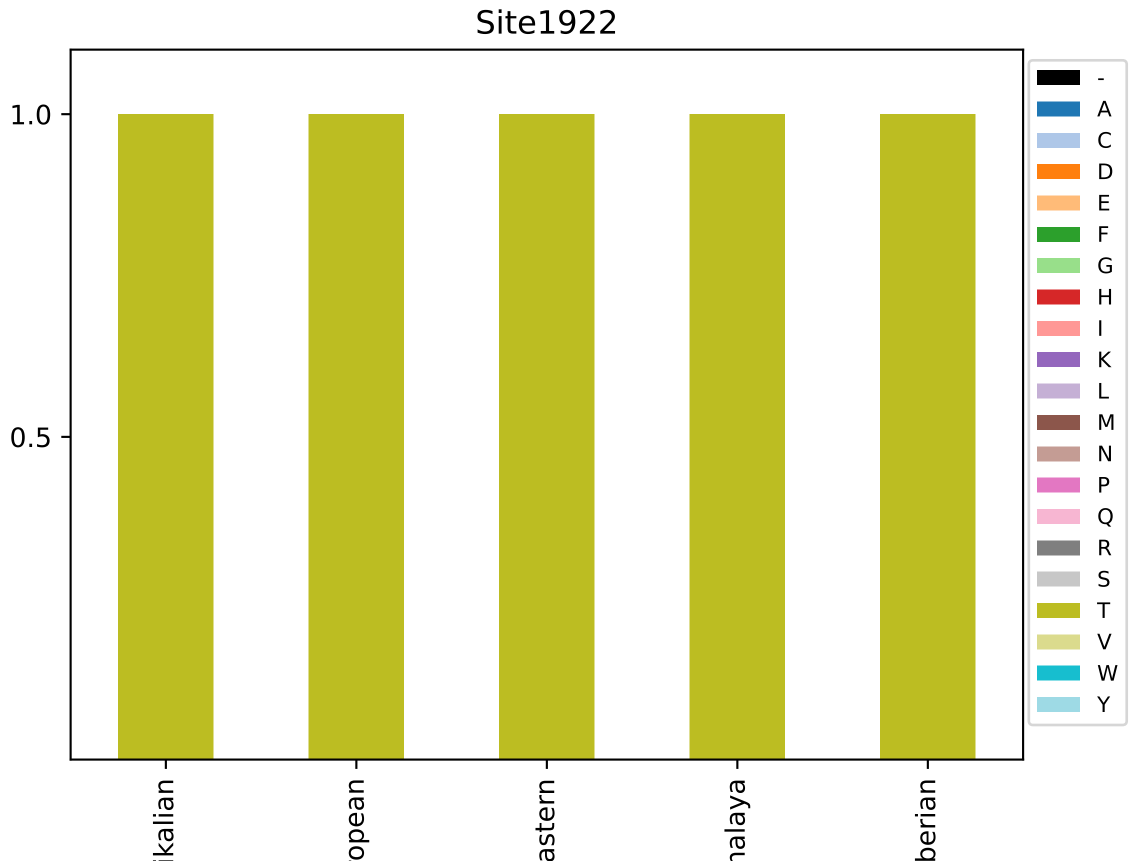

Site1922
1.0
0.5
-
A
C
D
E
F
G
H
I
K
L
M
N
P
Q
R
S
T
V
W
Y
ikalian
opean
astern
nalaya
berian

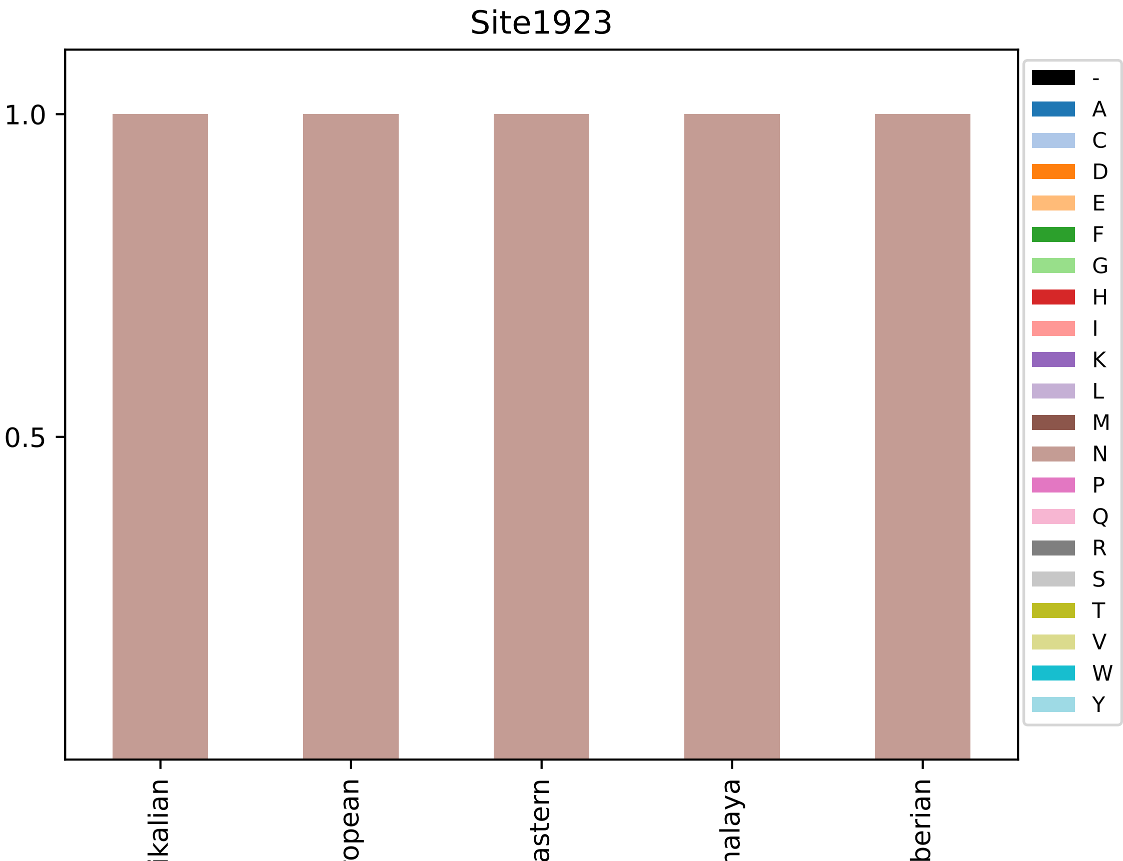
Site1923
1.0
0.5
-
A
C
D
E
F
G
H
I
K
L
M
N
P
Q
R
S
T
V
W
Y

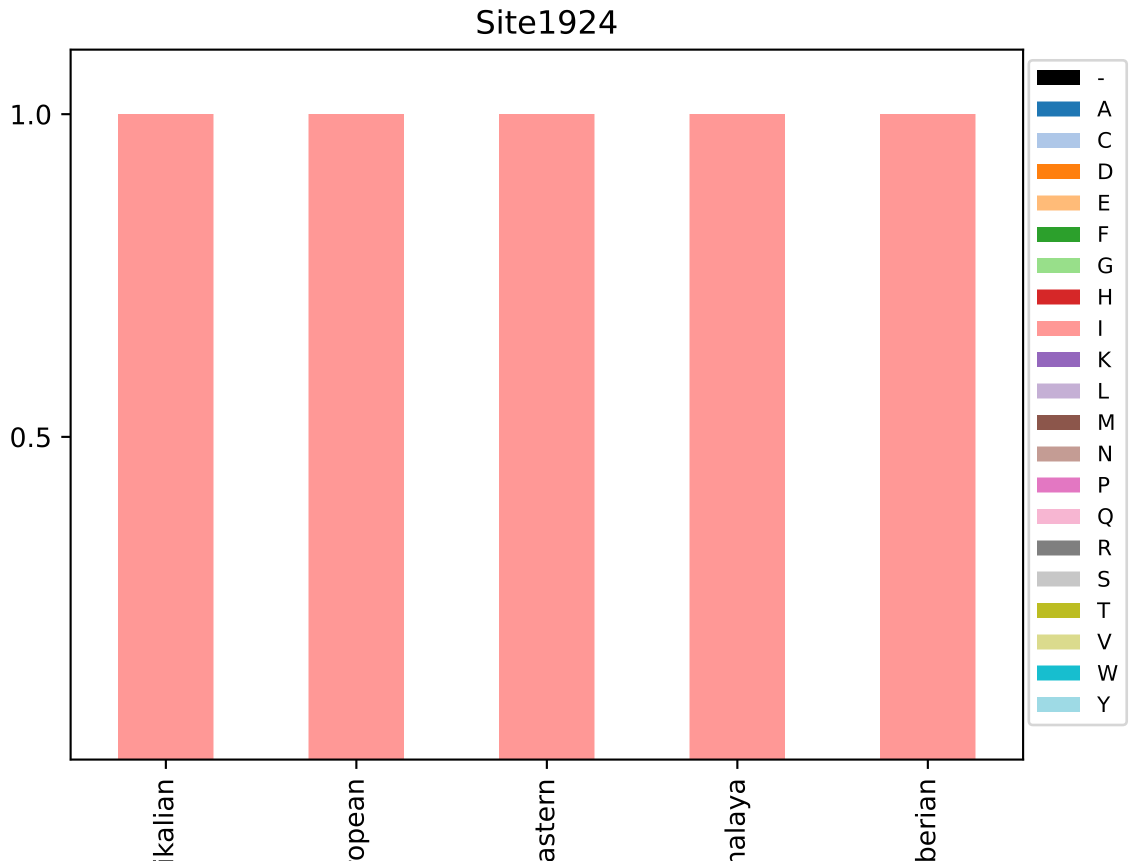
Site1924
1.0
0.5
-
A
C
D
E
F
G
H
I
K
L
M
N
P
Q
R
S
T
V
W
Y
ikalian
opean
astern
nalaya
berian

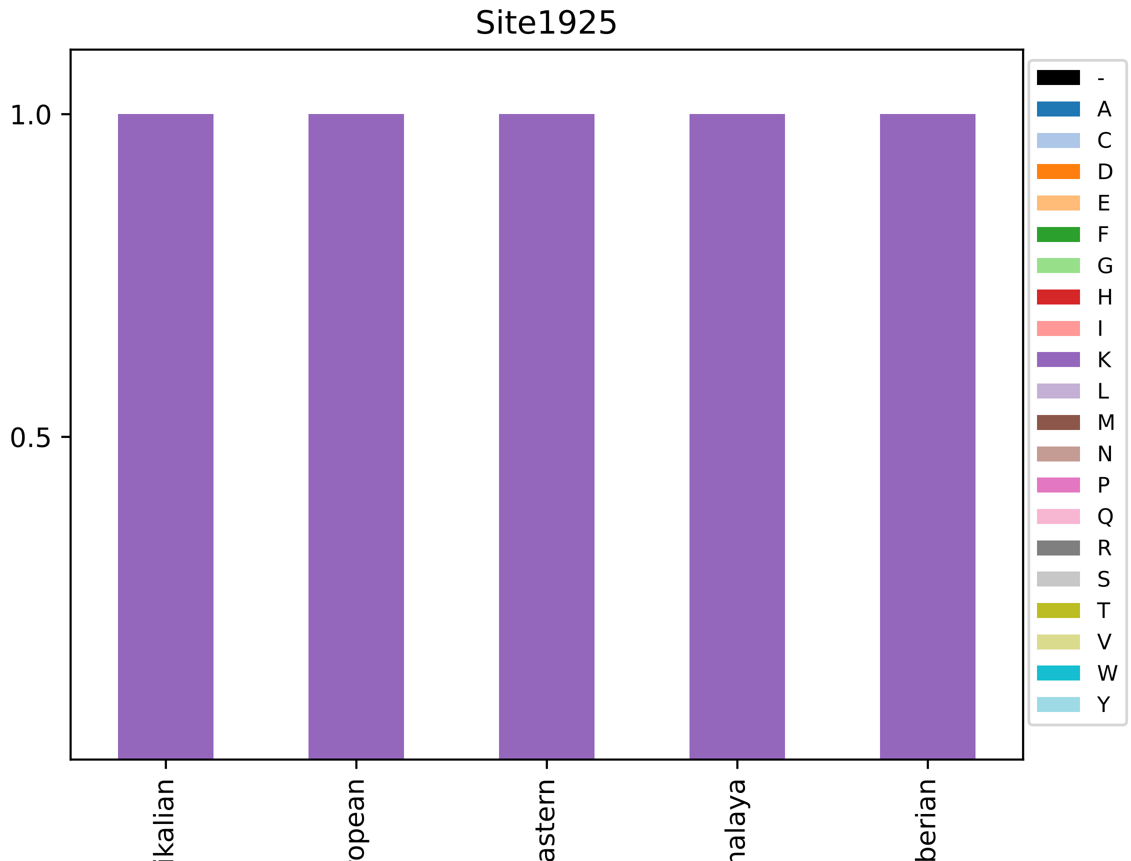

Site1925
1.0
0.5
-
A
C
D
E
F
G
H
I
K
L
M
N
P
Q
R
S
T
V
W
Y
ikalian
opean
astern
nalaya
berian

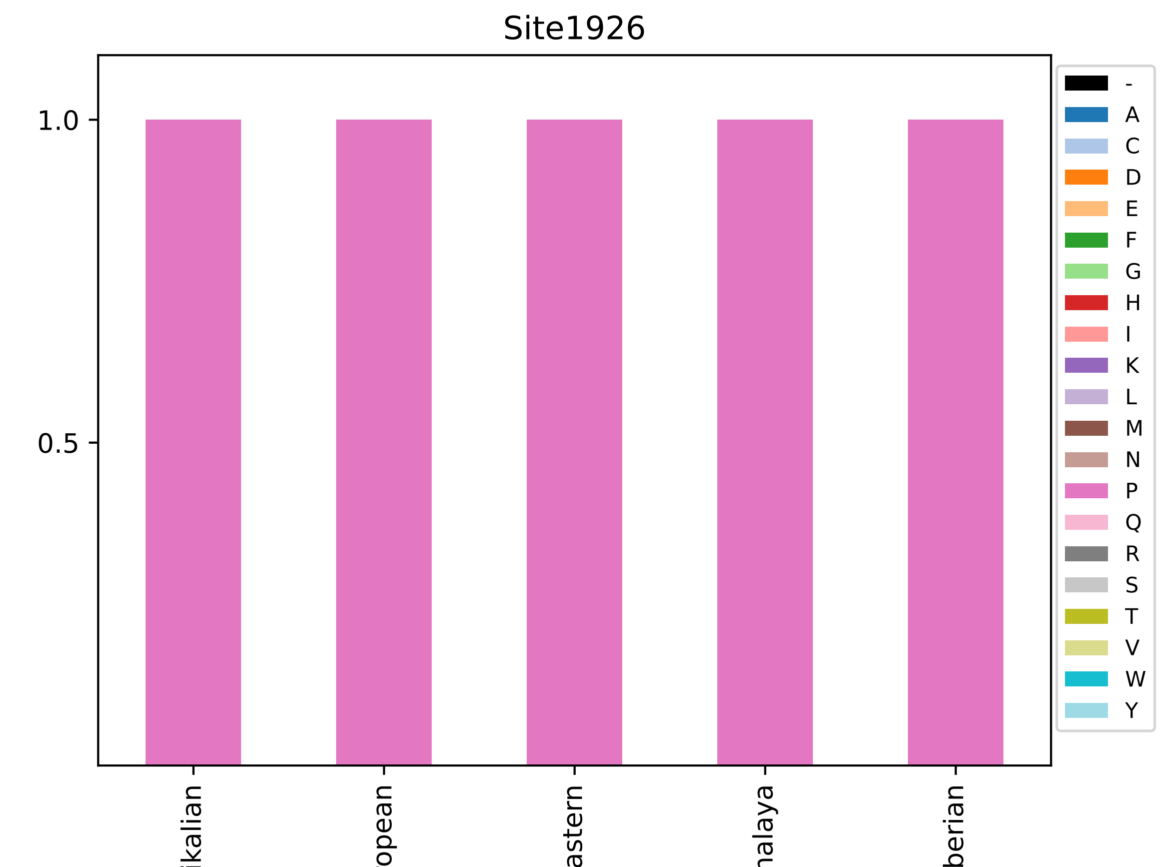

Site1926
1.0
0.5
-
A
C
D
E
F
G
H
I
K
L
M
N
P
Q
R
S
T
V
W
Y
ikalian
ropean
astern
nalaya
berian

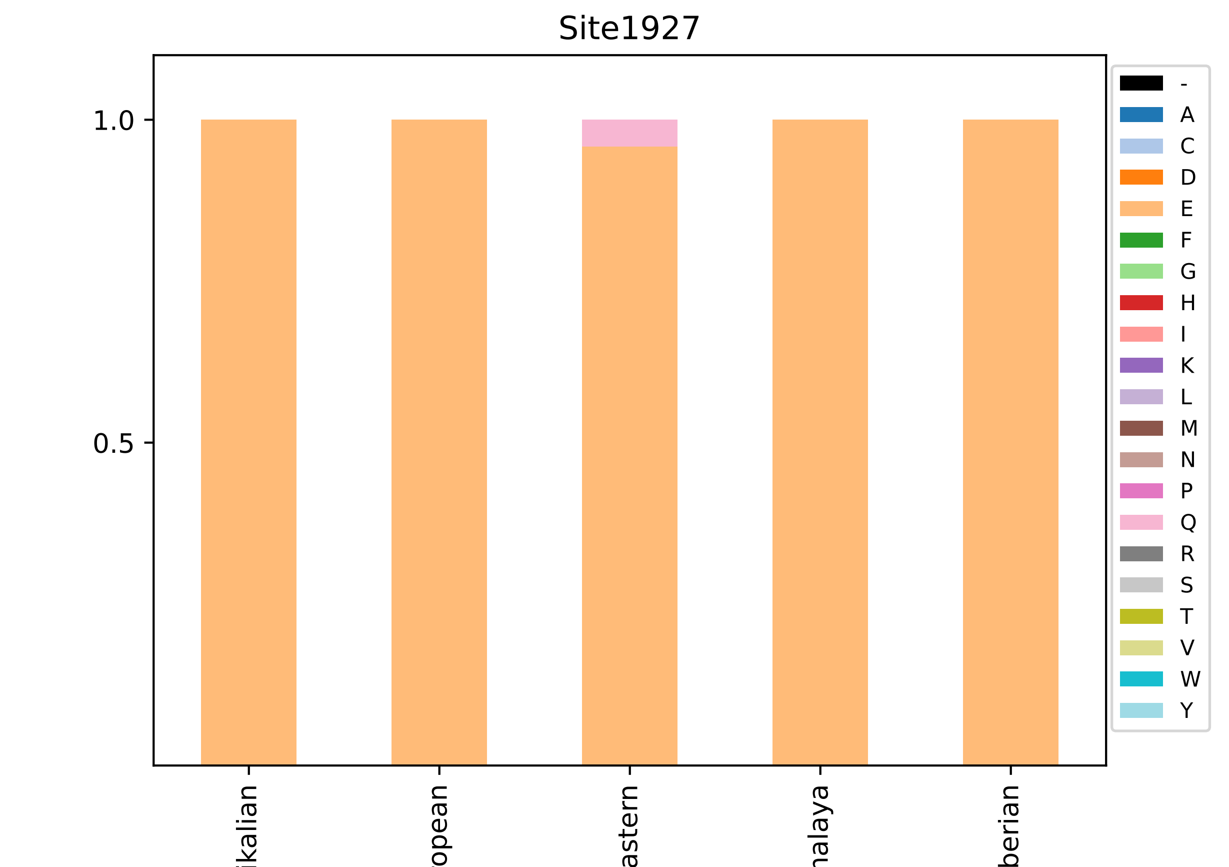
Site1927
1.0
0.5
-
A
C
D
E
F
G
H
I
K
L
M
N
P
Q
R
S
T
V
W
Y
ikalian
ropean
astern
nalaya
berian

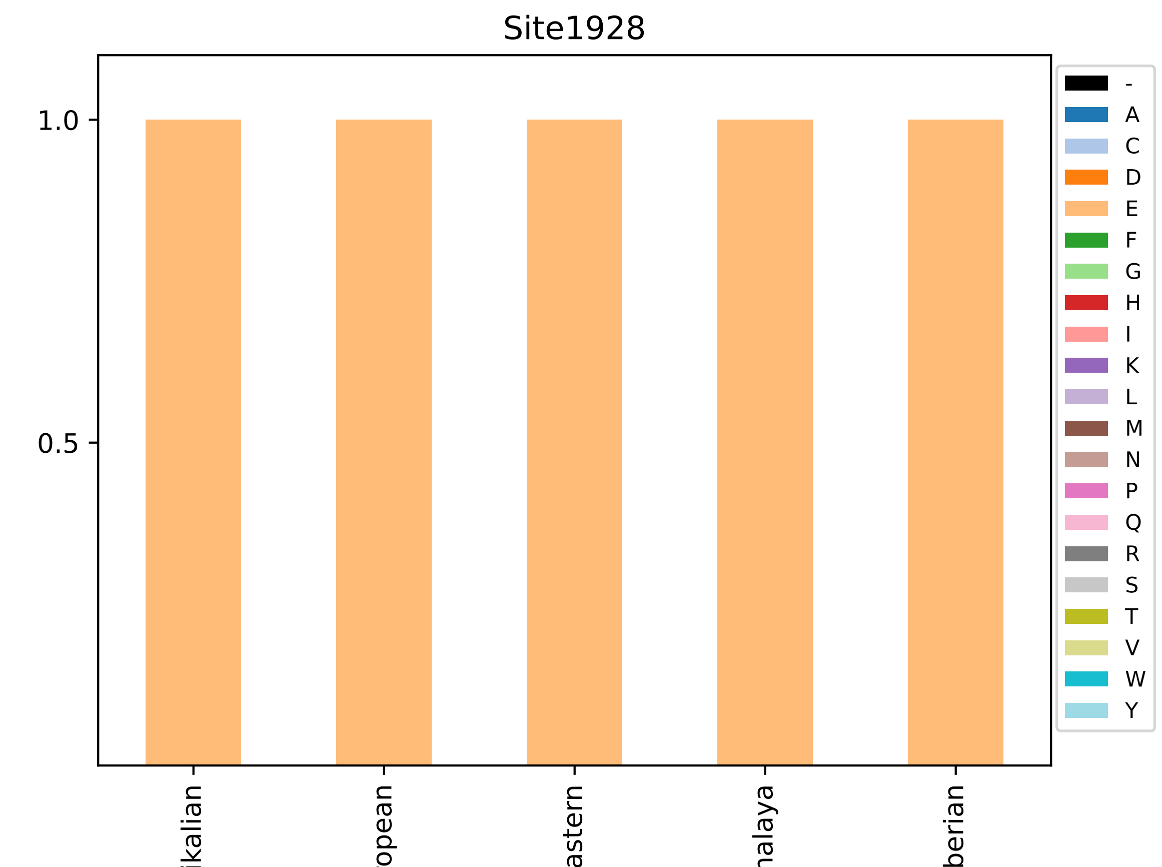
Site1928
1.0
0.5
-
A
C
D
E
F
G
H
I
K
L
M
N
P
Q
R
S
T
V
W
Y
ikalian
opean
astern
nalaya
berian

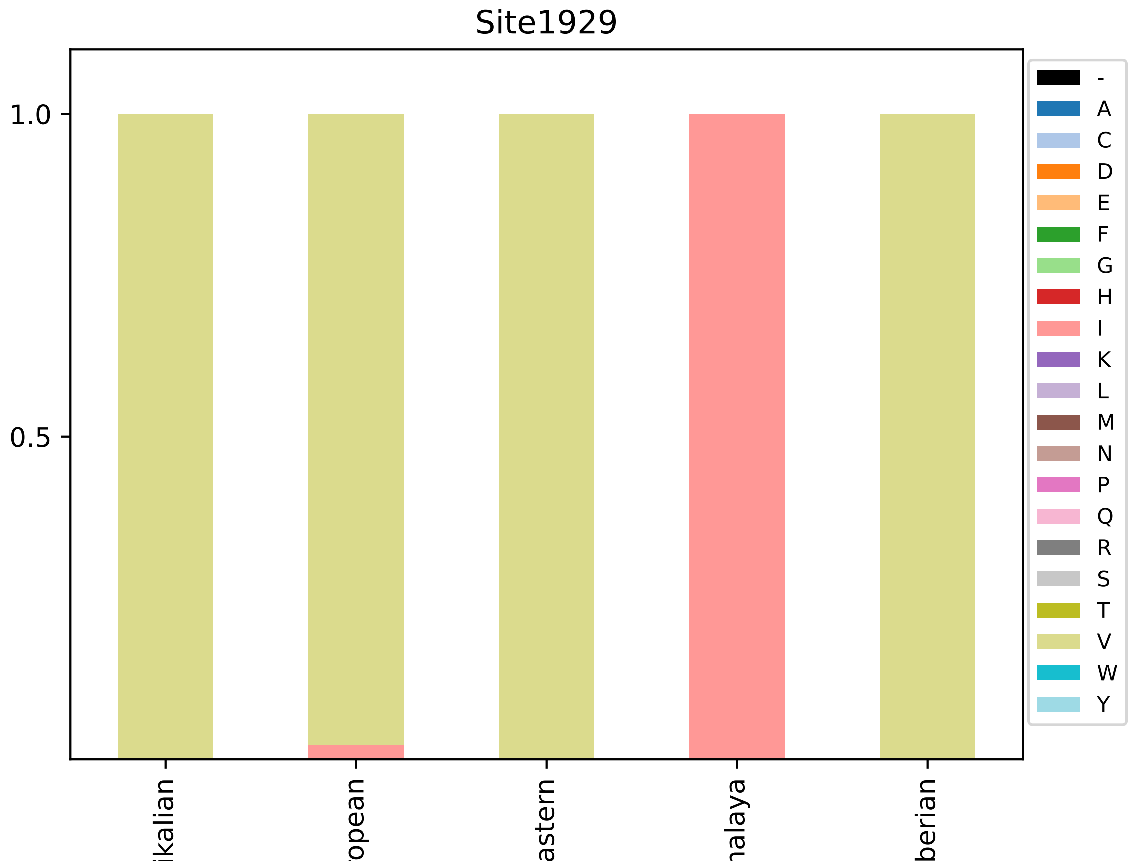
Site1929
1.0
0.5
-
A
C
D
E
F
G
H
I
K
L
M
N
P
Q
R
S
T
V
W
Y
ikalian
opean
astern
nalaya
berian

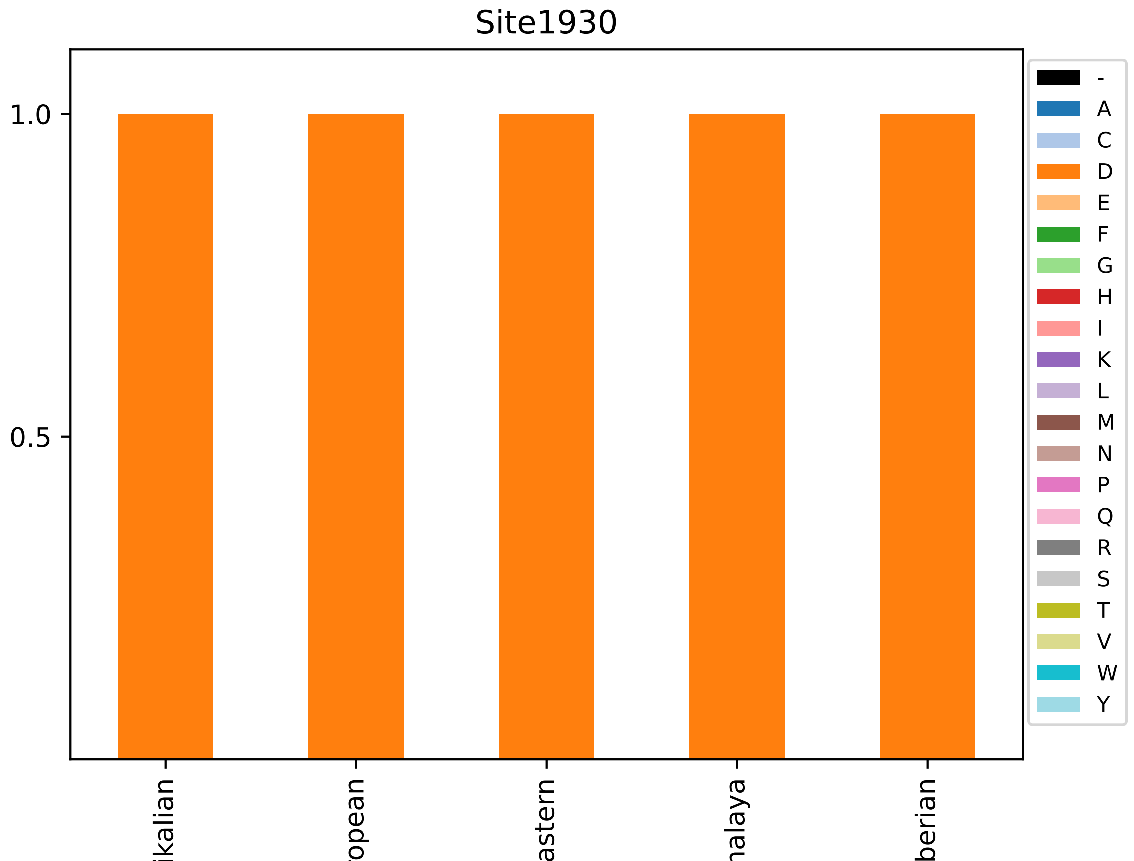

Site1930
1.0
0.5
-
A
C
D
E
F
G
H
I
K
L
M
N
P
Q
R
S
T
V
W
Y
ikalian
opean
astern
nalaya
berian

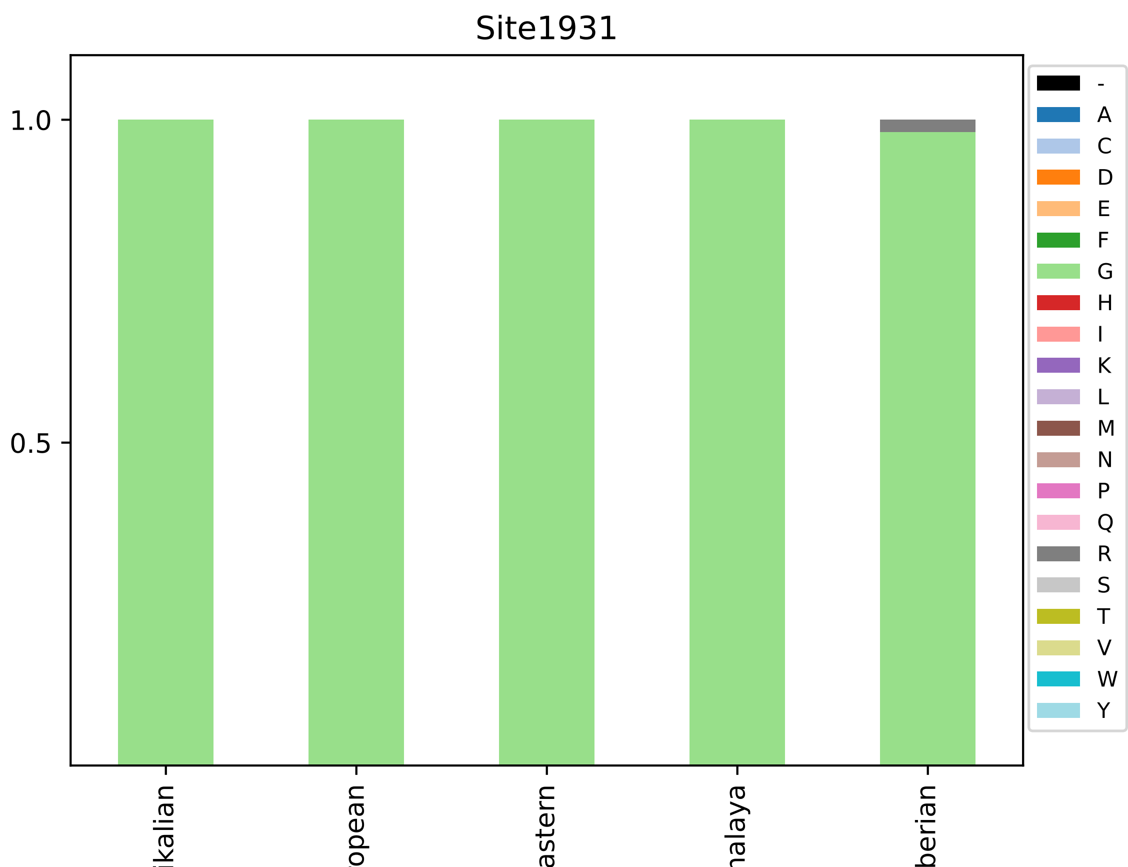
Site1931
1.0
0.5
ikalian
opean
astern
alaya
berian
-
A
C
D
E
F
G
H
I
K
L
M
N
P
Q
R
S
T
V
W
Y

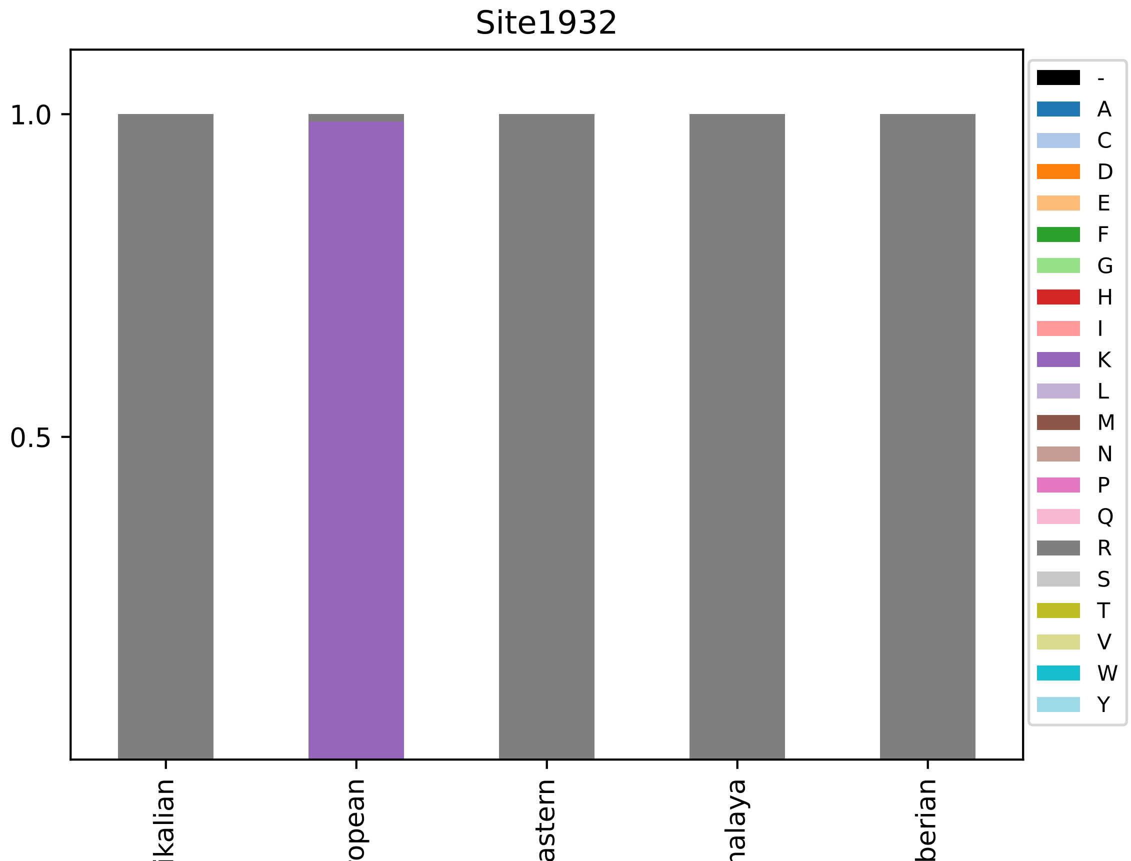
Site1932
1.0
0.5
-
A
C
D
E
F
G
H
I
K
L
M
N
P
Q
R
S
T
V
W
Y
ikalian
opean
astern
nalaya
berian

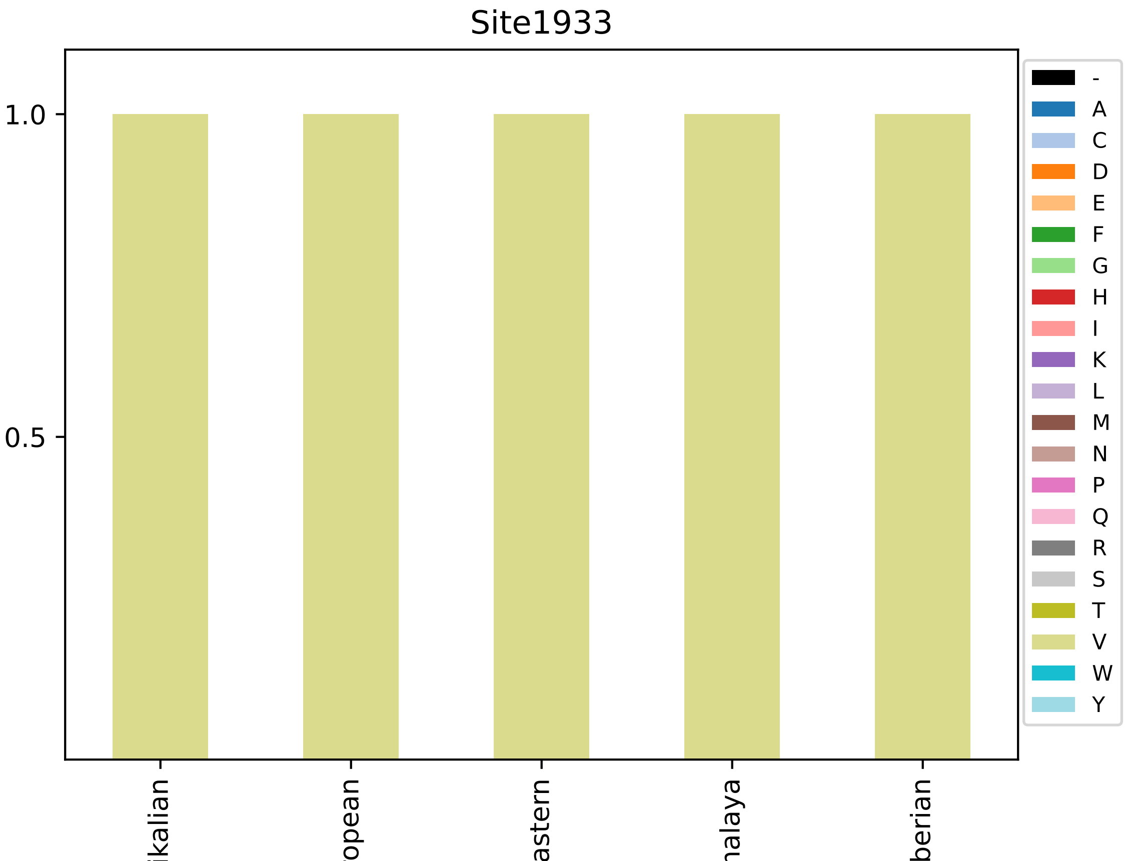

Site1933
1.0
0.5
-
A
C
D
E
F
G
H
I
K
L
M
N
P
Q
R
S
T
V
W
Y
ikalian
opean
astern
nalaya
berian

Site1934

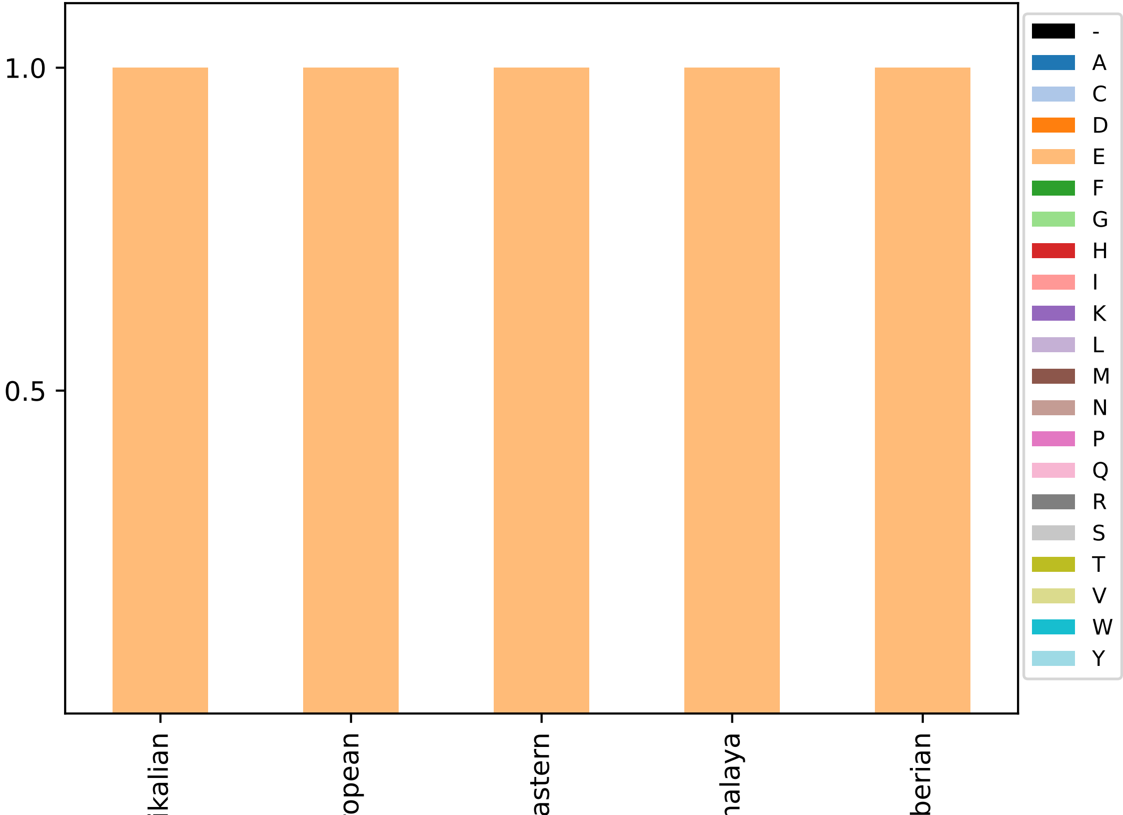
1.0
0.5
-
A
C
D
E
F
G
H
I
K
L
M
N
P
Q
R
S
T
V
W
Y
ikalian
opean
astern
nalaya
berian

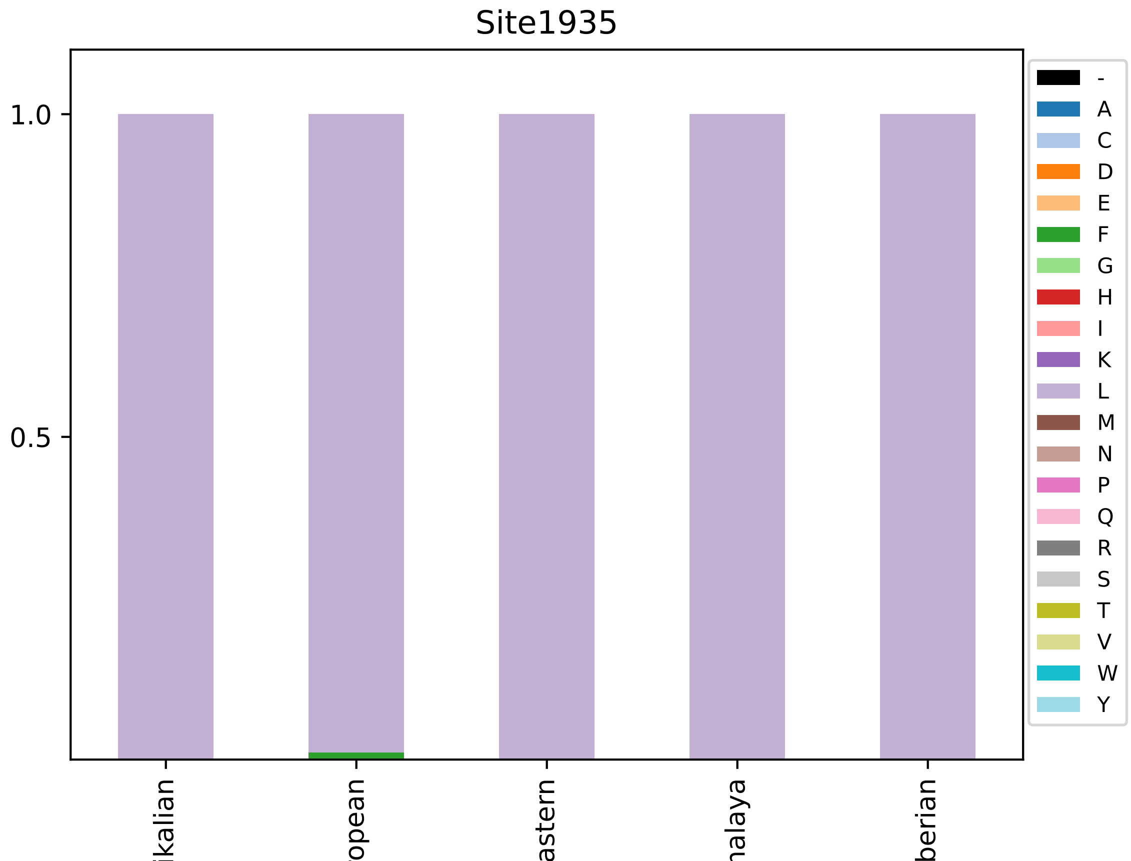
Site1935
1.0
0.5
-
A
C
D
E
F
G
H
I
K
L
M
N
P
Q
R
S
T
V
W
Y
ikalian
opean
astern
nalaya
berian

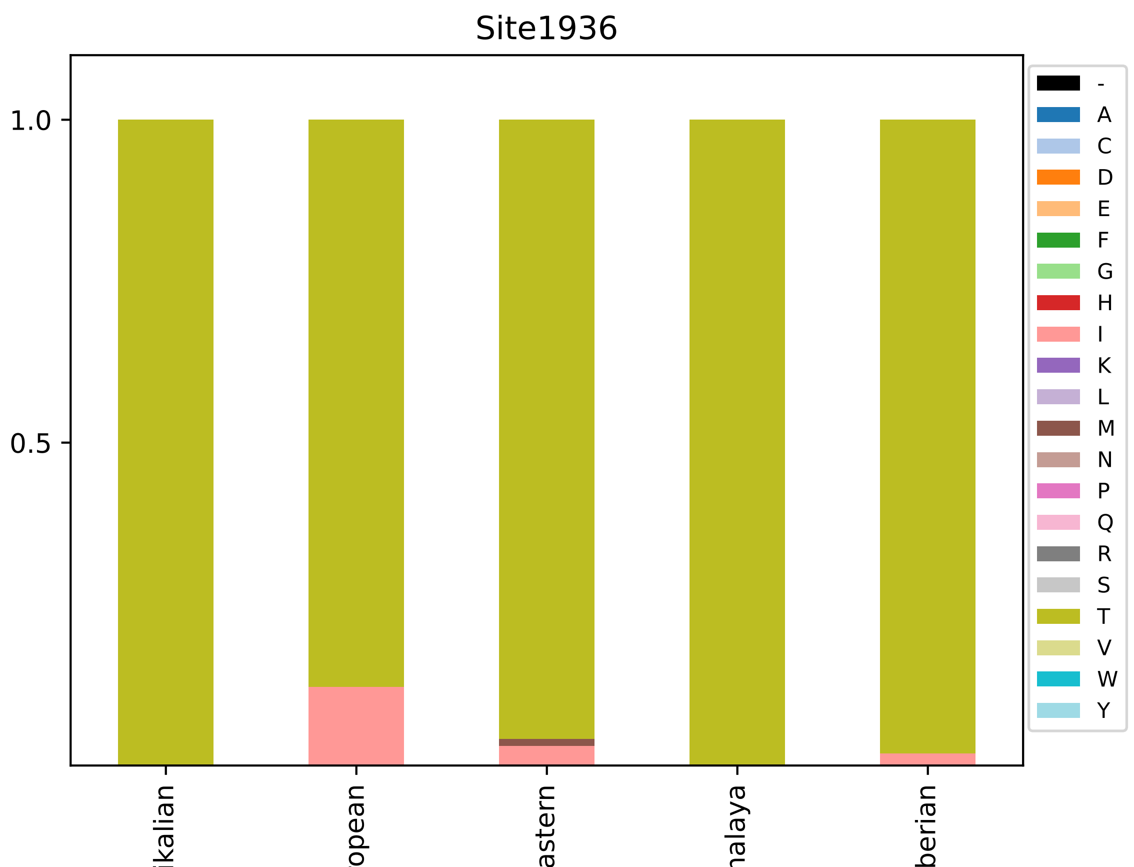

Site1936
1.0
0.5
-
A
C
D
E
F
G
H
I
K
L
M
N
P
Q
R
S
T
V
W
Y
ikalian
opean
astern
nalaya
berian

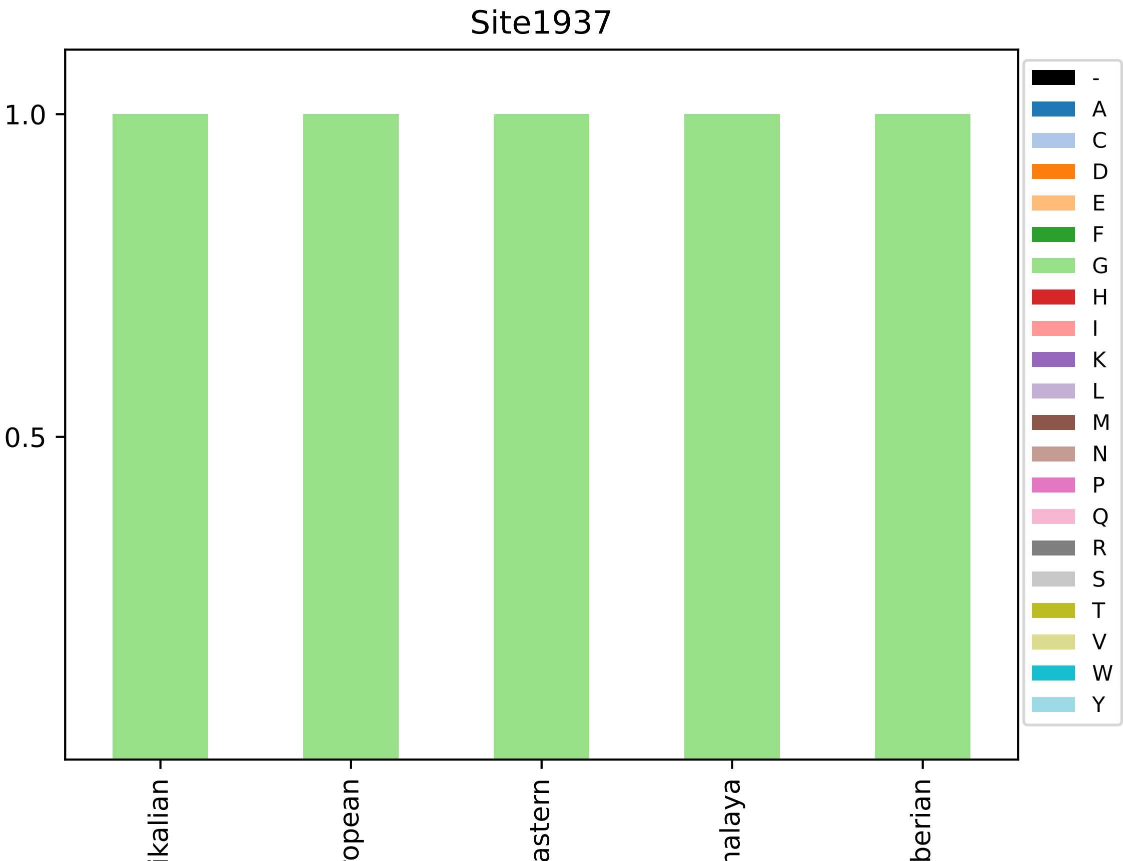
Site1937
1.0
0.5
-
A
C
D
E
F
G
H
I
K
L
M
N
P
Q
R
S
T
V
W
Y
ikalian
opean
astern
nalaya
berian

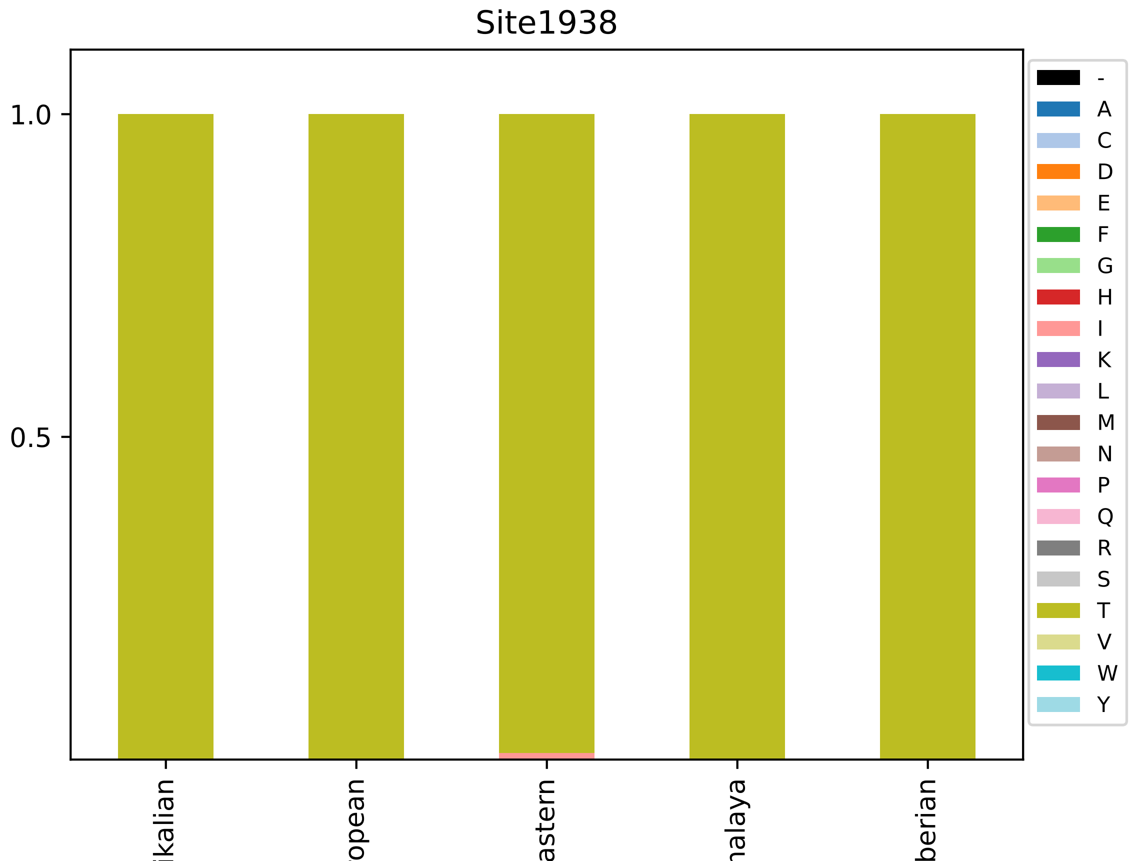

Site1938
1.0
0.5
-
A
C
D
E
F
G
H
I
K
L
M
N
P
Q
R
S
T
V
W
Y

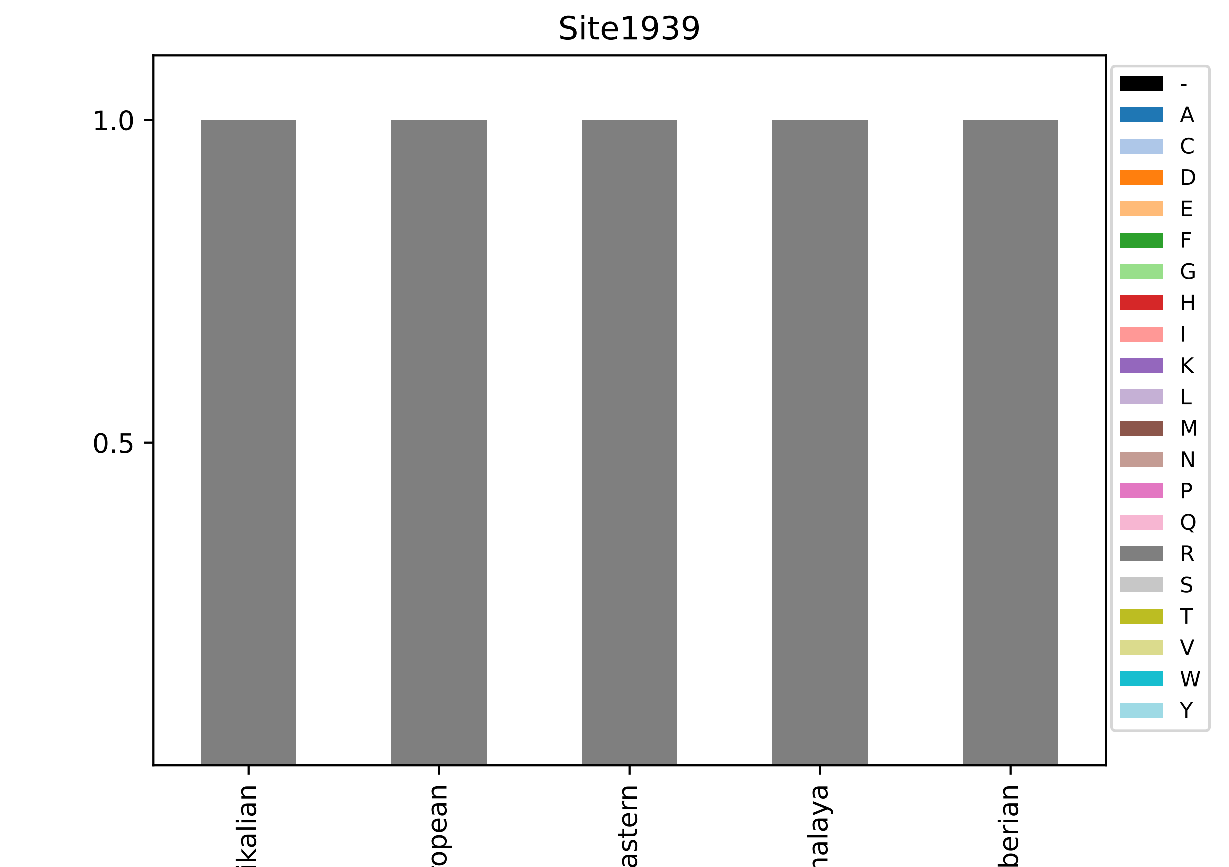

Site1939
1.0
0.5
-
A
C
D
E
F
G
H
I
K
L
M
N
P
Q
R
S
T
V
W
Y
ikalian
opean
astern
nalaya
berian

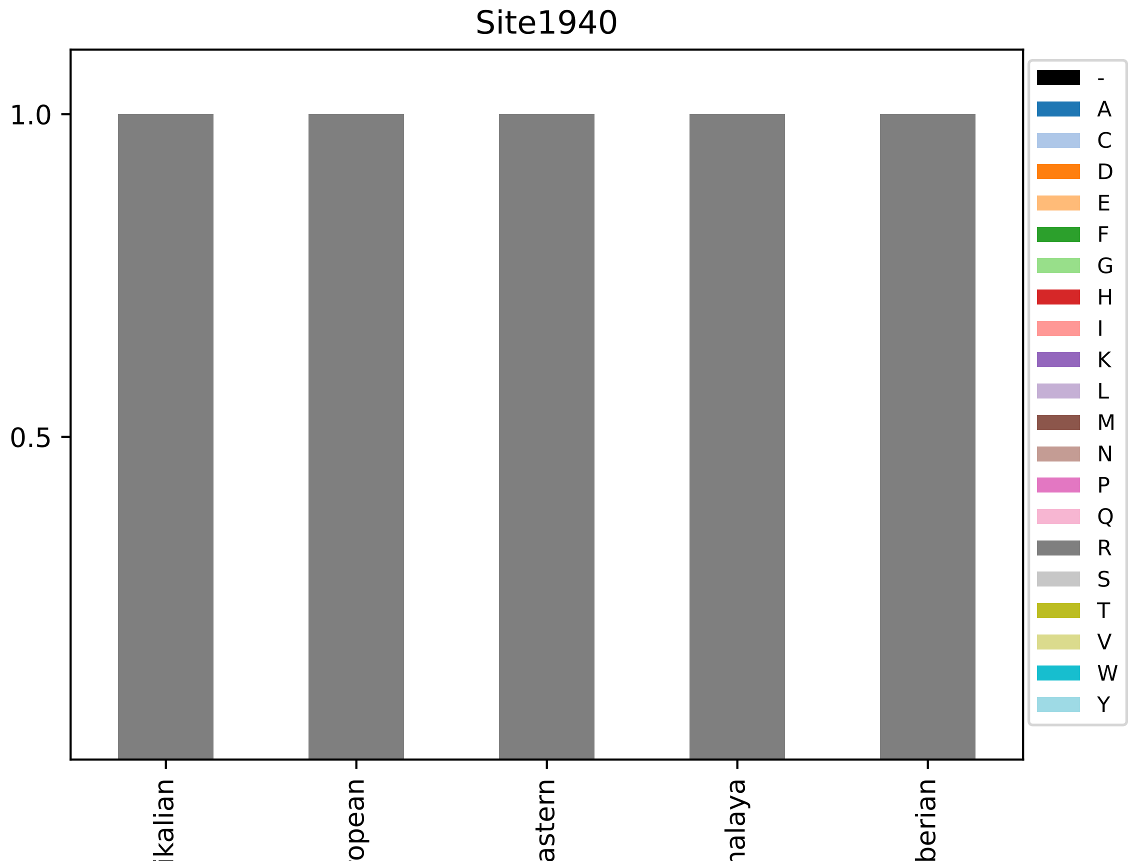
Site1940
1.0
0.5
-
A
C
D
E
F
G
H
I
K
L
M
N
P
Q
R
S
T
V
W
Y
ikalian
opean
astern
nalaya
berian

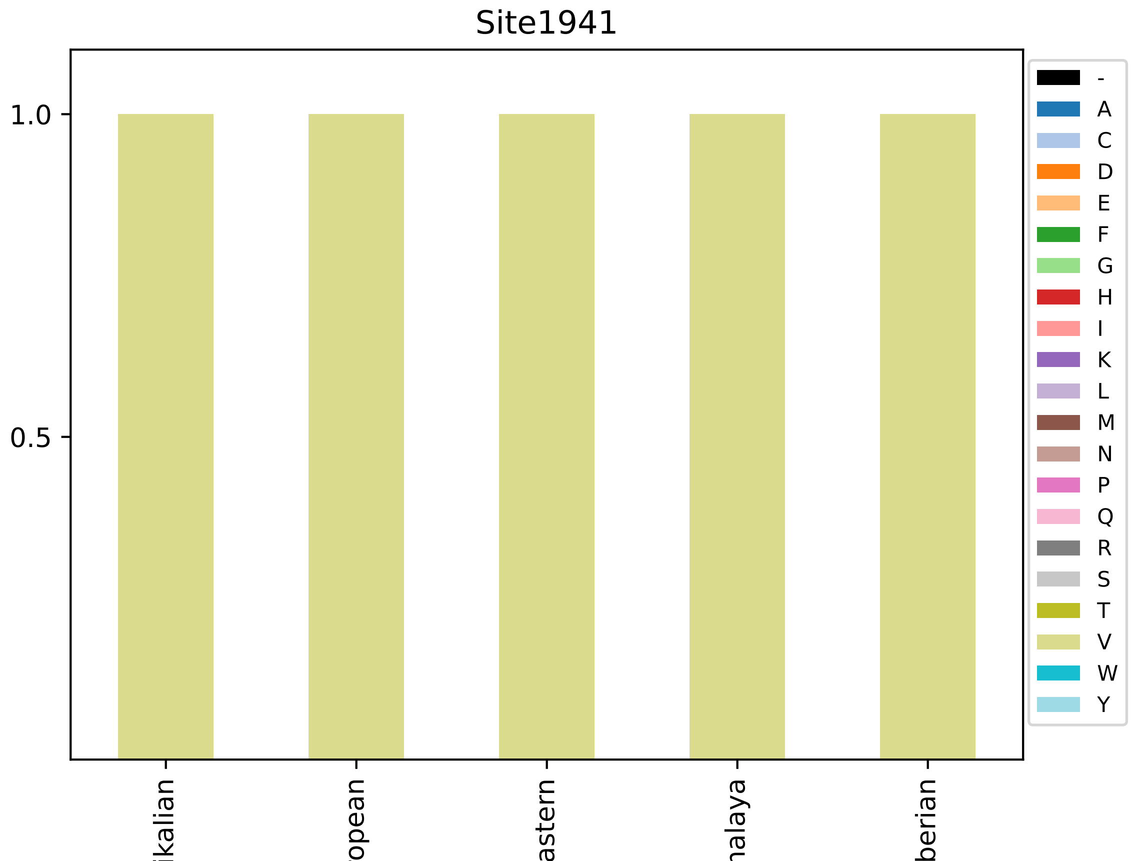

Site1941
1.0
0.5
-
A
C
D
E
F
G
H
I
K
L
M
N
P
Q
R
S
T
V
W
Y

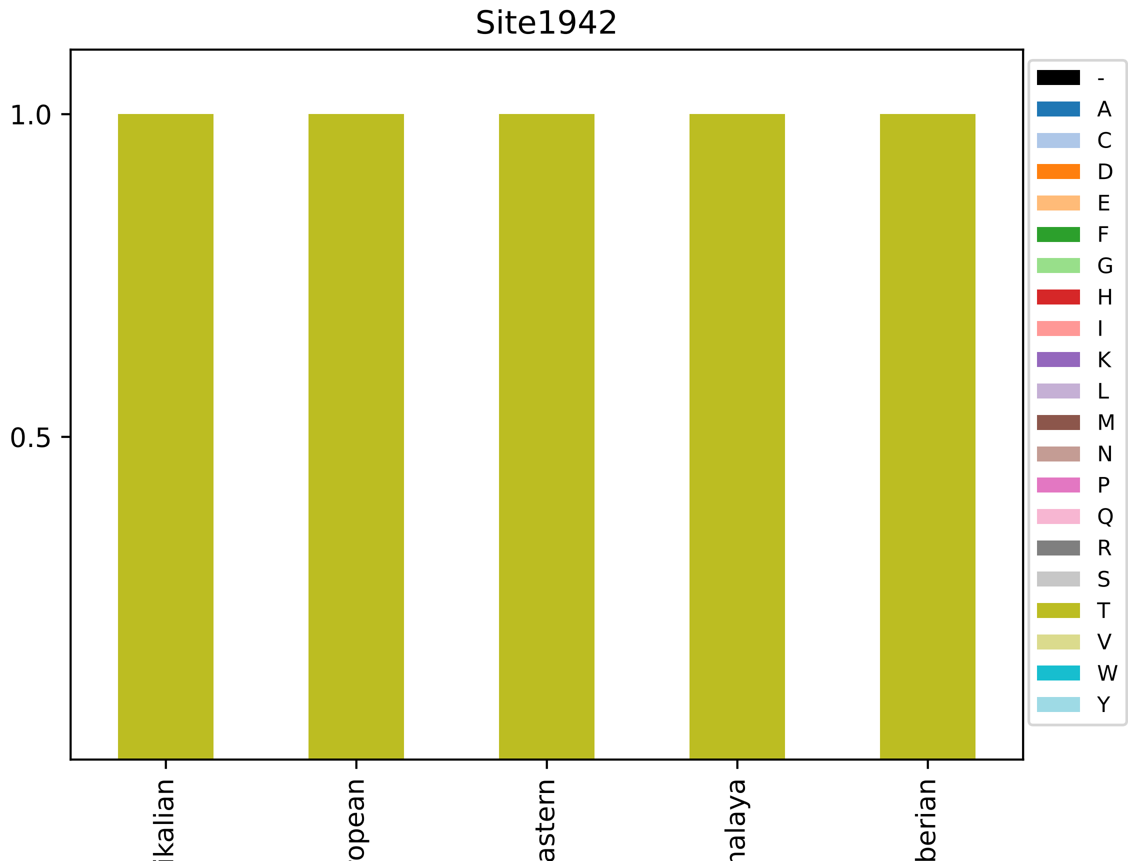

Site1942
1.0
0.5
-
A
C
D
E
F
G
H
I
K
L
M
N
P
Q
R
S
T
V
W
Y
ikalian
opean
astern
nalaya
berian

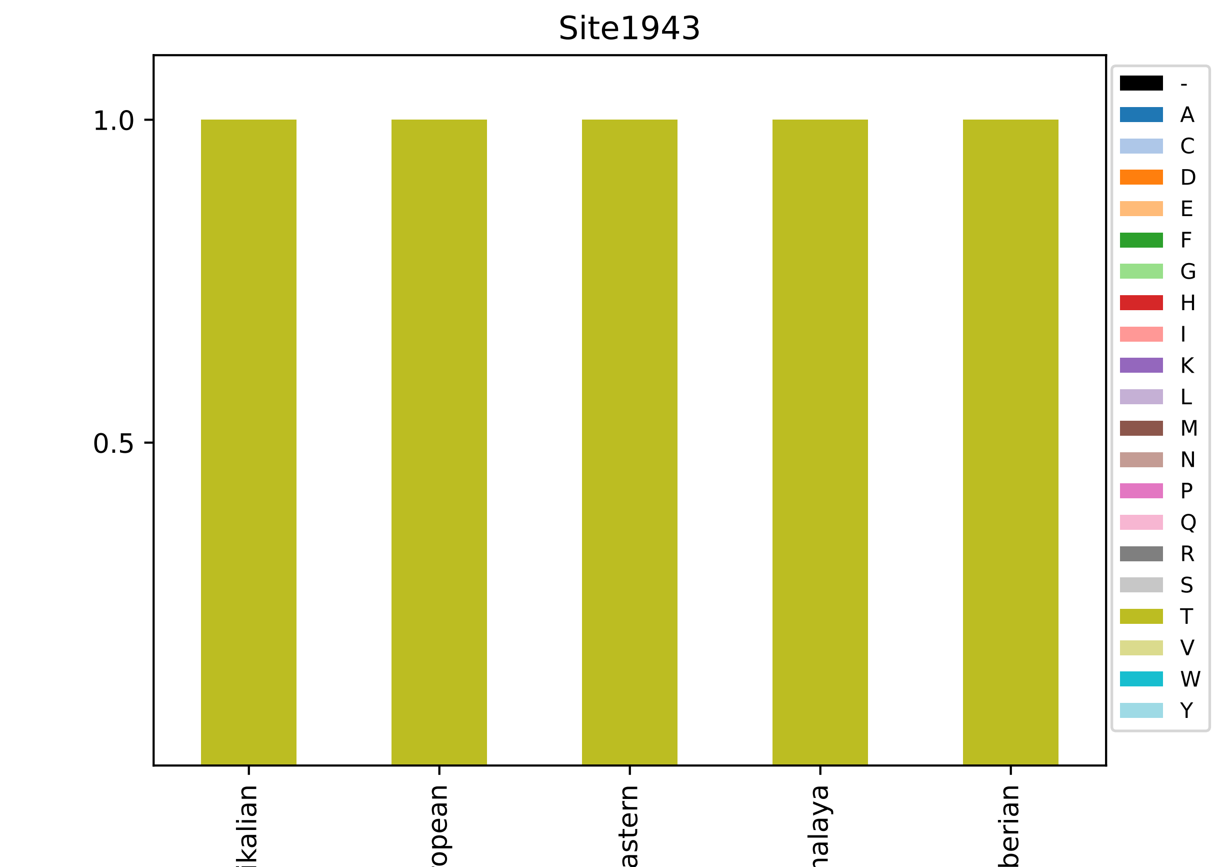
Site1943
1.0
0.5
-
A
C
D
E
F
G
H
I
K
L
M
N
P
Q
R
S
T
V
W
Y
ikalian
opean
astern
nalaya
berian

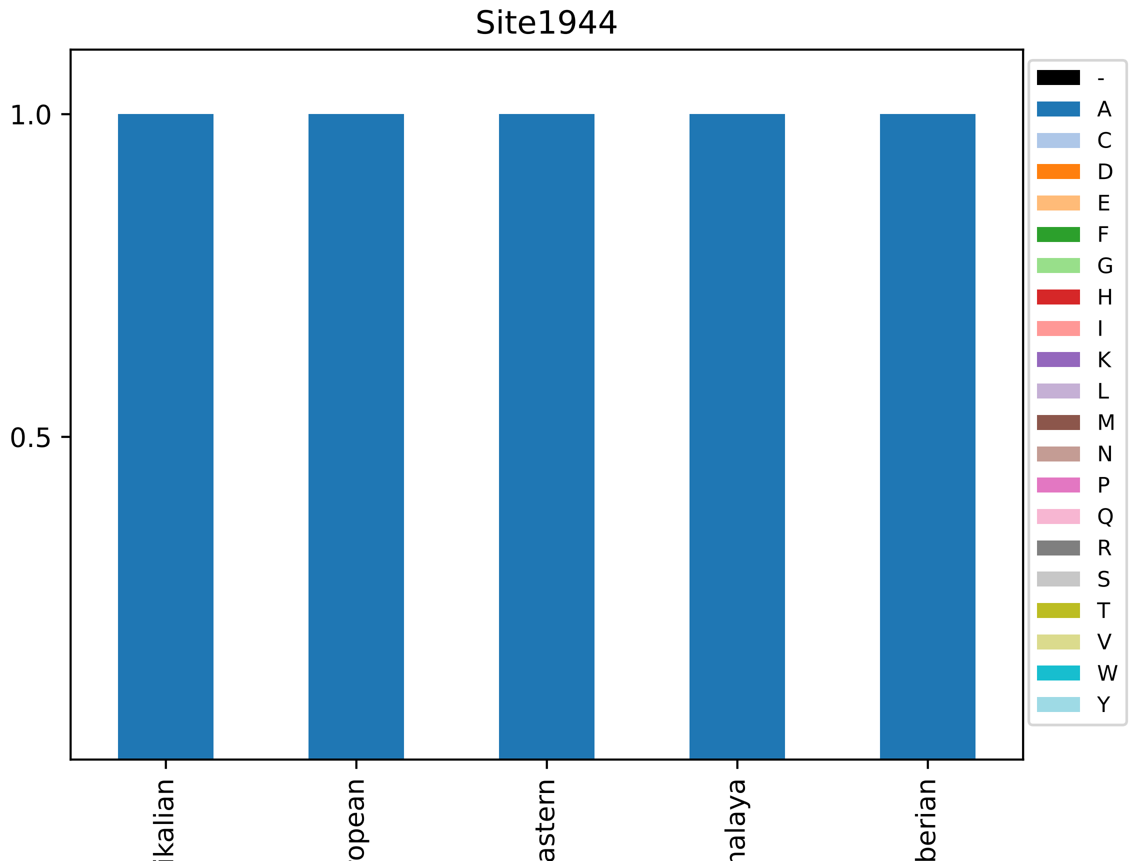
Site1944
1.0
0.5
-
A
C
D
E
F
G
H
I
K
L
M
N
P
Q
R
S
T
V
W
Y

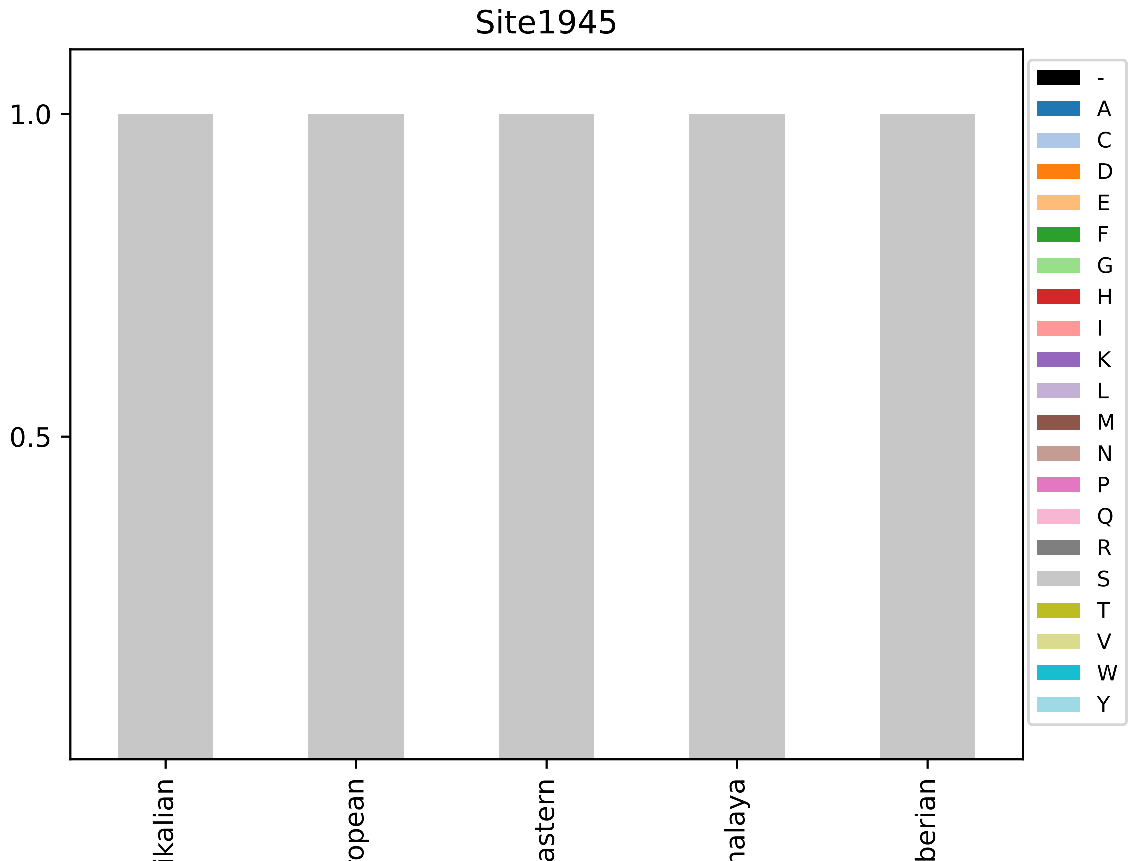
Site1945
1.0
0.5
-
A
C
D
E
F
G
H
I
K
L
M
N
P
Q
R
S
T
V
W
Y
ikalian
opean
astern
nalaya
berian

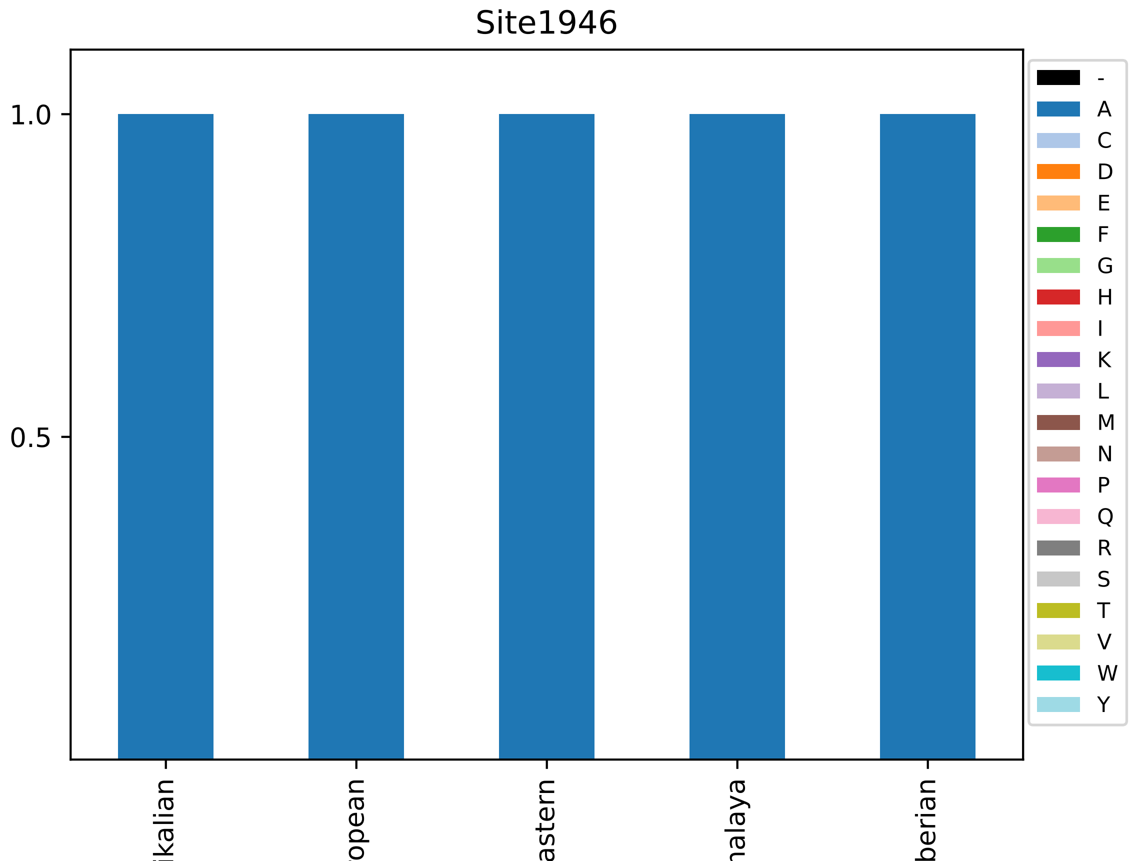
Site1946
1.0
0.5
-
A
C
D
E
F
G
H
I
K
L
M
N
P
Q
R
S
T
V
W
Y
ikalian
opean
astern
nalaya
berian

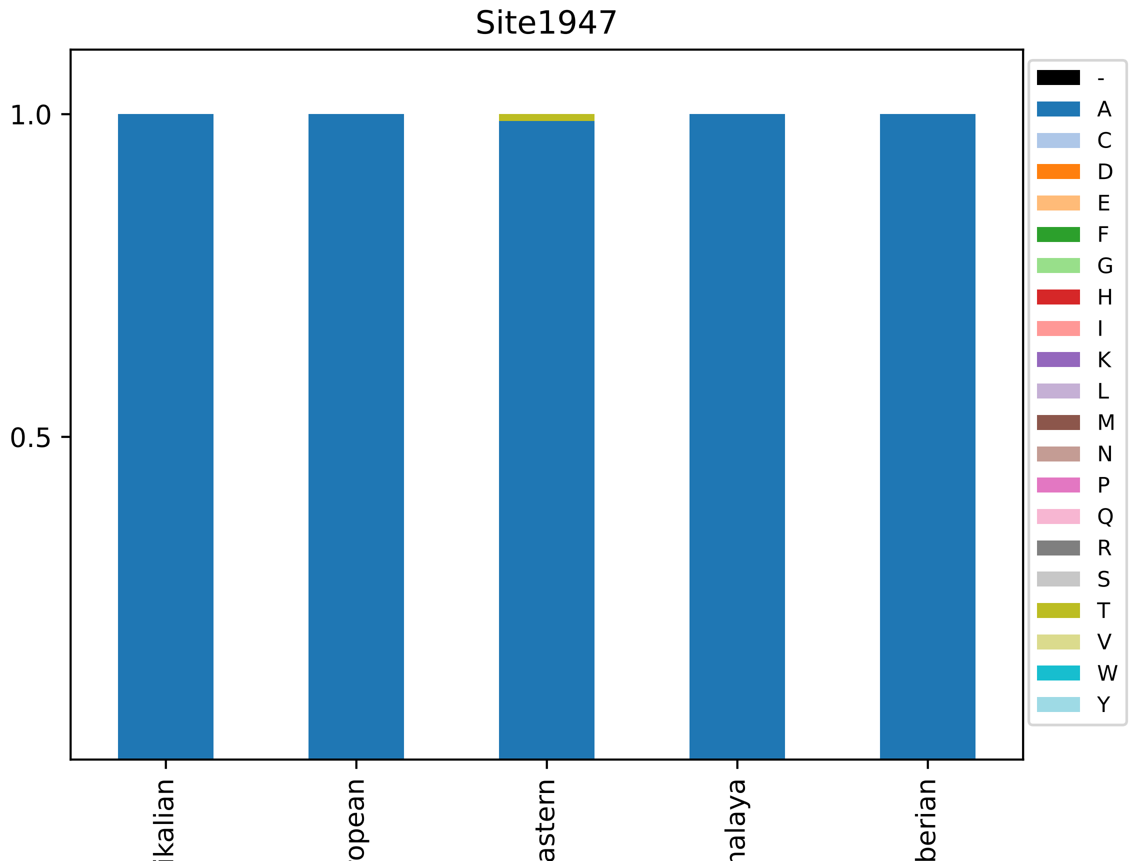
Site1947
1.0
0.5
ikalian
opean
astern
nalaya
berian
-
A
C
D
E
F
G
H
I
K
L
M
N
P
Q
R
S
T
V
W
Y

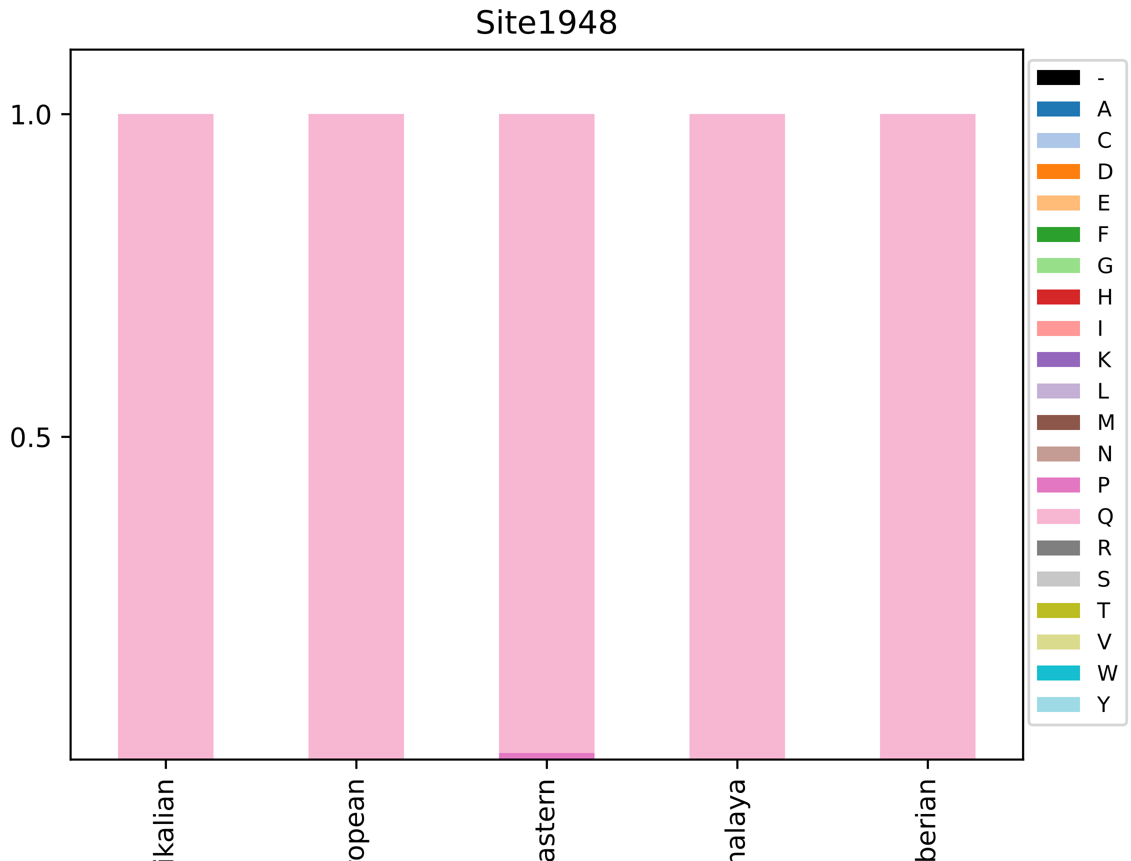
Site1948
1.0
0.5
-
A
C
D
E
F
G
H
I
K
L
M
N
P
Q
R
S
T
V
W
Y
ikalian
opean
astern
nalaya
berian

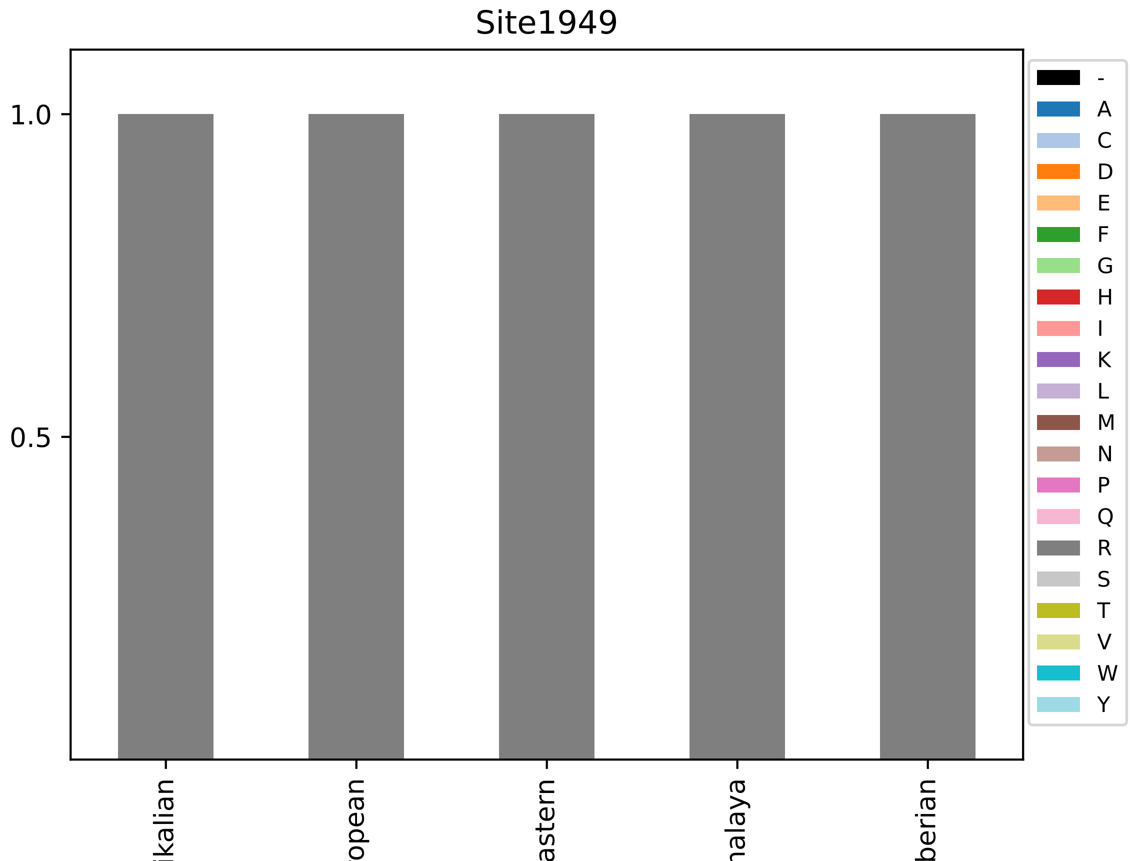

Site1949
1.0
0.5
-
A
C
D
E
F
G
H
I
K
L
M
N
P
Q
R
S
T
V
W
Y
ikalian
opean
astern
nalaya
berian

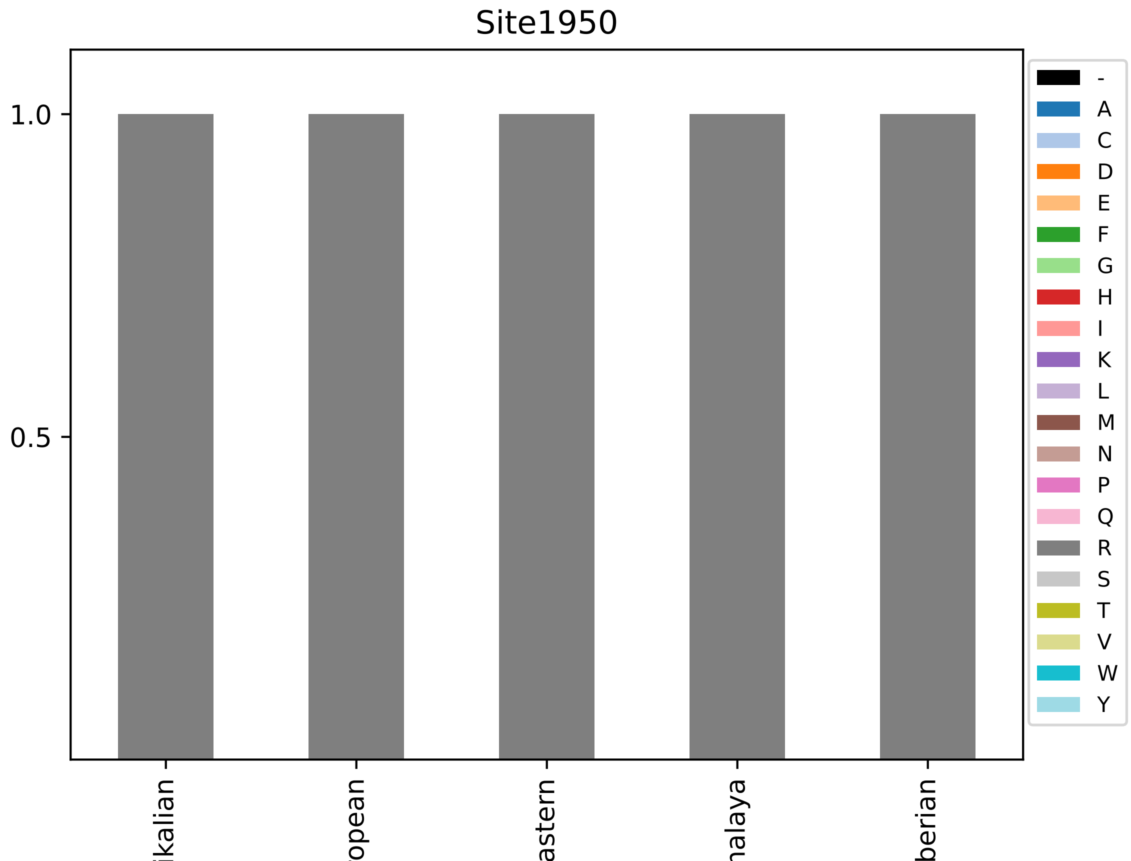
Site1950
1.0
0.5
-
A
C
D
E
F
G
H
I
K
L
M
N
P
Q
R
S
T
V
W
Y

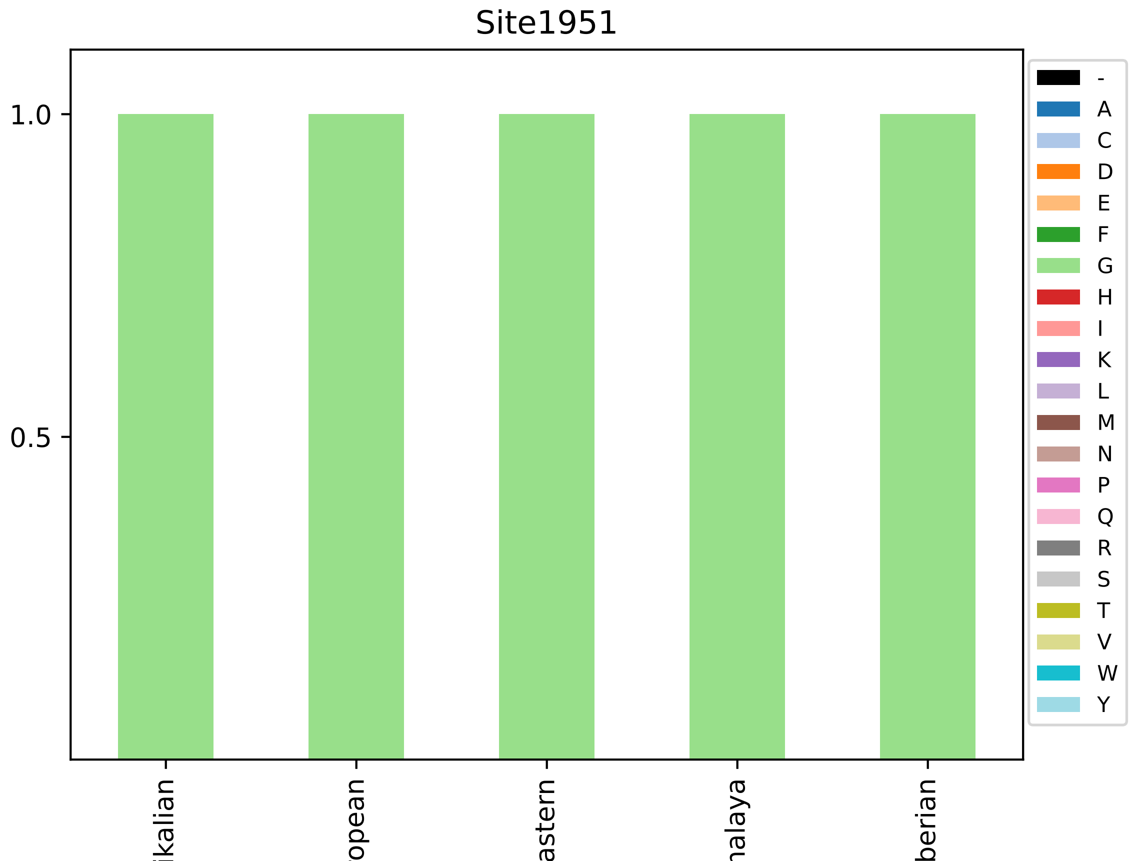
Site1951
1.0
0.5
-
A
C
D
E
F
G
H
I
K
L
M
N
P
Q
R
S
T
V
W
Y
ikalian
opean
astern
nalaya
berian

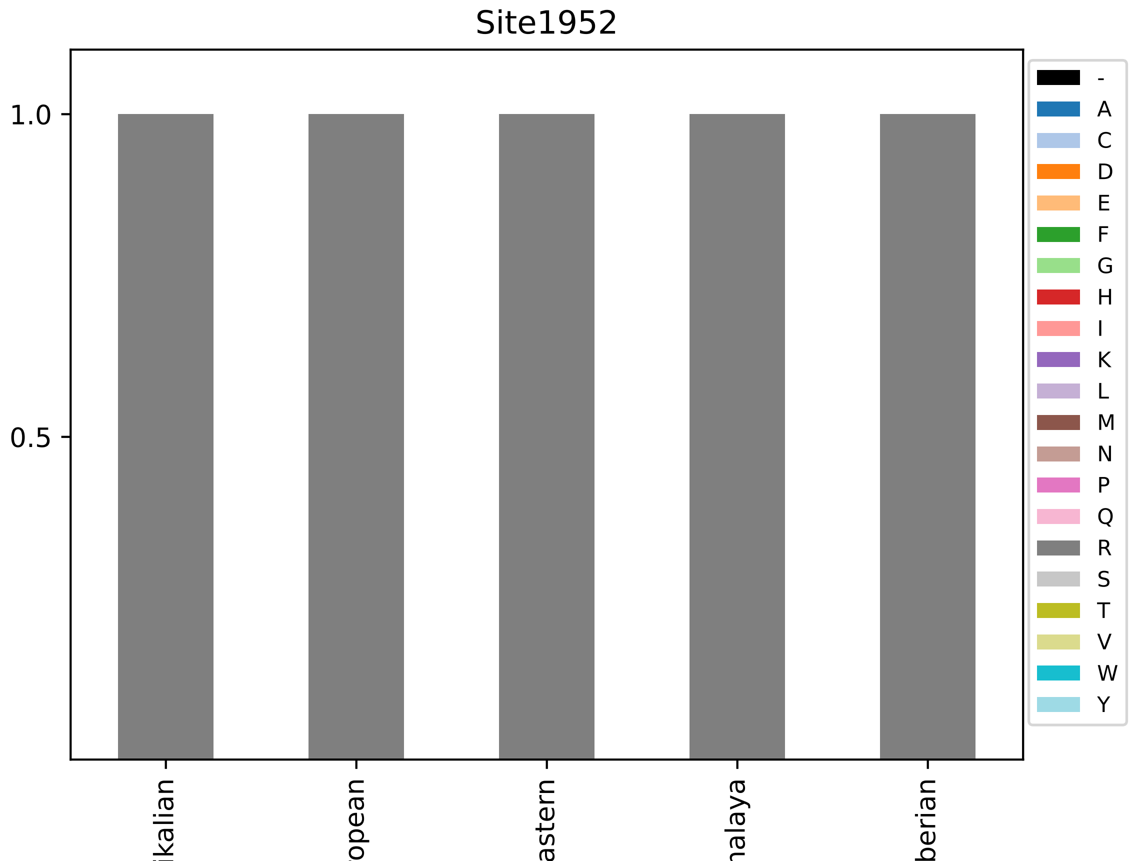
Site1952
1.0
0.5
ikalian
opean
astern
nalaya
berian
-
A
C
D
E
F
G
H
I
K
L
M
N
P
Q
R
S
T
V
W
Y

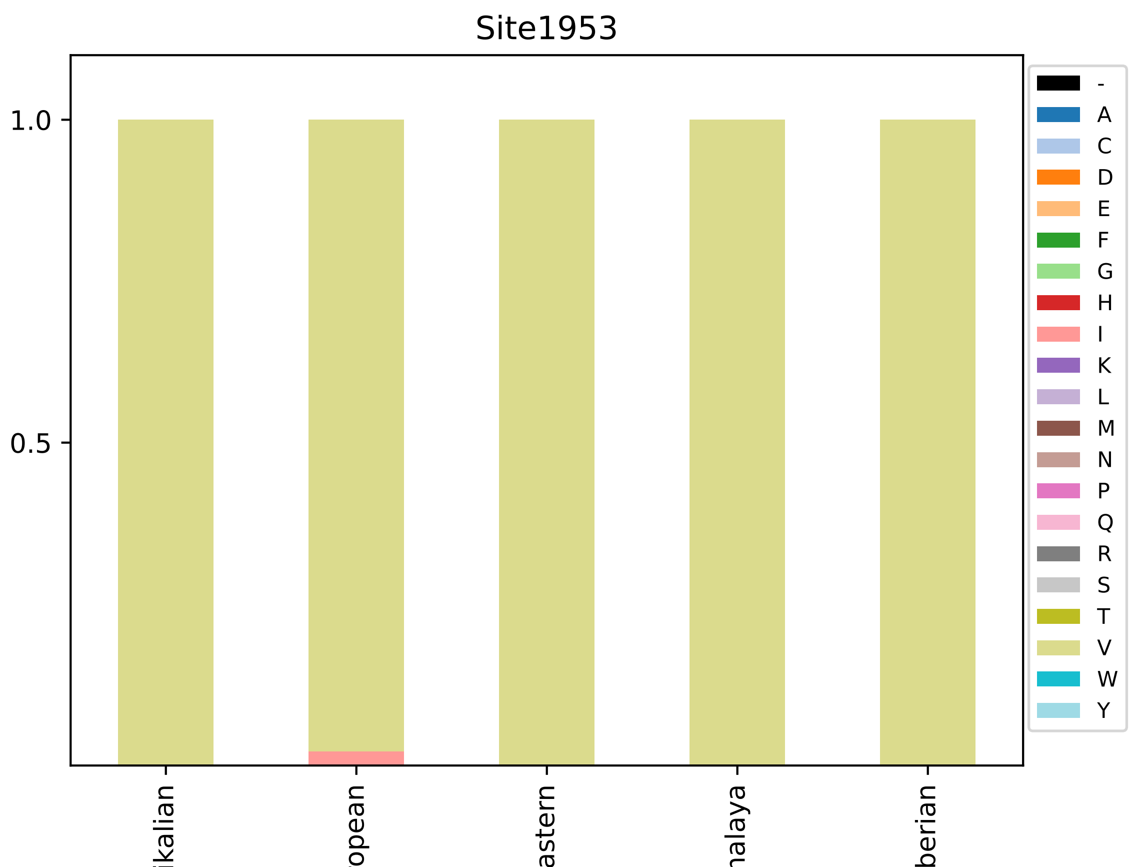
Site1953
1.0
0.5
-
A
C
D
E
F
G
H
I
K
L
M
N
P
Q
R
S
T
V
W
Y
ikalian
opean
astern
nalaya
berian

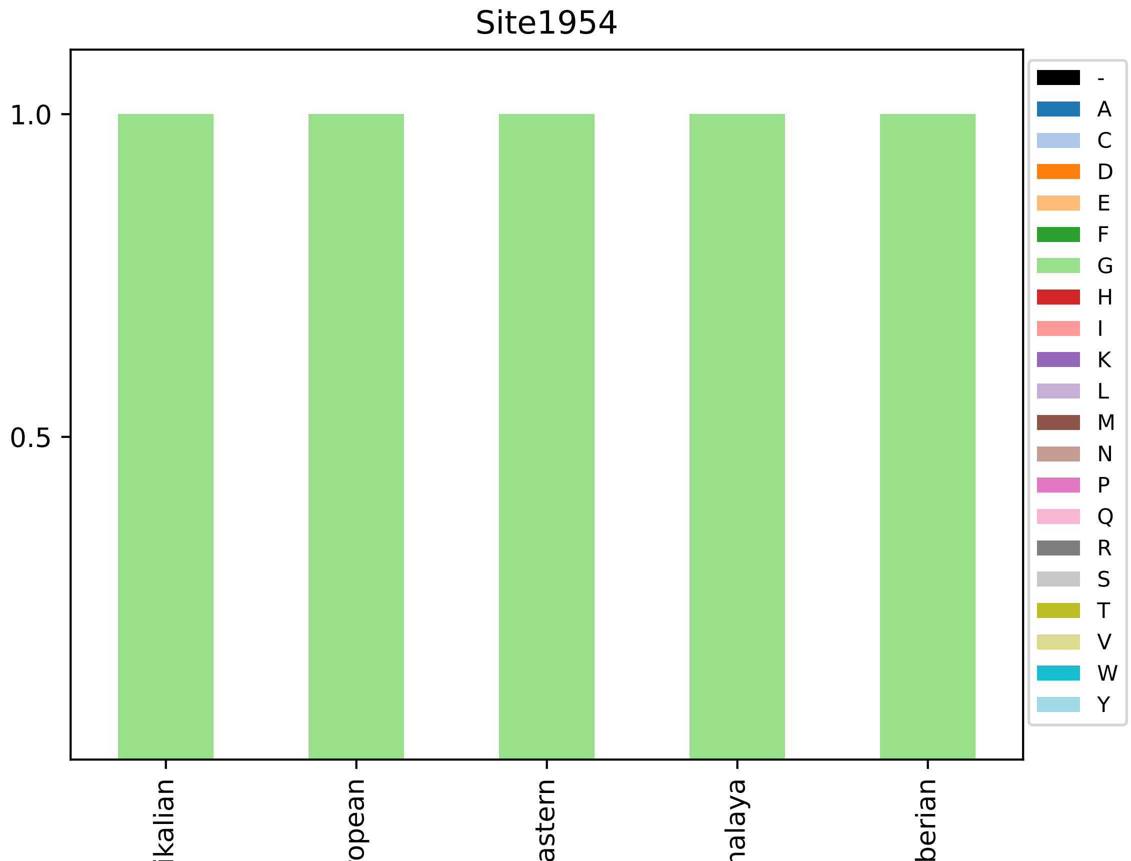

Site1954
1.0
0.5
-
A
C
D
E
F
G
H
I
K
L
M
N
P
Q
R
S
T
V
W
Y
ikalian
opean
astern
nalaya
berian

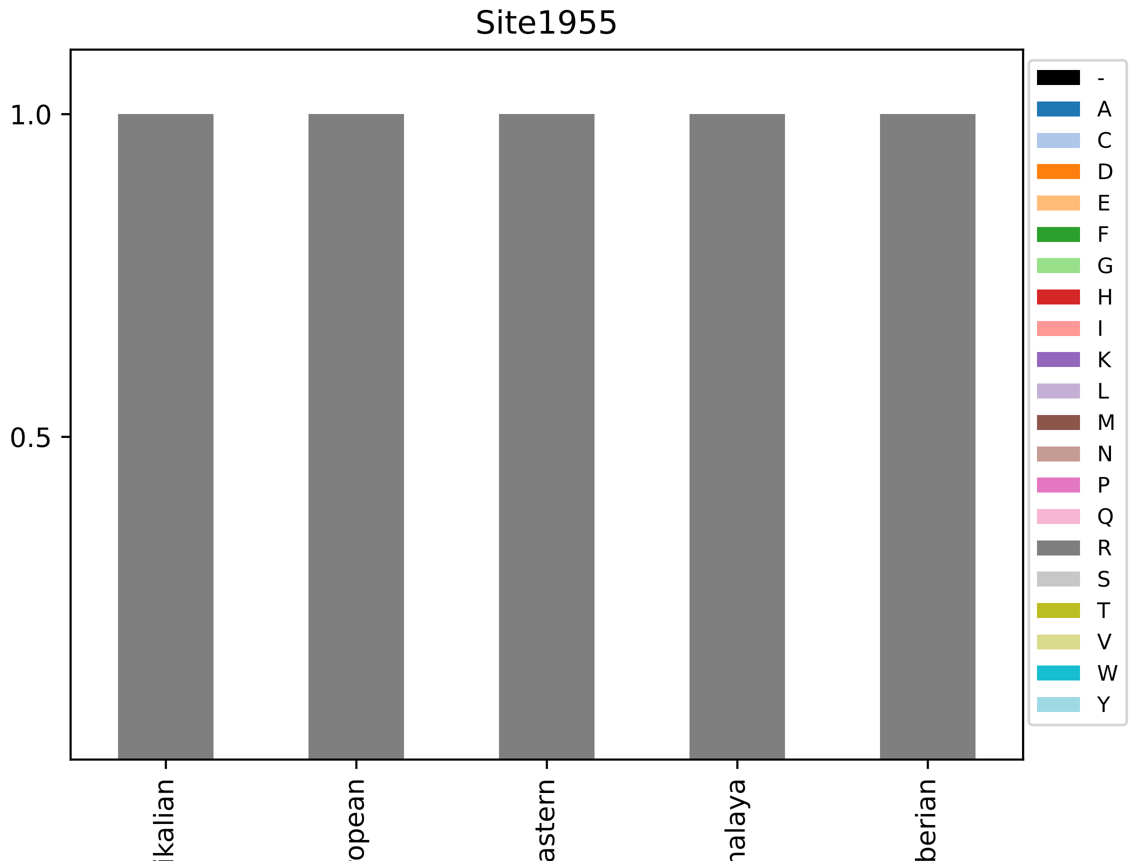
Site1955
1.0
0.5
-
A
C
D
E
F
G
H
I
K
L
M
N
P
Q
R
S
T
V
W
Y
ikalian
opean
astern
nalaya
berian

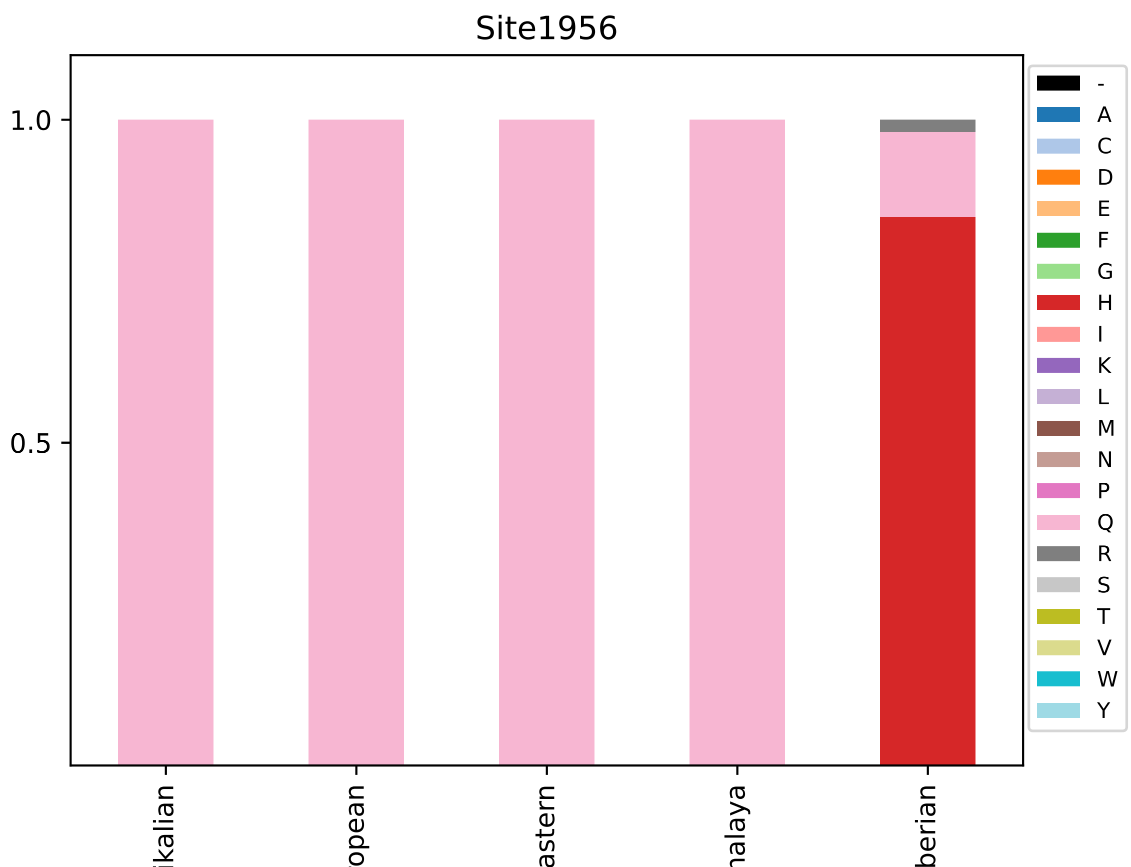
Site1956
1.0
0.5
ikalian
opean
astern
nalaya
berian
-
A
C
D
E
F
G
H
I
K
L
M
N
P
Q
R
S
T
V
W
Y

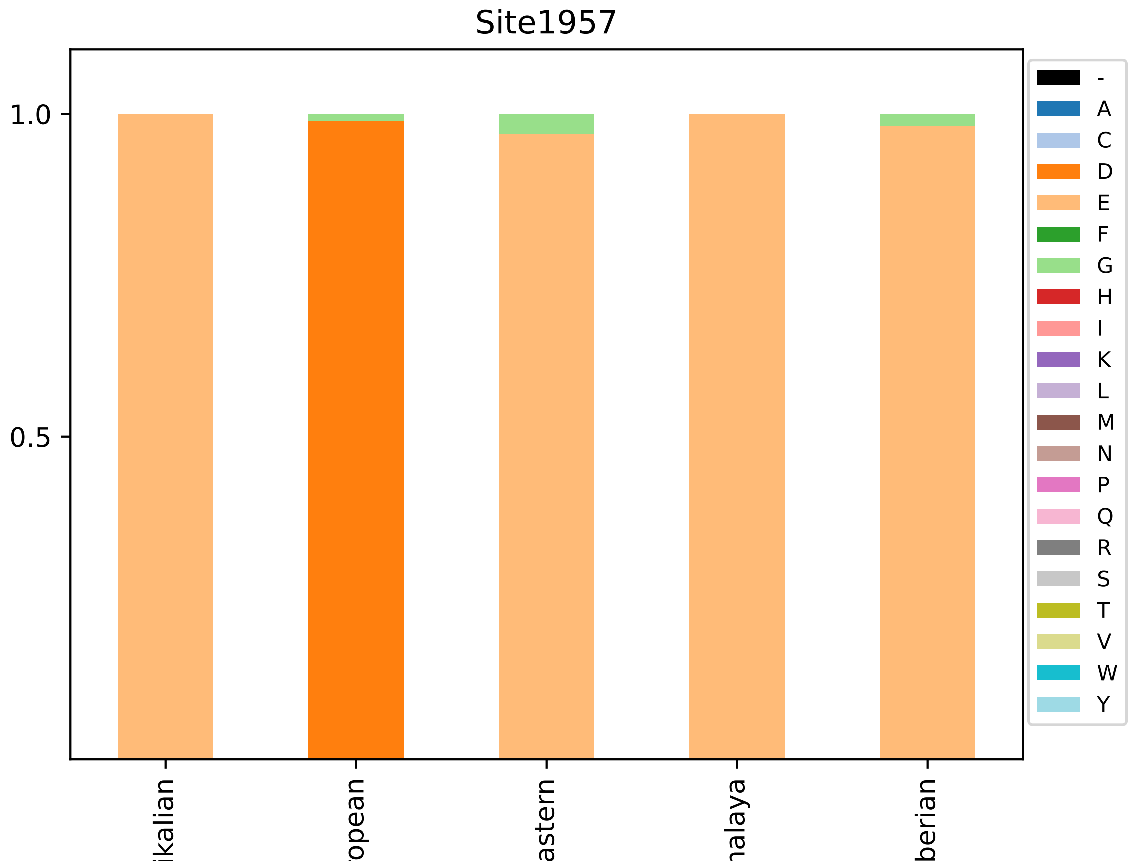

Site1957
1.0
0.5
ikalian
opean
astern
nalaya
berian
-
A
C
D
E
F
G
H
I
K
L
M
N
P
Q
R
S
T
V
W
Y

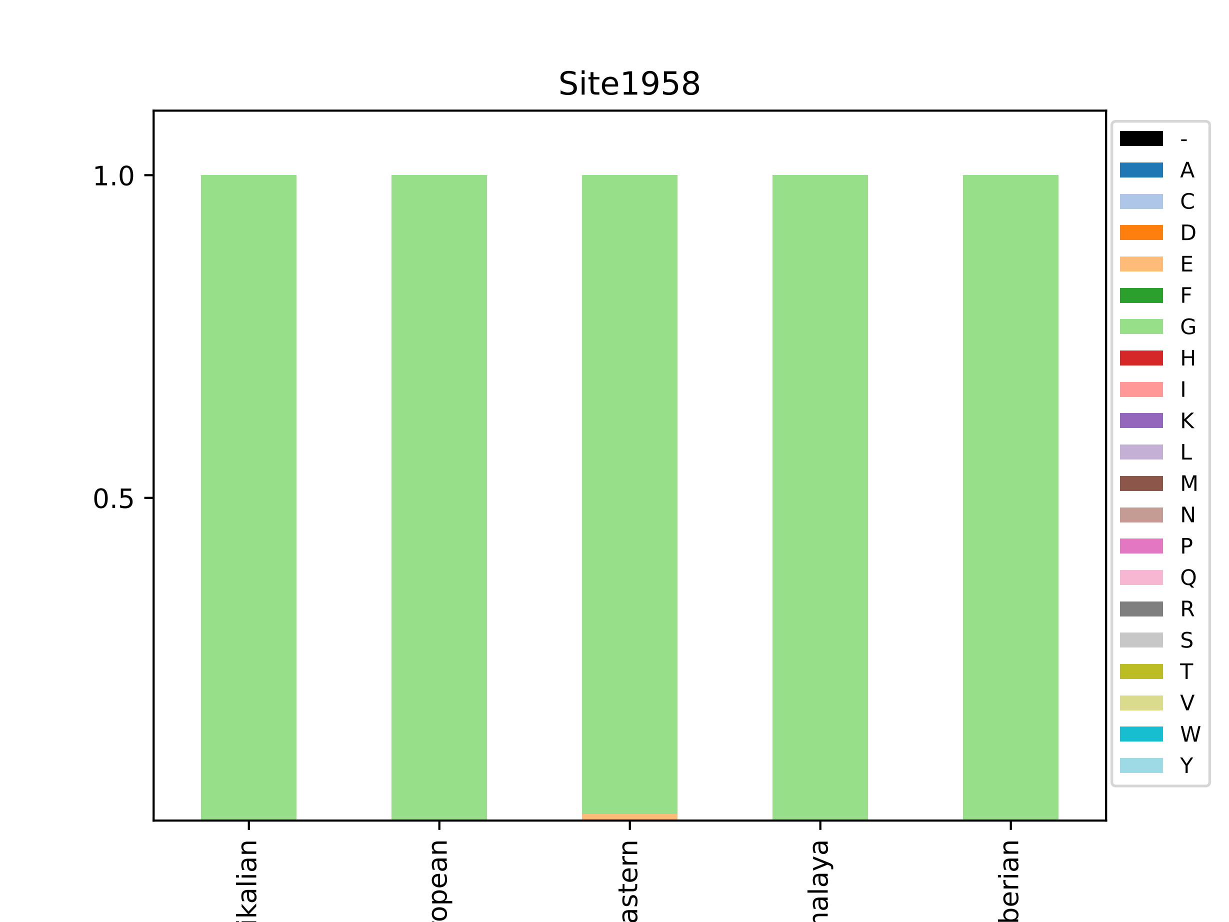

Site1958
1.0
0.5
-
A
C
D
E
F
G
H
I
K
L
M
N
P
Q
R
S
T
V
W
Y
ikalian
opean
astern
nalaya
berian

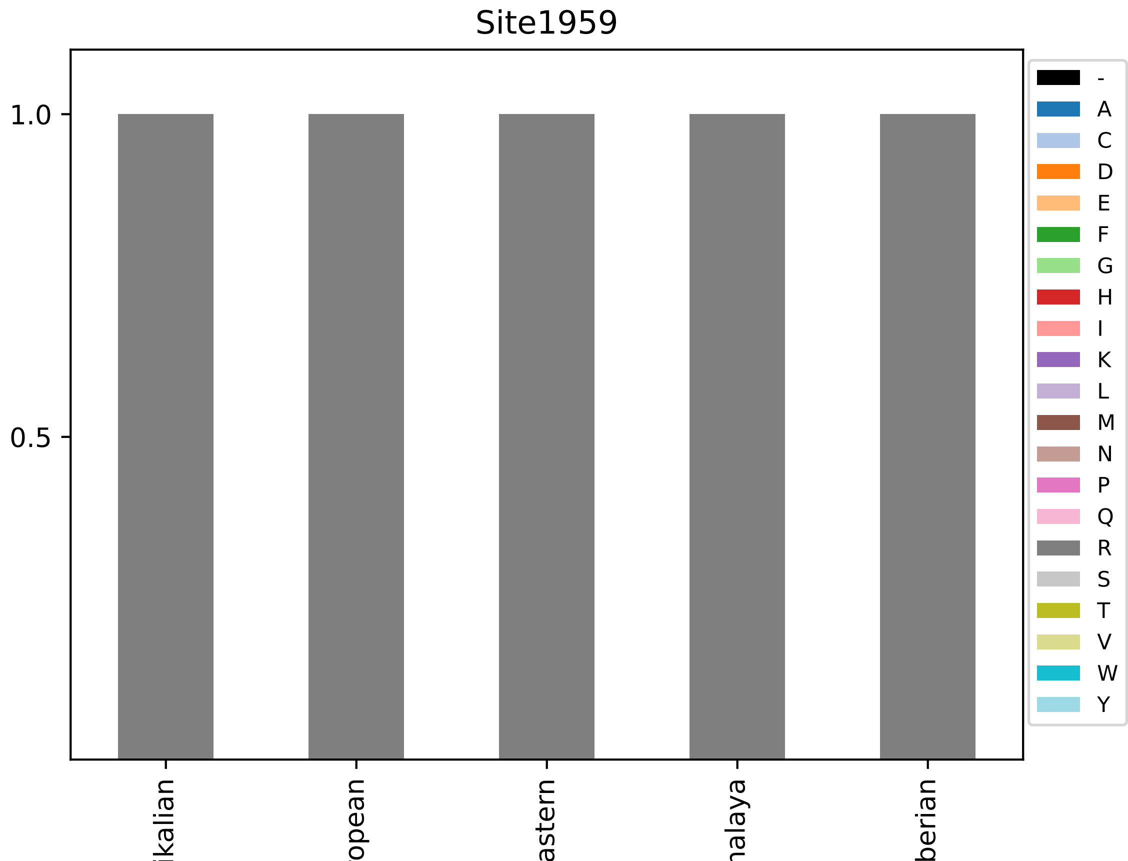

Site1959
1.0
0.5
-
A
C
D
E
F
G
H
I
K
L
M
N
P
Q
R
S
T
V
W
Y
ikalian
opean
astern
nalaya
berian

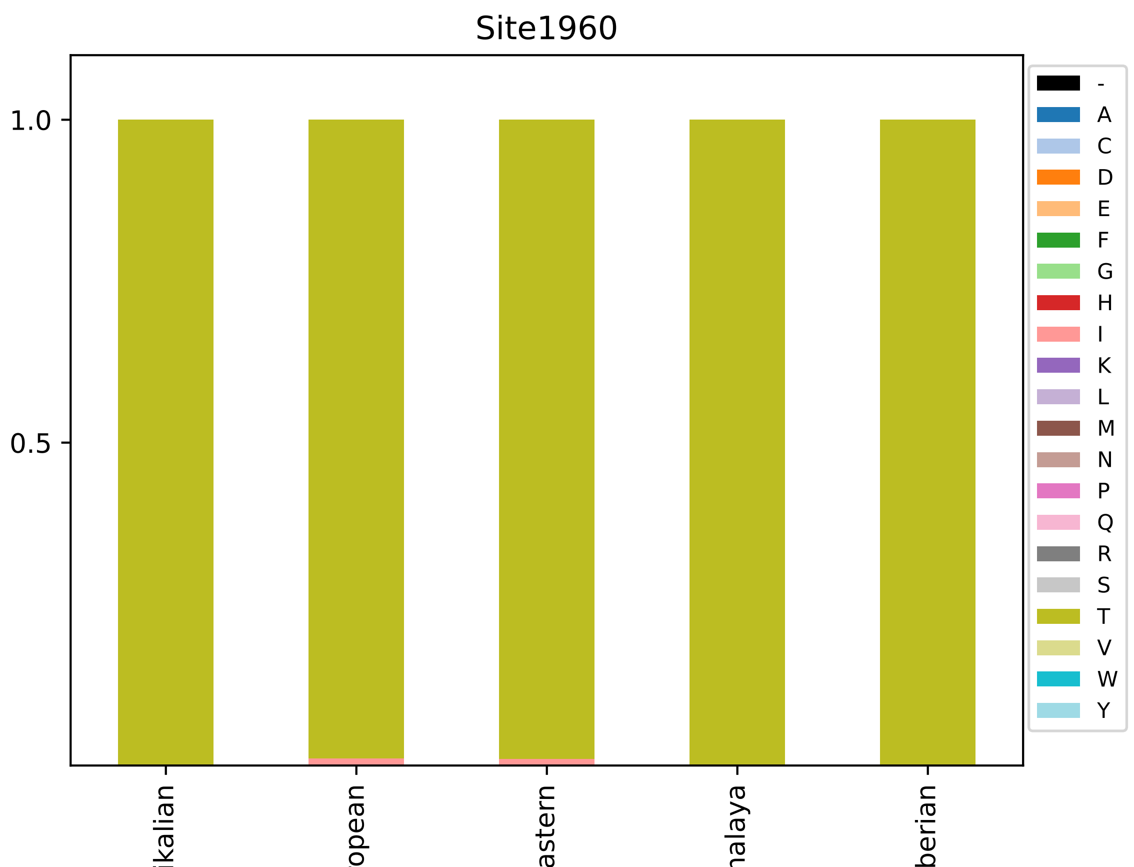
Site1960
1.0
0.5
ikalian
opean
astern
nalaya
berian
-
A
C
D
E
F
G
H
I
K
L
M
N
P
Q
R
S
T
V
W
Y

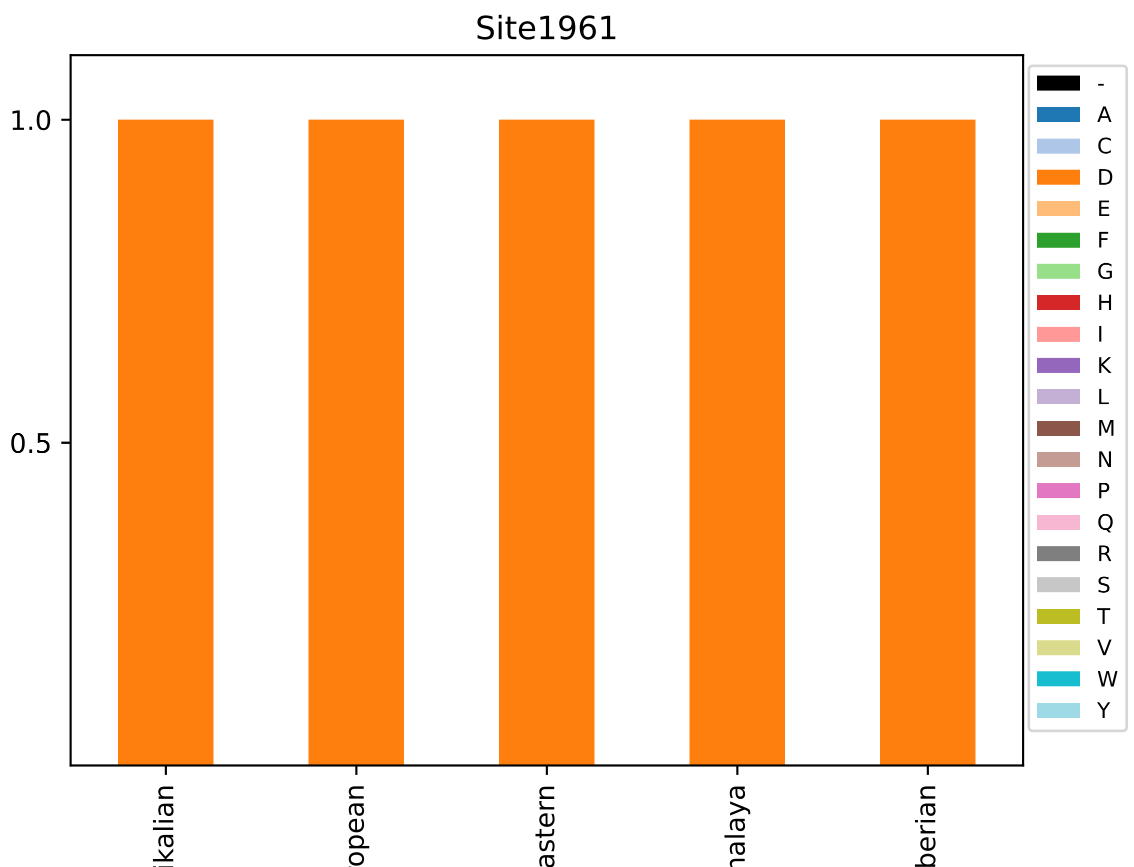
Site1961
1.0
0.5
-
A
C
D
E
F
G
H
I
K
L
M
N
P
Q
R
S
T
V
W
Y

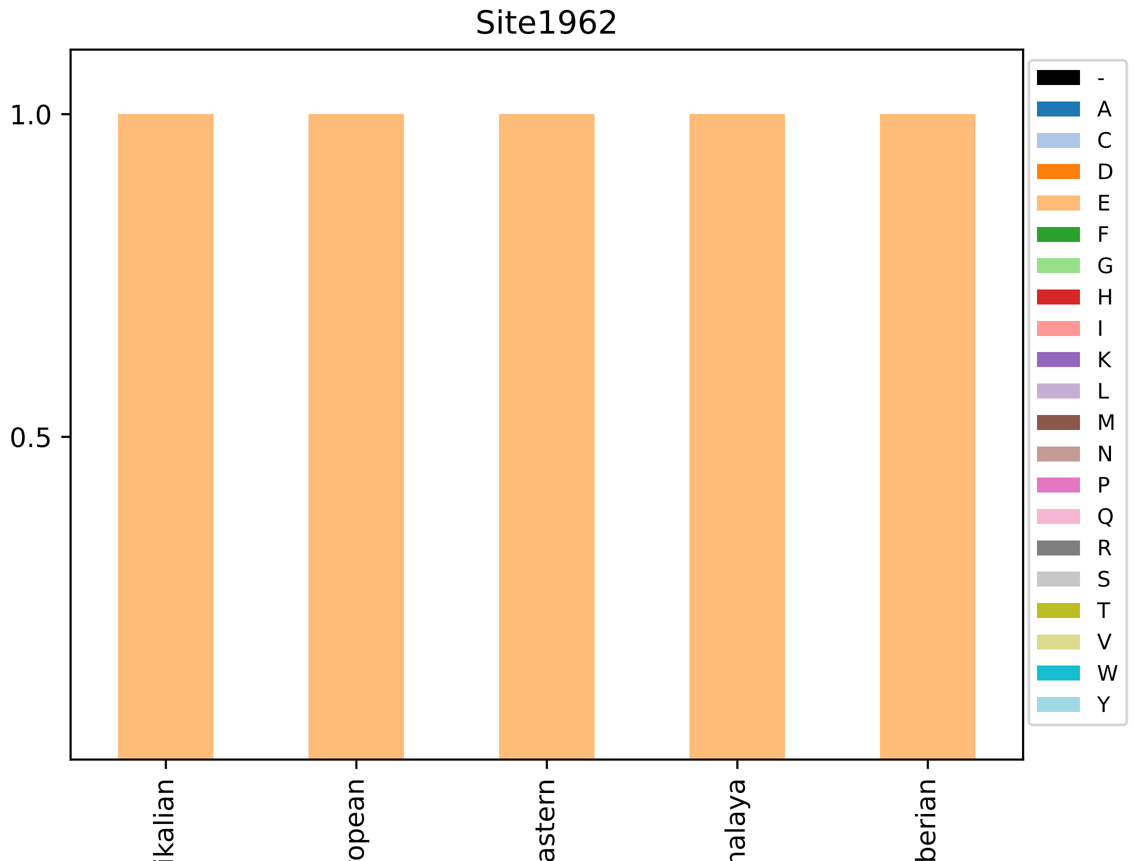

Site1962
1.0
0.5
-
A
C
D
E
F
G
H
I
K
L
M
N
P
Q
R
S
T
V
W
Y
ikalian
opean
astern
halaya
berian

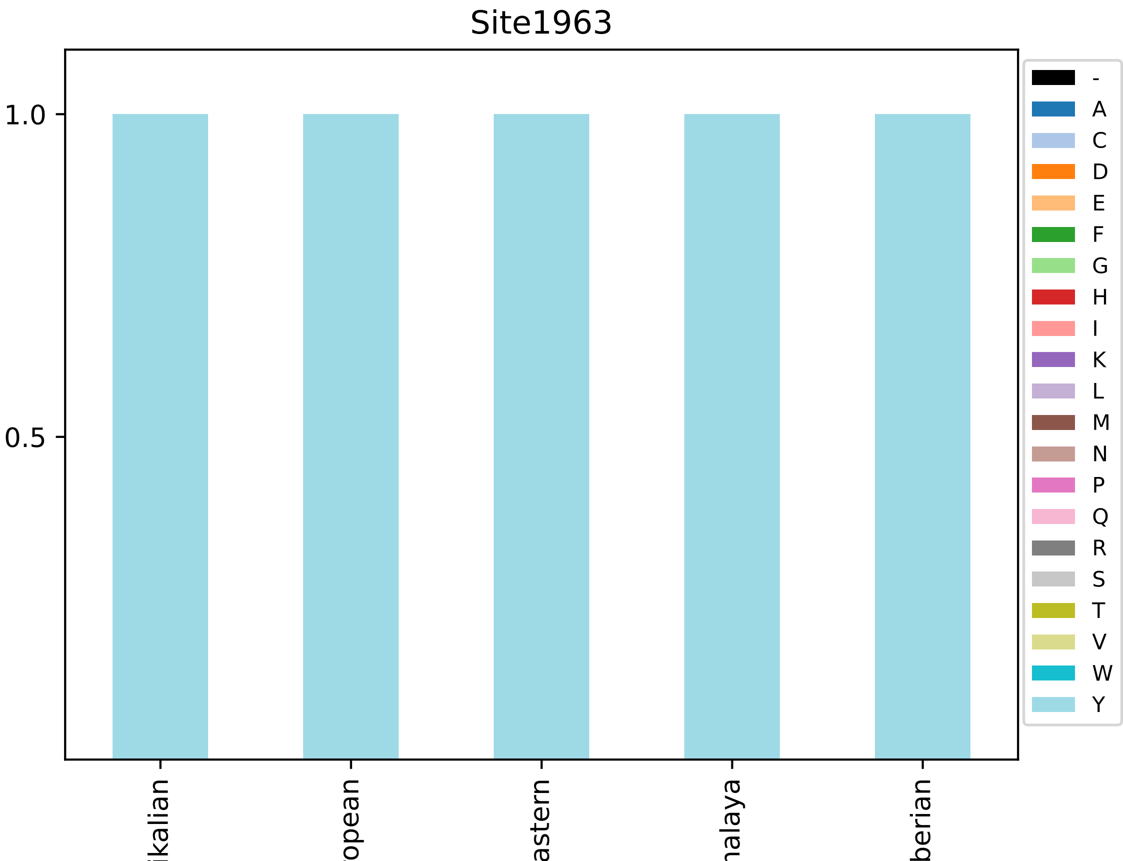
Site1963
1.0
0.5
-
A
C
D
E
F
G
H
I
K
L
M
N
P
Q
R
S
T
V
W
Y
ikalian
opean
astern
nalaya
berian

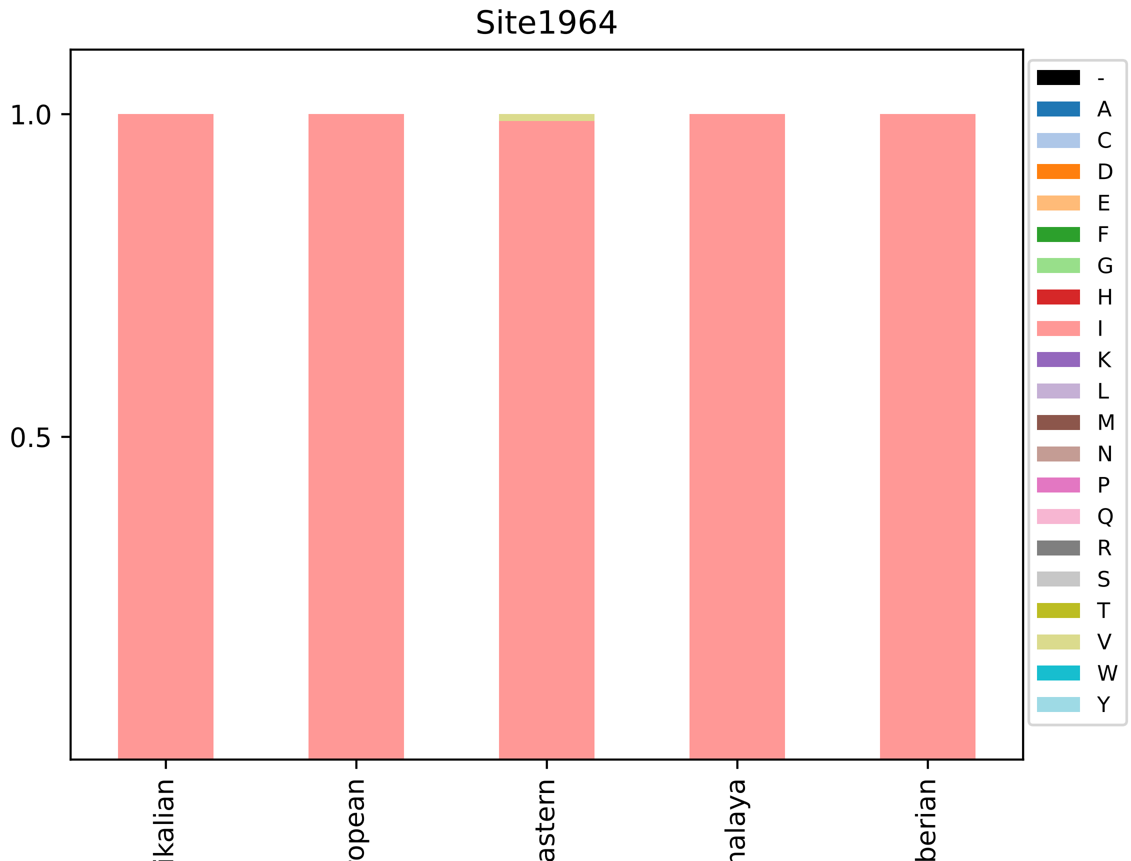

Site1964
1.0
0.5
-
A
C
D
E
F
G
H
I
K
L
M
N
P
Q
R
S
T
V
W
Y
ikalian
opean
astern
nalaya
berian

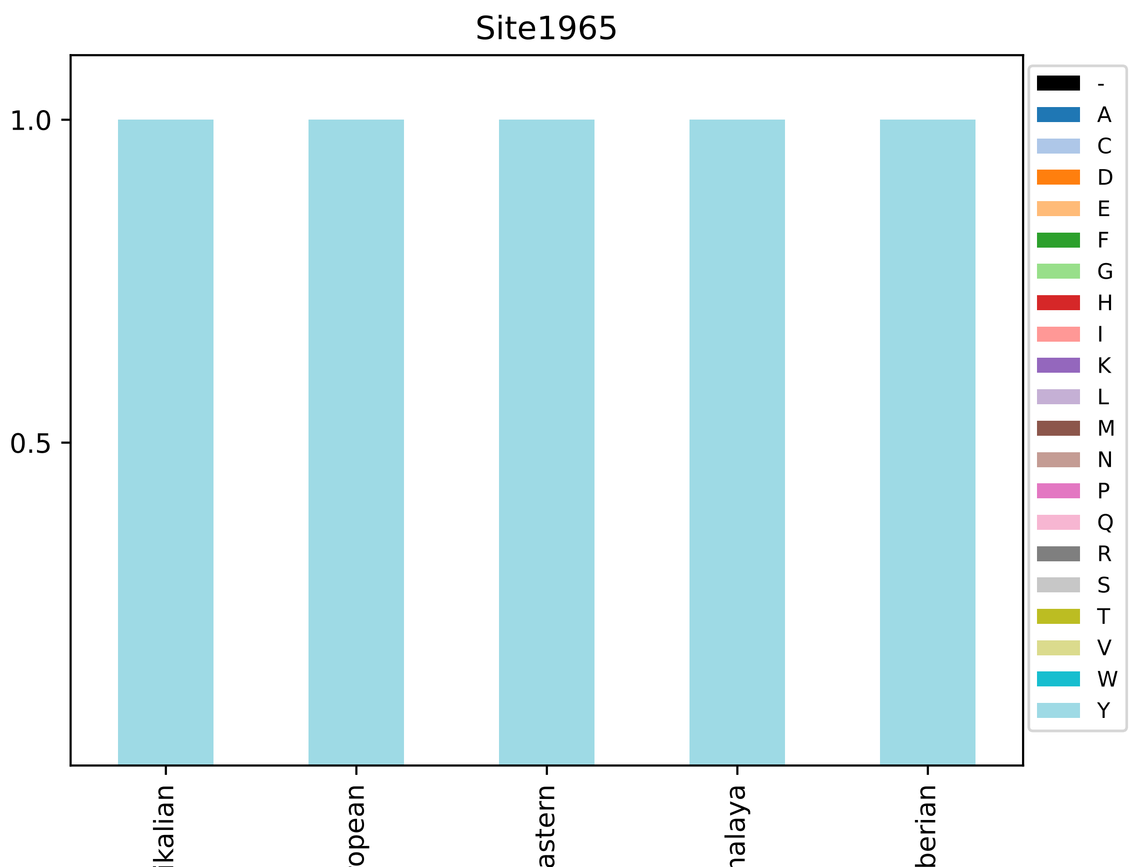

Site1965
1.0
0.5
-
A
C
D
E
F
G
H
I
K
L
M
N
P
Q
R
S
T
V
W
Y
ikalian
opean
astern
nalaya
berian

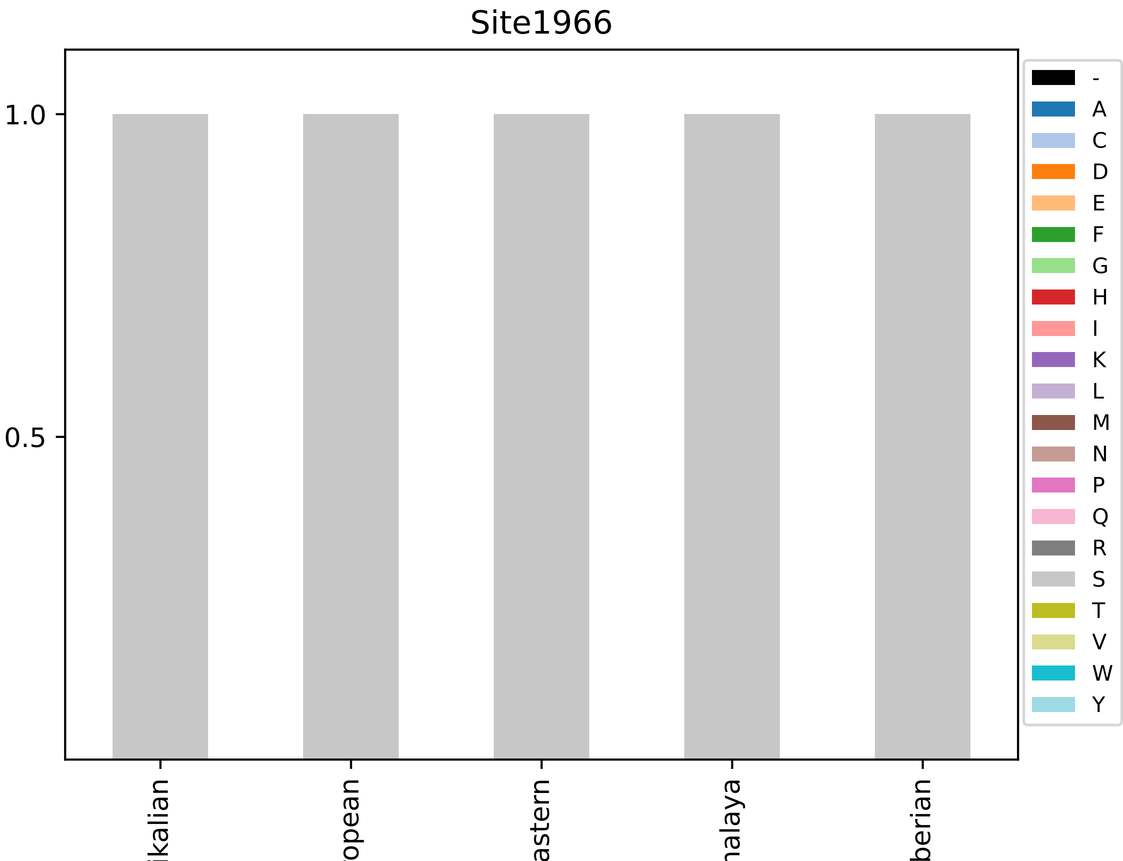
Site1966
1.0
0.5
-
A
C
D
E
F
G
H
I
K
L
M
N
P
Q
R
S
T
V
W
Y
ikalian
opean
astern
nalaya
berian

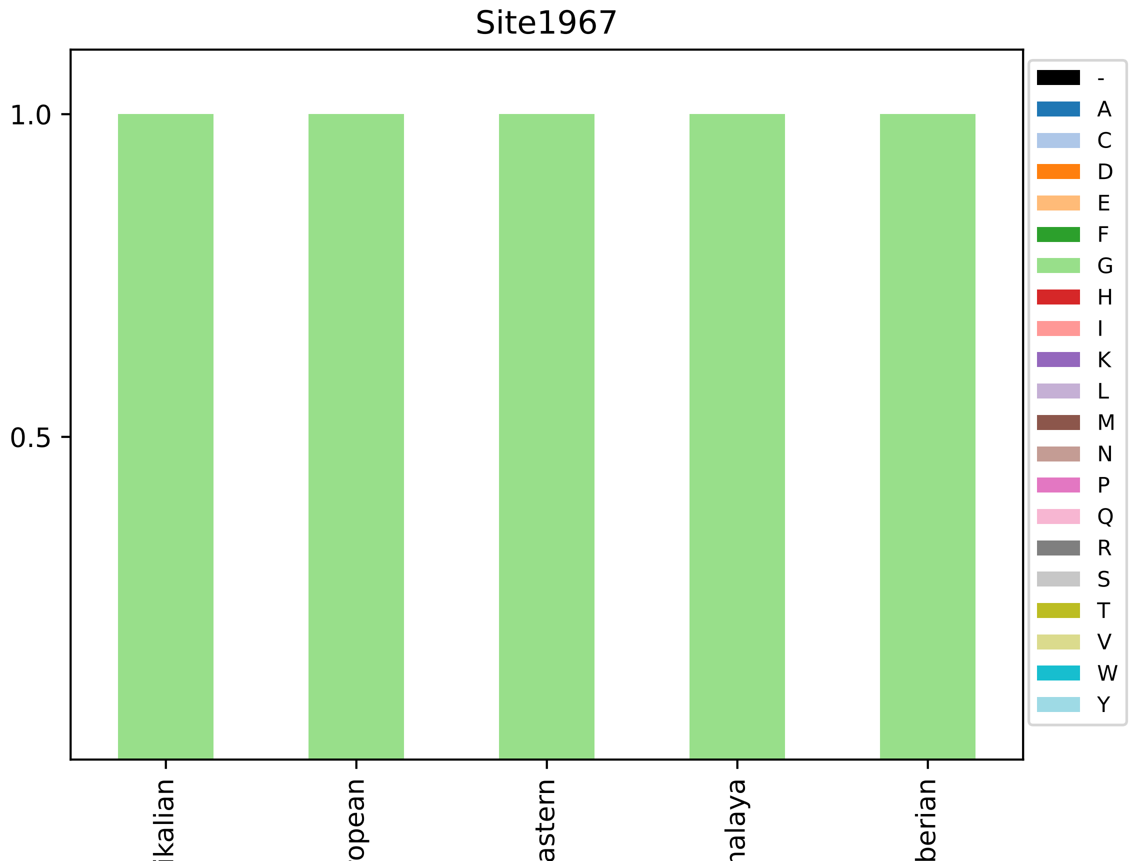
Site1967
1.0
0.5
-
A
C
D
E
F
G
H
I
K
L
M
N
P
Q
R
S
T
V
W
Y
ikalian
opean
astern
nalaya
berian

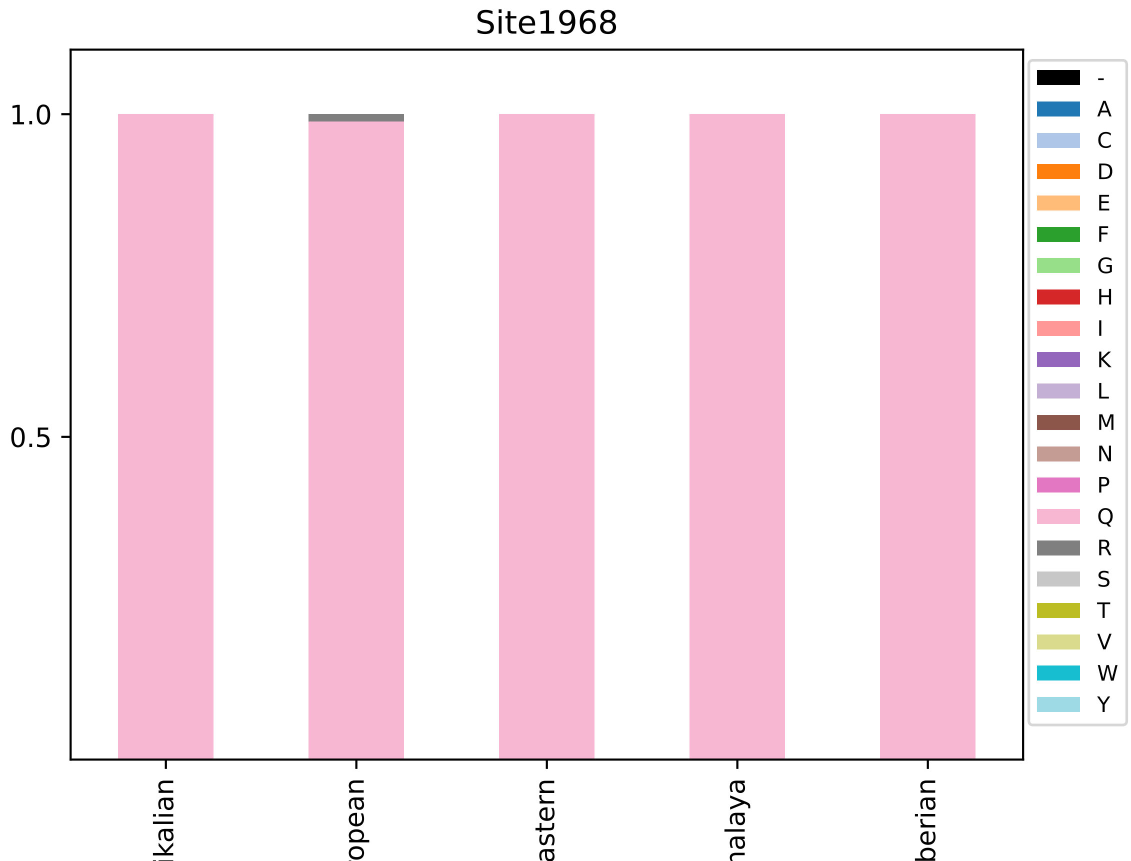

Site1968
1.0
0.5
-
A
C
D
E
F
G
H
I
K
L
M
N
P
Q
R
S
T
V
W
Y
ikalian
opean
astern
nalaya
berian

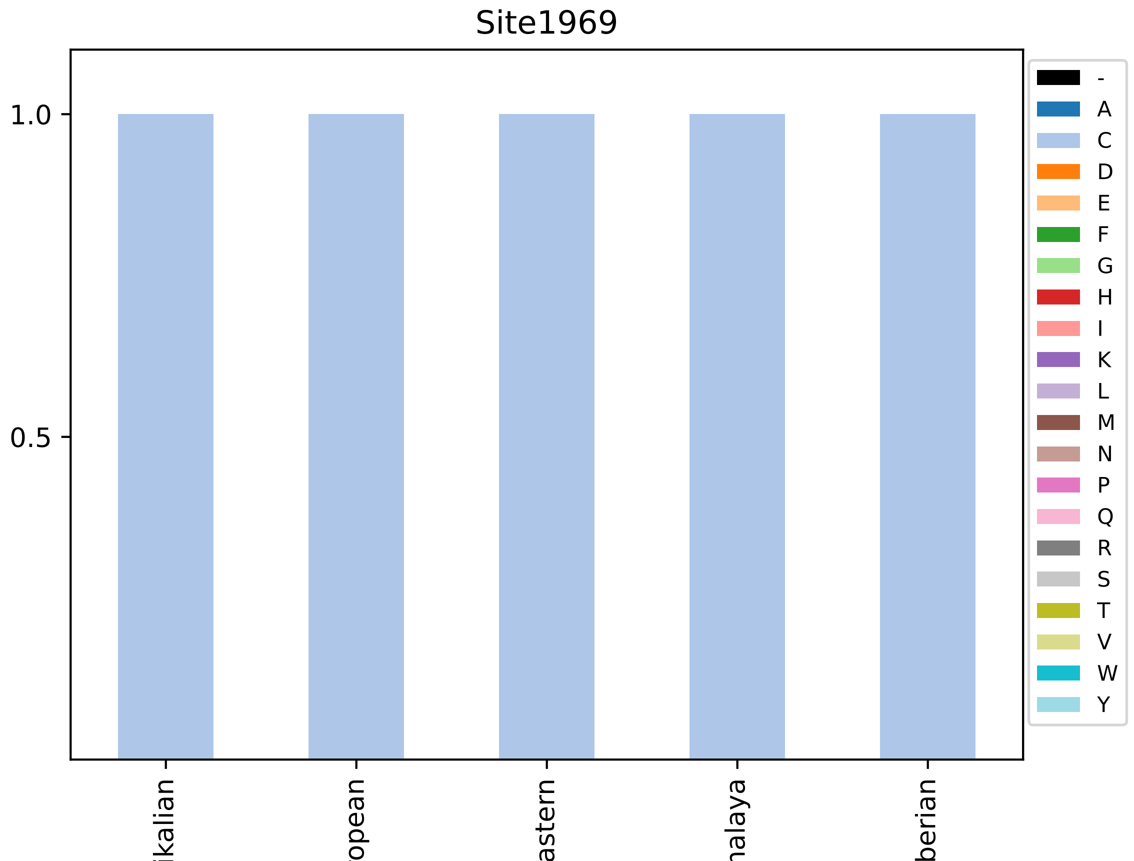

Site1969
1.0
0.5
-
A
C
D
E
F
G
H
I
K
L
M
N
P
Q
R
S
T
V
W
Y
ikalian
opean
astern
nalaya
berian

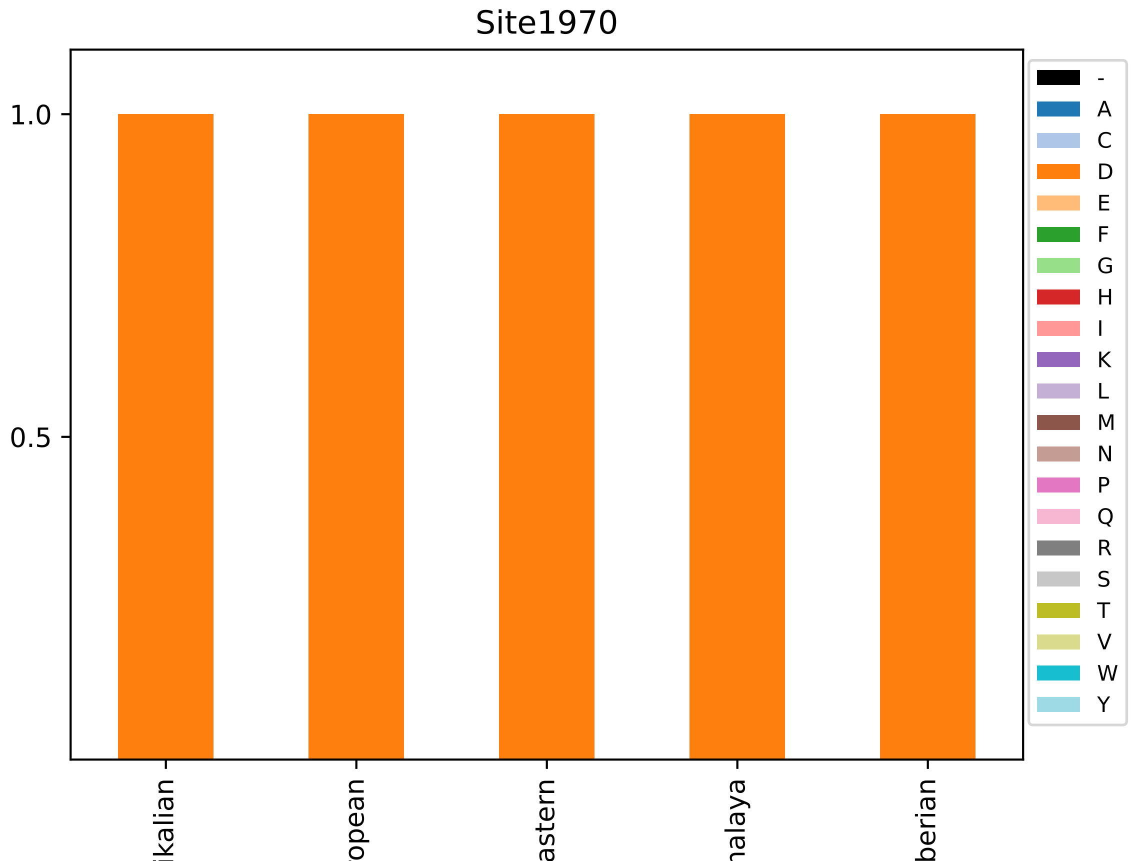

Site1970
1.0
0.5
-
A
C
D
E
F
G
H
I
K
L
M
N
P
Q
R
S
T
V
W
Y
ikalian
opean
astern
nalaya
berian

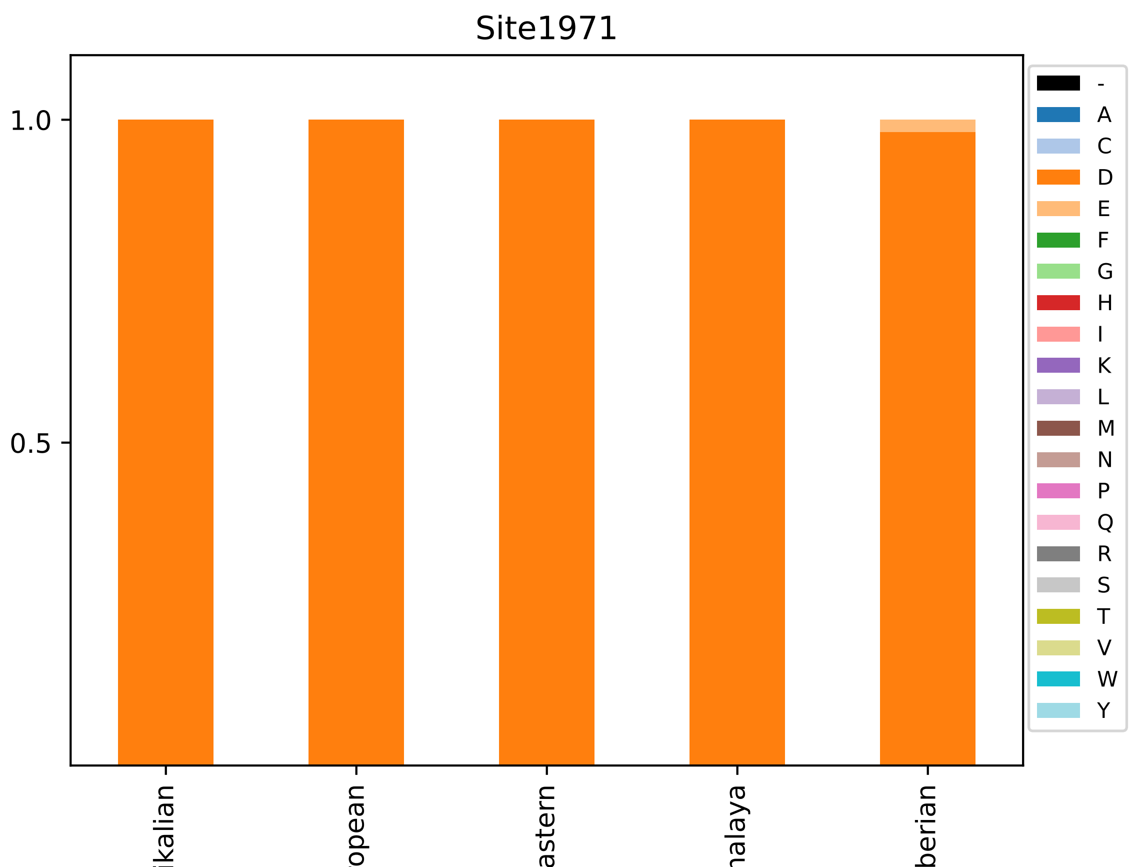

Site1971
1.0
0.5
-
A
C
D
E
F
G
H
I
K
L
M
N
P
Q
R
S
T
V
W
Y
ikalian
opean
astern
nalaya
berian

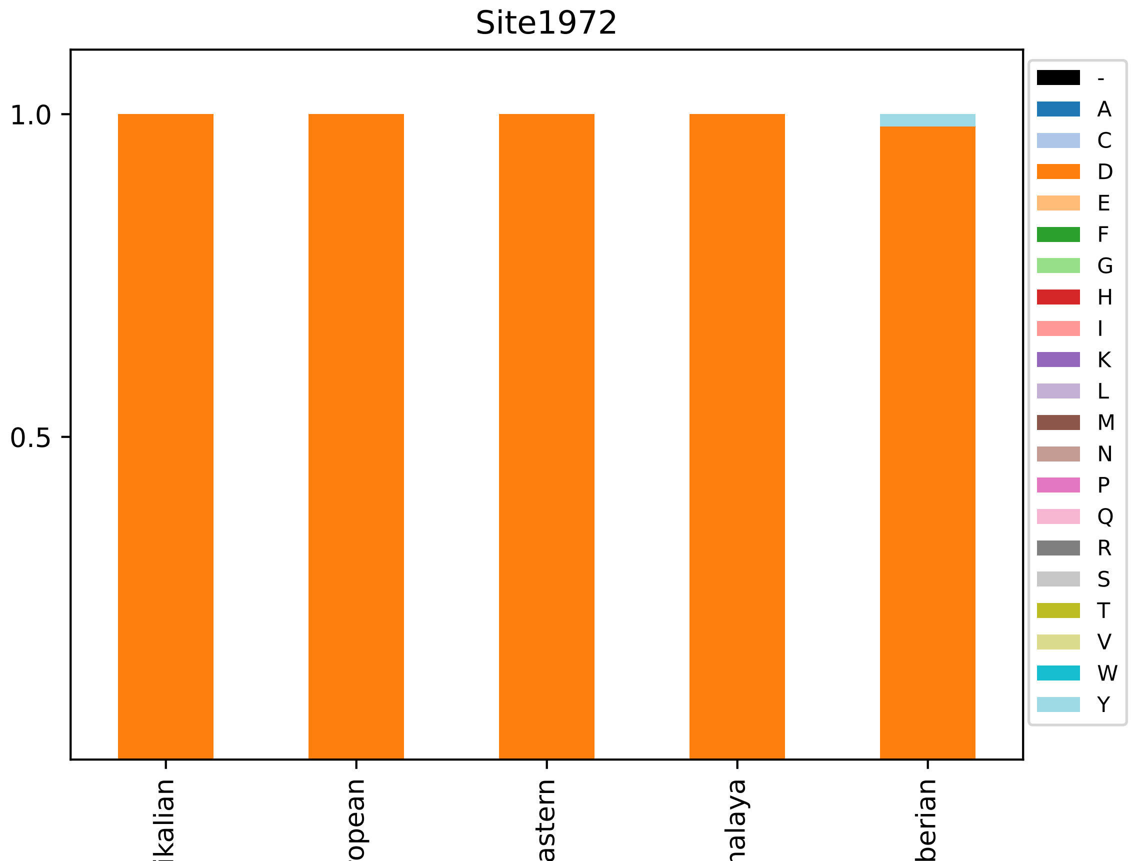
Site1972
1.0
0.5
-
A
C
D
E
F
G
H
I
K
L
M
N
P
Q
R
S
T
V
W
Y
ikalian
opean
astern
nalaya
berian

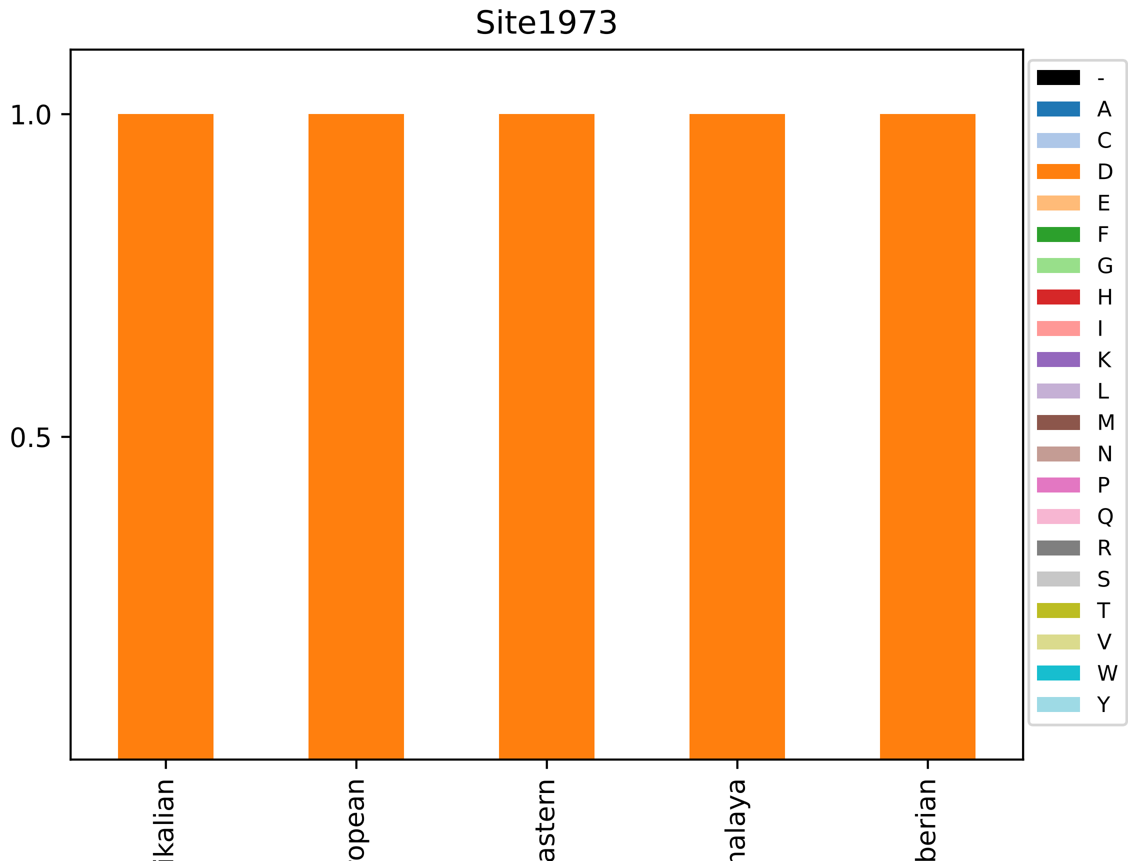

Site1973
1.0
0.5
-
A
C
D
E
F
G
H
I
K
L
M
N
P
Q
R
S
T
V
W
Y
ikalian
opean
astern
nalaya
berian

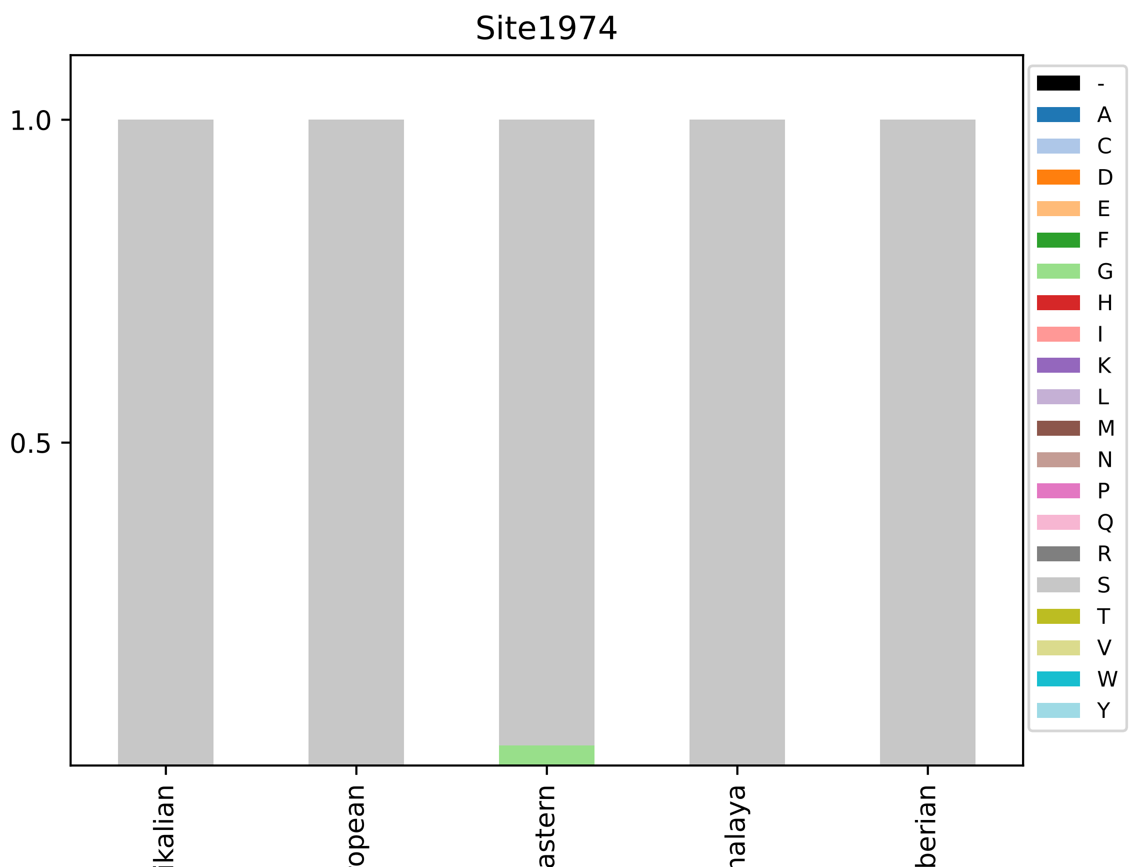
Site1974
1.0
0.5
-
A
C
D
E
F
G
H
I
K
L
M
N
P
Q
R
S
T
V
W
Y
ikalian
opean
astern
nalaya
berian

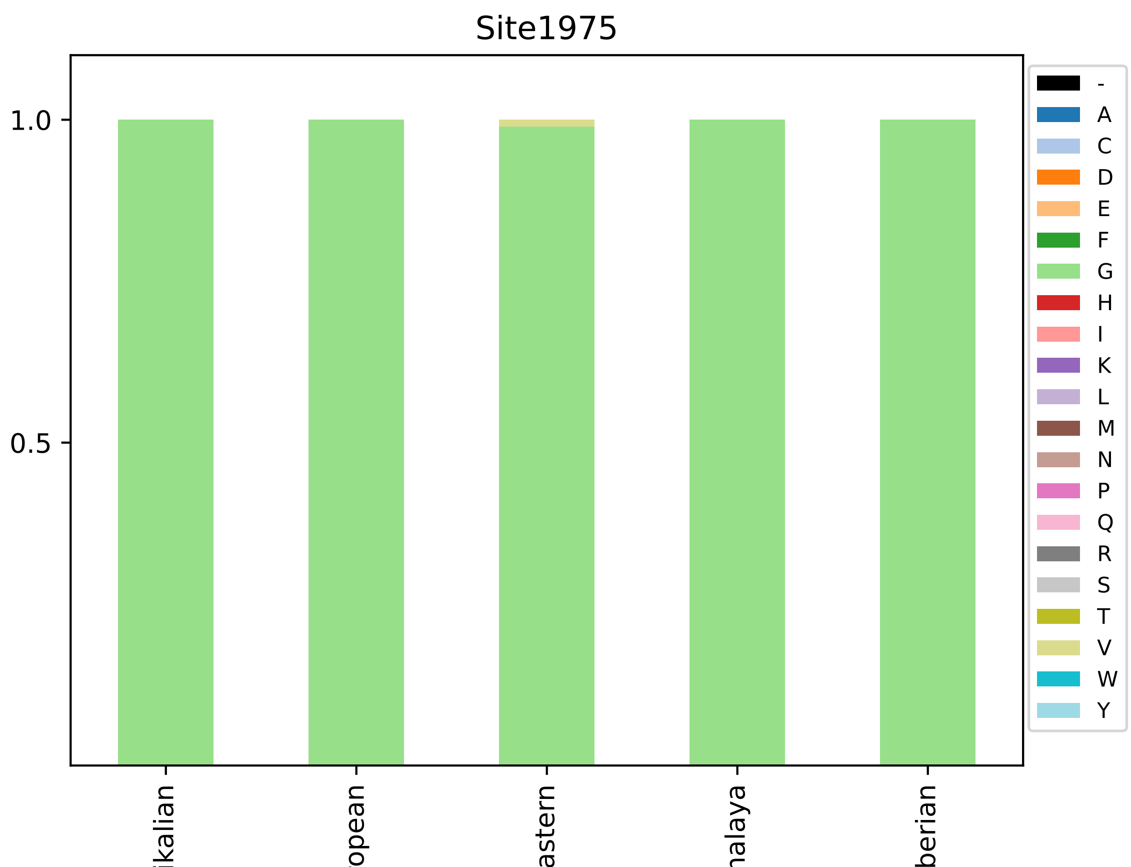

Site1975
1.0
0.5
-
A
C
D
E
F
G
H
I
K
L
M
N
P
Q
R
S
T
V
W
Y
ikalian
opean
astern
nalaya
berian

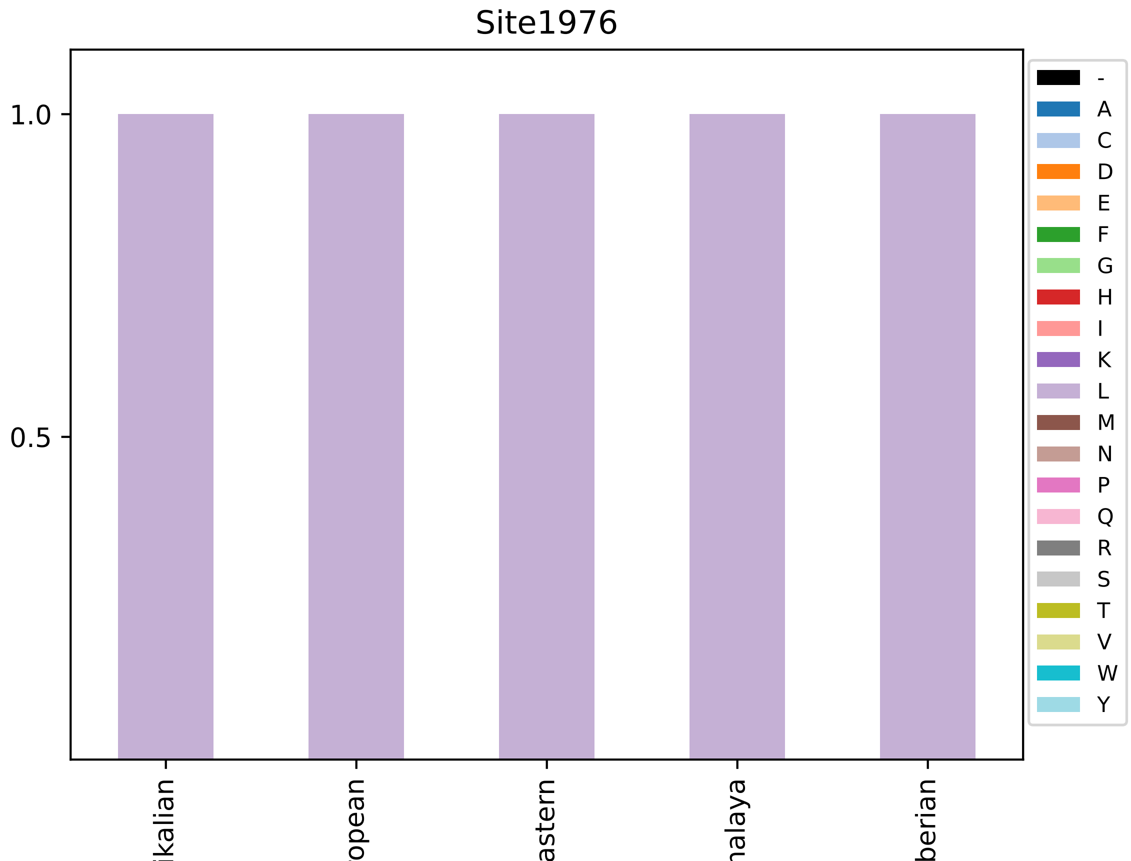

Site1976
1.0
0.5
ikalian
opean
astern
nalaya
berian
-
A
C
D
E
F
G
H
I
K
L
M
N
P
Q
R
S
T
V
W
Y

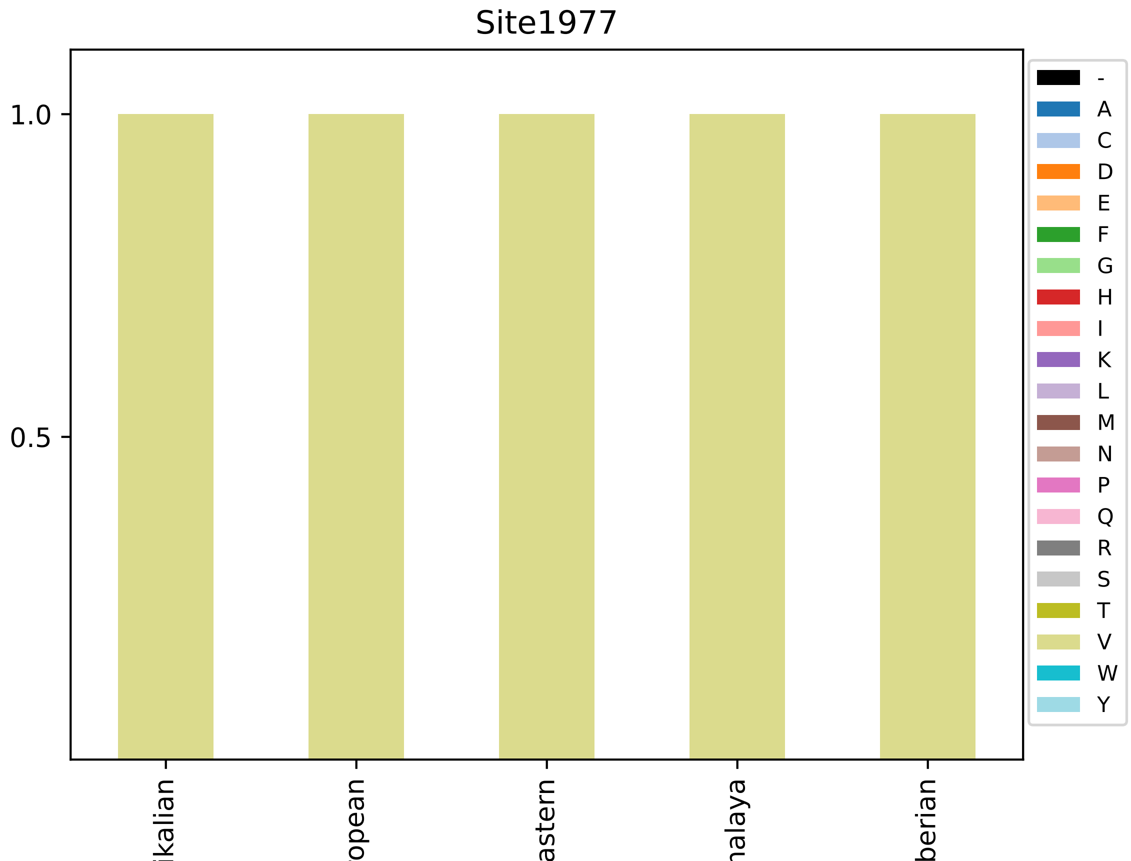

Site1977
1.0
0.5
-
A
C
D
E
F
G
H
I
K
L
M
N
P
Q
R
S
T
V
W
Y
ikalian
opean
astern
nalaya
berian

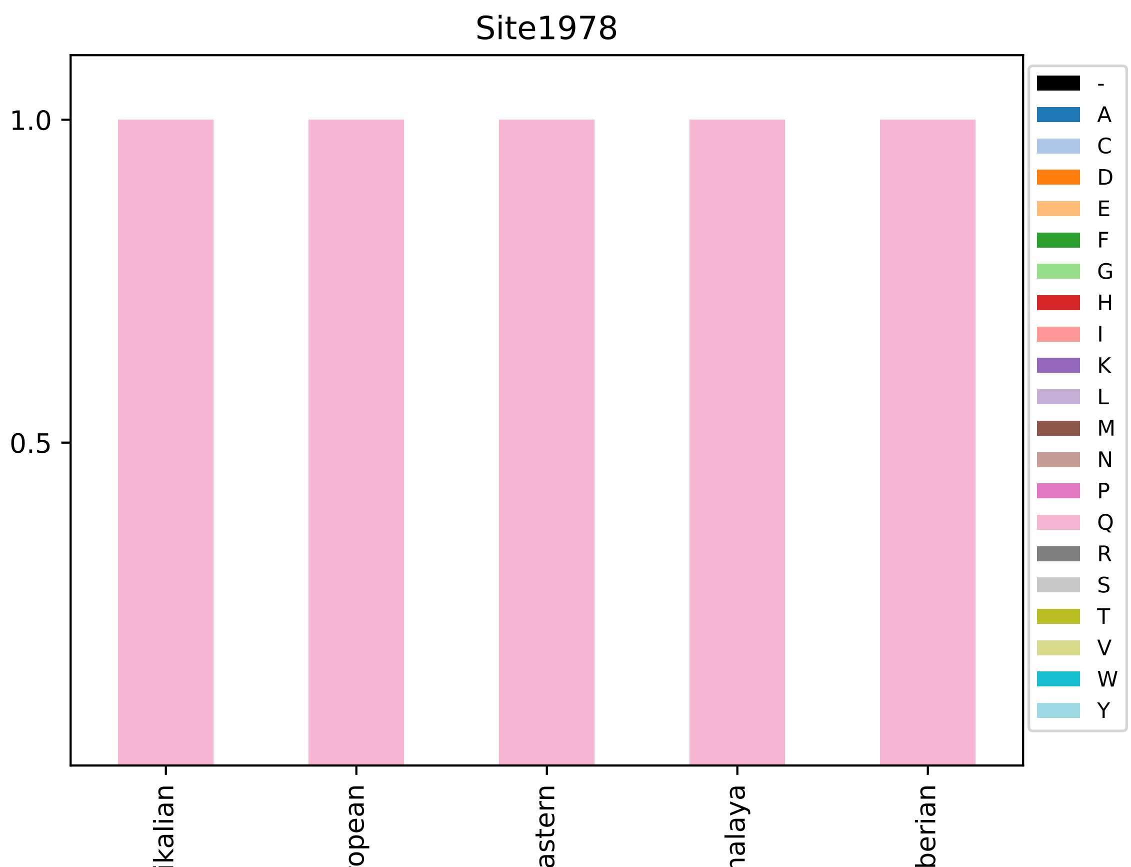

Site1978
1.0
0.5
-
A
C
D
E
F
G
H
I
K
L
M
N
P
Q
R
S
T
V
W
Y
ikalian
opean
astern
nalaya
berian

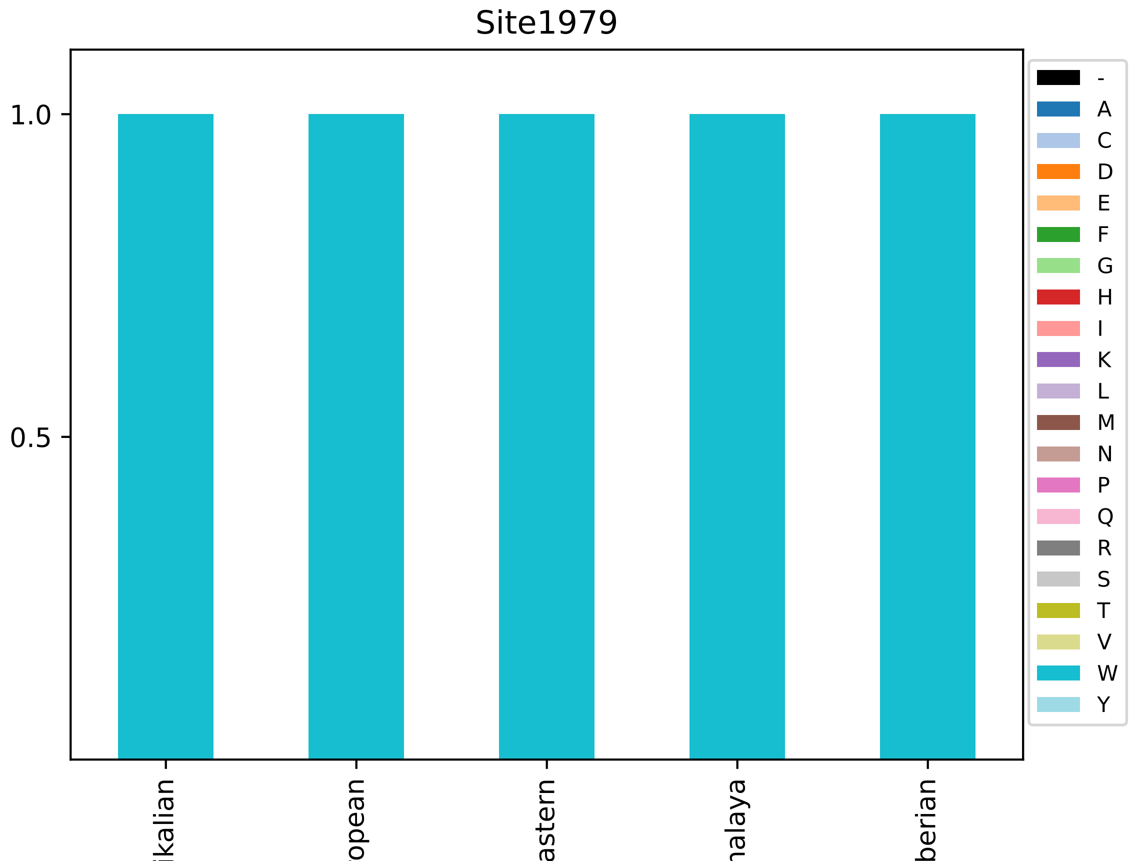

Site1979
1.0
0.5
ikalian
opean
astern
nalaya
berian
-
A
C
D
E
F
G
H
I
K
L
M
N
P
Q
R
S
T
V
W
Y

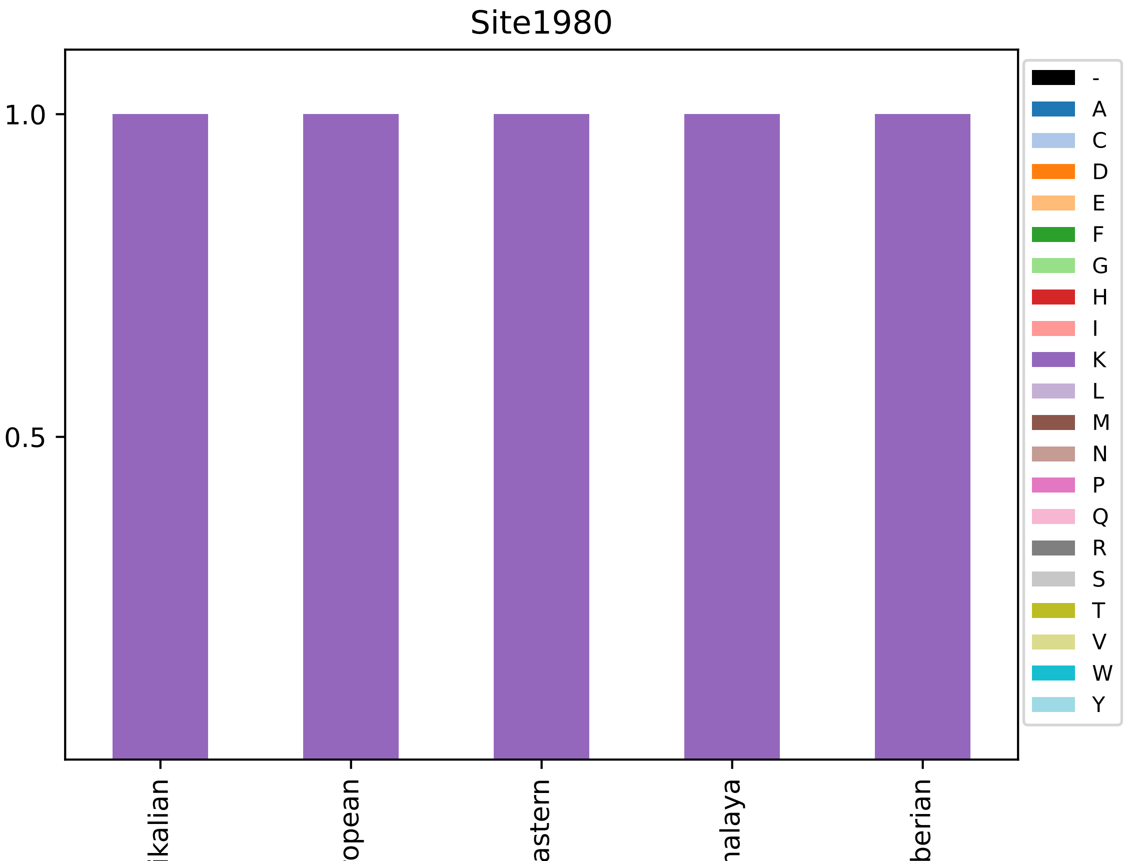

Site1980
1.0
0.5
-
A
C
D
E
F
G
H
I
K
L
M
N
P
Q
R
S
T
V
W
Y
ikalian
opean
astern
nalaya
berian

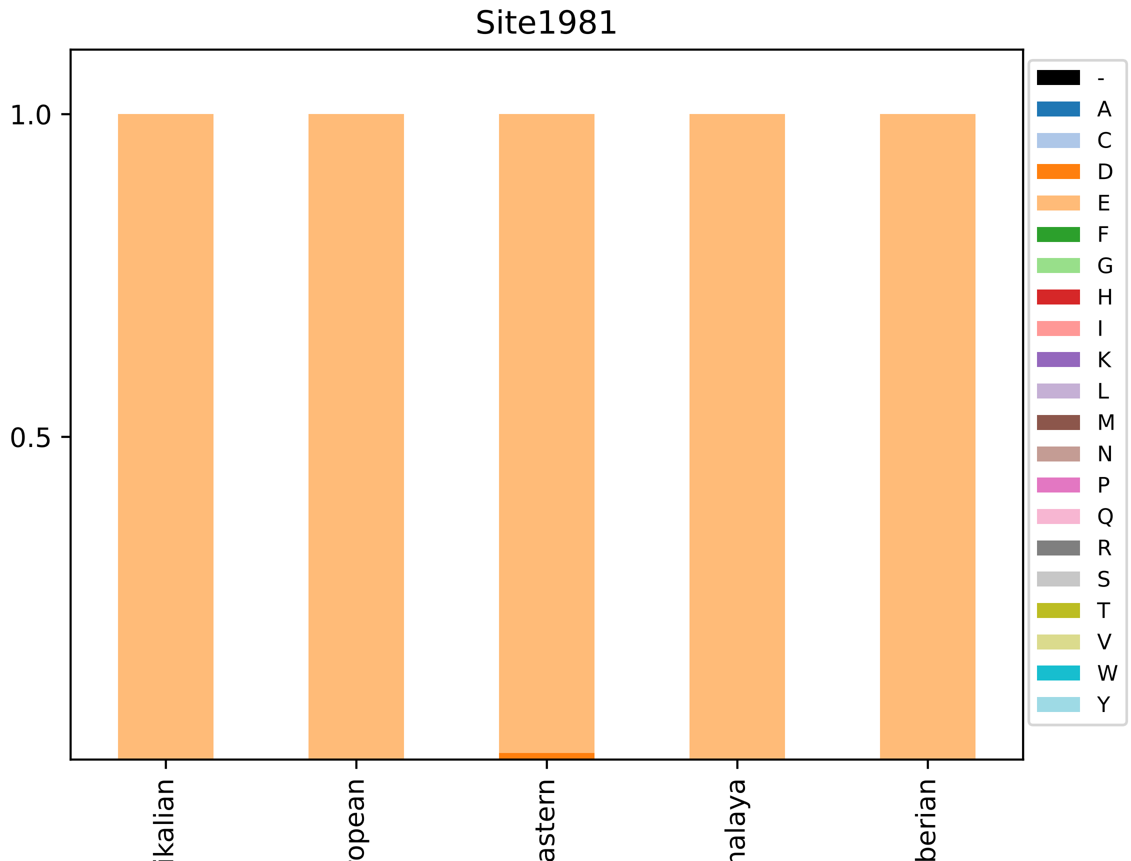
Site1981
1.0
0.5
-
A
C
D
E
F
G
H
I
K
L
M
N
P
Q
R
S
T
V
W
Y

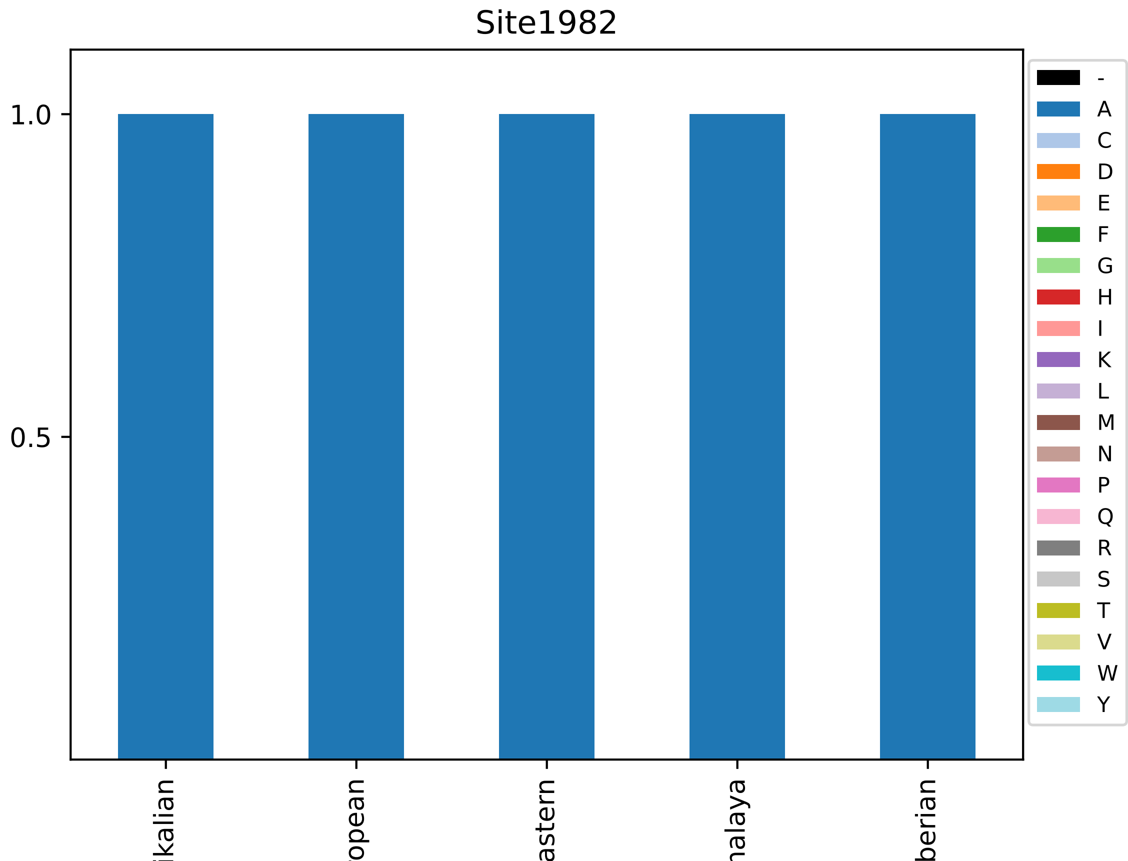

Site1982
1.0
0.5
-
A
C
D
E
F
G
H
I
K
L
M
N
P
Q
R
S
T
V
W
Y
ikalian
opean
astern
nalaya
berian

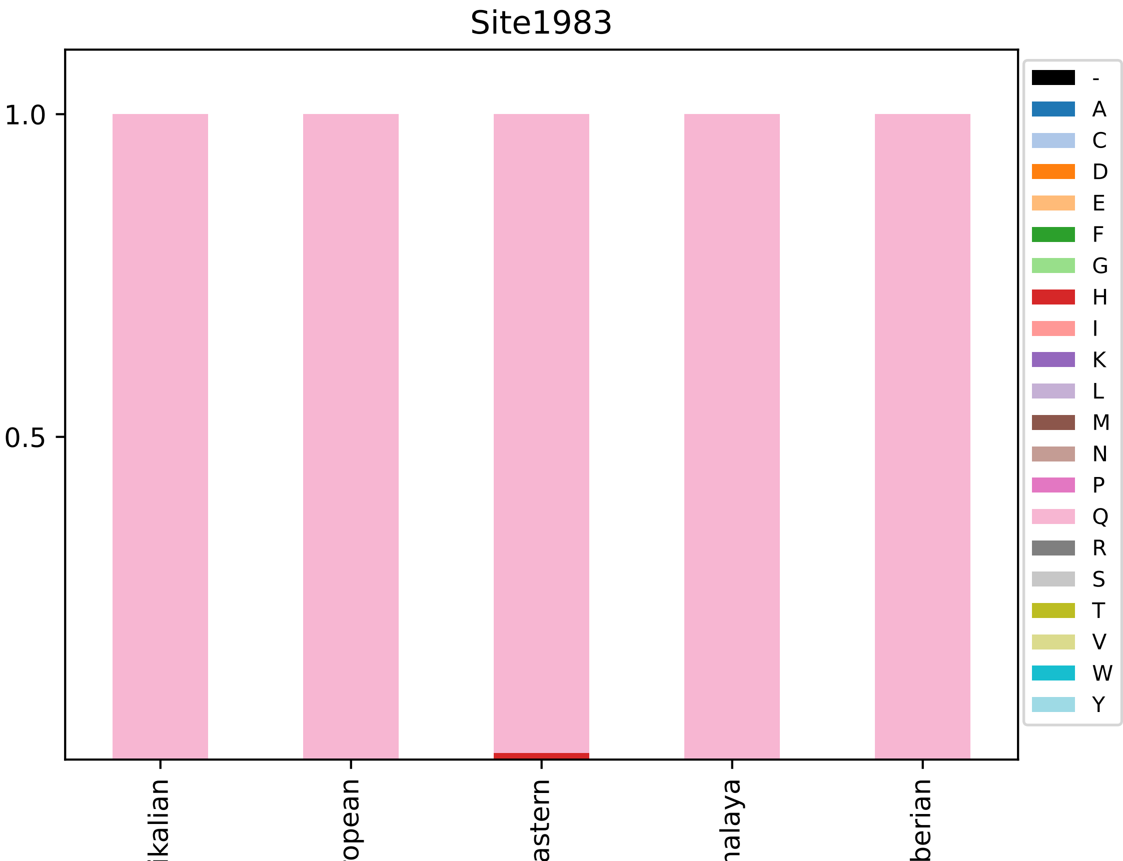
Site1983
1.0
0.5
-
A
C
D
E
F
G
H
I
K
L
M
N
P
Q
R
S
T
V
W
Y
ikalian
opean
astern
nalaya
berian

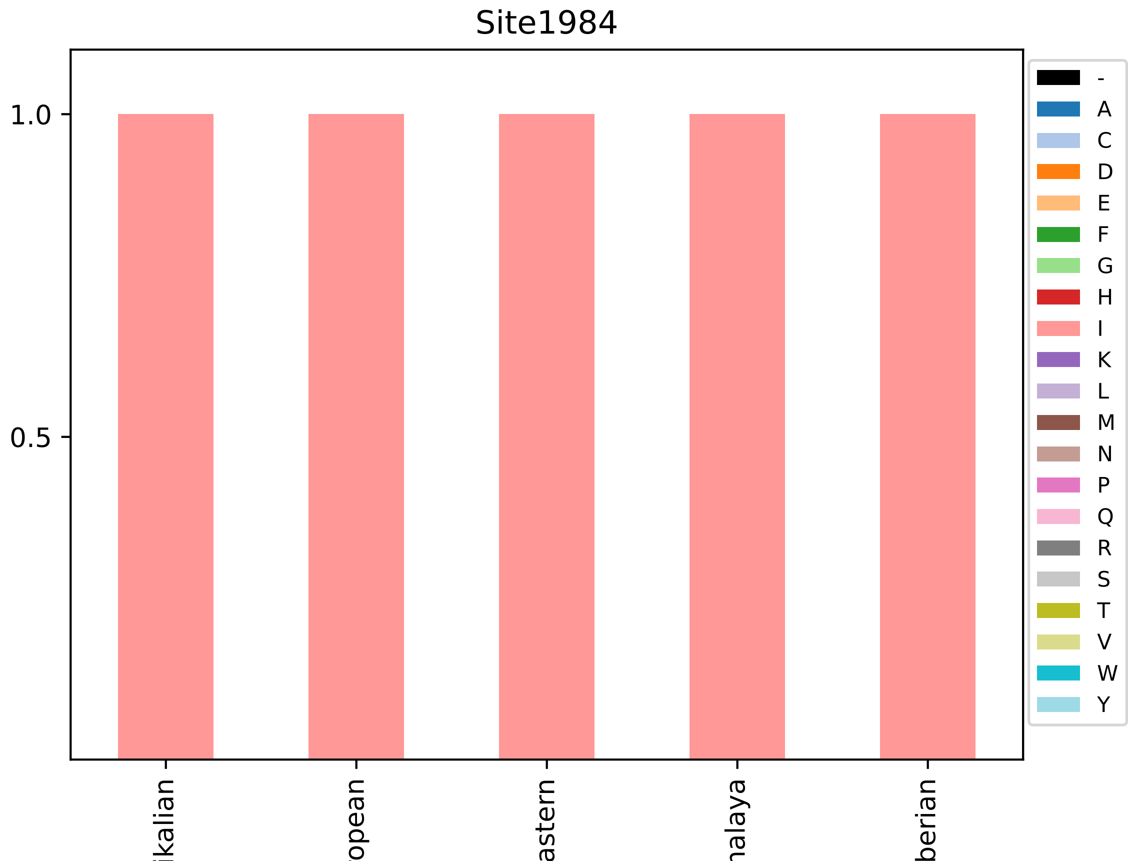

Site1984
1.0
0.5
-
A
C
D
E
F
G
H
I
K
L
M
N
P
Q
R
S
T
V
W
Y
ikalian
opean
astern
nalaya
berian

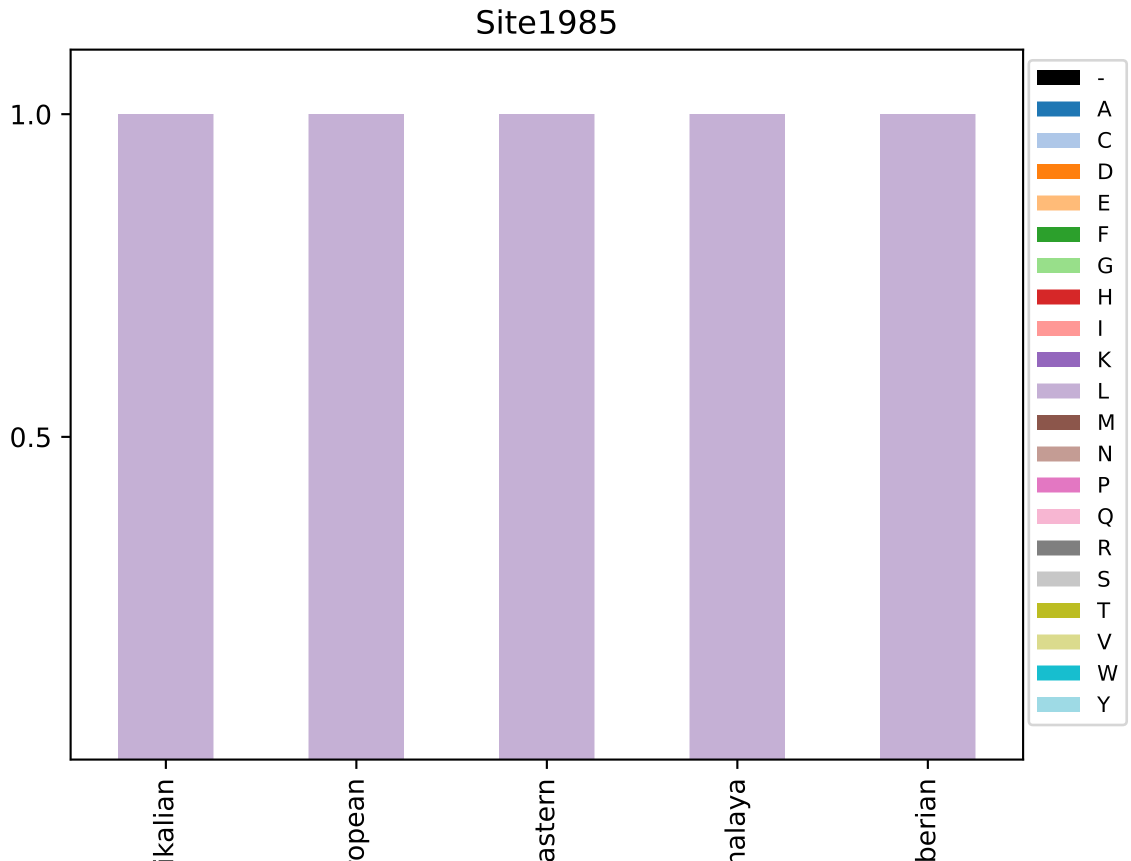

Site1985
1.0
0.5
-
A
C
D
E
F
G
H
I
K
L
M
N
P
Q
R
S
T
V
W
Y

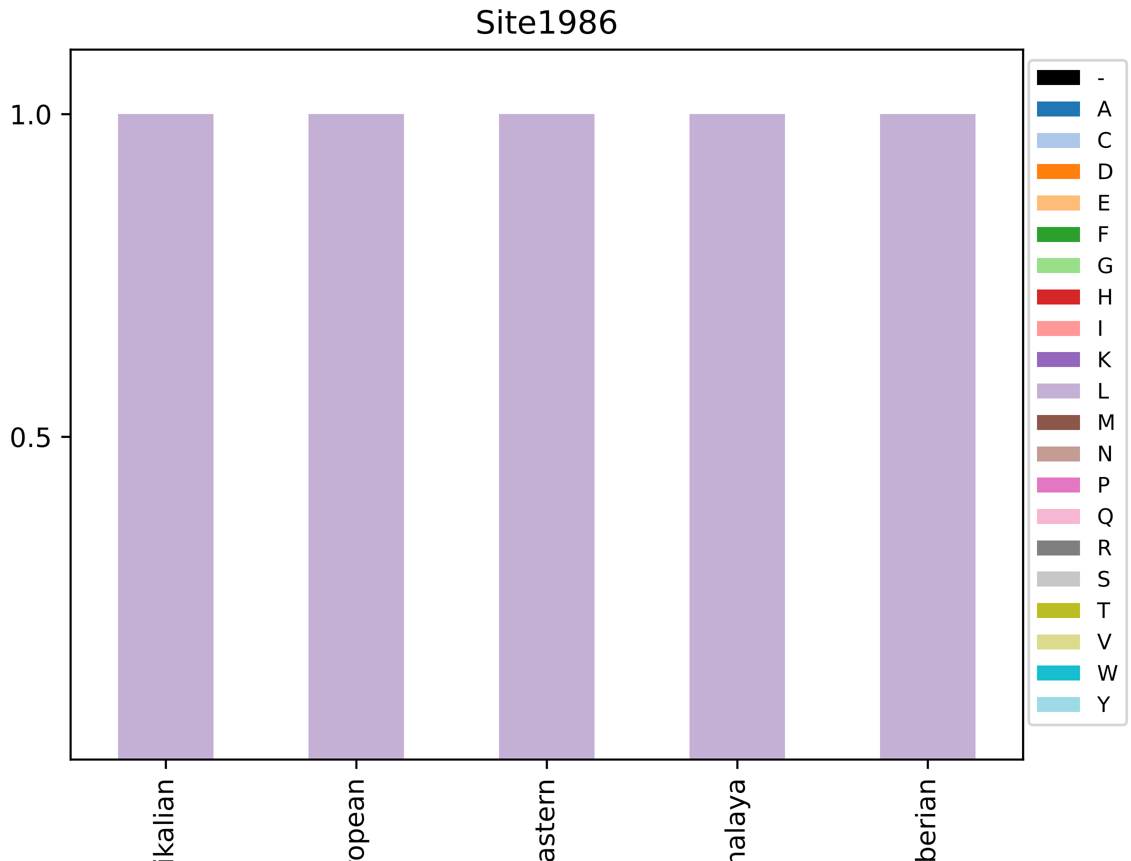
Site1986
1.0
0.5
-
A
C
D
E
F
G
H
I
K
L
M
N
P
Q
R
S
T
V
W
Y

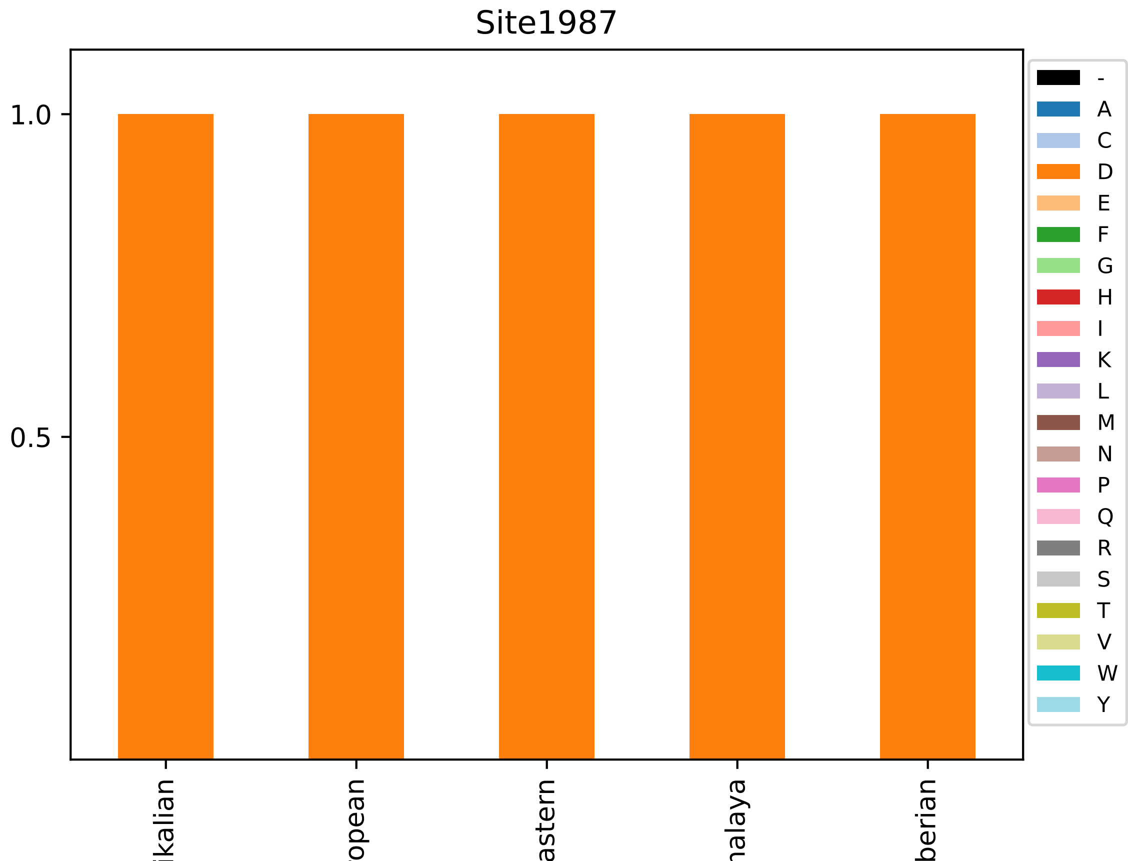
Site1987
1.0
0.5
-
A
C
D
E
F
G
H
I
K
L
M
N
P
Q
R
S
T
V
W
Y
ikalian
opean
astern
nalaya
berian

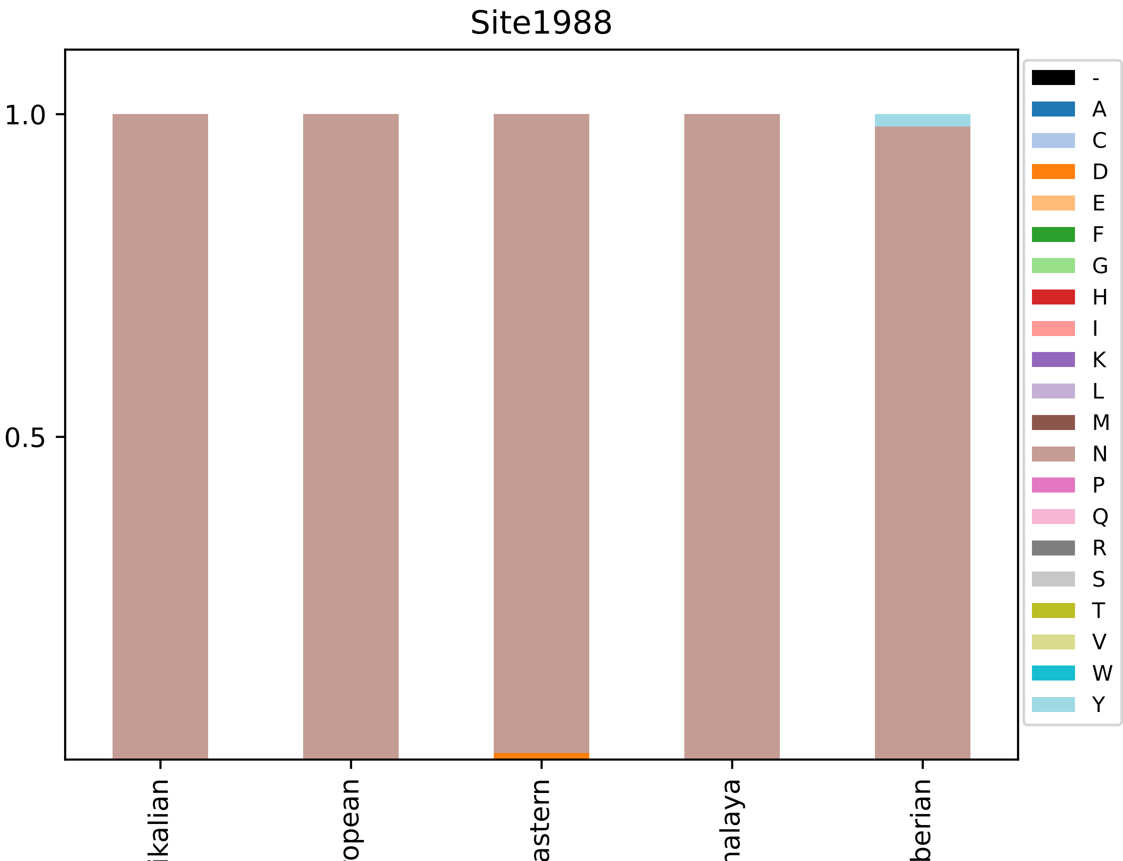

Site1988
1.0
0.5
-
A
C
D
E
F
G
H
I
K
L
M
N
P
Q
R
S
T
V
W
Y
ikalian
opean
astern
nalaya
berian

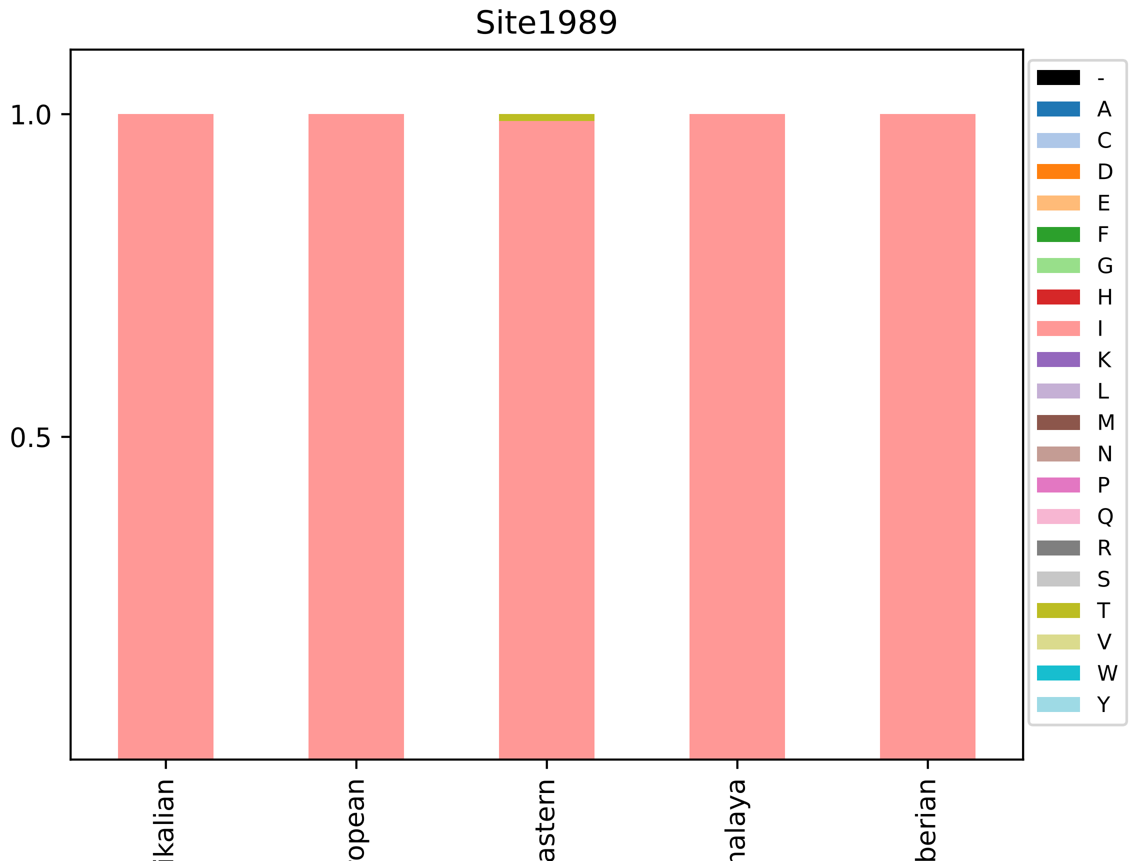
Site1989
1.0
0.5
-
A
C
D
E
F
G
H
I
K
L
M
N
P
Q
R
S
T
V
W
Y
ikalian
opean
astern
nalaya
berian

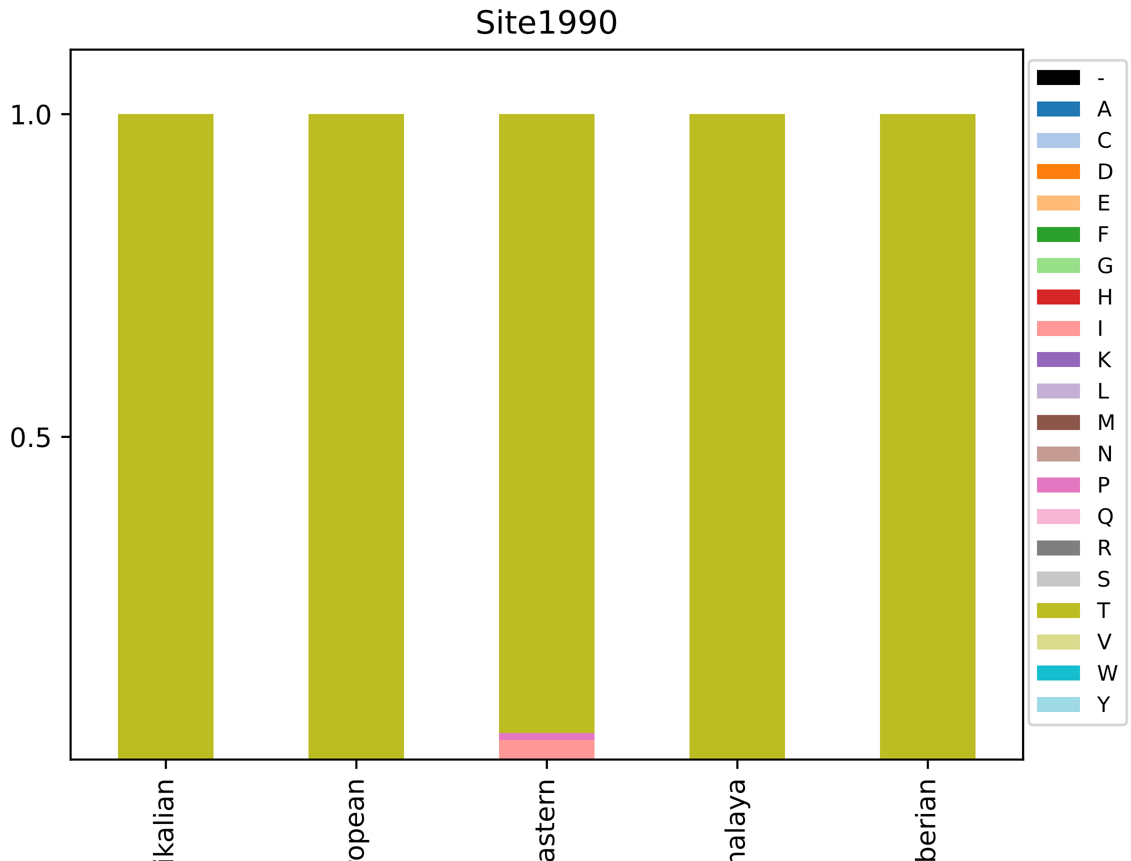

Site1990
1.0
0.5
ikalian
opean
astern
nalaya
berian
-
A
C
D
E
F
G
H
I
K
L
M
N
P
Q
R
S
T
V
W
Y

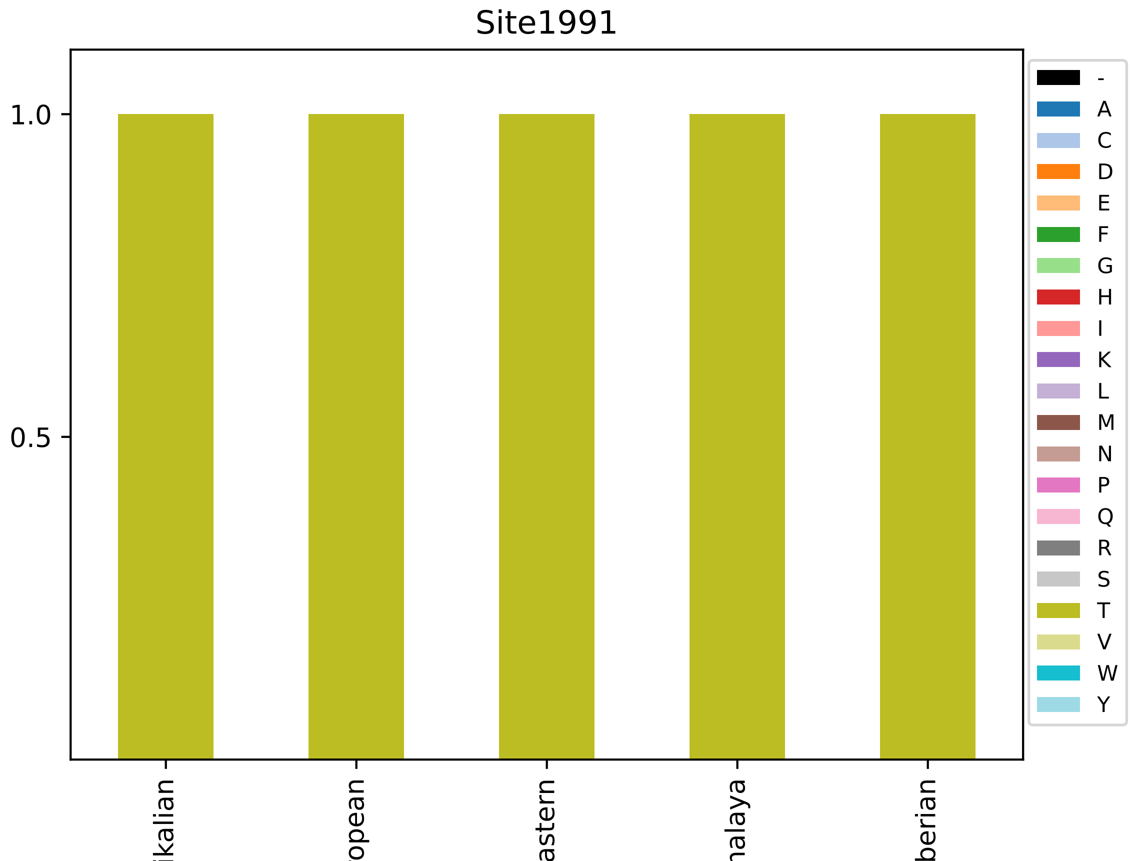

Site1991
1.0
0.5
ikalian
opean
astern
nalaya
berian
-
A
C
D
E
F
G
H
I
K
L
M
N
P
Q
R
S
T
V
W
Y

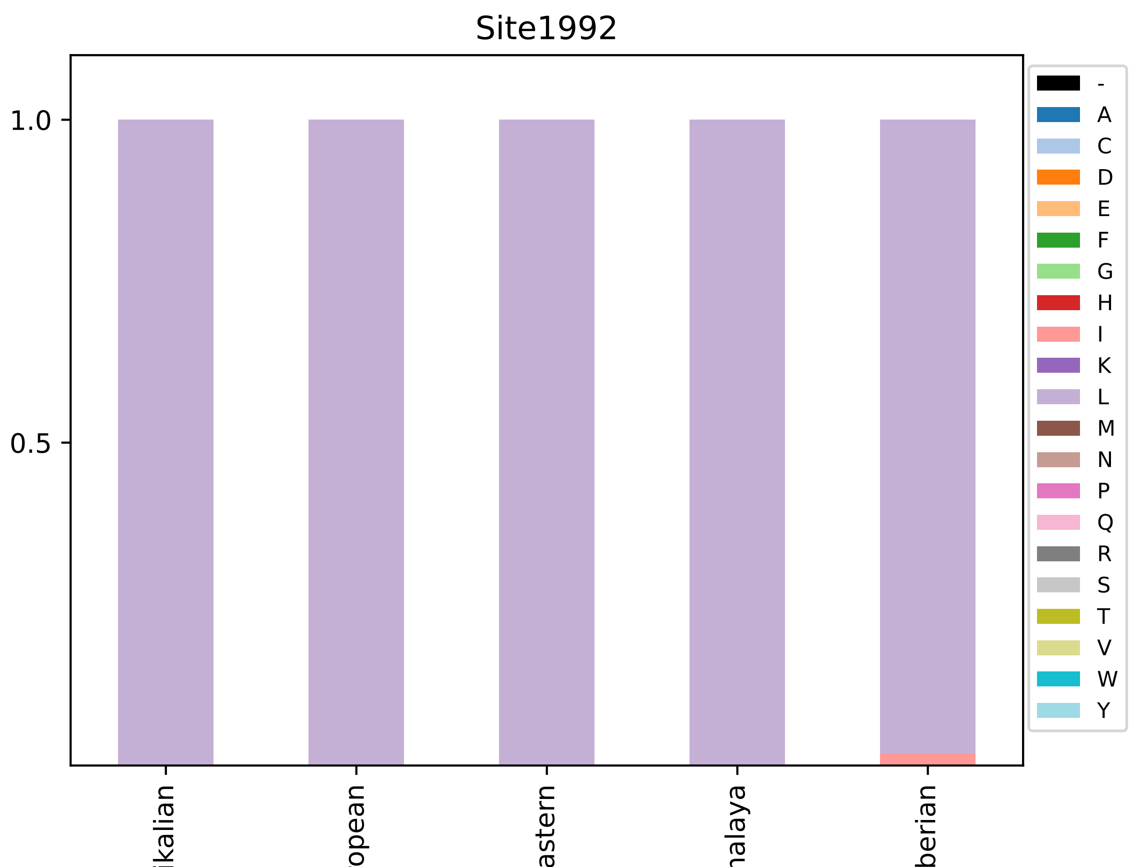

Site1992
1.0
0.5
-
A
C
D
E
F
G
H
I
K
L
M
N
P
Q
R
S
T
V
W
Y
ikalian
opean
astern
nalaya
berian

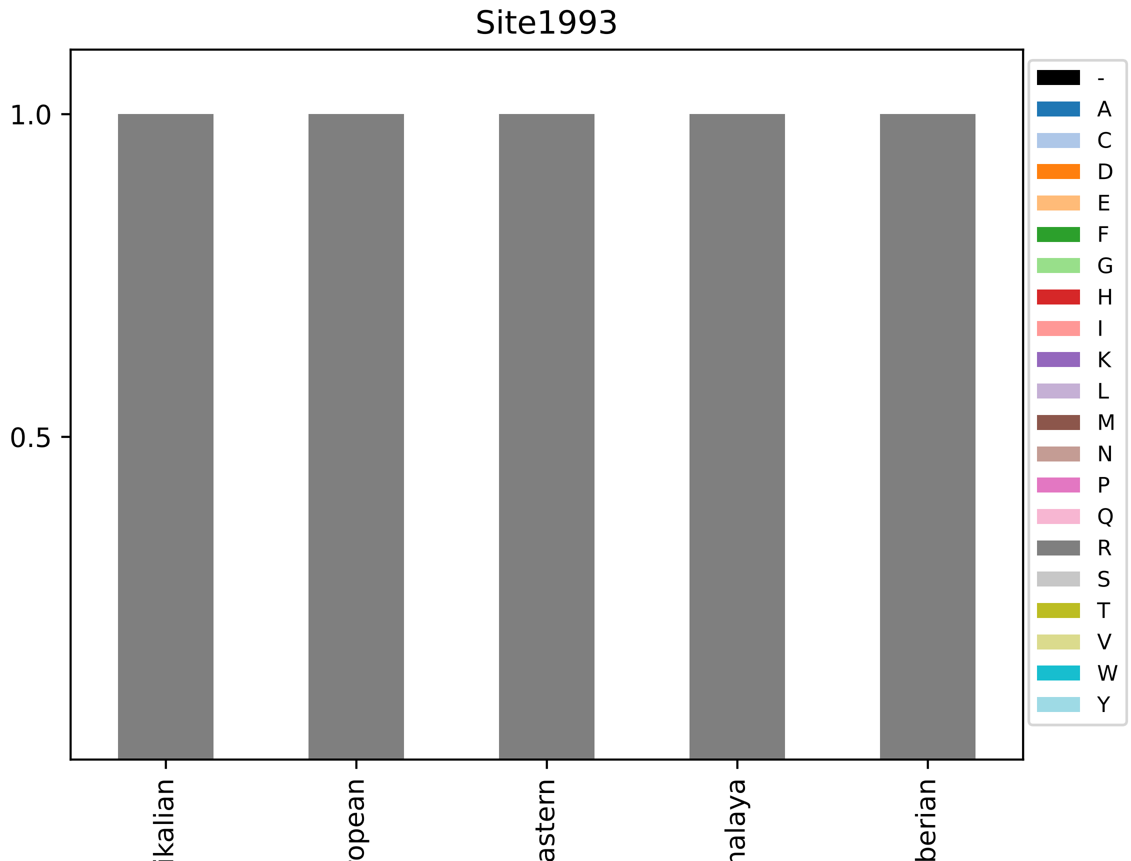

Site1993
1.0
0.5
-
A
C
D
E
F
G
H
I
K
L
M
N
P
Q
R
S
T
V
W
Y
ikalian
ropean
astern
nalaya
berian

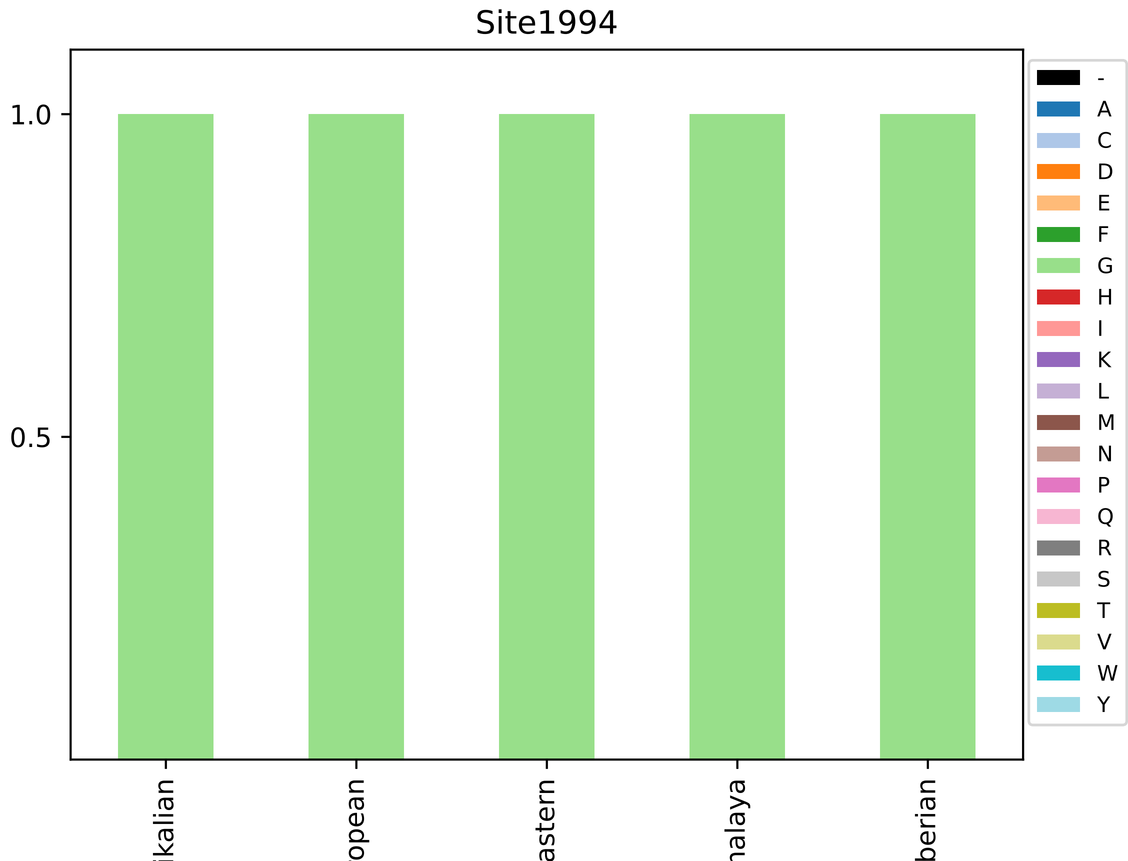

Site1994
1.0
0.5
-
A
C
D
E
F
G
H
I
K
L
M
N
P
Q
R
S
T
V
W
Y
ikalian
opean
astern
nalaya
berian

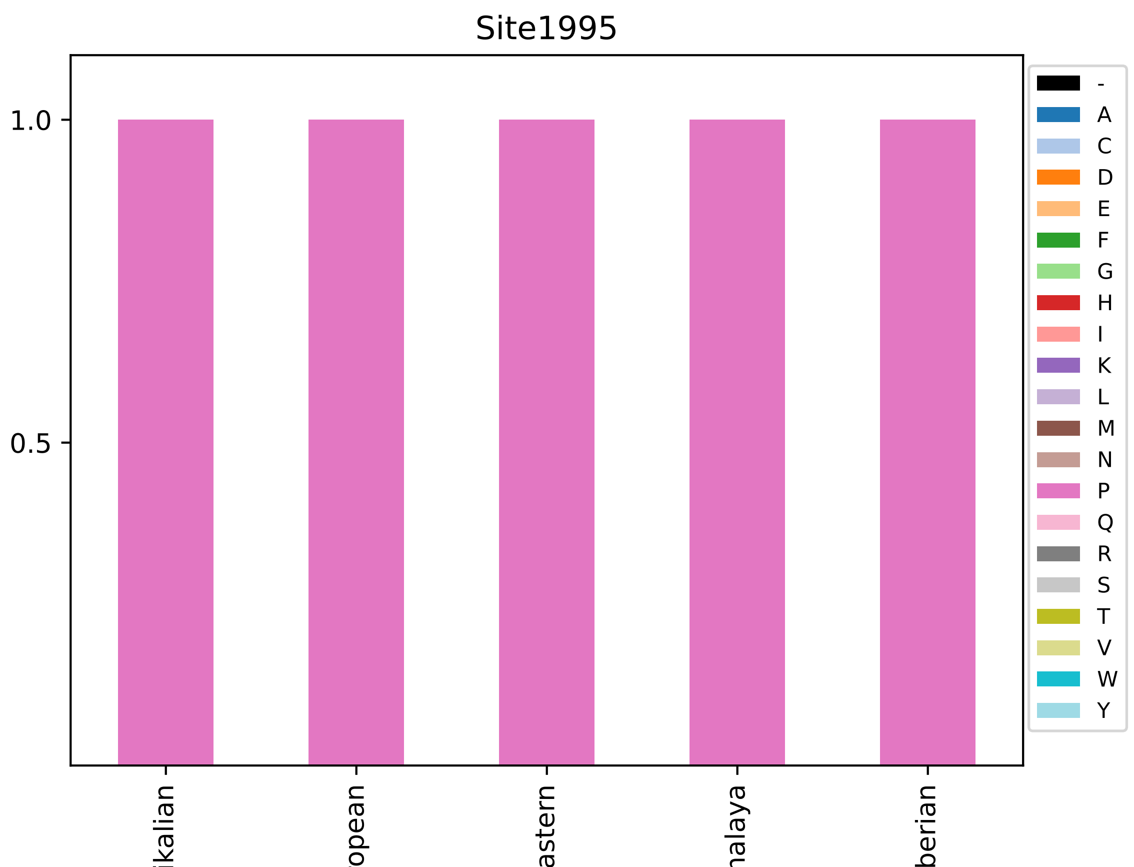
Site1995
1.0
0.5
-
A
C
D
E
F
G
H
I
K
L
M
N
P
Q
R
S
T
V
W
Y
ikalian
opean
astern
nalaya
berian

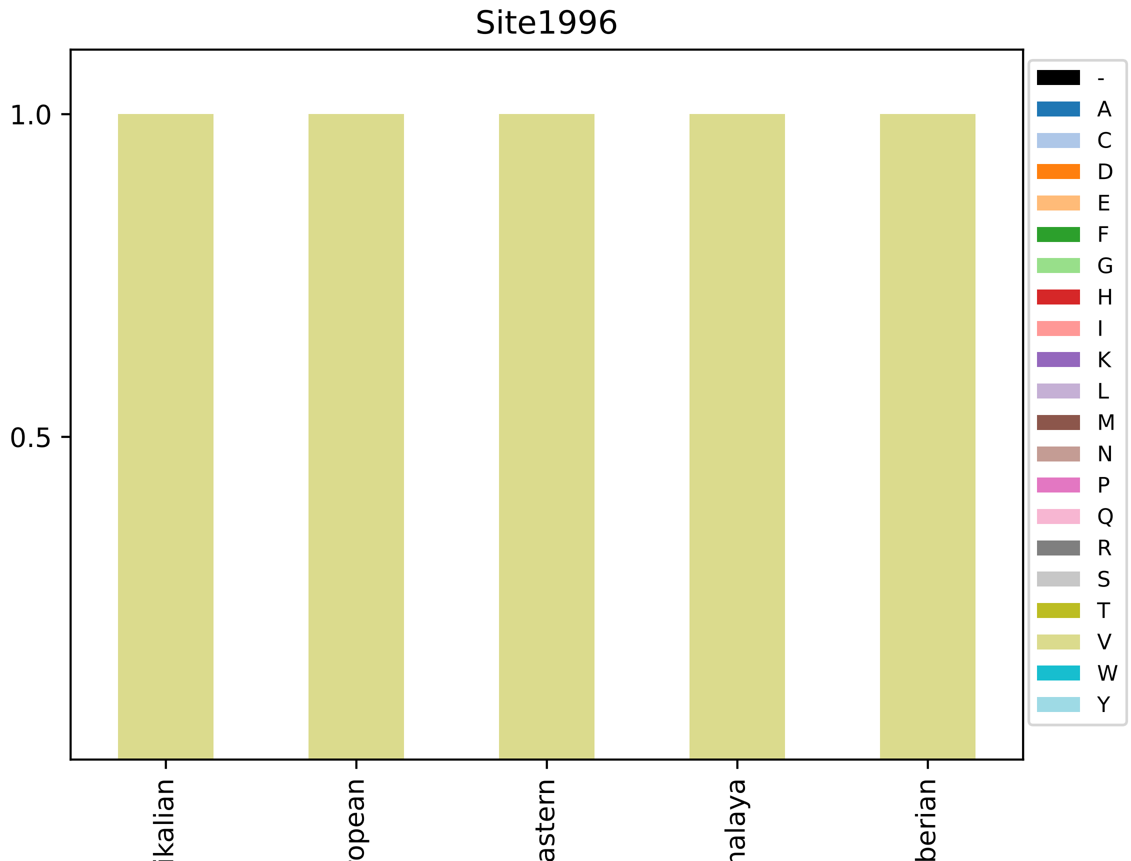
Site1996
1.0
0.5
ikalian
ropean
astern
nalaya
berian
-
A
C
D
E
F
G
H
I
K
L
M
N
P
Q
R
S
T
V
W
Y

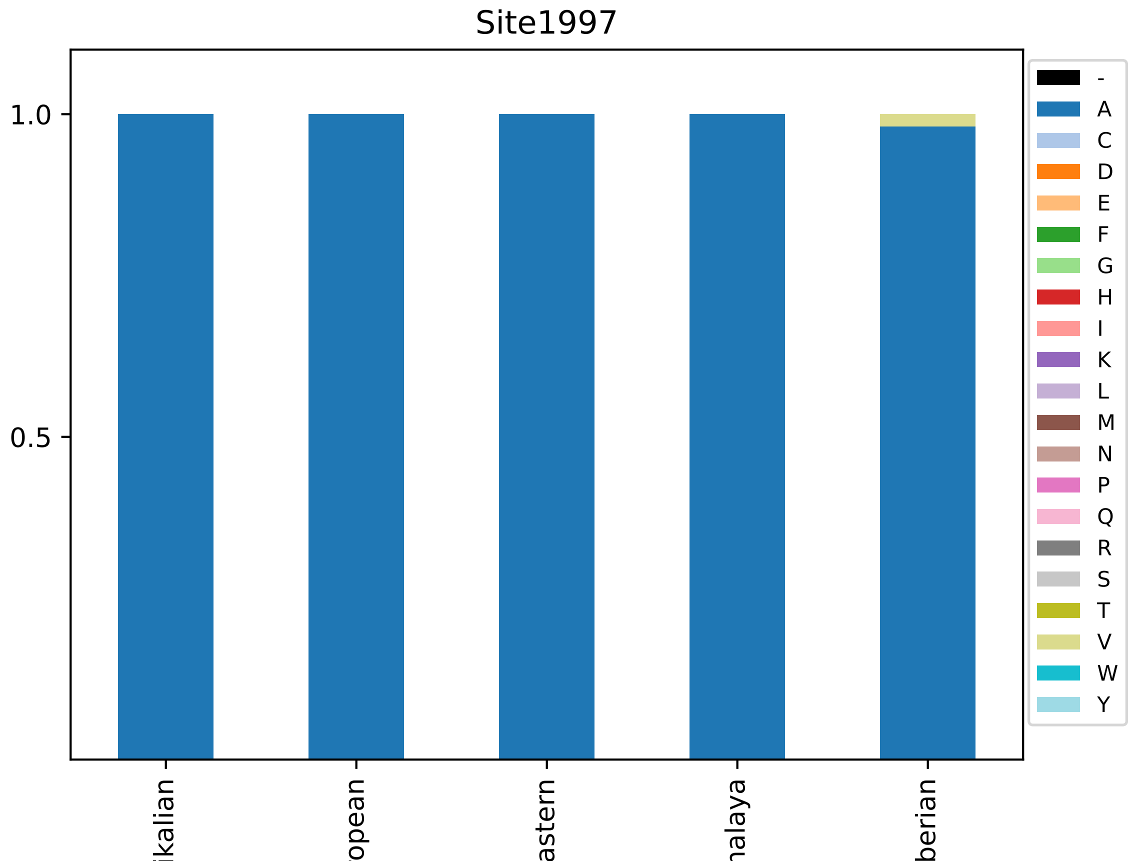
Site1997
1.0
0.5
-
A
C
D
E
F
G
H
I
K
L
M
N
P
Q
R
S
T
V
W
Y
ikalian
opean
astern
nalaya
berian

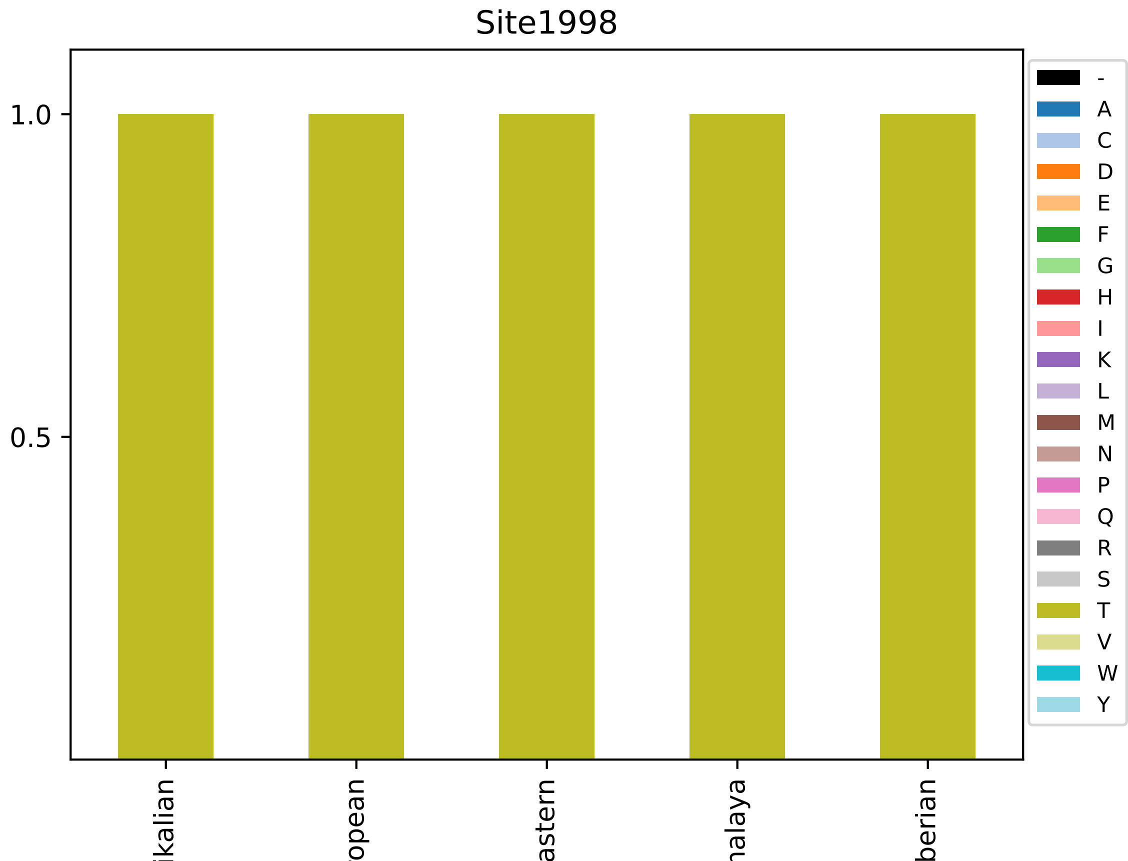

Site1998
1.0
0.5
-
A
C
D
E
F
G
H
I
K
L
M
N
P
Q
R
S
T
V
W
Y
ikalian
opean
astern
nalaya
berian

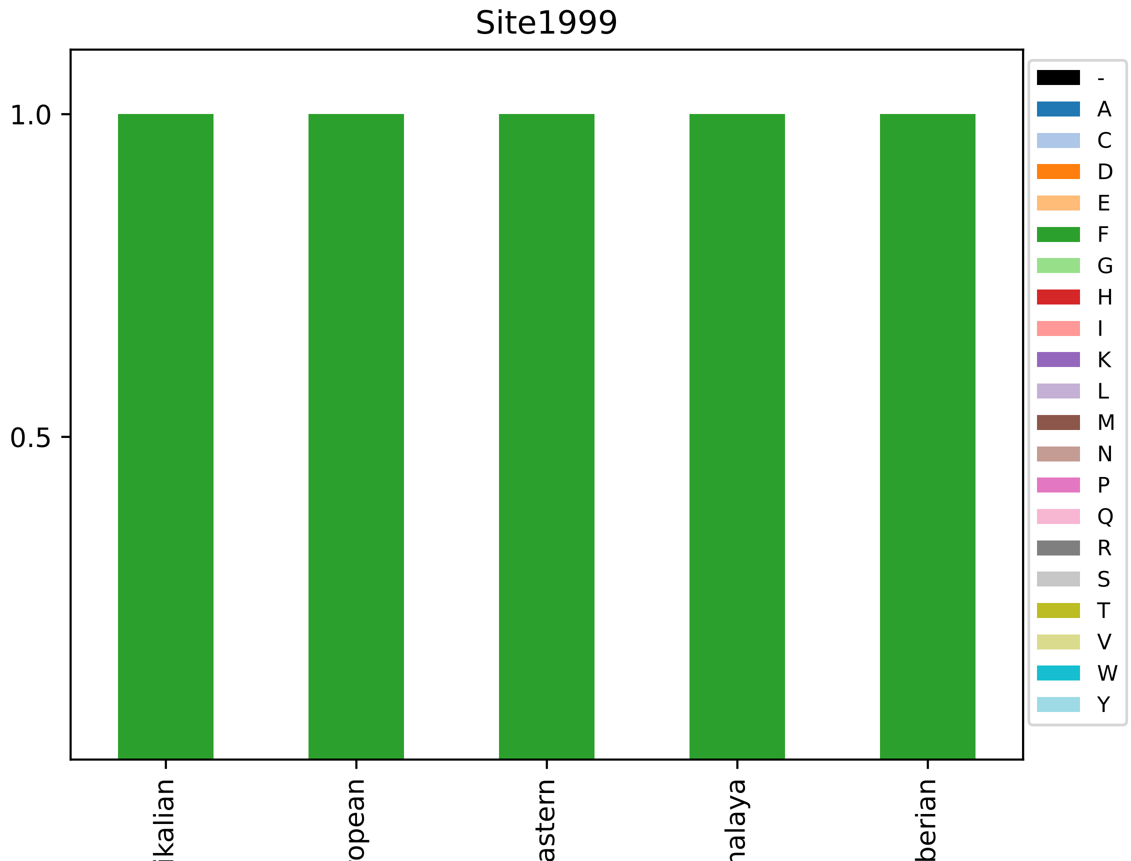
Site1999
1.0
0.5
-
A
C
D
E
F
G
H
I
K
L
M
N
P
Q
R
S
T
V
W
Y

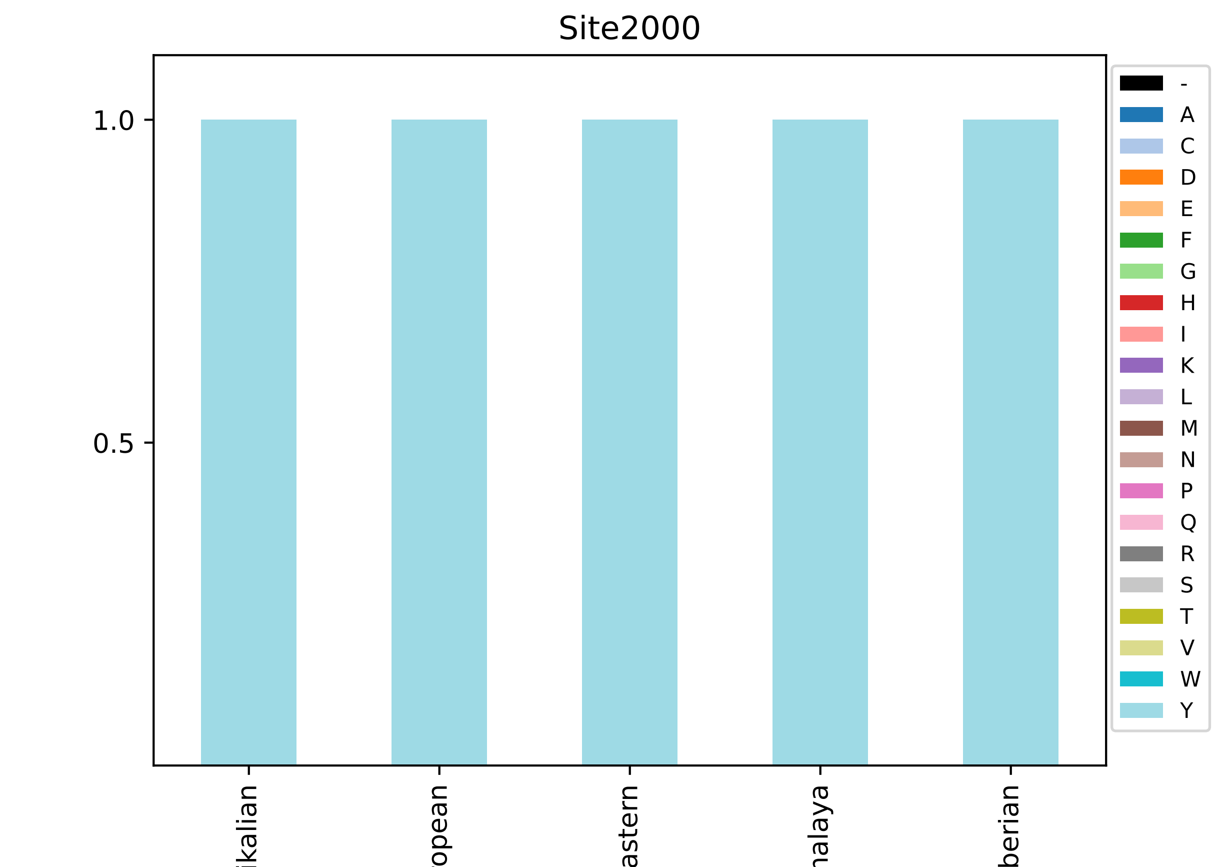
Site2000
1.0
0.5
-
A
C
D
E
F
G
H
I
K
L
M
N
P
Q
R
S
T
V
W
Y
ikalian
opean
astern
nalaya
berian

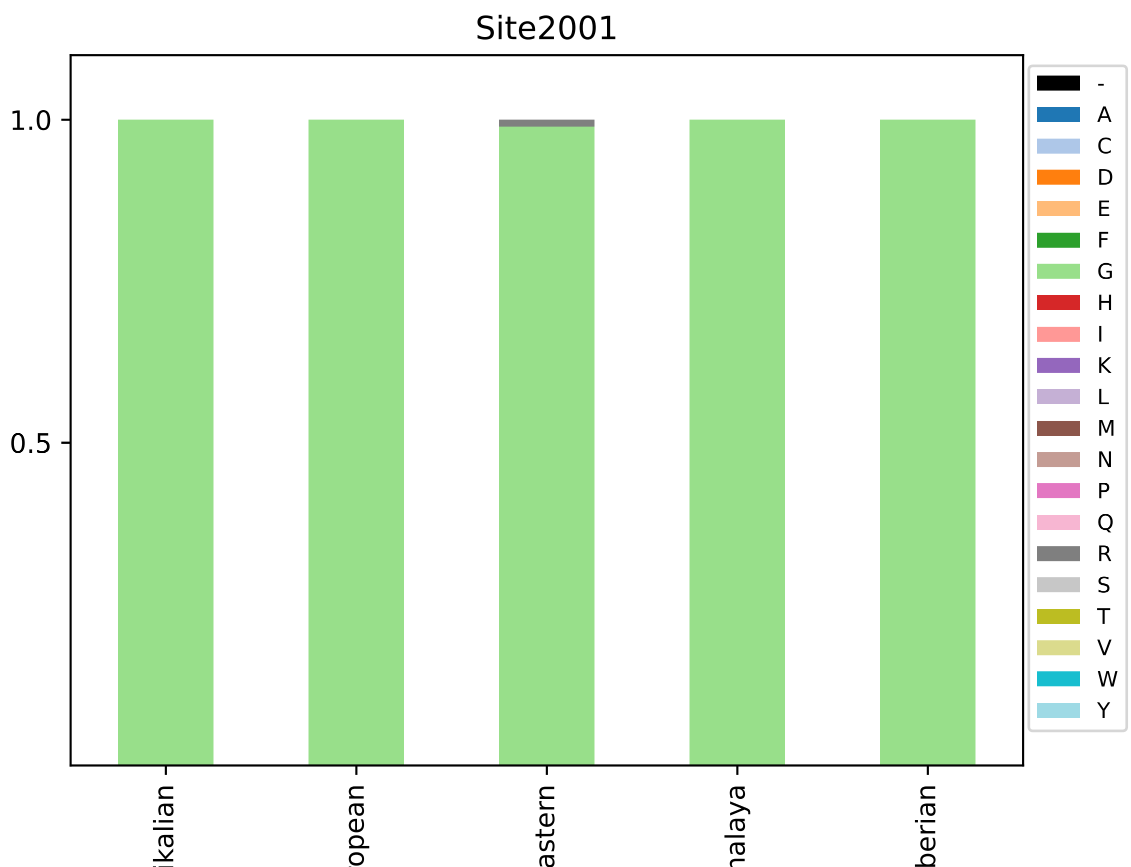

Site2001
1.0
0.5
-
A
C
D
E
F
G
H
I
K
L
M
N
P
Q
R
S
T
V
W
Y
ikalian
opean
astern
nalaya
berian

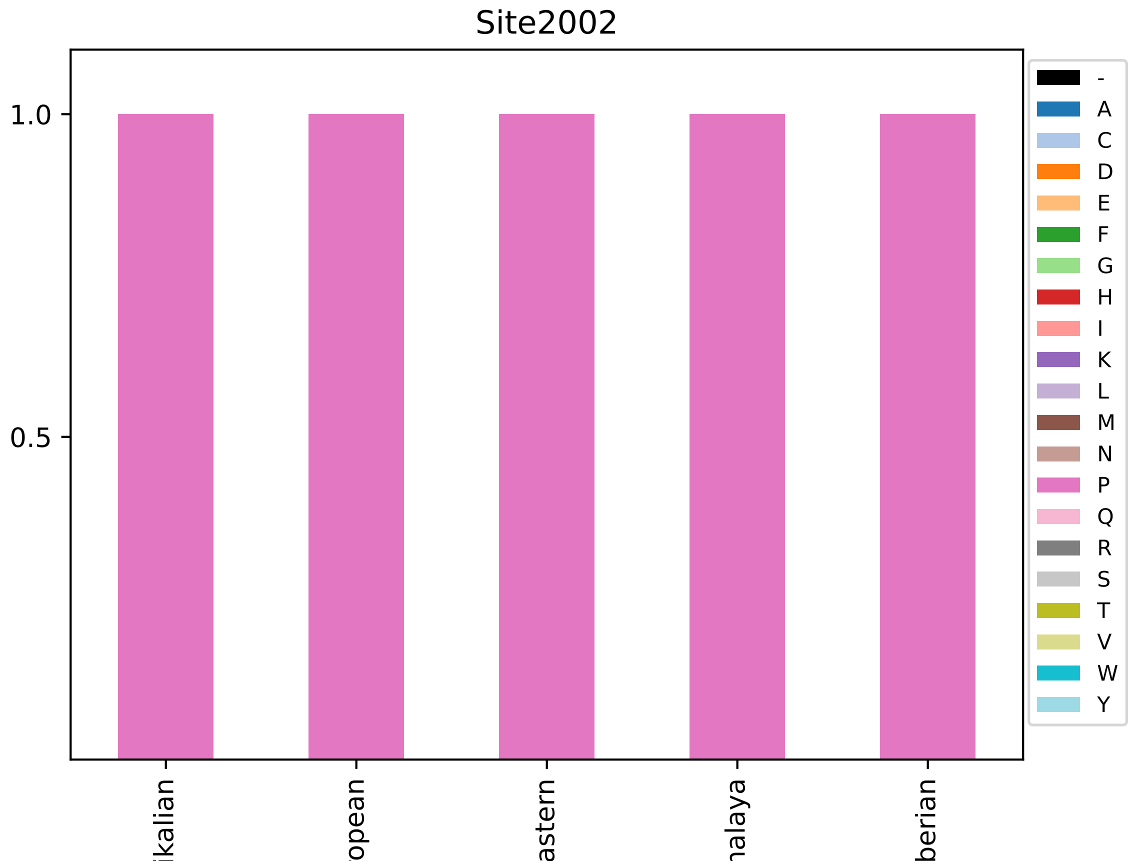
Site2002
1.0
0.5
-
A
C
D
E
F
G
H
I
K
L
M
N
P
Q
R
S
T
V
W
Y
ikalian
opean
astern
nalaya
berian

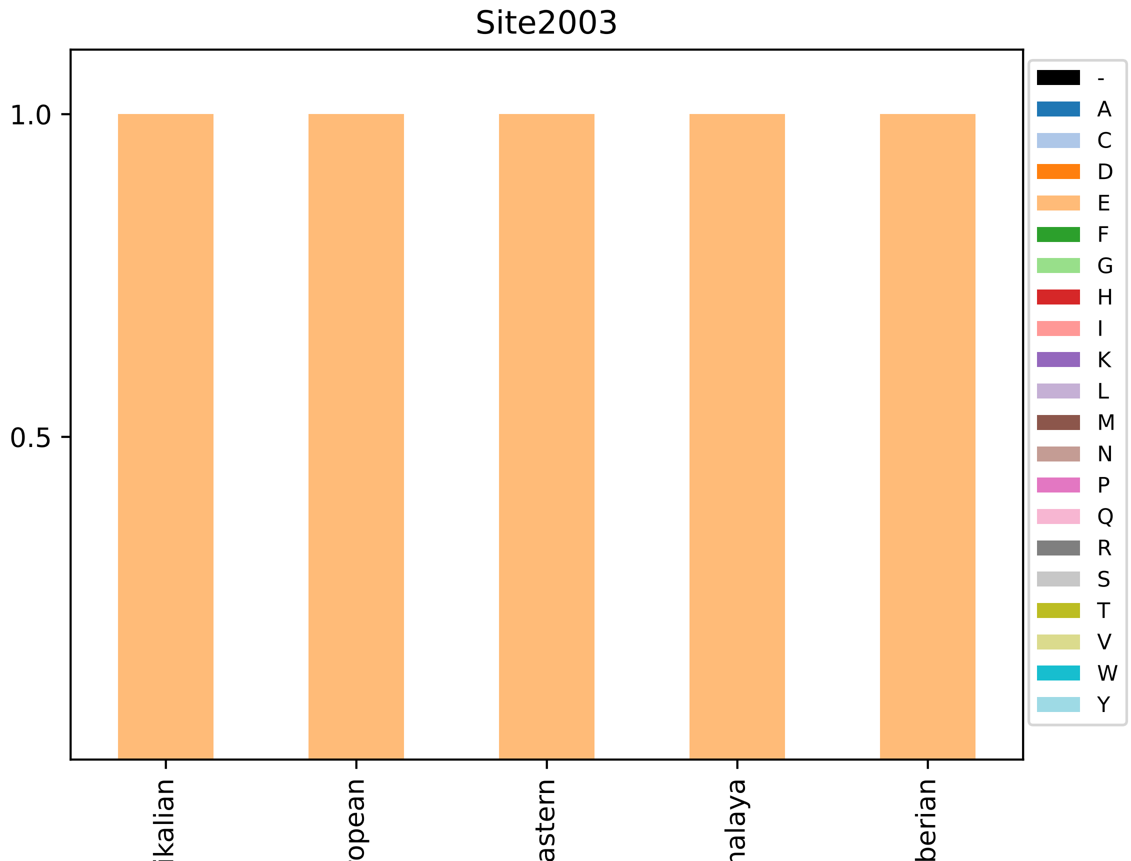

Site2003
1.0
0.5
-
A
C
D
E
F
G
H
I
K
L
M
N
P
Q
R
S
T
V
W
Y

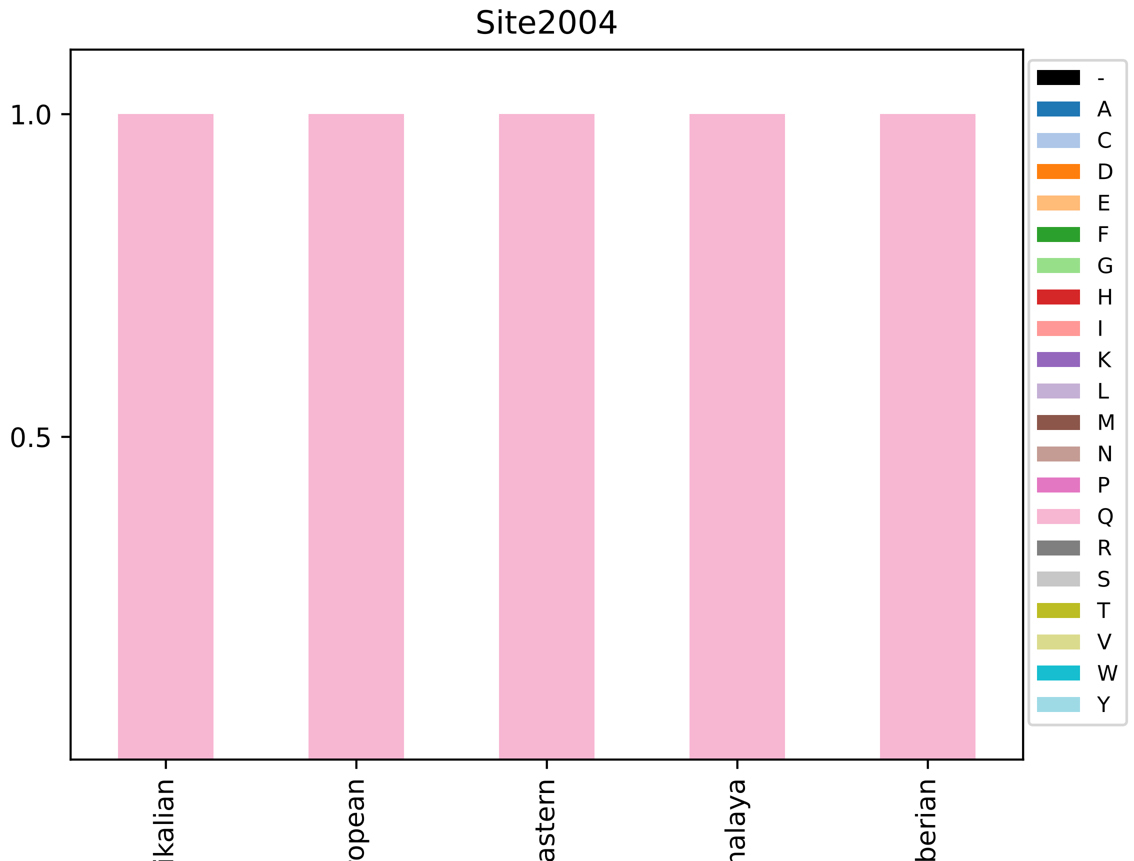

Site2004
1.0
0.5
-
A
C
D
E
F
G
H
I
K
L
M
N
P
Q
R
S
T
V
W
Y
ikalian
ropean
astern
nalaya
berian

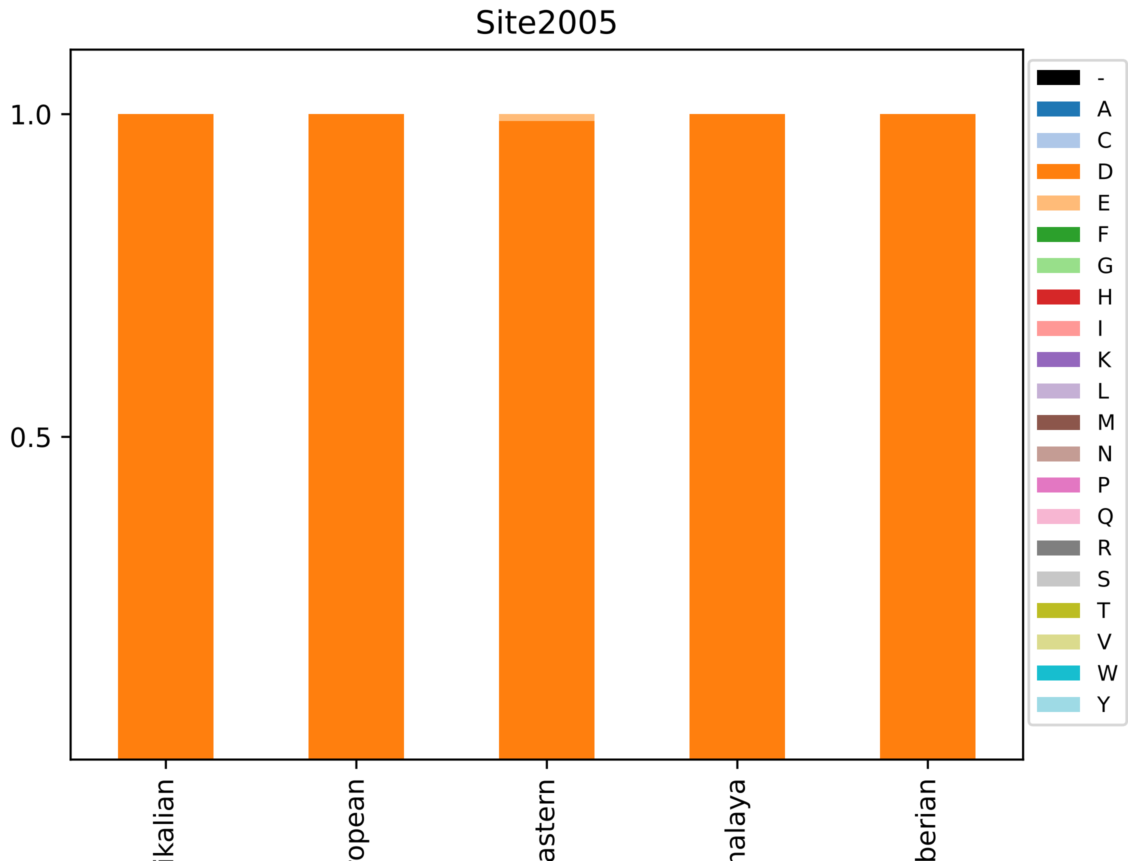

Site2005
1.0
0.5
-
A
C
D
E
F
G
H
I
K
L
M
N
P
Q
R
S
T
V
W
Y
ikalian
opean
astern
nalaya
berian

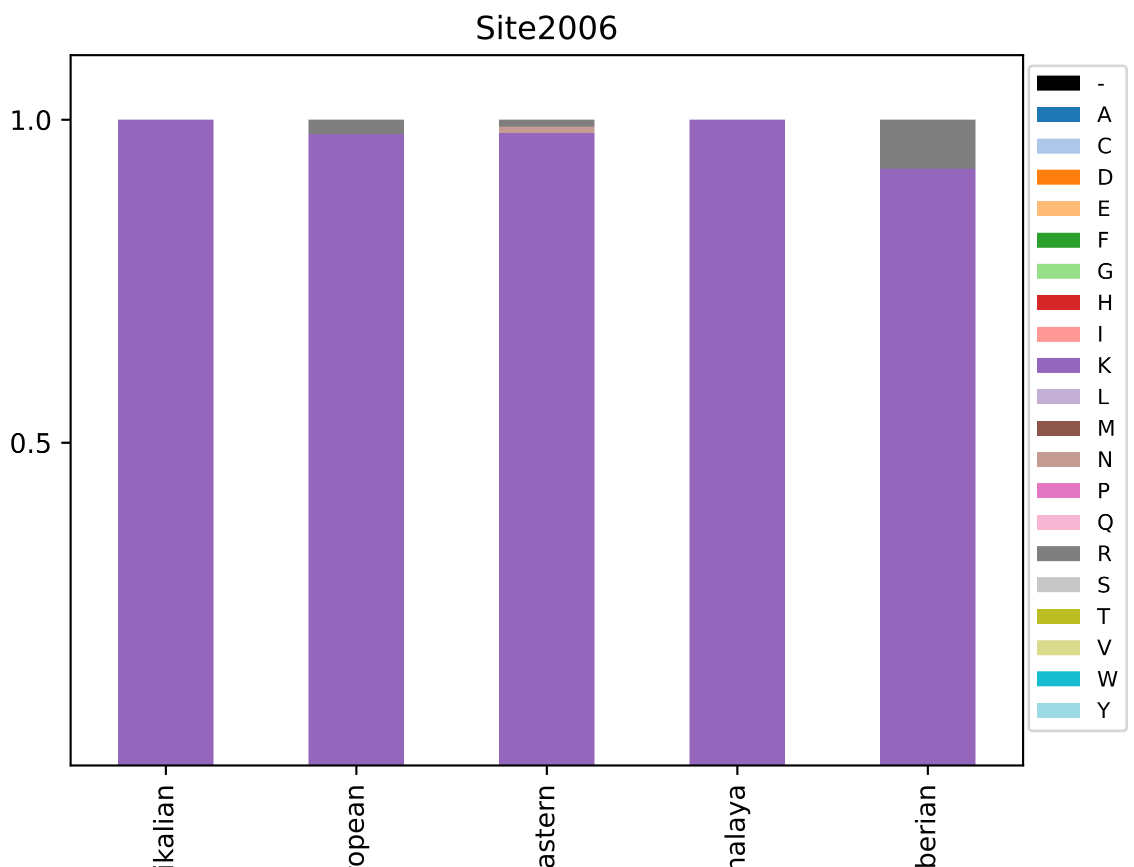

Site2006
1.0
0.5
-
A
C
D
E
F
G
H
I
K
L
M
N
P
Q
R
S
T
V
W
Y
ikalian
opean
astern
nalaya
berian

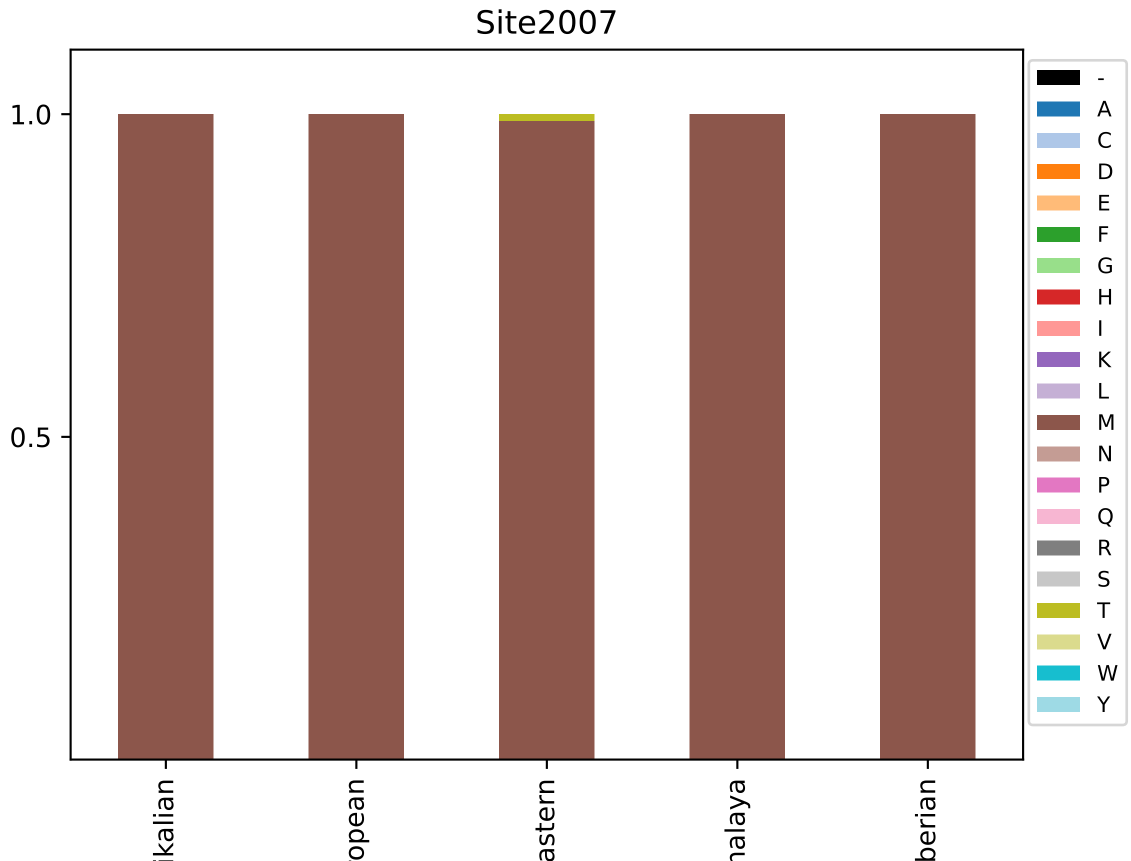

Site2007
1.0
0.5
-
A
C
D
E
F
G
H
I
K
L
M
N
P
Q
R
S
T
V
W
Y
ikalian
opean
astern
nalaya
berian

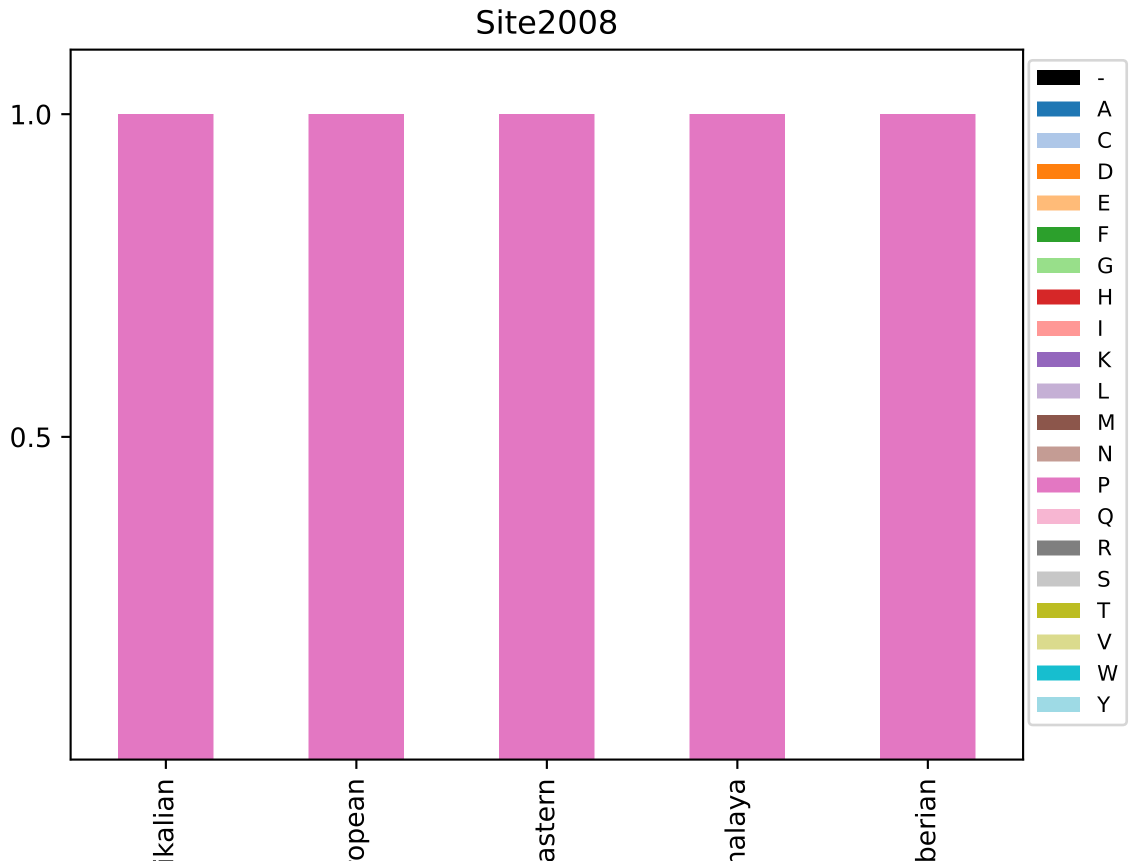
Site2008
1.0
0.5
ikalian
opean
astern
nalaya
berian
-
A
C
D
E
F
G
H
I
K
L
M
N
P
Q
R
S
T
V
W
Y

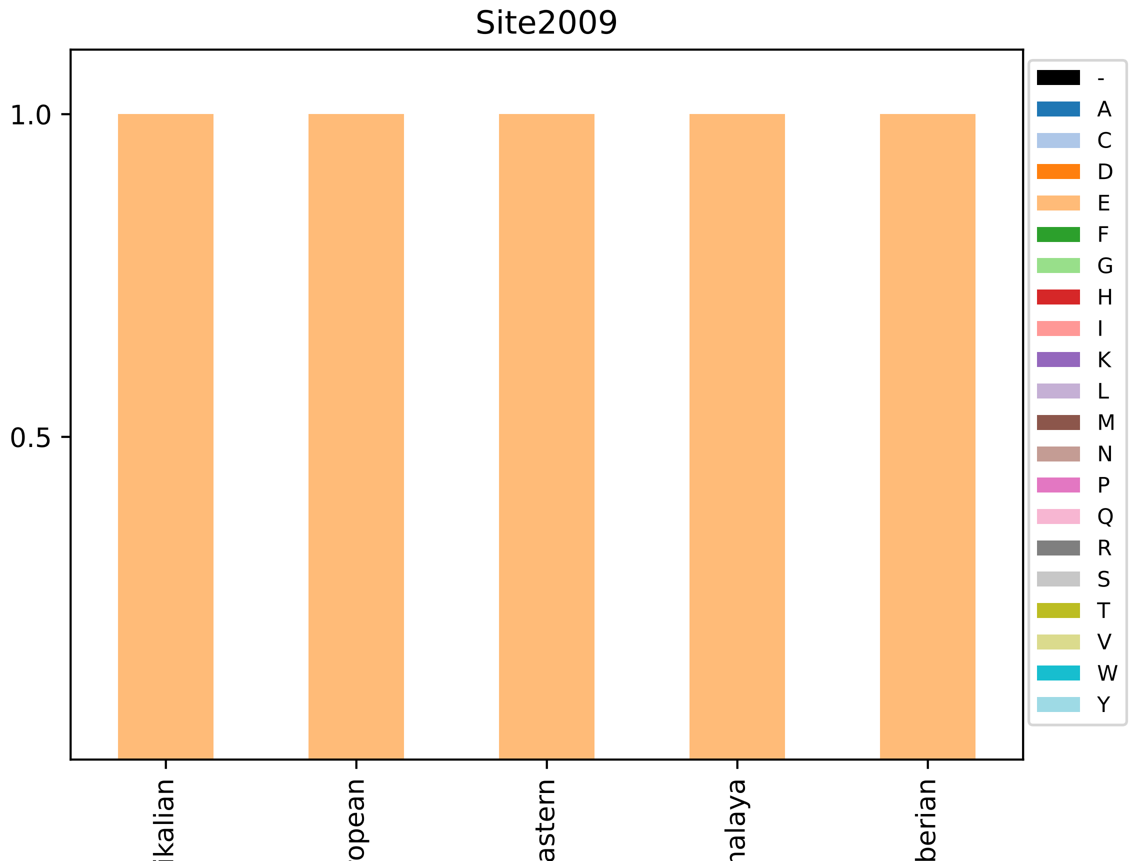

Site2009
1.0
0.5
-
A
C
D
E
F
G
H
I
K
L
M
N
P
Q
R
S
T
V
W
Y
ikalian
opean
astern
nalaya
berian

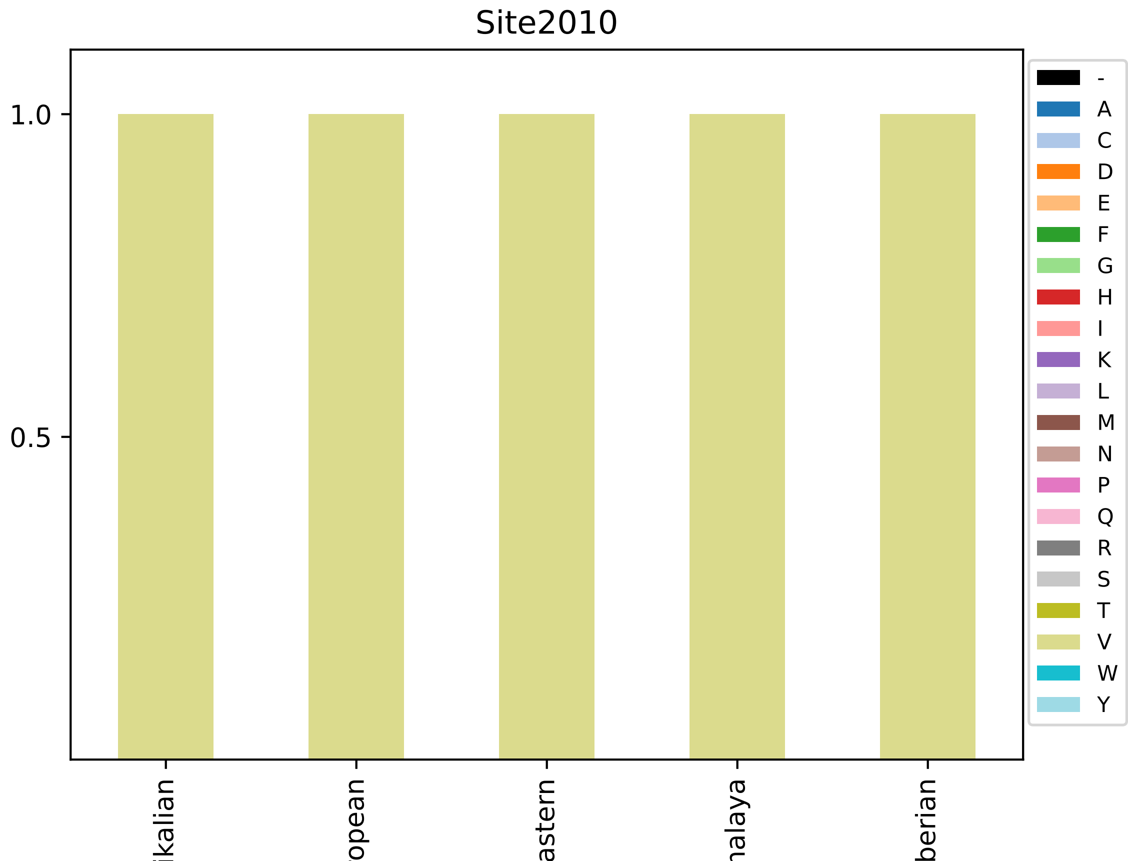

Site2010
1.0
0.5
-
A
C
D
E
F
G
H
I
K
L
M
N
P
Q
R
S
T
V
W
Y
ikalian
opean
astern
nalaya
berian

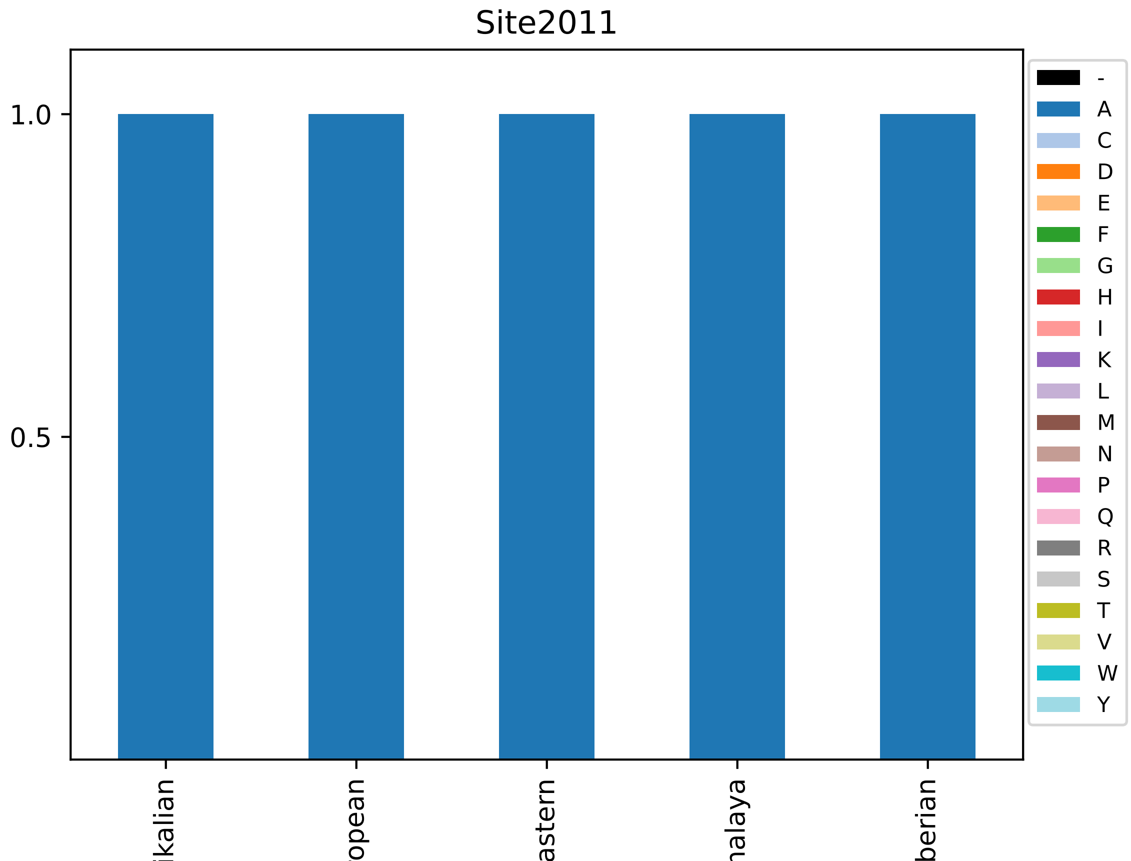

Site2011
1.0
0.5
ikalian
ropean
astern
nalaya
berian
-
A
C
D
E
F
G
H
I
K
L
M
N
P
Q
R
S
T
V
W
Y

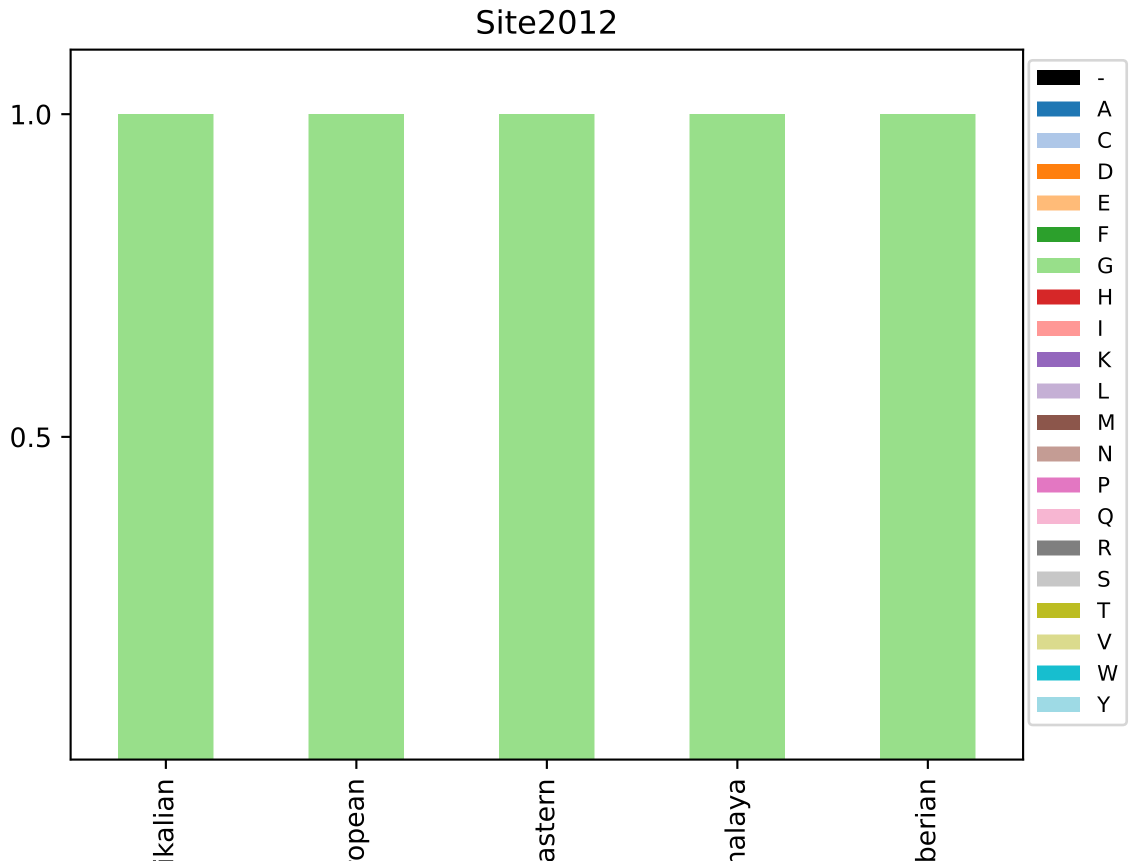
Site2012
1.0
0.5
-
A
C
D
E
F
G
H
I
K
L
M
N
P
Q
R
S
T
V
W
Y
ikalian
opean
astern
nalaya
berian

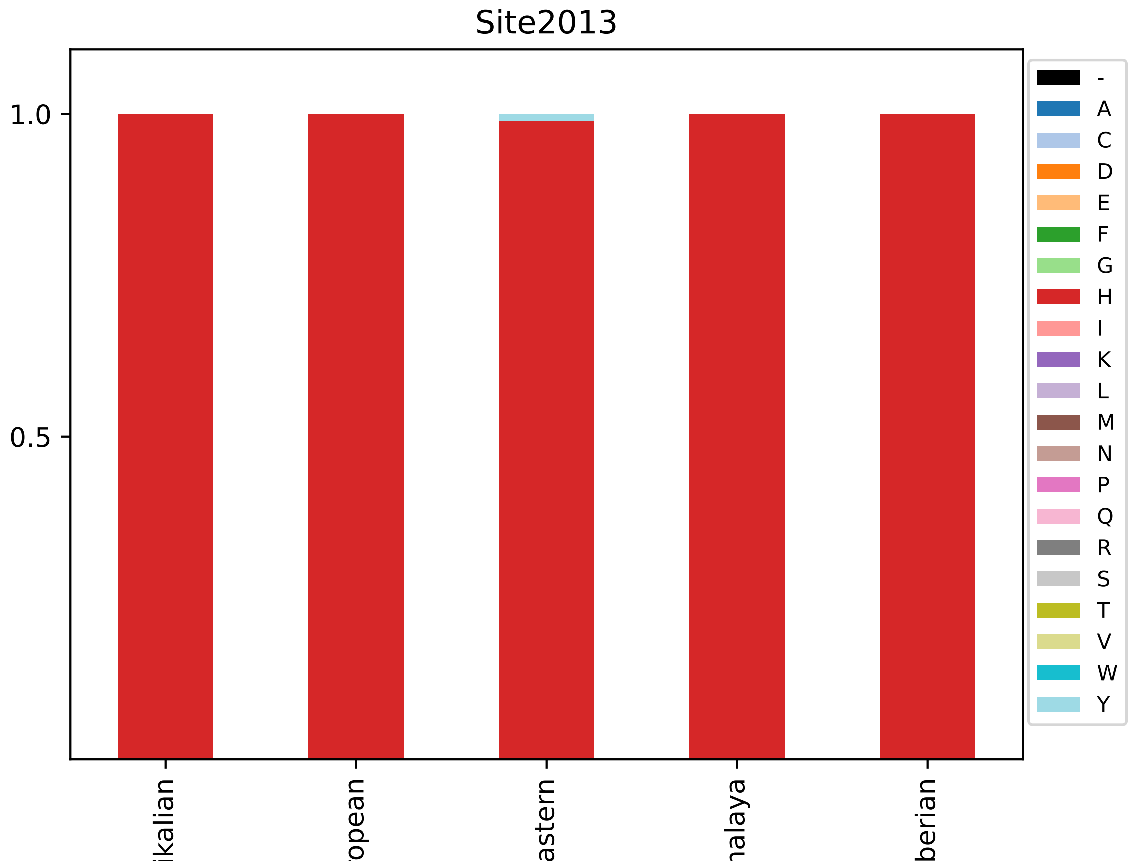
Site2013
1.0
0.5
-
A
C
D
E
F
G
H
I
K
L
M
N
P
Q
R
S
T
V
W
Y
ikalian
opean
astern
nalaya
berian

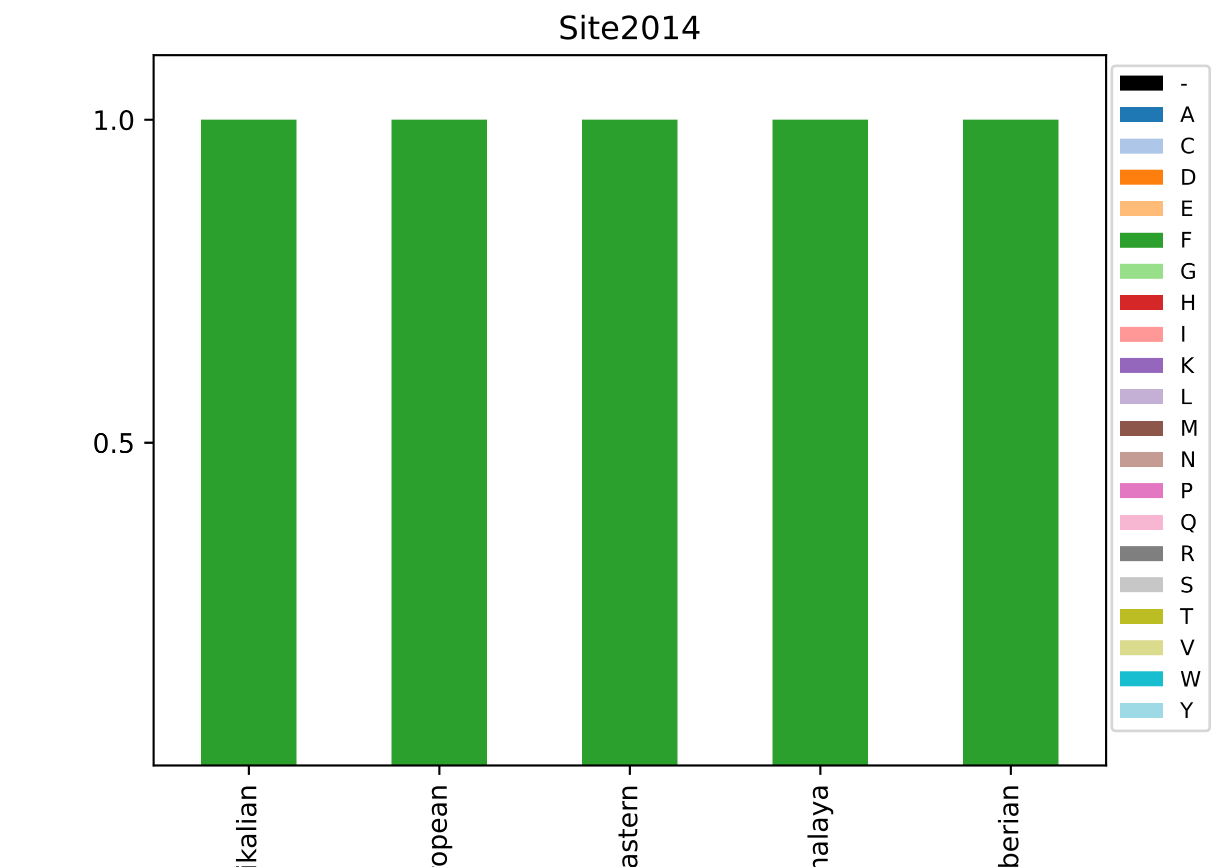

Site2014
1.0
0.5
-
A
C
D
E
F
G
H
I
K
L
M
N
P
Q
R
S
T
V
W
Y
ikalian
opean
astern
nalaya
berian

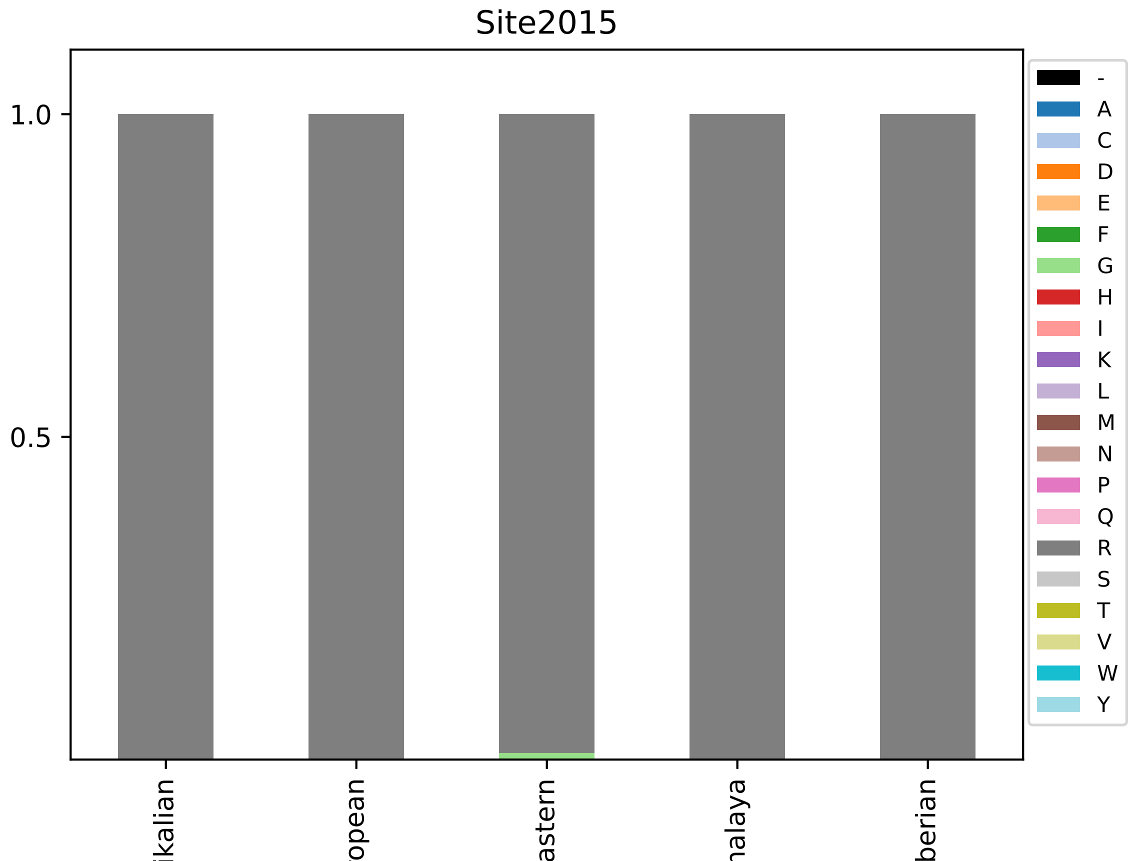

Site2015
1.0
0.5
-
A
C
D
E
F
G
H
I
K
L
M
N
P
Q
R
S
T
V
W
Y
ikalian
ropean
astern
nalaya
berian

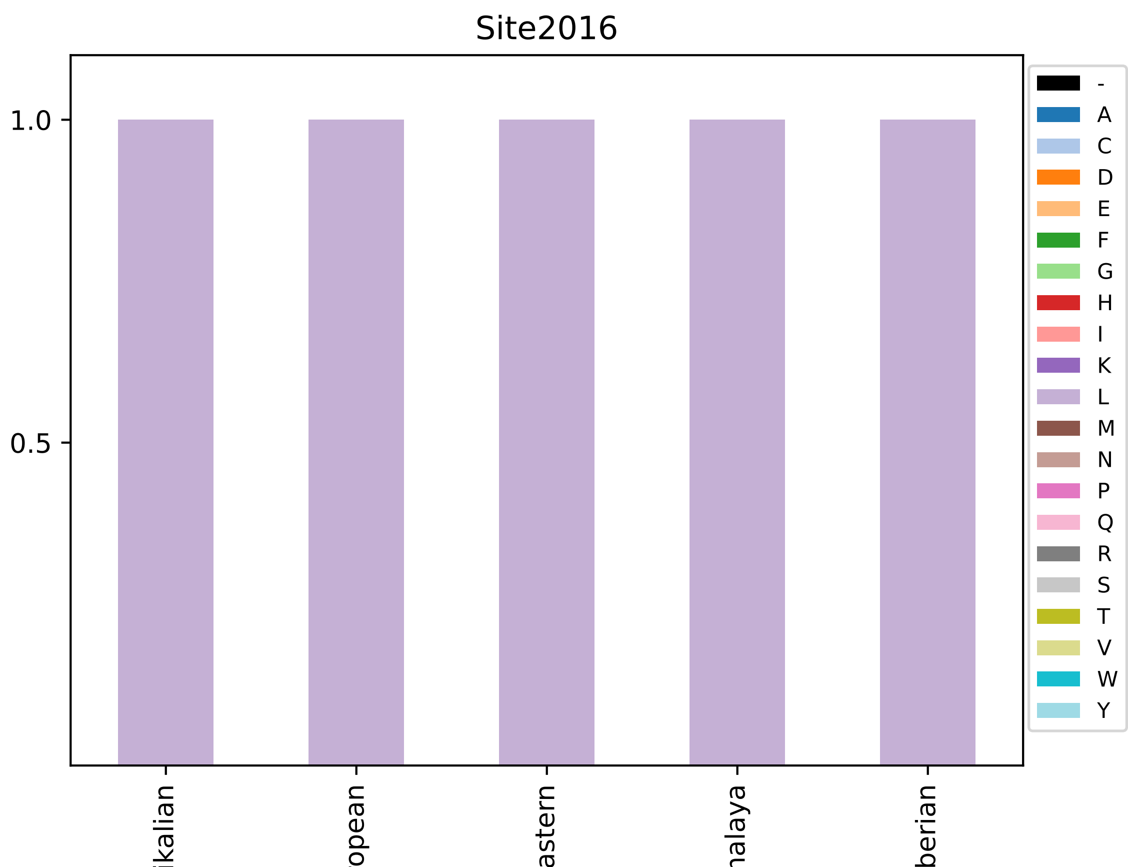
Site2016
1.0
0.5
-
A
C
D
E
F
G
H
I
K
L
M
N
P
Q
R
S
T
V
W
Y

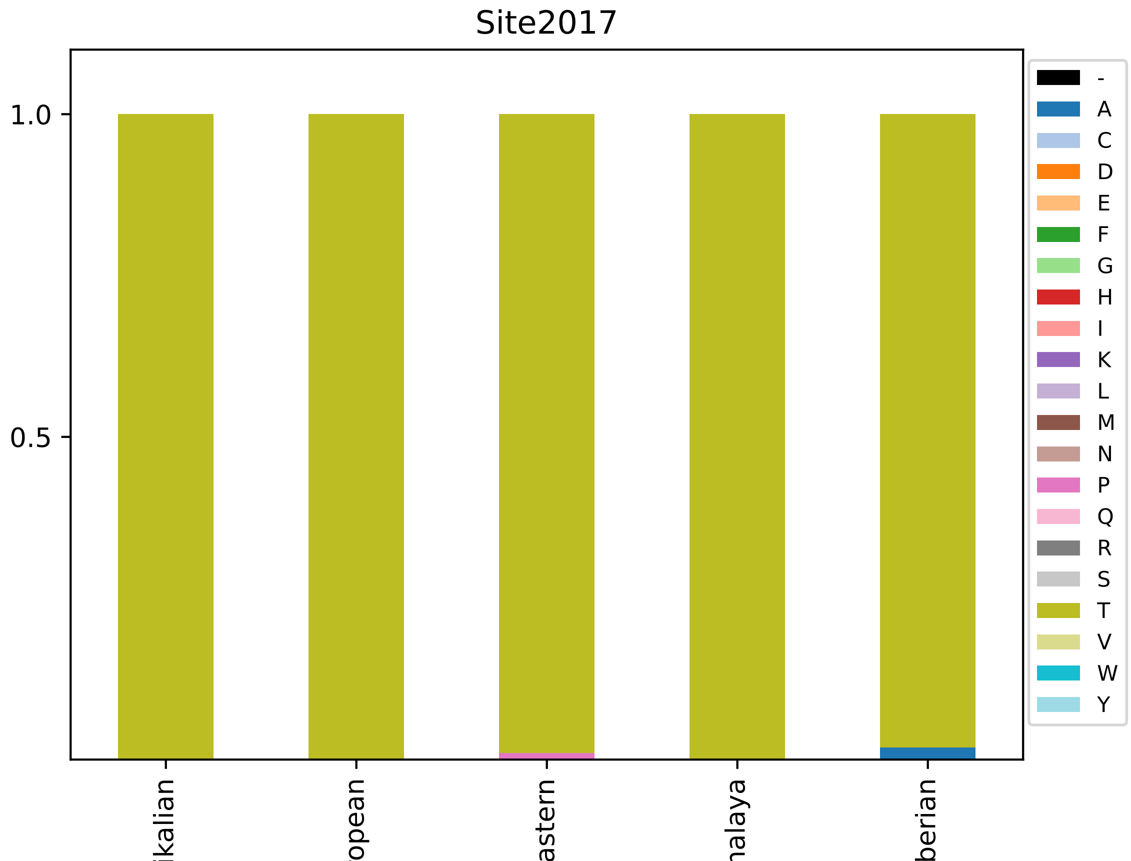

Site2017
1.0
0.5
-
A
C
D
E
F
G
H
I
K
L
M
N
P
Q
R
S
T
V
W
Y
ikalian
opean
astern
nalaya
berian

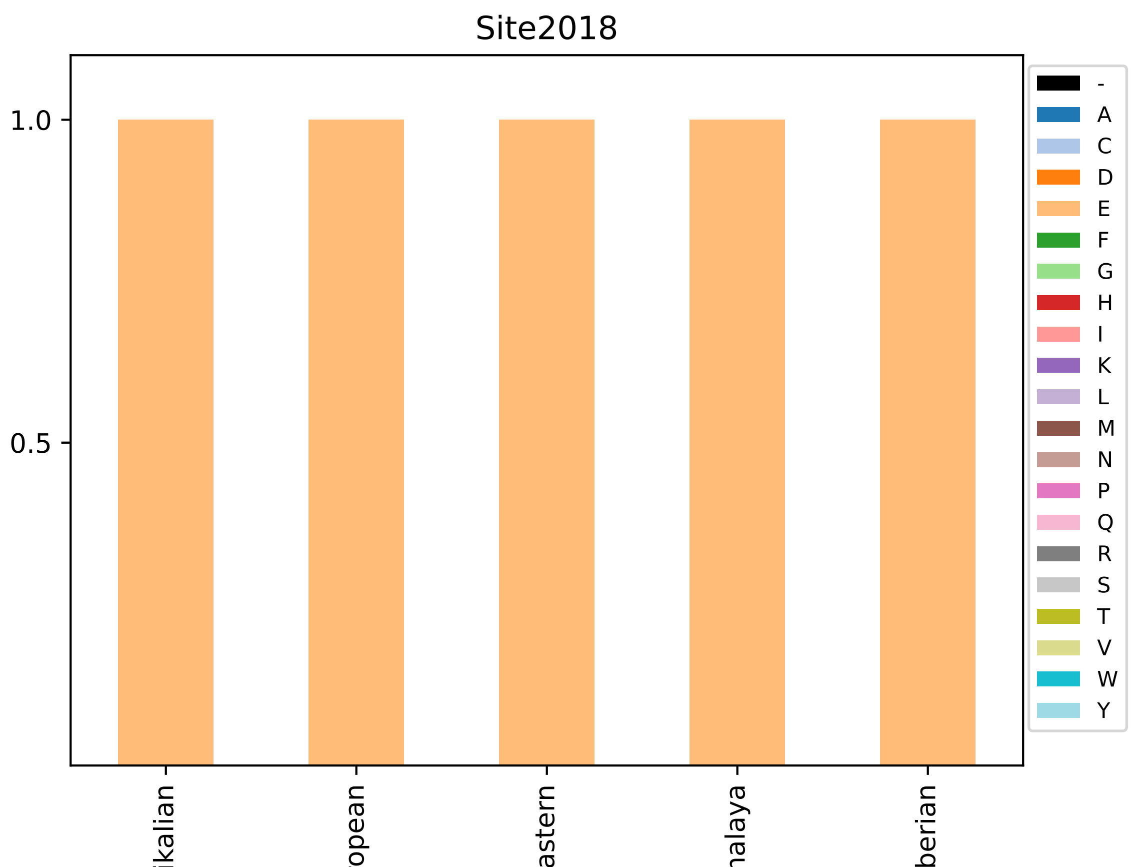

Site2018
1.0
0.5
-
A
C
D
E
F
G
H
I
K
L
M
N
P
Q
R
S
T
V
W
Y
ikalian
opean
astern
nalaya
berian

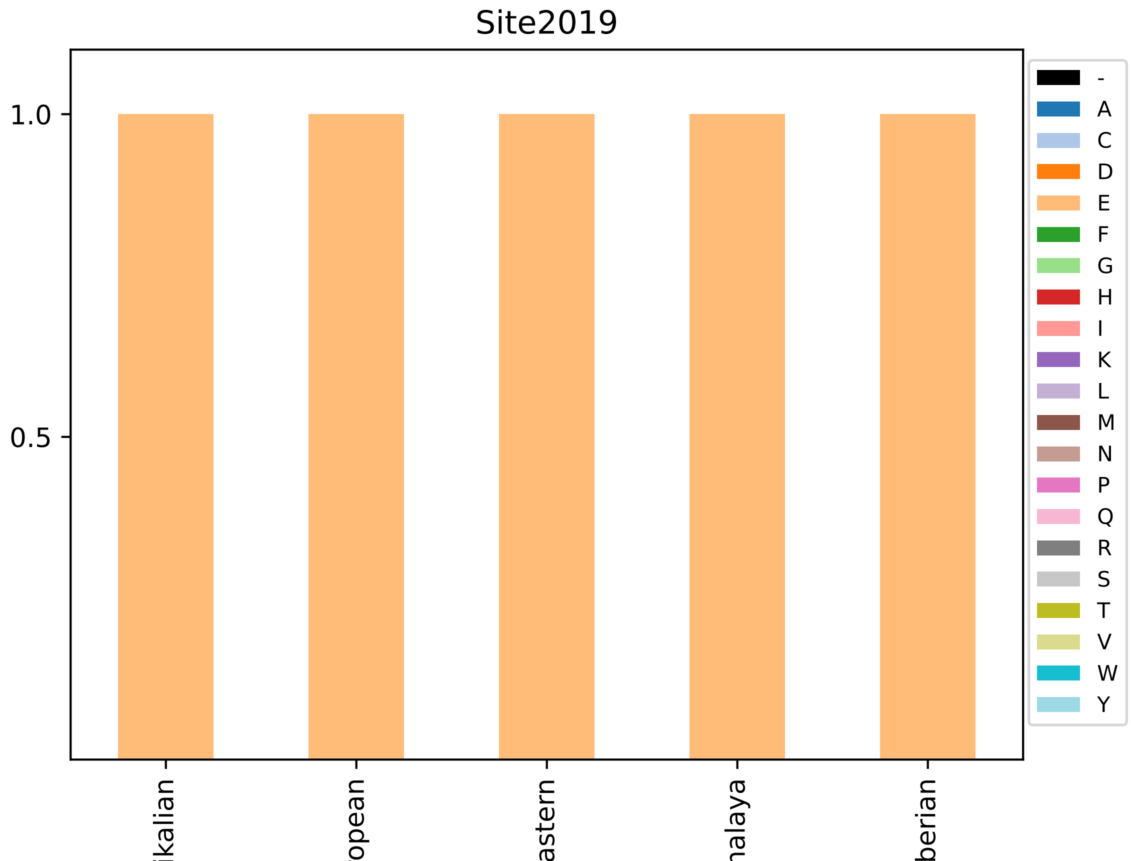

Site2019
1.0
0.5
-
A
C
D
E
F
G
H
I
K
L
M
N
P
Q
R
S
T
V
W
Y

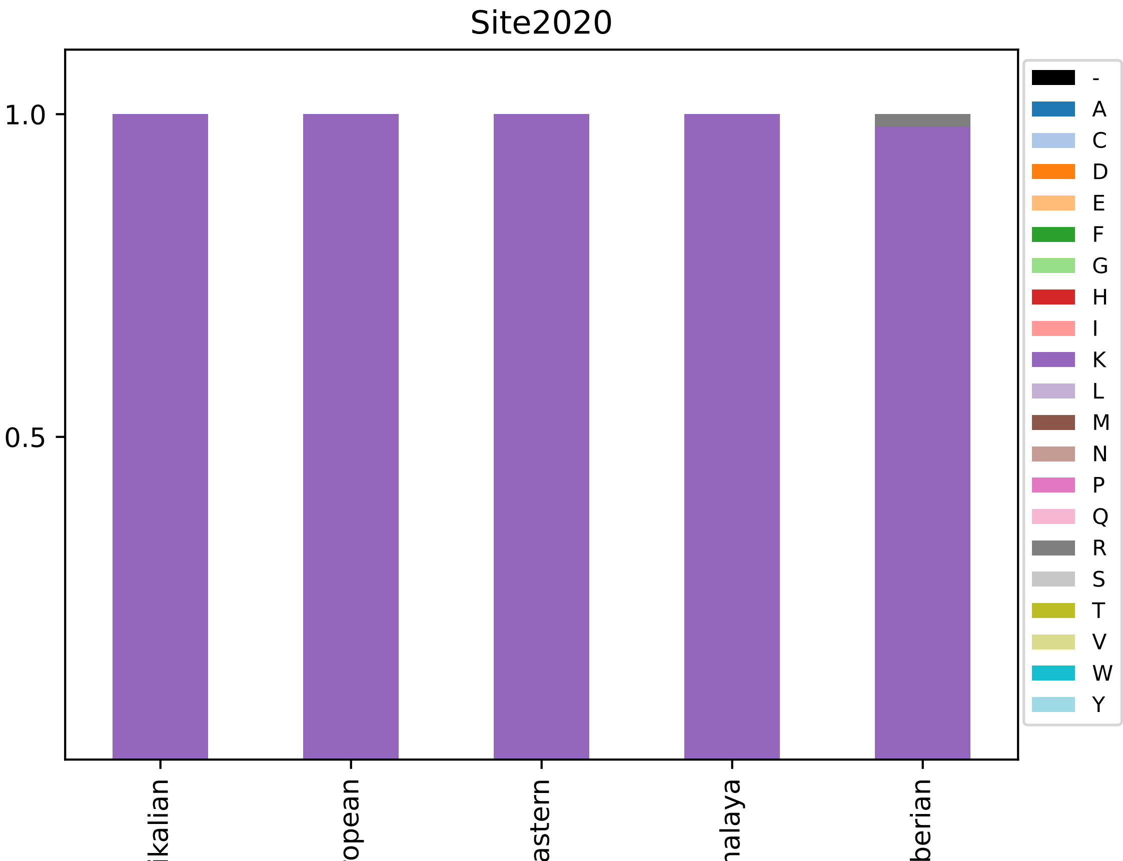
Site2020
1.0
0.5
-
A
C
D
E
F
G
H
I
K
L
M
N
P
Q
R
S
T
V
W
Y
ikalian
opean
astern
nalaya
berian

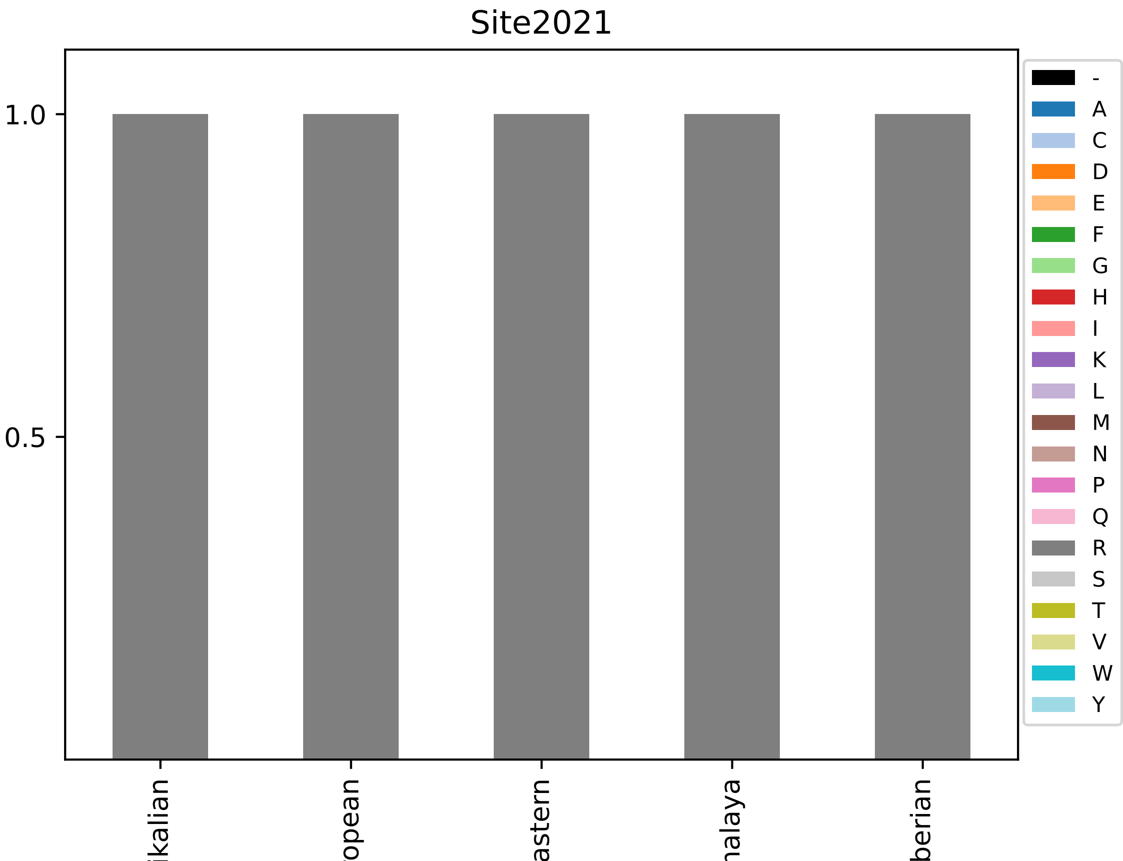
Site2021
1.0
0.5
ikalian
opean
astern
nalaya
berian
-
A
C
D
E
F
G
H
I
K
L
M
N
P
Q
R
S
T
V
W
Y

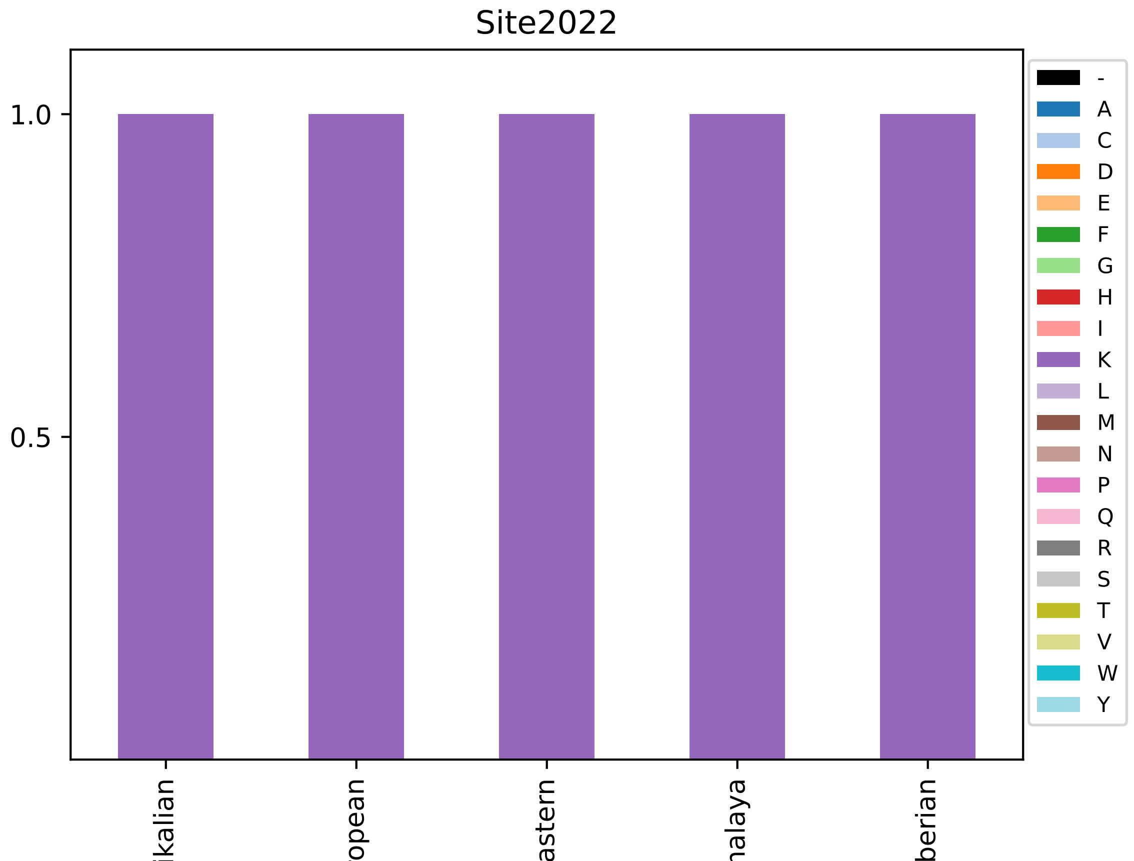

Site2022
1.0
0.5
-
A
C
D
E
F
G
H
I
K
L
M
N
P
Q
R
S
T
V
W
Y
ikalian
opean
astern
nalaya
berian

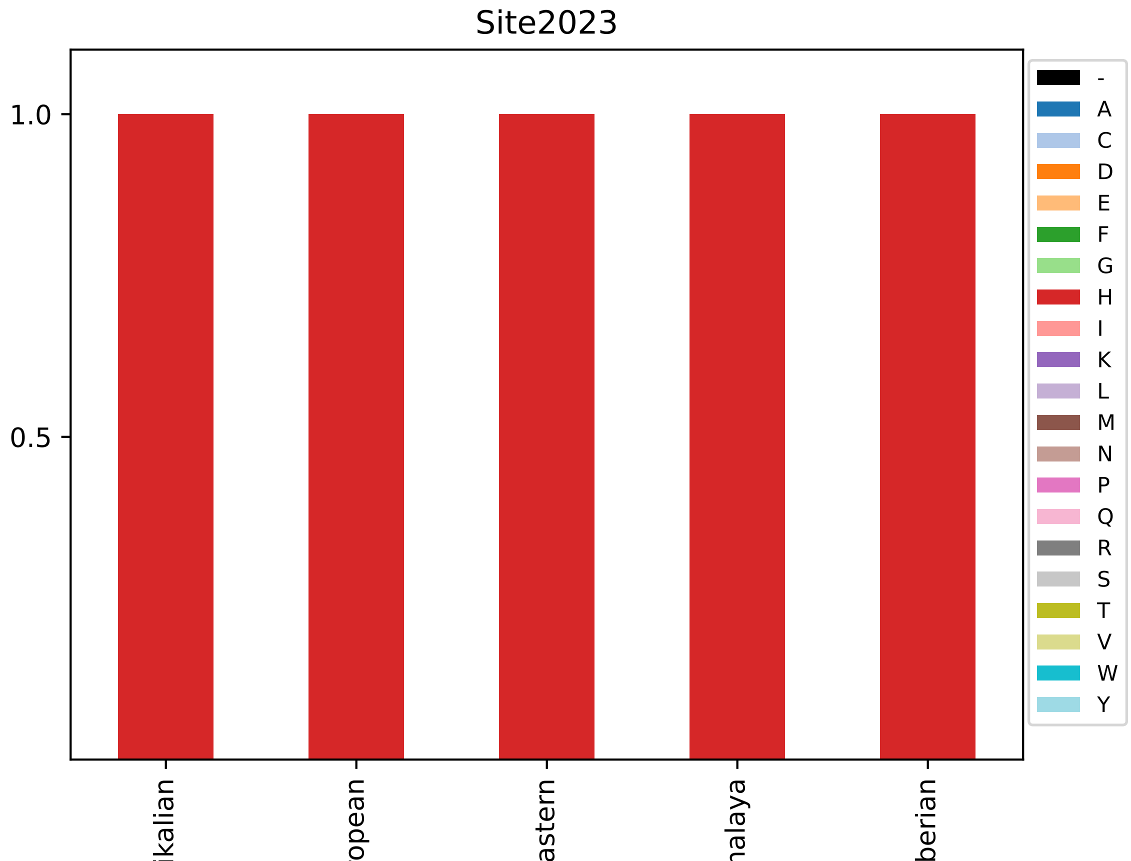
Site2023
1.0
0.5
-
A
C
D
E
F
G
H
I
K
L
M
N
P
Q
R
S
T
V
W
Y

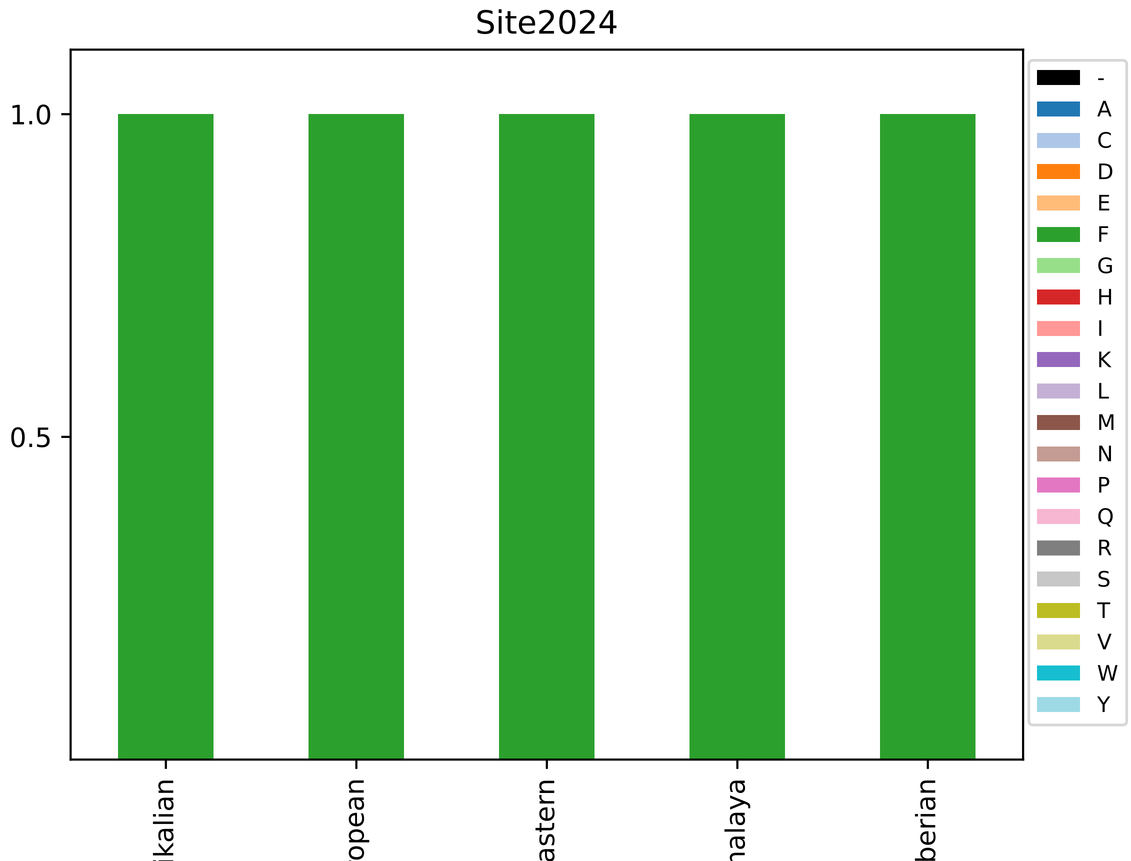

Site2024
1.0
0.5
-
A
C
D
E
F
G
H
I
K
L
M
N
P
Q
R
S
T
V
W
Y
ikalian
ropean
astern
halaya
berian

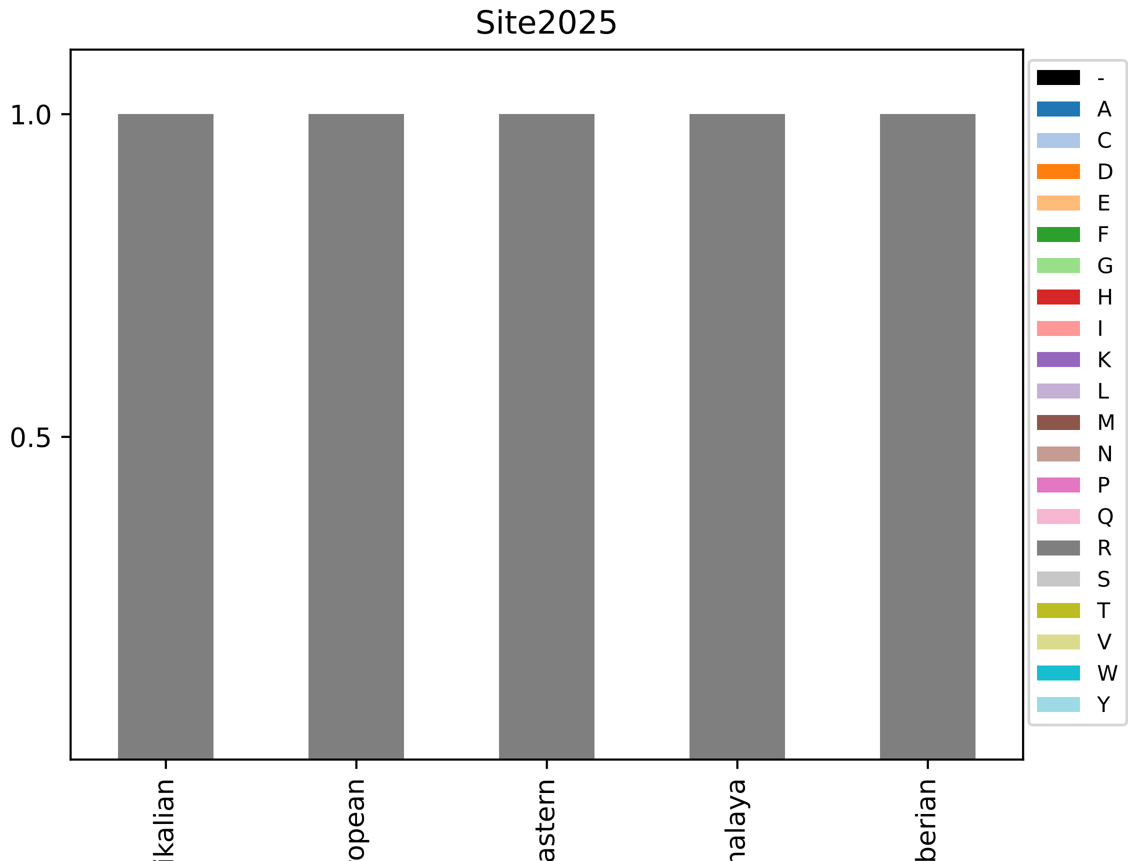

Site2025
1.0
0.5
-
A
C
D
E
F
G
H
I
K
L
M
N
P
Q
R
S
T
V
W
Y
ikalian
ropean
astern
nalaya
berian

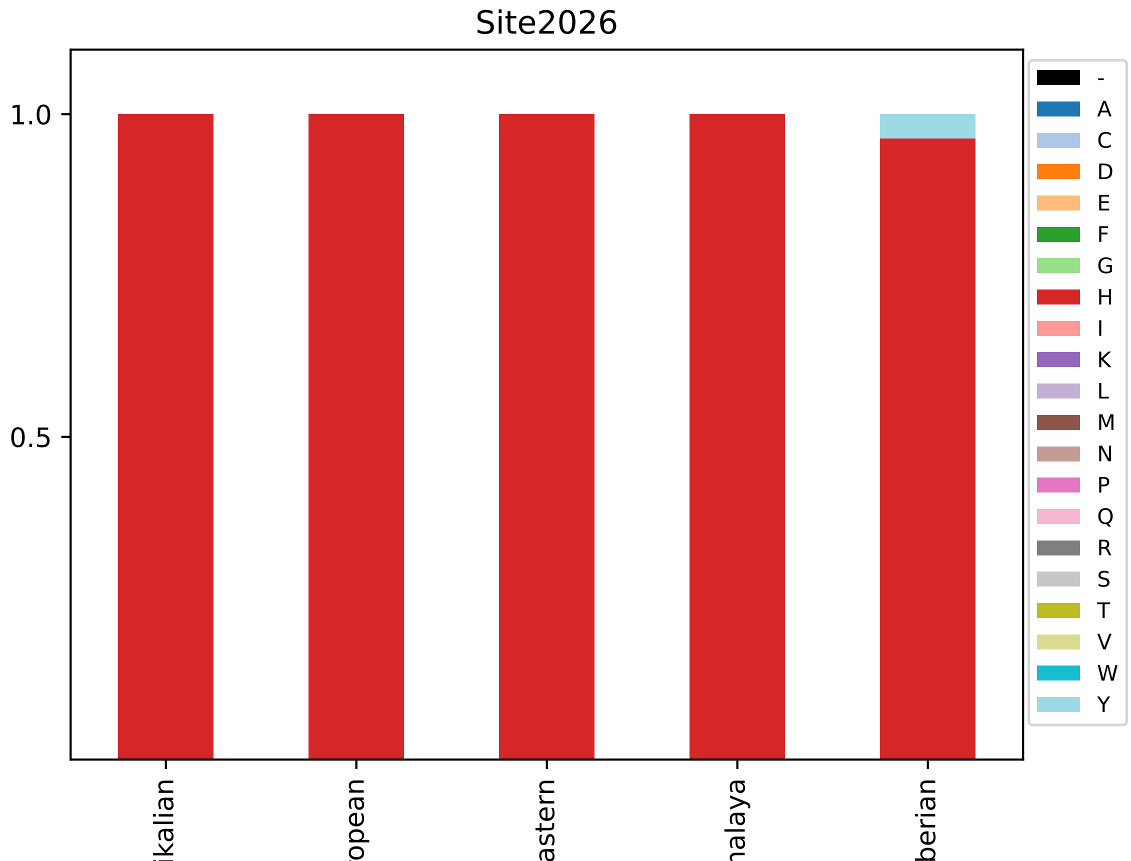

Site2026
1.0
0.5
-
A
C
D
E
F
G
H
I
K
L
M
N
P
Q
R
S
T
V
W
Y
ikalian
opean
astern
nalaya
berian

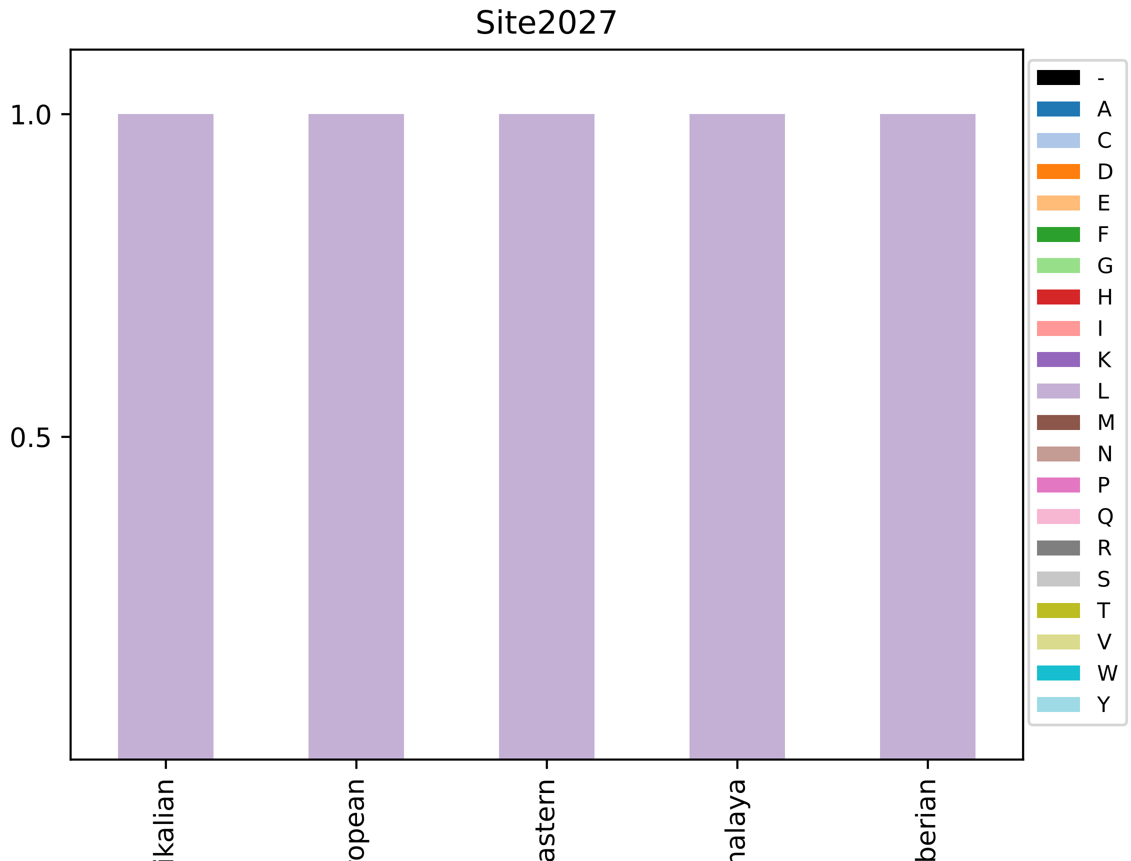

Site2027
1.0
0.5
-
A
C
D
E
F
G
H
I
K
L
M
N
P
Q
R
S
T
V
W
Y
ikalian
opean
astern
nalaya
berian

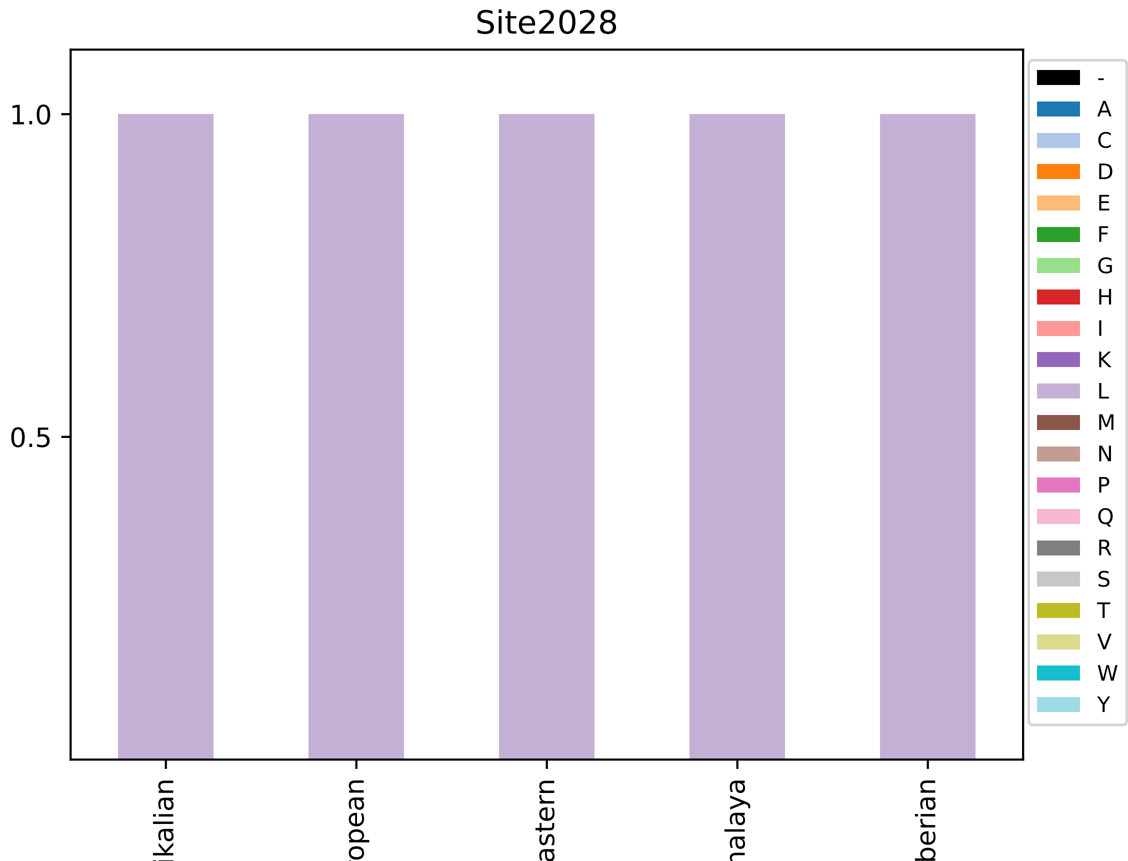

Site2028
1.0
0.5
ikalian
ropean
astern
nalaya
berian
-
A
C
D
E
F
G
H
I
K
L
M
N
P
Q
R
S
T
V
W
Y

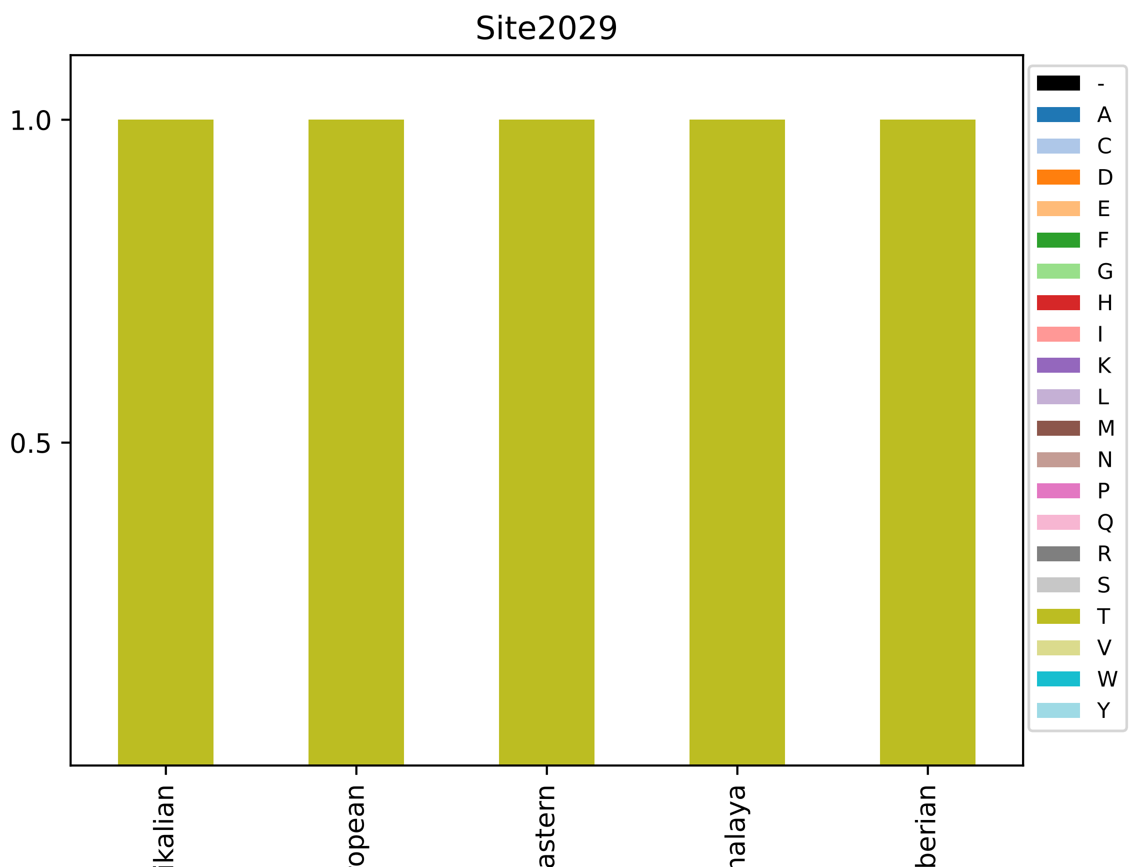

Site2029
1.0
0.5
-
A
C
D
E
F
G
H
I
K
L
M
N
P
Q
R
S
T
V
W
Y
ikalian
opean
astern
nalaya
berian

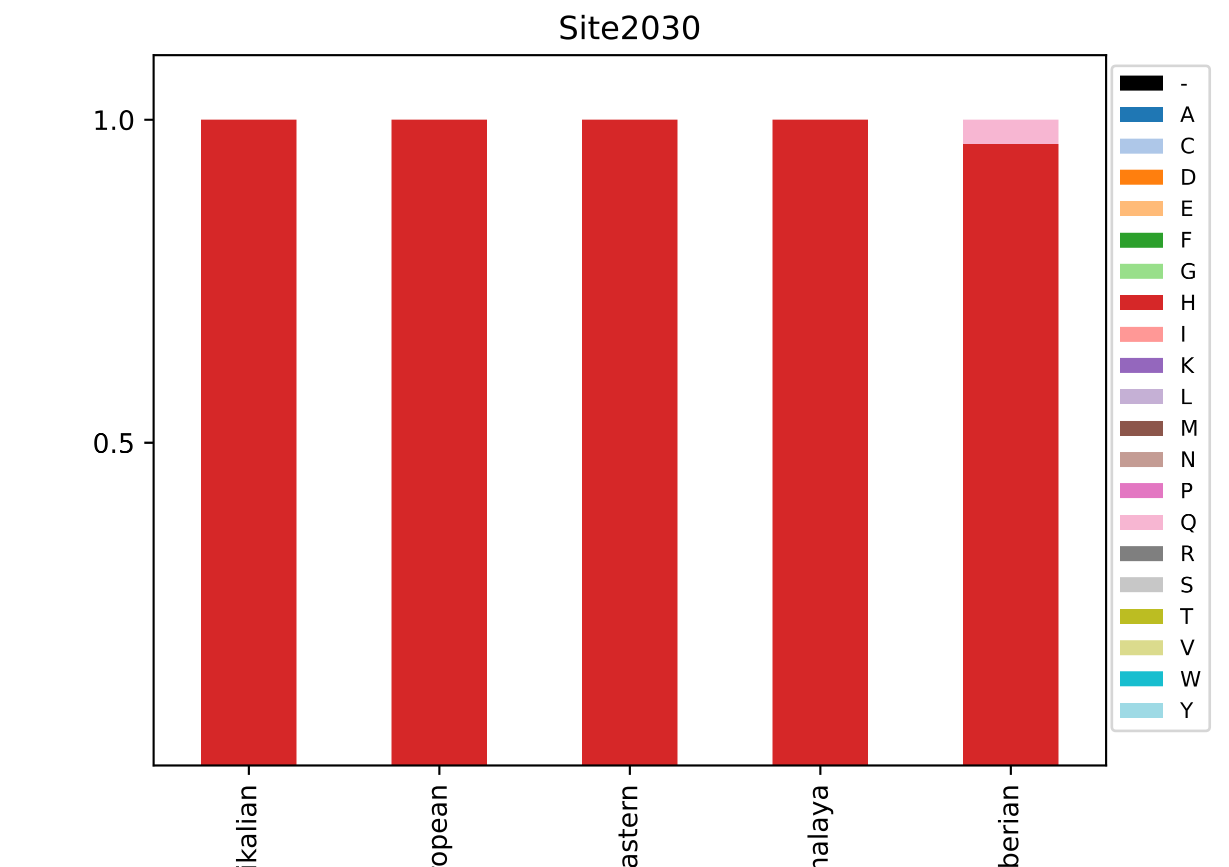

Site2030
1.0
0.5
ikalian
opean
astern
nalaya
berian
-
A
C
D
E
F
G
H
I
K
L
M
N
P
Q
R
S
T
V
W
Y

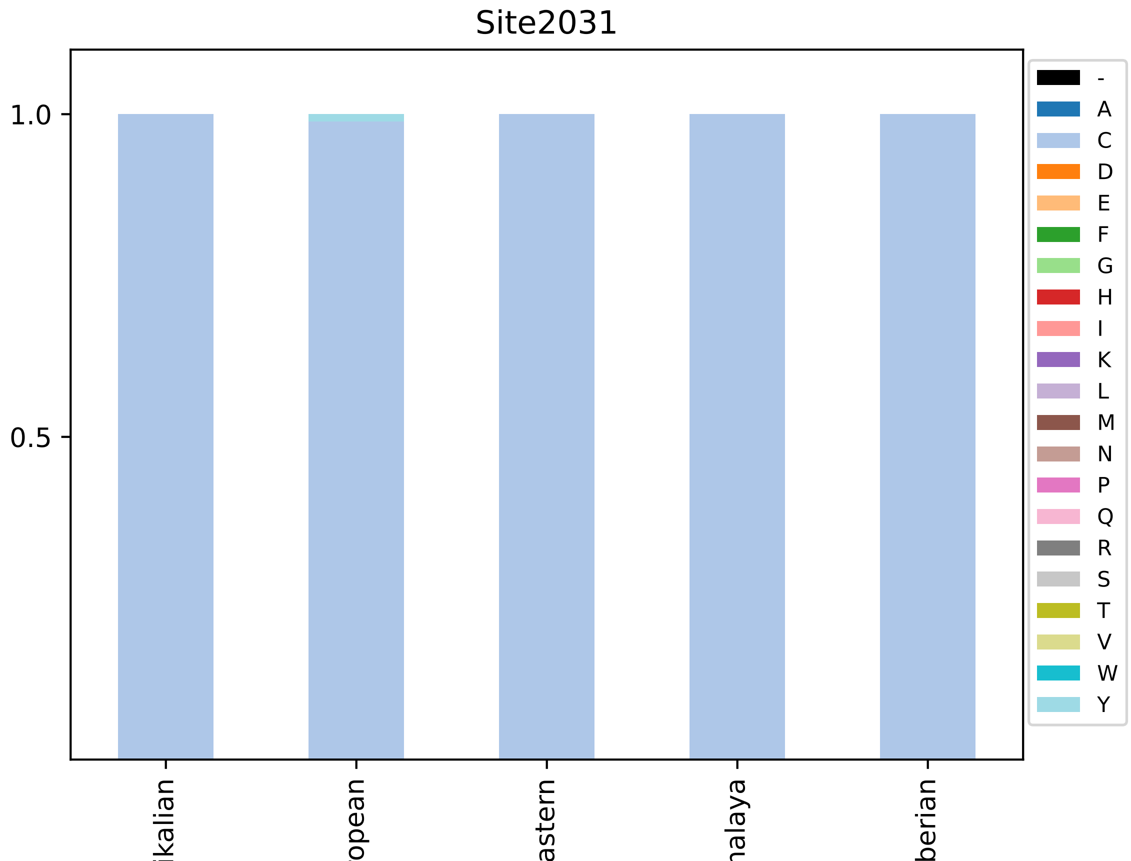
Site2031
1.0
0.5
ikalian
opean
astern
nalaya
berian
-
A
C
D
E
F
G
H
I
K
L
M
N
P
Q
R
S
T
V
W
Y

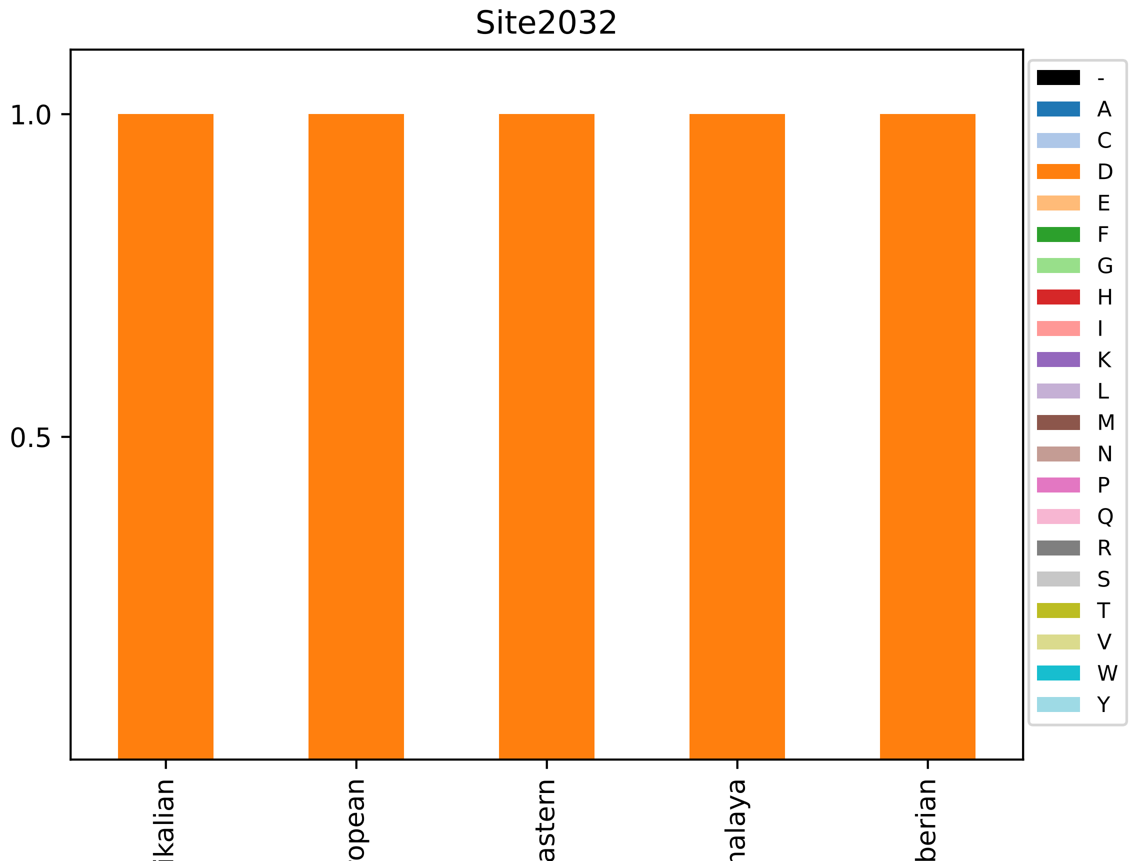
Site2032
1.0
0.5
-
A
C
D
E
F
G
H
I
K
L
M
N
P
Q
R
S
T
V
W
Y
ikalian
opean
astern
nalaya
berian

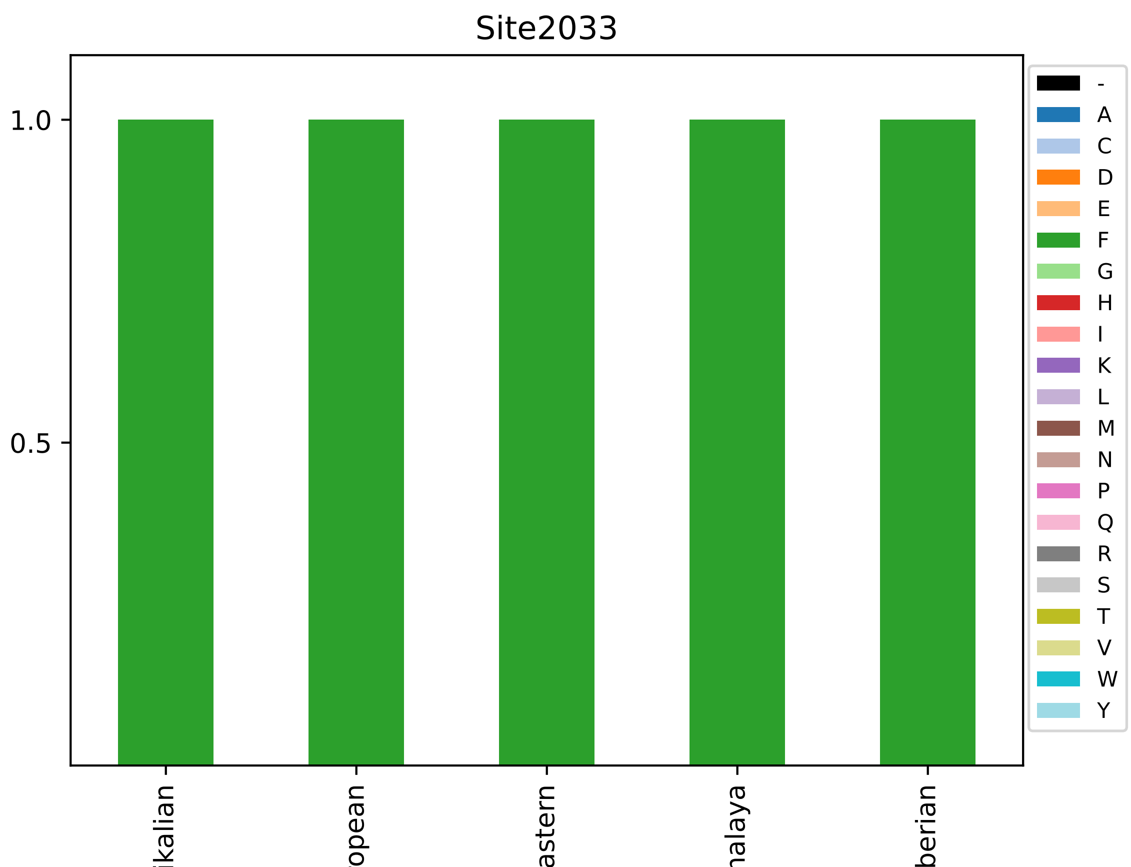

Site2033
1.0
0.5
-
A
C
D
E
F
G
H
I
K
L
M
N
P
Q
R
S
T
V
W
Y
ikalian
opean
astern
nalaya
berian

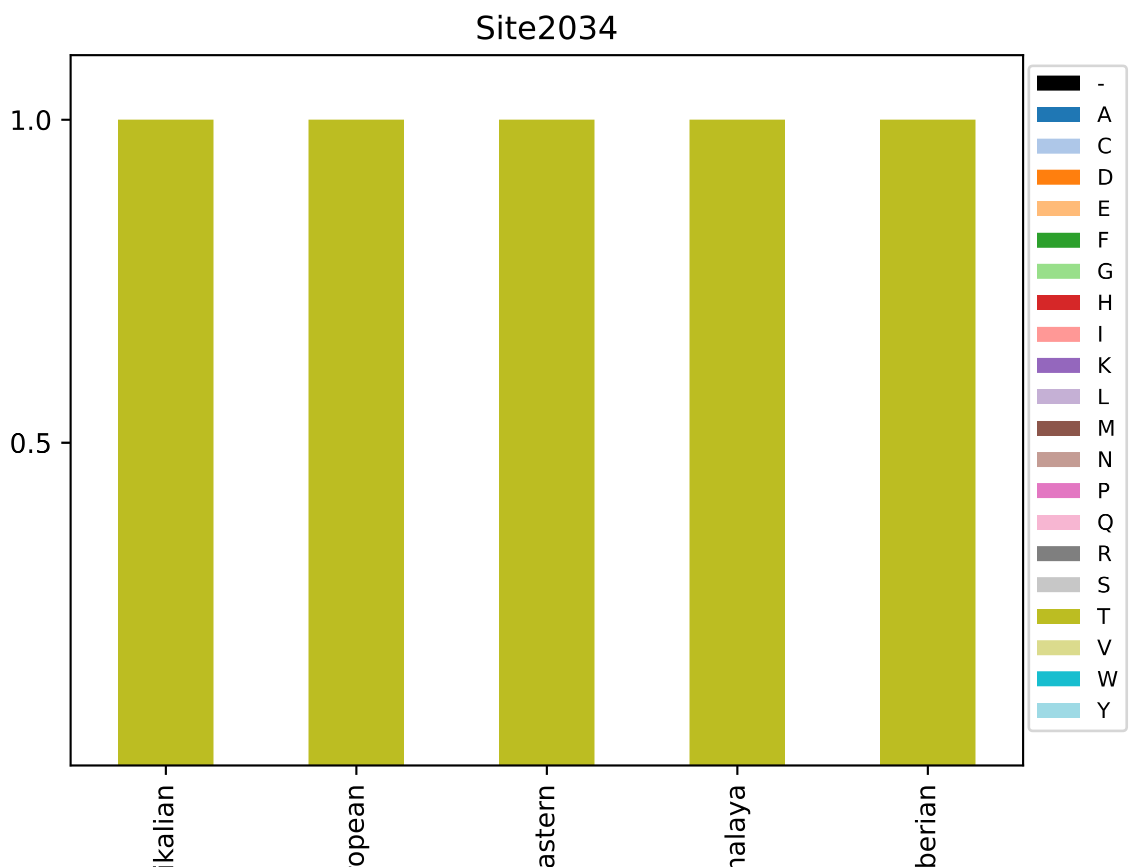

Site2034
1.0
0.5
-
A
C
D
E
F
G
H
I
K
L
M
N
P
Q
R
S
T
V
W
Y
ikalian
opean
astern
nalaya
berian

Site2035

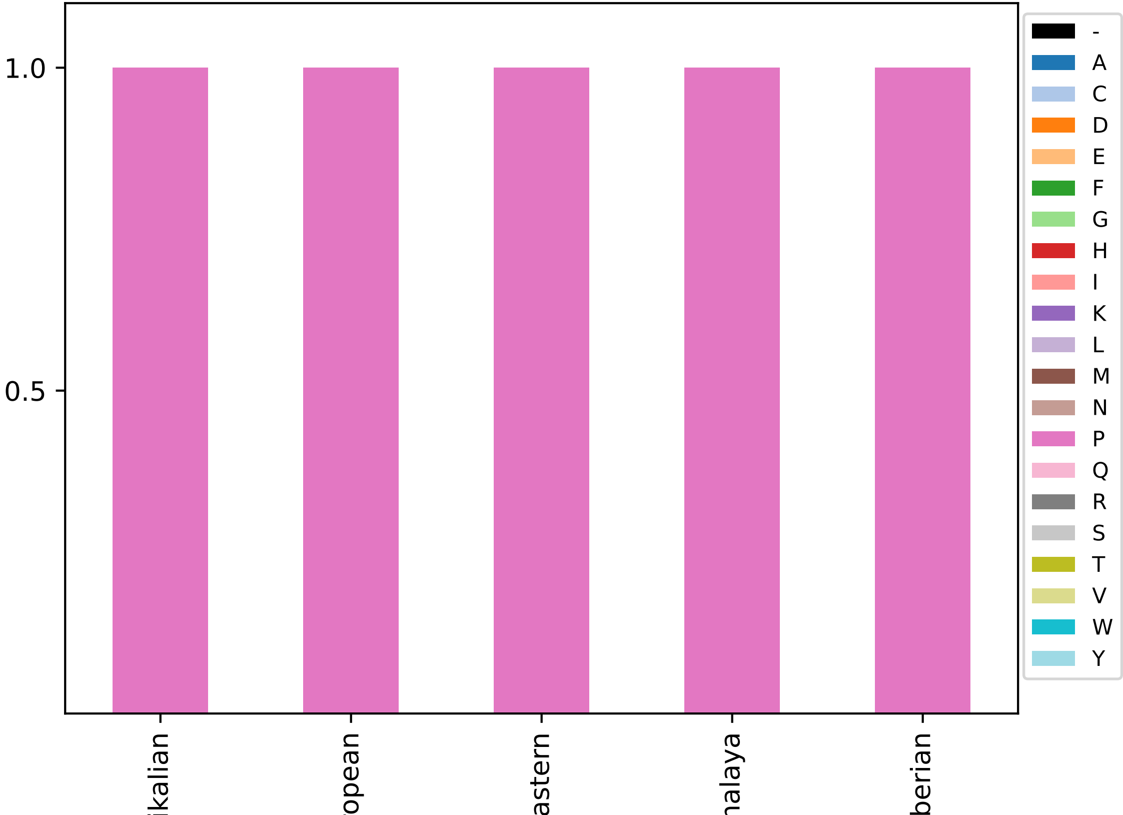

1.0
0.5
-
A
C
D
E
F
G
H
I
K
L
M
N
P
Q
R
S
T
V
W
Y
ikalian
opean
astern
nalaya
berian

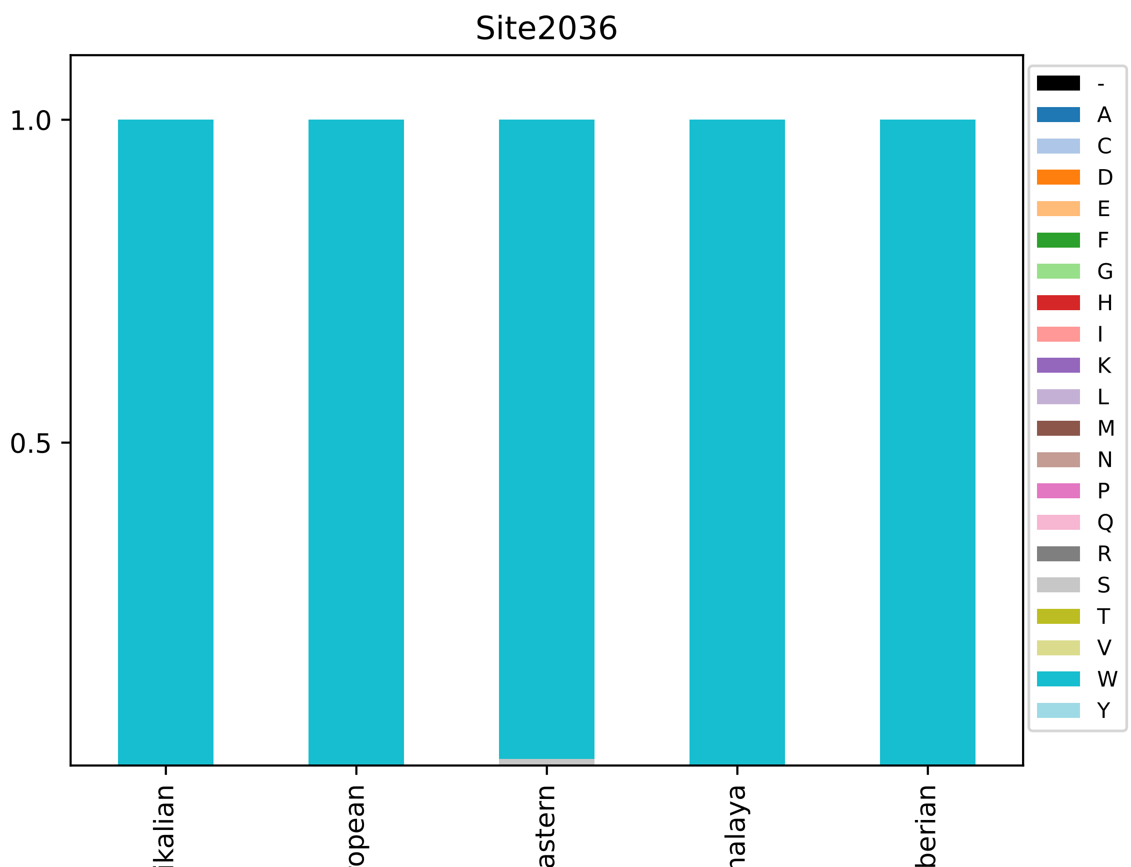

Site2036
1.0
0.5
-
A
C
D
E
F
G
H
I
K
L
M
N
P
Q
R
S
T
V
W
Y
ikalian
opean
astern
nalaya
berian

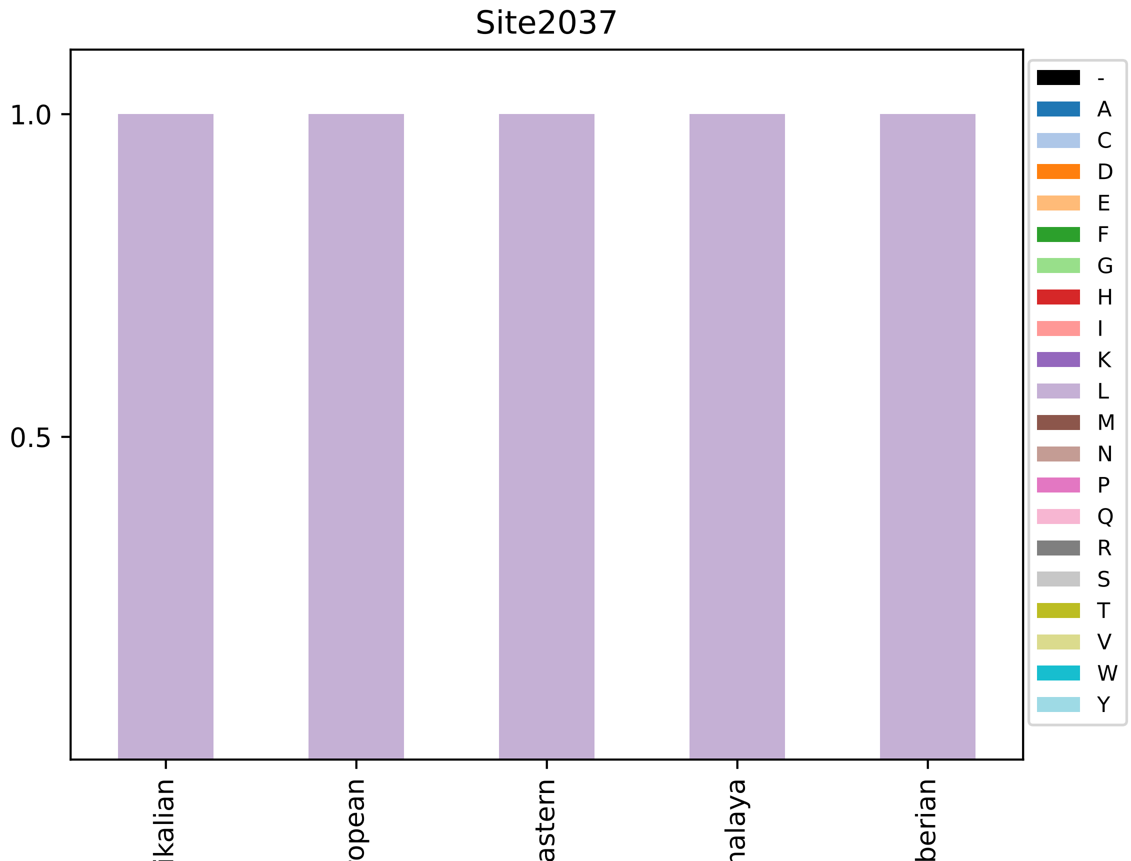
Site2037
1.0
0.5
-
A
C
D
E
F
G
H
I
K
L
M
N
P
Q
R
S
T
V
W
Y
ikalian
opean
astern
nalaya
berian

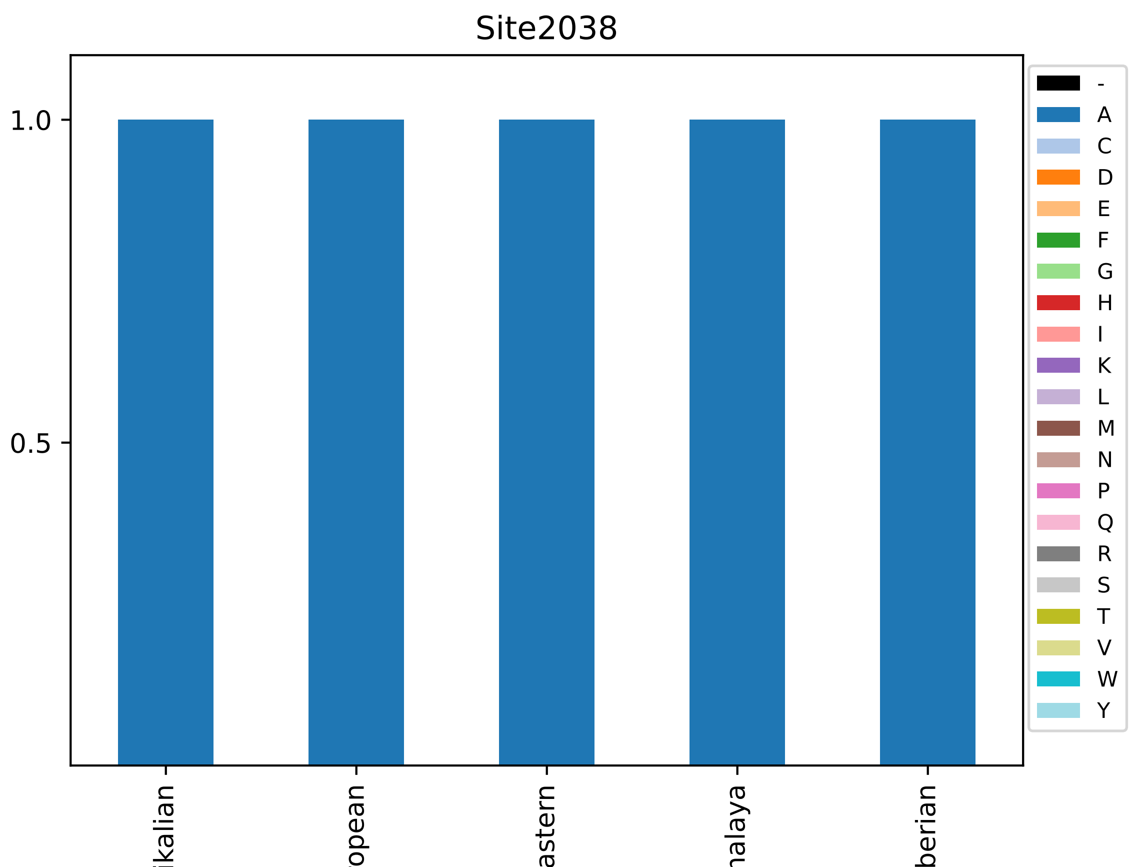
Site2038
1.0
0.5
ikalian
opean
astern
nalaya
berian
-
A
C
D
E
F
G
H
I
K
L
M
N
P
Q
R
S
T
V
W
Y

Site2039

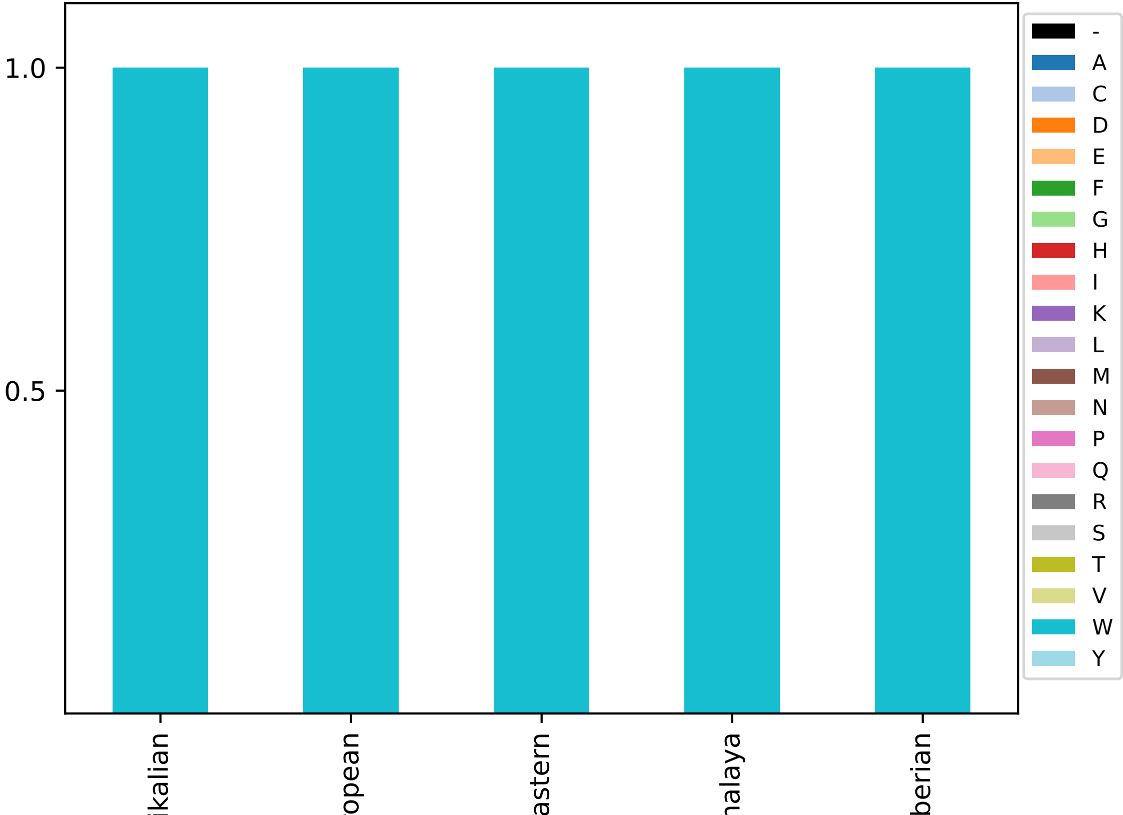

1.0
0.5
ikalian
opean
astern
nalaya
berian
-
A
C
D
E
F
G
H
I
K
L
M
N
P
Q
R
S
T
V
W
Y

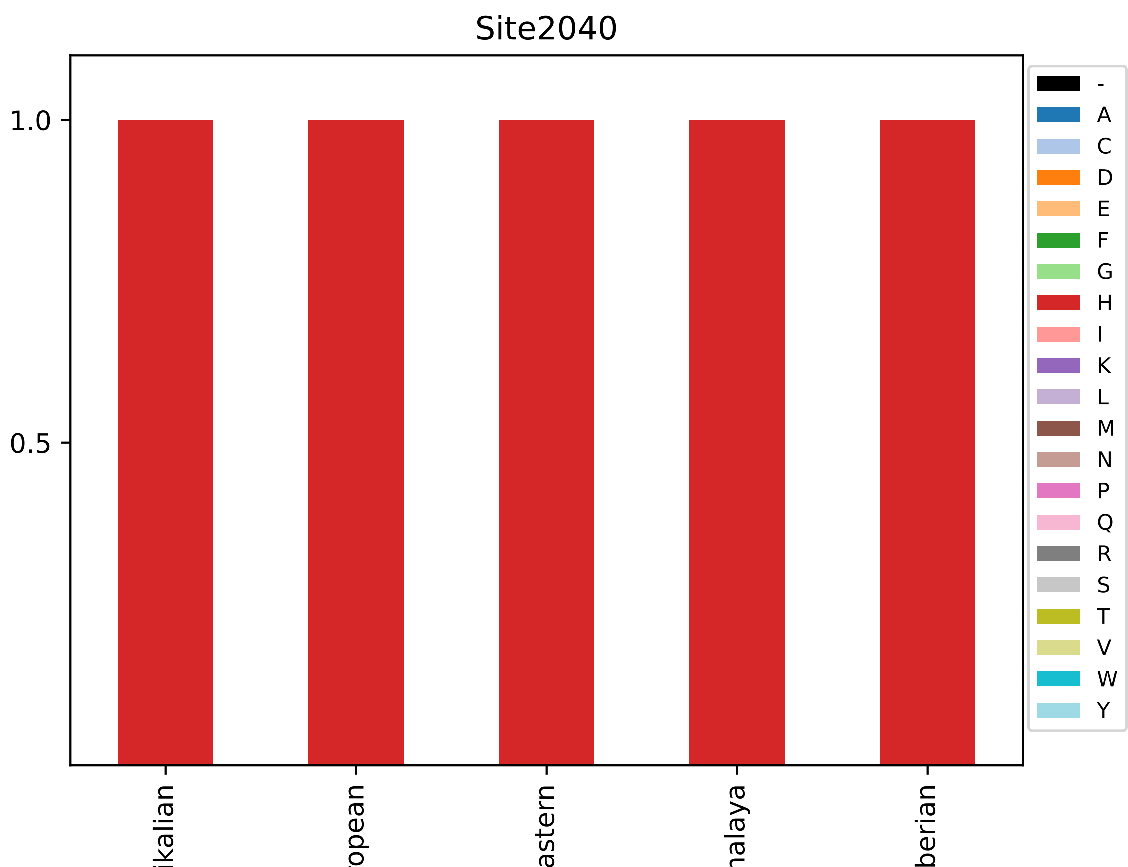

Site2040
1.0
0.5
-
A
C
D
E
F
G
H
I
K
L
M
N
P
Q
R
S
T
V
W
Y
ikalian
opean
astern
nalaya
berian

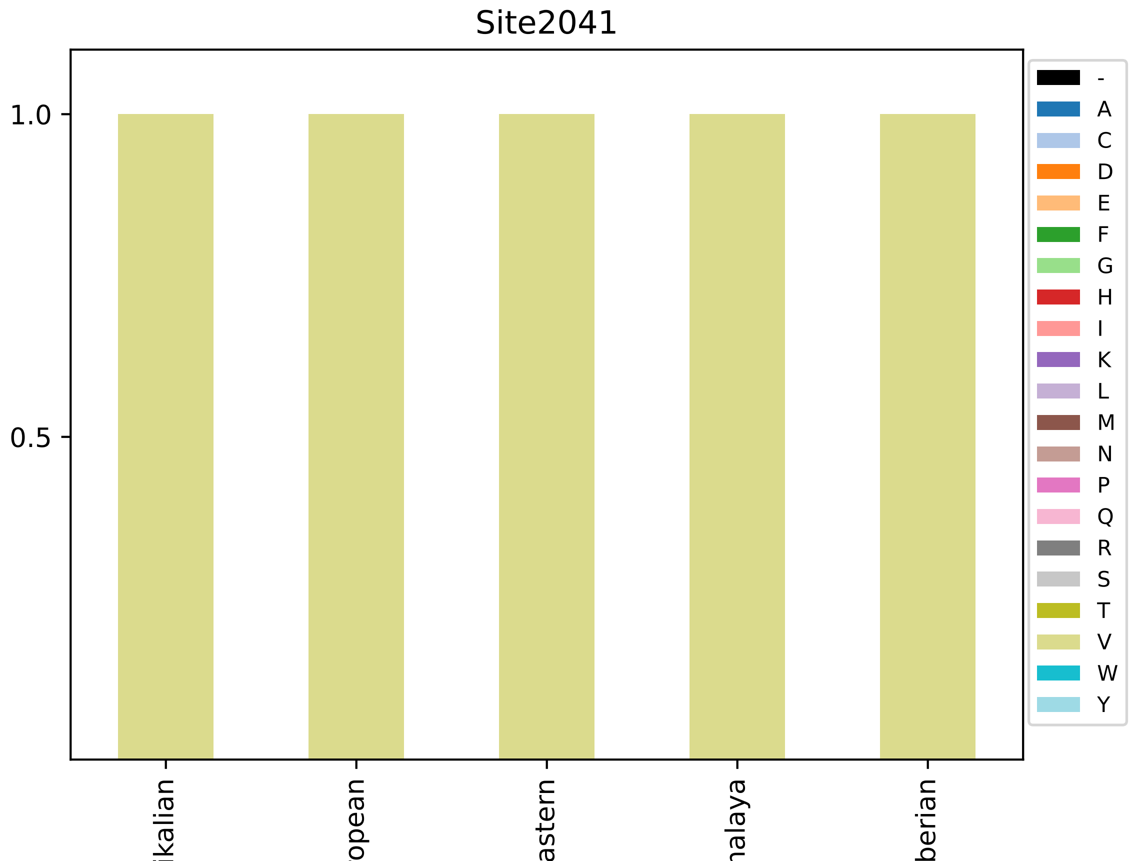

Site2041
1.0
0.5
-
A
C
D
E
F
G
H
I
K
L
M
N
P
Q
R
S
T
V
W
Y
ikalian
opean
astern
nalaya
berian

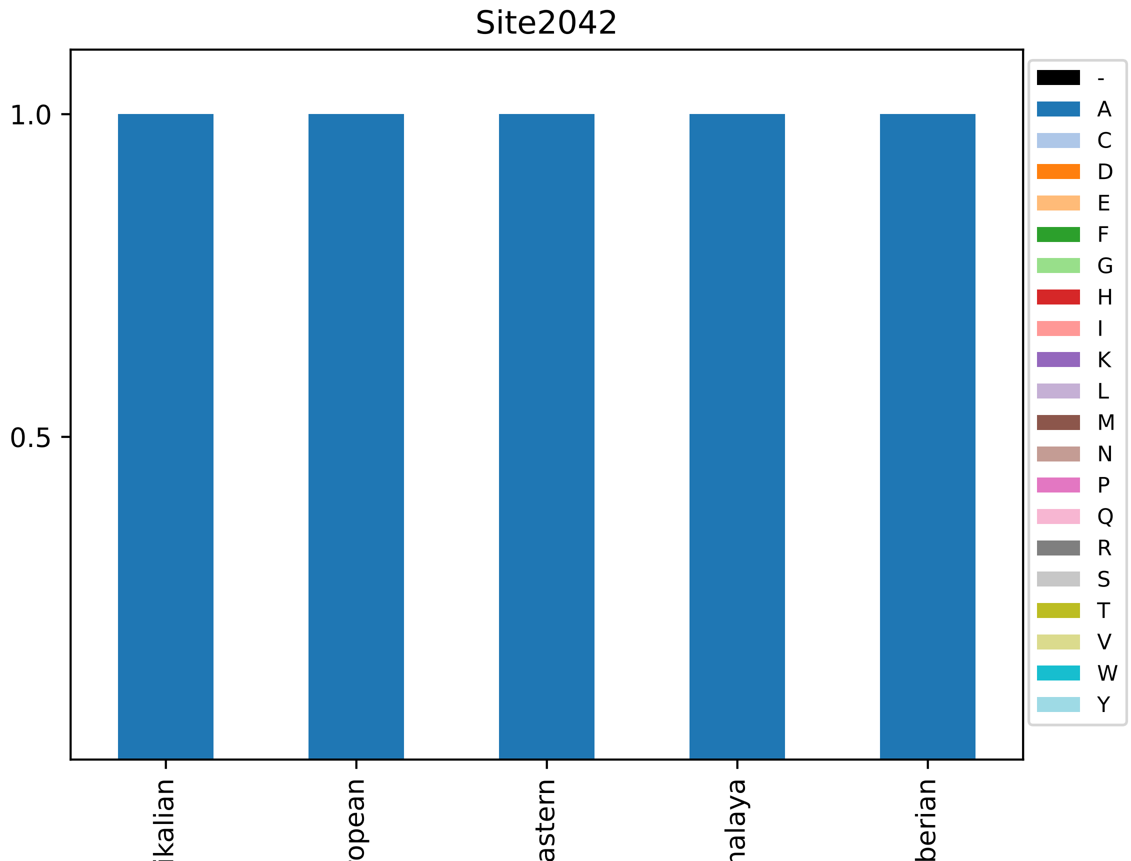
Site2042
1.0
0.5
-
A
C
D
E
F
G
H
I
K
L
M
N
P
Q
R
S
T
V
W
Y
ikalian
opean
astern
nalaya
berian

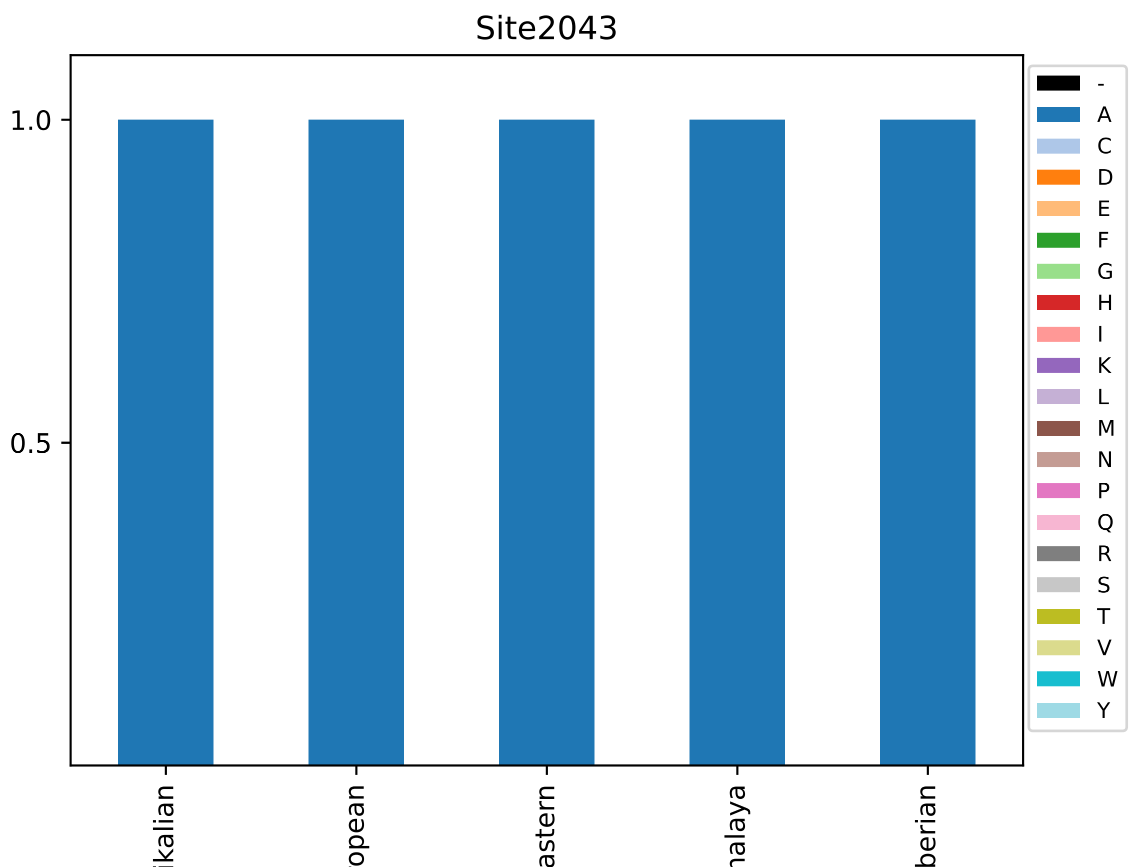

Site2043
1.0
0.5
-
A
C
D
E
F
G
H
I
K
L
M
N
P
Q
R
S
T
V
W
Y
ikalian
opean
astern
nalaya
berian

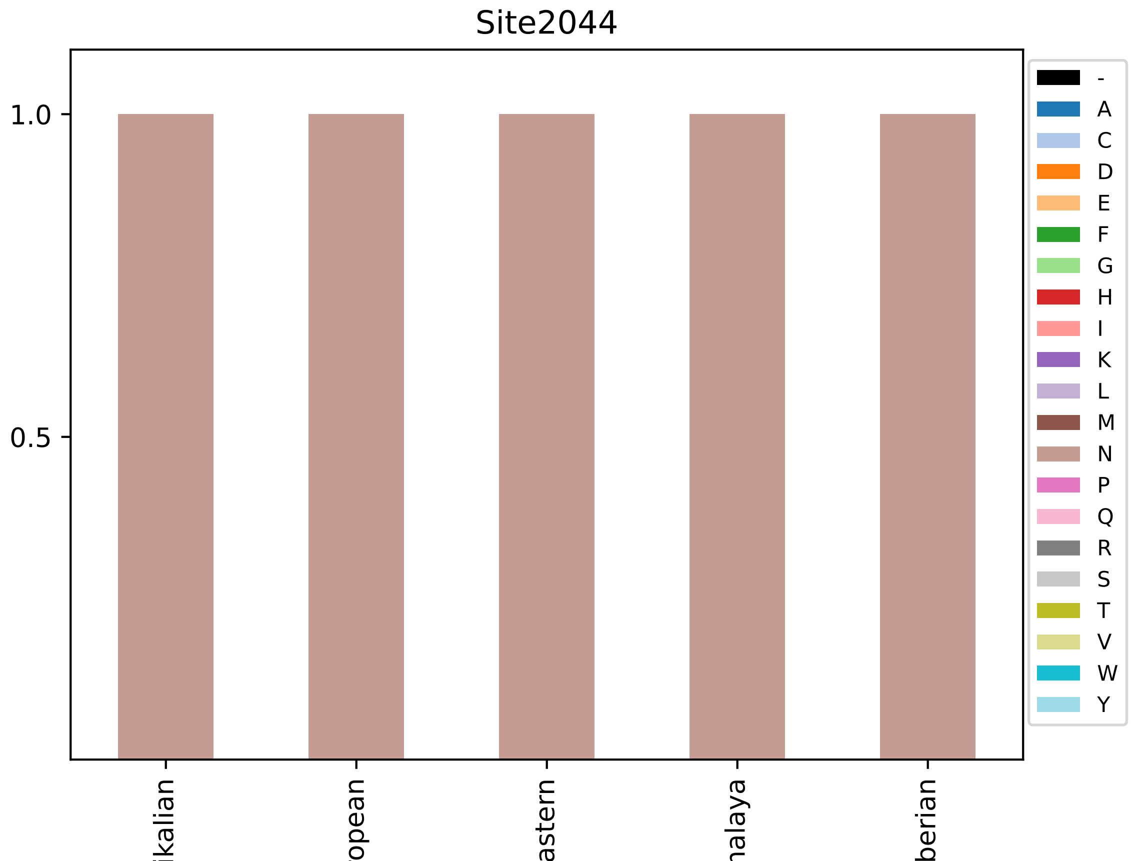
Site2044
1.0
0.5
-
A
C
D
E
F
G
H
I
K
L
M
N
P
Q
R
S
T
V
W
Y
ikalian
ropean
astern
nalaya
berian

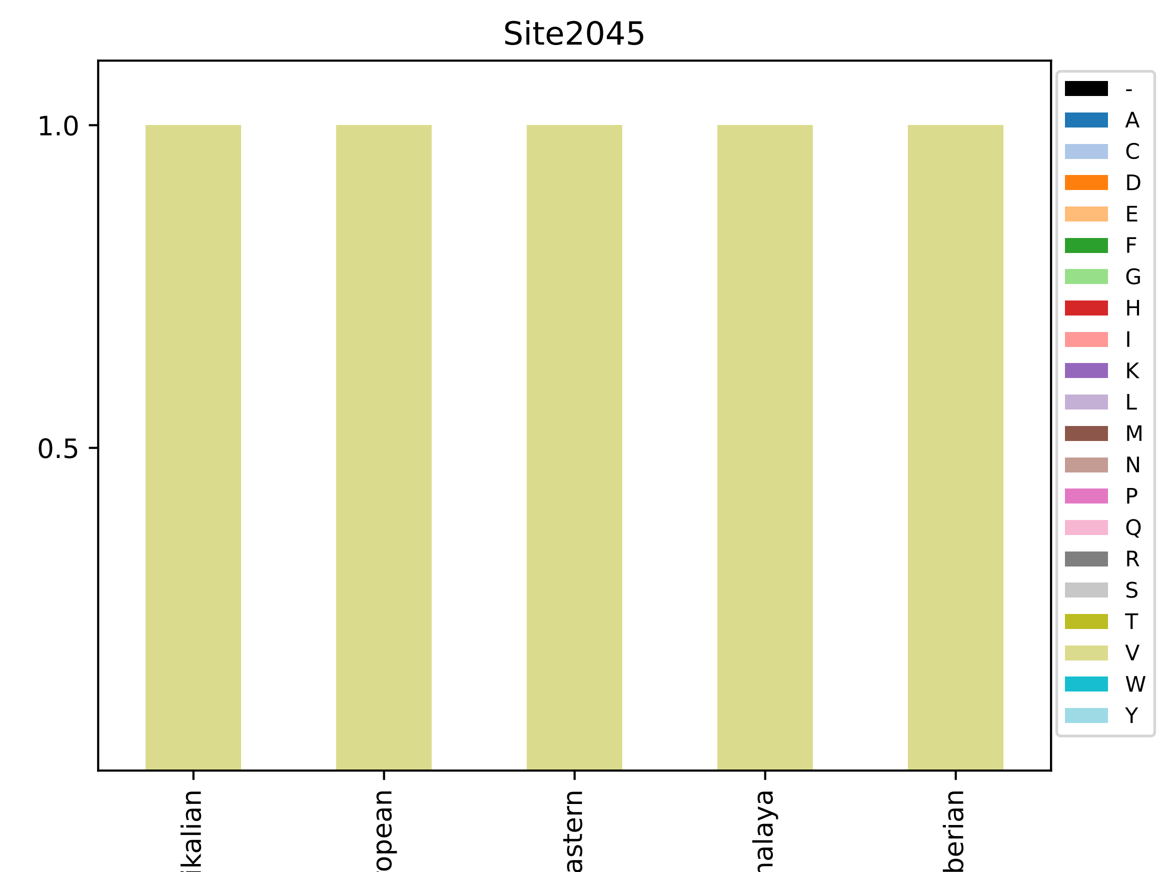

Site2045
1.0
0.5
ikalian
opean
astern
nalaya
berian
-
A
C
D
E
F
G
H
I
K
L
M
N
P
Q
R
S
T
V
W
Y

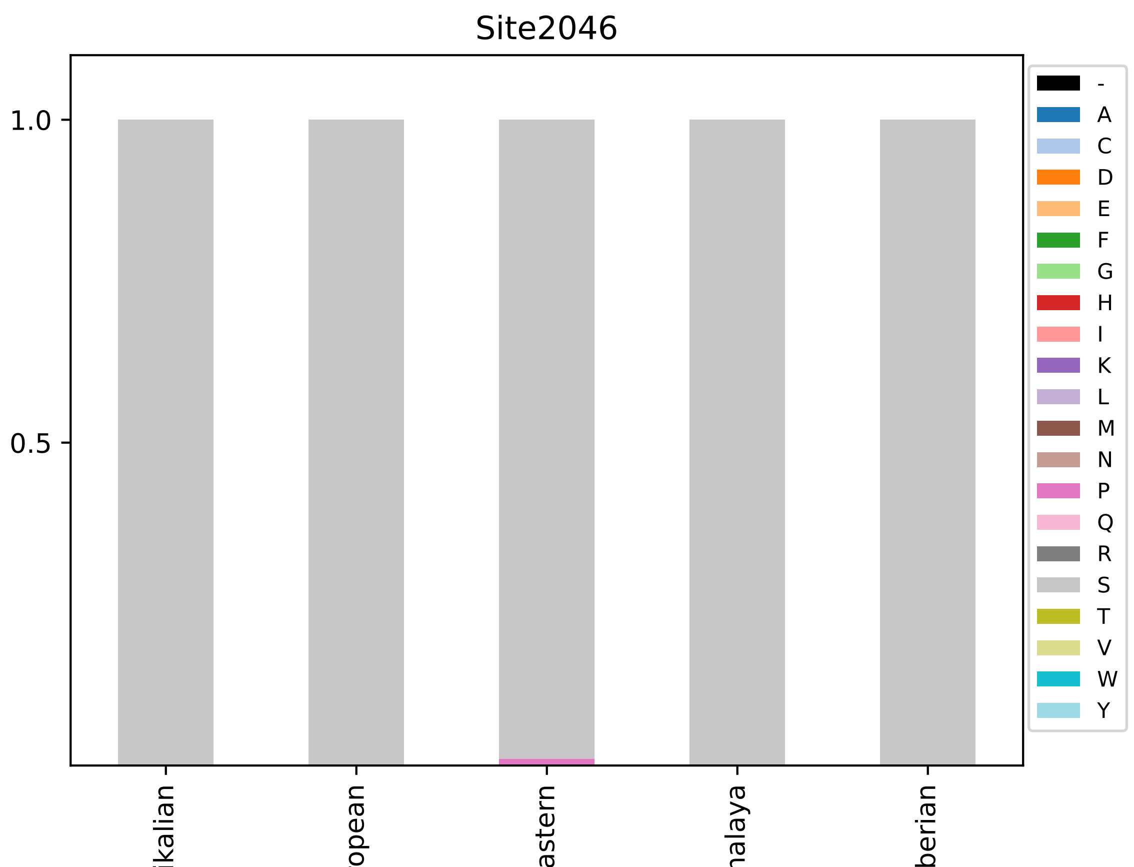
Site2046
1.0
0.5
-
A
C
D
E
F
G
H
I
K
L
M
N
P
Q
R
S
T
V
W
Y
ikalian
ropean
astern
nalaya
berian

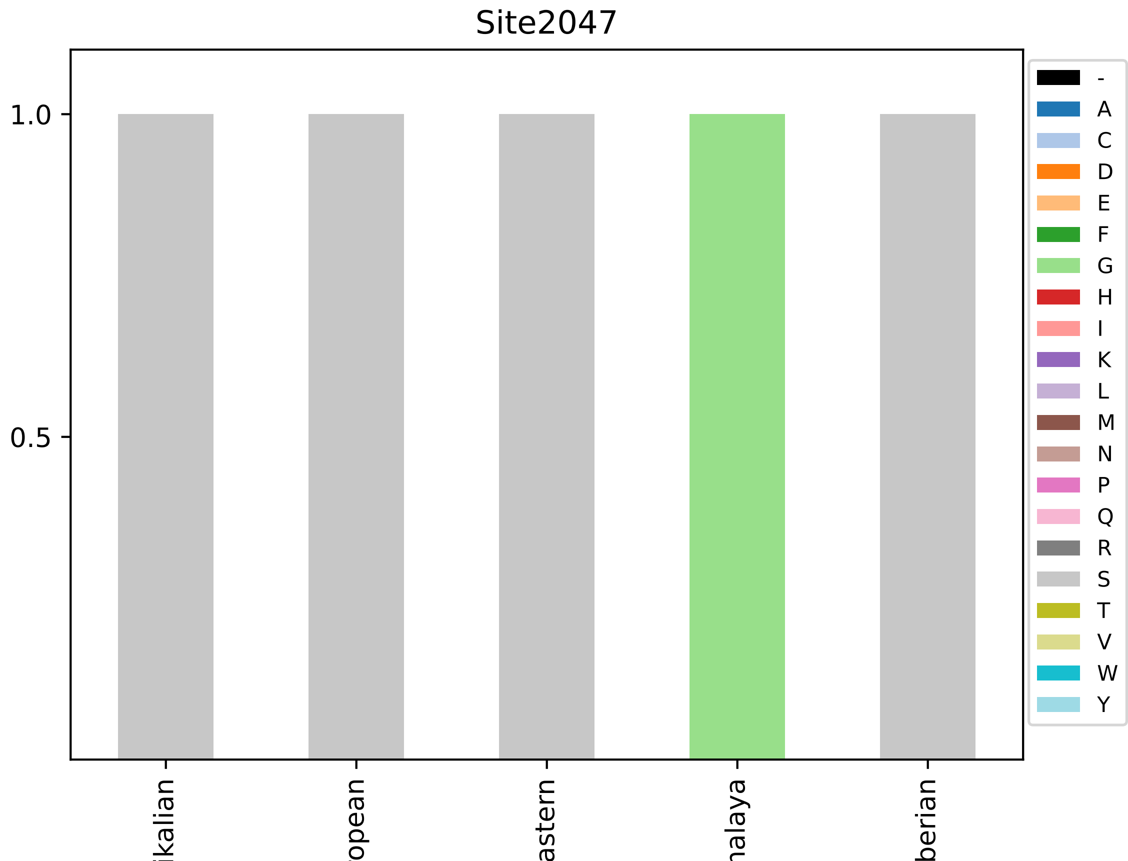
Site2047
1.0
0.5
ikalian
opean
astern
nalaya
berian
-
A
C
D
E
F
G
H
I
K
L
M
N
P
Q
R
S
T
V
W
Y

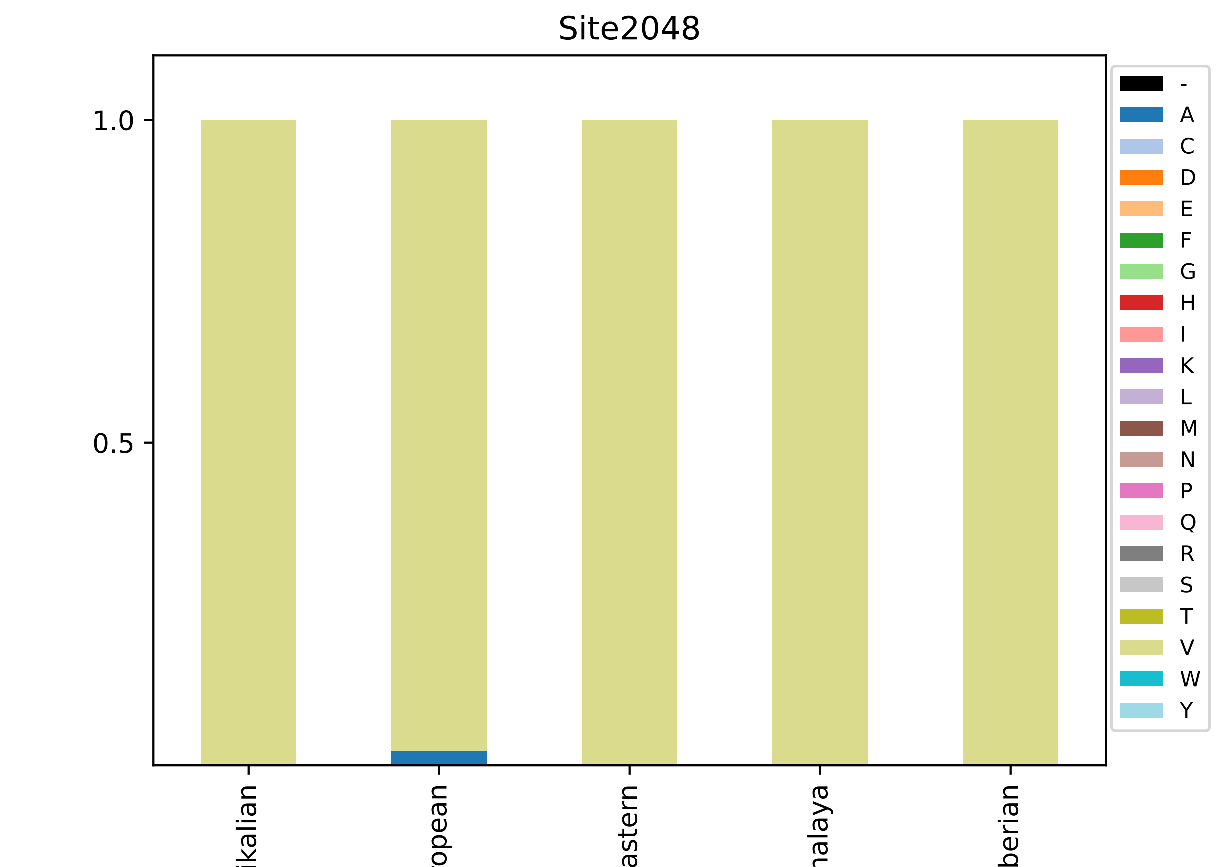

Site2048
1.0
0.5
-
A
C
D
E
F
G
H
I
K
L
M
N
P
Q
R
S
T
V
W
Y
ikalian
opean
astern
nalaya
berian

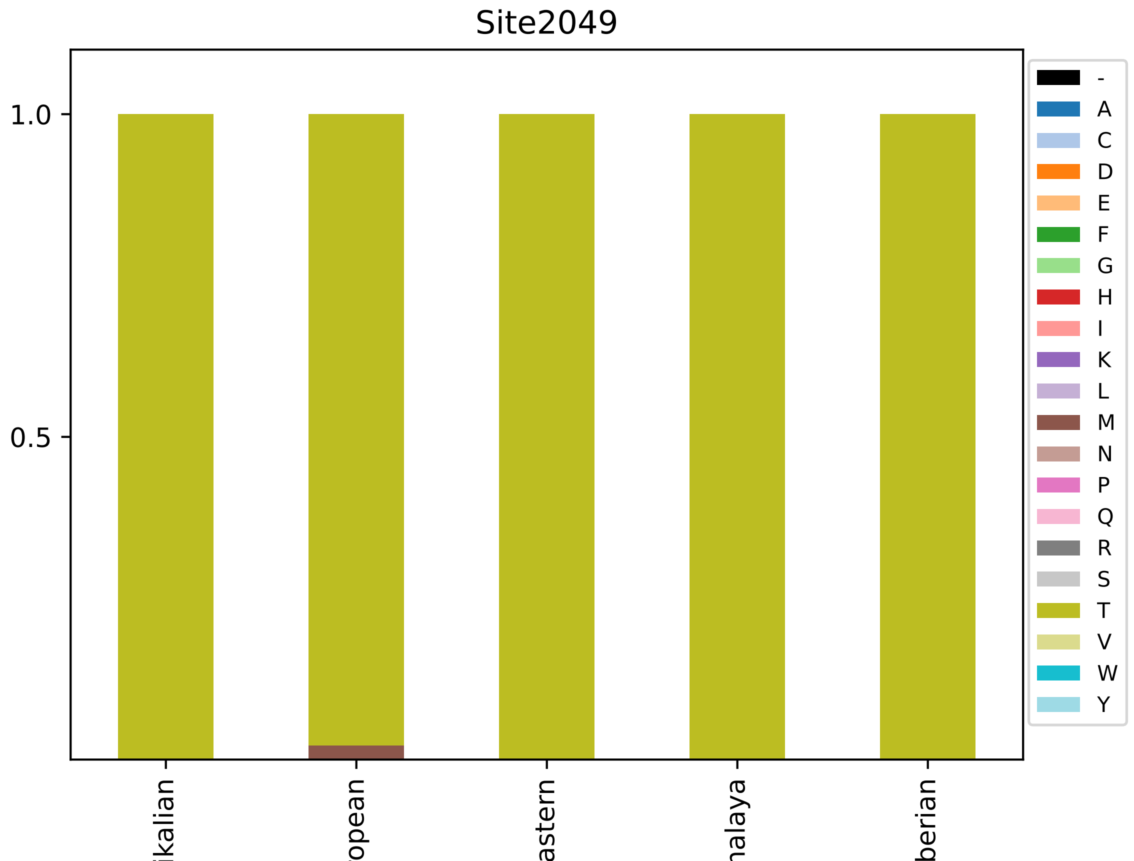
Site2049
1.0
0.5
-
A
C
D
E
F
G
H
I
K
L
M
N
P
Q
R
S
T
V
W
Y
ikalian
opean
astern
nalaya
berian

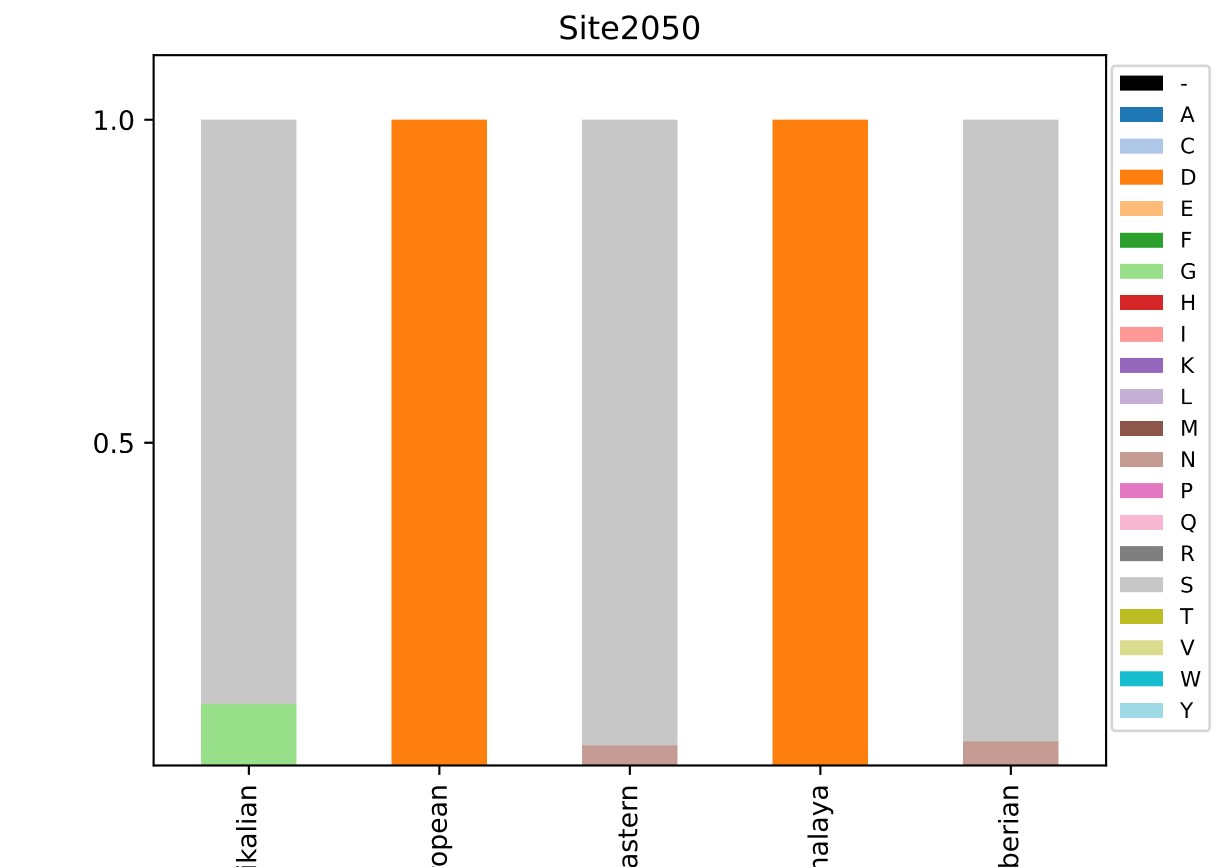
Site2050
1.0
0.5
-
A
C
D
E
F
G
H
I
K
L
M
N
P
Q
R
S
T
V
W
Y
ikalian
opean
astern
nalaya
berian

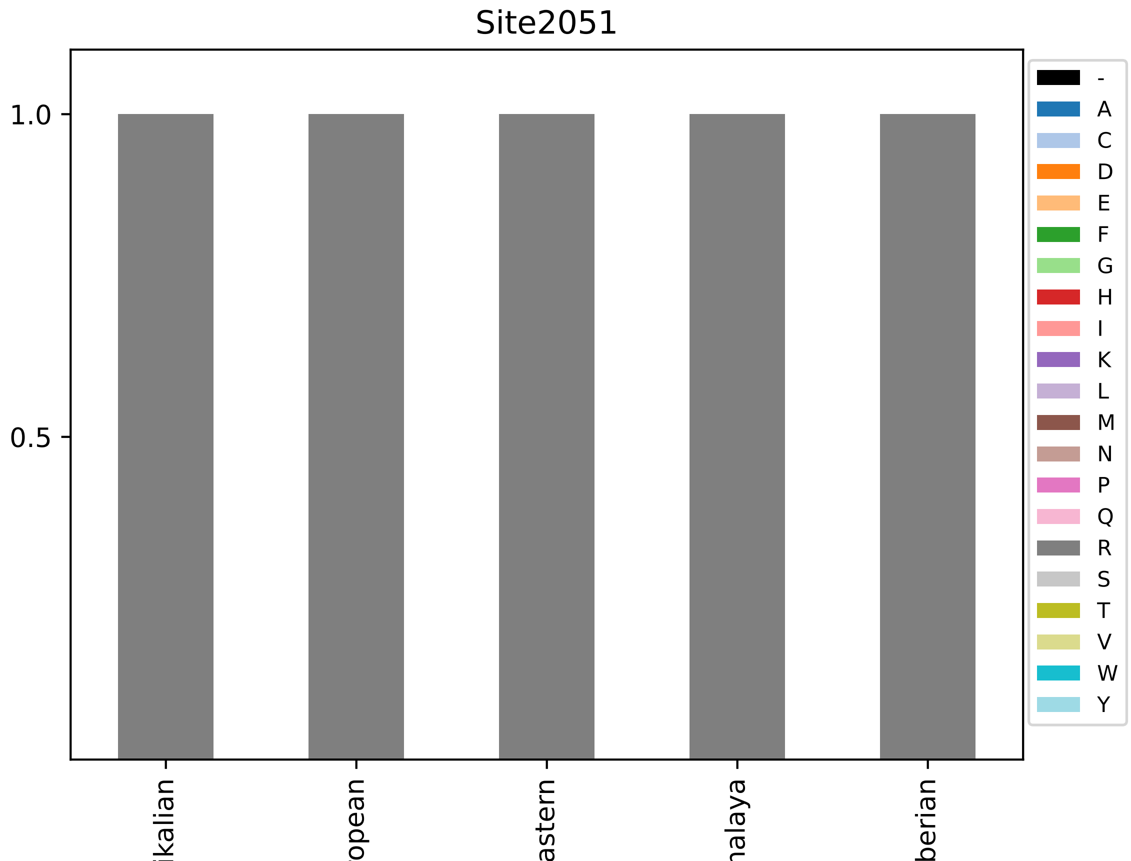

Site2051
1.0
0.5
-
A
C
D
E
F
G
H
I
K
L
M
N
P
Q
R
S
T
V
W
Y
ikalian
opean
astern
nalaya
berian

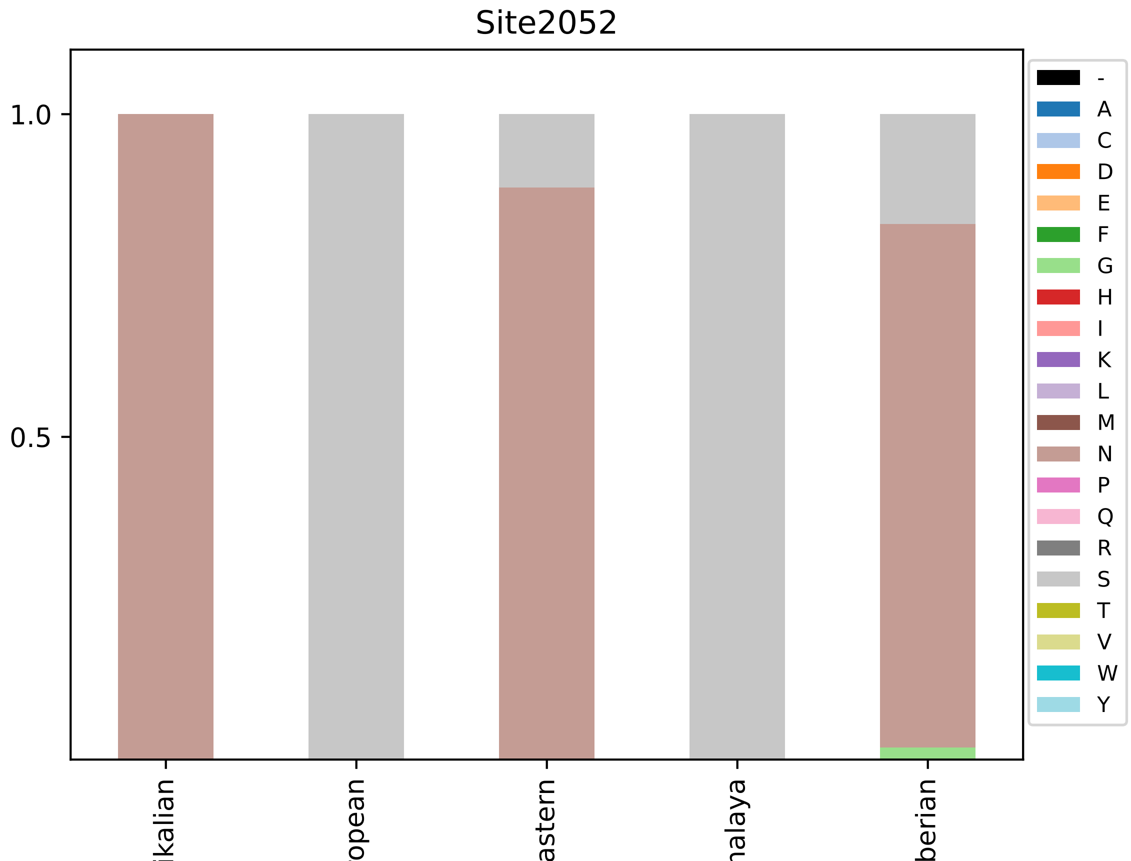

Site2052
1.0
0.5
-
A
C
D
E
F
G
H
I
K
L
M
N
P
Q
R
S
T
V
W
Y
ikalian
opean
astern
nalaya
berian

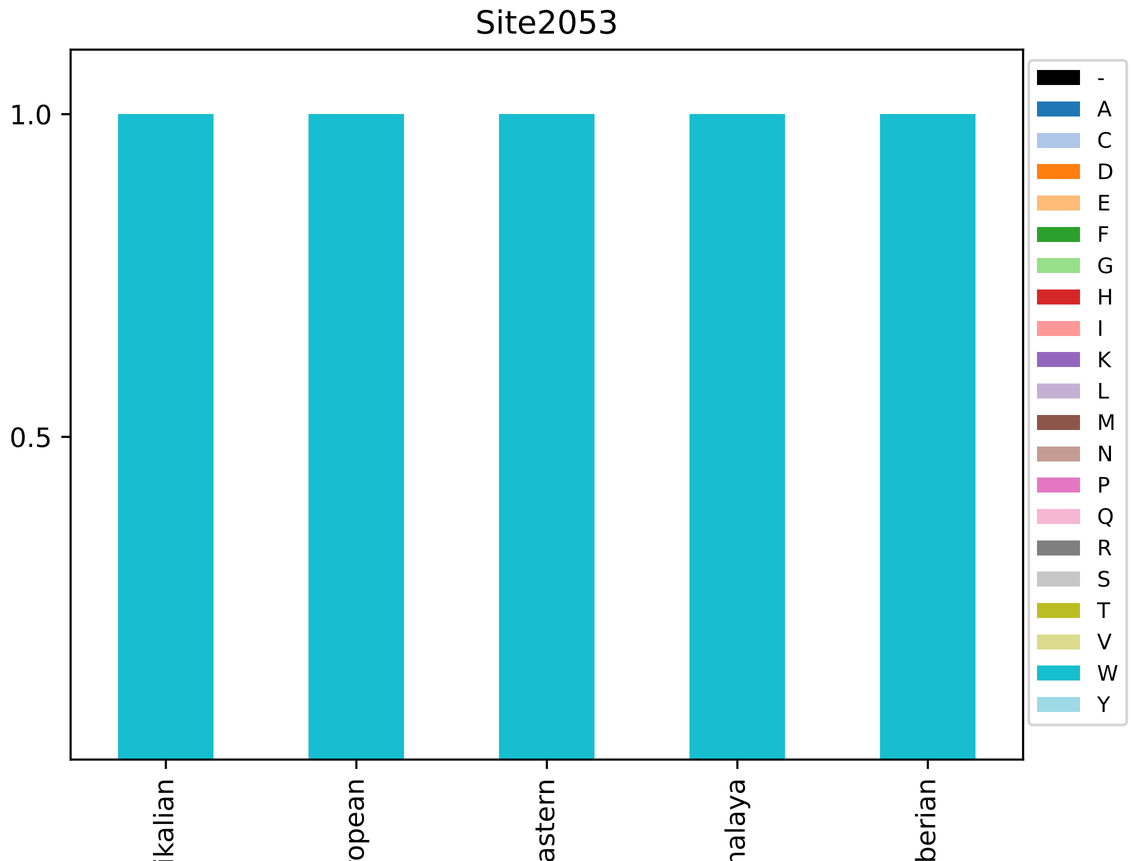

Site2053
1.0
0.5
ikalian
opean
astern
nalaya
berian
-
A
C
D
E
F
G
H
I
K
L
M
N
P
Q
R
S
T
V
W
Y

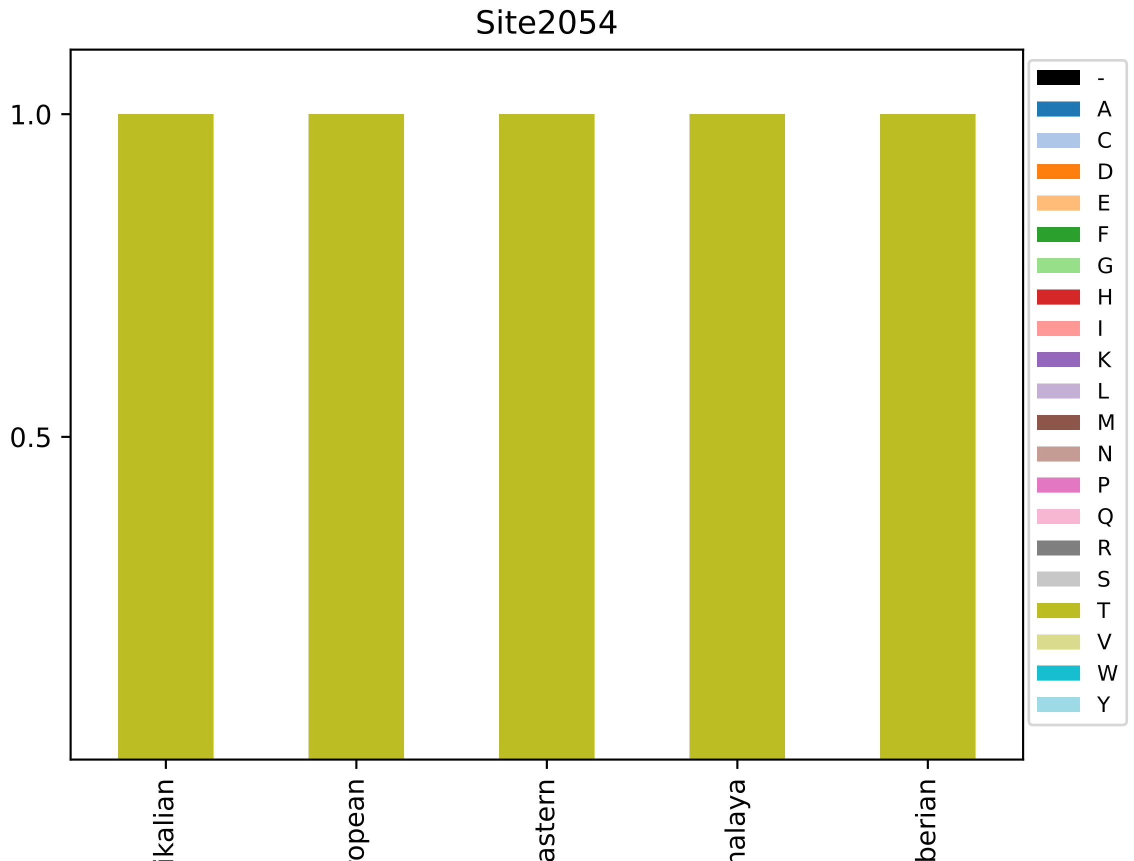
Site2054
1.0
0.5
-
A
C
D
E
F
G
H
I
K
L
M
N
P
Q
R
S
T
V
W
Y
ikalian
opean
astern
nalaya
berian

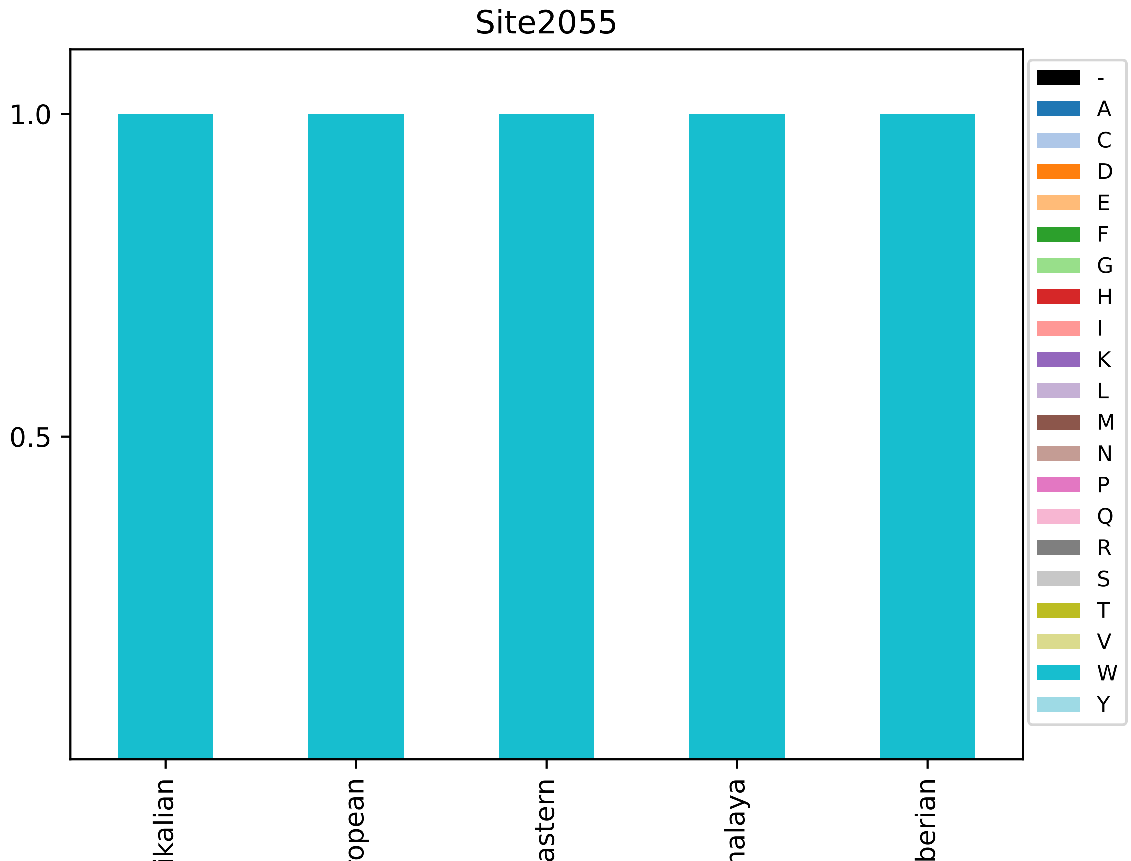
Site2055
1.0
0.5
-
A
C
D
E
F
G
H
I
K
L
M
N
P
Q
R
S
T
V
W
Y
ikalian
opean
astern
nalaya
berian

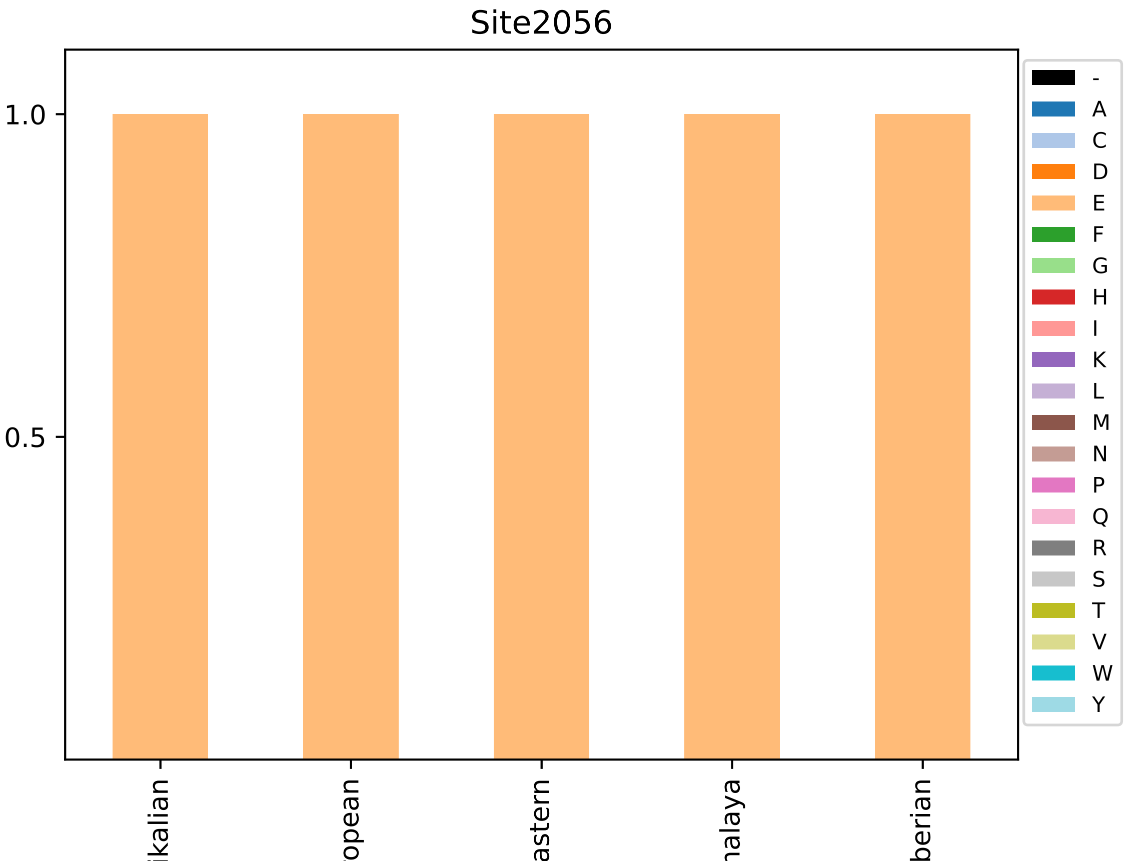

Site2056
1.0
0.5
-
A
C
D
E
F
G
H
I
K
L
M
N
P
Q
R
S
T
V
W
Y
ikalian
opean
astern
nalaya
berian

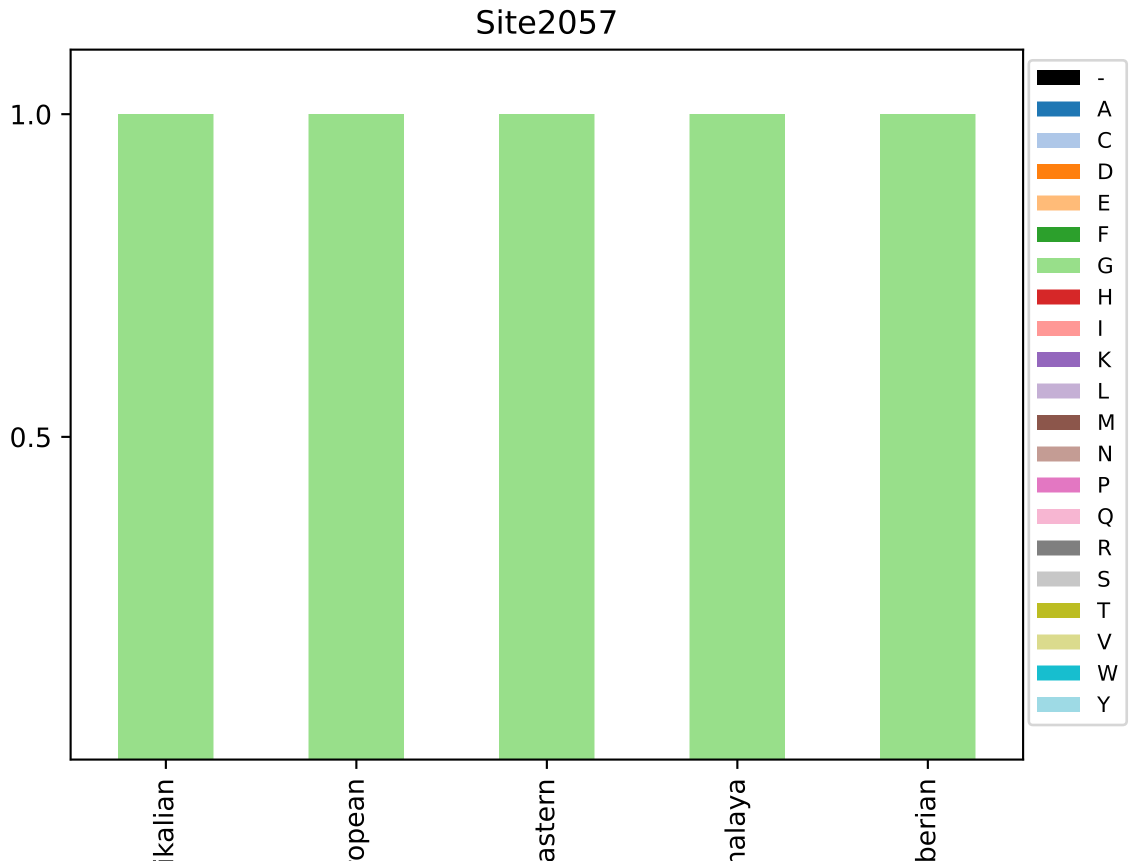

Site2057
1.0
0.5
-
A
C
D
E
F
G
H
I
K
L
M
N
P
Q
R
S
T
V
W
Y
ikalian
opean
astern
nalaya
berian

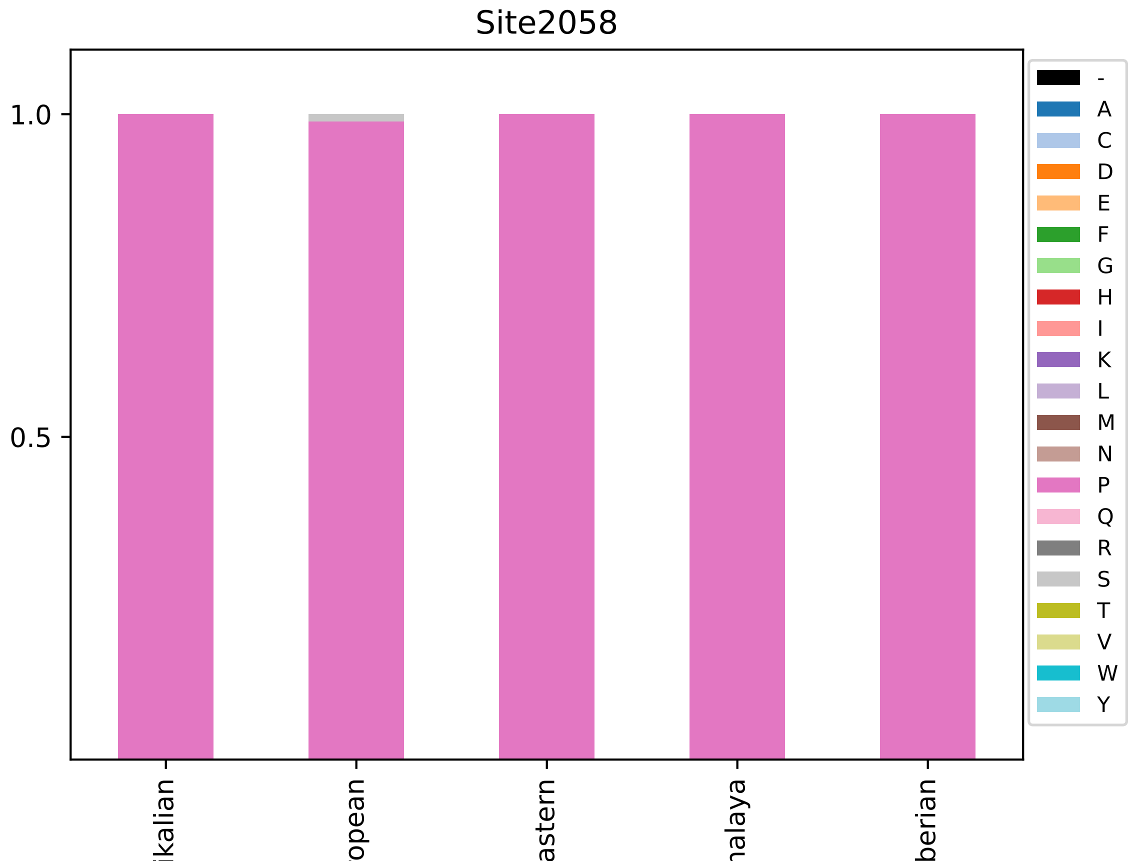

Site2058
1.0
0.5
-
A
C
D
E
F
G
H
I
K
L
M
N
P
Q
R
S
T
V
W
Y
ikalian
opean
astern
nalaya
berian

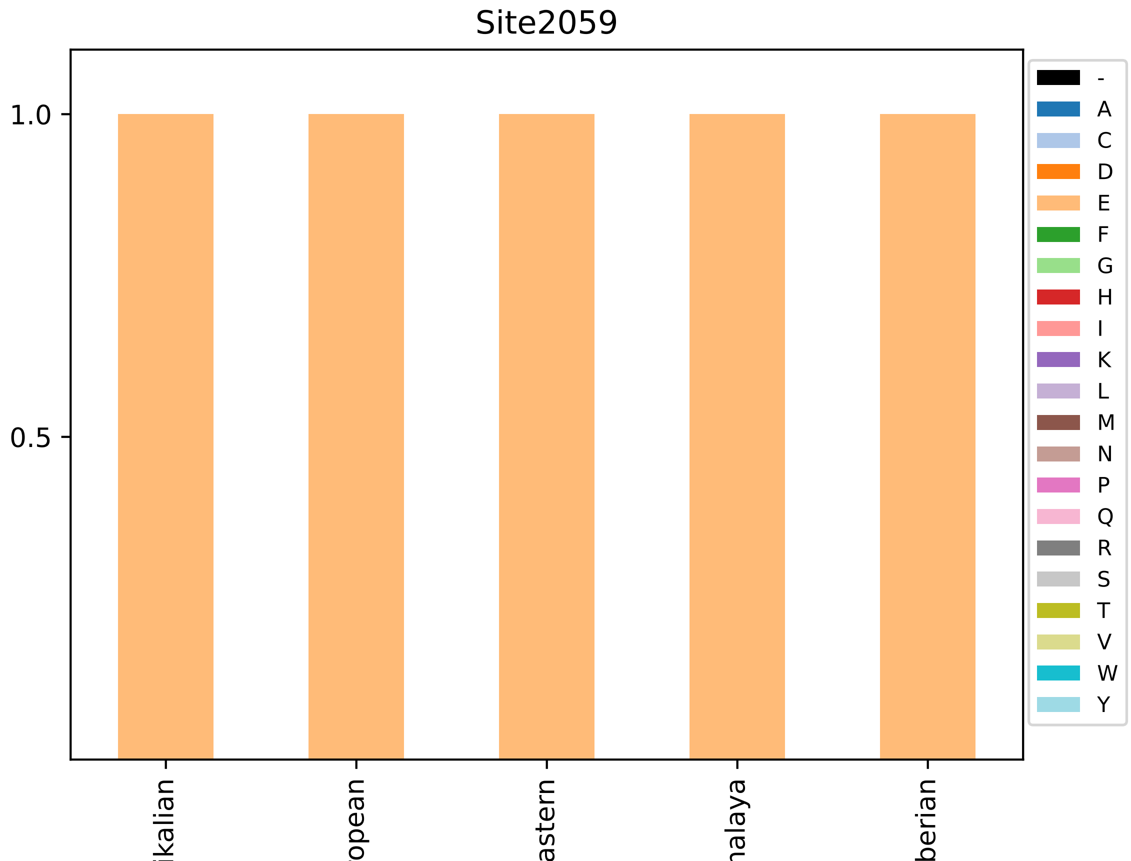
Site2059
1.0
0.5
ikalian
opean
astern
nalaya
berian
-
A
C
D
E
F
G
H
I
K
L
M
N
P
Q
R
S
T
V
W
Y

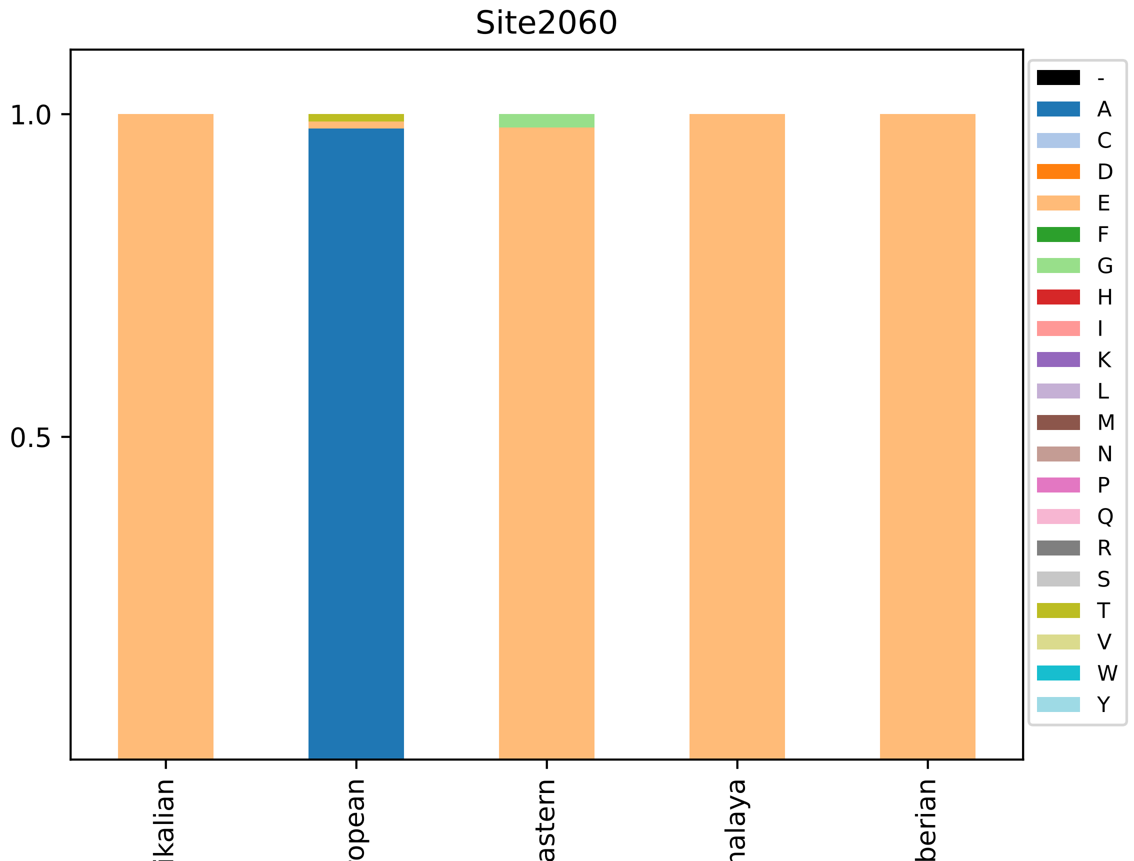

Site2060
1.0
0.5
-
A
C
D
E
F
G
H
I
K
L
M
N
P
Q
R
S
T
V
W
Y
ikalian
opean
astern
nalaya
berian

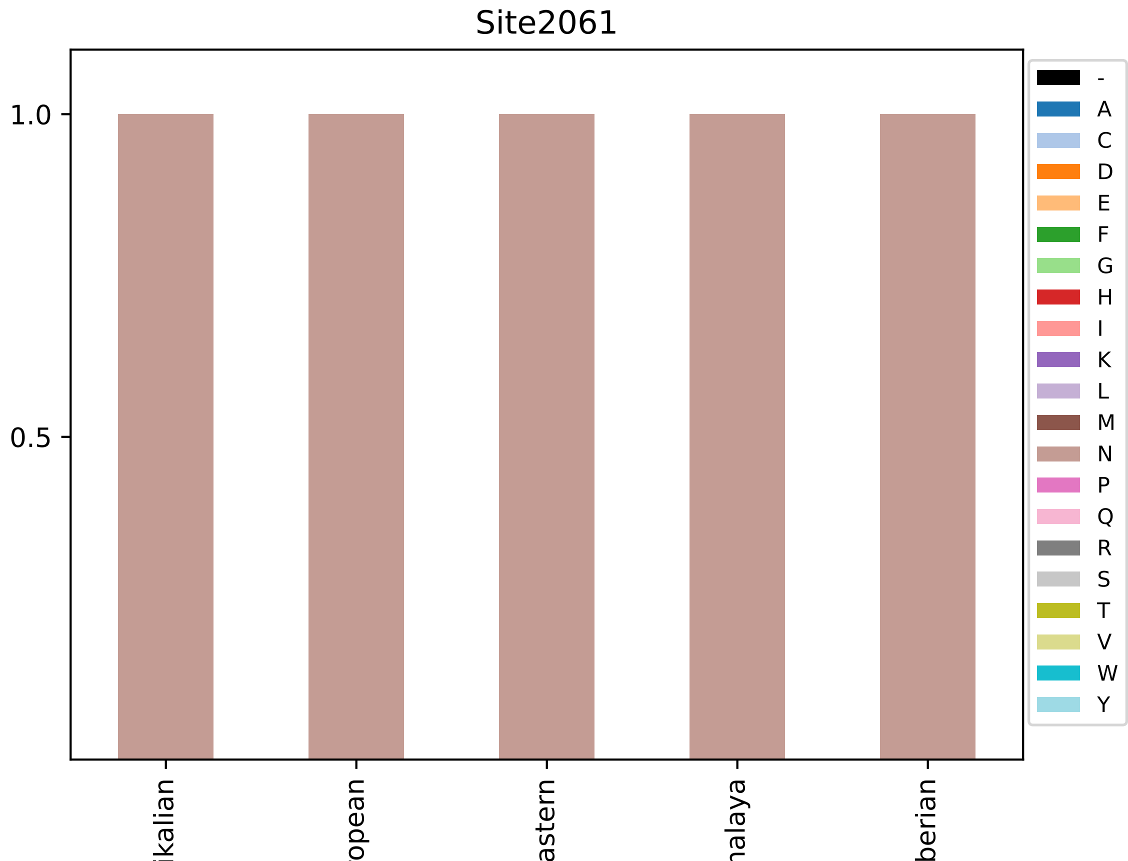

Site2061
1.0
0.5
ikalian
opean
astern
nalaya
berian
-
A
C
D
E
F
G
H
I
K
L
M
N
P
Q
R
S
T
V
W
Y

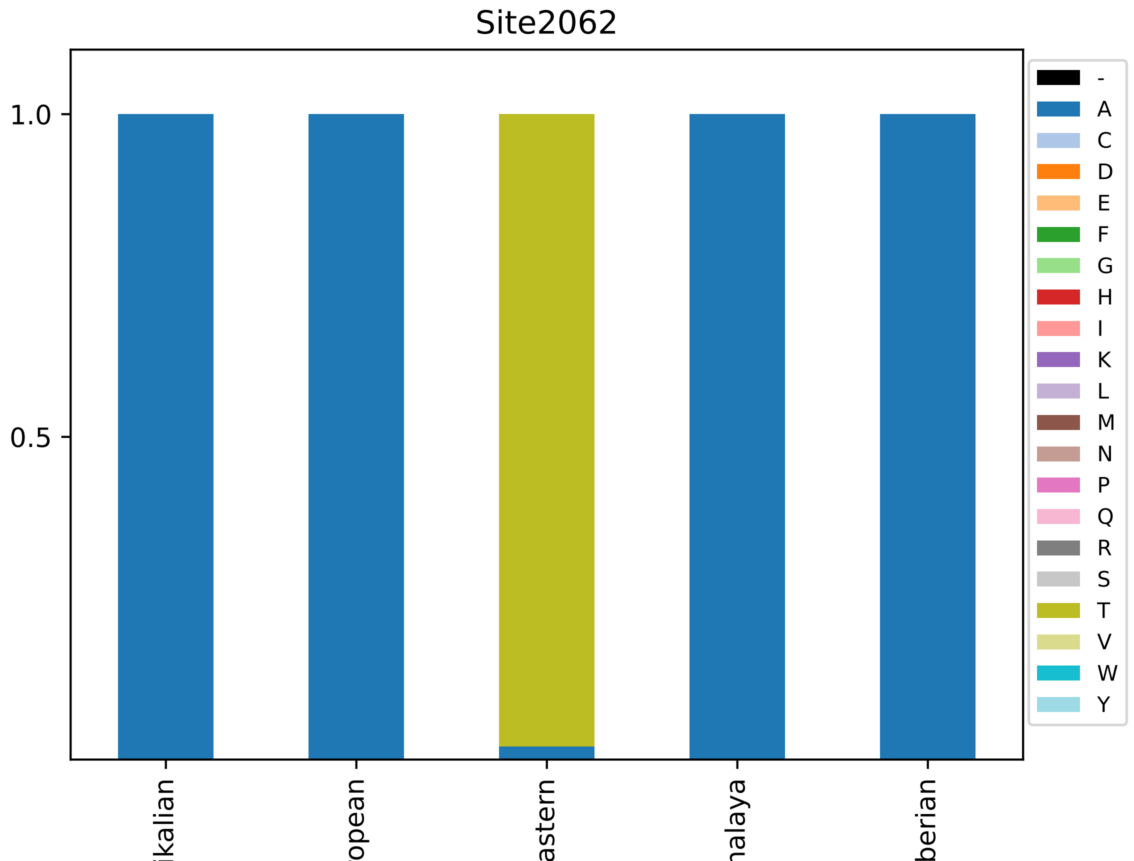

Site2062
1.0
0.5
ikalian
opean
astern
nalaya
berian
-
A
C
D
E
F
G
H
I
K
L
M
N
P
Q
R
S
T
V
W
Y

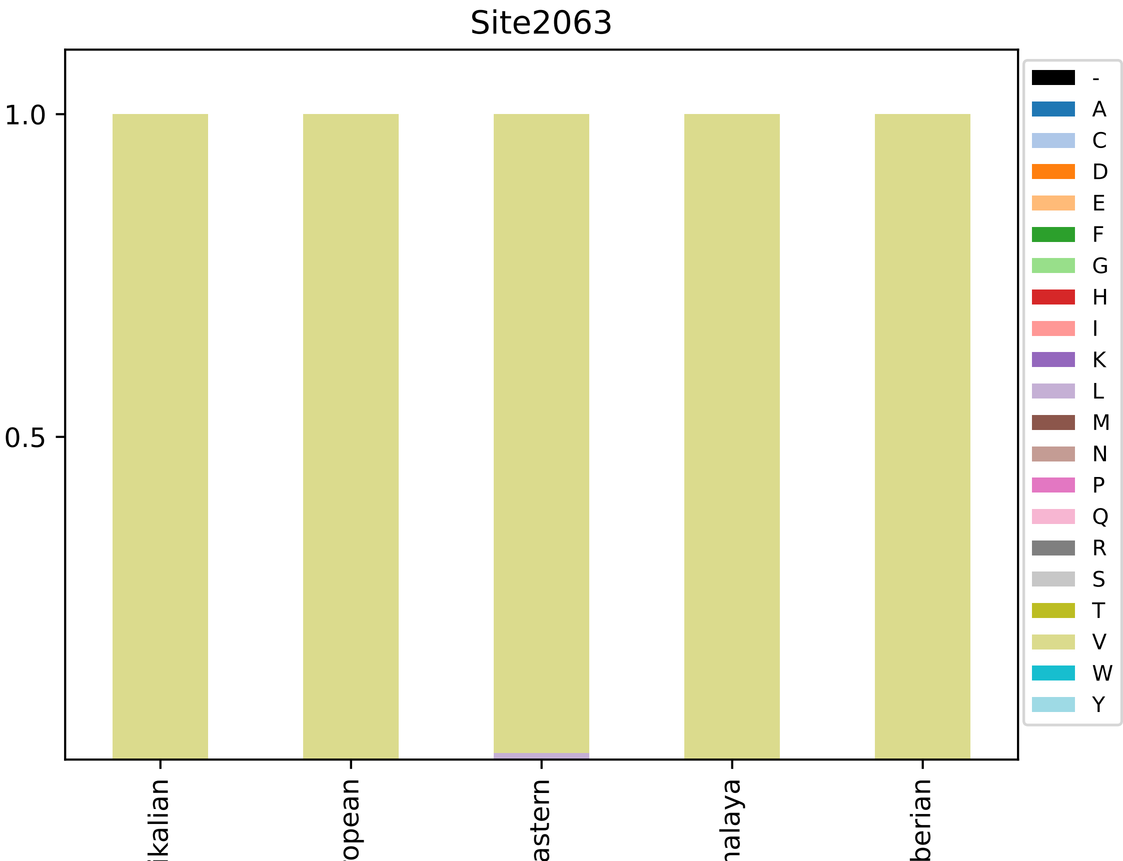
Site2063
1.0
0.5
-
A
C
D
E
F
G
H
I
K
L
M
N
P
Q
R
S
T
V
W
Y
ikalian
ropean
astern
nalaya
berian

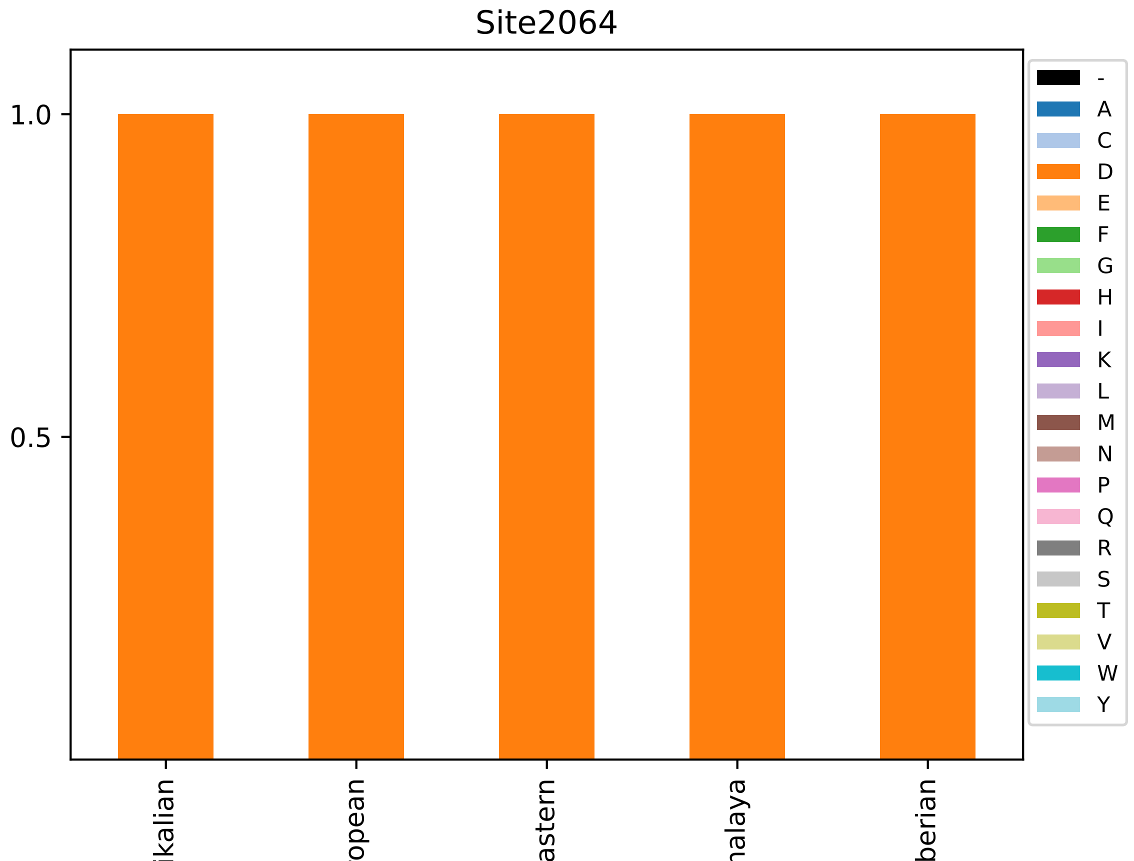
Site2064
1.0
0.5
ikalian
opean
astern
nalaya
berian
-
A
C
D
E
F
G
H
I
K
L
M
N
P
Q
R
S
T
V
W
Y

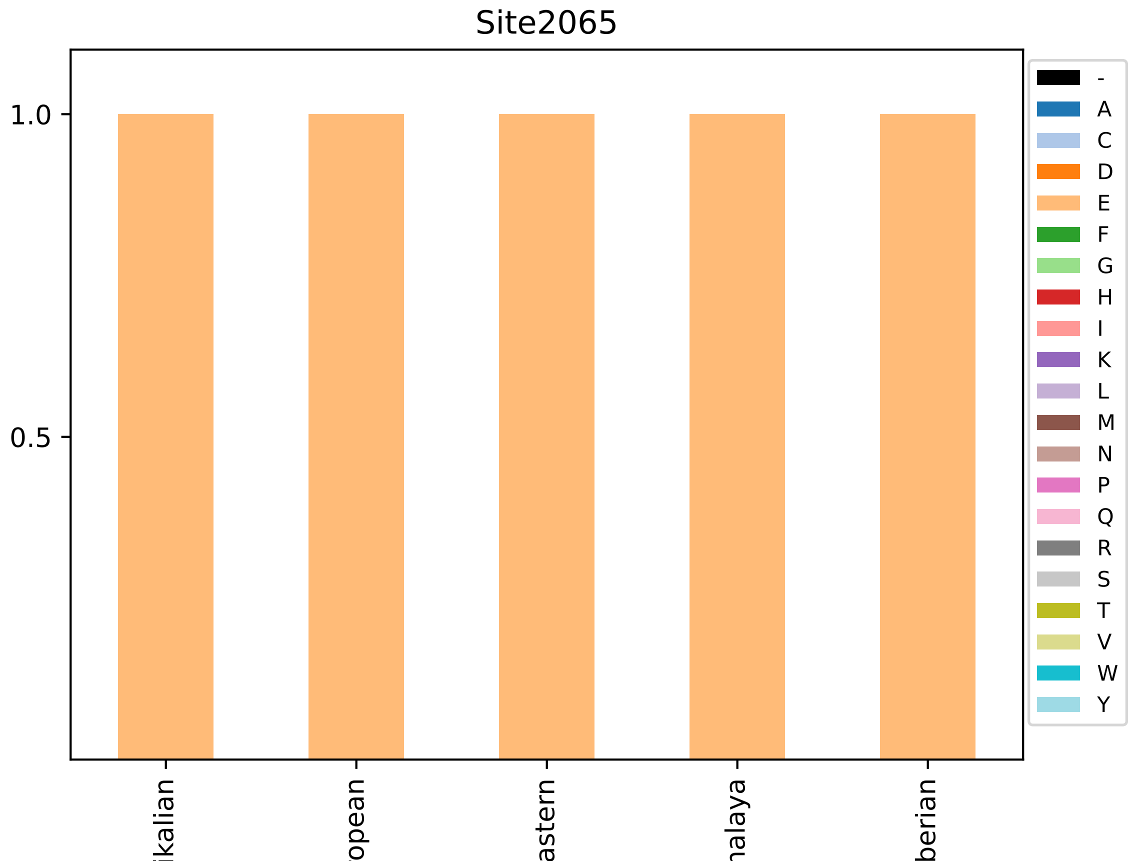

Site2065
1.0
0.5
-
A
C
D
E
F
G
H
I
K
L
M
N
P
Q
R
S
T
V
W
Y
ikalian
opean
astern
nalaya
berian

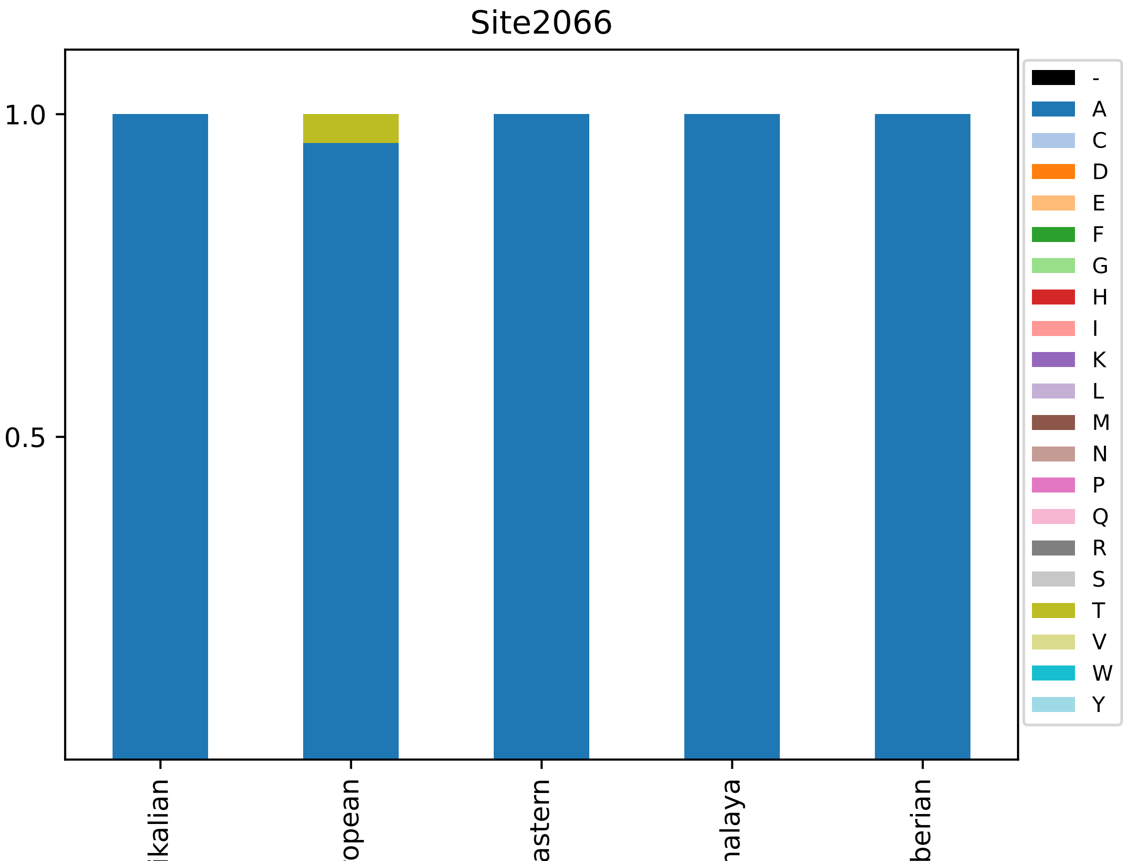

Site2066
1.0
0.5
-
A
C
D
E
F
G
H
I
K
L
M
N
P
Q
R
S
T
V
W
Y
ikalian
opean
astern
nalaya
berian

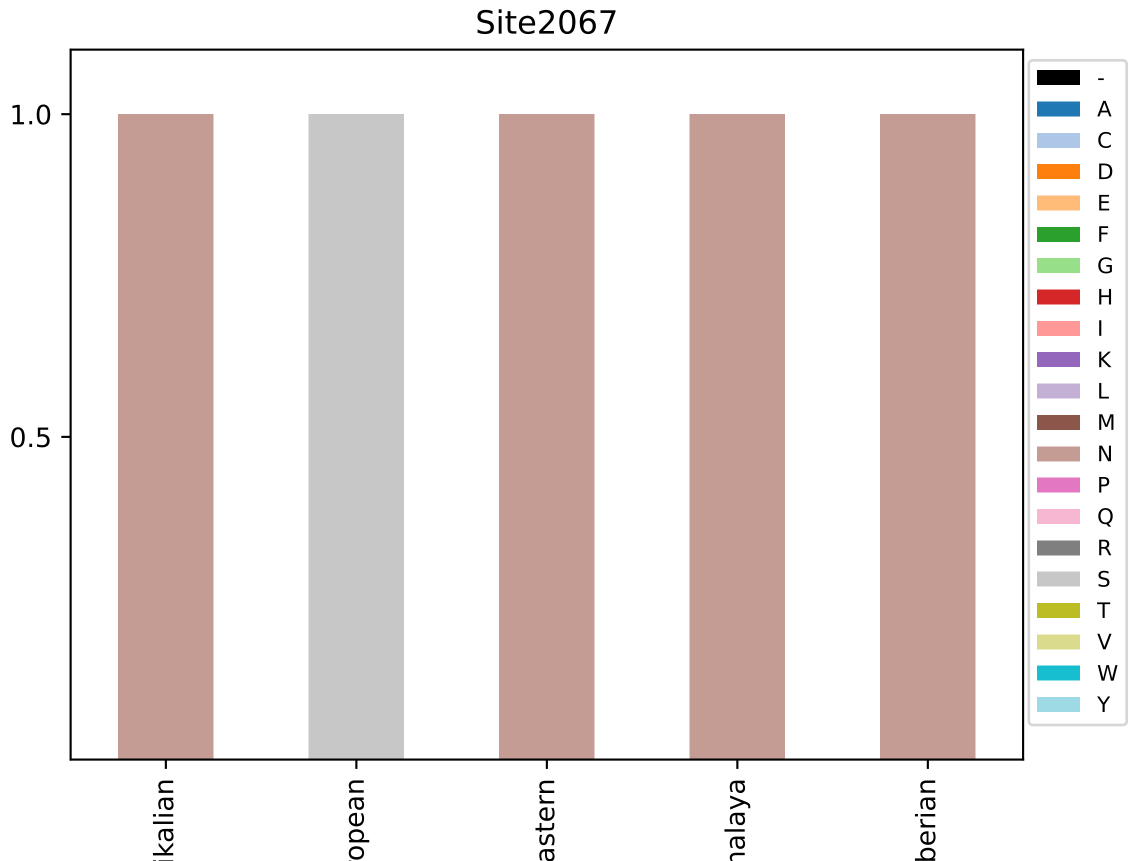
Site2067
1.0
0.5
ikalian
opean
astern
nalaya
berian
-
A
C
D
E
F
G
H
I
K
L
M
N
P
Q
R
S
T
V
W
Y

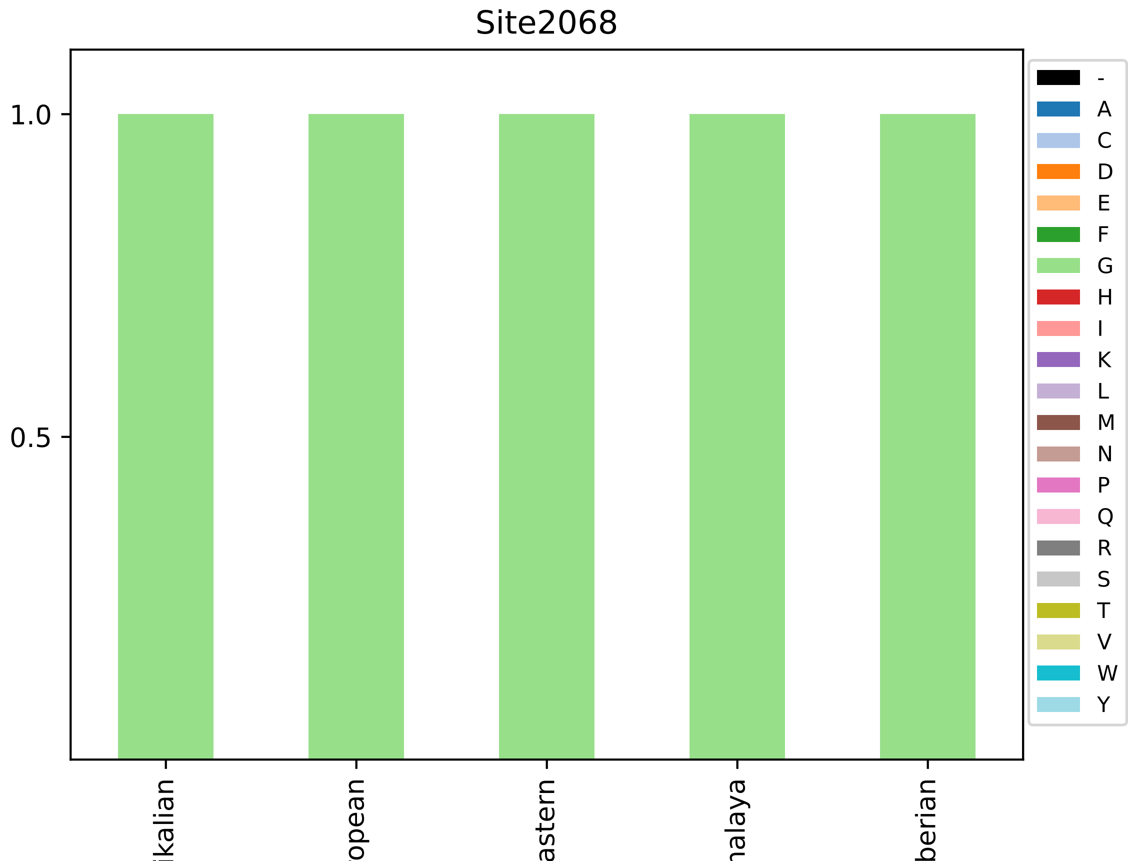
Site2068
1.0
0.5
-
A
C
D
E
F
G
H
I
K
L
M
N
P
Q
R
S
T
V
W
Y
ikalian
ropean
astern
nalaya
berian

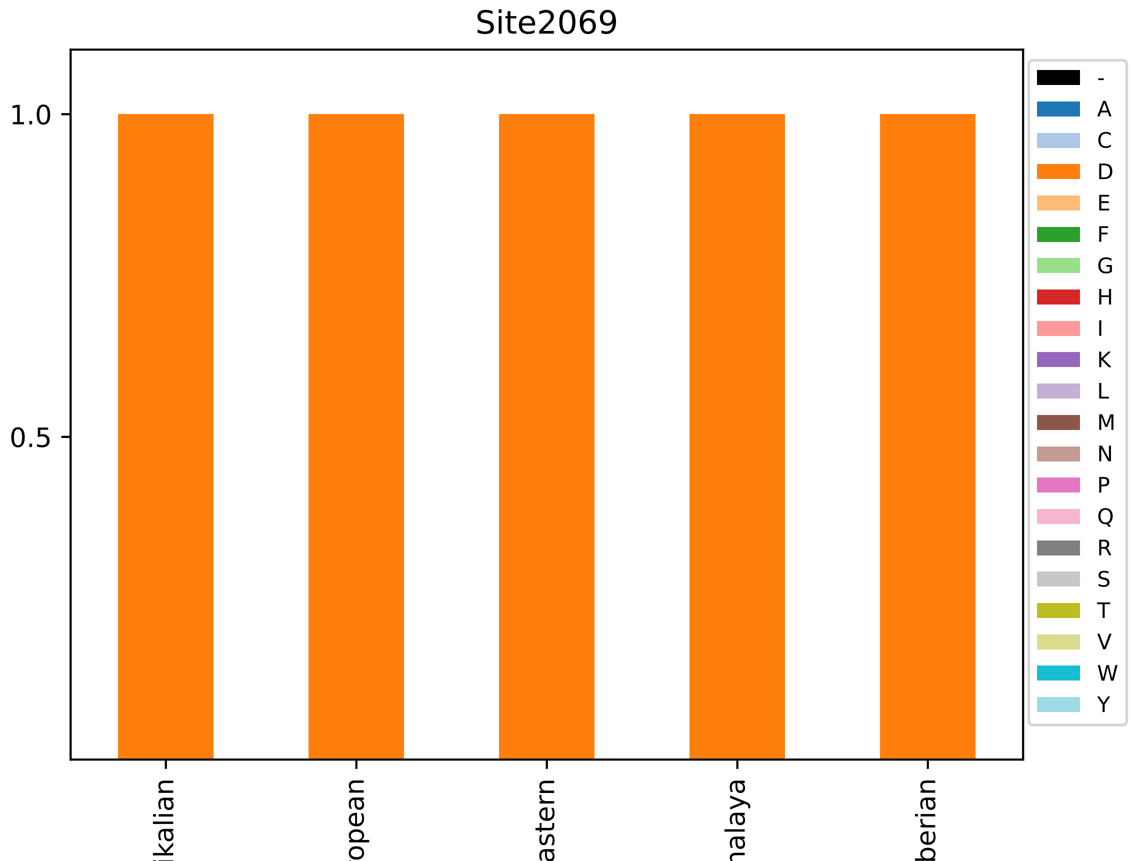
Site2069
1.0
0.5
-
A
C
D
E
F
G
H
I
K
L
M
N
P
Q
R
S
T
V
W
Y
ikalian
opean
astern
nalaya
berian

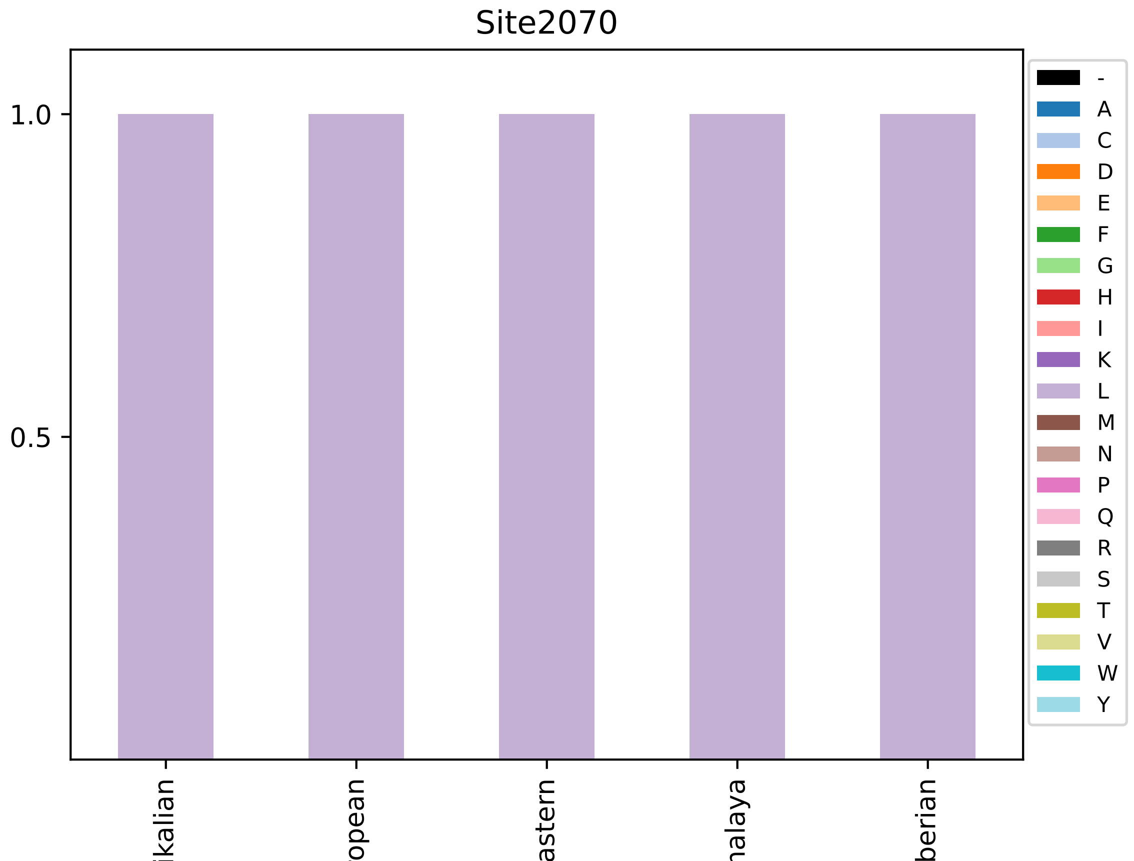

Site2070
1.0
0.5
-
A
C
D
E
F
G
H
I
K
L
M
N
P
Q
R
S
T
V
W
Y
ikalian
opean
astern
nalaya
berian

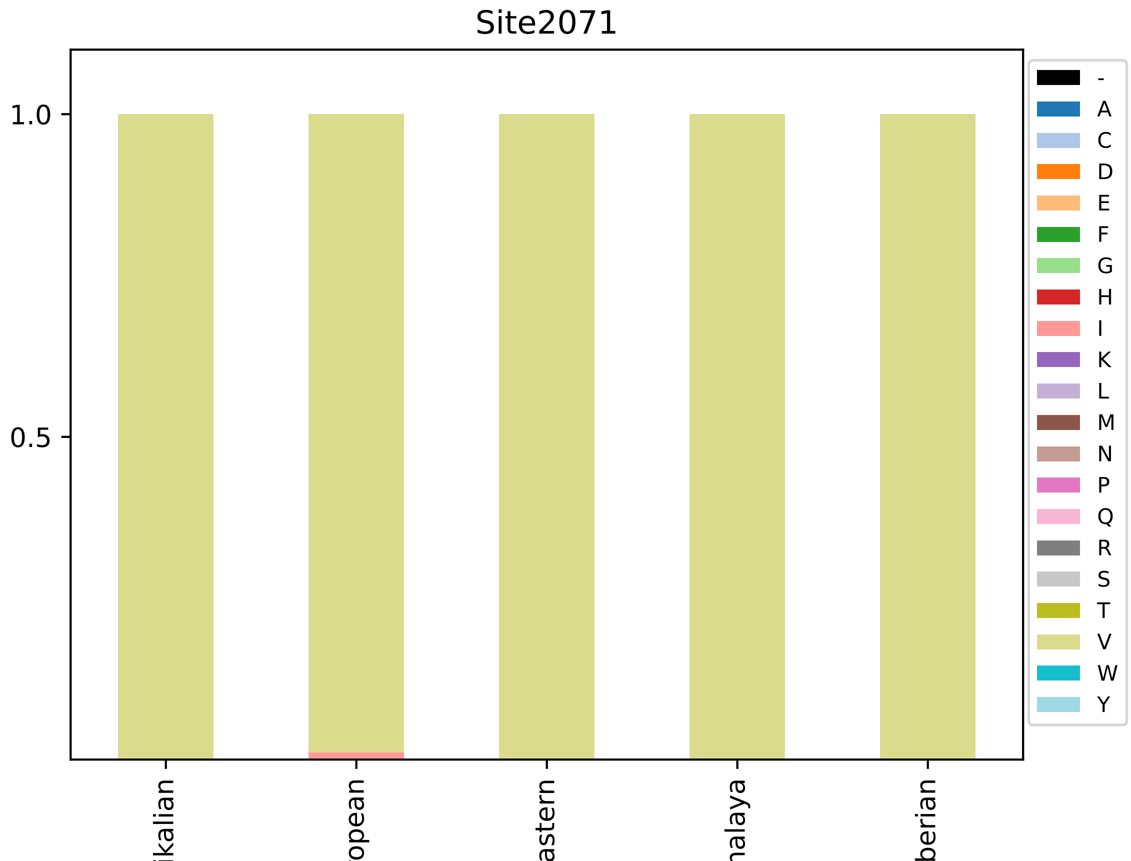
Site2071
1.0
0.5
ikalian
opean
astern
halaya
berian
-
A
C
D
E
F
G
H
I
K
L
M
N
P
Q
R
S
T
V
W
Y

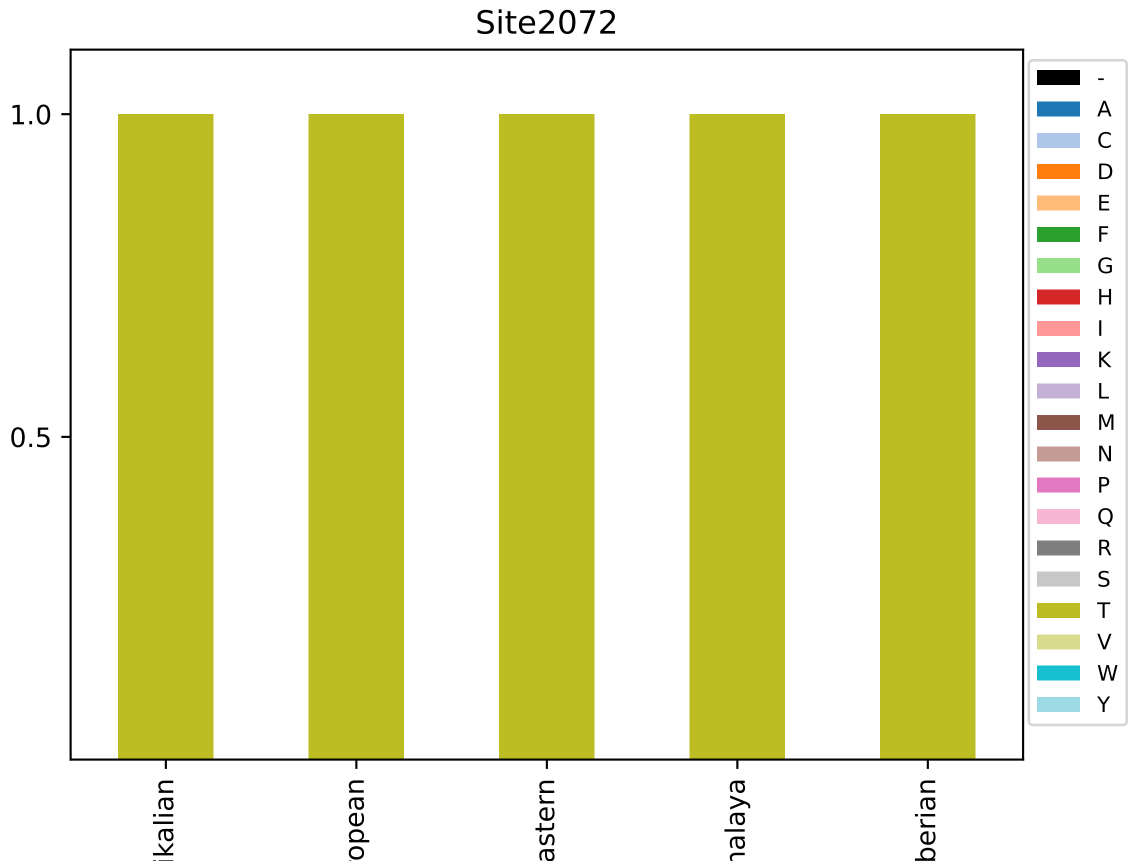
Site2072
1.0
0.5
ikalian
opean
astern
nalaya
berian
-
A
C
D
E
F
G
H
I
K
L
M
N
P
Q
R
S
T
V
W
Y

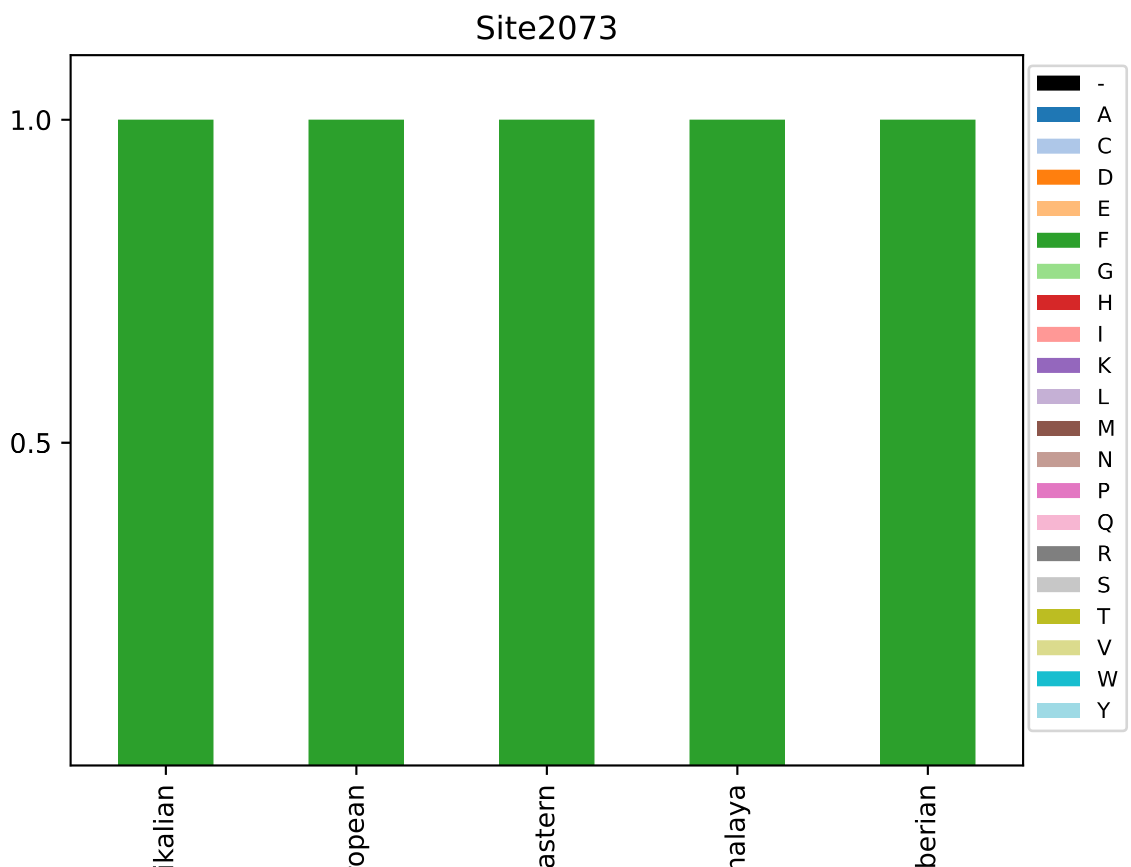

Site2073
1.0
0.5
-
A
C
D
E
F
G
H
I
K
L
M
N
P
Q
R
S
T
V
W
Y
ikalian
opean
astern
nalaya
berian

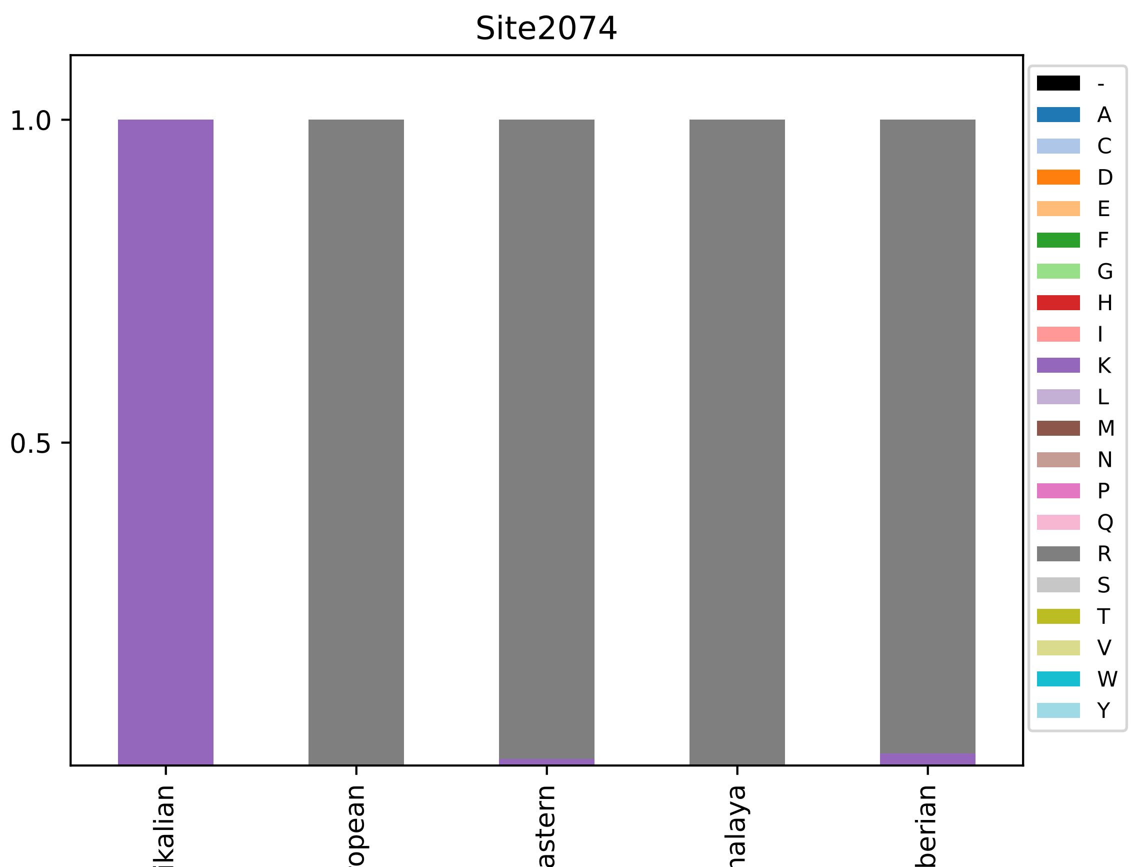

Site2074
1.0
0.5
-
A
C
D
E
F
G
H
I
K
L
M
N
P
Q
R
S
T
V
W
Y
ikalian
opean
astern
nalaya
berian

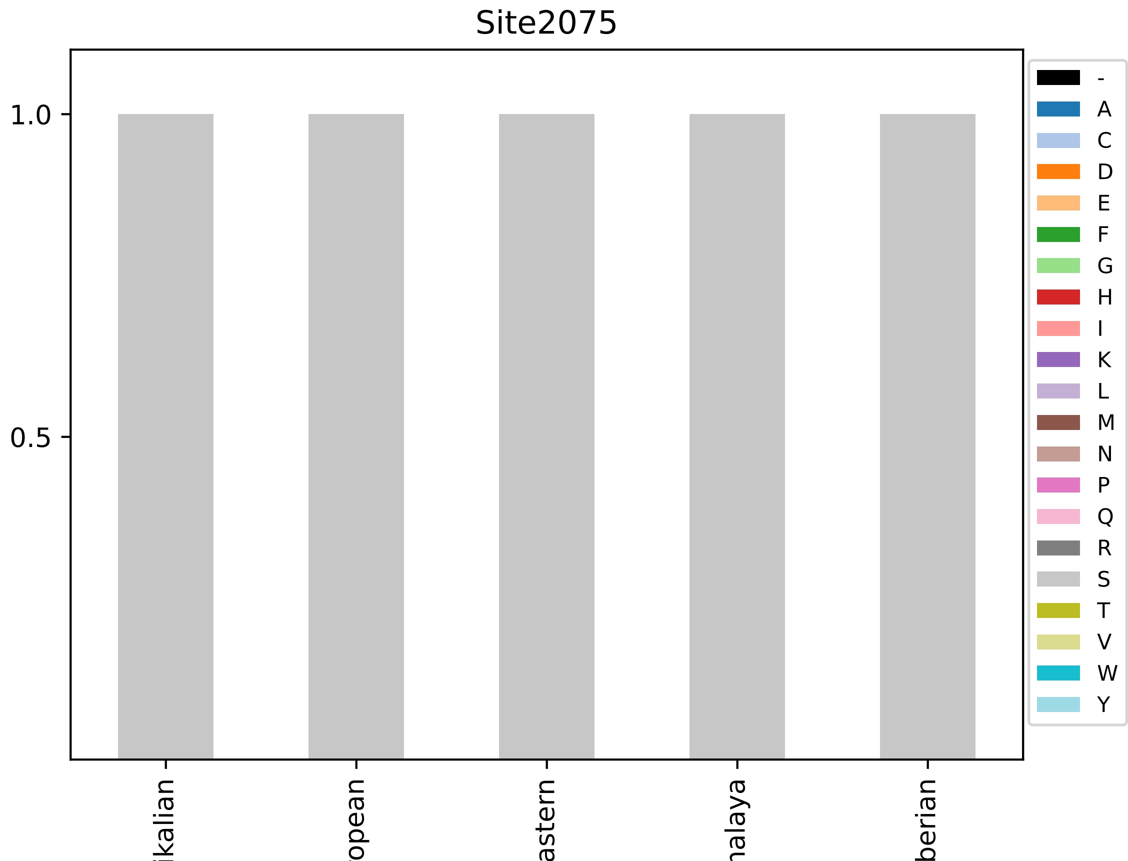

Site2075
1.0
0.5
-
A
C
D
E
F
G
H
I
K
L
M
N
P
Q
R
S
T
V
W
Y
ikalian
opean
astern
nalaya
berian

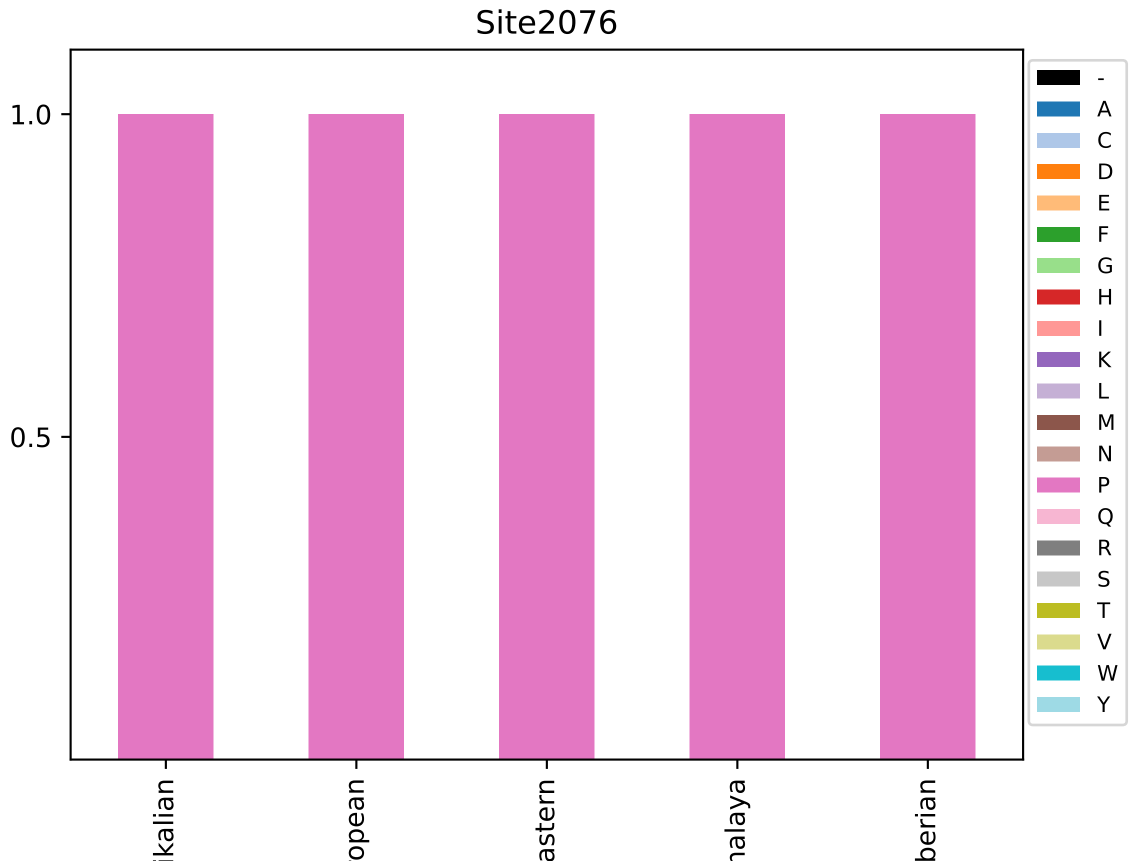

Site2076
1.0
0.5
-
A
C
D
E
F
G
H
I
K
L
M
N
P
Q
R
S
T
V
W
Y
ikalian
opean
astern
nalaya
berian

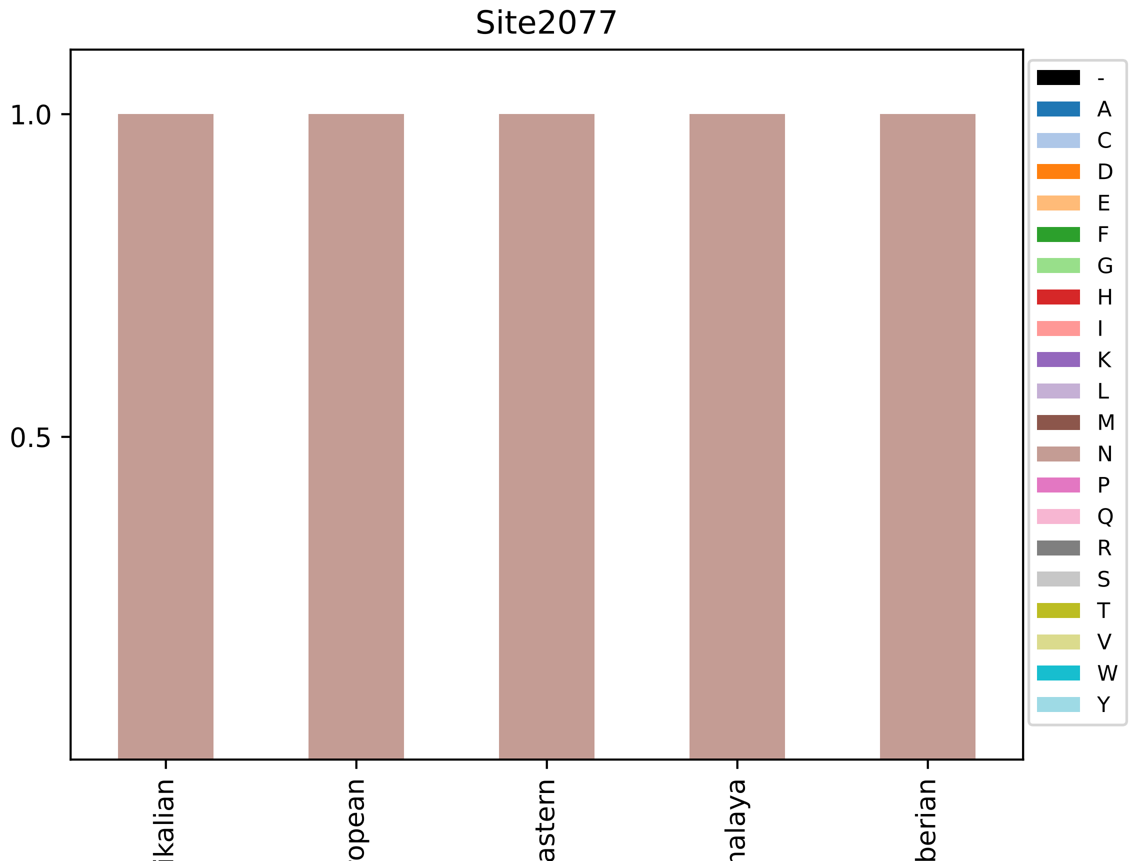

Site2077
1.0
0.5
-
A
C
D
E
F
G
H
I
K
L
M
N
P
Q
R
S
T
V
W
Y
ikalian
opean
astern
nalaya
berian

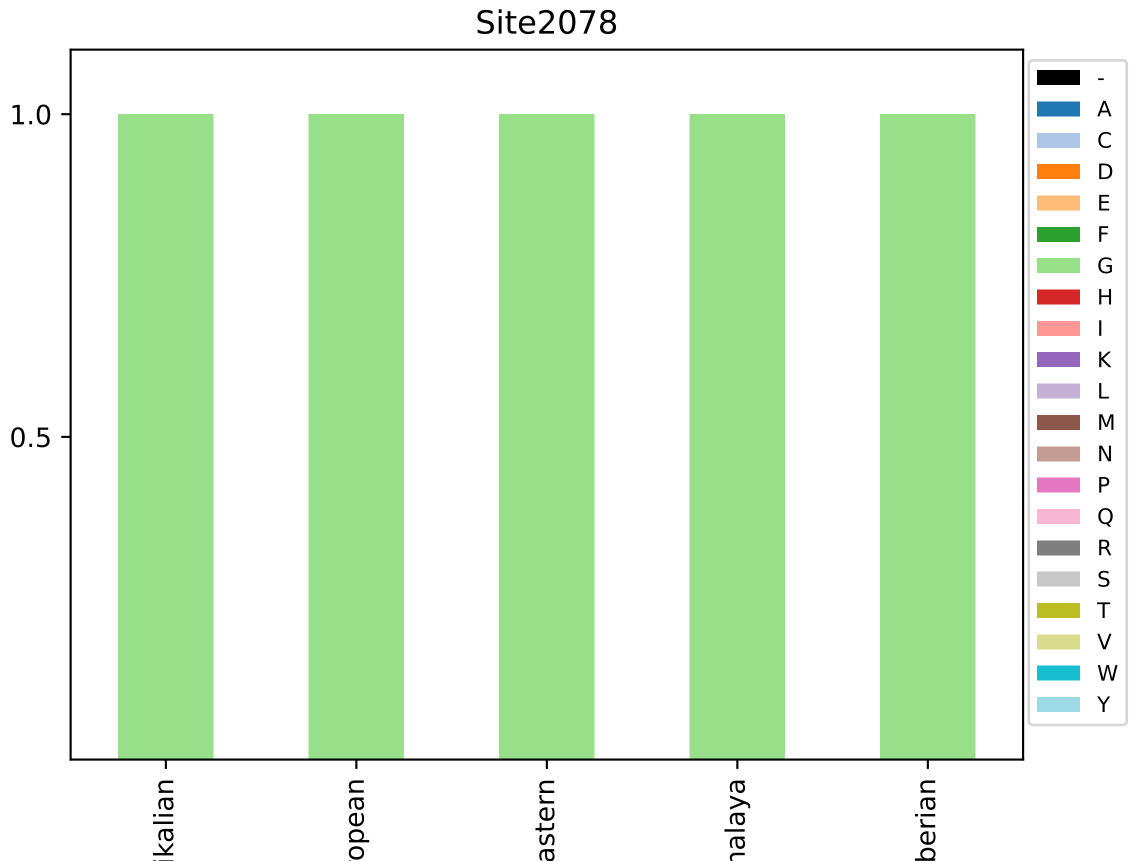
Site2078
1.0
0.5
-
A
C
D
E
F
G
H
I
K
L
M
N
P
Q
R
S
T
V
W
Y
ikalian
opean
astern
nalaya
berian

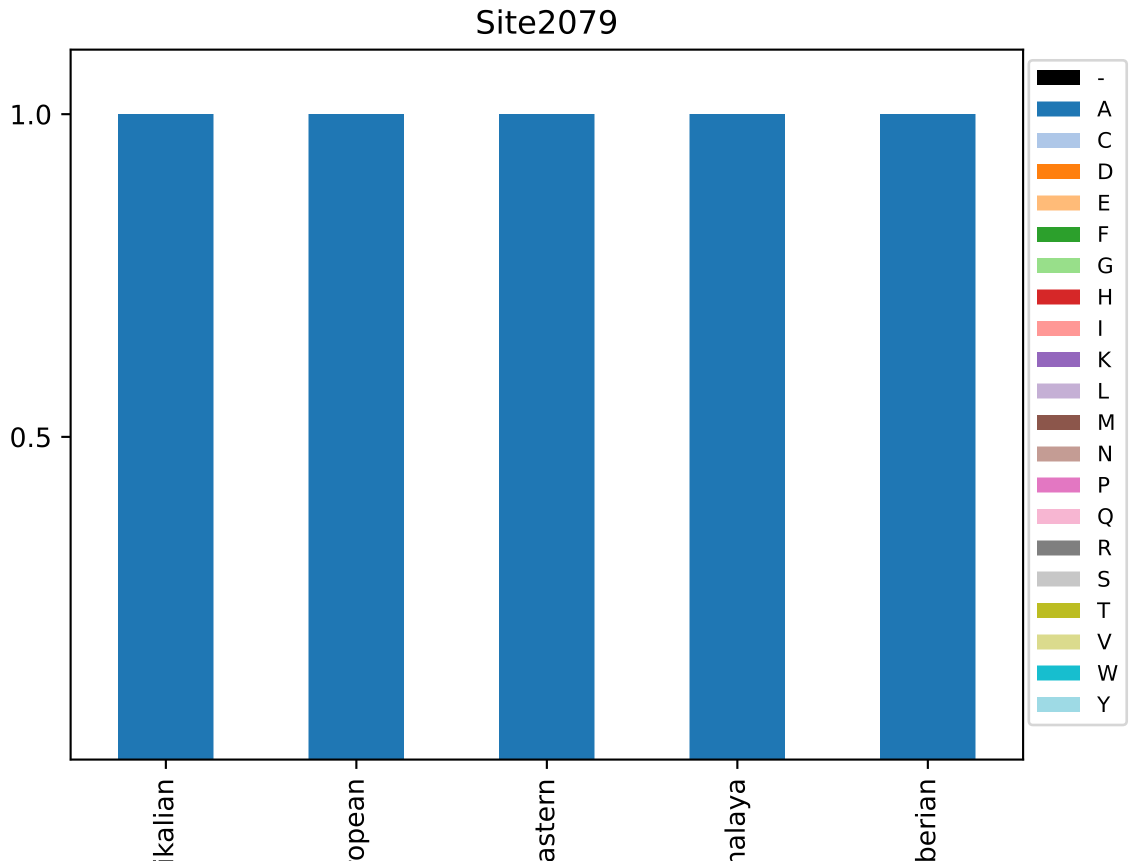

Site2079
1.0
0.5
-
A
C
D
E
F
G
H
I
K
L
M
N
P
Q
R
S
T
V
W
Y
ikalian
opean
astern
nalaya
berian

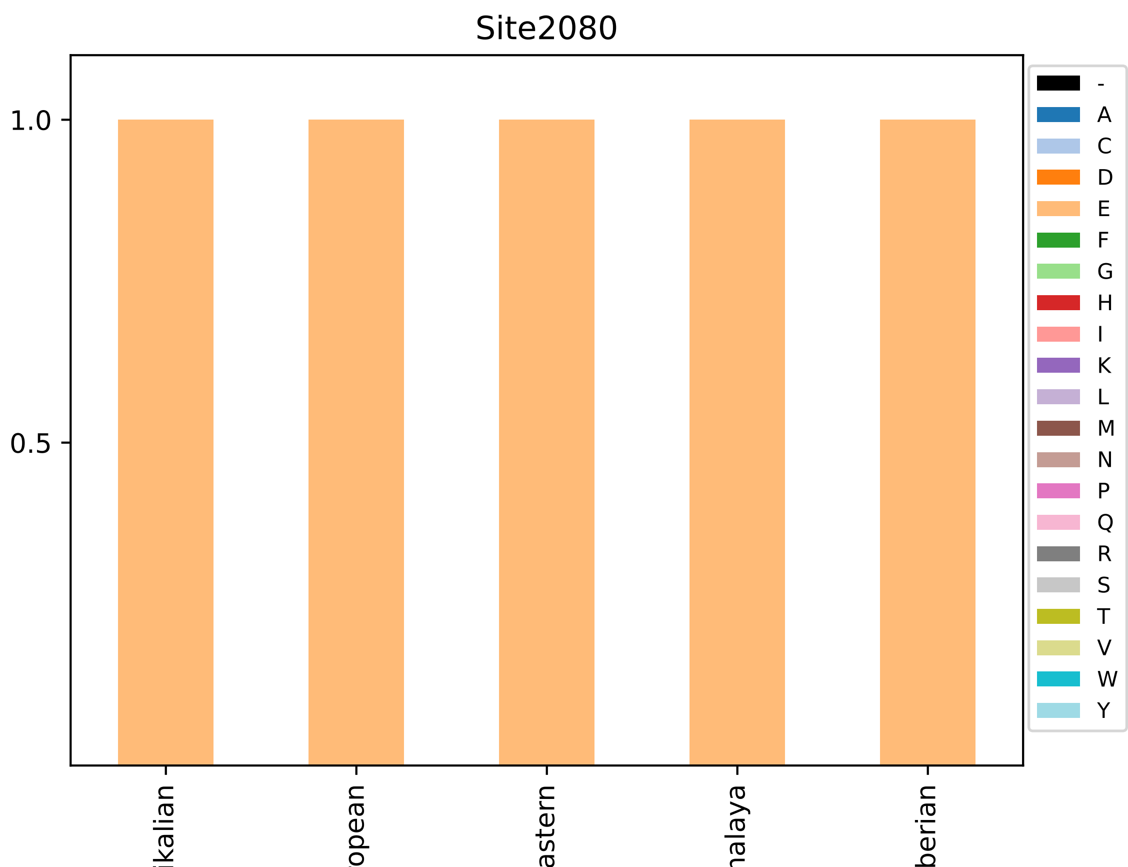
Site2080
1.0
0.5
-
A
C
D
E
F
G
H
I
K
L
M
N
P
Q
R
S
T
V
W
Y
ikalian
opean
astern
nalaya
berian

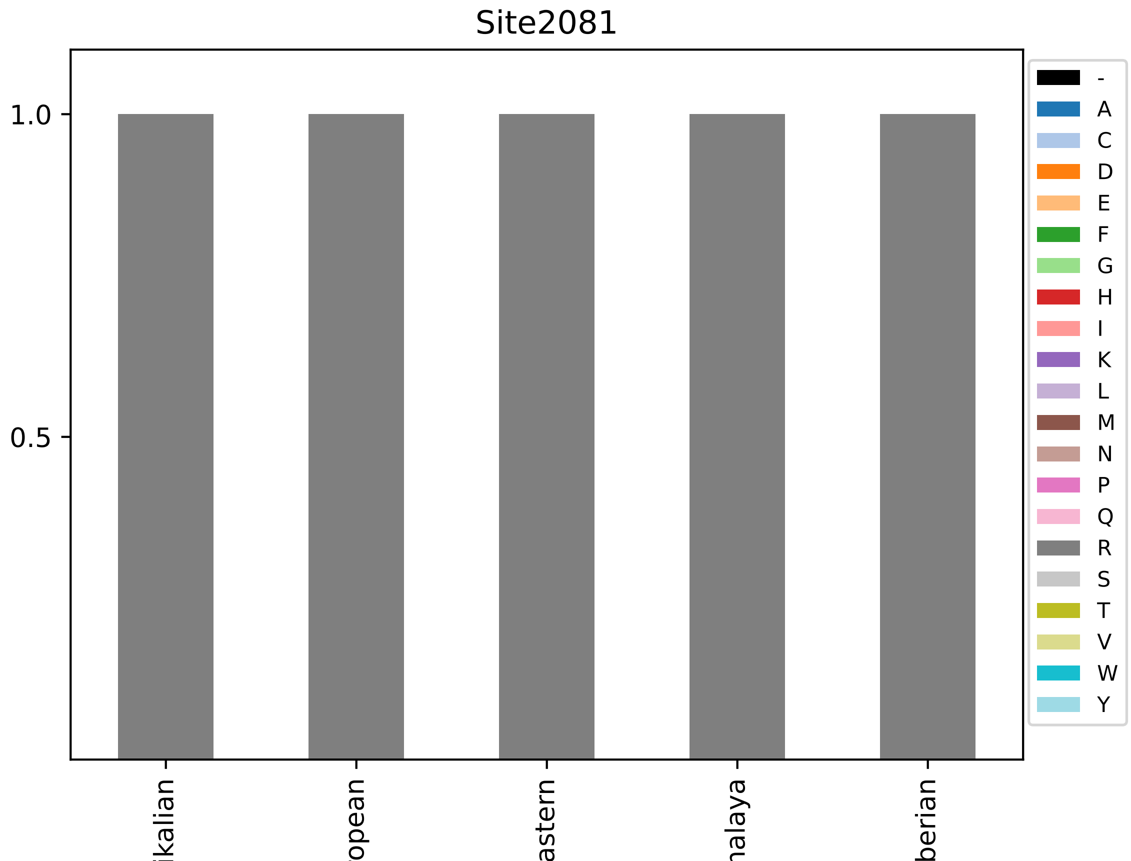

Site2081
1.0
0.5
-
A
C
D
E
F
G
H
I
K
L
M
N
P
Q
R
S
T
V
W
Y
ikalian
opean
astern
nalaya
berian

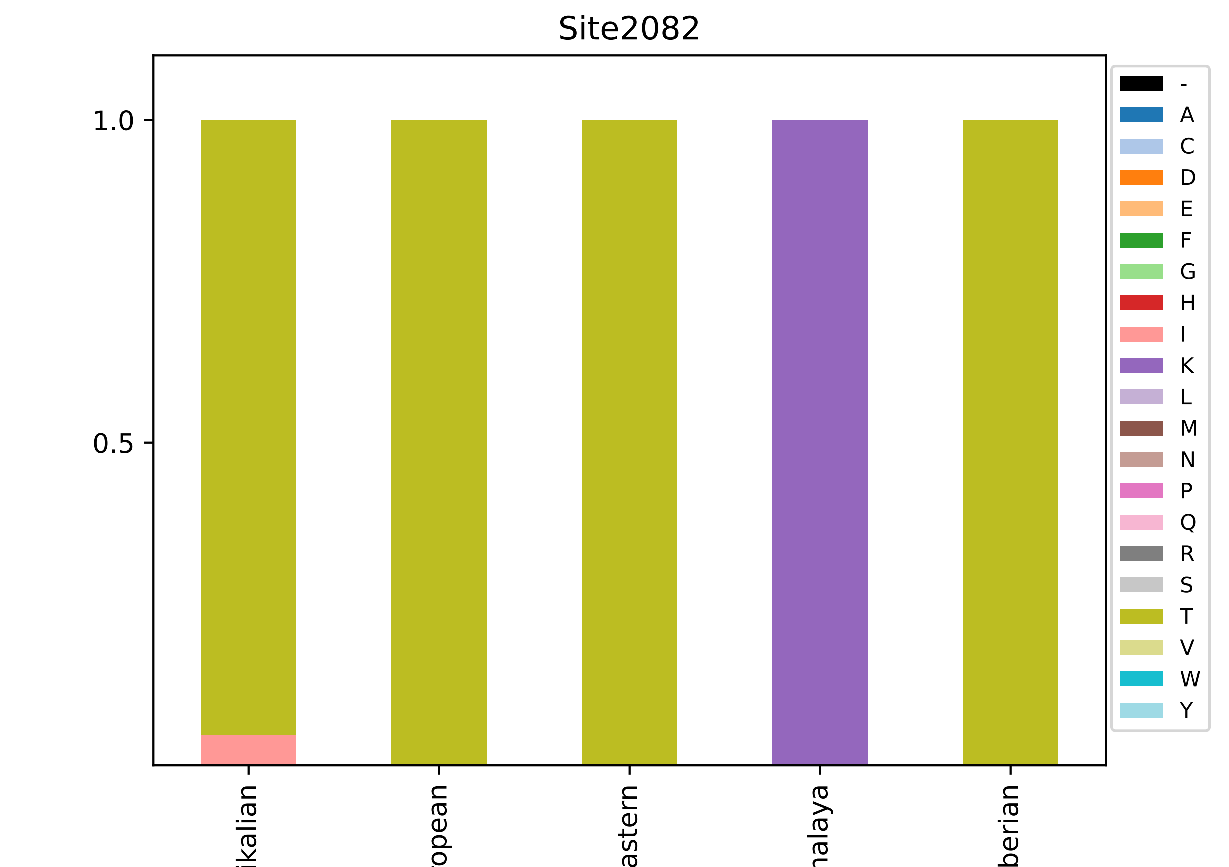
Site2082
1.0
0.5
-
A
C
D
E
F
G
H
I
K
L
M
N
P
Q
R
S
T
V
W
Y
ikalian
opean
astern
nalaya
berian

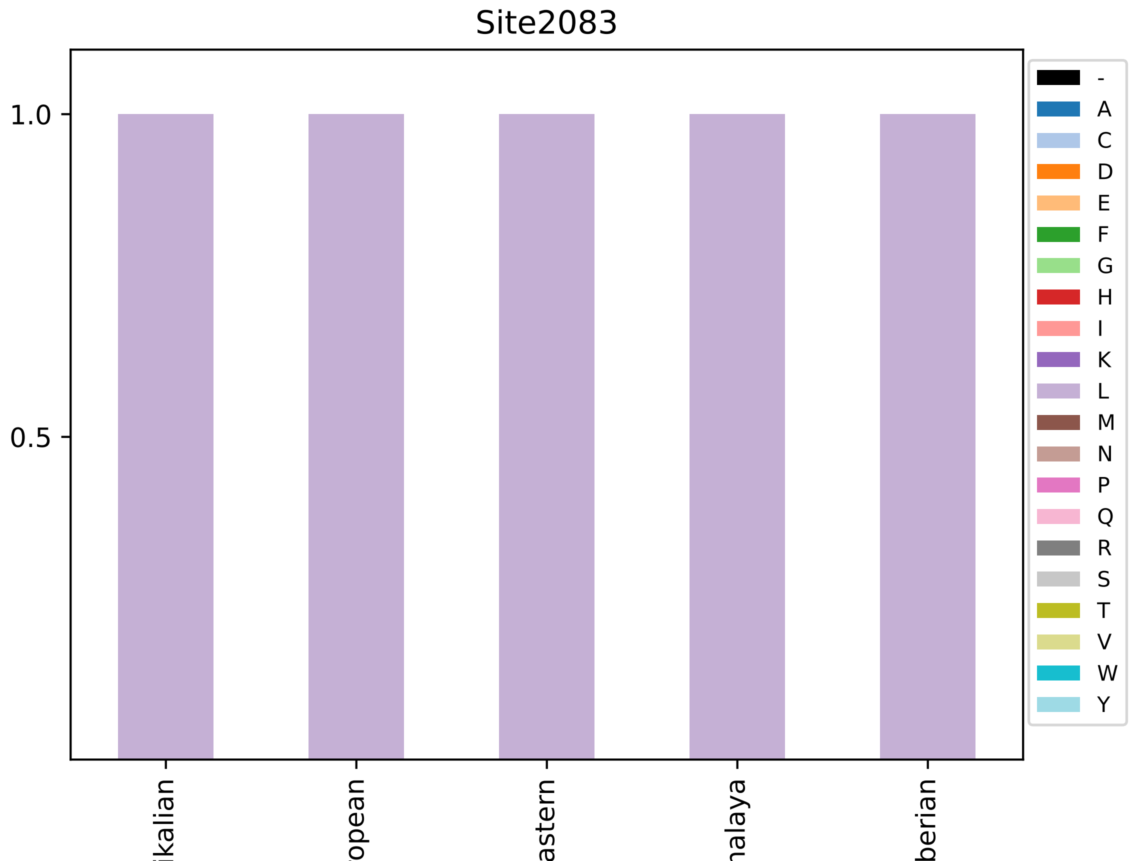

Site2083
1.0
0.5
-
A
C
D
E
F
G
H
I
K
L
M
N
P
Q
R
S
T
V
W
Y
ikalian
opean
astern
nalaya
berian

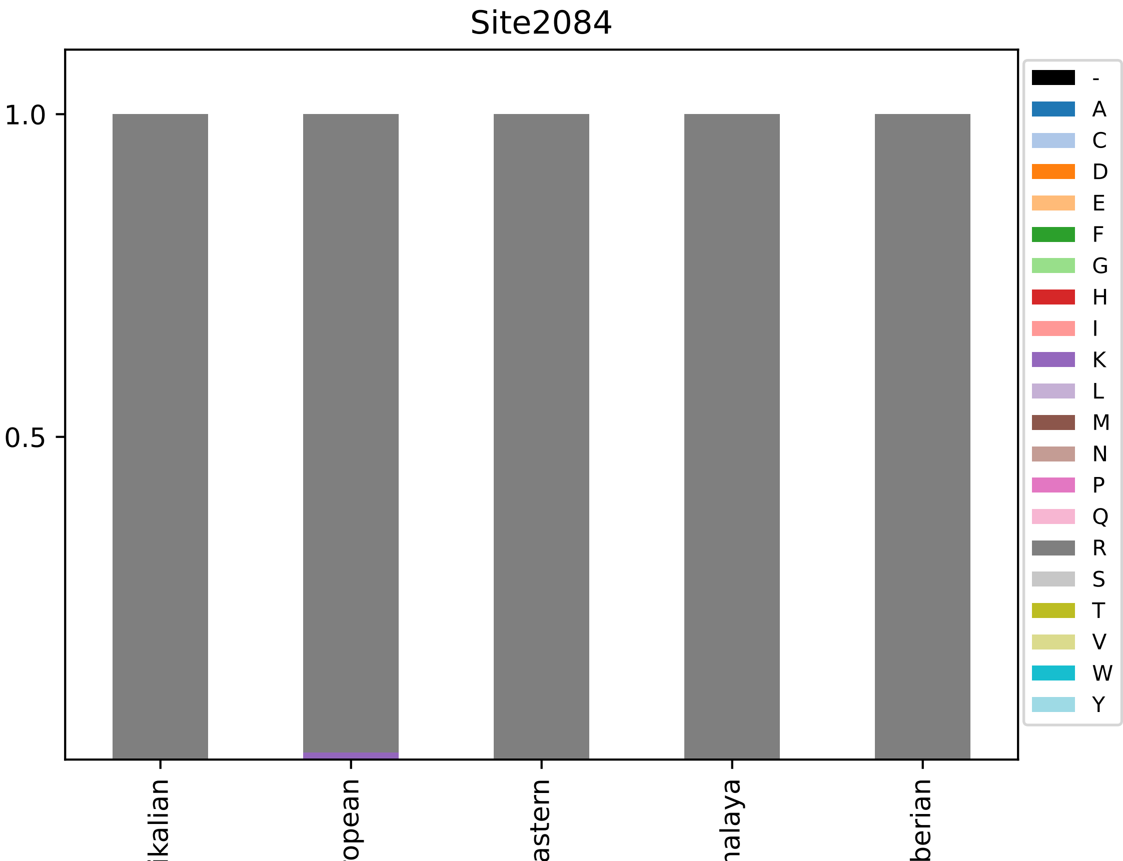
Site2084
1.0
0.5
-
A
C
D
E
F
G
H
I
K
L
M
N
P
Q
R
S
T
V
W
Y
ikalian
opean
astern
nalaya
berian

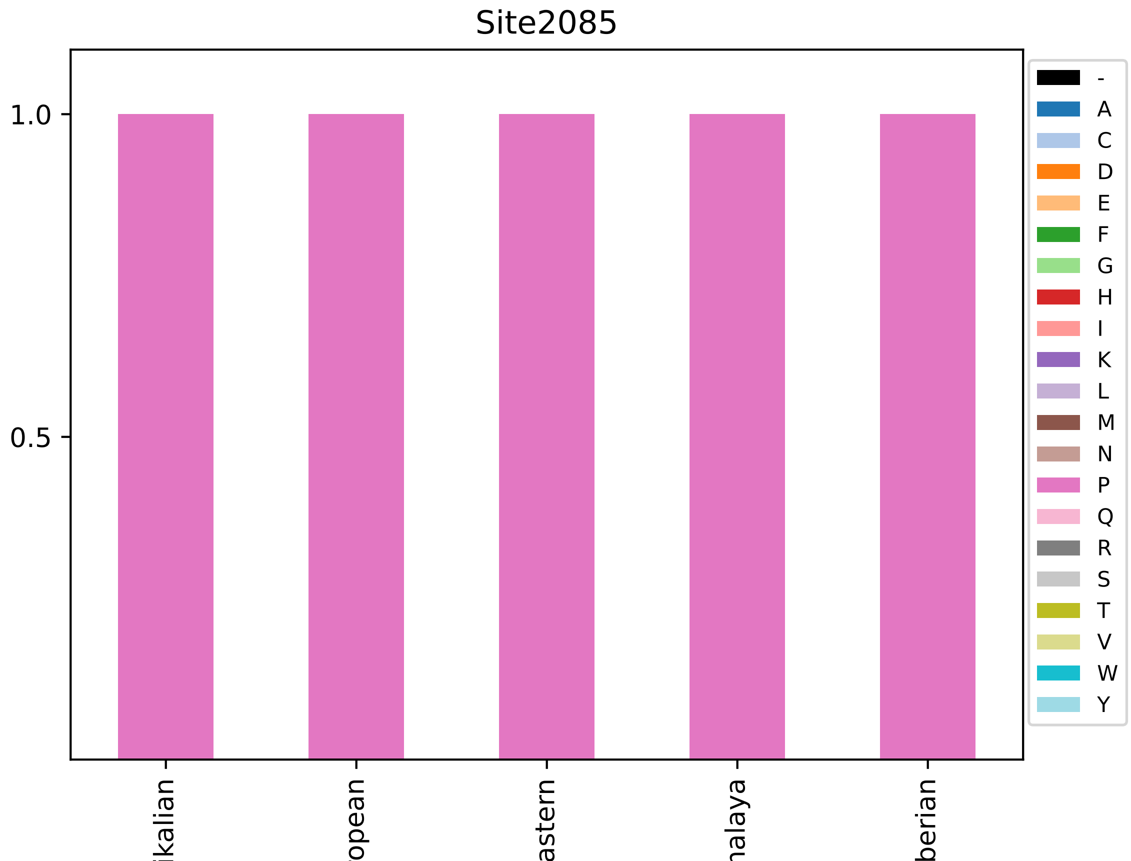
Site2085
1.0
0.5
-
A
C
D
E
F
G
H
I
K
L
M
N
P
Q
R
S
T
V
W
Y
ikalian
opean
astern
nalaya
berian

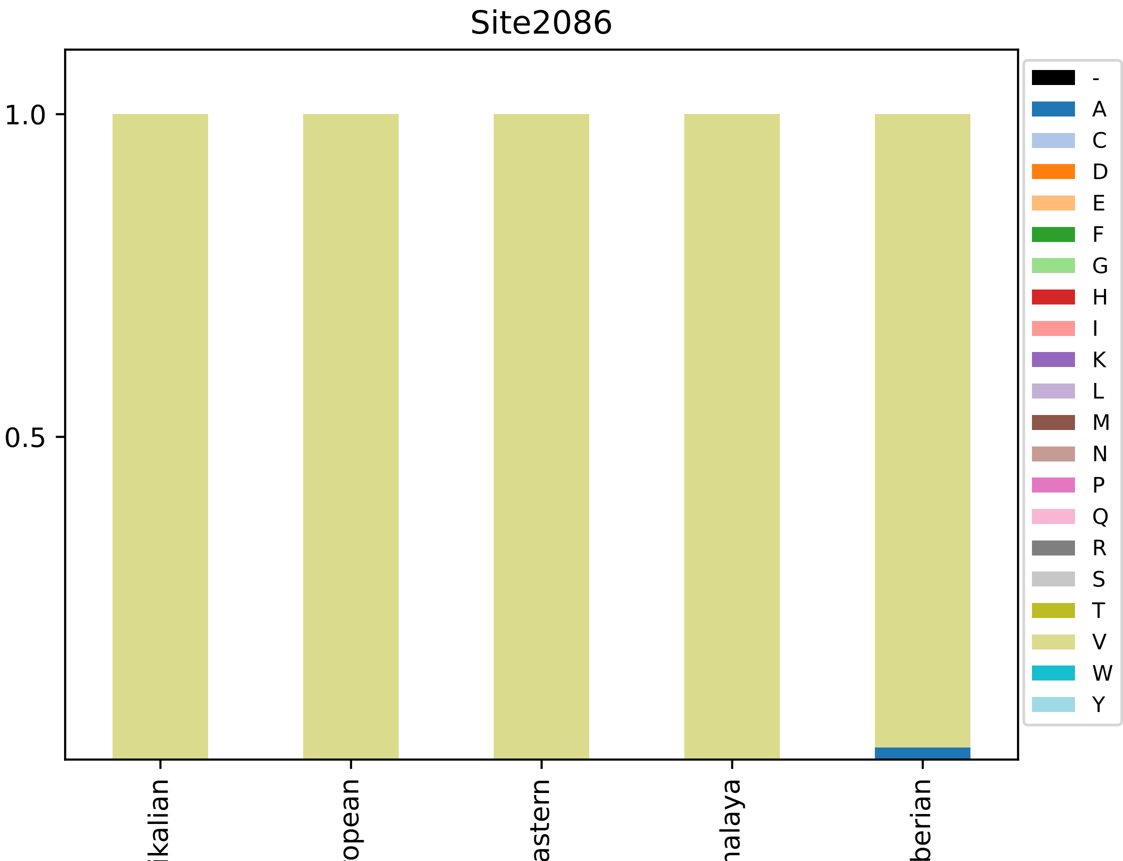
Site2086
1.0
0.5
-
A
C
D
E
F
G
H
I
K
L
M
N
P
Q
R
S
T
V
W
Y
ikalian
opean
astern
nalaya
berian

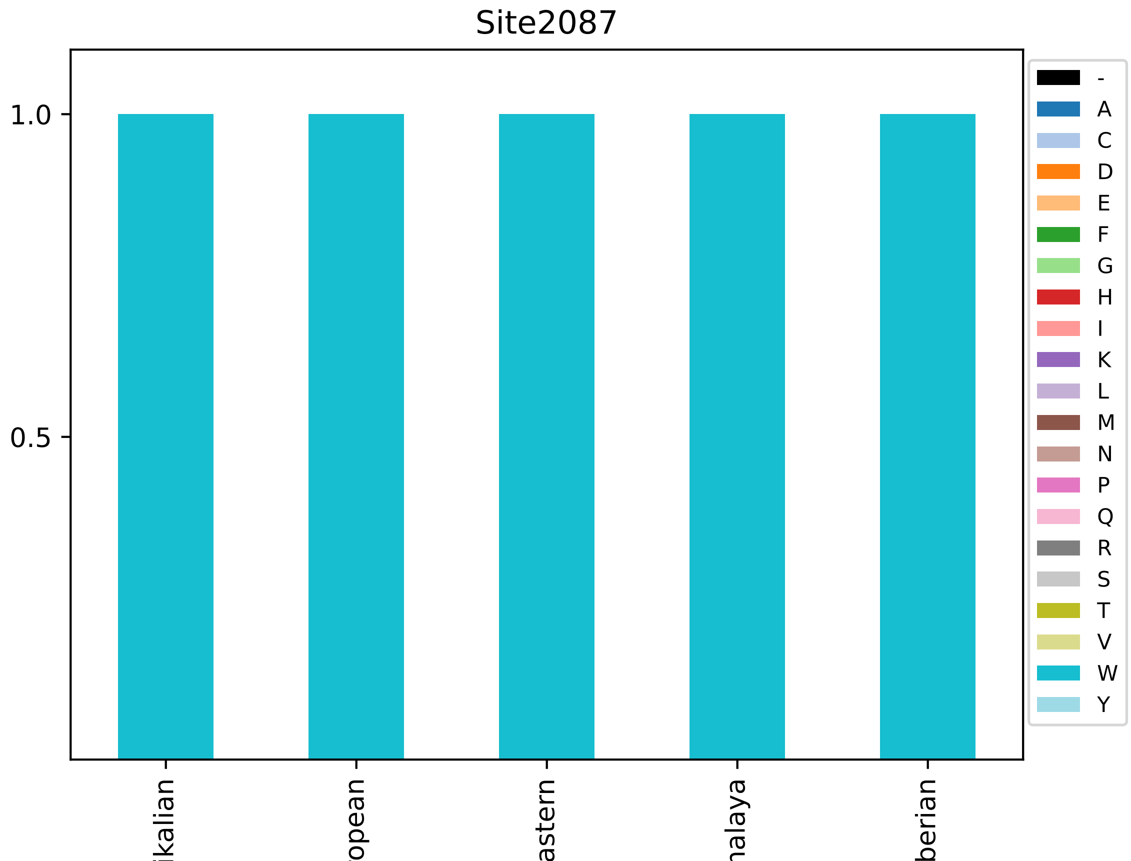

Site2087
1.0
0.5
-
A
C
D
E
F
G
H
I
K
L
M
N
P
Q
R
S
T
V
W
Y
ikalian
opean
astern
nalaya
berian

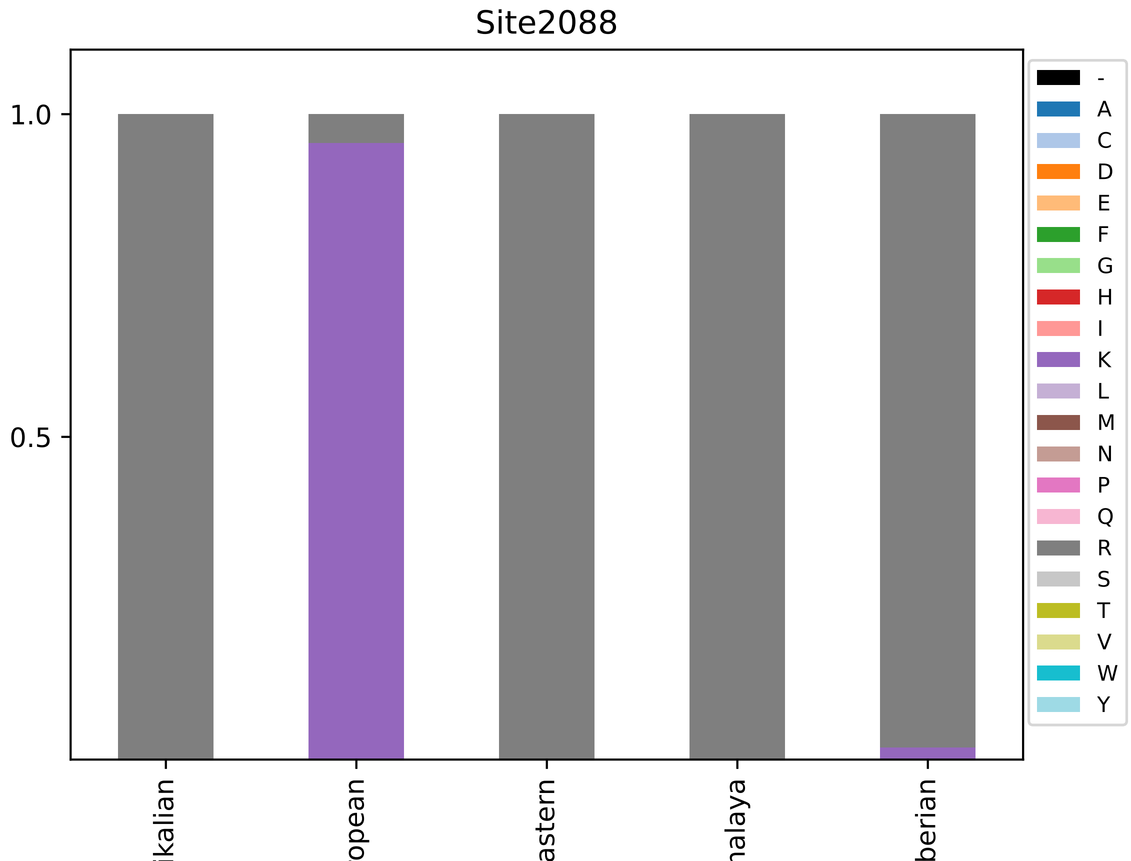

Site2088
1.0
0.5
-
A
C
D
E
F
G
H
I
K
L
M
N
P
Q
R
S
T
V
W
Y
ikalian
opean
astern
nalaya
berian

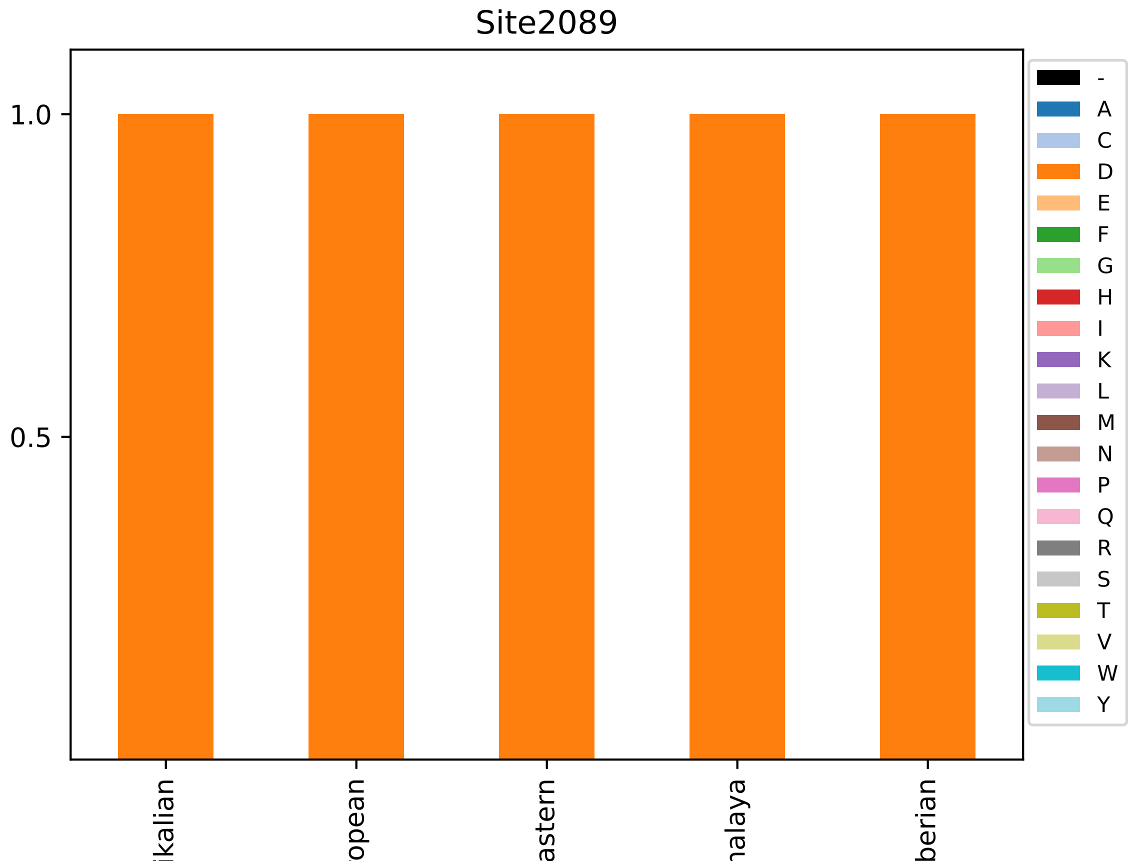

Site2089
1.0
0.5
-
A
C
D
E
F
G
H
I
K
L
M
N
P
Q
R
S
T
V
W
Y
ikalian
opean
astern
nalaya
berian

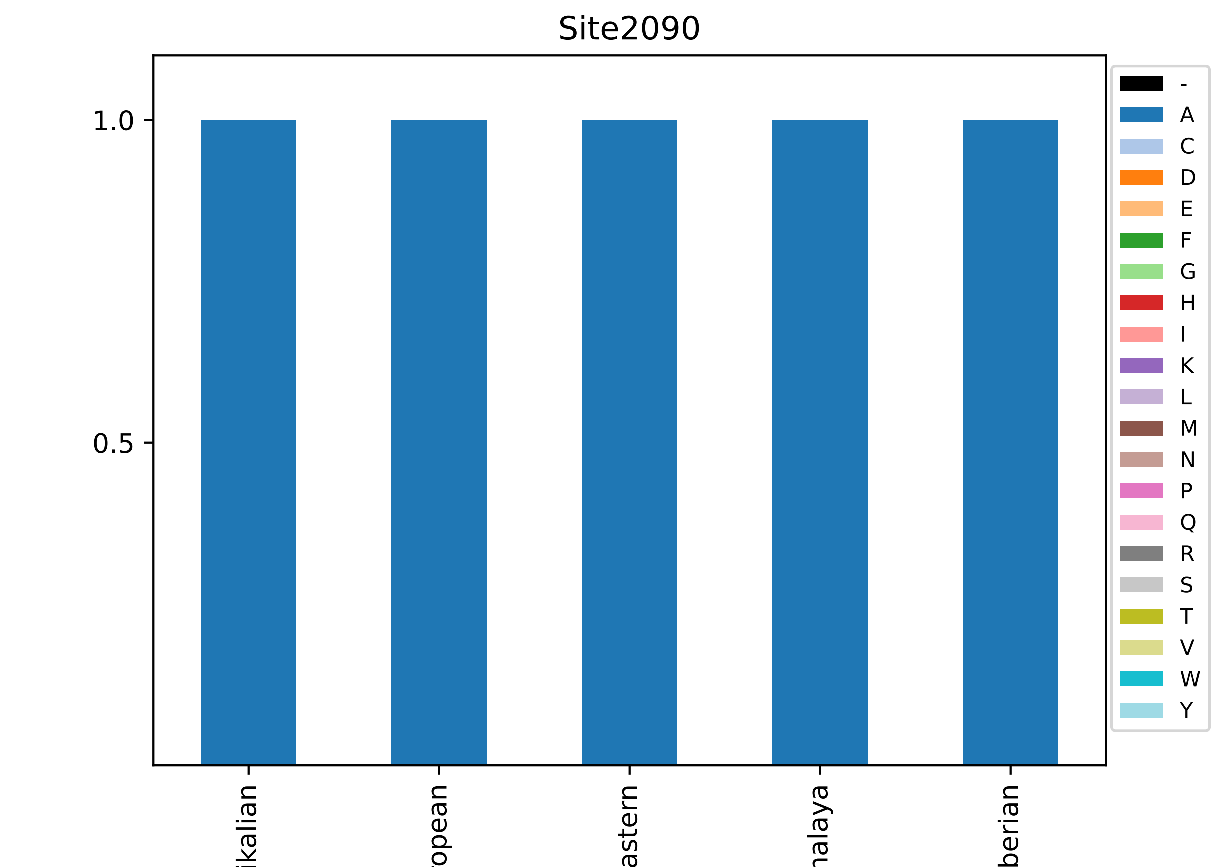
Site2090
1.0
0.5
-
A
C
D
E
F
G
H
I
K
L
M
N
P
Q
R
S
T
V
W
Y
ikalian
opean
astern
nalaya
berian

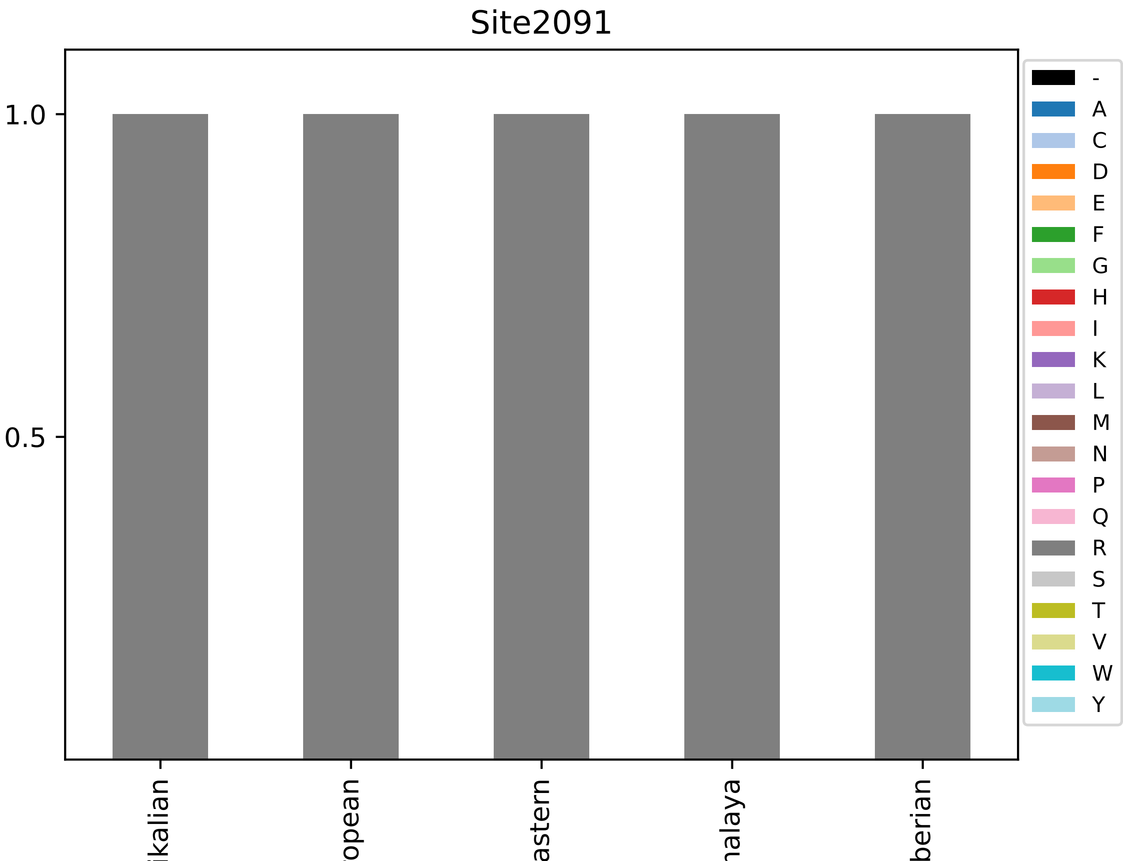

Site2091
1.0
0.5
-
A
C
D
E
F
G
H
I
K
L
M
N
P
Q
R
S
T
V
W
Y

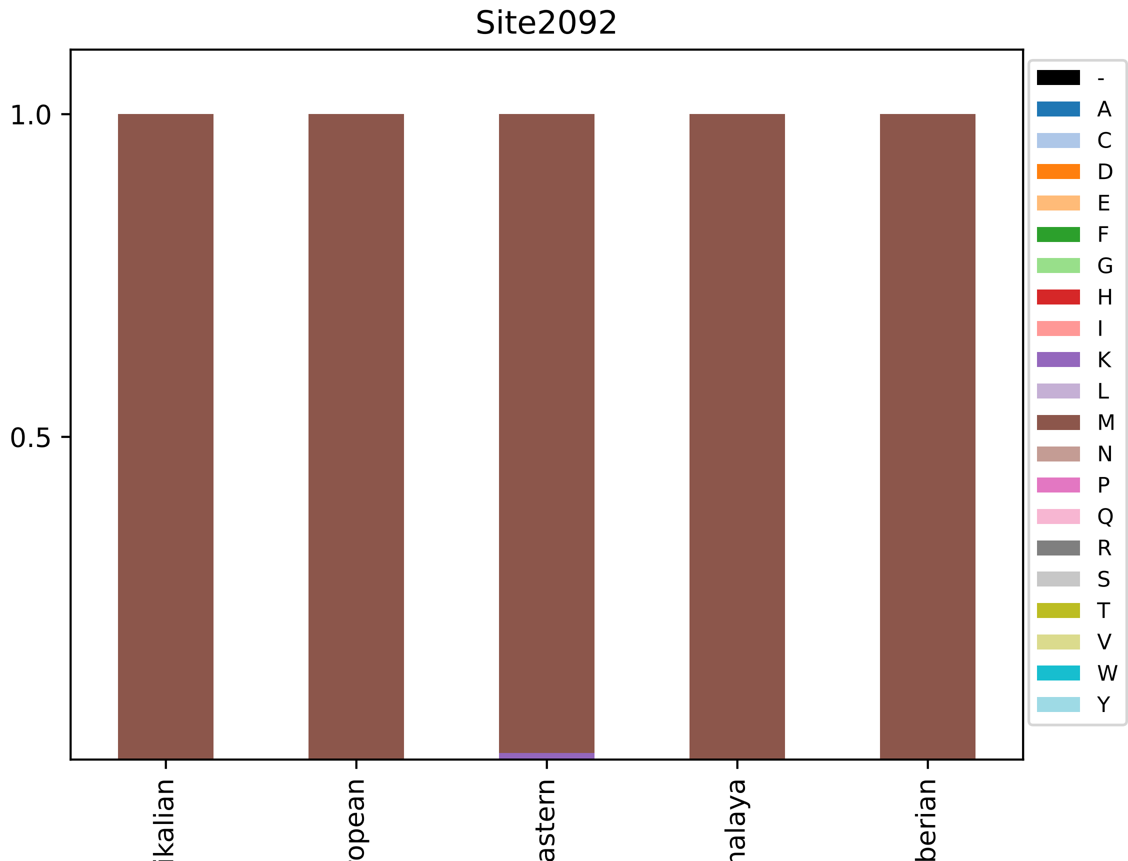

Site2092
1.0
0.5
-
A
C
D
E
F
G
H
I
K
L
M
N
P
Q
R
S
T
V
W
Y
ikalian
ropean
astern
nalaya
berian

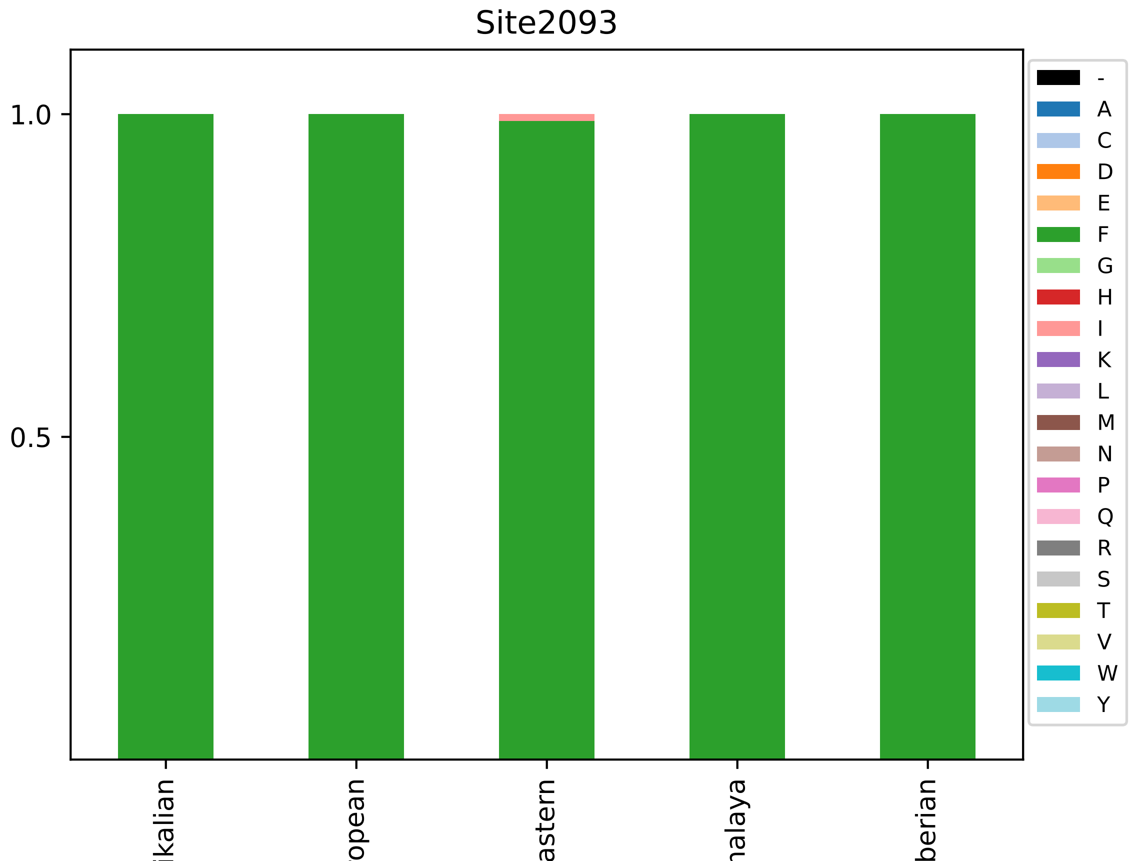
Site2093
1.0
0.5
-
A
C
D
E
F
G
H
I
K
L
M
N
P
Q
R
S
T
V
W
Y
ikalian
opean
astern
nalaya
berian

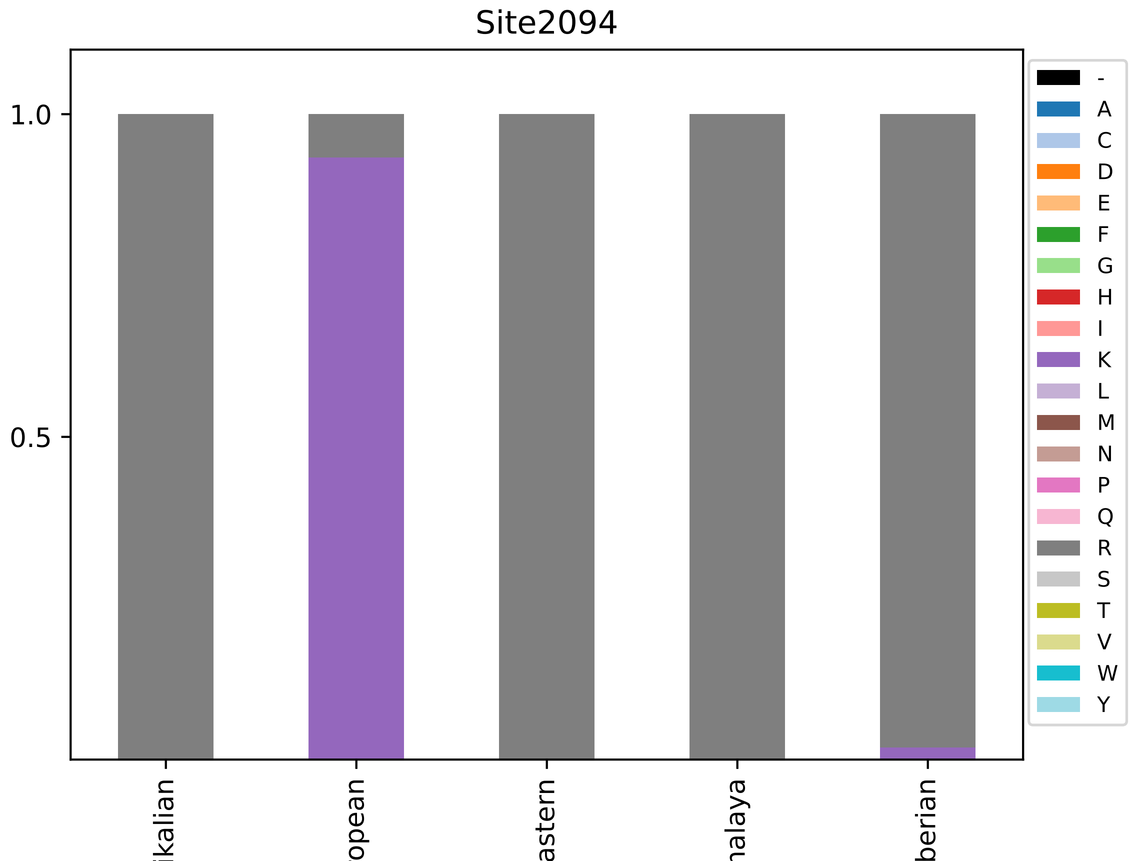
Site2094
1.0
0.5
-
A
C
D
E
F
G
H
I
K
L
M
N
P
Q
R
S
T
V
W
Y
ikalian
opean
astern
nalaya
berian

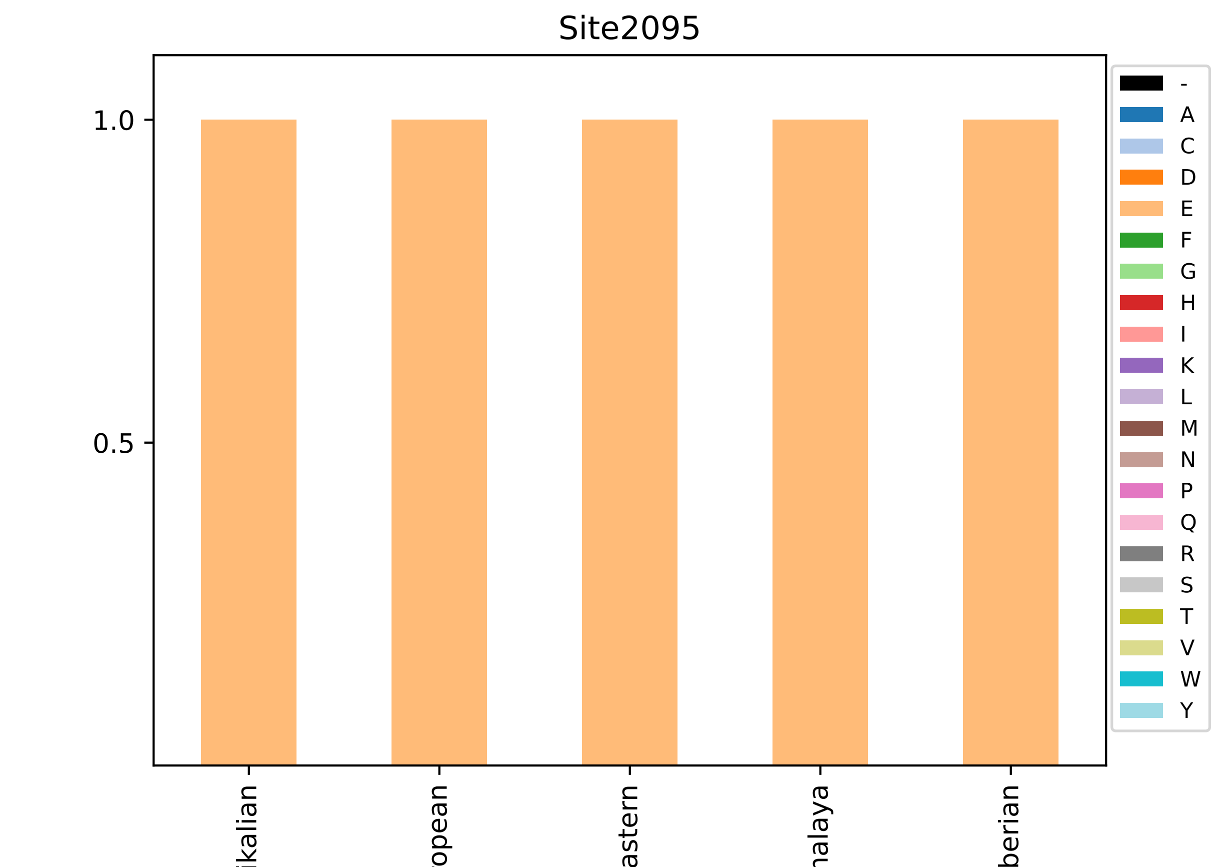
Site2095
1.0
0.5
ikalian
ropean
astern
nalaya
berian
-
A
C
D
E
F
G
H
I
K
L
M
N
P
Q
R
S
T
V
W
Y

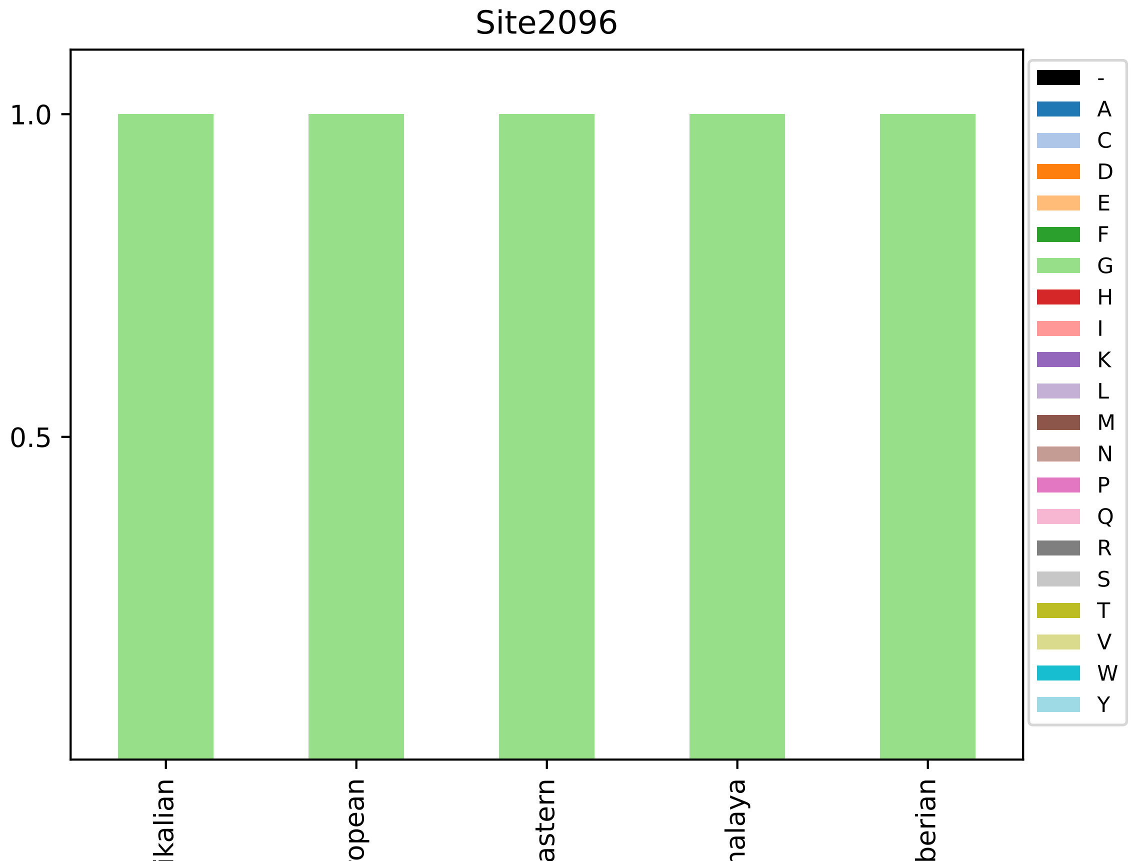
Site2096
1.0
0.5
-
A
C
D
E
F
G
H
I
K
L
M
N
P
Q
R
S
T
V
W
Y
ikalian
opean
astern
nalaya
berian

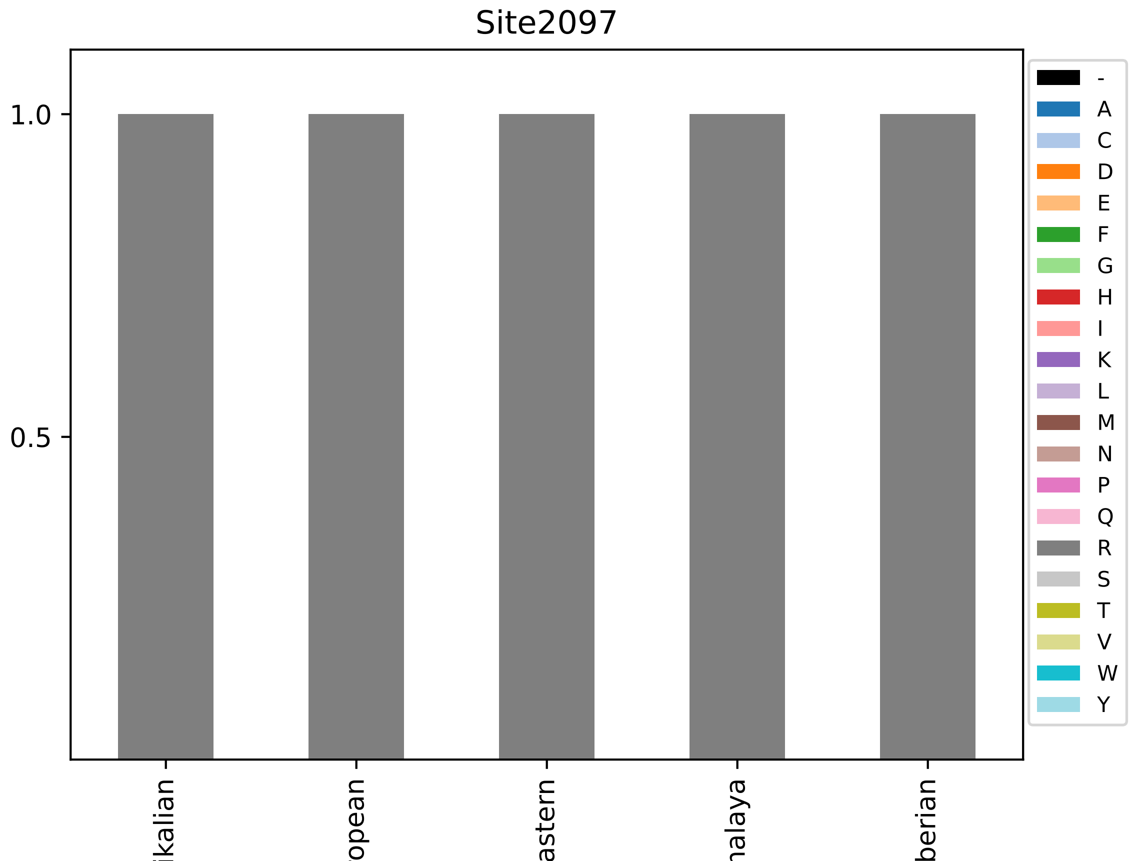

Site2097
1.0
0.5
-
A
C
D
E
F
G
H
I
K
L
M
N
P
Q
R
S
T
V
W
Y

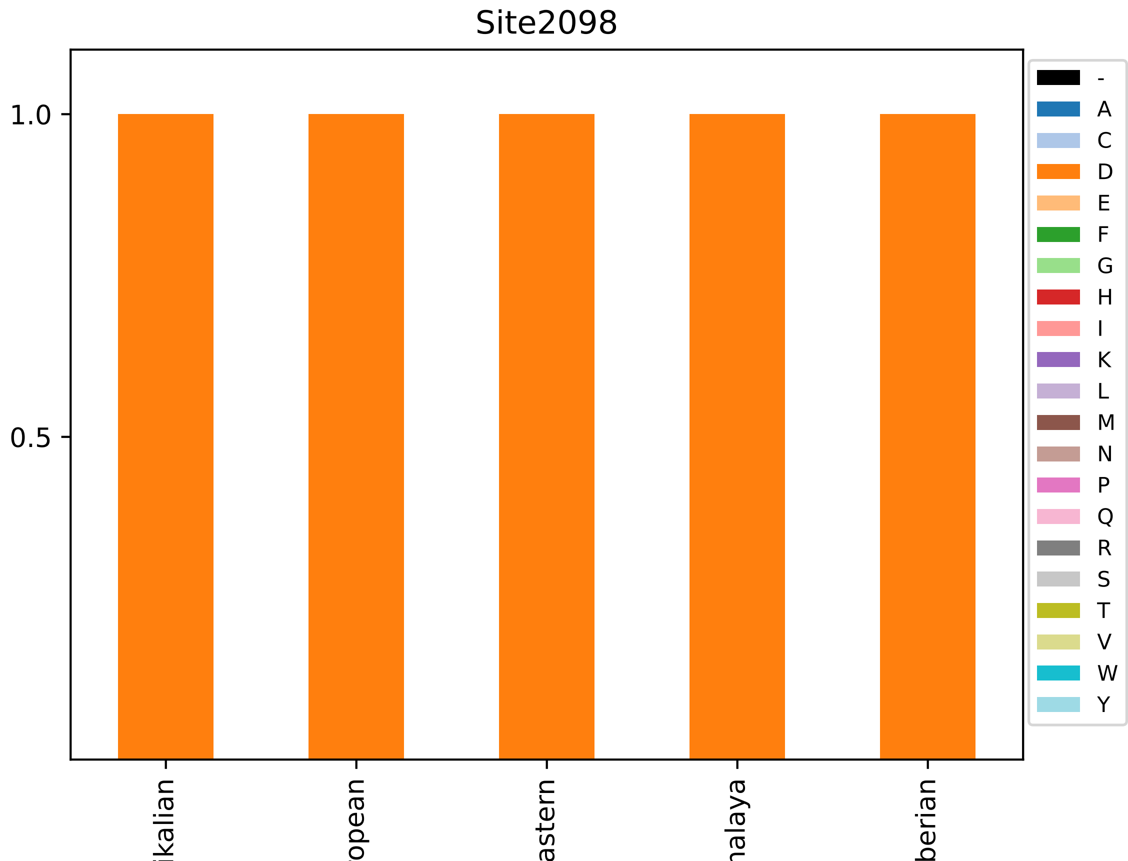

Site2098
1.0
0.5
-
A
C
D
E
F
G
H
I
K
L
M
N
P
Q
R
S
T
V
W
Y
ikalian
opean
astern
nalaya
berian

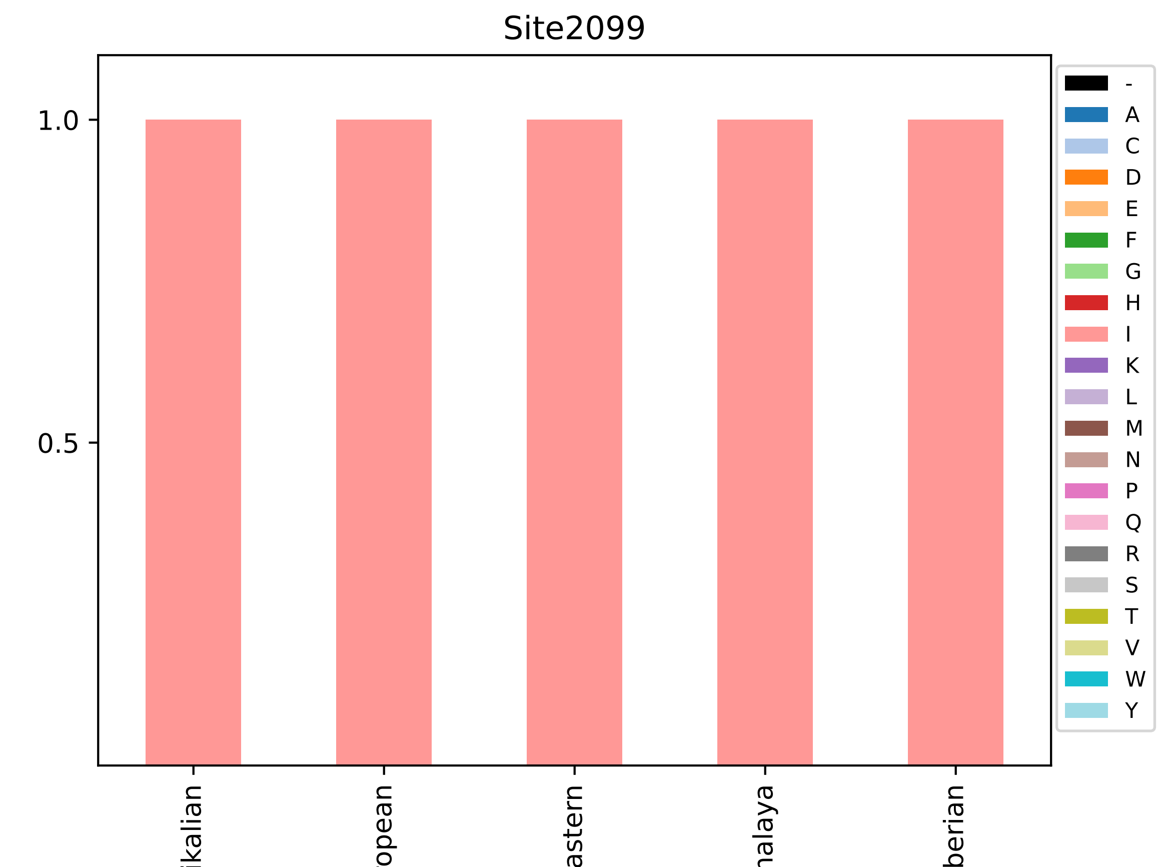

Site2099
1.0
0.5
-
A
C
D
E
F
G
H
I
K
L
M
N
P
Q
R
S
T
V
W
Y
ikalian
opean
astern
nalaya
berian

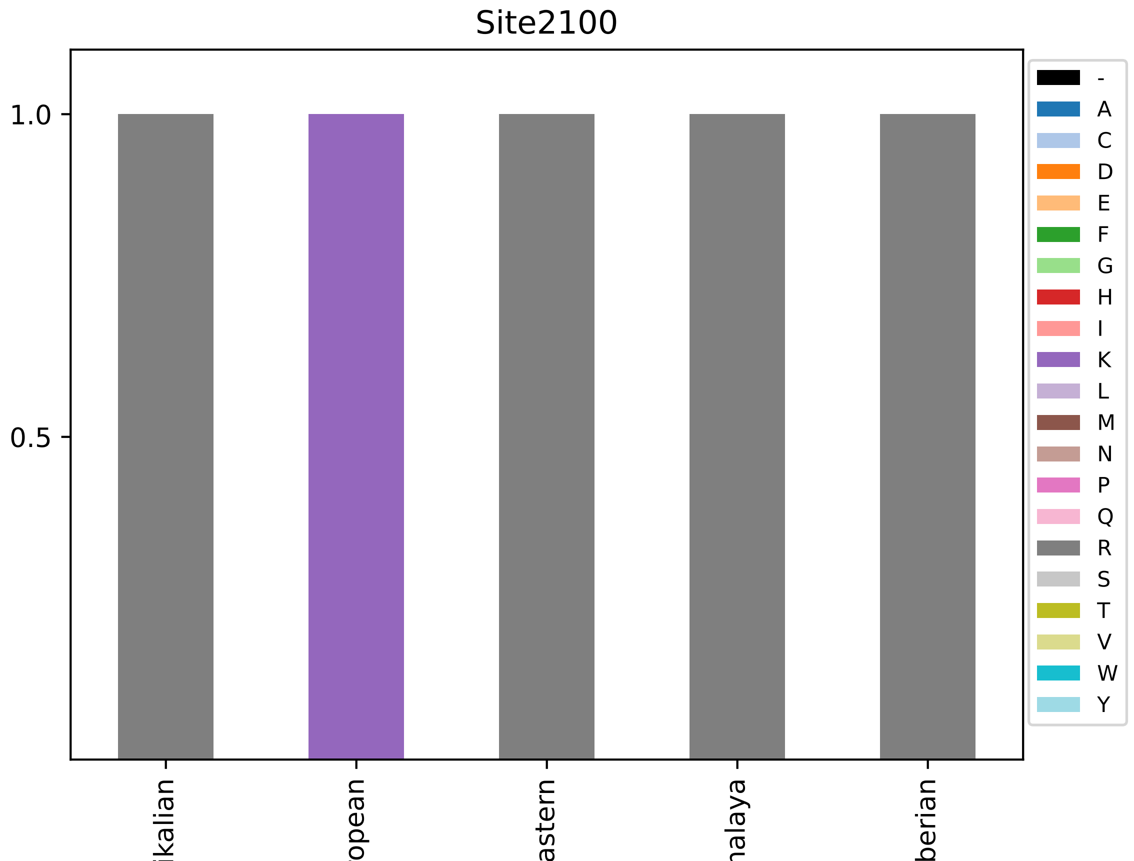
Site2100
1.0
0.5
-
A
C
D
E
F
G
H
I
K
L
M
N
P
Q
R
S
T
V
W
Y
ikalian
opean
astern
nalaya
berian

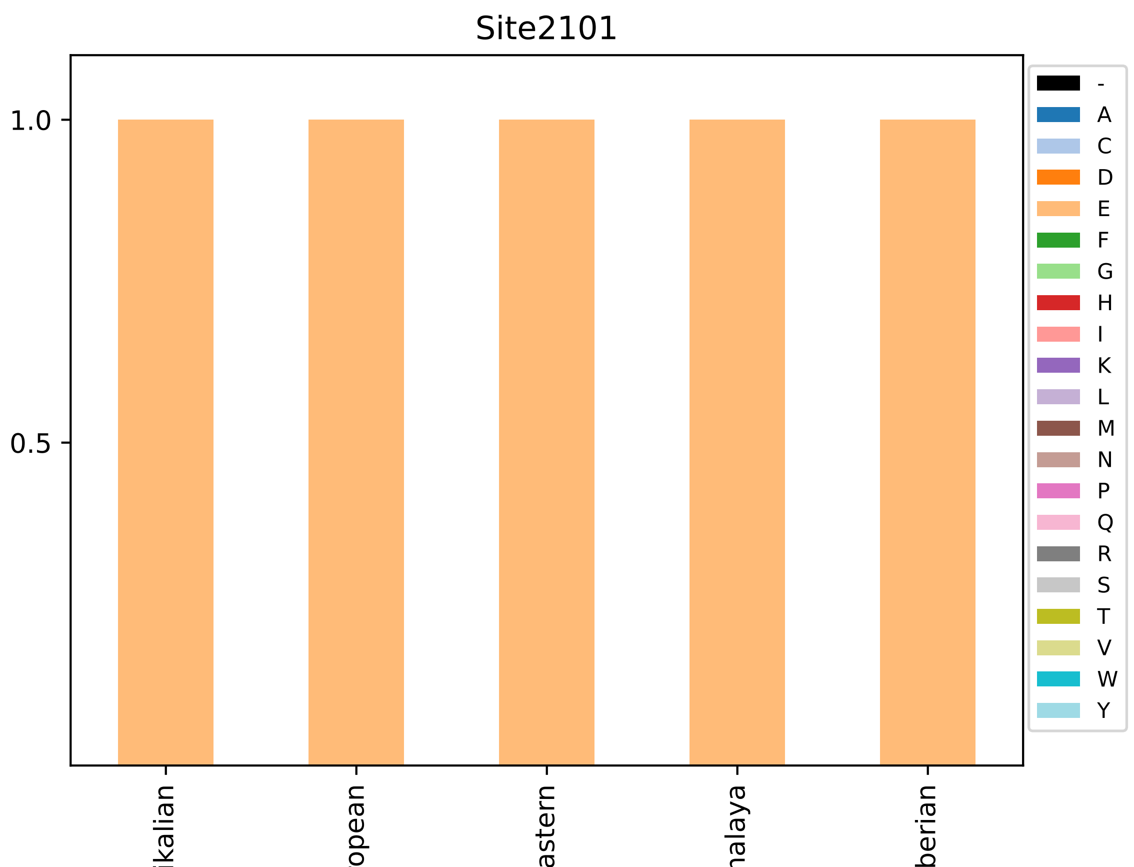
Site2101
1.0
0.5
-
A
C
D
E
F
G
H
I
K
L
M
N
P
Q
R
S
T
V
W
Y
ikalian
opean
astern
nalaya
berian

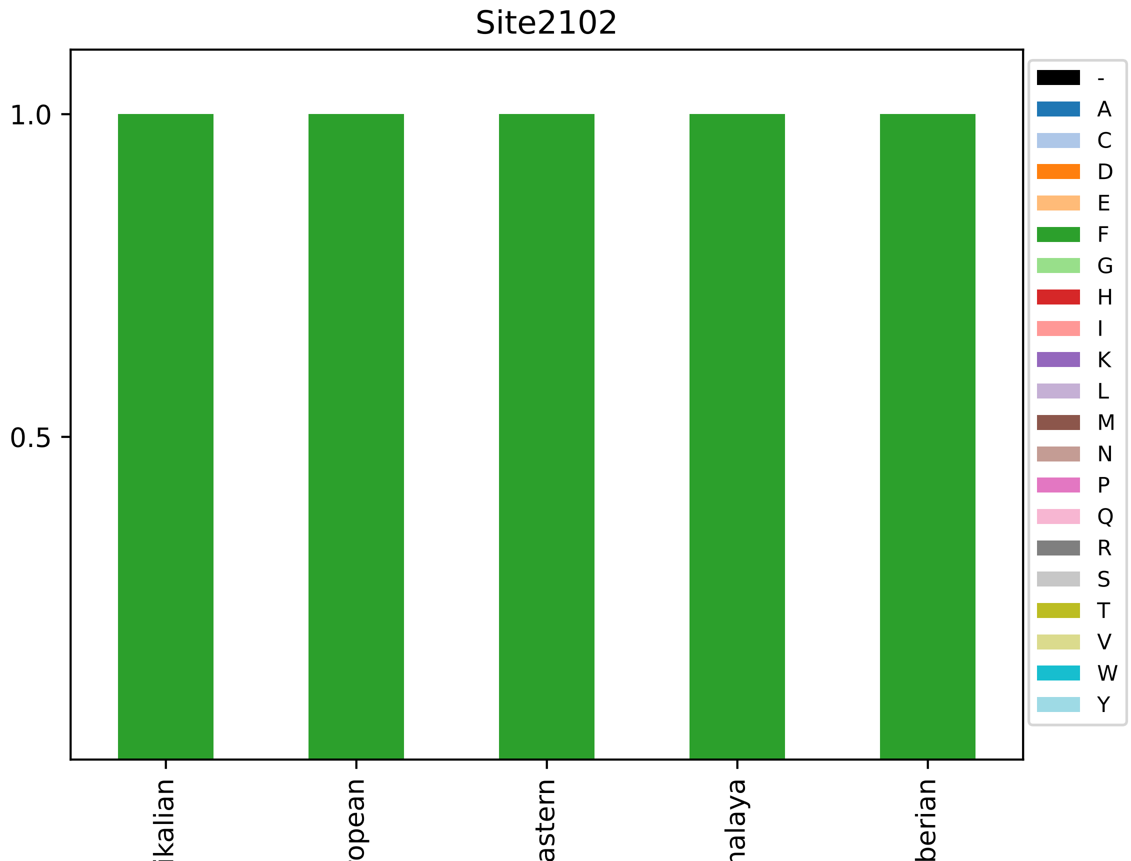

Site2102
1.0
0.5
-
A
C
D
E
F
G
H
I
K
L
M
N
P
Q
R
S
T
V
W
Y
ikalian
opean
astern
nalaya
berian

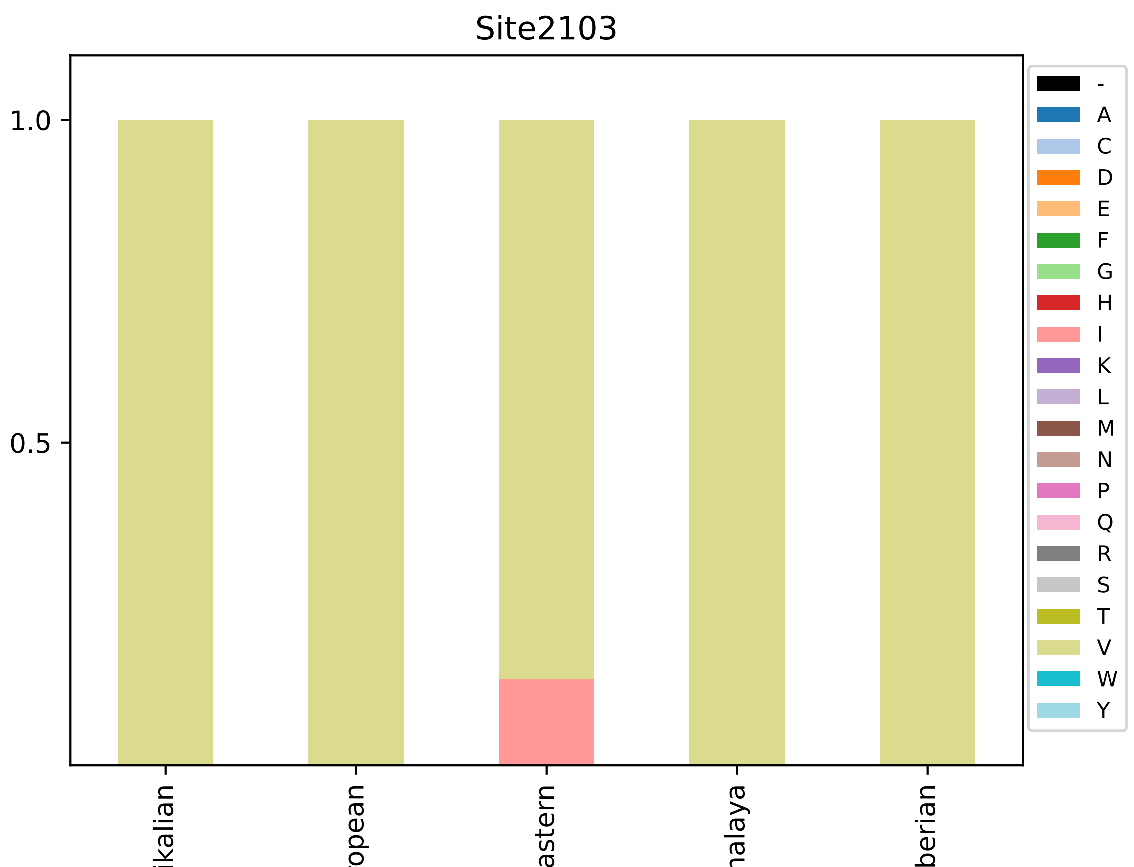
Site2103
1.0
0.5
-
A
C
D
E
F
G
H
I
K
L
M
N
P
Q
R
S
T
V
W
Y
ikalian
opean
astern
nalaya
berian

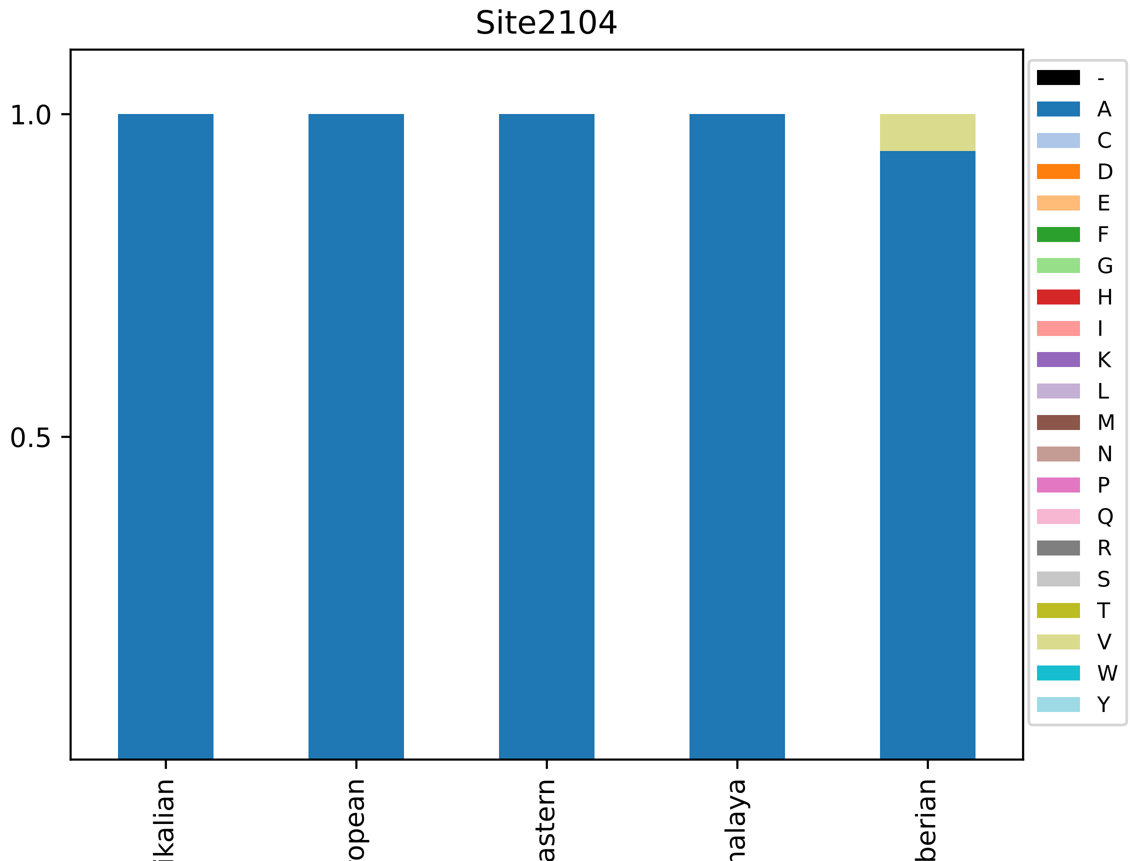

Site2104
1.0
0.5
-
A
C
D
E
F
G
H
I
K
L
M
N
P
Q
R
S
T
V
W
Y
ikalian
opean
astern
halaya
berian

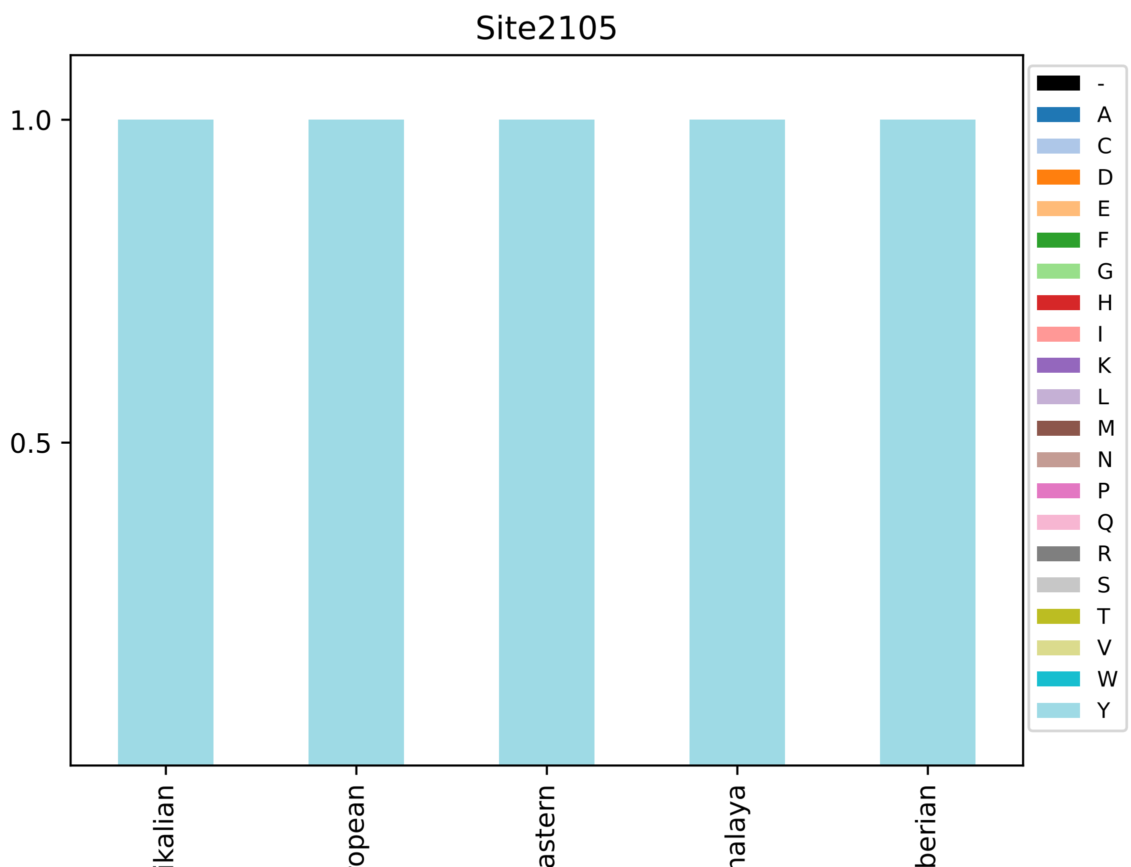

Site2105
1.0
0.5
ikalian
opean
astern
nalaya
berian
-
A
C
D
E
F
G
H
I
K
L
M
N
P
Q
R
S
T
V
W
Y

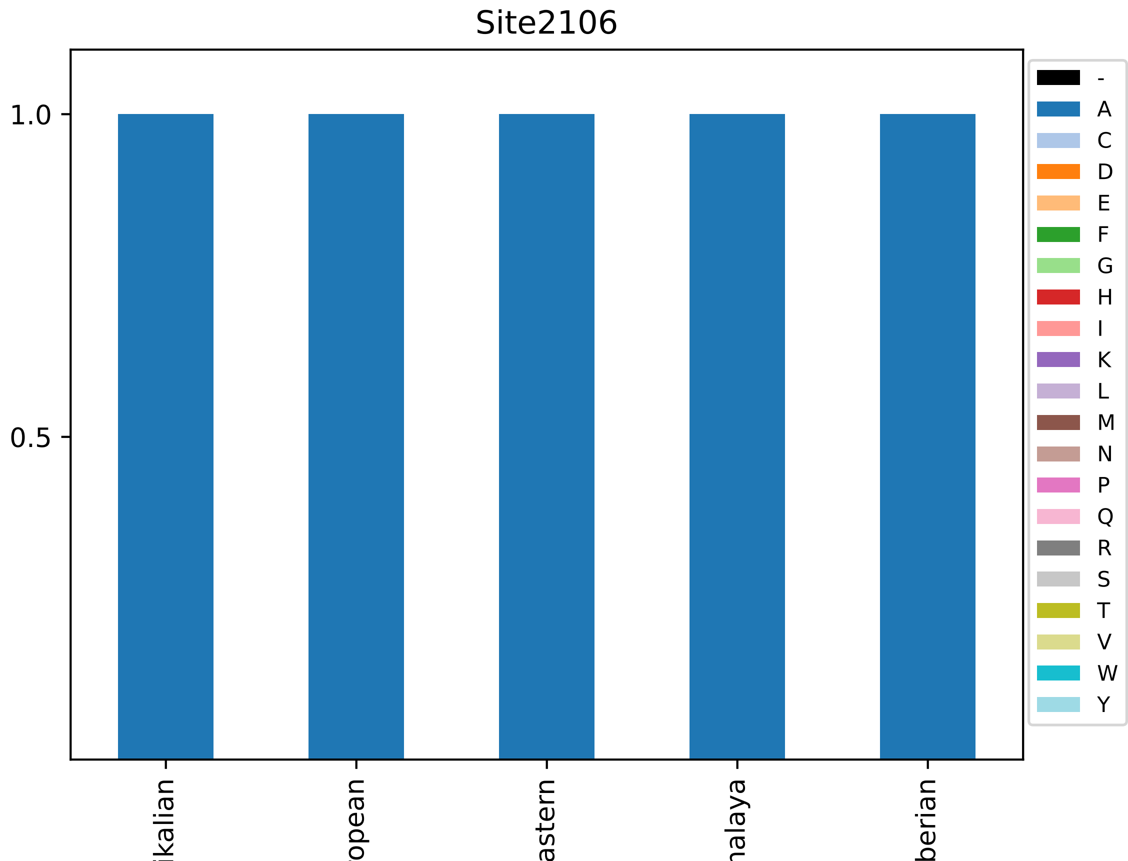
Site2106
1.0
0.5
ikalian
opean
astern
nalaya
berian
-
A
C
D
E
F
G
H
I
K
L
M
N
P
Q
R
S
T
V
W
Y

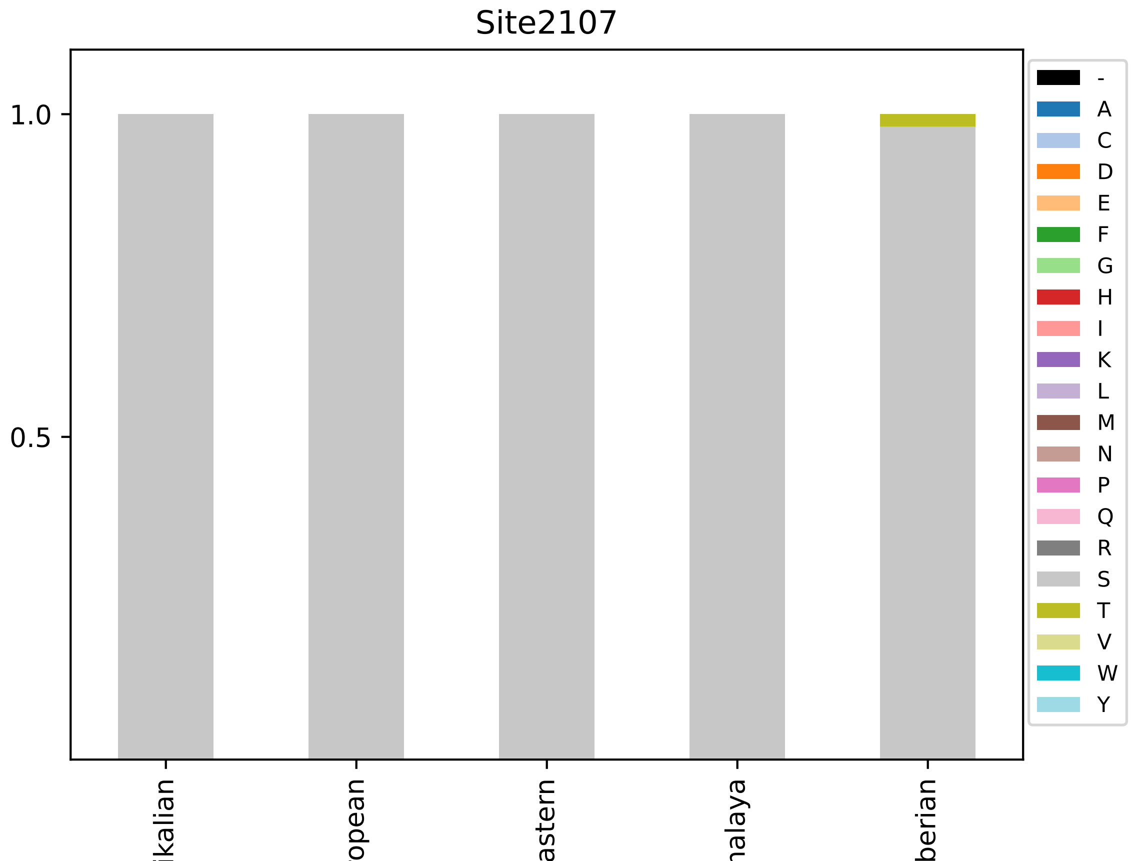
Site2107
1.0
0.5
-
A
C
D
E
F
G
H
I
K
L
M
N
P
Q
R
S
T
V
W
Y

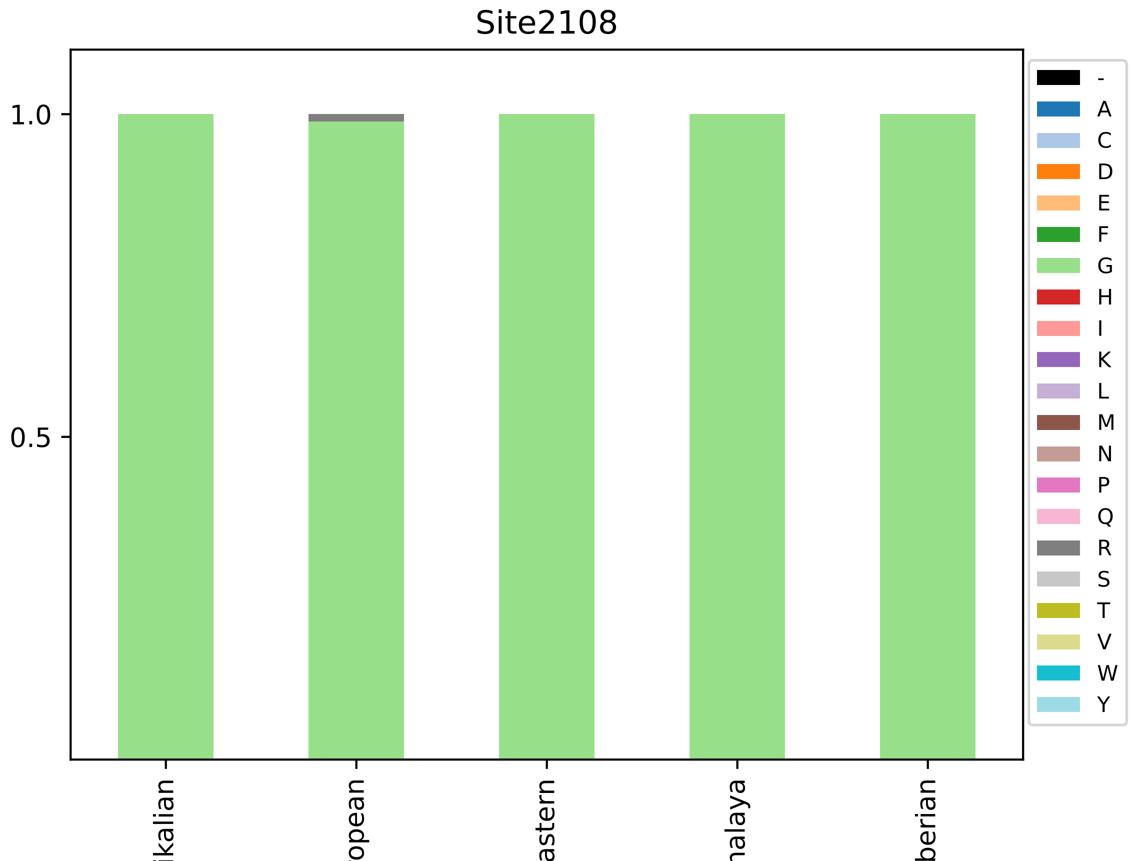

Site2108
1.0
0.5
-
A
C
D
E
F
G
H
I
K
L
M
N
P
Q
R
S
T
V
W
Y
ikalian
opean
astern
nalaya
berian

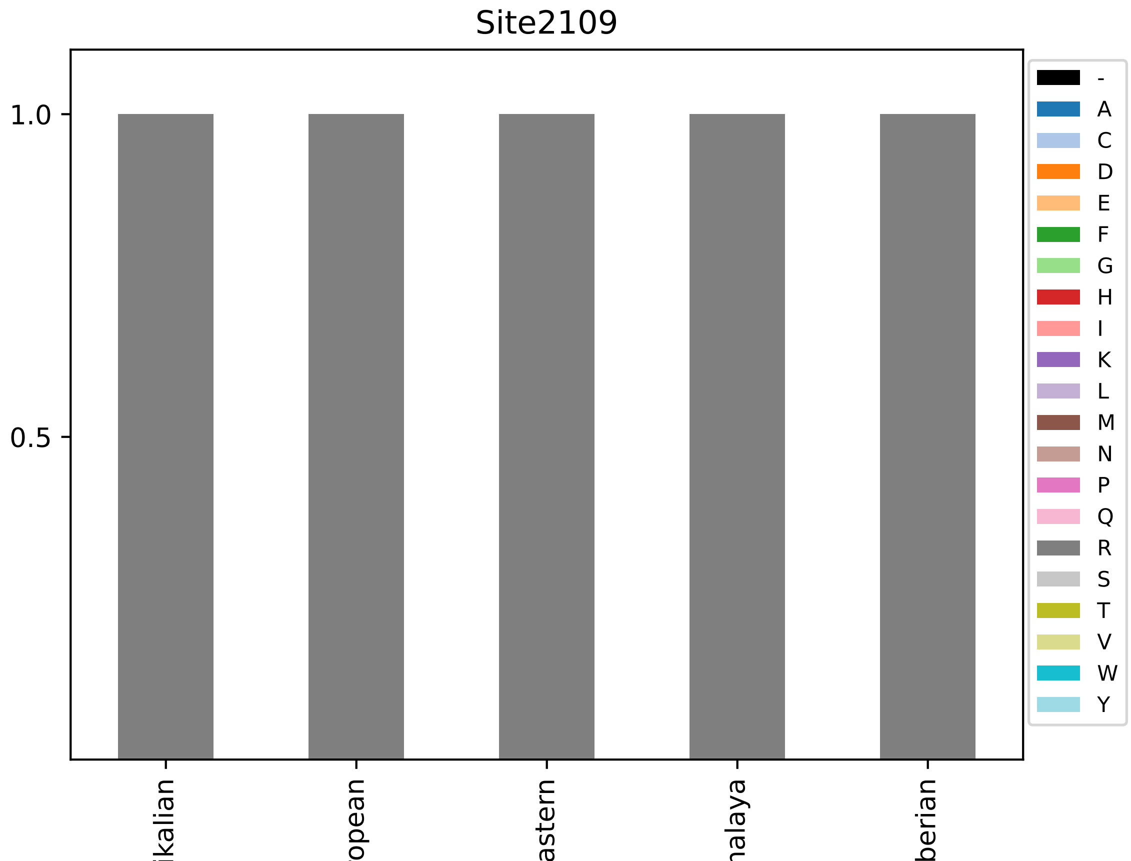

Site2109
1.0
0.5
-
A
C
D
E
F
G
H
I
K
L
M
N
P
Q
R
S
T
V
W
Y
ikalian
opean
astern
nalaya
berian

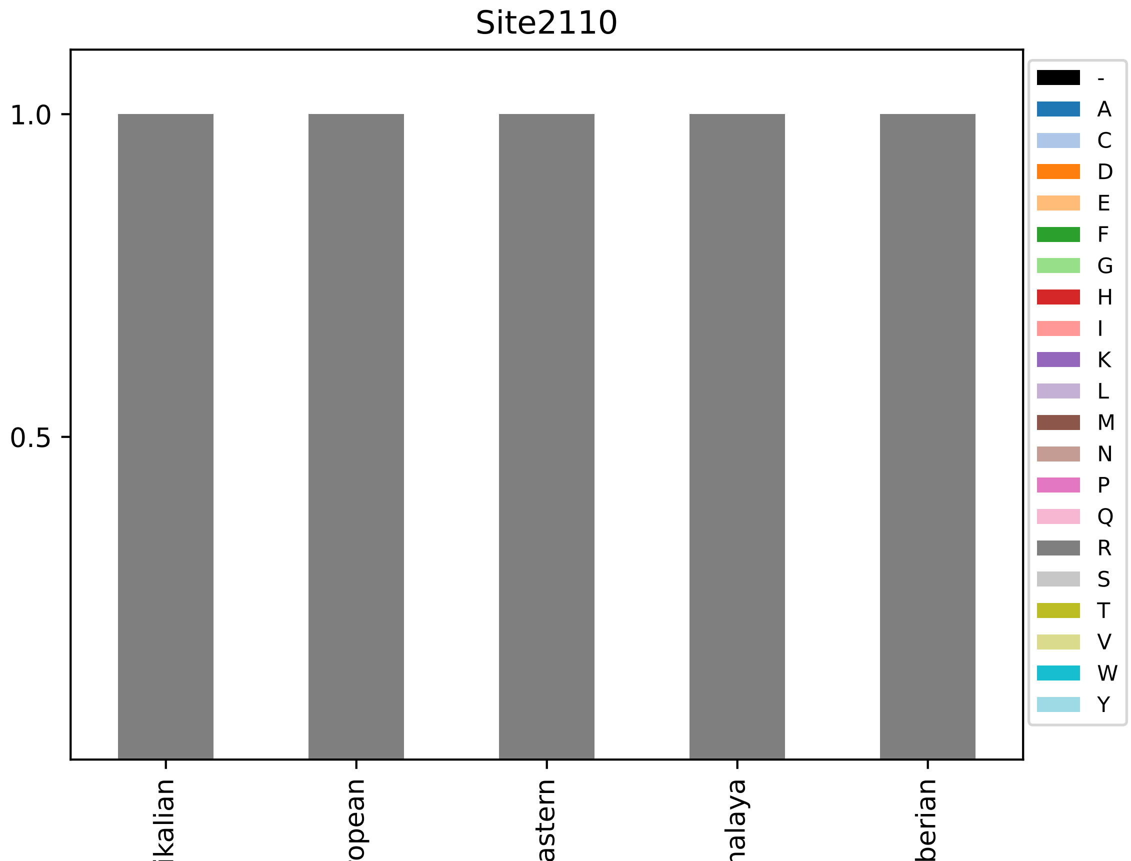
Site2110
1.0
0.5
ikalian
opean
astern
nalaya
berian
-
A
C
D
E
F
G
H
I
K
L
M
N
P
Q
R
S
T
V
W
Y

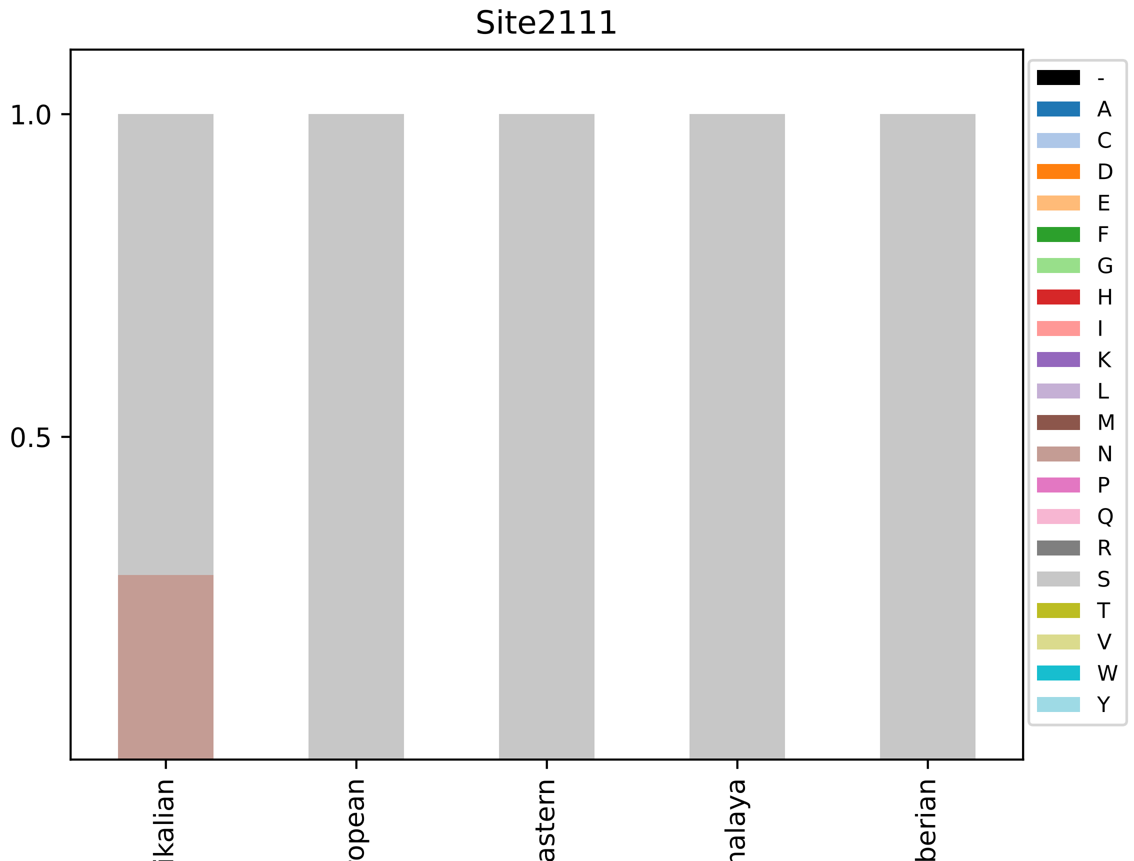
Site2111
1.0
0.5
-
A
C
D
E
F
G
H
I
K
L
M
N
P
Q
R
S
T
V
W
Y
ikalian
opean
astern
nalaya
berian

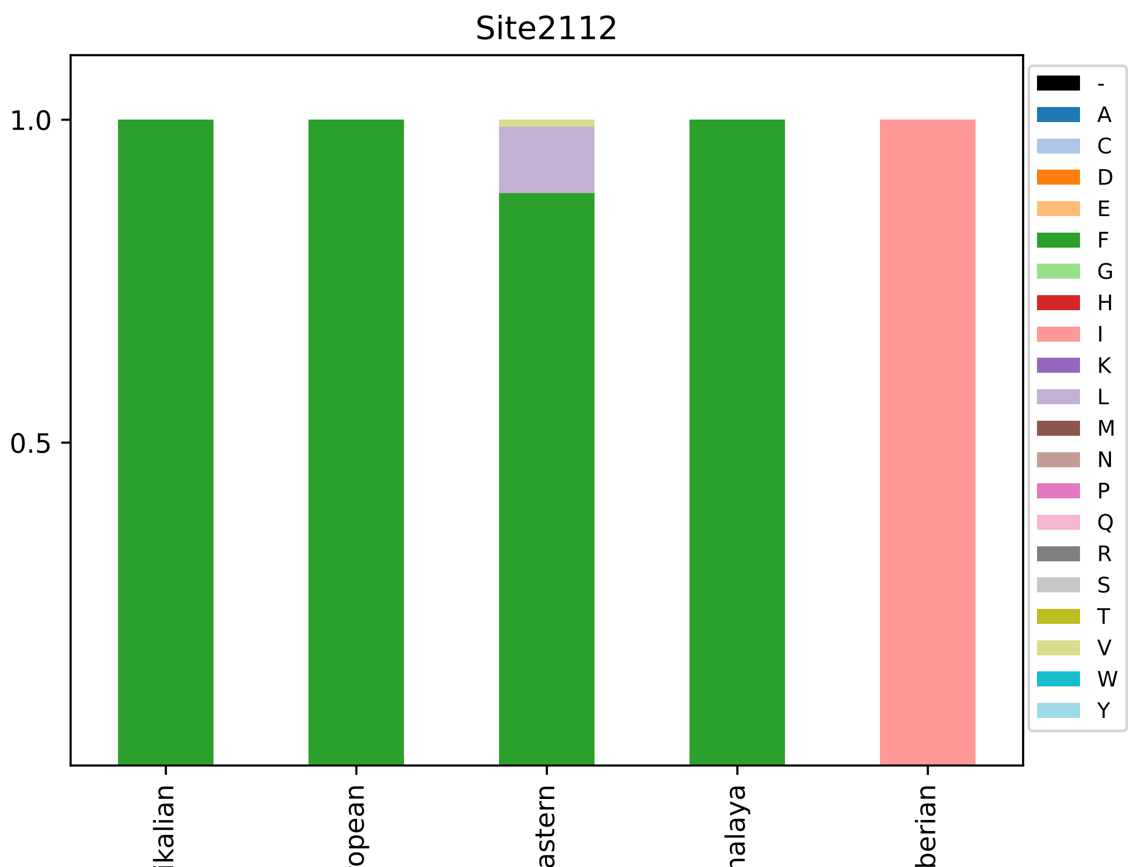

Site2112
1.0
0.5
-
A
C
D
E
F
G
H
I
K
L
M
N
P
Q
R
S
T
V
W
Y
ikalian
opean
astern
nalaya
berian

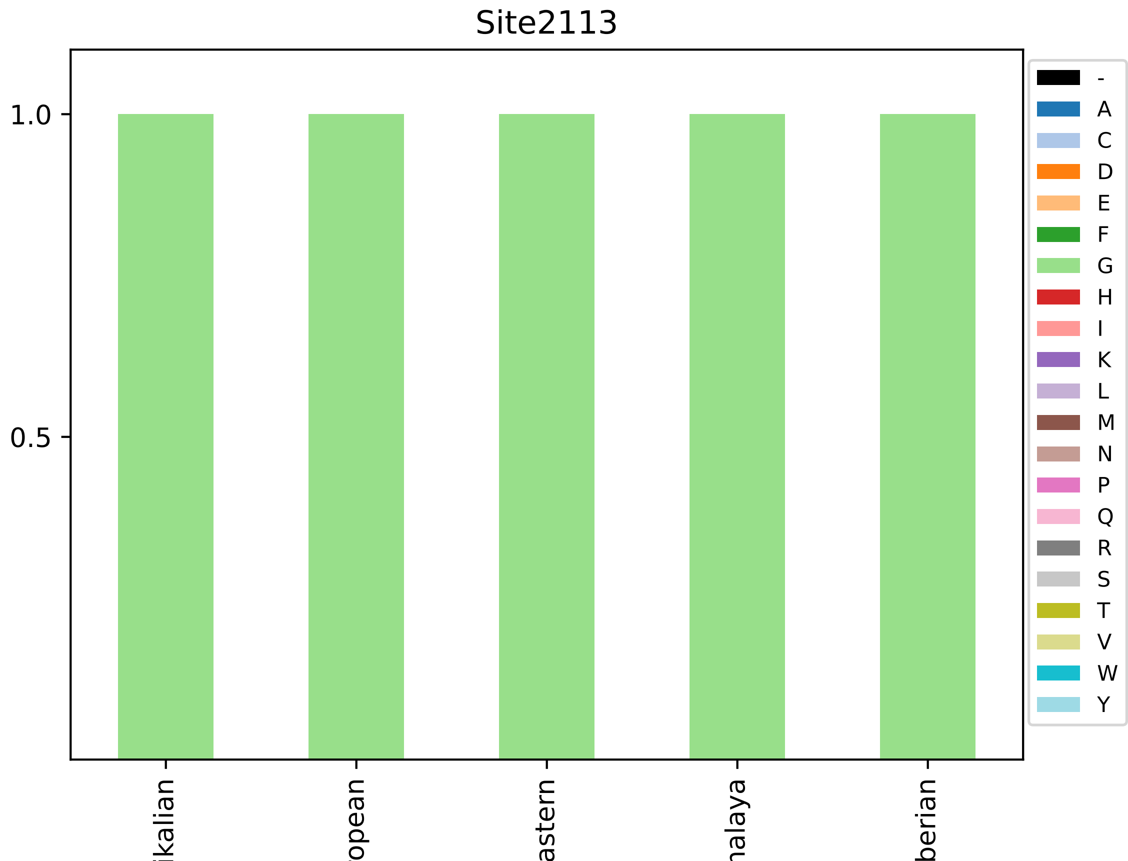
Site2113
1.0
0.5
-
A
C
D
E
F
G
H
I
K
L
M
N
P
Q
R
S
T
V
W
Y

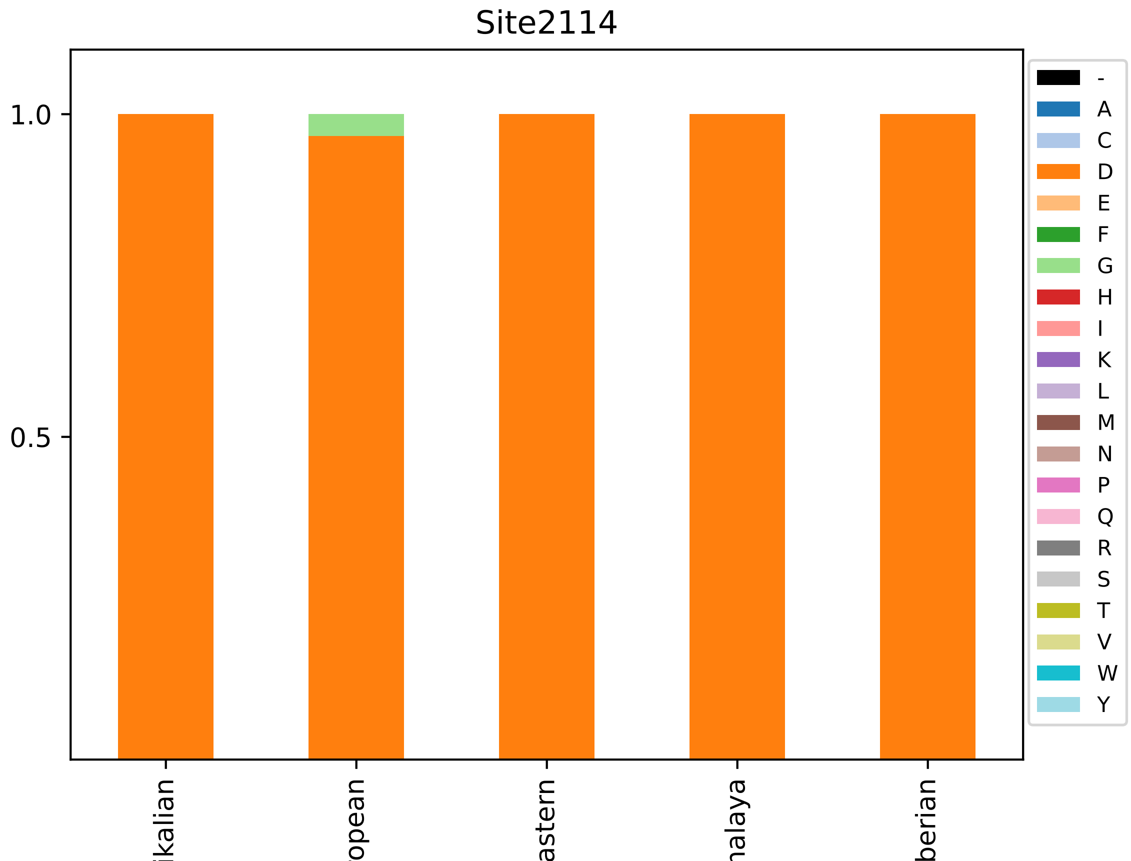
Site2114
1.0
0.5
ikalian
opean
astern
nalaya
berian
-
A
C
D
E
F
G
H
I
K
L
M
N
P
Q
R
S
T
V
W
Y

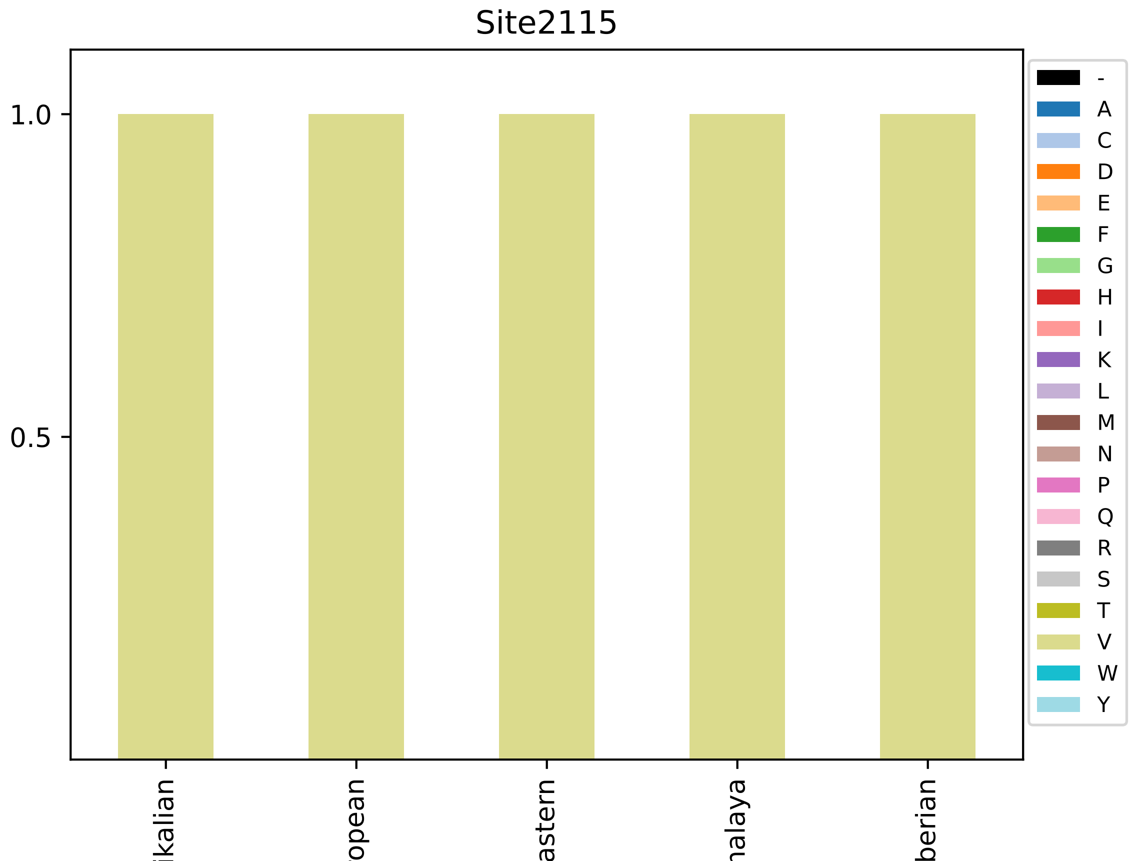
Site2115
1.0
0.5
-
A
C
D
E
F
G
H
I
K
L
M
N
P
Q
R
S
T
V
W
Y
ikalian
opean
astern
nalaya
berian

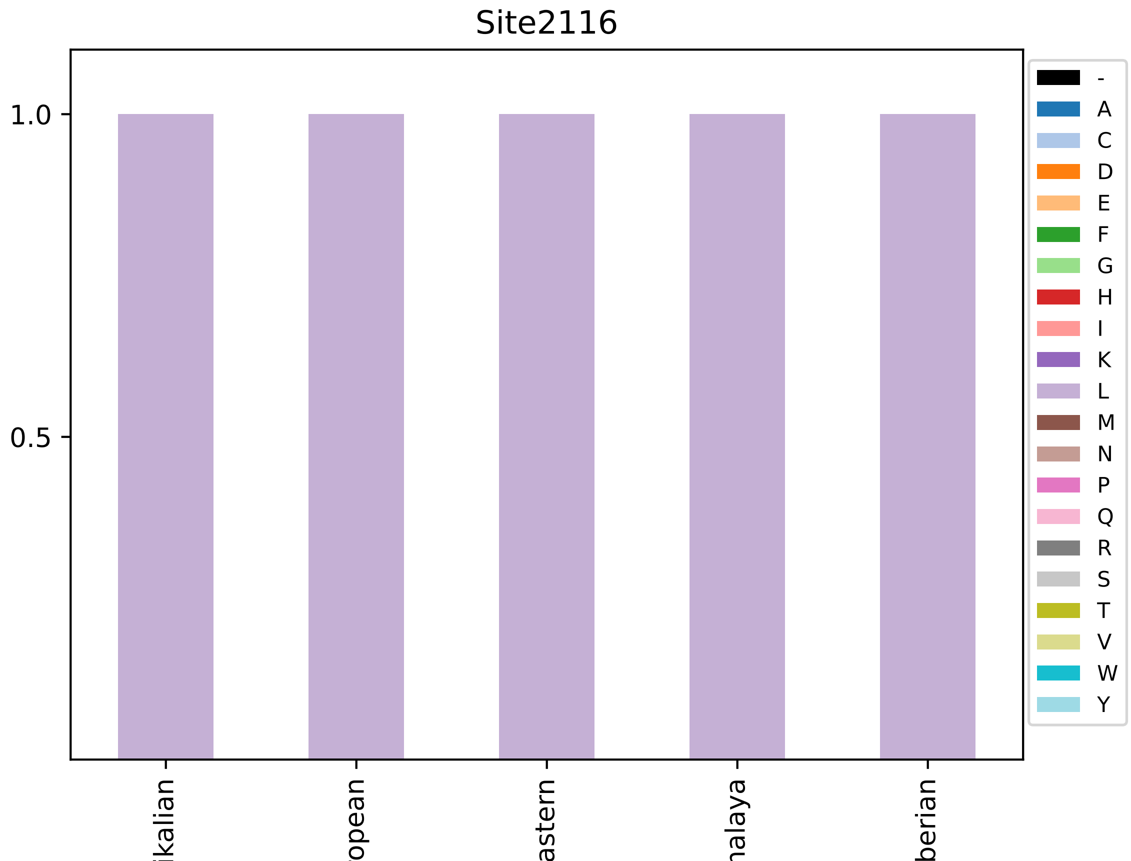

Site2116
1.0
0.5
-
A
C
D
E
F
G
H
I
K
L
M
N
P
Q
R
S
T
V
W
Y
ikalian
opean
astern
nalaya
berian

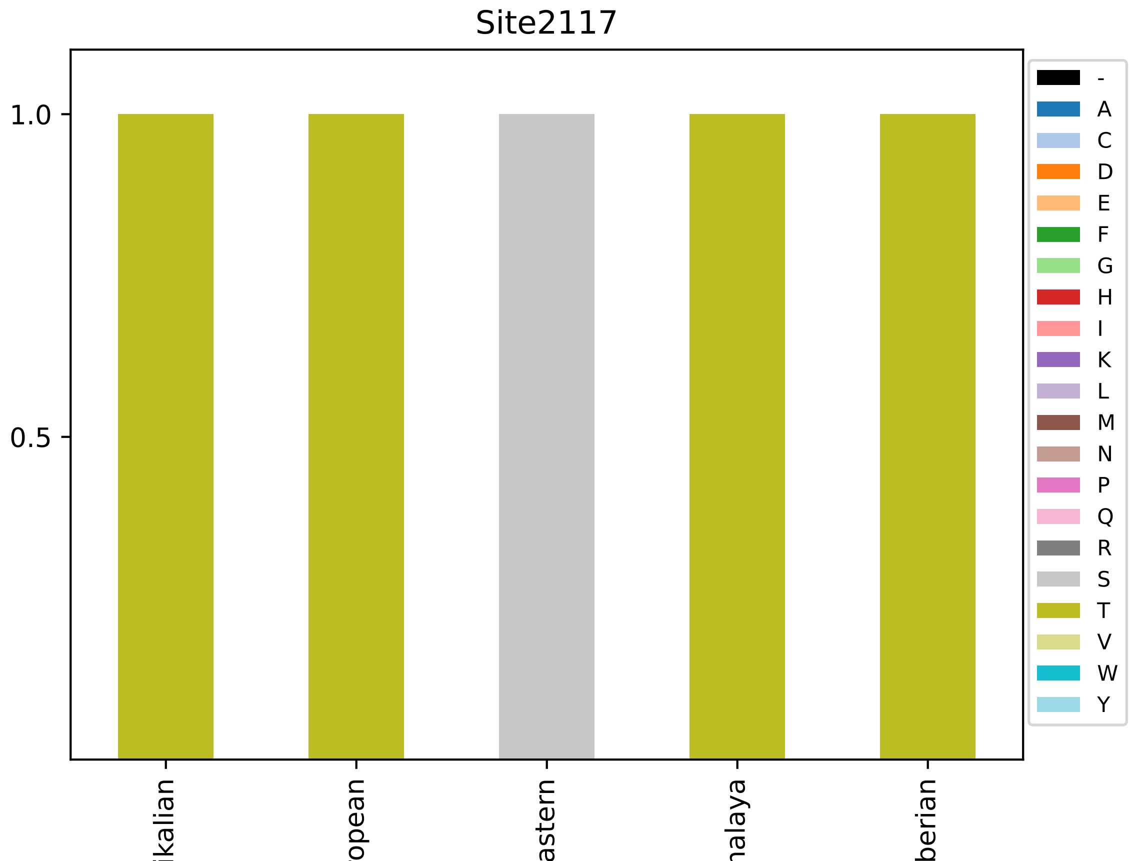

Site2117
1.0
0.5
ikalian
opean
astern
nalaya
berian
-
A
C
D
E
F
G
H
I
K
L
M
N
P
Q
R
S
T
V
W
Y

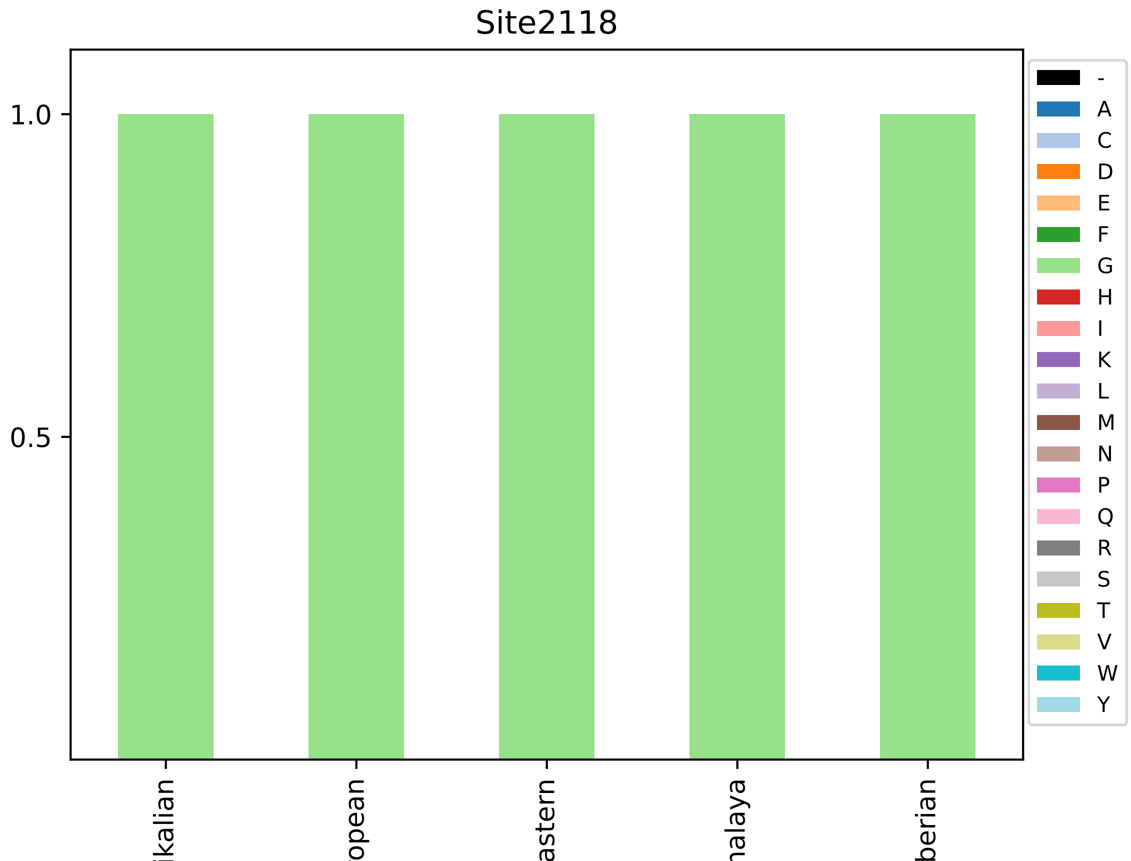
Site2118
1.0
0.5
-
A
C
D
E
F
G
H
I
K
L
M
N
P
Q
R
S
T
V
W
Y
ikalian
opean
astern
nalaya
berian

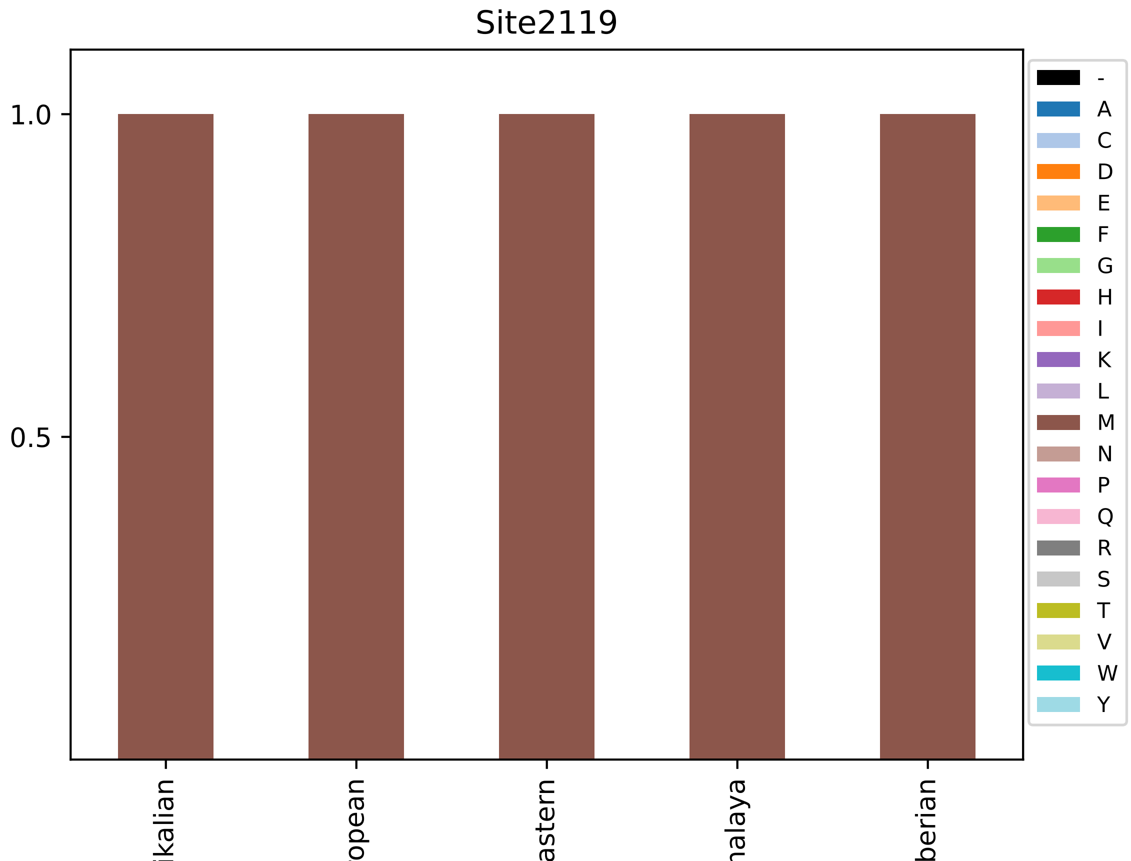

Site2119
1.0
0.5
-
A
C
D
E
F
G
H
I
K
L
M
N
P
Q
R
S
T
V
W
Y
ikalian
opean
astern
nalaya
berian

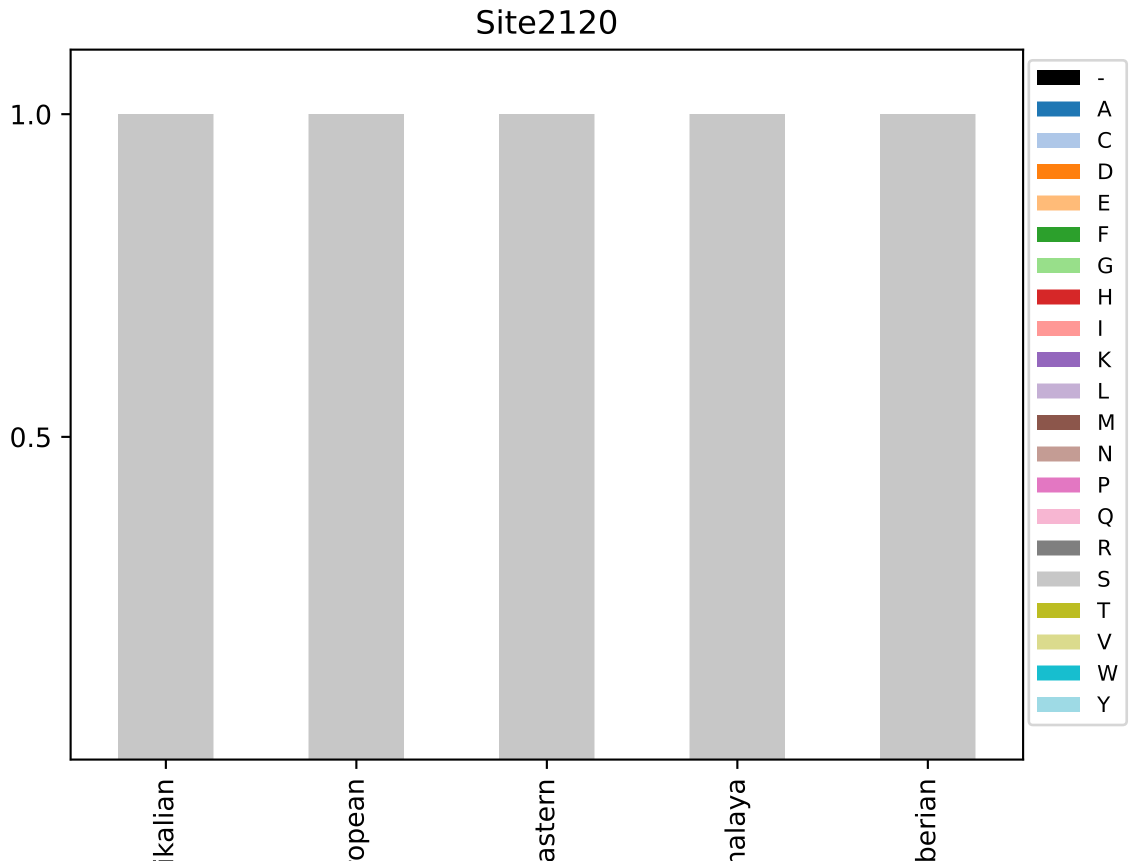
Site2120
1.0
0.5
-
A
C
D
E
F
G
H
I
K
L
M
N
P
Q
R
S
T
V
W
Y
ikalian
opean
astern
nalaya
berian

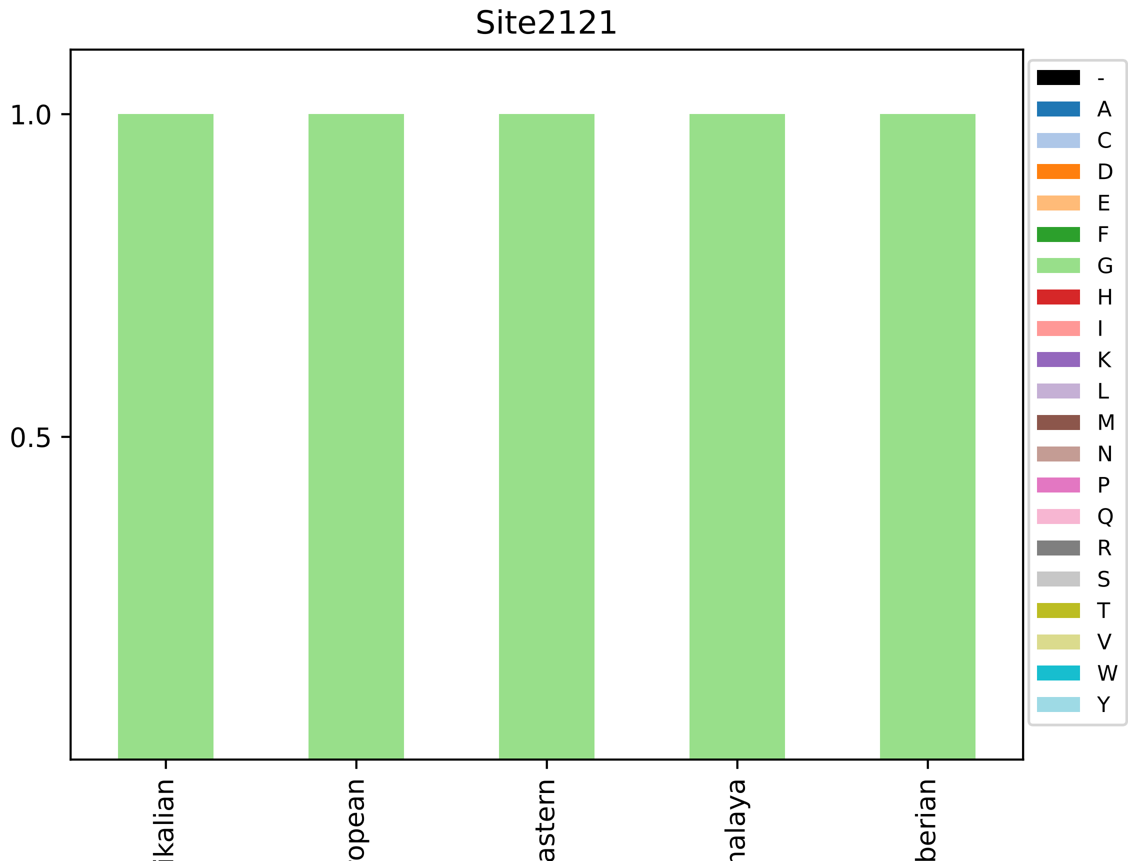
Site2121
1.0
0.5
-
A
C
D
E
F
G
H
I
K
L
M
N
P
Q
R
S
T
V
W
Y
ikalian
opean
astern
nalaya
berian

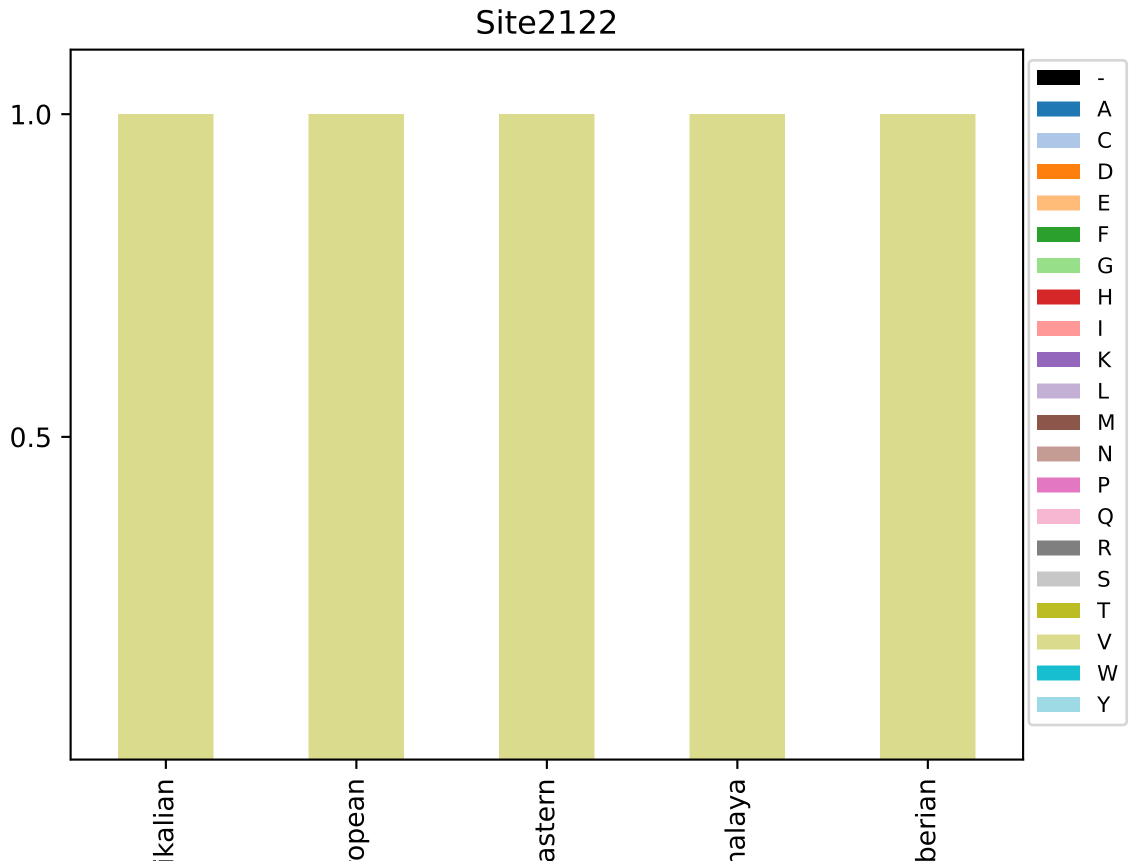
Site2122
1.0
0.5
ikalian
opean
astern
nalaya
berian
-
A
C
D
E
F
G
H
I
K
L
M
N
P
Q
R
S
T
V
W
Y

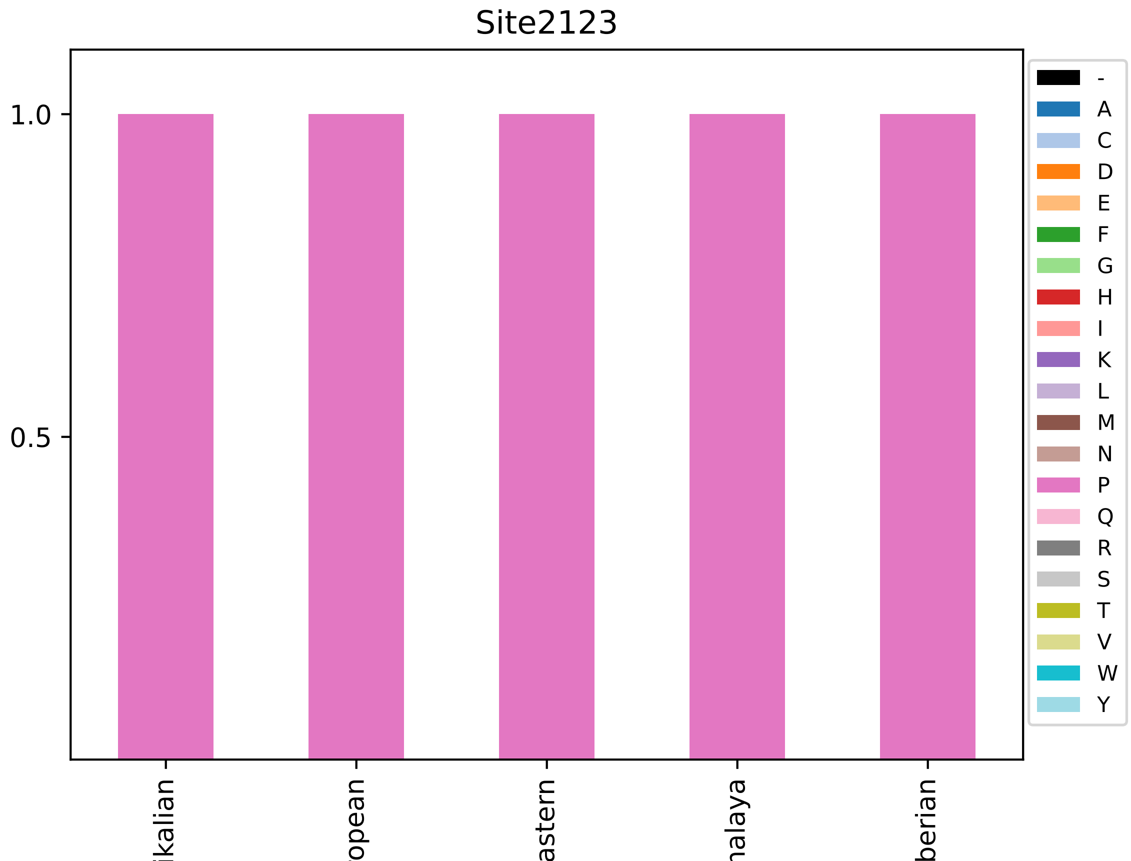

Site2123
1.0
0.5
-
A
C
D
E
F
G
H
I
K
L
M
N
P
Q
R
S
T
V
W
Y
ikalian
opean
astern
nalaya
berian

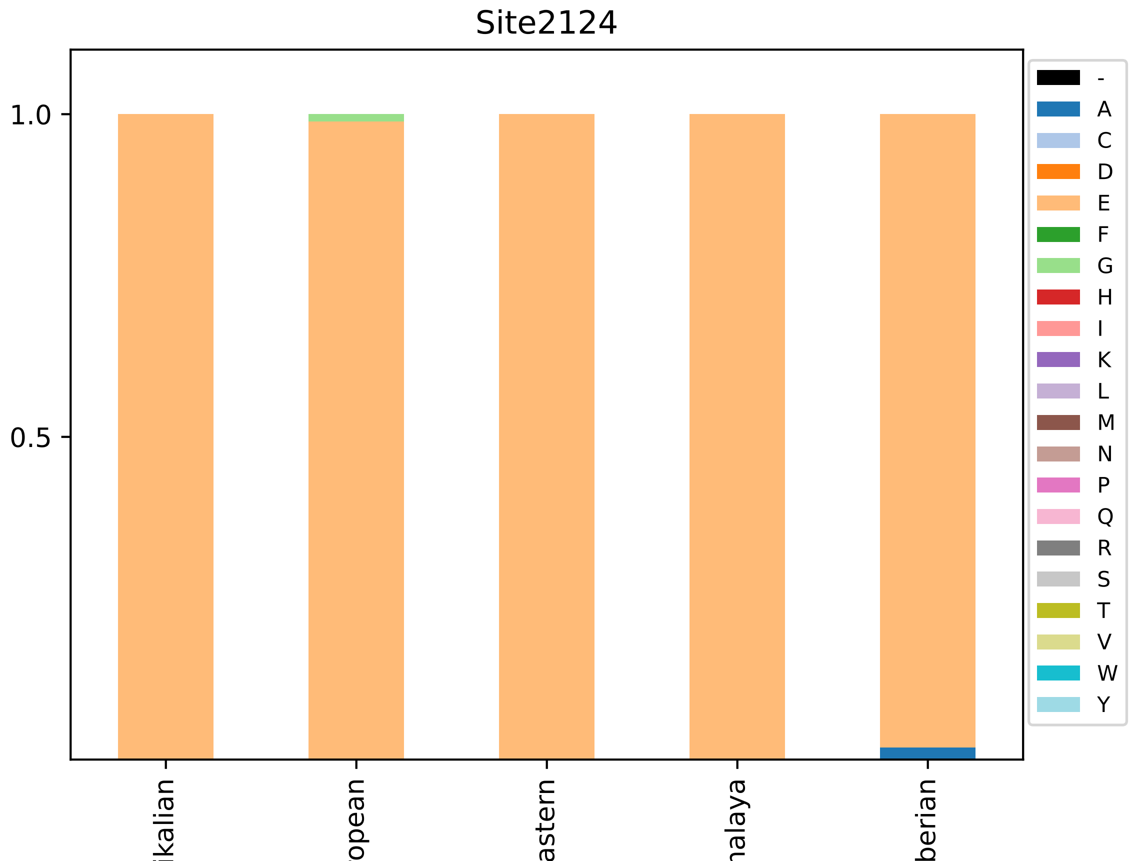

Site2124
1.0
0.5
-
A
C
D
E
F
G
H
I
K
L
M
N
P
Q
R
S
T
V
W
Y
ikalian
opean
astern
nalaya
berian

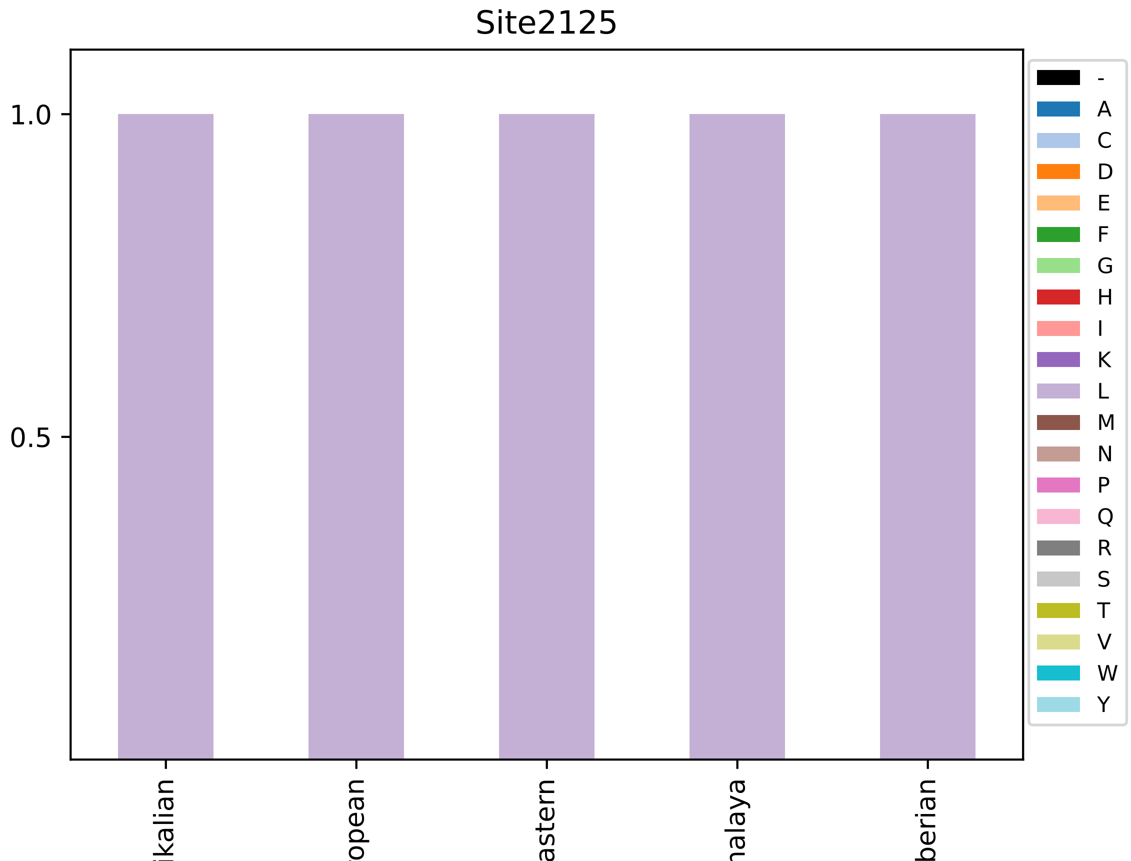

Site2125
1.0
0.5
-
A
C
D
E
F
G
H
I
K
L
M
N
P
Q
R
S
T
V
W
Y
ikalian
opean
astern
nalaya
berian

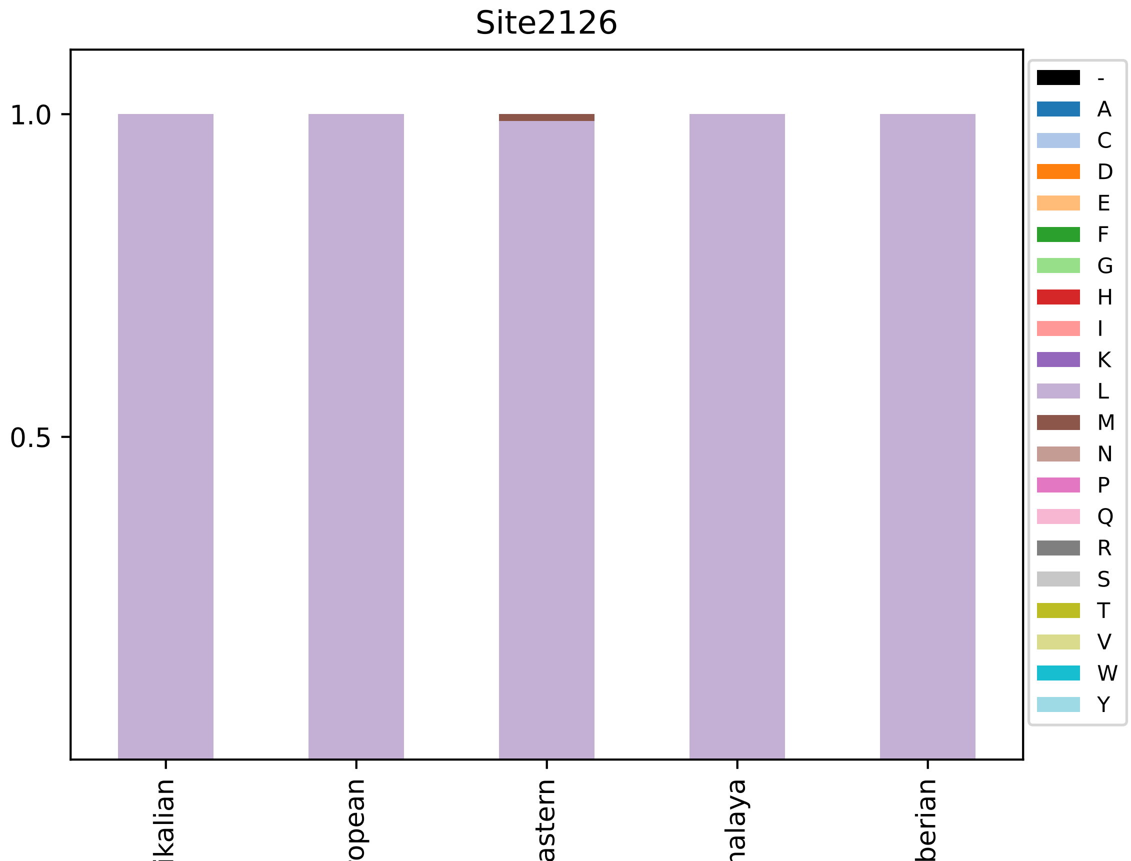
Site2126
1.0
0.5
-
A
C
D
E
F
G
H
I
K
L
M
N
P
Q
R
S
T
V
W
Y
ikalian
opean
astern
nalaya
berian

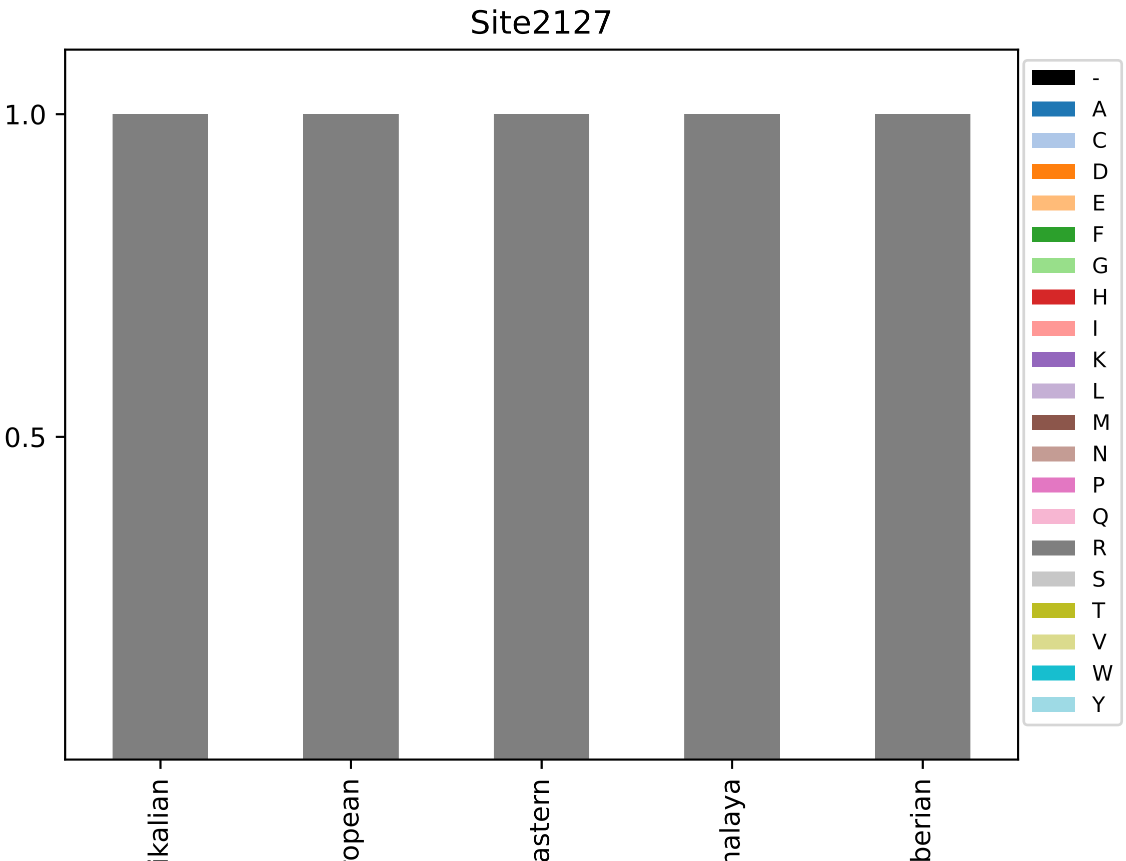

Site2127
1.0
0.5
-
A
C
D
E
F
G
H
I
K
L
M
N
P
Q
R
S
T
V
W
Y
ikalian
opean
astern
nalaya
berian

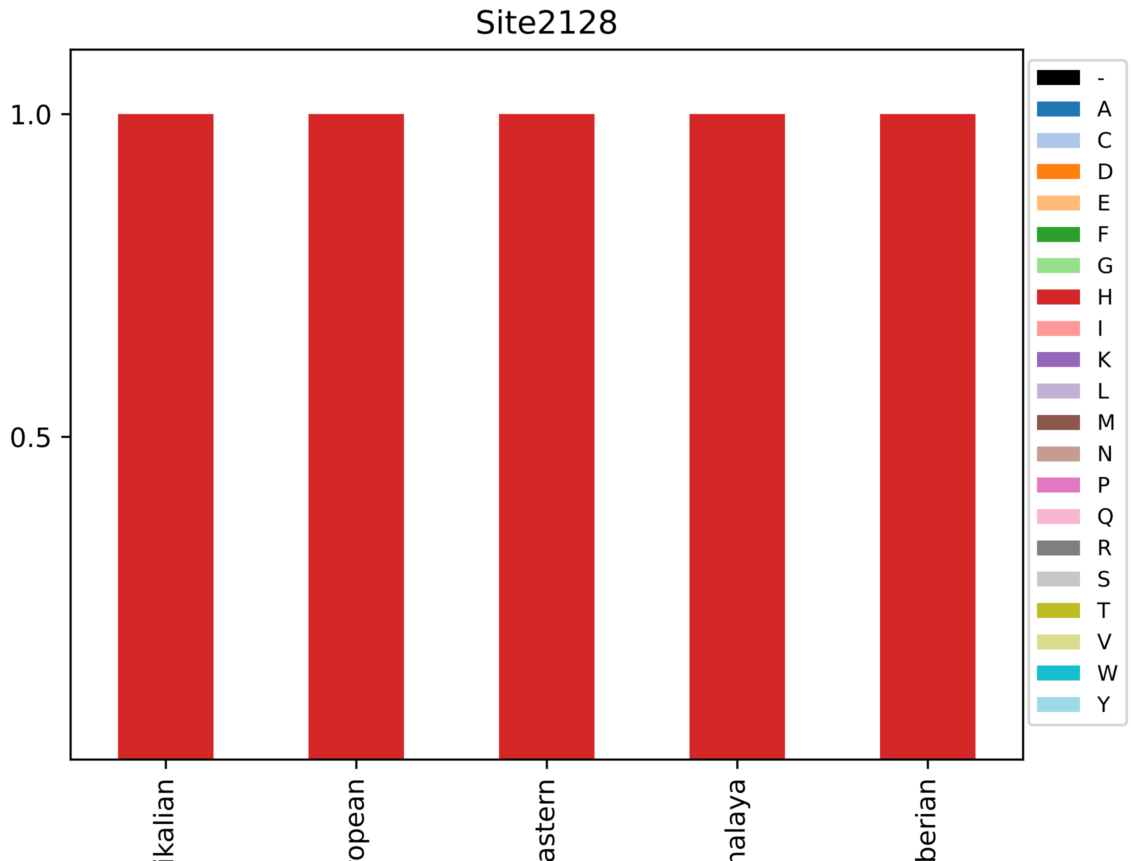

Site2128
1.0
0.5
-
A
C
D
E
F
G
H
I
K
L
M
N
P
Q
R
S
T
V
W
Y
ikalian
opean
astern
nalaya
berian

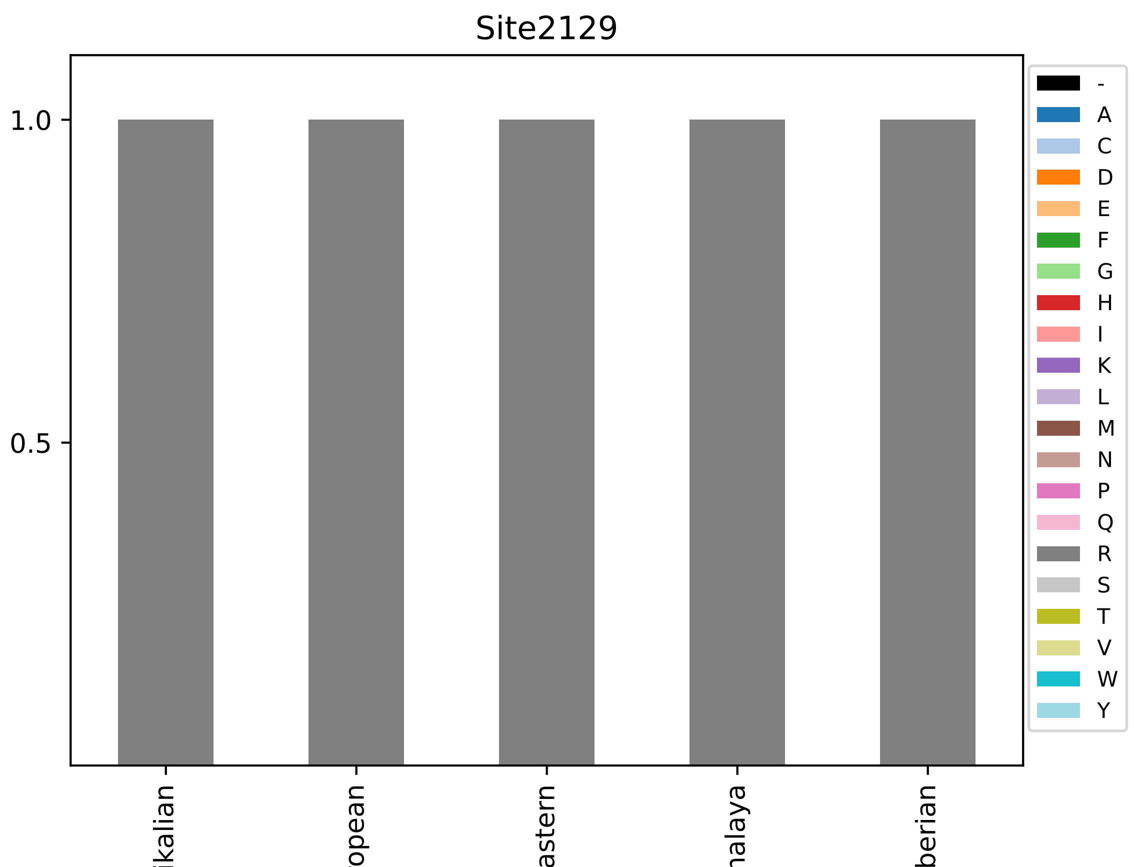
Site2129
1.0
0.5
-
A
C
D
E
F
G
H
I
K
L
M
N
P
Q
R
S
T
V
W
Y

Site2130

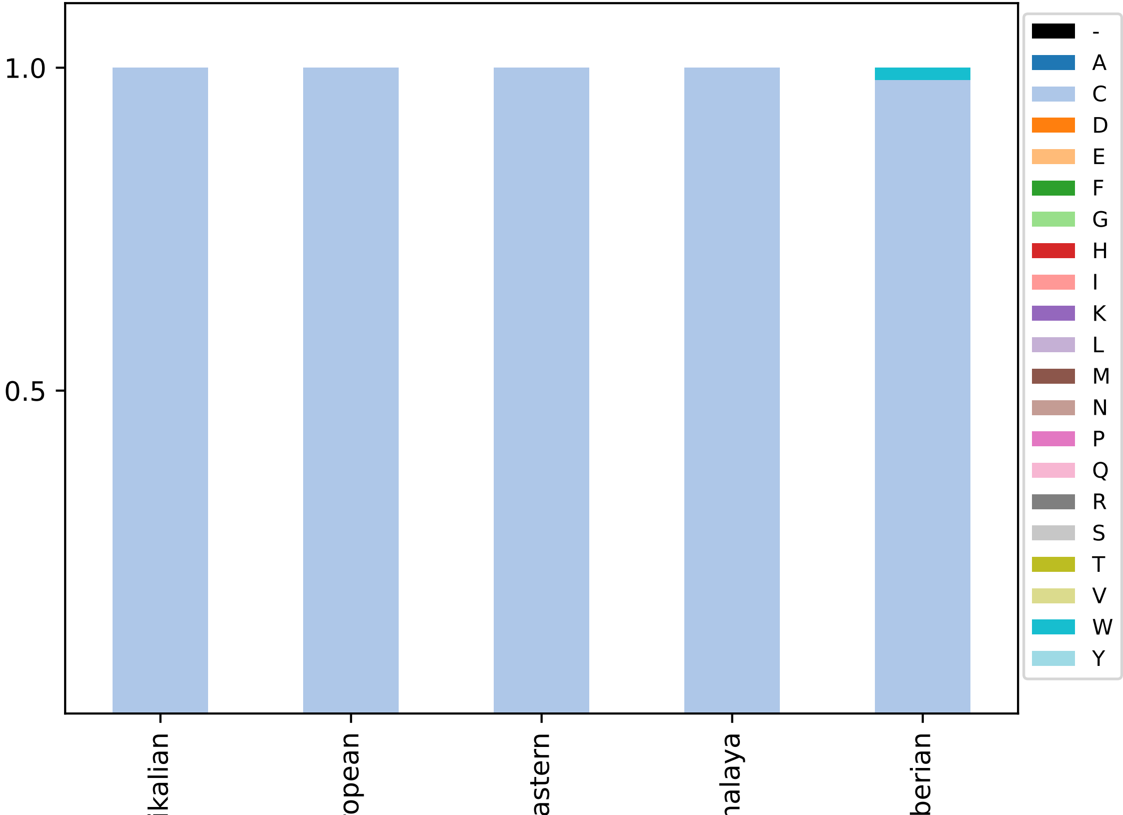
1.0
0.5
-
A
C
D
E
F
G
H
I
K
L
M
N
P
Q
R
S
T
V
W
Y
ikalian
opean
astern
nalaya
berian

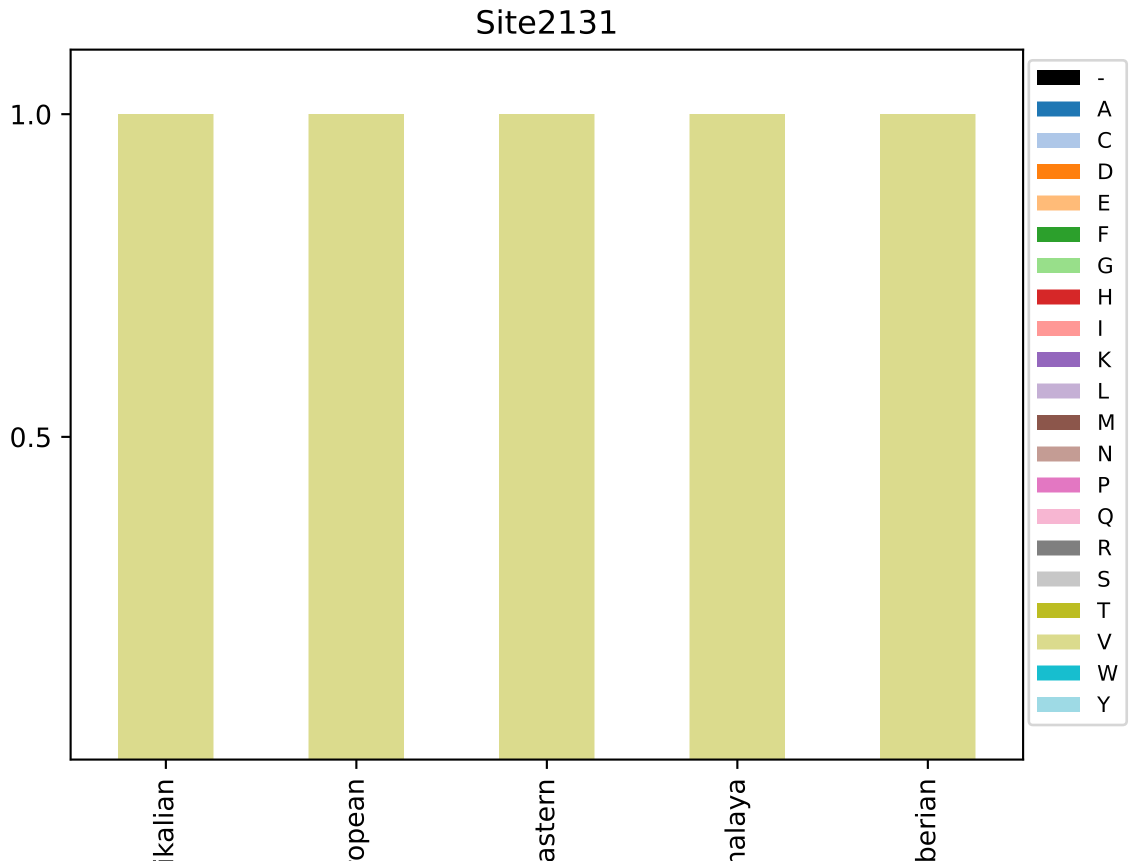
Site2131
1.0
0.5
-
A
C
D
E
F
G
H
I
K
L
M
N
P
Q
R
S
T
V
W
Y
ikalian
opean
astern
nalaya
berian

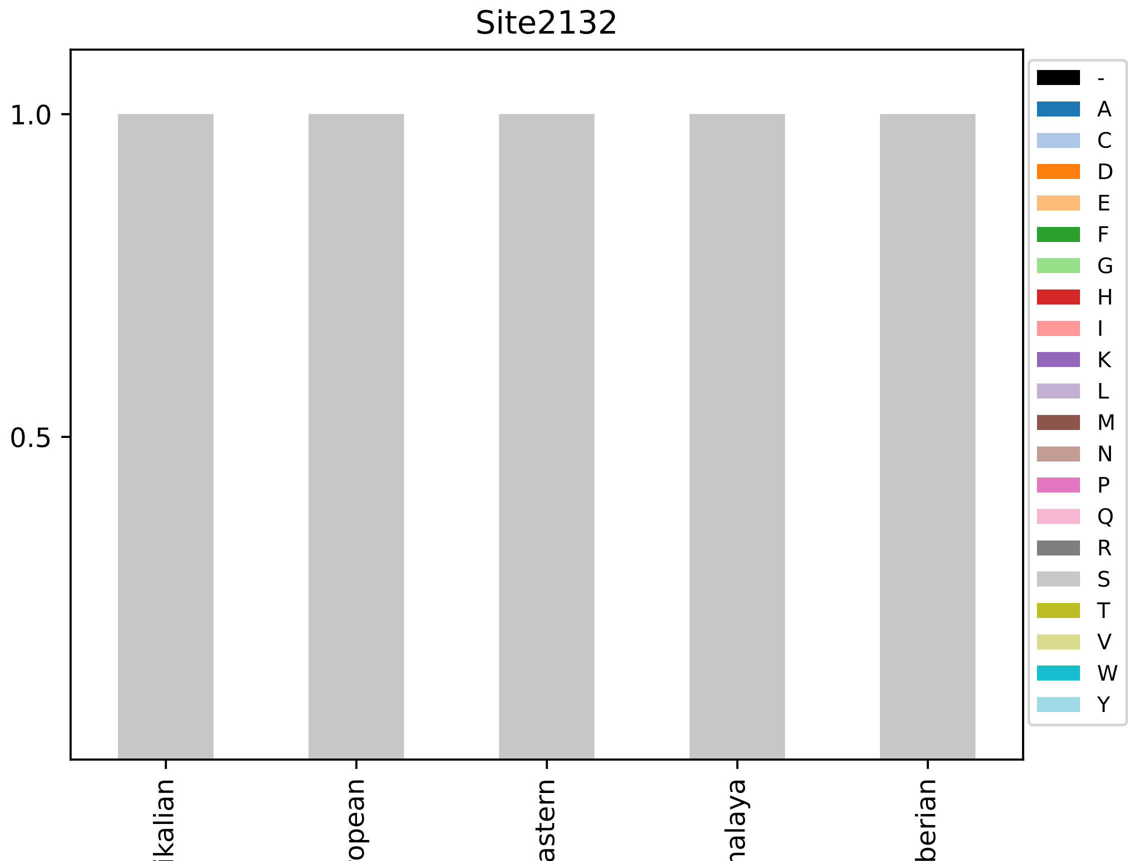

Site2132
1.0
0.5
-
A
C
D
E
F
G
H
I
K
L
M
N
P
Q
R
S
T
V
W
Y

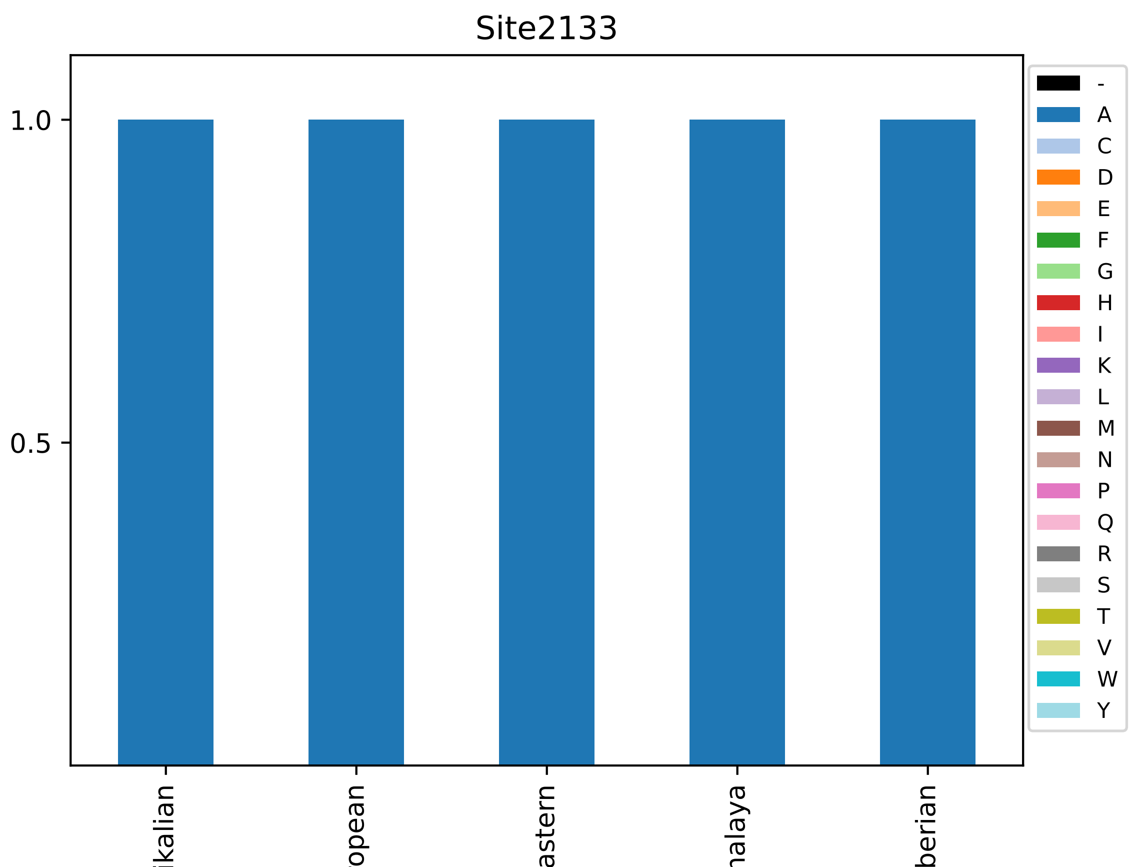

Site2133
1.0
0.5
-
A
C
D
E
F
G
H
I
K
L
M
N
P
Q
R
S
T
V
W
Y
ikalian
opean
astern
nalaya
berian

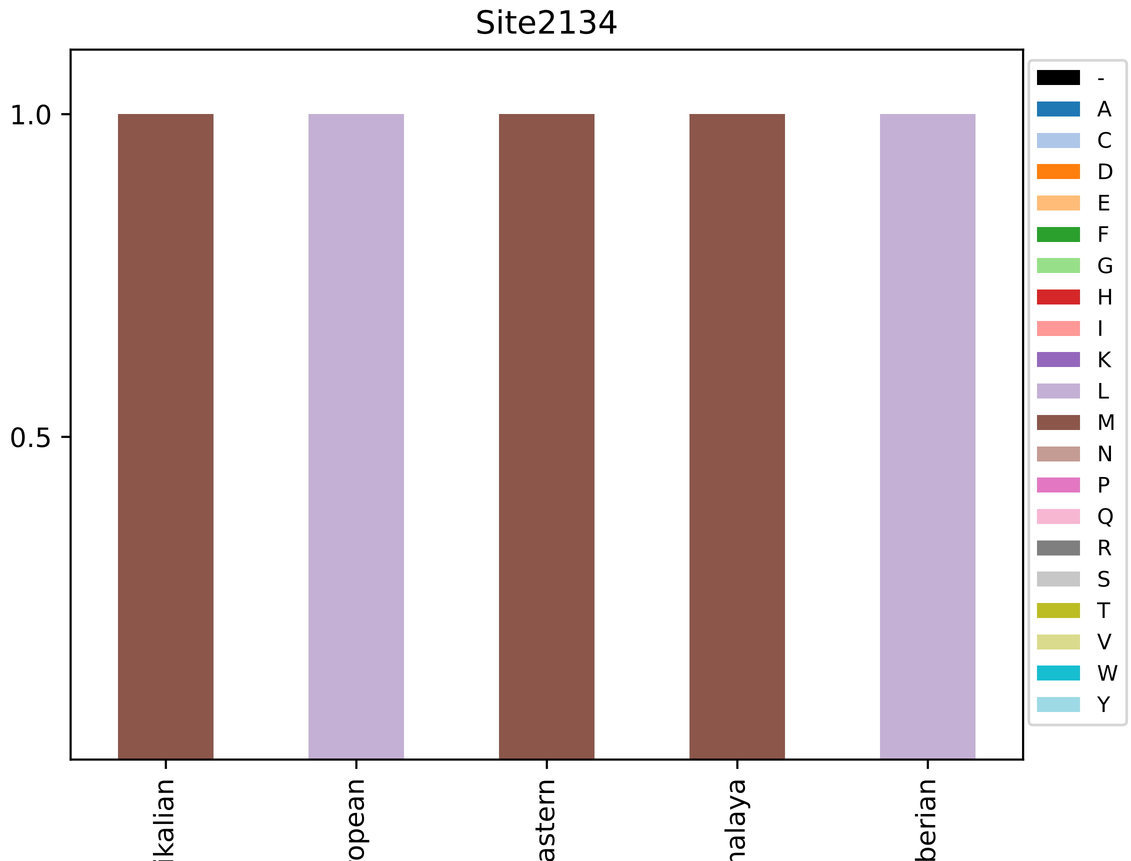
Site2134
1.0
0.5
ikalian
opean
astern
nalaya
berian
-
A
C
D
E
F
G
H
I
K
L
M
N
P
Q
R
S
T
V
W
Y

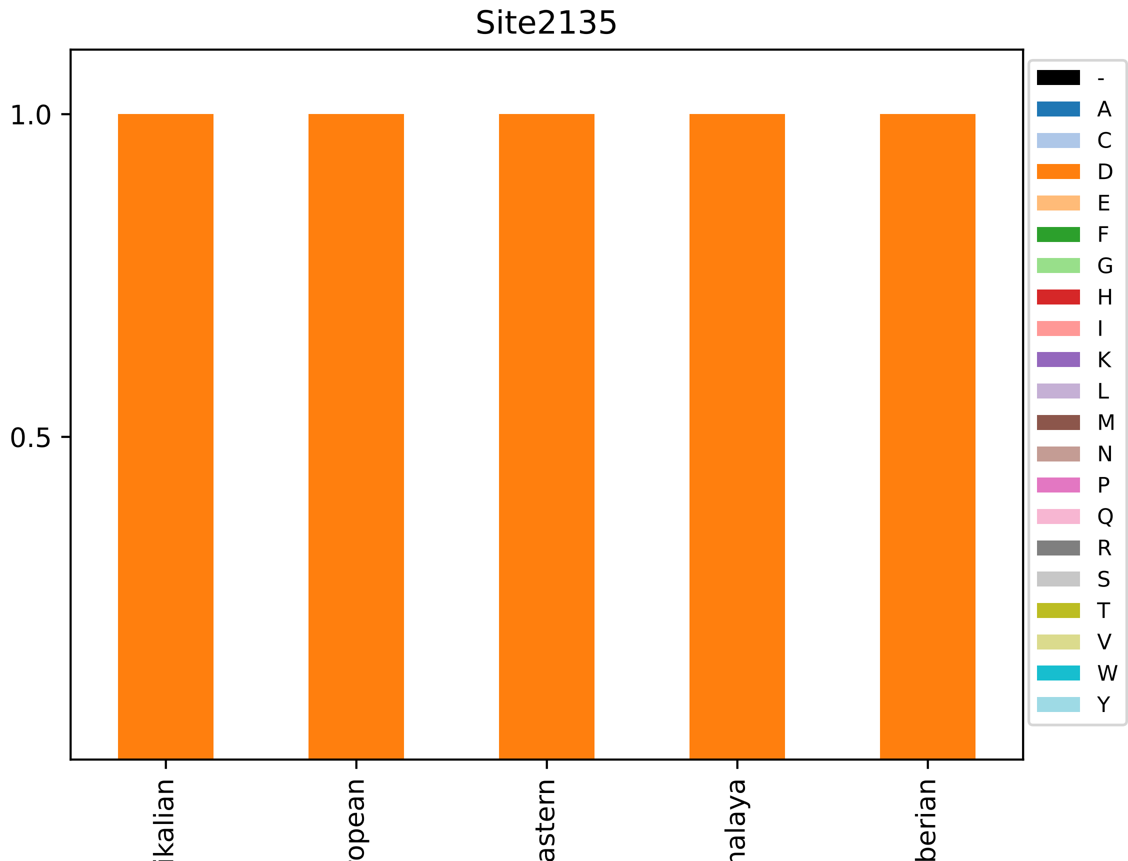
Site2135
1.0
0.5
-
A
C
D
E
F
G
H
I
K
L
M
N
P
Q
R
S
T
V
W
Y
ikalian
opean
astern
nalaya
berian

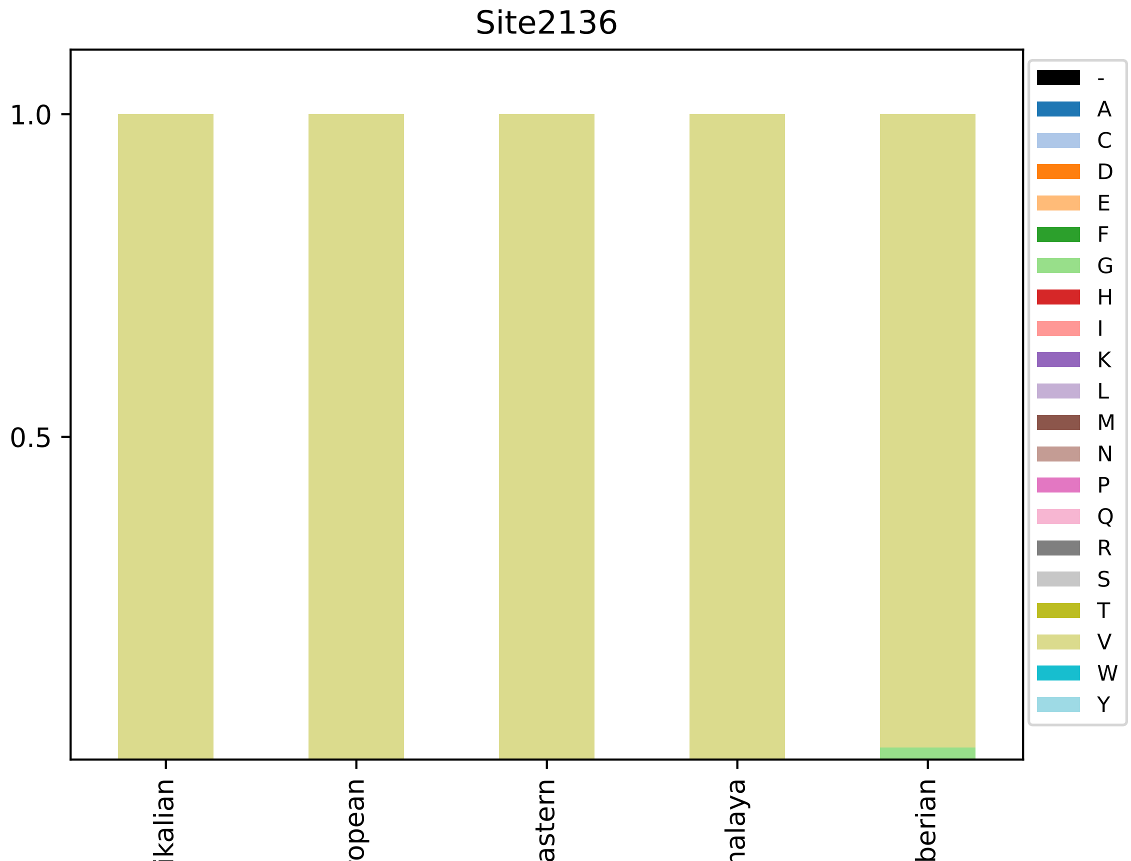
Site2136
1.0
0.5
-
A
C
D
E
F
G
H
I
K
L
M
N
P
Q
R
S
T
V
W
Y
ikalian
opean
astern
nalaya
berian

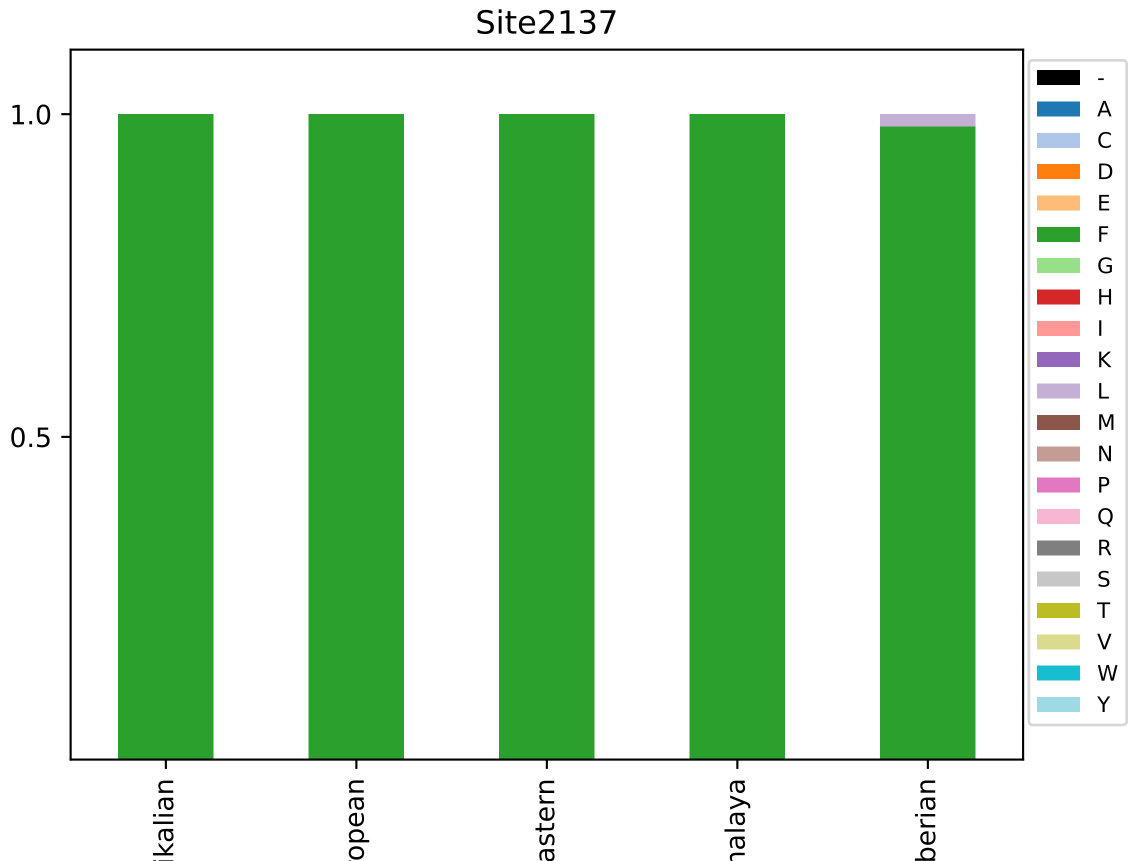
Site2137
1.0
0.5
-
A
C
D
E
F
G
H
I
K
L
M
N
P
Q
R
S
T
V
W
Y
ikalian
opean
astern
nalaya
berian

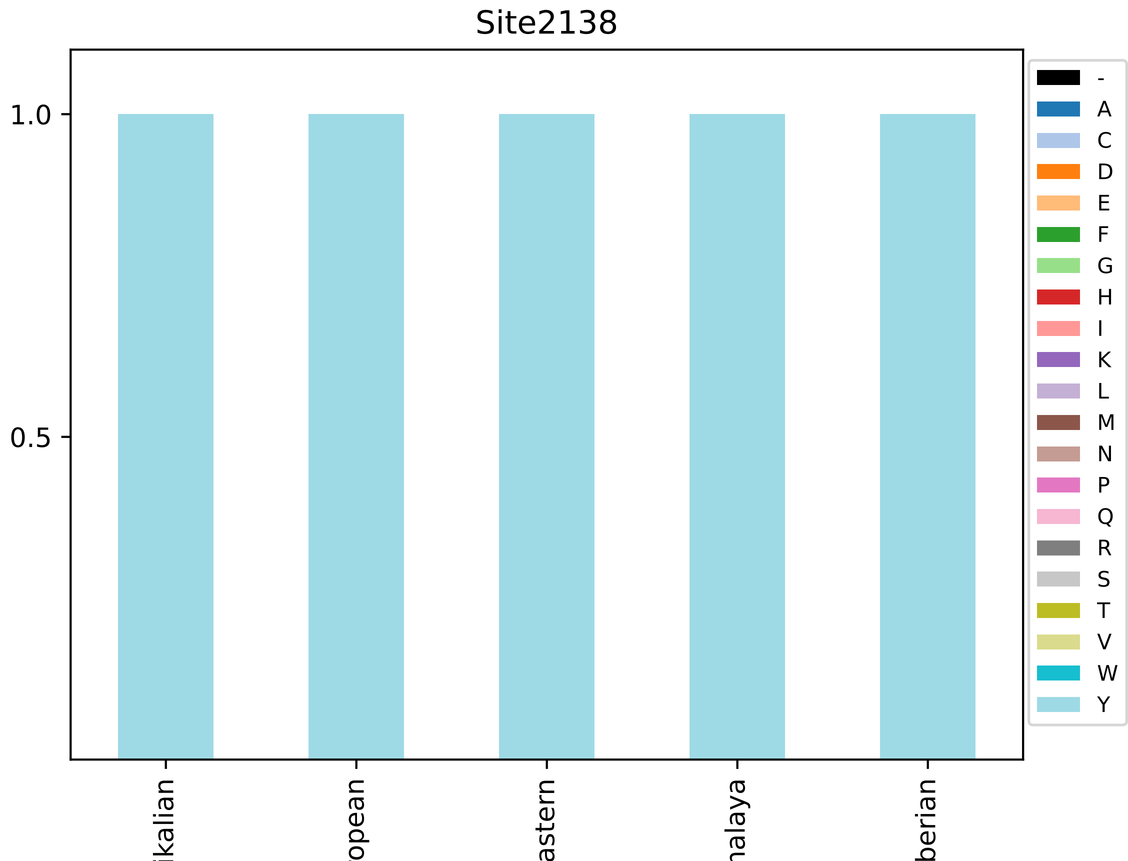

Site2138
1.0
0.5
-
A
C
D
E
F
G
H
I
K
L
M
N
P
Q
R
S
T
V
W
Y

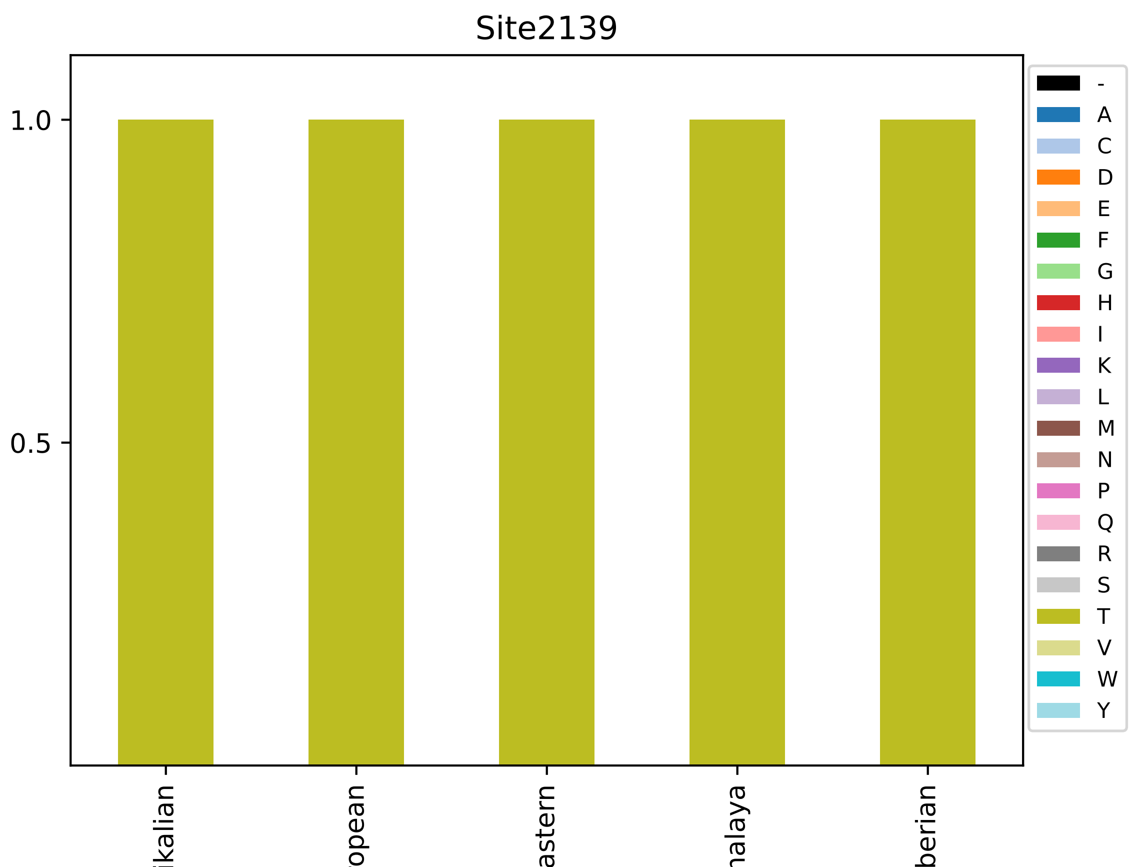

Site2139
1.0
0.5
-
A
C
D
E
F
G
H
I
K
L
M
N
P
Q
R
S
T
V
W
Y

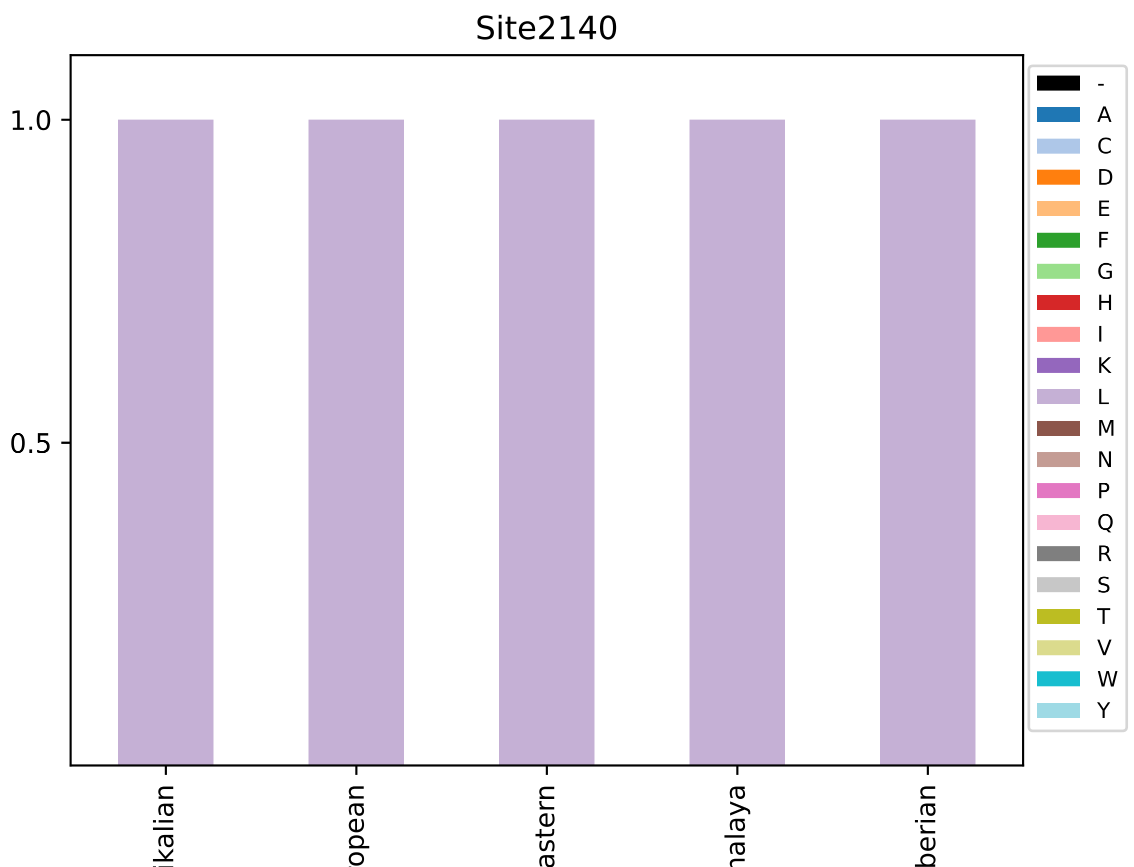
Site2140
1.0
0.5
-
A
C
D
E
F
G
H
I
K
L
M
N
P
Q
R
S
T
V
W
Y
ikalian
opean
astern
nalaya
berian

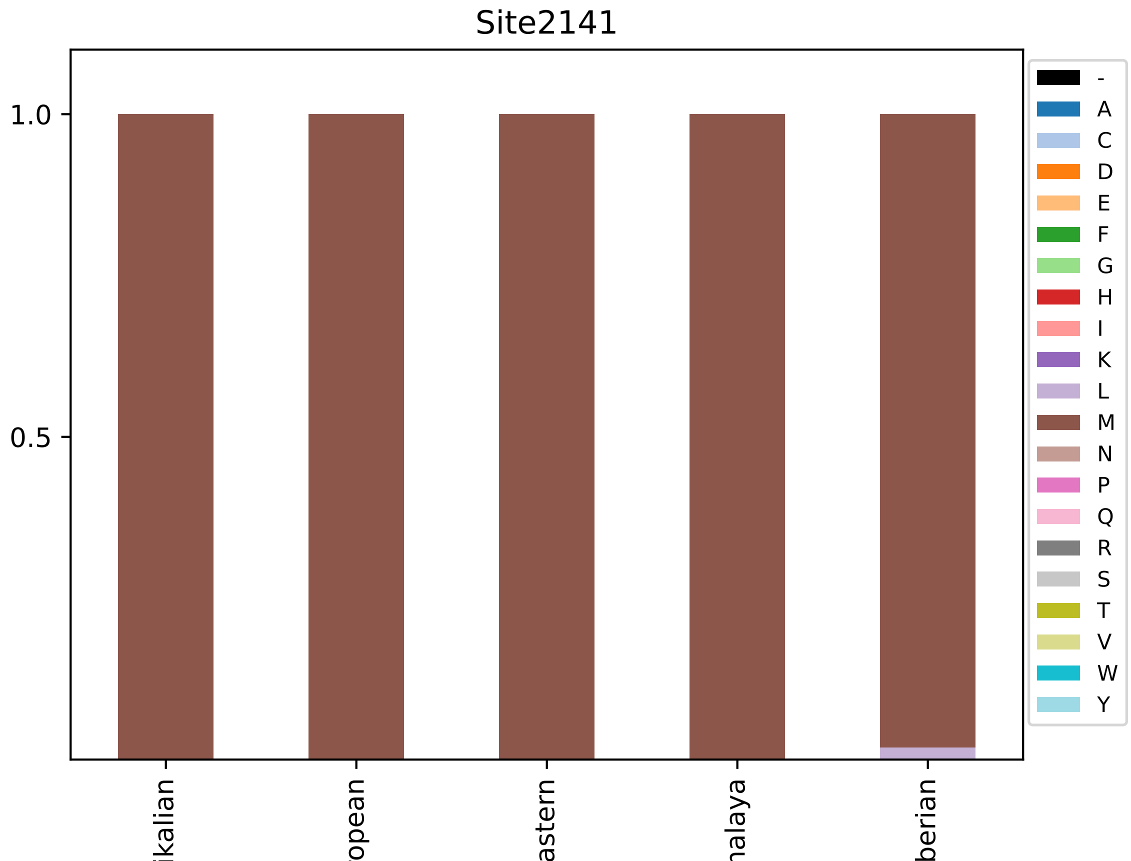

Site2141
1.0
0.5
-
A
C
D
E
F
G
H
I
K
L
M
N
P
Q
R
S
T
V
W
Y
ikalian
opean
astern
nalaya
berian

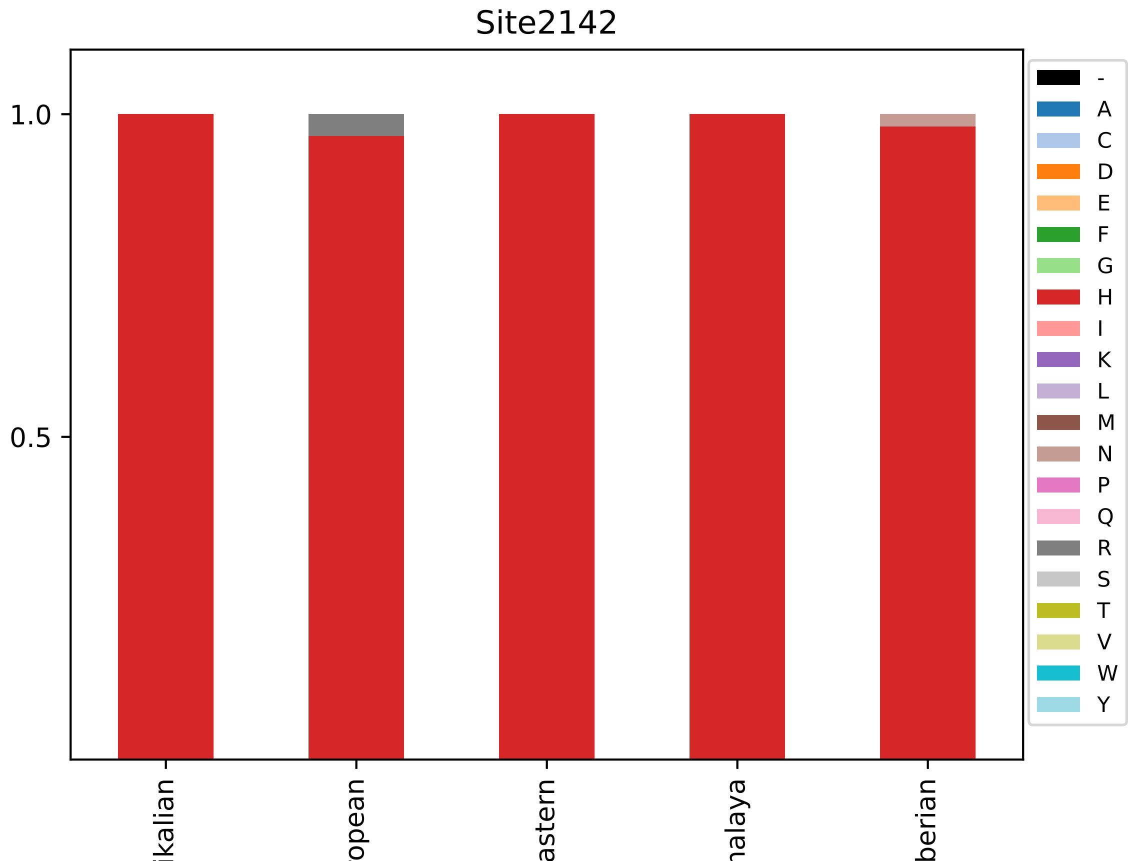
Site2142
1.0
0.5
-
A
C
D
E
F
G
H
I
K
L
M
N
P
Q
R
S
T
V
W
Y
ikalian
opean
astern
nalaya
berian

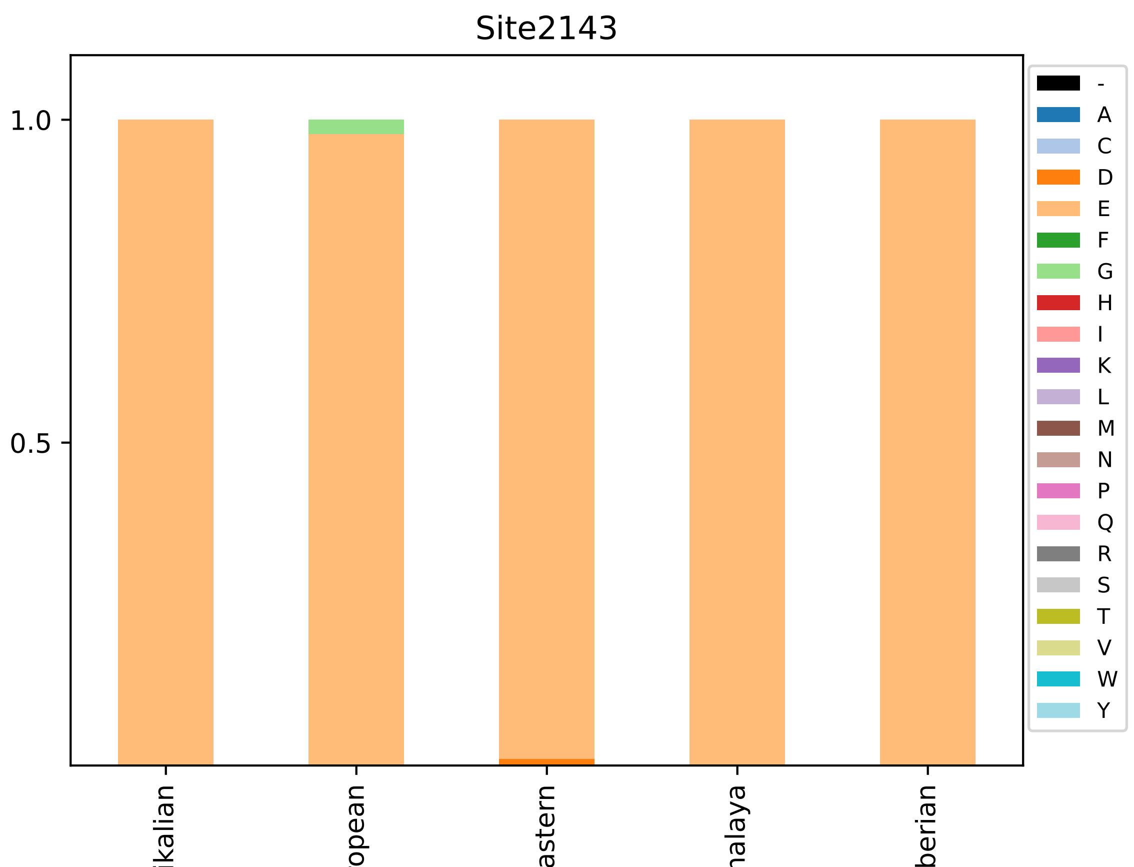
Site2143
1.0
0.5
-
A
C
D
E
F
G
H
I
K
L
M
N
P
Q
R
S
T
V
W
Y

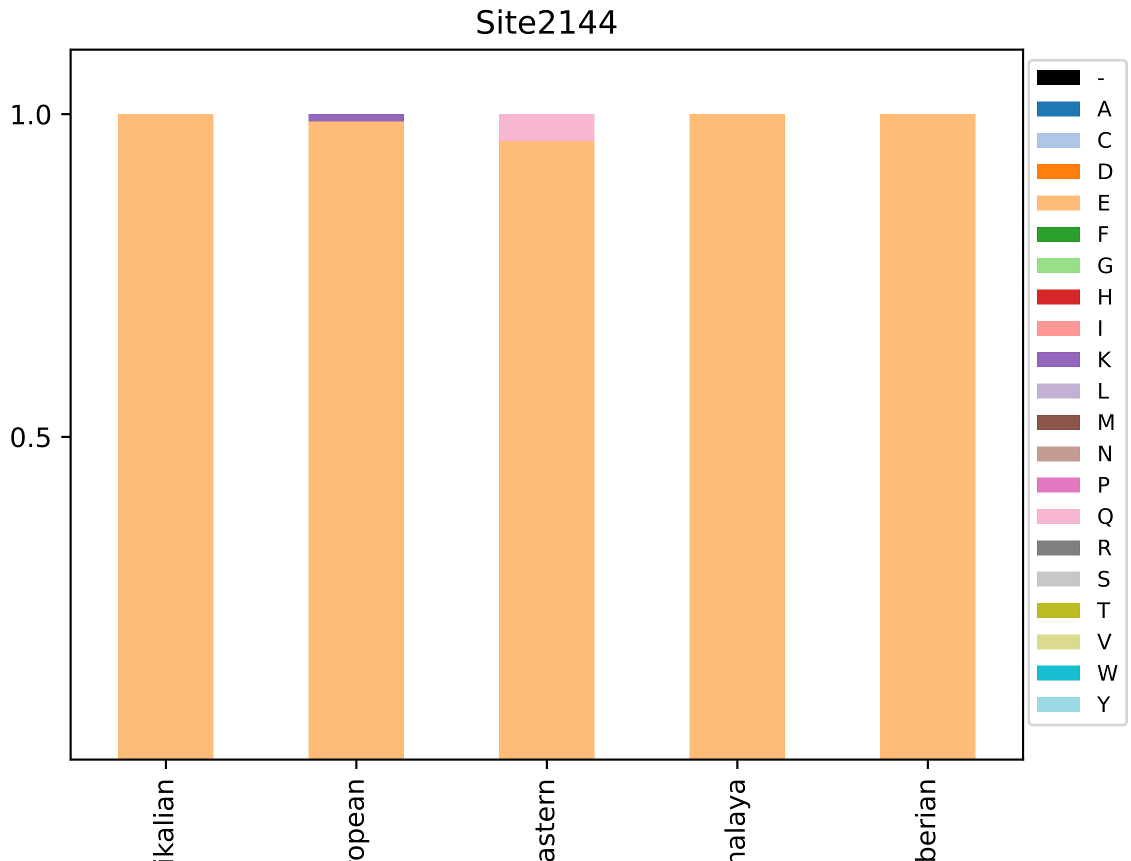

Site2144
1.0
0.5
ikalian
opean
astern
nalaya
berian
-
A
C
D
E
F
G
H
I
K
L
M
N
P
Q
R
S
T
V
W
Y

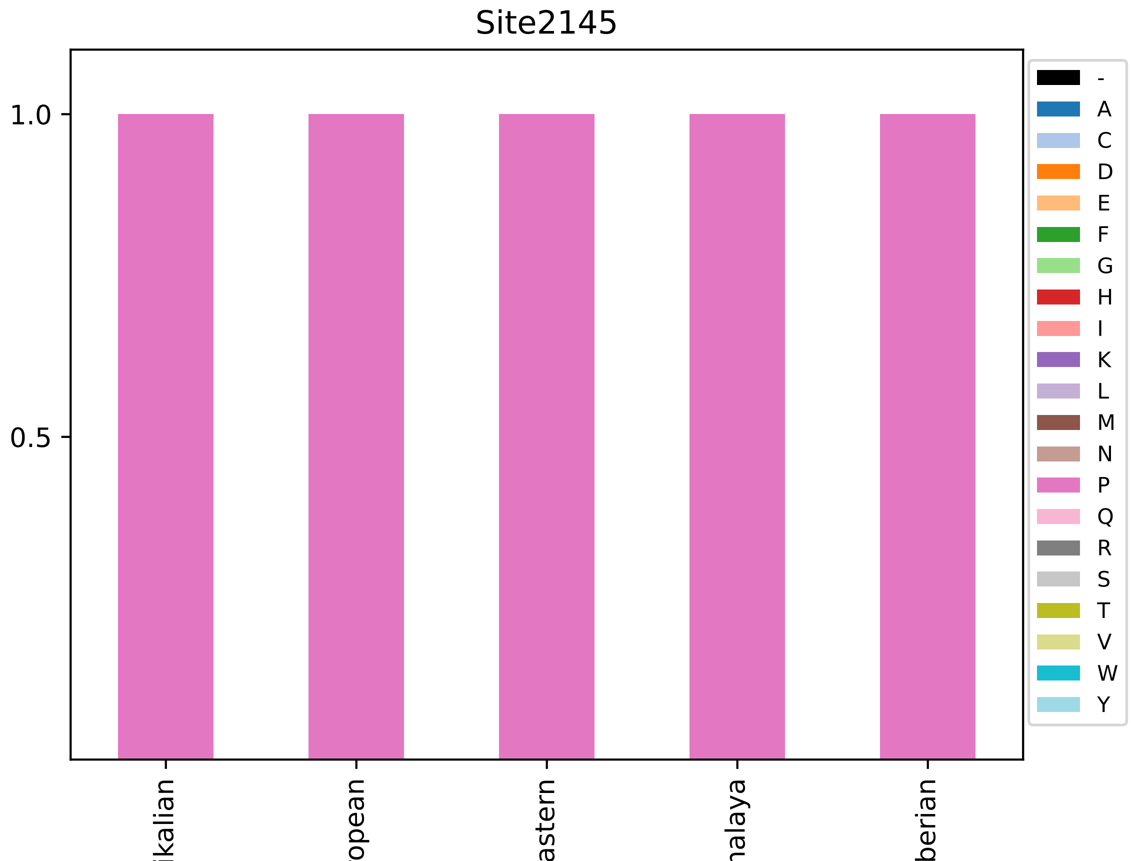

Site2145
1.0
0.5
-
A
C
D
E
F
G
H
I
K
L
M
N
P
Q
R
S
T
V
W
Y
ikalian
opean
astern
nalaya
berian

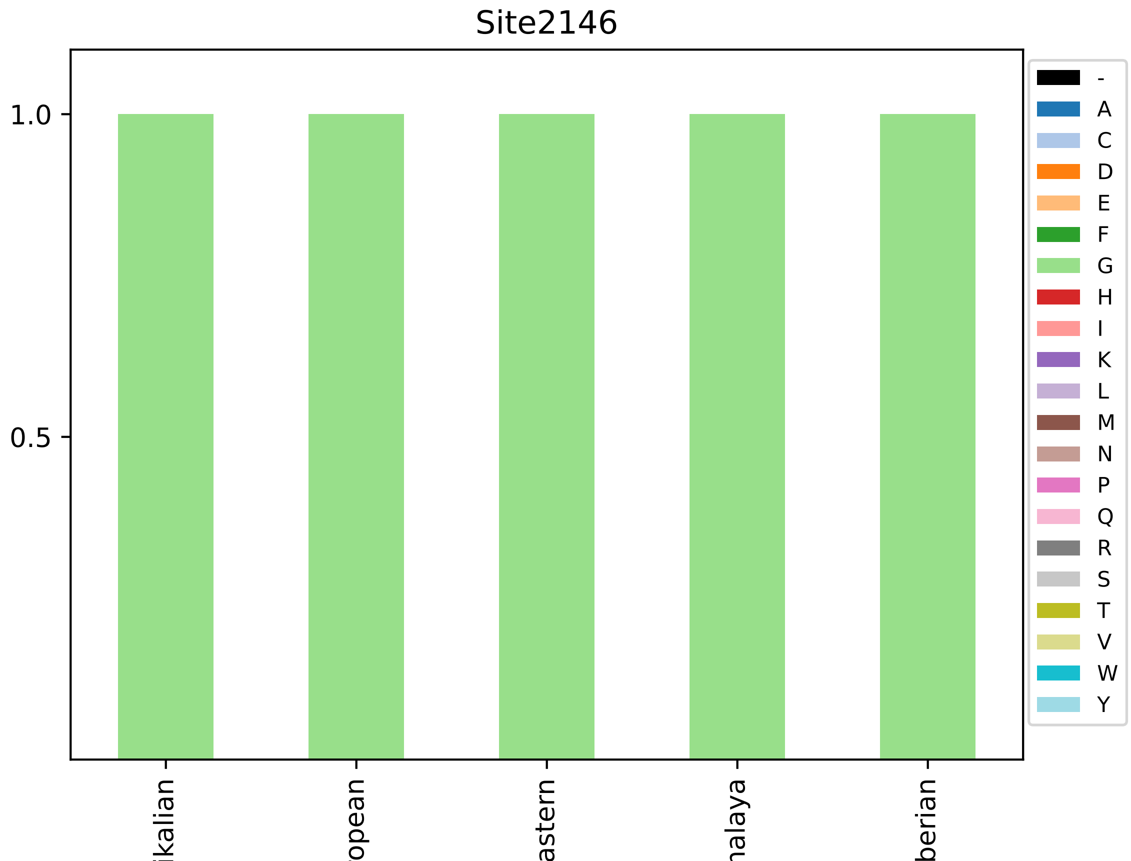

Site2146
1.0
0.5
-
A
C
D
E
F
G
H
I
K
L
M
N
P
Q
R
S
T
V
W
Y
ikalian
opean
astern
nalaya
berian

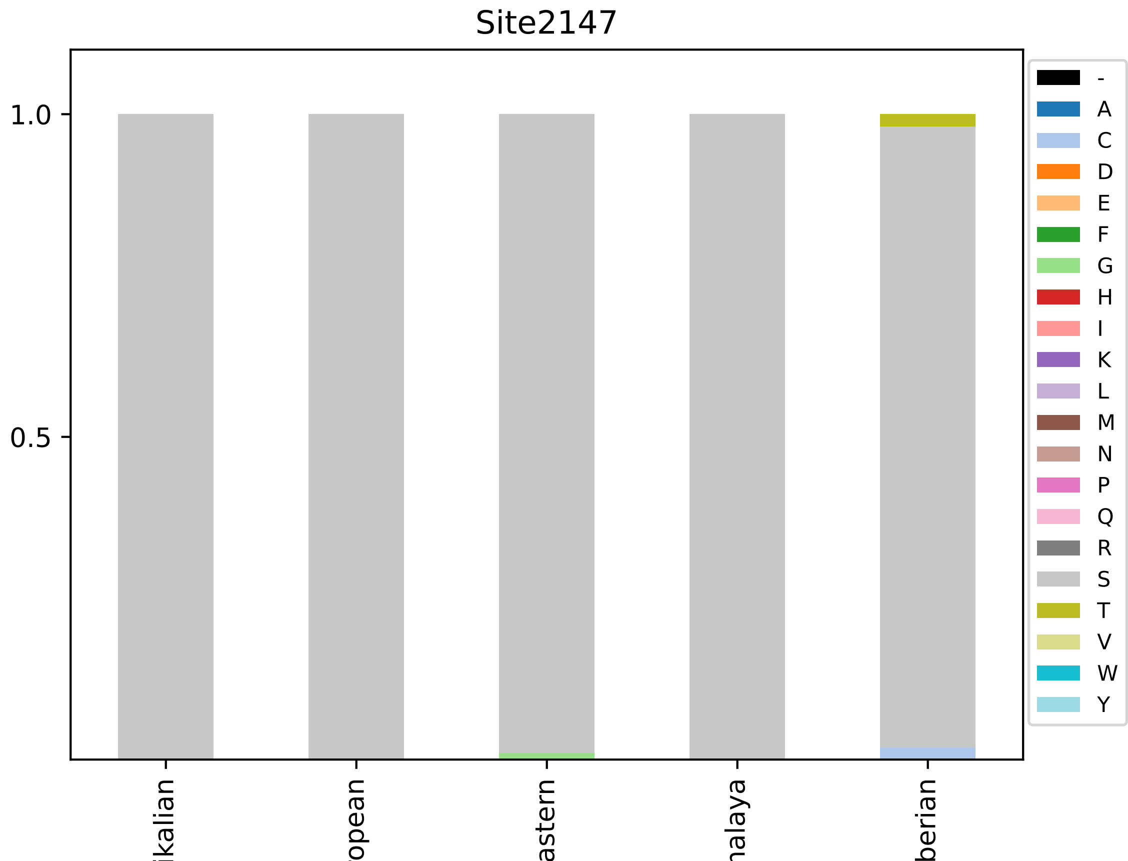
Site2147
1.0
0.5
-
A
C
D
E
F
G
H
I
K
L
M
N
P
Q
R
S
T
V
W
Y
ikalian
opean
astern
nalaya
berian

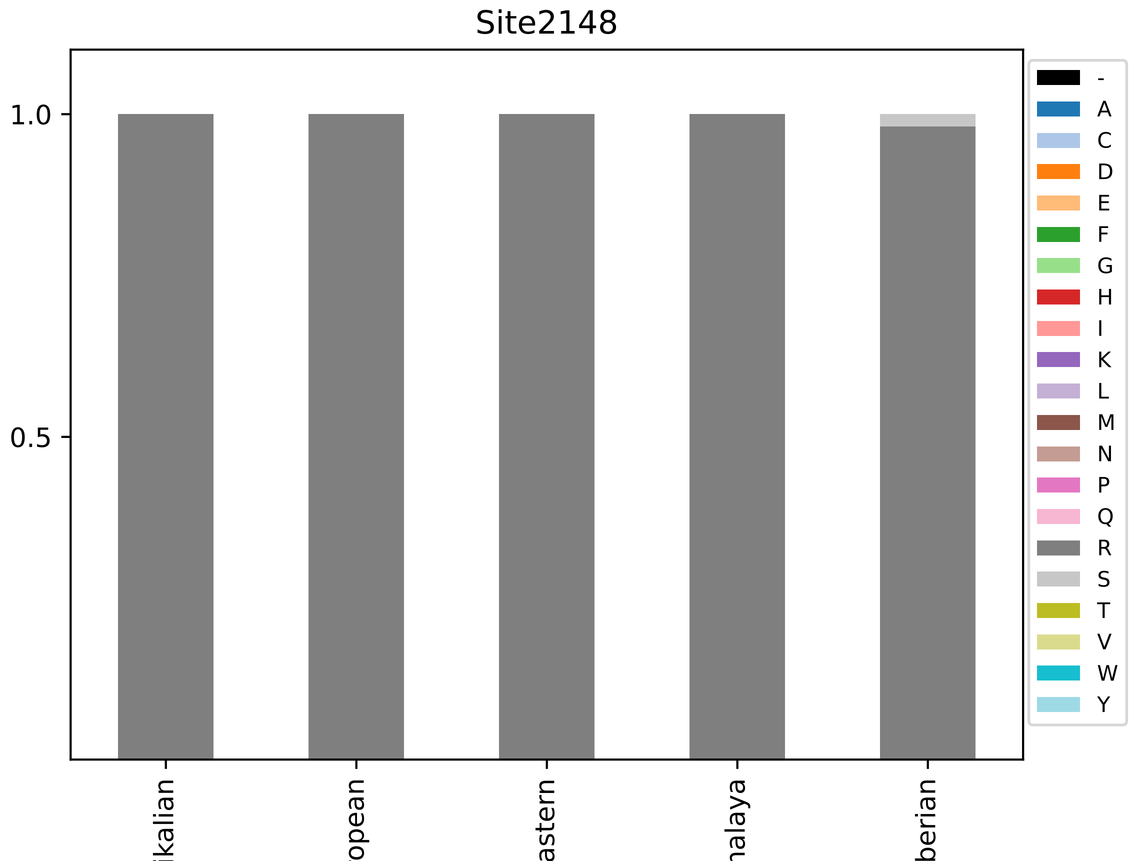
Site2148
1.0
0.5
-
A
C
D
E
F
G
H
I
K
L
M
N
P
Q
R
S
T
V
W
Y
ikalian
opean
astern
halaya
berian

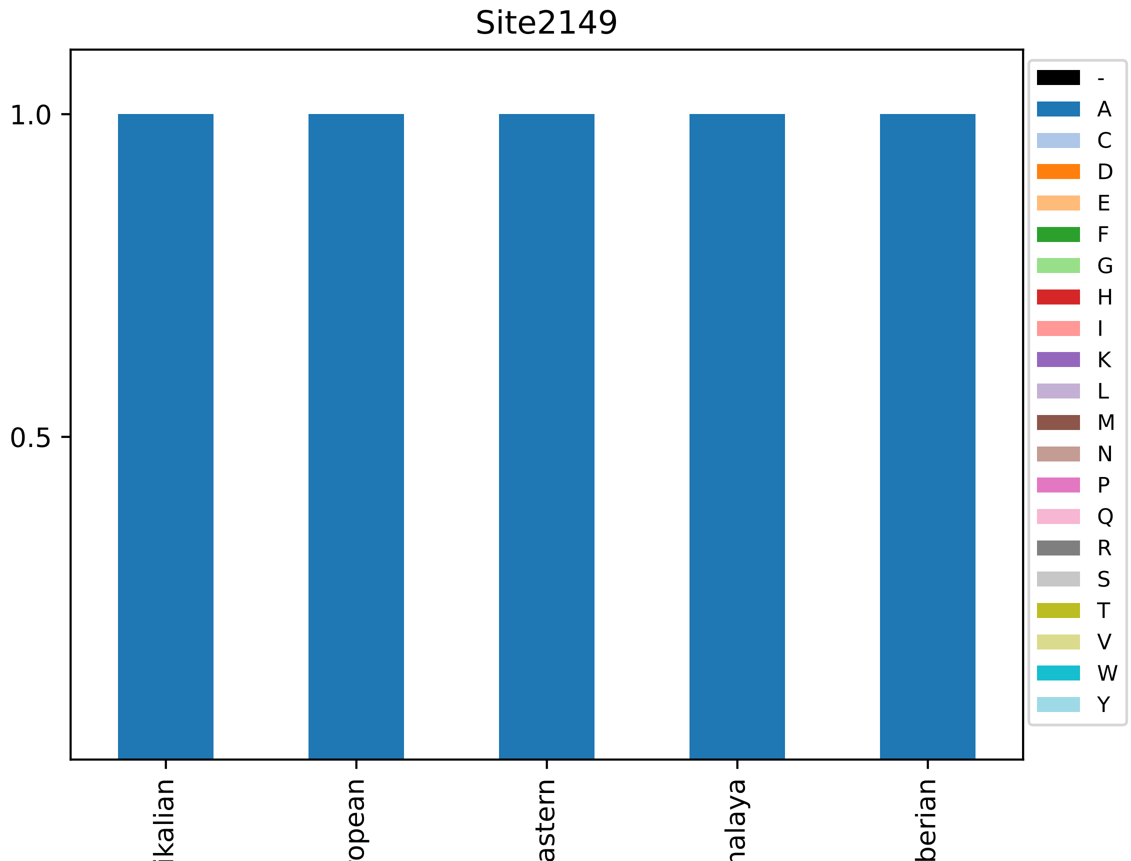

Site2149
1.0
0.5
-
A
C
D
E
F
G
H
I
K
L
M
N
P
Q
R
S
T
V
W
Y
ikalian
opean
astern
nalaya
berian

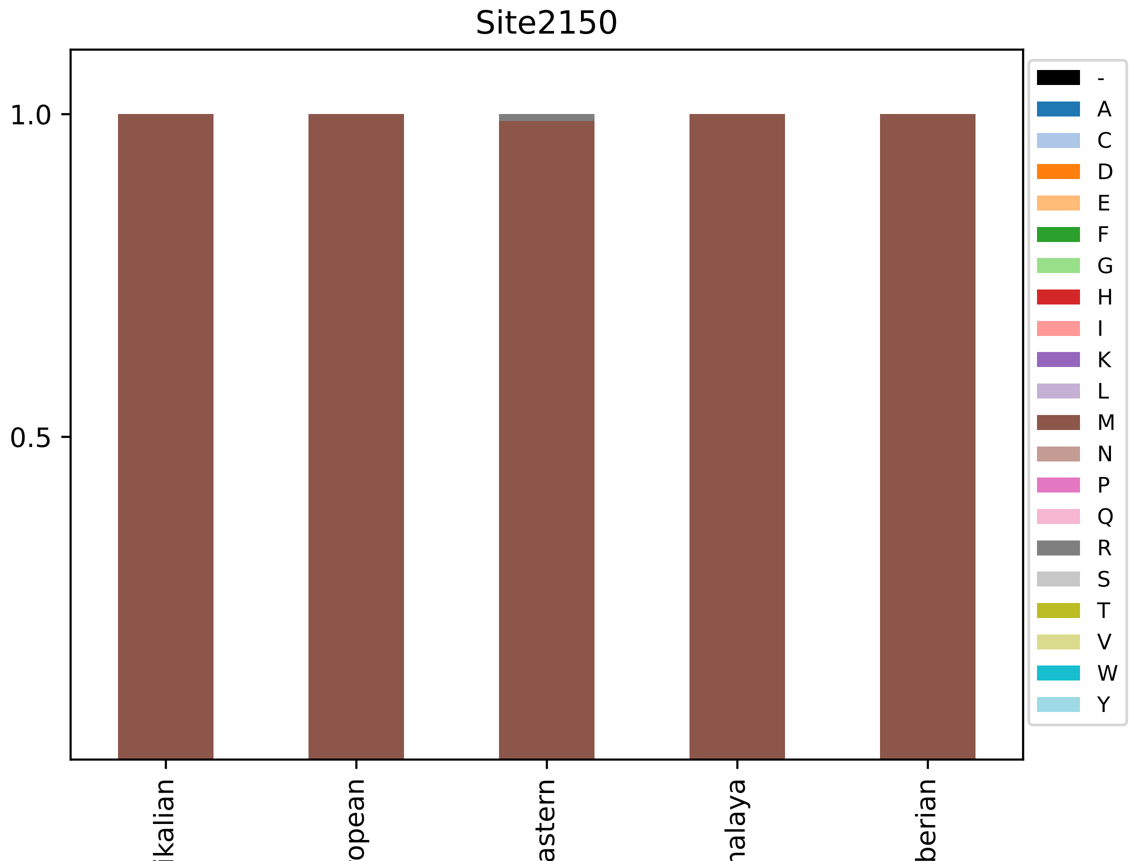
Site2150
1.0
0.5
ikalian
opean
astern
nalaya
berian
-
A
C
D
E
F
G
H
I
K
L
M
N
P
Q
R
S
T
V
W
Y

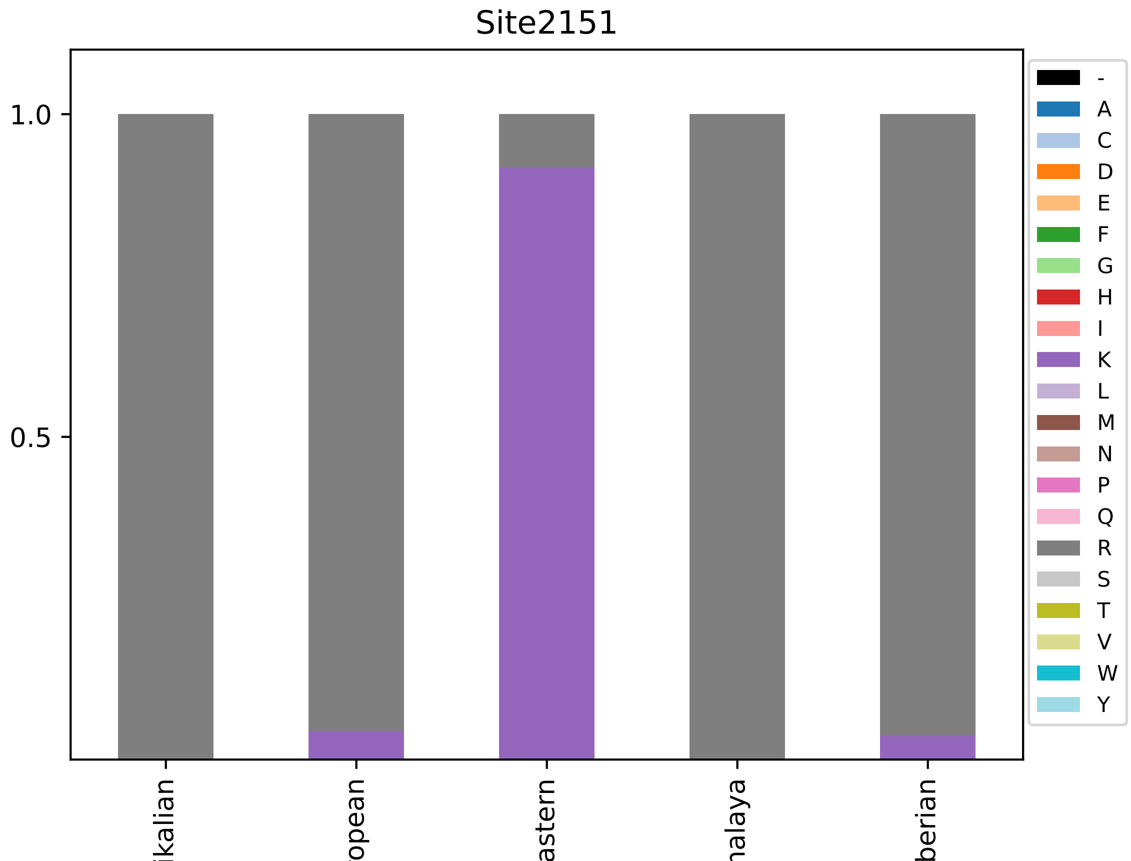
Site2151
1.0
0.5
-
A
C
D
E
F
G
H
I
K
L
M
N
P
Q
R
S
T
V
W
Y
ikalian
opean
astern
nalaya
berian

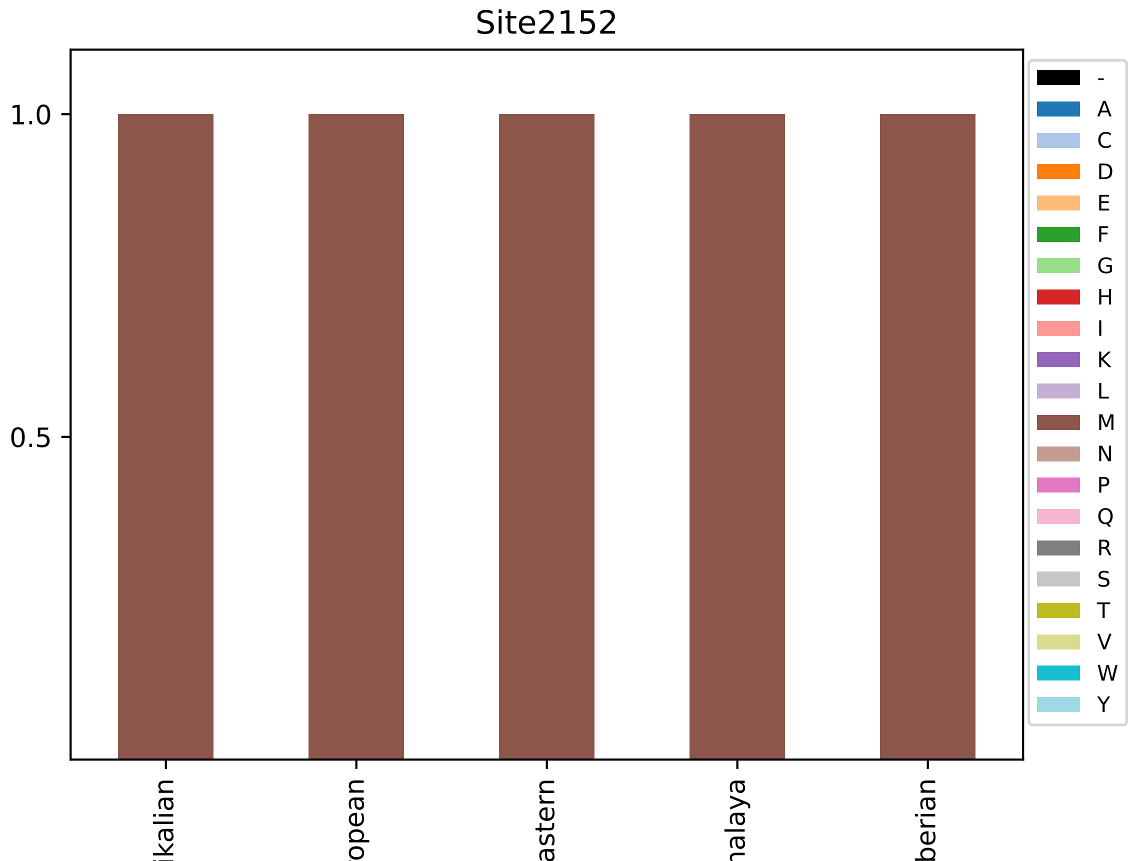

Site2152
1.0
0.5
-
A
C
D
E
F
G
H
I
K
L
M
N
P
Q
R
S
T
V
W
Y
ikalian
opean
astern
nalaya
berian

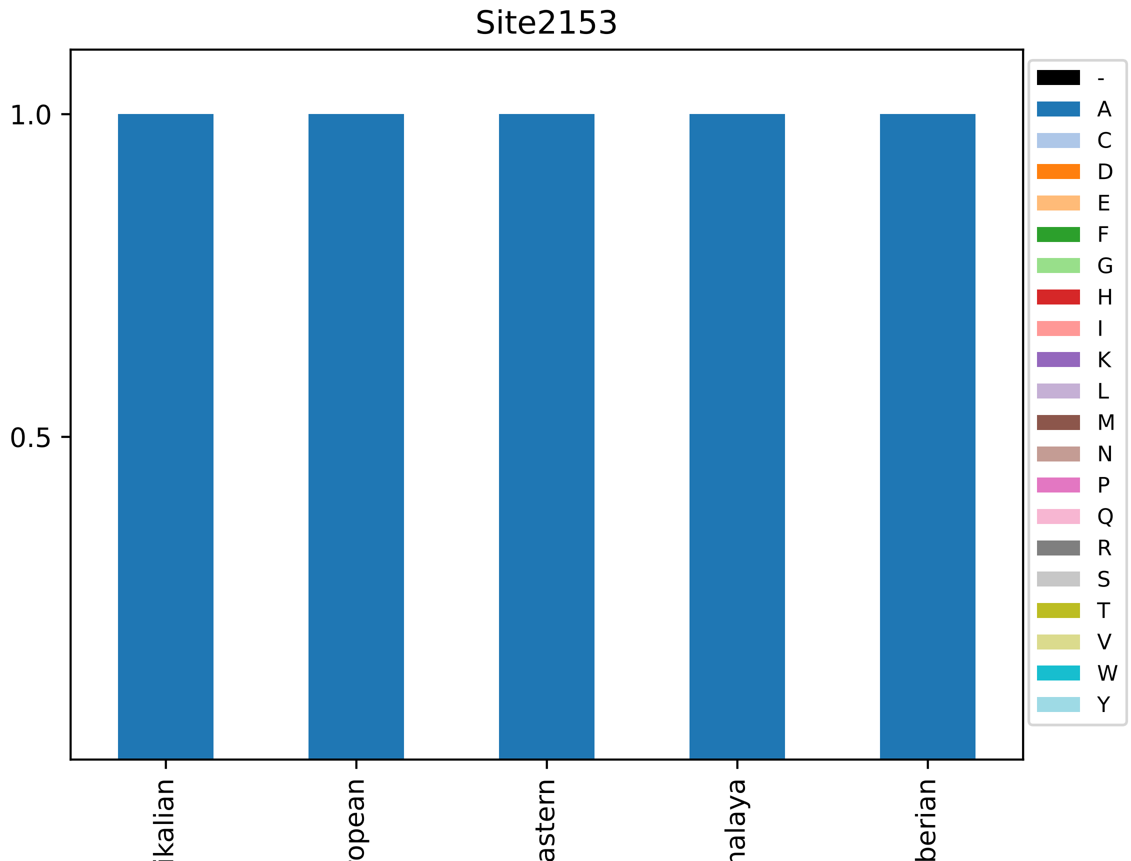

Site2153
1.0
0.5
-
A
C
D
E
F
G
H
I
K
L
M
N
P
Q
R
S
T
V
W
Y

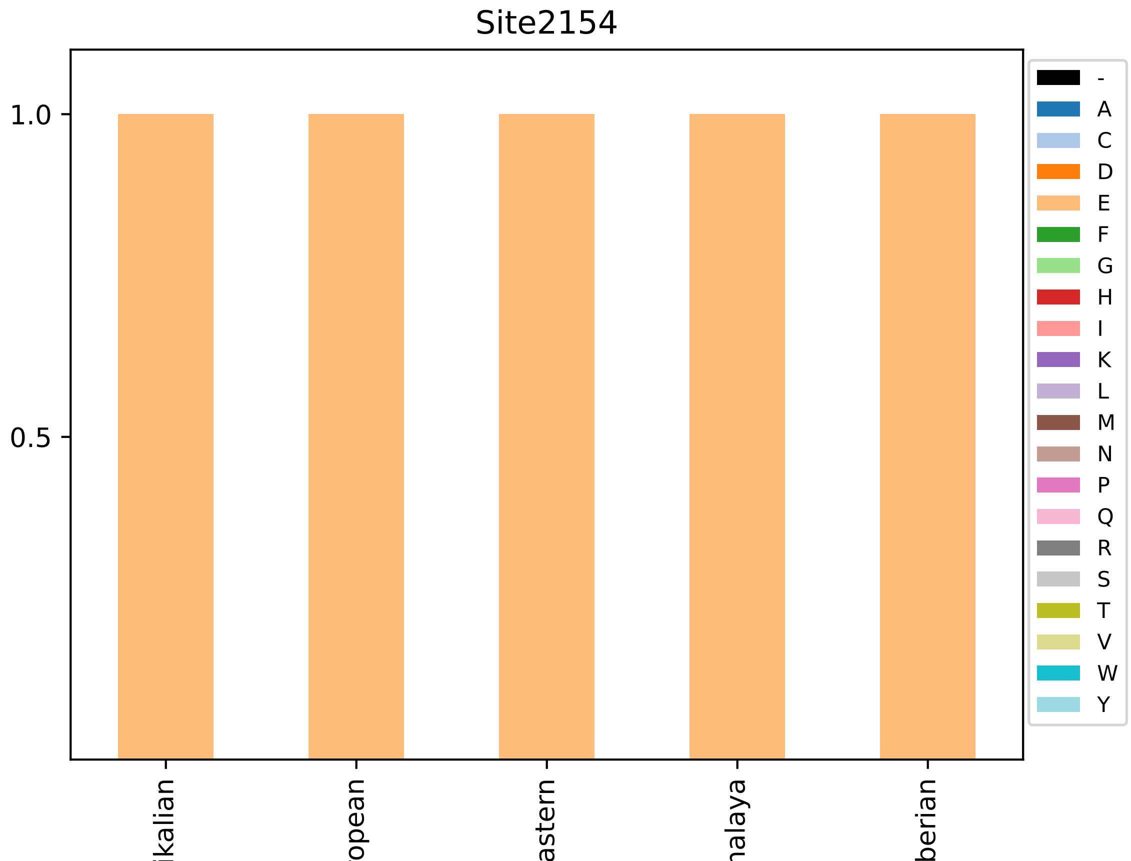
Site2154
1.0
0.5
ikalian
opean
astern
nalaya
berian
-
A
C
D
E
F
G
H
I
K
L
M
N
P
Q
R
S
T
V
W
Y

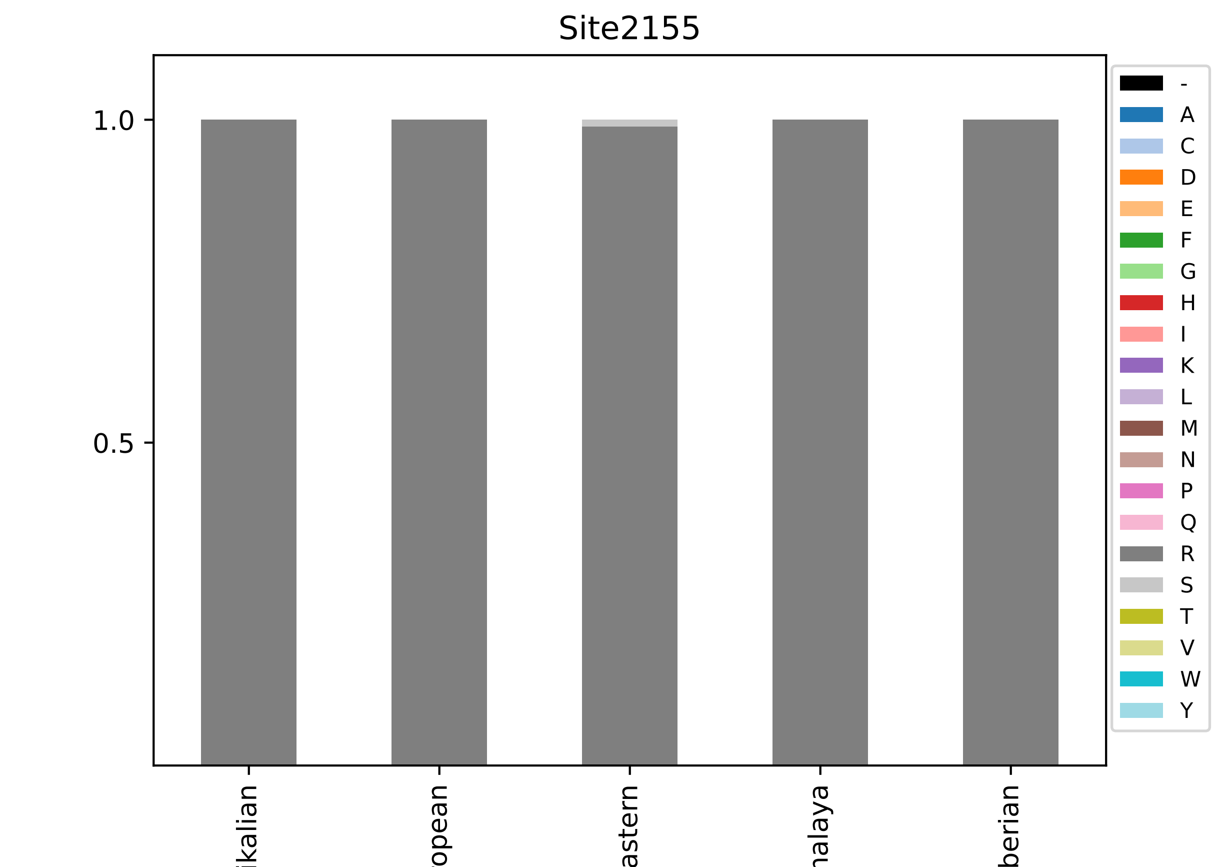
Site2155
1.0
0.5
-
A
C
D
E
F
G
H
I
K
L
M
N
P
Q
R
S
T
V
W
Y
ikalian
opean
astern
nalaya
berian

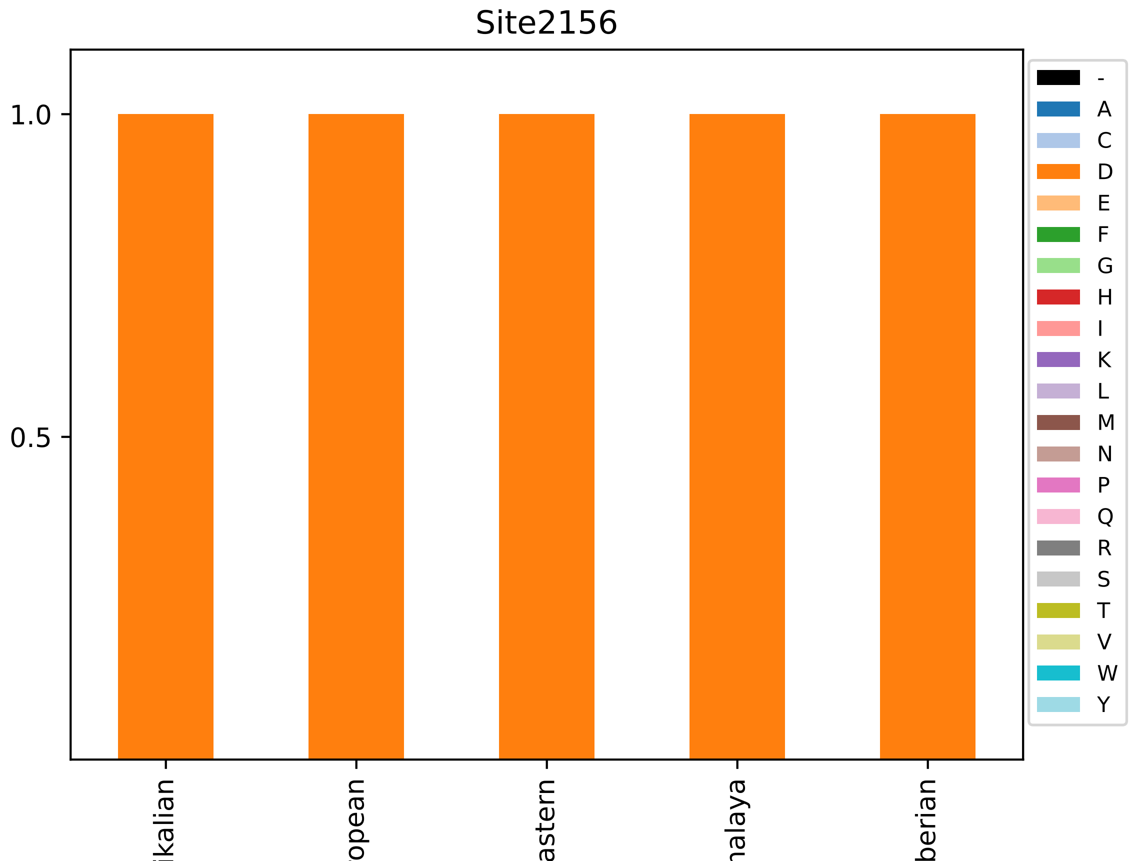

Site2156
1.0
0.5
-
A
C
D
E
F
G
H
I
K
L
M
N
P
Q
R
S
T
V
W
Y
ikalian
opean
astern
nalaya
berian

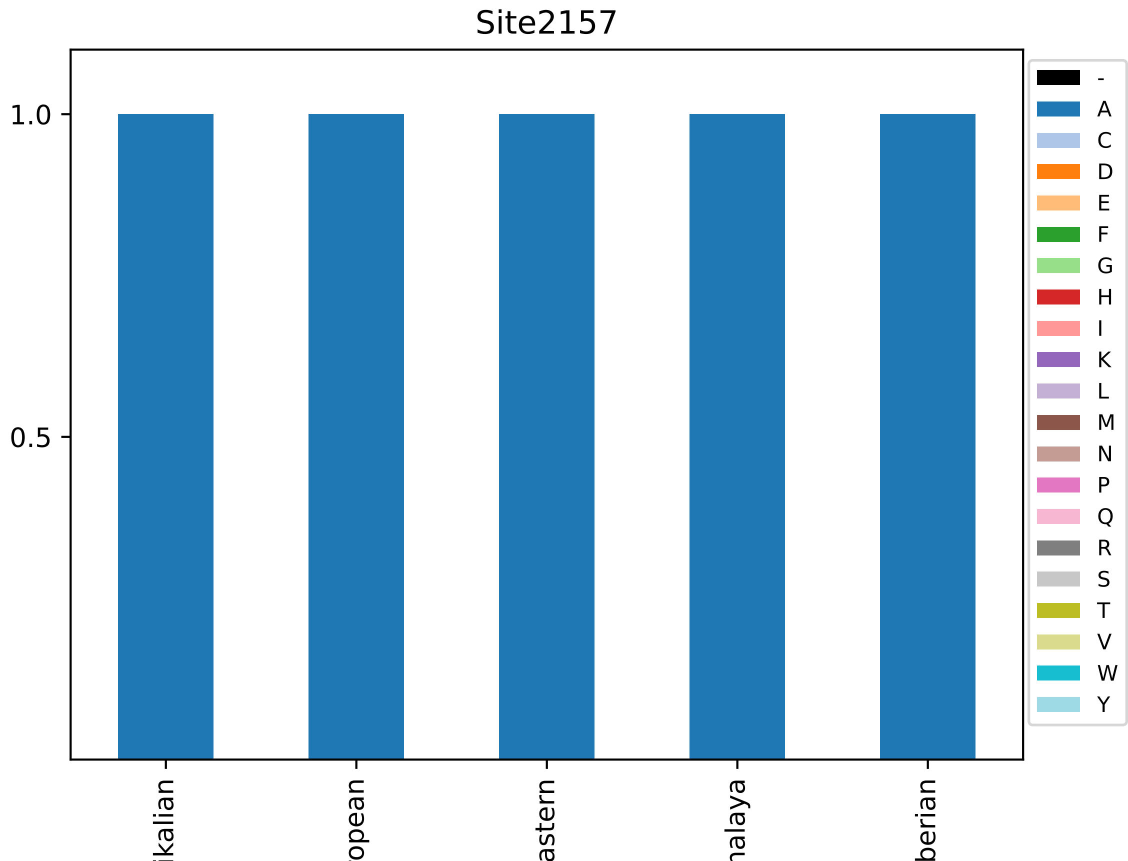

Site2157
1.0
0.5
ikalian
opean
astern
nalaya
berian
-
A
C
D
E
F
G
H
I
K
L
M
N
P
Q
R
S
T
V
W
Y

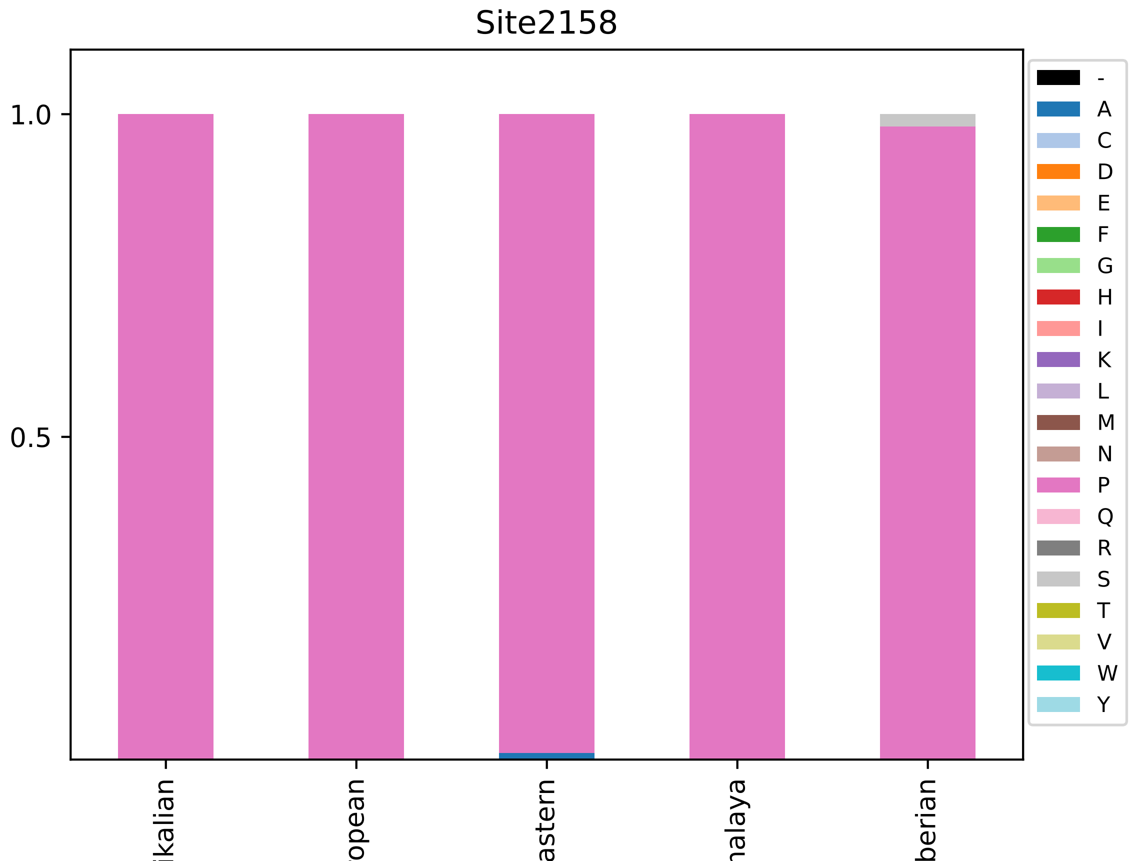

Site2158
1.0
0.5
ikalian
ropean
astern
nalaya
berian
-
A
C
D
E
F
G
H
I
K
L
M
N
P
Q
R
S
T
V
W
Y

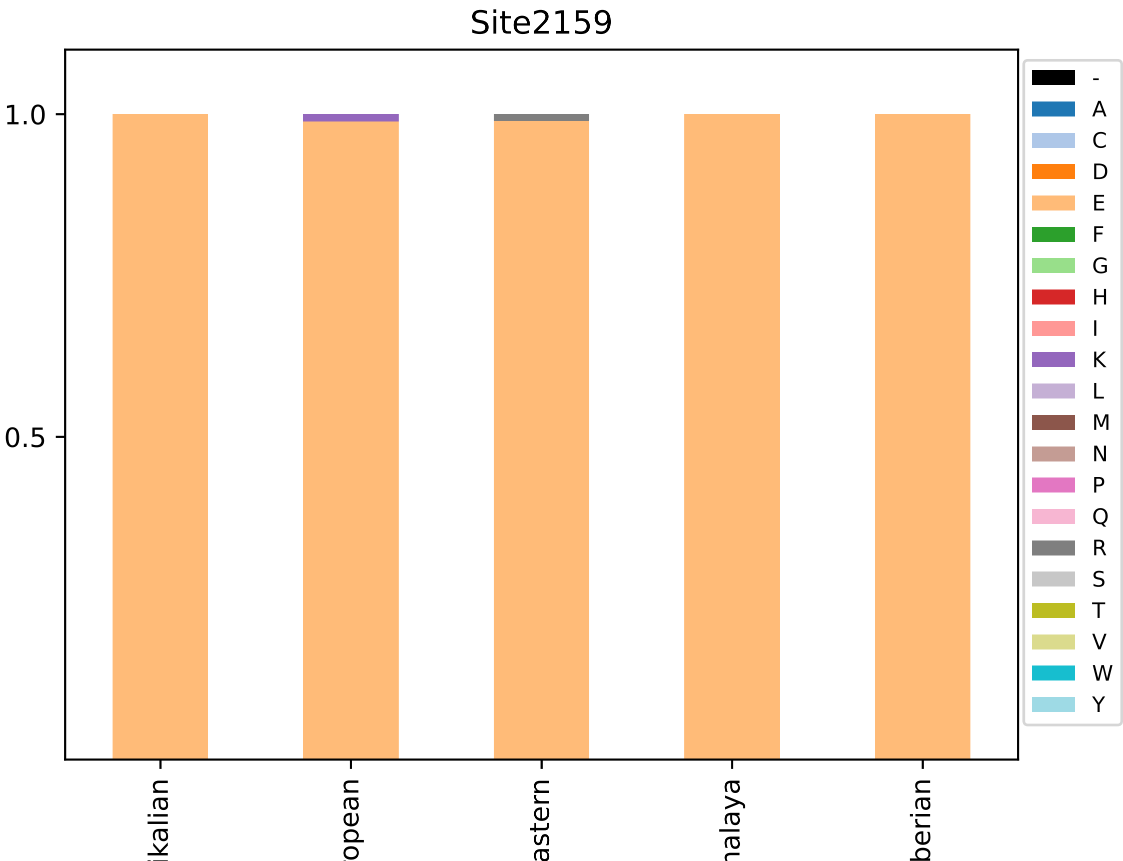
Site2159
1.0
0.5
-
A
C
D
E
F
G
H
I
K
L
M
N
P
Q
R
S
T
V
W
Y
ikalian
opean
astern
nalaya
berian

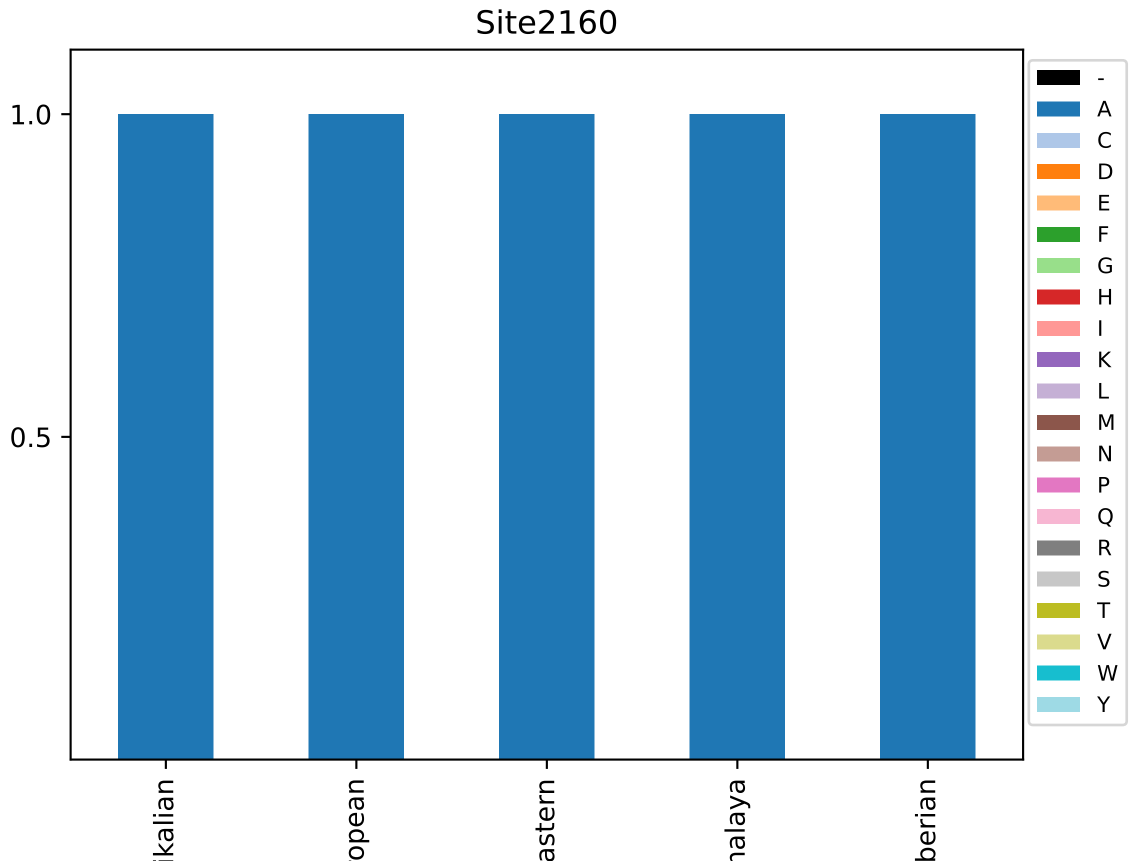

Site2160
1.0
0.5
-
A
C
D
E
F
G
H
I
K
L
M
N
P
Q
R
S
T
V
W
Y
ikalian
ropean
astern
nalaya
berian

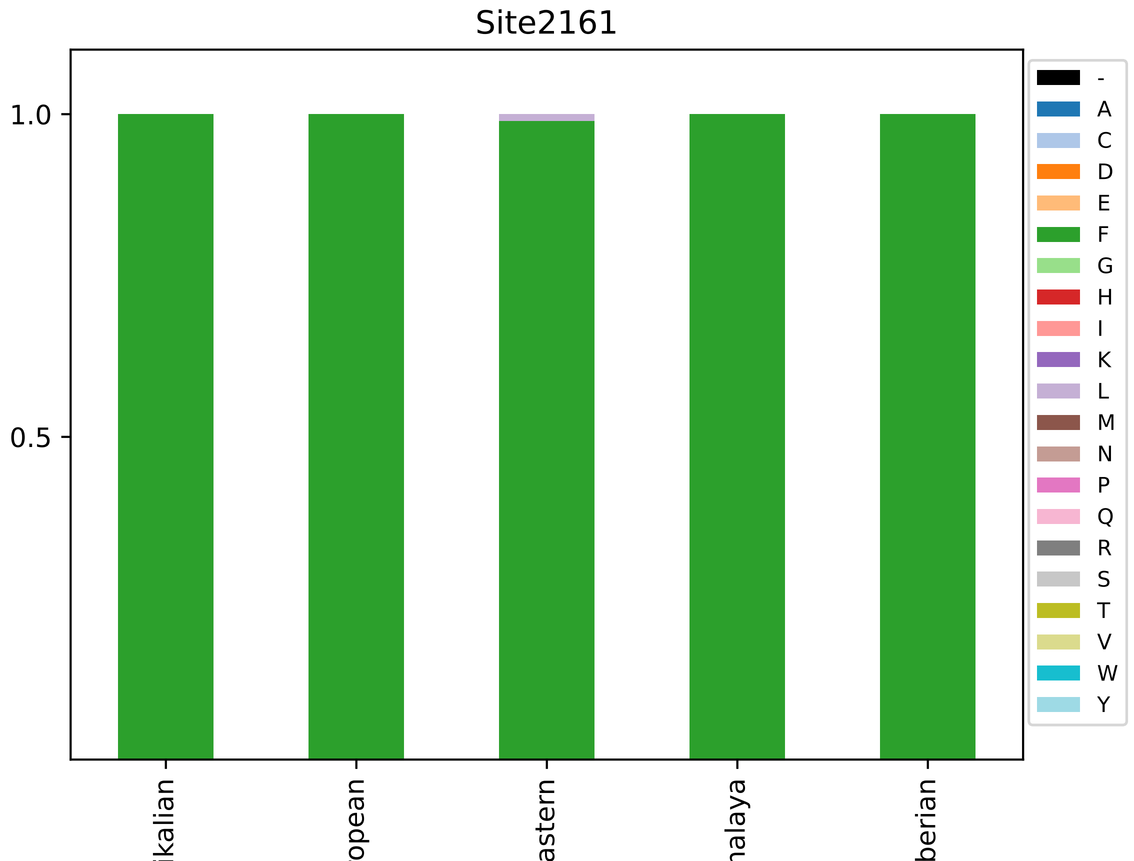

Site2161
1.0
0.5
-
A
C
D
E
F
G
H
I
K
L
M
N
P
Q
R
S
T
V
W
Y
ikalian
ropean
astern
nalaya
berian

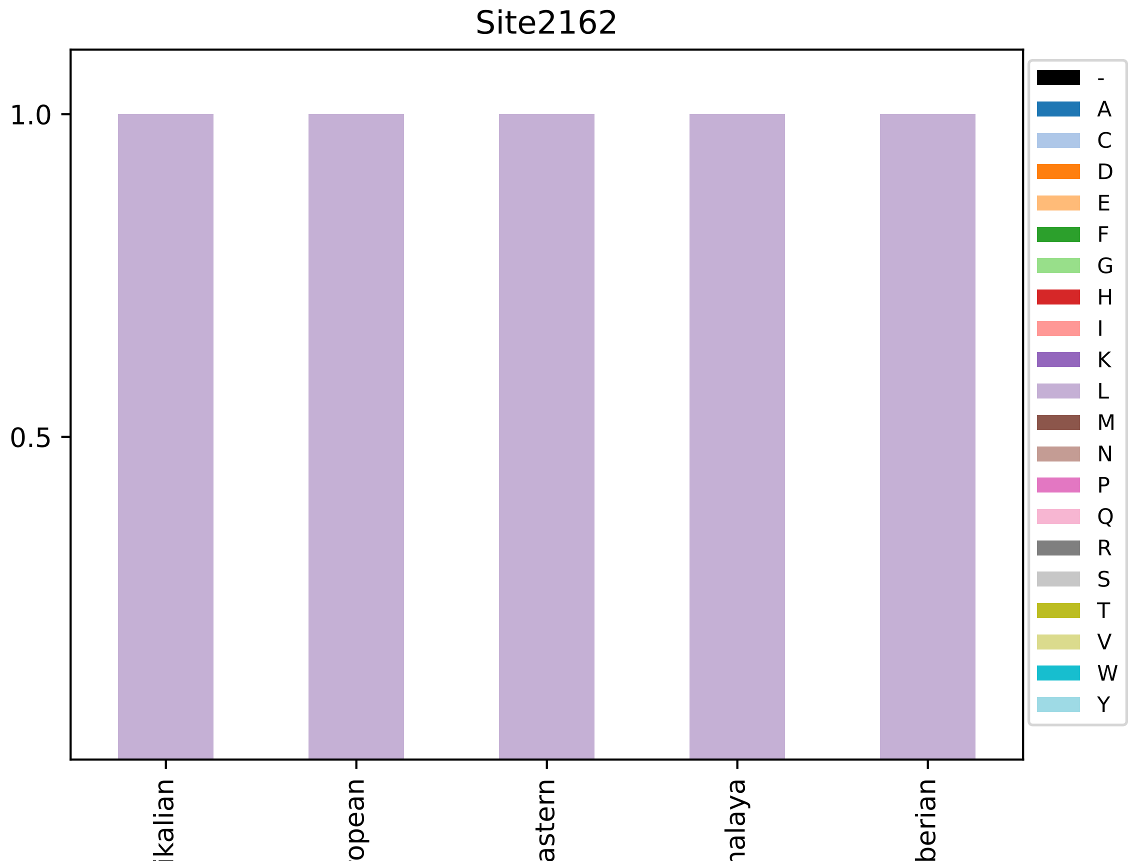

Site2162
1.0
0.5
-
A
C
D
E
F
G
H
I
K
L
M
N
P
Q
R
S
T
V
W
Y

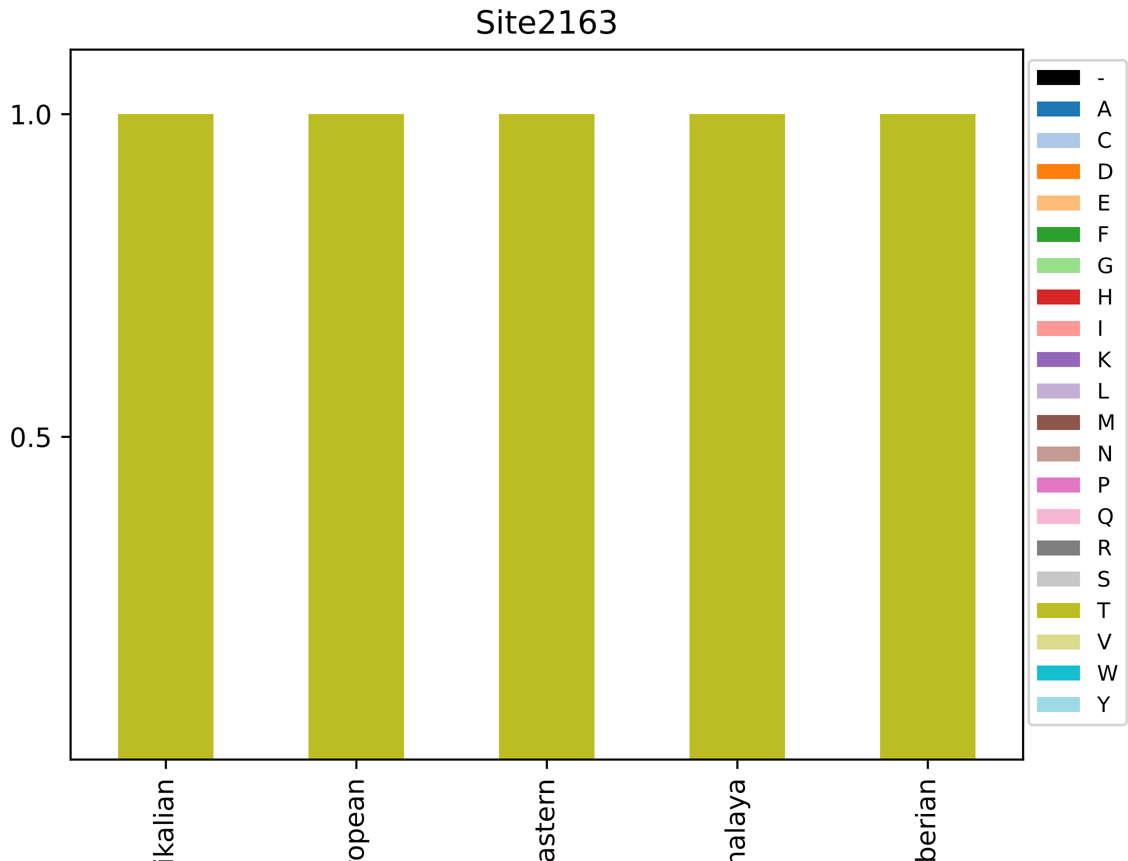

Site2163
1.0
0.5
ikalian
opean
astern
nalaya
berian
-
A
C
D
E
F
G
H
I
K
L
M
N
P
Q
R
S
T
V
W
Y

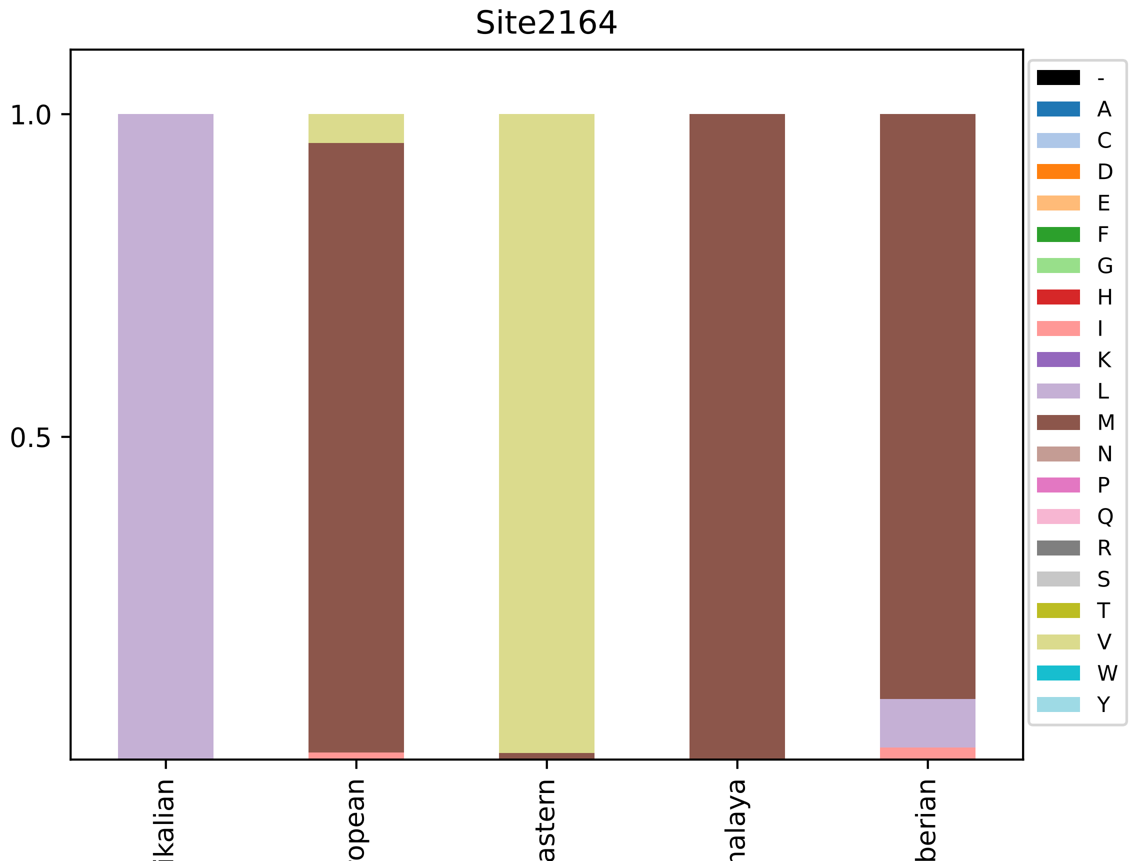
Site2164
1.0
0.5
-
A
C
D
E
F
G
H
I
K
L
M
N
P
Q
R
S
T
V
W
Y
ikalian
opean
astern
nalaya
berian

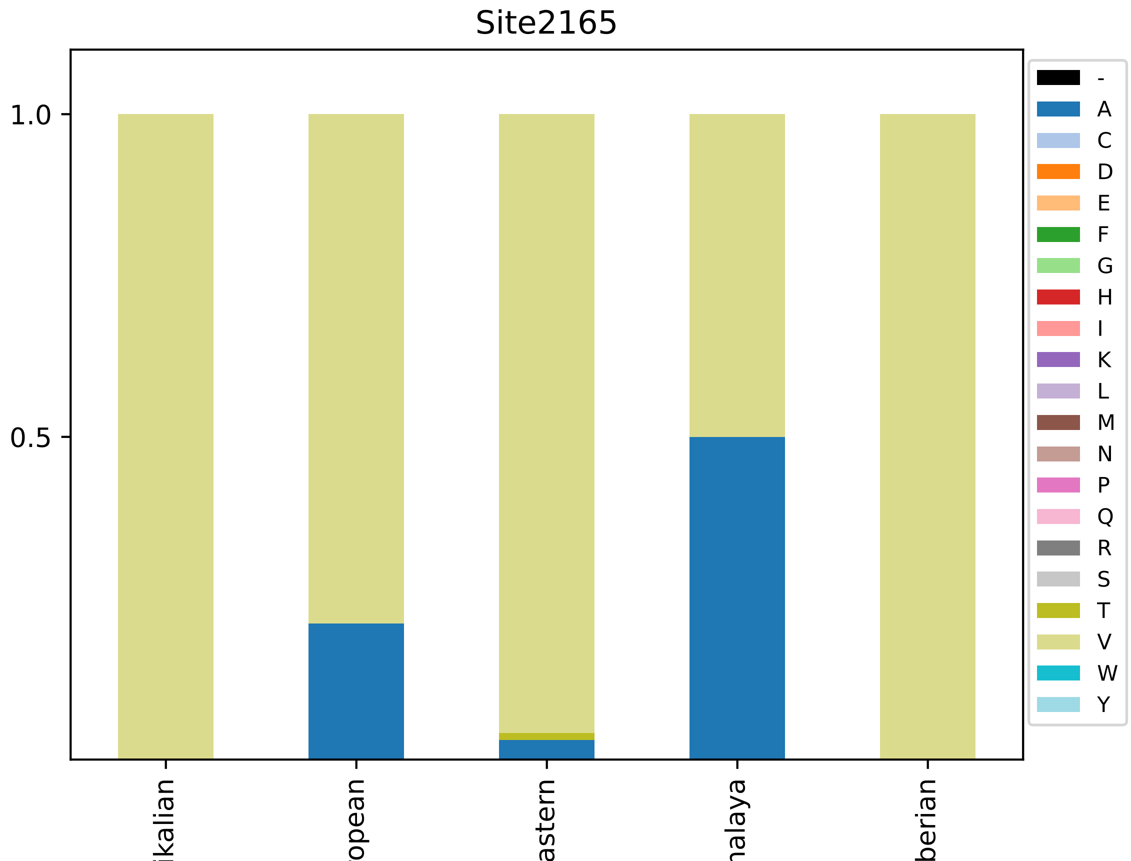
Site2165
1.0
0.5
-
A
C
D
E
F
G
H
I
K
L
M
N
P
Q
R
S
T
V
W
Y

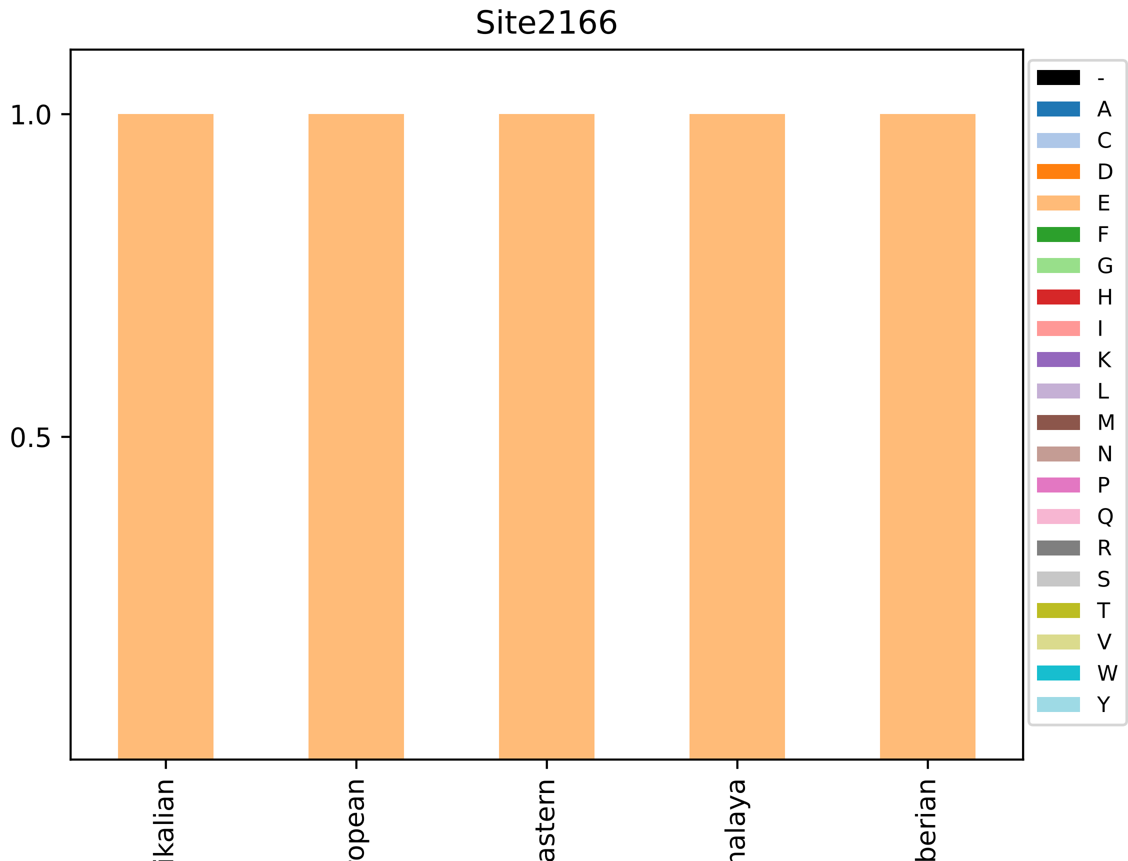
Site2166
1.0
0.5
-
A
C
D
E
F
G
H
I
K
L
M
N
P
Q
R
S
T
V
W
Y
ikalian
opean
astern
nalaya
berian

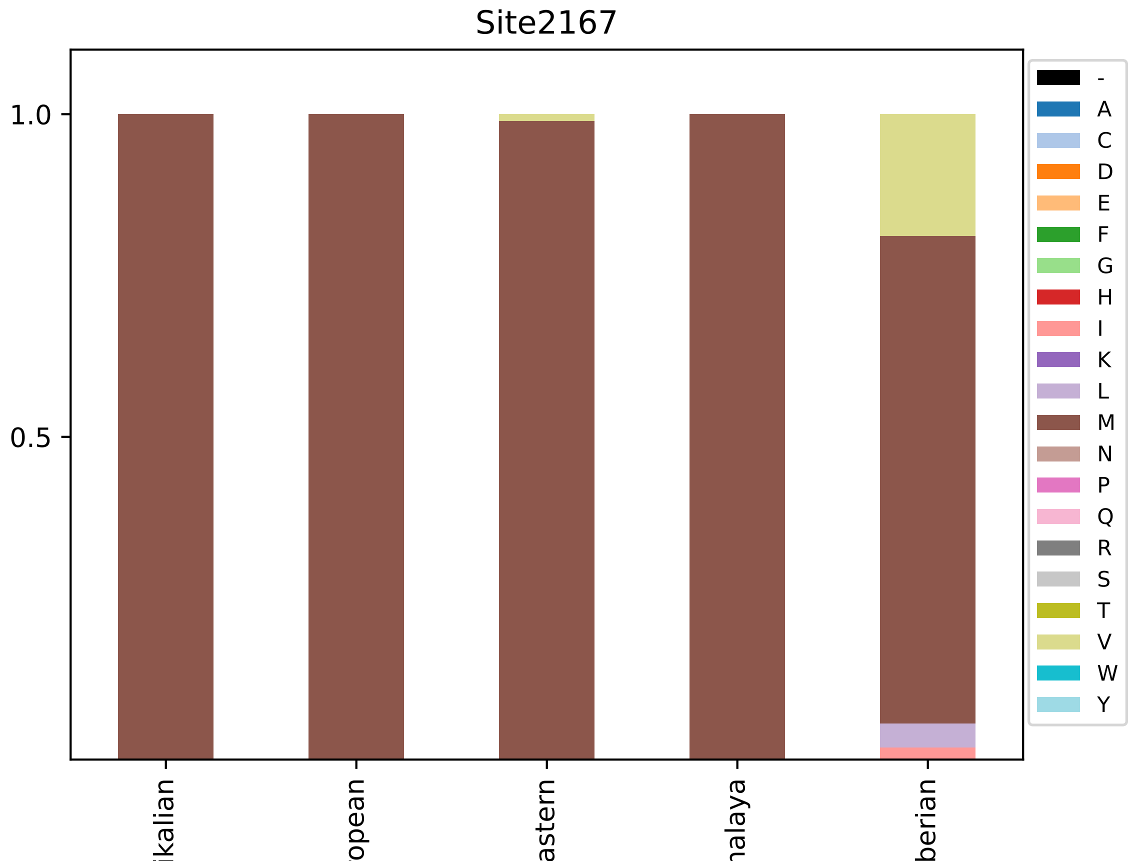

Site2167
1.0
0.5
ikalian
opean
astern
nalaya
berian
-
A
C
D
E
F
G
H
I
K
L
M
N
P
Q
R
S
T
V
W
Y

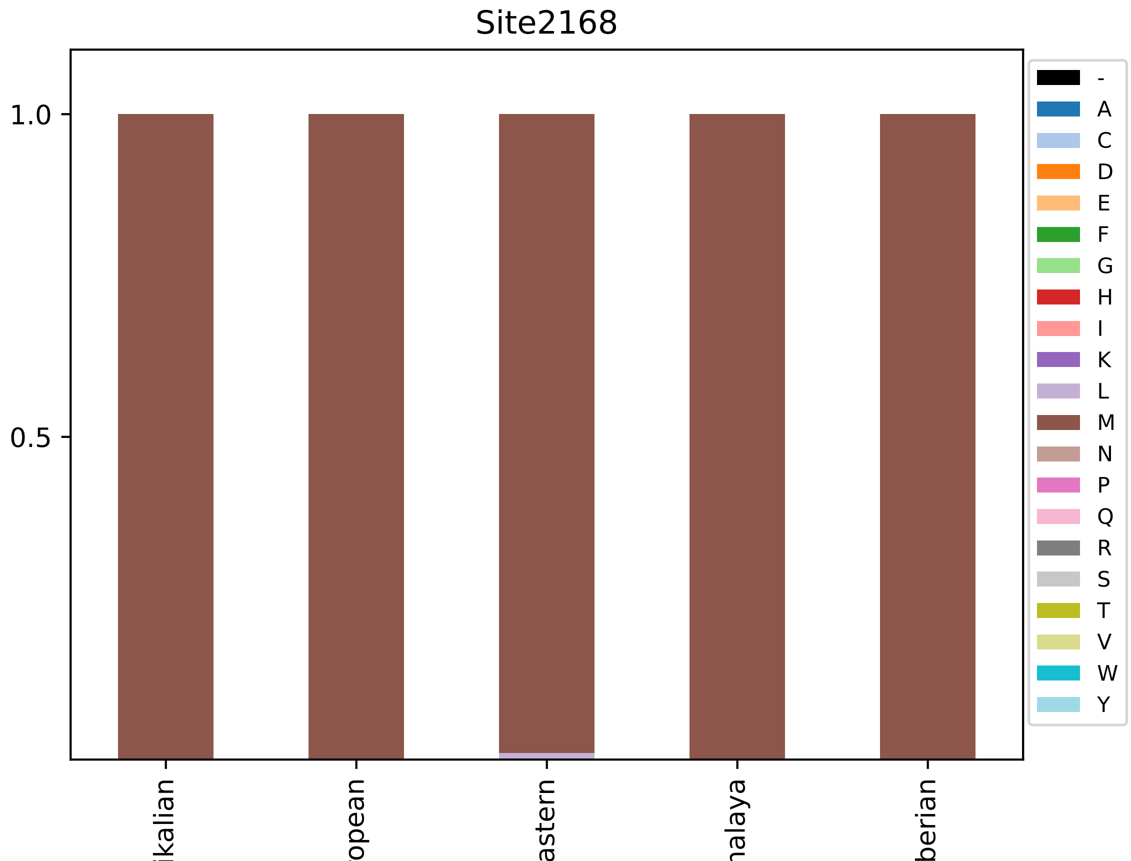

Site2168
1.0
0.5
-
A
C
D
E
F
G
H
I
K
L
M
N
P
Q
R
S
T
V
W
Y
ikalian
opean
astern
nalaya
berian

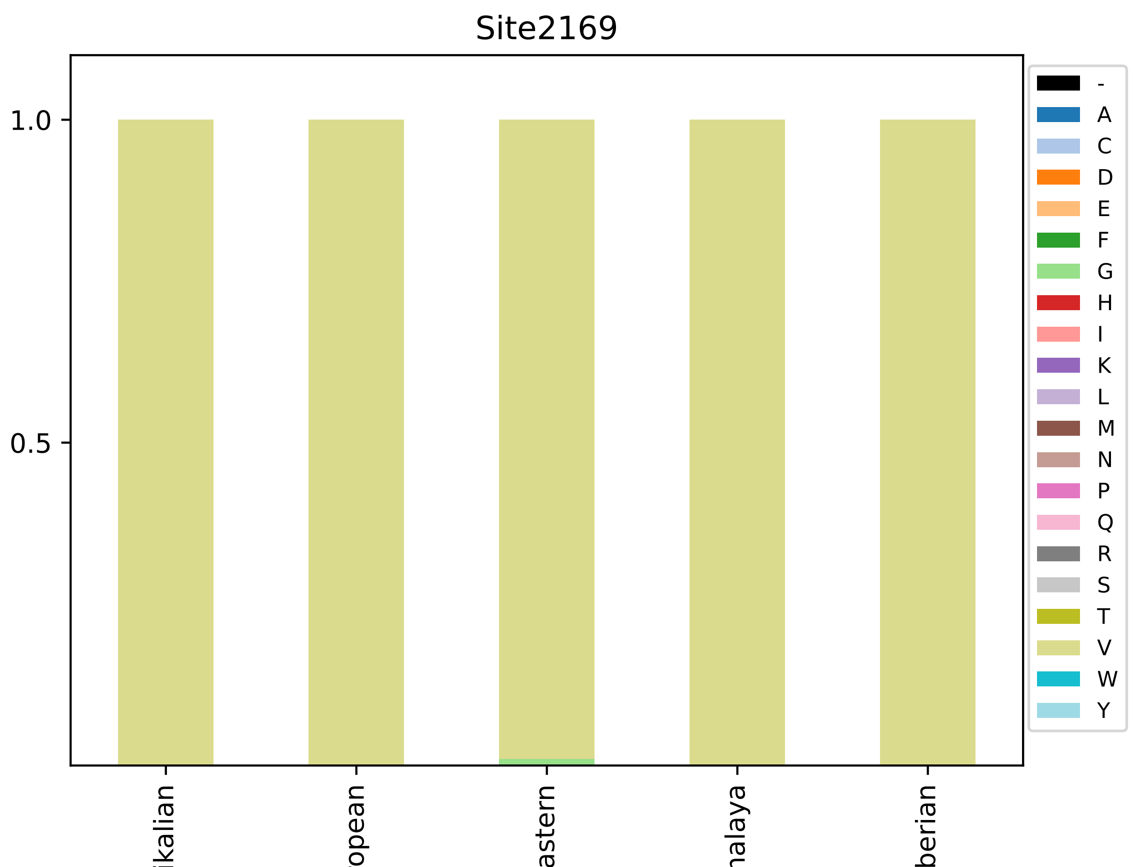
Site2169
1.0
0.5
-
A
C
D
E
F
G
H
I
K
L
M
N
P
Q
R
S
T
V
W
Y
ikalian
opean
astern
nalaya
berian

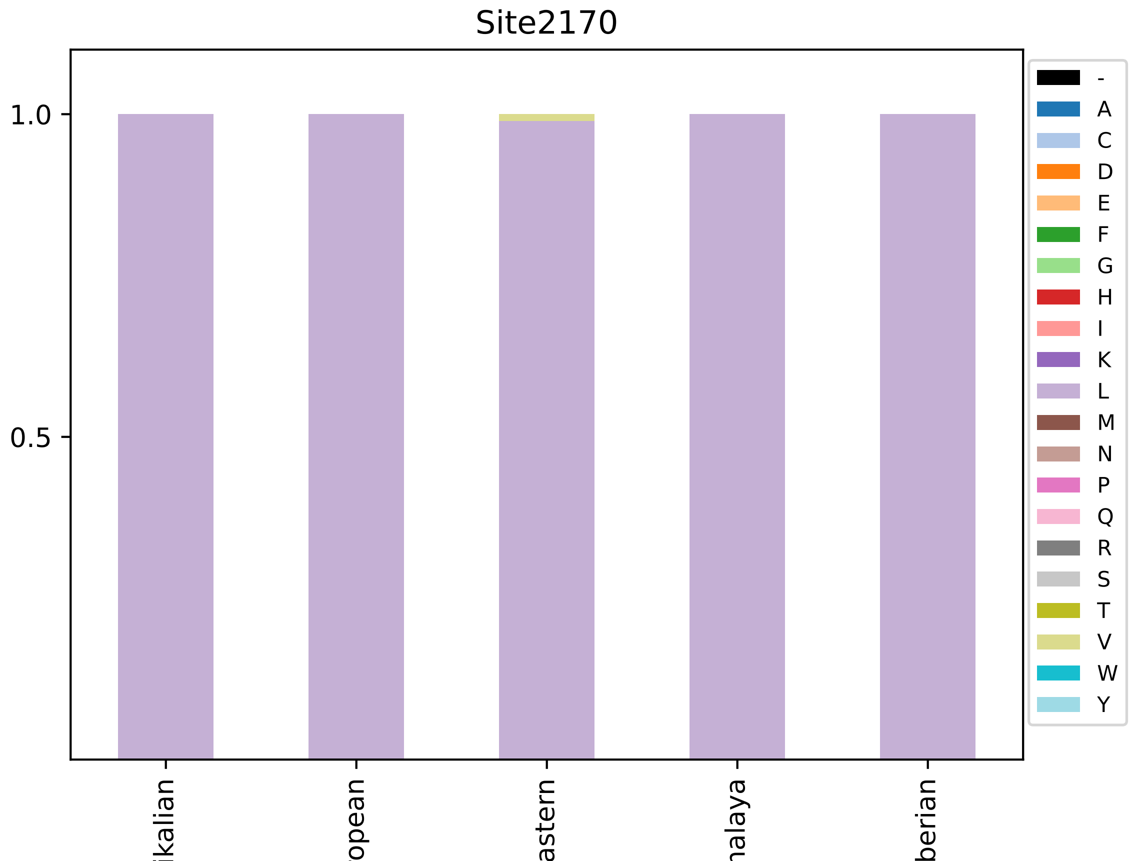

Site2170
1.0
0.5
-
A
C
D
E
F
G
H
I
K
L
M
N
P
Q
R
S
T
V
W
Y
ikalian
opean
astern
nalaya
berian

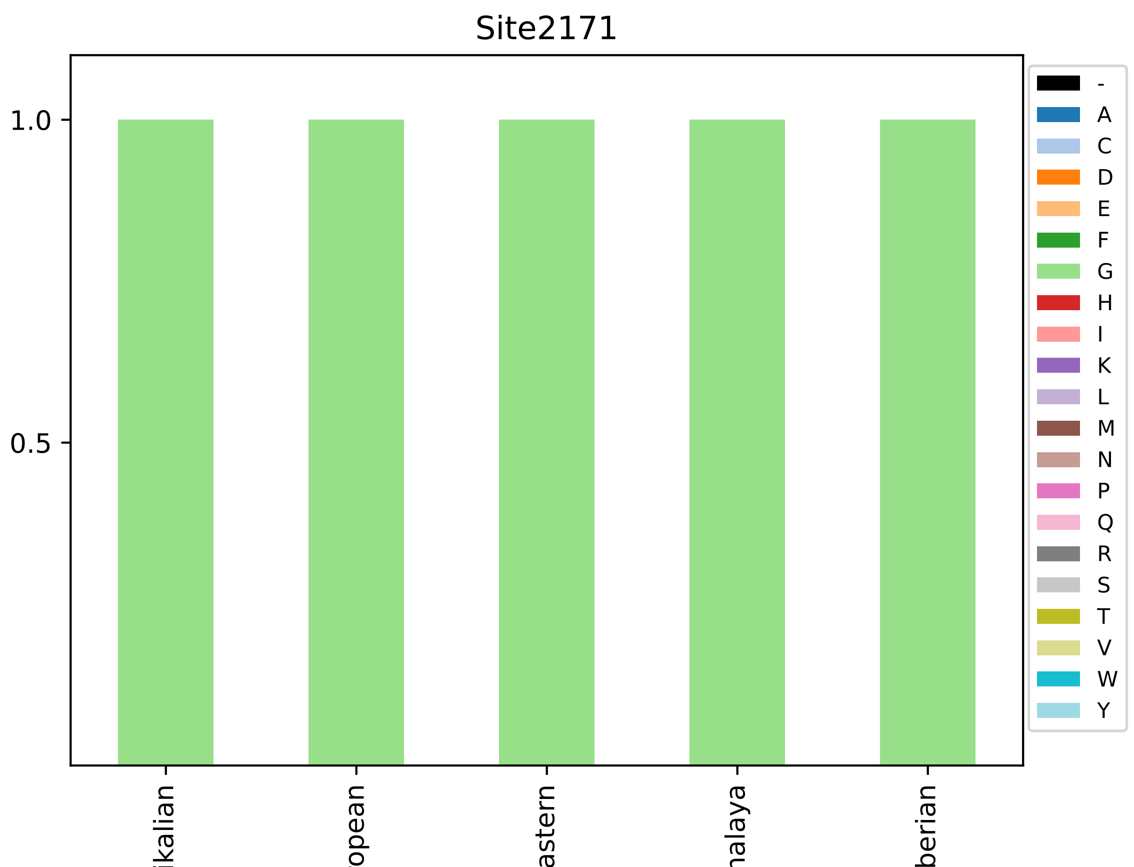
Site2171
1.0
0.5
-
A
C
D
E
F
G
H
I
K
L
M
N
P
Q
R
S
T
V
W
Y
ikalian
opean
astern
nalaya
berian

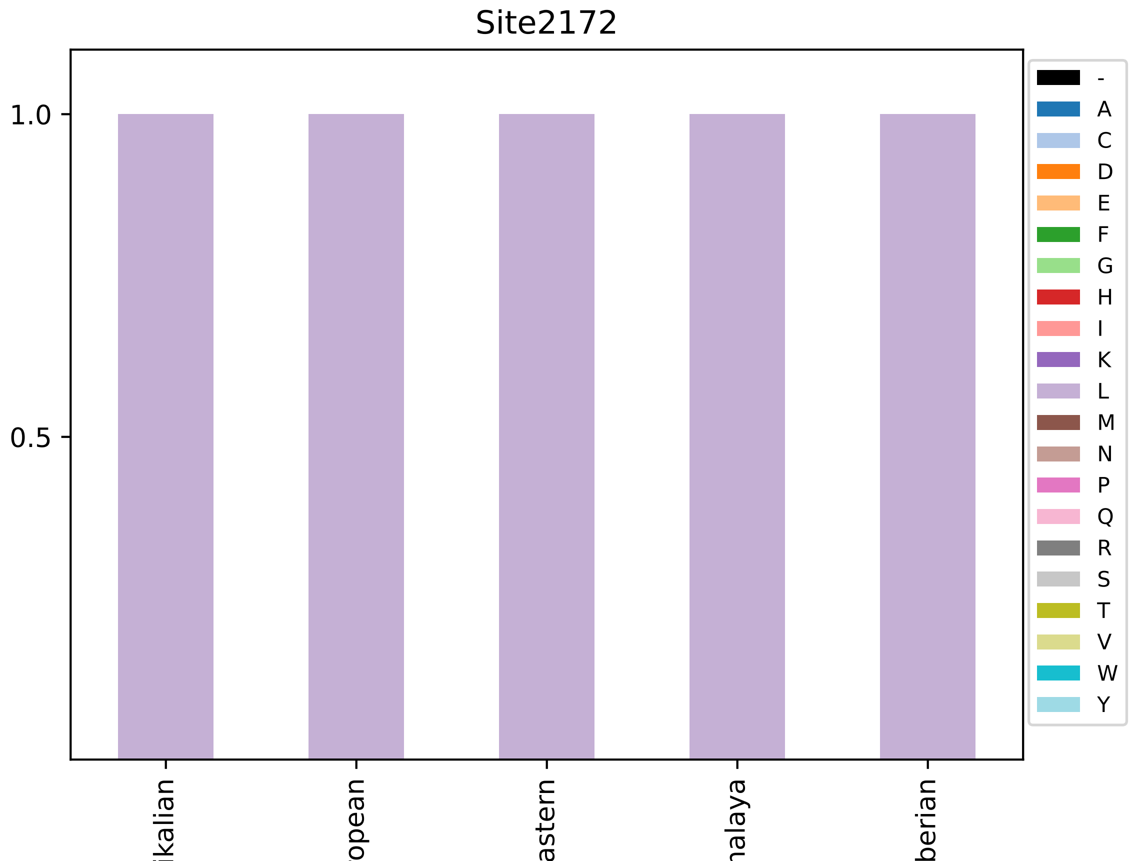

Site2172
1.0
0.5
-
A
C
D
E
F
G
H
I
K
L
M
N
P
Q
R
S
T
V
W
Y
ikalian
opean
astern
nalaya
berian

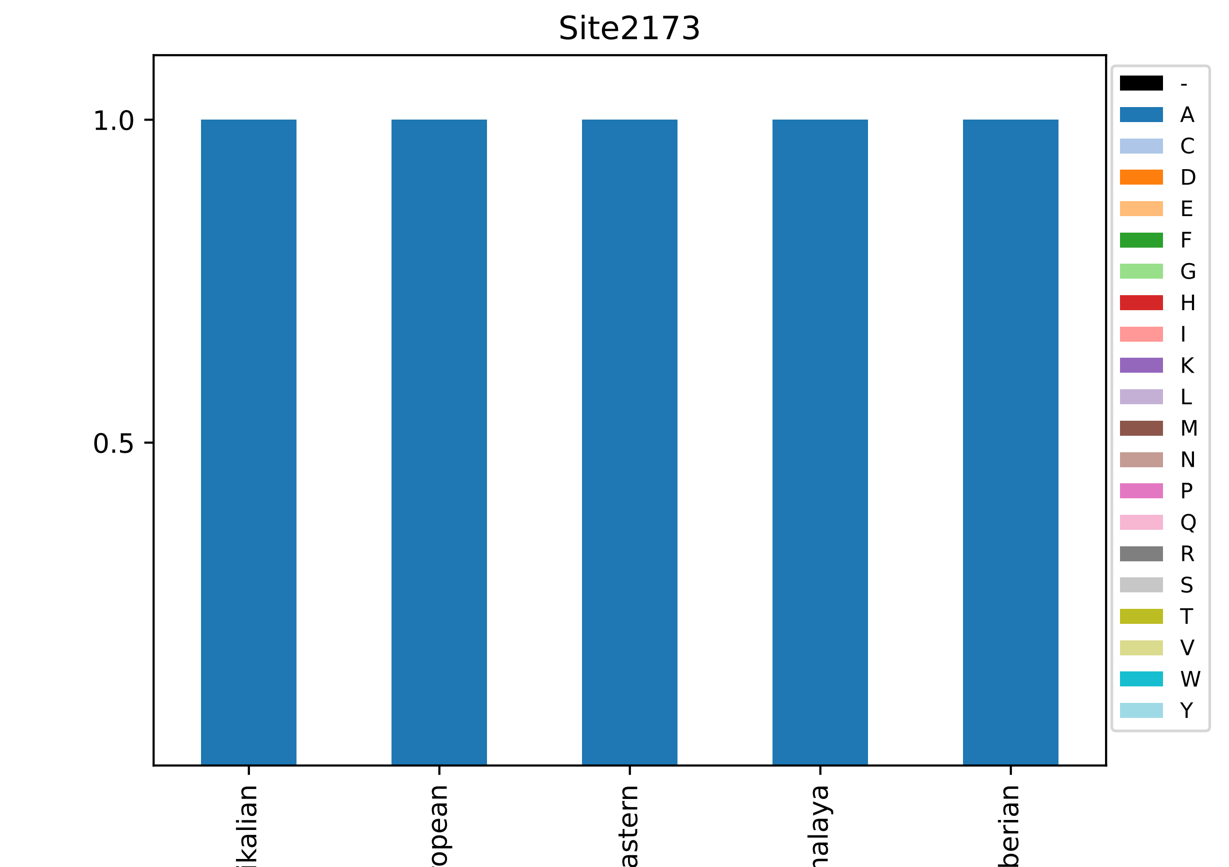

Site2173
1.0
0.5
-
A
C
D
E
F
G
H
I
K
L
M
N
P
Q
R
S
T
V
W
Y
ikalian
opean
astern
nalaya
berian

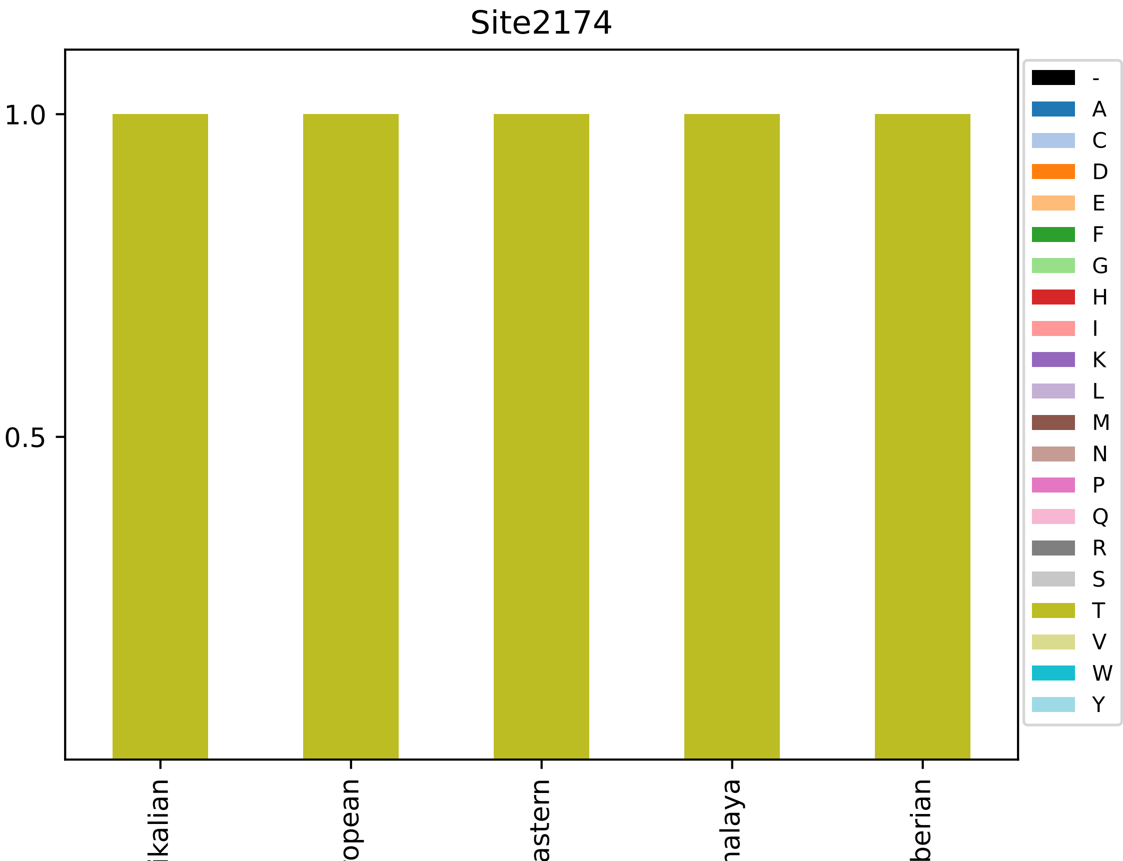

Site2174
1.0
0.5
-
A
C
D
E
F
G
H
I
K
L
M
N
P
Q
R
S
T
V
W
Y
ikalian
opean
astern
nalaya
berian

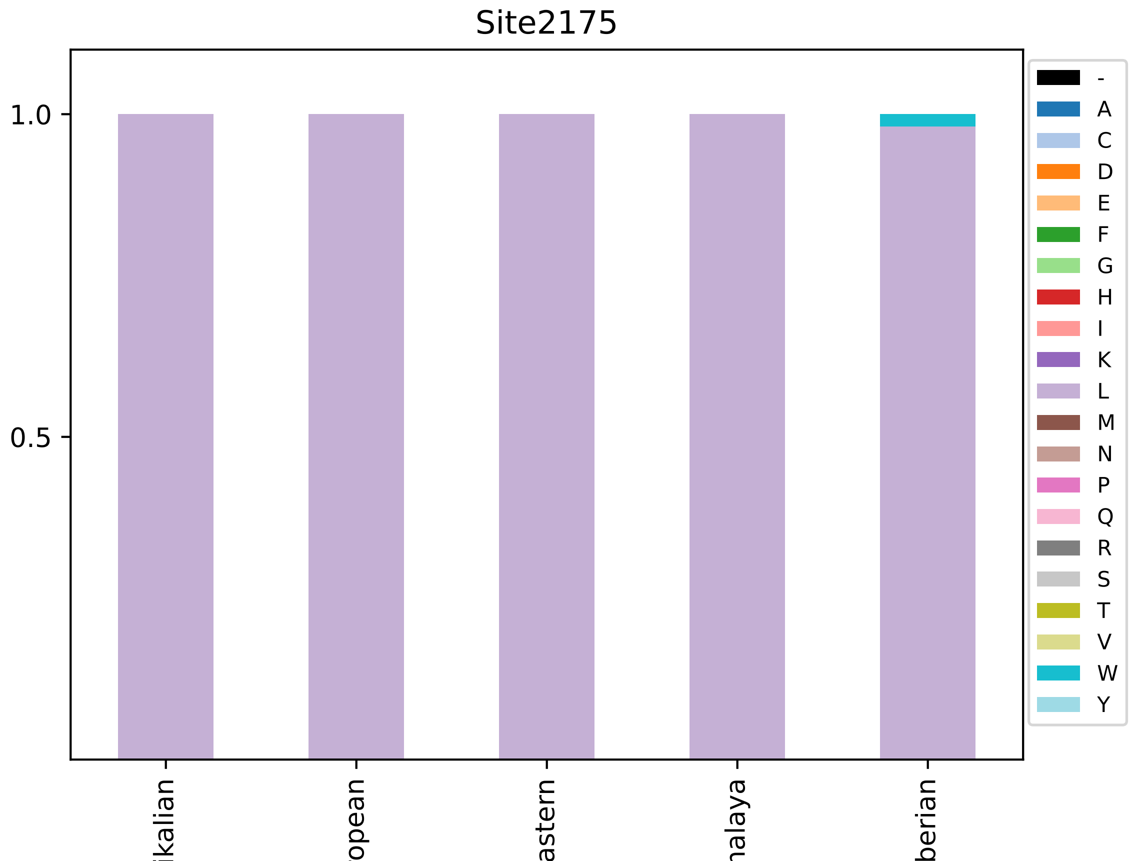

Site2175
1.0
0.5
-
A
C
D
E
F
G
H
I
K
L
M
N
P
Q
R
S
T
V
W
Y
ikalian
opean
astern
nalaya
berian

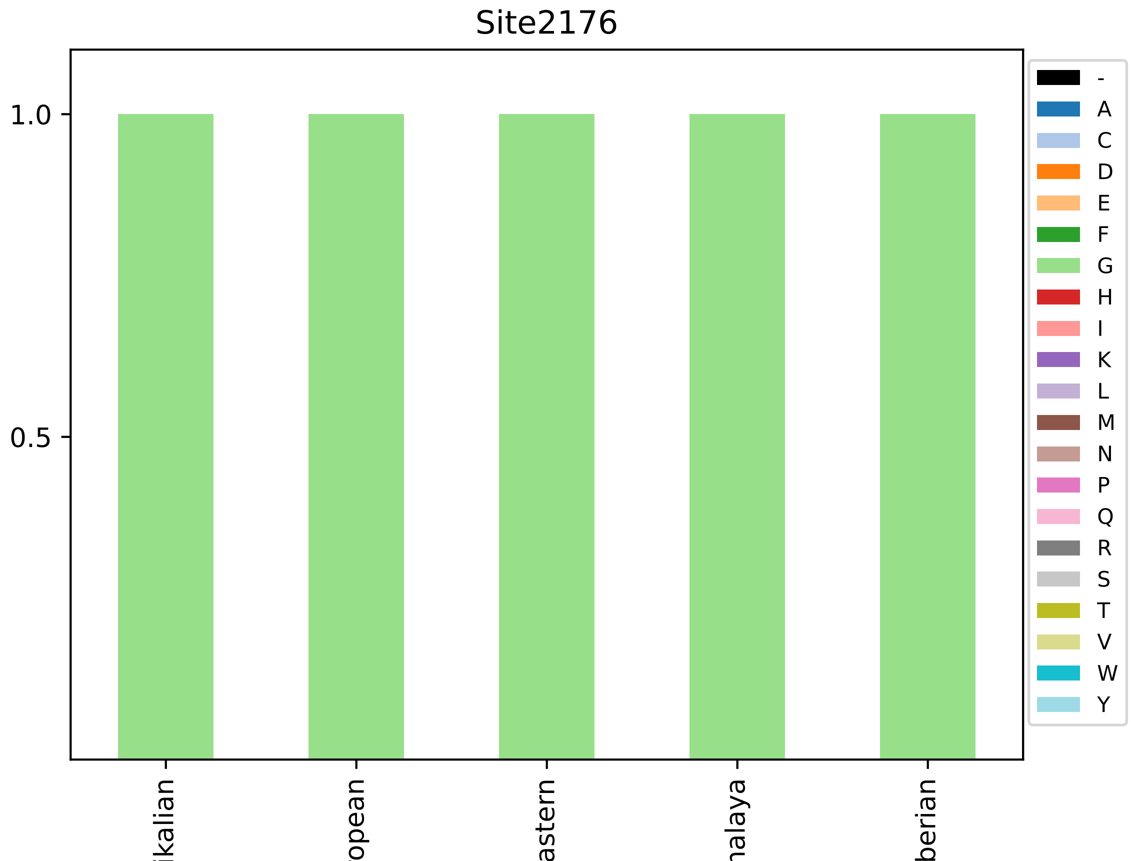
Site2176
1.0
0.5
-
A
C
D
E
F
G
H
I
K
L
M
N
P
Q
R
S
T
V
W
Y
ikalian
opean
astern
nalaya
berian

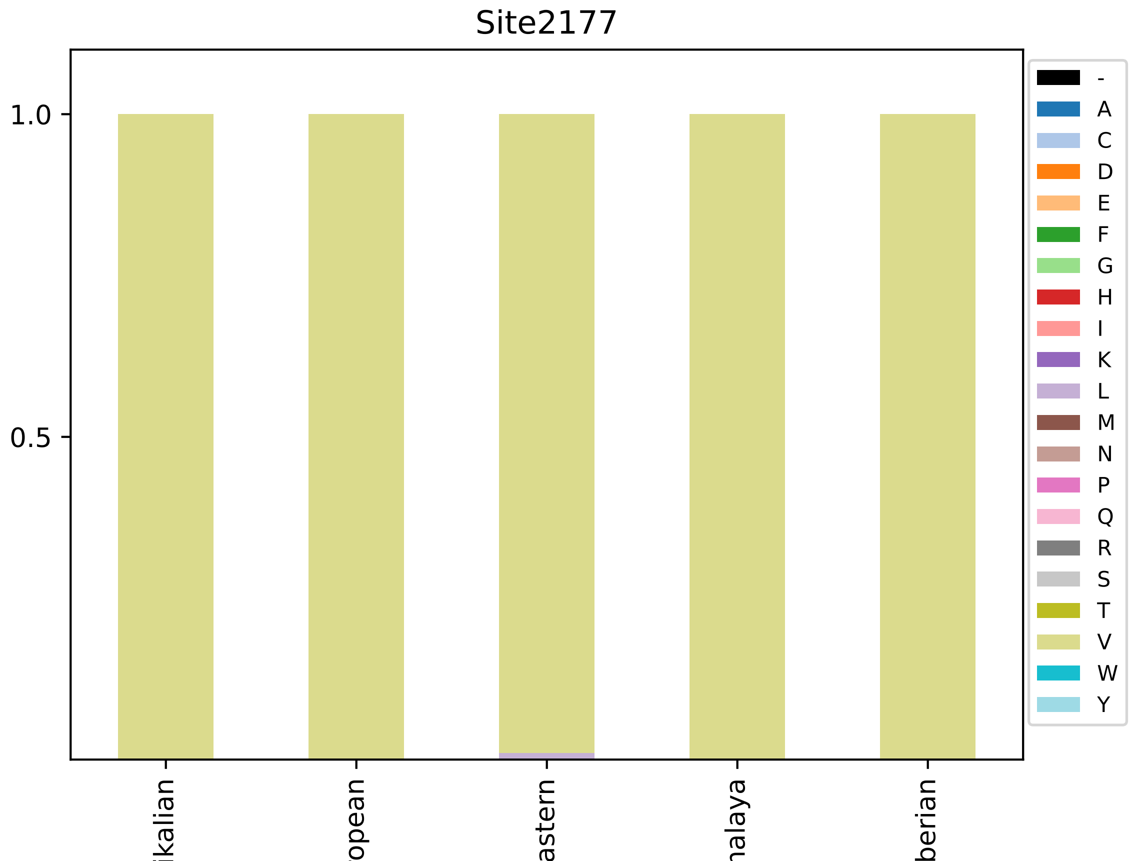
Site2177
1.0
0.5
-
A
C
D
E
F
G
H
I
K
L
M
N
P
Q
R
S
T
V
W
Y
ikalian
opean
astern
nalaya
berian

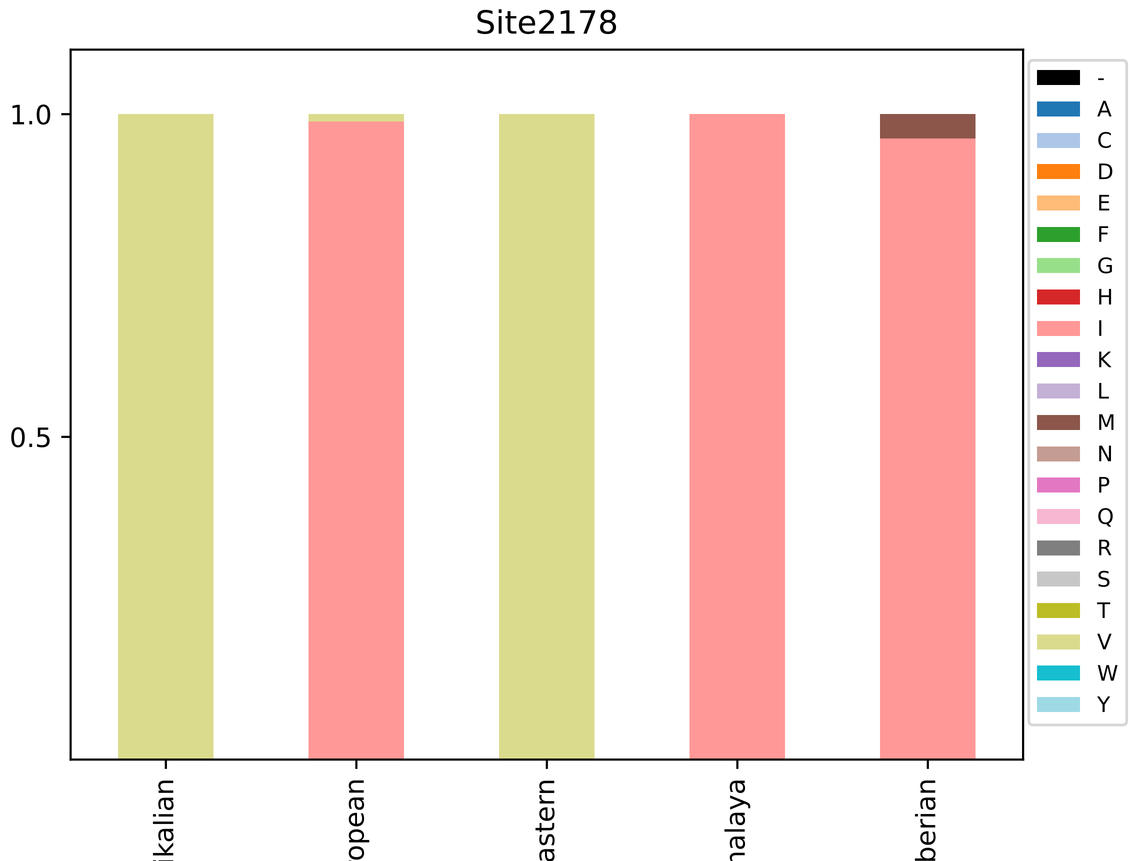
Site2178
1.0
0.5
-
A
C
D
E
F
G
H
I
K
L
M
N
P
Q
R
S
T
V
W
Y
ikalian
opean
astern
nalaya
berian

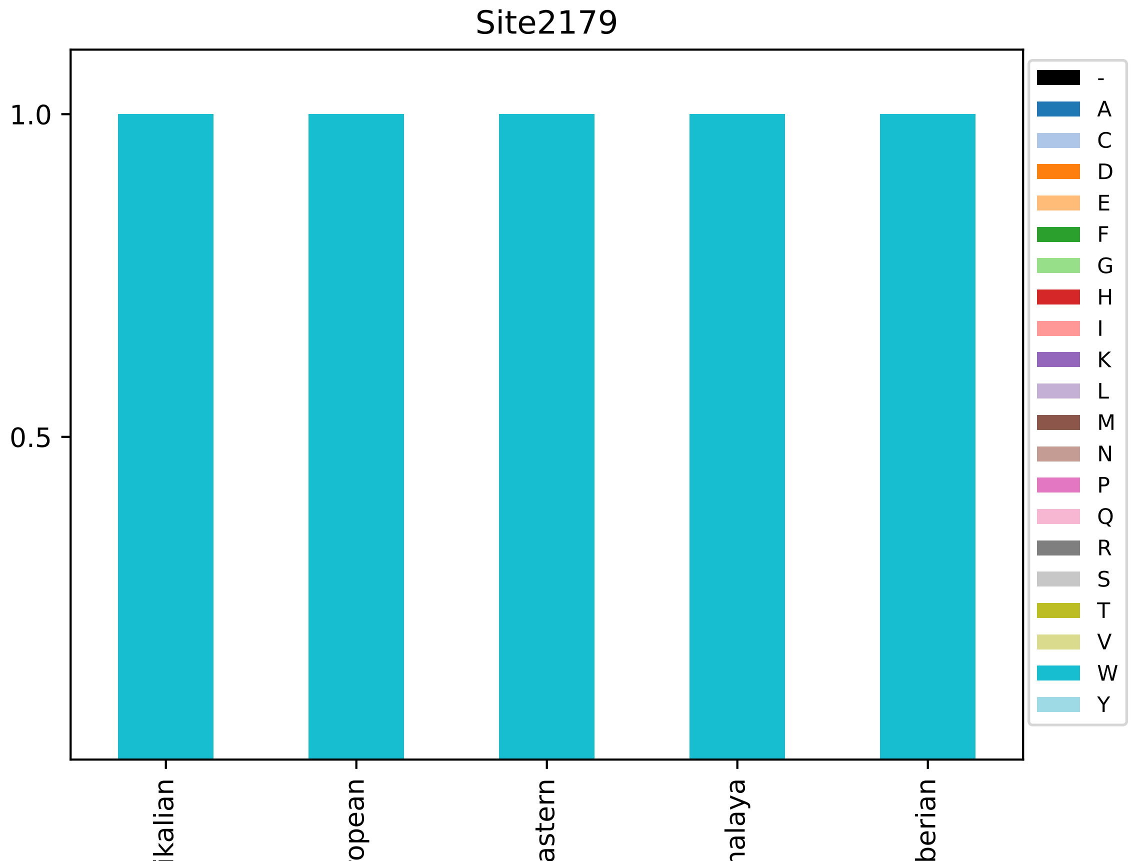
Site2179
1.0
0.5
ikalian
opean
astern
nalaya
berian
-
A
C
D
E
F
G
H
I
K
L
M
N
P
Q
R
S
T
V
W
Y

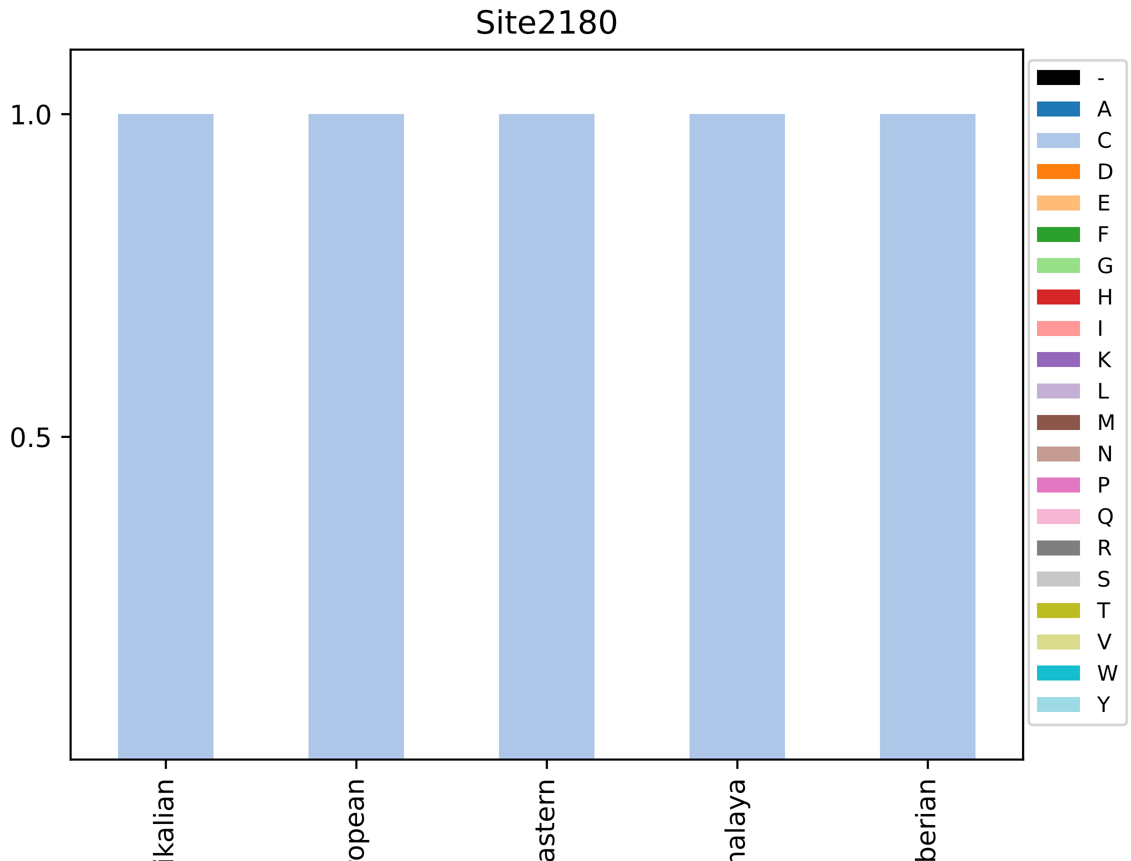
Site2180
1.0
0.5
-
A
C
D
E
F
G
H
I
K
L
M
N
P
Q
R
S
T
V
W
Y
ikalian
opean
astern
nalaya
berian

Site2181

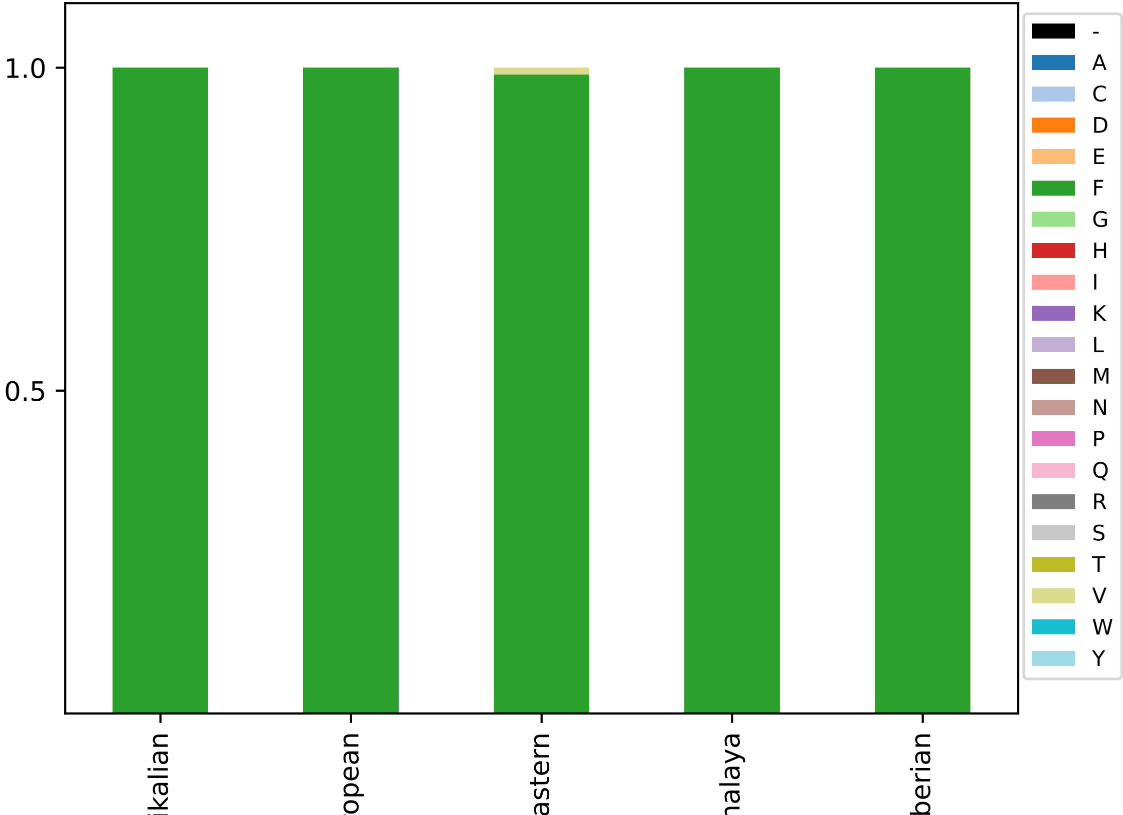
1.0
0.5
-
A
C
D
E
F
G
H
I
K
L
M
N
P
Q
R
S
T
V
W
Y
ikalian
opean
astern
nalaya
berian

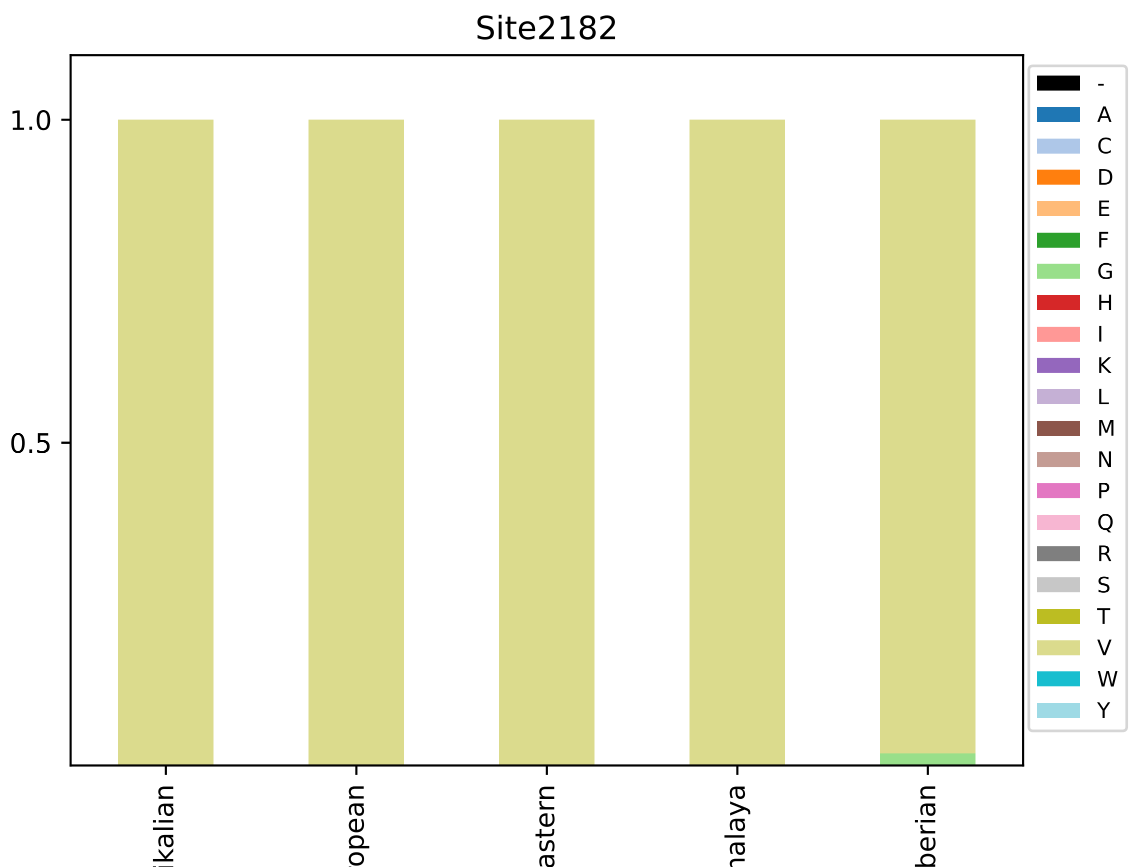
Site2182
1.0
0.5
-
A
C
D
E
F
G
H
I
K
L
M
N
P
Q
R
S
T
V
W
Y
ikalian
ropean
astern
nalaya
berian

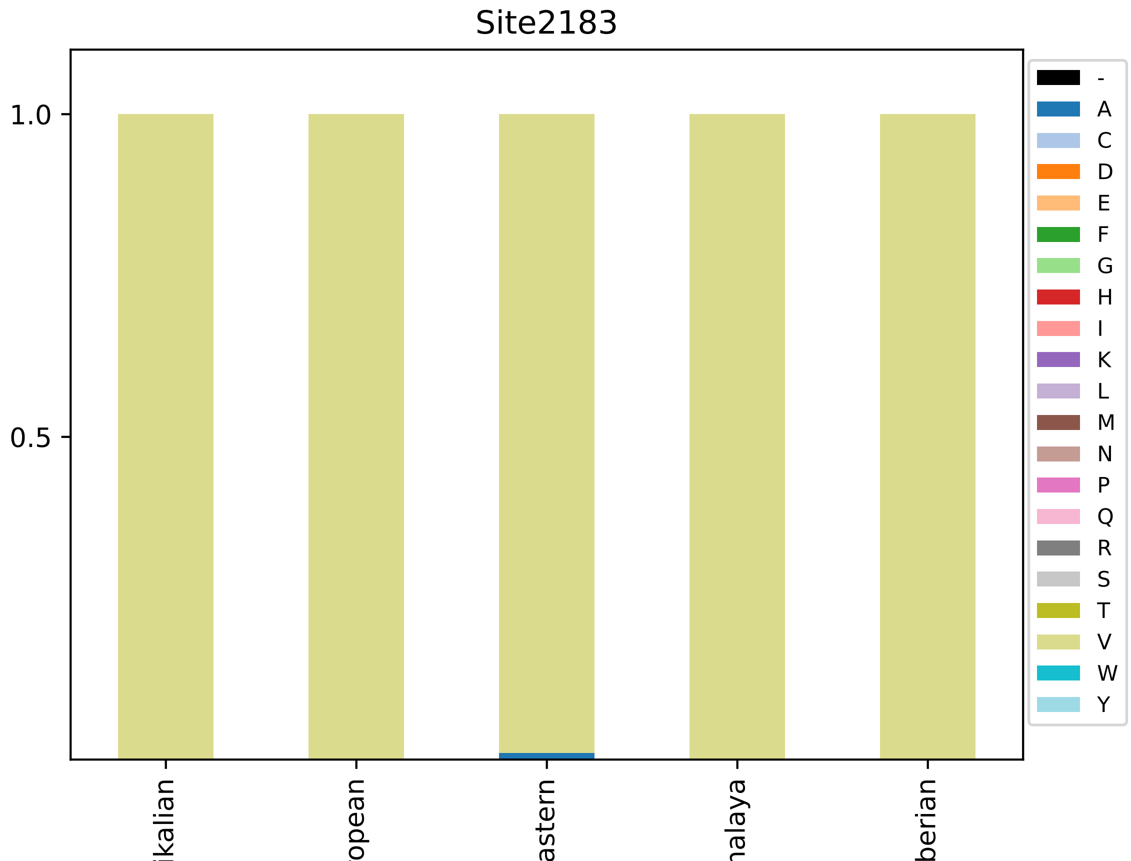

Site2183
1.0
0.5
-
A
C
D
E
F
G
H
I
K
L
M
N
P
Q
R
S
T
V
W
Y
ikalian
opean
astern
nalaya
berian

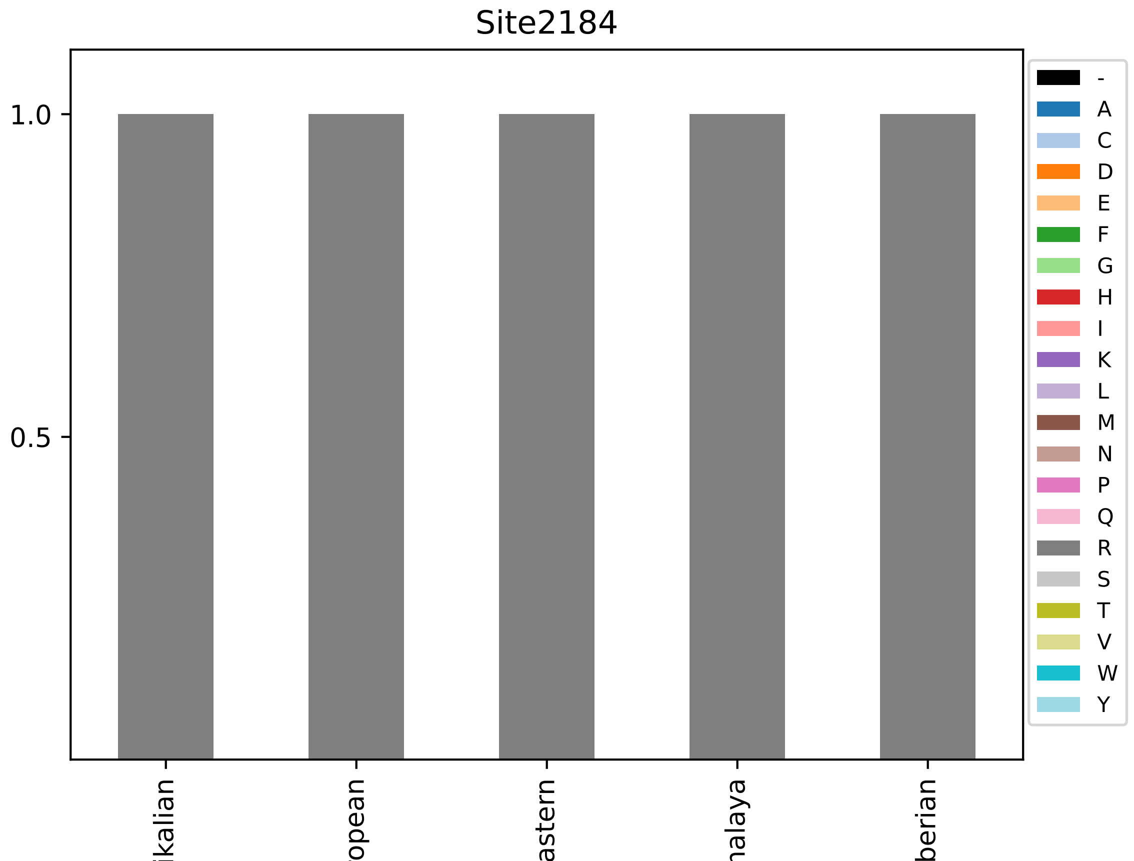

Site2184
1.0
0.5
-
A
C
D
E
F
G
H
I
K
L
M
N
P
Q
R
S
T
V
W
Y
ikalian
opean
astern
nalaya
berian

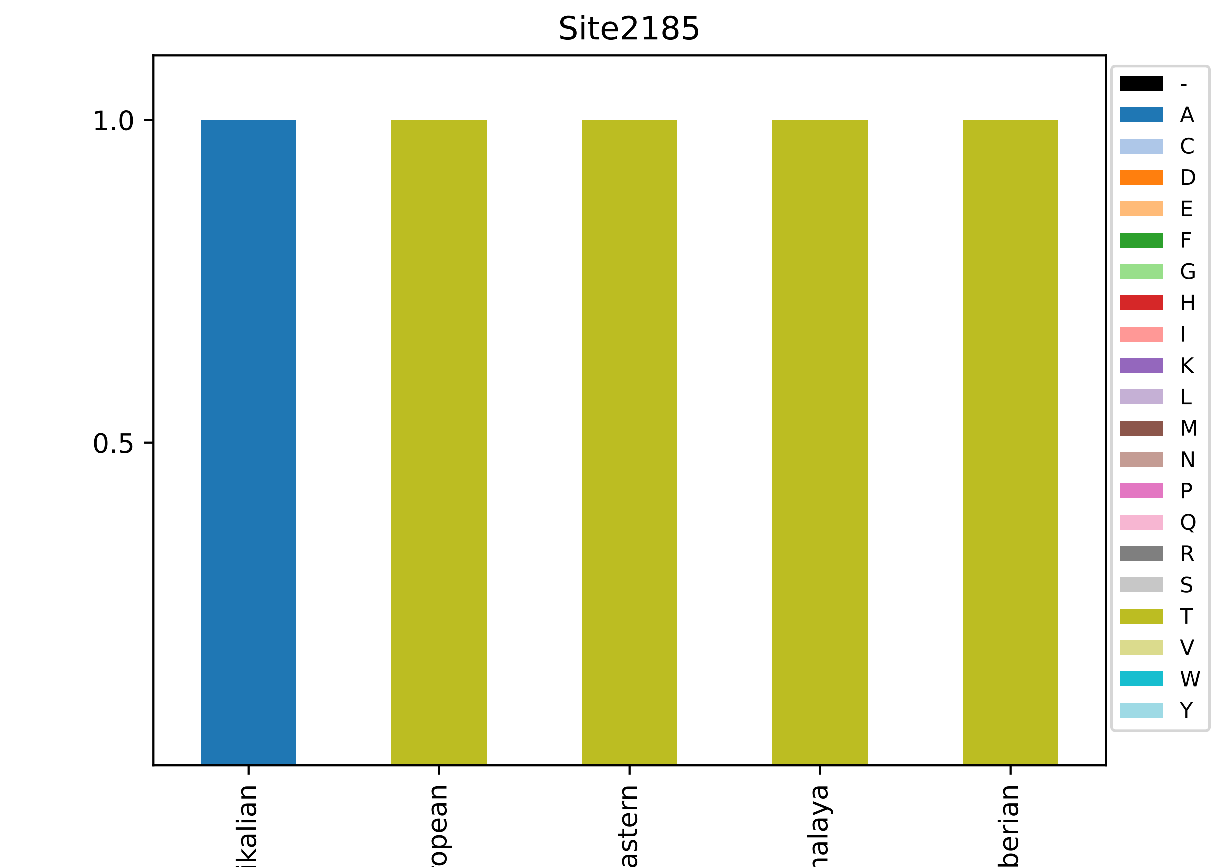
Site2185
1.0
0.5
ikalian
opean
astern
nalaya
berian
-
A
C
D
E
F
G
H
I
K
L
M
N
P
Q
R
S
T
V
W
Y

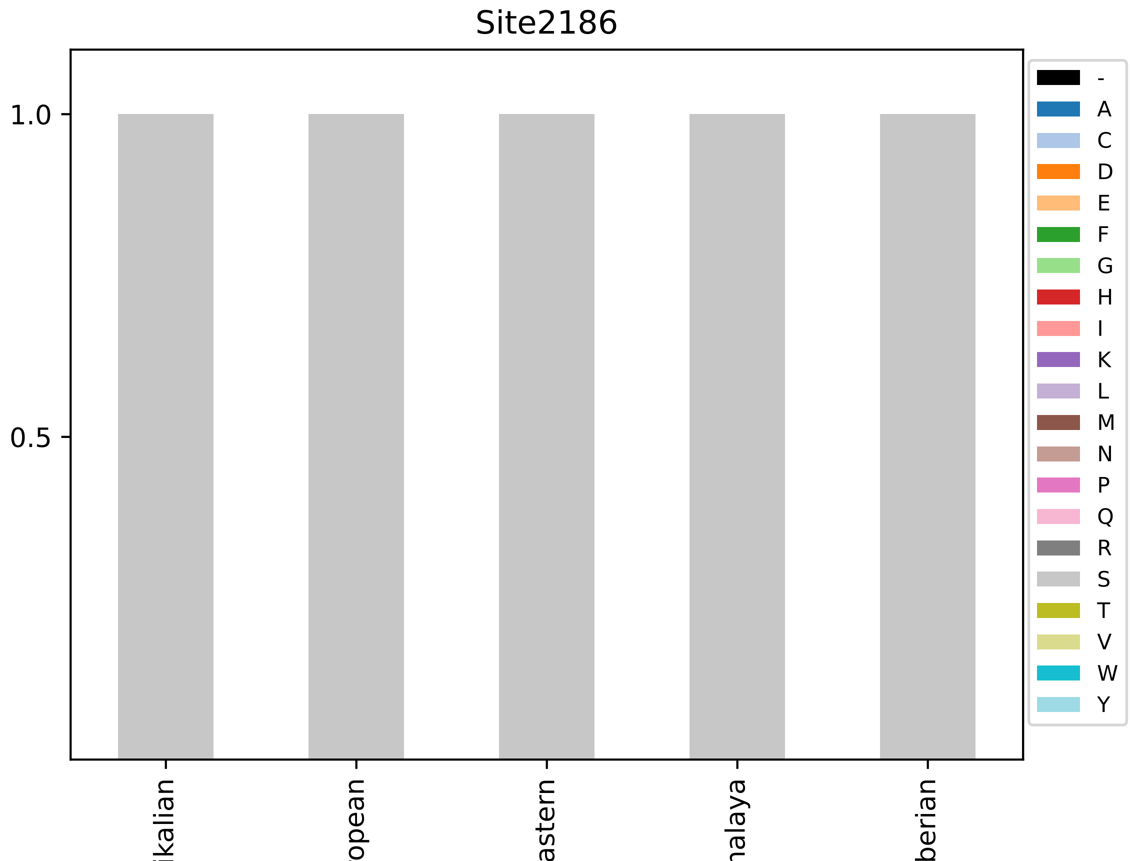
Site2186
1.0
0.5
ikalian
opean
astern
nalaya
berian
-
A
C
D
E
F
G
H
I
K
L
M
N
P
Q
R
S
T
V
W
Y

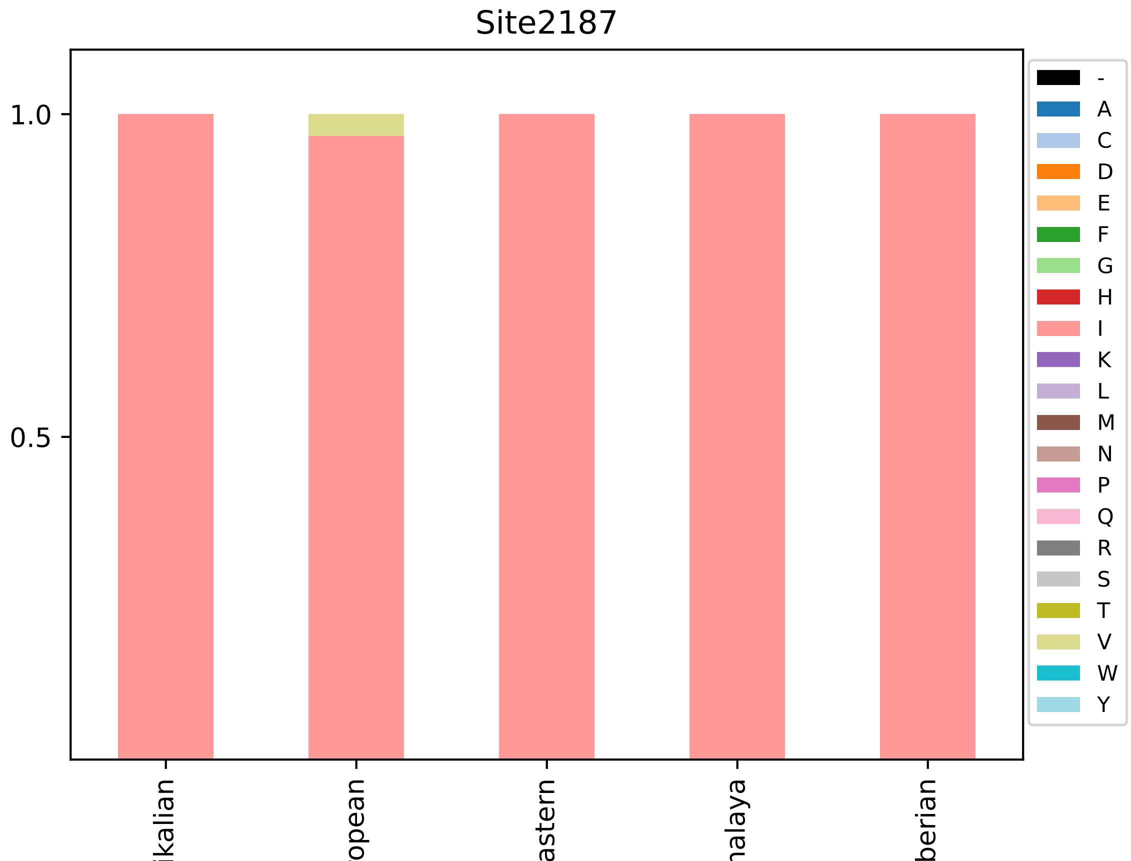
Site2187
1.0
0.5
-
A
C
D
E
F
G
H
I
K
L
M
N
P
Q
R
S
T
V
W
Y
ikalian
opean
astern
nalaya
berian

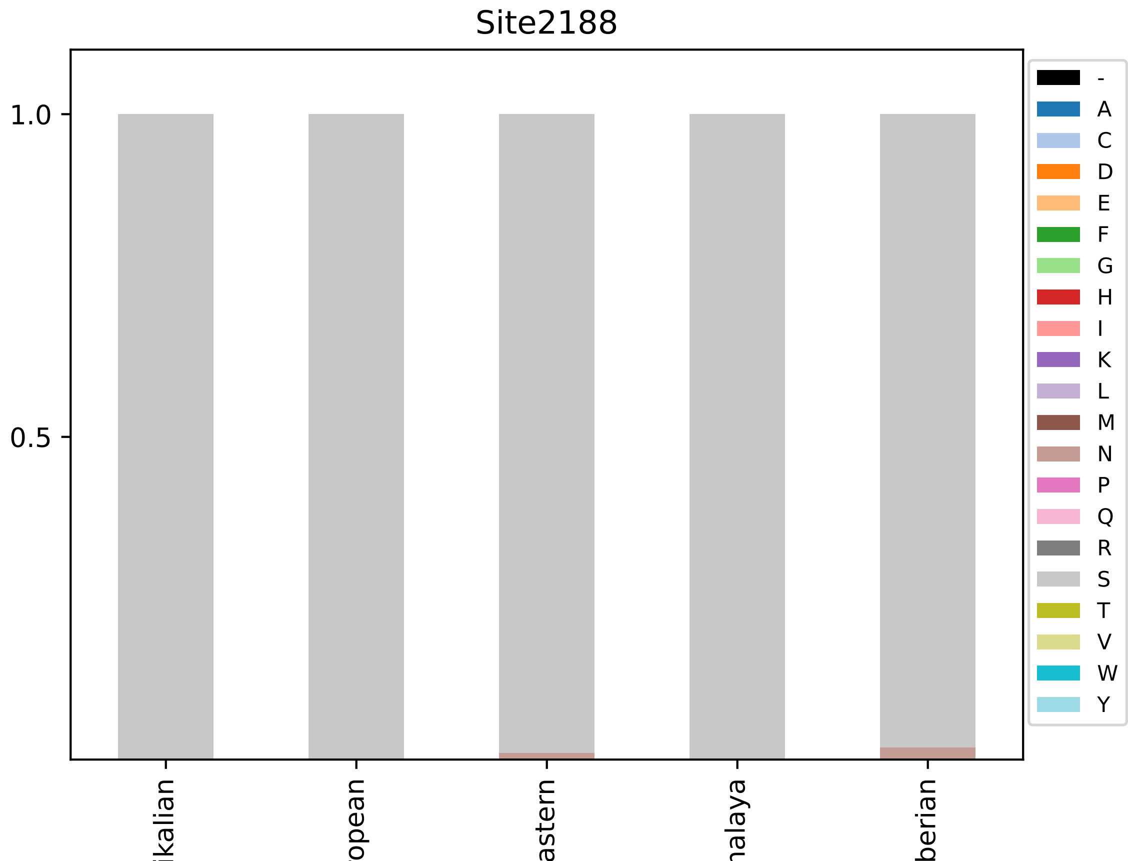

Site2188
1.0
0.5
-
A
C
D
E
F
G
H
I
K
L
M
N
P
Q
R
S
T
V
W
Y
ikalian
opean
astern
nalaya
berian

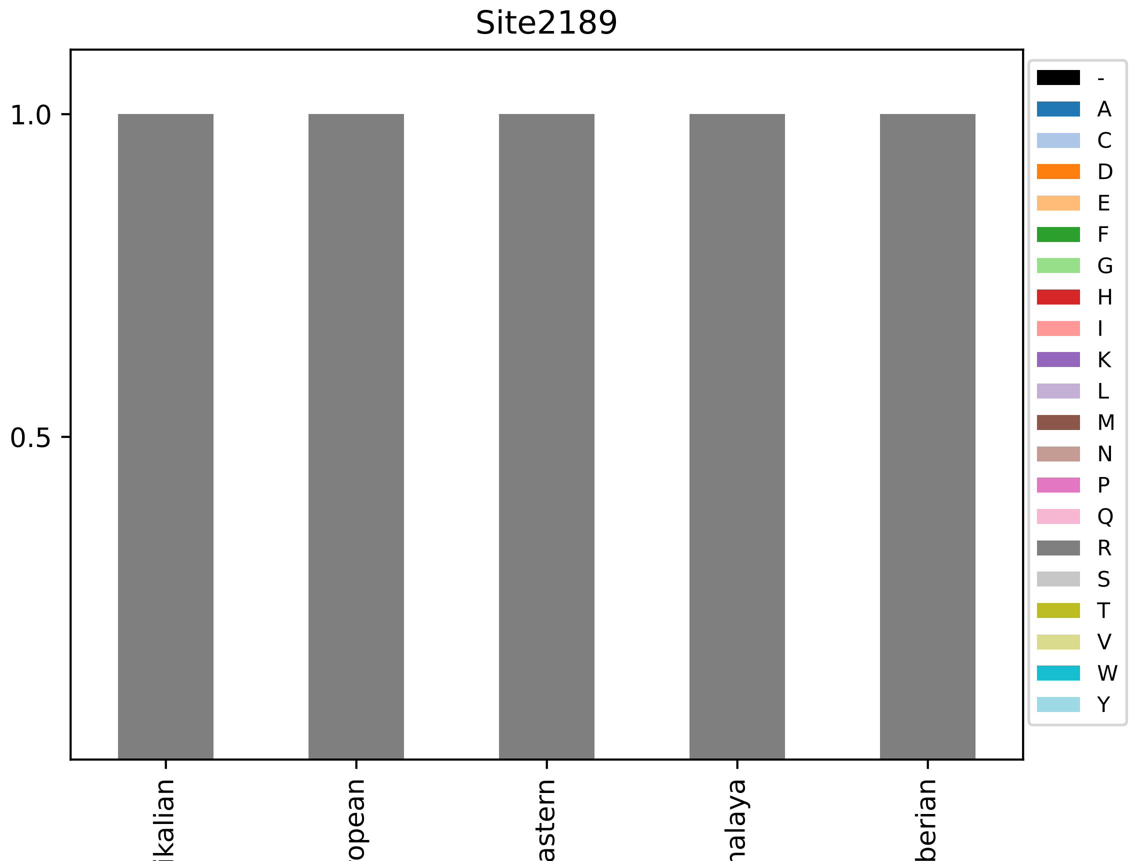
Site2189
1.0
0.5
-
A
C
D
E
F
G
H
I
K
L
M
N
P
Q
R
S
T
V
W
Y
ikalian
opean
astern
nalaya
berian

Site2190

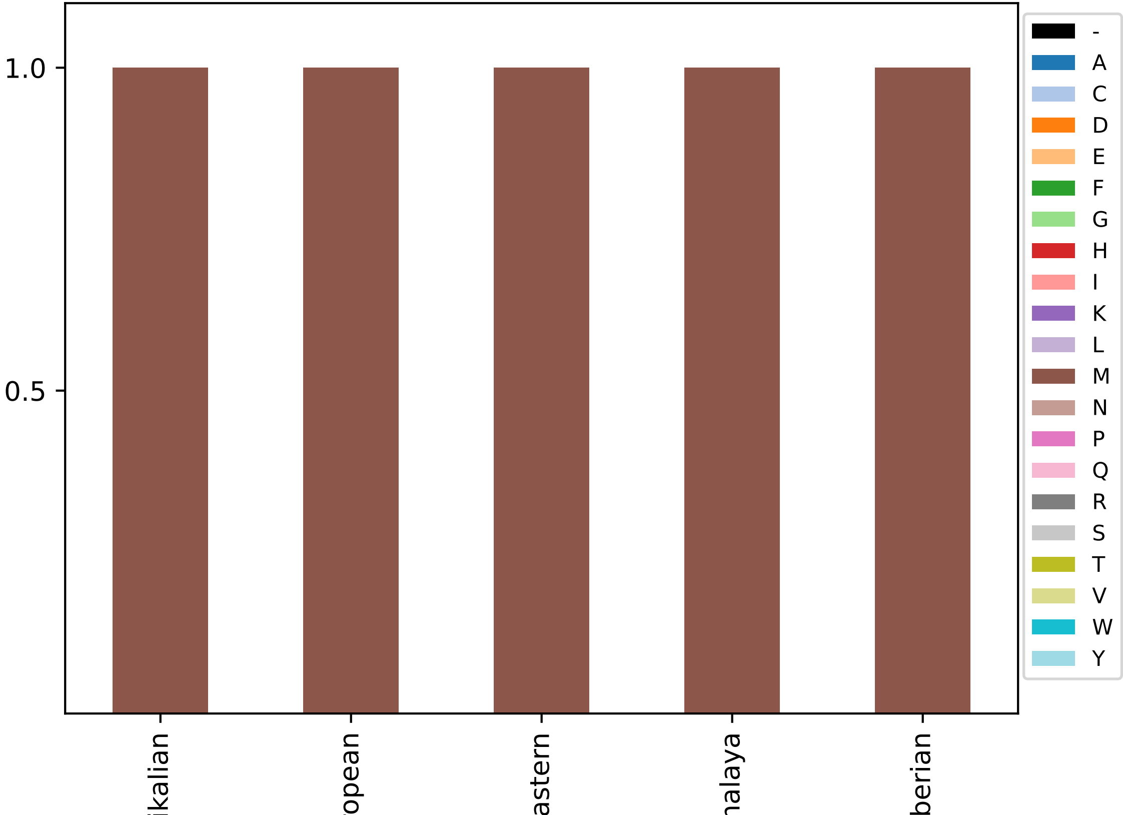

1.0
0.5
-
A
C
D
E
F
G
H
I
K
L
M
N
P
Q
R
S
T
V
W
Y
ikalian
opean
astern
nalaya
berian

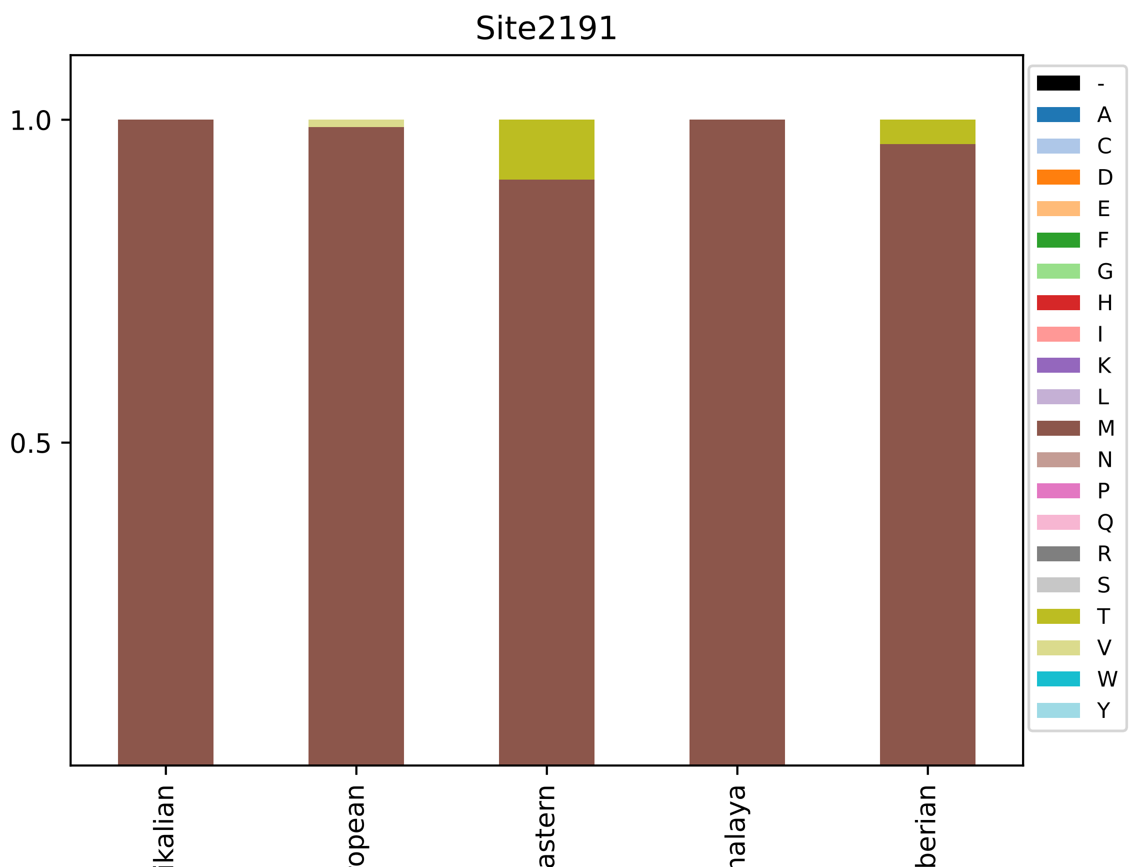

Site2191
1.0
0.5
-
A
C
D
E
F
G
H
I
K
L
M
N
P
Q
R
S
T
V
W
Y
ikalian
opean
astern
nalaya
berian

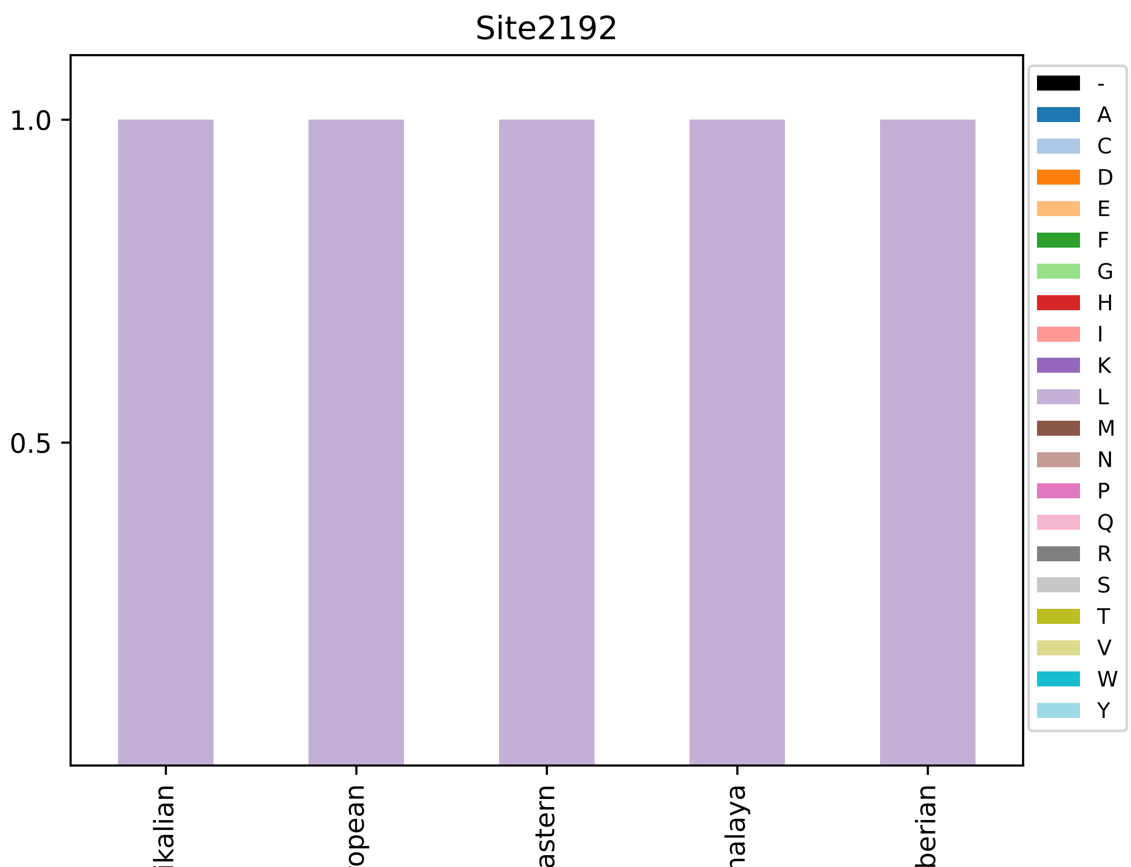

Site2192
1.0
0.5
ikalian
opean
astern
nalaya
berian
-
A
C
D
E
F
G
H
I
K
L
M
N
P
Q
R
S
T
V
W
Y

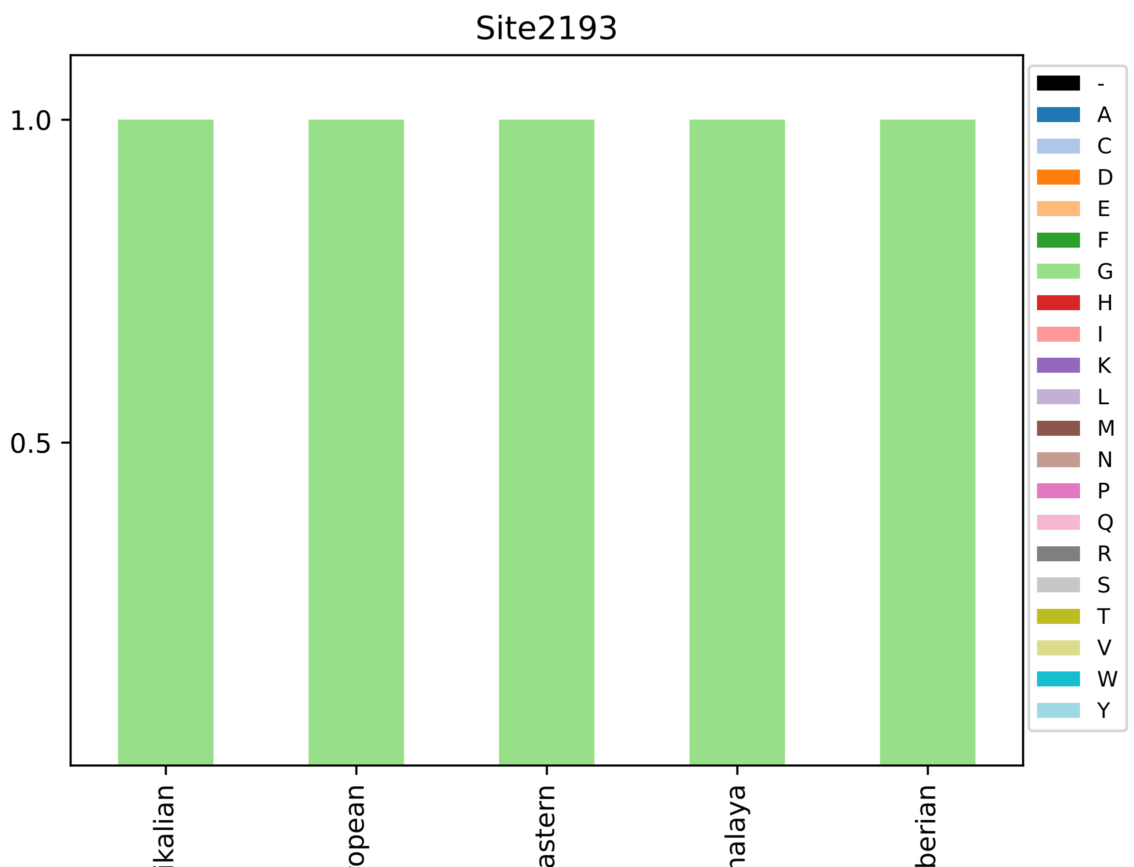

Site2193
1.0
0.5
ikalian
opean
astern
nalaya
berian
-
A
C
D
E
F
G
H
I
K
L
M
N
P
Q
R
S
T
V
W
Y

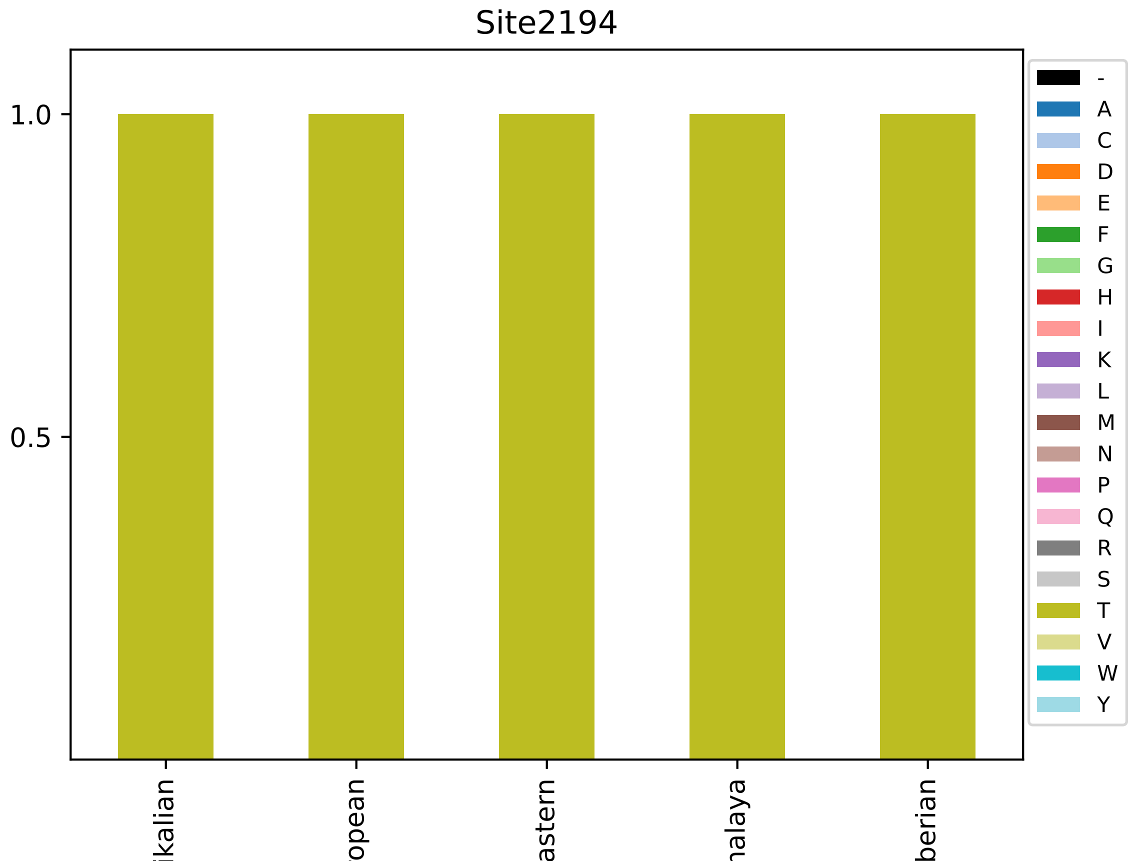
Site2194
1.0
0.5
-
A
C
D
E
F
G
H
I
K
L
M
N
P
Q
R
S
T
V
W
Y
ikalian
ropean
astern
nalaya
berian

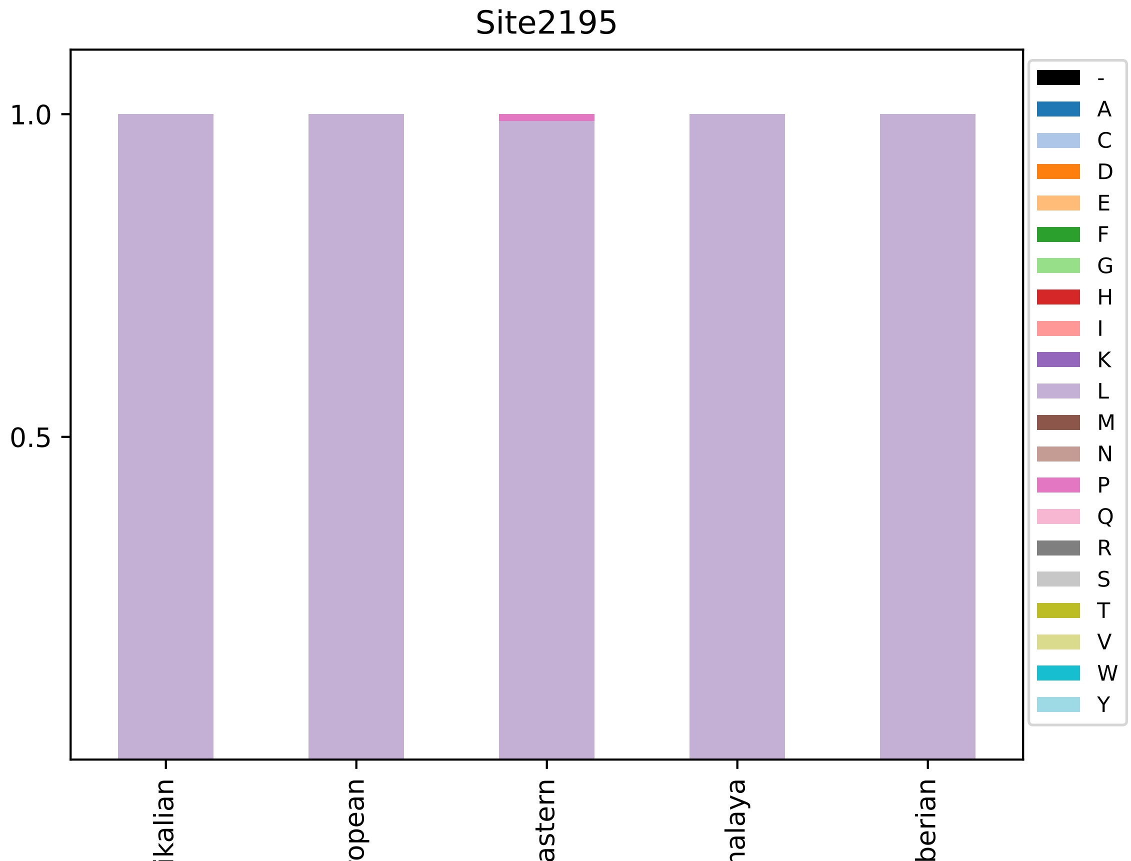
Site2195
1.0
0.5
ikalian
opean
astern
nalaya
berian
-
A
C
D
E
F
G
H
I
K
L
M
N
P
Q
R
S
T
V
W
Y

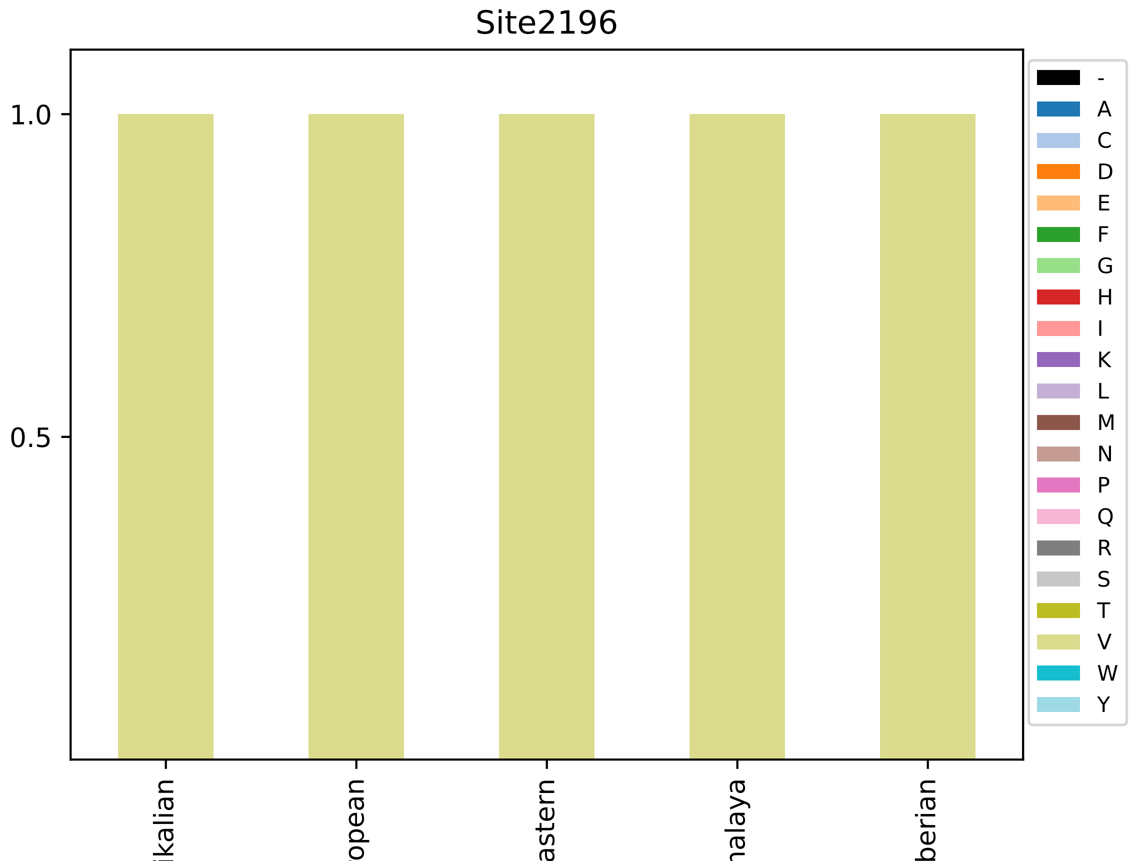

Site2196
1.0
0.5
ikalian
opean
astern
nalaya
berian
-
A
C
D
E
F
G
H
I
K
L
M
N
P
Q
R
S
T
V
W
Y

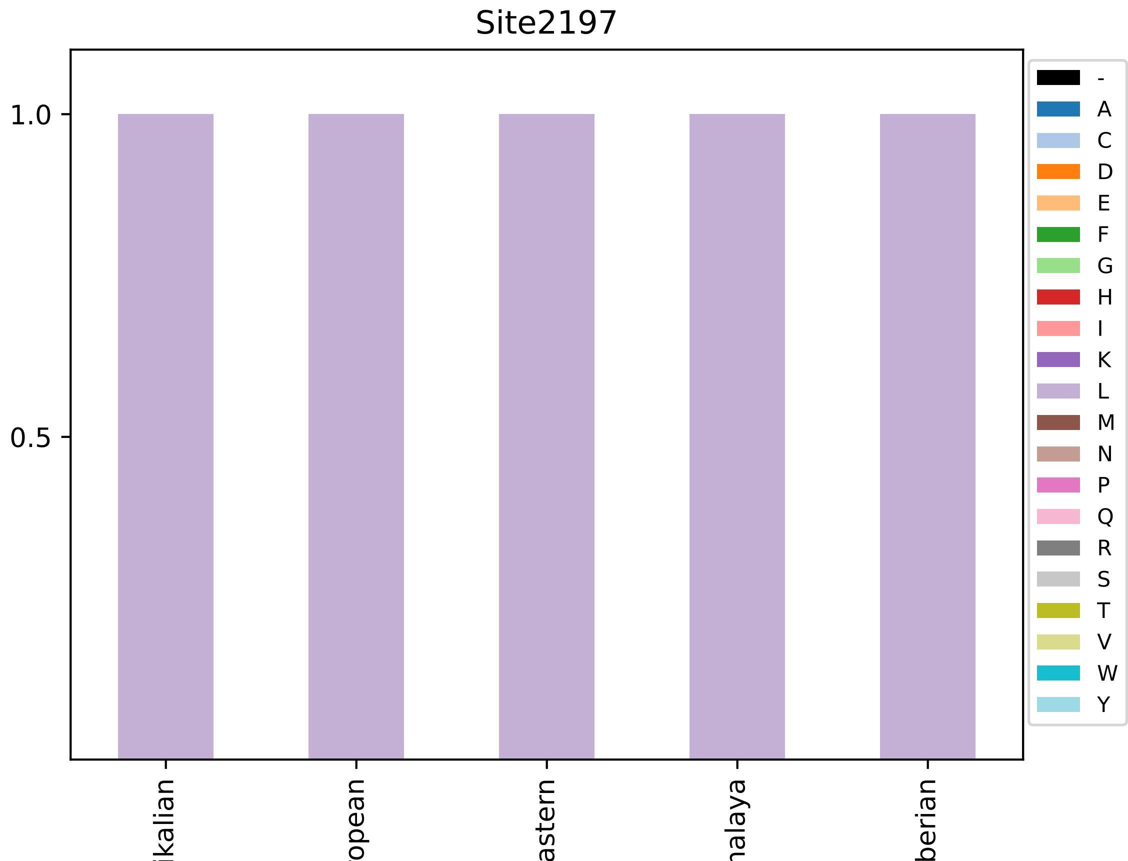

Site2197
1.0
0.5
ikalian
opean
astern
nalaya
berian
-
A
C
D
E
F
G
H
I
K
L
M
N
P
Q
R
S
T
V
W
Y

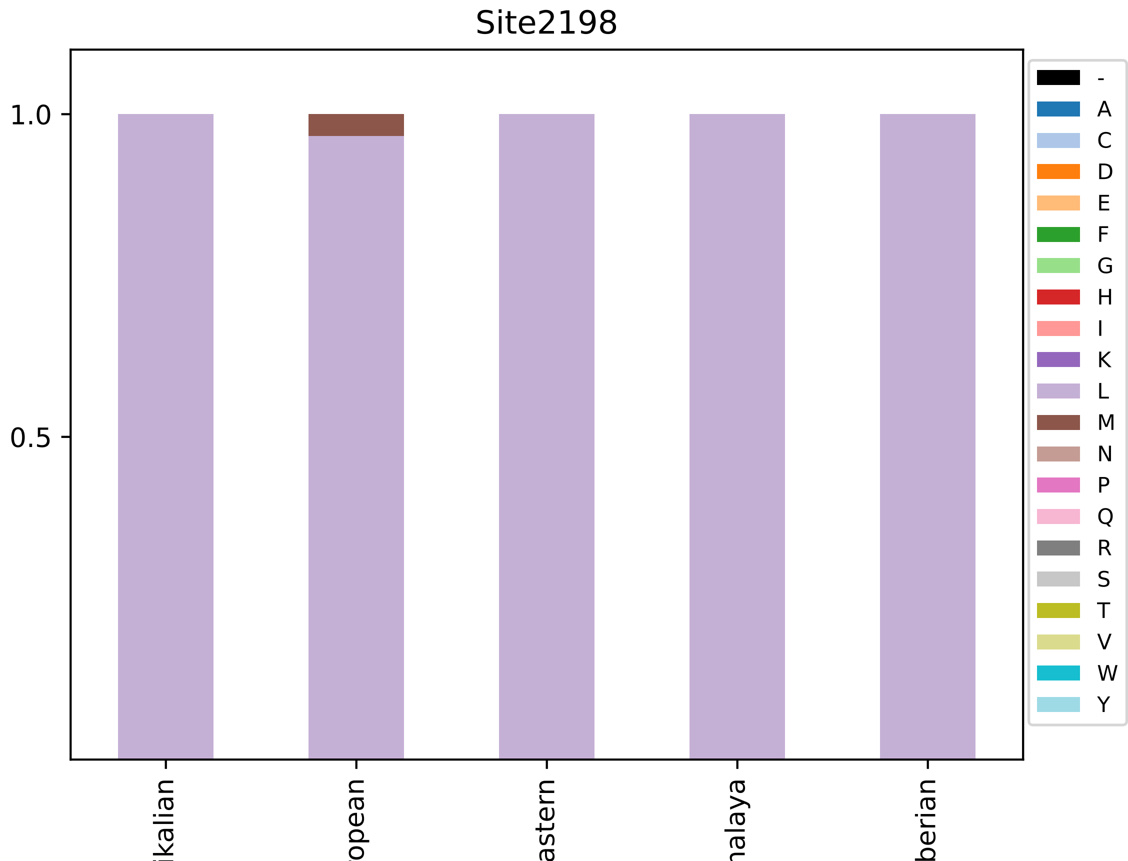
Site2198
1.0
0.5
-
A
C
D
E
F
G
H
I
K
L
M
N
P
Q
R
S
T
V
W
Y
ikalian
opean
astern
nalaya
berian

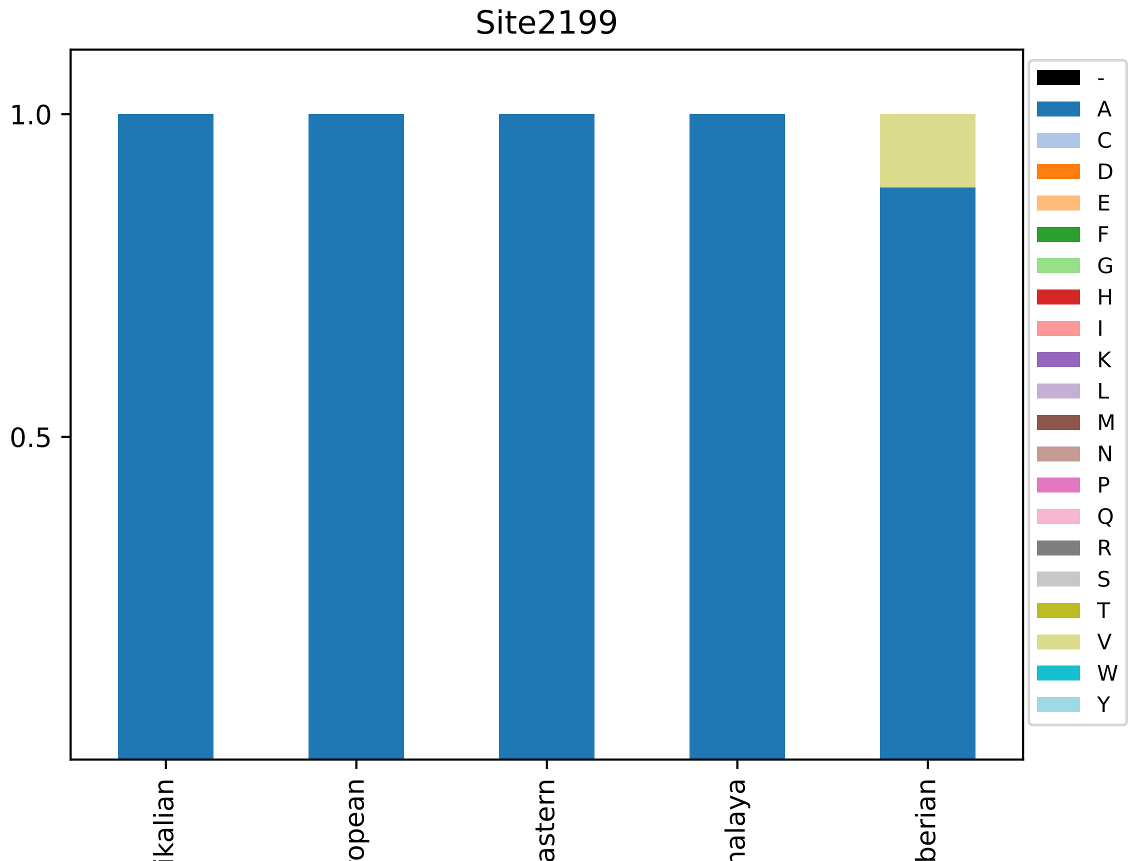
Site2199
1.0
0.5
-
A
C
D
E
F
G
H
I
K
L
M
N
P
Q
R
S
T
V
W
Y
ikalian
opean
astern
nalaya
berian

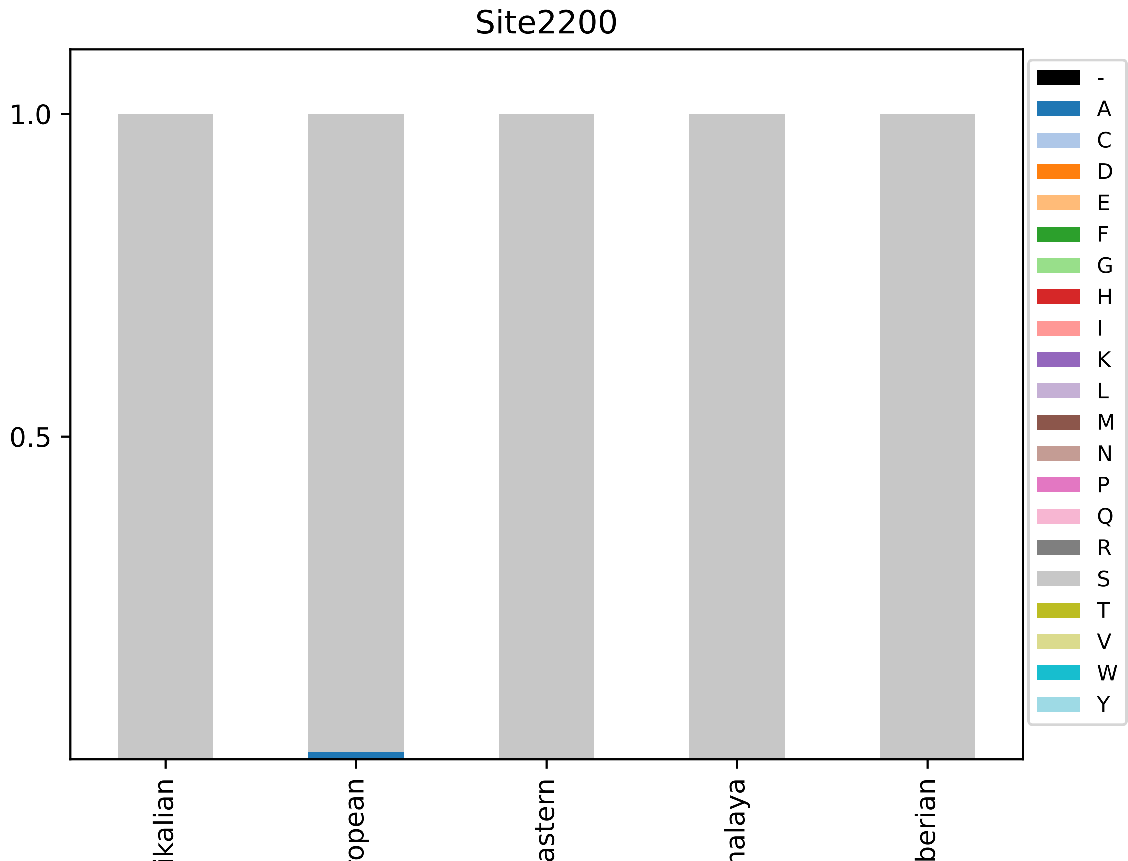

Site2200
1.0
0.5
-
A
C
D
E
F
G
H
I
K
L
M
N
P
Q
R
S
T
V
W
Y
ikalian
opean
astern
nalaya
berian

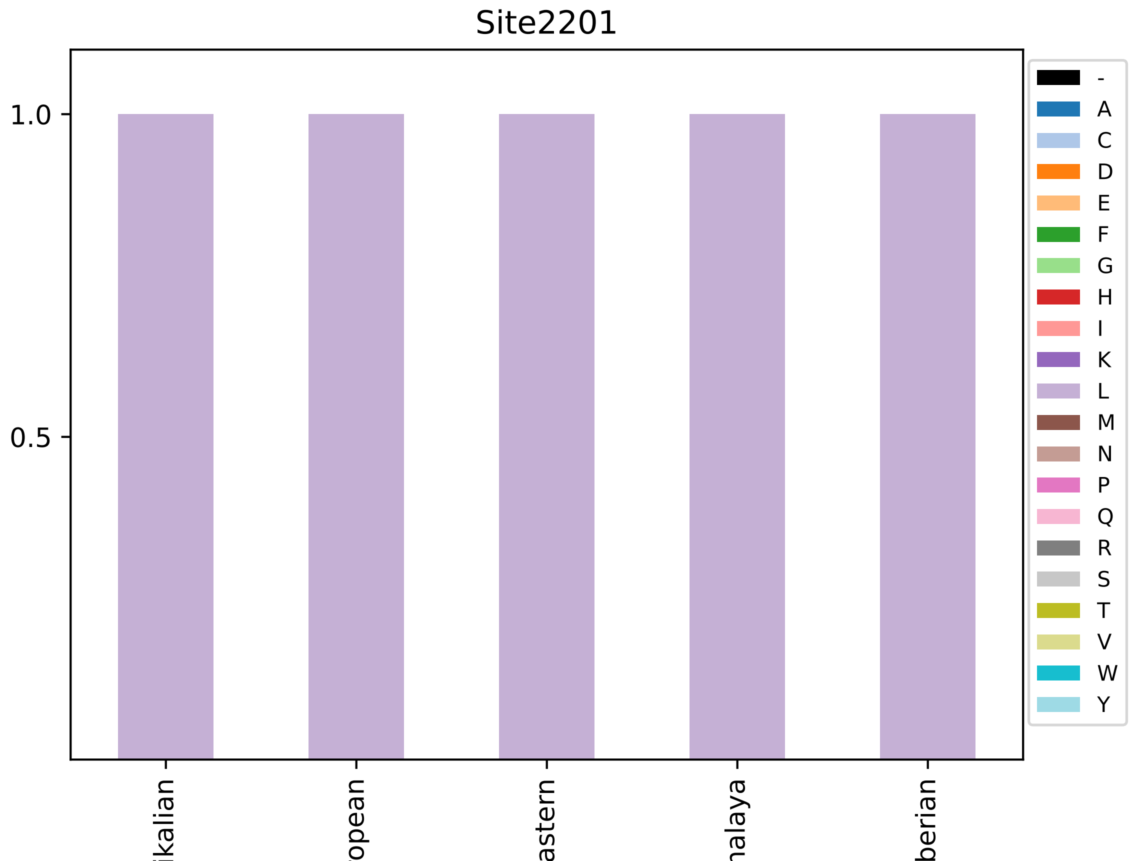

Site2201
1.0
0.5
ikalian
opean
astern
nalaya
berian
-
A
C
D
E
F
G
H
I
K
L
M
N
P
Q
R
S
T
V
W
Y

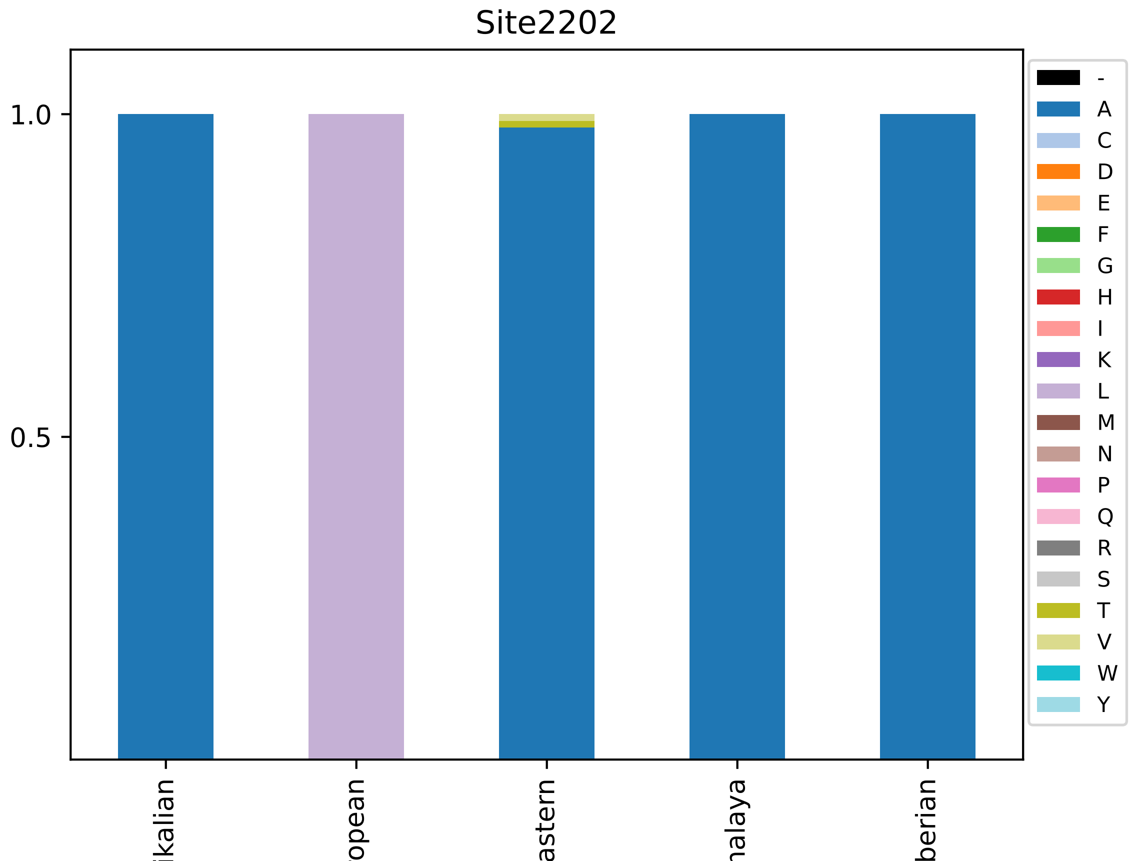

Site2202
1.0
0.5
ikalian
opean
astern
nalaya
berian
-
A
C
D
E
F
G
H
I
K
L
M
N
P
Q
R
S
T
V
W
Y

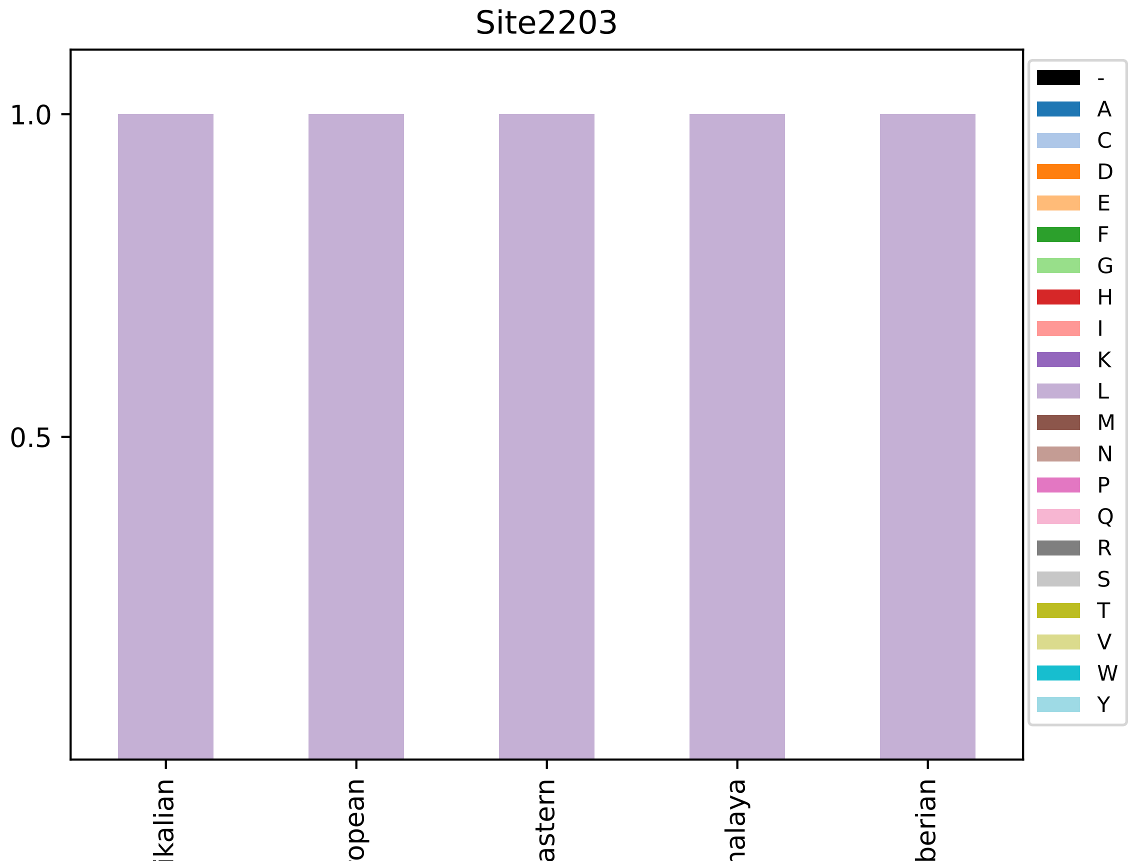

Site2203
1.0
0.5
-
A
C
D
E
F
G
H
I
K
L
M
N
P
Q
R
S
T
V
W
Y
ikalian
ropean
astern
nalaya
berian

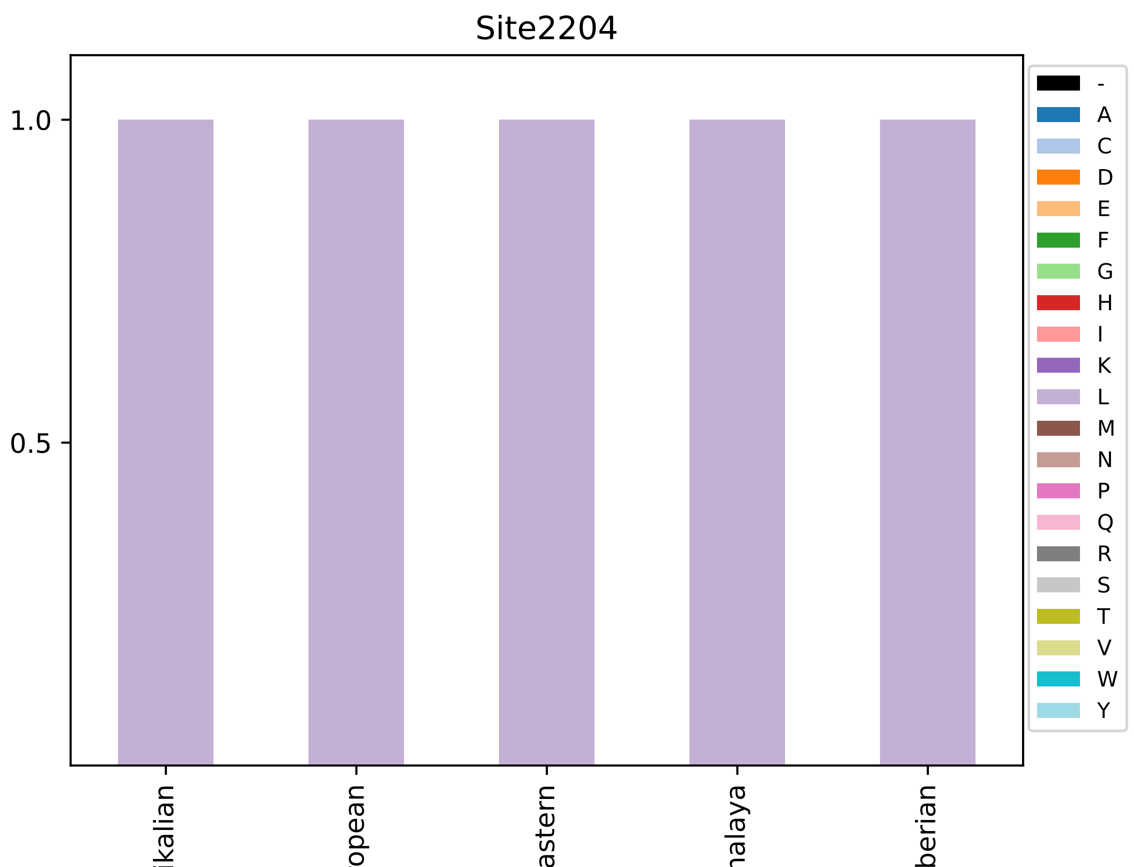
Site2204
1.0
0.5
-
A
C
D
E
F
G
H
I
K
L
M
N
P
Q
R
S
T
V
W
Y
ikalian
ropean
astern
nalaya
berian

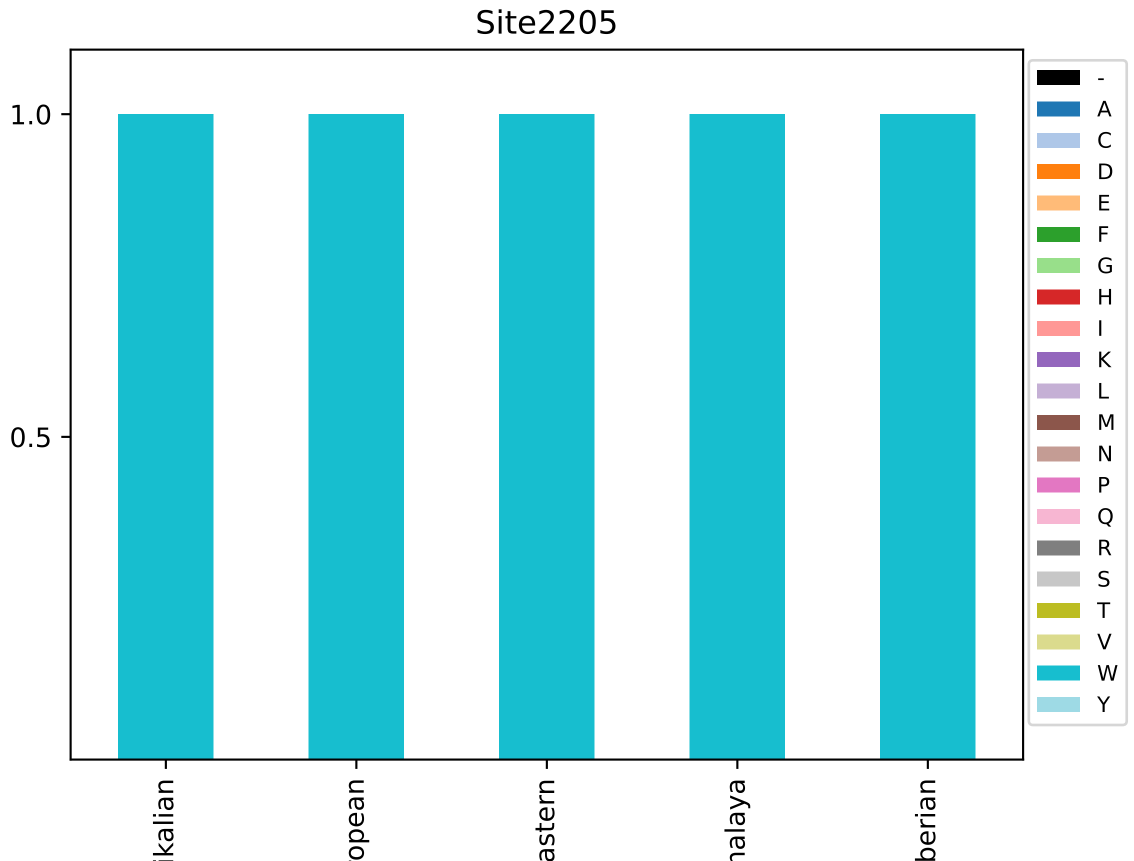

Site2205
1.0
0.5
-
A
C
D
E
F
G
H
I
K
L
M
N
P
Q
R
S
T
V
W
Y
ikalian
opean
astern
nalaya
berian

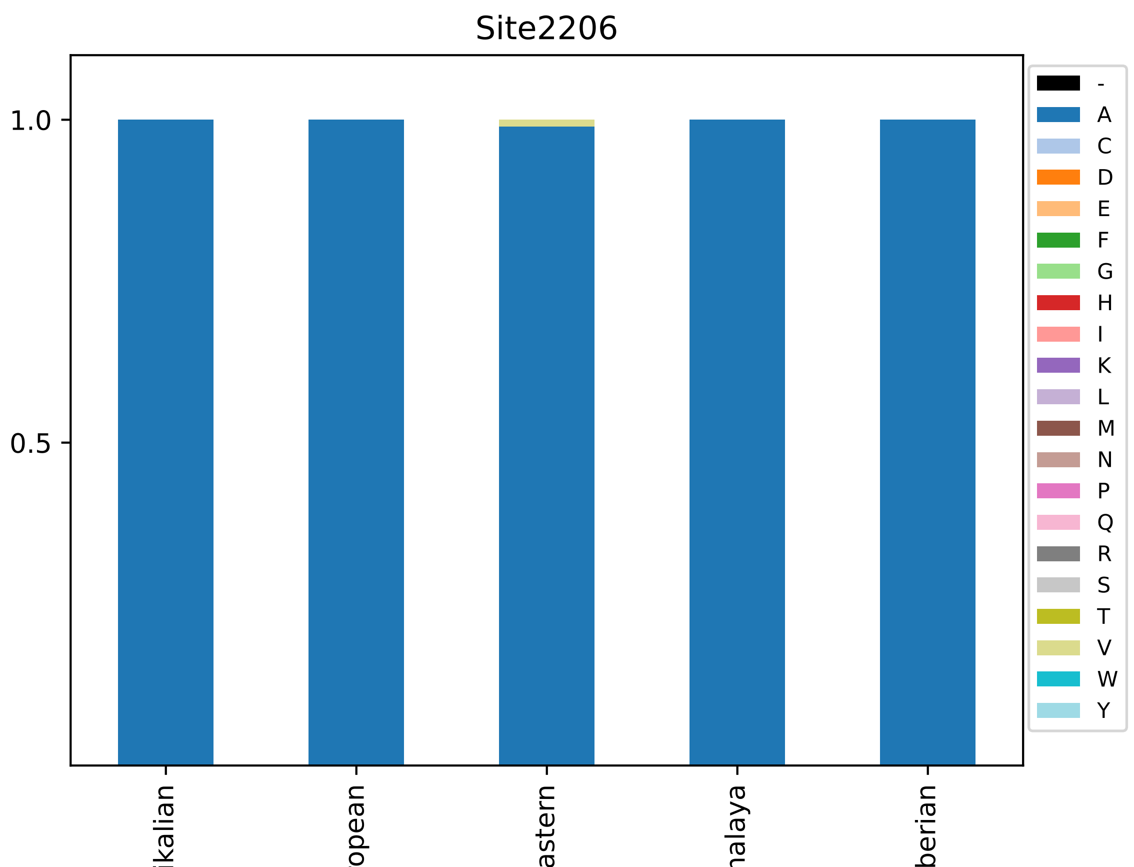
Site2206
1.0
0.5
-
A
C
D
E
F
G
H
I
K
L
M
N
P
Q
R
S
T
V
W
Y
ikalian
opean
astern
nalaya
berian

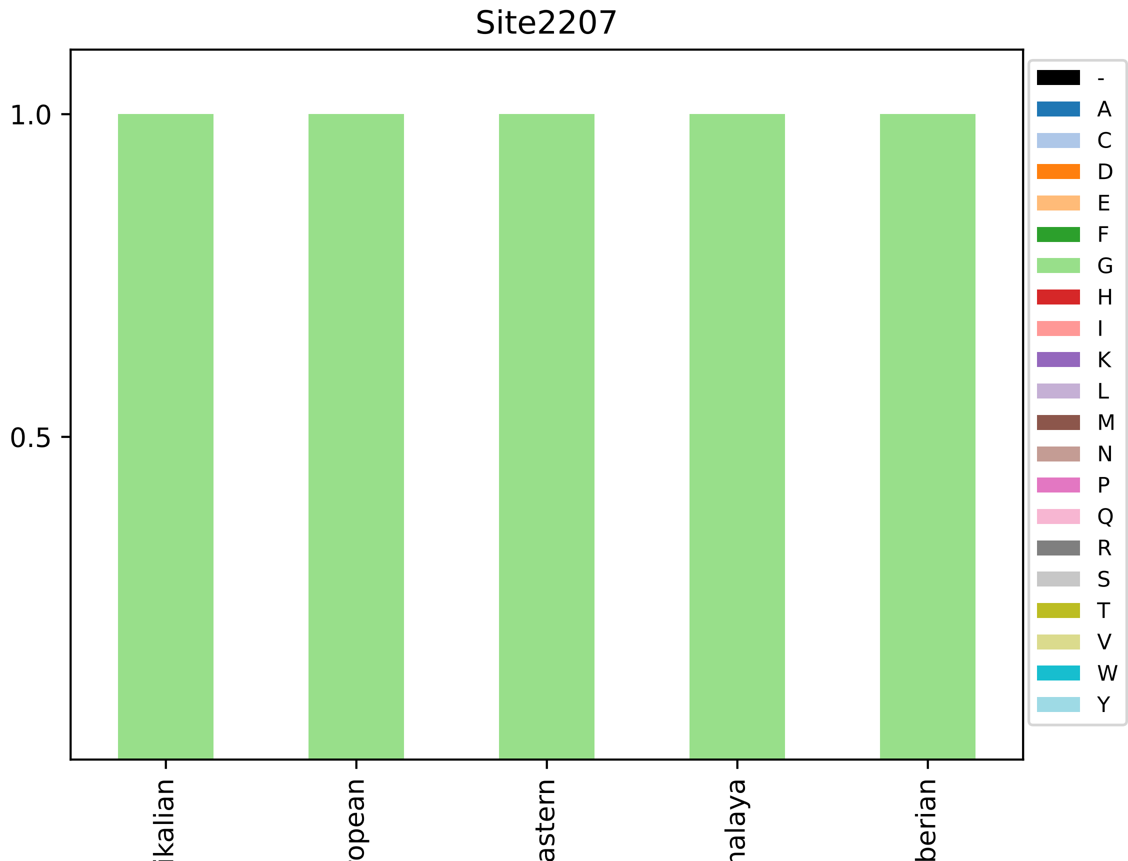

Site2207
1.0
0.5
ikalian
opean
astern
nalaya
berian
-
A
C
D
E
F
G
H
I
K
L
M
N
P
Q
R
S
T
V
W
Y

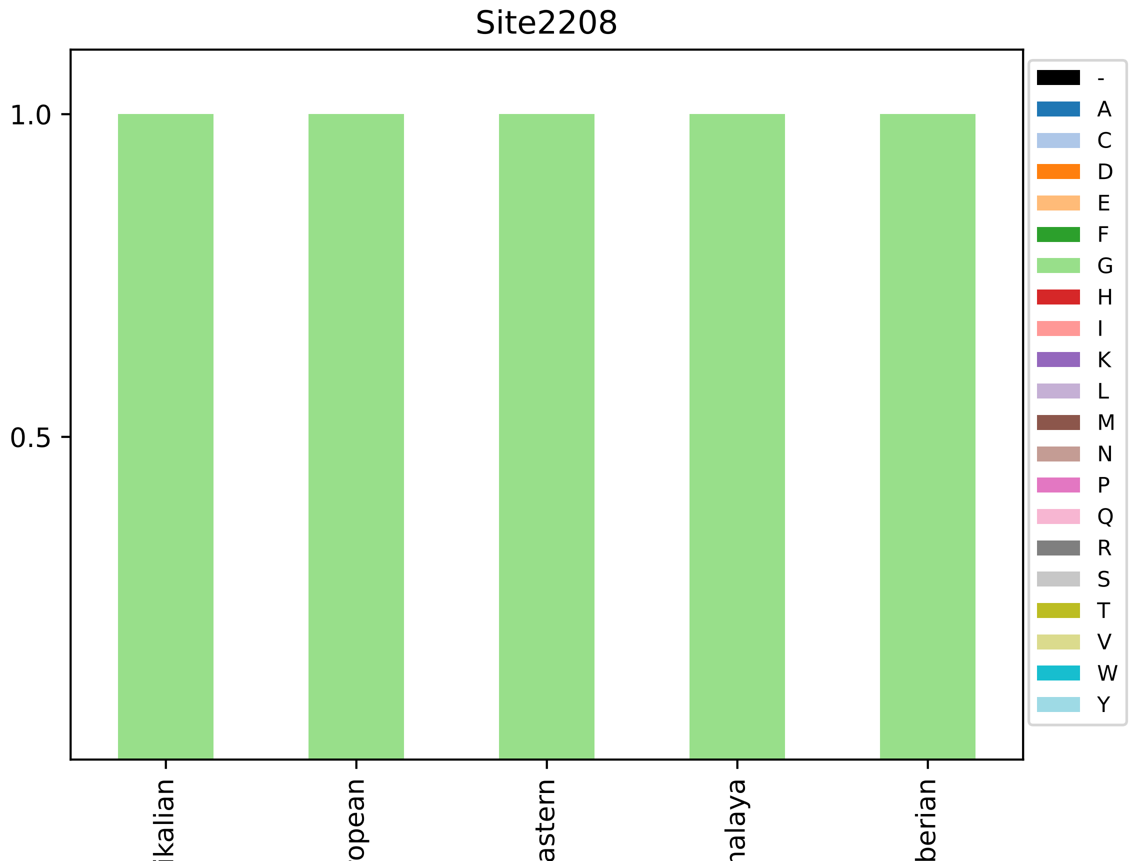

Site2208
1.0
0.5
-
A
C
D
E
F
G
H
I
K
L
M
N
P
Q
R
S
T
V
W
Y
ikalian
opean
astern
nalaya
berian

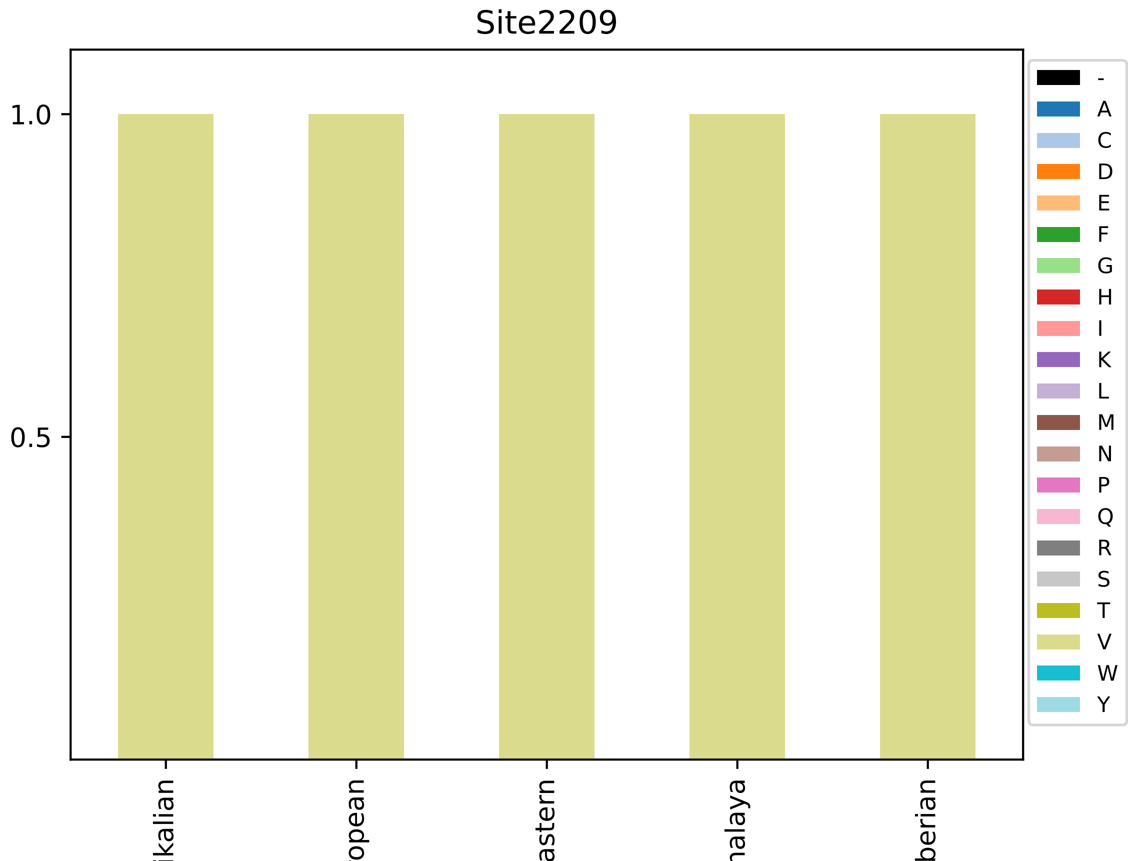

Site2209
1.0
0.5
-
A
C
D
E
F
G
H
I
K
L
M
N
P
Q
R
S
T
V
W
Y
ikalian
opean
astern
nalaya
berian

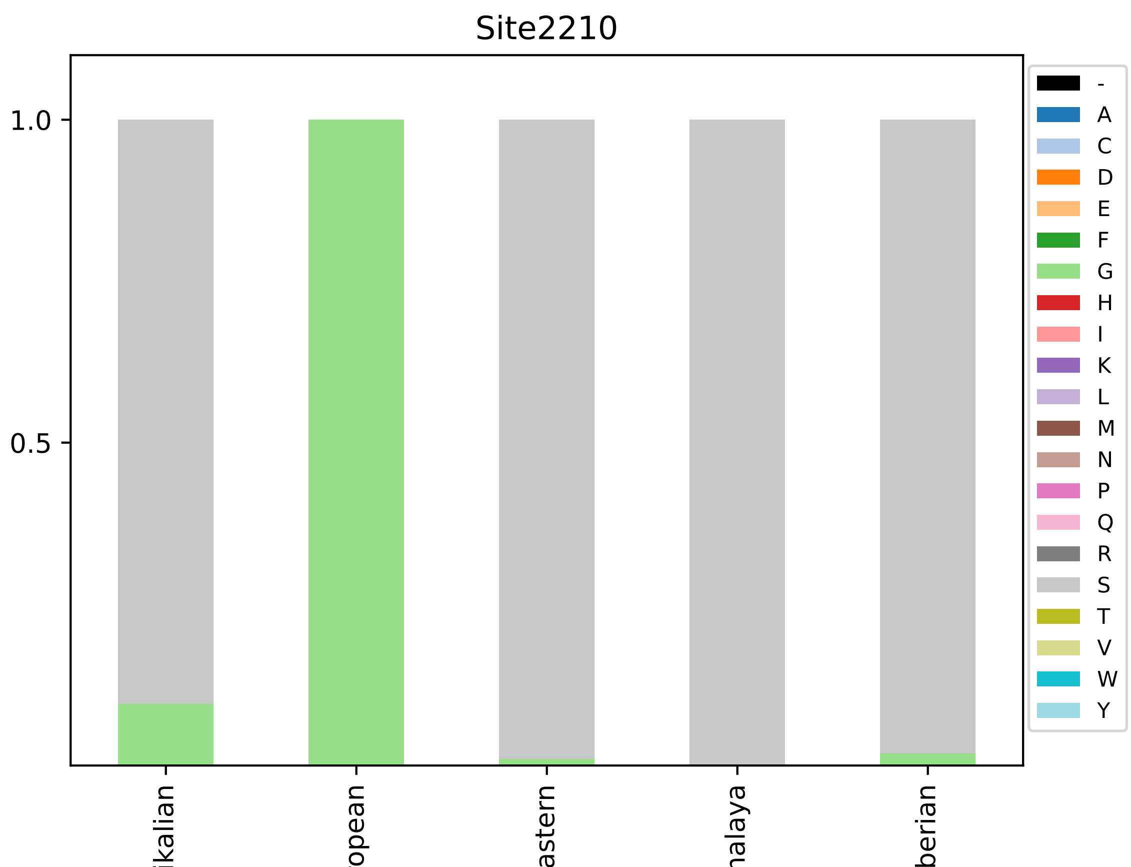

Site2210
1.0
0.5
-
A
C
D
E
F
G
H
I
K
L
M
N
P
Q
R
S
T
V
W
Y
ikalian
opean
astern
nalaya
berian

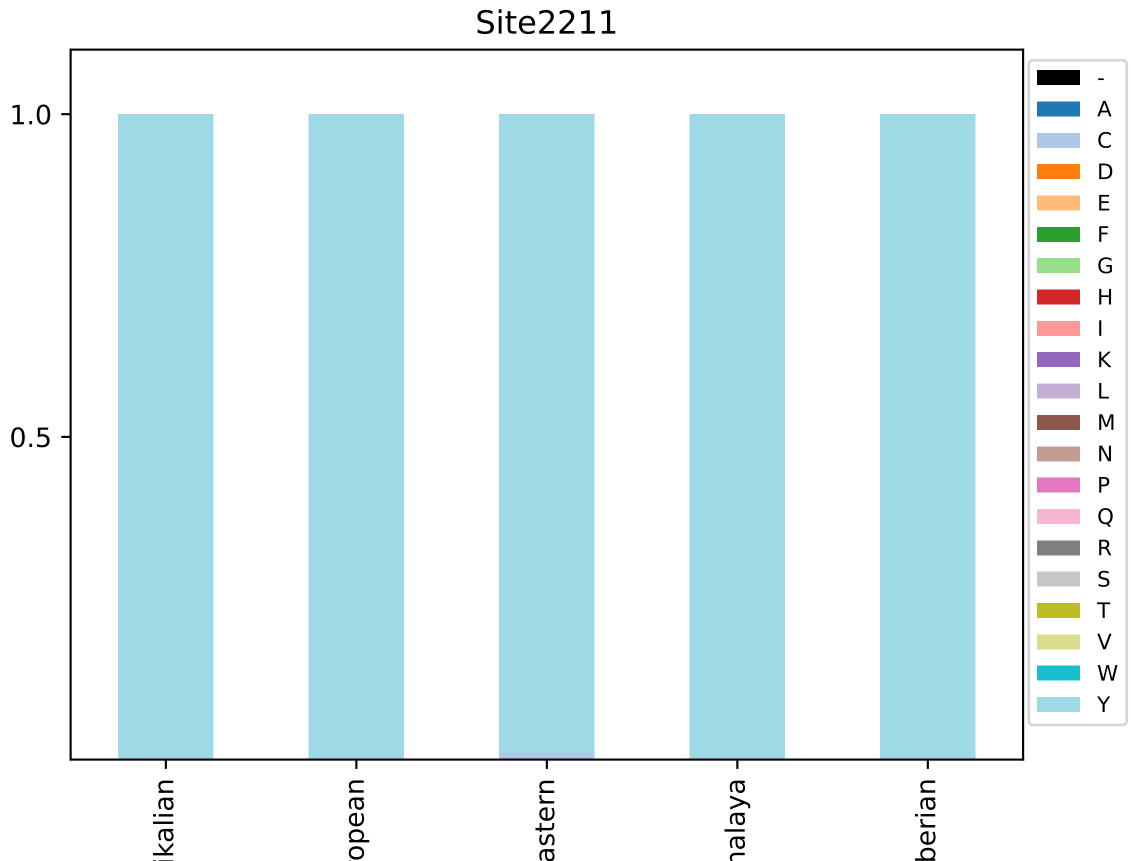

Site2211
1.0
0.5
ikalian
opean
astern
nalaya
berian
-
A
C
D
E
F
G
H
I
K
L
M
N
P
Q
R
S
T
V
W
Y

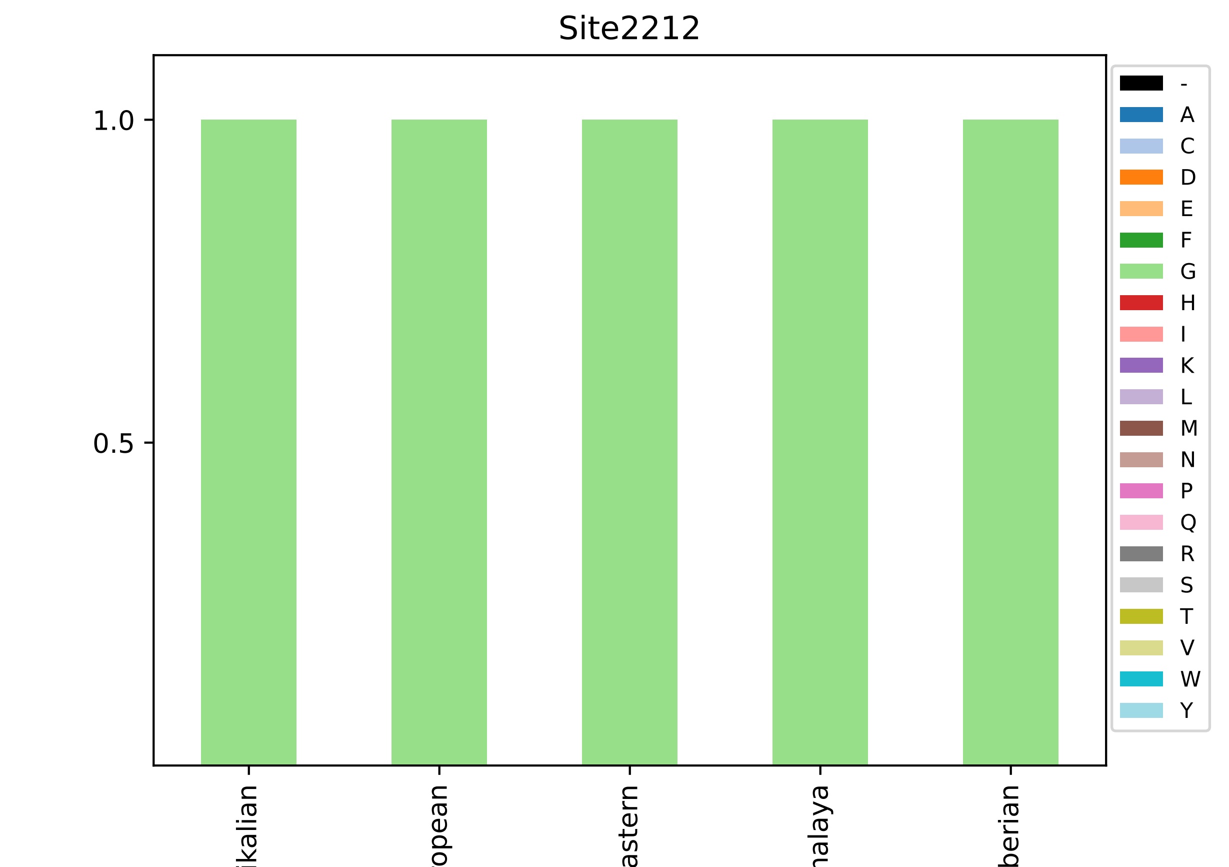

Site2212
1.0
0.5
-
A
C
D
E
F
G
H
I
K
L
M
N
P
Q
R
S
T
V
W
Y
ikalian
opean
astern
nalaya
berian

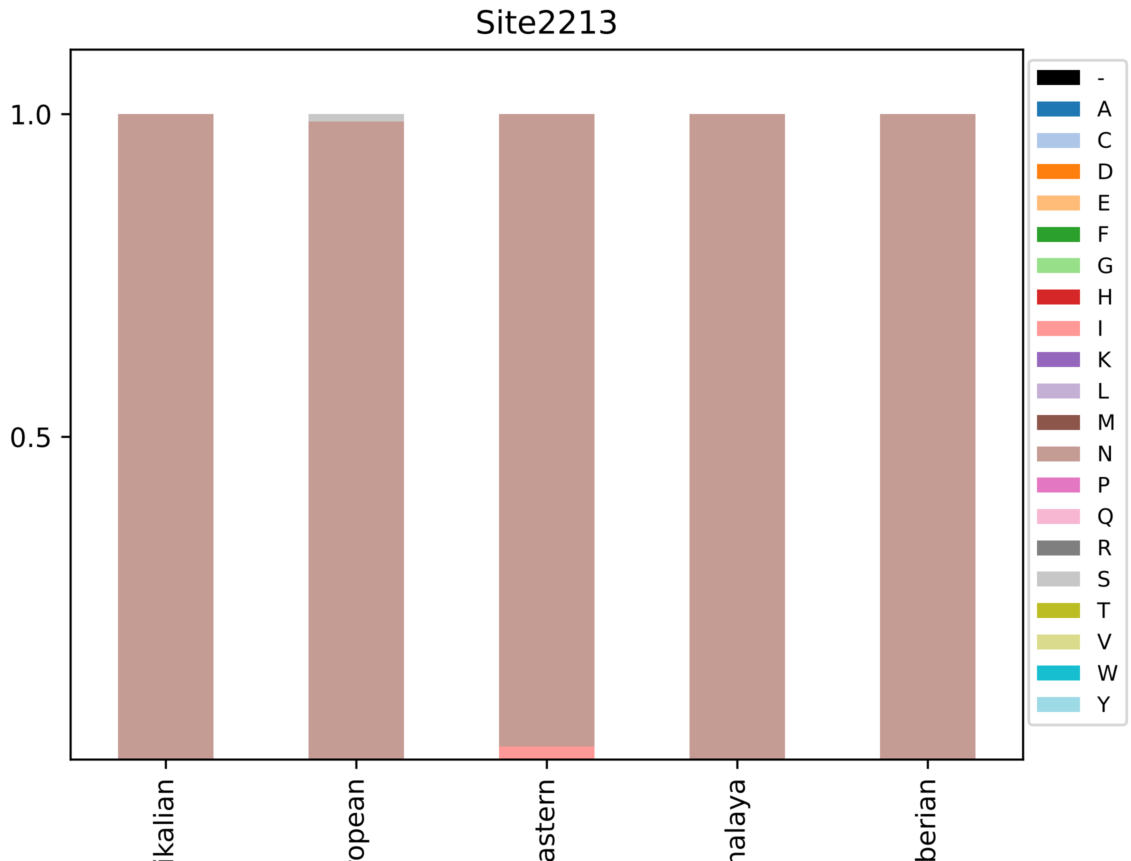
Site2213
1.0
0.5
ikalian
opean
astern
nalaya
berian
-
A
C
D
E
F
G
H
I
K
L
M
N
P
Q
R
S
T
V
W
Y

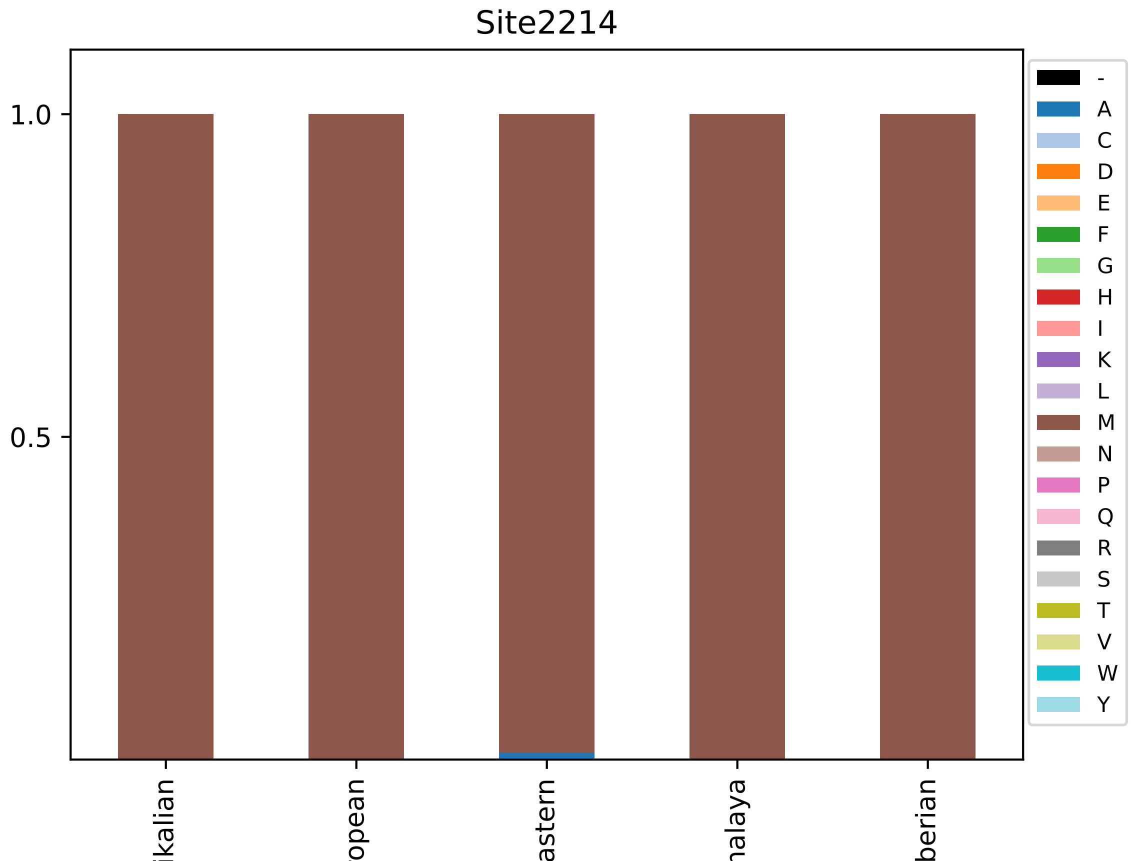

Site2214
1.0
0.5
-
A
C
D
E
F
G
H
I
K
L
M
N
P
Q
R
S
T
V
W
Y
ikalian
ropean
astern
nalaya
berian

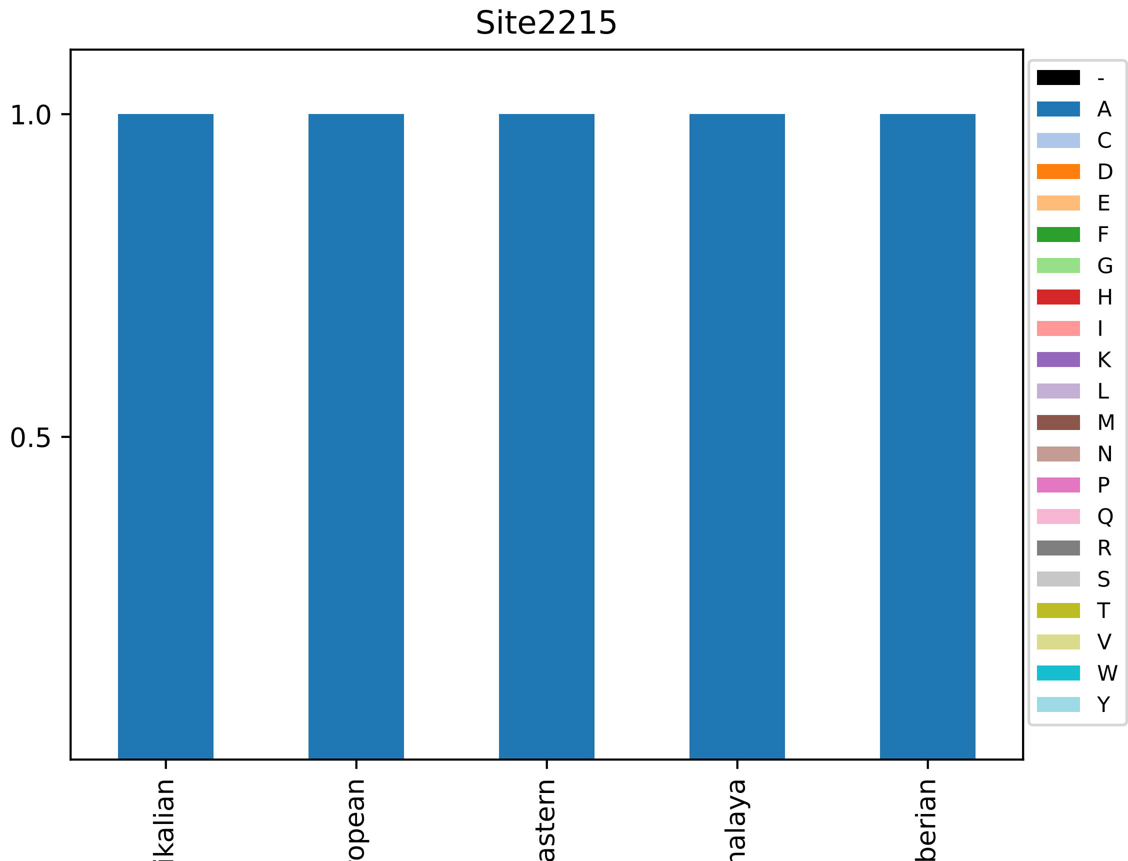

Site2215
1.0
0.5
-
A
C
D
E
F
G
H
I
K
L
M
N
P
Q
R
S
T
V
W
Y
ikalian
opean
astern
nalaya
berian

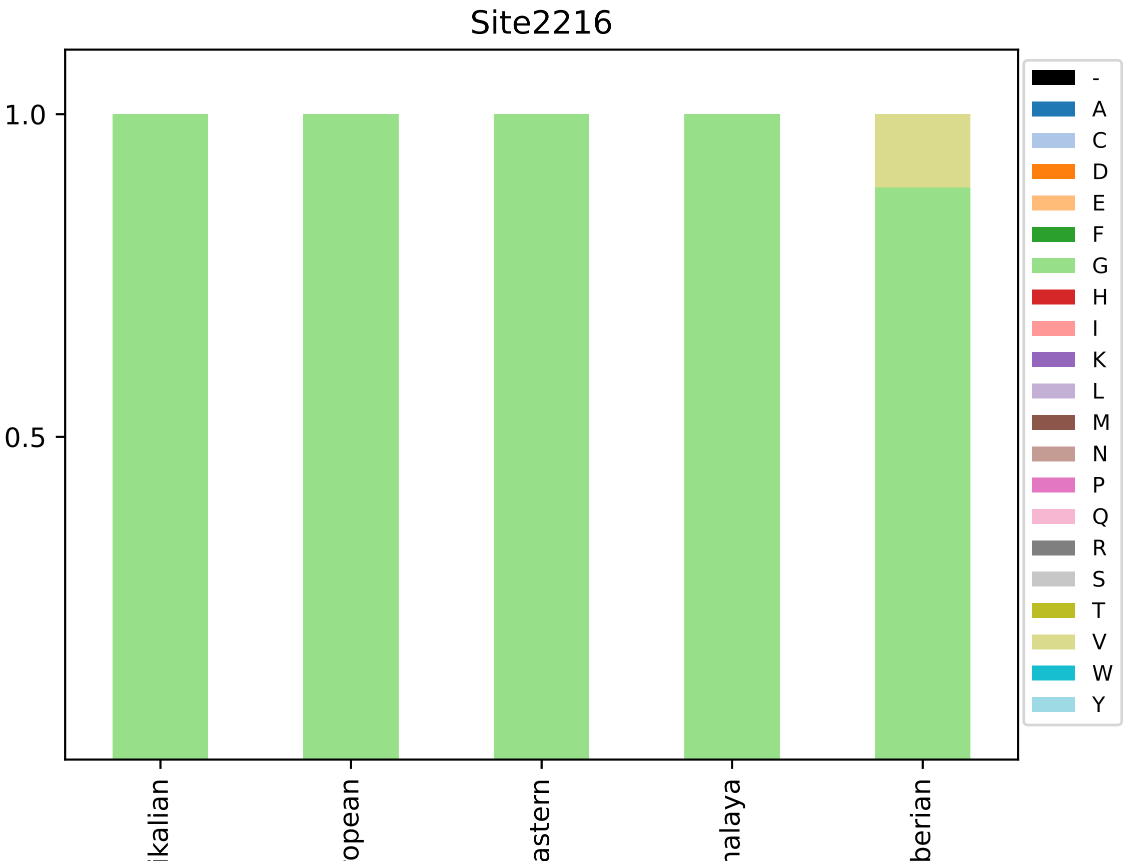
Site2216
1.0
0.5
-
A
C
D
E
F
G
H
I
K
L
M
N
P
Q
R
S
T
V
W
Y
ikalian
opean
astern
nalaya
berian

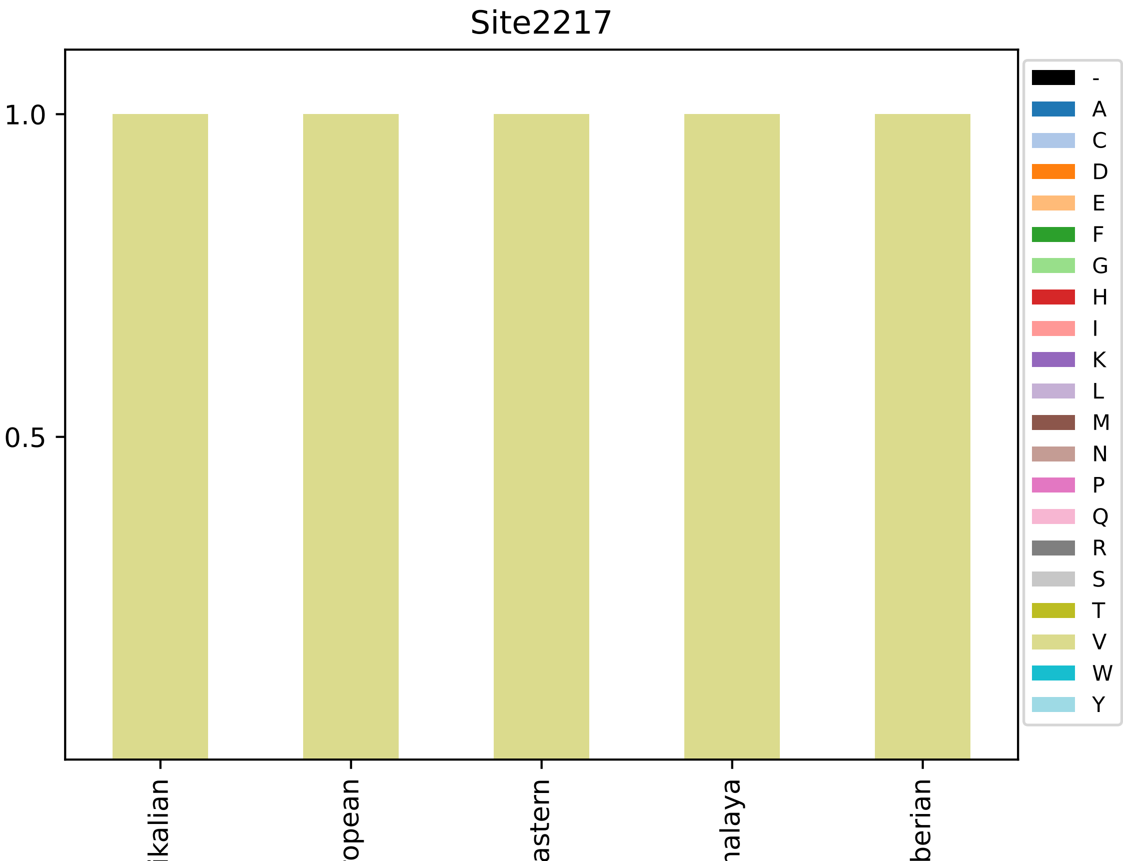
Site2217
1.0
0.5
-
A
C
D
E
F
G
H
I
K
L
M
N
P
Q
R
S
T
V
W
Y

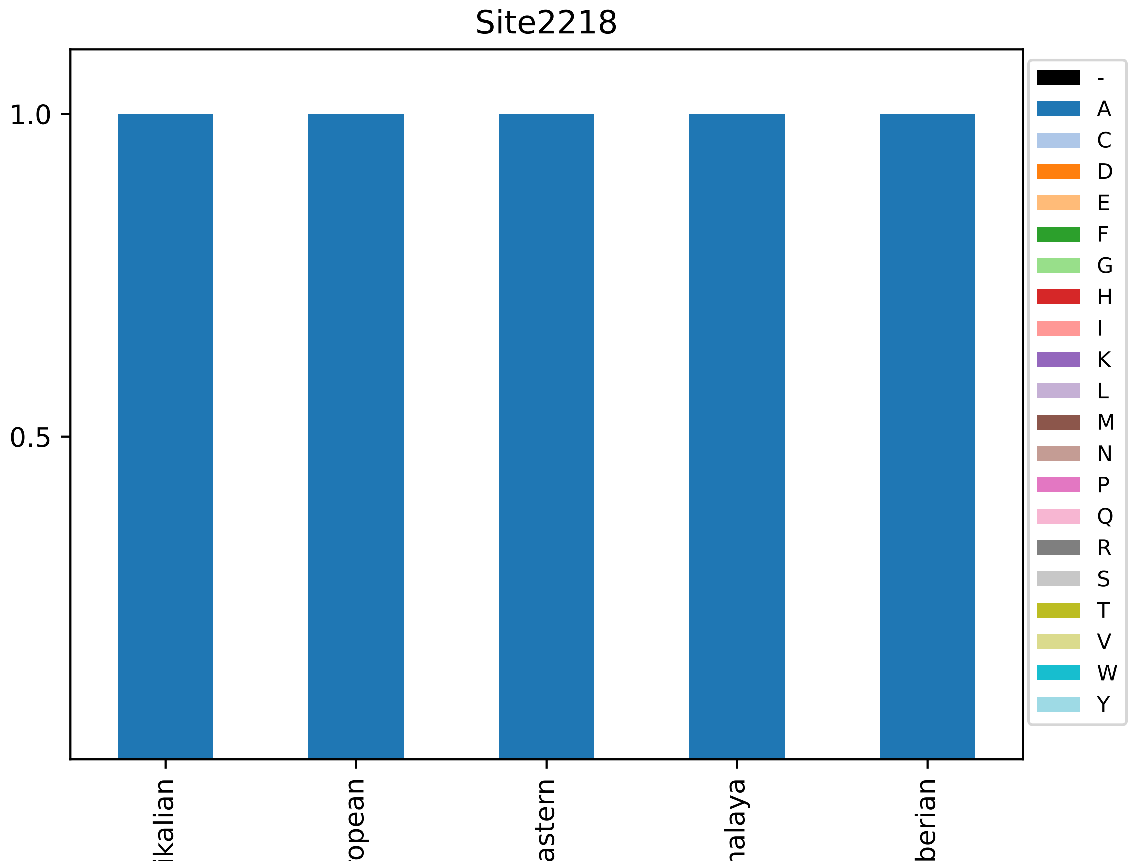
Site2218
1.0
0.5
-
A
C
D
E
F
G
H
I
K
L
M
N
P
Q
R
S
T
V
W
Y
ikalian
opean
astern
nalaya
berian

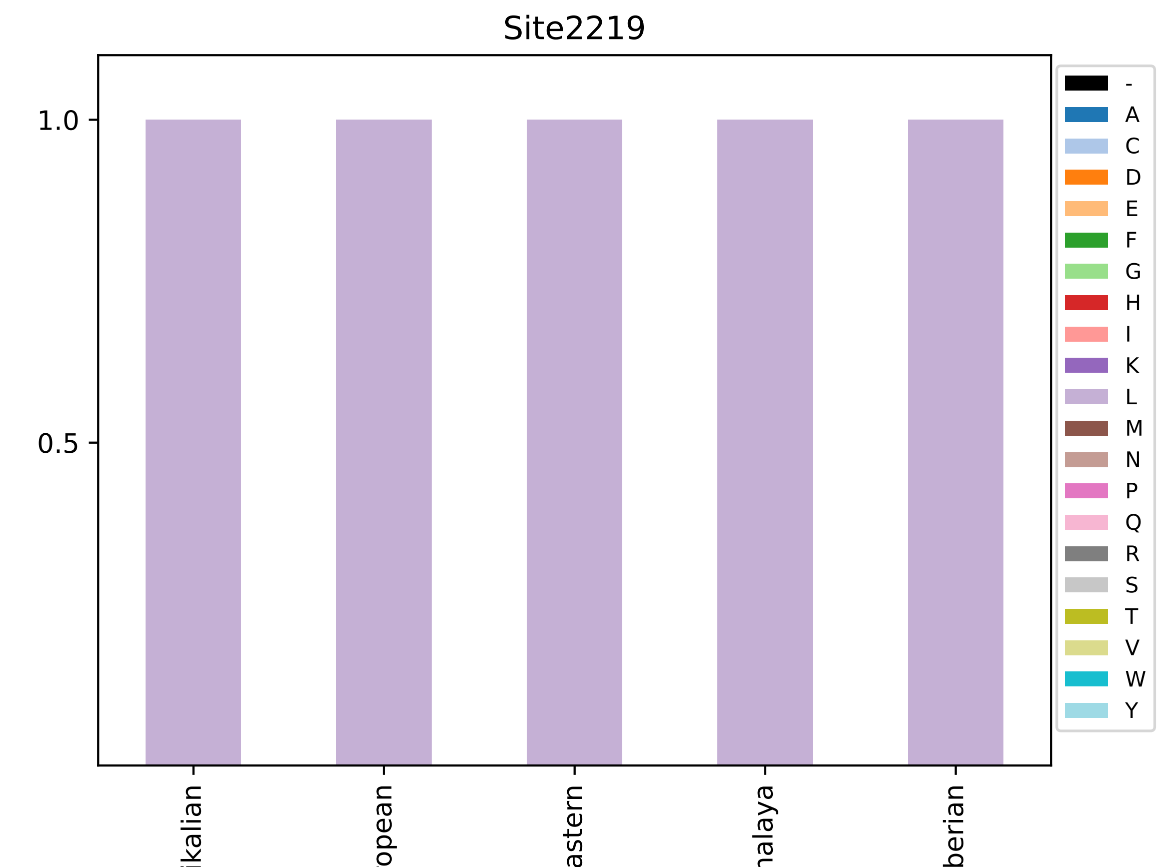

Site2219
1.0
0.5
-
A
C
D
E
F
G
H
I
K
L
M
N
P
Q
R
S
T
V
W
Y
ikalian
opean
astern
nalaya
berian

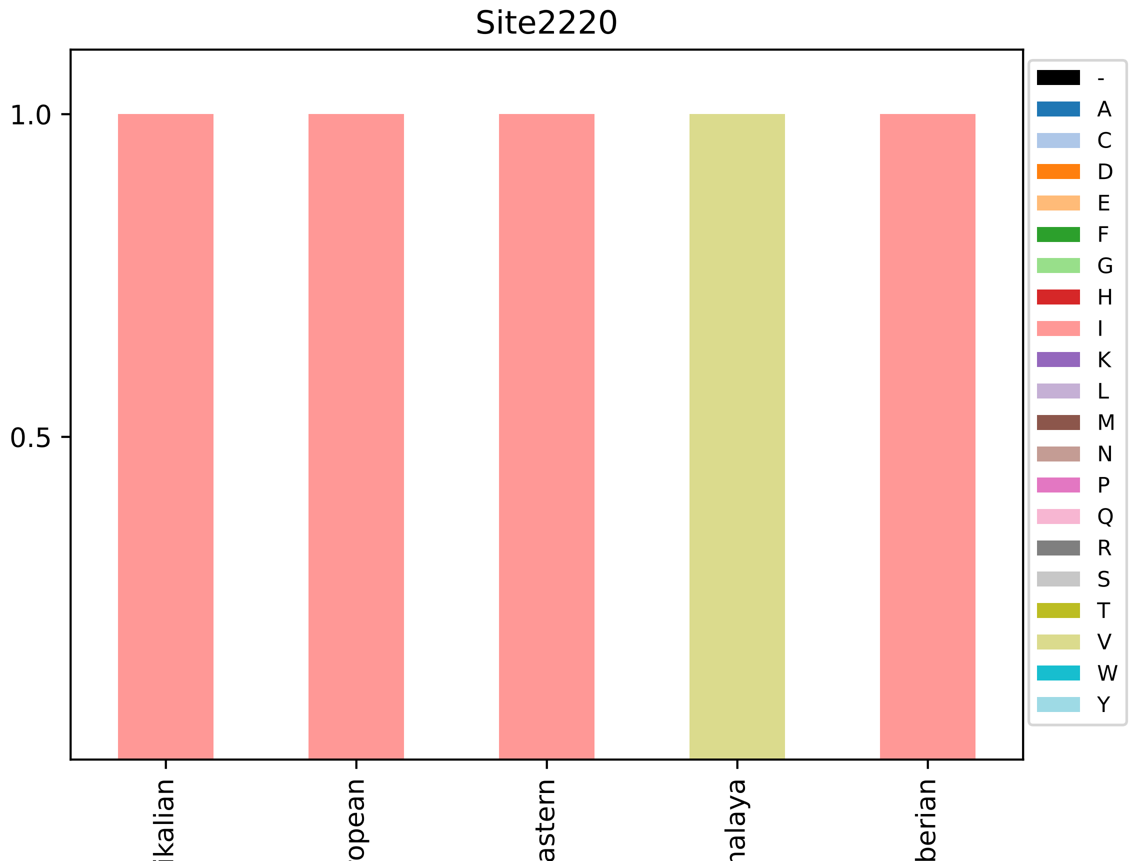

Site2220
1.0
0.5
-
A
C
D
E
F
G
H
I
K
L
M
N
P
Q
R
S
T
V
W
Y

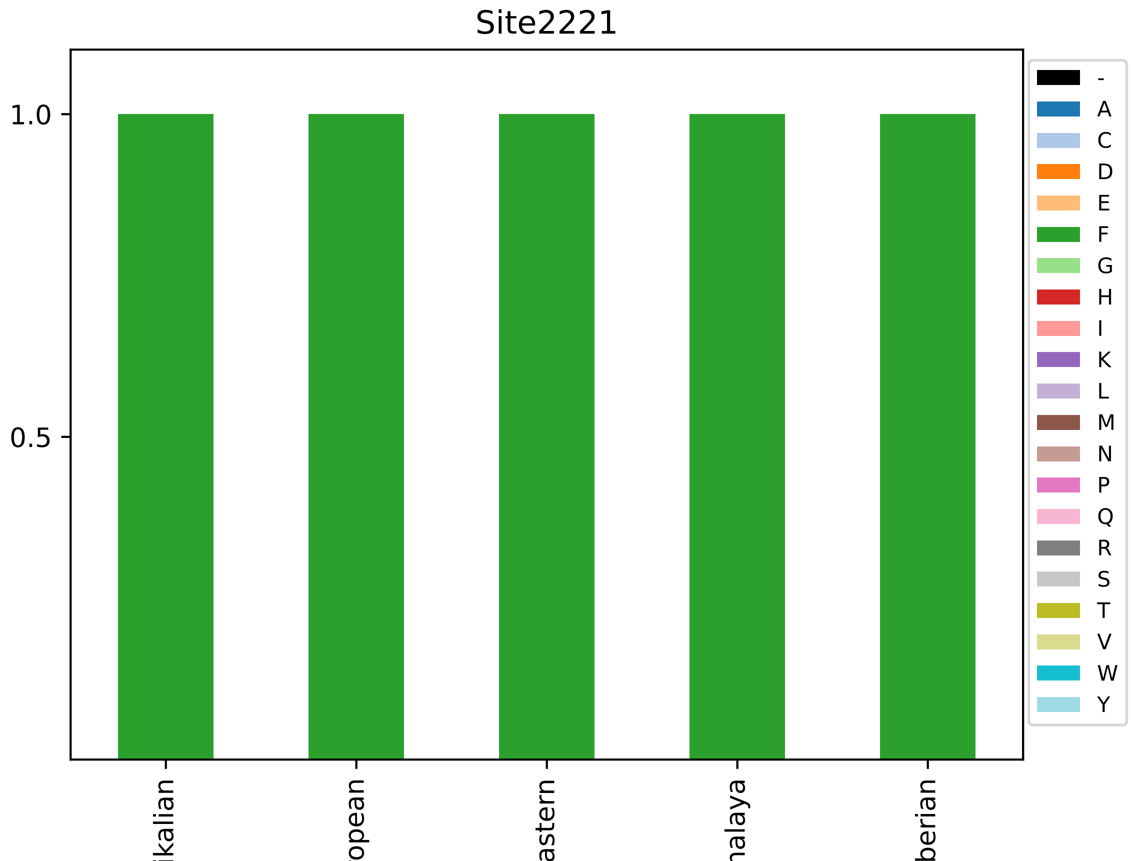

Site2221
1.0
0.5
-
A
C
D
E
F
G
H
I
K
L
M
N
P
Q
R
S
T
V
W
Y
ikalian
opean
astern
nalaya
berian

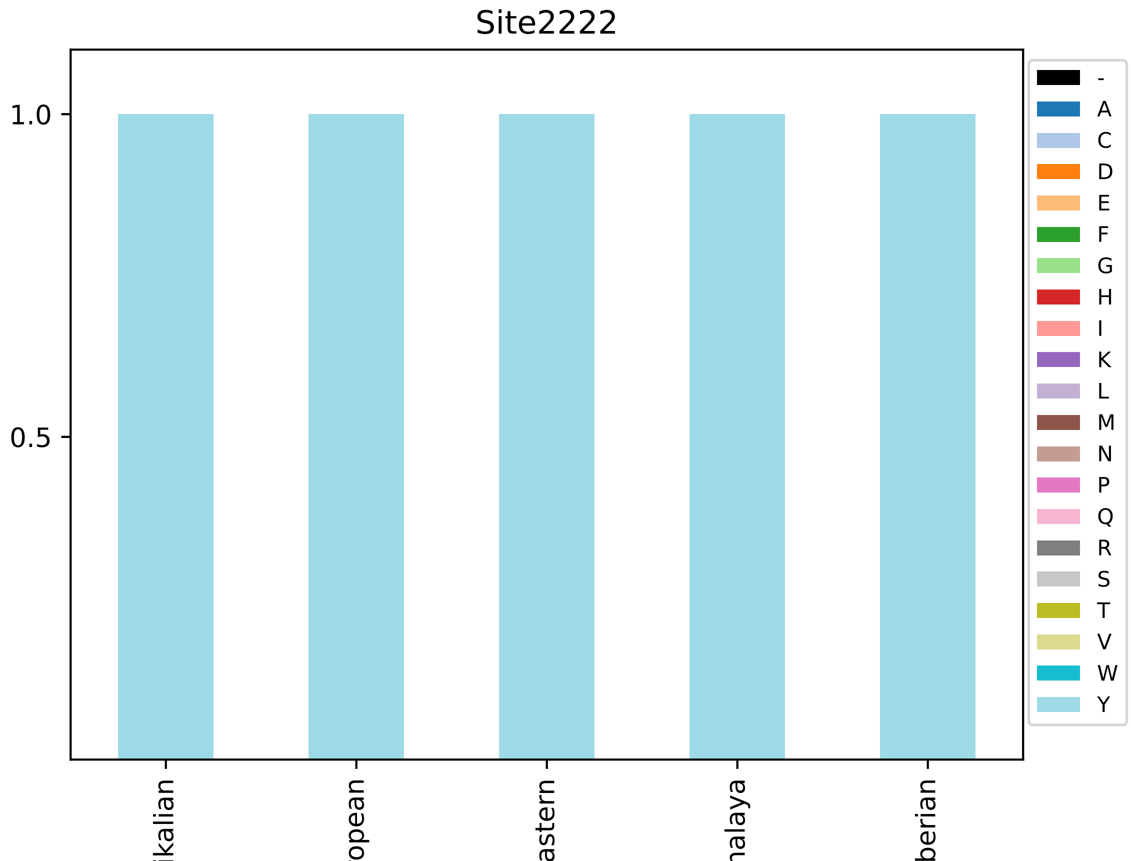

Site2222
1.0
0.5
-
A
C
D
E
F
G
H
I
K
L
M
N
P
Q
R
S
T
V
W
Y
ikalian
opean
astern
nalaya
berian

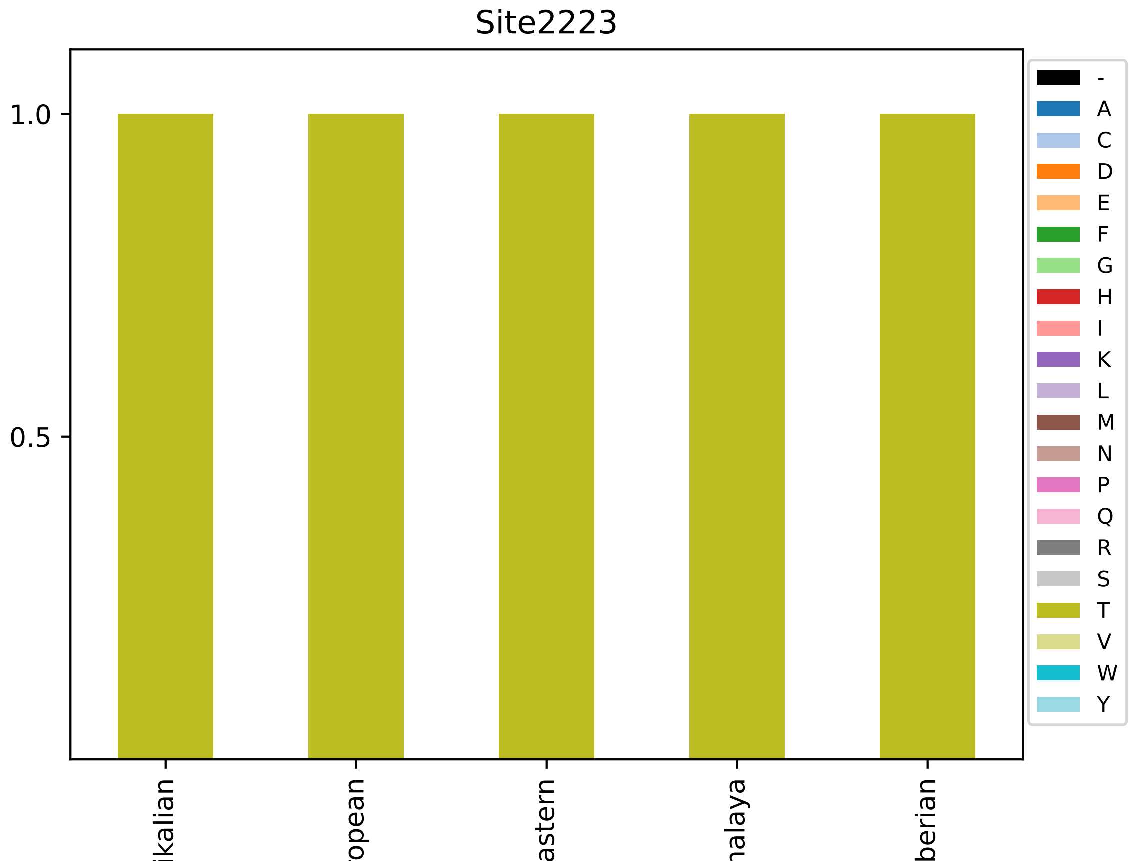

Site2223
1.0
0.5
ikalian
opean
astern
nalaya
berian
-
A
C
D
E
F
G
H
I
K
L
M
N
P
Q
R
S
T
V
W
Y

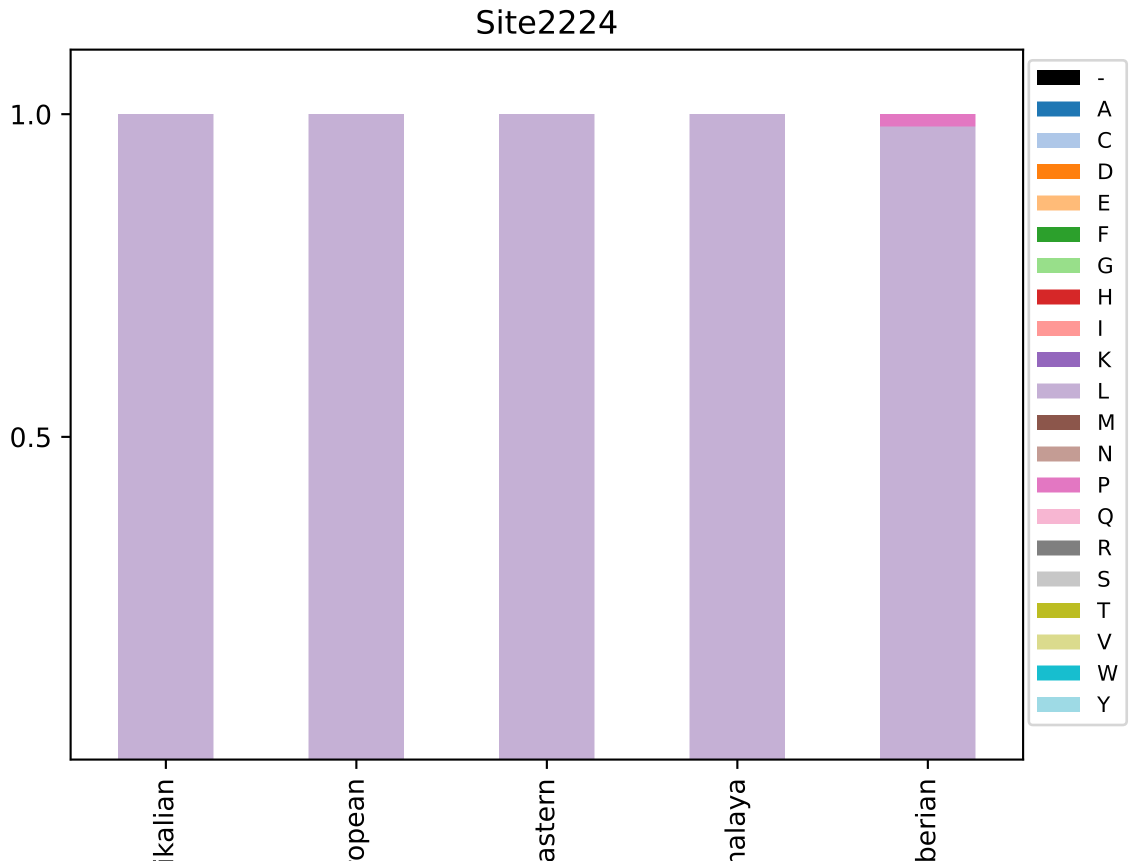
Site2224
1.0
0.5
-
A
C
D
E
F
G
H
I
K
L
M
N
P
Q
R
S
T
V
W
Y
ikalian
opean
astern
nalaya
berian

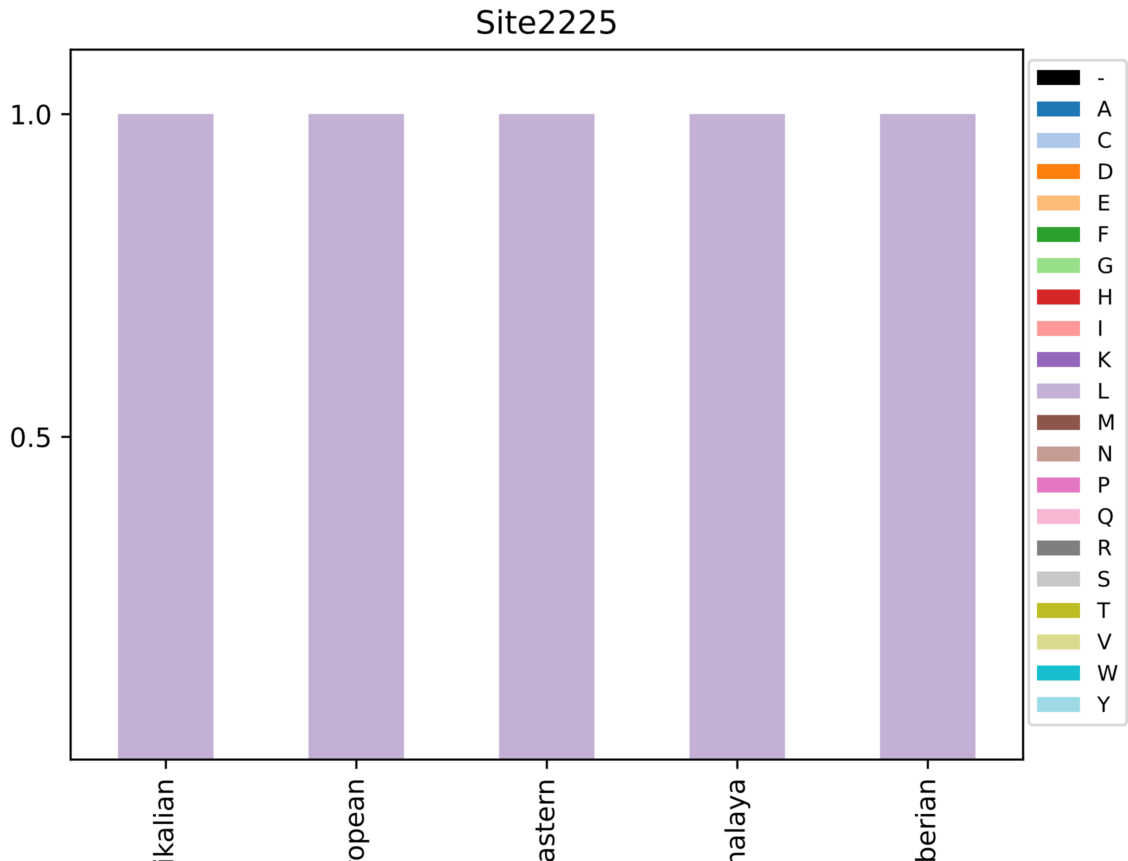

Site2225
1.0
0.5
-
A
C
D
E
F
G
H
I
K
L
M
N
P
Q
R
S
T
V
W
Y
ikalian
opean
astern
nalaya
berian

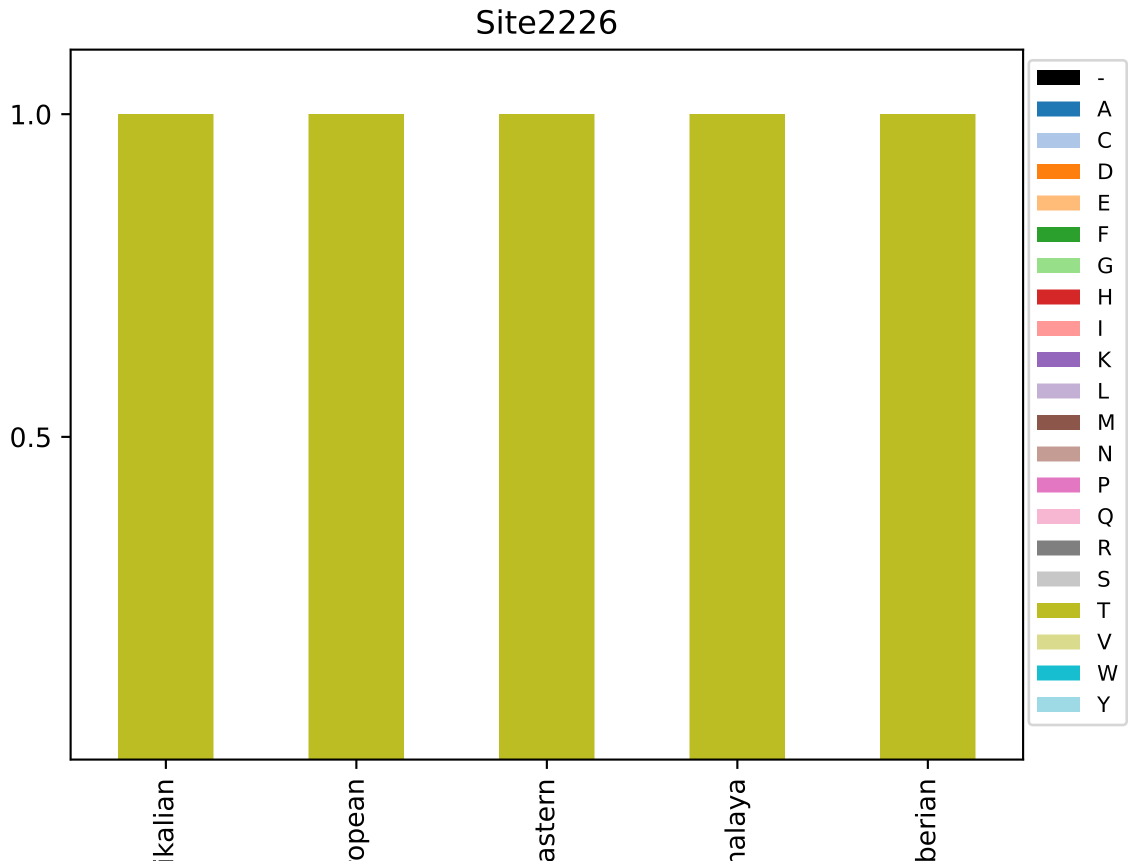

Site2226
1.0
0.5
-
A
C
D
E
F
G
H
I
K
L
M
N
P
Q
R
S
T
V
W
Y
ikalian
opean
astern
nalaya
berian

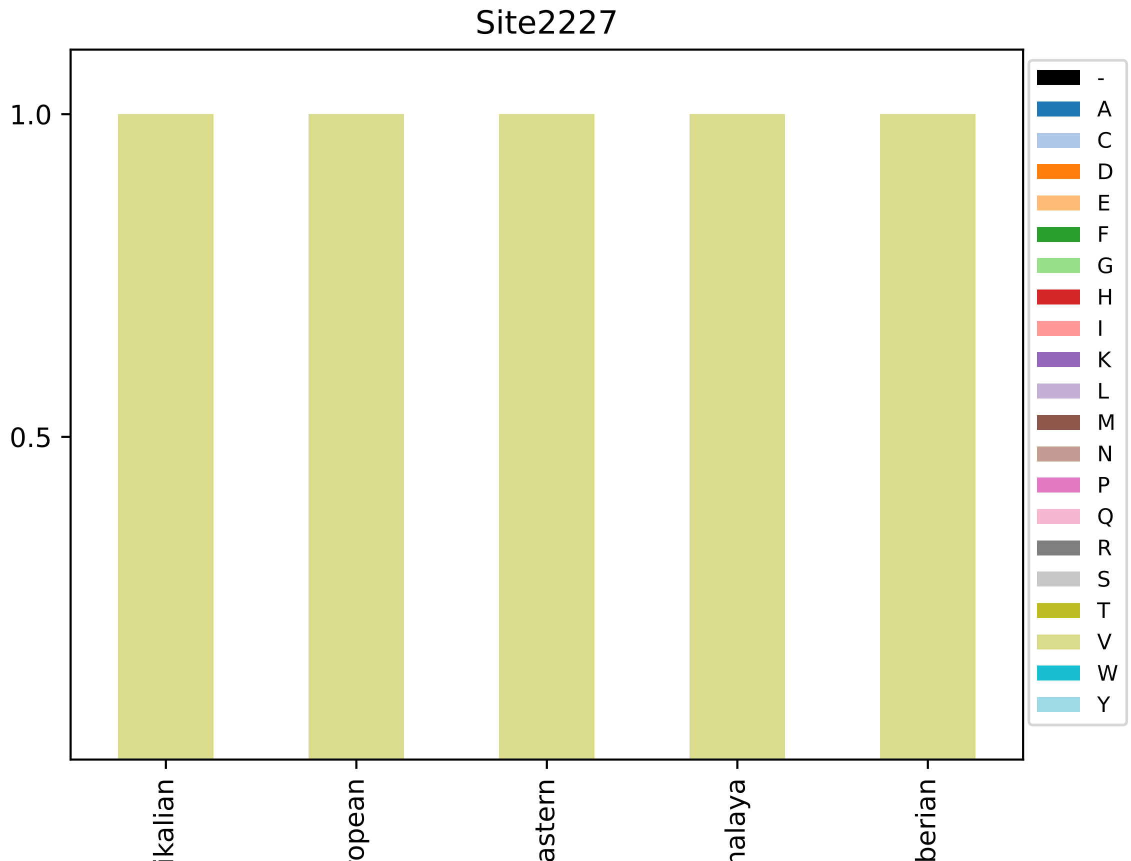

Site2227
1.0
0.5
-
A
C
D
E
F
G
H
I
K
L
M
N
P
Q
R
S
T
V
W
Y
ikalian
opean
astern
nalaya
berian

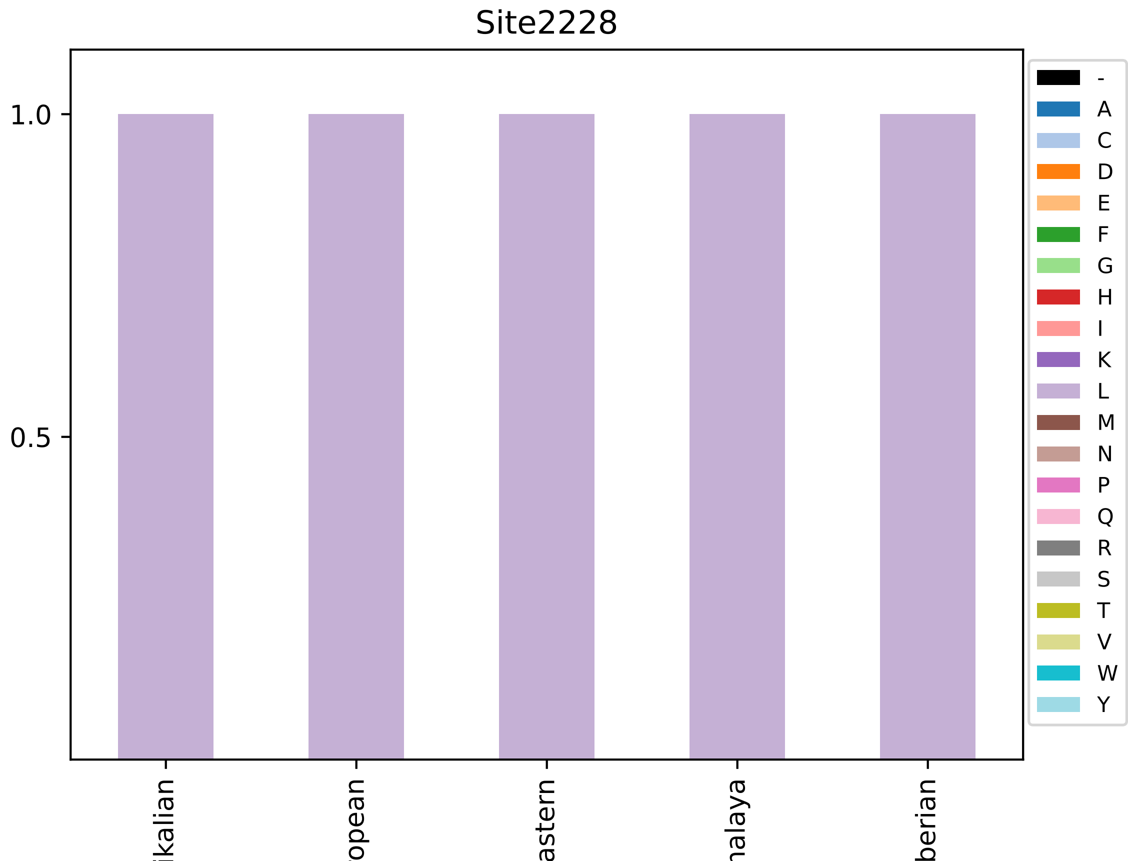

Site2228
1.0
0.5
-
A
C
D
E
F
G
H
I
K
L
M
N
P
Q
R
S
T
V
W
Y
ikalian
opean
astern
nalaya
berian

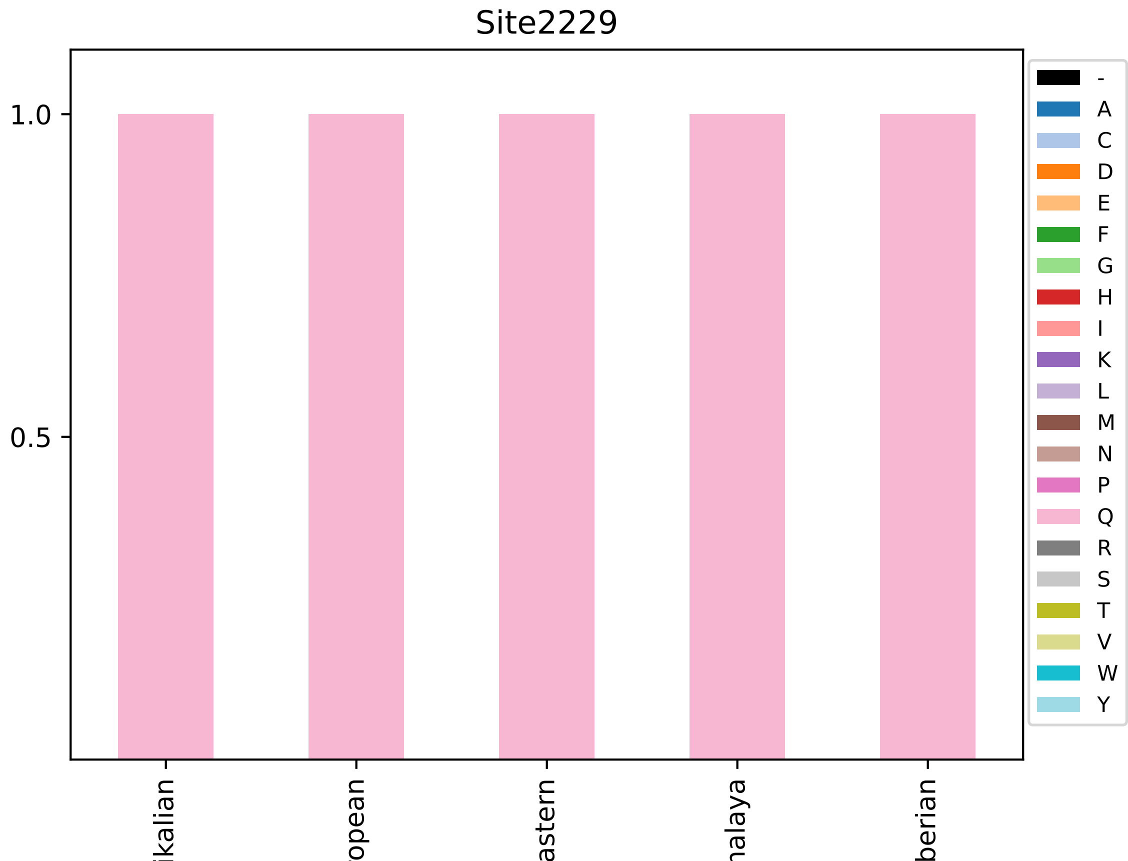
Site2229
1.0
0.5
-
A
C
D
E
F
G
H
I
K
L
M
N
P
Q
R
S
T
V
W
Y
ikalian
opean
astern
nalaya
berian

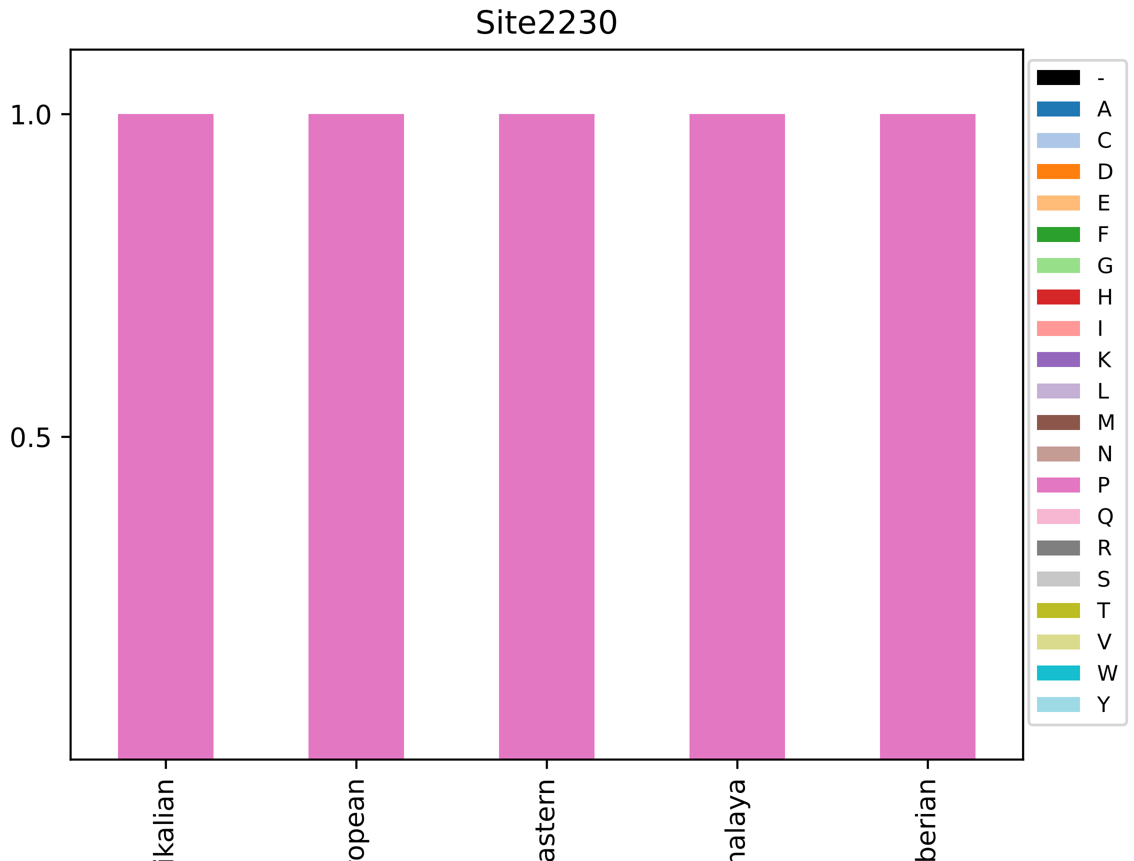
Site2230
1.0
0.5
-
A
C
D
E
F
G
H
I
K
L
M
N
P
Q
R
S
T
V
W
Y
ikalian
opean
astern
nalaya
berian

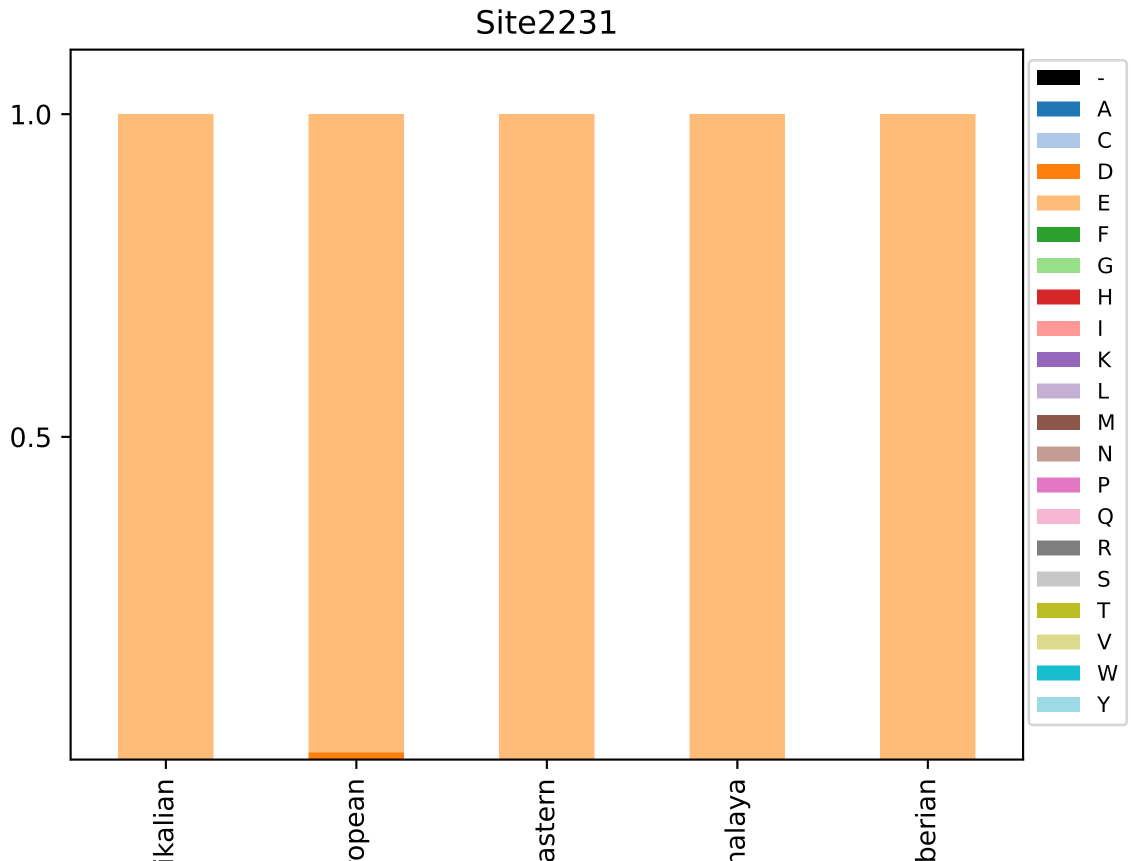
Site2231
1.0
0.5
-
A
C
D
E
F
G
H
I
K
L
M
N
P
Q
R
S
T
V
W
Y
ikalian
opean
astern
nalaya
berian

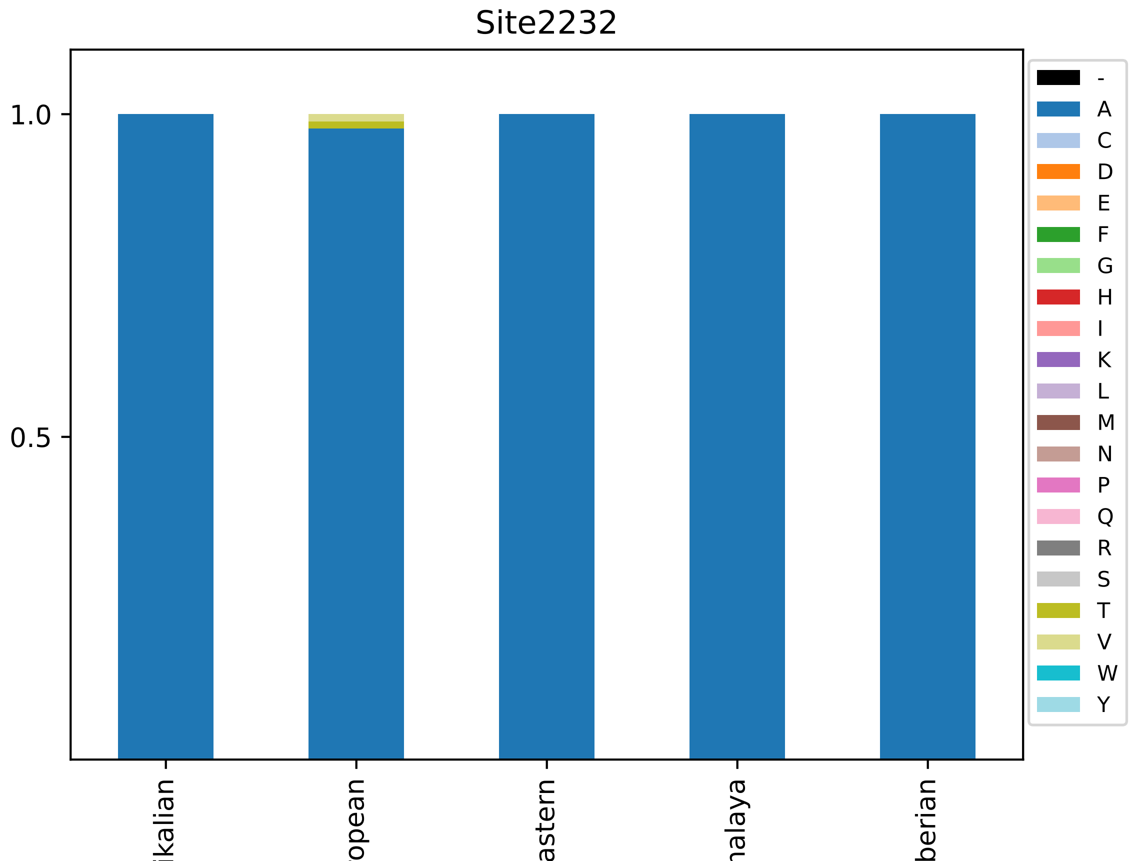
Site2232
1.0
0.5
ikalian
opean
astern
nalaya
berian
-
A
C
D
E
F
G
H
I
K
L
M
N
P
Q
R
S
T
V
W
Y

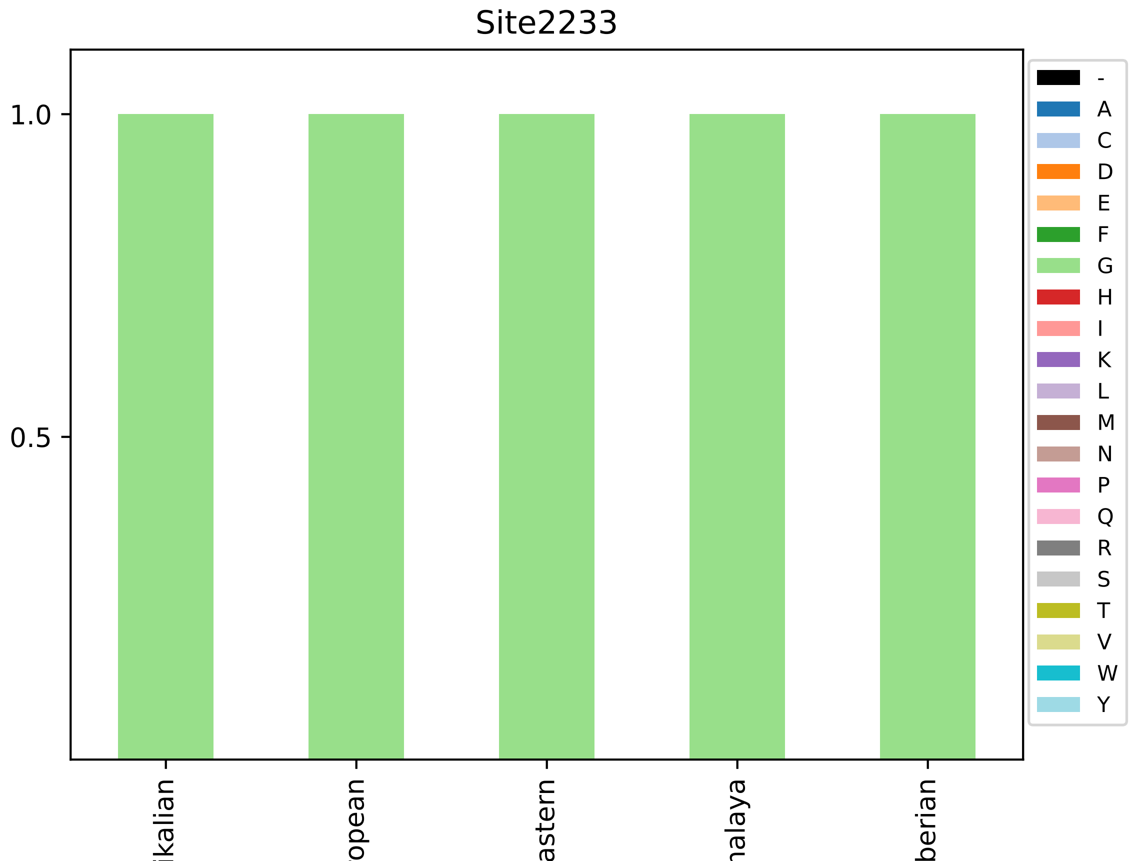
Site2233
1.0
0.5
-
A
C
D
E
F
G
H
I
K
L
M
N
P
Q
R
S
T
V
W
Y
ikalian
opean
astern
alaya
berian

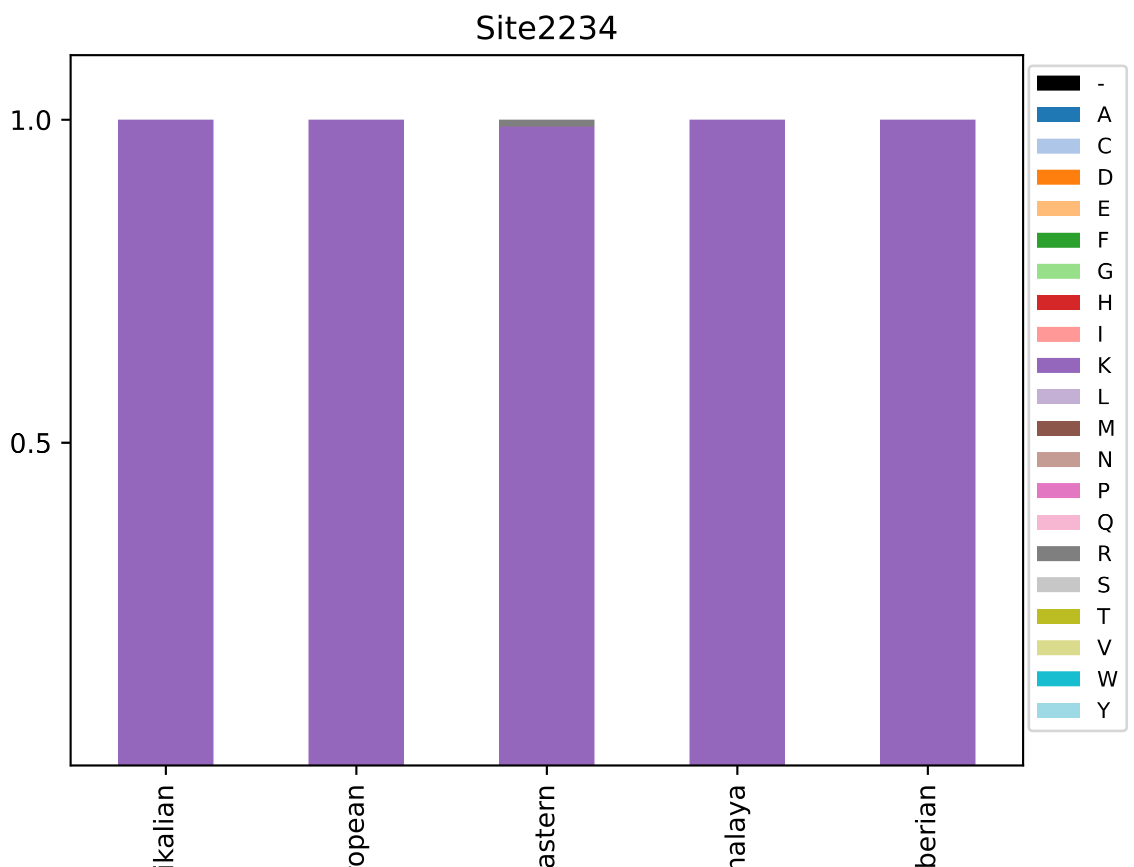

Site2234
1.0
0.5
-
A
C
D
E
F
G
H
I
K
L
M
N
P
Q
R
S
T
V
W
Y
ikalian
opean
astern
nalaya
berian

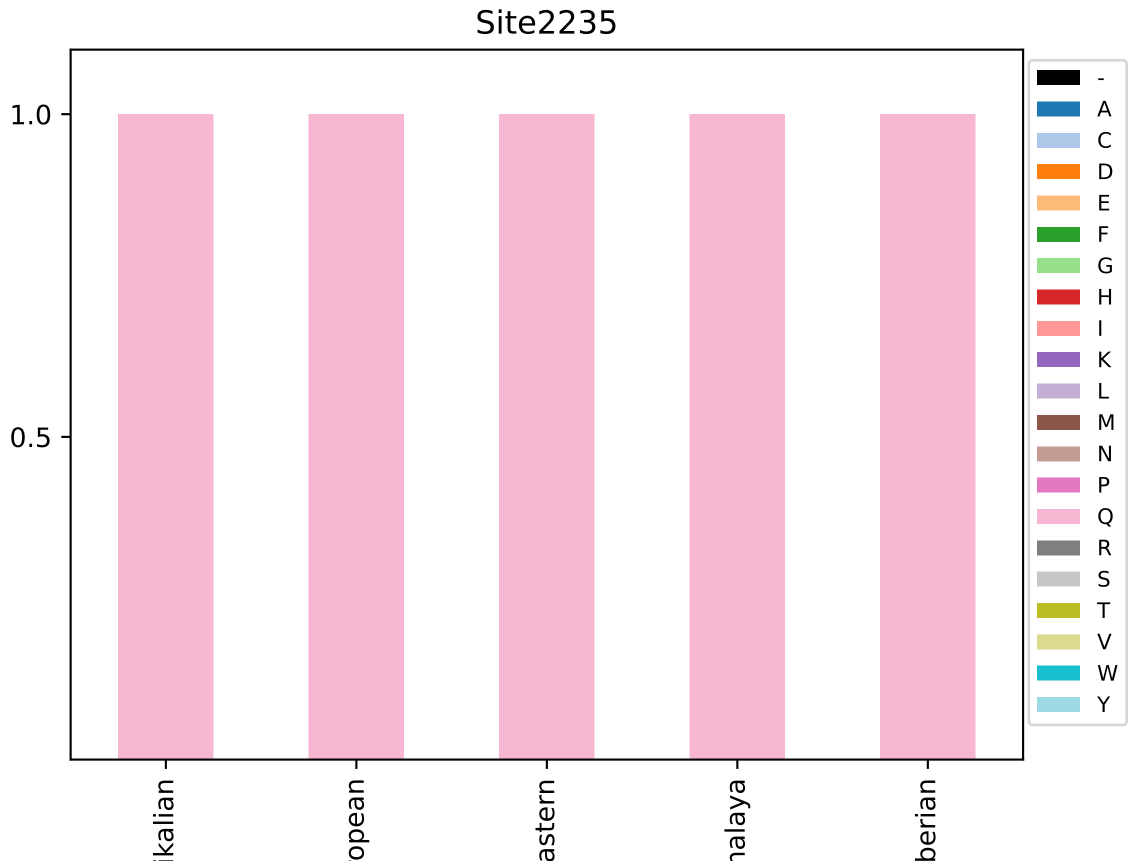
Site2235
1.0
0.5
-
A
C
D
E
F
G
H
I
K
L
M
N
P
Q
R
S
T
V
W
Y
ikalian
opean
astern
nalaya
berian

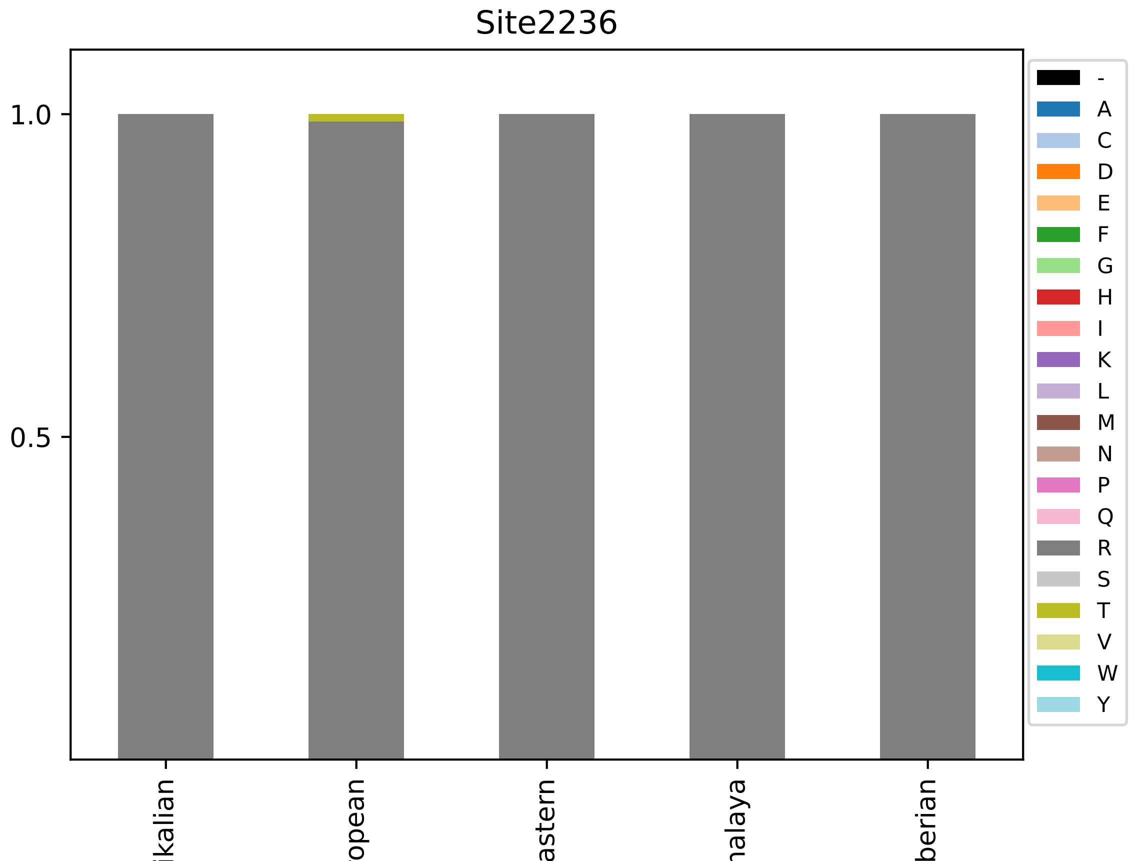

Site2236
1.0
0.5
-
A
C
D
E
F
G
H
I
K
L
M
N
P
Q
R
S
T
V
W
Y
ikalian
opean
astern
nalaya
berian

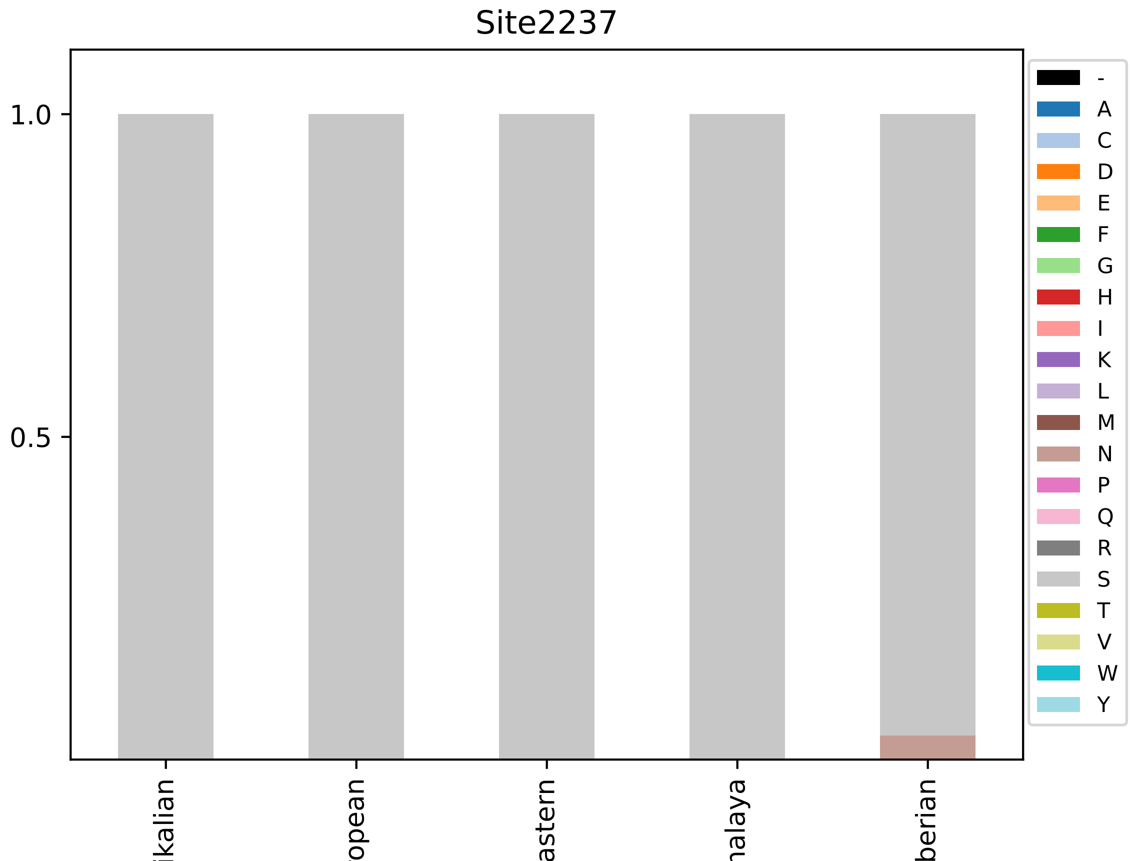

Site2237
1.0
0.5
-
A
C
D
E
F
G
H
I
K
L
M
N
P
Q
R
S
T
V
W
Y
ikalian
opean
astern
nalaya
berian

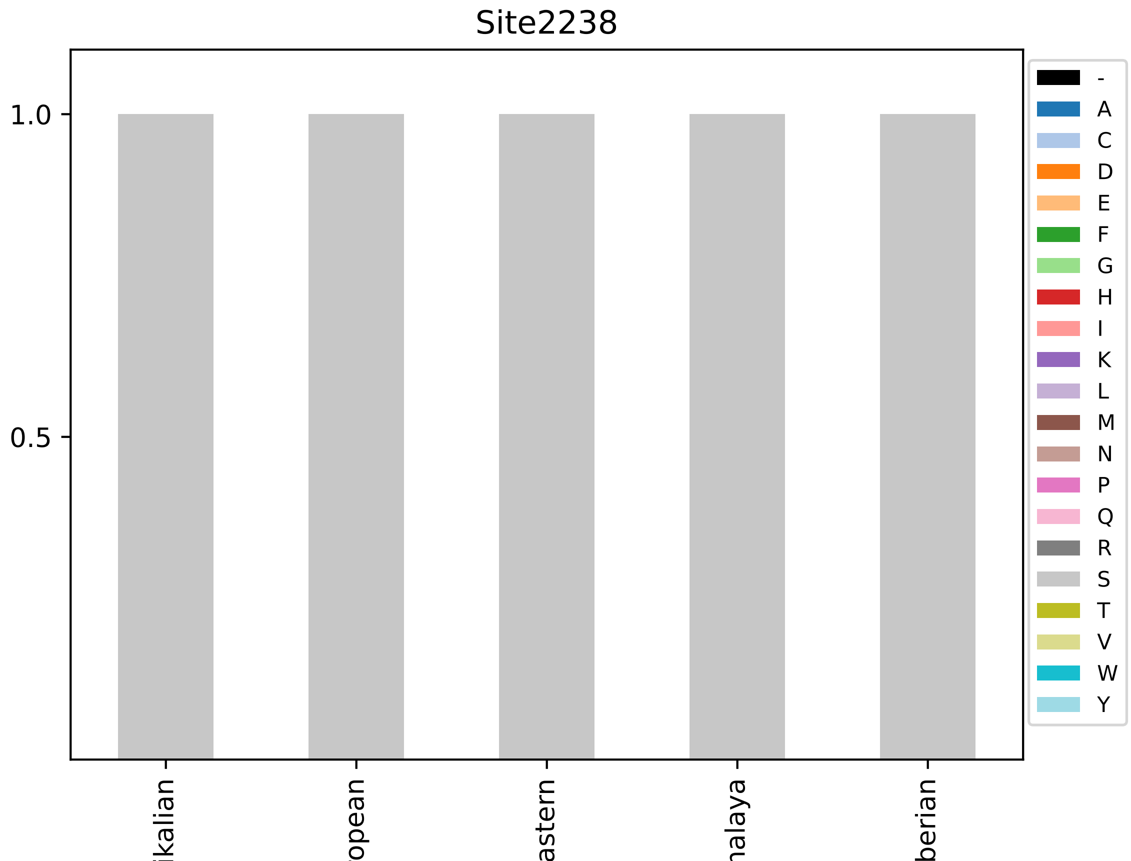

Site2238
1.0
0.5
-
A
C
D
E
F
G
H
I
K
L
M
N
P
Q
R
S
T
V
W
Y
ikalian
opean
astern
nalaya
berian

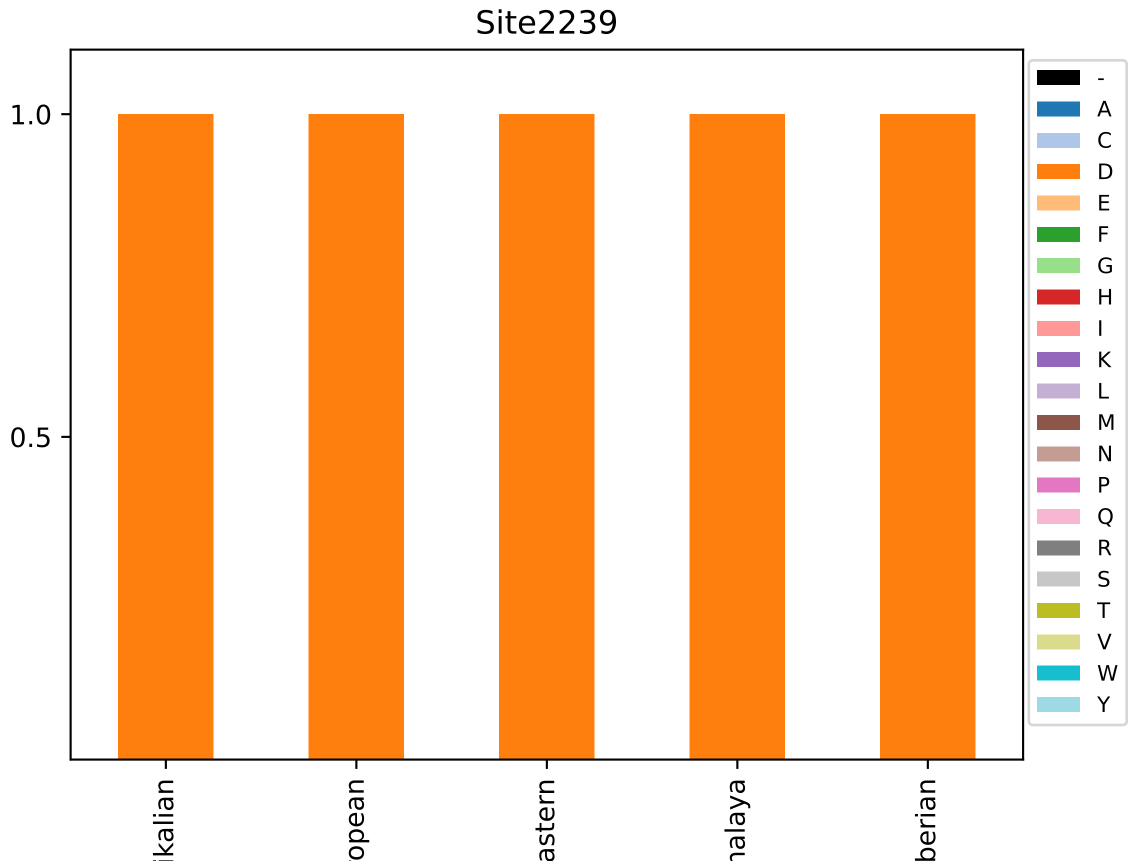

Site2239
1.0
0.5
ikalian
opean
astern
nalaya
berian
-
A
C
D
E
F
G
H
I
K
L
M
N
P
Q
R
S
T
V
W
Y

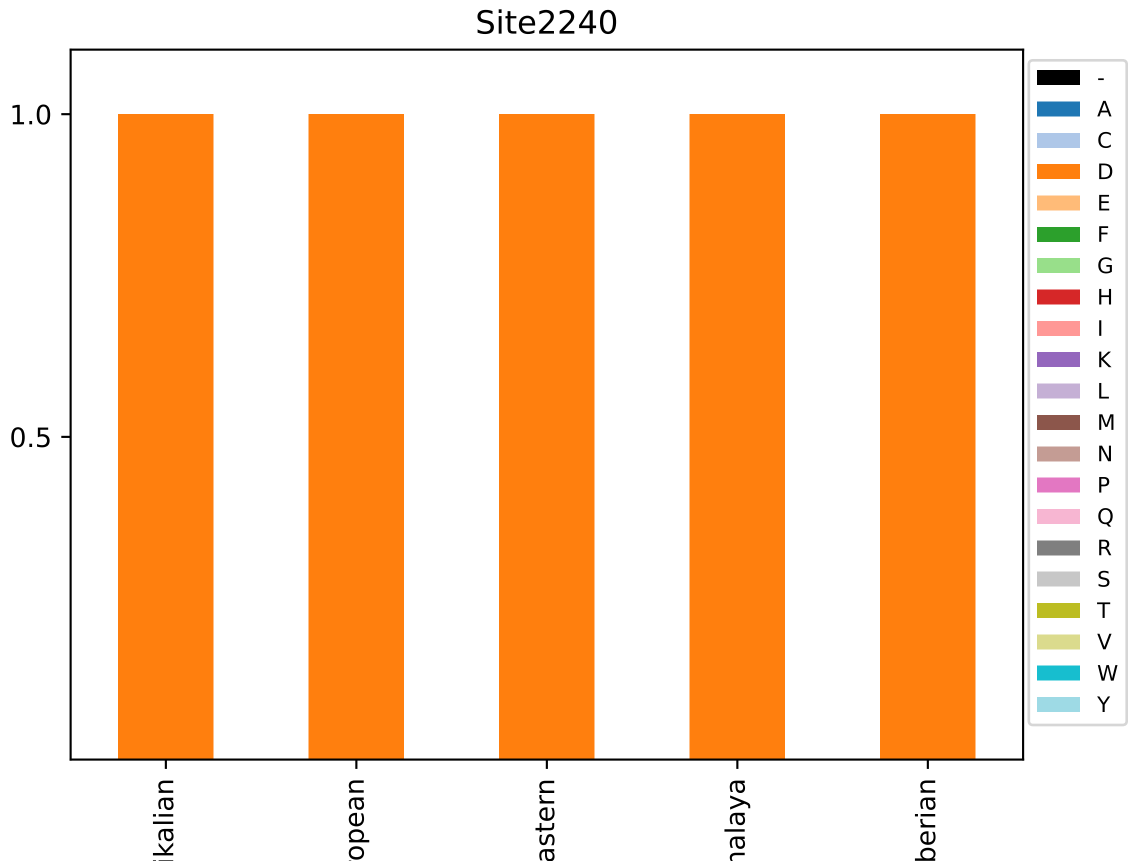

Site2240
1.0
0.5
-
A
C
D
E
F
G
H
I
K
L
M
N
P
Q
R
S
T
V
W
Y
ikalian
opean
astern
nalaya
berian

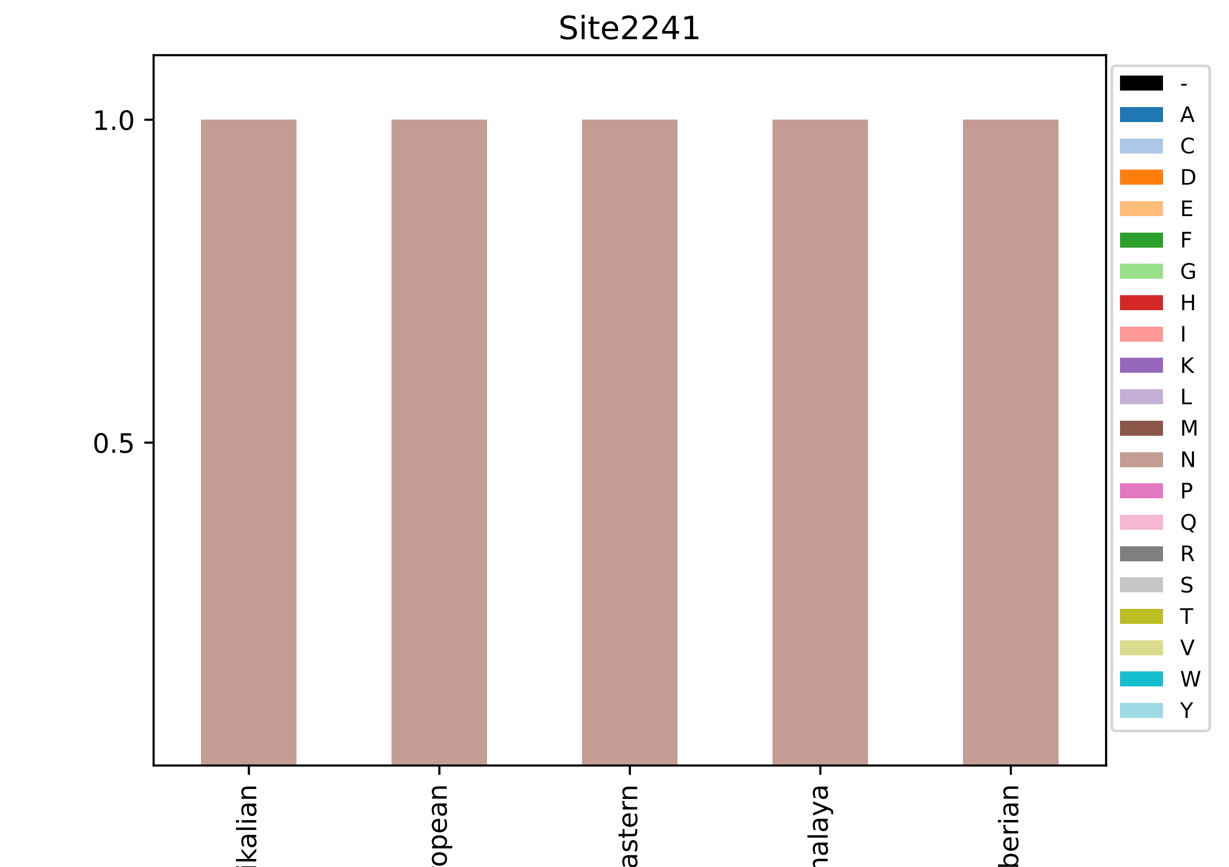
Site2241
1.0
0.5
-
A
C
D
E
F
G
H
I
K
L
M
N
P
Q
R
S
T
V
W
Y
ikalian
opean
astern
nalaya
berian

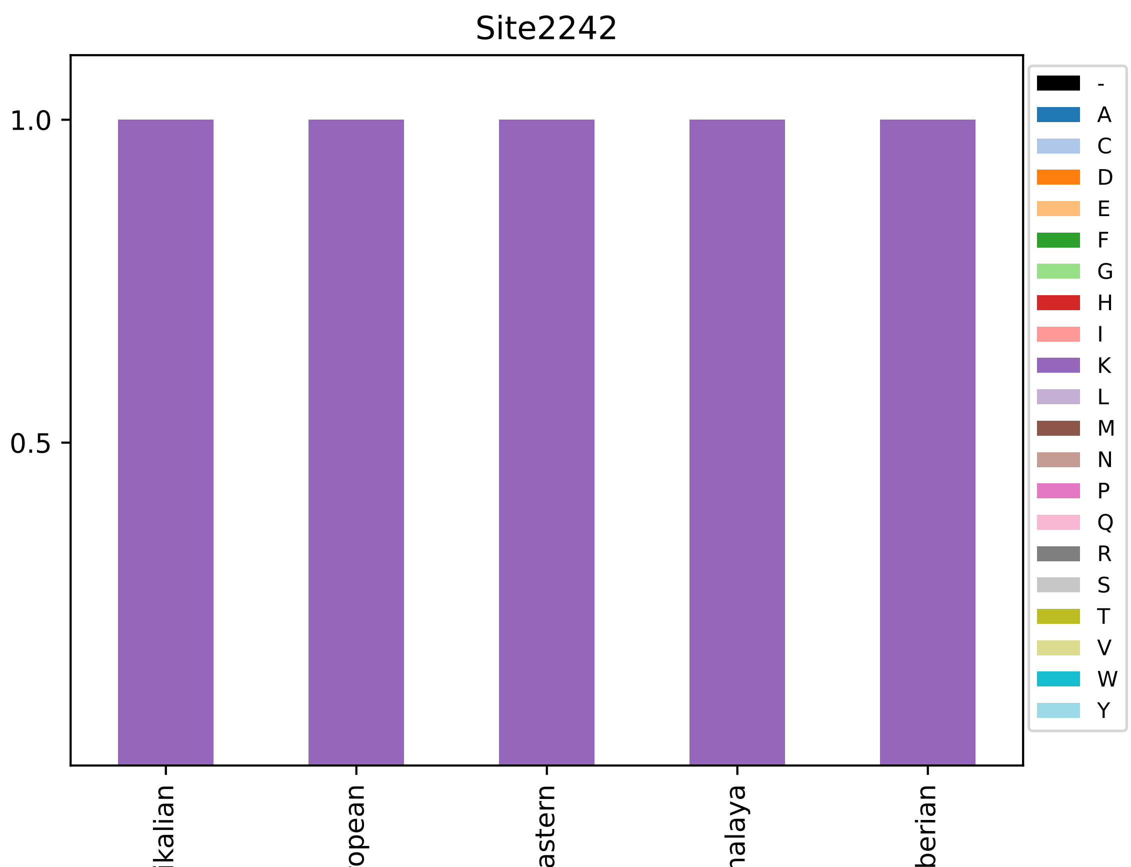

Site2242
1.0
0.5
-
A
C
D
E
F
G
H
I
K
L
M
N
P
Q
R
S
T
V
W
Y
ikalian
opean
astern
nalaya
berian

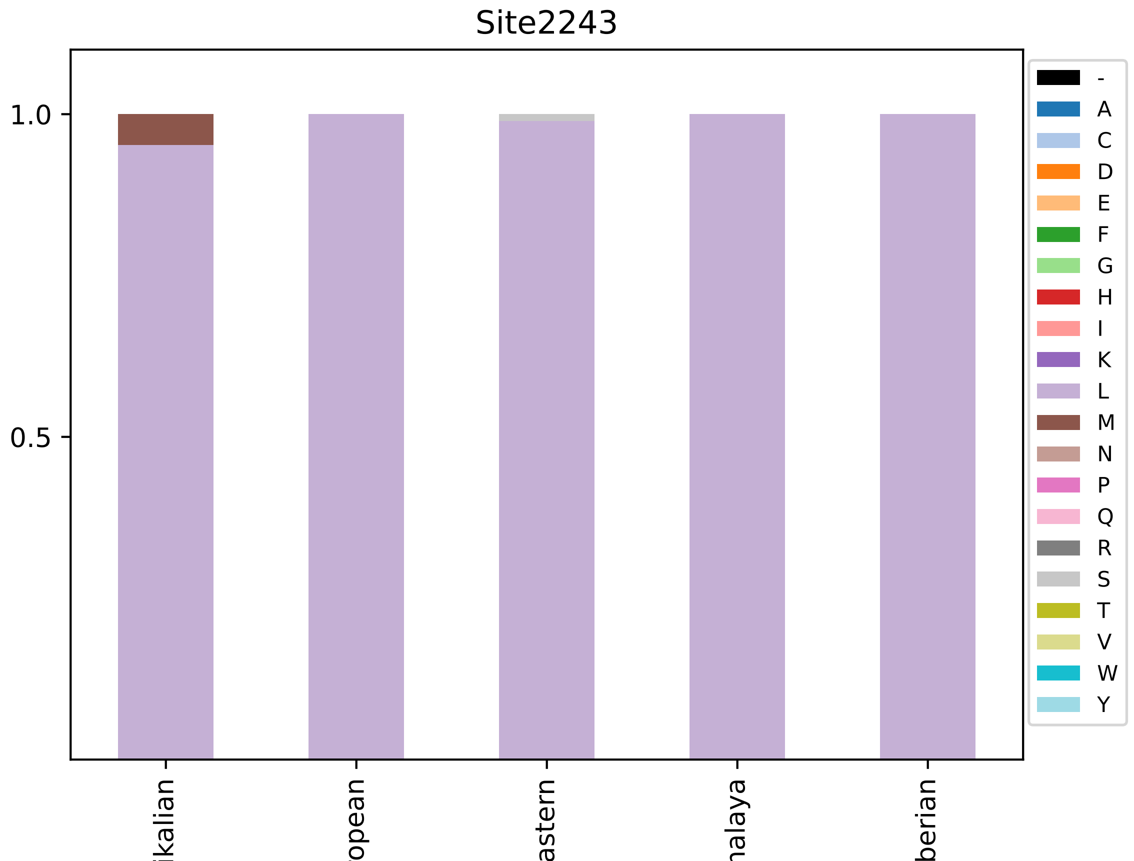
Site2243
1.0
0.5
-
A
C
D
E
F
G
H
I
K
L
M
N
P
Q
R
S
T
V
W
Y
ikalian
opean
astern
nalaya
berian

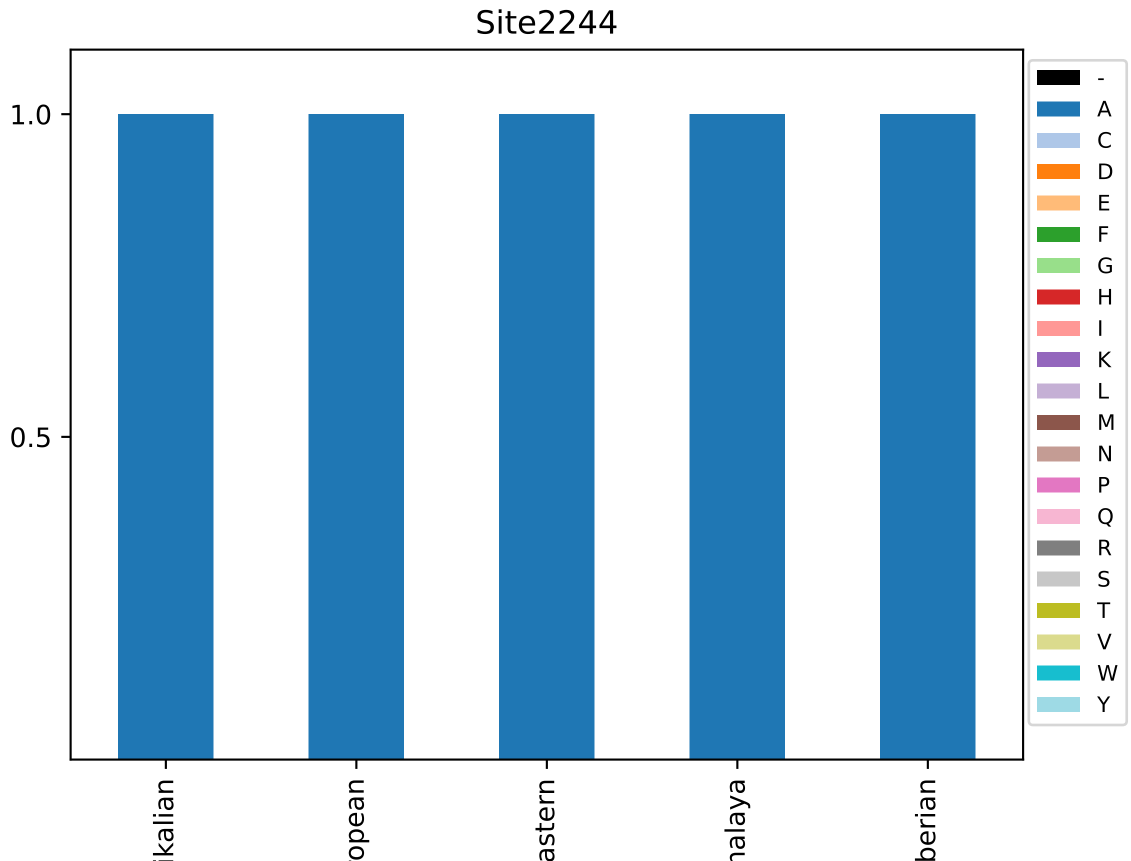
Site2244
1.0
0.5
ikalian
opean
astern
nalaya
berian
-
A
C
D
E
F
G
H
I
K
L
M
N
P
Q
R
S
T
V
W
Y

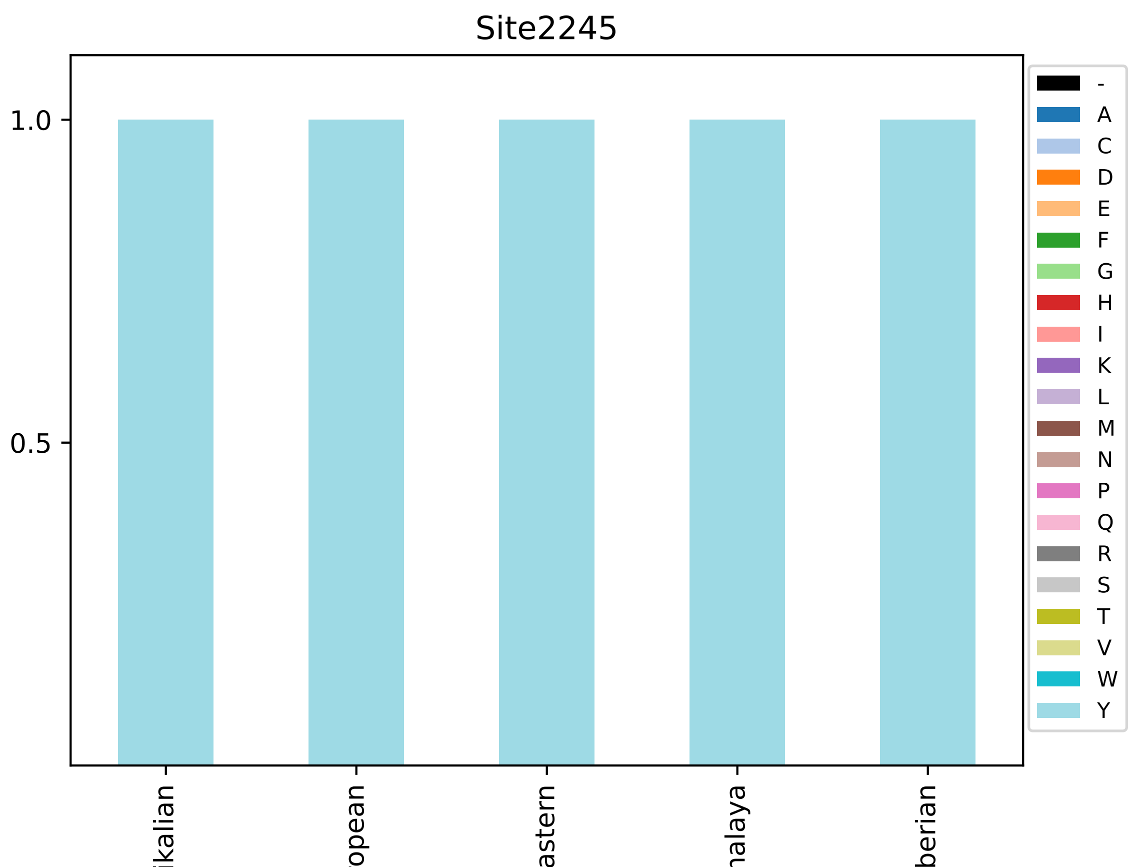

Site2245
1.0
0.5
ikalian
opean
astern
nalaya
berian
-
A
C
D
E
F
G
H
I
K
L
M
N
P
Q
R
S
T
V
W
Y

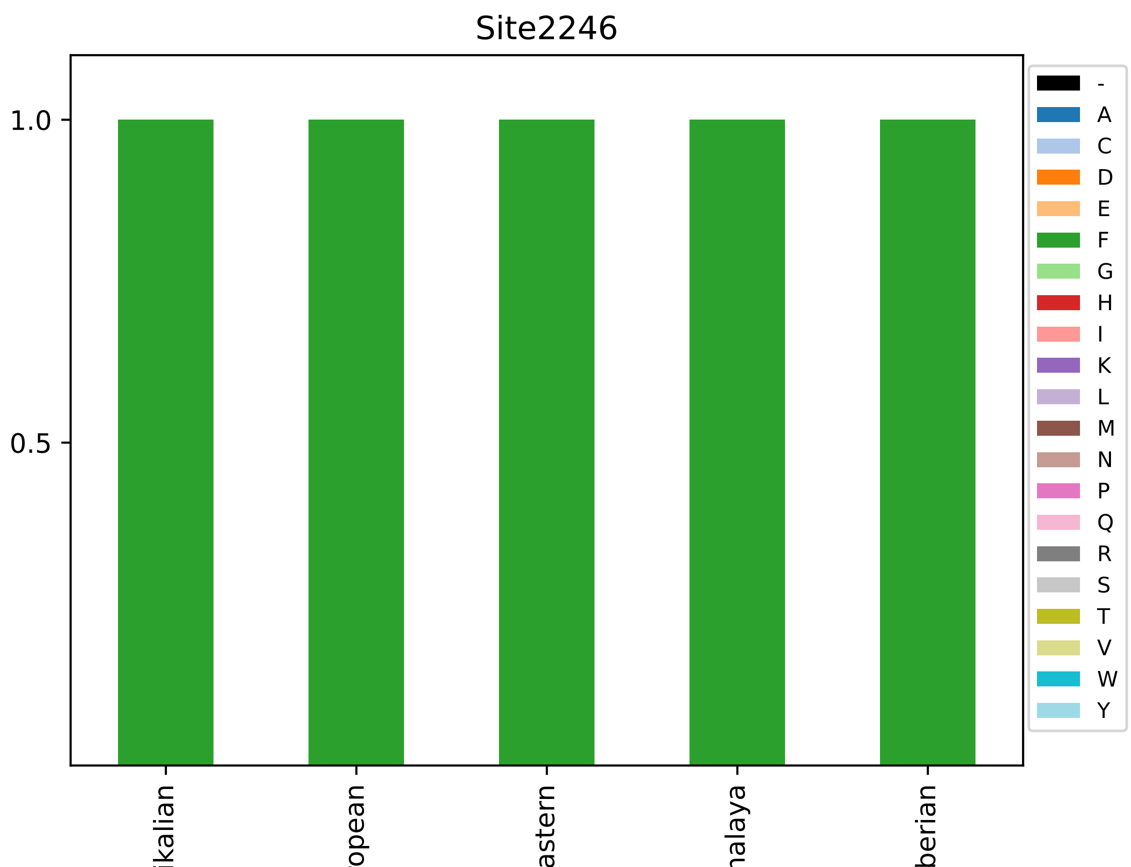

Site2246
1.0
0.5
-
A
C
D
E
F
G
H
I
K
L
M
N
P
Q
R
S
T
V
W
Y
ikalian
opean
astern
nalaya
berian

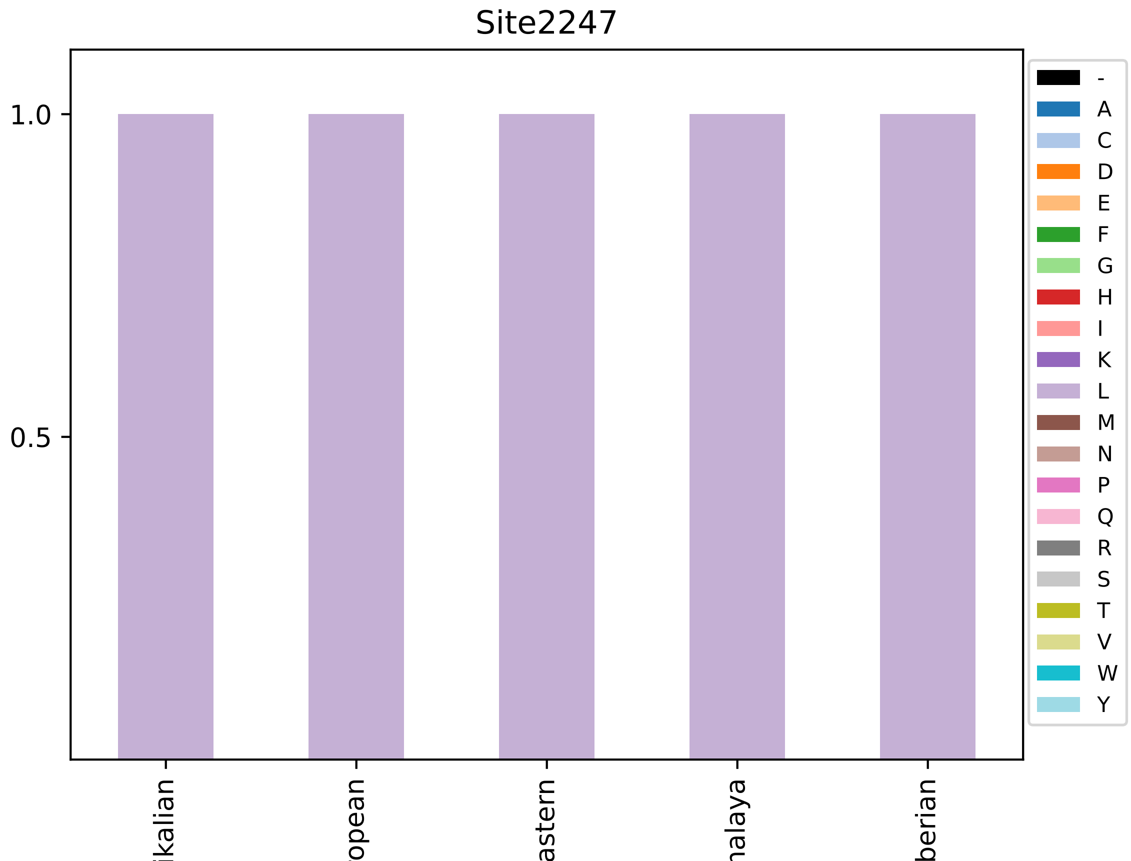

Site2247
1.0
0.5
ikalian
opean
astern
nalaya
berian
-
A
C
D
E
F
G
H
I
K
L
M
N
P
Q
R
S
T
V
W
Y

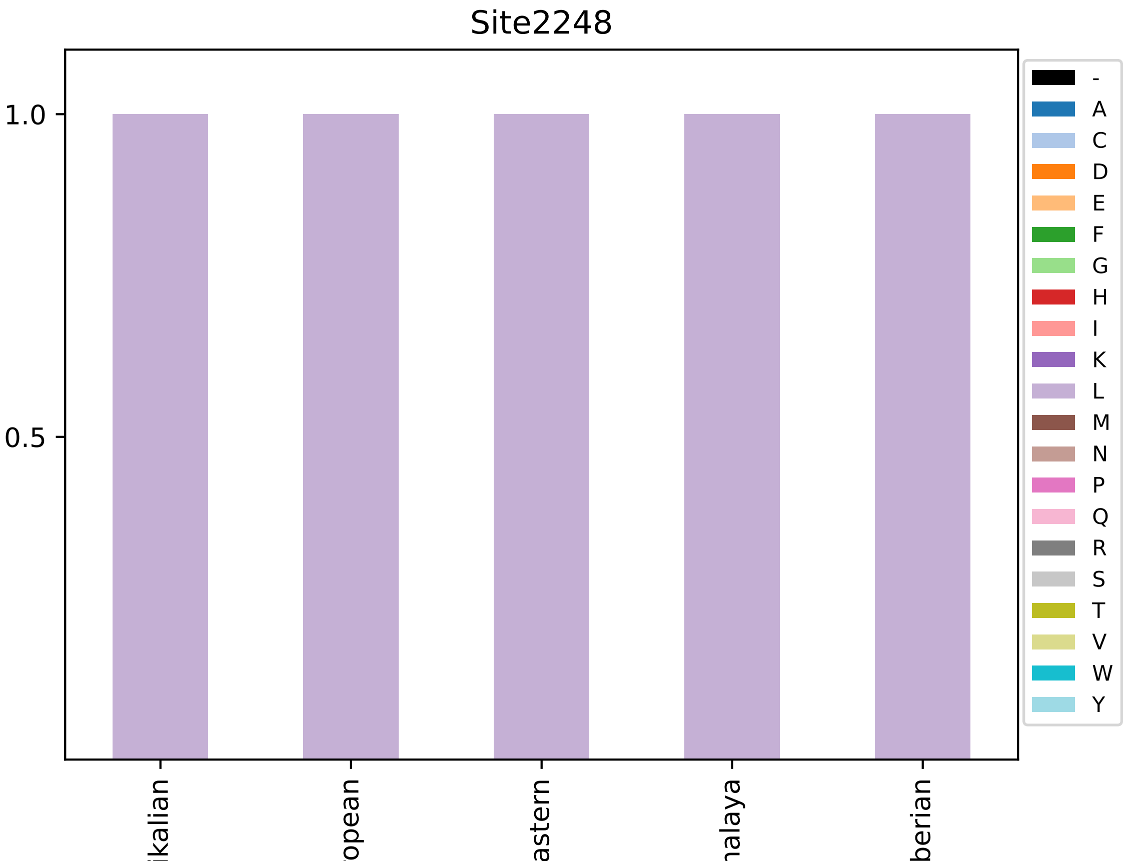

Site2248
1.0
0.5
-
A
C
D
E
F
G
H
I
K
L
M
N
P
Q
R
S
T
V
W
Y
ikalian
opean
astern
nalaya
berian

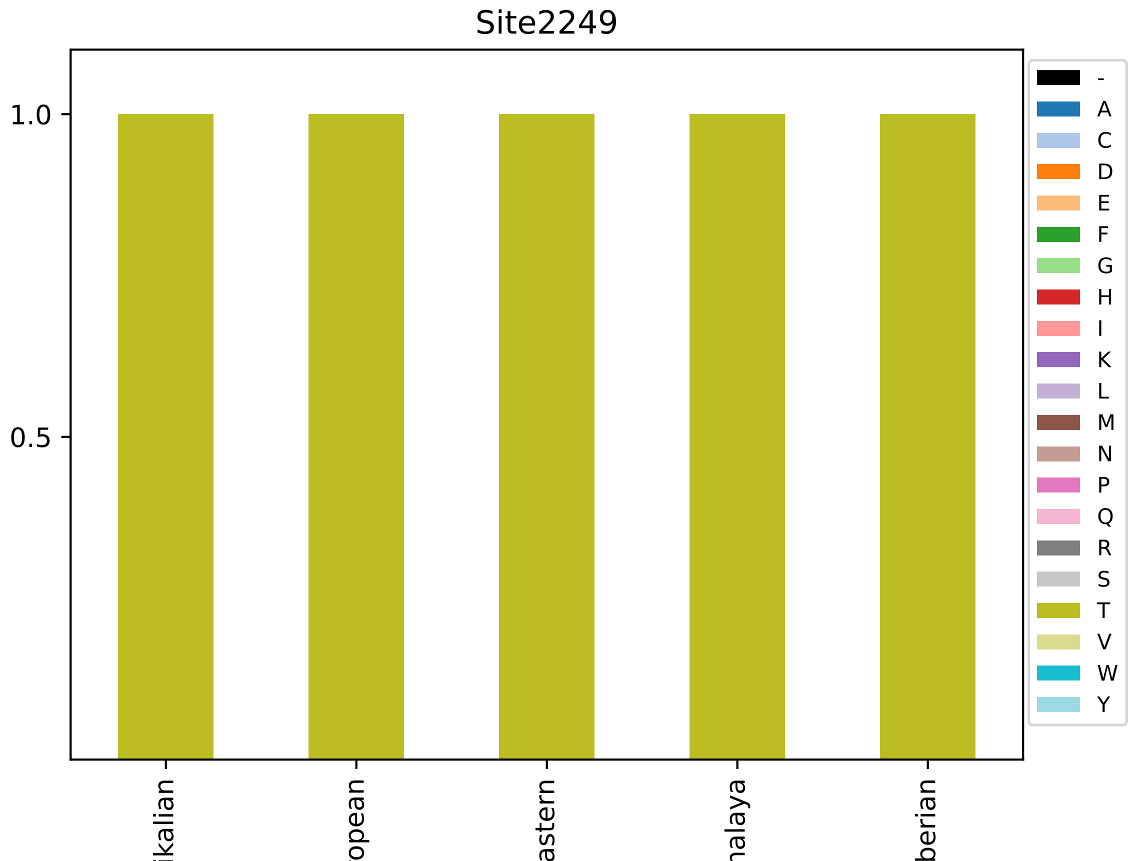
Site2249
1.0
0.5
-
A
C
D
E
F
G
H
I
K
L
M
N
P
Q
R
S
T
V
W
Y
ikalian
opean
astern
nalaya
berian

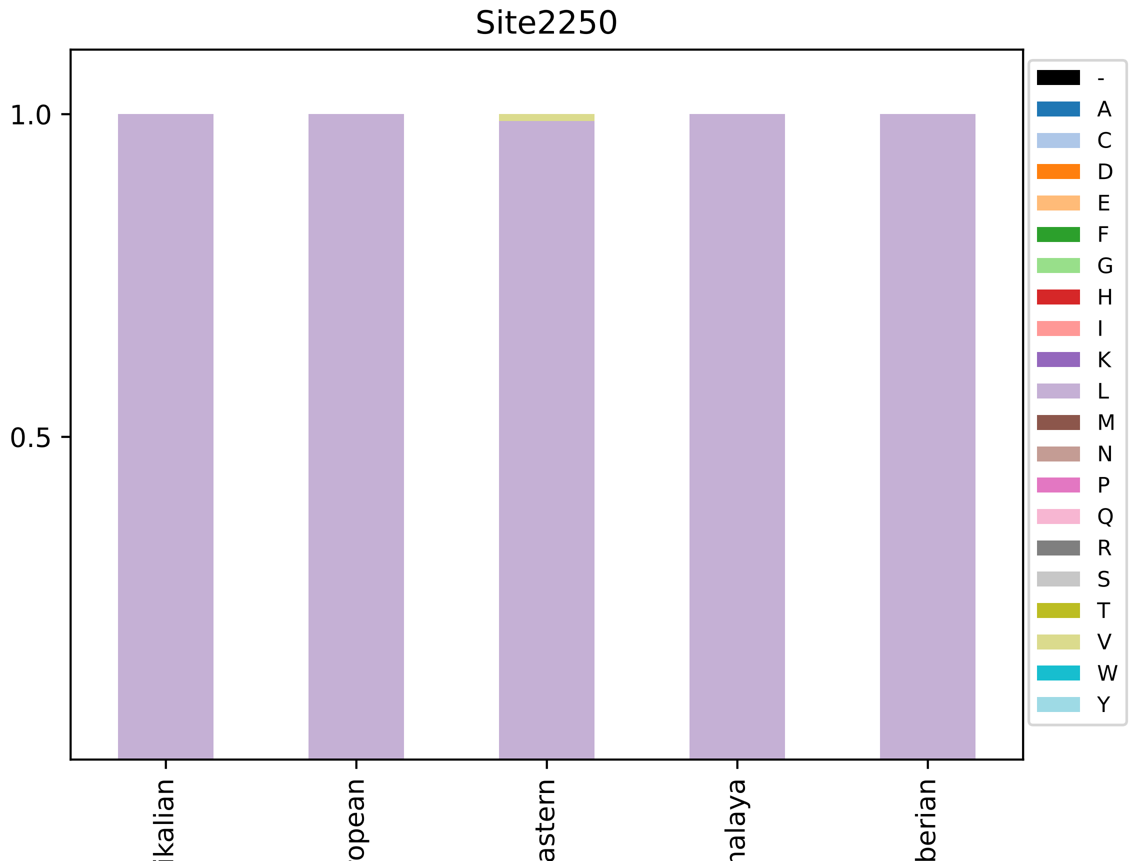
Site2250
1.0
0.5
-
A
C
D
E
F
G
H
I
K
L
M
N
P
Q
R
S
T
V
W
Y
ikalian
opean
astern
nalaya
berian

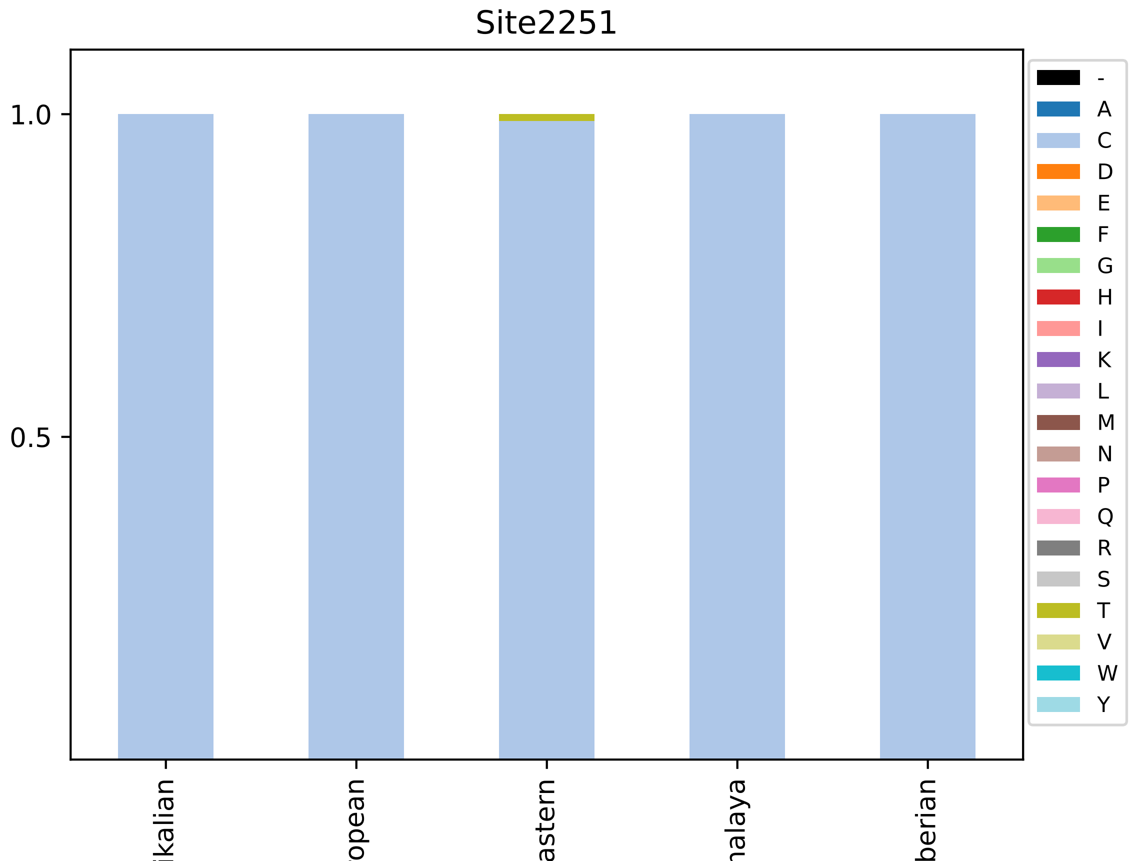
Site2251
1.0
0.5
-
A
C
D
E
F
G
H
I
K
L
M
N
P
Q
R
S
T
V
W
Y
ikalian
opean
astern
nalaya
berian

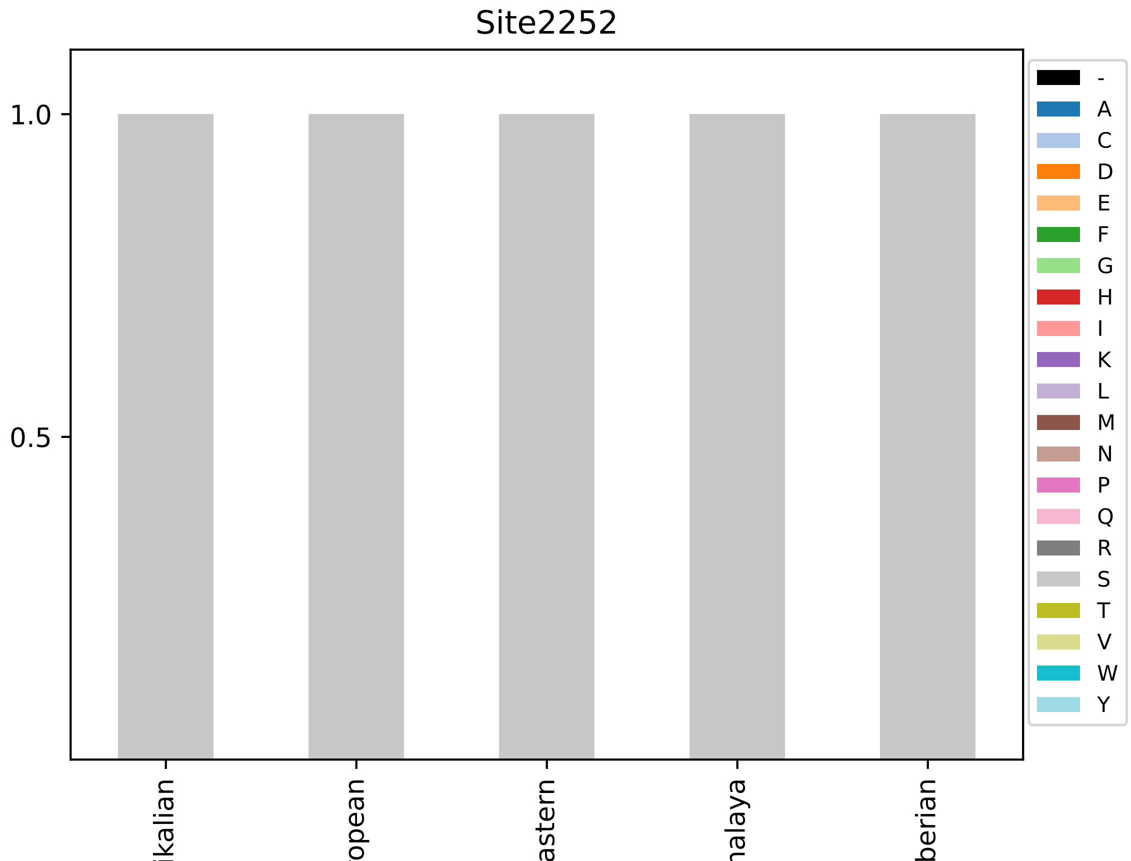
Site2252
1.0
0.5
-
A
C
D
E
F
G
H
I
K
L
M
N
P
Q
R
S
T
V
W
Y
ikalian
opean
astern
nalaya
berian

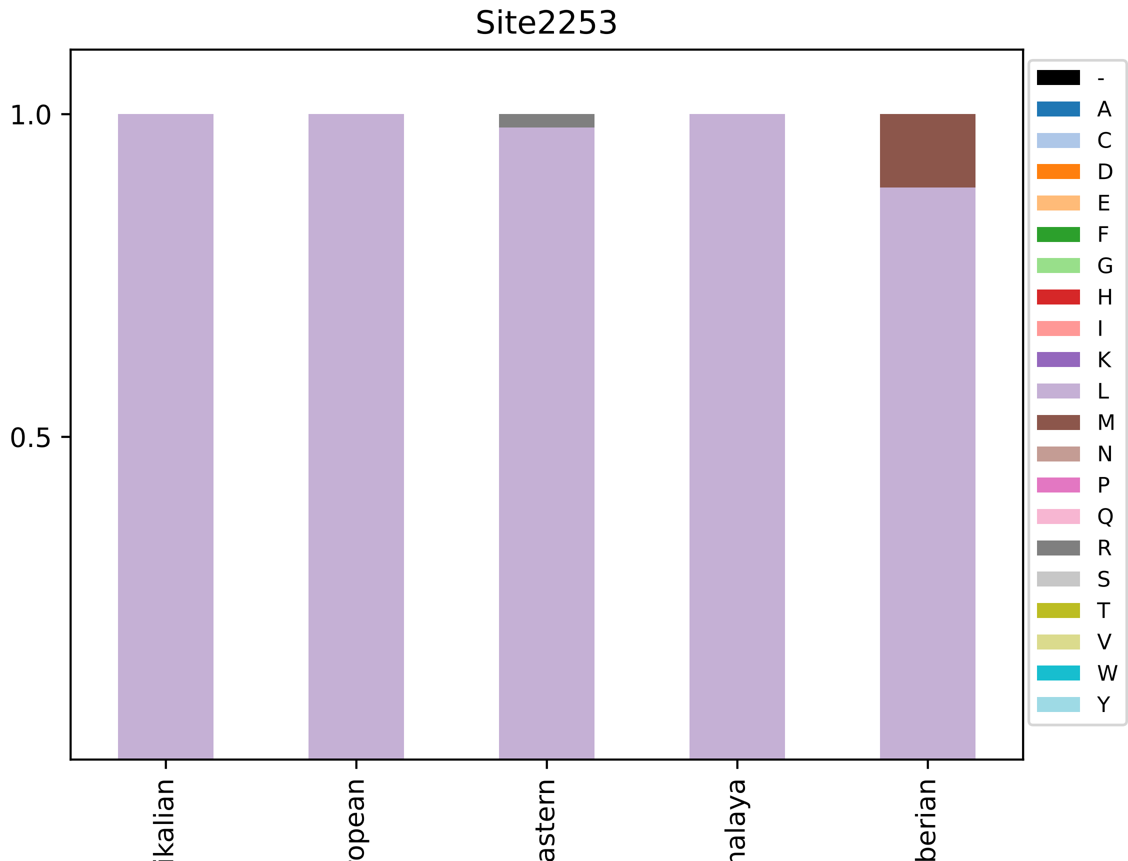

Site2253
1.0
0.5
-
A
C
D
E
F
G
H
I
K
L
M
N
P
Q
R
S
T
V
W
Y
ikalian
opean
astern
nalaya
berian

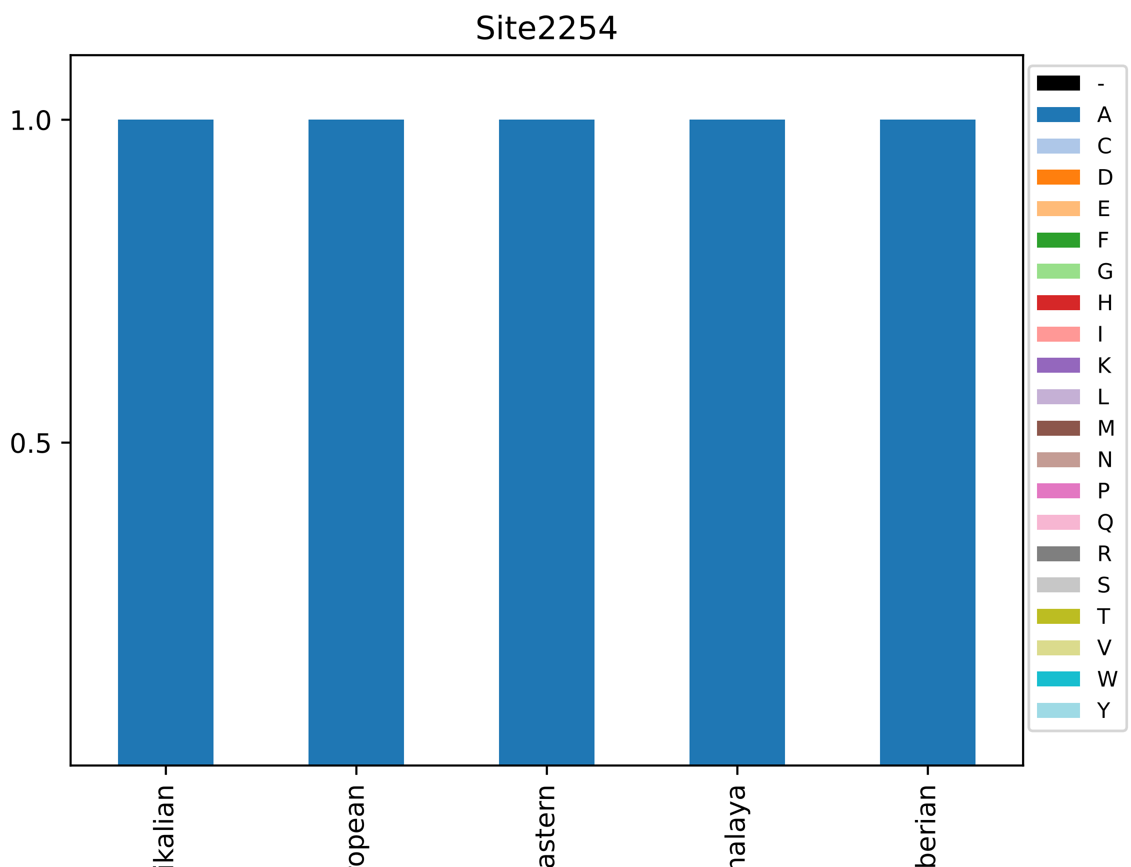

Site2254
1.0
0.5
-
A
C
D
E
F
G
H
I
K
L
M
N
P
Q
R
S
T
V
W
Y
ikalian
opean
astern
nalaya
berian

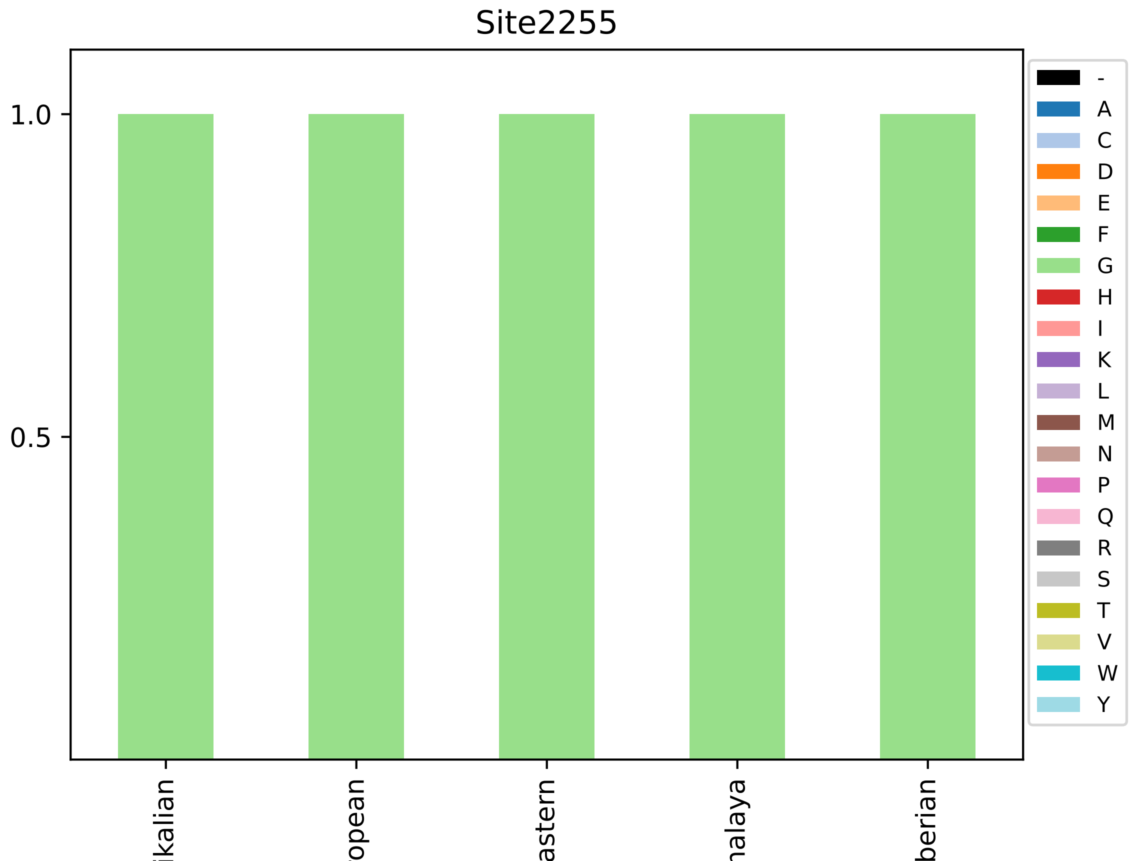

Site2255
1.0
0.5
ikalian
opean
astern
nalaya
berian
-
A
C
D
E
F
G
H
I
K
L
M
N
P
Q
R
S
T
V
W
Y

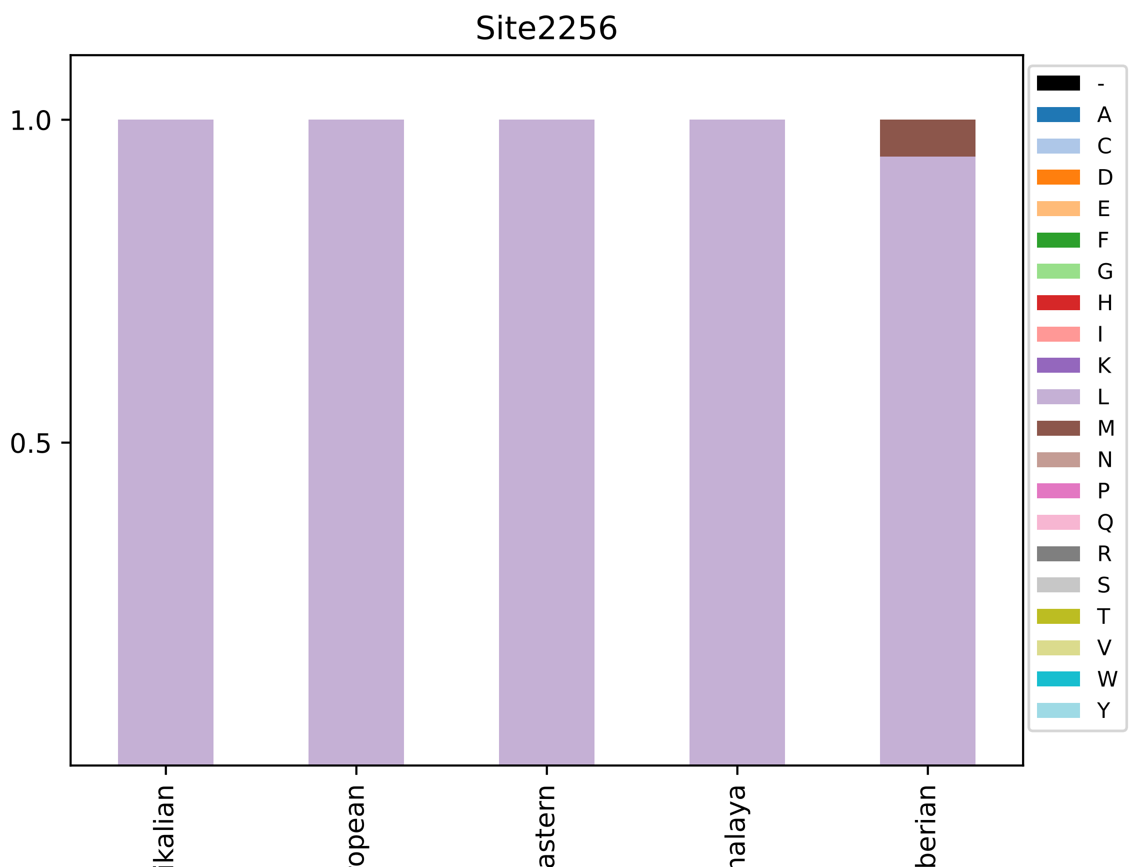
Site2256
1.0
0.5
-
A
C
D
E
F
G
H
I
K
L
M
N
P
Q
R
S
T
V
W
Y

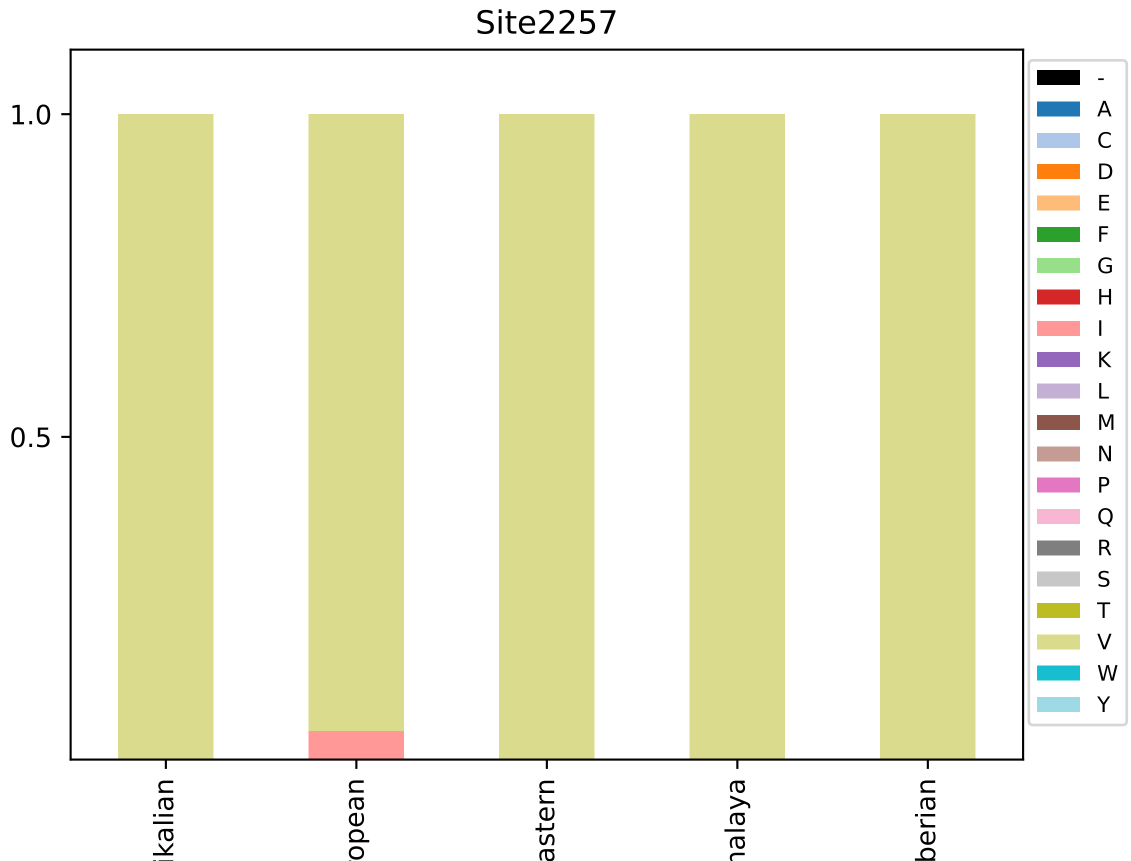
Site2257
1.0
0.5
-
A
C
D
E
F
G
H
I
K
L
M
N
P
Q
R
S
T
V
W
Y
ikalian
opean
astern
nalaya
berian

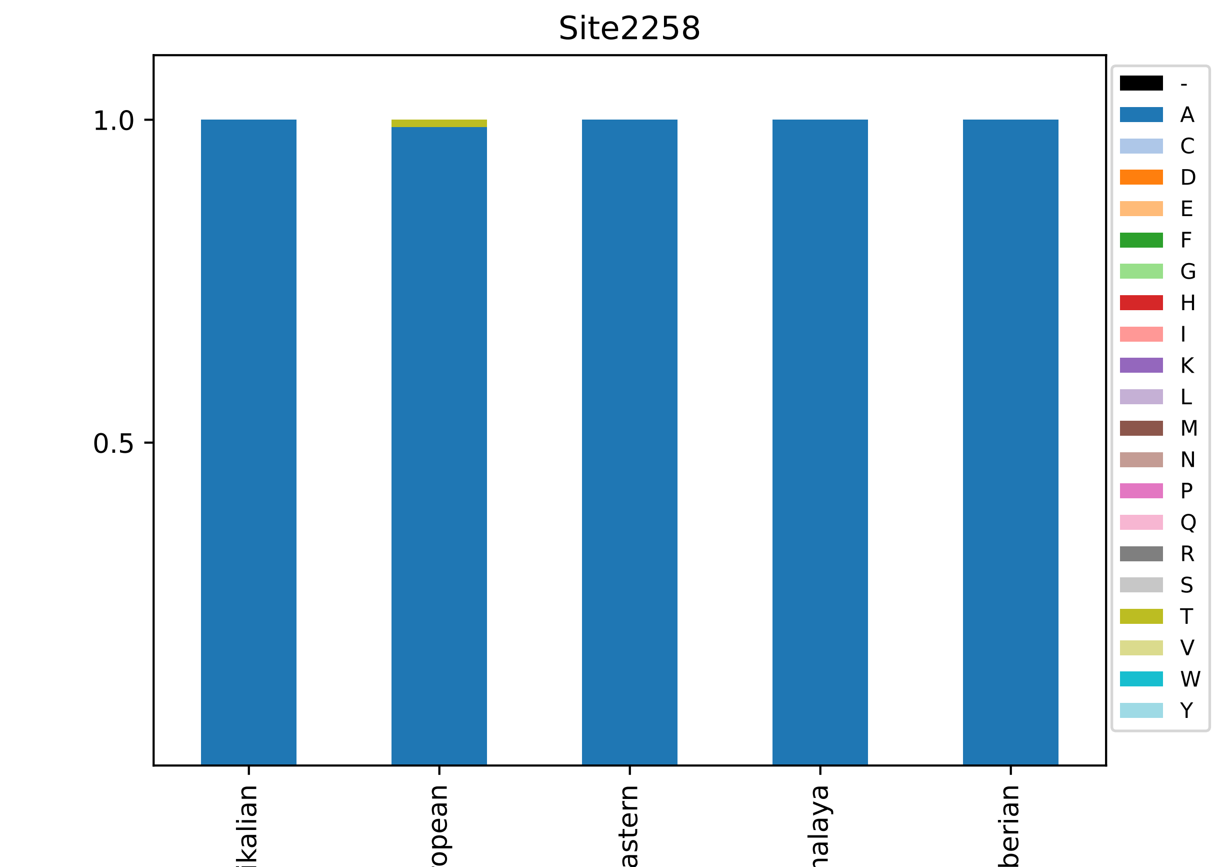
Site2258
1.0
0.5
-
A
C
D
E
F
G
H
I
K
L
M
N
P
Q
R
S
T
V
W
Y
ikalian
opean
astern
nalaya
berian

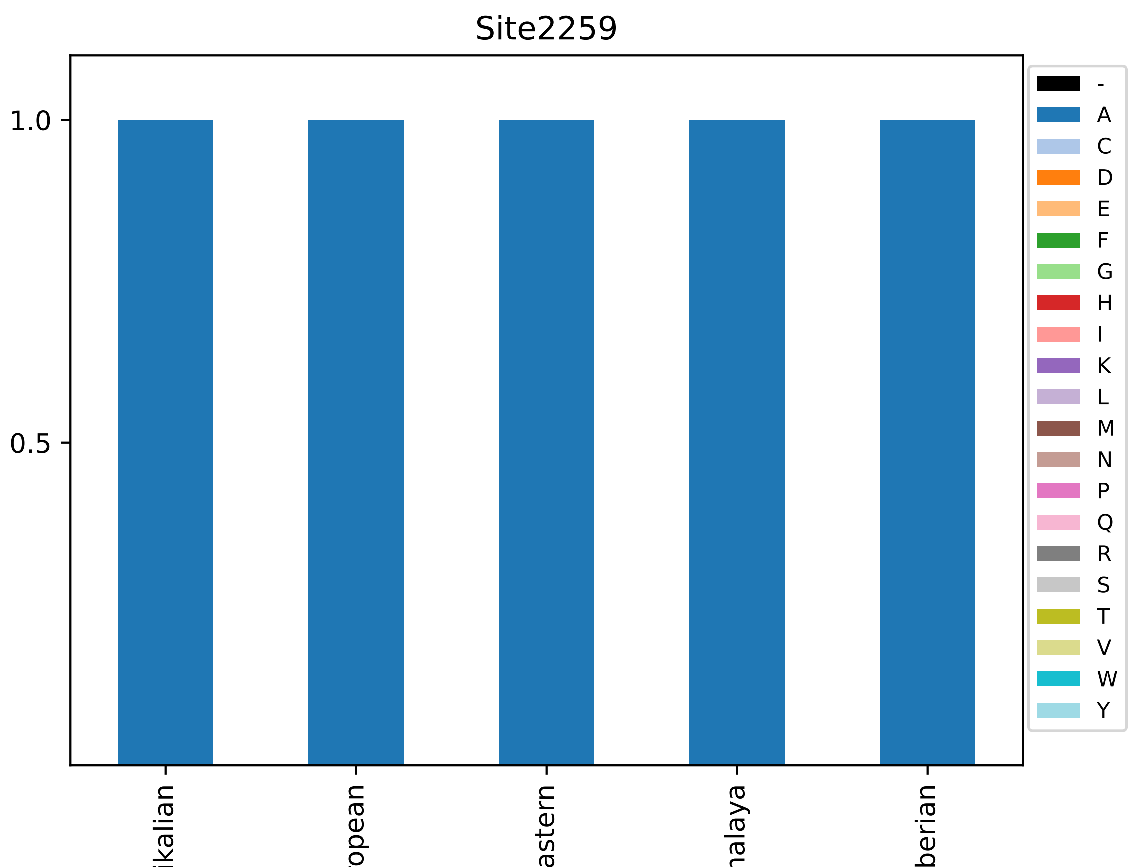
Site2259
1.0
0.5
-
A
C
D
E
F
G
H
I
K
L
M
N
P
Q
R
S
T
V
W
Y
ikalian
opean
astern
nalaya
berian

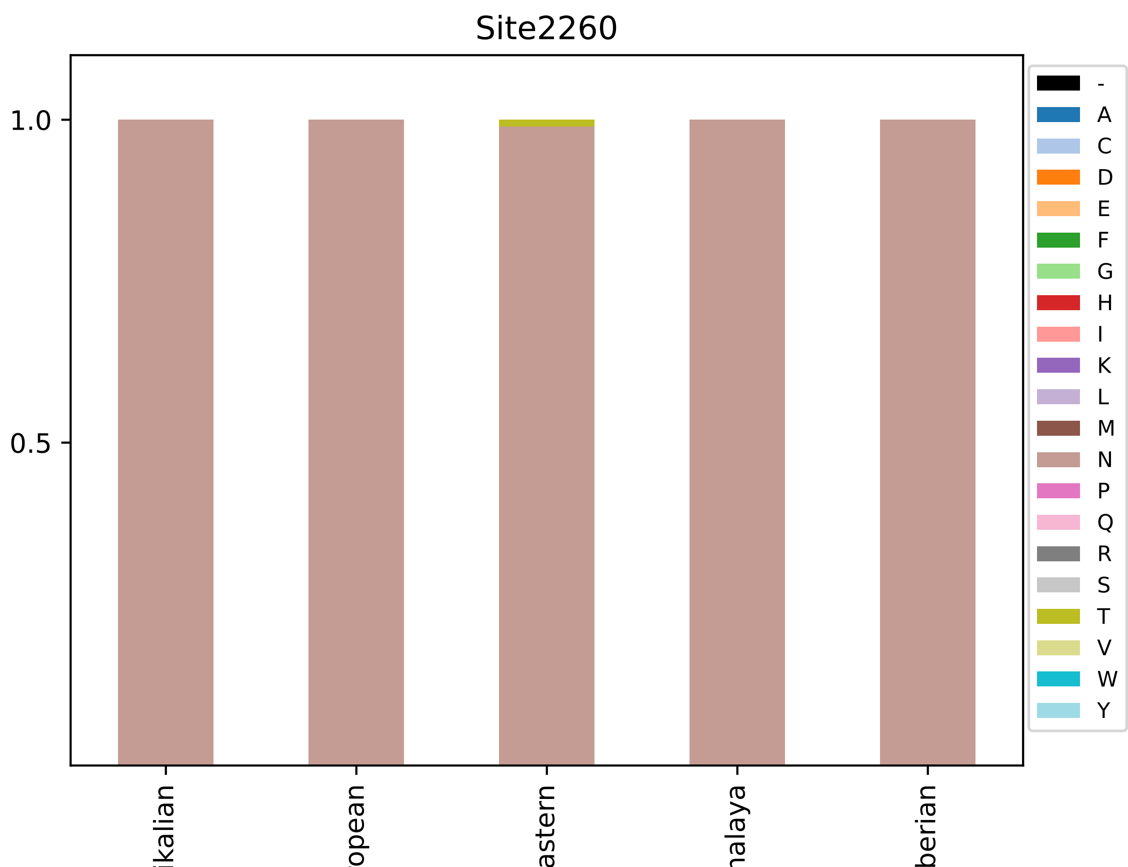

Site2260
1.0
0.5
-
A
C
D
E
F
G
H
I
K
L
M
N
P
Q
R
S
T
V
W
Y
ikalian
opean
astern
nalaya
berian

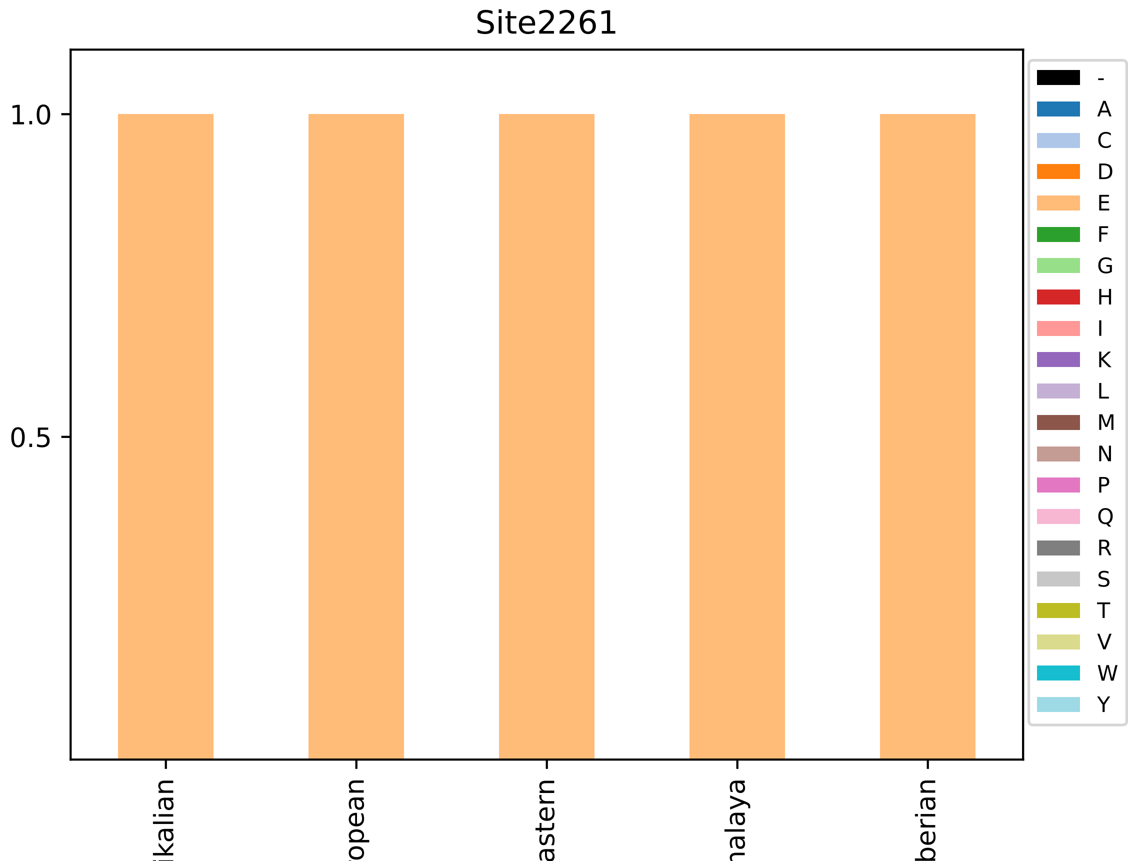

Site2261
1.0
0.5
-
A
C
D
E
F
G
H
I
K
L
M
N
P
Q
R
S
T
V
W
Y
ikalian
opean
astern
nalaya
berian

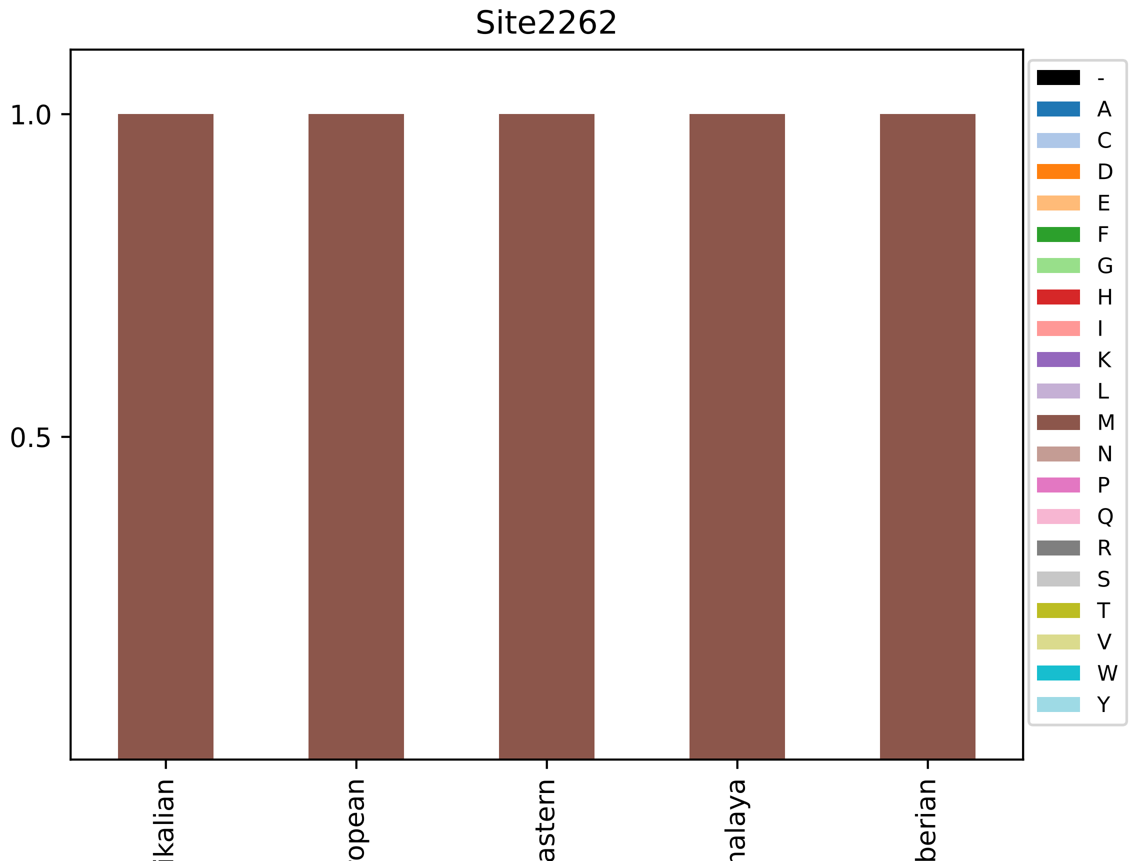
Site2262
1.0
0.5
-
A
C
D
E
F
G
H
I
K
L
M
N
P
Q
R
S
T
V
W
Y
ikalian
opean
astern
nalaya
berian

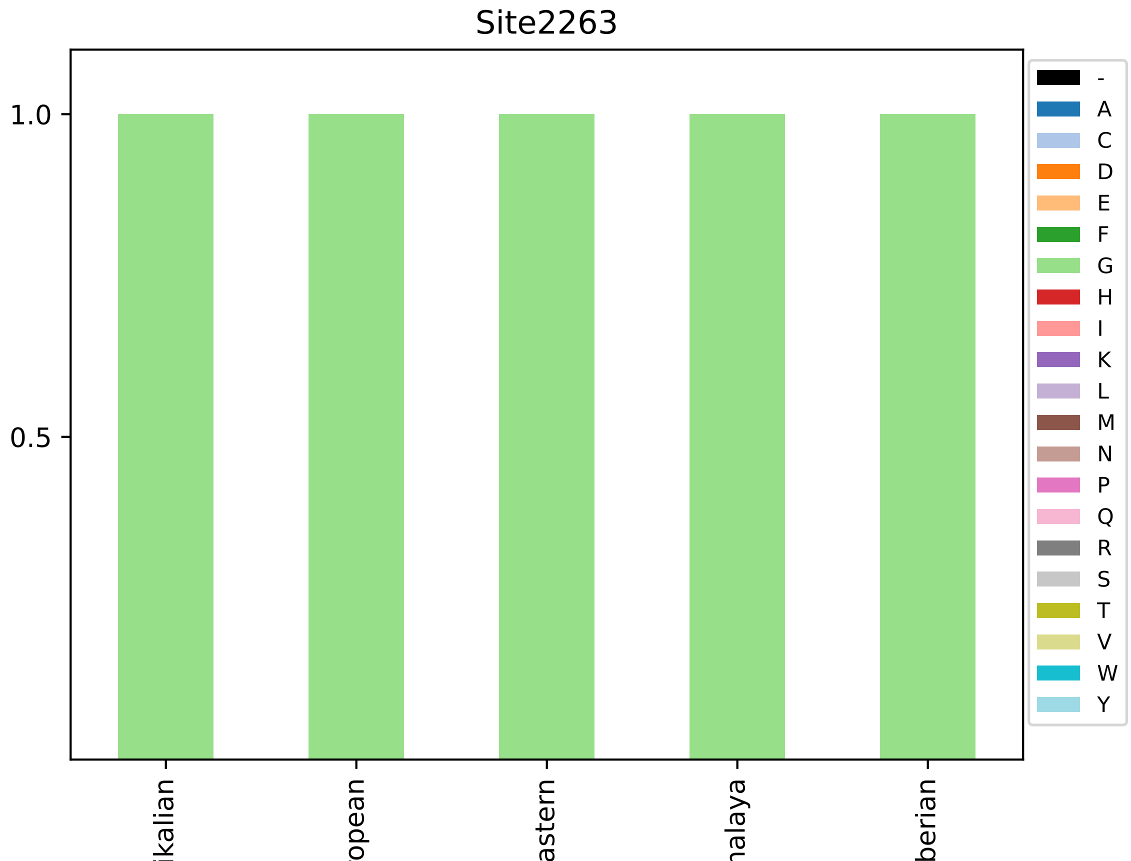

Site2263
1.0
0.5
ikalian
opean
astern
nalaya
berian
-
A
C
D
E
F
G
H
I
K
L
M
N
P
Q
R
S
T
V
W
Y

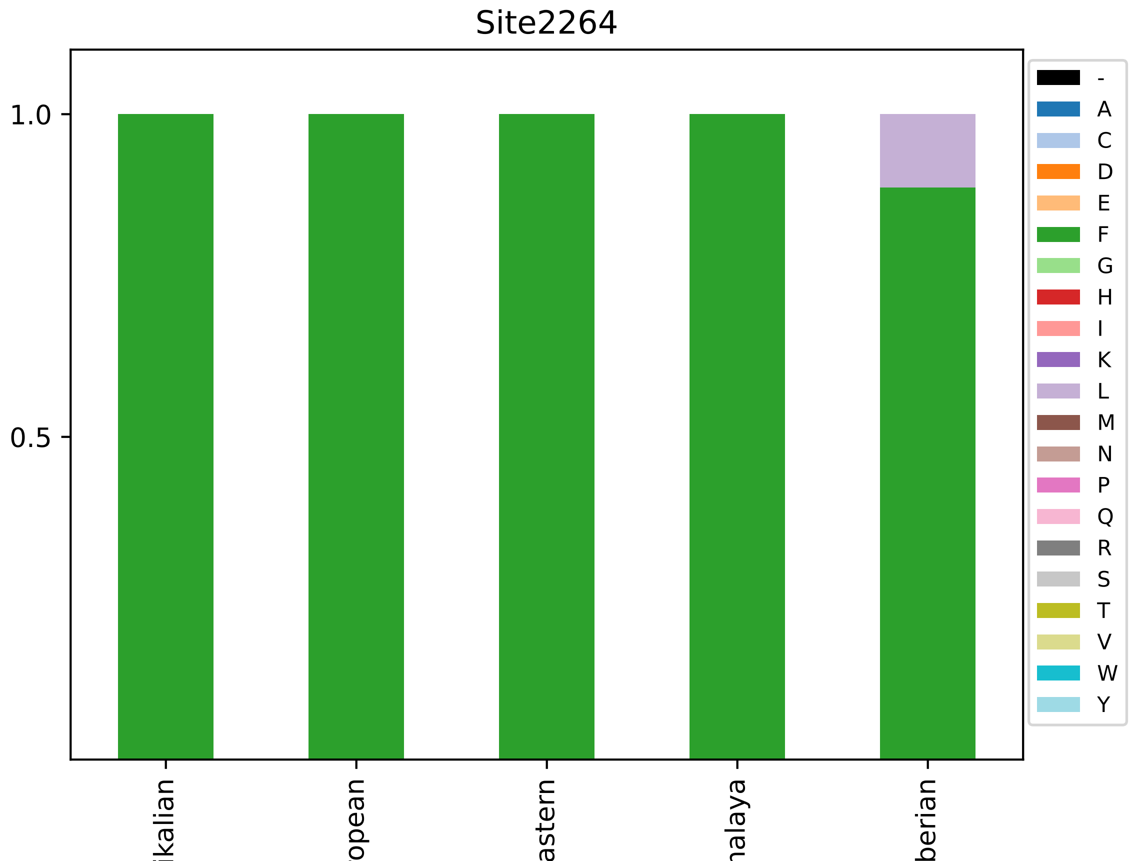

Site2264
1.0
0.5
-
A
C
D
E
F
G
H
I
K
L
M
N
P
Q
R
S
T
V
W
Y
ikalian
opean
astern
nalaya
berian

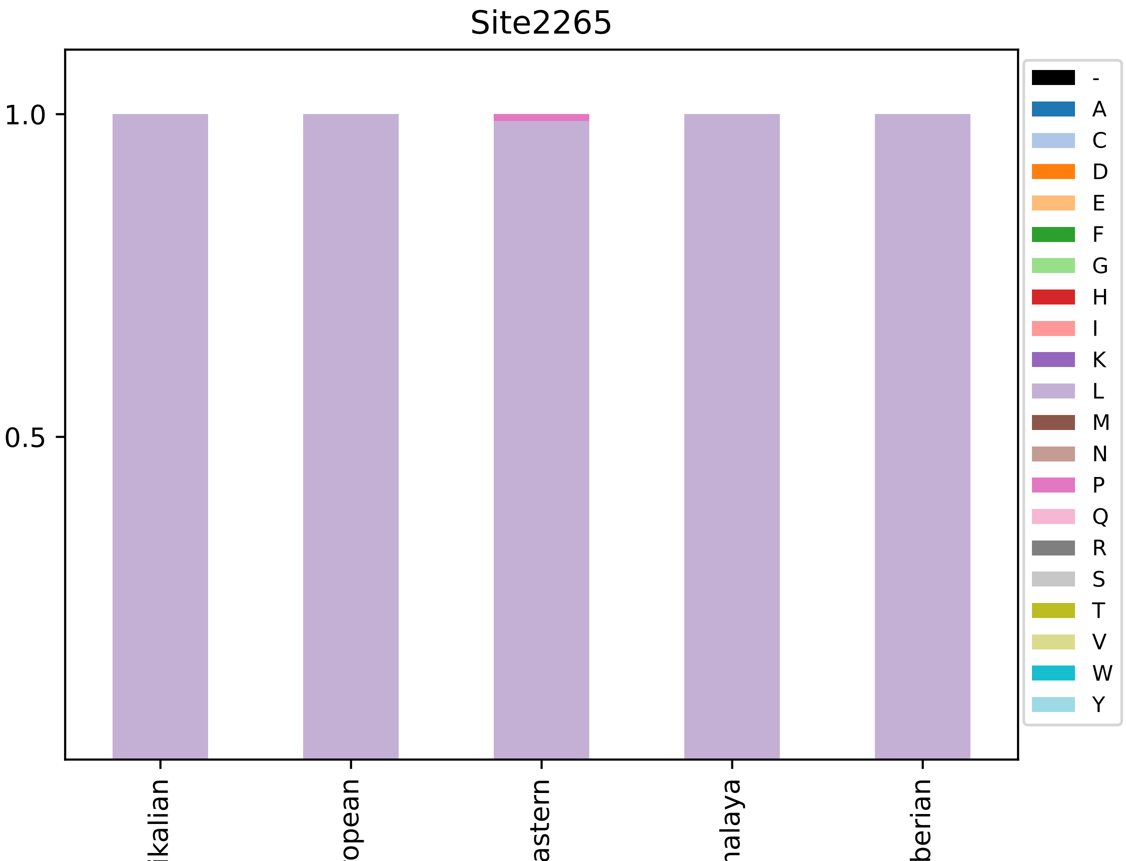
Site2265
1.0
0.5
-
A
C
D
E
F
G
H
I
K
L
M
N
P
Q
R
S
T
V
W
Y
ikalian
opean
astern
nalaya
berian

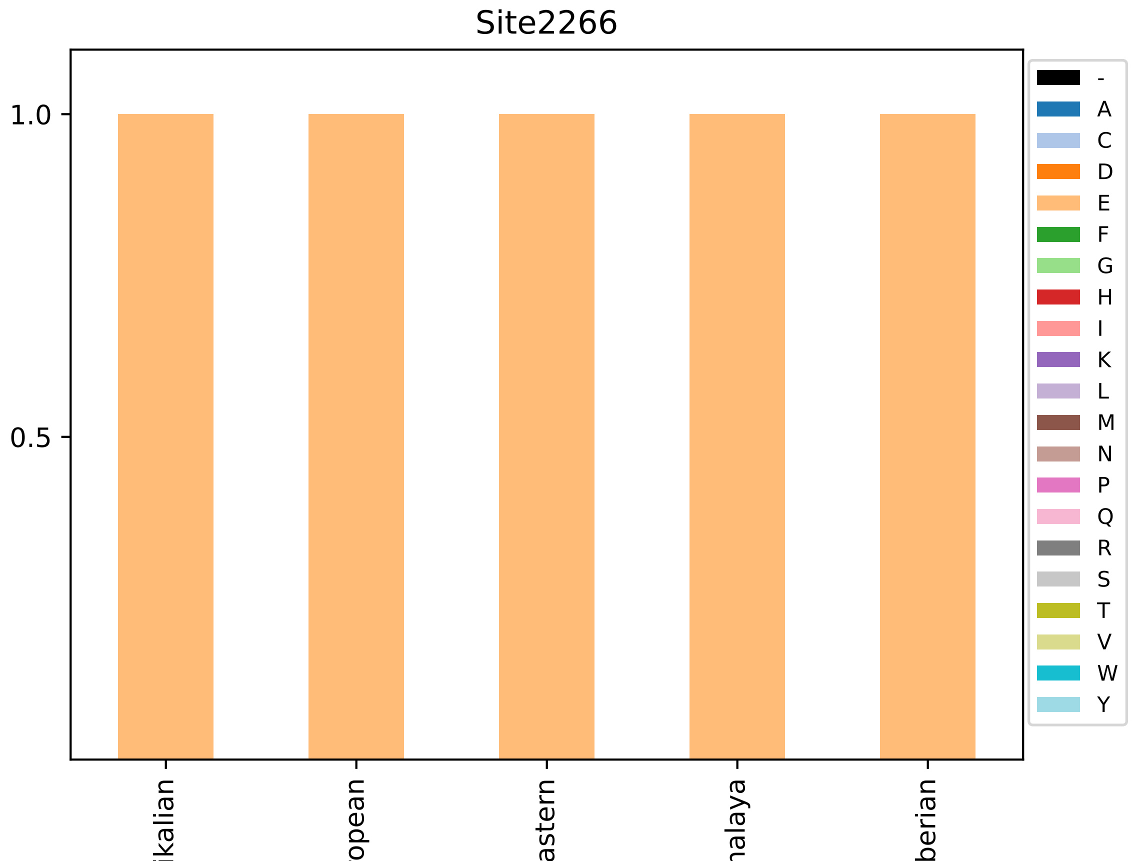
Site2266
1.0
0.5
-
A
C
D
E
F
G
H
I
K
L
M
N
P
Q
R
S
T
V
W
Y
ikalian
opean
astern
nalaya
berian

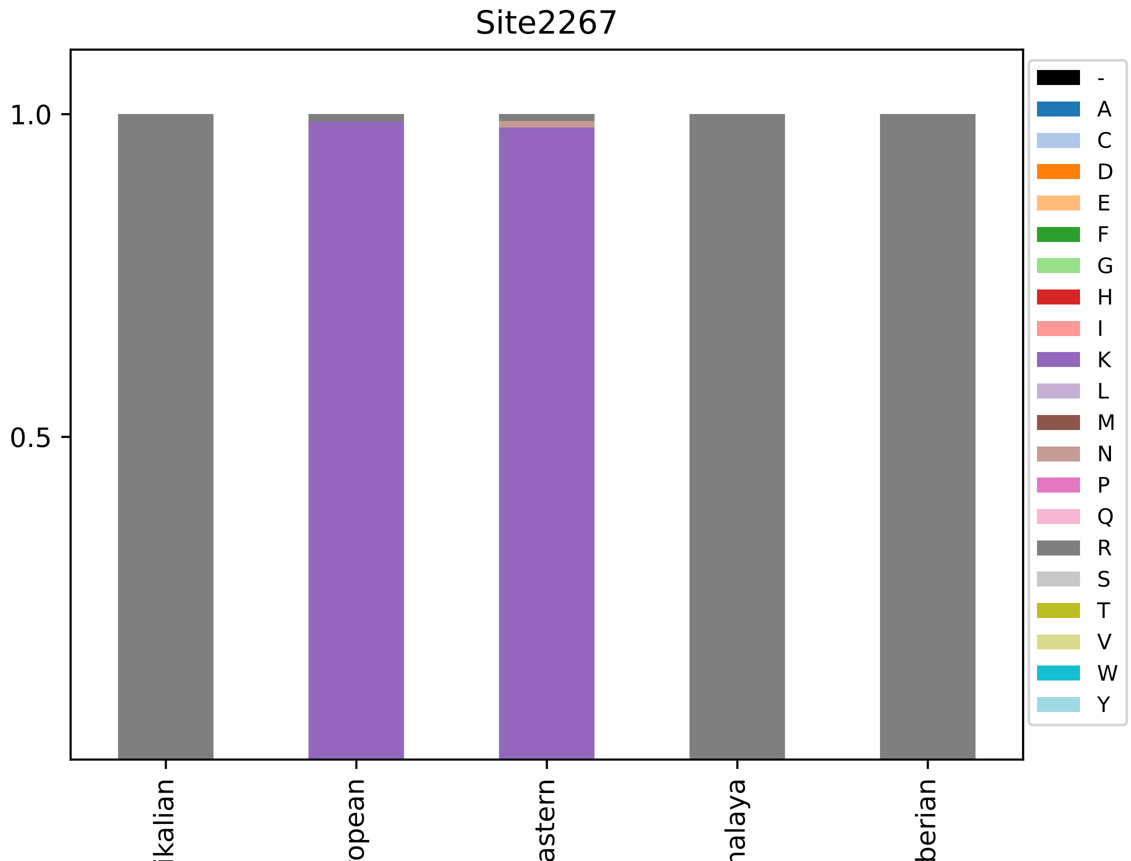
Site2267
1.0
0.5
-
A
C
D
E
F
G
H
I
K
L
M
N
P
Q
R
S
T
V
W
Y
ikalian
opean
astern
nalaya
berian

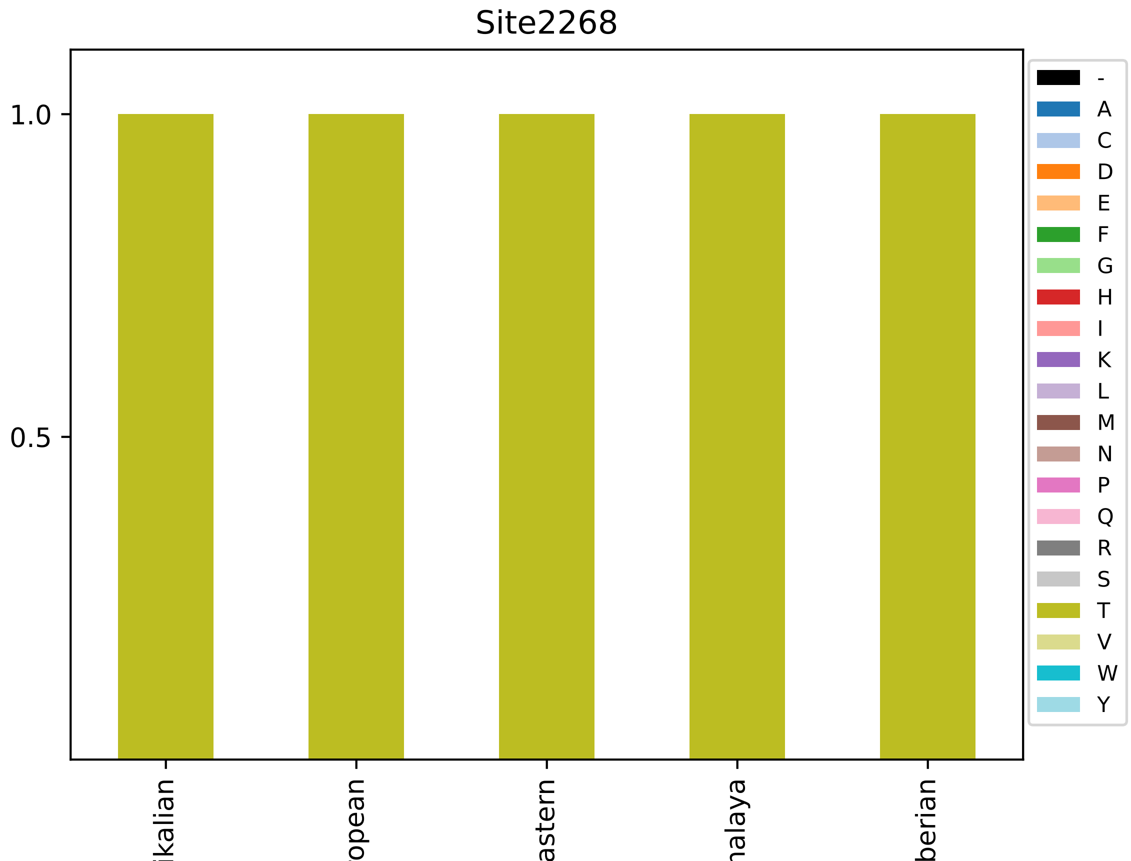

Site2268
1.0
0.5
-
A
C
D
E
F
G
H
I
K
L
M
N
P
Q
R
S
T
V
W
Y
ikalian
opean
astern
nalaya
berian

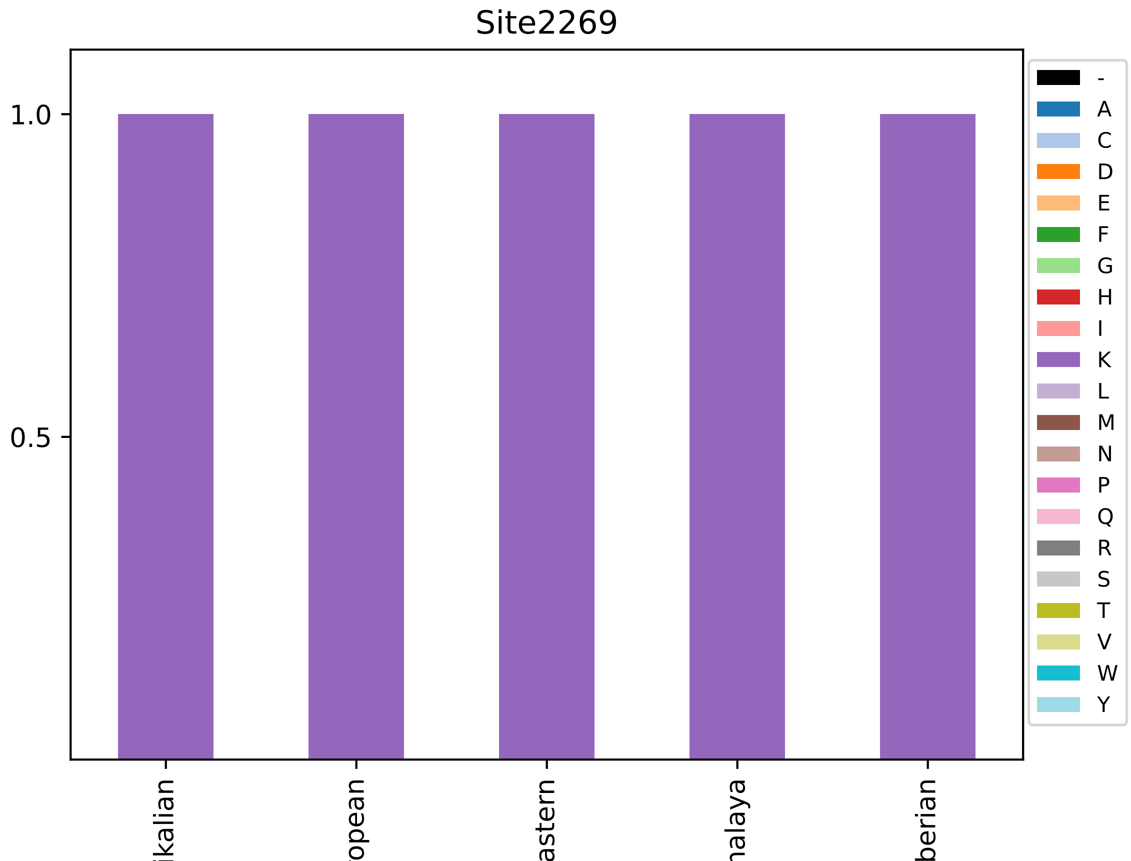
Site2269
1.0
0.5
-
A
C
D
E
F
G
H
I
K
L
M
N
P
Q
R
S
T
V
W
Y
ikalian
opean
astern
nalaya
berian

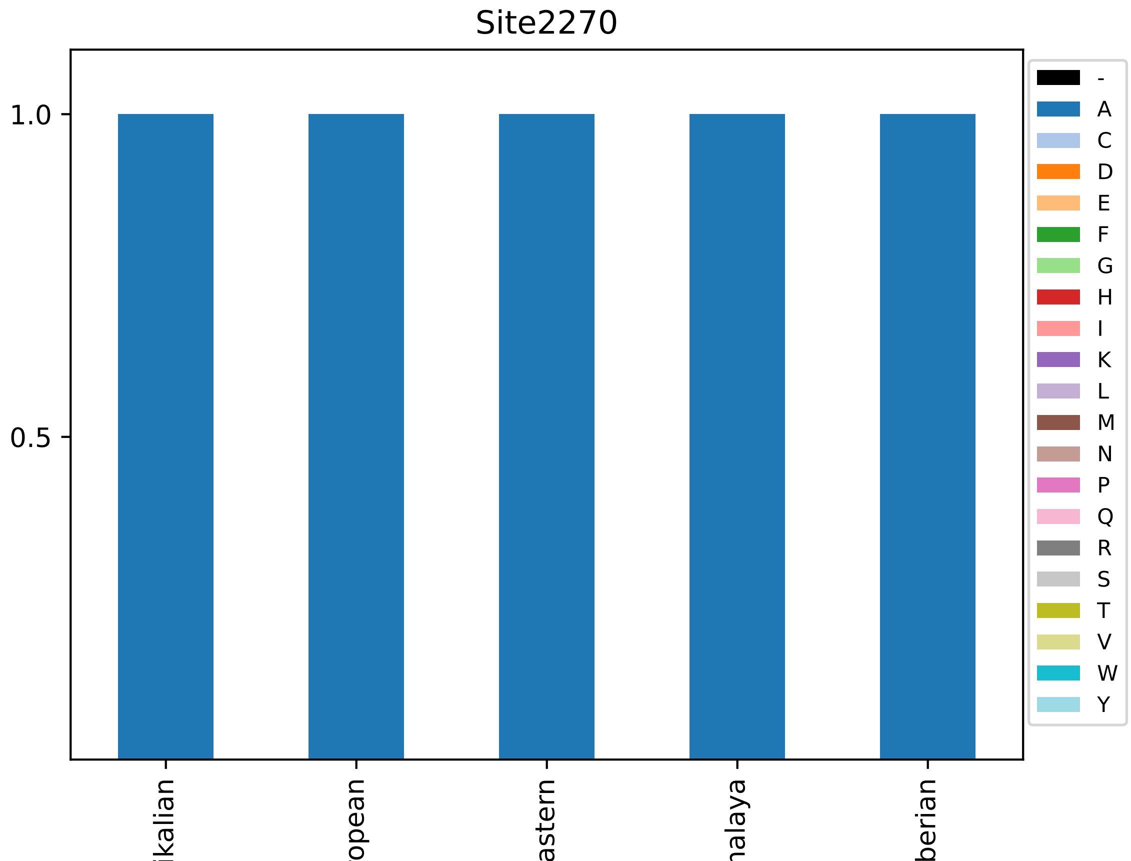
Site2270
1.0
0.5
-
A
C
D
E
F
G
H
I
K
L
M
N
P
Q
R
S
T
V
W
Y

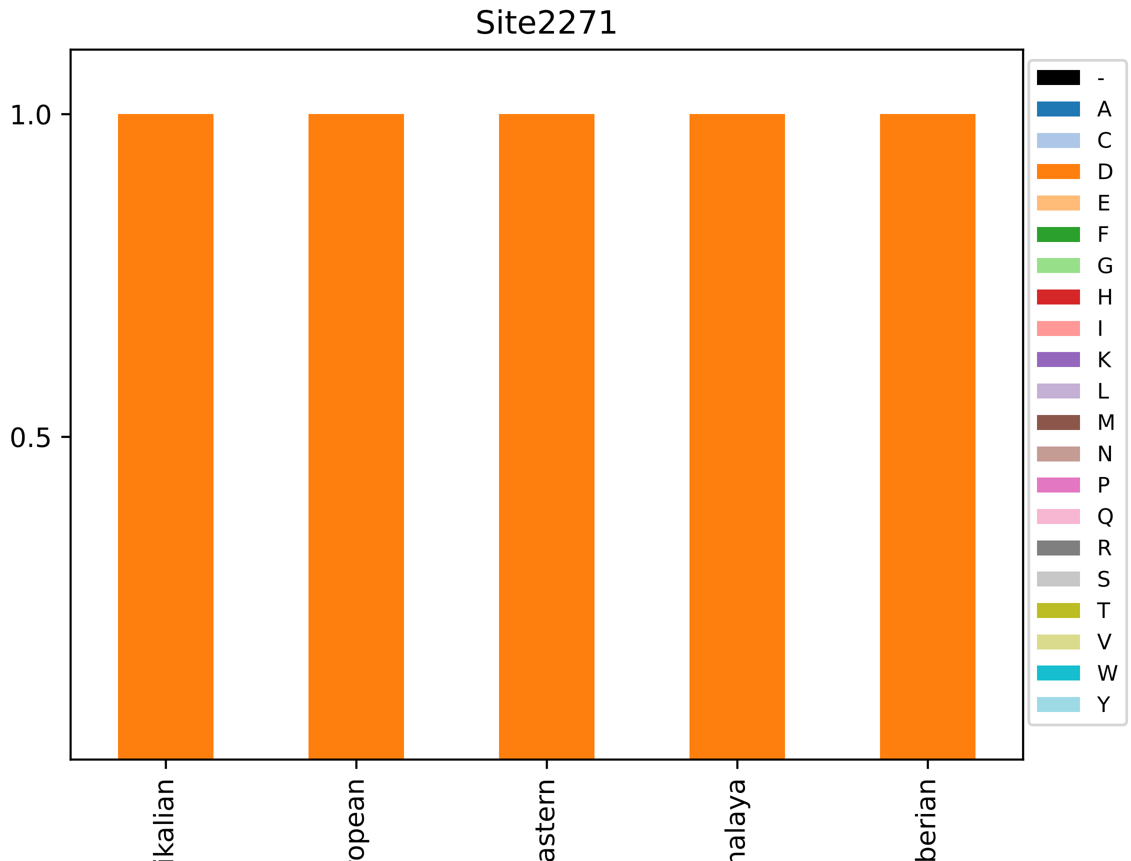
Site2271
1.0
0.5
-
A
C
D
E
F
G
H
I
K
L
M
N
P
Q
R
S
T
V
W
Y
ikalian
opean
astern
nalaya
berian

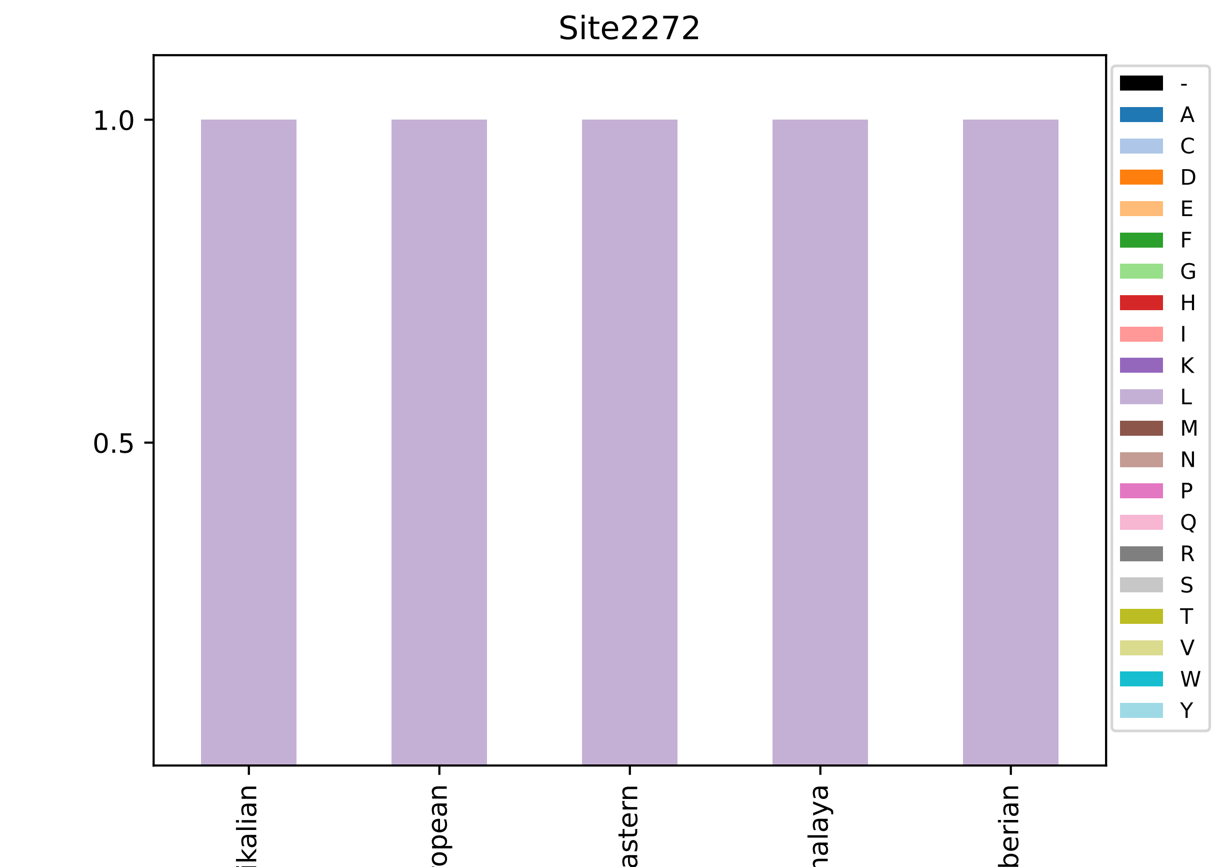
Site2272
1.0
0.5
-
A
C
D
E
F
G
H
I
K
L
M
N
P
Q
R
S
T
V
W
Y
ikalian
opean
astern
nalaya
berian

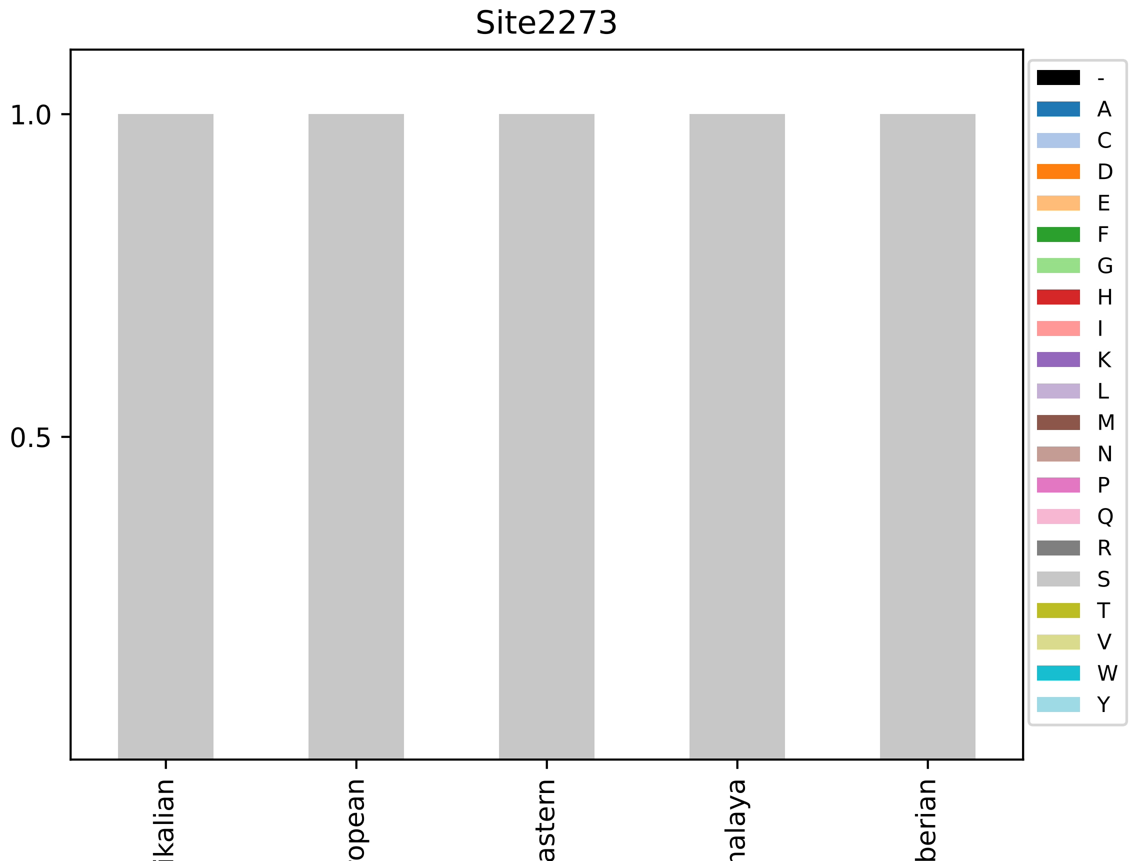

Site2273
1.0
0.5
-
A
C
D
E
F
G
H
I
K
L
M
N
P
Q
R
S
T
V
W
Y
ikalian
opean
astern
nalaya
berian

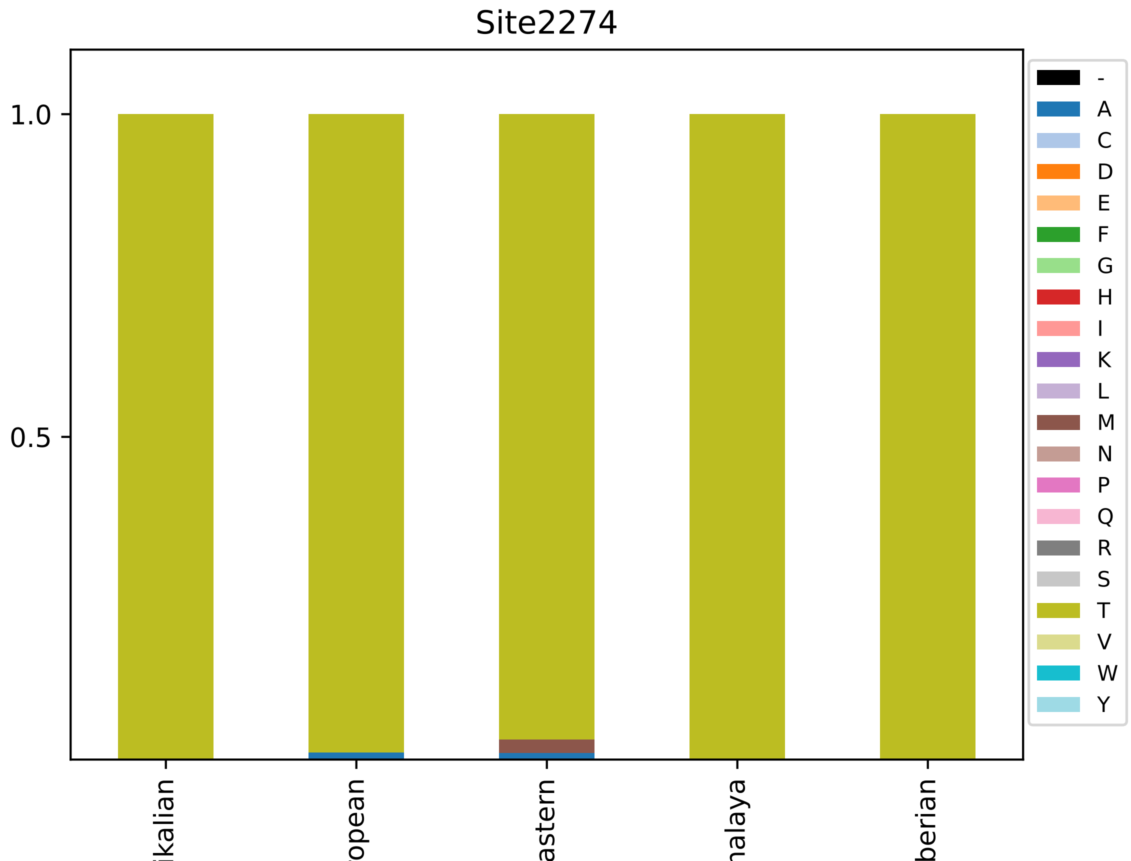

Site2274
1.0
0.5
ikalian
opean
astern
nalaya
berian
-
A
C
D
E
F
G
H
I
K
L
M
N
P
Q
R
S
T
V
W
Y

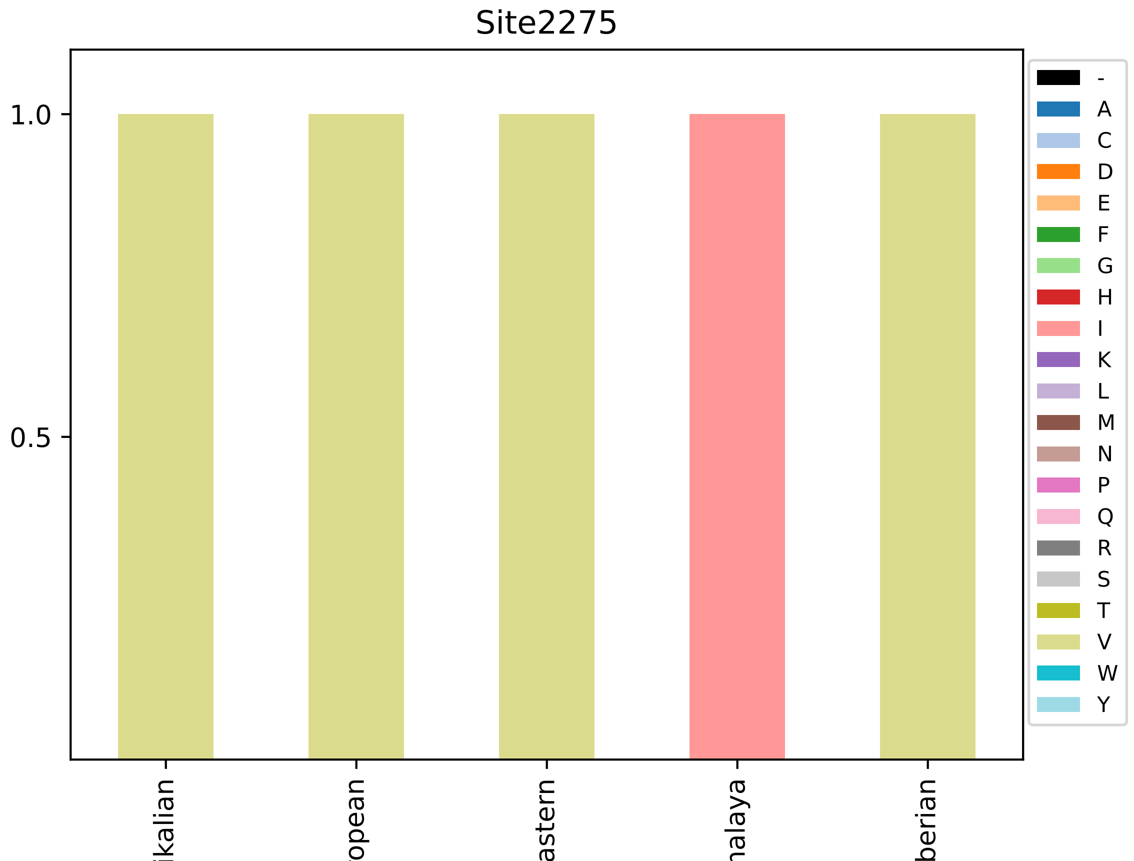
Site2275
1.0
0.5
-
A
C
D
E
F
G
H
I
K
L
M
N
P
Q
R
S
T
V
W
Y

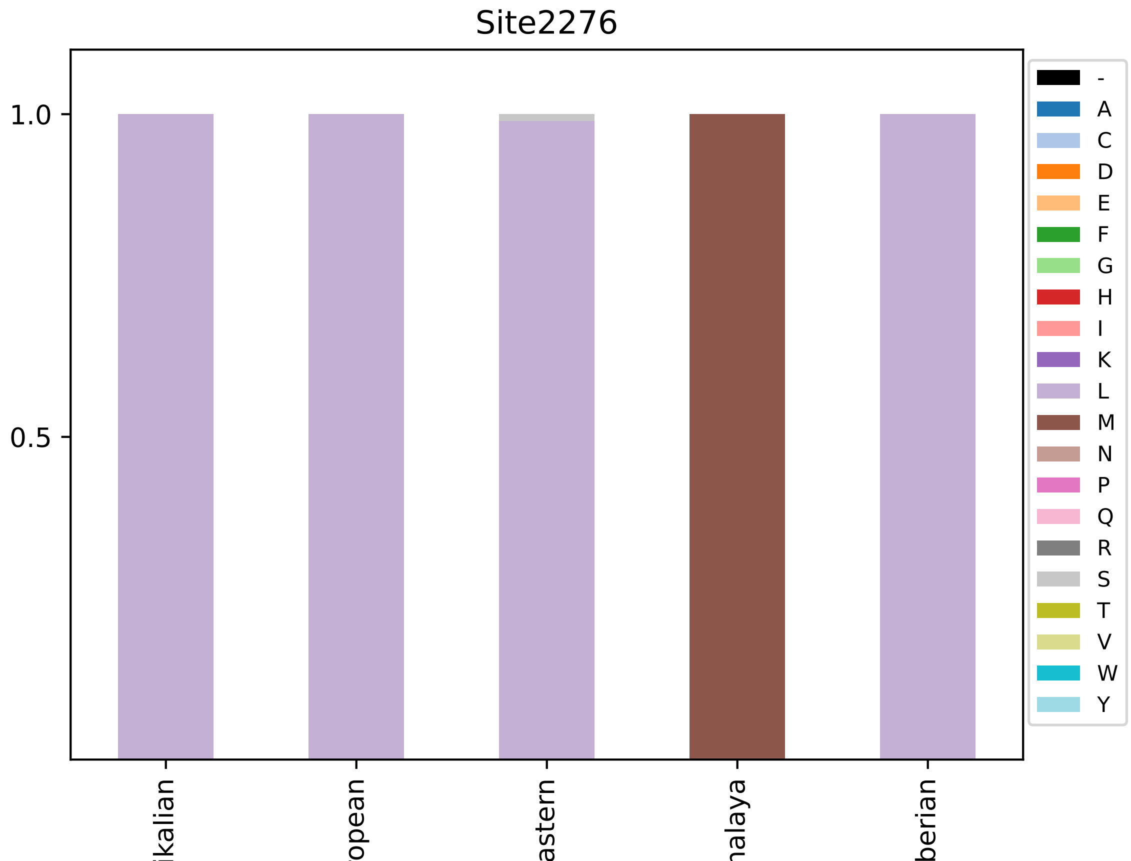
Site2276
1.0
0.5
-
A
C
D
E
F
G
H
I
K
L
M
N
P
Q
R
S
T
V
W
Y
ikalian
opean
astern
nalaya
berian

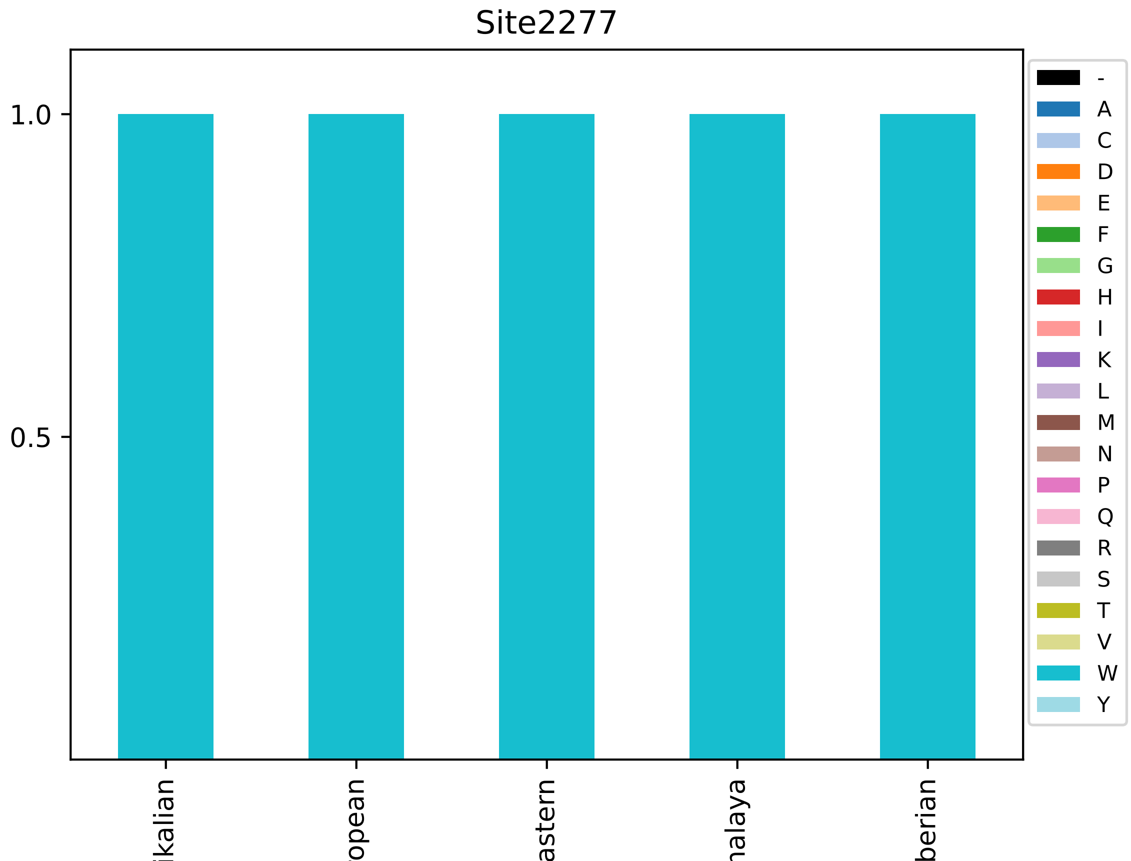
Site2277
1.0
0.5
-
A
C
D
E
F
G
H
I
K
L
M
N
P
Q
R
S
T
V
W
Y
ikalian
opean
astern
nalaya
berian

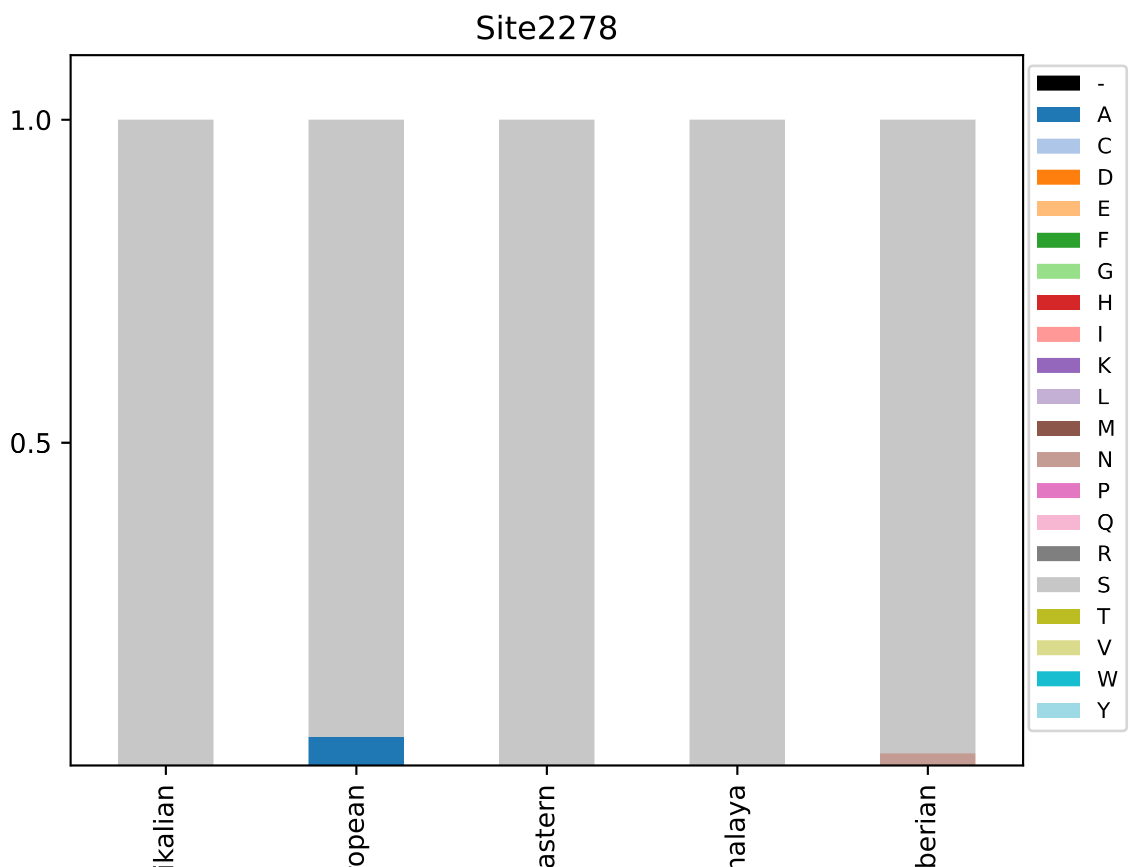
Site2278
1.0
0.5
-
A
C
D
E
F
G
H
I
K
L
M
N
P
Q
R
S
T
V
W
Y
ikalian
opean
astern
nalaya
berian

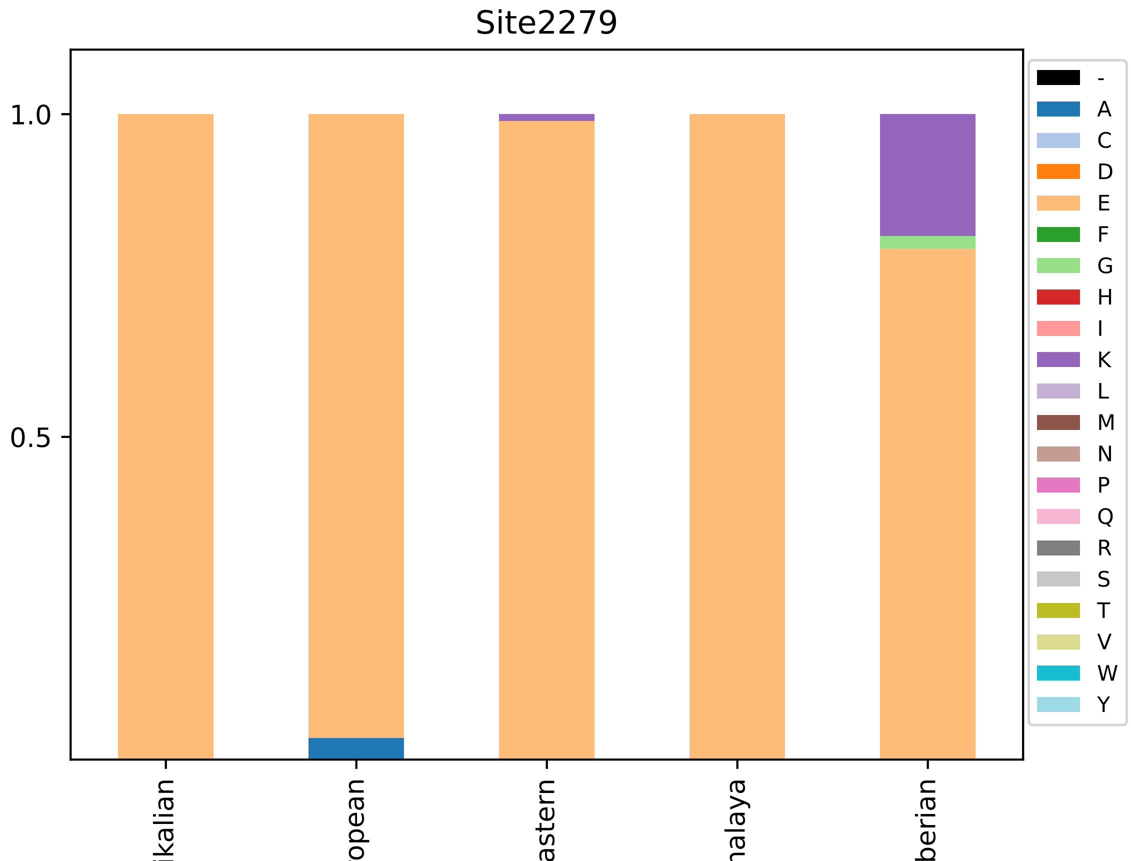

Site2279
1.0
0.5
ikalian
opean
astern
nalaya
berian
-
A
C
D
E
F
G
H
I
K
L
M
N
P
Q
R
S
T
V
W
Y

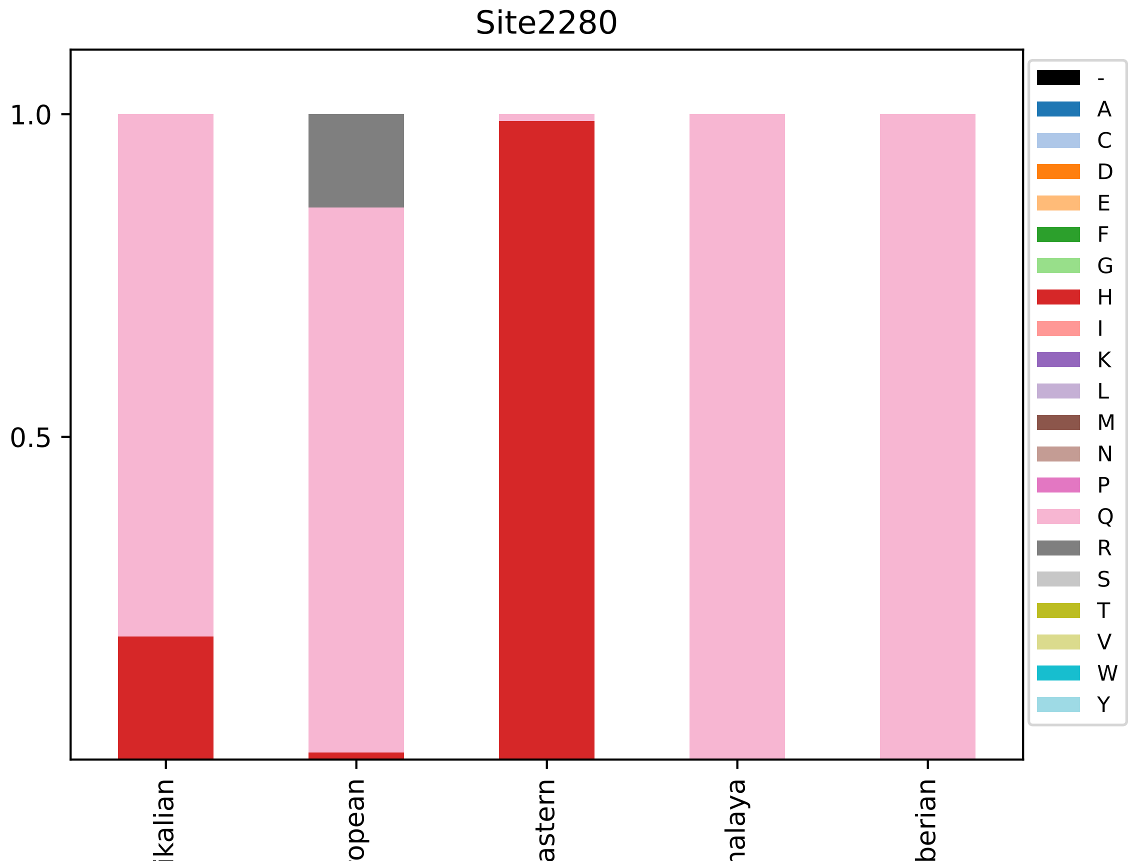
Site2280
1.0
0.5
-
A
C
D
E
F
G
H
I
K
L
M
N
P
Q
R
S
T
V
W
Y
ikalian
opean
astern
nalaya
berian

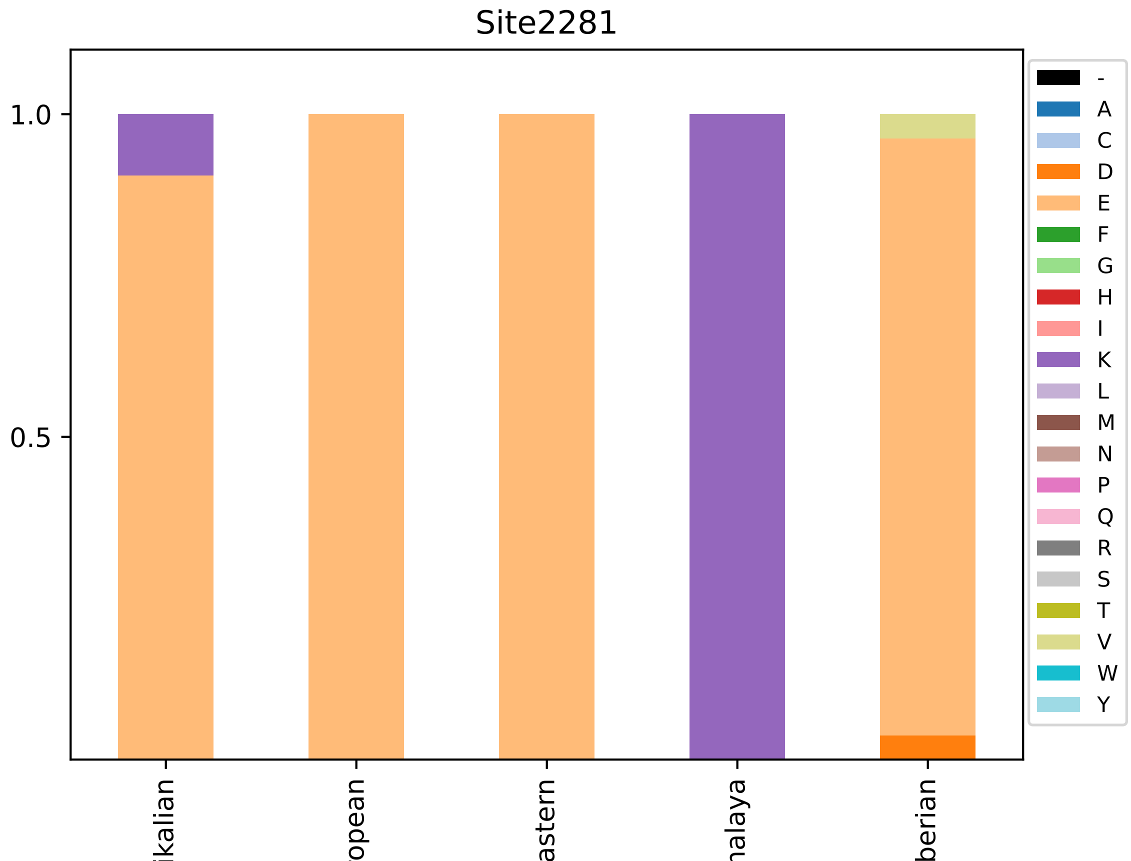

Site2281
1.0
0.5
-
A
C
D
E
F
G
H
I
K
L
M
N
P
Q
R
S
T
V
W
Y
ikalian
opean
astern
nalaya
berian

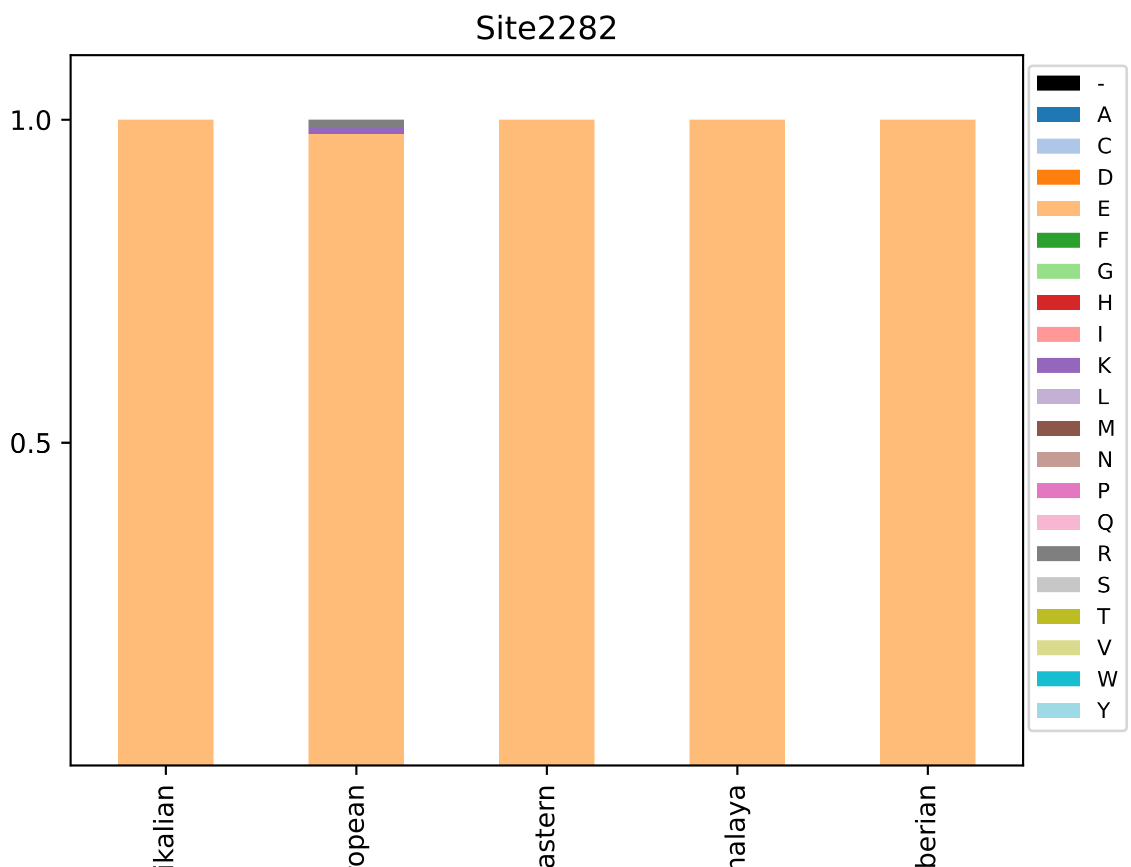

Site2282
1.0
0.5
-
A
C
D
E
F
G
H
I
K
L
M
N
P
Q
R
S
T
V
W
Y
ikalian
opean
astern
nalaya
berian

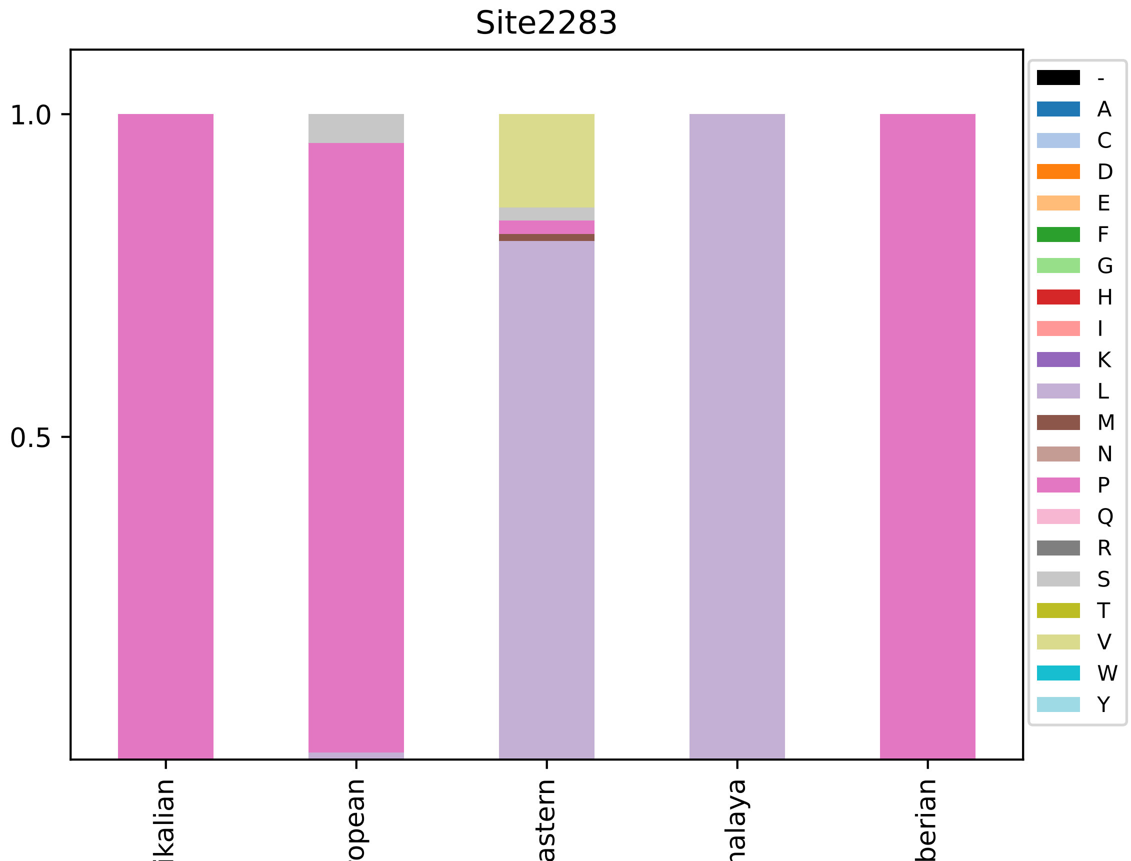

Site2283
1.0
0.5
-
A
C
D
E
F
G
H
I
K
L
M
N
P
Q
R
S
T
V
W
Y

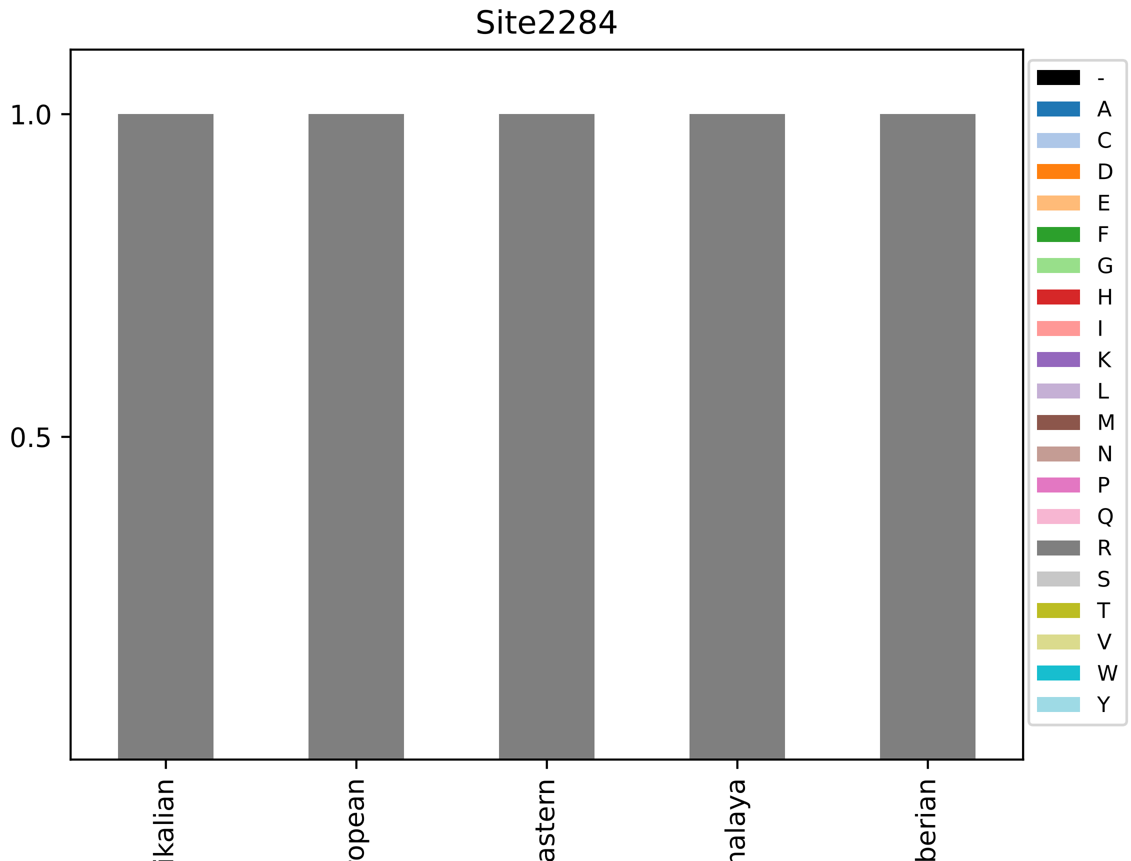
Site2284
1.0
0.5
-
A
C
D
E
F
G
H
I
K
L
M
N
P
Q
R
S
T
V
W
Y
ikalian
opean
astern
nalaya
berian

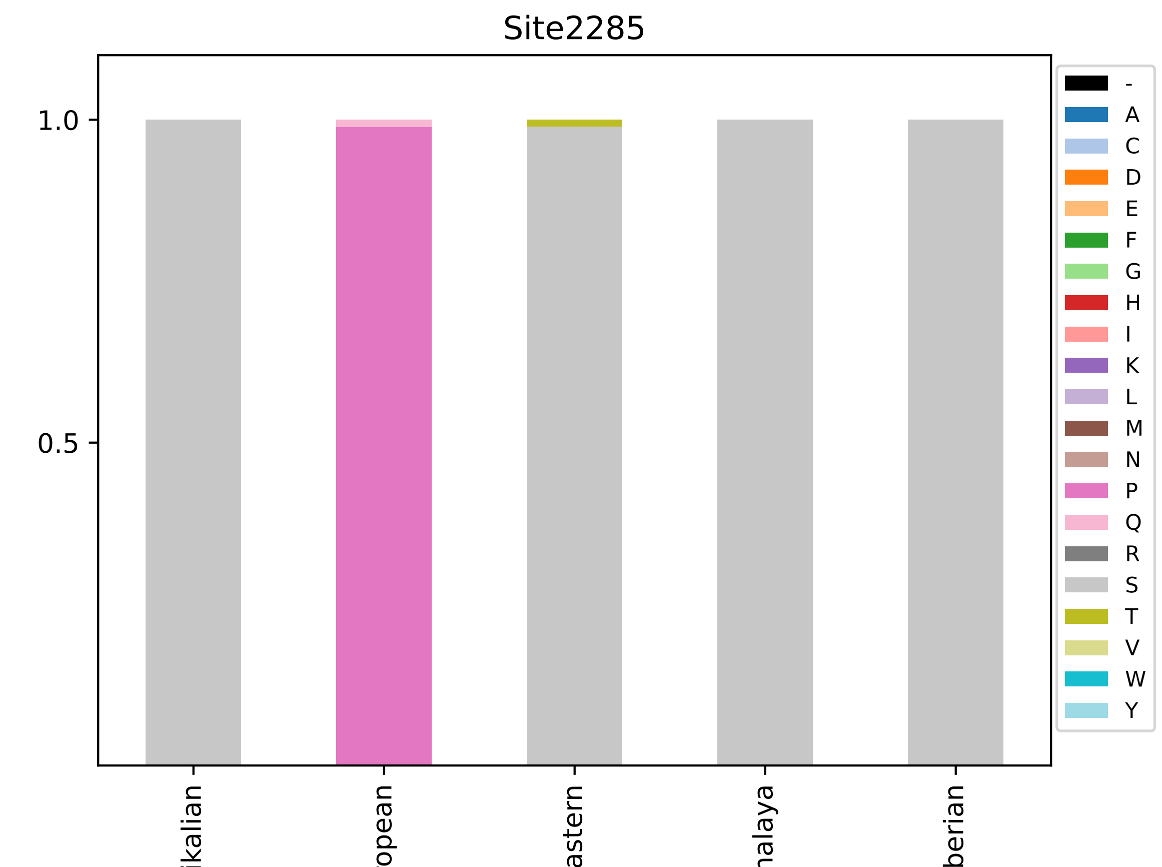

Site2285
1.0
0.5
-
A
C
D
E
F
G
H
I
K
L
M
N
P
Q
R
S
T
V
W
Y
ikalian
opean
astern
nalaya
berian

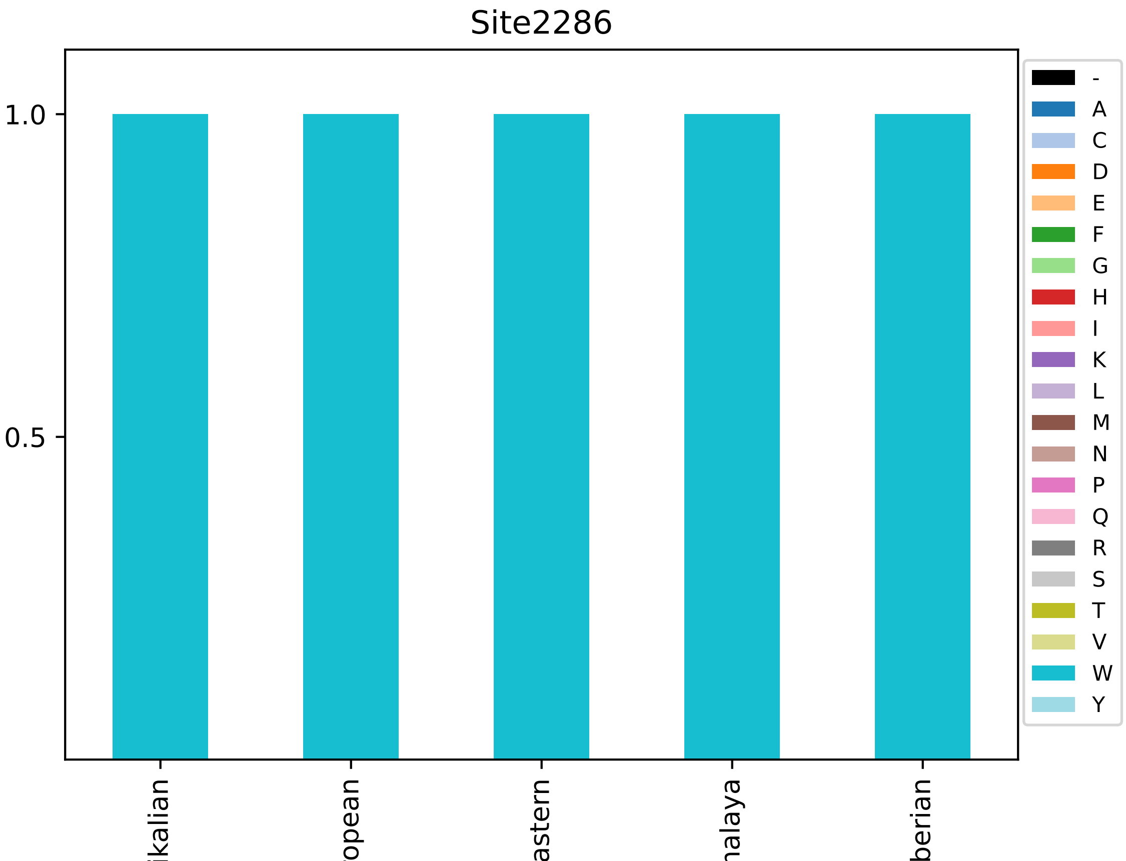
Site2286
1.0
0.5
-
A
C
D
E
F
G
H
I
K
L
M
N
P
Q
R
S
T
V
W
Y
ikalian
opean
astern
nalaya
berian

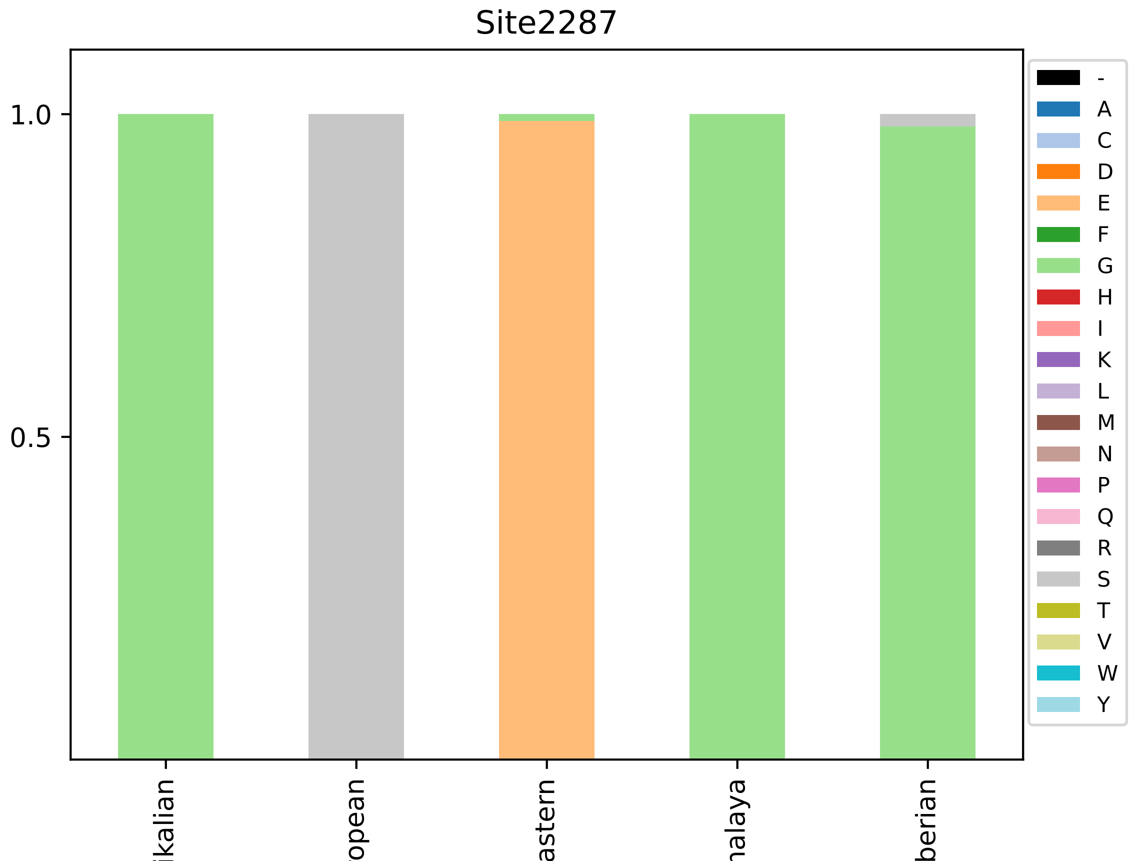
Site2287
1.0
0.5
-
A
C
D
E
F
G
H
I
K
L
M
N
P
Q
R
S
T
V
W
Y
ikalian
opean
astern
nalaya
berian

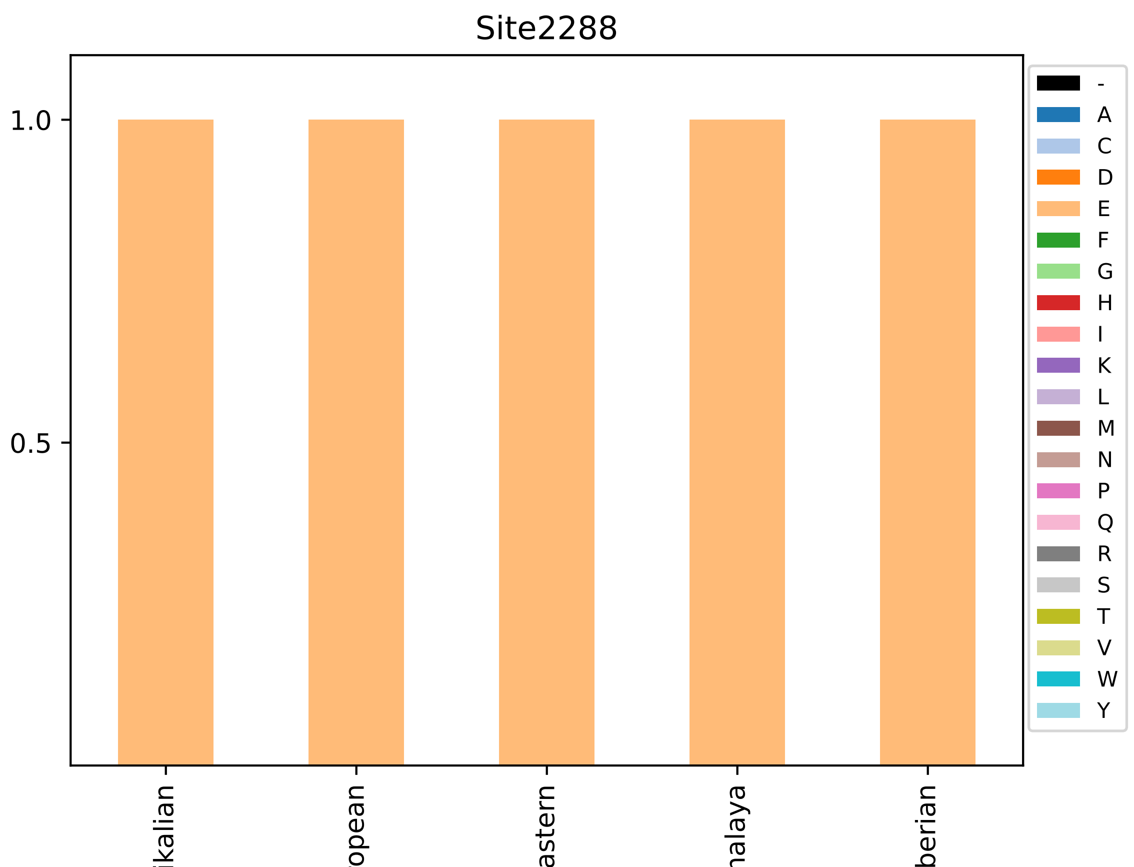

Site2288
1.0
0.5
-
A
C
D
E
F
G
H
I
K
L
M
N
P
Q
R
S
T
V
W
Y
ikalian
opean
astern
nalaya
berian

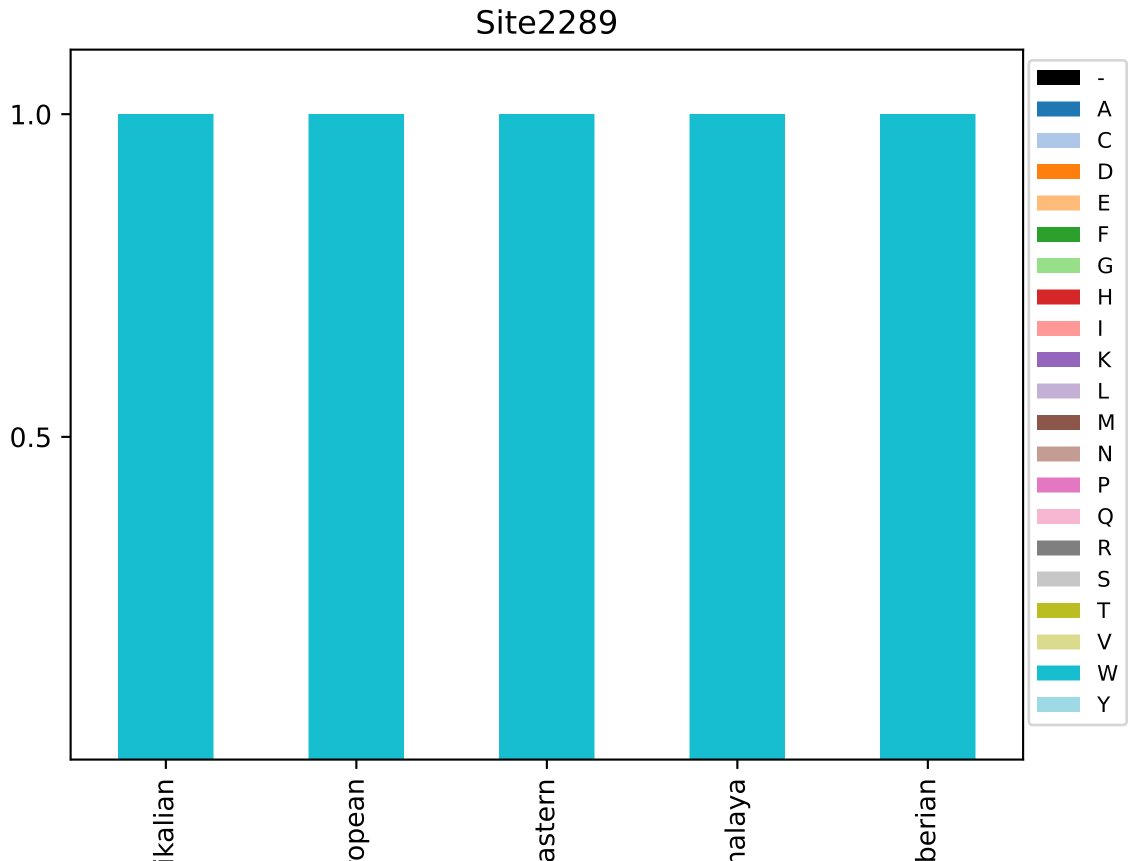
Site2289
1.0
0.5
-
A
C
D
E
F
G
H
I
K
L
M
N
P
Q
R
S
T
V
W
Y
ikalian
opean
astern
nalaya
berian

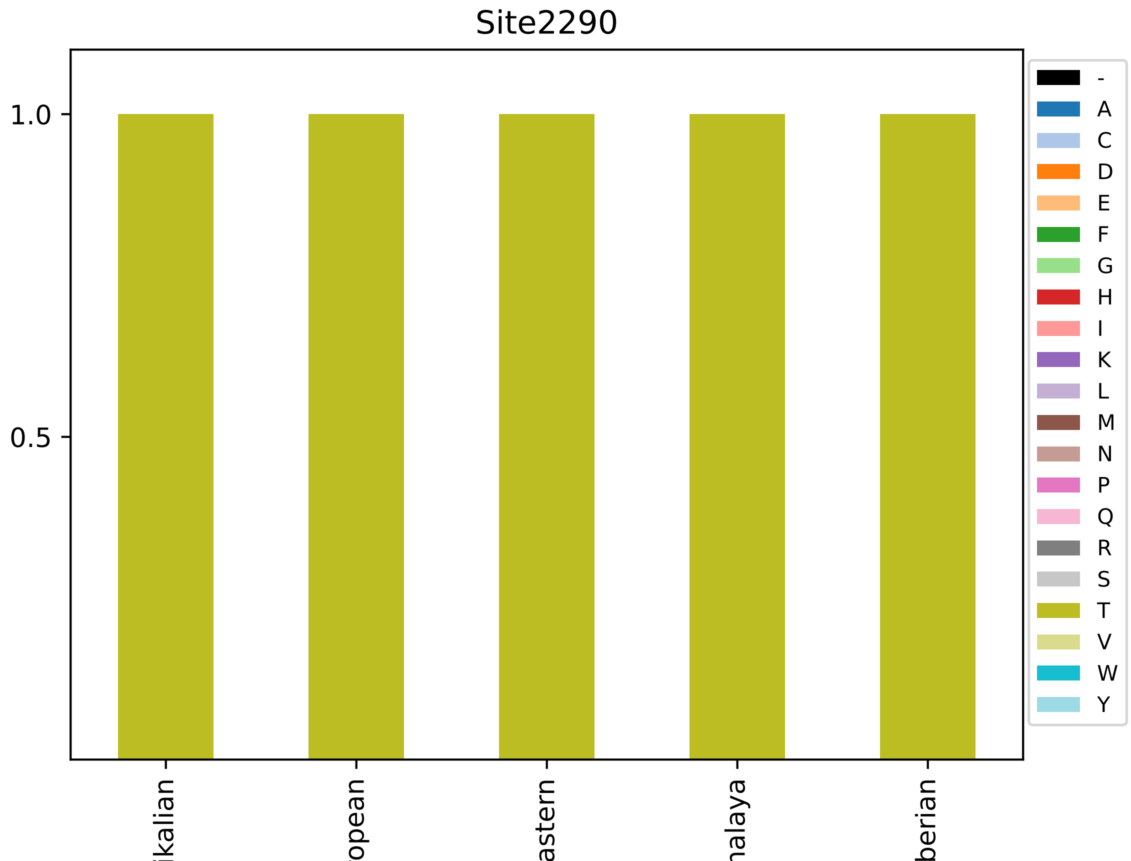
Site2290
1.0
0.5
-
A
C
D
E
F
G
H
I
K
L
M
N
P
Q
R
S
T
V
W
Y
ikalian
opean
astern
nalaya
berian

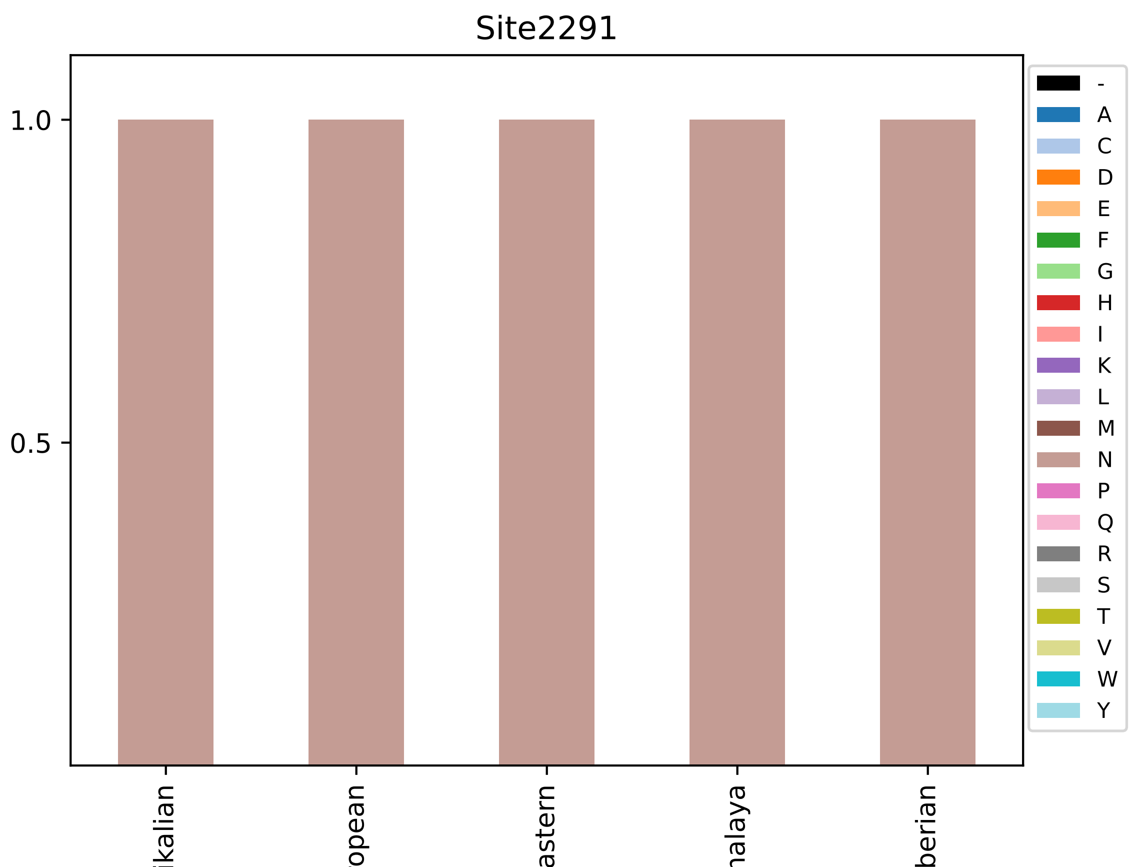

Site2291
1.0
0.5
-
A
C
D
E
F
G
H
I
K
L
M
N
P
Q
R
S
T
V
W
Y

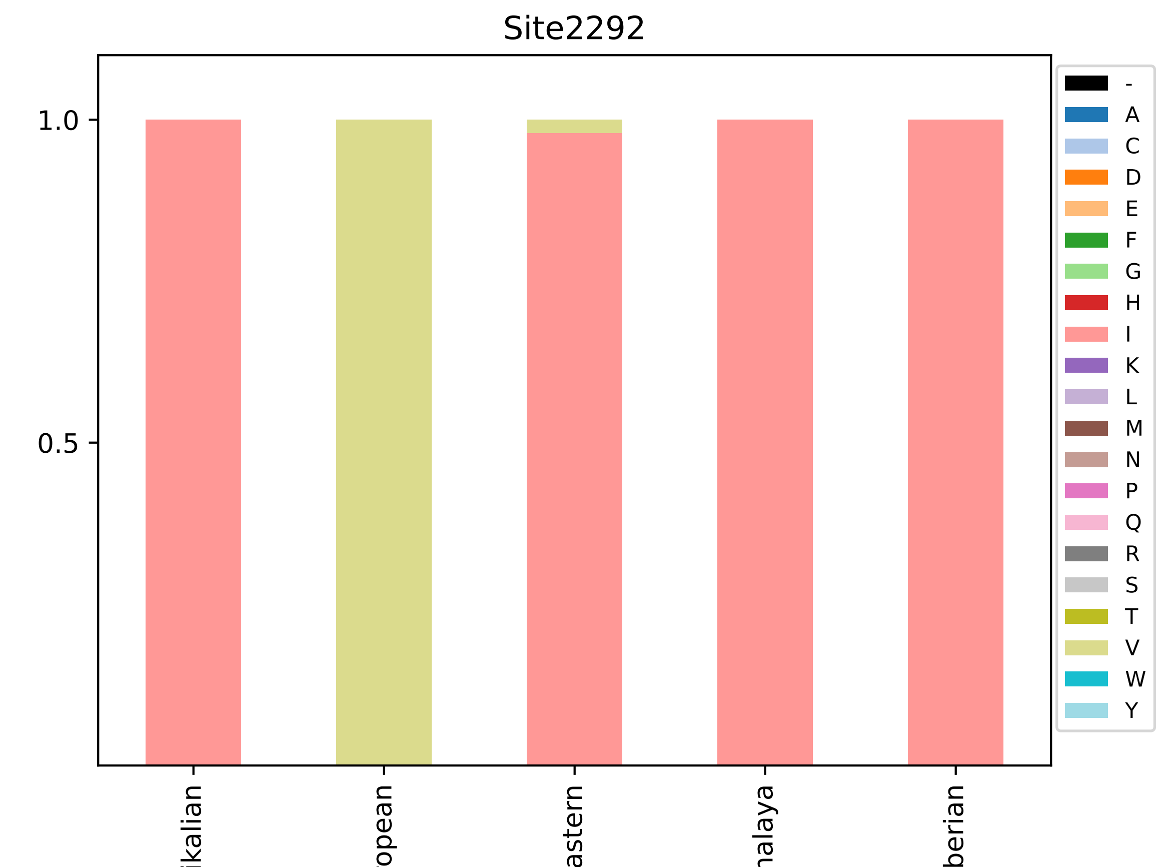

Site2292
1.0
0.5
-
A
C
D
E
F
G
H
I
K
L
M
N
P
Q
R
S
T
V
W
Y
ikalian
opean
astern
nalaya
berian

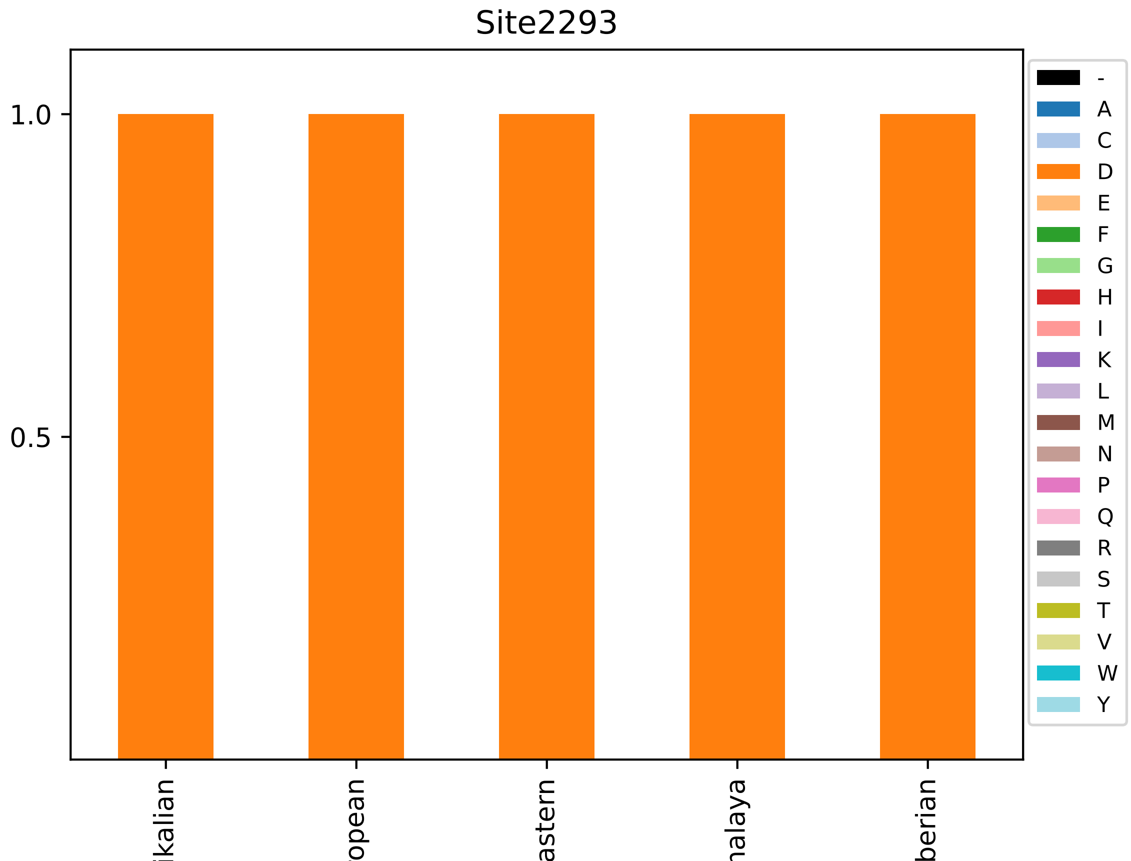

Site2293
1.0
0.5
-
A
C
D
E
F
G
H
I
K
L
M
N
P
Q
R
S
T
V
W
Y
ikalian
opean
astern
nalaya
berian

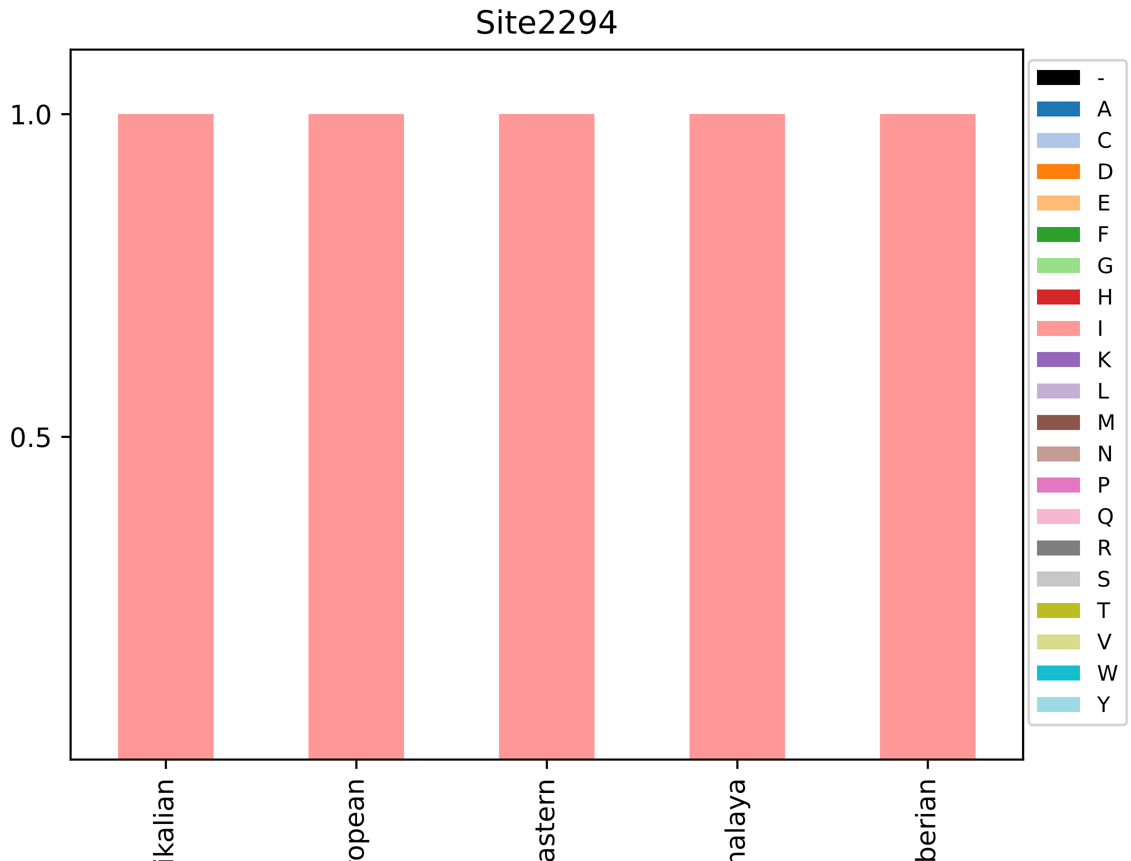
Site2294
1.0
0.5
-
A
C
D
E
F
G
H
I
K
L
M
N
P
Q
R
S
T
V
W
Y

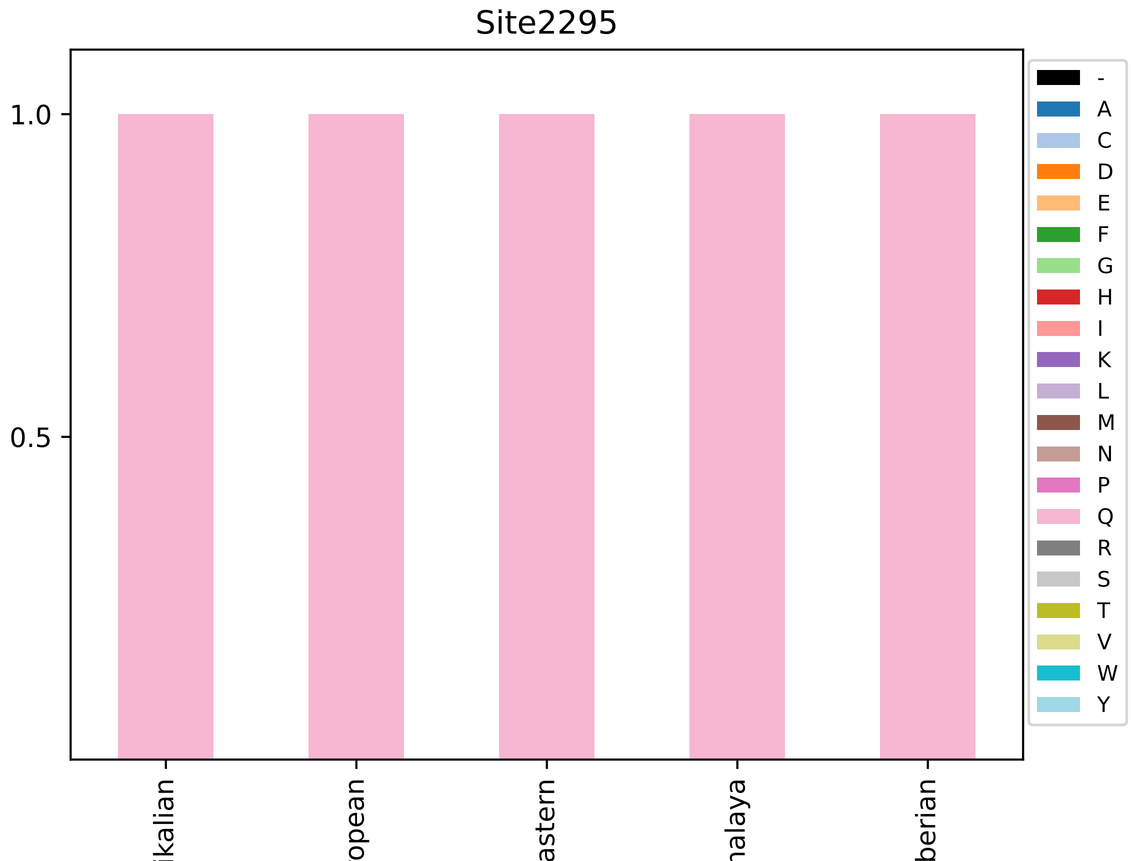
Site2295
1.0
0.5
ikalian
opean
astern
nalaya
berian
-
A
C
D
E
F
G
H
I
K
L
M
N
P
Q
R
S
T
V
W
Y

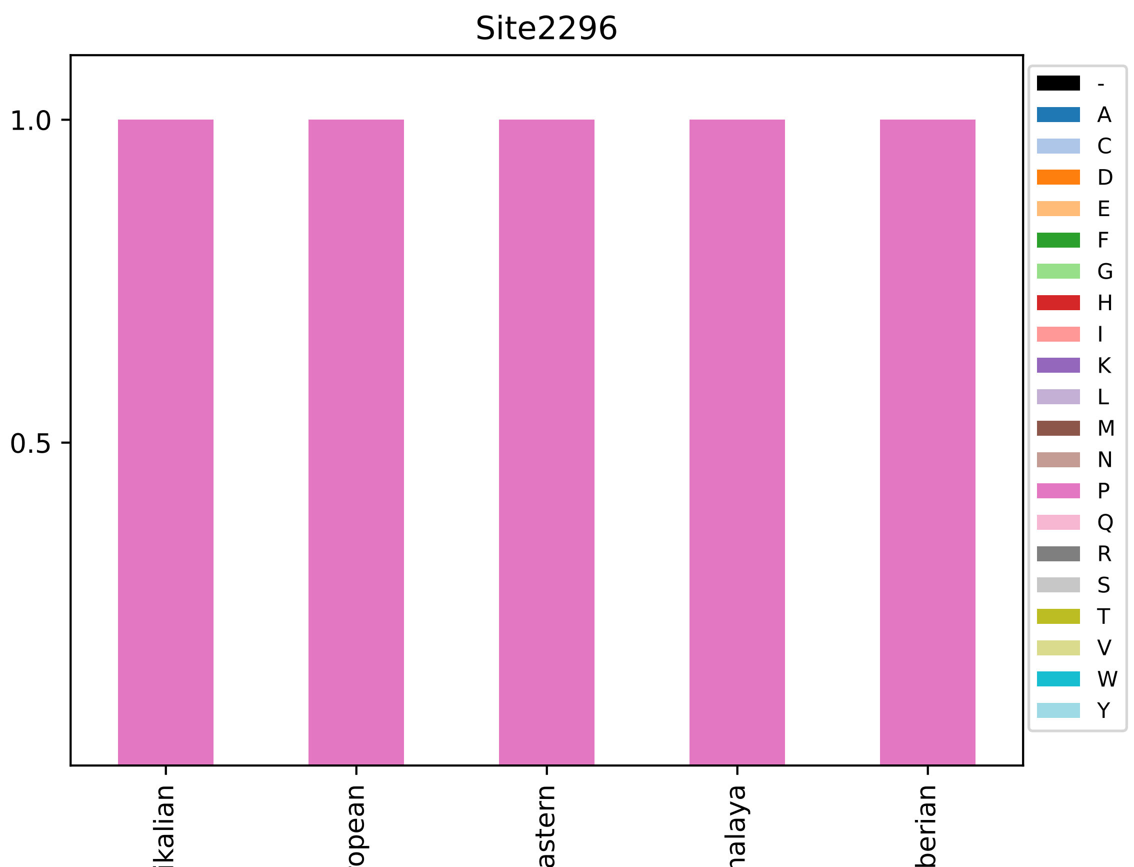

Site2296
1.0
0.5
ikalian
opean
astern
nalaya
berian
-
A
C
D
E
F
G
H
I
K
L
M
N
P
Q
R
S
T
V
W
Y

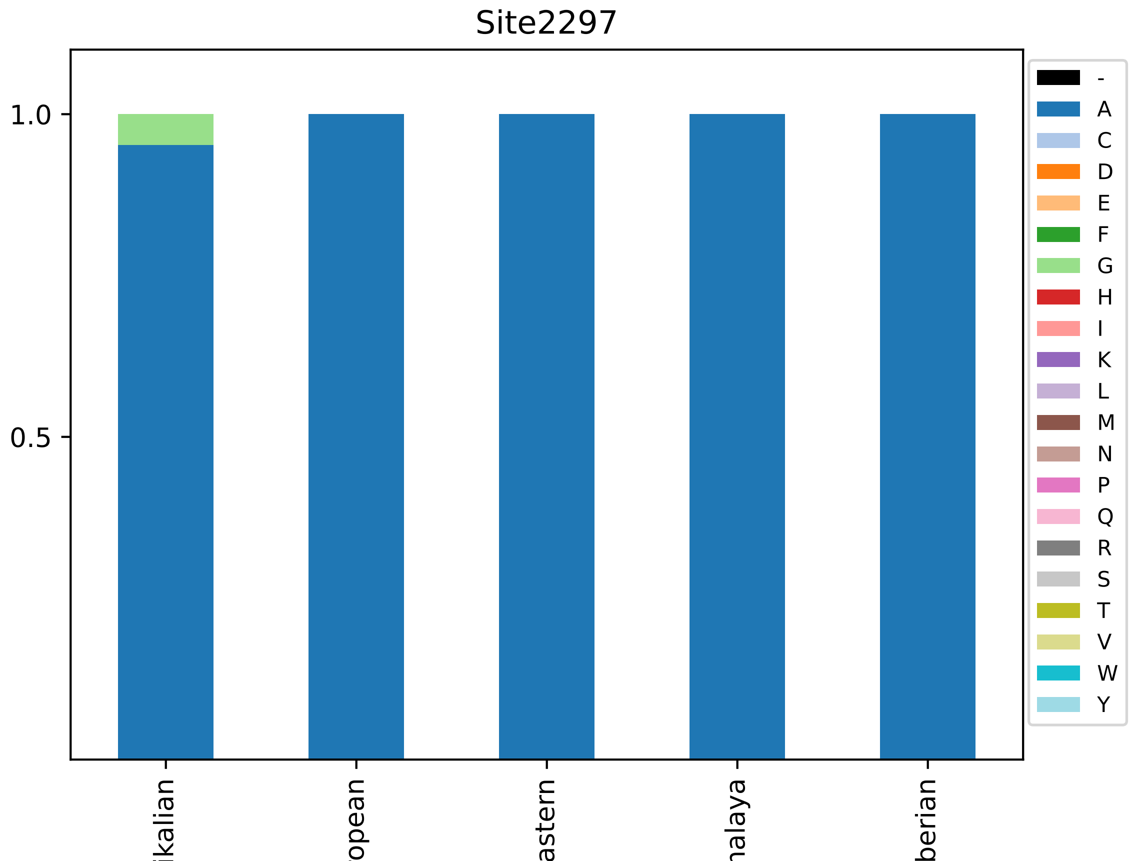
Site2297
1.0
0.5
-
A
C
D
E
F
G
H
I
K
L
M
N
P
Q
R
S
T
V
W
Y
ikalian
opean
astern
nalaya
berian

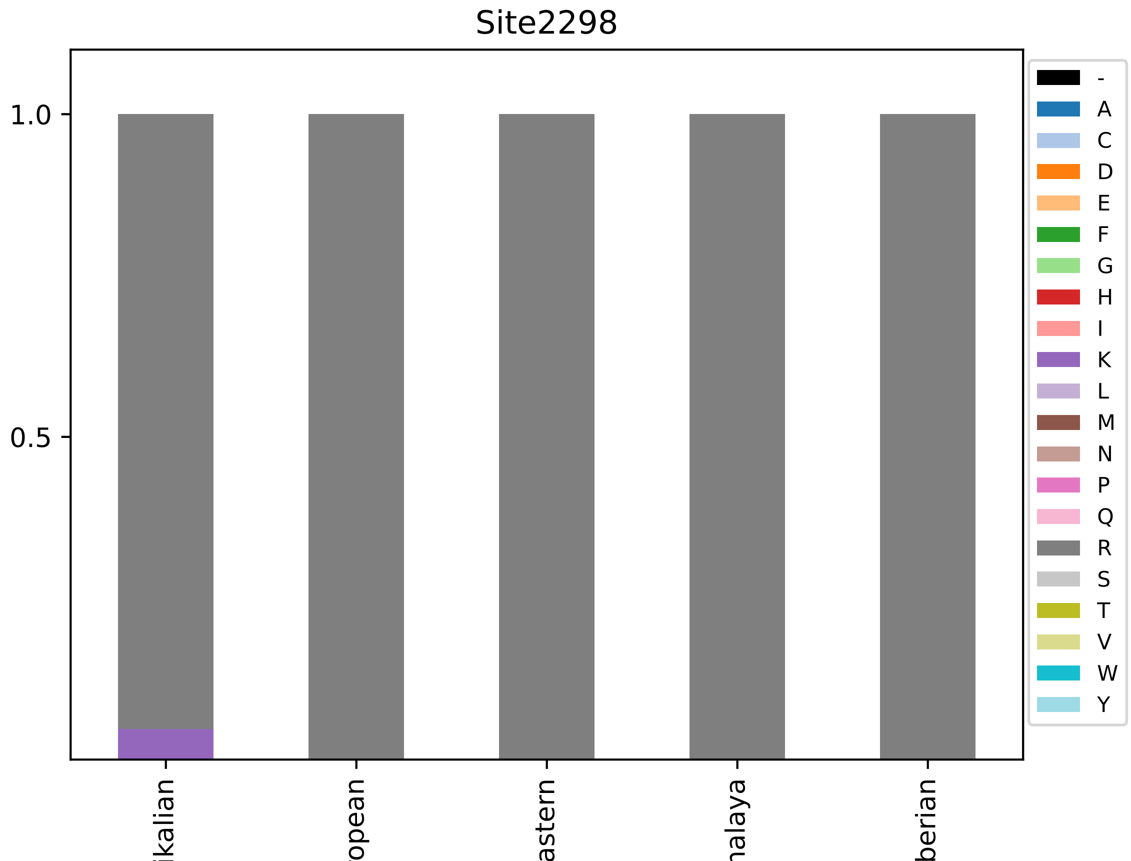
Site2298
1.0
0.5
-
A
C
D
E
F
G
H
I
K
L
M
N
P
Q
R
S
T
V
W
Y
ikalian
opean
astern
nalaya
berian

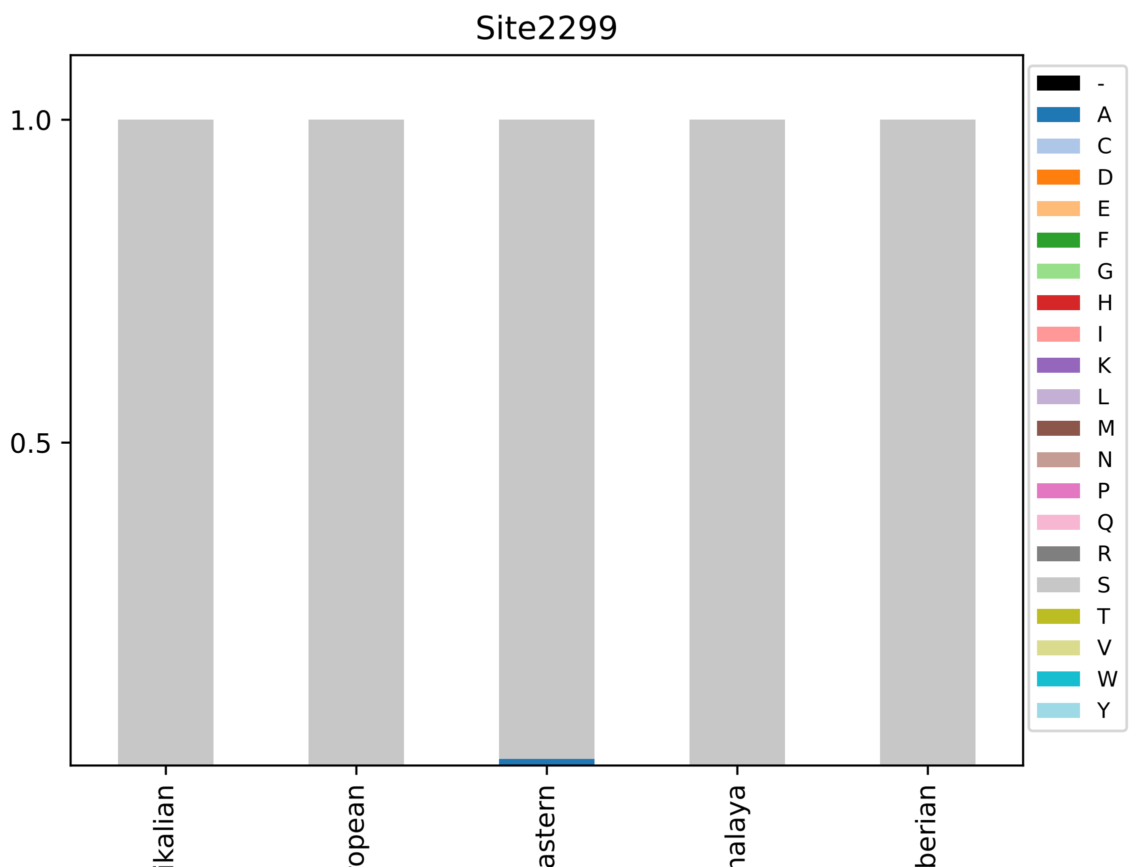
Site2299
1.0
0.5
-
A
C
D
E
F
G
H
I
K
L
M
N
P
Q
R
S
T
V
W
Y
ikalian
opean
astern
nalaya
berian

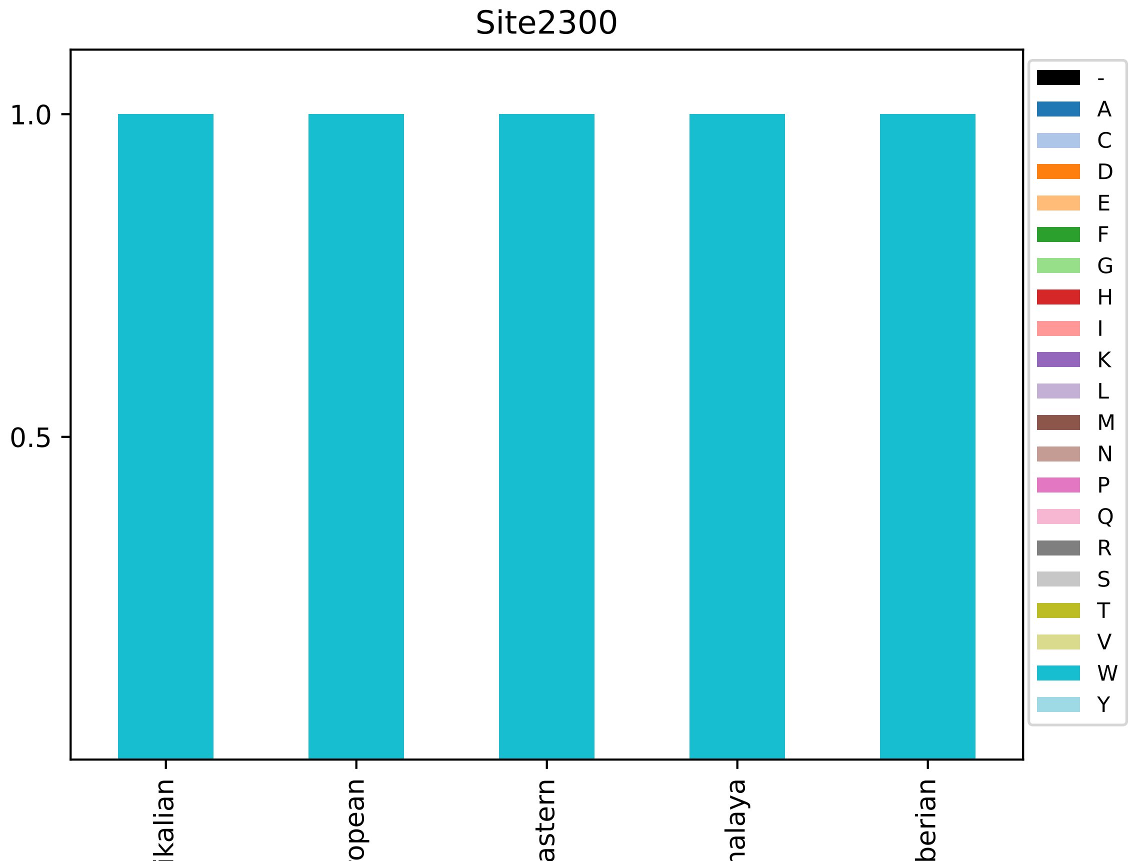

Site2300
1.0
0.5
-
A
C
D
E
F
G
H
I
K
L
M
N
P
Q
R
S
T
V
W
Y
ikalian
opean
astern
nalaya
berian

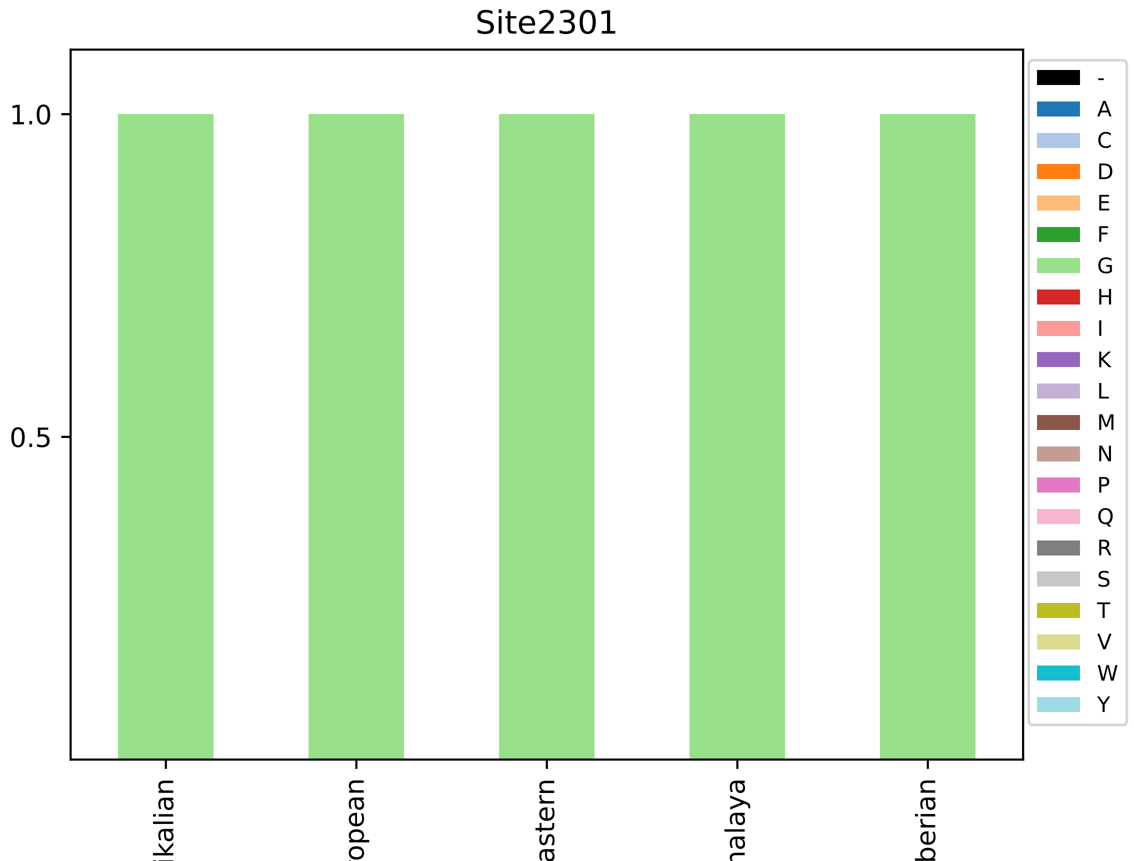
Site2301
1.0
0.5
-
A
C
D
E
F
G
H
I
K
L
M
N
P
Q
R
S
T
V
W
Y
ikalian
opean
astern
nalaya
berian

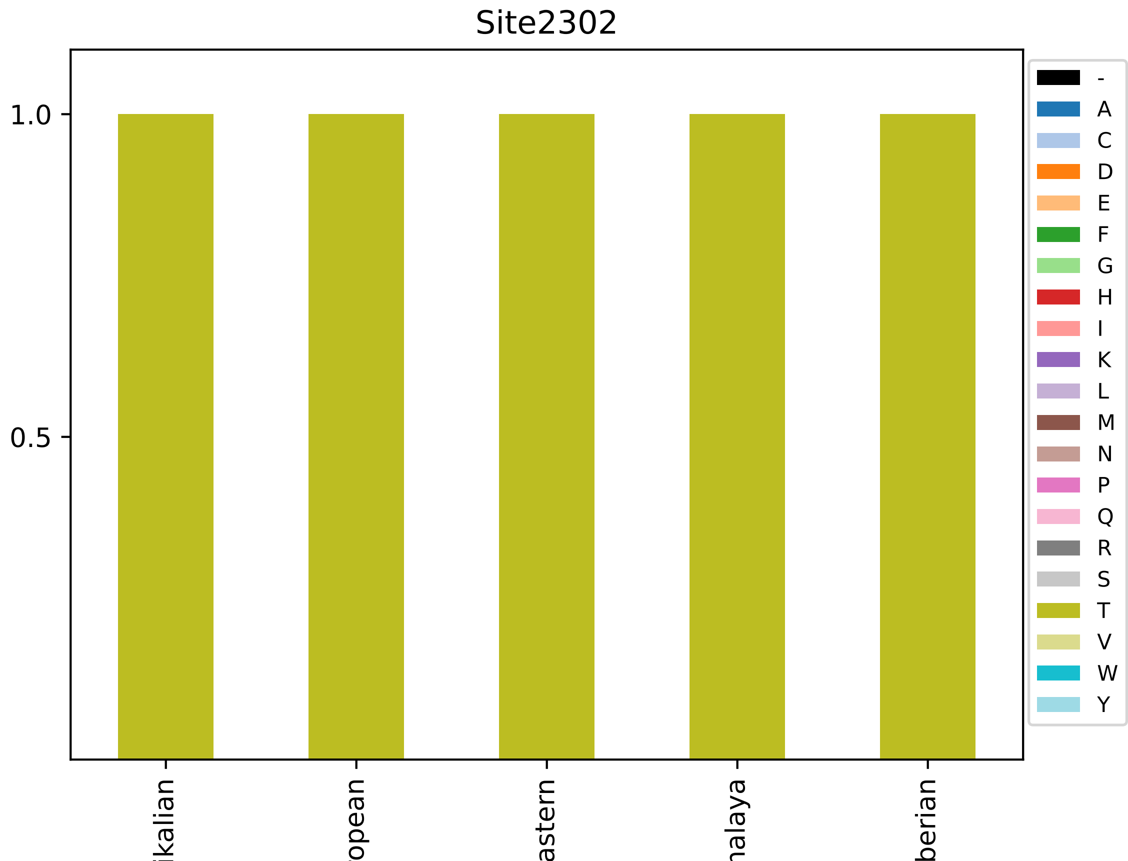
Site2302
1.0
0.5
-
A
C
D
E
F
G
H
I
K
L
M
N
P
Q
R
S
T
V
W
Y
ikalian
opean
astern
nalaya
berian

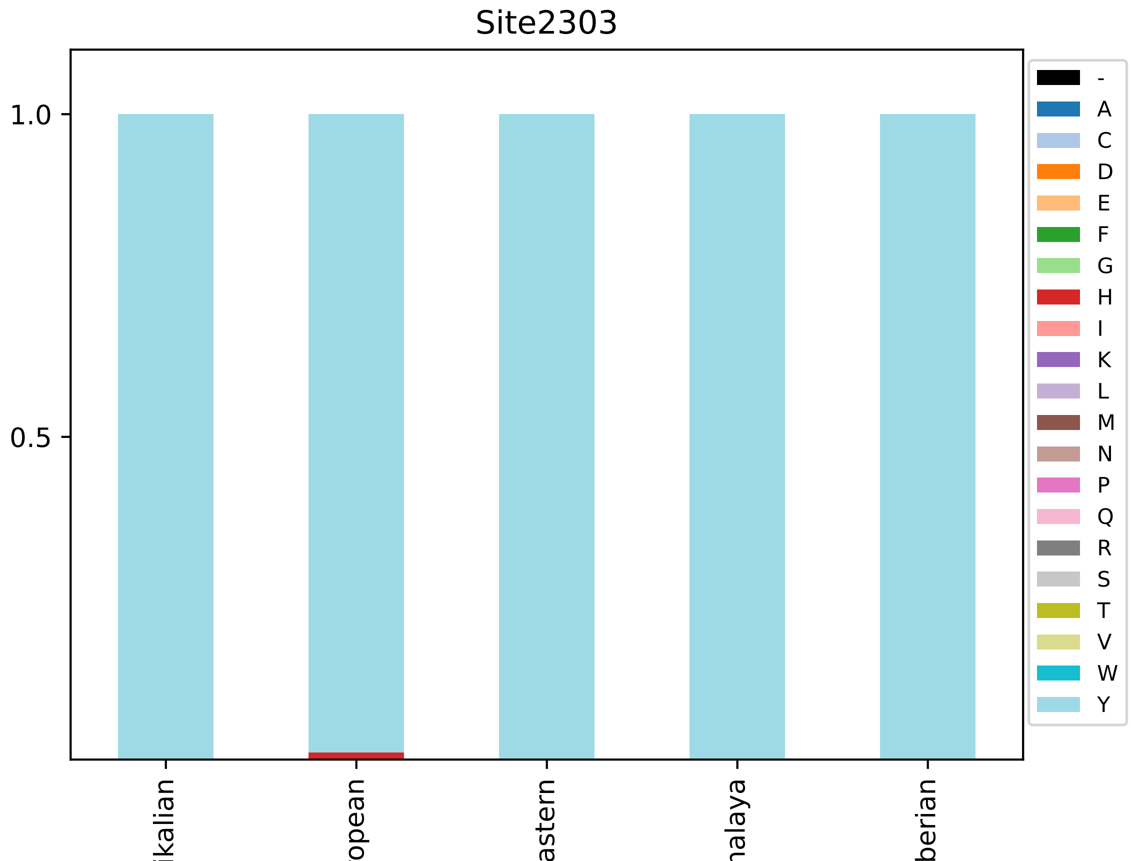
Site2303
1.0
0.5
-
A
C
D
E
F
G
H
I
K
L
M
N
P
Q
R
S
T
V
W
Y
ikalian
opean
astern
nalaya
berian

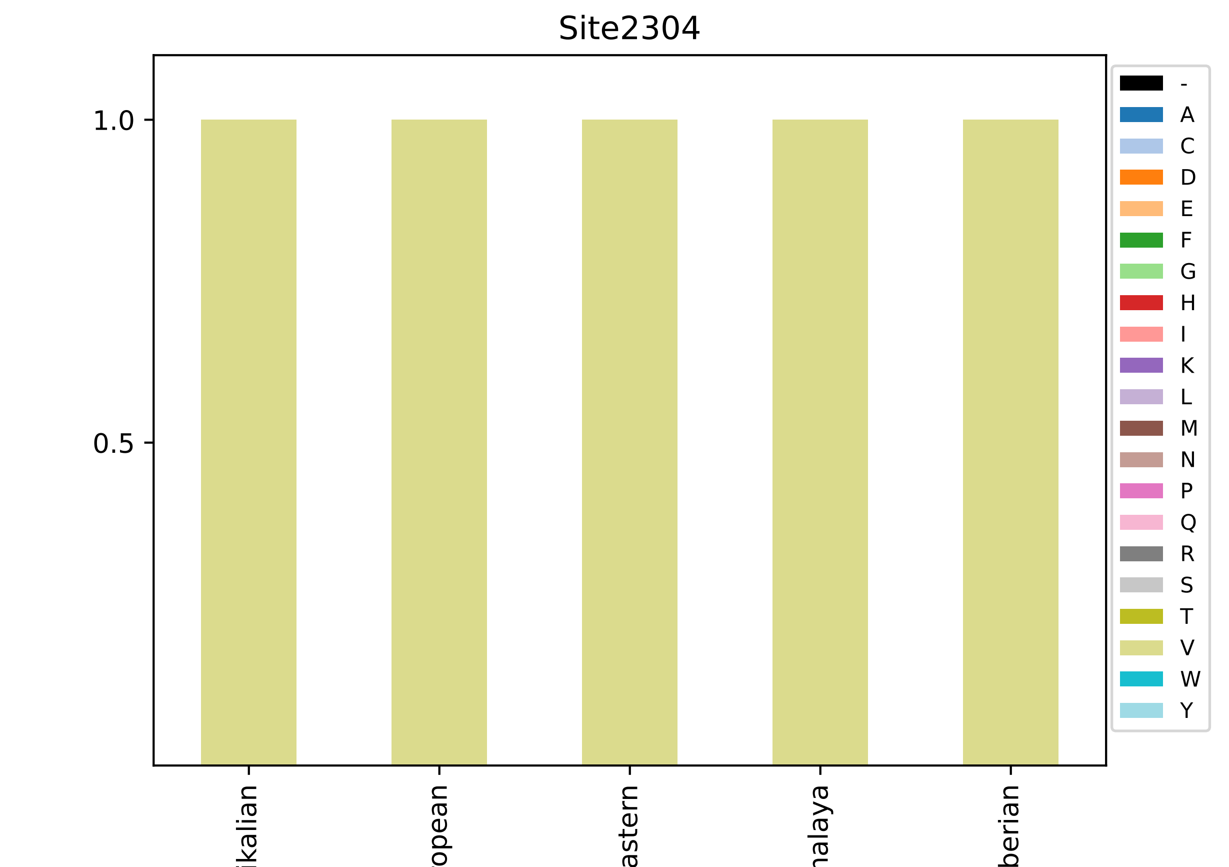

Site2304
1.0
0.5
-
A
C
D
E
F
G
H
I
K
L
M
N
P
Q
R
S
T
V
W
Y
ikalian
opean
astern
nalaya
berian

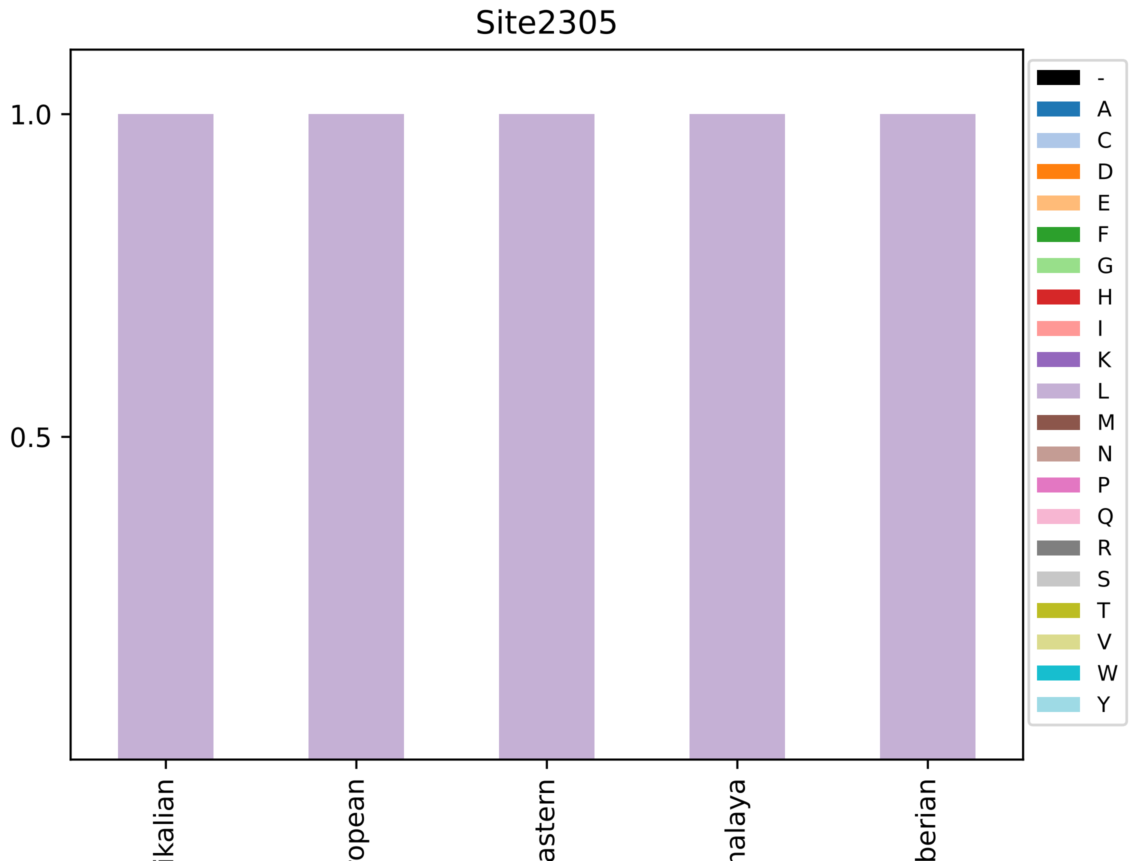
Site2305
1.0
0.5
-
A
C
D
E
F
G
H
I
K
L
M
N
P
Q
R
S
T
V
W
Y
ikalian
opean
astern
nalaya
berian

Site2306

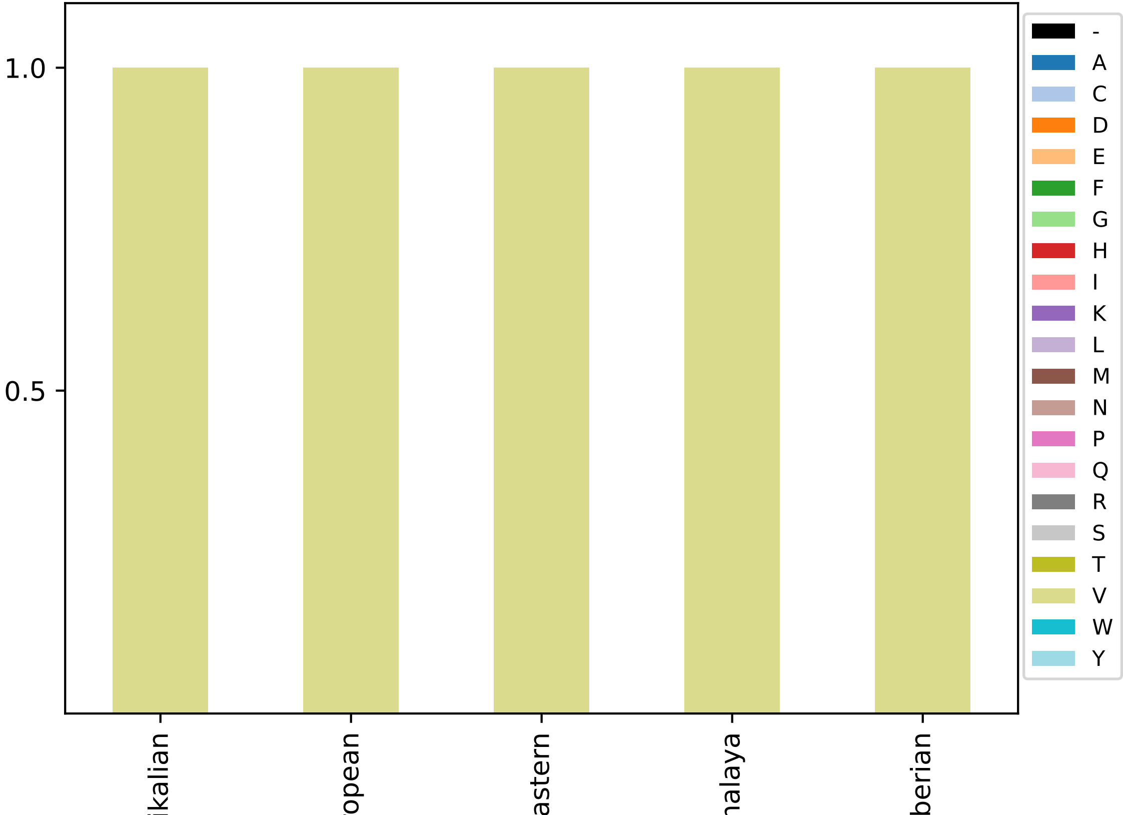

1.0
0.5
ikalian
ropean
astern
nalaya
berian
-
A
C
D
E
F
G
H
I
K
L
M
N
P
Q
R
S
T
V
W
Y

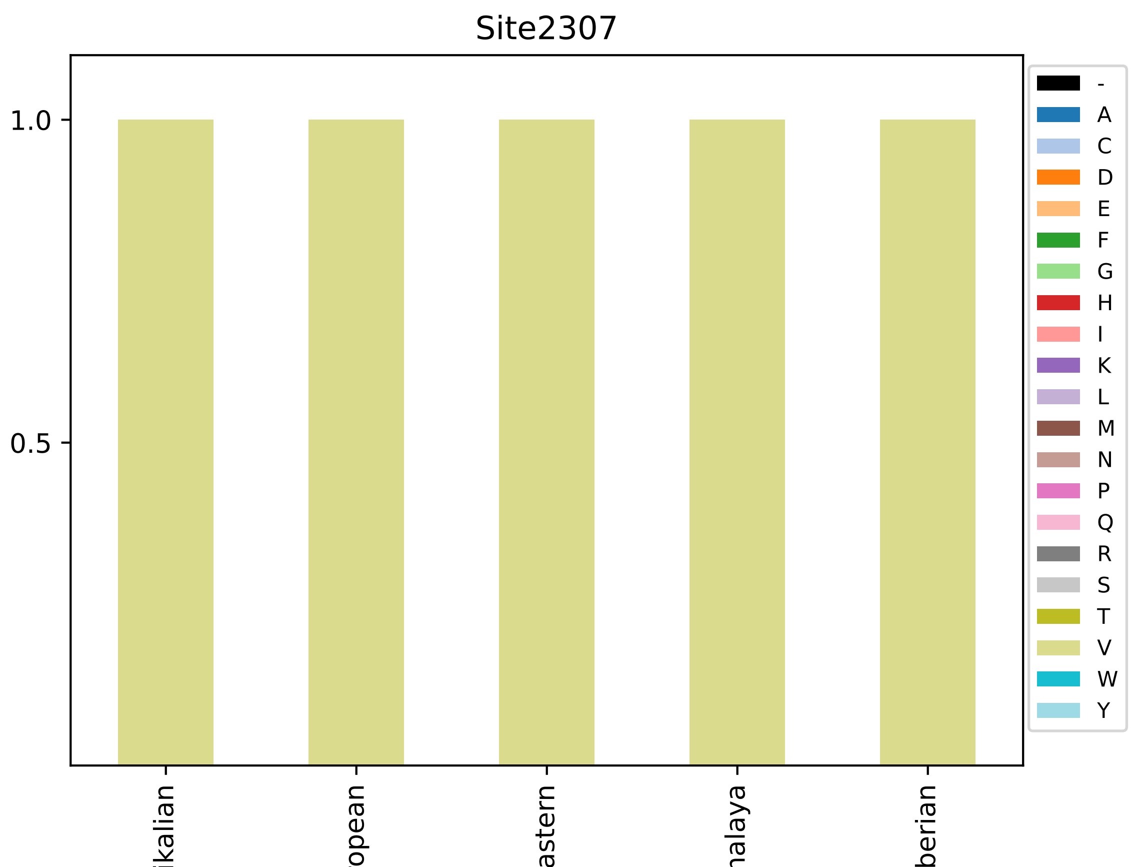
Site2307
1.0
0.5
ikalian
opean
astern
nalaya
berian
-
A
C
D
E
F
G
H
I
K
L
M
N
P
Q
R
S
T
V
W
Y

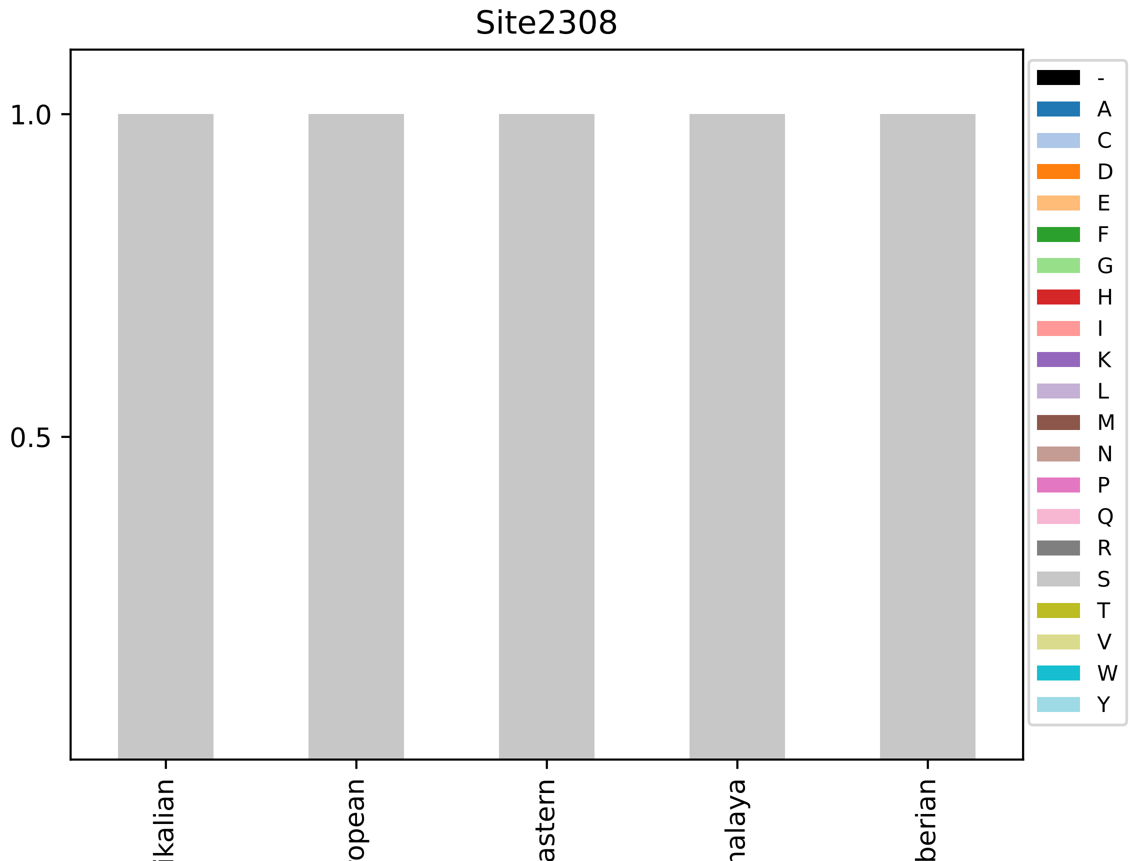

Site2308
1.0
0.5
-
A
C
D
E
F
G
H
I
K
L
M
N
P
Q
R
S
T
V
W
Y
ikalian
opean
astern
nalaya
berian

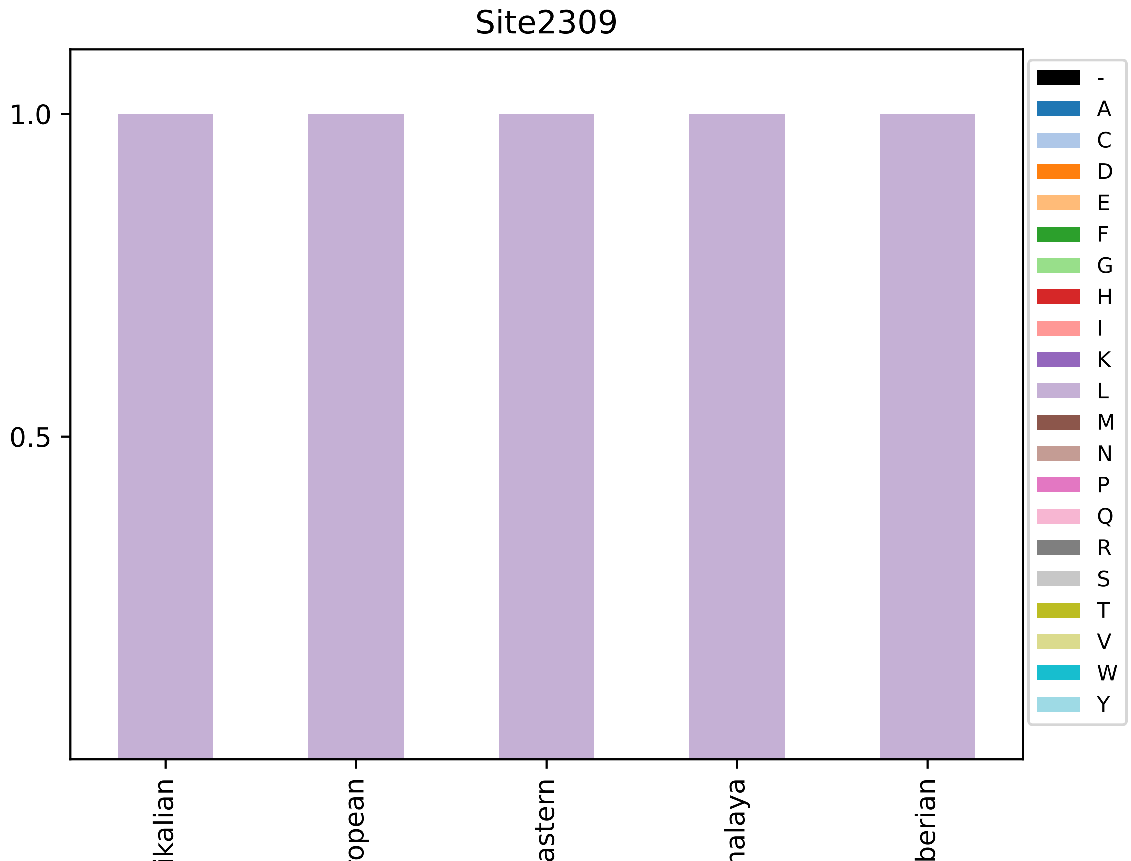
Site2309
1.0
0.5
-
A
C
D
E
F
G
H
I
K
L
M
N
P
Q
R
S
T
V
W
Y
ikalian
opean
astern
nalaya
berian

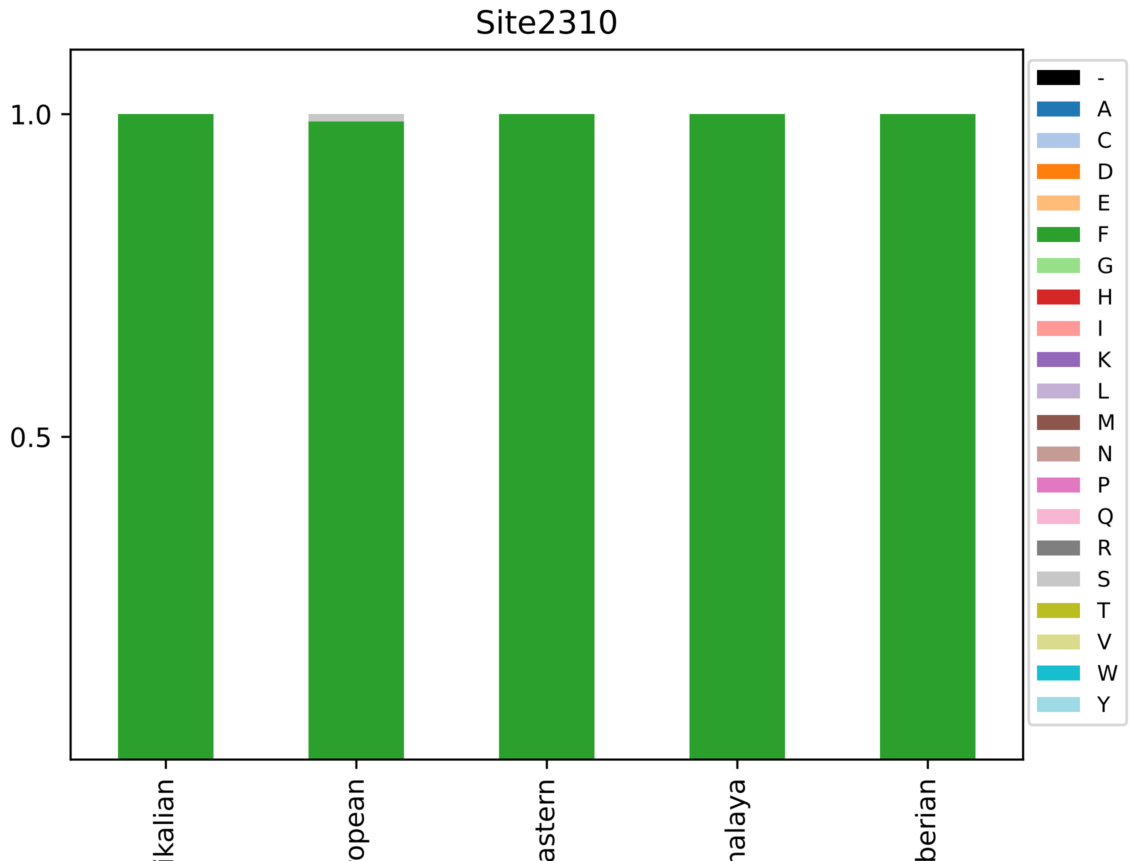
Site2310
1.0
0.5
-
A
C
D
E
F
G
H
I
K
L
M
N
P
Q
R
S
T
V
W
Y
ikalian
opean
astern
nalaya
berian

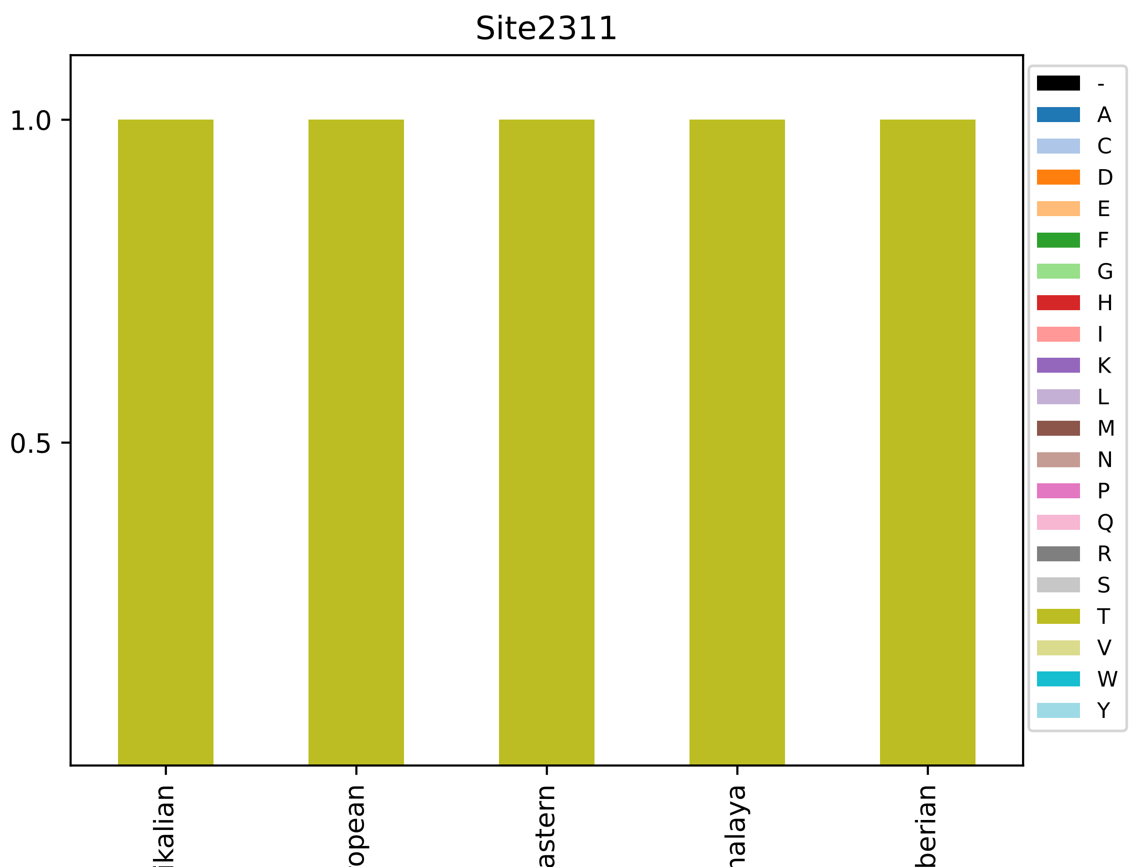
Site2311
1.0
0.5
-
A
C
D
E
F
G
H
I
K
L
M
N
P
Q
R
S
T
V
W
Y
ikalian
opean
astern
nalaya
berian

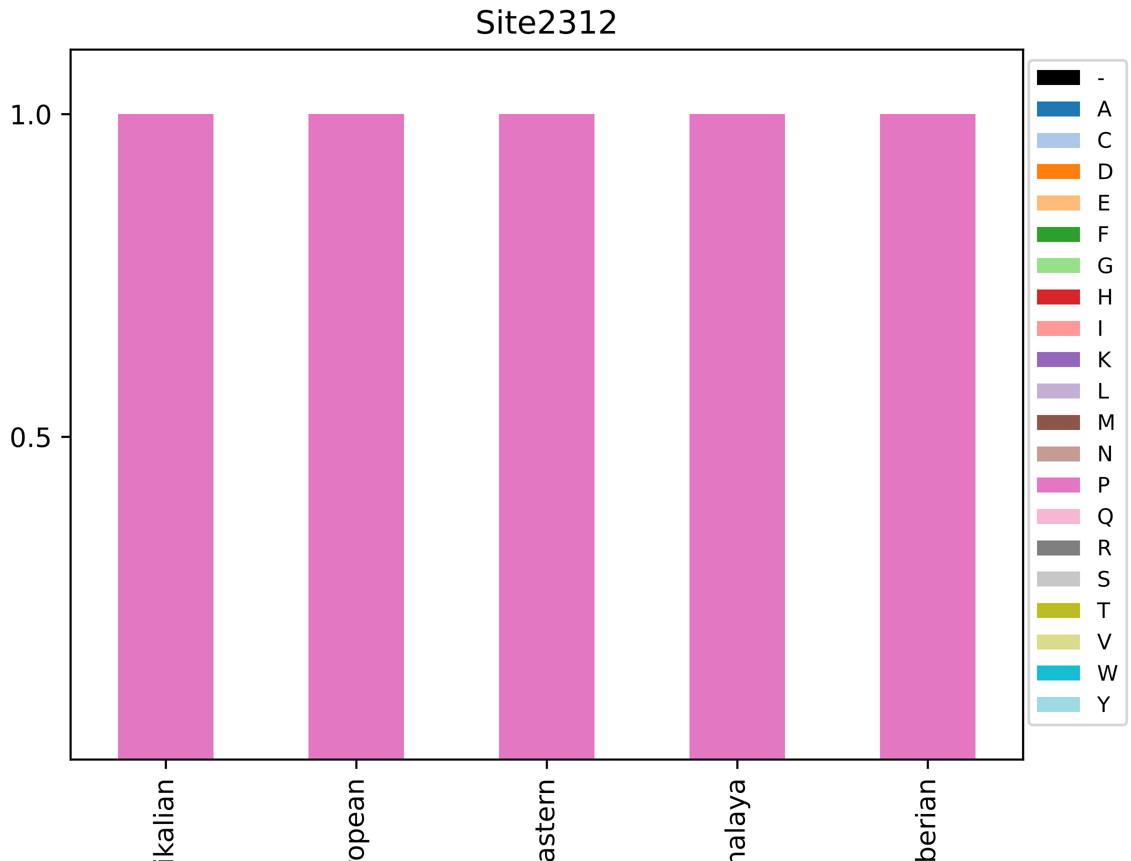

Site2312
1.0
0.5
-
A
C
D
E
F
G
H
I
K
L
M
N
P
Q
R
S
T
V
W
Y
ikalian
opean
astern
nalaya
berian

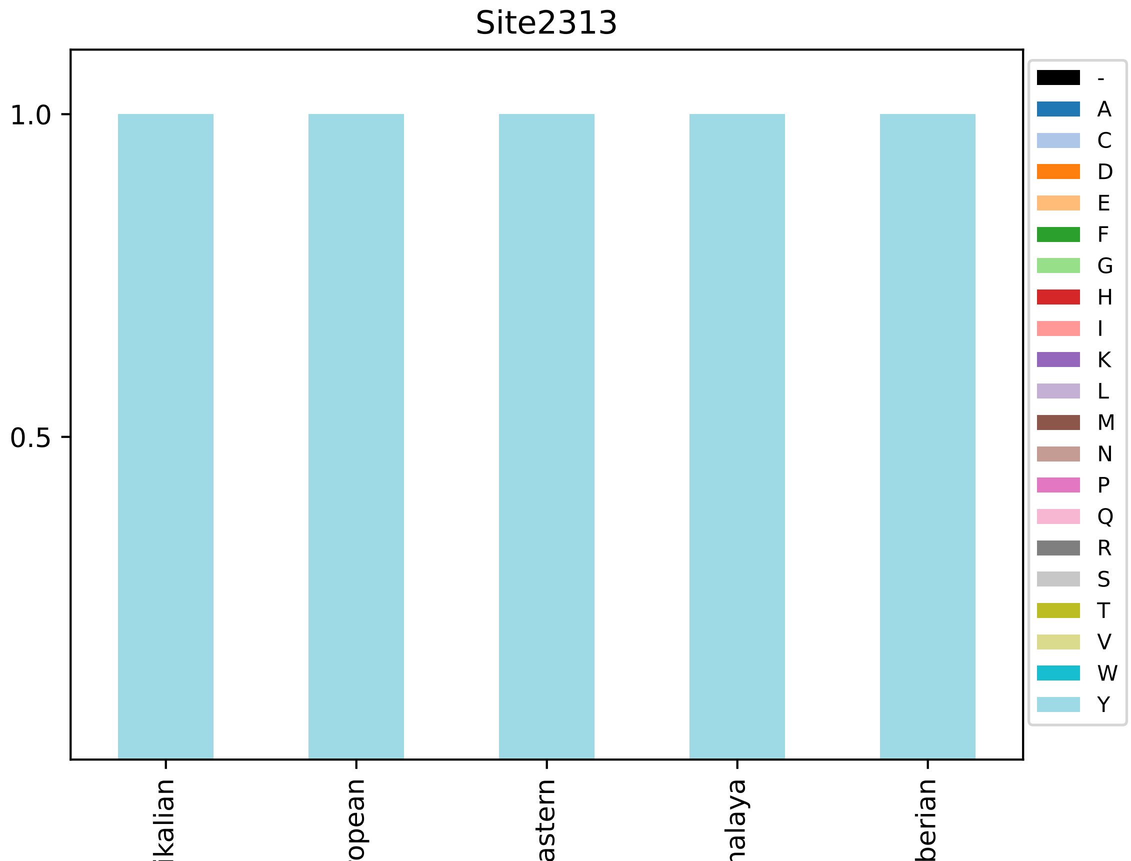

Site2313
1.0
0.5
-
A
C
D
E
F
G
H
I
K
L
M
N
P
Q
R
S
T
V
W
Y
ikalian
opean
astern
nalaya
berian

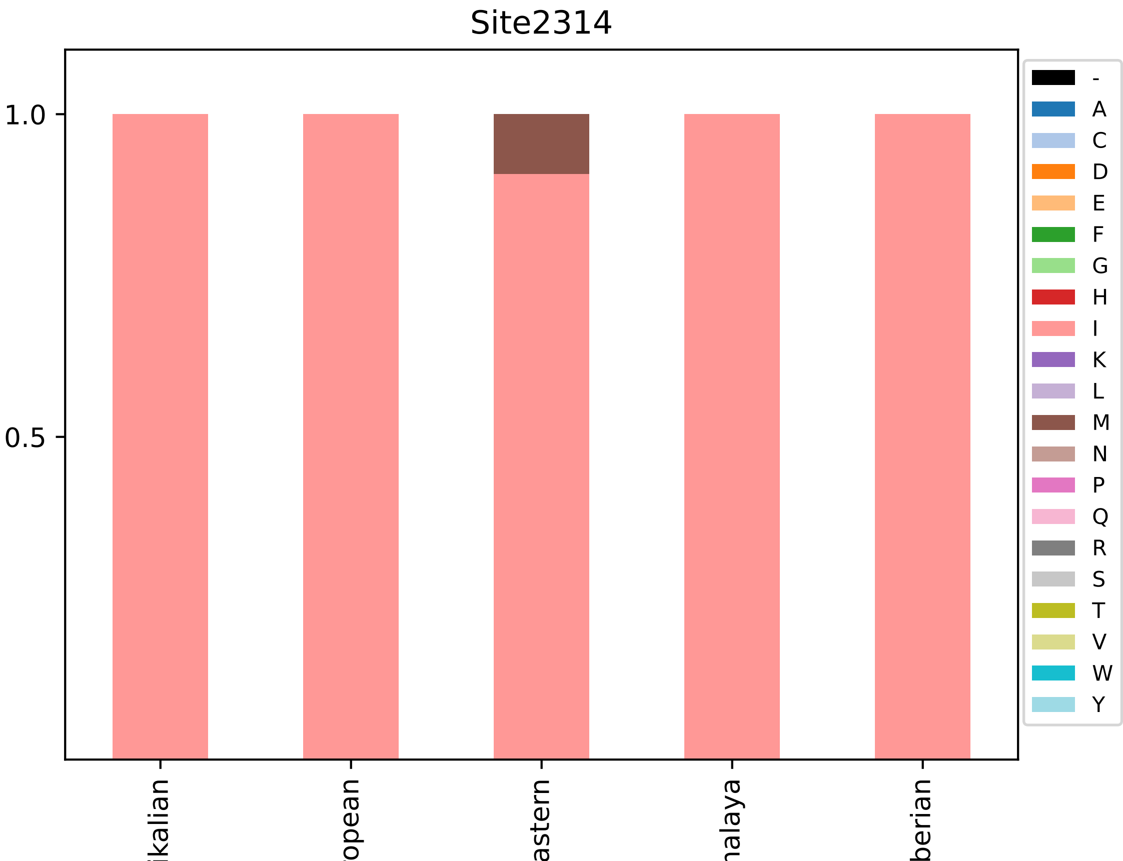
Site2314
1.0
0.5
-
A
C
D
E
F
G
H
I
K
L
M
N
P
Q
R
S
T
V
W
Y
ikalian
opean
astern
nalaya
berian

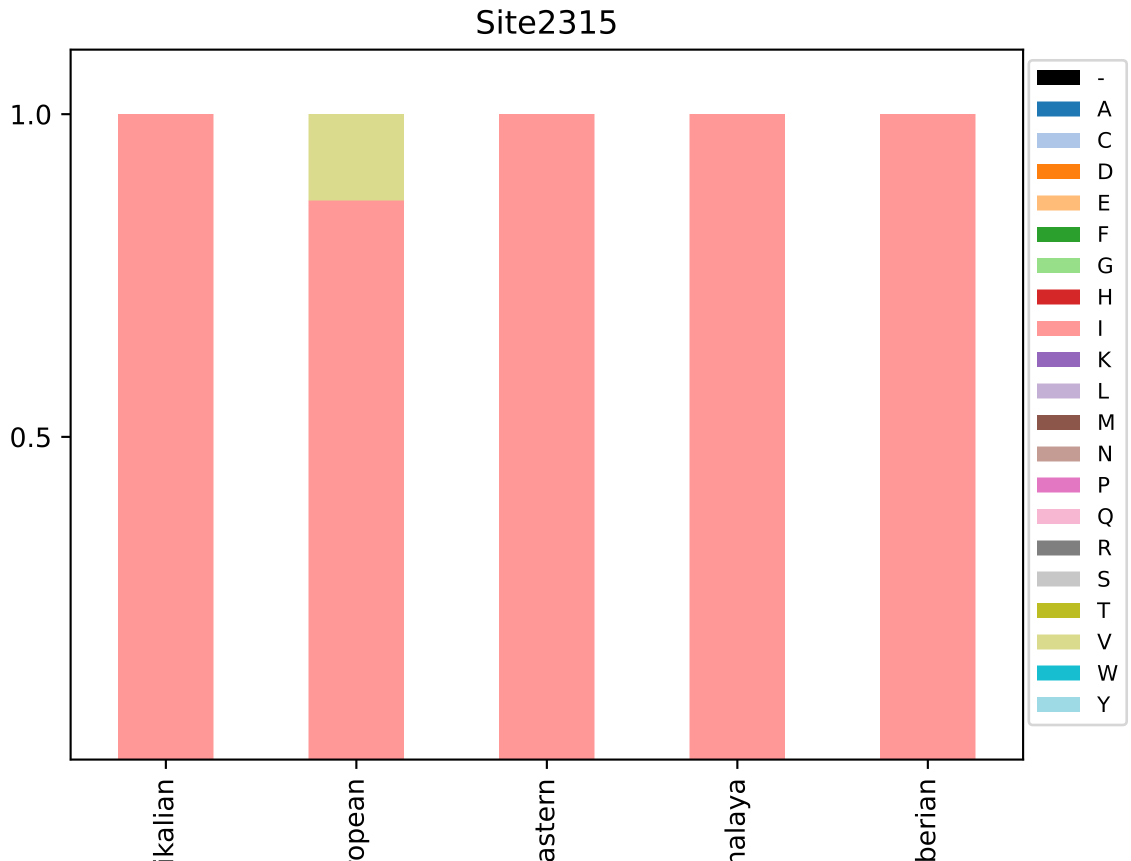
Site2315
1.0
0.5
-
A
C
D
E
F
G
H
I
K
L
M
N
P
Q
R
S
T
V
W
Y
ikalian
opean
astern
nalaya
berian

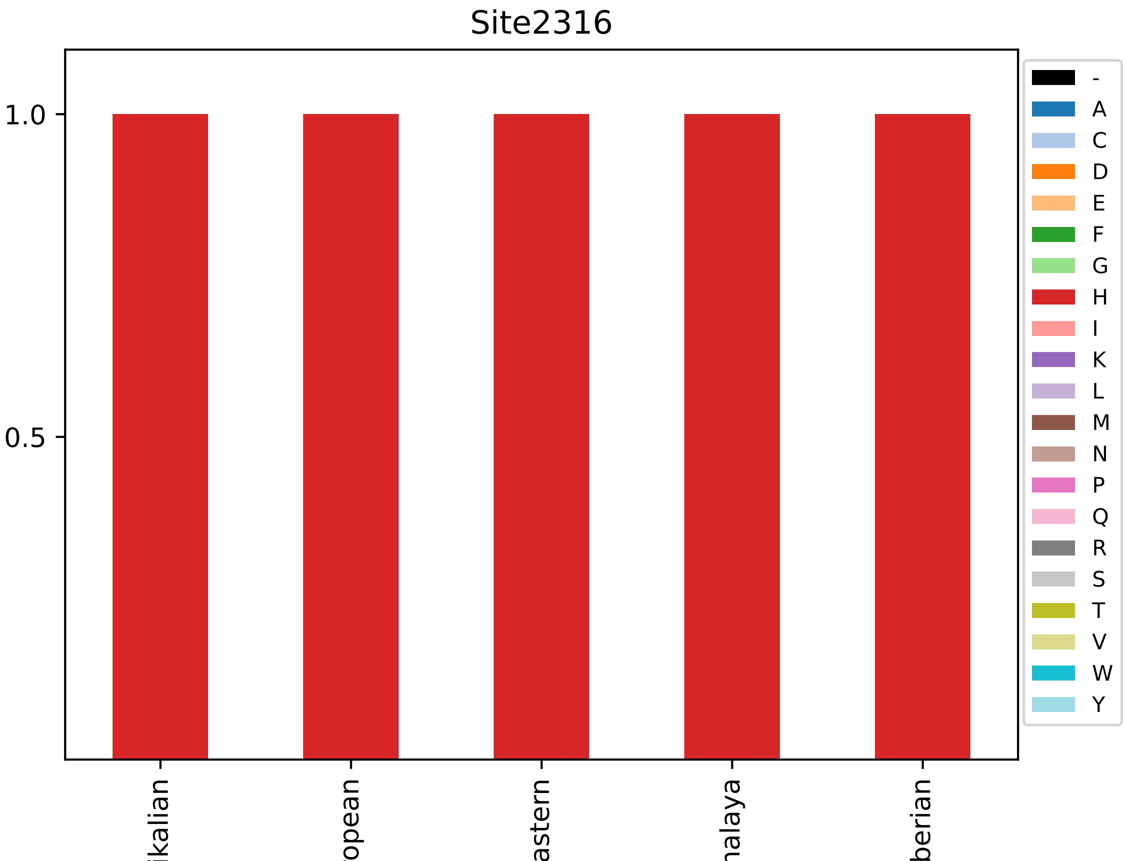

Site2316
1.0
0.5
-
A
C
D
E
F
G
H
I
K
L
M
N
P
Q
R
S
T
V
W
Y
ikalian
ropean
astern
malaya
berian

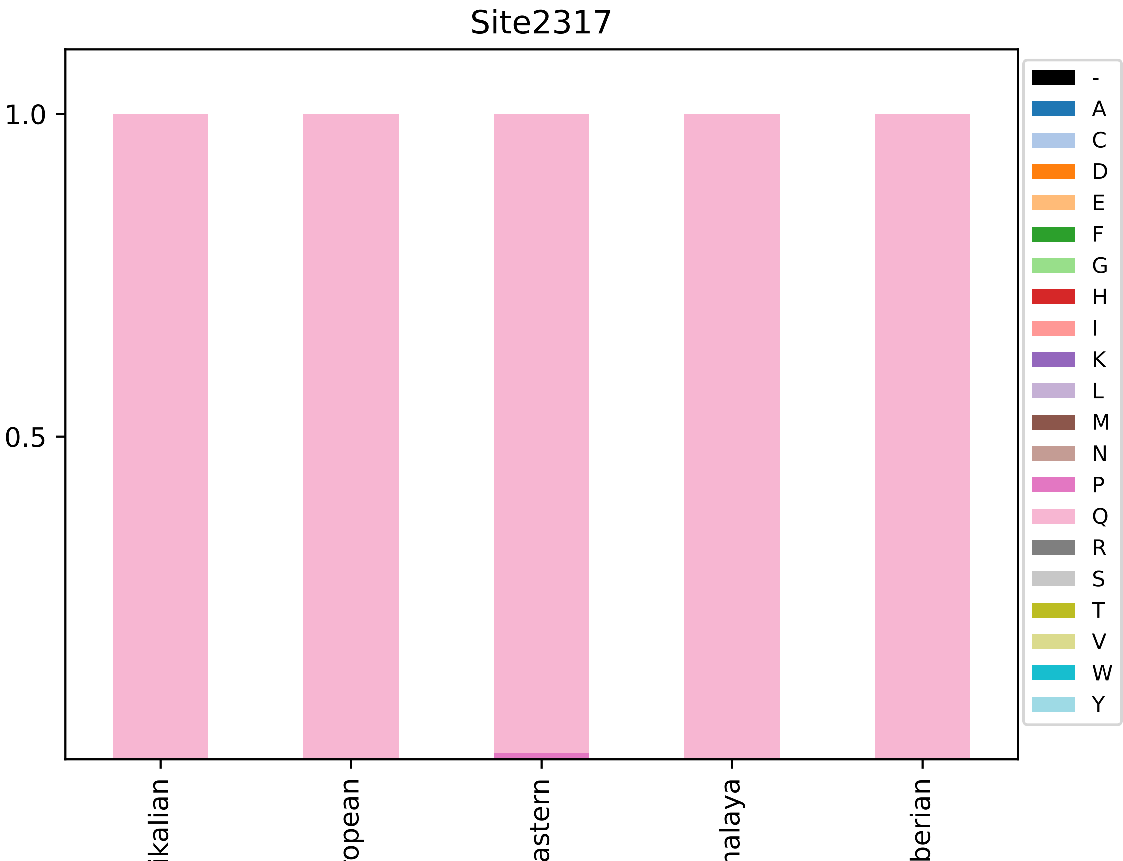

Site2317
1.0
0.5
ikalian
opean
astern
nalaya
berian
-
A
C
D
E
F
G
H
I
K
L
M
N
P
Q
R
S
T
V
W
Y

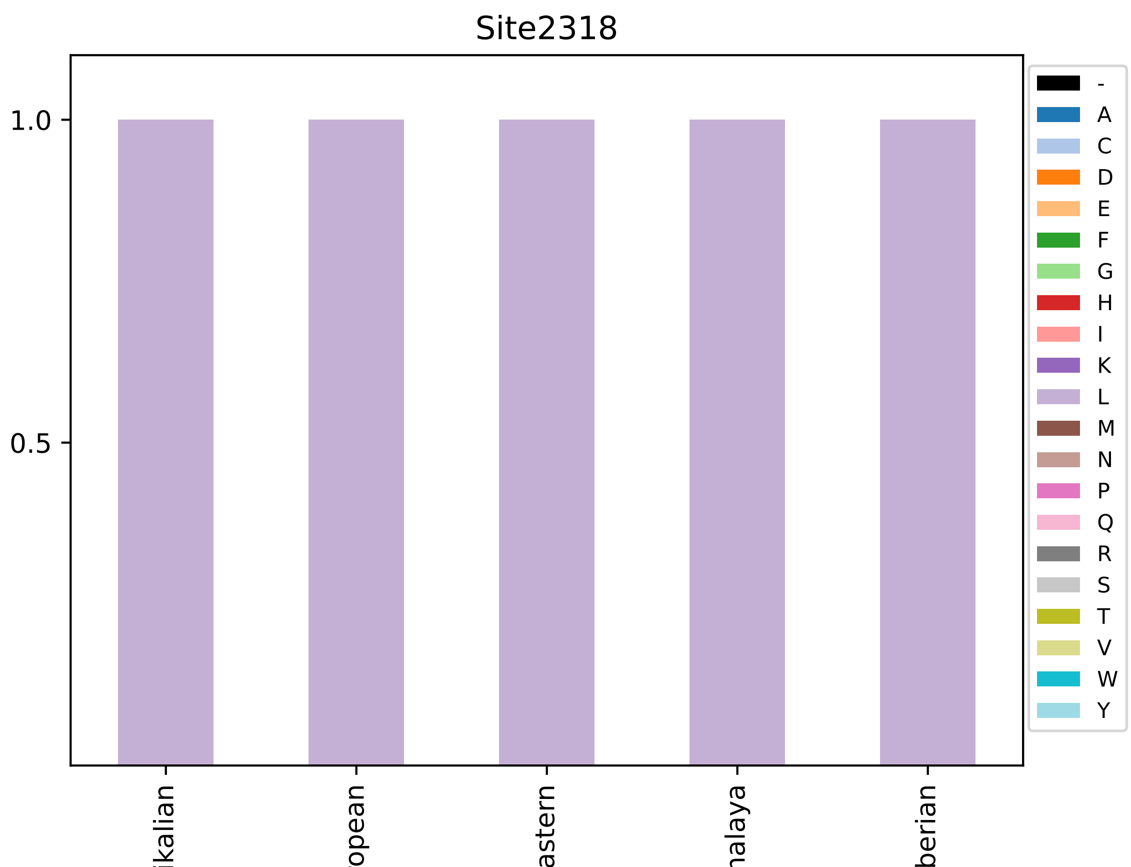

Site2318
1.0
0.5
-
A
C
D
E
F
G
H
I
K
L
M
N
P
Q
R
S
T
V
W
Y
ikalian
opean
astern
nalaya
berian

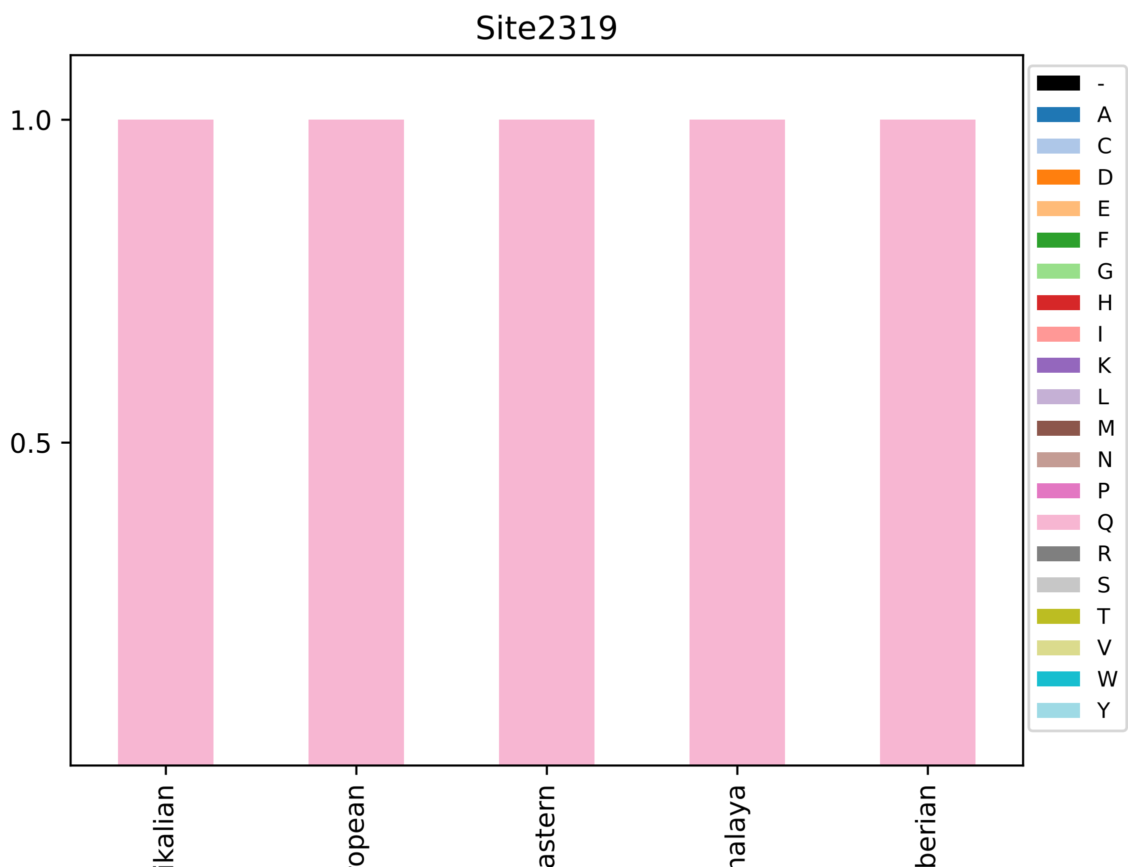

Site2319
1.0
0.5
-
A
C
D
E
F
G
H
I
K
L
M
N
P
Q
R
S
T
V
W
Y
ikalian
opean
astern
nalaya
berian

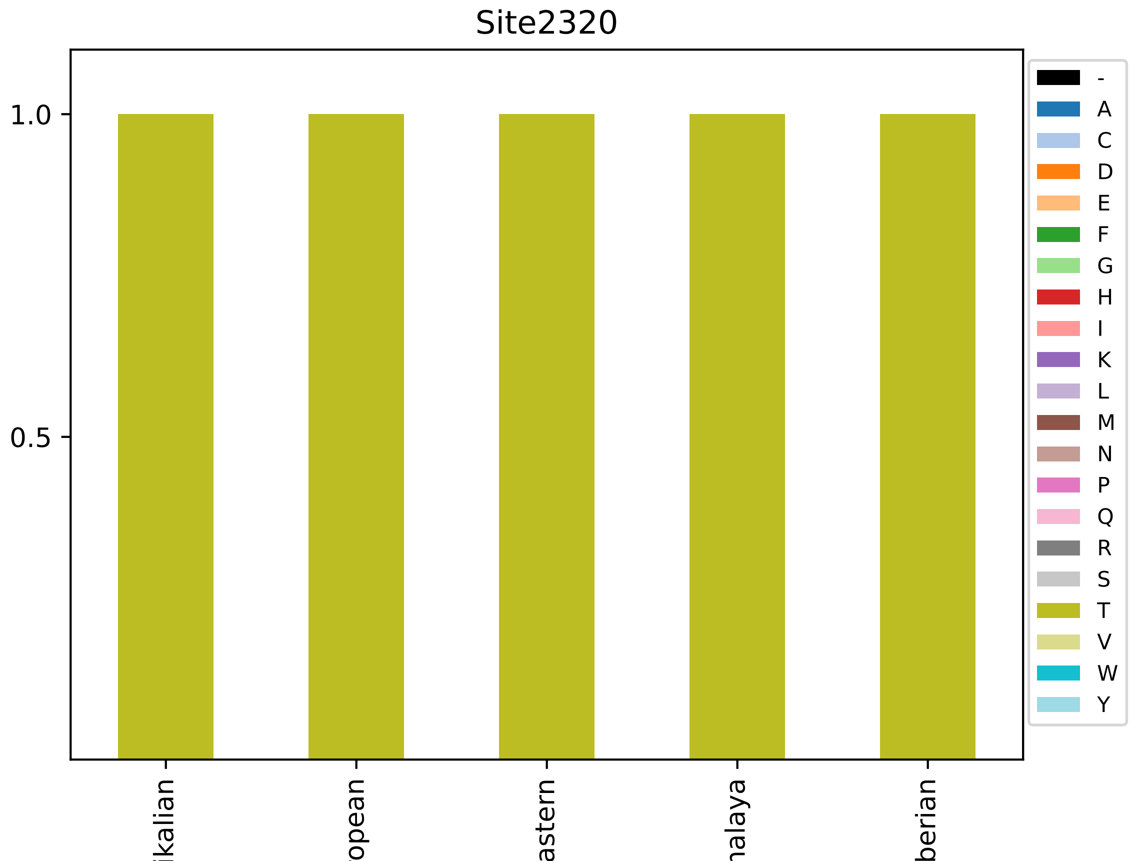
Site2320
1.0
0.5
-
A
C
D
E
F
G
H
I
K
L
M
N
P
Q
R
S
T
V
W
Y
ikalian
opean
astern
nalaya
berian

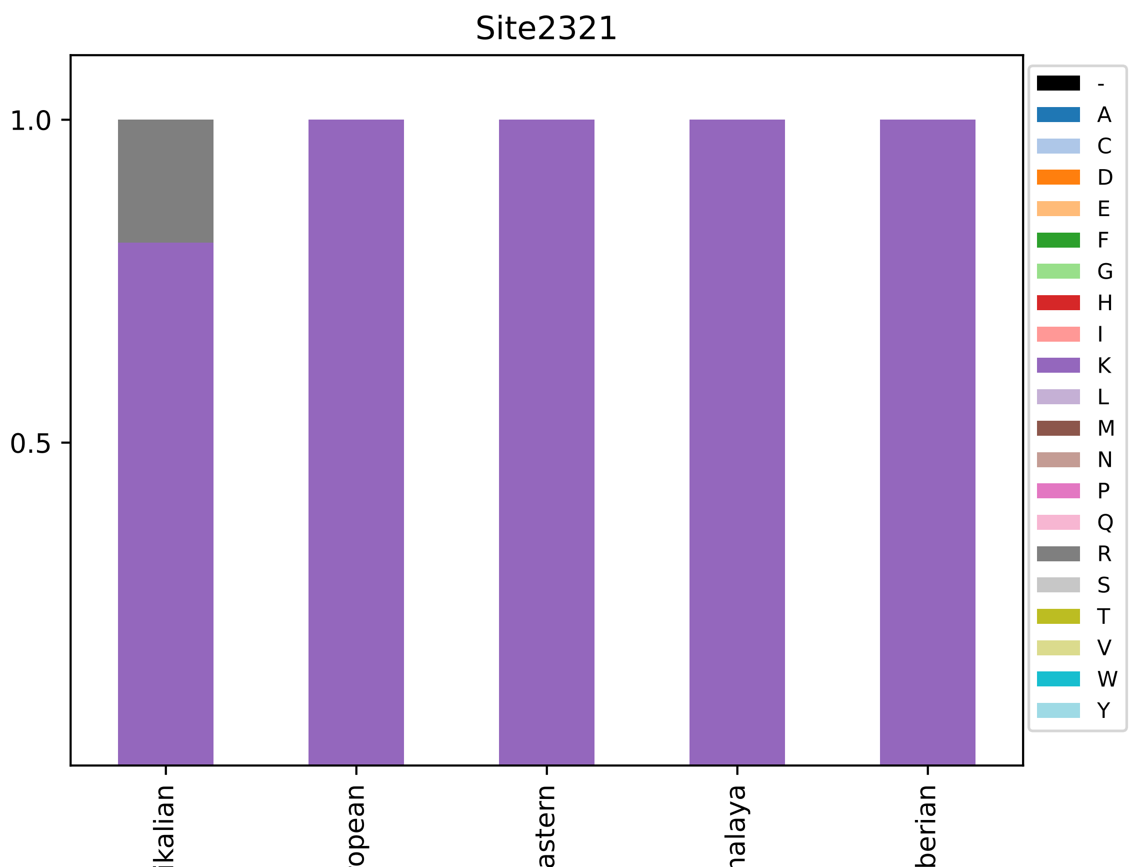

Site2321
1.0
0.5
-
A
C
D
E
F
G
H
I
K
L
M
N
P
Q
R
S
T
V
W
Y
ikalian
opean
astern
nalaya
berian

Site2322

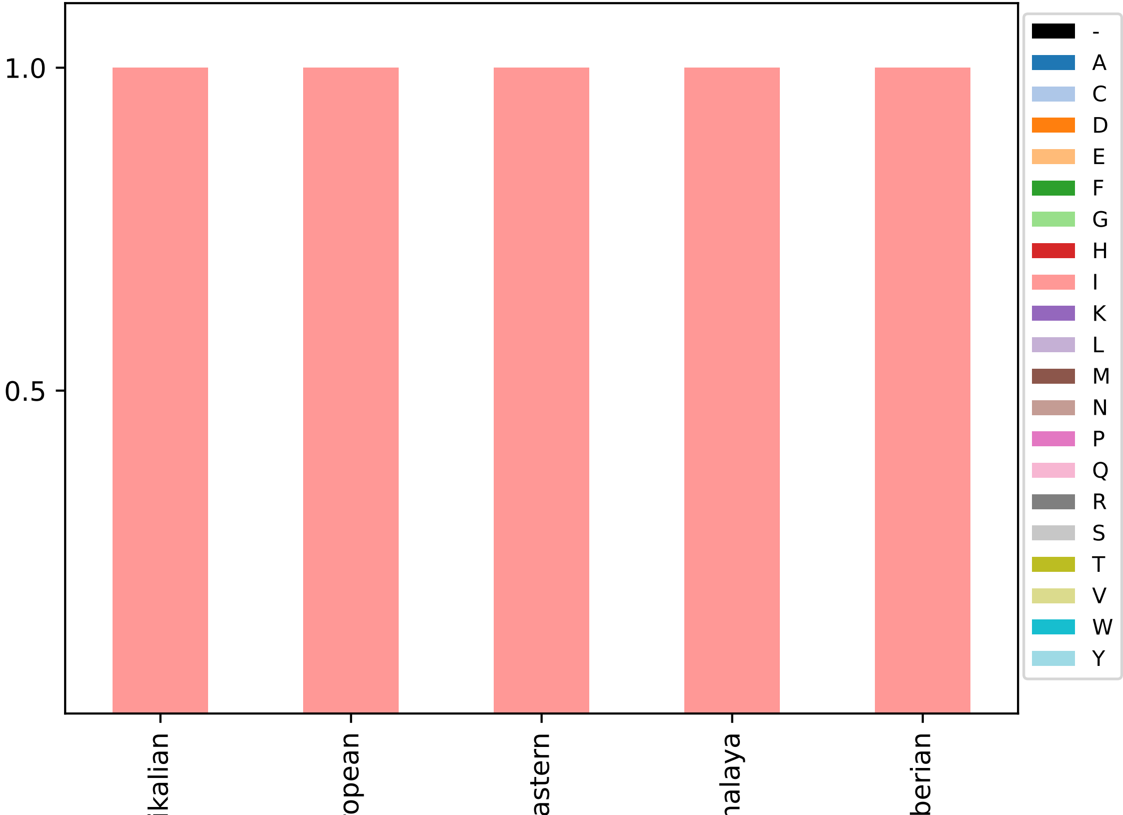
1.0
0.5
-
A
C
D
E
F
G
H
I
K
L
M
N
P
Q
R
S
T
V
W
Y
ikalian
opean
astern
nalaya
berian

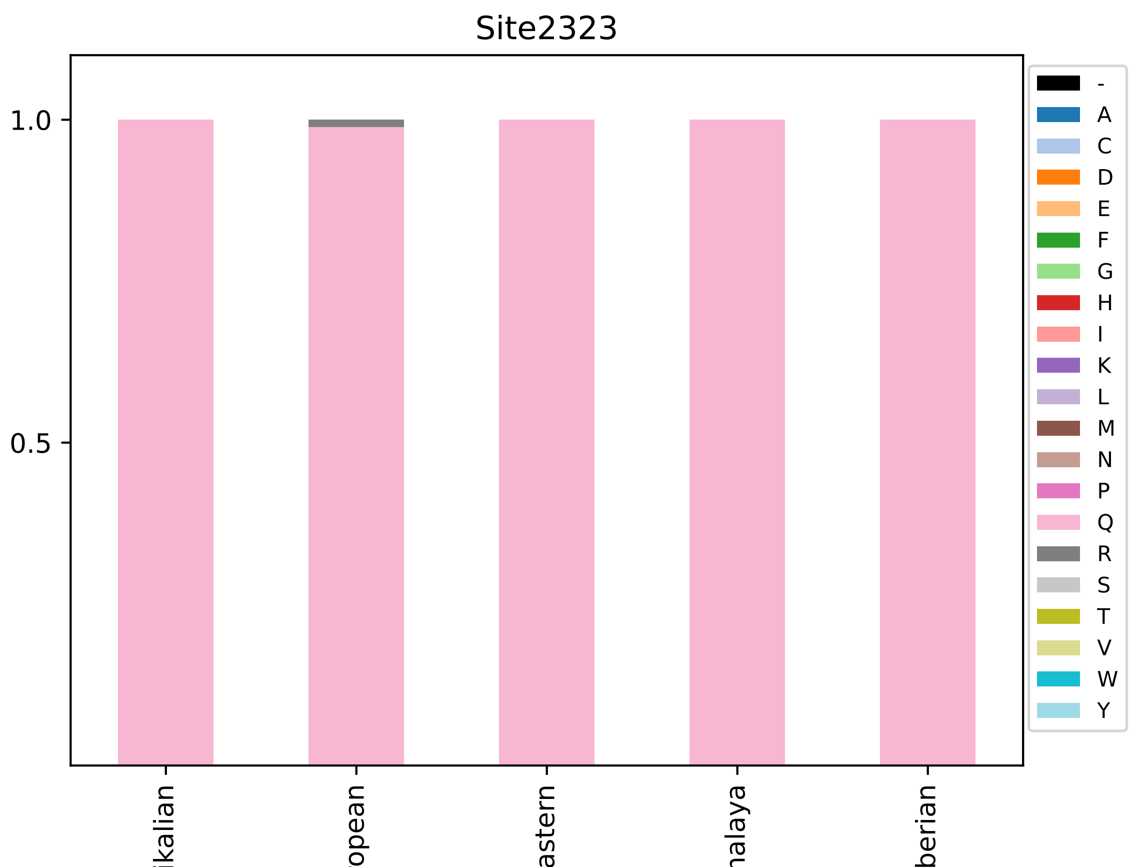
Site2323
1.0
0.5
-
A
C
D
E
F
G
H
I
K
L
M
N
P
Q
R
S
T
V
W
Y
ikalian
opean
astern
nalaya
berian

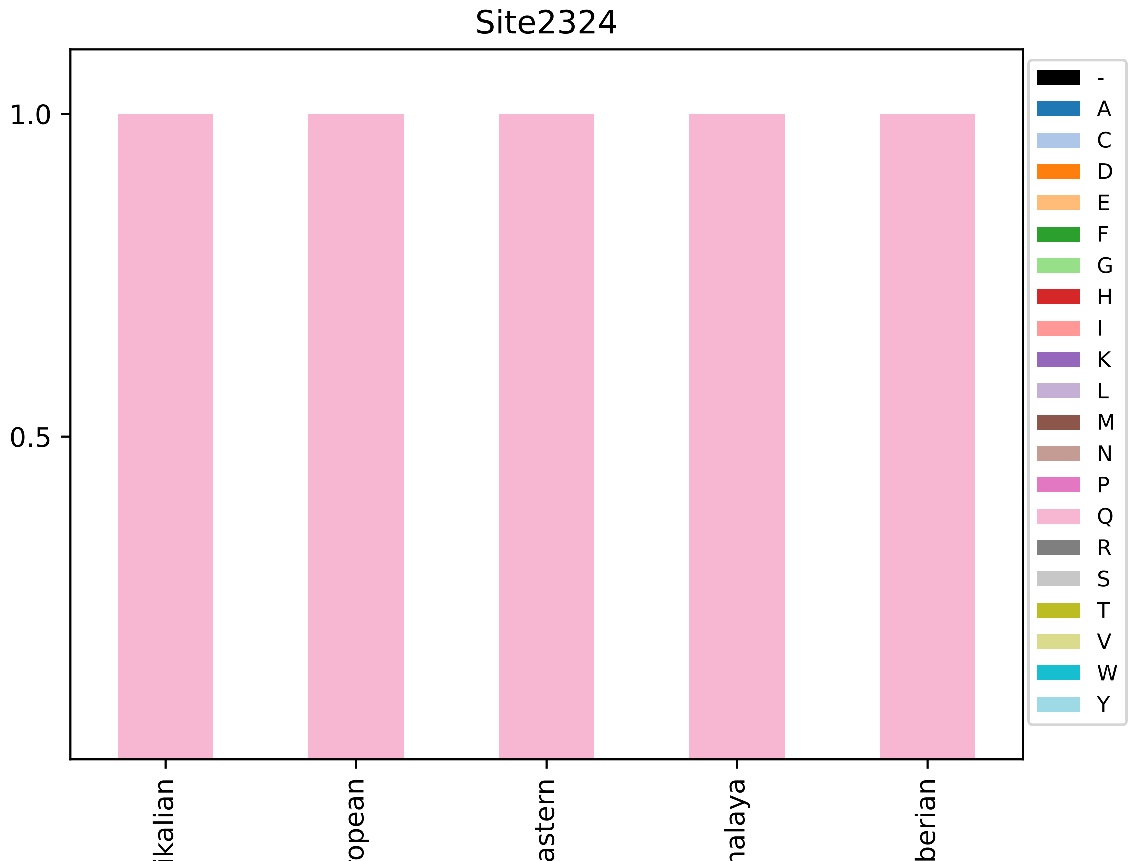

Site2324
1.0
0.5
-
A
C
D
E
F
G
H
I
K
L
M
N
P
Q
R
S
T
V
W
Y
ikalian
opean
astern
nalaya
berian

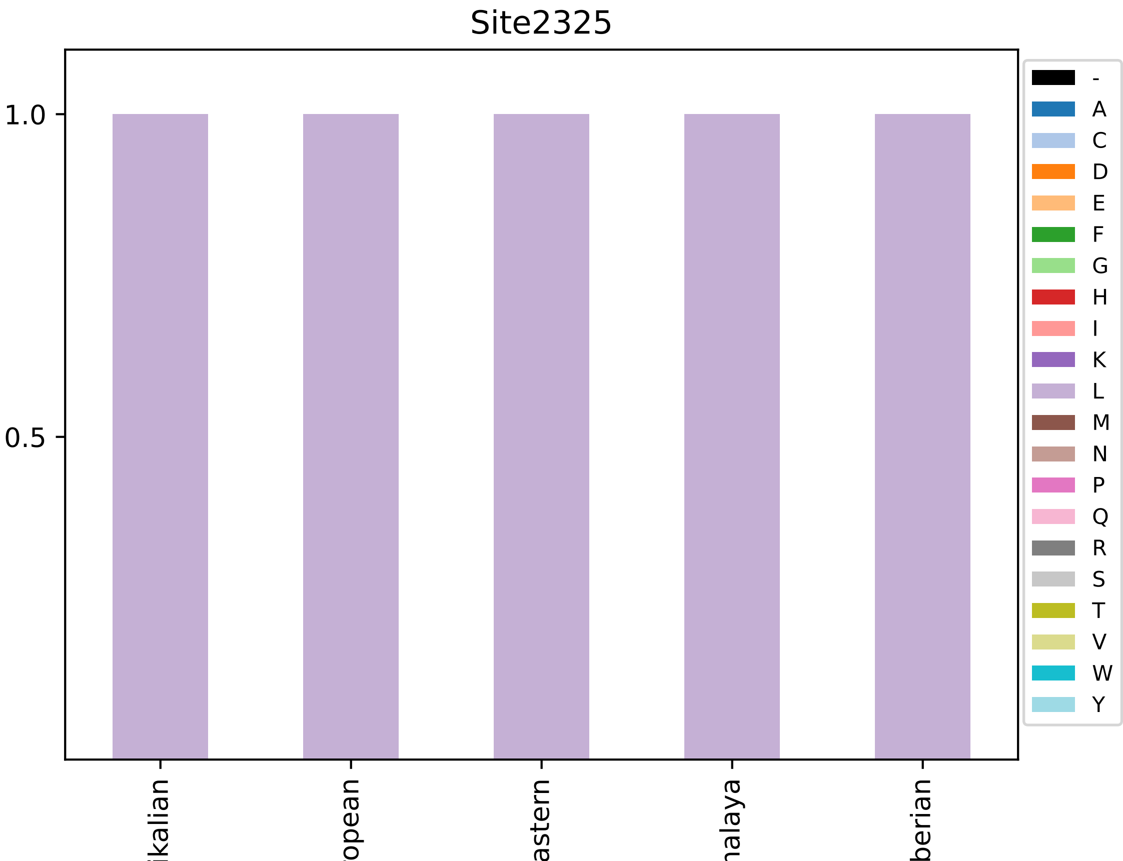

Site2325
1.0
0.5
-
A
C
D
E
F
G
H
I
K
L
M
N
P
Q
R
S
T
V
W
Y
ikalian
opean
astern
nalaya
berian

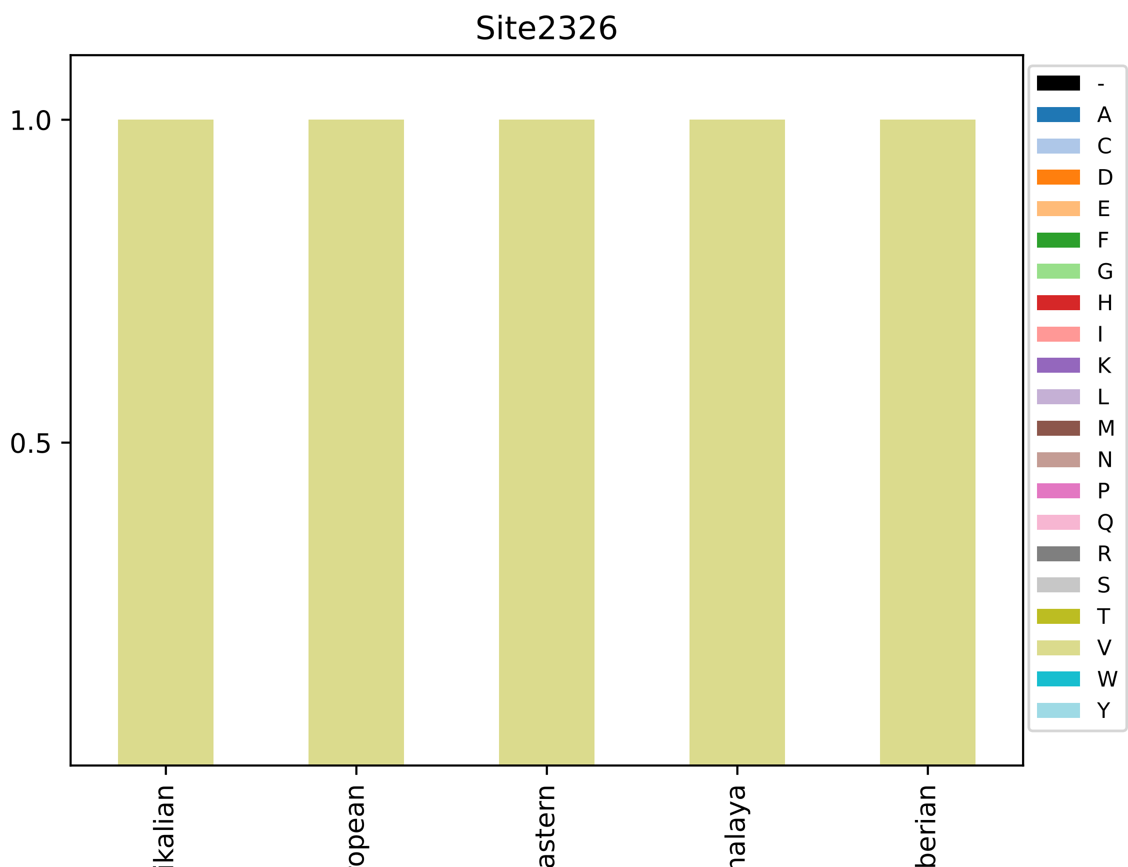

Site2326
1.0
0.5
ikalian
opean
astern
nalaya
berian
-
A
C
D
E
F
G
H
I
K
L
M
N
P
Q
R
S
T
V
W
Y

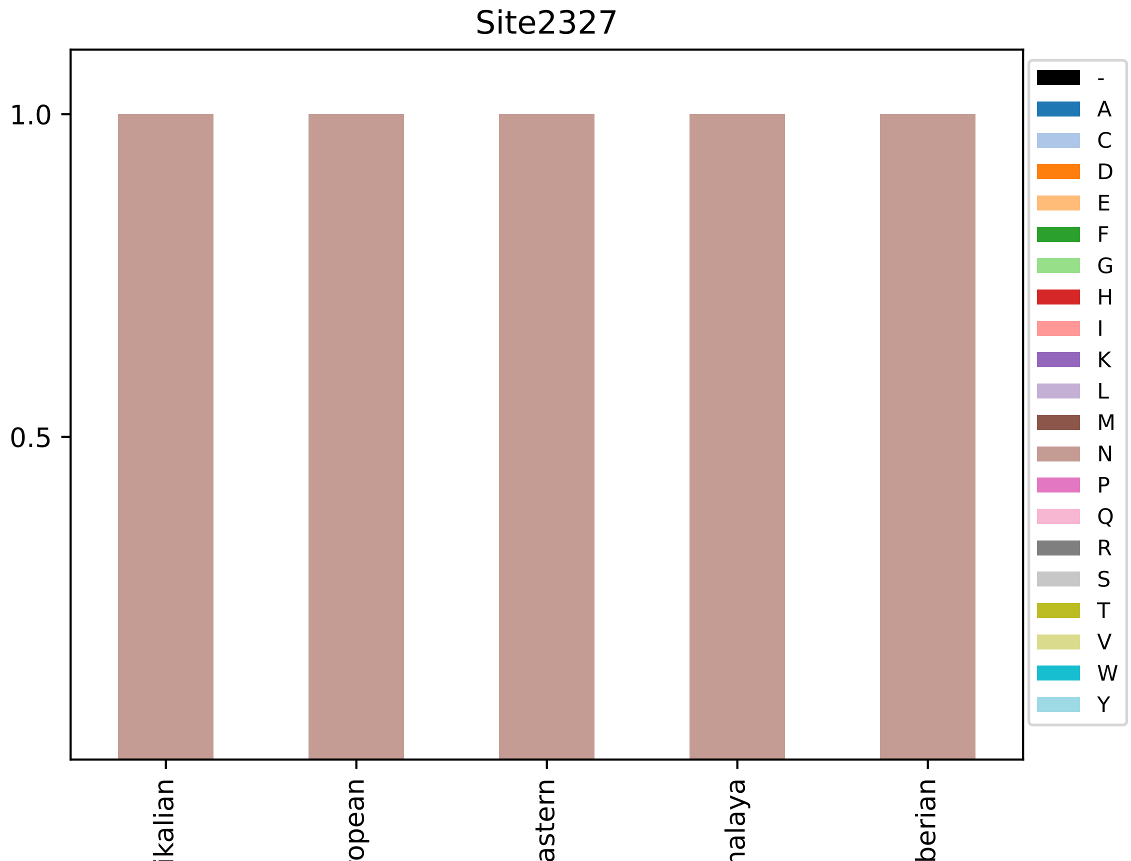
Site2327
1.0
0.5
-
A
C
D
E
F
G
H
I
K
L
M
N
P
Q
R
S
T
V
W
Y

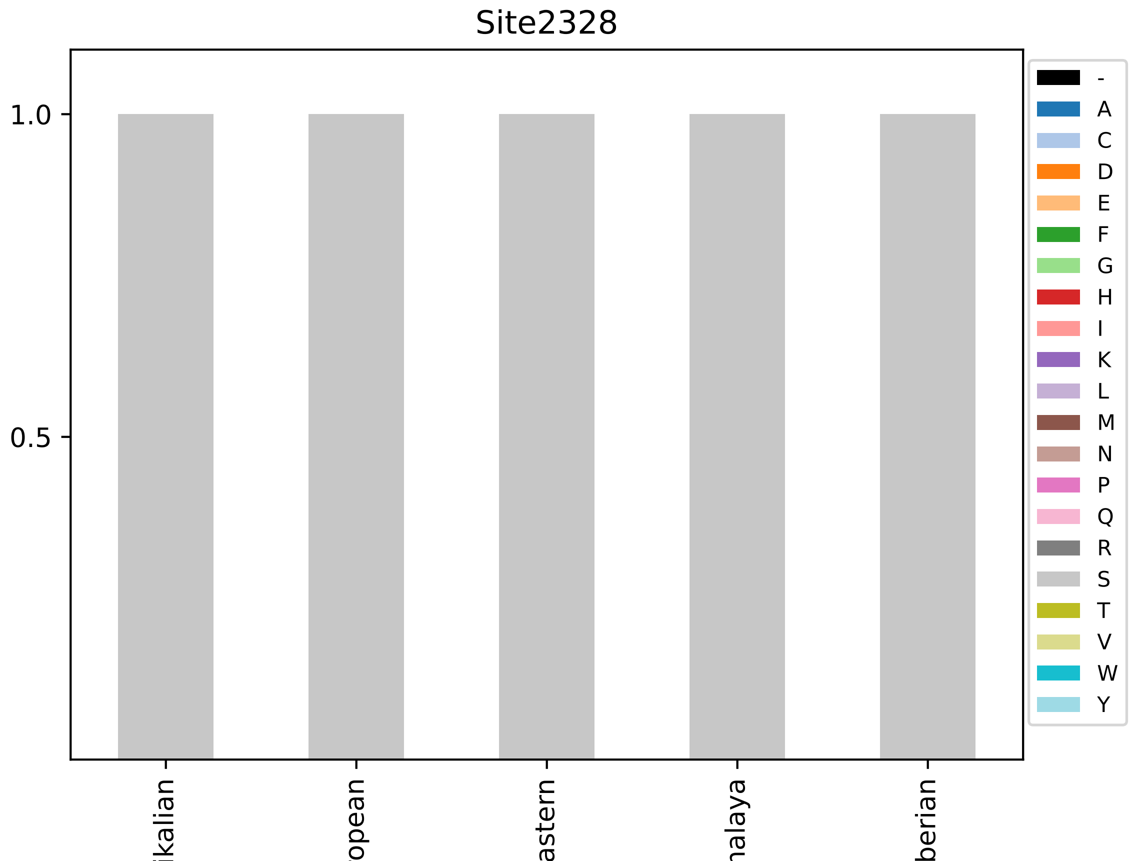
Site2328
1.0
0.5
-
A
C
D
E
F
G
H
I
K
L
M
N
P
Q
R
S
T
V
W
Y
ikalian
opean
astern
nalaya
berian

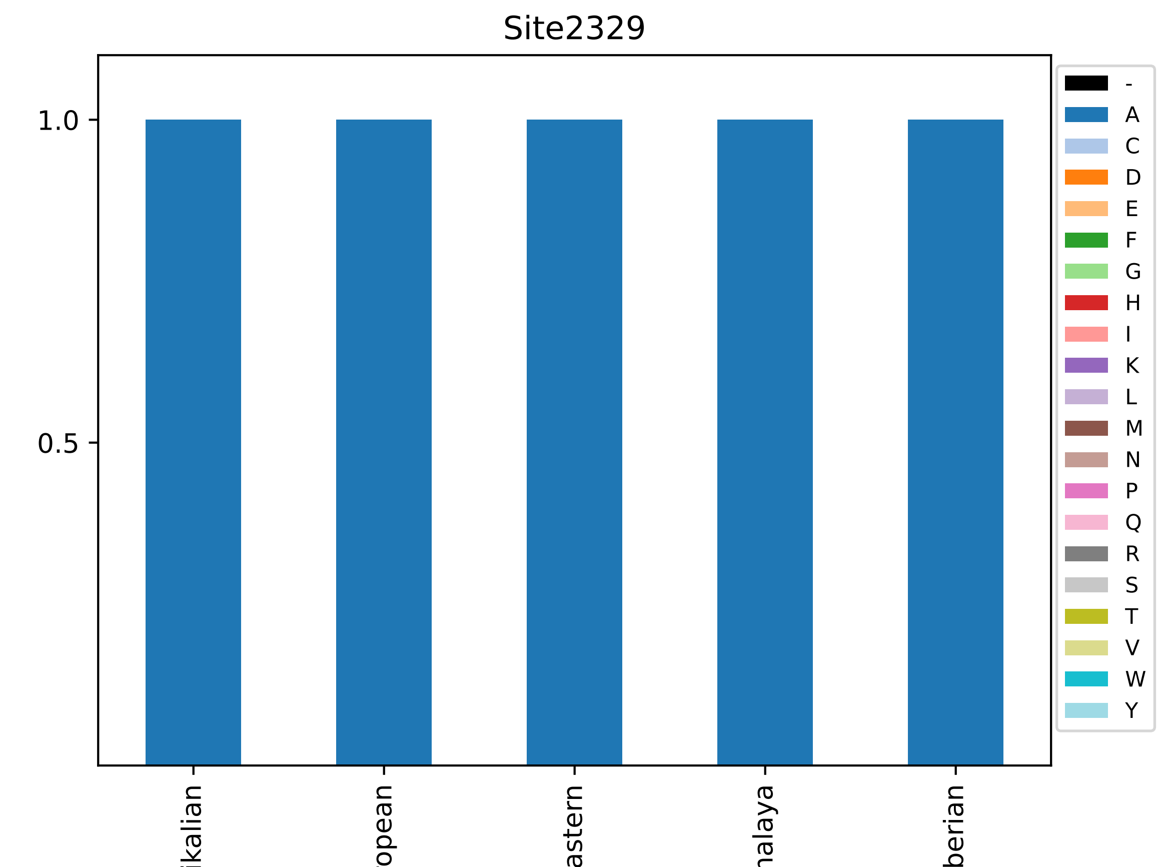

Site2329
1.0
0.5
-
A
C
D
E
F
G
H
I
K
L
M
N
P
Q
R
S
T
V
W
Y
ikalian
opean
astern
nalaya
berian

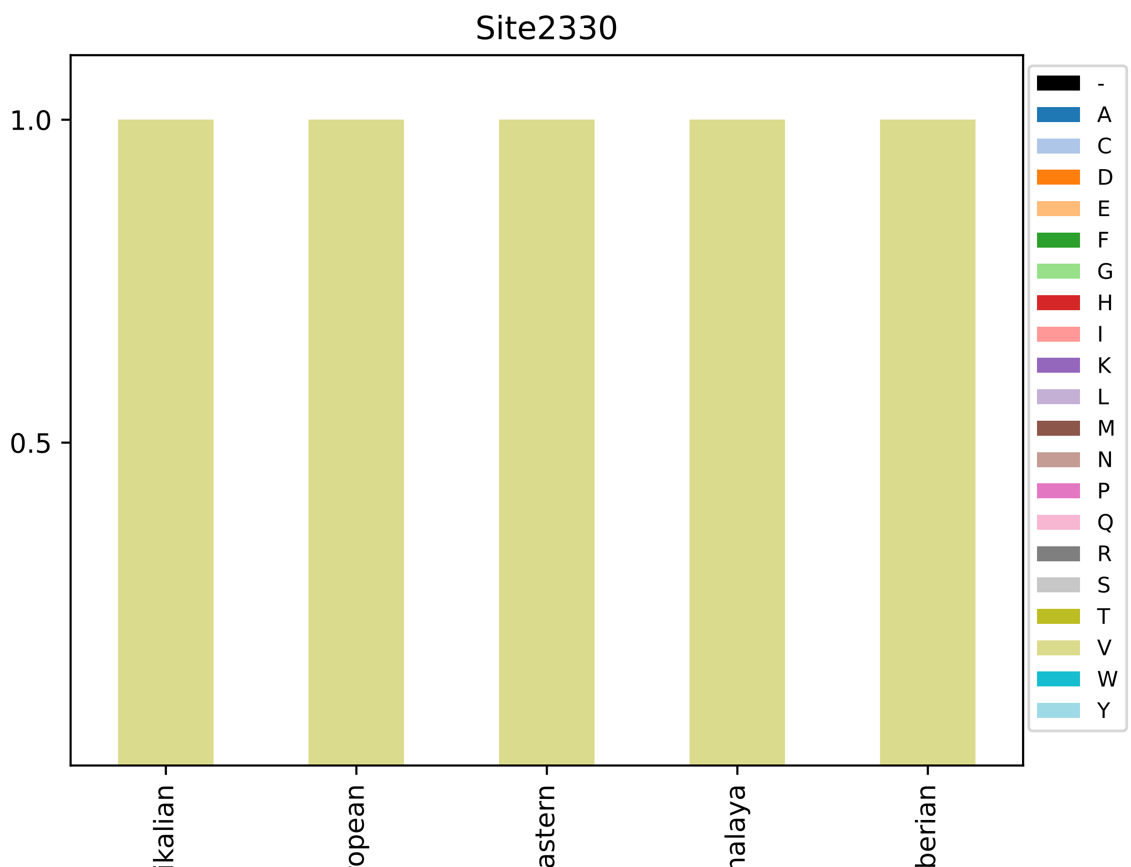
Site2330
1.0
0.5
-
A
C
D
E
F
G
H
I
K
L
M
N
P
Q
R
S
T
V
W
Y
ikalian
opean
astern
nalaya
berian

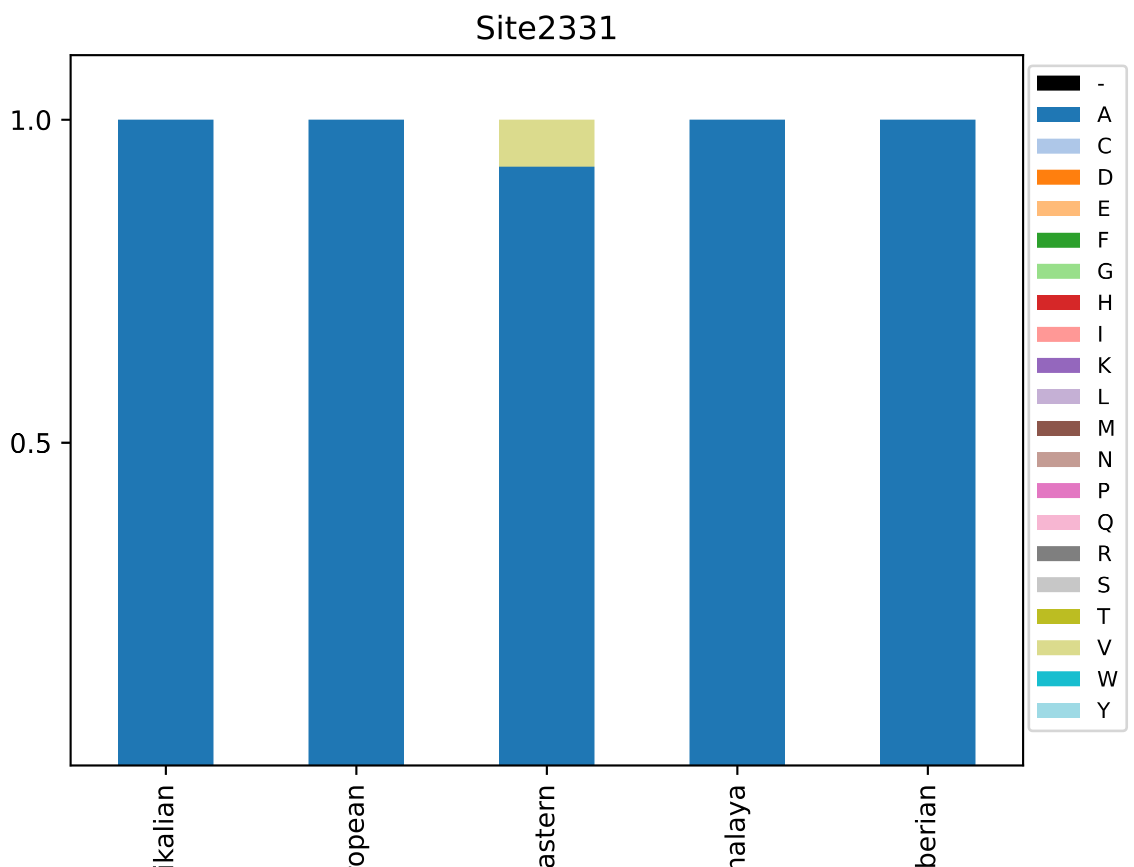
Site2331
1.0
0.5
ikalian
opean
astern
nalaya
berian
-
A
C
D
E
F
G
H
I
K
L
M
N
P
Q
R
S
T
V
W
Y

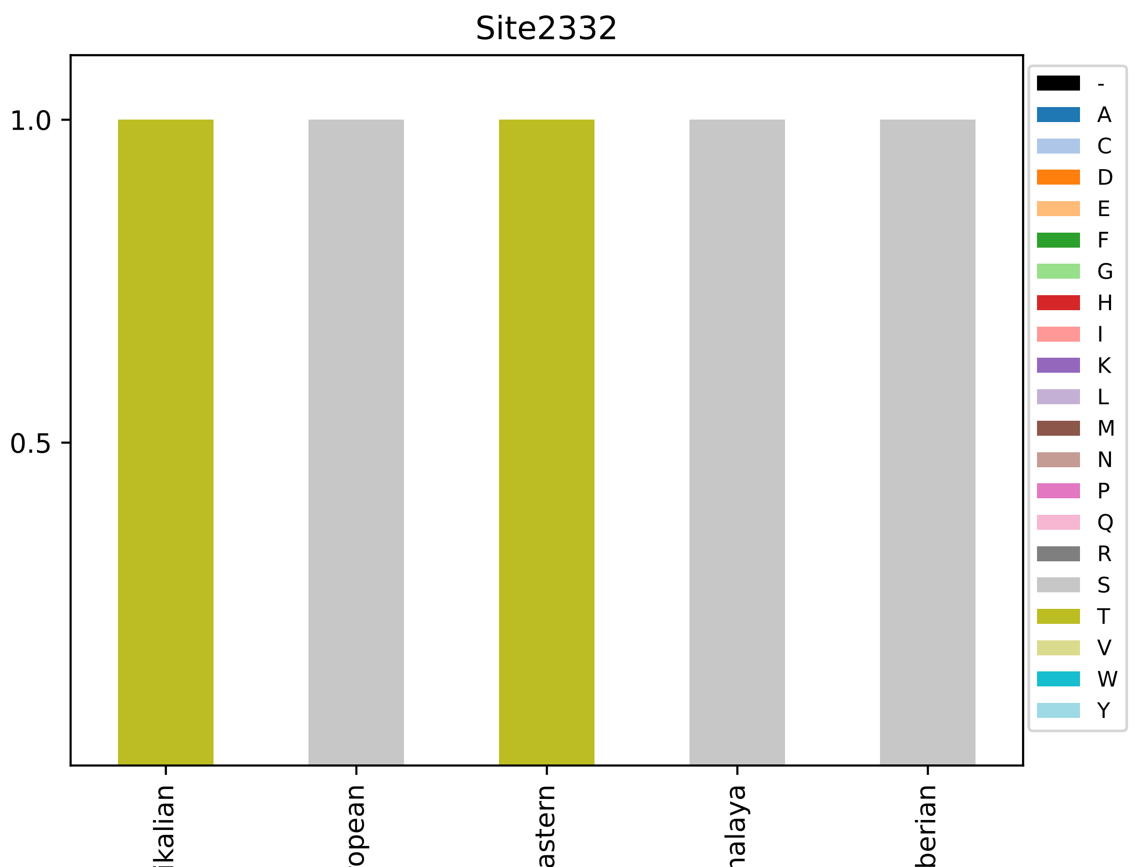

Site2332
1.0
0.5
-
A
C
D
E
F
G
H
I
K
L
M
N
P
Q
R
S
T
V
W
Y
ikalian
opean
astern
nalaya
berian

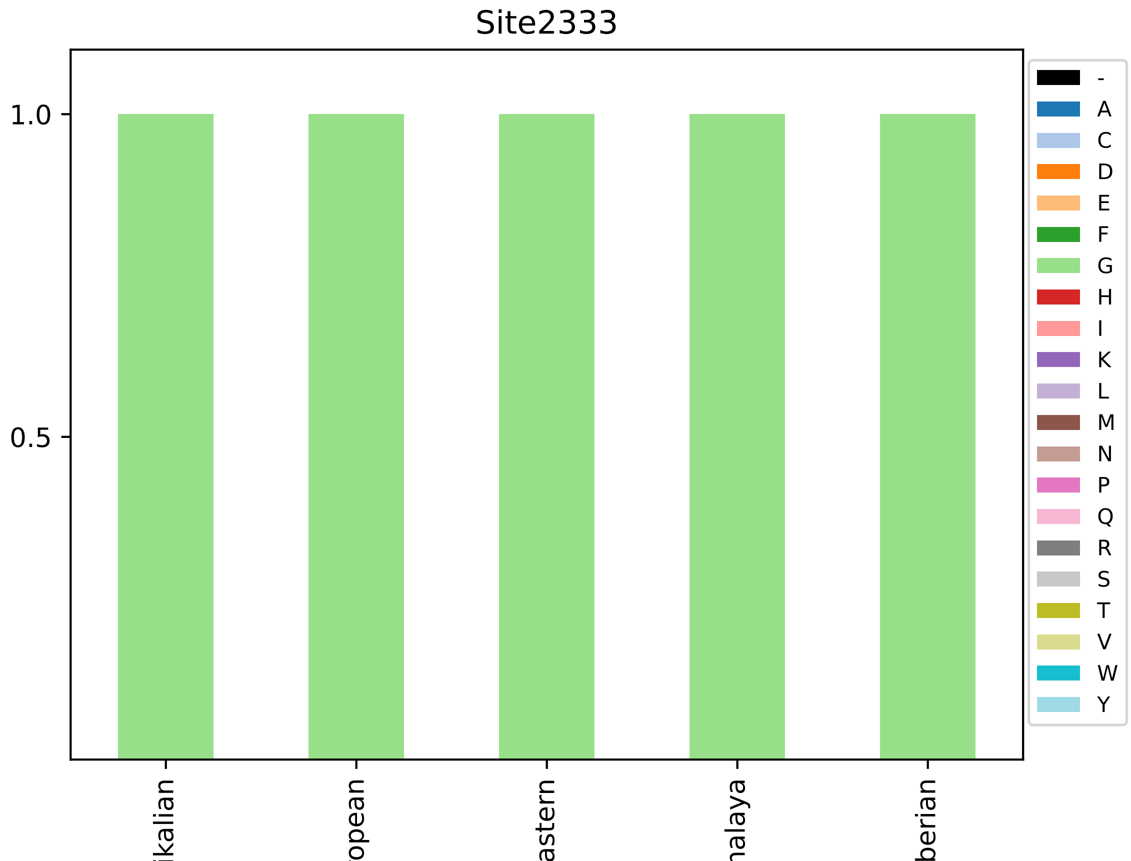
Site2333
1.0
0.5
-
A
C
D
E
F
G
H
I
K
L
M
N
P
Q
R
S
T
V
W
Y
ikalian
opean
astern
nalaya
berian

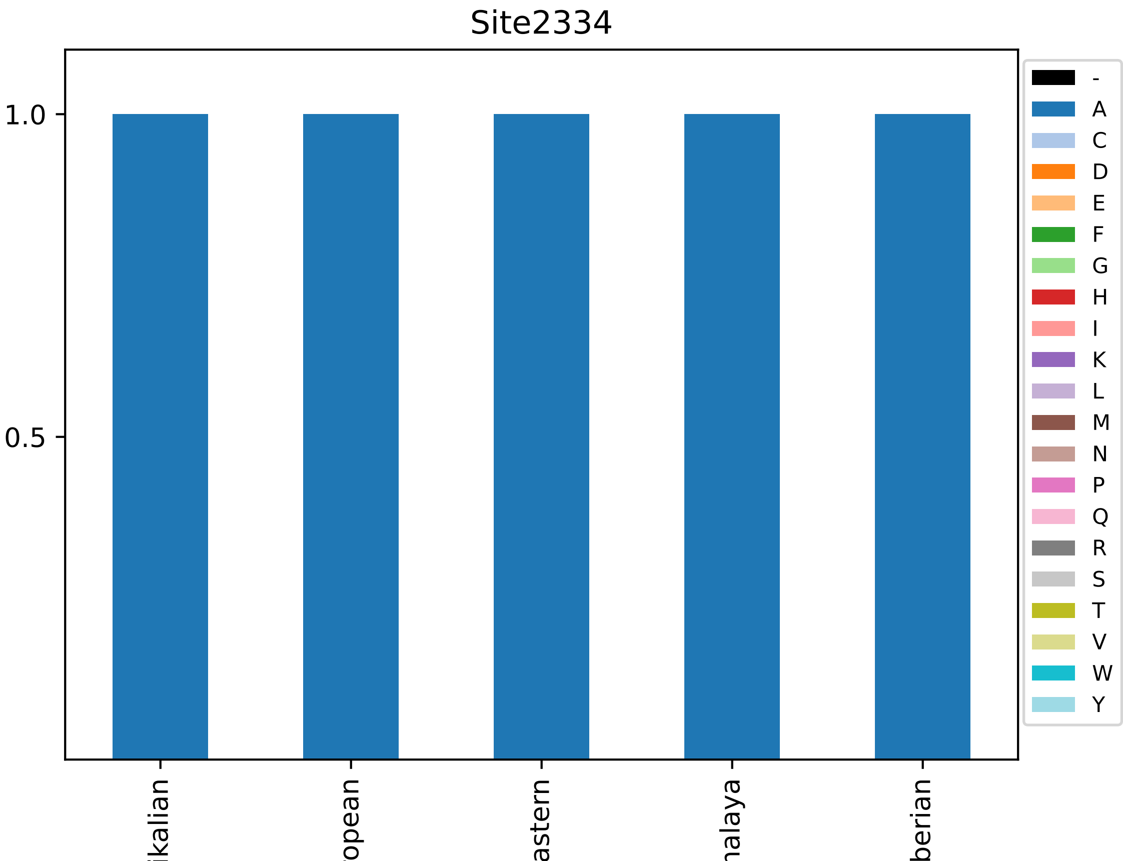
Site2334
1.0
0.5
-
A
C
D
E
F
G
H
I
K
L
M
N
P
Q
R
S
T
V
W
Y
ikalian
opean
astern
nalaya
berian

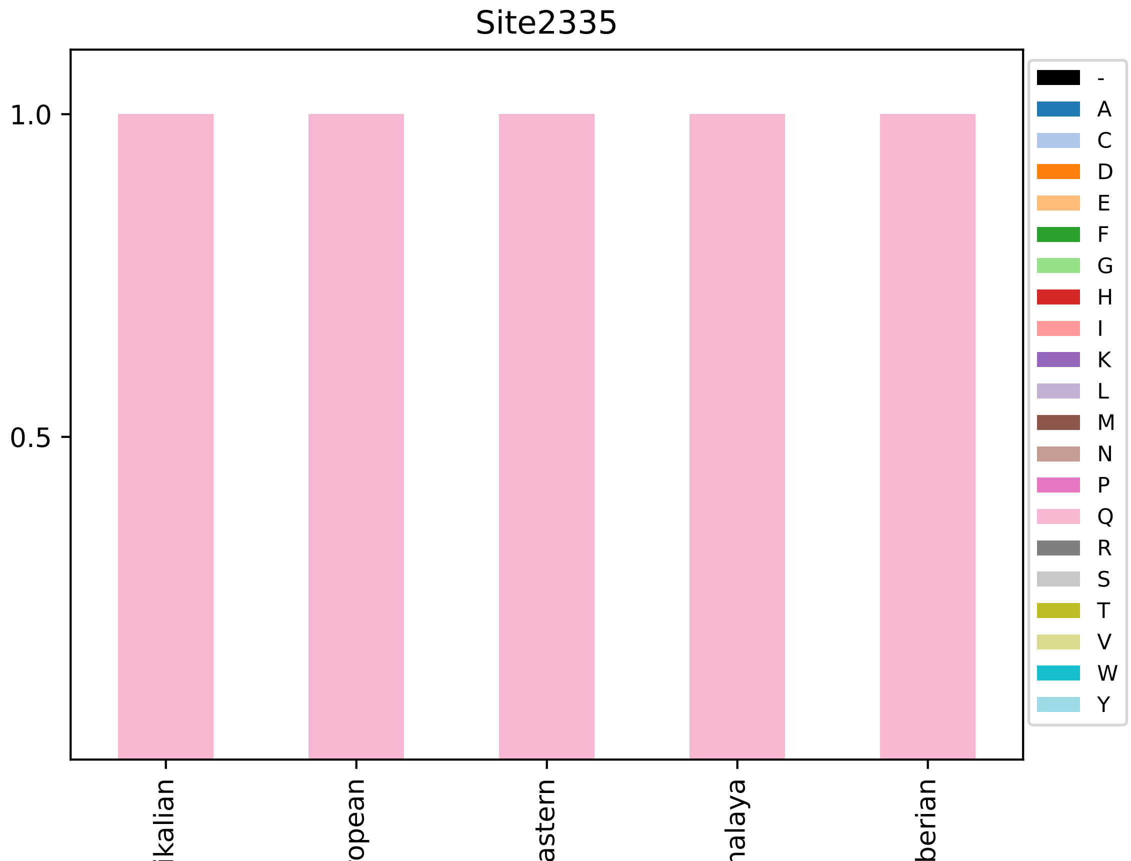
Site2335
1.0
0.5
-
A
C
D
E
F
G
H
I
K
L
M
N
P
Q
R
S
T
V
W
Y
ikalian
opean
astern
nalaya
berian

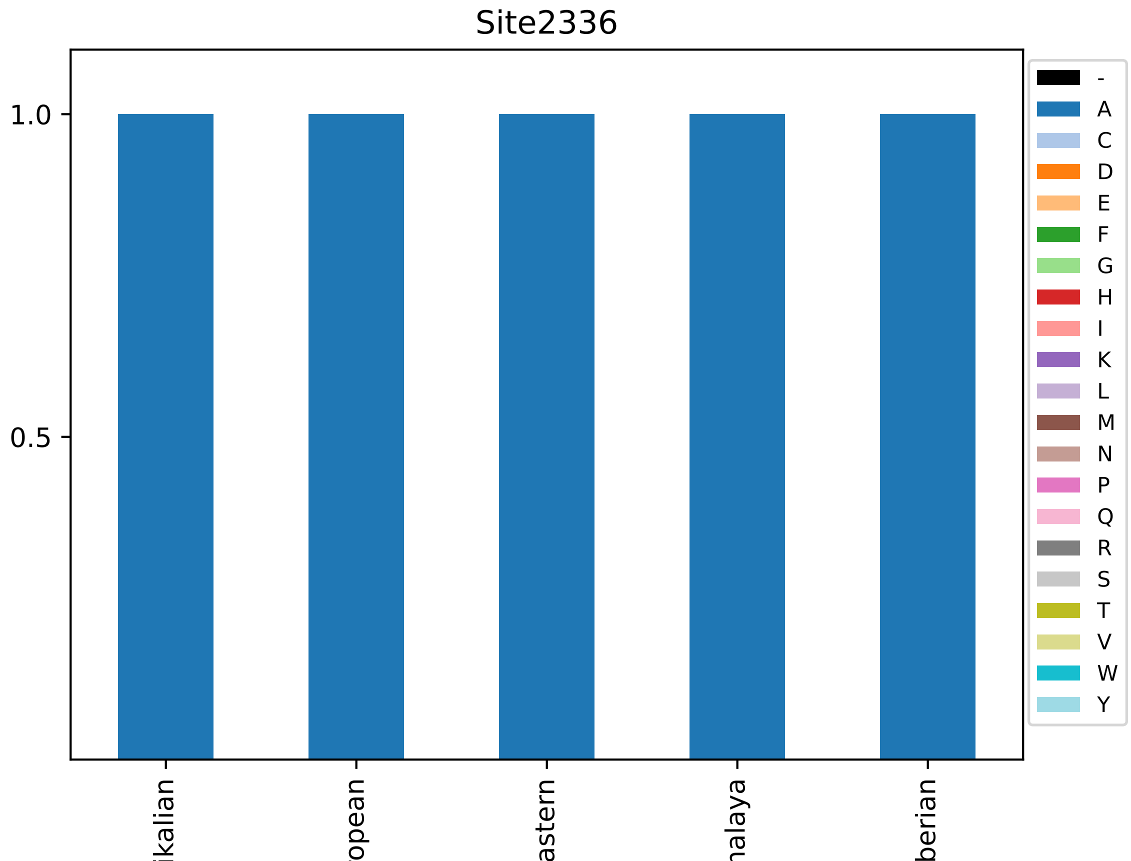

Site2336
1.0
0.5
ikalian
opean
astern
nalaya
berian
-
A
C
D
E
F
G
H
I
K
L
M
N
P
Q
R
S
T
V
W
Y

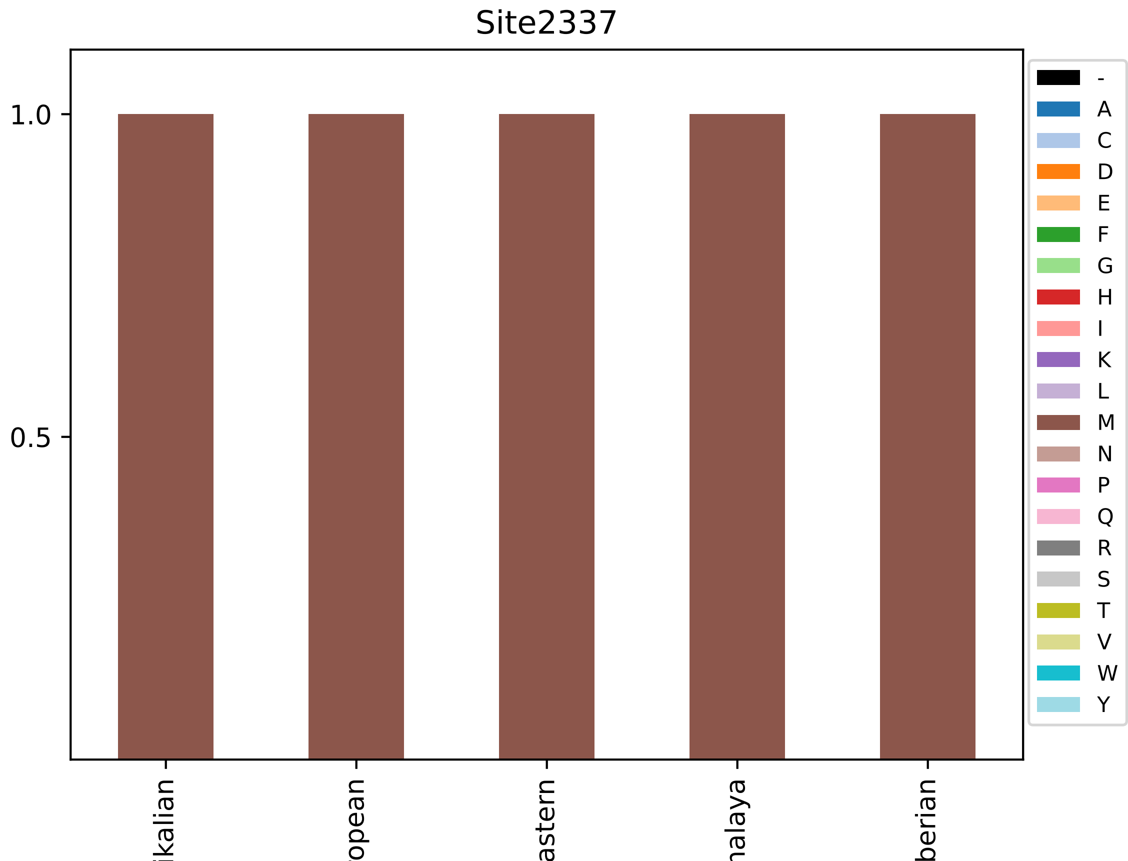
Site2337
1.0
0.5
-
A
C
D
E
F
G
H
I
K
L
M
N
P
Q
R
S
T
V
W
Y

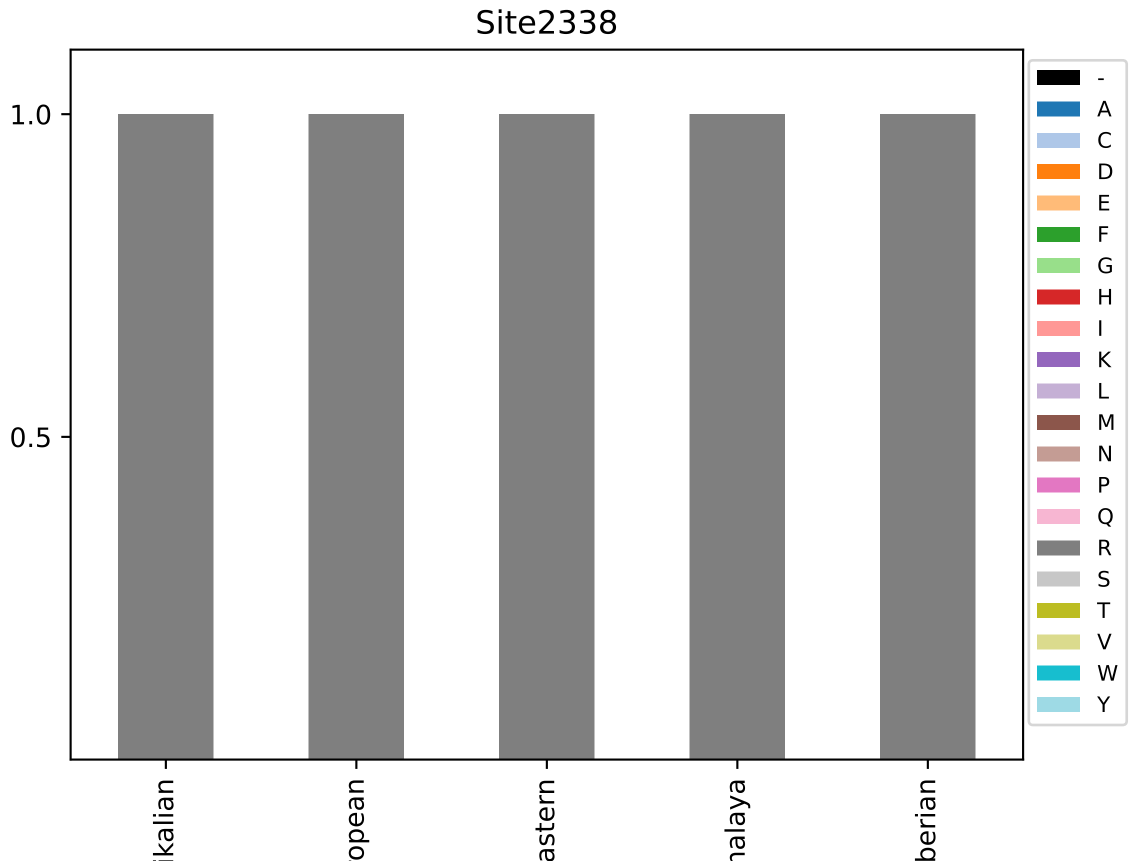

Site2338
1.0
0.5
-
A
C
D
E
F
G
H
I
K
L
M
N
P
Q
R
S
T
V
W
Y
ikalian
opean
astern
nalaya
berian

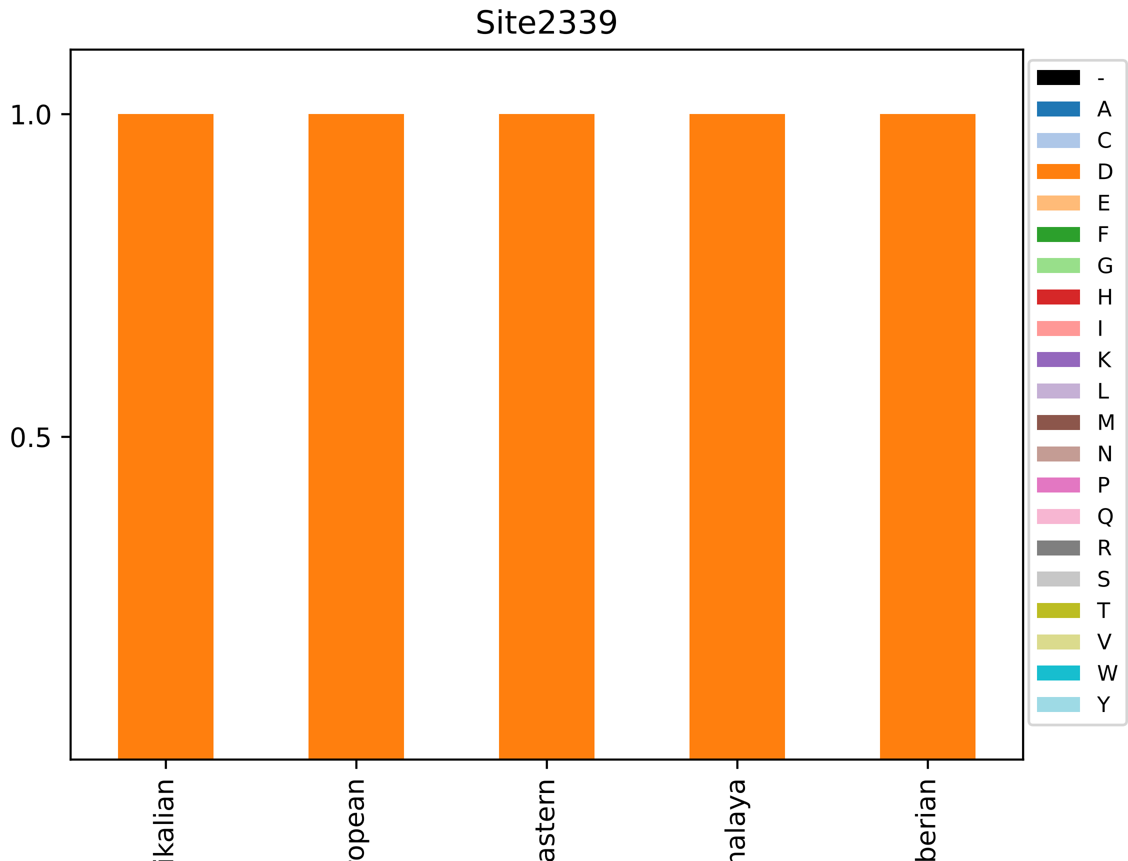

Site2339
1.0
0.5
ikalian
opean
astern
nalaya
berian
-
A
C
D
E
F
G
H
I
K
L
M
N
P
Q
R
S
T
V
W
Y

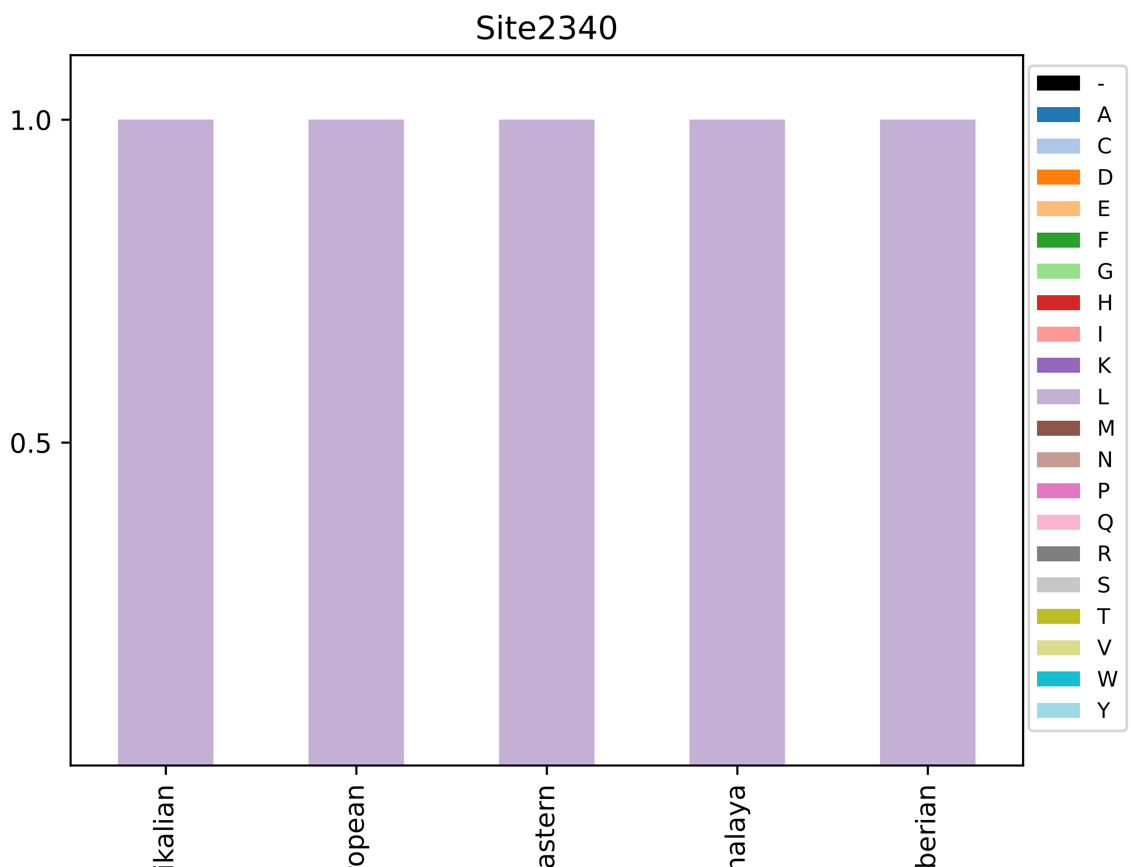

Site2340
1.0
0.5
-
A
C
D
E
F
G
H
I
K
L
M
N
P
Q
R
S
T
V
W
Y
ikalian
opean
astern
nalaya
berian

Site2341

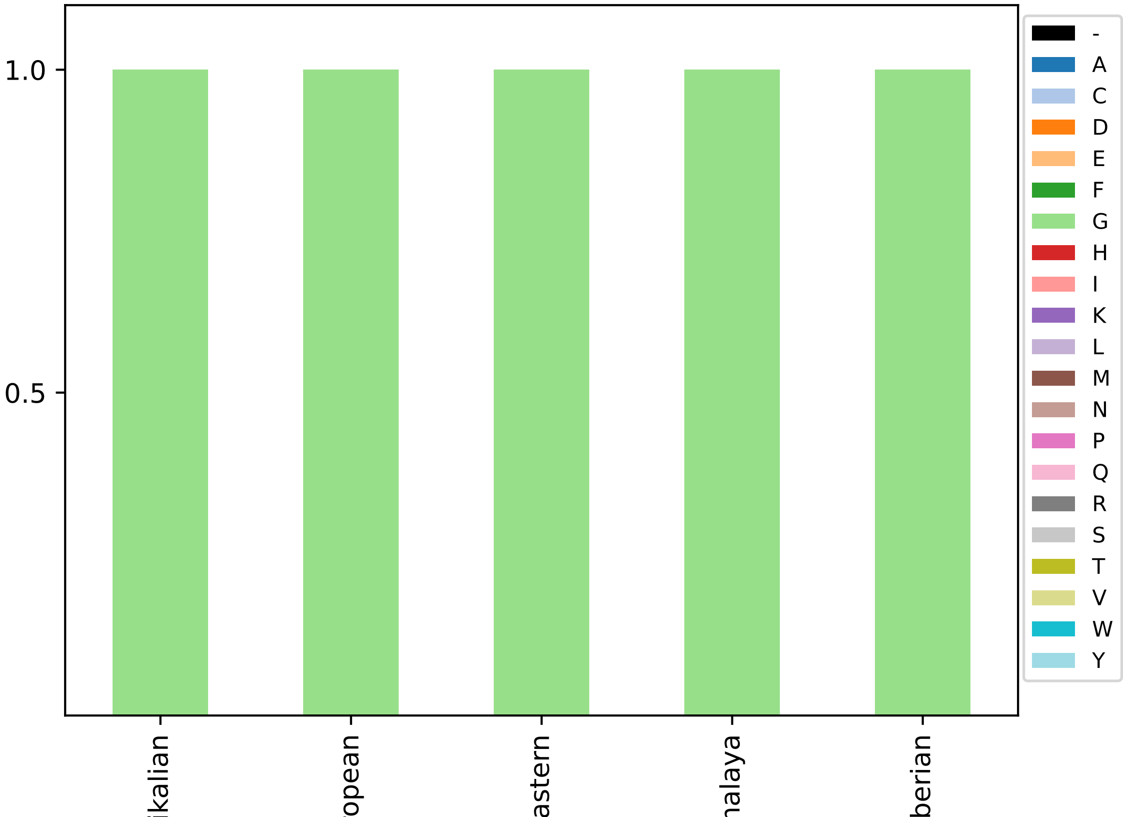
1.0
0.5
-
A
C
D
E
F
G
H
I
K
L
M
N
P
Q
R
S
T
V
W
Y
ikalian
opean
astern
nalaya
berian

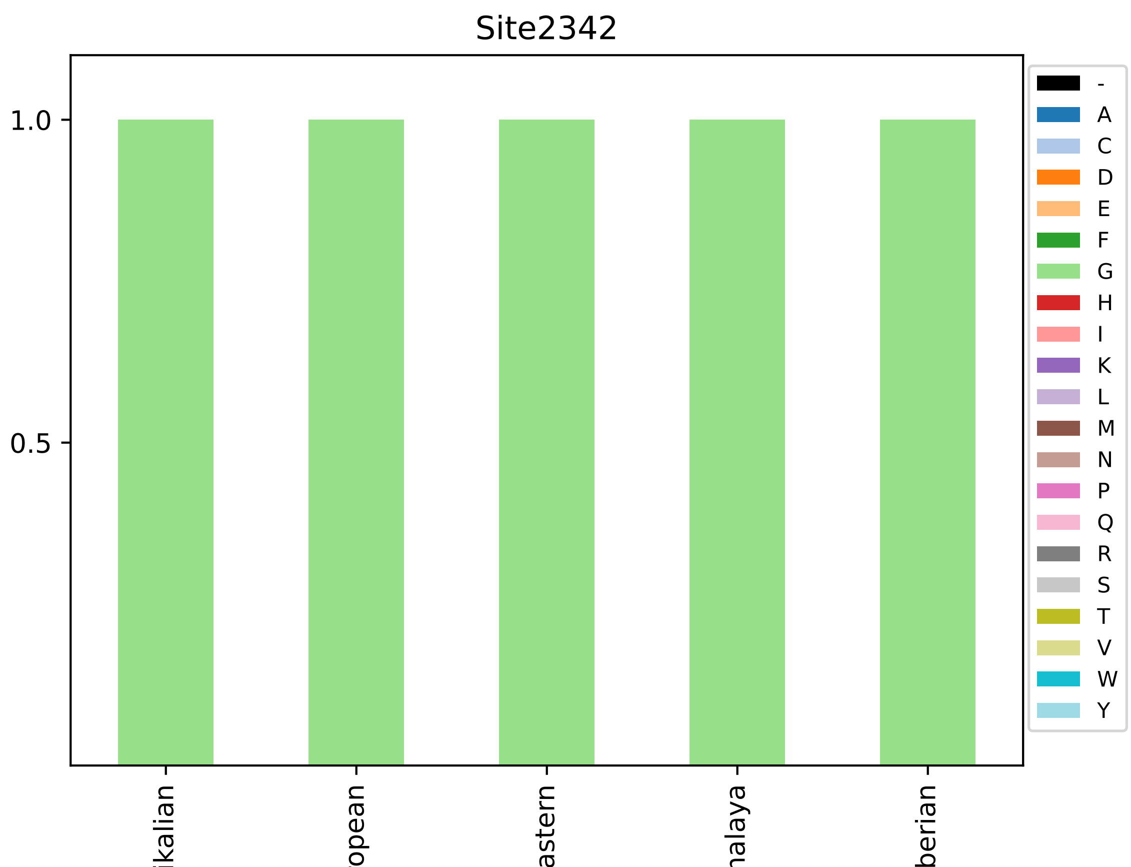
Site2342
1.0
0.5
-
A
C
D
E
F
G
H
I
K
L
M
N
P
Q
R
S
T
V
W
Y
ikalian
opean
astern
nalaya
berian

Site2343

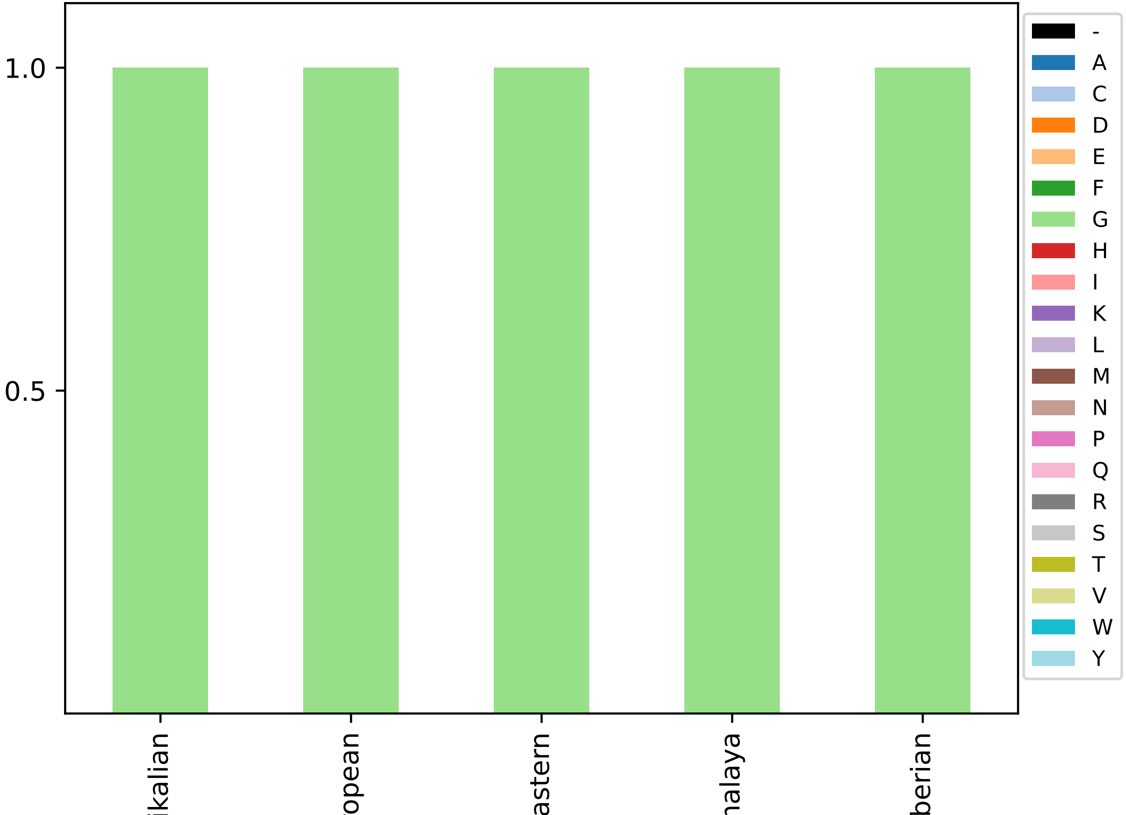

1.0
0.5
ikalian
opean
astern
nalaya
berian
-
A
C
D
E
F
G
H
I
K
L
M
N
P
Q
R
S
T
V
W
Y

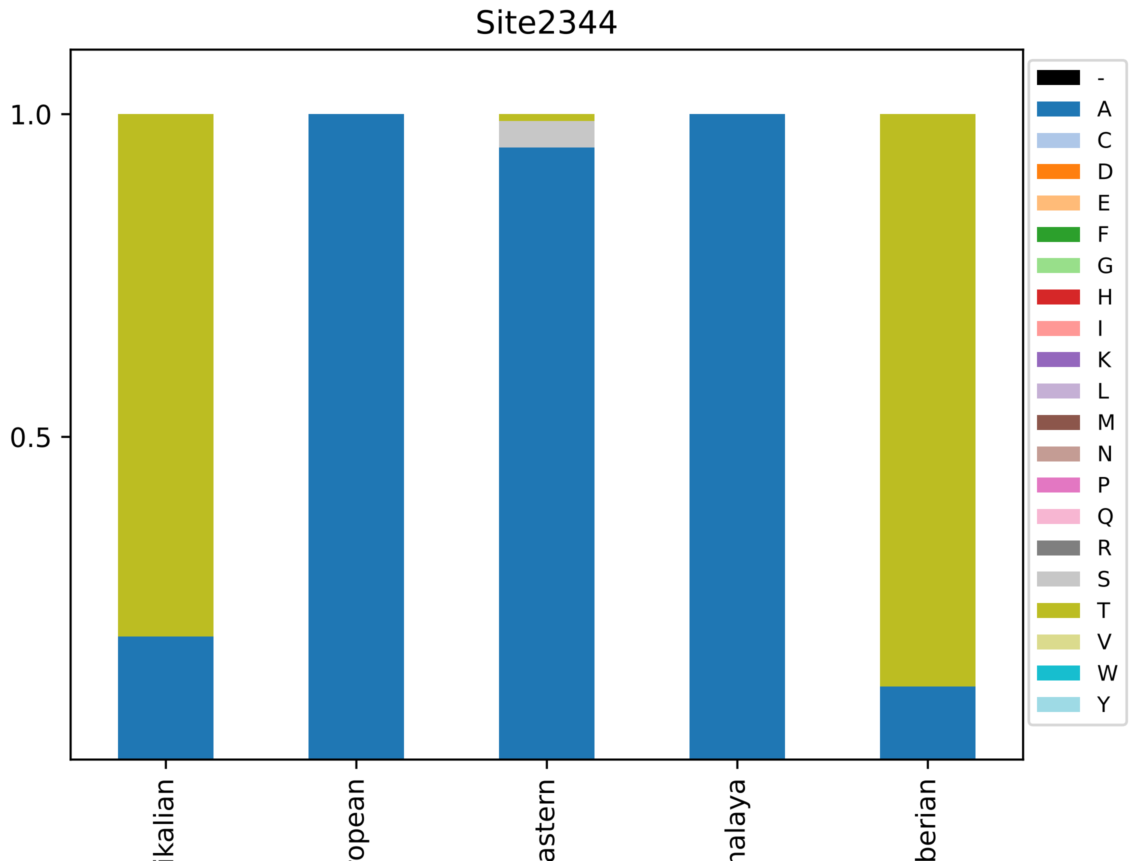

Site2344
1.0
0.5
-
A
C
D
E
F
G
H
I
K
L
M
N
P
Q
R
S
T
V
W
Y
ikalian
opean
astern
nalaya
berian

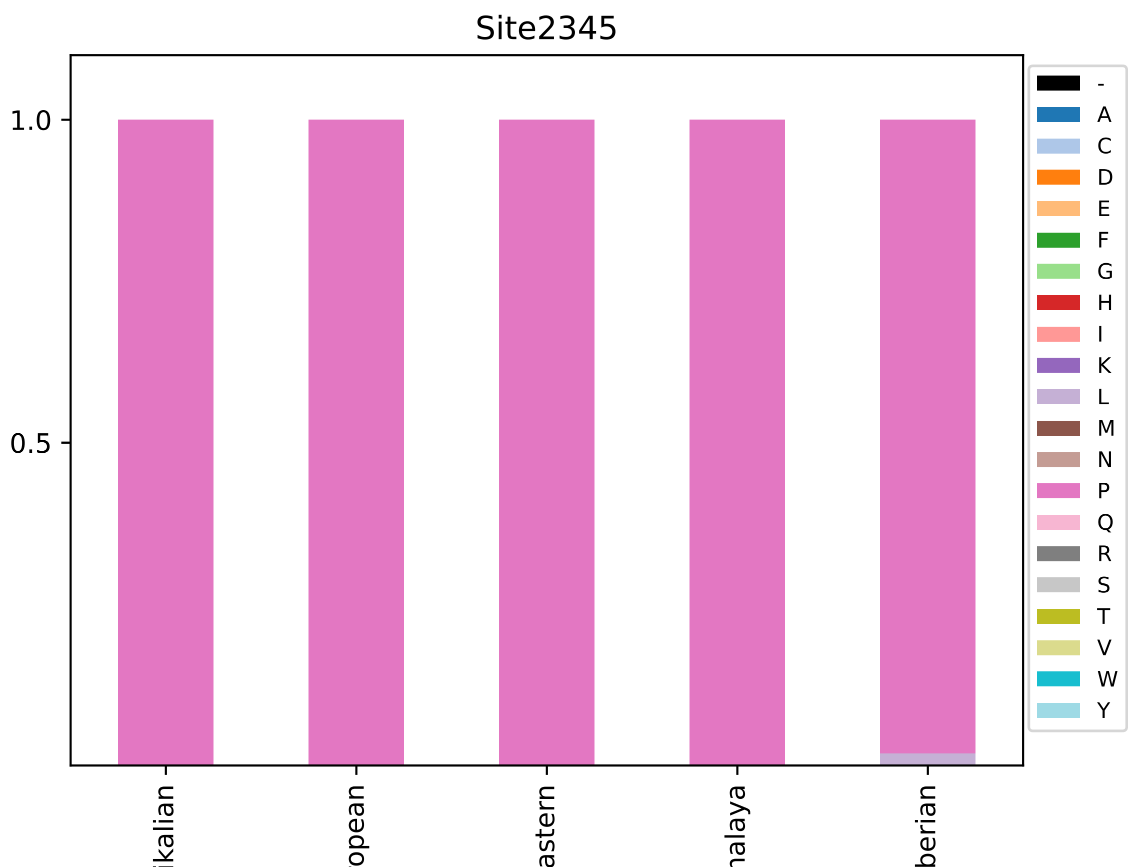
Site2345
1.0
0.5
ikalian
opean
astern
nalaya
berian
-
A
C
D
E
F
G
H
I
K
L
M
N
P
Q
R
S
T
V
W
Y

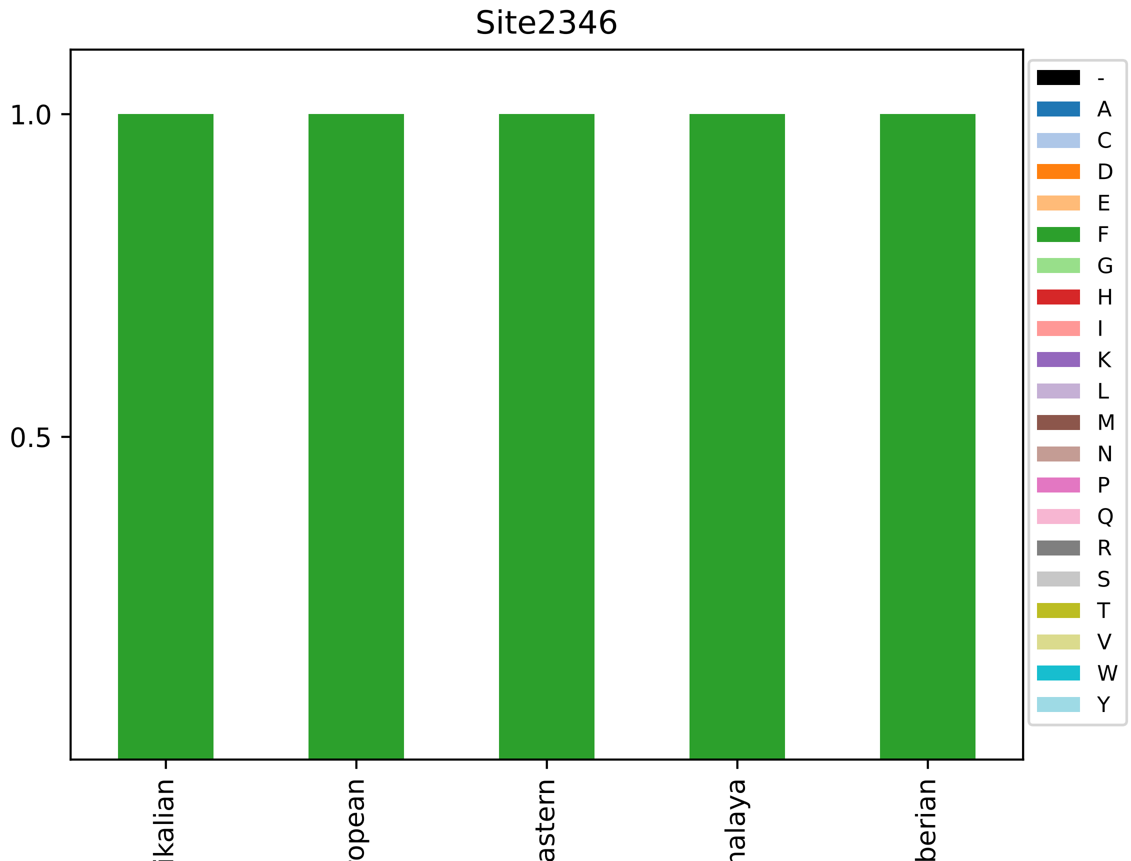
Site2346
1.0
0.5
-
A
C
D
E
F
G
H
I
K
L
M
N
P
Q
R
S
T
V
W
Y
ikalian
opean
astern
nalaya
berian

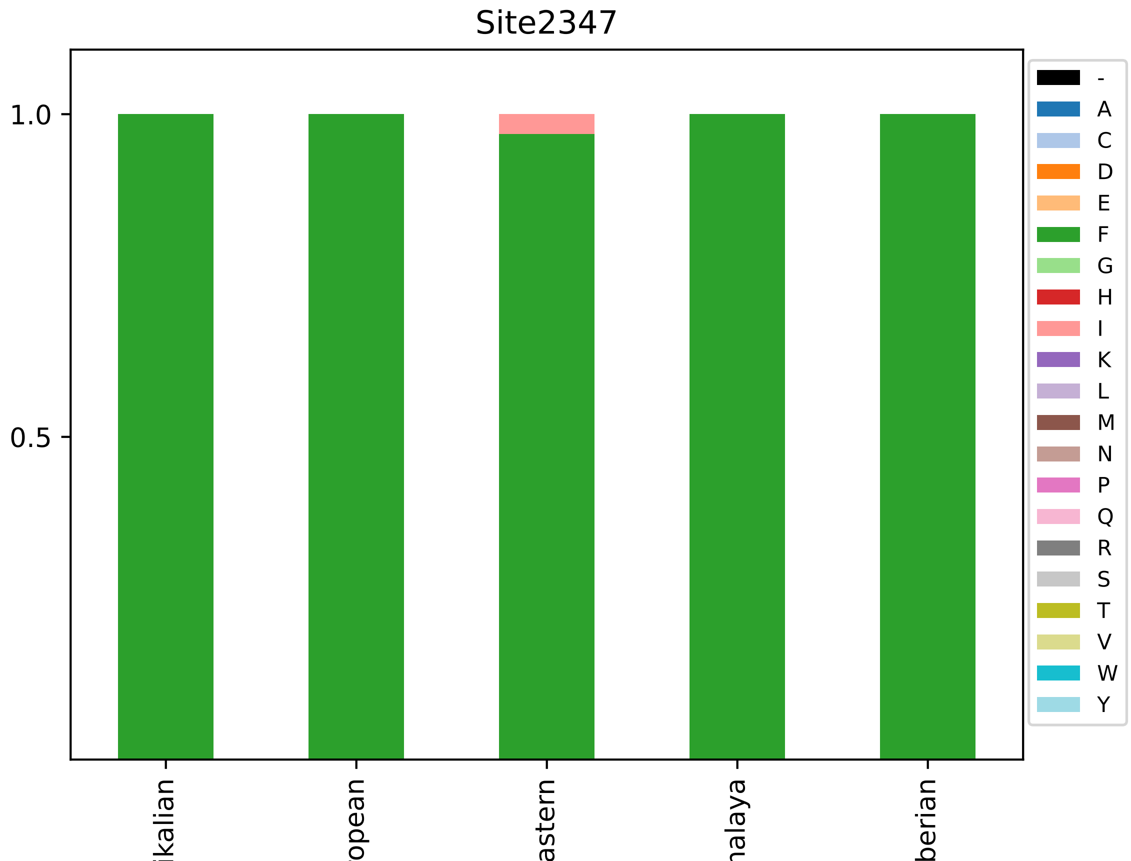
Site2347
1.0
0.5
-
A
C
D
E
F
G
H
I
K
L
M
N
P
Q
R
S
T
V
W
Y
ikalian
opean
astern
nalaya
berian

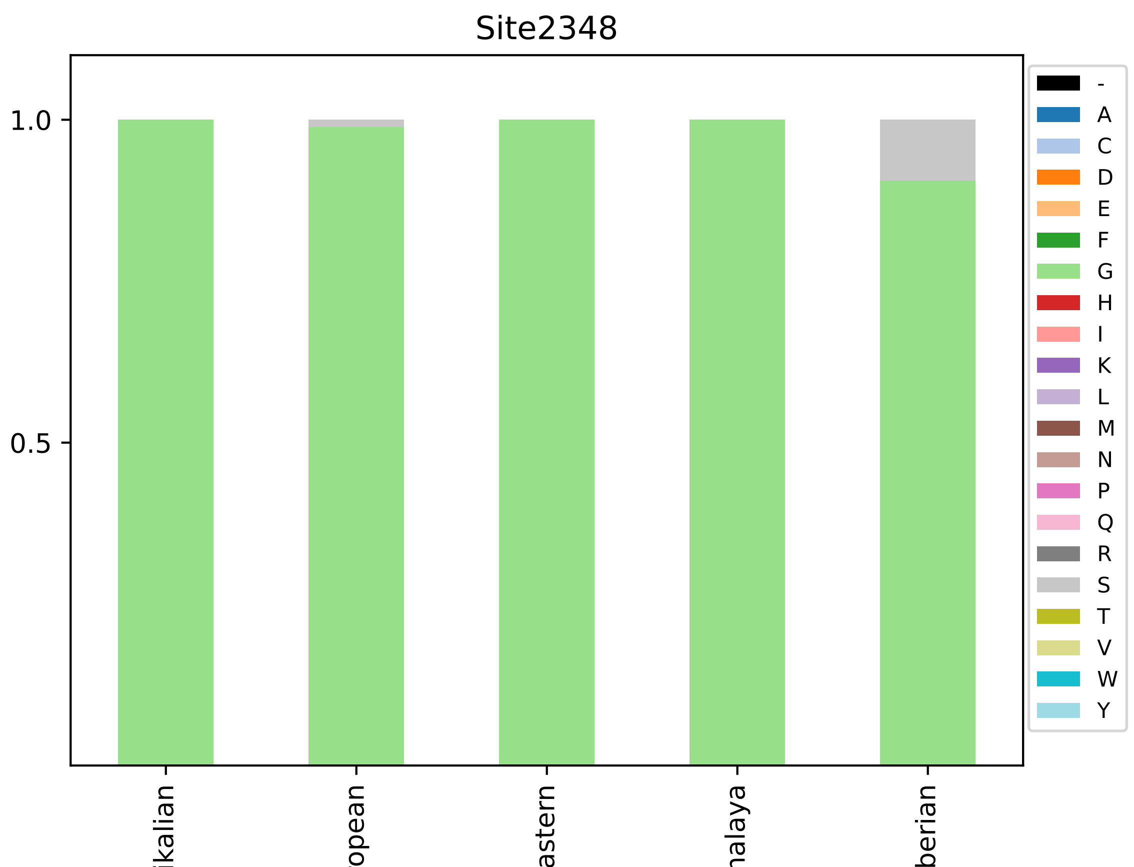

Site2348
1.0
0.5
ikalian
opean
astern
nalaya
berian
-
A
C
D
E
F
G
H
I
K
L
M
N
P
Q
R
S
T
V
W
Y

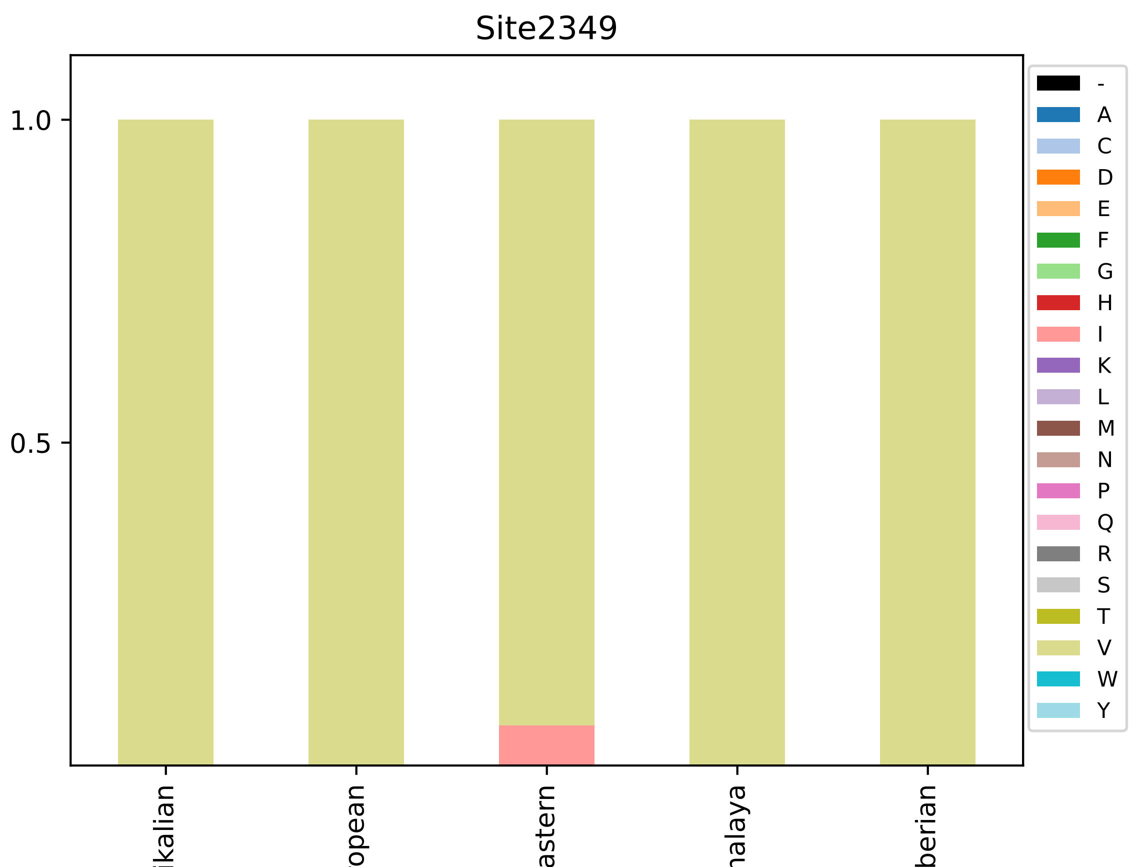

Site2349
1.0
0.5
-
A
C
D
E
F
G
H
I
K
L
M
N
P
Q
R
S
T
V
W
Y
ikalian
opean
astern
nalaya
berian

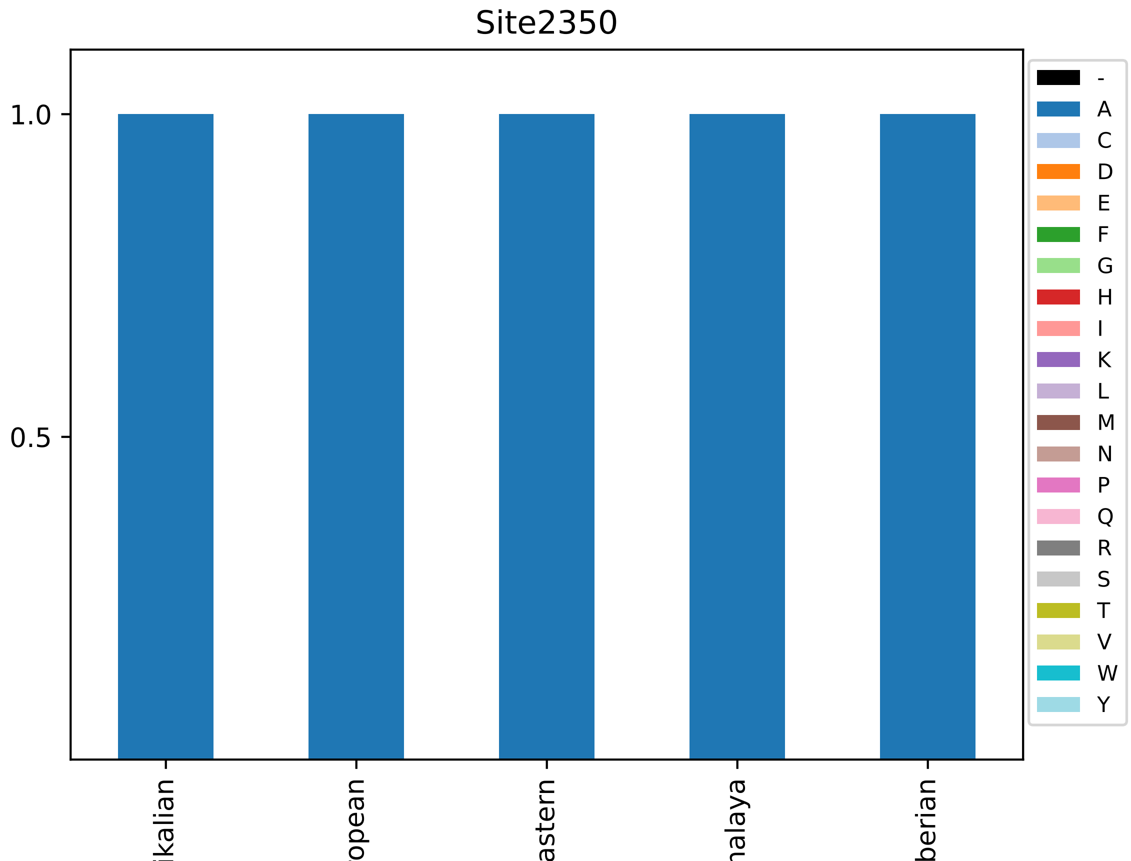

Site2350
1.0
0.5
ikalian
opean
astern
nalaya
berian
-
A
C
D
E
F
G
H
I
K
L
M
N
P
Q
R
S
T
V
W
Y

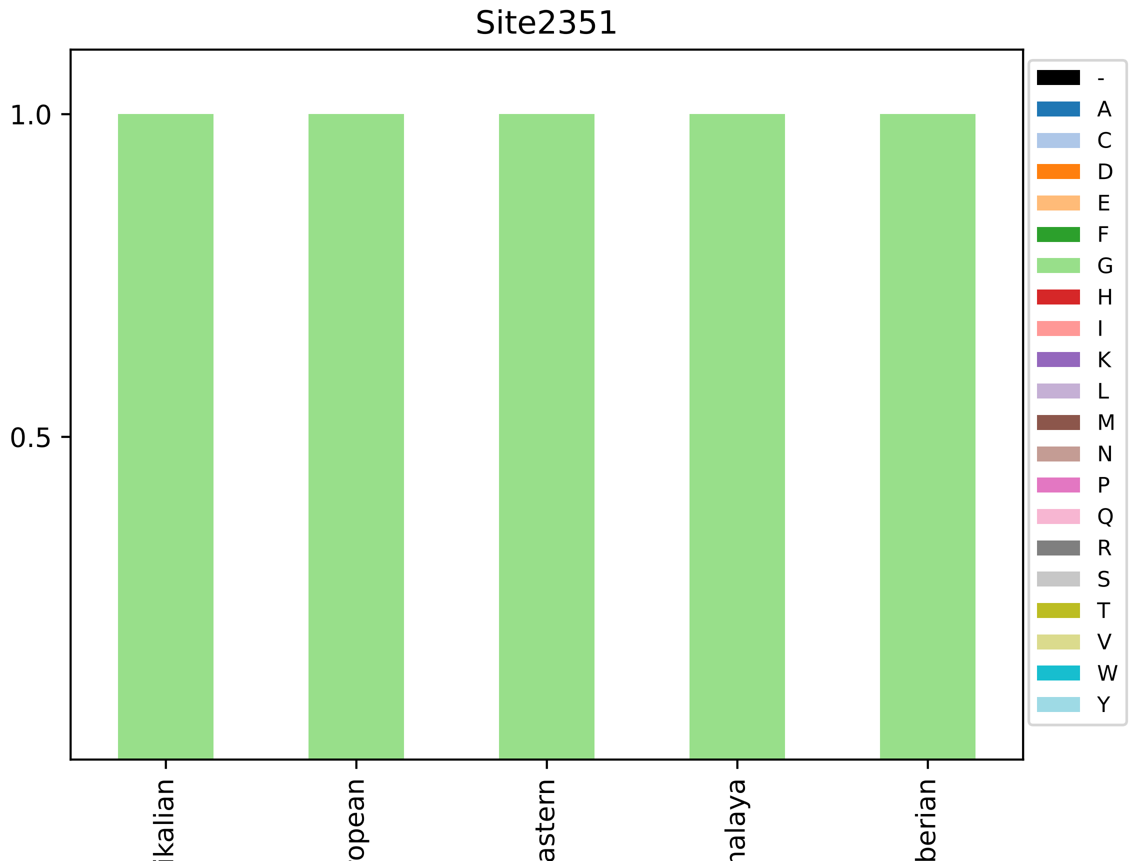
Site2351
1.0
0.5
-
A
C
D
E
F
G
H
I
K
L
M
N
P
Q
R
S
T
V
W
Y
ikalian
opean
astern
nalaya
berian

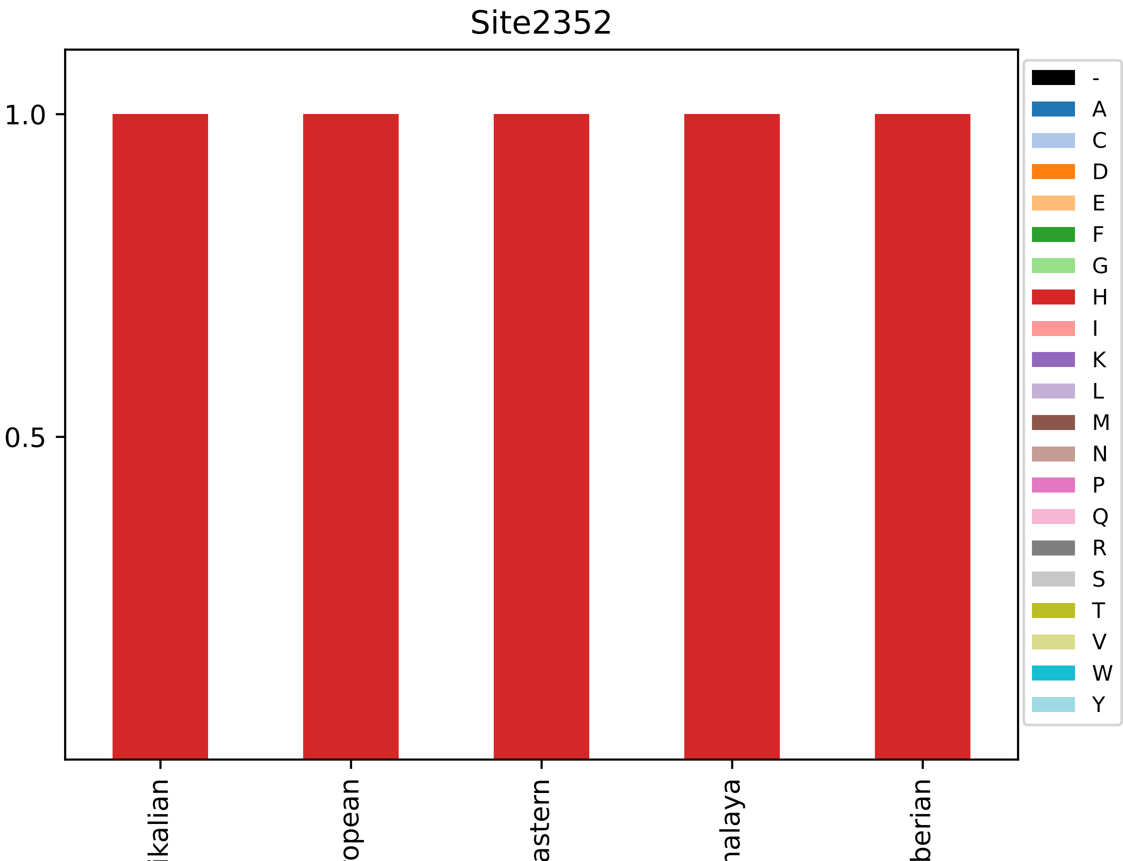

Site2352
1.0
0.5
-
A
C
D
E
F
G
H
I
K
L
M
N
P
Q
R
S
T
V
W
Y
ikalian
opean
astern
nalaya
berian

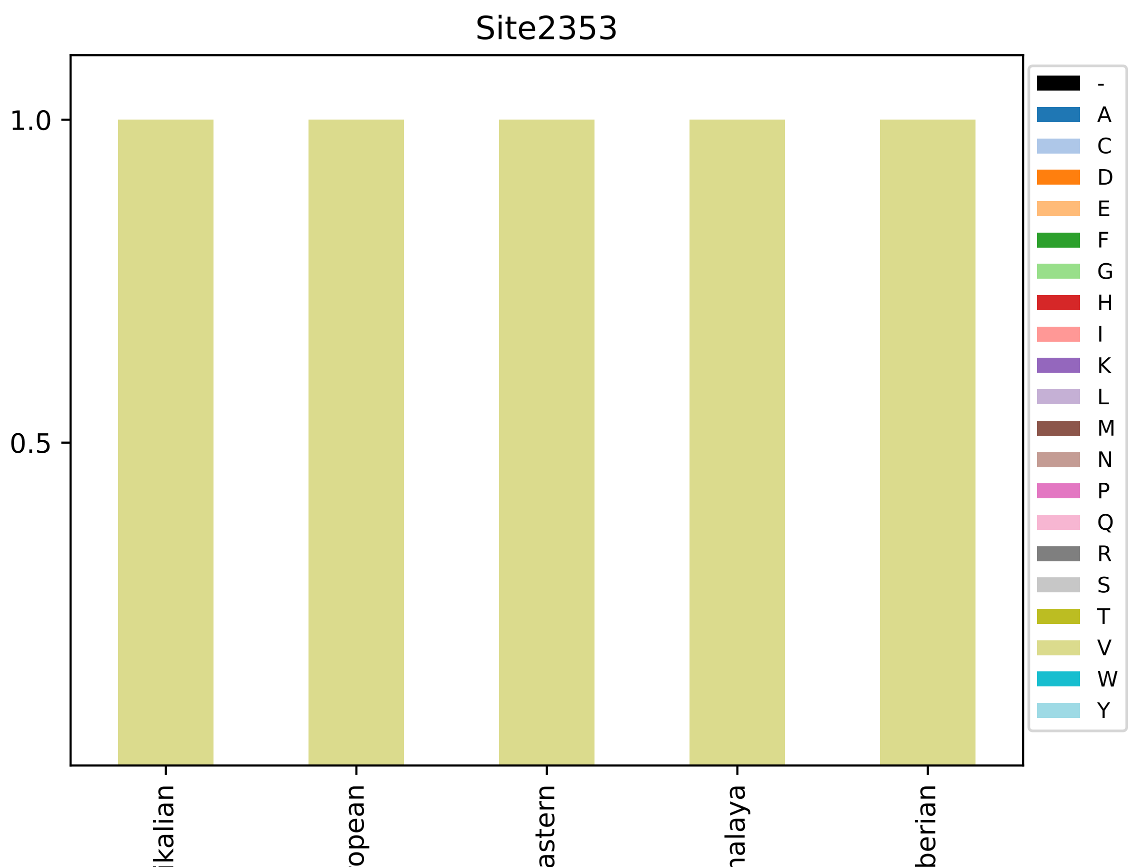
Site2353
1.0
0.5
-
A
C
D
E
F
G
H
I
K
L
M
N
P
Q
R
S
T
V
W
Y
ikalian
opean
astern
nalaya
berian

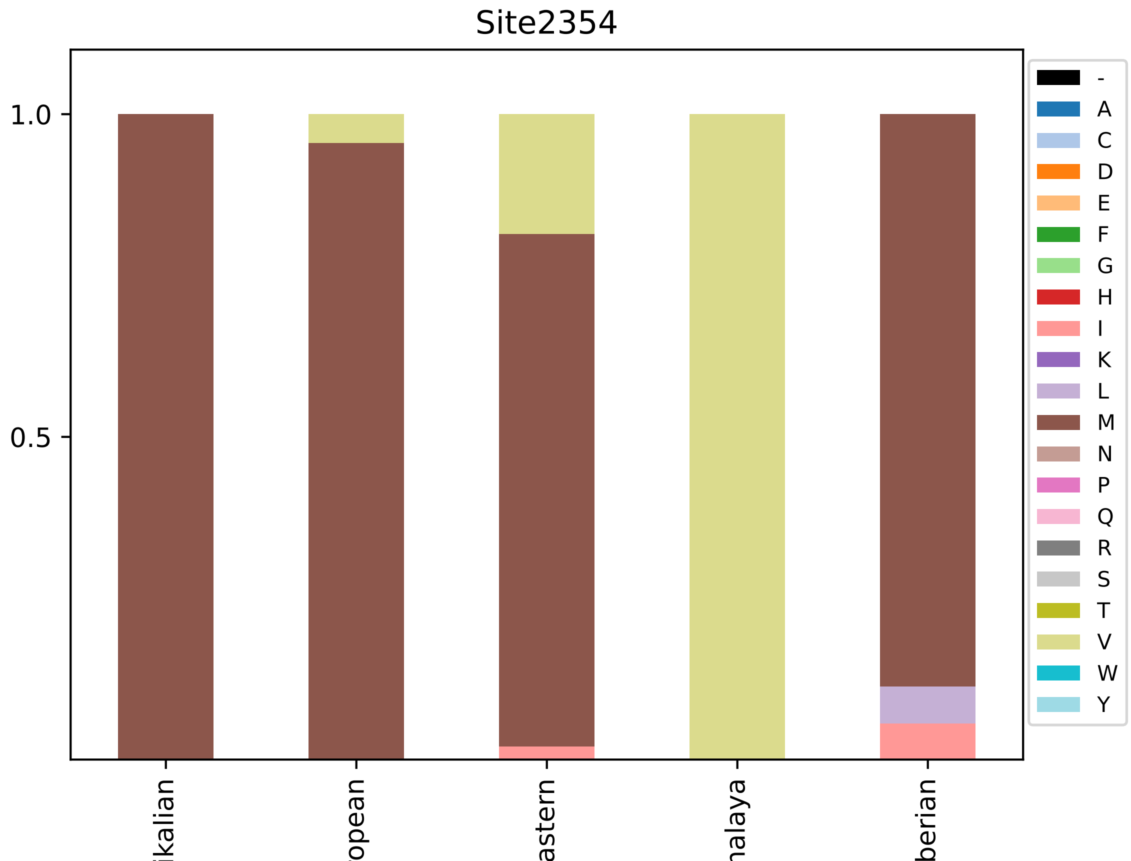
Site2354
1.0
0.5
-
A
C
D
E
F
G
H
I
K
L
M
N
P
Q
R
S
T
V
W
Y
ikalian
opean
astern
nalaya
berian

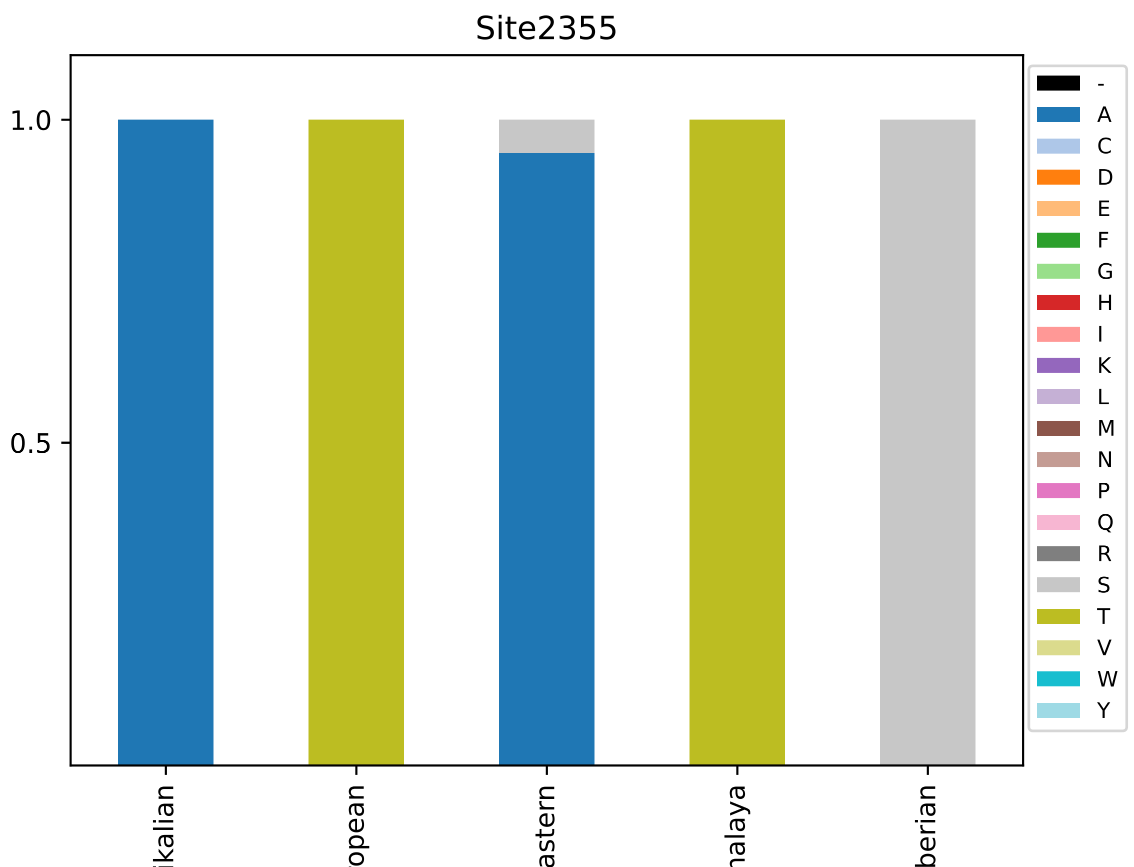

Site2355
1.0
0.5
ikalian
opean
astern
nalaya
berian
-
A
C
D
E
F
G
H
I
K
L
M
N
P
Q
R
S
T
V
W
Y

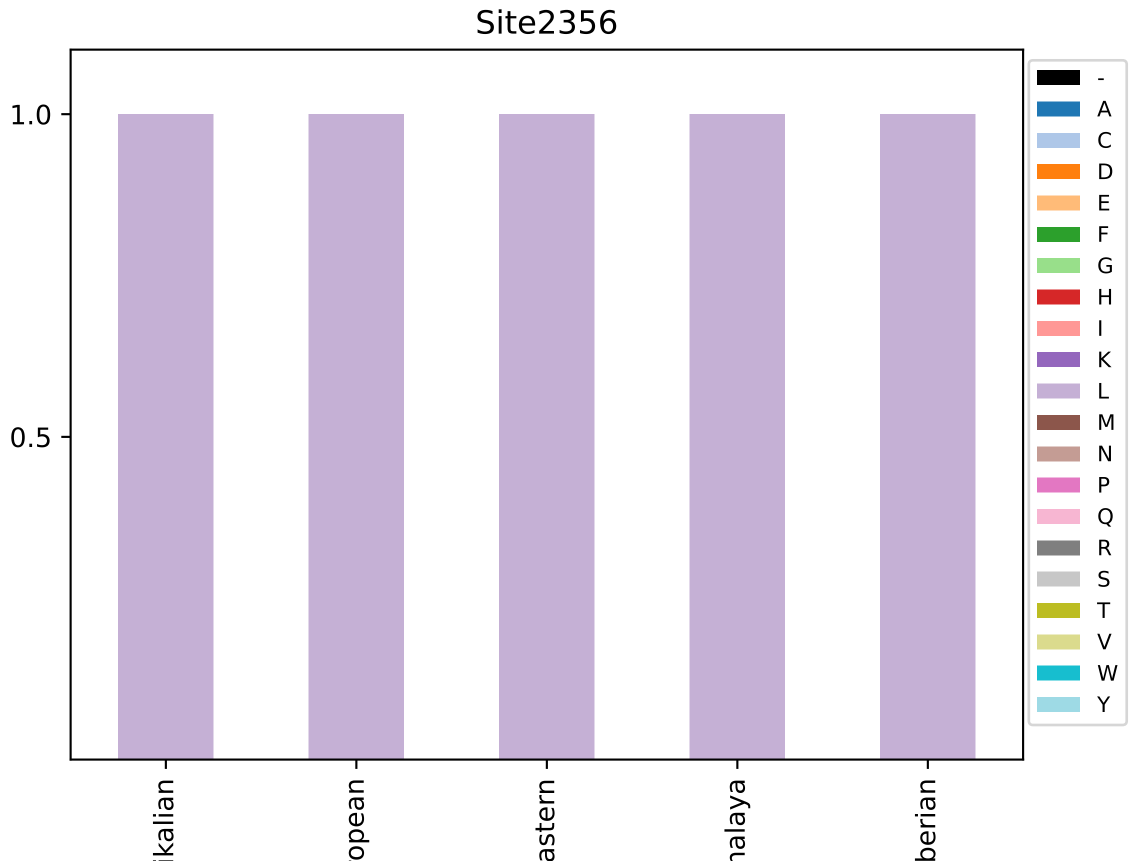
Site2356
1.0
0.5
ikalian
opean
astern
nalaya
berian
-
A
C
D
E
F
G
H
I
K
L
M
N
P
Q
R
S
T
V
W
Y

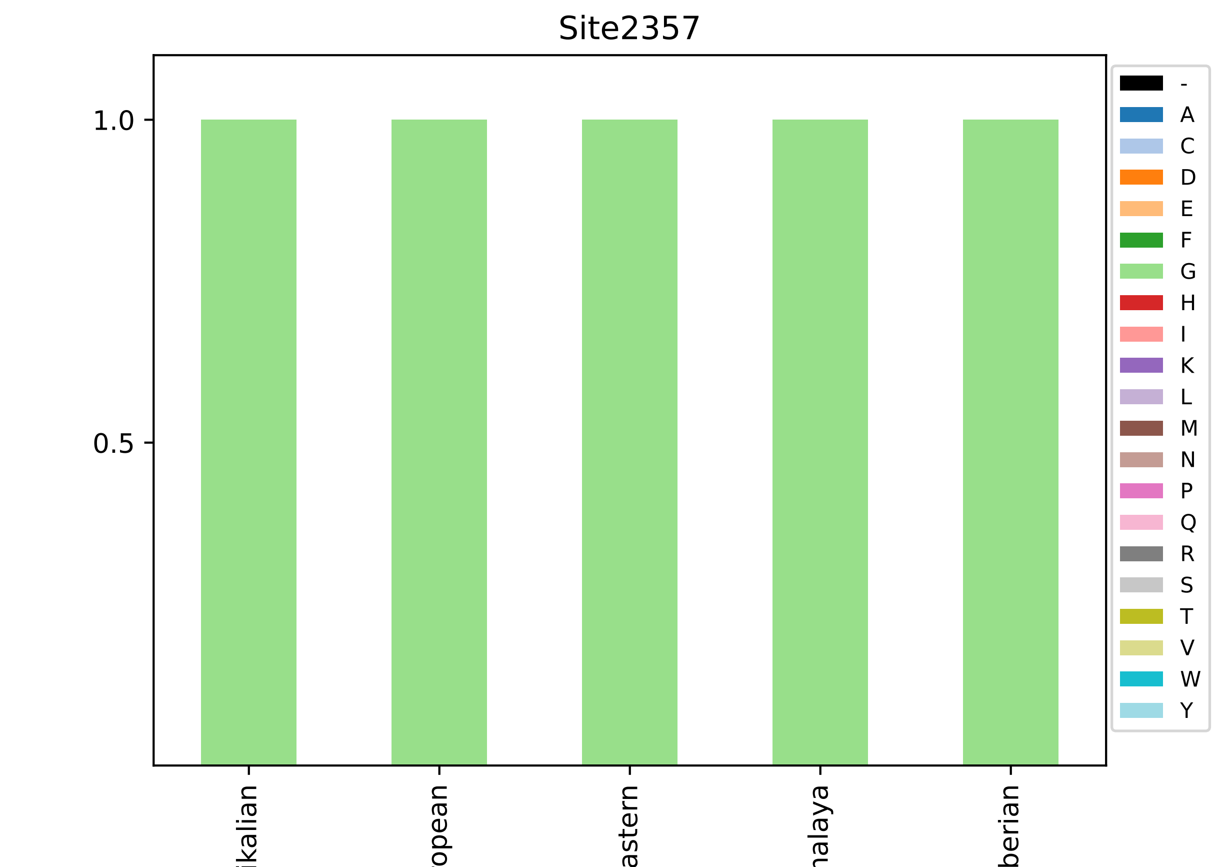

Site2357
1.0
0.5
ikalian
ropean
astern
nalaya
berian
-
A
C
D
E
F
G
H
I
K
L
M
N
P
Q
R
S
T
V
W
Y

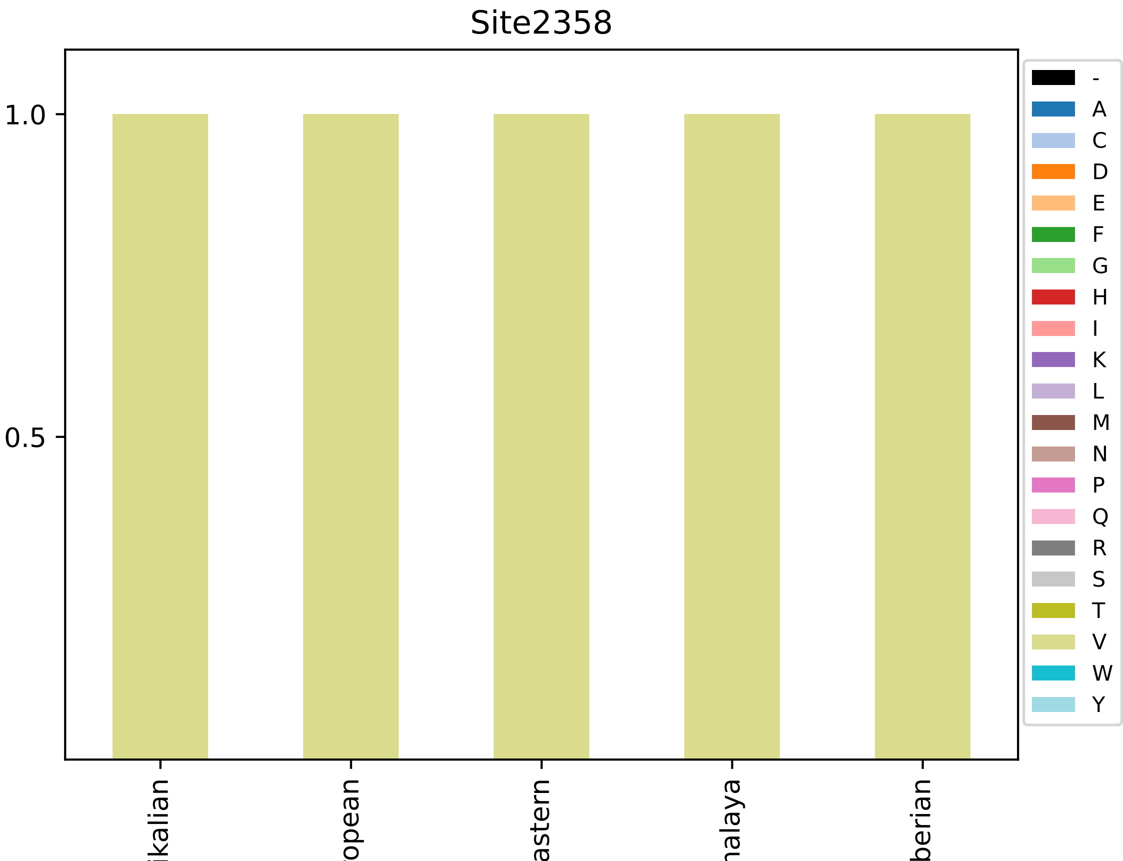

Site2358
1.0
0.5
-
A
C
D
E
F
G
H
I
K
L
M
N
P
Q
R
S
T
V
W
Y
ikalian
opean
astern
nalaya
berian

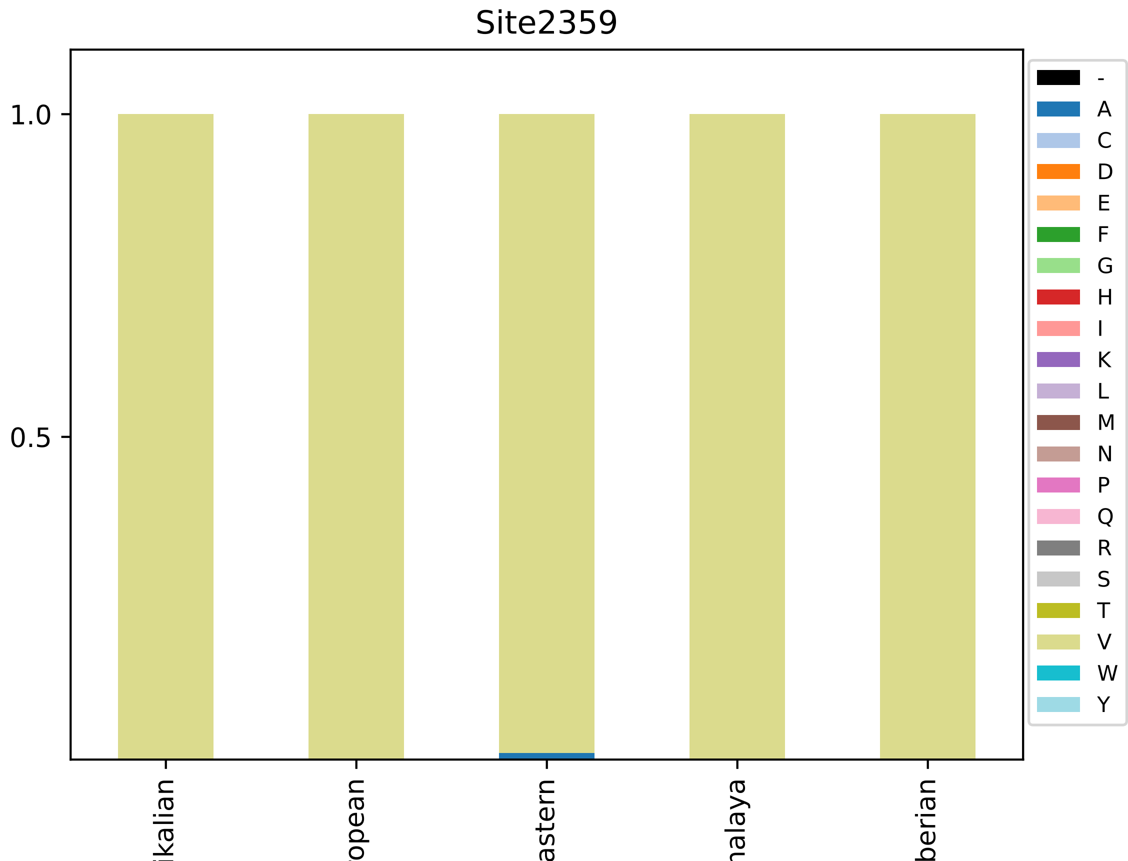
Site2359
1.0
0.5
-
A
C
D
E
F
G
H
I
K
L
M
N
P
Q
R
S
T
V
W
Y
ikalian
opean
astern
nalaya
berian

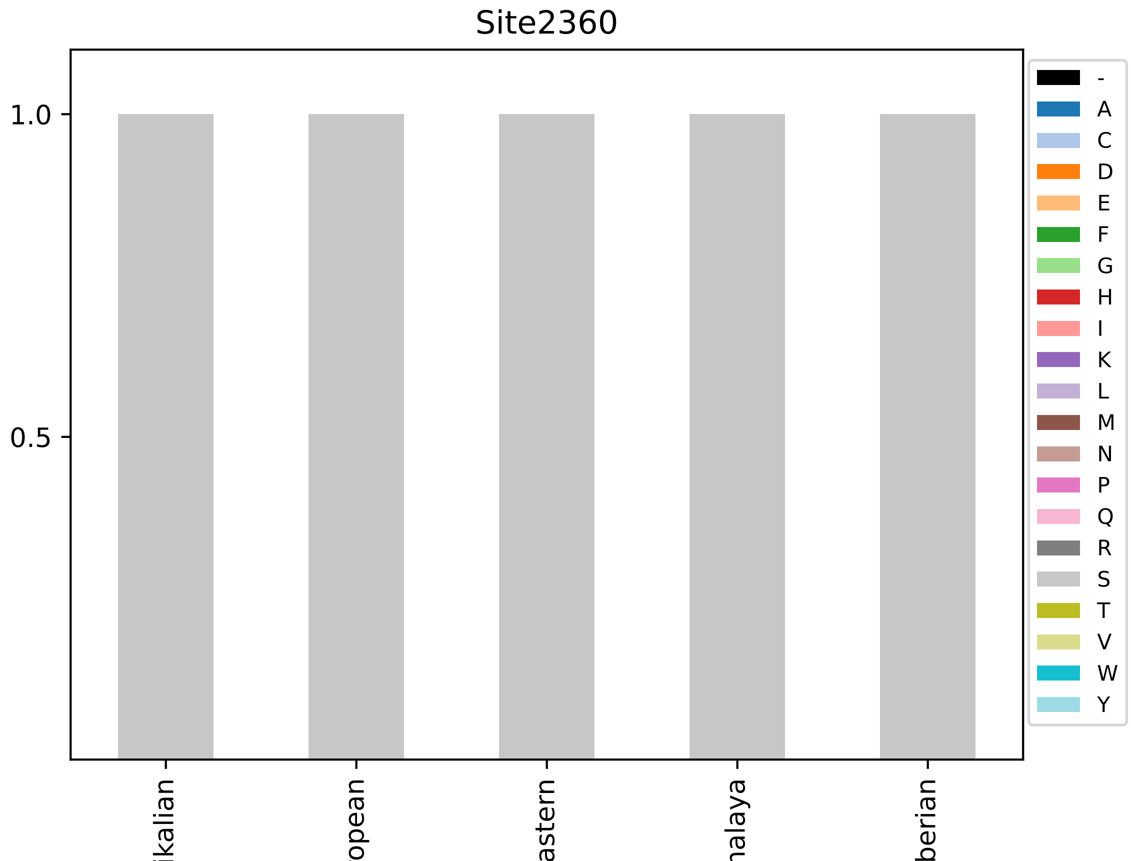

Site2360
1.0
0.5
-
A
C
D
E
F
G
H
I
K
L
M
N
P
Q
R
S
T
V
W
Y
ikalian
opean
astern
nalaya
berian

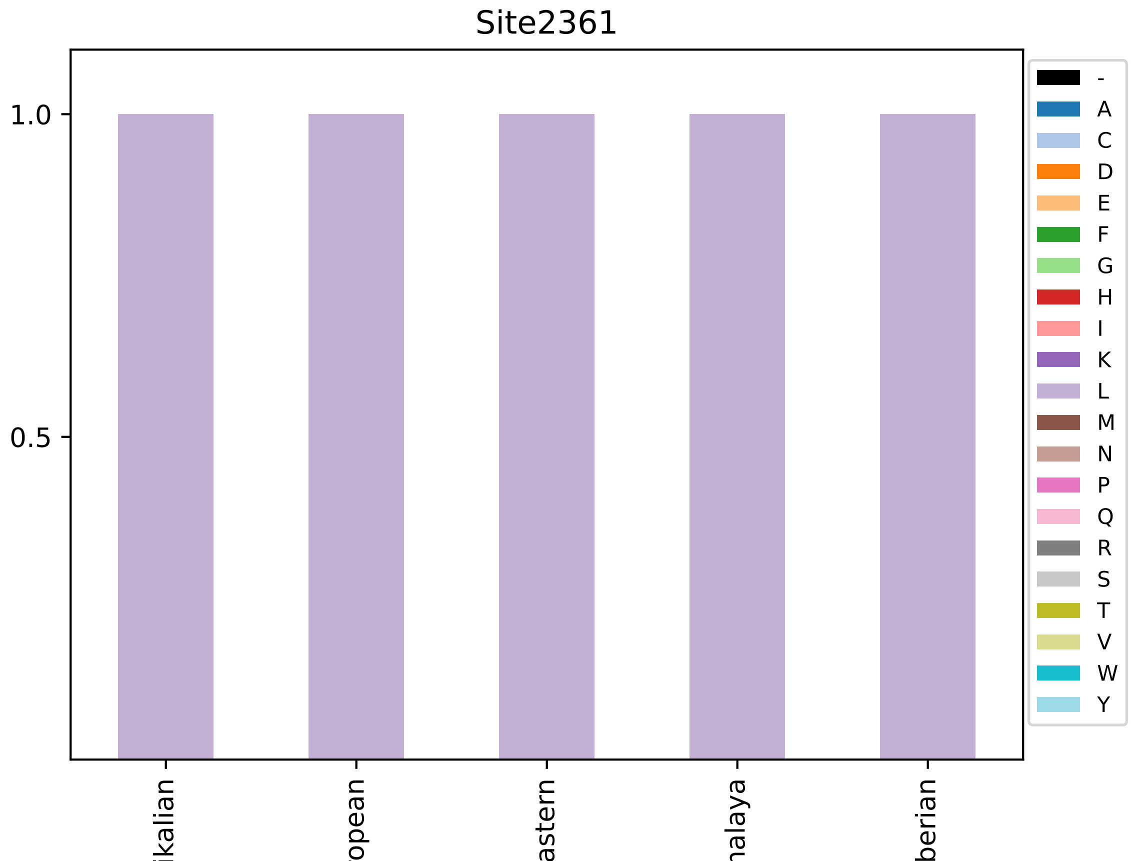
Site2361
1.0
0.5
ikalian
opean
astern
nalaya
berian
-
A
C
D
E
F
G
H
I
K
L
M
N
P
Q
R
S
T
V
W
Y

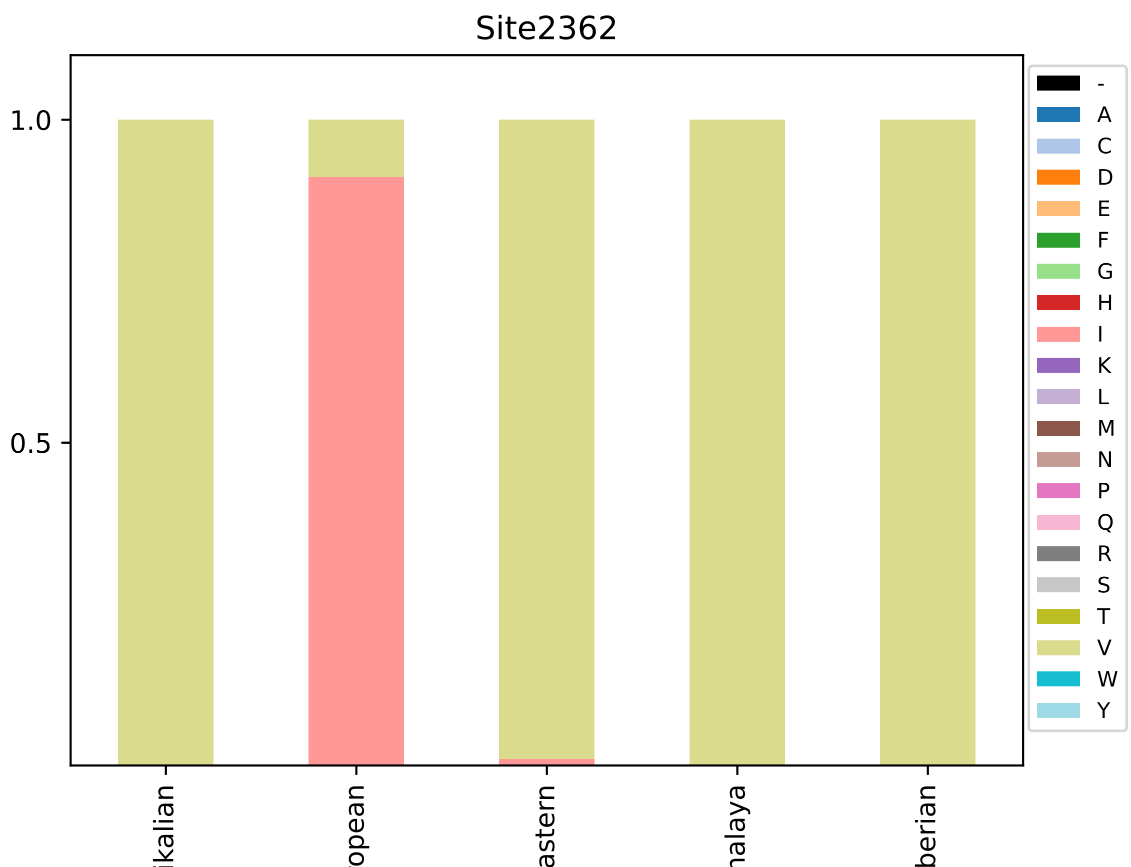
Site2362
1.0
0.5
ikalian
opean
astern
nalaya
berian
-
A
C
D
E
F
G
H
I
K
L
M
N
P
Q
R
S
T
V
W
Y

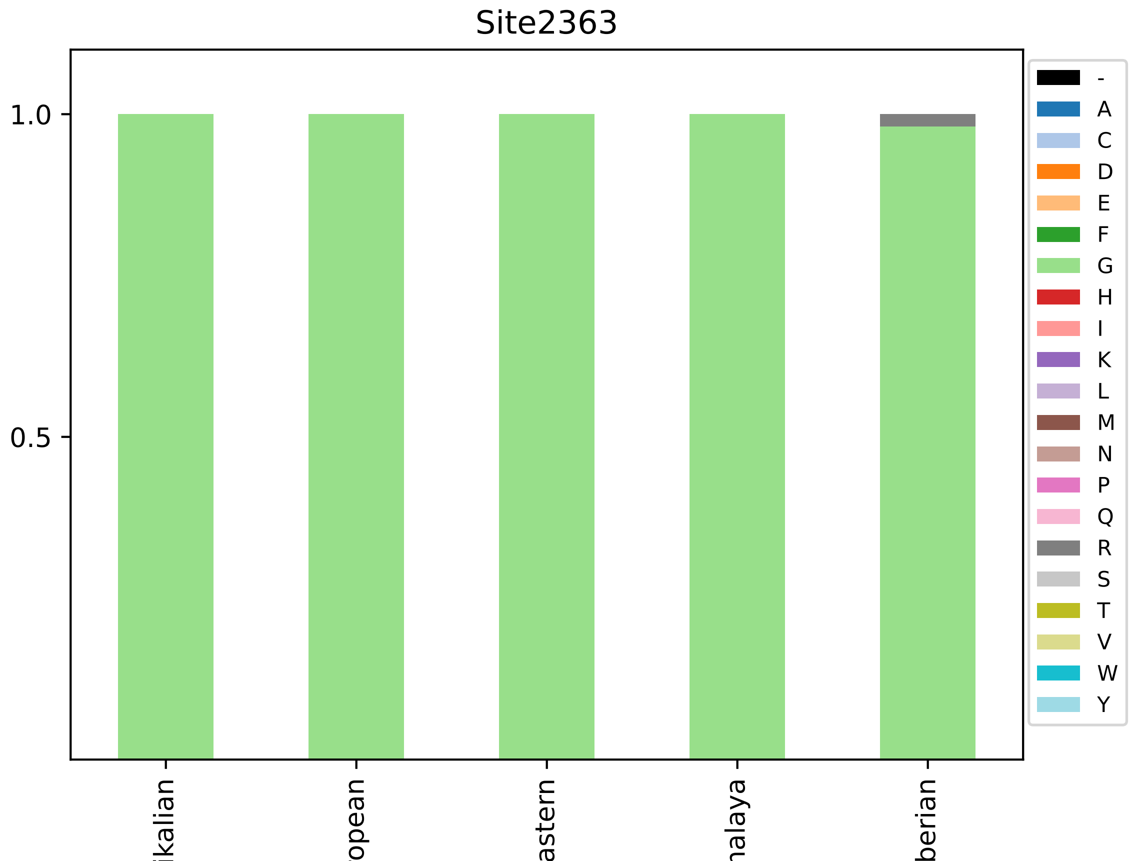
Site2363
1.0
0.5
ikalian
opean
astern
nalaya
berian
-
A
C
D
E
F
G
H
I
K
L
M
N
P
Q
R
S
T
V
W
Y

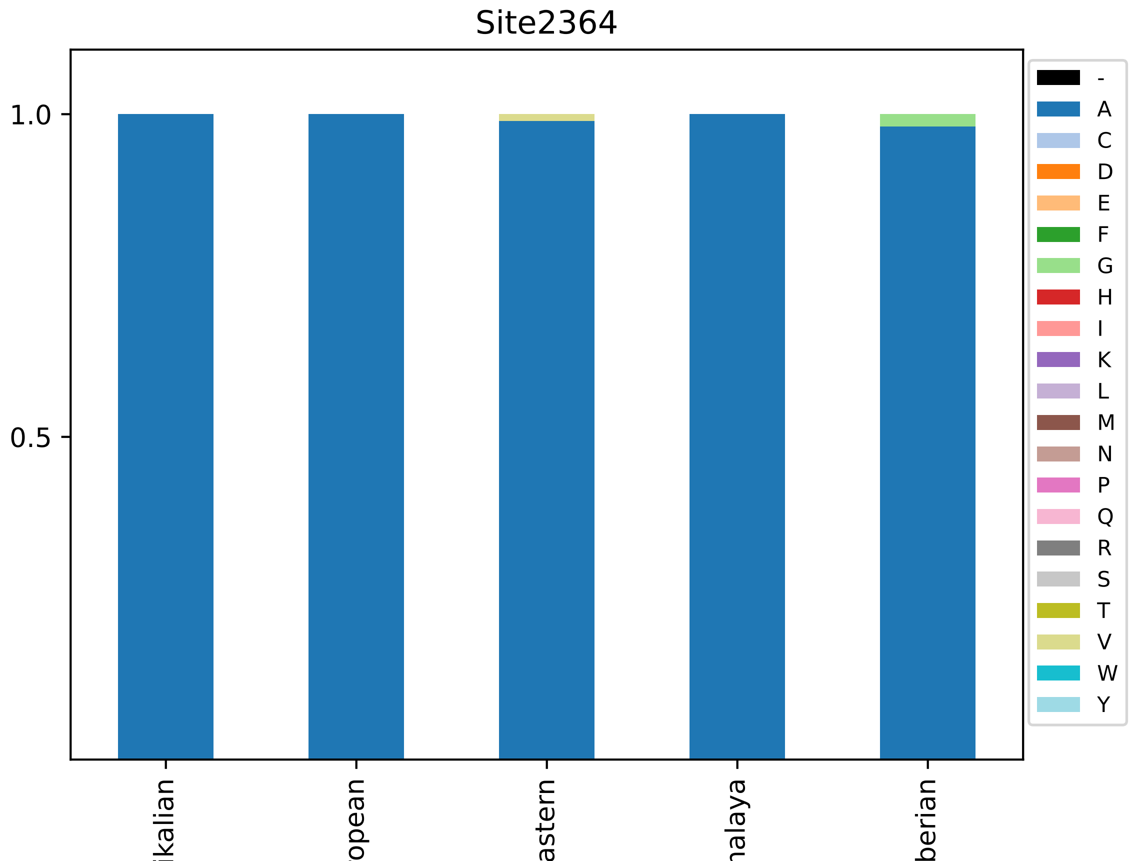
Site2364
1.0
0.5
-
A
C
D
E
F
G
H
I
K
L
M
N
P
Q
R
S
T
V
W
Y
ikalian
opean
astern
nalaya
berian

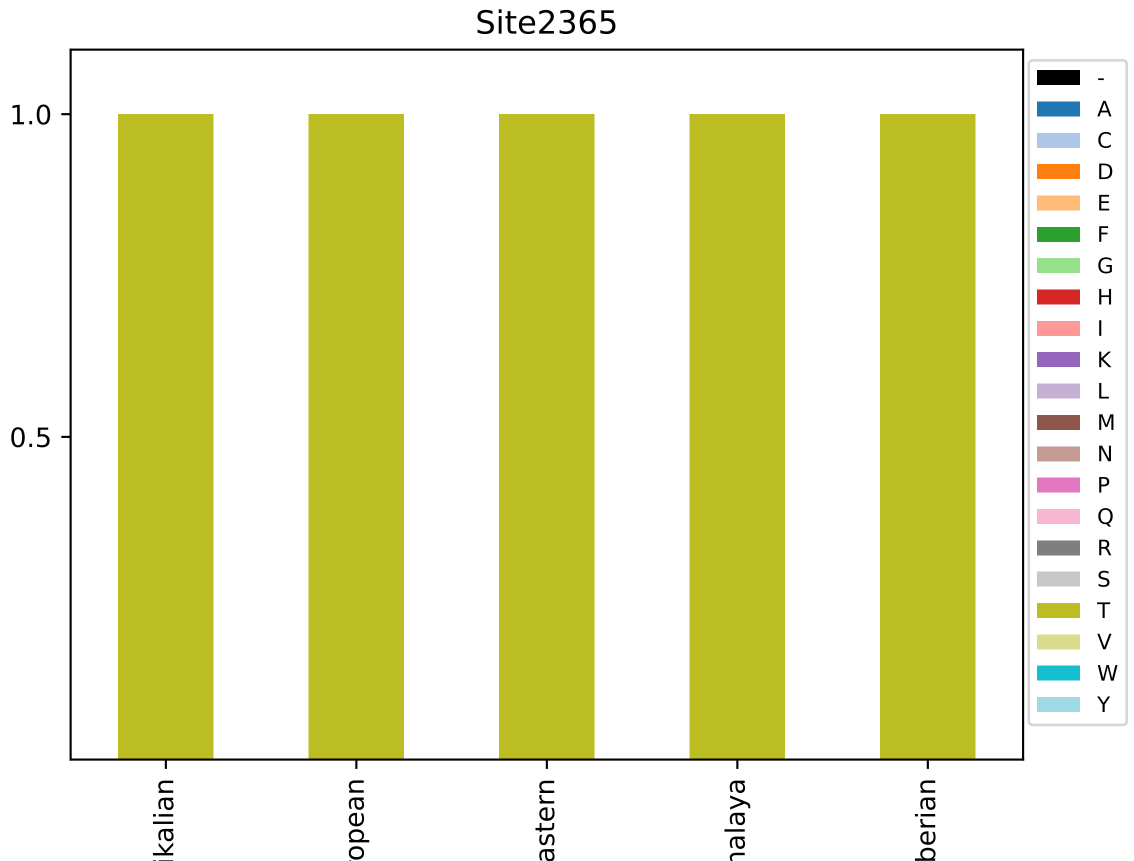
Site2365
1.0
0.5
-
A
C
D
E
F
G
H
I
K
L
M
N
P
Q
R
S
T
V
W
Y
ikalian
opean
astern
nalaya
berian

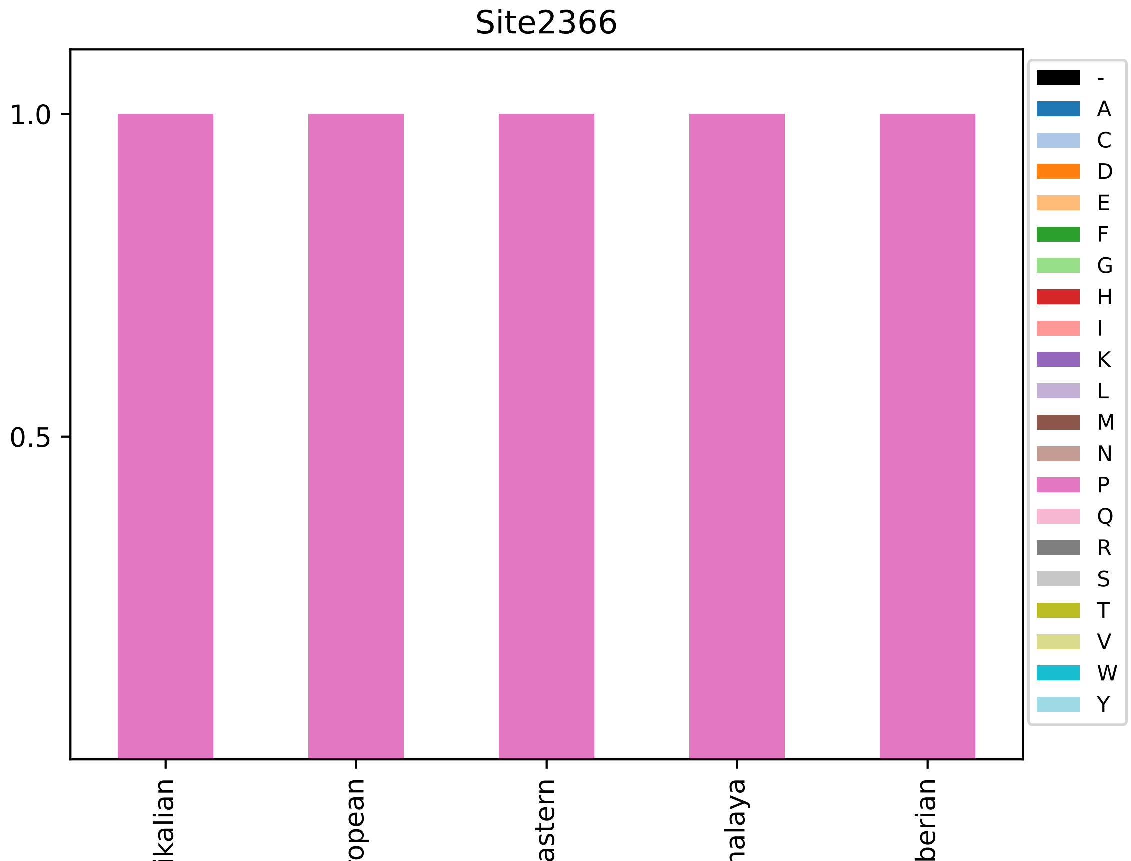
Site2366
1.0
0.5
-
A
C
D
E
F
G
H
I
K
L
M
N
P
Q
R
S
T
V
W
Y
ikalian
opean
astern
nalaya
berian

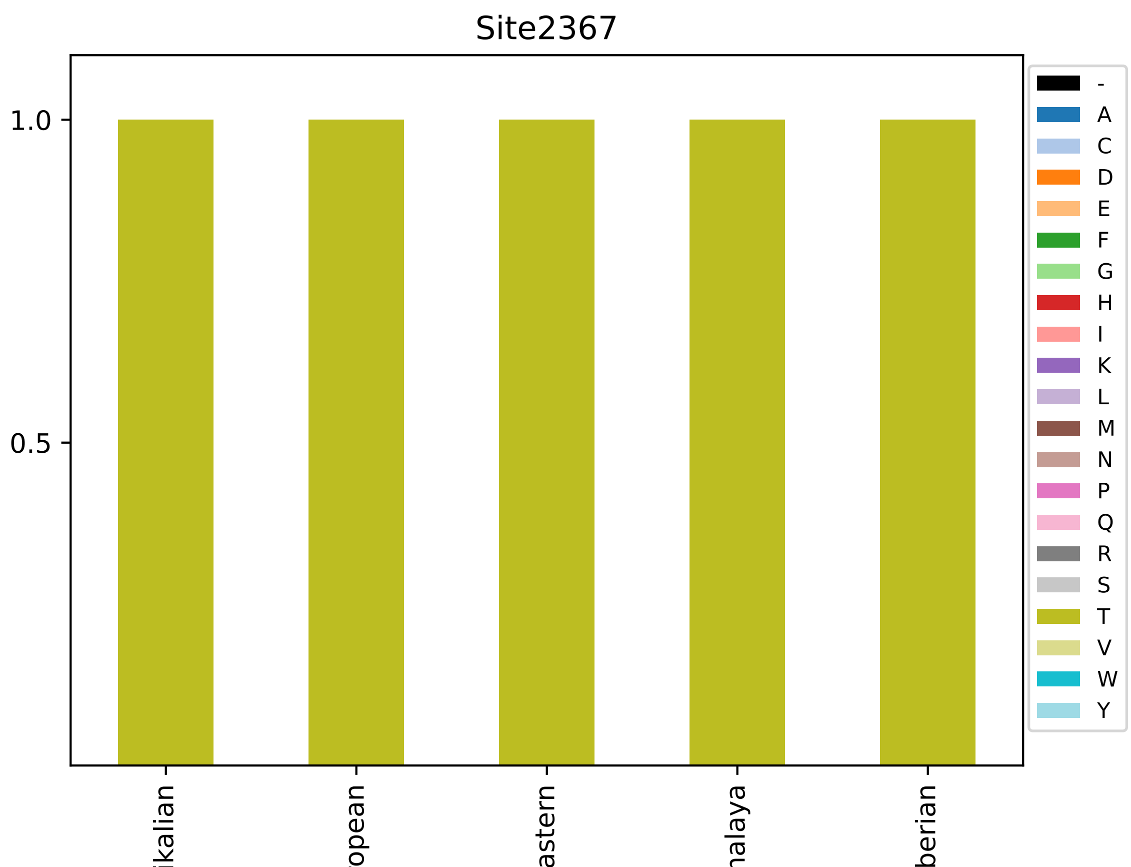
Site2367
1.0
0.5
-
A
C
D
E
F
G
H
I
K
L
M
N
P
Q
R
S
T
V
W
Y
ikalian
opean
astern
nalaya
berian

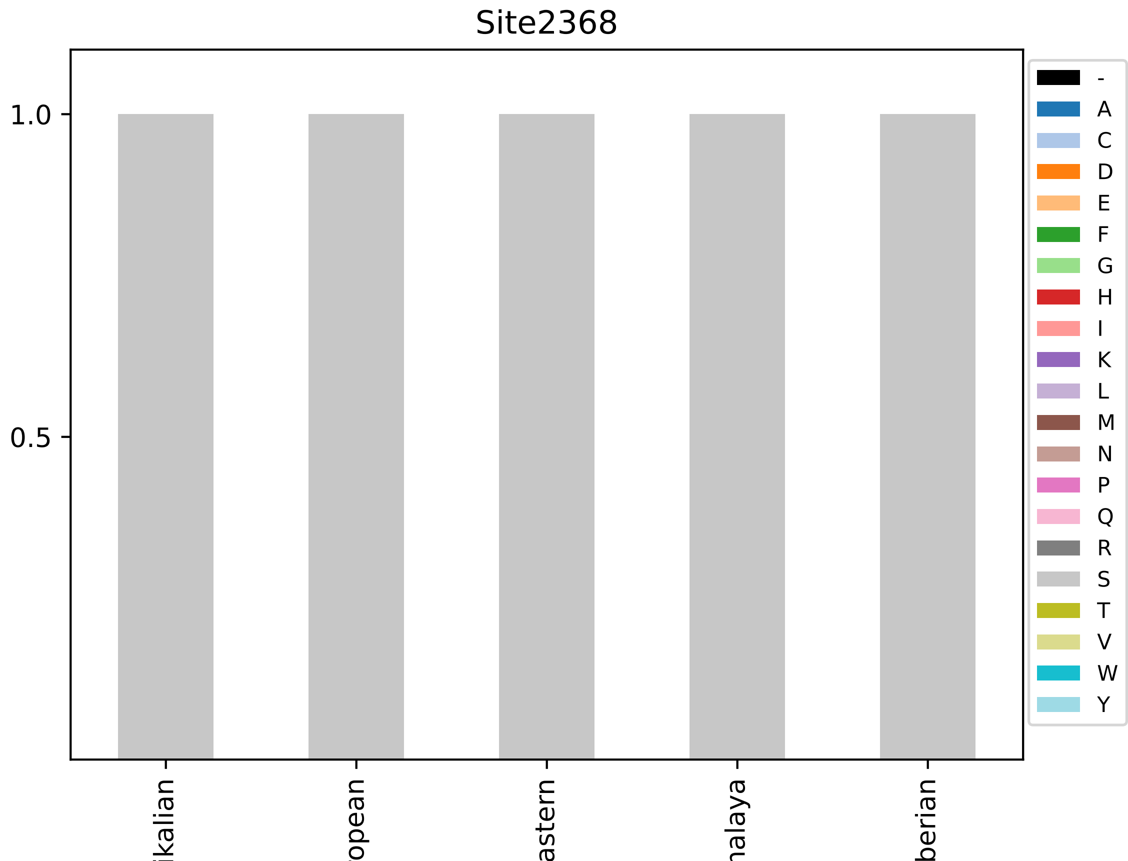
Site2368
1.0
0.5
-
A
C
D
E
F
G
H
I
K
L
M
N
P
Q
R
S
T
V
W
Y
ikalian
opean
astern
nalaya
berian

Site2369

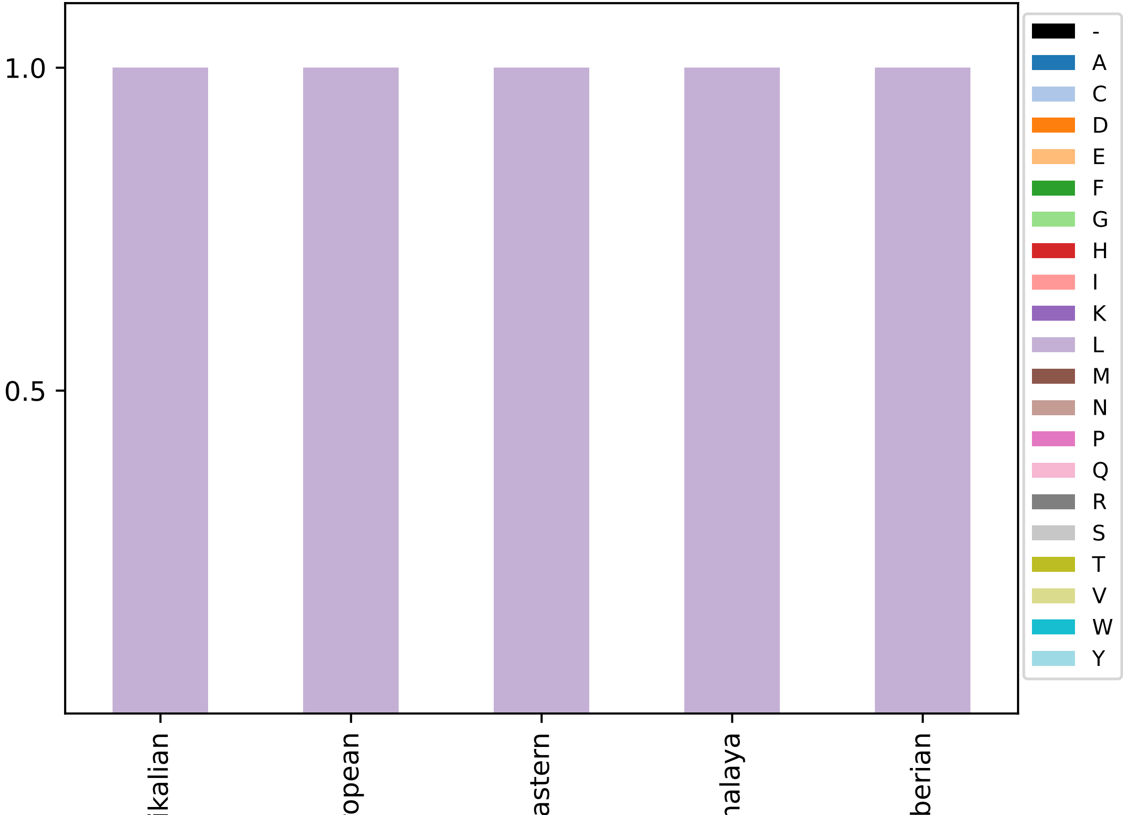

1.0
0.5
-
A
C
D
E
F
G
H
I
K
L
M
N
P
Q
R
S
T
V
W
Y
ikalian
opean
astern
nalaya
berian

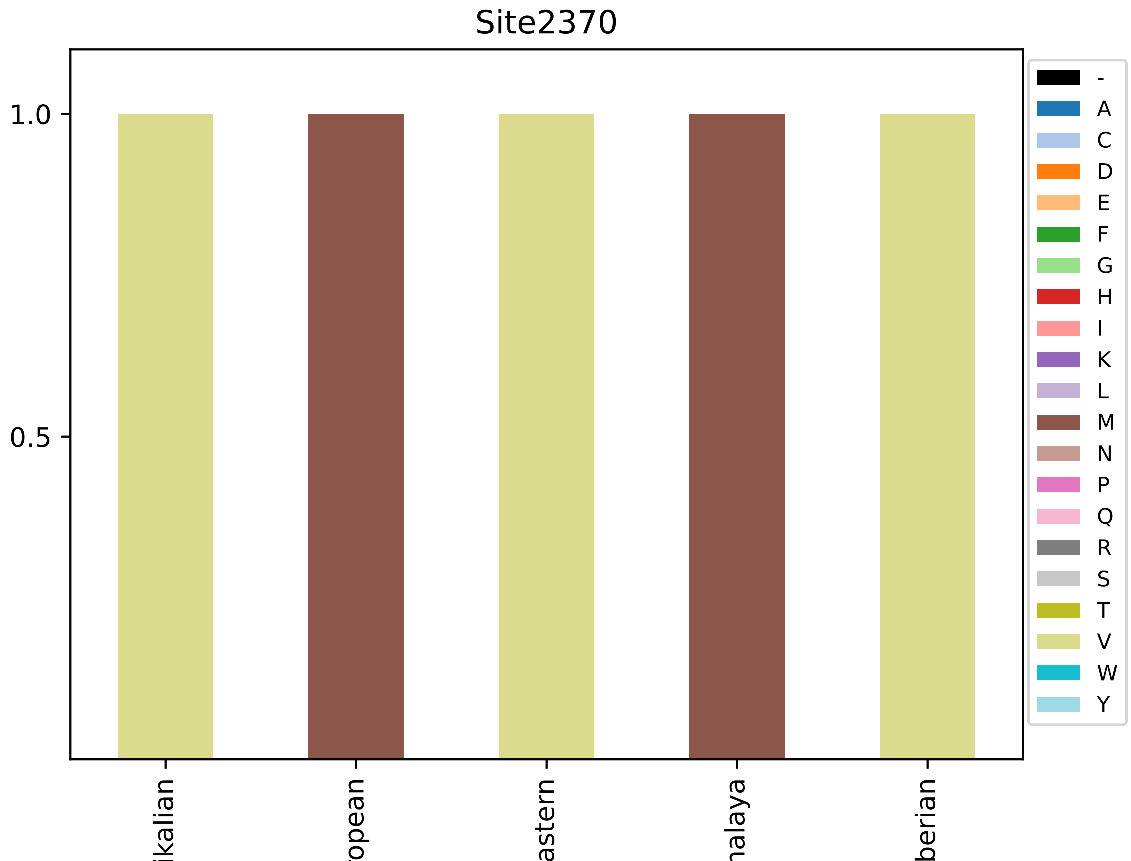

Site2370
1.0
0.5
-
A
C
D
E
F
G
H
I
K
L
M
N
P
Q
R
S
T
V
W
Y
ikalian
opean
astern
nalaya
berian

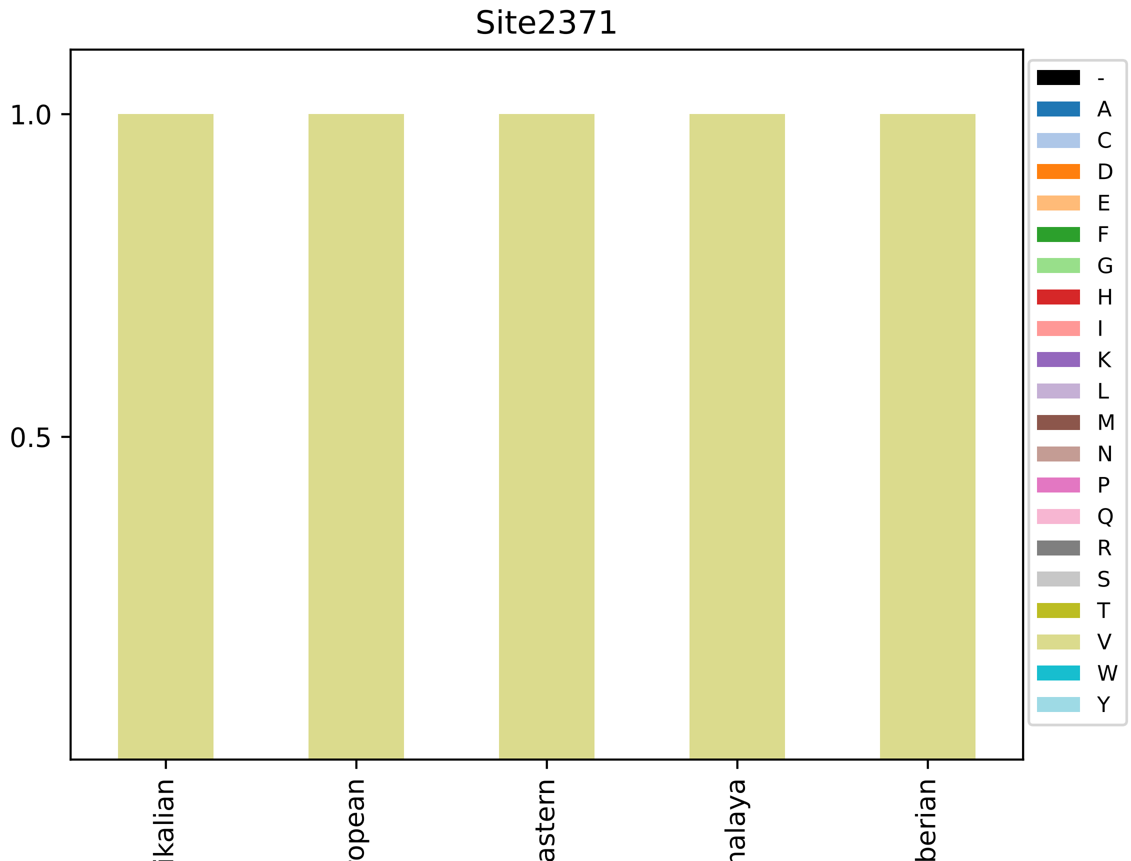

Site2371
1.0
0.5
-
A
C
D
E
F
G
H
I
K
L
M
N
P
Q
R
S
T
V
W
Y
ikalian
opean
astern
nalaya
berian

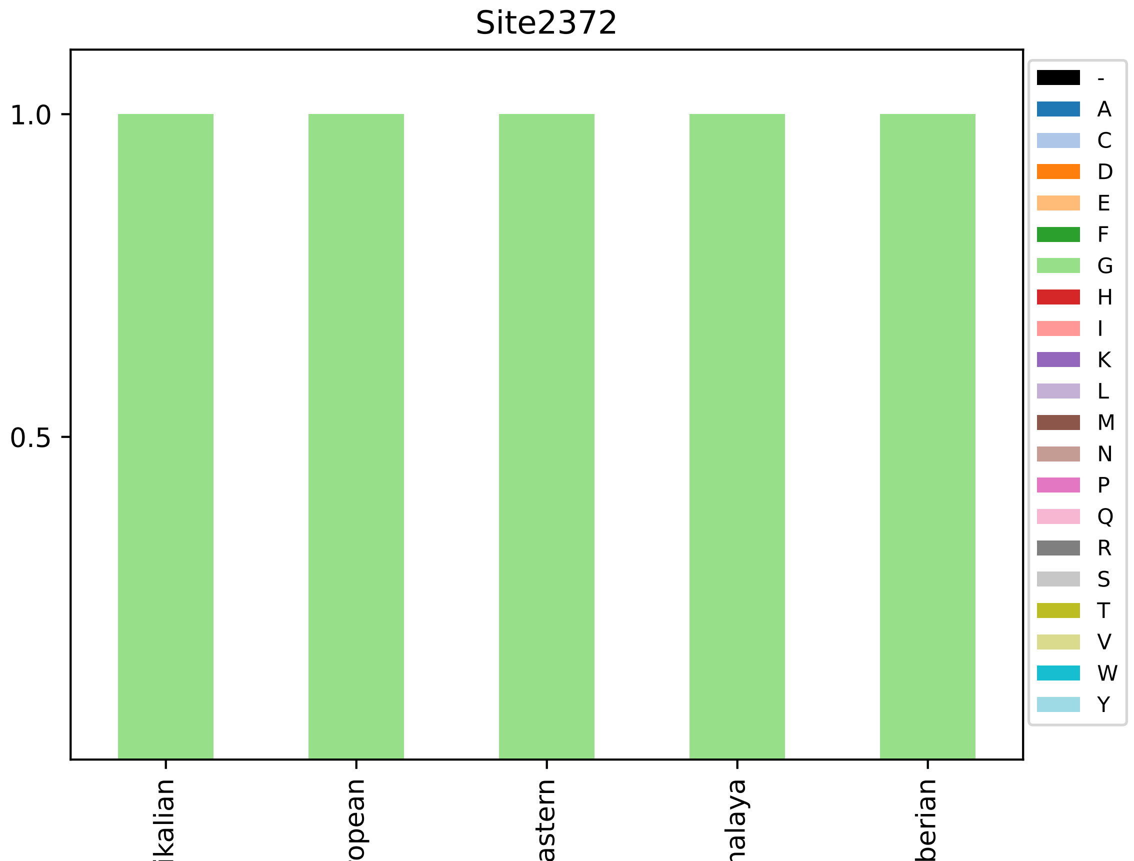

Site2372
1.0
0.5
-
A
C
D
E
F
G
H
I
K
L
M
N
P
Q
R
S
T
V
W
Y
ikalian
opean
astern
nalaya
berian

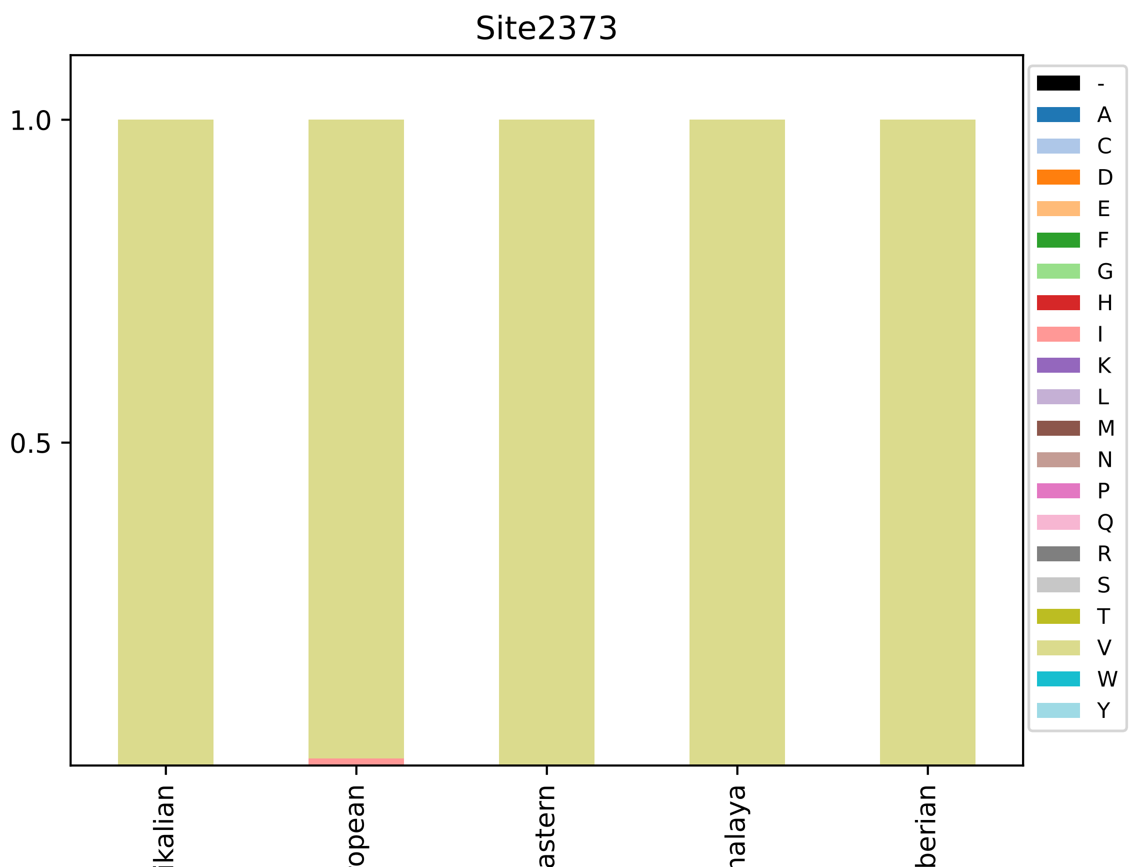
Site2373
1.0
0.5
-
A
C
D
E
F
G
H
I
K
L
M
N
P
Q
R
S
T
V
W
Y
ikalian
opean
astern
nalaya
berian

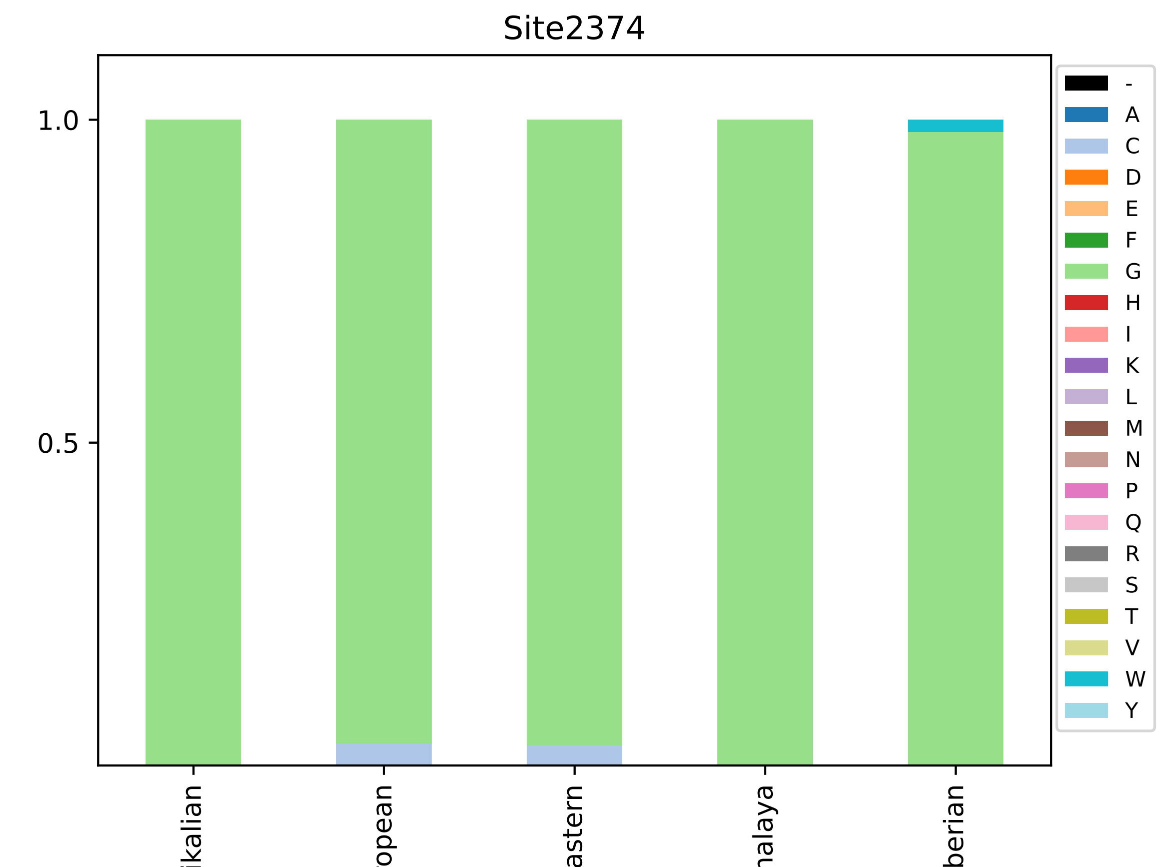
Site2374
1.0
0.5
ikalian
opean
astern
halaya
berian
-
A
C
D
E
F
G
H
I
K
L
M
N
P
Q
R
S
T
V
W
Y

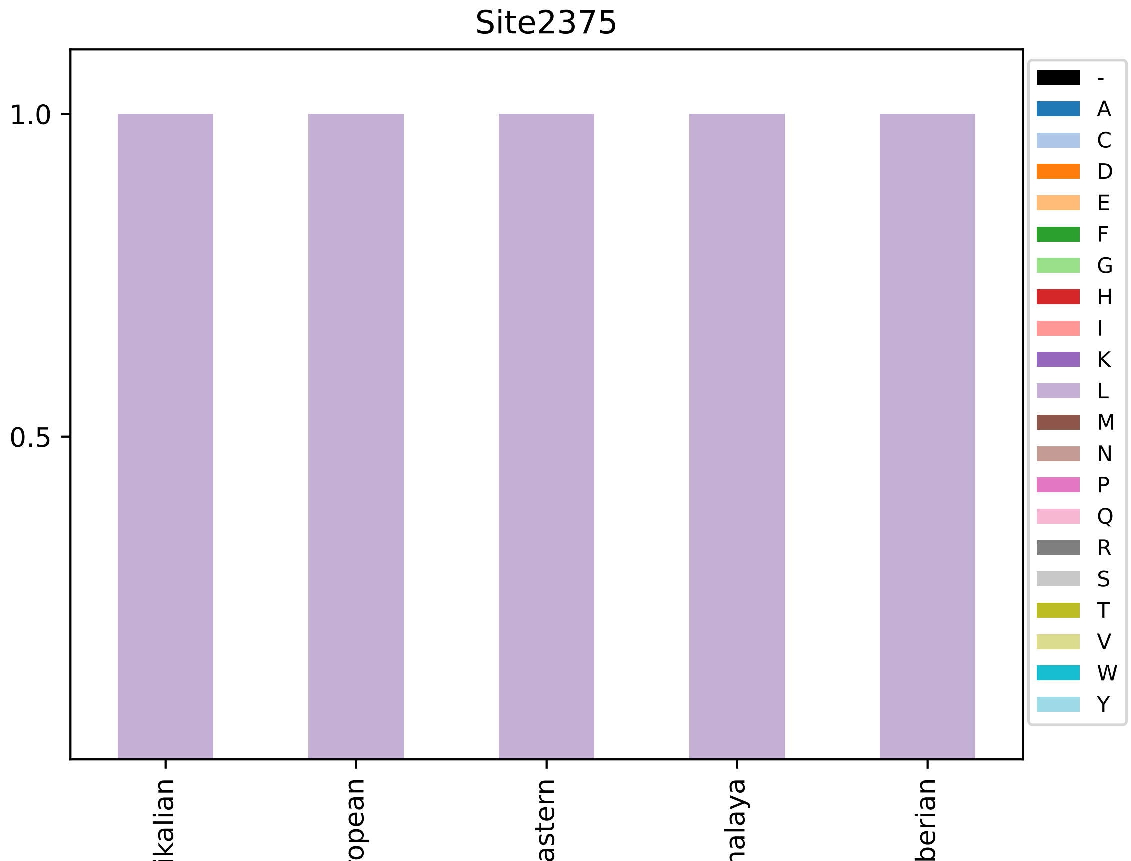

Site2375
1.0
0.5
-
A
C
D
E
F
G
H
I
K
L
M
N
P
Q
R
S
T
V
W
Y
ikalian
opean
astern
nalaya
berian

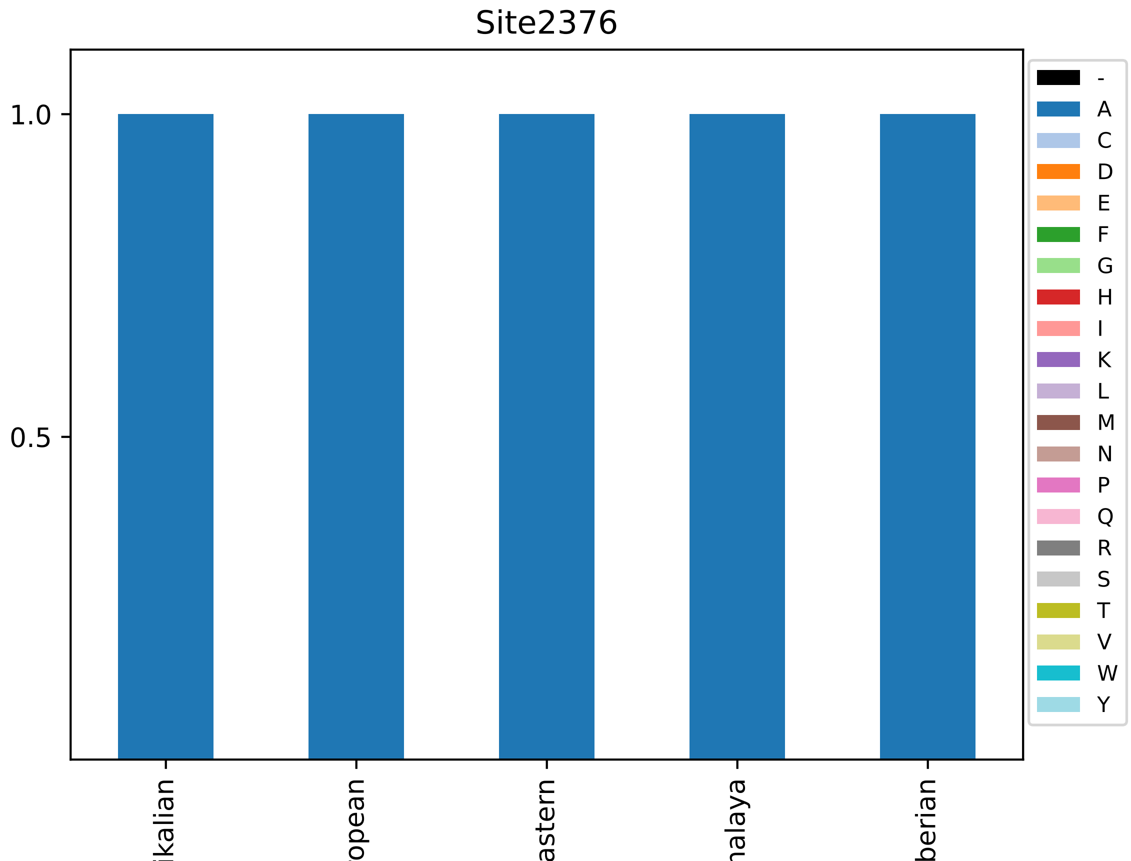
Site2376
1.0
0.5
-
A
C
D
E
F
G
H
I
K
L
M
N
P
Q
R
S
T
V
W
Y

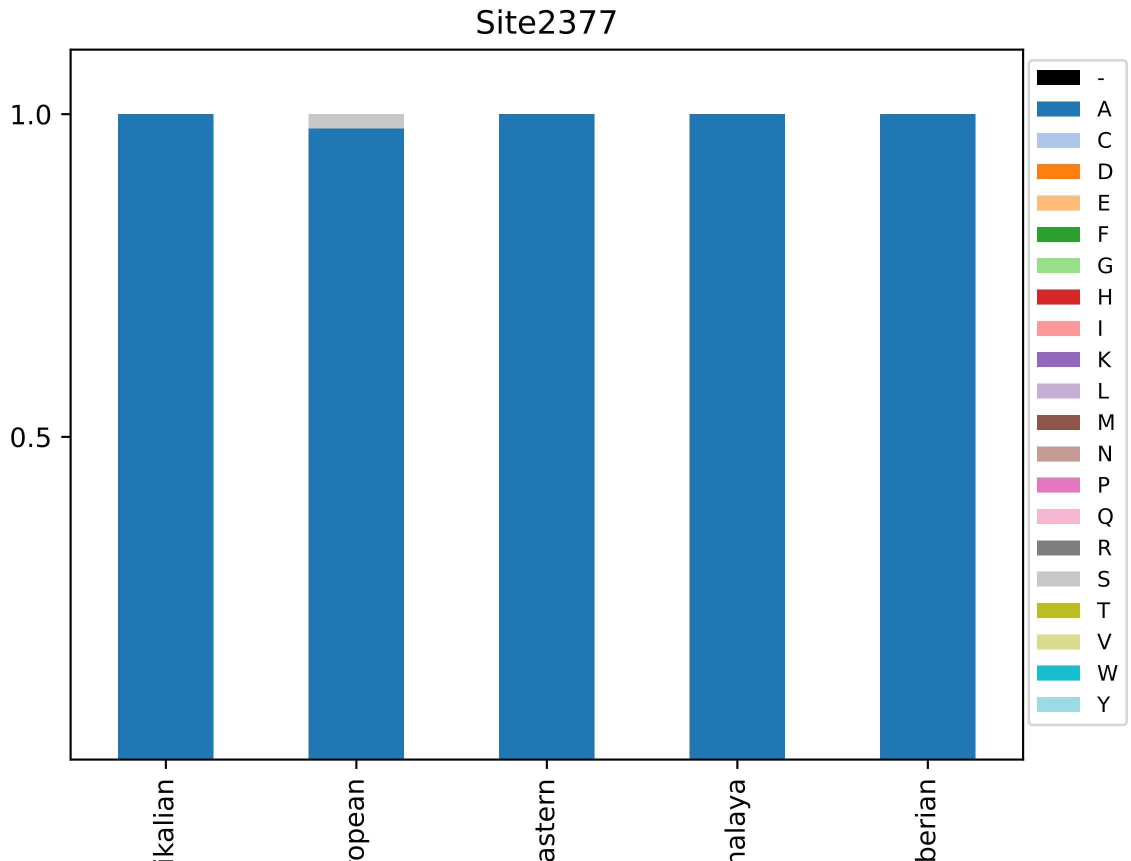
Site2377
1.0
0.5
-
A
C
D
E
F
G
H
I
K
L
M
N
P
Q
R
S
T
V
W
Y
ikalian
opean
astern
nalaya
berian

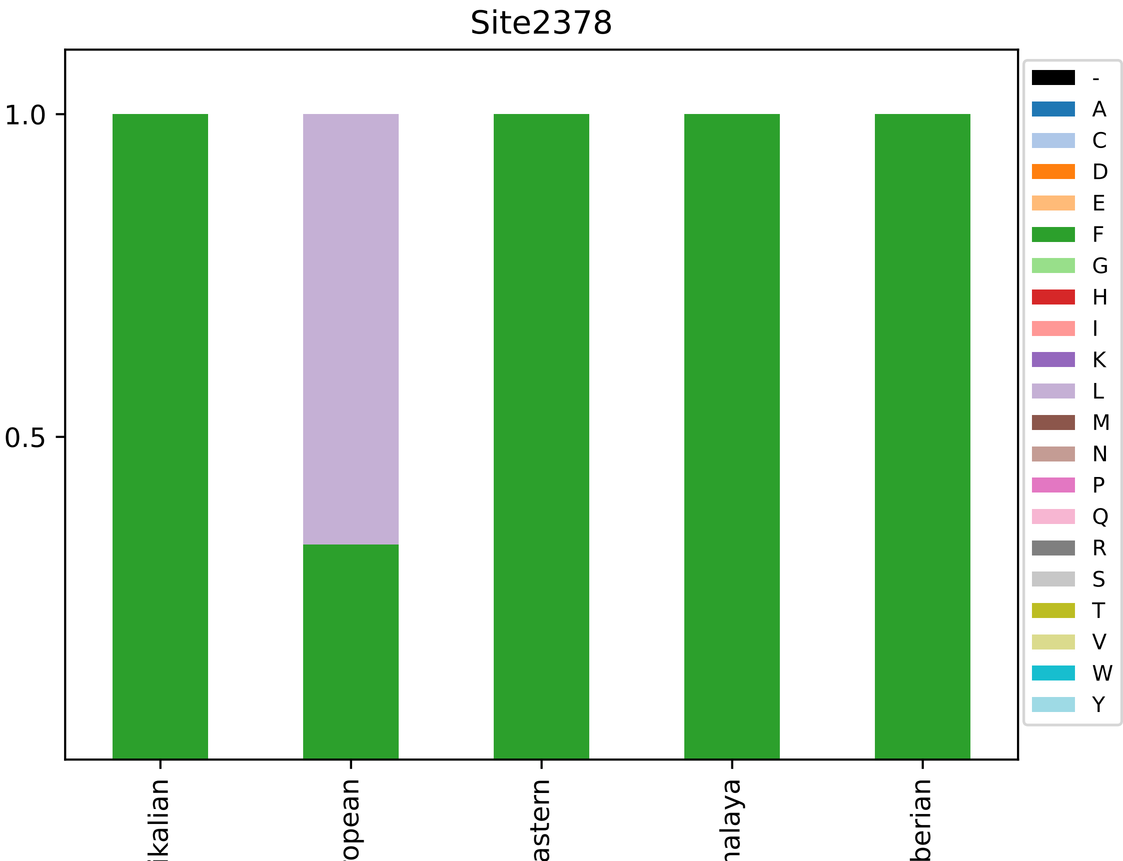
Site2378
1.0
0.5
ikalian
opean
astern
nalaya
berian
-
A
C
D
E
F
G
H
I
K
L
M
N
P
Q
R
S
T
V
W
Y

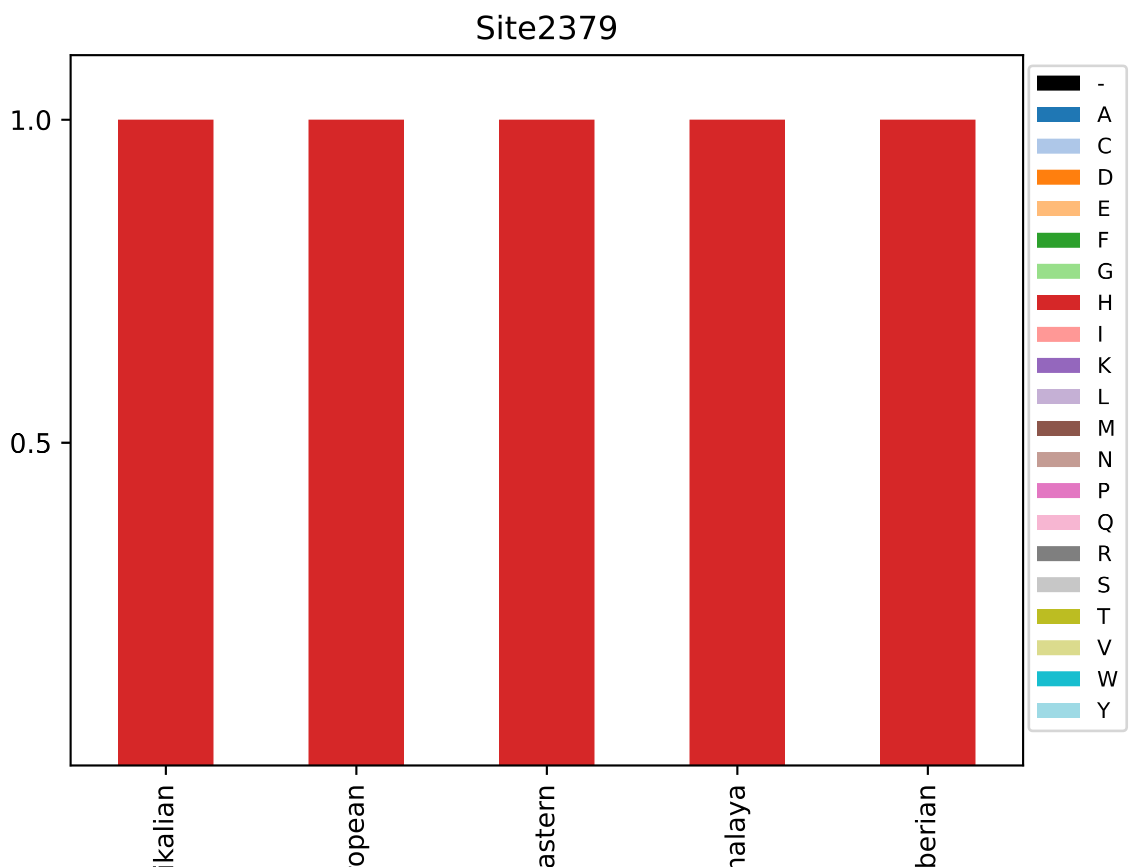
Site2379
1.0
0.5
ikalian
opean
astern
nalaya
berian
-
A
C
D
E
F
G
H
I
K
L
M
N
P
Q
R
S
T
V
W
Y

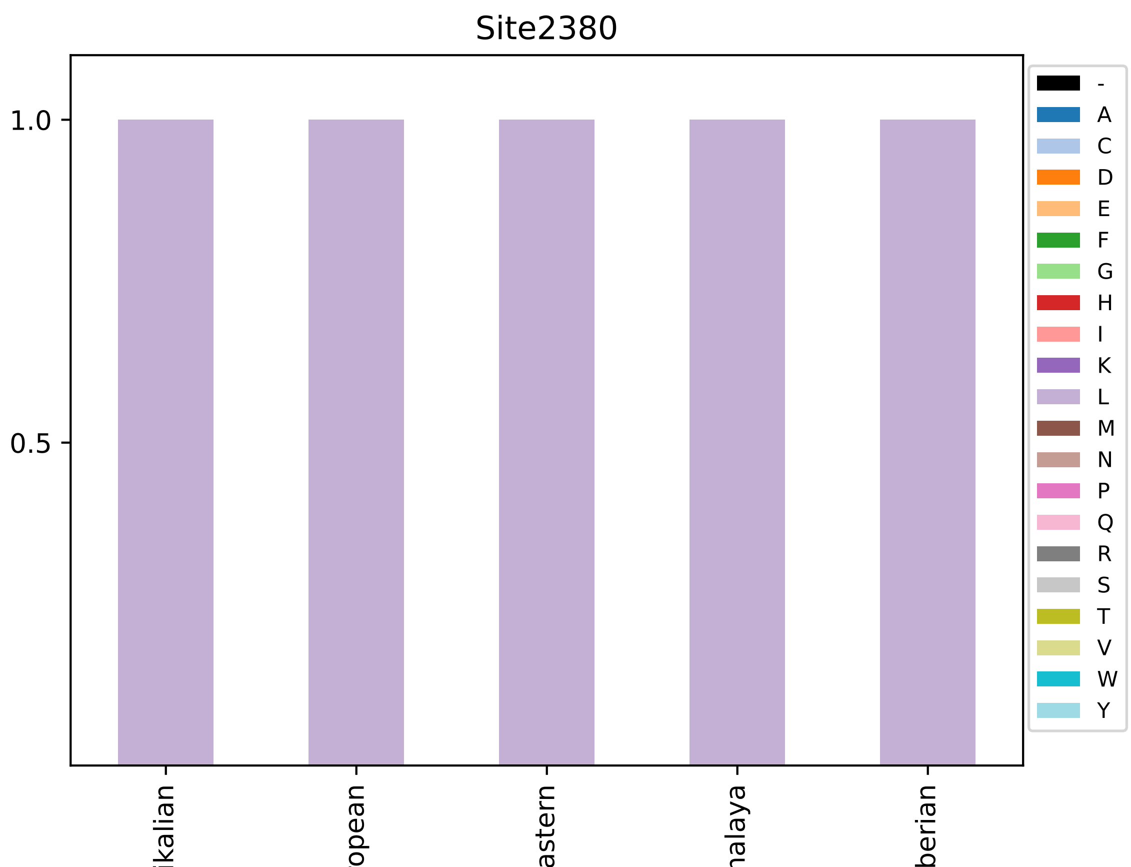

Site2380
1.0
0.5
-
A
C
D
E
F
G
H
I
K
L
M
N
P
Q
R
S
T
V
W
Y
ikalian
opean
astern
nalaya
berian

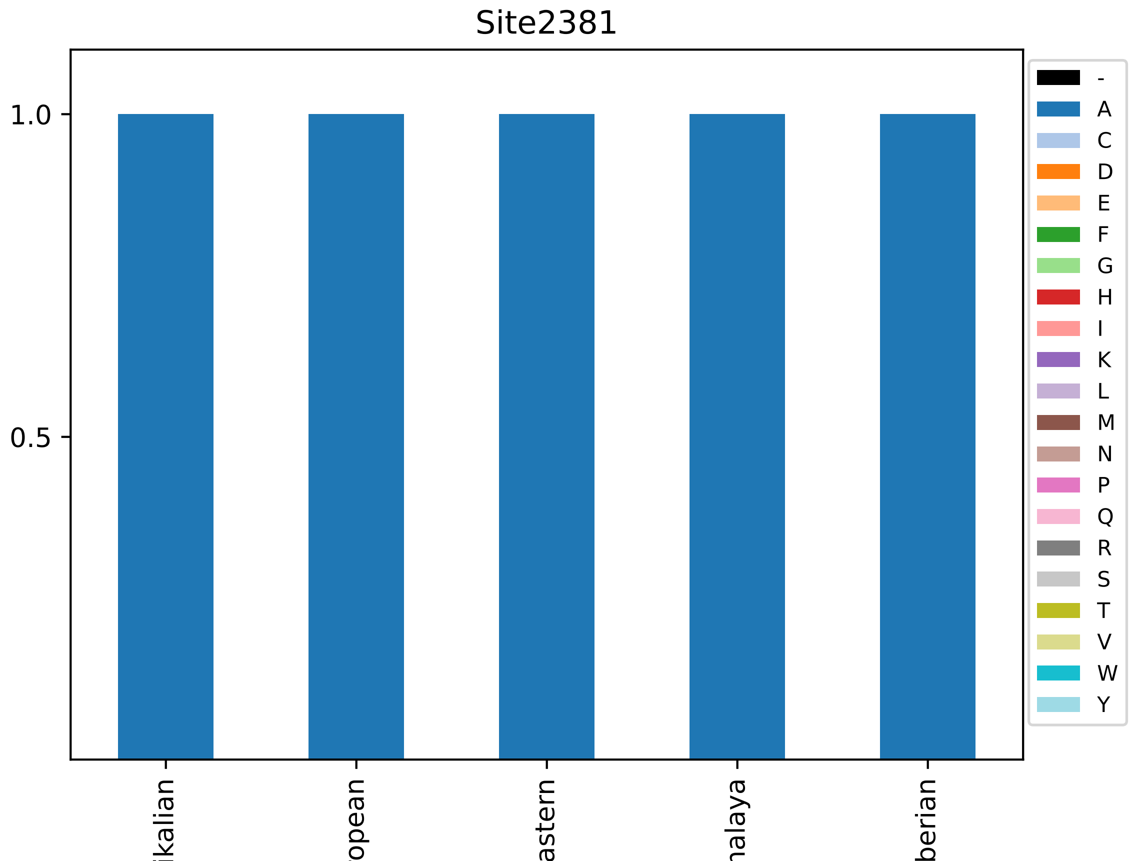
Site2381
1.0
0.5
ikalian
opean
astern
nalaya
berian
-
A
C
D
E
F
G
H
I
K
L
M
N
P
Q
R
S
T
V
W
Y

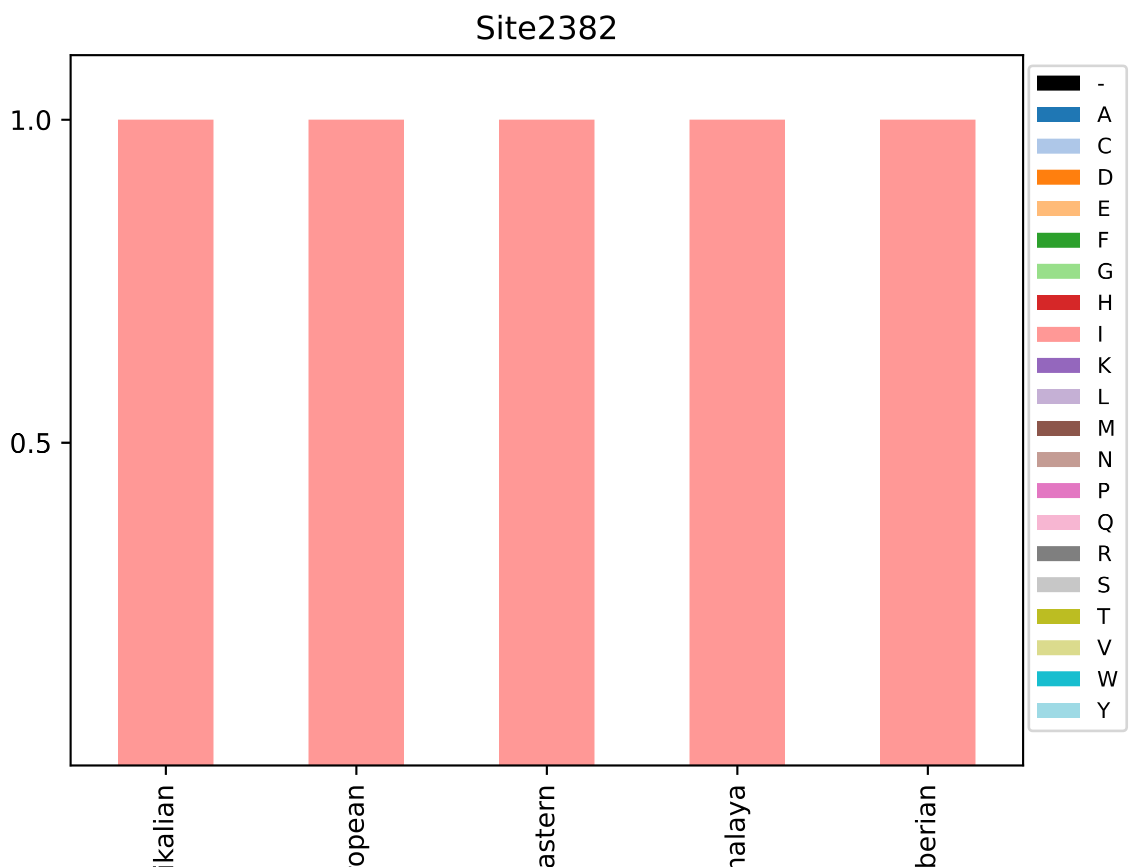
Site2382
1.0
0.5
-
A
C
D
E
F
G
H
I
K
L
M
N
P
Q
R
S
T
V
W
Y
ikalian
opean
astern
nalaya
berian

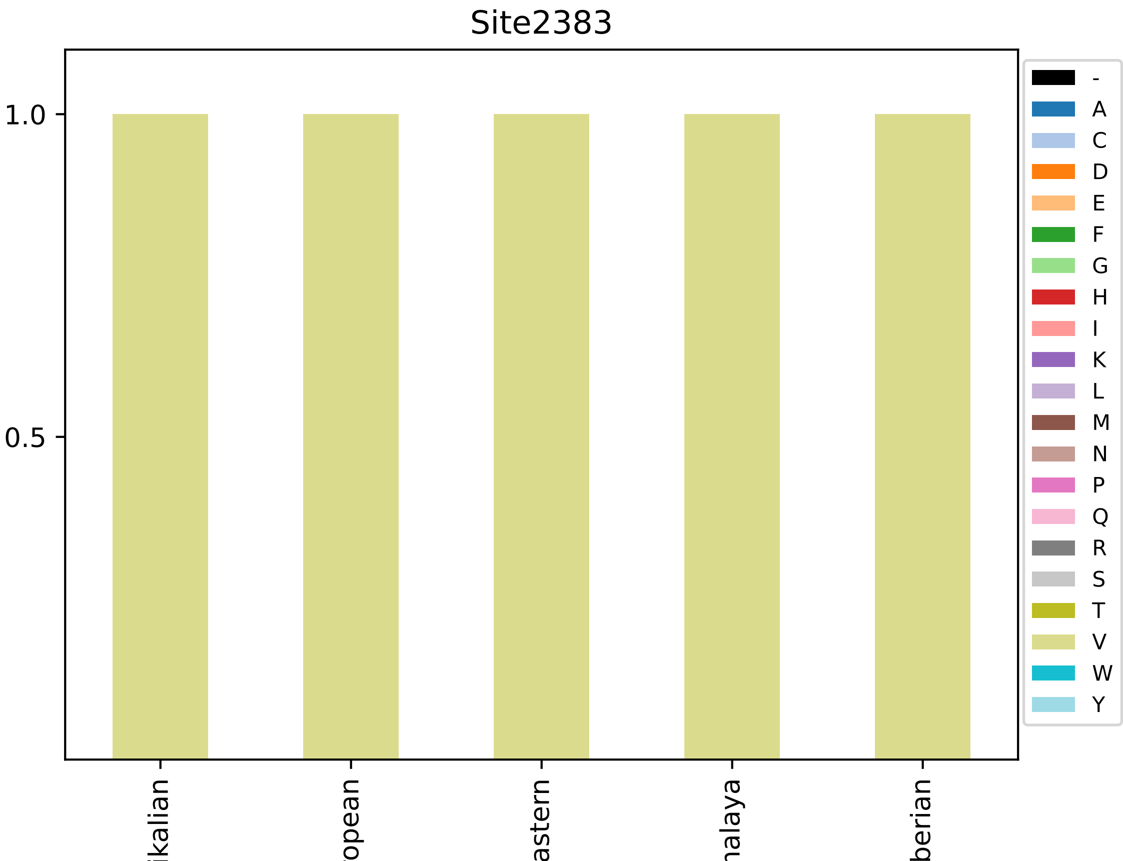
Site2383
1.0
0.5
-
A
C
D
E
F
G
H
I
K
L
M
N
P
Q
R
S
T
V
W
Y
ikalian
opean
astern
nalaya
berian

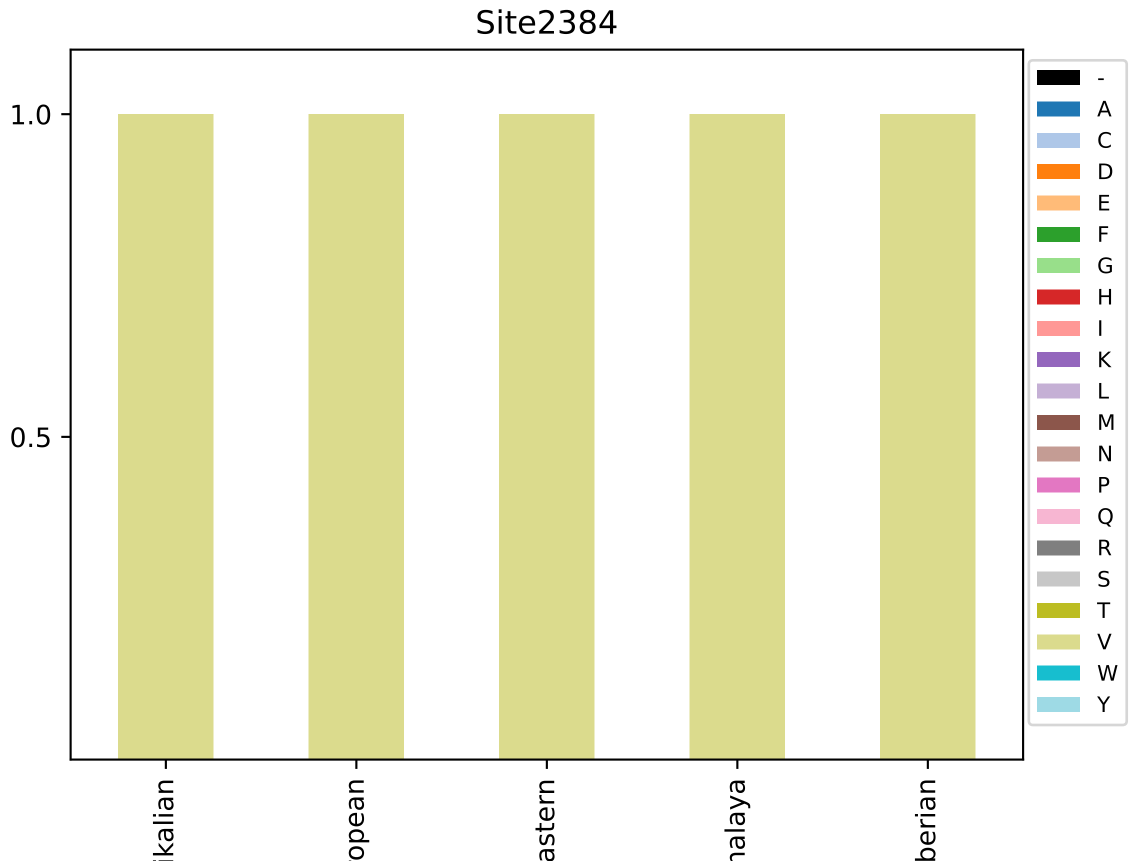

Site2384
1.0
0.5
-
A
C
D
E
F
G
H
I
K
L
M
N
P
Q
R
S
T
V
W
Y
ikalian
opean
astern
nalaya
berian

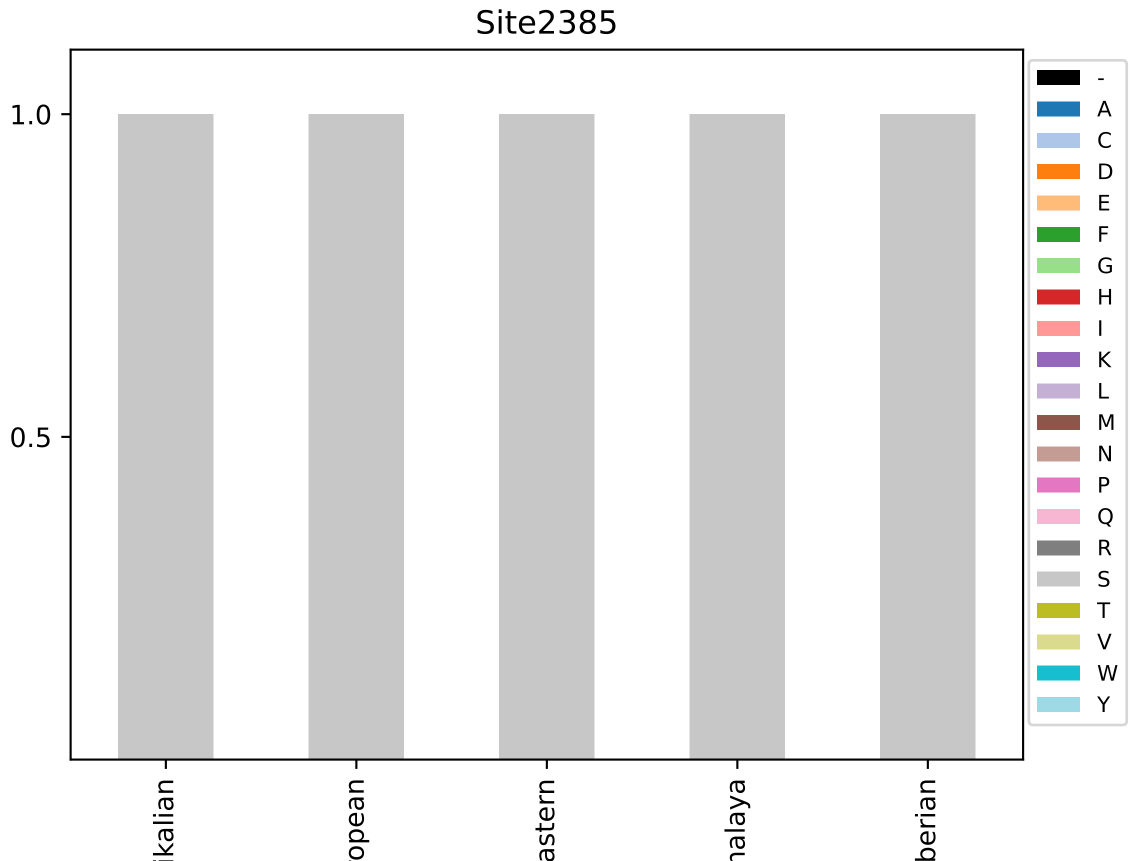

Site2385
1.0
0.5
ikalian
opean
astern
nalaya
berian
-
A
C
D
E
F
G
H
I
K
L
M
N
P
Q
R
S
T
V
W
Y

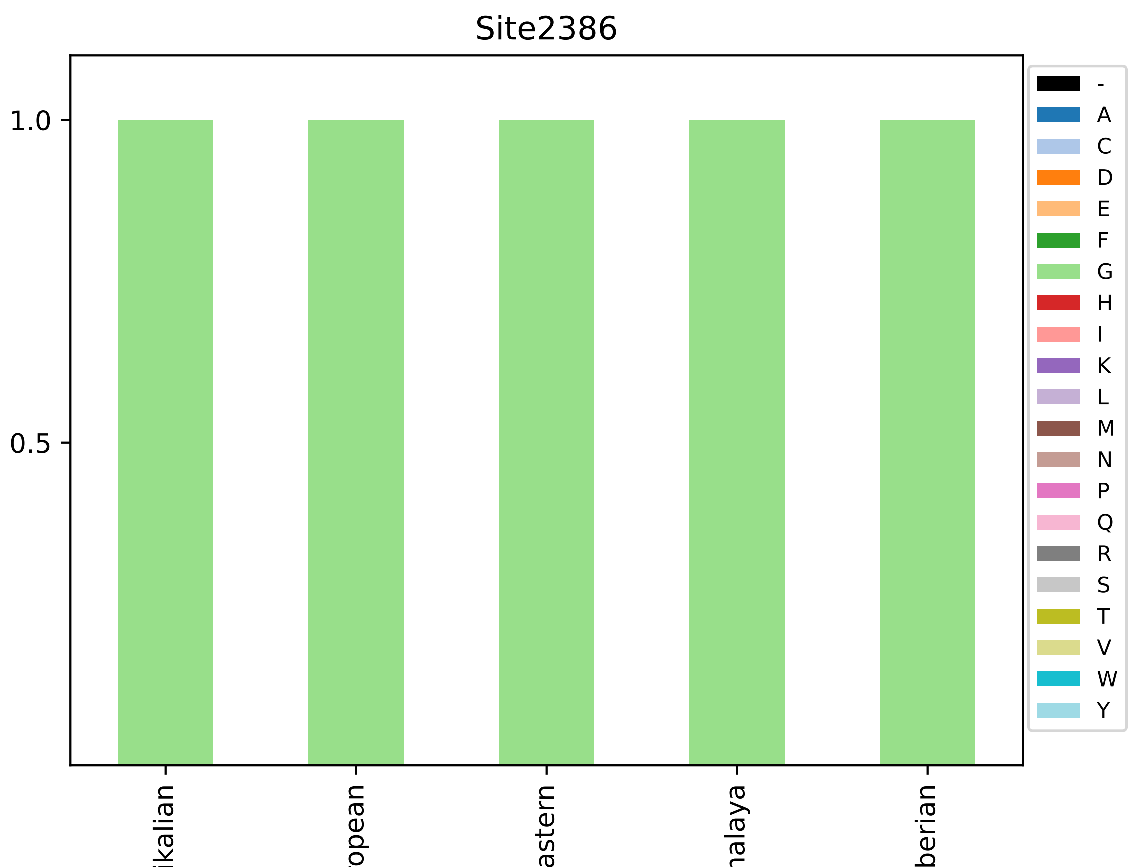

Site2386
1.0
0.5
-
A
C
D
E
F
G
H
I
K
L
M
N
P
Q
R
S
T
V
W
Y
ikalian
opean
astern
nalaya
berian

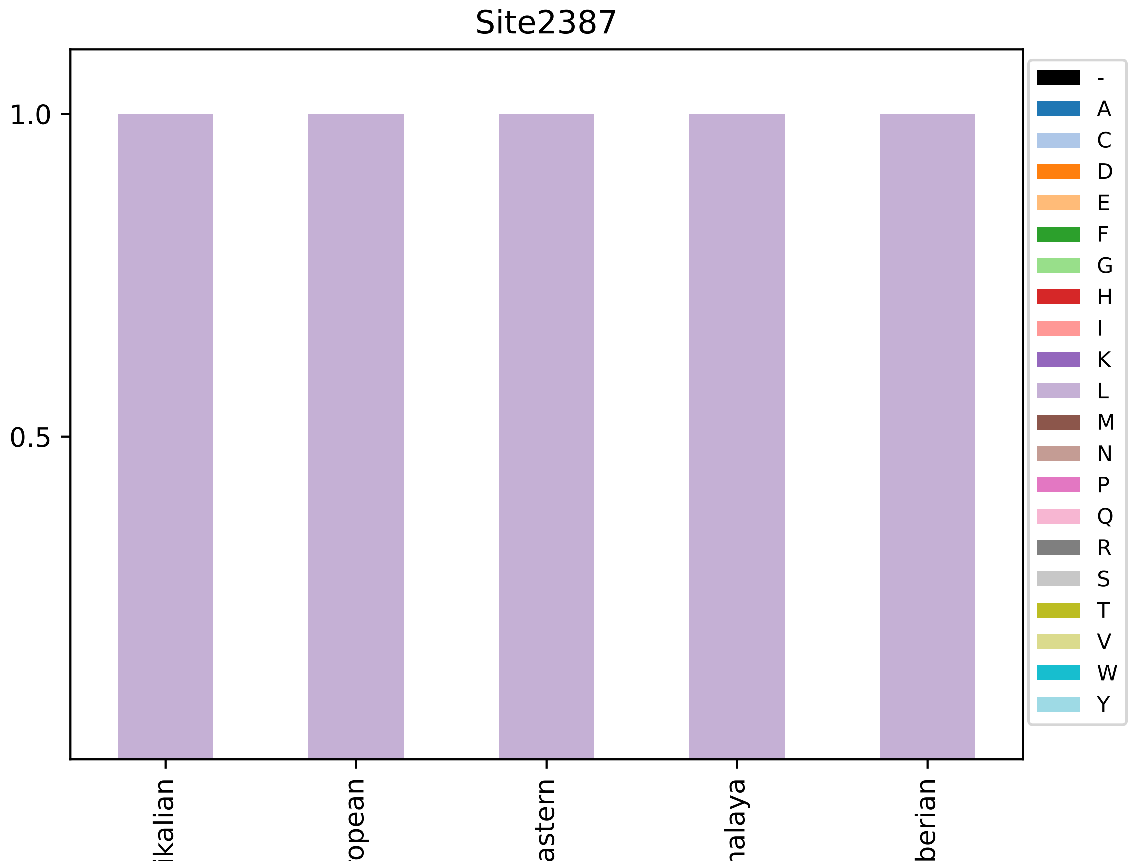
Site2387
1.0
0.5
-
A
C
D
E
F
G
H
I
K
L
M
N
P
Q
R
S
T
V
W
Y
ikalian
opean
astern
nalaya
berian

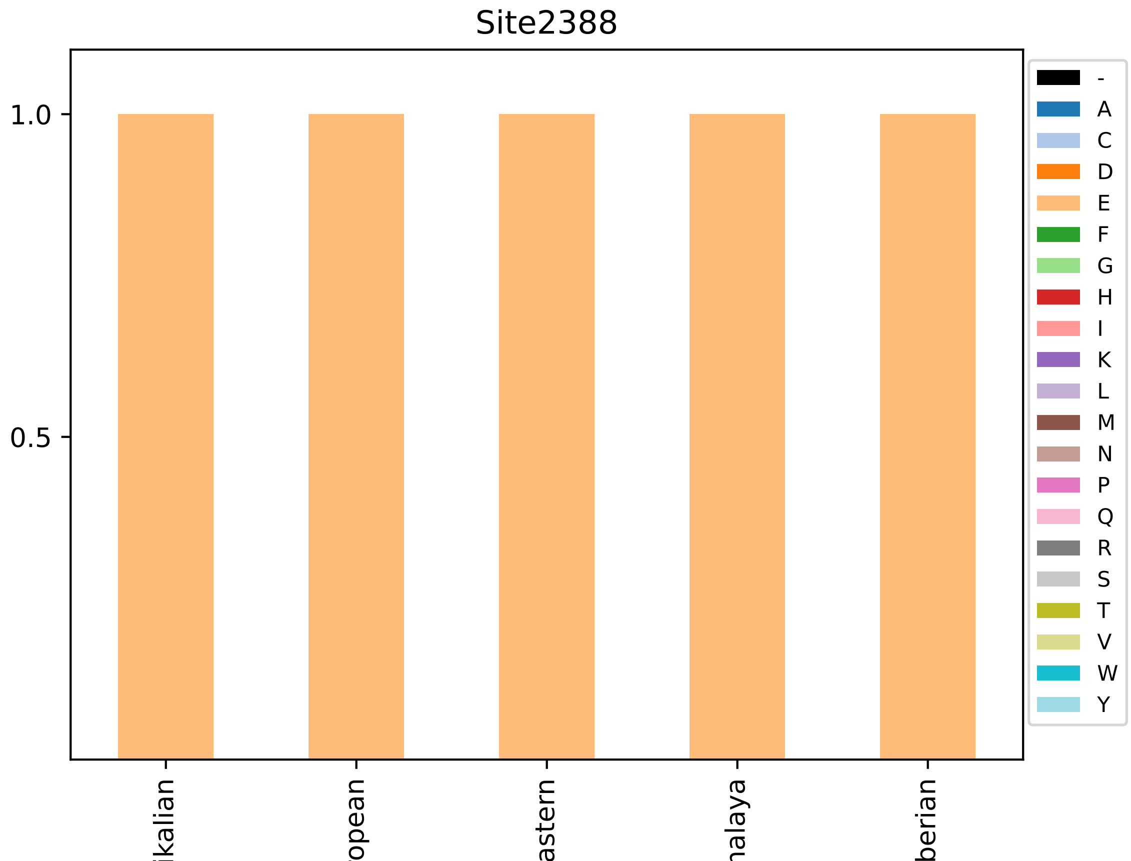
Site2388
1.0
0.5
-
A
C
D
E
F
G
H
I
K
L
M
N
P
Q
R
S
T
V
W
Y
ikalian
opean
astern
nalaya
berian

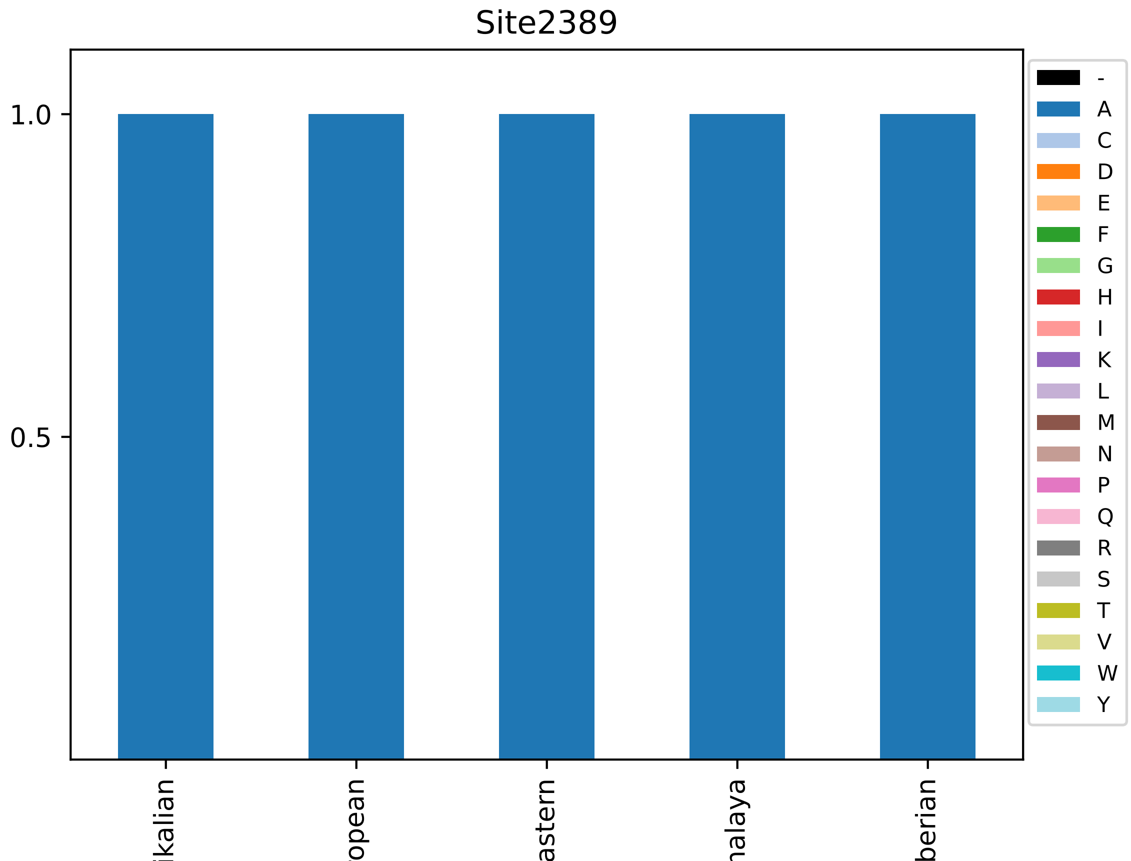
Site2389
1.0
0.5
-
A
C
D
E
F
G
H
I
K
L
M
N
P
Q
R
S
T
V
W
Y
ikalian
opean
astern
nalaya
berian

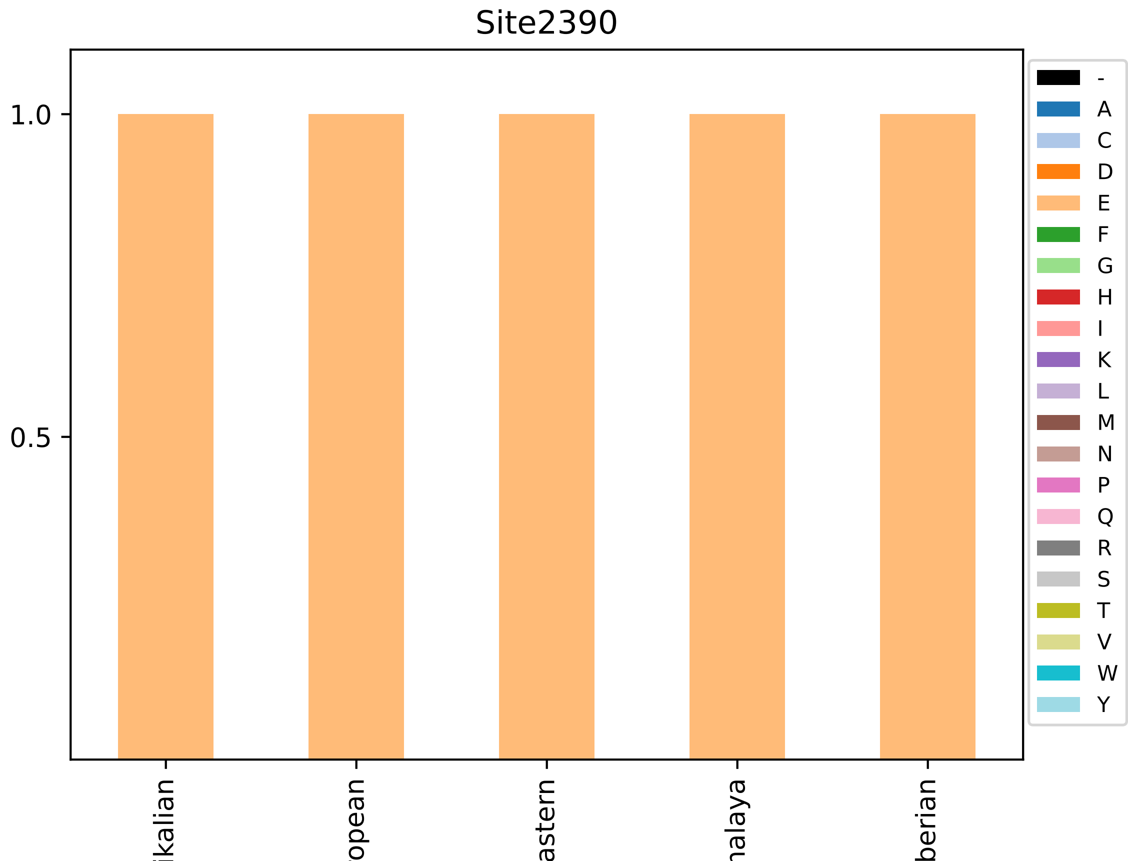

Site2390
1.0
0.5
-
A
C
D
E
F
G
H
I
K
L
M
N
P
Q
R
S
T
V
W
Y
ikalian
opean
astern
nalaya
berian

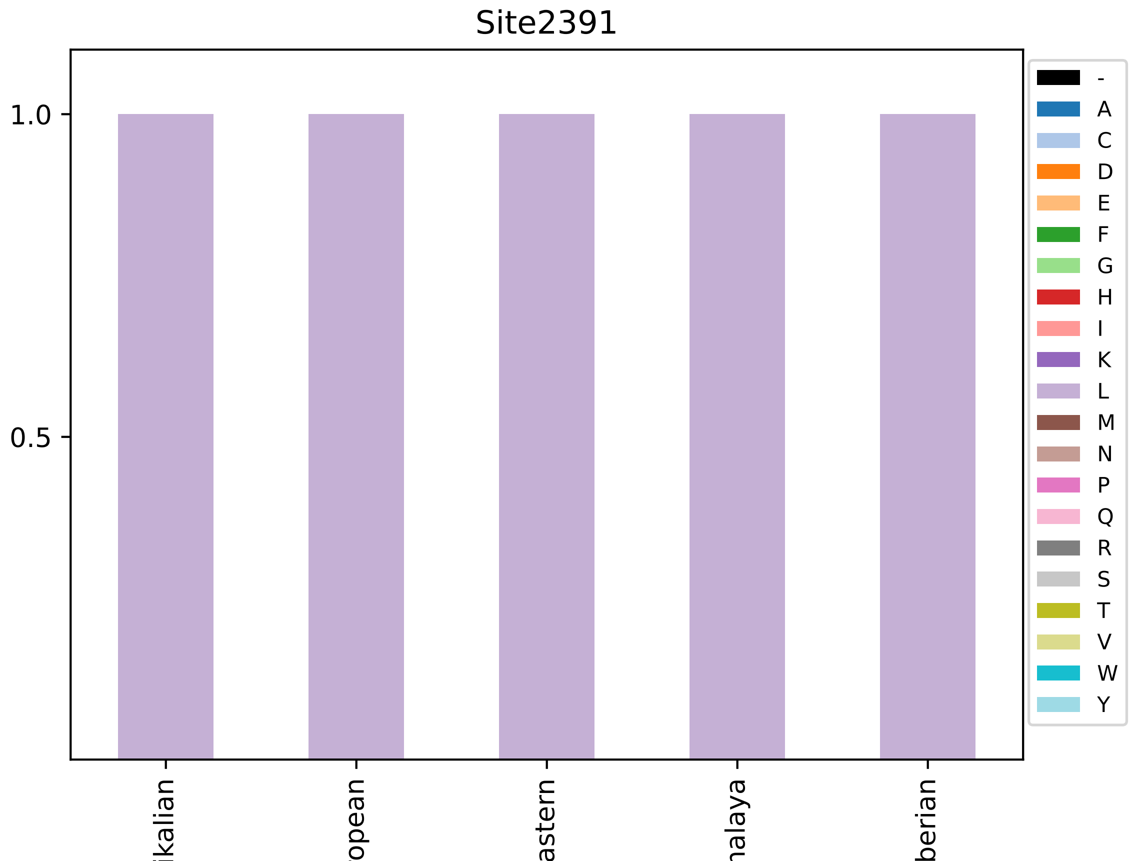

Site2391
1.0
0.5
-
A
C
D
E
F
G
H
I
K
L
M
N
P
Q
R
S
T
V
W
Y

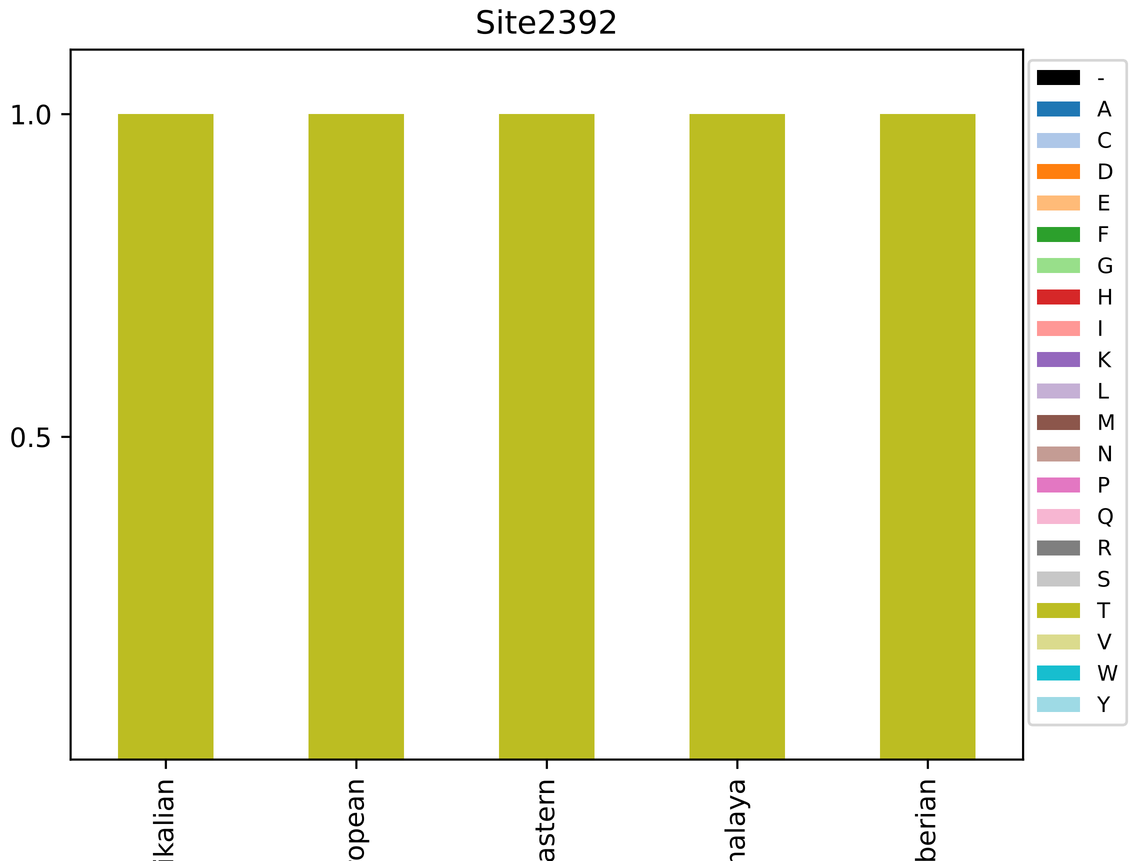
Site2392
1.0
0.5
-
A
C
D
E
F
G
H
I
K
L
M
N
P
Q
R
S
T
V
W
Y
ikalian
opean
astern
nalaya
berian

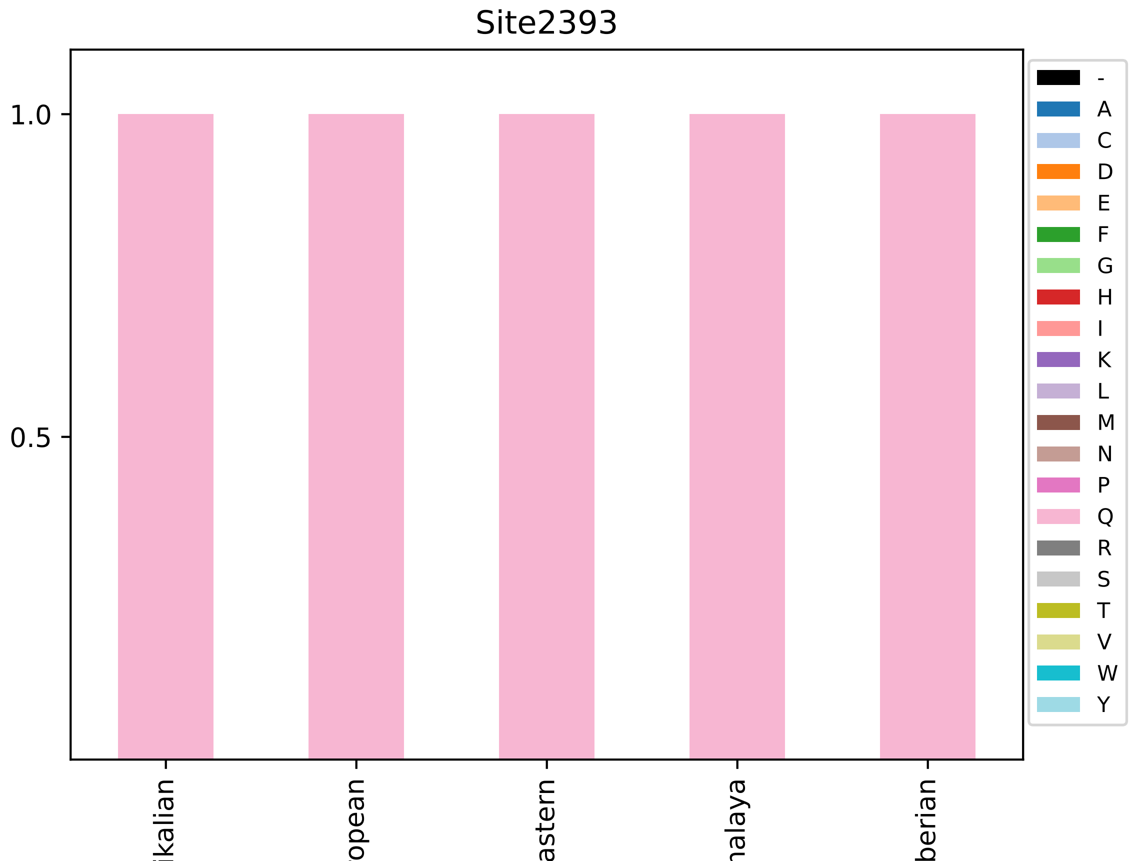
Site2393
1.0
0.5
-
A
C
D
E
F
G
H
I
K
L
M
N
P
Q
R
S
T
V
W
Y
ikalian
opean
astern
nalaya
berian

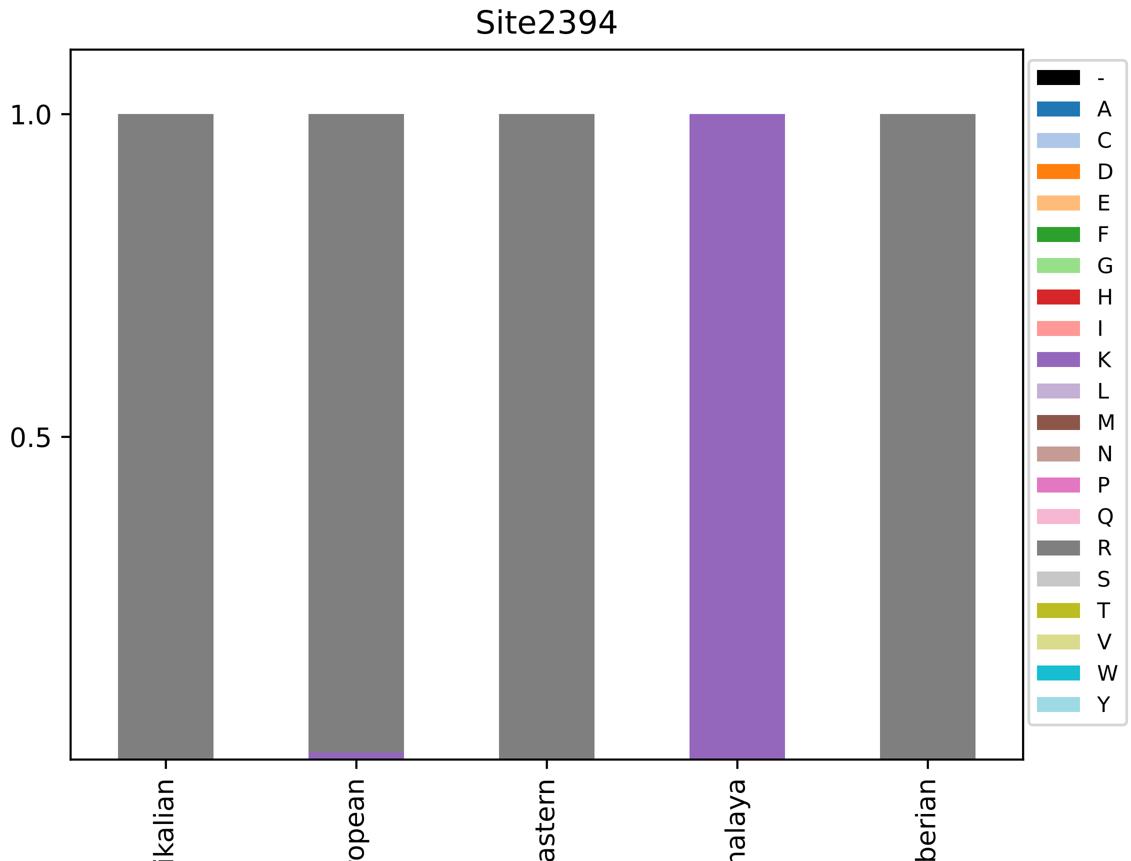

Site2394
1.0
0.5
-
A
C
D
E
F
G
H
I
K
L
M
N
P
Q
R
S
T
V
W
Y
ikalian
opean
astern
nalaya
berian

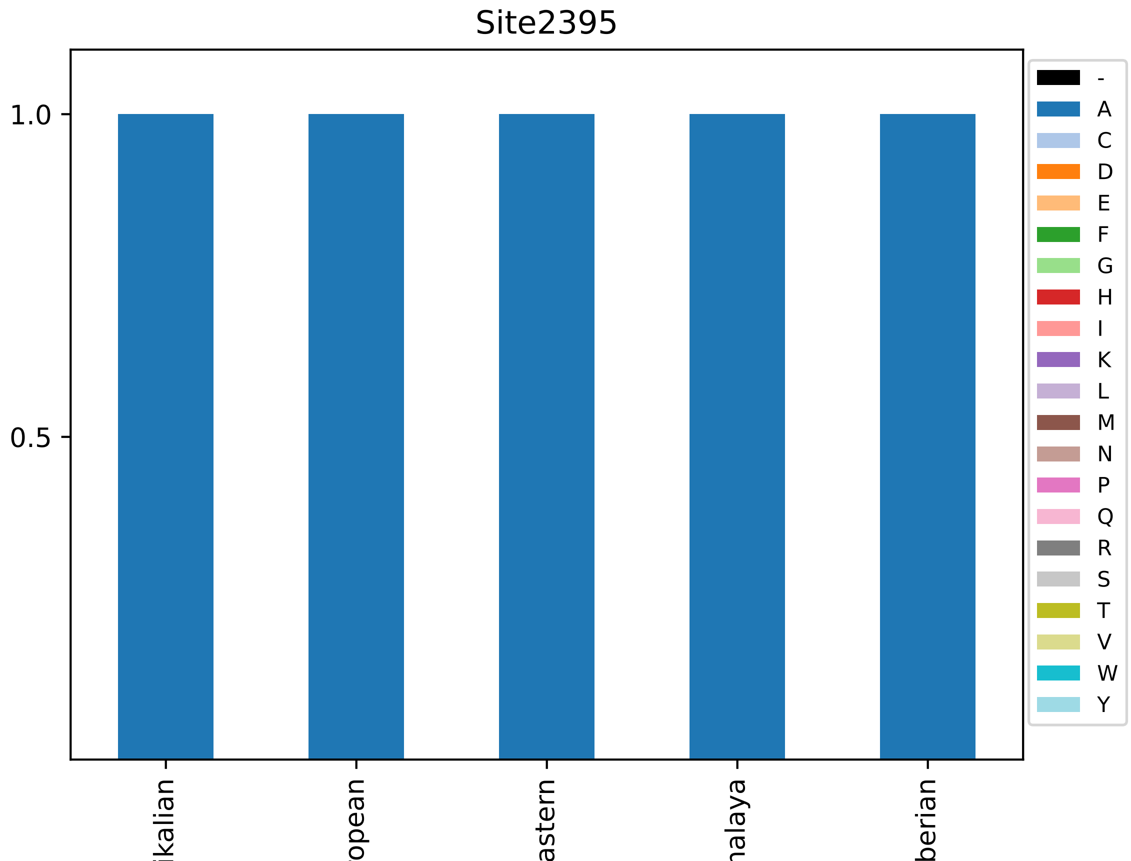

Site2395
1.0
0.5
ikalian
opean
astern
nalaya
berian
-
A
C
D
E
F
G
H
I
K
L
M
N
P
Q
R
S
T
V
W
Y

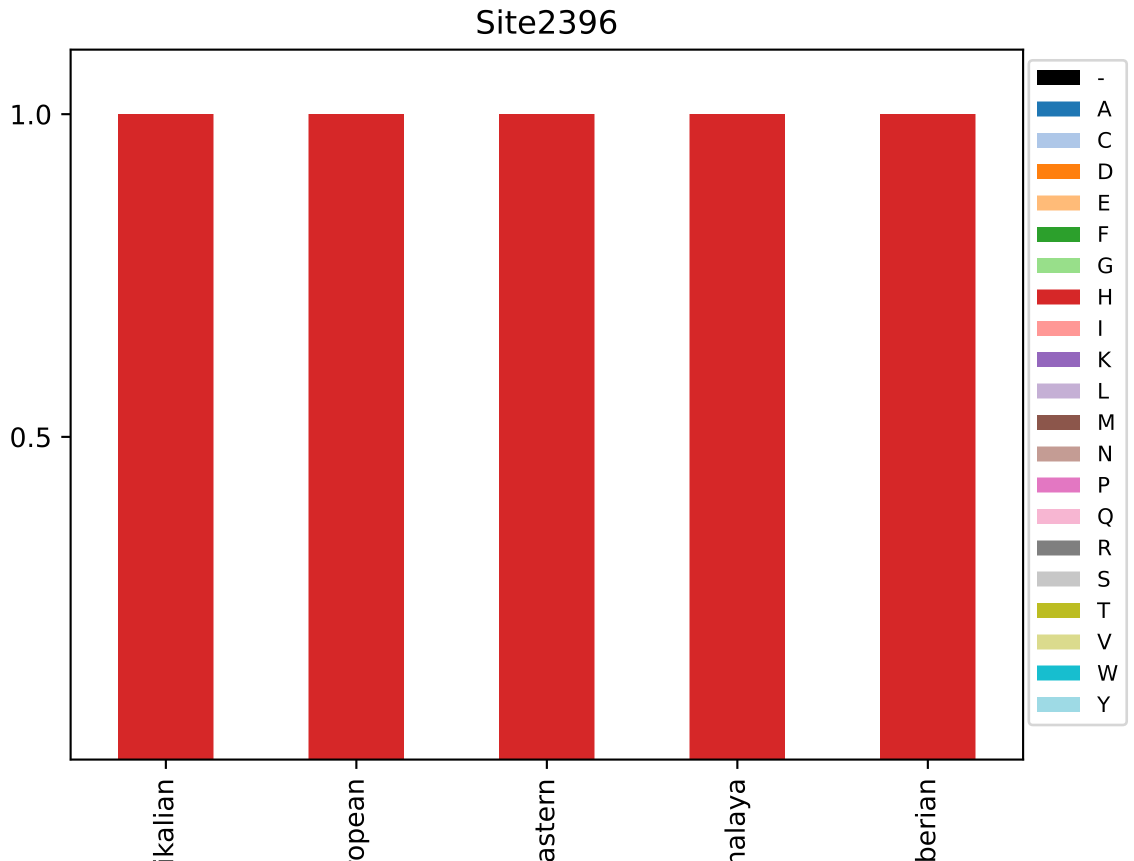

Site2396
1.0
0.5
ikalian
opean
astern
nalaya
berian
-
A
C
D
E
F
G
H
I
K
L
M
N
P
Q
R
S
T
V
W
Y

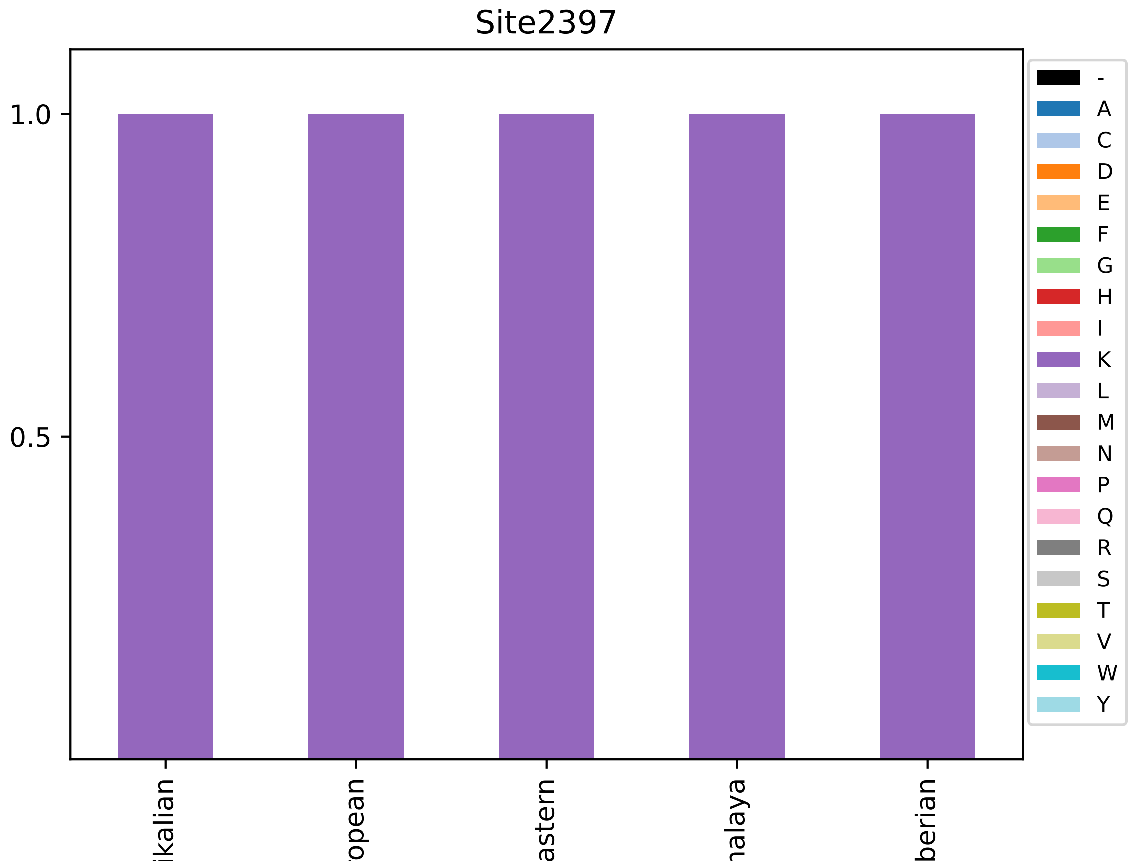

Site2397
1.0
0.5
-
A
C
D
E
F
G
H
I
K
L
M
N
P
Q
R
S
T
V
W
Y
ikalian
ropean
astern
nalaya
berian

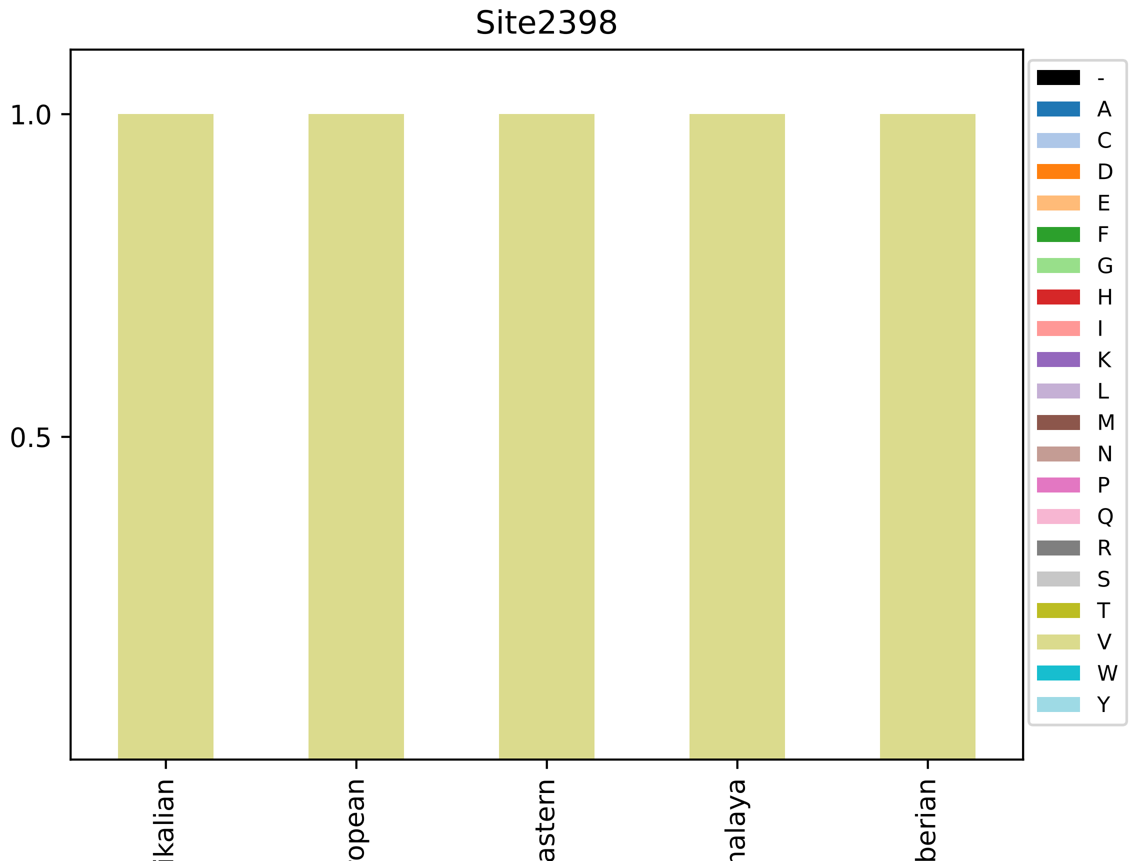
Site2398
1.0
0.5
-
A
C
D
E
F
G
H
I
K
L
M
N
P
Q
R
S
T
V
W
Y
ikalian
opean
astern
nalaya
berian

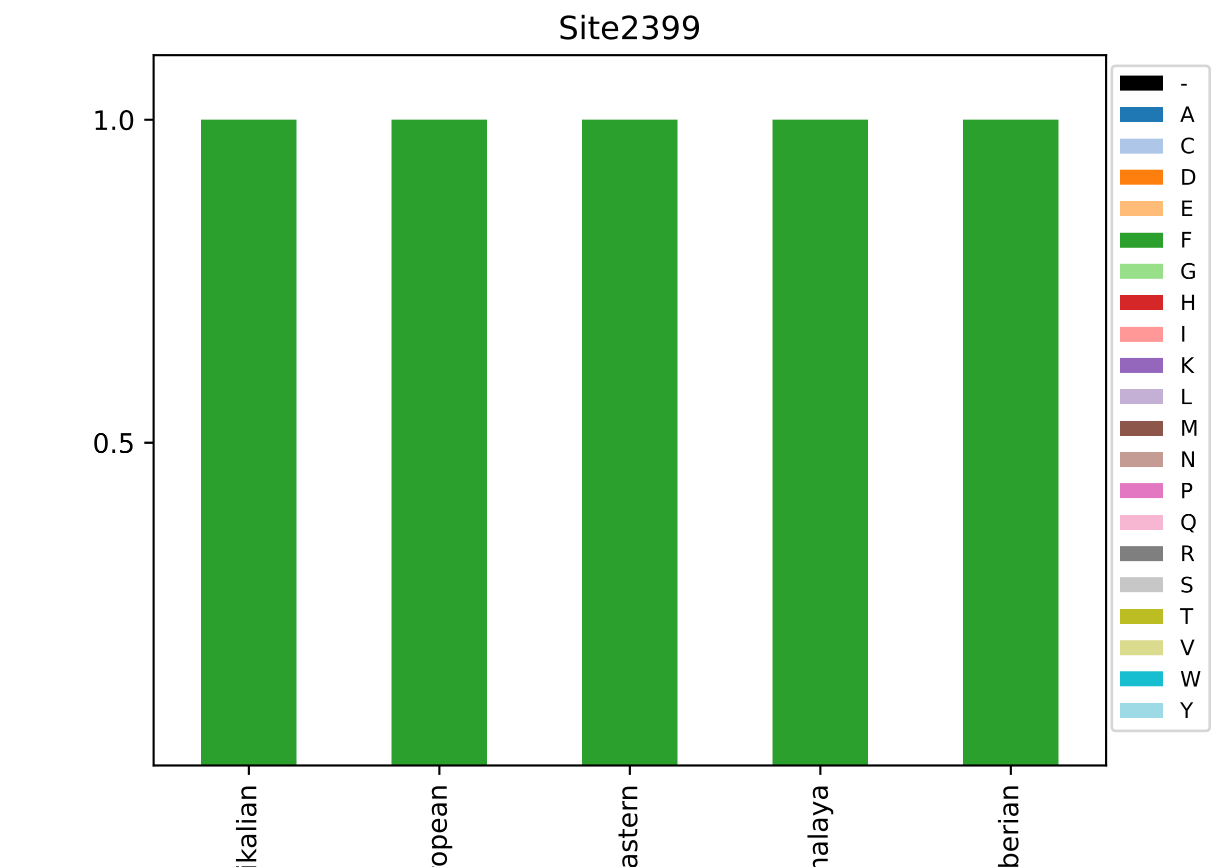
Site2399
1.0
0.5
-
A
C
D
E
F
G
H
I
K
L
M
N
P
Q
R
S
T
V
W
Y
ikalian
ropean
astern
nalaya
berian

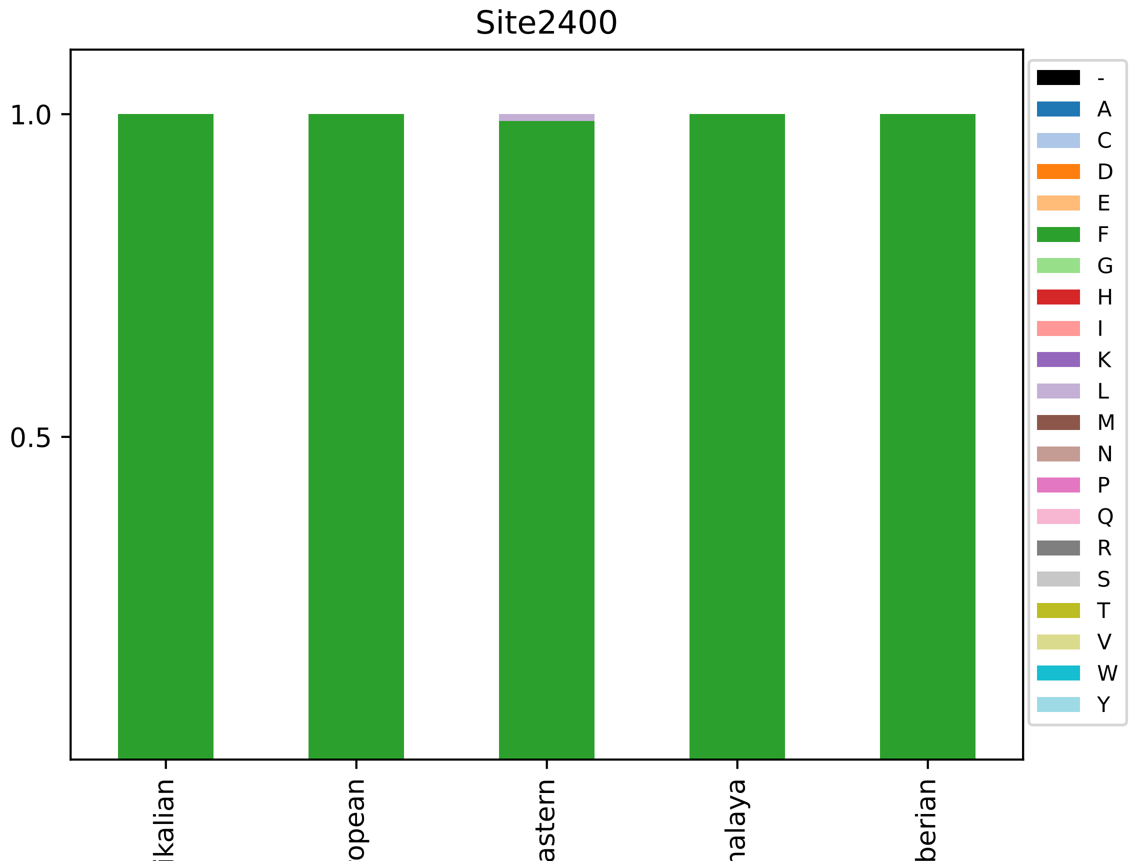

Site2400
1.0
0.5
ikalian
opean
astern
nalaya
berian
-
A
C
D
E
F
G
H
I
K
L
M
N
P
Q
R
S
T
V
W
Y

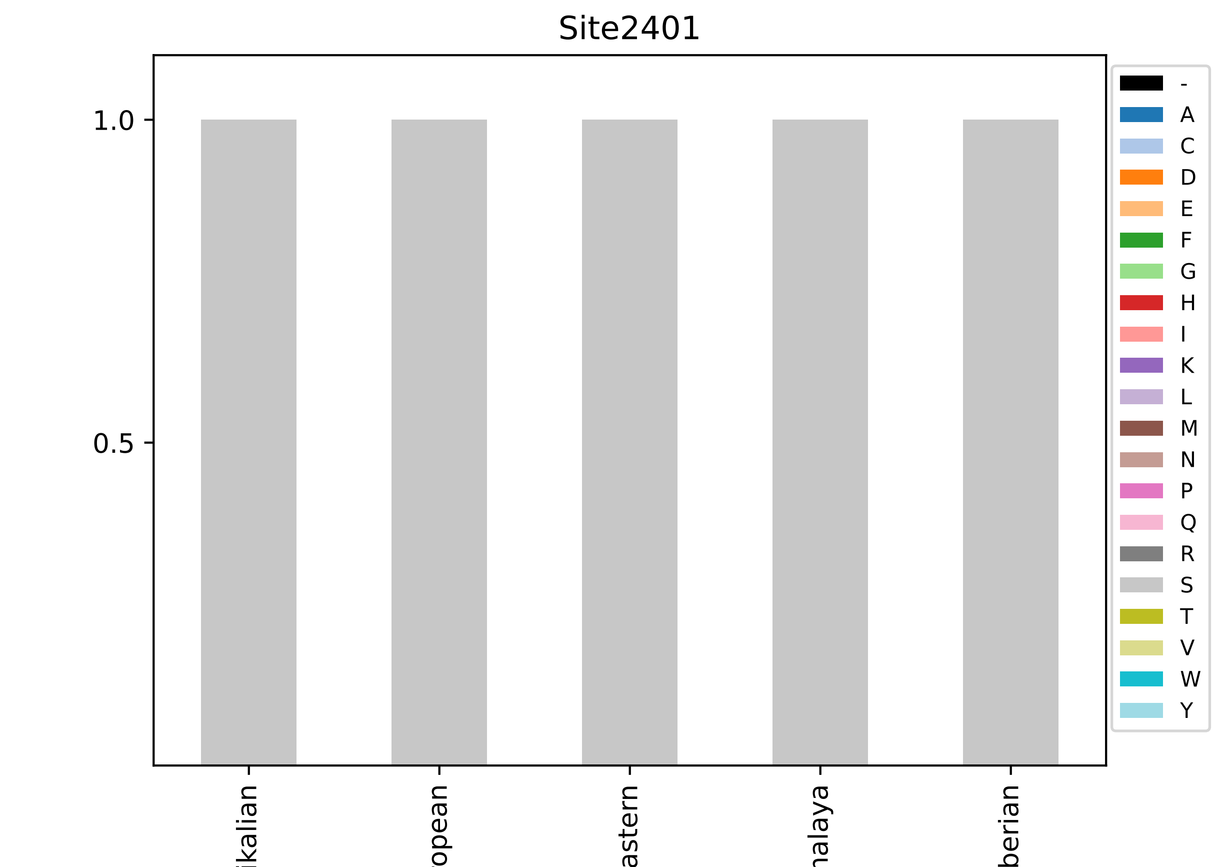

Site2401
1.0
0.5
ikalian
opean
astern
nalaya
berian
-
A
C
D
E
F
G
H
I
K
L
M
N
P
Q
R
S
T
V
W
Y

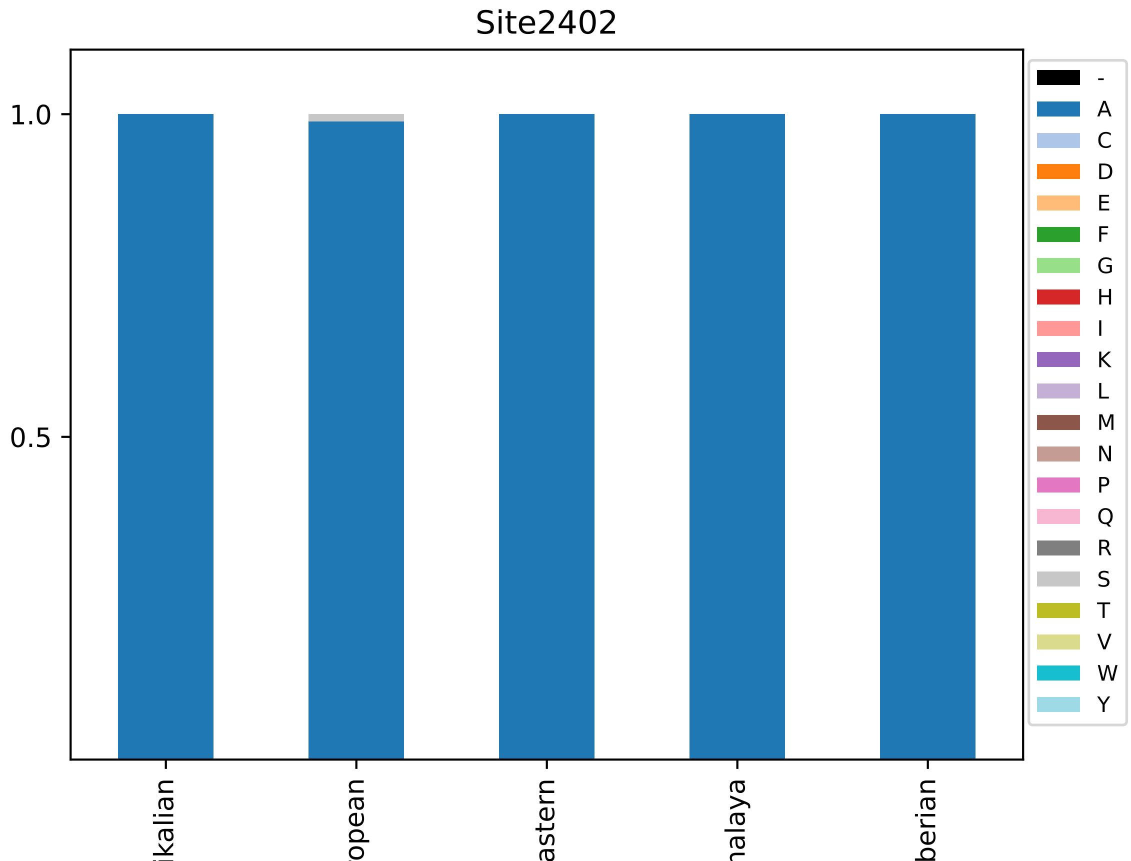

Site2402
1.0
0.5
-
A
C
D
E
F
G
H
I
K
L
M
N
P
Q
R
S
T
V
W
Y
ikalian
opean
astern
nalaya
berian

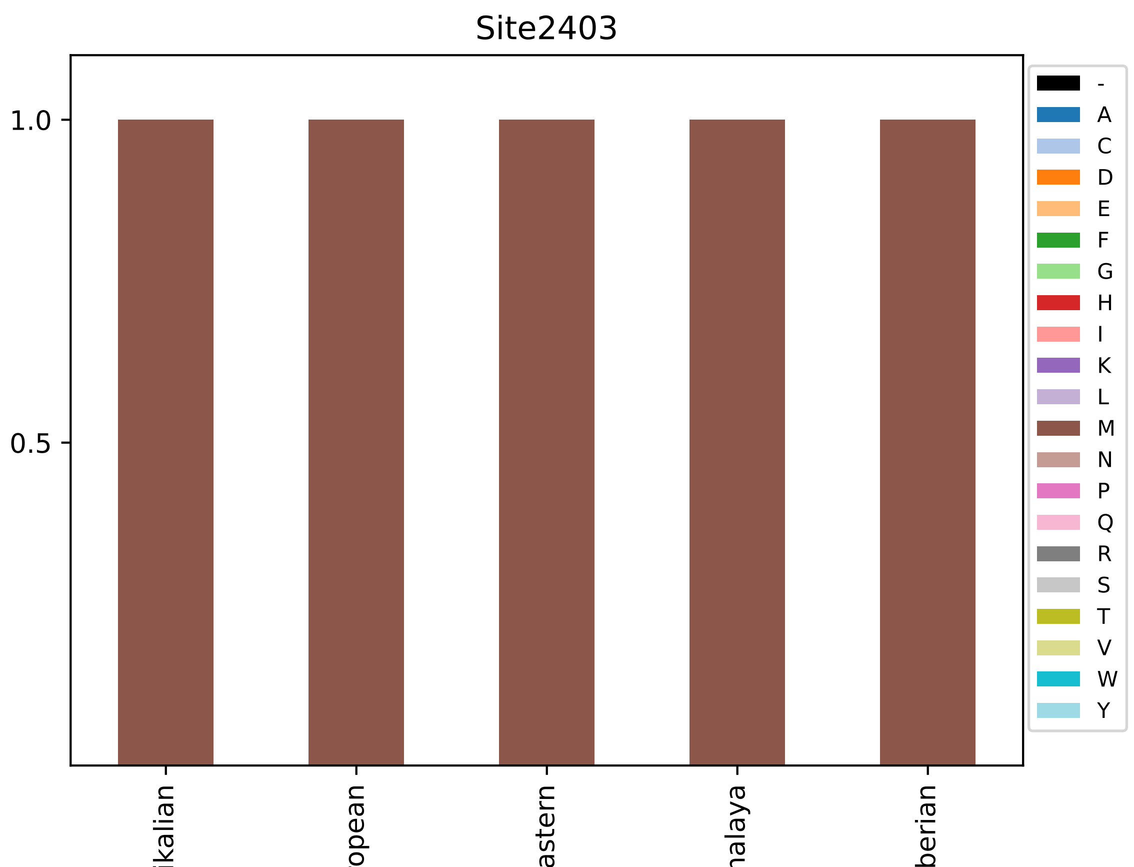

Site2403
1.0
0.5
-
A
C
D
E
F
G
H
I
K
L
M
N
P
Q
R
S
T
V
W
Y
ikalian
opean
astern
nalaya
berian

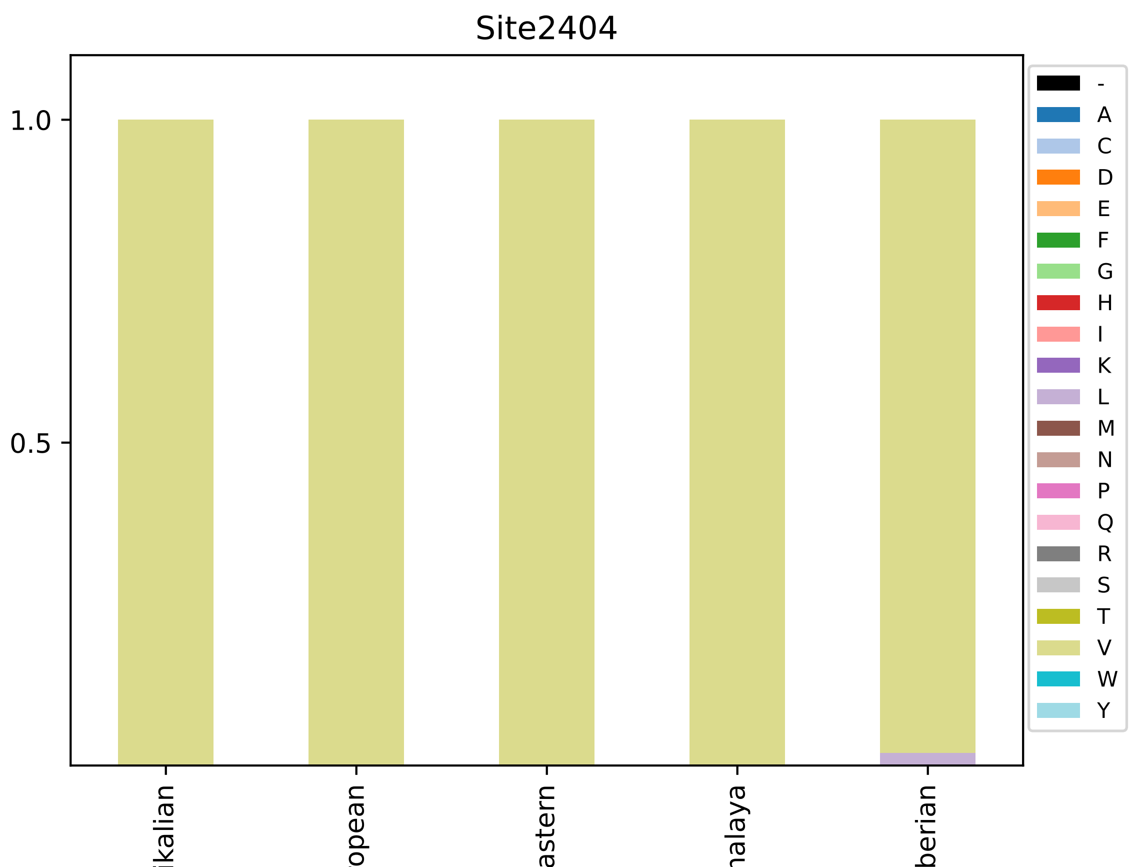
Site2404
1.0
0.5
-
A
C
D
E
F
G
H
I
K
L
M
N
P
Q
R
S
T
V
W
Y
ikalian
opean
astern
nalaya
berian

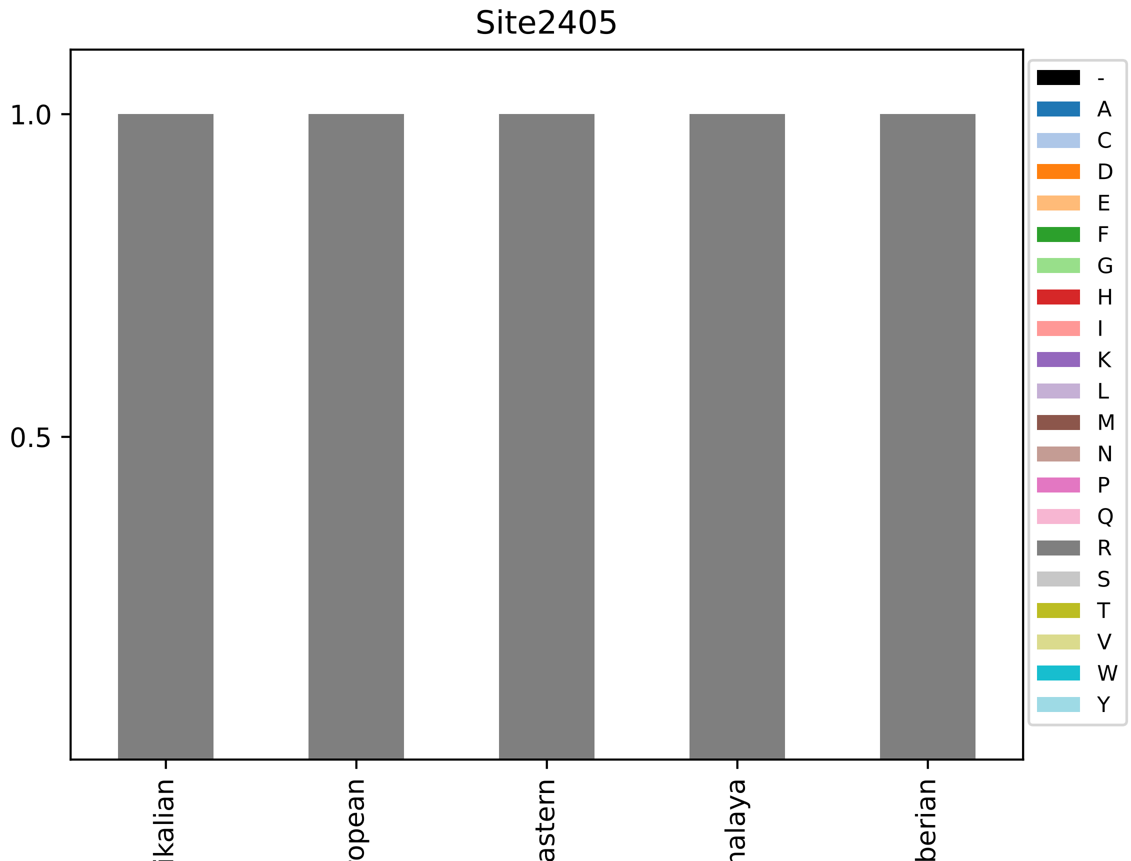
Site2405
1.0
0.5
-
A
C
D
E
F
G
H
I
K
L
M
N
P
Q
R
S
T
V
W
Y
ikalian
opean
astern
nalaya
berian

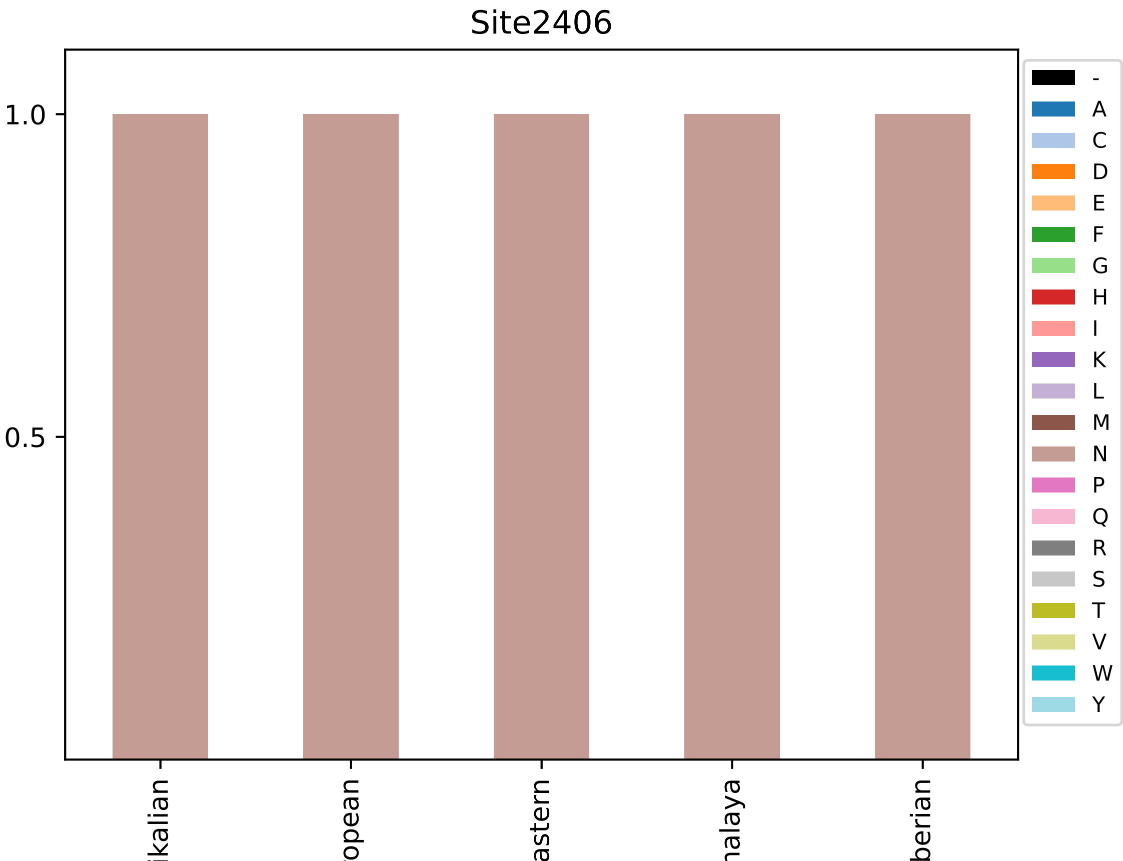

Site2406
1.0
0.5
-
A
C
D
E
F
G
H
I
K
L
M
N
P
Q
R
S
T
V
W
Y
ikalian
opean
astern
nalaya
berian

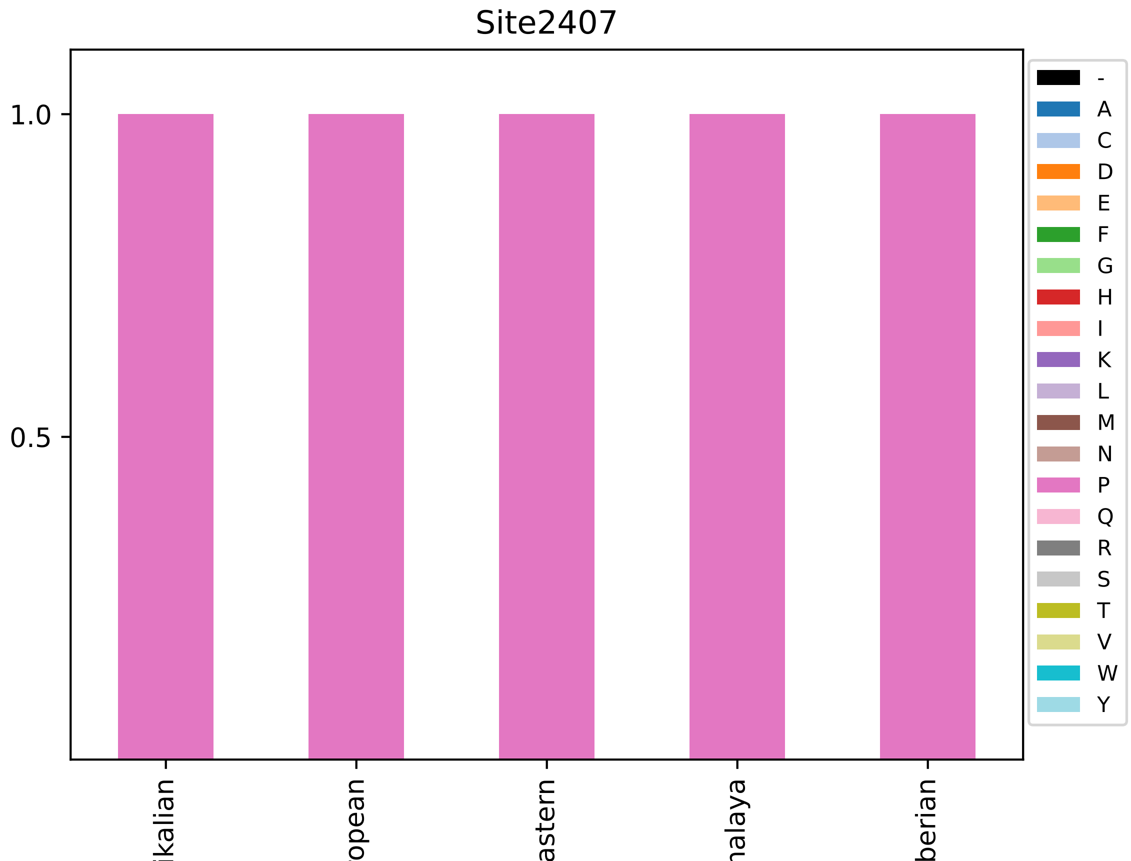
Site2407
1.0
0.5
-
A
C
D
E
F
G
H
I
K
L
M
N
P
Q
R
S
T
V
W
Y
ikalian
ropean
astern
nalaya
berian

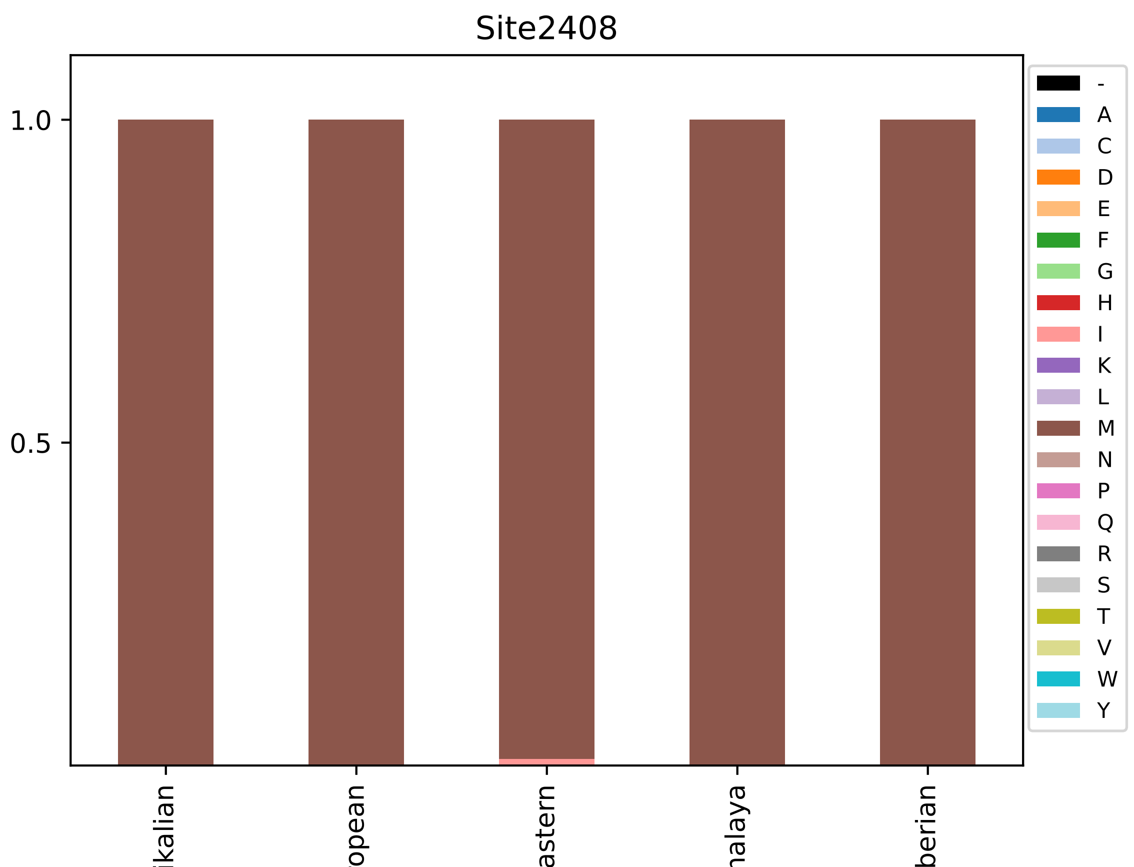
Site2408
1.0
0.5
ikalian
opean
astern
nalaya
berian
-
A
C
D
E
F
G
H
I
K
L
M
N
P
Q
R
S
T
V
W
Y

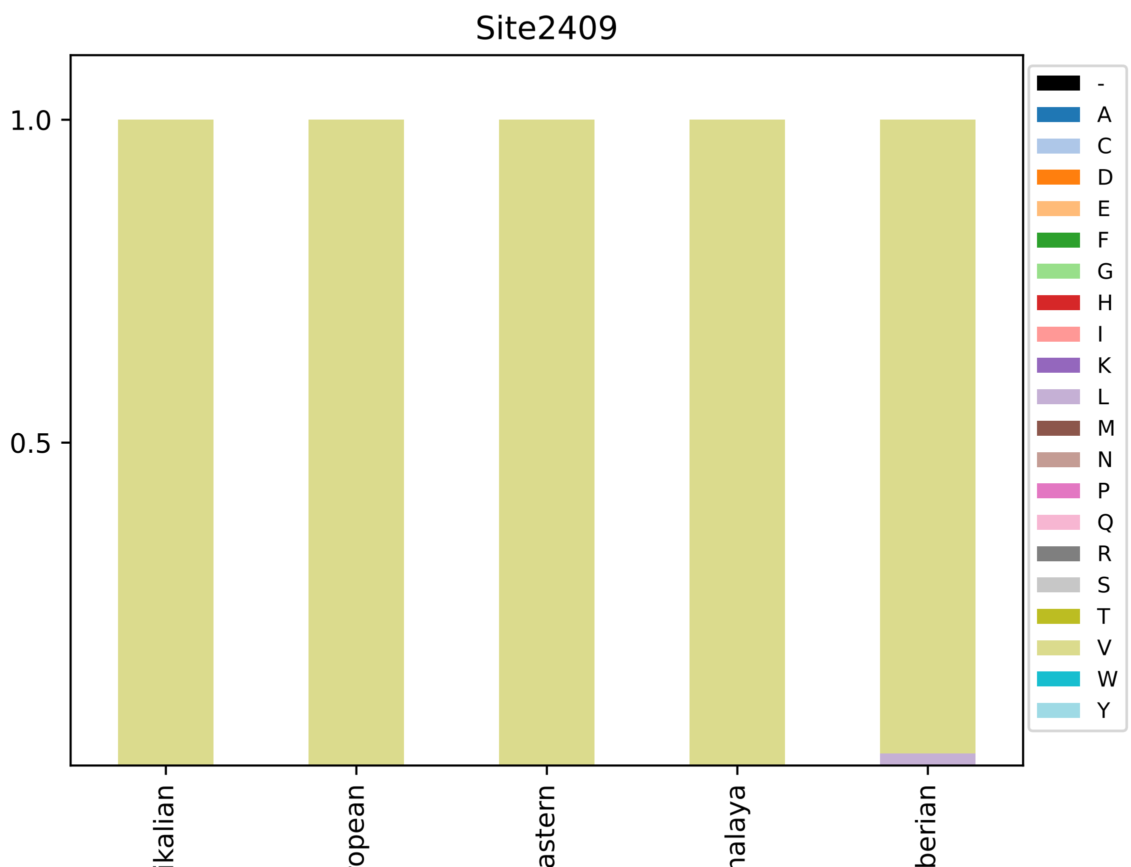

Site2409
1.0
0.5
ikalian
opean
astern
nalaya
berian
-
A
C
D
E
F
G
H
I
K
L
M
N
P
Q
R
S
T
V
W
Y

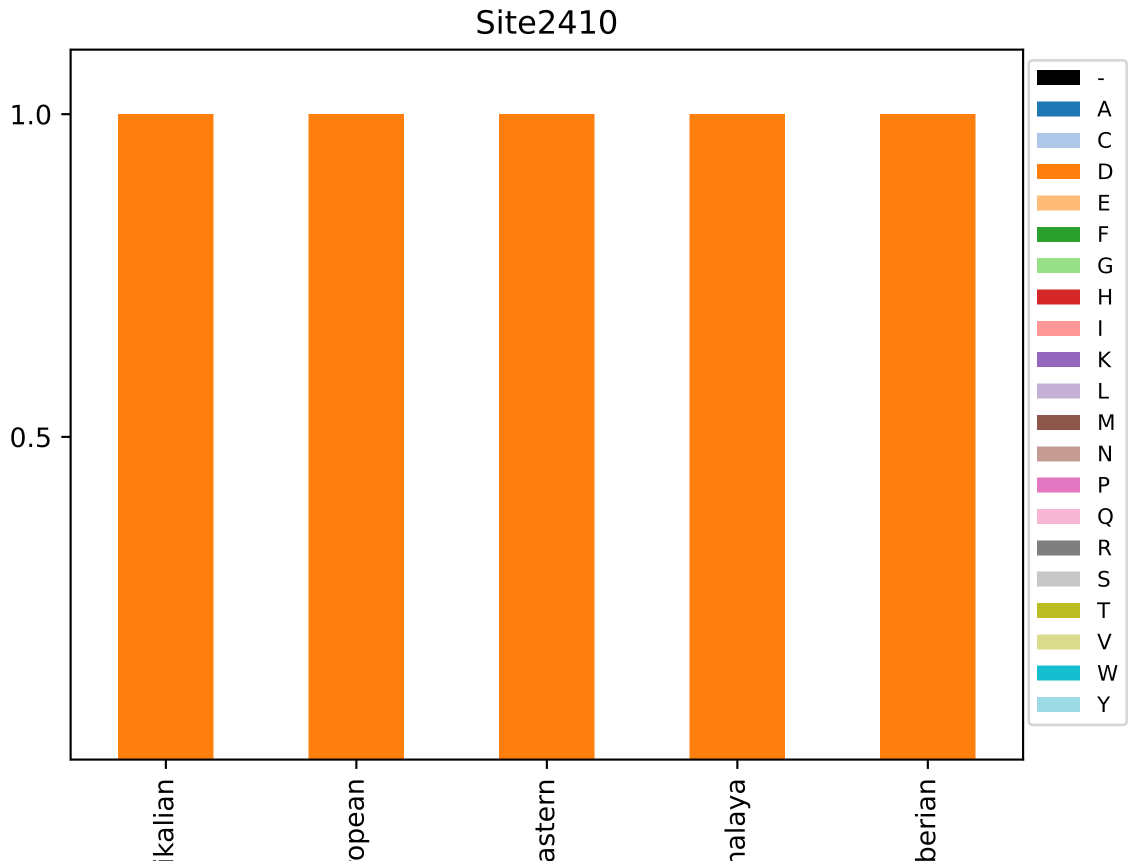
Site2410
1.0
0.5
-
A
C
D
E
F
G
H
I
K
L
M
N
P
Q
R
S
T
V
W
Y
ikalian
opean
astern
nalaya
berian

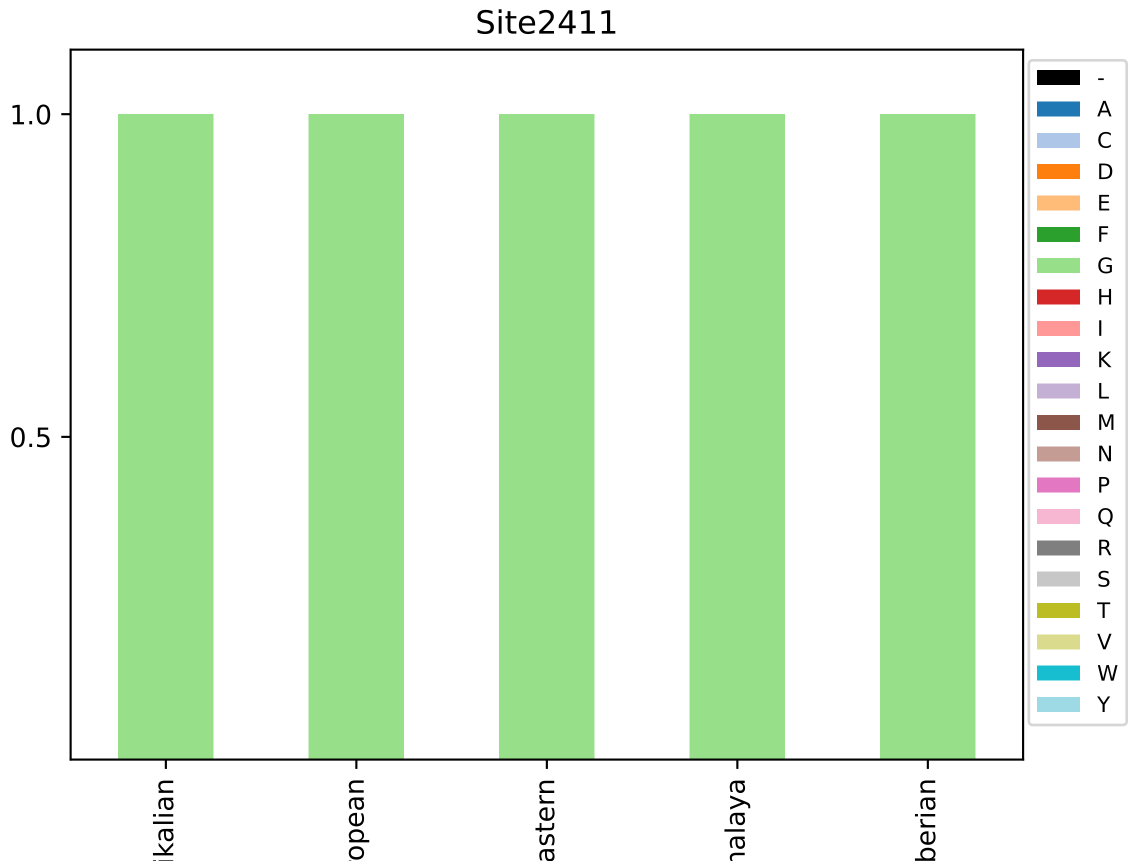

Site2411
1.0
0.5
-
A
C
D
E
F
G
H
I
K
L
M
N
P
Q
R
S
T
V
W
Y
ikalian
ropean
astern
nalaya
berian

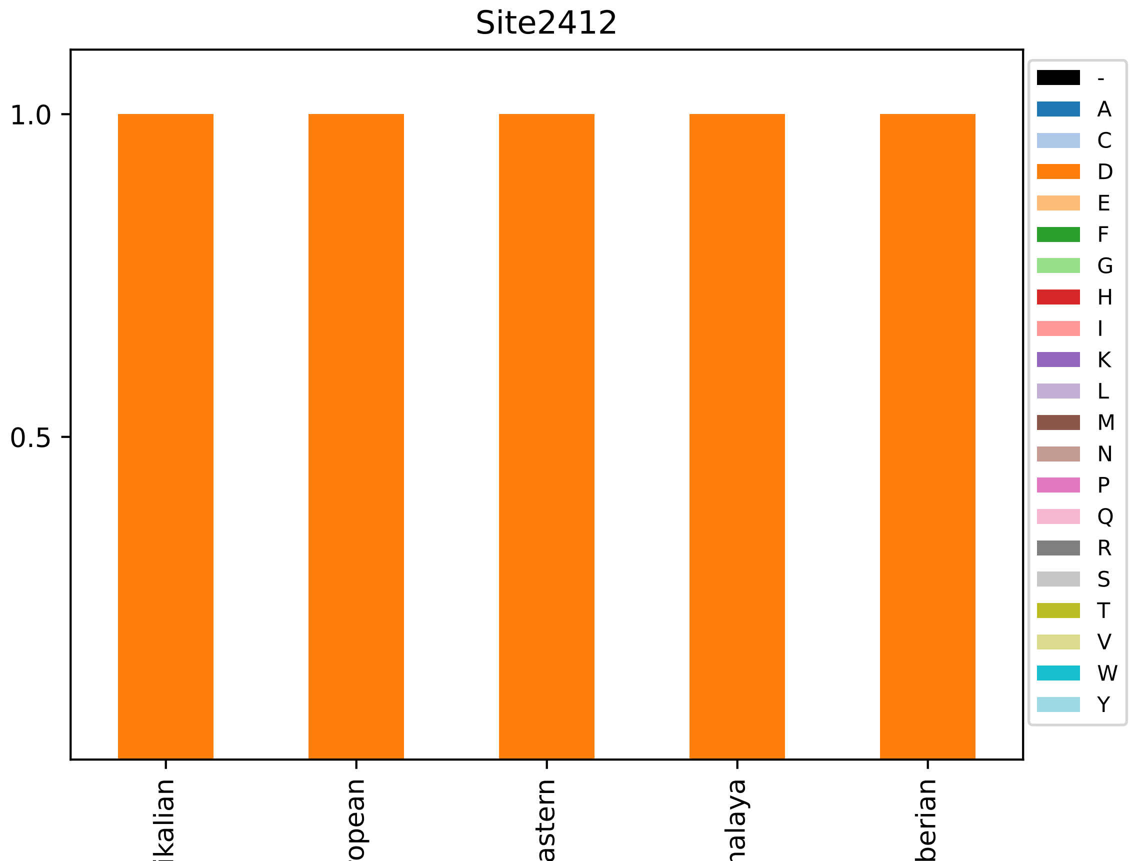
Site2412
1.0
0.5
-
A
C
D
E
F
G
H
I
K
L
M
N
P
Q
R
S
T
V
W
Y
ikalian
opean
astern
nalaya
berian

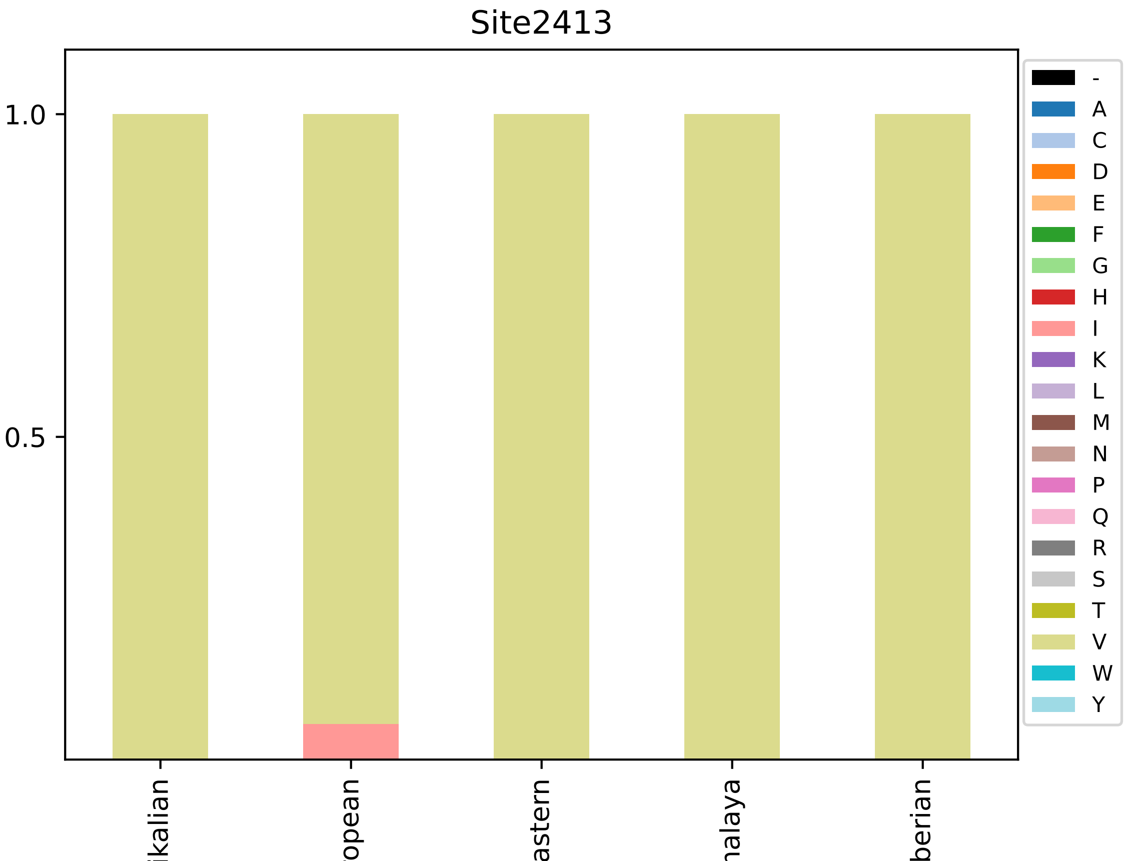
Site2413
1.0
0.5
-
A
C
D
E
F
G
H
I
K
L
M
N
P
Q
R
S
T
V
W
Y
ikalian
ropean
astern
nalaya
berian

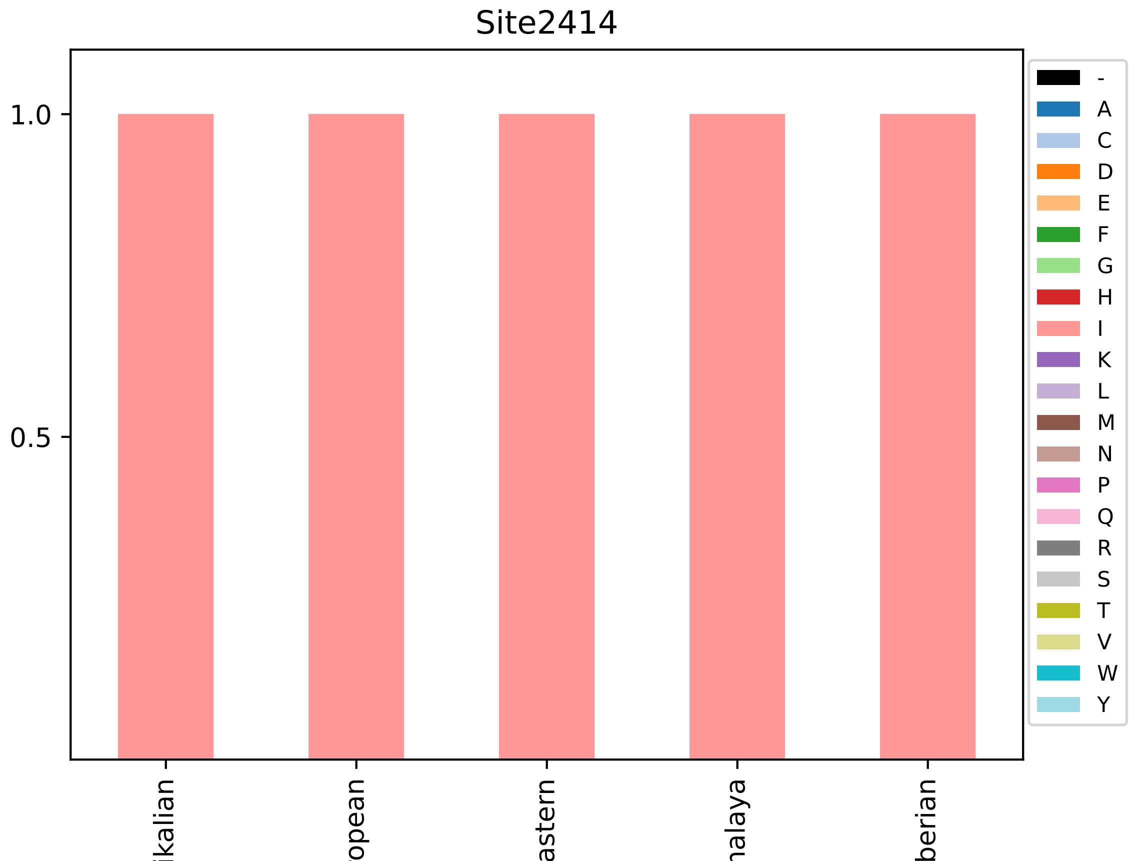
Site2414
1.0
0.5
-
A
C
D
E
F
G
H
I
K
L
M
N
P
Q
R
S
T
V
W
Y
ikalian
ropean
astern
nalaya
berian

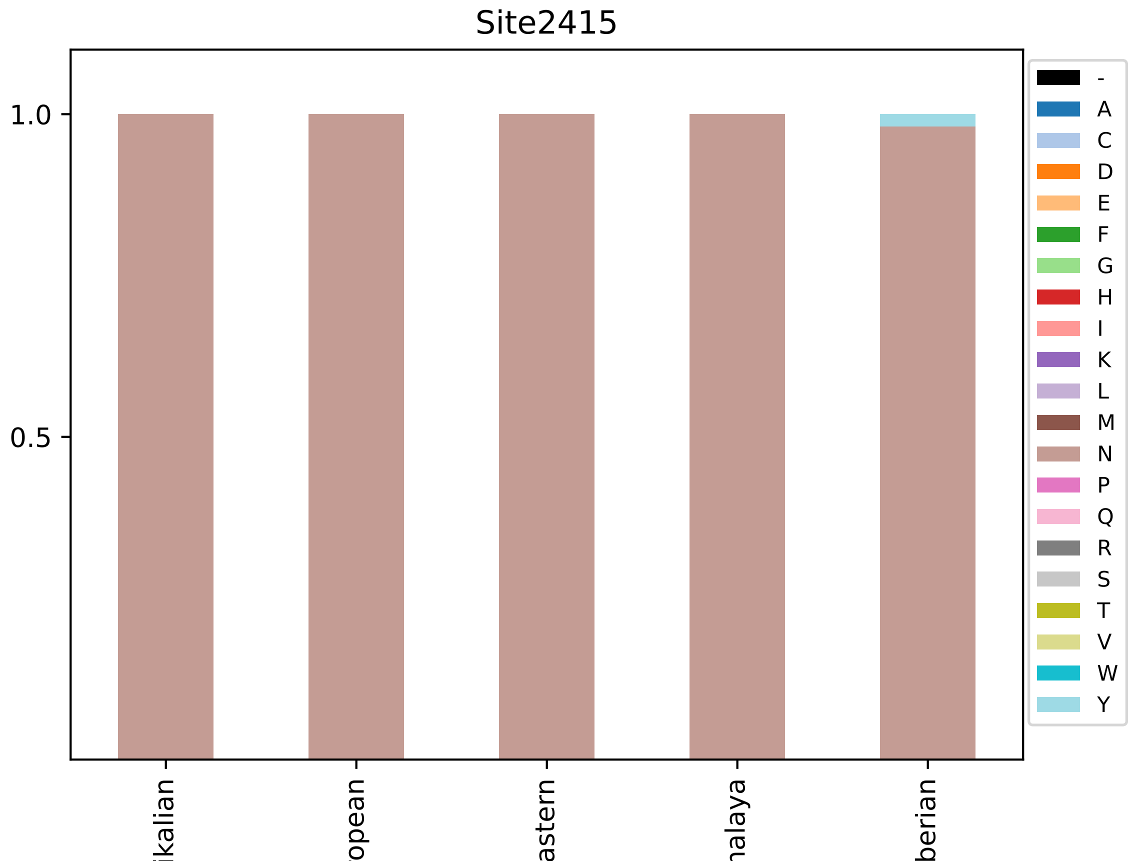
Site2415
1.0
0.5
-
A
C
D
E
F
G
H
I
K
L
M
N
P
Q
R
S
T
V
W
Y
ikalian
opean
astern
nalaya
berian

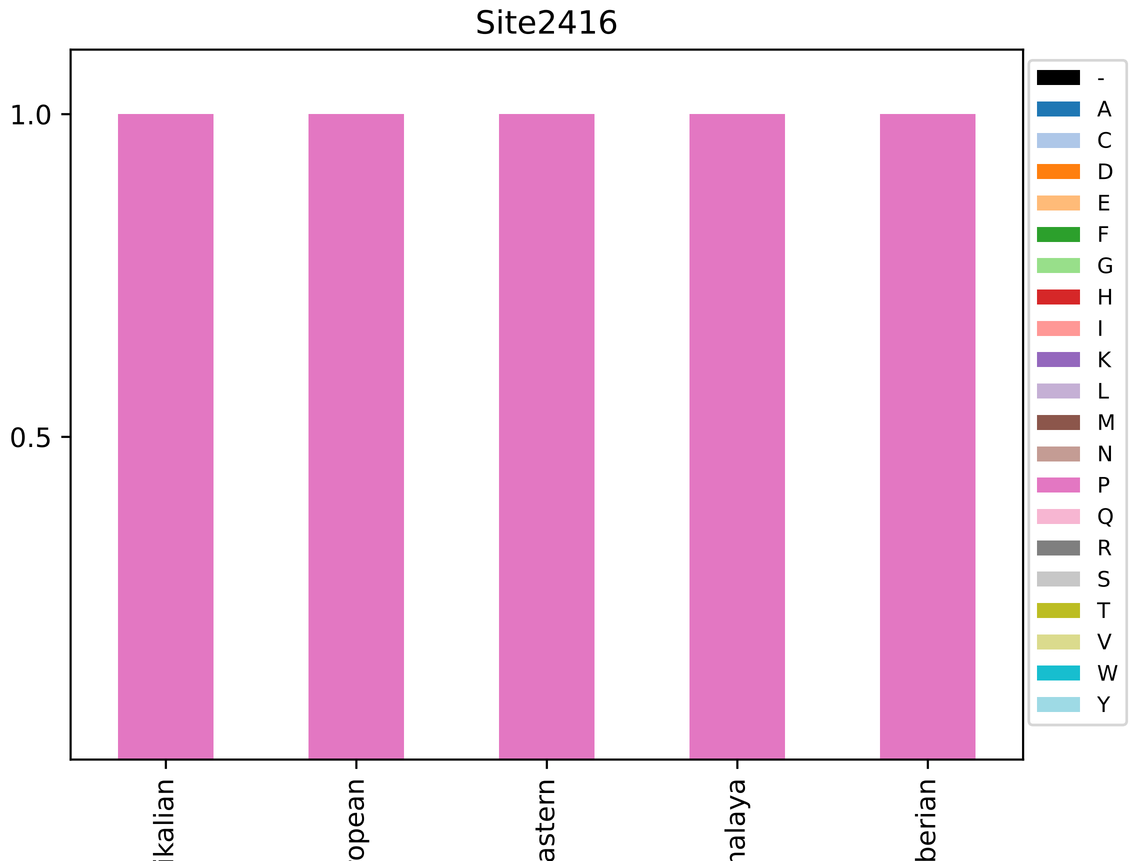
Site2416
1.0
0.5
-
A
C
D
E
F
G
H
I
K
L
M
N
P
Q
R
S
T
V
W
Y
ikalian
opean
astern
nalaya
berian

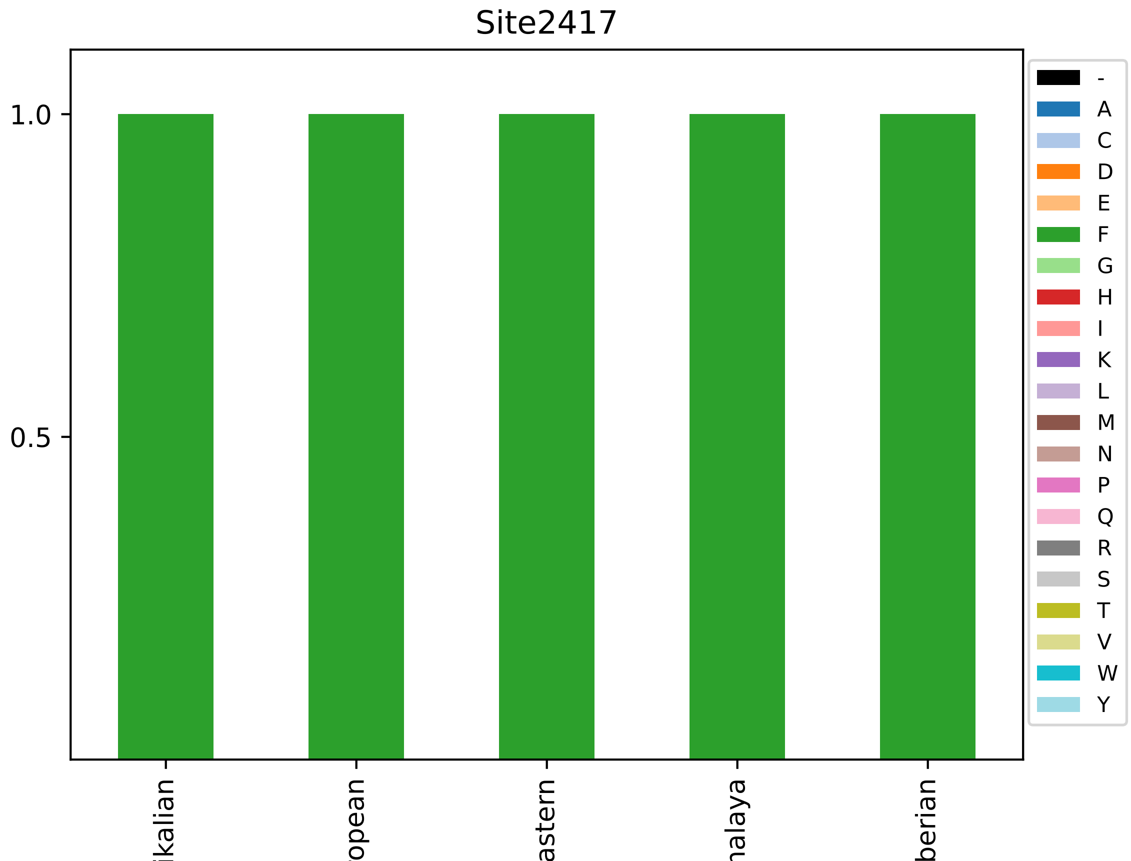
Site2417
1.0
0.5
-
A
C
D
E
F
G
H
I
K
L
M
N
P
Q
R
S
T
V
W
Y
ikalian
opean
astern
nalaya
berian

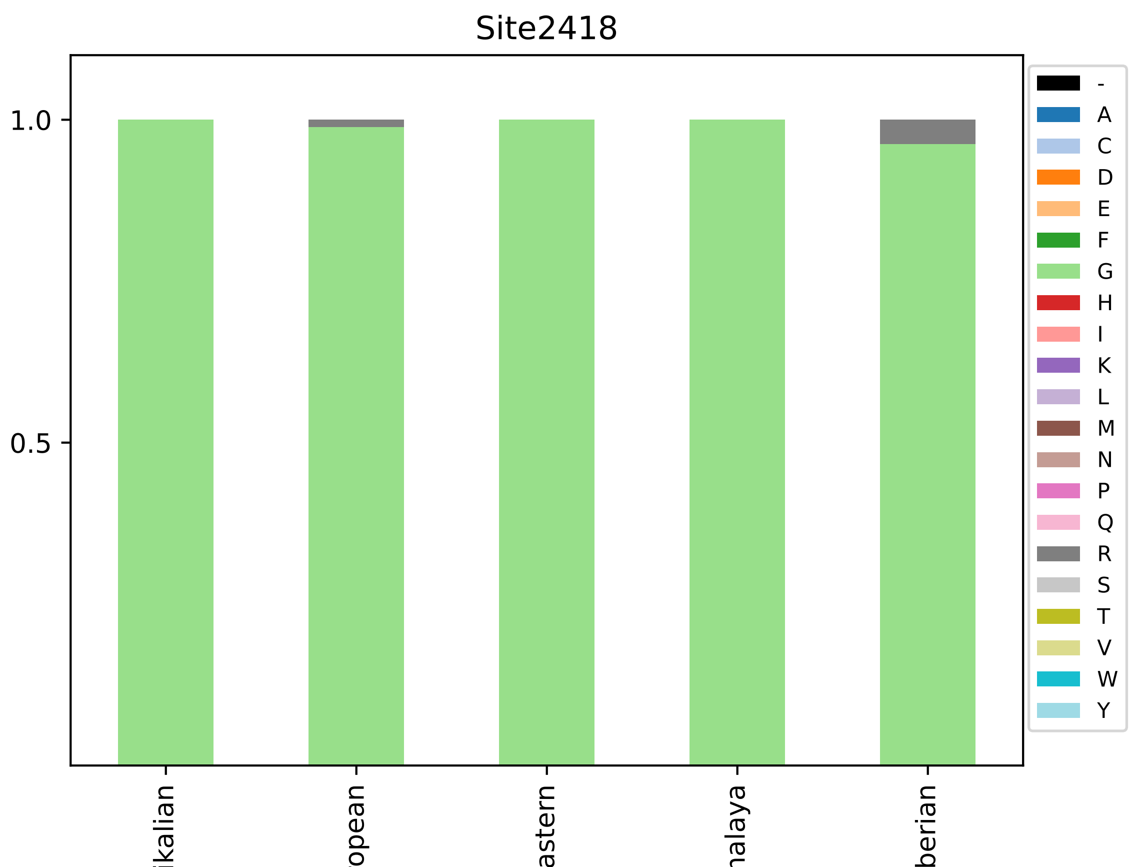

Site2418
1.0
0.5
-
A
C
D
E
F
G
H
I
K
L
M
N
P
Q
R
S
T
V
W
Y

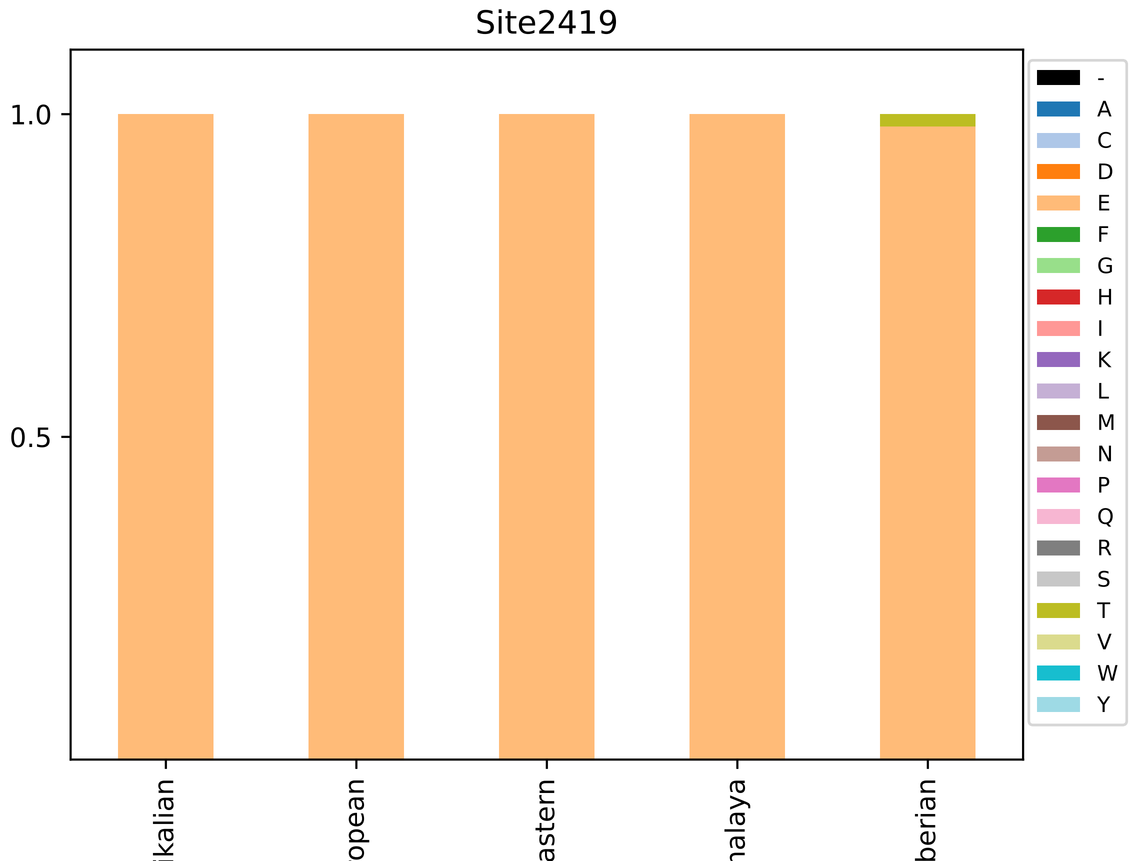
Site2419
1.0
0.5
-
A
C
D
E
F
G
H
I
K
L
M
N
P
Q
R
S
T
V
W
Y

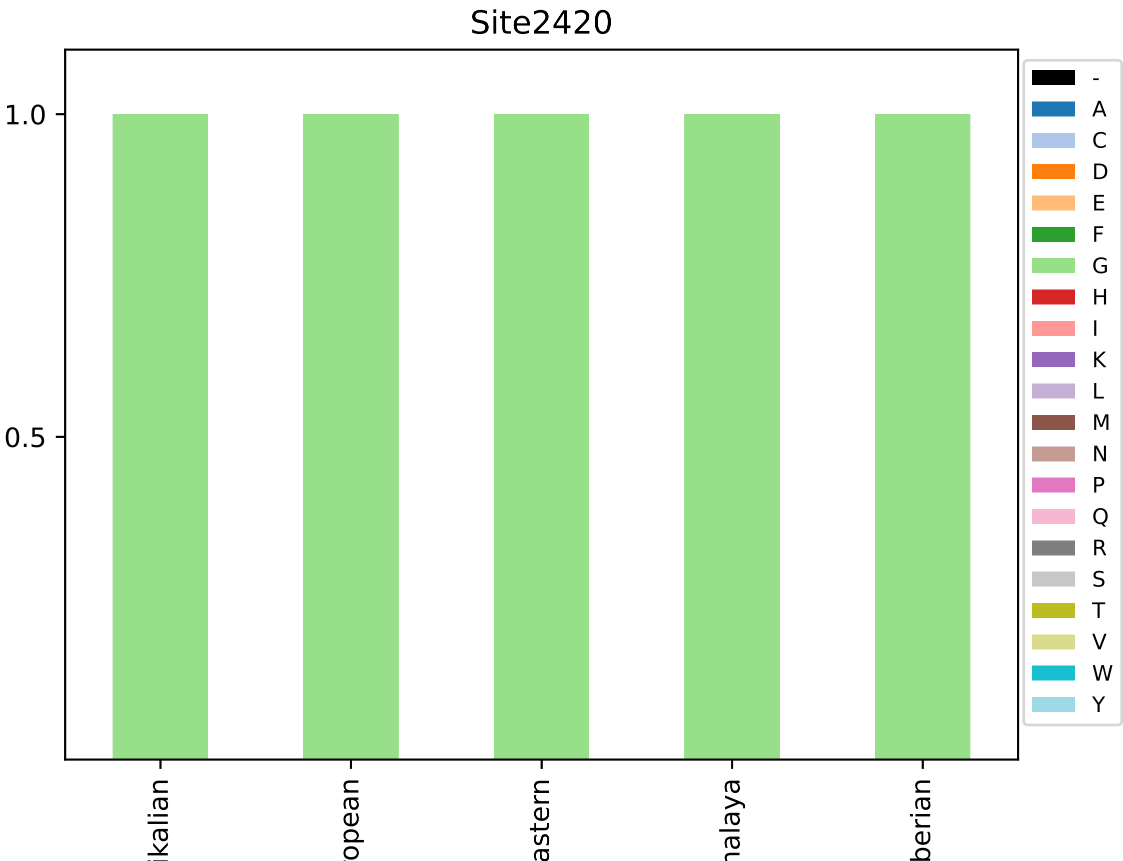

Site2420
1.0
0.5
ikalian
opean
astern
nalaya
berian
-
A
C
D
E
F
G
H
I
K
L
M
N
P
Q
R
S
T
V
W
Y

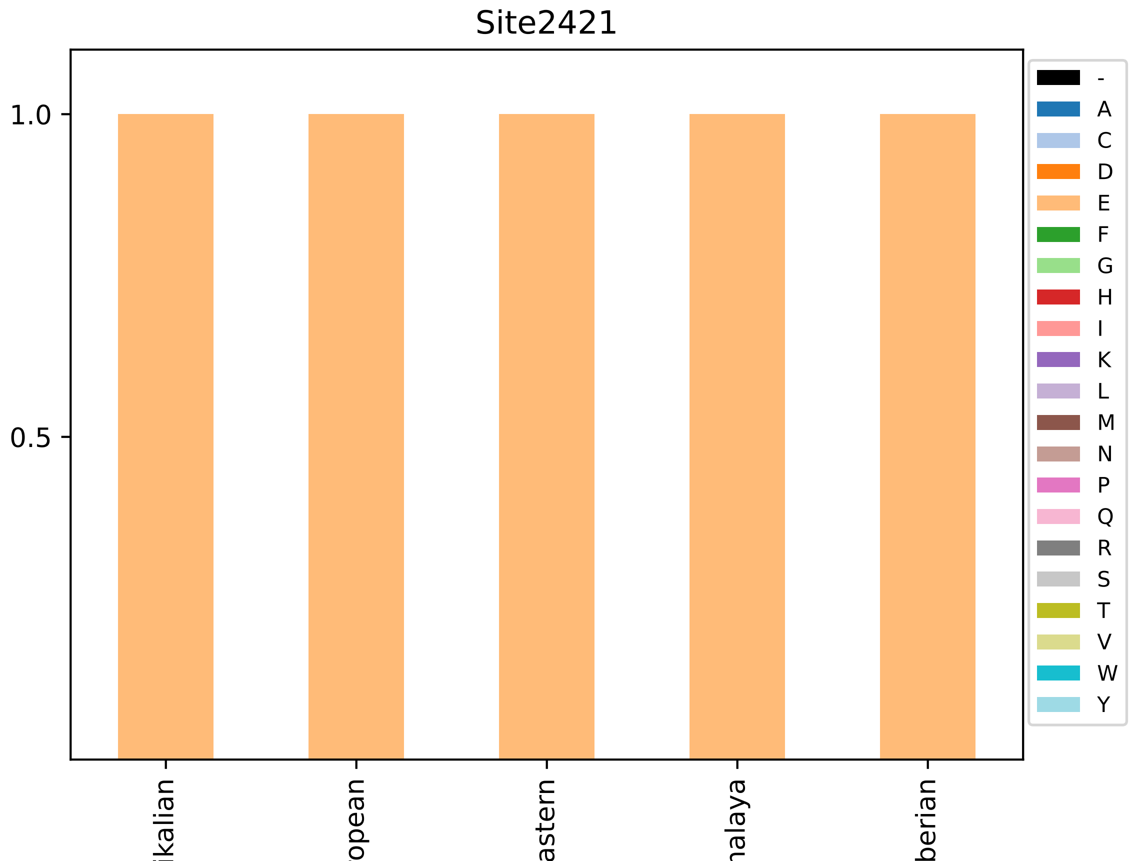
Site2421
1.0
0.5
-
A
C
D
E
F
G
H
I
K
L
M
N
P
Q
R
S
T
V
W
Y
ikalian
opean
astern
nalaya
berian

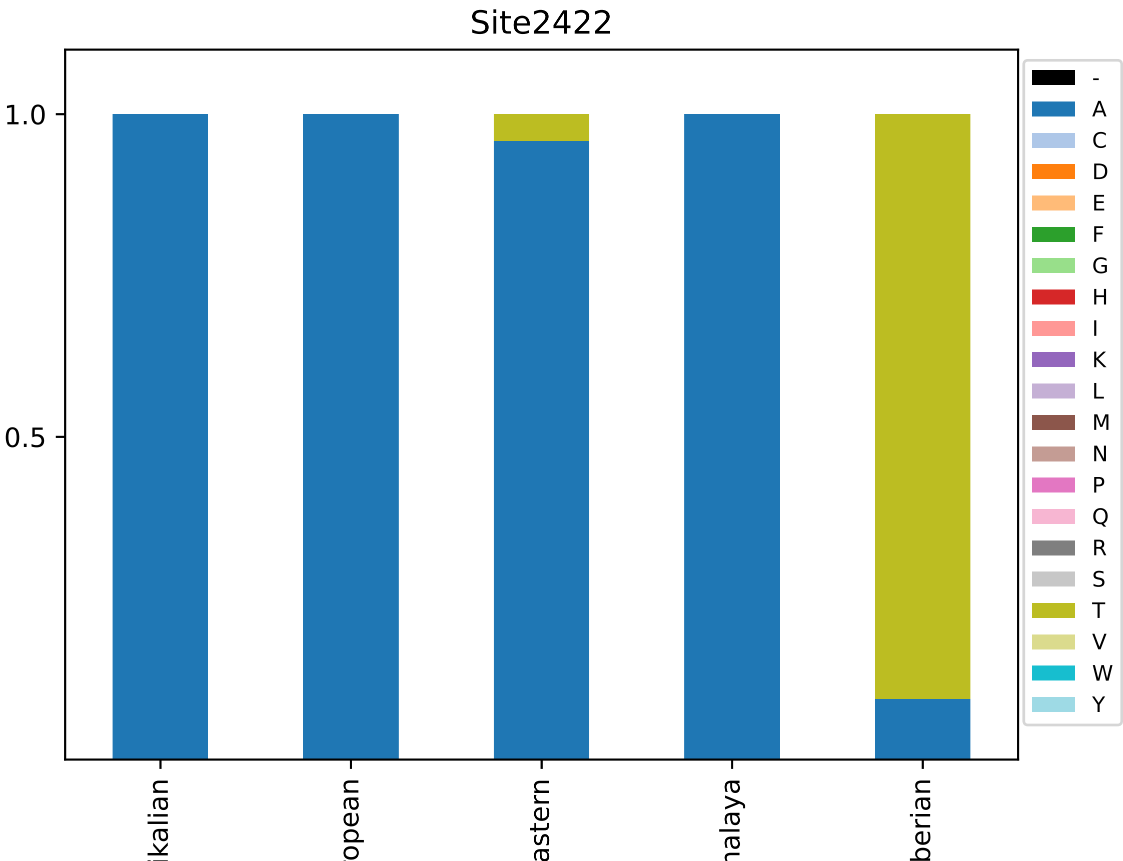

Site2422
1.0
0.5
-
A
C
D
E
F
G
H
I
K
L
M
N
P
Q
R
S
T
V
W
Y
ikalian
opean
astern
alaya
berian

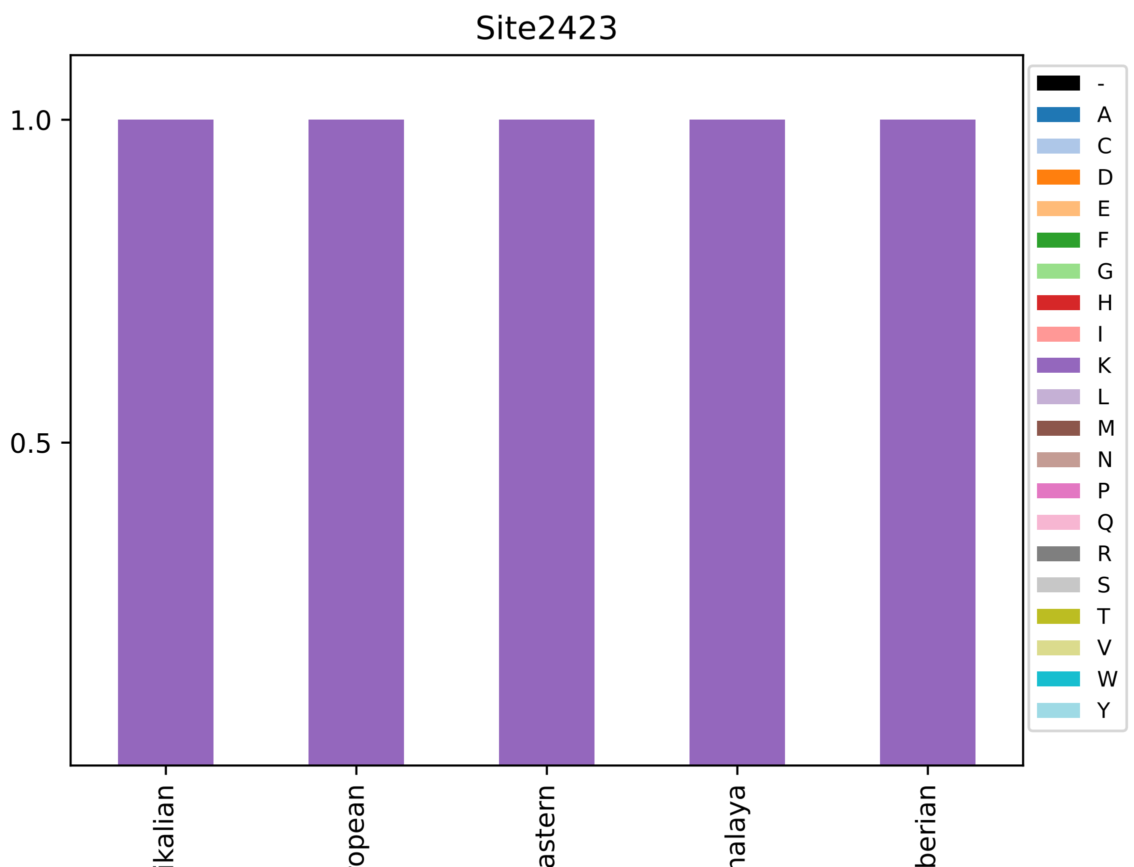

Site2423
1.0
0.5
-
A
C
D
E
F
G
H
I
K
L
M
N
P
Q
R
S
T
V
W
Y
ikalian
opean
astern
nalaya
berian

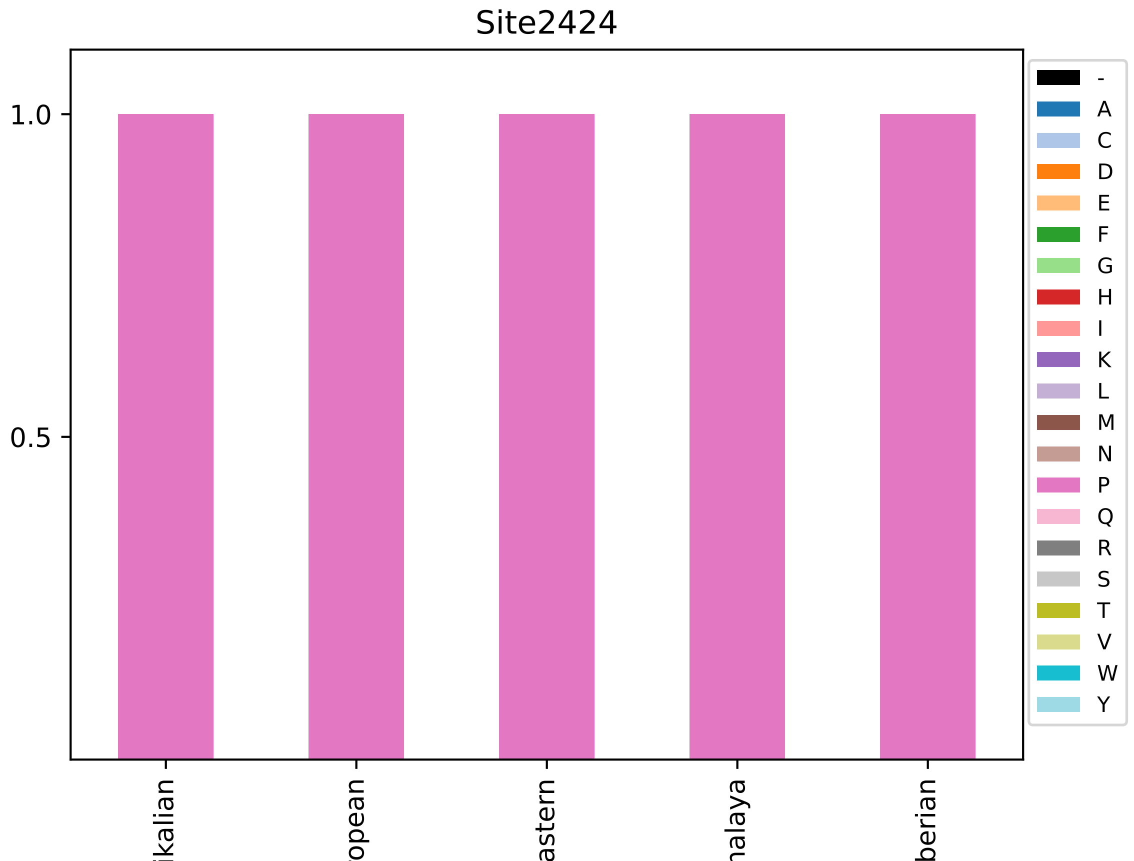

Site2424
1.0
0.5
-
A
C
D
E
F
G
H
I
K
L
M
N
P
Q
R
S
T
V
W
Y
ikalian
ropean
astern
nalaya
berian

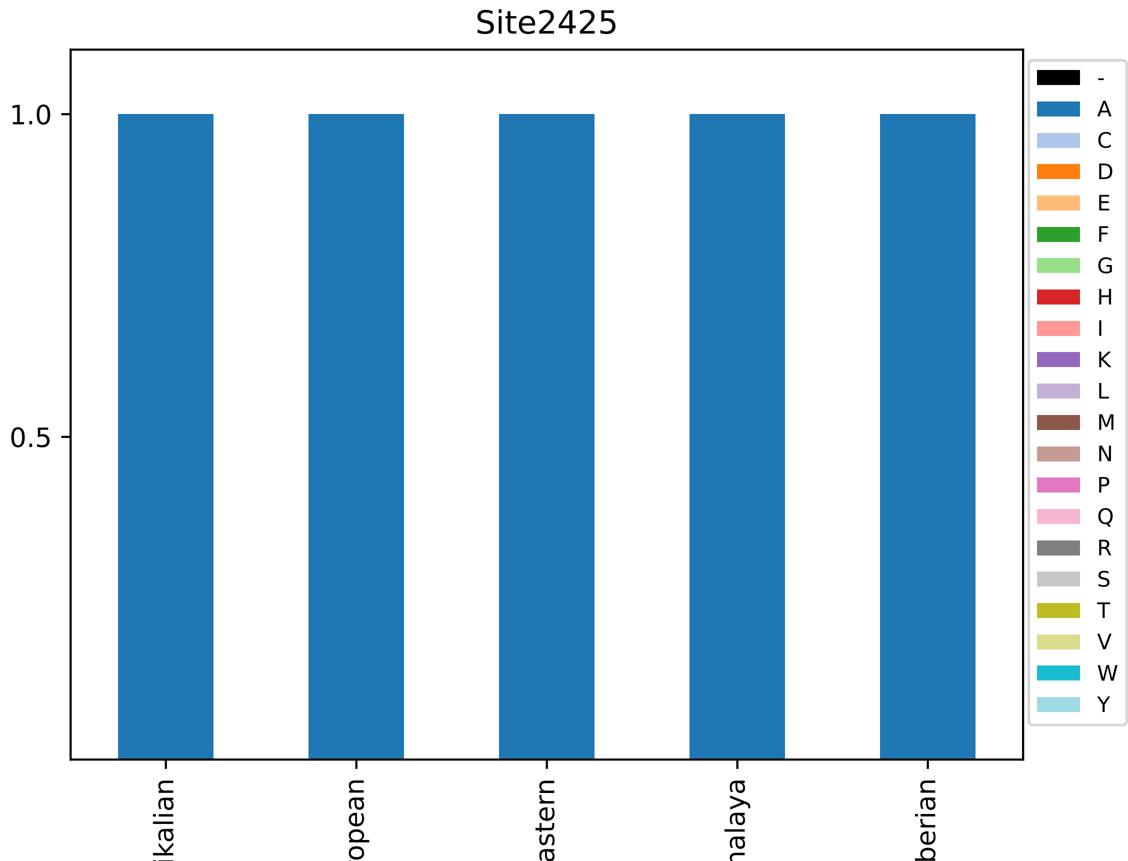

Site2425
1.0
0.5
-
A
C
D
E
F
G
H
I
K
L
M
N
P
Q
R
S
T
V
W
Y
ikalian
opean
astern
nalaya
berian

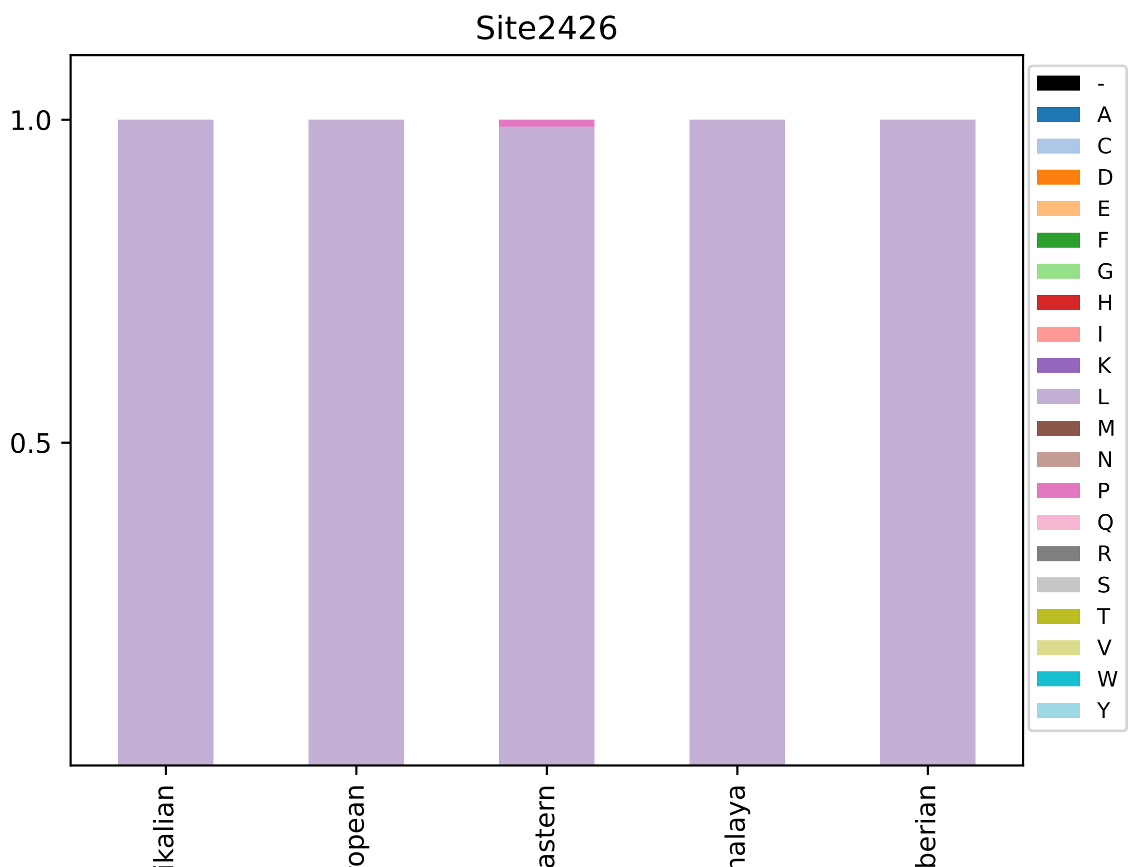

Site2426
1.0
0.5
-
A
C
D
E
F
G
H
I
K
L
M
N
P
Q
R
S
T
V
W
Y
ikalian
opean
astern
nalaya
berian

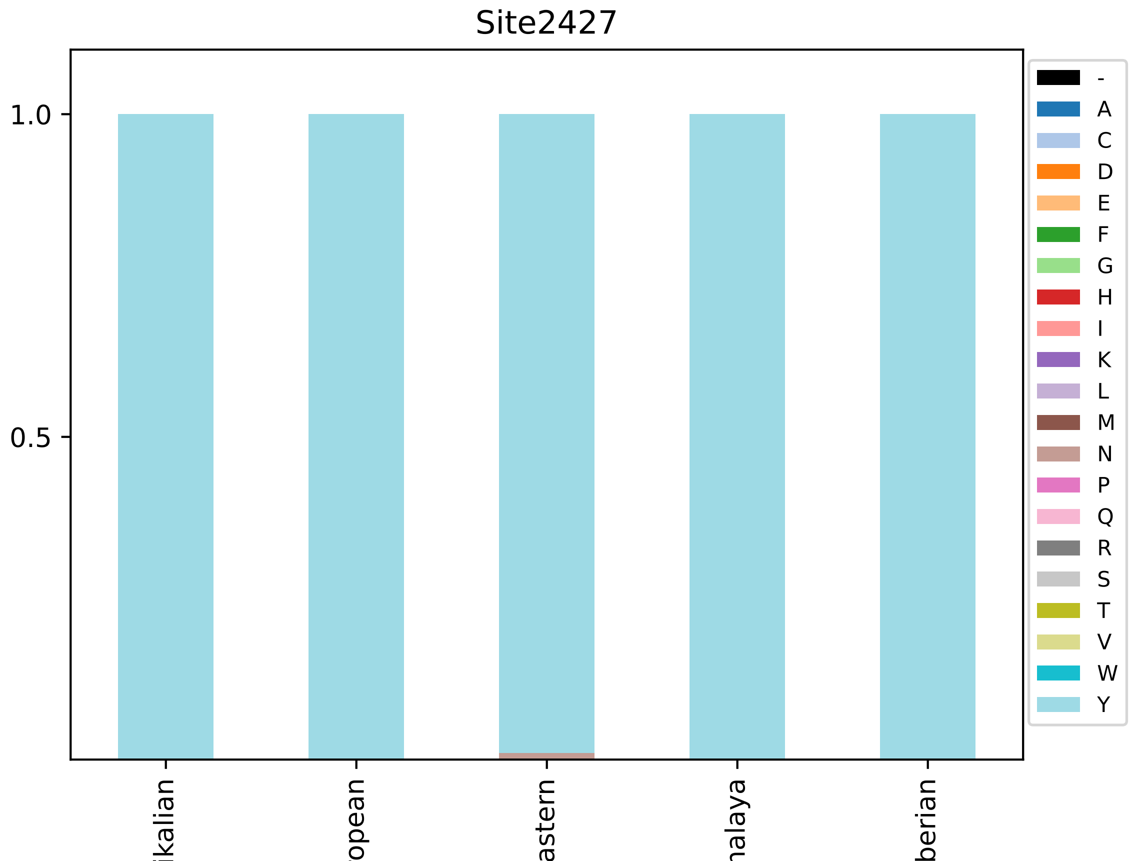

Site2427
1.0
0.5
ikalian
opean
astern
nalaya
berian
-
A
C
D
E
F
G
H
I
K
L
M
N
P
Q
R
S
T
V
W
Y

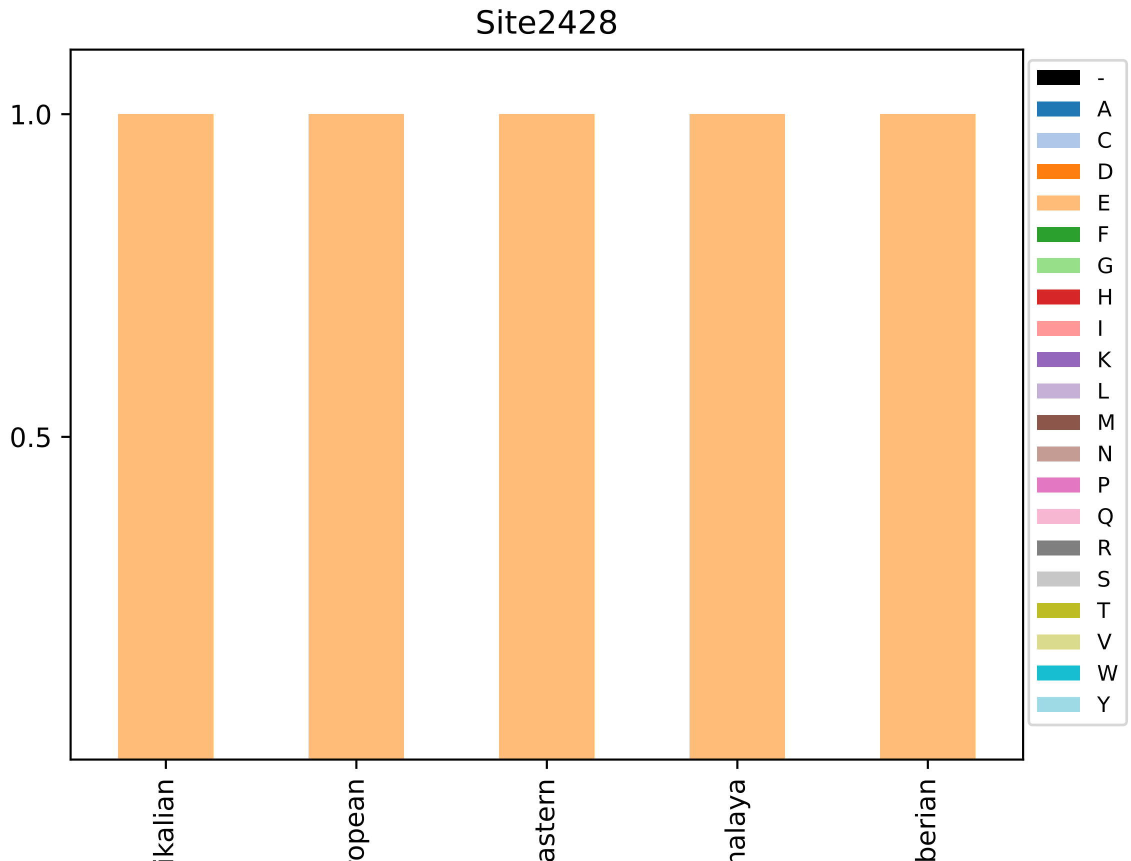
Site2428
1.0
0.5
-
A
C
D
E
F
G
H
I
K
L
M
N
P
Q
R
S
T
V
W
Y
ikalian
opean
astern
nalaya
berian

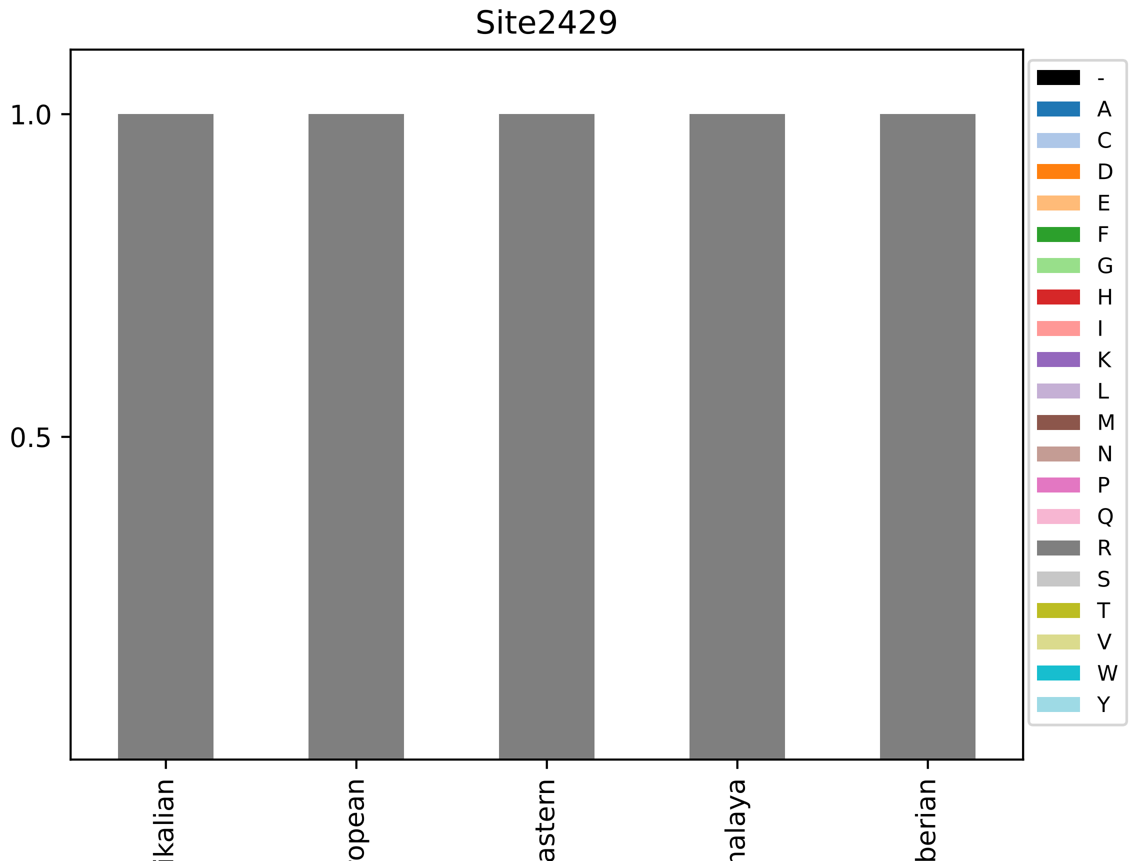
Site2429
1.0
0.5
-
A
C
D
E
F
G
H
I
K
L
M
N
P
Q
R
S
T
V
W
Y
ikalian
opean
astern
nalaya
berian

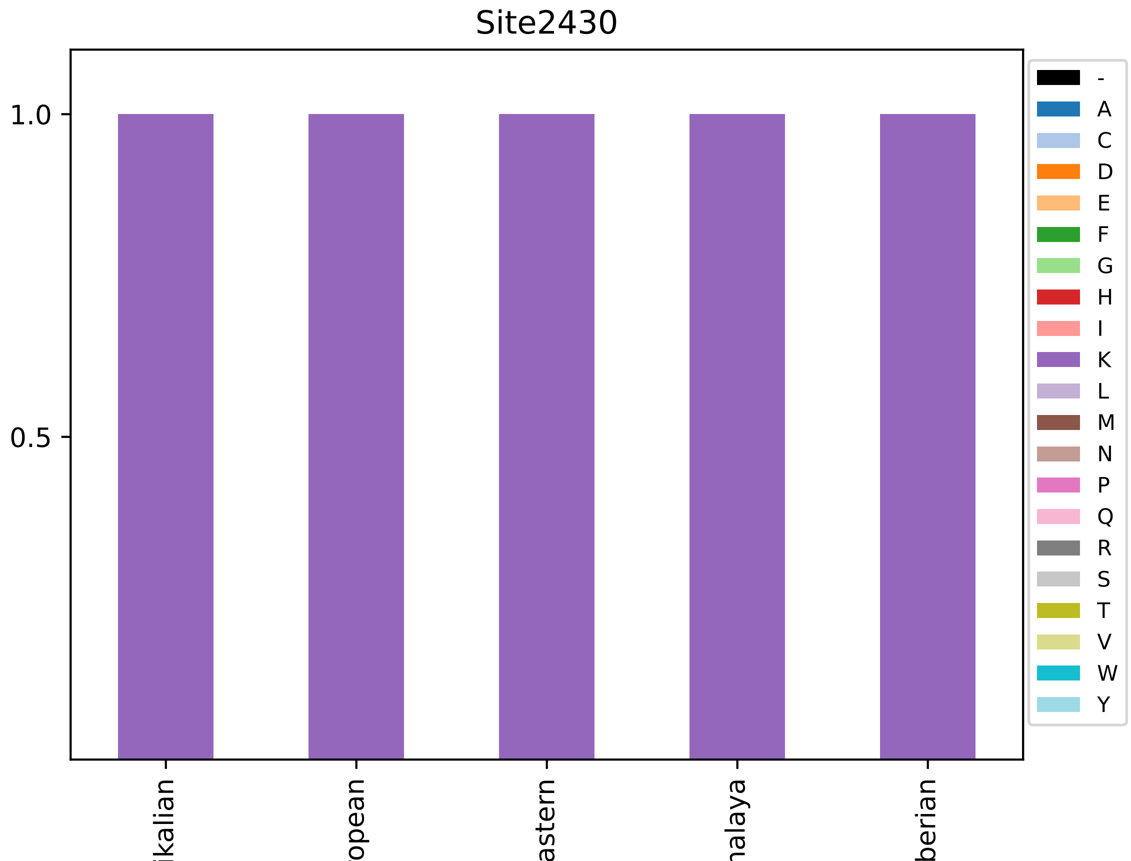

Site2430
1.0
0.5
-
A
C
D
E
F
G
H
I
K
L
M
N
P
Q
R
S
T
V
W
Y
ikalian
opean
astern
nalaya
berian

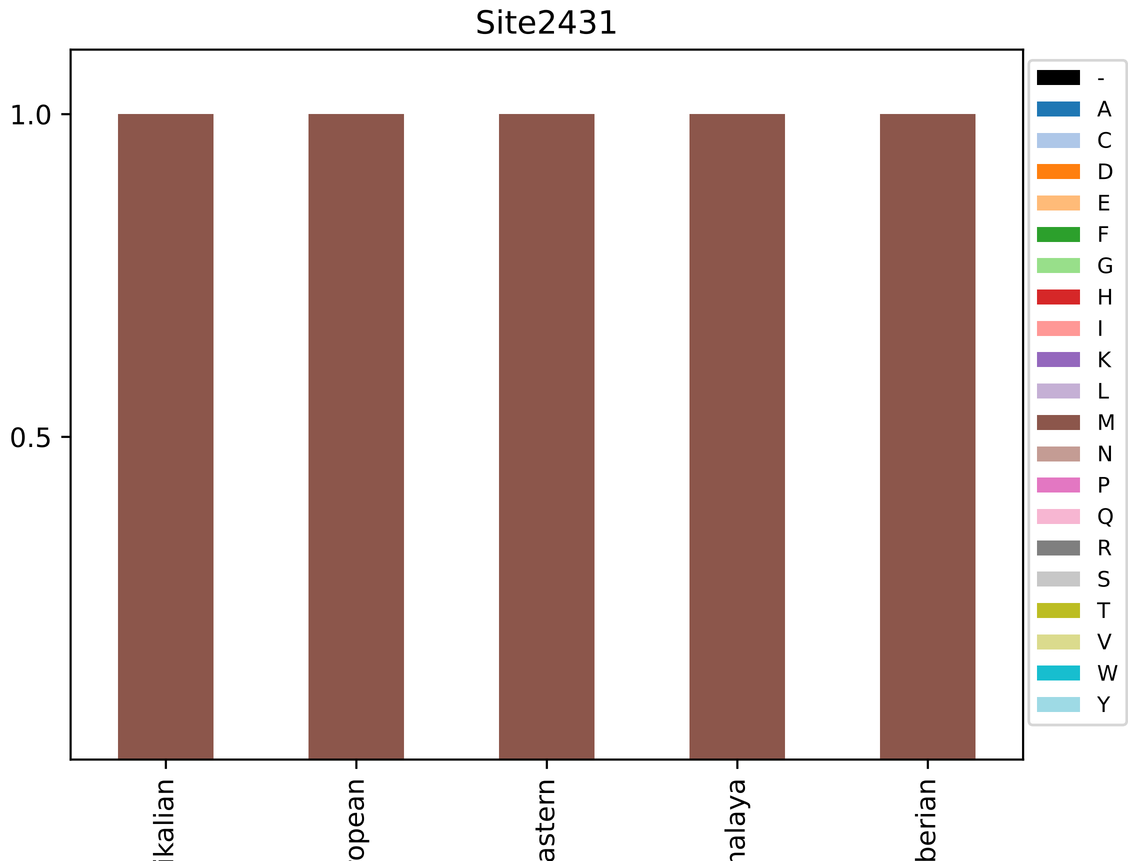
Site2431
1.0
0.5
-
A
C
D
E
F
G
H
I
K
L
M
N
P
Q
R
S
T
V
W
Y

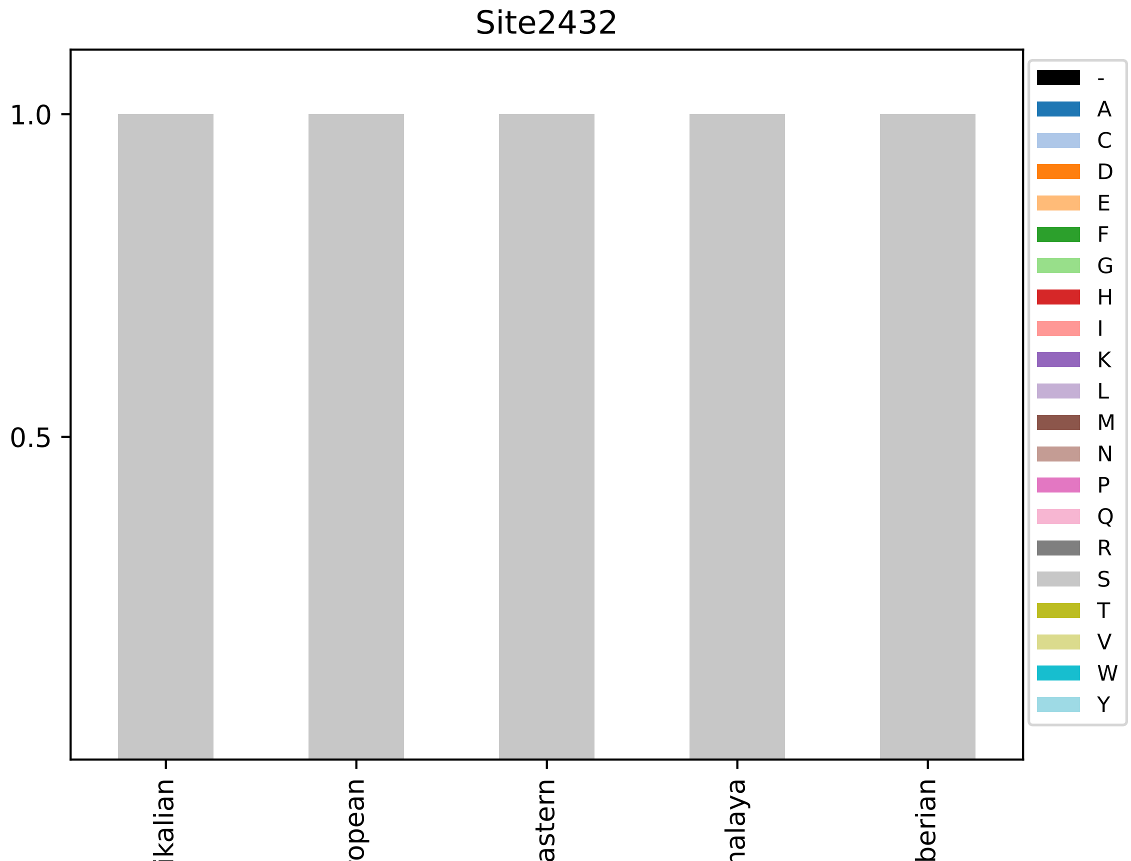

Site2432
1.0
0.5
-
A
C
D
E
F
G
H
I
K
L
M
N
P
Q
R
S
T
V
W
Y
ikalian
opean
astern
nalaya
berian

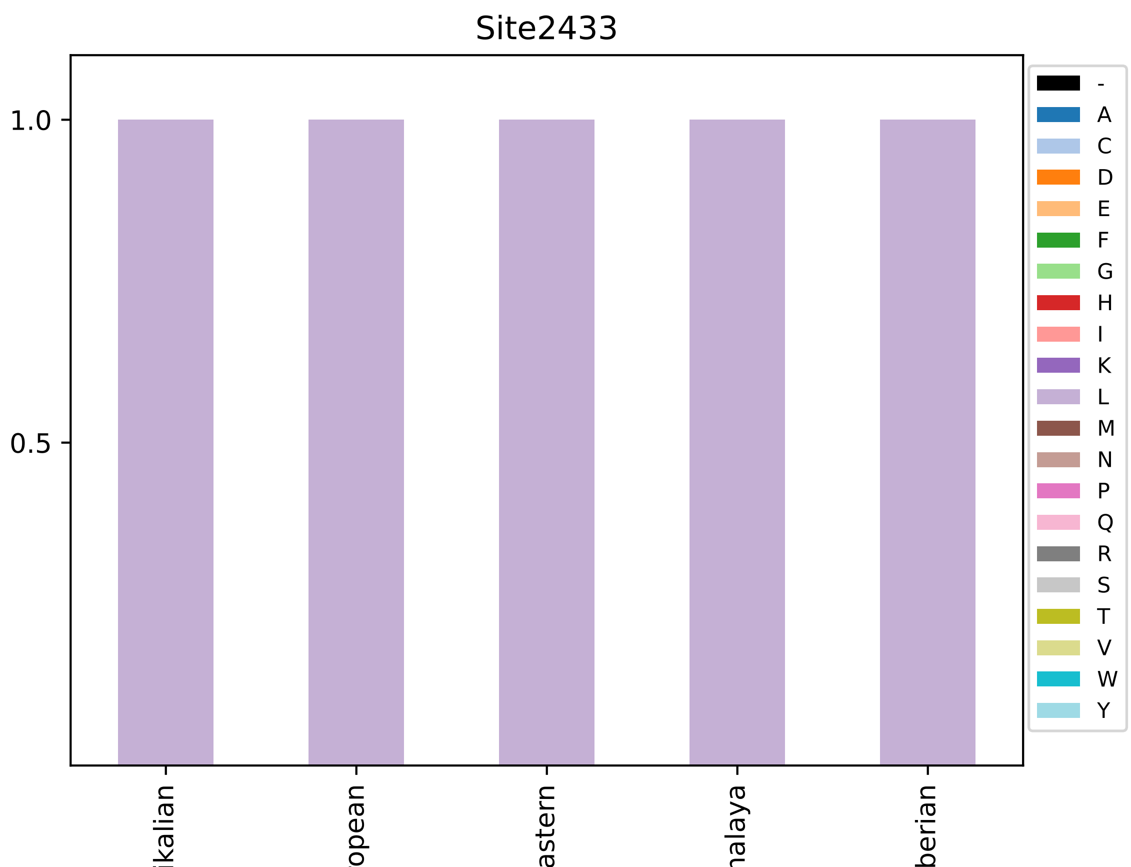
Site2433
1.0
0.5
-
A
C
D
E
F
G
H
I
K
L
M
N
P
Q
R
S
T
V
W
Y
ikalian
ropean
astern
nalaya
berian

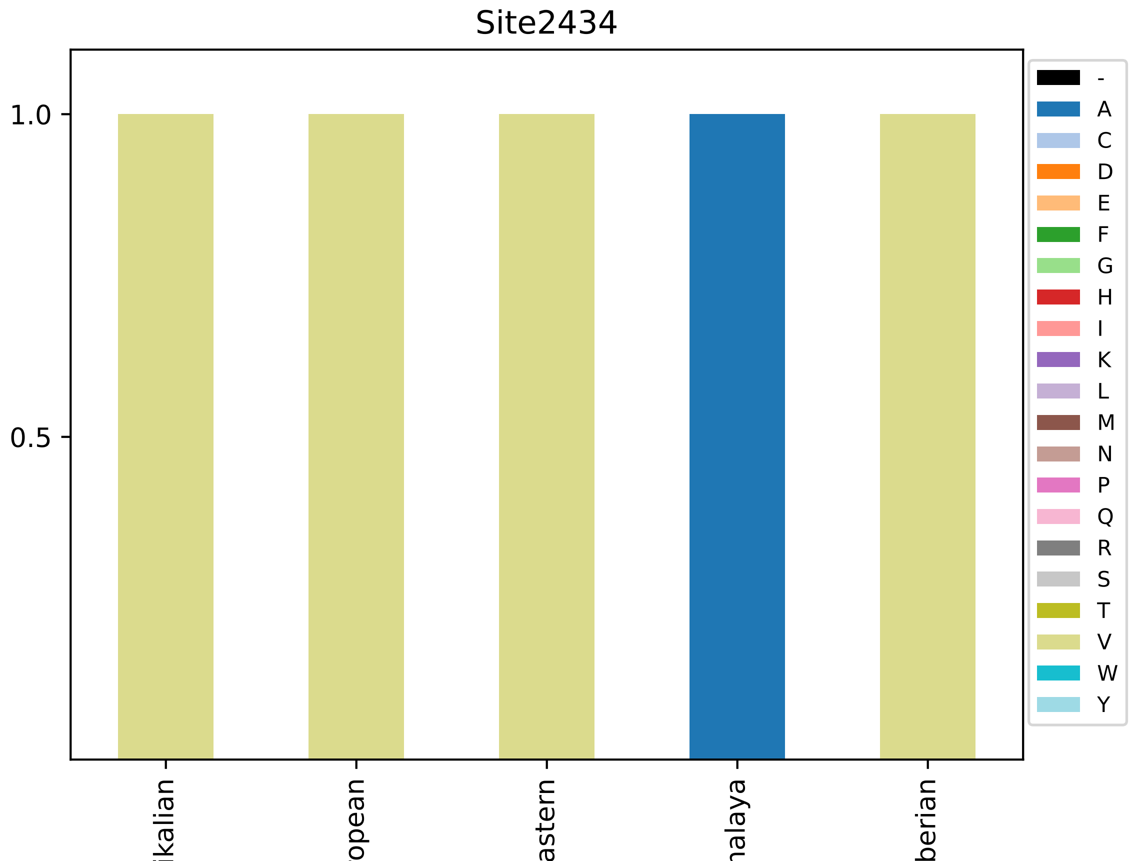

Site2434
1.0
0.5
ikalian
ropean
astern
halaya
berian
-
A
C
D
E
F
G
H
I
K
L
M
N
P
Q
R
S
T
V
W
Y

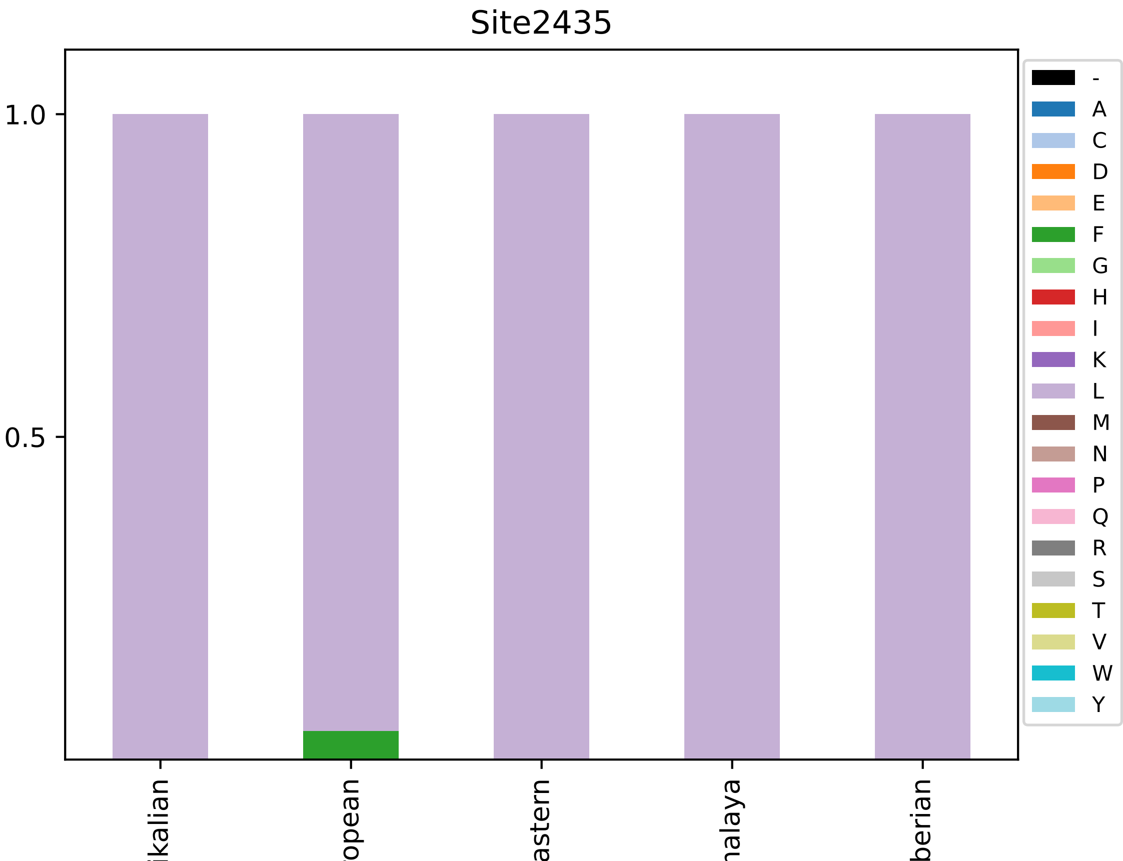

Site2435
1.0
0.5
-
A
C
D
E
F
G
H
I
K
L
M
N
P
Q
R
S
T
V
W
Y
ikalian
ropean
astern
nalaya
berian

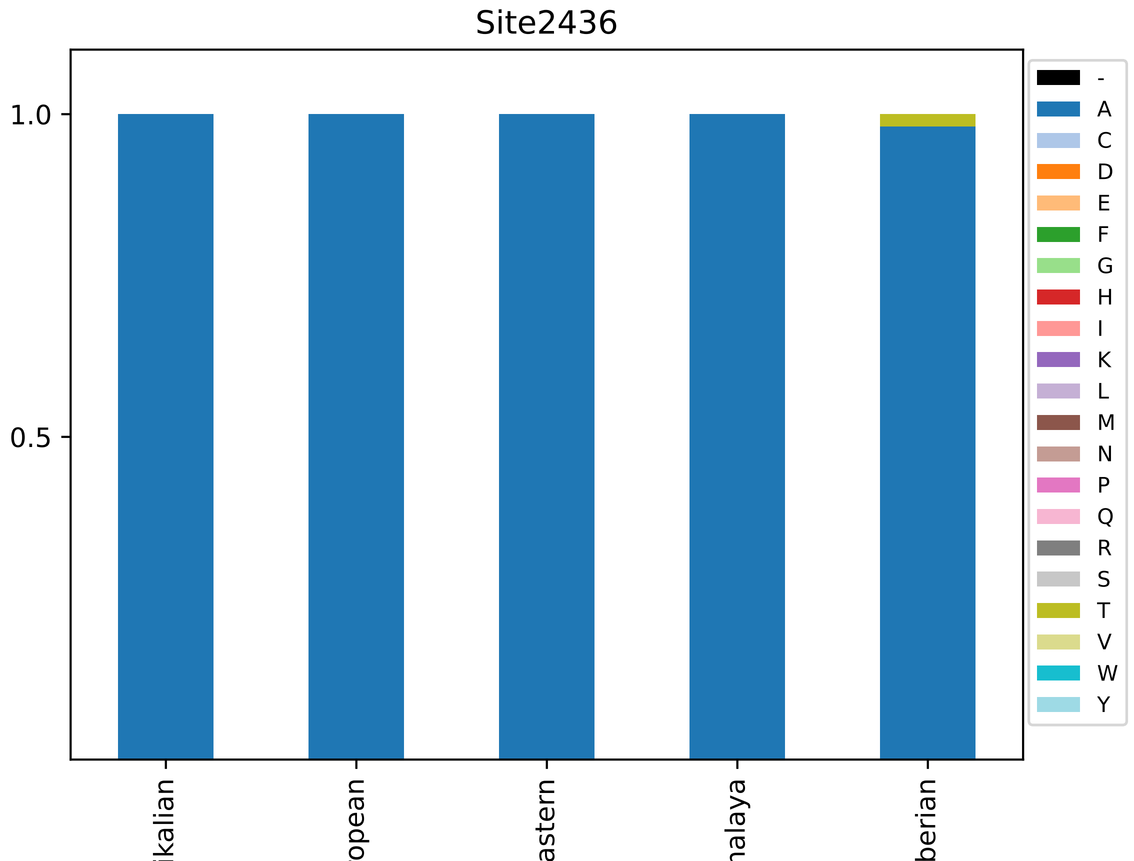
Site2436
1.0
0.5
ikalian
opean
astern
halaya
berian
-
A
C
D
E
F
G
H
I
K
L
M
N
P
Q
R
S
T
V
W
Y

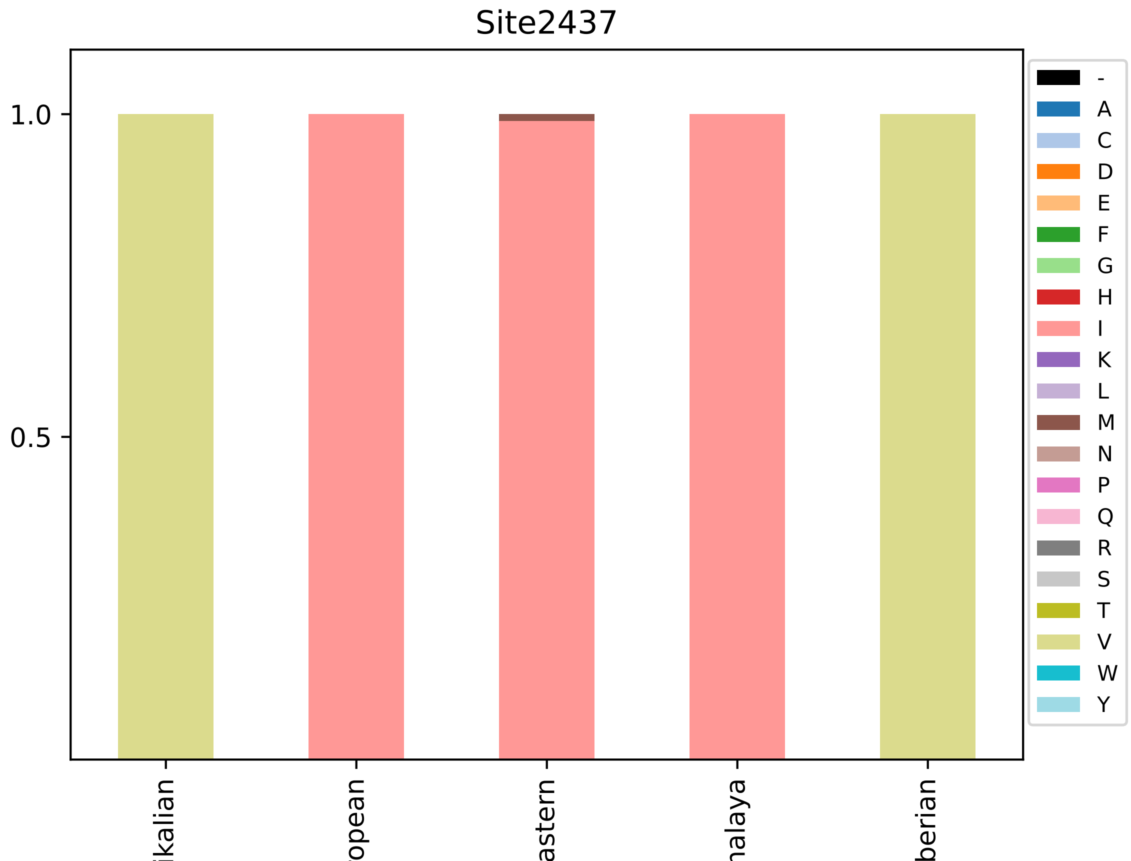
Site2437
1.0
0.5
-
A
C
D
E
F
G
H
I
K
L
M
N
P
Q
R
S
T
V
W
Y
ikalian
opean
astern
nalaya
berian

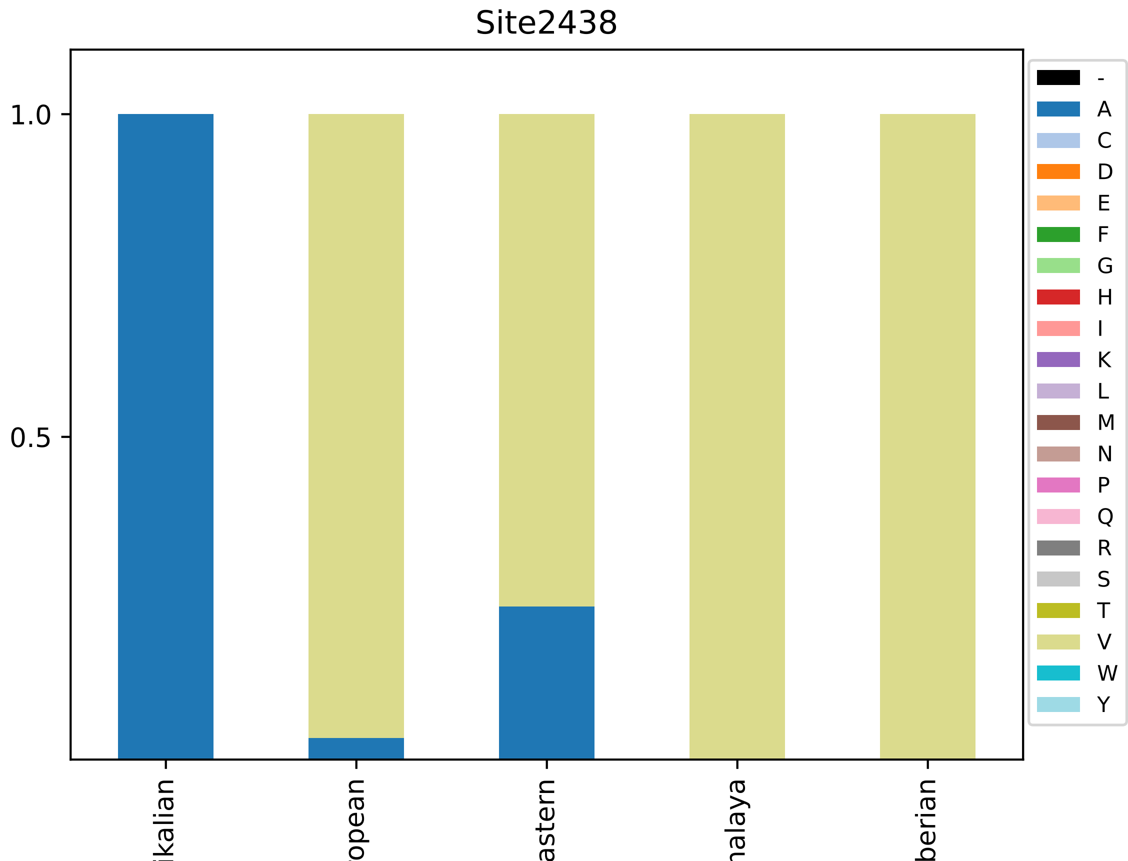

Site2438
1.0
0.5
-
A
C
D
E
F
G
H
I
K
L
M
N
P
Q
R
S
T
V
W
Y

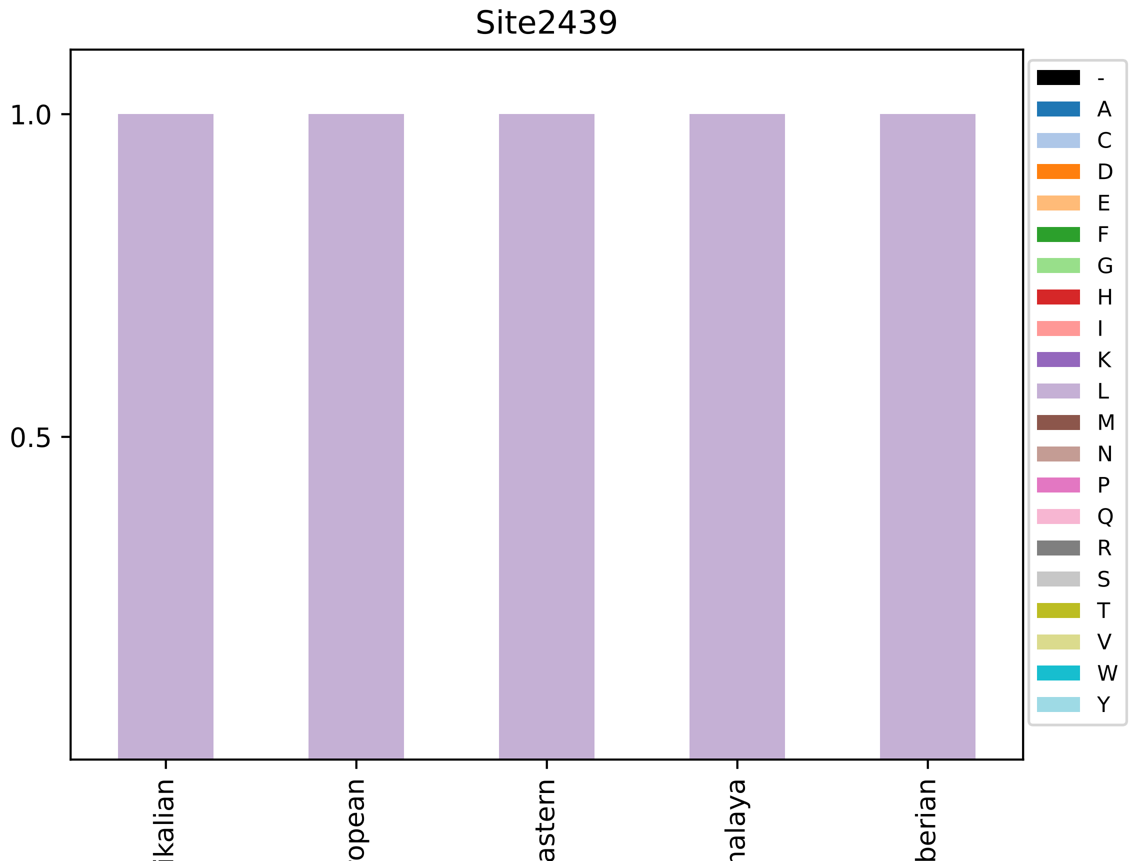

Site2439
1.0
0.5
-
A
C
D
E
F
G
H
I
K
L
M
N
P
Q
R
S
T
V
W
Y
ikalian
opean
astern
nalaya
berian

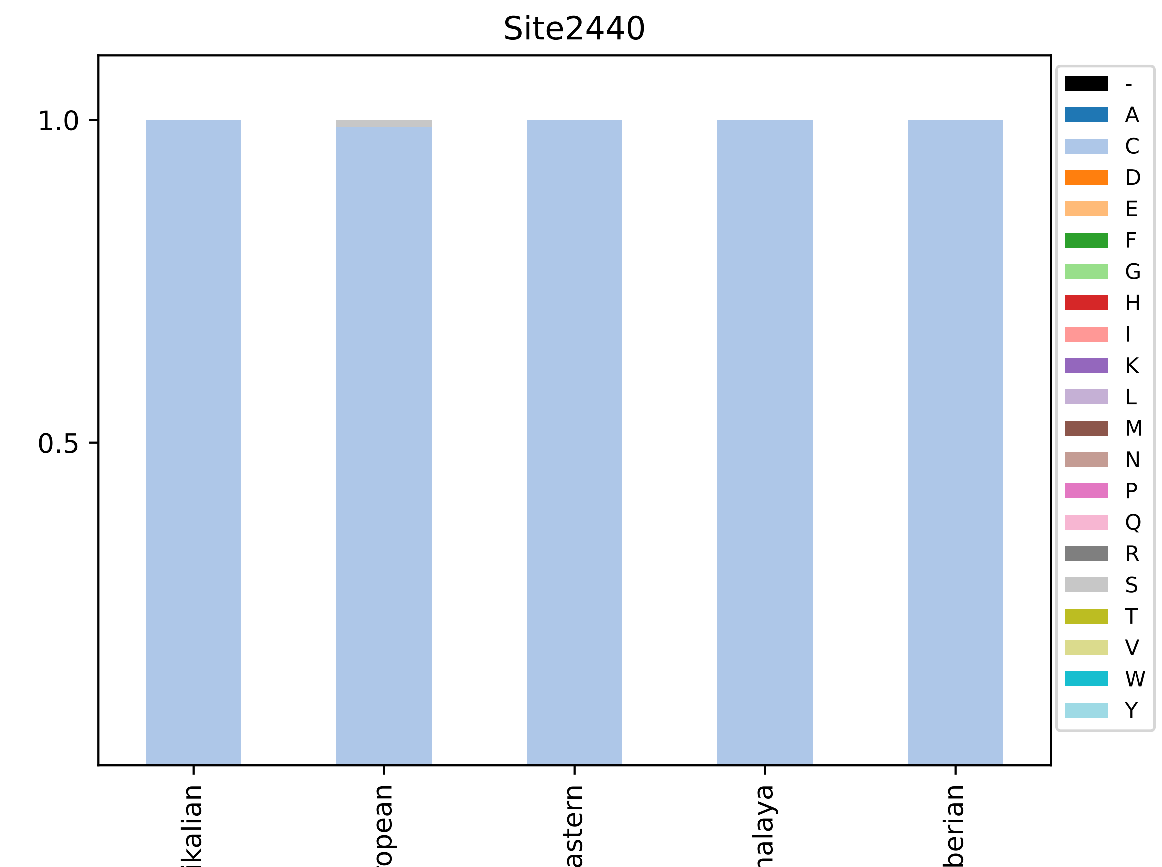
Site2440
1.0
0.5
ikalian
opean
astern
nalaya
berian
-
A
C
D
E
F
G
H
I
K
L
M
N
P
Q
R
S
T
V
W
Y

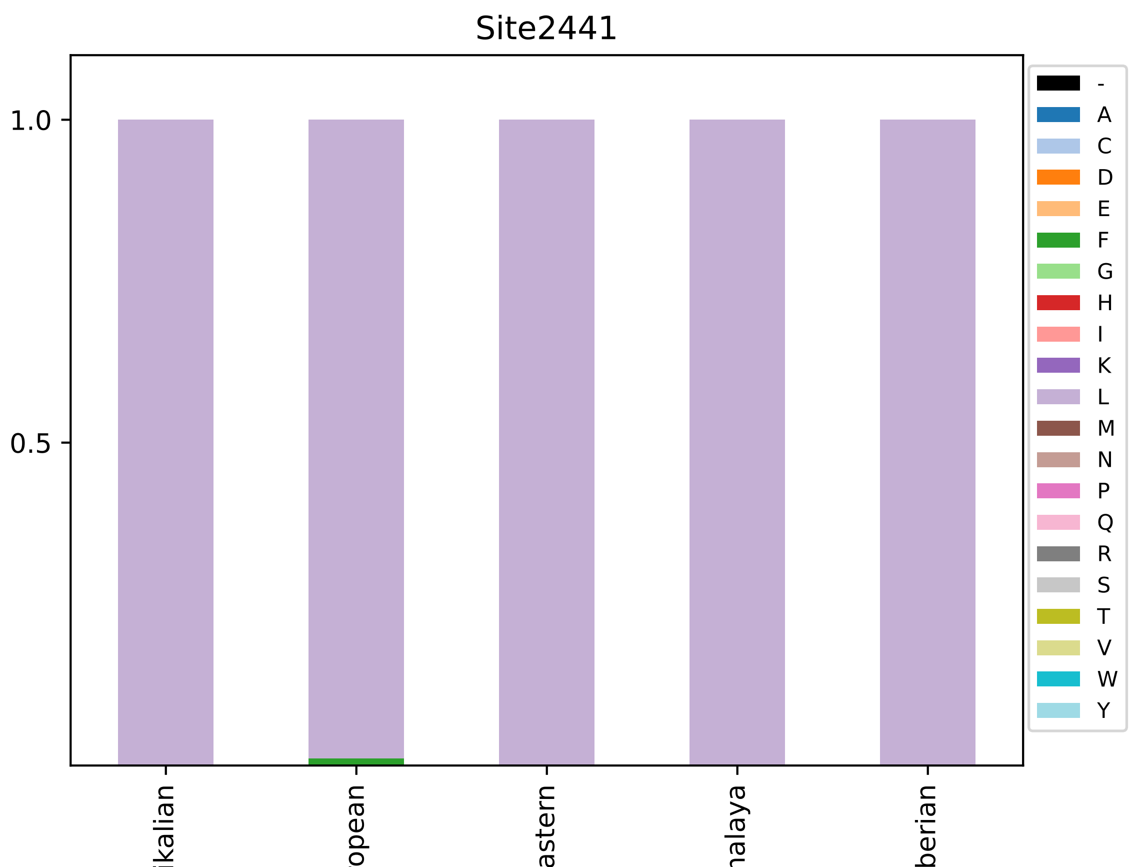
Site2441
1.0
0.5
-
A
C
D
E
F
G
H
I
K
L
M
N
P
Q
R
S
T
V
W
Y
ikalian
opean
astern
nalaya
berian

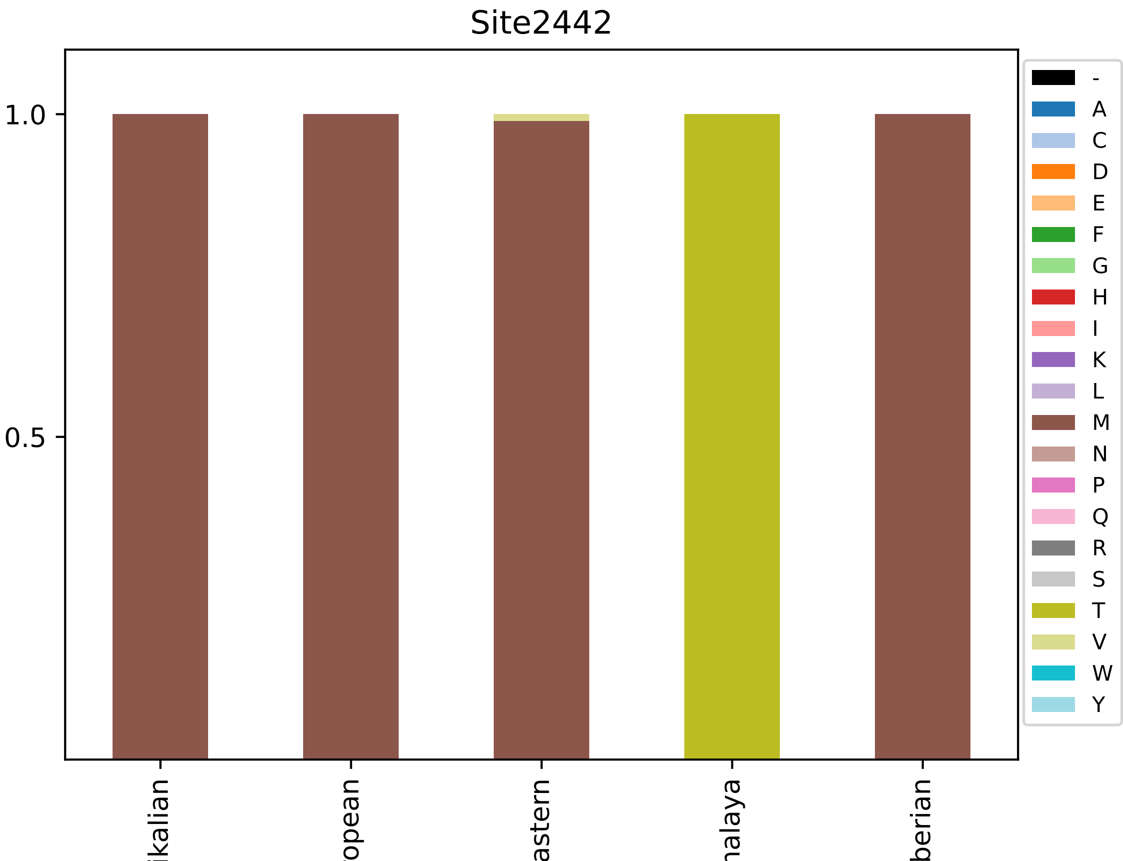

Site2442
1.0
0.5
-
A
C
D
E
F
G
H
I
K
L
M
N
P
Q
R
S
T
V
W
Y
ikalian
opean
astern
nalaya
berian

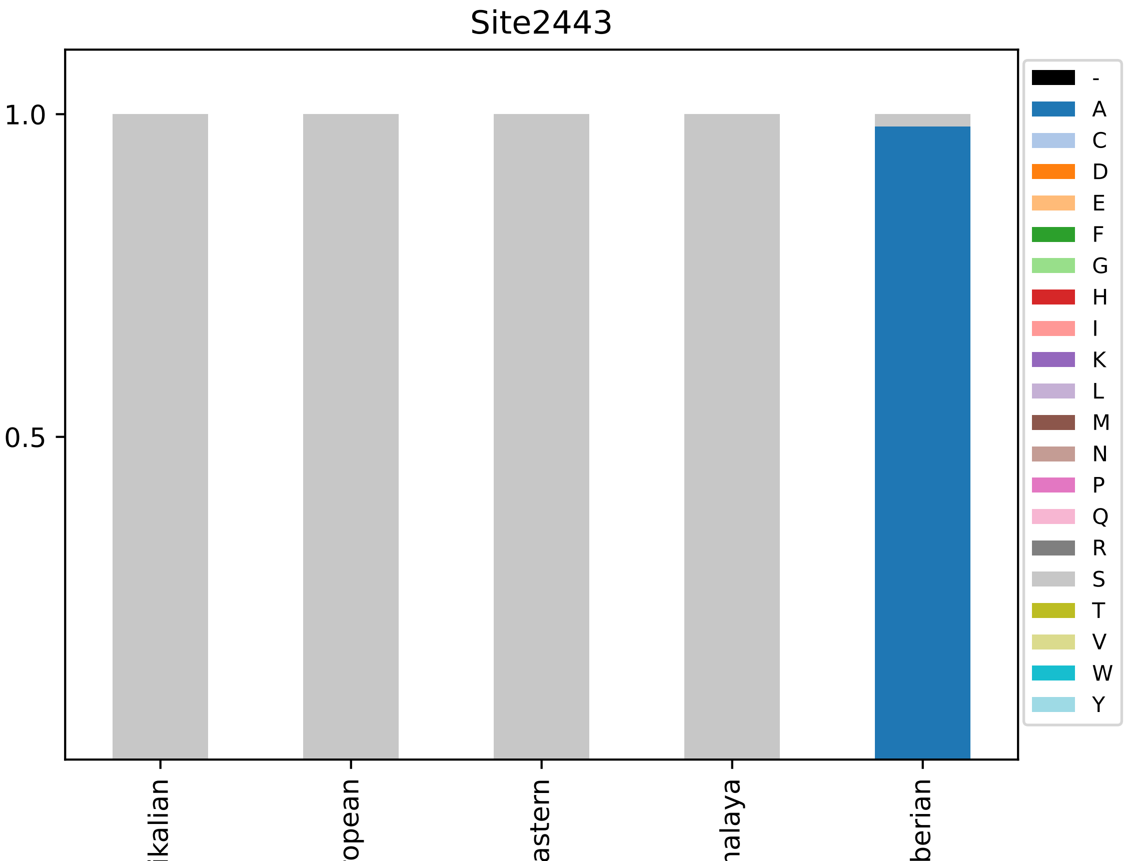

Site2443
1.0
0.5
-
A
C
D
E
F
G
H
I
K
L
M
N
P
Q
R
S
T
V
W
Y
ikalian
opean
astern
nalaya
berian

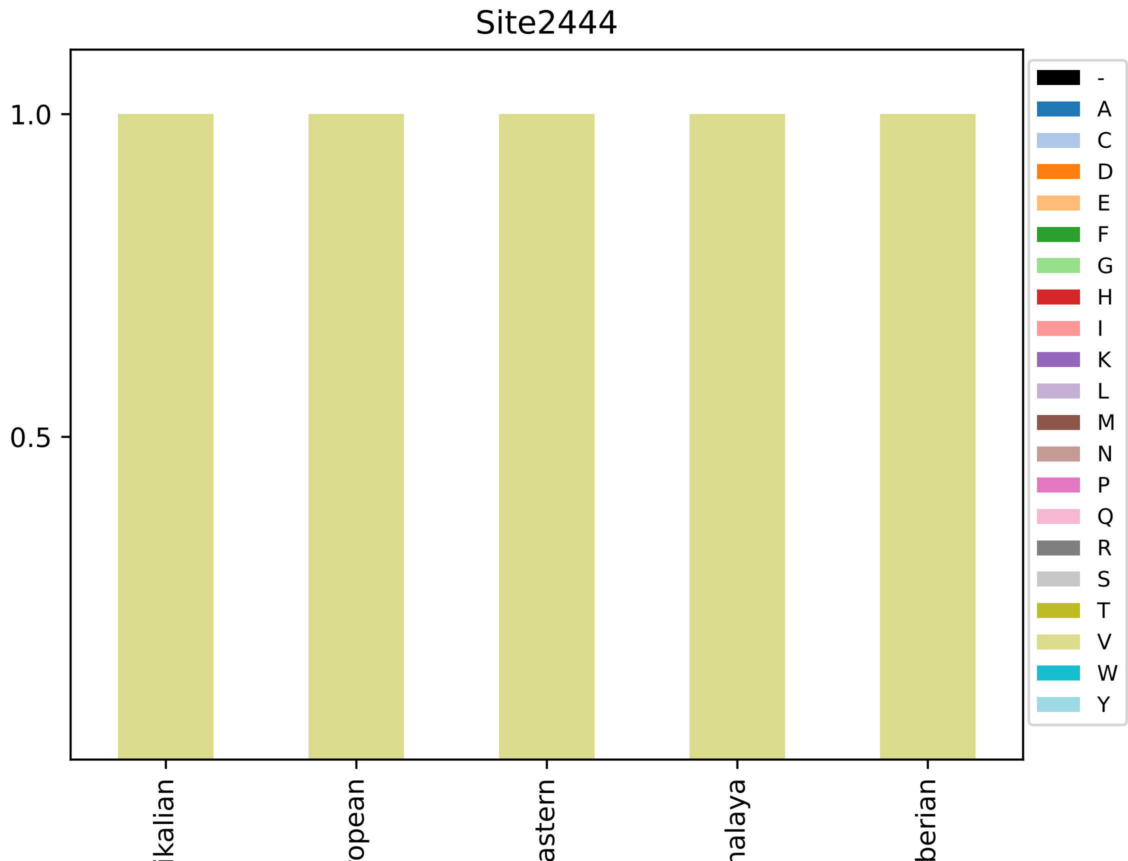
Site2444
1.0
0.5
-
A
C
D
E
F
G
H
I
K
L
M
N
P
Q
R
S
T
V
W
Y
ikalian
opean
astern
nalaya
berian

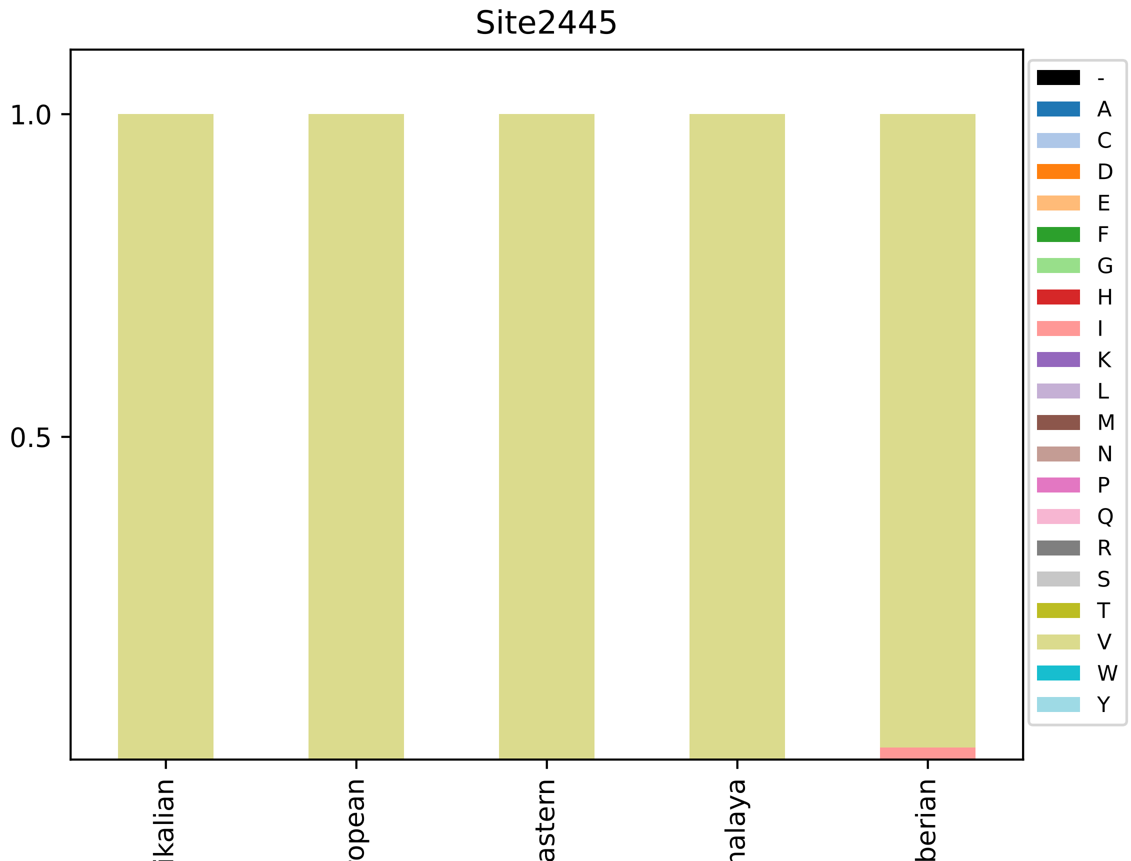

Site2445
1.0
0.5
ikalian
opean
astern
halaya
berian
-
A
C
D
E
F
G
H
I
K
L
M
N
P
Q
R
S
T
V
W
Y

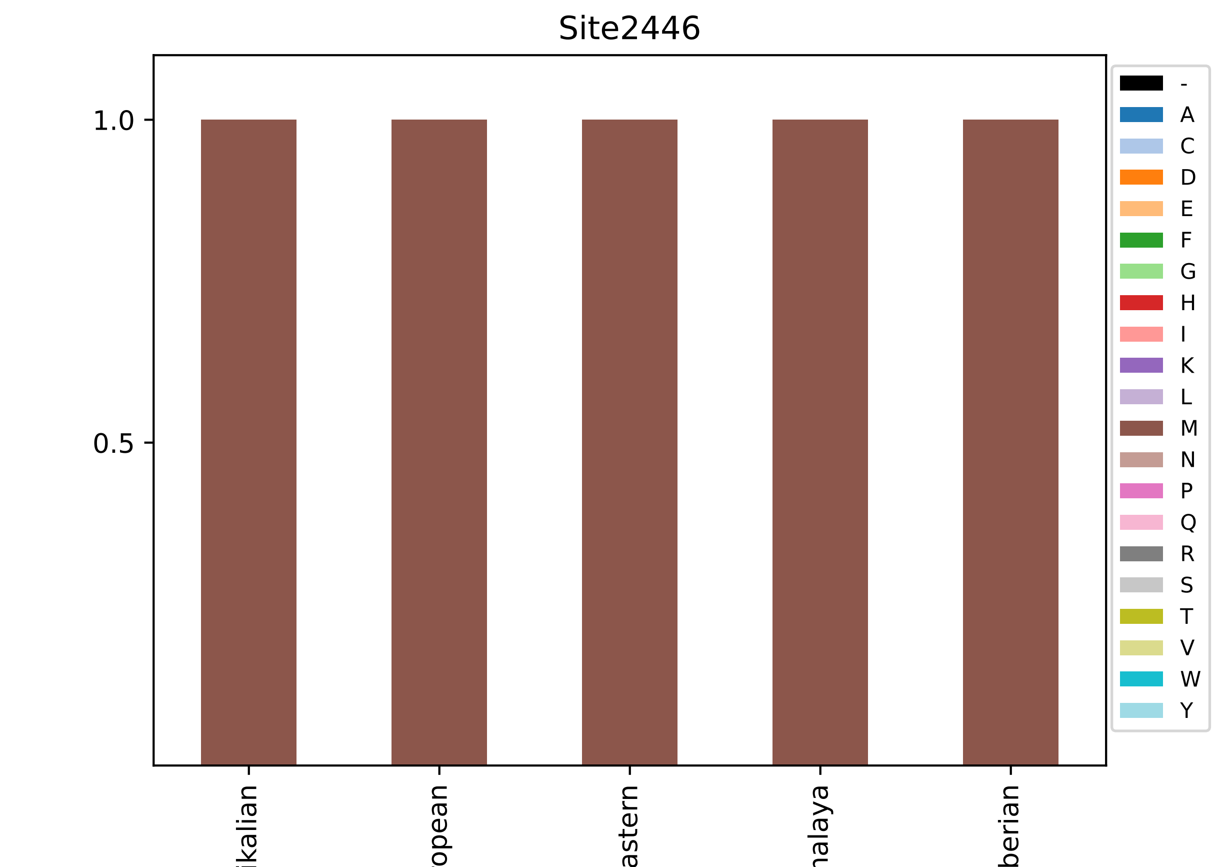

Site2446
1.0
0.5
-
A
C
D
E
F
G
H
I
K
L
M
N
P
Q
R
S
T
V
W
Y
ikalian
opean
astern
nalaya
berian

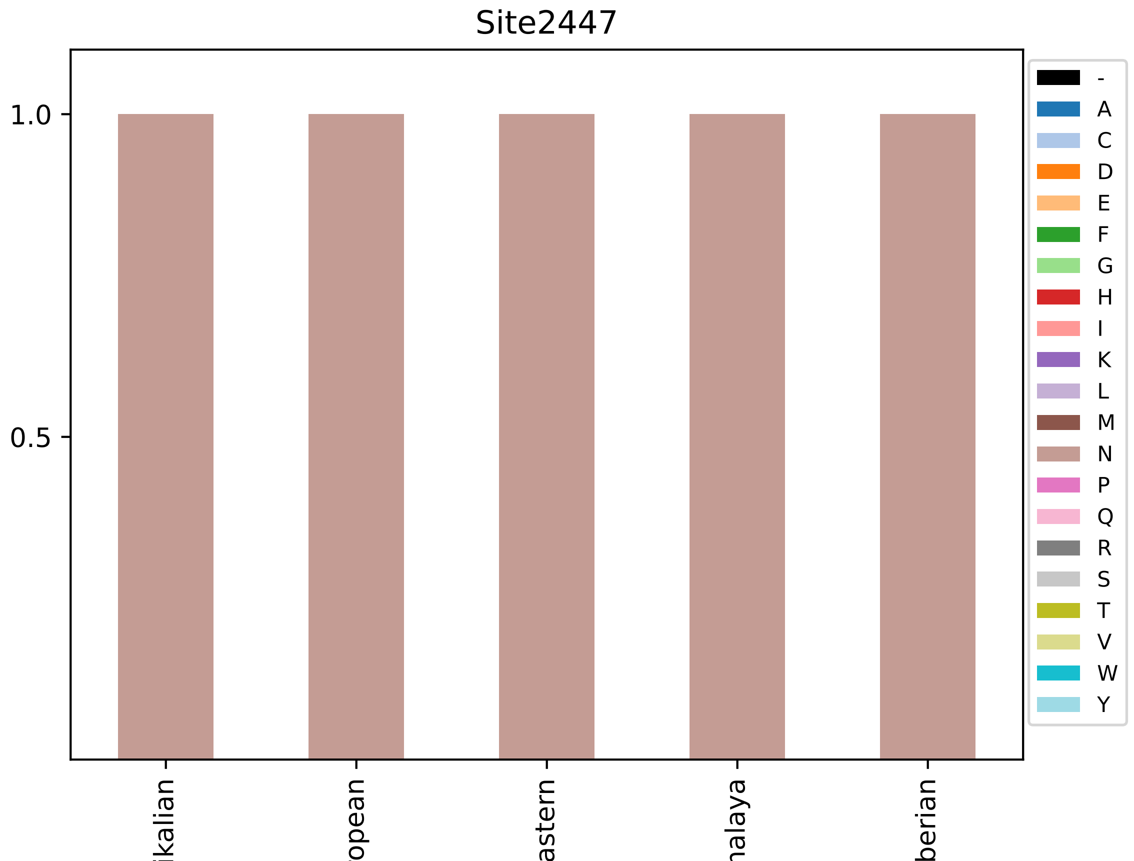
Site2447
1.0
0.5
ikalian
opean
astern
nalaya
berian
-
A
C
D
E
F
G
H
I
K
L
M
N
P
Q
R
S
T
V
W
Y

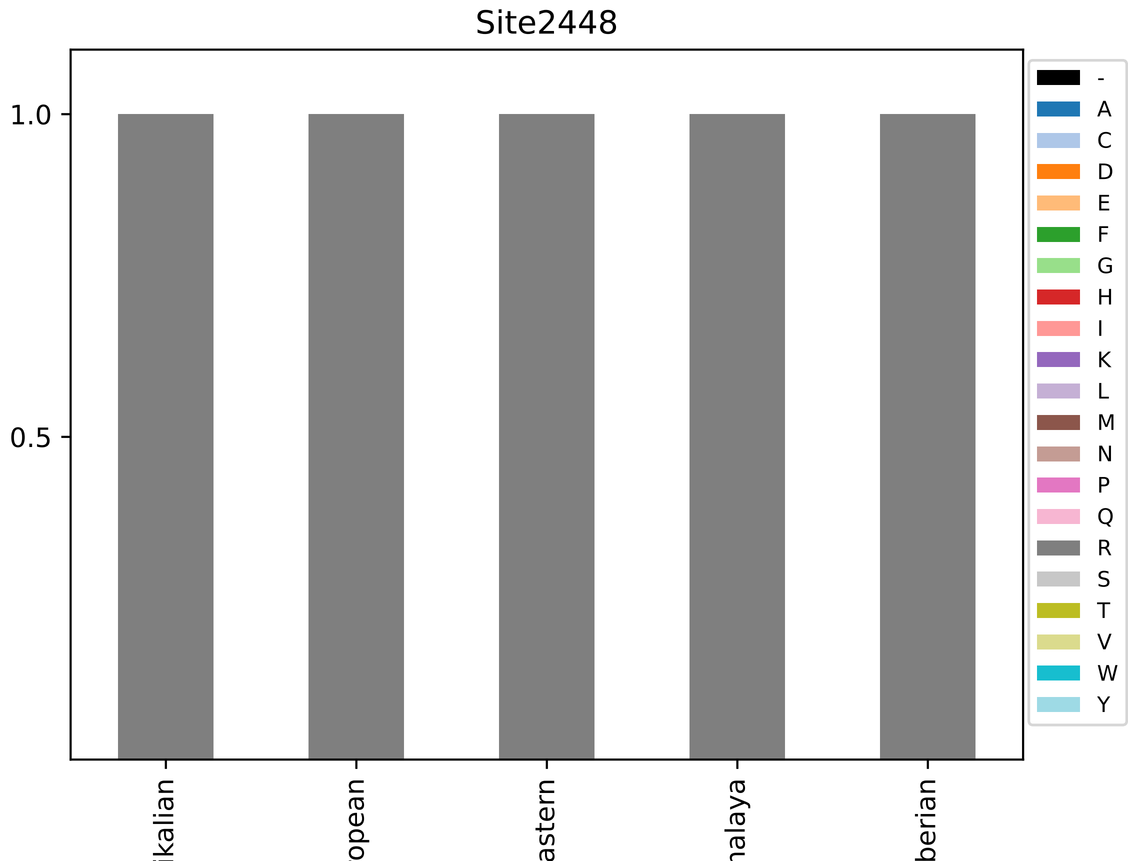

Site2448
1.0
0.5
-
A
C
D
E
F
G
H
I
K
L
M
N
P
Q
R
S
T
V
W
Y
ikalian
opean
astern
nalaya
berian

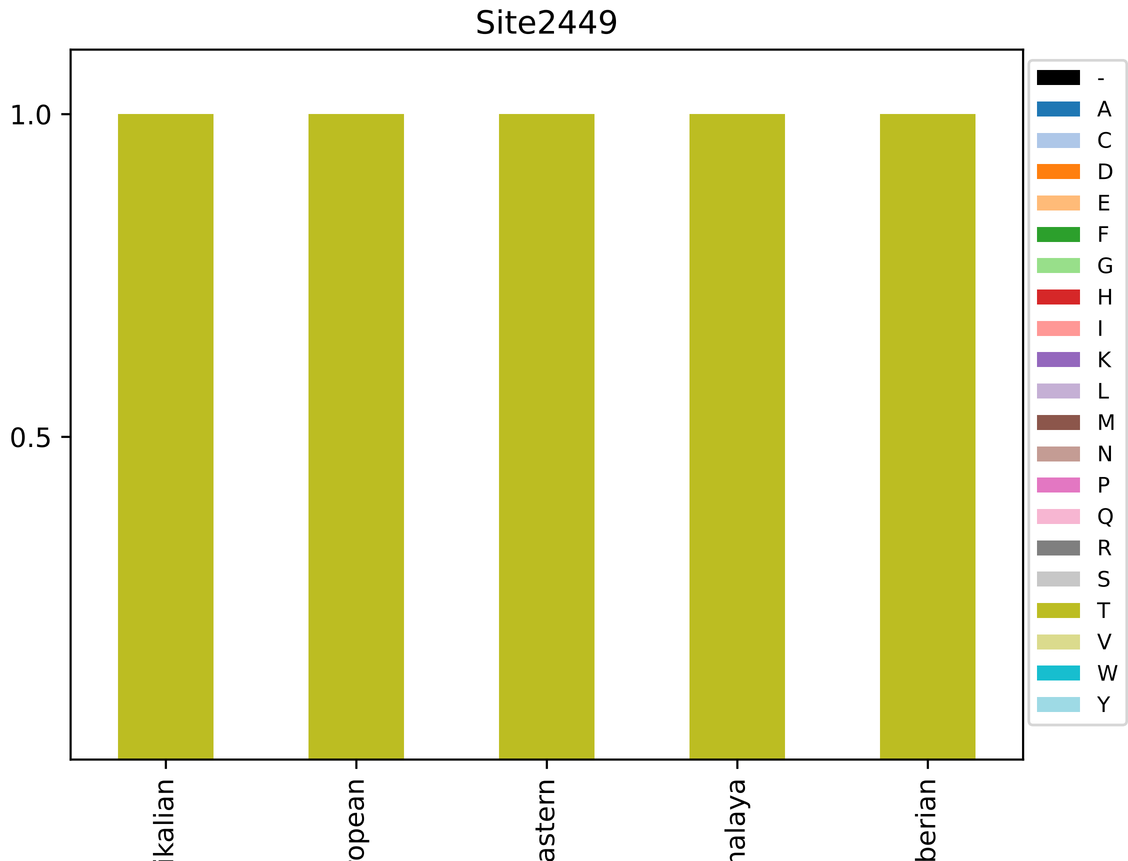
Site2449
1.0
0.5
-
A
C
D
E
F
G
H
I
K
L
M
N
P
Q
R
S
T
V
W
Y
ikalian
opean
astern
nalaya
berian

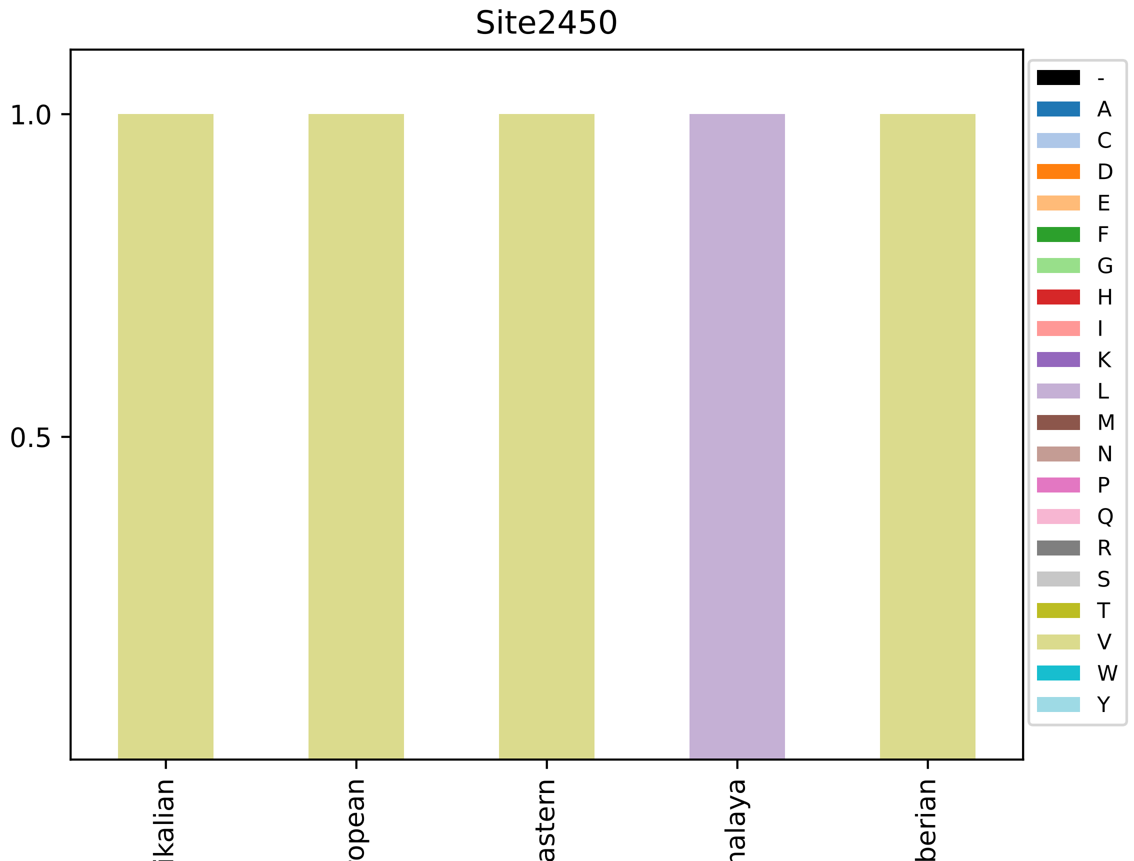

Site2450
1.0
0.5
-
A
C
D
E
F
G
H
I
K
L
M
N
P
Q
R
S
T
V
W
Y
ikalian
opean
astern
nalaya
berian

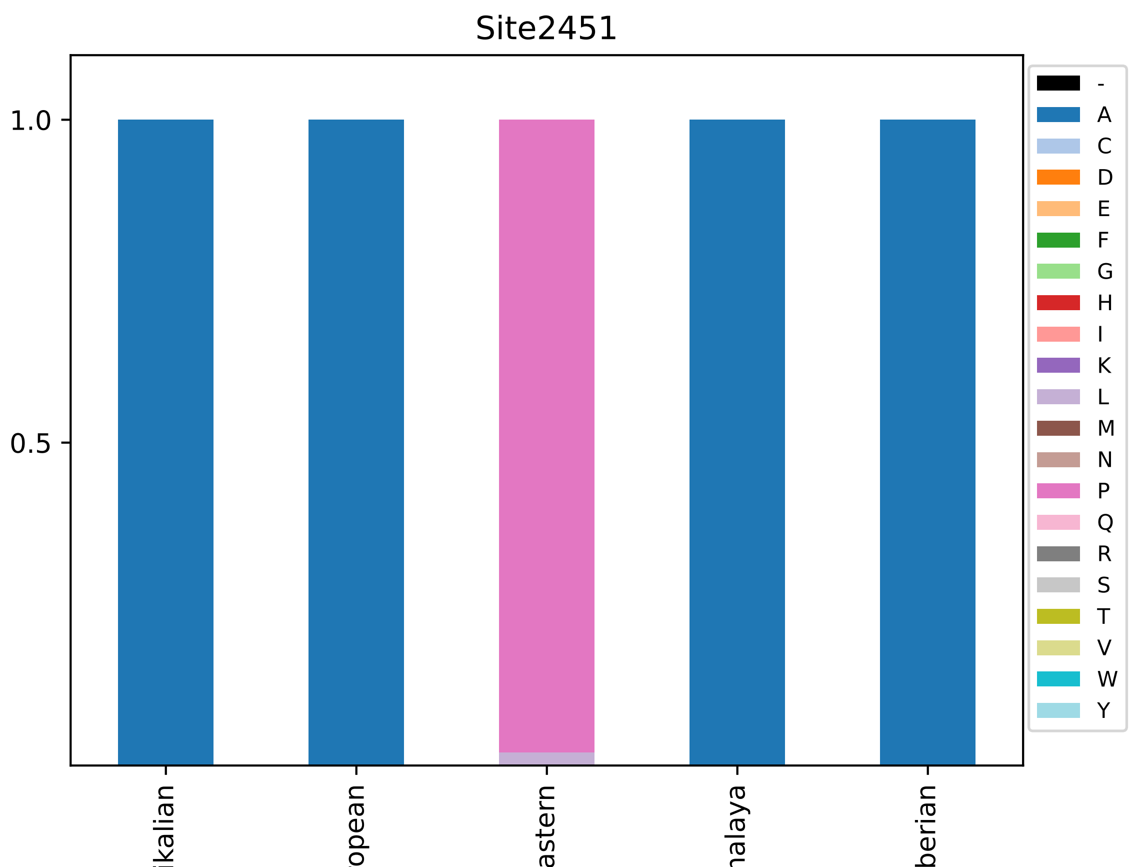

Site2451
1.0
0.5
-
A
C
D
E
F
G
H
I
K
L
M
N
P
Q
R
S
T
V
W
Y
ikalian
opean
astern
nalaya
berian

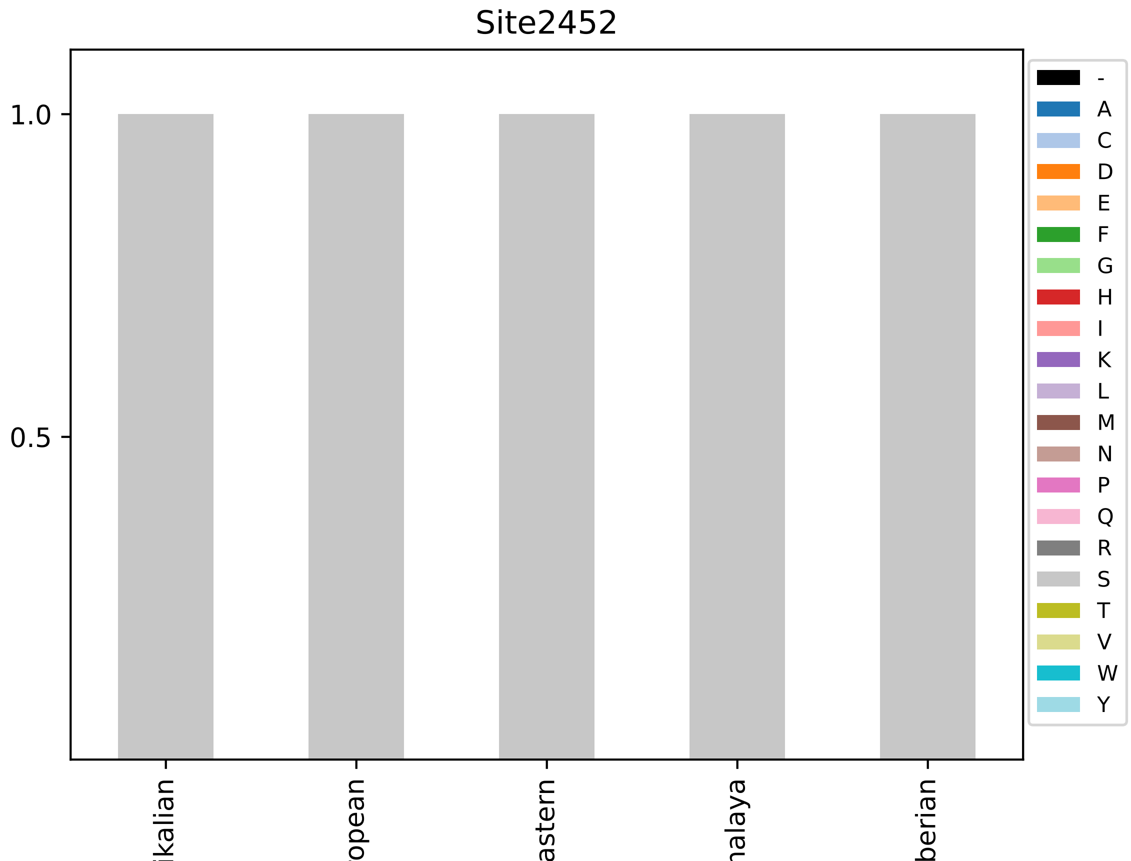

Site2452
1.0
0.5
-
A
C
D
E
F
G
H
I
K
L
M
N
P
Q
R
S
T
V
W
Y
ikalian
opean
astern
halaya
berian

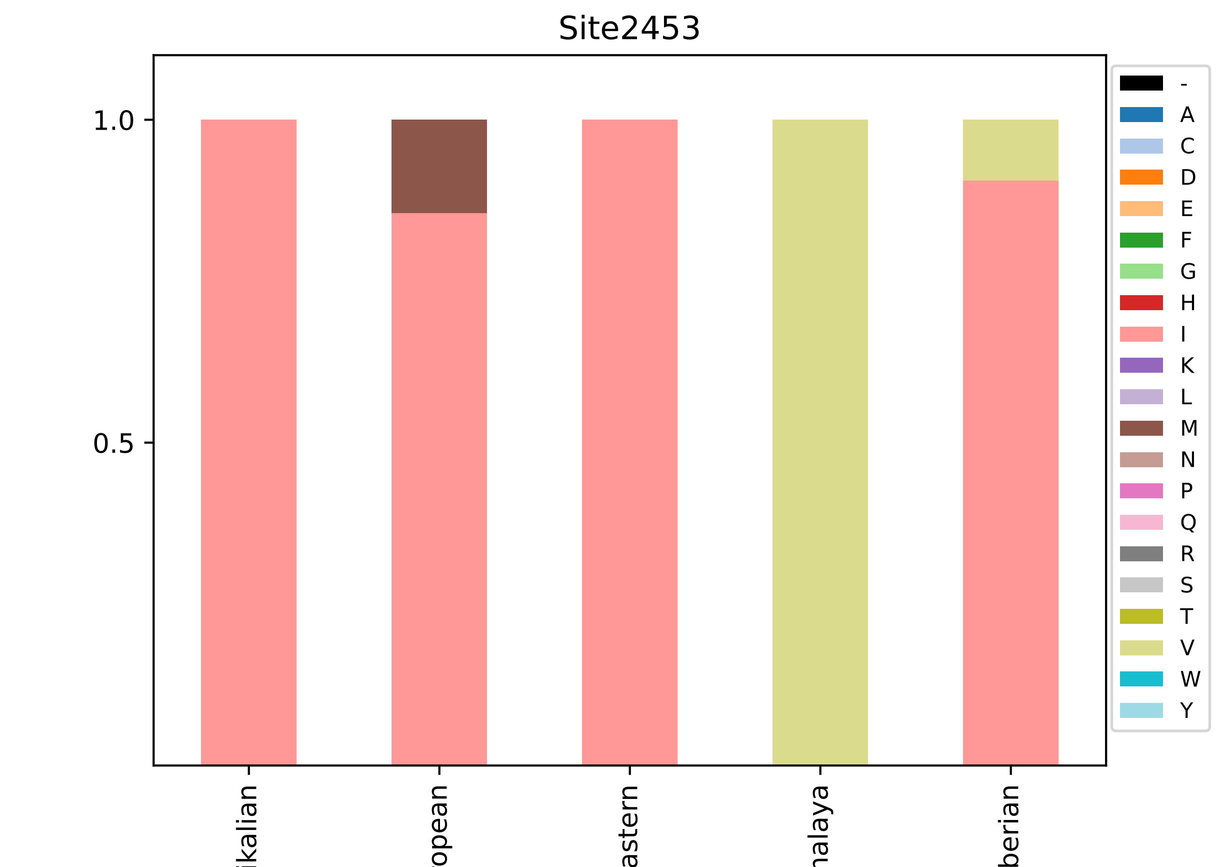
Site2453
1.0
0.5
ikalian
opean
astern
nalaya
berian
-
A
C
D
E
F
G
H
I
K
L
M
N
P
Q
R
S
T
V
W
Y

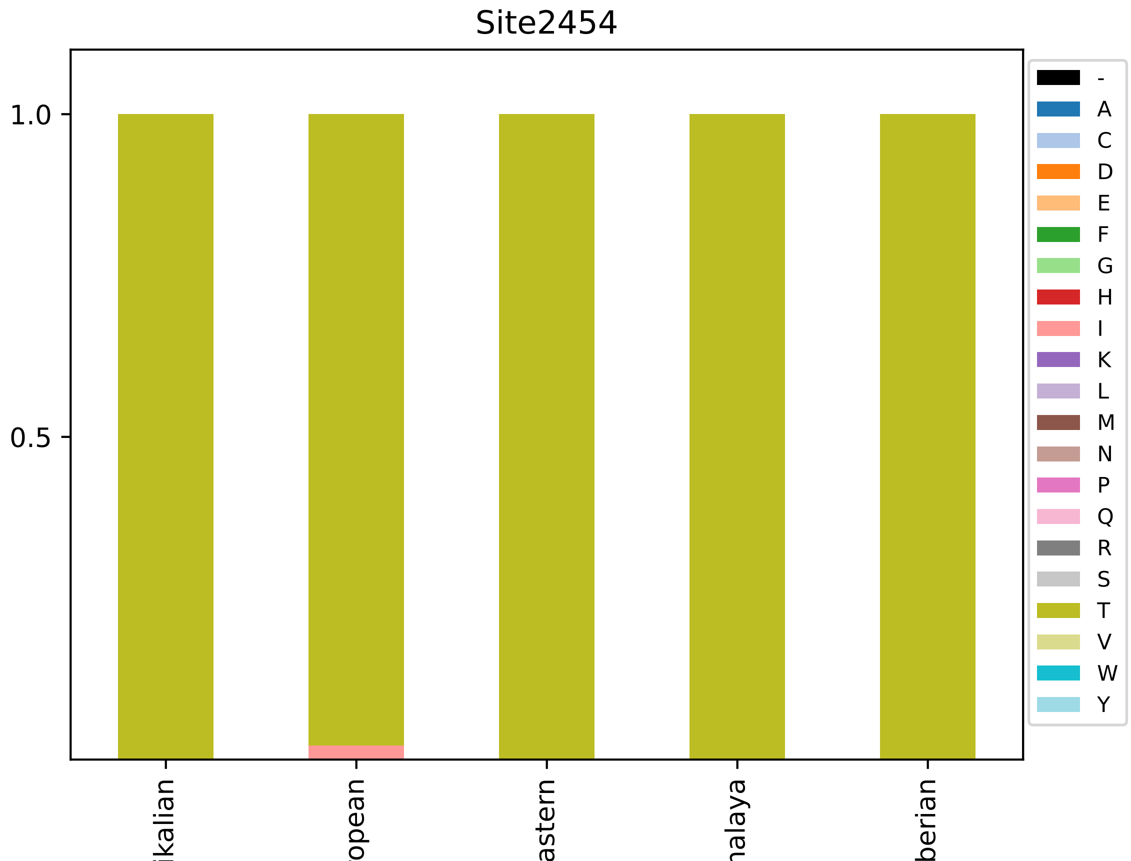

Site2454
1.0
0.5
-
A
C
D
E
F
G
H
I
K
L
M
N
P
Q
R
S
T
V
W
Y
ikalian
opean
astern
nalaya
berian

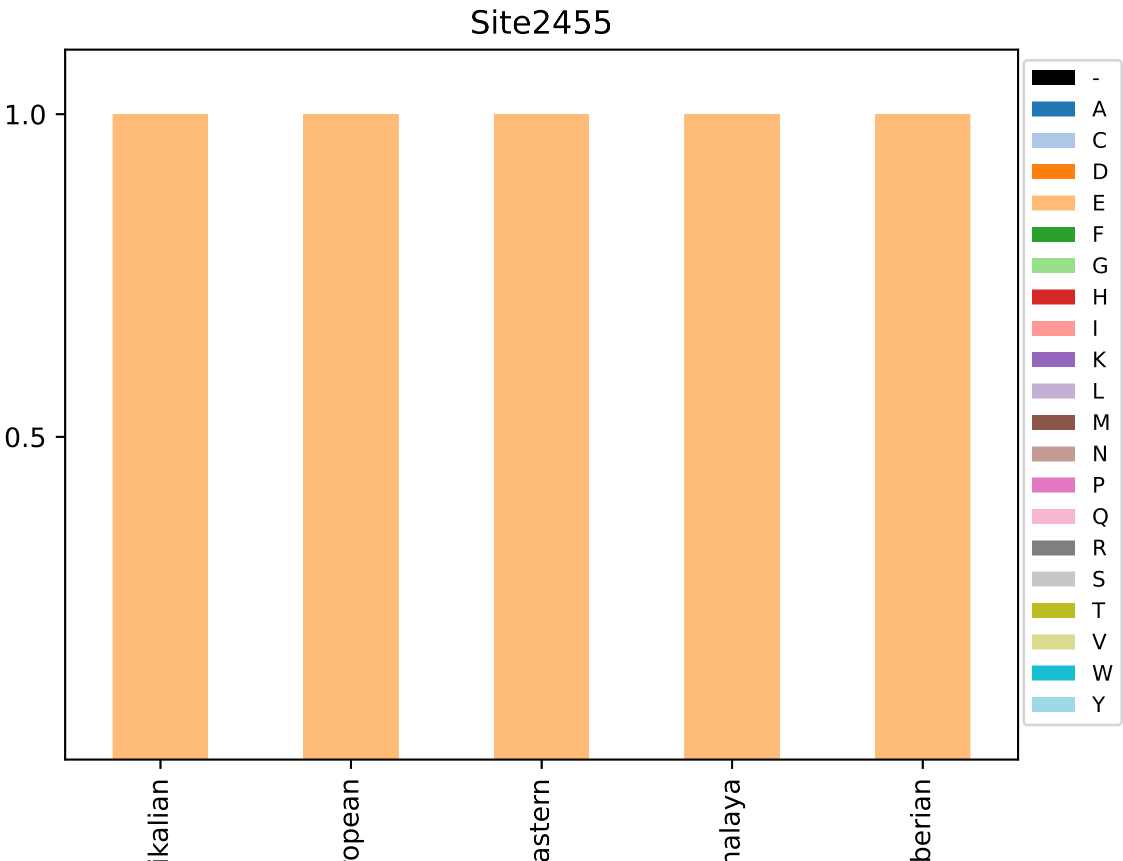
Site2455
1.0
0.5
-
A
C
D
E
F
G
H
I
K
L
M
N
P
Q
R
S
T
V
W
Y
ikalian
opean
astern
nalaya
berian

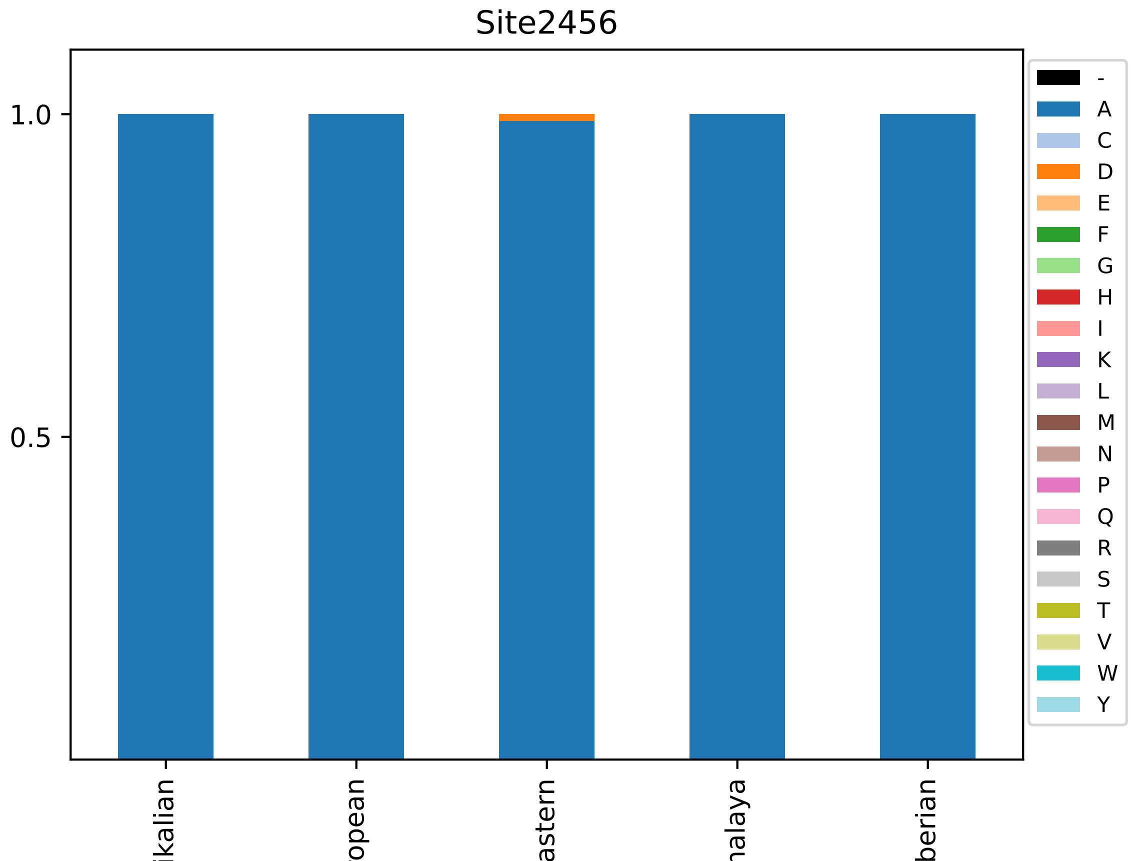

Site2456
1.0
0.5
-
A
C
D
E
F
G
H
I
K
L
M
N
P
Q
R
S
T
V
W
Y
ikalian
opean
astern
nalaya
berian

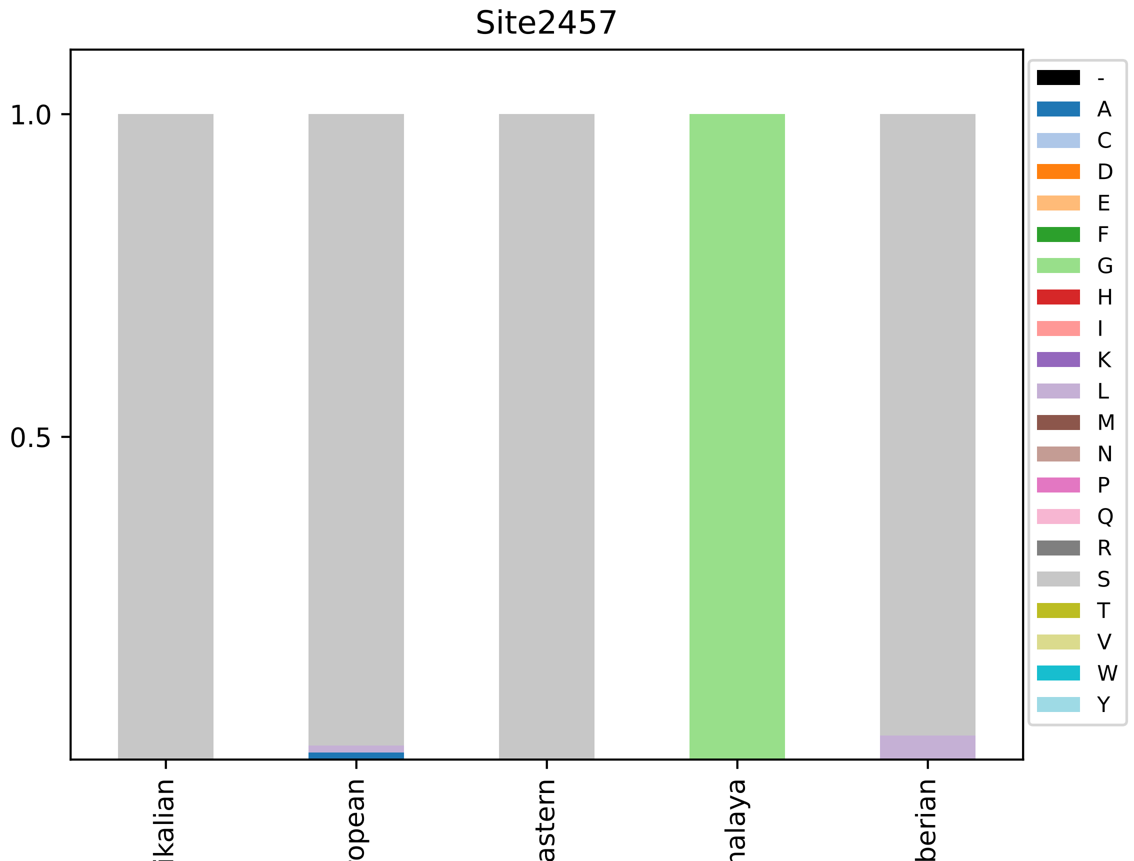

Site2457
1.0
0.5
-
A
C
D
E
F
G
H
I
K
L
M
N
P
Q
R
S
T
V
W
Y
ikalian
opean
astern
nalaya
berian

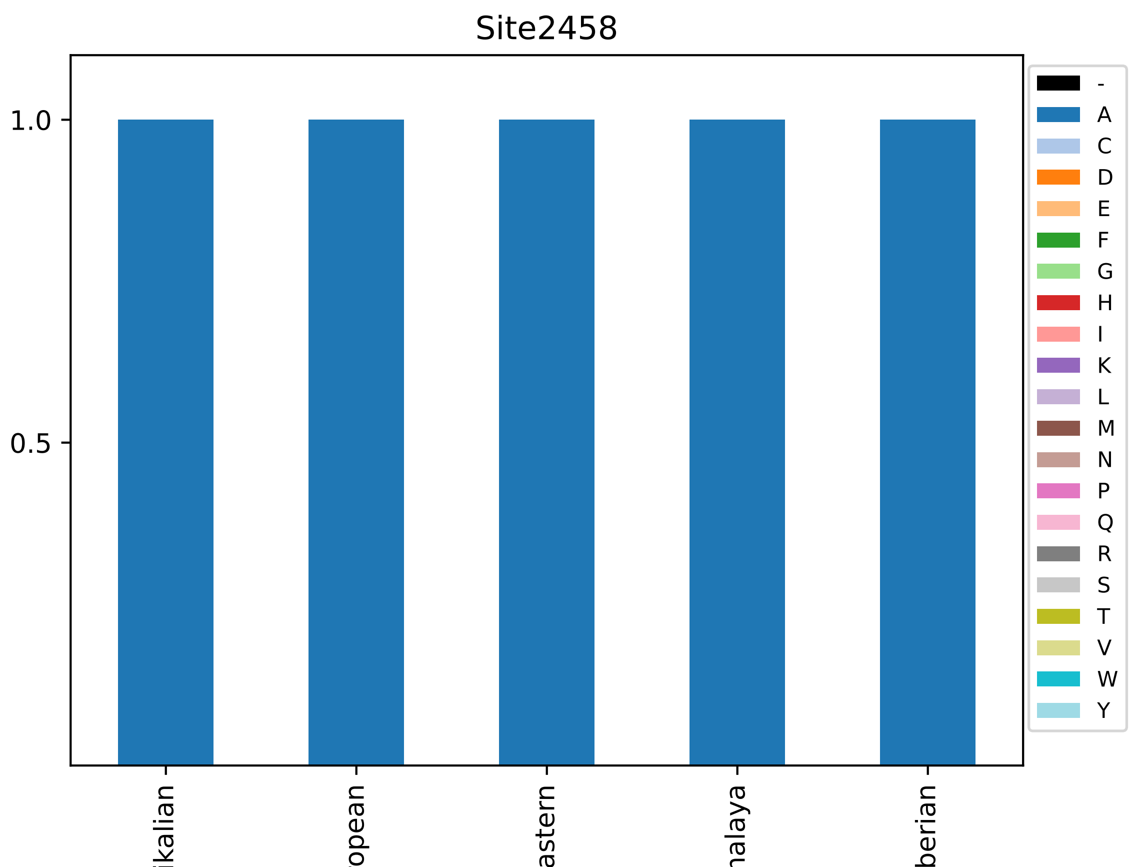
Site2458
1.0
0.5
-
A
C
D
E
F
G
H
I
K
L
M
N
P
Q
R
S
T
V
W
Y
ikalian
opean
astern
nalaya
berian

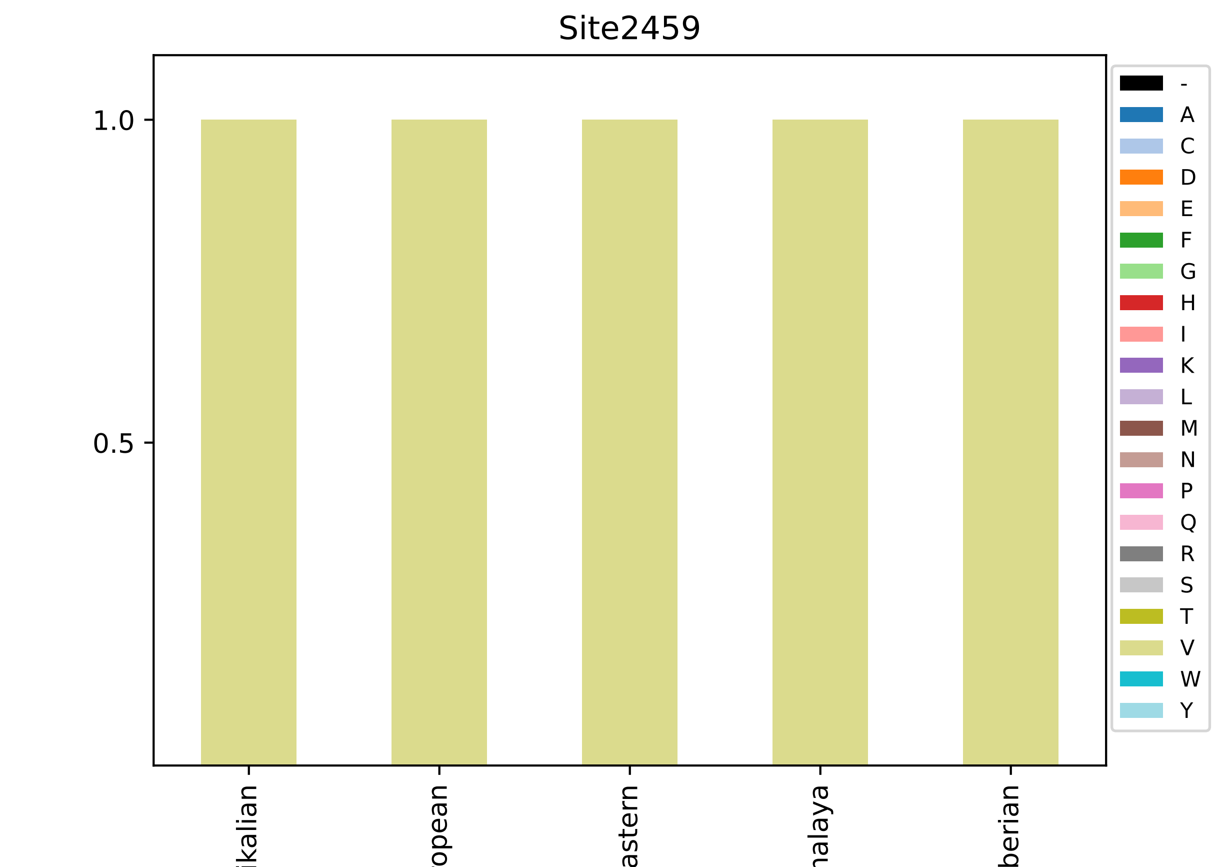
Site2459
1.0
0.5
-
A
C
D
E
F
G
H
I
K
L
M
N
P
Q
R
S
T
V
W
Y
ikalian
opean
astern
nalaya
berian

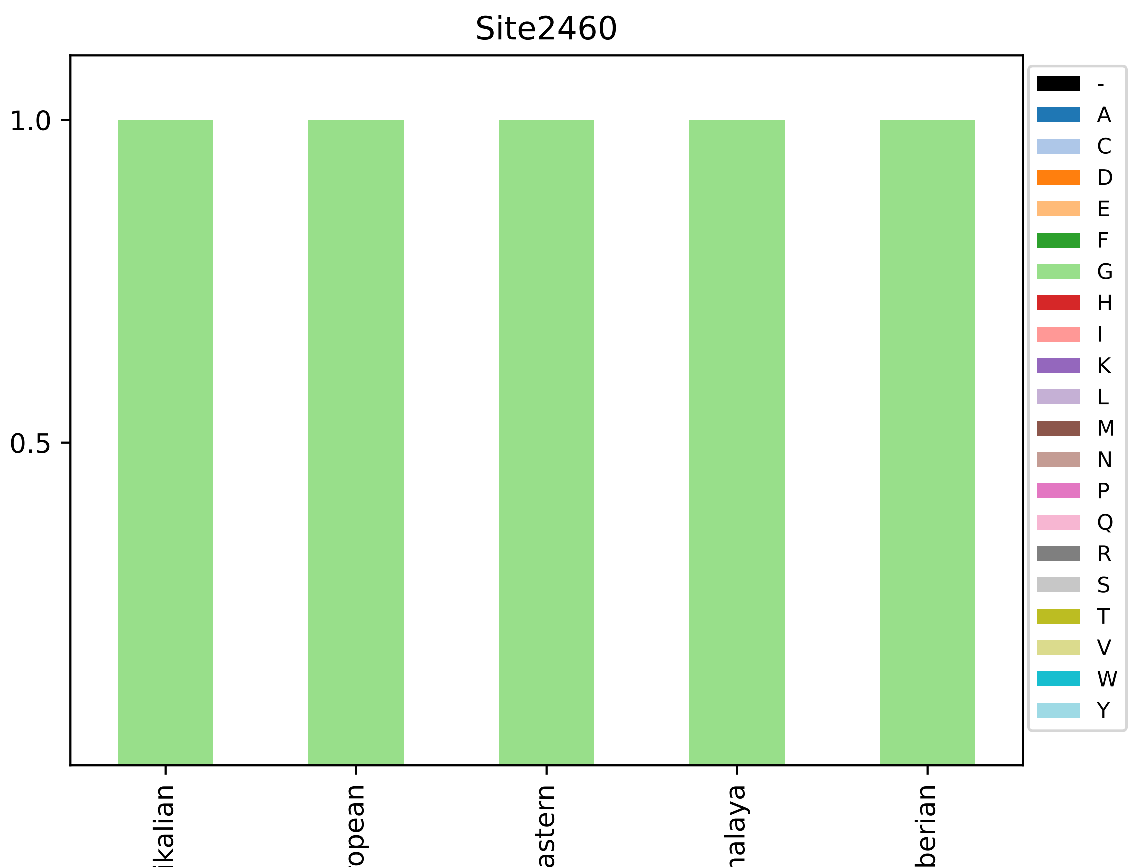

Site2460
1.0
0.5
ikalian
opean
astern
nalaya
berian
-
A
C
D
E
F
G
H
I
K
L
M
N
P
Q
R
S
T
V
W
Y

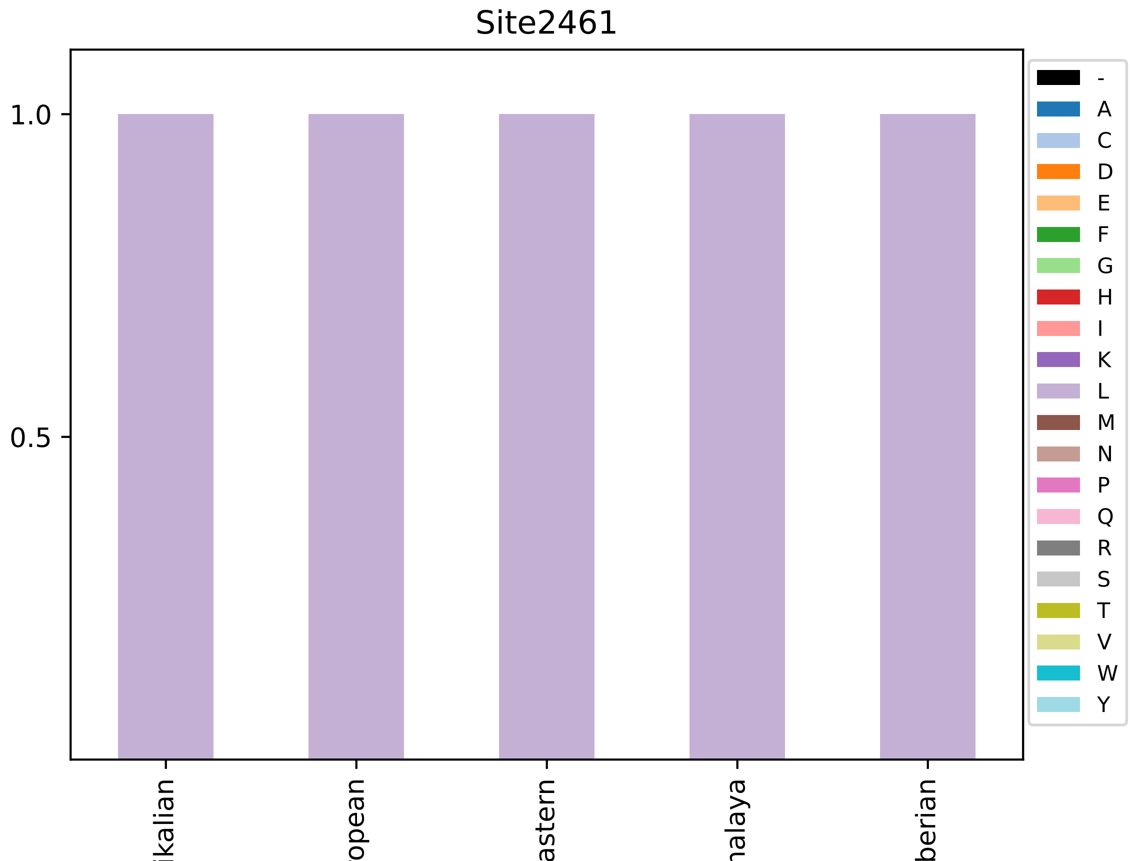

Site2461
1.0
0.5
-
A
C
D
E
F
G
H
I
K
L
M
N
P
Q
R
S
T
V
W
Y
ikalian
opean
astern
nalaya
berian

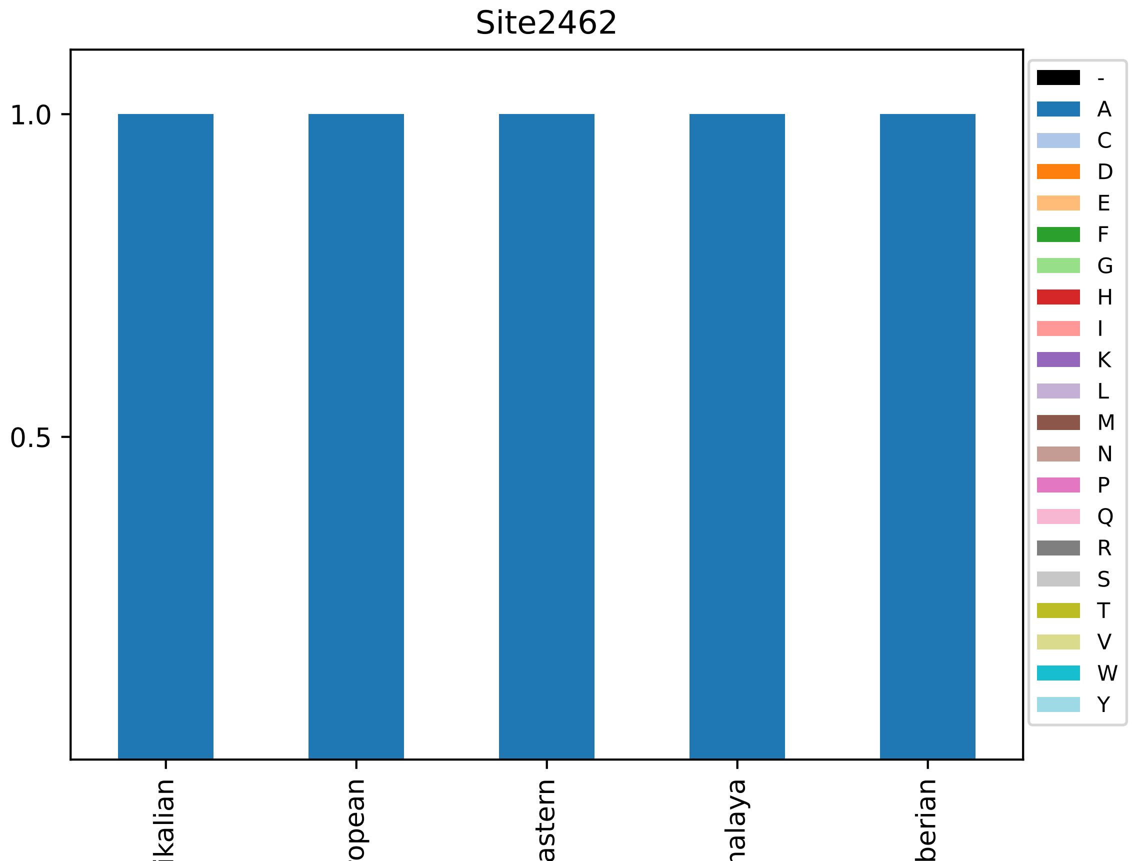

Site2462
1.0
0.5
-
A
C
D
E
F
G
H
I
K
L
M
N
P
Q
R
S
T
V
W
Y
ikalian
ropean
astern
nalaya
berian

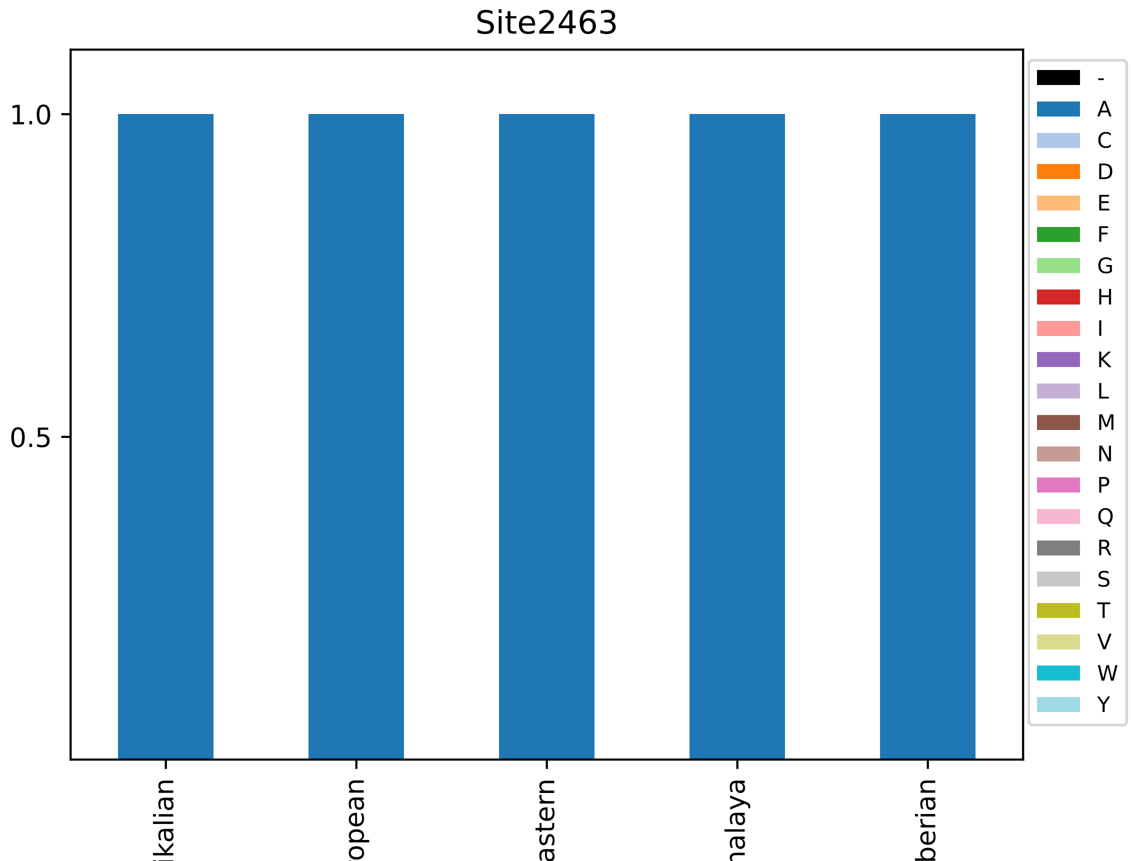
Site2463
1.0
0.5
-
A
C
D
E
F
G
H
I
K
L
M
N
P
Q
R
S
T
V
W
Y
ikalian
opean
astern
nalaya
berian

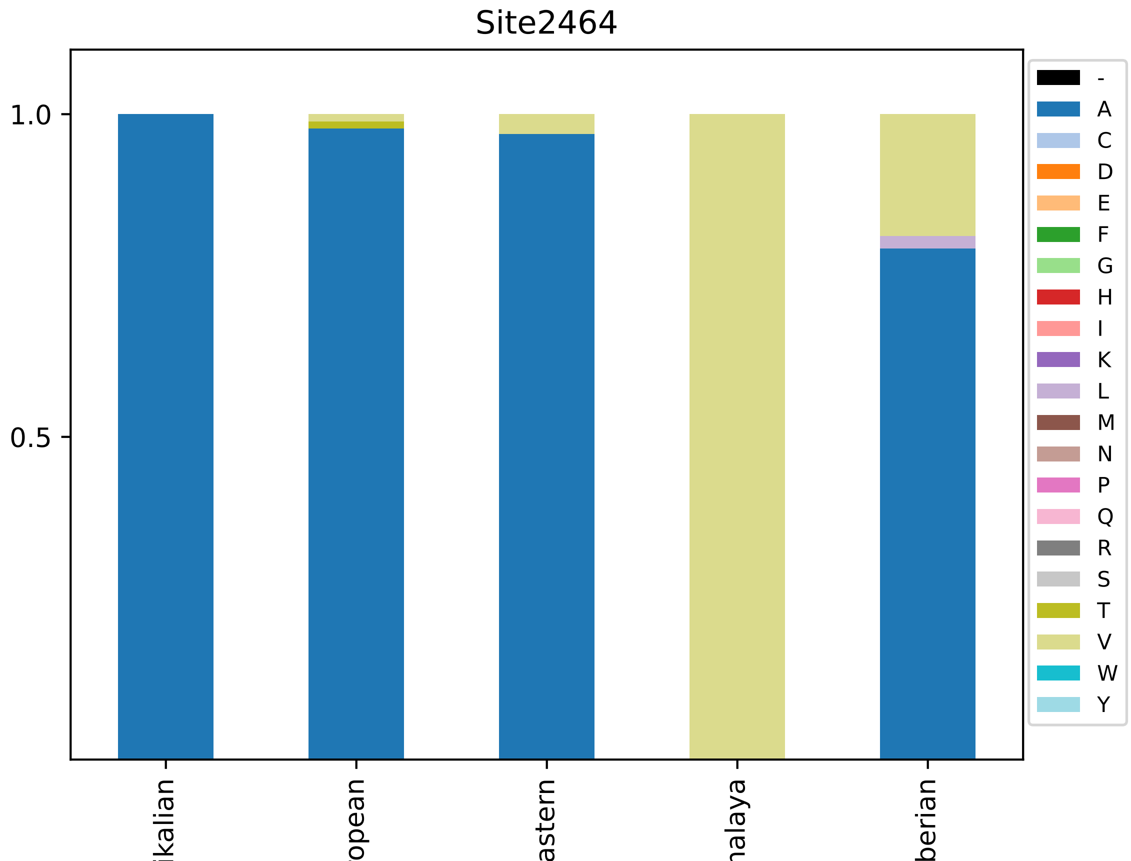
Site2464
1.0
0.5
-
A
C
D
E
F
G
H
I
K
L
M
N
P
Q
R
S
T
V
W
Y
ikalian
opean
astern
nalaya
berian

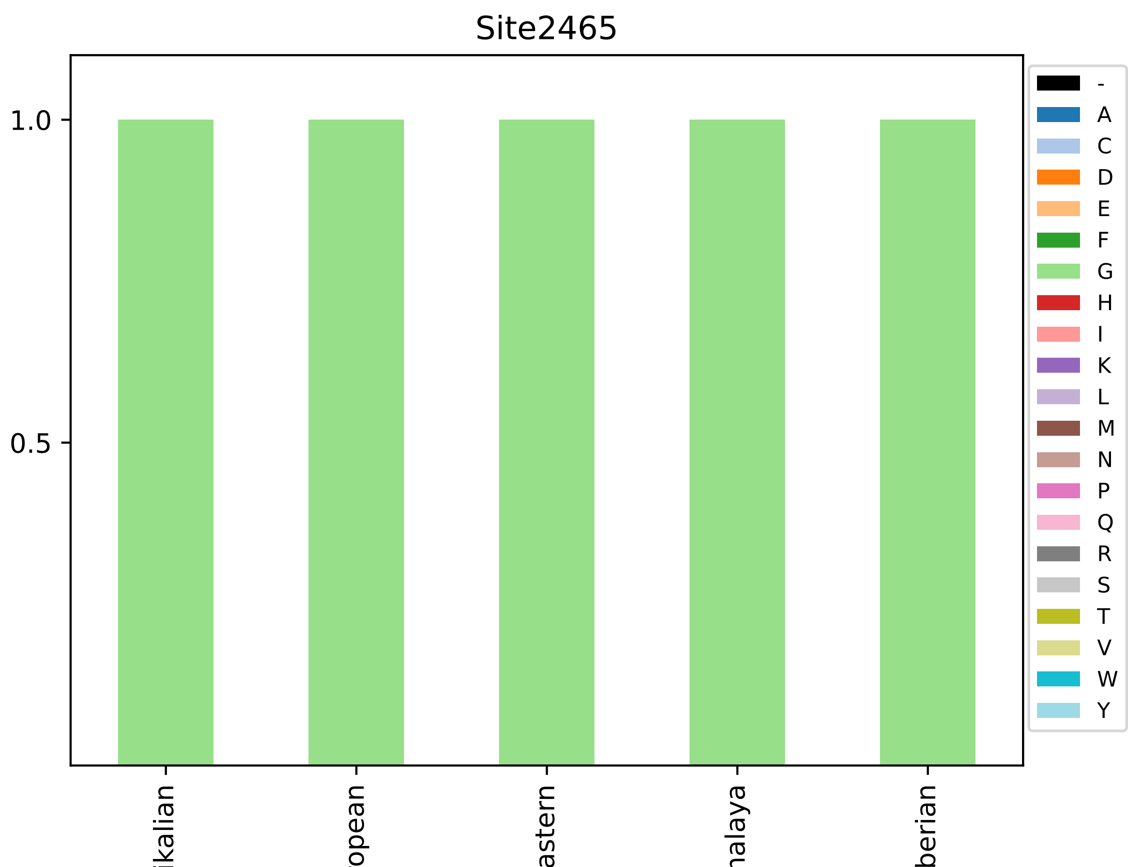

Site2465
1.0
0.5
-
A
C
D
E
F
G
H
I
K
L
M
N
P
Q
R
S
T
V
W
Y
ikalian
opean
astern
nalaya
berian

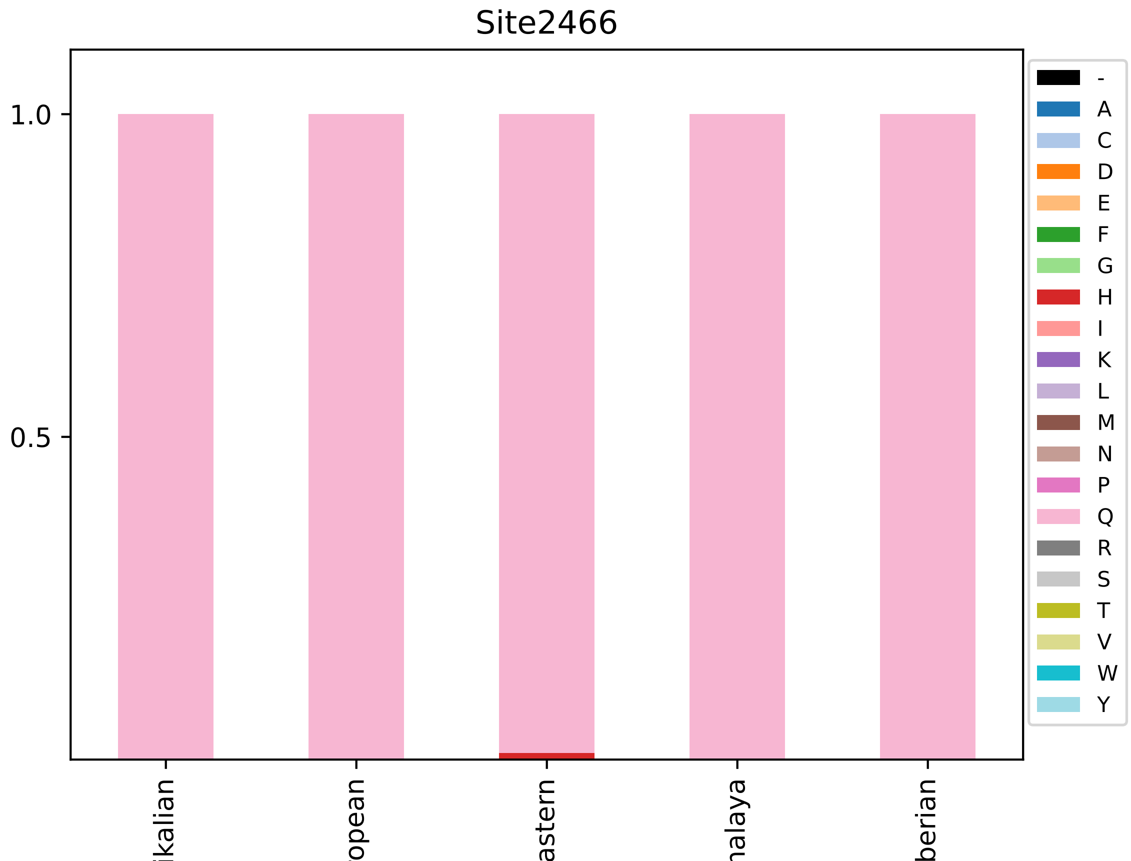

Site2466
1.0
0.5
-
A
C
D
E
F
G
H
I
K
L
M
N
P
Q
R
S
T
V
W
Y
ikalian
opean
astern
nalaya
berian

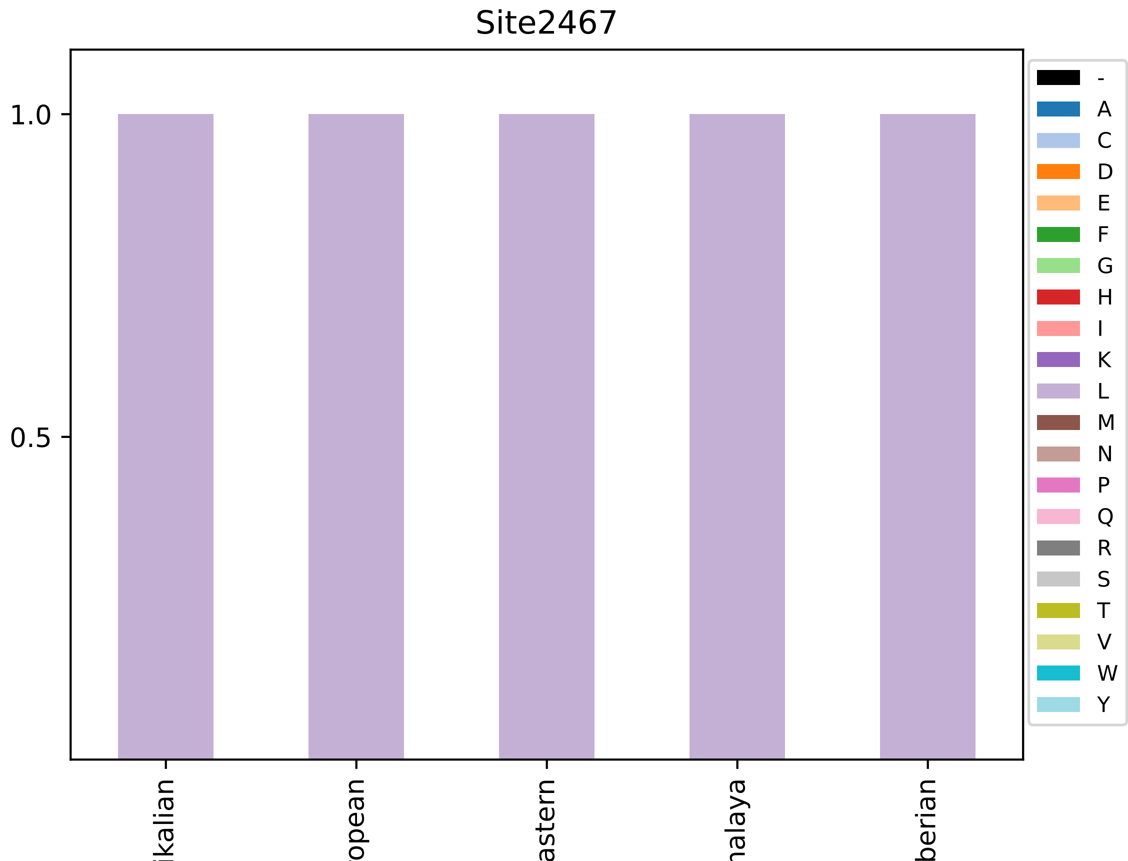
Site2467
1.0
0.5
-
A
C
D
E
F
G
H
I
K
L
M
N
P
Q
R
S
T
V
W
Y
ikalian
ropean
astern
nalaya
berian

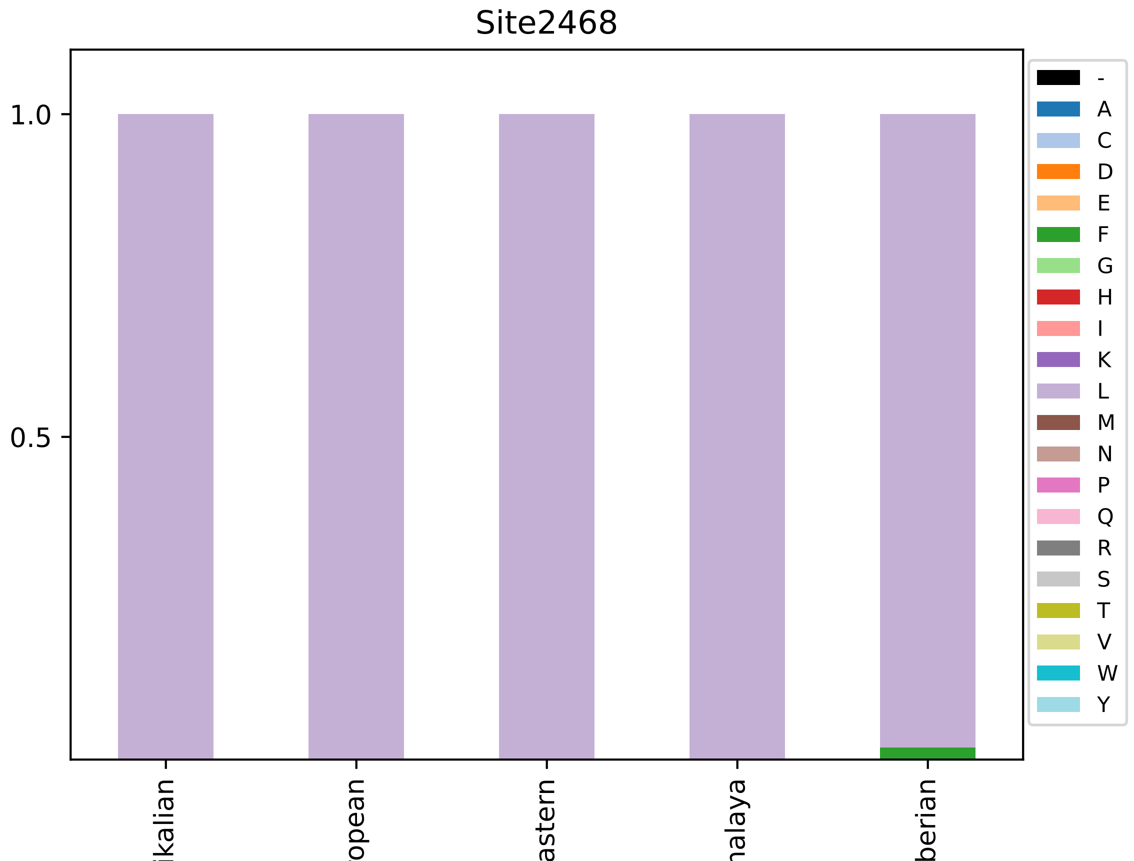

Site2468
1.0
0.5
-
A
C
D
E
F
G
H
I
K
L
M
N
P
Q
R
S
T
V
W
Y
ikalian
opean
astern
nalaya
berian

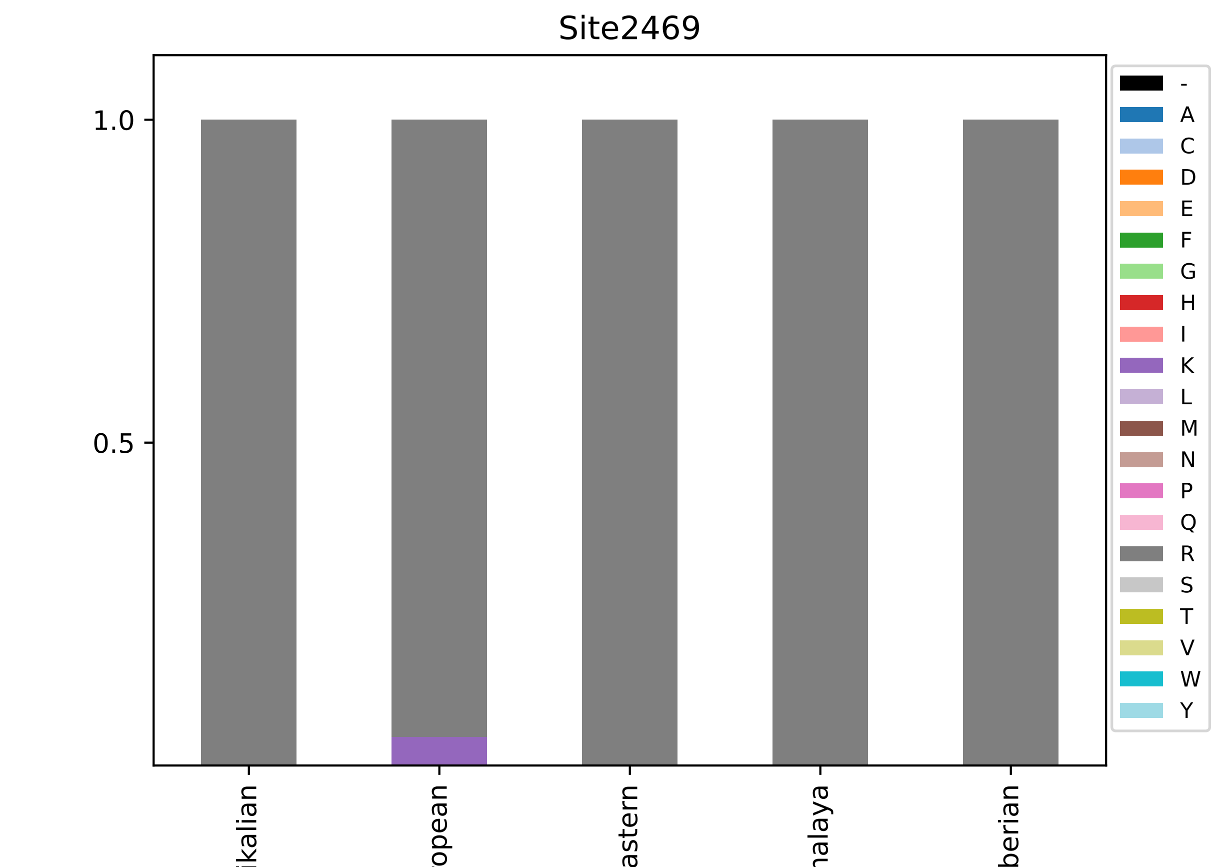
Site2469
1.0
0.5
-
A
C
D
E
F
G
H
I
K
L
M
N
P
Q
R
S
T
V
W
Y
ikalian
opean
astern
nalaya
berian

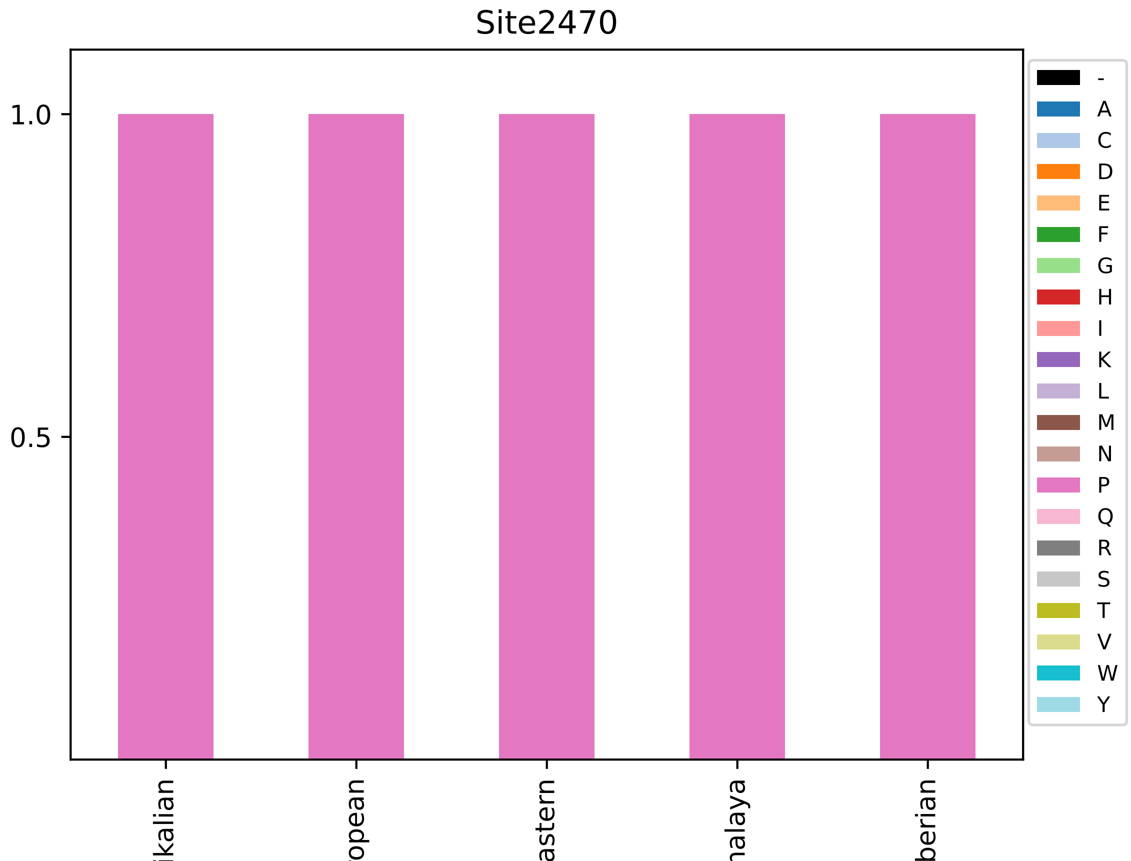

Site2470
1.0
0.5
-
A
C
D
E
F
G
H
I
K
L
M
N
P
Q
R
S
T
V
W
Y
ikalian
opean
astern
nalaya
berian

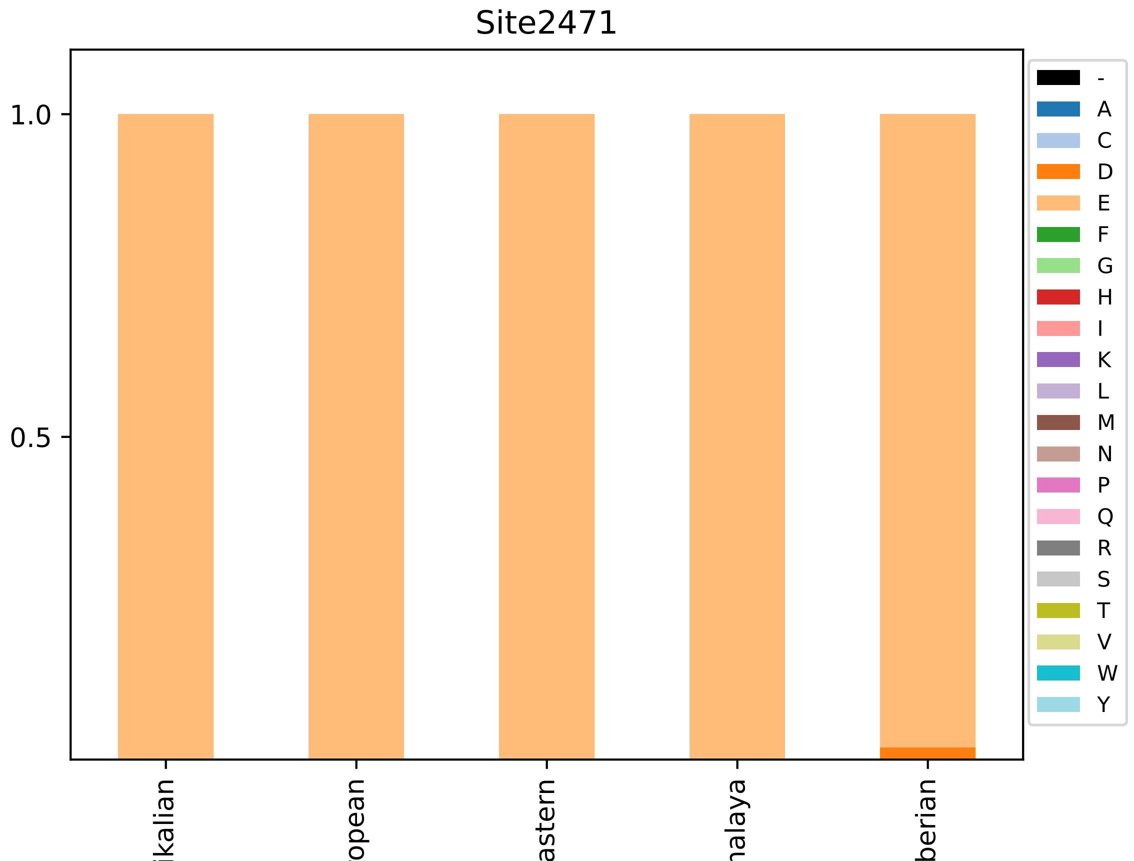

Site2471
1.0
0.5
-
A
C
D
E
F
G
H
I
K
L
M
N
P
Q
R
S
T
V
W
Y
ikalian
opean
astern
nalaya
berian

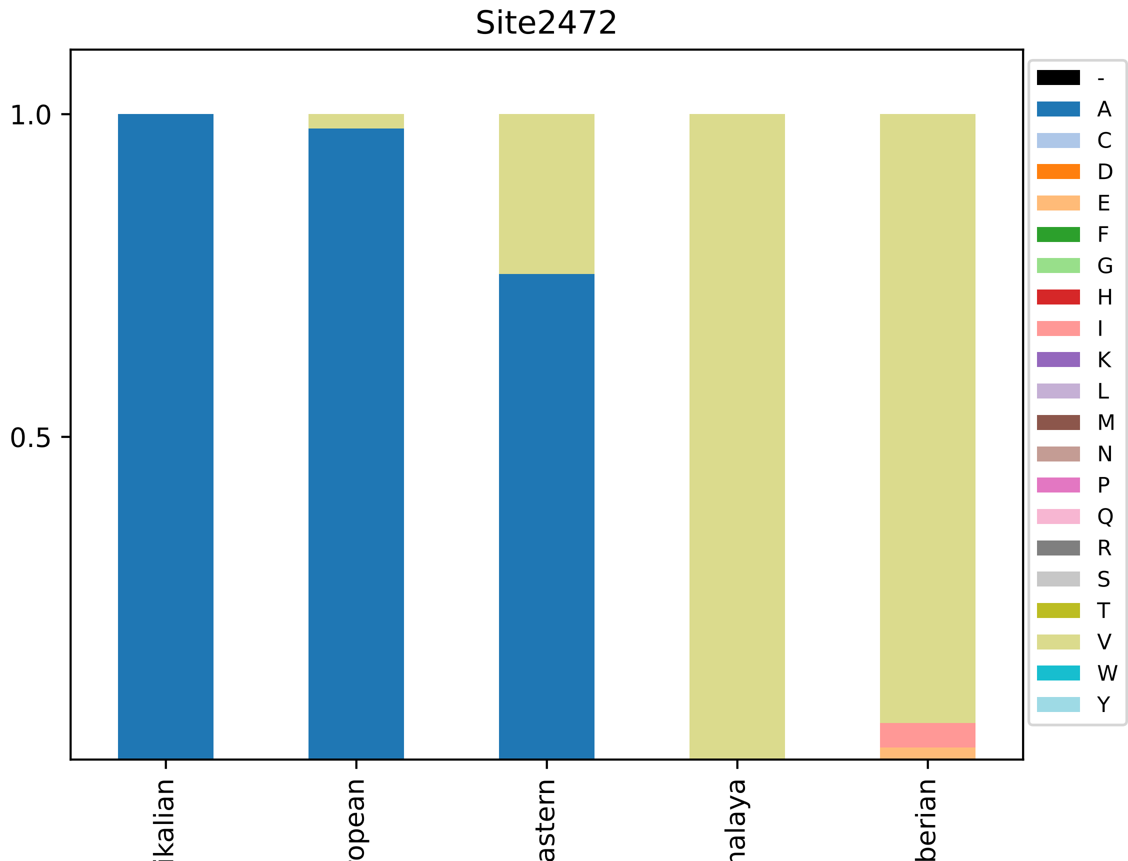

Site2472
1.0
0.5
-
A
C
D
E
F
G
H
I
K
L
M
N
P
Q
R
S
T
V
W
Y
ikalian
opean
astern
nalaya
berian

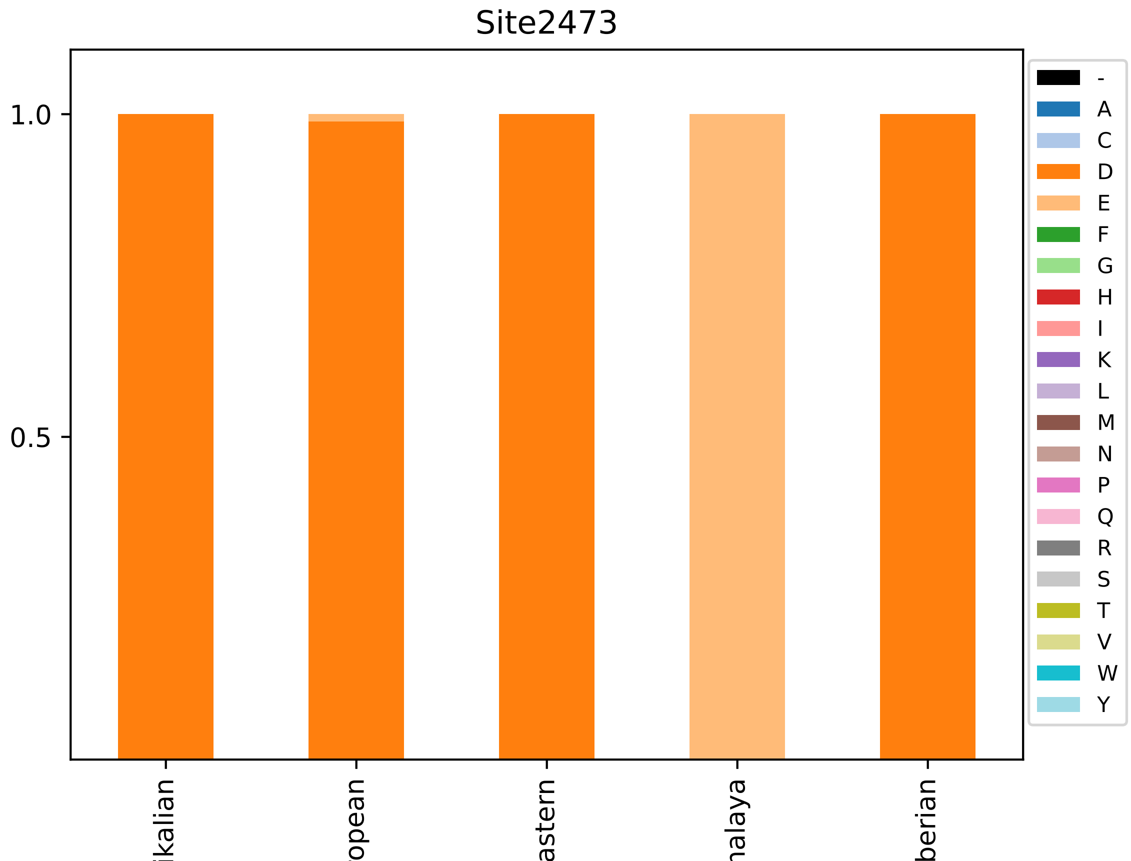

Site2473
1.0
0.5
-
A
C
D
E
F
G
H
I
K
L
M
N
P
Q
R
S
T
V
W
Y
ikalian
opean
astern
nalaya
berian

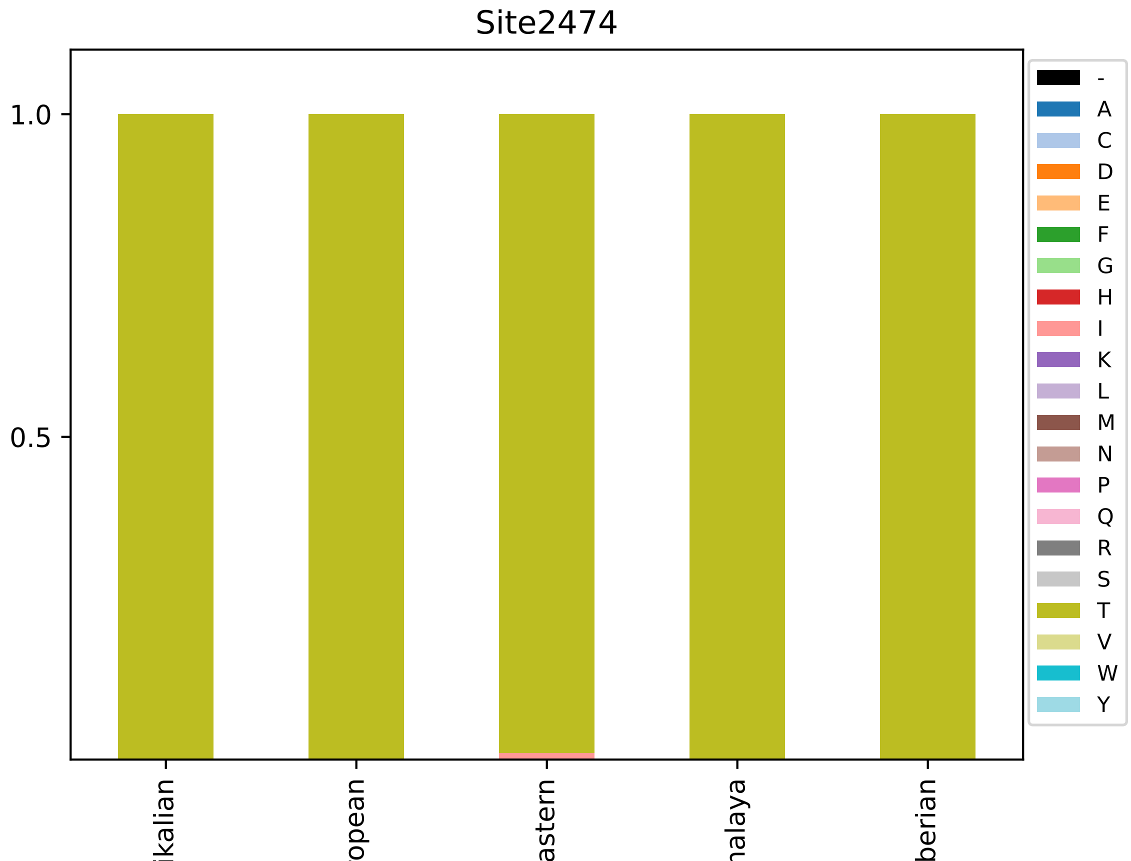

Site2474
1.0
0.5
-
A
C
D
E
F
G
H
I
K
L
M
N
P
Q
R
S
T
V
W
Y
ikalian
opean
astern
nalaya
berian

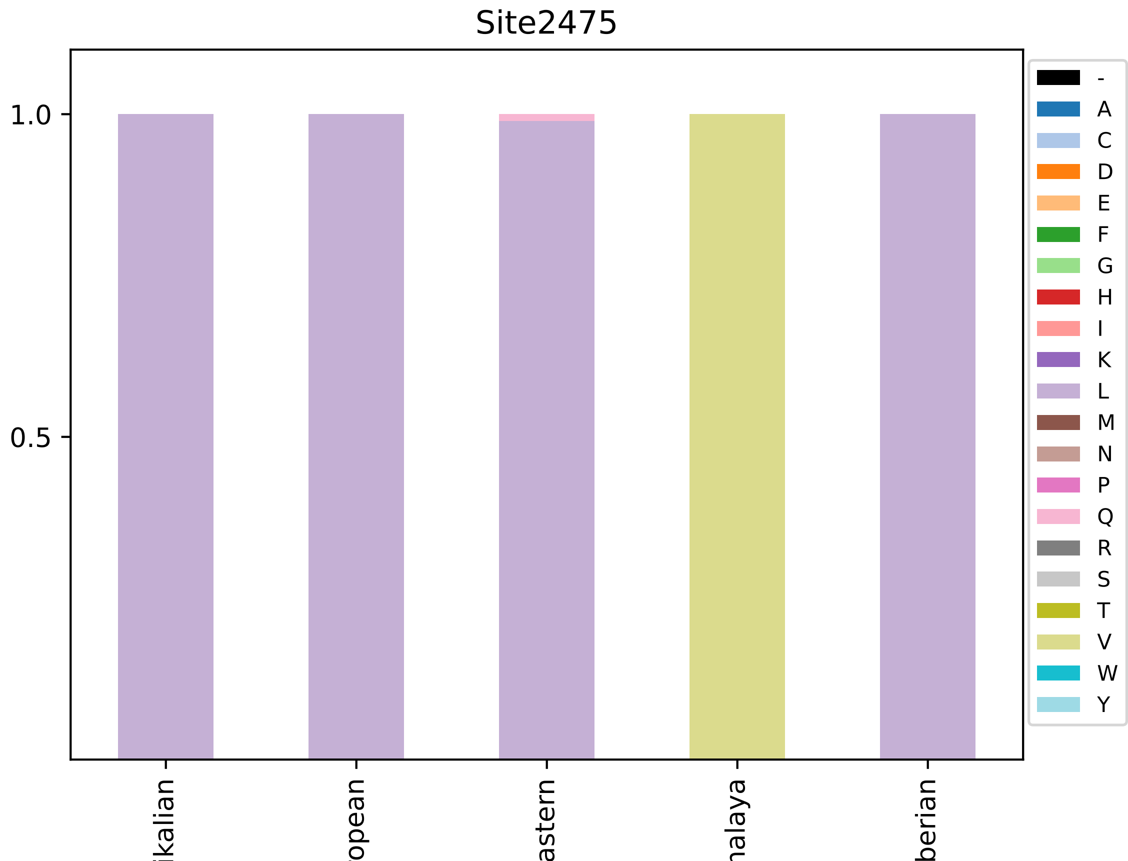
Site2475
1.0
0.5
-
A
C
D
E
F
G
H
I
K
L
M
N
P
Q
R
S
T
V
W
Y
ikalian
ropean
astern
nalaya
berian

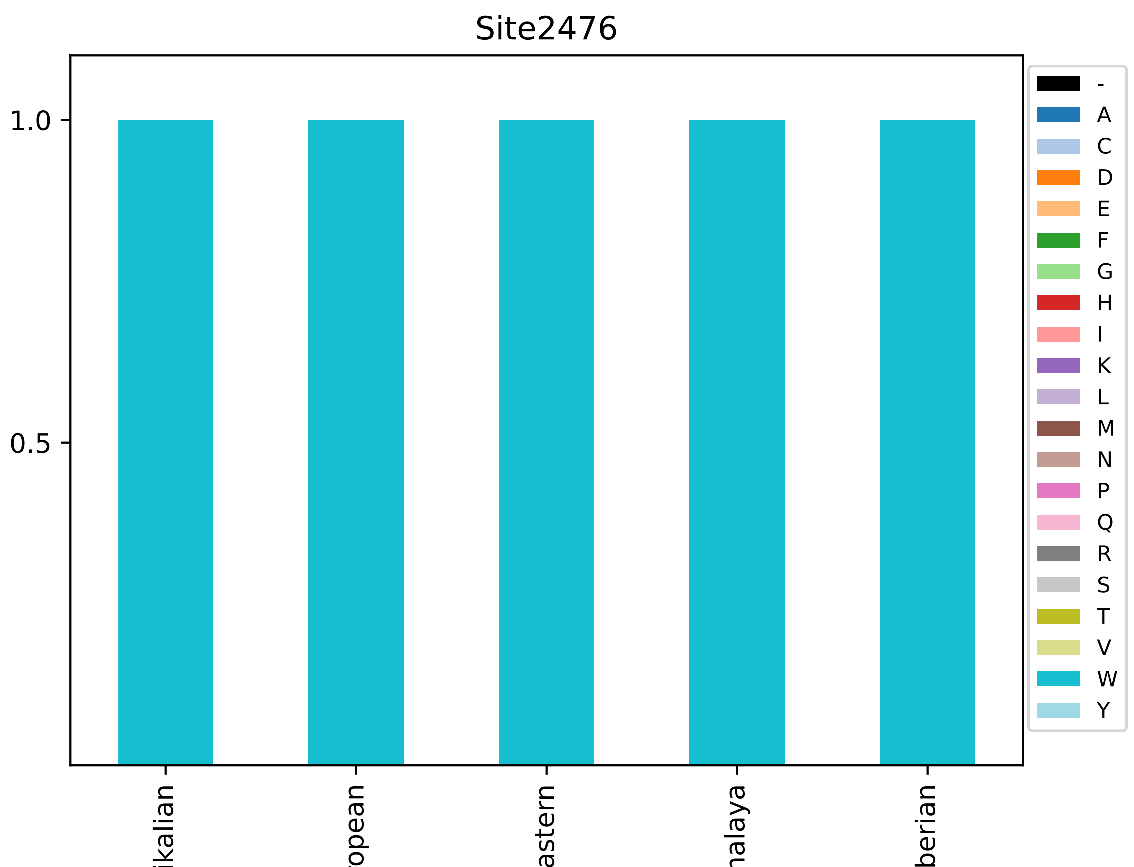

Site2476
1.0
0.5
-
A
C
D
E
F
G
H
I
K
L
M
N
P
Q
R
S
T
V
W
Y
ikalian
opean
astern
nalaya
berian

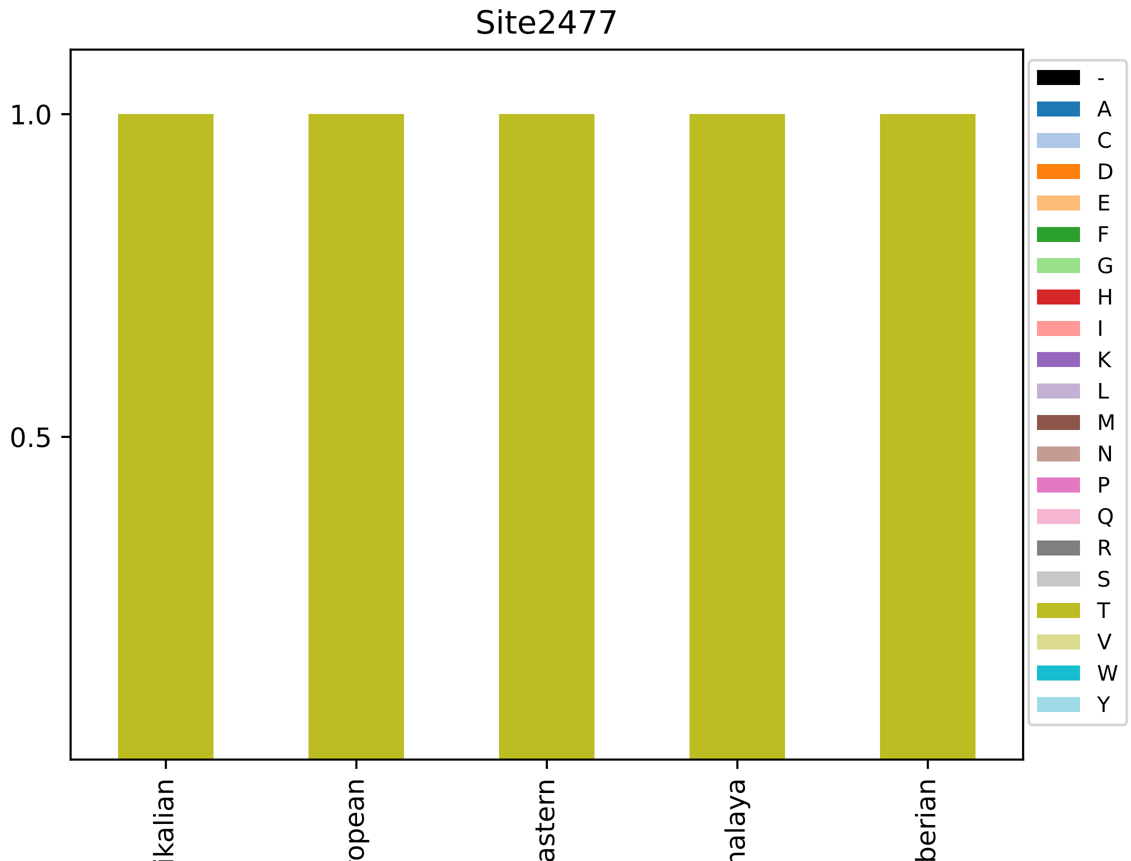
Site2477
1.0
0.5
ikalian
opean
astern
nalaya
berian
-
A
C
D
E
F
G
H
I
K
L
M
N
P
Q
R
S
T
V
W
Y

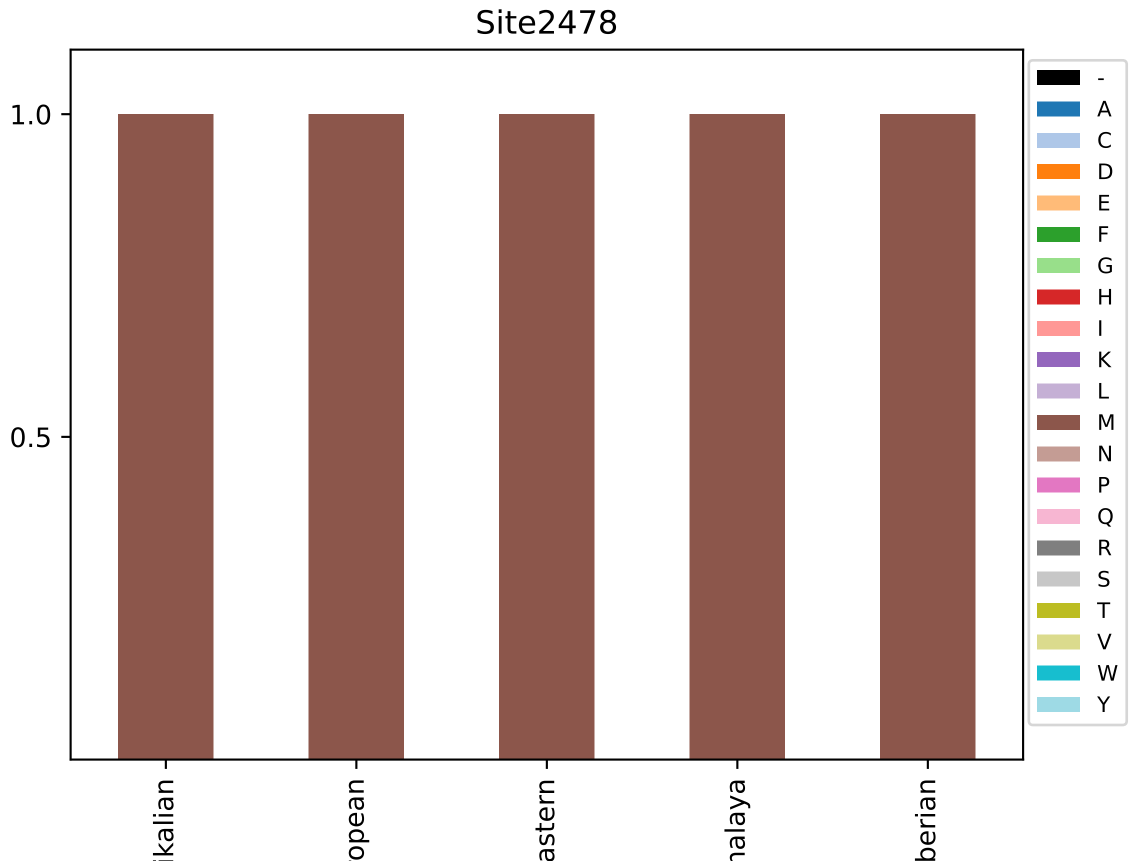
Site2478
1.0
0.5
-
A
C
D
E
F
G
H
I
K
L
M
N
P
Q
R
S
T
V
W
Y
ikalian
opean
astern
nalaya
berian

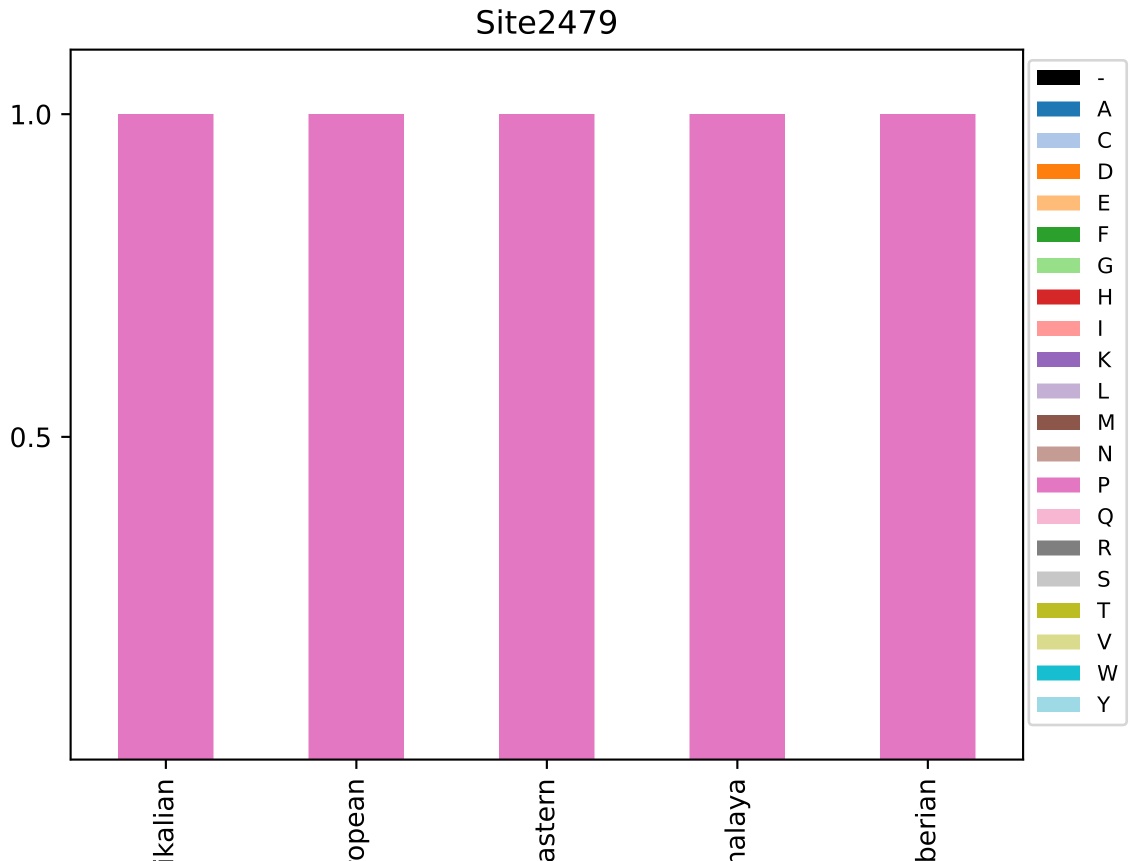
Site2479
1.0
0.5
-
A
C
D
E
F
G
H
I
K
L
M
N
P
Q
R
S
T
V
W
Y
ikalian
opean
astern
nalaya
berian

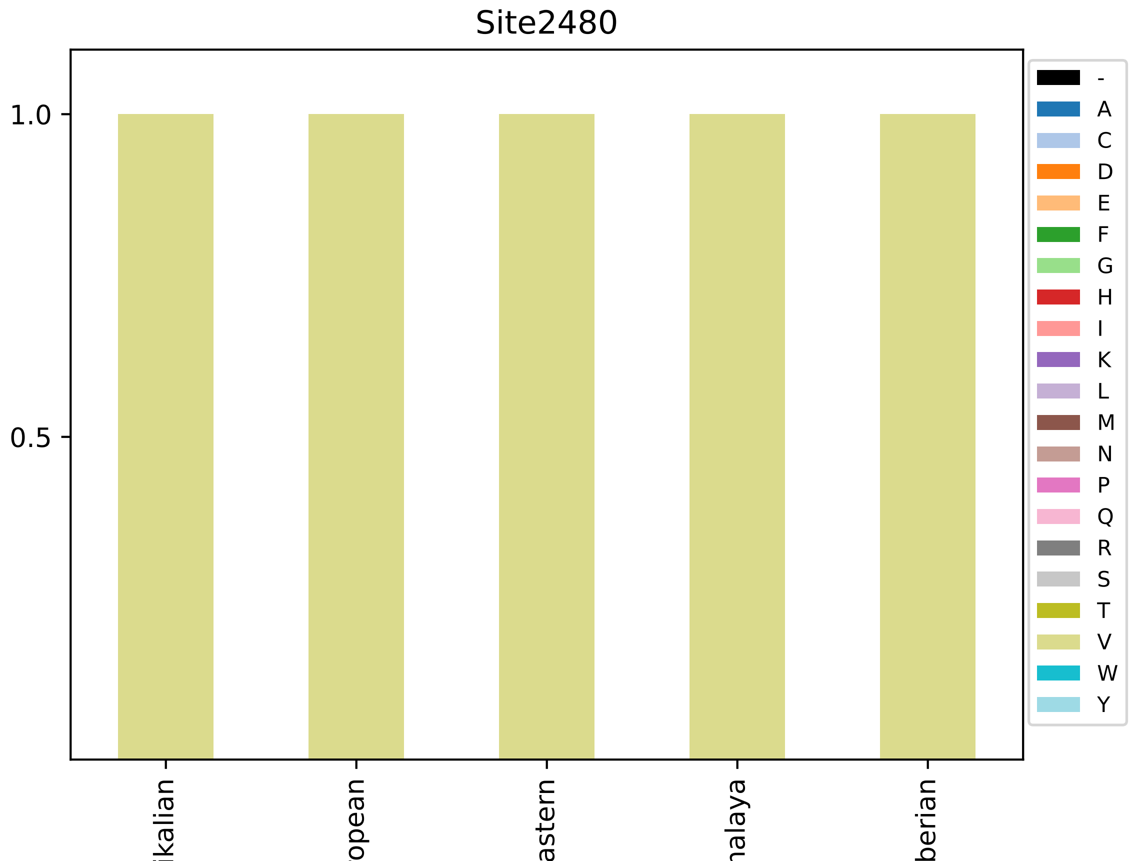
Site2480
1.0
0.5
-
A
C
D
E
F
G
H
I
K
L
M
N
P
Q
R
S
T
V
W
Y
ikalian
opean
astern
nalaya
berian

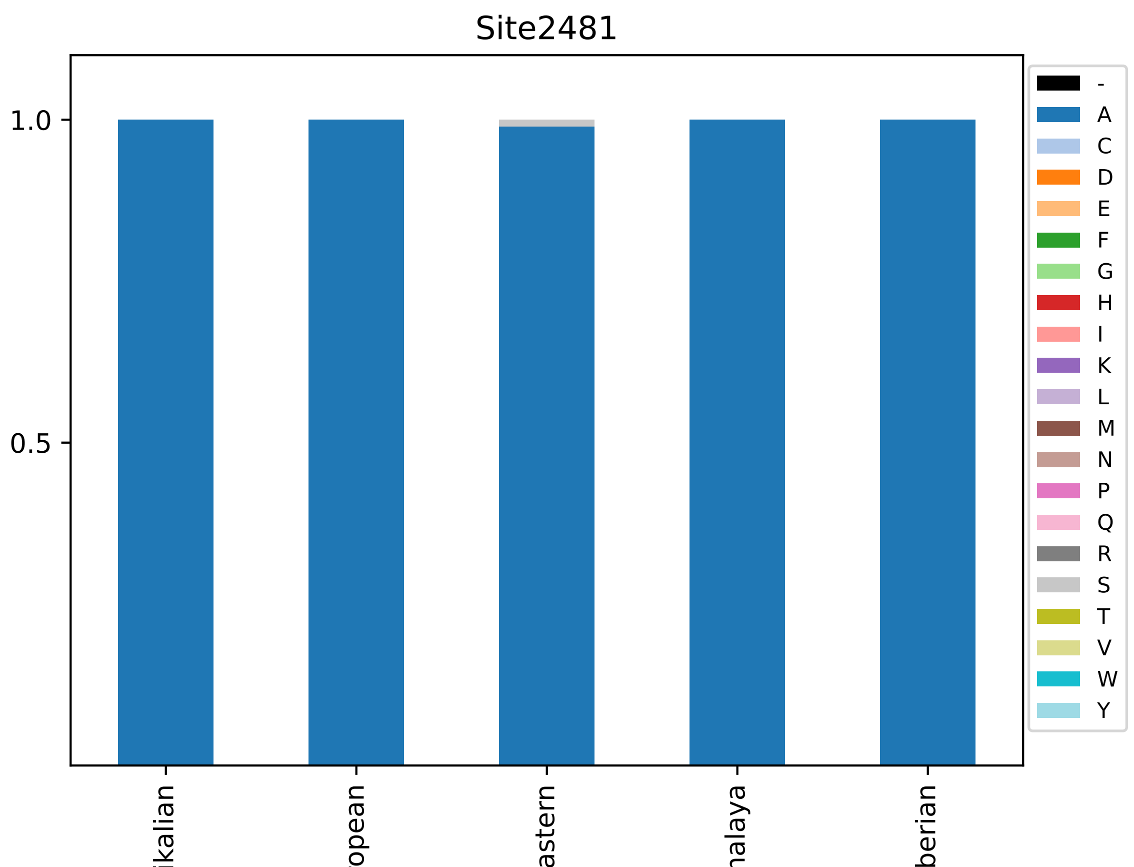
Site2481
1.0
0.5
-
A
C
D
E
F
G
H
I
K
L
M
N
P
Q
R
S
T
V
W
Y
ikalian
opean
astern
nalaya
berian

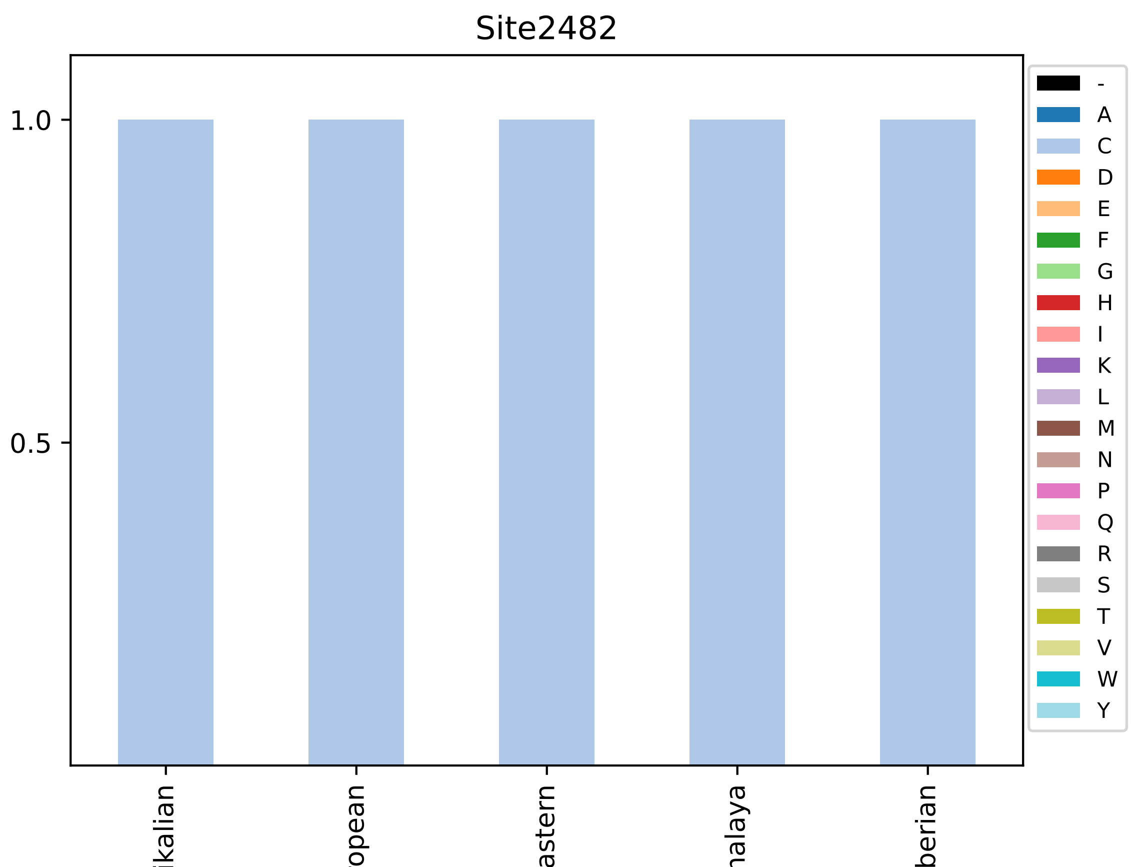

Site2482
1.0
0.5
-
A
C
D
E
F
G
H
I
K
L
M
N
P
Q
R
S
T
V
W
Y
ikalian
opean
astern
nalaya
berian

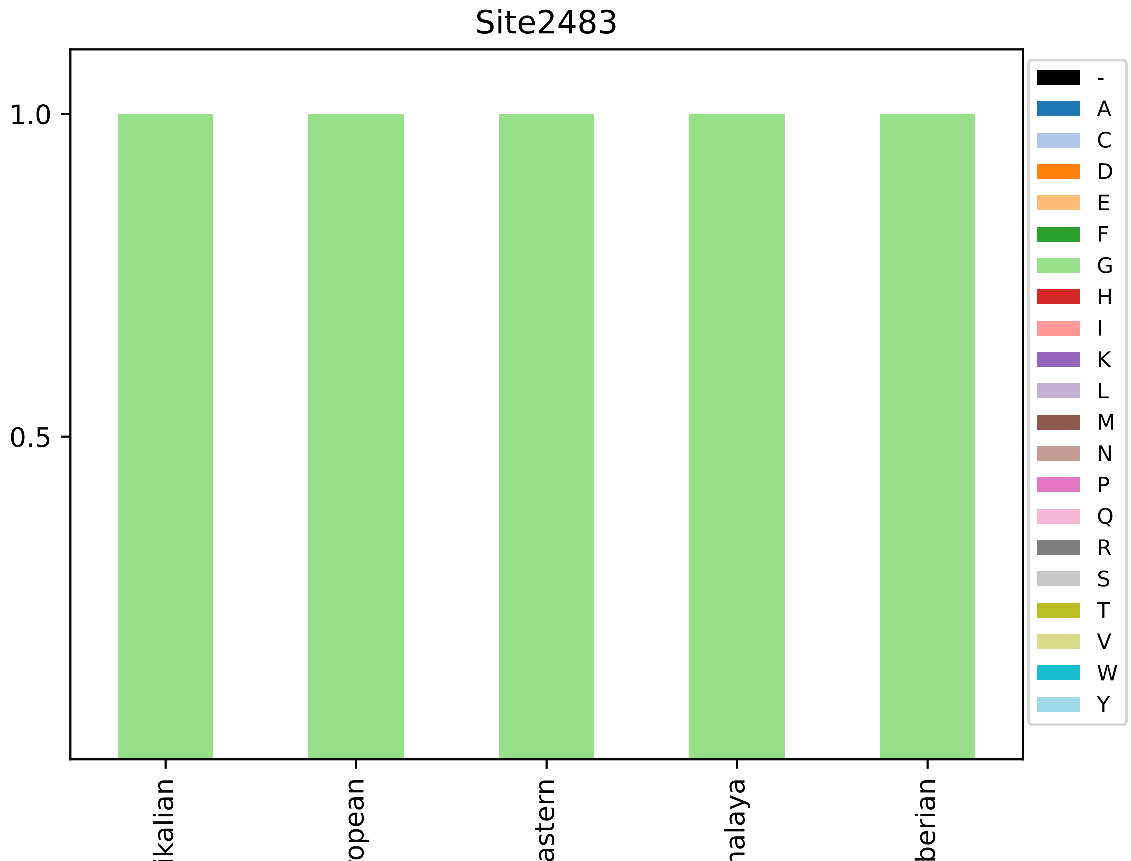
Site2483
1.0
0.5
-
A
C
D
E
F
G
H
I
K
L
M
N
P
Q
R
S
T
V
W
Y
ikalian
opean
astern
nalaya
berian

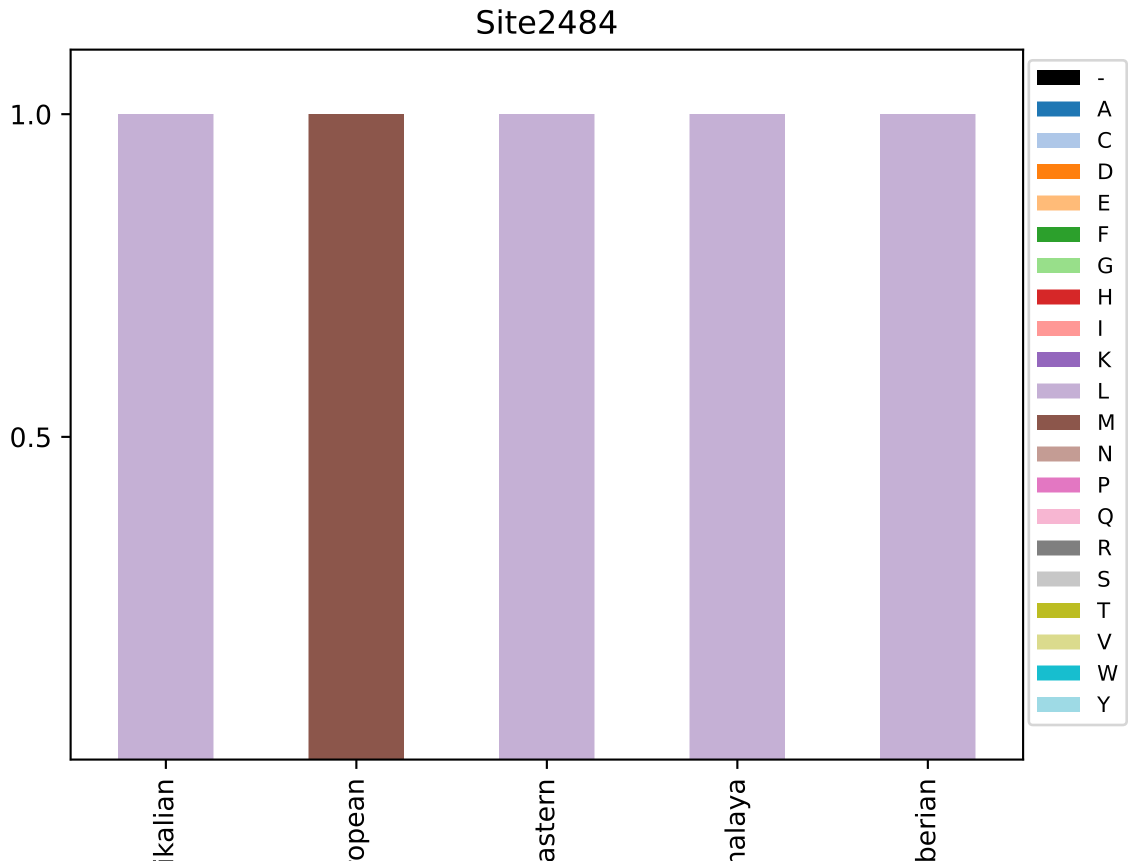

Site2484
1.0
0.5
-
A
C
D
E
F
G
H
I
K
L
M
N
P
Q
R
S
T
V
W
Y
ikalian
opean
astern
nalaya
berian

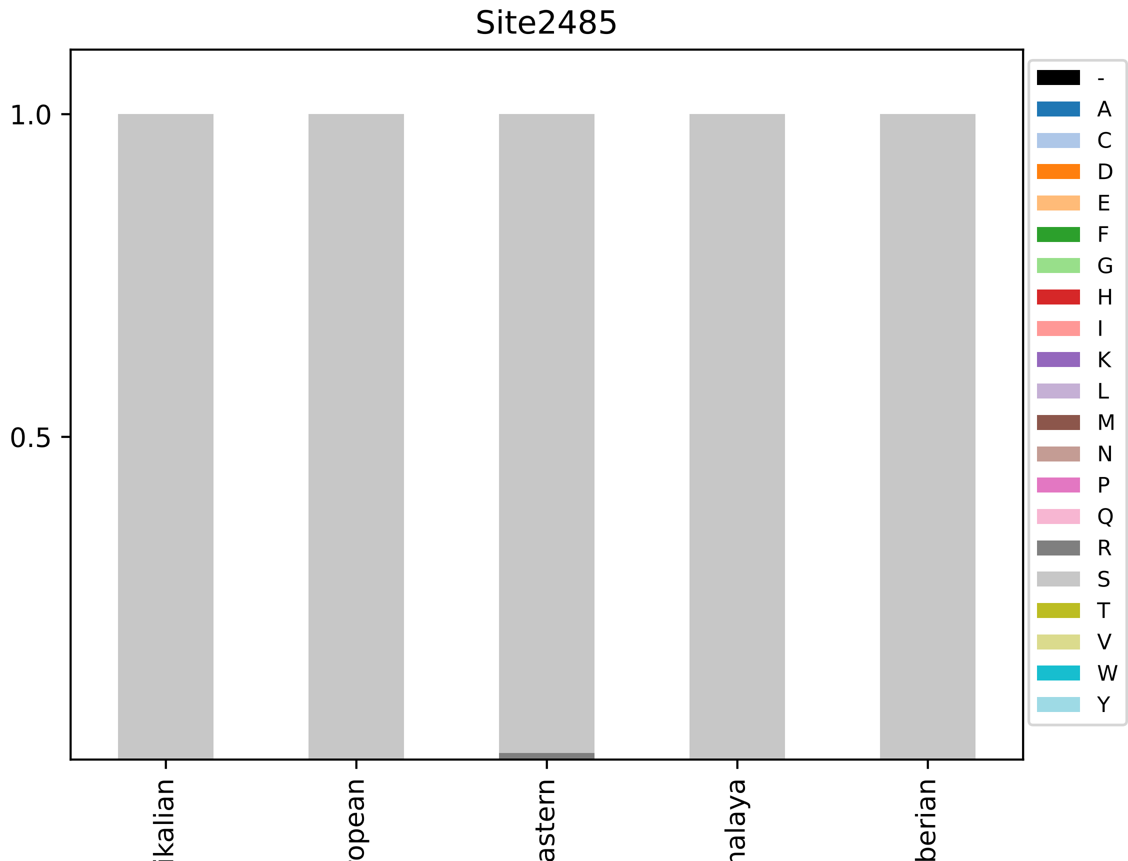

Site2485
1.0
0.5
-
A
C
D
E
F
G
H
I
K
L
M
N
P
Q
R
S
T
V
W
Y
ikalian
opean
astern
nalaya
berian

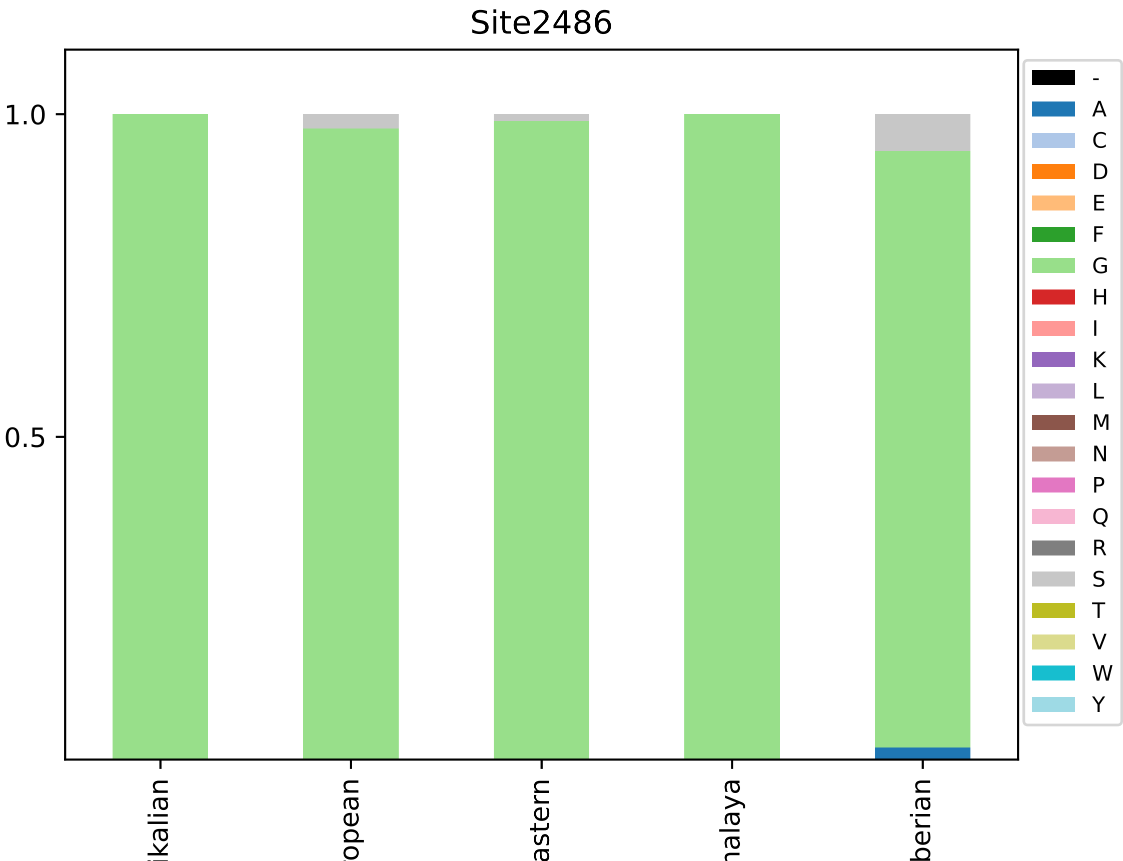
Site2486
1.0
0.5
-
A
C
D
E
F
G
H
I
K
L
M
N
P
Q
R
S
T
V
W
Y
ikalian
opean
astern
nalaya
berian

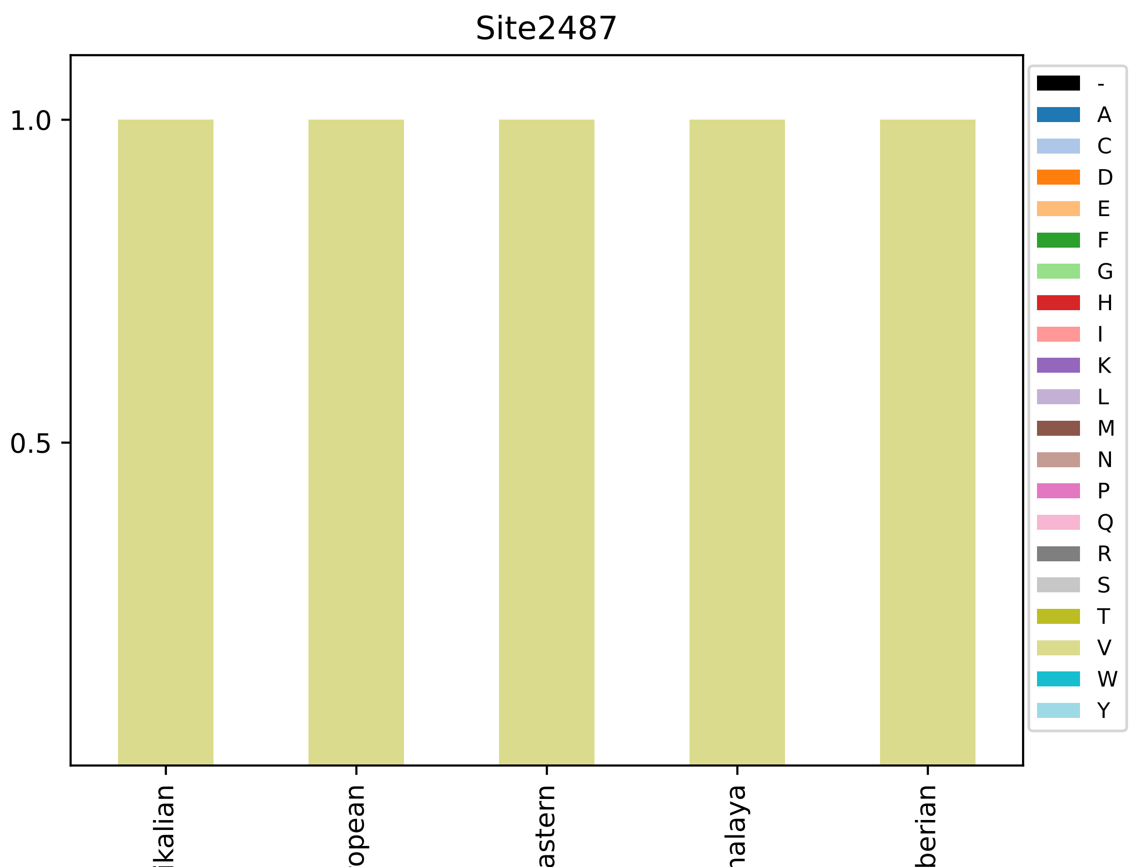

Site2487
1.0
0.5
-
A
C
D
E
F
G
H
I
K
L
M
N
P
Q
R
S
T
V
W
Y
ikalian
opean
astern
nalaya
berian

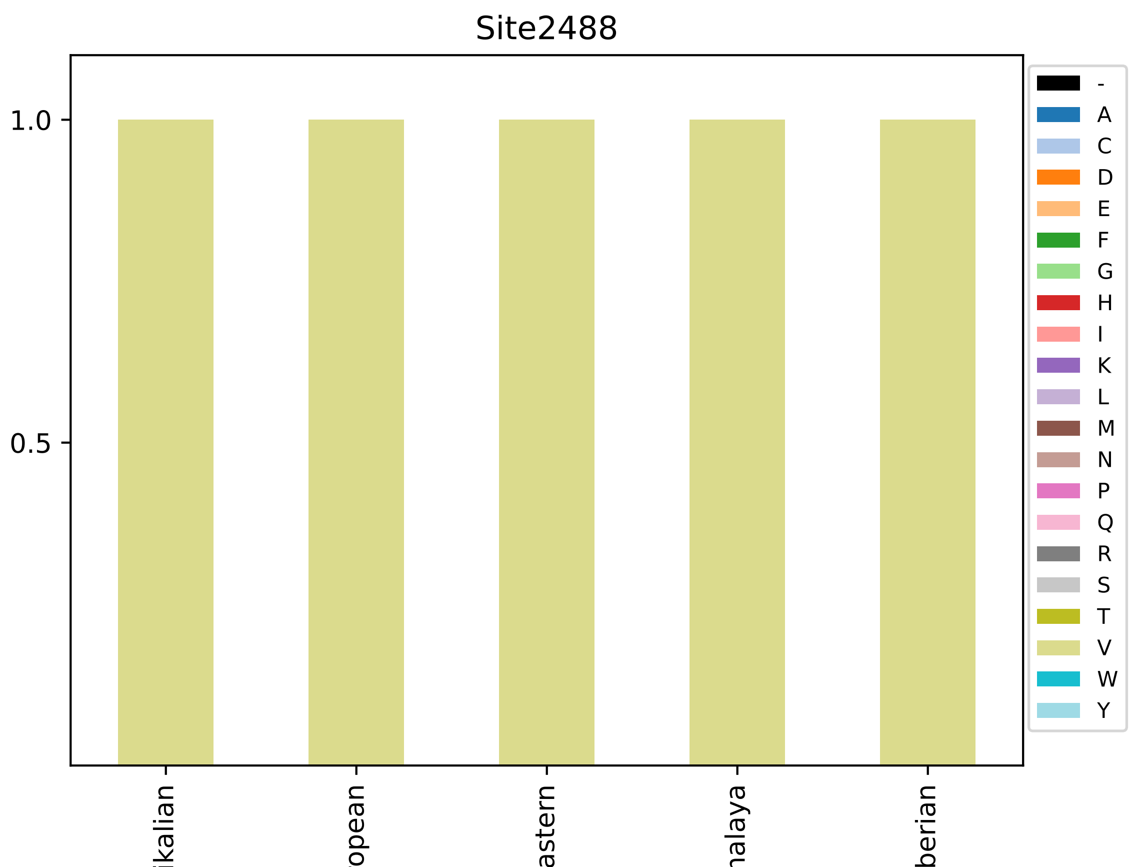
Site2488
1.0
0.5
ikalian
opean
astern
nalaya
berian
-
A
C
D
E
F
G
H
I
K
L
M
N
P
Q
R
S
T
V
W
Y

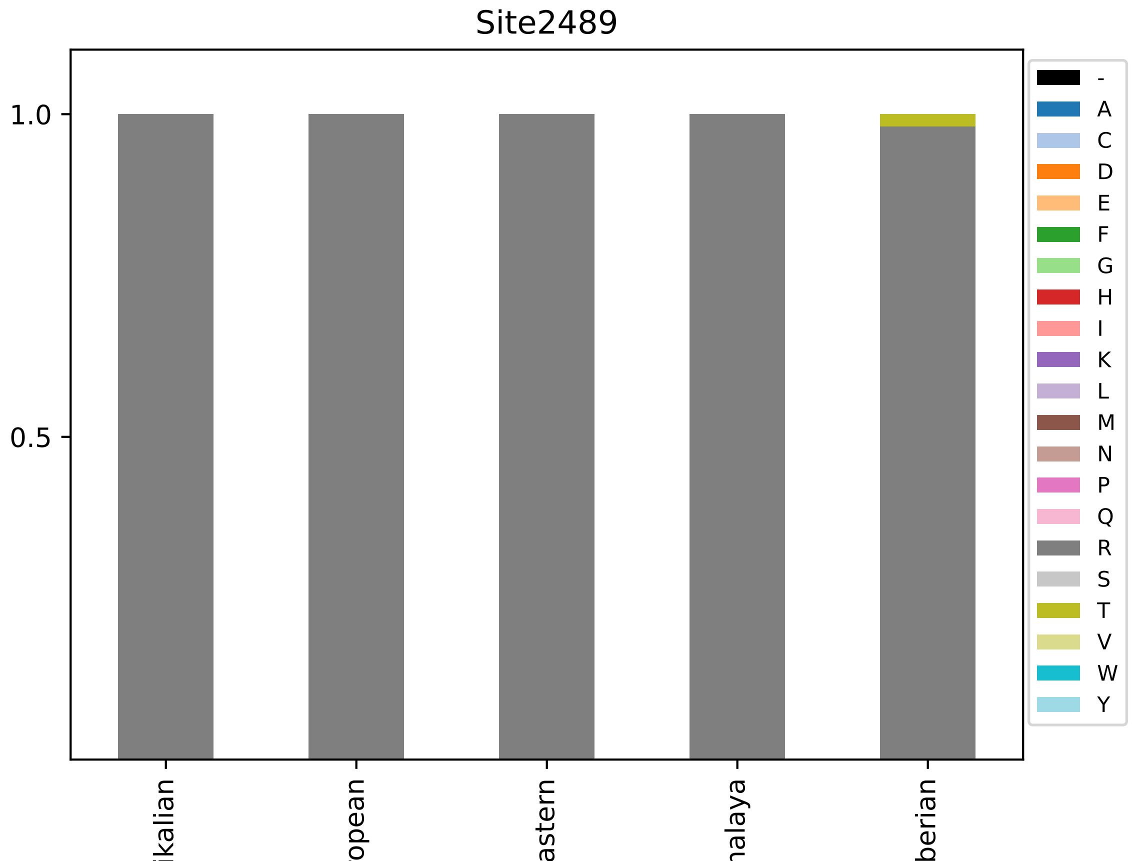
Site2489
1.0
0.5
-
A
C
D
E
F
G
H
I
K
L
M
N
P
Q
R
S
T
V
W
Y
ikalian
opean
astern
nalaya
berian

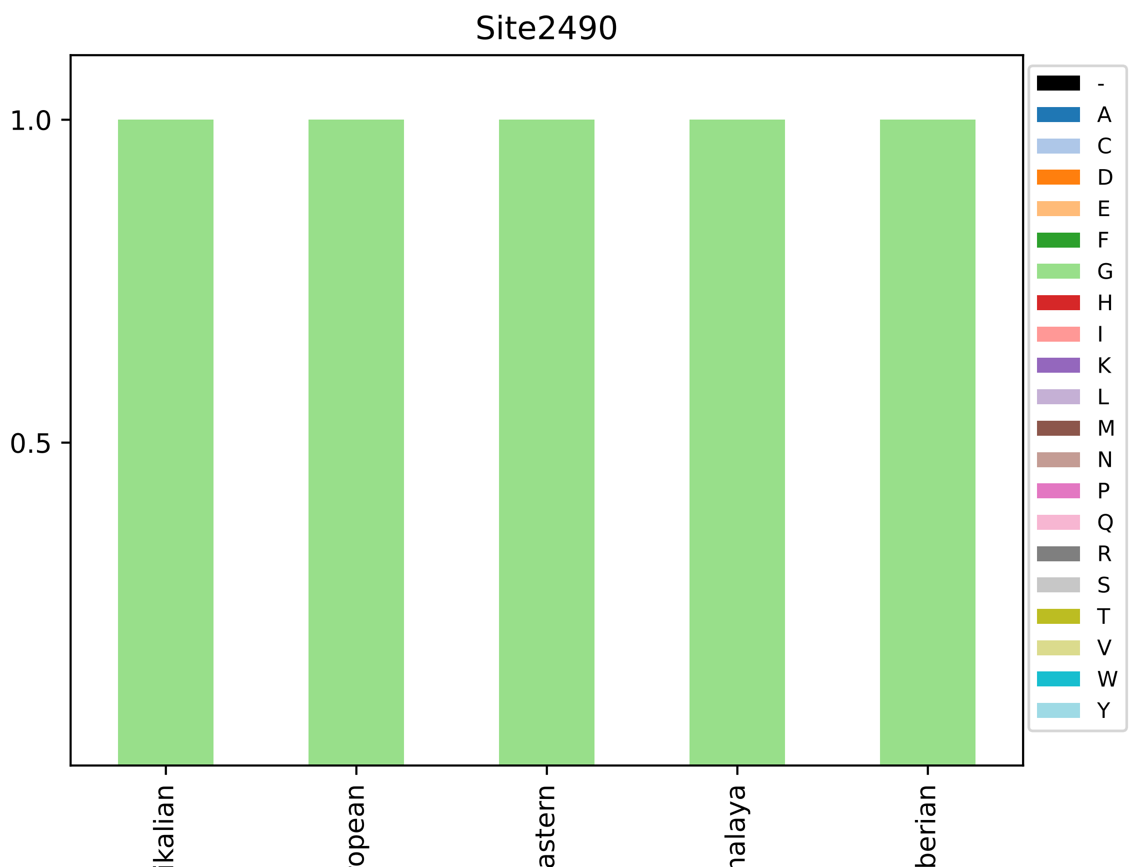

Site2490
1.0
0.5
ikalian
opean
astern
nalaya
berian
-
A
C
D
E
F
G
H
I
K
L
M
N
P
Q
R
S
T
V
W
Y

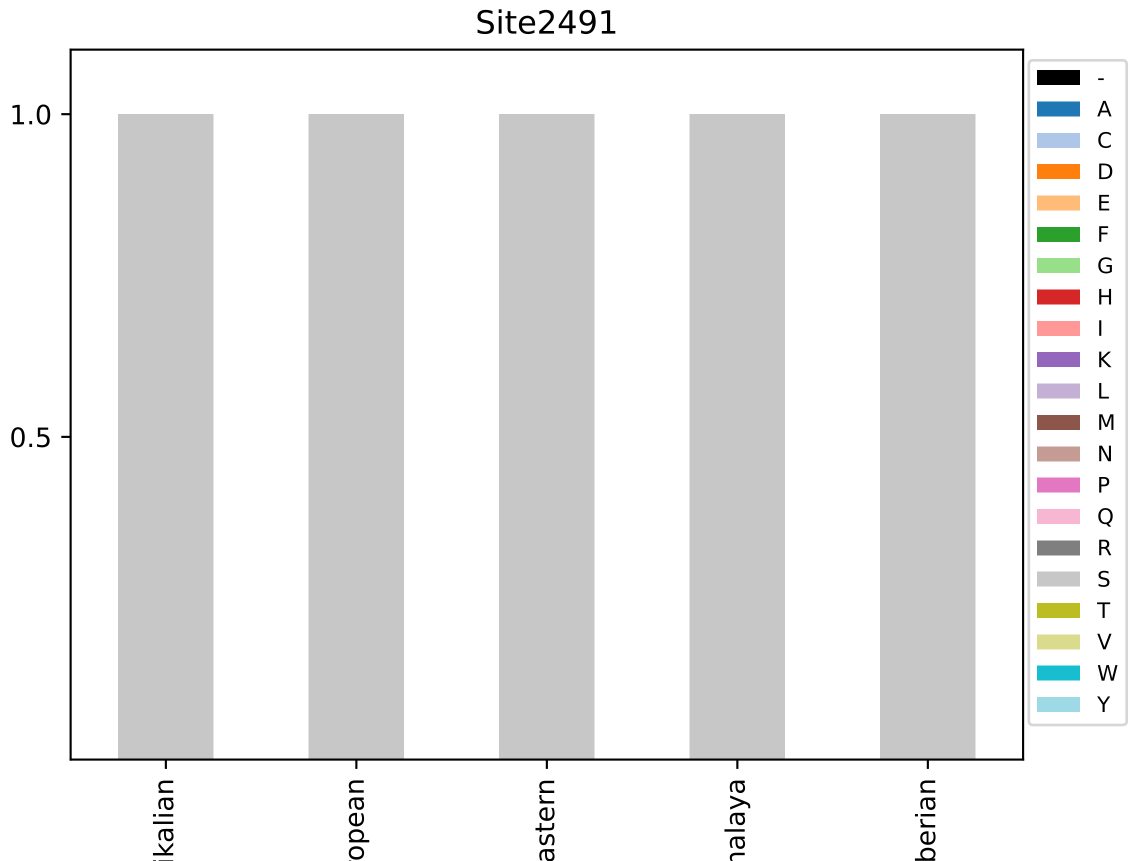
Site2491
1.0
0.5
-
A
C
D
E
F
G
H
I
K
L
M
N
P
Q
R
S
T
V
W
Y
ikalian
opean
astern
nalaya
berian

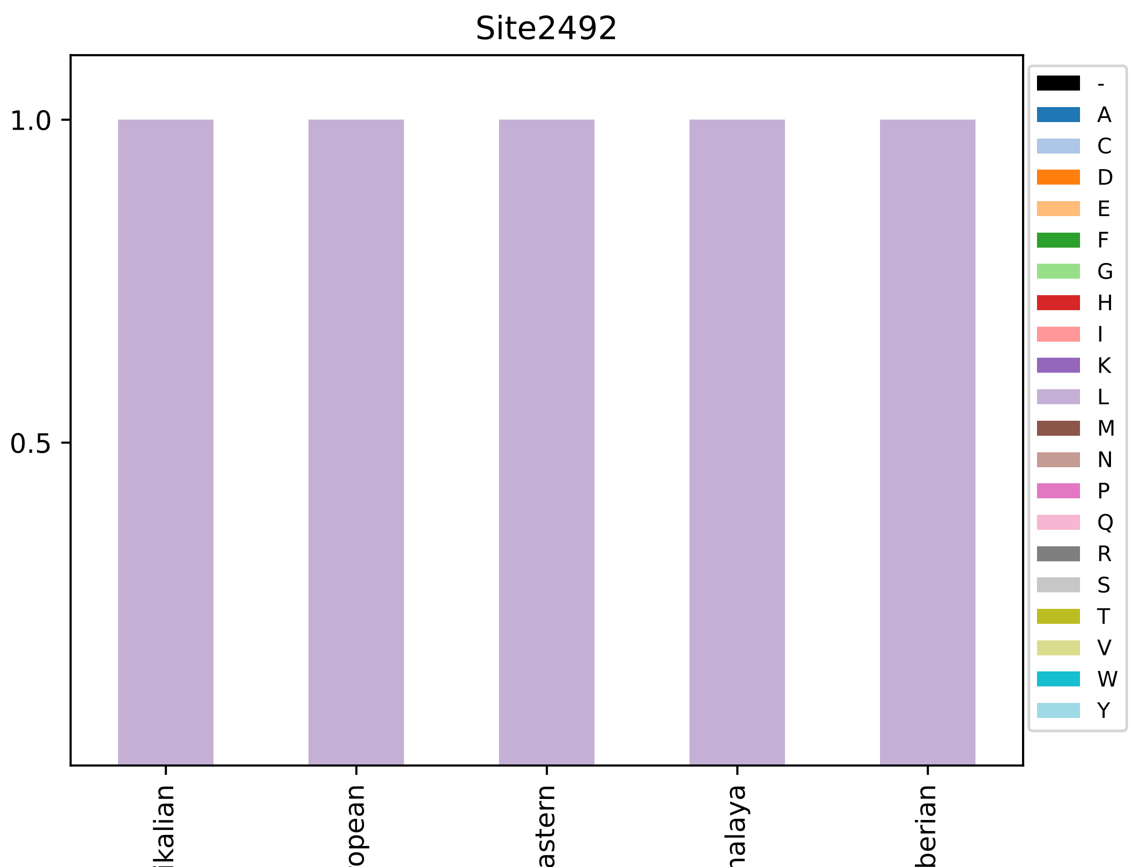

Site2492
1.0
0.5
-
A
C
D
E
F
G
H
I
K
L
M
N
P
Q
R
S
T
V
W
Y
ikalian
opean
astern
nalaya
berian

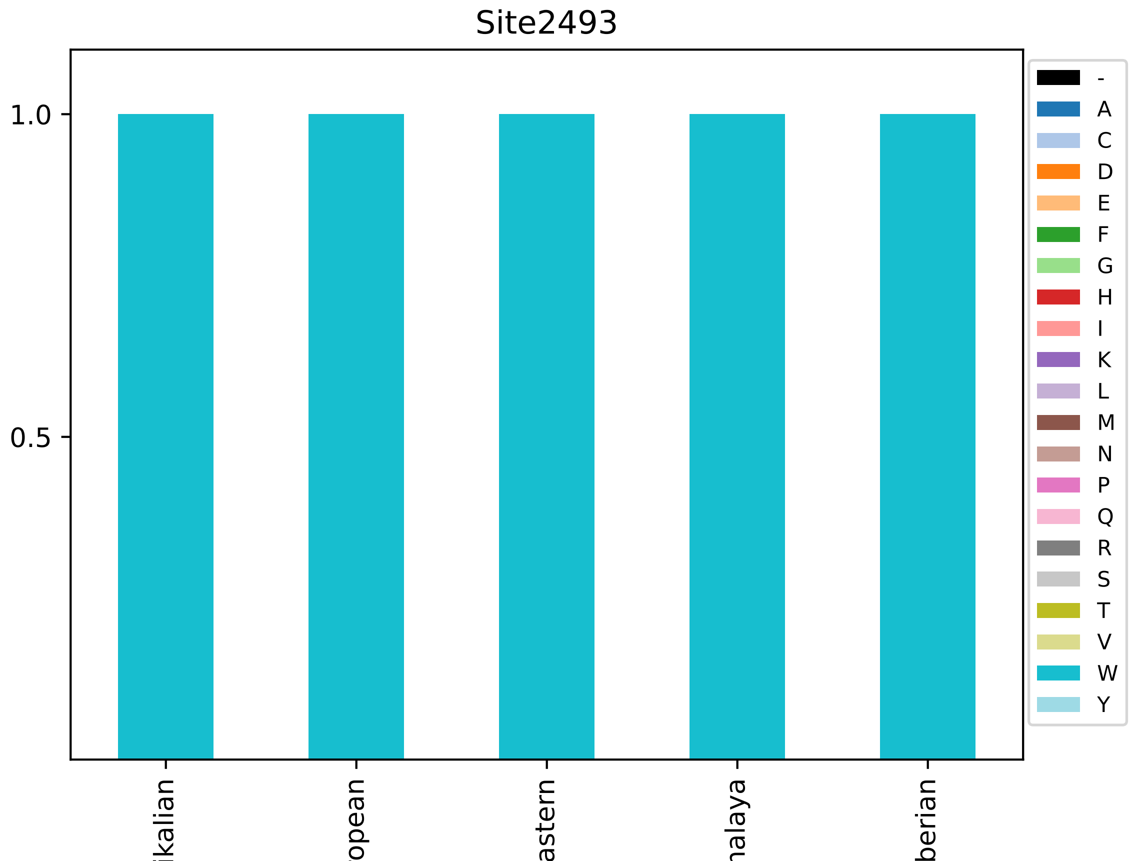

Site2493
1.0
0.5
-
A
C
D
E
F
G
H
I
K
L
M
N
P
Q
R
S
T
V
W
Y
ikalian
opean
astern
nalaya
berian

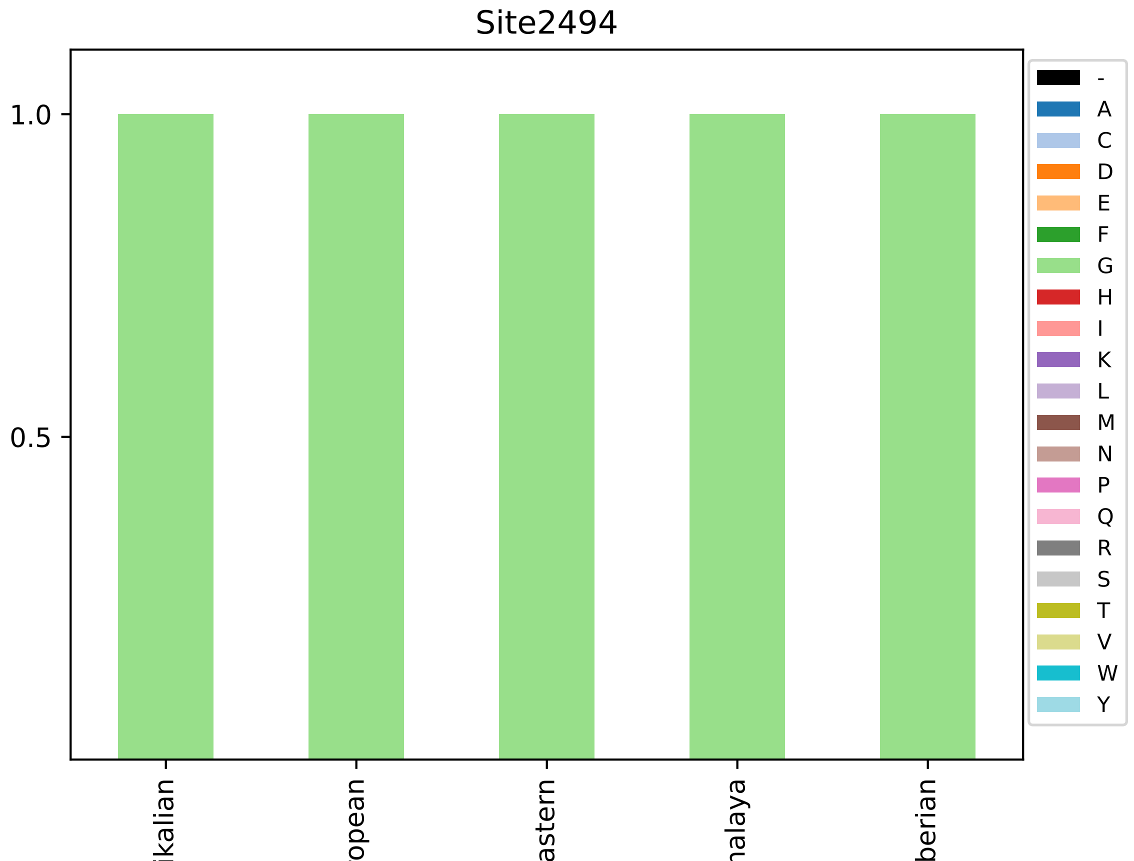
Site2494
1.0
0.5
-
A
C
D
E
F
G
H
I
K
L
M
N
P
Q
R
S
T
V
W
Y
ikalian
opean
astern
nalaya
berian

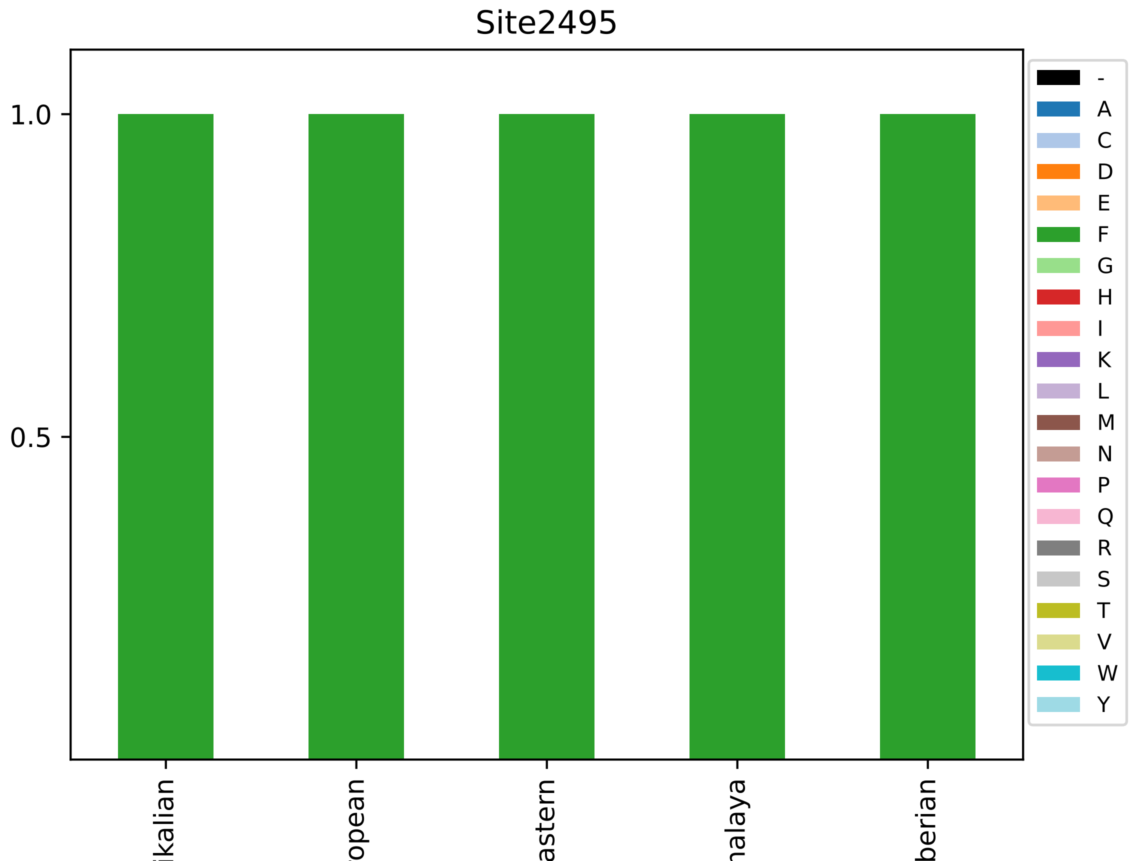

Site2495
1.0
0.5
-
A
C
D
E
F
G
H
I
K
L
M
N
P
Q
R
S
T
V
W
Y

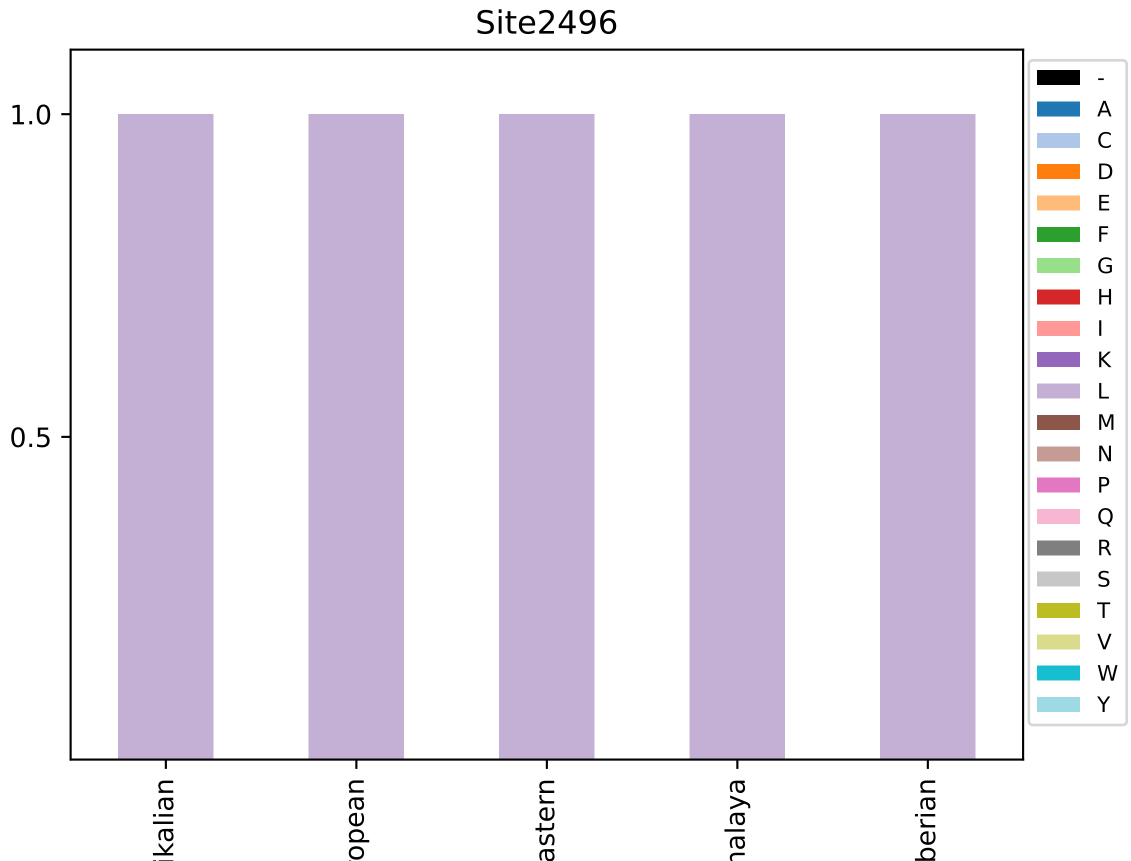

Site2496
1.0
0.5
-
A
C
D
E
F
G
H
I
K
L
M
N
P
Q
R
S
T
V
W
Y
ikalian
opean
astern
nalaya
berian

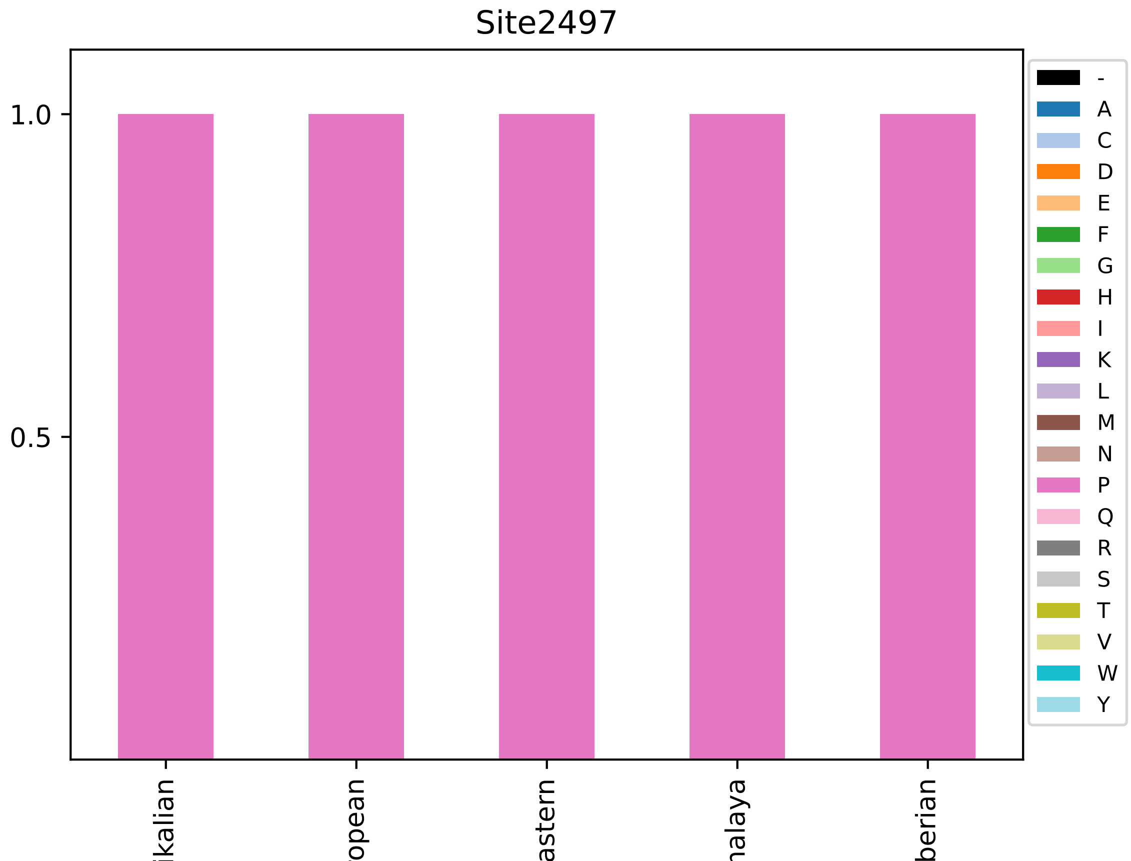
Site2497
1.0
0.5
ikalian
opean
astern
nalaya
berian
-
A
C
D
E
F
G
H
I
K
L
M
N
P
Q
R
S
T
V
W
Y

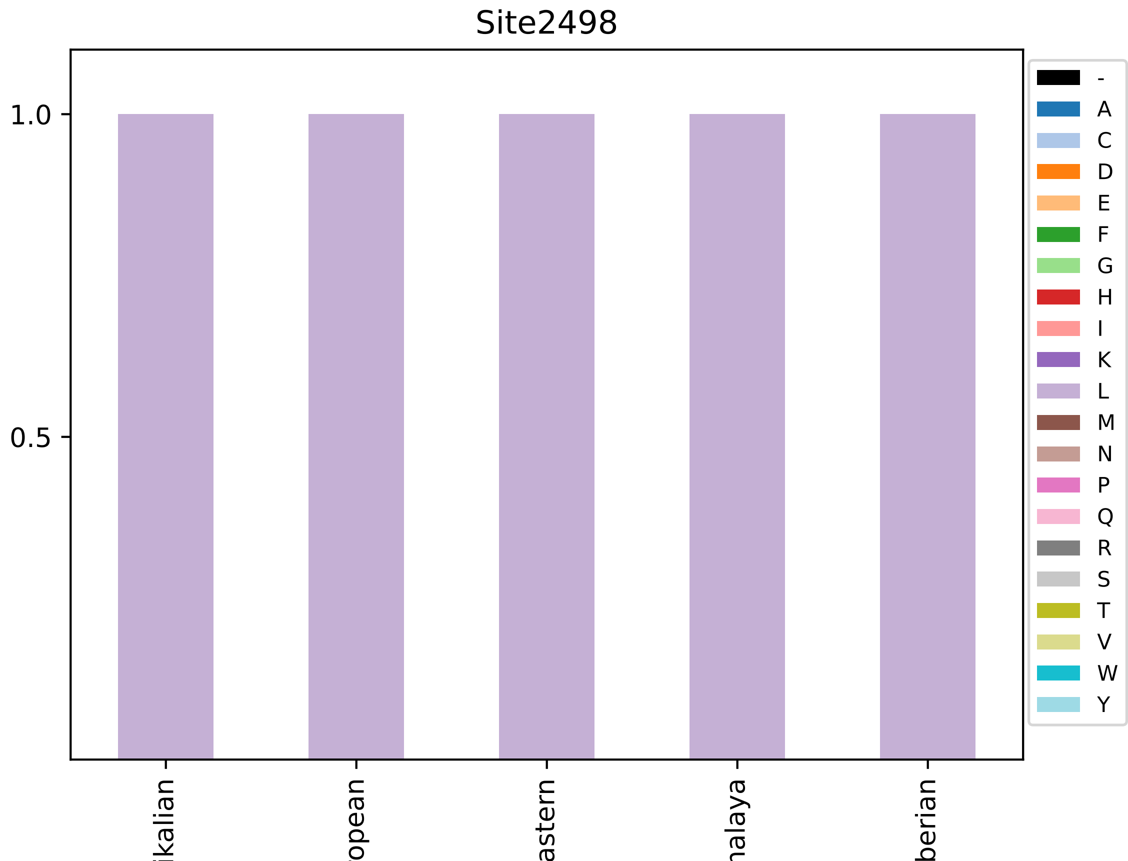

Site2498
1.0
0.5
-
A
C
D
E
F
G
H
I
K
L
M
N
P
Q
R
S
T
V
W
Y
ikalian
ropean
astern
nalaya
berian

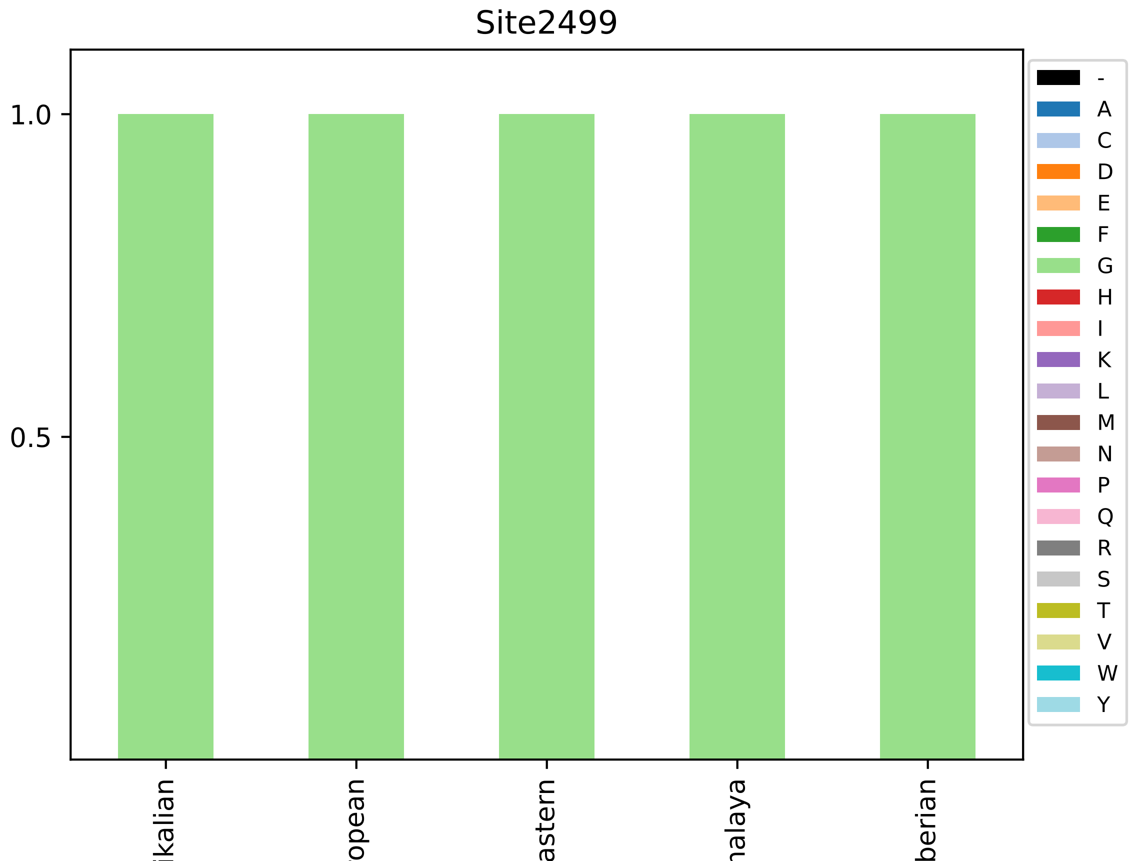

Site2499
1.0
0.5
ikalian
opean
astern
nalaya
berian
-
A
C
D
E
F
G
H
I
K
L
M
N
P
Q
R
S
T
V
W
Y

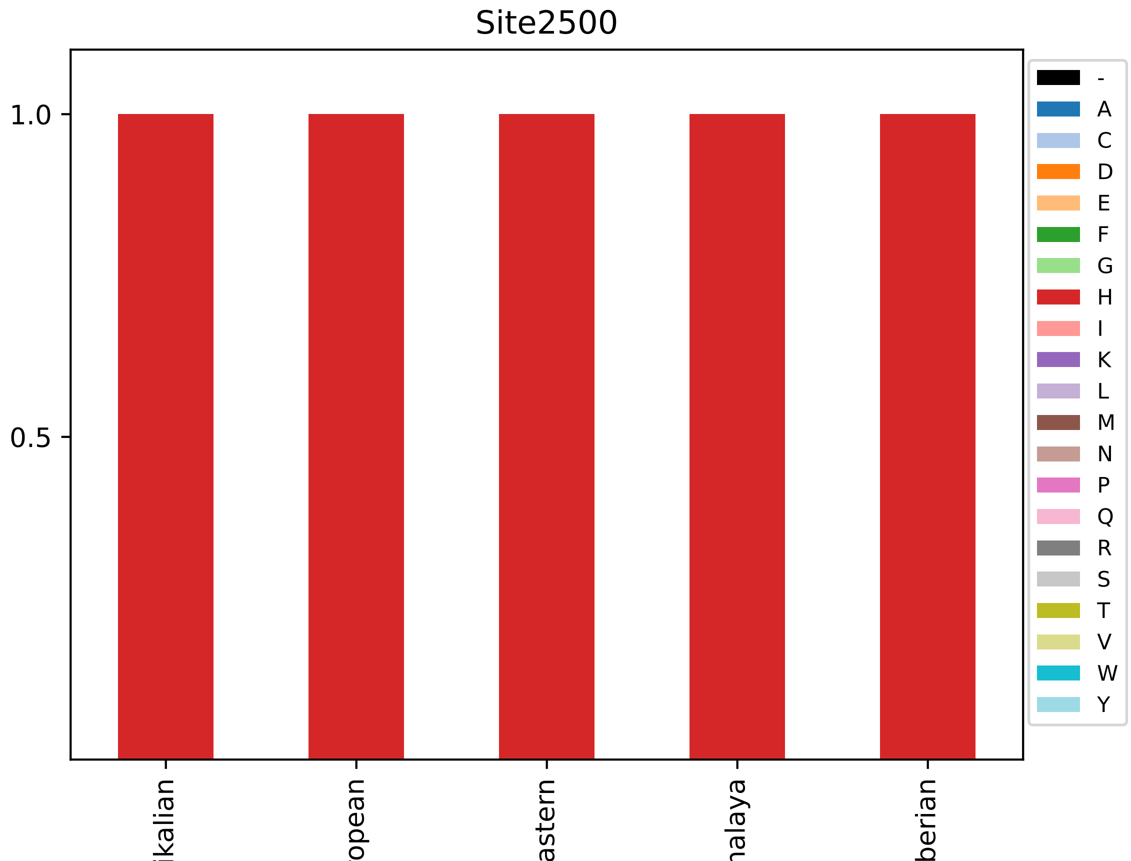
Site2500
1.0
0.5
-
A
C
D
E
F
G
H
I
K
L
M
N
P
Q
R
S
T
V
W
Y
ikalian
opean
astern
nalaya
berian

Site2501

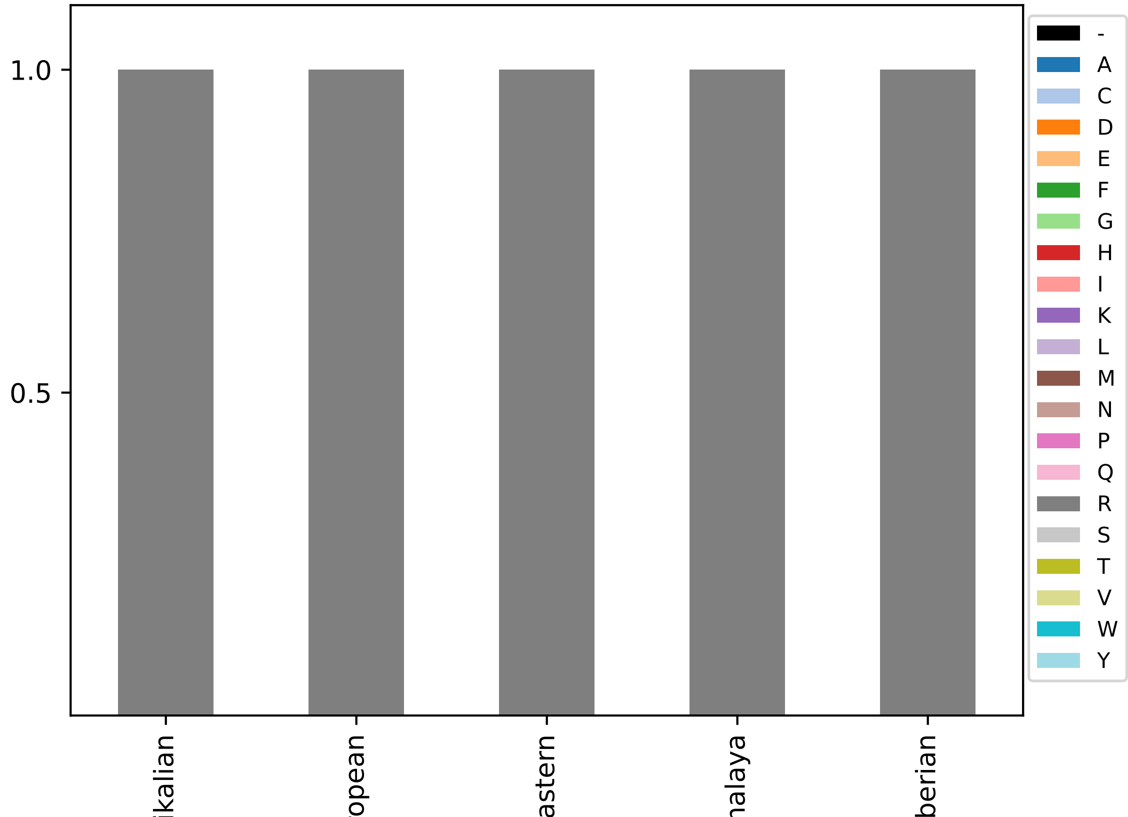

1.0
0.5
-
A
C
D
E
F
G
H
I
K
L
M
N
P
Q
R
S
T
V
W
Y
ikalian
opean
astern
nalaya
berian

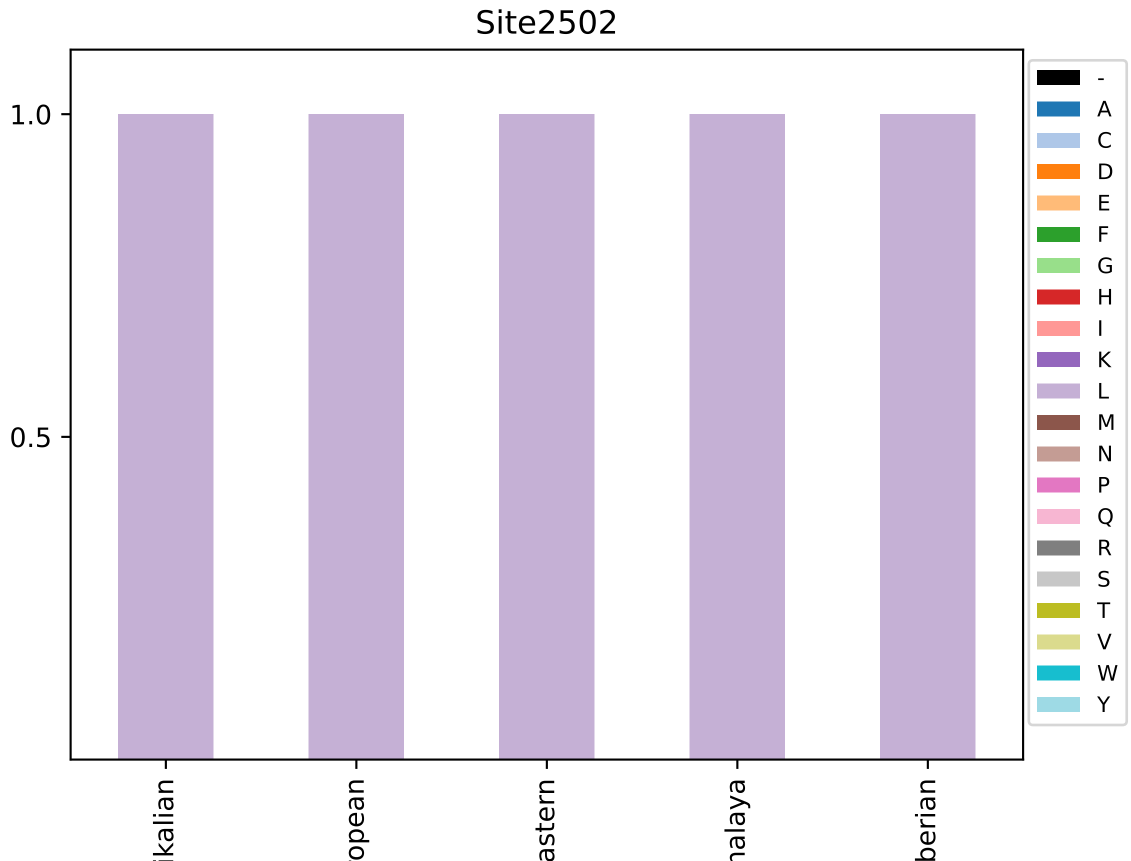

Site2502
1.0
0.5
-
A
C
D
E
F
G
H
I
K
L
M
N
P
Q
R
S
T
V
W
Y

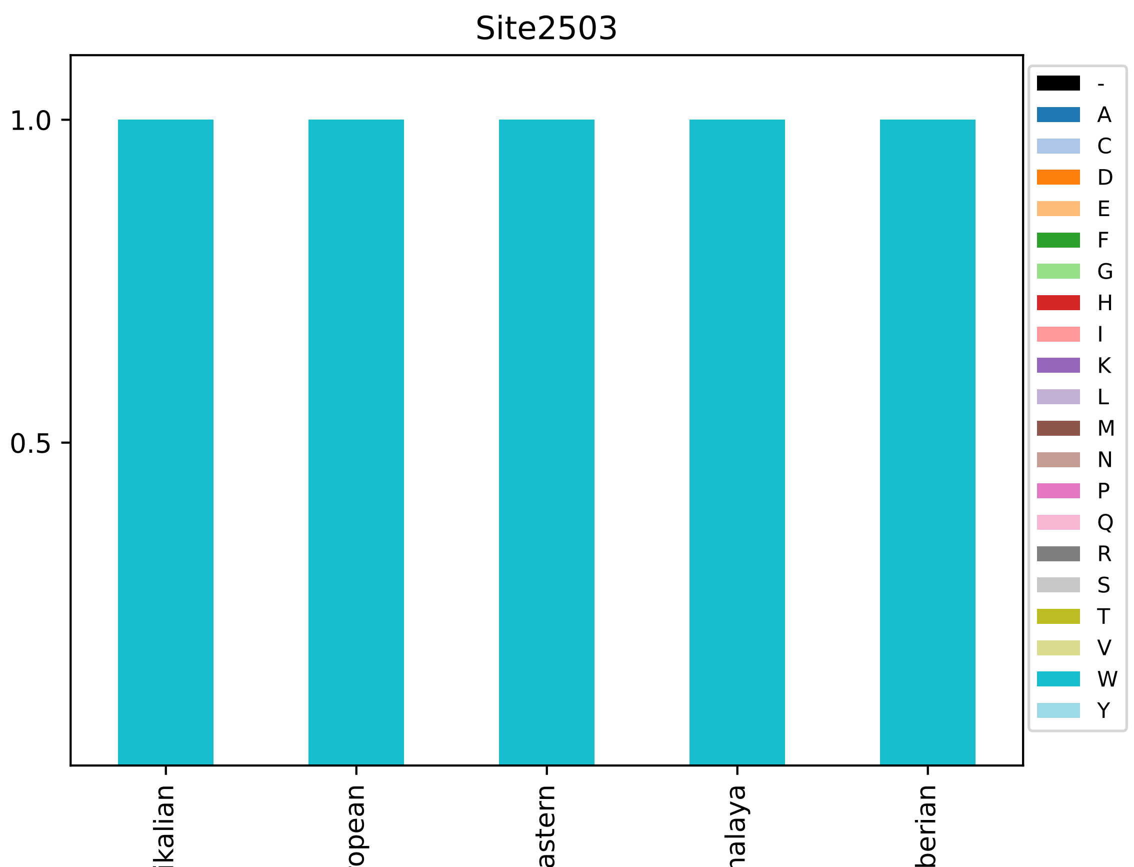

Site2503
1.0
0.5
-
A
C
D
E
F
G
H
I
K
L
M
N
P
Q
R
S
T
V
W
Y
ikalian
opean
astern
nalaya
berian

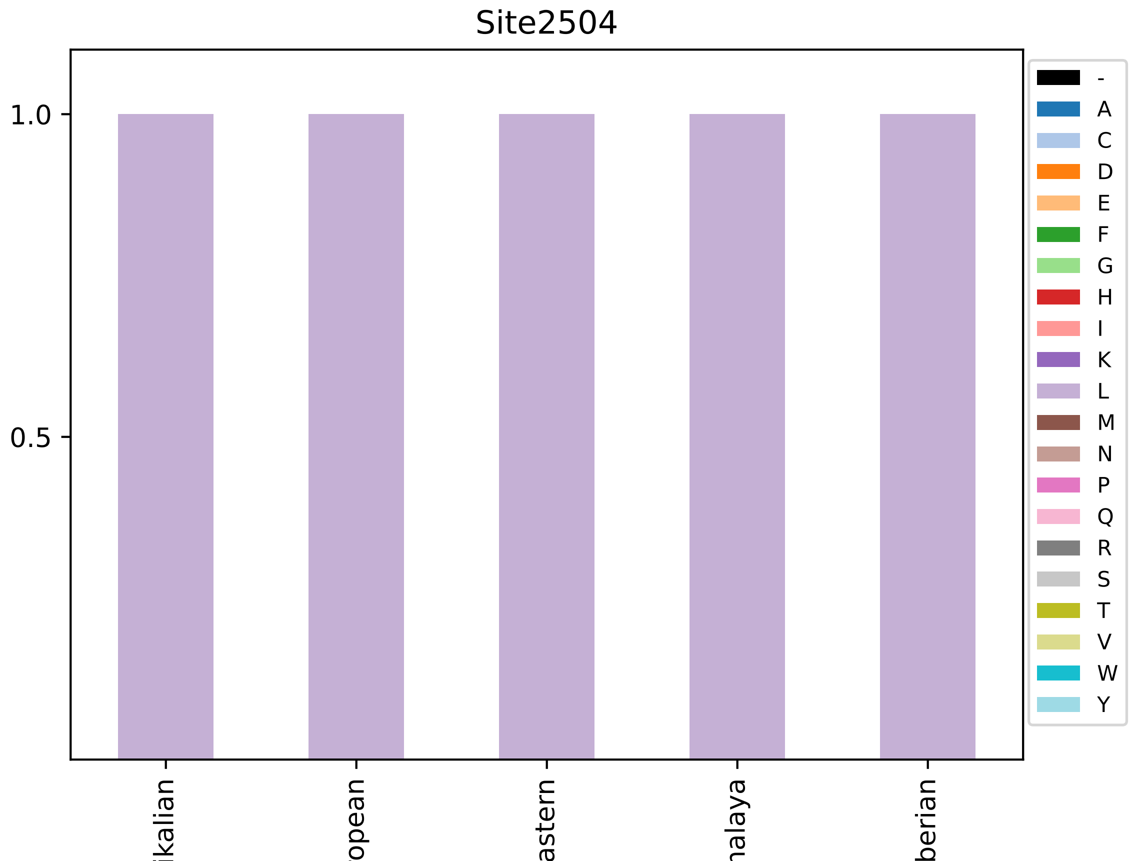
Site2504
1.0
0.5
-
A
C
D
E
F
G
H
I
K
L
M
N
P
Q
R
S
T
V
W
Y
ikalian
opean
astern
nalaya
berian

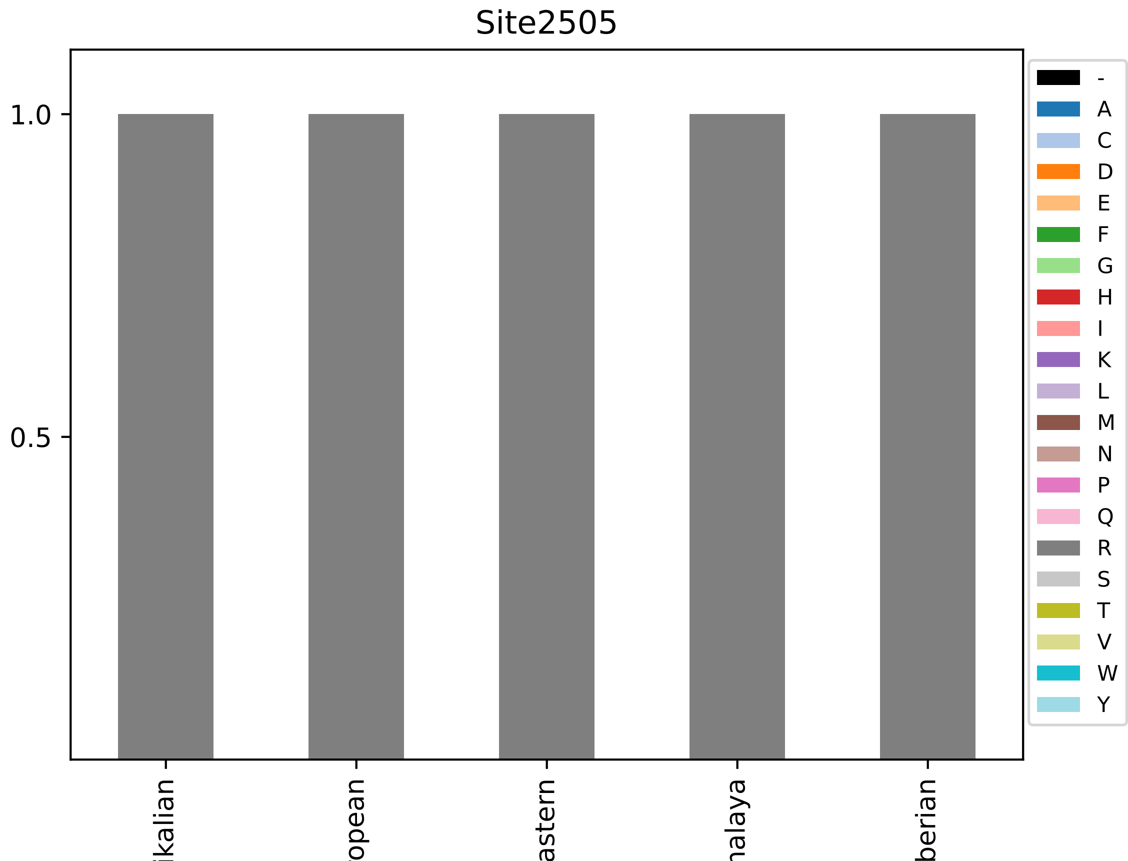

Site2505
1.0
0.5
-
A
C
D
E
F
G
H
I
K
L
M
N
P
Q
R
S
T
V
W
Y
ikalian
opean
astern
nalaya
berian

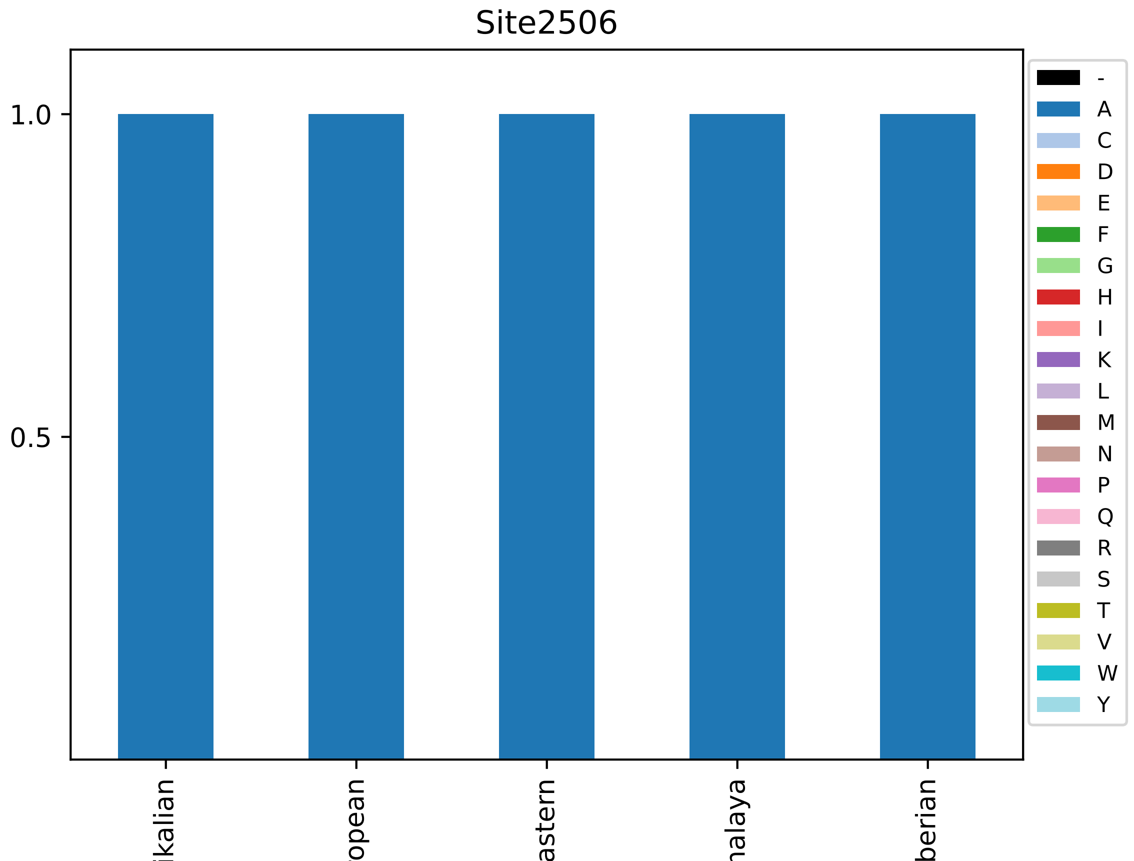

Site2506
1.0
0.5
ikalian
opean
astern
nalaya
berian
-
A
C
D
E
F
G
H
I
K
L
M
N
P
Q
R
S
T
V
W
Y

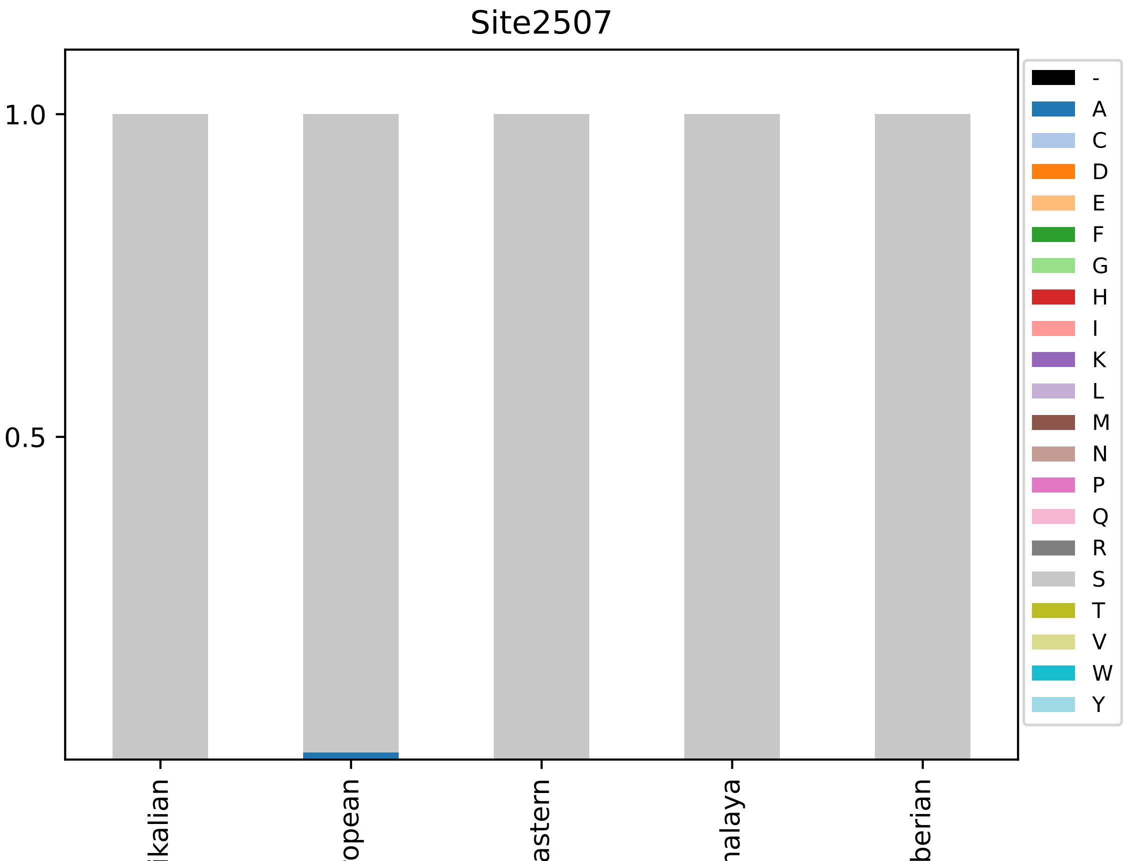

Site2507
1.0
0.5
-
A
C
D
E
F
G
H
I
K
L
M
N
P
Q
R
S
T
V
W
Y
ikalian
opean
astern
nalaya
berian

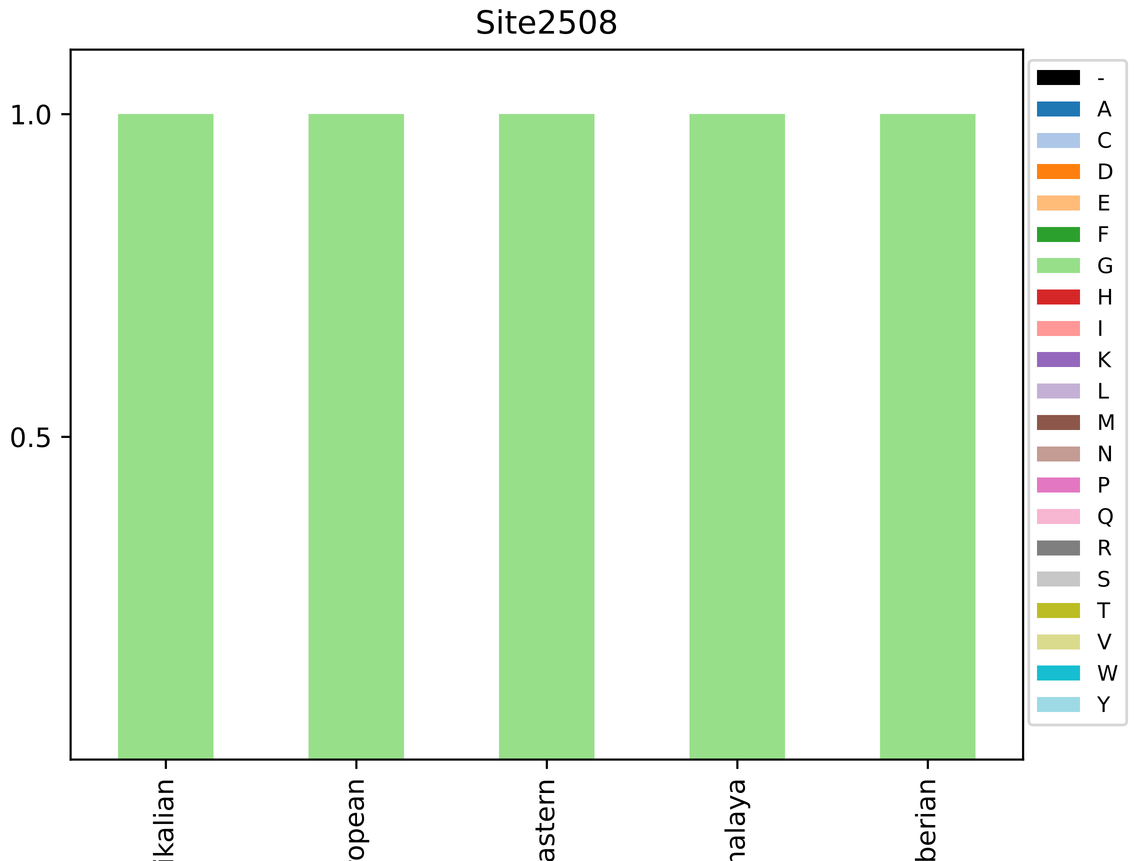
Site2508
1.0
0.5
-
A
C
D
E
F
G
H
I
K
L
M
N
P
Q
R
S
T
V
W
Y
ikalian
opean
astern
nalaya
berian

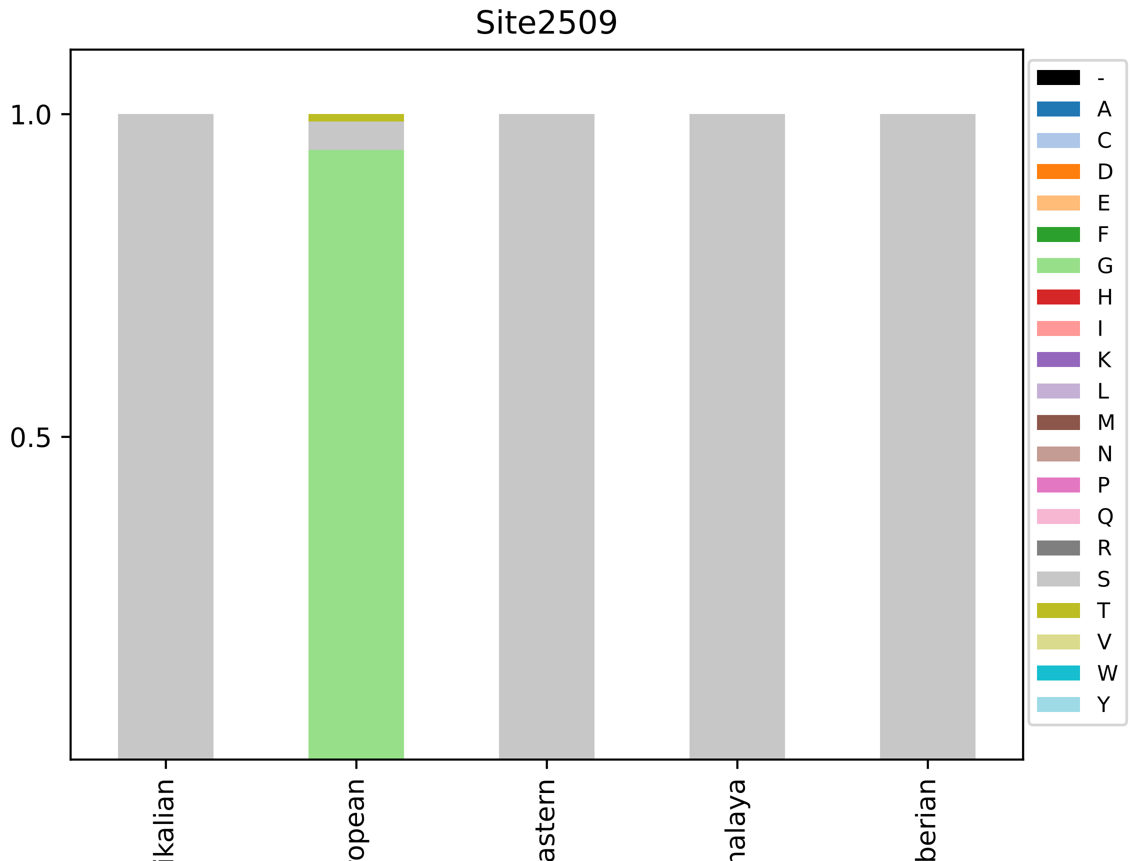
Site2509
1.0
0.5
-
A
C
D
E
F
G
H
I
K
L
M
N
P
Q
R
S
T
V
W
Y
ikalian
opean
astern
nalaya
berian

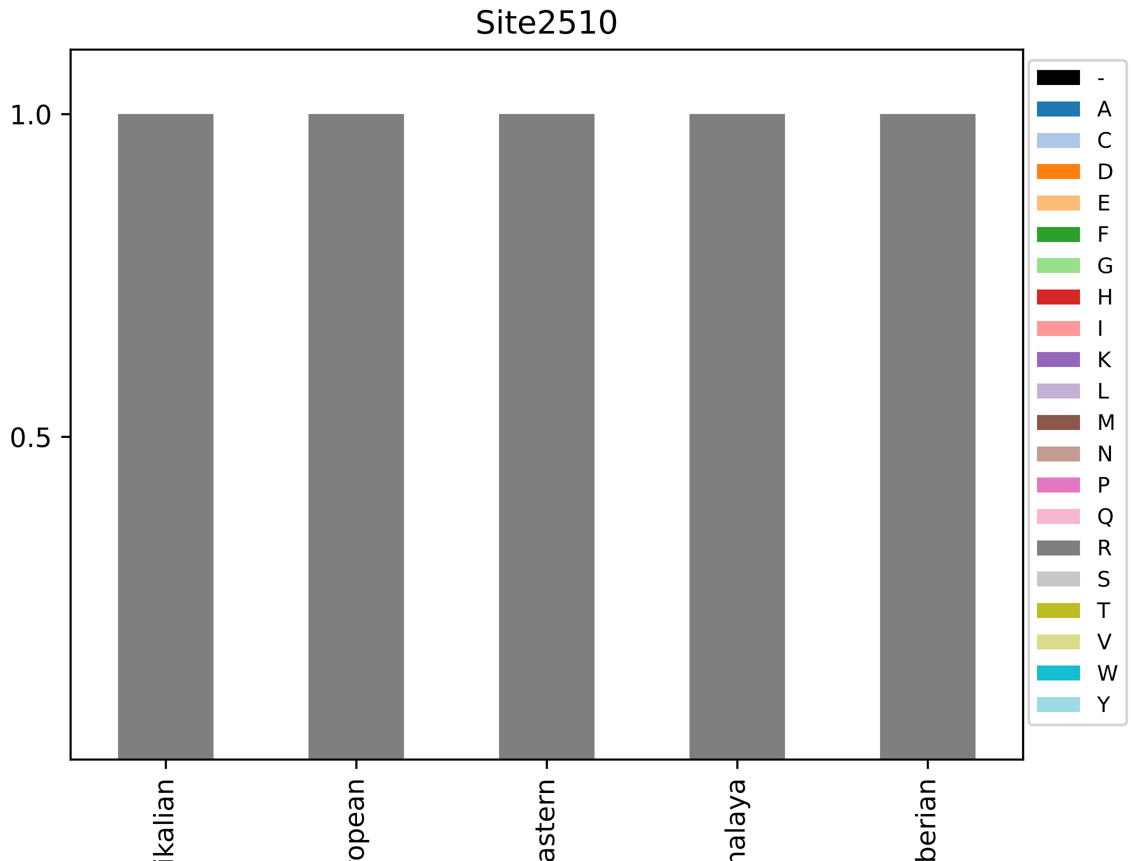

Site2510
1.0
0.5
-
A
C
D
E
F
G
H
I
K
L
M
N
P
Q
R
S
T
V
W
Y
ikalian
opean
astern
nalaya
berian

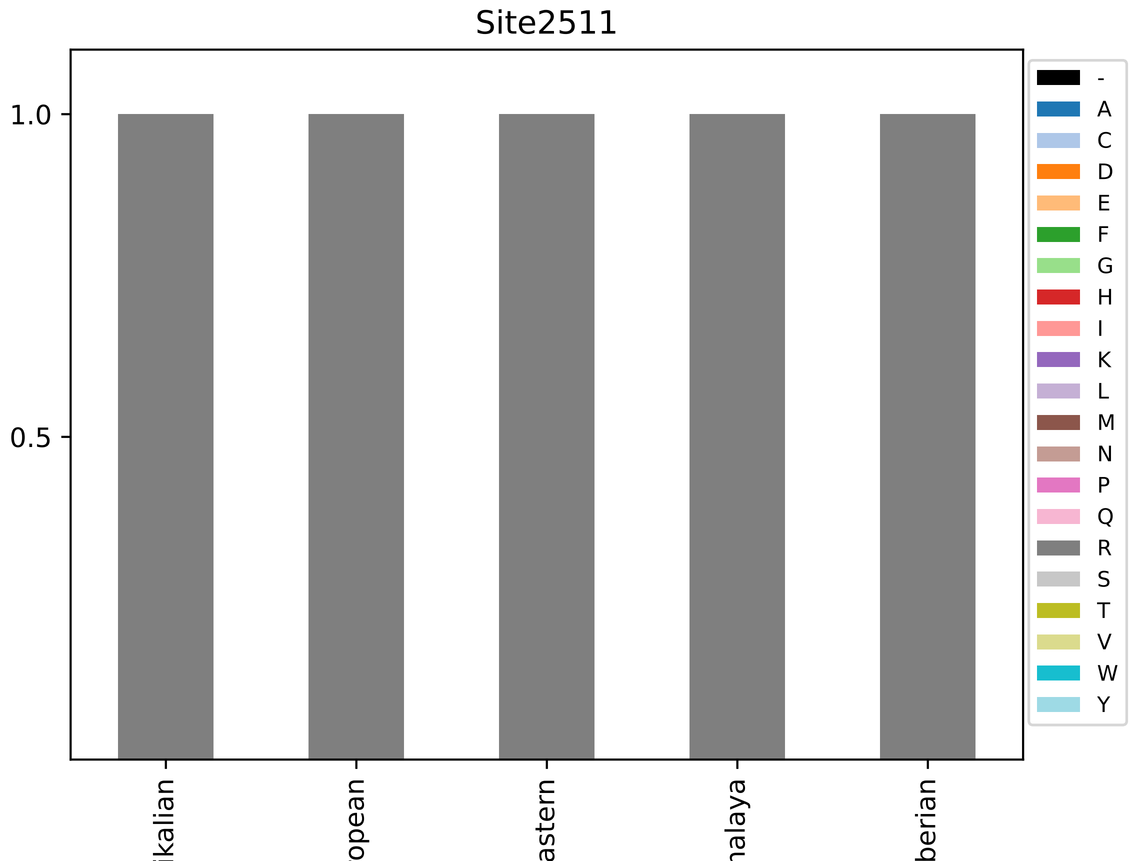
Site2511
1.0
0.5
-
A
C
D
E
F
G
H
I
K
L
M
N
P
Q
R
S
T
V
W
Y
ikalian
opean
astern
nalaya
berian

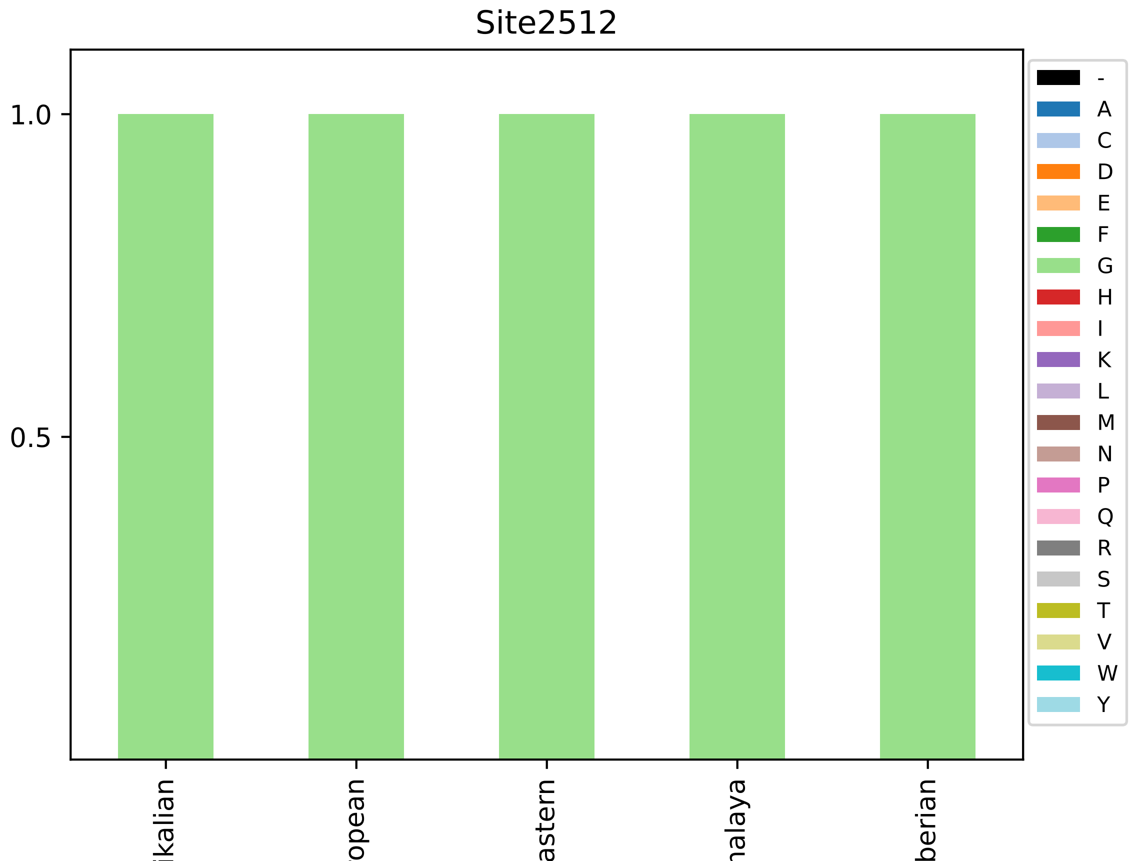
Site2512
1.0
0.5
-
A
C
D
E
F
G
H
I
K
L
M
N
P
Q
R
S
T
V
W
Y
ikalian
opean
astern
nalaya
berian

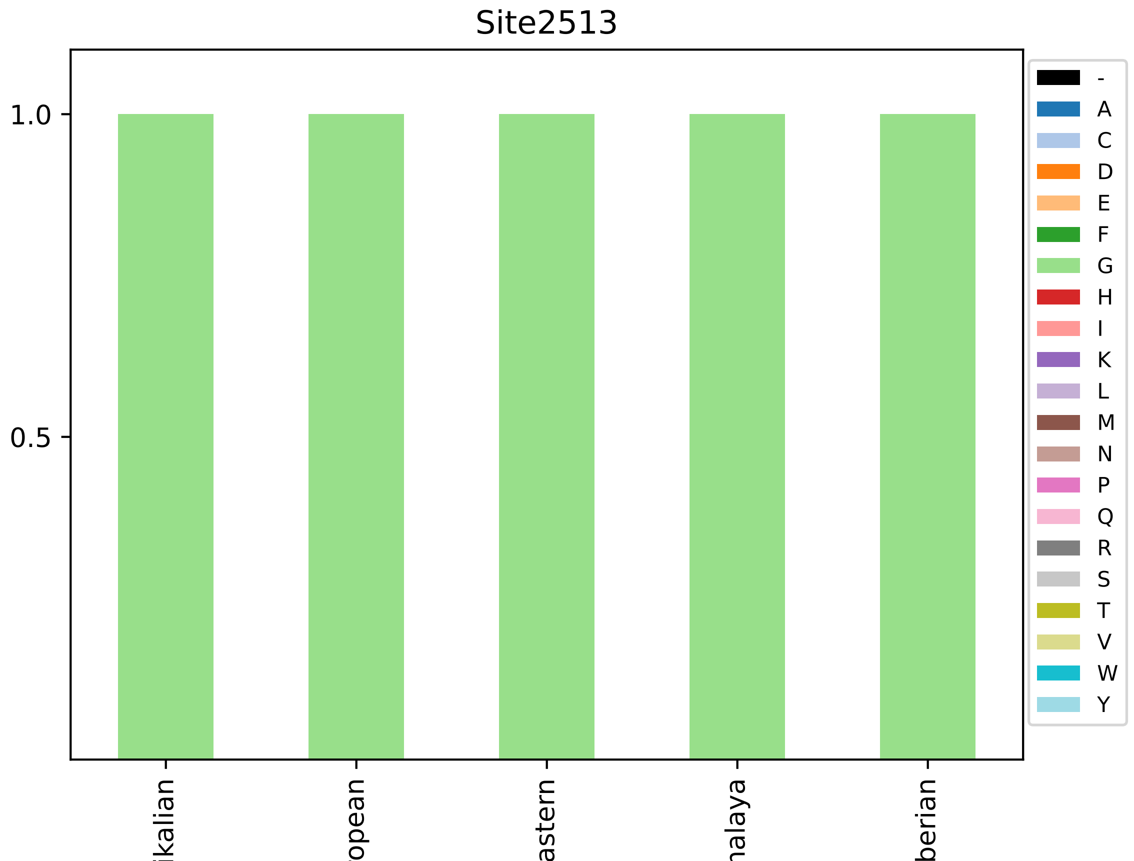

Site2513
1.0
0.5
ikalian
opean
astern
nalaya
berian
-
A
C
D
E
F
G
H
I
K
L
M
N
P
Q
R
S
T
V
W
Y

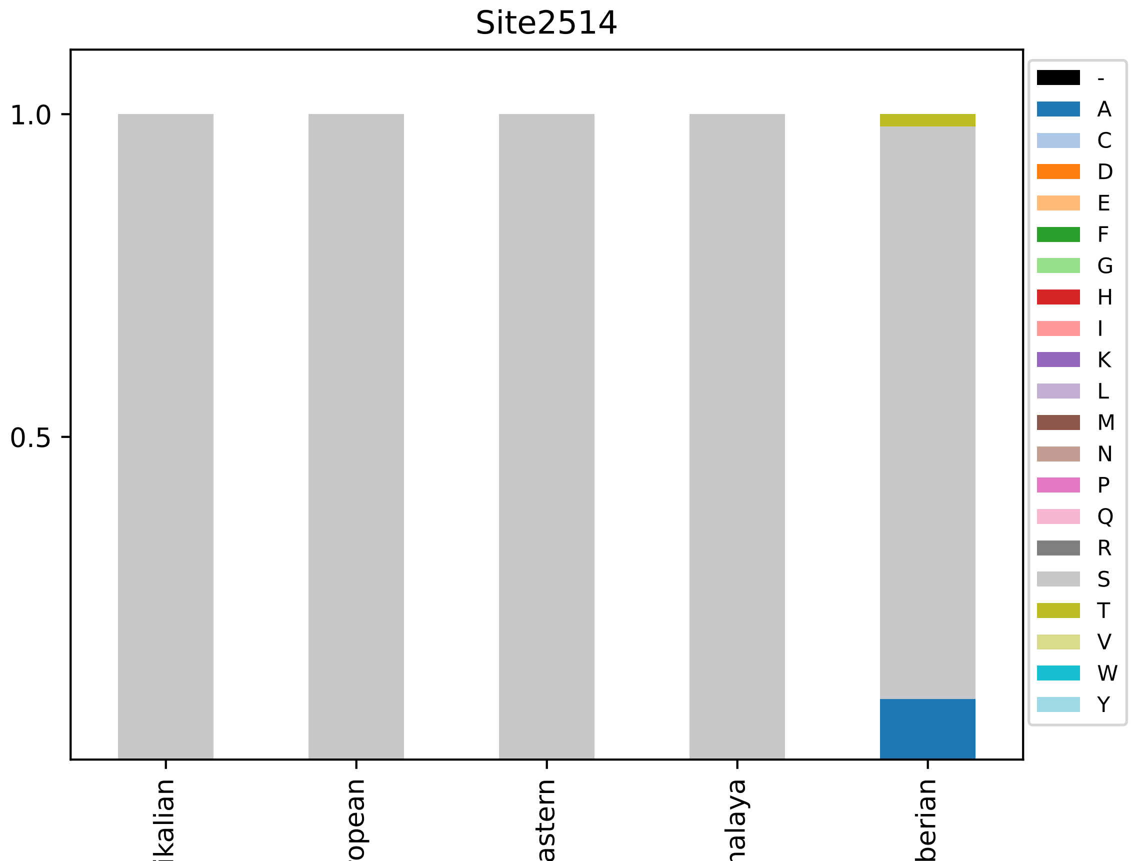
Site2514
1.0
0.5
-
A
C
D
E
F
G
H
I
K
L
M
N
P
Q
R
S
T
V
W
Y
ikalian
opean
astern
nalaya
berian

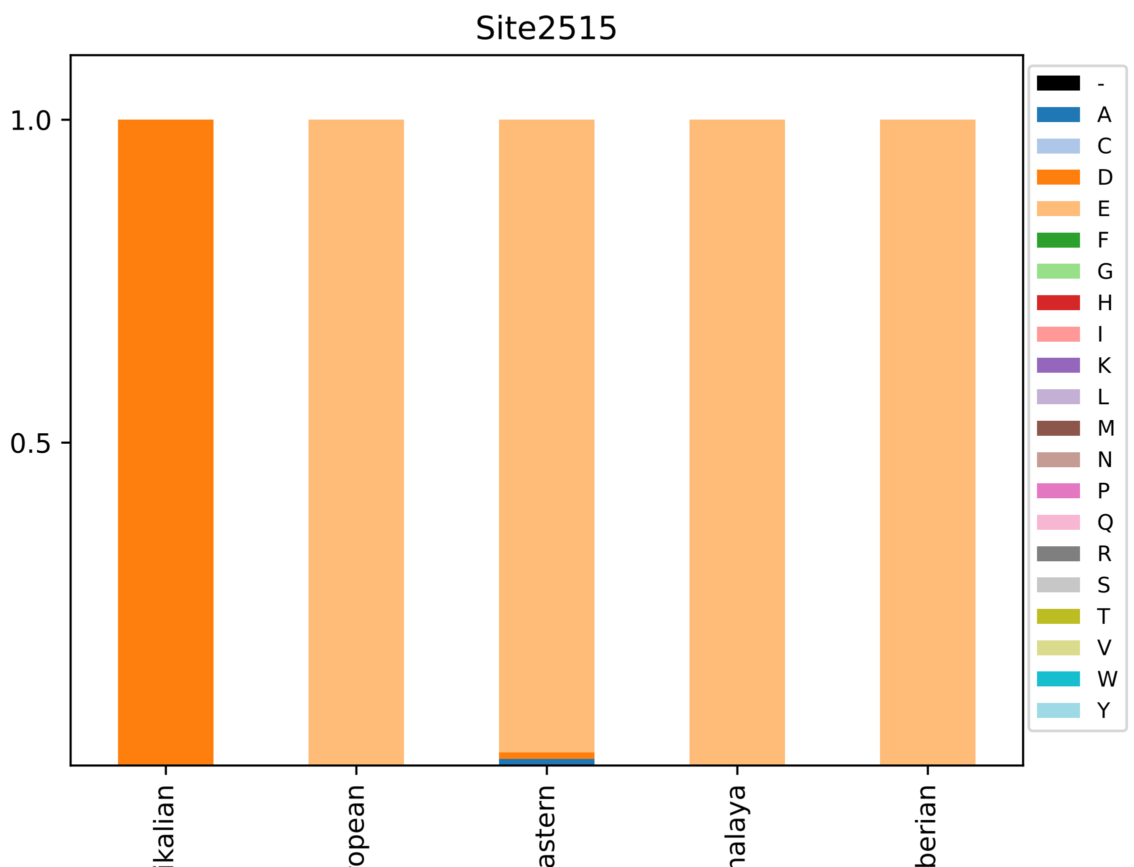
Site2515
1.0
0.5
-
A
C
D
E
F
G
H
I
K
L
M
N
P
Q
R
S
T
V
W
Y

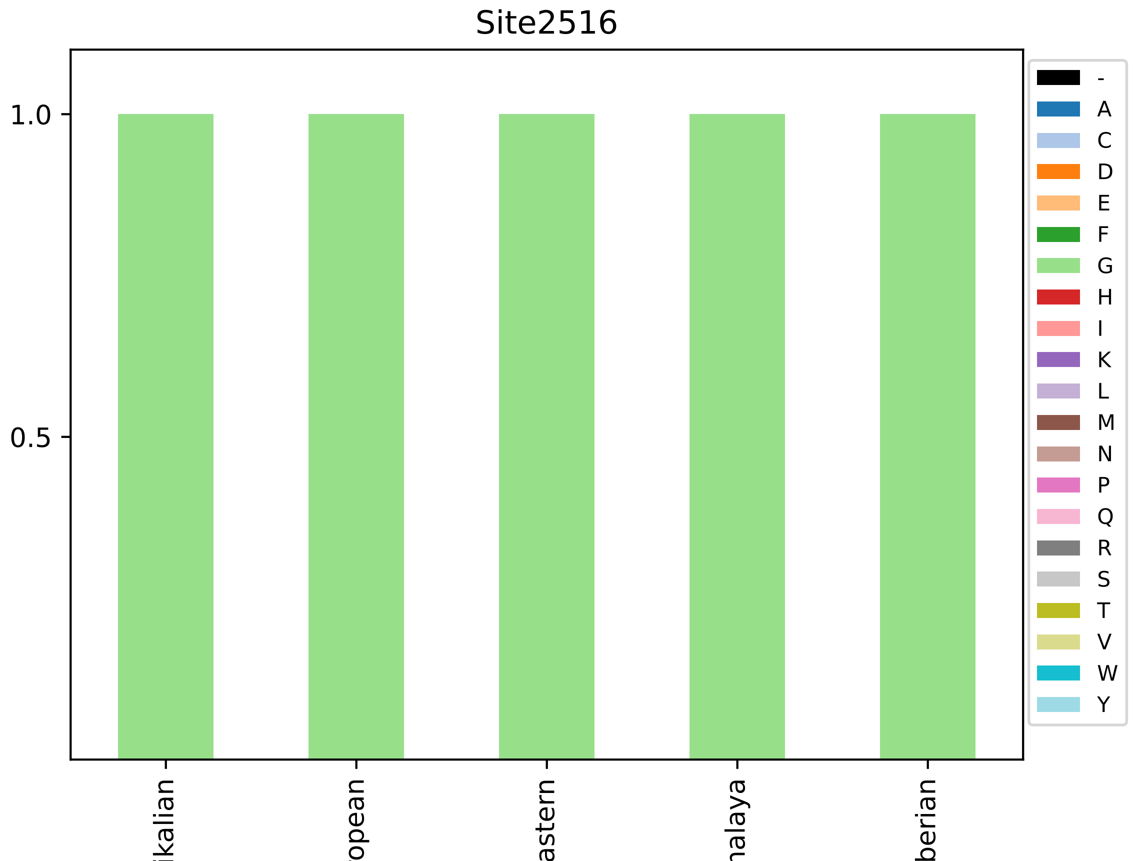

Site2516
1.0
0.5
-
A
C
D
E
F
G
H
I
K
L
M
N
P
Q
R
S
T
V
W
Y
ikalian
opean
astern
nalaya
berian

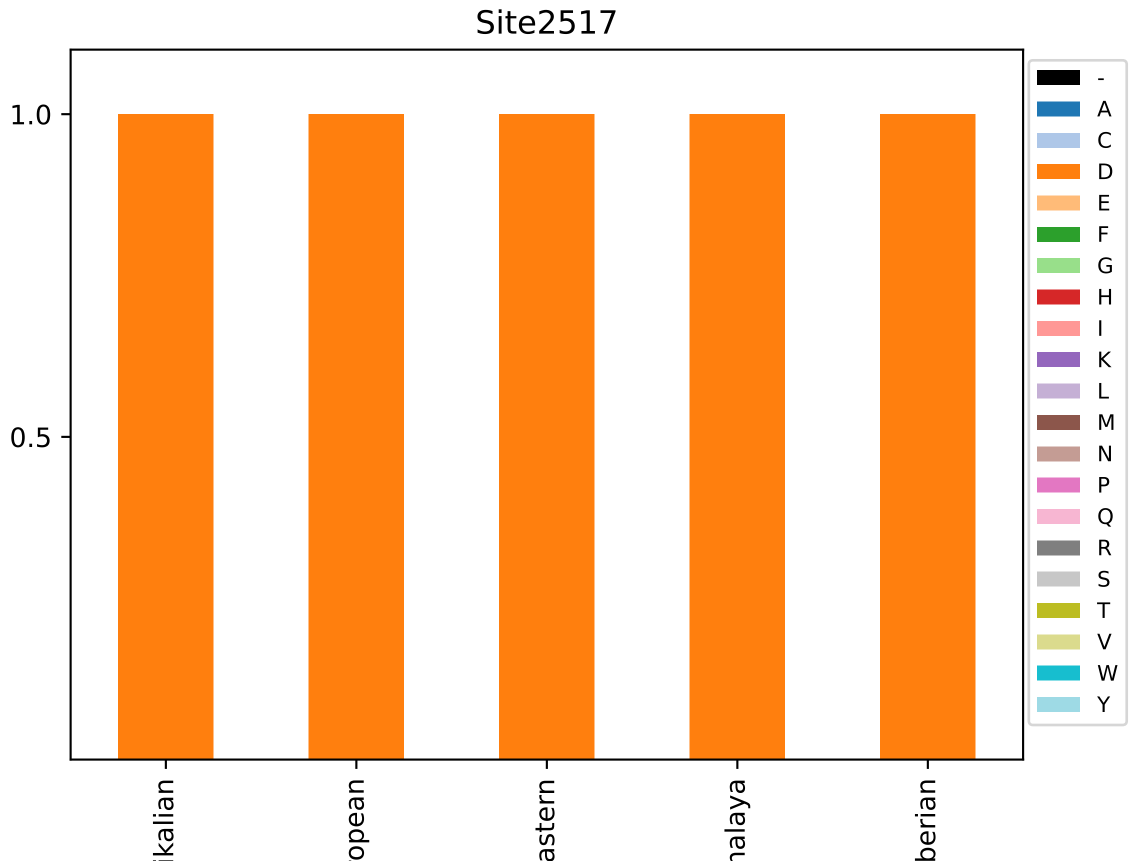

Site2517
1.0
0.5
-
A
C
D
E
F
G
H
I
K
L
M
N
P
Q
R
S
T
V
W
Y
ikalian
ropean
astern
nalaya
berian

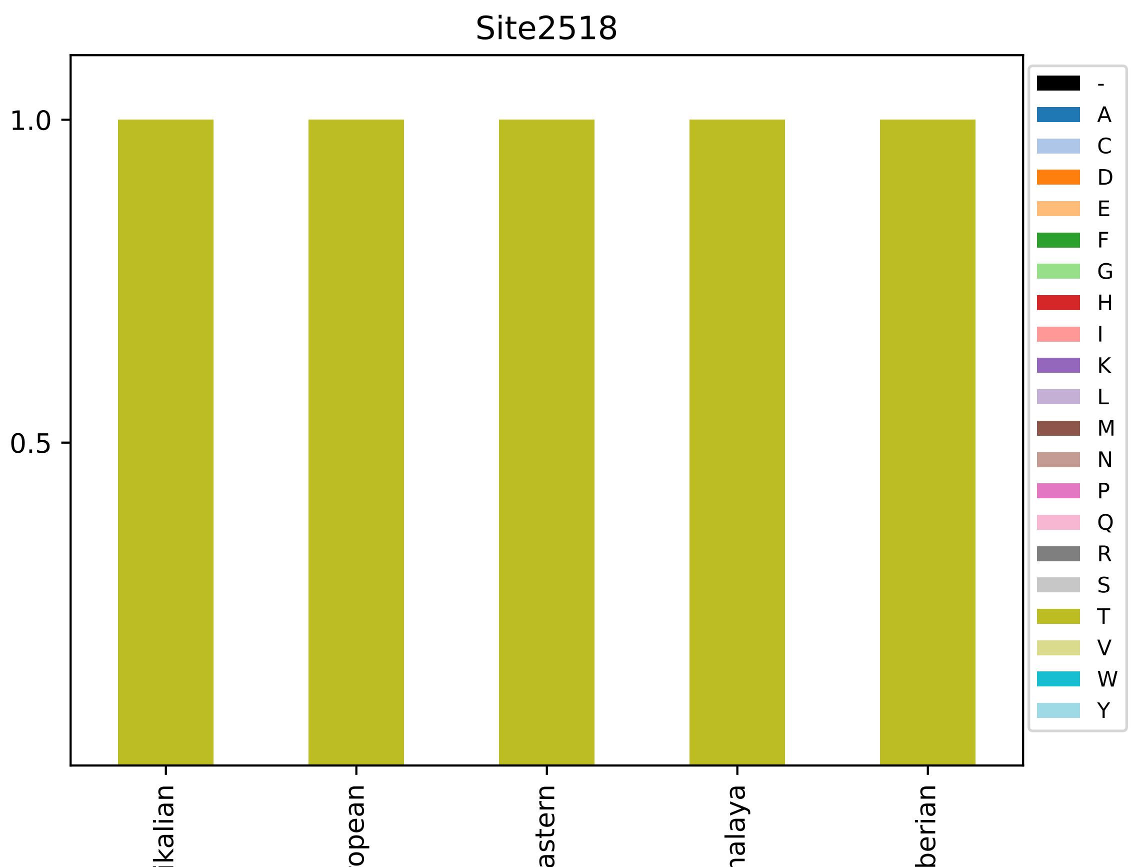
Site2518
1.0
0.5
-
A
C
D
E
F
G
H
I
K
L
M
N
P
Q
R
S
T
V
W
Y
ikalian
opean
astern
nalaya
berian

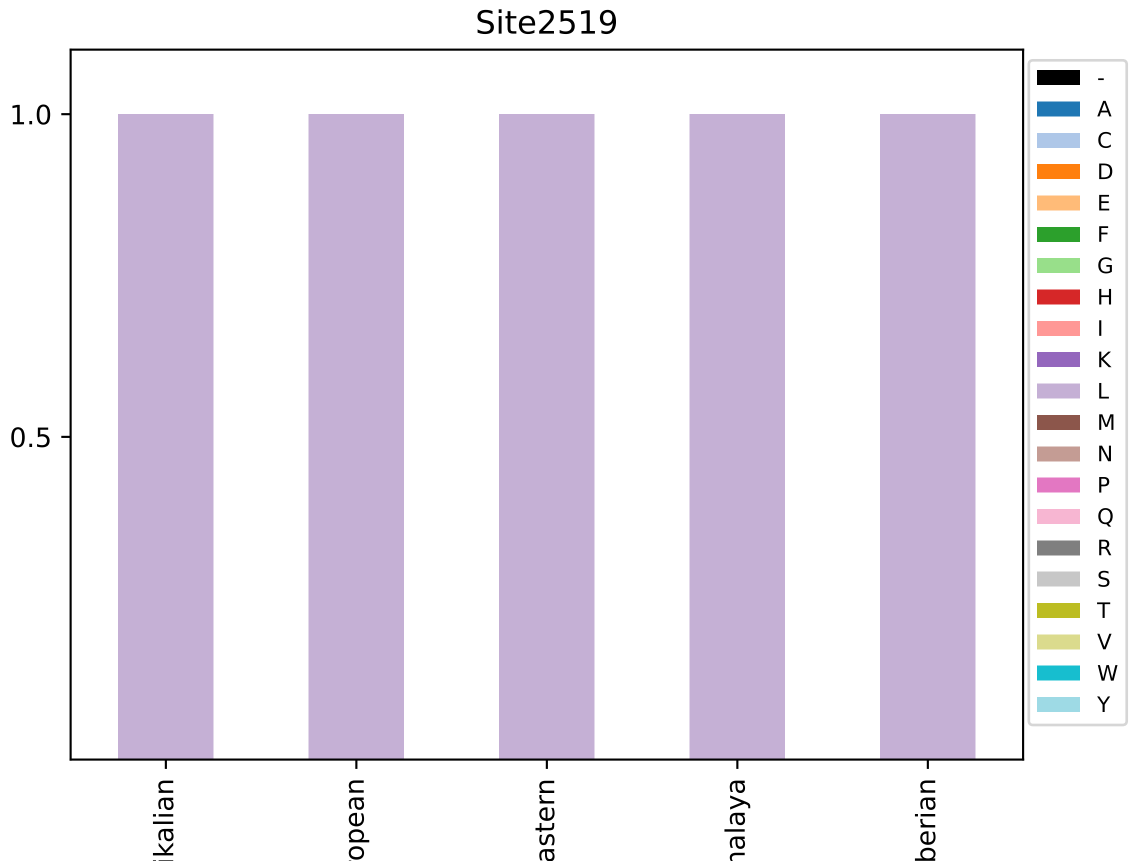
Site2519
1.0
0.5
-
A
C
D
E
F
G
H
I
K
L
M
N
P
Q
R
S
T
V
W
Y

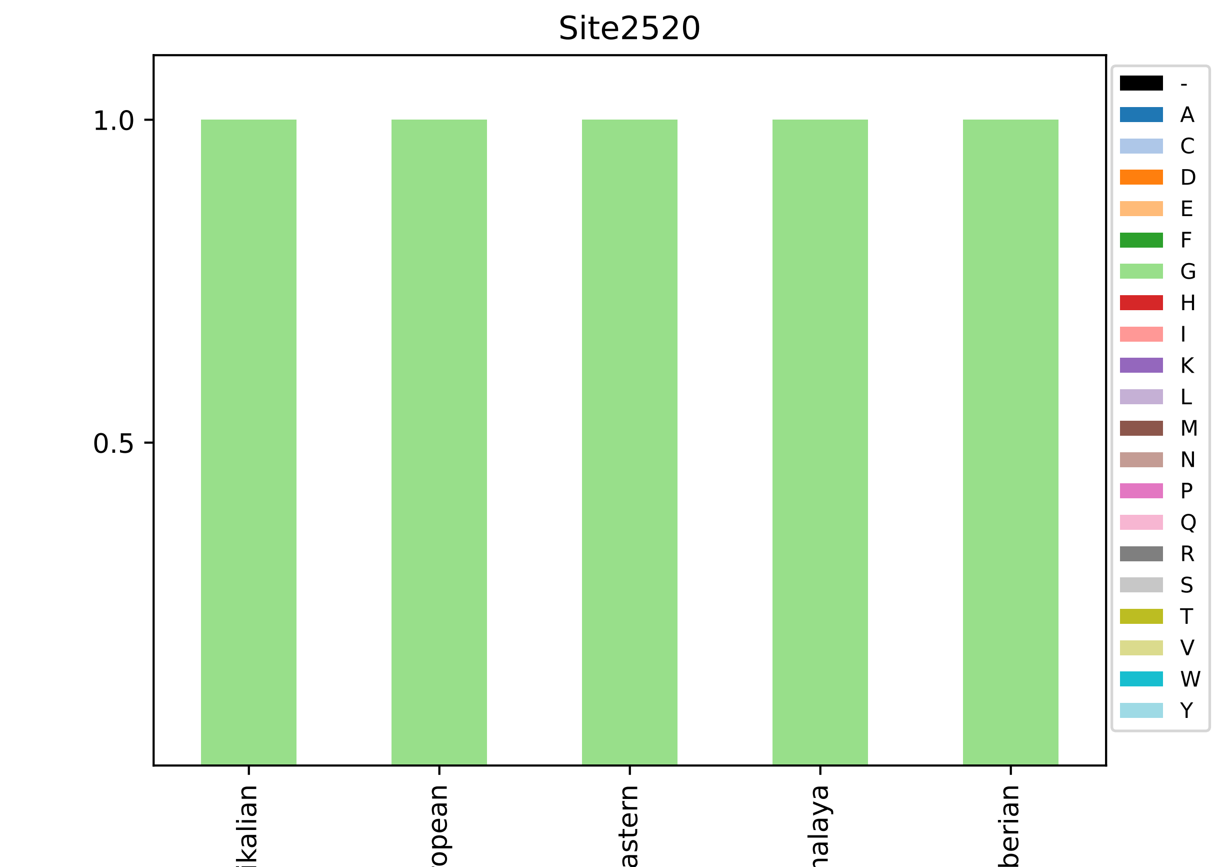
Site2520
1.0
0.5
-
A
C
D
E
F
G
H
I
K
L
M
N
P
Q
R
S
T
V
W
Y
ikalian
opean
astern
nalaya
berian

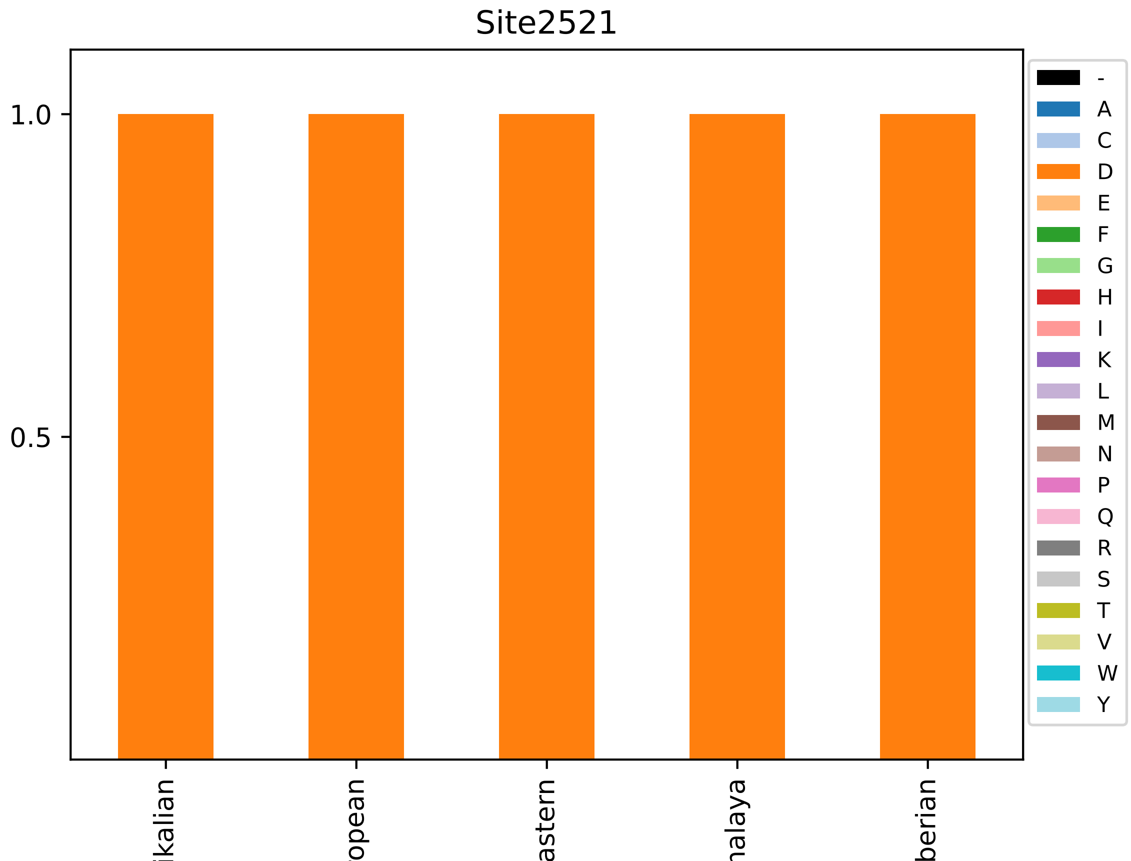
Site2521
1.0
0.5
-
A
C
D
E
F
G
H
I
K
L
M
N
P
Q
R
S
T
V
W
Y
ikalian
opean
astern
nalaya
berian

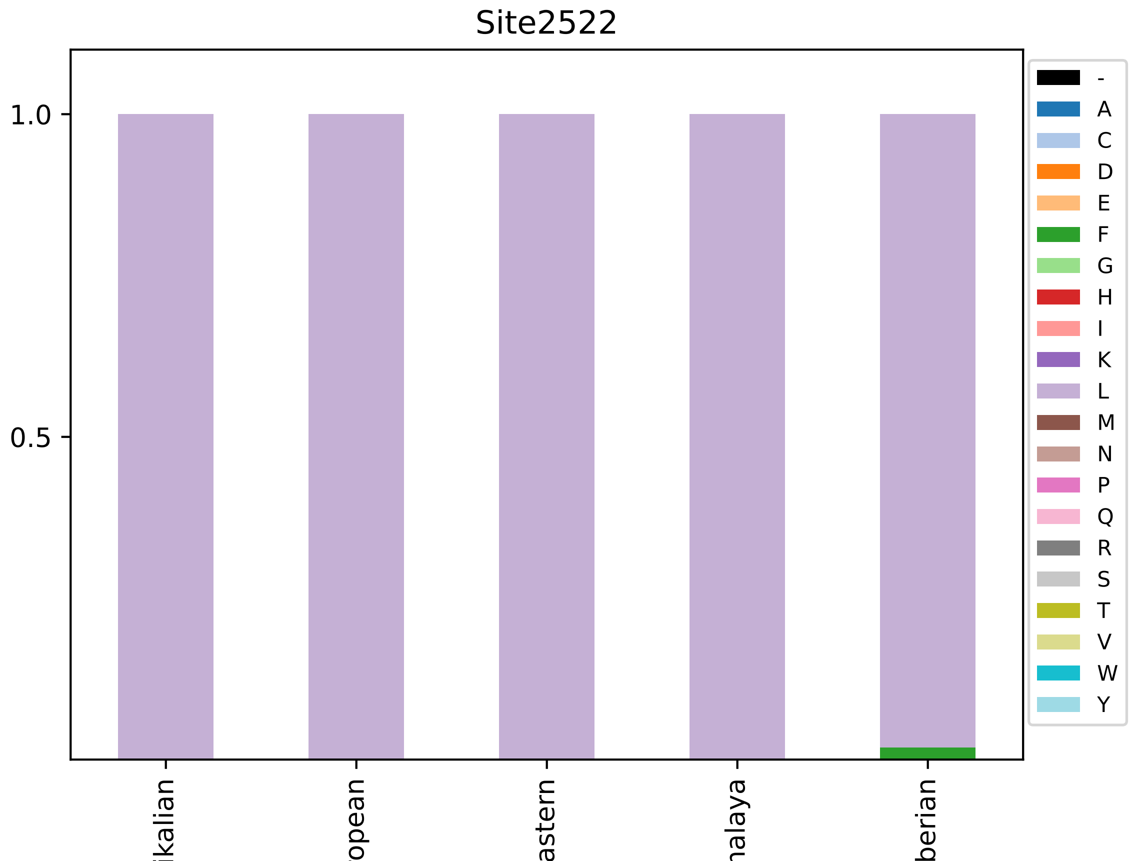

Site2522
1.0
0.5
-
A
C
D
E
F
G
H
I
K
L
M
N
P
Q
R
S
T
V
W
Y
ikalian
opean
astern
nalaya
berian

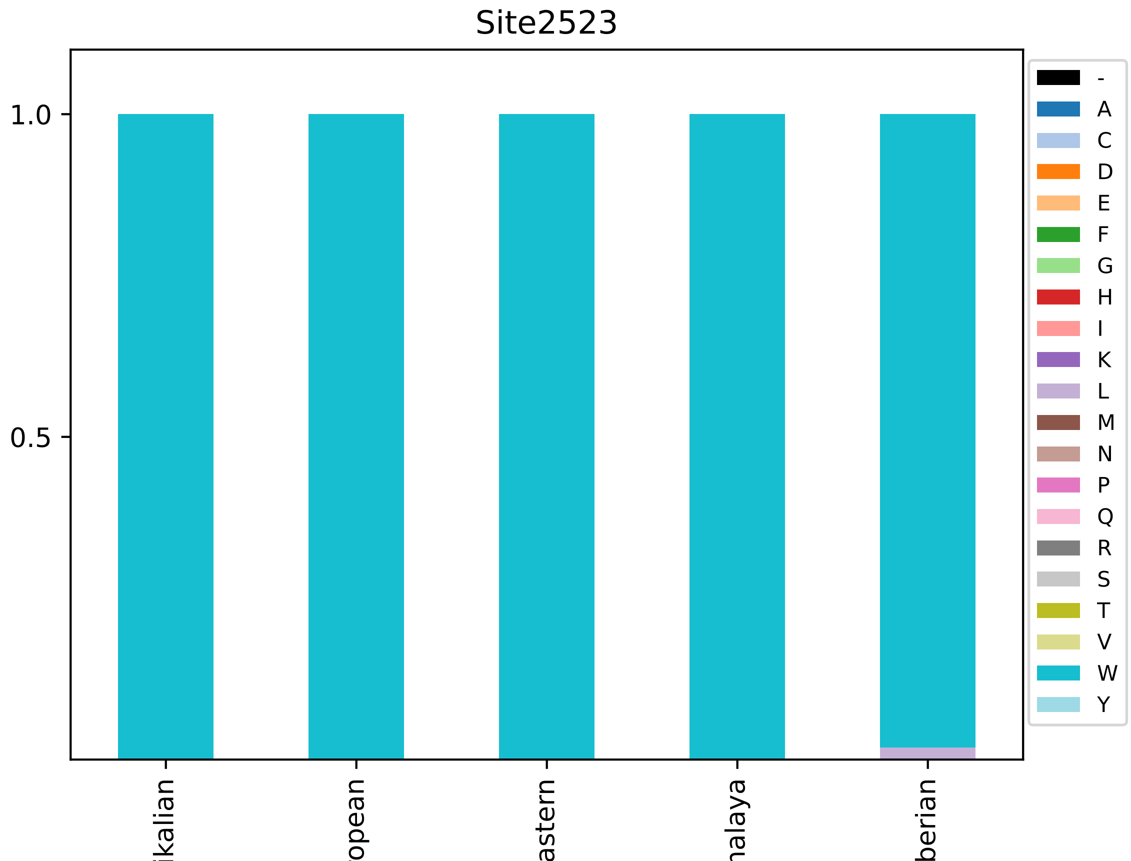
Site2523
1.0
0.5
-
A
C
D
E
F
G
H
I
K
L
M
N
P
Q
R
S
T
V
W
Y
ikalian
opean
astern
halaya
berian

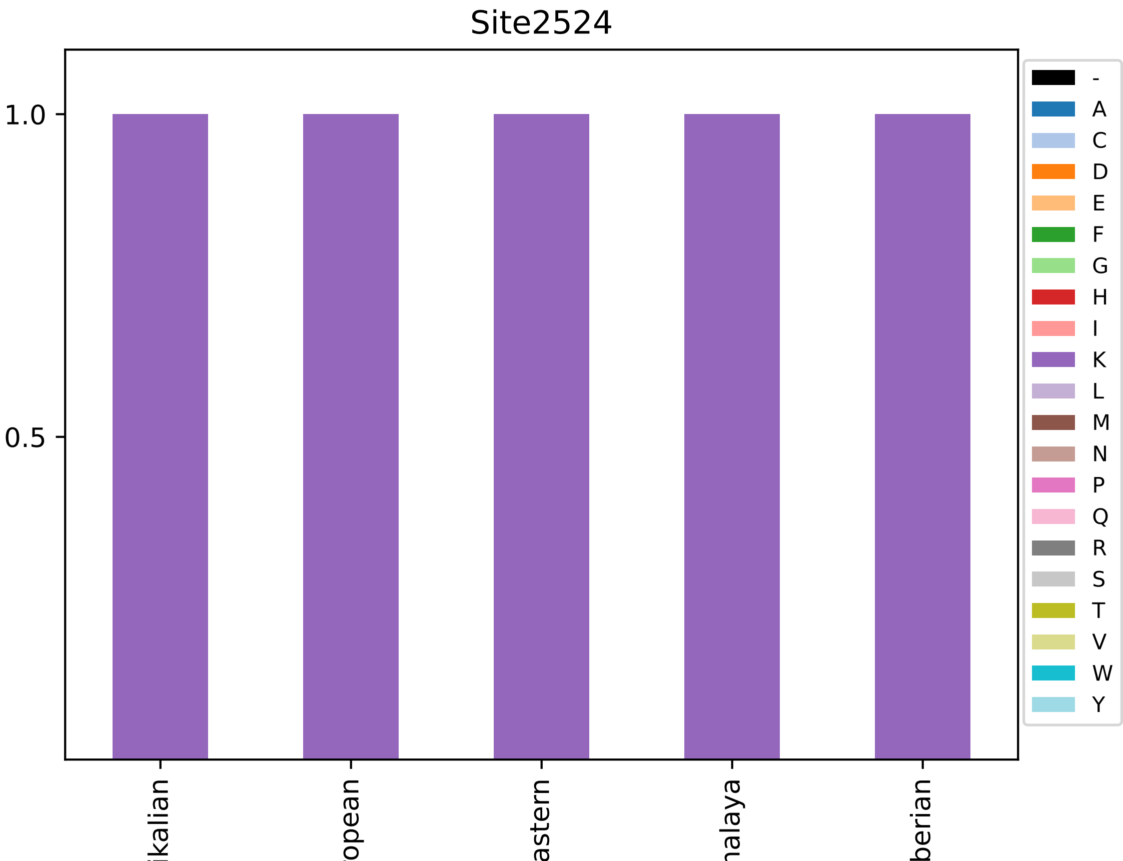
Site2524
1.0
0.5
-
A
C
D
E
F
G
H
I
K
L
M
N
P
Q
R
S
T
V
W
Y
ikalian
opean
astern
nalaya
berian

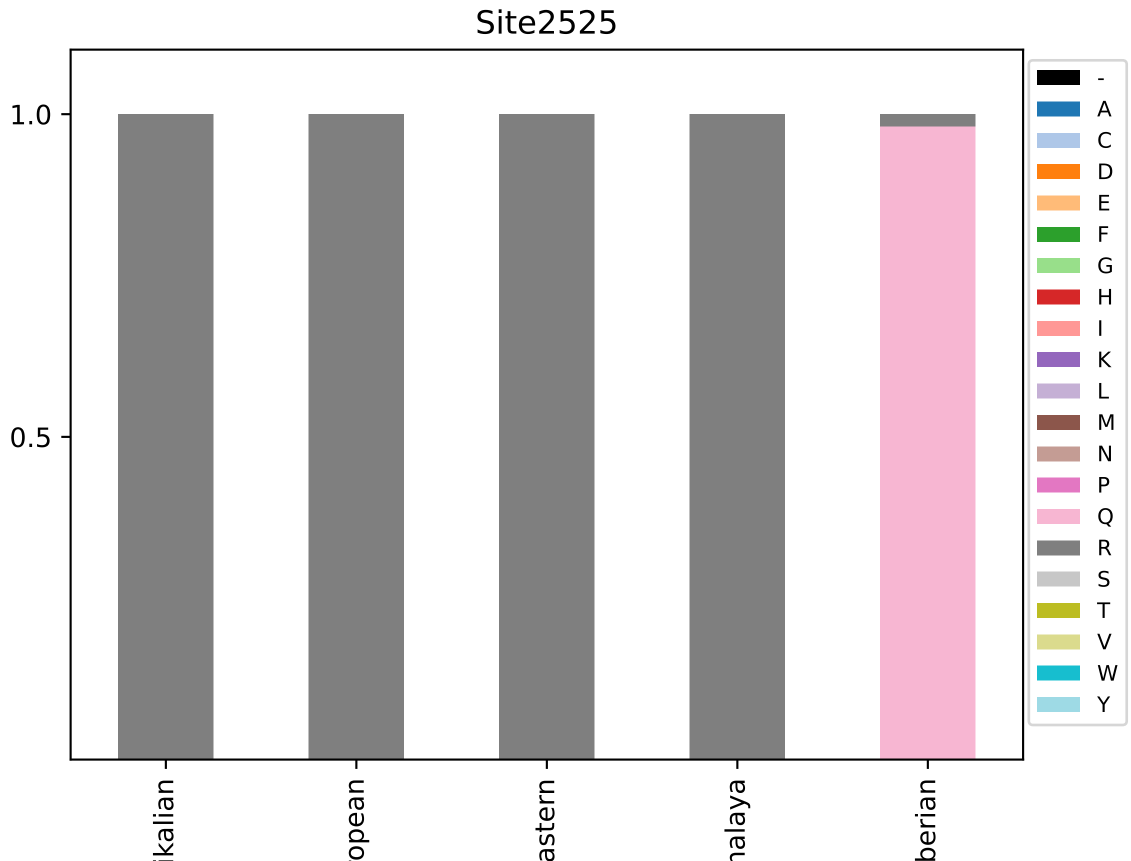

Site2525
1.0
0.5
-
A
C
D
E
F
G
H
I
K
L
M
N
P
Q
R
S
T
V
W
Y
ikalian
opean
astern
nalaya
berian

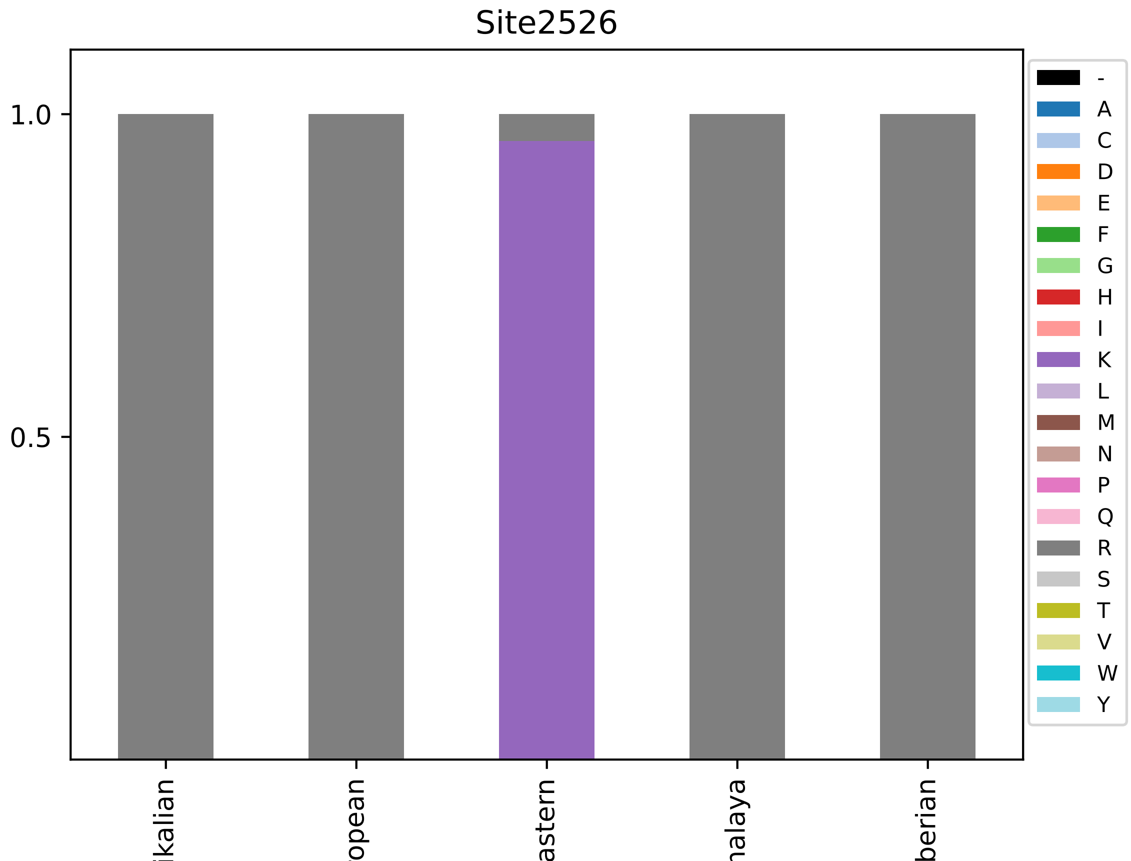
Site2526
1.0
0.5
-
A
C
D
E
F
G
H
I
K
L
M
N
P
Q
R
S
T
V
W
Y
ikalian
opean
astern
nalaya
berian

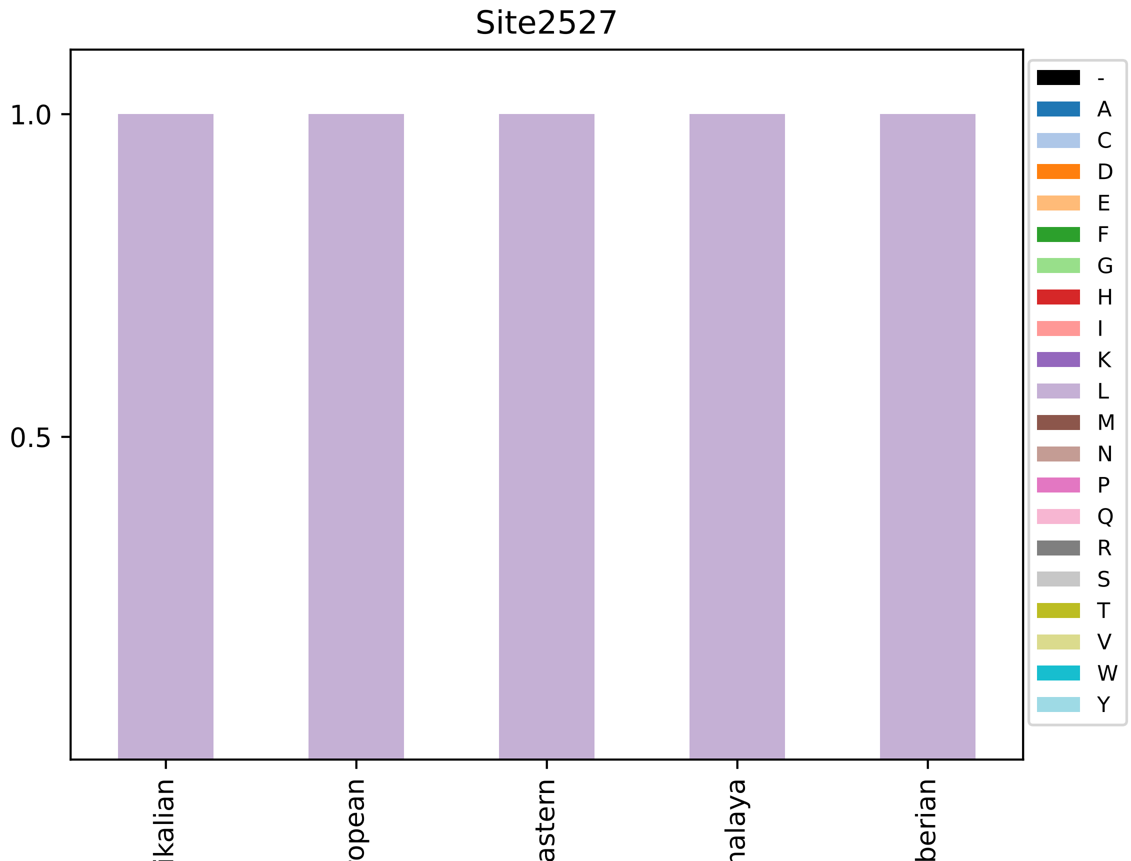
Site2527
1.0
0.5
-
A
C
D
E
F
G
H
I
K
L
M
N
P
Q
R
S
T
V
W
Y
ikalian
opean
astern
nalaya
berian

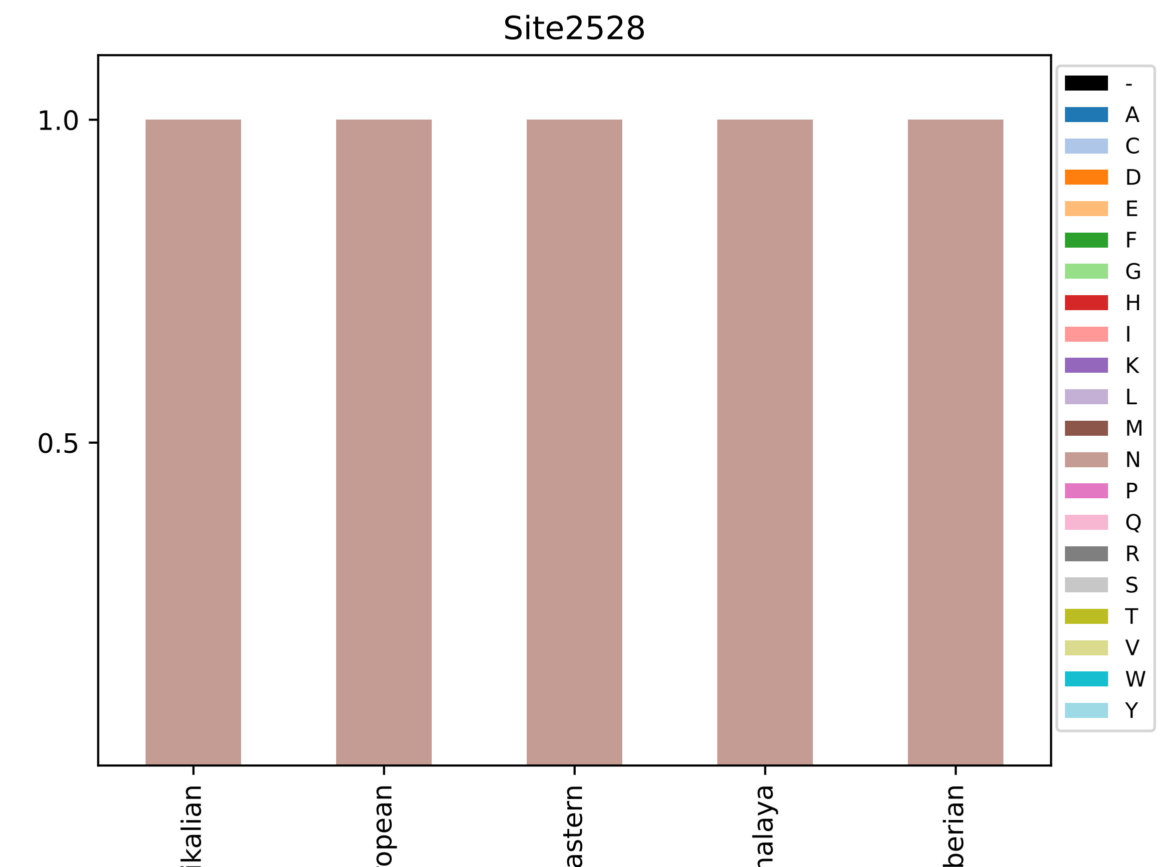

Site2528
1.0
0.5
ikalian
opean
astern
nalaya
berian
-
A
C
D
E
F
G
H
I
K
L
M
N
P
Q
R
S
T
V
W
Y

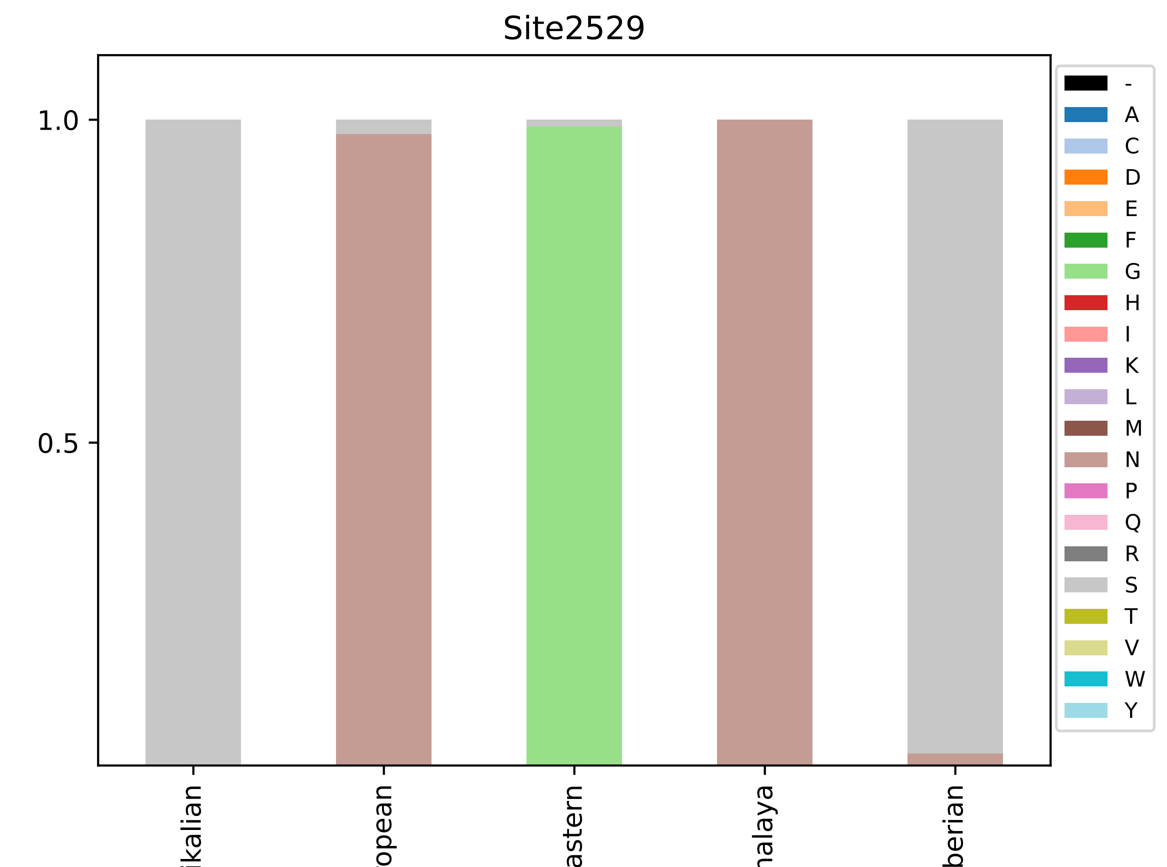
Site2529
1.0
0.5
ikalian
ropean
astern
nalaya
berian
-
A
C
D
E
F
G
H
I
K
L
M
N
P
Q
R
S
T
V
W
Y

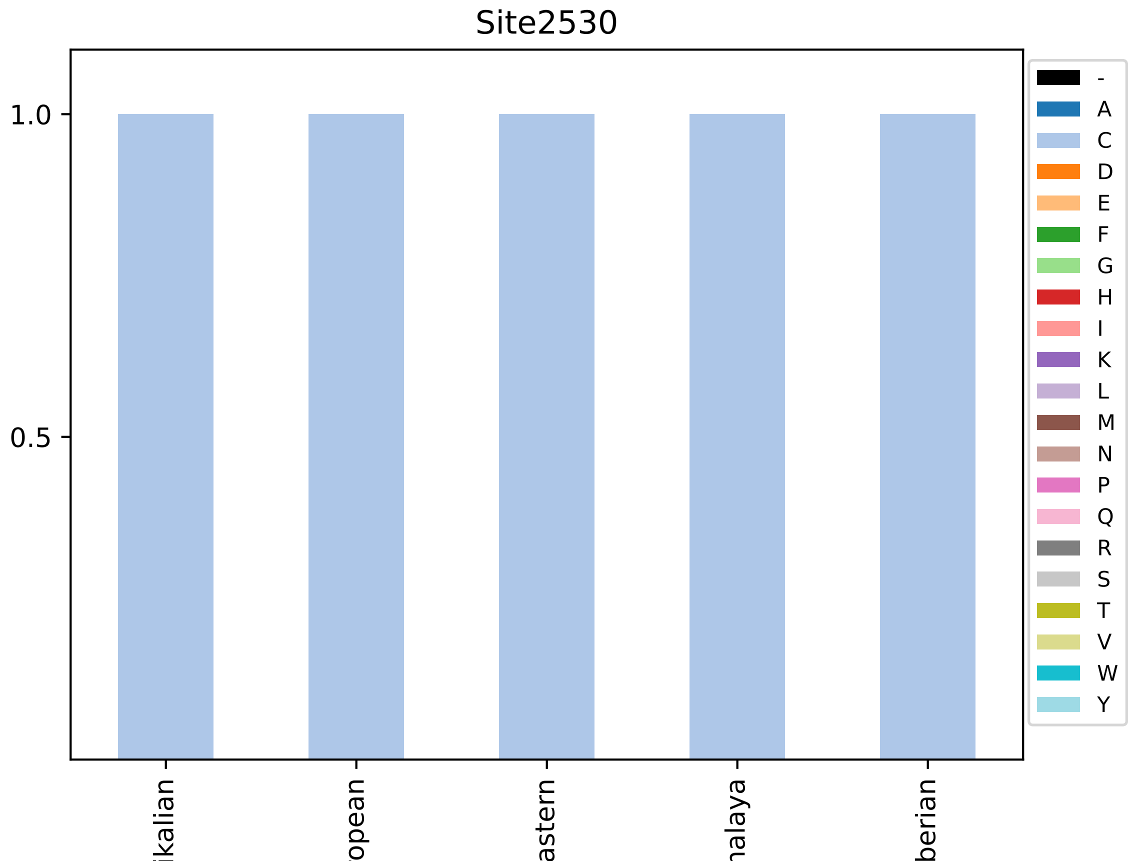

Site2530
1.0
0.5
-
A
C
D
E
F
G
H
I
K
L
M
N
P
Q
R
S
T
V
W
Y
ikalian
opean
astern
nalaya
berian

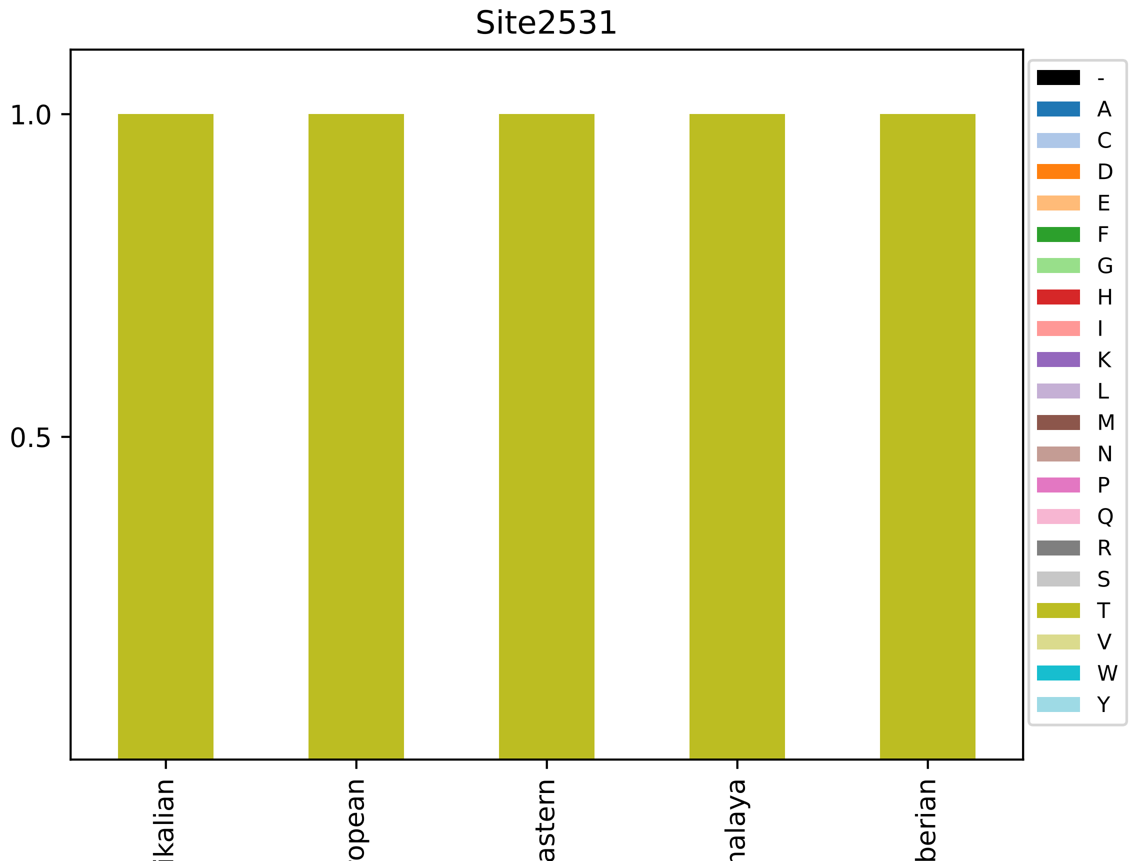

Site2531
1.0
0.5
-
A
C
D
E
F
G
H
I
K
L
M
N
P
Q
R
S
T
V
W
Y
ikalian
opean
astern
nalaya
berian

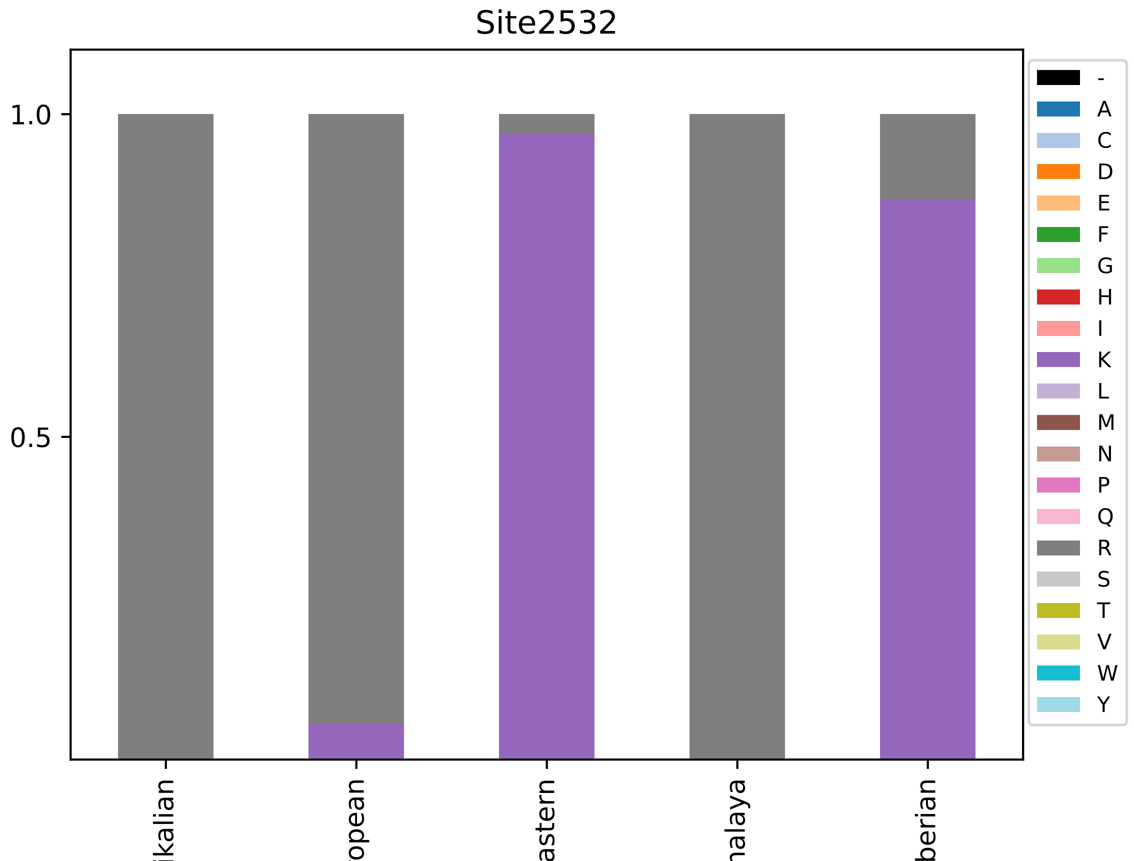
Site2532
1.0
0.5
ikalian
opean
astern
nalaya
berian
-
A
C
D
E
F
G
H
I
K
L
M
N
P
Q
R
S
T
V
W
Y

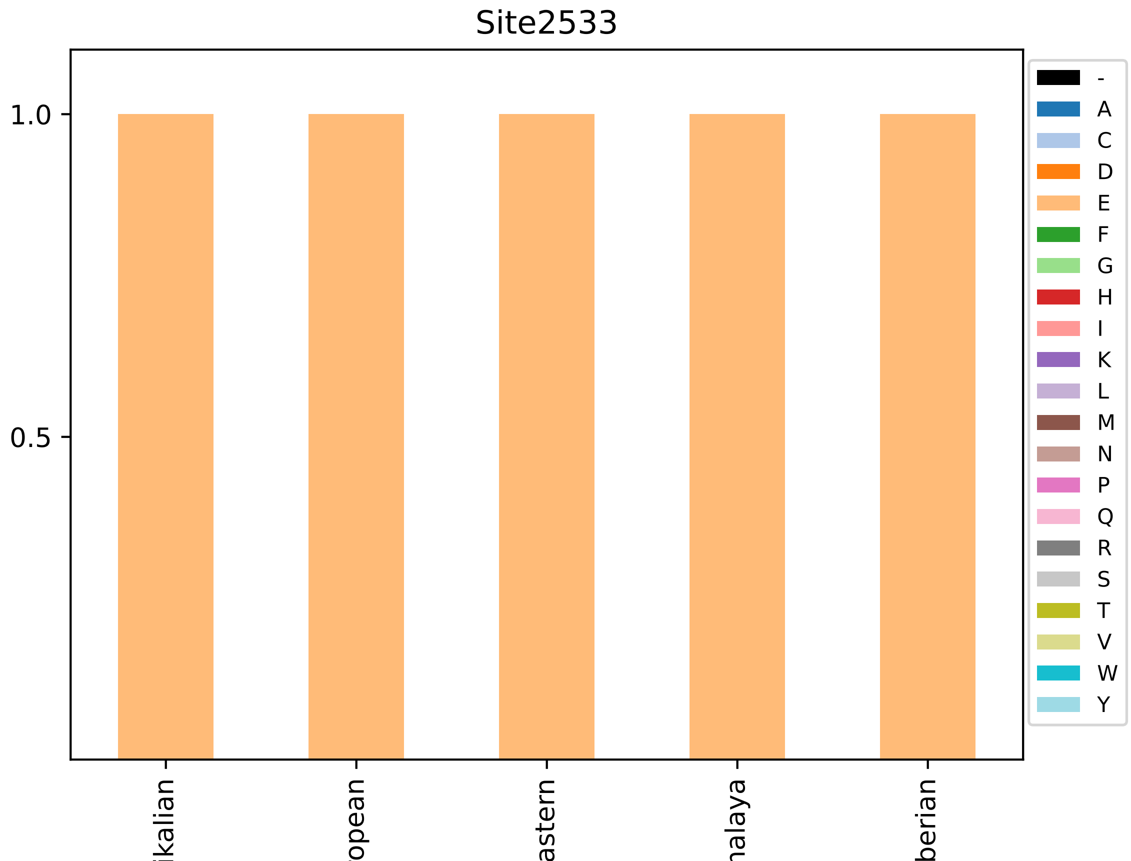

Site2533
1.0
0.5
-
A
C
D
E
F
G
H
I
K
L
M
N
P
Q
R
S
T
V
W
Y
ikalian
opean
astern
nalaya
berian

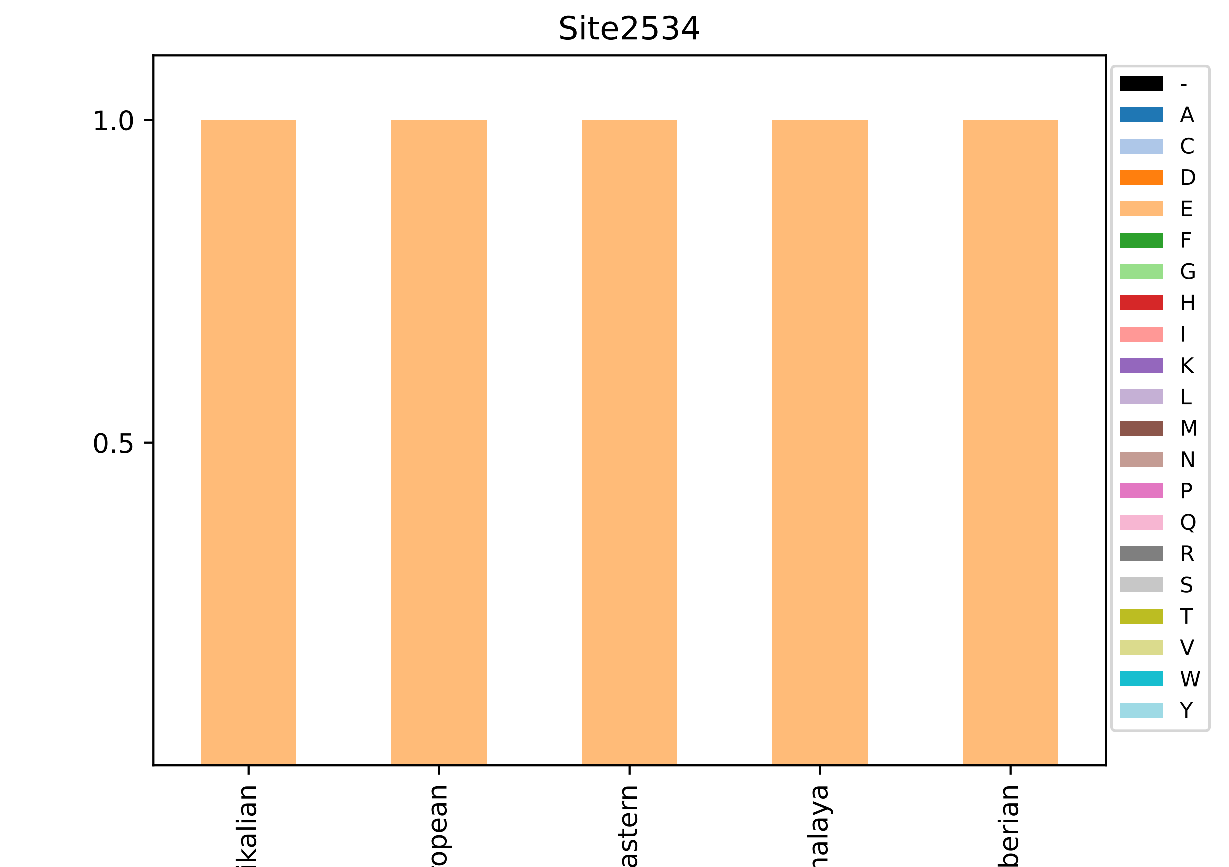
Site2534
1.0
0.5
-
A
C
D
E
F
G
H
I
K
L
M
N
P
Q
R
S
T
V
W
Y
ikalian
opean
astern
nalaya
berian

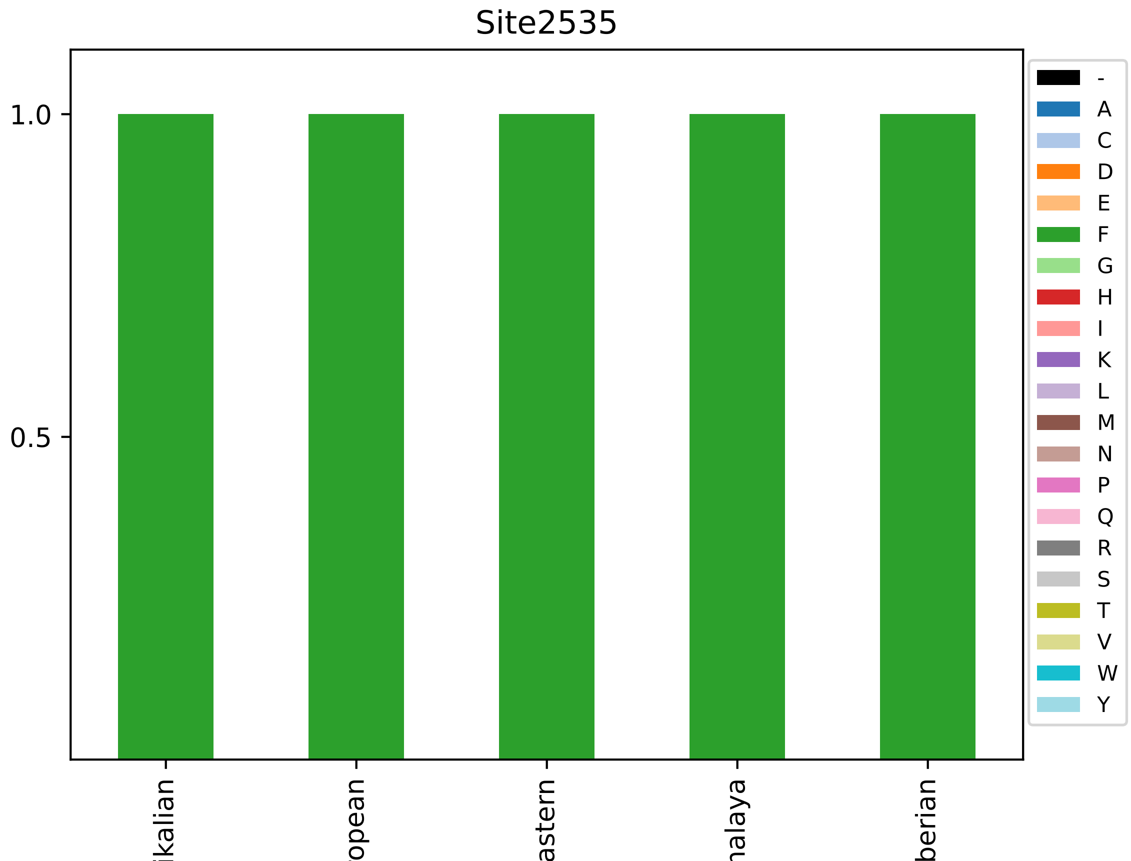
Site2535
1.0
0.5
-
A
C
D
E
F
G
H
I
K
L
M
N
P
Q
R
S
T
V
W
Y
ikalian
opean
astern
nalaya
berian

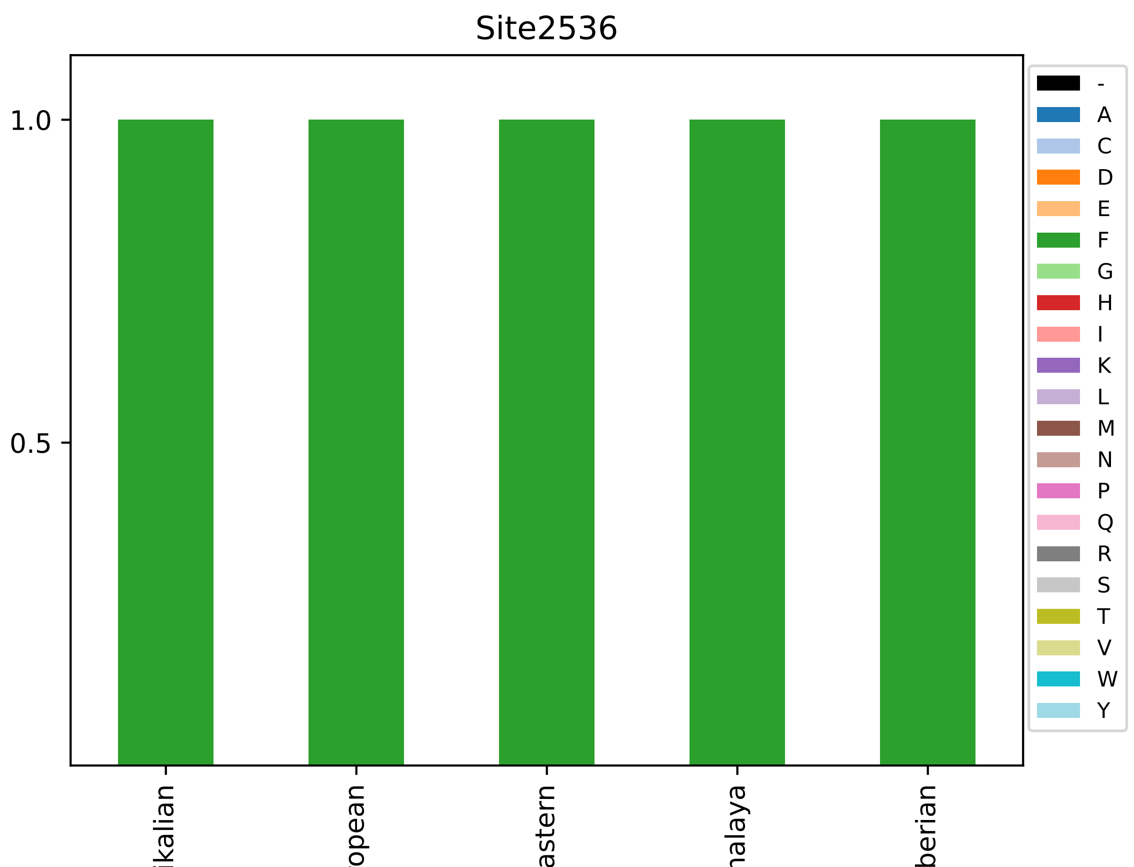
Site2536
1.0
0.5
ikalian
opean
astern
nalaya
berian
-
A
C
D
E
F
G
H
I
K
L
M
N
P
Q
R
S
T
V
W
Y

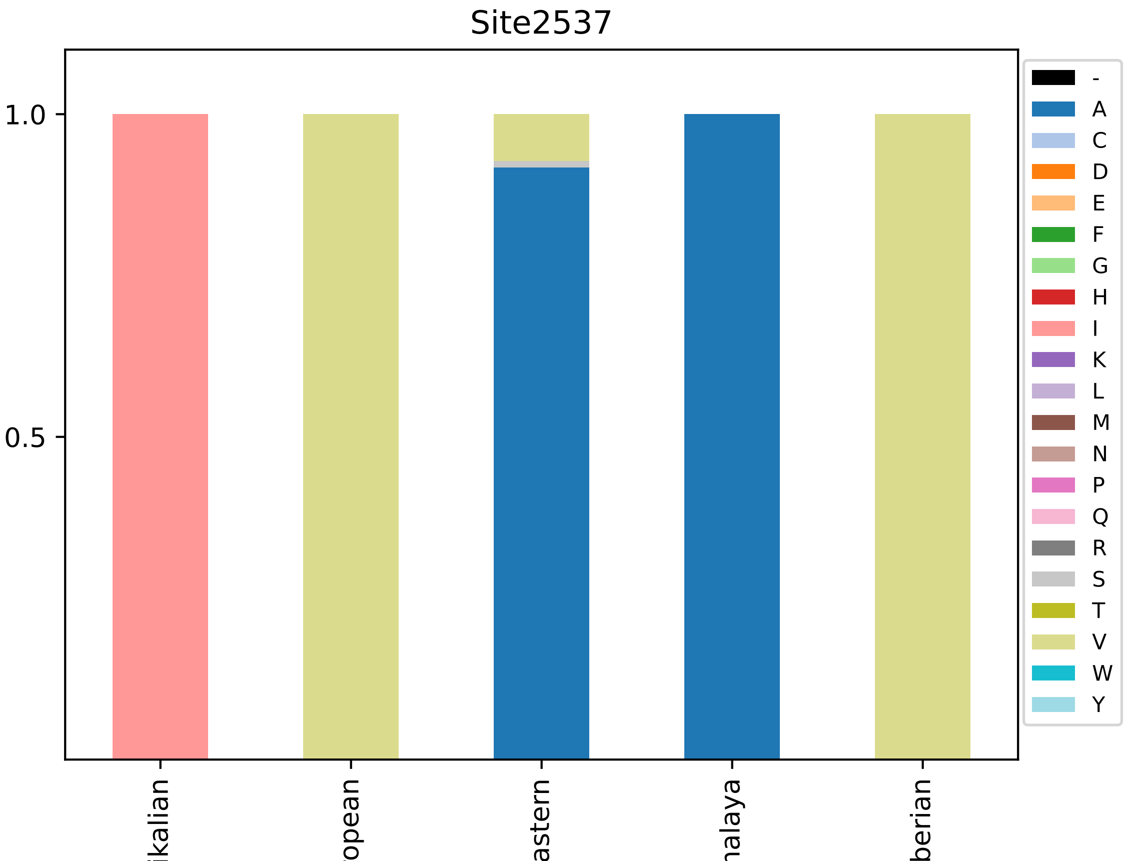

Site2537
1.0
0.5
-
A
C
D
E
F
G
H
I
K
L
M
N
P
Q
R
S
T
V
W
Y
ikalian
opean
astern
nalaya
berian

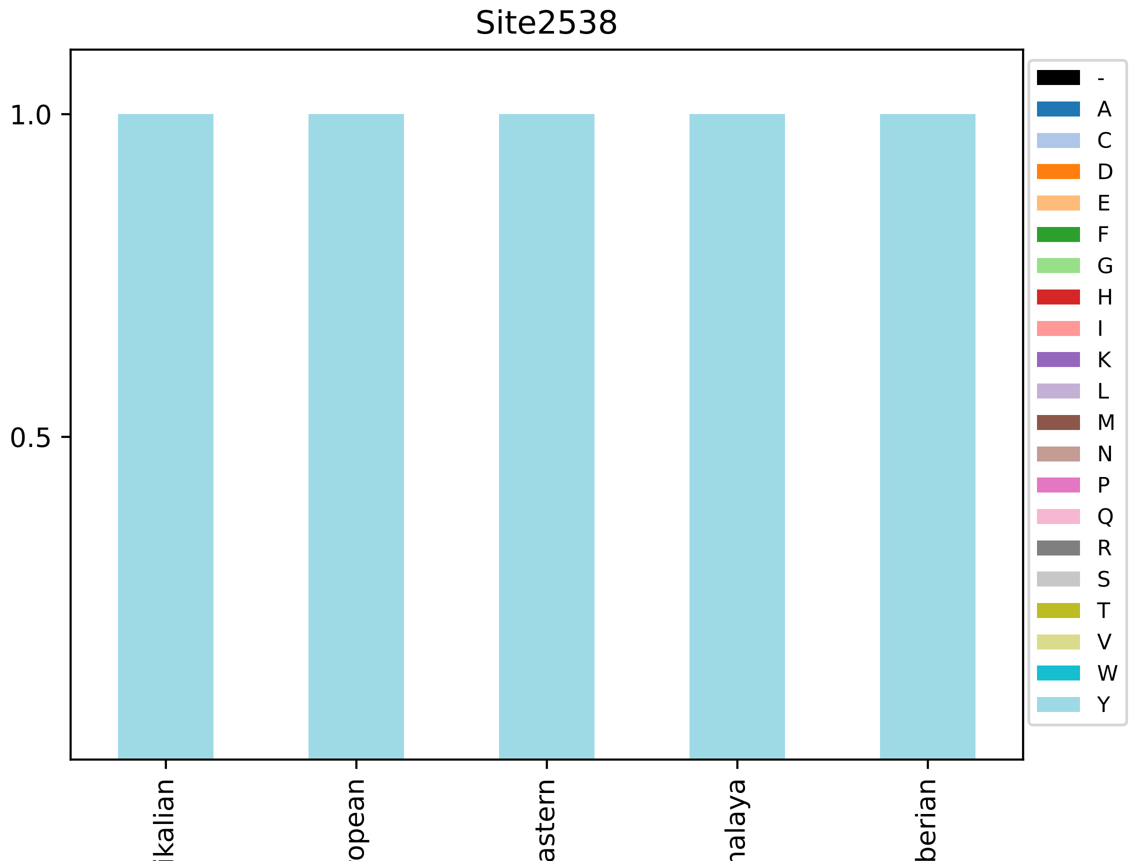

Site2538
1.0
0.5
-
A
C
D
E
F
G
H
I
K
L
M
N
P
Q
R
S
T
V
W
Y
ikalian
opean
astern
nalaya
berian

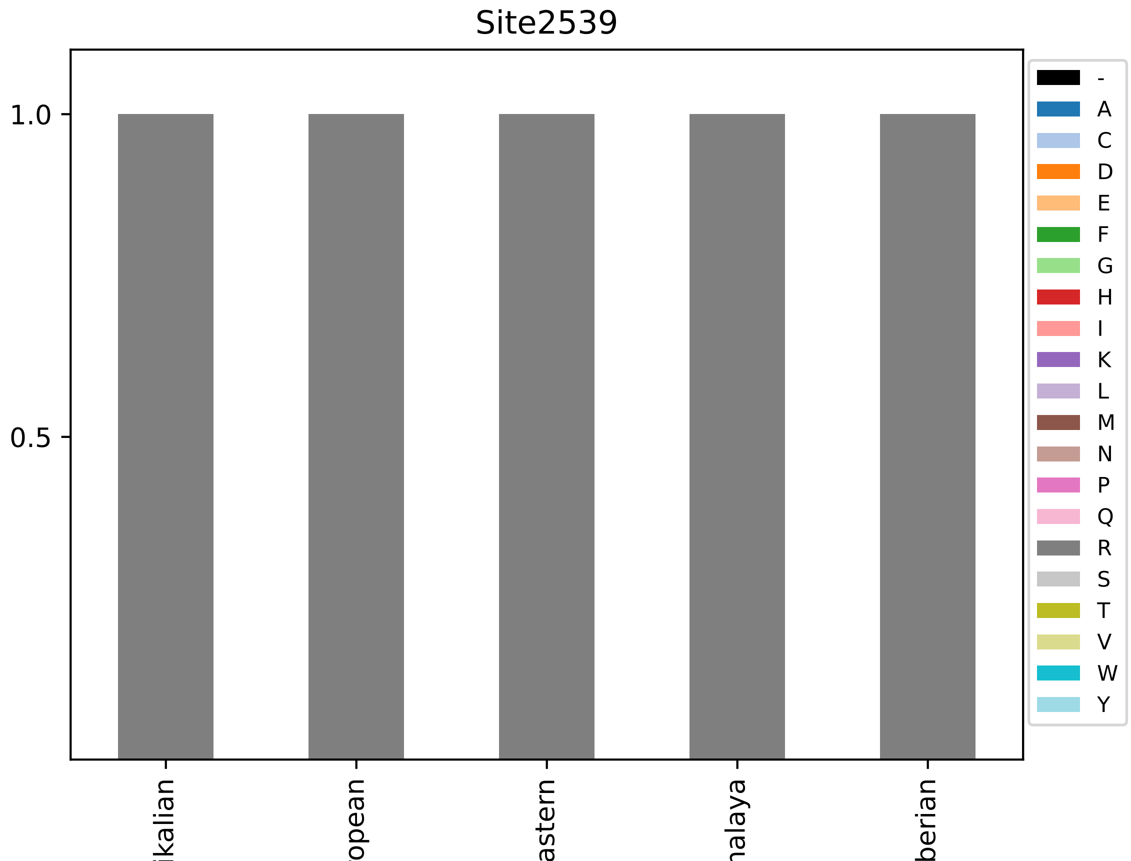
Site2539
1.0
0.5
-
A
C
D
E
F
G
H
I
K
L
M
N
P
Q
R
S
T
V
W
Y
ikalian
opean
astern
nalaya
berian

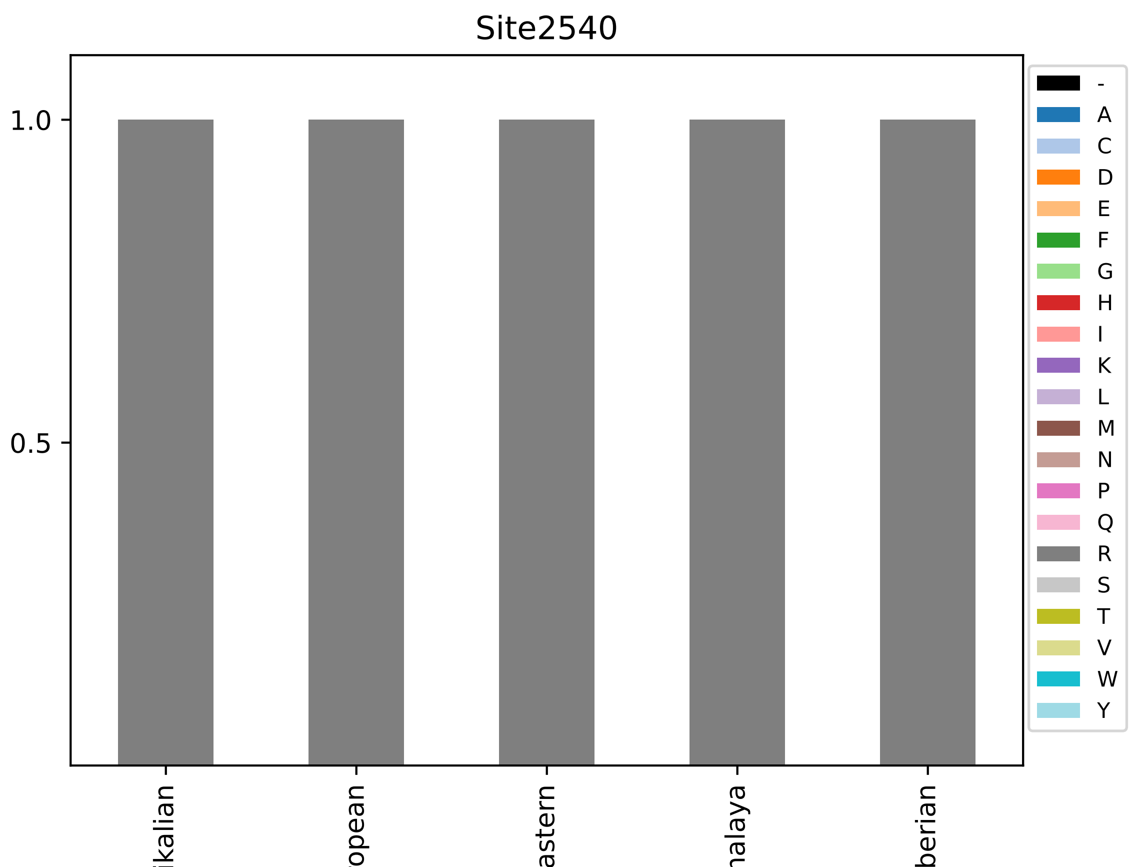
Site2540
1.0
0.5
-
A
C
D
E
F
G
H
I
K
L
M
N
P
Q
R
S
T
V
W
Y
ikalian
opean
astern
nalaya
berian

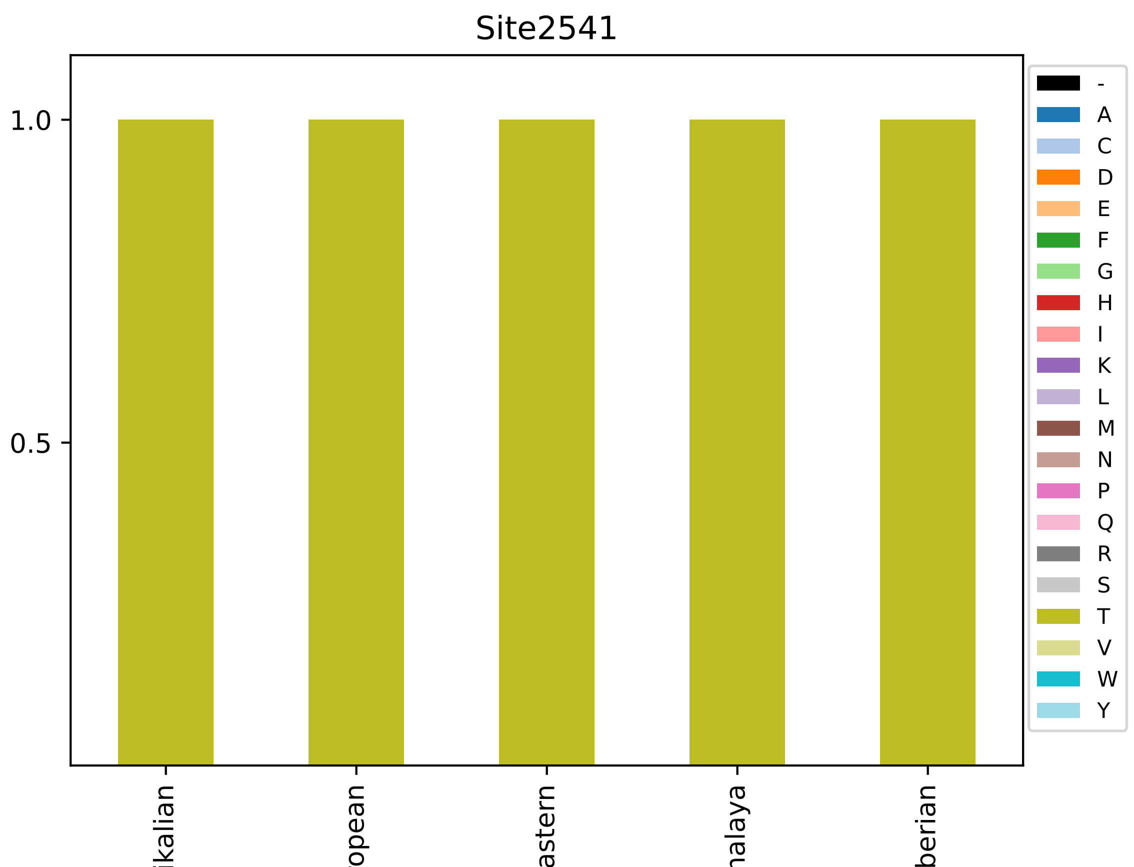
Site2541
1.0
0.5
-
A
C
D
E
F
G
H
I
K
L
M
N
P
Q
R
S
T
V
W
Y
ikalian
opean
astern
nalaya
berian

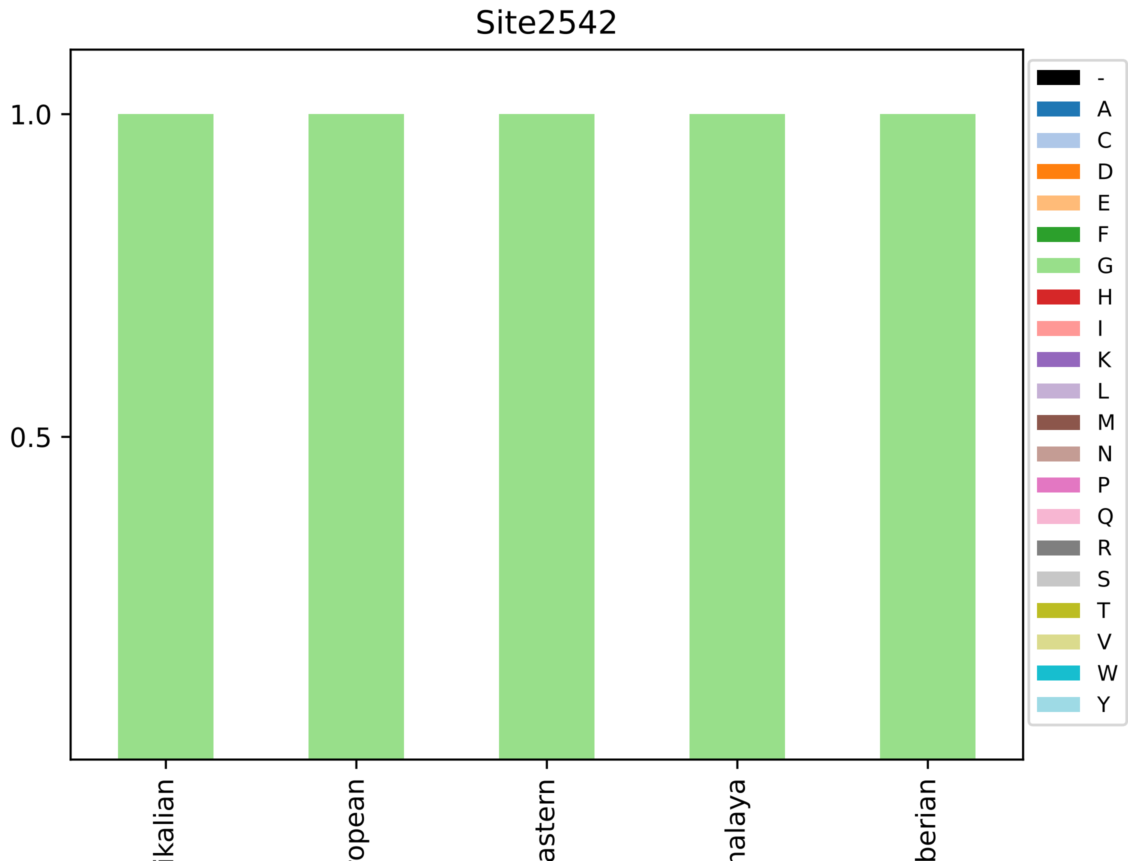

Site2542
1.0
0.5
-
A
C
D
E
F
G
H
I
K
L
M
N
P
Q
R
S
T
V
W
Y
ikalian
opean
astern
nalaya
berian

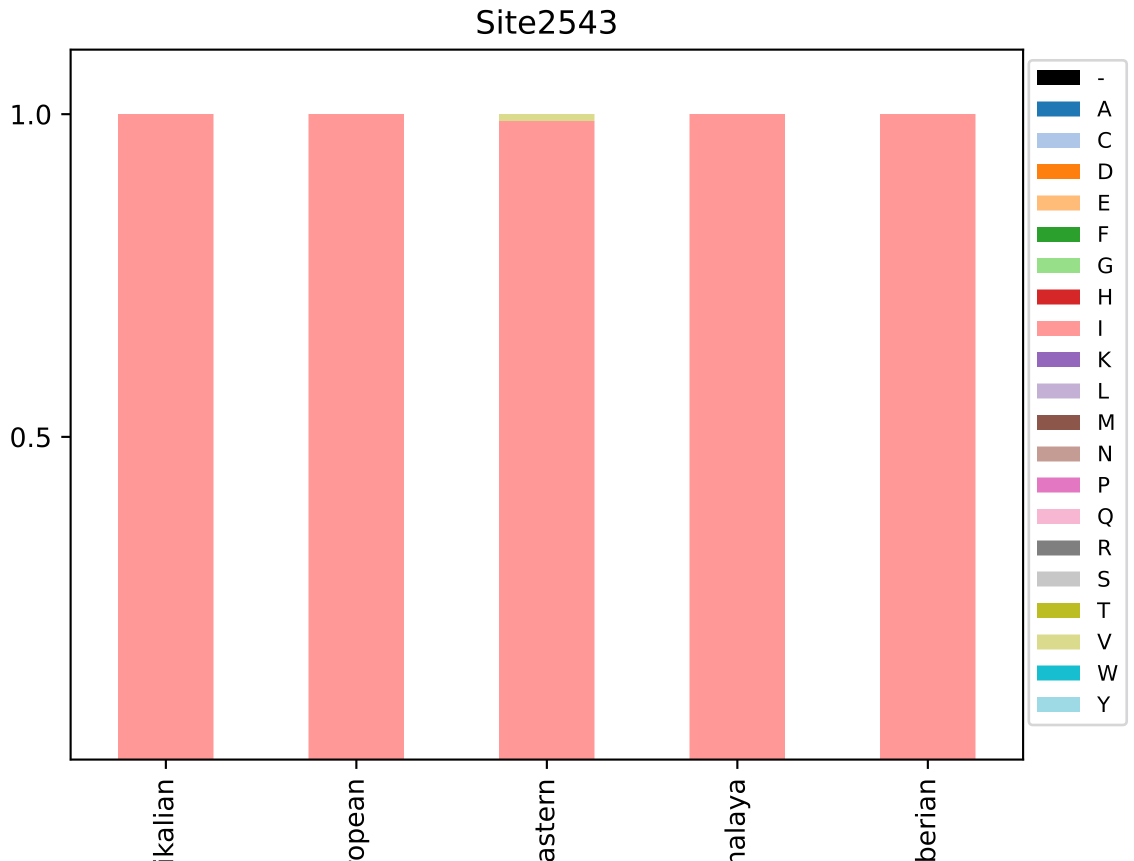

Site2543
1.0
0.5
-
A
C
D
E
F
G
H
I
K
L
M
N
P
Q
R
S
T
V
W
Y
ikalian
opean
astern
nalaya
berian

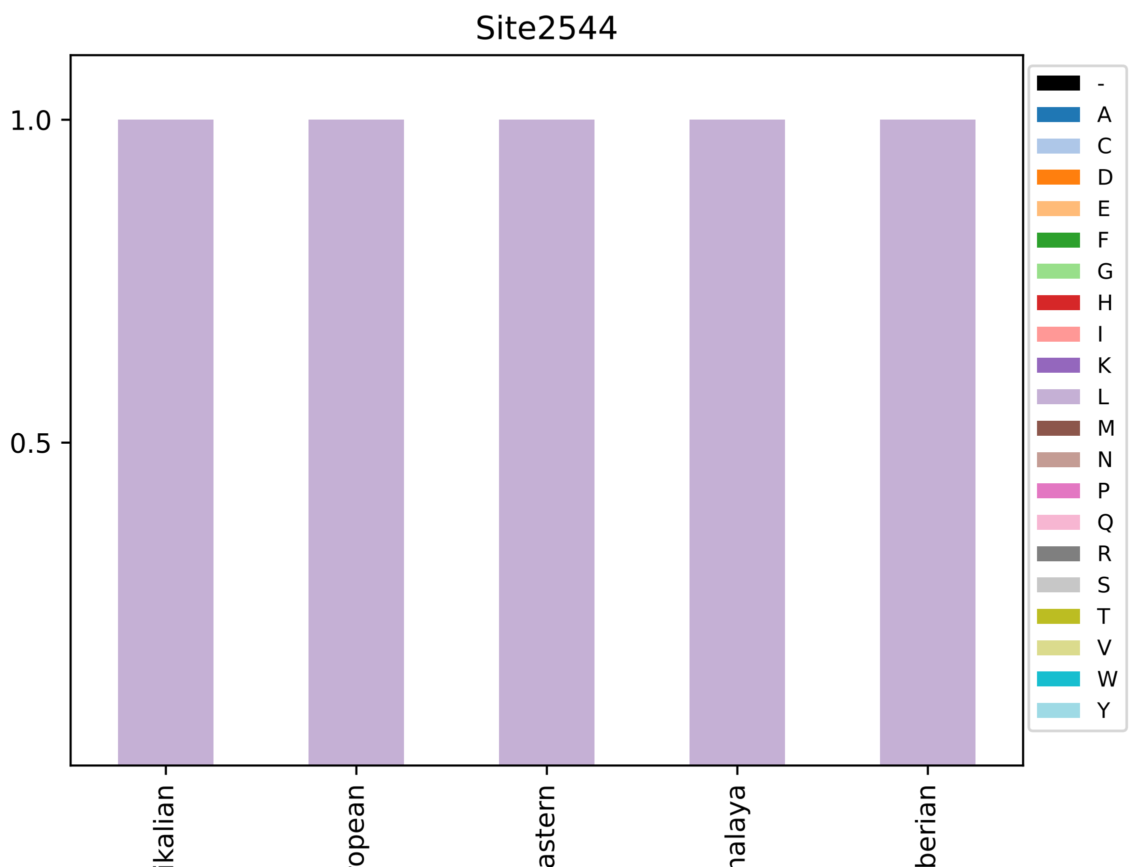
Site2544
1.0
0.5
-
A
C
D
E
F
G
H
I
K
L
M
N
P
Q
R
S
T
V
W
Y
ikalian
opean
astern
nalaya
berian

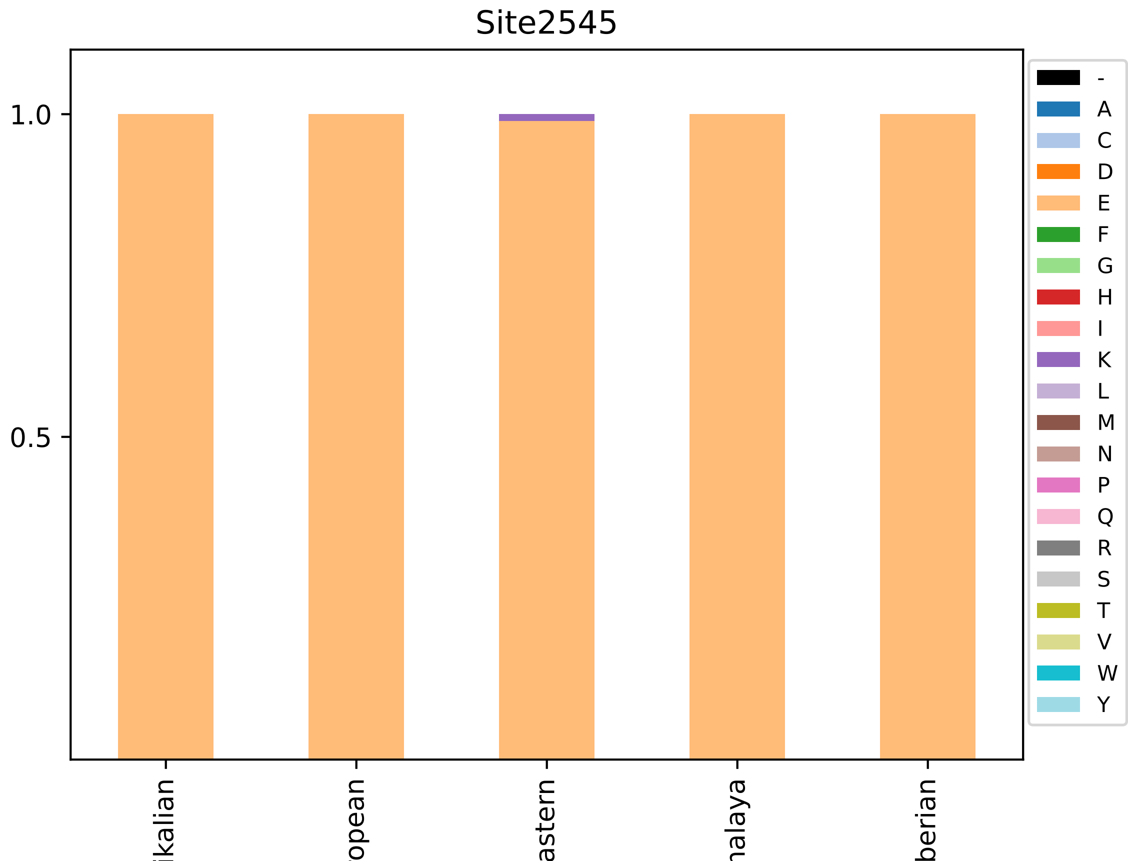

Site2545
1.0
0.5
-
A
C
D
E
F
G
H
I
K
L
M
N
P
Q
R
S
T
V
W
Y
ikalian
opean
astern
nalaya
berian

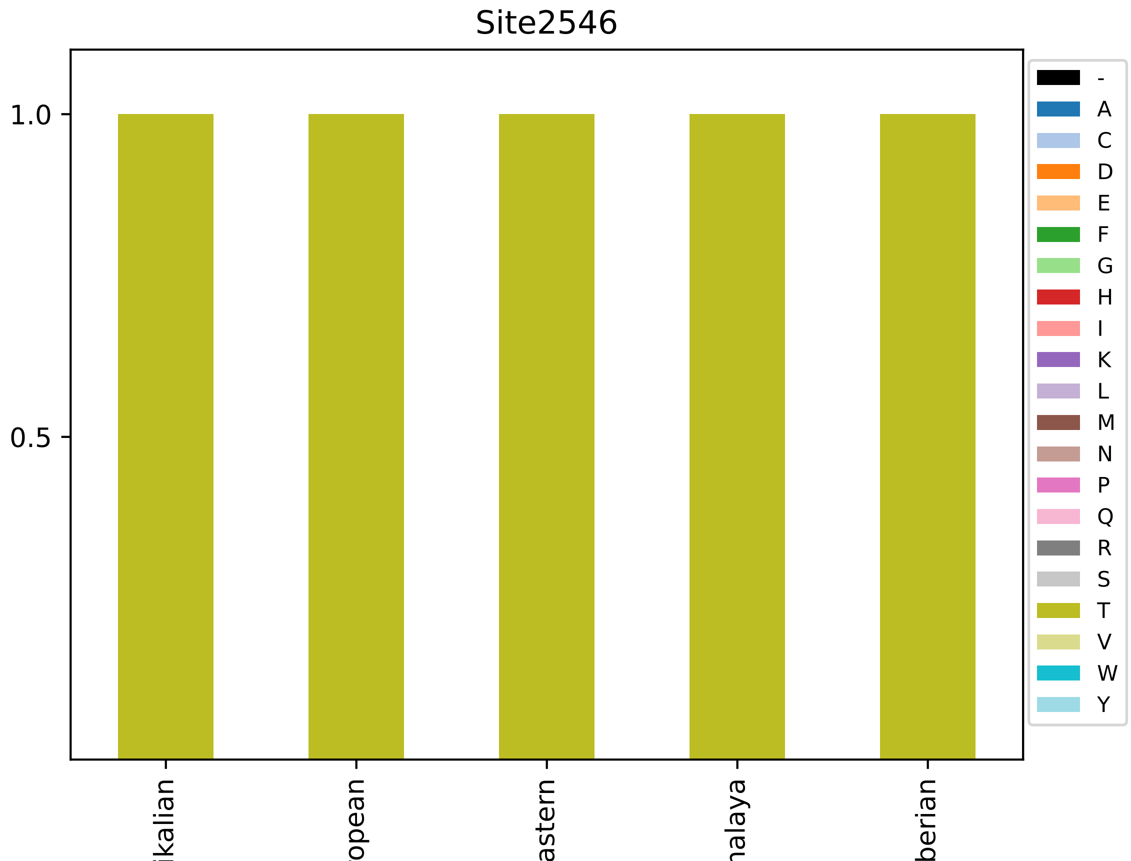

Site2546
1.0
0.5
-
A
C
D
E
F
G
H
I
K
L
M
N
P
Q
R
S
T
V
W
Y
ikalian
opean
astern
nalaya
berian

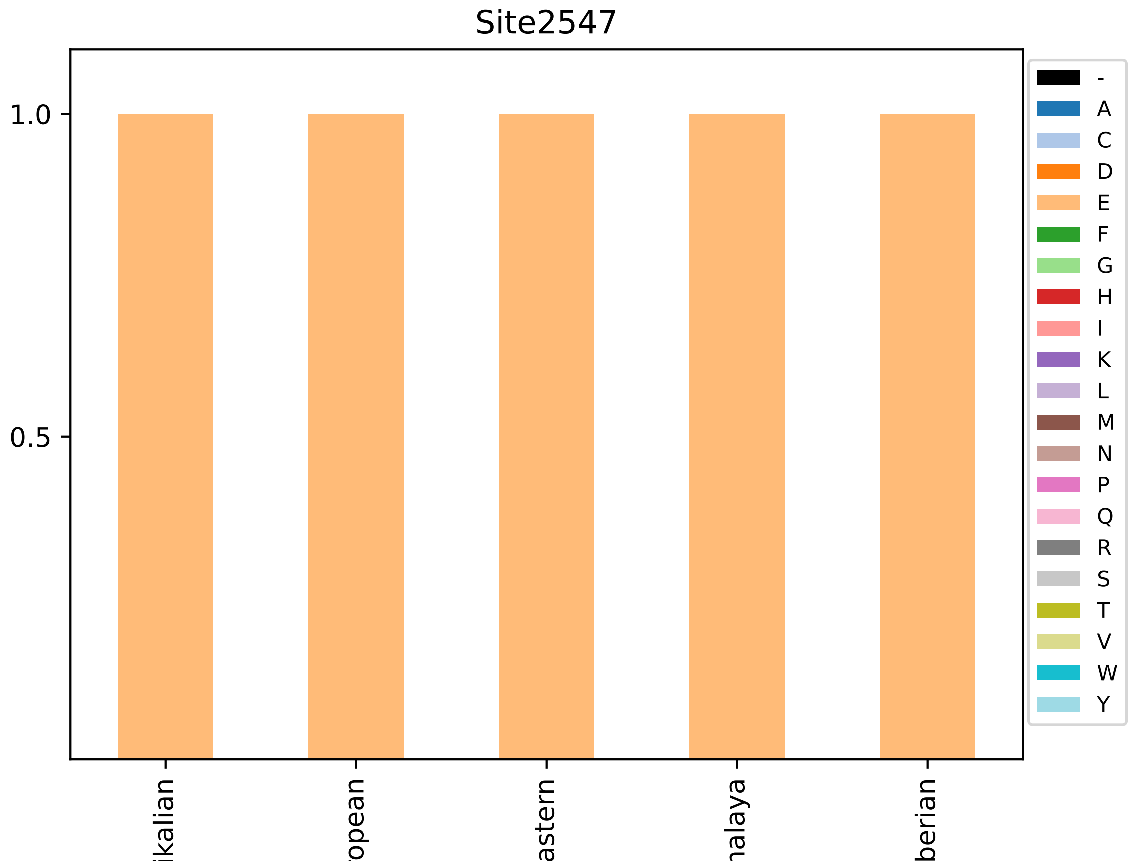
Site2547
1.0
0.5
-
A
C
D
E
F
G
H
I
K
L
M
N
P
Q
R
S
T
V
W
Y

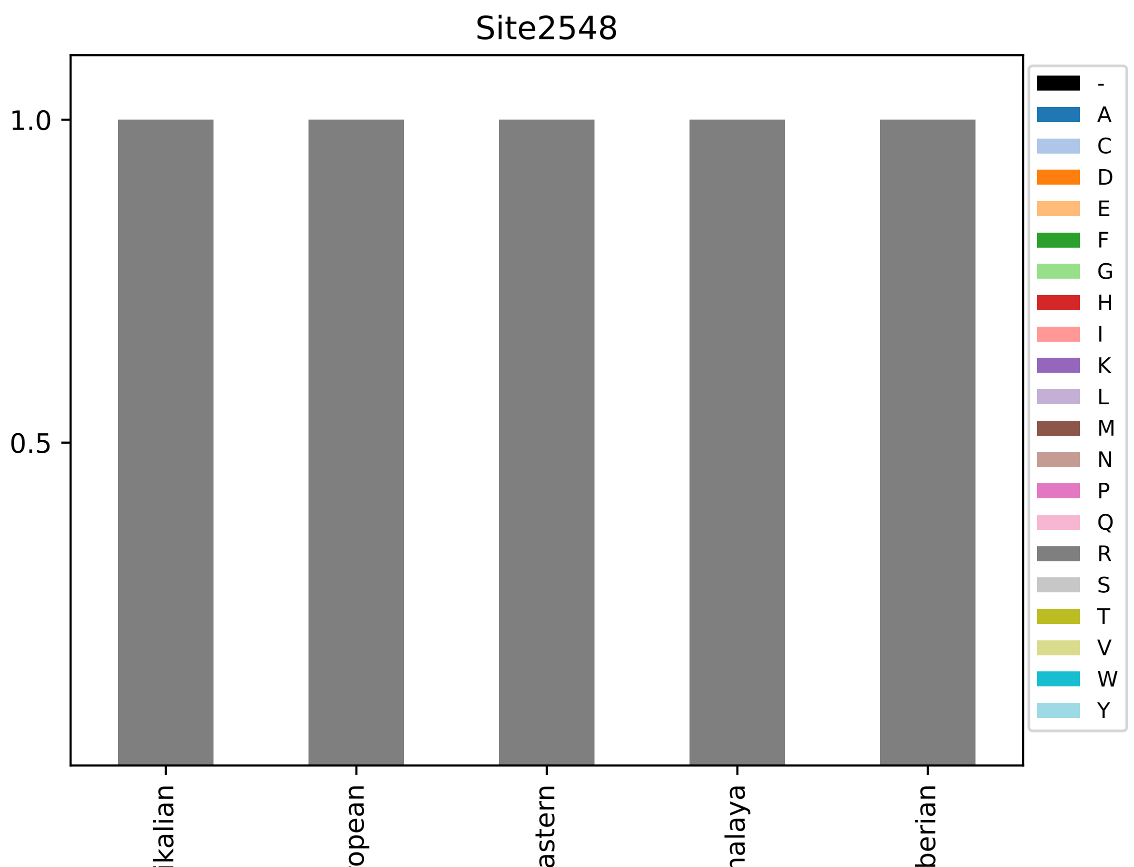

Site2548
1.0
0.5
-
A
C
D
E
F
G
H
I
K
L
M
N
P
Q
R
S
T
V
W
Y
ikalian
opean
astern
nalaya
berian

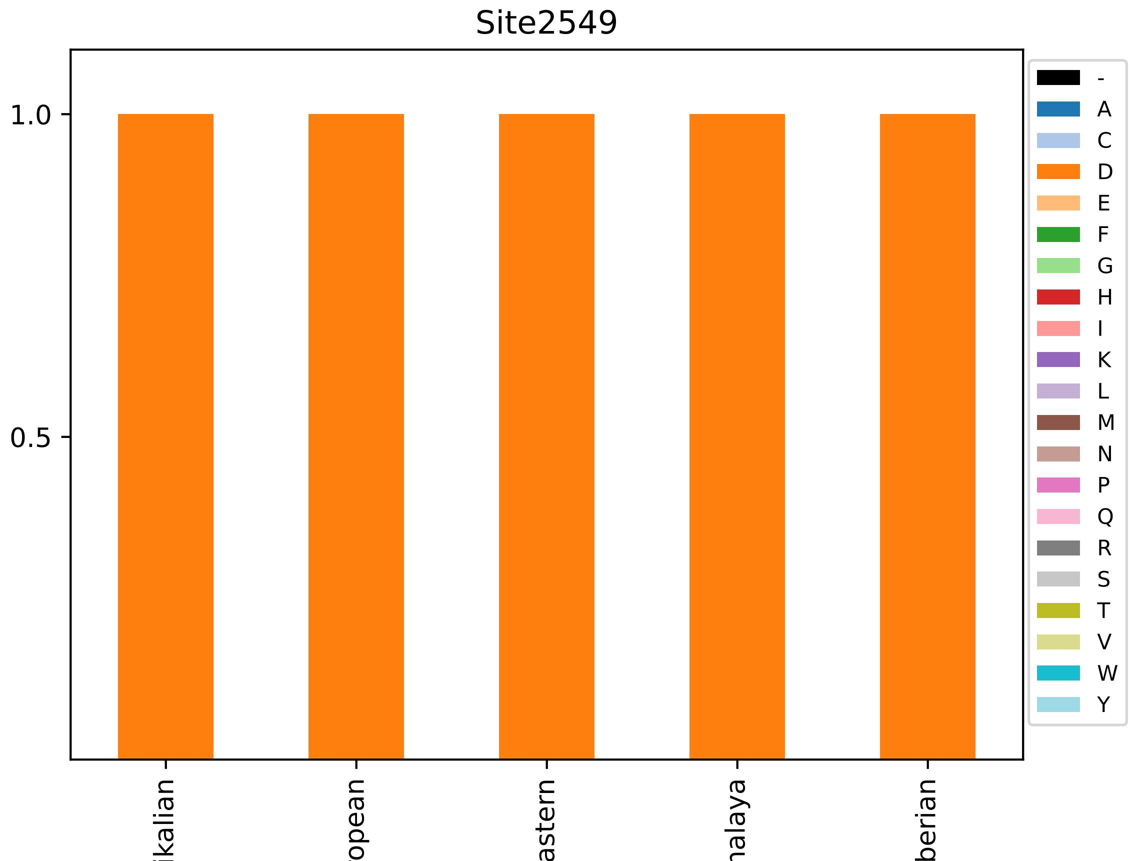
Site2549
1.0
0.5
-
A
C
D
E
F
G
H
I
K
L
M
N
P
Q
R
S
T
V
W
Y
ikalian
opean
astern
nalaya
berian

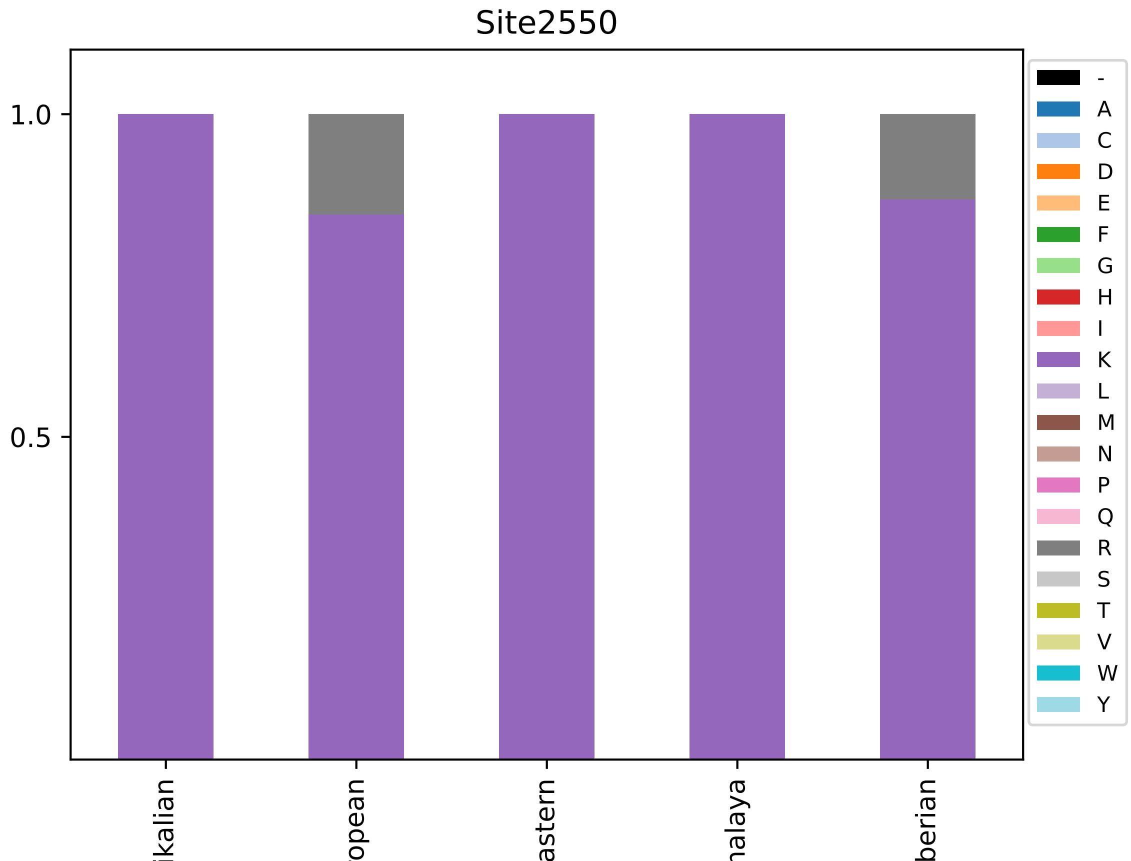

Site2550
1.0
0.5
-
A
C
D
E
F
G
H
I
K
L
M
N
P
Q
R
S
T
V
W
Y
ikalian
opean
astern
nalaya
berian

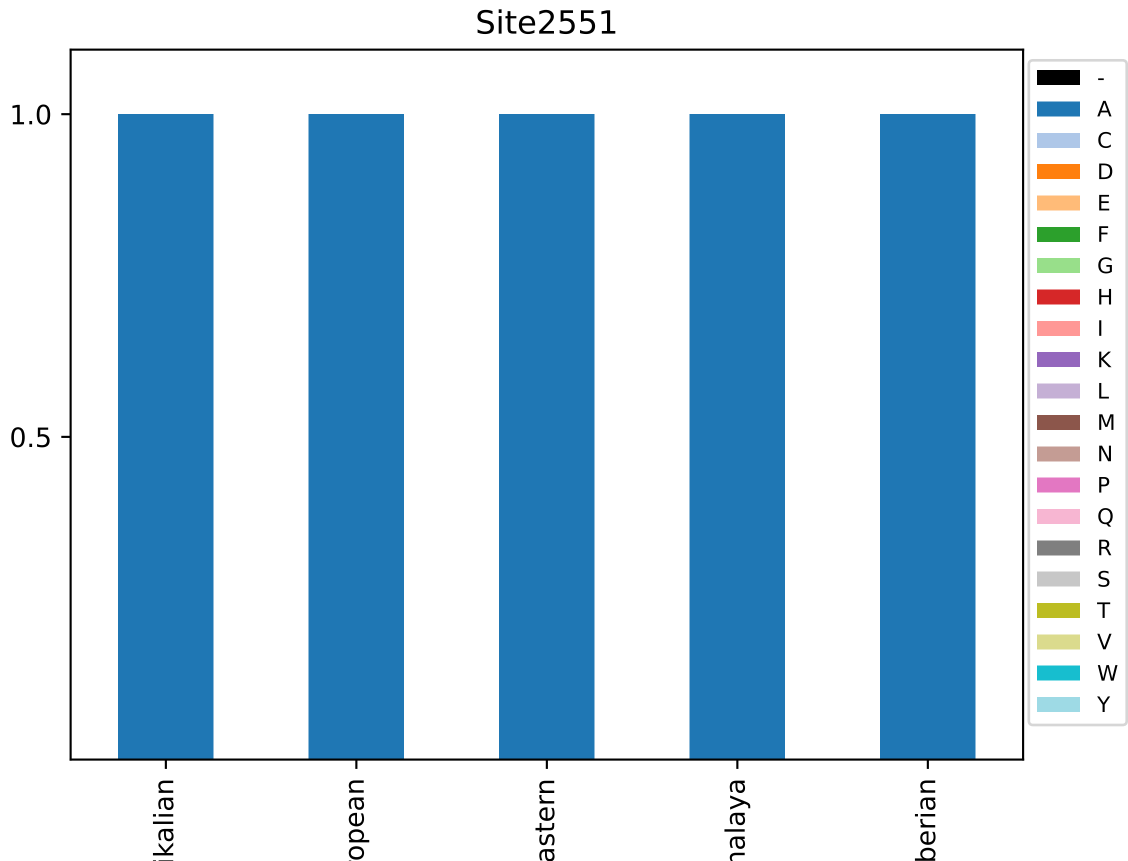

Site2551
1.0
0.5
-
A
C
D
E
F
G
H
I
K
L
M
N
P
Q
R
S
T
V
W
Y
ikalian
opean
astern
nalaya
berian

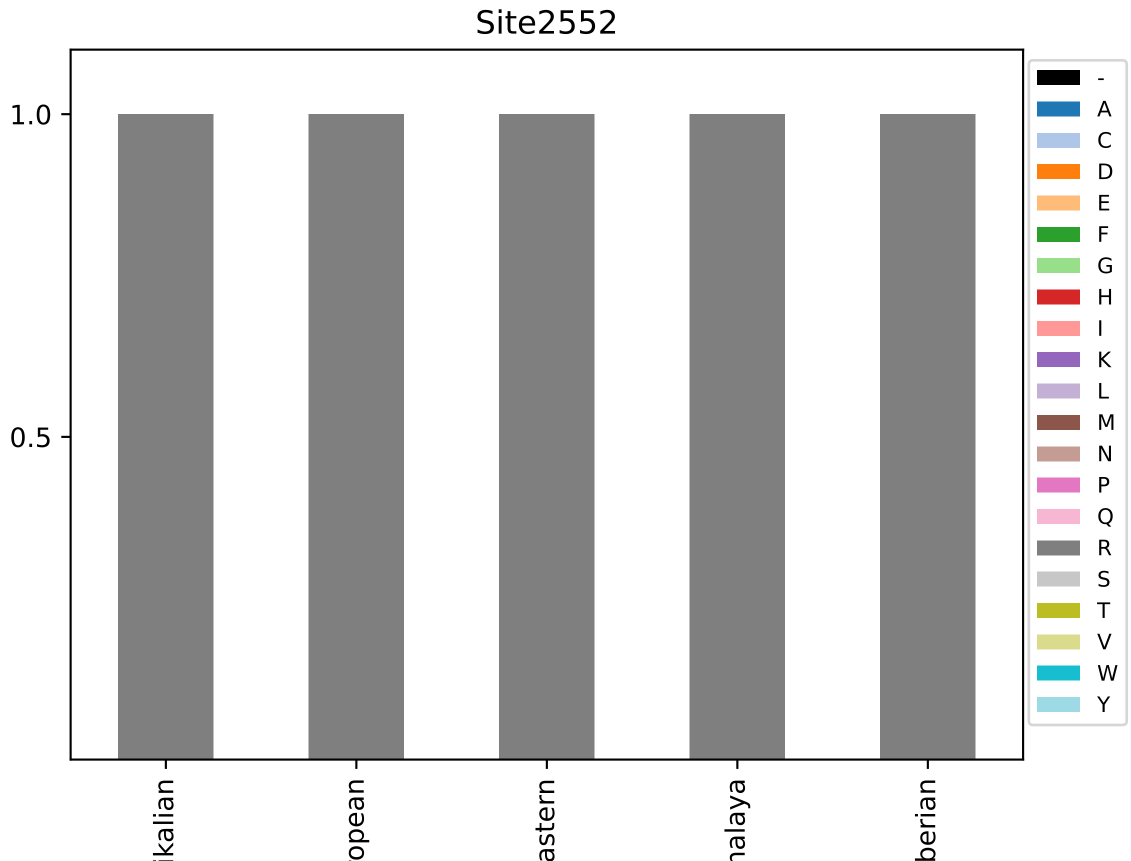

Site2552
1.0
0.5
-
A
C
D
E
F
G
H
I
K
L
M
N
P
Q
R
S
T
V
W
Y
ikalian
opean
astern
halaya
berian

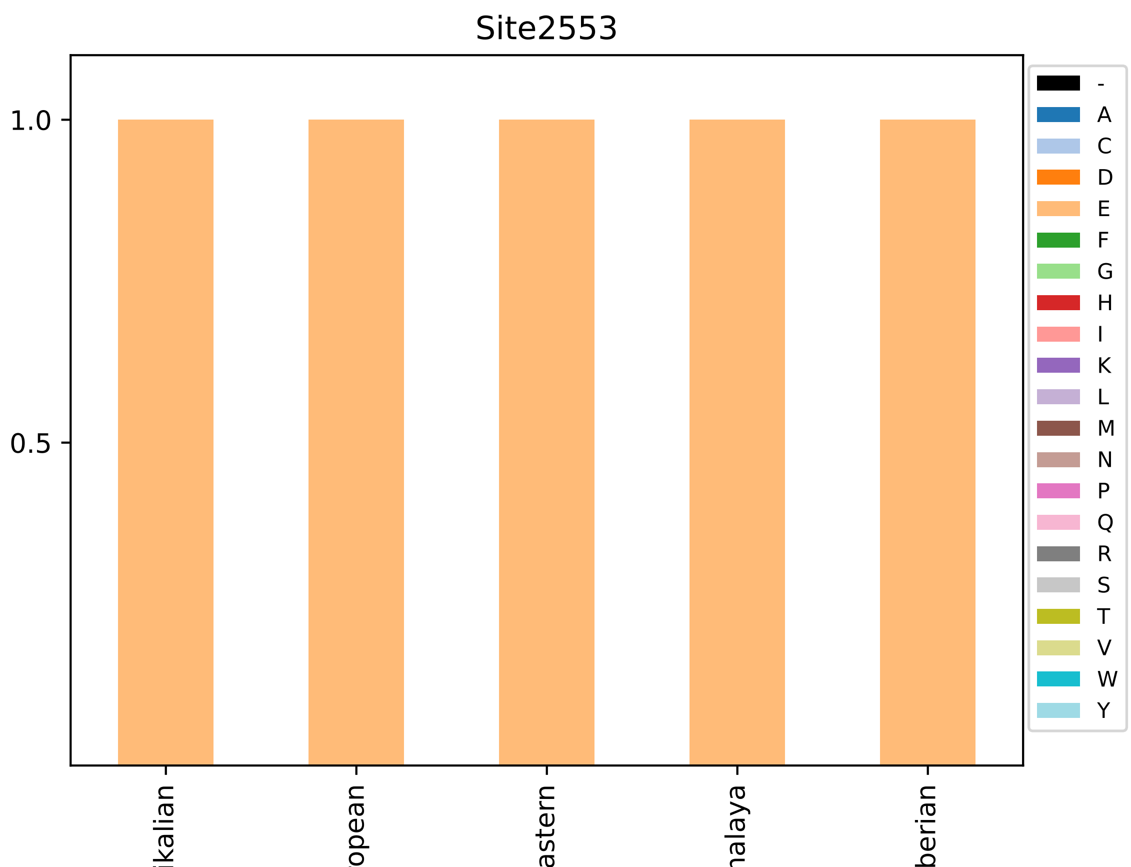
Site2553
1.0
0.5
-
A
C
D
E
F
G
H
I
K
L
M
N
P
Q
R
S
T
V
W
Y
ikalian
opean
astern
nalaya
berian

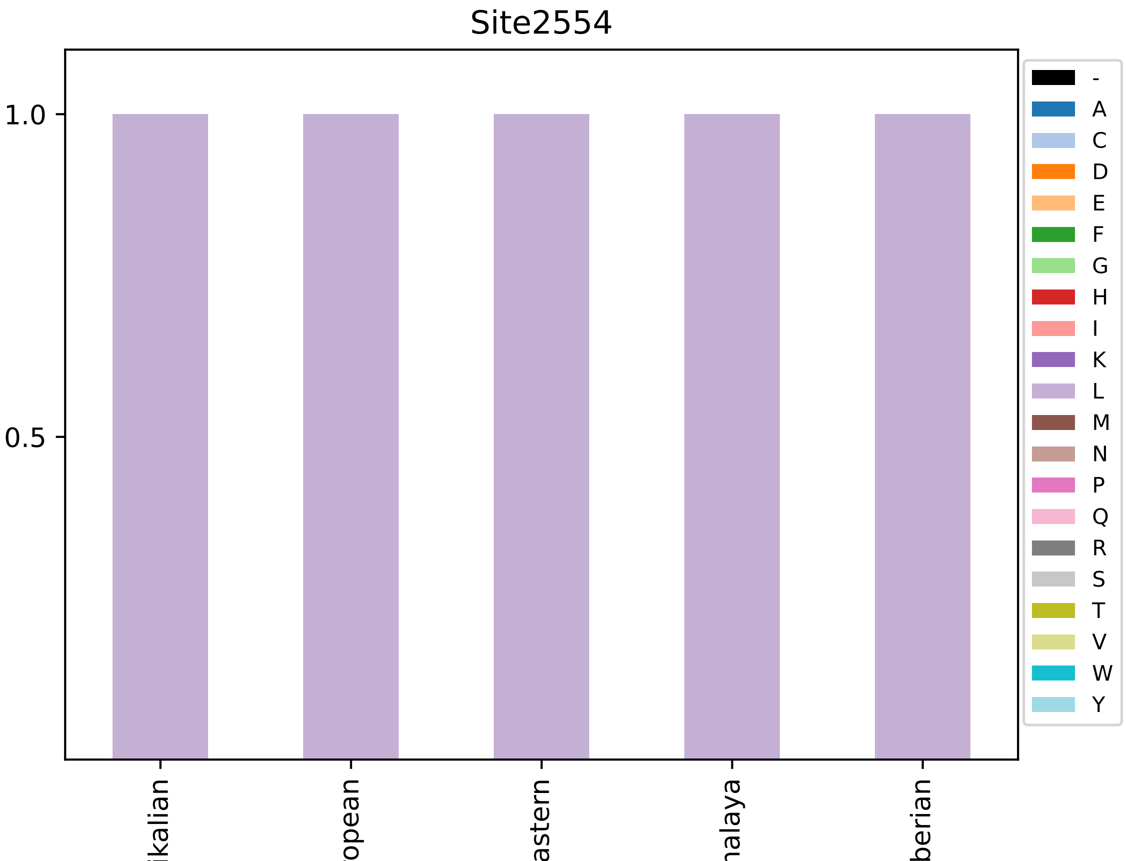
Site2554
1.0
0.5
-
A
C
D
E
F
G
H
I
K
L
M
N
P
Q
R
S
T
V
W
Y
ikalian
opean
astern
nalaya
berian

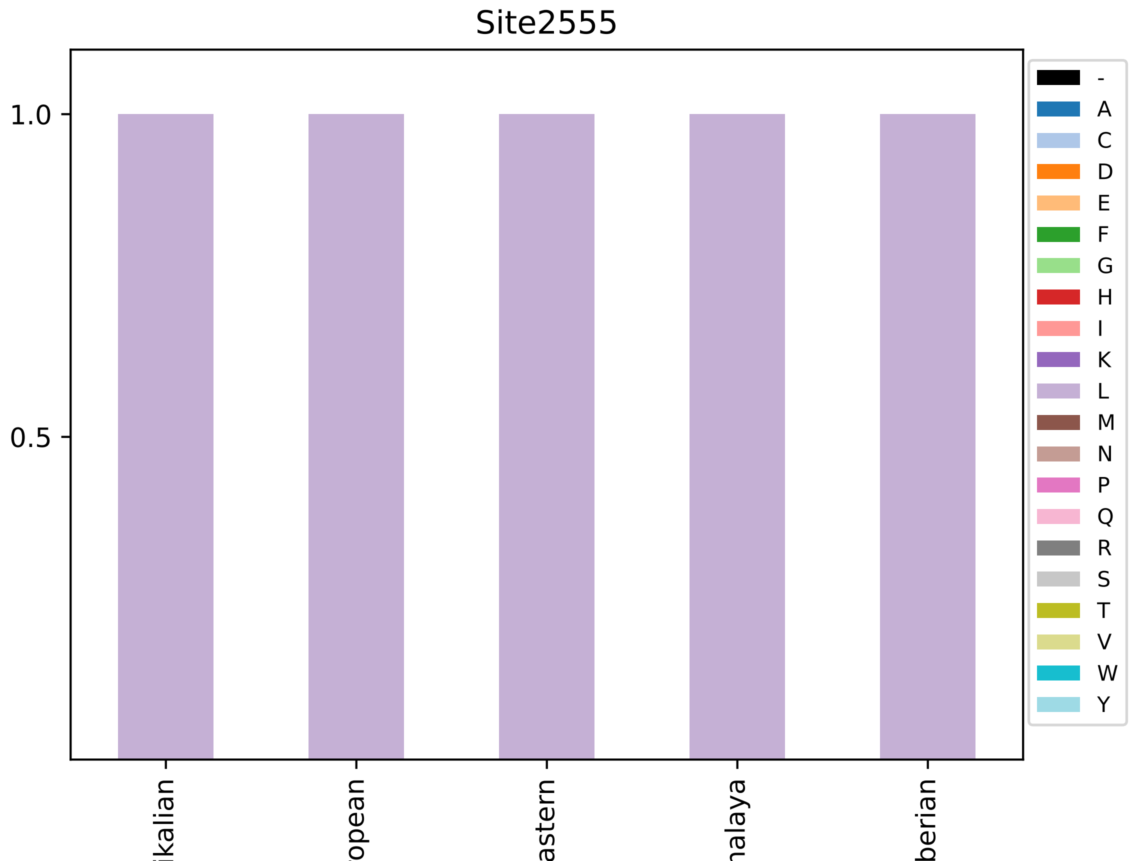
Site2555
1.0
0.5
-
A
C
D
E
F
G
H
I
K
L
M
N
P
Q
R
S
T
V
W
Y
ikalian
opean
astern
nalaya
berian

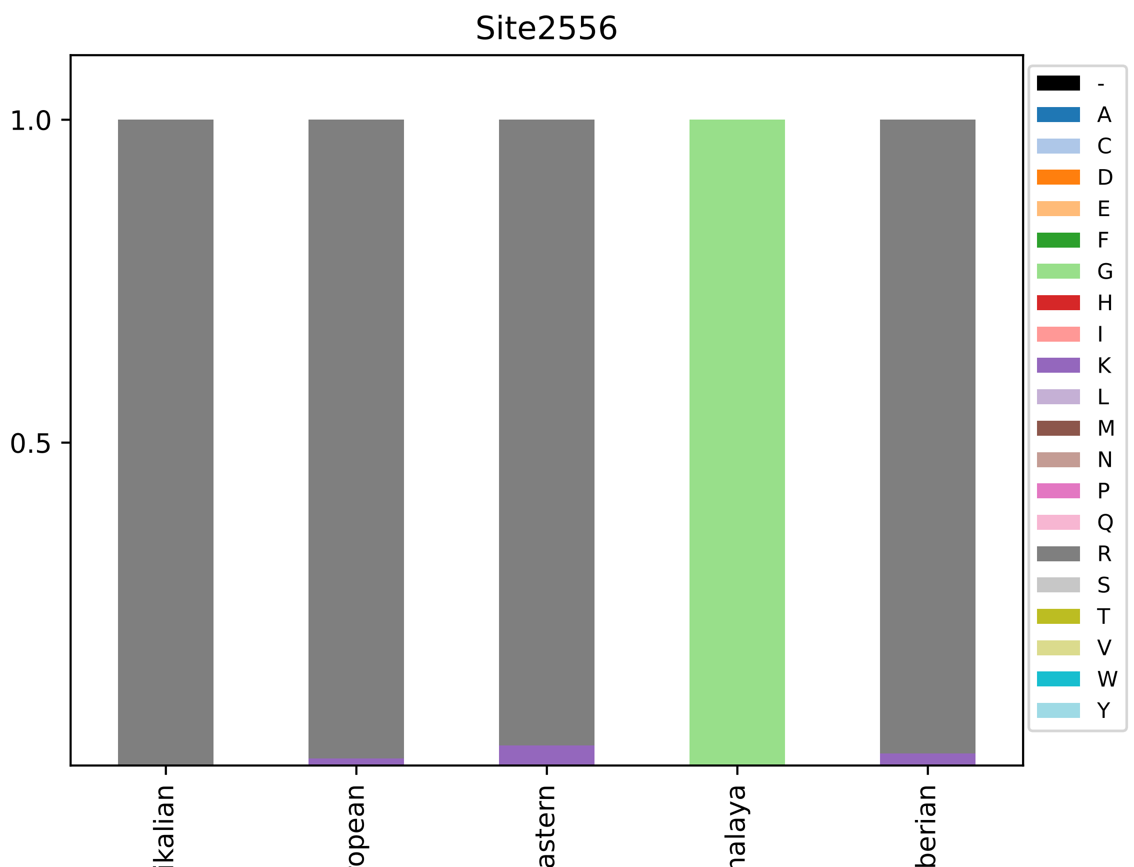

Site2556
1.0
0.5
ikalian
opean
astern
nalaya
berian
-
A
C
D
E
F
G
H
I
K
L
M
N
P
Q
R
S
T
V
W
Y

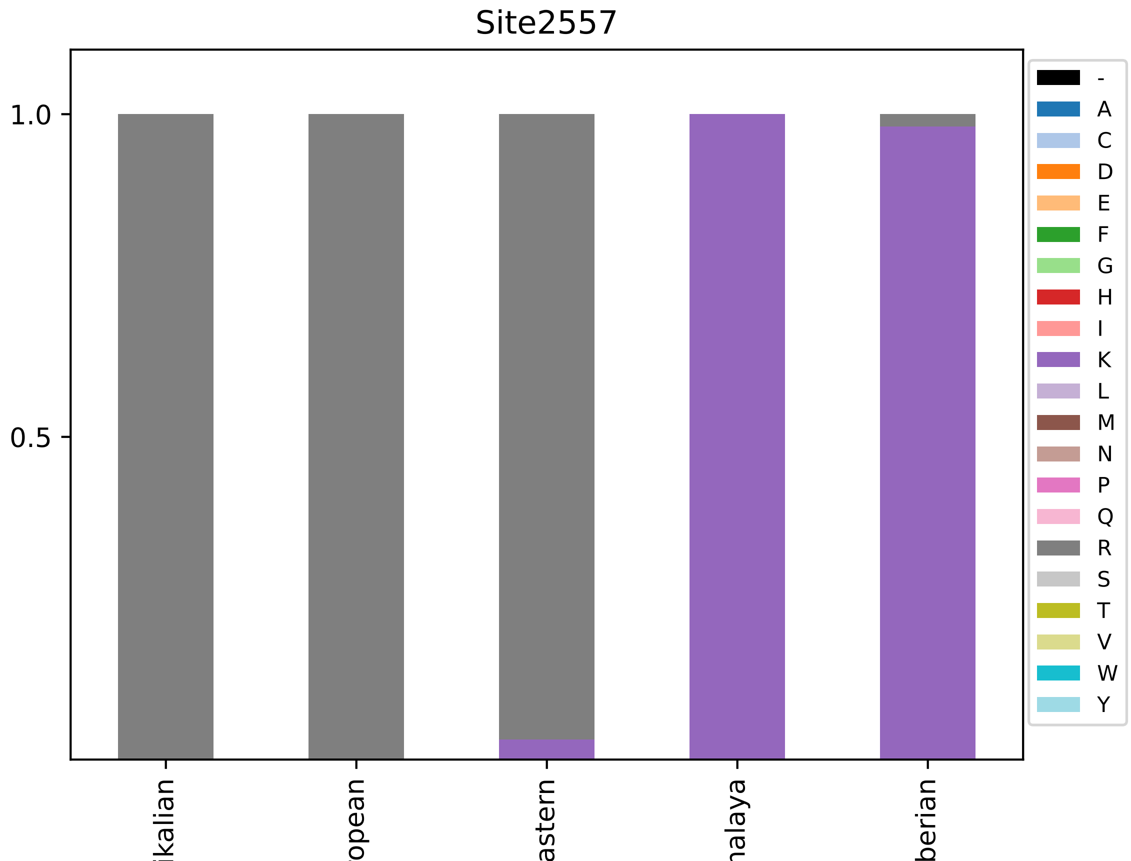

Site2557
1.0
0.5
-
A
C
D
E
F
G
H
I
K
L
M
N
P
Q
R
S
T
V
W
Y
ikalian
opean
astern
nalaya
berian

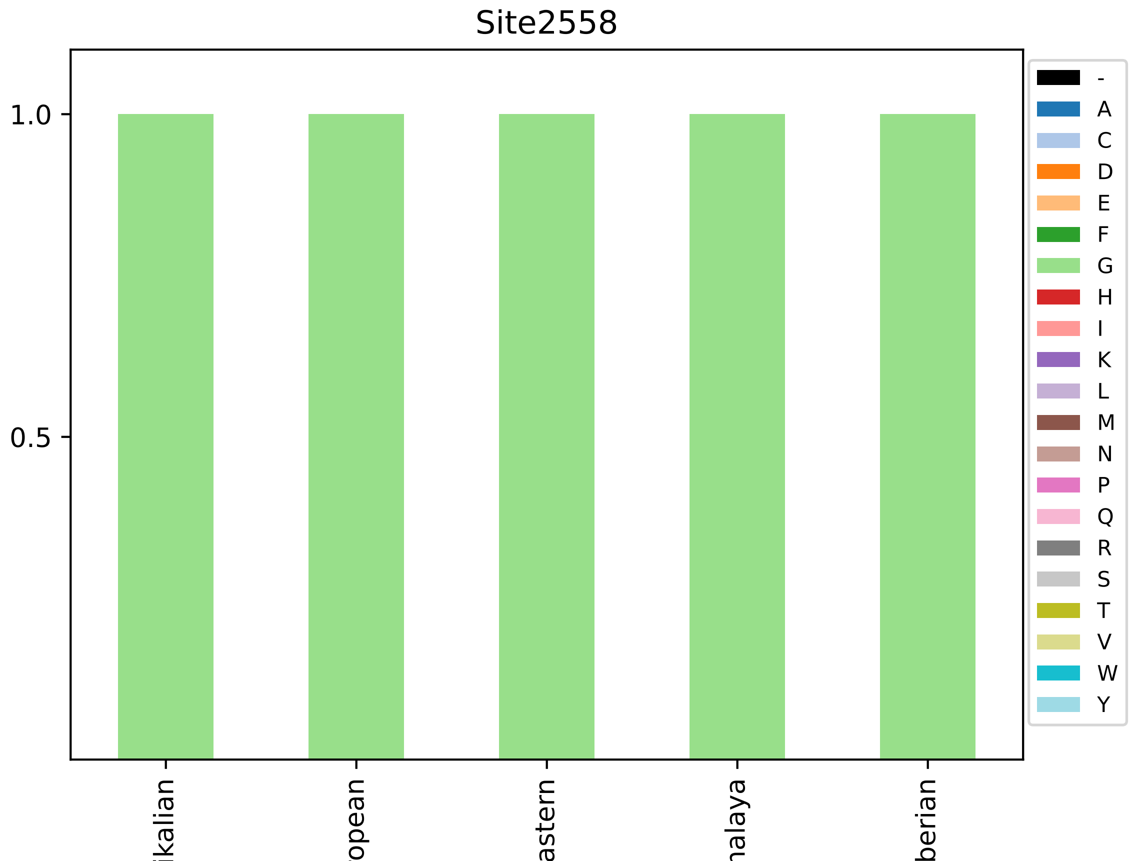
Site2558
1.0
0.5
-
A
C
D
E
F
G
H
I
K
L
M
N
P
Q
R
S
T
V
W
Y
ikalian
opean
astern
nalaya
berian

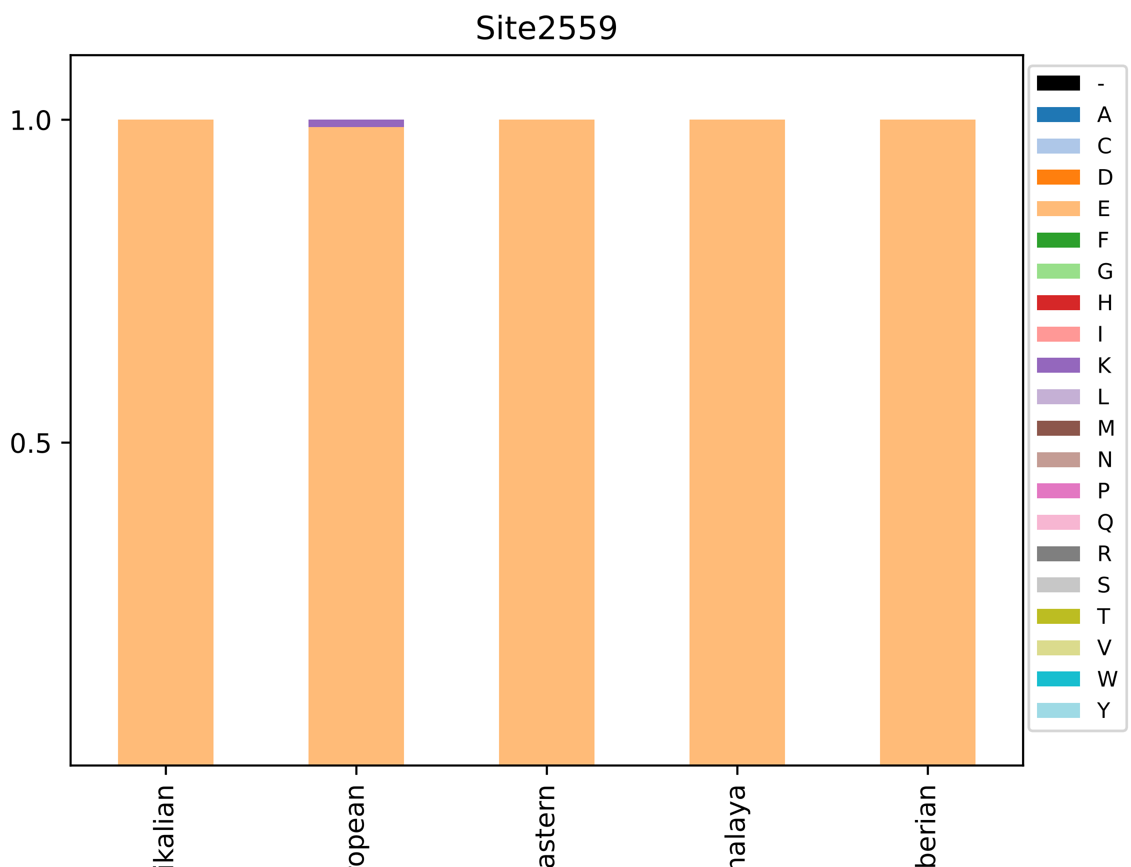

Site2559
1.0
0.5
-
A
C
D
E
F
G
H
I
K
L
M
N
P
Q
R
S
T
V
W
Y
ikalian
opean
astern
nalaya
berian

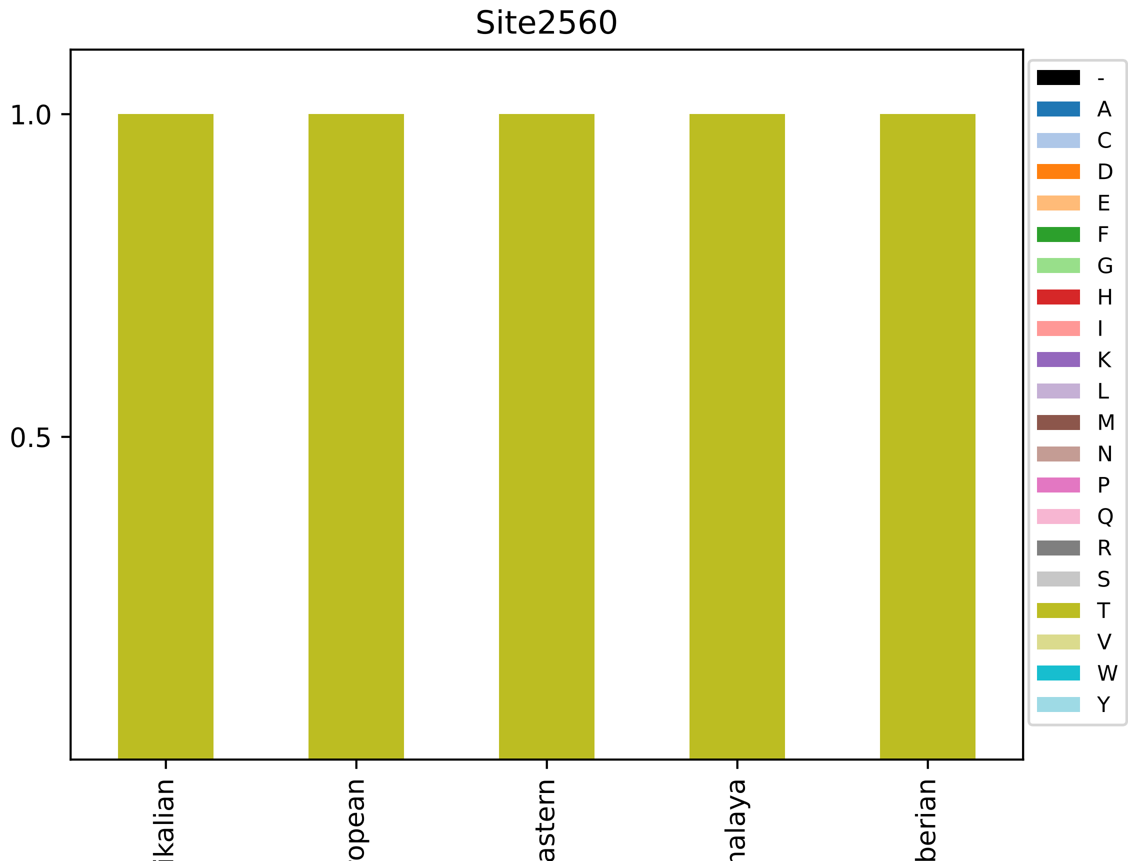

Site2560
1.0
0.5
-
A
C
D
E
F
G
H
I
K
L
M
N
P
Q
R
S
T
V
W
Y
ikalian
ropean
astern
nalaya
berian

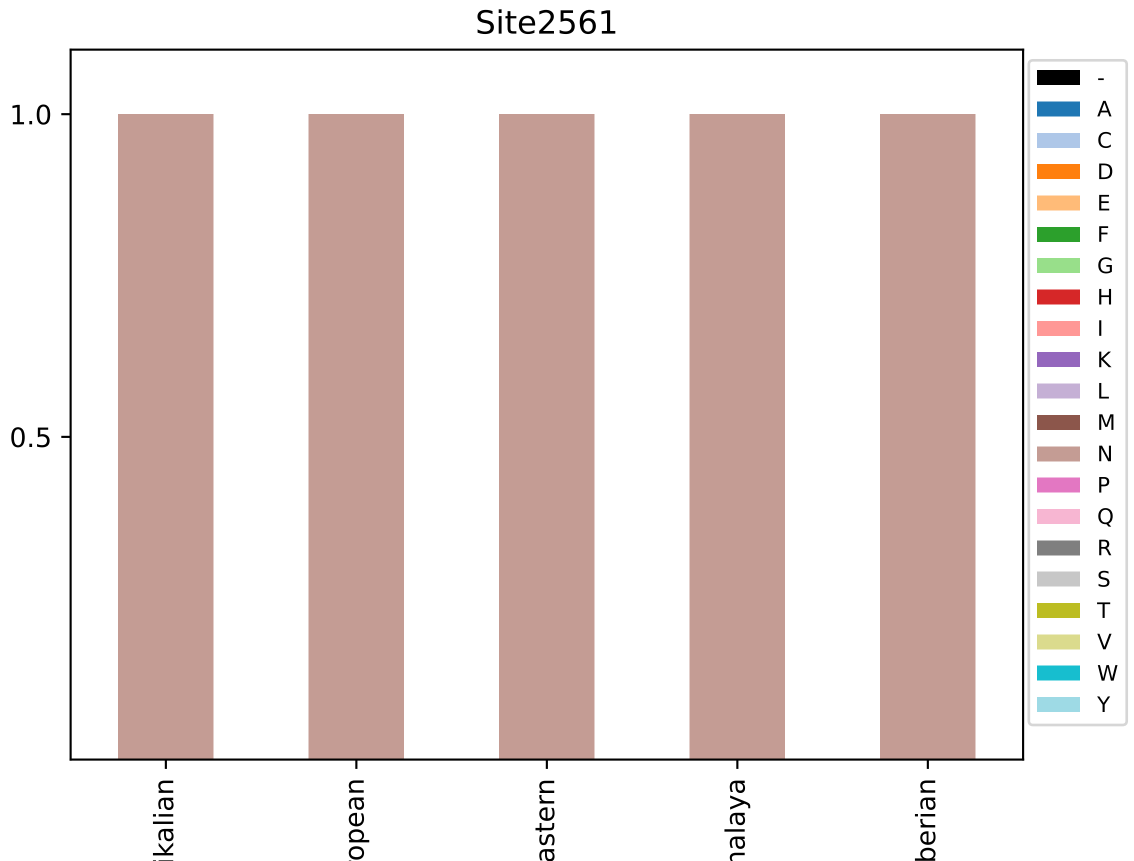
Site2561
1.0
0.5
-
A
C
D
E
F
G
H
I
K
L
M
N
P
Q
R
S
T
V
W
Y

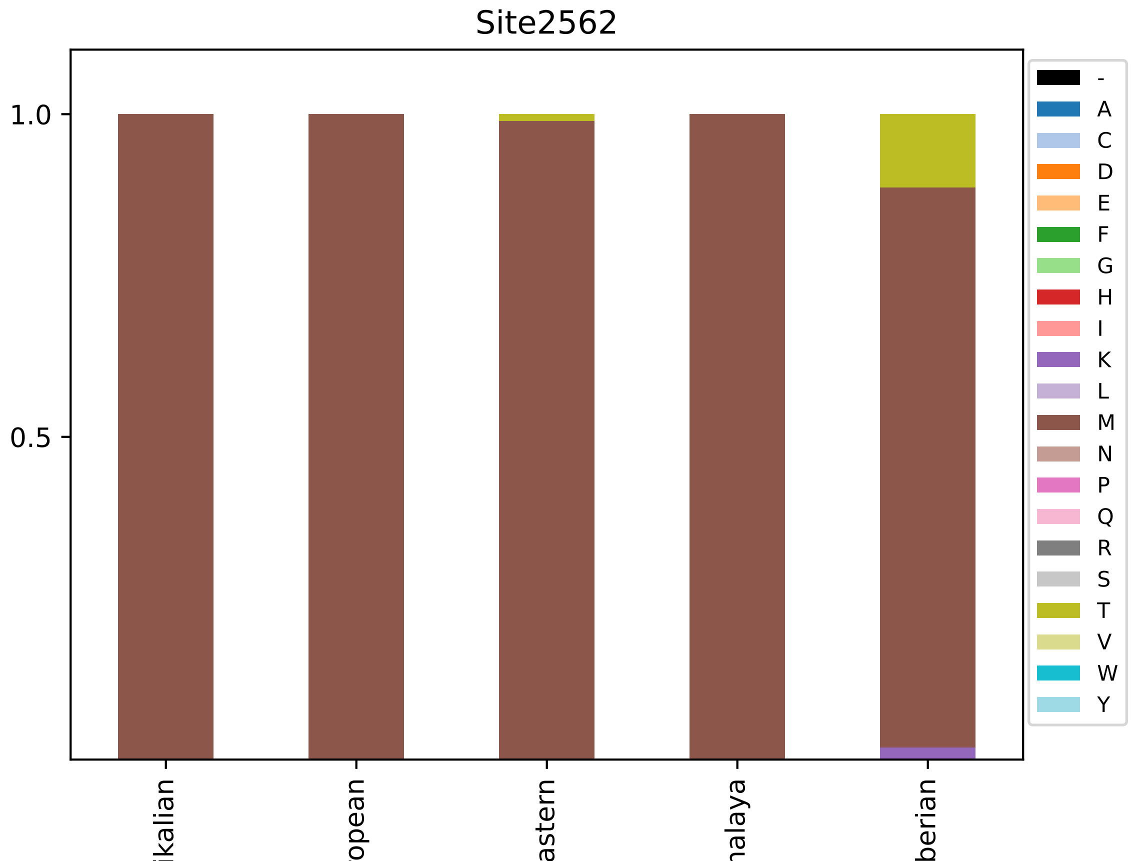

Site2562
1.0
0.5
-
A
C
D
E
F
G
H
I
K
L
M
N
P
Q
R
S
T
V
W
Y
ikalian
opean
astern
nalaya
berian

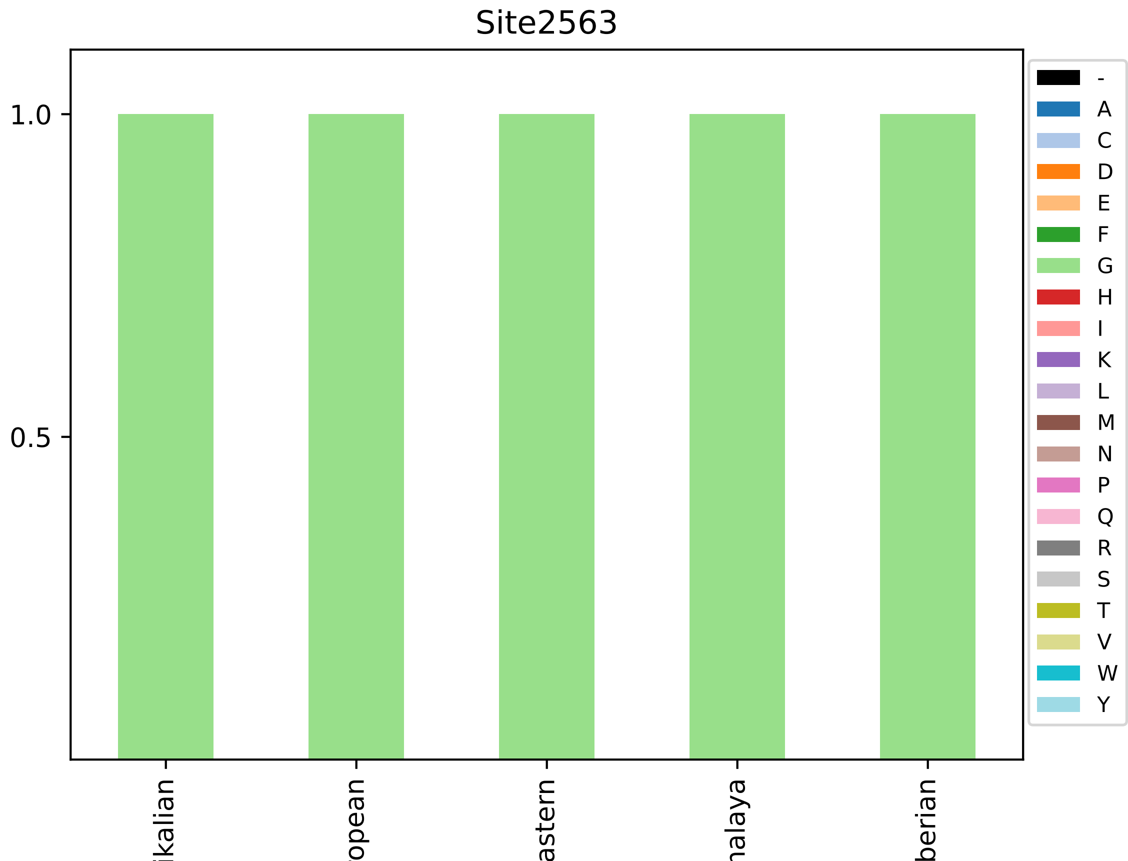

Site2563
1.0
0.5
-
A
C
D
E
F
G
H
I
K
L
M
N
P
Q
R
S
T
V
W
Y
ikalian
opean
astern
nalaya
berian

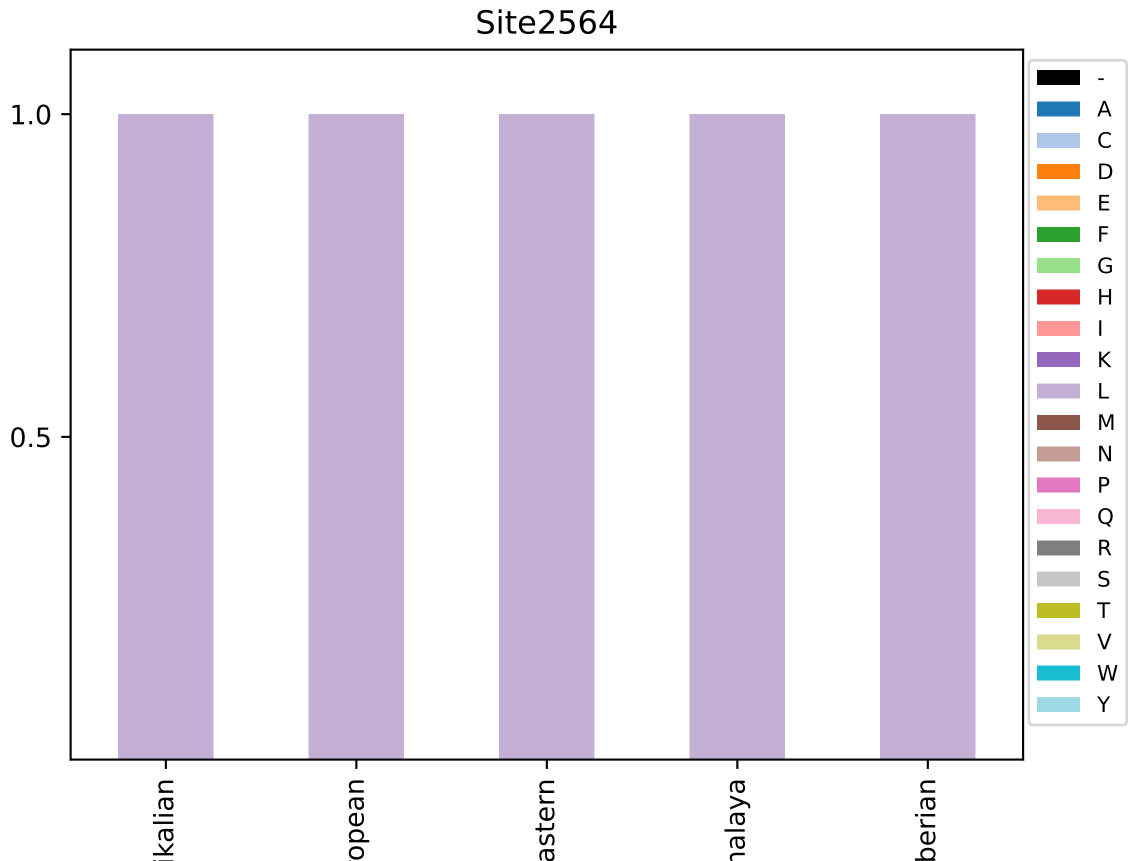
Site2564
1.0
0.5
ikalian
opean
astern
nalaya
berian
-
A
C
D
E
F
G
H
I
K
L
M
N
P
Q
R
S
T
V
W
Y

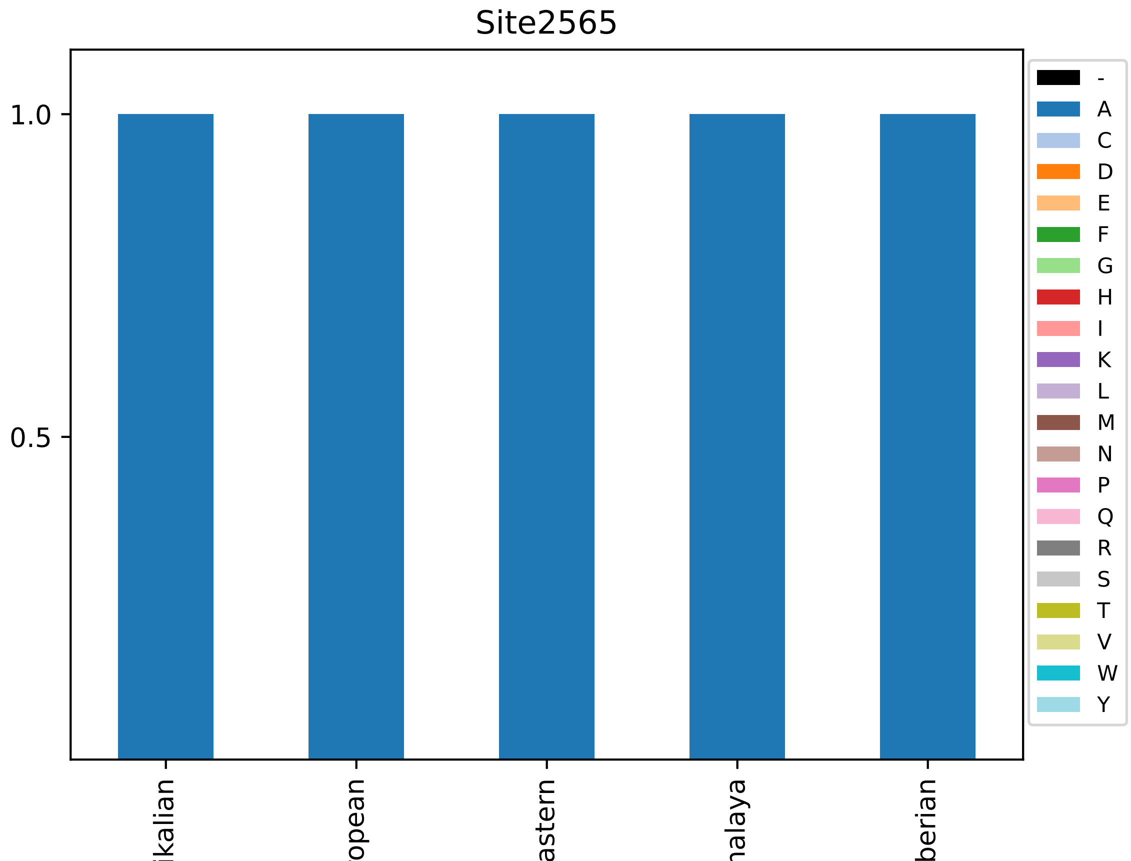
Site2565
1.0
0.5
-
A
C
D
E
F
G
H
I
K
L
M
N
P
Q
R
S
T
V
W
Y
ikalian
opean
astern
nalaya
berian

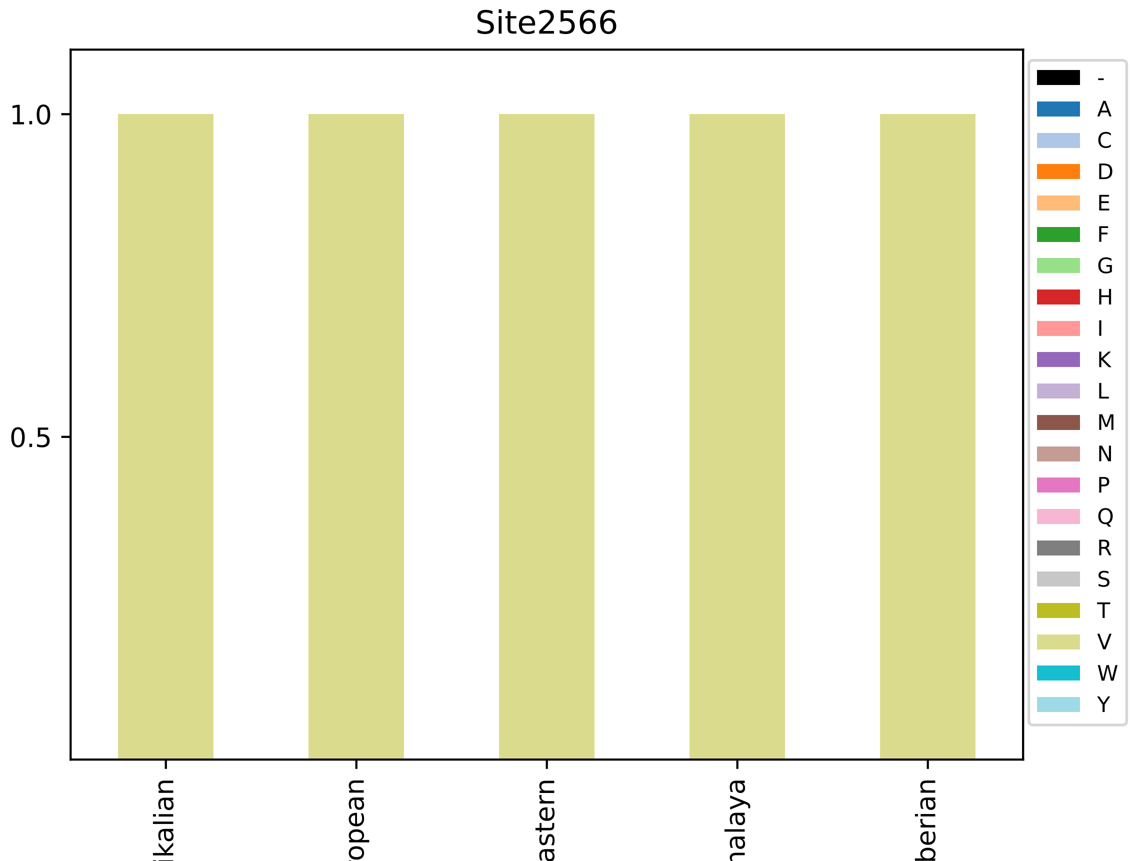
Site2566
1.0
0.5
-
A
C
D
E
F
G
H
I
K
L
M
N
P
Q
R
S
T
V
W
Y
ikalian
opean
astern
nalaya
berian

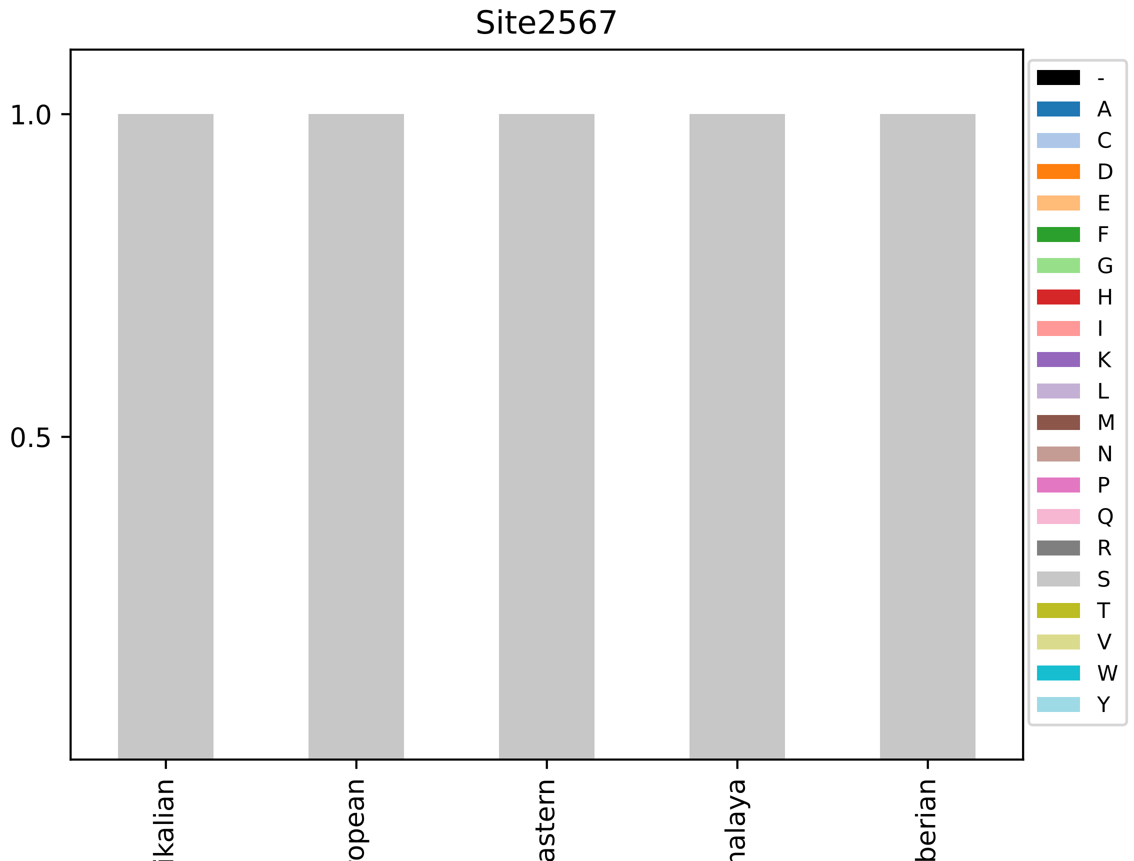

Site2567
1.0
0.5
ikalian
opean
astern
nalaya
berian
-
A
C
D
E
F
G
H
I
K
L
M
N
P
Q
R
S
T
V
W
Y

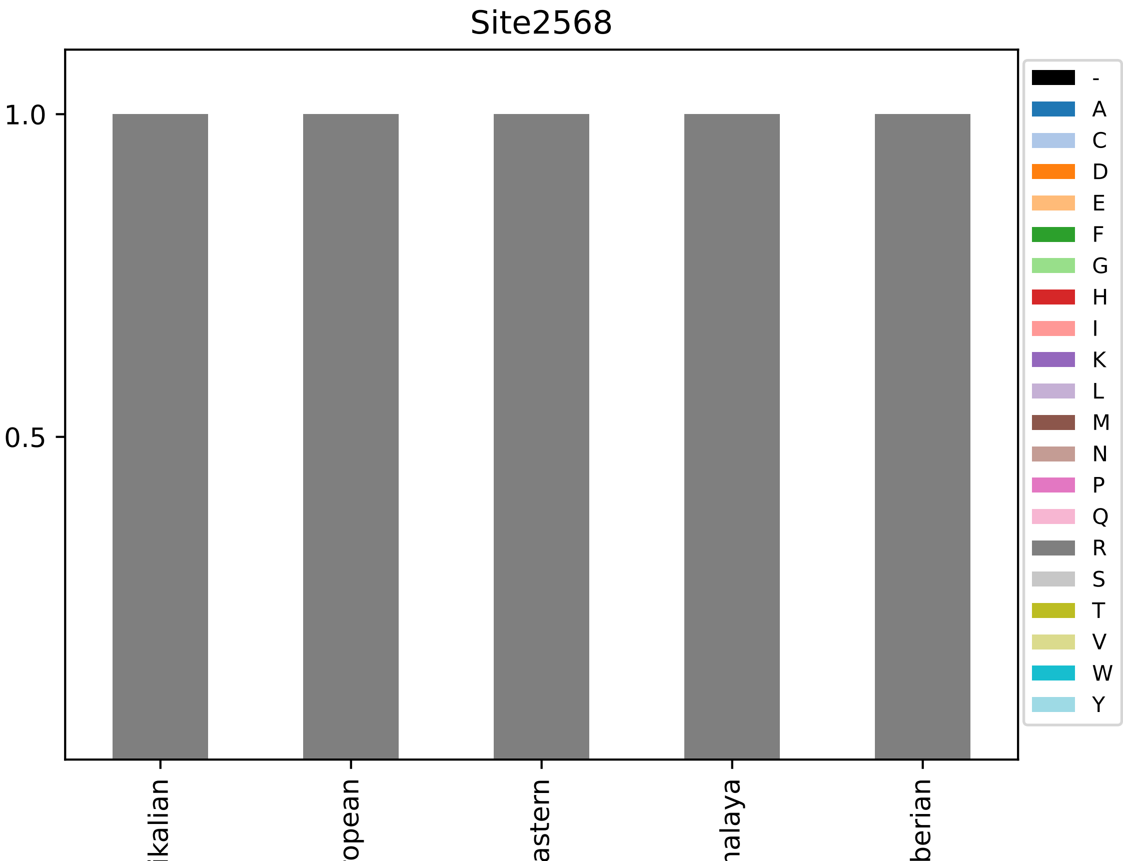
Site2568
1.0
0.5
-
A
C
D
E
F
G
H
I
K
L
M
N
P
Q
R
S
T
V
W
Y
ikalian
opean
astern
nalaya
berian

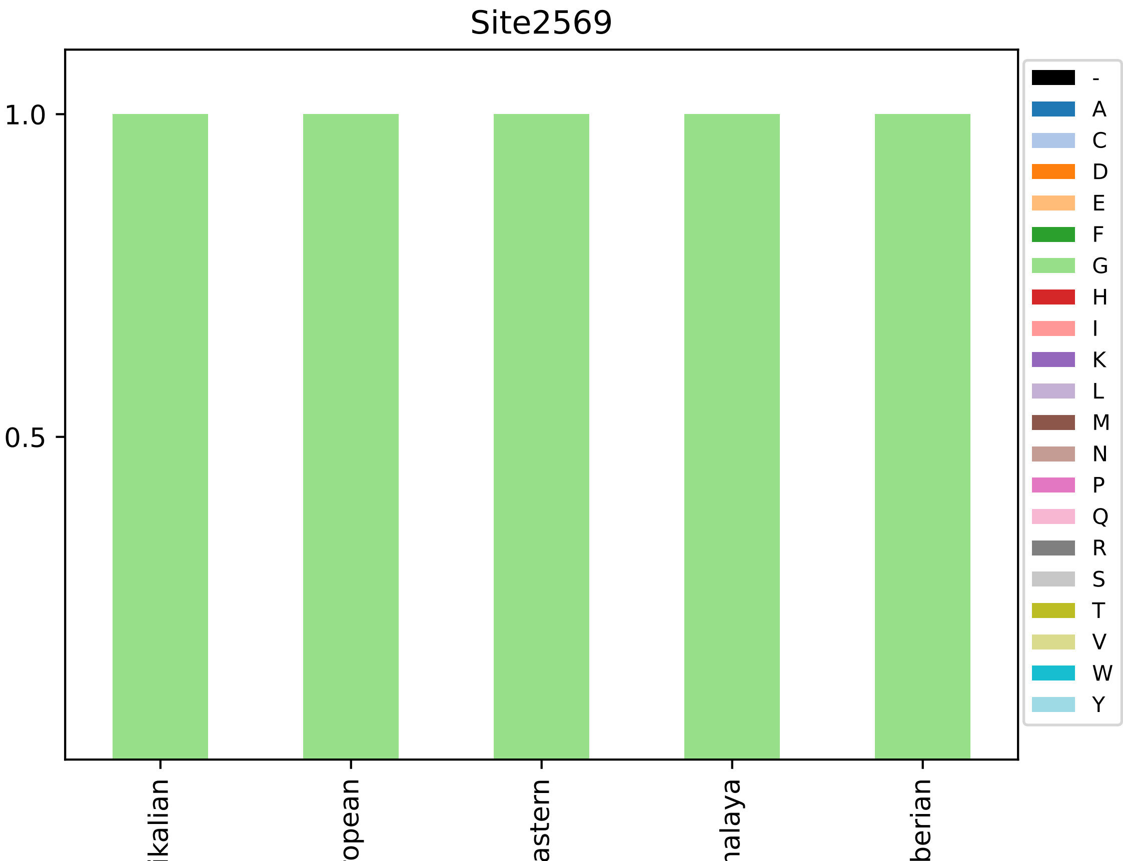

Site2569
1.0
0.5
ikalian
opean
astern
nalaya
berian
-
A
C
D
E
F
G
H
I
K
L
M
N
P
Q
R
S
T
V
W
Y

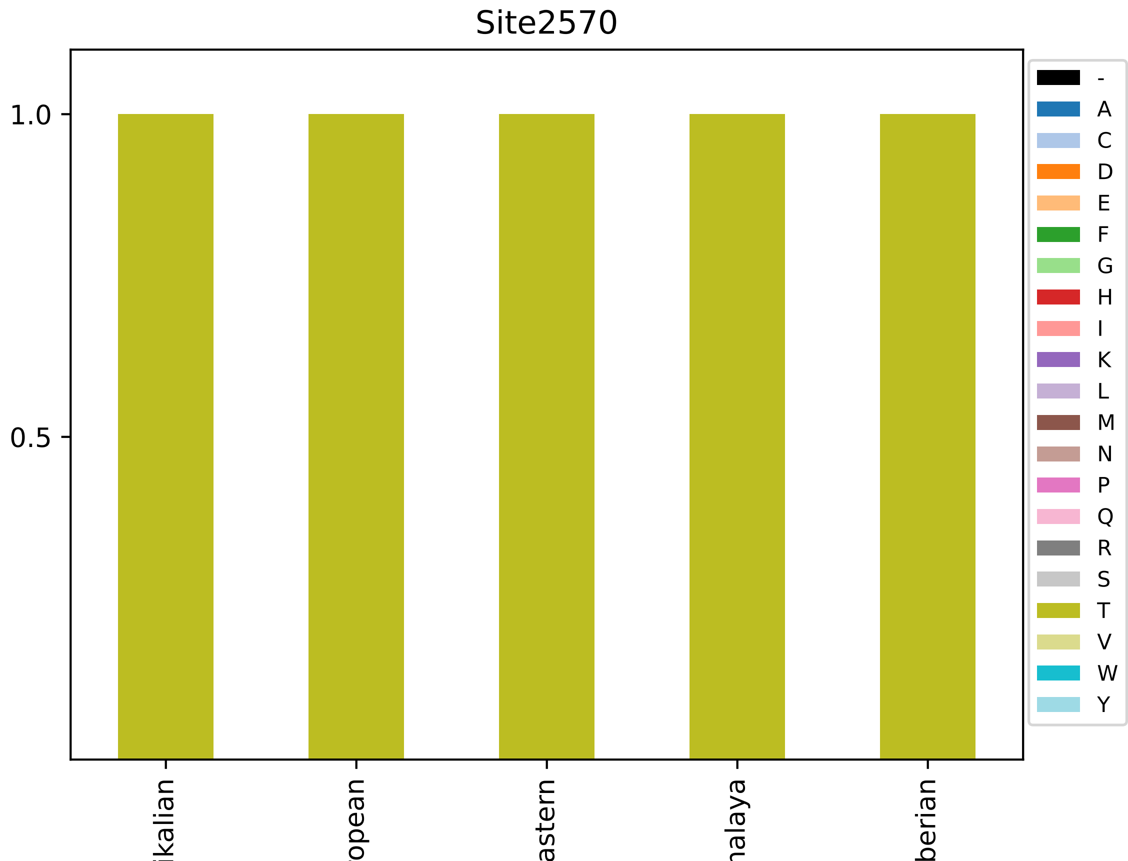

Site2570
1.0
0.5
-
A
C
D
E
F
G
H
I
K
L
M
N
P
Q
R
S
T
V
W
Y
ikalian
ropean
astern
nalaya
berian

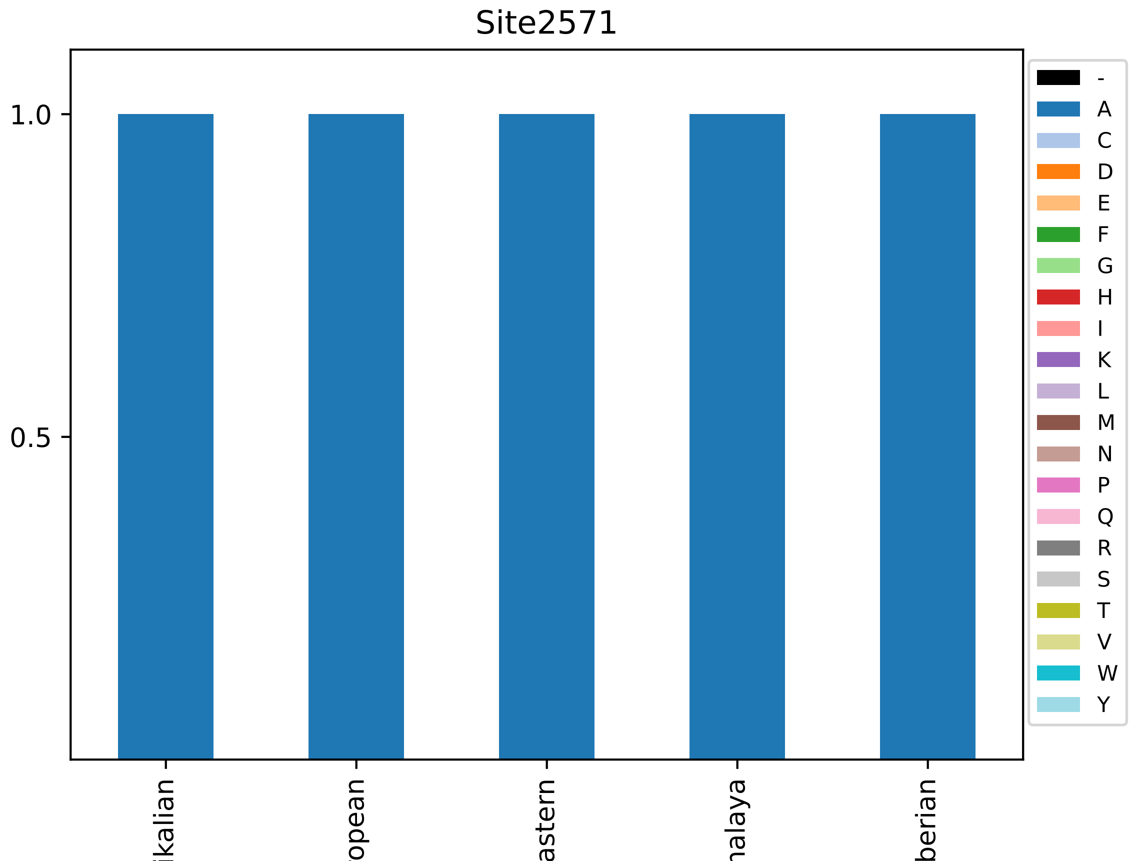
Site2571
1.0
0.5
-
A
C
D
E
F
G
H
I
K
L
M
N
P
Q
R
S
T
V
W
Y
ikalian
opean
astern
nalaya
berian

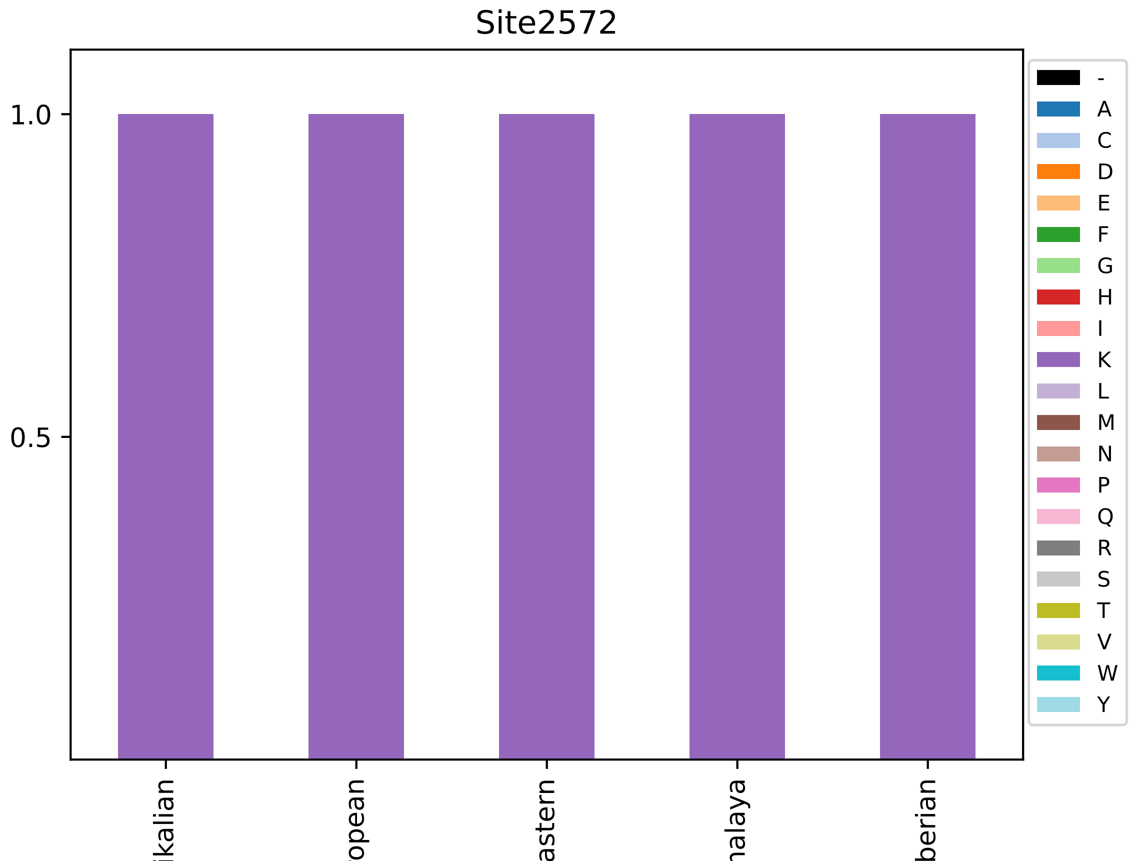
Site2572
1.0
0.5
-
A
C
D
E
F
G
H
I
K
L
M
N
P
Q
R
S
T
V
W
Y
ikalian
opean
astern
nalaya
berian

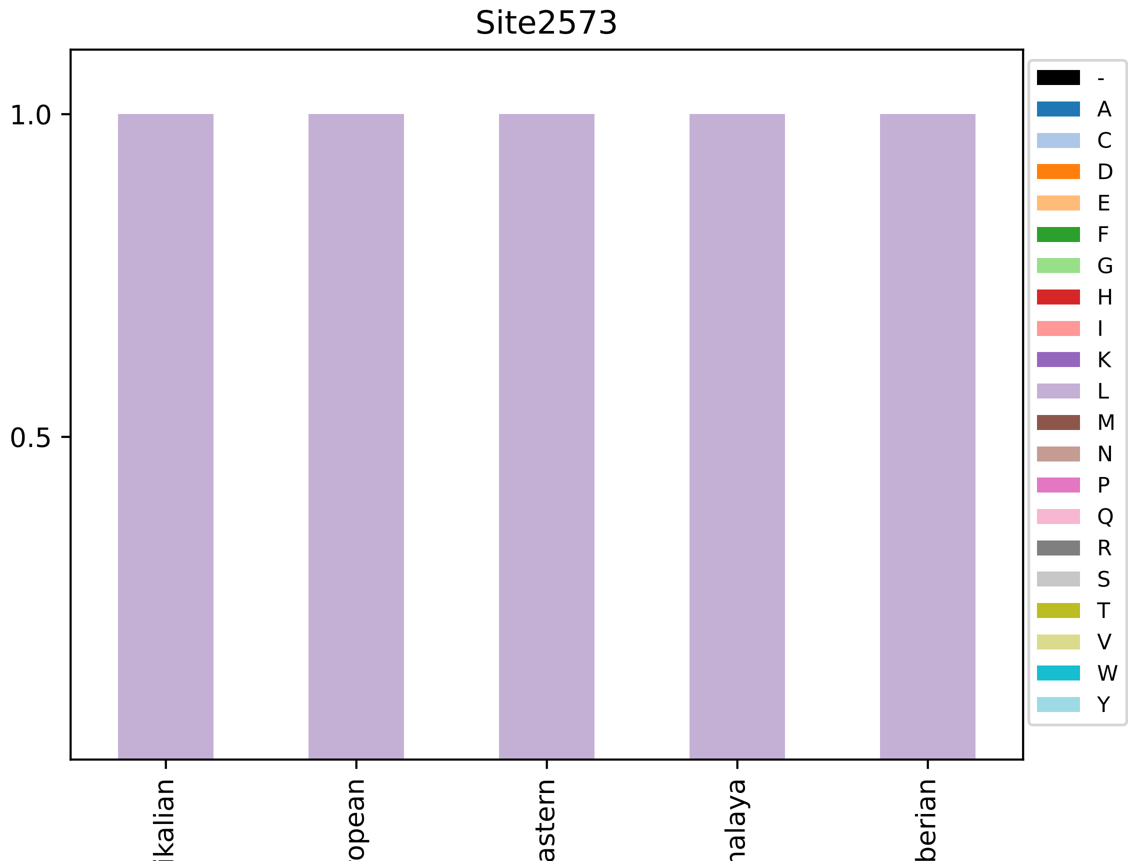

Site2573
1.0
0.5
-
A
C
D
E
F
G
H
I
K
L
M
N
P
Q
R
S
T
V
W
Y
ikalian
opean
astern
nalaya
berian

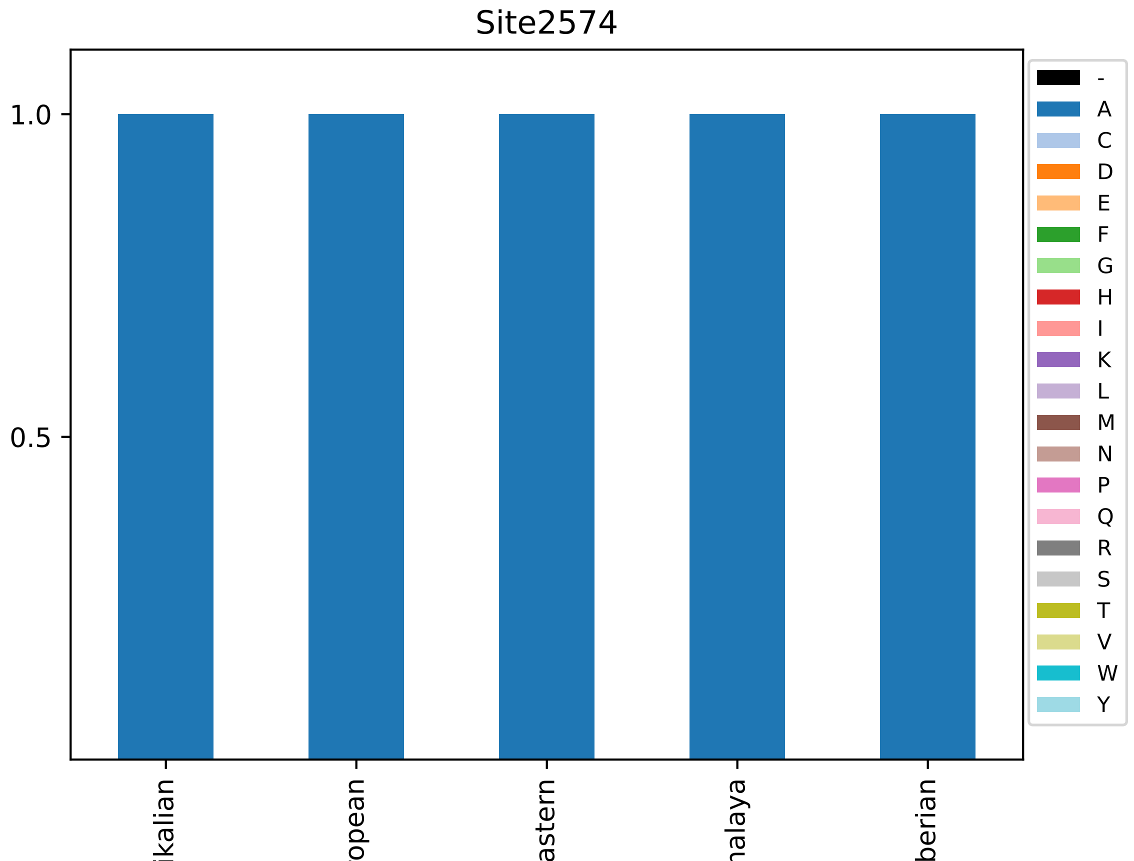
Site2574
1.0
0.5
-
A
C
D
E
F
G
H
I
K
L
M
N
P
Q
R
S
T
V
W
Y
ikalian
opean
astern
nalaya
berian

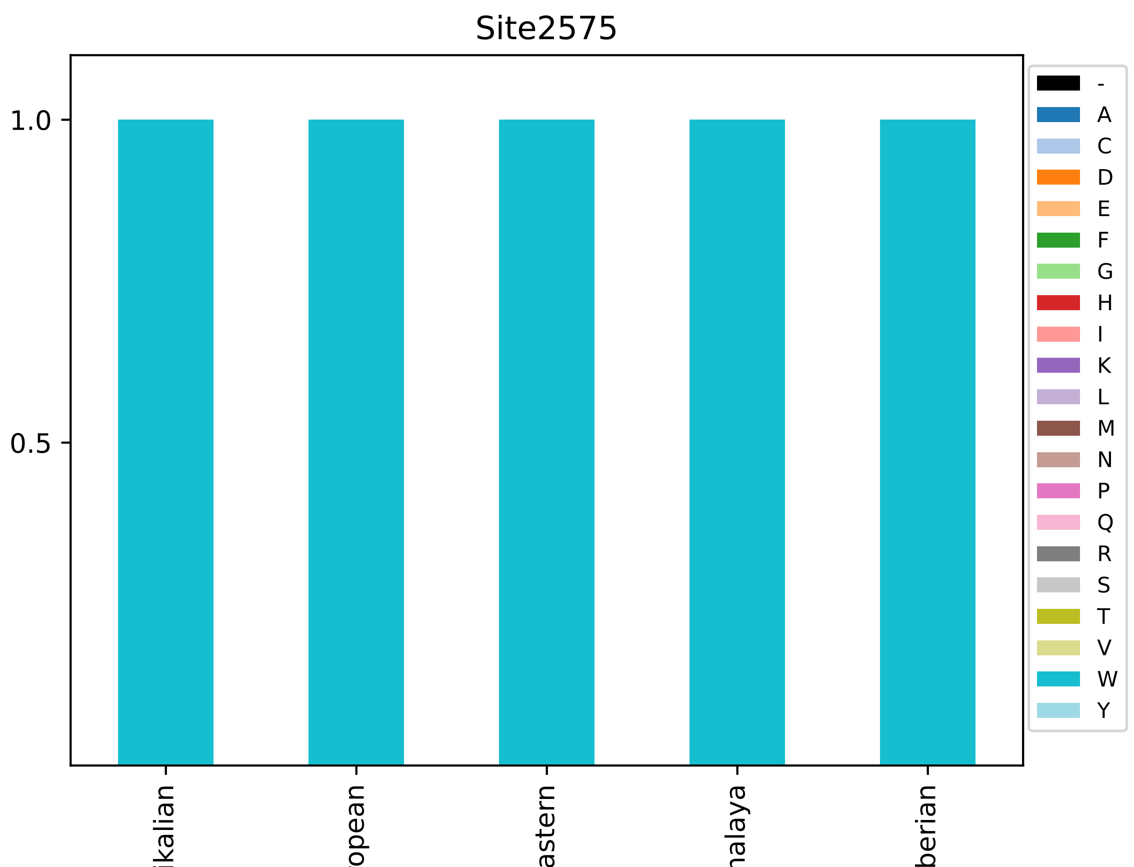
Site2575
1.0
0.5
-
A
C
D
E
F
G
H
I
K
L
M
N
P
Q
R
S
T
V
W
Y
ikalian
opean
astern
nalaya
berian

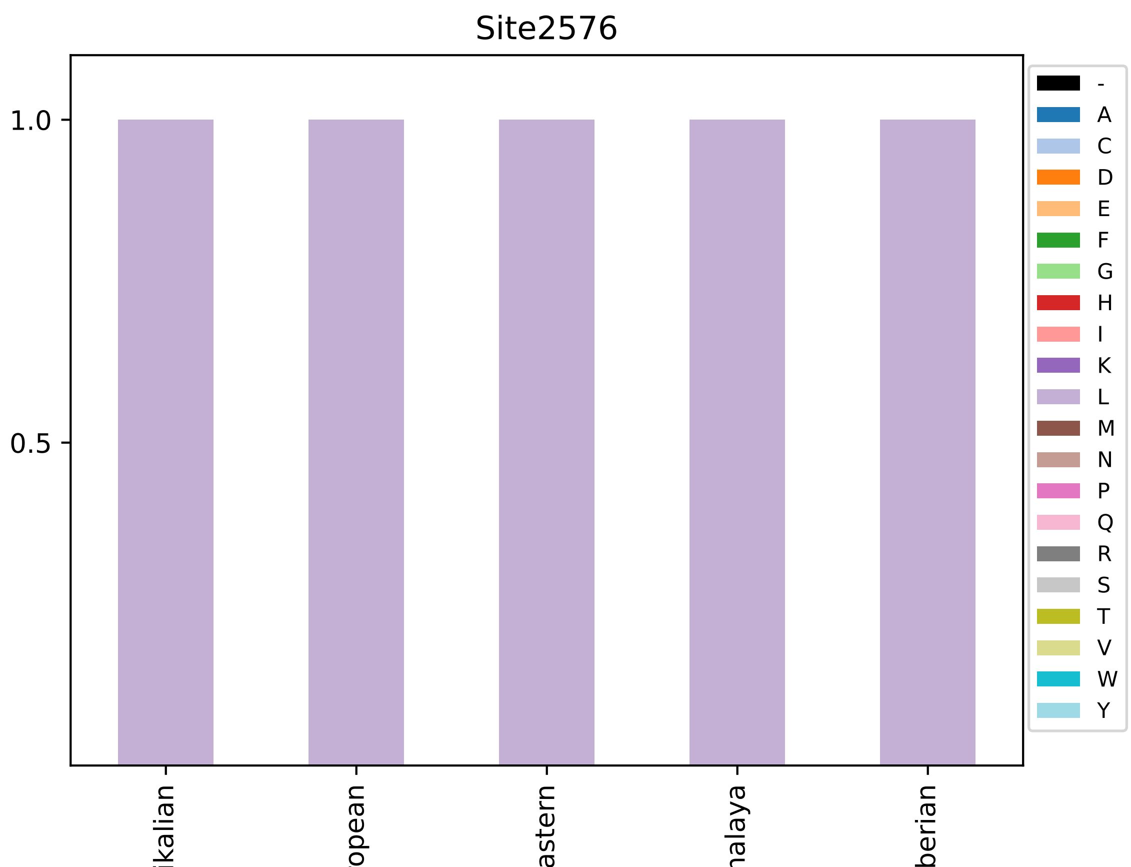

Site2576
1.0
0.5
ikalian
opean
astern
nalaya
berian
-
A
C
D
E
F
G
H
I
K
L
M
N
P
Q
R
S
T
V
W
Y

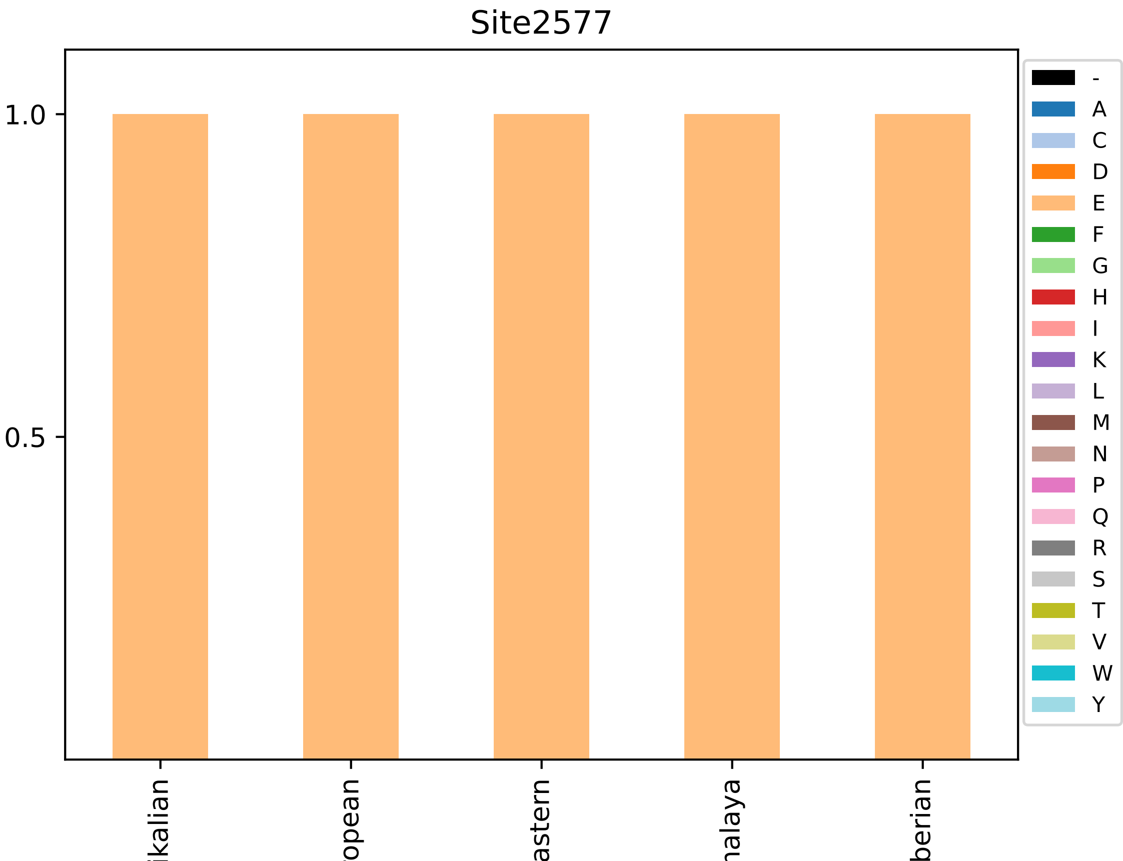
Site2577
1.0
0.5
-
A
C
D
E
F
G
H
I
K
L
M
N
P
Q
R
S
T
V
W
Y
ikalian
opean
astern
nalaya
berian

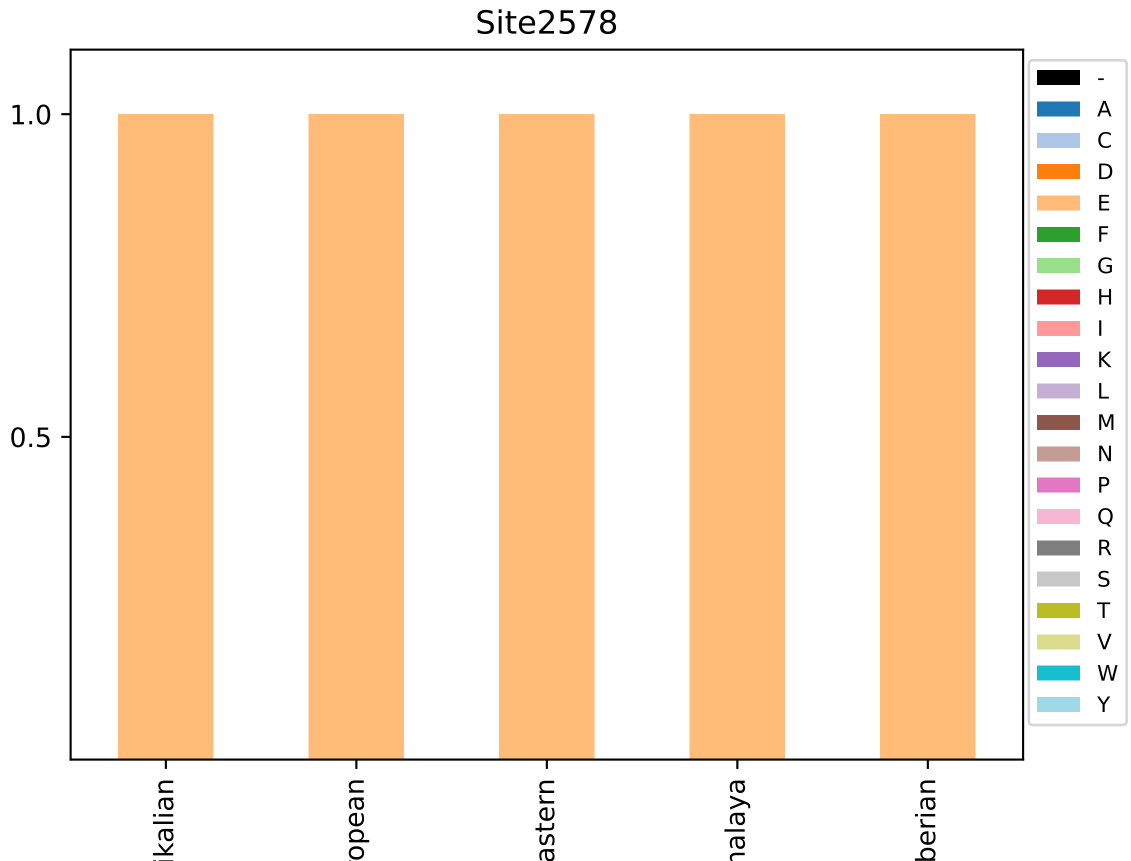

Site2578
1.0
0.5
-
A
C
D
E
F
G
H
I
K
L
M
N
P
Q
R
S
T
V
W
Y
ikalian
opean
astern
nalaya
berian

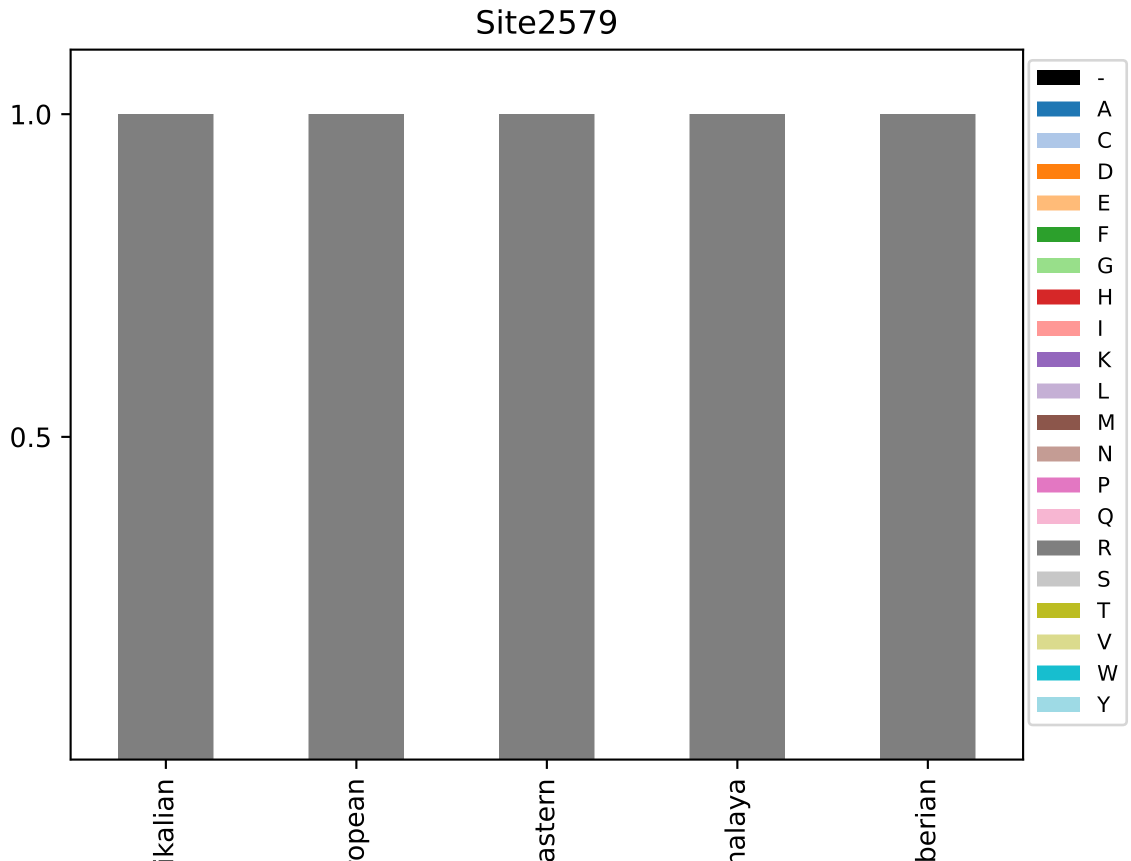
Site2579
1.0
0.5
-
A
C
D
E
F
G
H
I
K
L
M
N
P
Q
R
S
T
V
W
Y
ikalian
opean
astern
nalaya
berian

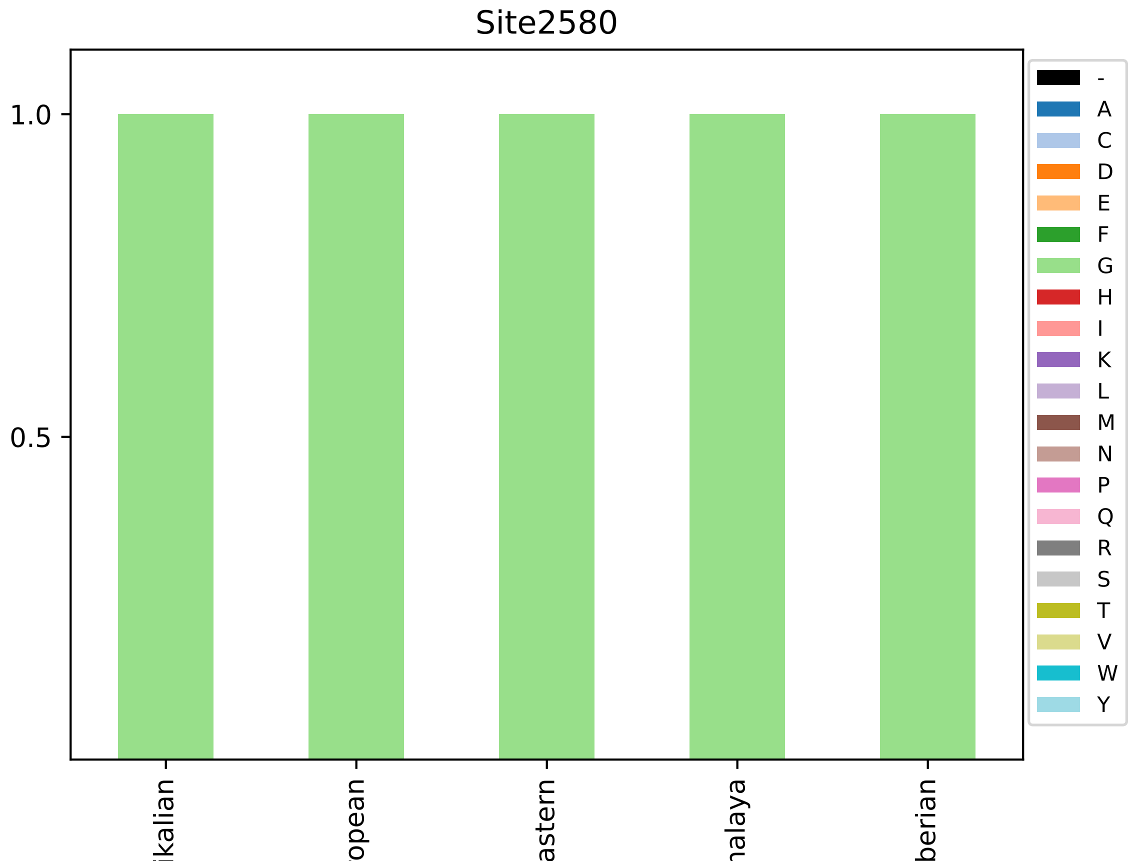

Site2580
1.0
0.5
-
A
C
D
E
F
G
H
I
K
L
M
N
P
Q
R
S
T
V
W
Y
ikalian
opean
astern
nalaya
berian

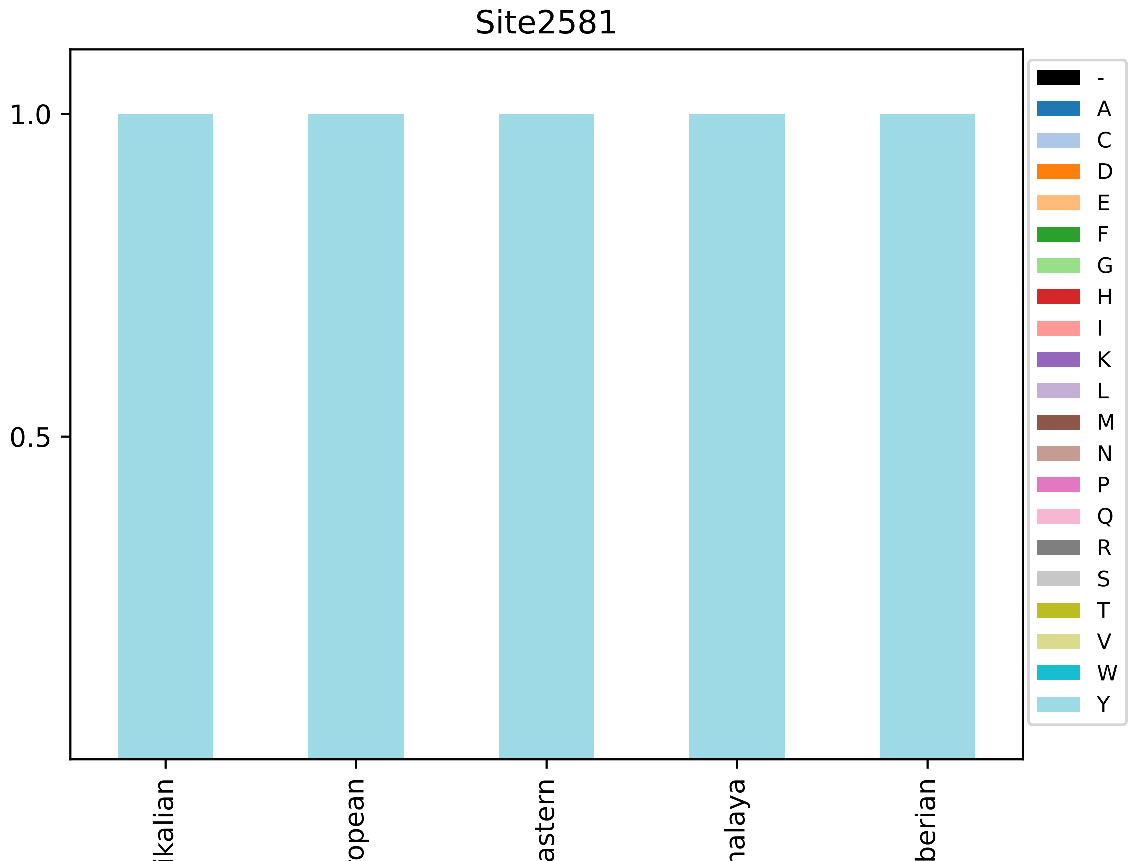

Site2581
1.0
0.5
-
A
C
D
E
F
G
H
I
K
L
M
N
P
Q
R
S
T
V
W
Y
ikalian
opean
astern
nalaya
berian

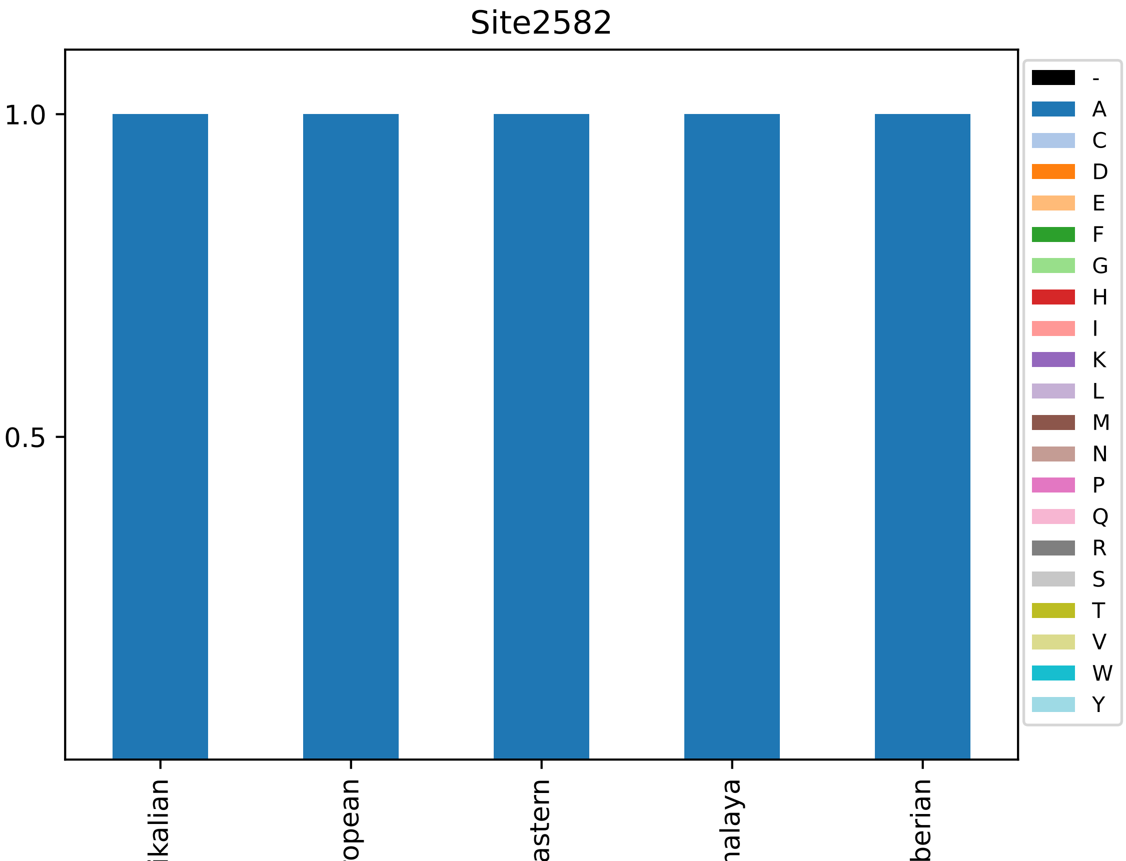

Site2582
1.0
0.5
-
A
C
D
E
F
G
H
I
K
L
M
N
P
Q
R
S
T
V
W
Y
ikalian
opean
astern
nalaya
berian

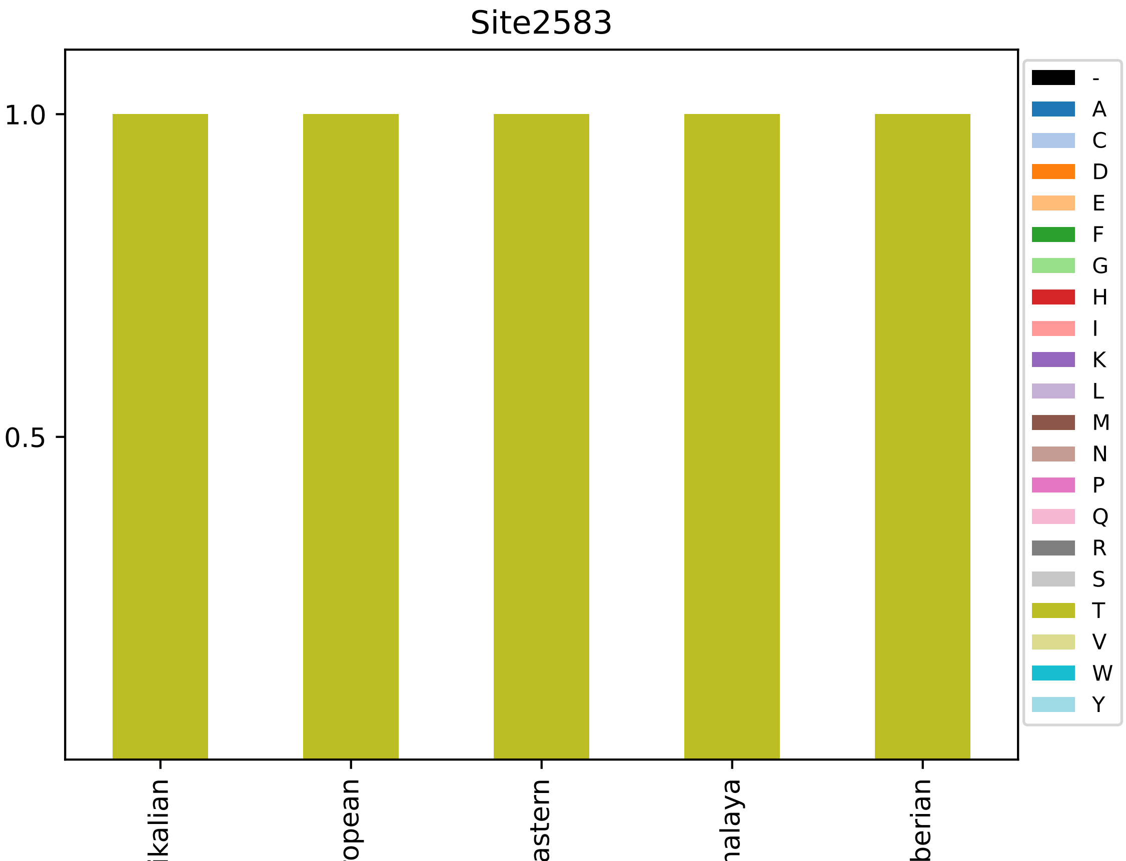

Site2583
1.0
0.5
-
A
C
D
E
F
G
H
I
K
L
M
N
P
Q
R
S
T
V
W
Y
ikalian
opean
astern
nalaya
berian

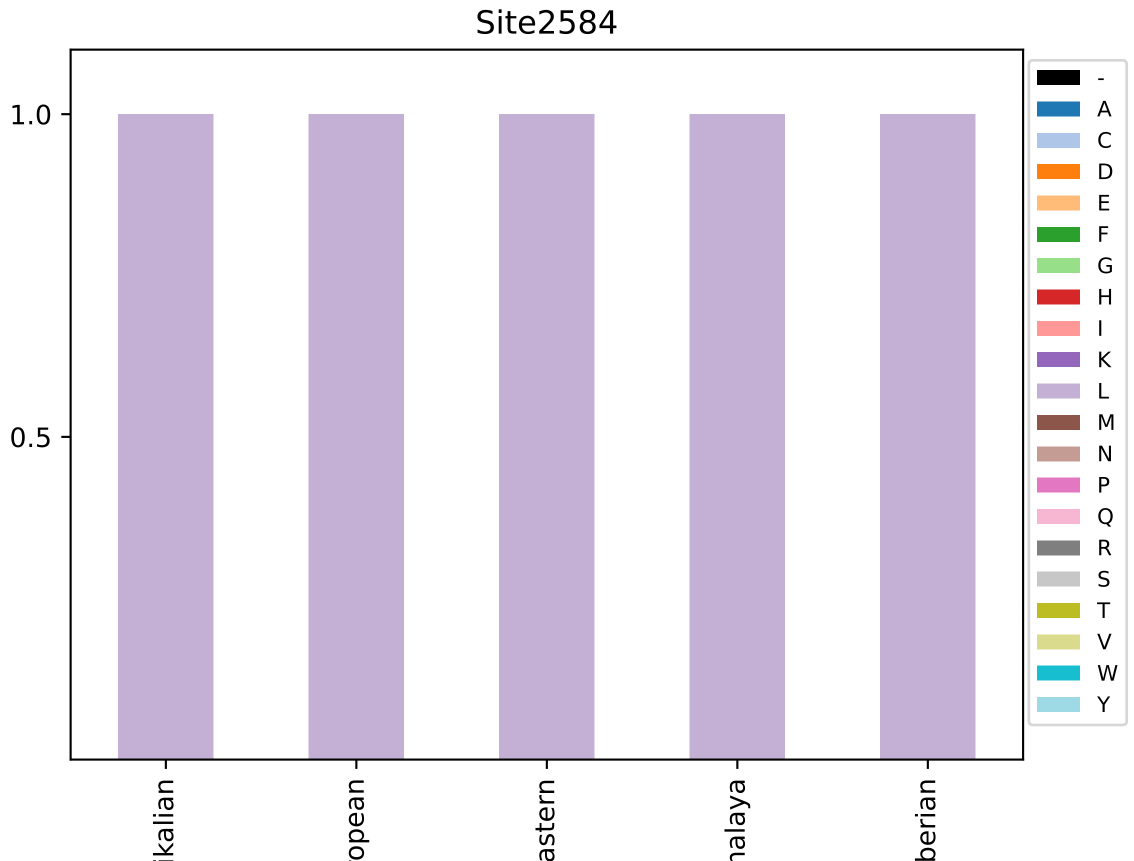

Site2584
1.0
0.5
-
A
C
D
E
F
G
H
I
K
L
M
N
P
Q
R
S
T
V
W
Y
ikalian
opean
astern
halaya
berian

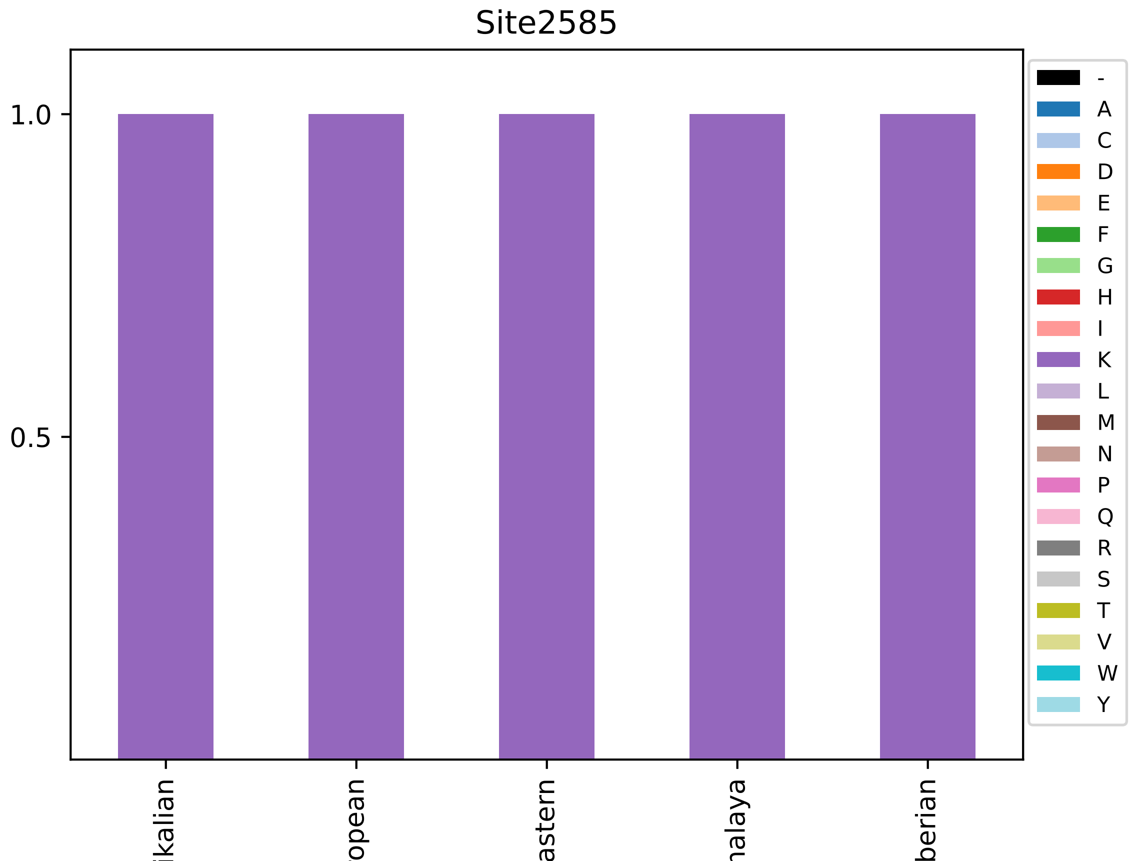

Site2585
1.0
0.5
ikalian
opean
astern
nalaya
berian
-
A
C
D
E
F
G
H
I
K
L
M
N
P
Q
R
S
T
V
W
Y

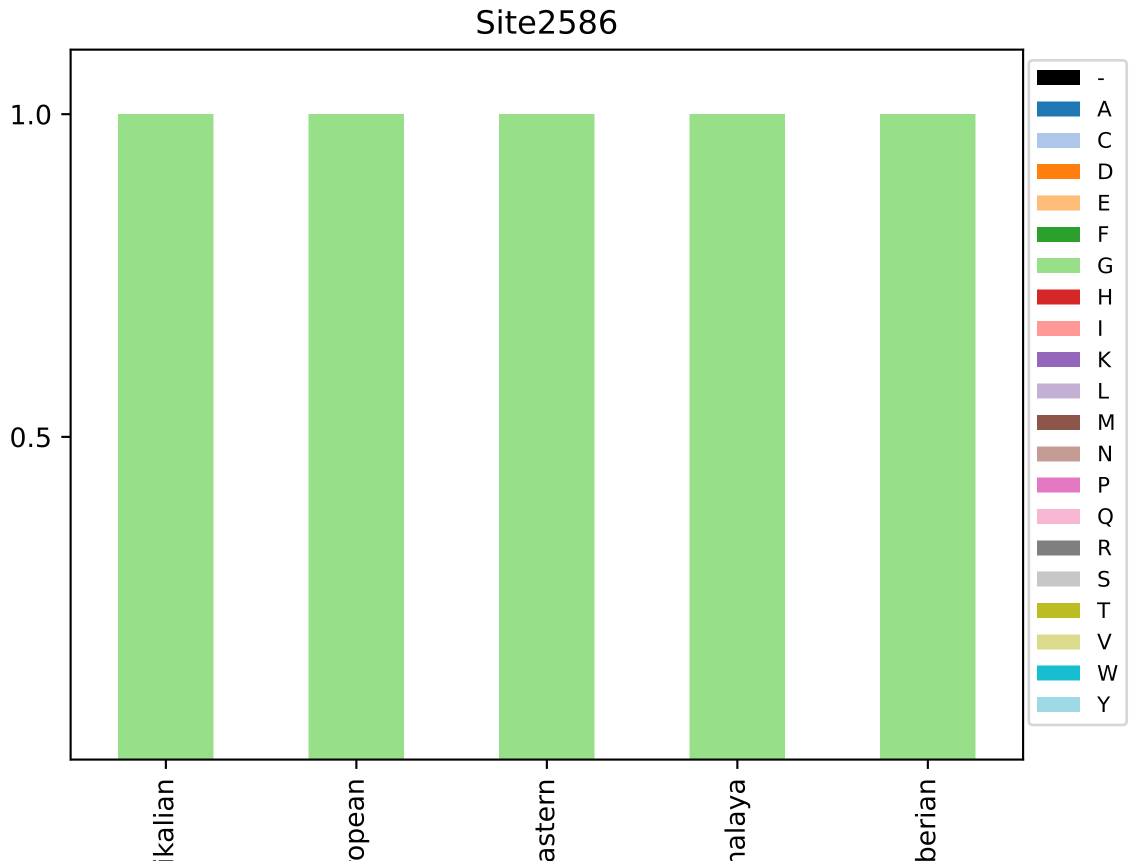

Site2586
1.0
0.5
-
A
C
D
E
F
G
H
I
K
L
M
N
P
Q
R
S
T
V
W
Y
ikalian
opean
astern
nalaya
berian

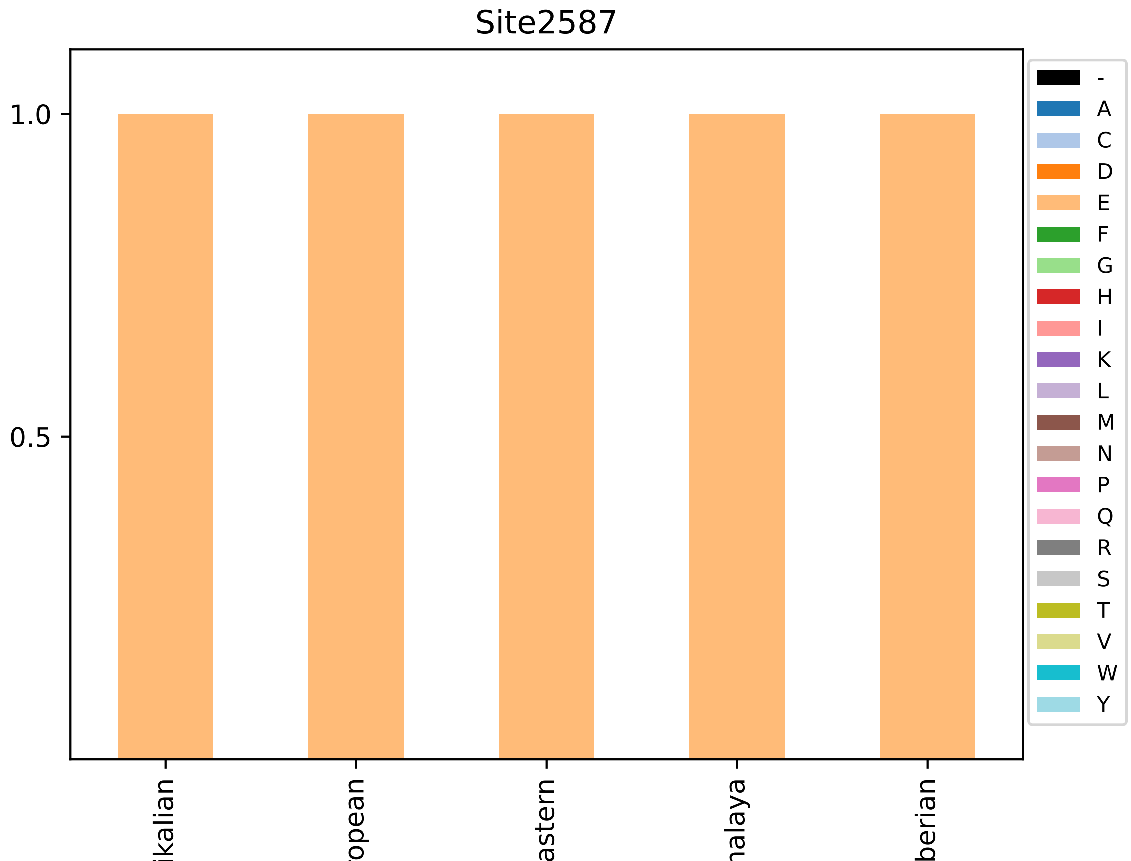

Site2587
1.0
0.5
-
A
C
D
E
F
G
H
I
K
L
M
N
P
Q
R
S
T
V
W
Y

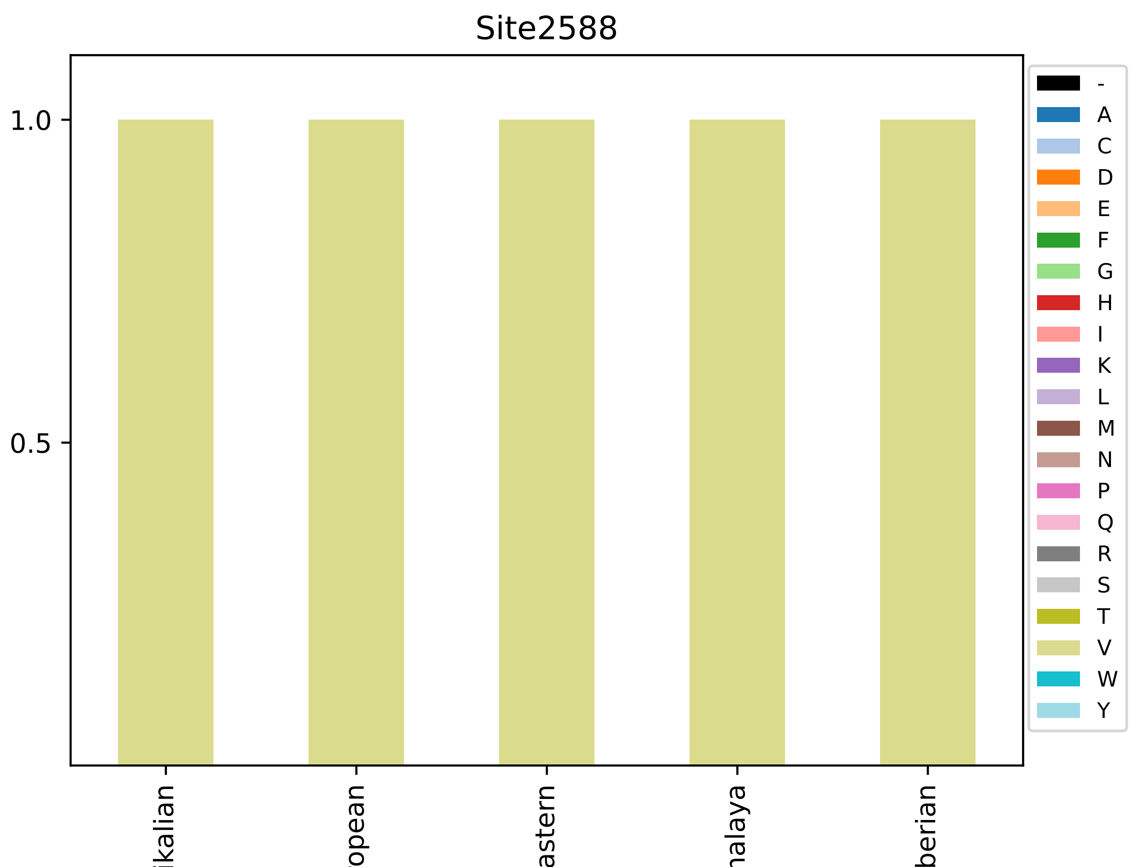

Site2588
1.0
0.5
-
A
C
D
E
F
G
H
I
K
L
M
N
P
Q
R
S
T
V
W
Y
ikalian
opean
astern
nalaya
berian

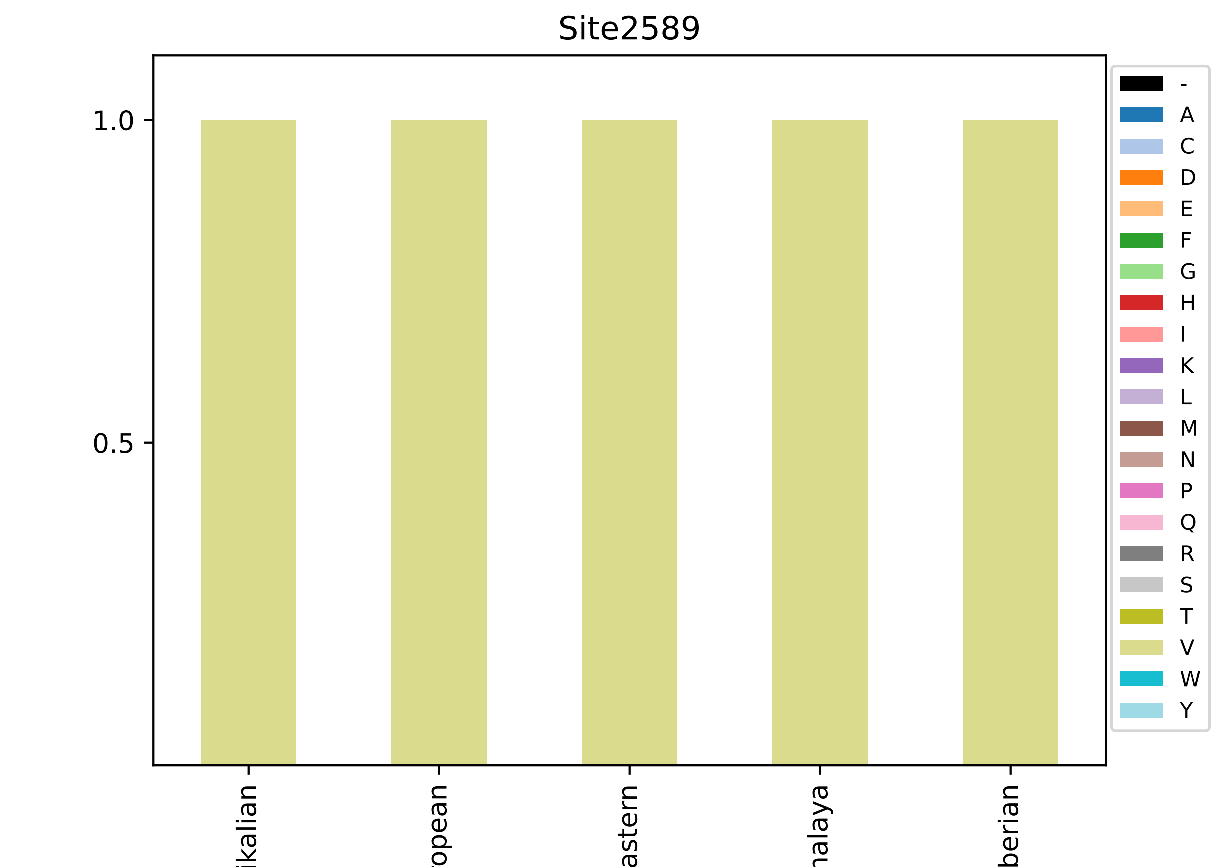
Site2589
1.0
0.5
ikalian
opean
astern
nalaya
berian
-
A
C
D
E
F
G
H
I
K
L
M
N
P
Q
R
S
T
V
W
Y

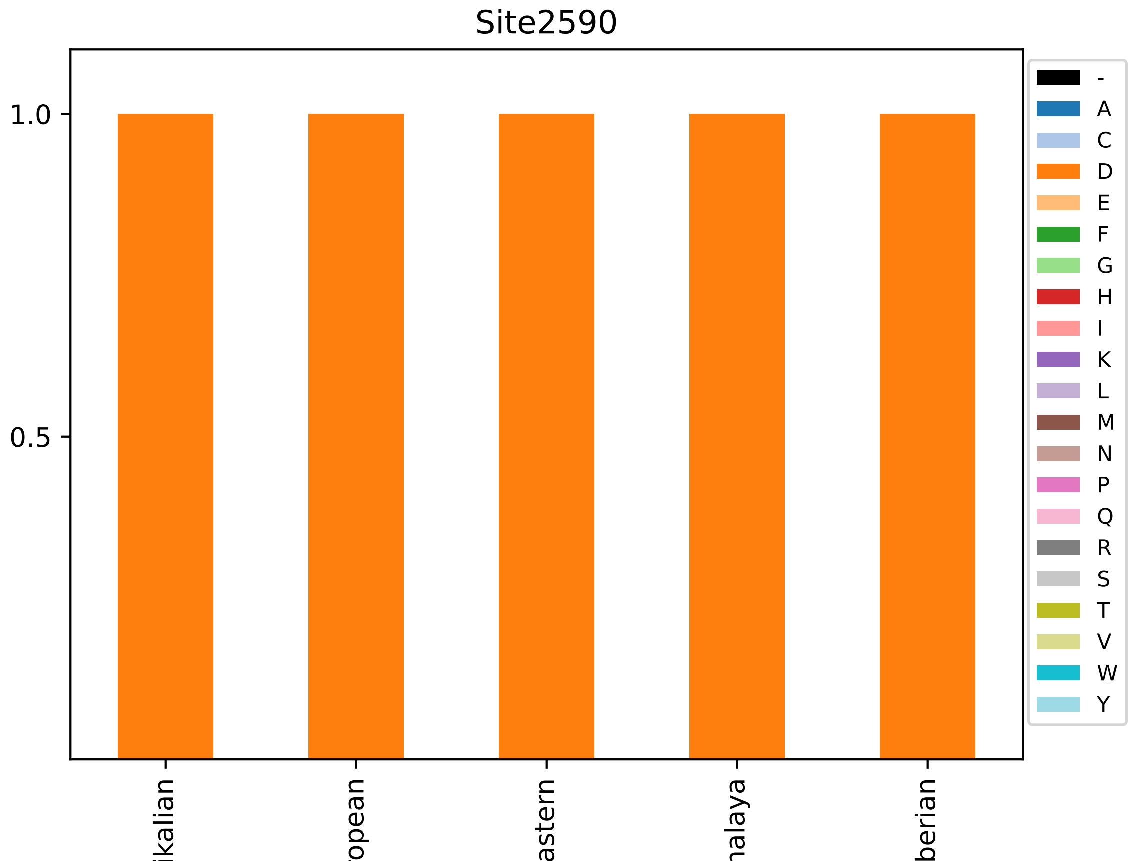

Site2590
1.0
0.5
-
A
C
D
E
F
G
H
I
K
L
M
N
P
Q
R
S
T
V
W
Y
ikalian
opean
astern
nalaya
berian

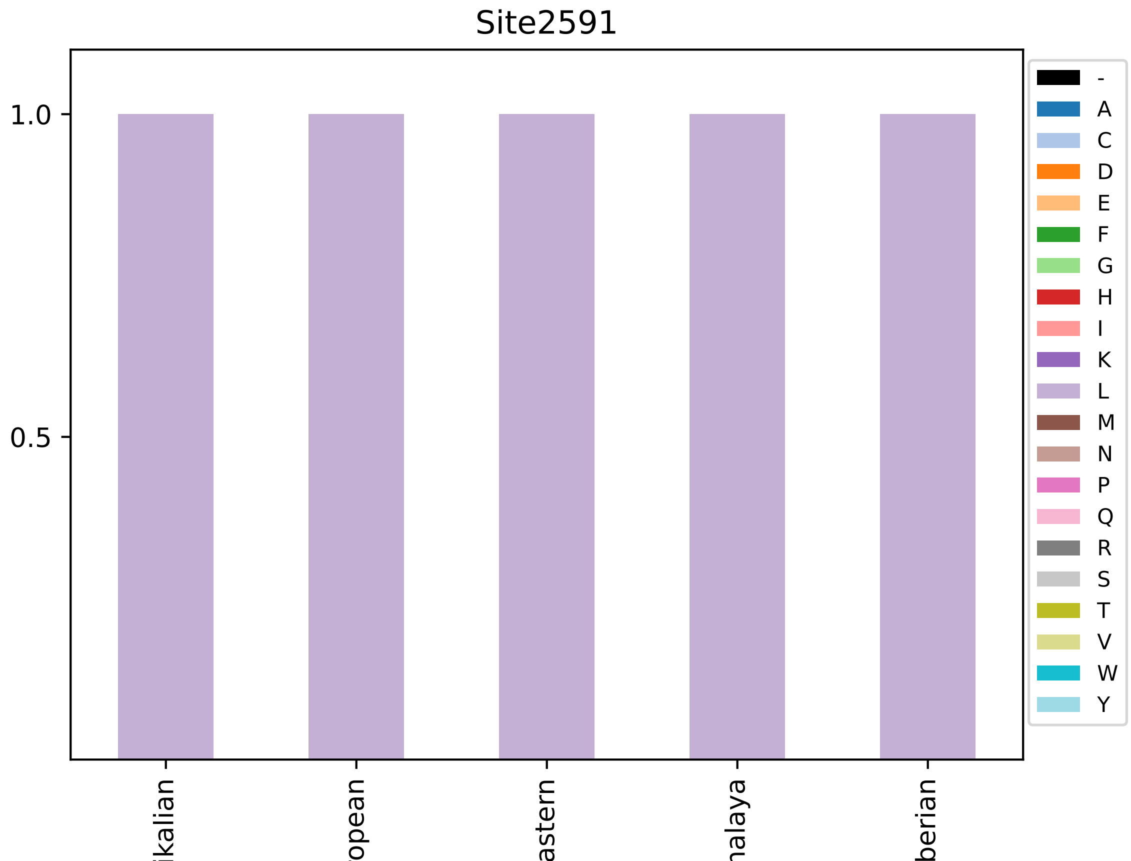
Site2591
1.0
0.5
-
A
C
D
E
F
G
H
I
K
L
M
N
P
Q
R
S
T
V
W
Y
ikalian
opean
astern
nalaya
berian

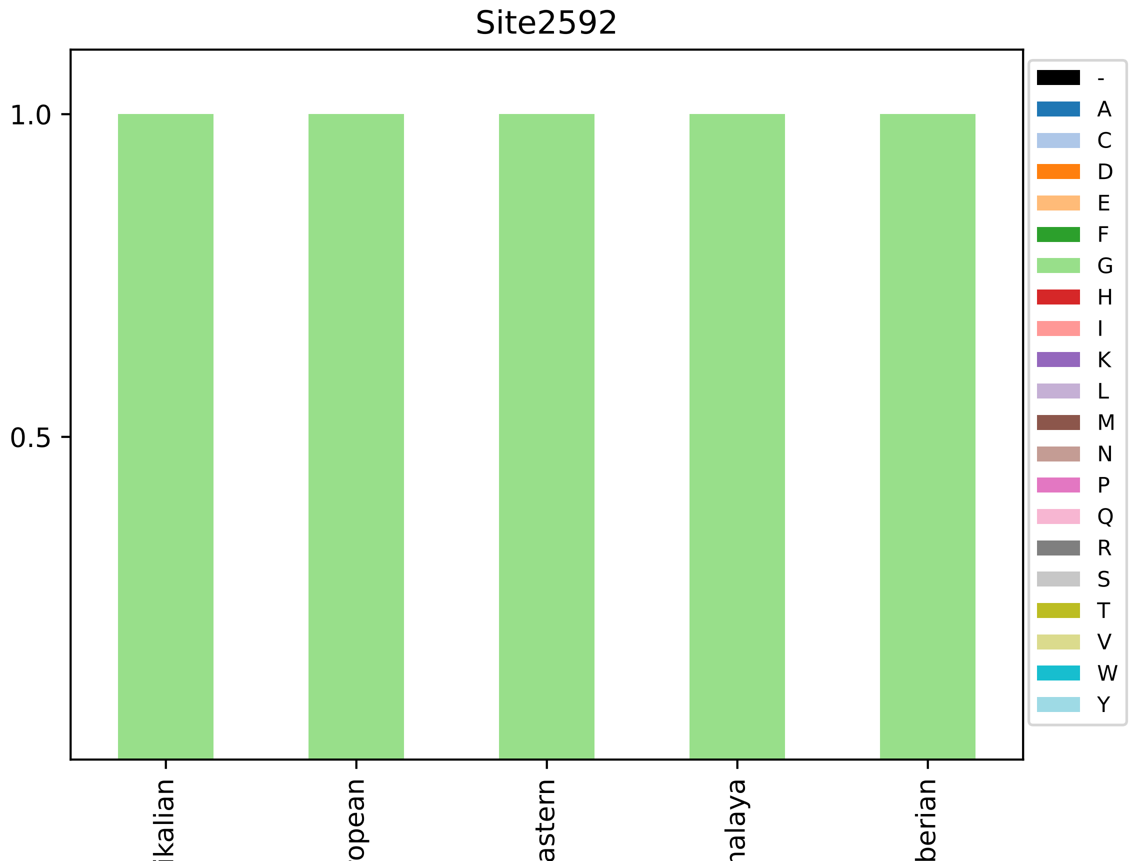

Site2592
1.0
0.5
-
A
C
D
E
F
G
H
I
K
L
M
N
P
Q
R
S
T
V
W
Y
ikalian
opean
astern
nalaya
berian

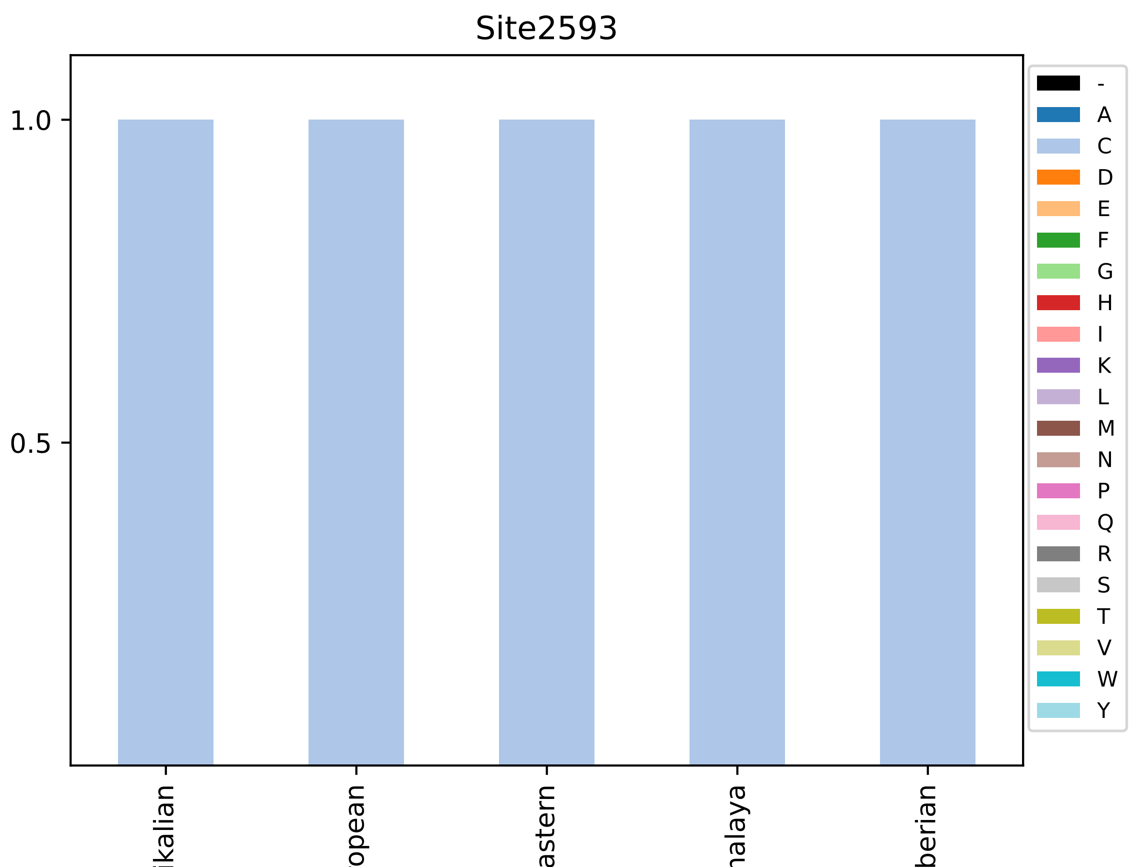

Site2593
1.0
0.5
-
A
C
D
E
F
G
H
I
K
L
M
N
P
Q
R
S
T
V
W
Y
ikalian
opean
astern
nalaya
berian

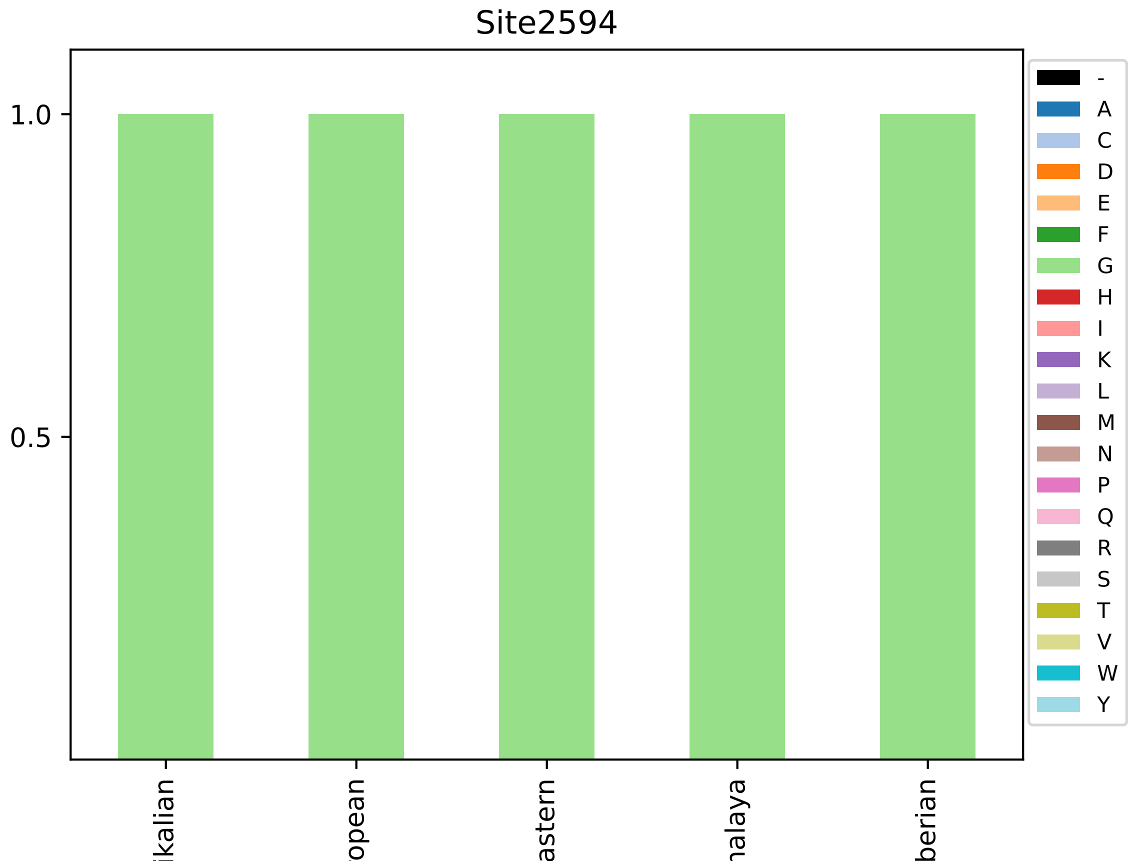

Site2594
1.0
0.5
ikalian
opean
astern
nalaya
berian
-
A
C
D
E
F
G
H
I
K
L
M
N
P
Q
R
S
T
V
W
Y

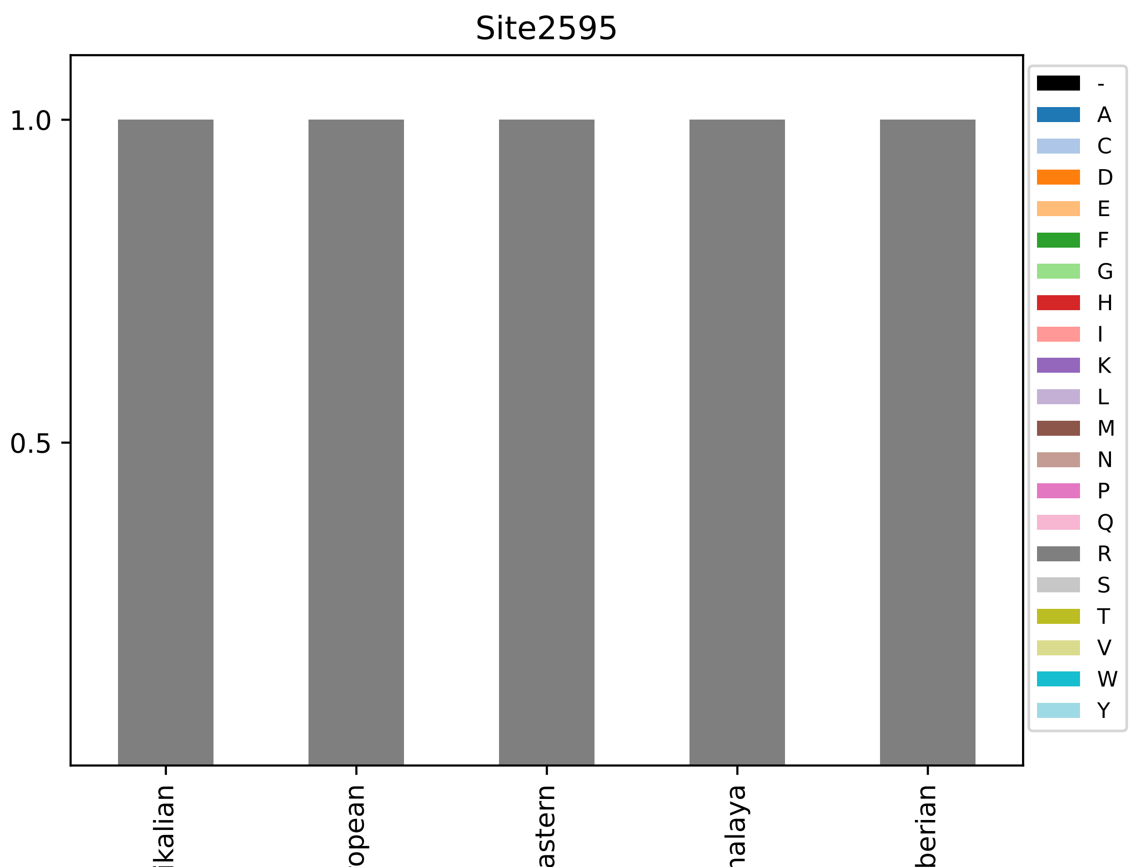
Site2595
1.0
0.5
-
A
C
D
E
F
G
H
I
K
L
M
N
P
Q
R
S
T
V
W
Y
ikalian
opean
astern
nalaya
berian

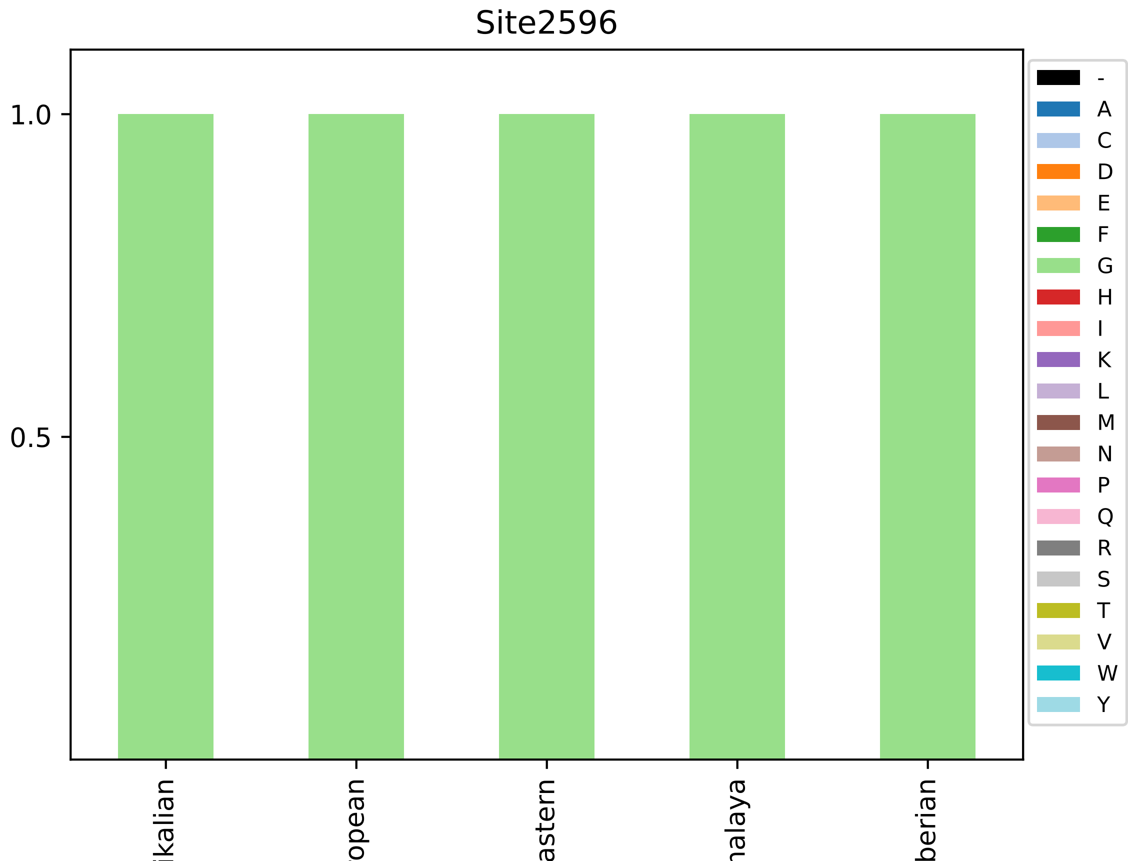

Site2596
1.0
0.5
-
A
C
D
E
F
G
H
I
K
L
M
N
P
Q
R
S
T
V
W
Y
ikalian
opean
astern
nalaya
berian

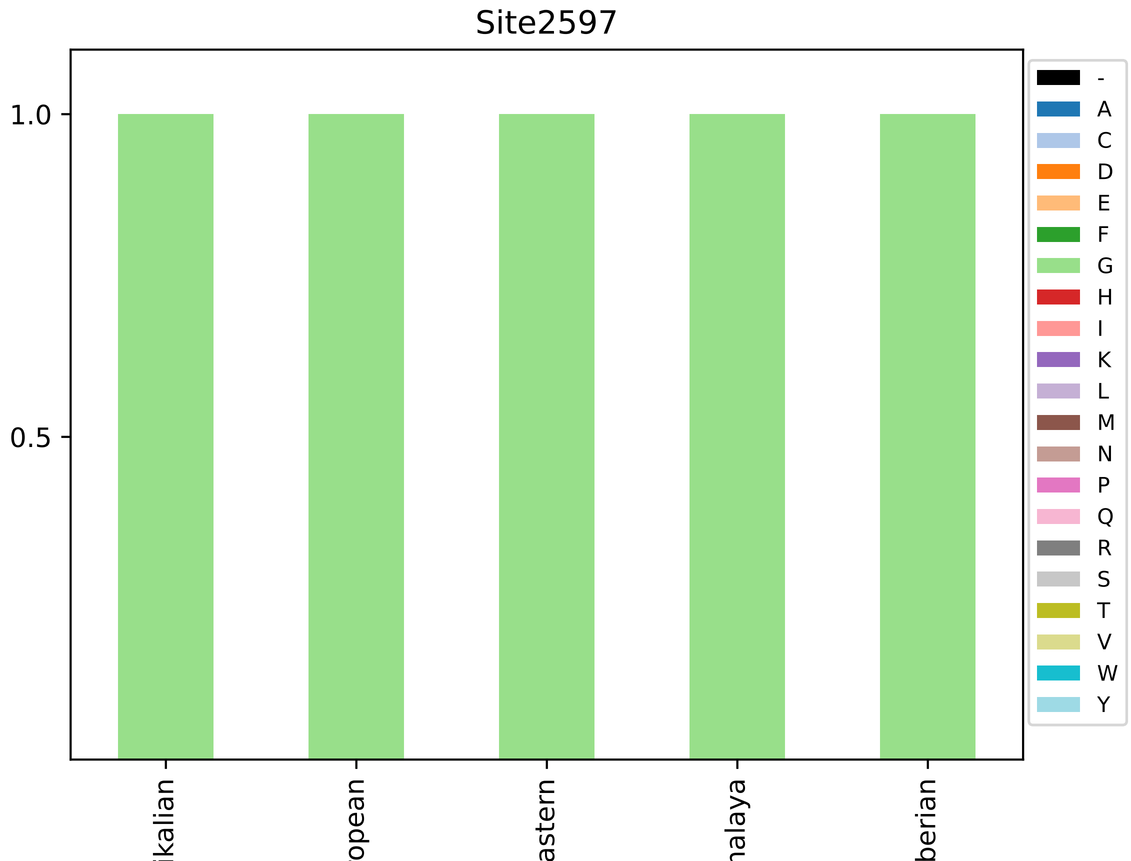
Site2597
1.0
0.5
-
A
C
D
E
F
G
H
I
K
L
M
N
P
Q
R
S
T
V
W
Y
ikalian
opean
astern
alaya
berian

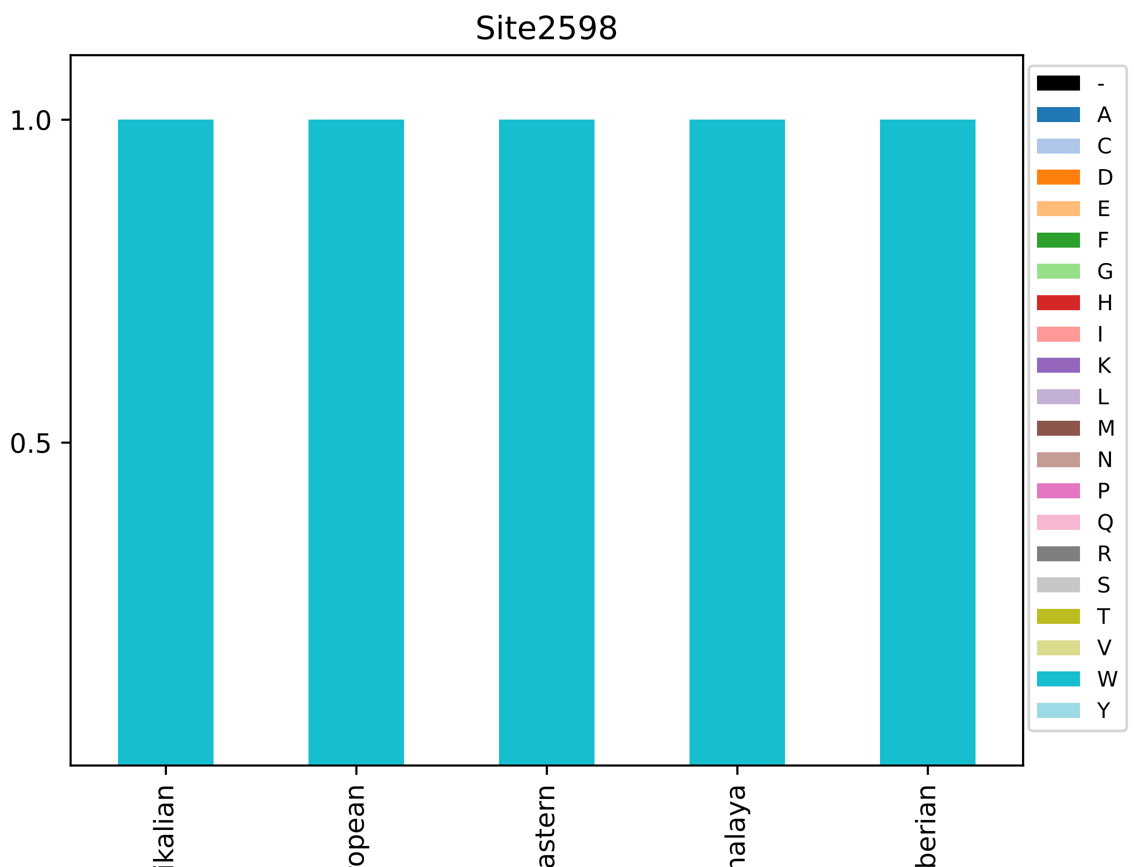

Site2598
1.0
0.5
-
A
C
D
E
F
G
H
I
K
L
M
N
P
Q
R
S
T
V
W
Y
ikalian
opean
astern
nalaya
berian

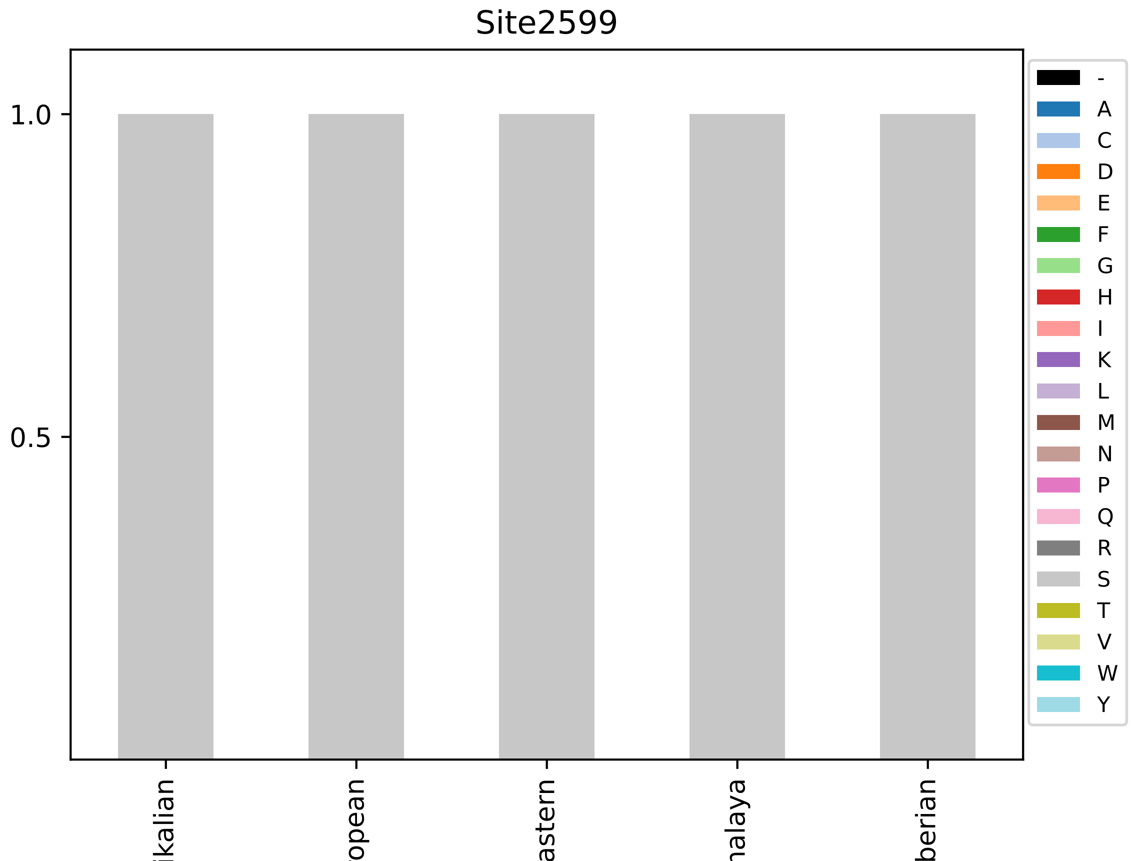

Site2599
1.0
0.5
ikalian
opean
astern
nalaya
berian
-
A
C
D
E
F
G
H
I
K
L
M
N
P
Q
R
S
T
V
W
Y

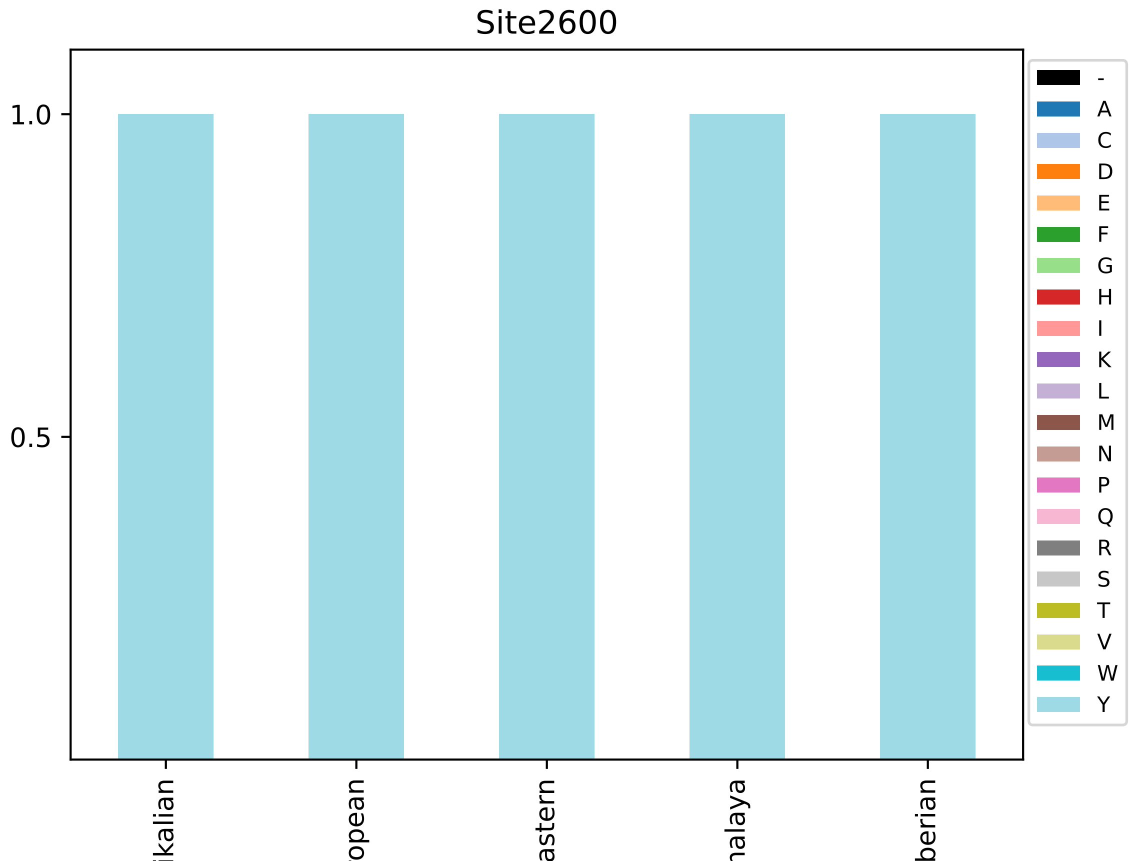

Site2600
1.0
0.5
-
A
C
D
E
F
G
H
I
K
L
M
N
P
Q
R
S
T
V
W
Y
ikalian
opean
astern
nalaya
berian

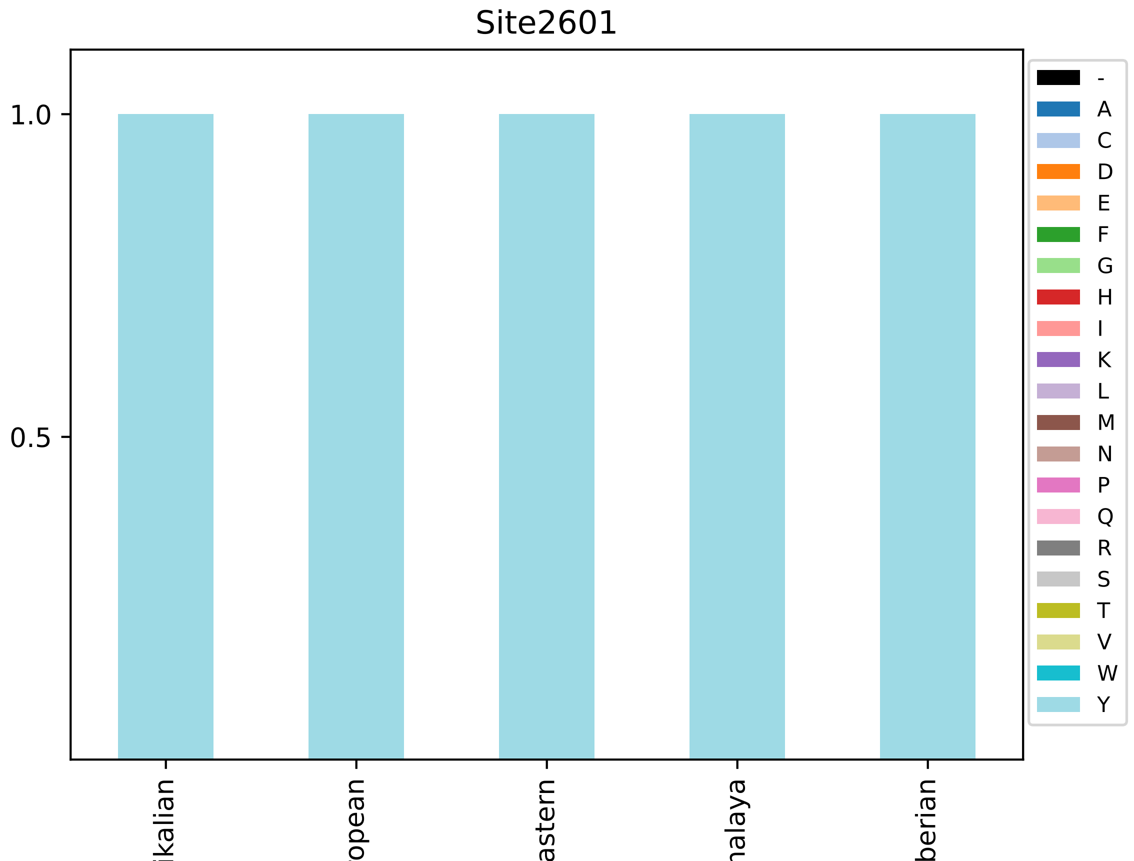

Site2601
1.0
0.5
ikalian
opean
astern
nalaya
berian
-
A
C
D
E
F
G
H
I
K
L
M
N
P
Q
R
S
T
V
W
Y

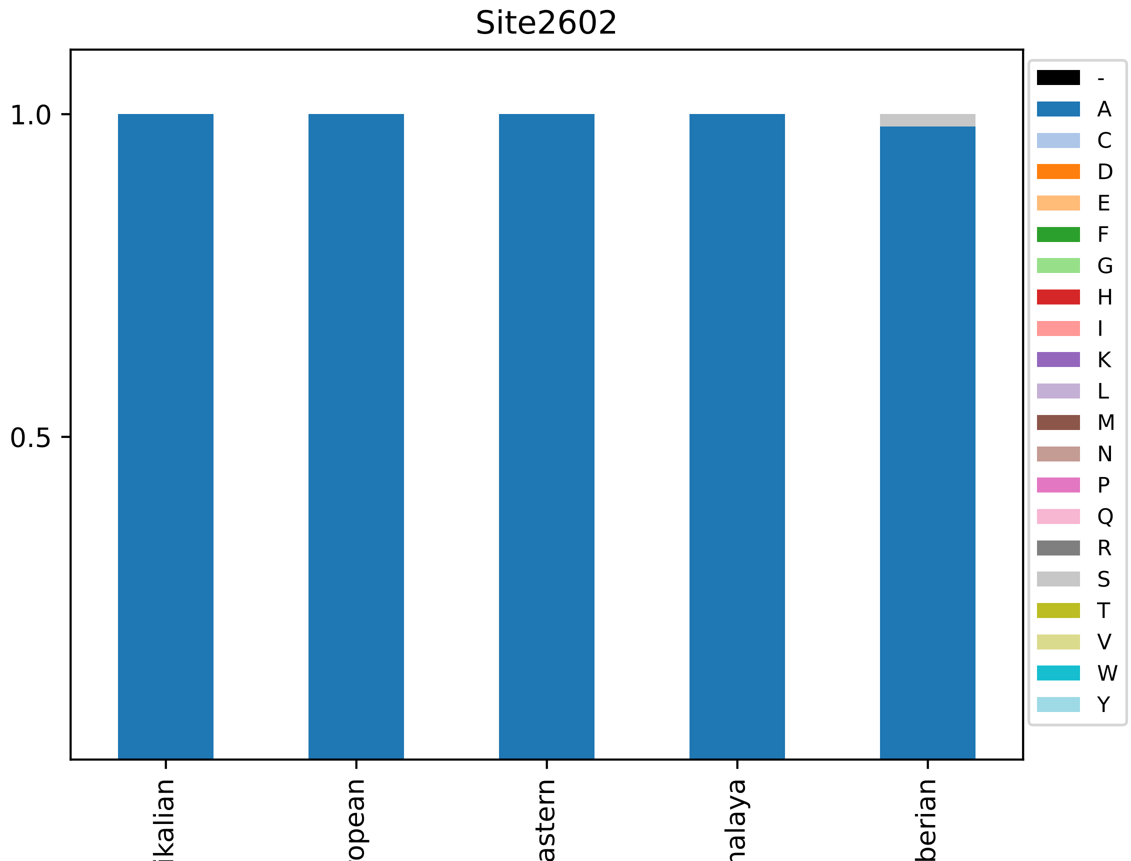

Site2602
1.0
0.5
-
A
C
D
E
F
G
H
I
K
L
M
N
P
Q
R
S
T
V
W
Y
ikalian
ropean
astern
nalaya
berian

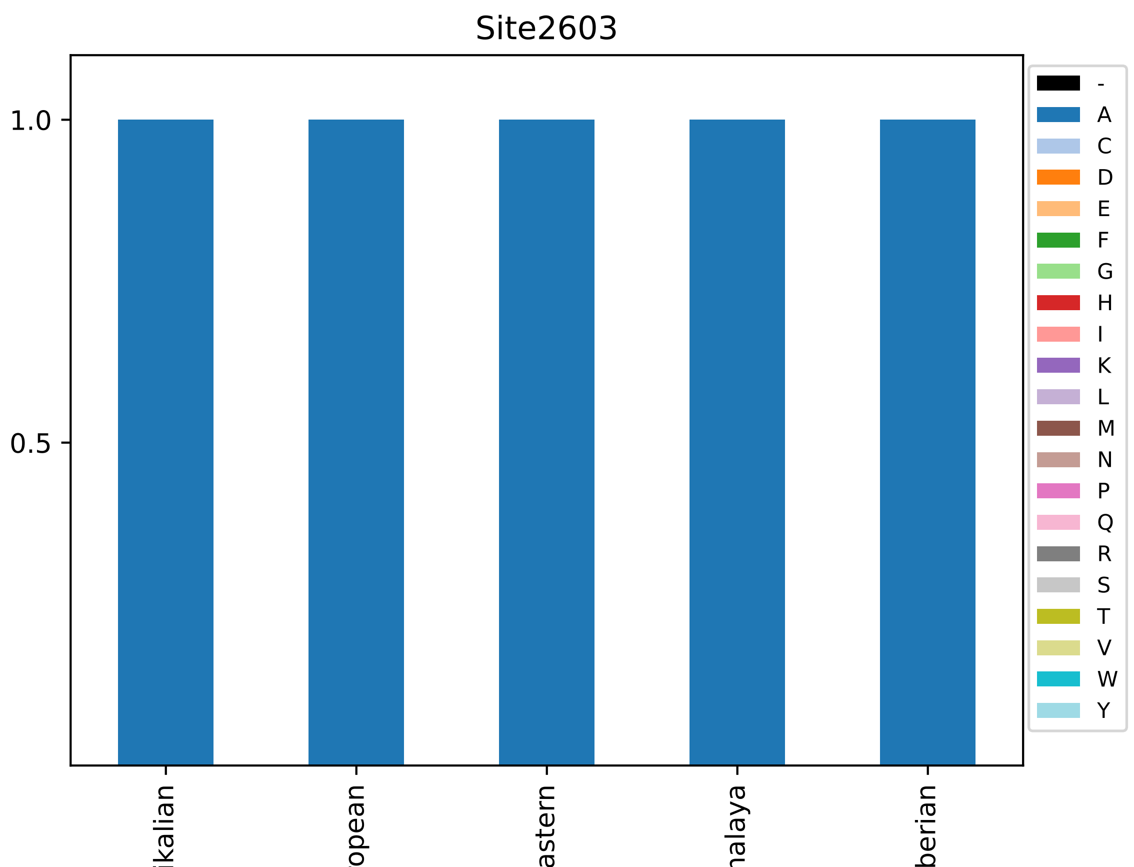
Site2603
1.0
0.5
ikalian
opean
astern
nalaya
berian
-
A
C
D
E
F
G
H
I
K
L
M
N
P
Q
R
S
T
V
W
Y

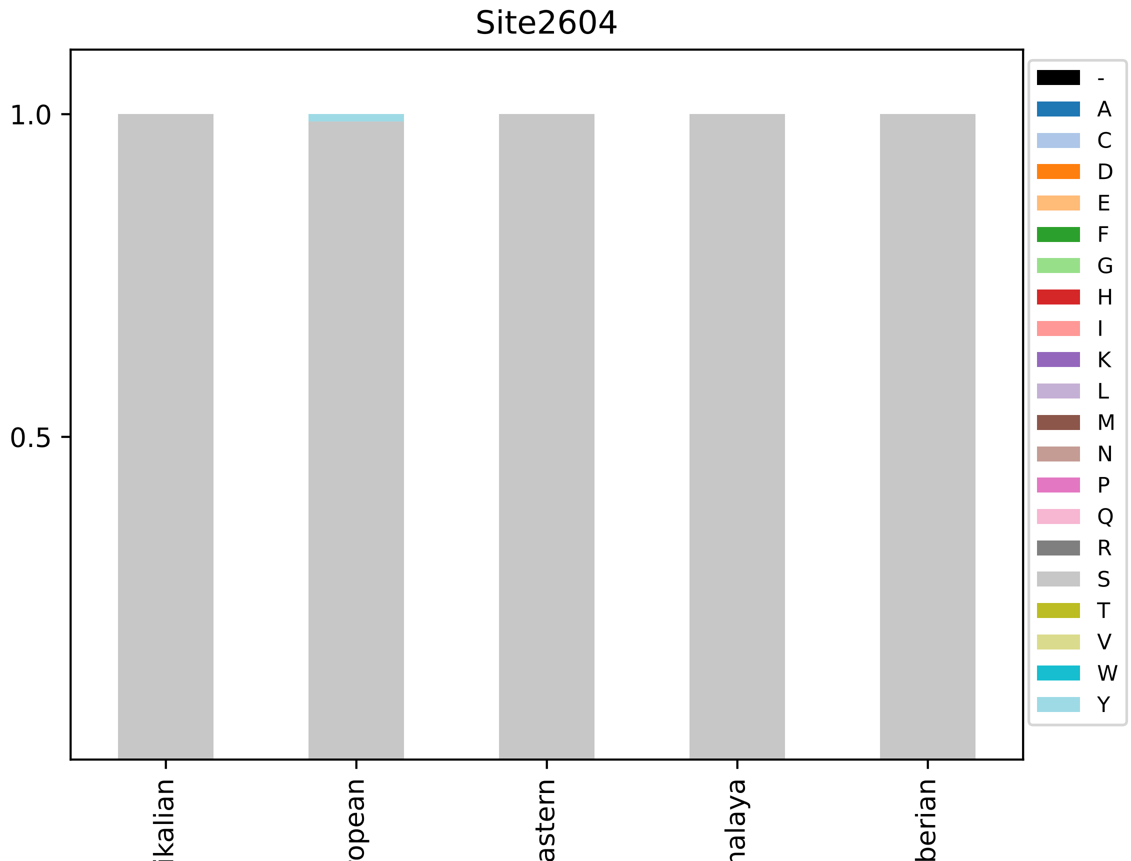

Site2604
1.0
0.5
ikalian
opean
astern
nalaya
berian
-
A
C
D
E
F
G
H
I
K
L
M
N
P
Q
R
S
T
V
W
Y

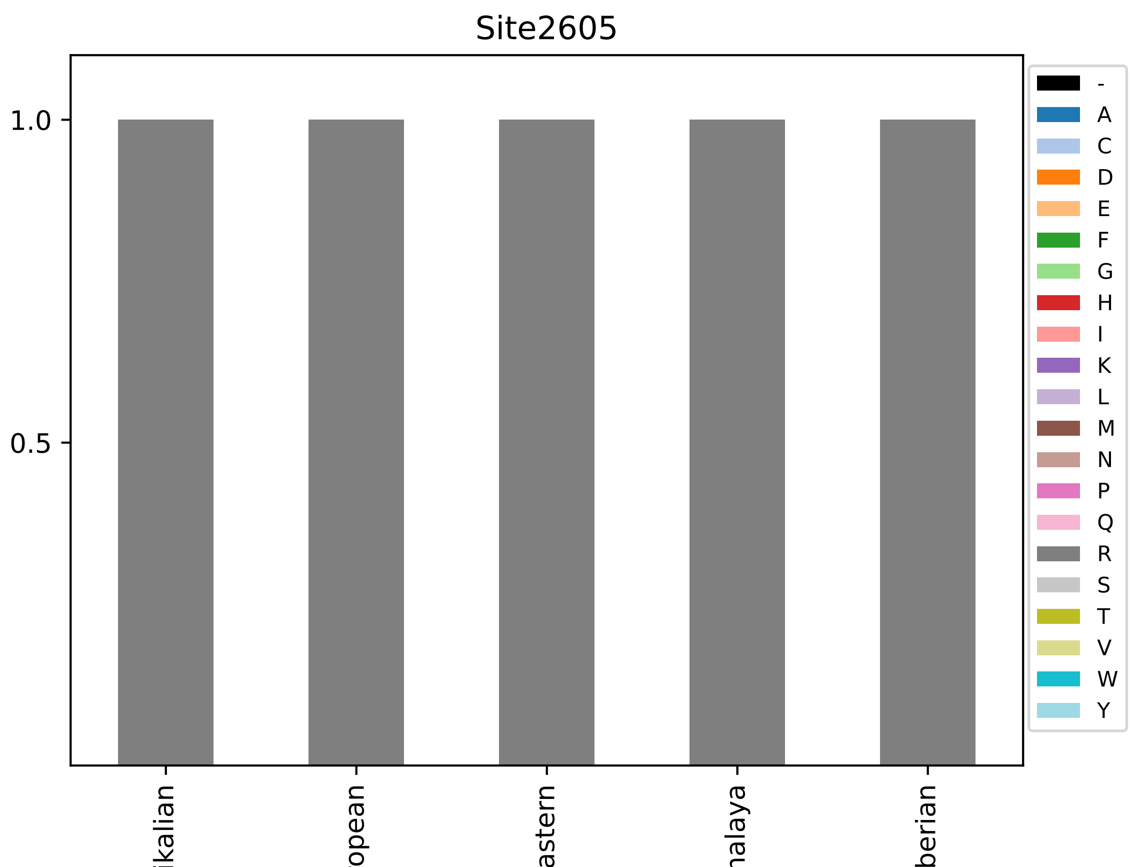

Site2605
1.0
0.5
-
A
C
D
E
F
G
H
I
K
L
M
N
P
Q
R
S
T
V
W
Y
ikalian
opean
astern
halaya
berian

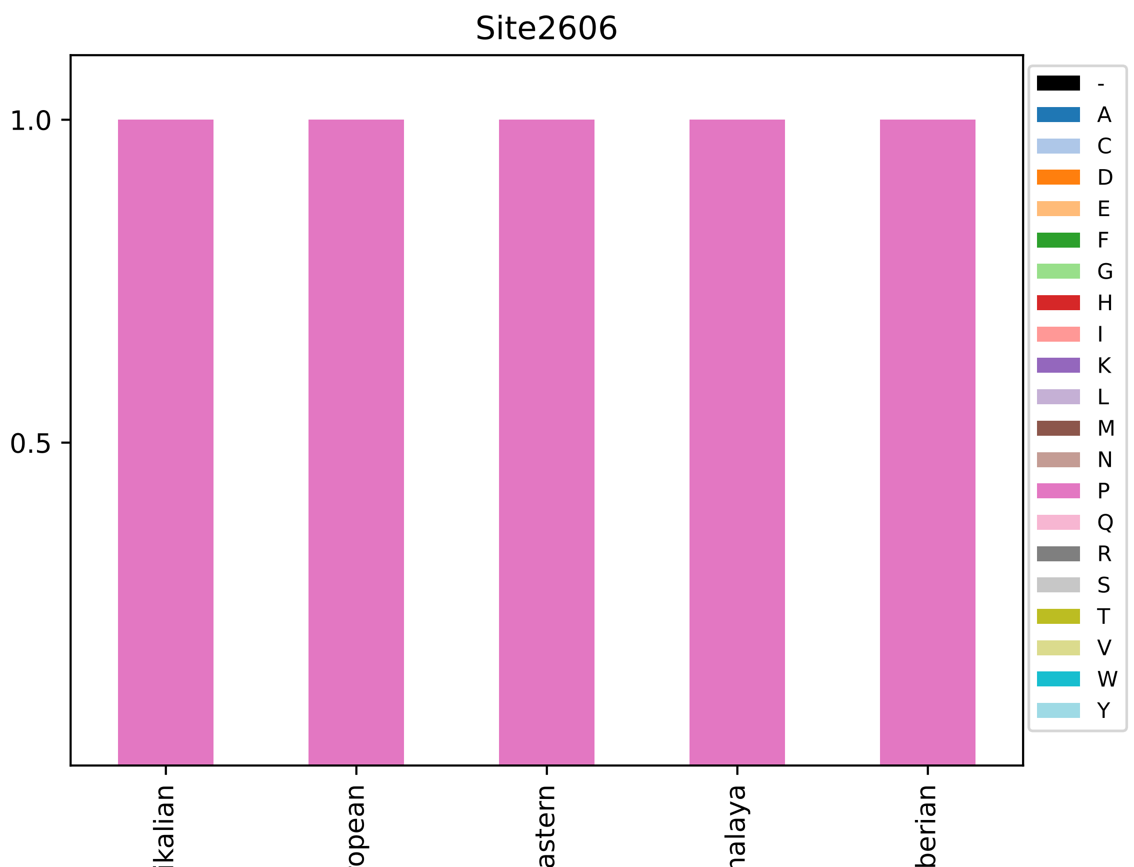
Site2606
1.0
0.5
ikalian
opean
astern
nalaya
berian
-
A
C
D
E
F
G
H
I
K
L
M
N
P
Q
R
S
T
V
W
Y

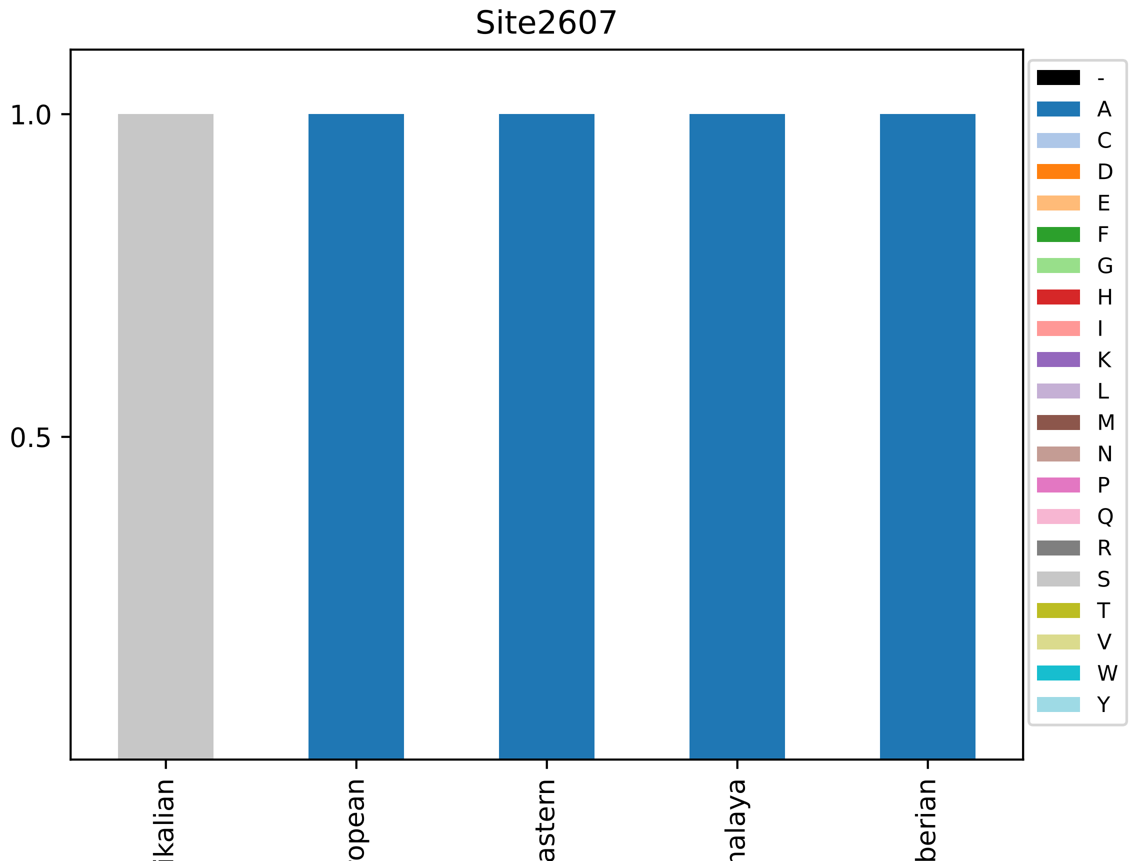

Site2607
1.0
0.5
-
A
C
D
E
F
G
H
I
K
L
M
N
P
Q
R
S
T
V
W
Y
ikalian
opean
astern
nalaya
berian

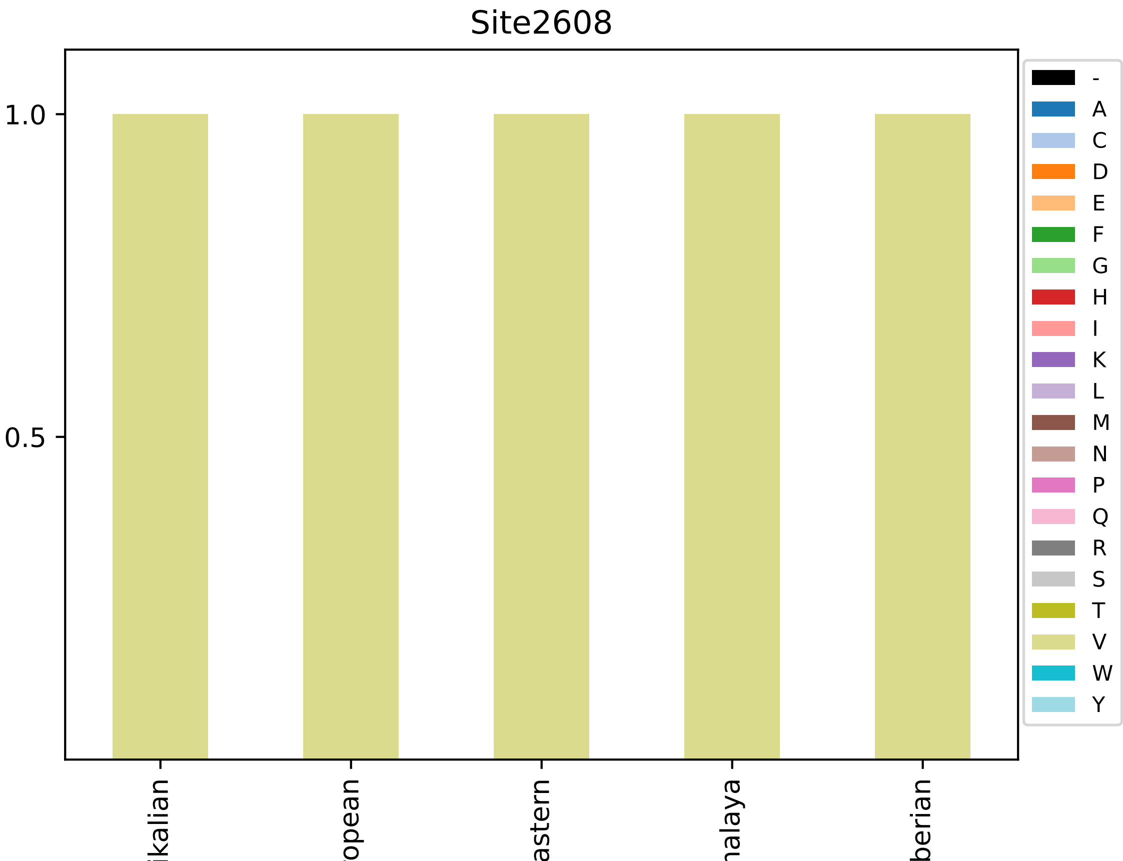
Site2608
1.0
0.5
-
A
C
D
E
F
G
H
I
K
L
M
N
P
Q
R
S
T
V
W
Y
ikalian
opean
astern
nalaya
berian

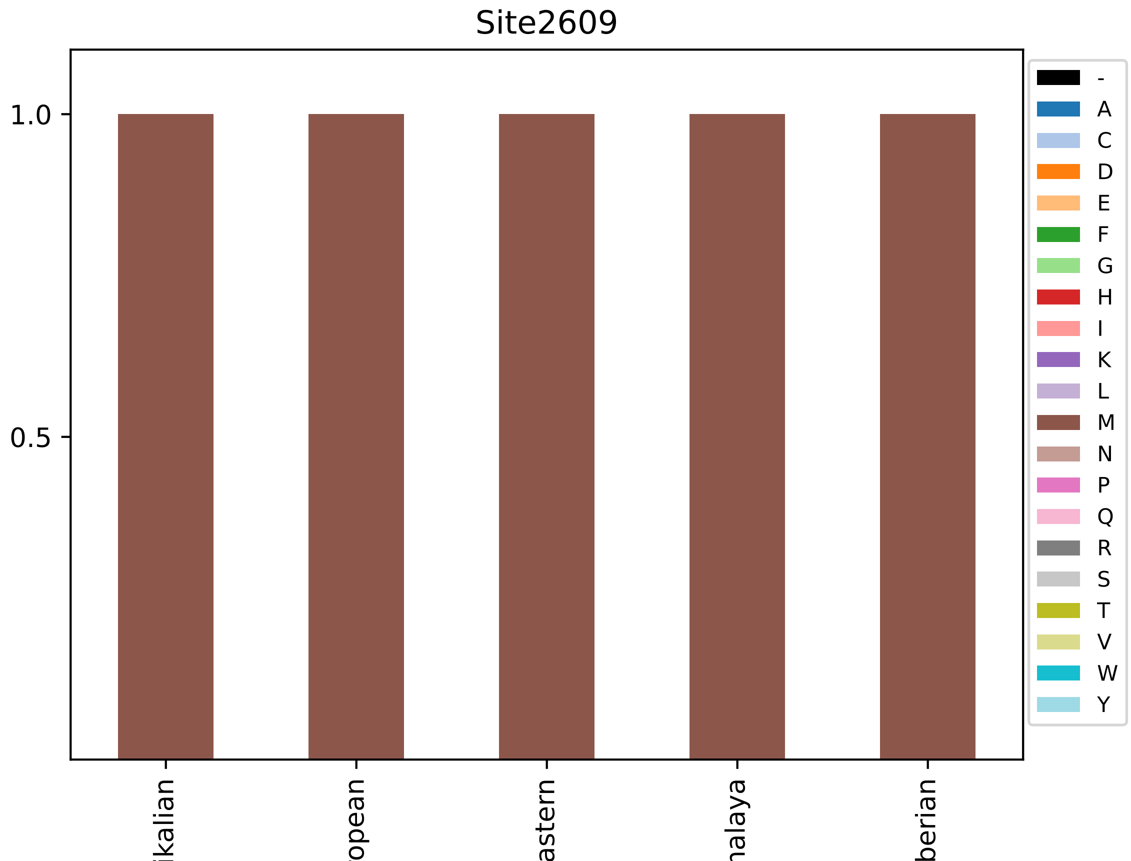

Site2609
1.0
0.5
-
A
C
D
E
F
G
H
I
K
L
M
N
P
Q
R
S
T
V
W
Y
ikalian
opean
astern
nalaya
berian

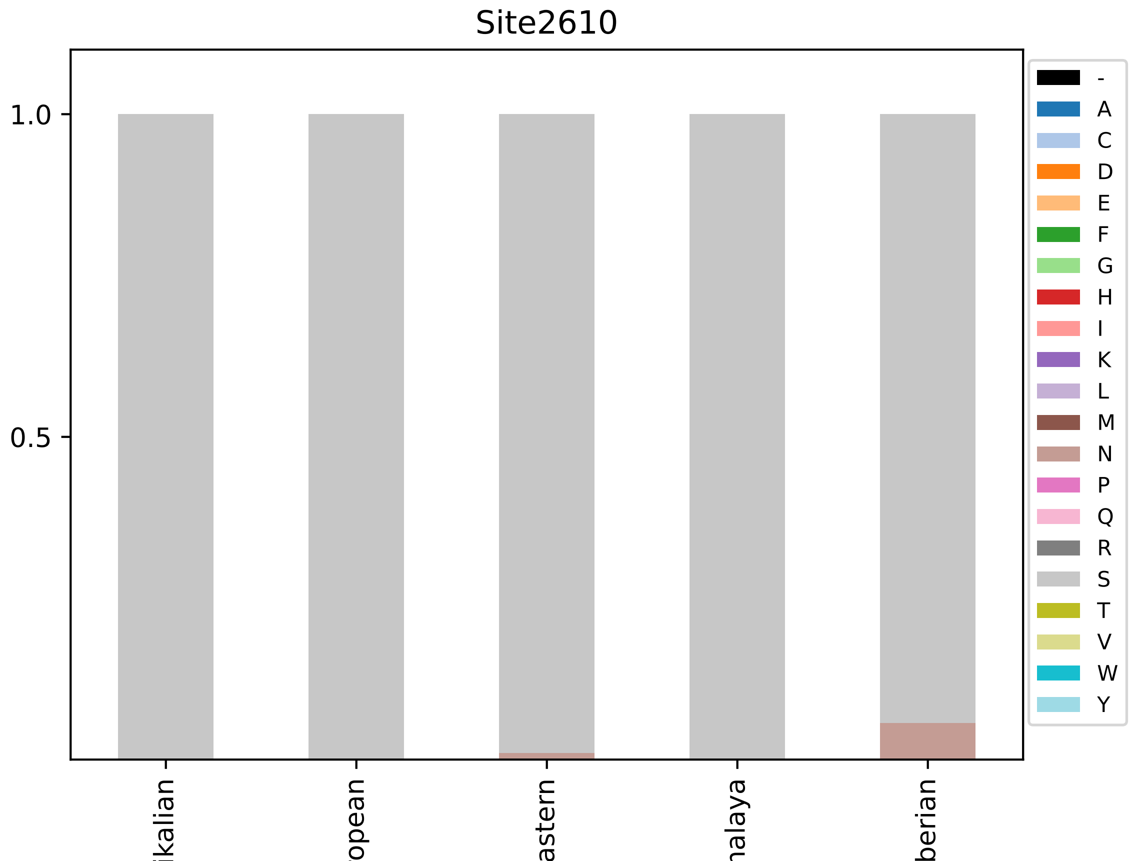

Site2610
1.0
0.5
-
A
C
D
E
F
G
H
I
K
L
M
N
P
Q
R
S
T
V
W
Y
ikalian
opean
astern
nalaya
berian

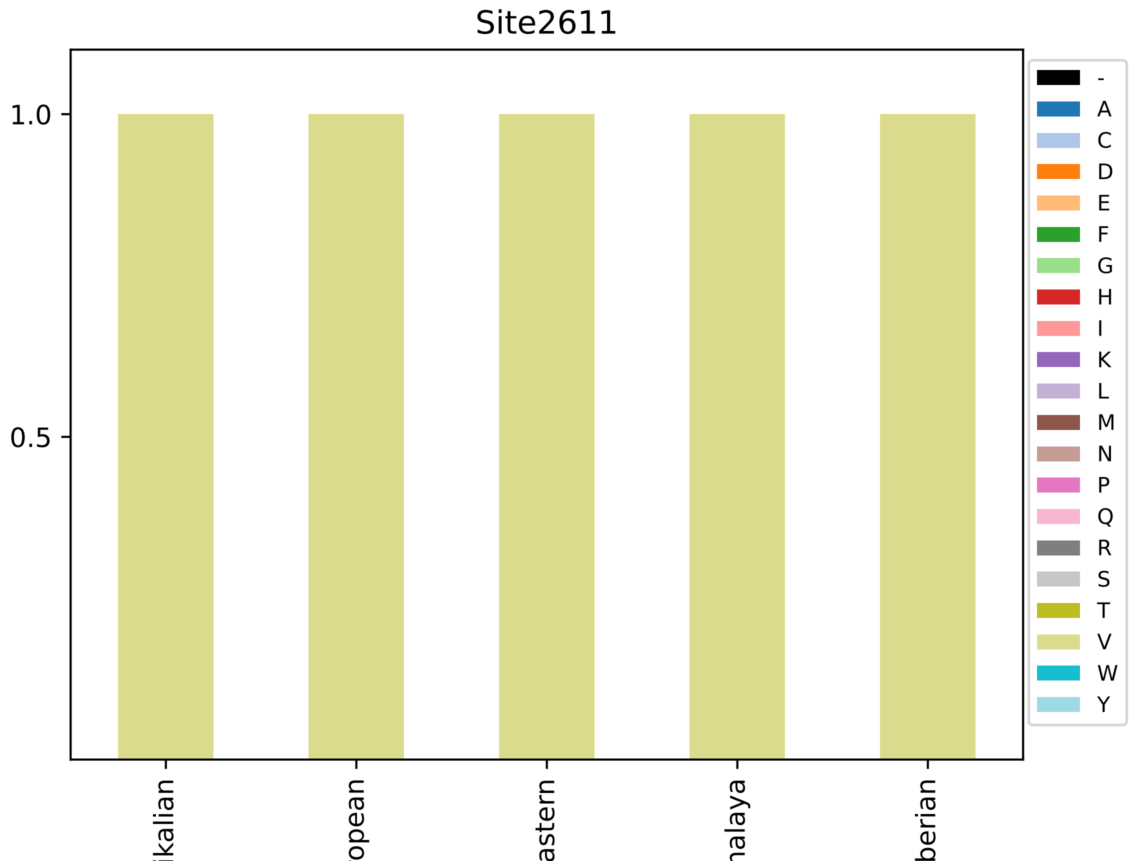
Site2611
1.0
0.5
ikalian
ropean
astern
nalaya
berian
-
A
C
D
E
F
G
H
I
K
L
M
N
P
Q
R
S
T
V
W
Y

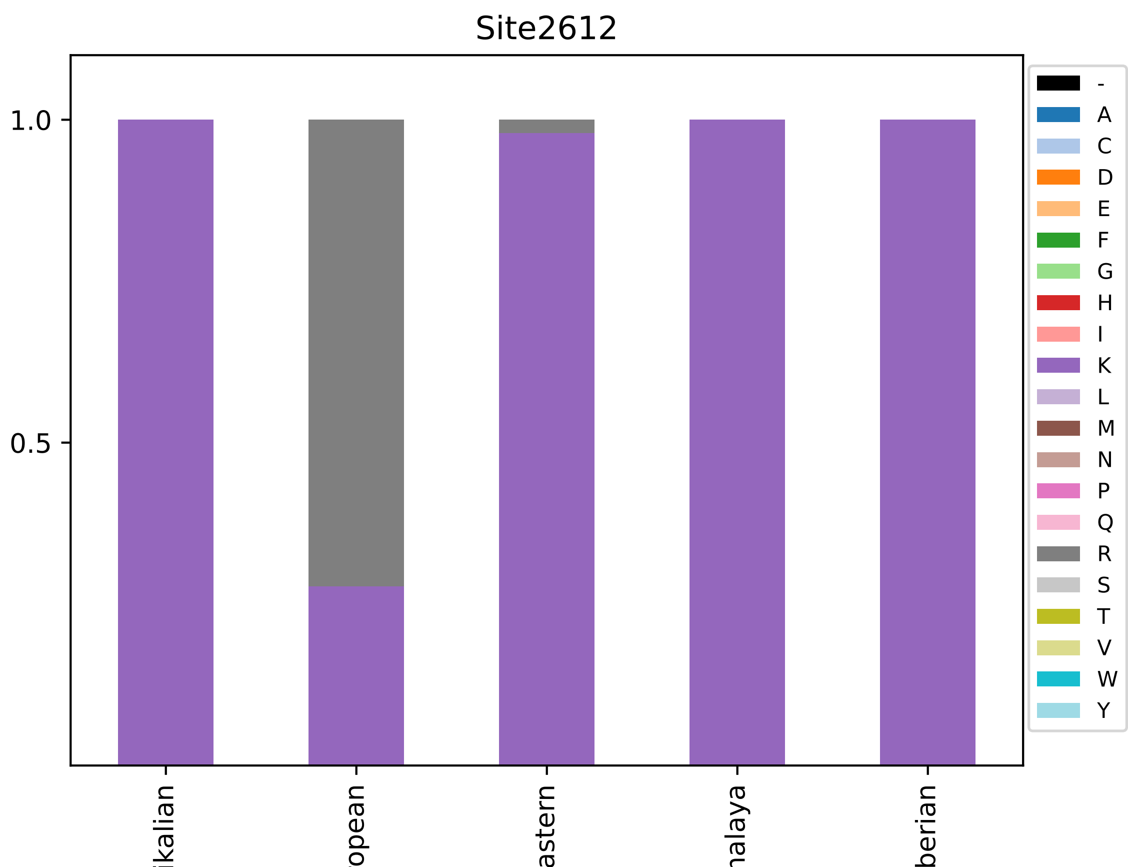

Site2612
1.0
0.5
-
A
C
D
E
F
G
H
I
K
L
M
N
P
Q
R
S
T
V
W
Y
ikalian
opean
astern
nalaya
berian

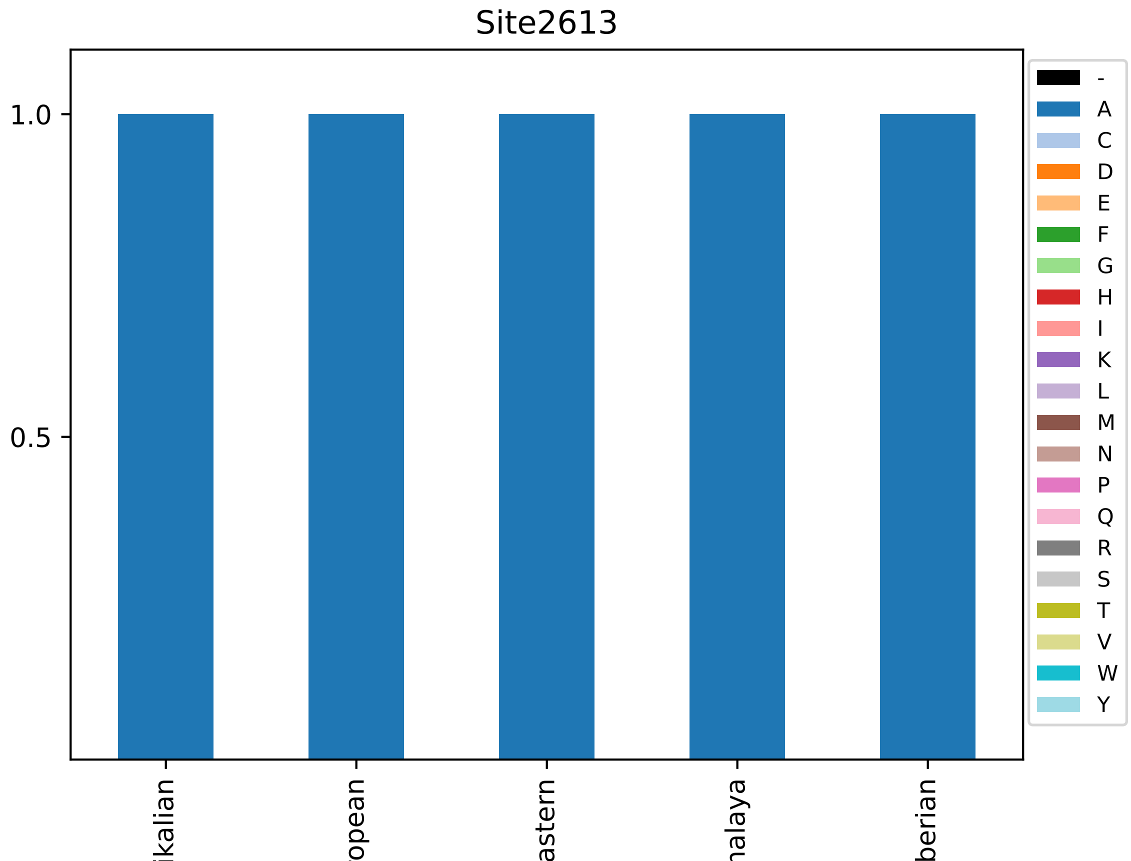
Site2613
1.0
0.5
-
A
C
D
E
F
G
H
I
K
L
M
N
P
Q
R
S
T
V
W
Y
ikalian
opean
astern
nalaya
berian

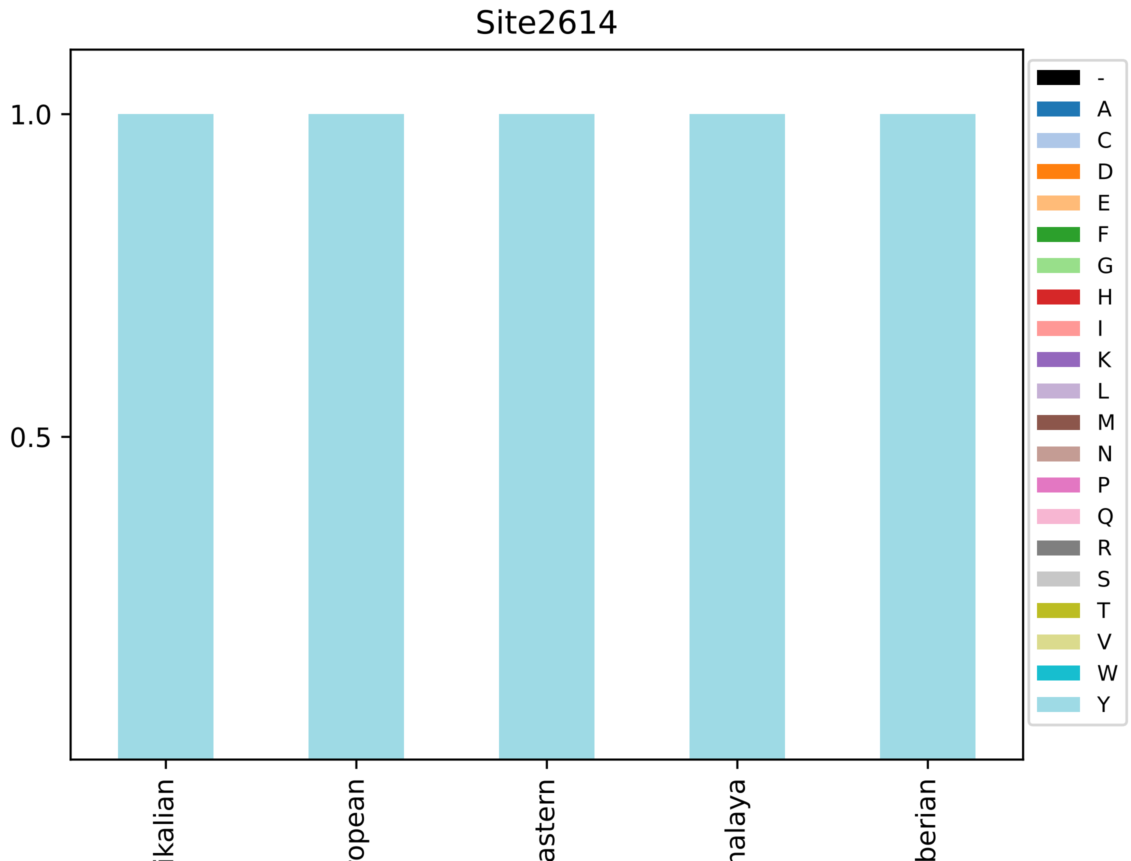
Site2614
1.0
0.5
-
A
C
D
E
F
G
H
I
K
L
M
N
P
Q
R
S
T
V
W
Y
ikalian
opean
astern
nalaya
berian

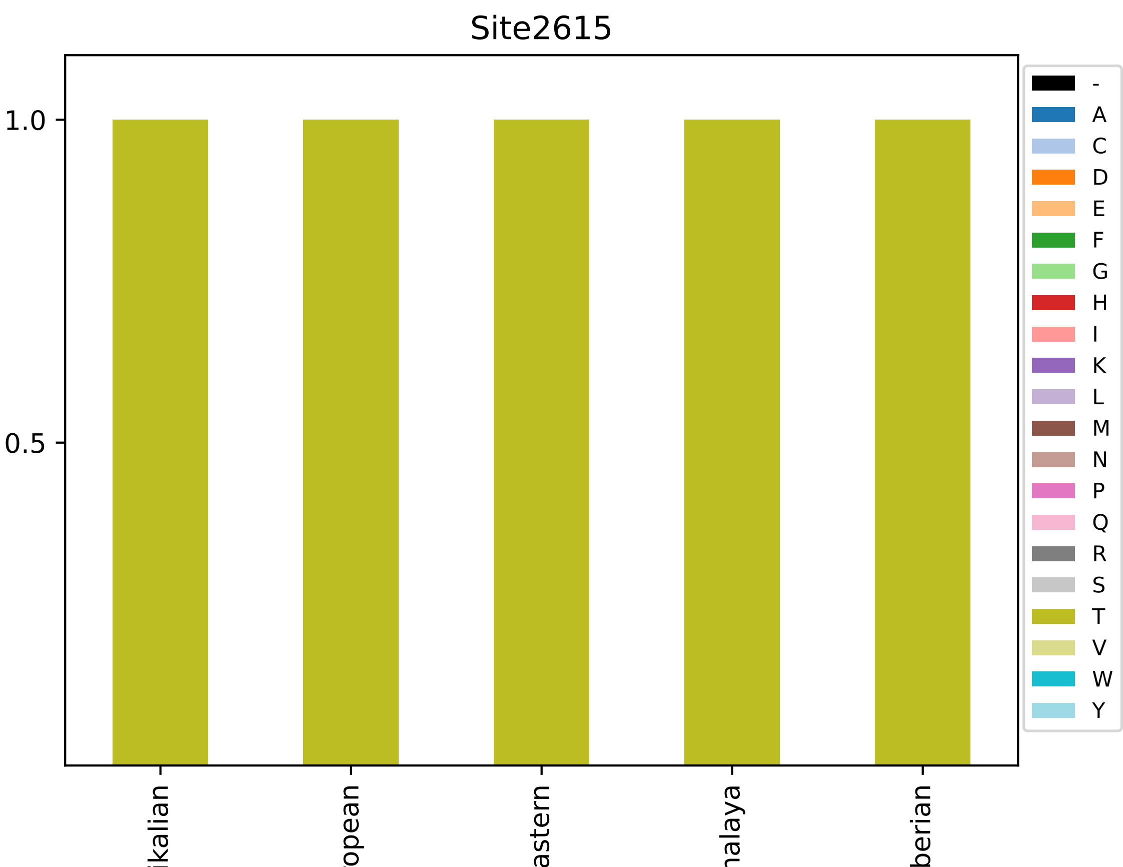

Site2615
1.0
0.5
-
A
C
D
E
F
G
H
I
K
L
M
N
P
Q
R
S
T
V
W
Y
ikalian
opean
astern
nalaya
berian

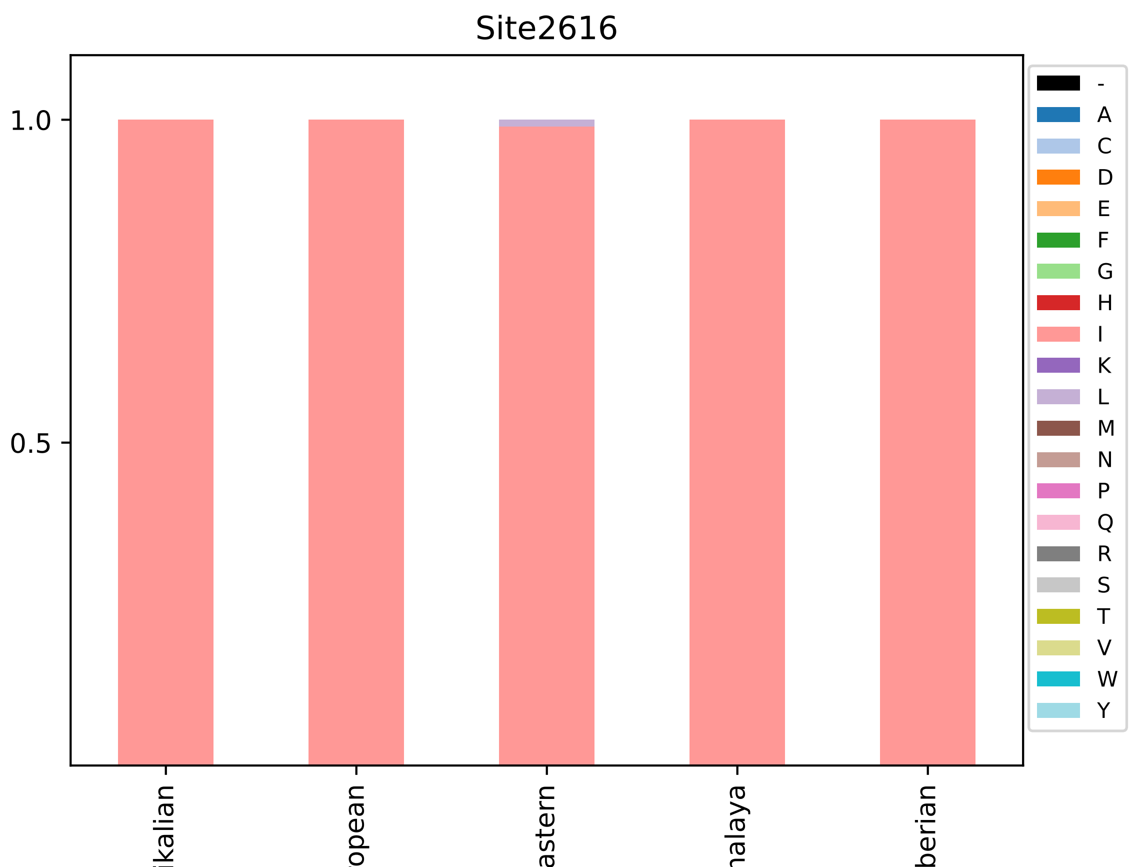

Site2616
1.0
0.5
ikalian
ropean
astern
nalaya
berian
-
A
C
D
E
F
G
H
I
K
L
M
N
P
Q
R
S
T
V
W
Y

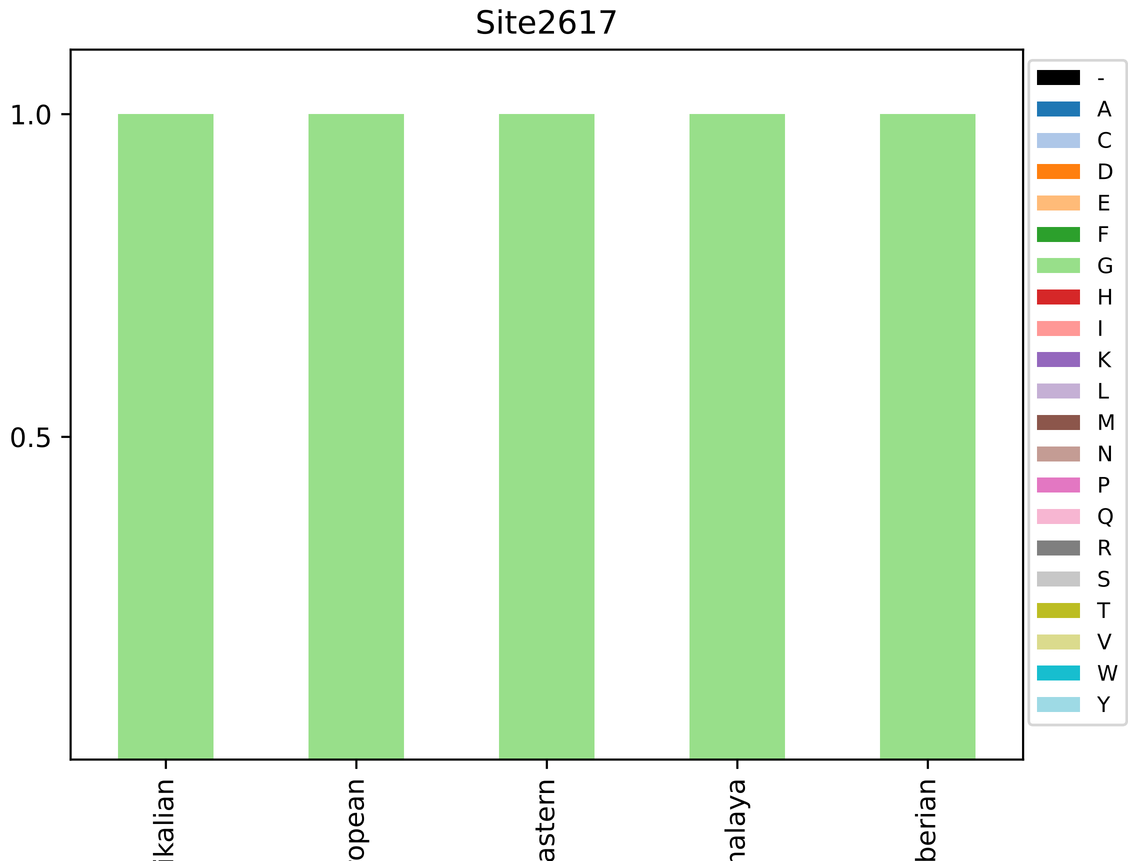
Site2617
1.0
0.5
ikalian
opean
astern
nalaya
berian
-
A
C
D
E
F
G
H
I
K
L
M
N
P
Q
R
S
T
V
W
Y

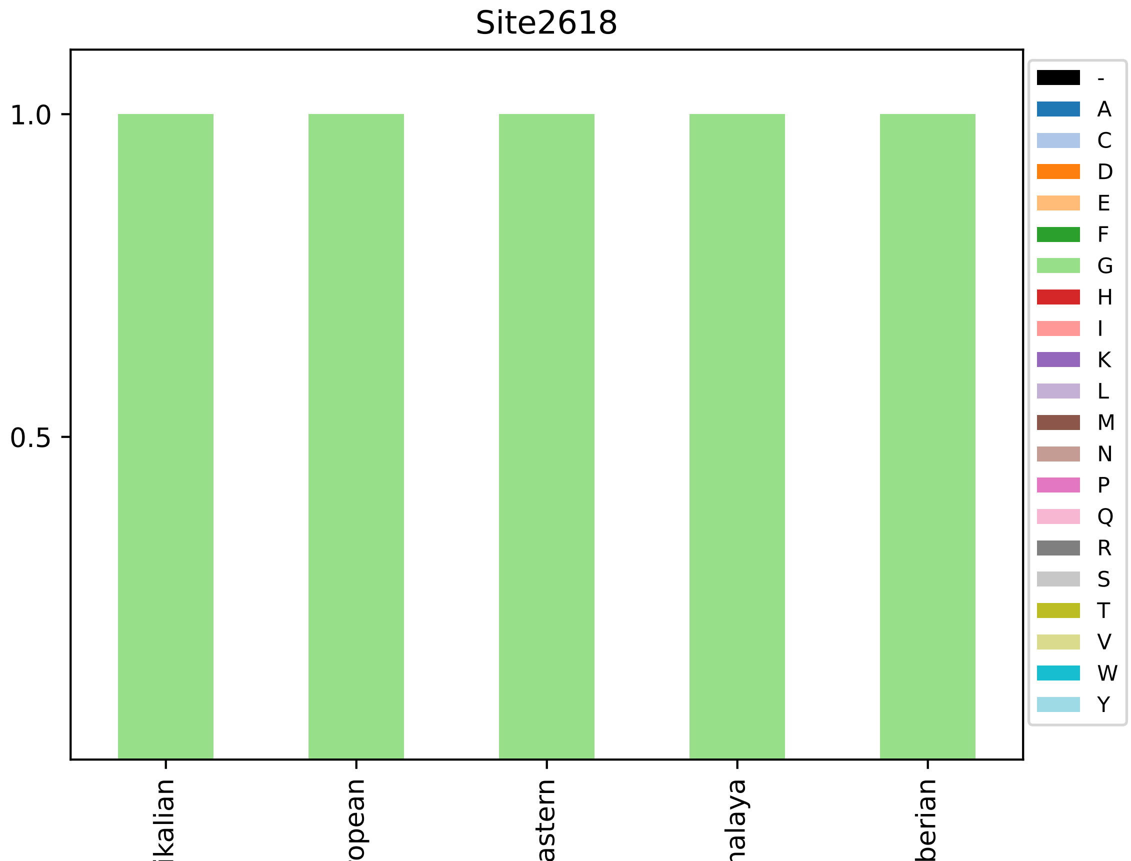

Site2618
1.0
0.5
ikalian
opean
astern
nalaya
berian
-
A
C
D
E
F
G
H
I
K
L
M
N
P
Q
R
S
T
V
W
Y

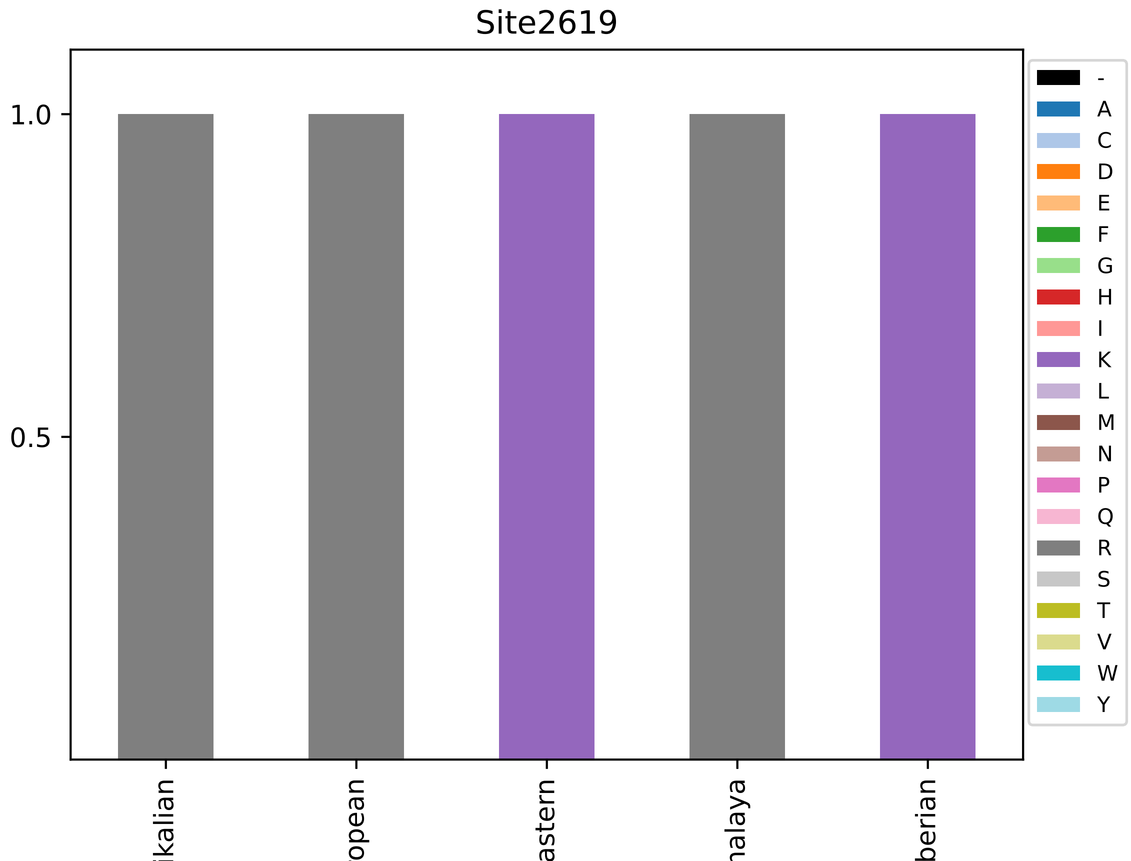

Site2619
1.0
0.5
-
A
C
D
E
F
G
H
I
K
L
M
N
P
Q
R
S
T
V
W
Y
ikalian
opean
astern
nalaya
berian

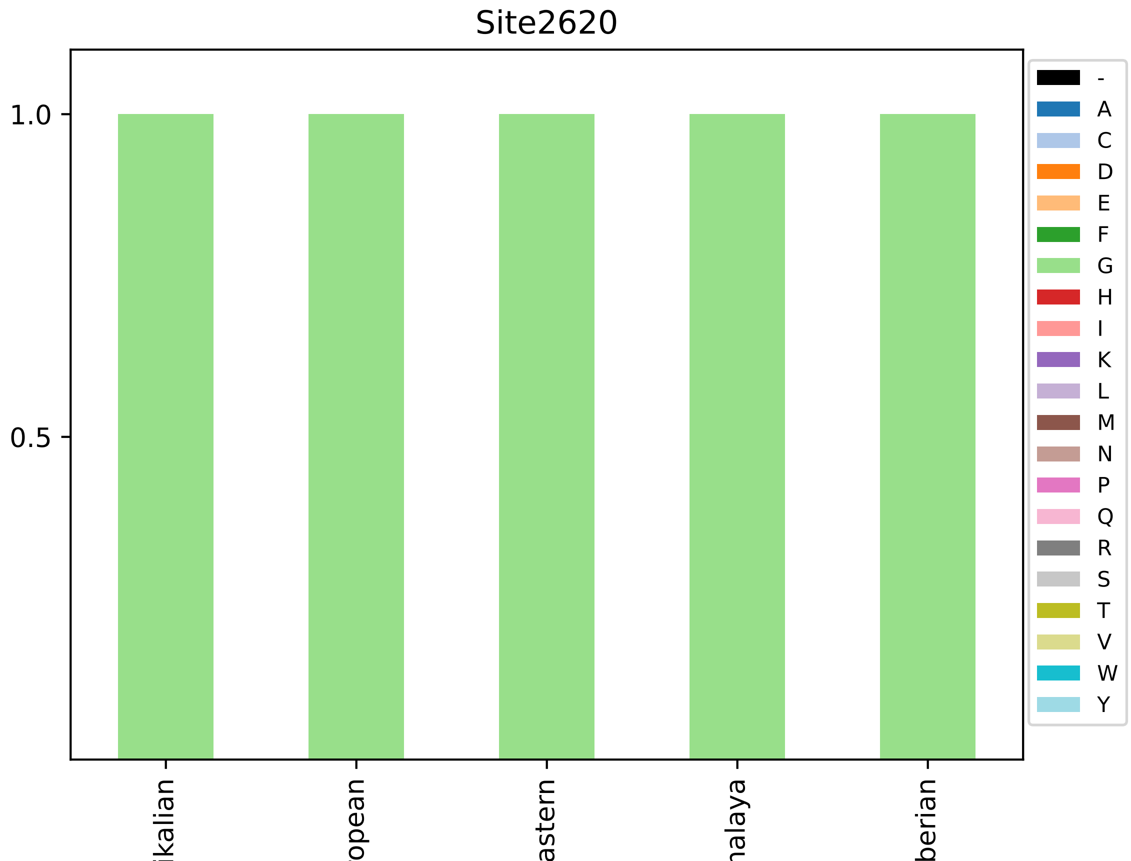
Site2620
1.0
0.5
-
A
C
D
E
F
G
H
I
K
L
M
N
P
Q
R
S
T
V
W
Y
ikalian
opean
astern
nalaya
berian

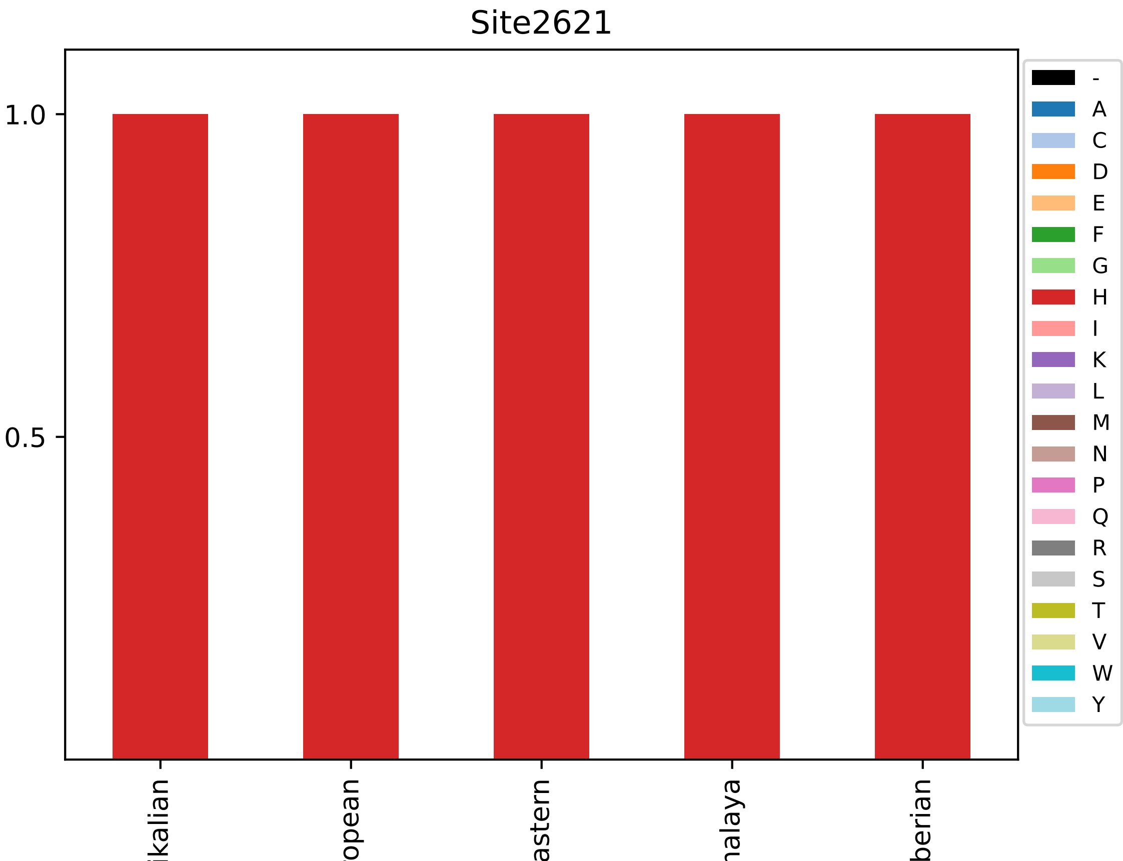

Site2621
1.0
0.5
-
A
C
D
E
F
G
H
I
K
L
M
N
P
Q
R
S
T
V
W
Y

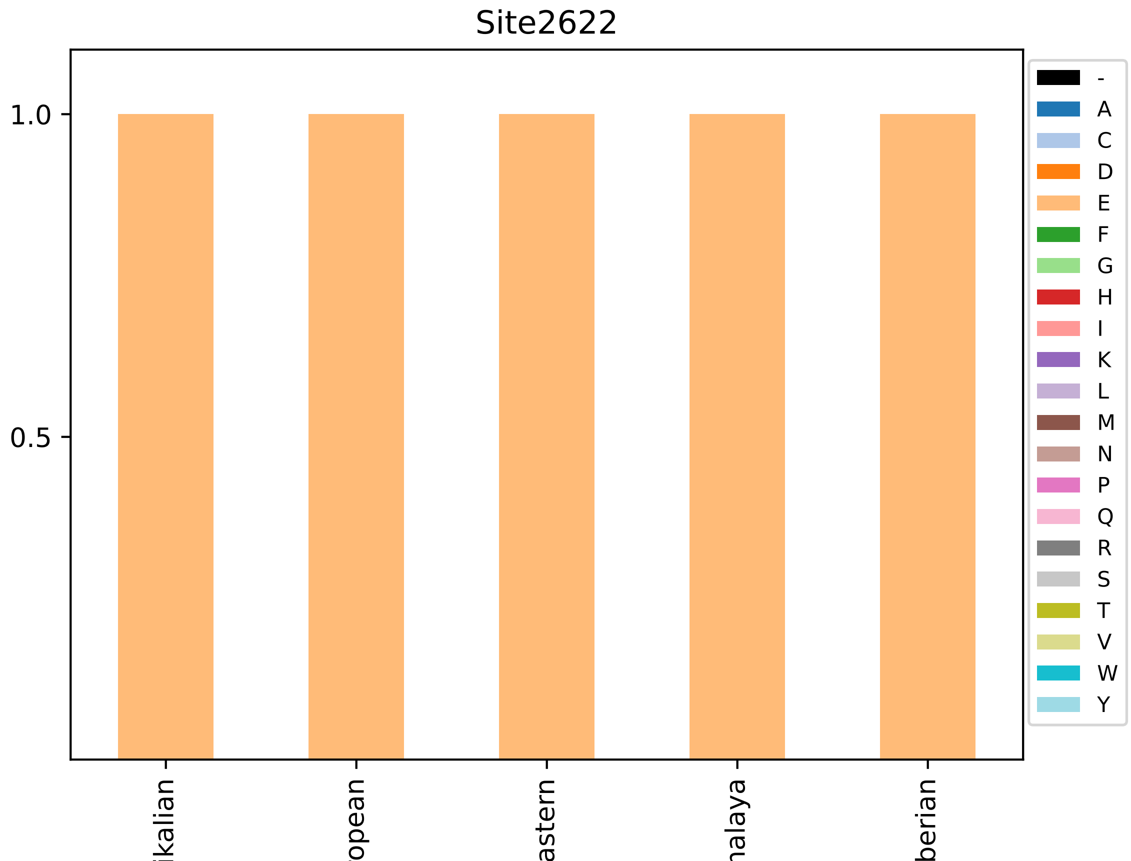

Site2622
1.0
0.5
-
A
C
D
E
F
G
H
I
K
L
M
N
P
Q
R
S
T
V
W
Y

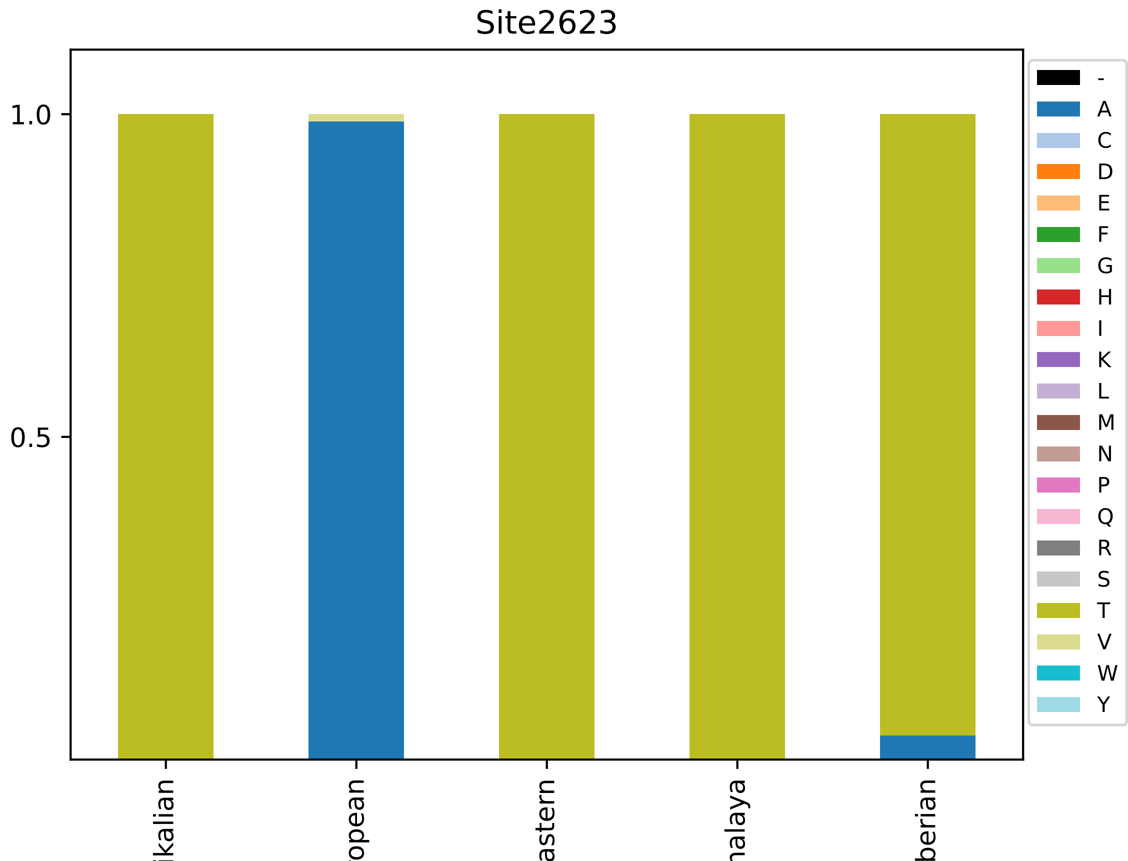

Site2623
1.0
0.5
ikalian
opean
astern
nalaya
berian
-
A
C
D
E
F
G
H
I
K
L
M
N
P
Q
R
S
T
V
W
Y

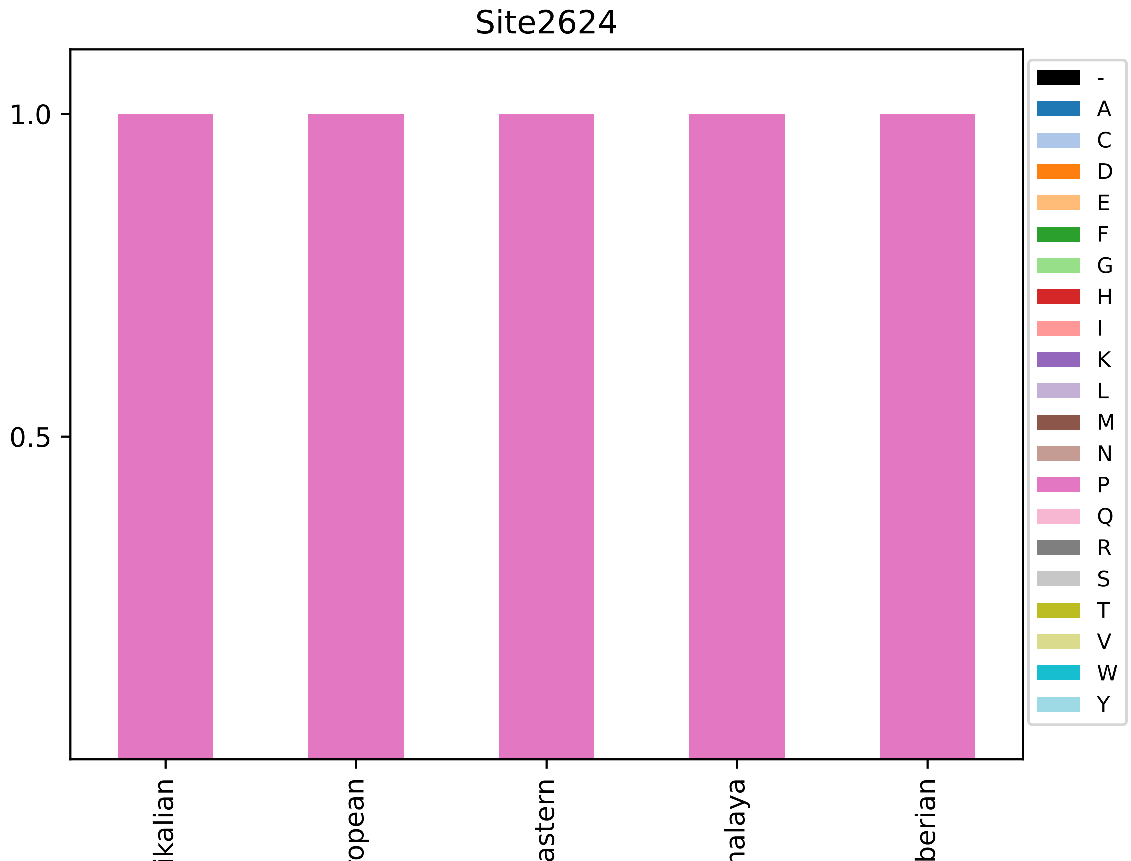

Site2624
1.0
0.5
-
A
C
D
E
F
G
H
I
K
L
M
N
P
Q
R
S
T
V
W
Y
ikalian
opean
astern
nalaya
berian

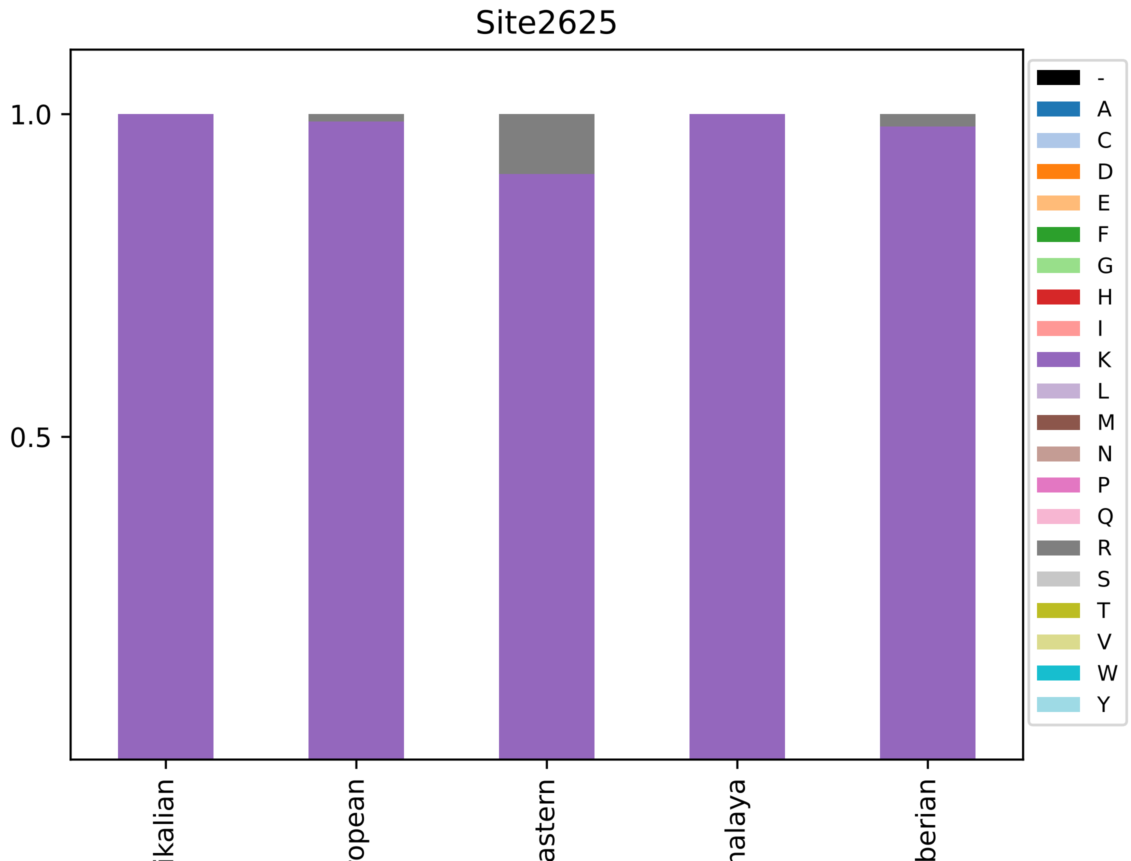

Site2625
1.0
0.5
-
A
C
D
E
F
G
H
I
K
L
M
N
P
Q
R
S
T
V
W
Y
ikalian
opean
astern
nalaya
berian

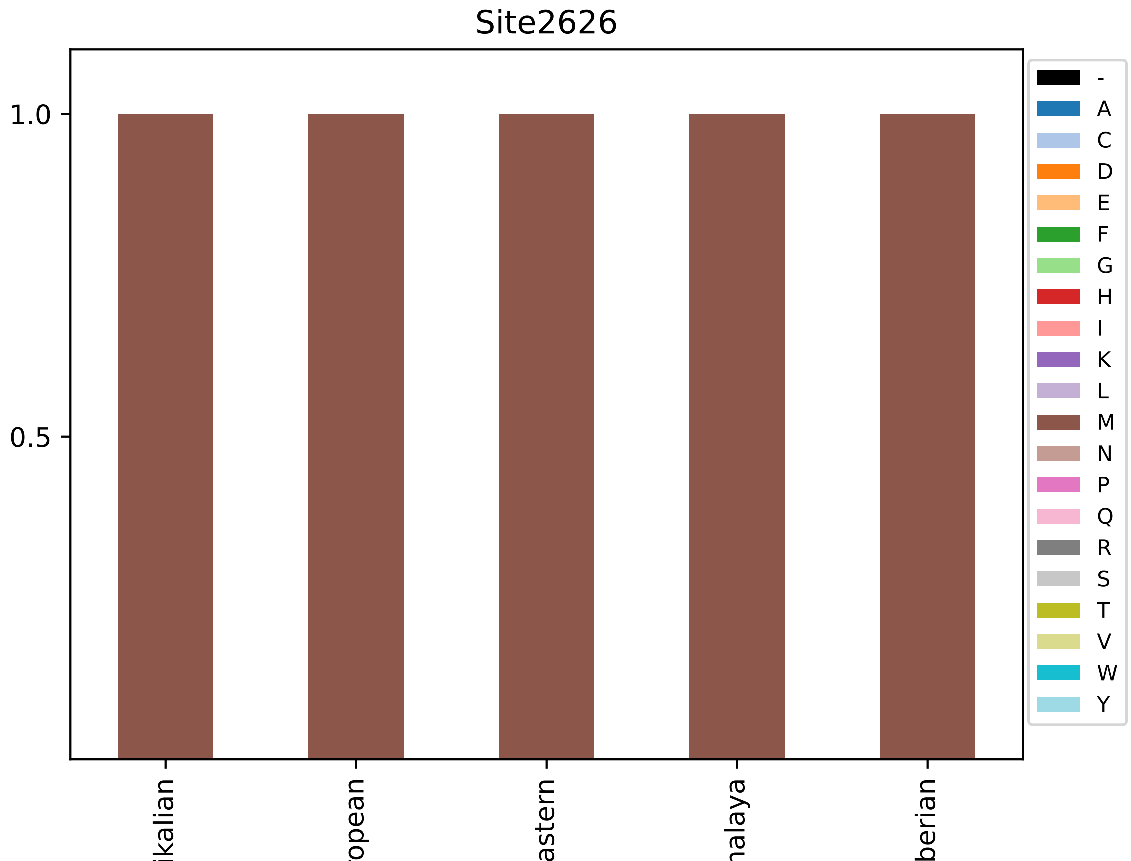

Site2626
1.0
0.5
-
A
C
D
E
F
G
H
I
K
L
M
N
P
Q
R
S
T
V
W
Y
ikalian
opean
astern
nalaya
berian

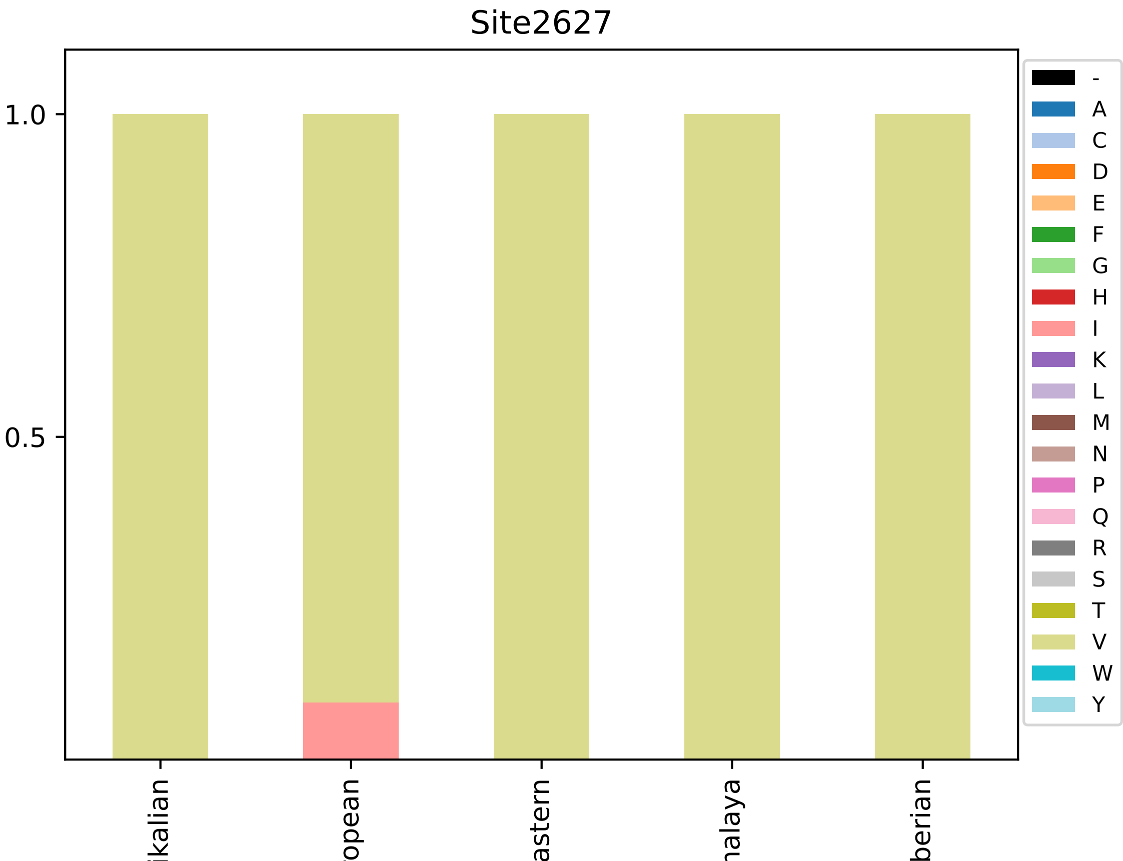

Site2627
1.0
0.5
-
A
C
D
E
F
G
H
I
K
L
M
N
P
Q
R
S
T
V
W
Y
ikalian
opean
astern
nalaya
berian

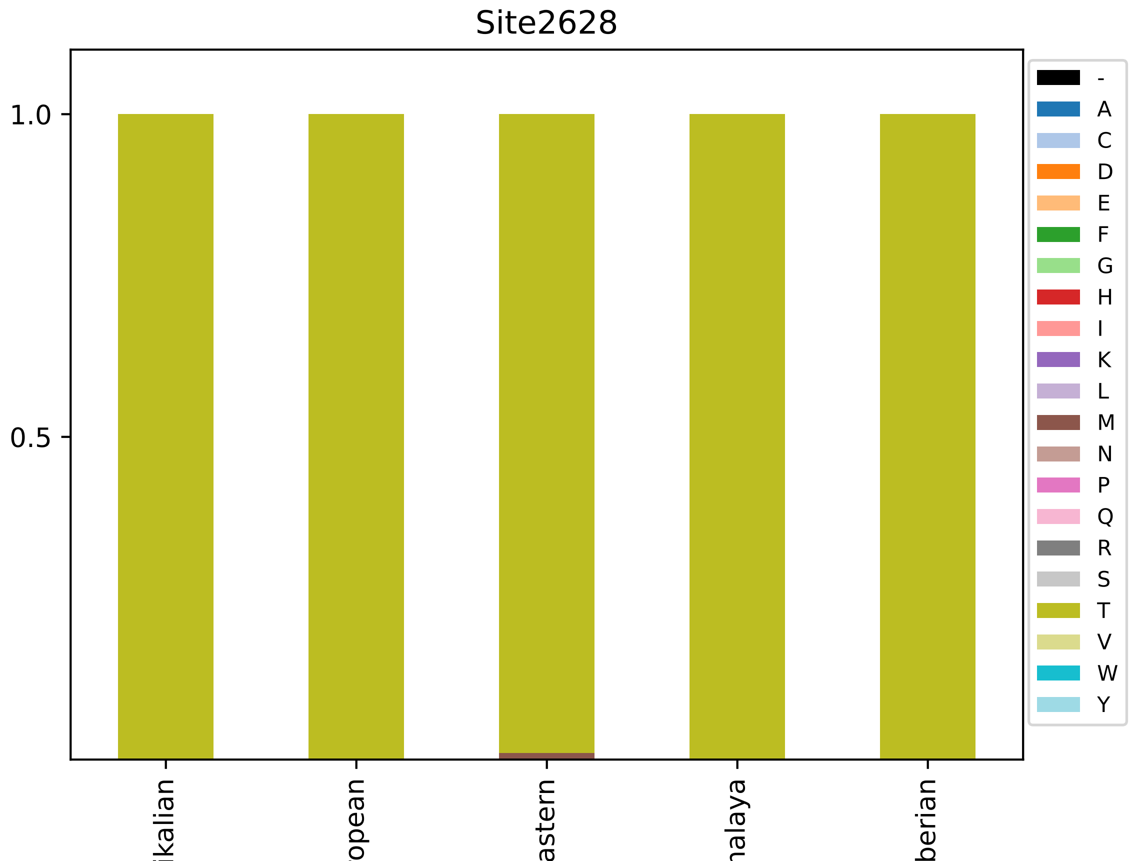
Site2628
1.0
0.5
ikalian
opean
astern
nalaya
berian
-
A
C
D
E
F
G
H
I
K
L
M
N
P
Q
R
S
T
V
W
Y

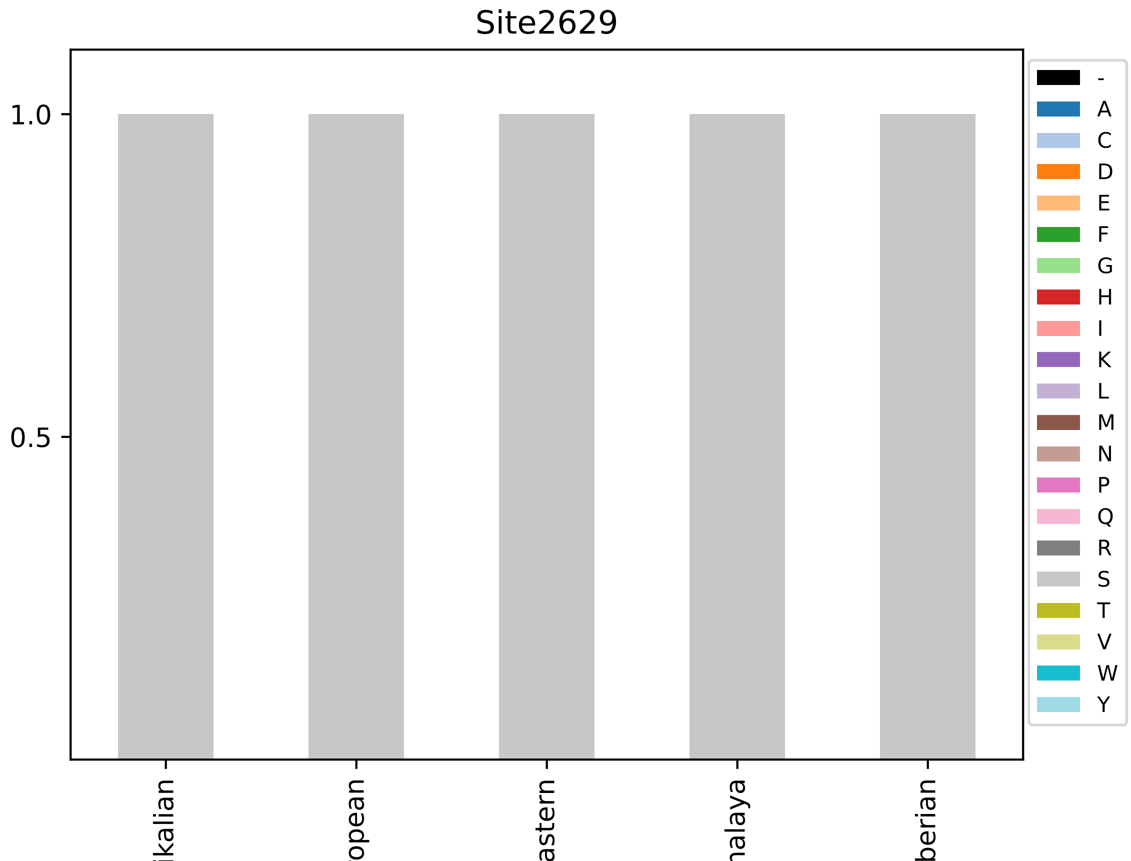

Site2629
1.0
0.5
ikalian
opean
astern
nalaya
berian
-
A
C
D
E
F
G
H
I
K
L
M
N
P
Q
R
S
T
V
W
Y

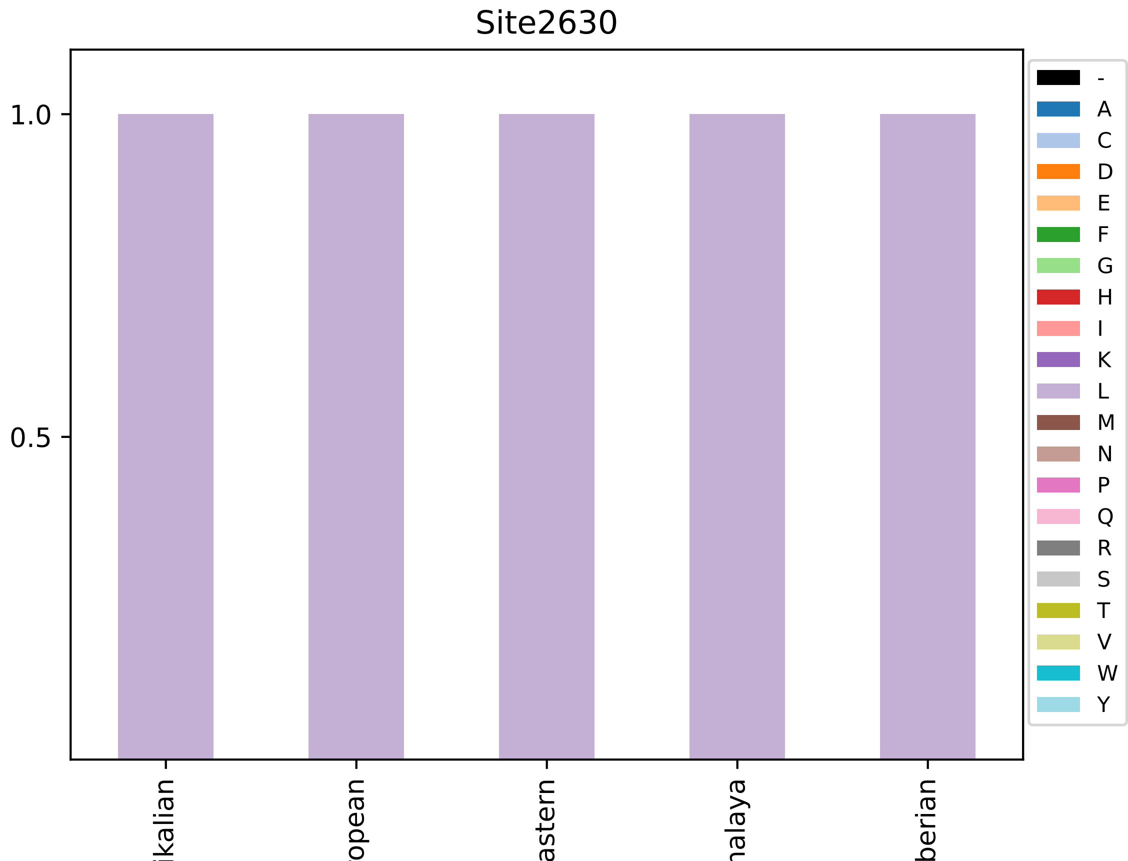
Site2630
1.0
0.5
-
A
C
D
E
F
G
H
I
K
L
M
N
P
Q
R
S
T
V
W
Y
ikalian
opean
astern
nalaya
berian

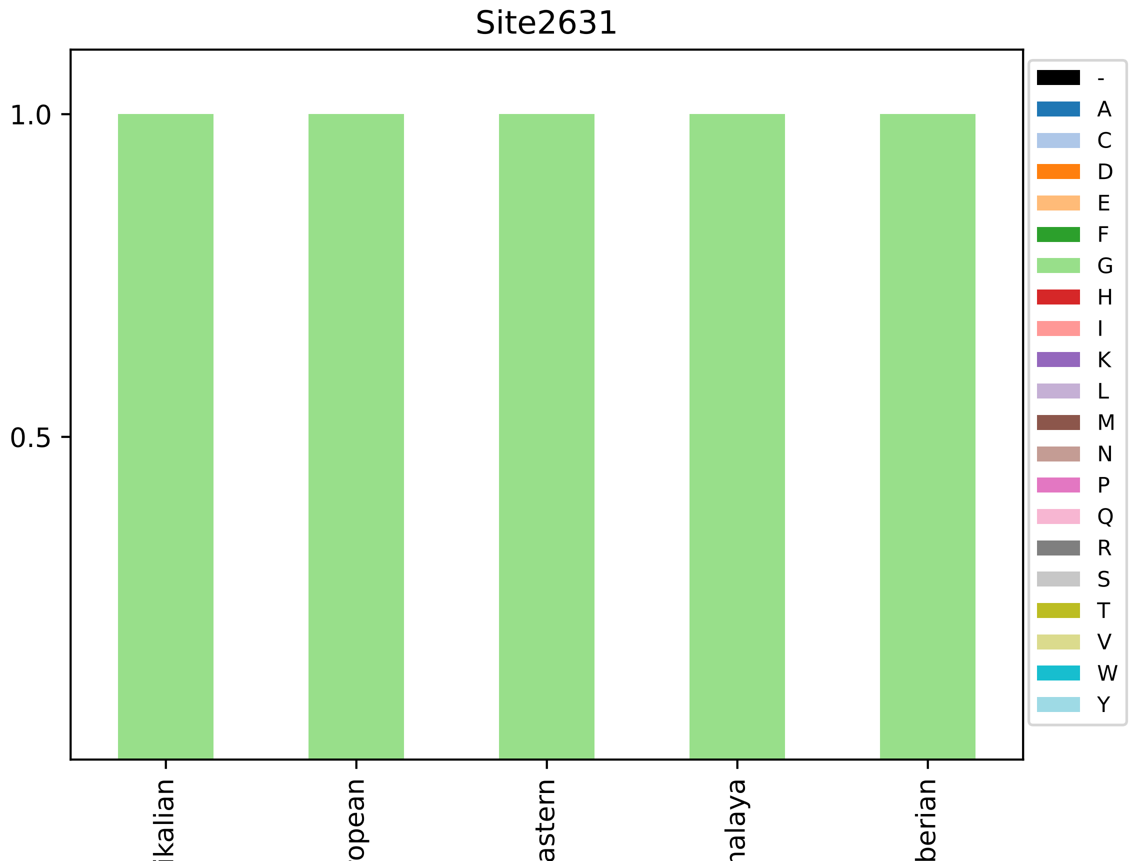

Site2631
1.0
0.5
-
A
C
D
E
F
G
H
I
K
L
M
N
P
Q
R
S
T
V
W
Y
ikalian
opean
astern
nalaya
berian

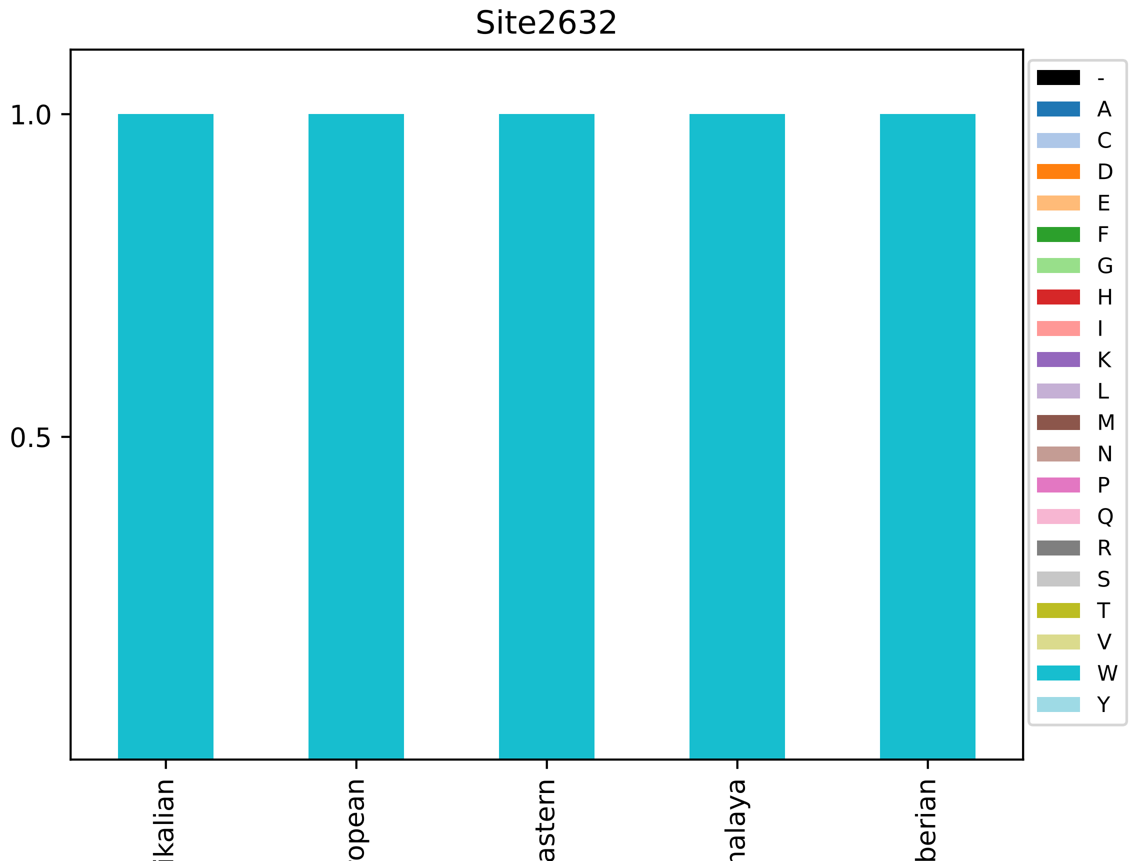
Site2632
1.0
0.5
-
A
C
D
E
F
G
H
I
K
L
M
N
P
Q
R
S
T
V
W
Y
ikalian
opean
astern
nalaya
berian

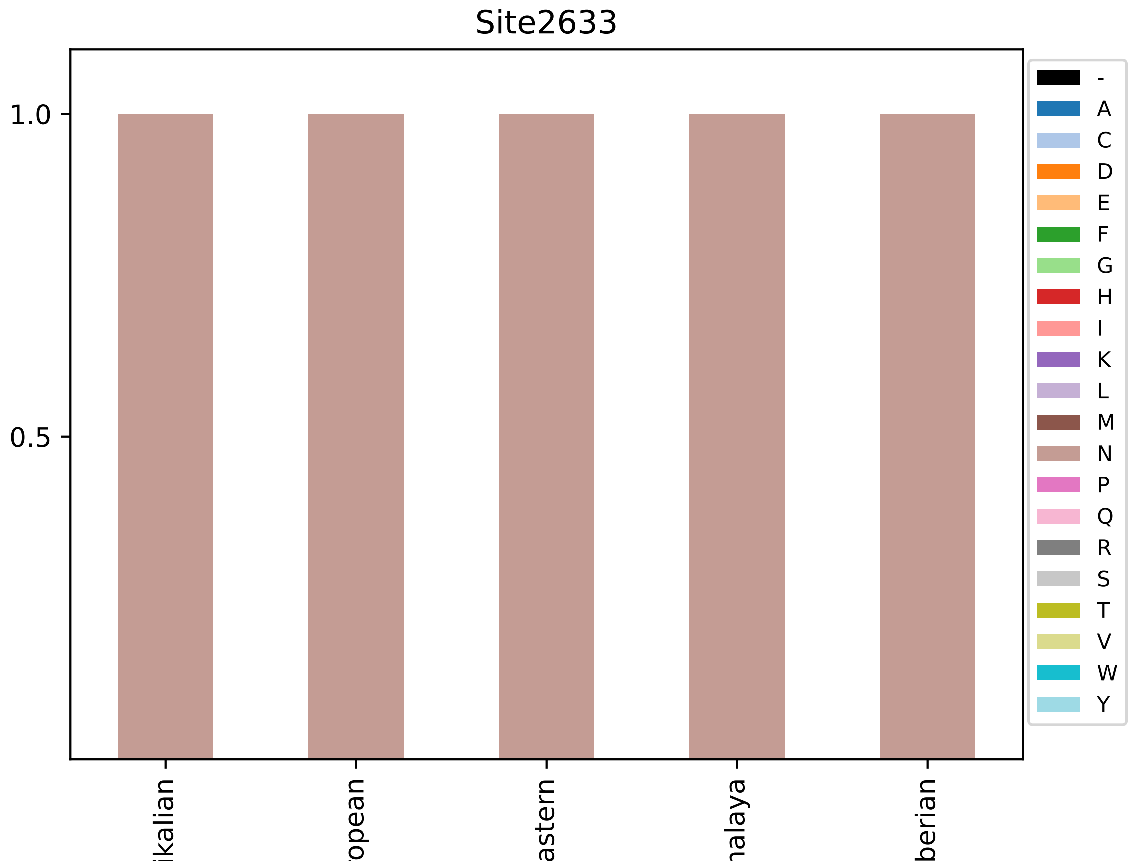
Site2633
1.0
0.5
-
A
C
D
E
F
G
H
I
K
L
M
N
P
Q
R
S
T
V
W
Y
ikalian
opean
astern
nalaya
berian

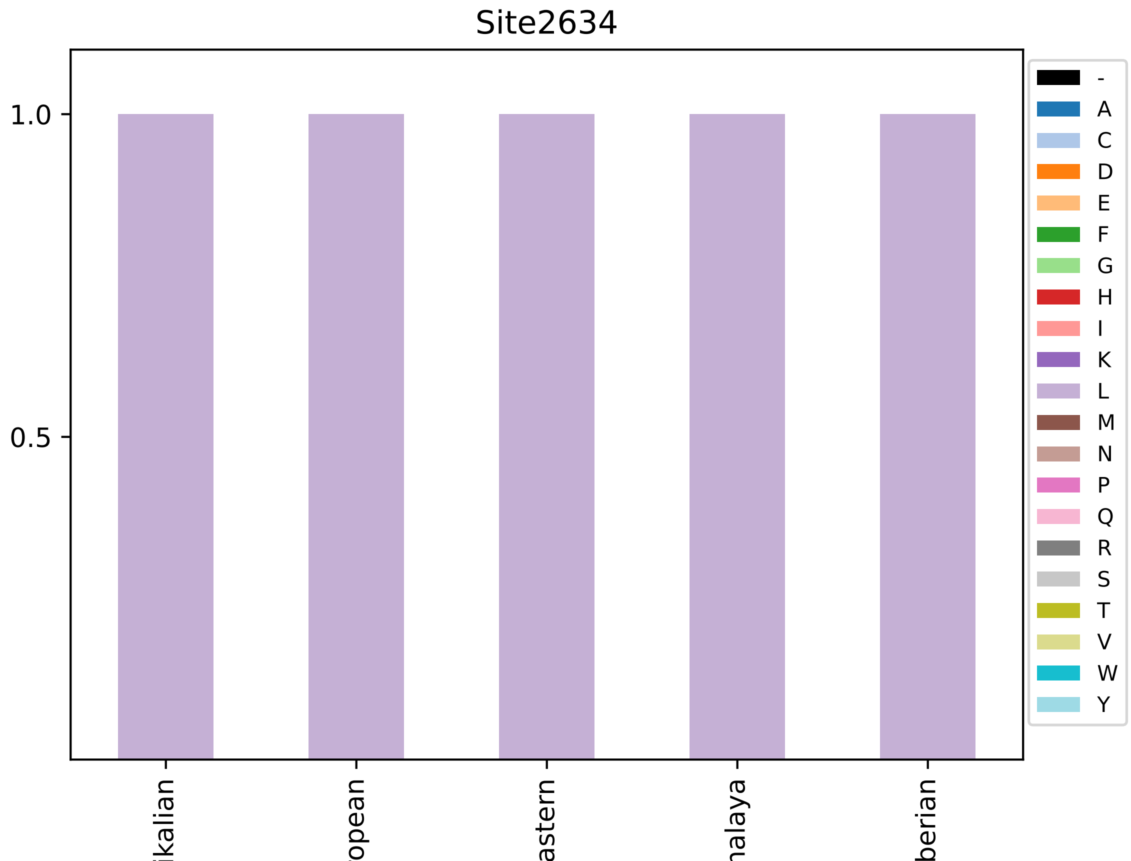

Site2634
1.0
0.5
ikalian
ropean
astern
nalaya
berian
-
A
C
D
E
F
G
H
I
K
L
M
N
P
Q
R
S
T
V
W
Y

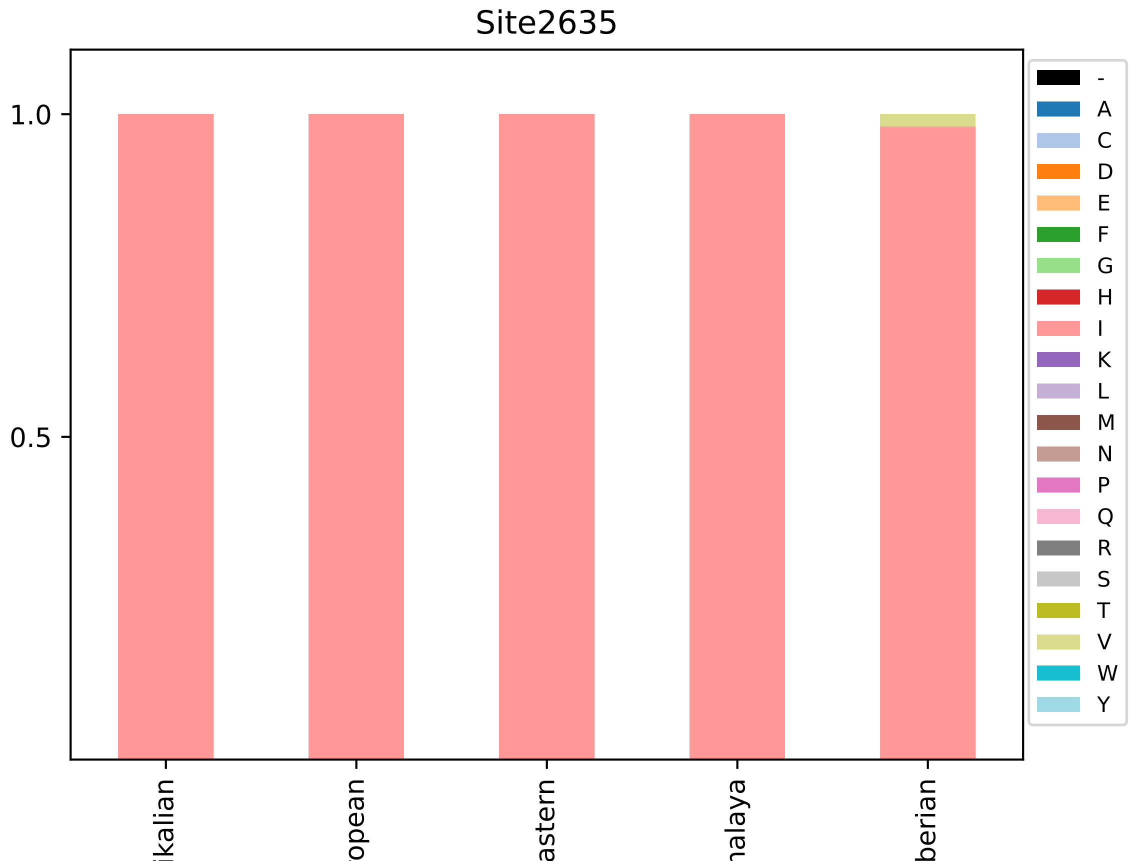

Site2635
1.0
0.5
-
A
C
D
E
F
G
H
I
K
L
M
N
P
Q
R
S
T
V
W
Y
ikalian
opean
astern
nalaya
berian

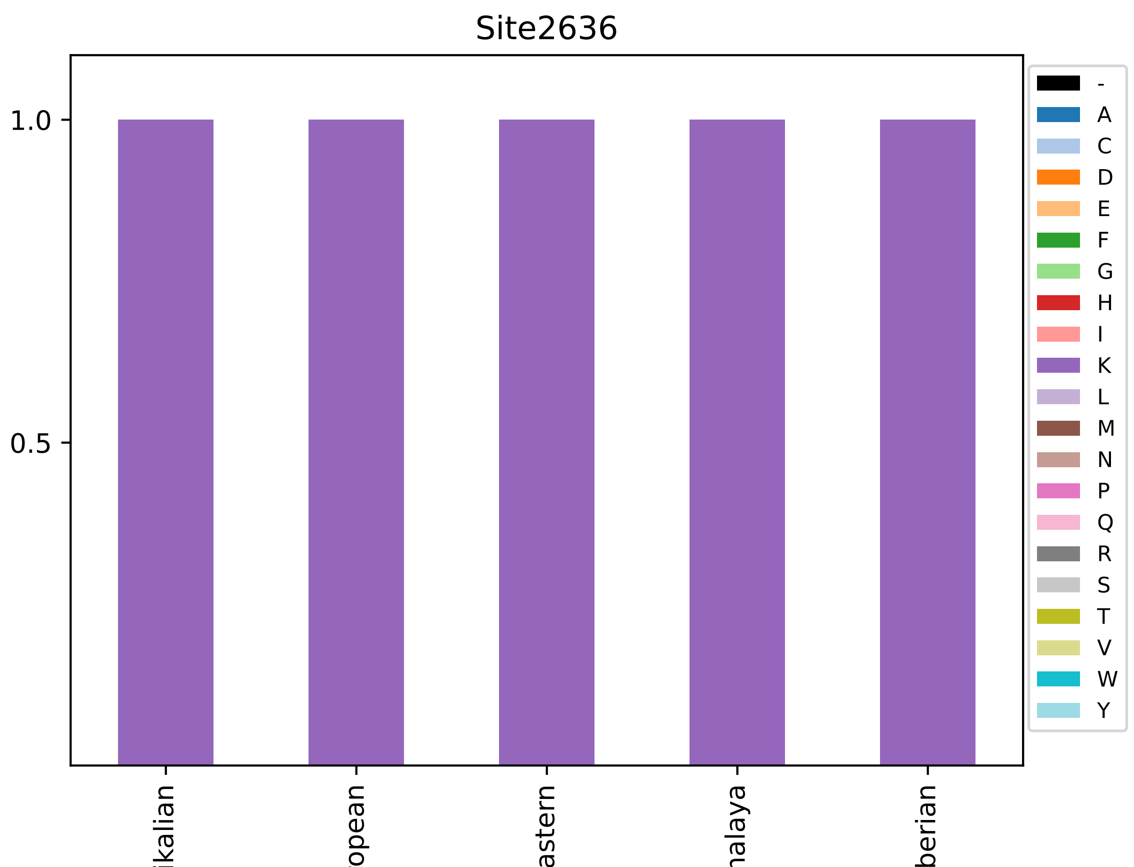
Site2636
1.0
0.5
-
A
C
D
E
F
G
H
I
K
L
M
N
P
Q
R
S
T
V
W
Y
ikalian
ropean
astern
nalaya
berian

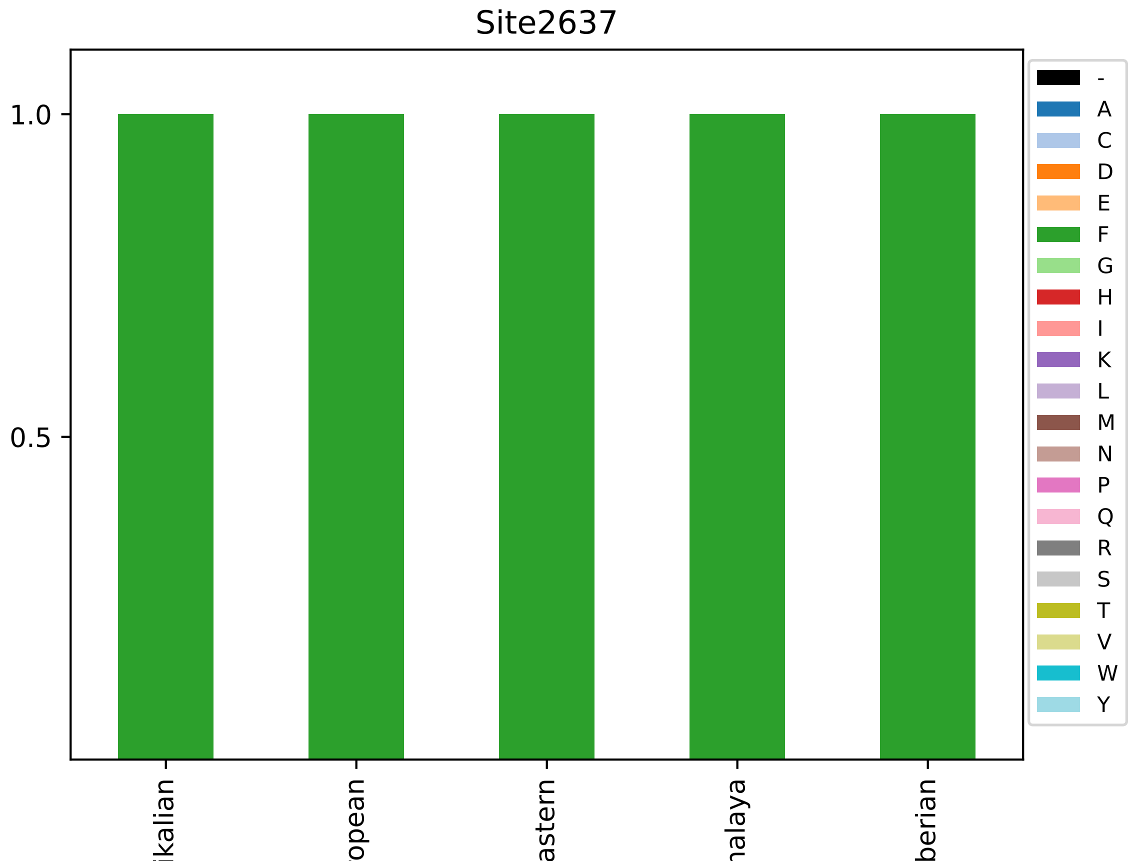
Site2637
1.0
0.5
-
A
C
D
E
F
G
H
I
K
L
M
N
P
Q
R
S
T
V
W
Y
ikalian
opean
astern
nalaya
berian

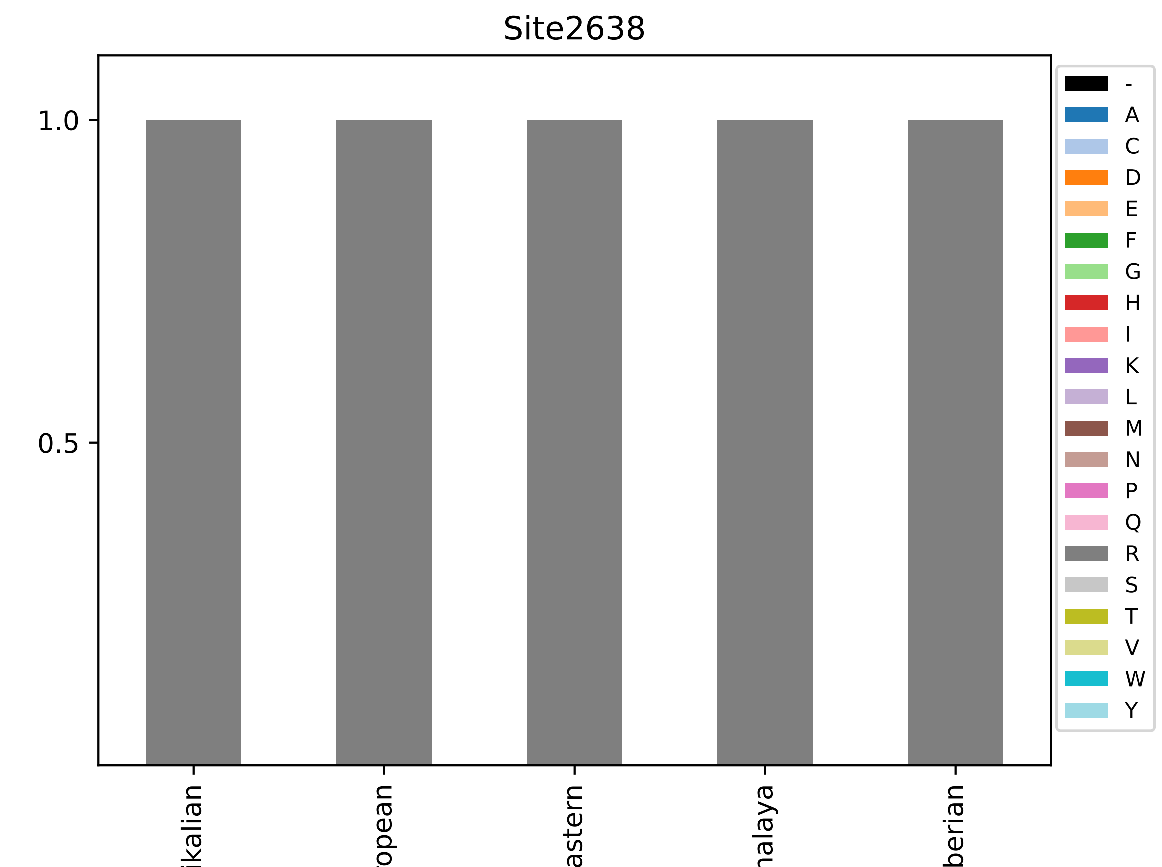

Site2638
1.0
0.5
ikalian
opean
astern
nalaya
berian
-
A
C
D
E
F
G
H
I
K
L
M
N
P
Q
R
S
T
V
W
Y

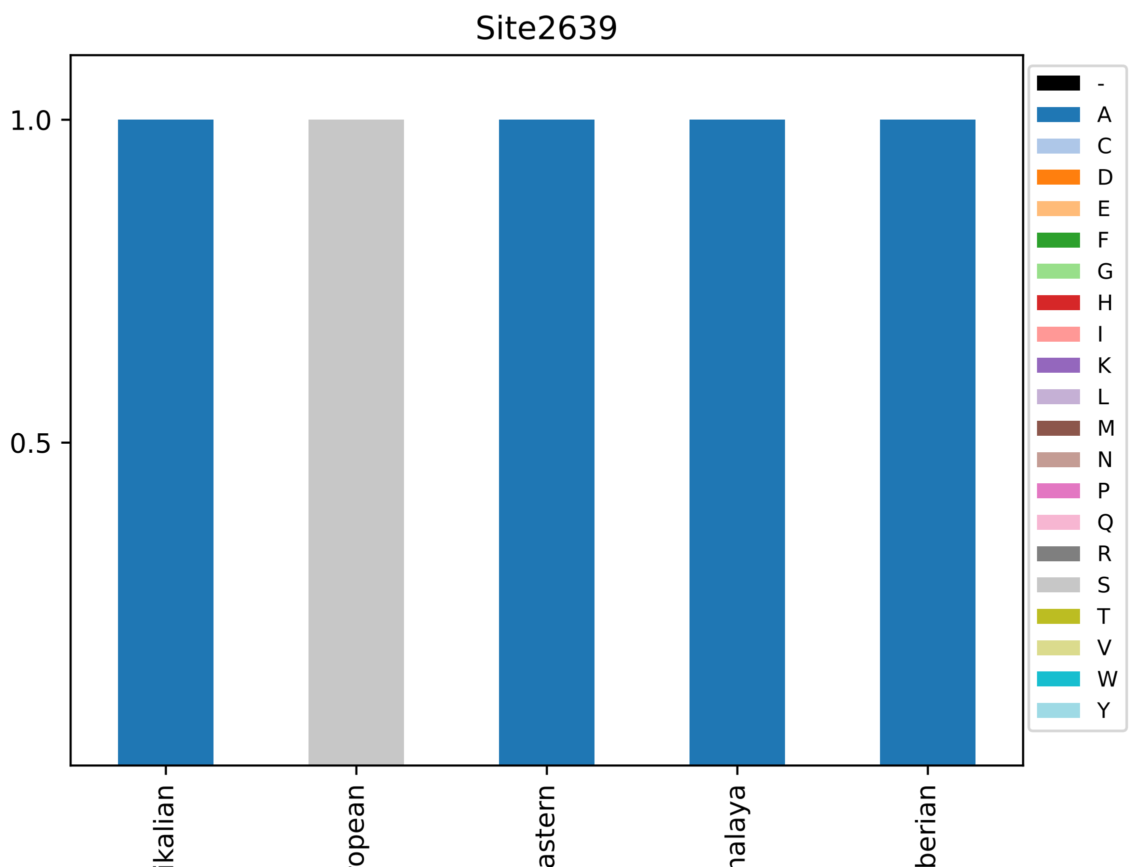
Site2639
1.0
0.5
ikalian
opean
astern
nalaya
berian
-
A
C
D
E
F
G
H
I
K
L
M
N
P
Q
R
S
T
V
W
Y

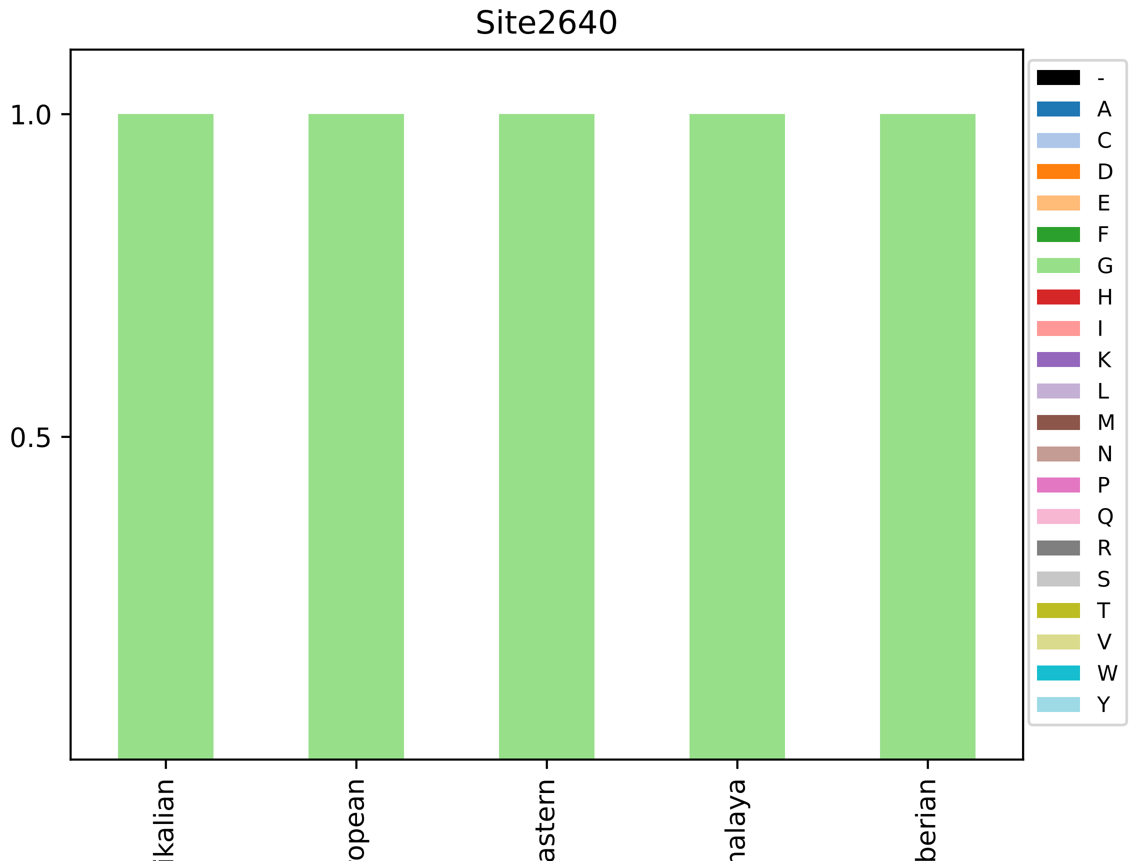

Site2640
1.0
0.5
-
A
C
D
E
F
G
H
I
K
L
M
N
P
Q
R
S
T
V
W
Y
ikalian
ropean
astern
nalaya
berian

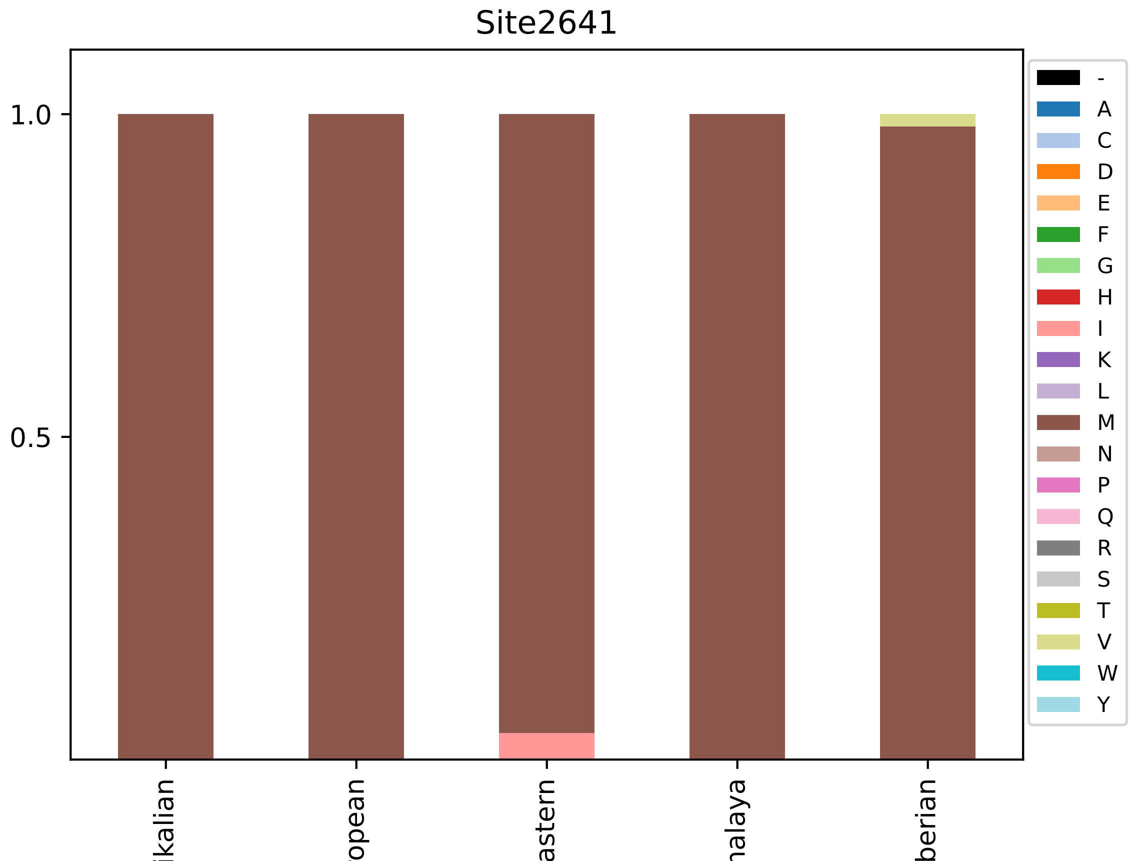

Site2641
1.0
0.5
-
A
C
D
E
F
G
H
I
K
L
M
N
P
Q
R
S
T
V
W
Y
ikalian
opean
astern
nalaya
berian

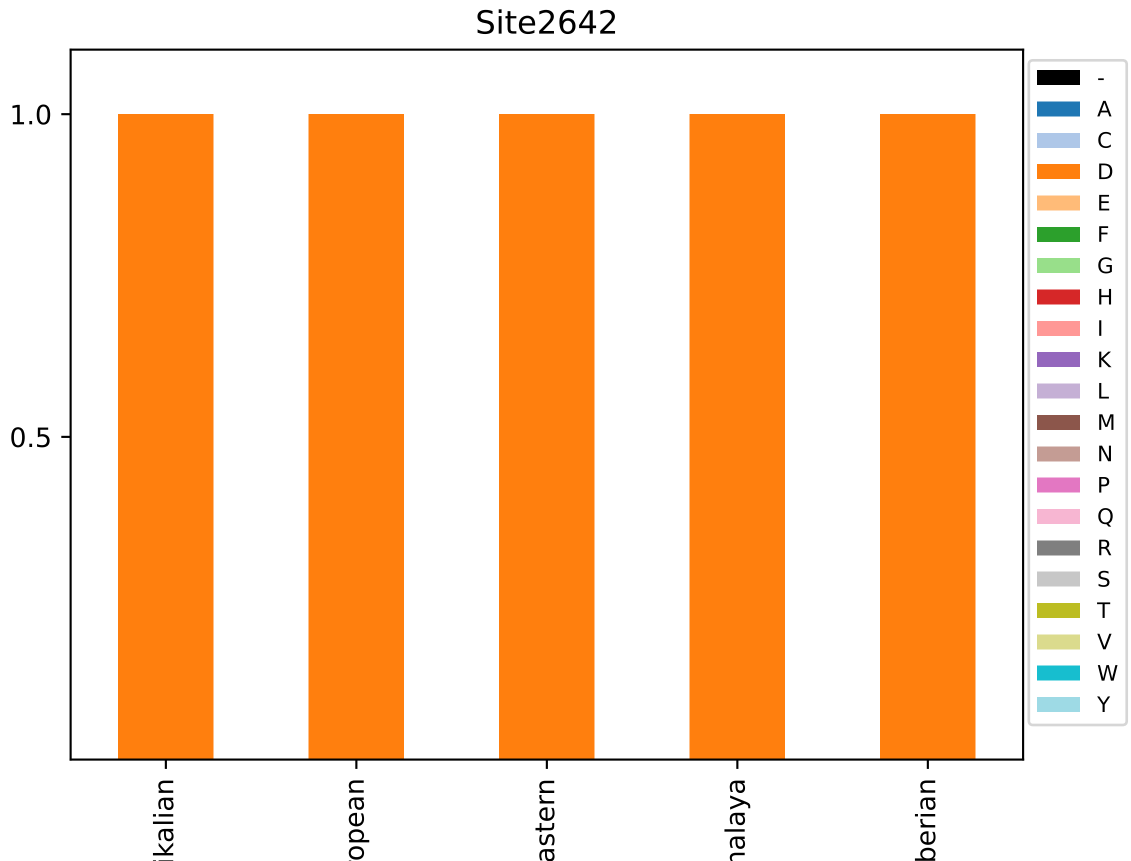
Site2642
1.0
0.5
-
A
C
D
E
F
G
H
I
K
L
M
N
P
Q
R
S
T
V
W
Y
ikalian
opean
astern
nalaya
berian

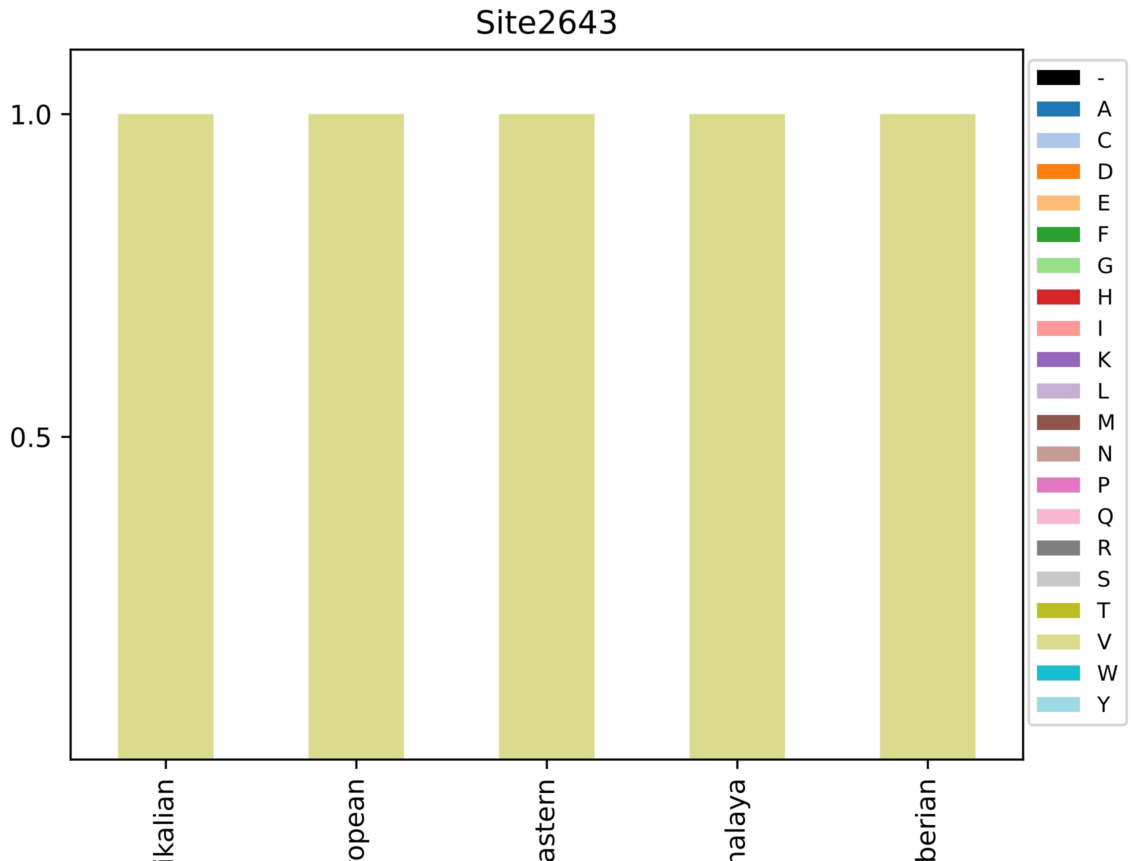

Site2643
1.0
0.5
ikalian
ropean
astern
nalaya
berian
-
A
C
D
E
F
G
H
I
K
L
M
N
P
Q
R
S
T
V
W
Y

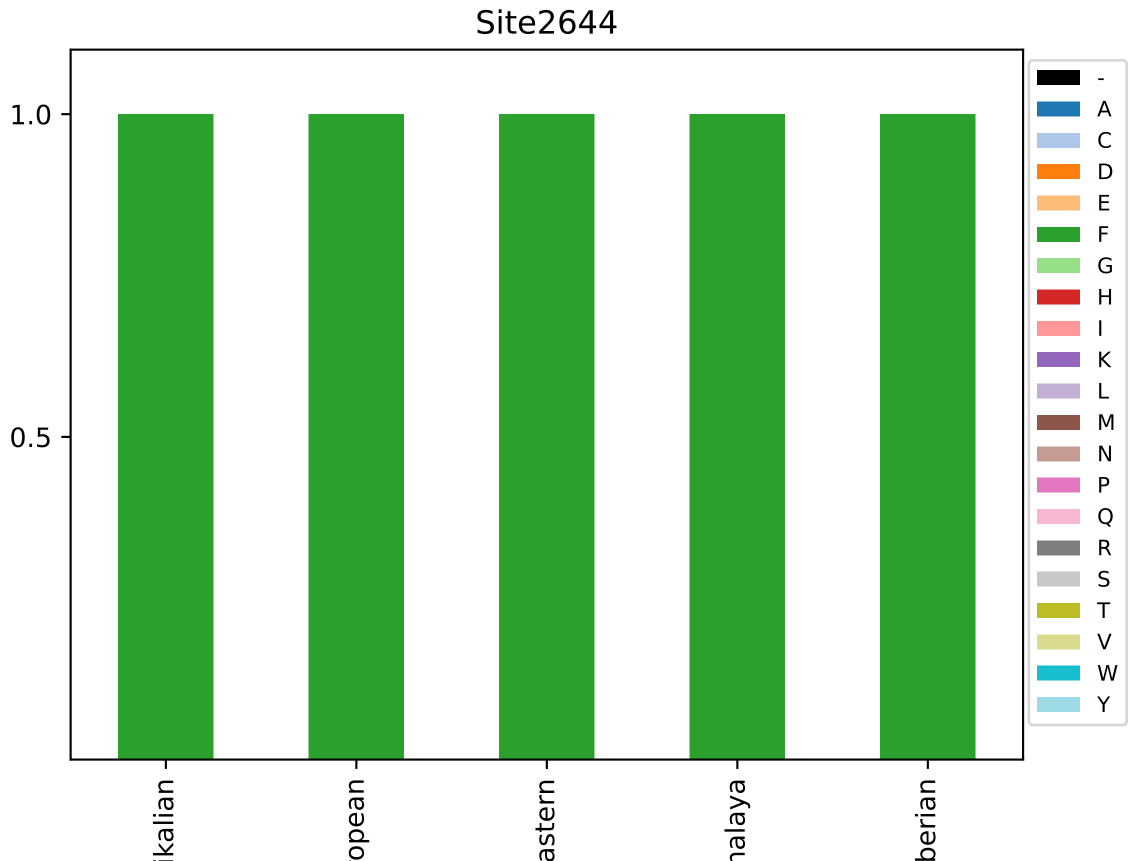

Site2644
1.0
0.5
-
A
C
D
E
F
G
H
I
K
L
M
N
P
Q
R
S
T
V
W
Y
ikalian
ropean
astern
nalaya
berian

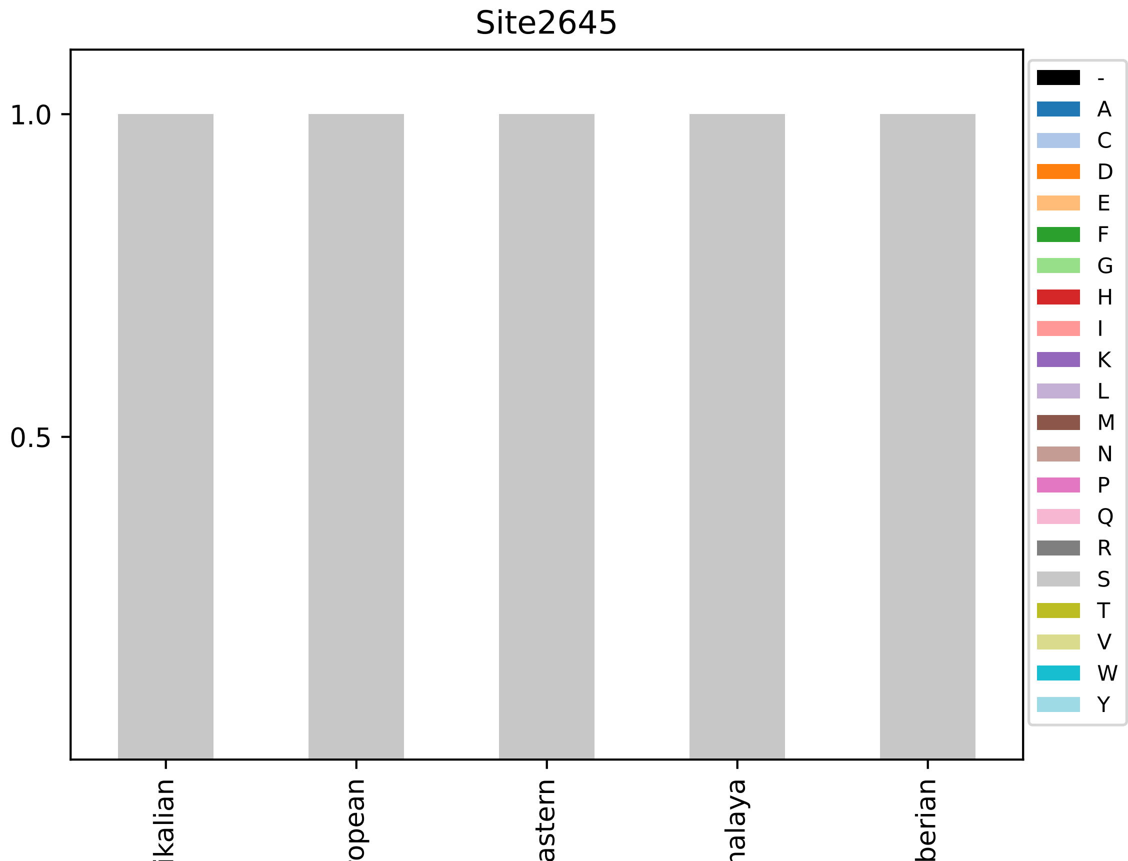

Site2645
1.0
0.5
-
A
C
D
E
F
G
H
I
K
L
M
N
P
Q
R
S
T
V
W
Y
ikalian
opean
astern
nalaya
berian

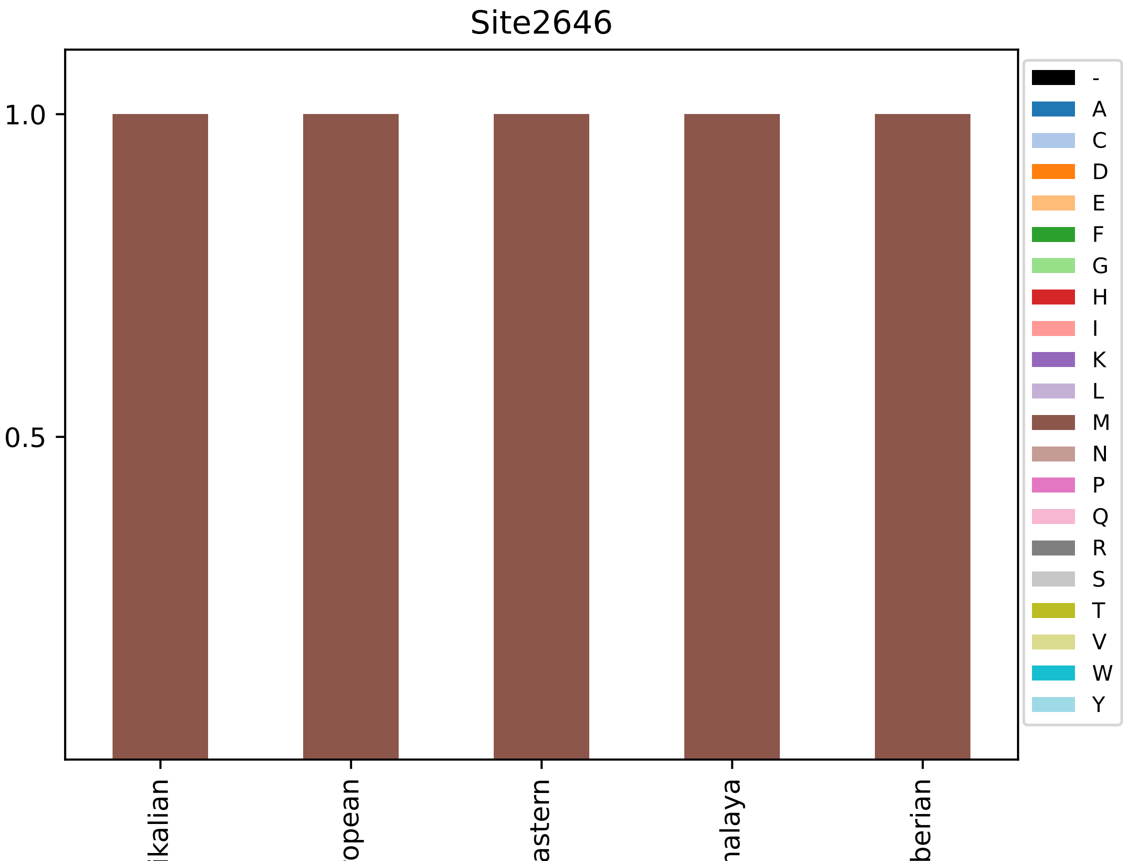
Site2646
1.0
0.5
-
A
C
D
E
F
G
H
I
K
L
M
N
P
Q
R
S
T
V
W
Y

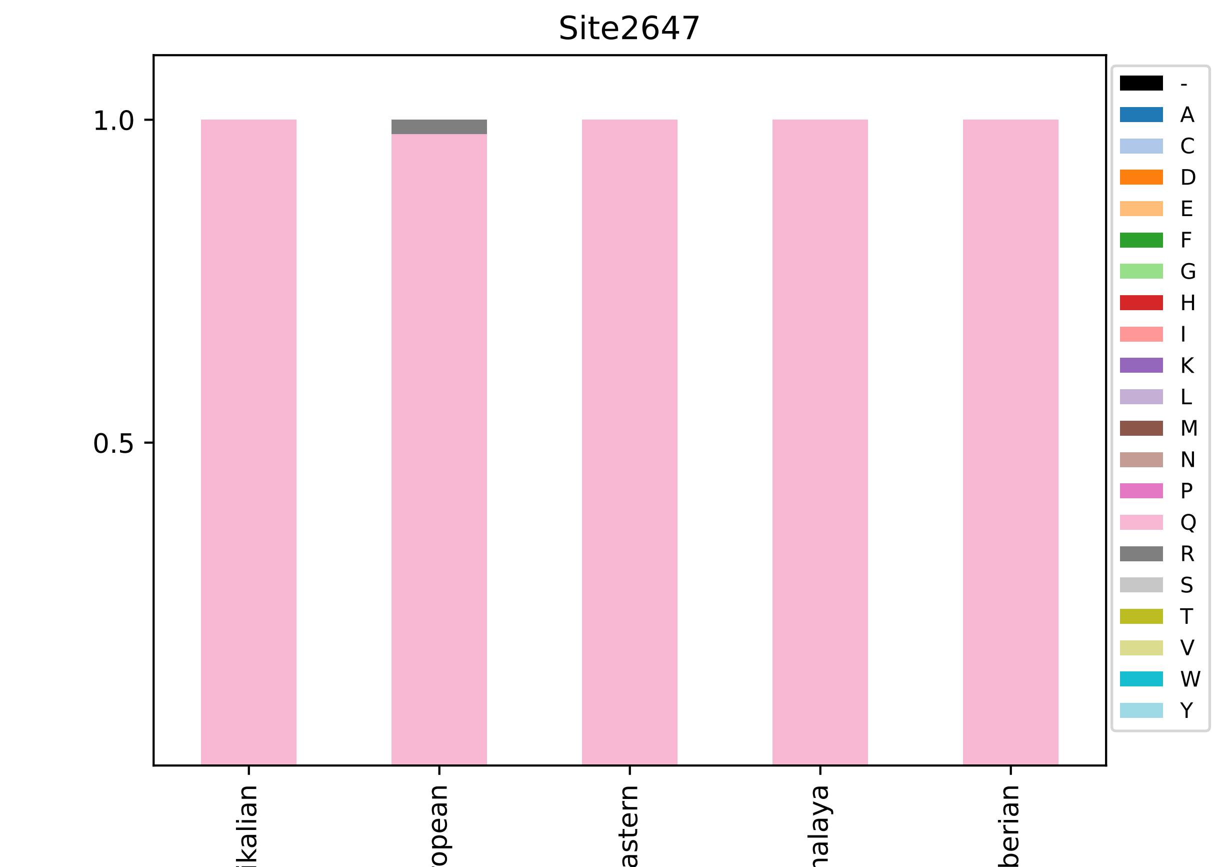
Site2647
1.0
0.5
-
A
C
D
E
F
G
H
I
K
L
M
N
P
Q
R
S
T
V
W
Y
ikalian
opean
astern
nalaya
berian

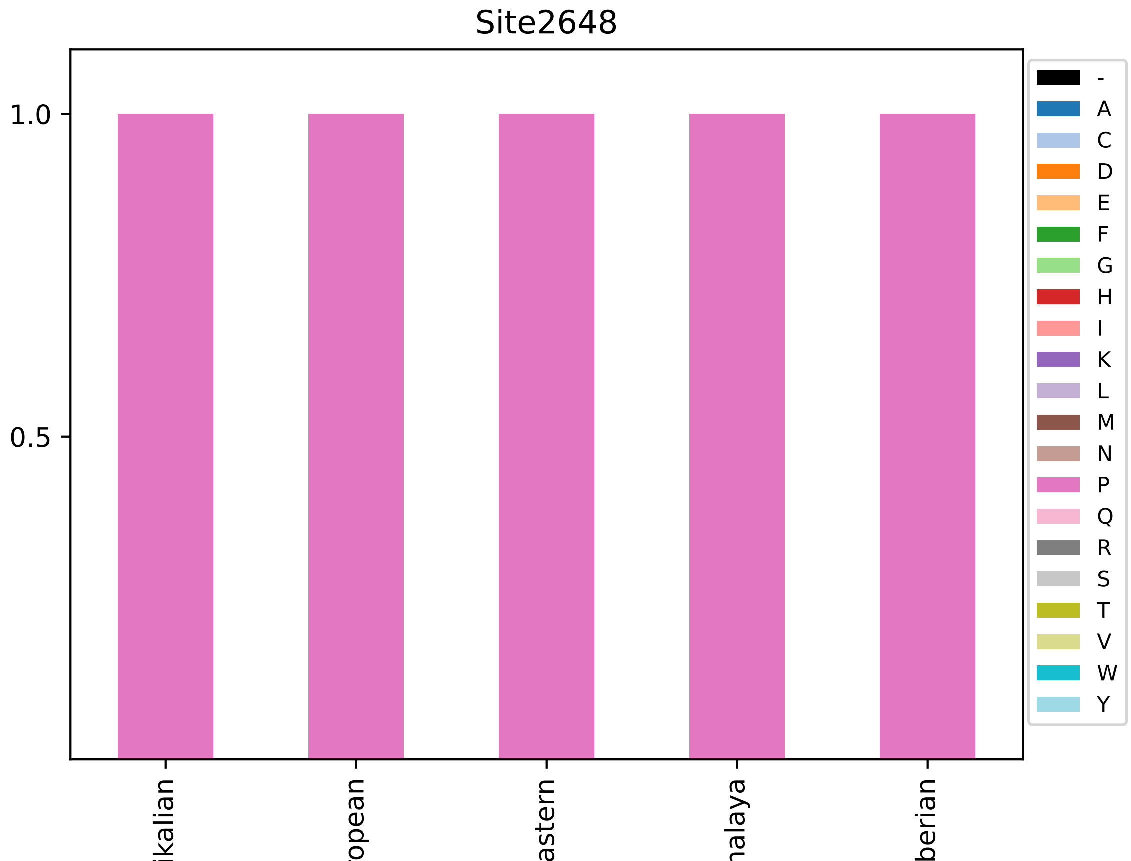

Site2648
1.0
0.5
-
A
C
D
E
F
G
H
I
K
L
M
N
P
Q
R
S
T
V
W
Y
ikalian
opean
astern
nalaya
berian

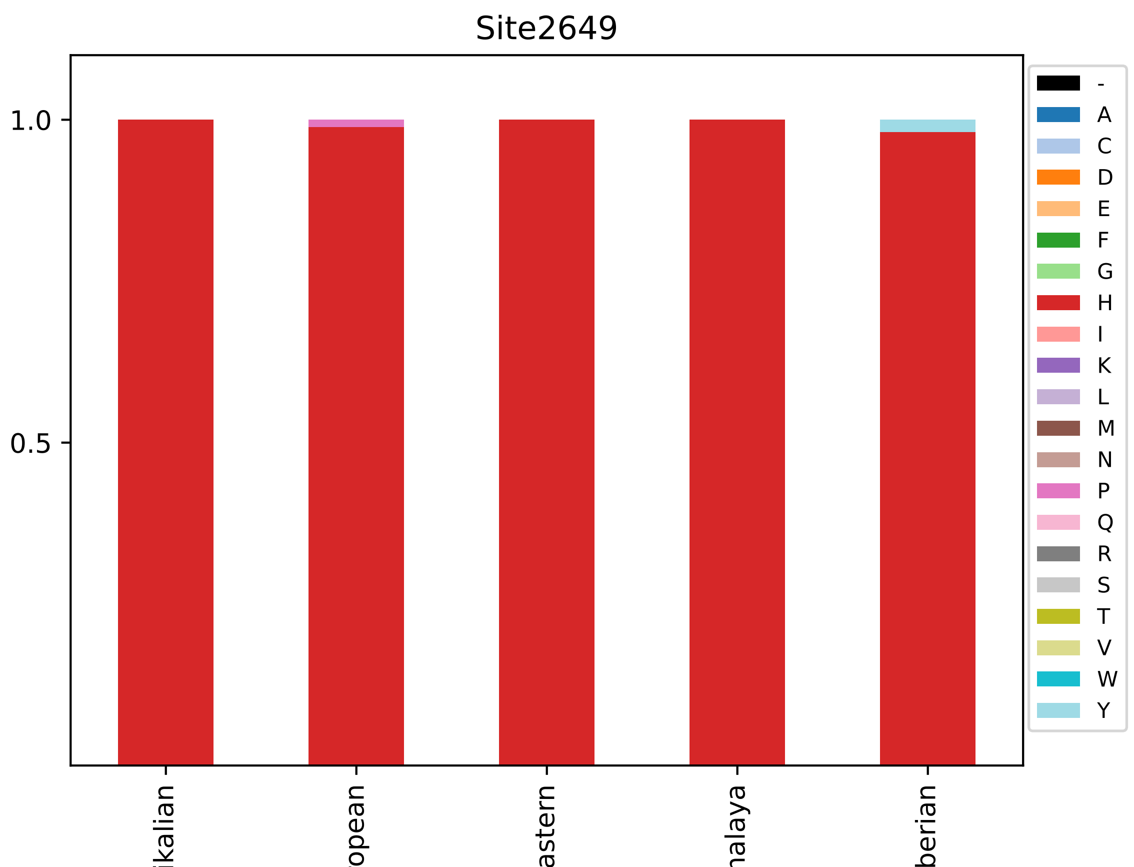

Site2649
1.0
0.5
-
A
C
D
E
F
G
H
I
K
L
M
N
P
Q
R
S
T
V
W
Y
ikalian
opean
astern
nalaya
berian

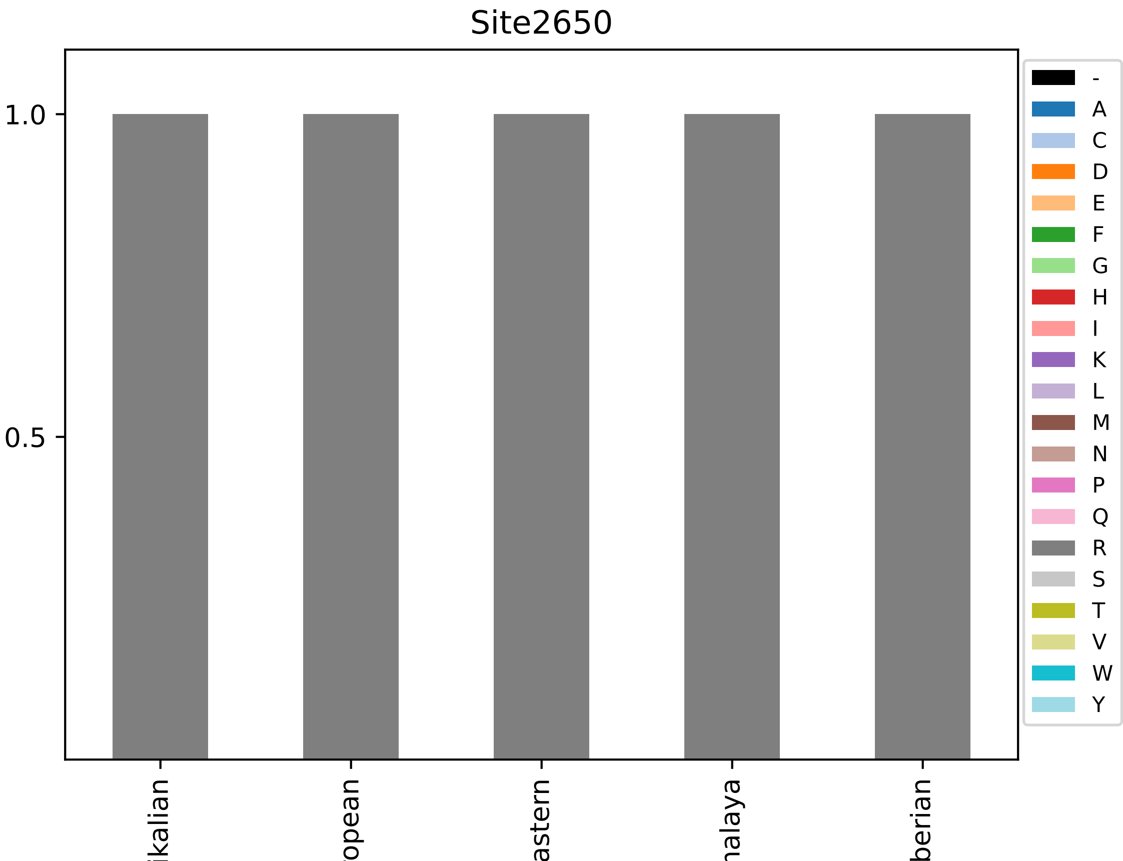
Site2650
1.0
0.5
-
A
C
D
E
F
G
H
I
K
L
M
N
P
Q
R
S
T
V
W
Y
ikalian
opean
astern
nalaya
berian

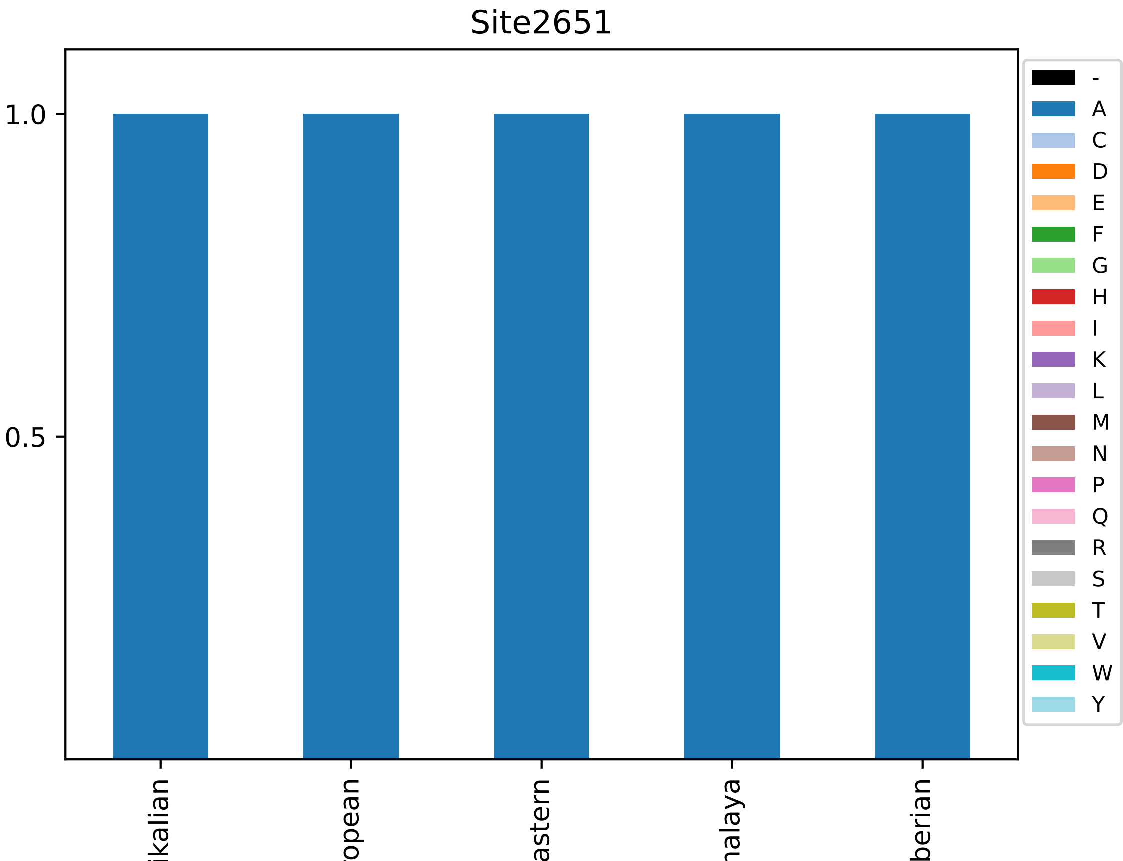

Site2651
1.0
0.5
-
A
C
D
E
F
G
H
I
K
L
M
N
P
Q
R
S
T
V
W
Y
ikalian
opean
astern
nalaya
berian

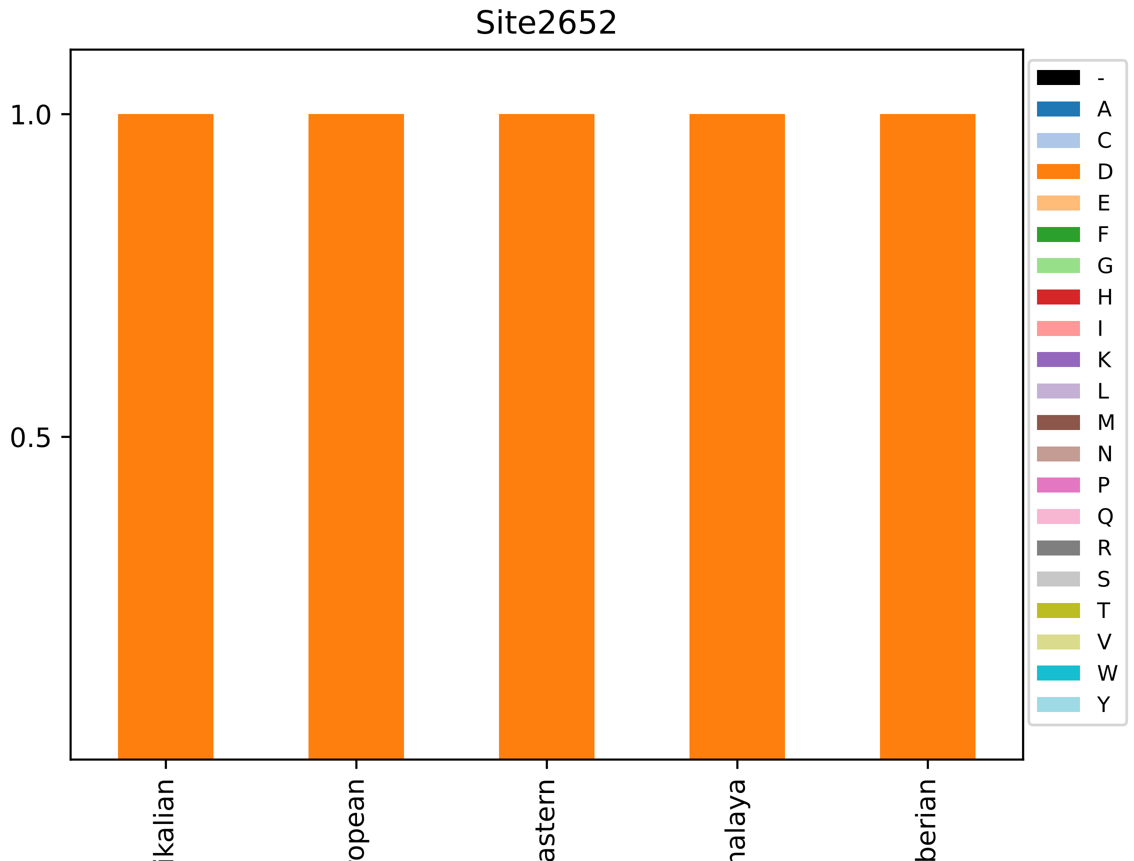

Site2652
1.0
0.5
-
A
C
D
E
F
G
H
I
K
L
M
N
P
Q
R
S
T
V
W
Y
ikalian
opean
astern
nalaya
berian

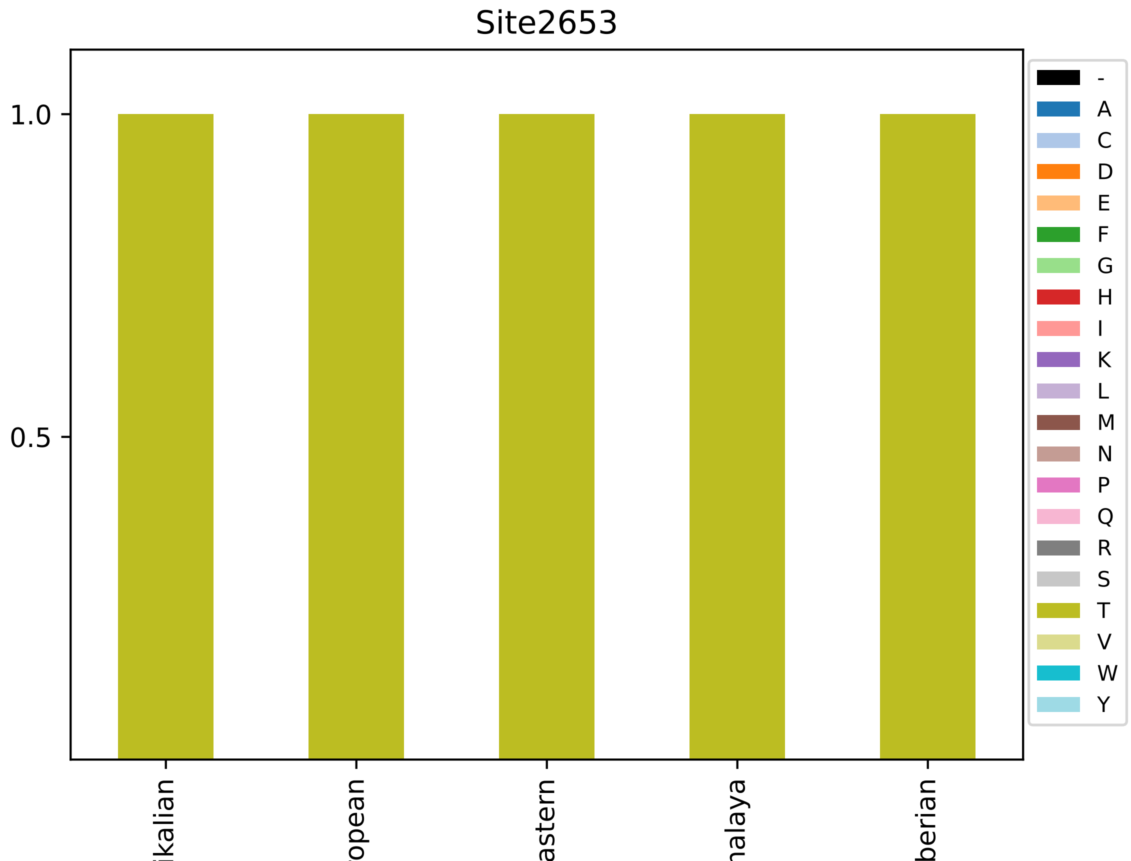

Site2653
1.0
0.5
-
A
C
D
E
F
G
H
I
K
L
M
N
P
Q
R
S
T
V
W
Y
ikalian
opean
astern
nalaya
berian

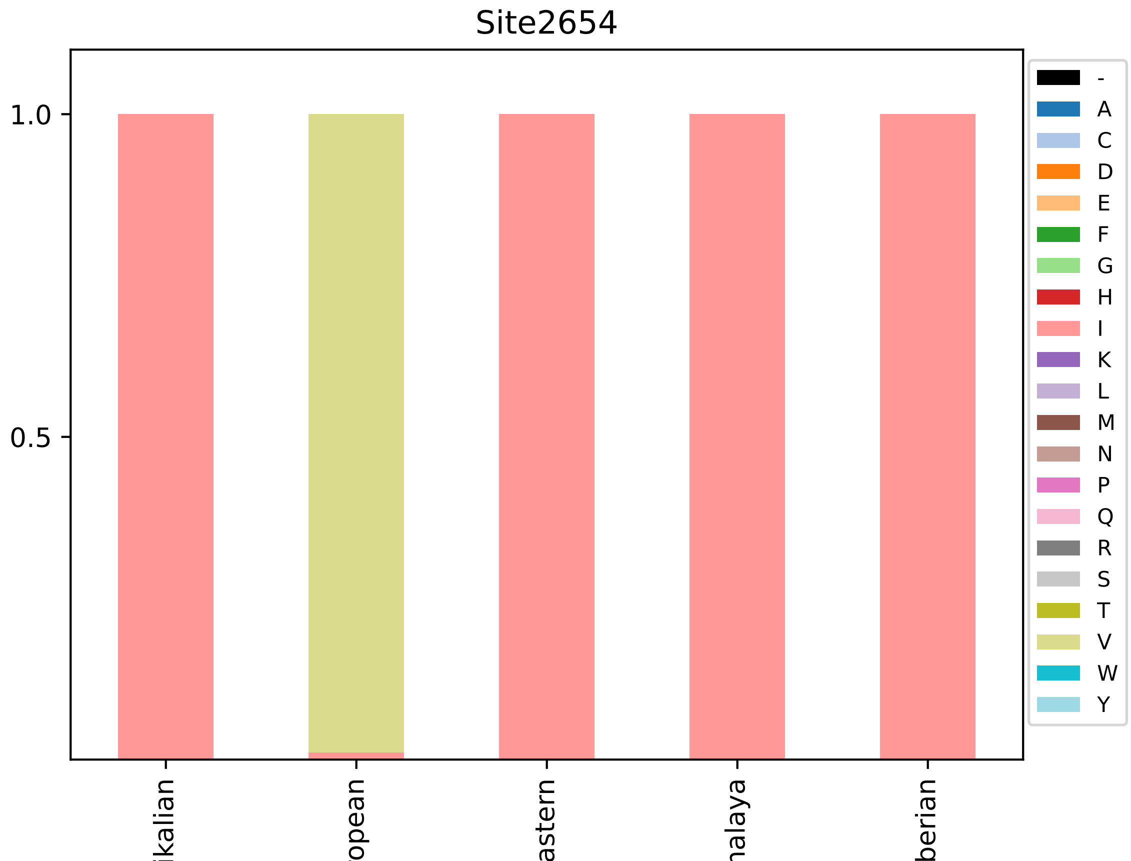
Site2654
1.0
0.5
-
A
C
D
E
F
G
H
I
K
L
M
N
P
Q
R
S
T
V
W
Y
ikalian
opean
astern
nalaya
berian

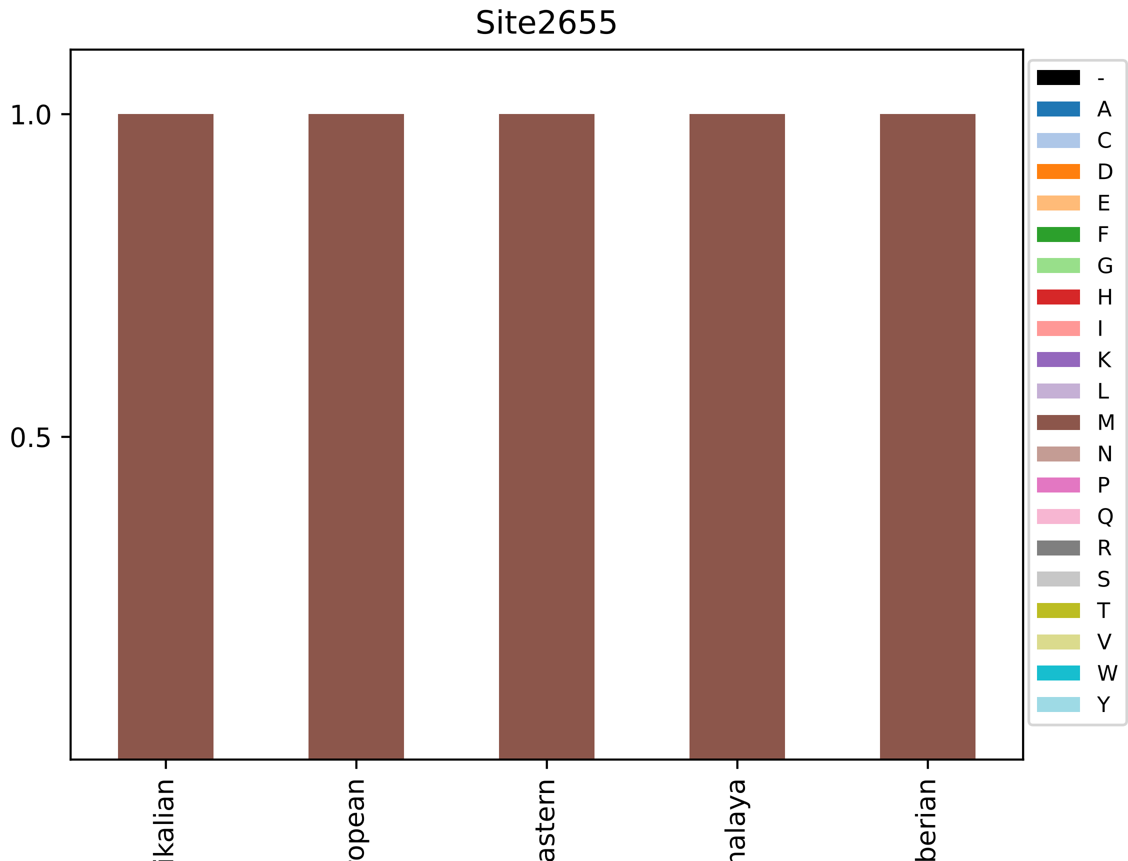

Site2655
1.0
0.5
-
A
C
D
E
F
G
H
I
K
L
M
N
P
Q
R
S
T
V
W
Y
ikalian
opean
astern
nalaya
berian

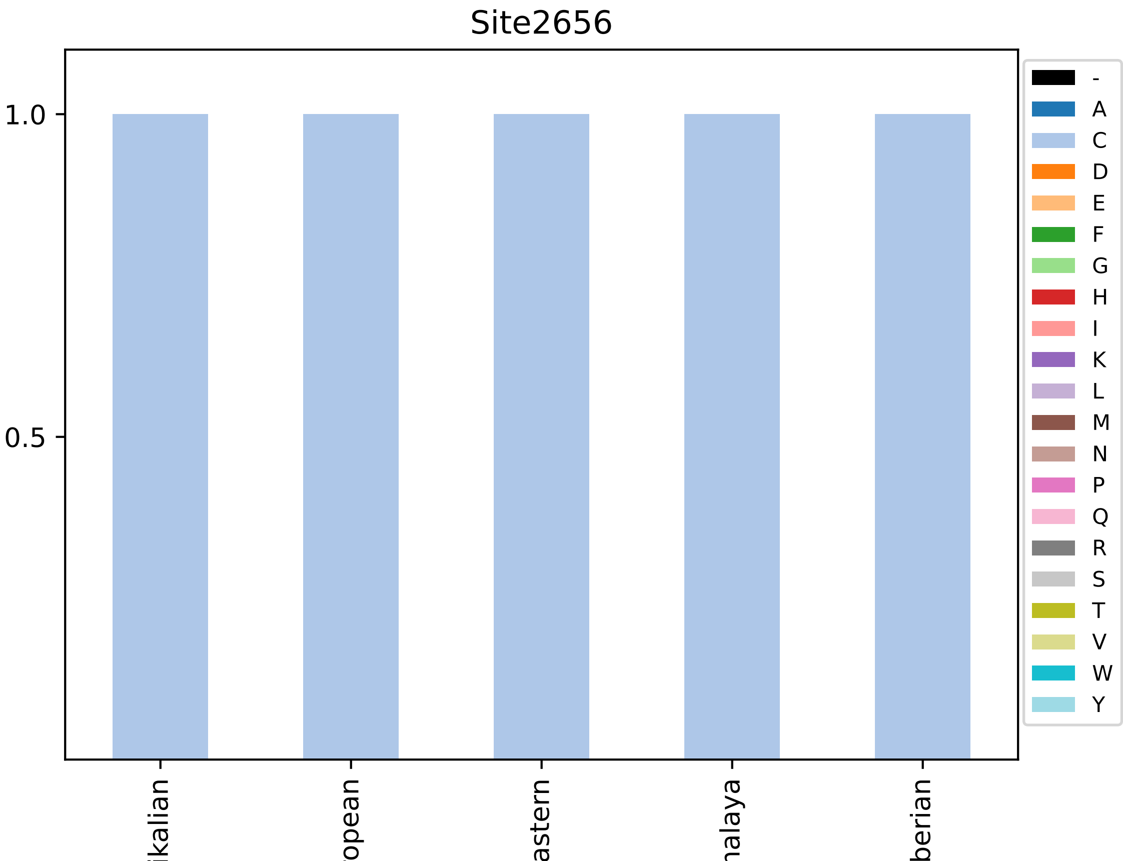
Site2656
1.0
0.5
ikalian
opean
astern
nalaya
berian
-
A
C
D
E
F
G
H
I
K
L
M
N
P
Q
R
S
T
V
W
Y

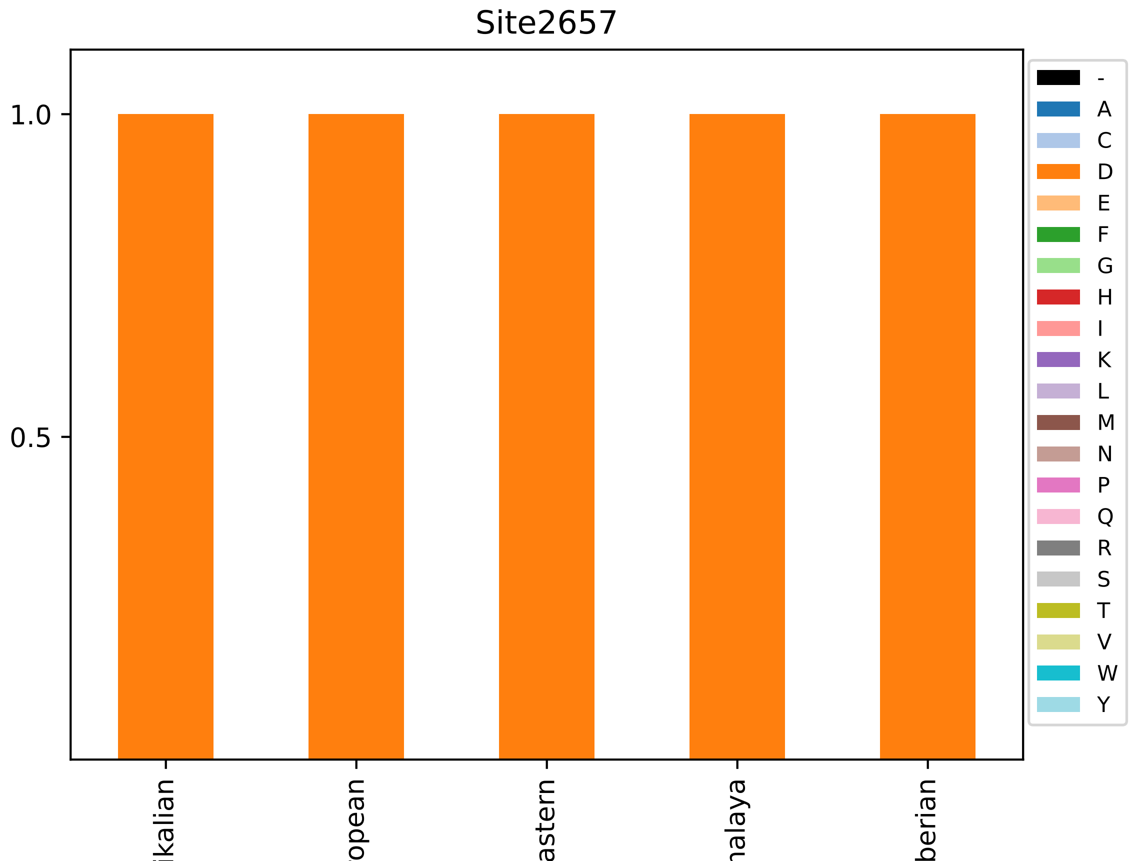

Site2657
1.0
0.5
-
A
C
D
E
F
G
H
I
K
L
M
N
P
Q
R
S
T
V
W
Y
ikalian
opean
astern
nalaya
berian

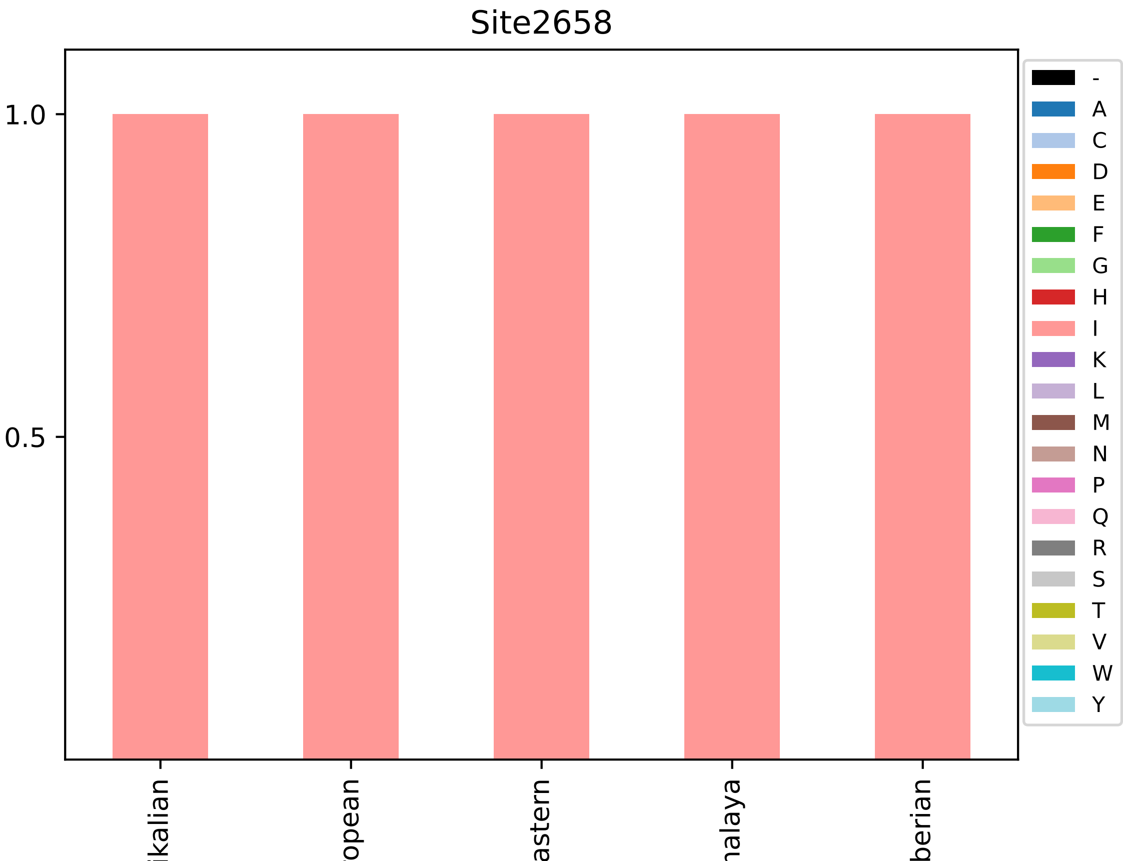

Site2658
1.0
0.5
-
A
C
D
E
F
G
H
I
K
L
M
N
P
Q
R
S
T
V
W
Y
ikalian
opean
astern
nalaya
berian

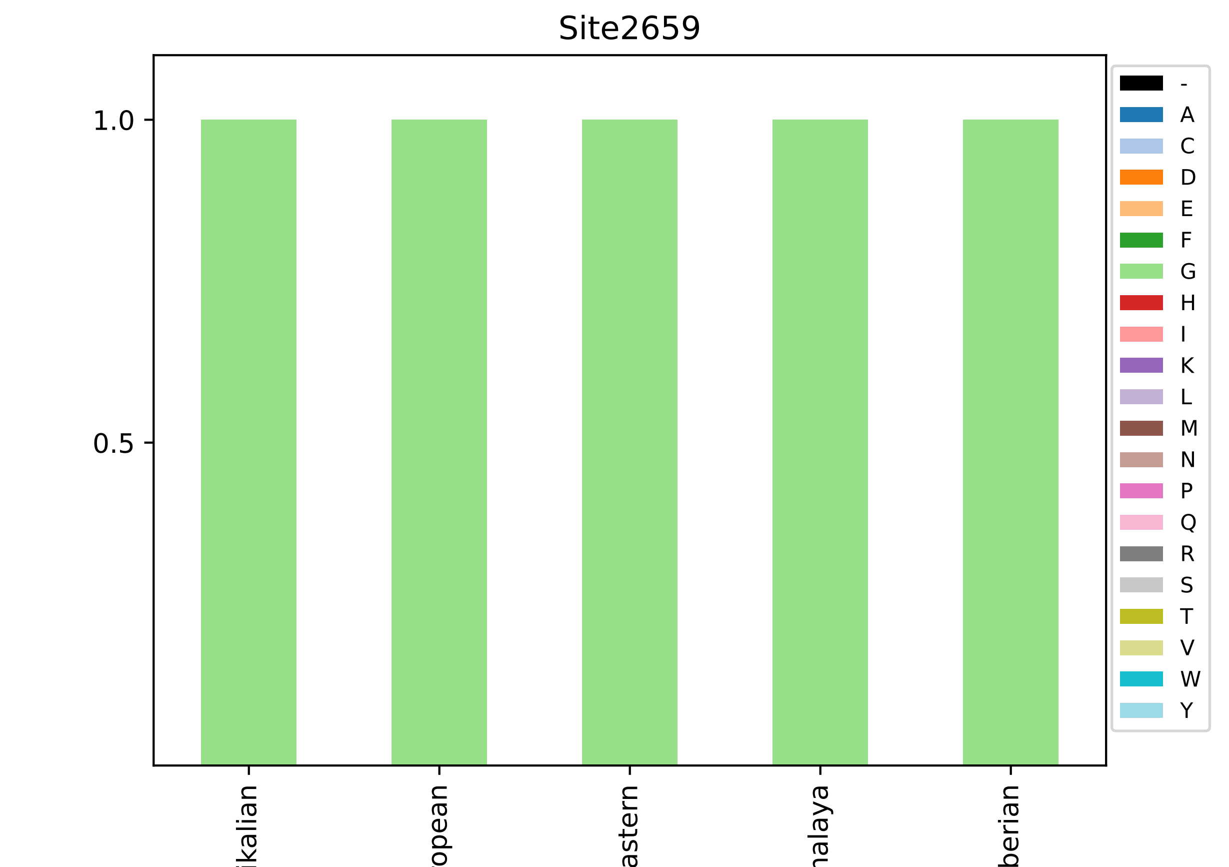
Site2659
1.0
0.5
-
A
C
D
E
F
G
H
I
K
L
M
N
P
Q
R
S
T
V
W
Y
ikalian
opean
astern
nalaya
berian

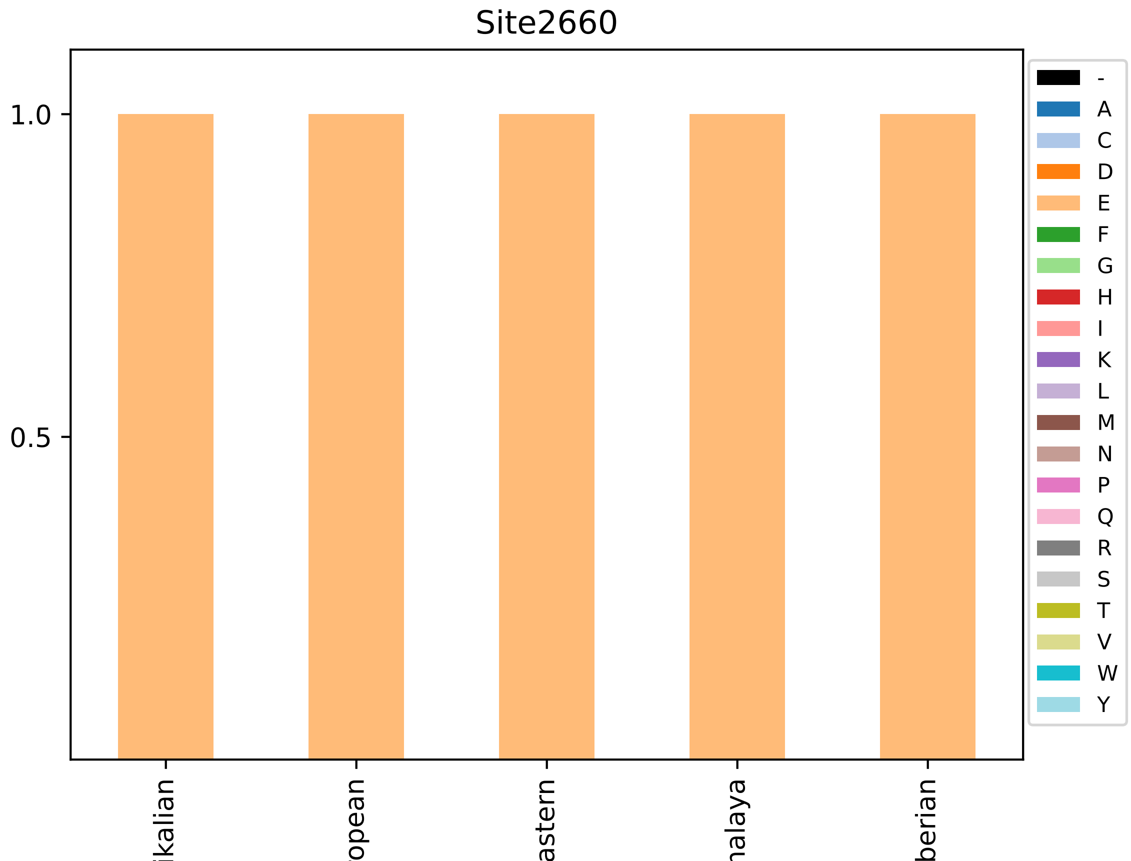

Site2660
1.0
0.5
-
A
C
D
E
F
G
H
I
K
L
M
N
P
Q
R
S
T
V
W
Y

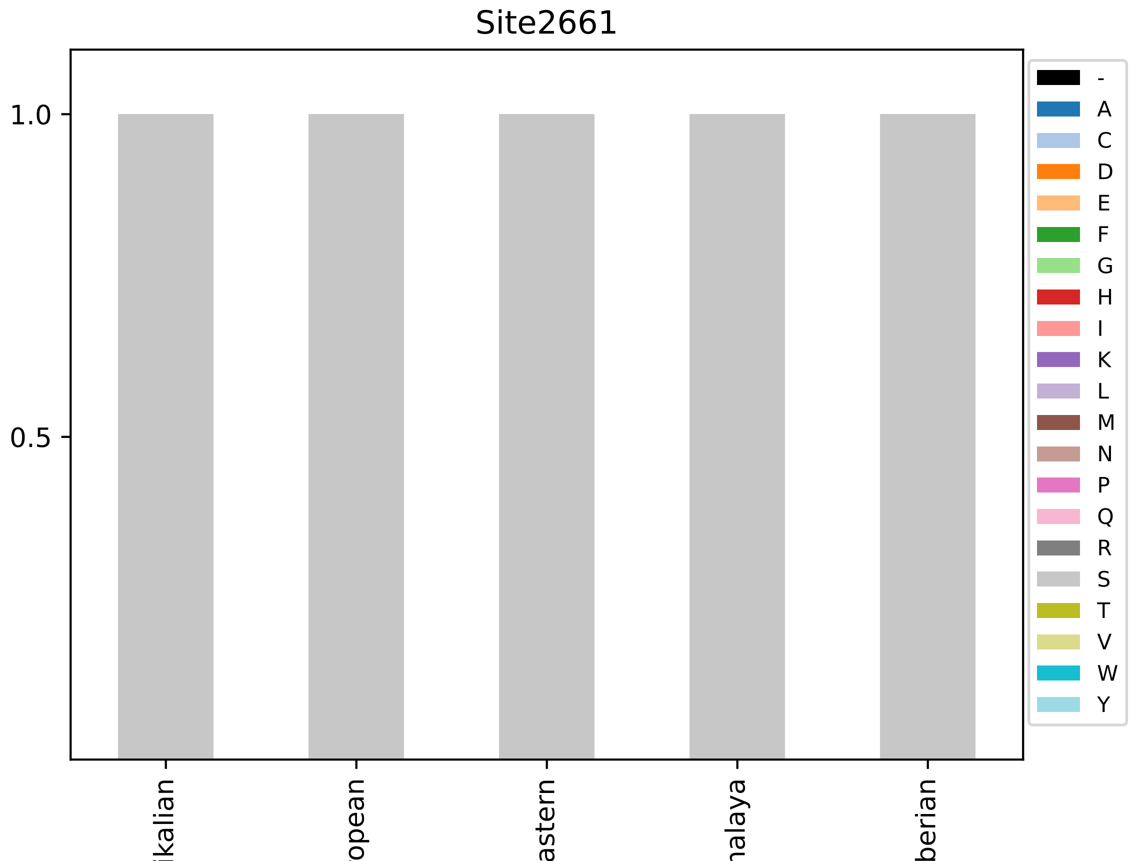
Site2661
1.0
0.5
-
A
C
D
E
F
G
H
I
K
L
M
N
P
Q
R
S
T
V
W
Y
ikalian
opean
astern
nalaya
berian

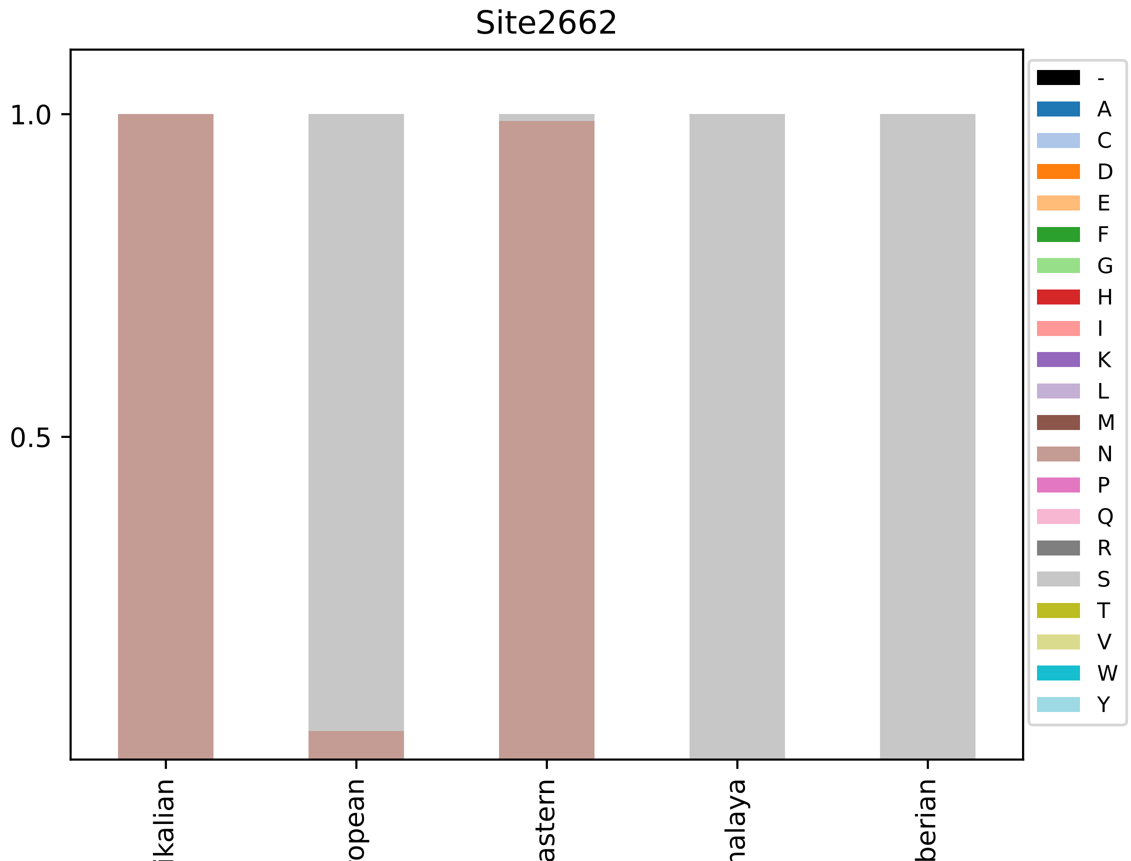

Site2662
1.0
0.5
-
A
C
D
E
F
G
H
I
K
L
M
N
P
Q
R
S
T
V
W
Y
ikalian
opean
astern
nalaya
berian

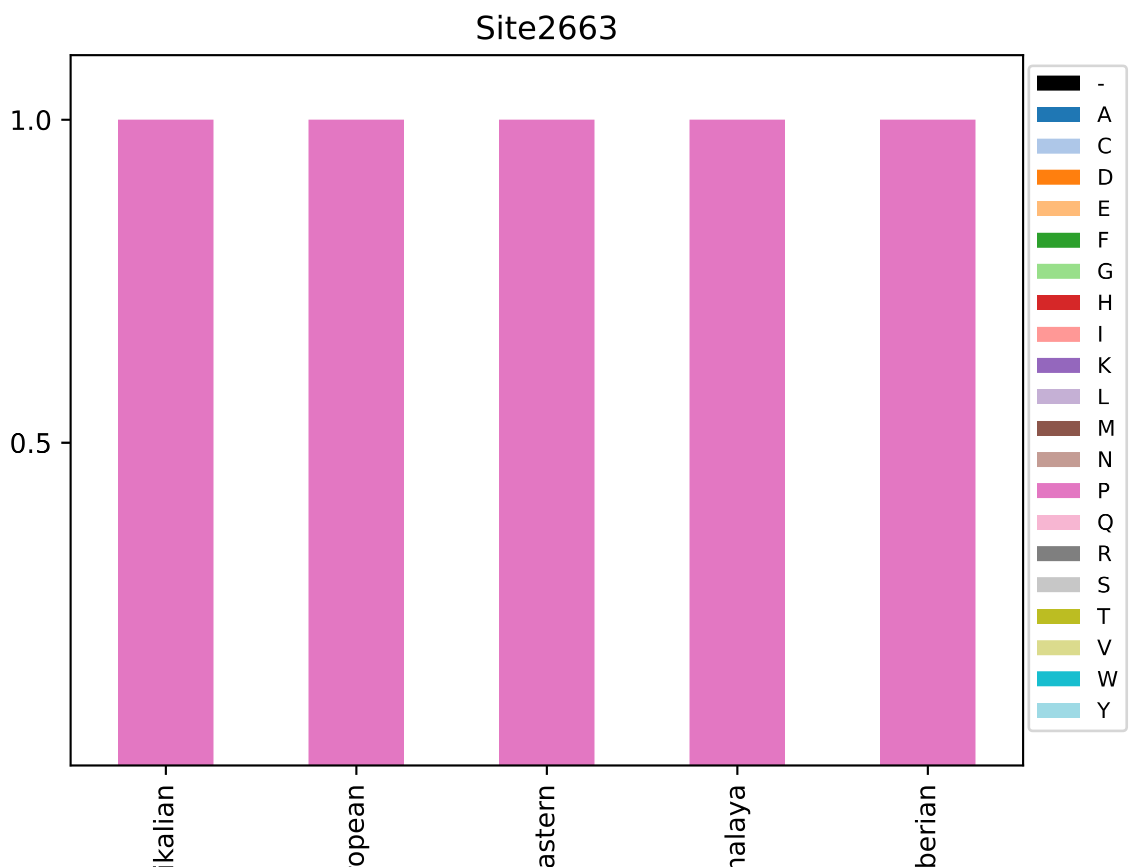

Site2663
1.0
0.5
-
A
C
D
E
F
G
H
I
K
L
M
N
P
Q
R
S
T
V
W
Y
ikalian
opean
astern
nalaya
berian

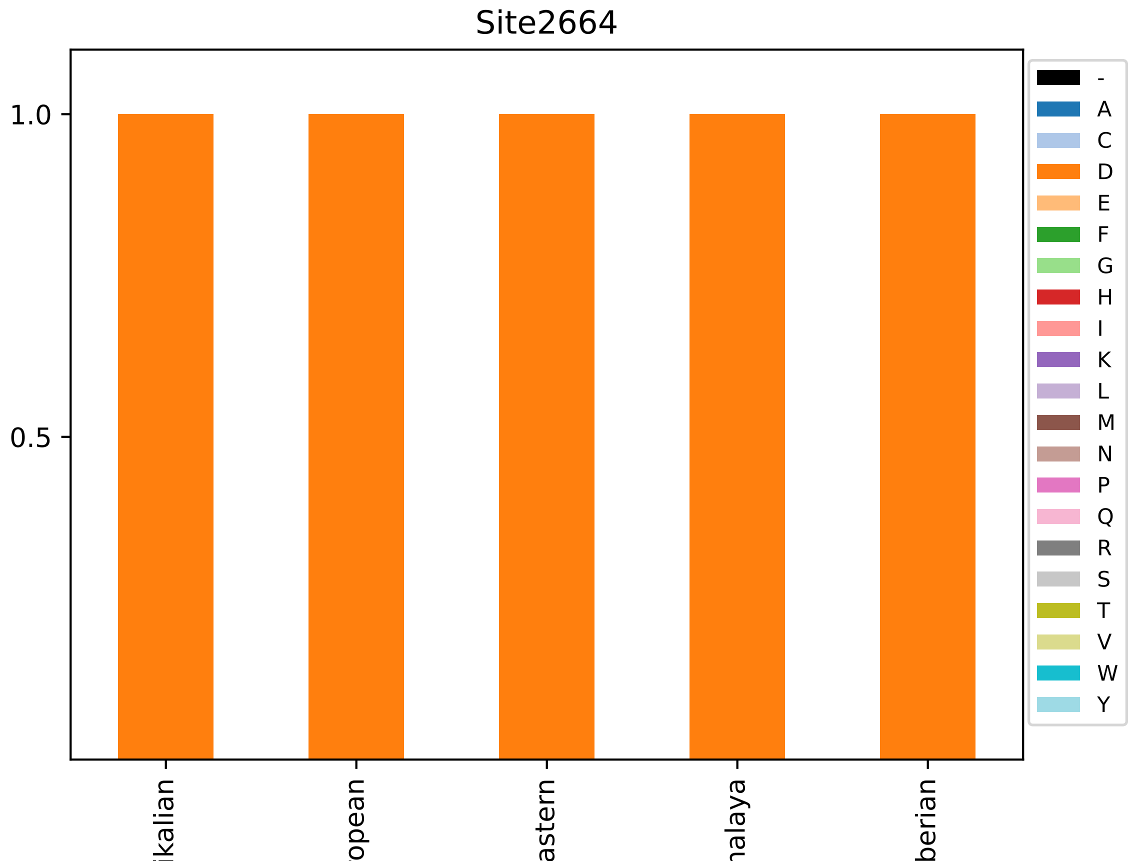

Site2664
1.0
0.5
-
A
C
D
E
F
G
H
I
K
L
M
N
P
Q
R
S
T
V
W
Y
ikalian
opean
astern
nalaya
berian

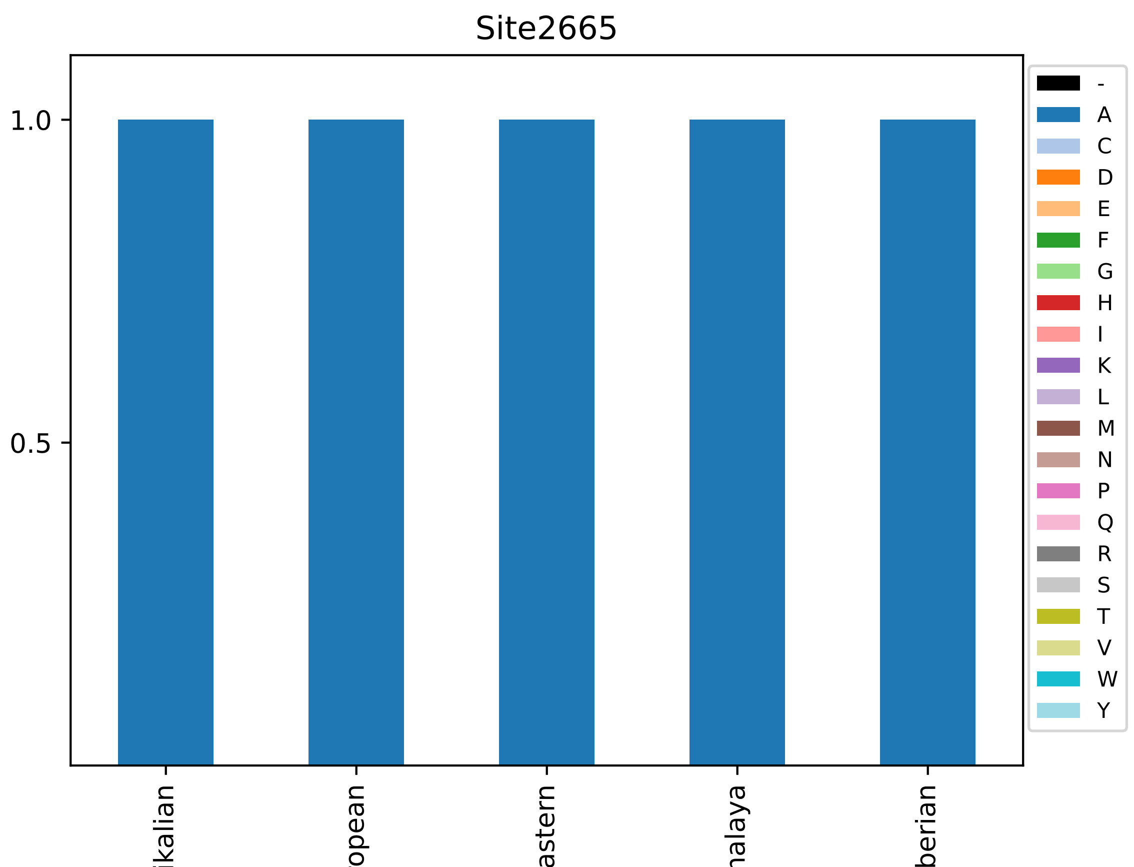
Site2665
1.0
0.5
ikalian
ropean
astern
nalaya
berian
-
A
C
D
E
F
G
H
I
K
L
M
N
P
Q
R
S
T
V
W
Y

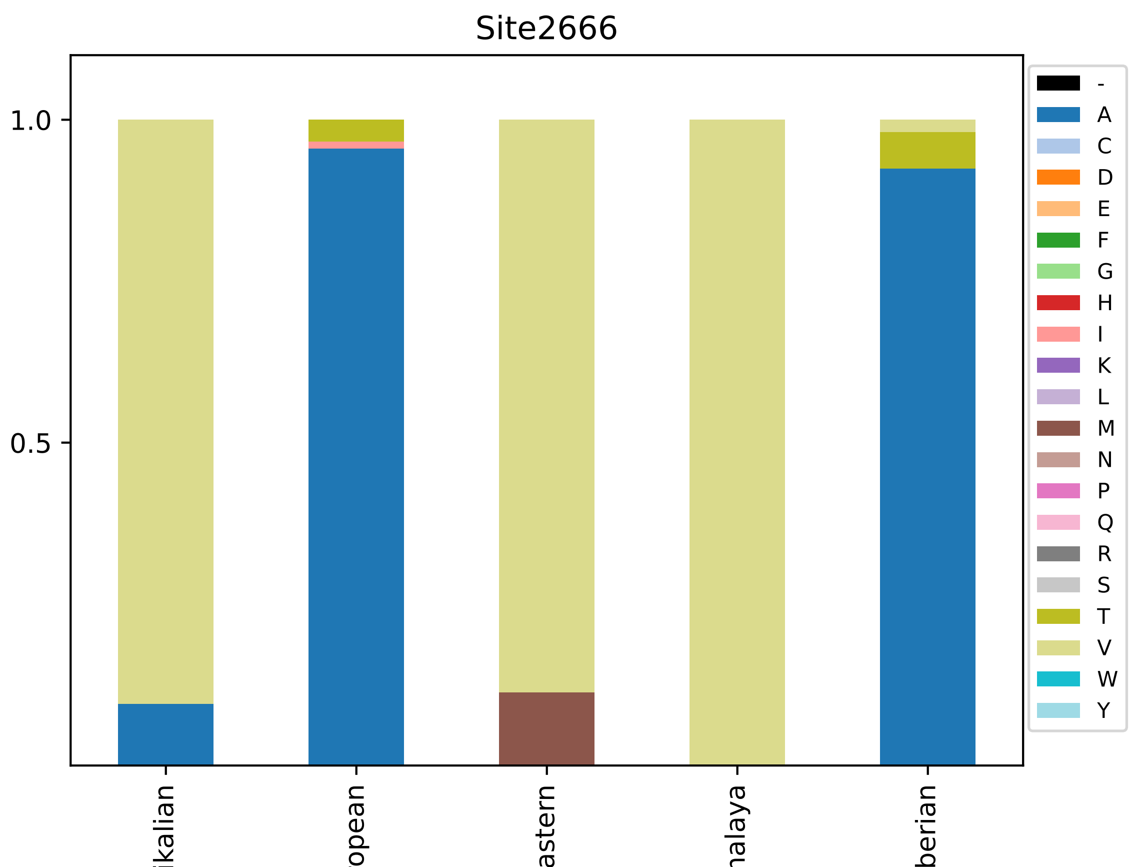
Site2666
1.0
0.5
-
A
C
D
E
F
G
H
I
K
L
M
N
P
Q
R
S
T
V
W
Y

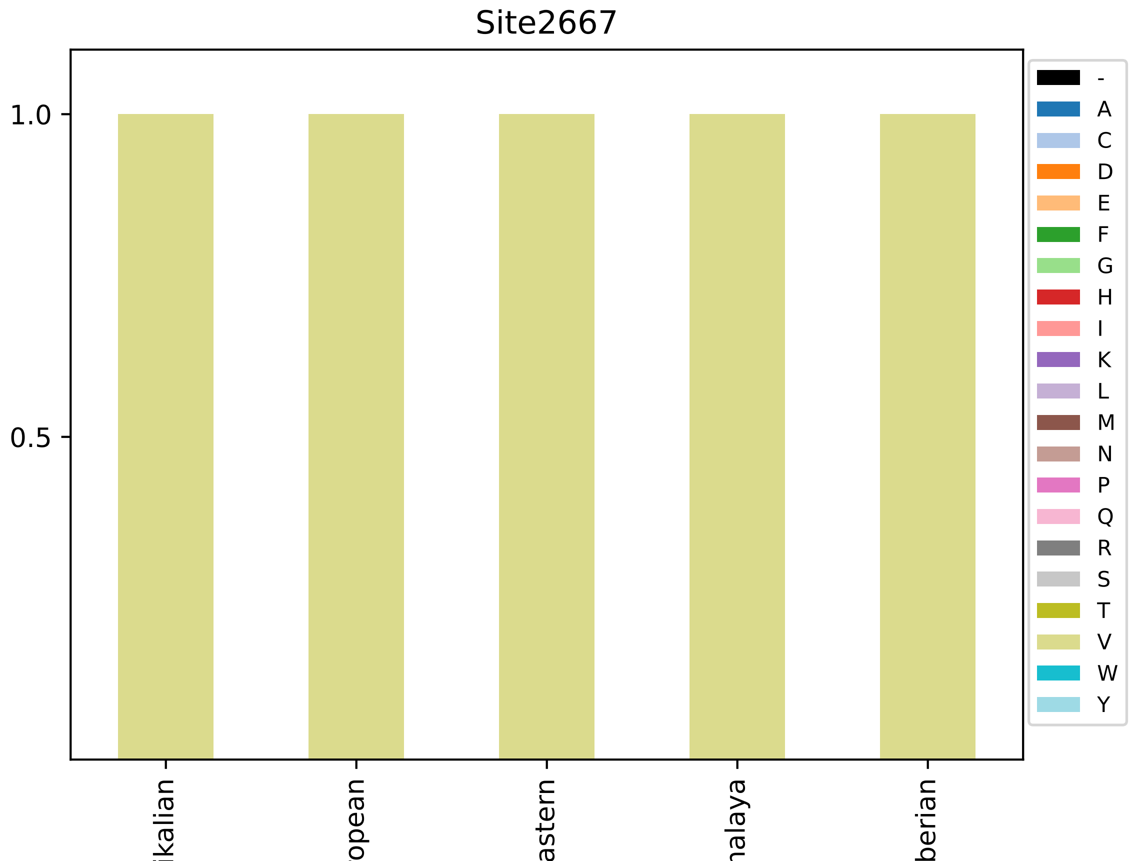
Site2667
1.0
0.5
-
A
C
D
E
F
G
H
I
K
L
M
N
P
Q
R
S
T
V
W
Y
ikalian
opean
astern
nalaya
berian

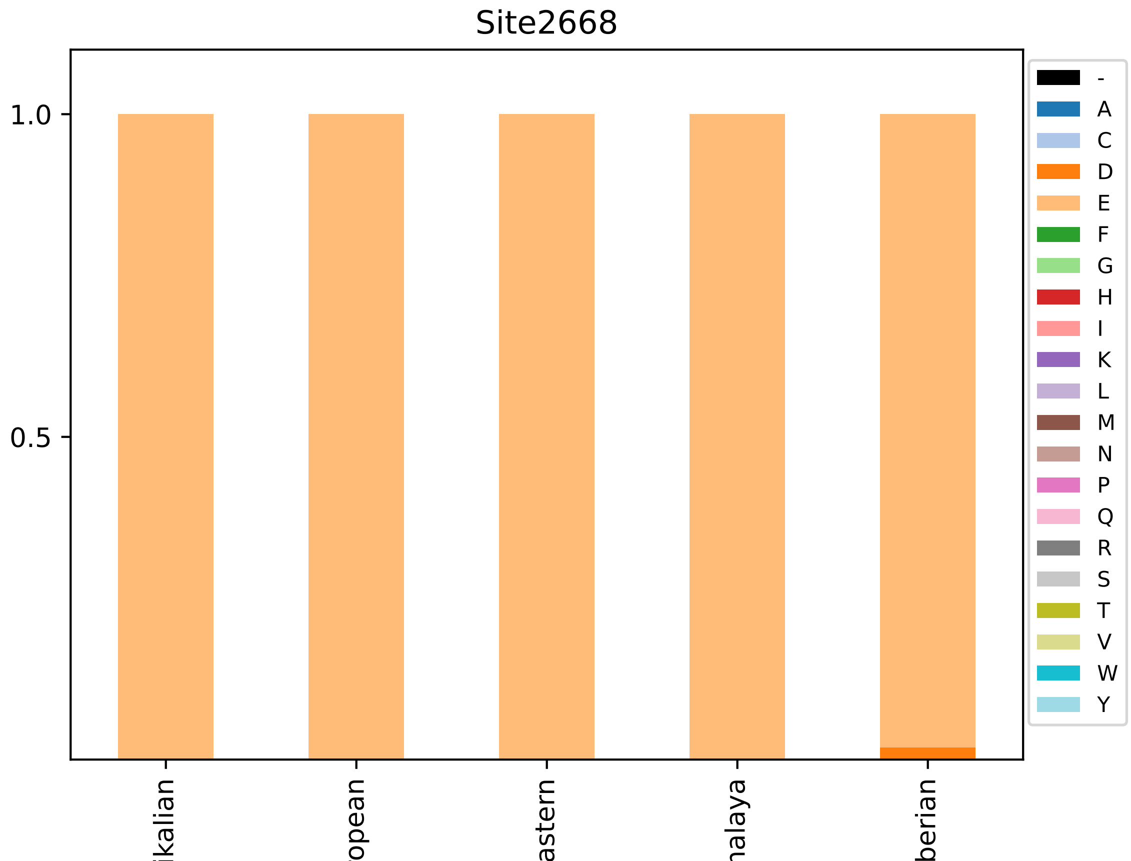

Site2668
1.0
0.5
-
A
C
D
E
F
G
H
I
K
L
M
N
P
Q
R
S
T
V
W
Y
ikalian
opean
astern
halaya
berian

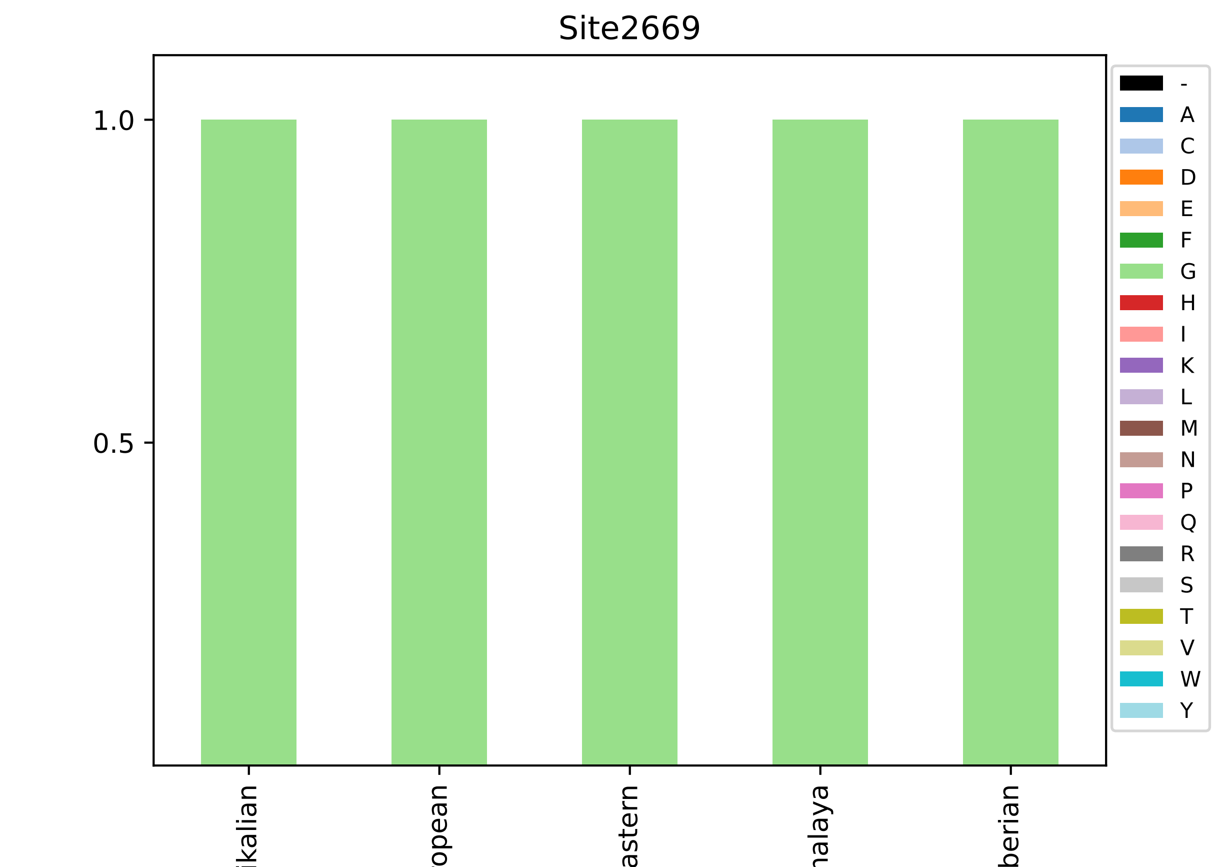

Site2669
1.0
0.5
-
A
C
D
E
F
G
H
I
K
L
M
N
P
Q
R
S
T
V
W
Y
ikalian
opean
astern
nalaya
berian

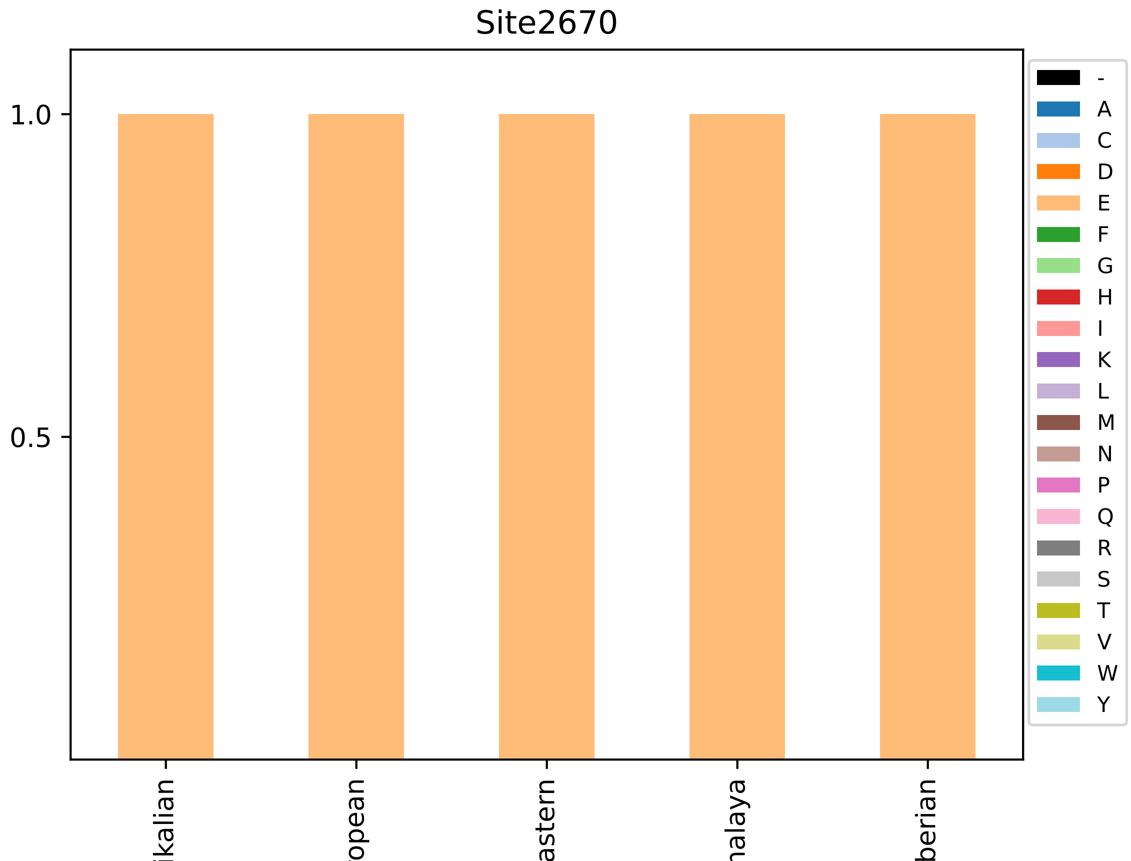
Site2670
1.0
0.5
-
A
C
D
E
F
G
H
I
K
L
M
N
P
Q
R
S
T
V
W
Y
ikalian
opean
astern
nalaya
berian

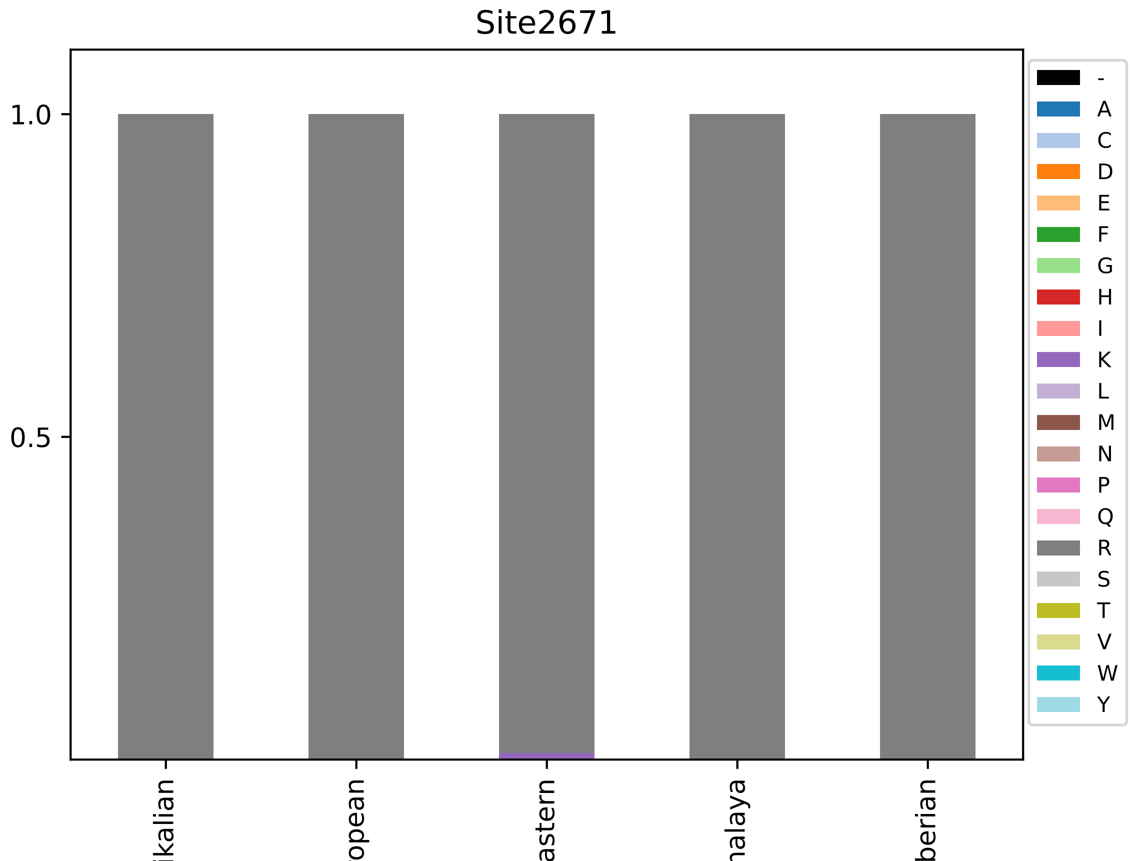

Site2671
1.0
0.5
-
A
C
D
E
F
G
H
I
K
L
M
N
P
Q
R
S
T
V
W
Y
ikalian
opean
astern
nalaya
berian

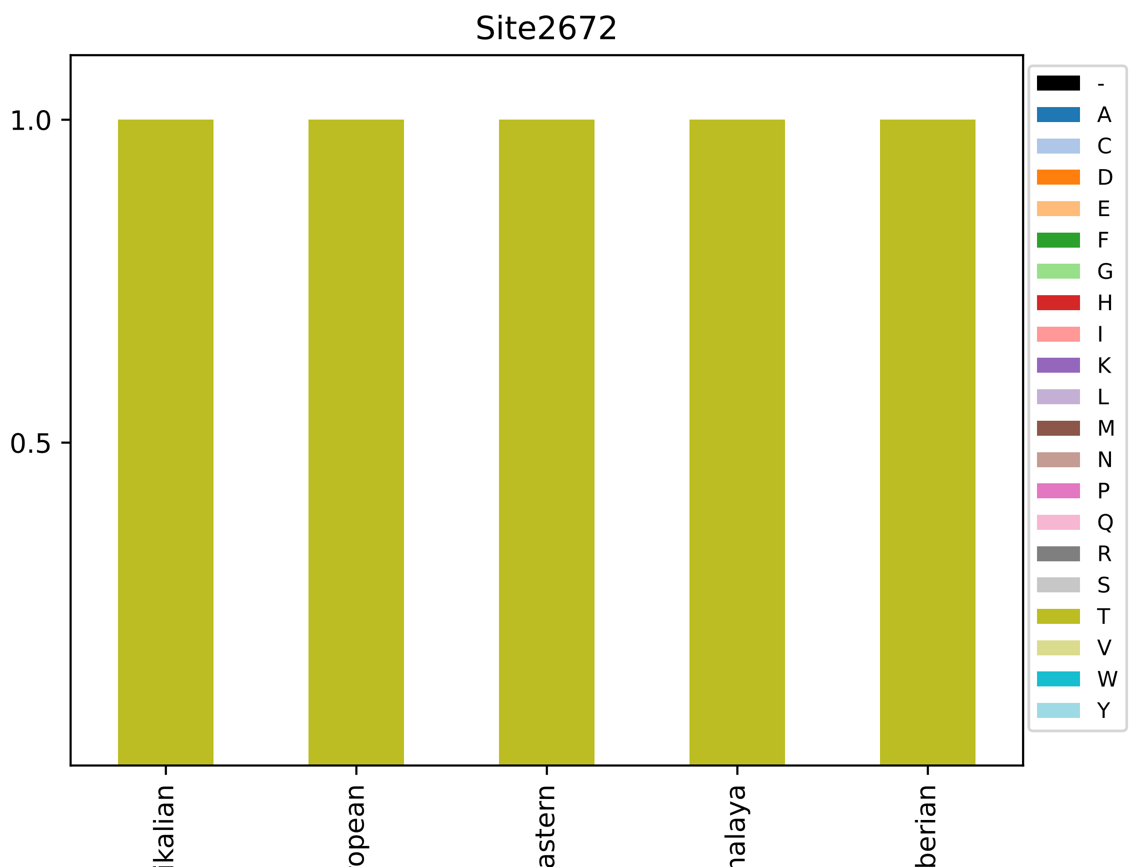
Site2672
1.0
0.5
-
A
C
D
E
F
G
H
I
K
L
M
N
P
Q
R
S
T
V
W
Y
ikalian
opean
astern
nalaya
berian

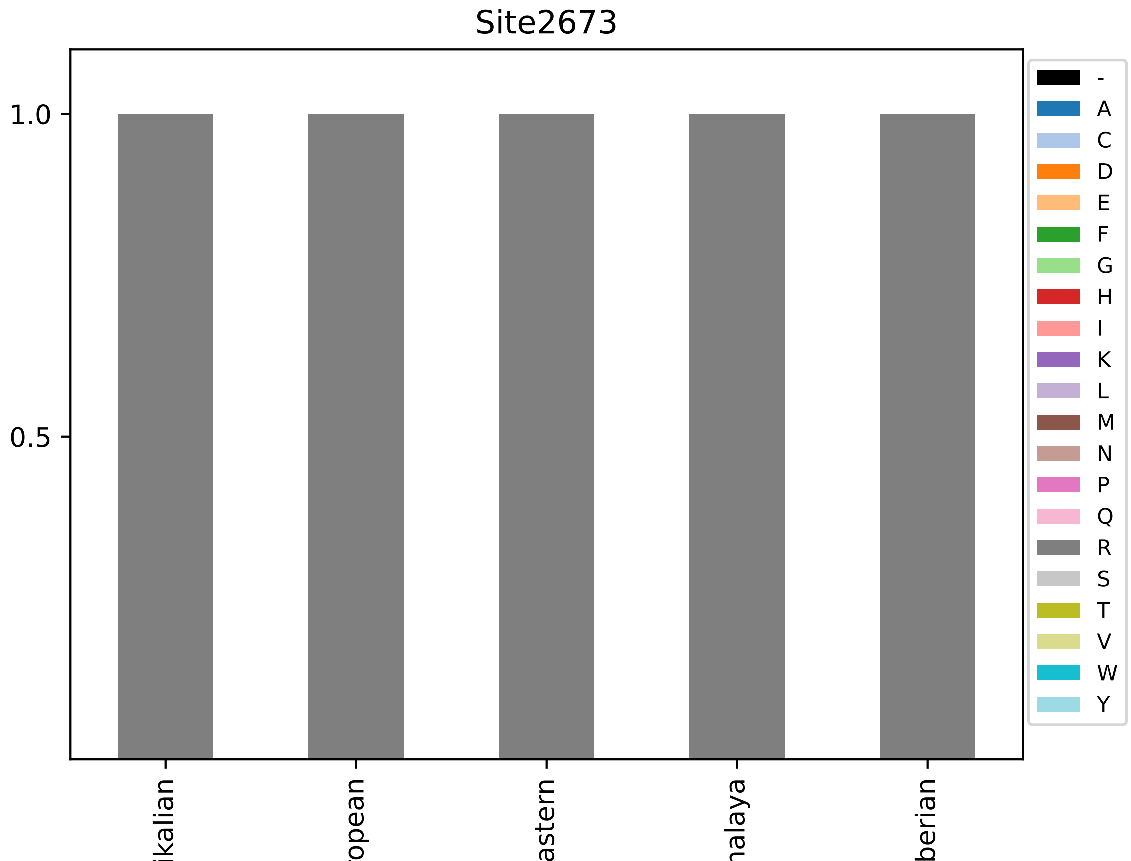

Site2673
1.0
0.5
-
A
C
D
E
F
G
H
I
K
L
M
N
P
Q
R
S
T
V
W
Y

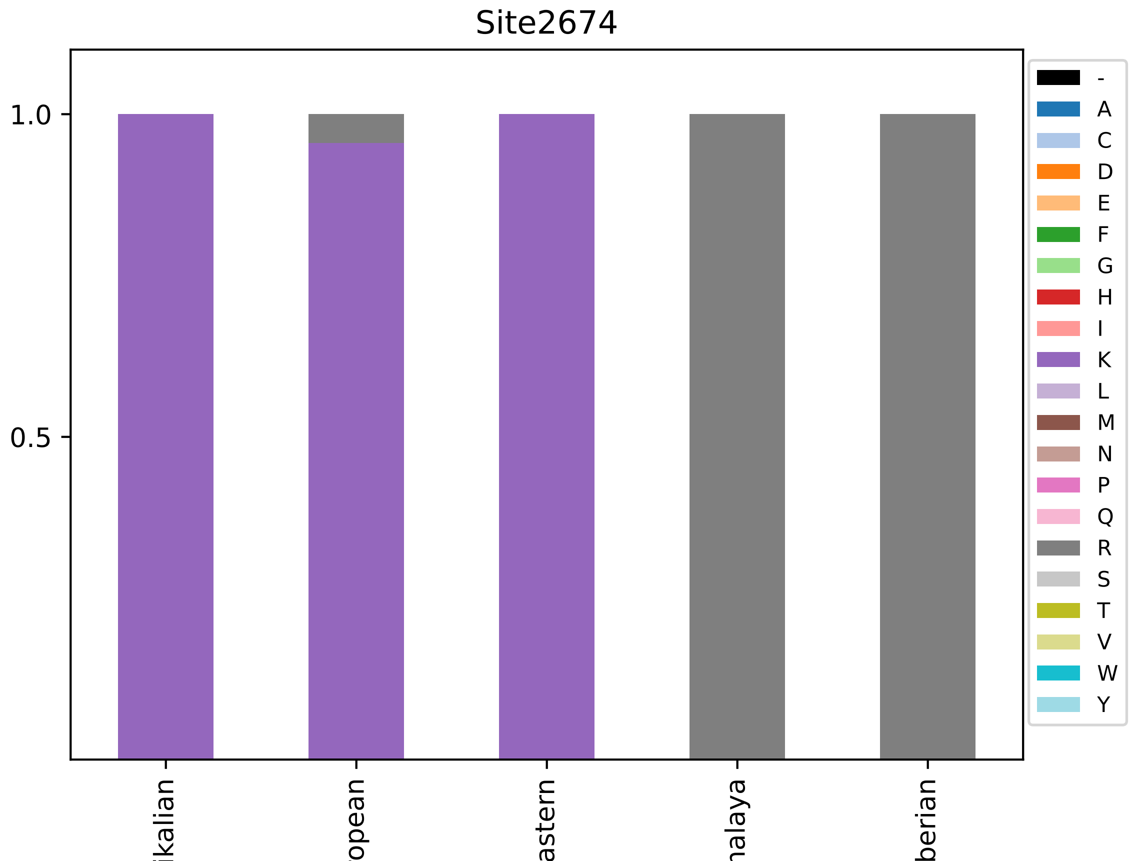
Site2674
1.0
0.5
-
A
C
D
E
F
G
H
I
K
L
M
N
P
Q
R
S
T
V
W
Y
ikalian
opean
astern
nalaya
berian

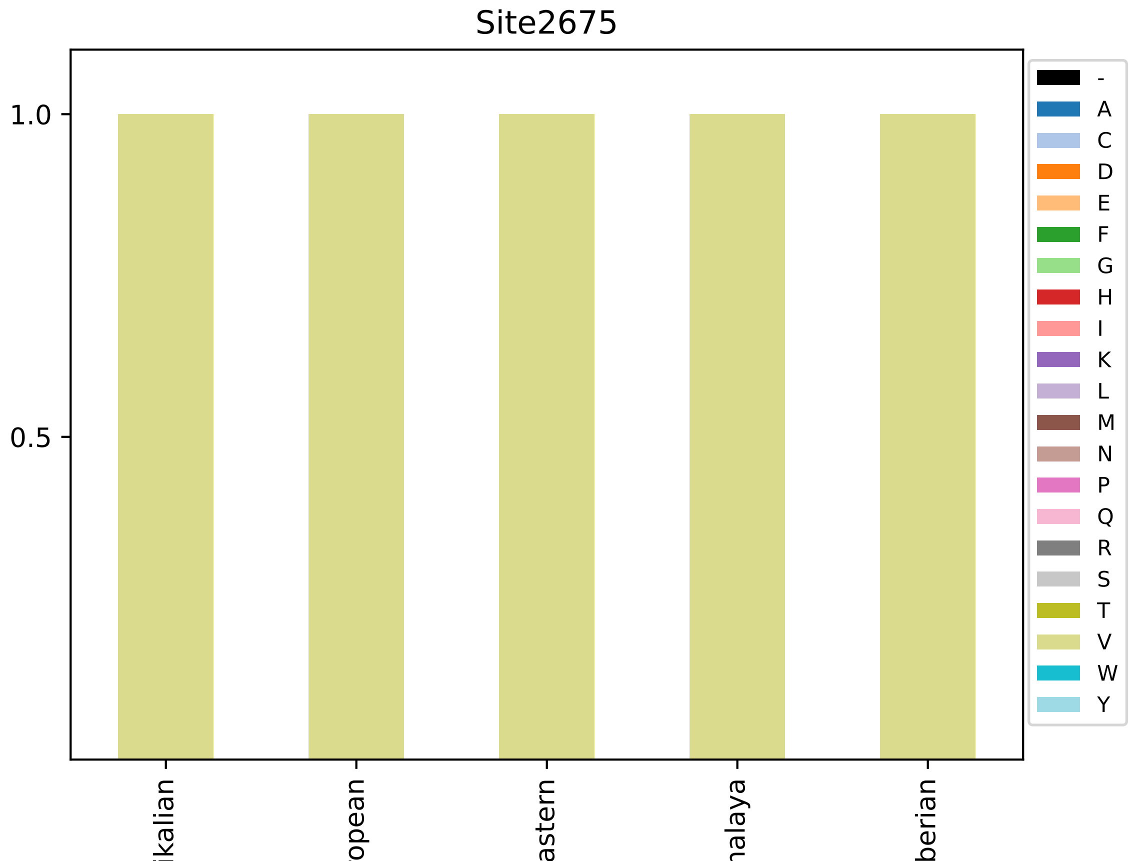
Site2675
1.0
0.5
ikalian
opean
astern
nalaya
berian
-
A
C
D
E
F
G
H
I
K
L
M
N
P
Q
R
S
T
V
W
Y

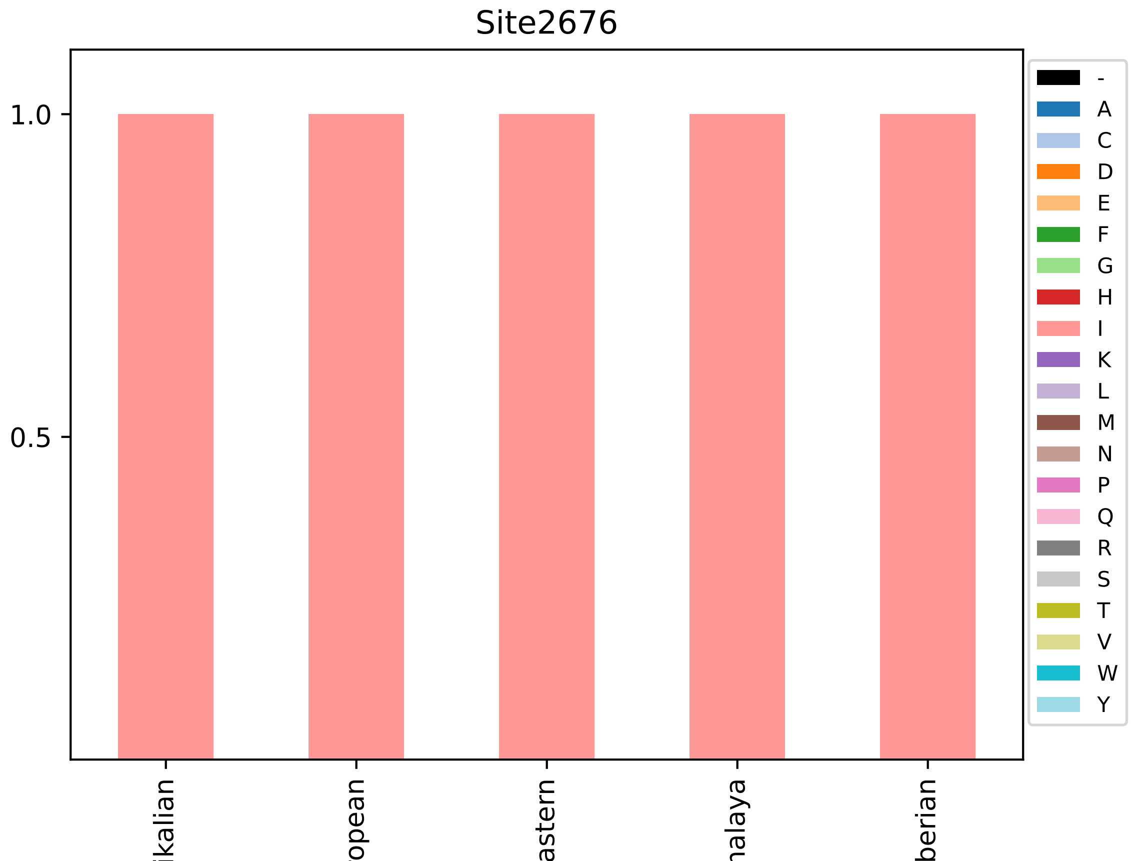
Site2676
1.0
0.5
ikalian
opean
astern
nalaya
berian
-
A
C
D
E
F
G
H
I
K
L
M
N
P
Q
R
S
T
V
W
Y

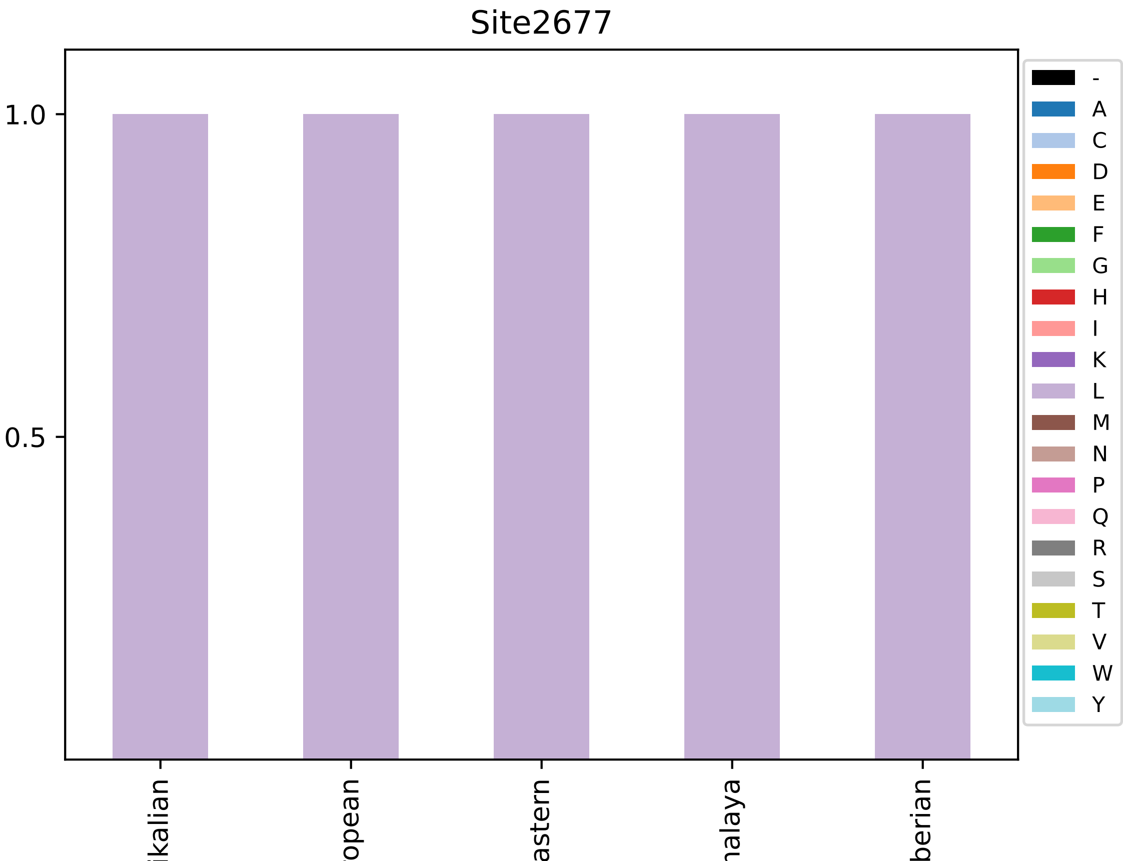
Site2677
1.0
0.5
-
A
C
D
E
F
G
H
I
K
L
M
N
P
Q
R
S
T
V
W
Y
ikalian
opean
astern
nalaya
berian

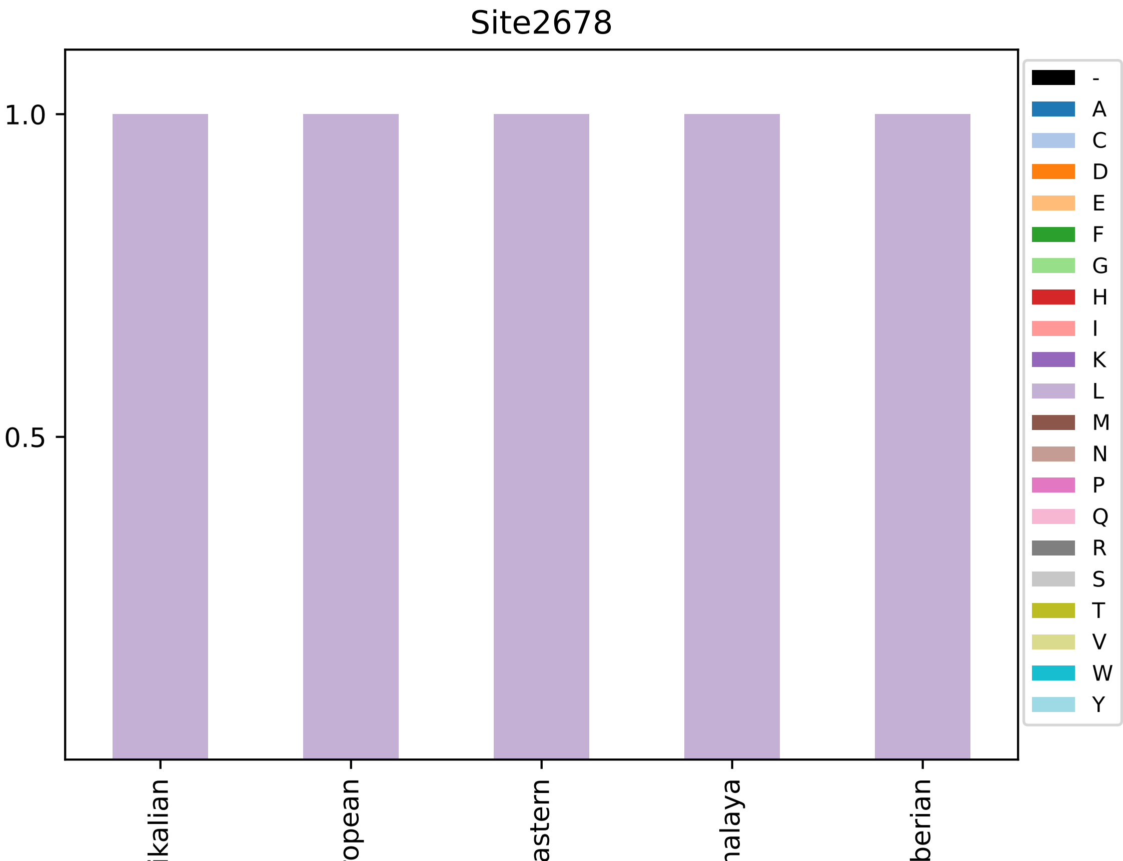

Site2678
1.0
0.5
-
A
C
D
E
F
G
H
I
K
L
M
N
P
Q
R
S
T
V
W
Y
ikalian
opean
astern
nalaya
berian

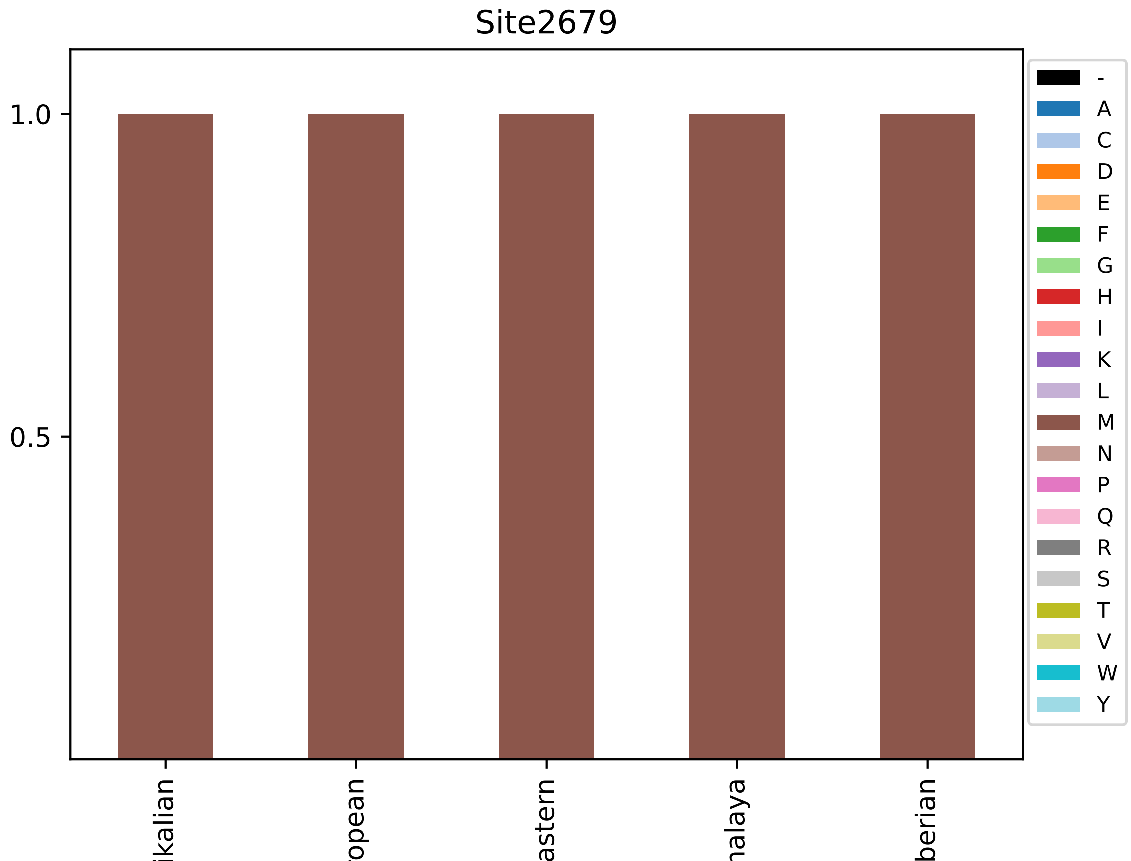
Site2679
1.0
0.5
-
A
C
D
E
F
G
H
I
K
L
M
N
P
Q
R
S
T
V
W
Y
ikalian
opean
astern
nalaya
berian

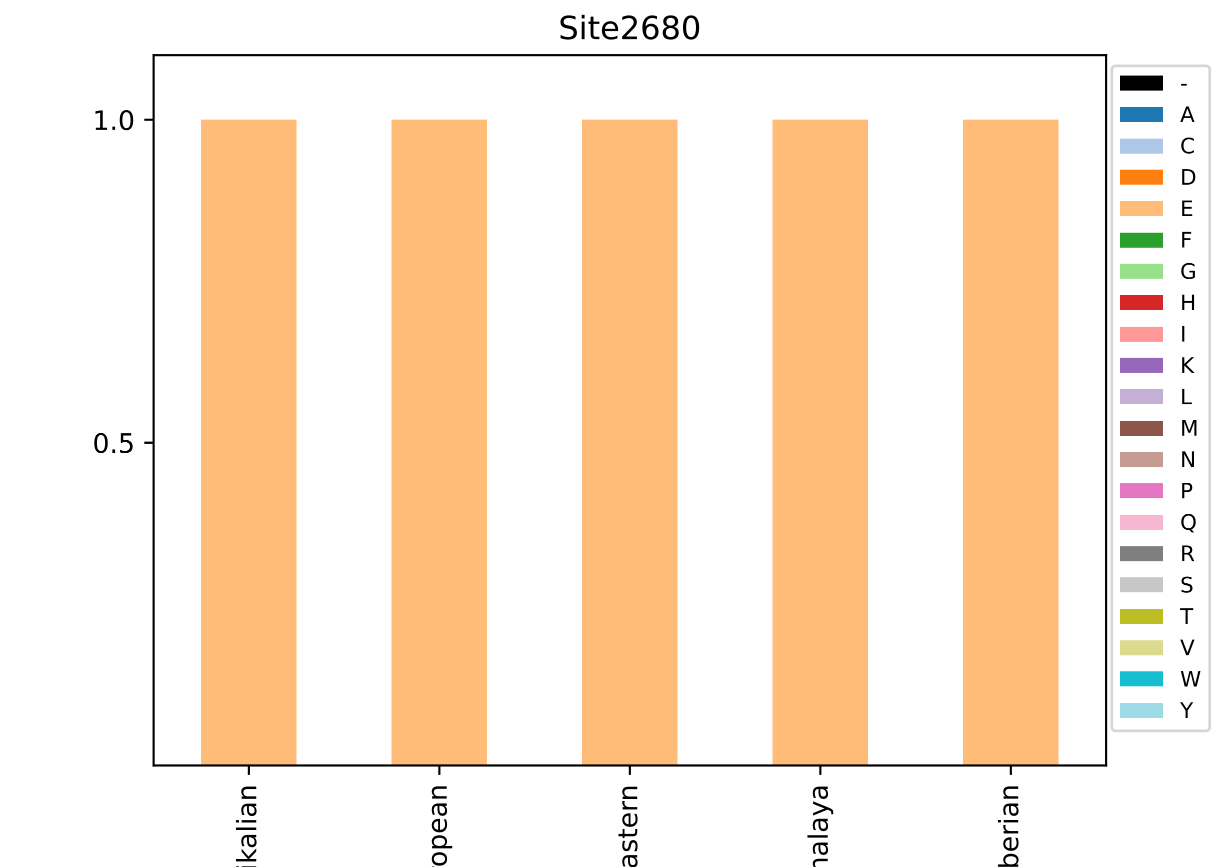

Site2680
1.0
0.5
-
A
C
D
E
F
G
H
I
K
L
M
N
P
Q
R
S
T
V
W
Y

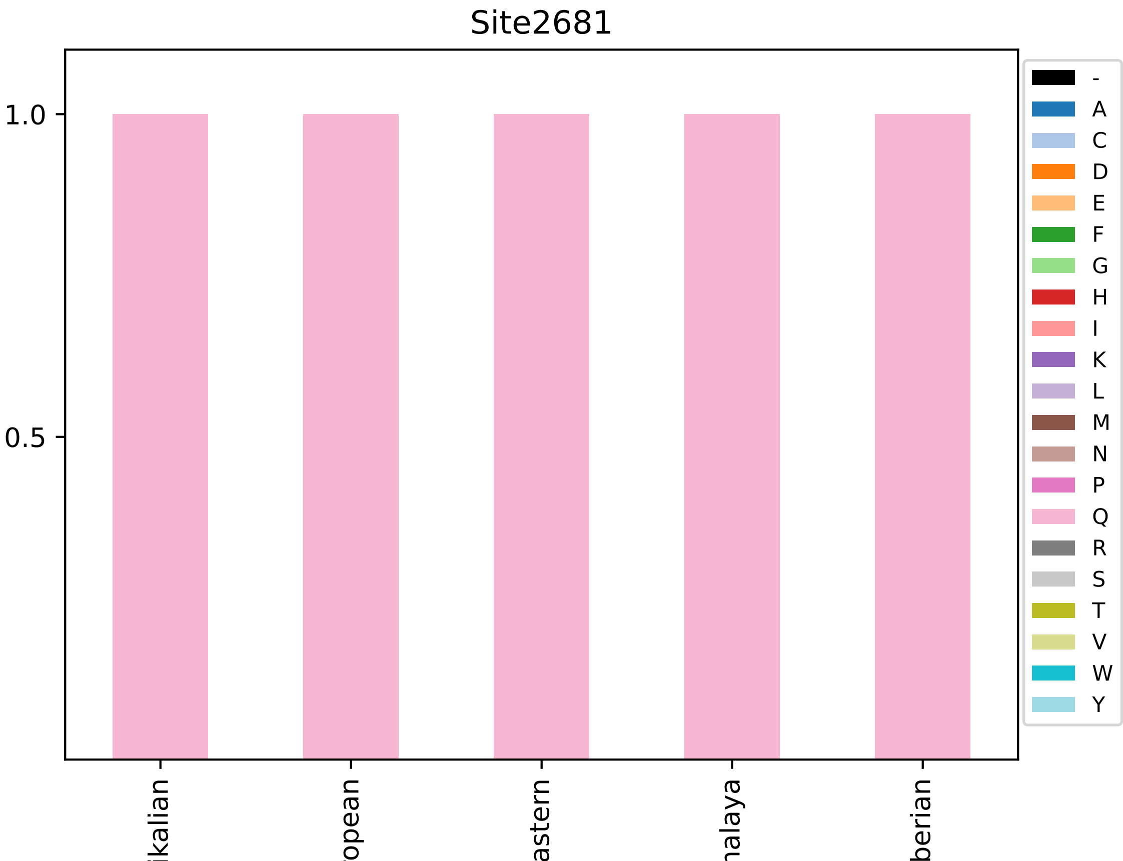

Site2681
1.0
0.5
-
A
C
D
E
F
G
H
I
K
L
M
N
P
Q
R
S
T
V
W
Y
ikalian
opean
astern
nalaya
berian

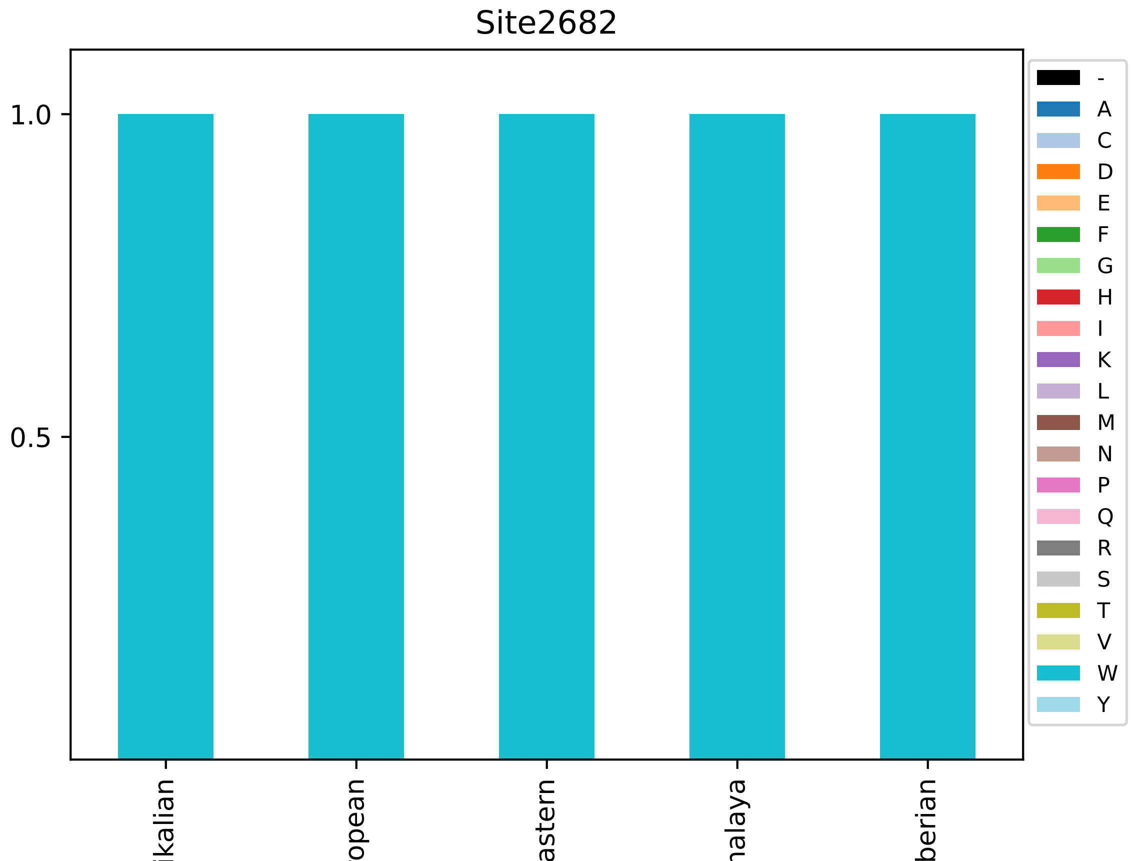
Site2682
1.0
0.5
-
A
C
D
E
F
G
H
I
K
L
M
N
P
Q
R
S
T
V
W
Y
ikalian
opean
astern
nalaya
berian

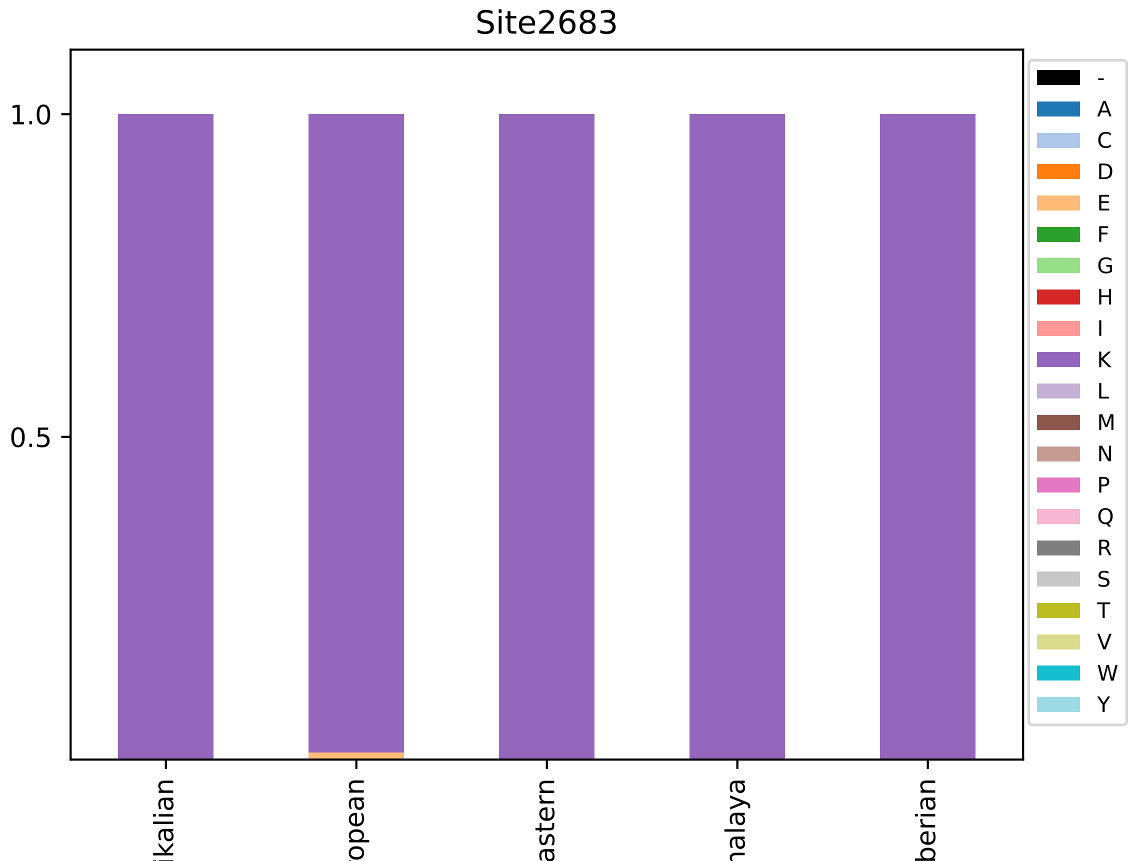
Site2683
1.0
0.5
-
A
C
D
E
F
G
H
I
K
L
M
N
P
Q
R
S
T
V
W
Y
ikalian
opean
astern
nalaya
berian

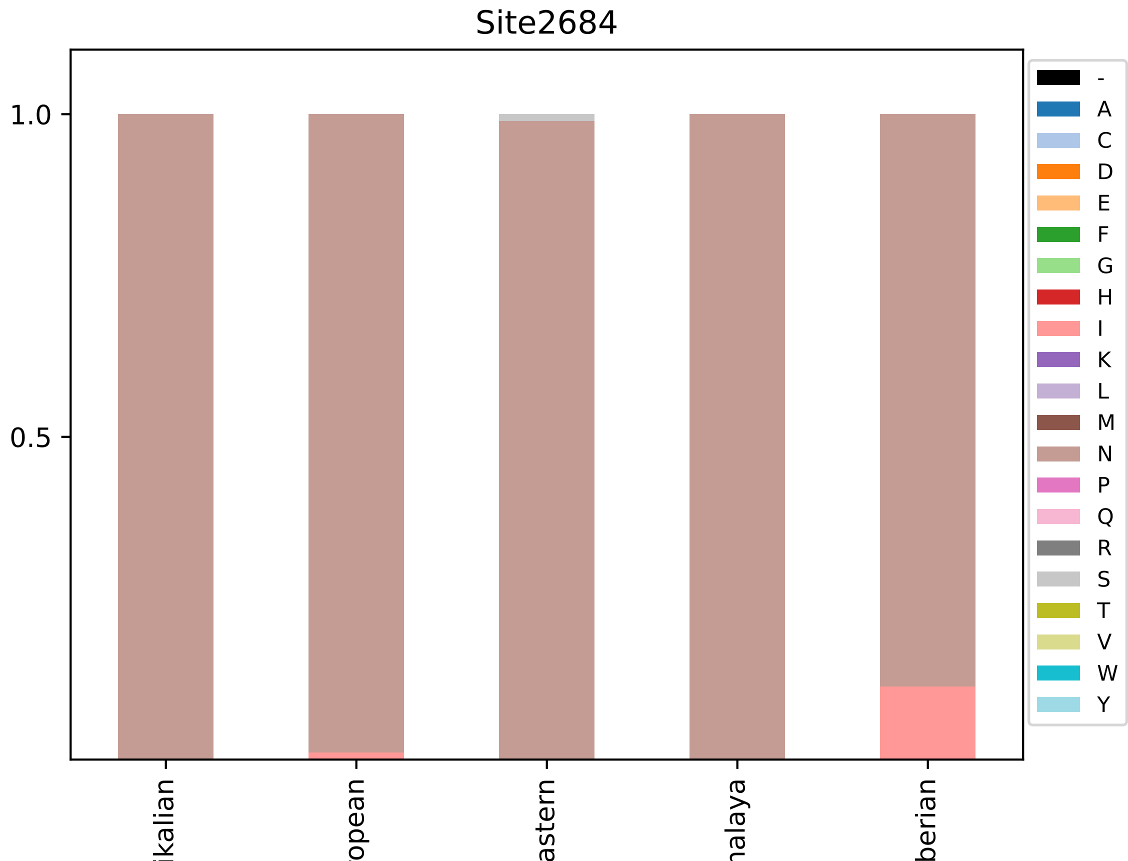
Site2684
1.0
0.5
-
A
C
D
E
F
G
H
I
K
L
M
N
P
Q
R
S
T
V
W
Y
ikalian
ropean
astern
nalaya
berian

Site2685

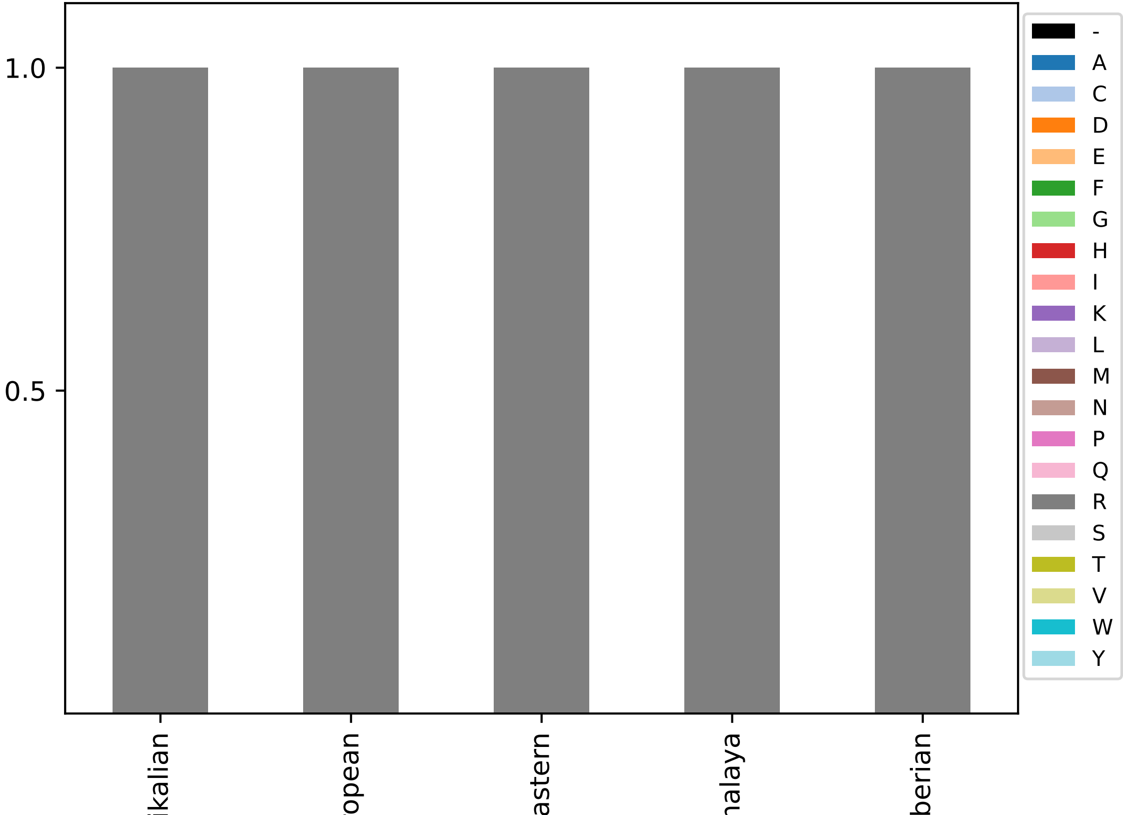

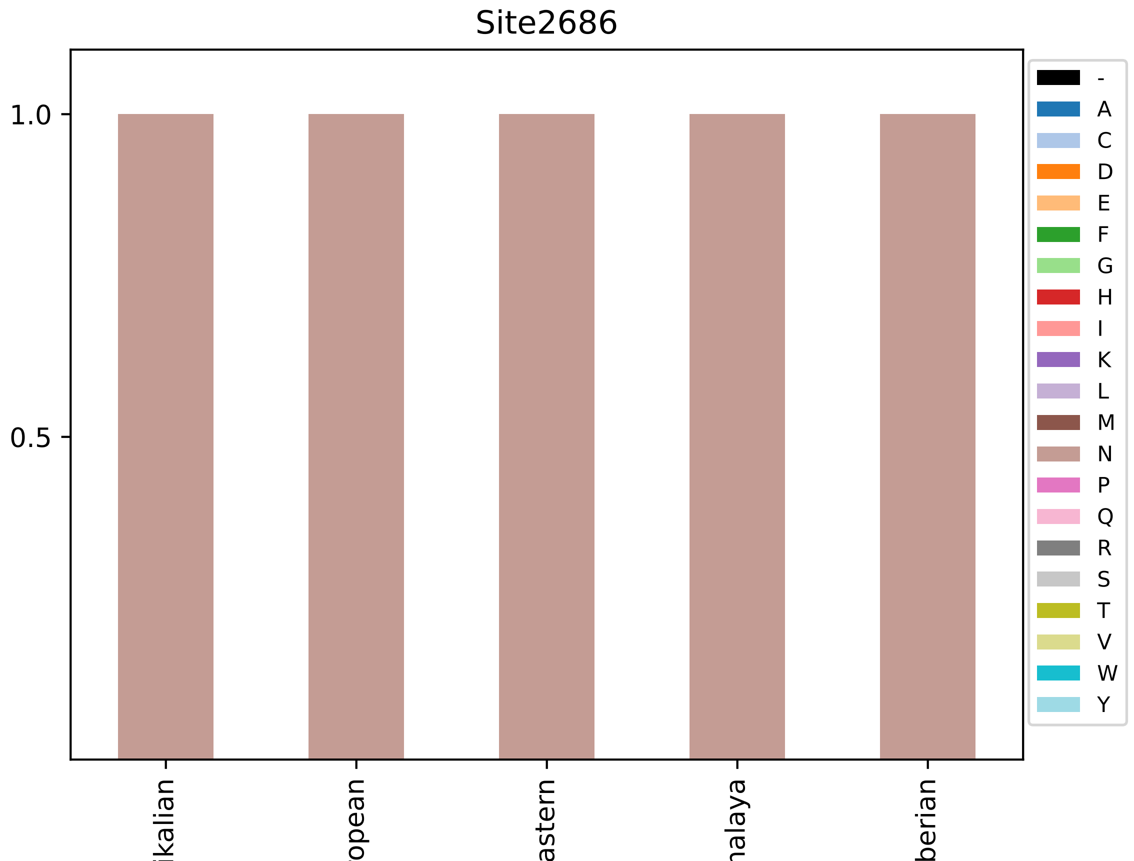

Site2686
1.0
0.5
-
A
C
D
E
F
G
H
I
K
L
M
N
P
Q
R
S
T
V
W
Y
ikalian
opean
astern
nalaya
berian

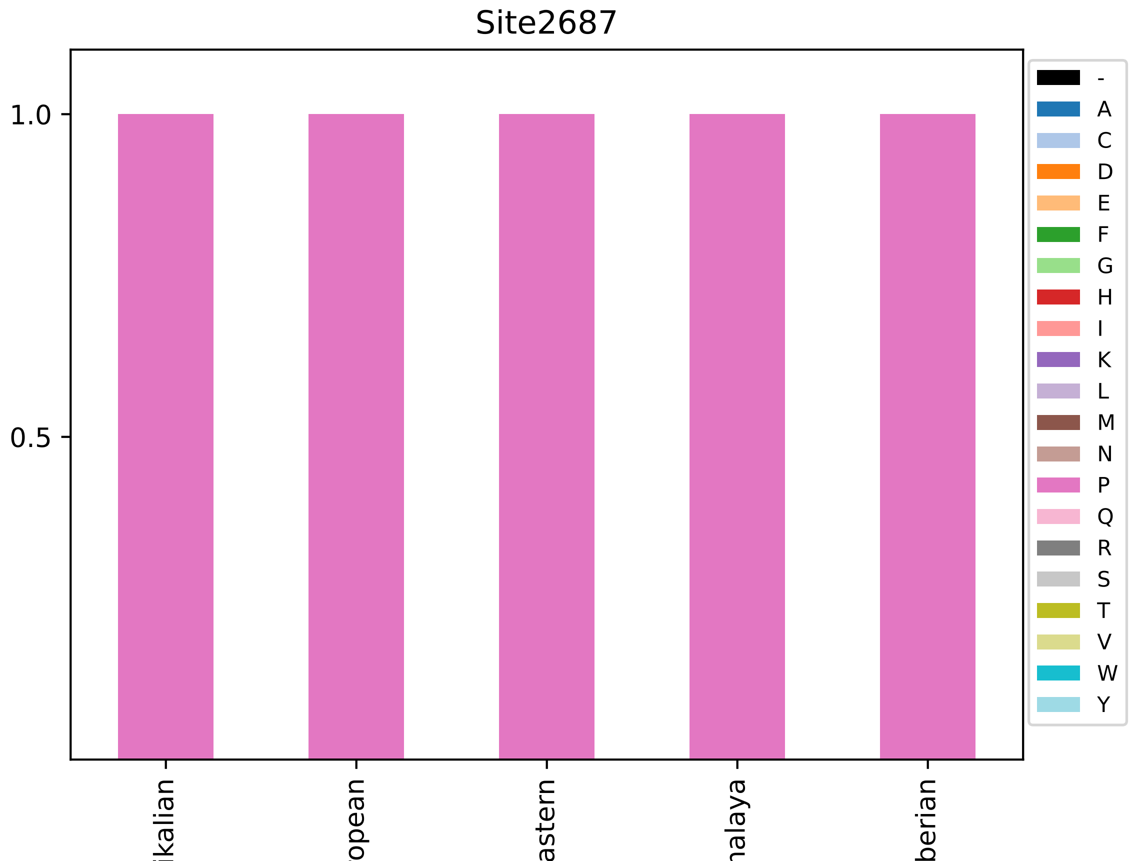

Site2687
1.0
0.5
-
A
C
D
E
F
G
H
I
K
L
M
N
P
Q
R
S
T
V
W
Y
ikalian
opean
astern
nalaya
berian

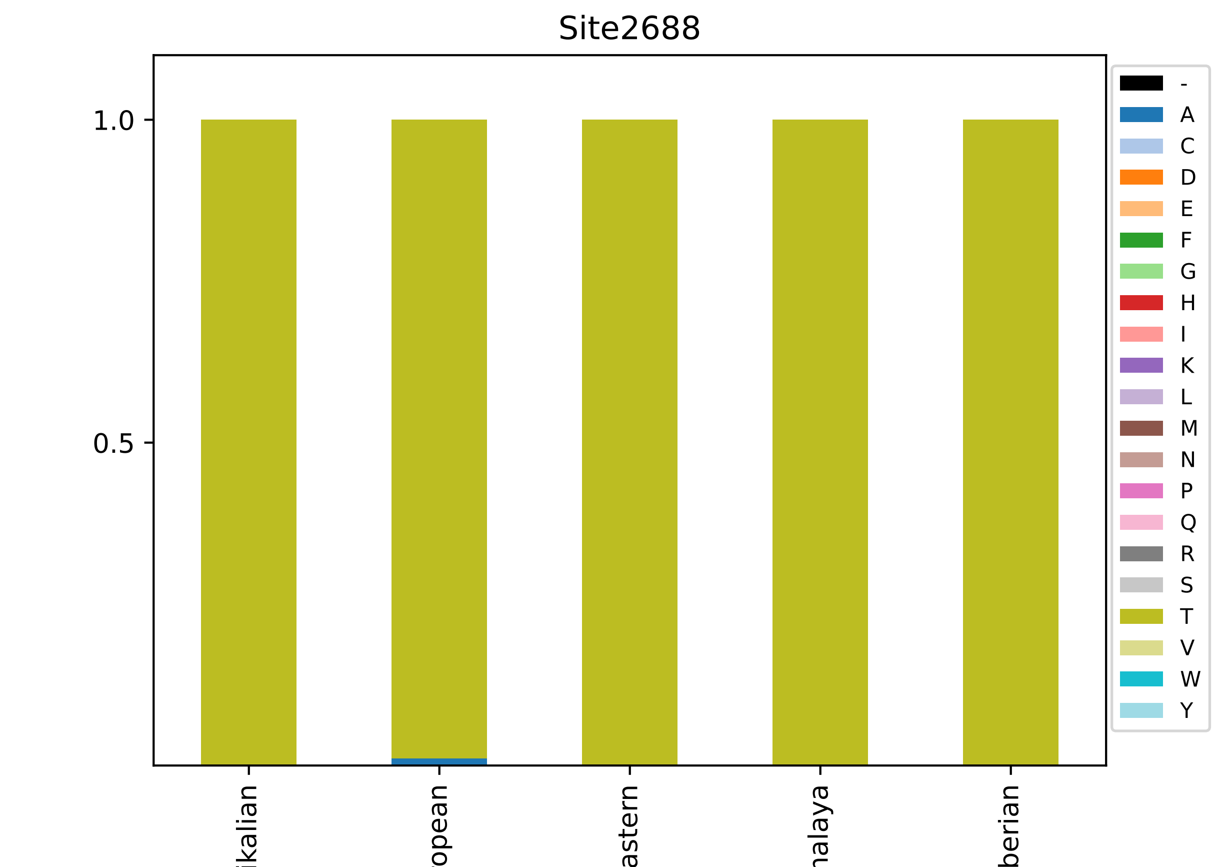

Site2688
1.0
0.5
-
A
C
D
E
F
G
H
I
K
L
M
N
P
Q
R
S
T
V
W
Y
ikalian
opean
astern
nalaya
berian

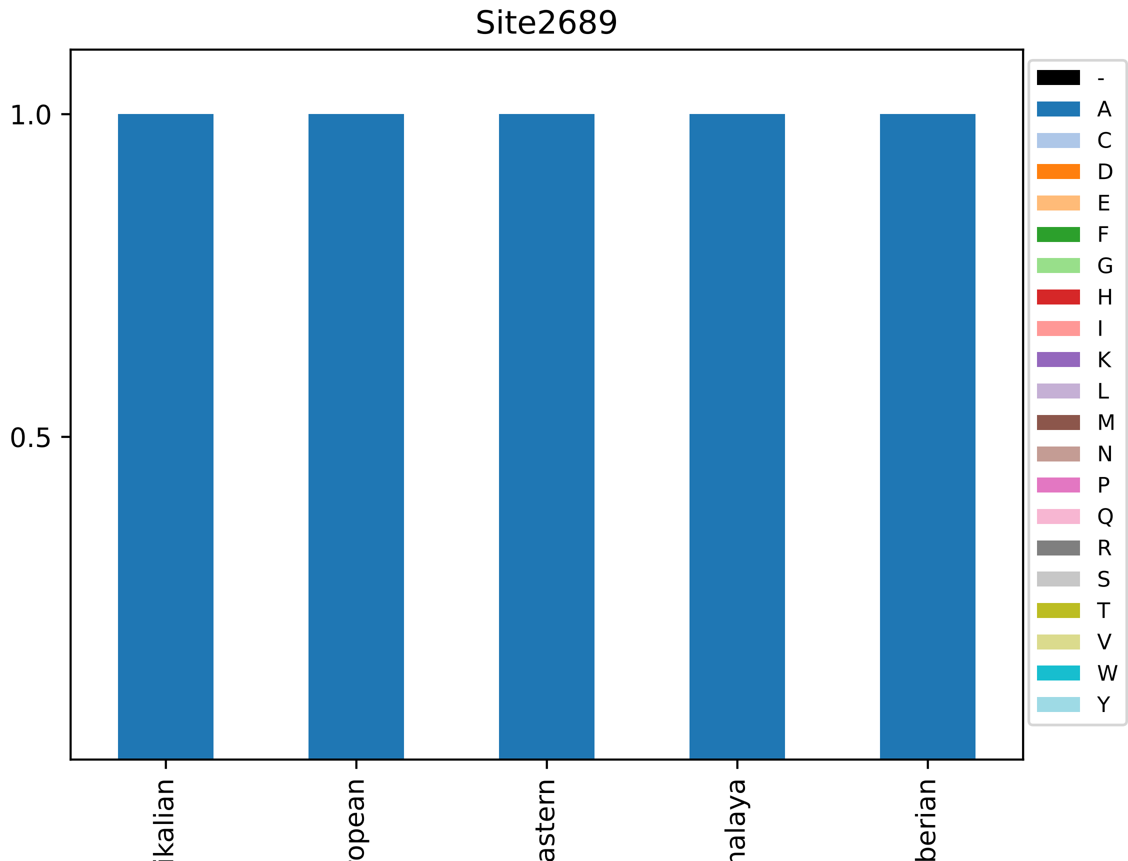
Site2689
1.0
0.5
ikalian
opean
astern
nalaya
berian
-
A
C
D
E
F
G
H
I
K
L
M
N
P
Q
R
S
T
V
W
Y

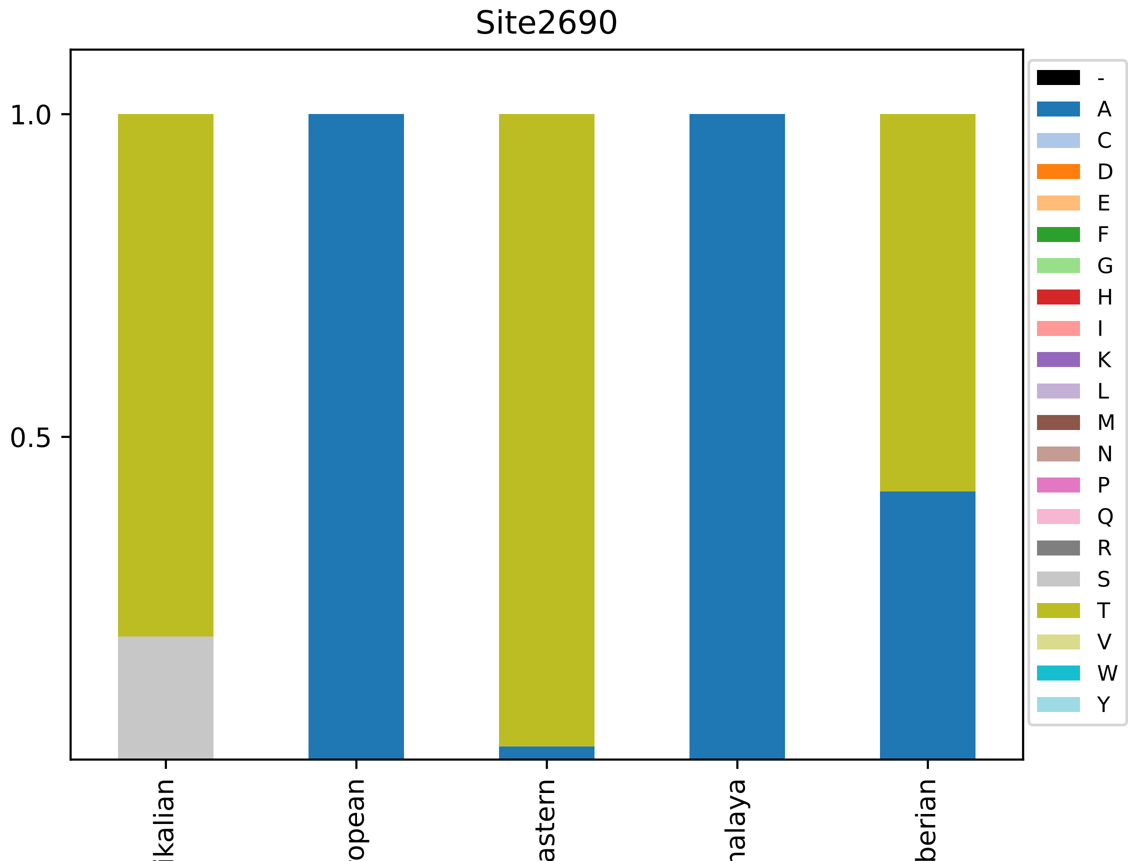
Site2690
1.0
0.5
ikalian
opean
astern
nalaya
berian
-
A
C
D
E
F
G
H
I
K
L
M
N
P
Q
R
S
T
V
W
Y

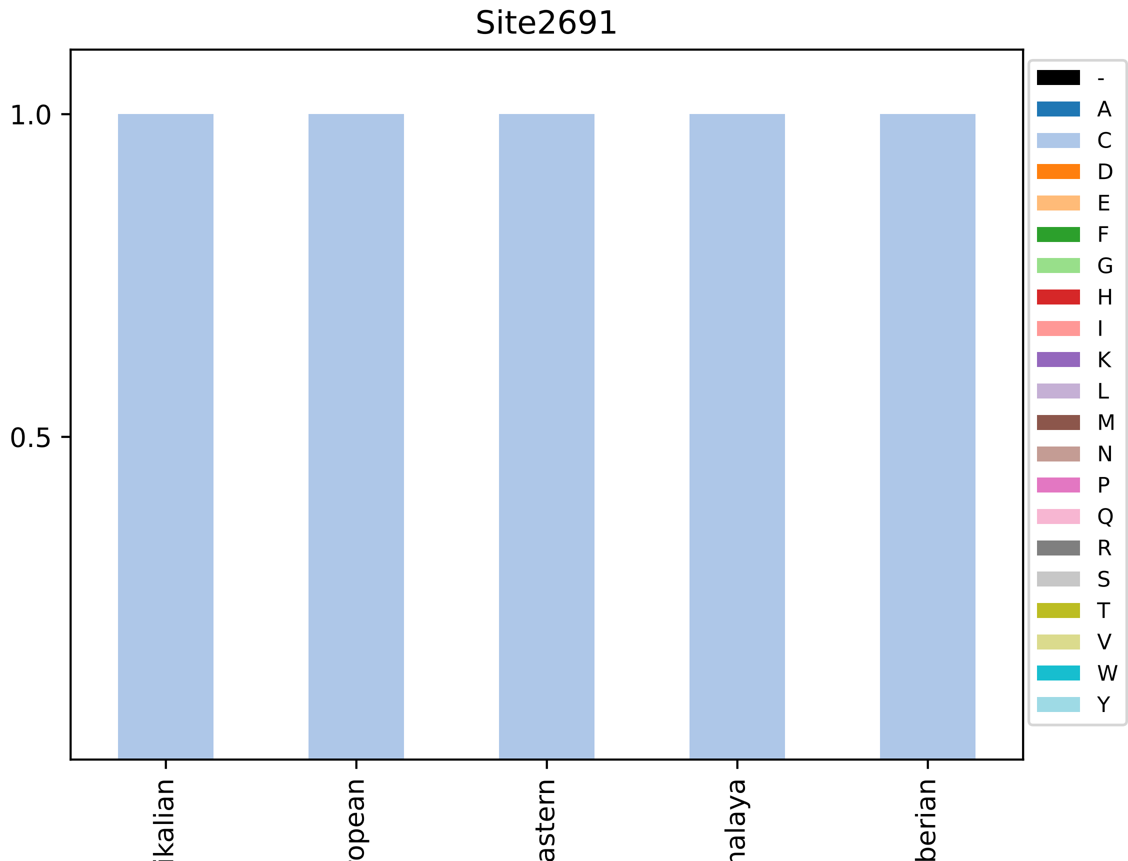
Site2691
1.0
0.5
-
A
C
D
E
F
G
H
I
K
L
M
N
P
Q
R
S
T
V
W
Y
ikalian
opean
astern
nalaya
berian

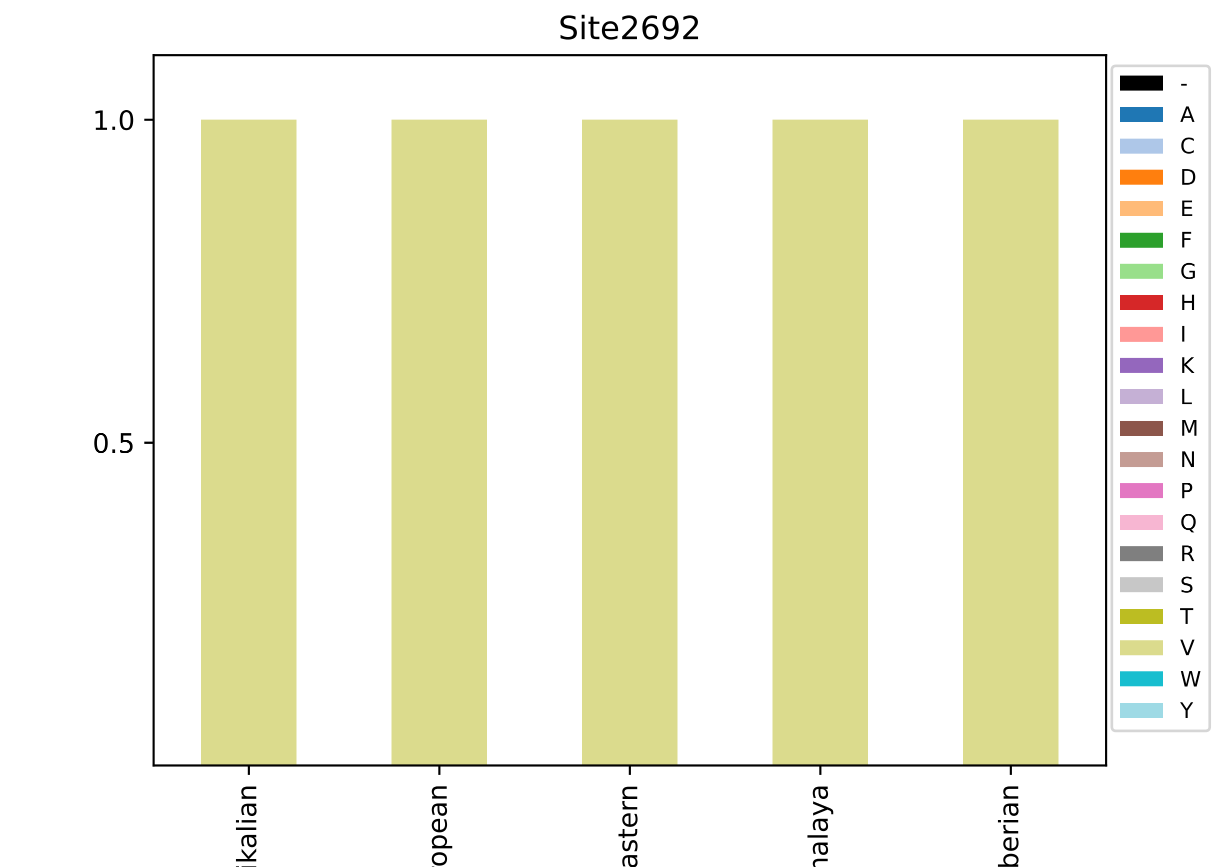

Site2692
1.0
0.5
-
A
C
D
E
F
G
H
I
K
L
M
N
P
Q
R
S
T
V
W
Y
ikalian
opean
astern
nalaya
berian

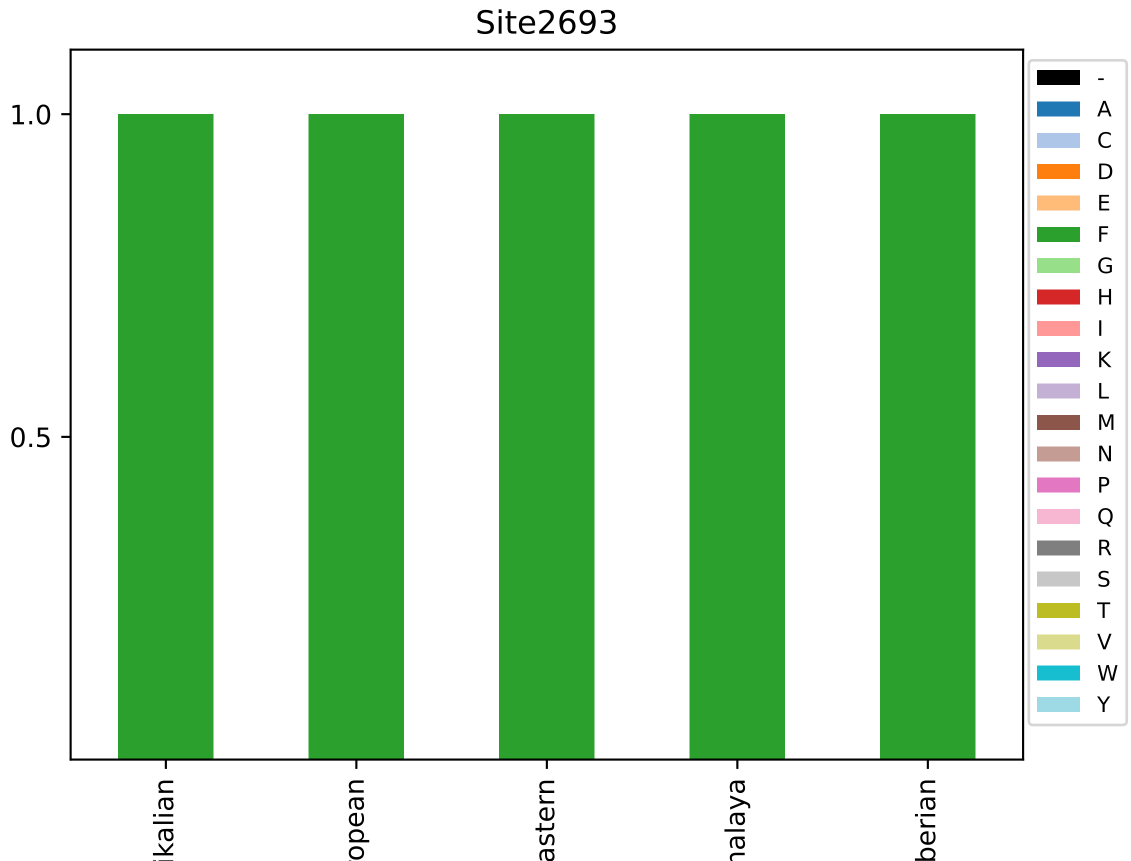

Site2693
1.0
0.5
ikalian
opean
astern
nalaya
berian
-
A
C
D
E
F
G
H
I
K
L
M
N
P
Q
R
S
T
V
W
Y

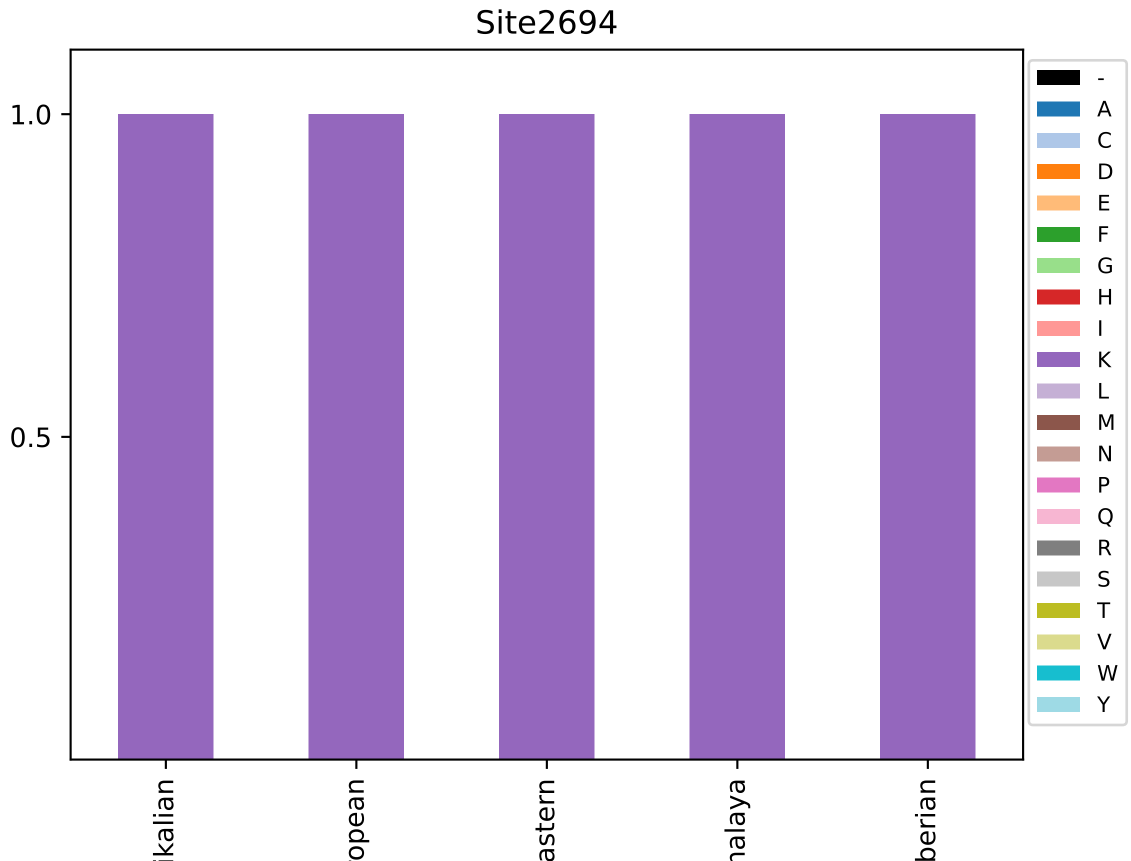

Site2694
1.0
0.5
-
A
C
D
E
F
G
H
I
K
L
M
N
P
Q
R
S
T
V
W
Y
ikalian
opean
astern
nalaya
berian

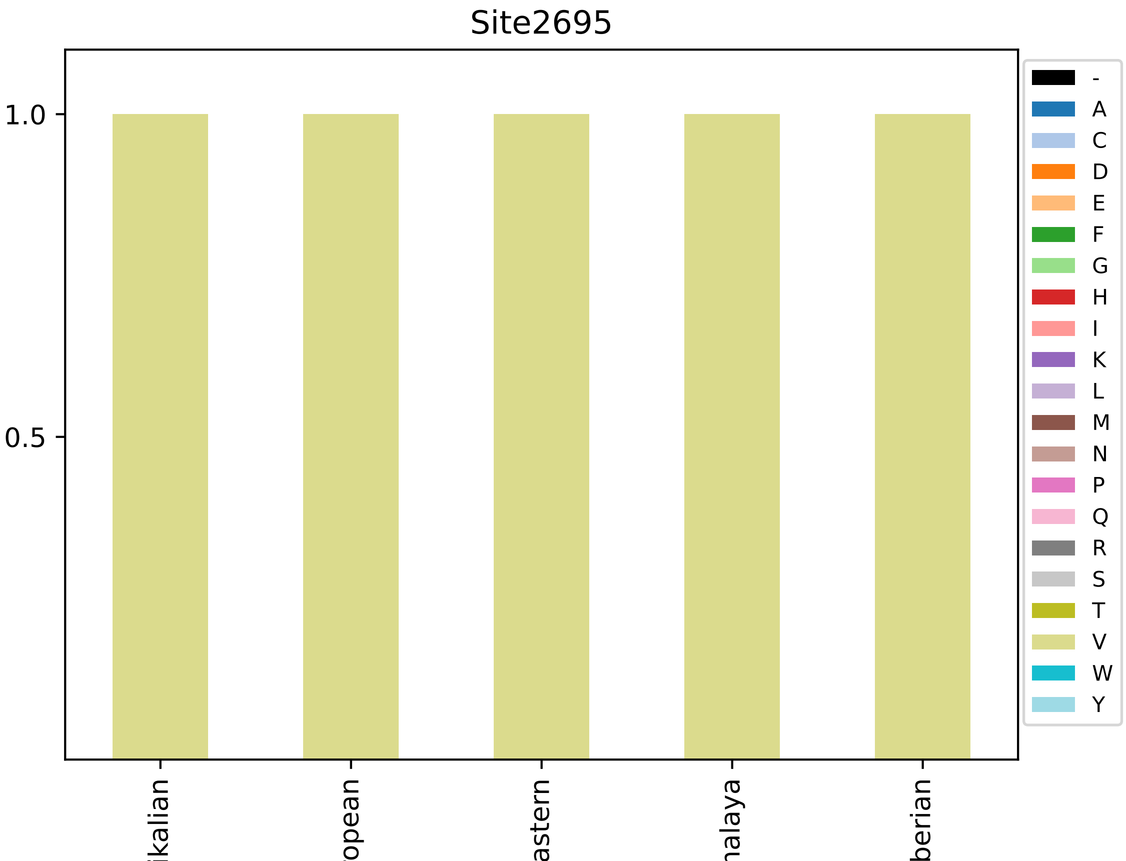
Site2695
1.0
0.5
-
A
C
D
E
F
G
H
I
K
L
M
N
P
Q
R
S
T
V
W
Y
ikalian
opean
astern
nalaya
berian

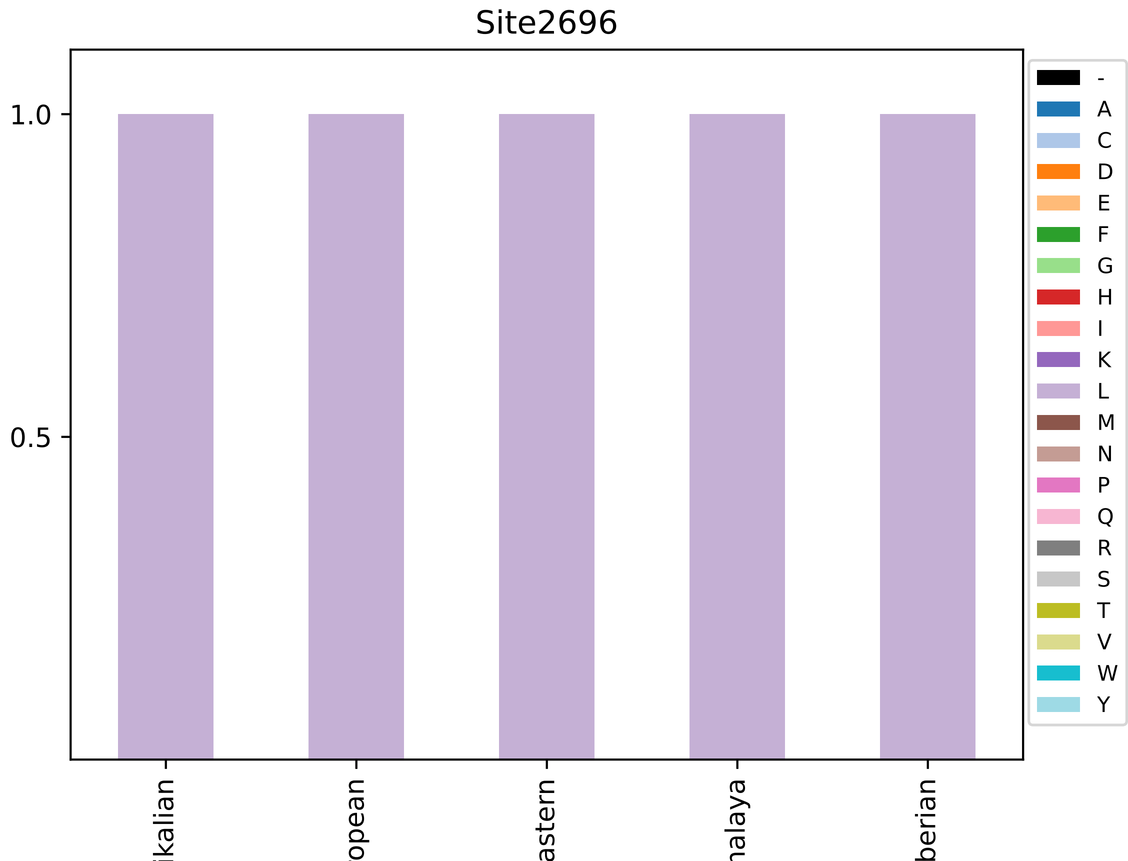

Site2696
1.0
0.5
ikalian
opean
astern
nalaya
berian
-
A
C
D
E
F
G
H
I
K
L
M
N
P
Q
R
S
T
V
W
Y

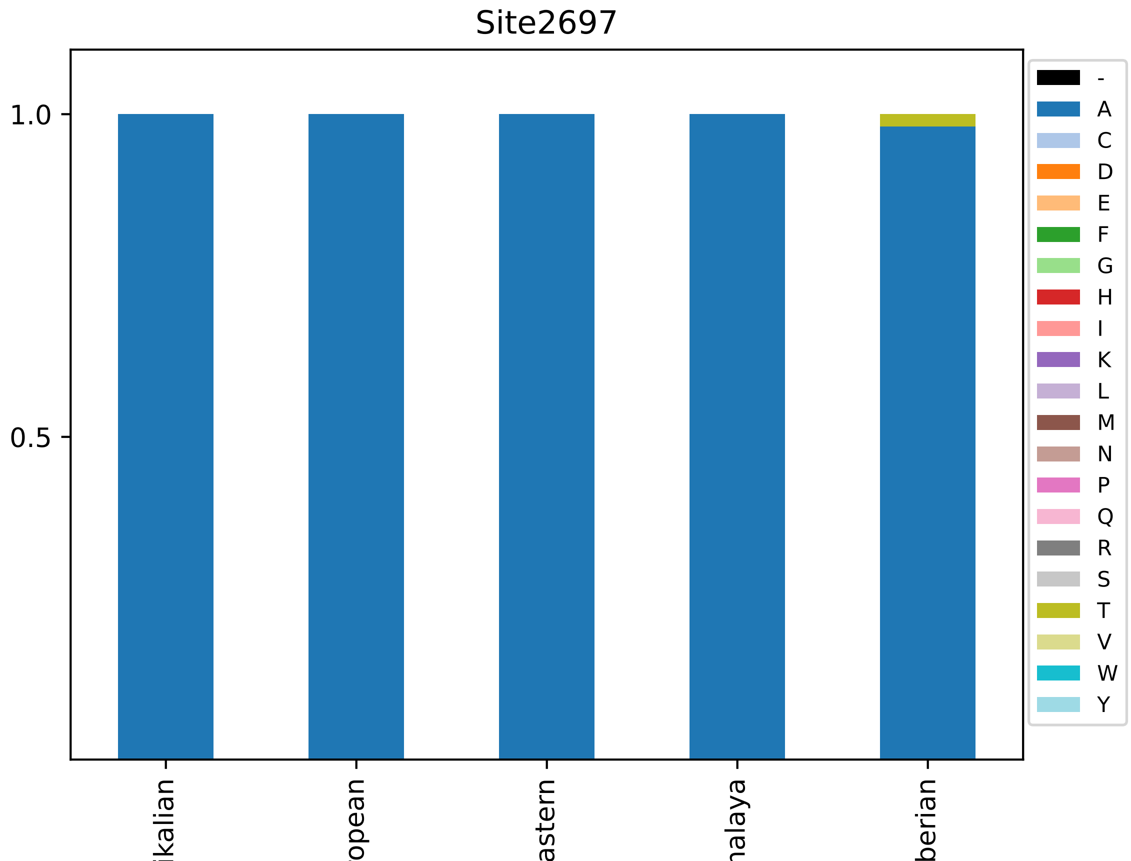
Site2697
1.0
0.5
-
A
C
D
E
F
G
H
I
K
L
M
N
P
Q
R
S
T
V
W
Y
ikalian
opean
astern
nalaya
berian

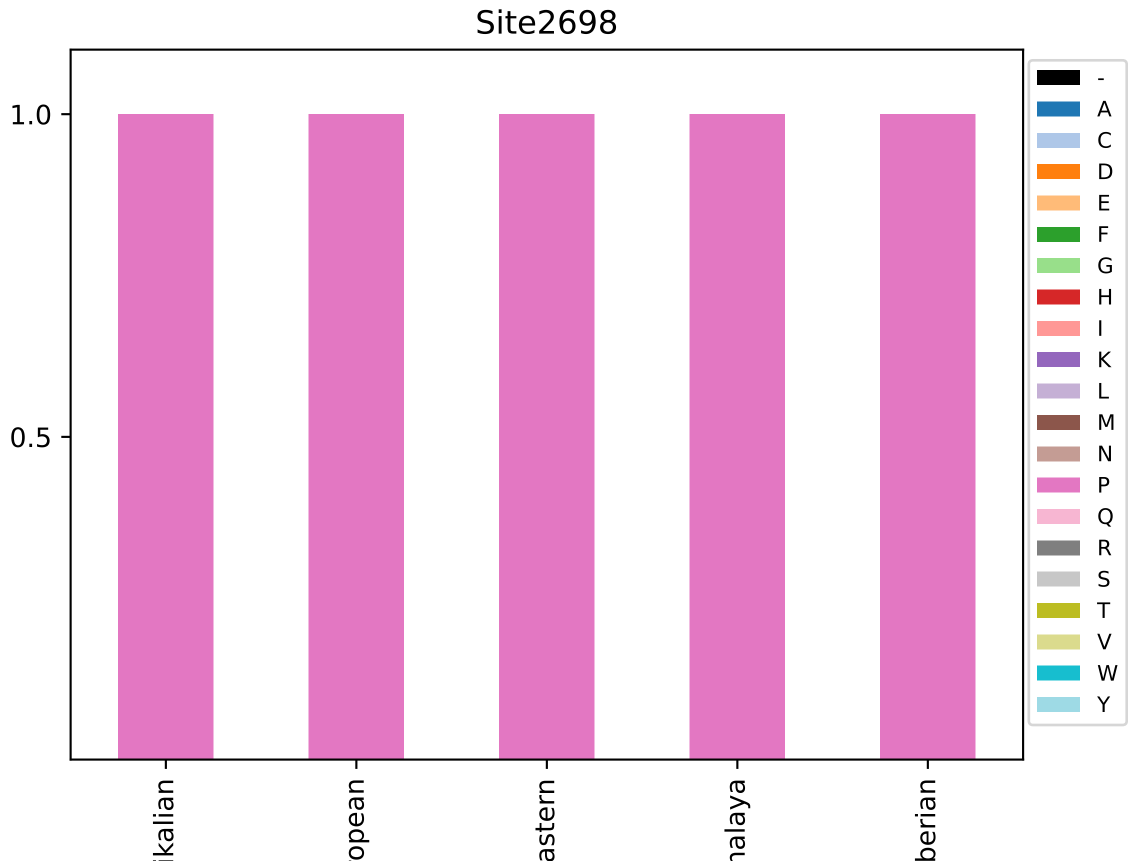

Site2698
1.0
0.5
-
A
C
D
E
F
G
H
I
K
L
M
N
P
Q
R
S
T
V
W
Y
ikalian
opean
astern
nalaya
berian

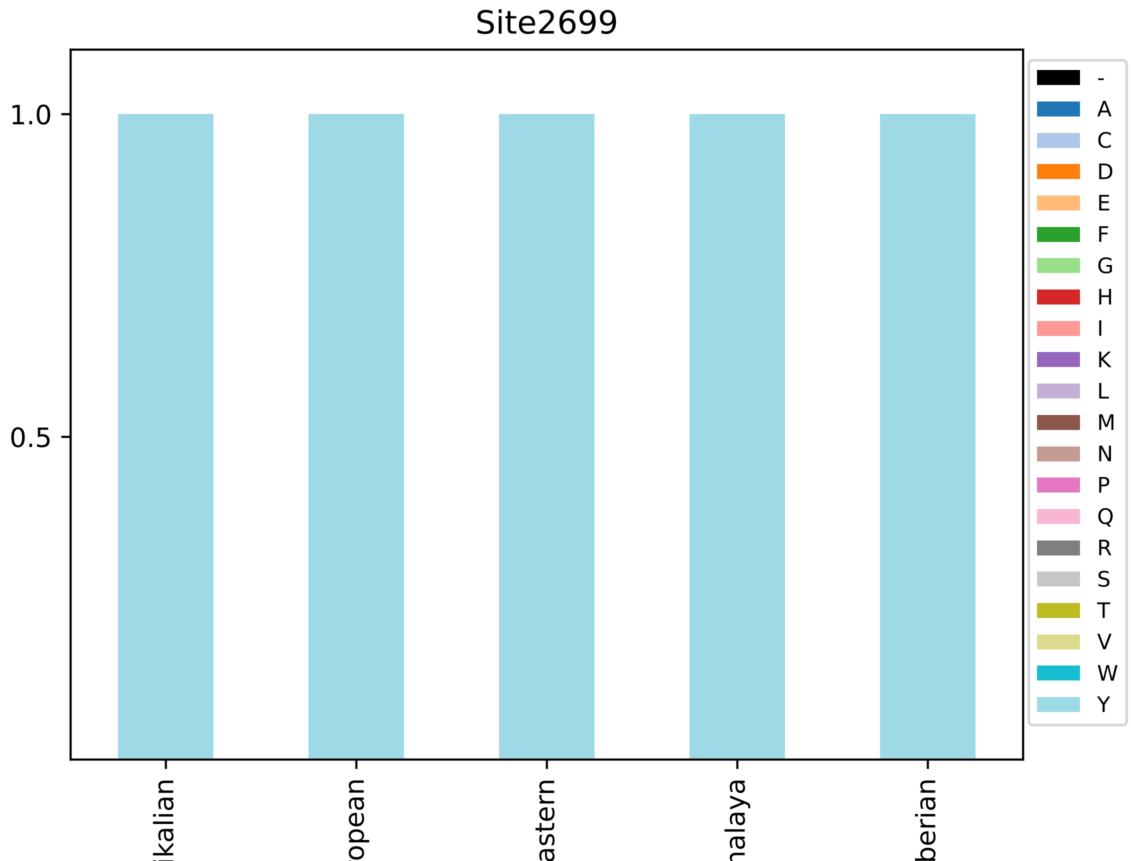

Site2699
1.0
0.5
-
A
C
D
E
F
G
H
I
K
L
M
N
P
Q
R
S
T
V
W
Y
ikalian
opean
astern
nalaya
berian

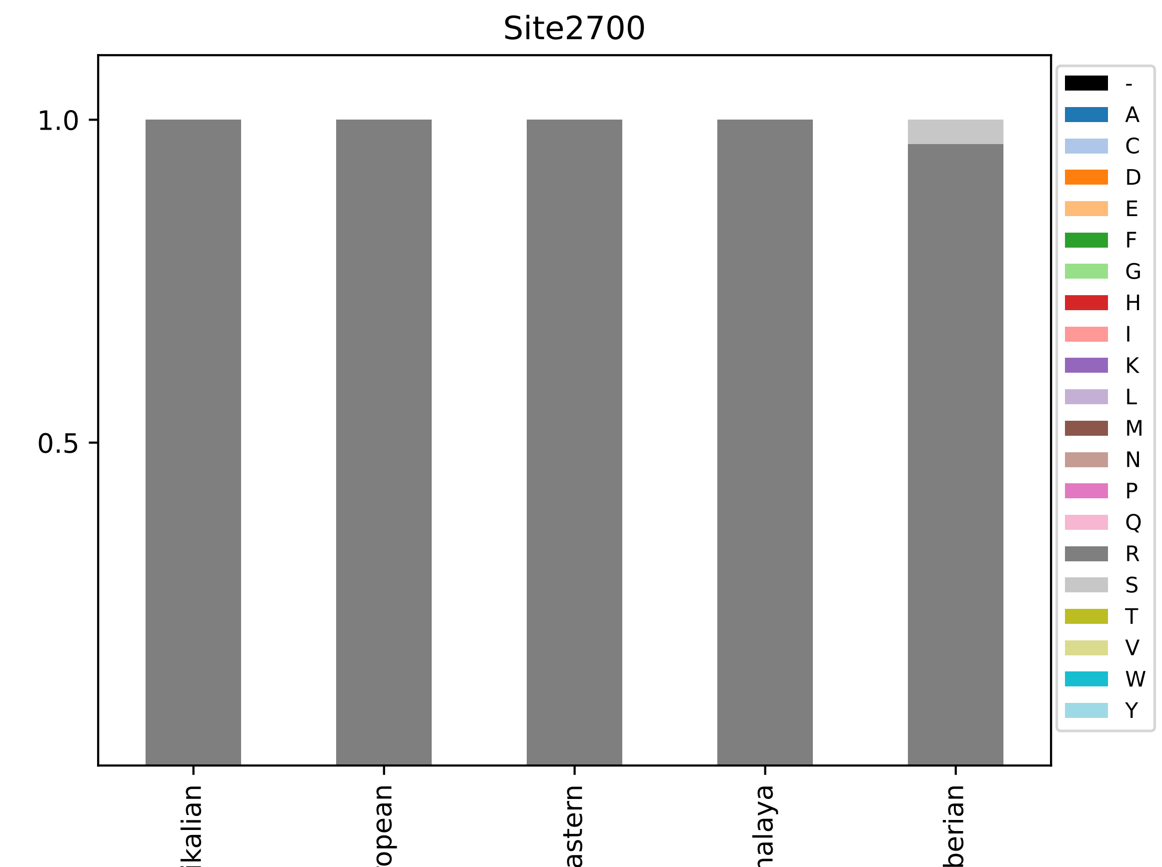

Site2700
1.0
0.5
ikalian
opean
astern
nalaya
berian
-
A
C
D
E
F
G
H
I
K
L
M
N
P
Q
R
S
T
V
W
Y

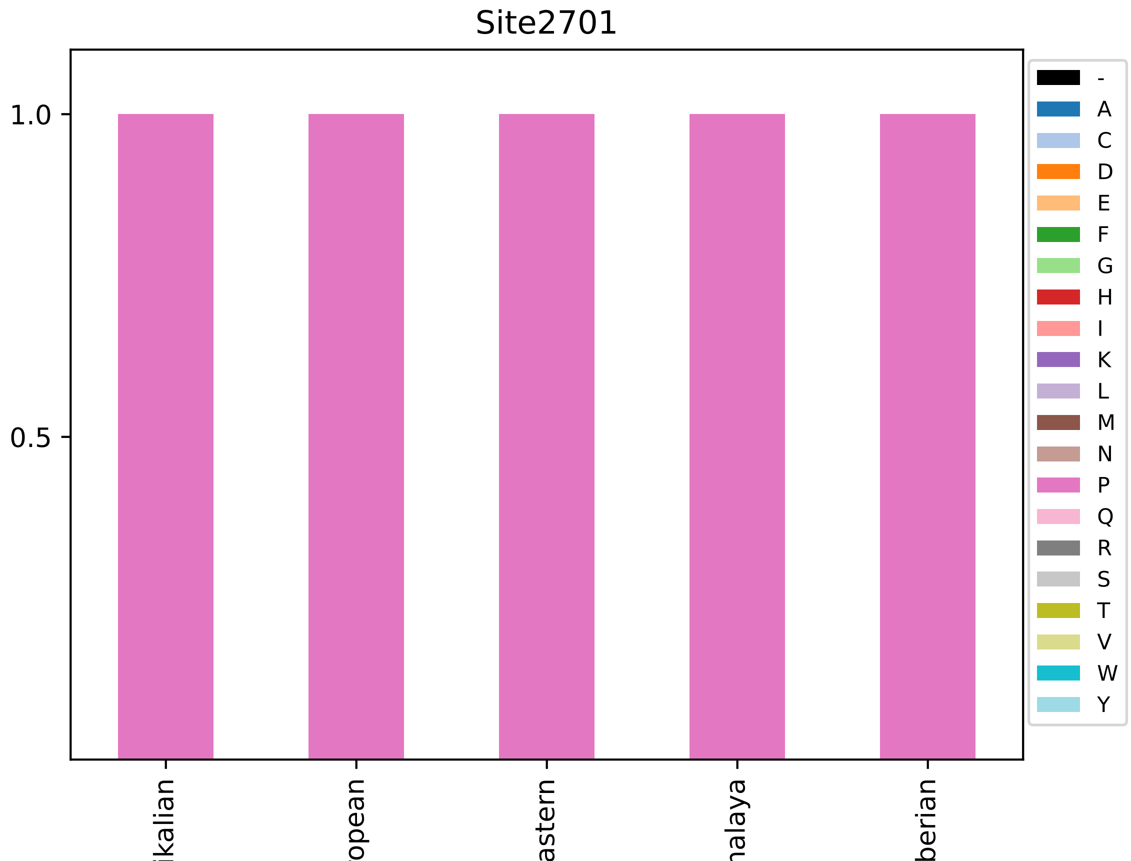

Site2701
1.0
0.5
ikalian
opean
astern
nalaya
berian
-
A
C
D
E
F
G
H
I
K
L
M
N
P
Q
R
S
T
V
W
Y

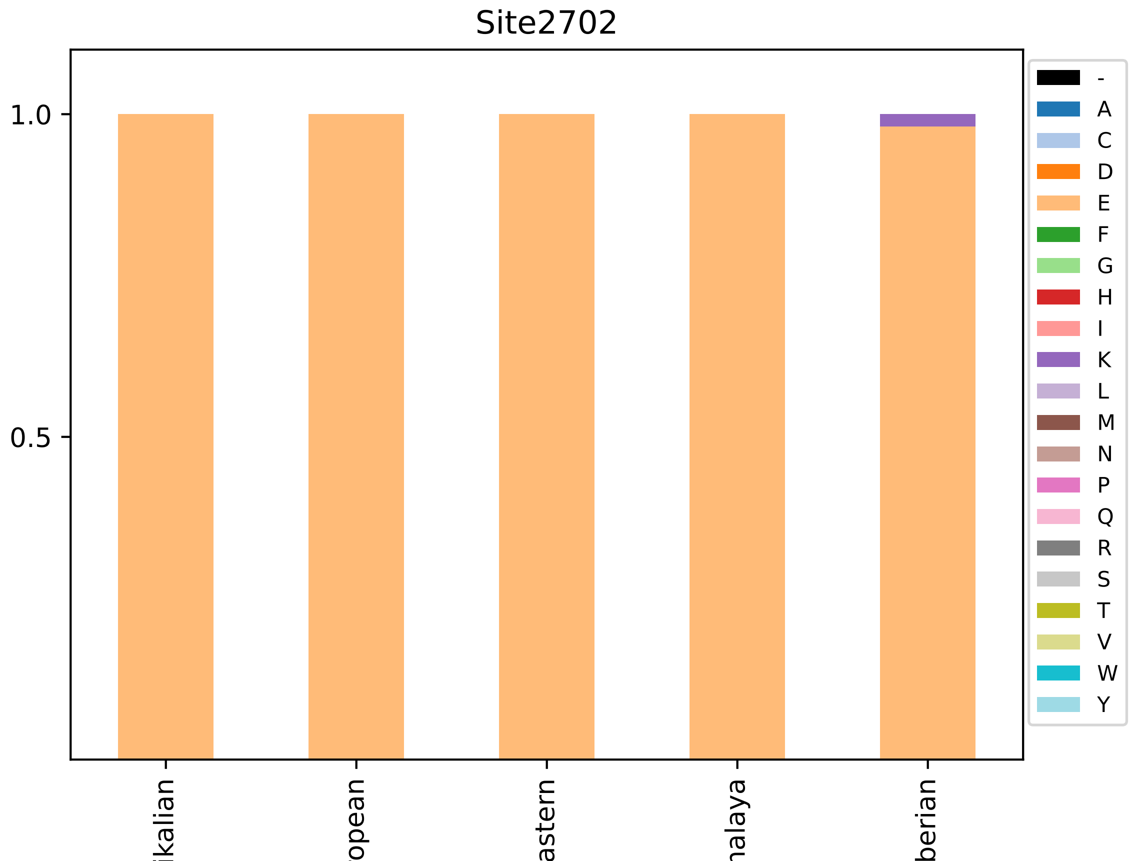

Site2702
1.0
0.5
-
A
C
D
E
F
G
H
I
K
L
M
N
P
Q
R
S
T
V
W
Y
ikalian
opean
astern
nalaya
berian

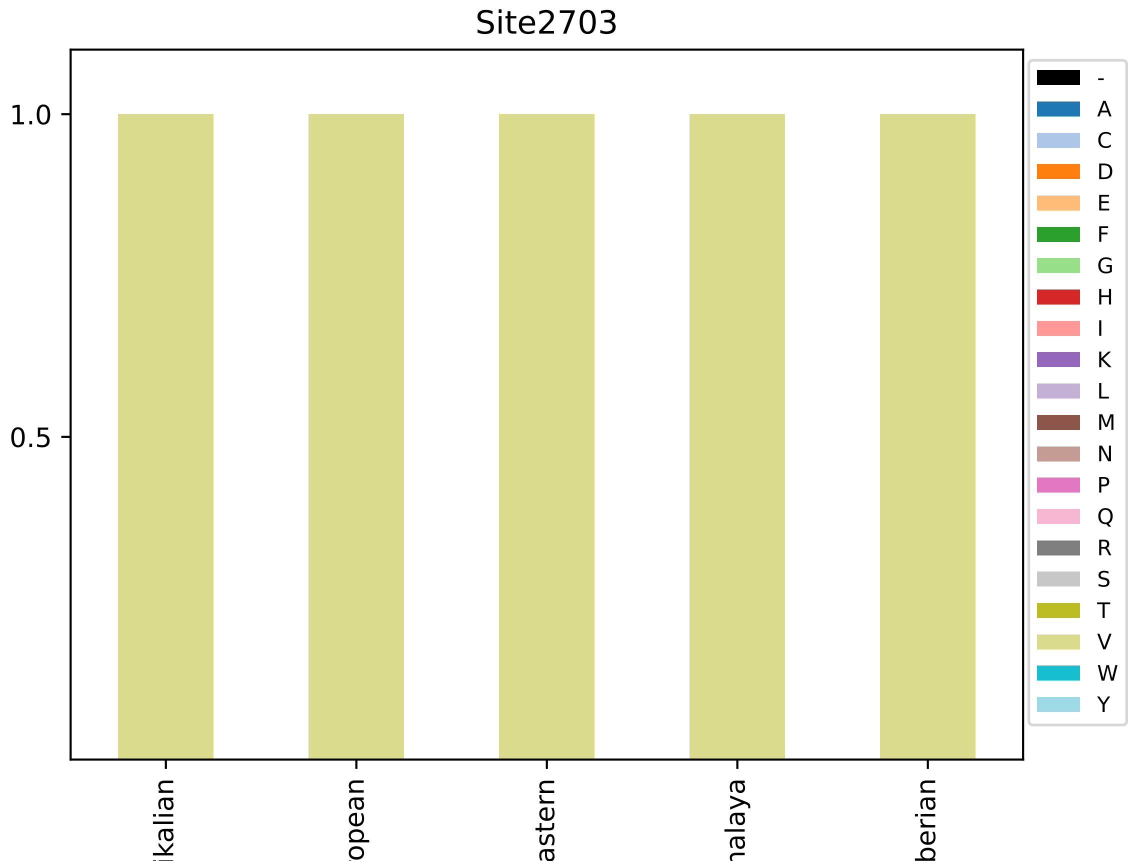

Site2703
1.0
0.5
ikalian
opean
astern
nalaya
berian
-
A
C
D
E
F
G
H
I
K
L
M
N
P
Q
R
S
T
V
W
Y

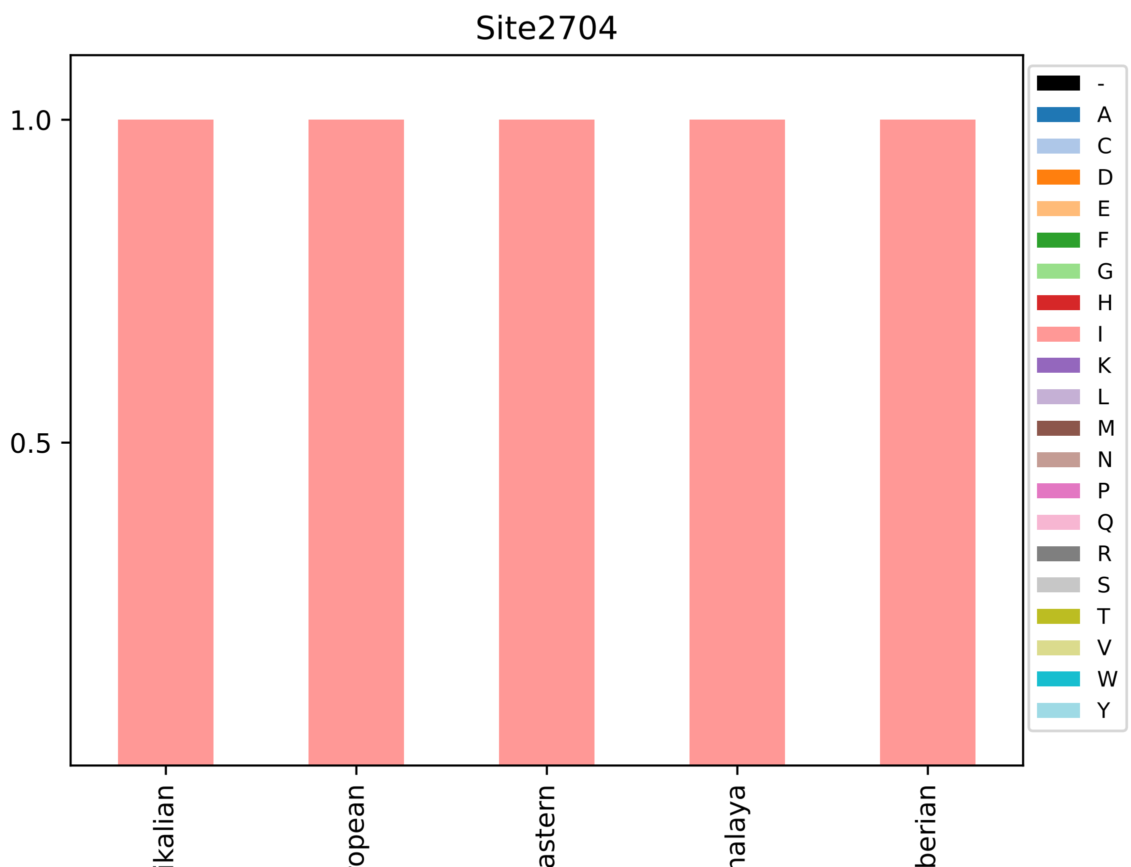

Site2704
1.0
0.5
-
A
C
D
E
F
G
H
I
K
L
M
N
P
Q
R
S
T
V
W
Y
ikalian
opean
astern
nalaya
berian

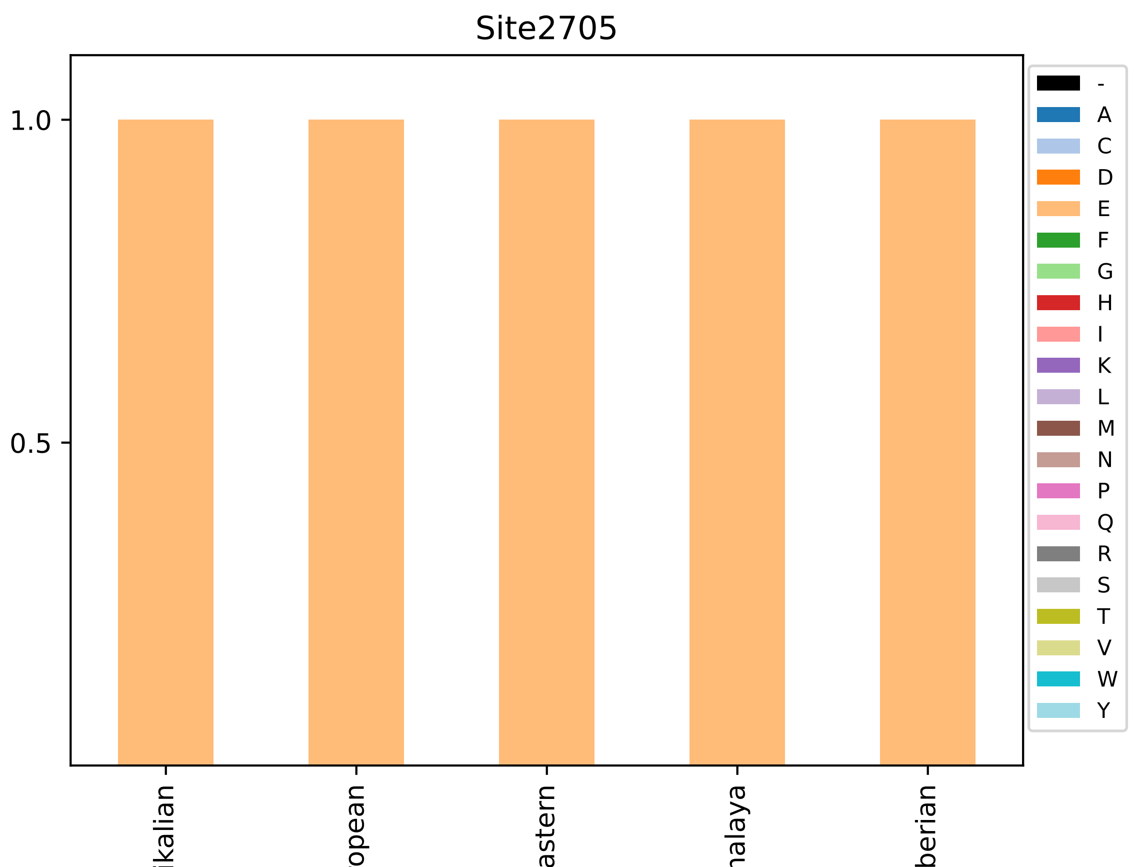

Site2705
1.0
0.5
-
A
C
D
E
F
G
H
I
K
L
M
N
P
Q
R
S
T
V
W
Y
ikalian
opean
astern
nalaya
berian

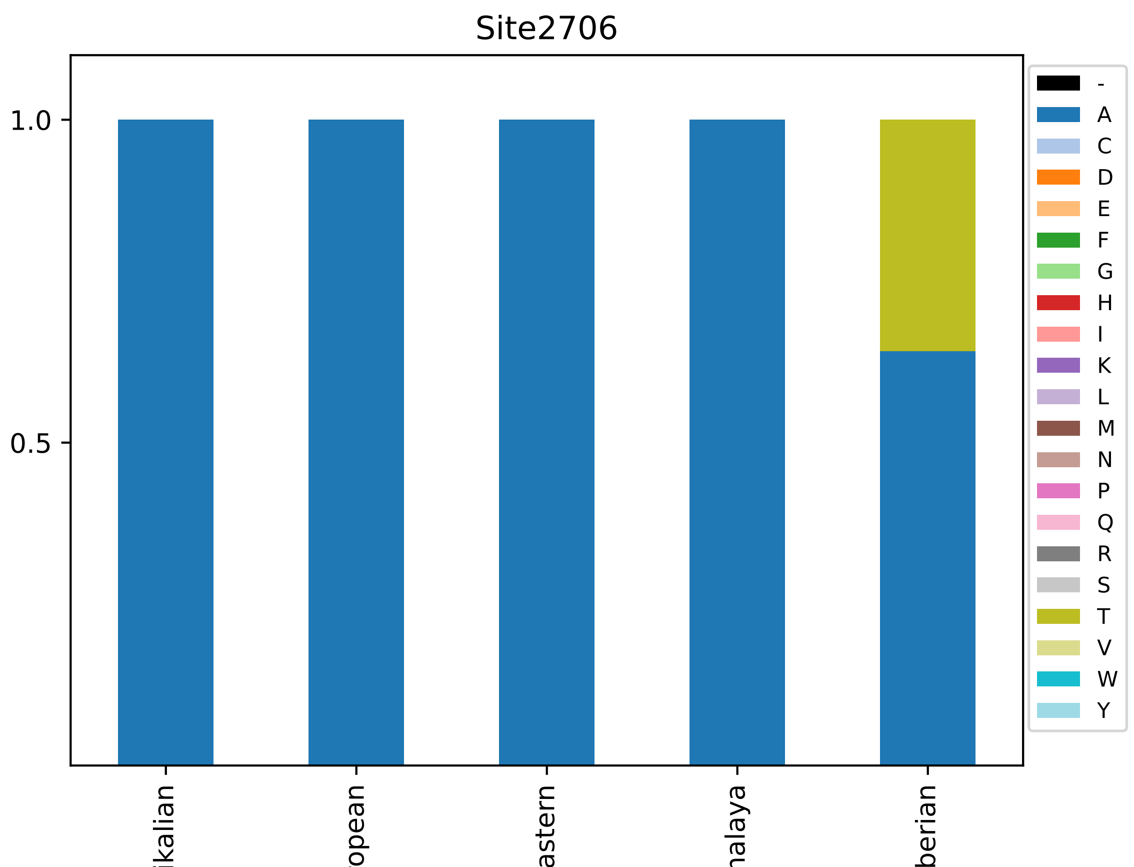
Site2706
1.0
0.5
-
A
C
D
E
F
G
H
I
K
L
M
N
P
Q
R
S
T
V
W
Y
ikalian
opean
astern
nalaya
berian

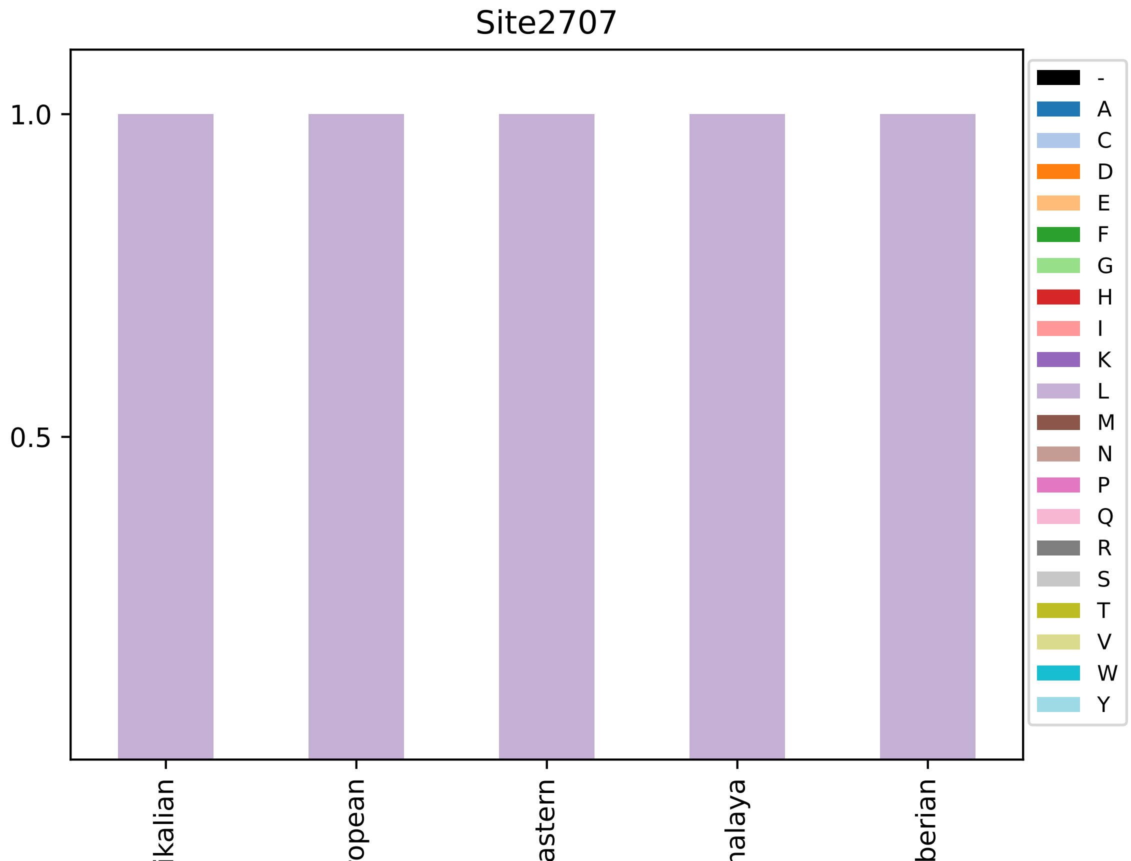
Site2707
1.0
0.5
-
A
C
D
E
F
G
H
I
K
L
M
N
P
Q
R
S
T
V
W
Y
ikalian
opean
astern
nalaya
berian

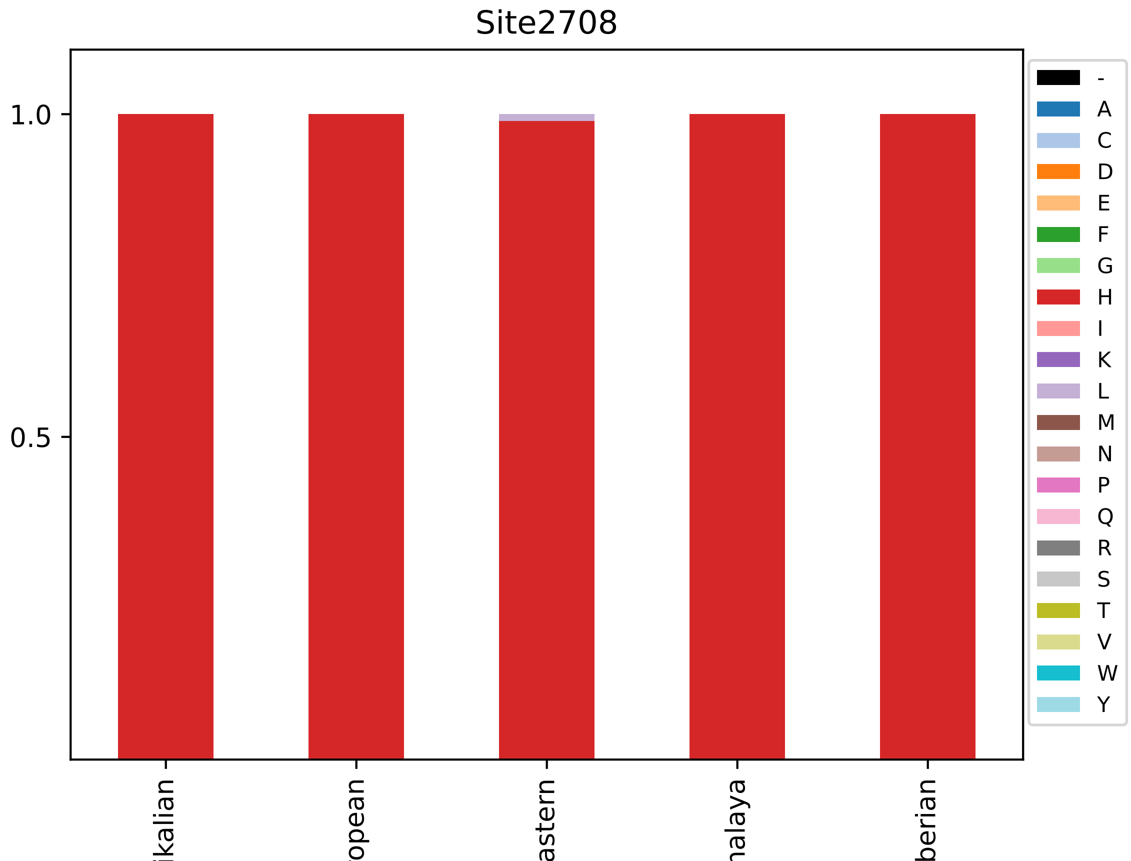

Site2708
1.0
0.5
-
A
C
D
E
F
G
H
I
K
L
M
N
P
Q
R
S
T
V
W
Y
ikalian
opean
astern
nalaya
berian

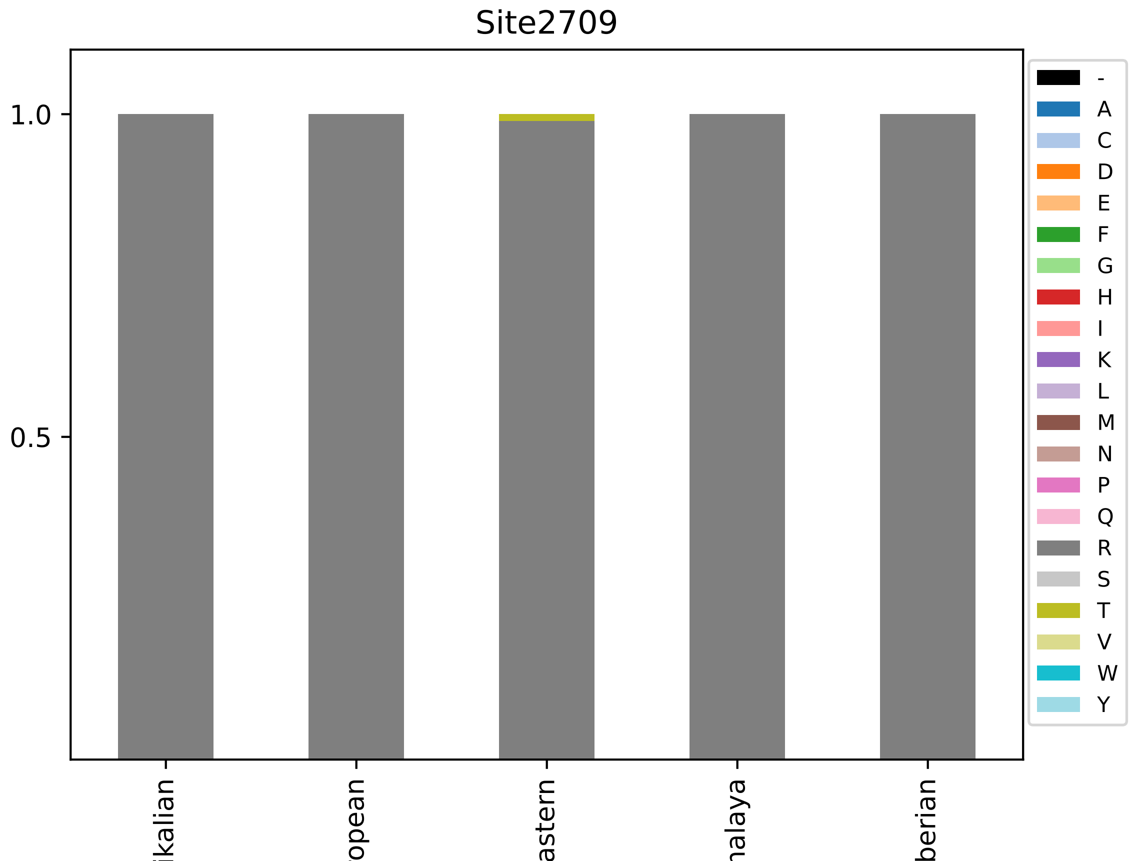

Site2709
1.0
0.5
-
A
C
D
E
F
G
H
I
K
L
M
N
P
Q
R
S
T
V
W
Y
ikalian
opean
astern
nalaya
berian

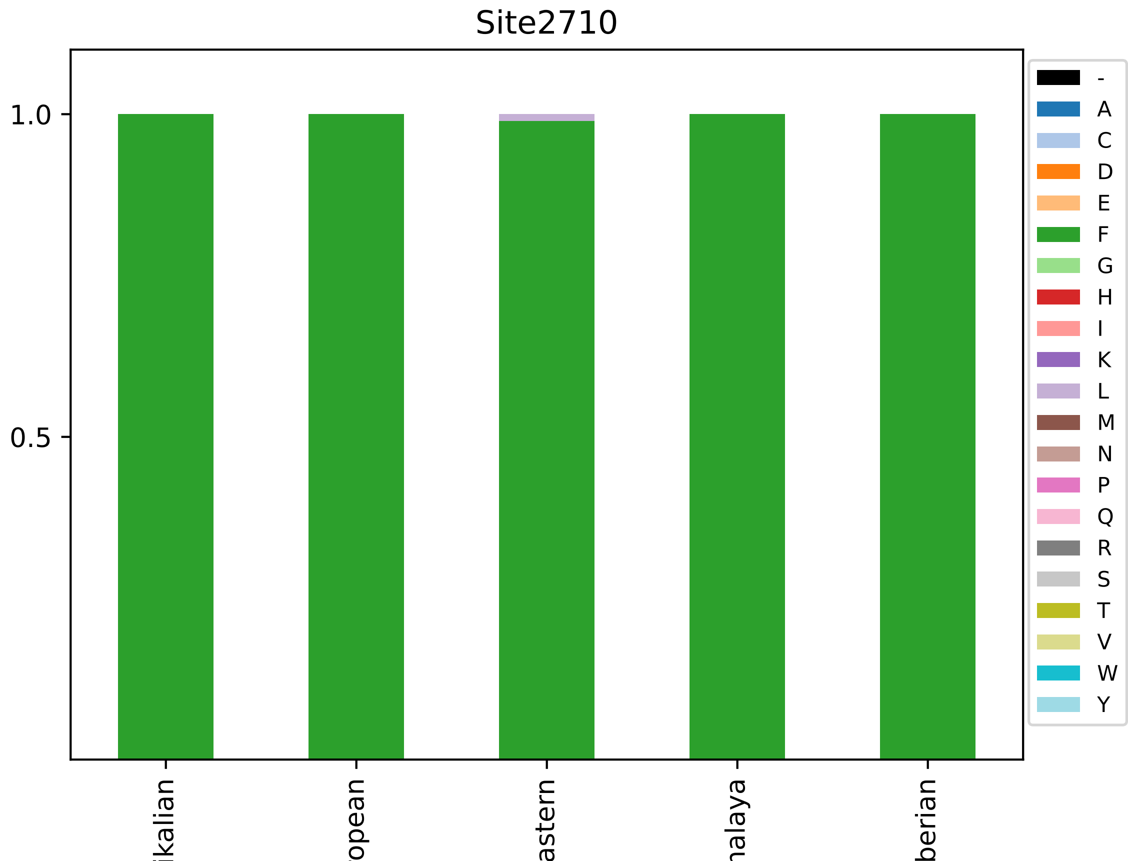

Site2710
1.0
0.5
-
A
C
D
E
F
G
H
I
K
L
M
N
P
Q
R
S
T
V
W
Y
ikalian
opean
astern
nalaya
berian

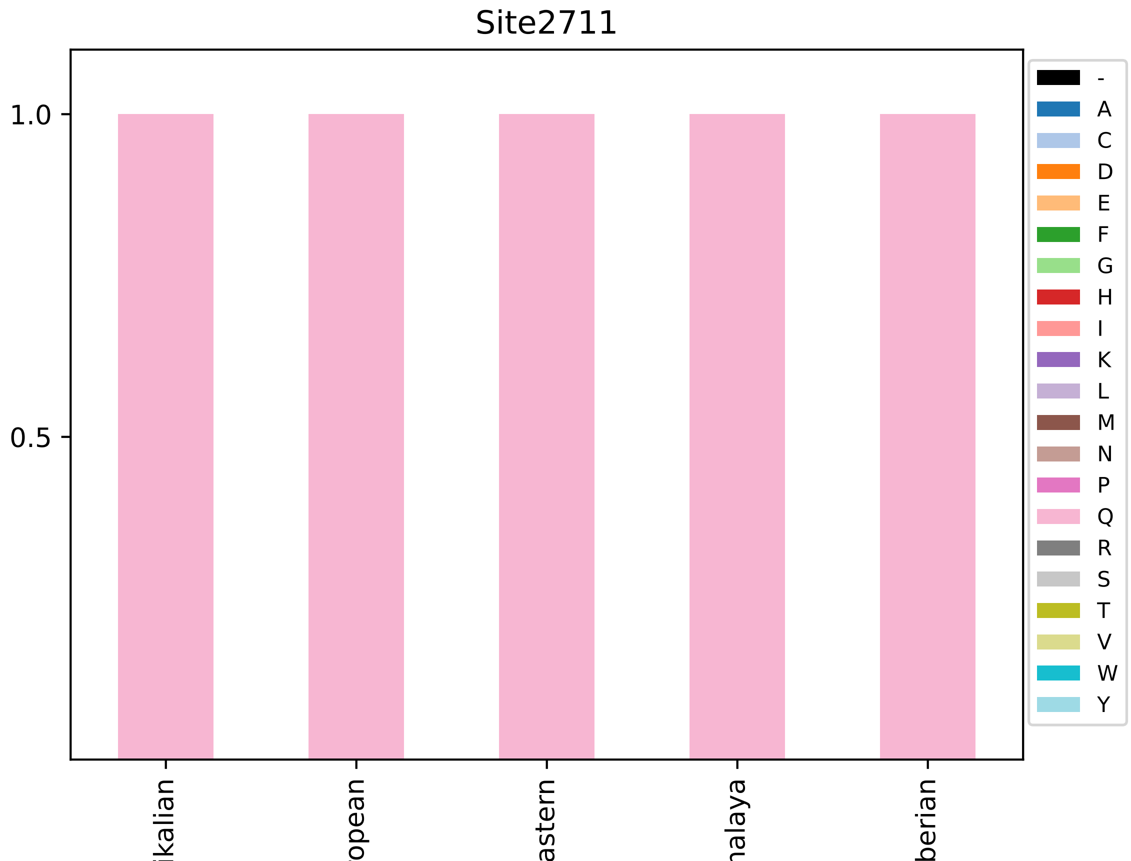
Site2711
1.0
0.5
-
A
C
D
E
F
G
H
I
K
L
M
N
P
Q
R
S
T
V
W
Y
ikalian
opean
astern
nalaya
berian

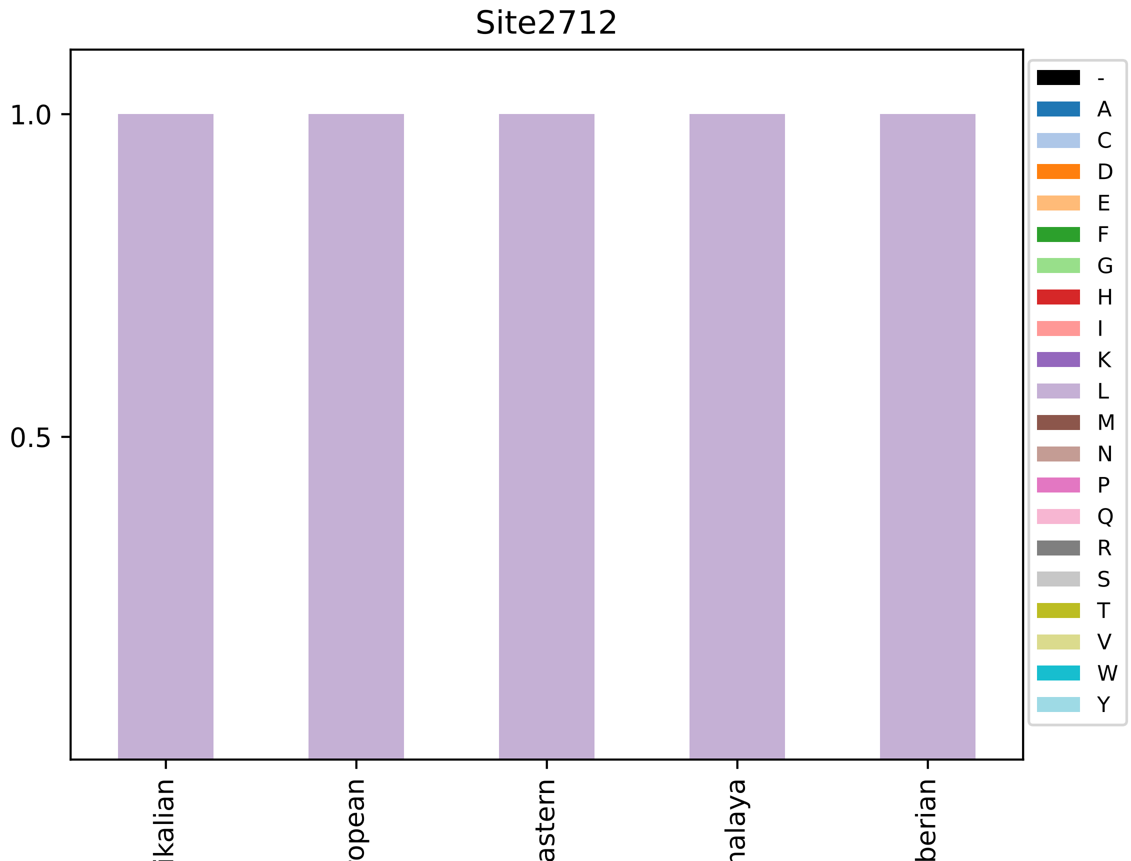
Site2712
1.0
0.5
-
A
C
D
E
F
G
H
I
K
L
M
N
P
Q
R
S
T
V
W
Y
ikalian
opean
astern
nalaya
berian

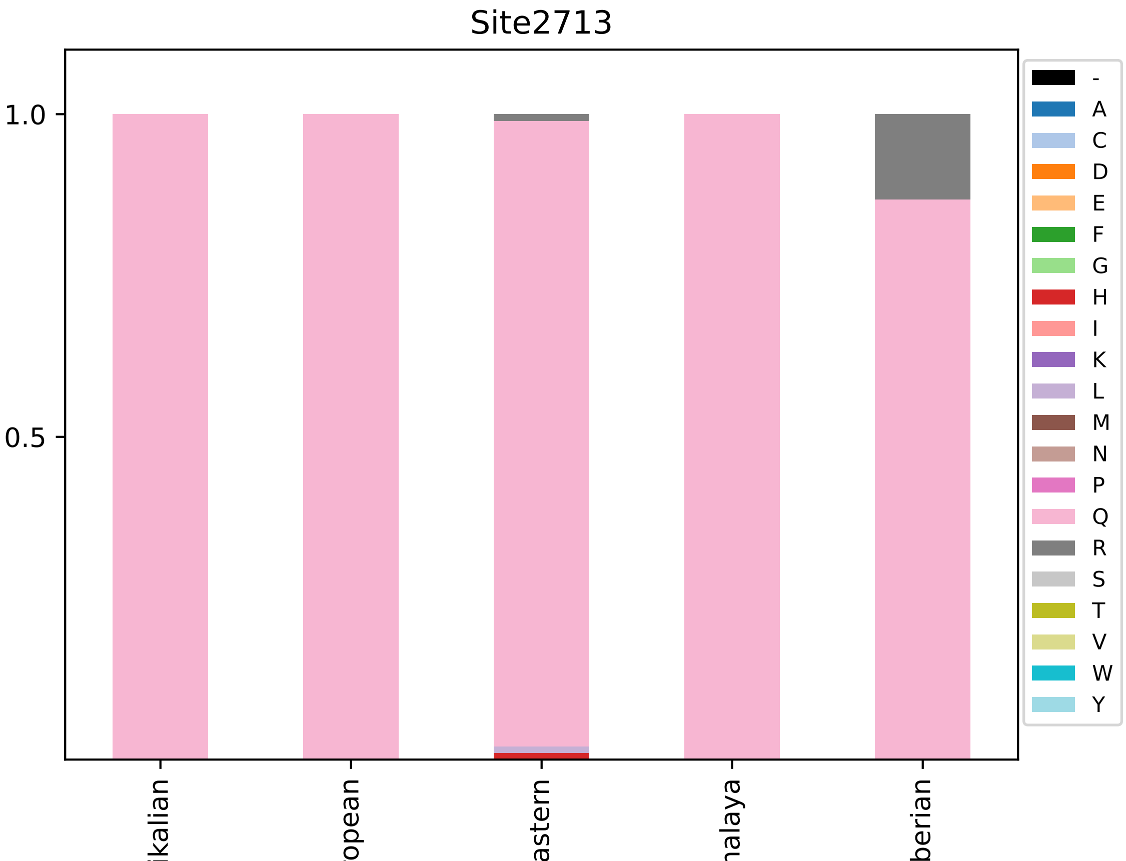
Site2713
1.0
0.5
-
A
C
D
E
F
G
H
I
K
L
M
N
P
Q
R
S
T
V
W
Y
ikalian
opean
astern
nalaya
berian

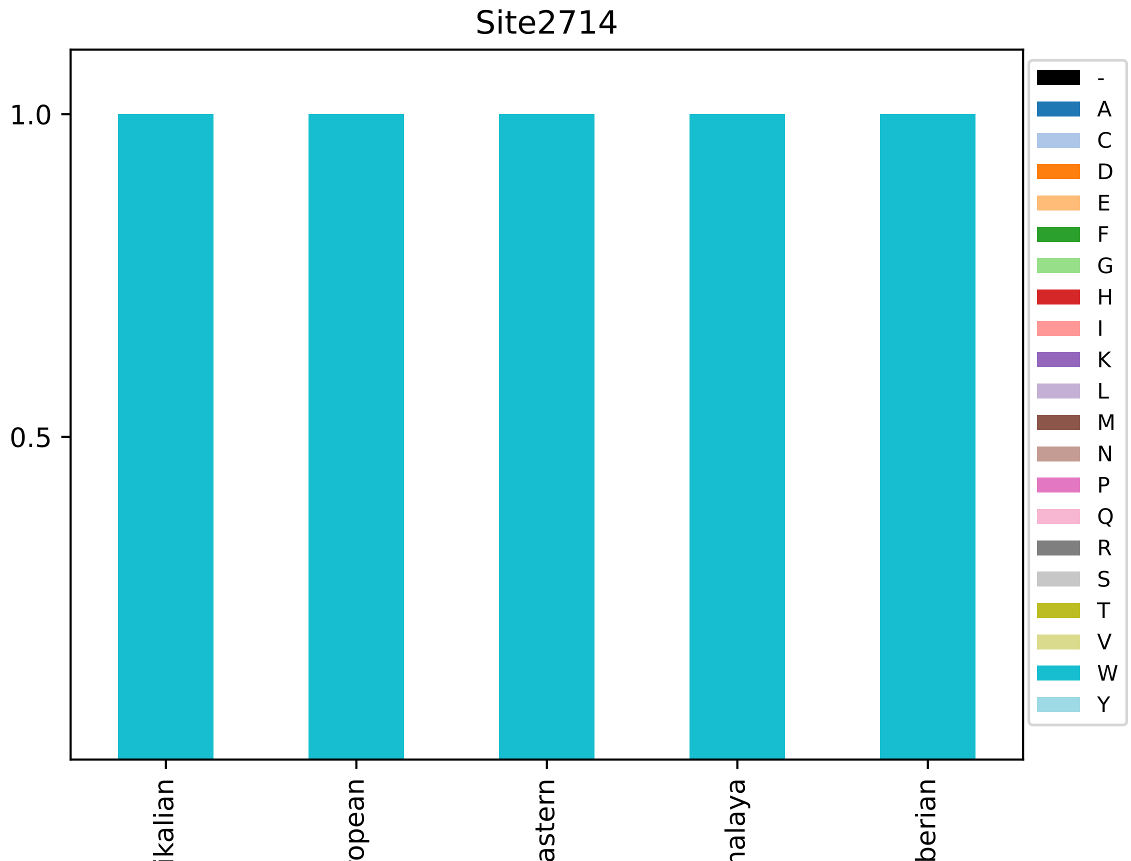

Site2714
1.0
0.5
-
A
C
D
E
F
G
H
I
K
L
M
N
P
Q
R
S
T
V
W
Y
ikalian
opean
astern
nalaya
berian

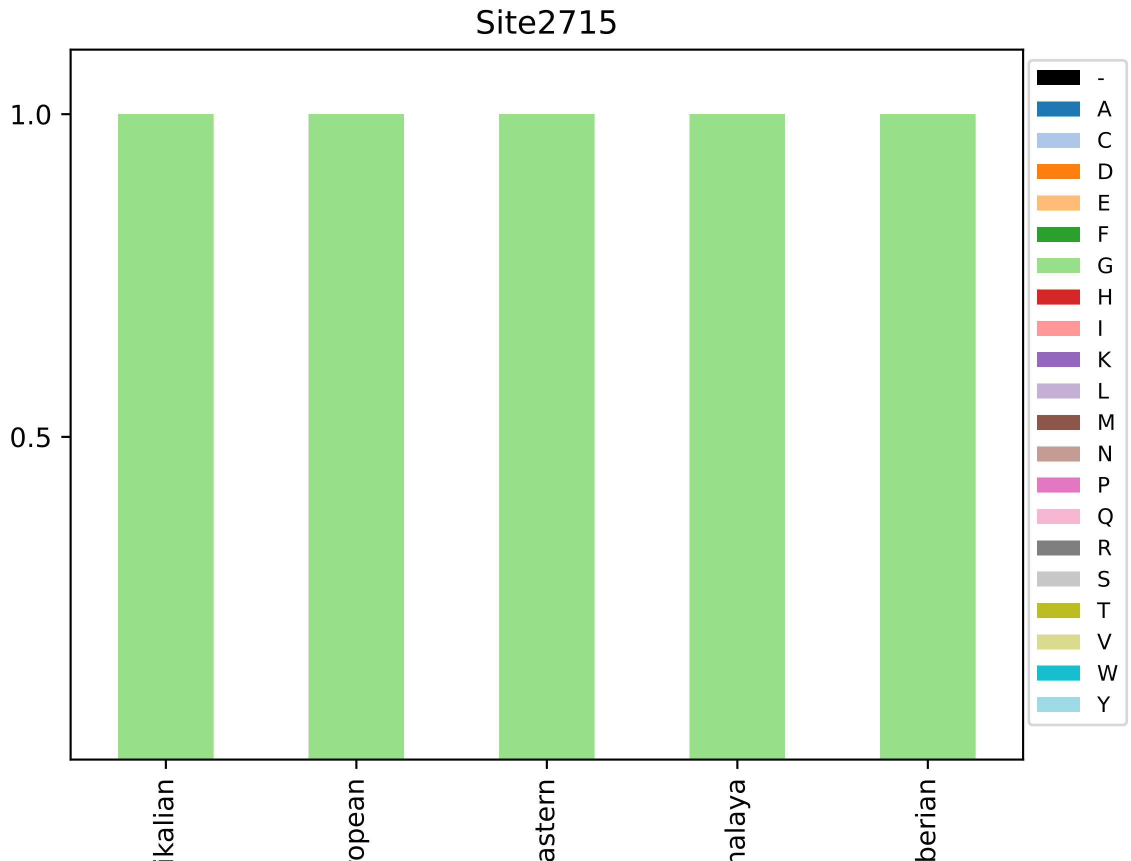

Site2715
1.0
0.5
-
A
C
D
E
F
G
H
I
K
L
M
N
P
Q
R
S
T
V
W
Y

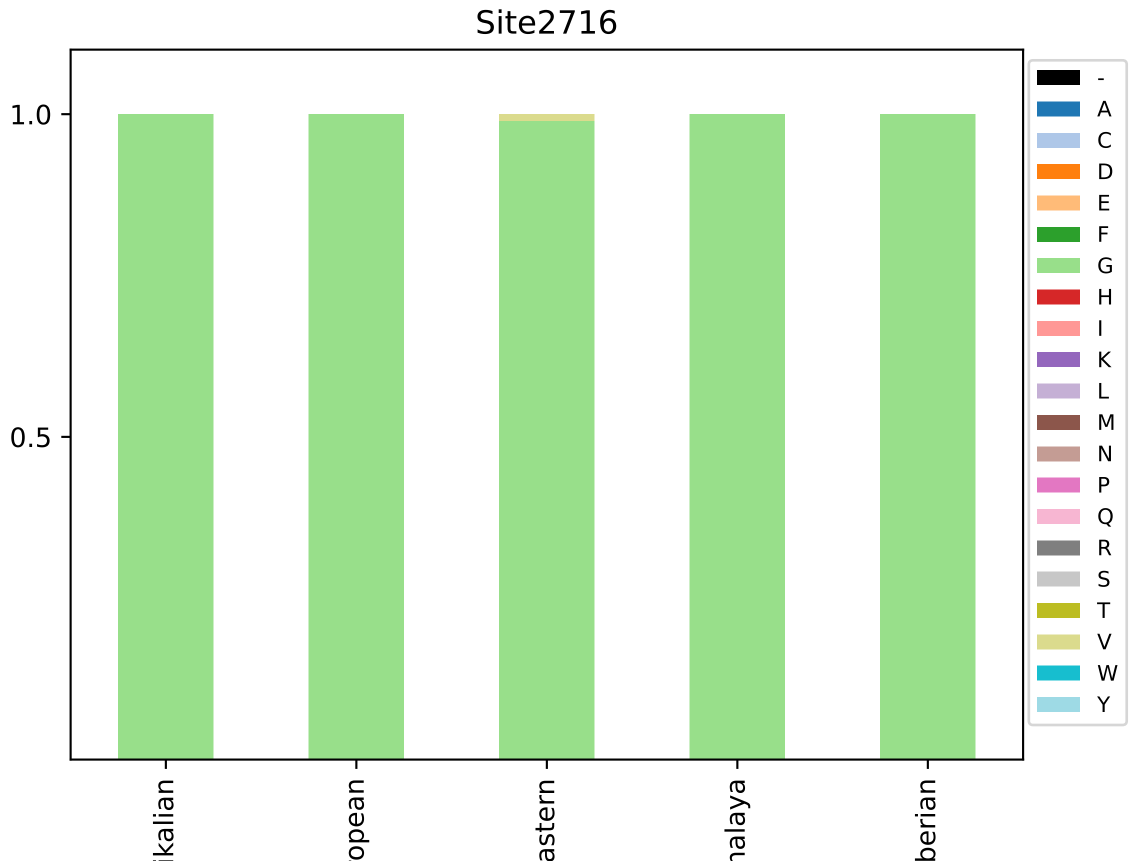

Site2716
1.0
0.5
-
A
C
D
E
F
G
H
I
K
L
M
N
P
Q
R
S
T
V
W
Y
ikalian
opean
astern
nalaya
berian

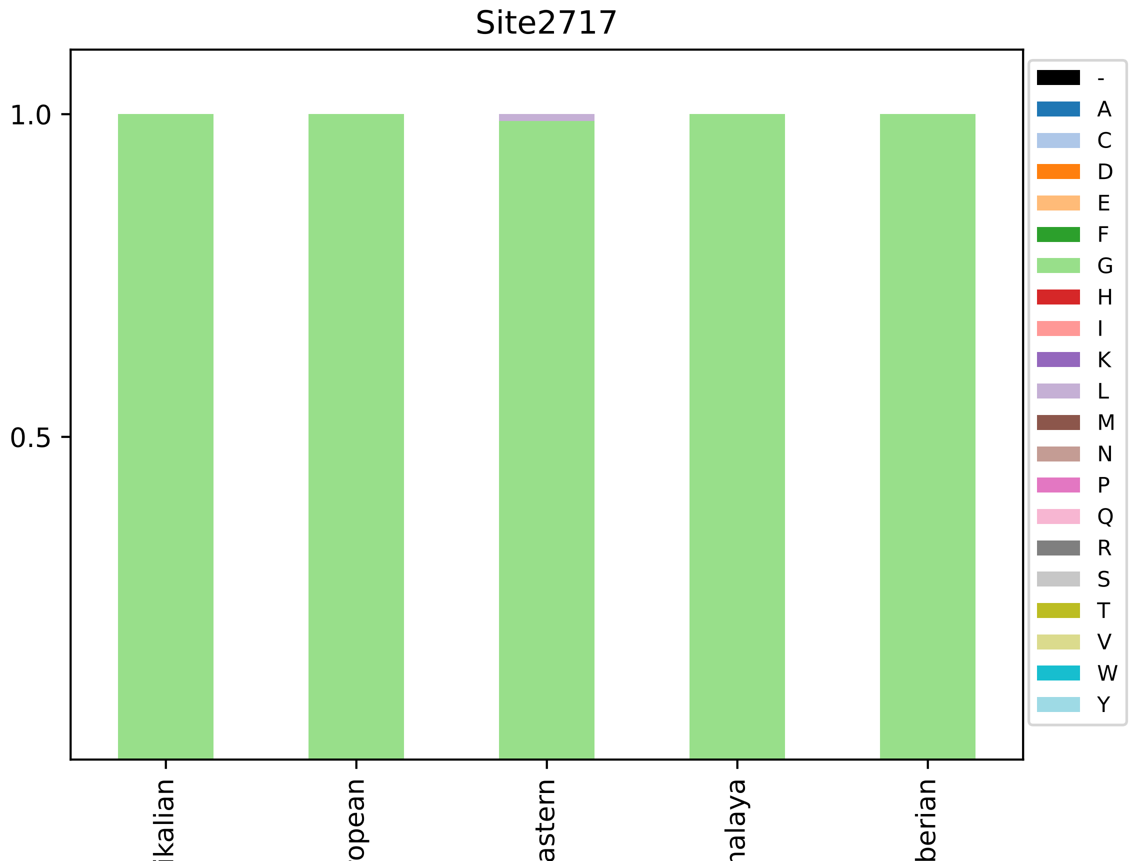

Site2717
1.0
0.5
-
A
C
D
E
F
G
H
I
K
L
M
N
P
Q
R
S
T
V
W
Y
ikalian
ropean
astern
nalaya
berian

Site2718

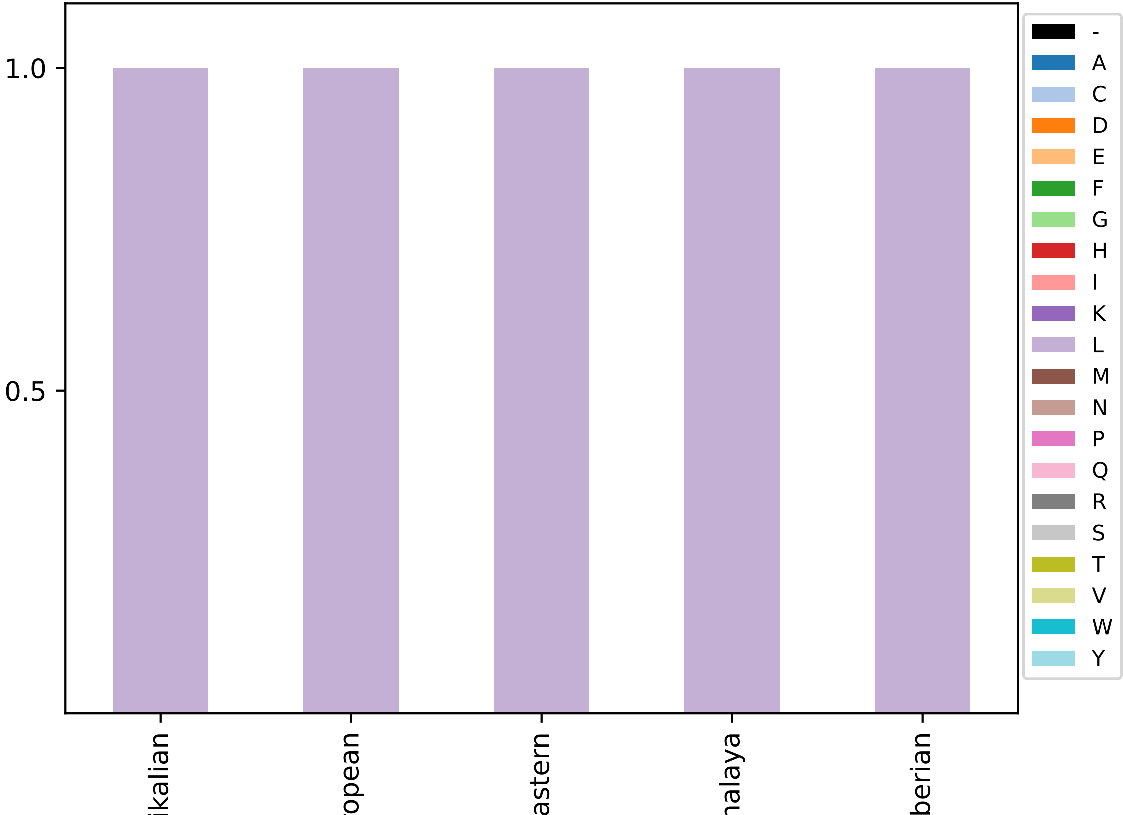

1.0
0.5
-
A
C
D
E
F
G
H
I
K
L
M
N
P
Q
R
S
T
V
W
Y
ikalian
opean
astern
nalaya
berian

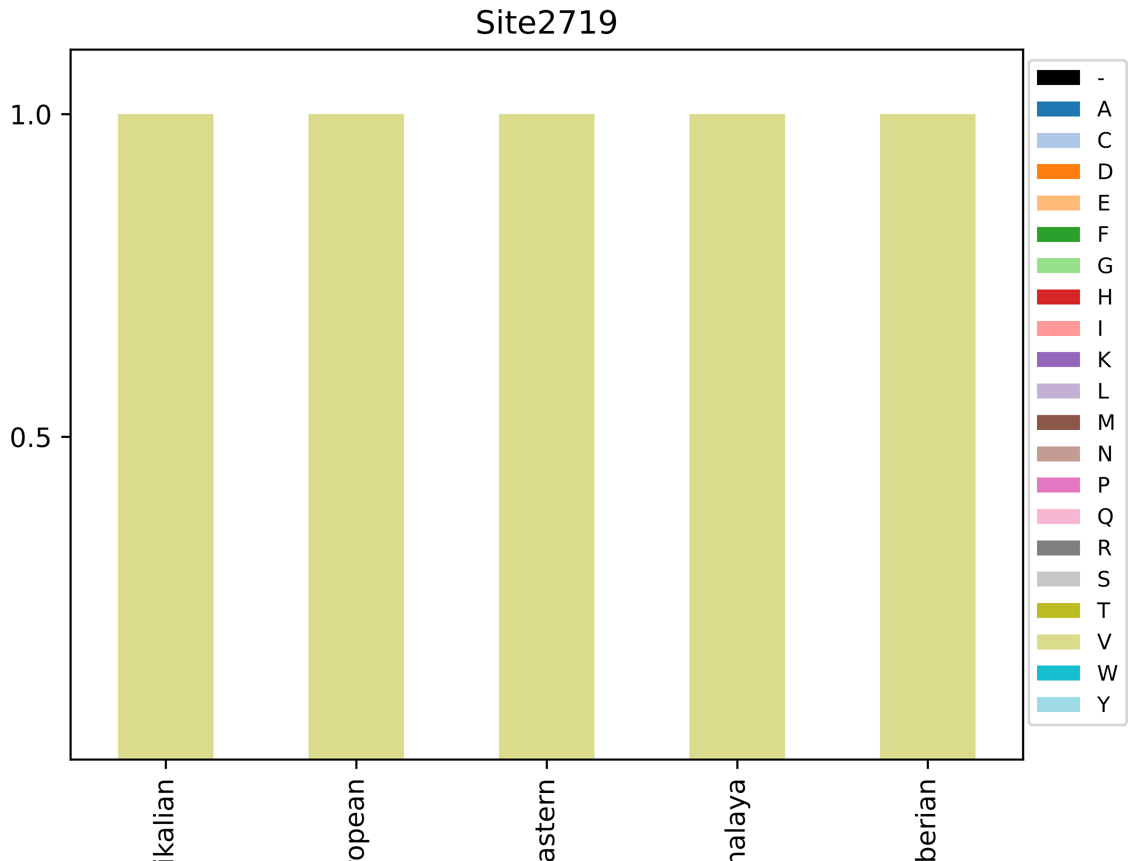
Site2719
1.0
0.5
ikalian
opean
astern
nalaya
berian
-
A
C
D
E
F
G
H
I
K
L
M
N
P
Q
R
S
T
V
W
Y

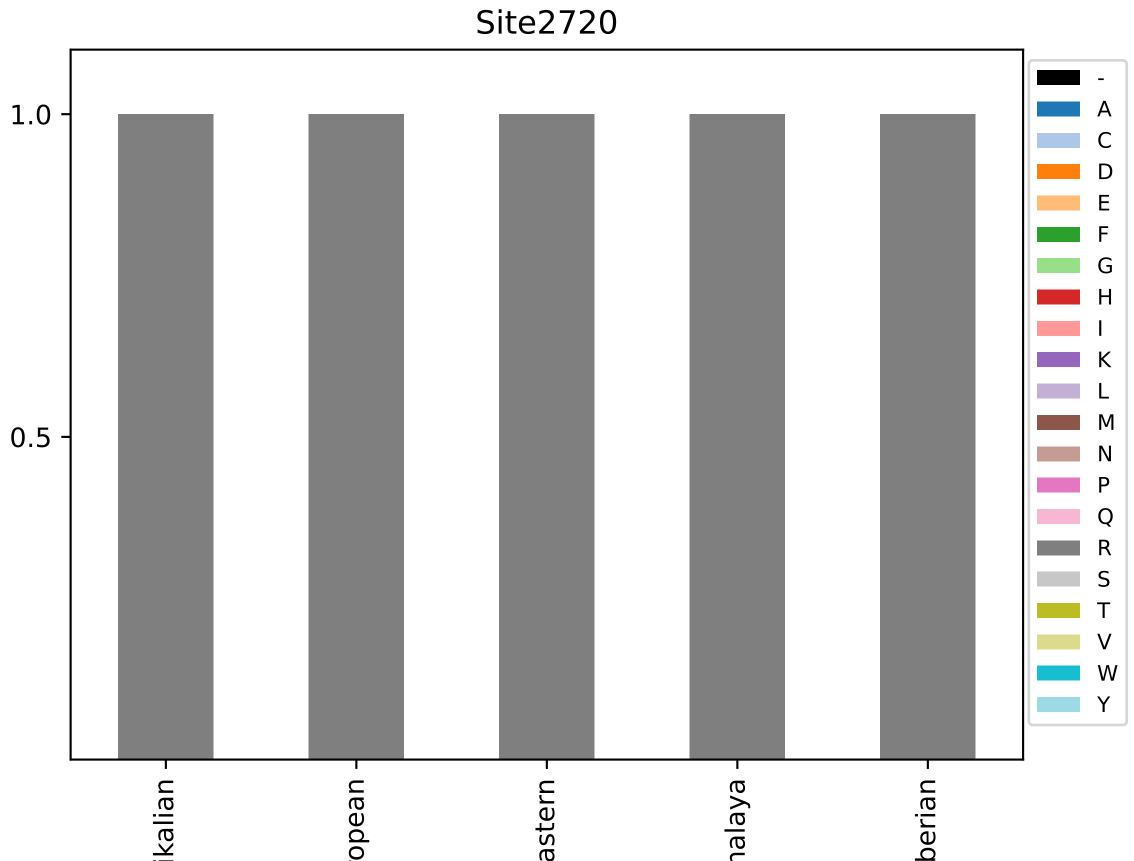

Site2720
1.0
0.5
-
A
C
D
E
F
G
H
I
K
L
M
N
P
Q
R
S
T
V
W
Y
ikalian
opean
astern
nalaya
berian

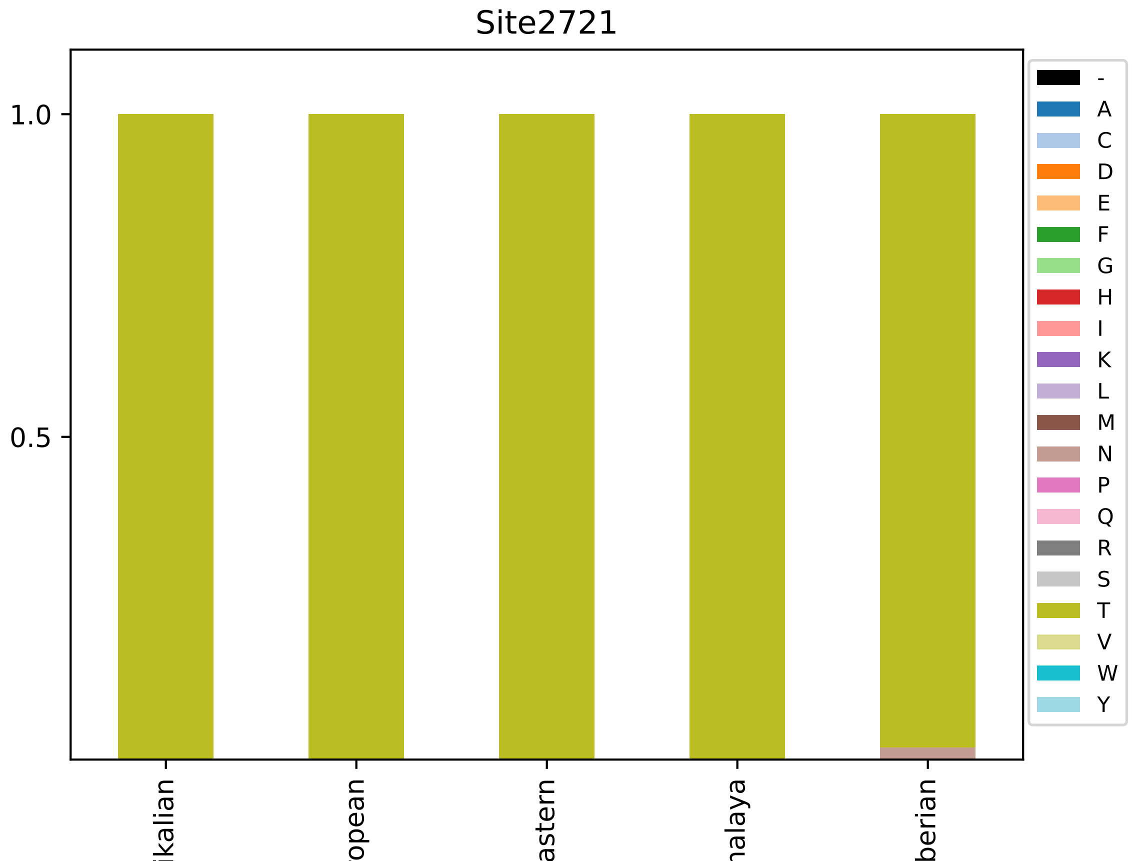

Site2721
1.0
0.5
ikalian
opean
astern
nalaya
berian
-
A
C
D
E
F
G
H
I
K
L
M
N
P
Q
R
S
T
V
W
Y

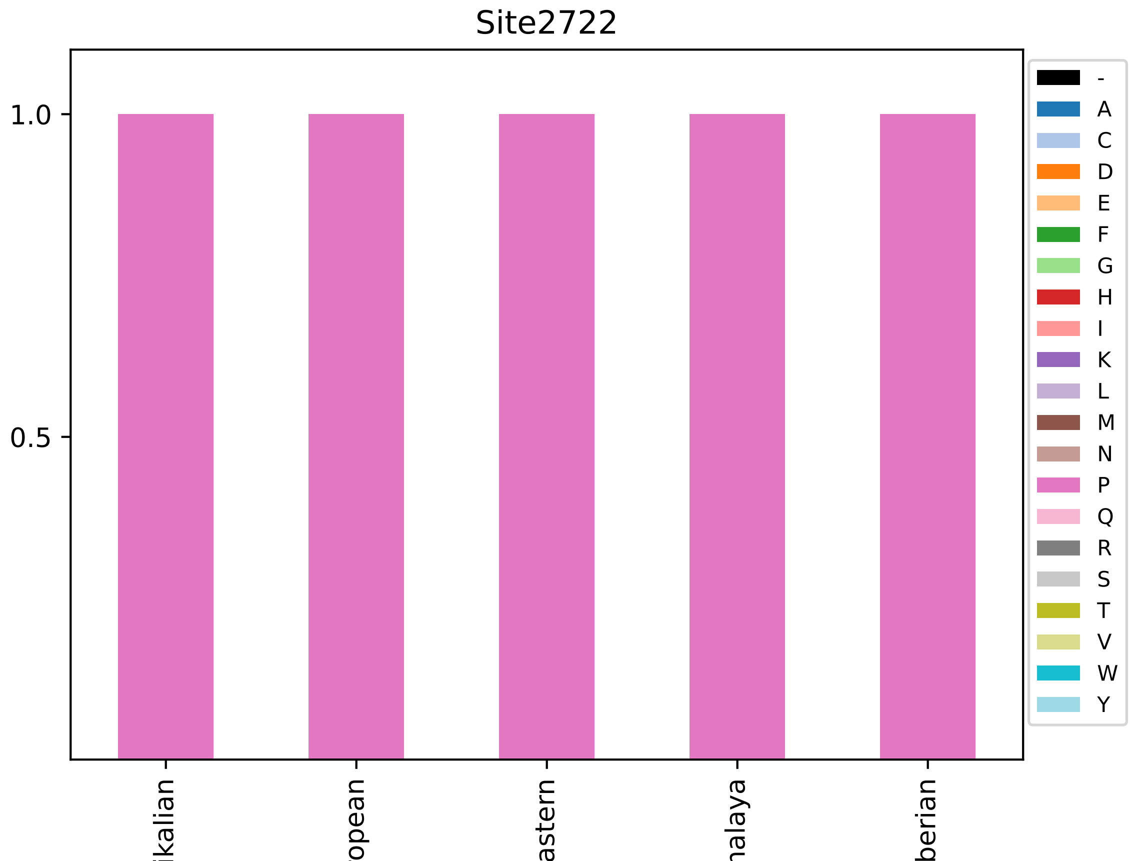
Site2722
1.0
0.5
-
A
C
D
E
F
G
H
I
K
L
M
N
P
Q
R
S
T
V
W
Y
ikalian
opean
astern
nalaya
berian

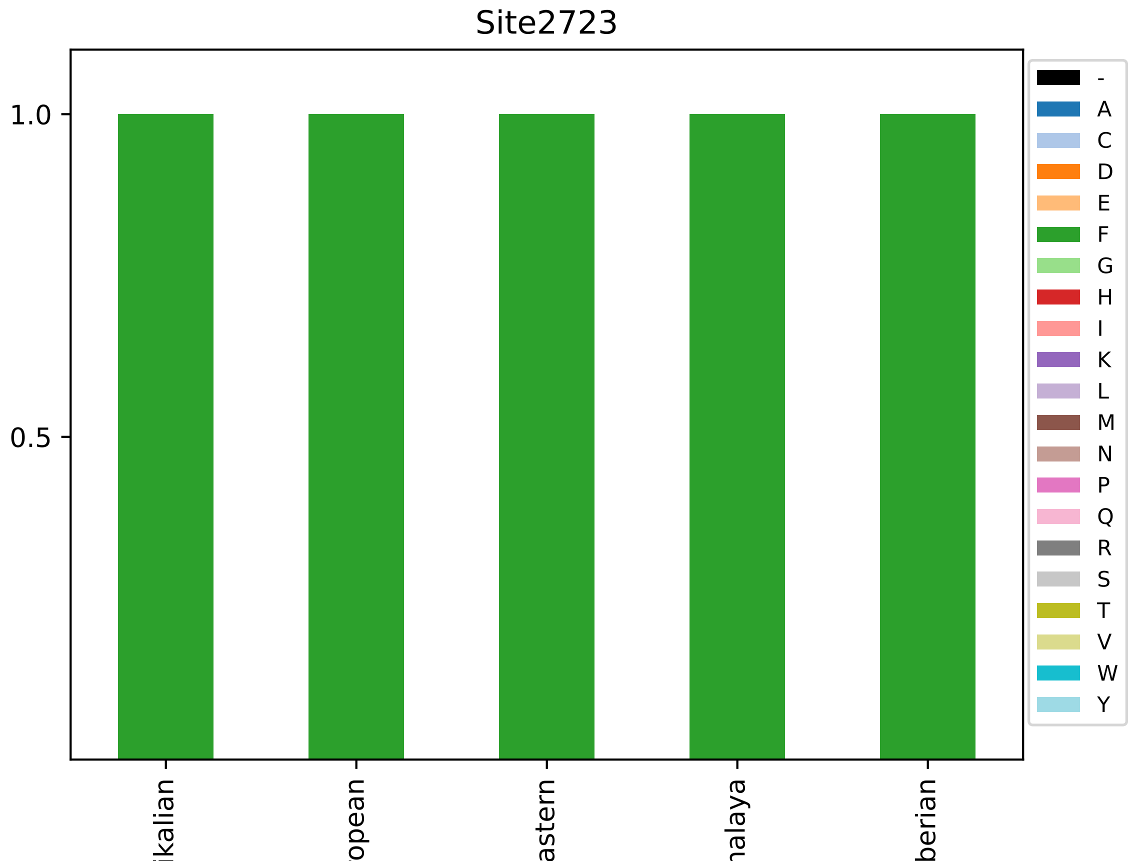
Site2723
1.0
0.5
-
A
C
D
E
F
G
H
I
K
L
M
N
P
Q
R
S
T
V
W
Y
ikalian
opean
astern
nalaya
berian

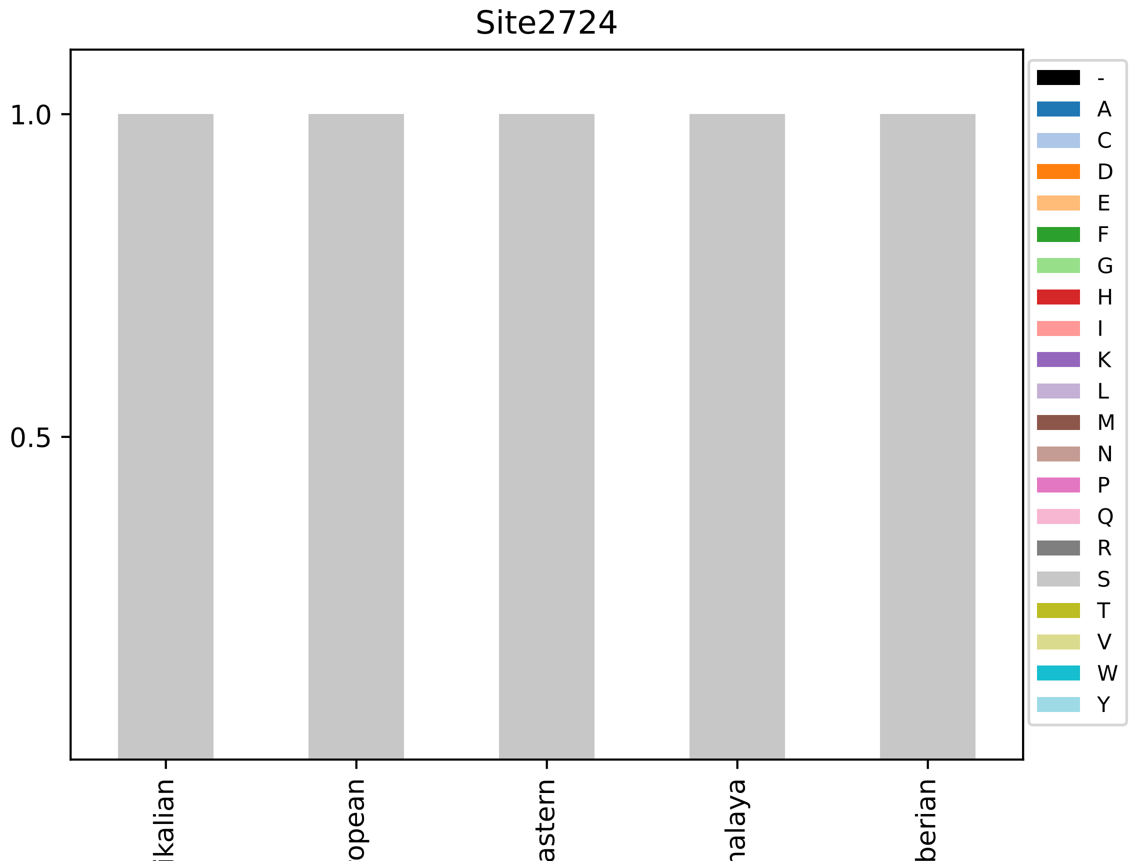
Site2724
1.0
0.5
-
A
C
D
E
F
G
H
I
K
L
M
N
P
Q
R
S
T
V
W
Y
ikalian
opean
astern
nalaya
berian

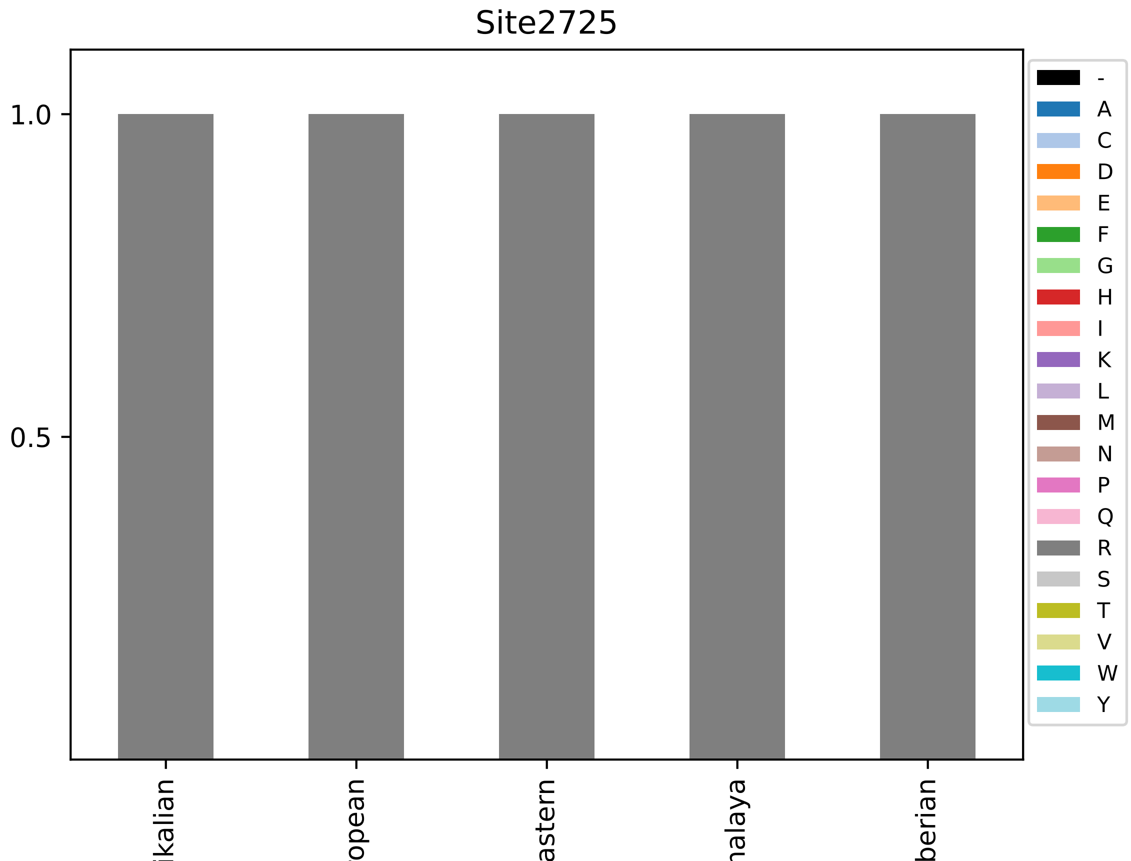
Site2725
1.0
0.5
-
A
C
D
E
F
G
H
I
K
L
M
N
P
Q
R
S
T
V
W
Y
ikalian
opean
astern
alaya
berian

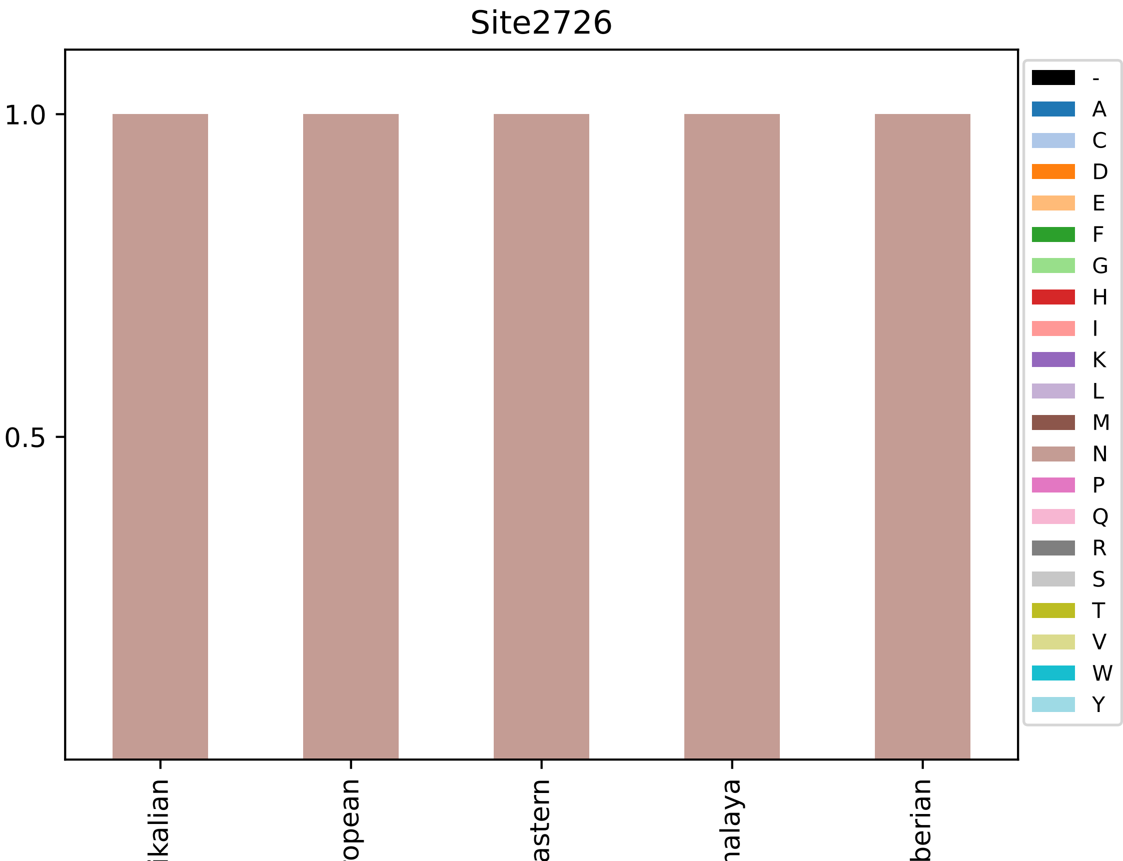

Site2726
1.0
0.5
-
A
C
D
E
F
G
H
I
K
L
M
N
P
Q
R
S
T
V
W
Y
ikalian
opean
astern
nalaya
berian

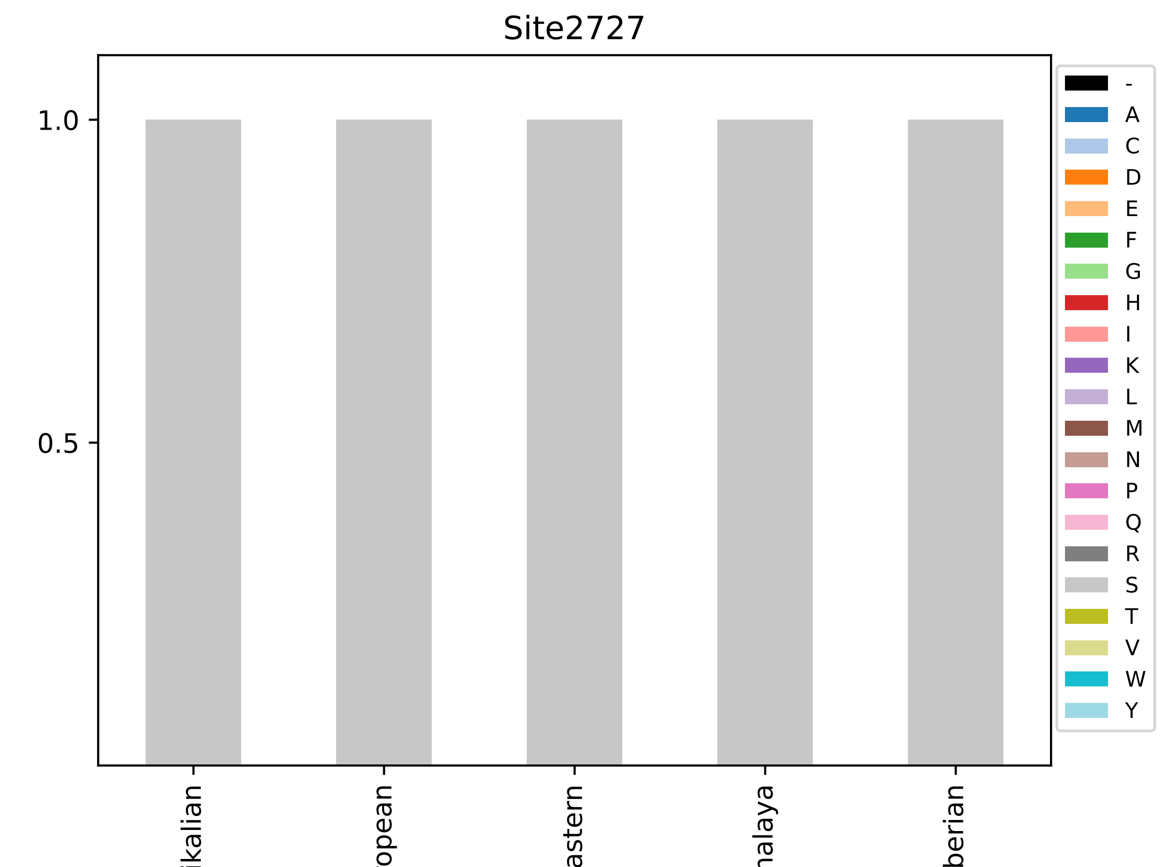

Site2727
1.0
0.5
ikalian
ropean
astern
nalaya
berian
-
A
C
D
E
F
G
H
I
K
L
M
N
P
Q
R
S
T
V
W
Y

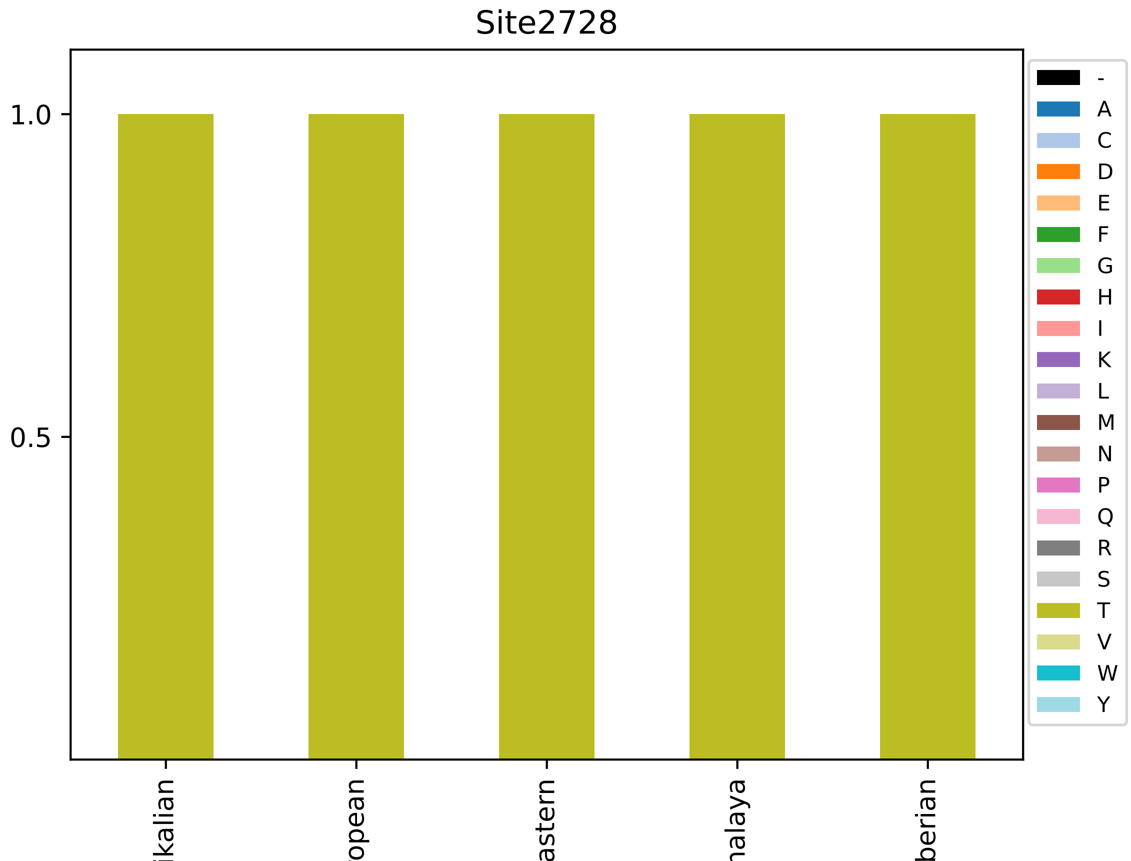
Site2728
1.0
0.5
-
A
C
D
E
F
G
H
I
K
L
M
N
P
Q
R
S
T
V
W
Y
ikalian
opean
astern
halaya
berian

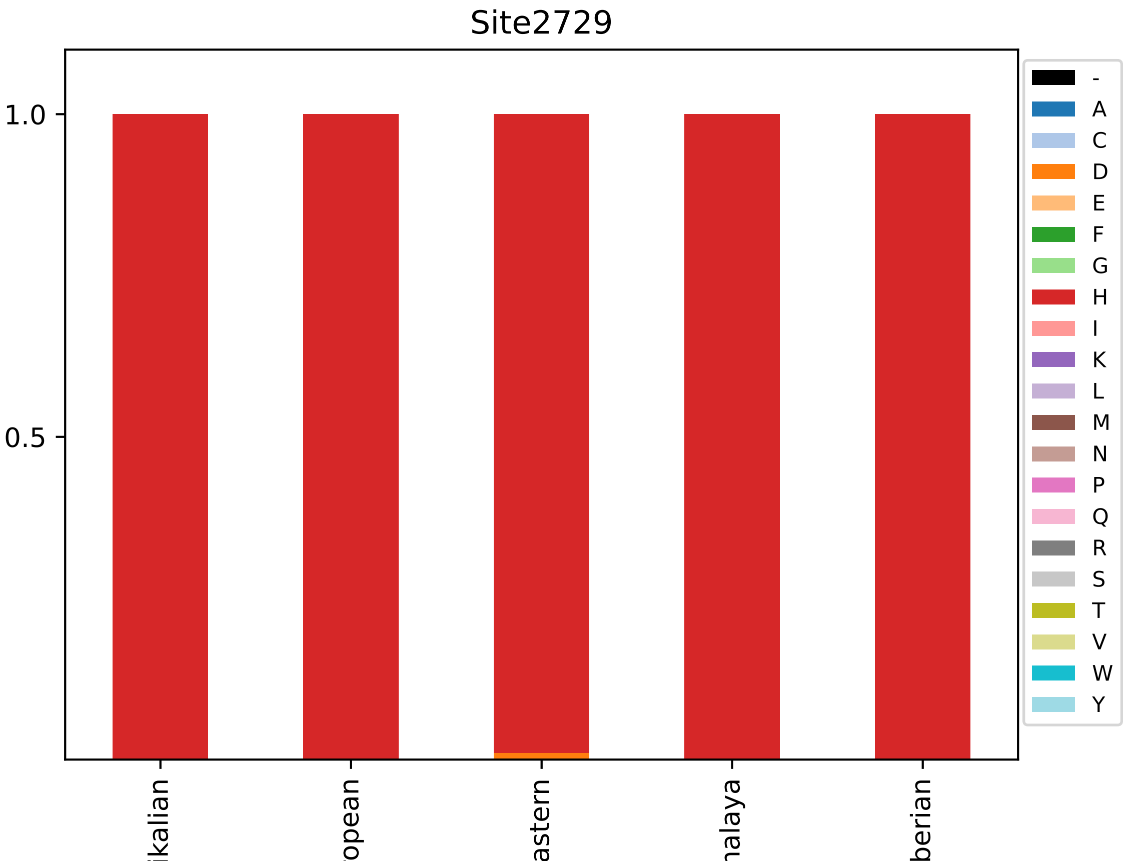
Site2729
1.0
0.5
-
A
C
D
E
F
G
H
I
K
L
M
N
P
Q
R
S
T
V
W
Y

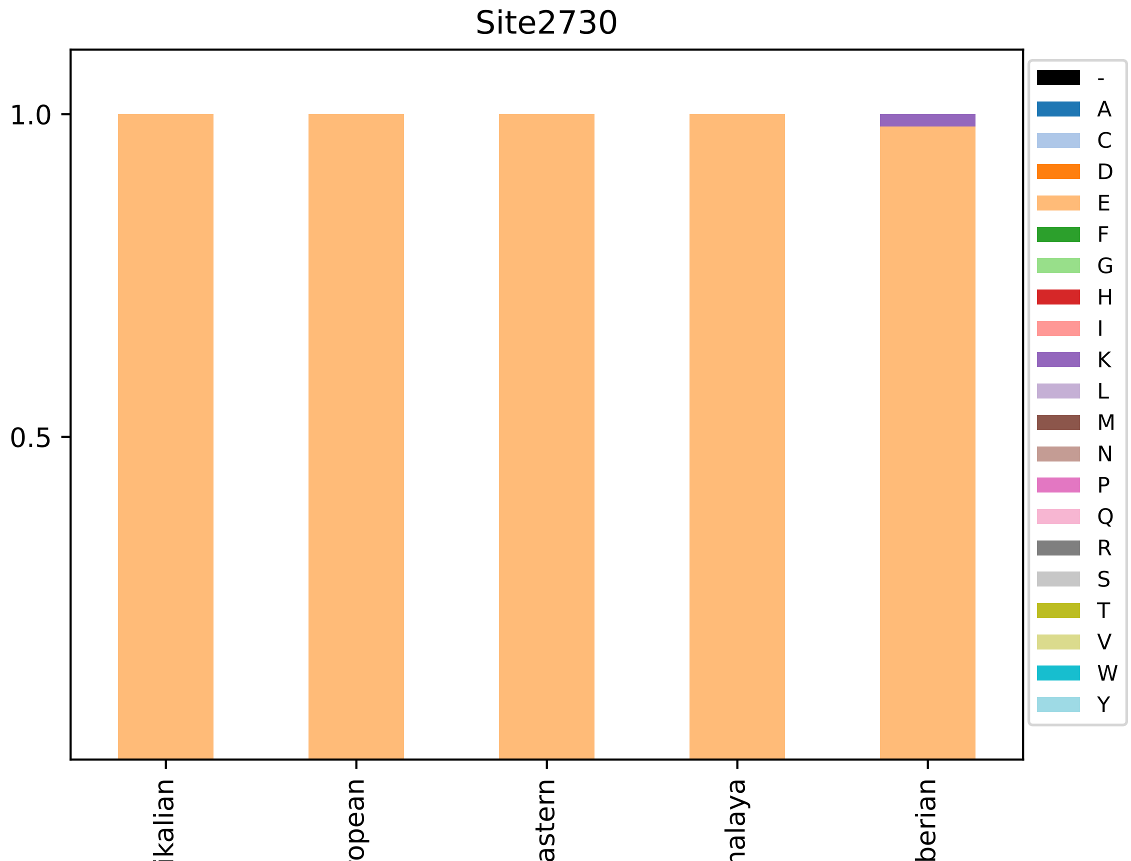
Site2730
1.0
0.5
-
A
C
D
E
F
G
H
I
K
L
M
N
P
Q
R
S
T
V
W
Y
ikalian
opean
astern
nalaya
berian

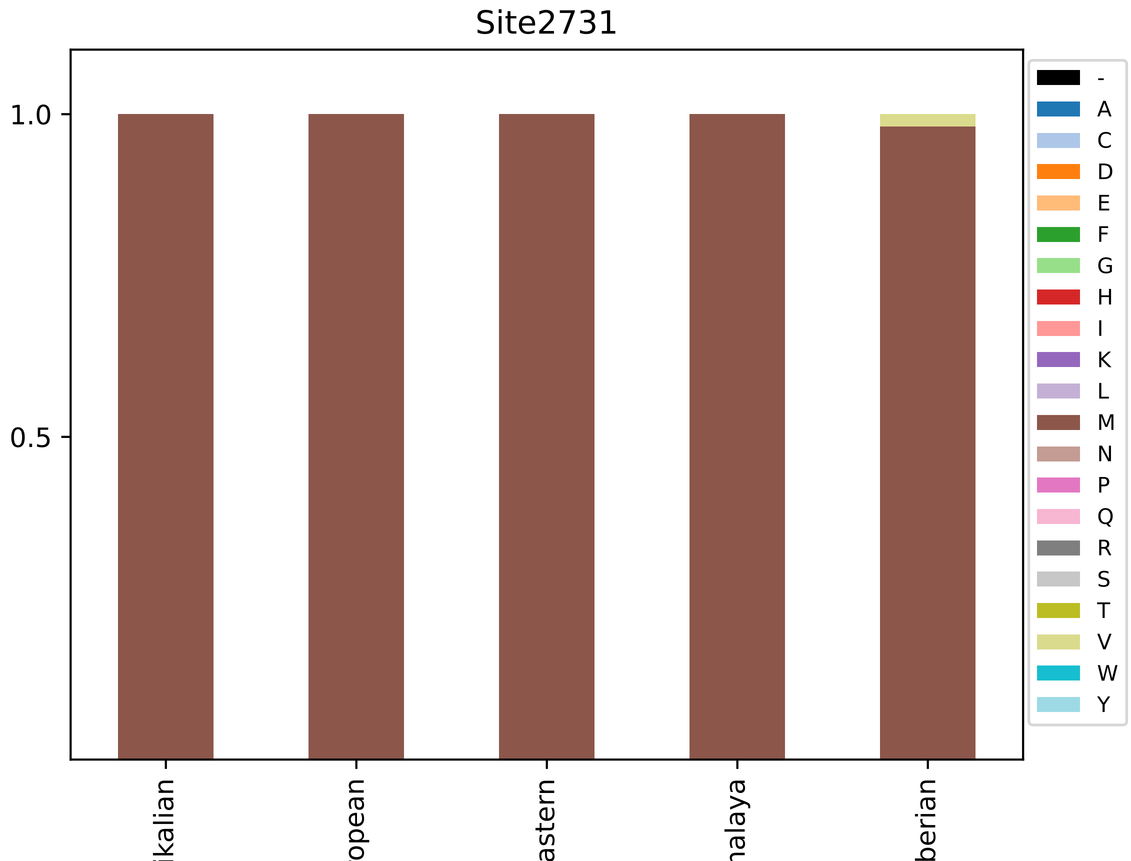

Site2731
1.0
0.5
-
A
C
D
E
F
G
H
I
K
L
M
N
P
Q
R
S
T
V
W
Y
ikalian
opean
astern
nalaya
berian

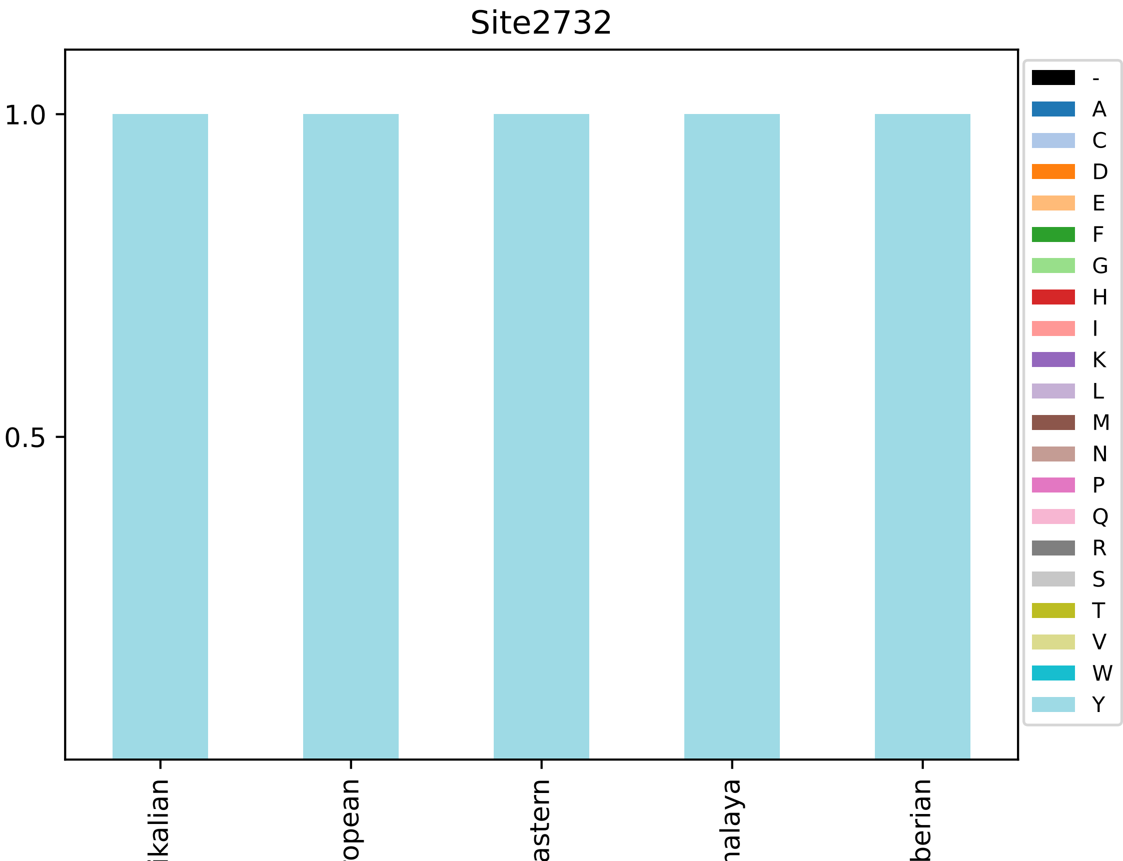
Site2732
1.0
0.5
-
A
C
D
E
F
G
H
I
K
L
M
N
P
Q
R
S
T
V
W
Y

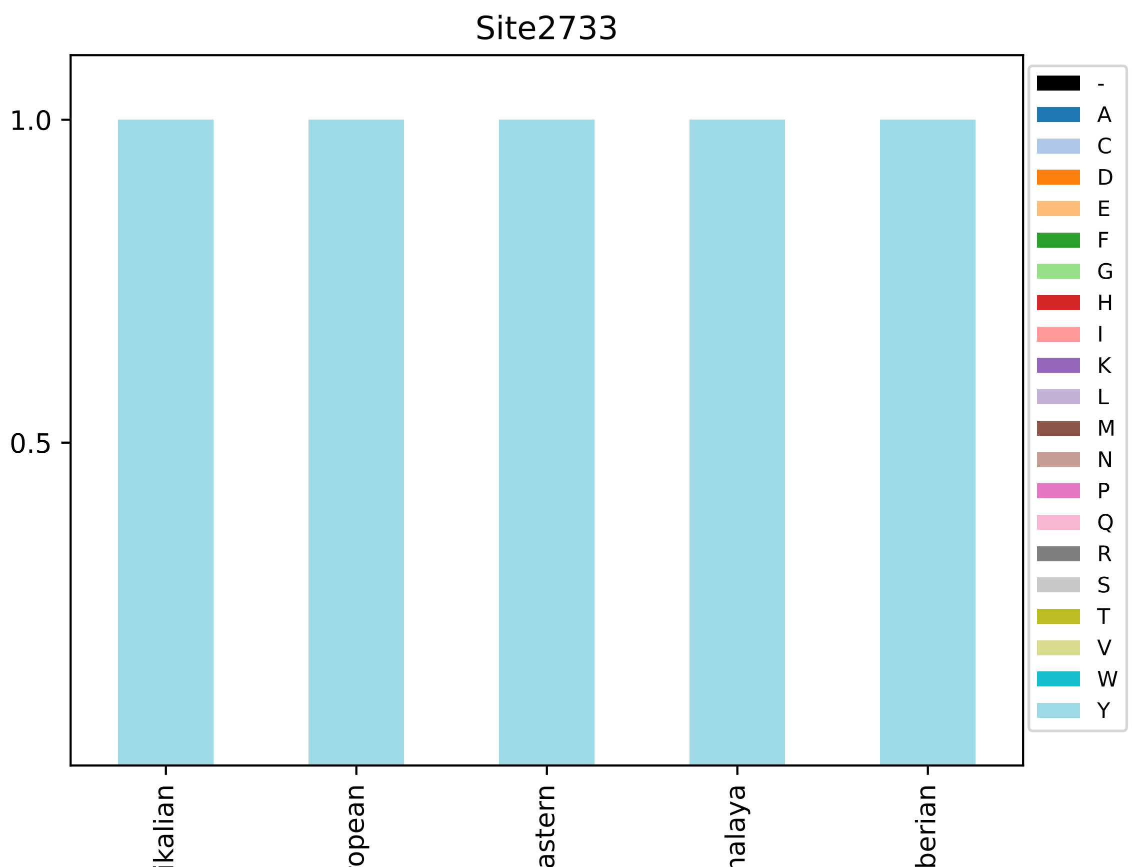

Site2733
1.0
0.5
-
A
C
D
E
F
G
H
I
K
L
M
N
P
Q
R
S
T
V
W
Y
ikalian
opean
astern
halaya
berian

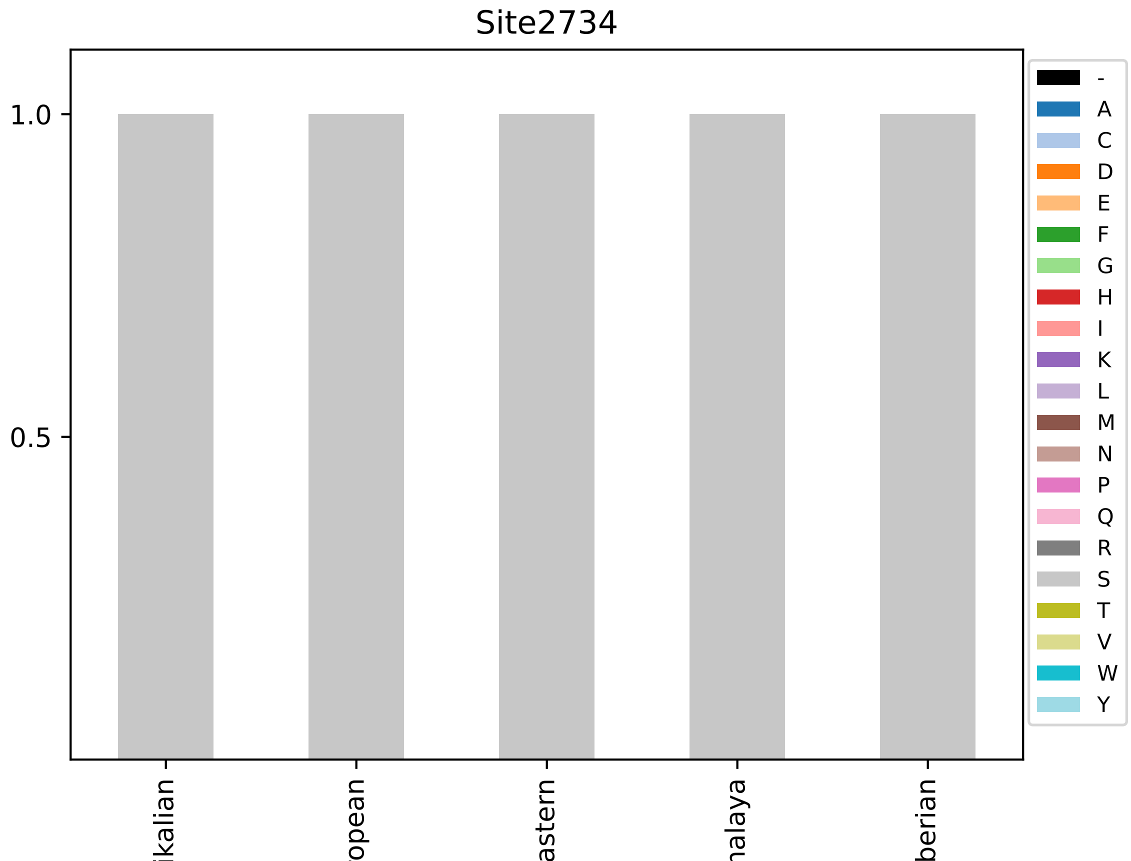
Site2734
1.0
0.5
-
A
C
D
E
F
G
H
I
K
L
M
N
P
Q
R
S
T
V
W
Y
ikalian
opean
astern
nalaya
berian

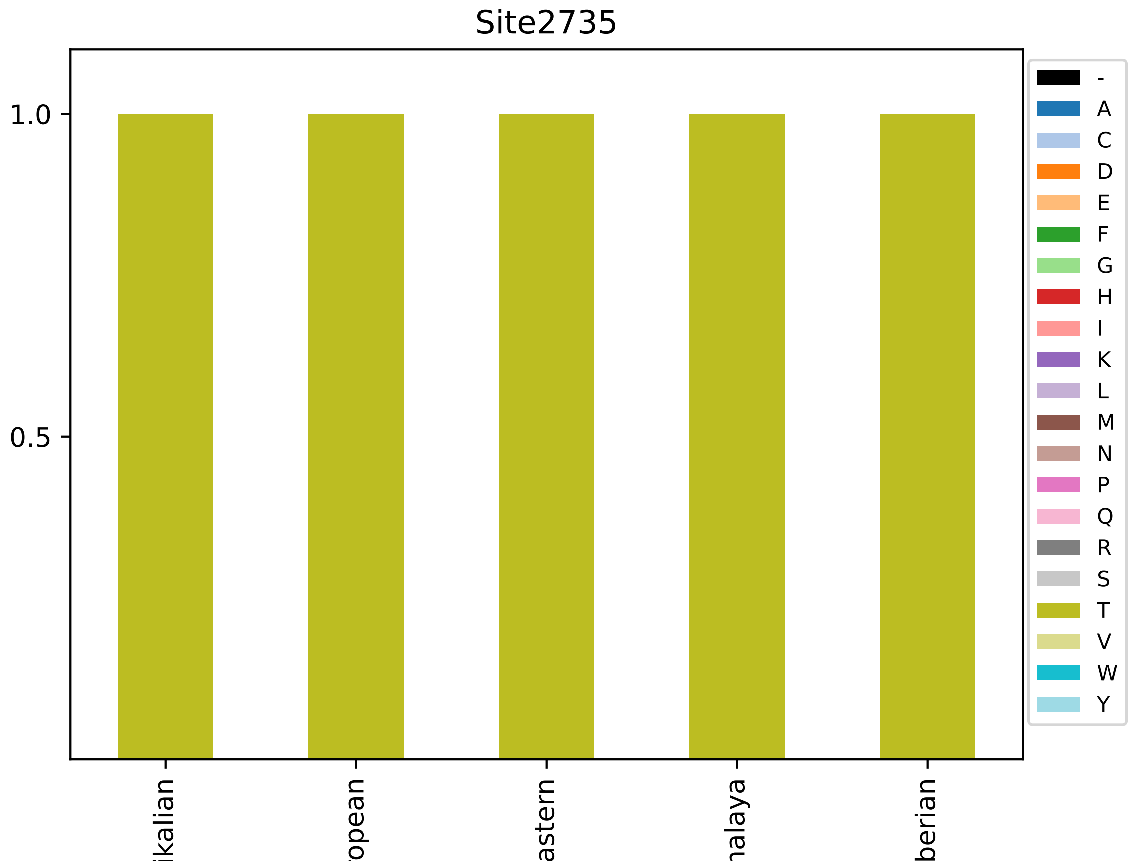
Site2735
1.0
0.5
-
A
C
D
E
F
G
H
I
K
L
M
N
P
Q
R
S
T
V
W
Y
ikalian
opean
astern
nalaya
berian

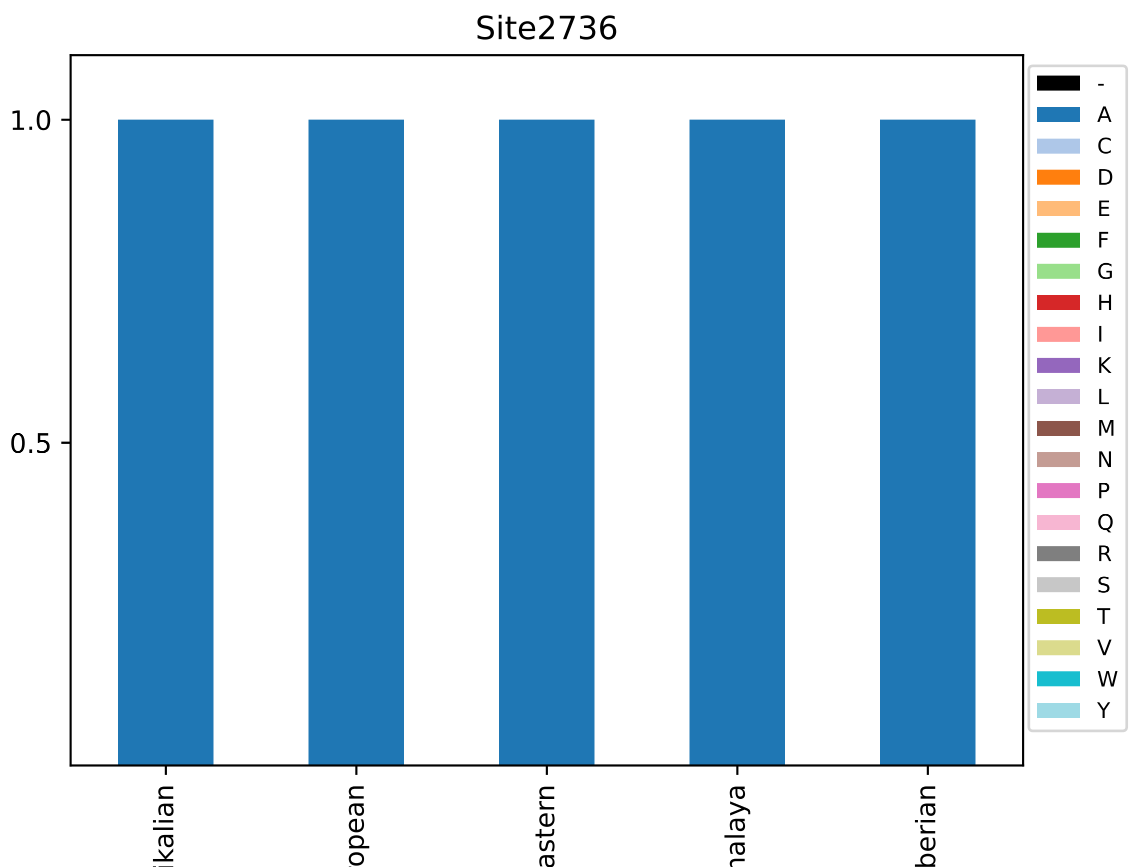

Site2736
1.0
0.5
-
A
C
D
E
F
G
H
I
K
L
M
N
P
Q
R
S
T
V
W
Y
ikalian
opean
astern
nalaya
berian

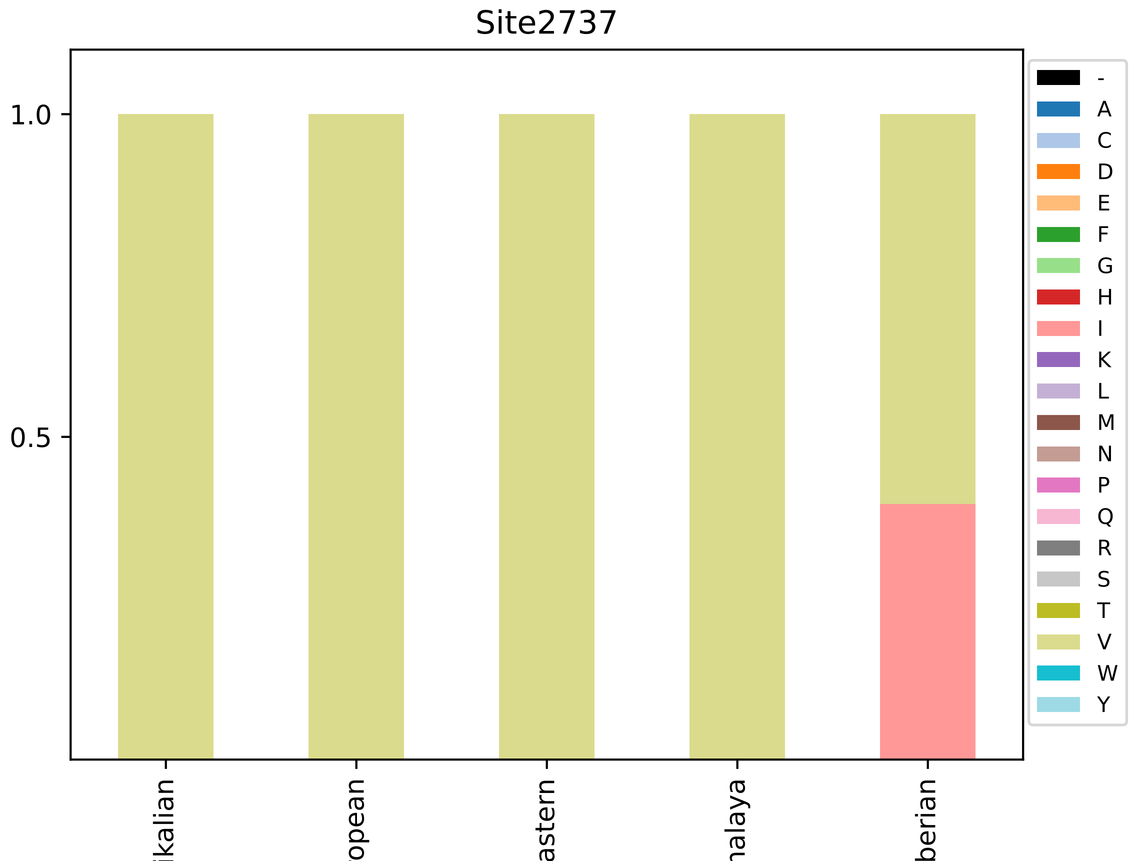
Site2737
1.0
0.5
-
A
C
D
E
F
G
H
I
K
L
M
N
P
Q
R
S
T
V
W
Y
ikalian
opean
astern
nalaya
berian

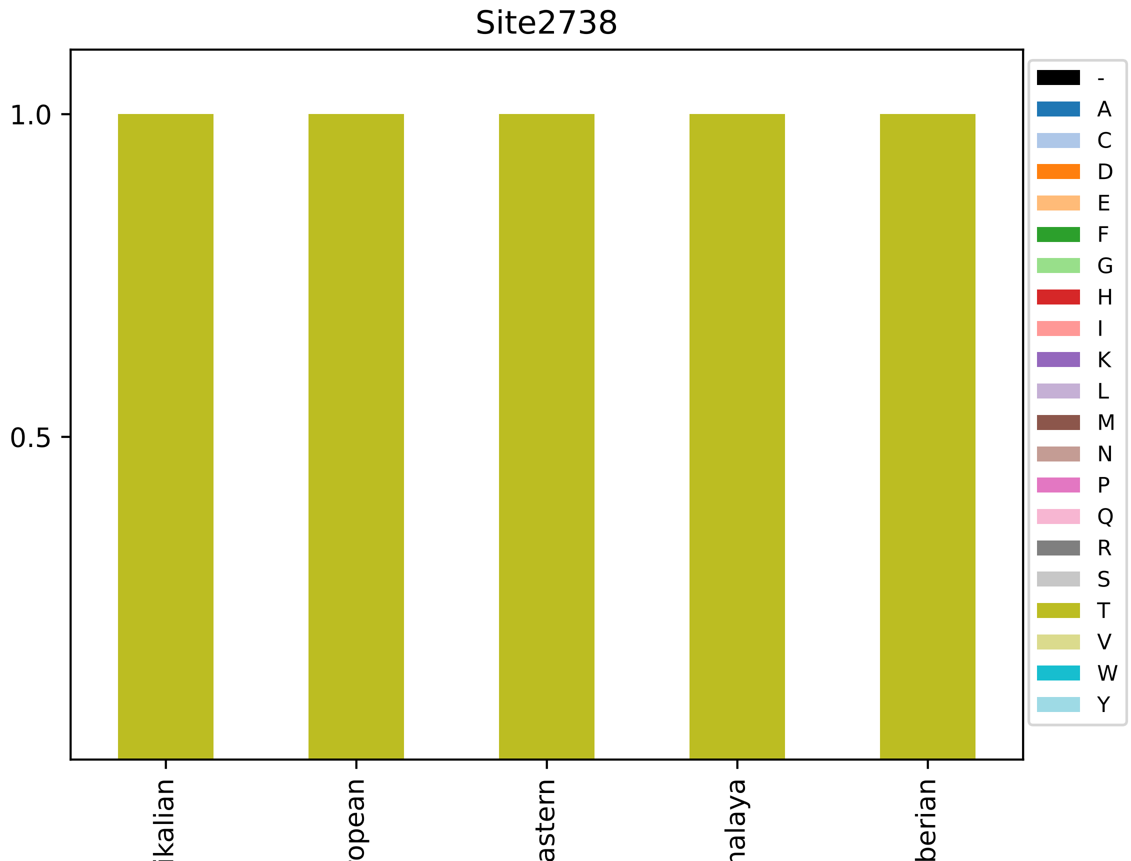
Site2738
1.0
0.5
ikalian
opean
astern
nalaya
berian
-
A
C
D
E
F
G
H
I
K
L
M
N
P
Q
R
S
T
V
W
Y

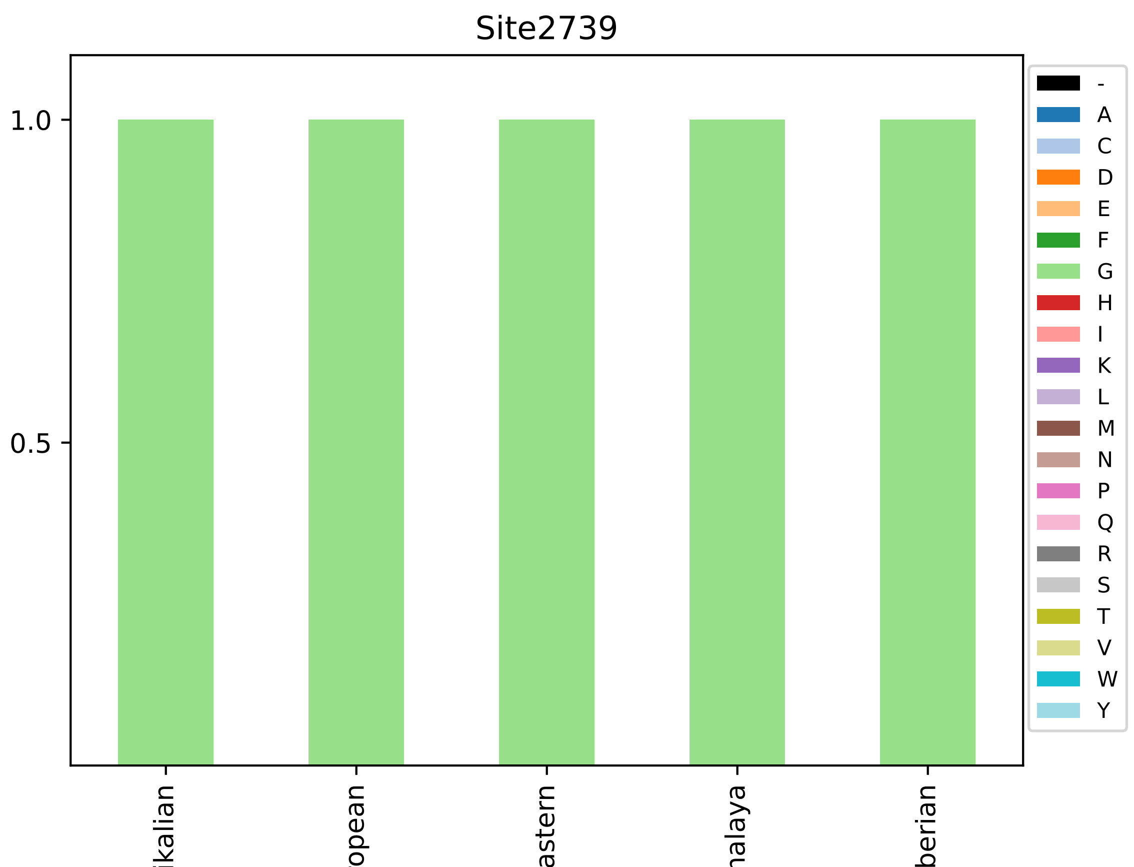

Site2739
1.0
0.5
-
A
C
D
E
F
G
H
I
K
L
M
N
P
Q
R
S
T
V
W
Y
ikalian
opean
astern
nalaya
berian

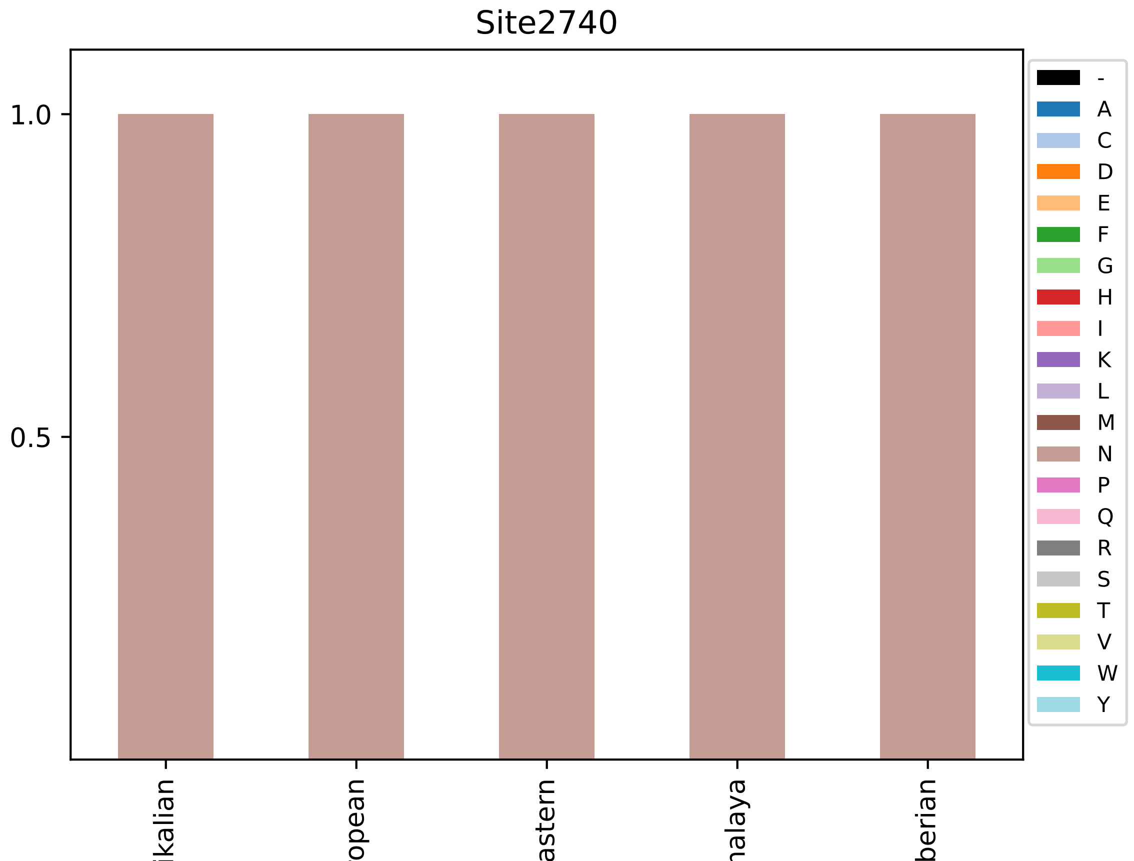
Site2740
1.0
0.5
ikalian
opean
astern
nalaya
berian
-
A
C
D
E
F
G
H
I
K
L
M
N
P
Q
R
S
T
V
W
Y

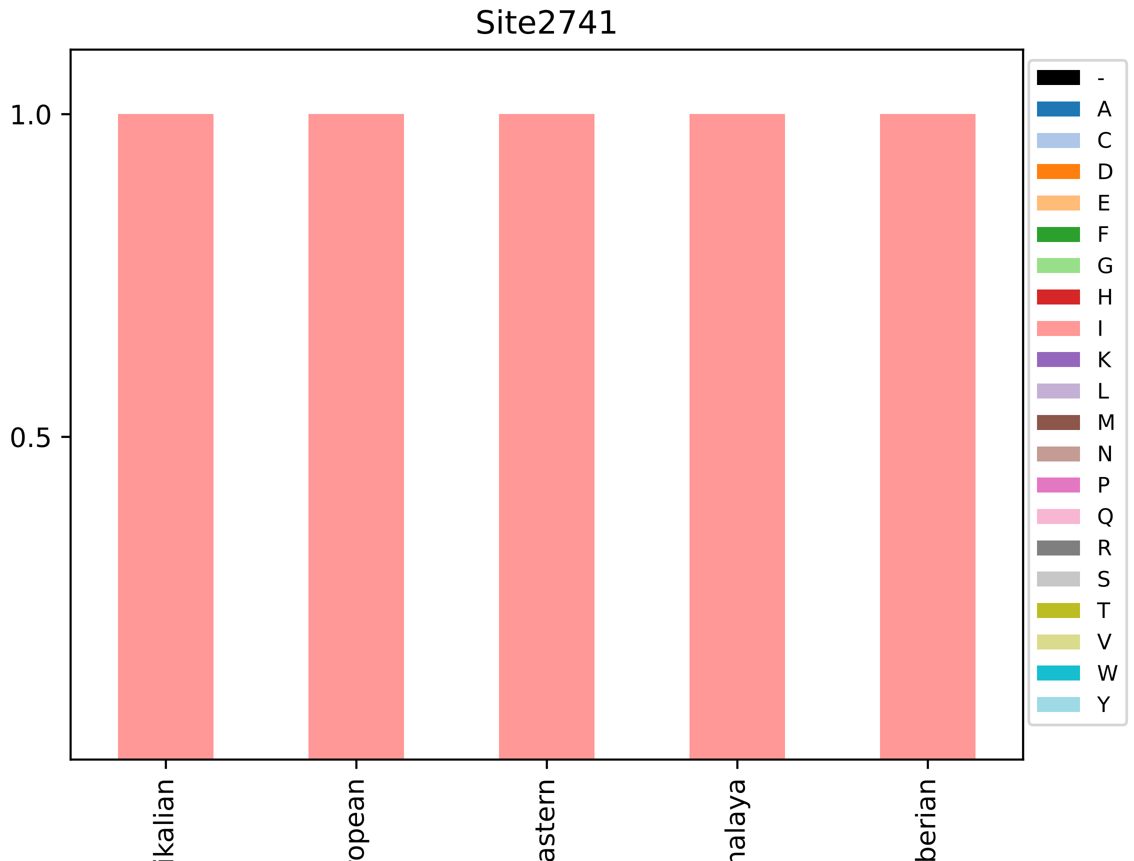

Site2741
1.0
0.5
-
A
C
D
E
F
G
H
I
K
L
M
N
P
Q
R
S
T
V
W
Y
ikalian
opean
astern
nalaya
berian

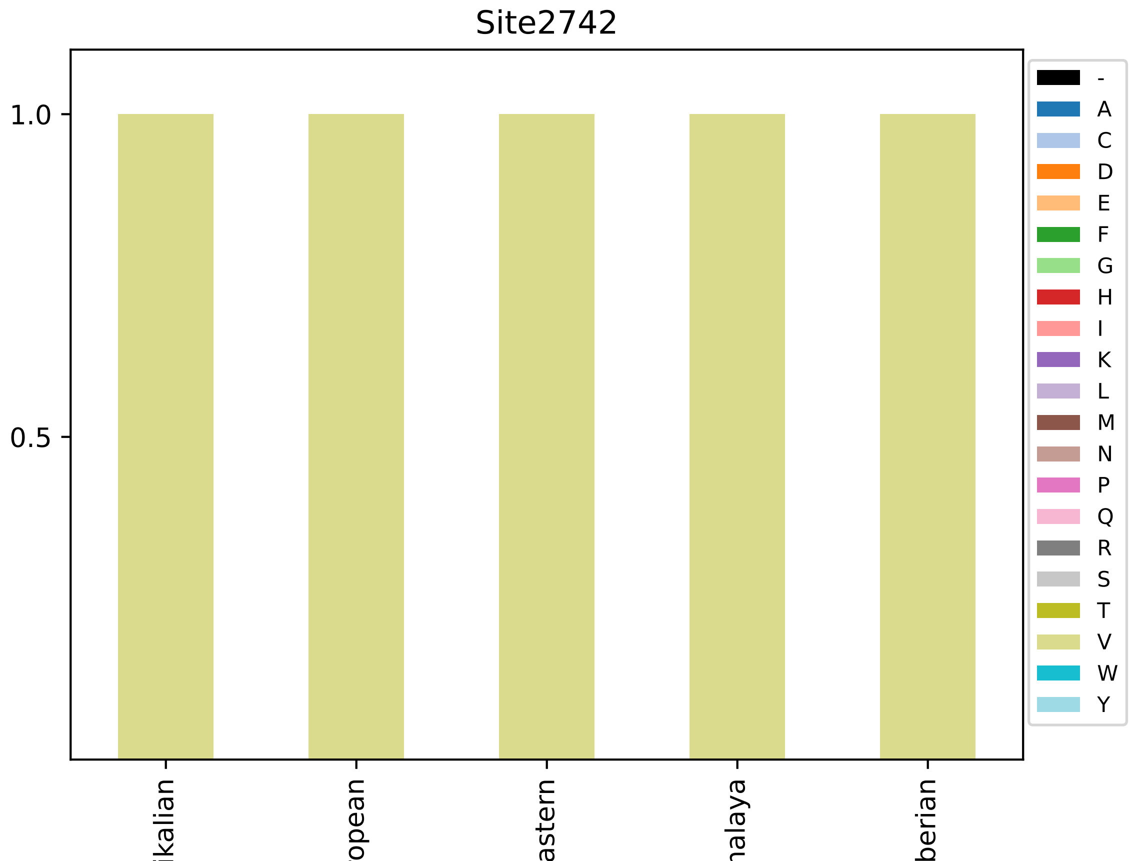

Site2742
1.0
0.5
-
A
C
D
E
F
G
H
I
K
L
M
N
P
Q
R
S
T
V
W
Y
ikalian
opean
astern
nalaya
berian

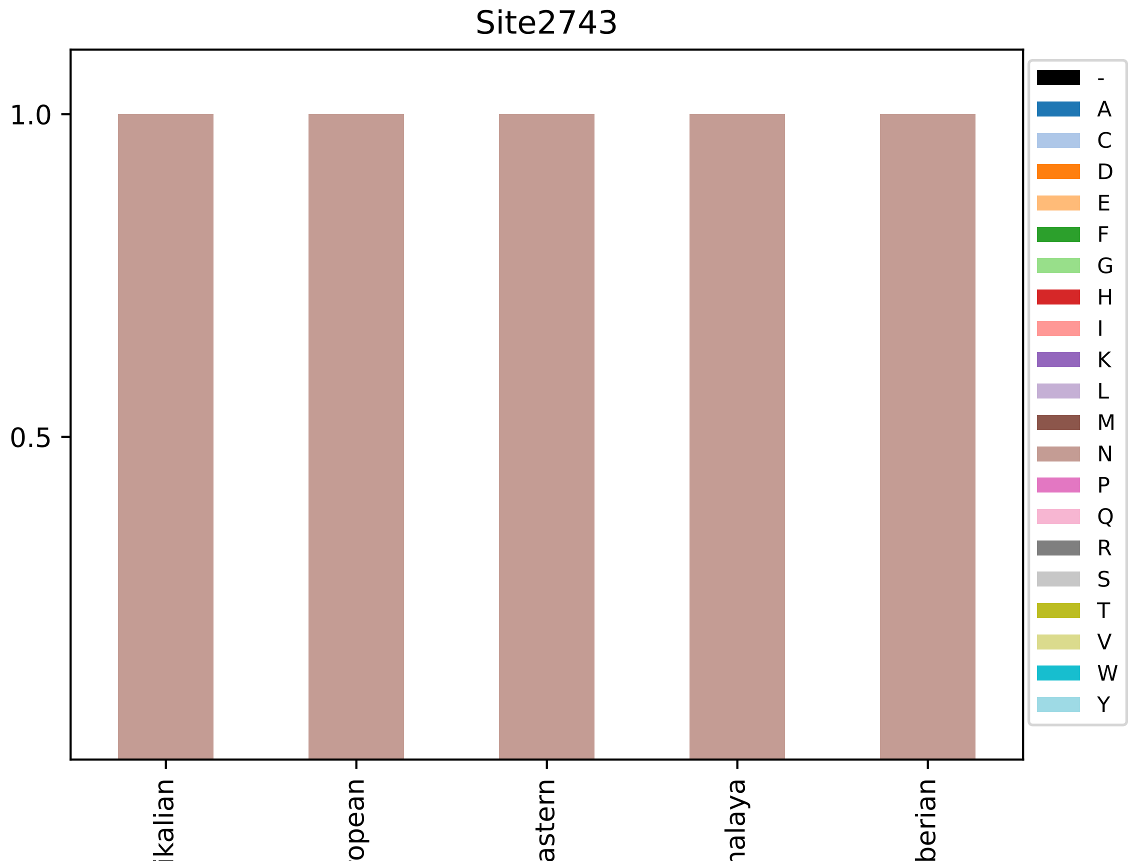
Site2743
1.0
0.5
-
A
C
D
E
F
G
H
I
K
L
M
N
P
Q
R
S
T
V
W
Y
ikalian
opean
astern
nalaya
berian

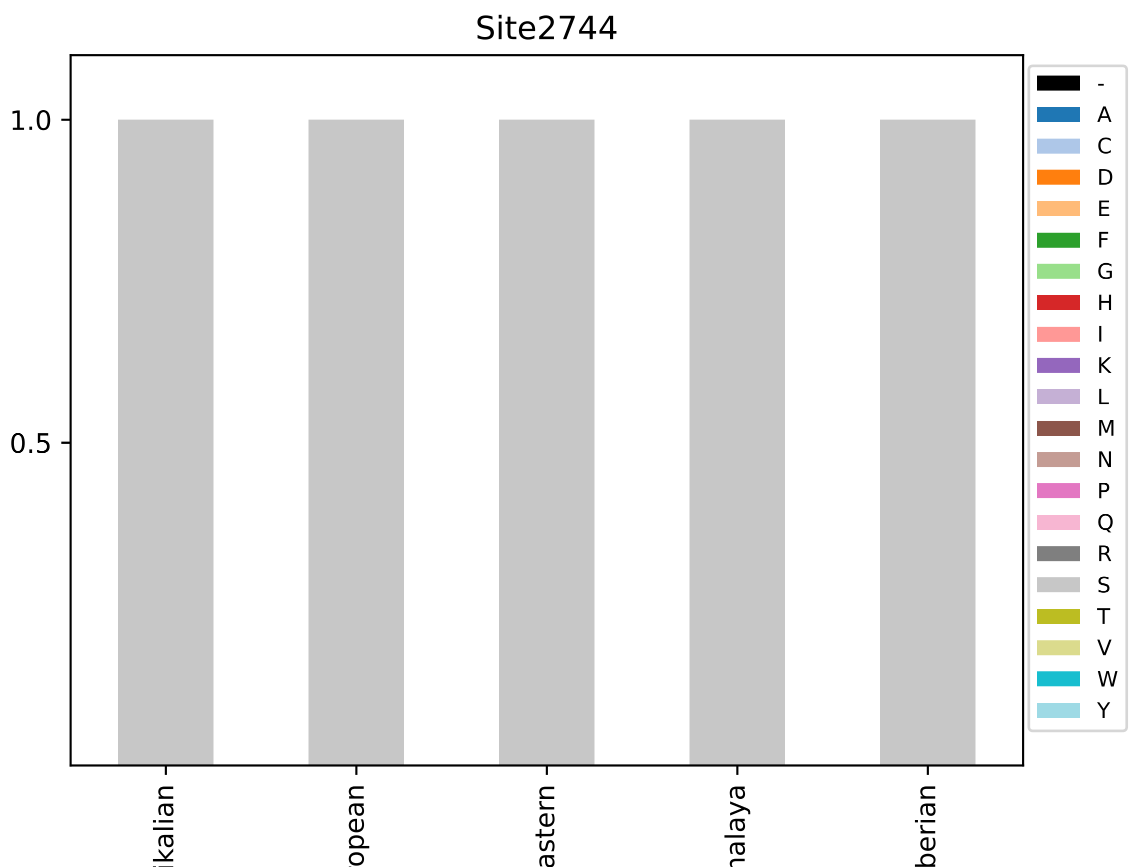
Site2744
1.0
0.5
-
A
C
D
E
F
G
H
I
K
L
M
N
P
Q
R
S
T
V
W
Y
ikalian
opean
astern
nalaya
berian

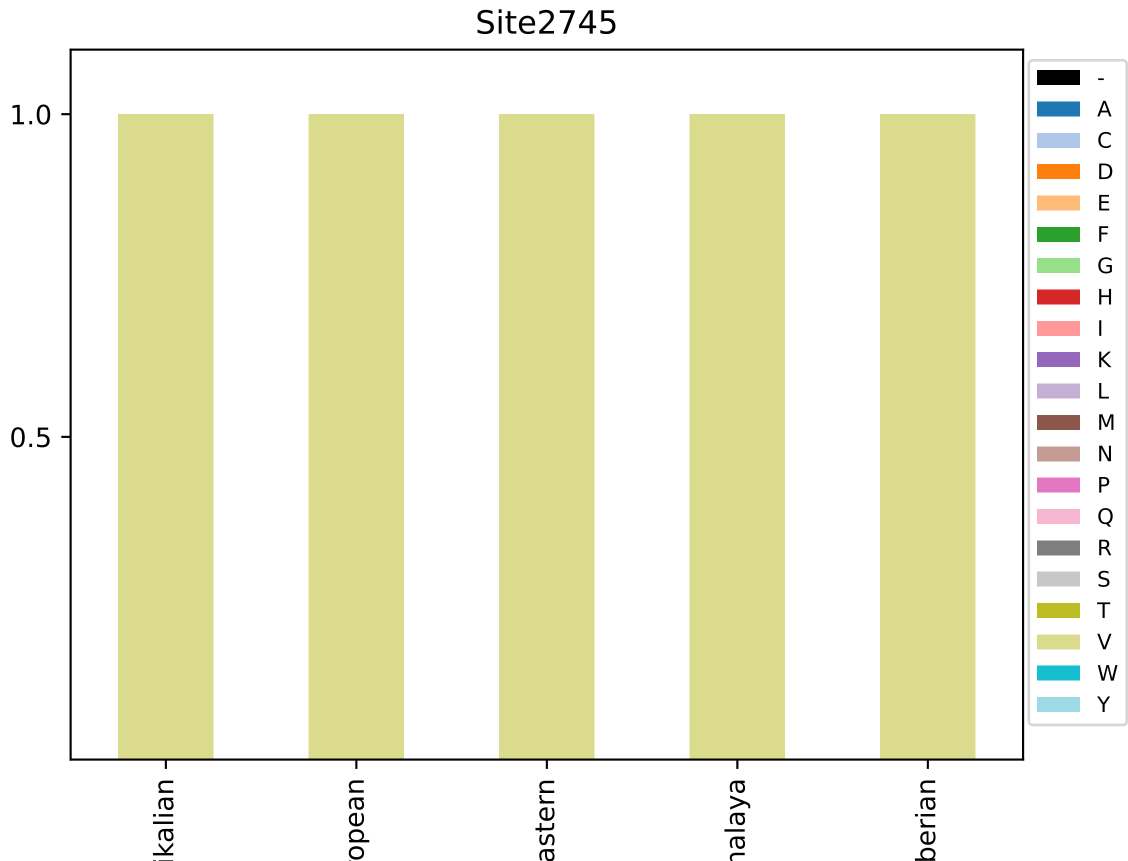
Site2745
1.0
0.5
-
A
C
D
E
F
G
H
I
K
L
M
N
P
Q
R
S
T
V
W
Y
ikalian
opean
astern
nalaya
berian

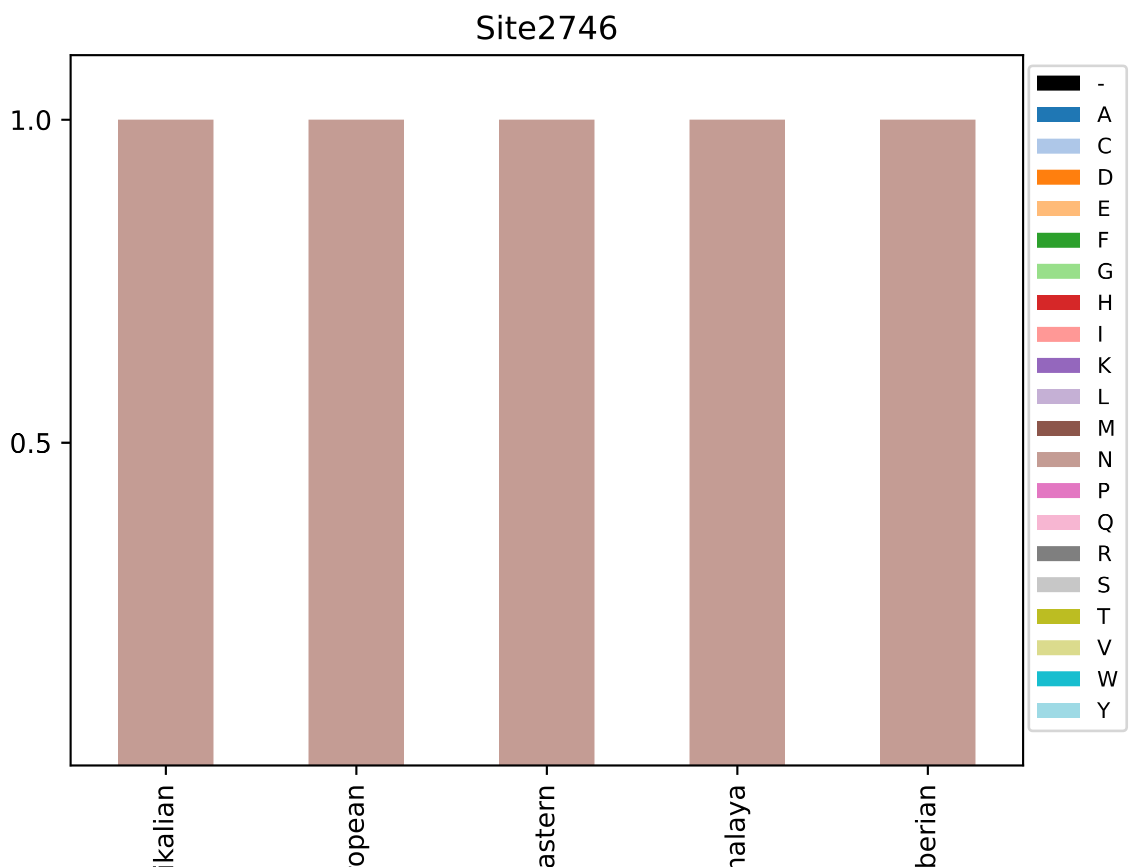
Site2746
1.0
0.5
-
A
C
D
E
F
G
H
I
K
L
M
N
P
Q
R
S
T
V
W
Y
ikalian
opean
astern
nalaya
berian

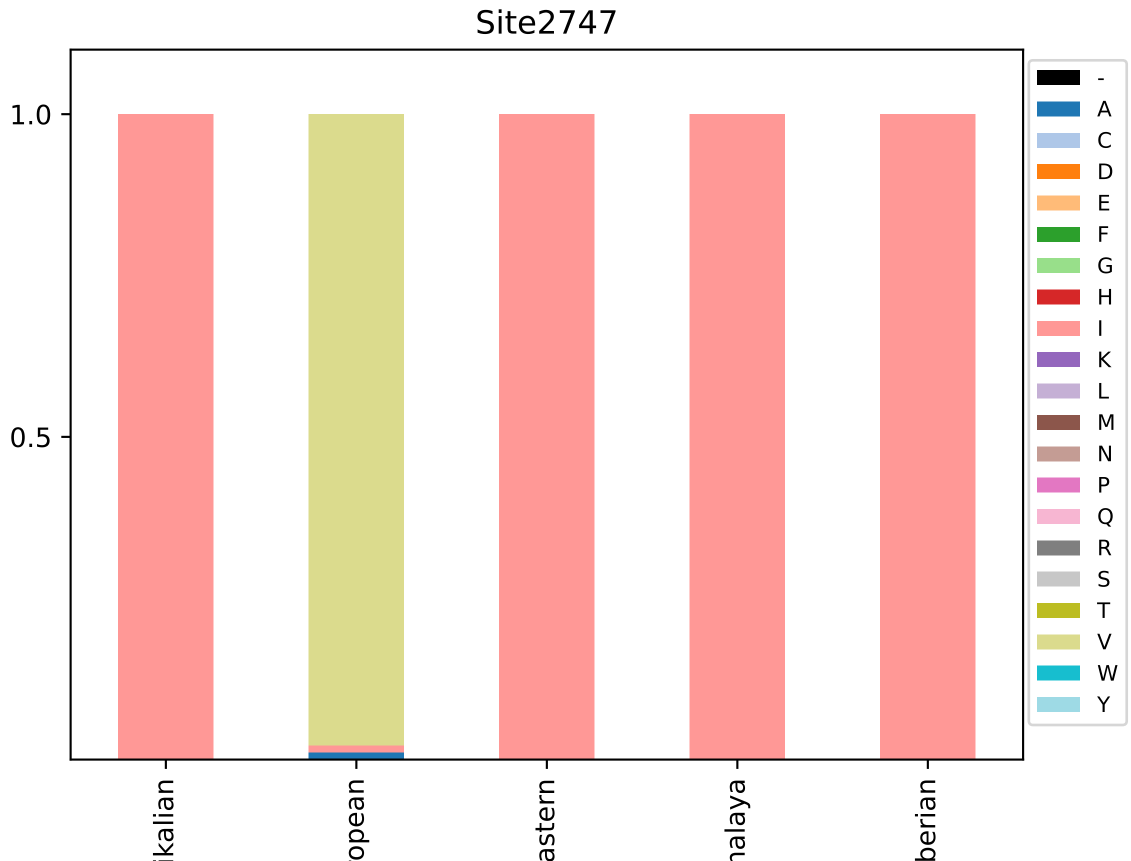

Site2747
1.0
0.5
ikalian
opean
astern
nalaya
berian
-
A
C
D
E
F
G
H
I
K
L
M
N
P
Q
R
S
T
V
W
Y

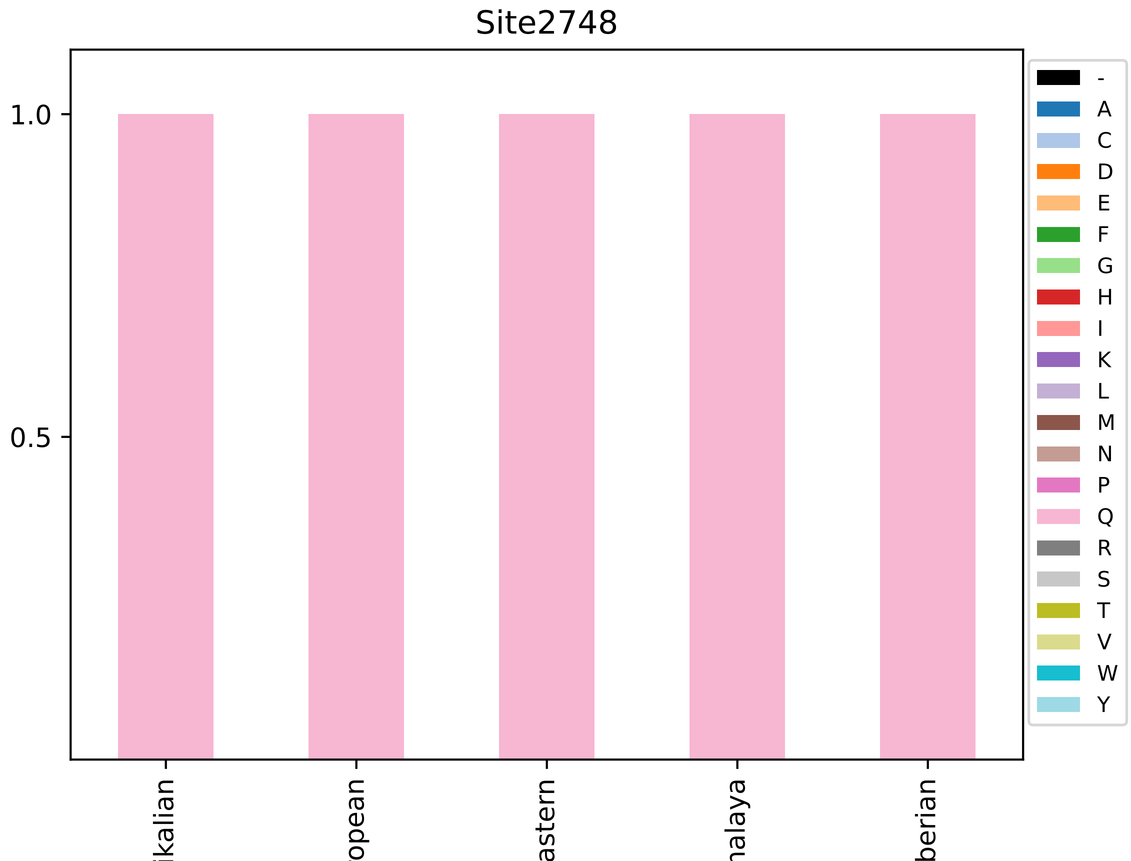

Site2748
1.0
0.5
-
A
C
D
E
F
G
H
I
K
L
M
N
P
Q
R
S
T
V
W
Y
ikalian
opean
astern
nalaya
berian

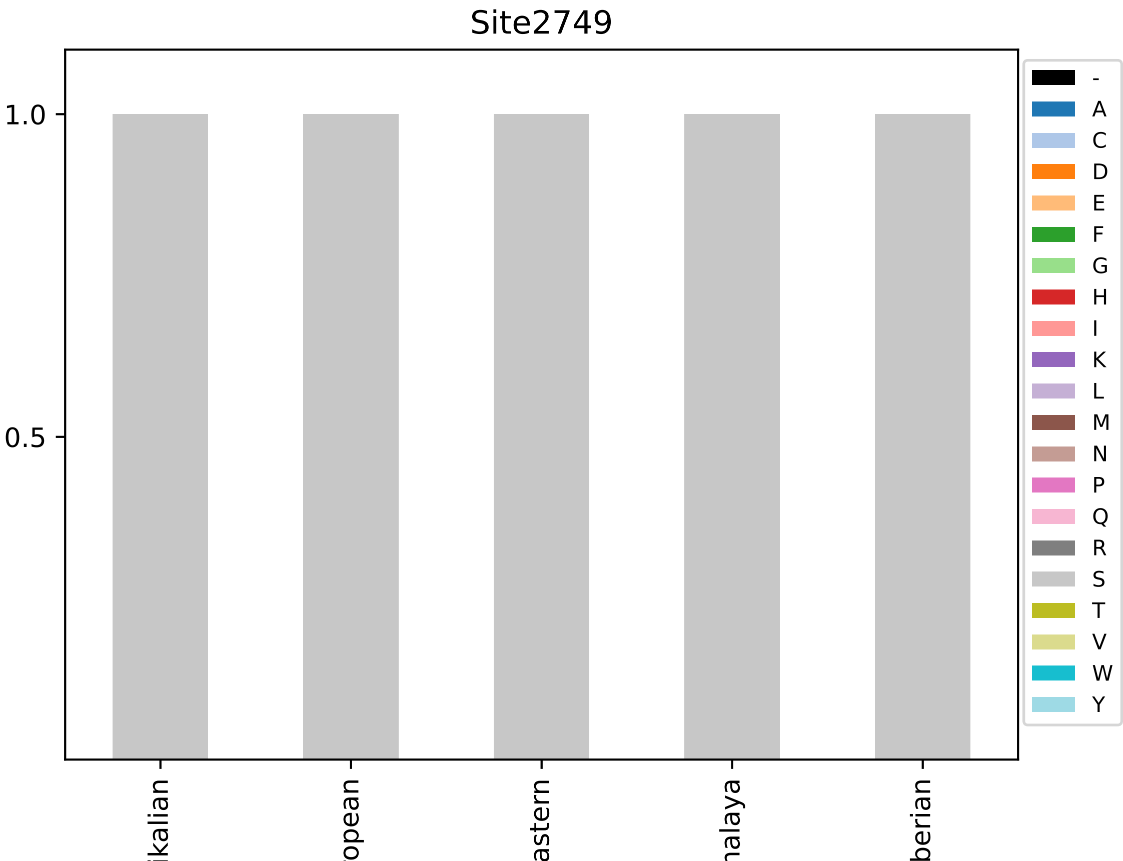
Site2749
1.0
0.5
-
A
C
D
E
F
G
H
I
K
L
M
N
P
Q
R
S
T
V
W
Y

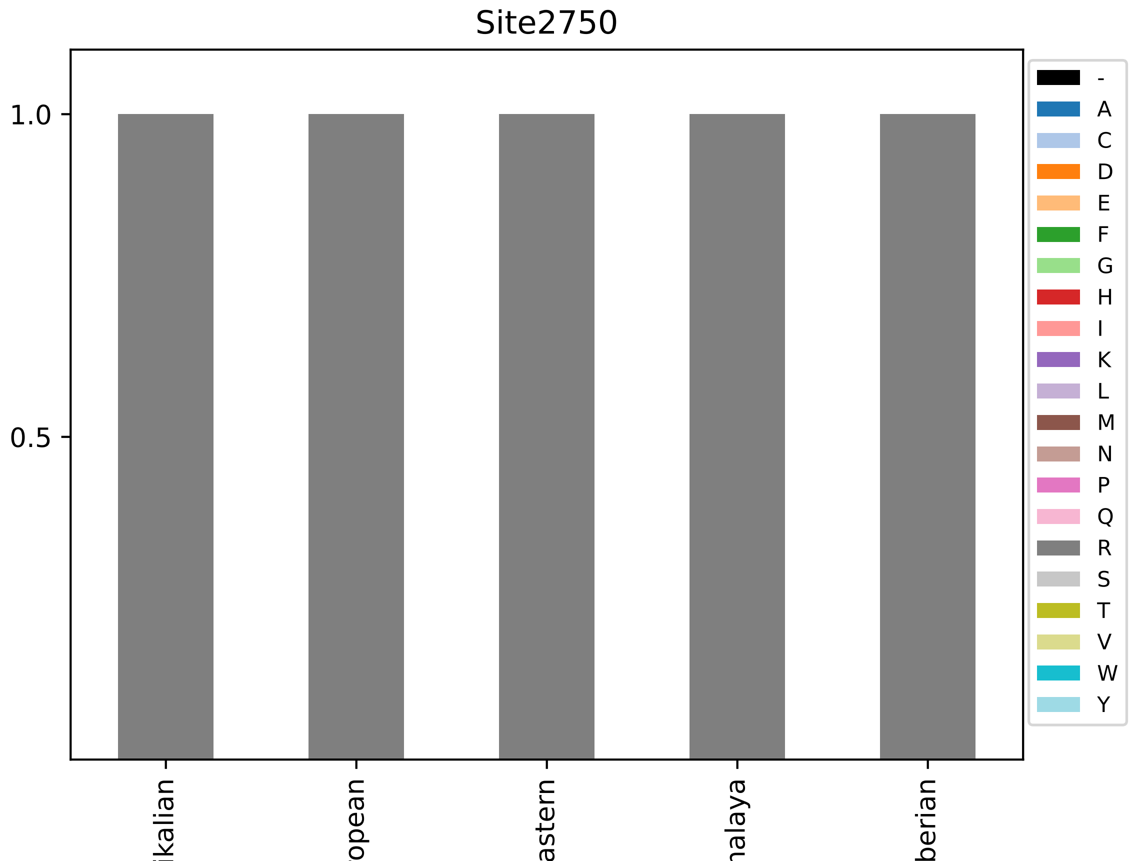
Site2750
1.0
0.5
-
A
C
D
E
F
G
H
I
K
L
M
N
P
Q
R
S
T
V
W
Y
ikalian
opean
astern
nalaya
berian

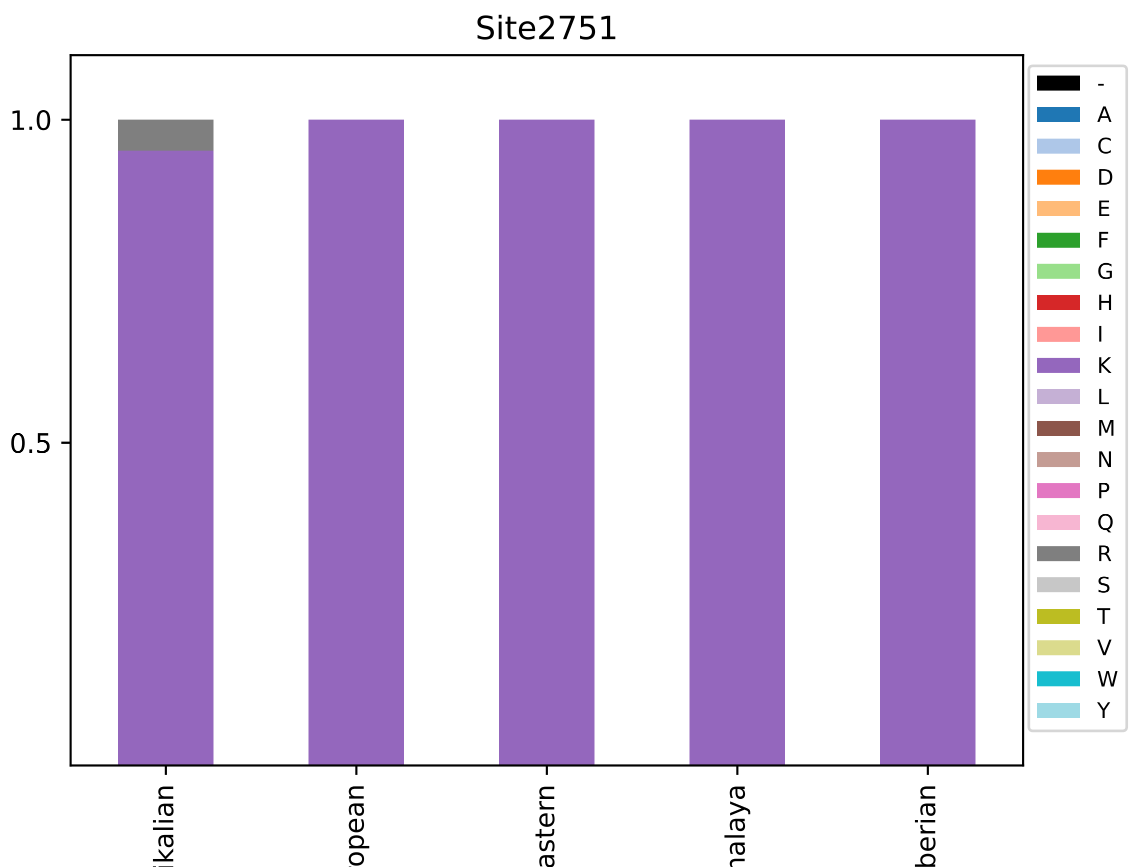
Site2751
1.0
0.5
ikalian
opean
astern
nalaya
berian
-
A
C
D
E
F
G
H
I
K
L
M
N
P
Q
R
S
T
V
W
Y

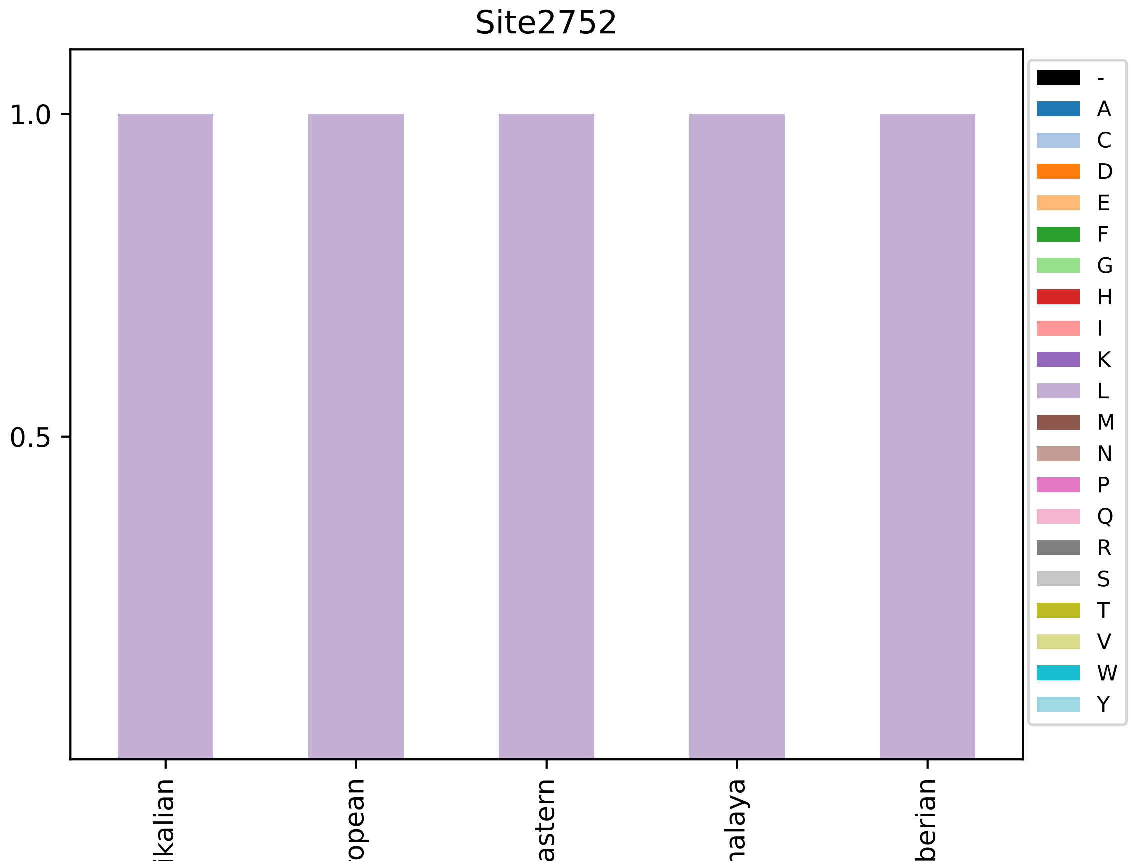
Site2752
1.0
0.5
ikalian
opean
astern
nalaya
berian
-
A
C
D
E
F
G
H
I
K
L
M
N
P
Q
R
S
T
V
W
Y

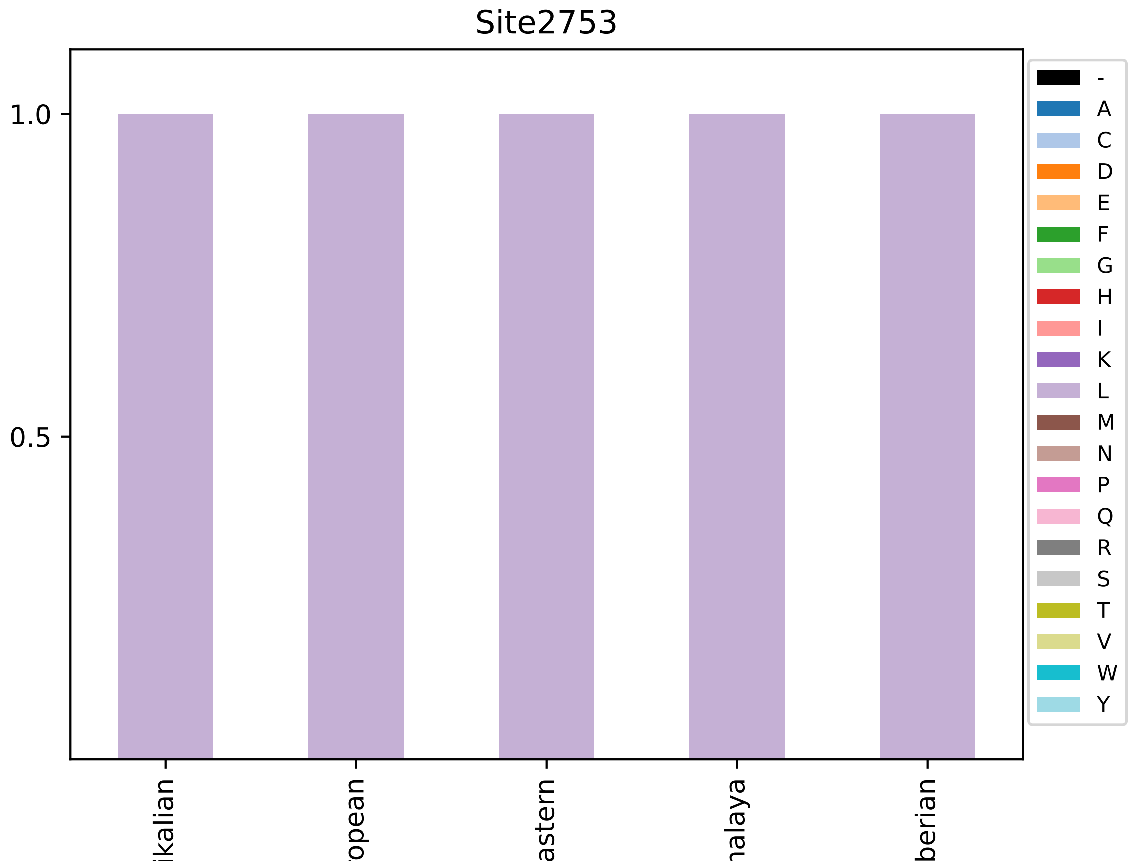
Site2753
1.0
0.5
-
A
C
D
E
F
G
H
I
K
L
M
N
P
Q
R
S
T
V
W
Y
ikalian
opean
astern
nalaya
berian

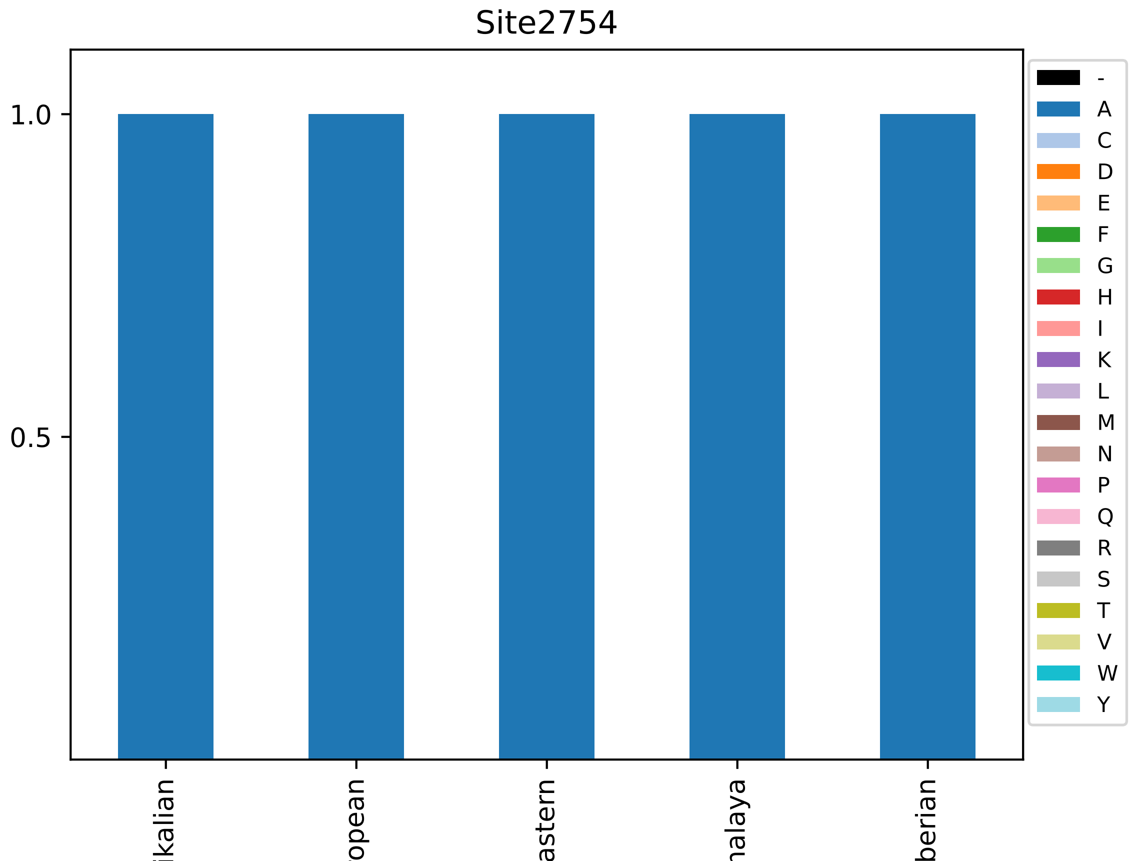

Site2754
1.0
0.5
-
A
C
D
E
F
G
H
I
K
L
M
N
P
Q
R
S
T
V
W
Y
ikalian
opean
astern
nalaya
berian

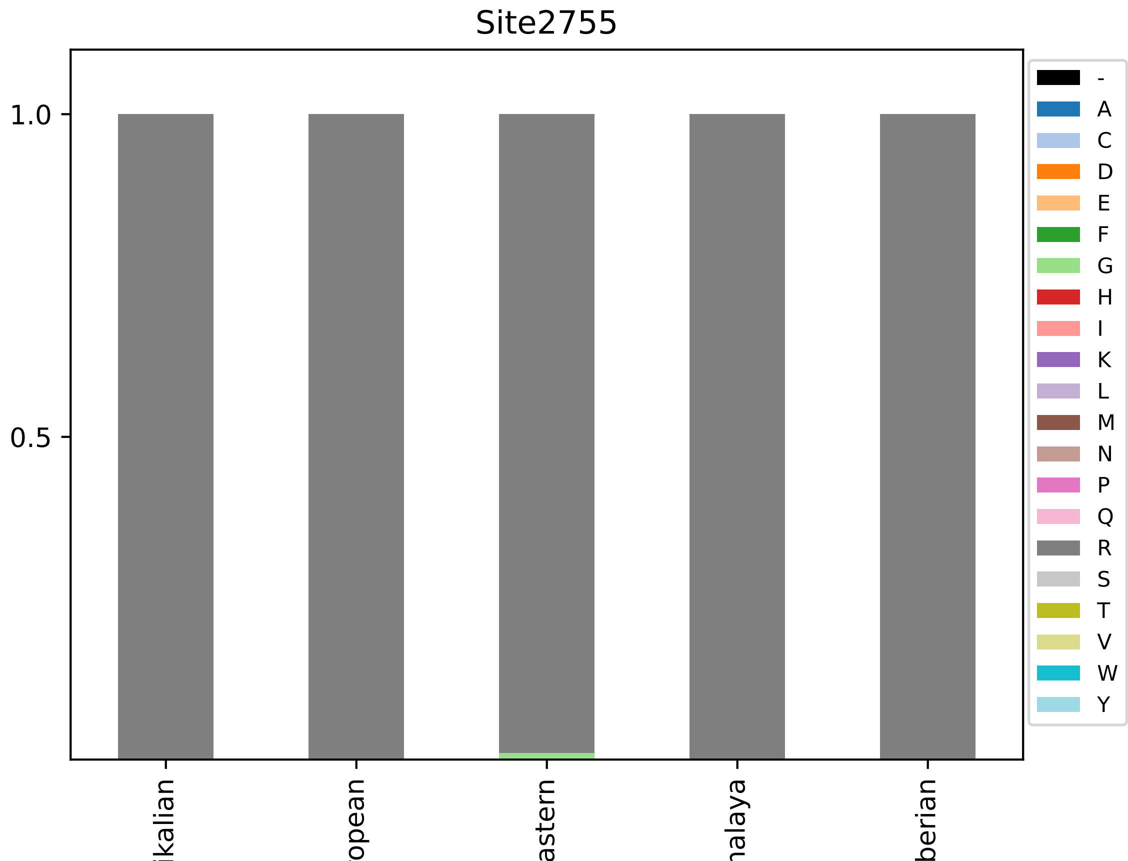

Site2755
1.0
0.5
-
A
C
D
E
F
G
H
I
K
L
M
N
P
Q
R
S
T
V
W
Y
ikalian
opean
astern
nalaya
berian

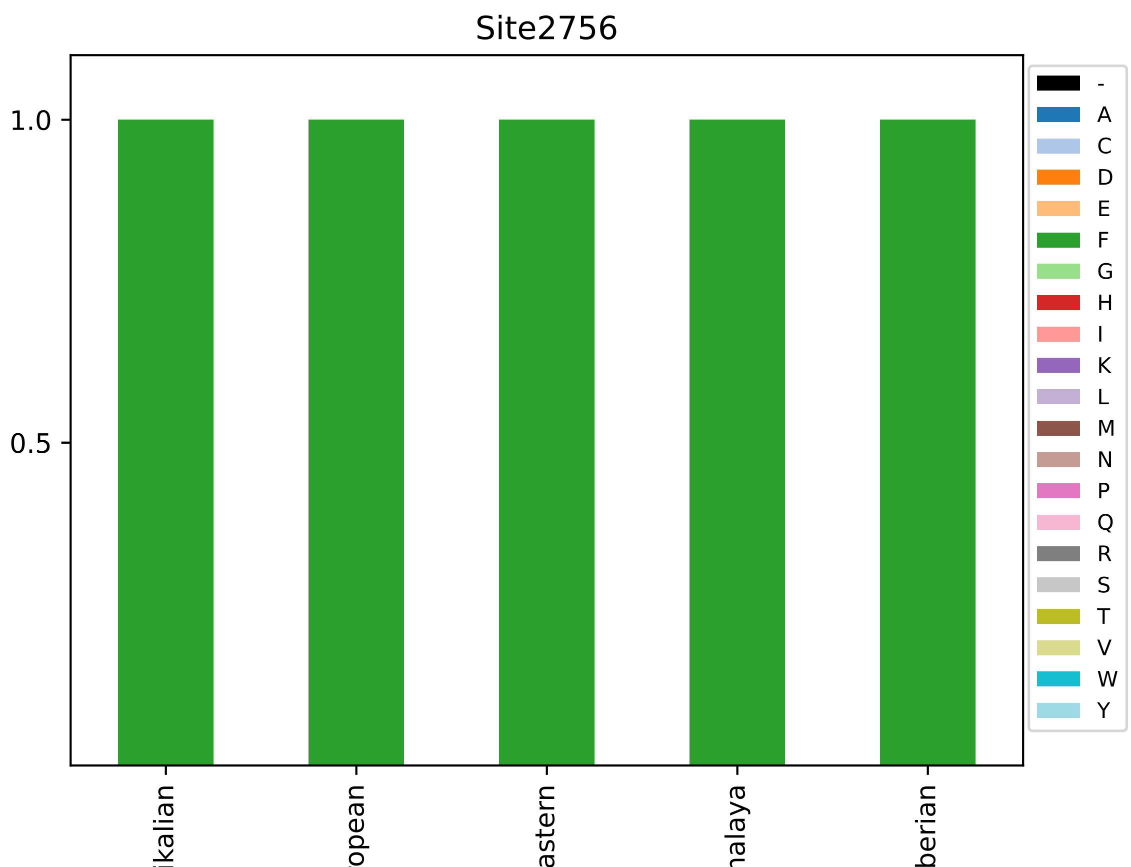
Site2756
1.0
0.5
-
A
C
D
E
F
G
H
I
K
L
M
N
P
Q
R
S
T
V
W
Y

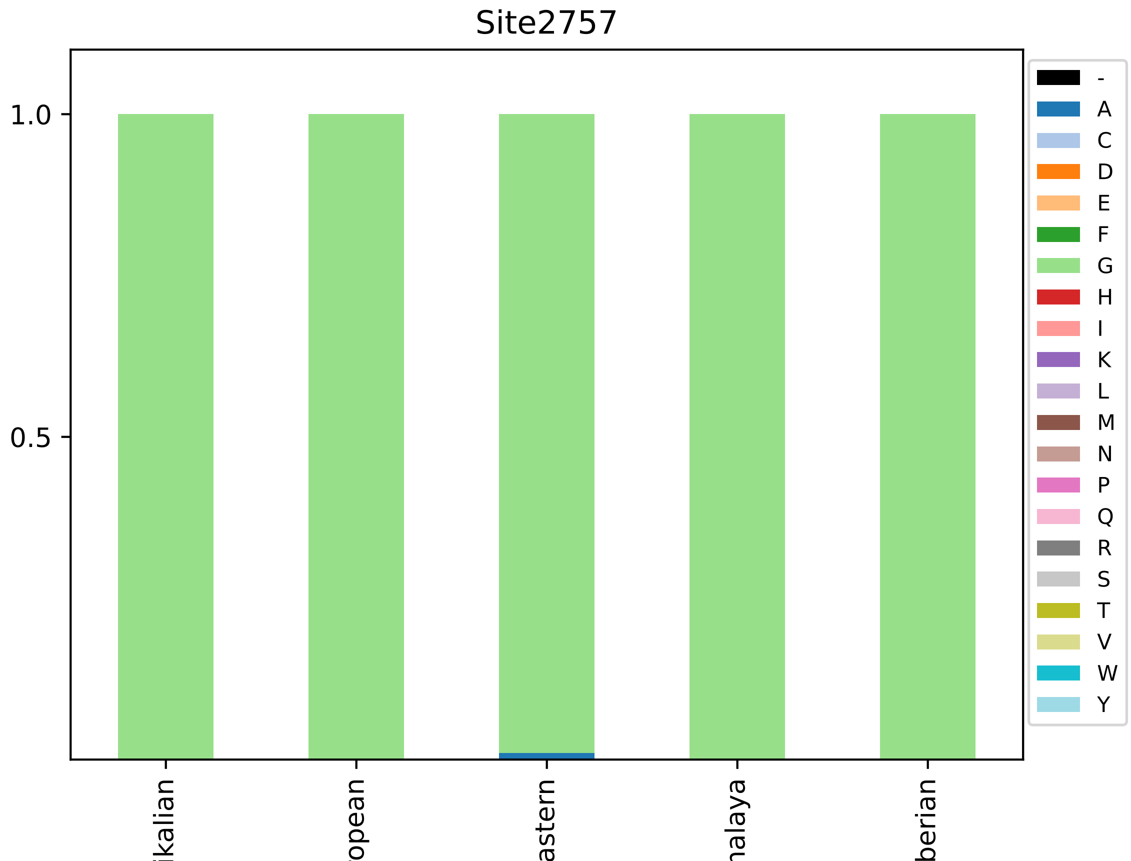
Site2757
1.0
0.5
-
A
C
D
E
F
G
H
I
K
L
M
N
P
Q
R
S
T
V
W
Y
ikalian
opean
astern
nalaya
berian

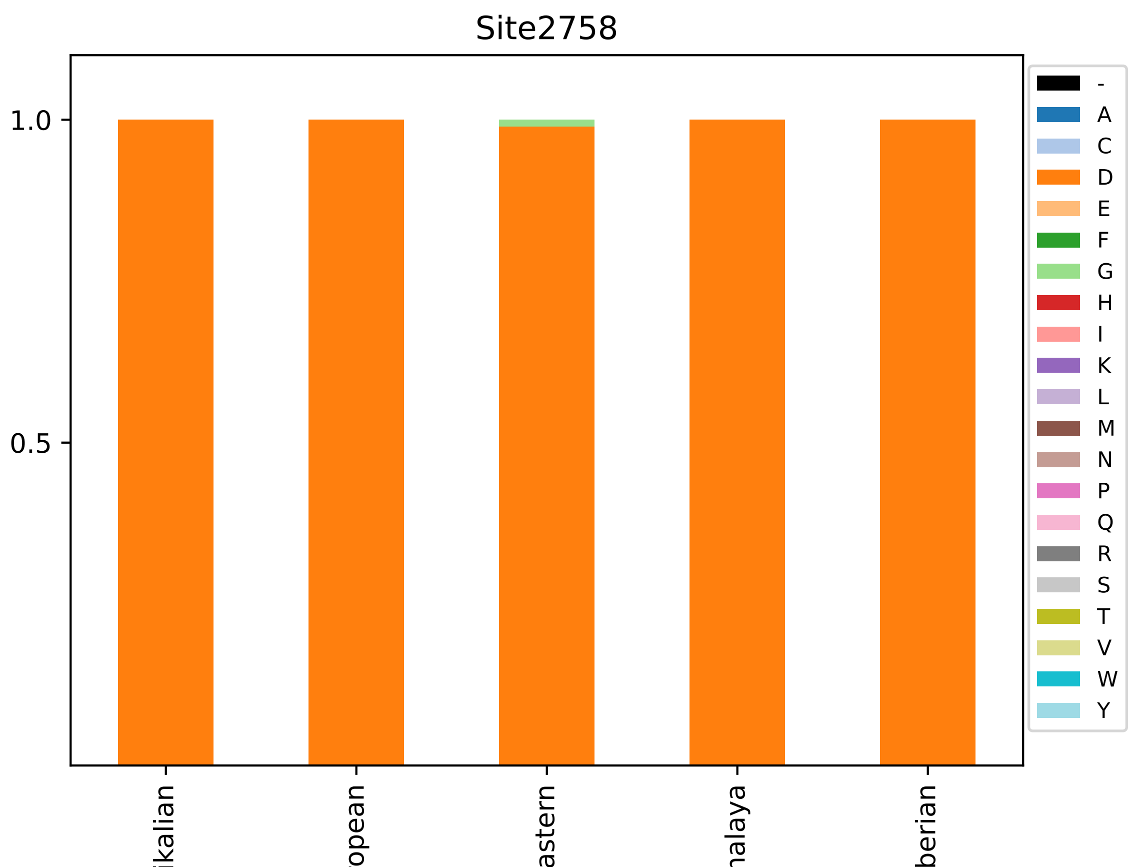

Site2758
1.0
0.5
-
A
C
D
E
F
G
H
I
K
L
M
N
P
Q
R
S
T
V
W
Y
ikalian
opean
astern
nalaya
berian

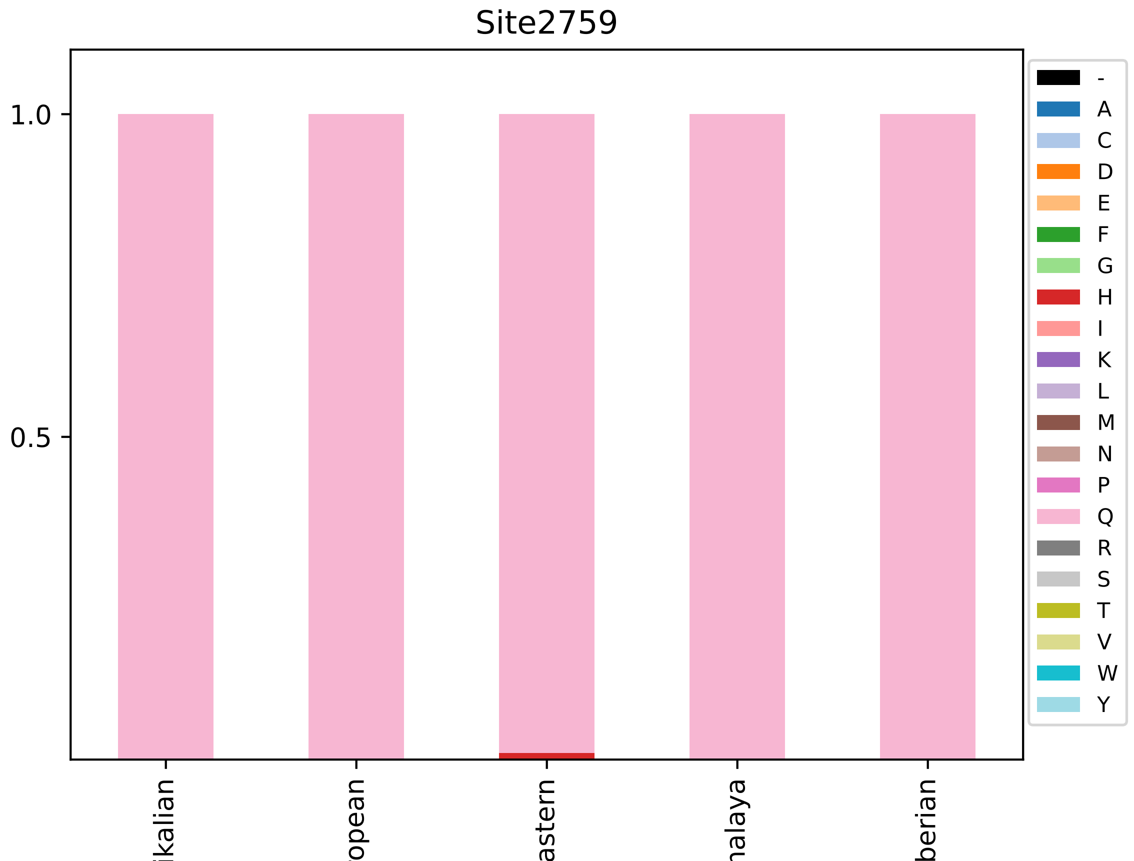

Site2759
1.0
0.5
-
A
C
D
E
F
G
H
I
K
L
M
N
P
Q
R
S
T
V
W
Y
ikalian
opean
astern
nalaya
berian

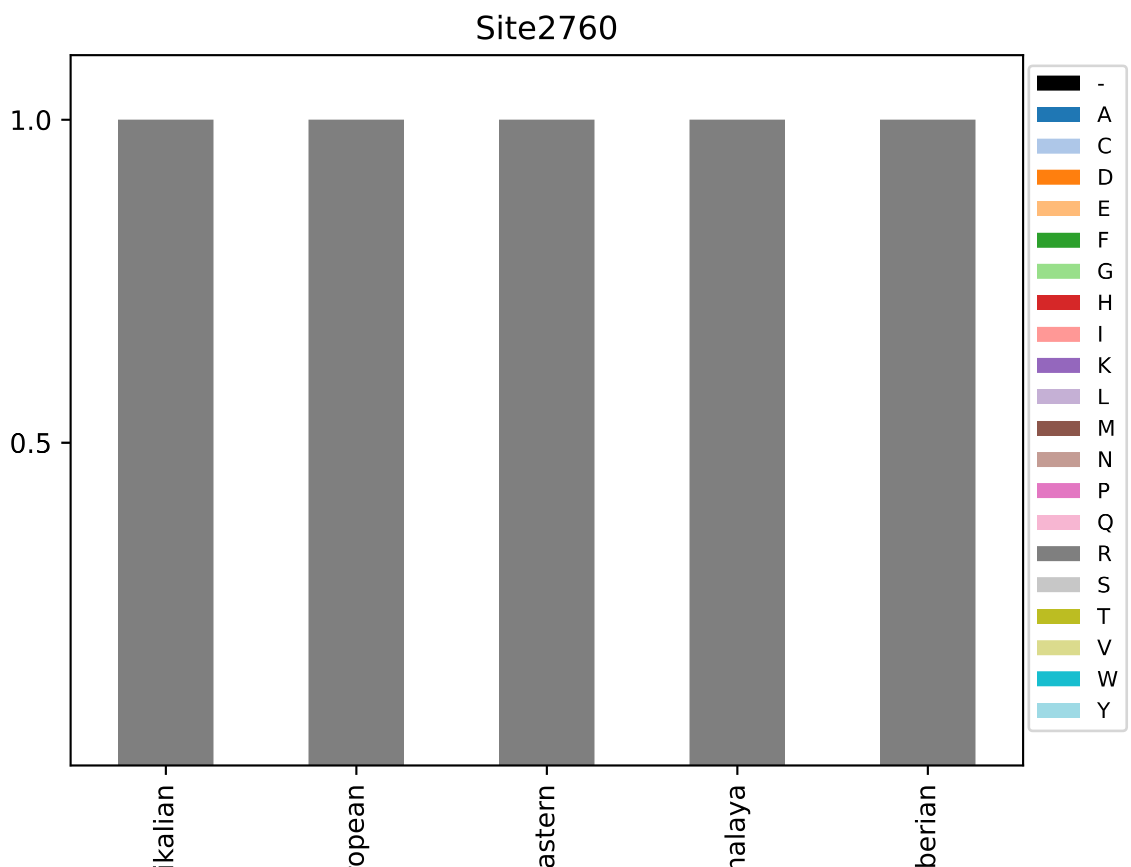
Site2760
1.0
0.5
-
A
C
D
E
F
G
H
I
K
L
M
N
P
Q
R
S
T
V
W
Y
ikalian
ropean
astern
nalaya
berian

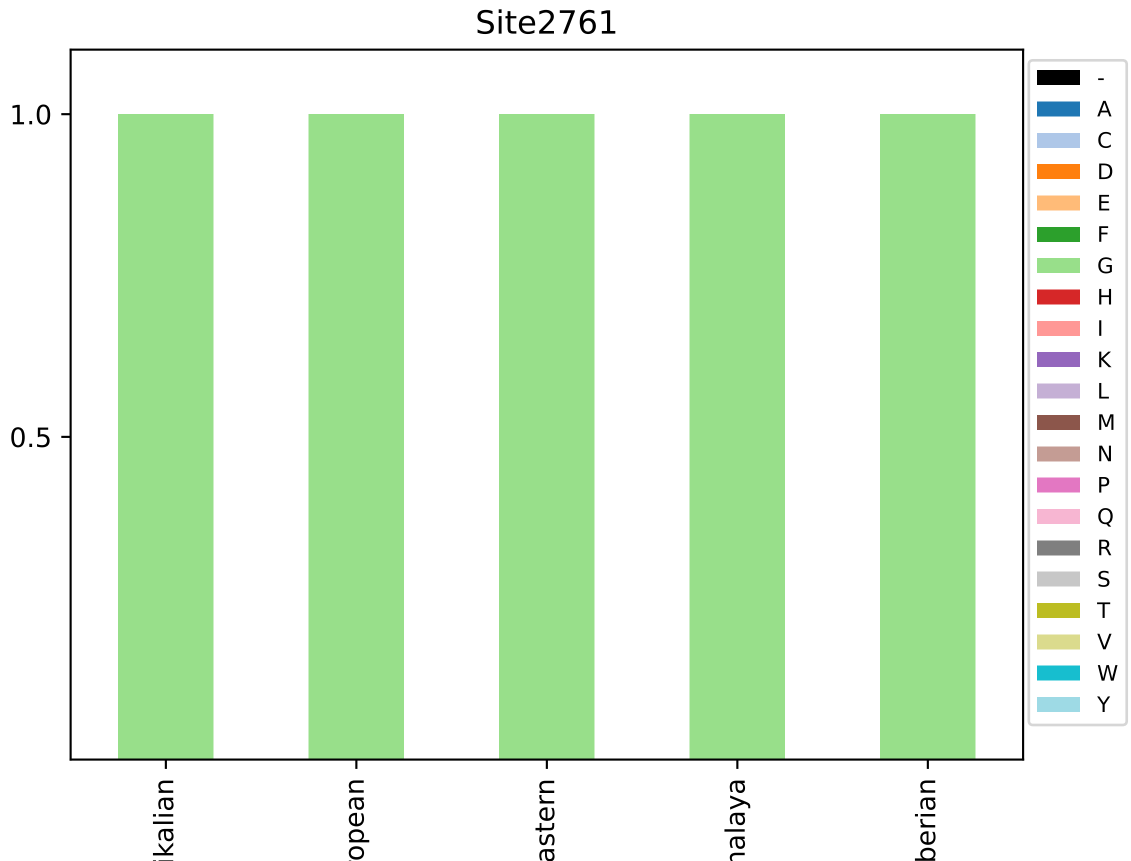
Site2761
1.0
0.5
-
A
C
D
E
F
G
H
I
K
L
M
N
P
Q
R
S
T
V
W
Y
ikalian
opean
astern
nalaya
berian

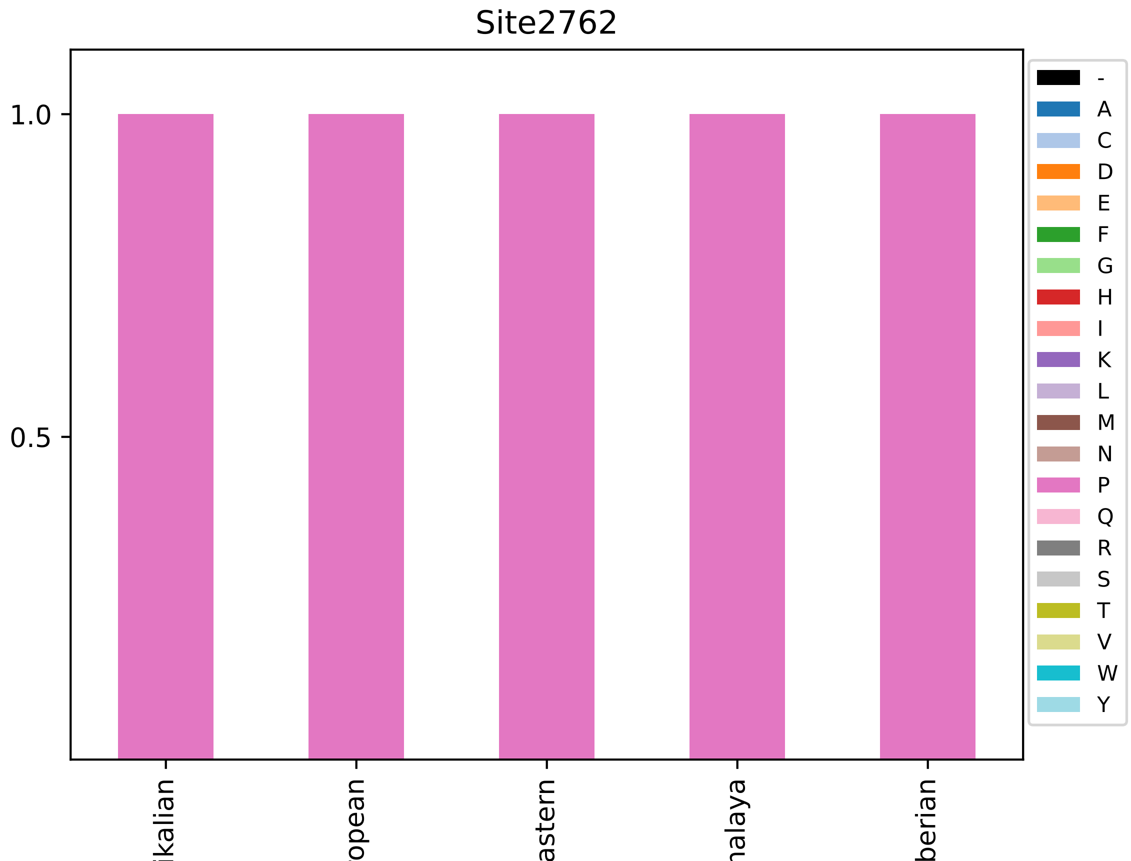
Site2762
1.0
0.5
-
A
C
D
E
F
G
H
I
K
L
M
N
P
Q
R
S
T
V
W
Y
ikalian
opean
astern
nalaya
berian

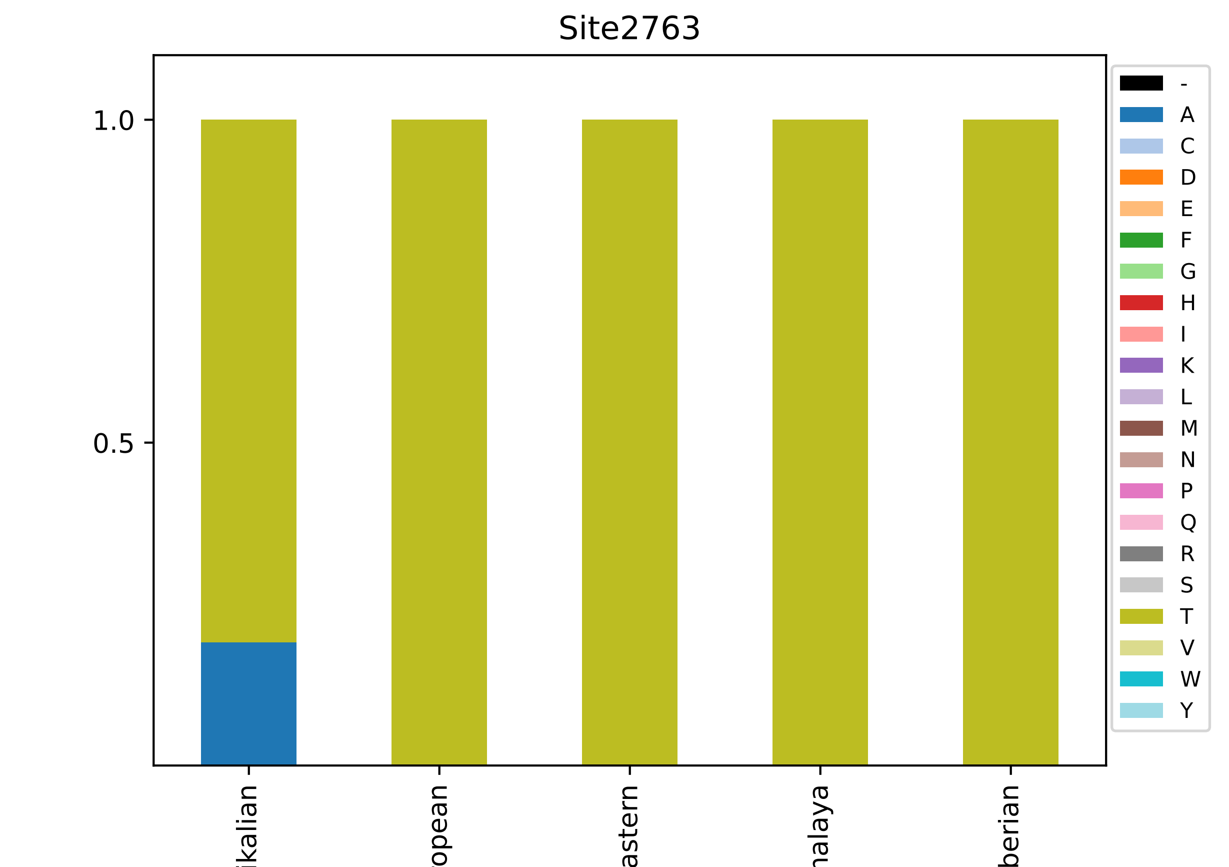

Site2763
1.0
0.5
-
A
C
D
E
F
G
H
I
K
L
M
N
P
Q
R
S
T
V
W
Y
ikalian
opean
astern
nalaya
berian

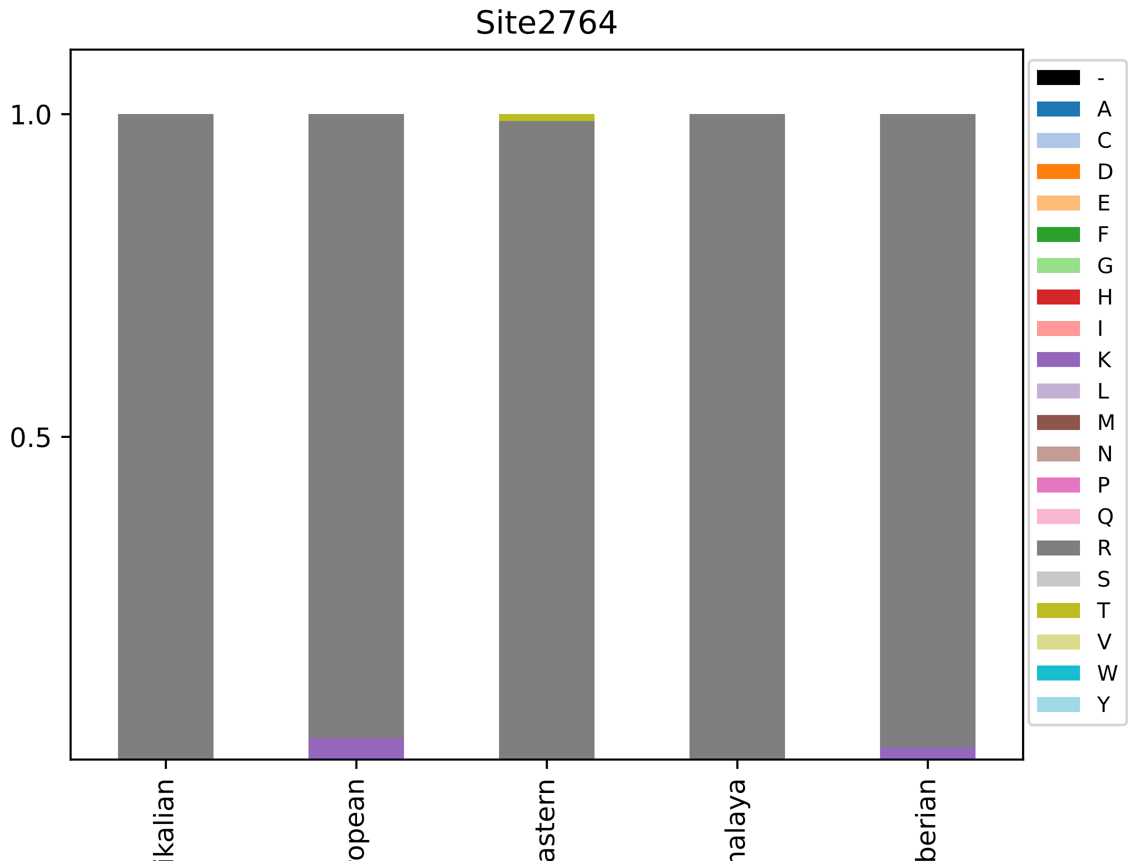

Site2764
1.0
0.5
-
A
C
D
E
F
G
H
I
K
L
M
N
P
Q
R
S
T
V
W
Y
ikalian
opean
astern
nalaya
berian

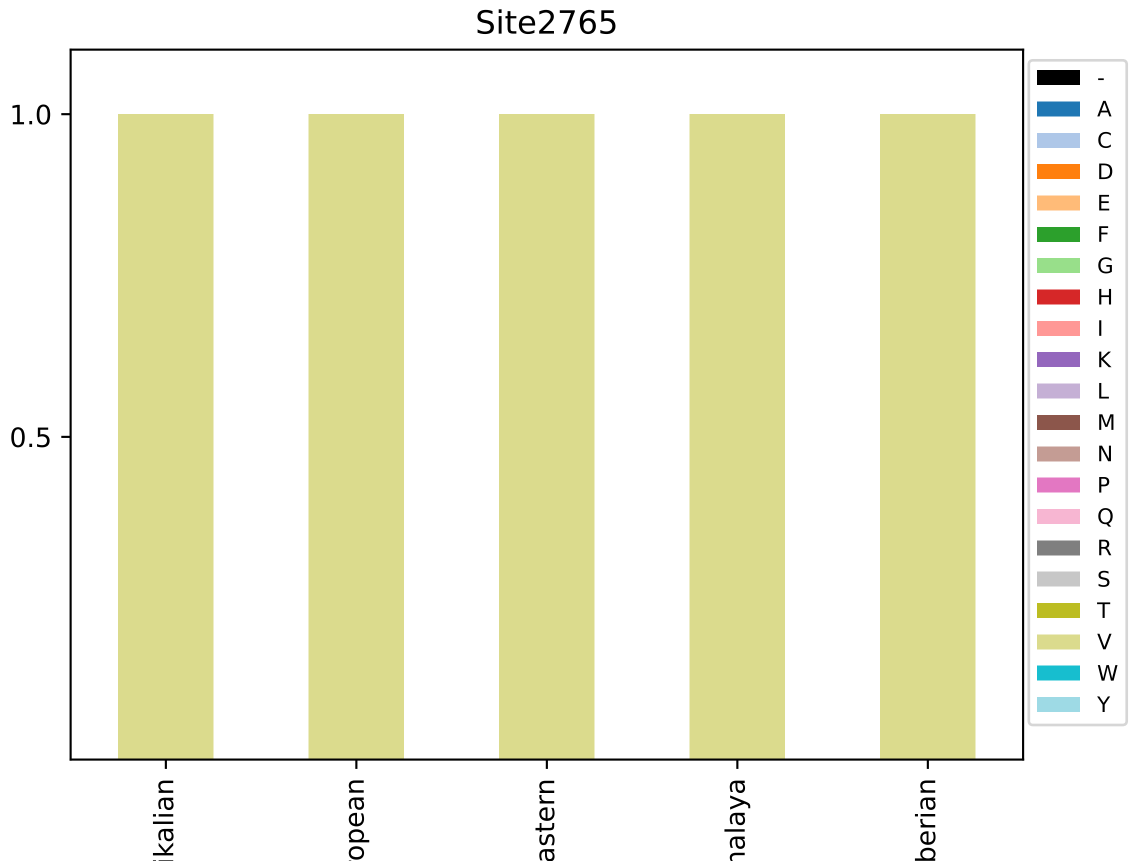
Site2765
1.0
0.5
ikalian
opean
astern
nalaya
berian
-
A
C
D
E
F
G
H
I
K
L
M
N
P
Q
R
S
T
V
W
Y

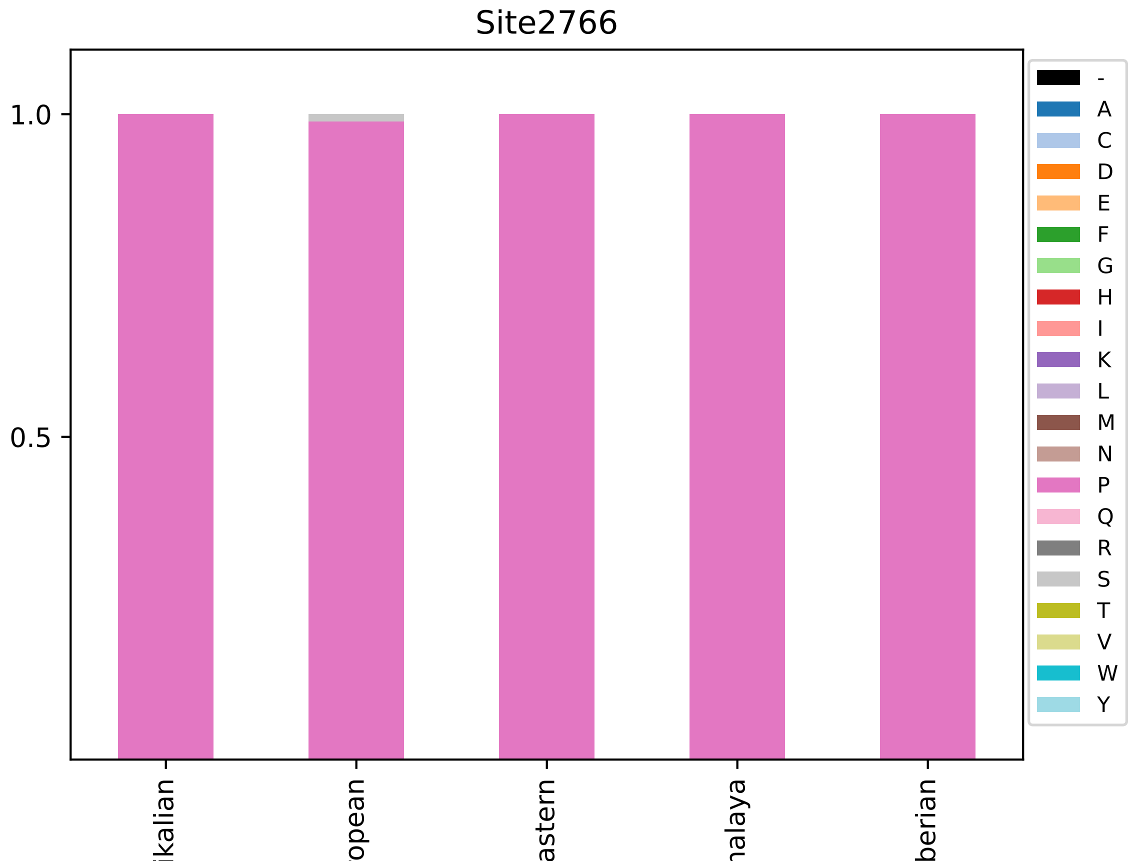

Site2766
1.0
0.5
-
A
C
D
E
F
G
H
I
K
L
M
N
P
Q
R
S
T
V
W
Y
ikalian
opean
astern
nalaya
berian

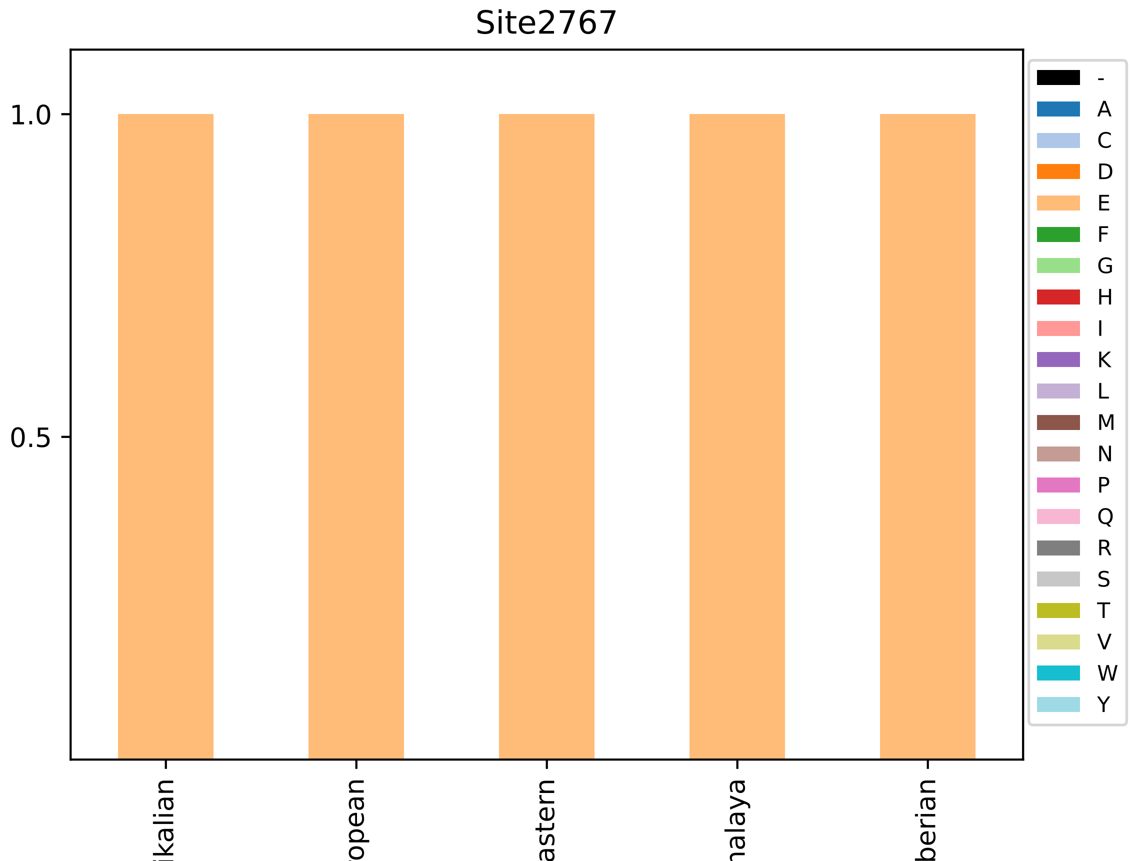
Site2767
1.0
0.5
-
A
C
D
E
F
G
H
I
K
L
M
N
P
Q
R
S
T
V
W
Y
ikalian
opean
astern
nalaya
berian

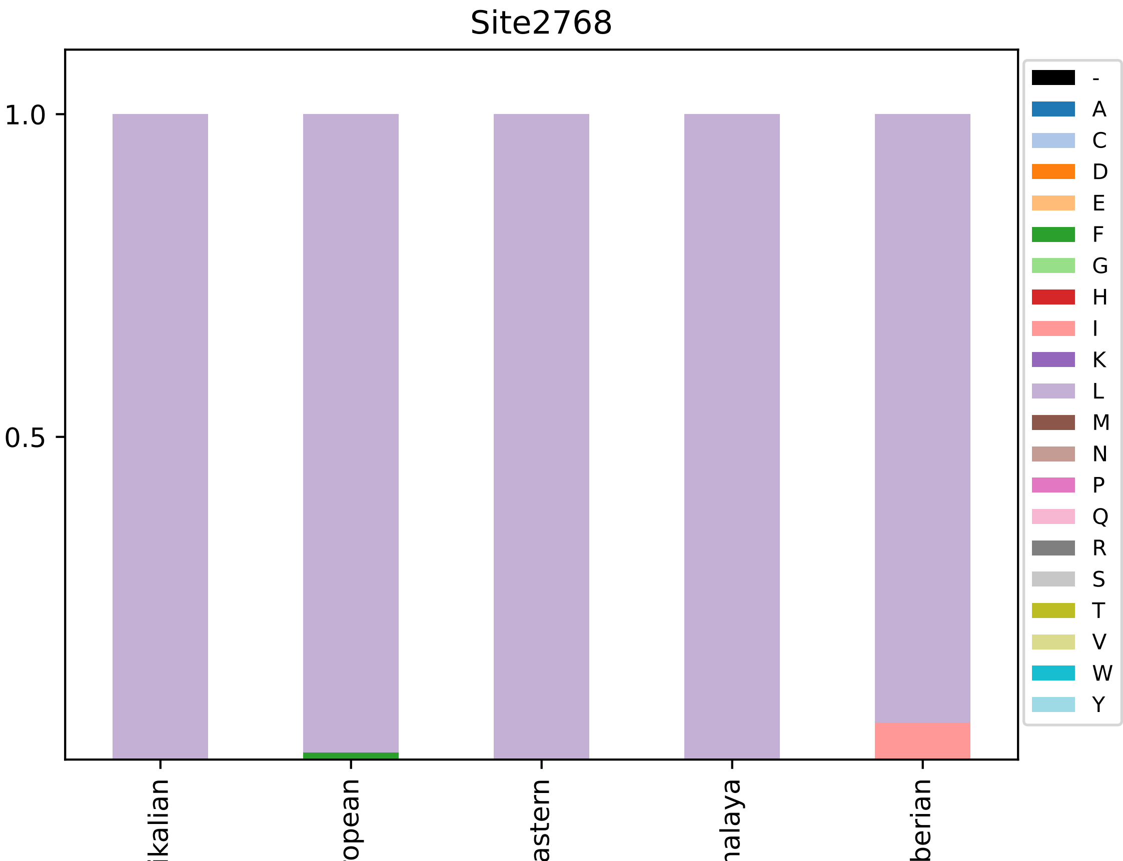

Site2768
1.0
0.5
-
A
C
D
E
F
G
H
I
K
L
M
N
P
Q
R
S
T
V
W
Y
ikalian
opean
astern
nalaya
berian

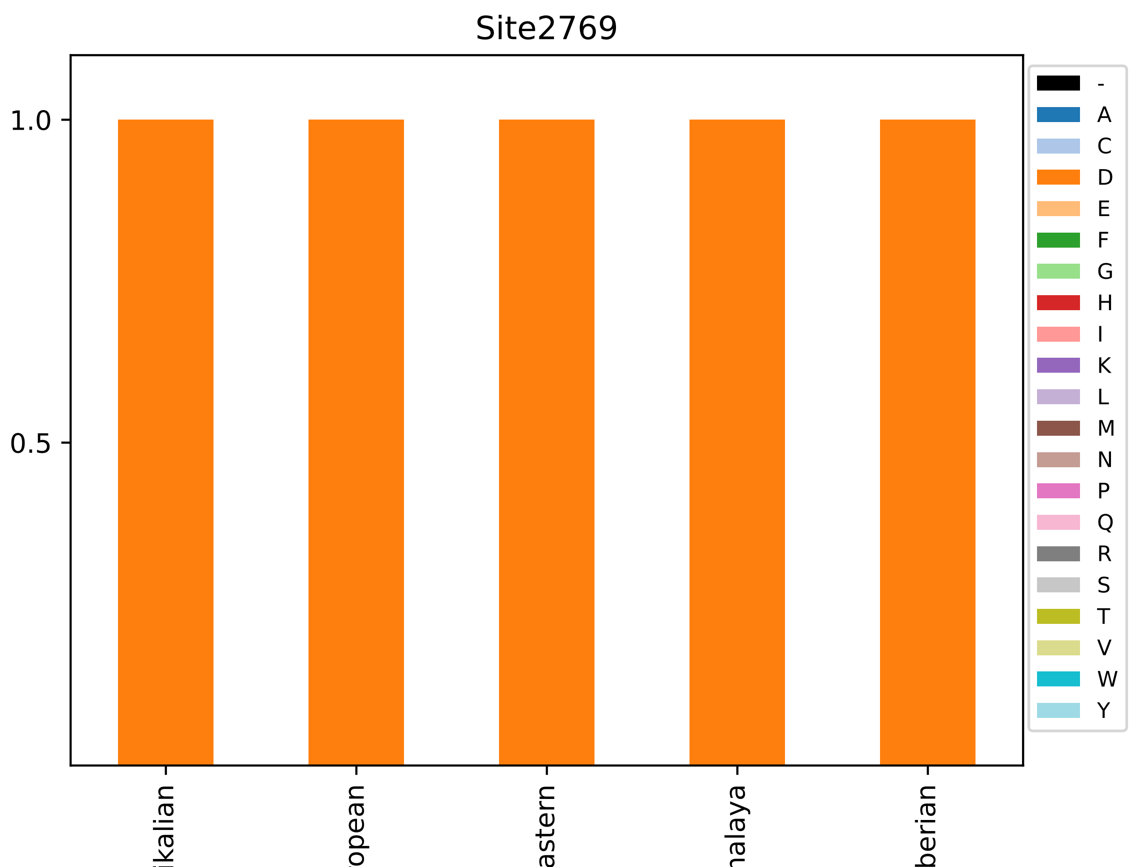

Site2769
1.0
0.5
-
A
C
D
E
F
G
H
I
K
L
M
N
P
Q
R
S
T
V
W
Y
ikalian
opean
astern
nalaya
berian

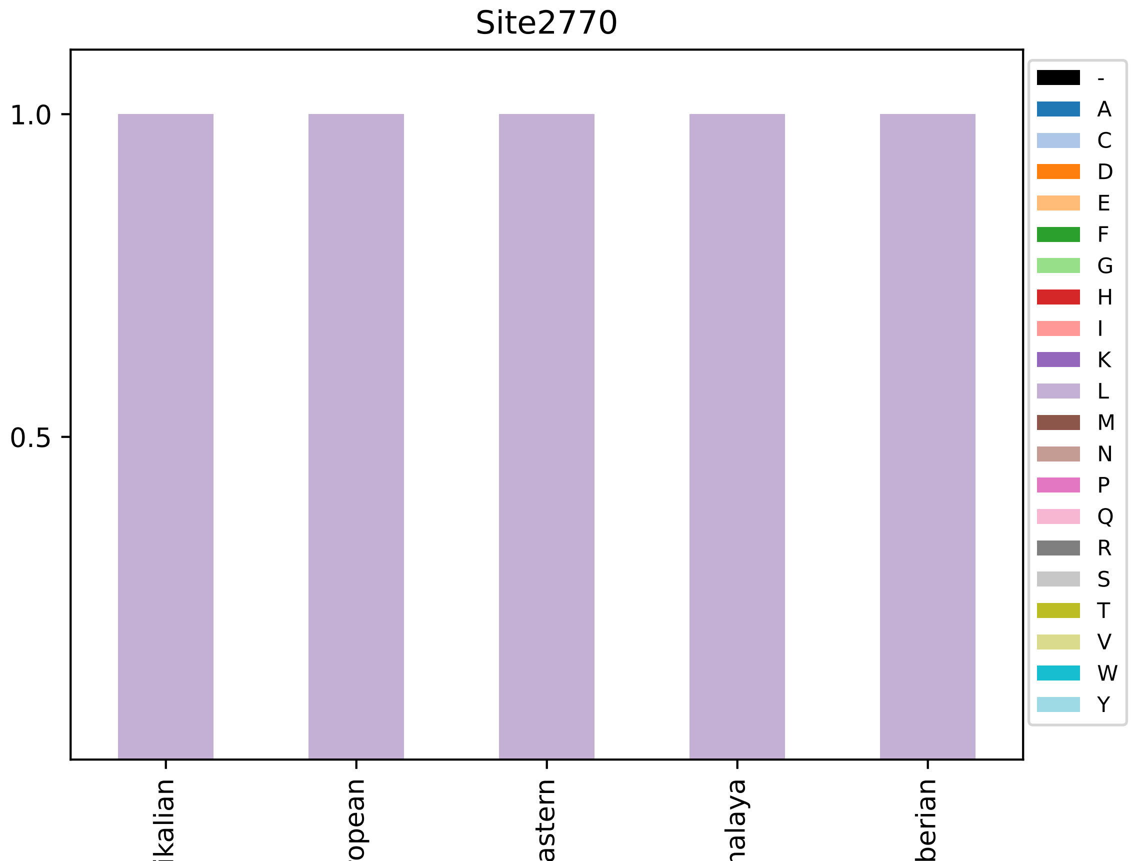
Site2770
1.0
0.5
-
A
C
D
E
F
G
H
I
K
L
M
N
P
Q
R
S
T
V
W
Y
ikalian
opean
astern
nalaya
berian

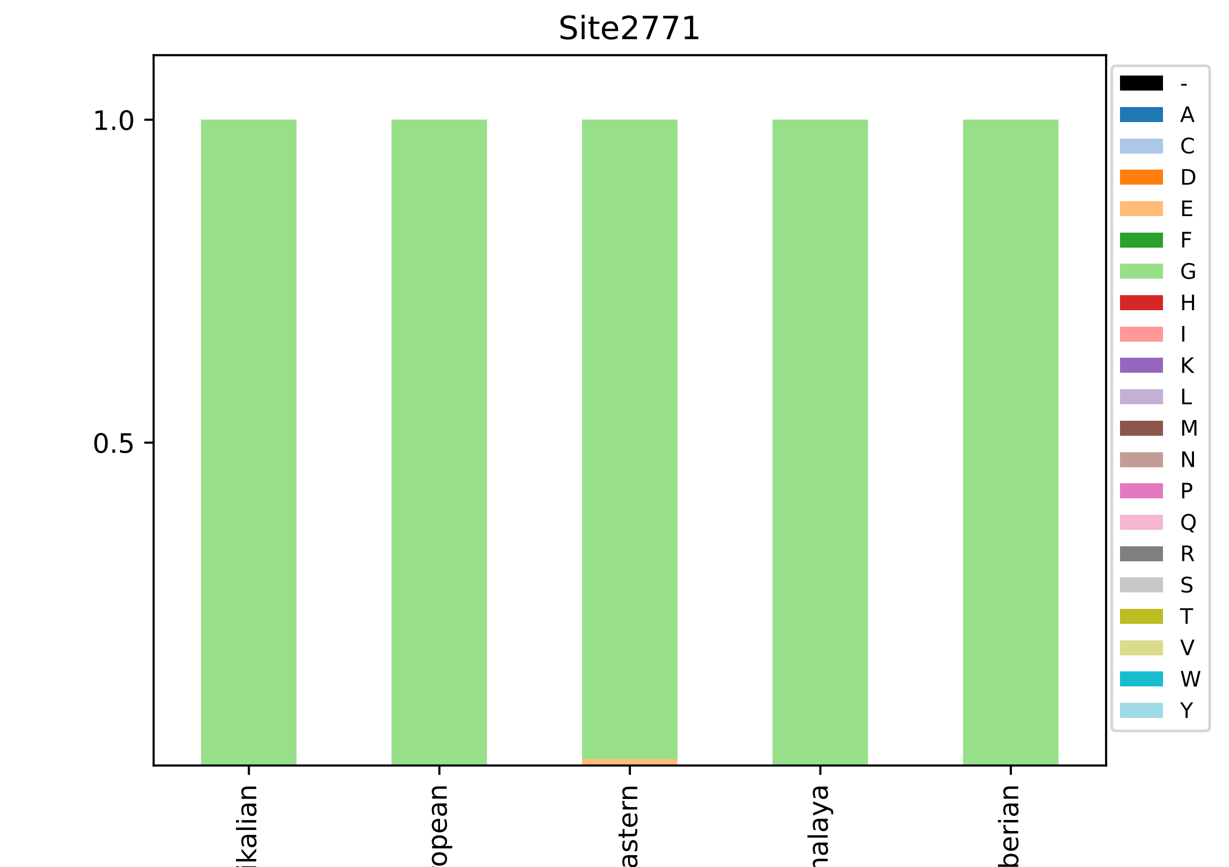
Site2771
1.0
0.5
-
A
C
D
E
F
G
H
I
K
L
M
N
P
Q
R
S
T
V
W
Y
ikalian
opean
astern
nalaya
berian

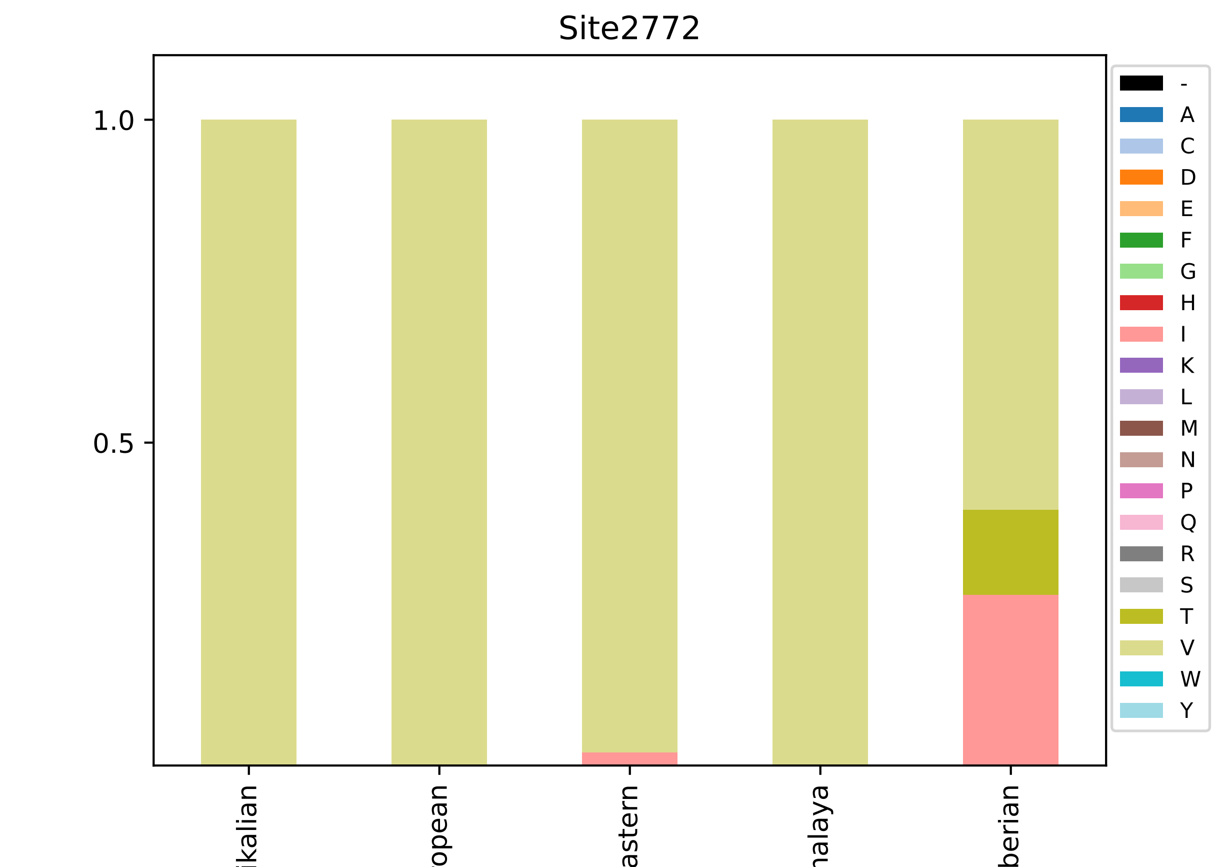

Site2772
1.0
0.5
-
A
C
D
E
F
G
H
I
K
L
M
N
P
Q
R
S
T
V
W
Y
ikalian
opean
astern
nalaya
berian

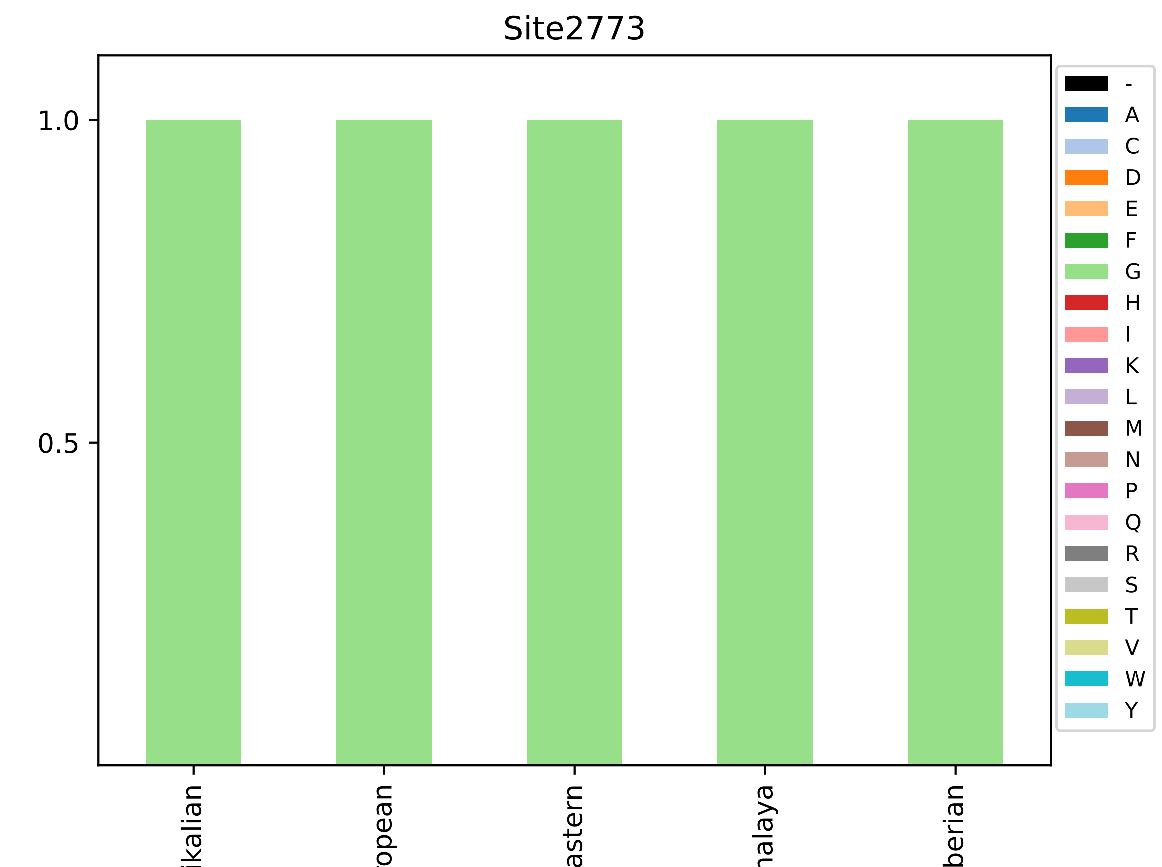
Site2773
1.0
0.5
-
A
C
D
E
F
G
H
I
K
L
M
N
P
Q
R
S
T
V
W
Y
ikalian
opean
astern
nalaya
berian

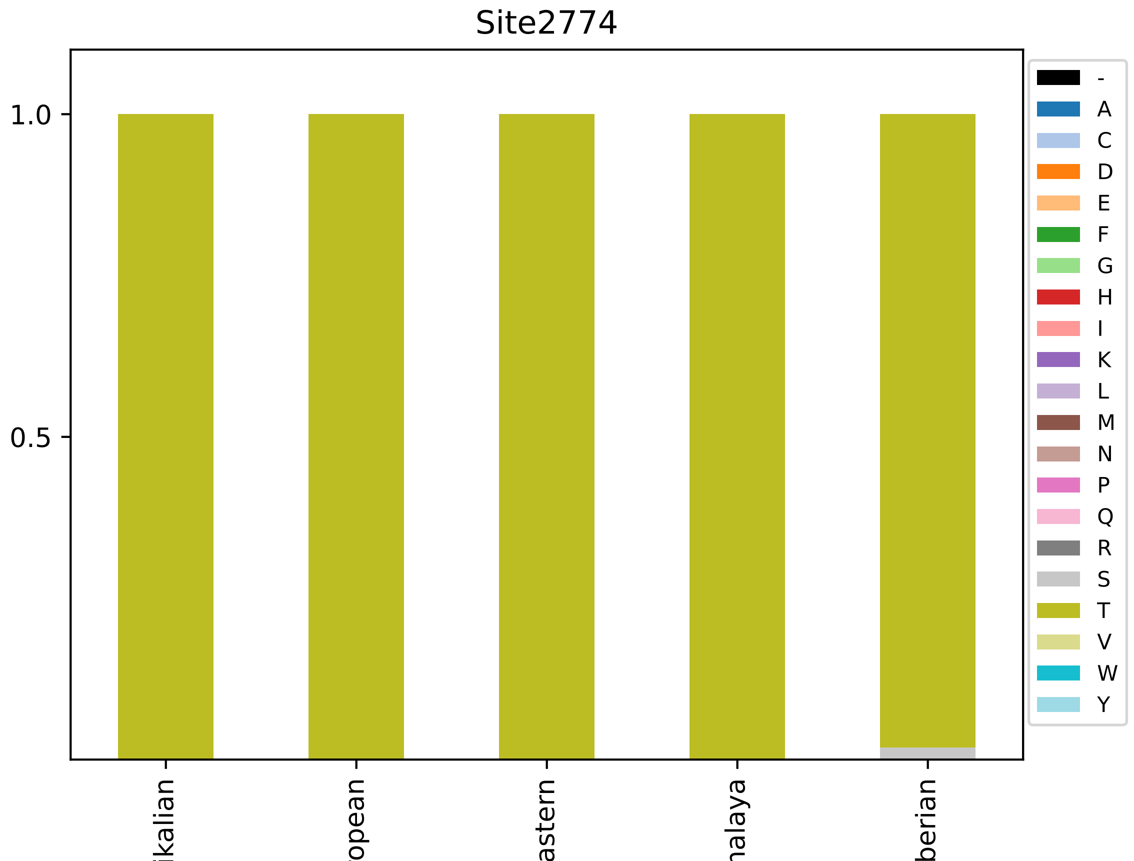

Site2774
1.0
0.5
ikalian
opean
astern
nalaya
berian
-
A
C
D
E
F
G
H
I
K
L
M
N
P
Q
R
S
T
V
W
Y

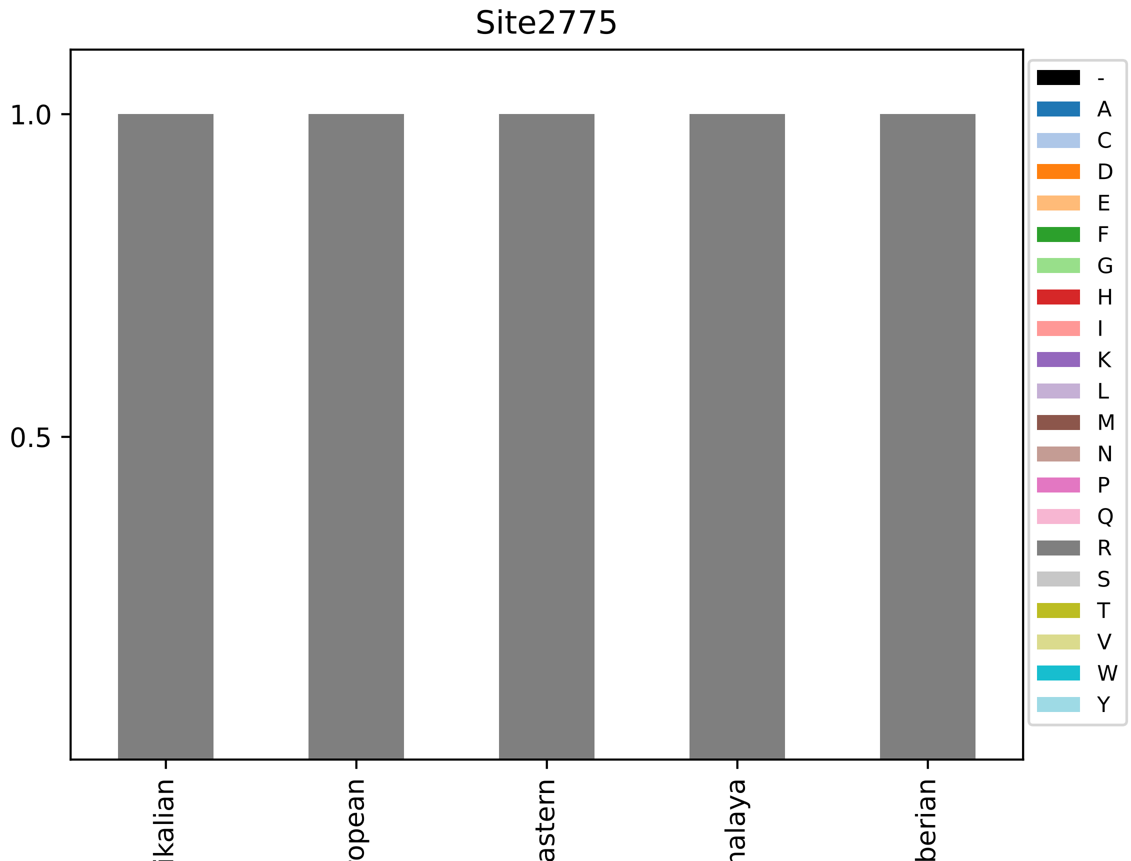

Site2775
1.0
0.5
ikalian
opean
astern
nalaya
berian
-
A
C
D
E
F
G
H
I
K
L
M
N
P
Q
R
S
T
V
W
Y

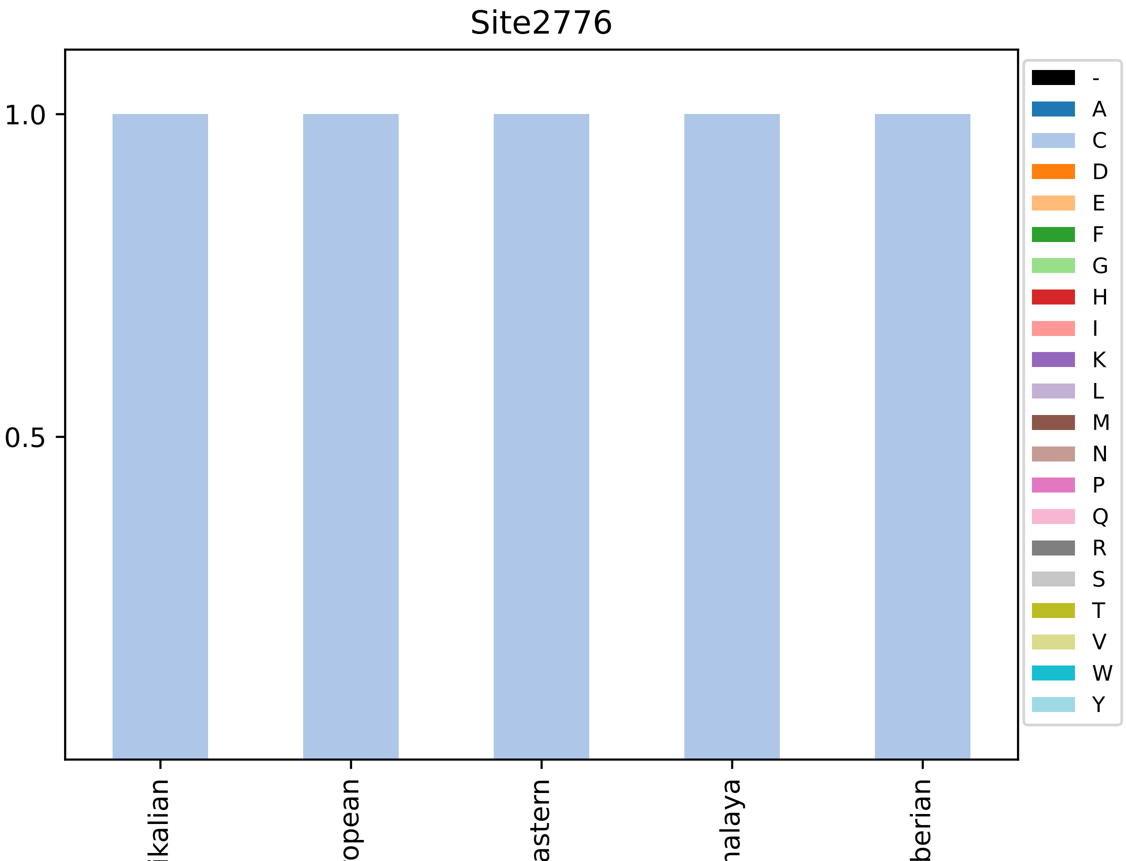

Site2776
1.0
0.5
-
A
C
D
E
F
G
H
I
K
L
M
N
P
Q
R
S
T
V
W
Y

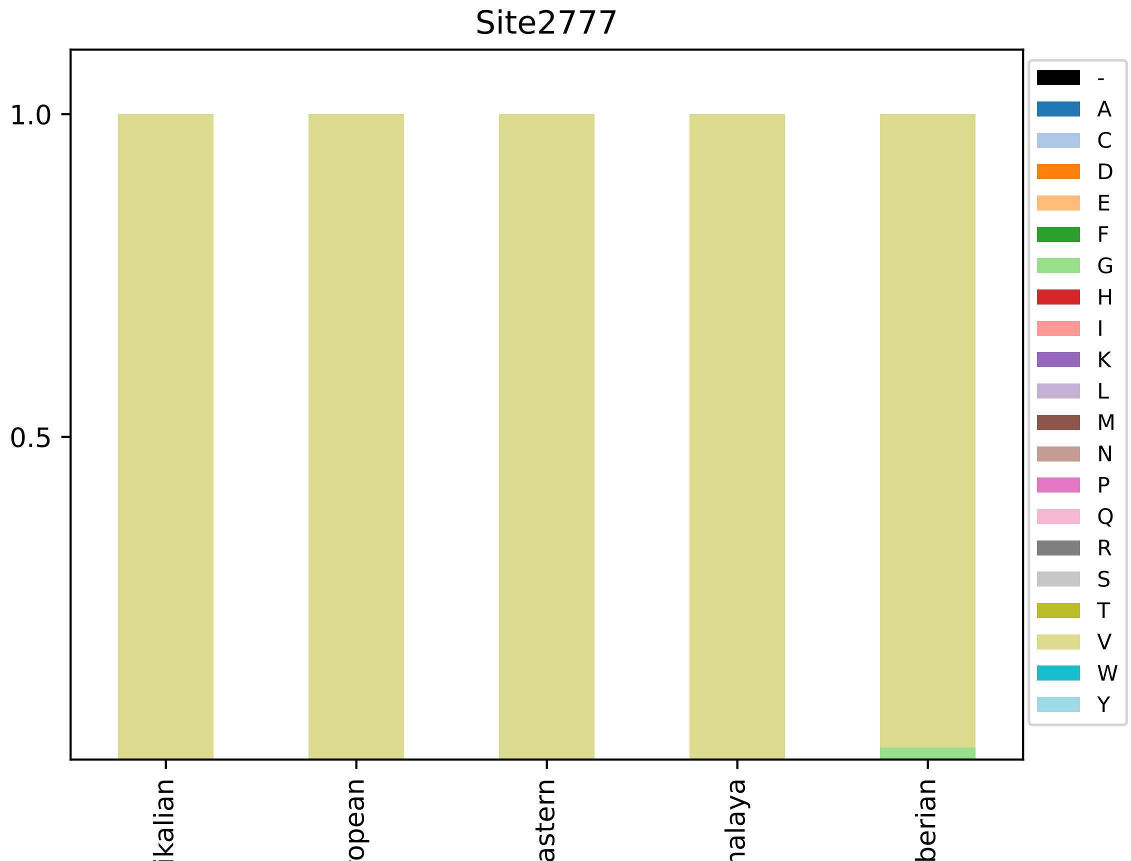
Site2777
1.0
0.5
-
A
C
D
E
F
G
H
I
K
L
M
N
P
Q
R
S
T
V
W
Y
ikalian
opean
astern
nalaya
berian

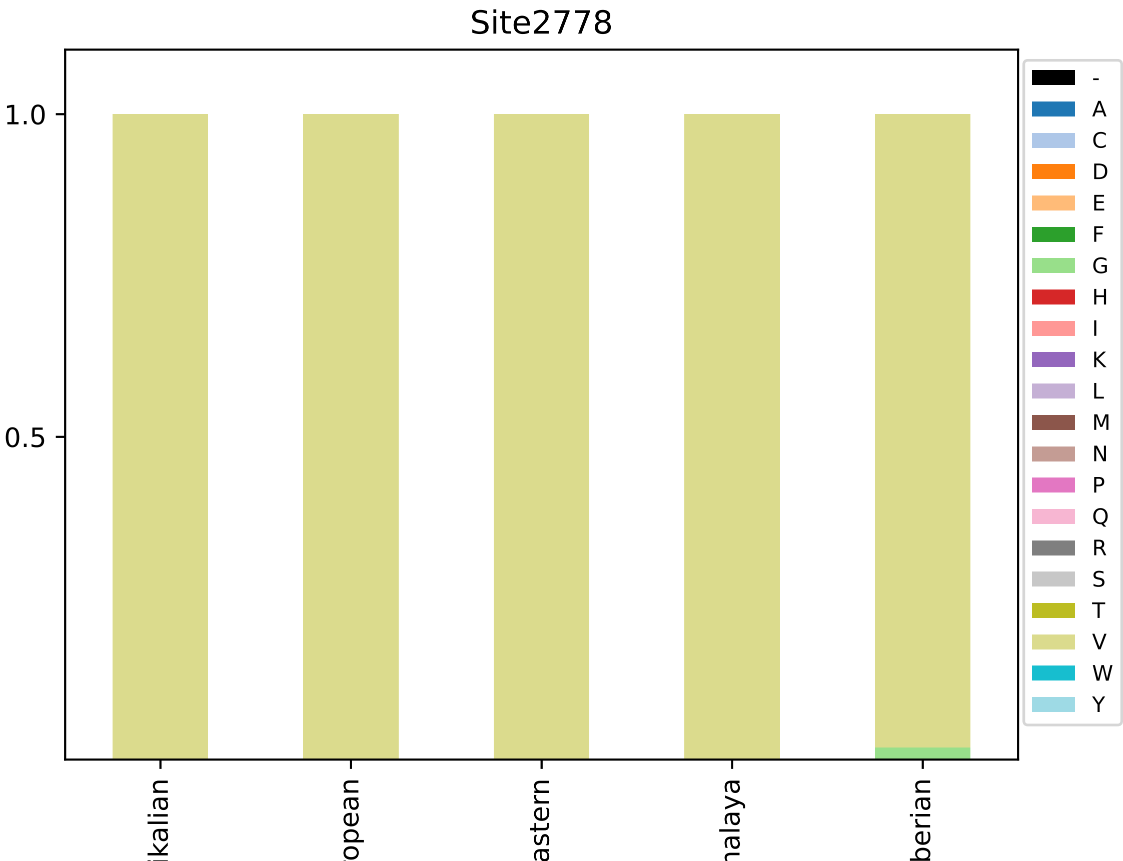

Site2778
1.0
0.5
-
A
C
D
E
F
G
H
I
K
L
M
N
P
Q
R
S
T
V
W
Y
ikalian
opean
astern
halaya
berian

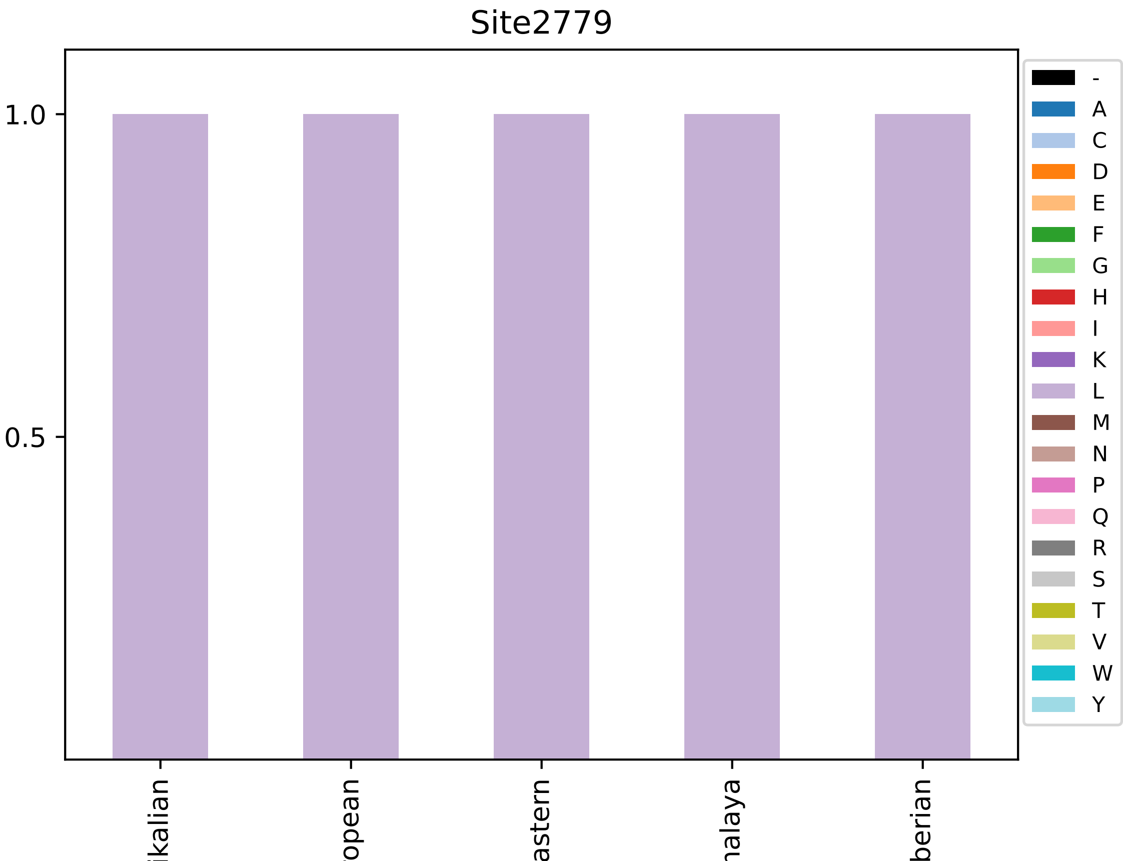
Site2779
1.0
0.5
-
A
C
D
E
F
G
H
I
K
L
M
N
P
Q
R
S
T
V
W
Y
ikalian
opean
astern
nalaya
berian

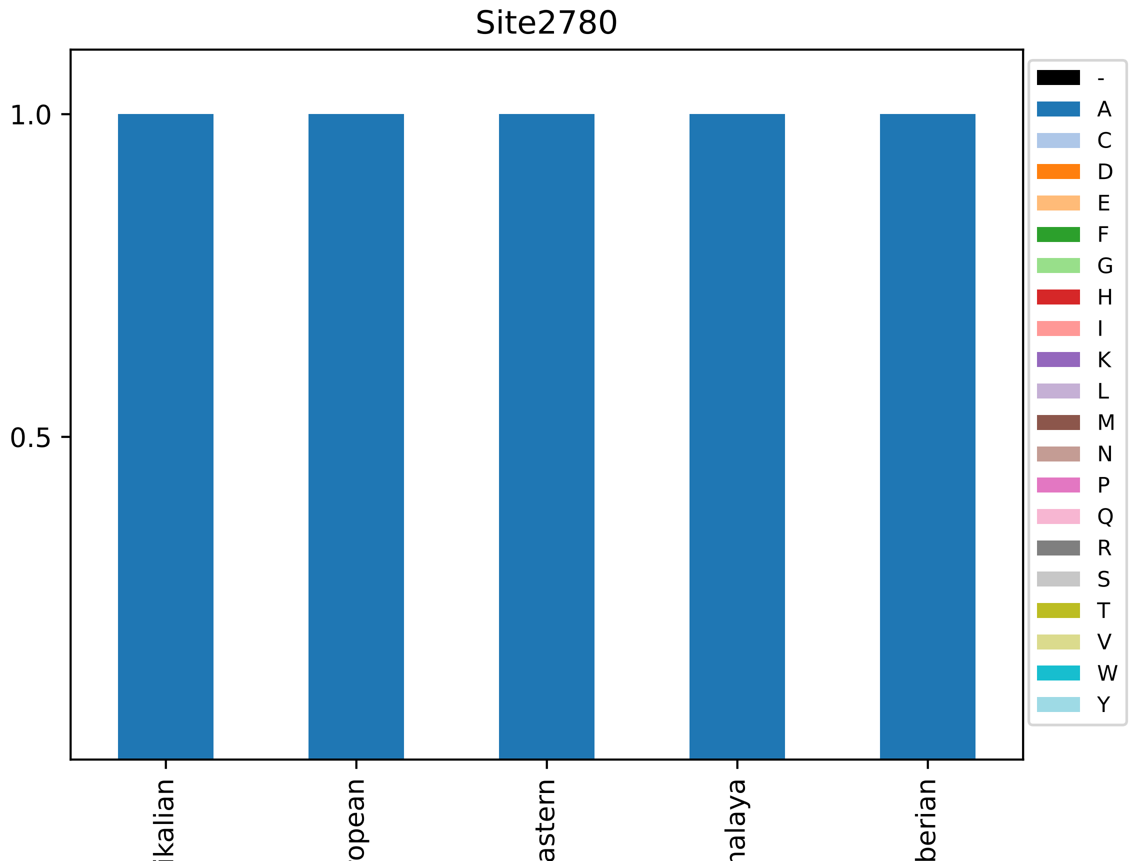

Site2780
1.0
0.5
-
A
C
D
E
F
G
H
I
K
L
M
N
P
Q
R
S
T
V
W
Y
ikalian
opean
astern
nalaya
berian

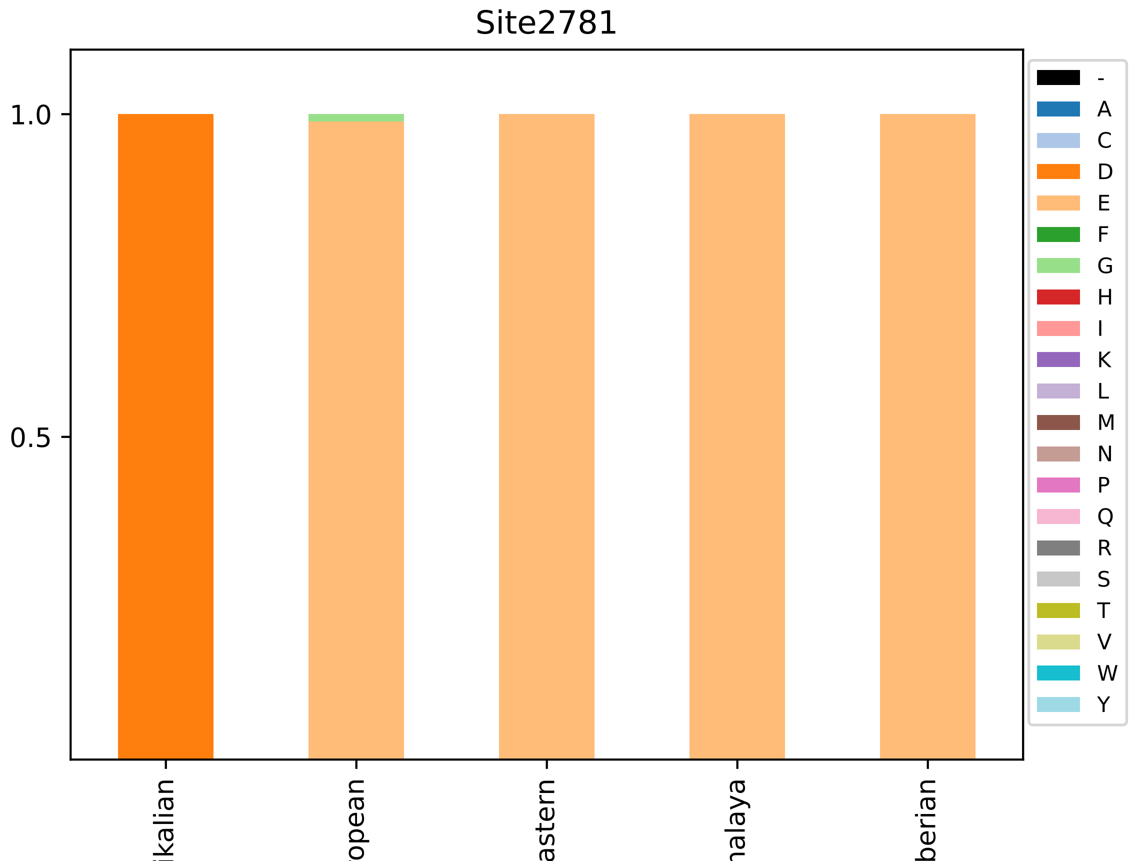
Site2781
1.0
0.5
-
A
C
D
E
F
G
H
I
K
L
M
N
P
Q
R
S
T
V
W
Y
ikalian
opean
astern
nalaya
berian

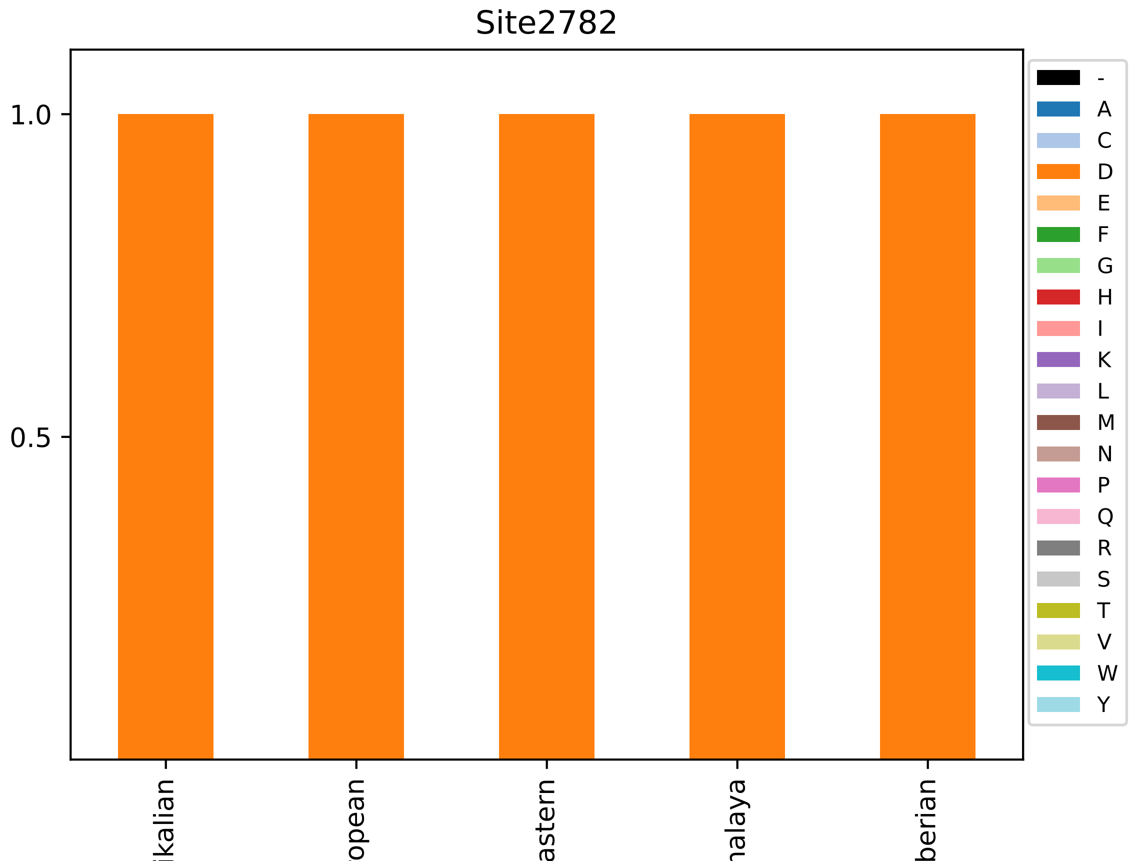

Site2782
1.0
0.5
-
A
C
D
E
F
G
H
I
K
L
M
N
P
Q
R
S
T
V
W
Y
ikalian
opean
astern
nalaya
berian

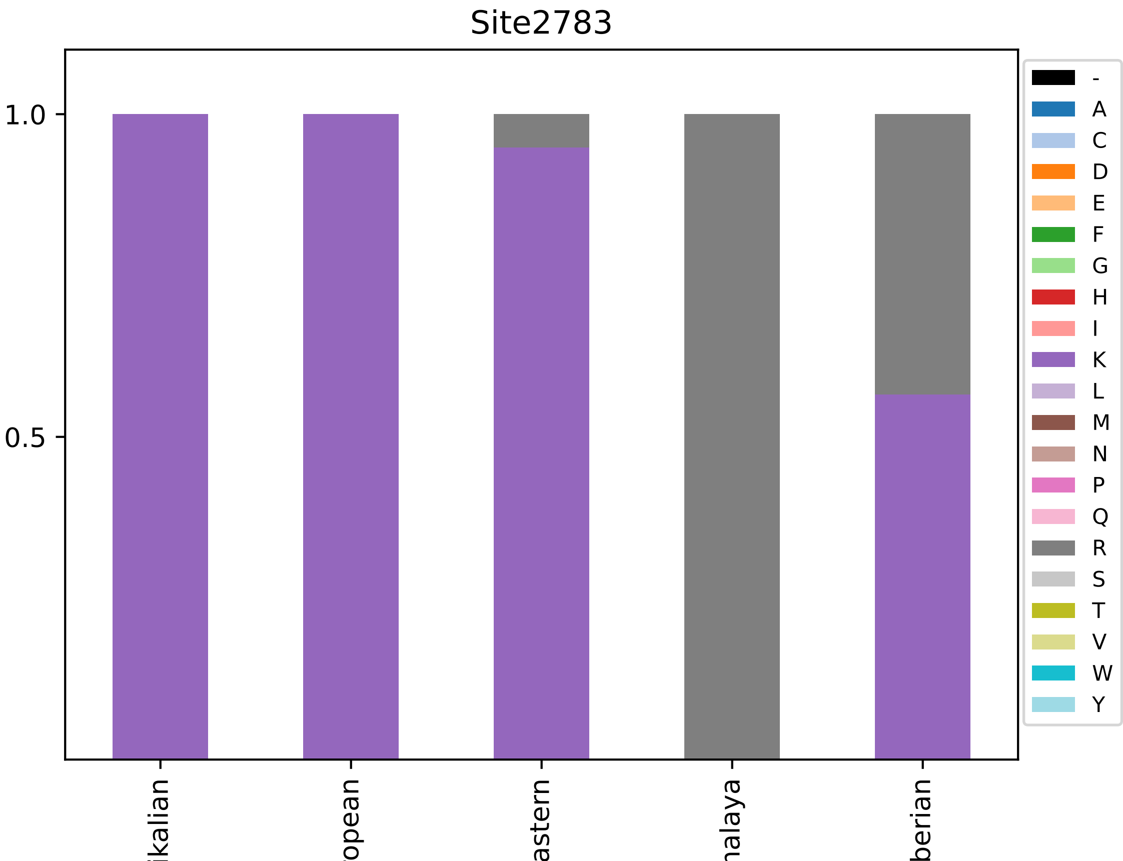

Site2783
1.0
0.5
-
A
C
D
E
F
G
H
I
K
L
M
N
P
Q
R
S
T
V
W
Y
ikalian
opean
astern
nalaya
berian

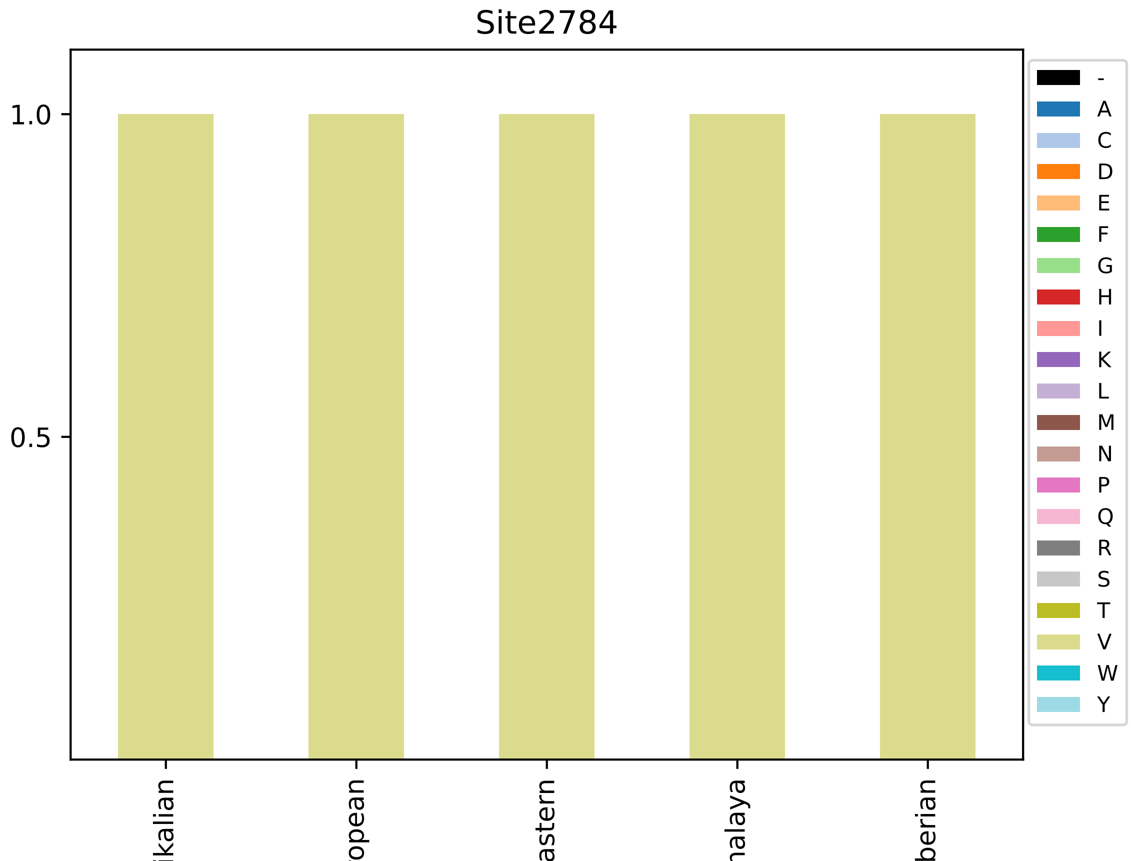

Site2784
1.0
0.5
-
A
C
D
E
F
G
H
I
K
L
M
N
P
Q
R
S
T
V
W
Y
ikalian
opean
astern
nalaya
berian

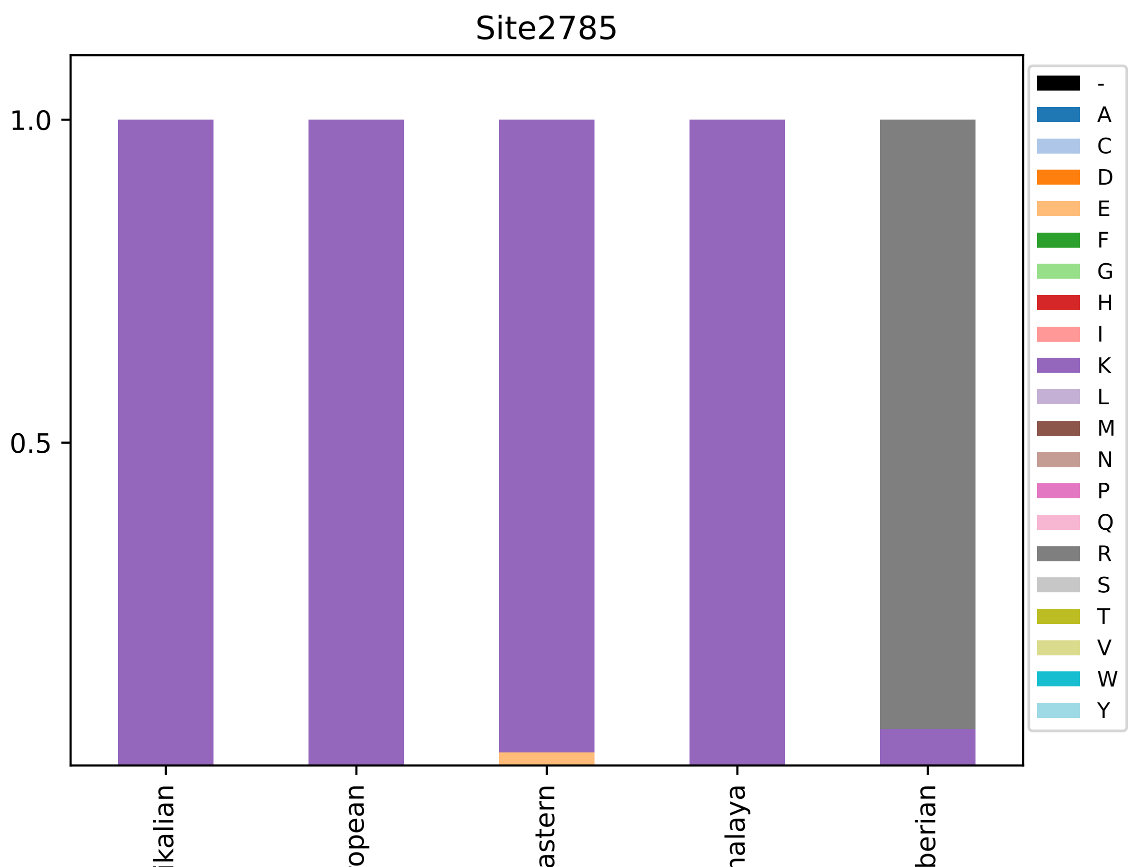

Site2785
1.0
0.5
ikalian
opean
astern
nalaya
berian
-
A
C
D
E
F
G
H
I
K
L
M
N
P
Q
R
S
T
V
W
Y

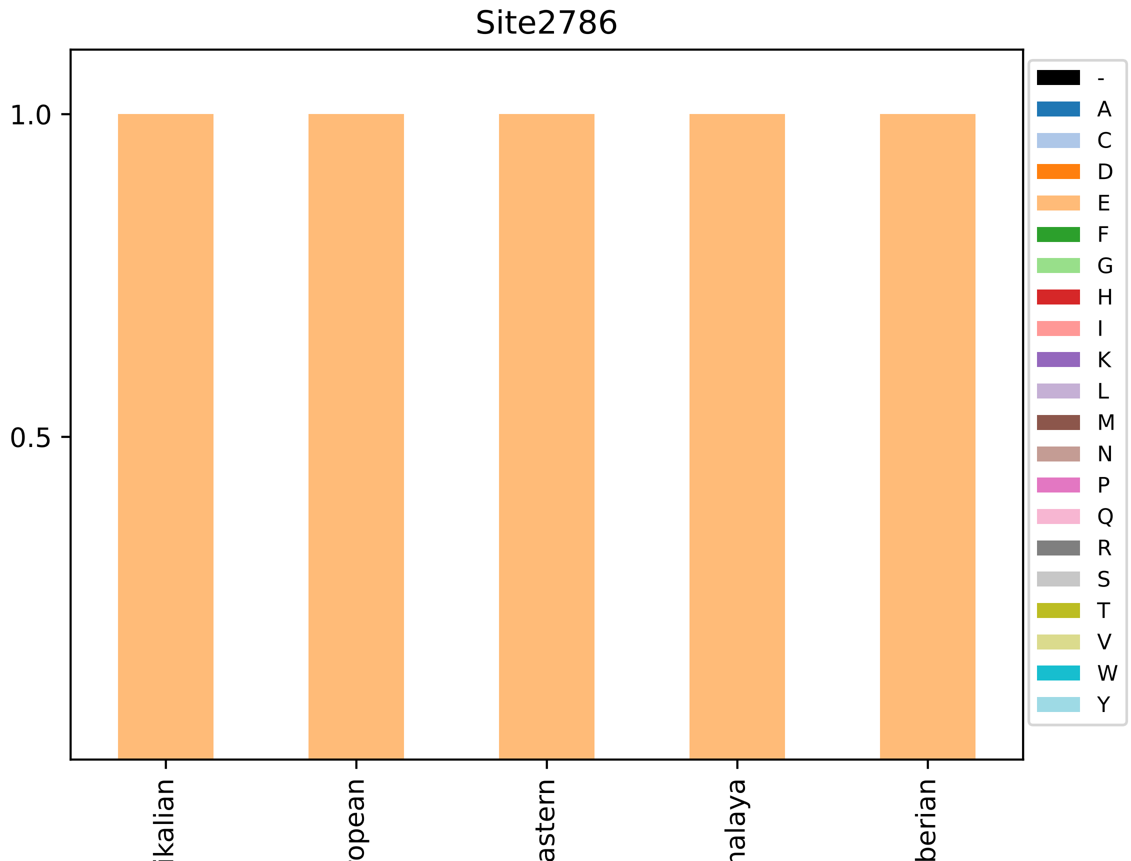
Site2786
1.0
0.5
-
A
C
D
E
F
G
H
I
K
L
M
N
P
Q
R
S
T
V
W
Y

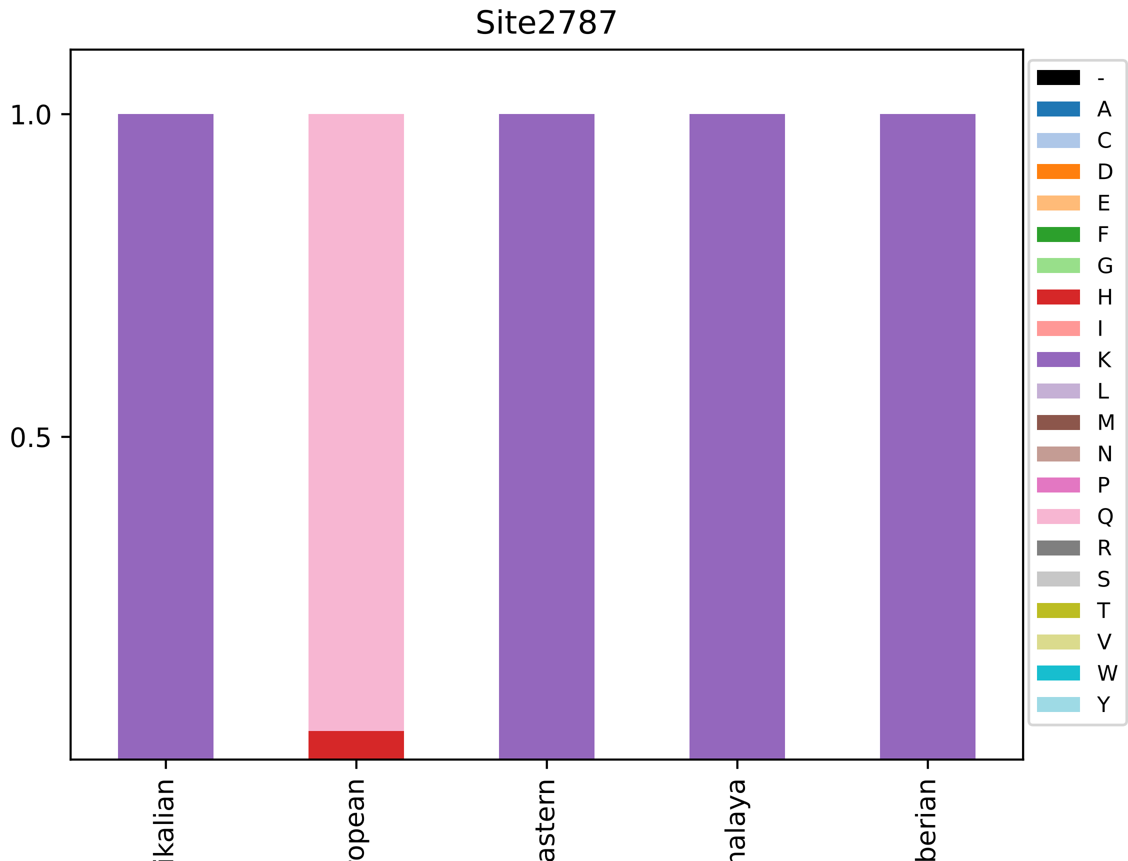
Site2787
1.0
0.5
-
A
C
D
E
F
G
H
I
K
L
M
N
P
Q
R
S
T
V
W
Y
ikalian
opean
astern
nalaya
berian

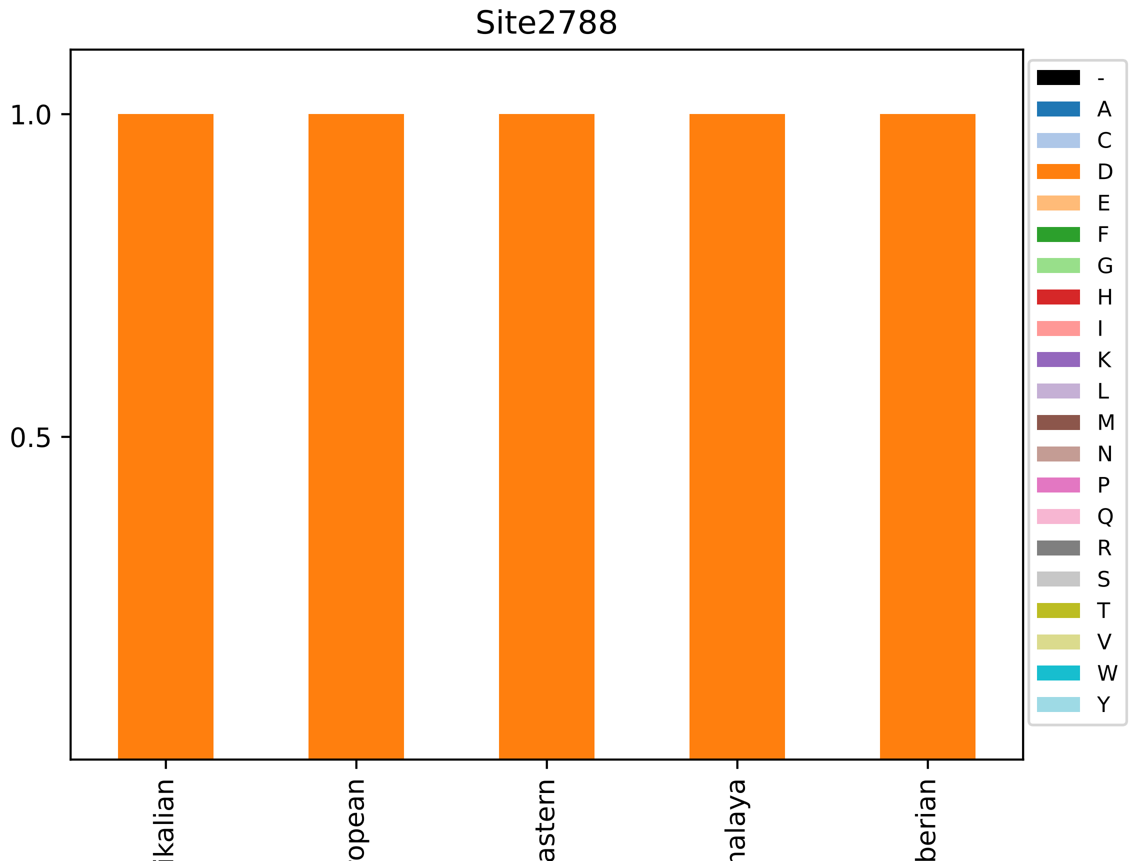
Site2788
1.0
0.5
ikalian
opean
astern
nalaya
berian
-
A
C
D
E
F
G
H
I
K
L
M
N
P
Q
R
S
T
V
W
Y

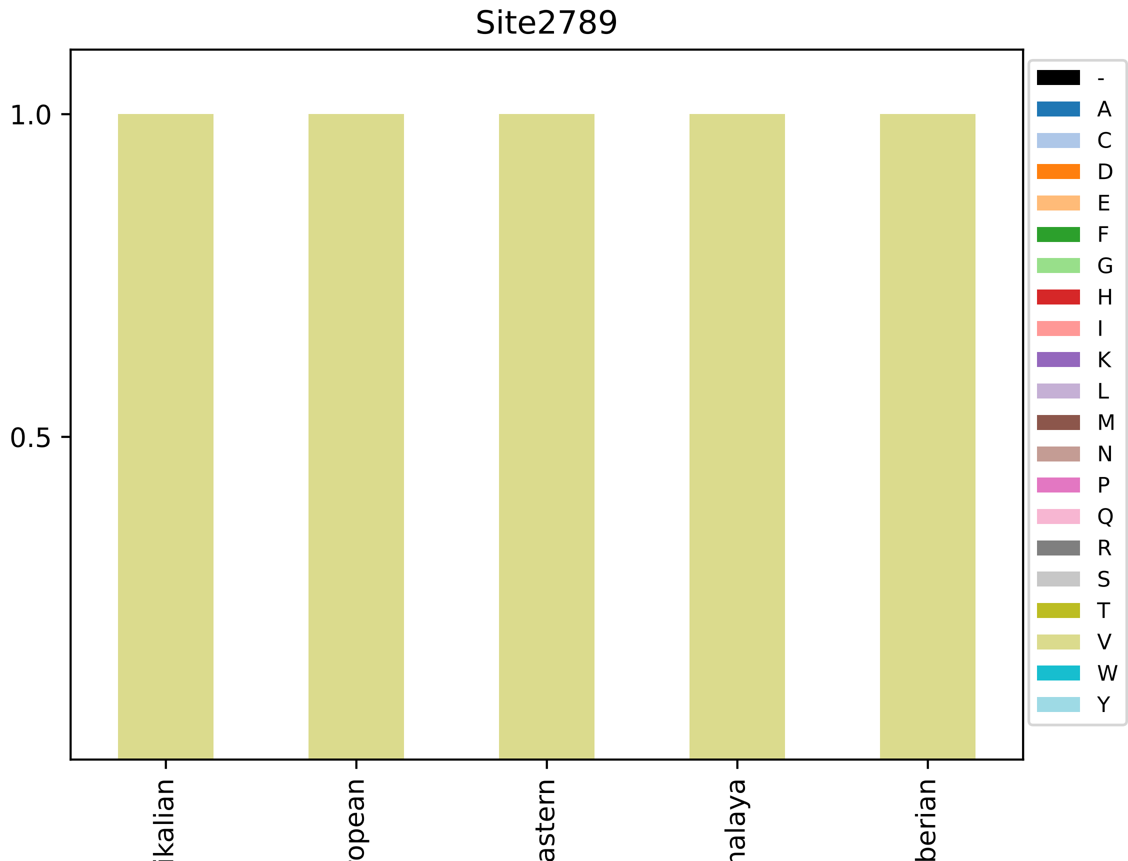

Site2789
1.0
0.5
-
A
C
D
E
F
G
H
I
K
L
M
N
P
Q
R
S
T
V
W
Y
ikalian
opean
astern
nalaya
berian

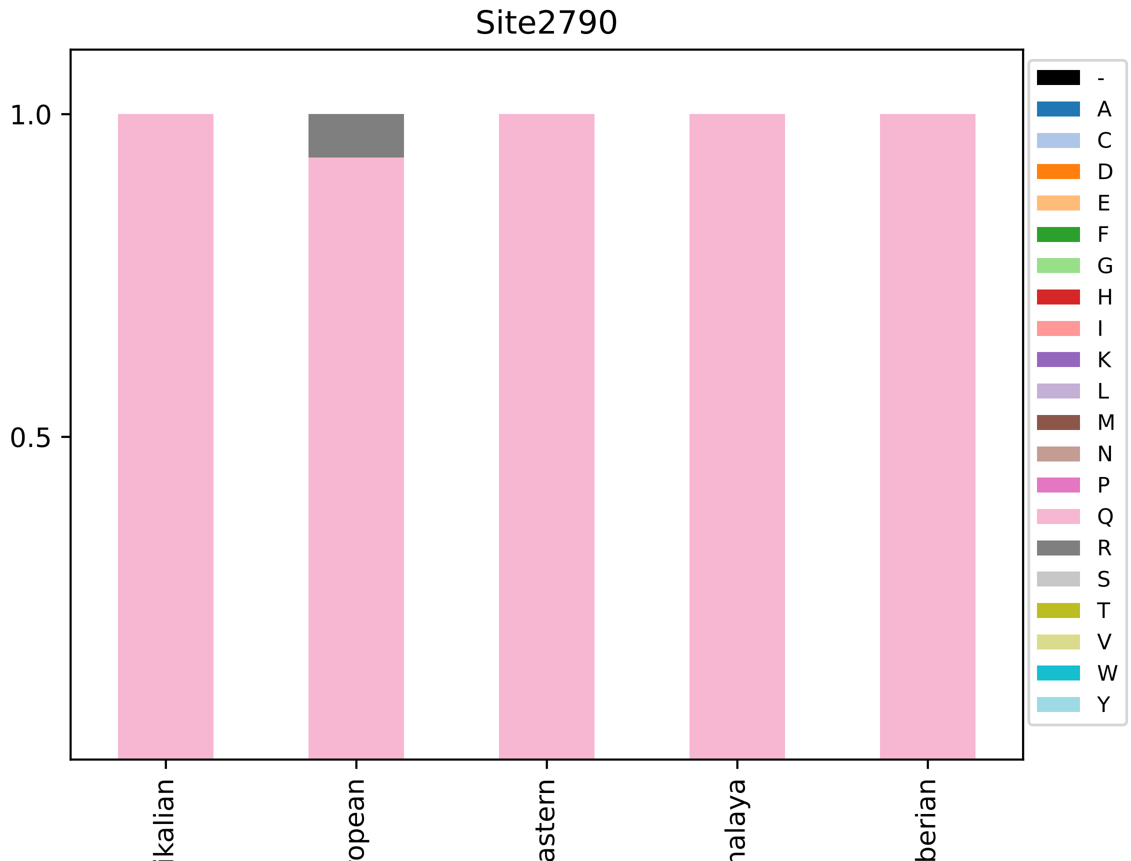
Site2790
1.0
0.5
-
A
C
D
E
F
G
H
I
K
L
M
N
P
Q
R
S
T
V
W
Y
ikalian
ropean
astern
nalaya
berian

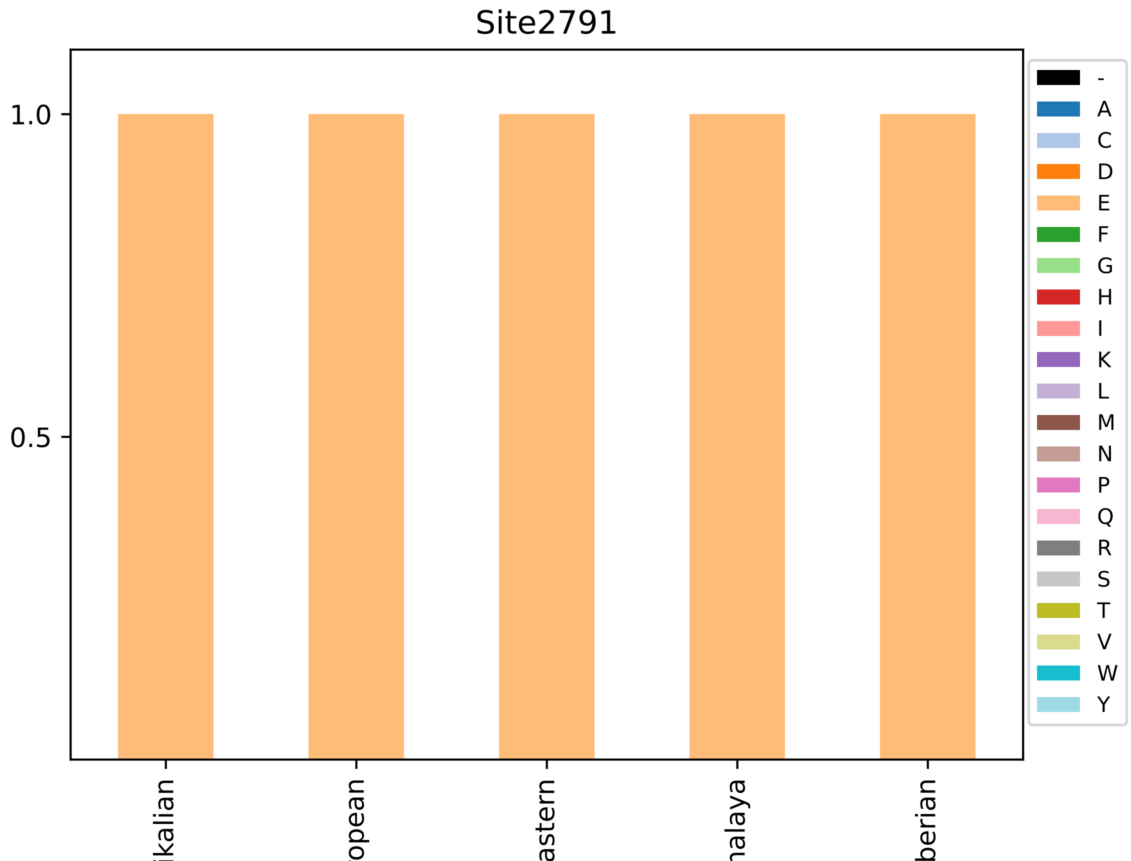
Site2791
1.0
0.5
-
A
C
D
E
F
G
H
I
K
L
M
N
P
Q
R
S
T
V
W
Y
ikalian
opean
astern
nalaya
berian

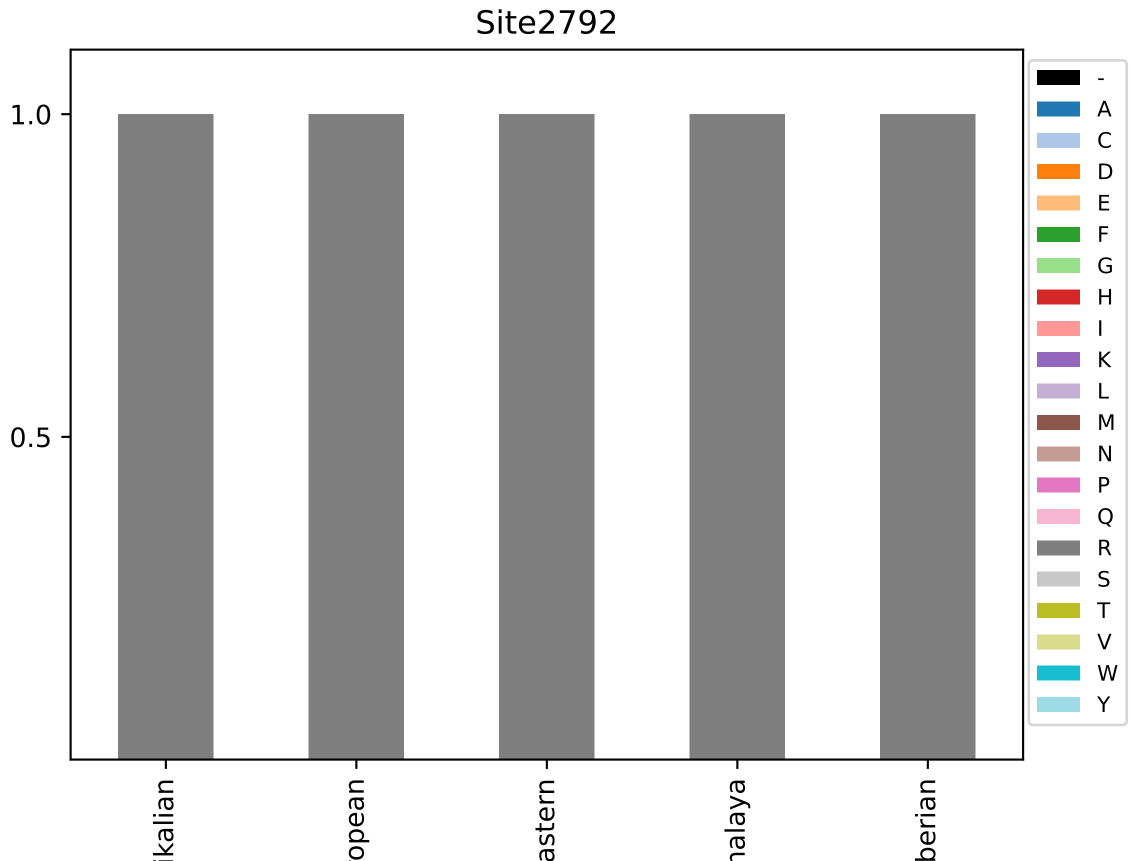
Site2792
1.0
0.5
-
A
C
D
E
F
G
H
I
K
L
M
N
P
Q
R
S
T
V
W
Y
ikalian
opean
astern
nalaya
berian

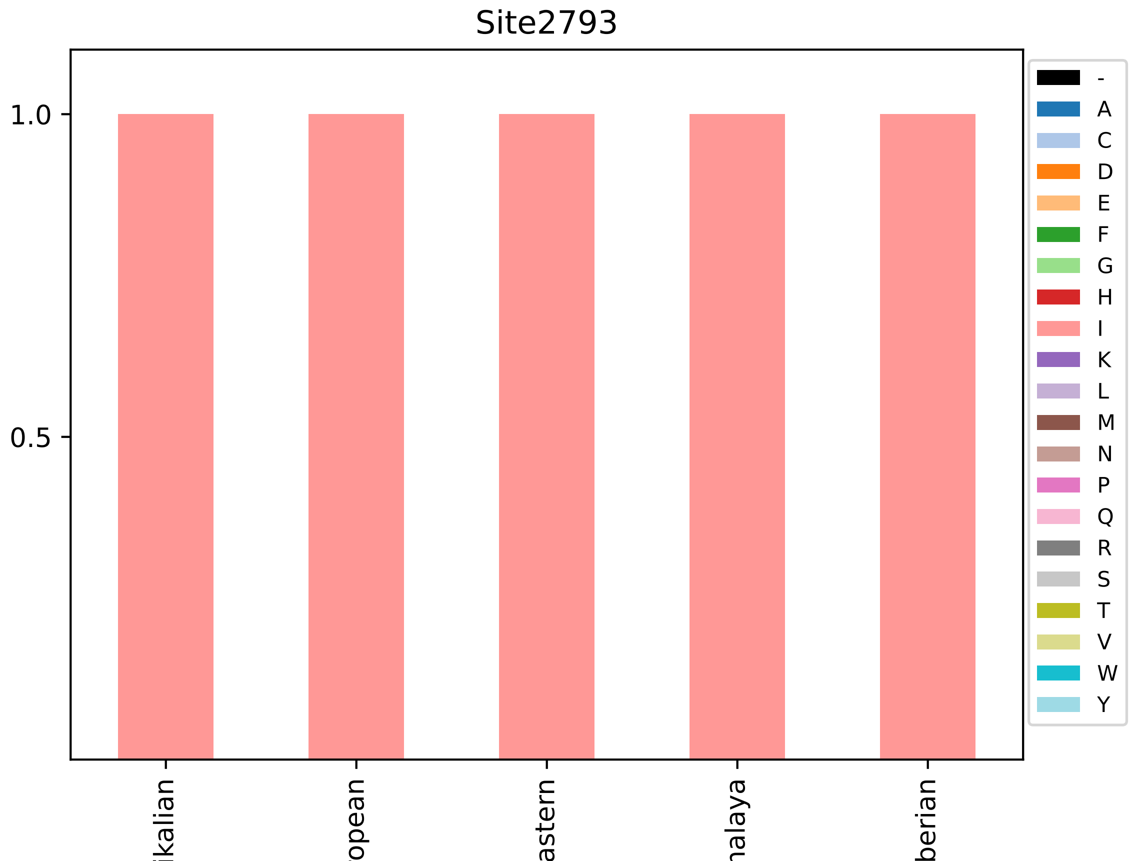

Site2793
1.0
0.5
-
A
C
D
E
F
G
H
I
K
L
M
N
P
Q
R
S
T
V
W
Y
ikalian
opean
astern
nalaya
berian

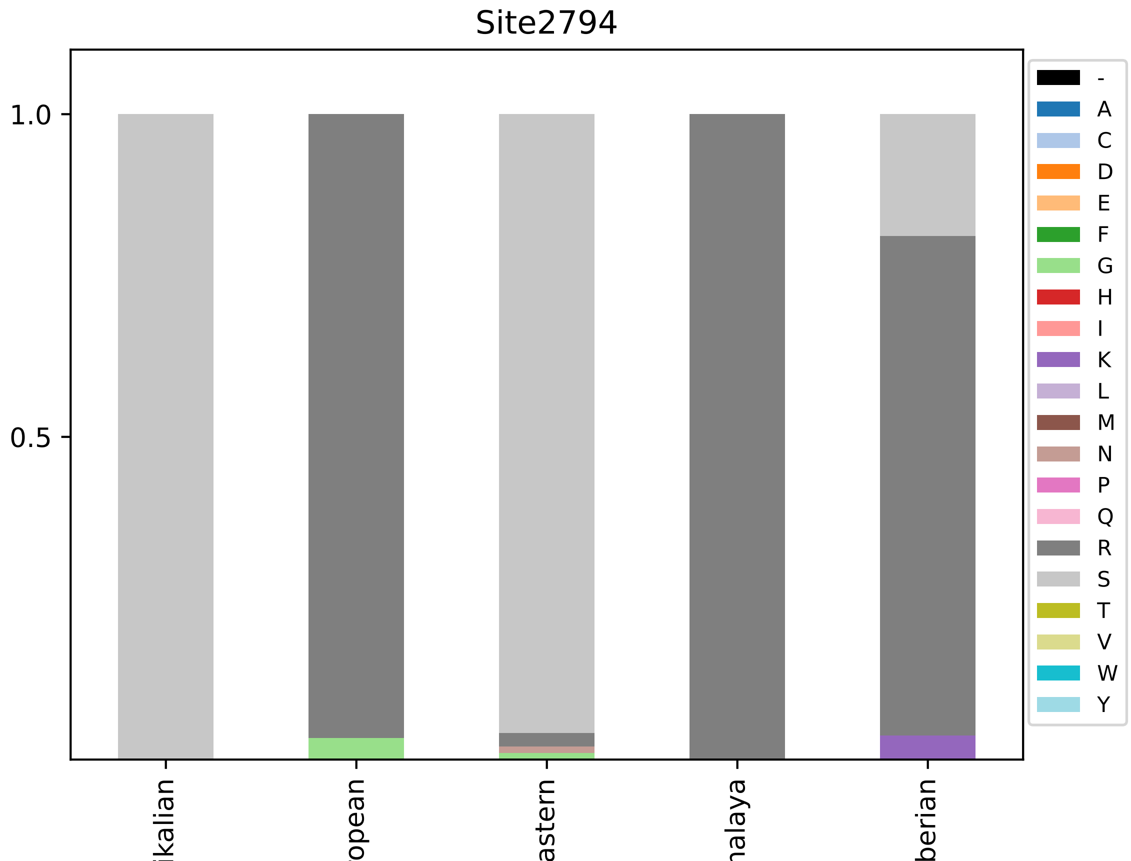
Site2794
1.0
0.5
-
A
C
D
E
F
G
H
I
K
L
M
N
P
Q
R
S
T
V
W
Y
ikalian
opean
astern
nalaya
berian

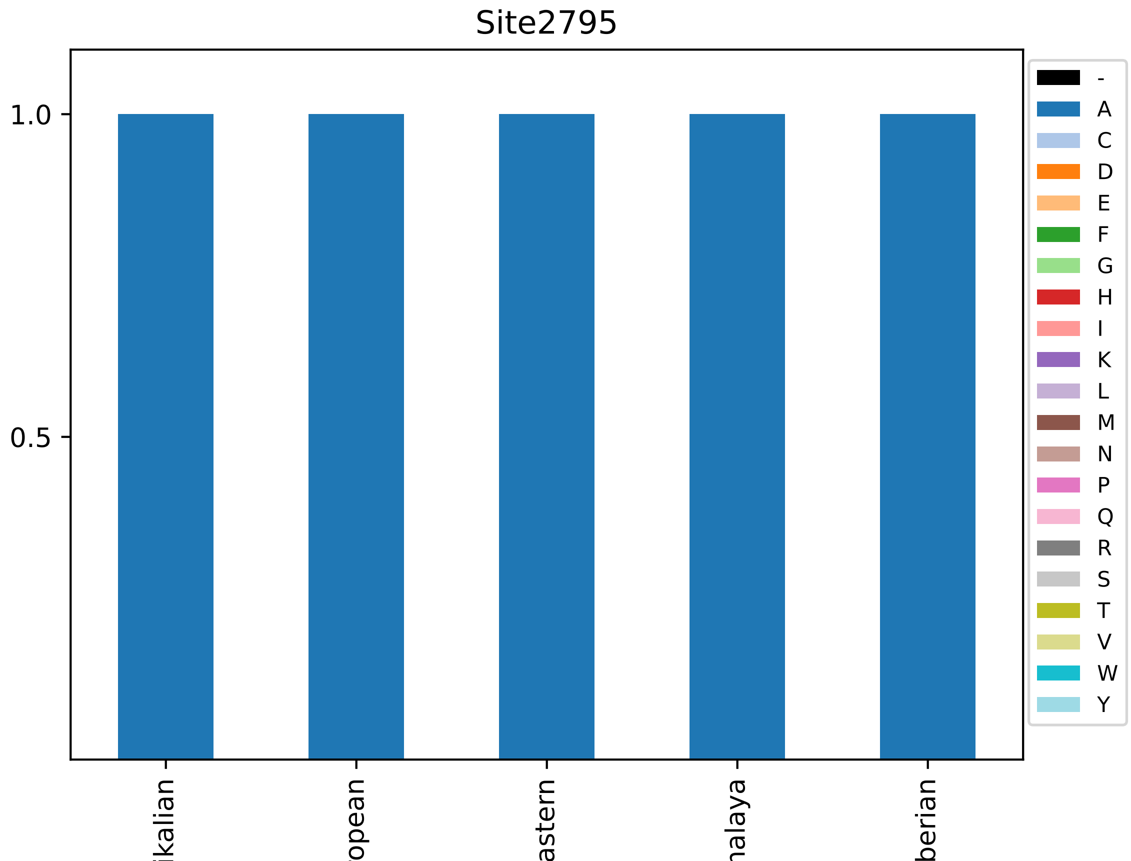

Site2795
1.0
0.5
-
A
C
D
E
F
G
H
I
K
L
M
N
P
Q
R
S
T
V
W
Y
ikalian
opean
astern
nalaya
berian

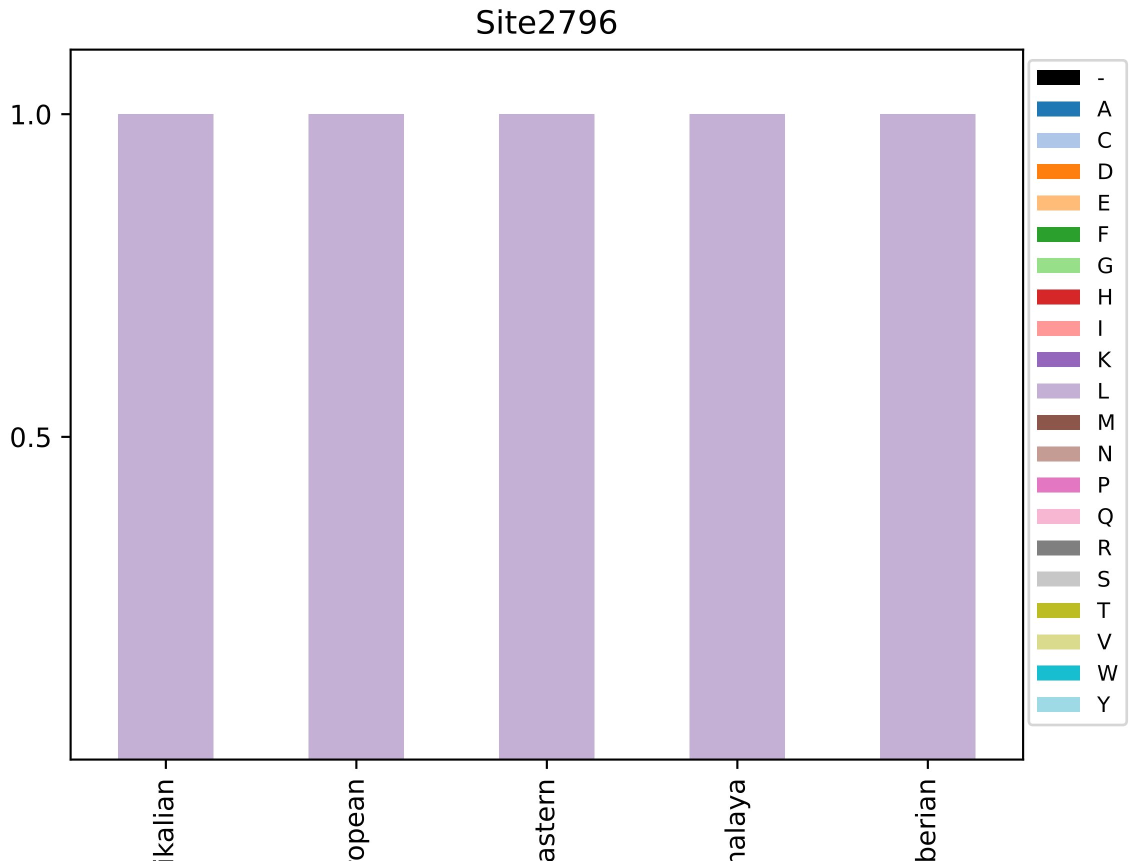

Site2796
1.0
0.5
-
A
C
D
E
F
G
H
I
K
L
M
N
P
Q
R
S
T
V
W
Y
ikalian
opean
astern
nalaya
berian

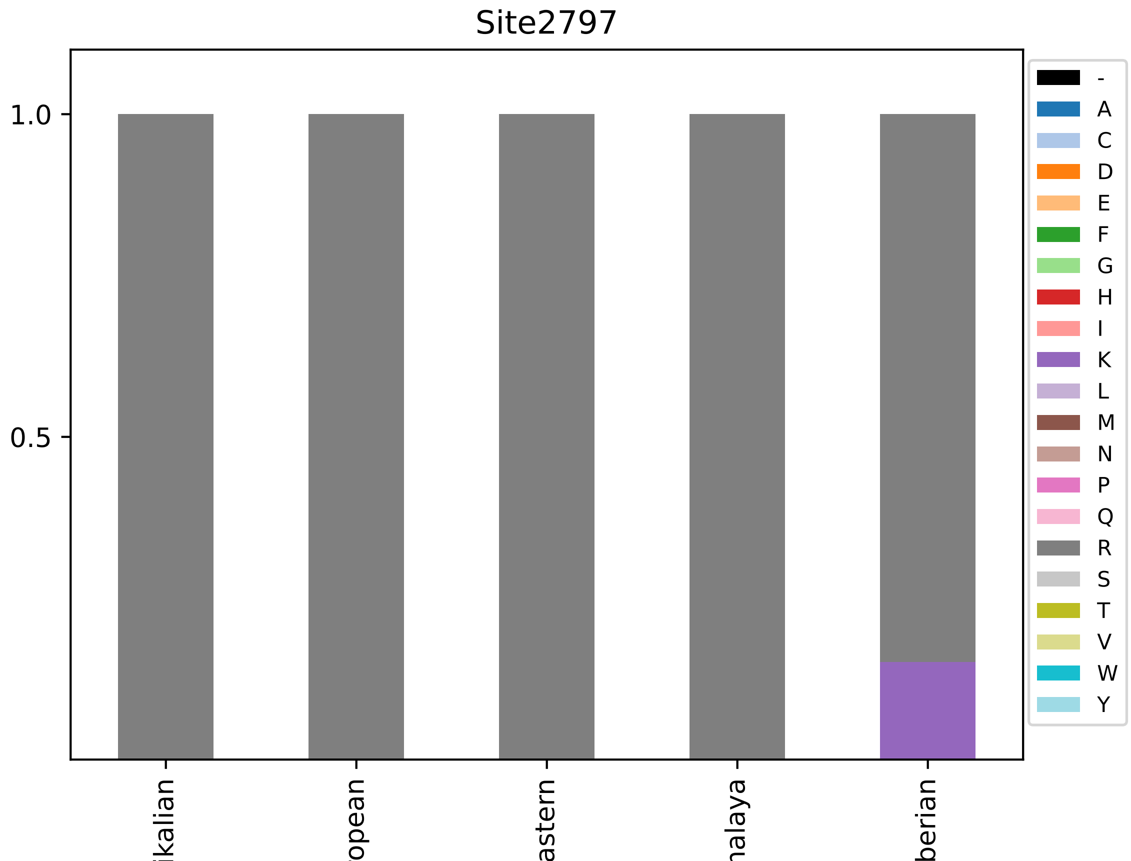

Site2797
1.0
0.5
-
A
C
D
E
F
G
H
I
K
L
M
N
P
Q
R
S
T
V
W
Y
ikalian
opean
astern
nalaya
berian

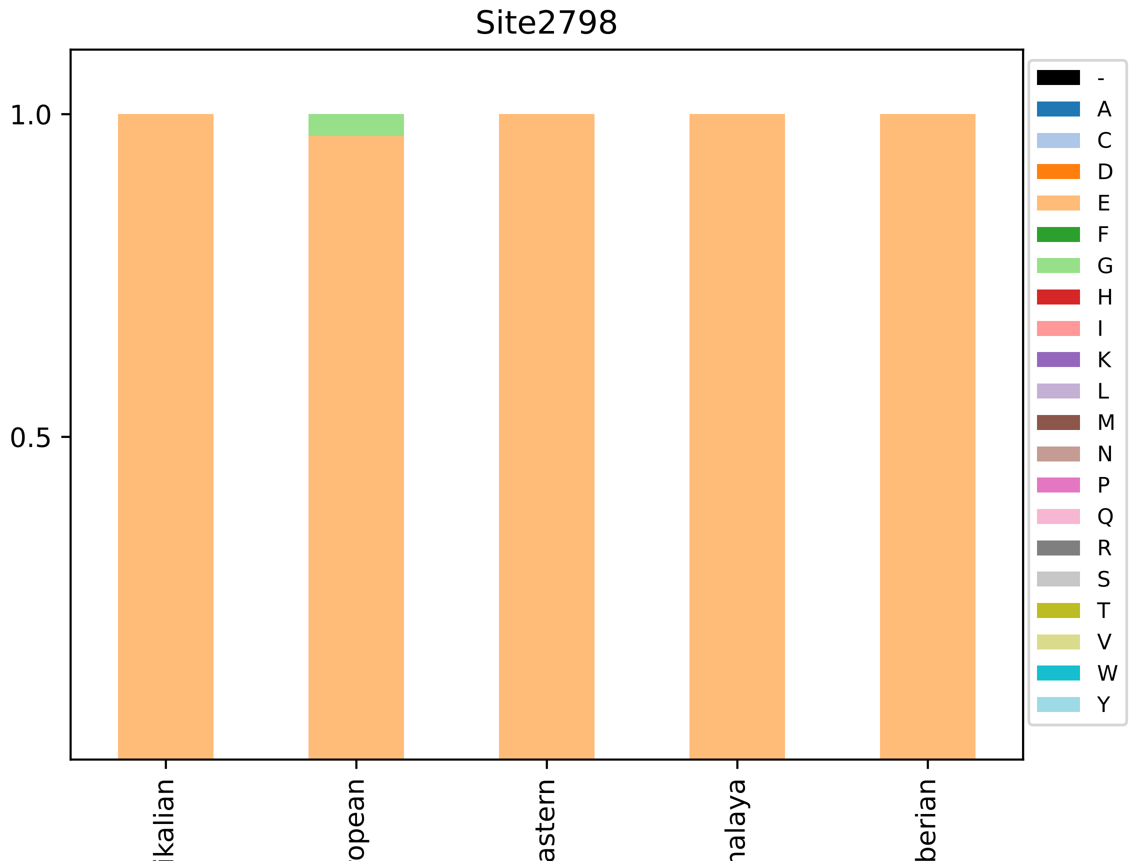
Site2798
1.0
0.5
-
A
C
D
E
F
G
H
I
K
L
M
N
P
Q
R
S
T
V
W
Y
ikalian
opean
astern
nalaya
berian

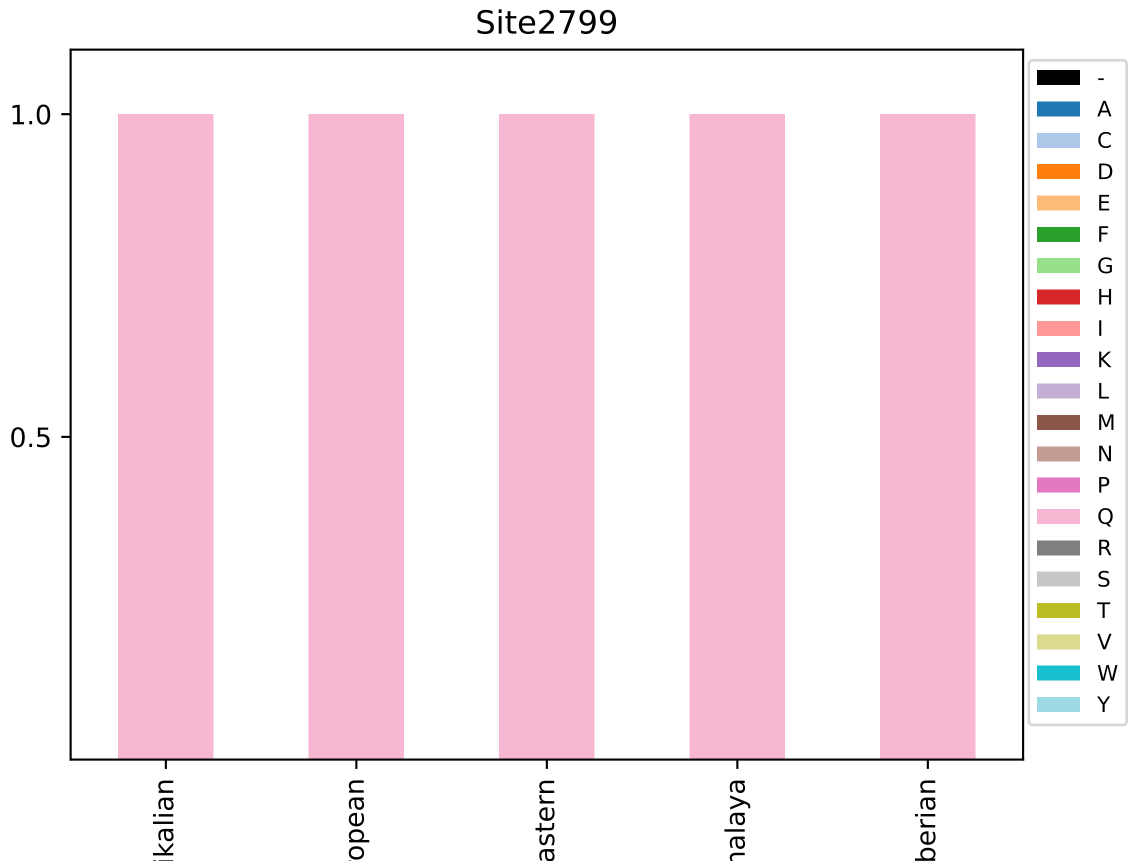

Site2799
1.0
0.5
-
A
C
D
E
F
G
H
I
K
L
M
N
P
Q
R
S
T
V
W
Y
ikalian
opean
astern
nalaya
berian

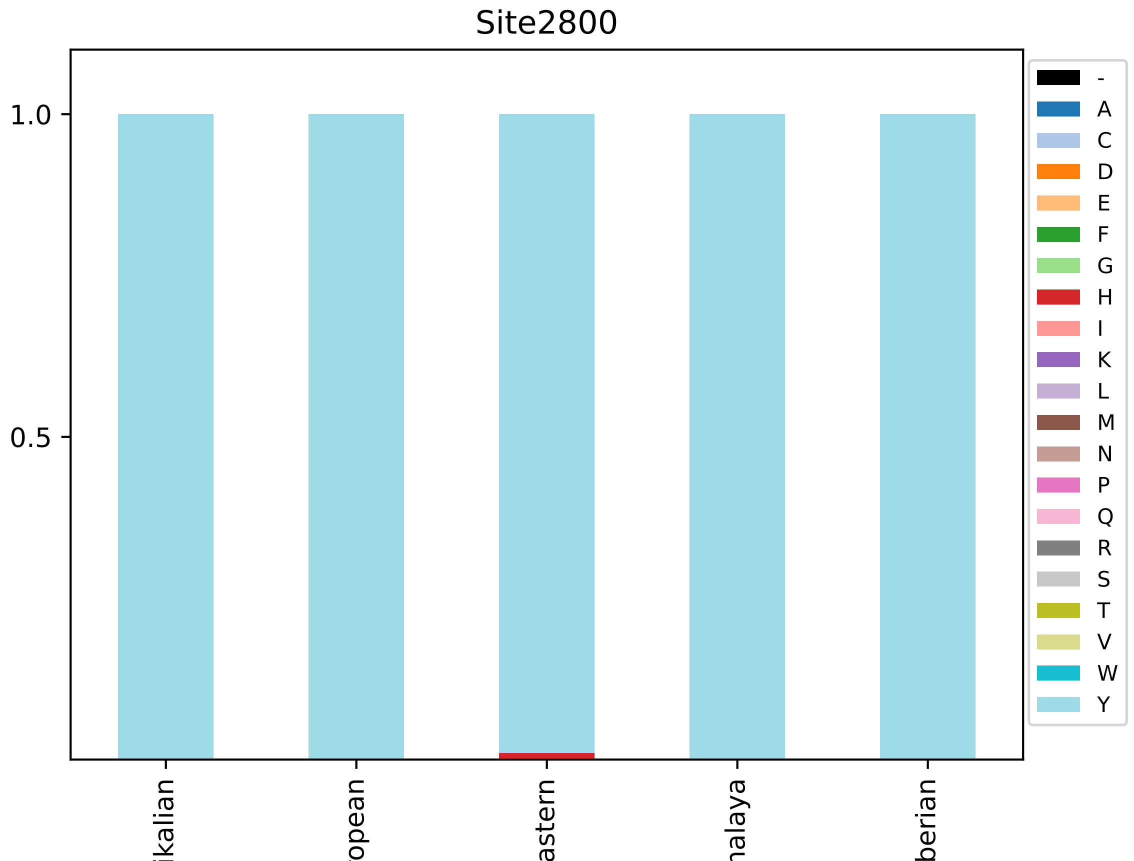
Site2800
1.0
0.5
-
A
C
D
E
F
G
H
I
K
L
M
N
P
Q
R
S
T
V
W
Y
ikalian
opean
astern
nalaya
berian

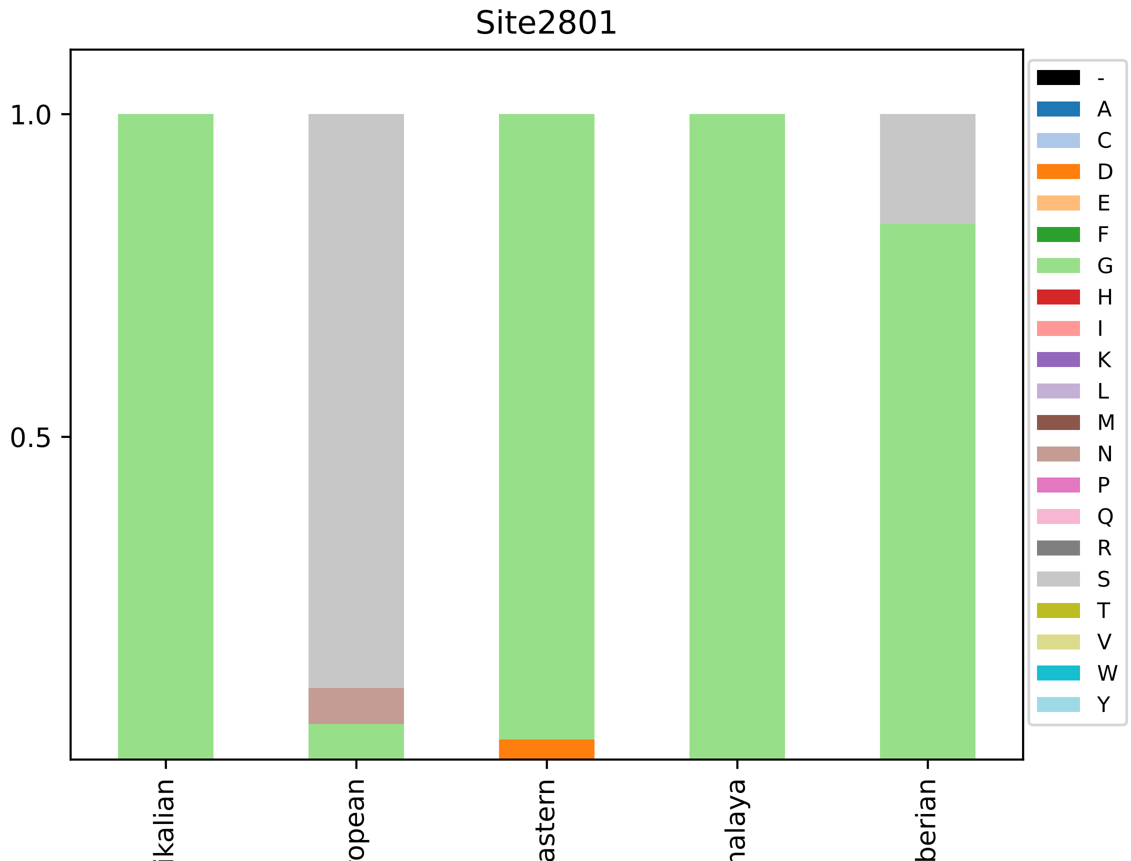
Site2801
1.0
0.5
ikalian
opean
astern
nalaya
berian
-
A
C
D
E
F
G
H
I
K
L
M
N
P
Q
R
S
T
V
W
Y

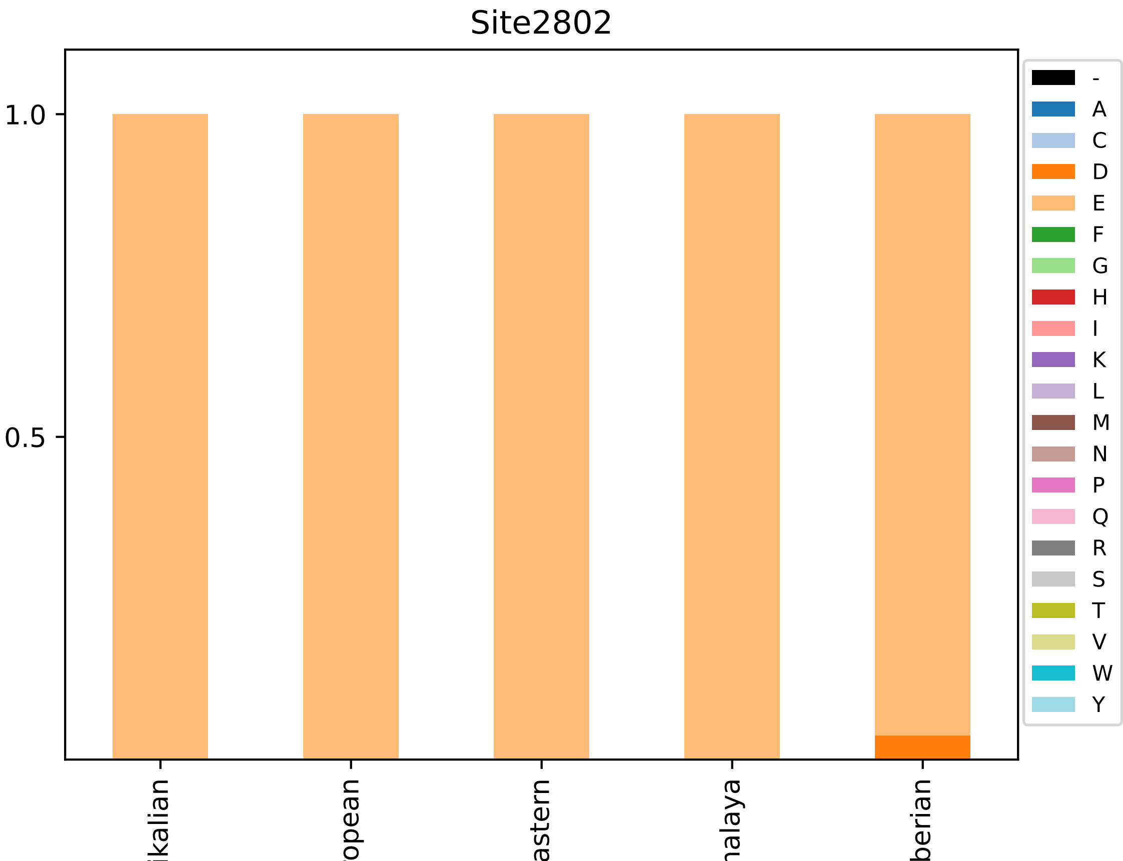

Site2802
1.0
0.5
-
A
C
D
E
F
G
H
I
K
L
M
N
P
Q
R
S
T
V
W
Y

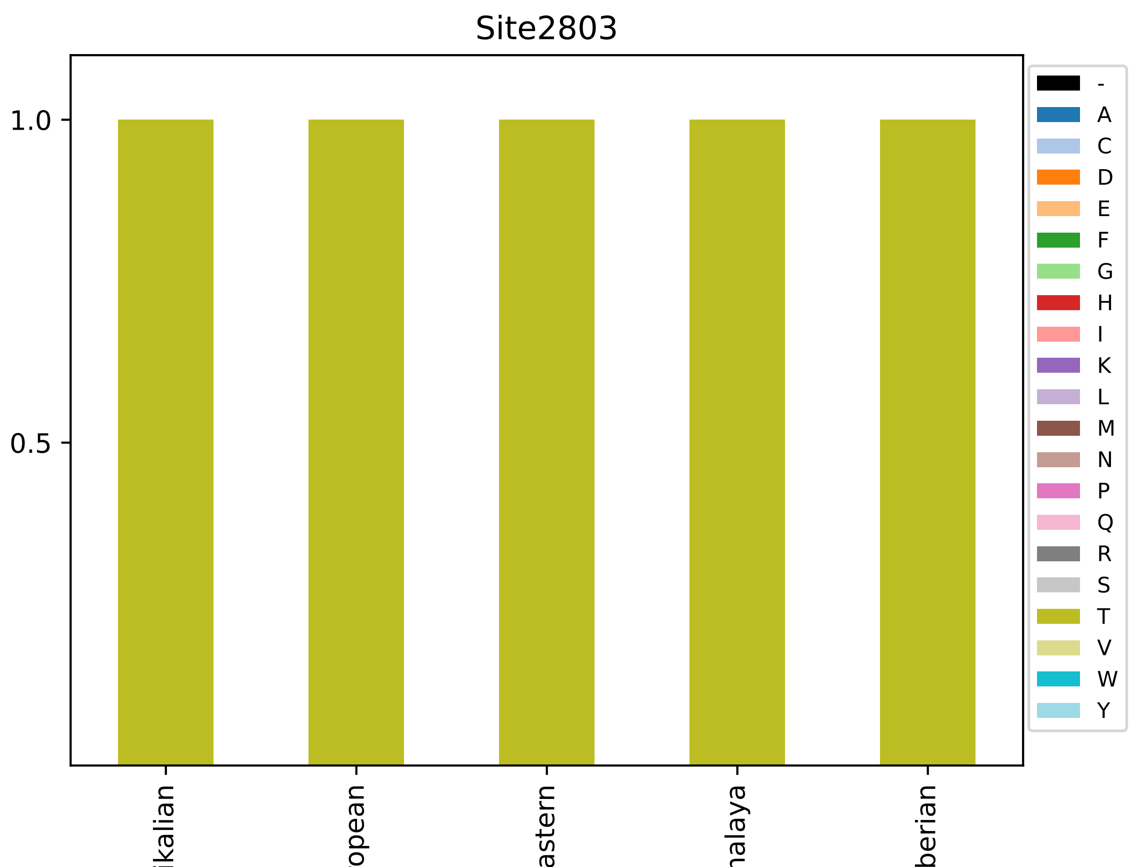

Site2803
1.0
0.5
-
A
C
D
E
F
G
H
I
K
L
M
N
P
Q
R
S
T
V
W
Y
ikalian
opean
astern
nalaya
berian

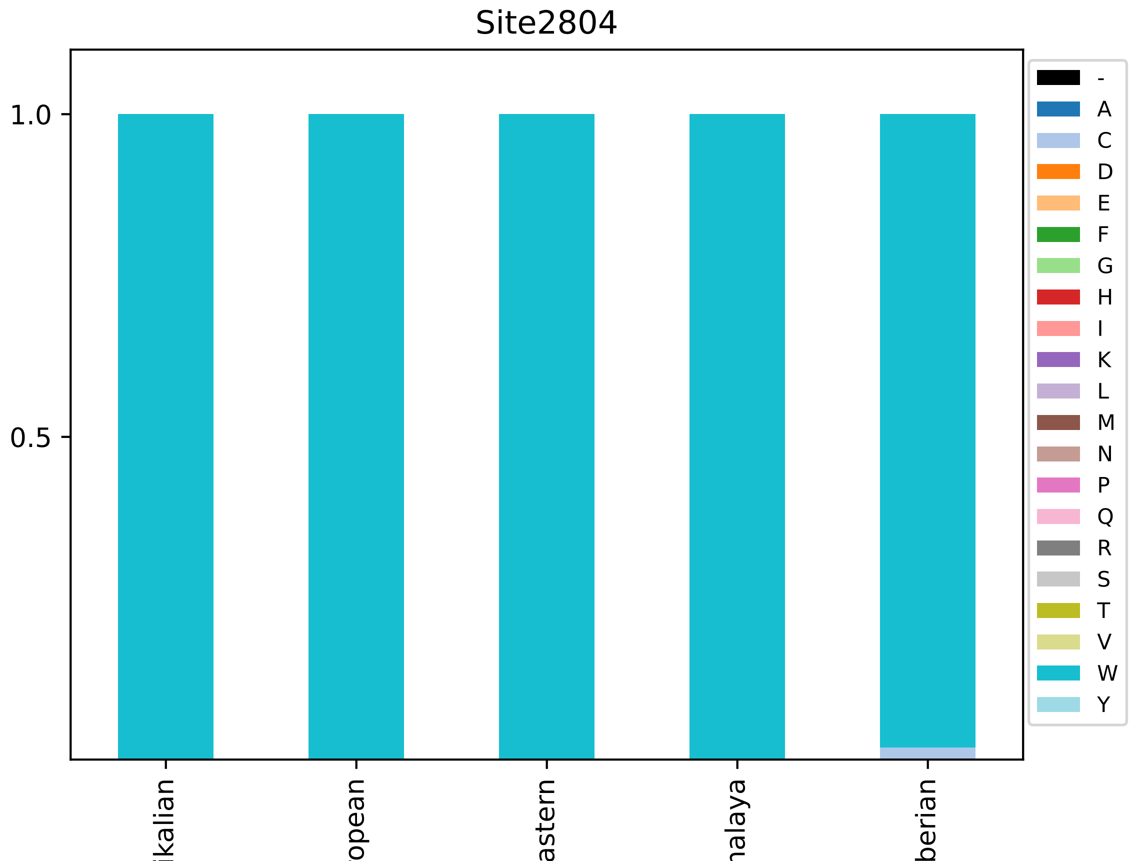

Site2804
1.0
0.5
-
A
C
D
E
F
G
H
I
K
L
M
N
P
Q
R
S
T
V
W
Y
ikalian
opean
astern
nalaya
berian

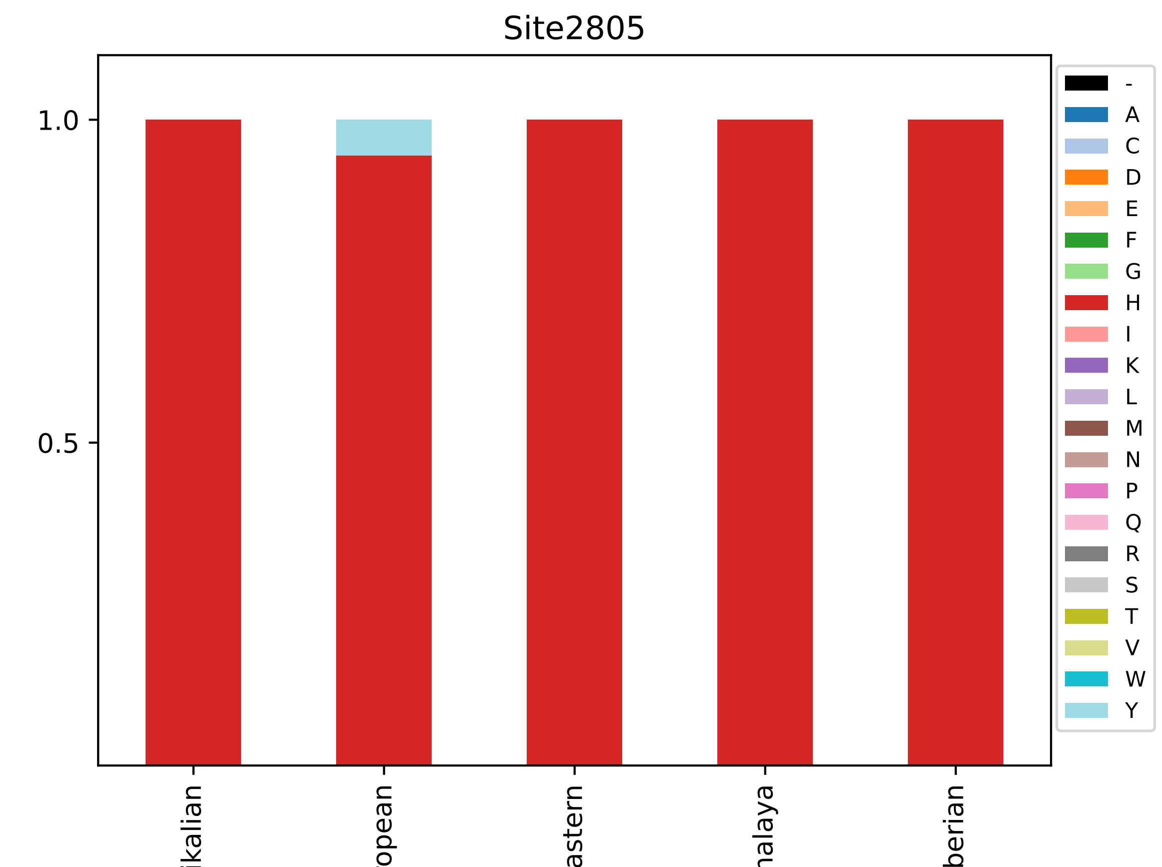

Site2805
1.0
0.5
-
A
C
D
E
F
G
H
I
K
L
M
N
P
Q
R
S
T
V
W
Y
ikalian
opean
astern
nalaya
berian

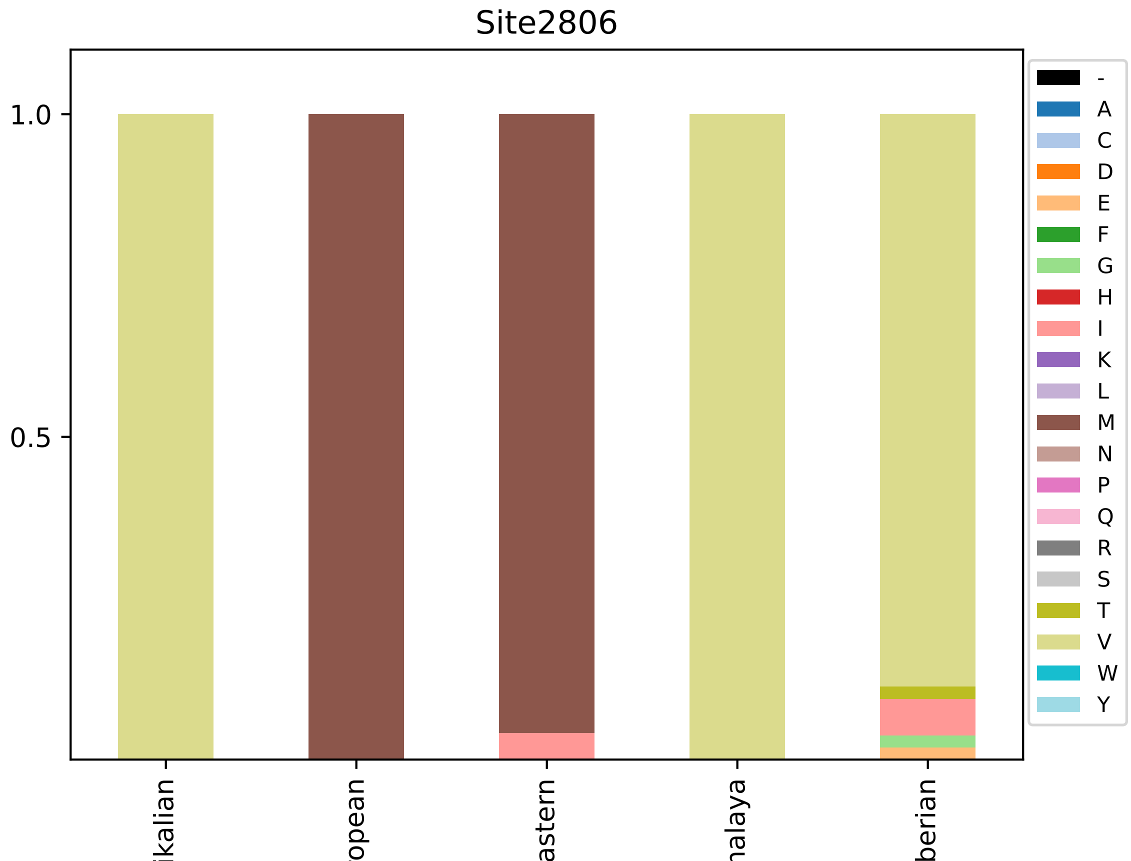

Site2806
1.0
0.5
-
A
C
D
E
F
G
H
I
K
L
M
N
P
Q
R
S
T
V
W
Y
ikalian
opean
astern
nalaya
berian

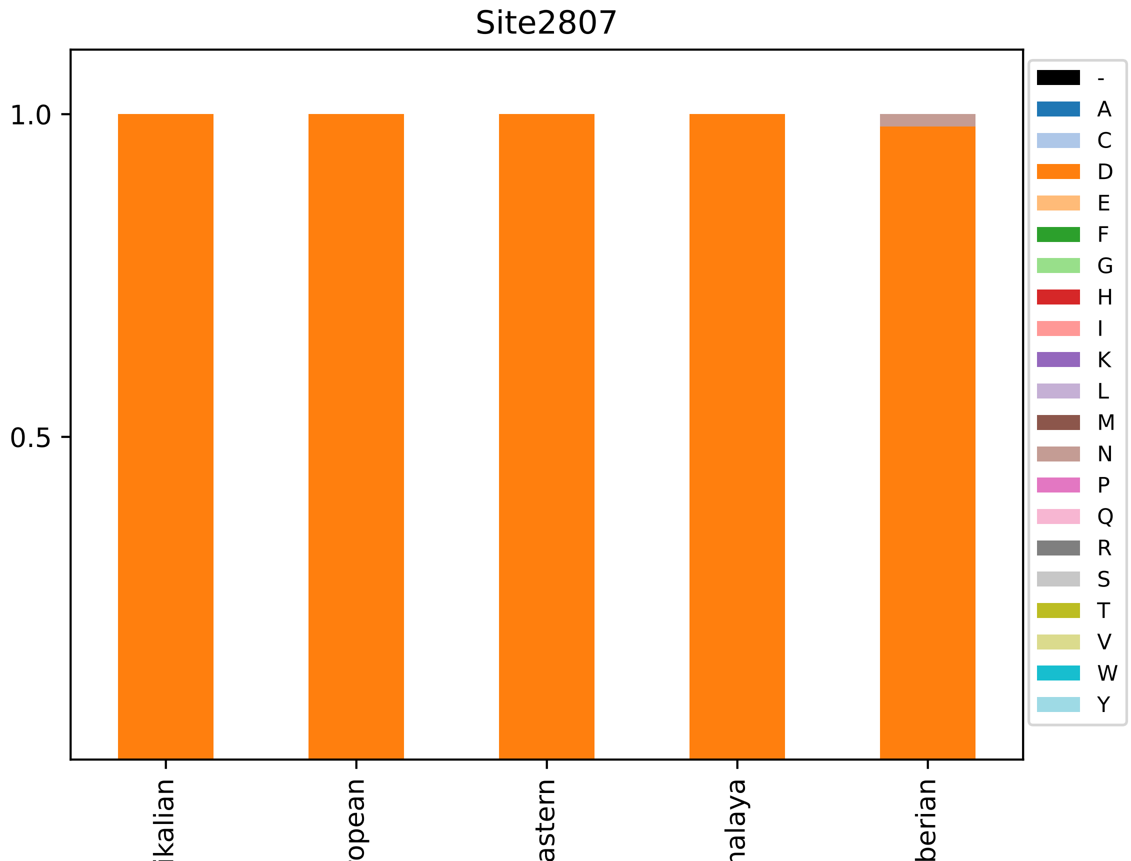

Site2807
1.0
0.5
ikalian
opean
astern
nalaya
berian
-
A
C
D
E
F
G
H
I
K
L
M
N
P
Q
R
S
T
V
W
Y

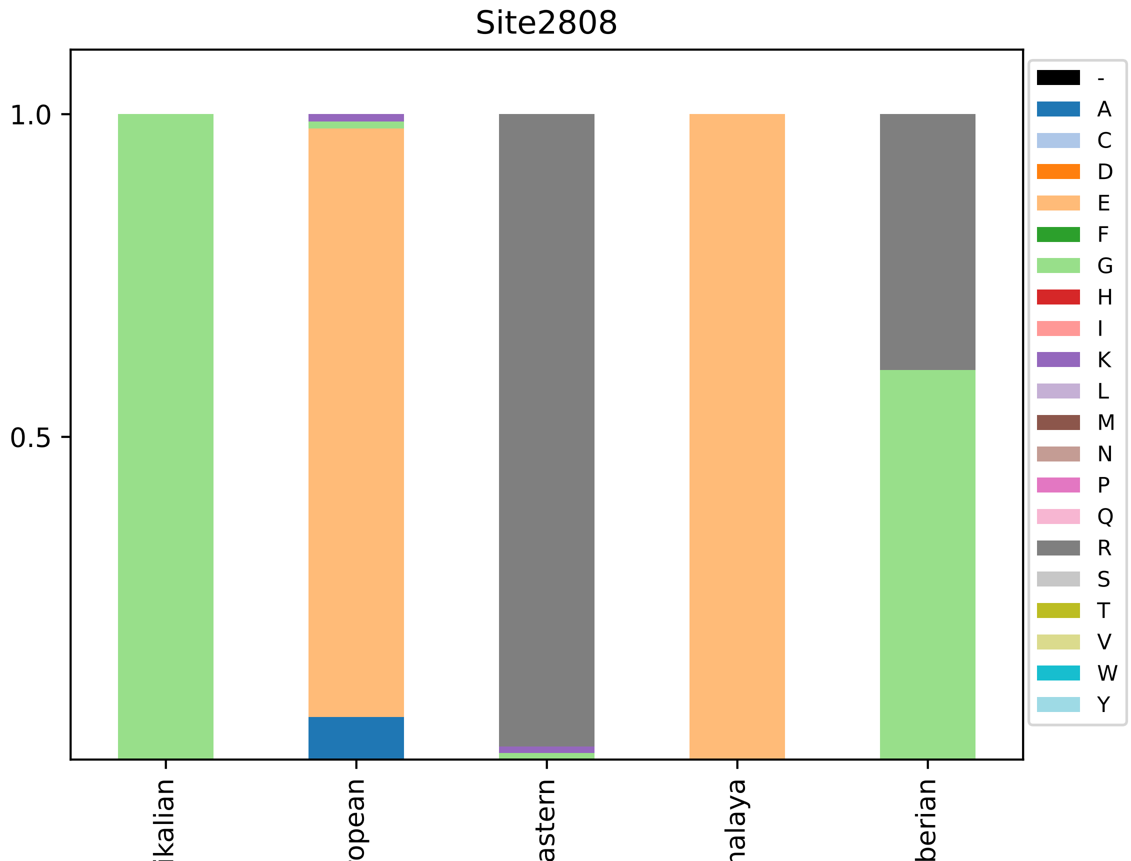
Site2808
1.0
0.5
-
A
C
D
E
F
G
H
I
K
L
M
N
P
Q
R
S
T
V
W
Y
ikalian
opean
astern
nalaya
berian

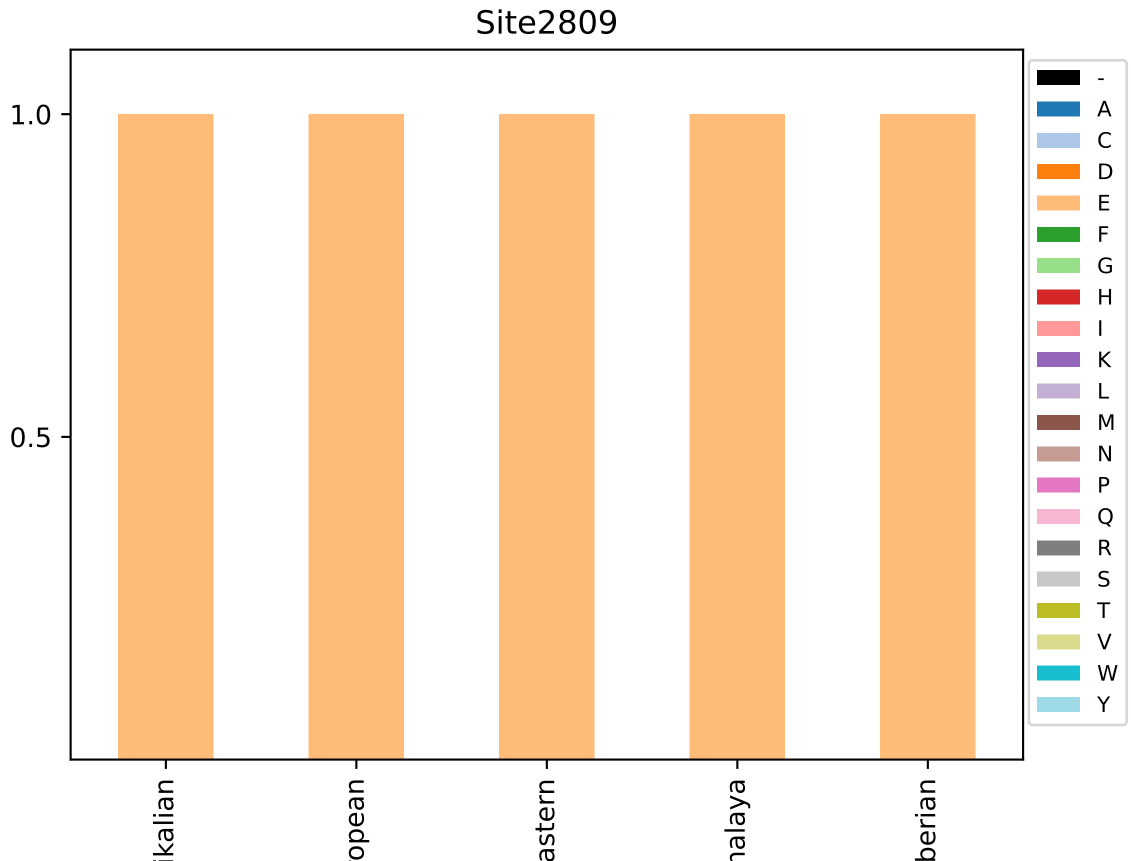
Site2809
1.0
0.5
-
A
C
D
E
F
G
H
I
K
L
M
N
P
Q
R
S
T
V
W
Y
ikalian
opean
astern
nalaya
berian

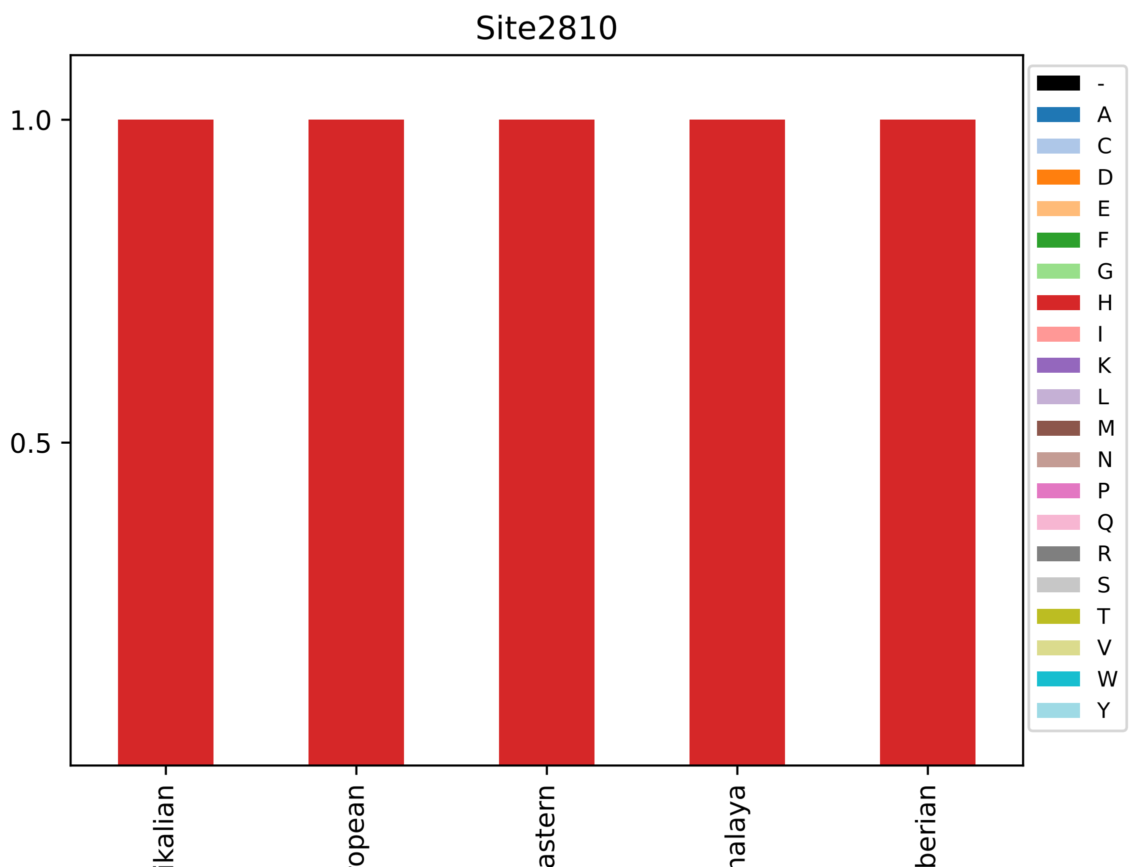

Site2810
1.0
0.5
-
A
C
D
E
F
G
H
I
K
L
M
N
P
Q
R
S
T
V
W
Y
ikalian
opean
astern
nalaya
berian

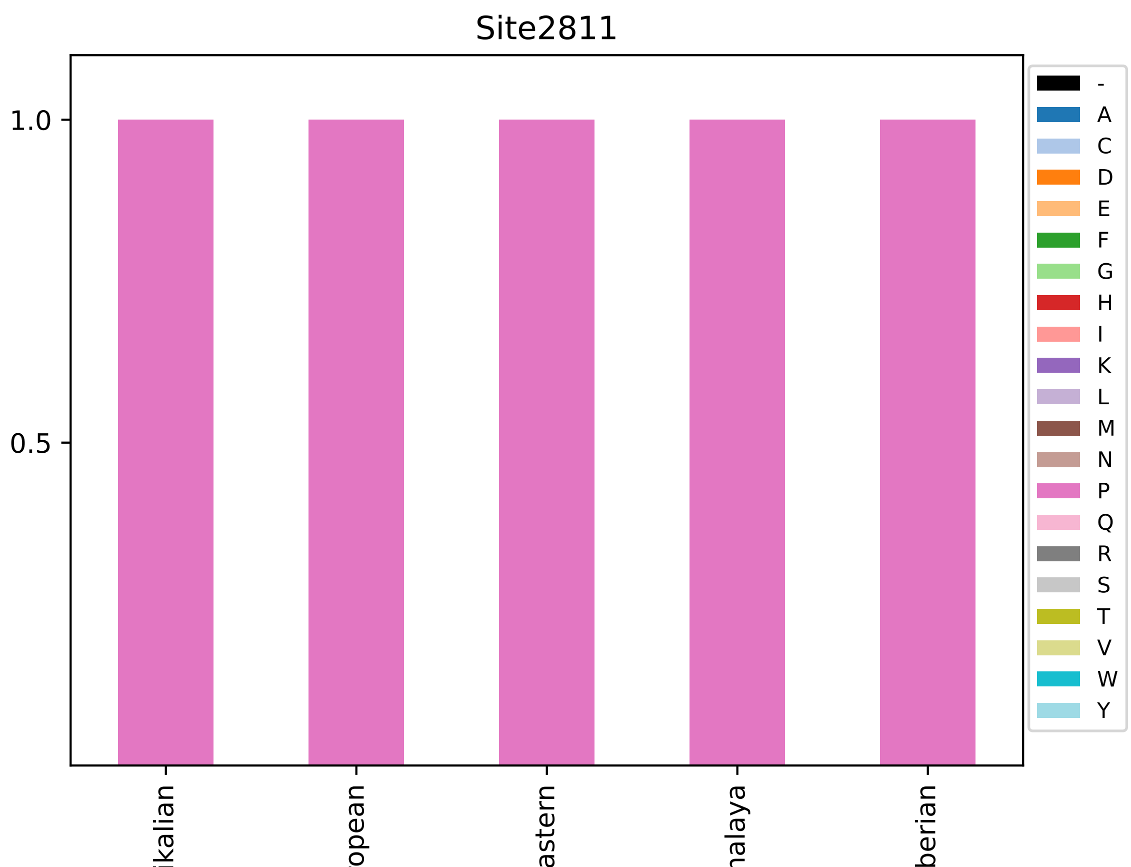

Site2811
1.0
0.5
ikalian
opean
astern
nalaya
berian
-
A
C
D
E
F
G
H
I
K
L
M
N
P
Q
R
S
T
V
W
Y

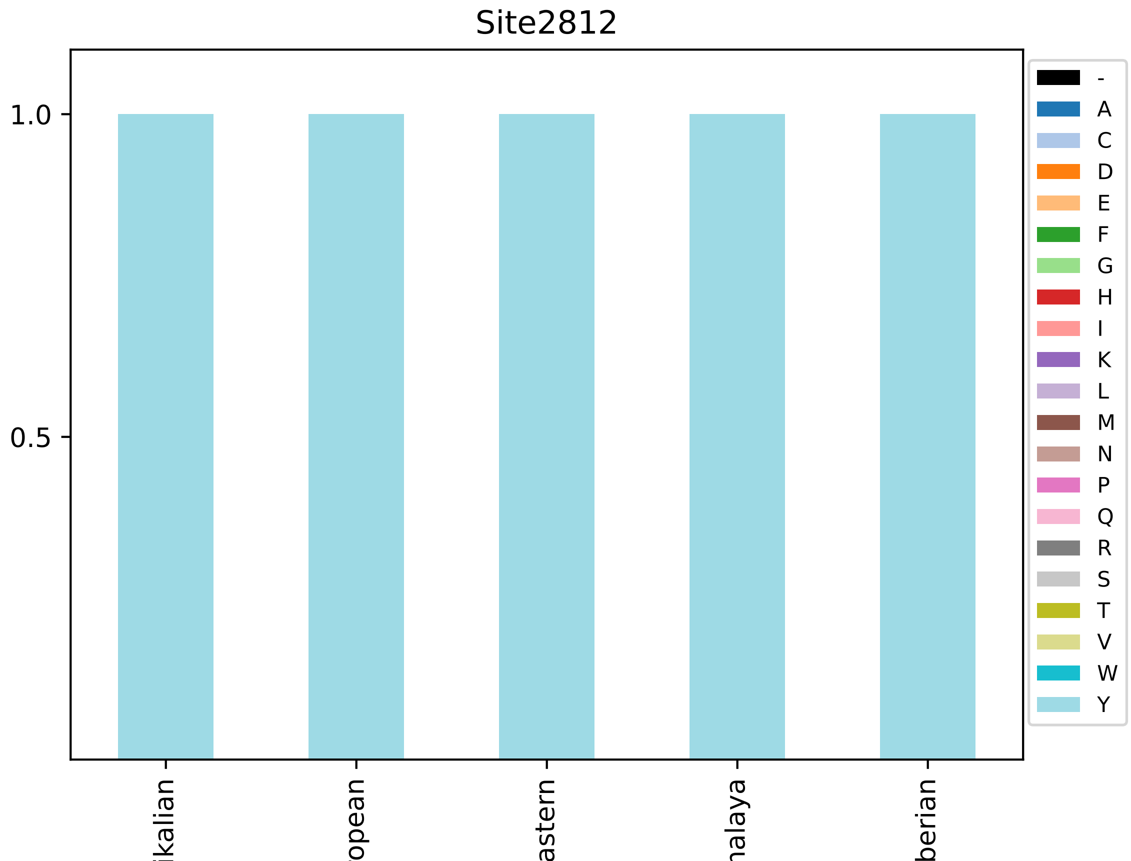

Site2812
1.0
0.5
-
A
C
D
E
F
G
H
I
K
L
M
N
P
Q
R
S
T
V
W
Y
ikalian
opean
astern
nalaya
berian

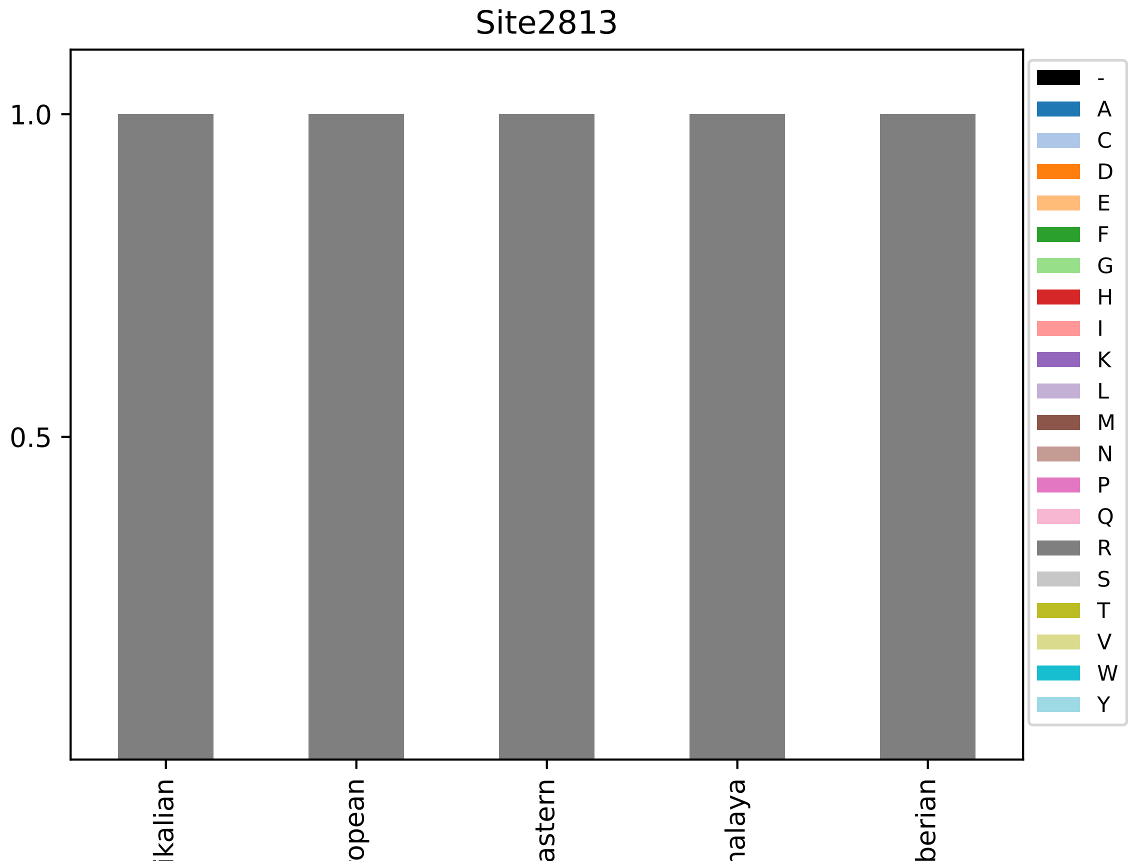
Site2813
1.0
0.5
-
A
C
D
E
F
G
H
I
K
L
M
N
P
Q
R
S
T
V
W
Y
ikalian
opean
astern
nalaya
berian

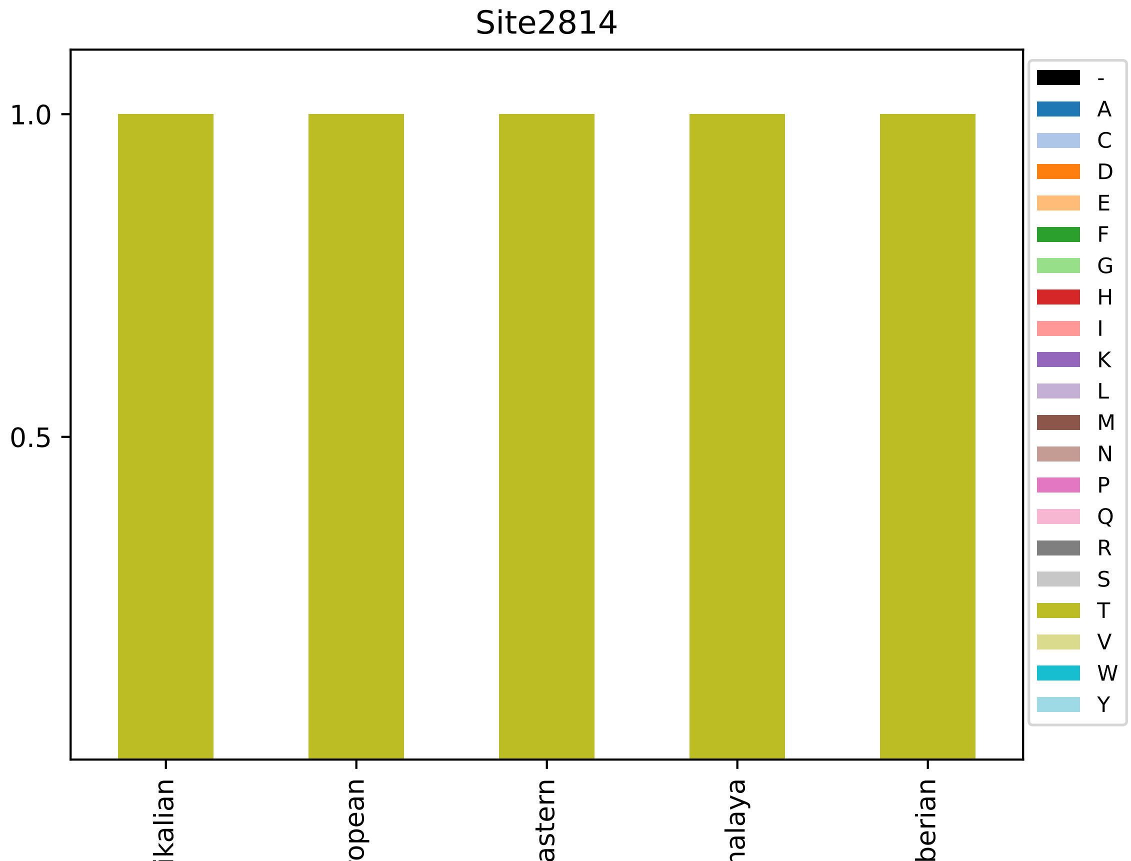

Site2814
1.0
0.5
ikalian
opean
astern
nalaya
berian
-
A
C
D
E
F
G
H
I
K
L
M
N
P
Q
R
S
T
V
W
Y

Site2815

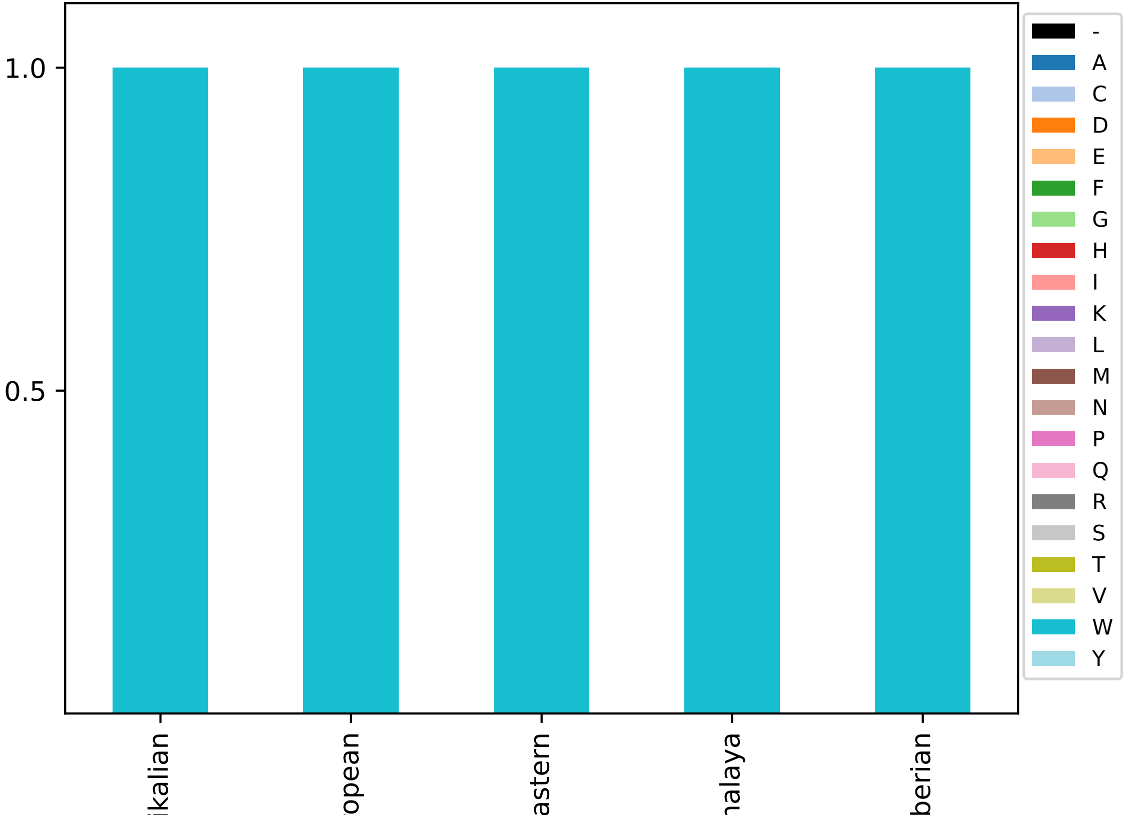

1.0
0.5
-
A
C
D
E
F
G
H
I
K
L
M
N
P
Q
R
S
T
V
W
Y
ikalian
opean
astern
nalaya
berian

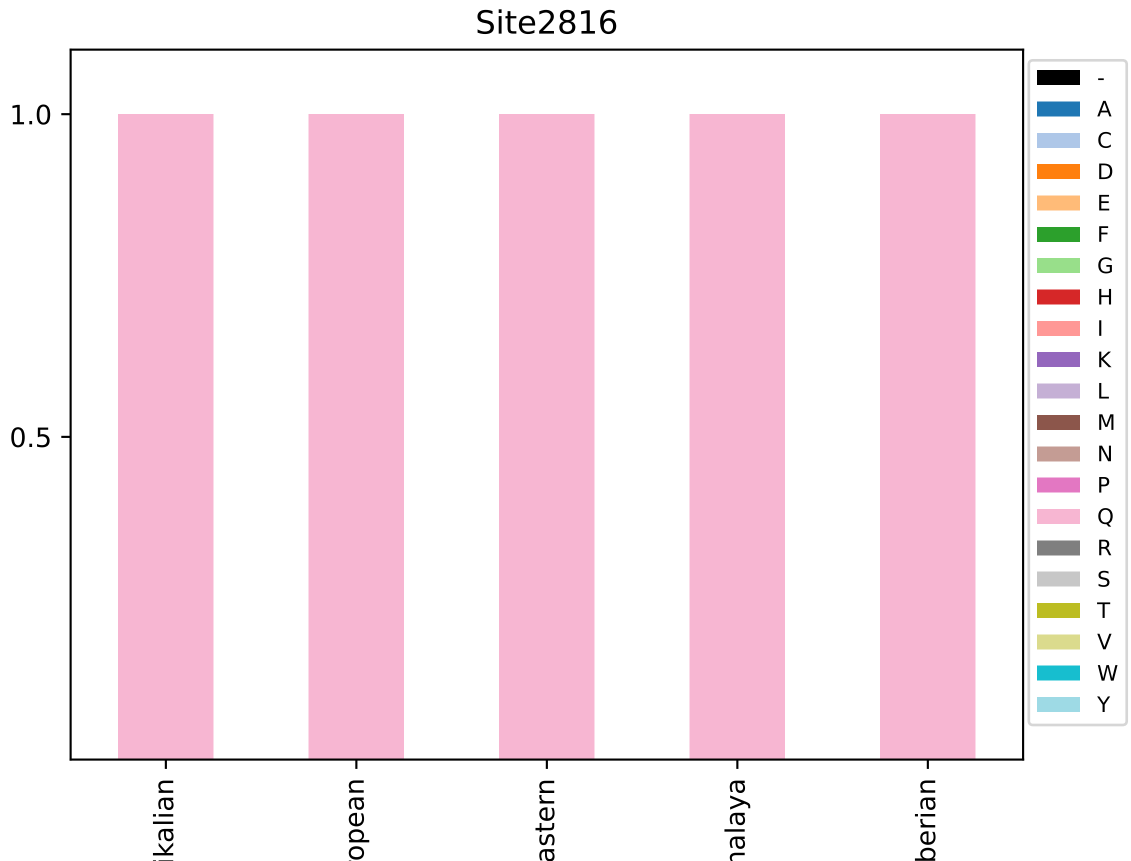

Site2816
1.0
0.5
-
A
C
D
E
F
G
H
I
K
L
M
N
P
Q
R
S
T
V
W
Y
ikalian
opean
astern
nalaya
berian

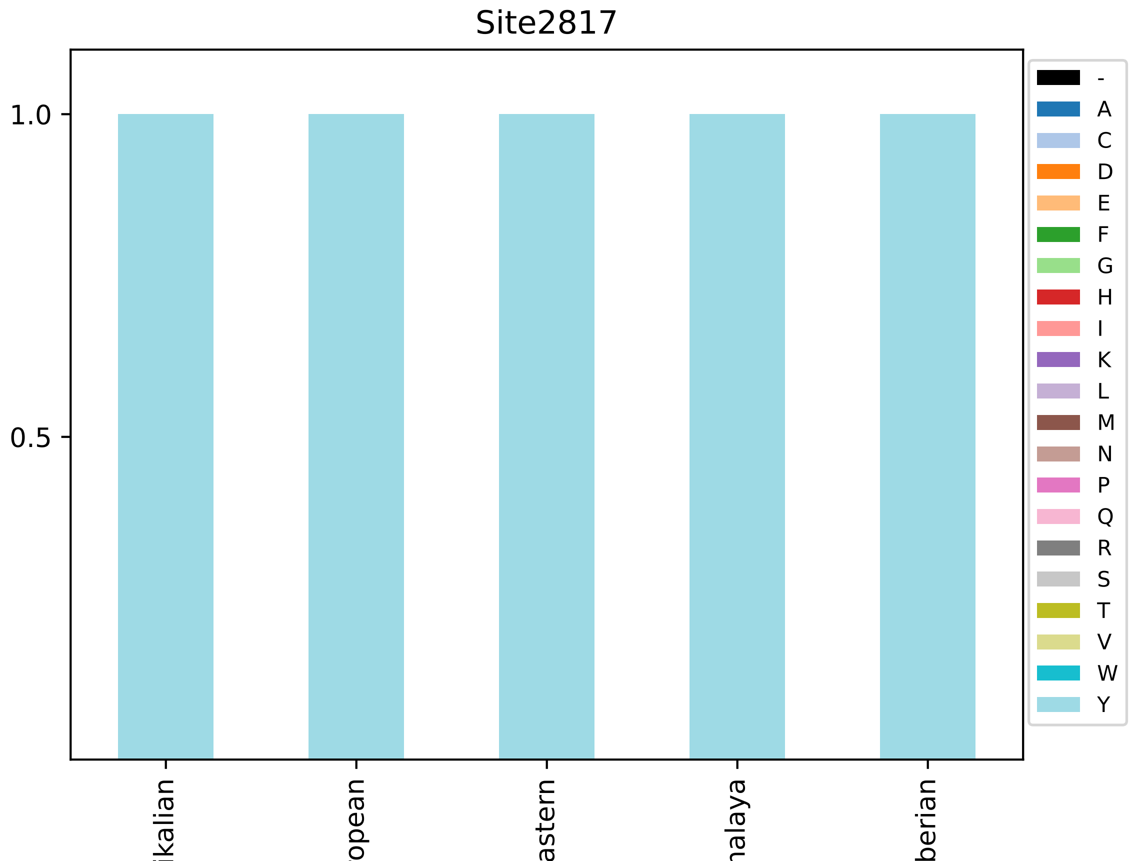
Site2817
1.0
0.5
-
A
C
D
E
F
G
H
I
K
L
M
N
P
Q
R
S
T
V
W
Y
ikalian
opean
astern
nalaya
berian

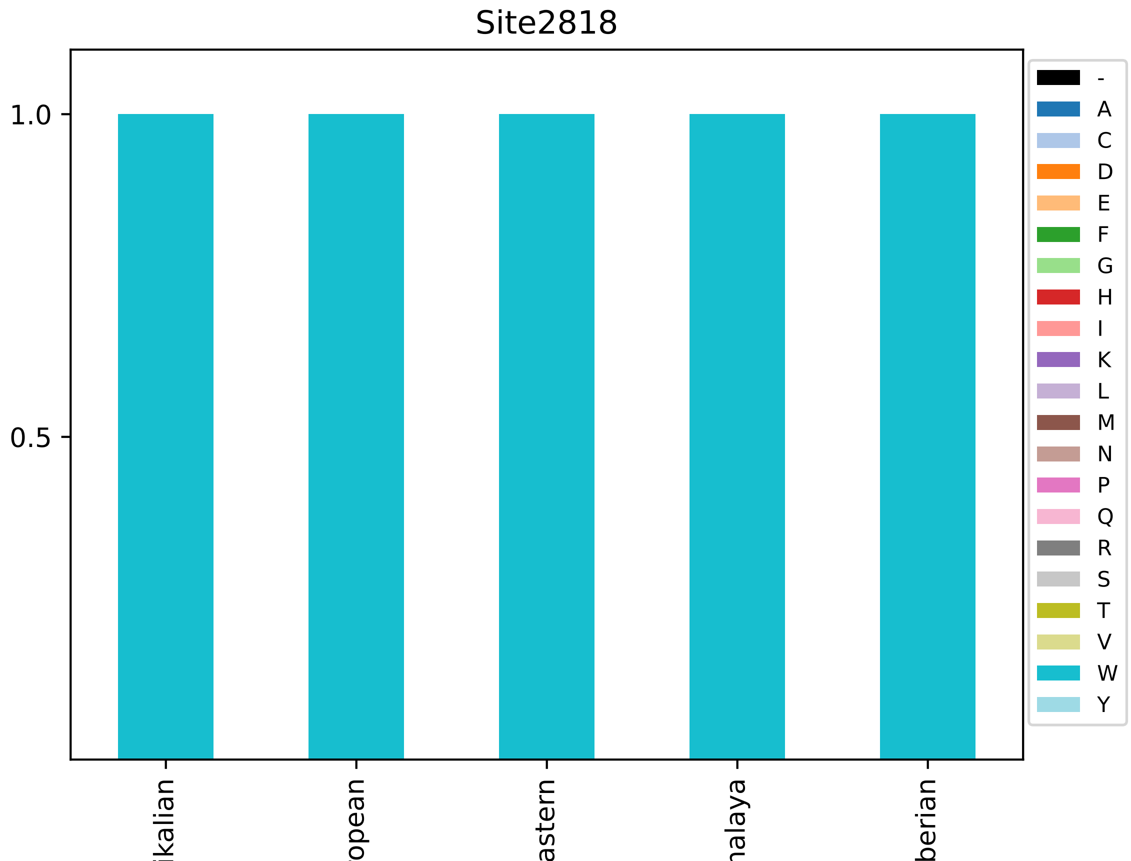

Site2818
1.0
0.5
-
A
C
D
E
F
G
H
I
K
L
M
N
P
Q
R
S
T
V
W
Y
ikalian
opean
astern
nalaya
berian

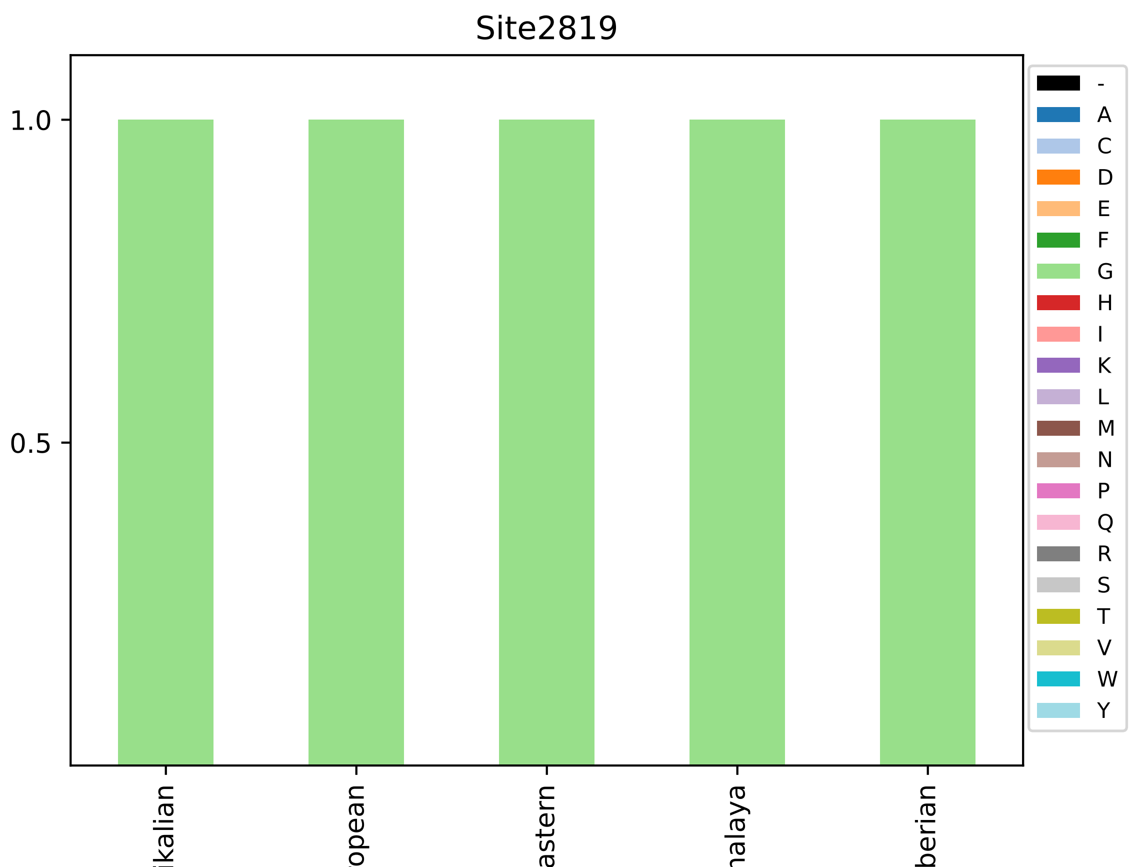
Site2819
1.0
0.5
ikalian
opean
astern
nalaya
berian
-
A
C
D
E
F
G
H
I
K
L
M
N
P
Q
R
S
T
V
W
Y

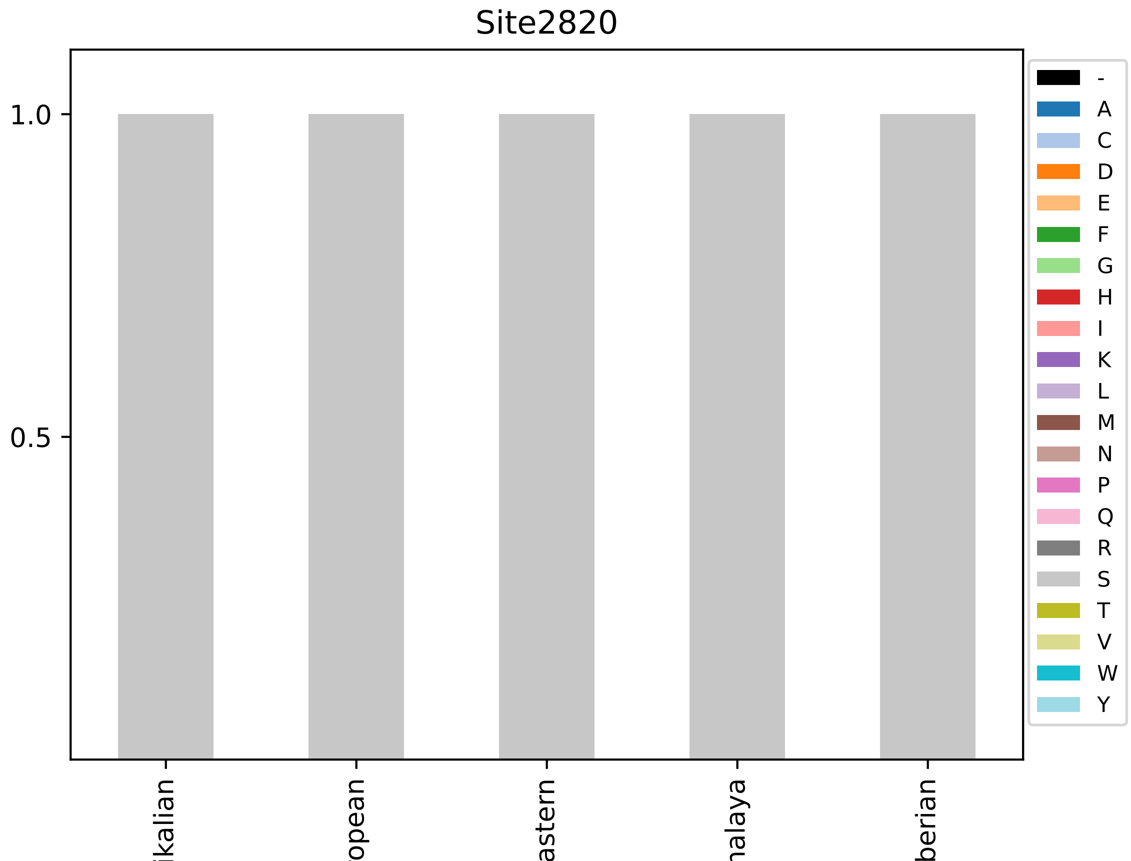
Site2820
1.0
0.5
ikalian
opean
astern
nalaya
berian
-
A
C
D
E
F
G
H
I
K
L
M
N
P
Q
R
S
T
V
W
Y

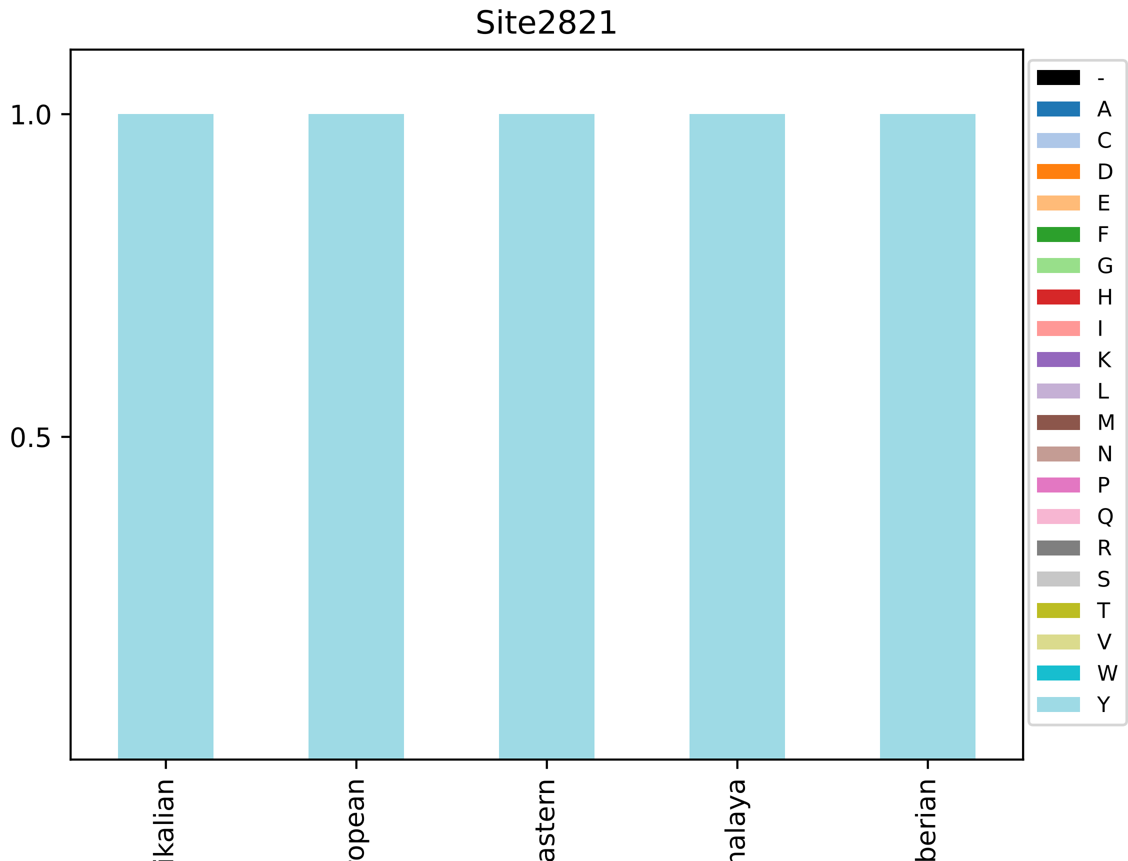

Site2821
1.0
0.5
ikalian
opean
astern
nalaya
berian
-
A
C
D
E
F
G
H
I
K
L
M
N
P
Q
R
S
T
V
W
Y

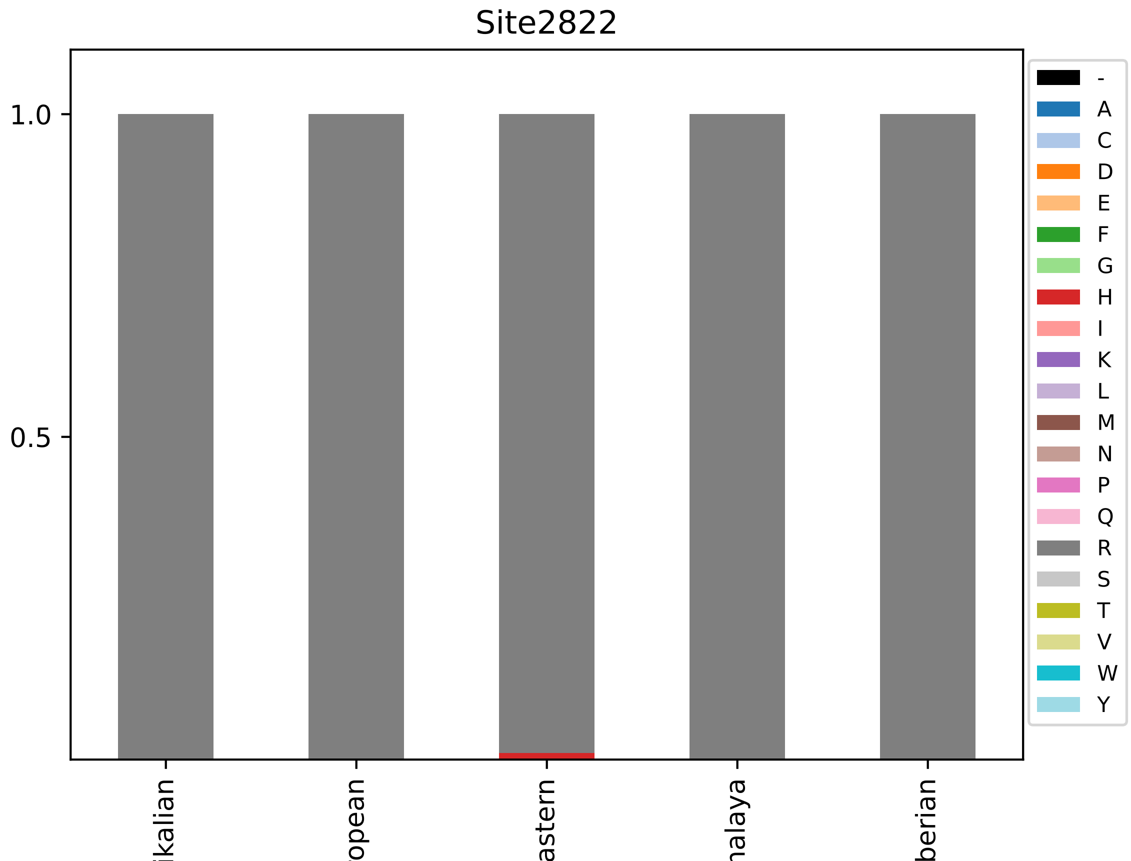

Site2822
1.0
0.5
ikalian
opean
astern
nalaya
berian
-
A
C
D
E
F
G
H
I
K
L
M
N
P
Q
R
S
T
V
W
Y

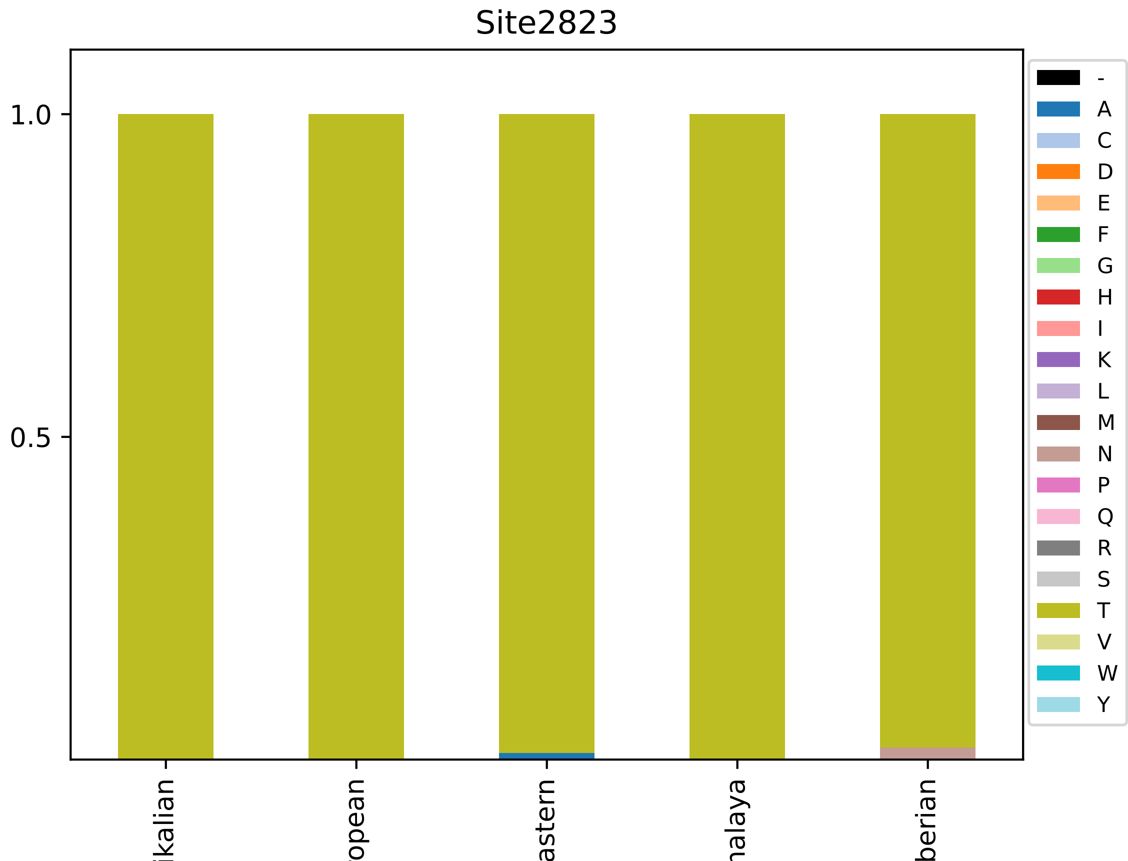

Site2823
1.0
0.5
-
A
C
D
E
F
G
H
I
K
L
M
N
P
Q
R
S
T
V
W
Y
ikalian
opean
astern
nalaya
berian

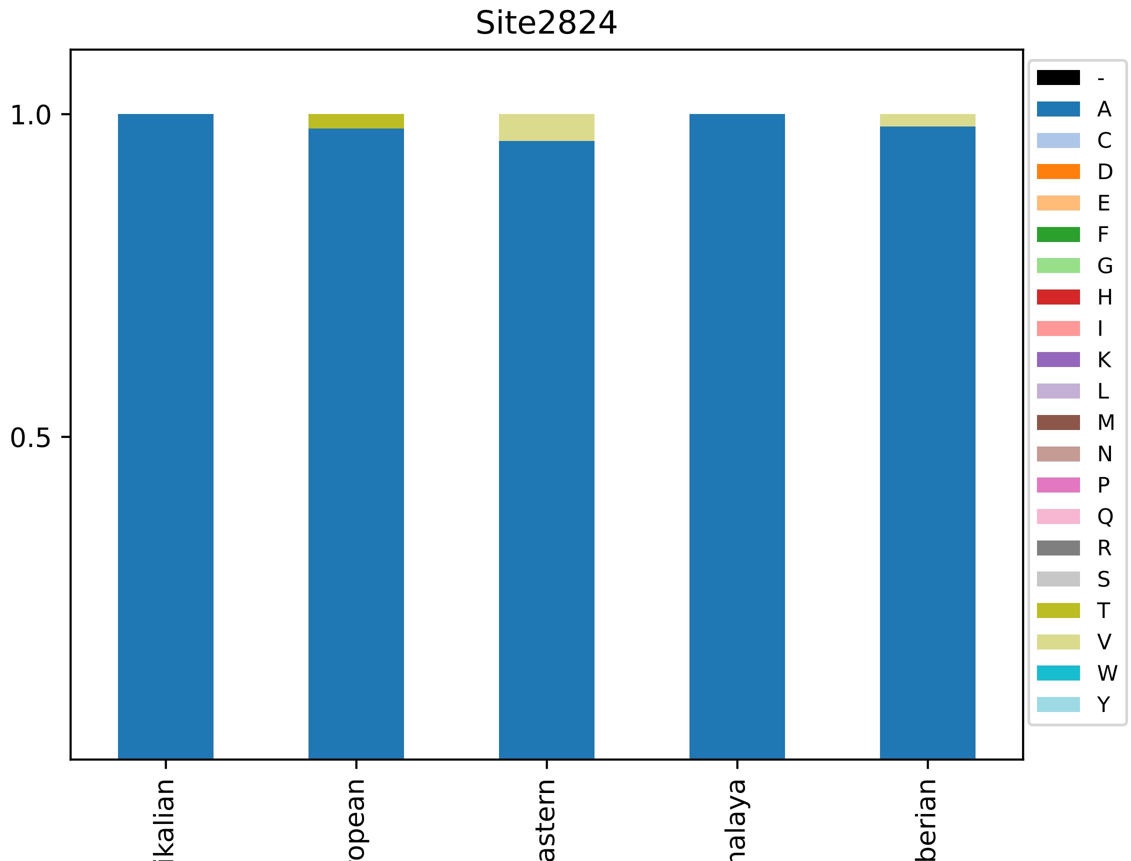
Site2824
1.0
0.5
-
A
C
D
E
F
G
H
I
K
L
M
N
P
Q
R
S
T
V
W
Y
ikalian
opean
astern
nalaya
berian

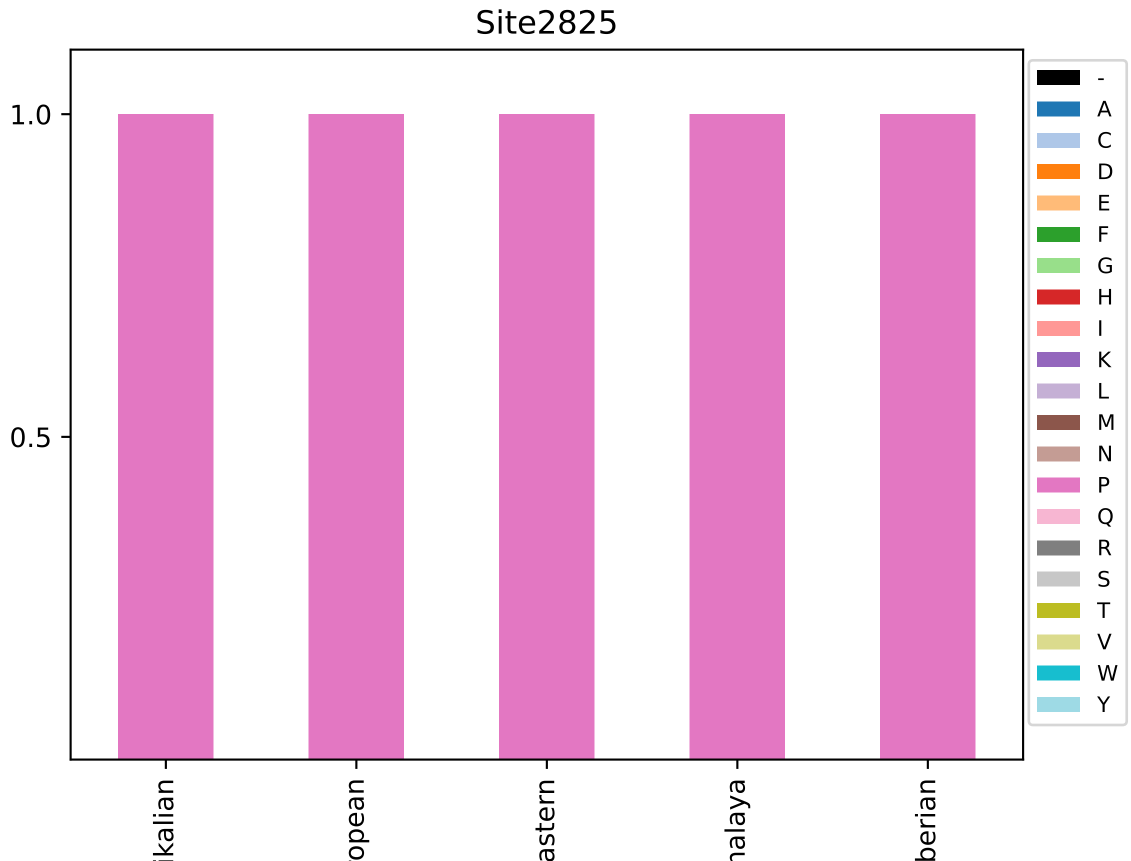
Site2825
1.0
0.5
ikalian
opean
astern
nalaya
berian
-
A
C
D
E
F
G
H
I
K
L
M
N
P
Q
R
S
T
V
W
Y

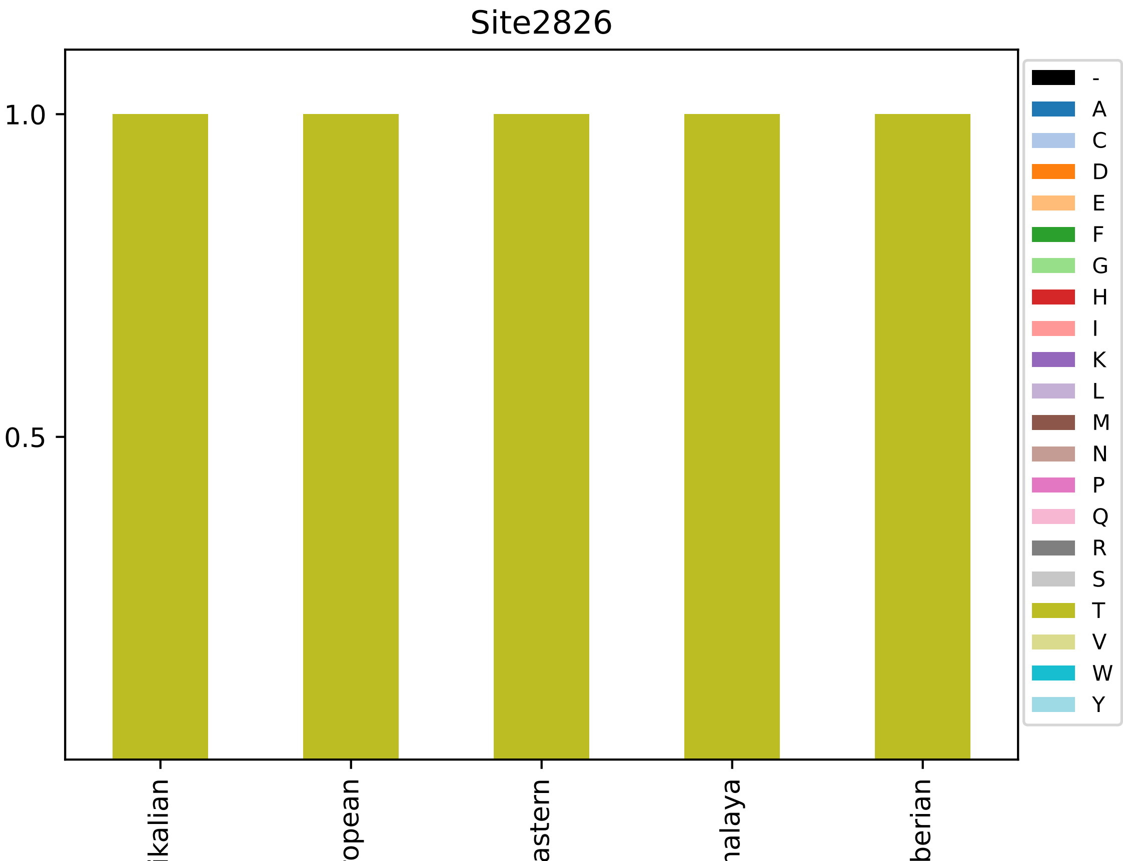

Site2826
1.0
0.5
-
A
C
D
E
F
G
H
I
K
L
M
N
P
Q
R
S
T
V
W
Y
ikalian
opean
astern
nalaya
berian

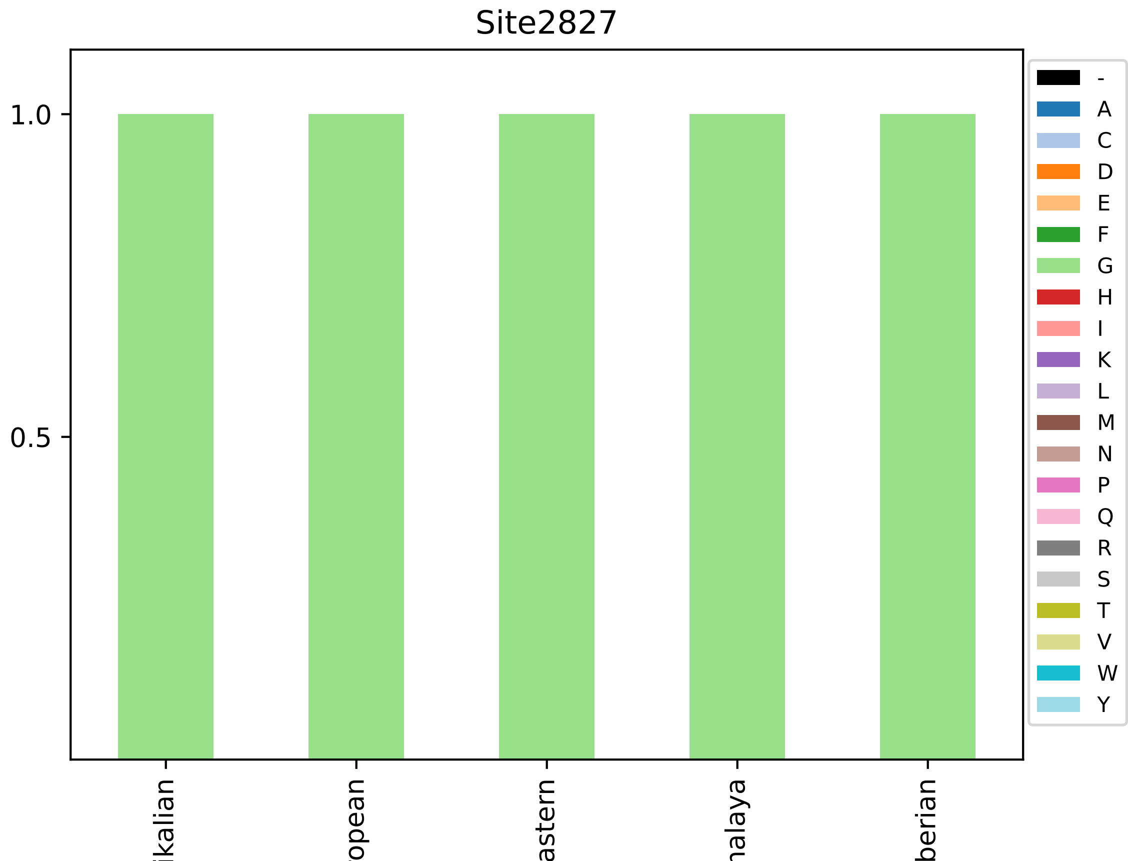

Site2827
1.0
0.5
-
A
C
D
E
F
G
H
I
K
L
M
N
P
Q
R
S
T
V
W
Y
ikalian
opean
astern
nalaya
berian

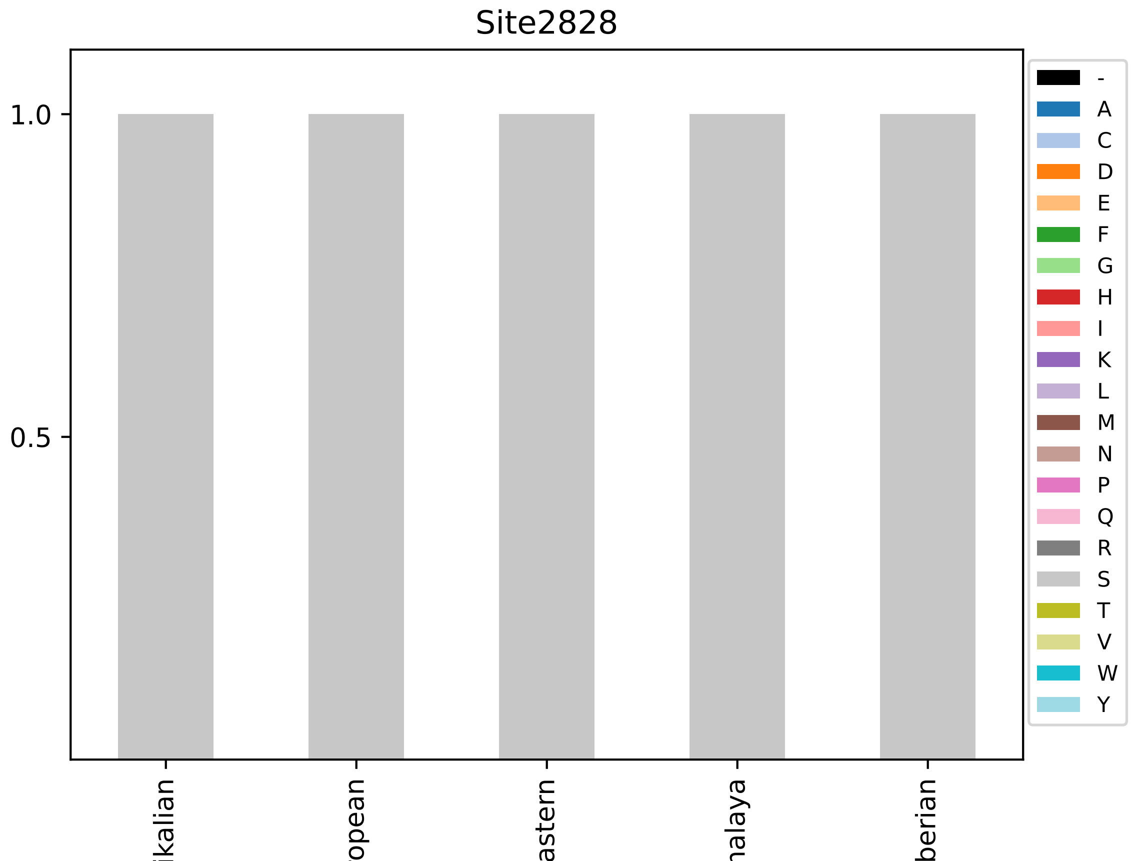

Site2828
1.0
0.5
-
A
C
D
E
F
G
H
I
K
L
M
N
P
Q
R
S
T
V
W
Y
ikalian
opean
astern
nalaya
berian

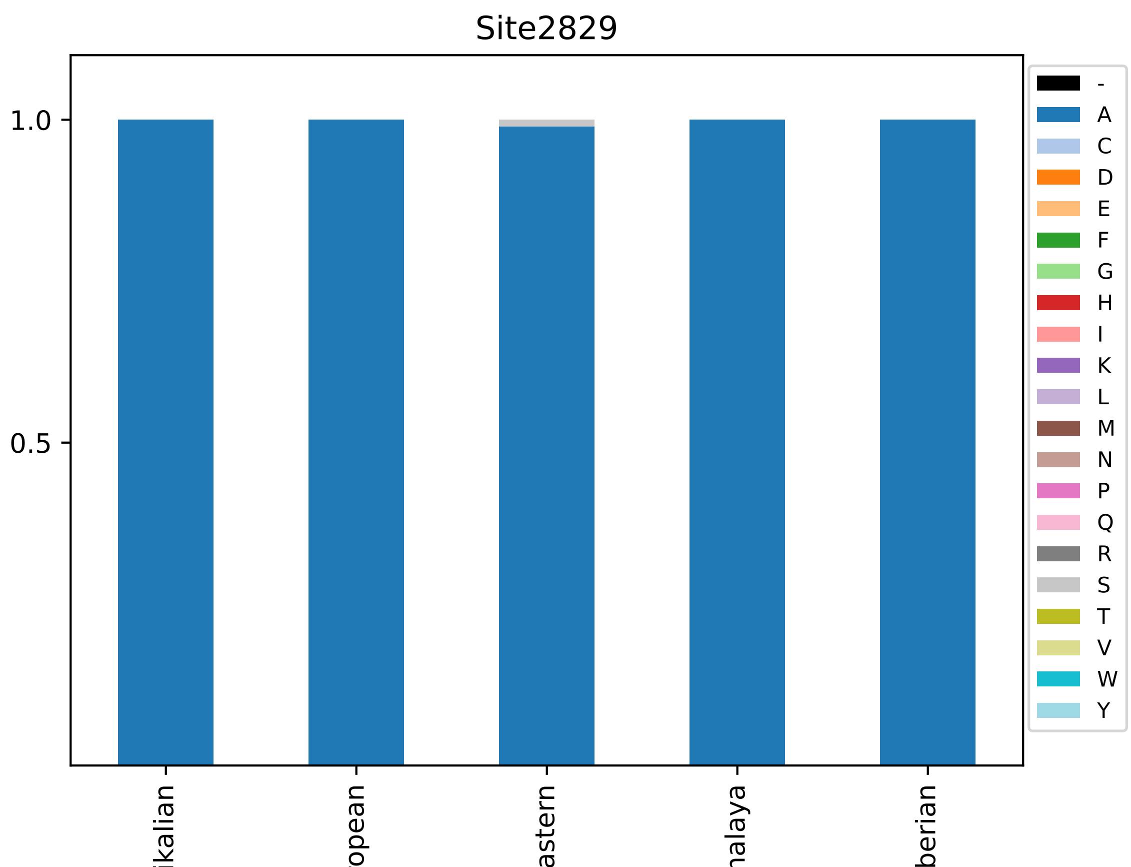

Site2829
1.0
0.5
ikalian
opean
astern
nalaya
berian
-
A
C
D
E
F
G
H
I
K
L
M
N
P
Q
R
S
T
V
W
Y

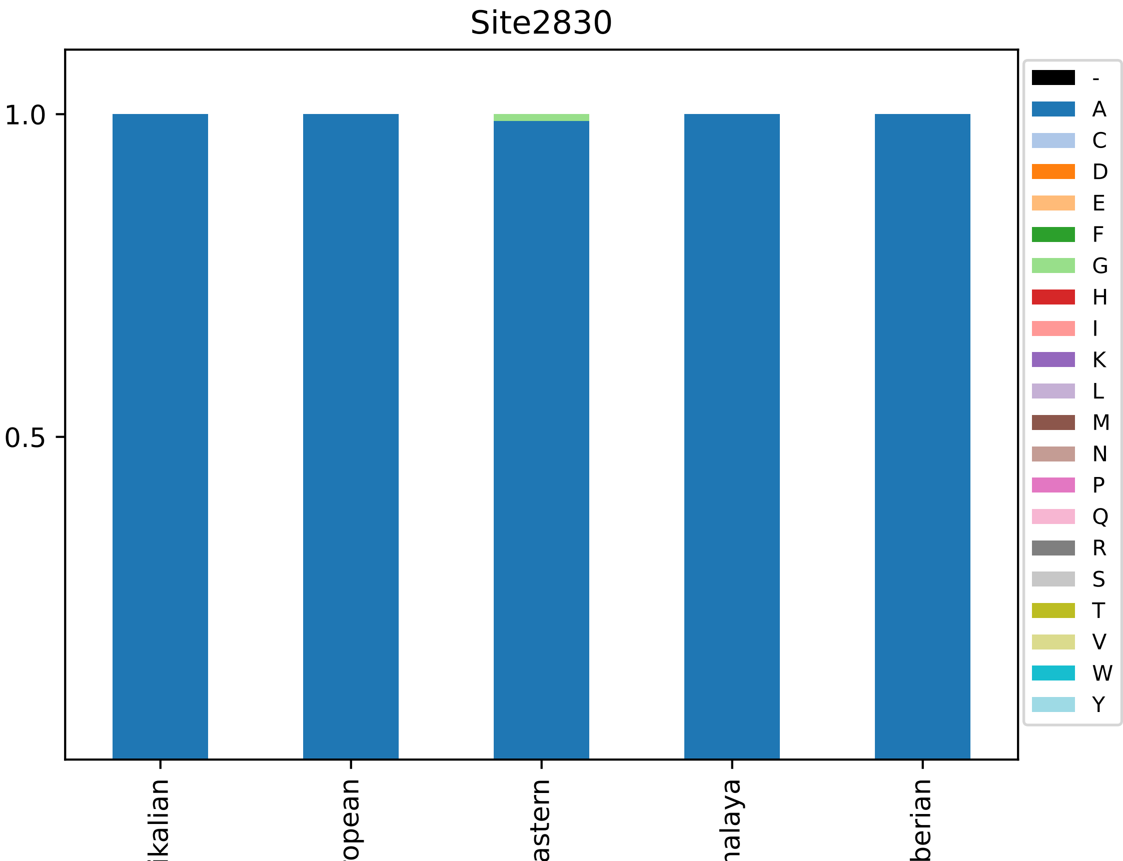

Site2830
1.0
0.5
ikalian
opean
astern
nalaya
berian
-
A
C
D
E
F
G
H
I
K
L
M
N
P
Q
R
S
T
V
W
Y

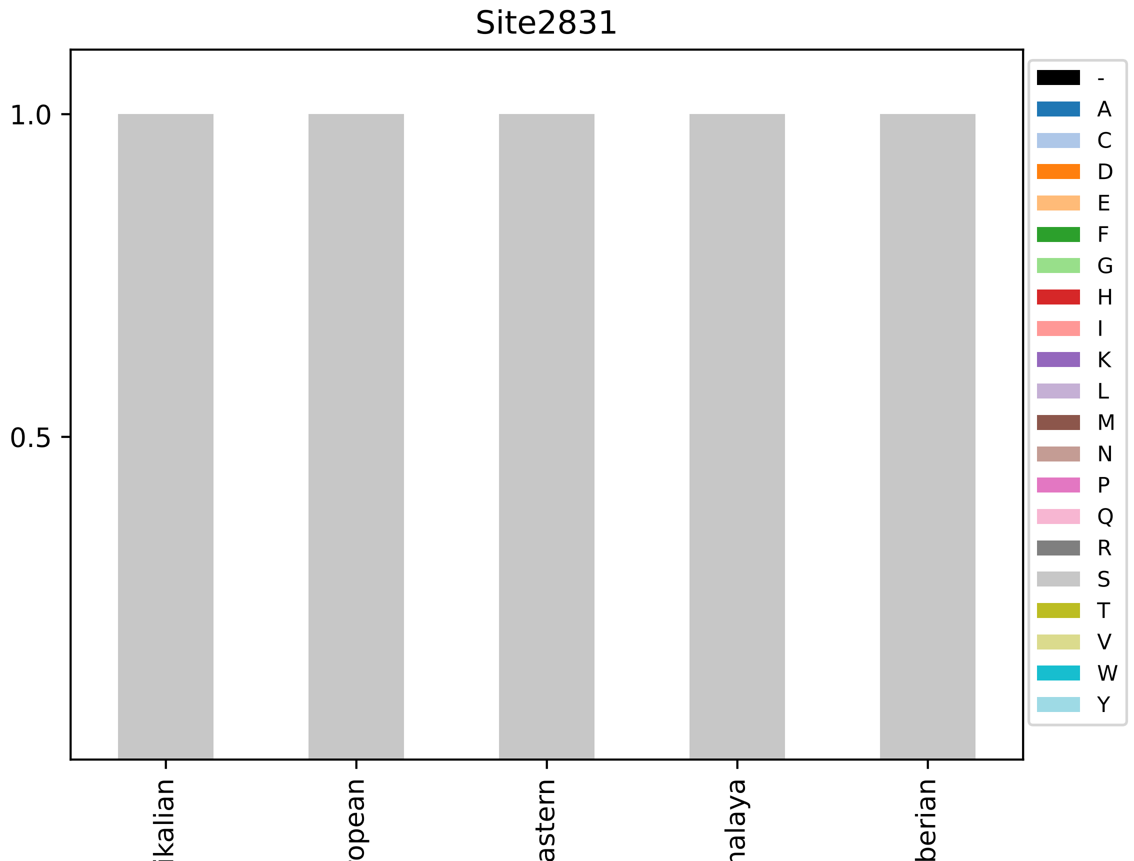
Site2831
1.0
0.5
-
A
C
D
E
F
G
H
I
K
L
M
N
P
Q
R
S
T
V
W
Y
ikalian
opean
astern
nalaya
berian

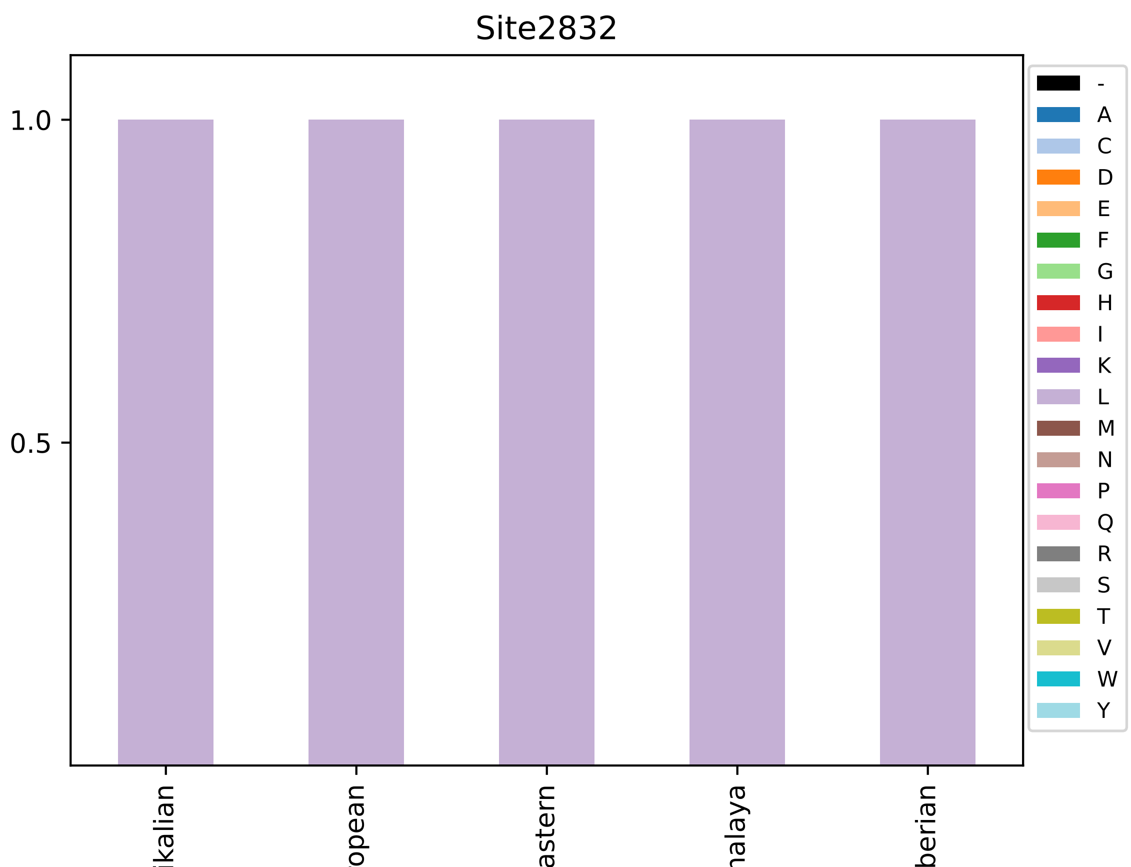
Site2832
1.0
0.5
-
A
C
D
E
F
G
H
I
K
L
M
N
P
Q
R
S
T
V
W
Y
ikalian
opean
astern
nalaya
berian

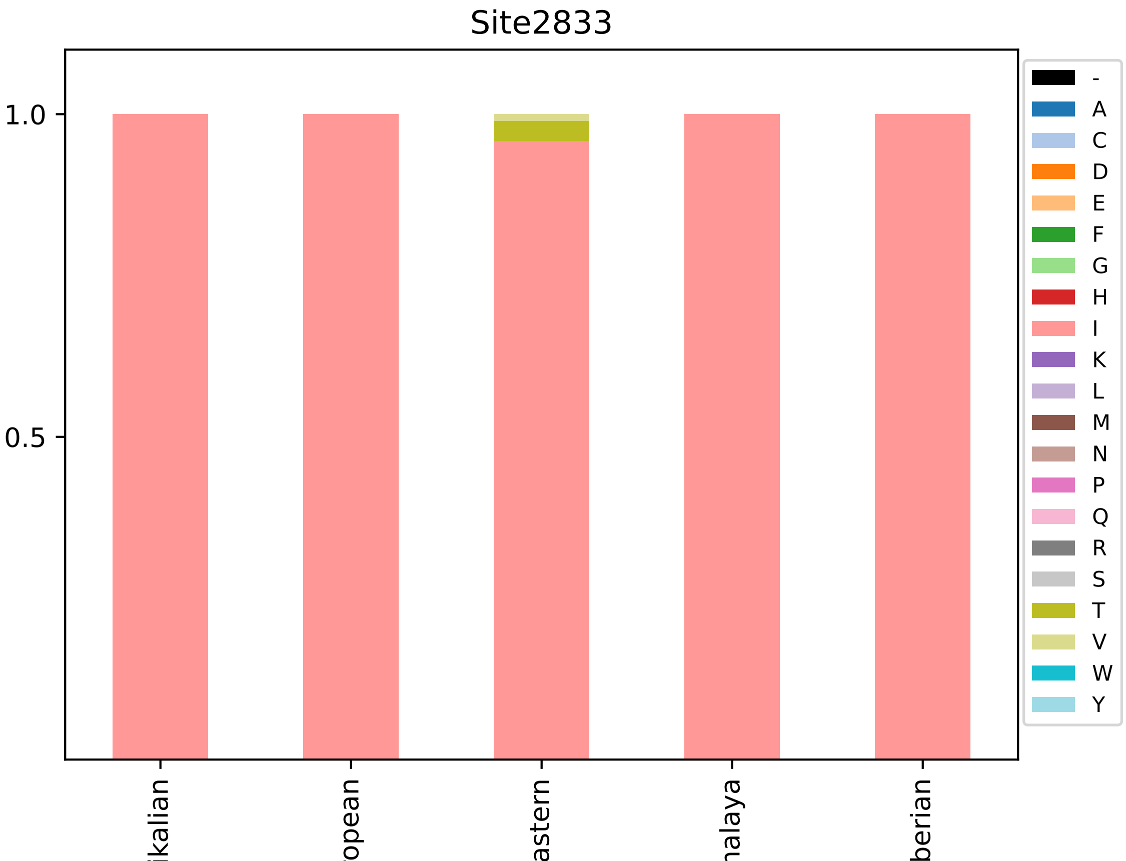
Site2833
1.0
0.5
-
A
C
D
E
F
G
H
I
K
L
M
N
P
Q
R
S
T
V
W
Y
ikalian
opean
astern
nalaya
berian

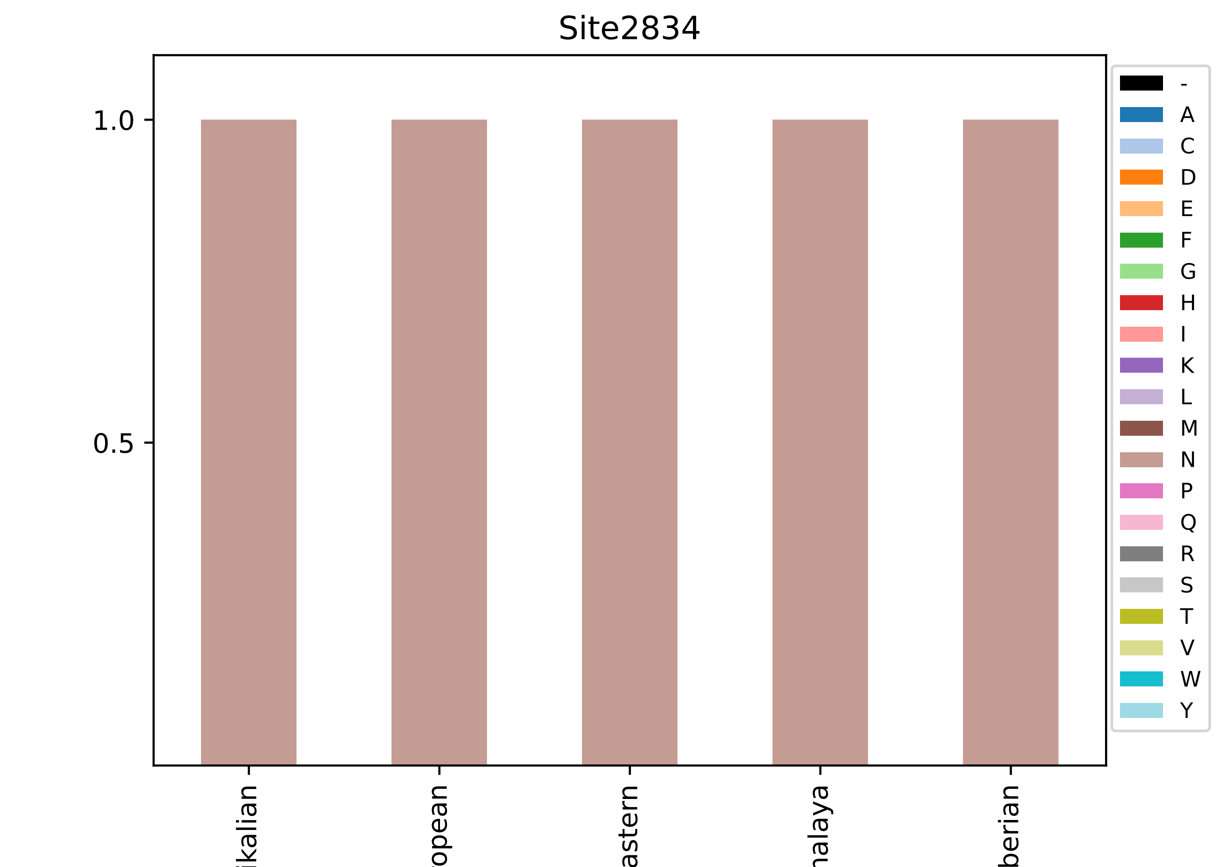

Site2834
1.0
0.5
-
A
C
D
E
F
G
H
I
K
L
M
N
P
Q
R
S
T
V
W
Y
ikalian
opean
astern
nalaya
berian

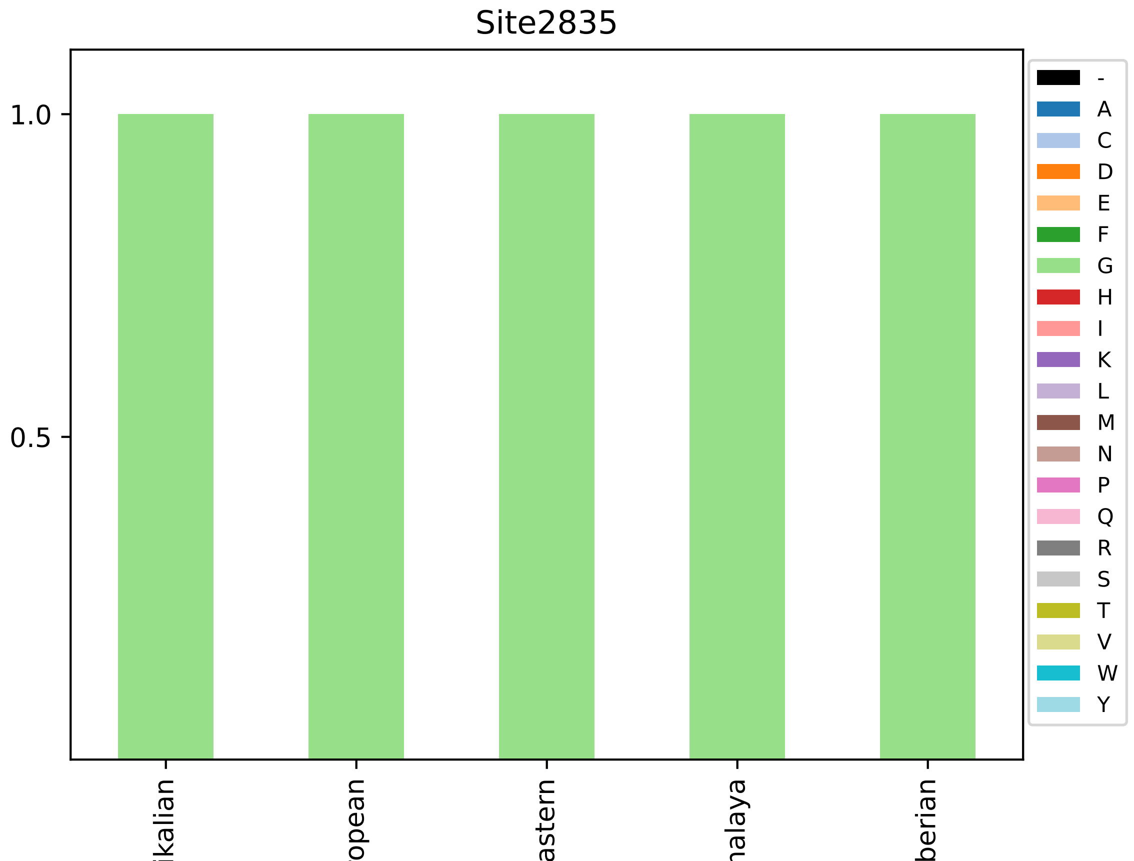
Site2835
1.0
0.5
-
A
C
D
E
F
G
H
I
K
L
M
N
P
Q
R
S
T
V
W
Y
ikalian
opean
astern
nalaya
berian

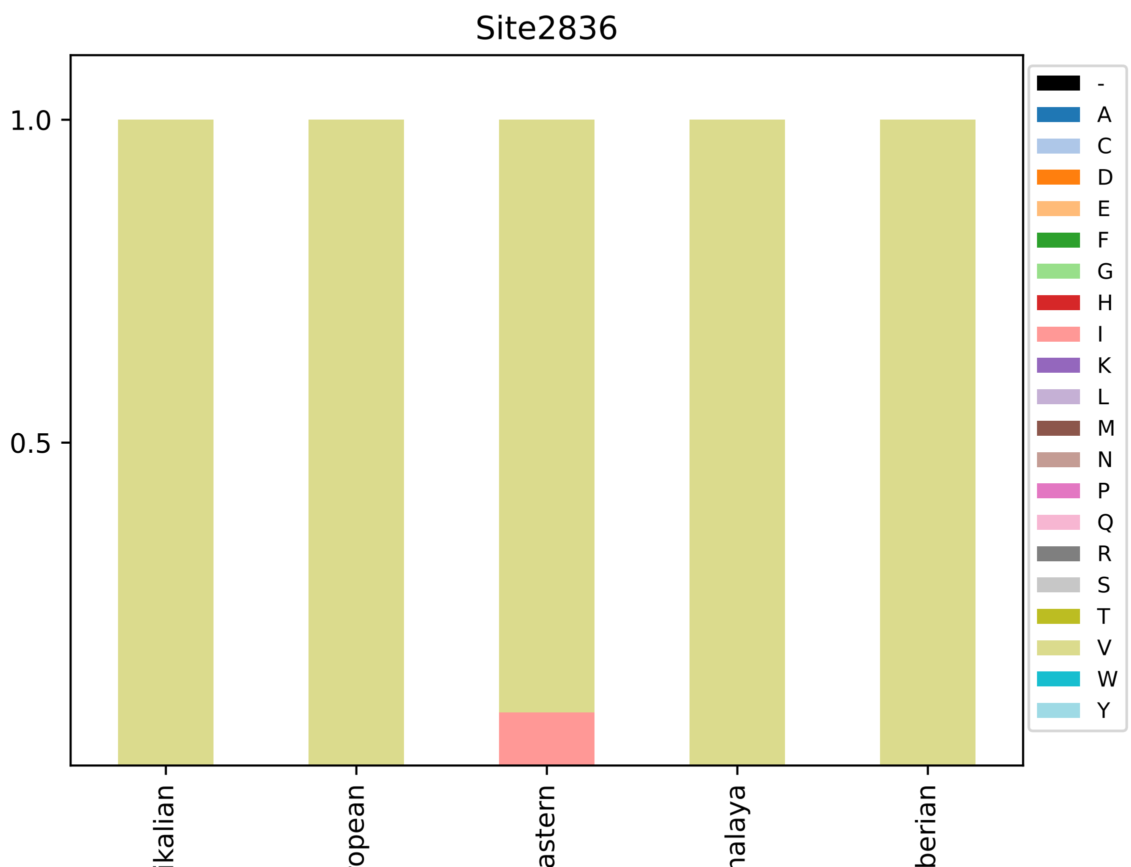

Site2836
1.0
0.5
-
A
C
D
E
F
G
H
I
K
L
M
N
P
Q
R
S
T
V
W
Y
ikalian
opean
astern
nalaya
berian

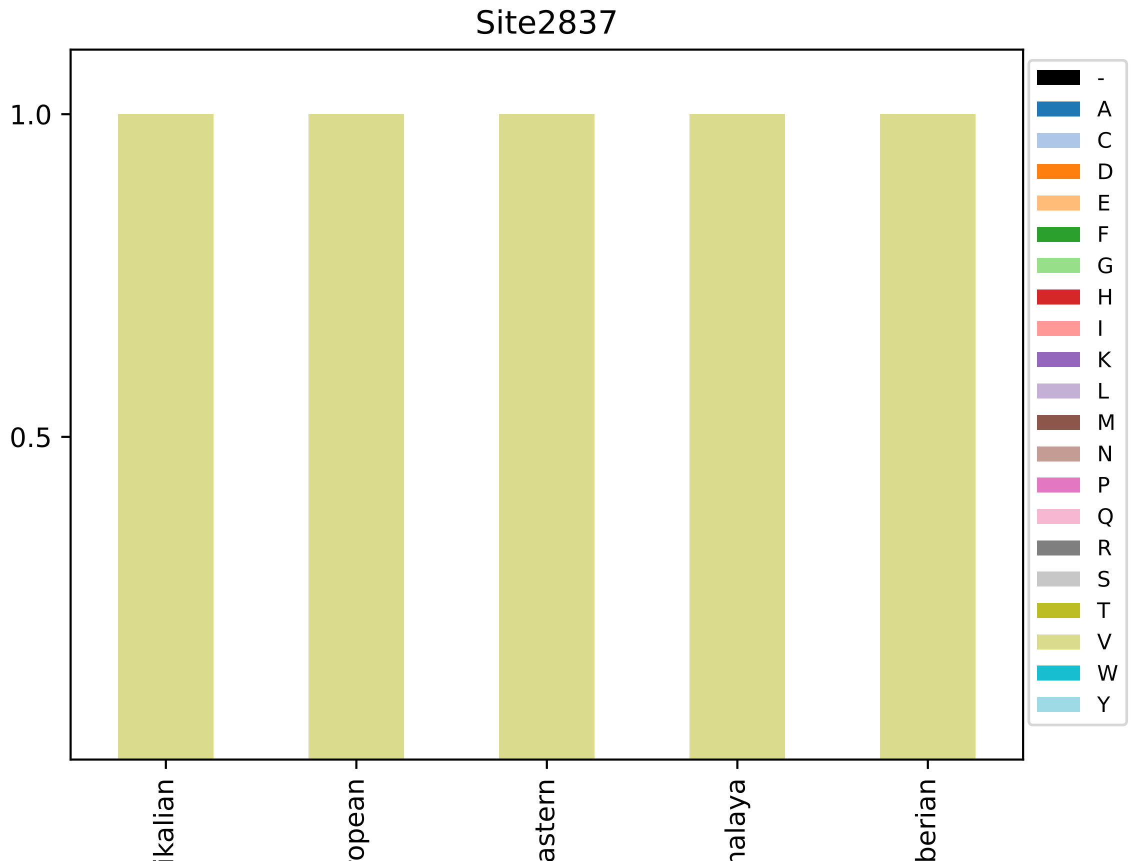
Site2837
1.0
0.5
ikalian
opean
astern
nalaya
berian
-
A
C
D
E
F
G
H
I
K
L
M
N
P
Q
R
S
T
V
W
Y

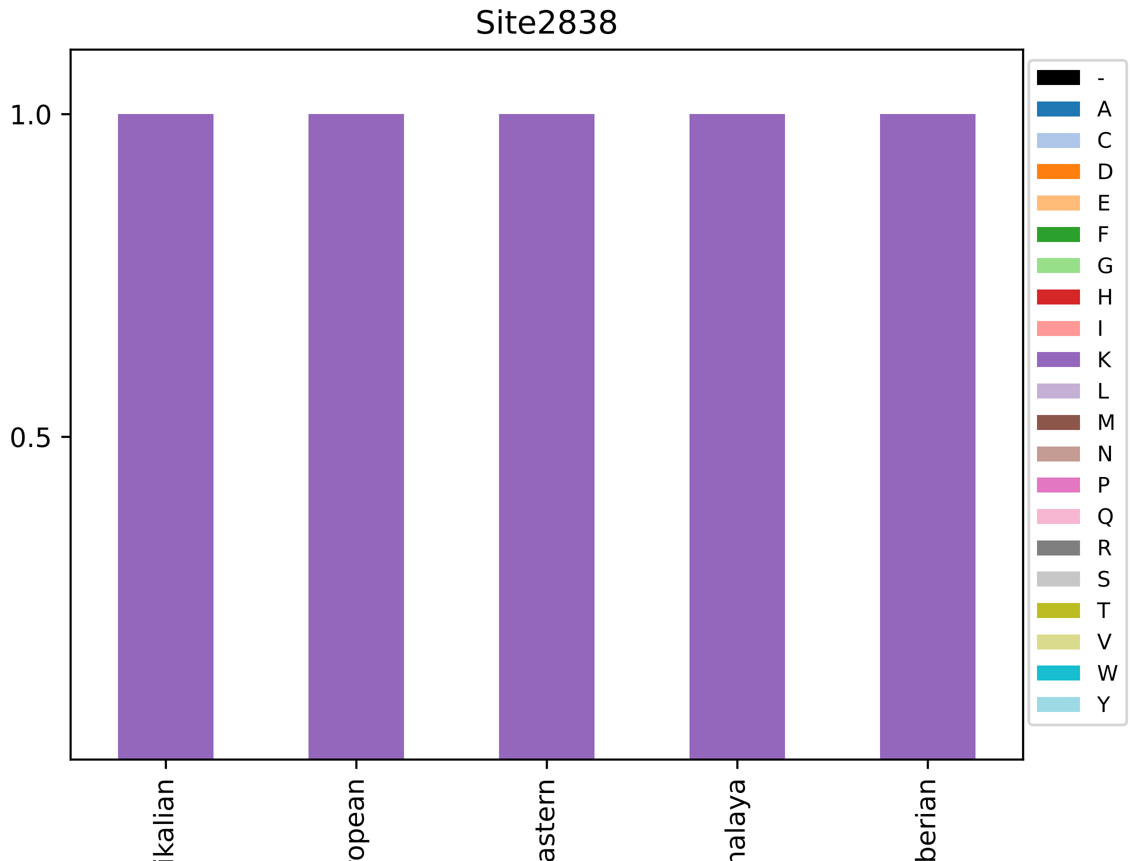
Site2838
1.0
0.5
-
A
C
D
E
F
G
H
I
K
L
M
N
P
Q
R
S
T
V
W
Y
ikalian
opean
astern
nalaya
berian

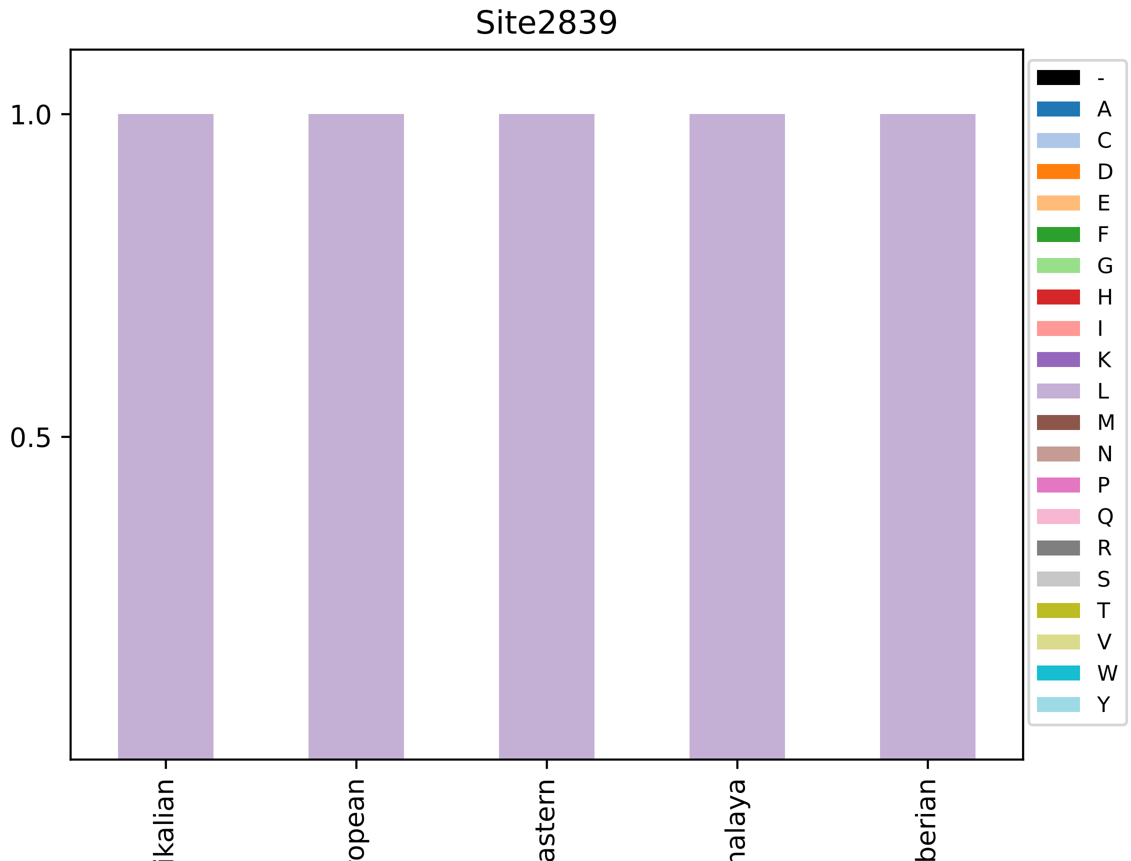

Site2839
1.0
0.5
-
A
C
D
E
F
G
H
I
K
L
M
N
P
Q
R
S
T
V
W
Y
ikalian
opean
astern
nalaya
berian

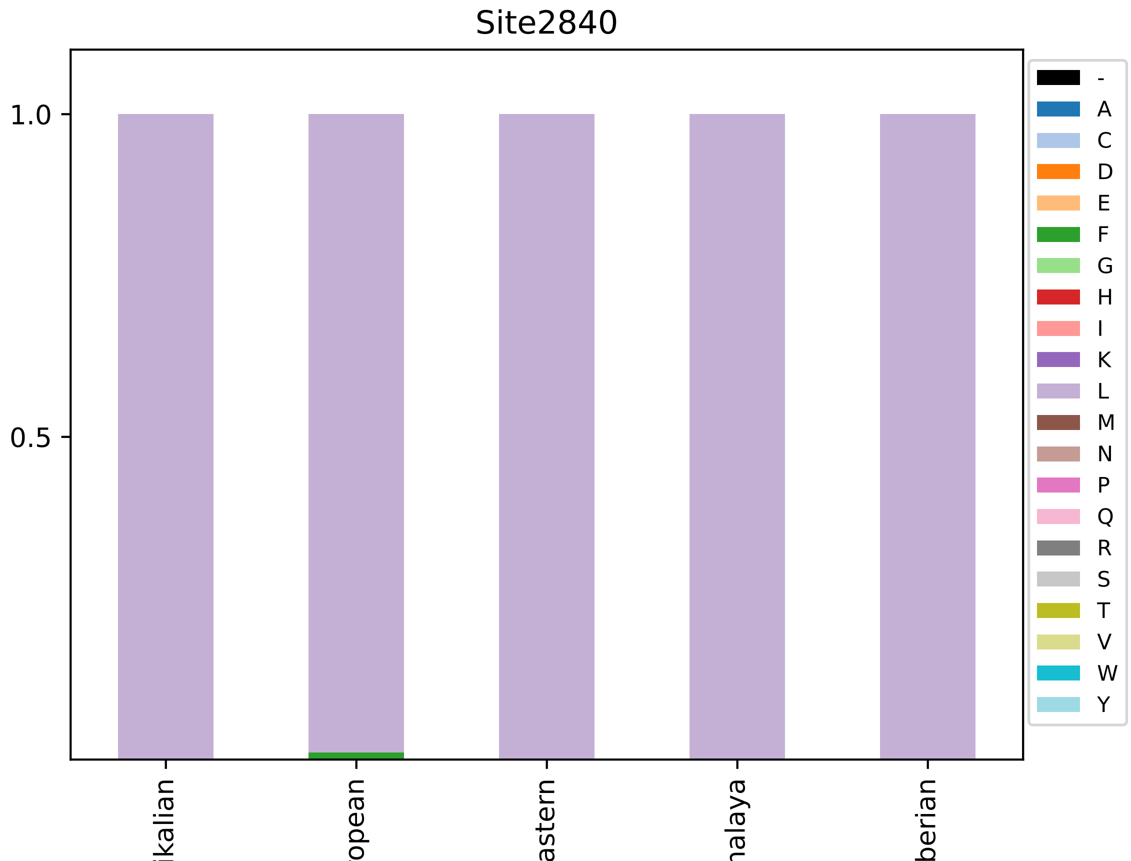
Site2840
1.0
0.5
-
A
C
D
E
F
G
H
I
K
L
M
N
P
Q
R
S
T
V
W
Y
ikalian
ropean
astern
nalaya
berian

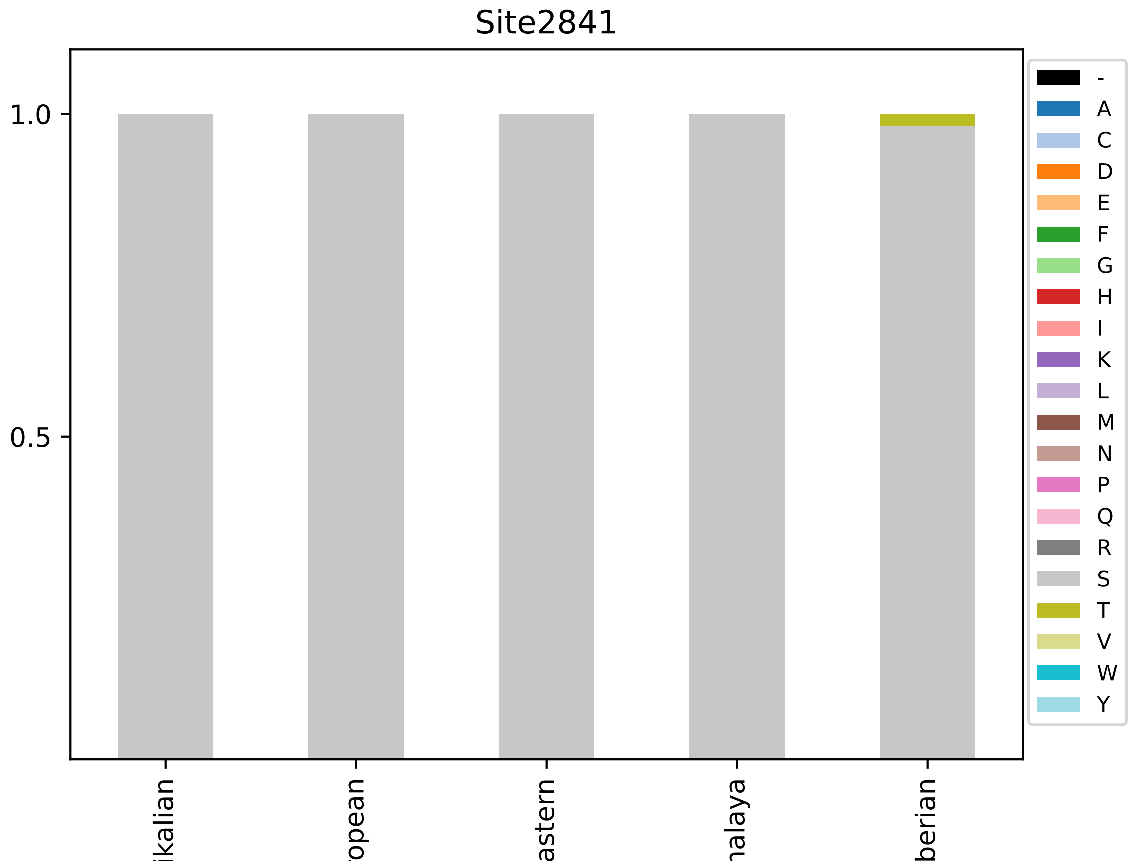

Site2841
1.0
0.5
ikalian
opean
astern
nalaya
berian
-
A
C
D
E
F
G
H
I
K
L
M
N
P
Q
R
S
T
V
W
Y

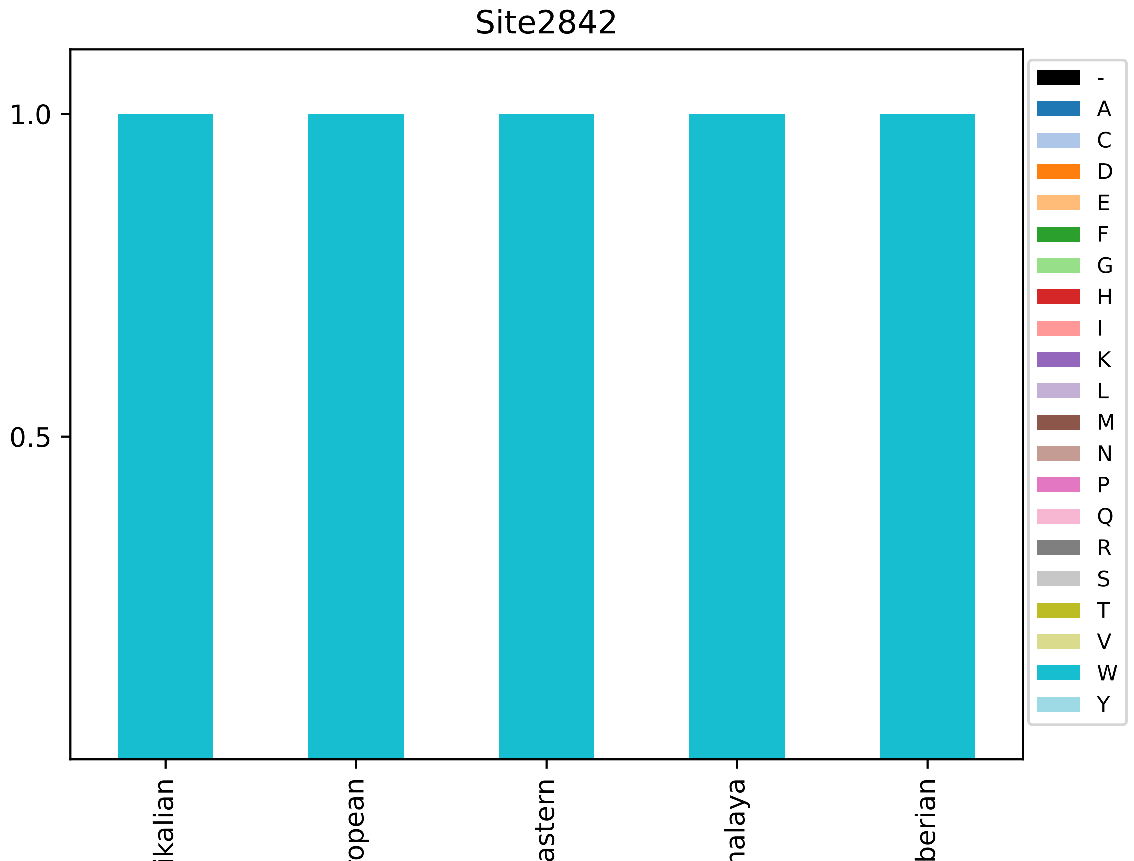

Site2842
1.0
0.5
-
A
C
D
E
F
G
H
I
K
L
M
N
P
Q
R
S
T
V
W
Y
ikalian
opean
astern
nalaya
berian

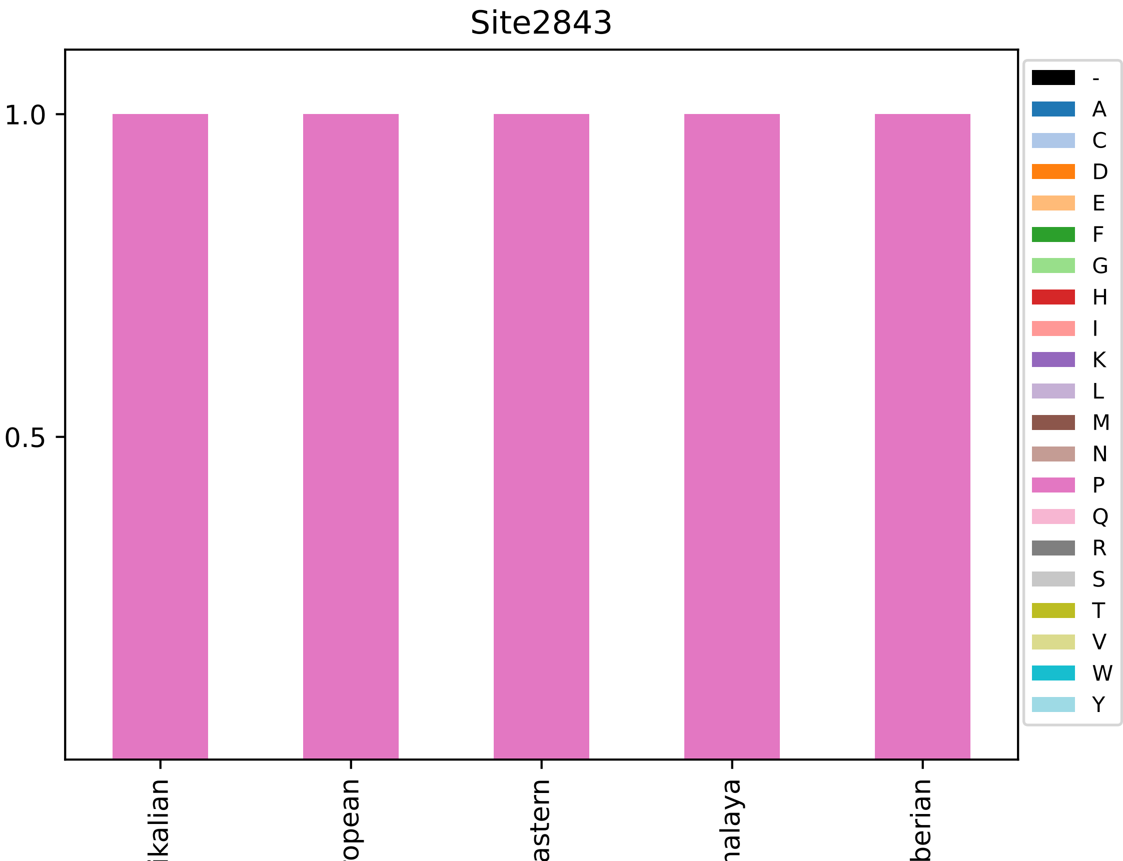

Site2843
1.0
0.5
-
A
C
D
E
F
G
H
I
K
L
M
N
P
Q
R
S
T
V
W
Y
ikalian
opean
astern
nalaya
berian

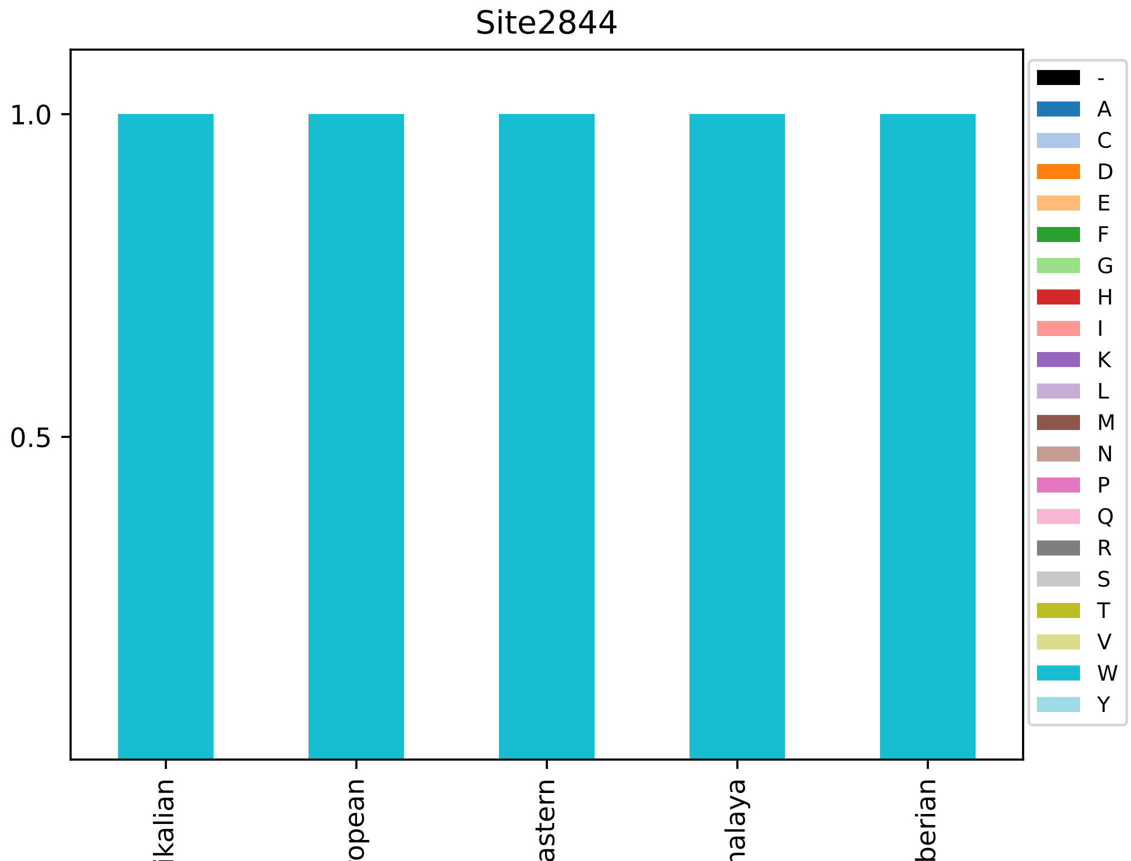
Site2844
1.0
0.5
-
A
C
D
E
F
G
H
I
K
L
M
N
P
Q
R
S
T
V
W
Y
ikalian
opean
astern
nalaya
berian

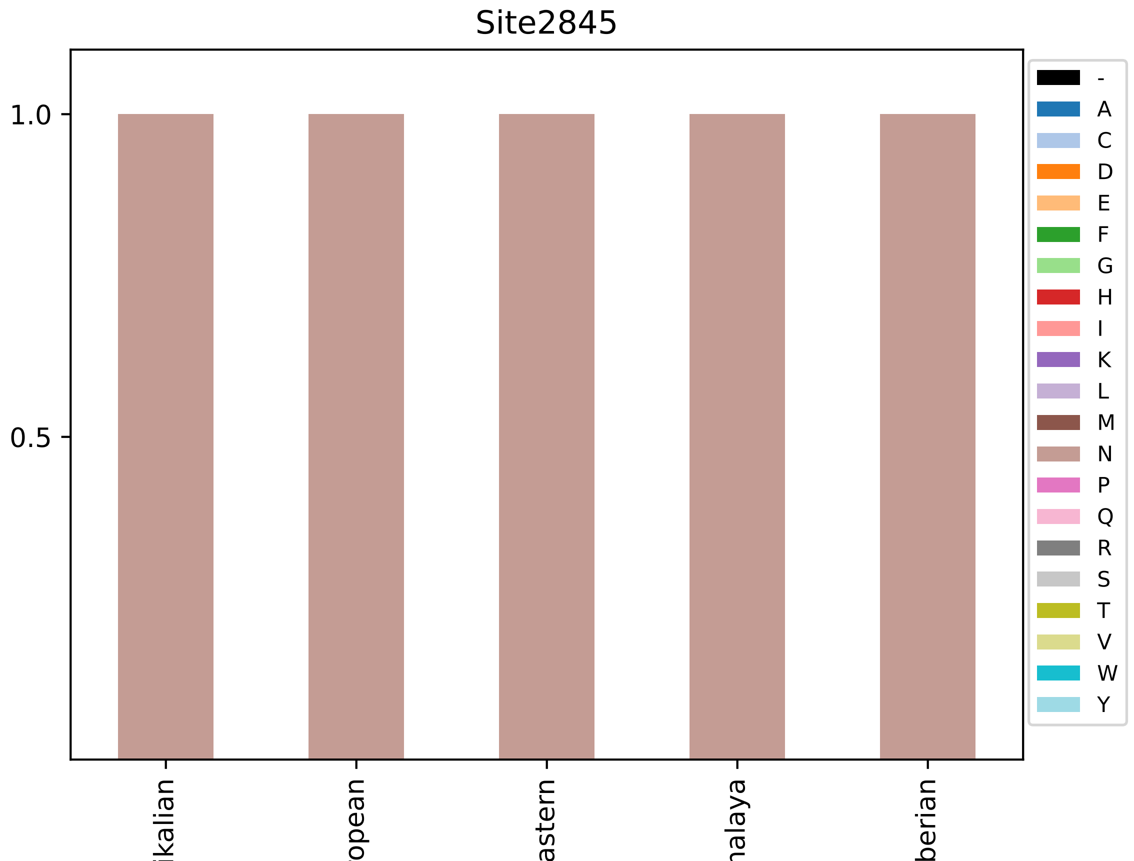
Site2845
1.0
0.5
-
A
C
D
E
F
G
H
I
K
L
M
N
P
Q
R
S
T
V
W
Y
ikalian
opean
astern
nalaya
berian

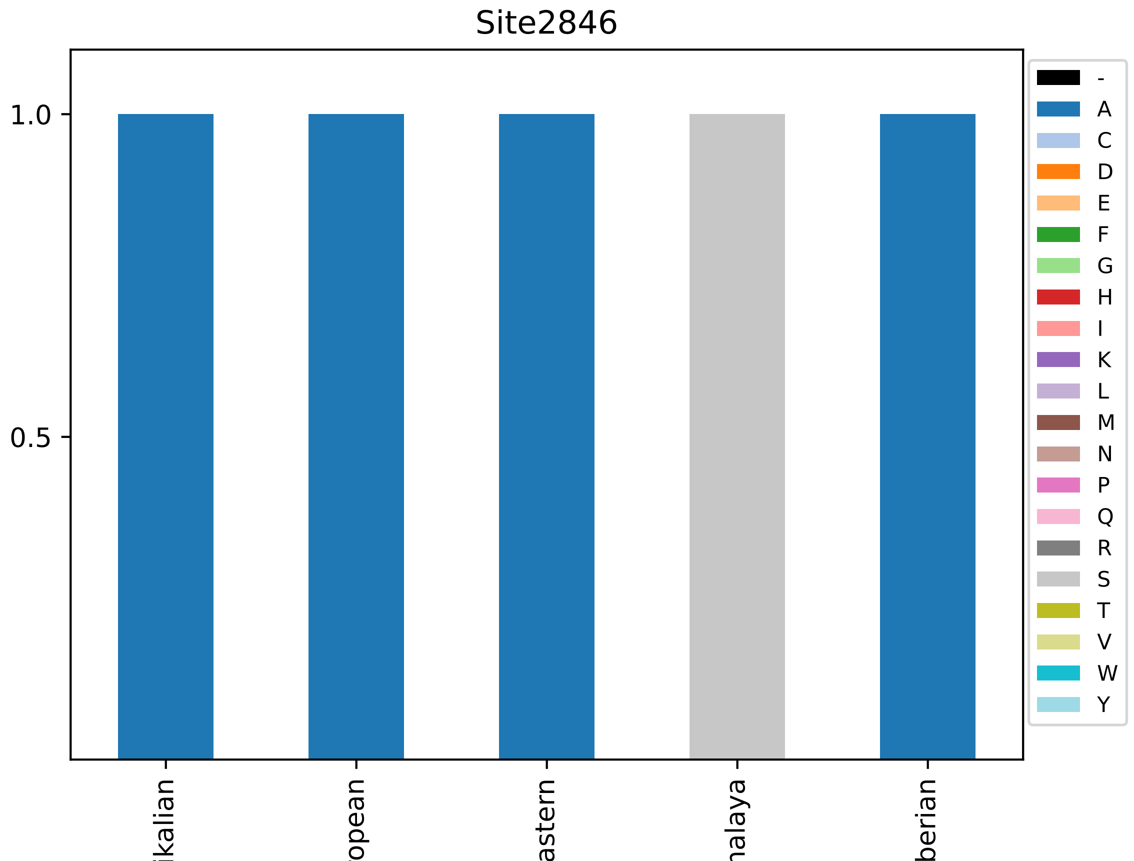
Site2846
1.0
0.5
ikalian
opean
astern
nalaya
berian
-
A
C
D
E
F
G
H
I
K
L
M
N
P
Q
R
S
T
V
W
Y

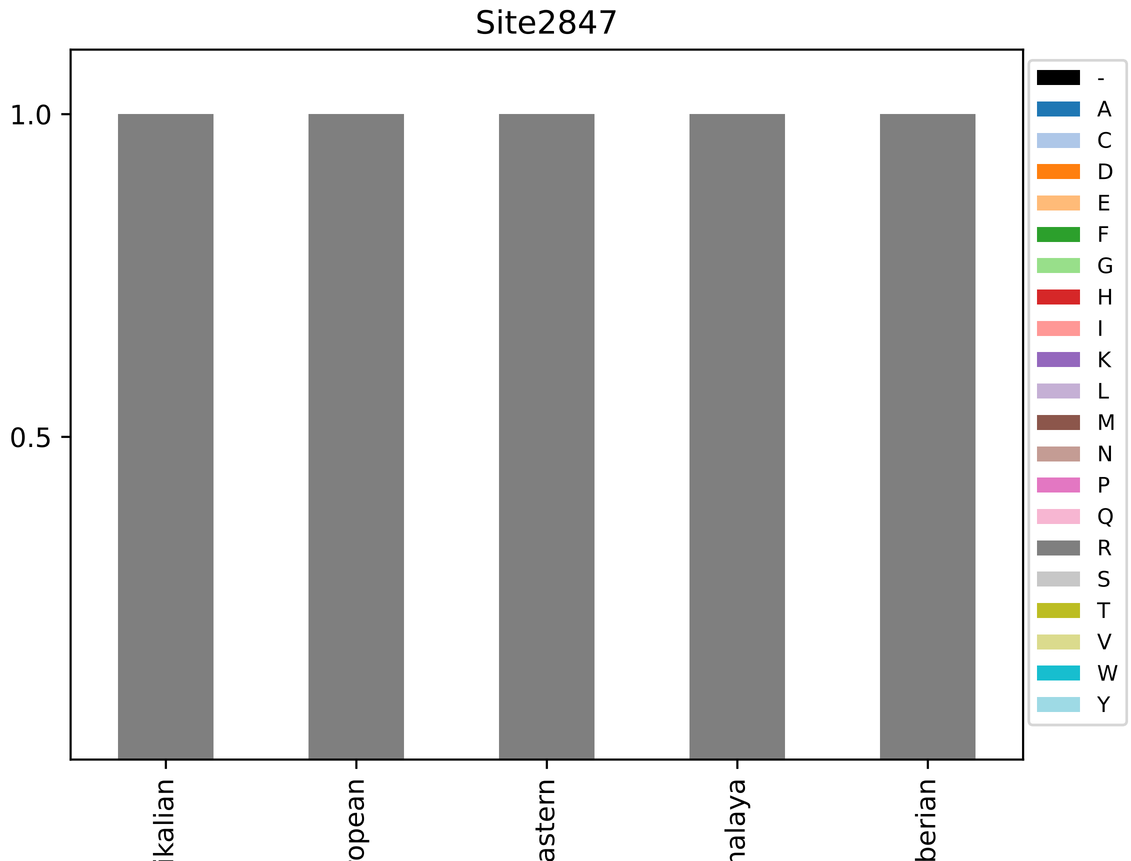

Site2847
1.0
0.5
-
A
C
D
E
F
G
H
I
K
L
M
N
P
Q
R
S
T
V
W
Y
ikalian
opean
astern
nalaya
berian

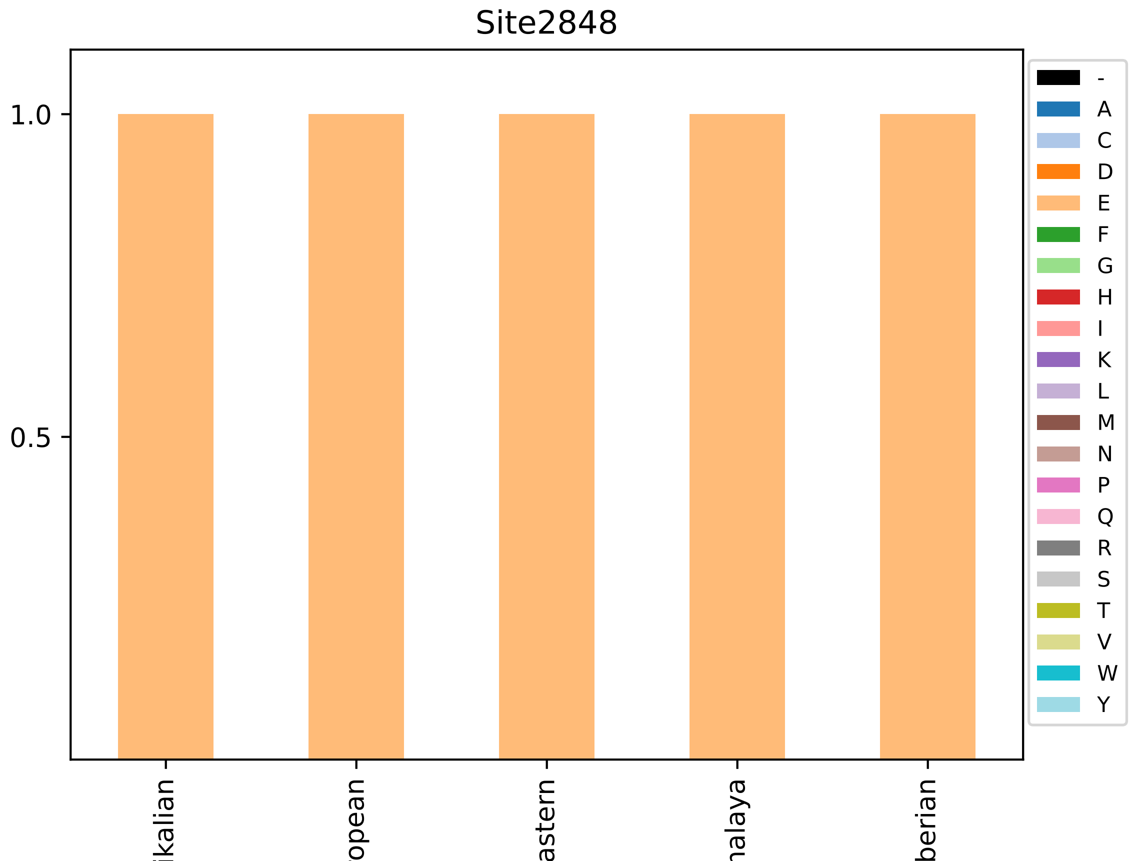
Site2848
1.0
0.5
-
A
C
D
E
F
G
H
I
K
L
M
N
P
Q
R
S
T
V
W
Y
ikalian
opean
astern
nalaya
berian

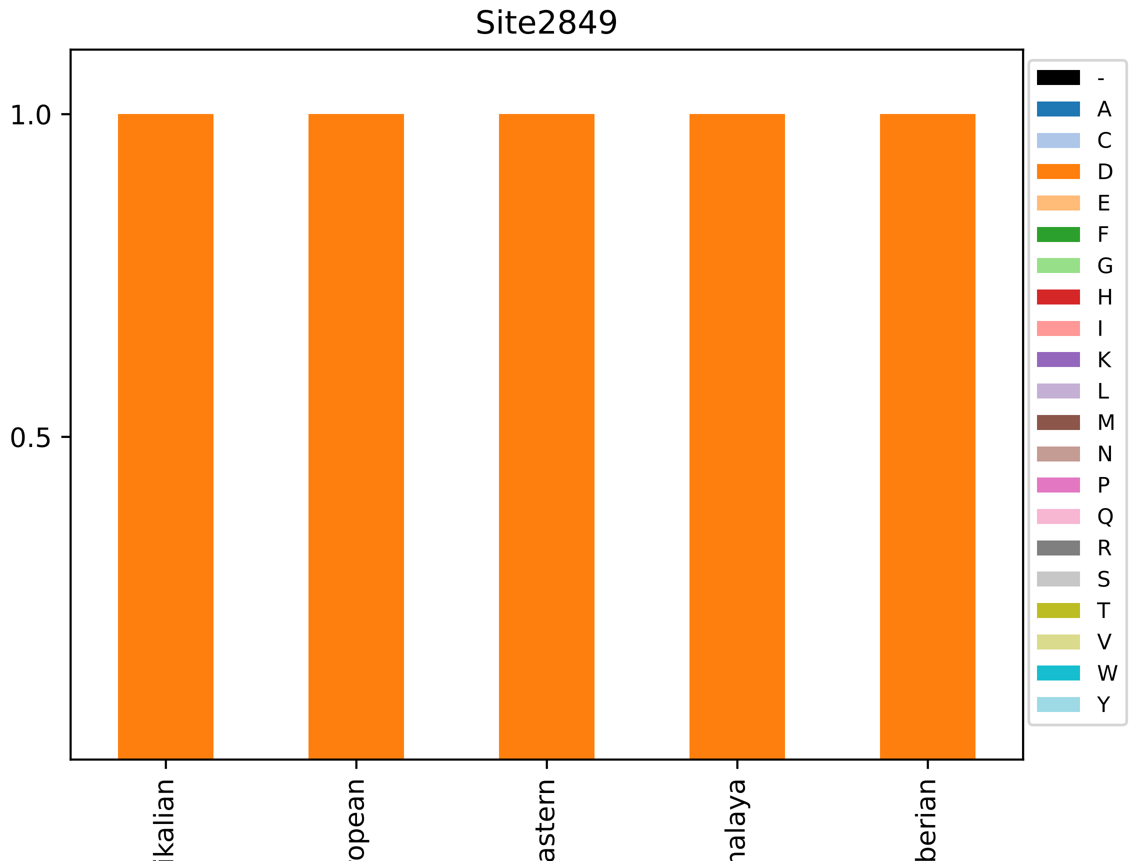

Site2849
1.0
0.5
ikalian
opean
astern
nalaya
berian
-
A
C
D
E
F
G
H
I
K
L
M
N
P
Q
R
S
T
V
W
Y

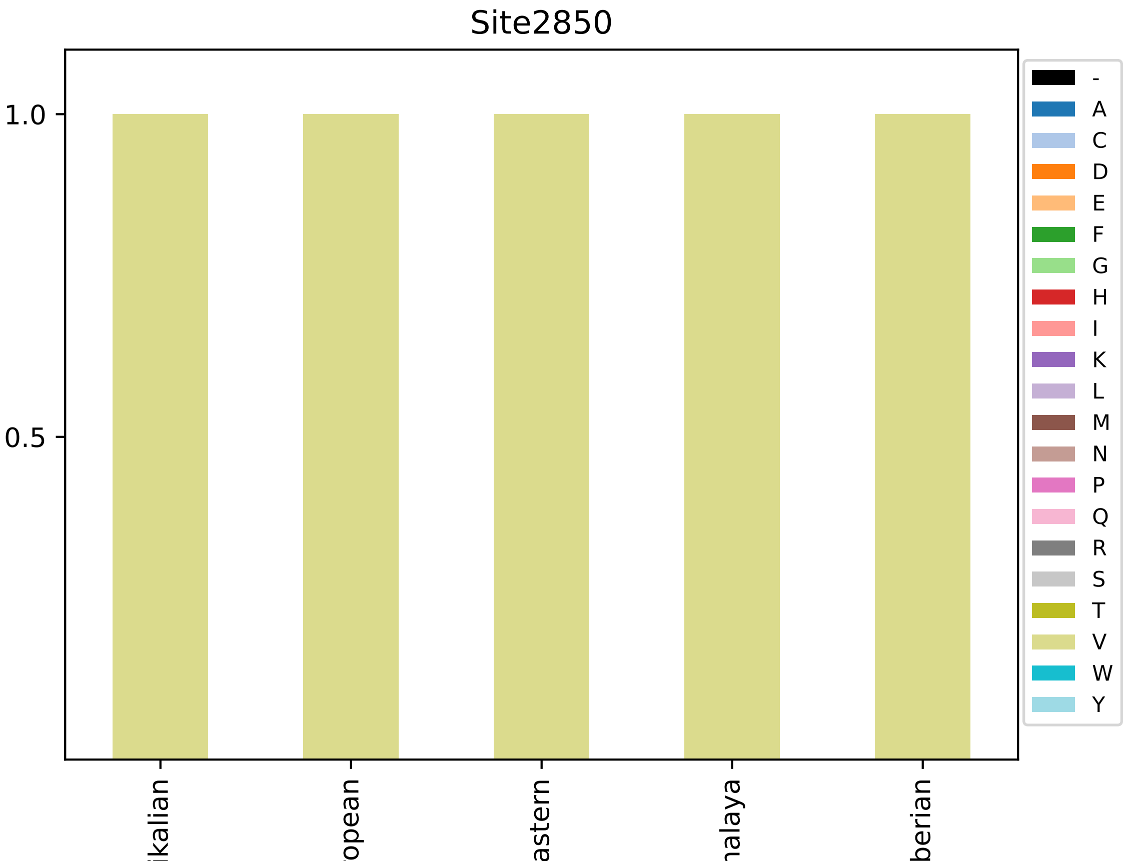

Site2850
1.0
0.5
ikalian
opean
astern
nalaya
berian
-
A
C
D
E
F
G
H
I
K
L
M
N
P
Q
R
S
T
V
W
Y

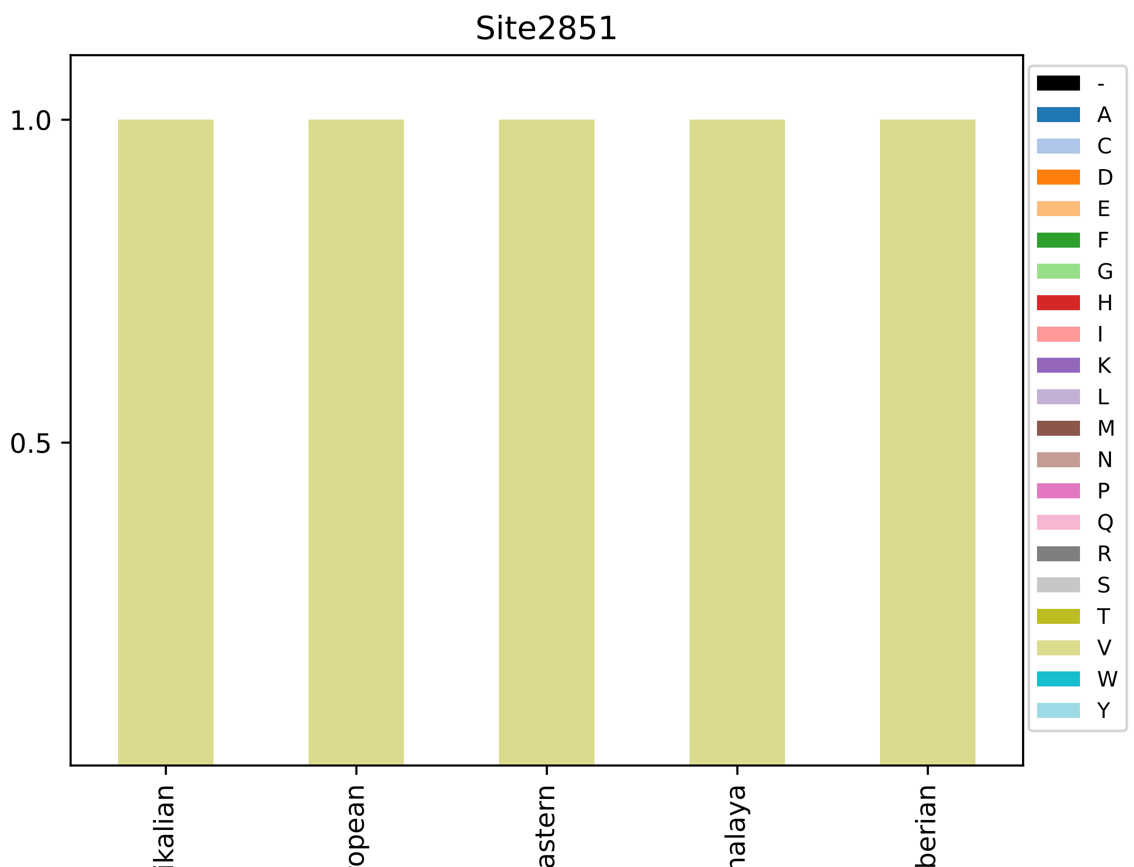
Site2851
1.0
0.5
-
A
C
D
E
F
G
H
I
K
L
M
N
P
Q
R
S
T
V
W
Y
ikalian
opean
astern
nalaya
berian

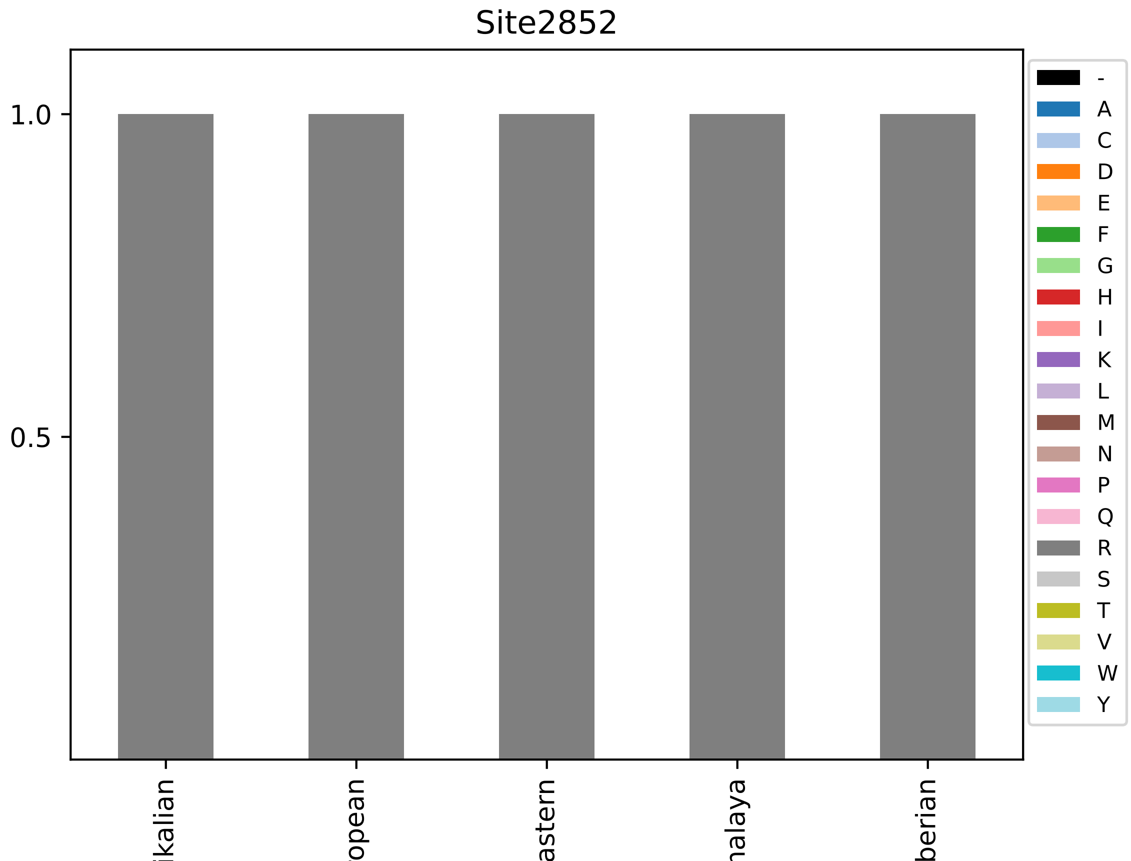
Site2852
1.0
0.5
ikalian
opean
astern
nalaya
berian
-
A
C
D
E
F
G
H
I
K
L
M
N
P
Q
R
S
T
V
W
Y

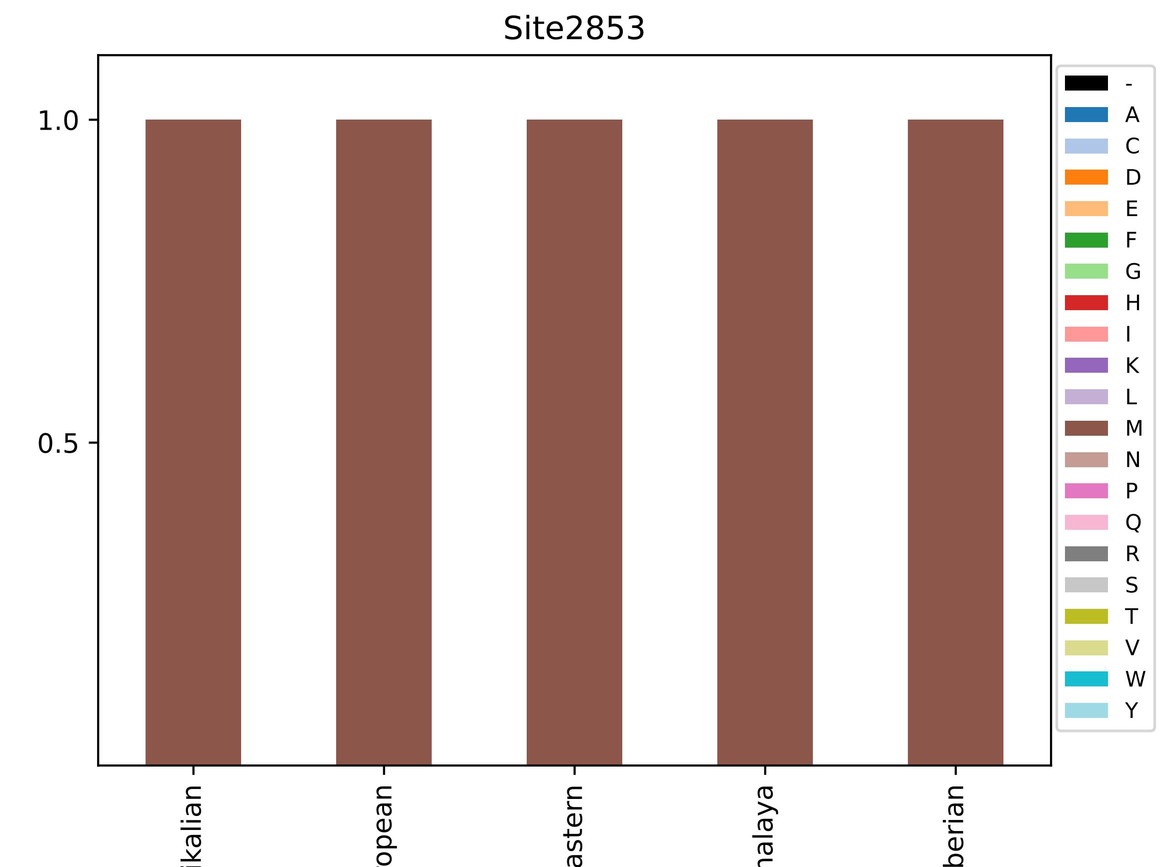
Site2853
1.0
0.5
-
A
C
D
E
F
G
H
I
K
L
M
N
P
Q
R
S
T
V
W
Y
ikalian
opean
astern
nalaya
berian

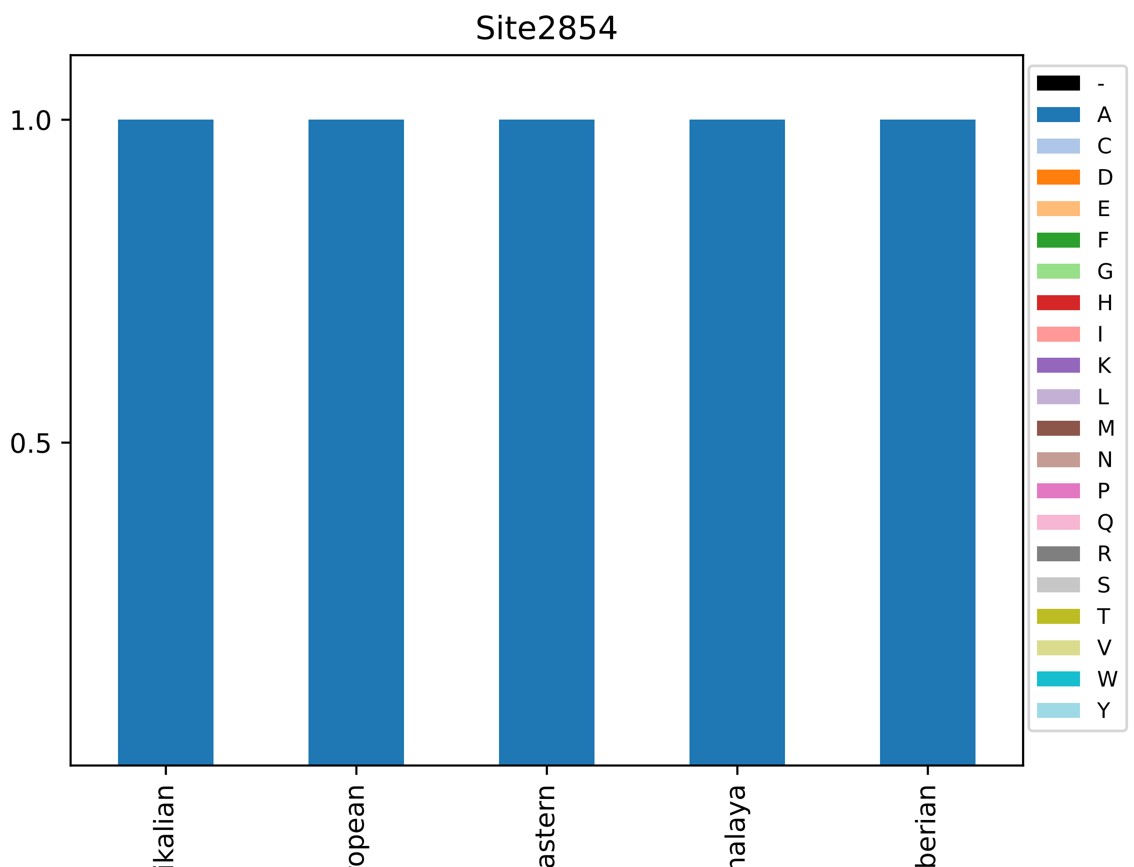

Site2854
1.0
0.5
ikalian
opean
astern
nalaya
berian
-
A
C
D
E
F
G
H
I
K
L
M
N
P
Q
R
S
T
V
W
Y

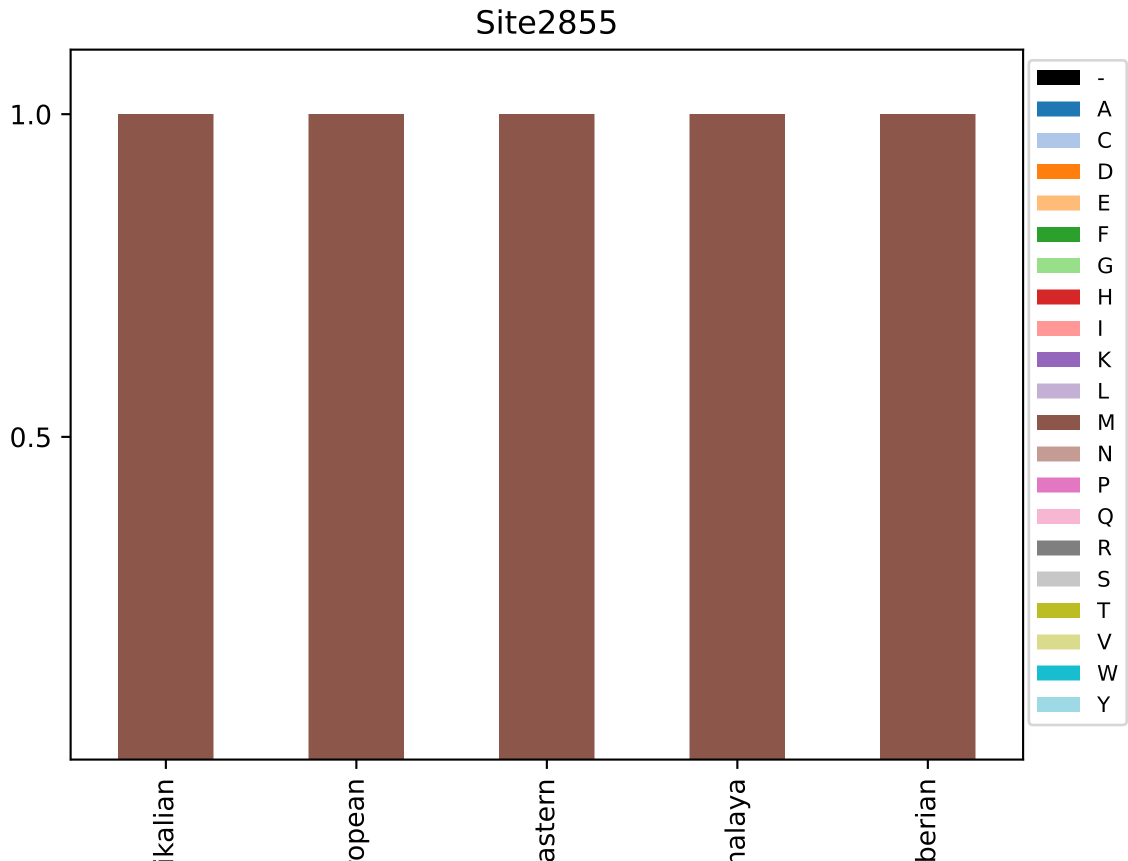

Site2855
1.0
0.5
-
A
C
D
E
F
G
H
I
K
L
M
N
P
Q
R
S
T
V
W
Y
ikalian
opean
astern
nalaya
berian

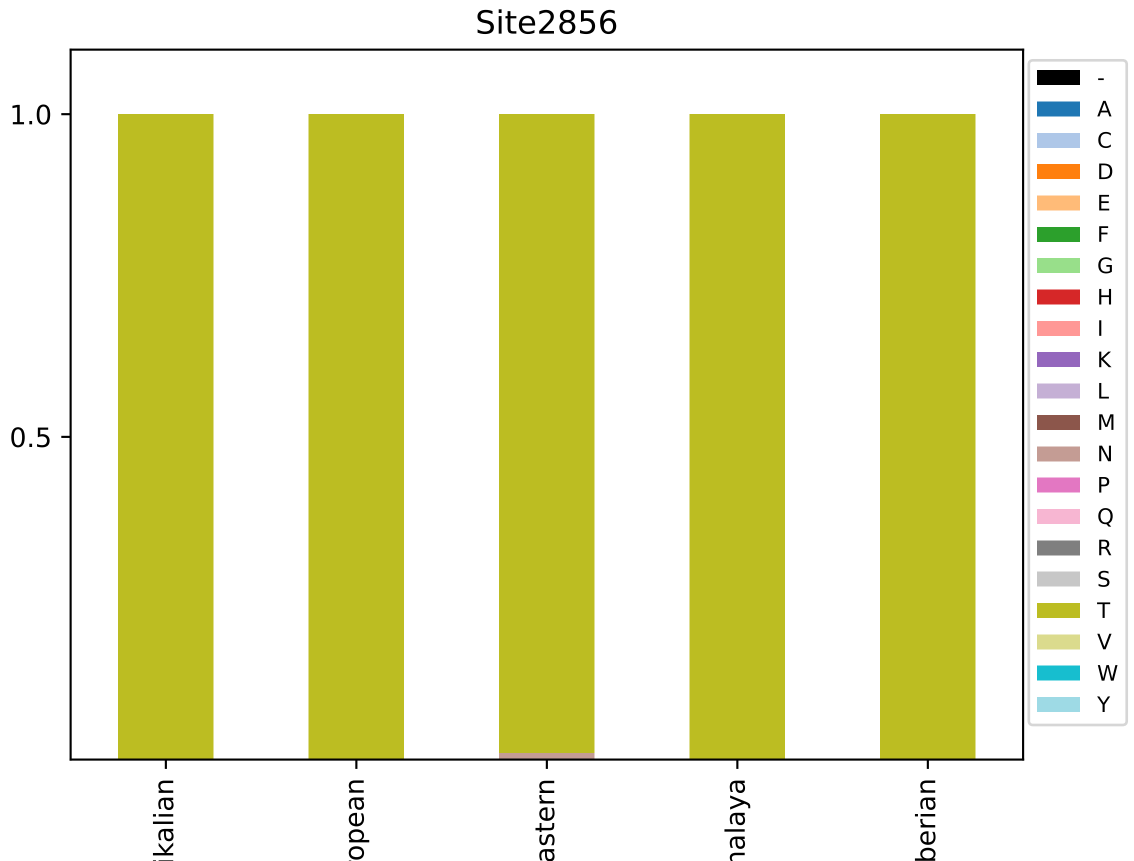

Site2856
1.0
0.5
-
A
C
D
E
F
G
H
I
K
L
M
N
P
Q
R
S
T
V
W
Y
ikalian
opean
astern
nalaya
berian

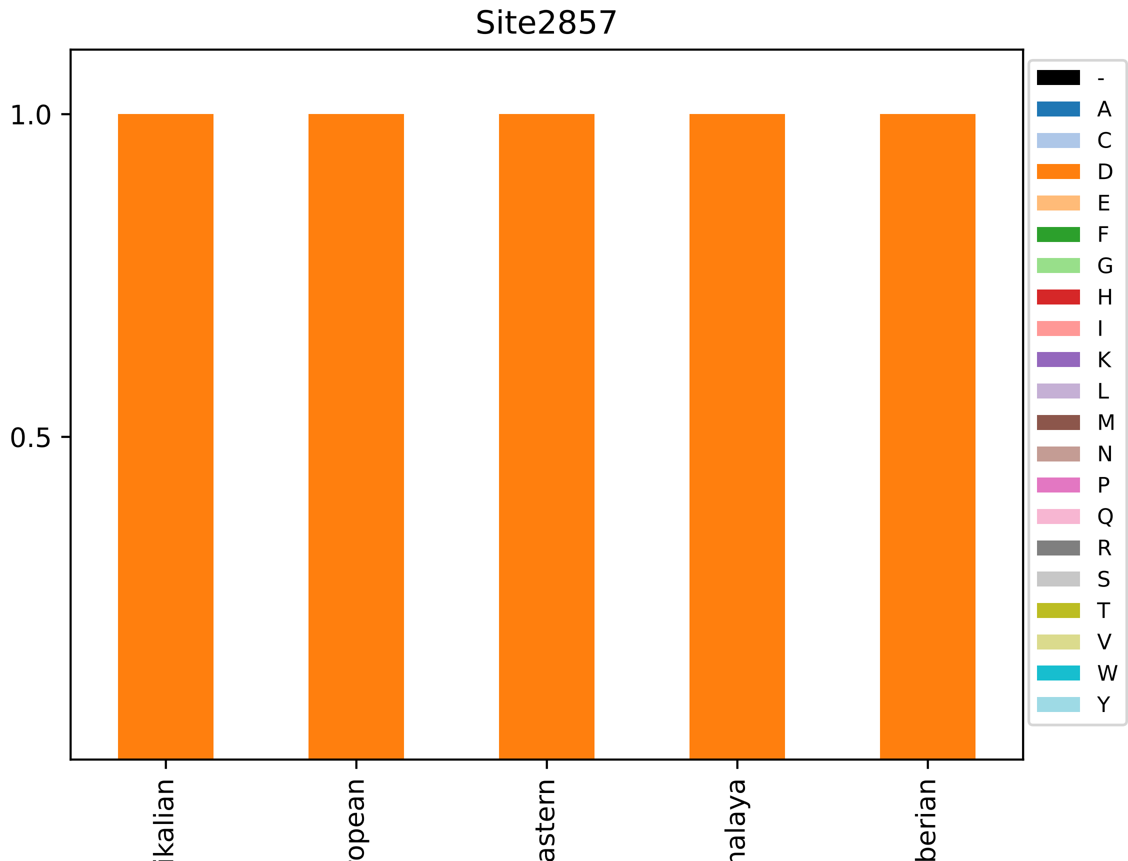

Site2857
1.0
0.5
-
A
C
D
E
F
G
H
I
K
L
M
N
P
Q
R
S
T
V
W
Y
ikalian
opean
astern
nalaya
berian

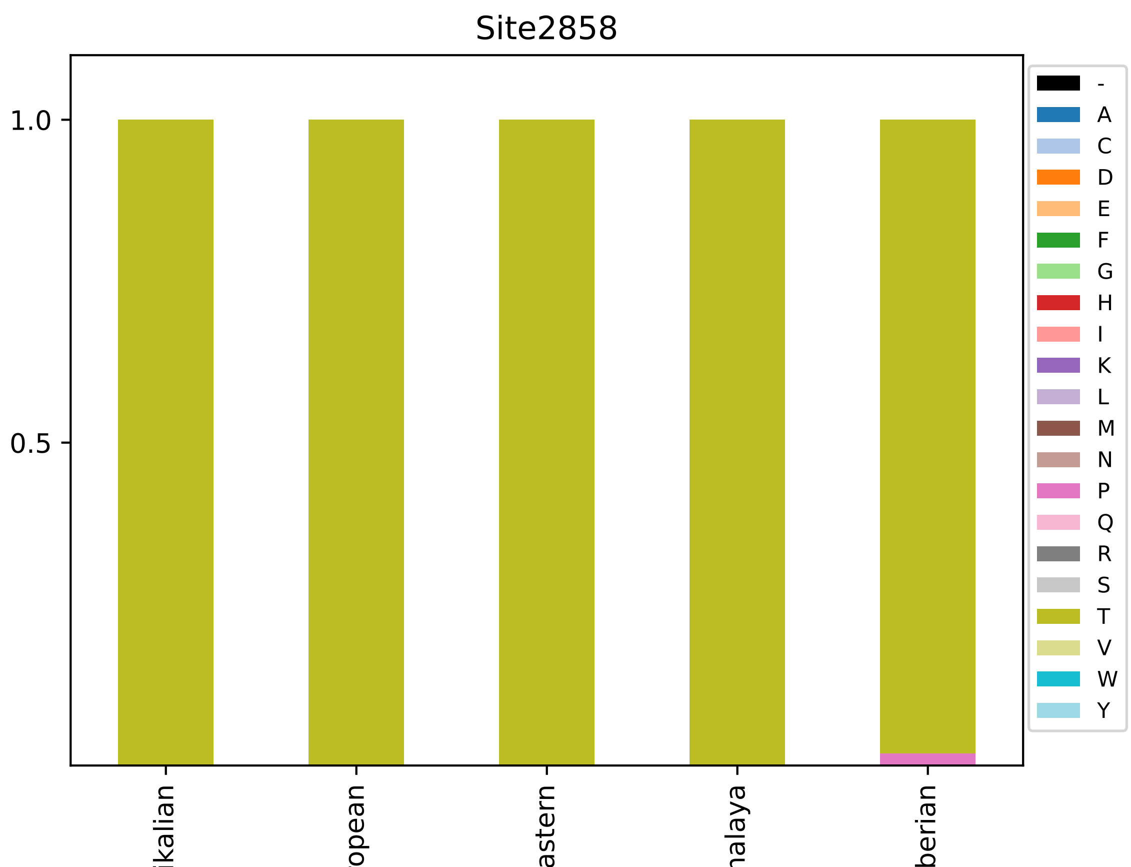

Site2858
1.0
0.5
-
A
C
D
E
F
G
H
I
K
L
M
N
P
Q
R
S
T
V
W
Y
ikalian
opean
astern
nalaya
berian

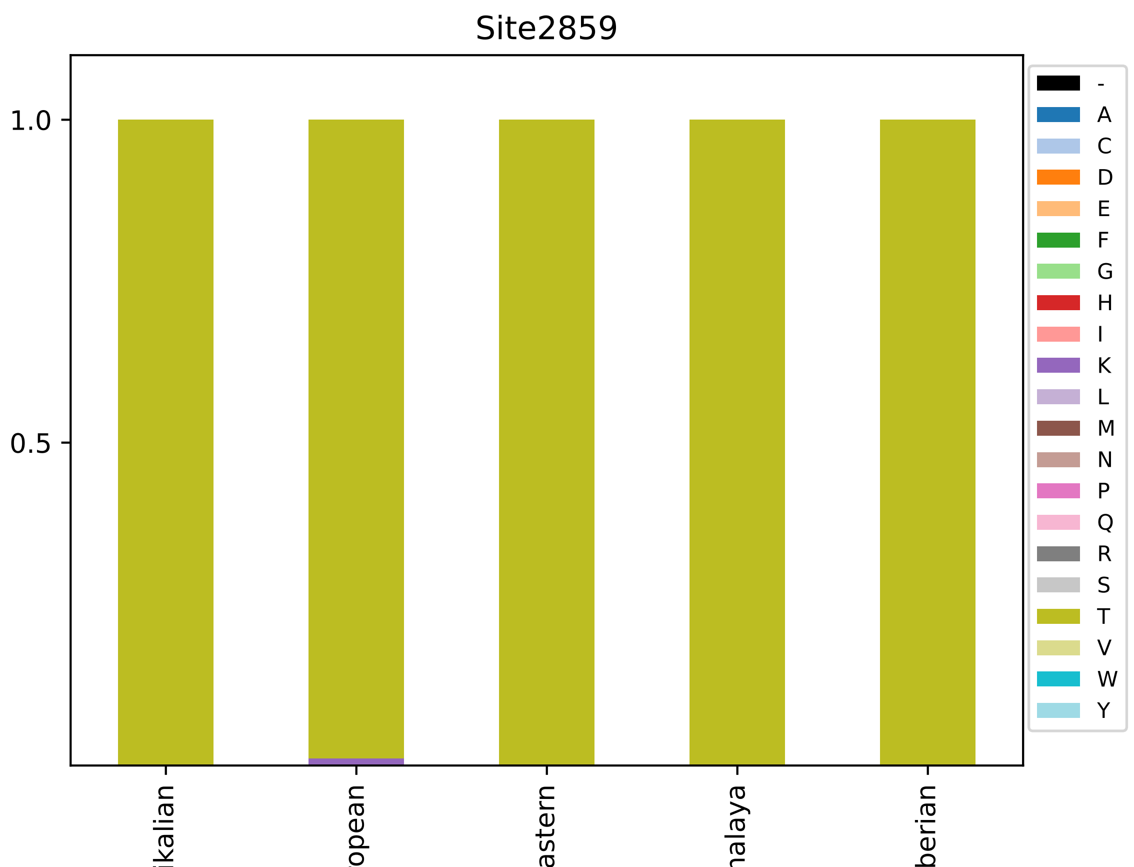

Site2859
1.0
0.5
-
A
C
D
E
F
G
H
I
K
L
M
N
P
Q
R
S
T
V
W
Y

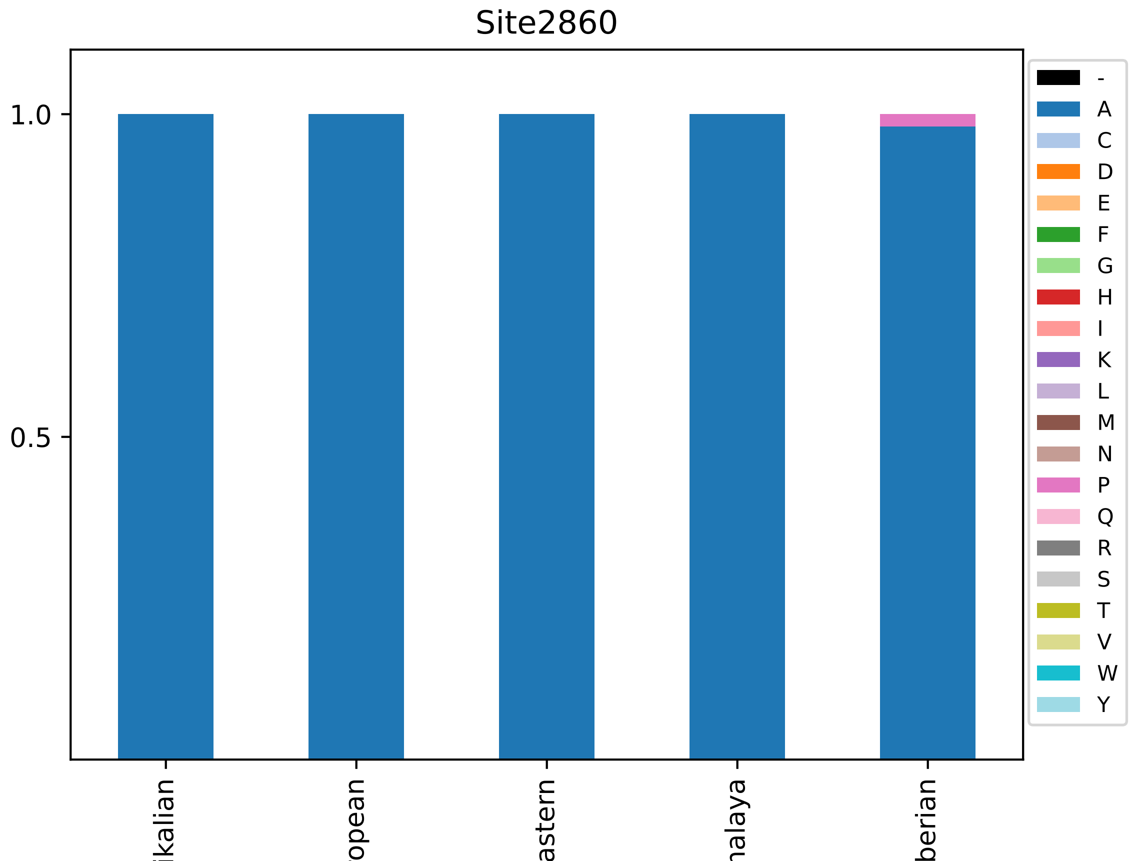
Site2860
1.0
0.5
-
A
C
D
E
F
G
H
I
K
L
M
N
P
Q
R
S
T
V
W
Y
ikalian
opean
astern
nalaya
berian

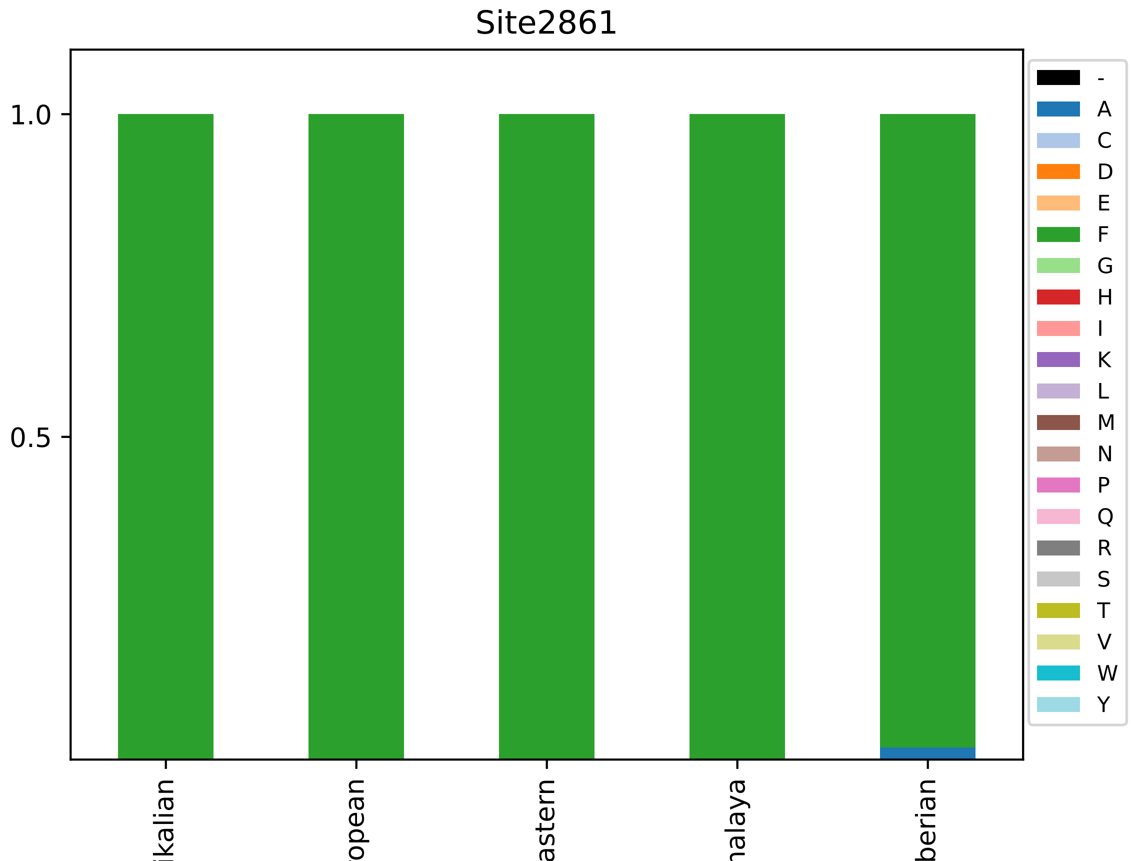
Site2861
1.0
0.5
-
A
C
D
E
F
G
H
I
K
L
M
N
P
Q
R
S
T
V
W
Y
ikalian
opean
astern
nalaya
berian

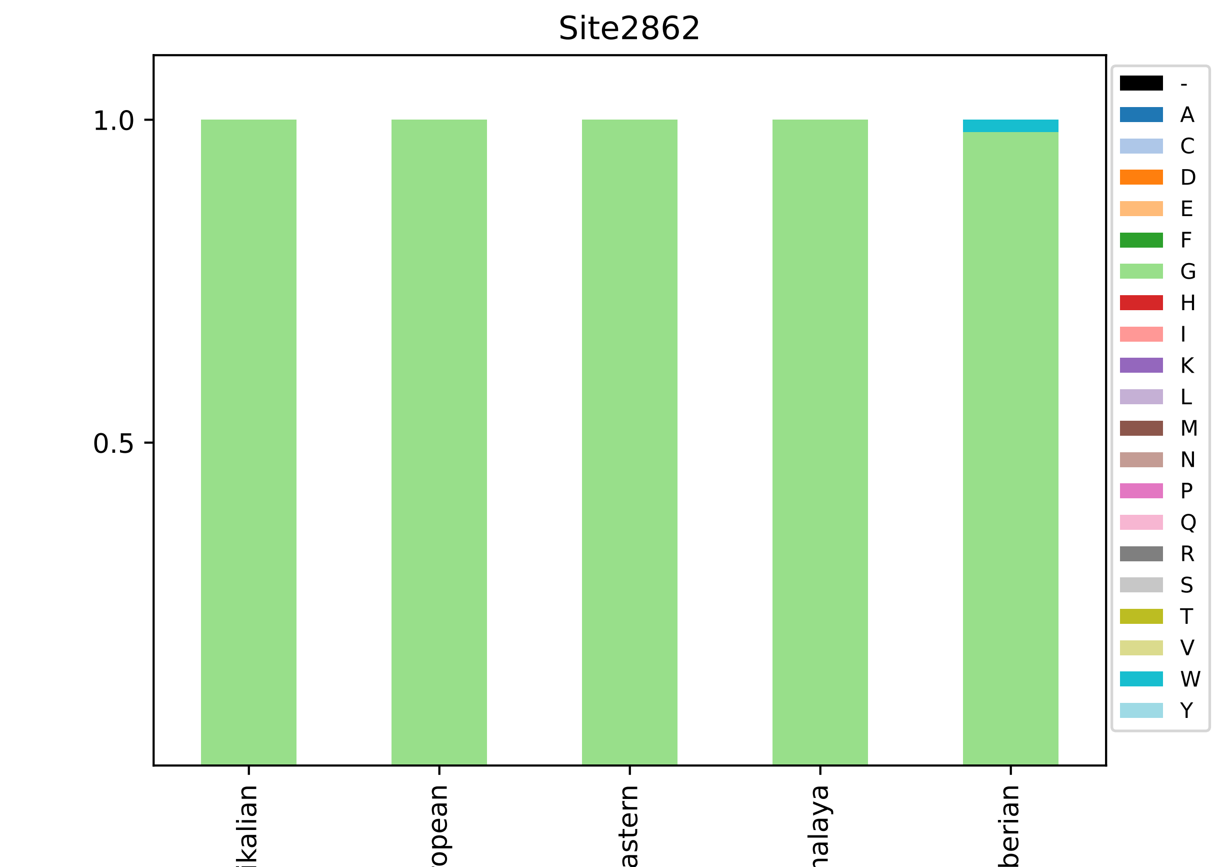

Site2862
1.0
0.5
-
A
C
D
E
F
G
H
I
K
L
M
N
P
Q
R
S
T
V
W
Y
ikalian
opean
astern
nalaya
berian

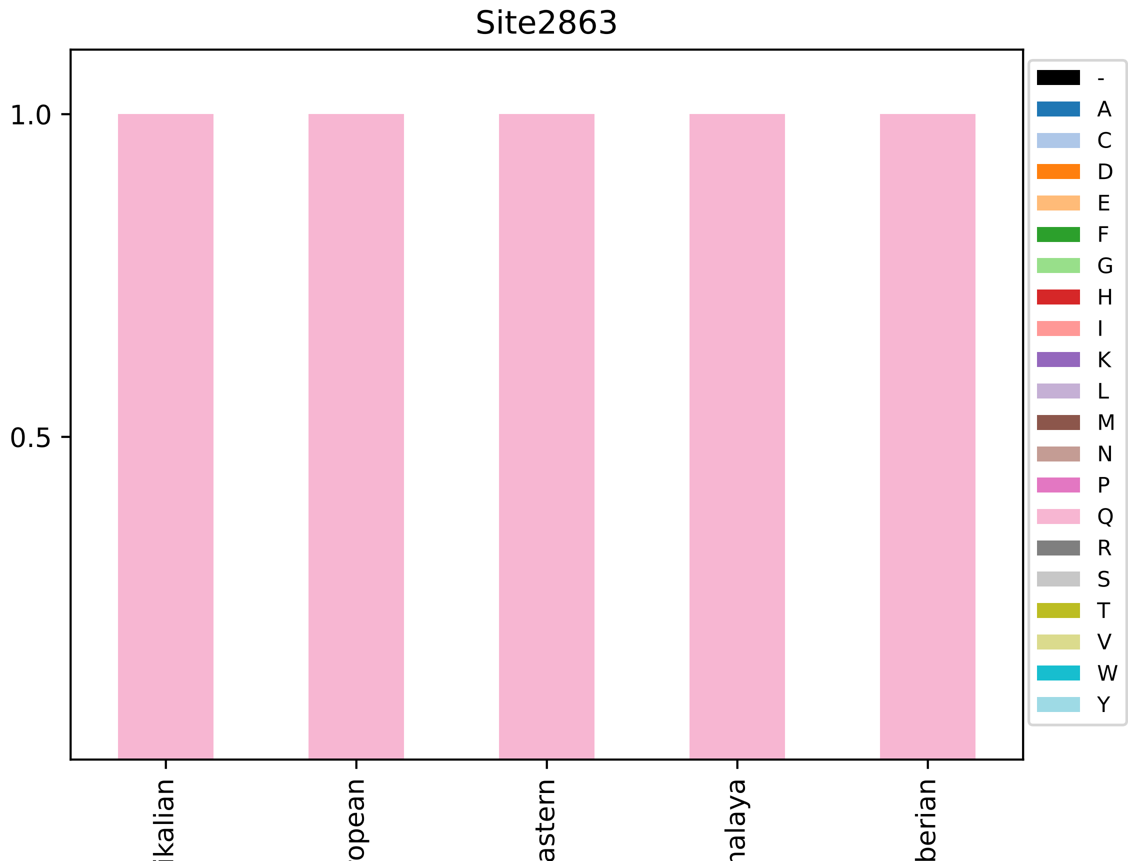
Site2863
1.0
0.5
-
A
C
D
E
F
G
H
I
K
L
M
N
P
Q
R
S
T
V
W
Y
ikalian
opean
astern
nalaya
berian

Site2864

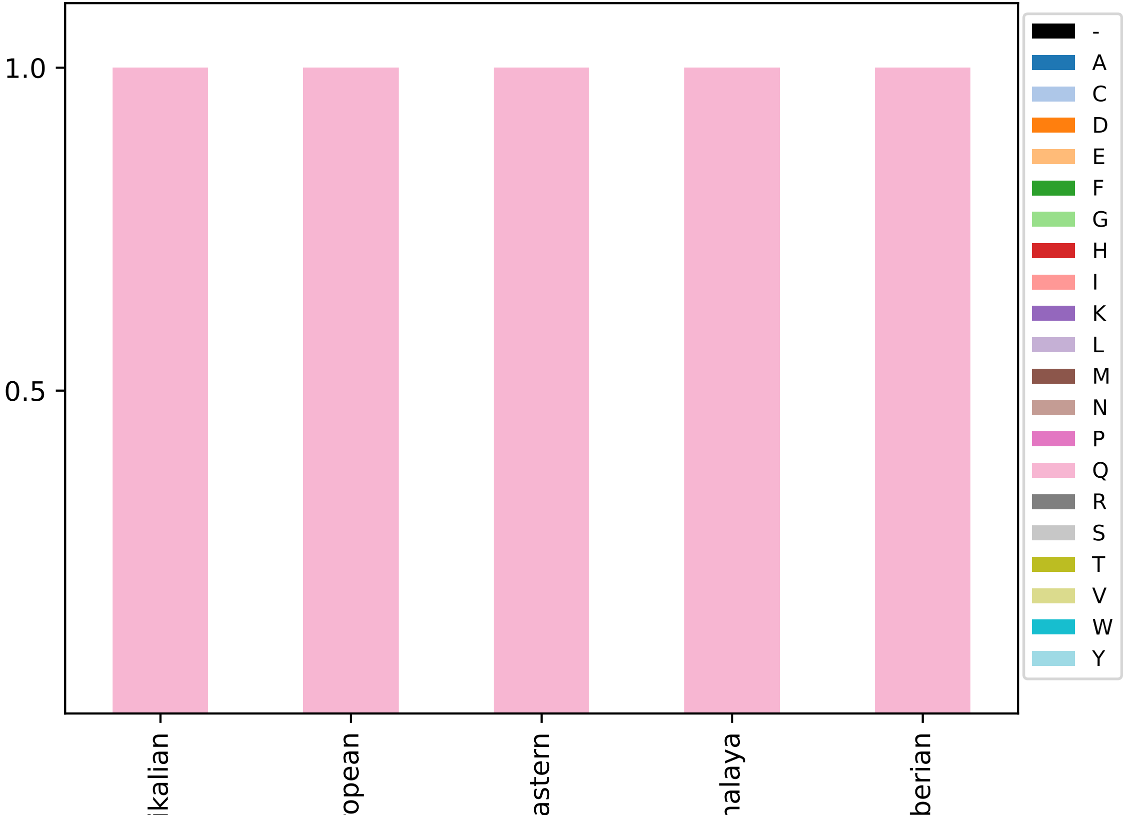

1.0
0.5
-
A
C
D
E
F
G
H
I
K
L
M
N
P
Q
R
S
T
V
W
Y
ikalian
opean
astern
nalaya
berian

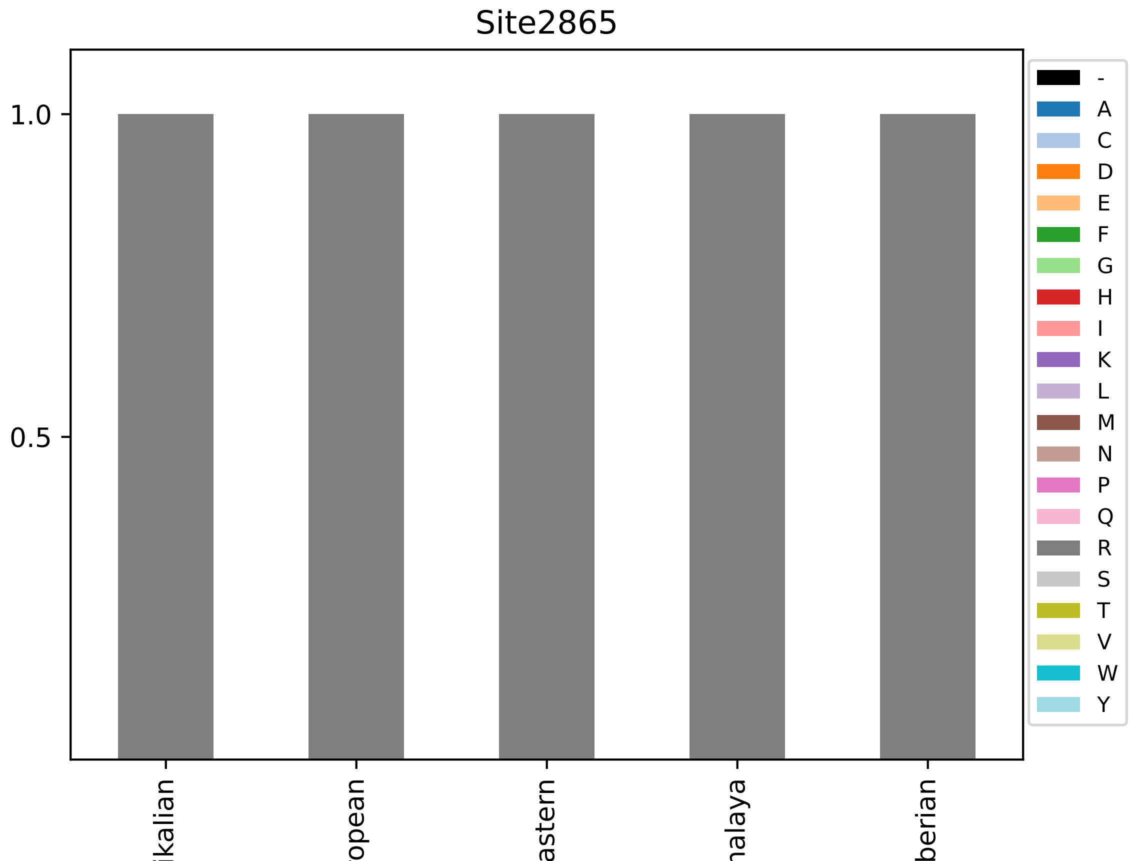
Site2865
1.0
0.5
-
A
C
D
E
F
G
H
I
K
L
M
N
P
Q
R
S
T
V
W
Y
ikalian
opean
astern
nalaya
berian

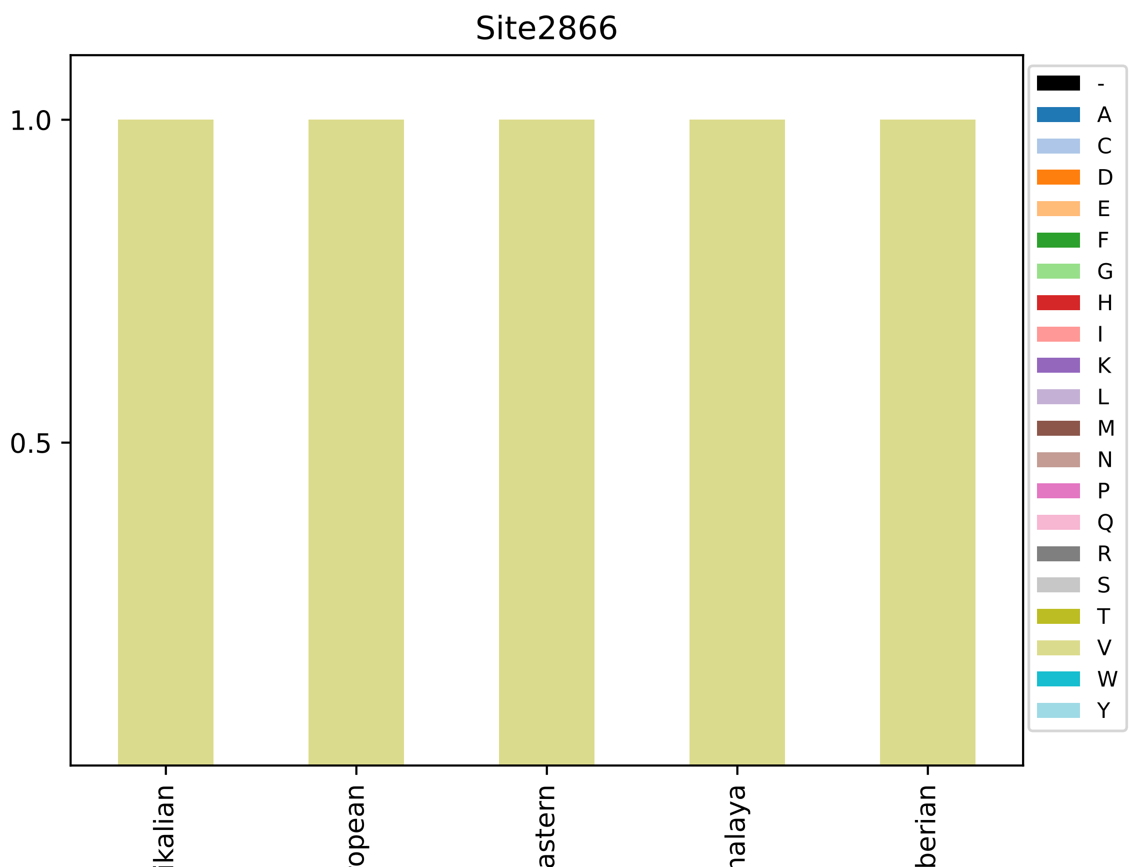

Site2866
1.0
0.5
-
A
C
D
E
F
G
H
I
K
L
M
N
P
Q
R
S
T
V
W
Y
ikalian
opean
astern
nalaya
berian

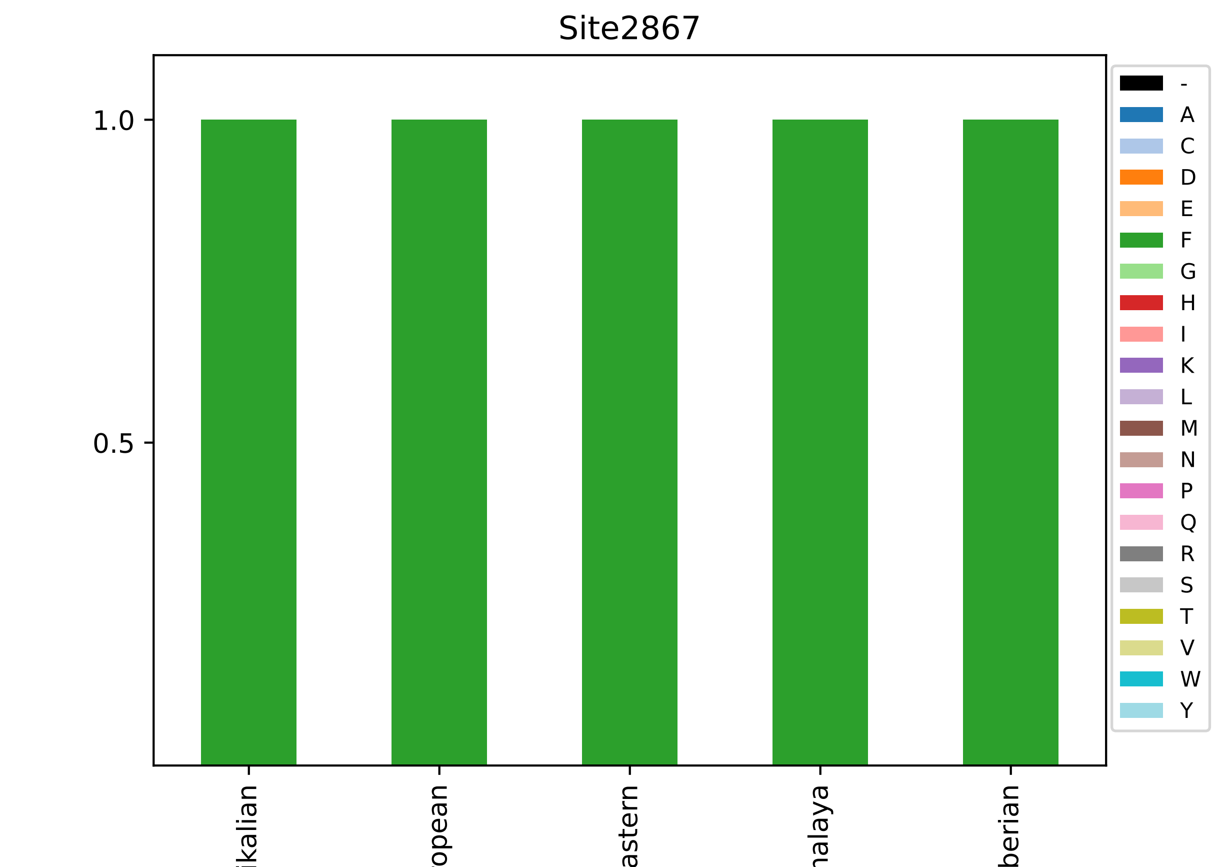

Site2867
1.0
0.5
-
A
C
D
E
F
G
H
I
K
L
M
N
P
Q
R
S
T
V
W
Y
ikalian
opean
astern
nalaya
berian

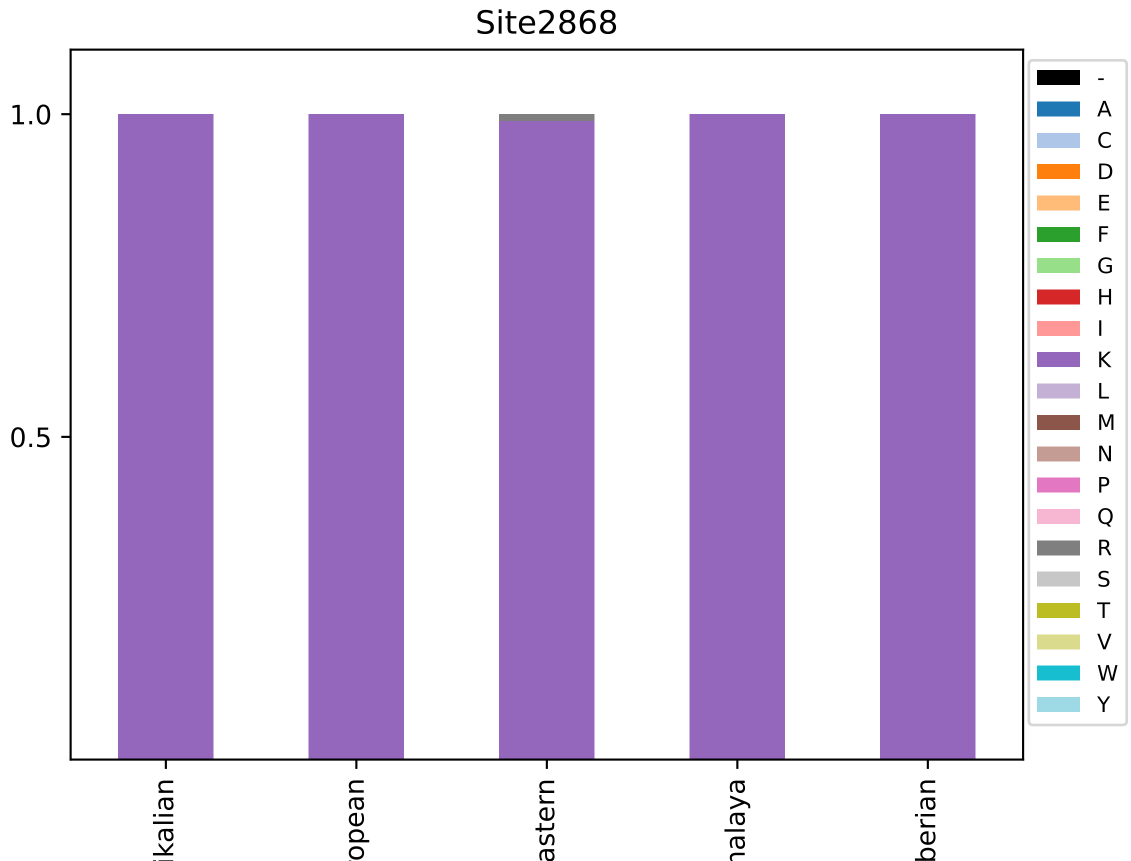

Site2868
1.0
0.5
ikalian
opean
astern
nalaya
berian
-
A
C
D
E
F
G
H
I
K
L
M
N
P
Q
R
S
T
V
W
Y

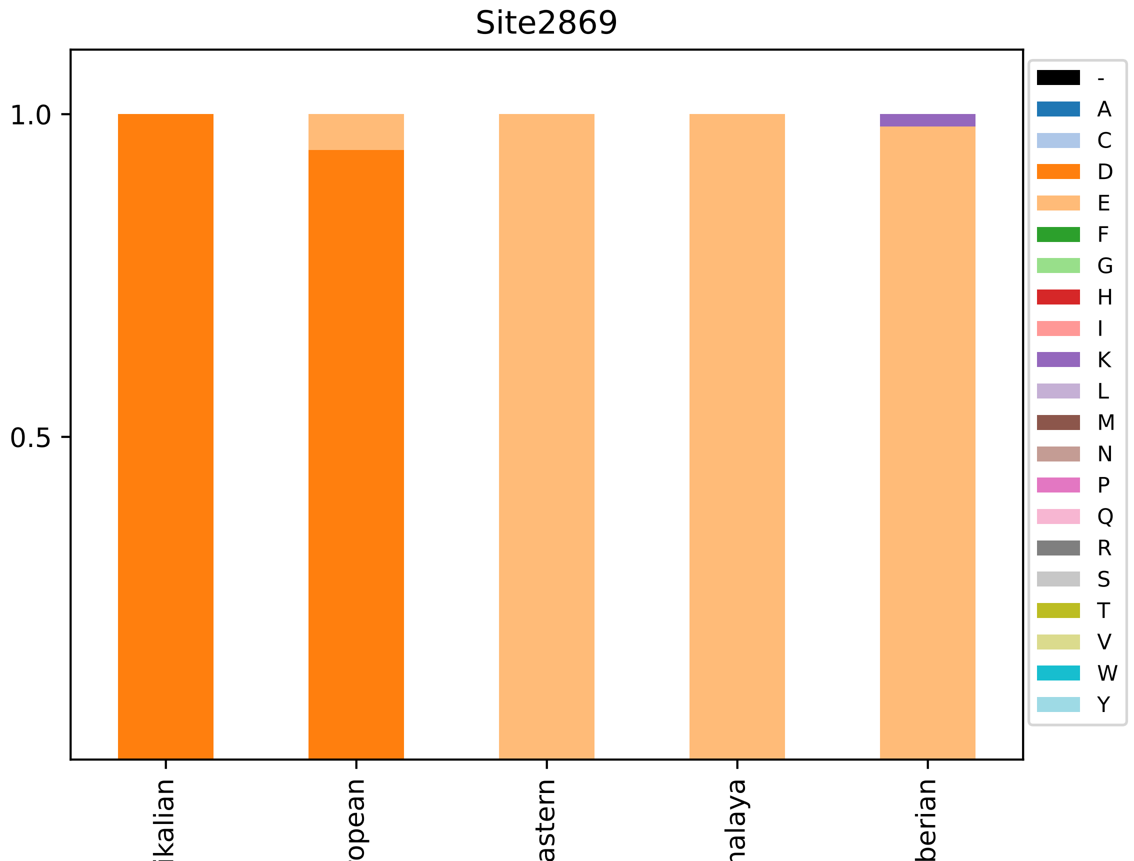
Site2869
1.0
0.5
-
A
C
D
E
F
G
H
I
K
L
M
N
P
Q
R
S
T
V
W
Y
ikalian
opean
astern
nalaya
berian

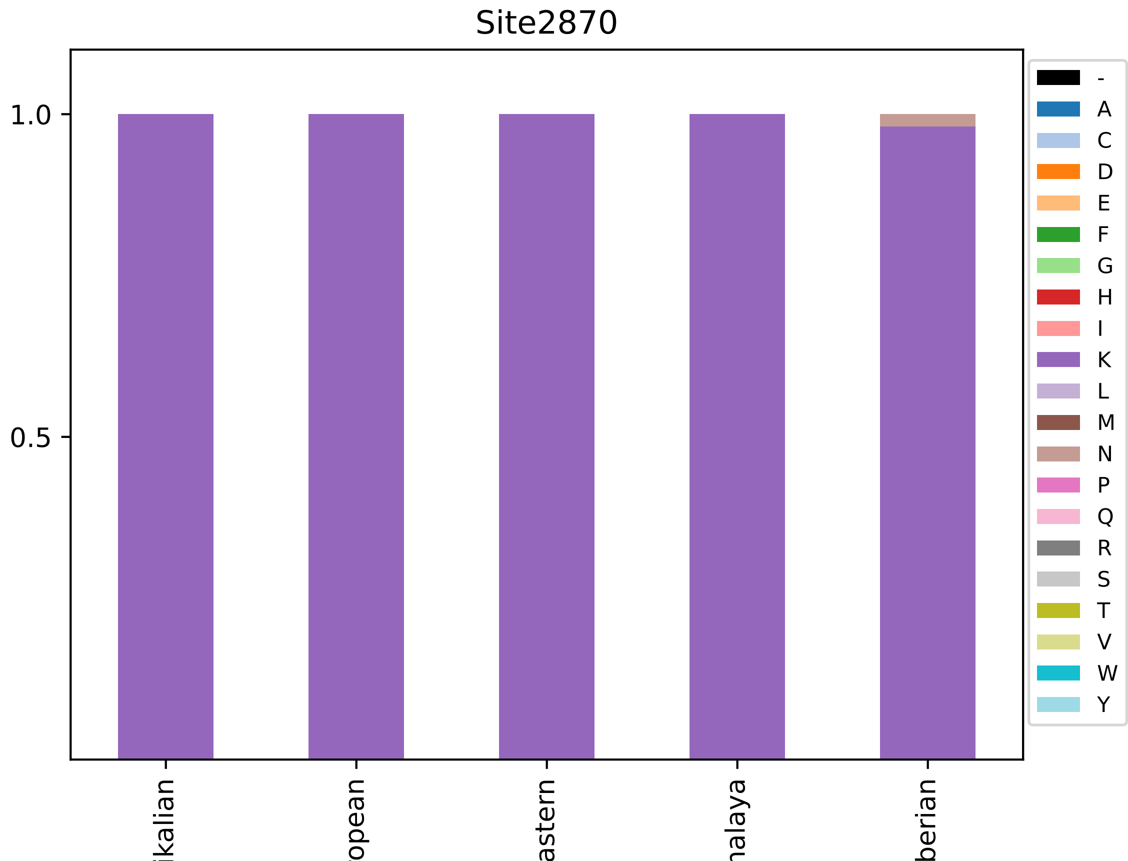

Site2870
1.0
0.5
-
A
C
D
E
F
G
H
I
K
L
M
N
P
Q
R
S
T
V
W
Y
ikalian
opean
astern
nalaya
berian

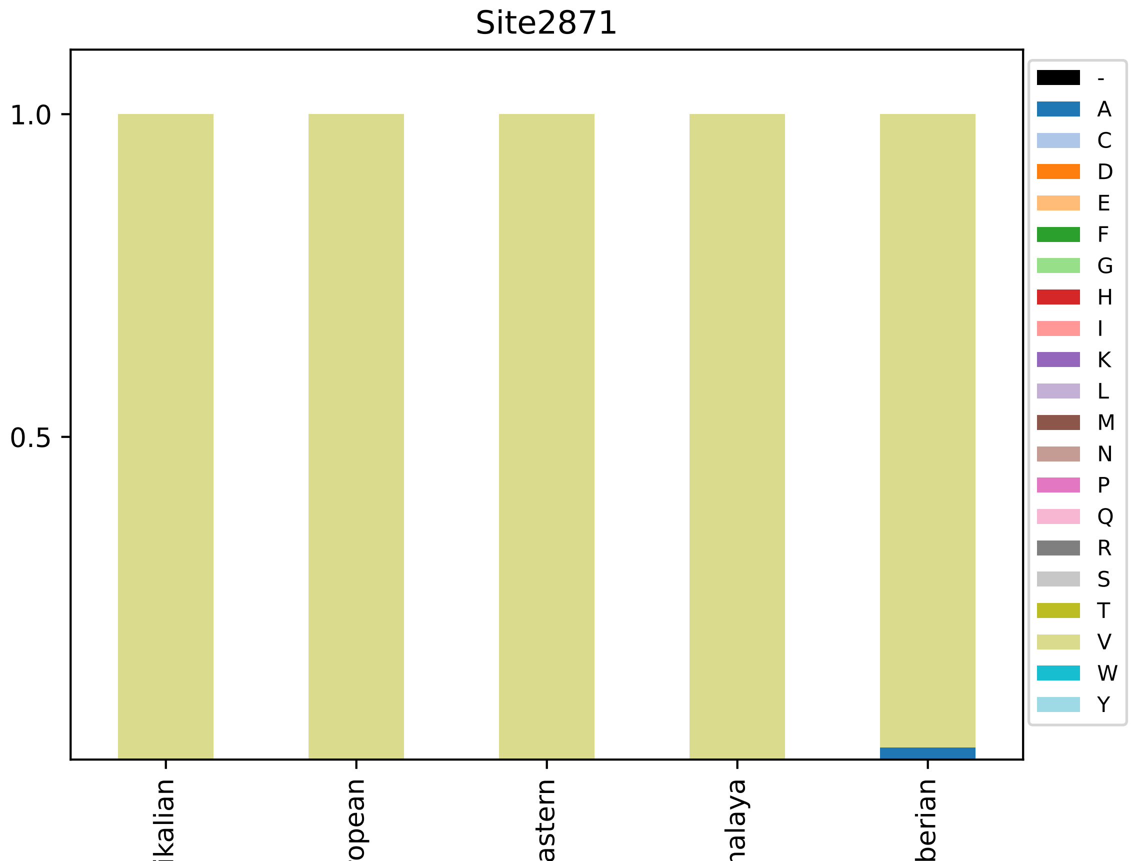
Site2871
1.0
0.5
-
A
C
D
E
F
G
H
I
K
L
M
N
P
Q
R
S
T
V
W
Y
ikalian
opean
astern
nalaya
berian

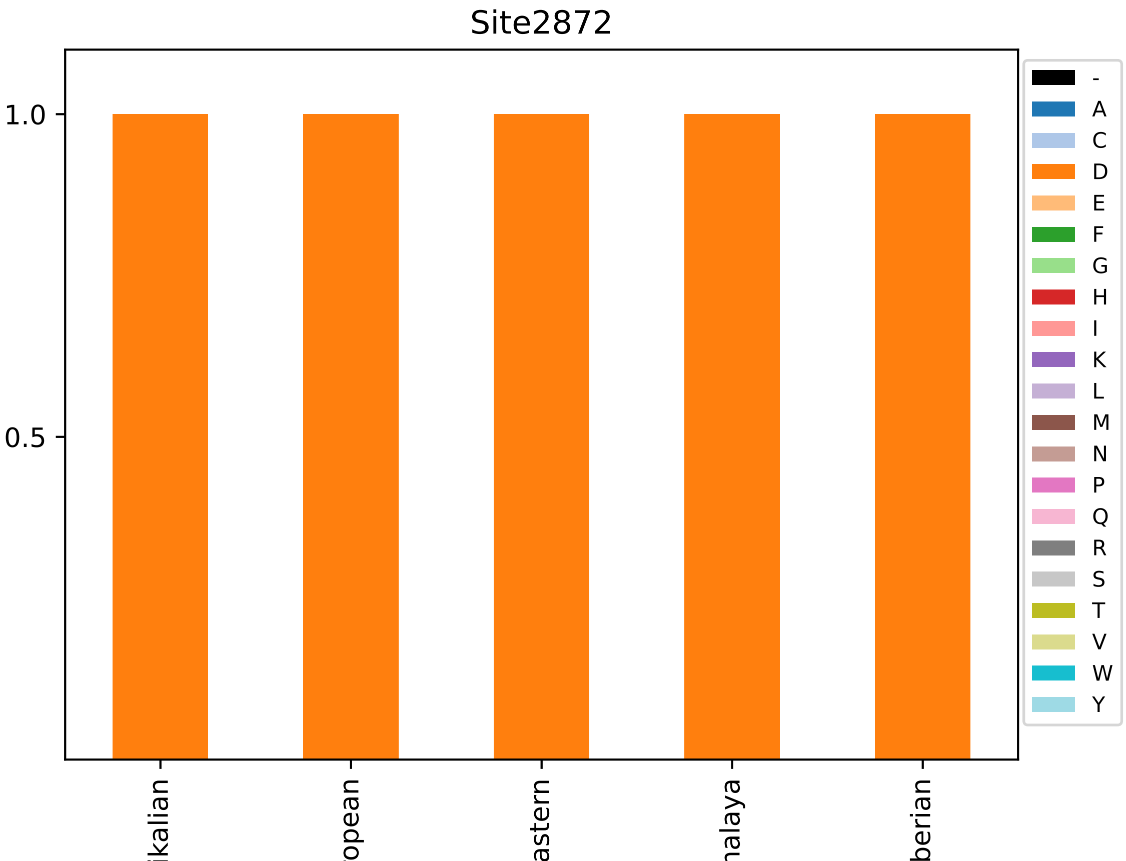
Site2872
1.0
0.5
-
A
C
D
E
F
G
H
I
K
L
M
N
P
Q
R
S
T
V
W
Y

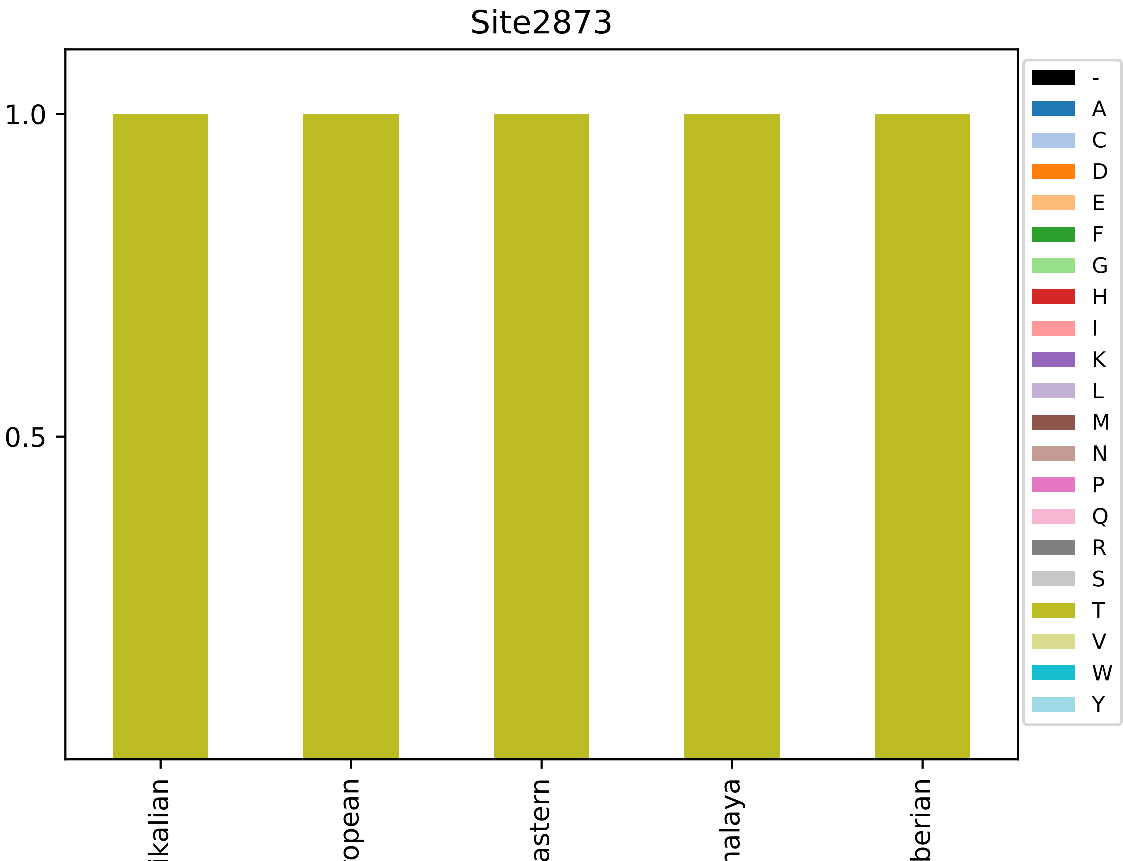
Site2873
1.0
0.5
-
A
C
D
E
F
G
H
I
K
L
M
N
P
Q
R
S
T
V
W
Y
ikalian
opean
astern
nalaya
berian

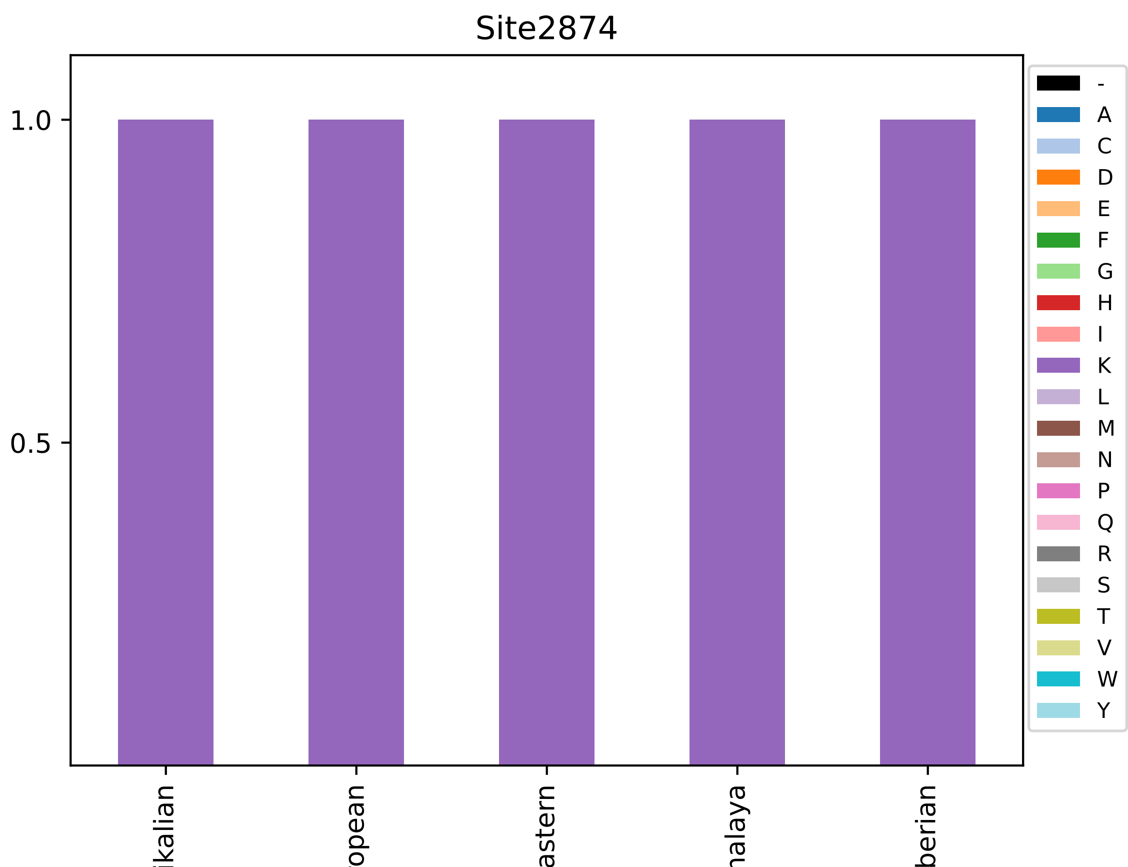

Site2874
1.0
0.5
-
A
C
D
E
F
G
H
I
K
L
M
N
P
Q
R
S
T
V
W
Y
ikalian
opean
astern
nalaya
berian

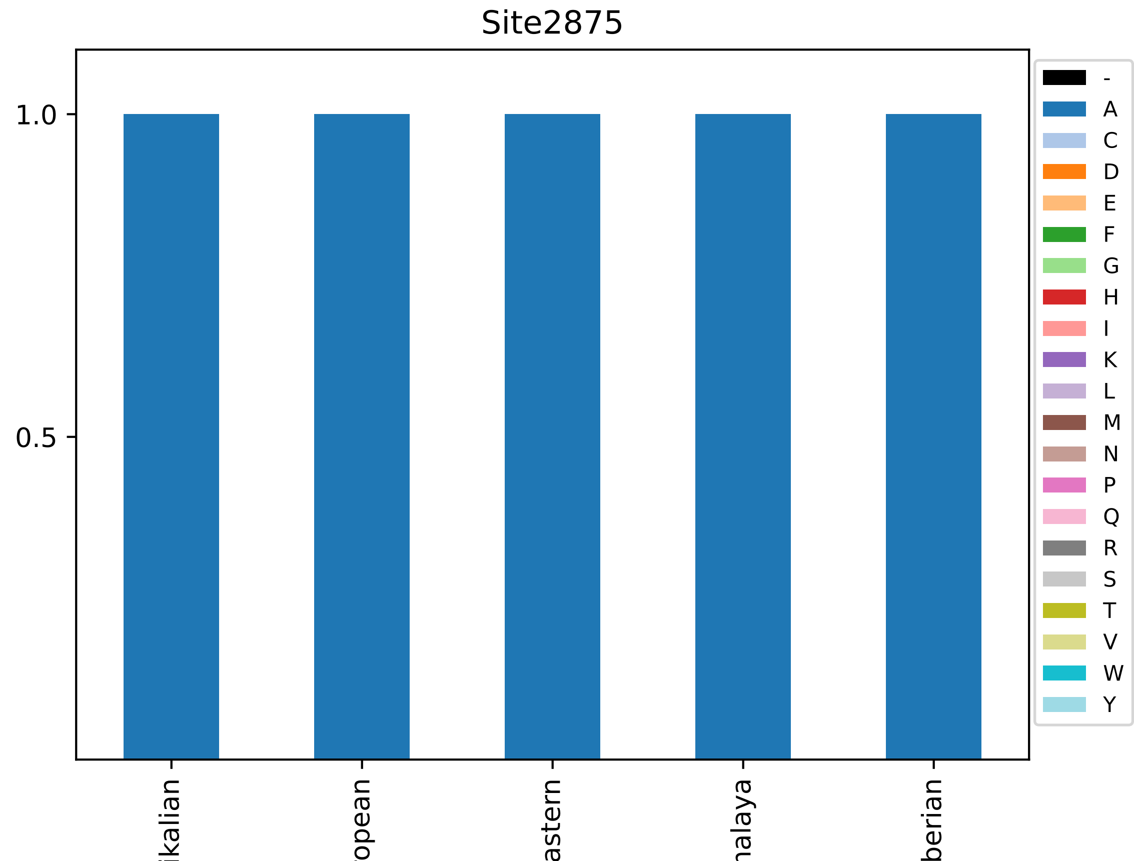
Site2875
1.0
0.5
ikalian
opean
astern
nalaya
berian
-
A
C
D
E
F
G
H
I
K
L
M
N
P
Q
R
S
T
V
W
Y

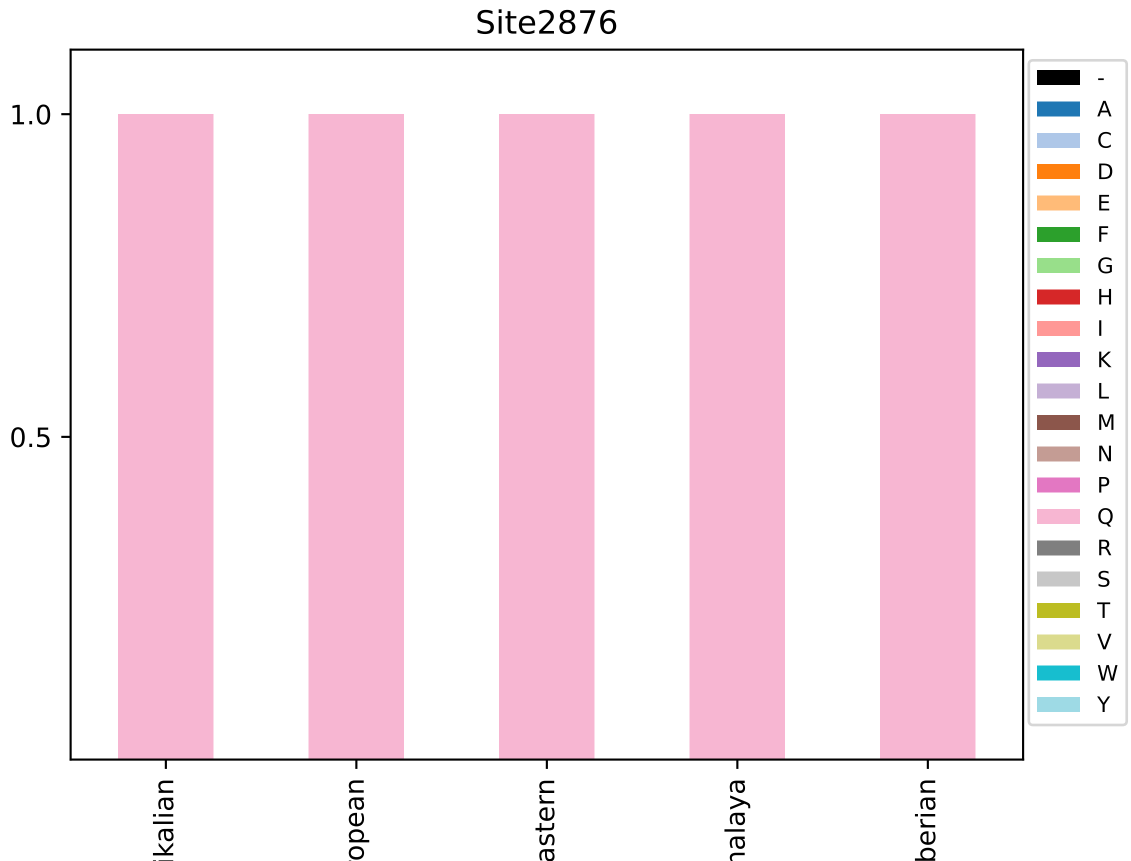
Site2876
1.0
0.5
-
A
C
D
E
F
G
H
I
K
L
M
N
P
Q
R
S
T
V
W
Y
ikalian
opean
astern
nalaya
berian

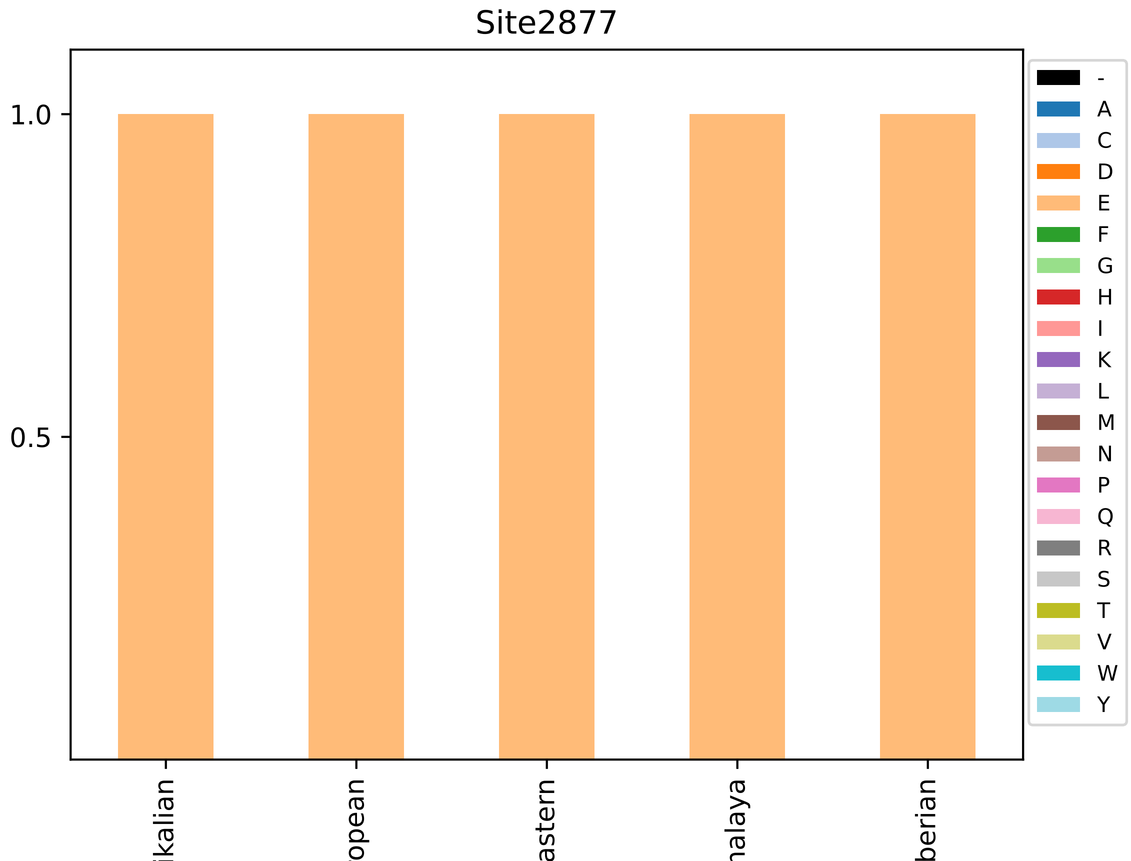
Site2877
1.0
0.5
-
A
C
D
E
F
G
H
I
K
L
M
N
P
Q
R
S
T
V
W
Y
ikalian
opean
astern
nalaya
berian

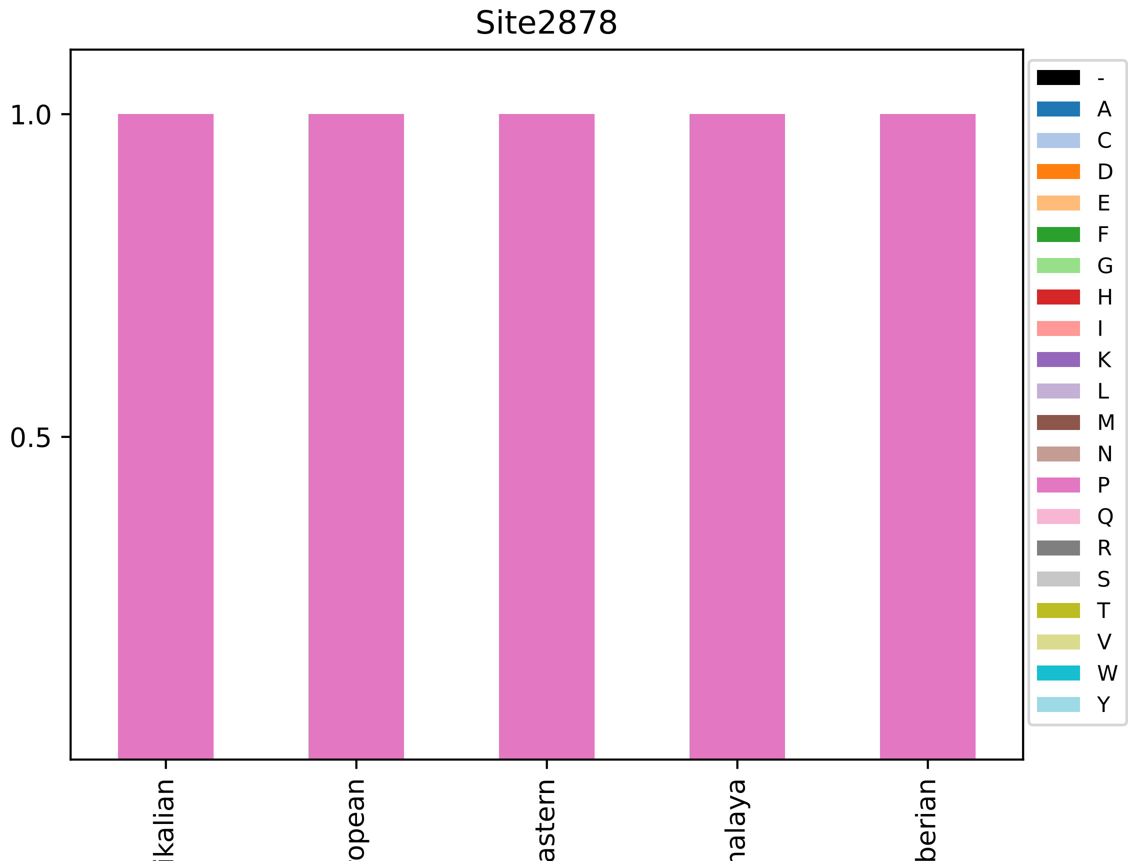

Site2878
1.0
0.5
-
A
C
D
E
F
G
H
I
K
L
M
N
P
Q
R
S
T
V
W
Y
ikalian
opean
astern
nalaya
berian

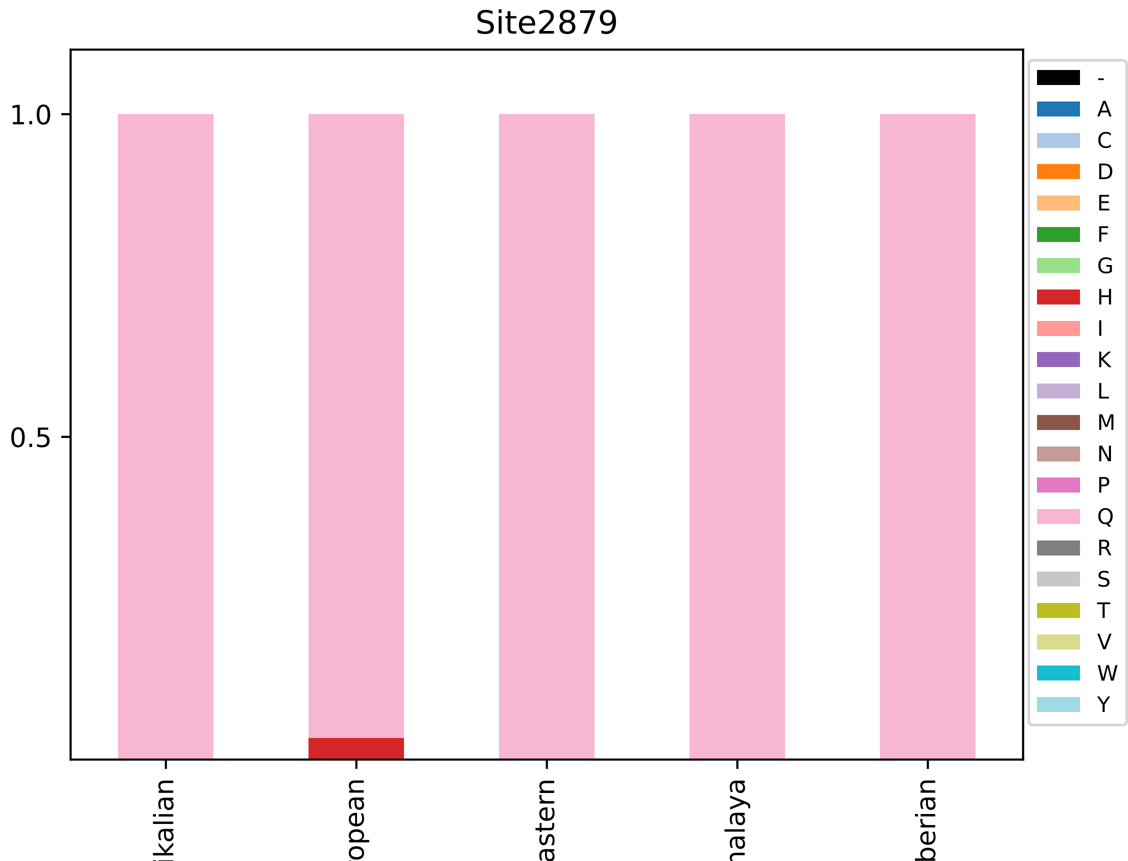

Site2879
1.0
0.5
ikalian
opean
astern
nalaya
berian
-
A
C
D
E
F
G
H
I
K
L
M
N
P
Q
R
S
T
V
W
Y

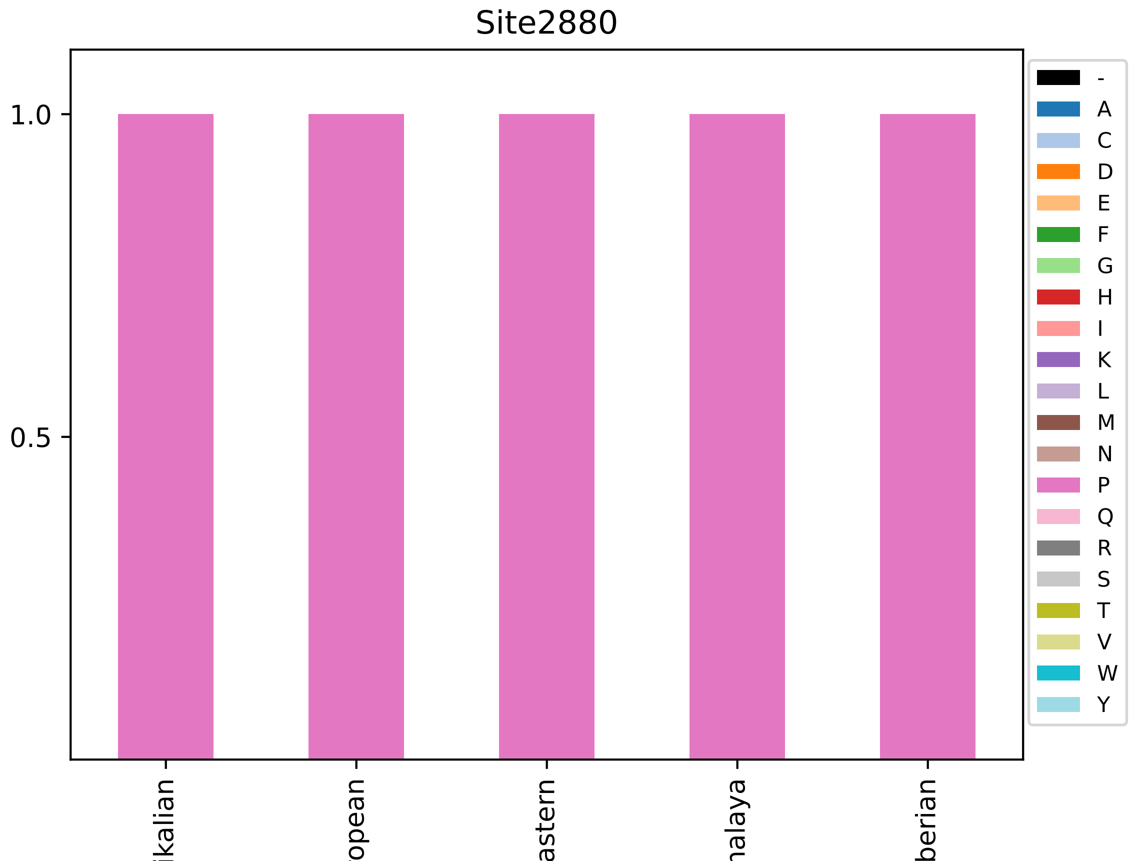

Site2880
1.0
0.5
-
A
C
D
E
F
G
H
I
K
L
M
N
P
Q
R
S
T
V
W
Y

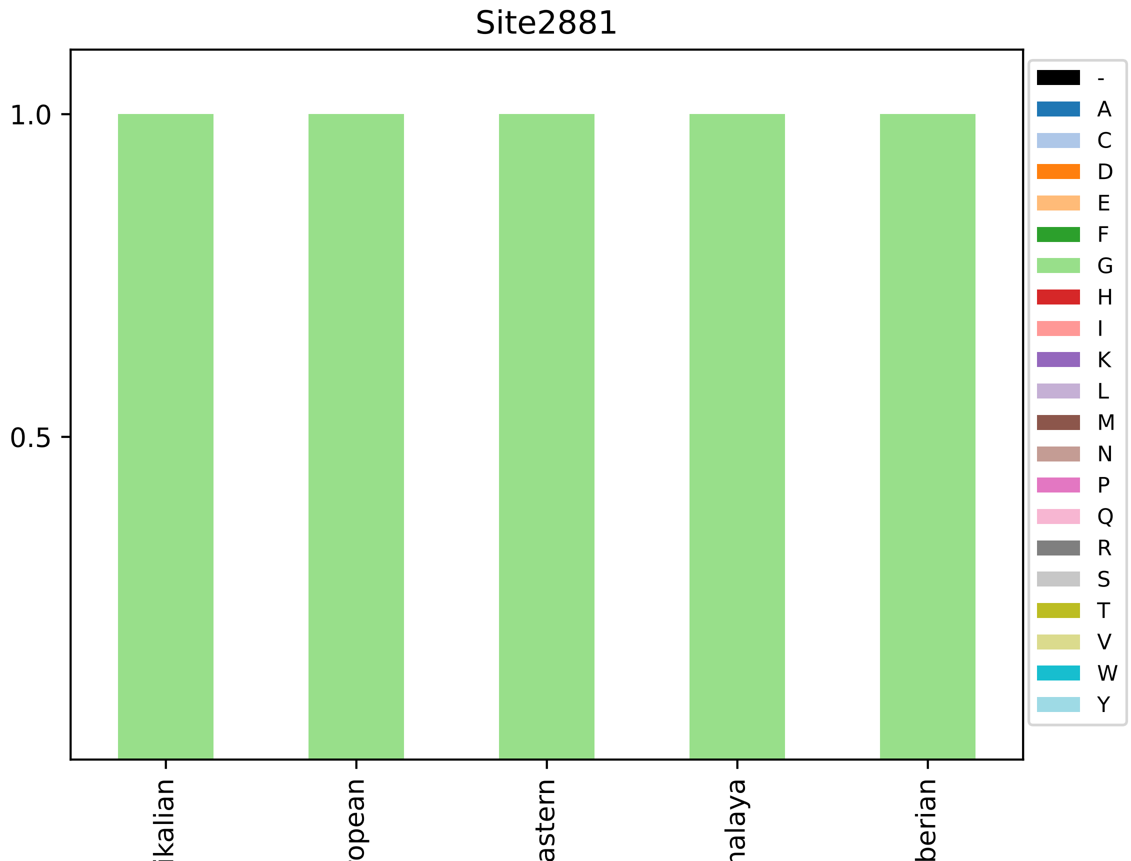
Site2881
1.0
0.5
-
A
C
D
E
F
G
H
I
K
L
M
N
P
Q
R
S
T
V
W
Y
ikalian
opean
astern
nalaya
berian

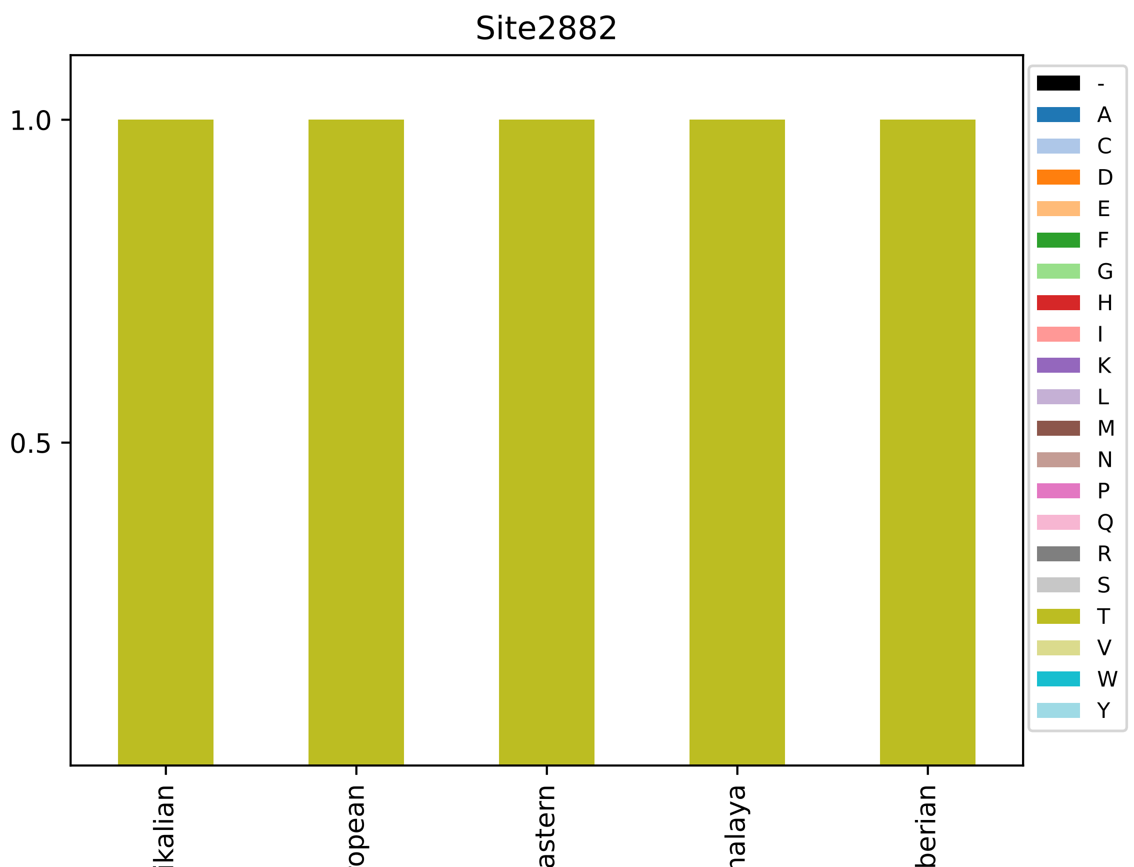

Site2882
1.0
0.5
ikalian
opean
astern
nalaya
berian
-
A
C
D
E
F
G
H
I
K
L
M
N
P
Q
R
S
T
V
W
Y

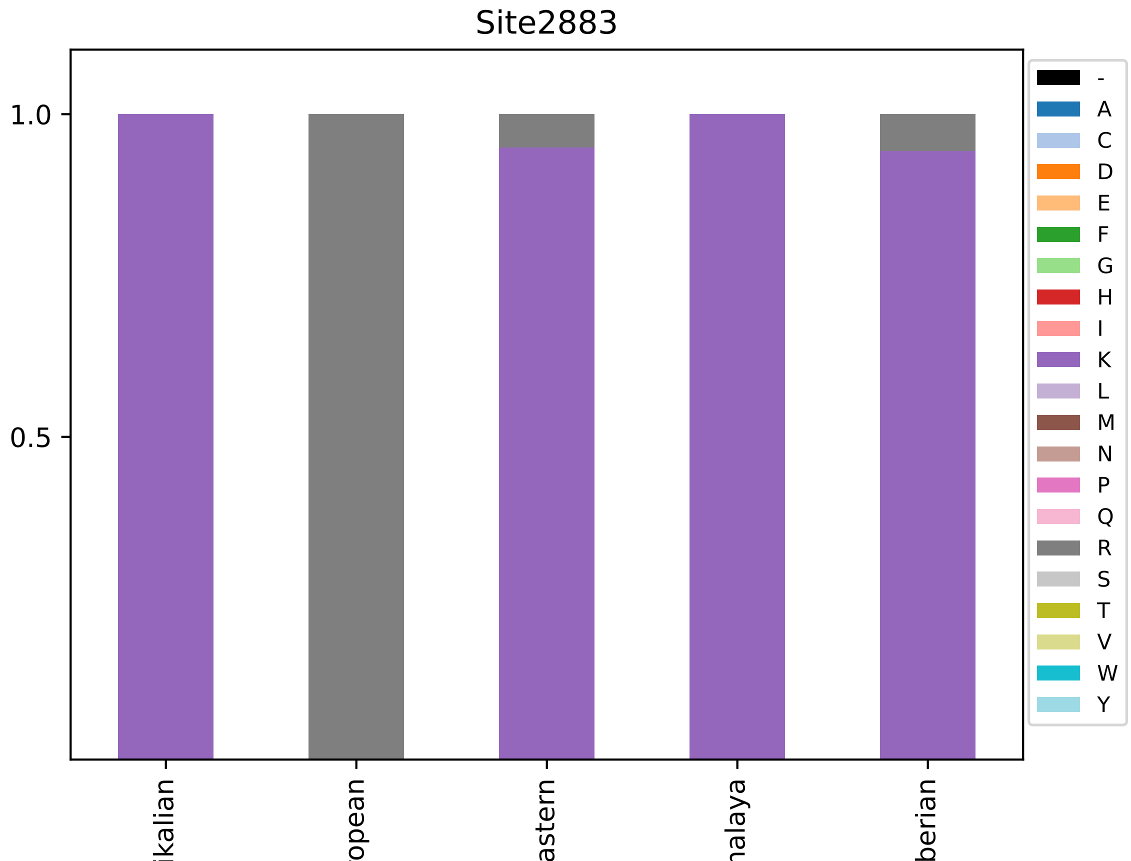

Site2883
1.0
0.5
-
A
C
D
E
F
G
H
I
K
L
M
N
P
Q
R
S
T
V
W
Y
ikalian
opean
astern
nalaya
berian

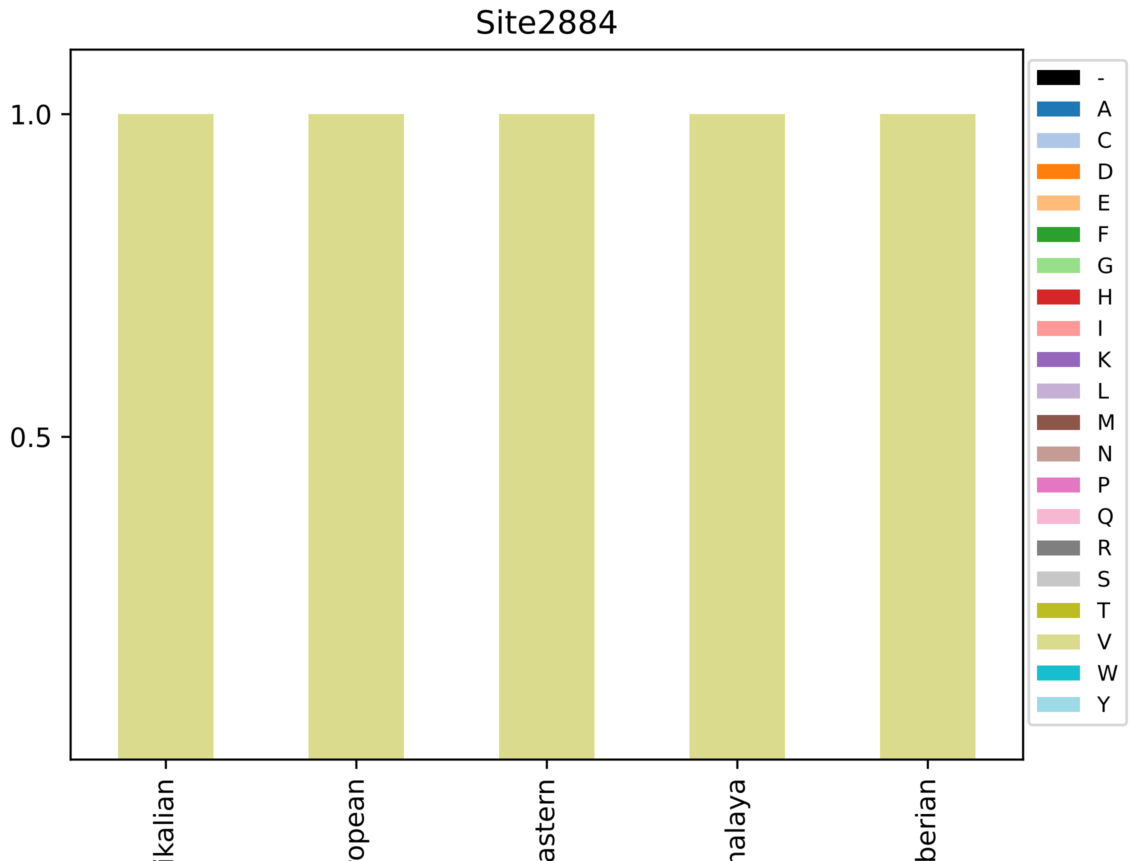

Site2884
1.0
0.5
-
A
C
D
E
F
G
H
I
K
L
M
N
P
Q
R
S
T
V
W
Y
ikalian
opean
astern
nalaya
berian

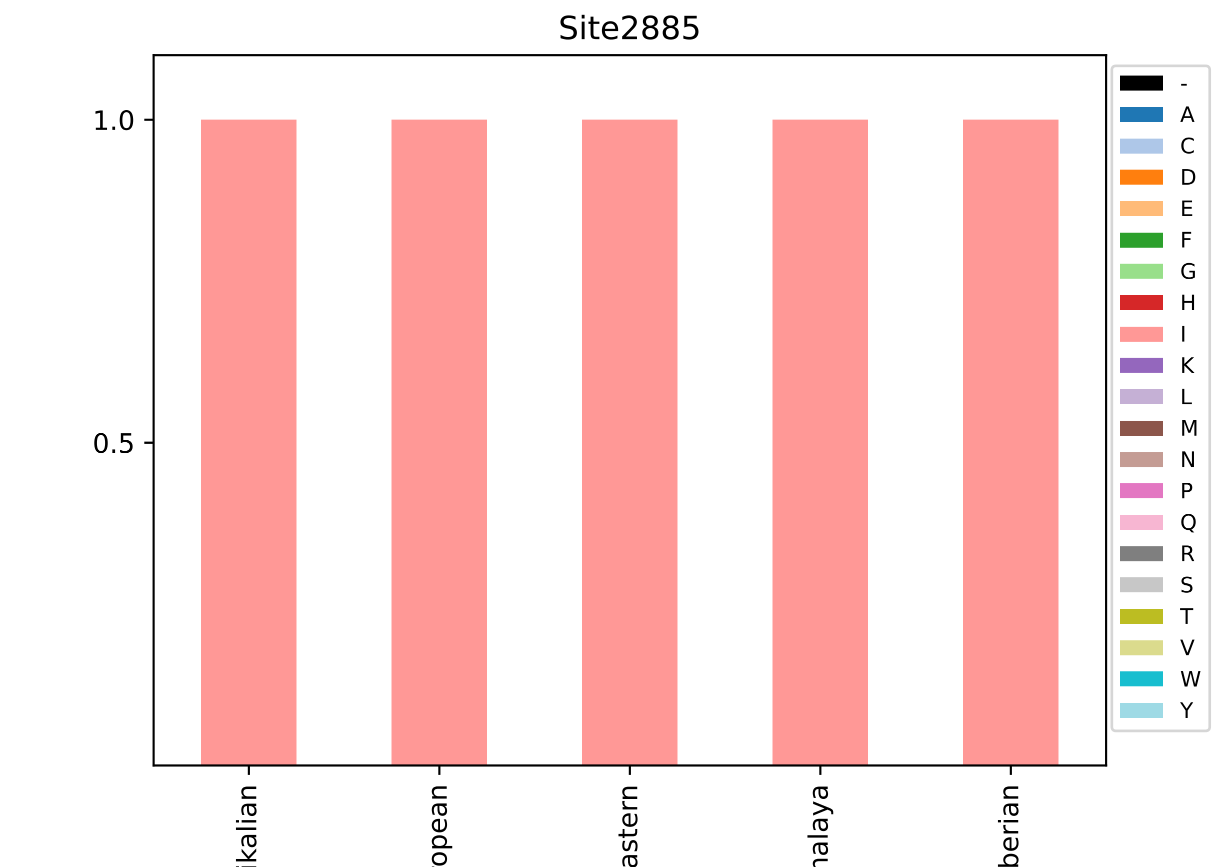

Site2885
1.0
0.5
-
A
C
D
E
F
G
H
I
K
L
M
N
P
Q
R
S
T
V
W
Y
ikalian
opean
astern
nalaya
berian

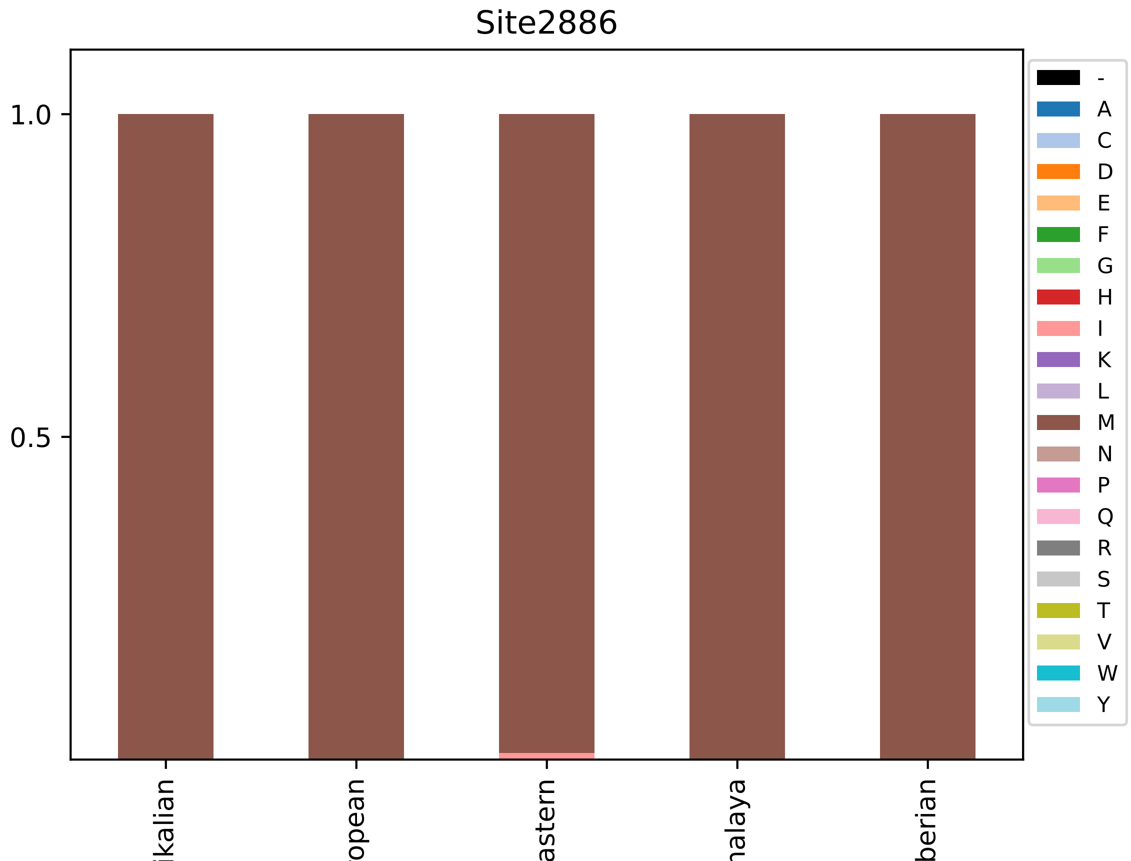
Site2886
1.0
0.5
-
A
C
D
E
F
G
H
I
K
L
M
N
P
Q
R
S
T
V
W
Y
ikalian
opean
astern
nalaya
berian

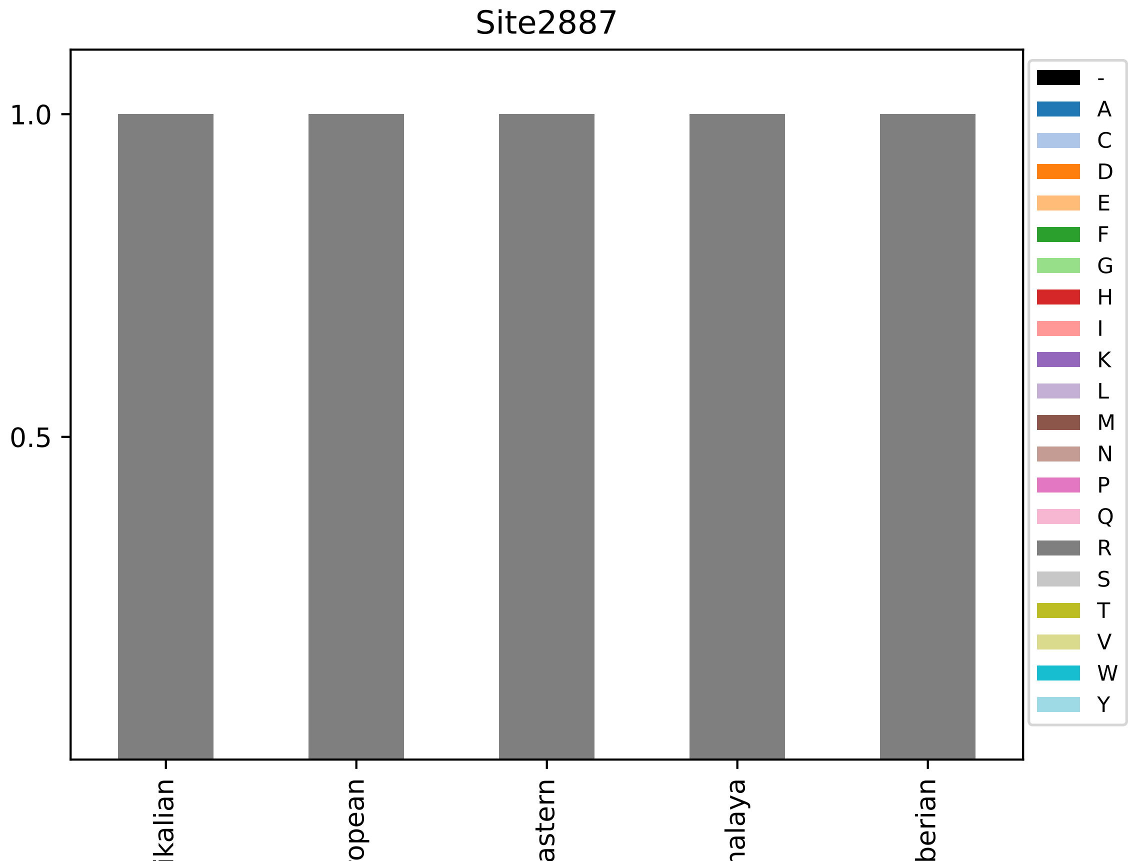
Site2887
1.0
0.5
-
A
C
D
E
F
G
H
I
K
L
M
N
P
Q
R
S
T
V
W
Y

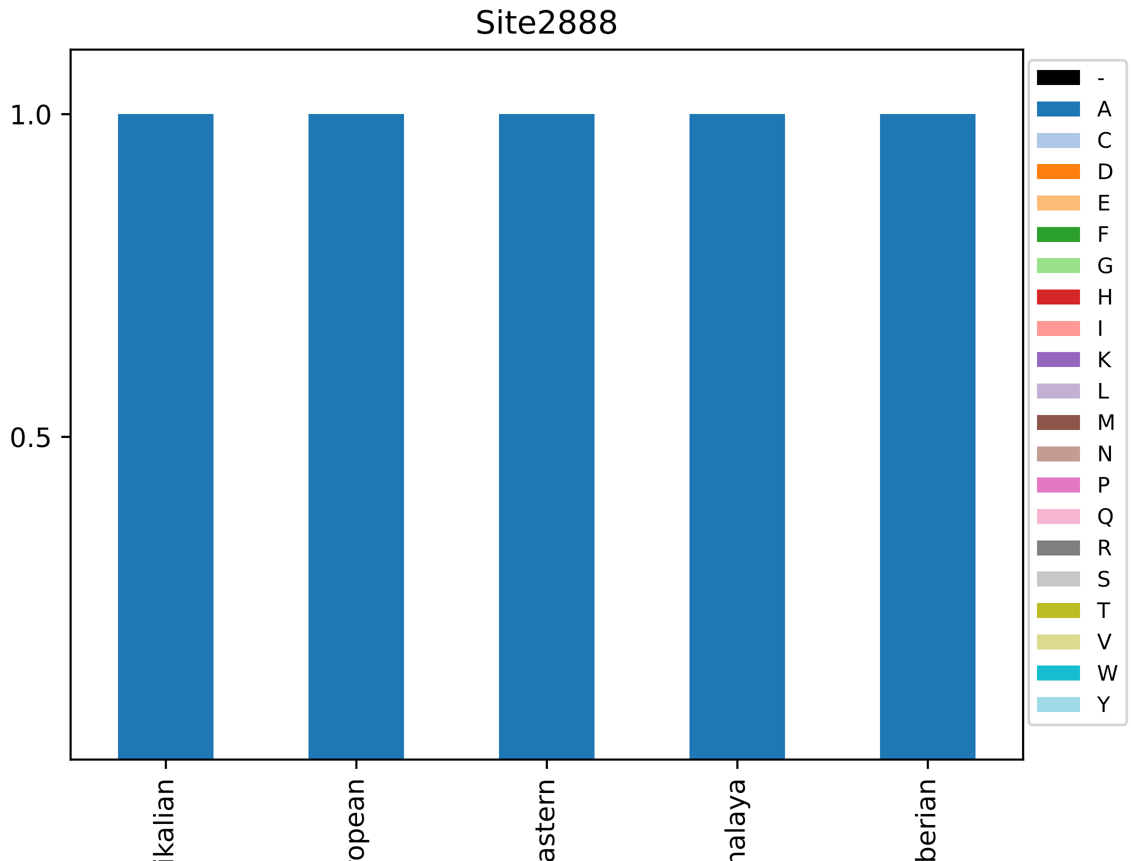
Site2888
1.0
0.5
-
A
C
D
E
F
G
H
I
K
L
M
N
P
Q
R
S
T
V
W
Y
ikalian
opean
astern
nalaya
berian

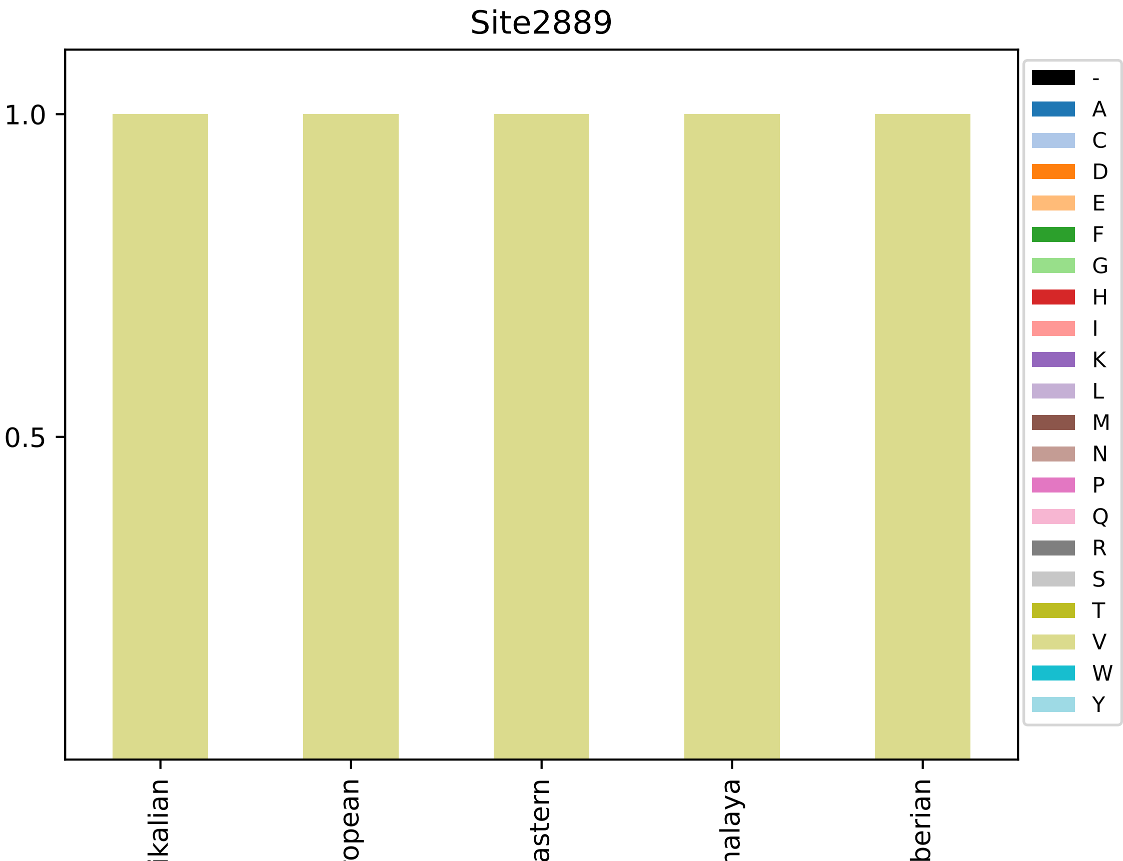
Site2889
1.0
0.5
ikalian
opean
astern
nalaya
berian
-
A
C
D
E
F
G
H
I
K
L
M
N
P
Q
R
S
T
V
W
Y

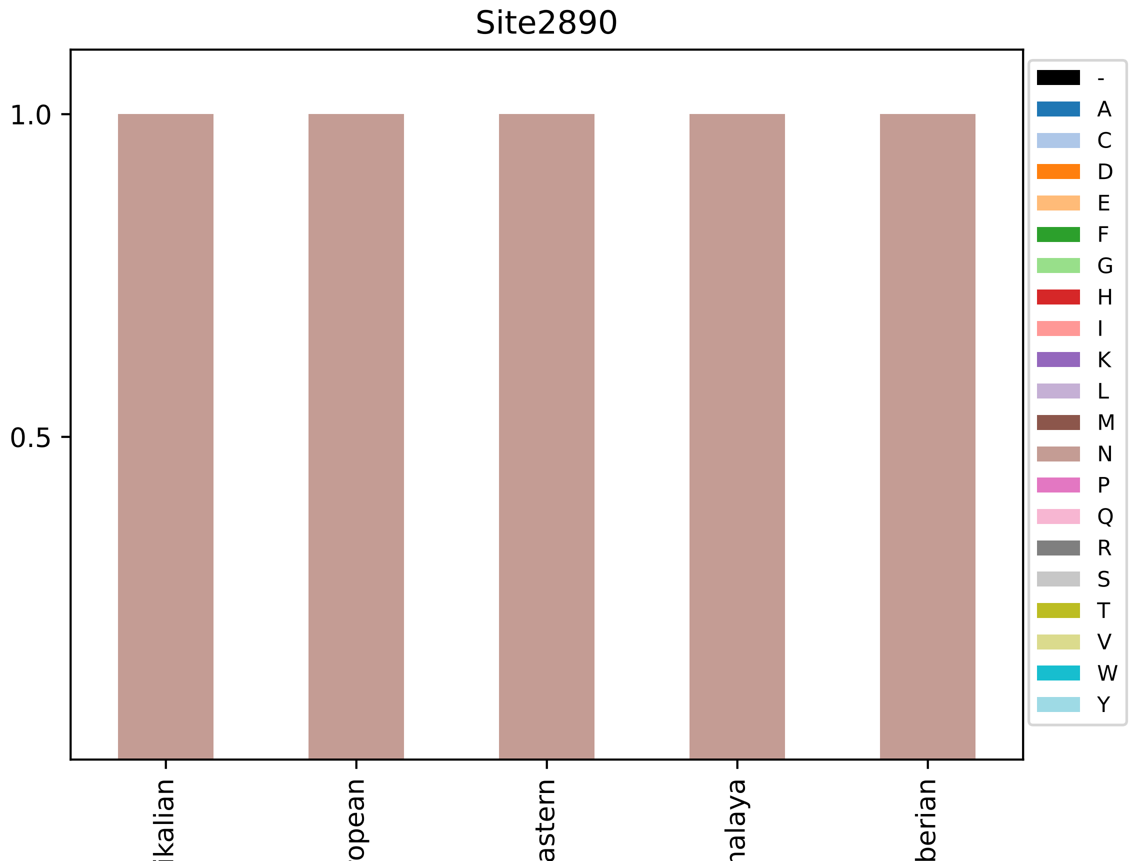

Site2890
1.0
0.5
ikalian
opean
astern
nalaya
berian
-
A
C
D
E
F
G
H
I
K
L
M
N
P
Q
R
S
T
V
W
Y

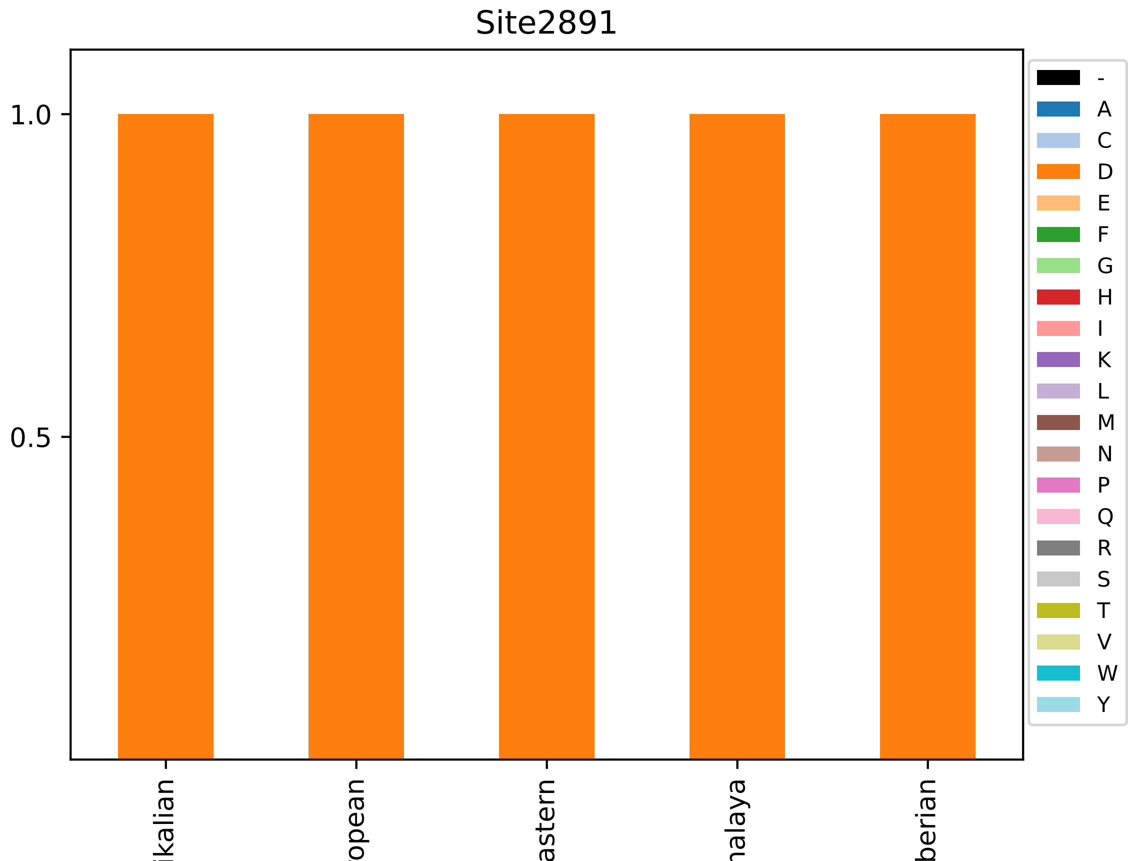

Site2891
1.0
0.5
-
A
C
D
E
F
G
H
I
K
L
M
N
P
Q
R
S
T
V
W
Y
ikalian
opean
astern
nalaya
berian

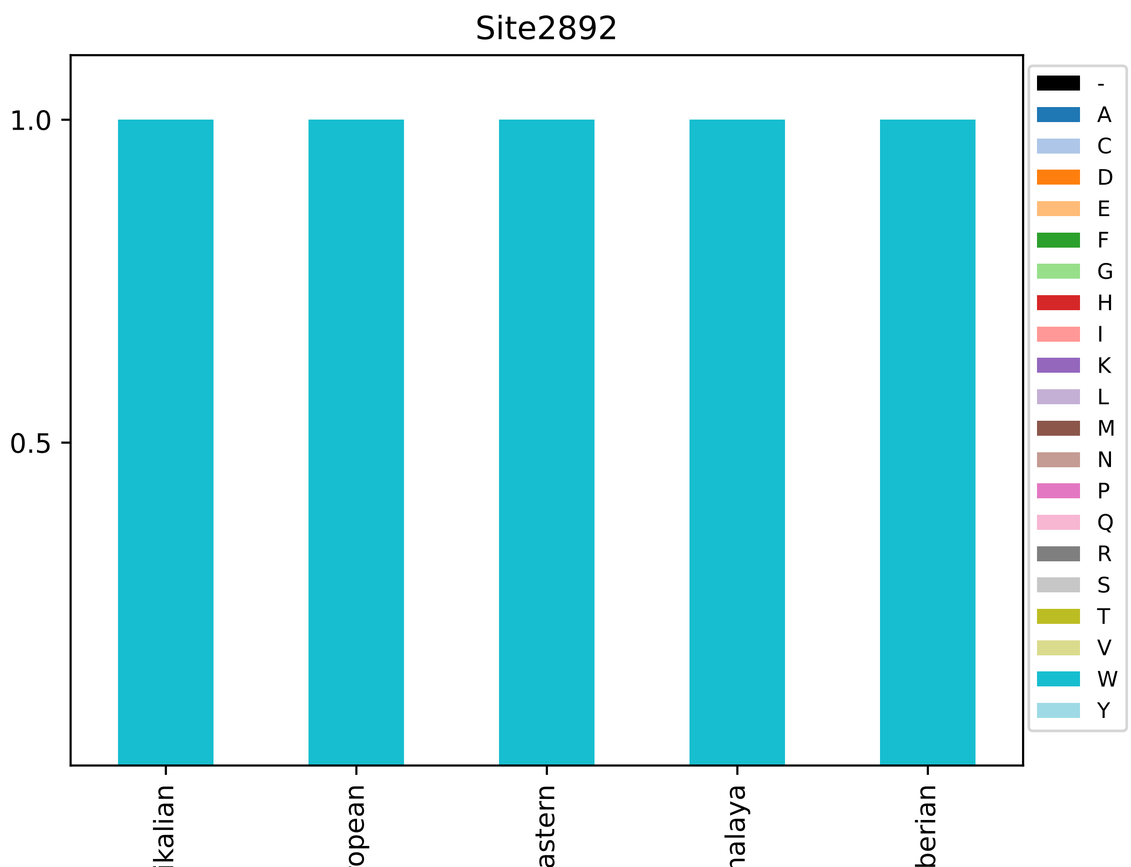
Site2892
1.0
0.5
-
A
C
D
E
F
G
H
I
K
L
M
N
P
Q
R
S
T
V
W
Y

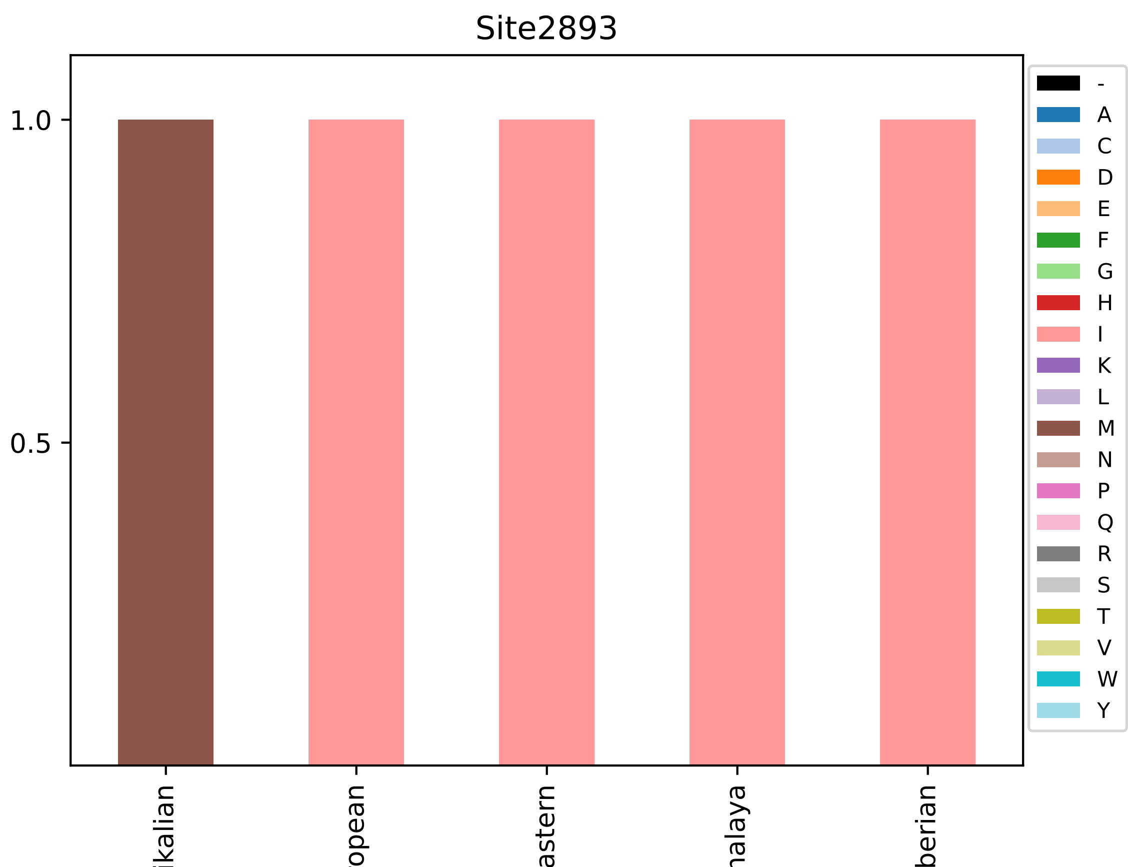
Site2893
1.0
0.5
-
A
C
D
E
F
G
H
I
K
L
M
N
P
Q
R
S
T
V
W
Y

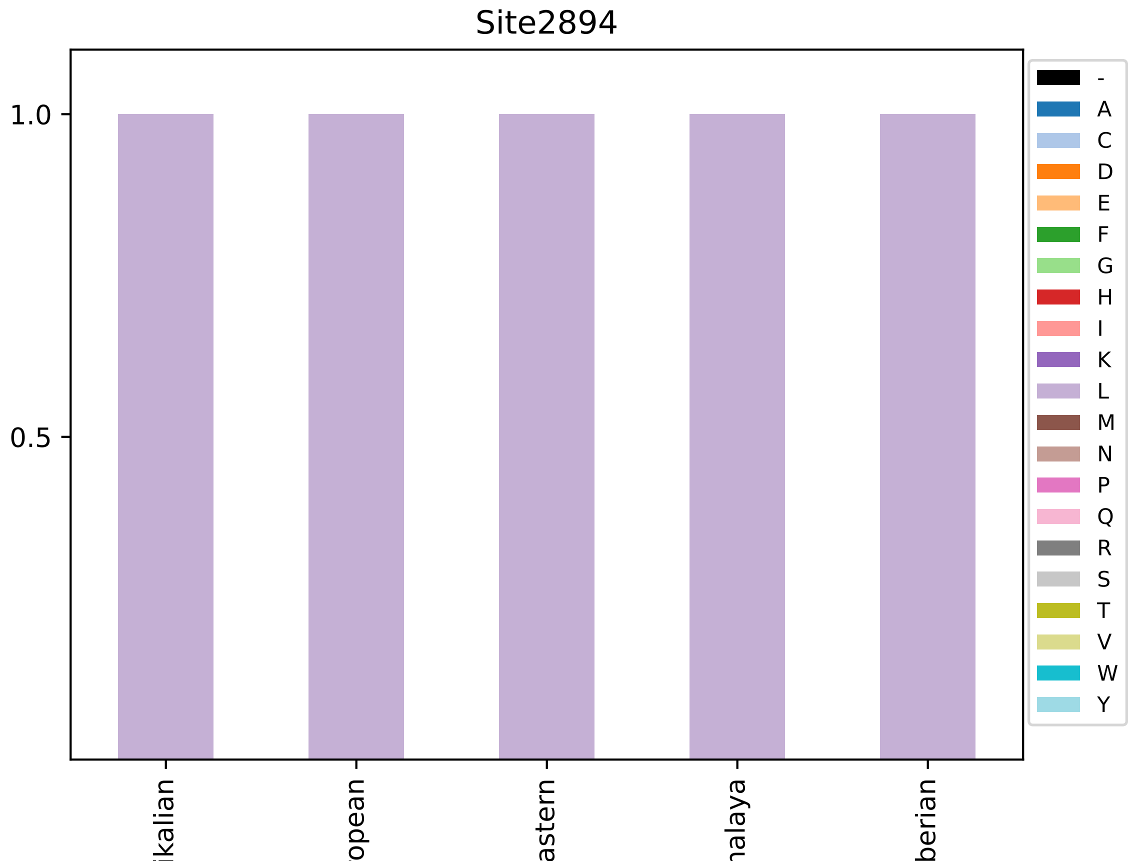

Site2894
1.0
0.5
-
A
C
D
E
F
G
H
I
K
L
M
N
P
Q
R
S
T
V
W
Y
ikalian
opean
astern
nalaya
berian

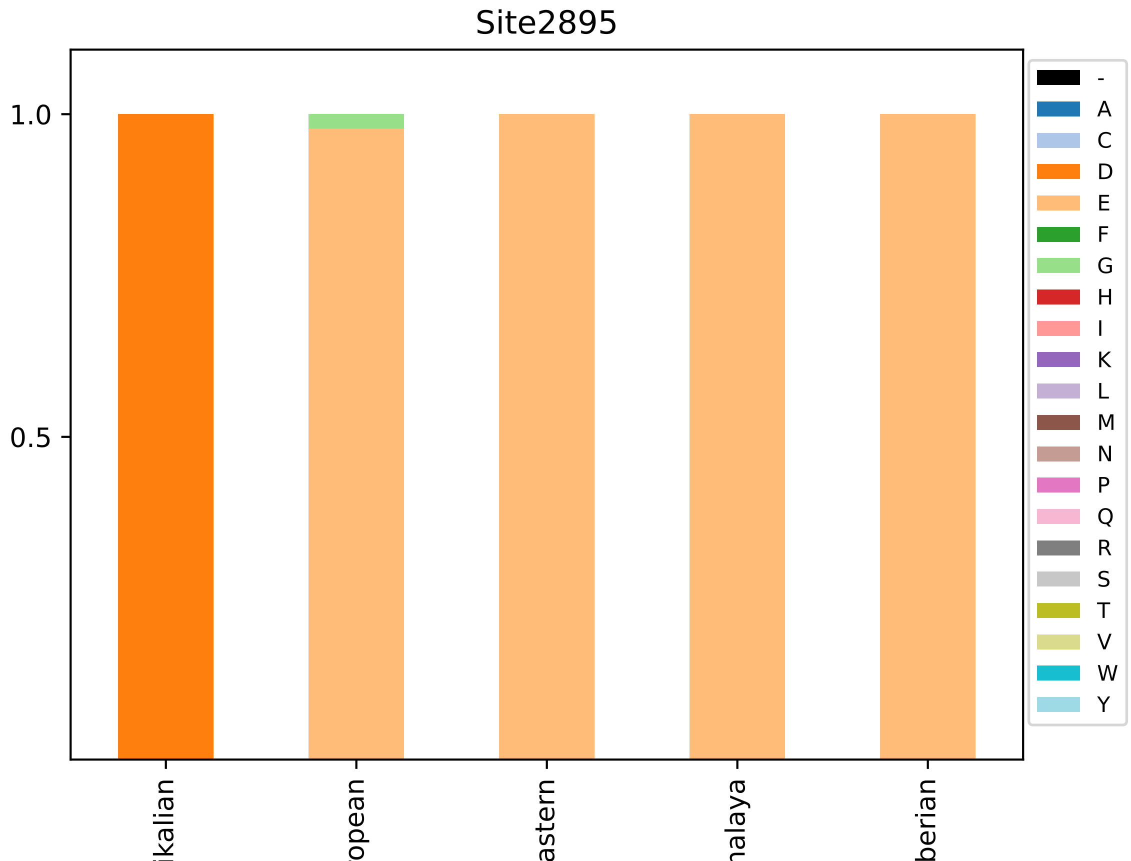

Site2895
1.0
0.5
ikalian
opean
astern
nalaya
berian
-
A
C
D
E
F
G
H
I
K
L
M
N
P
Q
R
S
T
V
W
Y

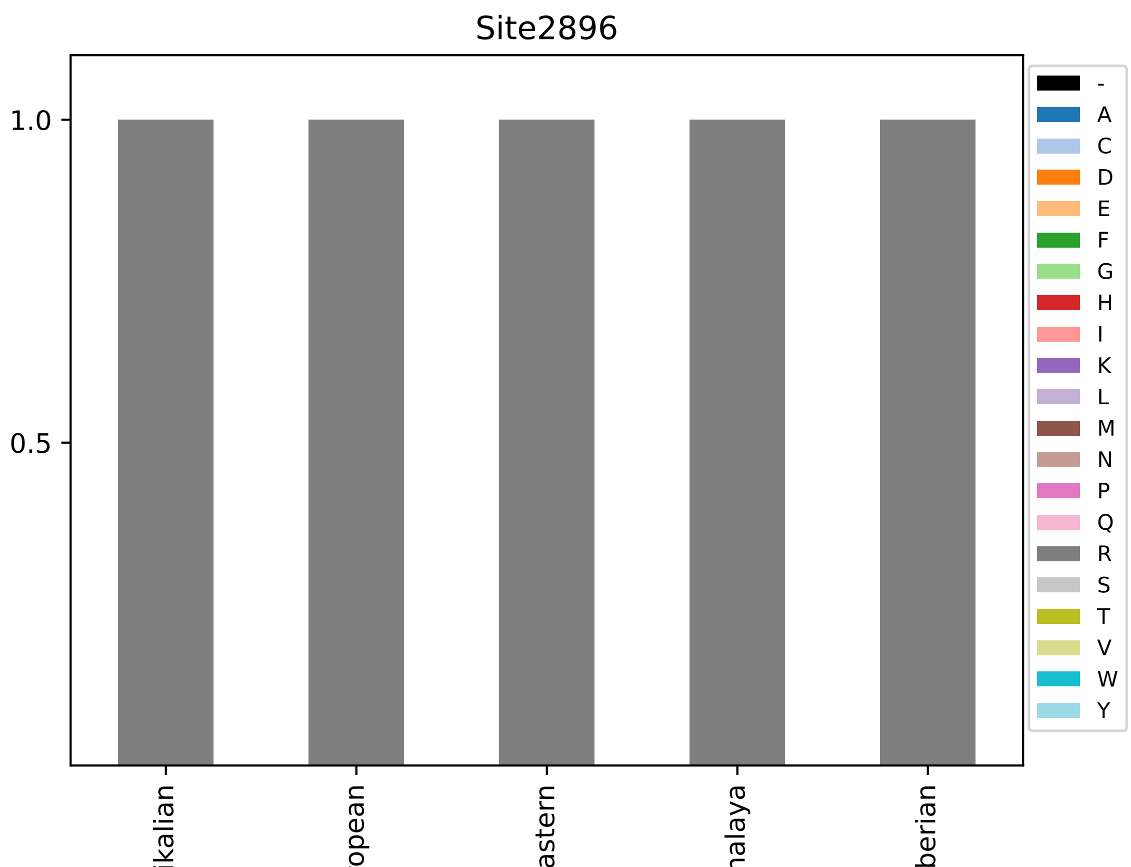

Site2896
1.0
0.5
-
A
C
D
E
F
G
H
I
K
L
M
N
P
Q
R
S
T
V
W
Y
ikalian
opean
astern
nalaya
berian

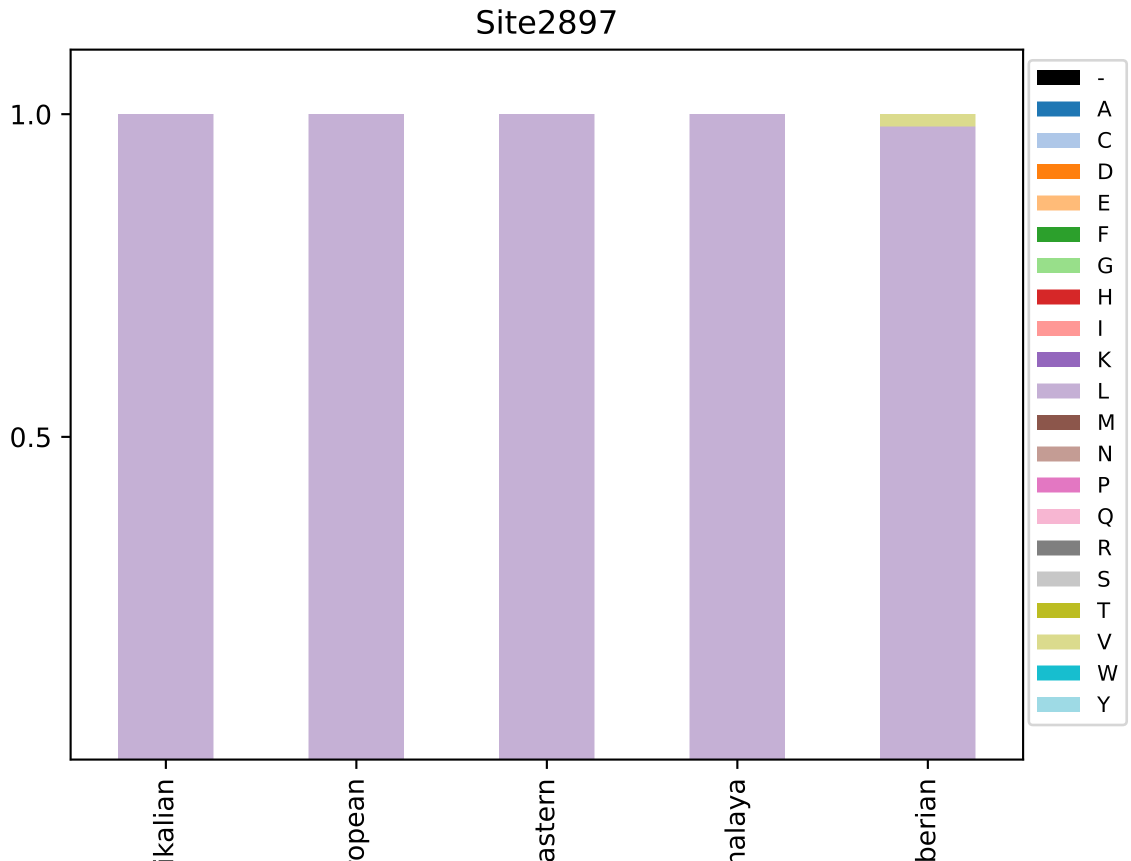
Site2897
1.0
0.5
ikalian
opean
astern
nalaya
berian
-
A
C
D
E
F
G
H
I
K
L
M
N
P
Q
R
S
T
V
W
Y

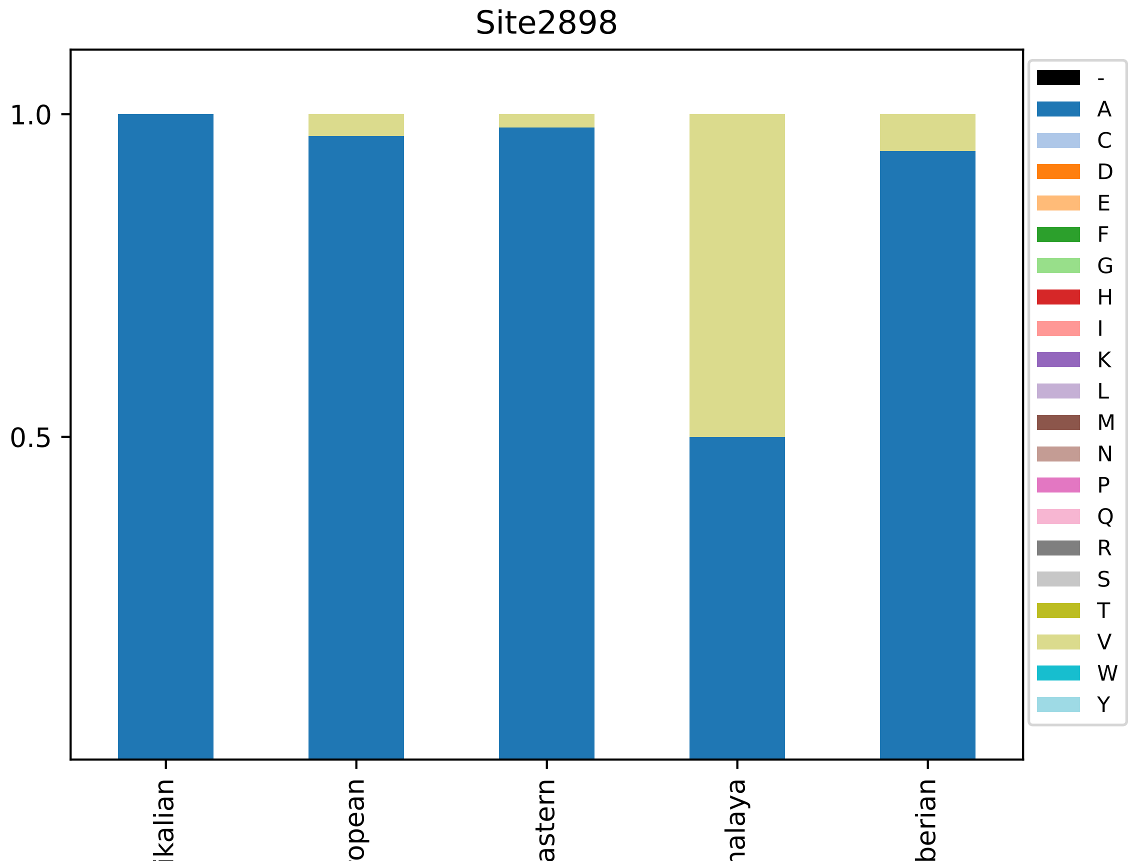
Site2898
1.0
0.5
-
A
C
D
E
F
G
H
I
K
L
M
N
P
Q
R
S
T
V
W
Y
ikalian
opean
astern
nalaya
berian

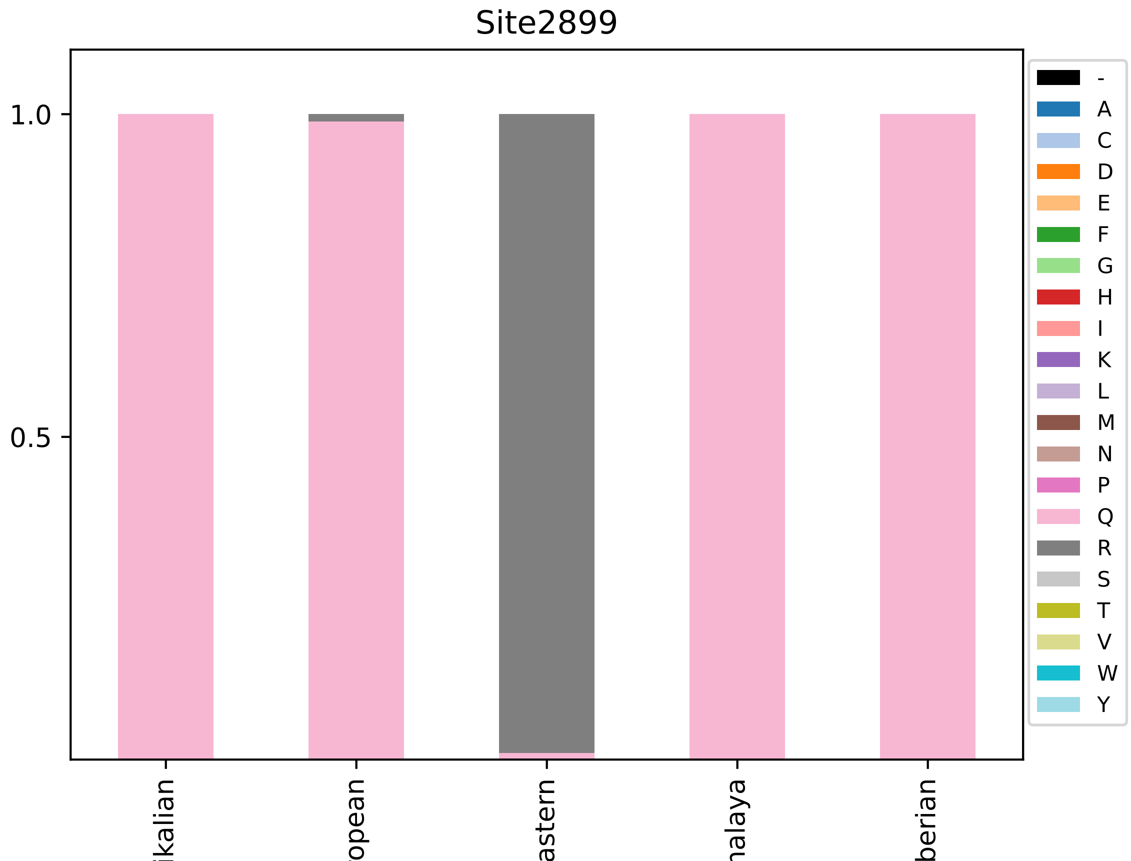
Site2899
1.0
0.5
ikalian
opean
astern
nalaya
berian
-
A
C
D
E
F
G
H
I
K
L
M
N
P
Q
R
S
T
V
W
Y

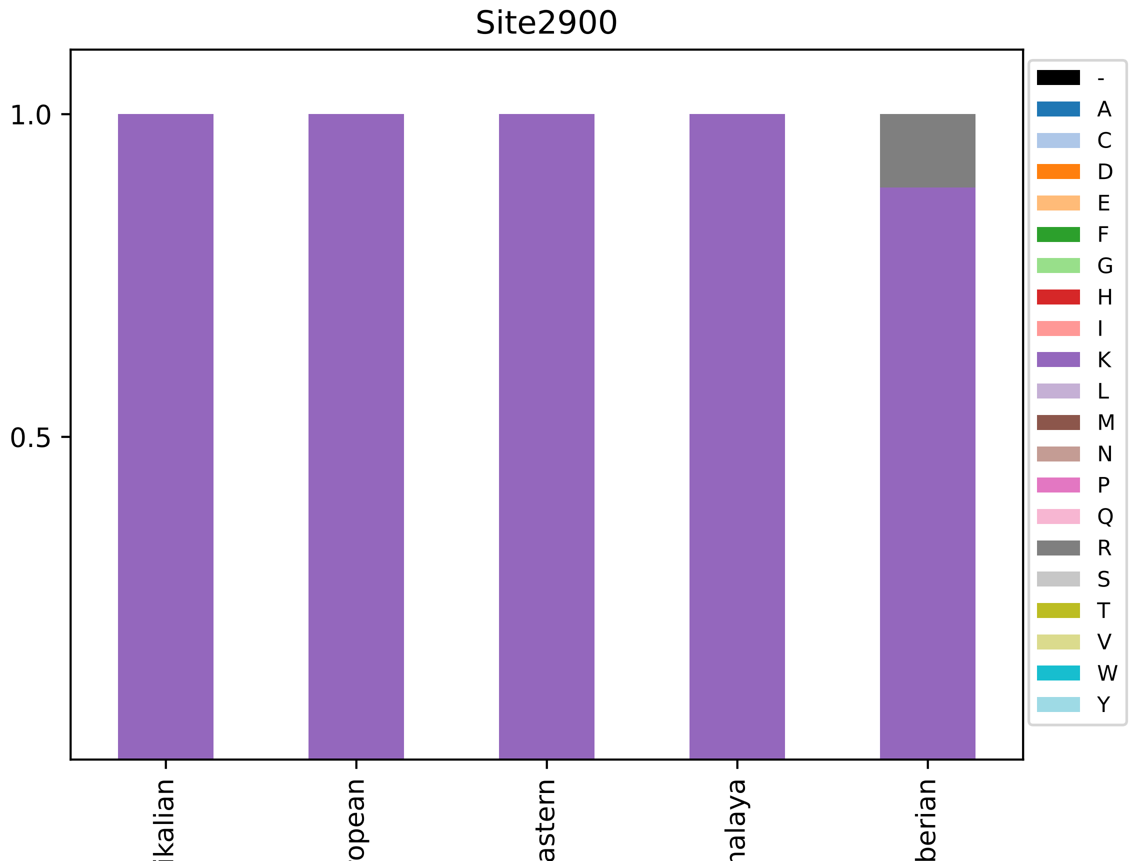
Site2900
1.0
0.5
-
A
C
D
E
F
G
H
I
K
L
M
N
P
Q
R
S
T
V
W
Y

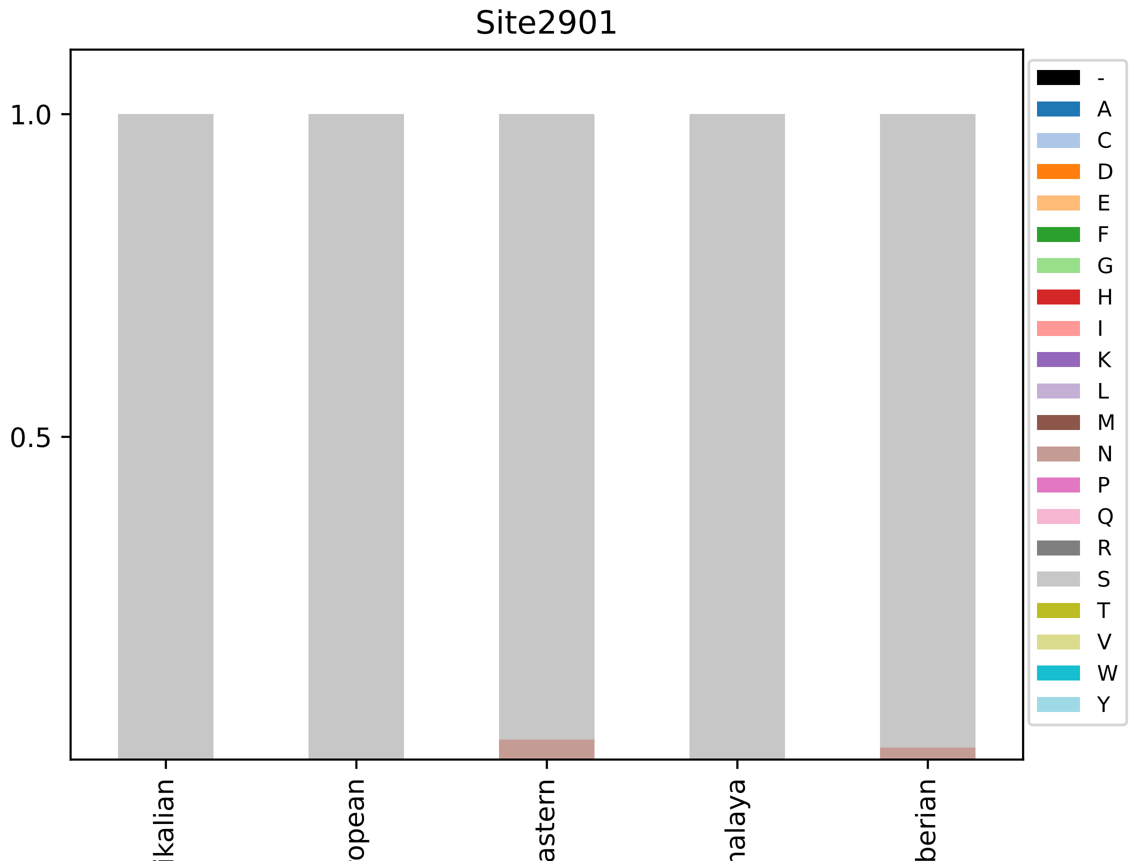
Site2901
1.0
0.5
-
A
C
D
E
F
G
H
I
K
L
M
N
P
Q
R
S
T
V
W
Y
ikalian
opean
astern
nalaya
berian

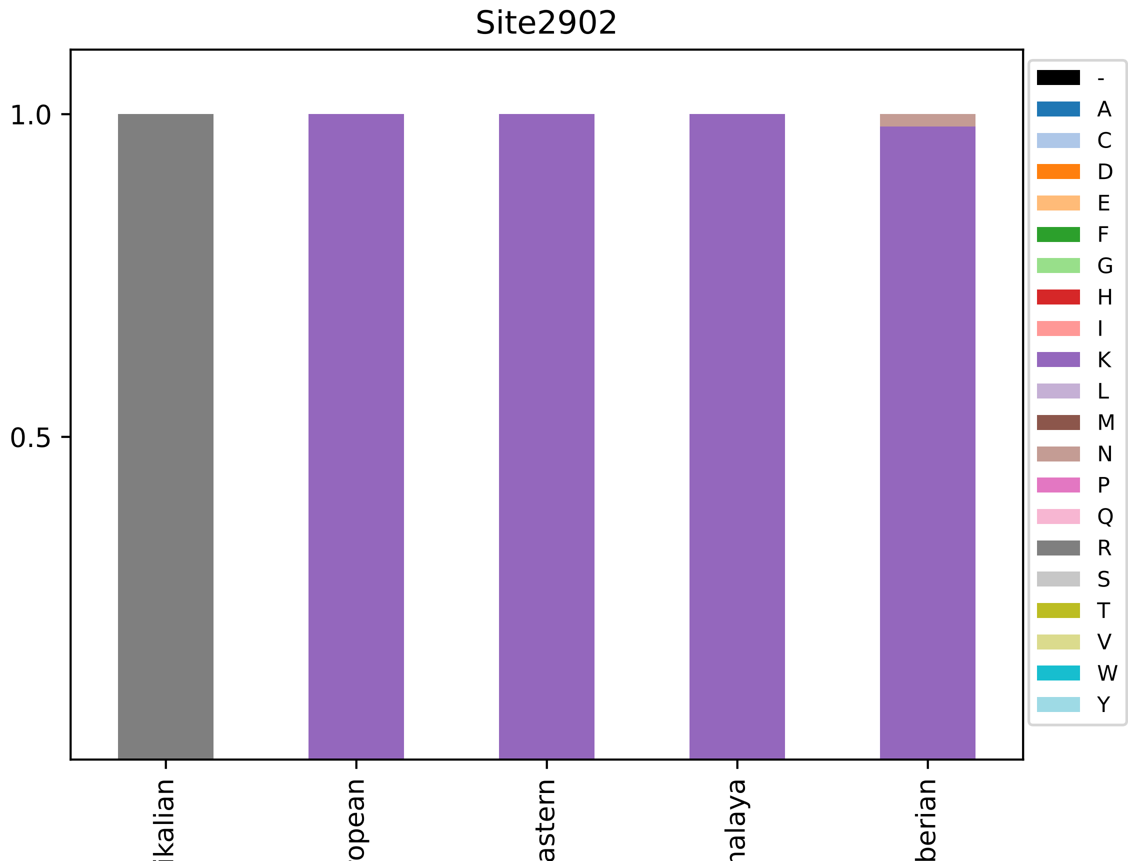

Site2902
1.0
0.5
-
A
C
D
E
F
G
H
I
K
L
M
N
P
Q
R
S
T
V
W
Y
ikalian
opean
astern
nalaya
berian

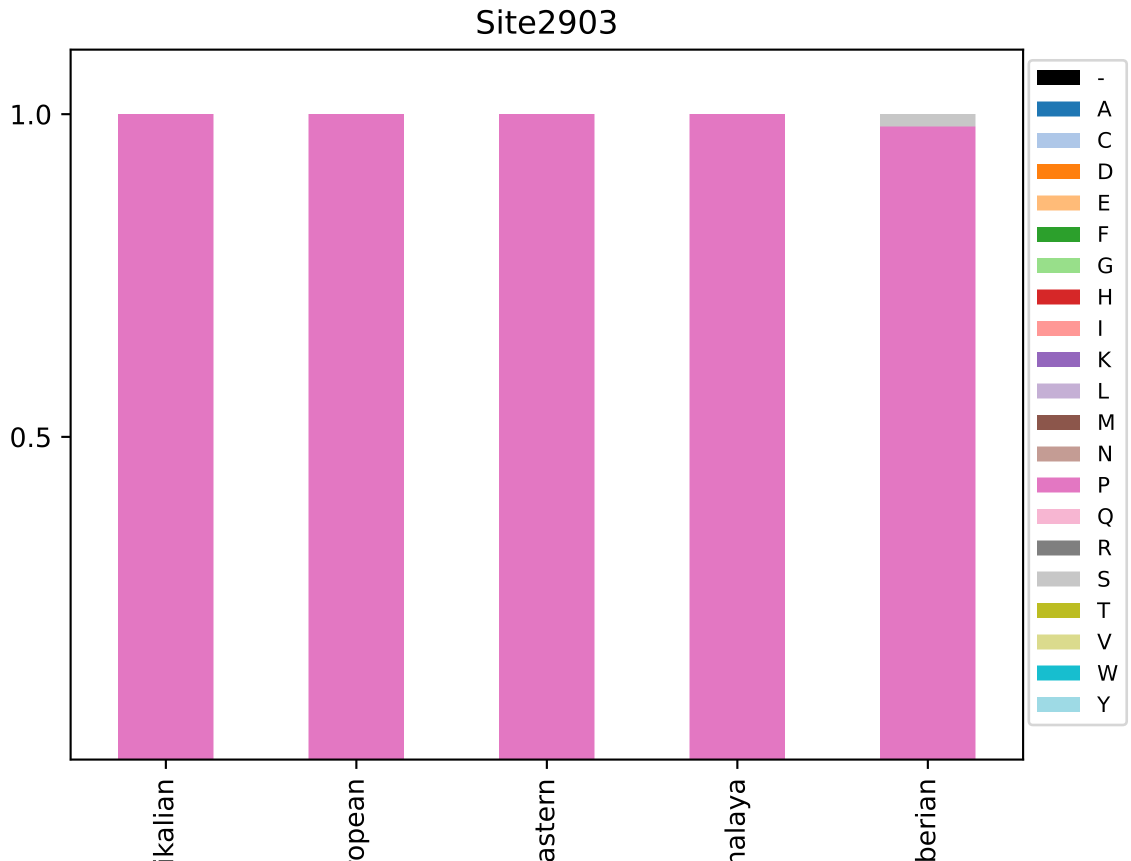

Site2903
1.0
0.5
-
A
C
D
E
F
G
H
I
K
L
M
N
P
Q
R
S
T
V
W
Y
ikalian
opean
astern
nalaya
berian

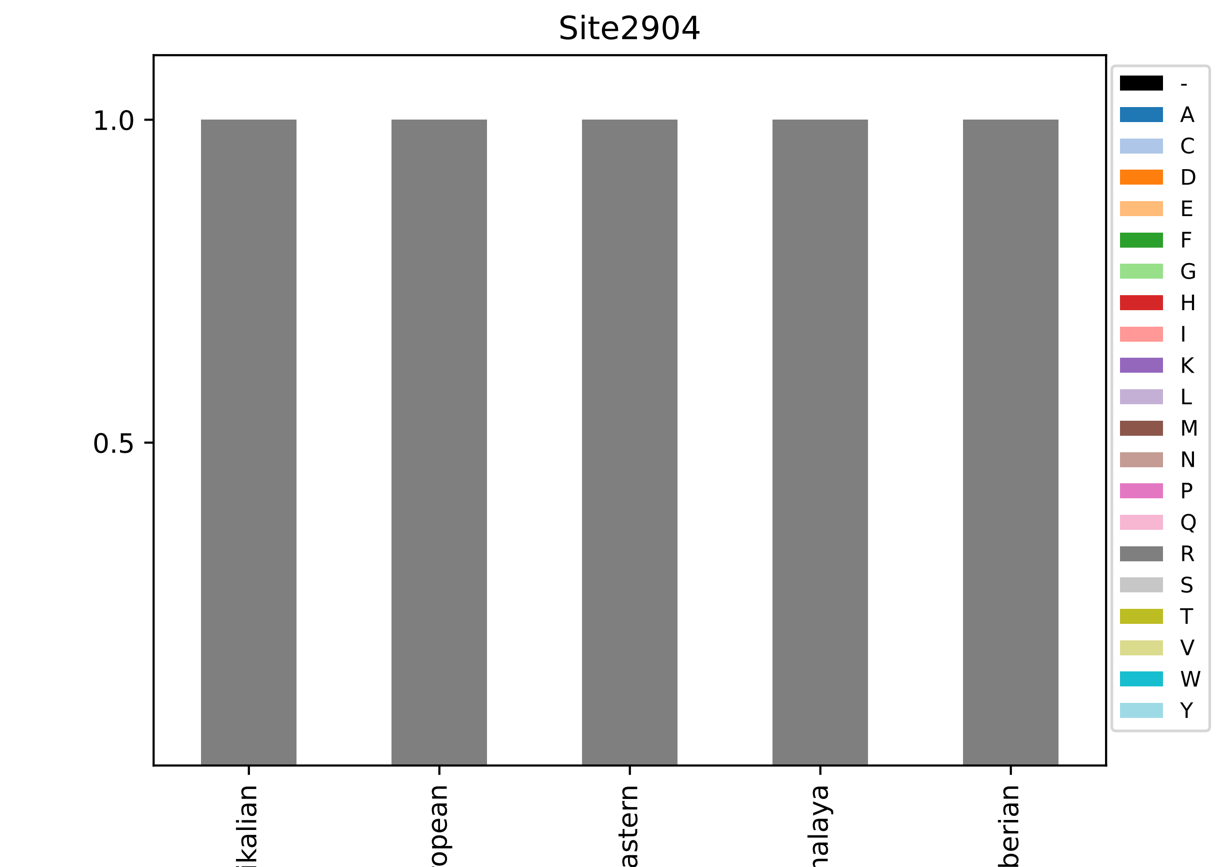
Site2904
1.0
0.5
ikalian
opean
astern
nalaya
berian
-
A
C
D
E
F
G
H
I
K
L
M
N
P
Q
R
S
T
V
W
Y

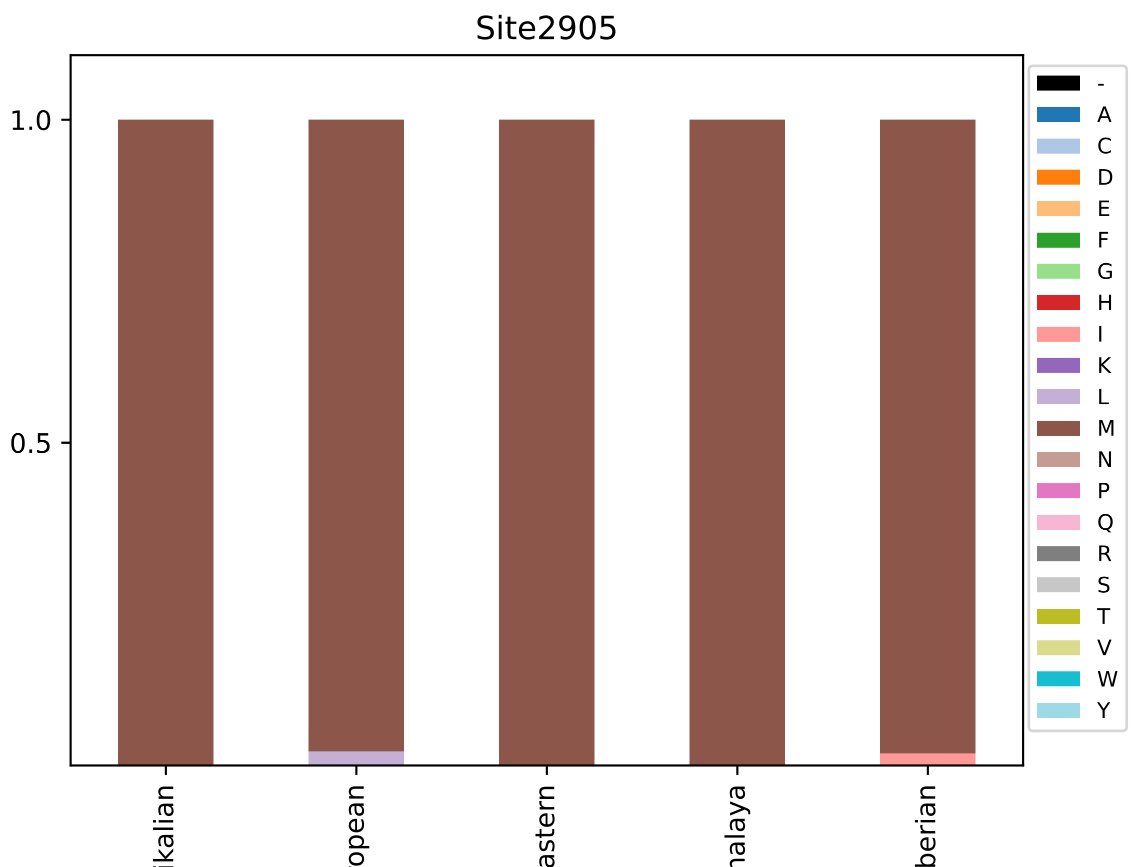

Site2905
1.0
0.5
-
A
C
D
E
F
G
H
I
K
L
M
N
P
Q
R
S
T
V
W
Y
ikalian
opean
astern
nalaya
berian

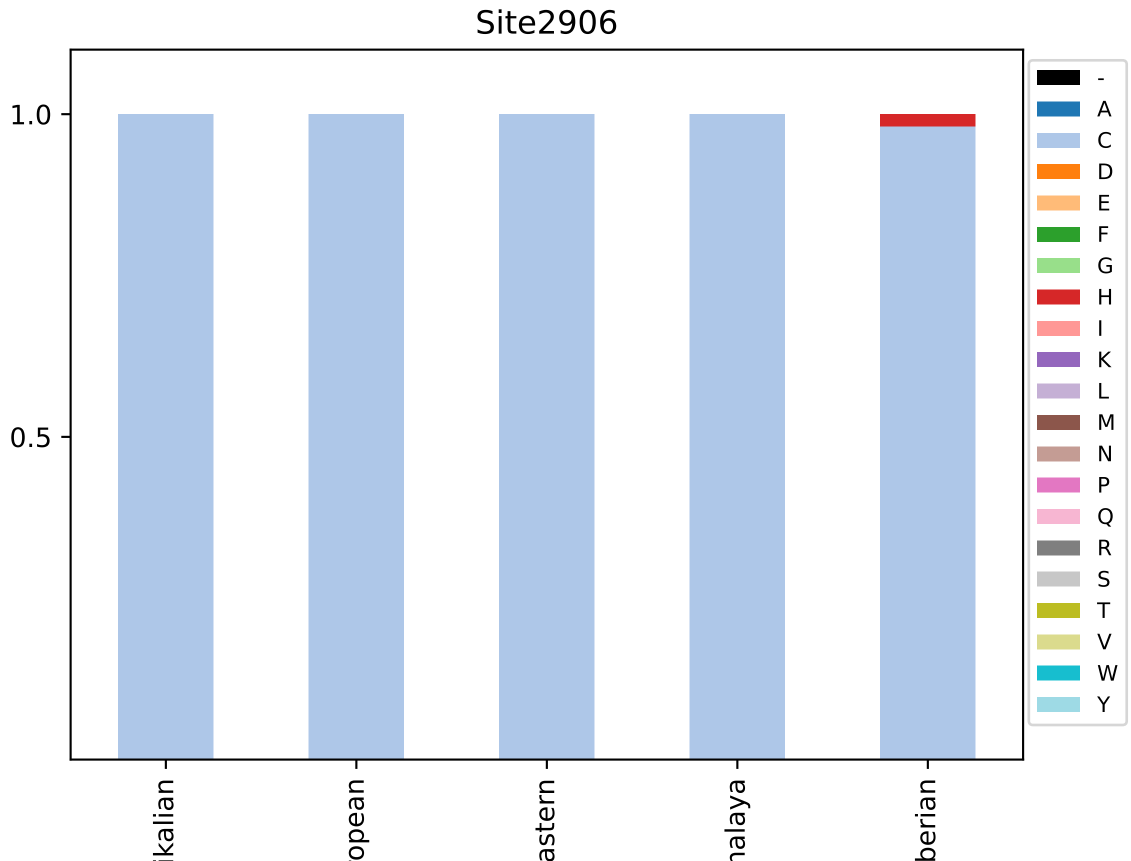

Site2906
1.0
0.5
-
A
C
D
E
F
G
H
I
K
L
M
N
P
Q
R
S
T
V
W
Y
ikalian
opean
astern
nalaya
berian

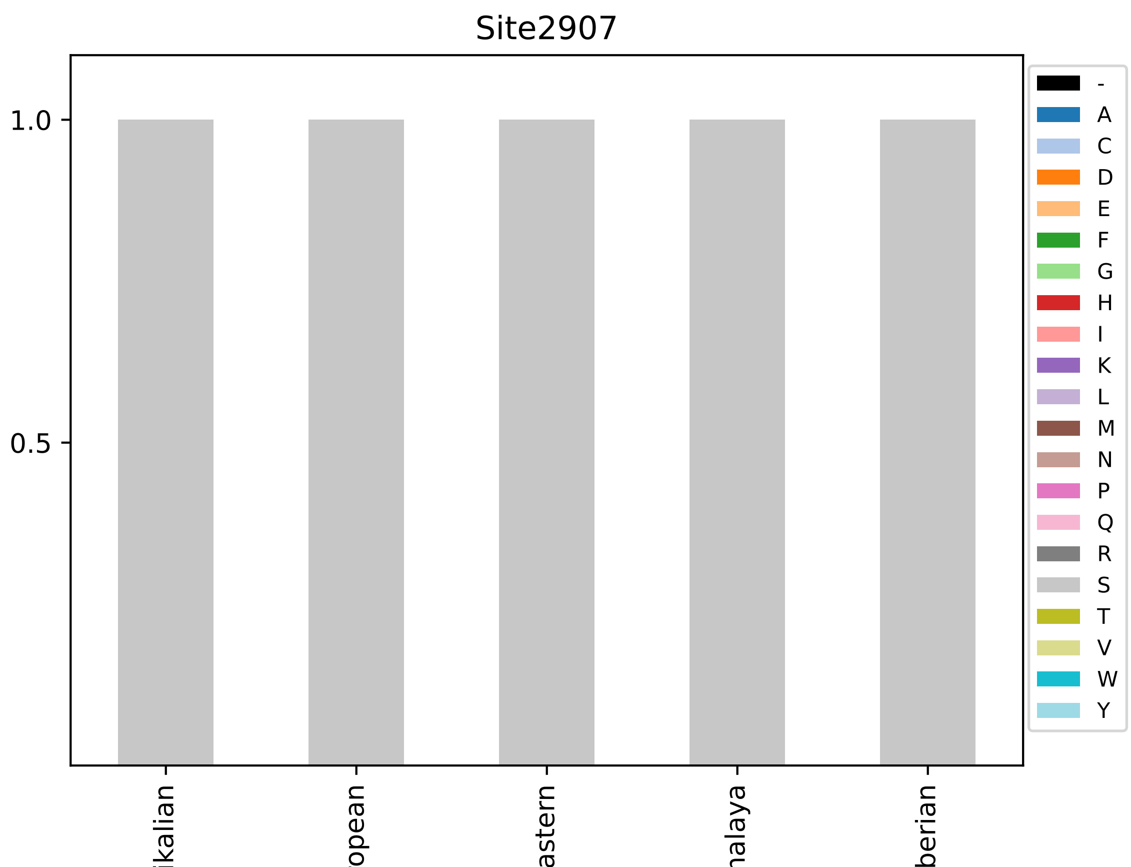

Site2907
1.0
0.5
-
A
C
D
E
F
G
H
I
K
L
M
N
P
Q
R
S
T
V
W
Y
ikalian
opean
astern
nalaya
berian

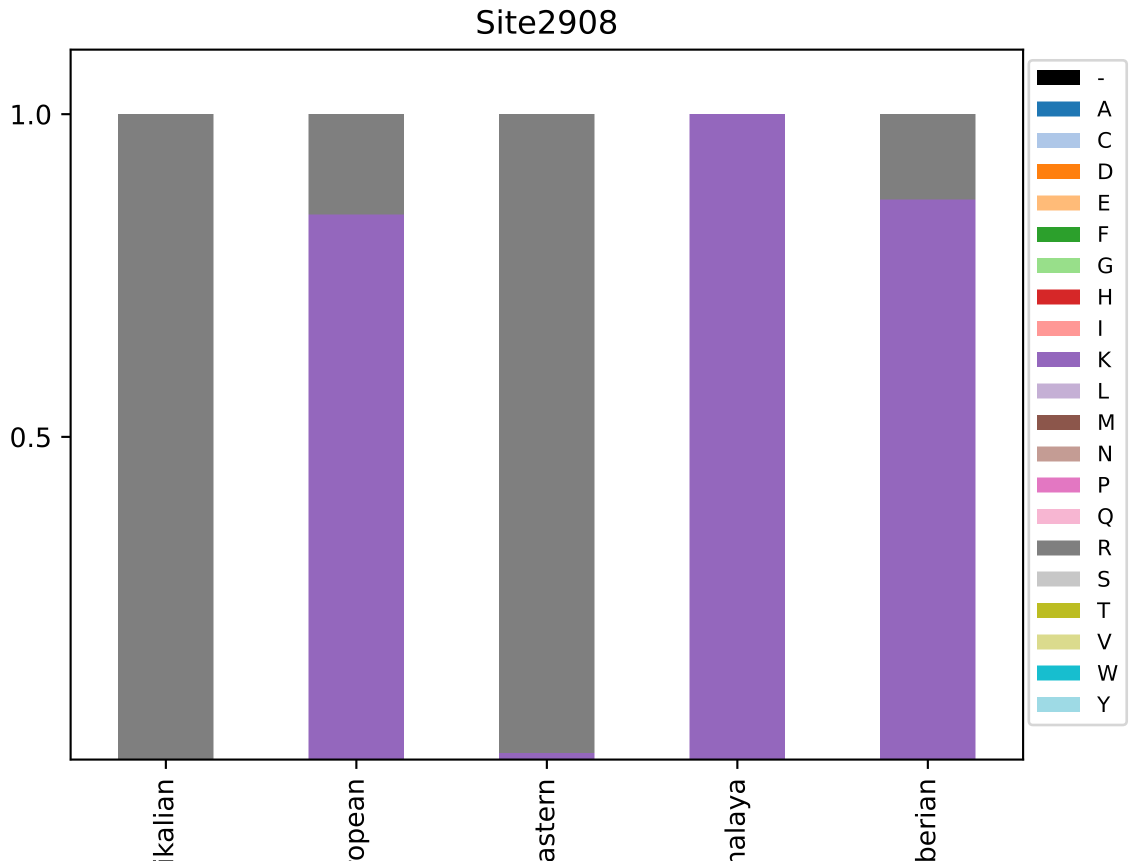

Site2908
1.0
0.5
-
A
C
D
E
F
G
H
I
K
L
M
N
P
Q
R
S
T
V
W
Y
ikalian
opean
astern
nalaya
berian

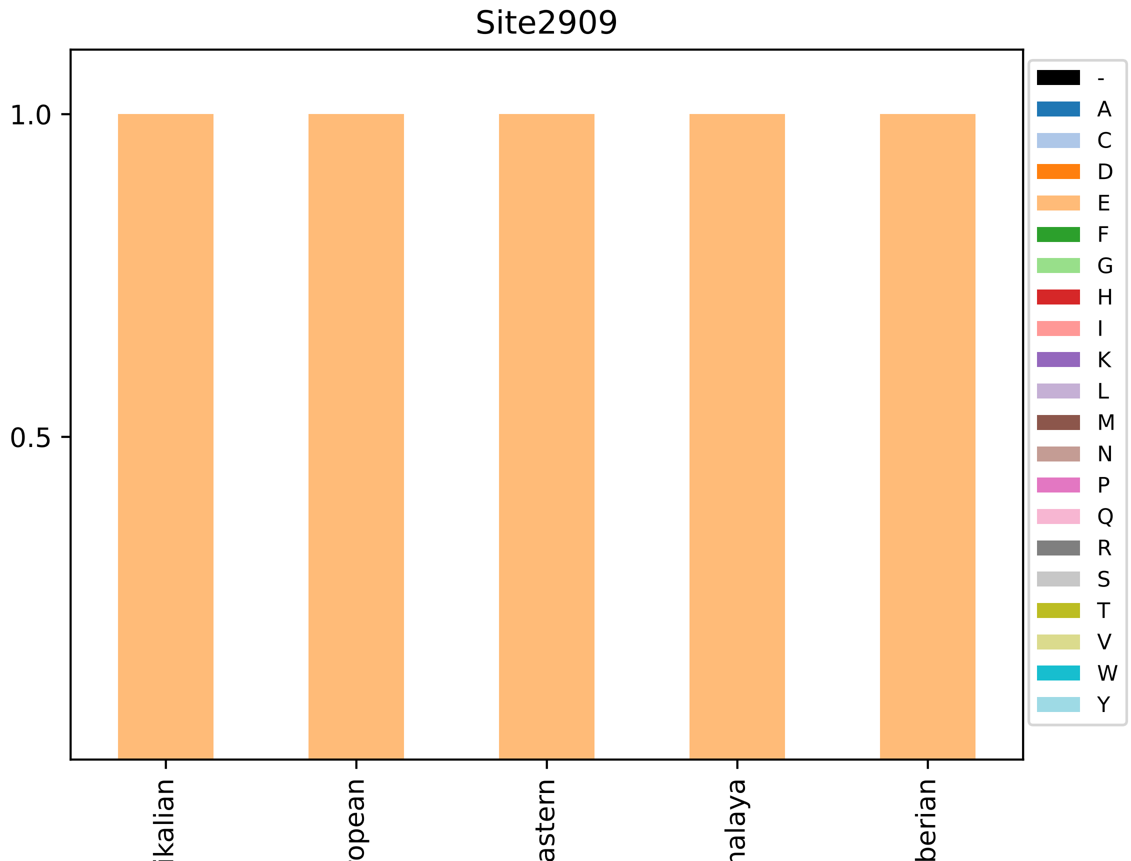

Site2909
1.0
0.5
-
A
C
D
E
F
G
H
I
K
L
M
N
P
Q
R
S
T
V
W
Y
ikalian
opean
astern
nalaya
berian

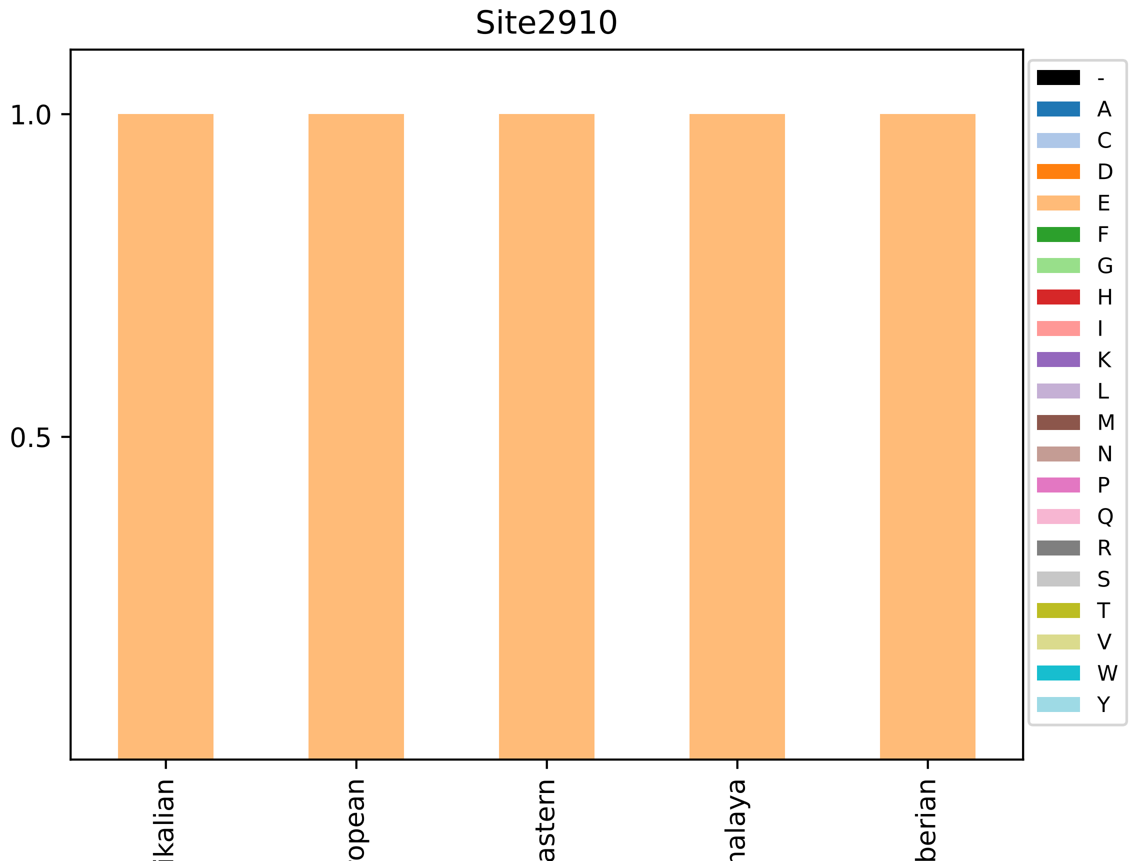
Site2910
1.0
0.5
-
A
C
D
E
F
G
H
I
K
L
M
N
P
Q
R
S
T
V
W
Y
ikalian
opean
astern
nalaya
berian

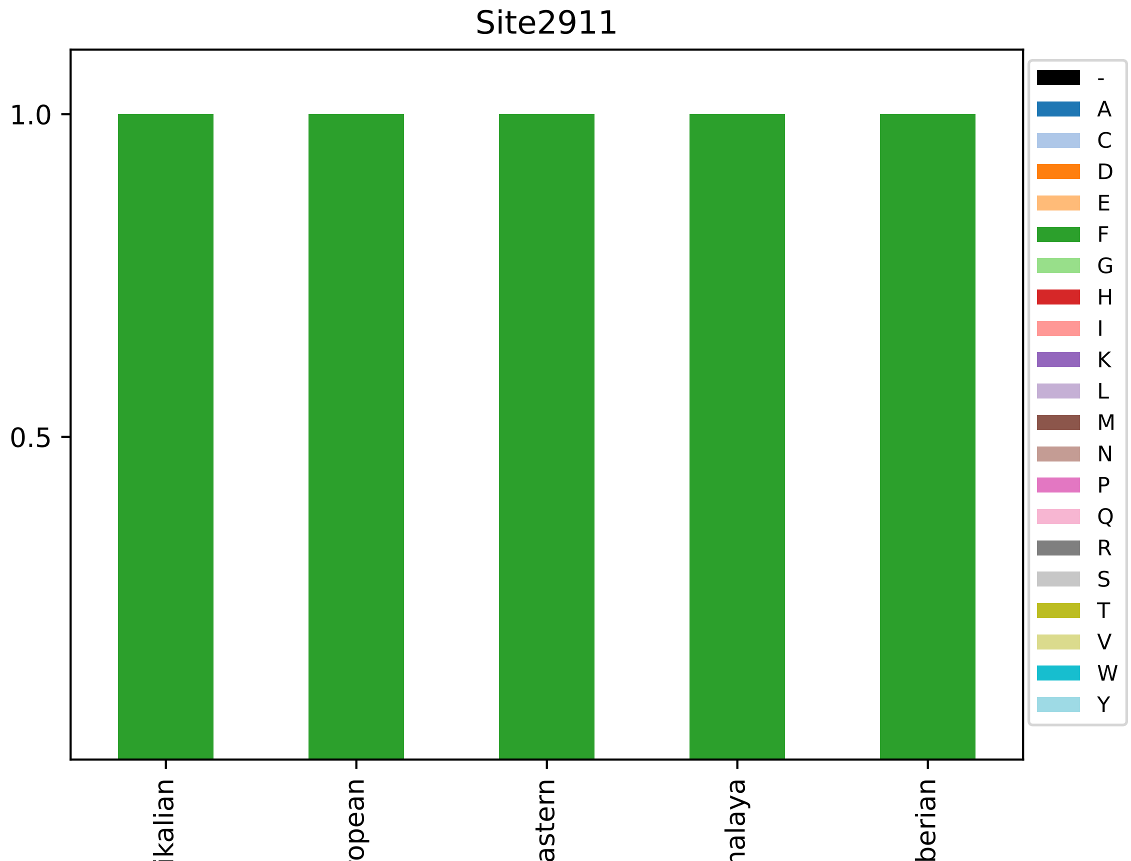
Site2911
1.0
0.5
-
A
C
D
E
F
G
H
I
K
L
M
N
P
Q
R
S
T
V
W
Y
ikalian
opean
astern
nalaya
berian

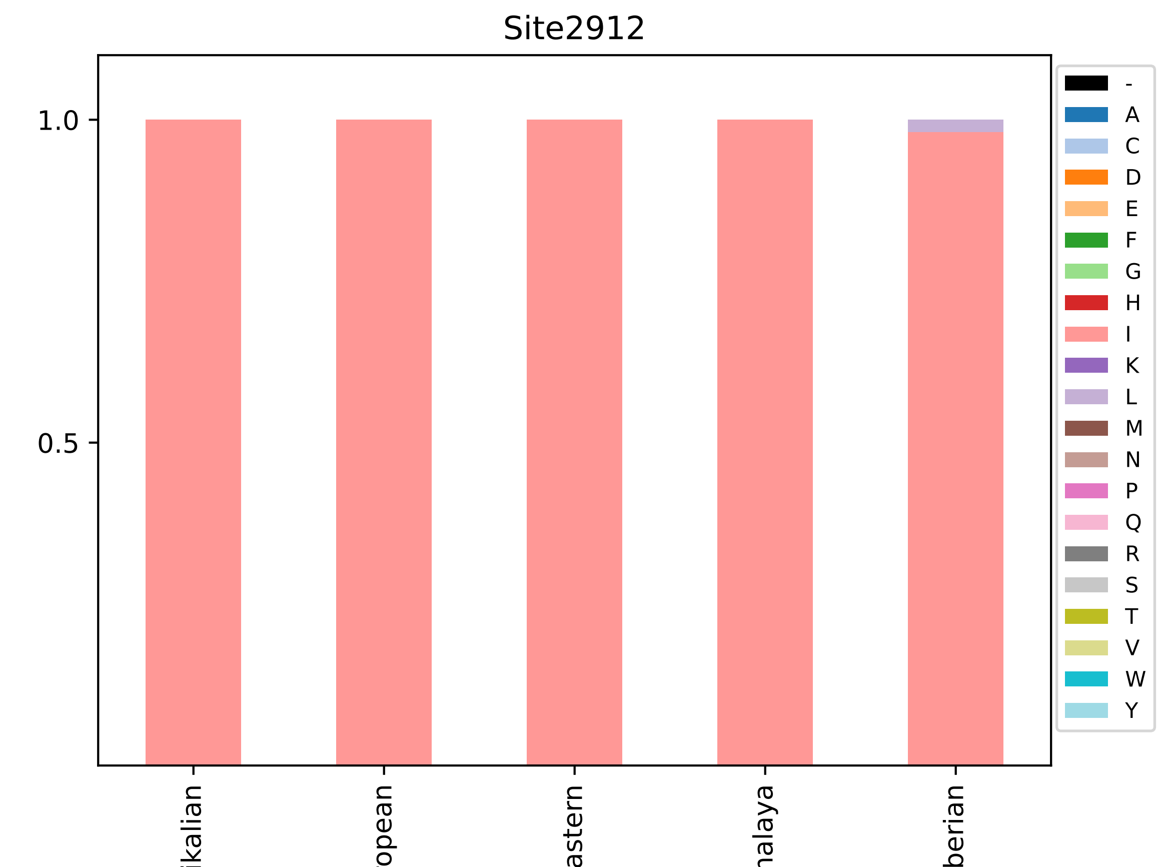

Site2912
1.0
0.5
-
A
C
D
E
F
G
H
I
K
L
M
N
P
Q
R
S
T
V
W
Y
ikalian
opean
astern
nalaya
berian

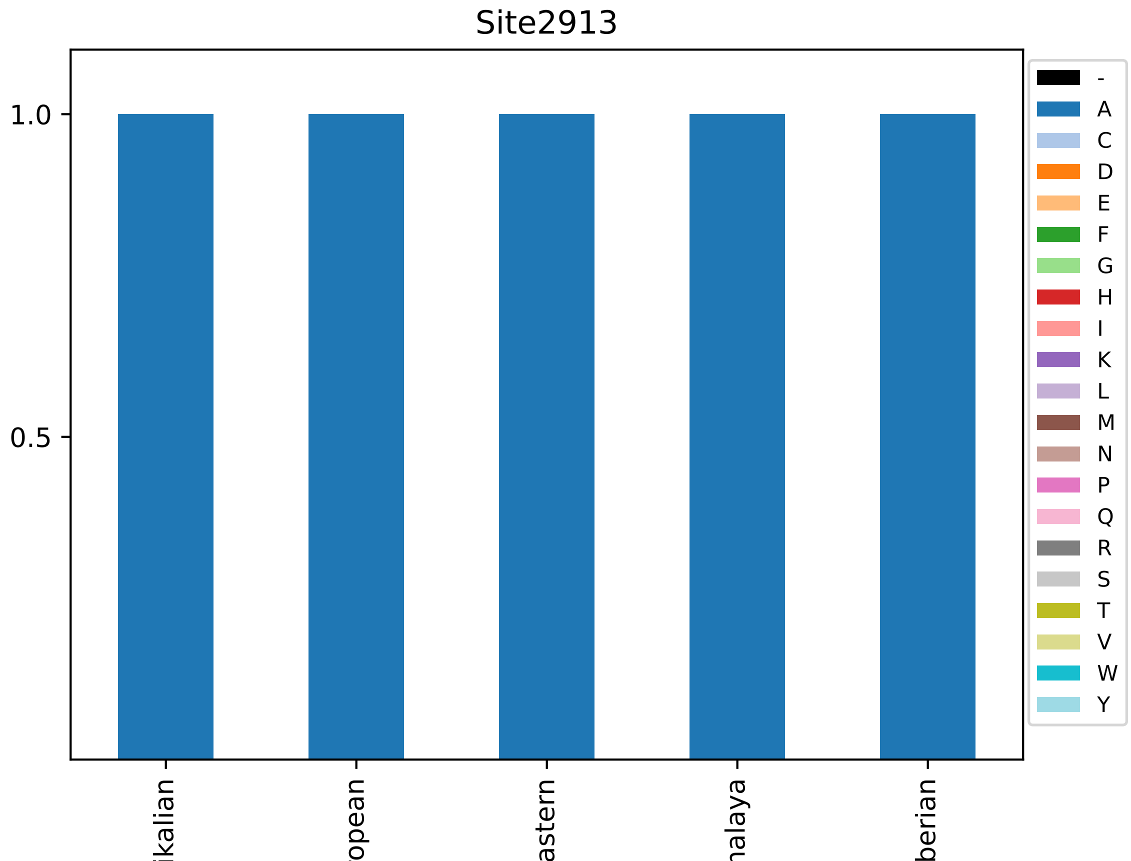
Site2913
1.0
0.5
-
A
C
D
E
F
G
H
I
K
L
M
N
P
Q
R
S
T
V
W
Y
ikalian
opean
astern
nalaya
berian

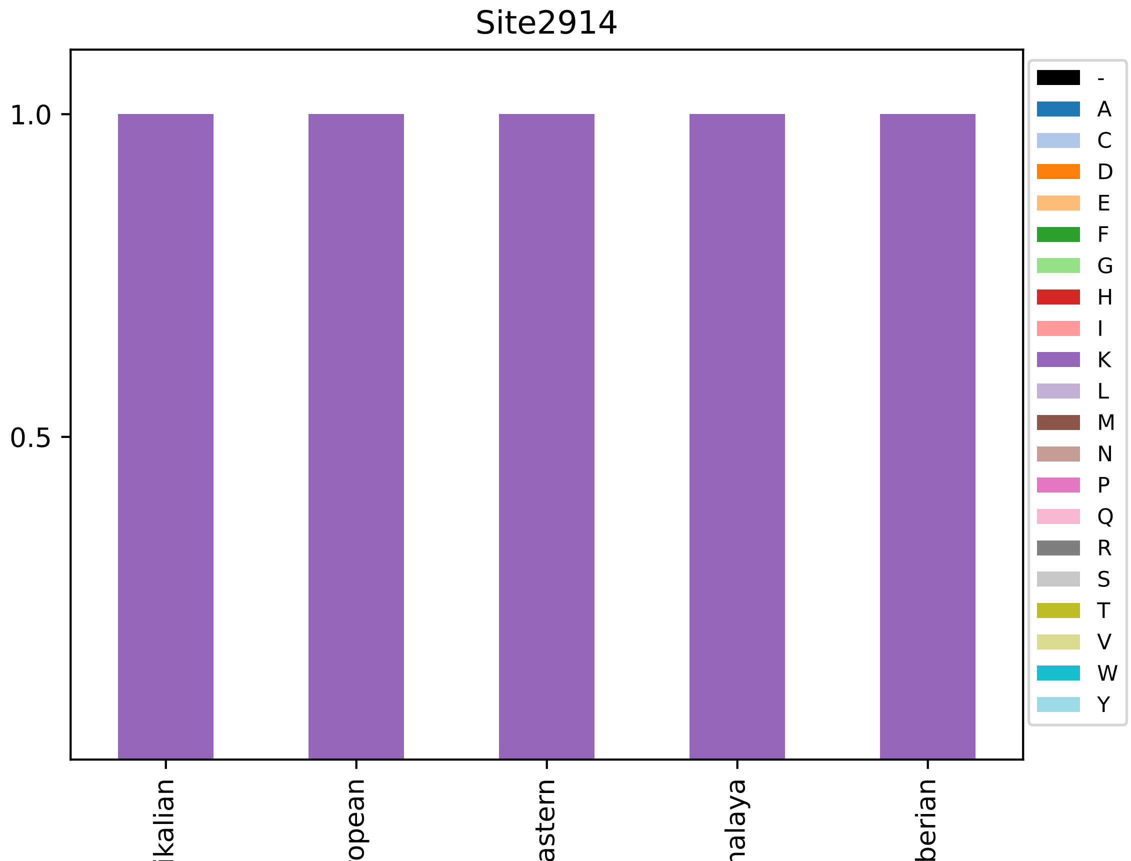

Site2914
1.0
0.5
-
A
C
D
E
F
G
H
I
K
L
M
N
P
Q
R
S
T
V
W
Y
ikalian
opean
astern
nalaya
berian

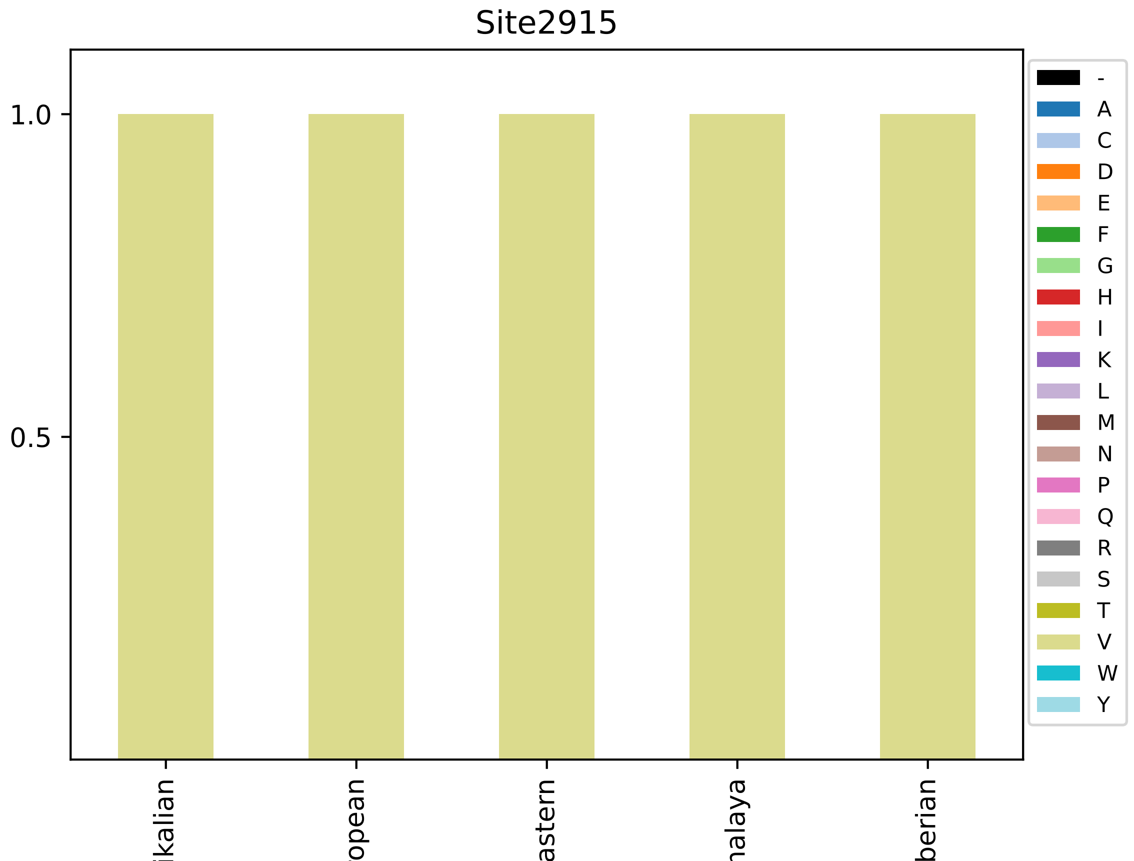
Site2915
1.0
0.5
-
A
C
D
E
F
G
H
I
K
L
M
N
P
Q
R
S
T
V
W
Y

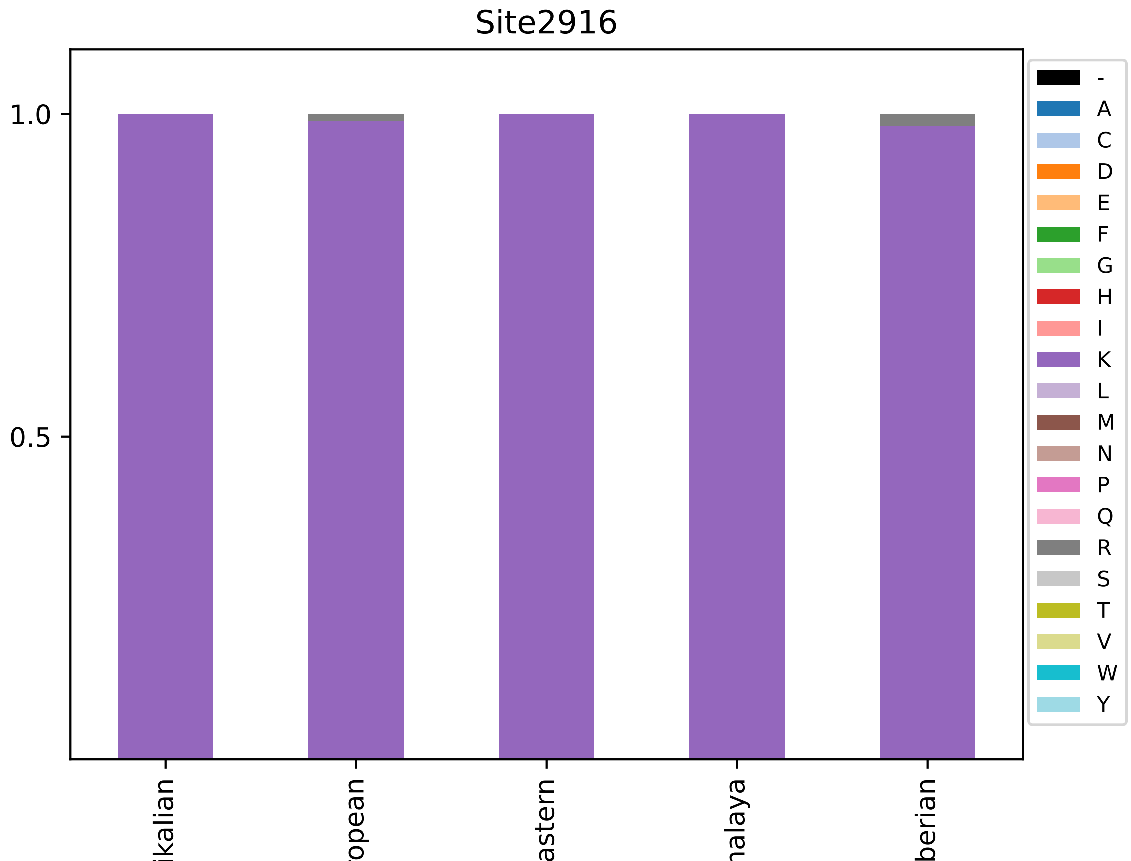
Site2916
1.0
0.5
ikalian
opean
astern
nalaya
berian
-
A
C
D
E
F
G
H
I
K
L
M
N
P
Q
R
S
T
V
W
Y

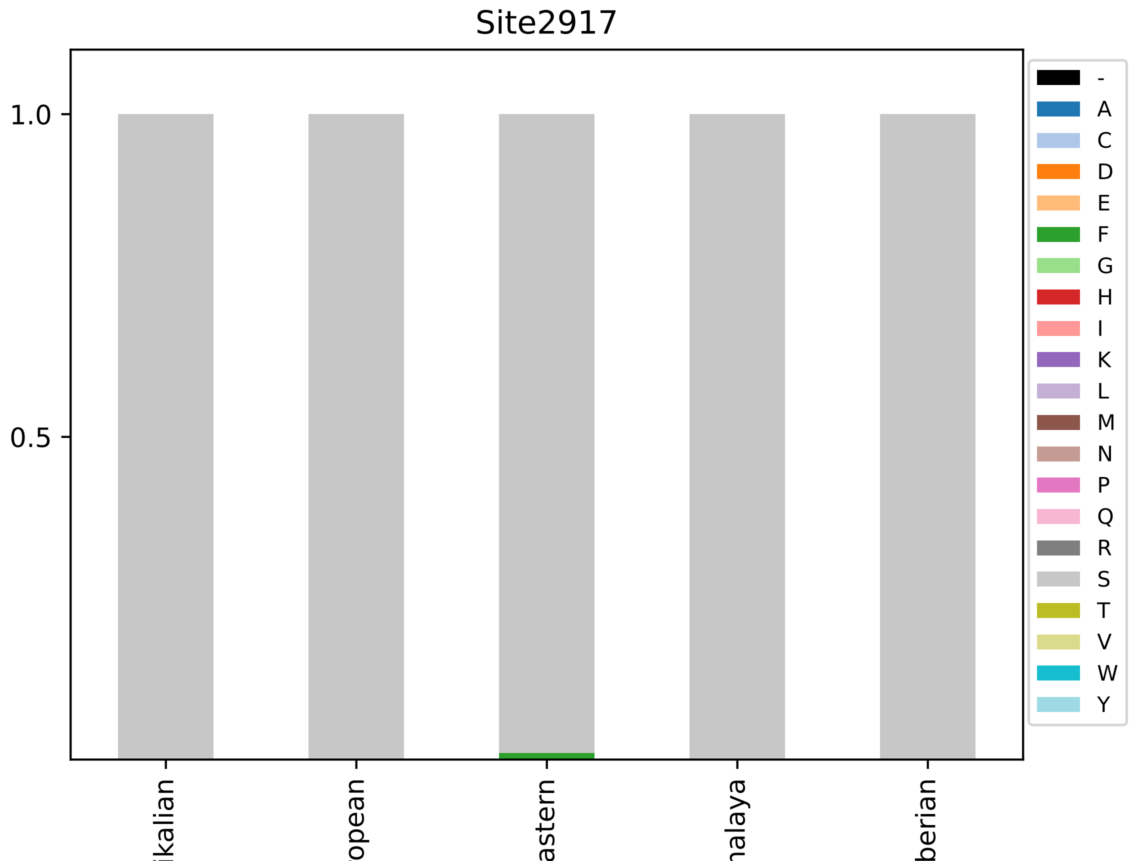

Site2917
1.0
0.5
-
A
C
D
E
F
G
H
I
K
L
M
N
P
Q
R
S
T
V
W
Y
ikalian
opean
astern
nalaya
berian

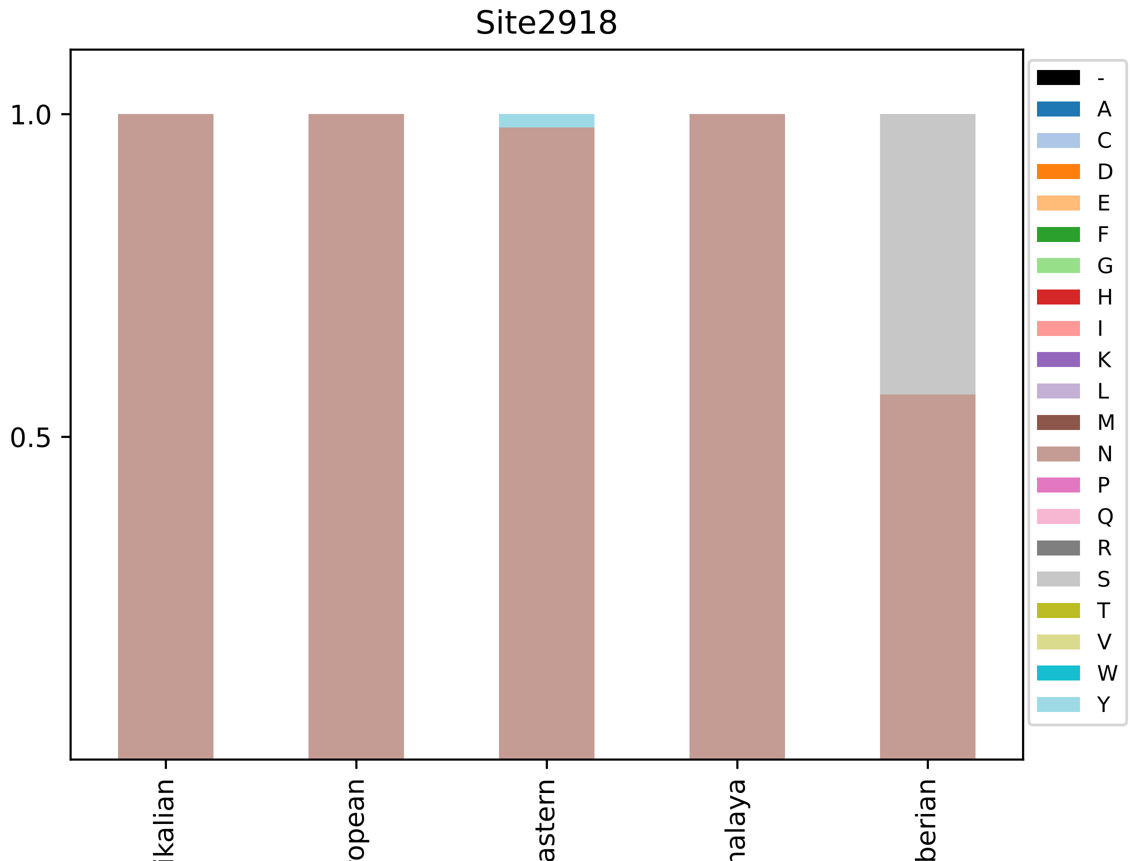
Site2918
1.0
0.5
-
A
C
D
E
F
G
H
I
K
L
M
N
P
Q
R
S
T
V
W
Y
ikalian
opean
astern
nalaya
berian

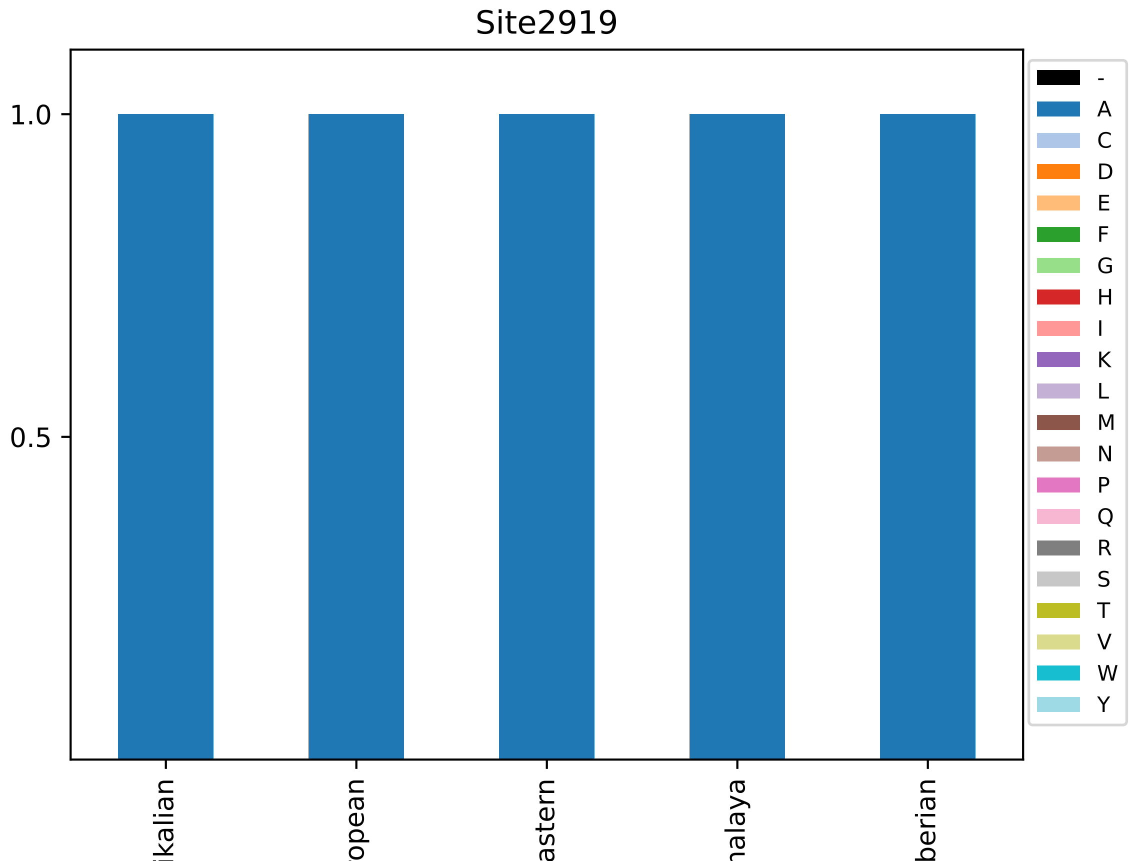
Site2919
1.0
0.5
-
A
C
D
E
F
G
H
I
K
L
M
N
P
Q
R
S
T
V
W
Y

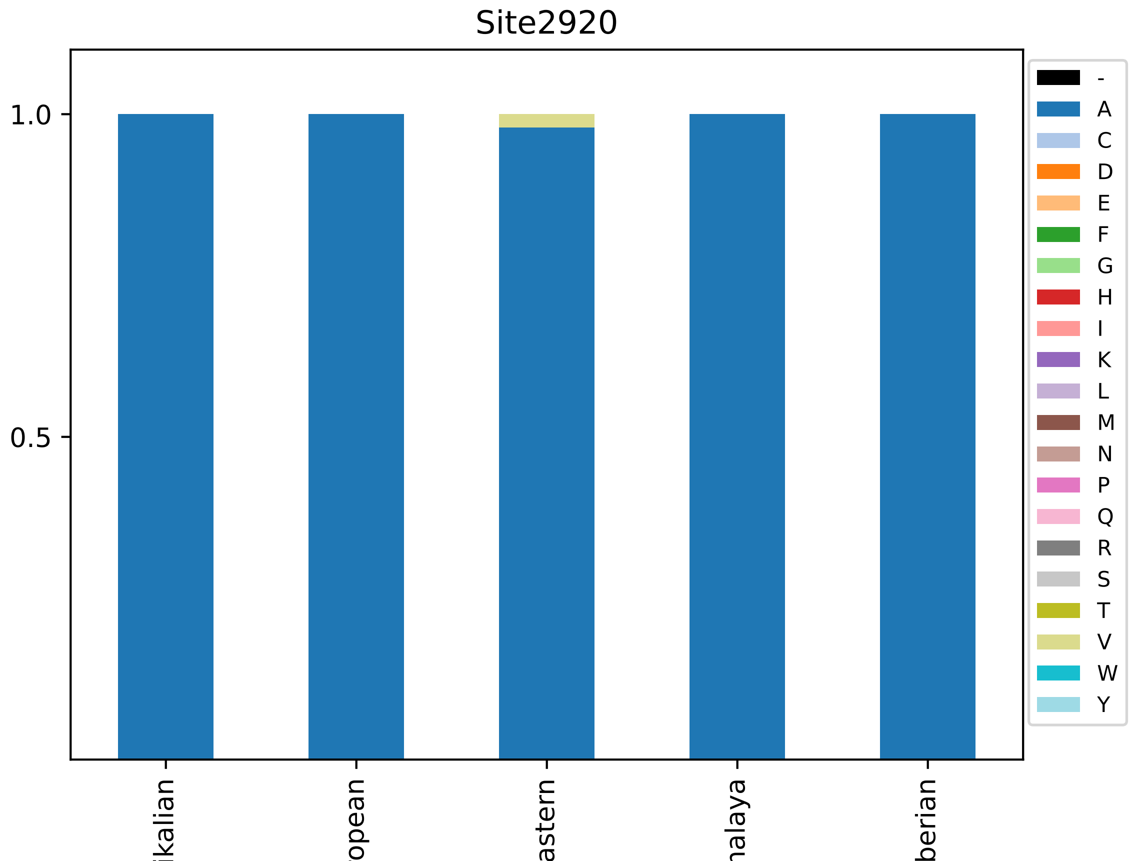

Site2920
1.0
0.5
-
A
C
D
E
F
G
H
I
K
L
M
N
P
Q
R
S
T
V
W
Y
ikalian
opean
astern
nalaya
berian

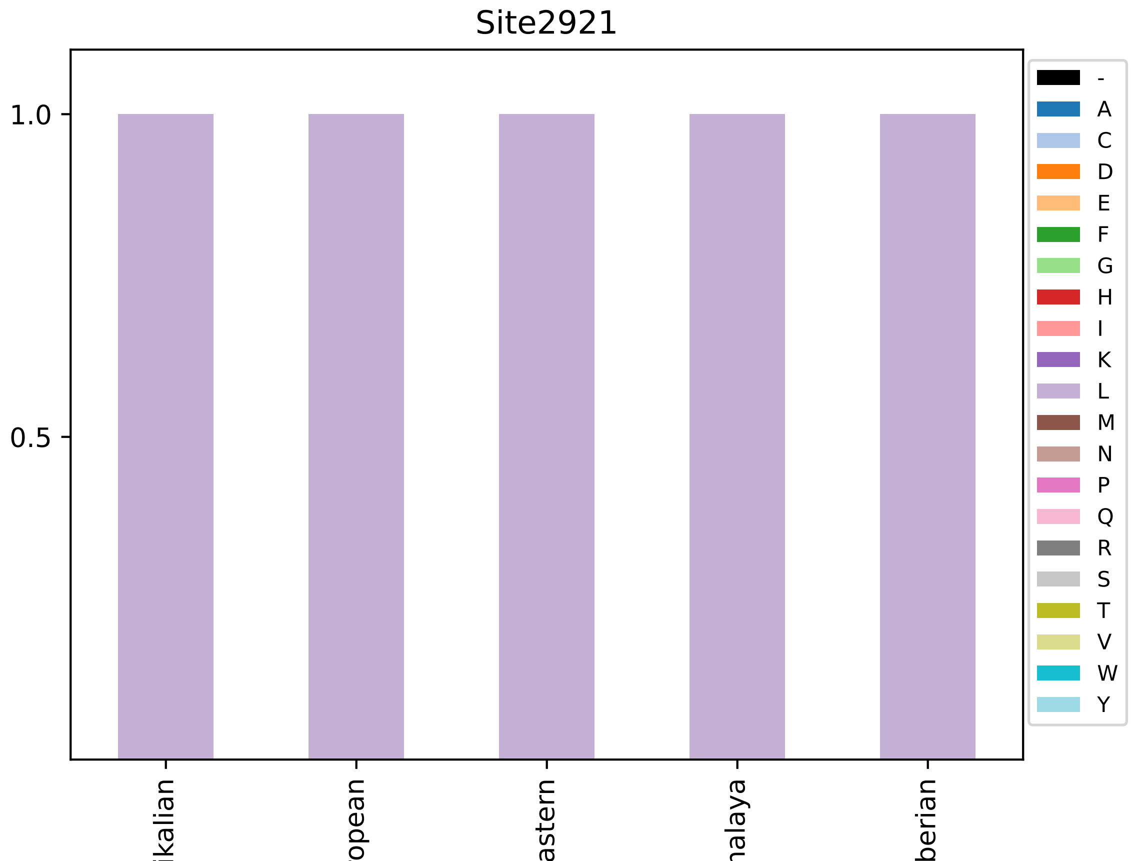
Site2921
1.0
0.5
-
A
C
D
E
F
G
H
I
K
L
M
N
P
Q
R
S
T
V
W
Y
ikalian
opean
astern
nalaya
berian

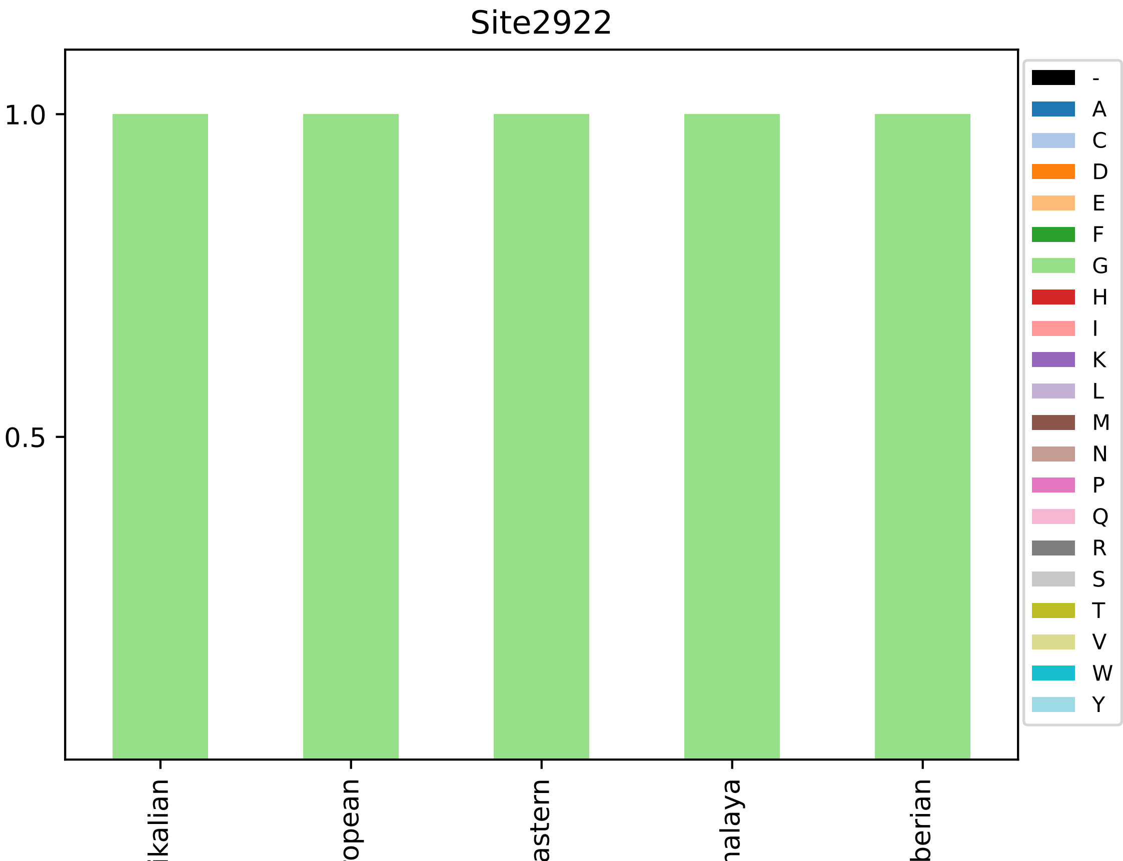

Site2922
1.0
0.5
-
A
C
D
E
F
G
H
I
K
L
M
N
P
Q
R
S
T
V
W
Y
ikalian
opean
astern
nalaya
berian

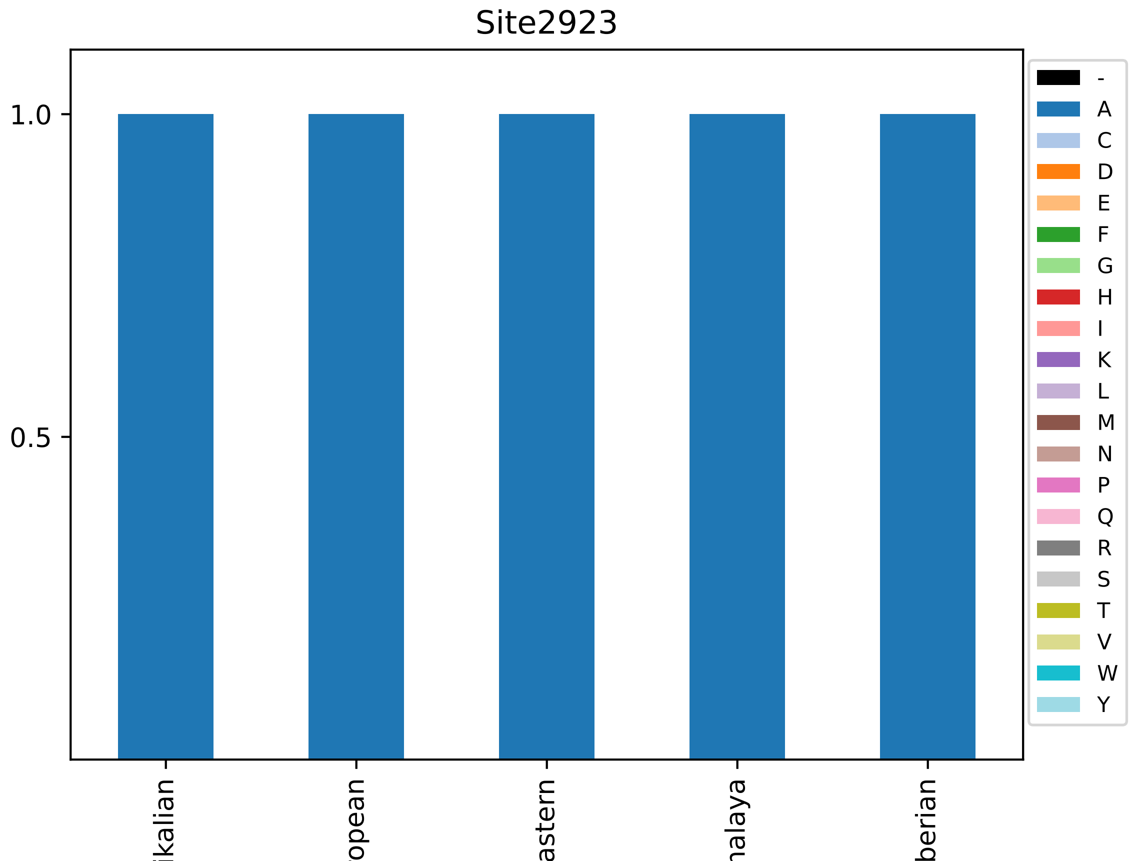
Site2923
1.0
0.5
-
A
C
D
E
F
G
H
I
K
L
M
N
P
Q
R
S
T
V
W
Y
ikalian
opean
astern
nalaya
berian

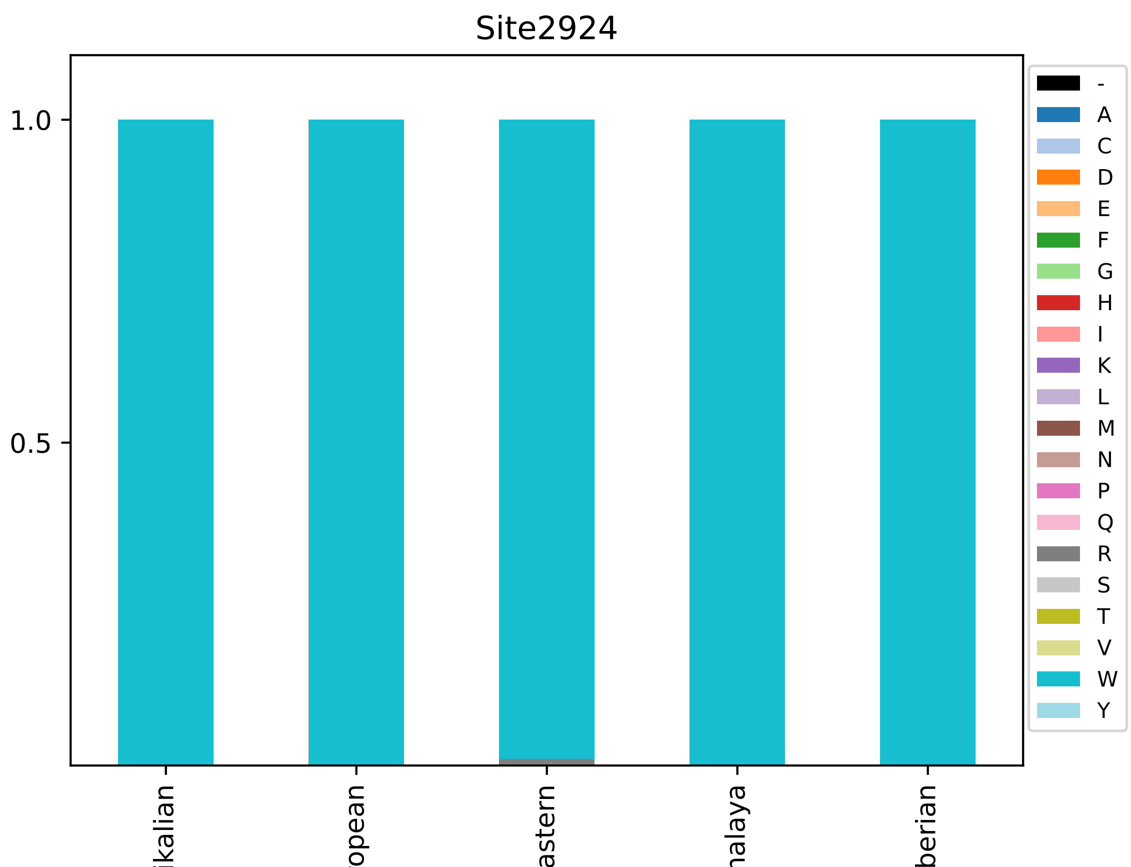
Site2924
1.0
0.5
-
A
C
D
E
F
G
H
I
K
L
M
N
P
Q
R
S
T
V
W
Y
ikalian
opean
astern
nalaya
berian

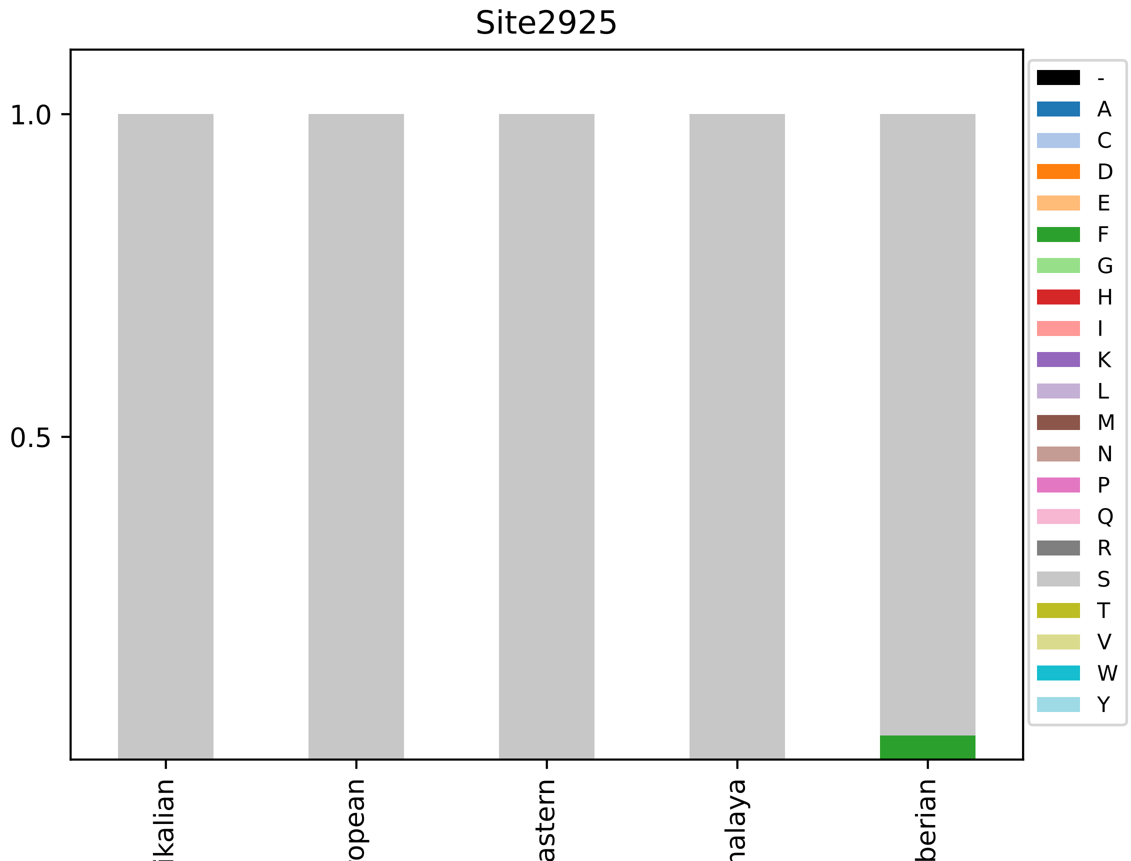

Site2925
1.0
0.5
-
A
C
D
E
F
G
H
I
K
L
M
N
P
Q
R
S
T
V
W
Y
ikalian
opean
astern
nalaya
berian

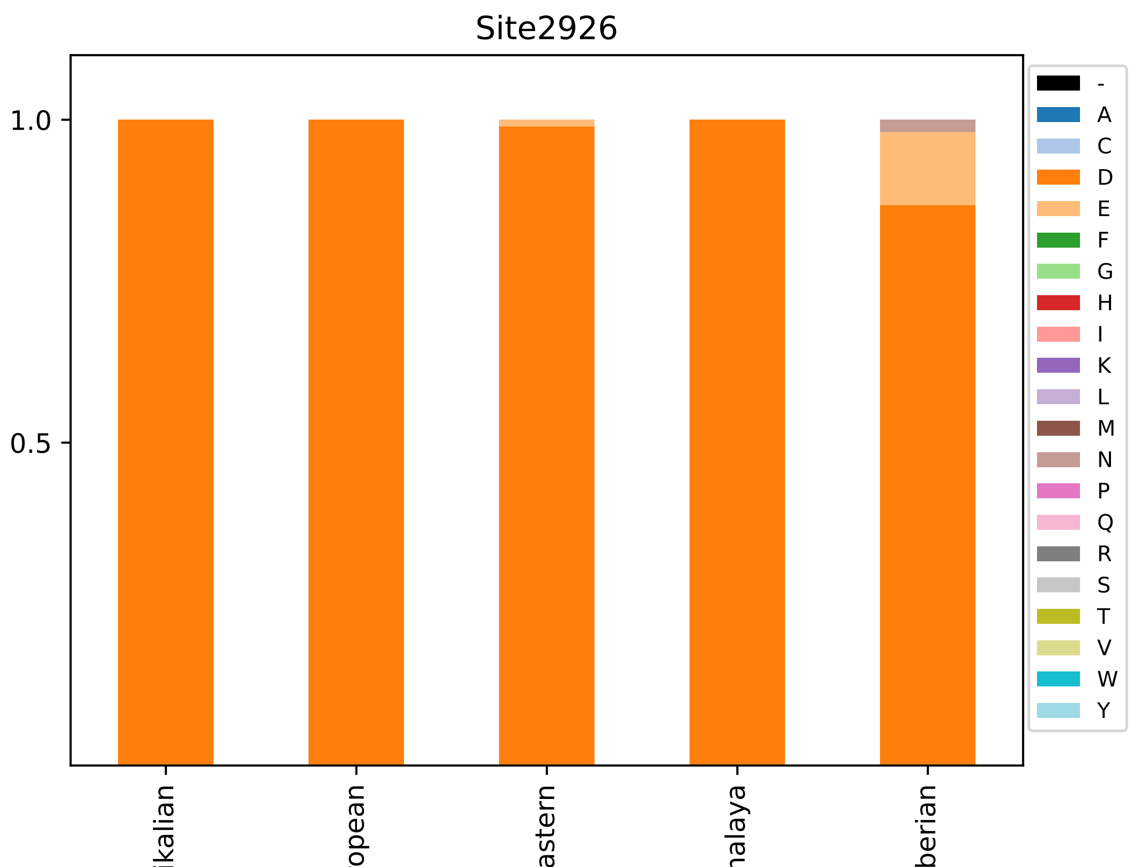
Site2926
1.0
0.5
ikalian
opean
astern
nalaya
berian
-
A
C
D
E
F
G
H
I
K
L
M
N
P
Q
R
S
T
V
W
Y

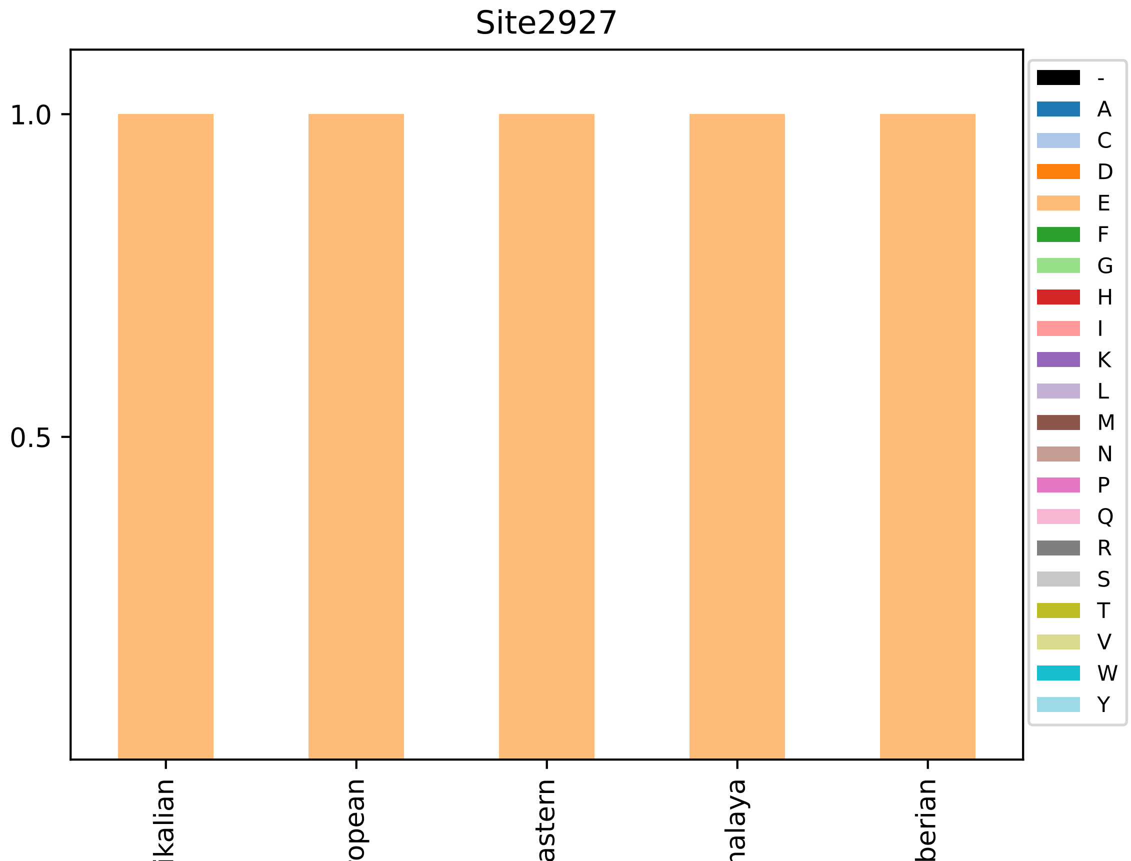
Site2927
1.0
0.5
-
A
C
D
E
F
G
H
I
K
L
M
N
P
Q
R
S
T
V
W
Y
ikalian
opean
astern
nalaya
berian

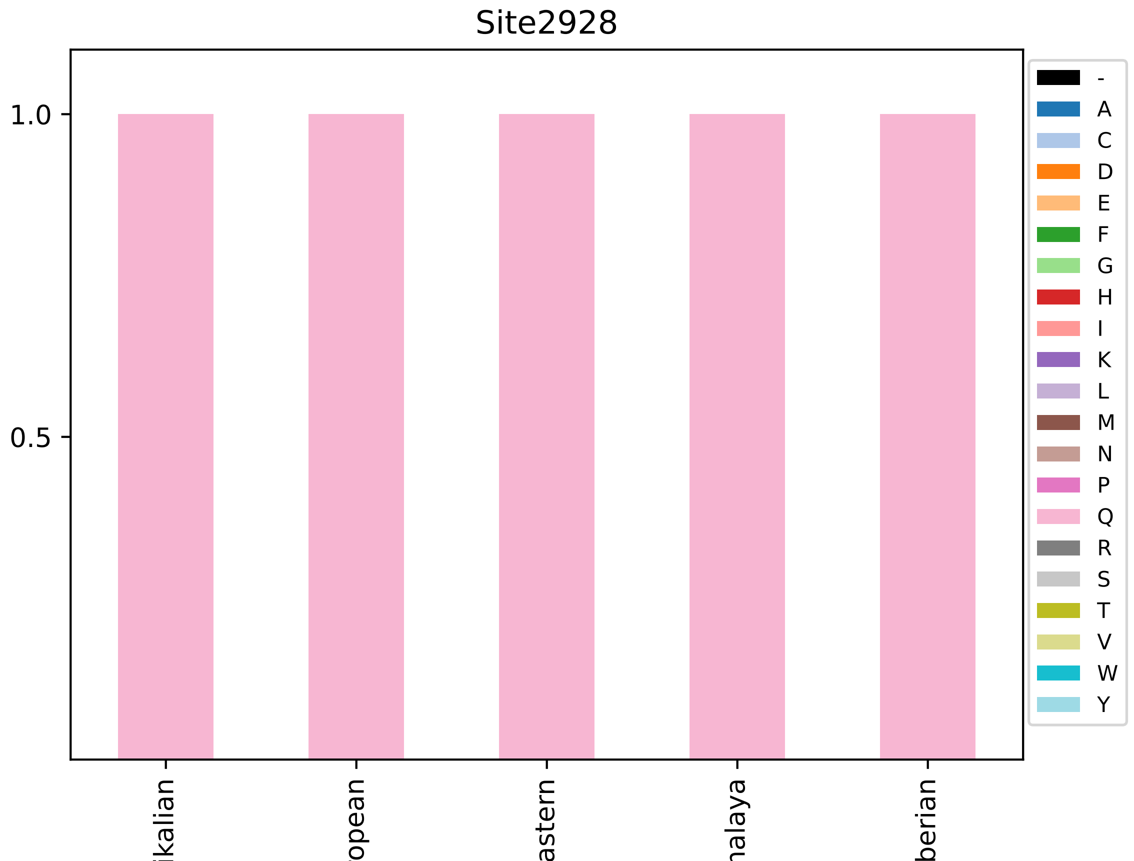
Site2928
1.0
0.5
ikalian
ropean
astern
nalaya
berian
-
A
C
D
E
F
G
H
I
K
L
M
N
P
Q
R
S
T
V
W
Y

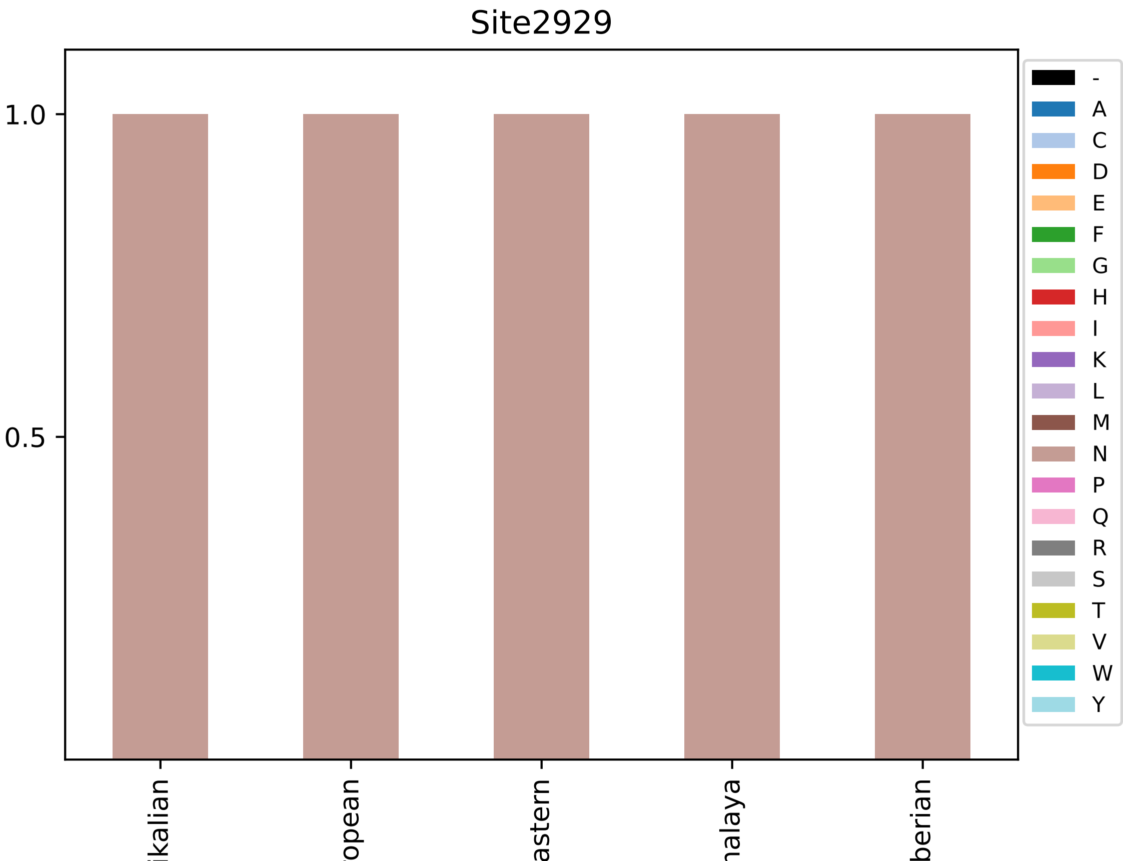
Site2929
1.0
0.5
-
A
C
D
E
F
G
H
I
K
L
M
N
P
Q
R
S
T
V
W
Y
ikalian
opean
astern
nalaya
berian

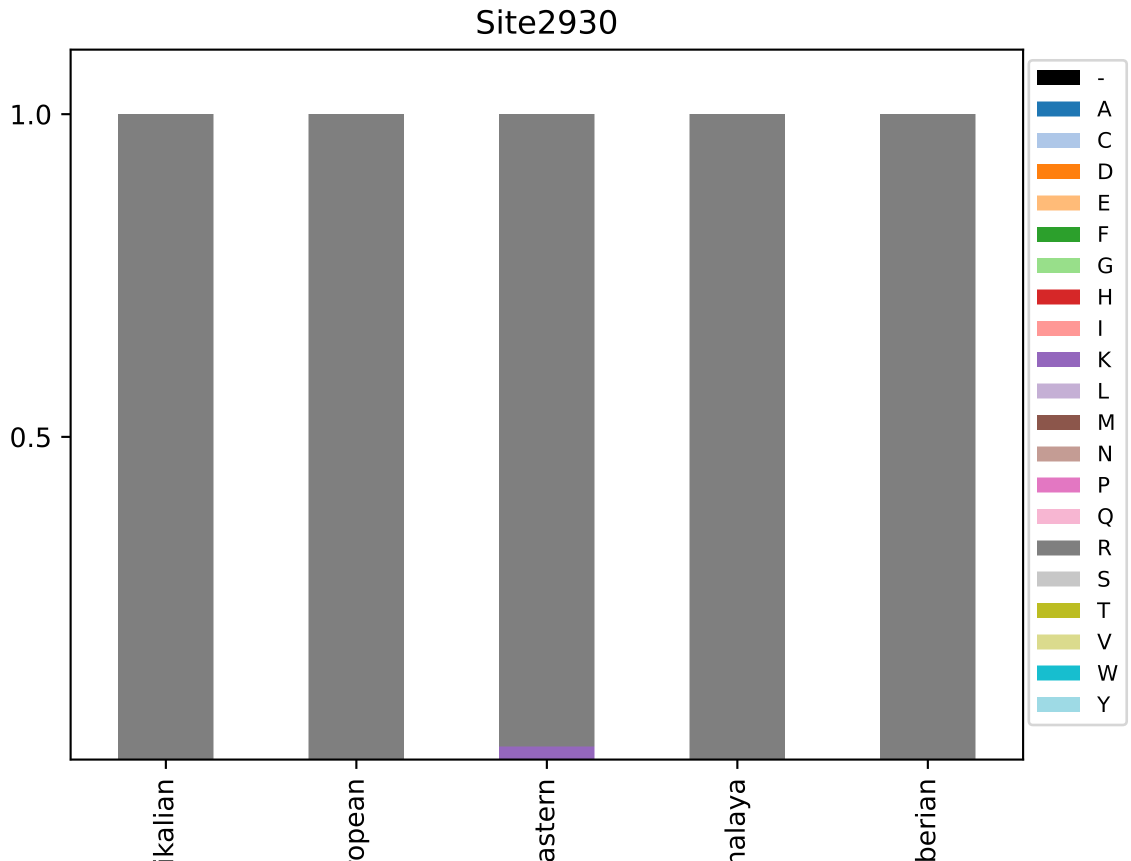

Site2930
1.0
0.5
-
A
C
D
E
F
G
H
I
K
L
M
N
P
Q
R
S
T
V
W
Y
ikalian
opean
astern
nalaya
berian

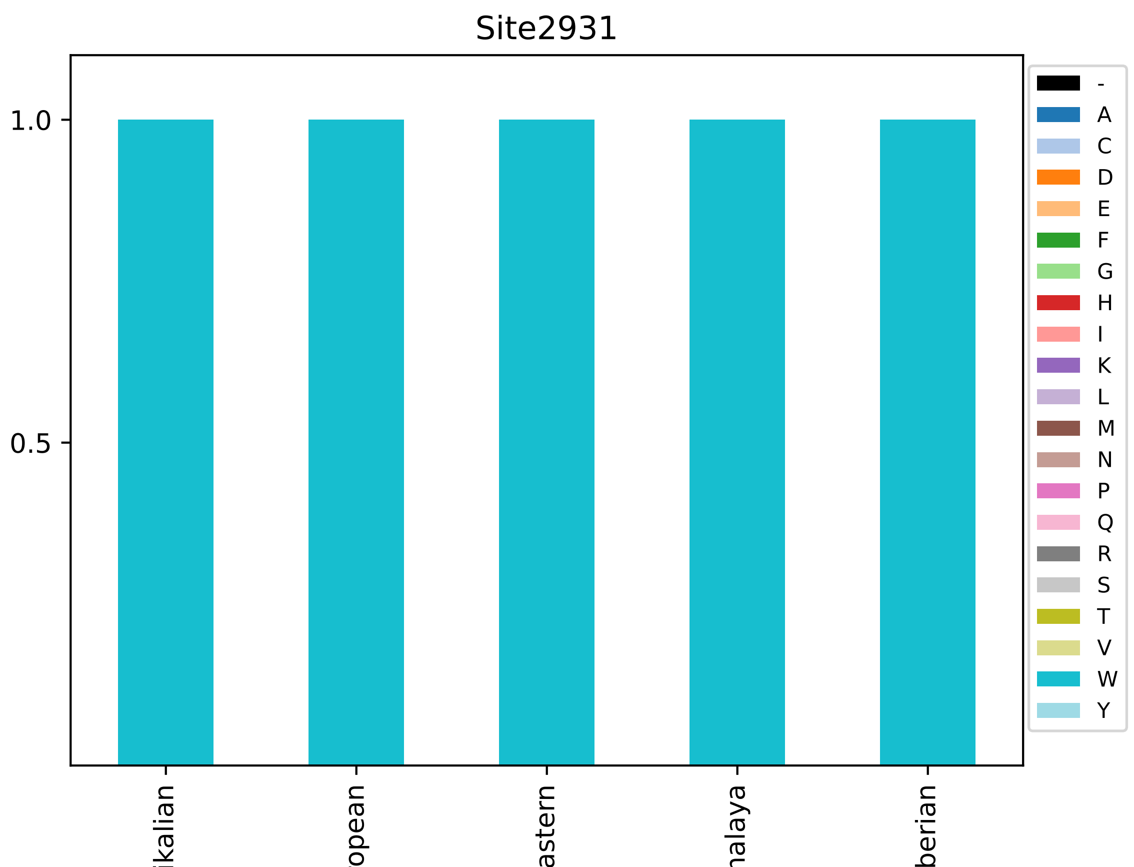

Site2931
1.0
0.5
-
A
C
D
E
F
G
H
I
K
L
M
N
P
Q
R
S
T
V
W
Y
ikalian
ropean
astern
nalaya
berian

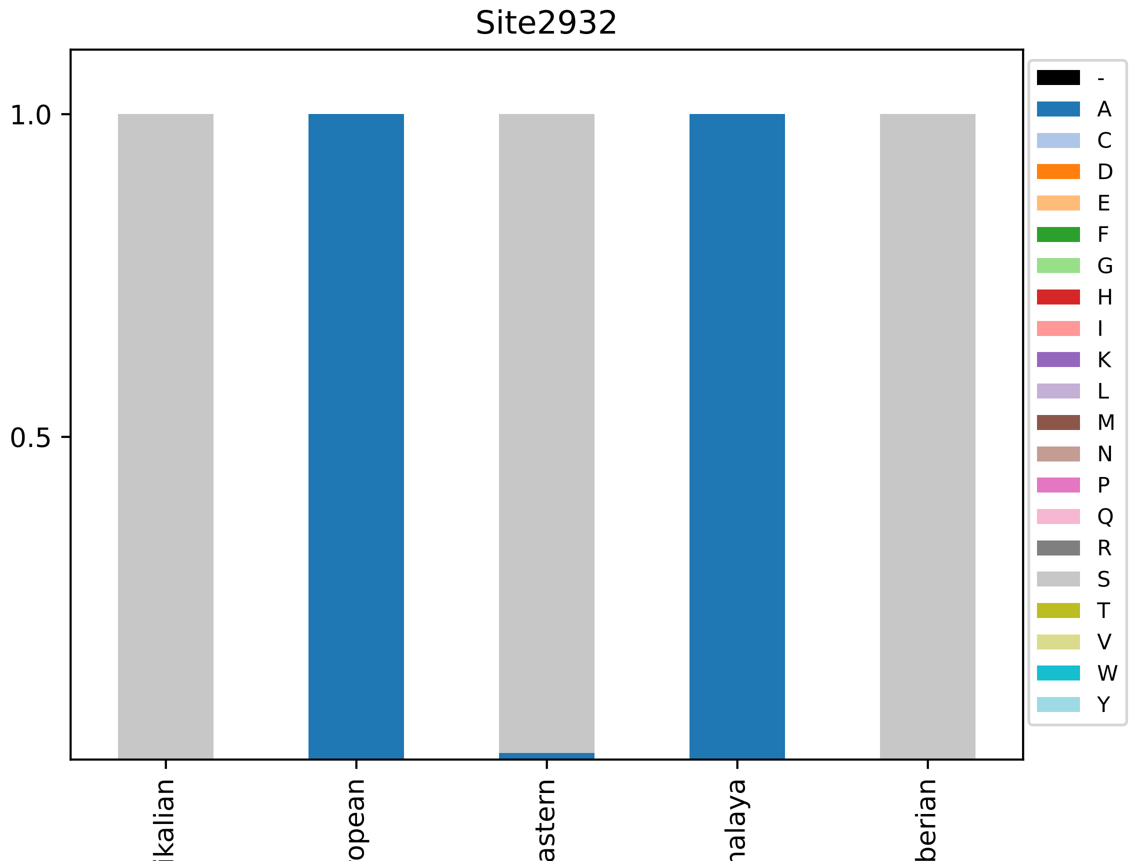

Site2932
1.0
0.5
ikalian
opean
astern
nalaya
berian
-
A
C
D
E
F
G
H
I
K
L
M
N
P
Q
R
S
T
V
W
Y

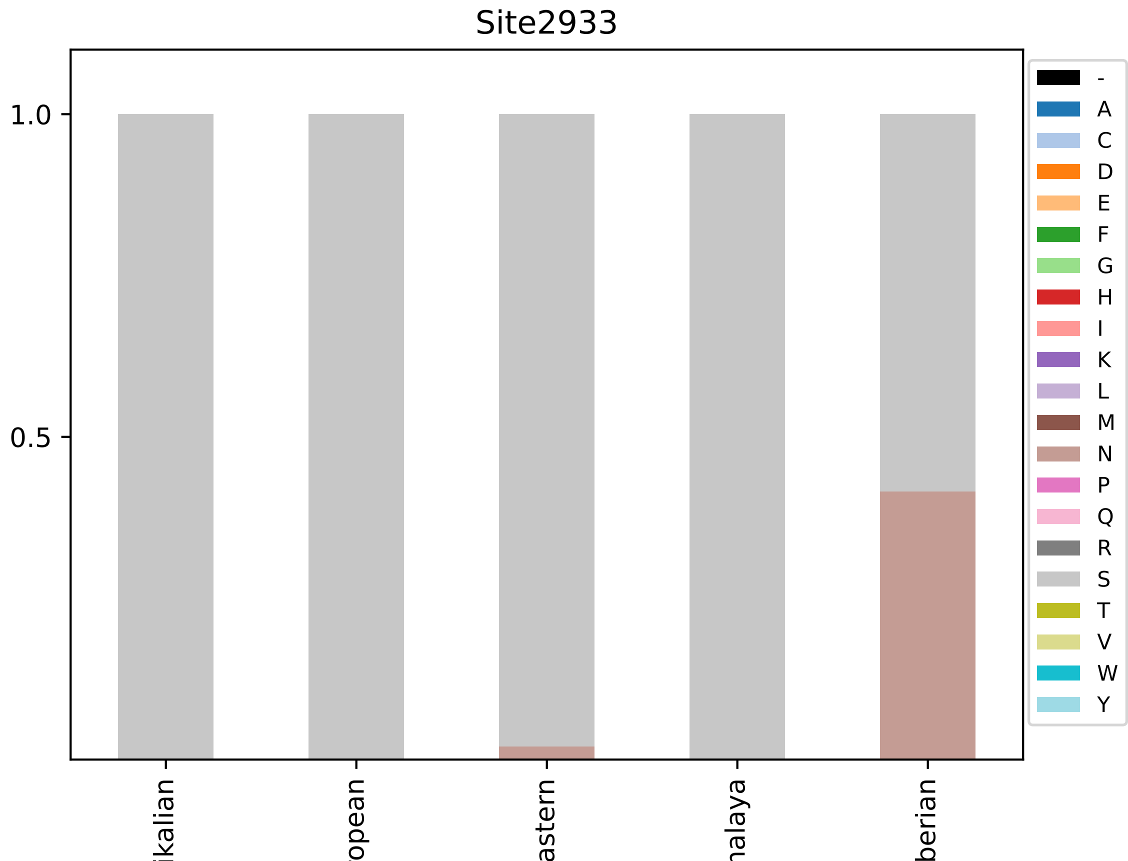

Site2933
1.0
0.5
-
A
C
D
E
F
G
H
I
K
L
M
N
P
Q
R
S
T
V
W
Y
ikalian
opean
astern
nalaya
berian

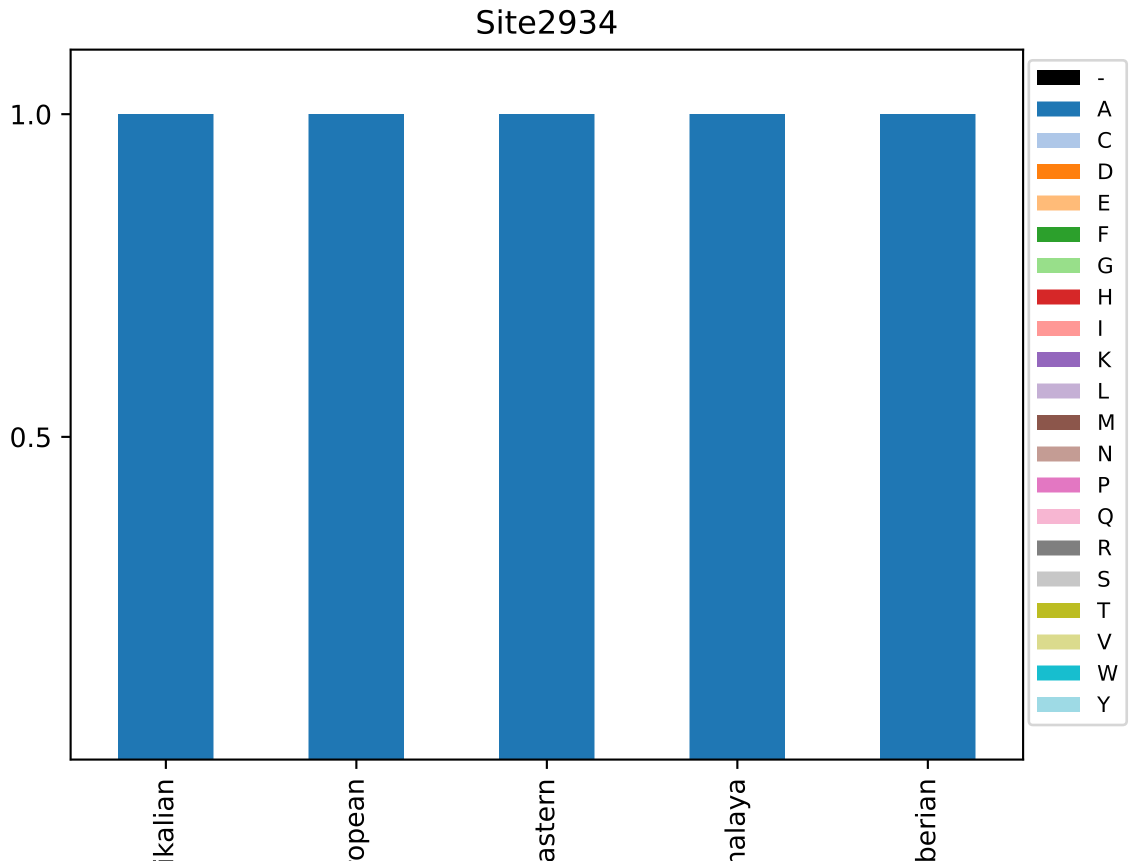
Site2934
1.0
0.5
-
A
C
D
E
F
G
H
I
K
L
M
N
P
Q
R
S
T
V
W
Y

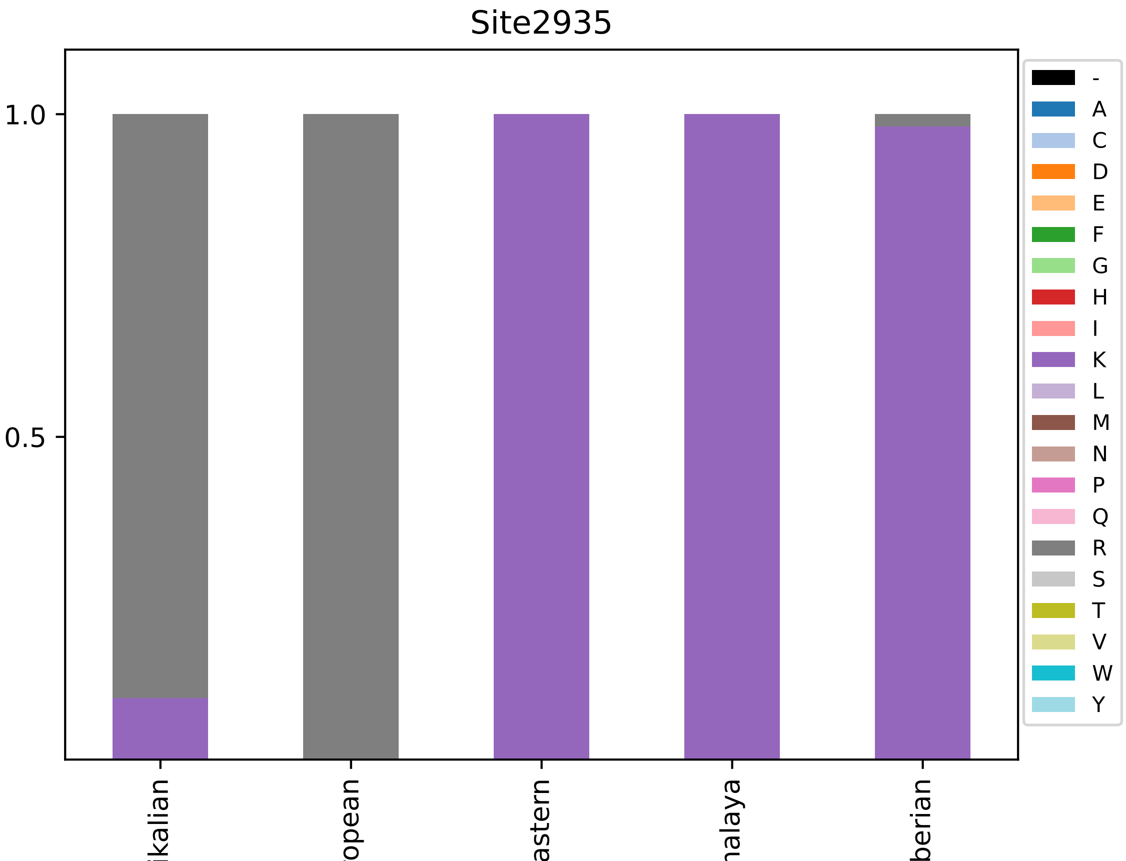
Site2935
1.0
0.5
-
A
C
D
E
F
G
H
I
K
L
M
N
P
Q
R
S
T
V
W
Y
ikalian
opean
astern
nalaya
berian

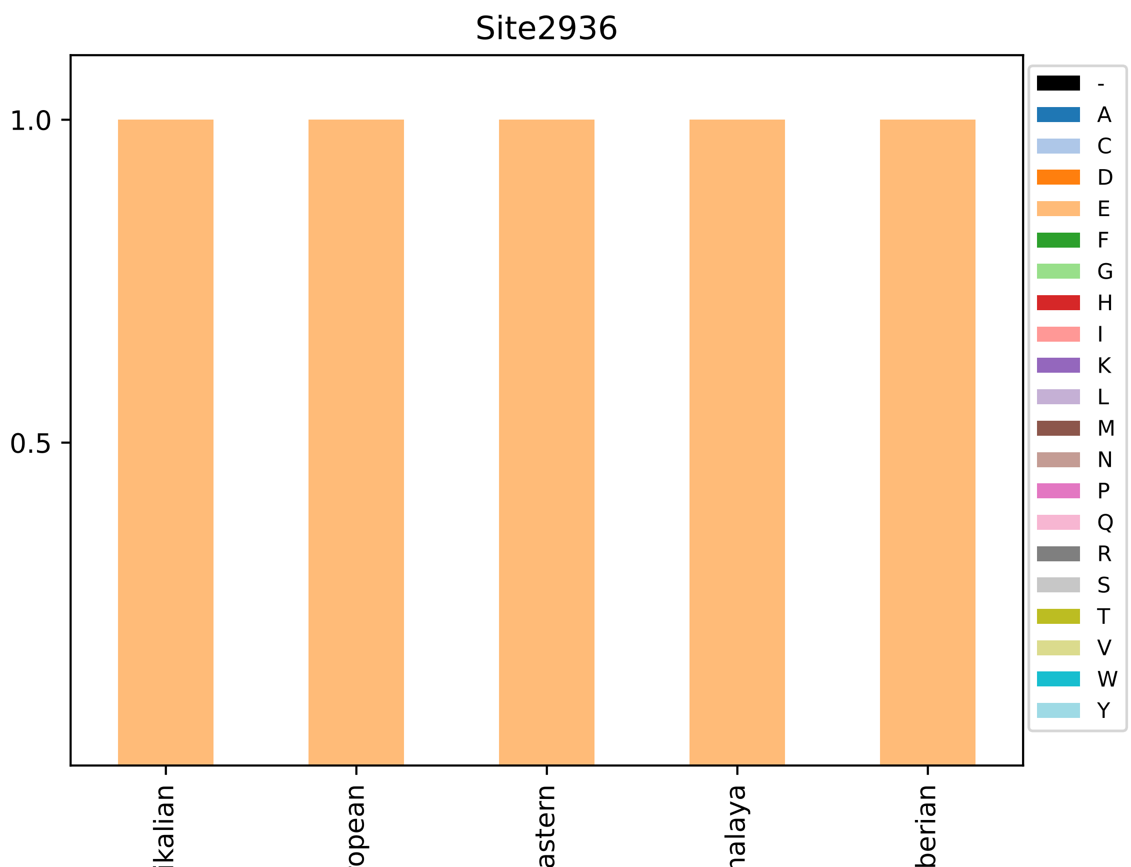
Site2936
1.0
0.5
ikalian
opean
astern
nalaya
berian
-
A
C
D
E
F
G
H
I
K
L
M
N
P
Q
R
S
T
V
W
Y

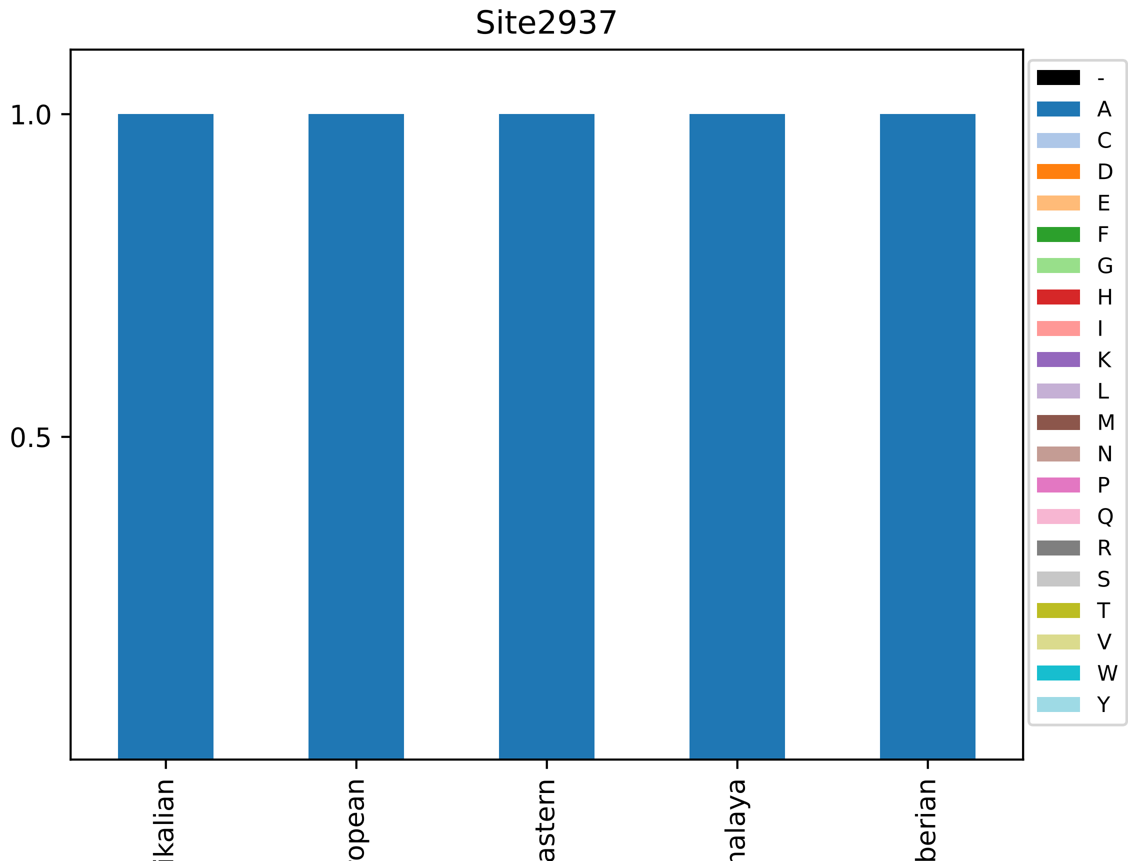

Site2937
1.0
0.5
ikalian
opean
astern
nalaya
berian
-
A
C
D
E
F
G
H
I
K
L
M
N
P
Q
R
S
T
V
W
Y

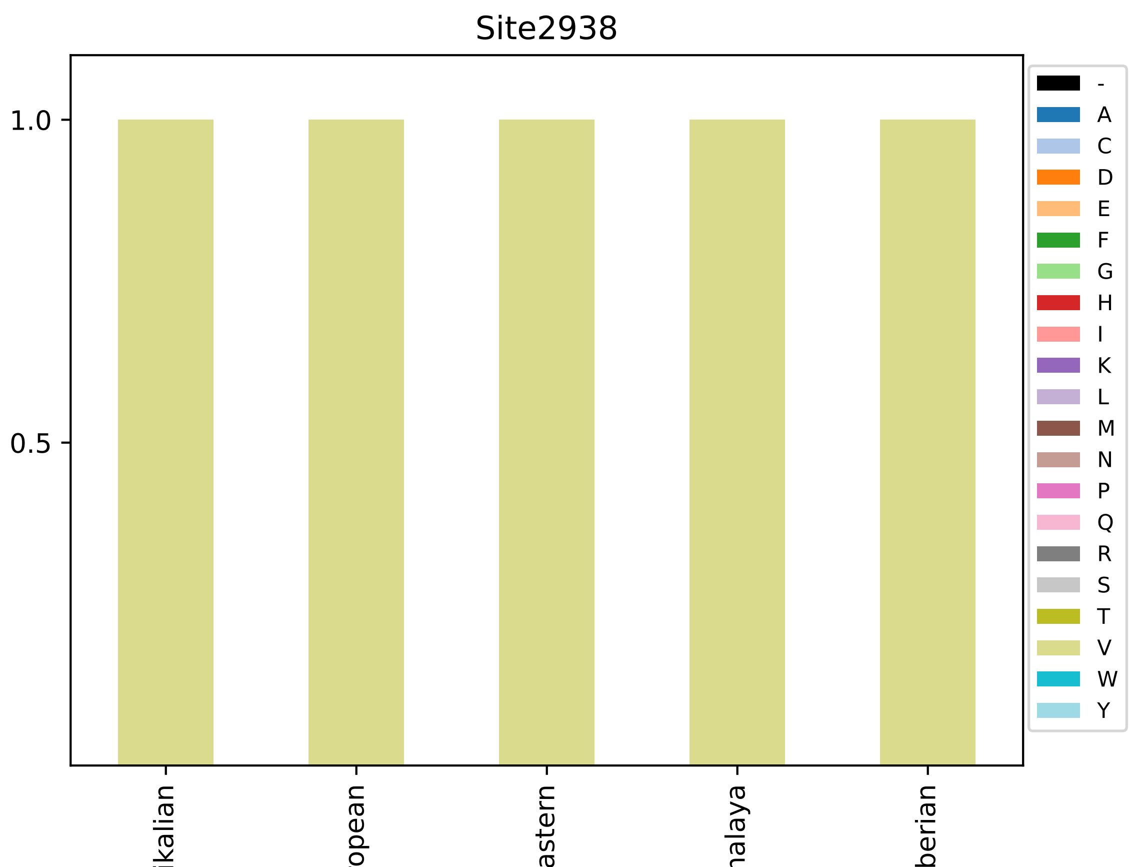
Site2938
1.0
0.5
ikalian
opean
astern
nalaya
berian
-
A
C
D
E
F
G
H
I
K
L
M
N
P
Q
R
S
T
V
W
Y

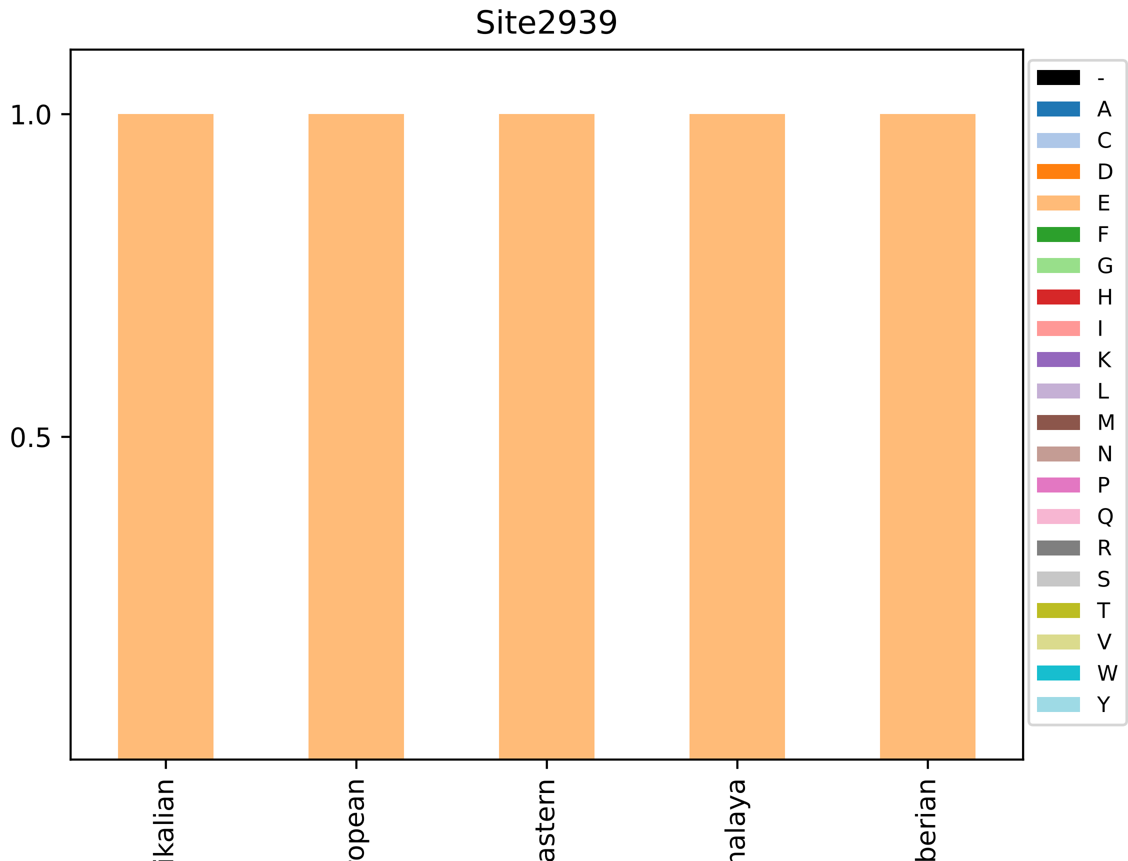
Site2939
1.0
0.5
-
A
C
D
E
F
G
H
I
K
L
M
N
P
Q
R
S
T
V
W
Y
ikalian
opean
astern
nalaya
berian

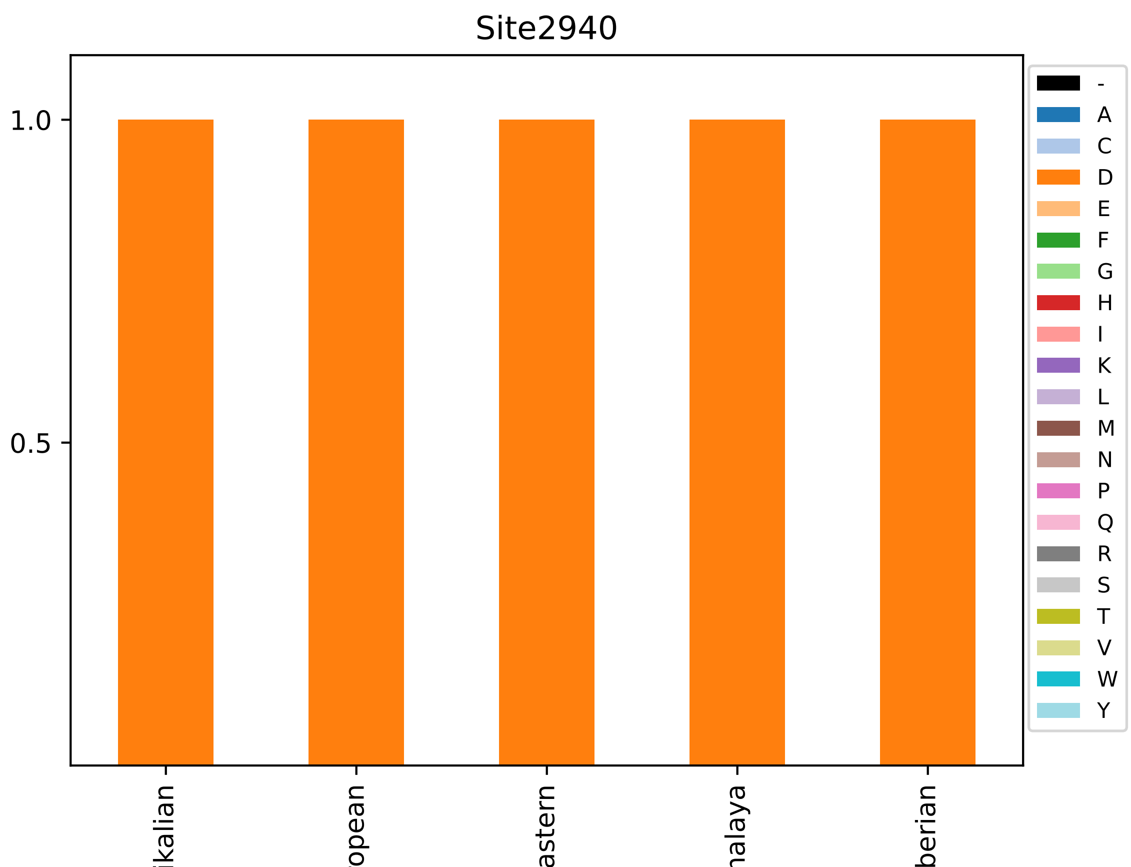
Site2940
1.0
0.5
ikalian
opean
astern
nalaya
berian
-
A
C
D
E
F
G
H
I
K
L
M
N
P
Q
R
S
T
V
W
Y

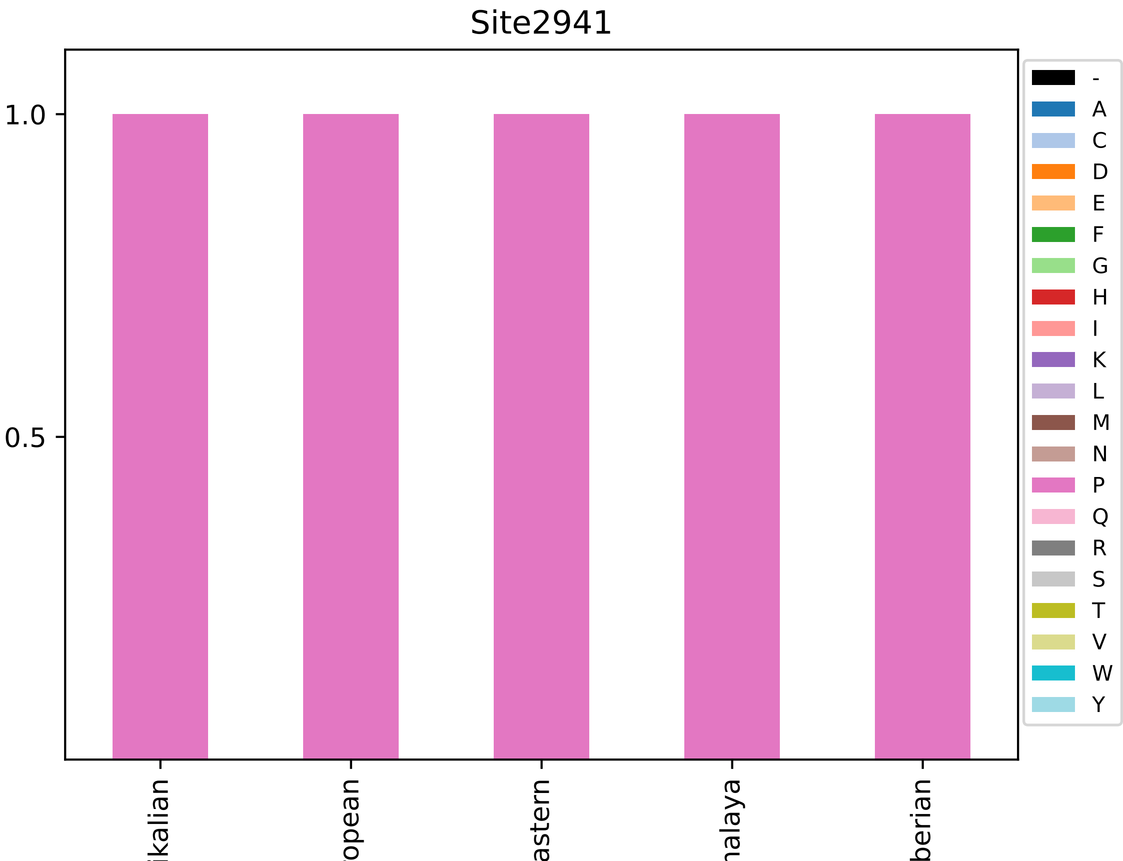
Site2941
1.0
0.5
-
A
C
D
E
F
G
H
I
K
L
M
N
P
Q
R
S
T
V
W
Y

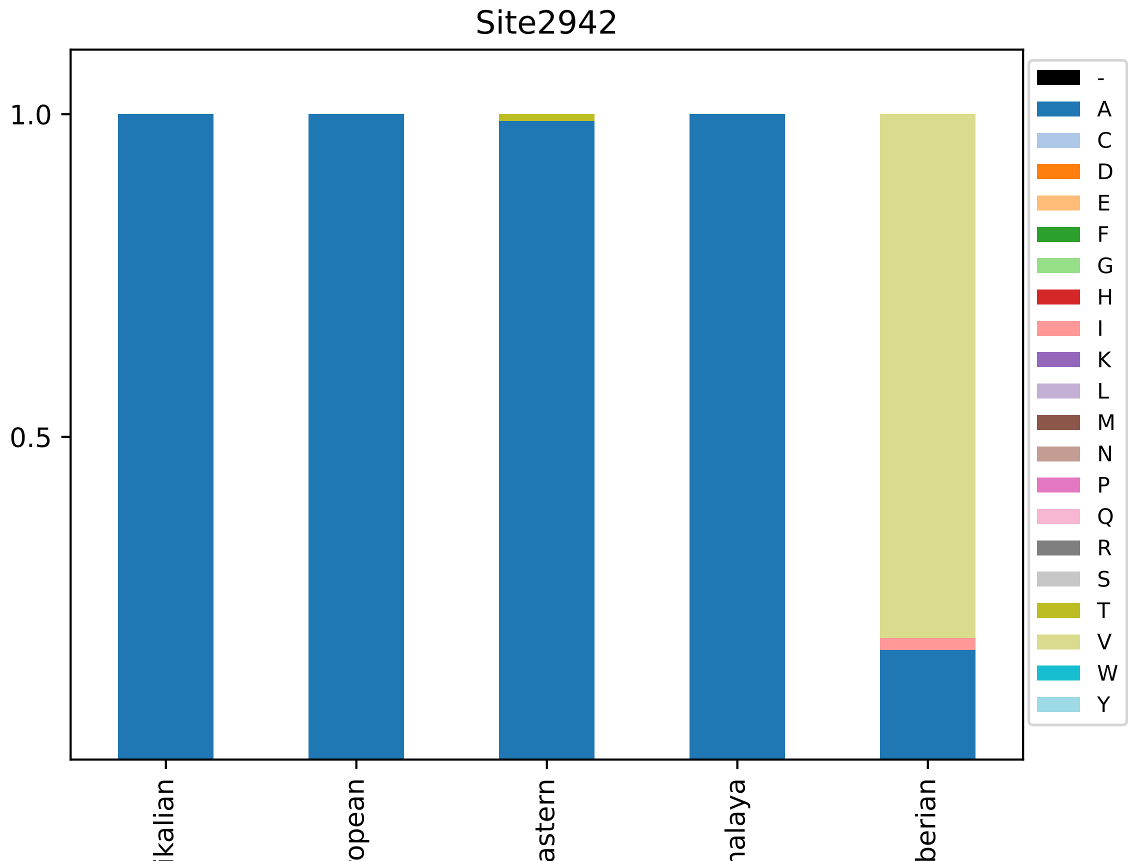

Site2942
1.0
0.5
-
A
C
D
E
F
G
H
I
K
L
M
N
P
Q
R
S
T
V
W
Y
ikalian
opean
astern
nalaya
berian

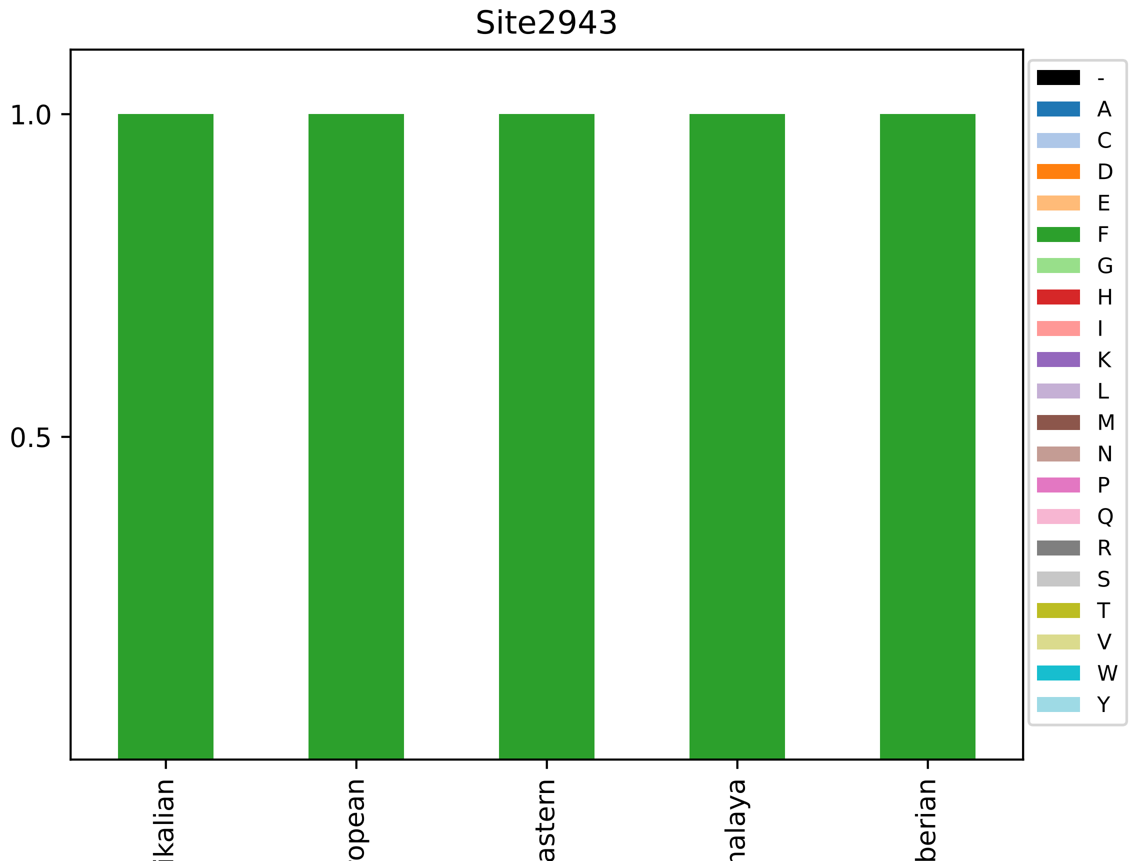

Site2943
1.0
0.5
-
A
C
D
E
F
G
H
I
K
L
M
N
P
Q
R
S
T
V
W
Y
ikalian
opean
astern
nalaya
berian

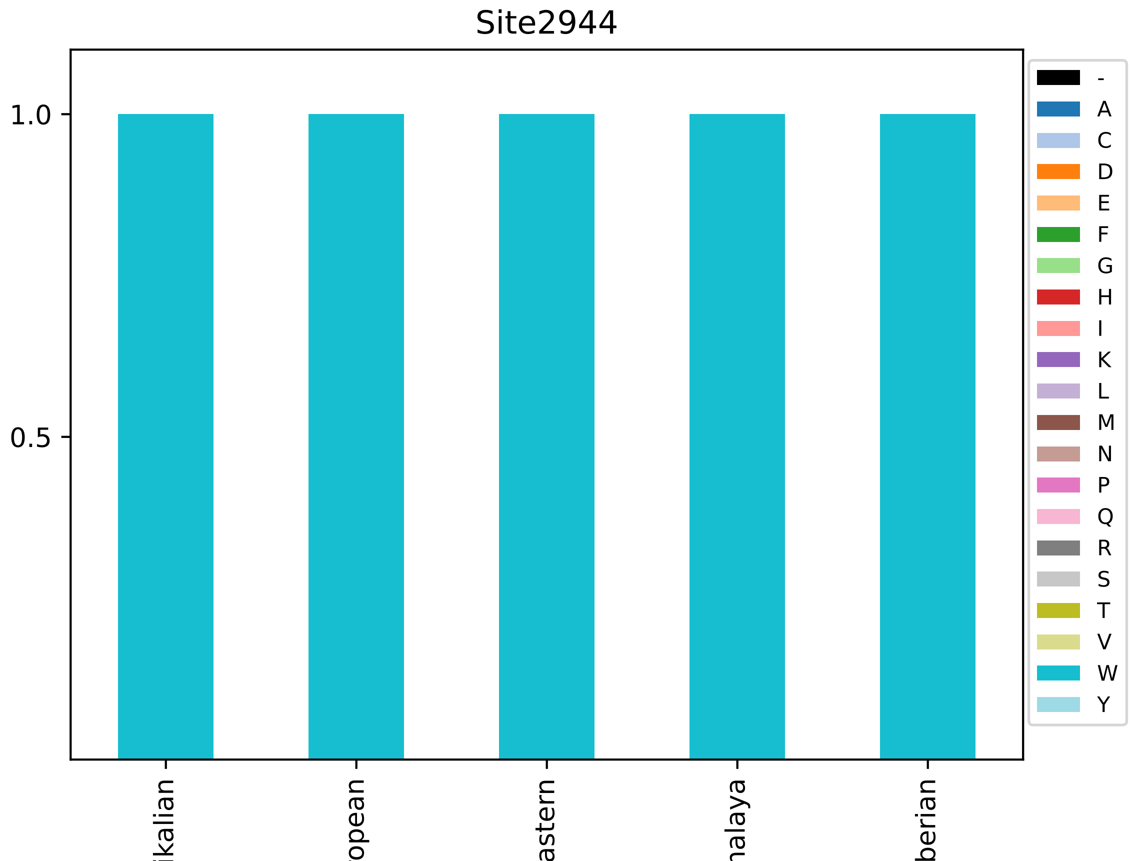
Site2944
1.0
0.5
-
A
C
D
E
F
G
H
I
K
L
M
N
P
Q
R
S
T
V
W
Y
ikalian
opean
astern
nalaya
berian

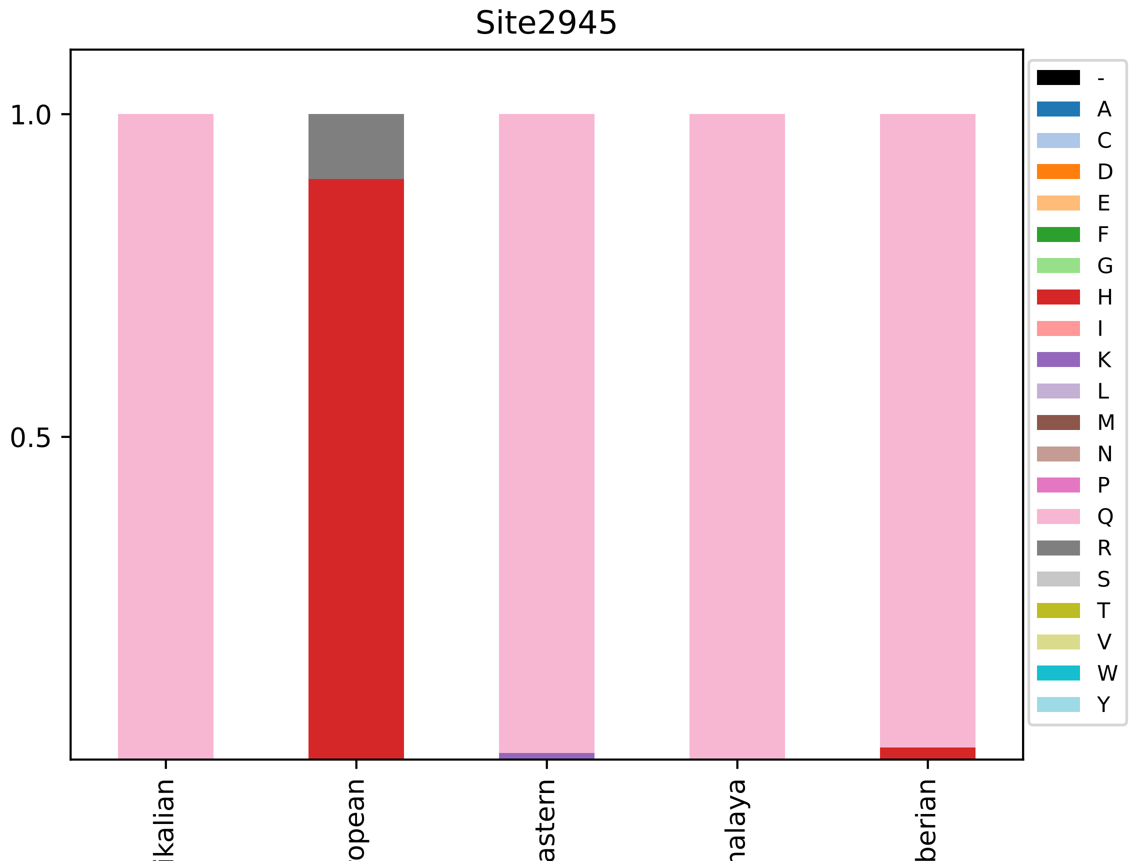
Site2945
1.0
0.5
-
A
C
D
E
F
G
H
I
K
L
M
N
P
Q
R
S
T
V
W
Y
ikalian
opean
astern
nalaya
berian

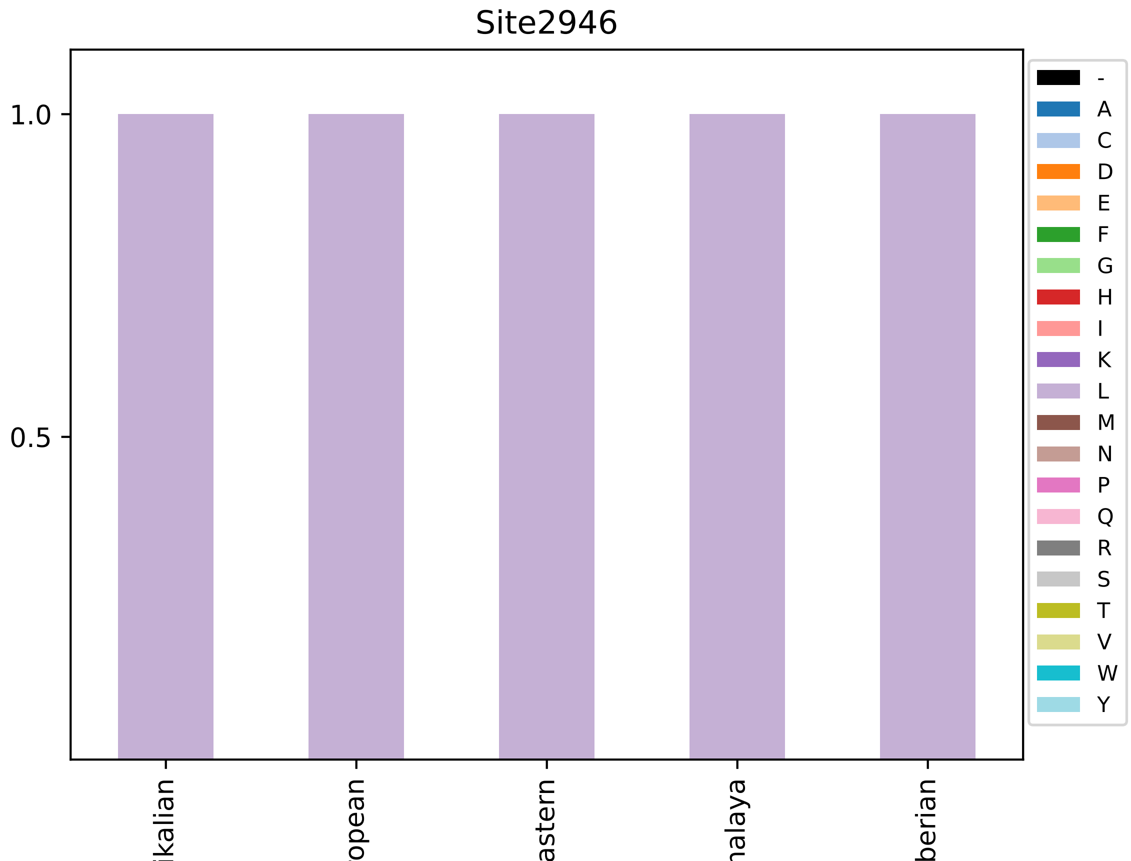
Site2946
1.0
0.5
-
A
C
D
E
F
G
H
I
K
L
M
N
P
Q
R
S
T
V
W
Y
ikalian
opean
astern
nalaya
berian

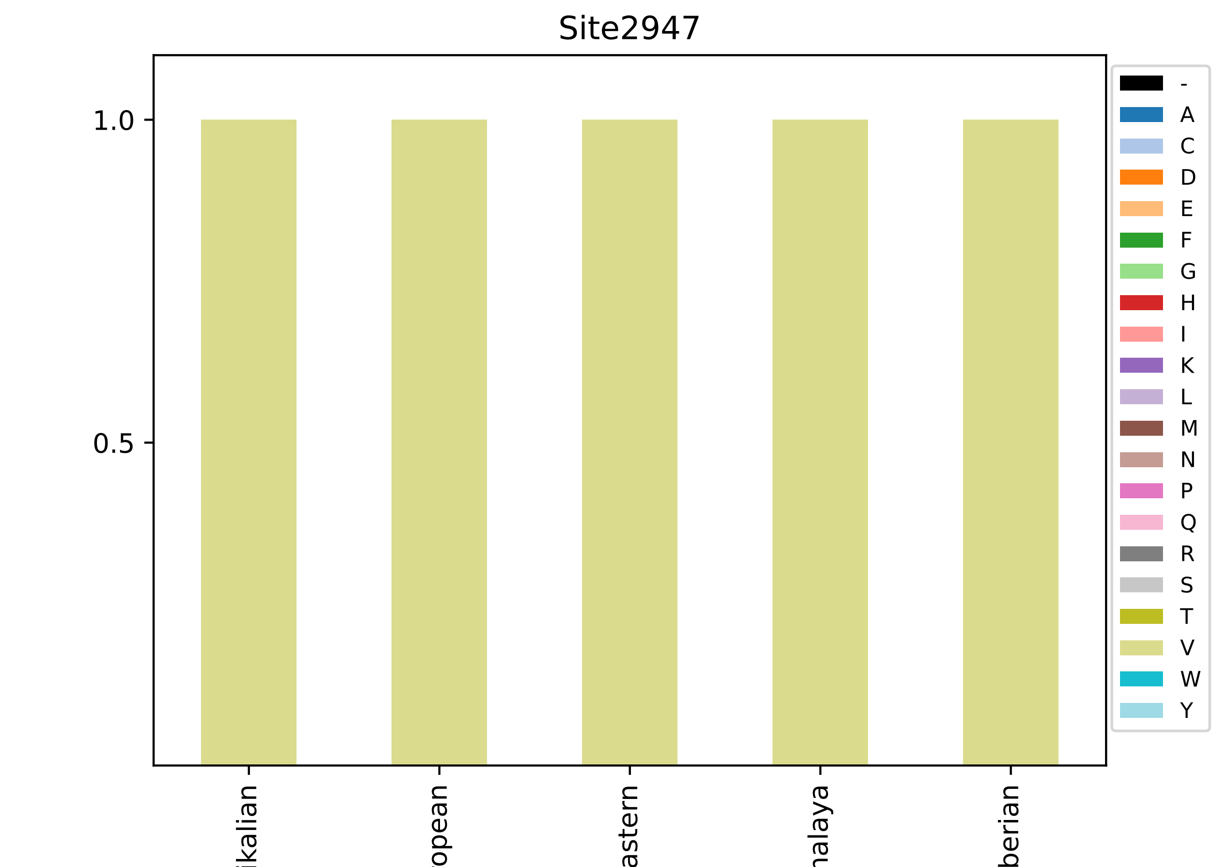
Site2947
1.0
0.5
-
A
C
D
E
F
G
H
I
K
L
M
N
P
Q
R
S
T
V
W
Y

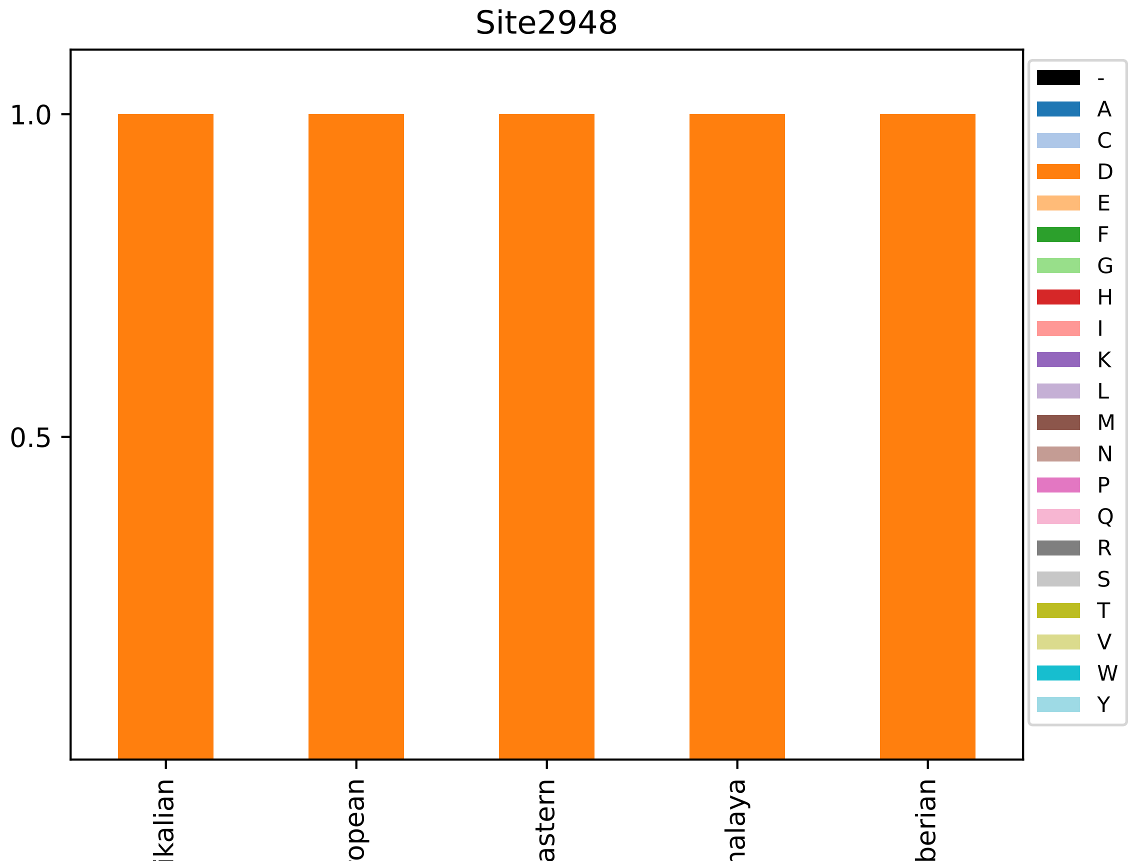
Site2948
1.0
0.5
-
A
C
D
E
F
G
H
I
K
L
M
N
P
Q
R
S
T
V
W
Y
ikalian
opean
astern
nalaya
berian

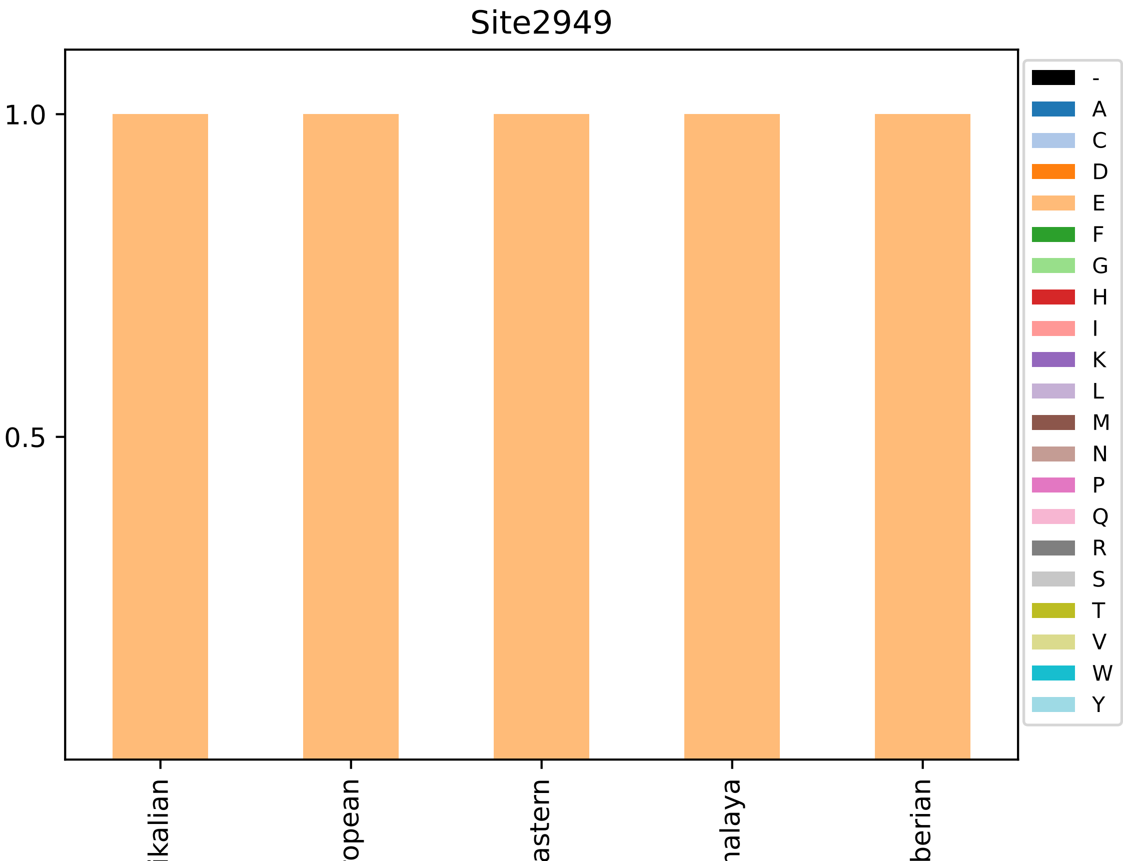

Site2949
1.0
0.5
-
A
C
D
E
F
G
H
I
K
L
M
N
P
Q
R
S
T
V
W
Y
ikalian
opean
astern
nalaya
berian

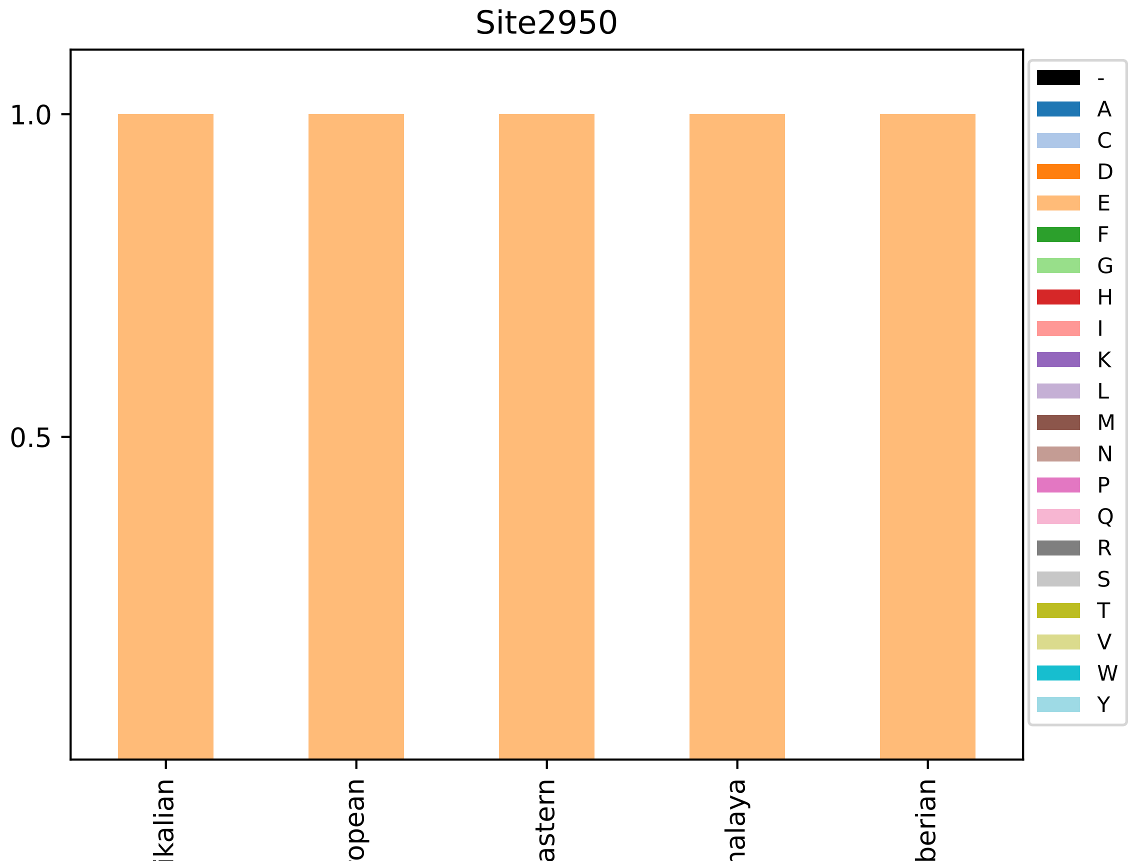
Site2950
1.0
0.5
ikalian
opean
astern
nalaya
berian
-
A
C
D
E
F
G
H
I
K
L
M
N
P
Q
R
S
T
V
W
Y

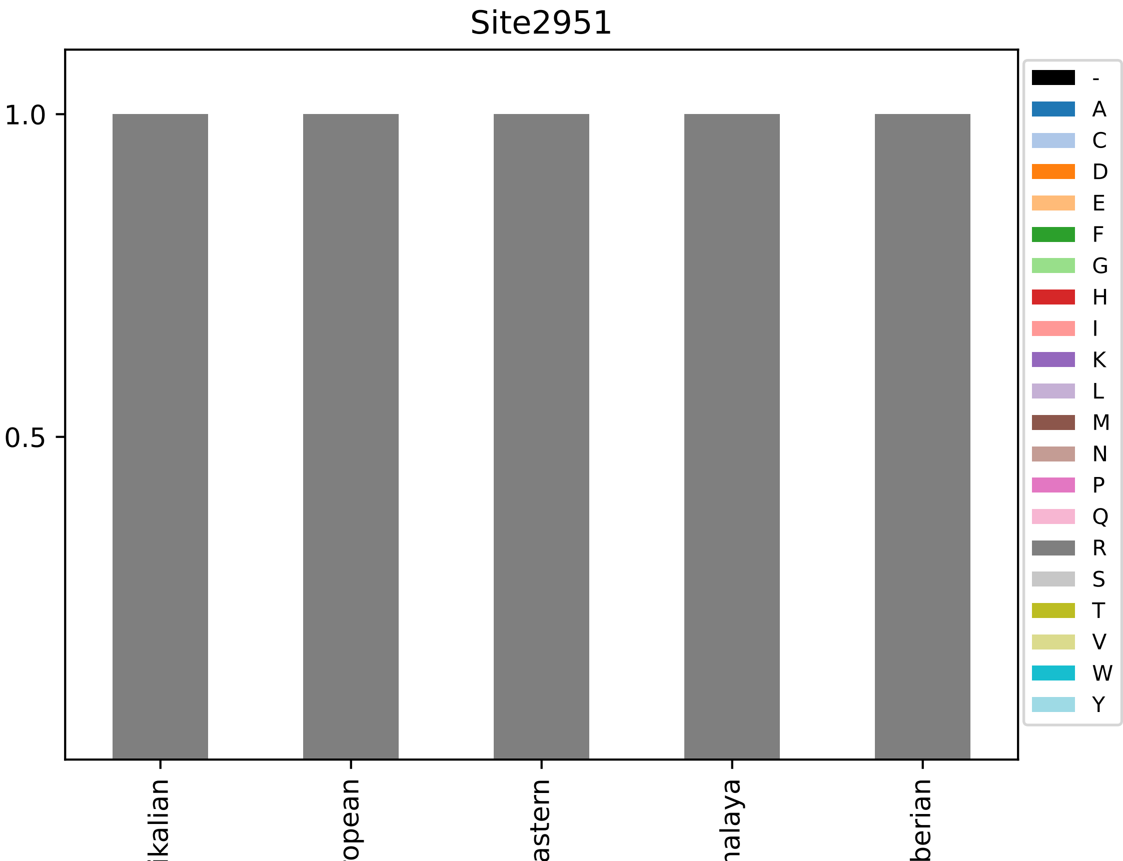

Site2951
1.0
0.5
-
A
C
D
E
F
G
H
I
K
L
M
N
P
Q
R
S
T
V
W
Y
ikalian
ropean
astern
nalaya
berian

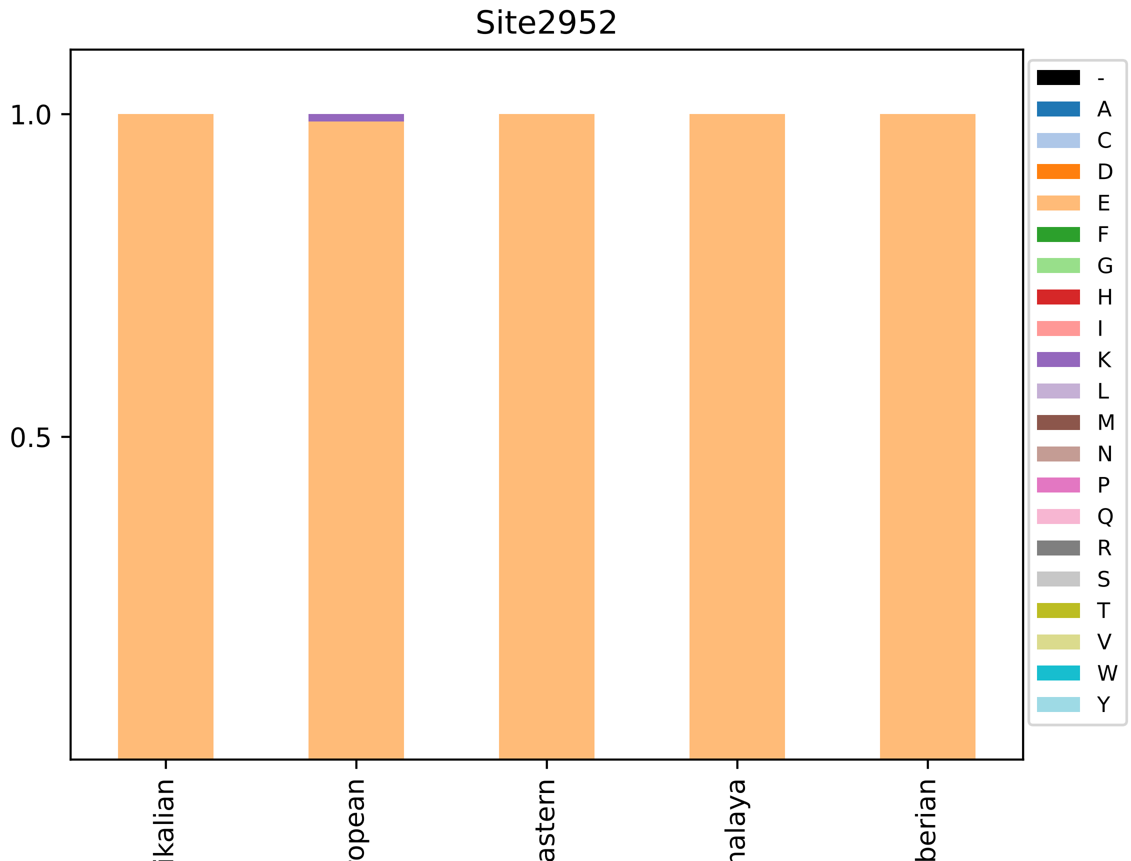
Site2952
1.0
0.5
-
A
C
D
E
F
G
H
I
K
L
M
N
P
Q
R
S
T
V
W
Y
ikalian
opean
astern
nalaya
berian

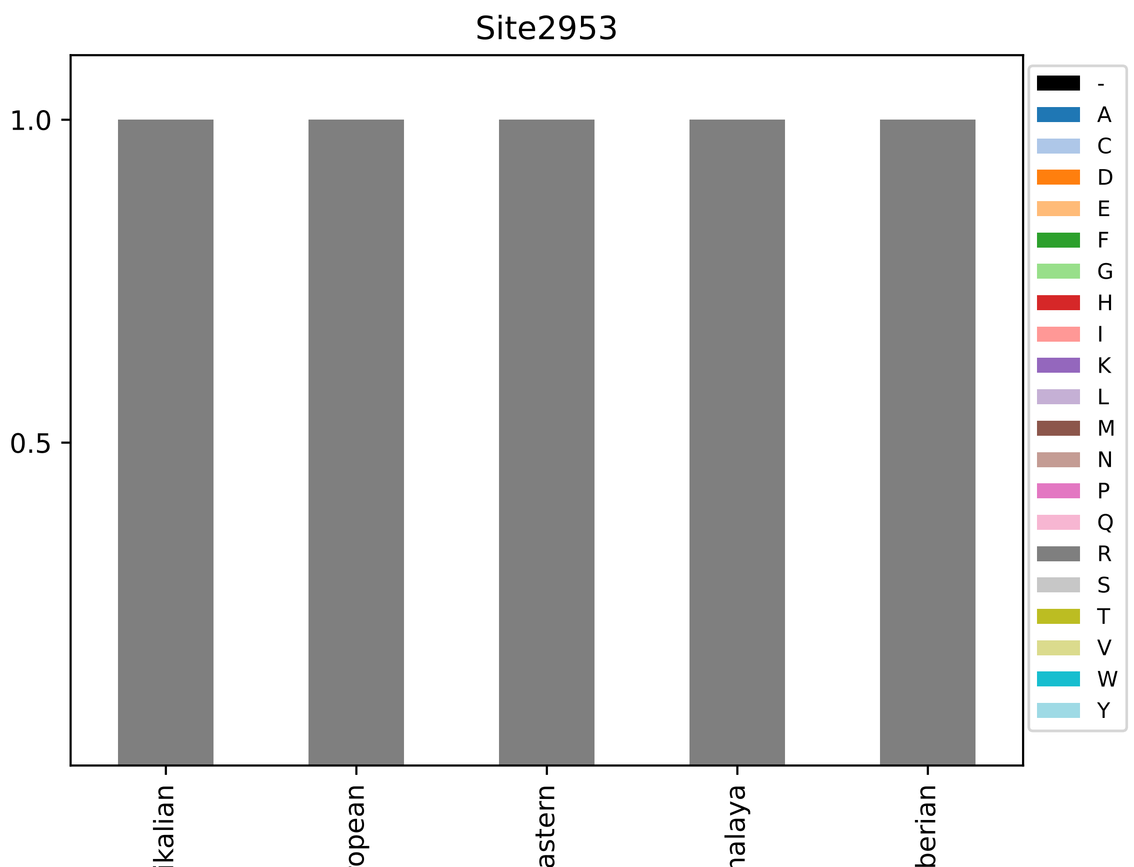
Site2953
1.0
0.5
-
A
C
D
E
F
G
H
I
K
L
M
N
P
Q
R
S
T
V
W
Y
ikalian
opean
astern
nalaya
berian

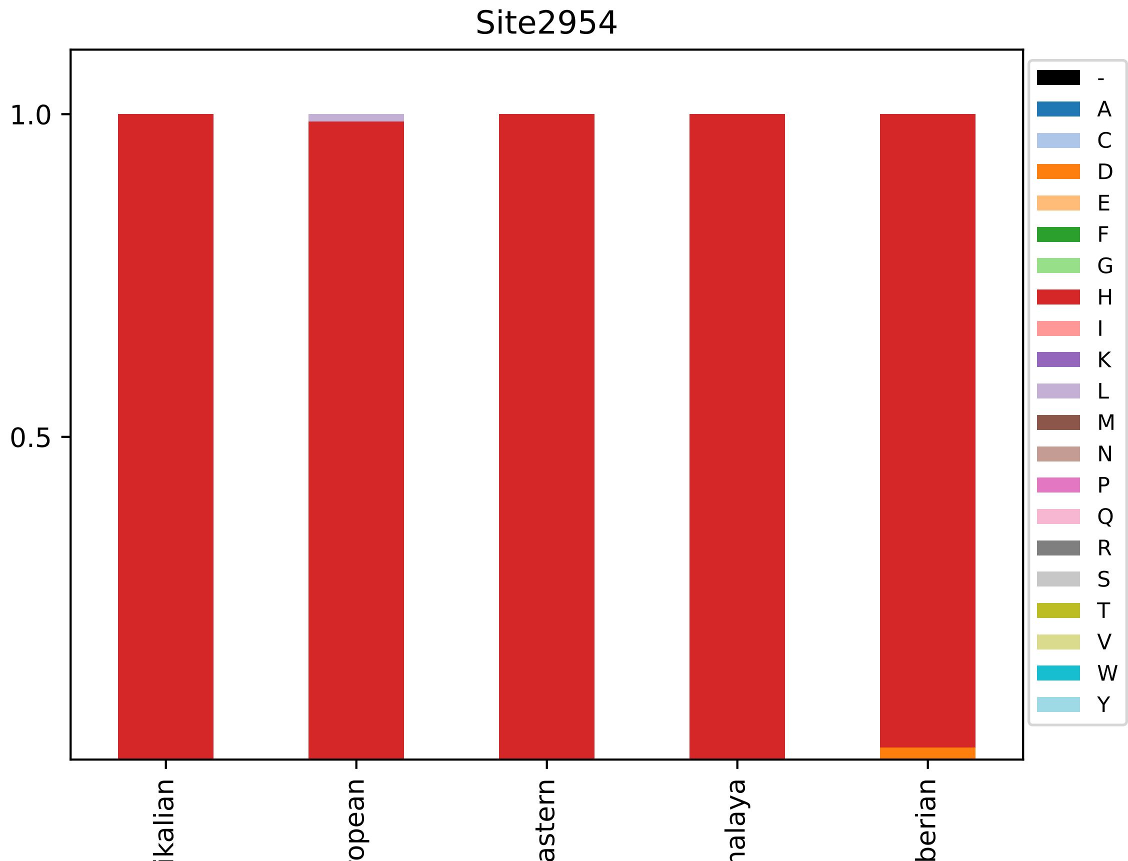
Site2954
1.0
0.5
-
A
C
D
E
F
G
H
I
K
L
M
N
P
Q
R
S
T
V
W
Y
ikalian
opean
astern
nalaya
berian

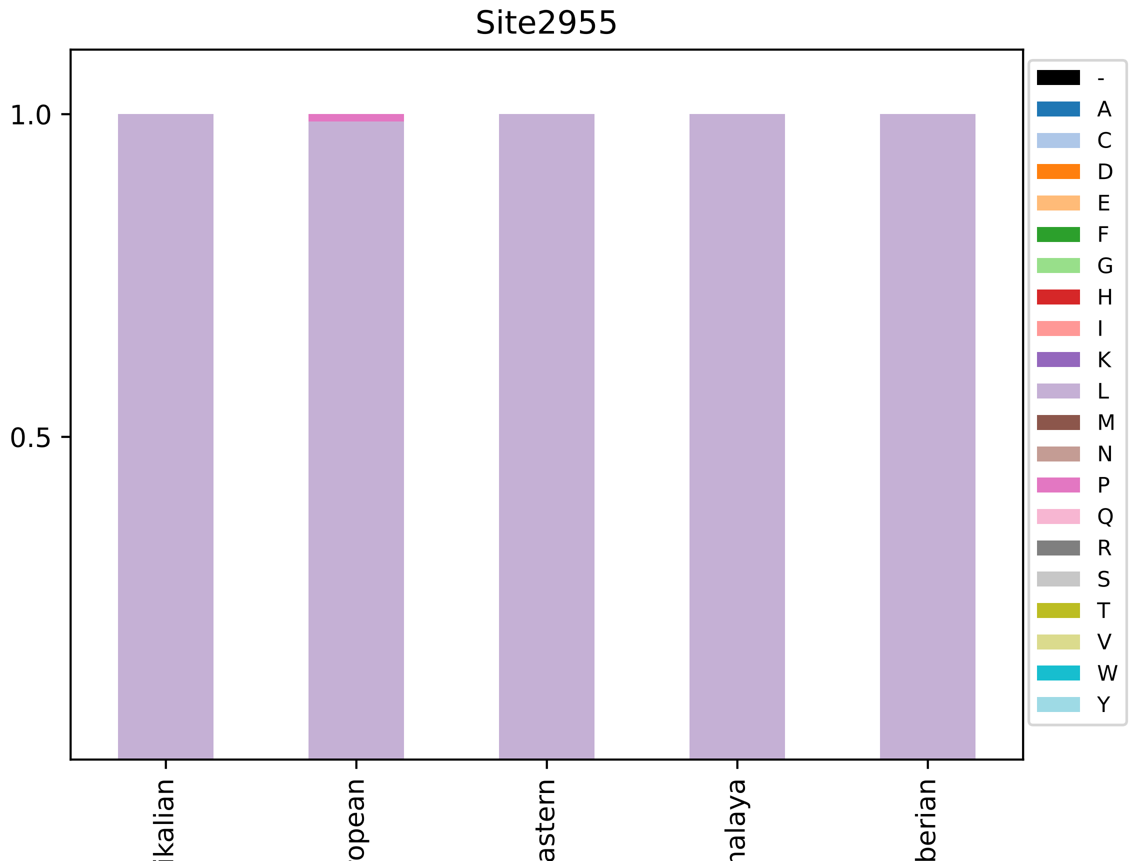

Site2955
1.0
0.5
ikalian
opean
astern
nalaya
berian
-
A
C
D
E
F
G
H
I
K
L
M
N
P
Q
R
S
T
V
W
Y

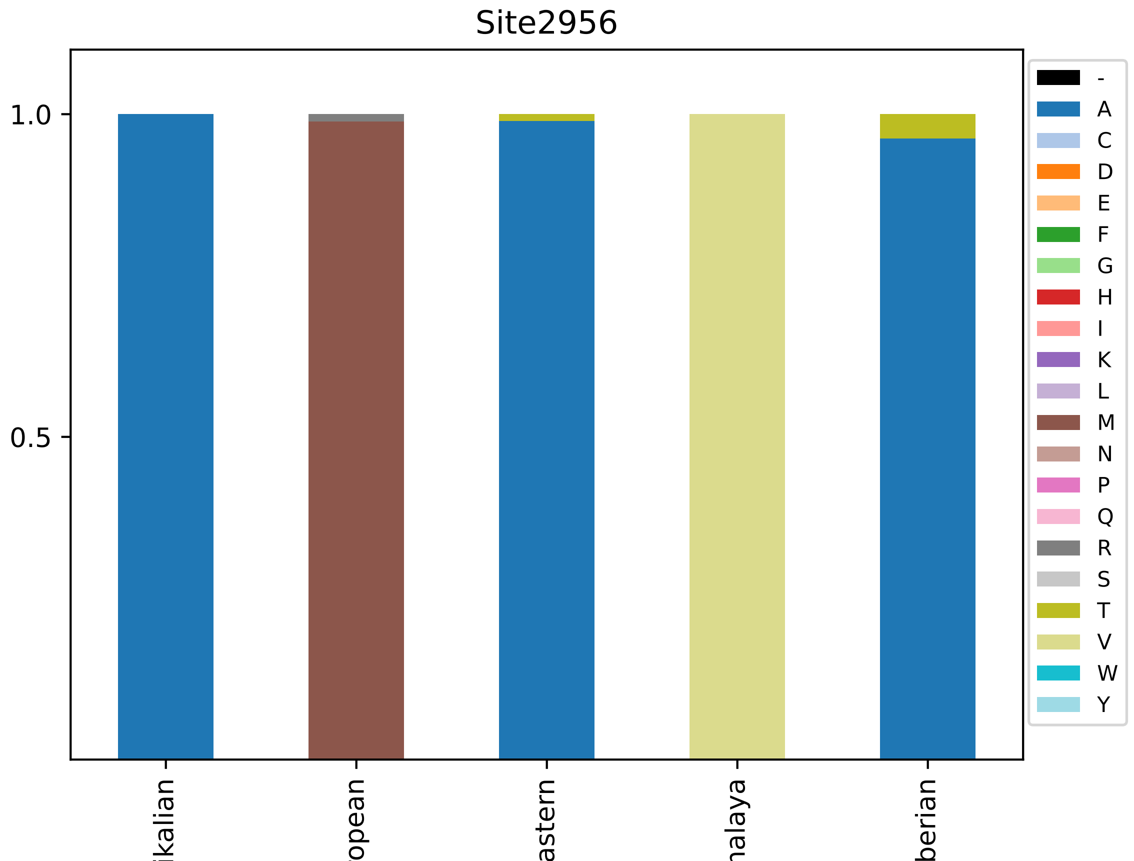

Site2956
1.0
0.5
-
A
C
D
E
F
G
H
I
K
L
M
N
P
Q
R
S
T
V
W
Y
ikalian
ropean
astern
nalaya
berian

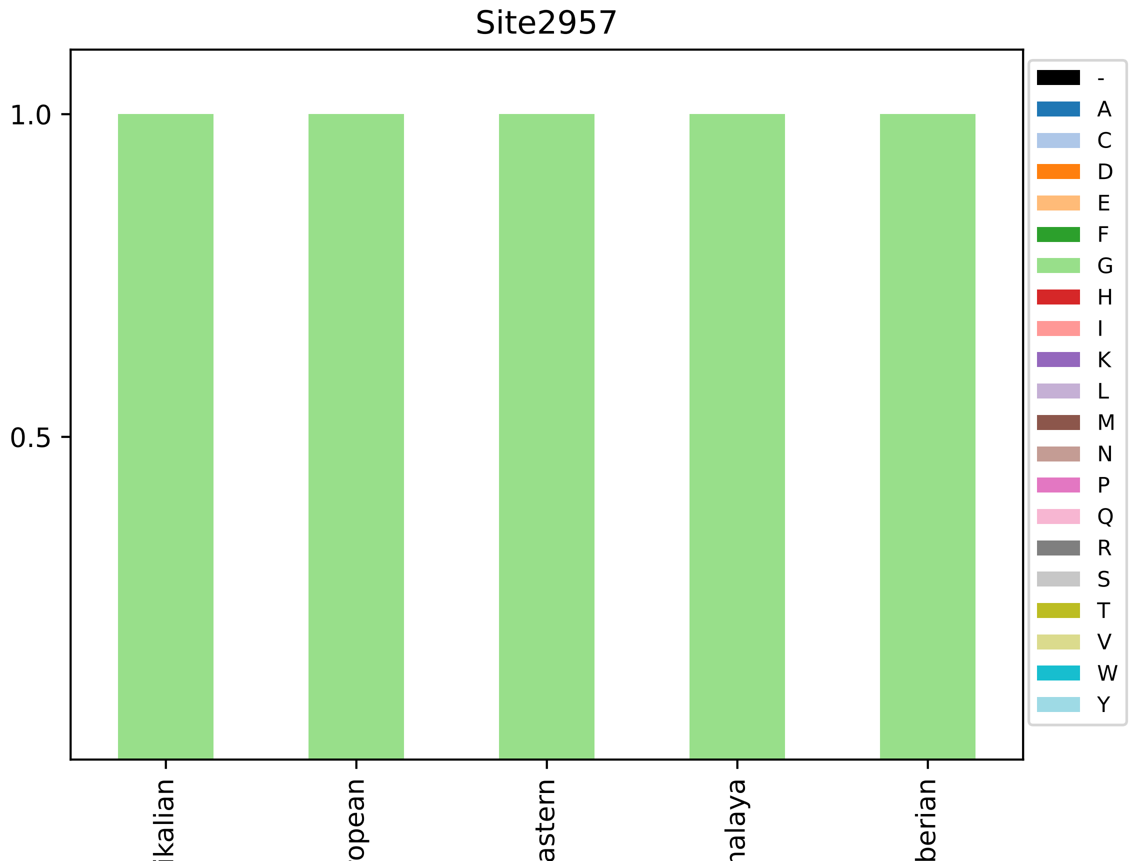

Site2957
1.0
0.5
-
A
C
D
E
F
G
H
I
K
L
M
N
P
Q
R
S
T
V
W
Y
ikalian
opean
astern
nalaya
berian

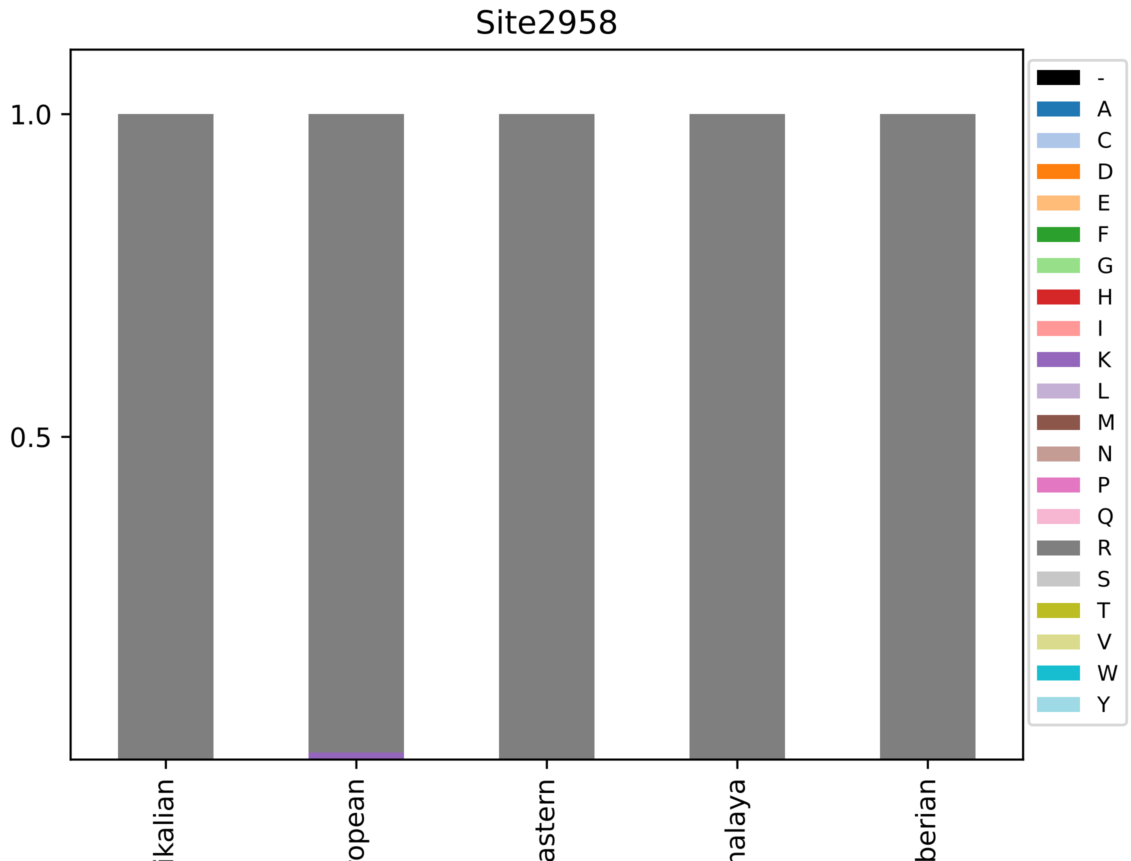
Site2958
1.0
0.5
-
A
C
D
E
F
G
H
I
K
L
M
N
P
Q
R
S
T
V
W
Y
ikalian
opean
astern
nalaya
berian

Site2959

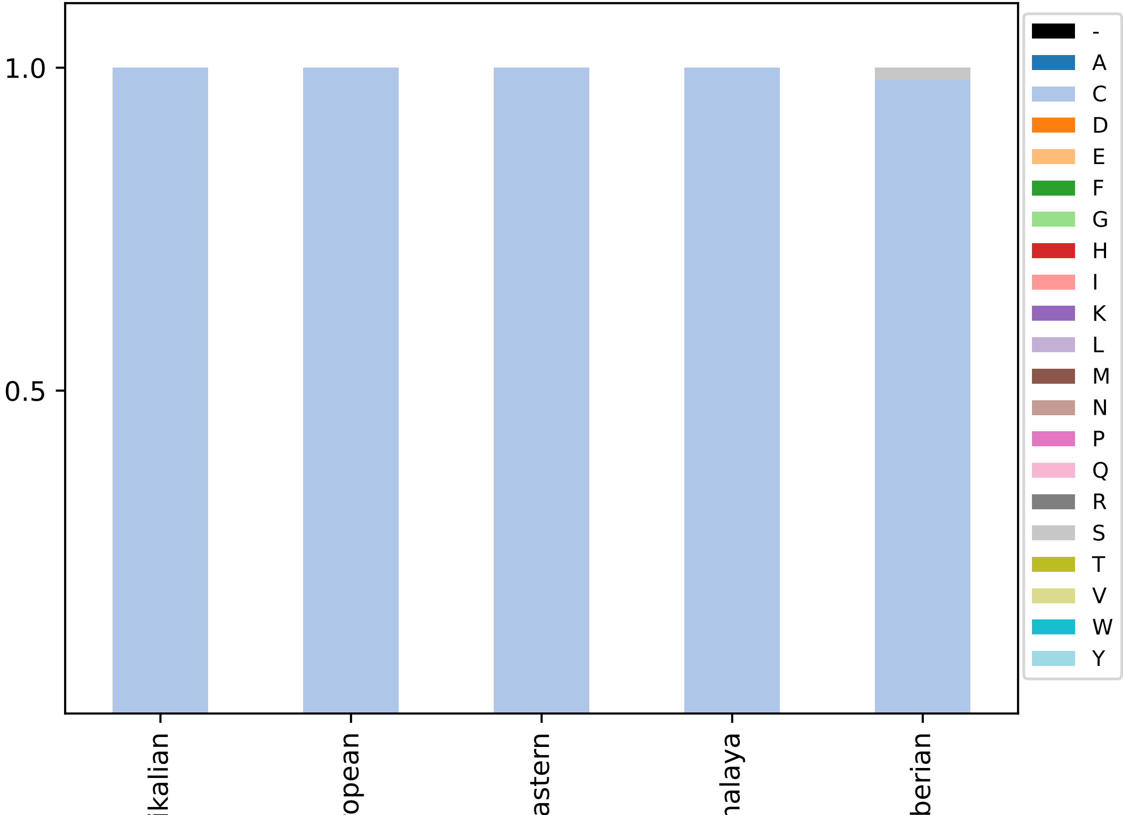
1.0
0.5
ikalian
opean
astern
nalaya
berian
-
A
C
D
E
F
G
H
I
K
L
M
N
P
Q
R
S
T
V
W
Y

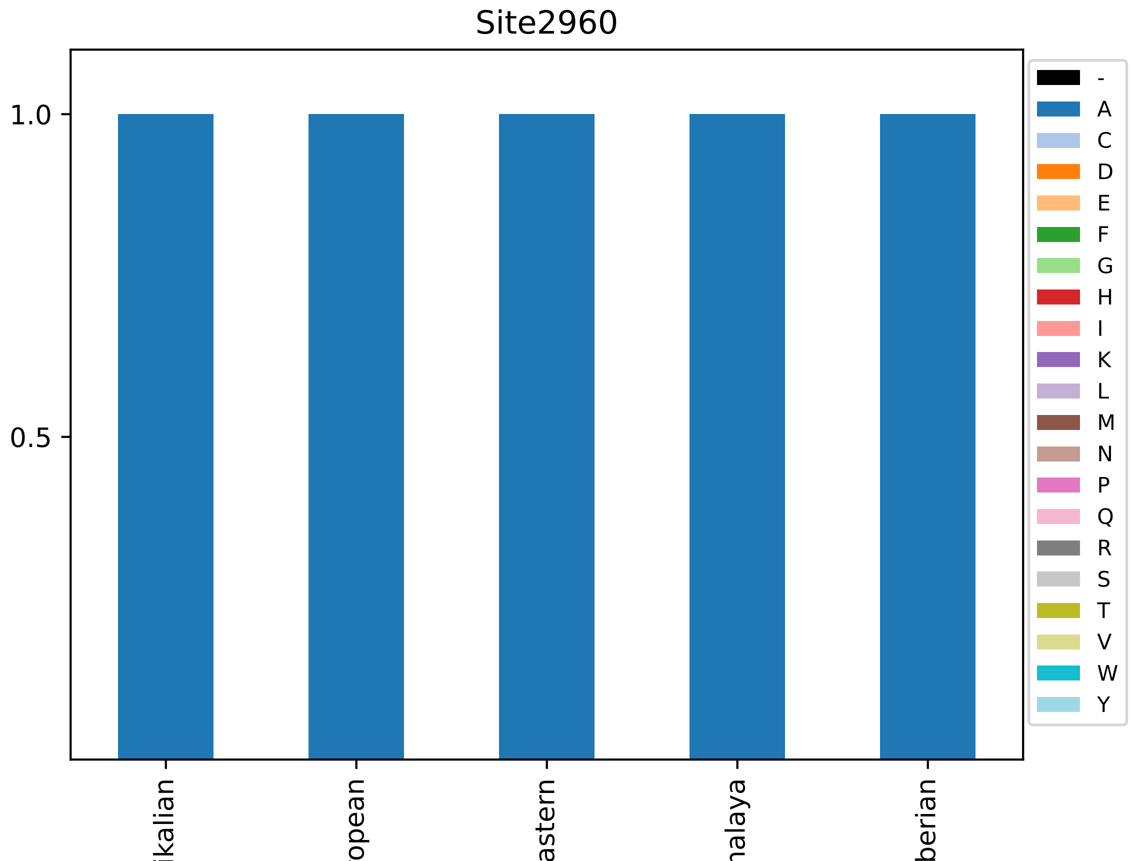

Site2960
1.0
0.5
ikalian
ropean
astern
nalaya
berian
-
A
C
D
E
F
G
H
I
K
L
M
N
P
Q
R
S
T
V
W
Y

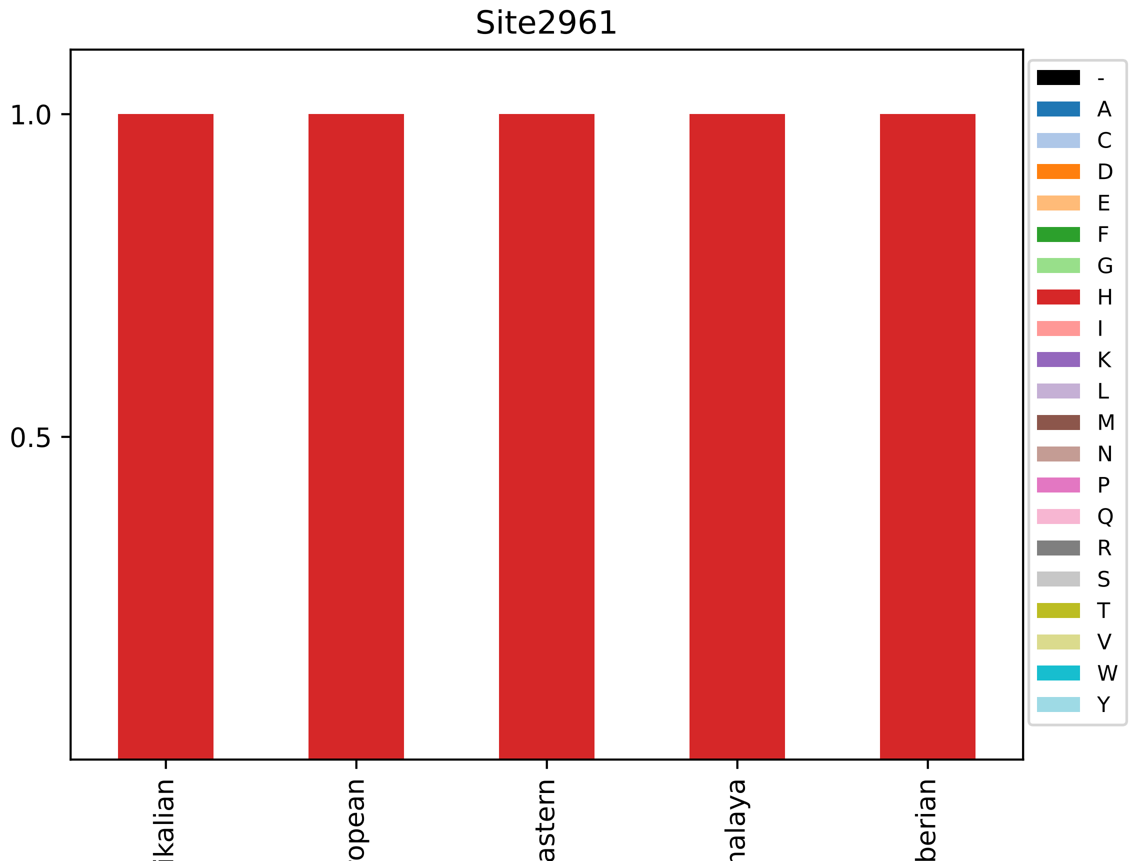
Site2961
1.0
0.5
-
A
C
D
E
F
G
H
I
K
L
M
N
P
Q
R
S
T
V
W
Y
ikalian
opean
astern
nalaya
berian

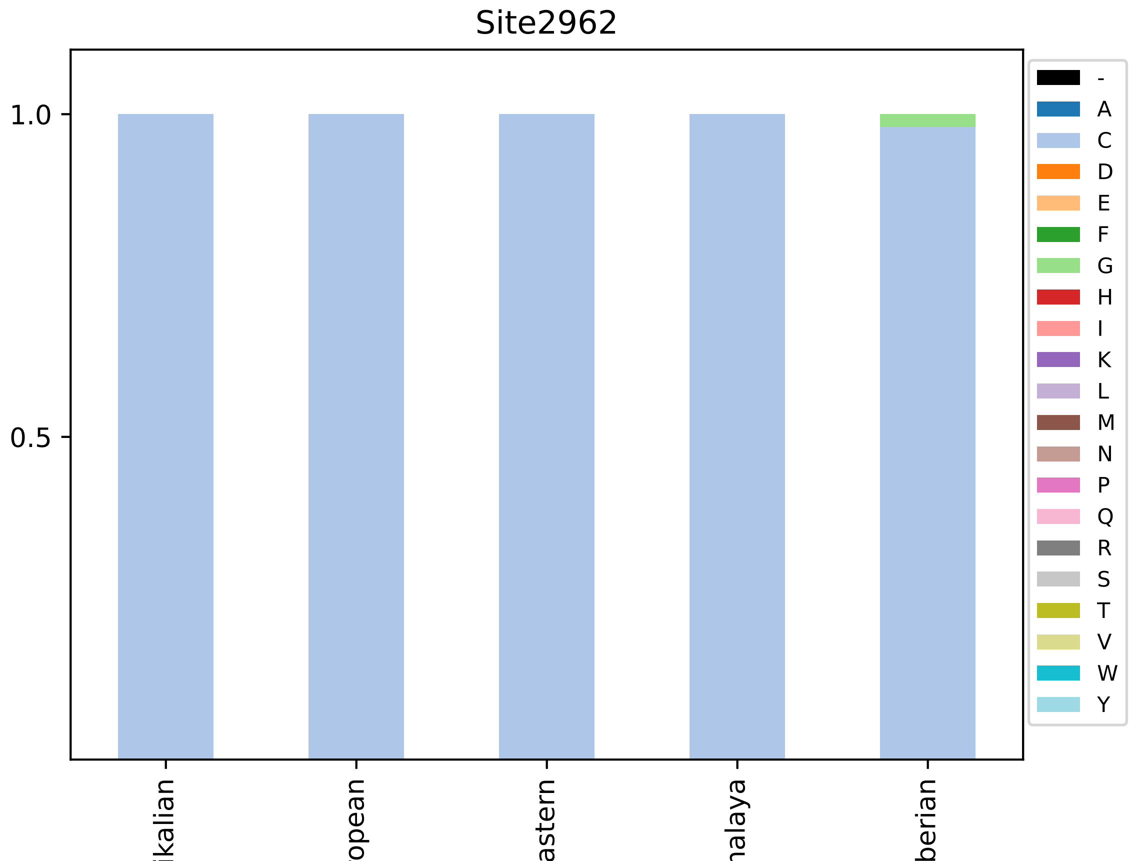

Site2962
1.0
0.5
-
A
C
D
E
F
G
H
I
K
L
M
N
P
Q
R
S
T
V
W
Y
ikalian
opean
astern
nalaya
berian

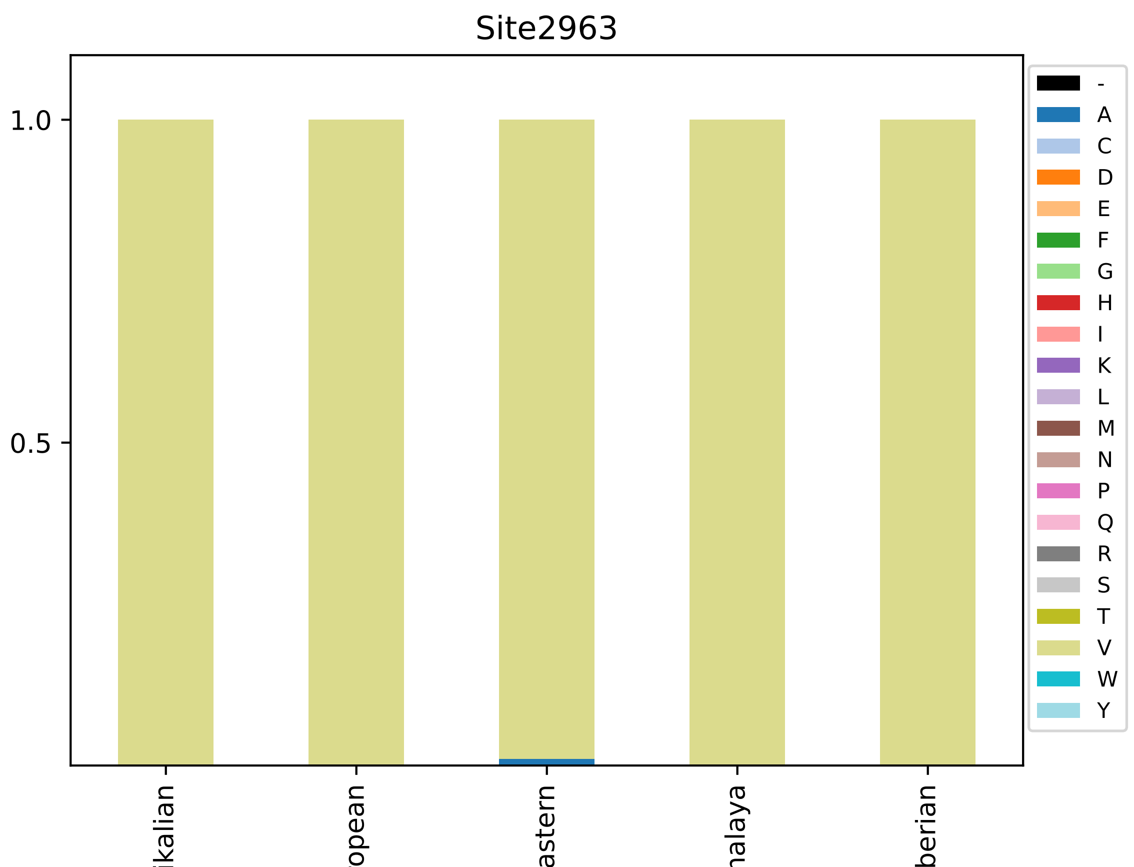

Site2963
1.0
0.5
-
A
C
D
E
F
G
H
I
K
L
M
N
P
Q
R
S
T
V
W
Y
ikalian
opean
astern
nalaya
berian

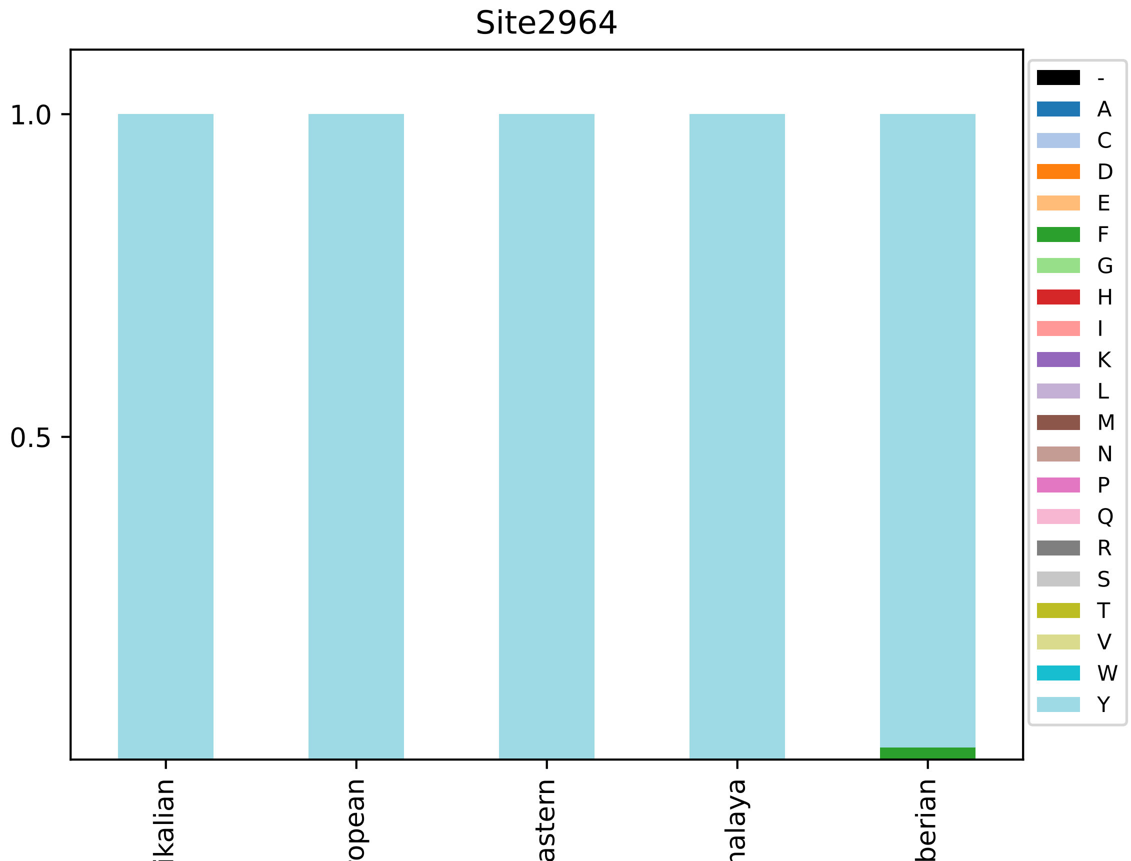

Site2964
1.0
0.5
ikalian
opean
astern
nalaya
berian
-
A
C
D
E
F
G
H
I
K
L
M
N
P
Q
R
S
T
V
W
Y

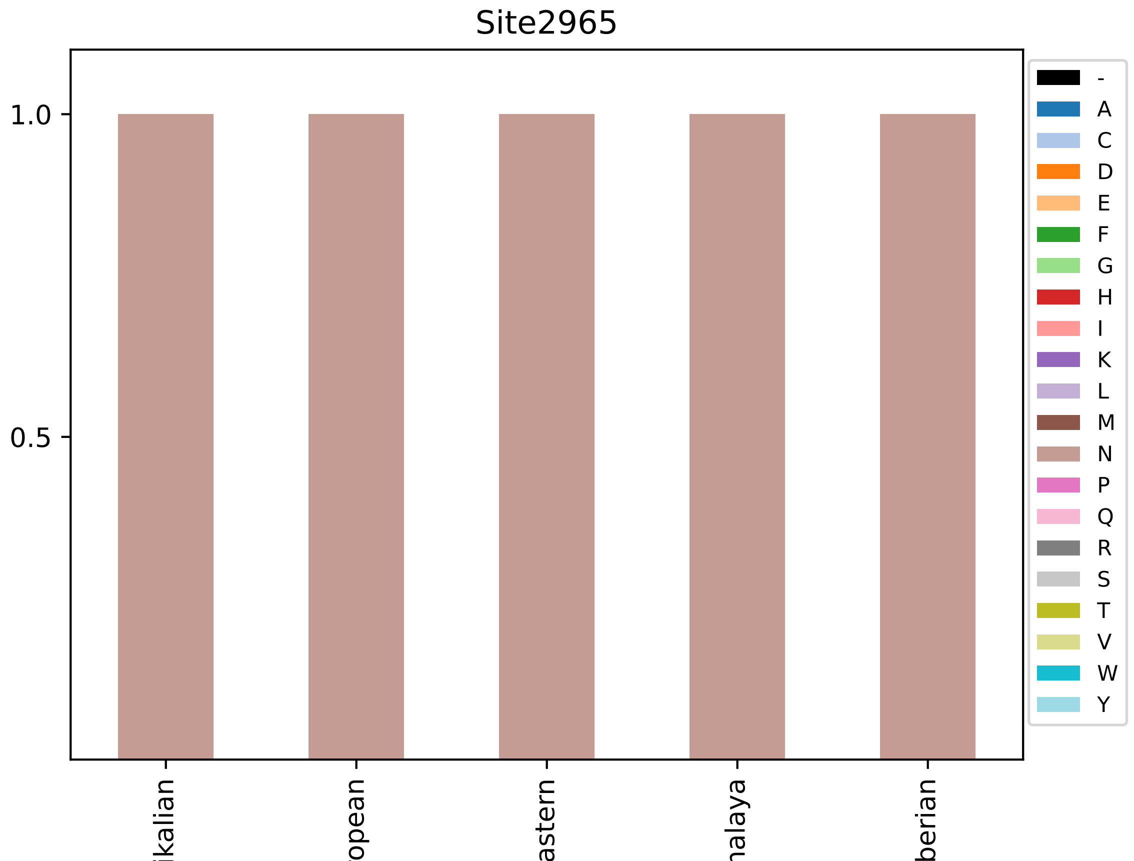
Site2965
1.0
0.5
-
A
C
D
E
F
G
H
I
K
L
M
N
P
Q
R
S
T
V
W
Y

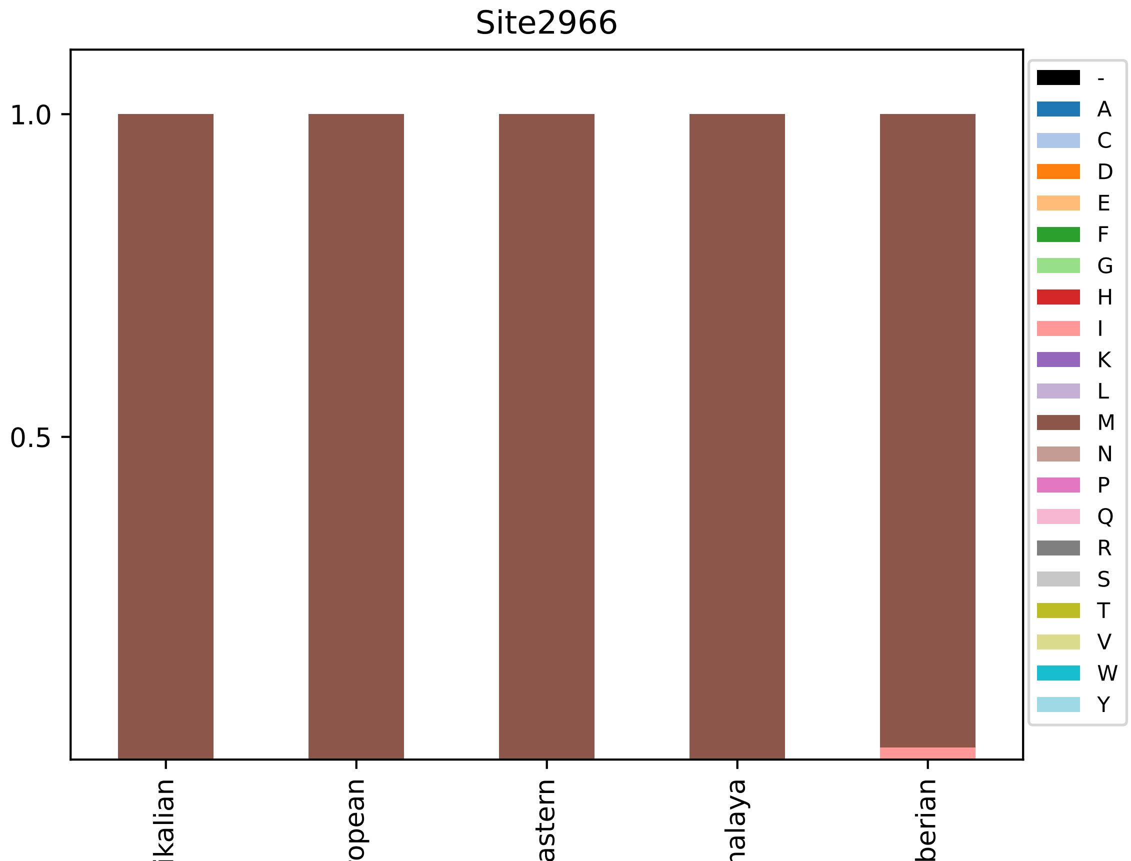

Site2966
1.0
0.5
-
A
C
D
E
F
G
H
I
K
L
M
N
P
Q
R
S
T
V
W
Y
ikalian
opean
astern
nalaya
berian

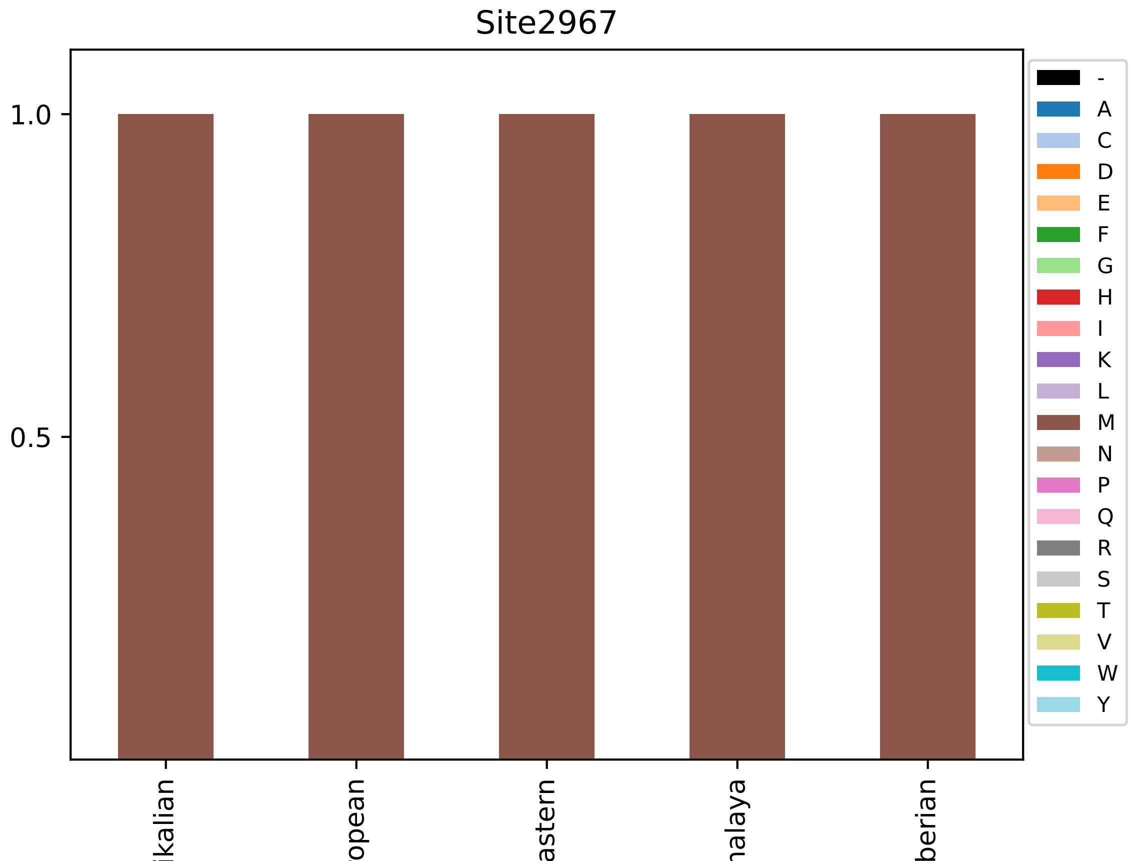
Site2967
1.0
0.5
-
A
C
D
E
F
G
H
I
K
L
M
N
P
Q
R
S
T
V
W
Y
ikalian
opean
astern
nalaya
berian

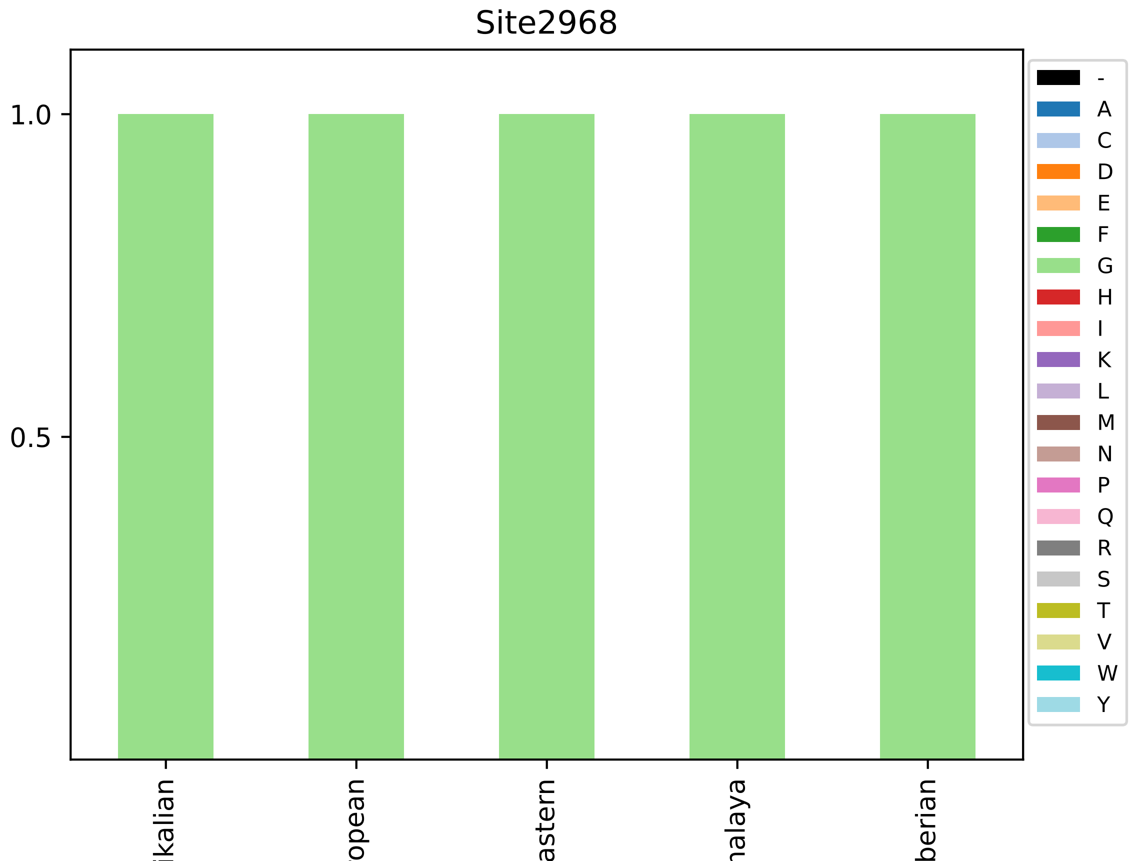
Site2968
1.0
0.5
-
A
C
D
E
F
G
H
I
K
L
M
N
P
Q
R
S
T
V
W
Y
ikalian
opean
astern
nalaya
berian

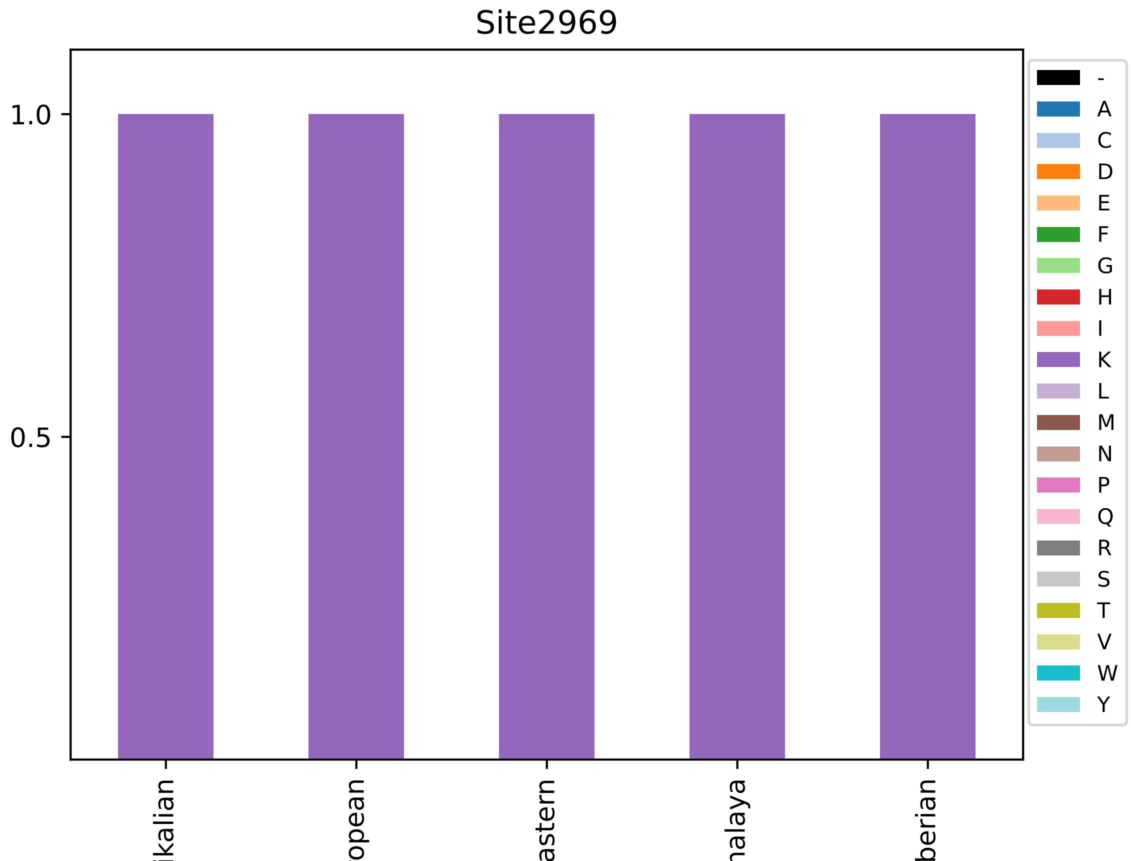
Site2969
1.0
0.5
-
A
C
D
E
F
G
H
I
K
L
M
N
P
Q
R
S
T
V
W
Y
ikalian
opean
astern
nalaya
berian

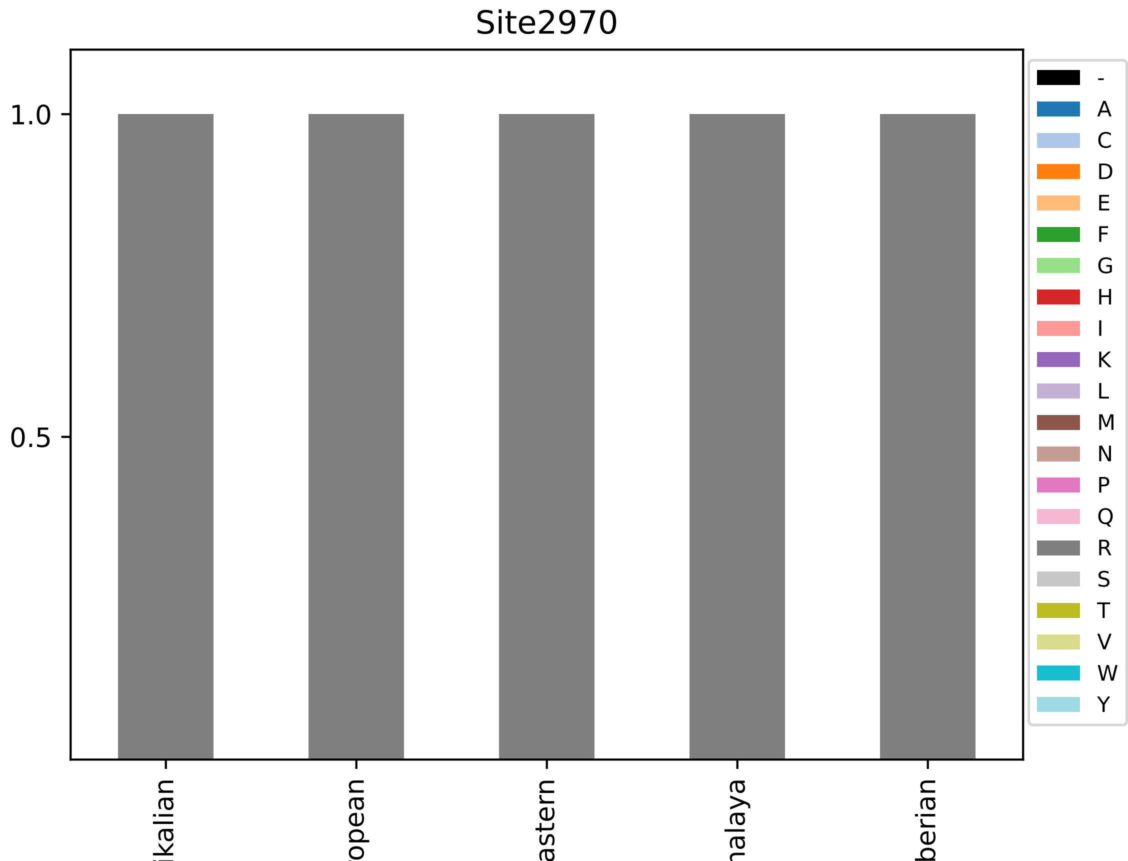

Site2970
1.0
0.5
-
A
C
D
E
F
G
H
I
K
L
M
N
P
Q
R
S
T
V
W
Y
ikalian
ropean
astern
nalaya
berian

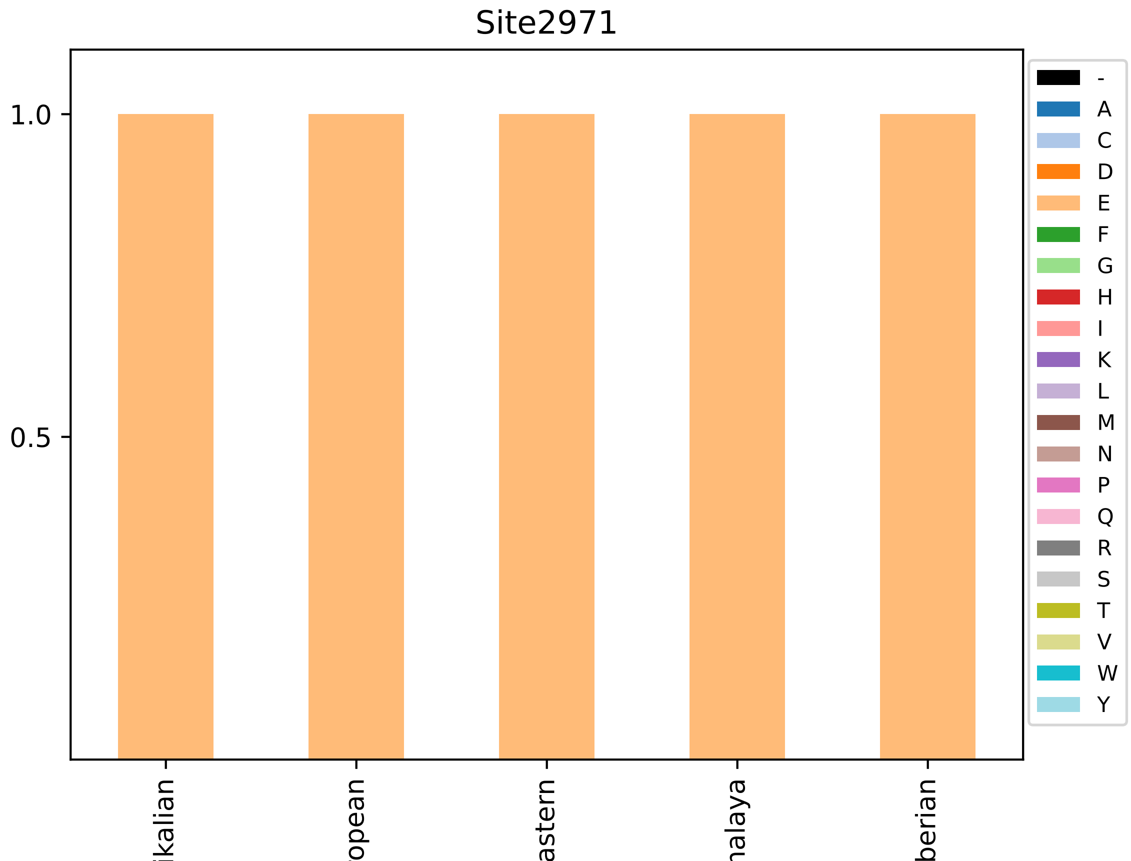

Site2971
1.0
0.5
-
A
C
D
E
F
G
H
I
K
L
M
N
P
Q
R
S
T
V
W
Y
ikalian
opean
astern
nalaya
berian

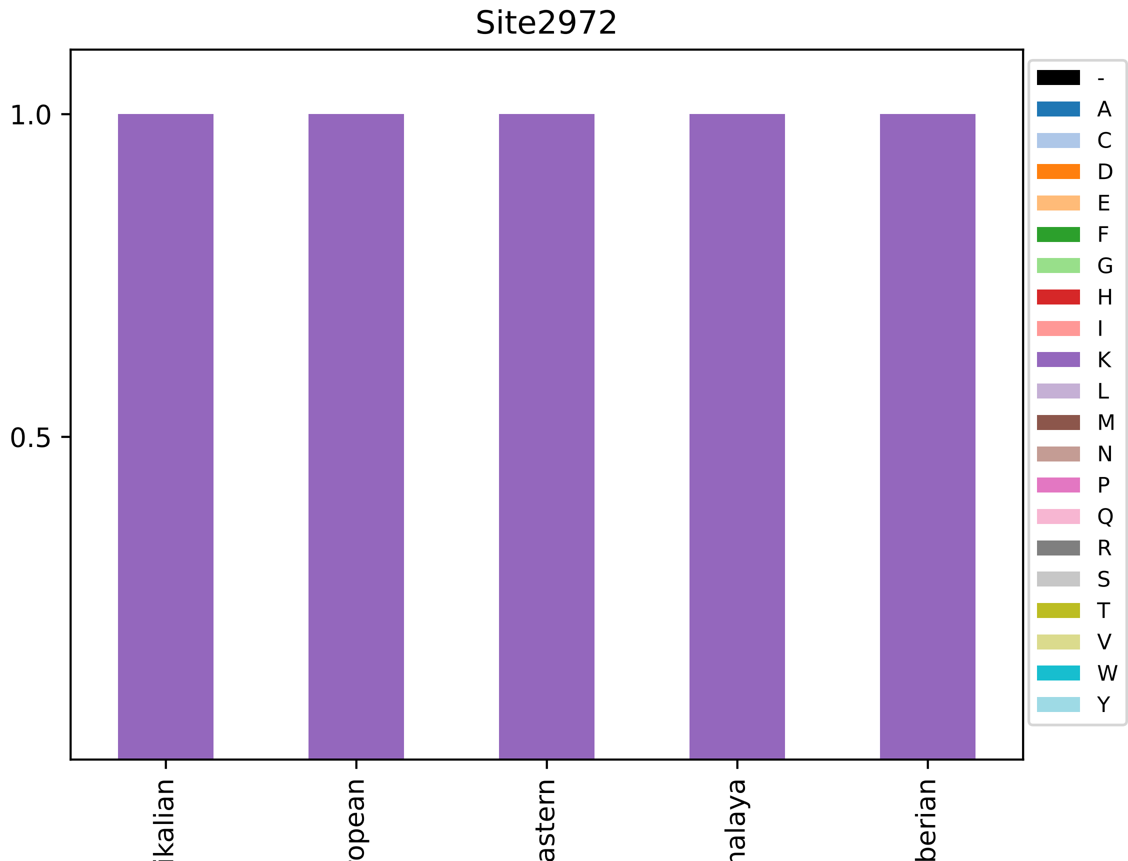

Site2972
1.0
0.5
ikalian
opean
astern
nalaya
berian
-
A
C
D
E
F
G
H
I
K
L
M
N
P
Q
R
S
T
V
W
Y

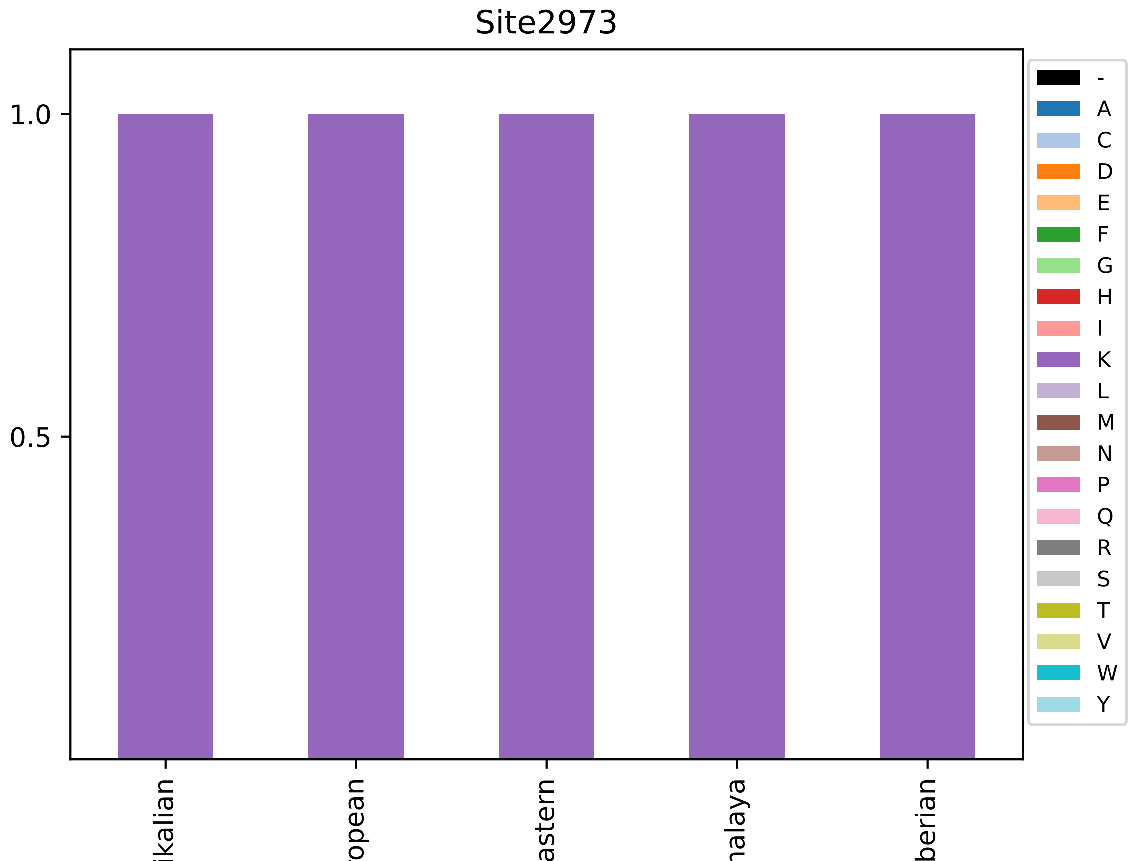
Site2973
1.0
0.5
-
A
C
D
E
F
G
H
I
K
L
M
N
P
Q
R
S
T
V
W
Y
ikalian
opean
astern
nalaya
berian

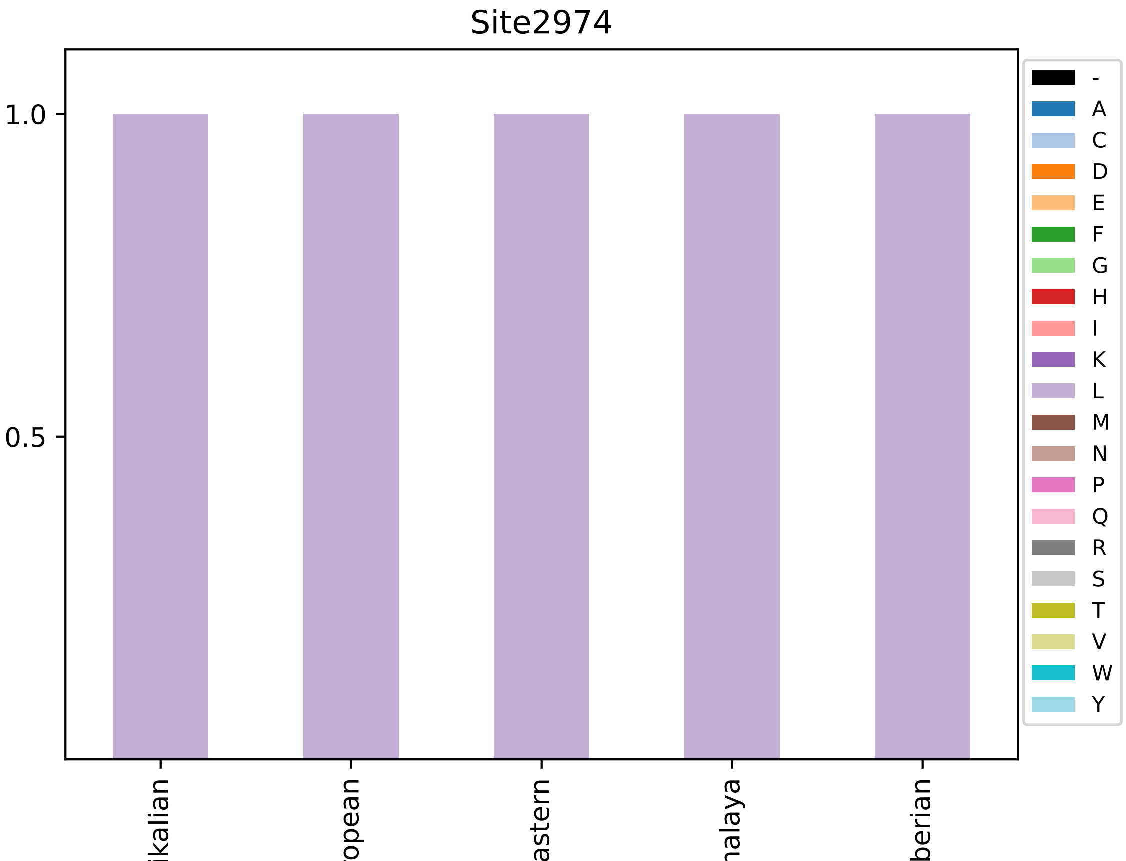

Site2974
1.0
0.5
-
A
C
D
E
F
G
H
I
K
L
M
N
P
Q
R
S
T
V
W
Y
ikalian
opean
astern
nalaya
berian

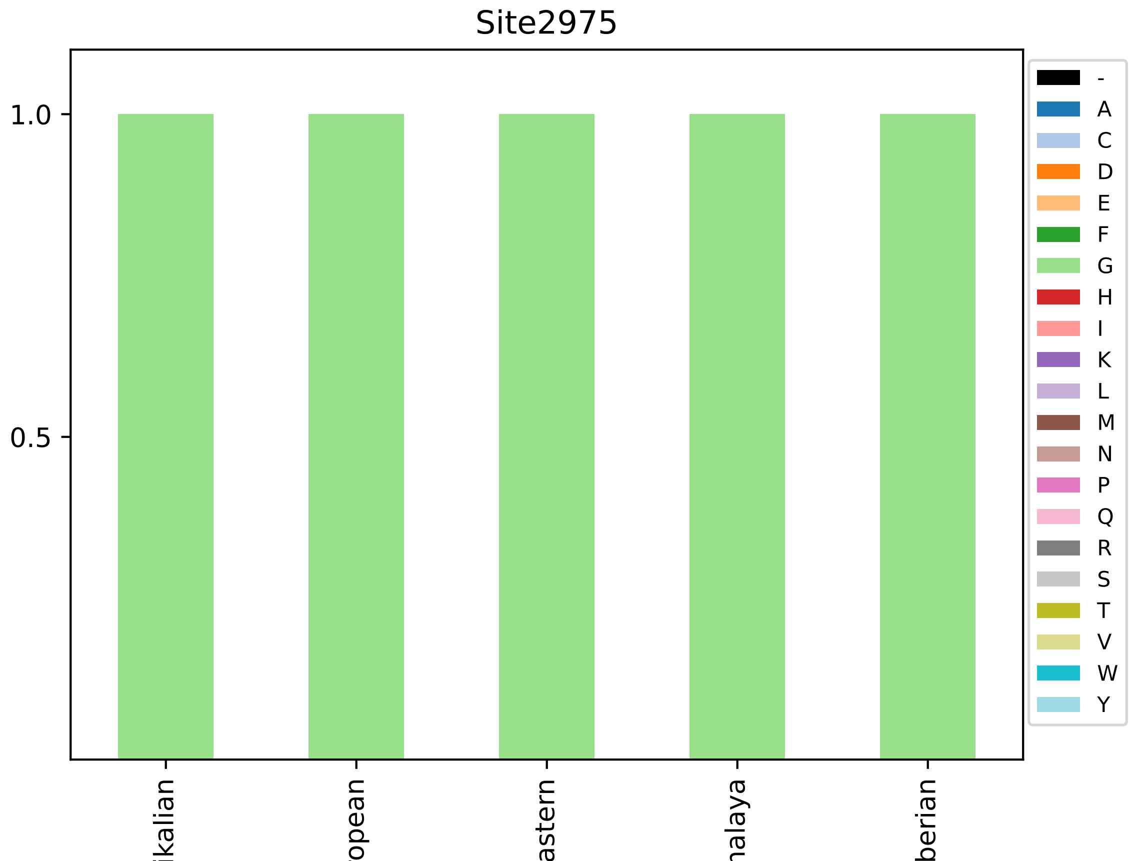

Site2975
1.0
0.5
ikalian
opean
astern
nalaya
berian
-
A
C
D
E
F
G
H
I
K
L
M
N
P
Q
R
S
T
V
W
Y

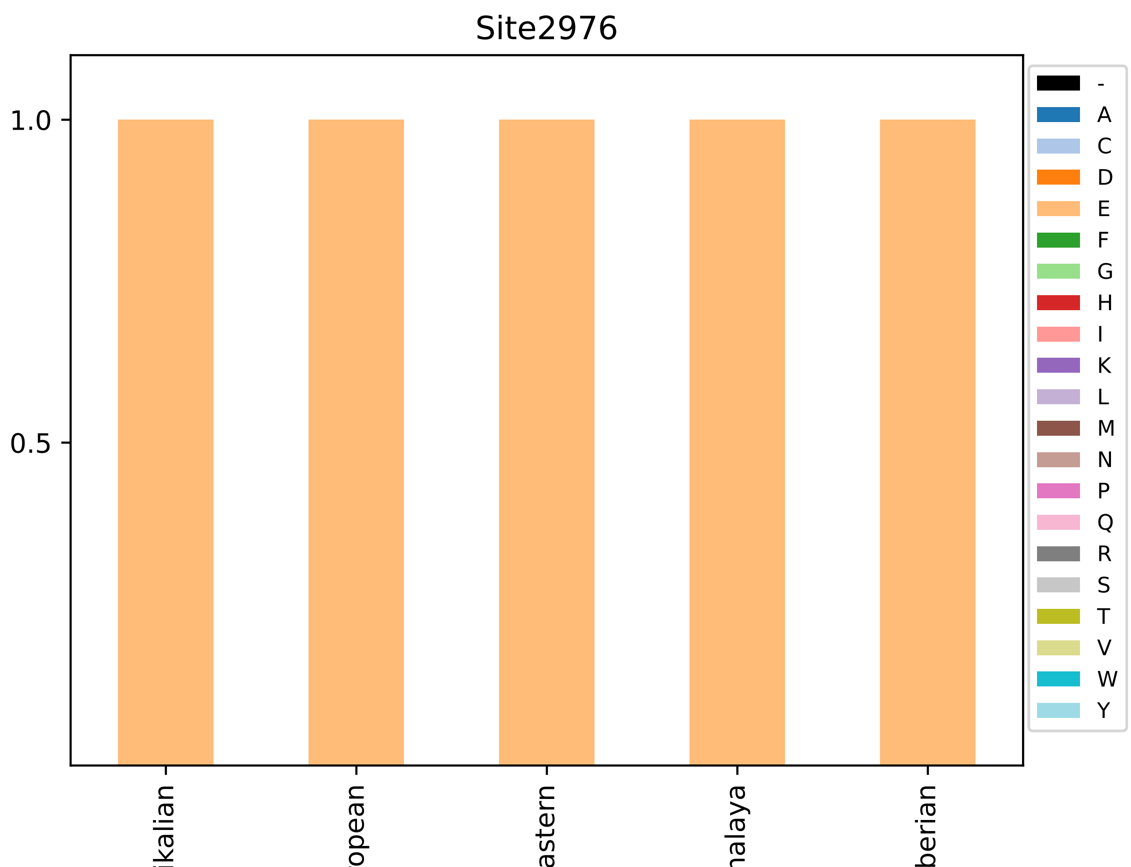

Site2976
1.0
0.5
ikalian
ropean
astern
nalaya
berian
-
A
C
D
E
F
G
H
I
K
L
M
N
P
Q
R
S
T
V
W
Y

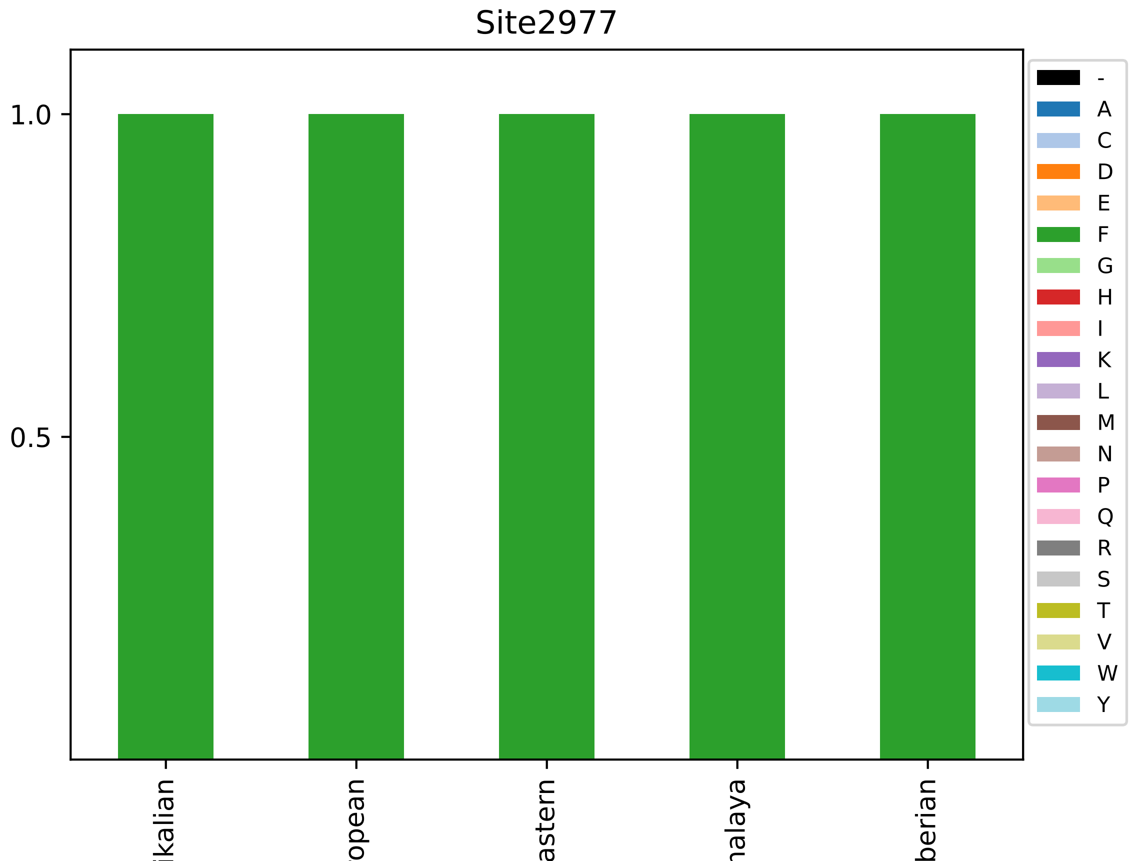

Site2977
1.0
0.5
ikalian
opean
astern
nalaya
berian
-
A
C
D
E
F
G
H
I
K
L
M
N
P
Q
R
S
T
V
W
Y

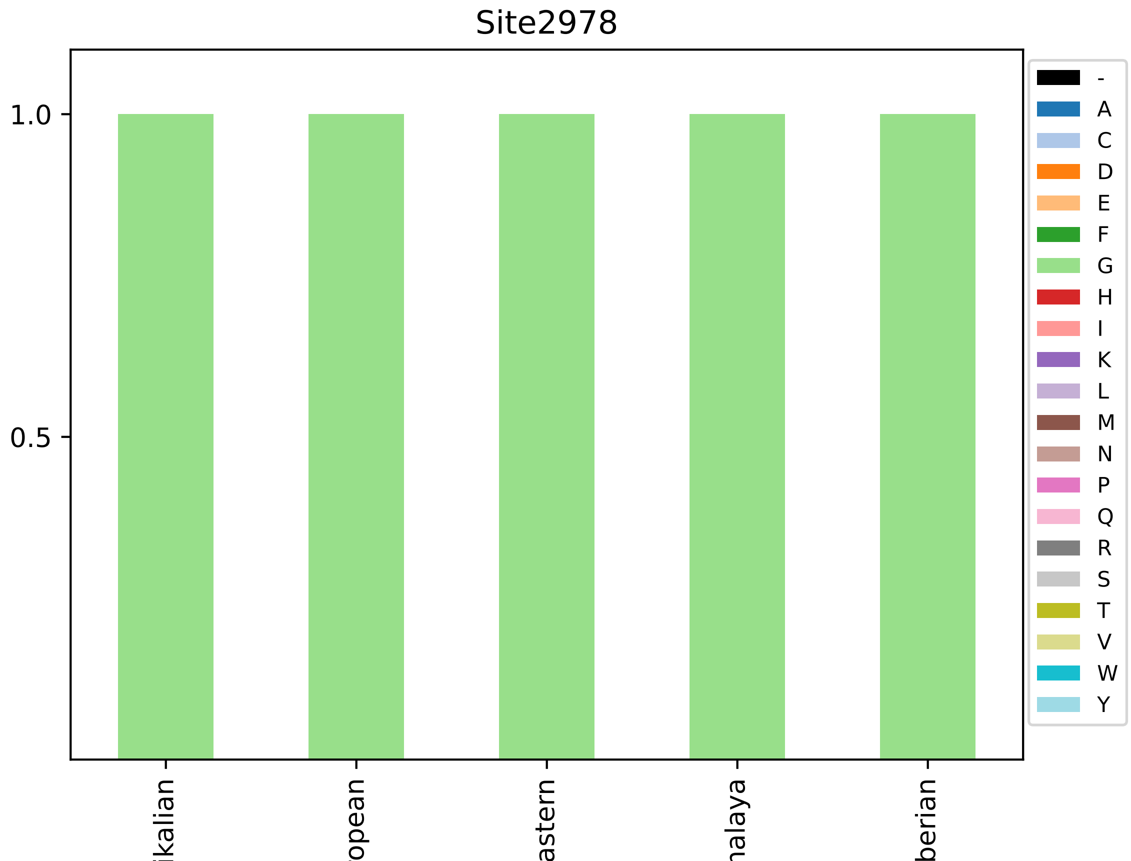
Site2978
1.0
0.5
ikalian
opean
astern
nalaya
berian
-
A
C
D
E
F
G
H
I
K
L
M
N
P
Q
R
S
T
V
W
Y

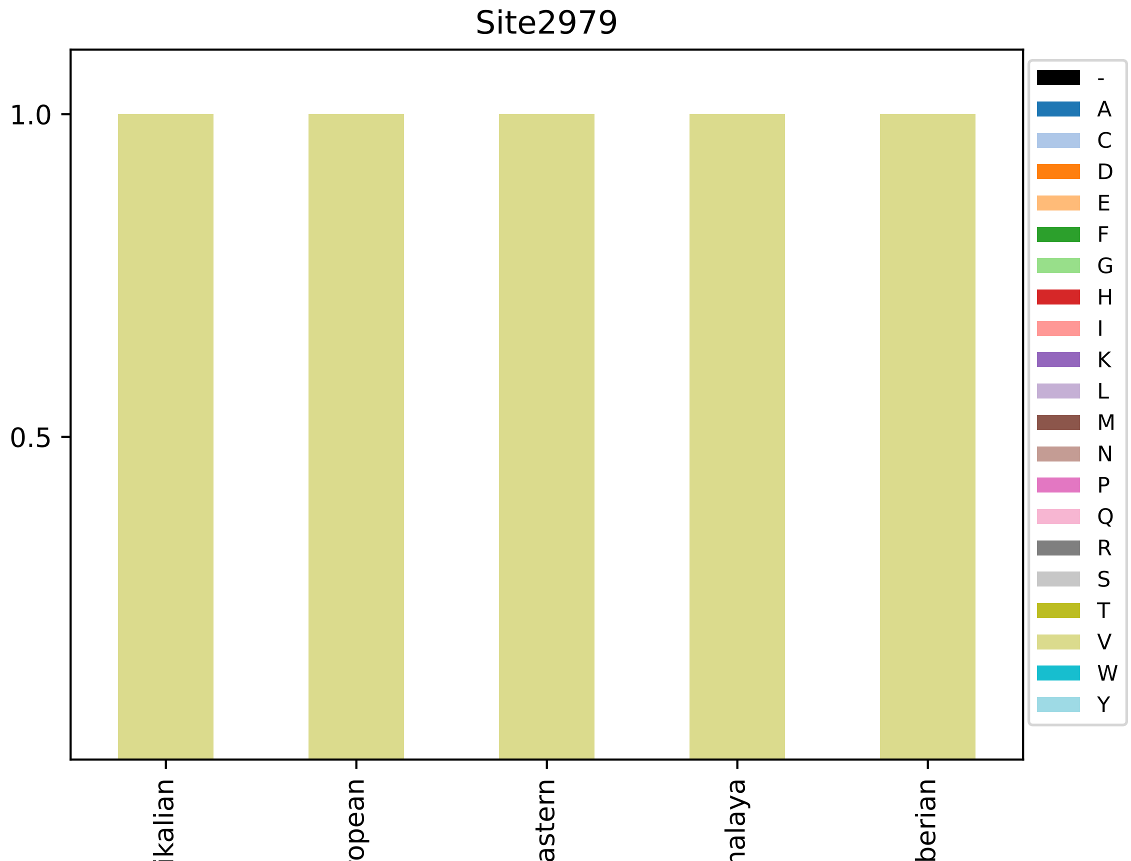

Site2979
1.0
0.5
ikalian
opean
astern
nalaya
berian
-
A
C
D
E
F
G
H
I
K
L
M
N
P
Q
R
S
T
V
W
Y

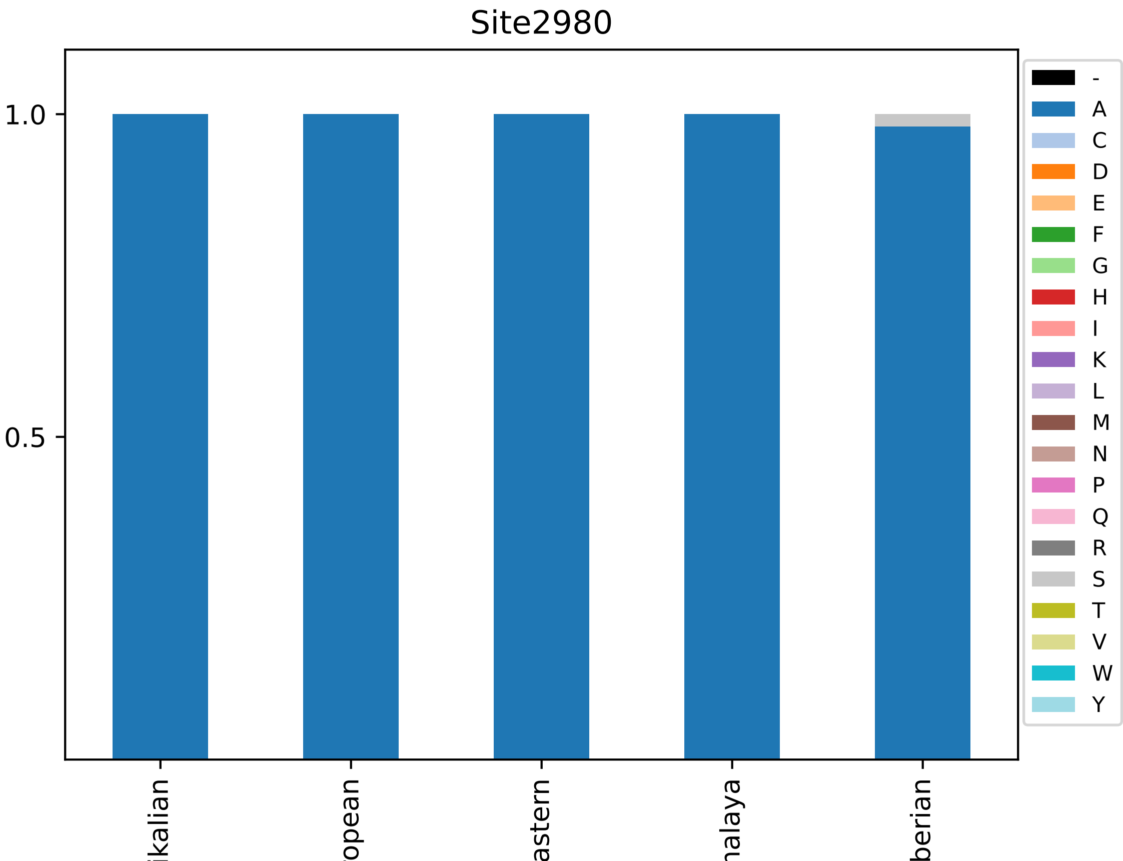

Site2980
1.0
0.5
ikalian
opean
astern
nalaya
berian
-
A
C
D
E
F
G
H
I
K
L
M
N
P
Q
R
S
T
V
W
Y

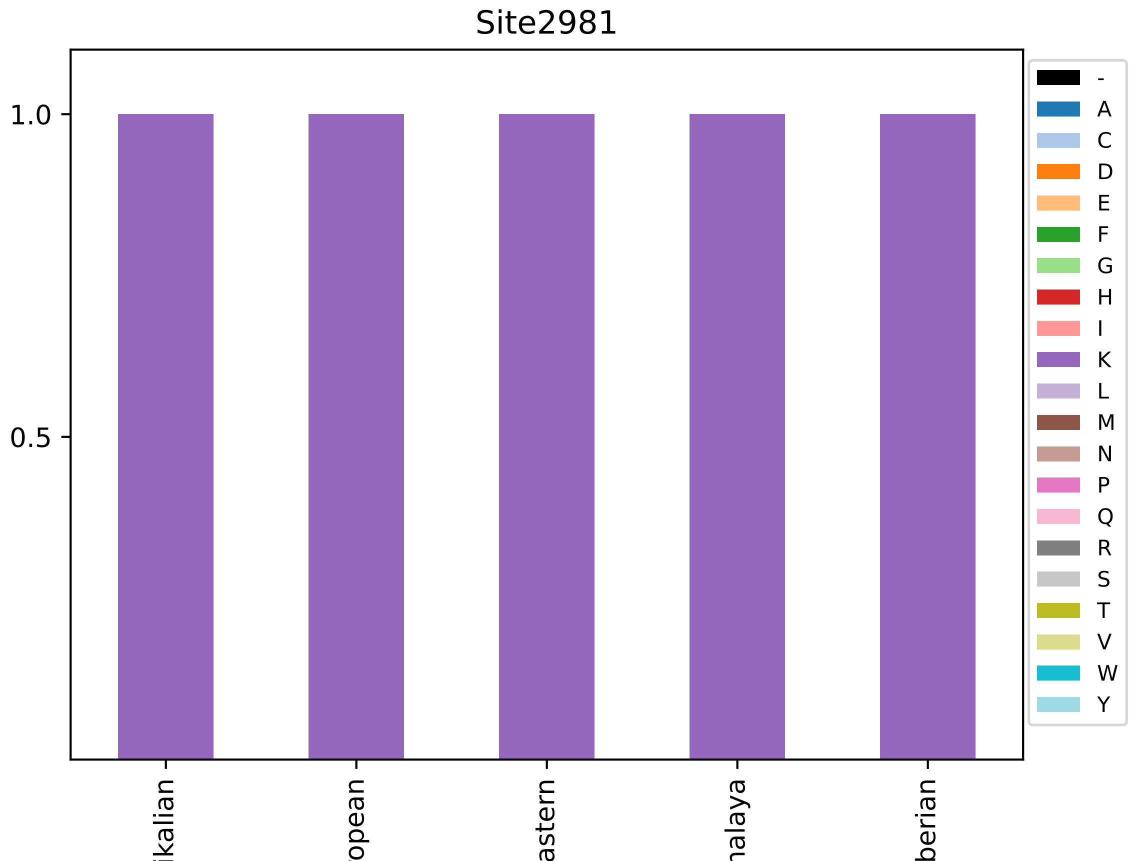
Site2981
1.0
0.5
-
A
C
D
E
F
G
H
I
K
L
M
N
P
Q
R
S
T
V
W
Y
ikalian
opean
astern
nalaya
berian

Site2982

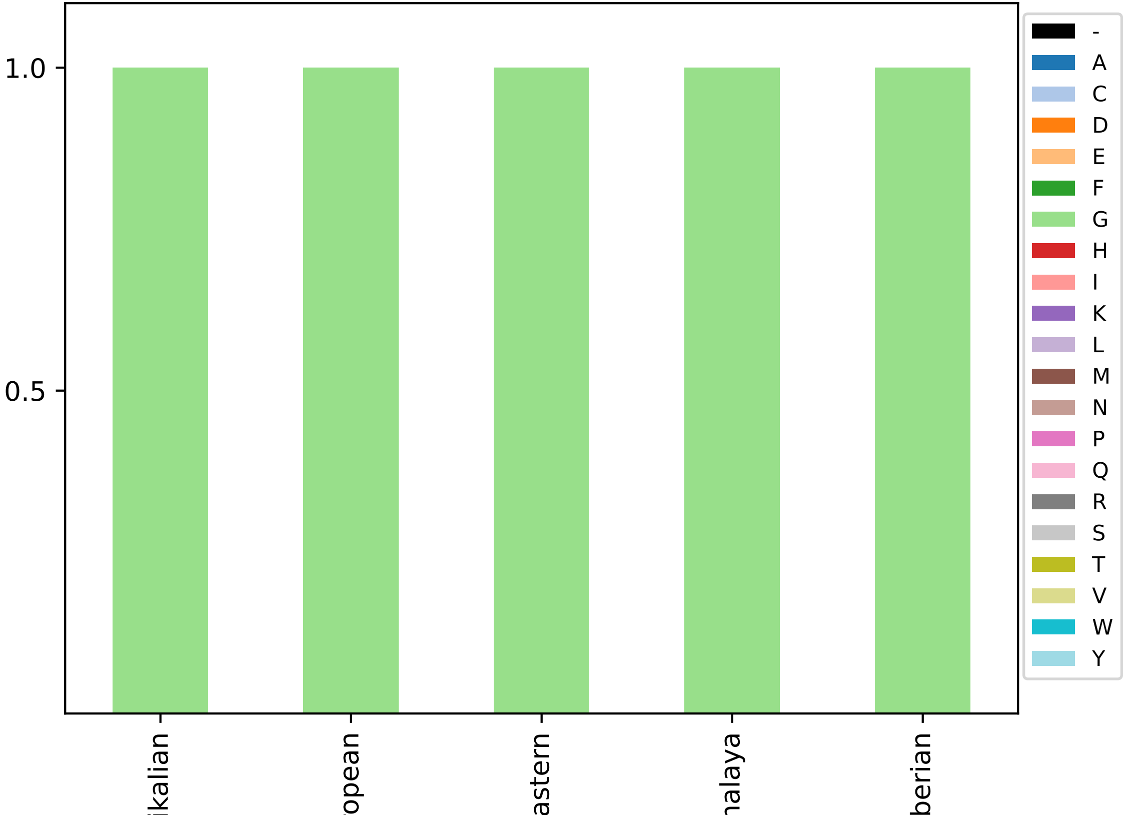

1.0
0.5
-
A
C
D
E
F
G
H
I
K
L
M
N
P
Q
R
S
T
V
W
Y
ikalian
opean
astern
nalaya
berian

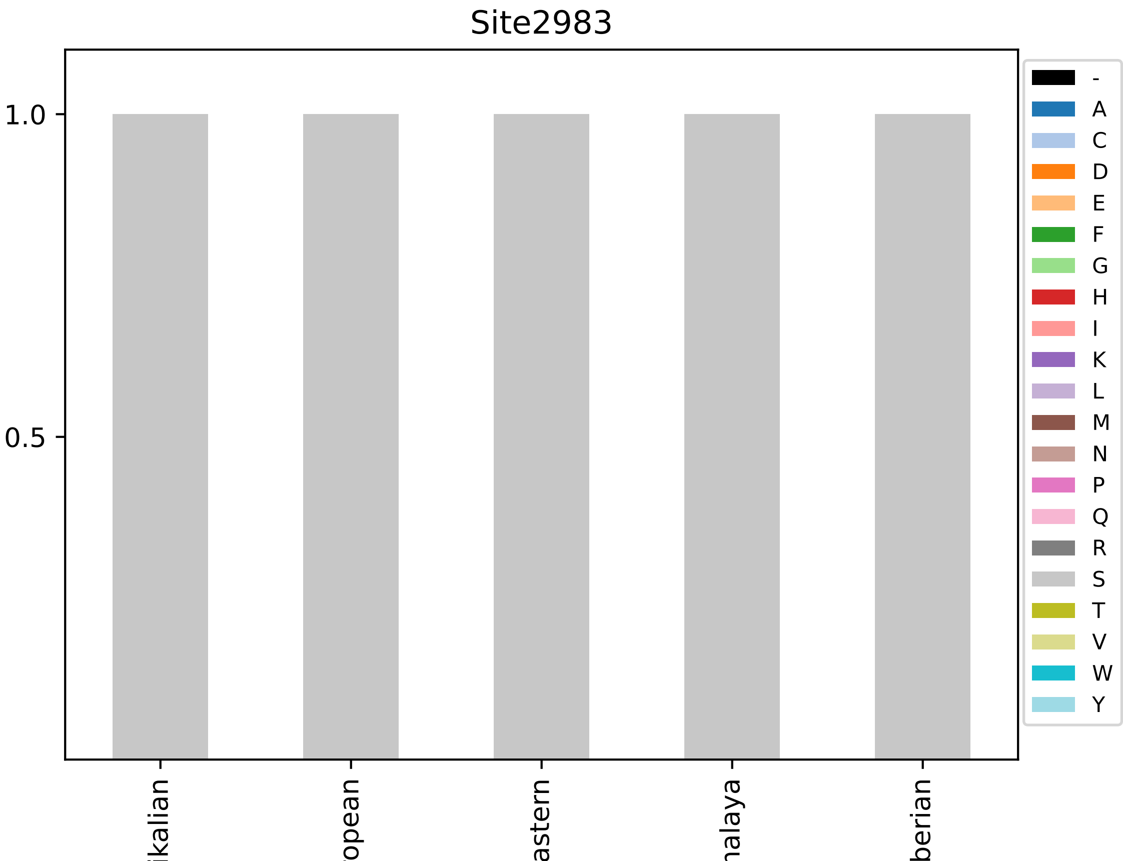

Site2983
1.0
0.5
-
A
C
D
E
F
G
H
I
K
L
M
N
P
Q
R
S
T
V
W
Y
ikalian
opean
astern
nalaya
berian

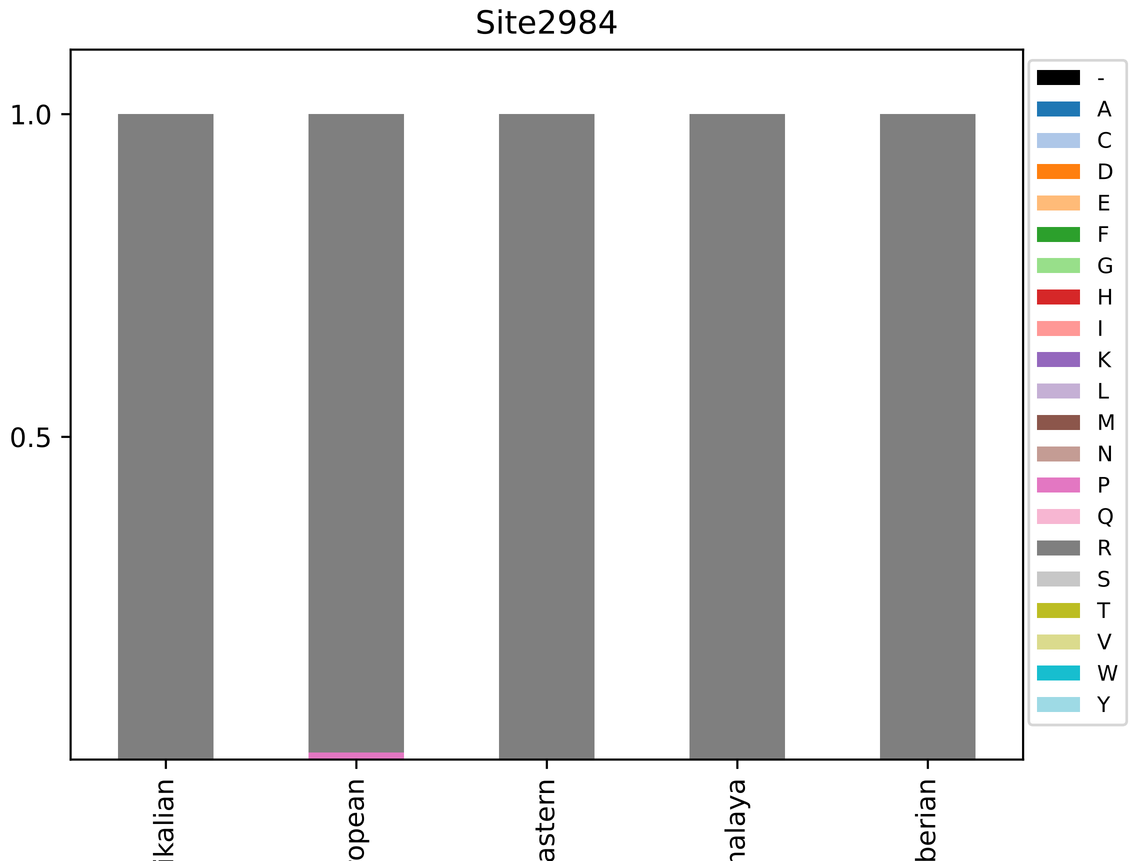
Site2984
1.0
0.5
-
A
C
D
E
F
G
H
I
K
L
M
N
P
Q
R
S
T
V
W
Y
ikalian
opean
astern
nalaya
berian

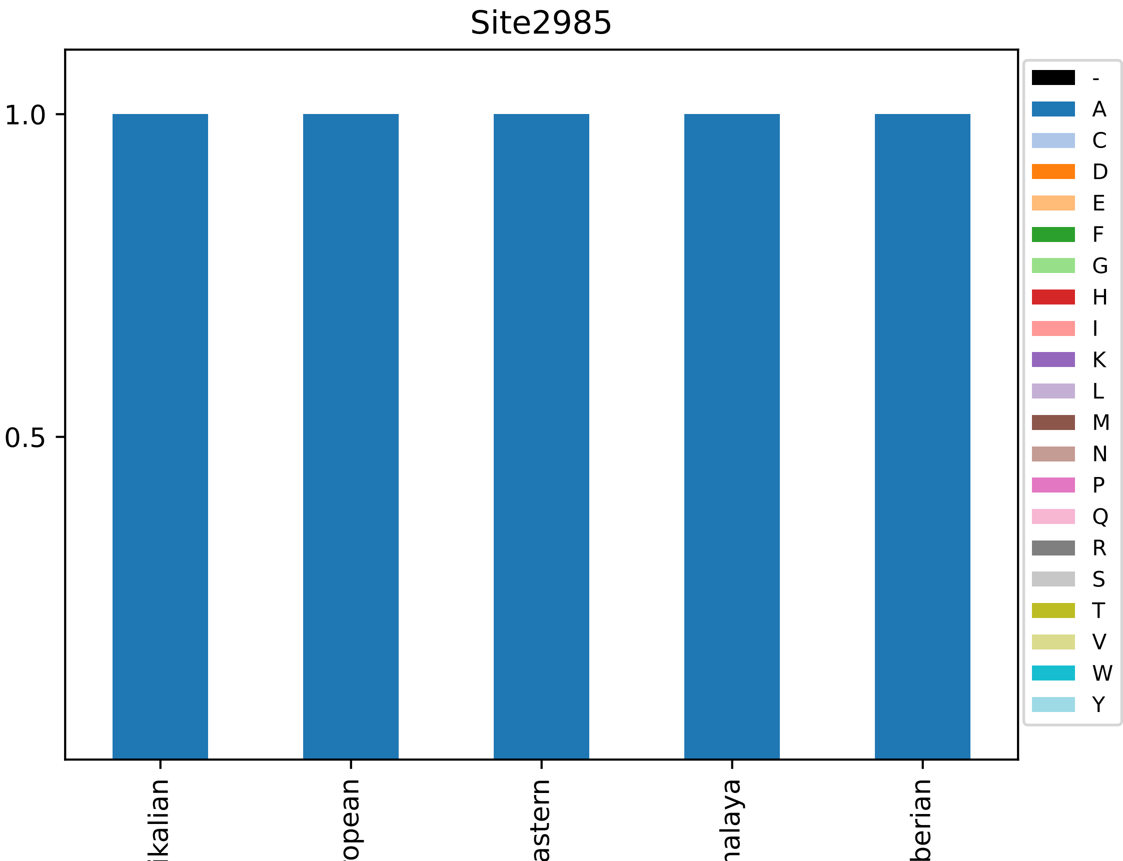

Site2985
1.0
0.5
-
A
C
D
E
F
G
H
I
K
L
M
N
P
Q
R
S
T
V
W
Y
ikalian
opean
astern
nalaya
berian

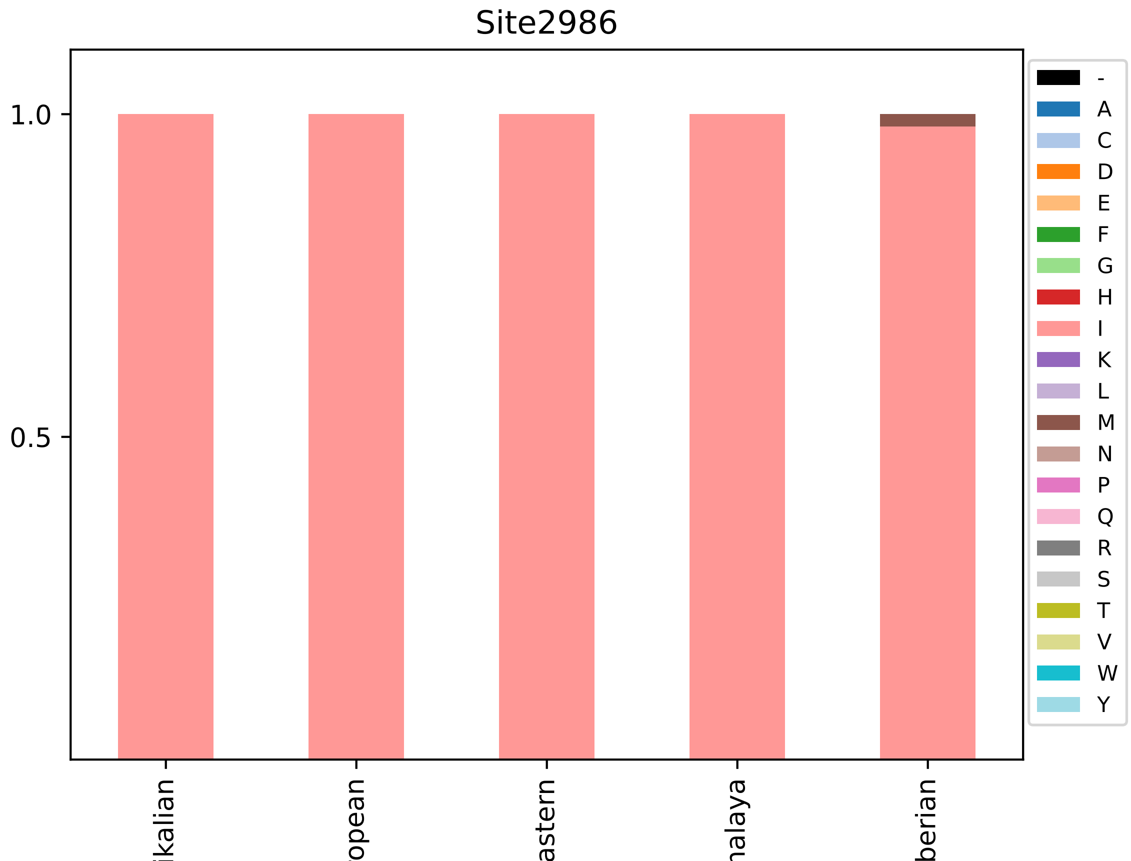
Site2986
1.0
0.5
-
A
C
D
E
F
G
H
I
K
L
M
N
P
Q
R
S
T
V
W
Y
ikalian
ropean
astern
nalaya
berian

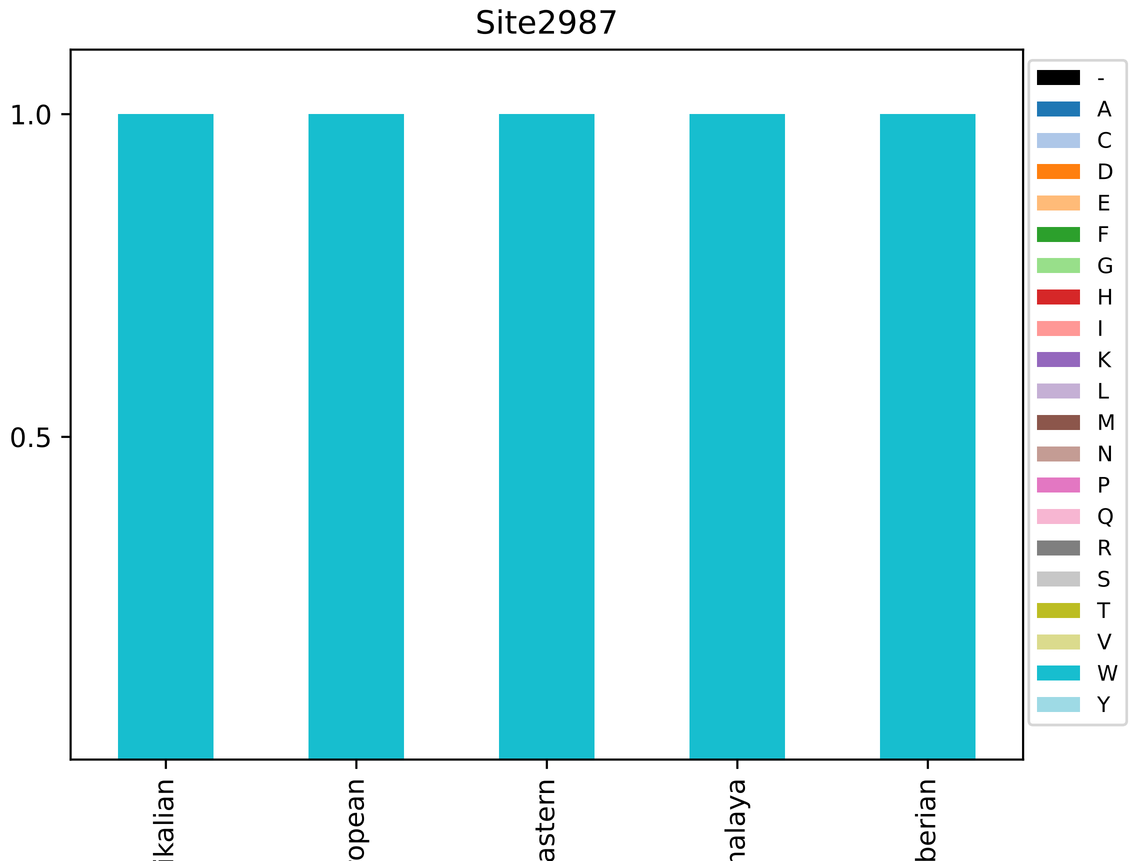

Site2987
1.0
0.5
-
A
C
D
E
F
G
H
I
K
L
M
N
P
Q
R
S
T
V
W
Y
ikalian
opean
astern
nalaya
berian

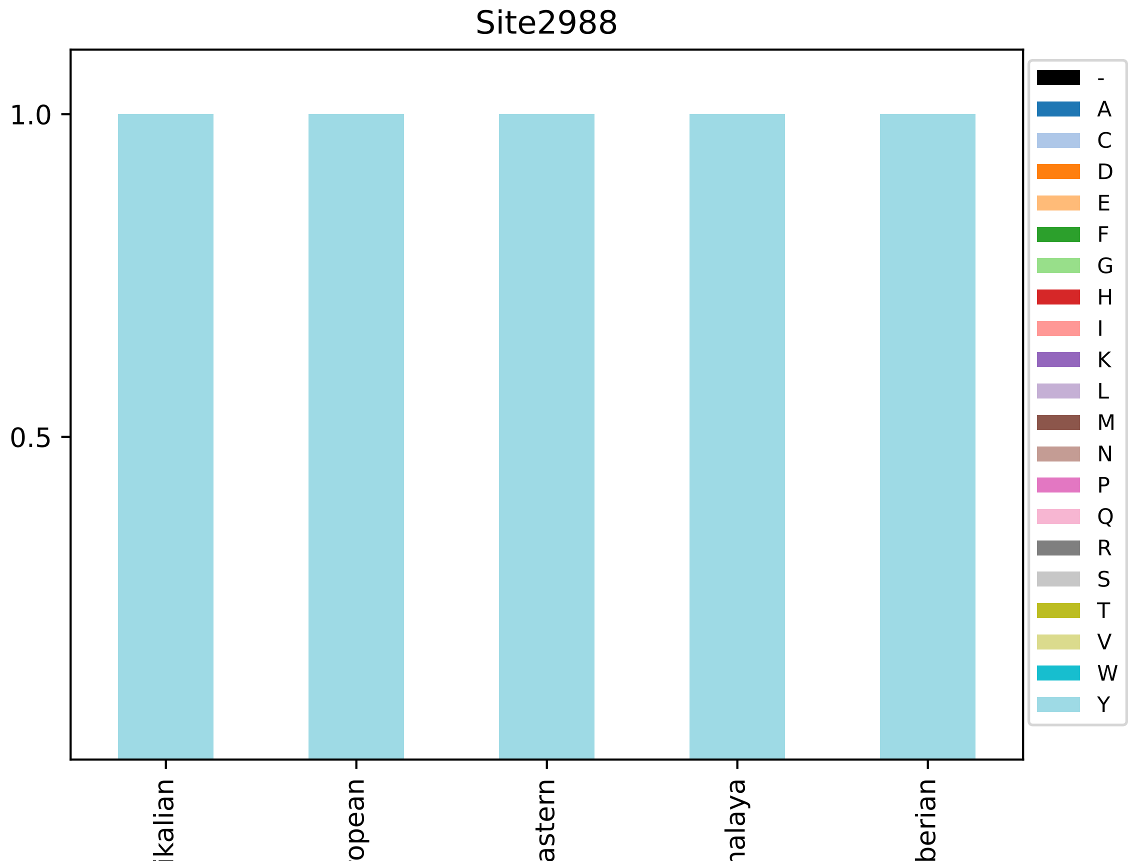

Site2988
1.0
0.5
ikalian
opean
astern
nalaya
berian
-
A
C
D
E
F
G
H
I
K
L
M
N
P
Q
R
S
T
V
W
Y

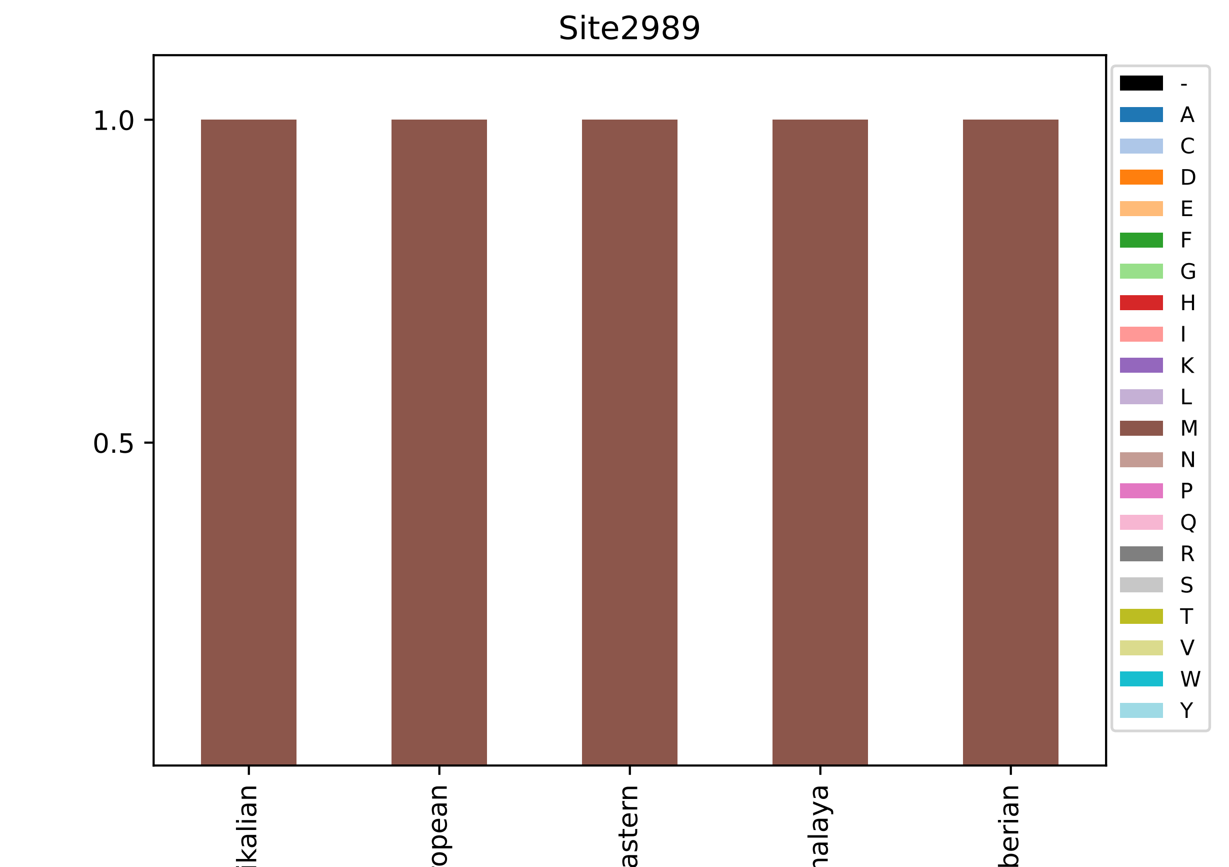
Site2989
1.0
0.5
-
A
C
D
E
F
G
H
I
K
L
M
N
P
Q
R
S
T
V
W
Y
ikalian
opean
astern
nalaya
berian

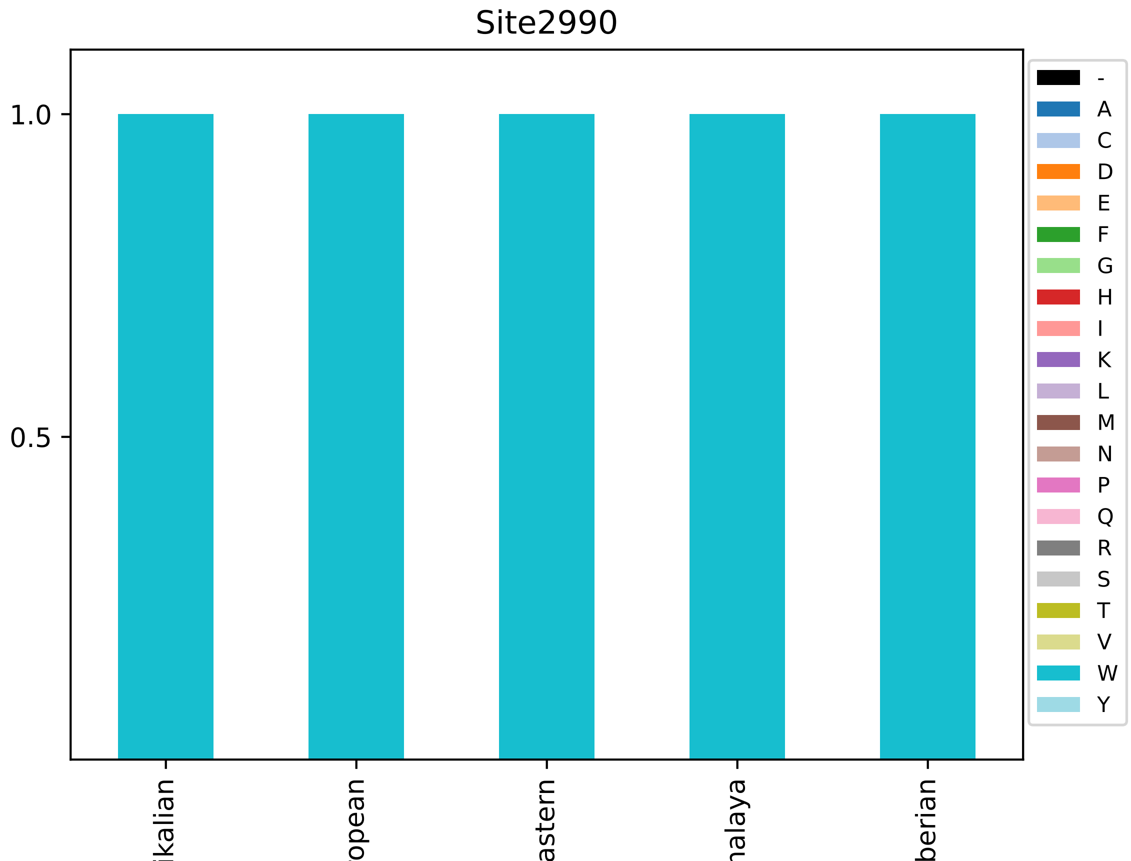

Site2990
1.0
0.5
-
A
C
D
E
F
G
H
I
K
L
M
N
P
Q
R
S
T
V
W
Y
ikalian
opean
astern
nalaya
berian

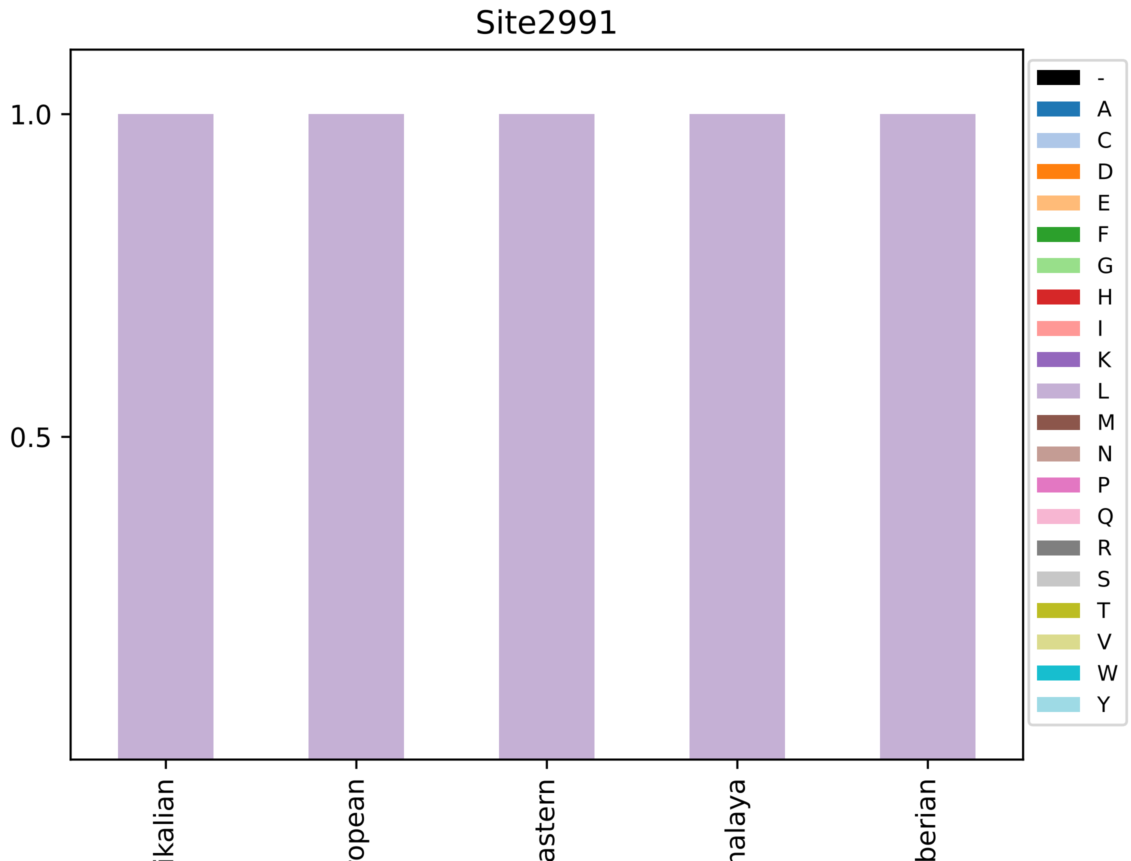

Site2991
1.0
0.5
-
A
C
D
E
F
G
H
I
K
L
M
N
P
Q
R
S
T
V
W
Y
ikalian
ropean
astern
nalaya
berian

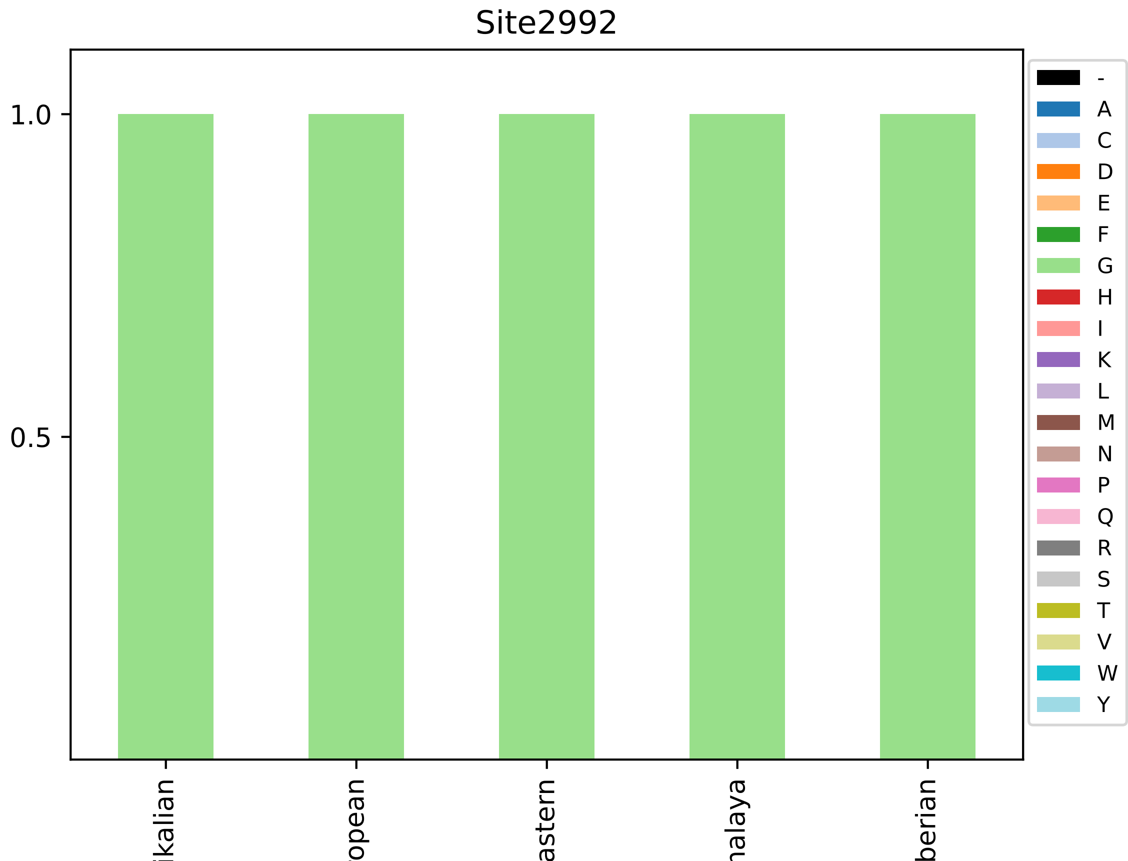
Site2992
1.0
0.5
ikalian
opean
astern
nalaya
berian
-
A
C
D
E
F
G
H
I
K
L
M
N
P
Q
R
S
T
V
W
Y

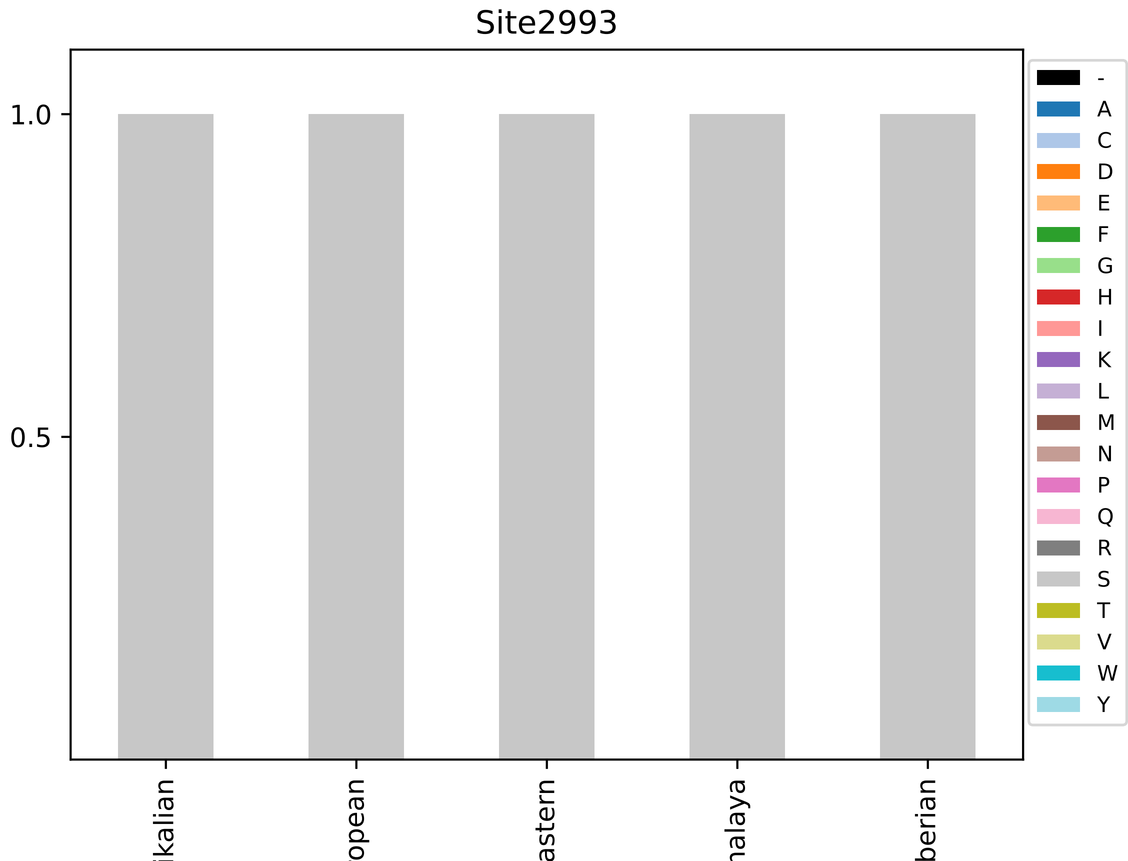
Site2993
1.0
0.5
-
A
C
D
E
F
G
H
I
K
L
M
N
P
Q
R
S
T
V
W
Y
ikalian
opean
astern
nalaya
berian

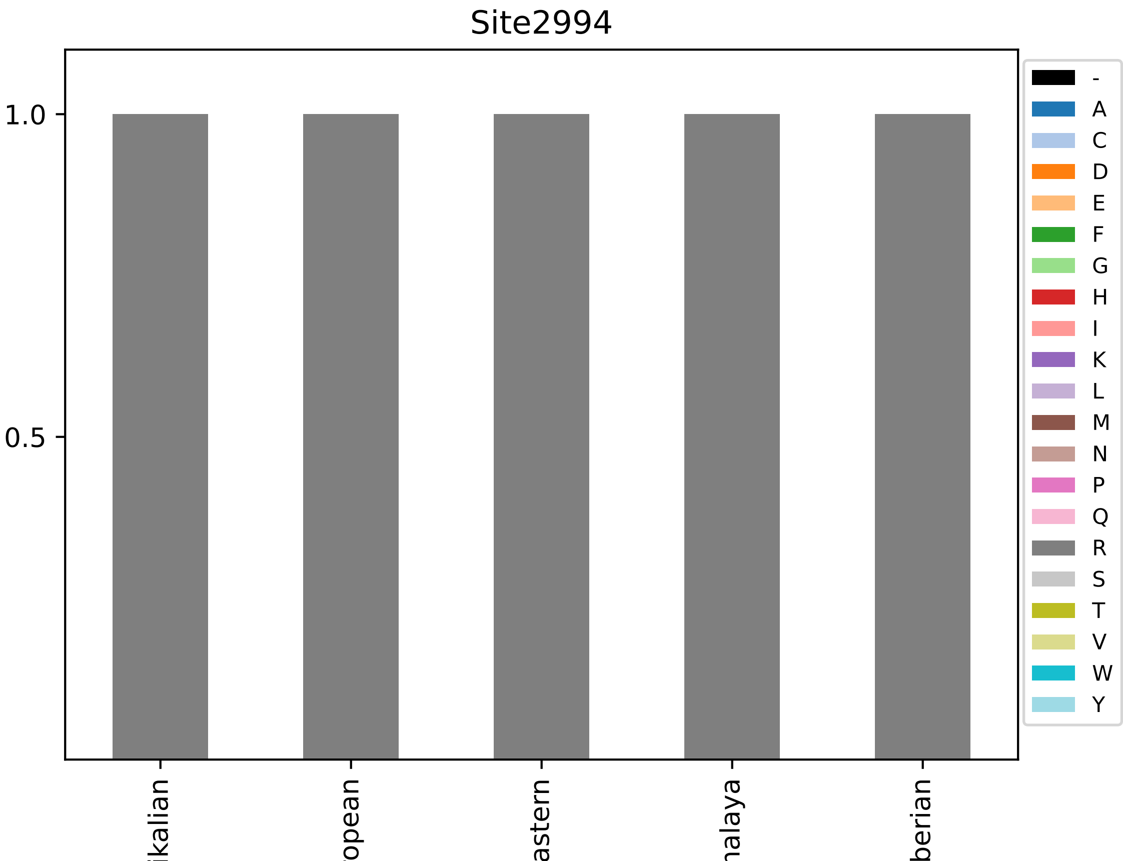

Site2994
1.0
0.5
-
A
C
D
E
F
G
H
I
K
L
M
N
P
Q
R
S
T
V
W
Y
ikalian
opean
astern
nalaya
berian

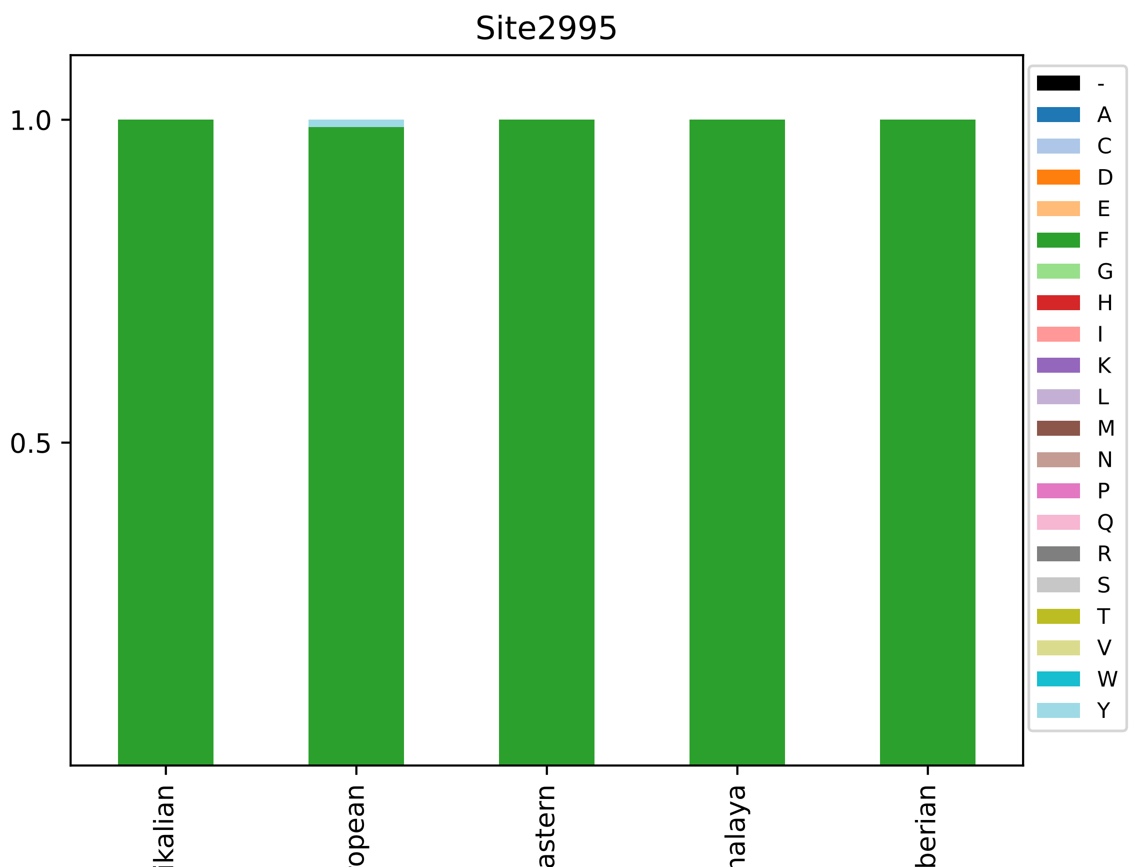
Site2995
1.0
0.5
-
A
C
D
E
F
G
H
I
K
L
M
N
P
Q
R
S
T
V
W
Y
ikalian
opean
astern
nalaya
berian

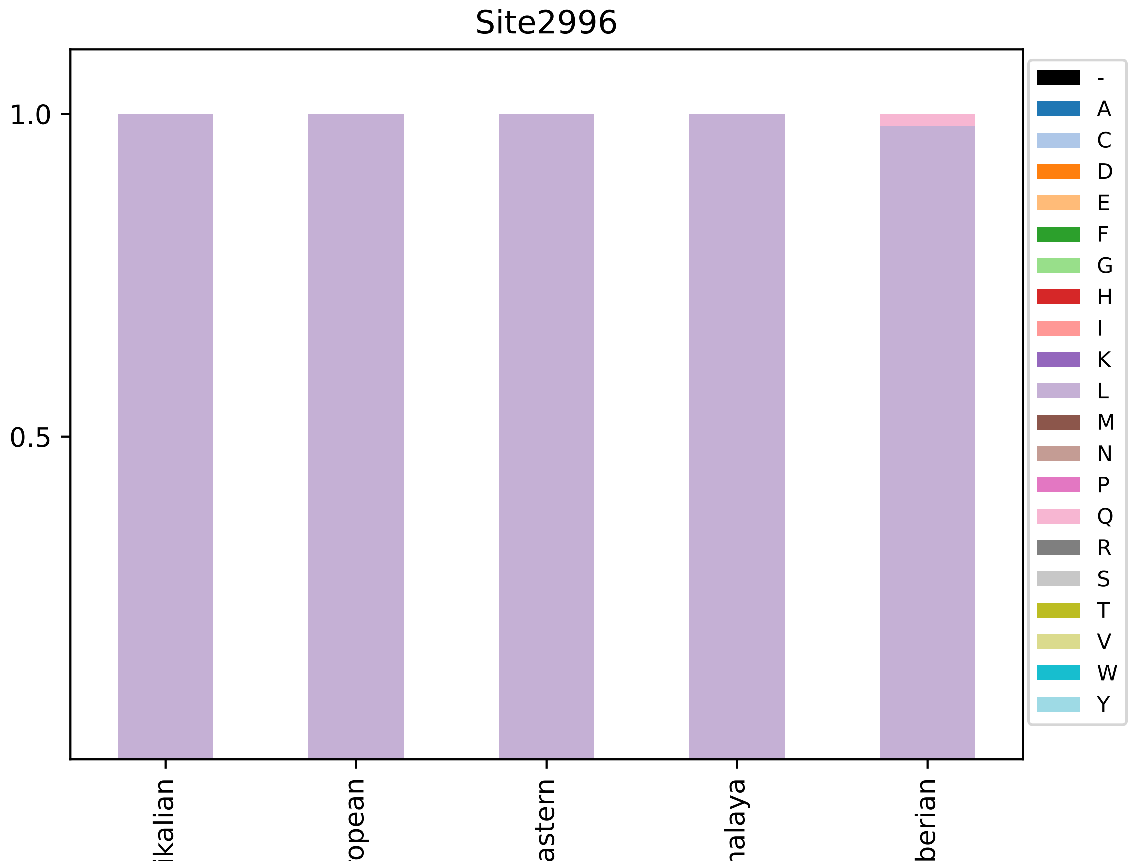

Site2996
1.0
0.5
-
A
C
D
E
F
G
H
I
K
L
M
N
P
Q
R
S
T
V
W
Y
ikalian
opean
astern
nalaya
berian

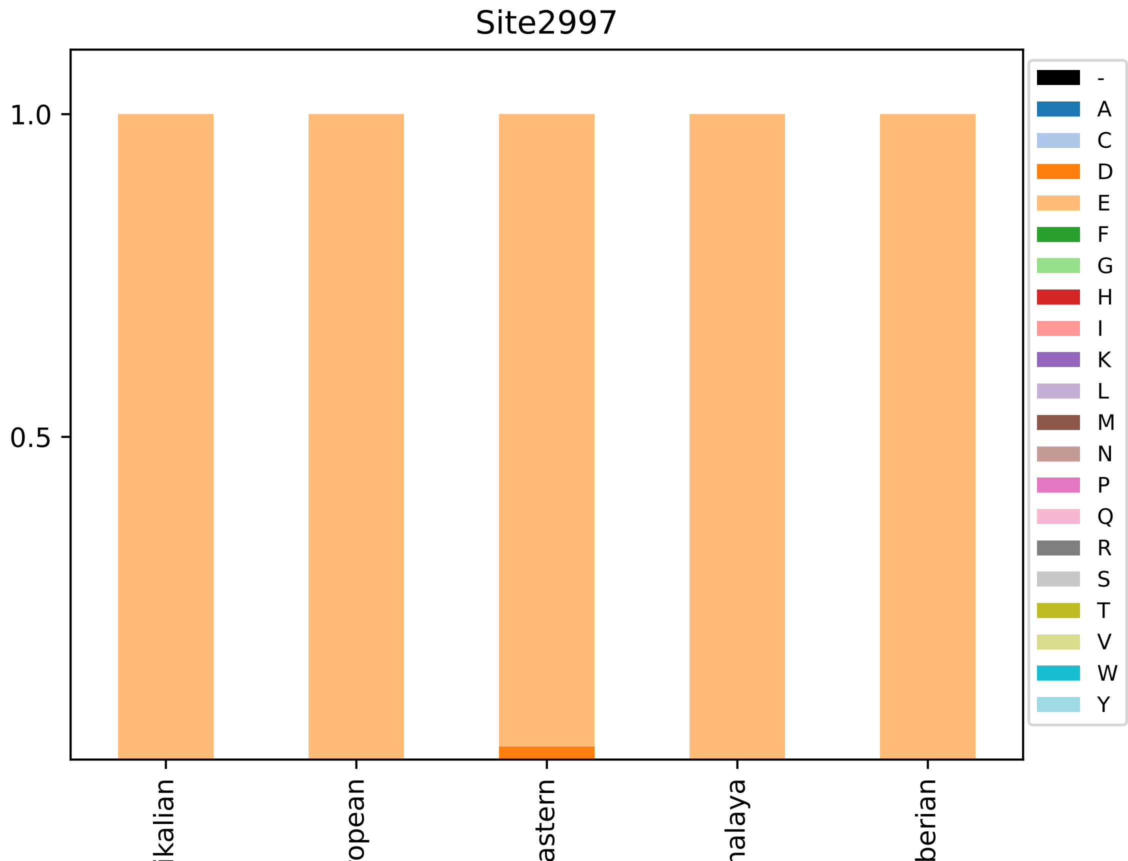
Site2997
1.0
0.5
-
A
C
D
E
F
G
H
I
K
L
M
N
P
Q
R
S
T
V
W
Y
ikalian
opean
astern
nalaya
berian

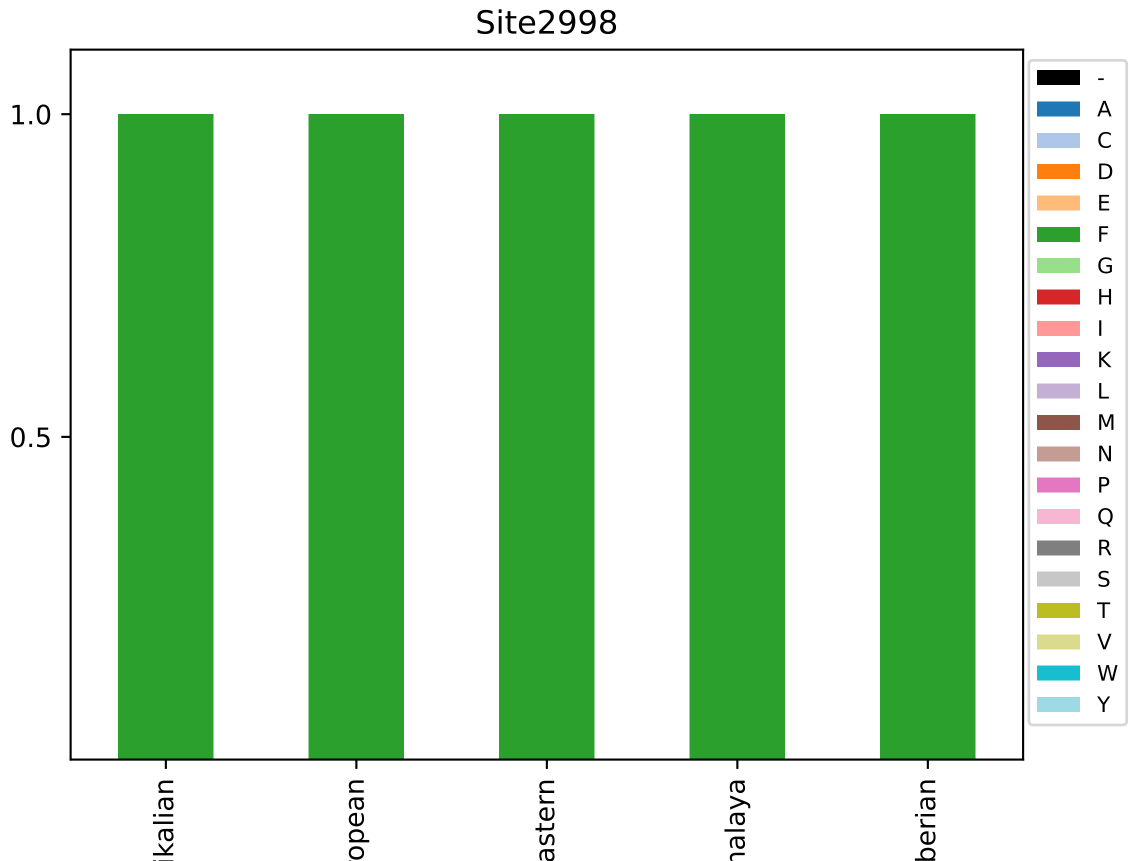
Site2998
1.0
0.5
ikalian
ropean
astern
nalaya
berian
-
A
C
D
E
F
G
H
I
K
L
M
N
P
Q
R
S
T
V
W
Y

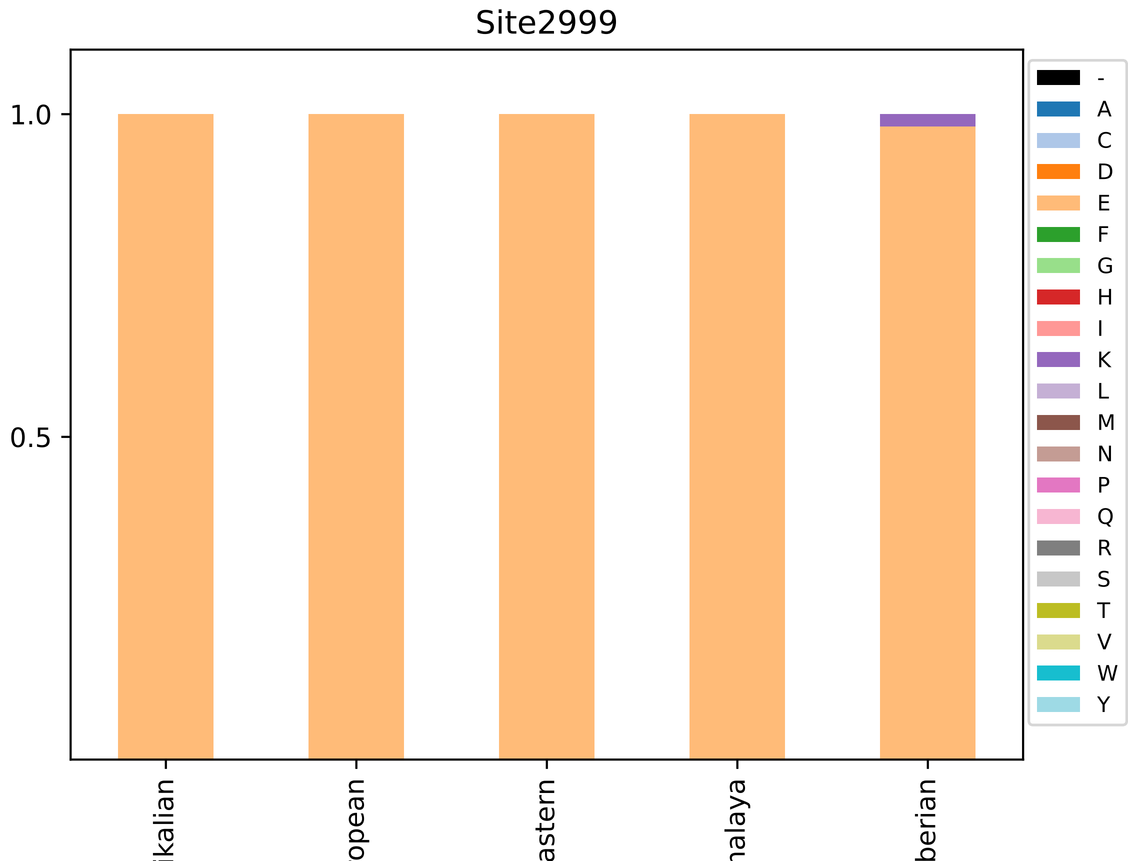

Site2999
1.0
0.5
-
A
C
D
E
F
G
H
I
K
L
M
N
P
Q
R
S
T
V
W
Y
ikalian
opean
astern
nalaya
berian

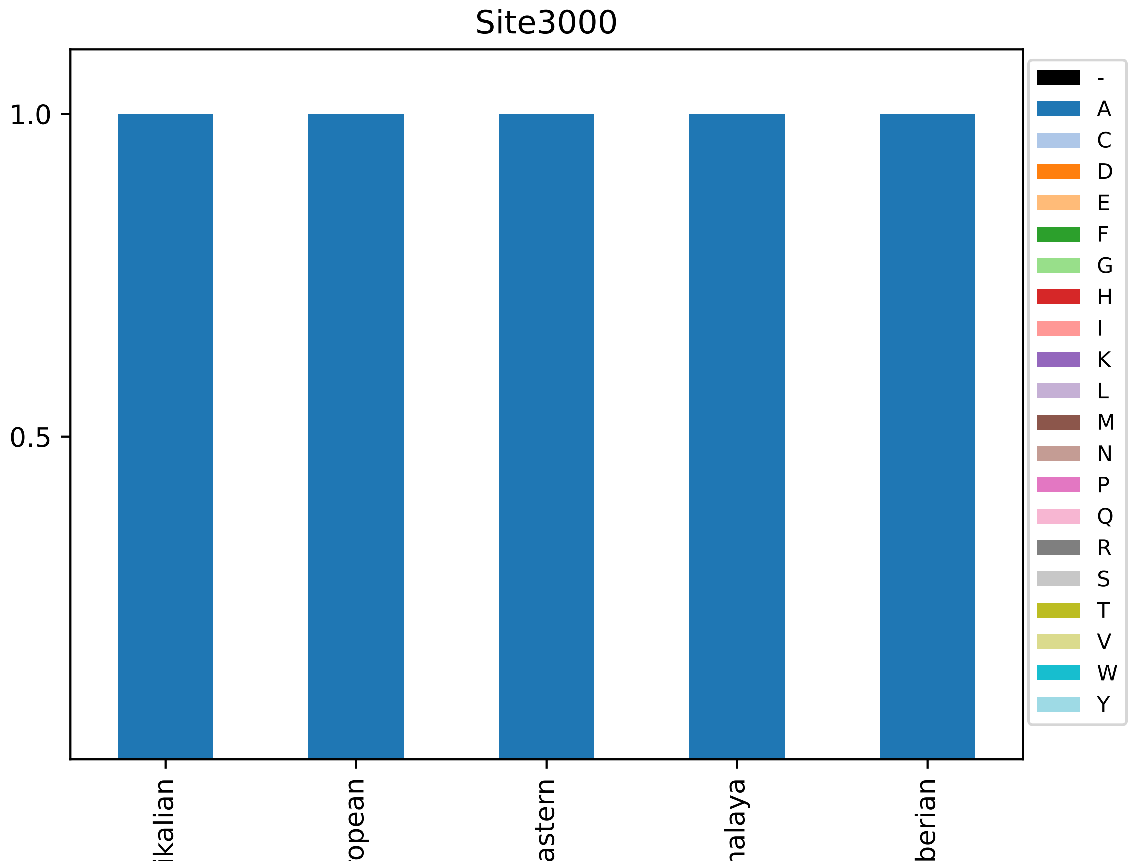

Site3000
1.0
0.5
-
A
C
D
E
F
G
H
I
K
L
M
N
P
Q
R
S
T
V
W
Y
ikalian
opean
astern
nalaya
berian

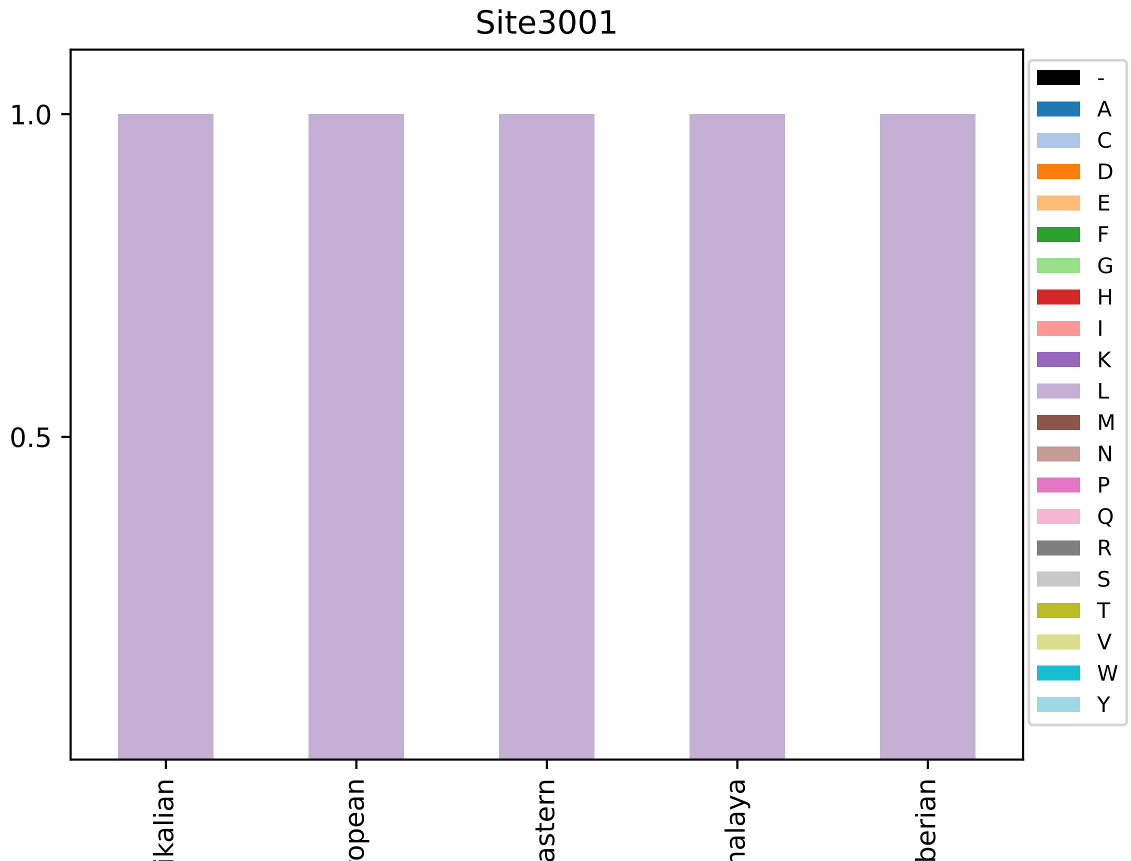
Site3001
1.0
0.5
-
A
C
D
E
F
G
H
I
K
L
M
N
P
Q
R
S
T
V
W
Y
ikalian
opean
astern
nalaya
berian

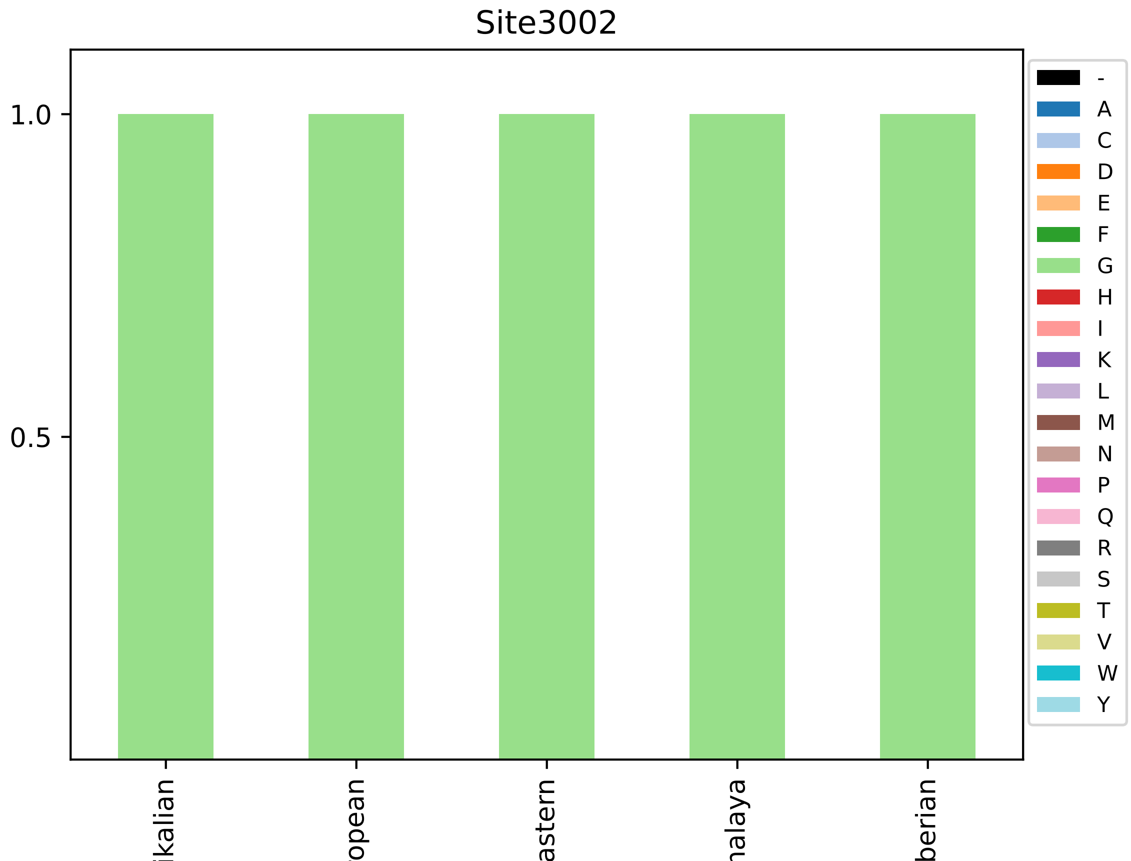
Site3002
1.0
0.5
-
A
C
D
E
F
G
H
I
K
L
M
N
P
Q
R
S
T
V
W
Y

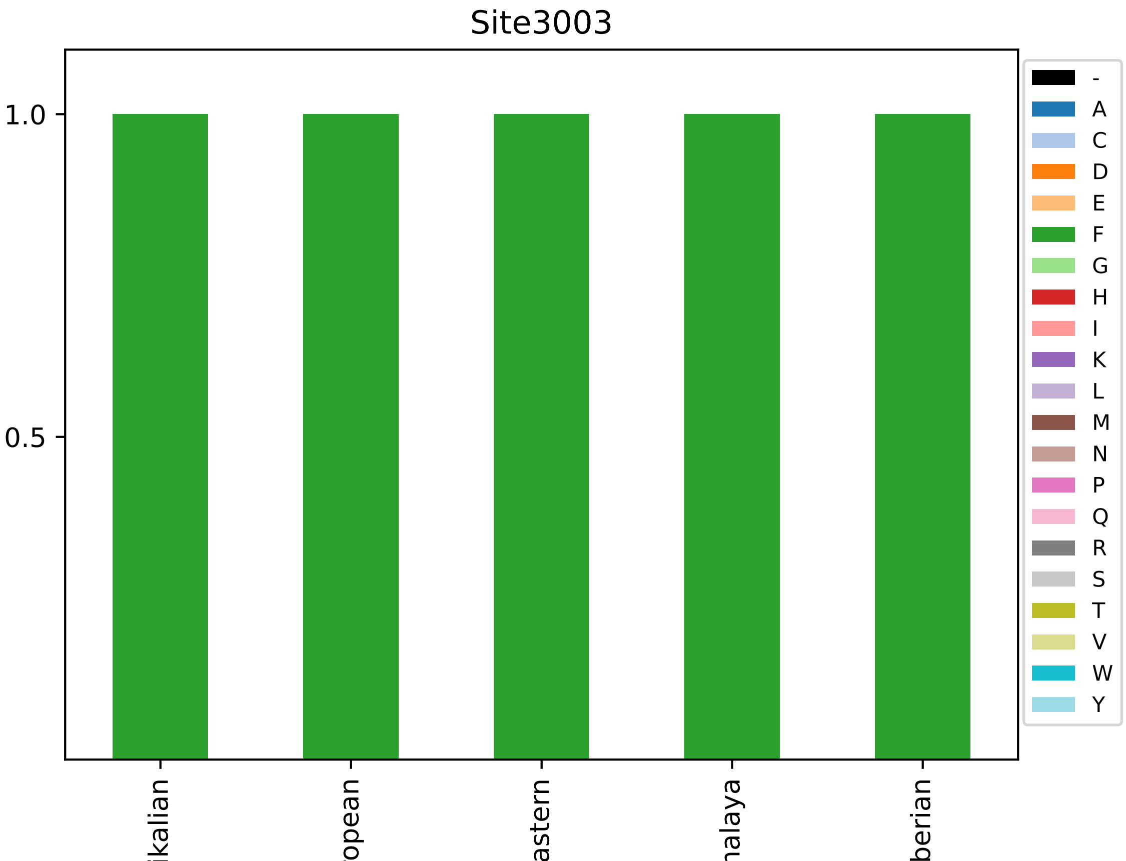

Site3003
1.0
0.5
-
A
C
D
E
F
G
H
I
K
L
M
N
P
Q
R
S
T
V
W
Y
ikalian
opean
astern
nalaya
berian

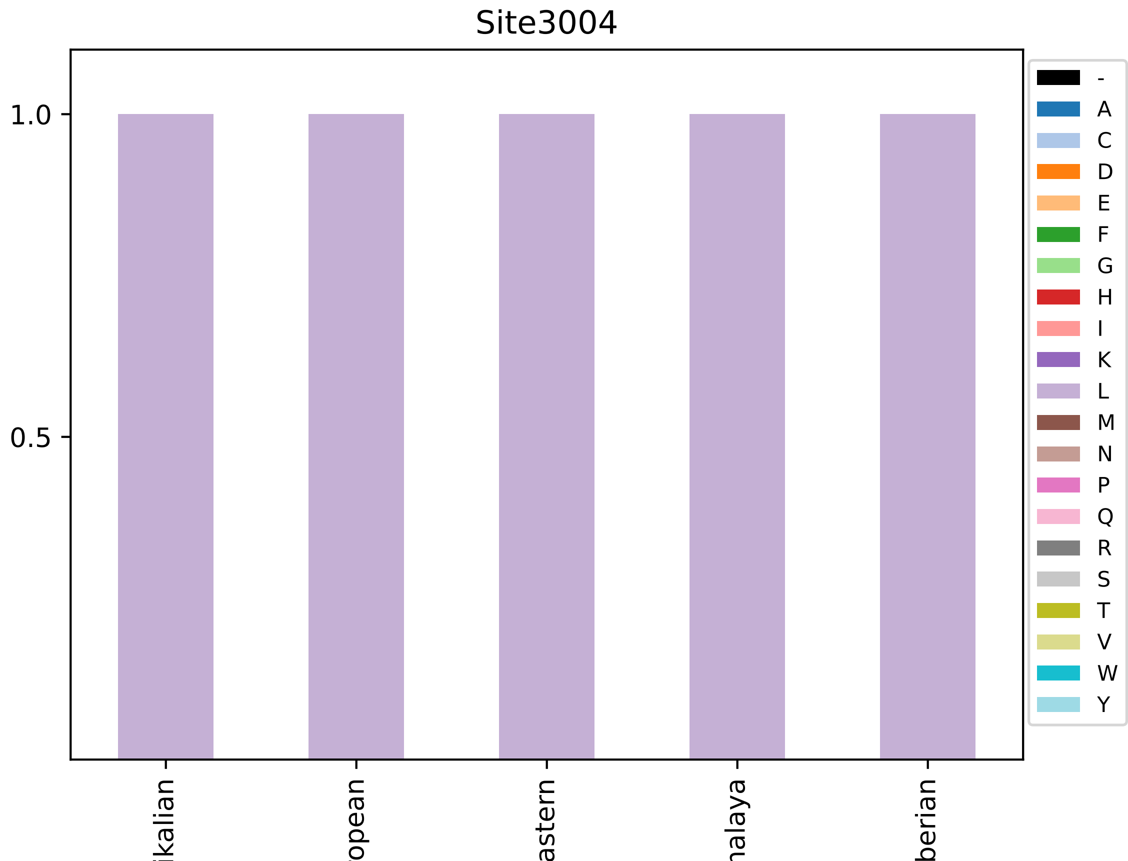

Site3004
1.0
0.5
-
A
C
D
E
F
G
H
I
K
L
M
N
P
Q
R
S
T
V
W
Y
ikalian
opean
astern
nalaya
berian

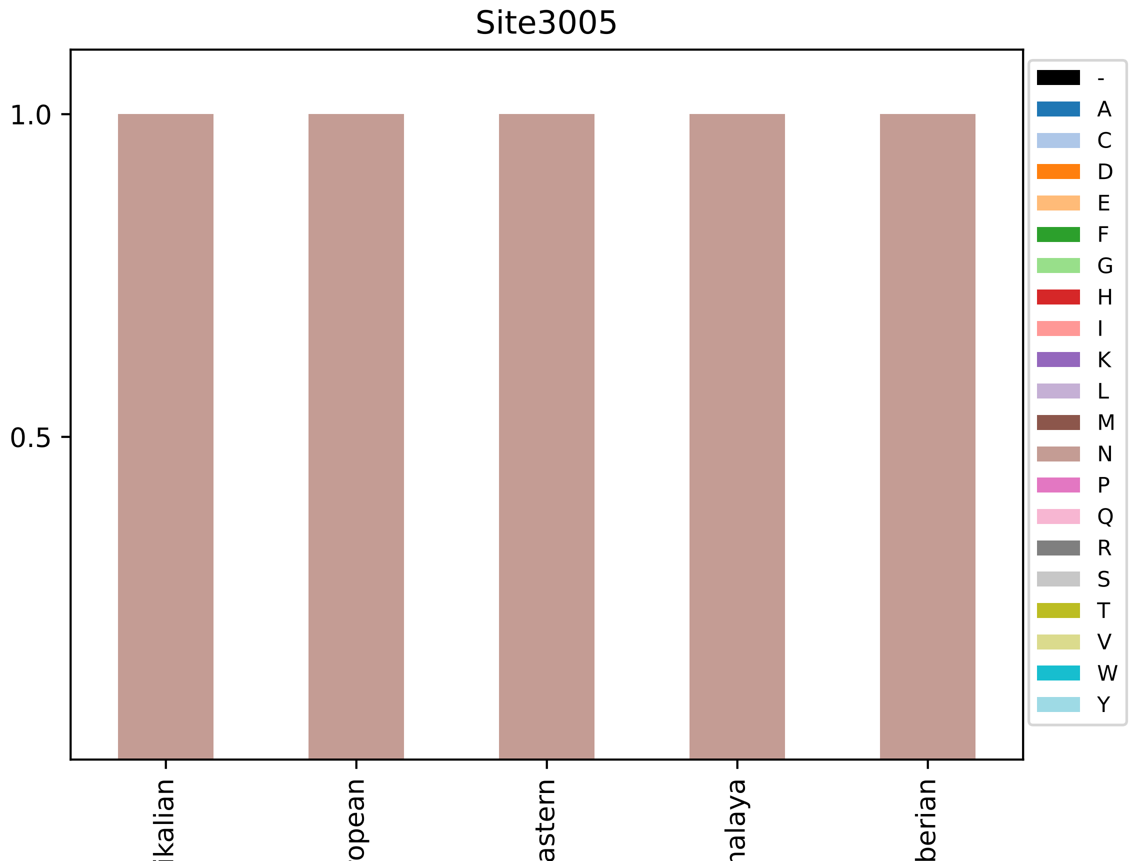

Site3005
1.0
0.5
-
A
C
D
E
F
G
H
I
K
L
M
N
P
Q
R
S
T
V
W
Y
ikalian
opean
astern
nalaya
berian

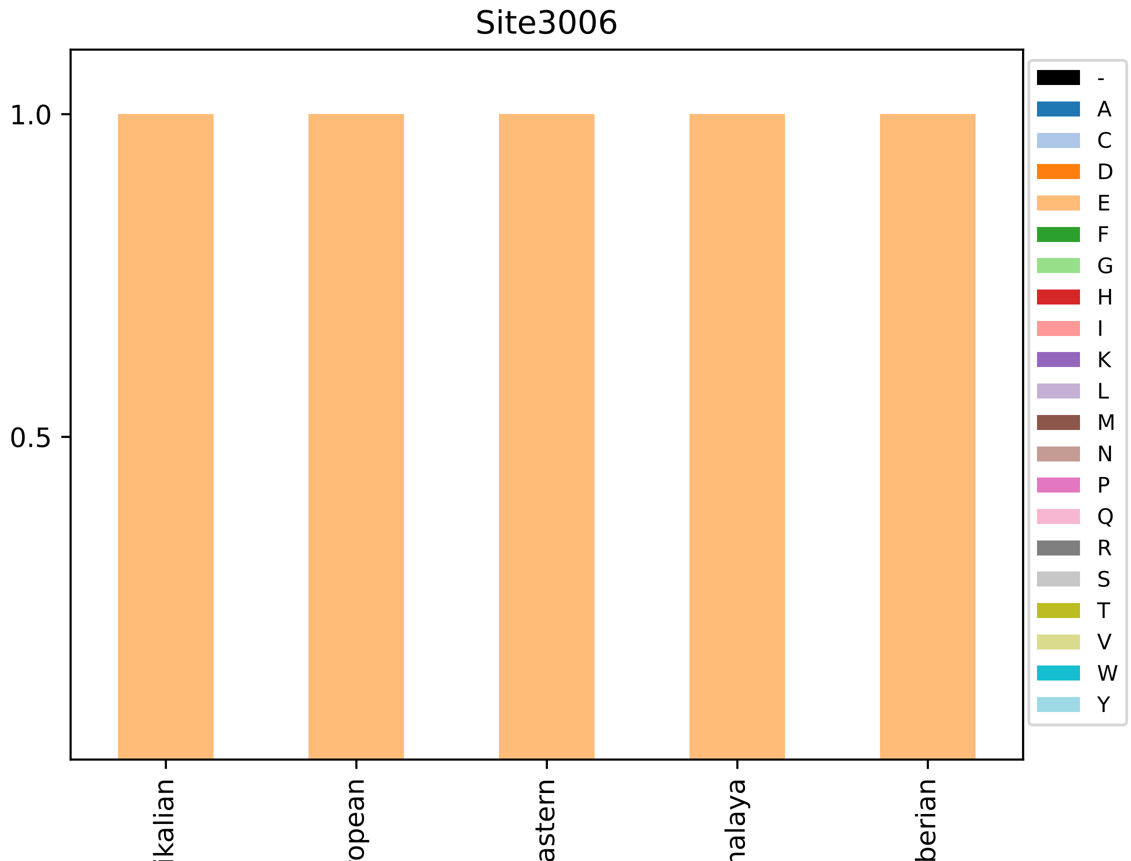
Site3006
1.0
0.5
-
A
C
D
E
F
G
H
I
K
L
M
N
P
Q
R
S
T
V
W
Y
ikalian
ropean
astern
nalaya
berian

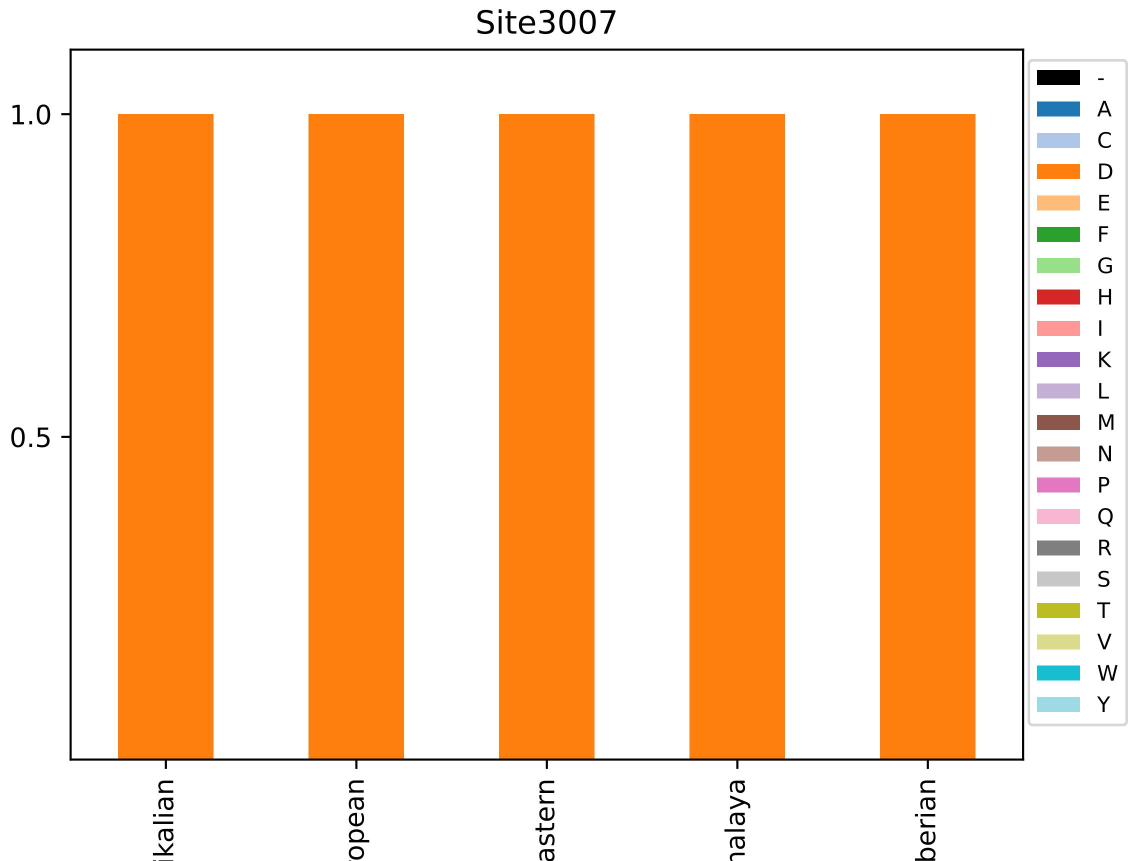

Site3007
1.0
0.5
-
A
C
D
E
F
G
H
I
K
L
M
N
P
Q
R
S
T
V
W
Y
ikalian
opean
astern
nalaya
berian

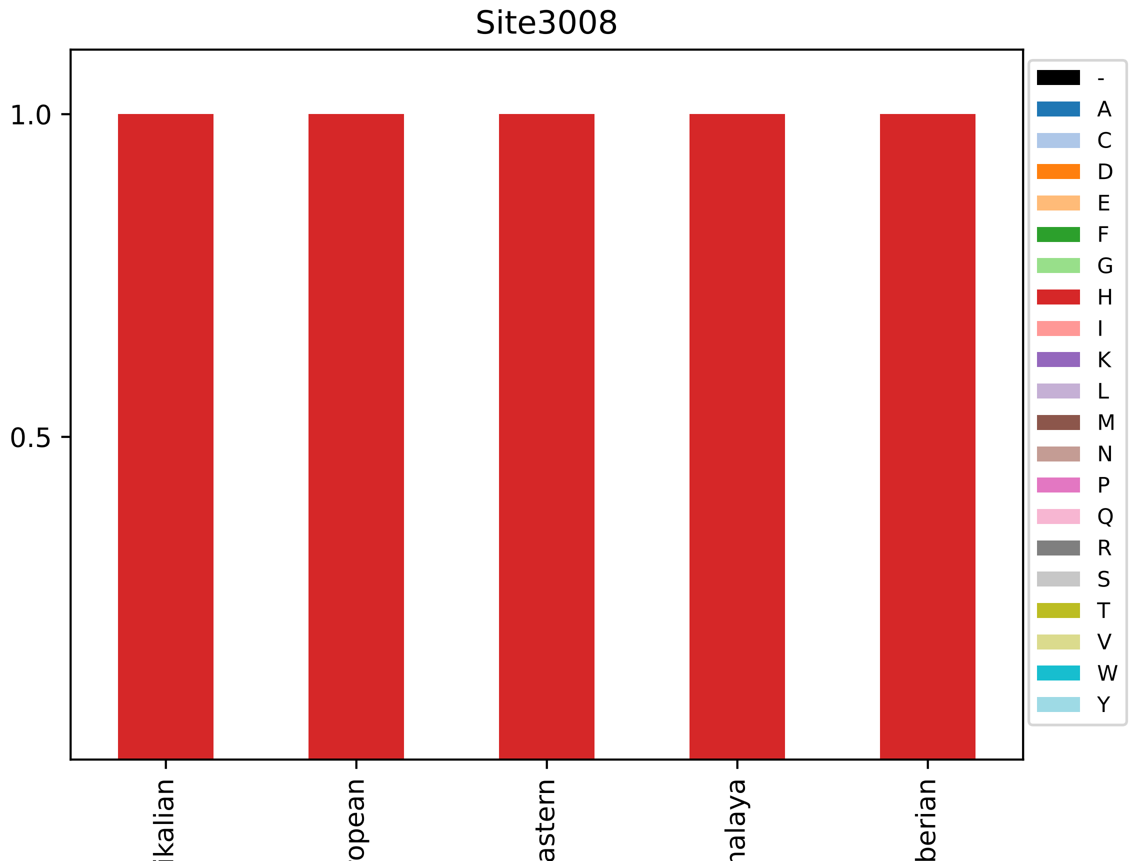

Site3008
1.0
0.5
-
A
C
D
E
F
G
H
I
K
L
M
N
P
Q
R
S
T
V
W
Y
ikalian
opean
astern
nalaya
berian

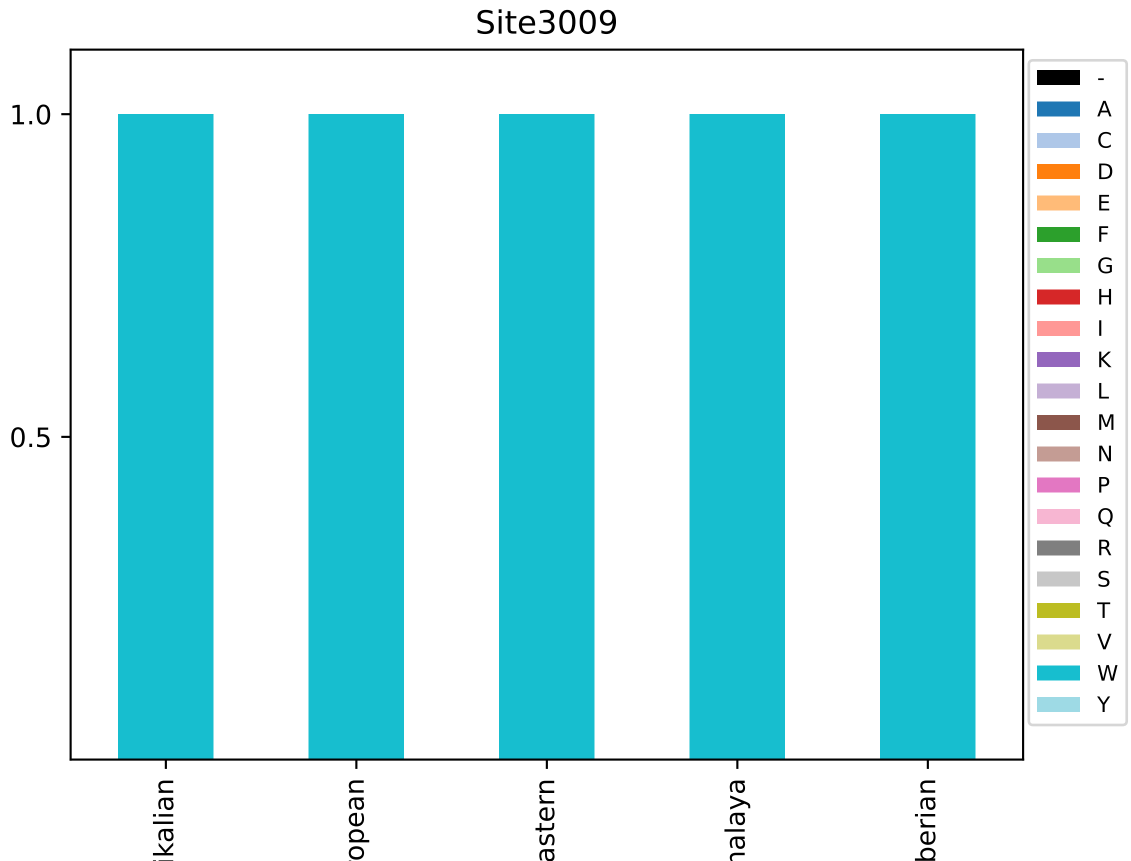
Site3009
1.0
0.5
-
A
C
D
E
F
G
H
I
K
L
M
N
P
Q
R
S
T
V
W
Y
ikalian
opean
astern
nalaya
berian

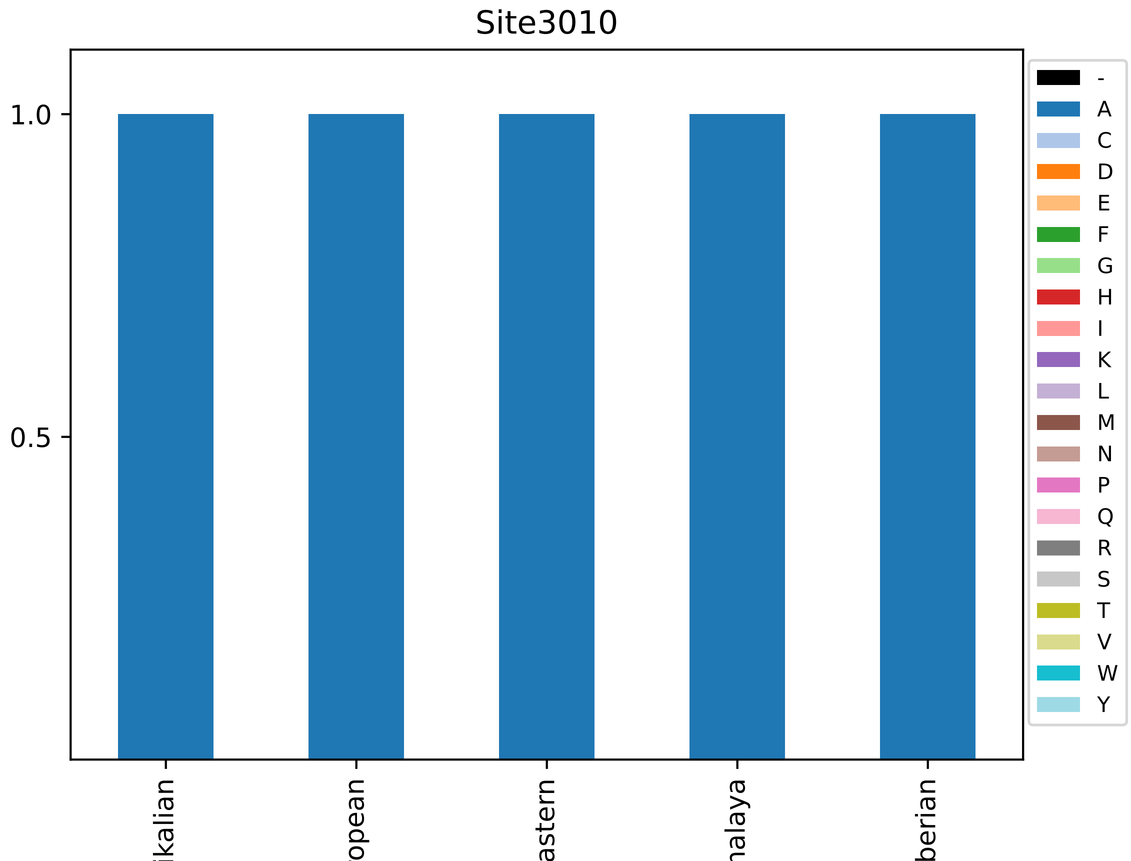

Site3010
1.0
0.5
-
A
C
D
E
F
G
H
I
K
L
M
N
P
Q
R
S
T
V
W
Y
ikalian
opean
astern
nalaya
berian

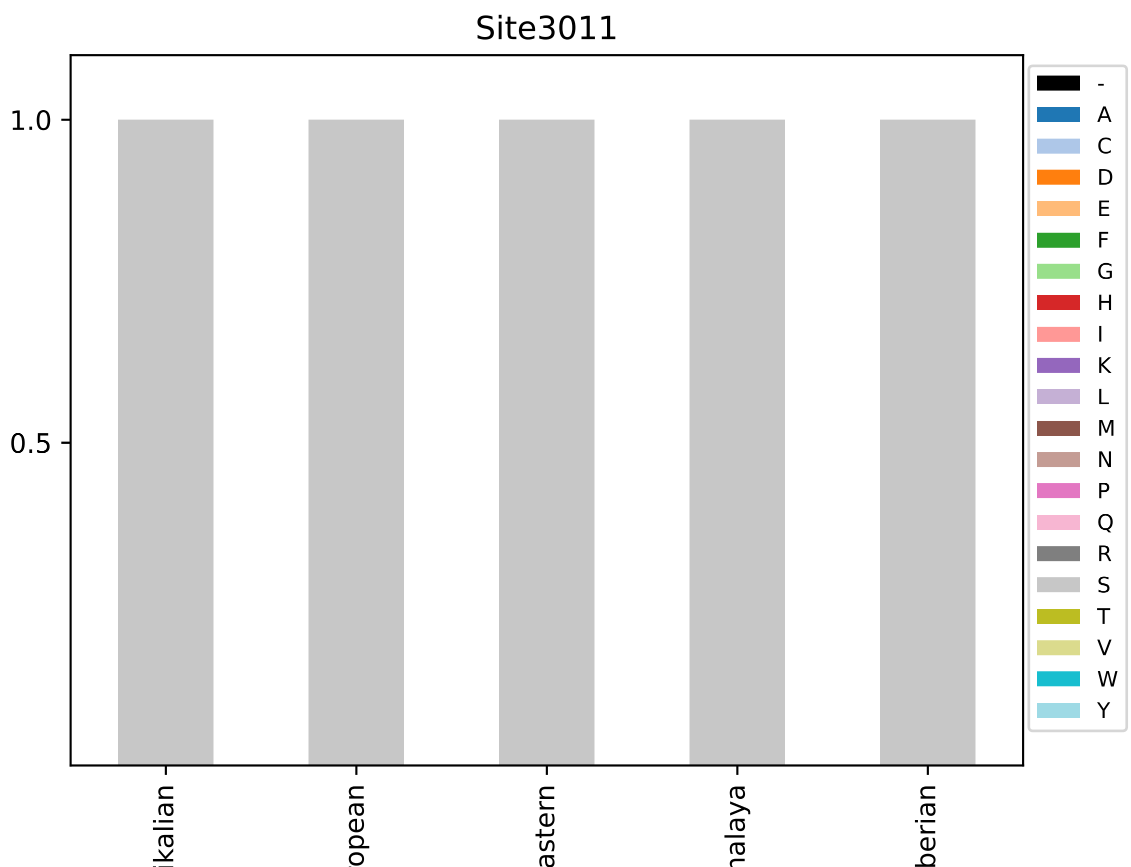
Site3011
1.0
0.5
ikalian
ropean
astern
malaya
berian
-
A
C
D
E
F
G
H
I
K
L
M
N
P
Q
R
S
T
V
W
Y

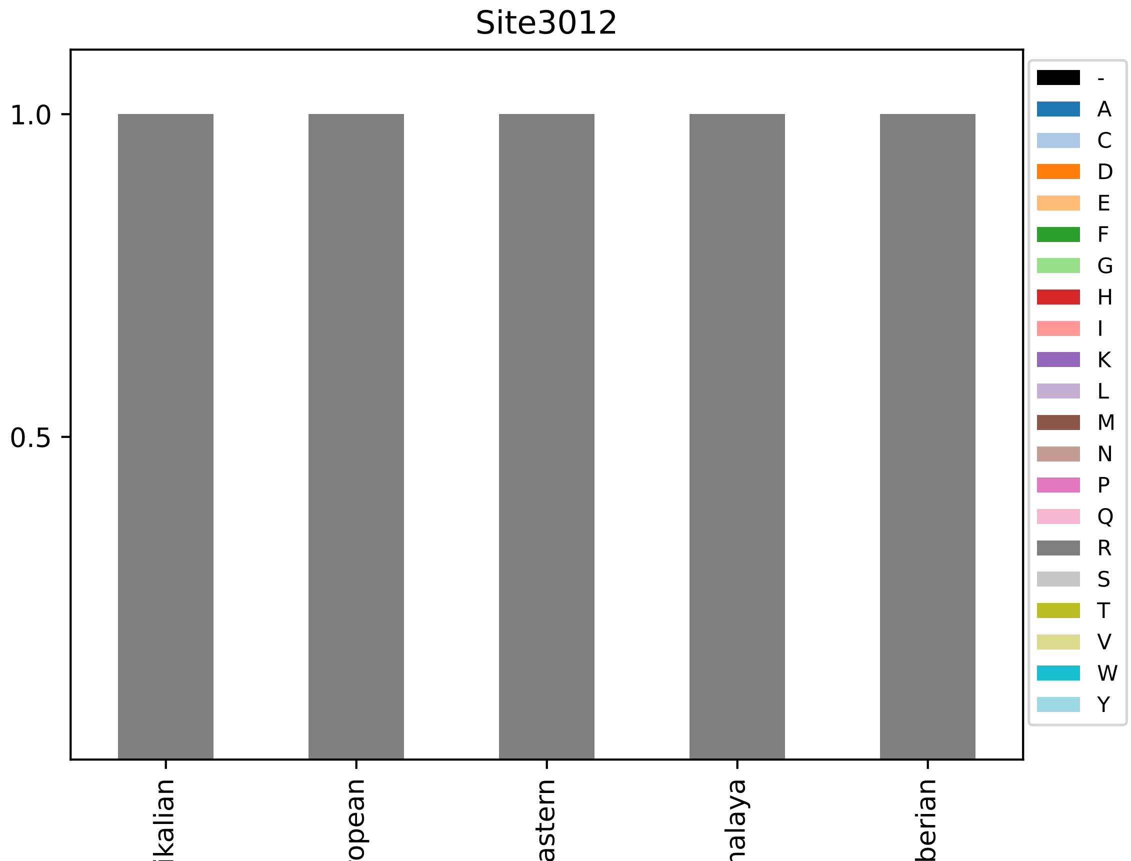

Site3012
1.0
0.5
-
A
C
D
E
F
G
H
I
K
L
M
N
P
Q
R
S
T
V
W
Y
ikalian
opean
astern
nalaya
berian

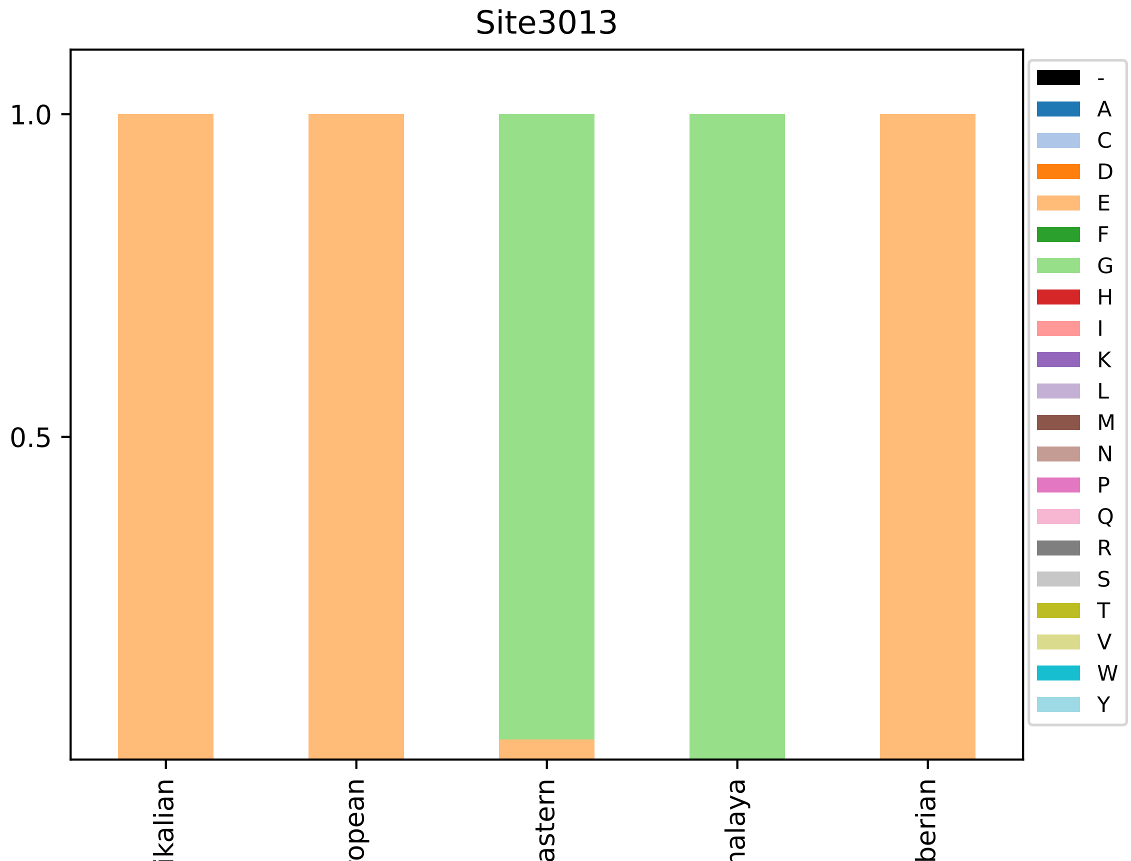
Site3013
1.0
0.5
-
A
C
D
E
F
G
H
I
K
L
M
N
P
Q
R
S
T
V
W
Y
ikalian
opean
astern
nalaya
berian

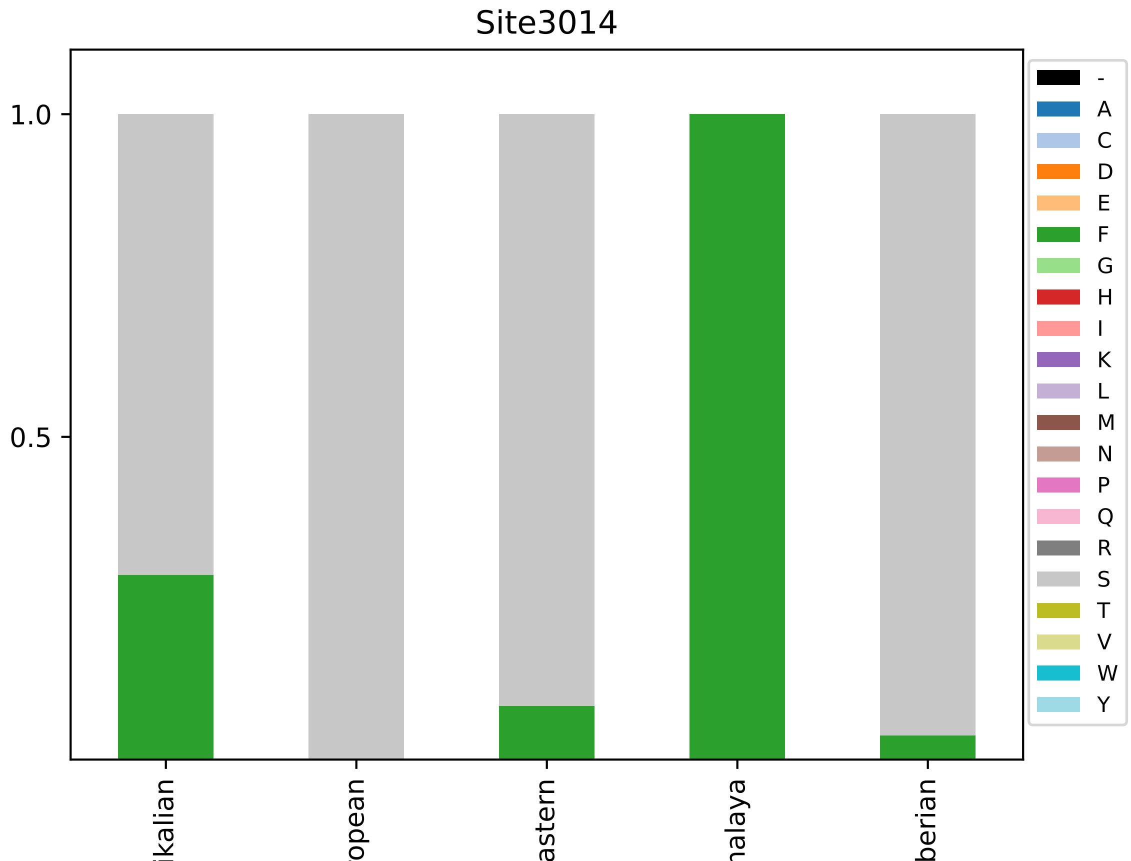
Site3014
1.0
0.5
-
A
C
D
E
F
G
H
I
K
L
M
N
P
Q
R
S
T
V
W
Y
ikalian
opean
astern
nalaya
berian

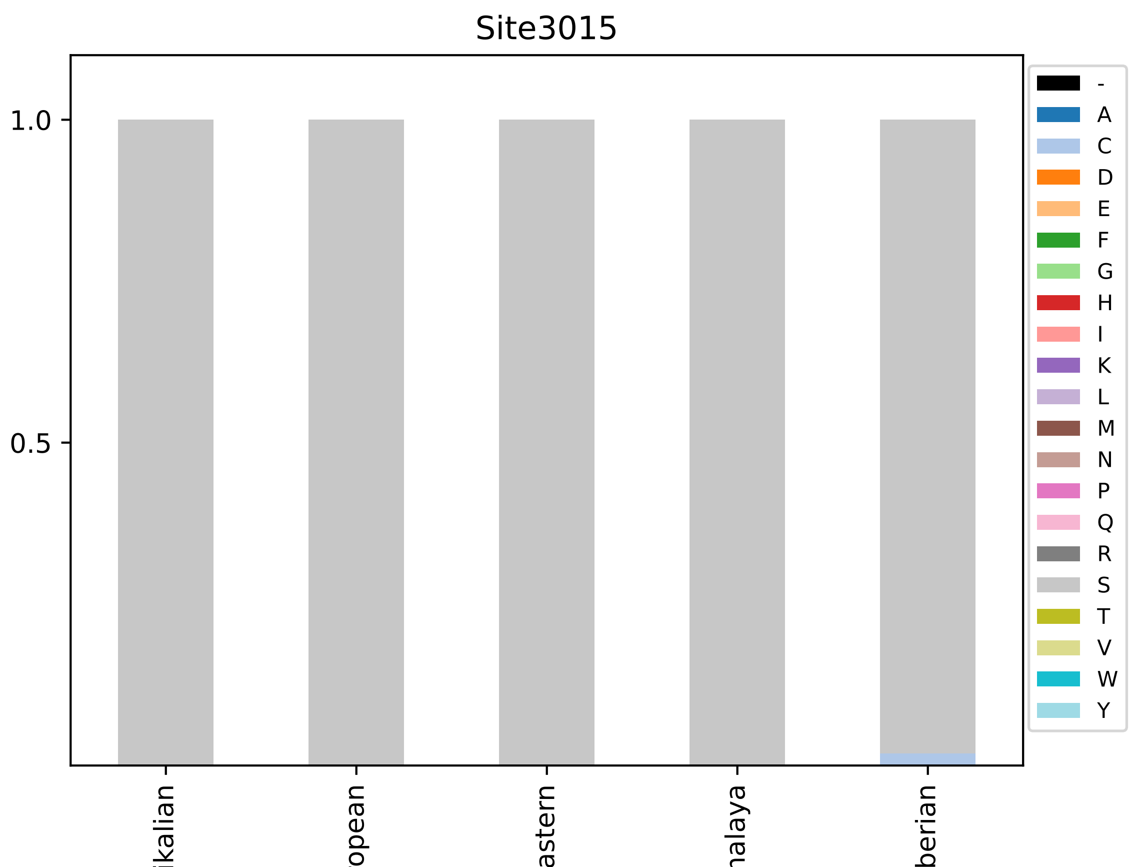

Site3015
1.0
0.5
-
A
C
D
E
F
G
H
I
K
L
M
N
P
Q
R
S
T
V
W
Y
ikalian
opean
astern
nalaya
berian

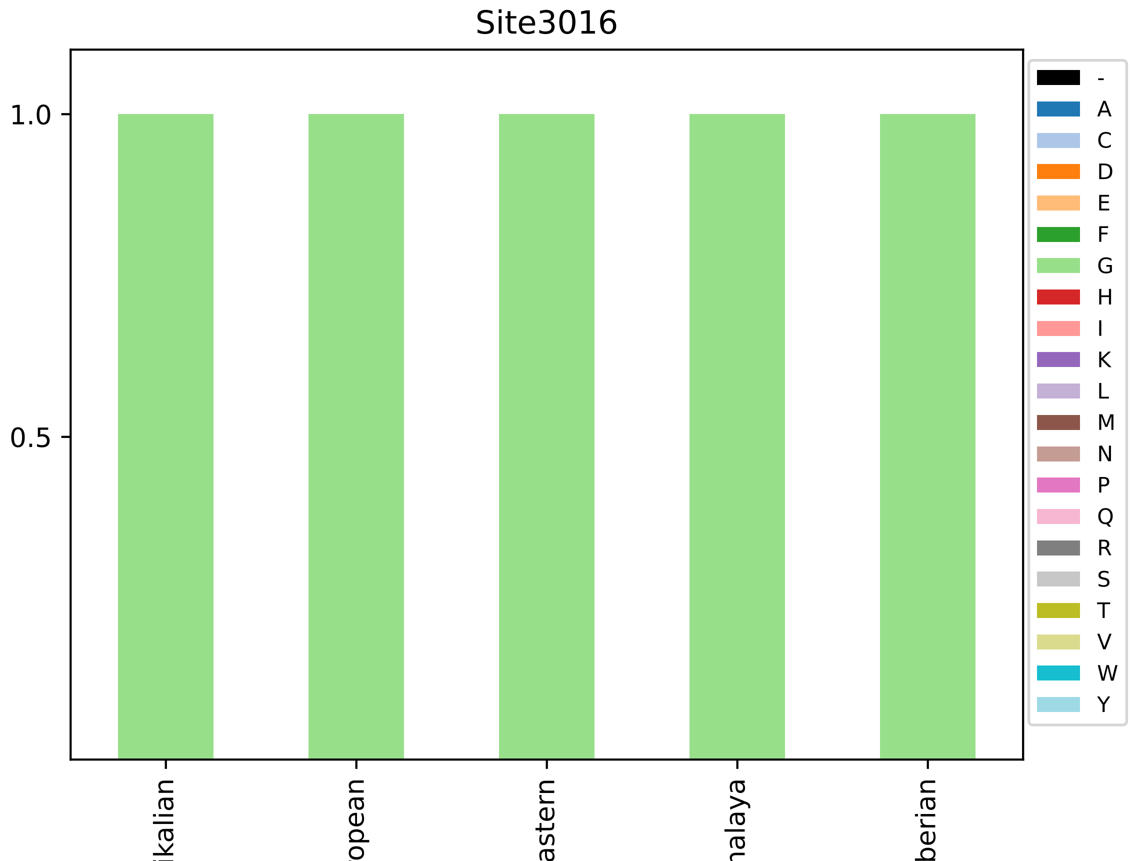

Site3016
1.0
0.5
-
A
C
D
E
F
G
H
I
K
L
M
N
P
Q
R
S
T
V
W
Y
ikalian
opean
astern
nalaya
berian

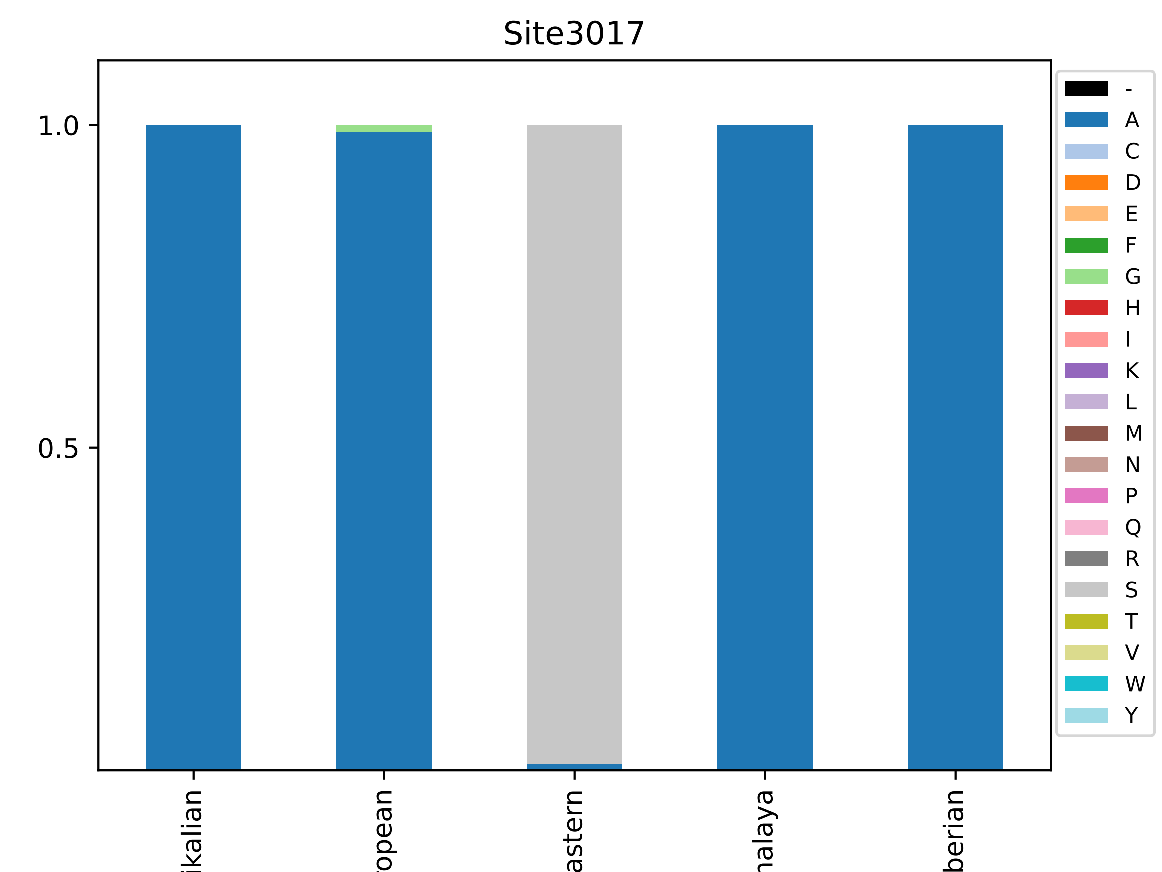
Site3017
1.0
0.5
-
A
C
D
E
F
G
H
I
K
L
M
N
P
Q
R
S
T
V
W
Y
ikalian
opean
astern
nalaya
berian

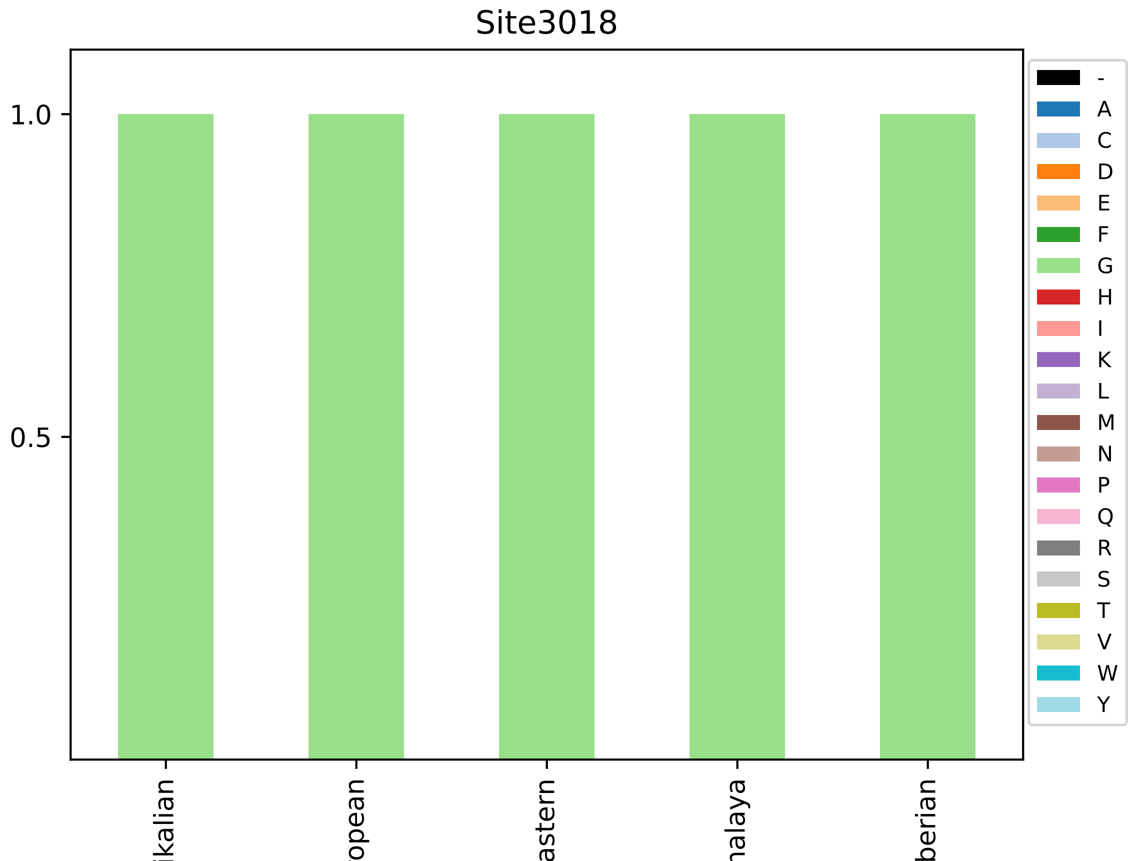

Site3018
1.0
0.5
-
A
C
D
E
F
G
H
I
K
L
M
N
P
Q
R
S
T
V
W
Y
ikalian
opean
astern
nalaya
berian

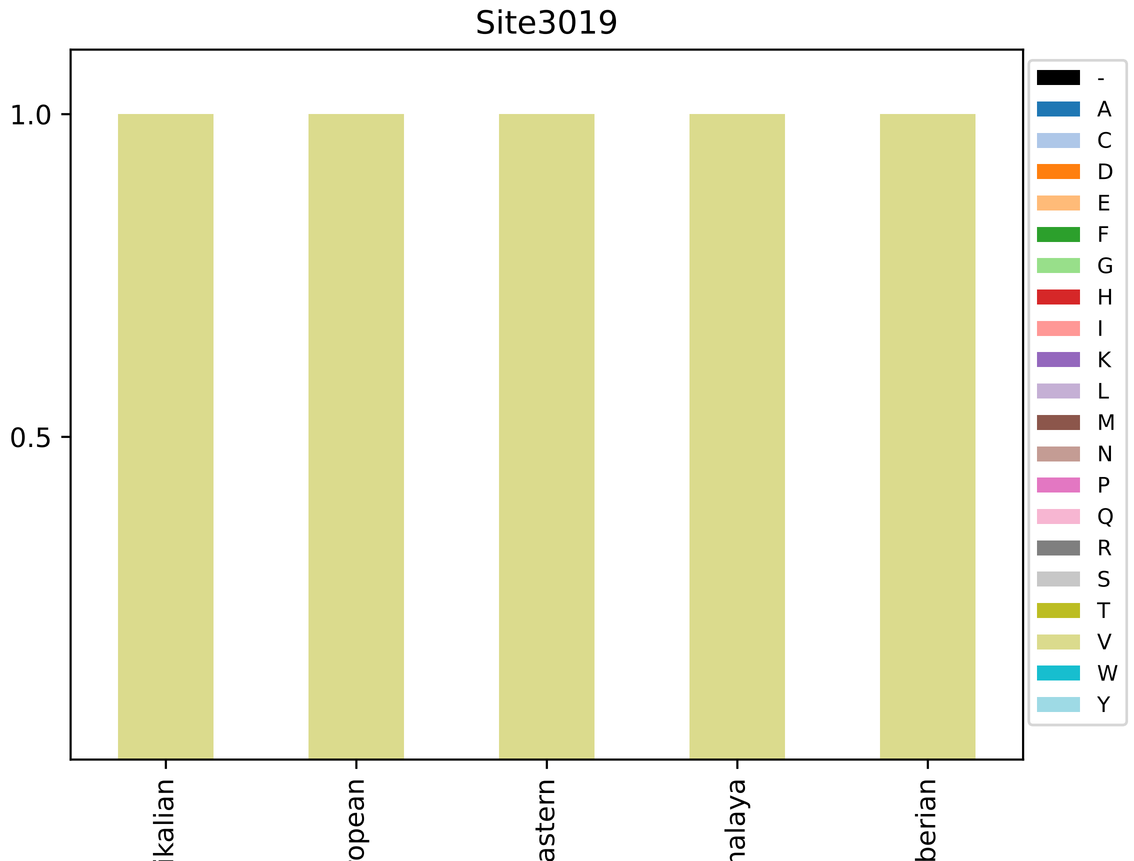
Site3019
1.0
0.5
-
A
C
D
E
F
G
H
I
K
L
M
N
P
Q
R
S
T
V
W
Y
ikalian
opean
astern
nalaya
berian

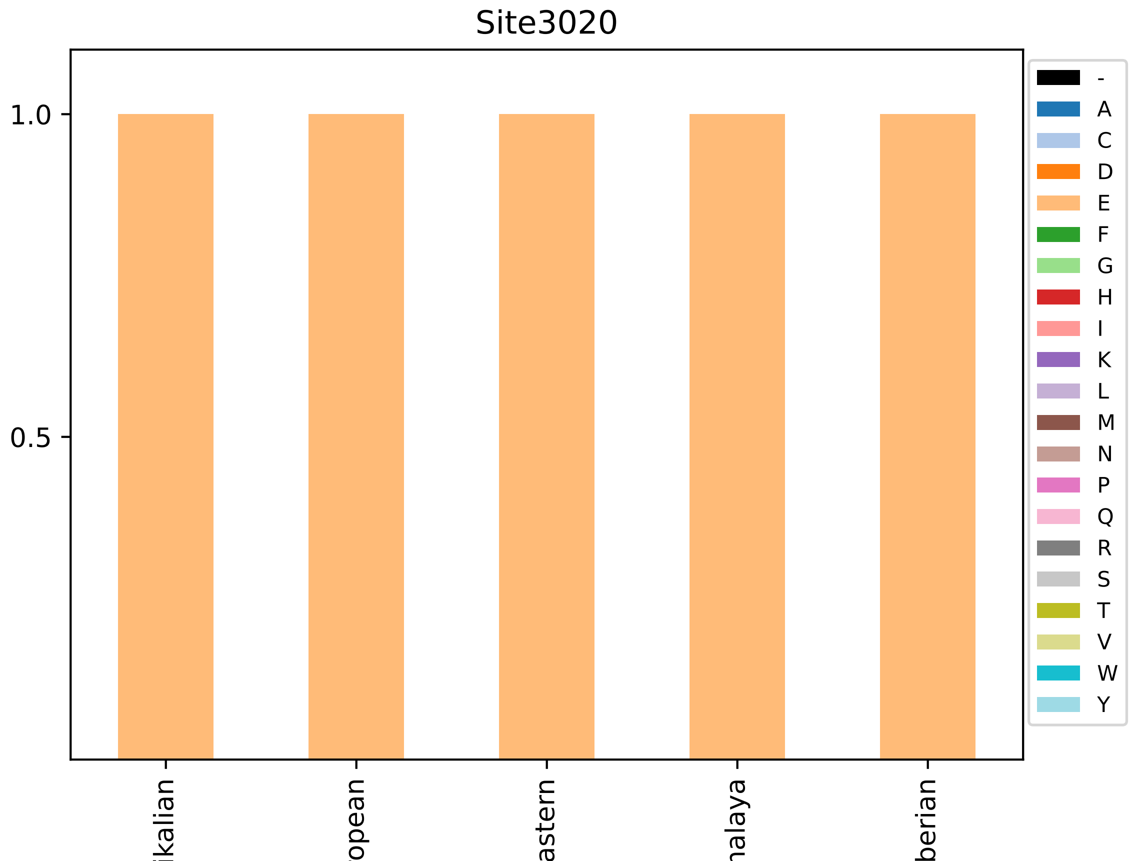
Site3020
1.0
0.5
-
A
C
D
E
F
G
H
I
K
L
M
N
P
Q
R
S
T
V
W
Y
ikalian
opean
astern
nalaya
berian

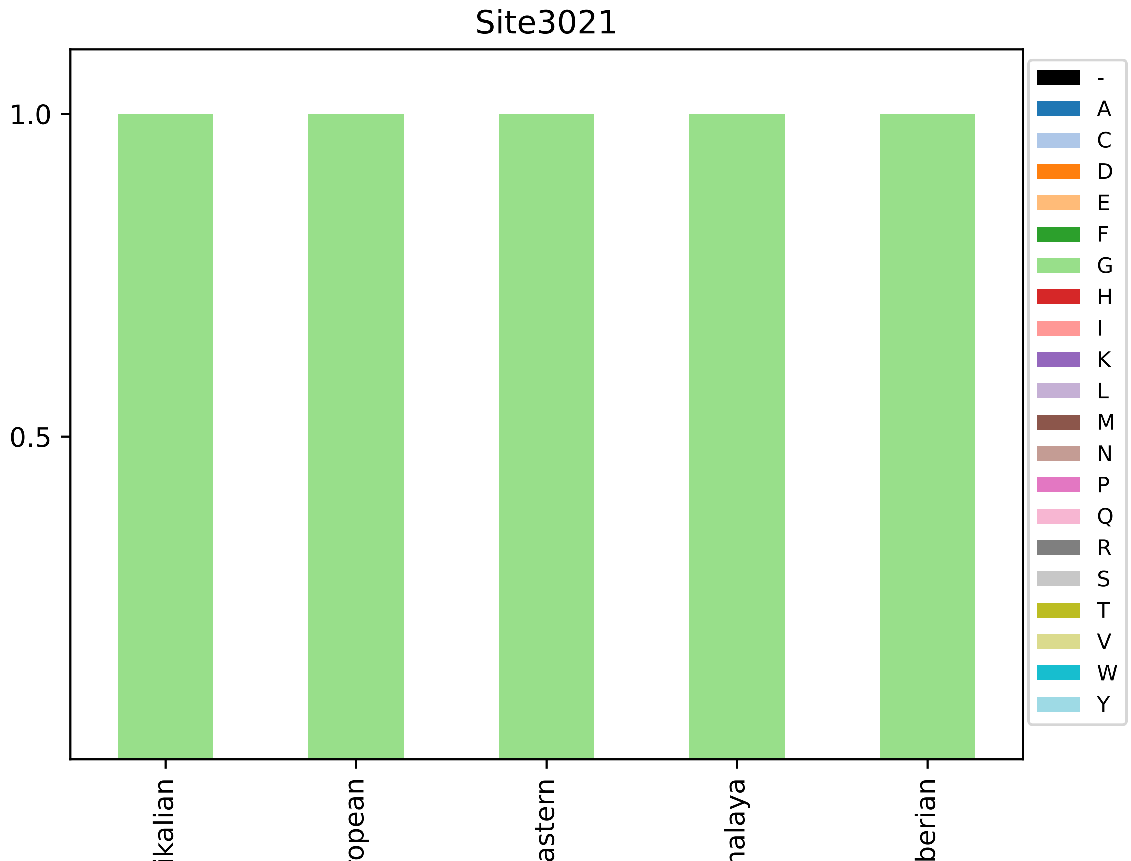
Site3021
1.0
0.5
-
A
C
D
E
F
G
H
I
K
L
M
N
P
Q
R
S
T
V
W
Y
ikalian
opean
astern
nalaya
berian

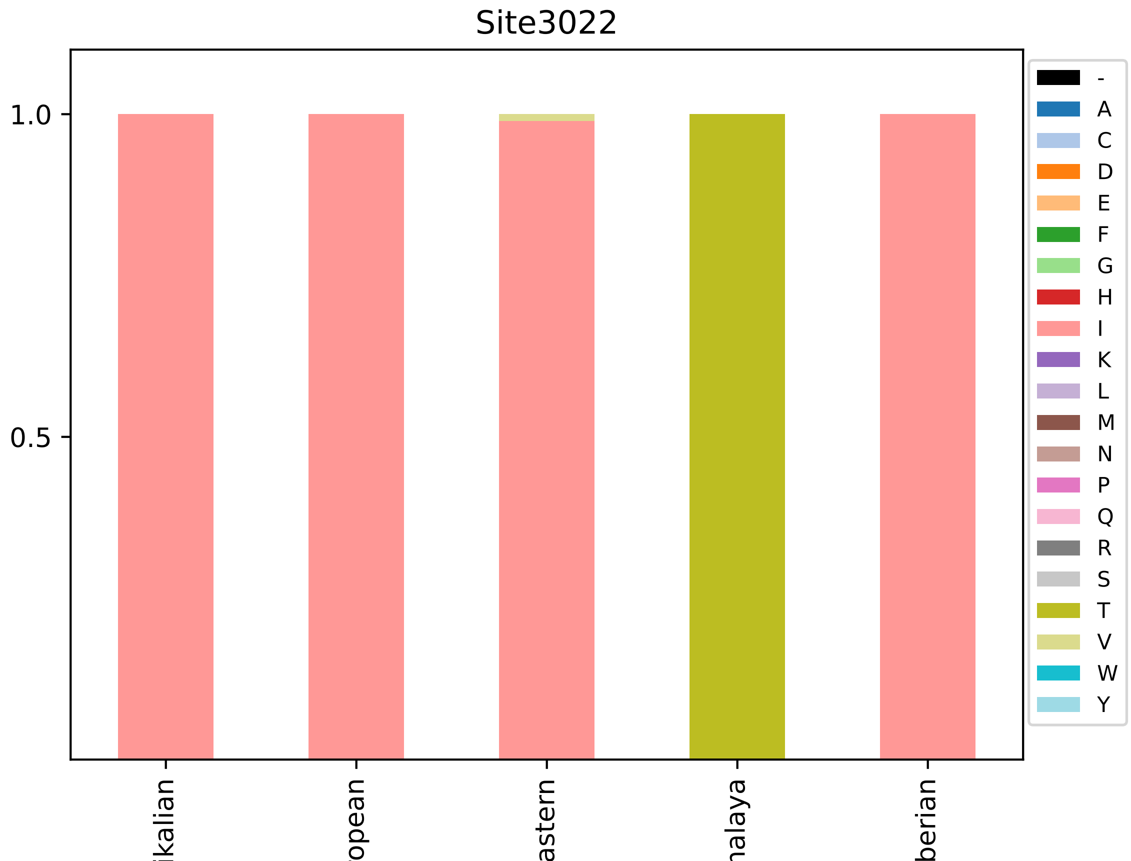
Site3022
1.0
0.5
-
A
C
D
E
F
G
H
I
K
L
M
N
P
Q
R
S
T
V
W
Y
ikalian
opean
astern
nalaya
berian

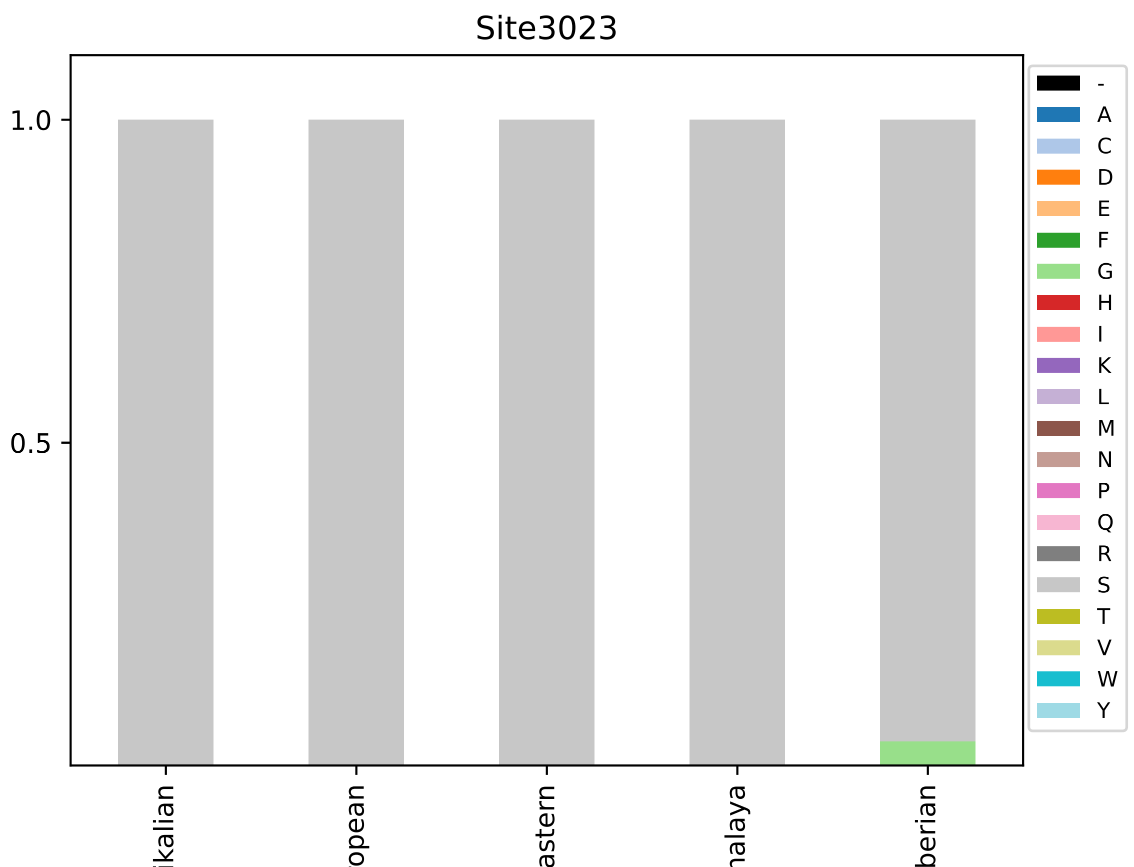
Site3023
1.0
0.5
ikalian
opean
astern
nalaya
berian
-
A
C
D
E
F
G
H
I
K
L
M
N
P
Q
R
S
T
V
W
Y

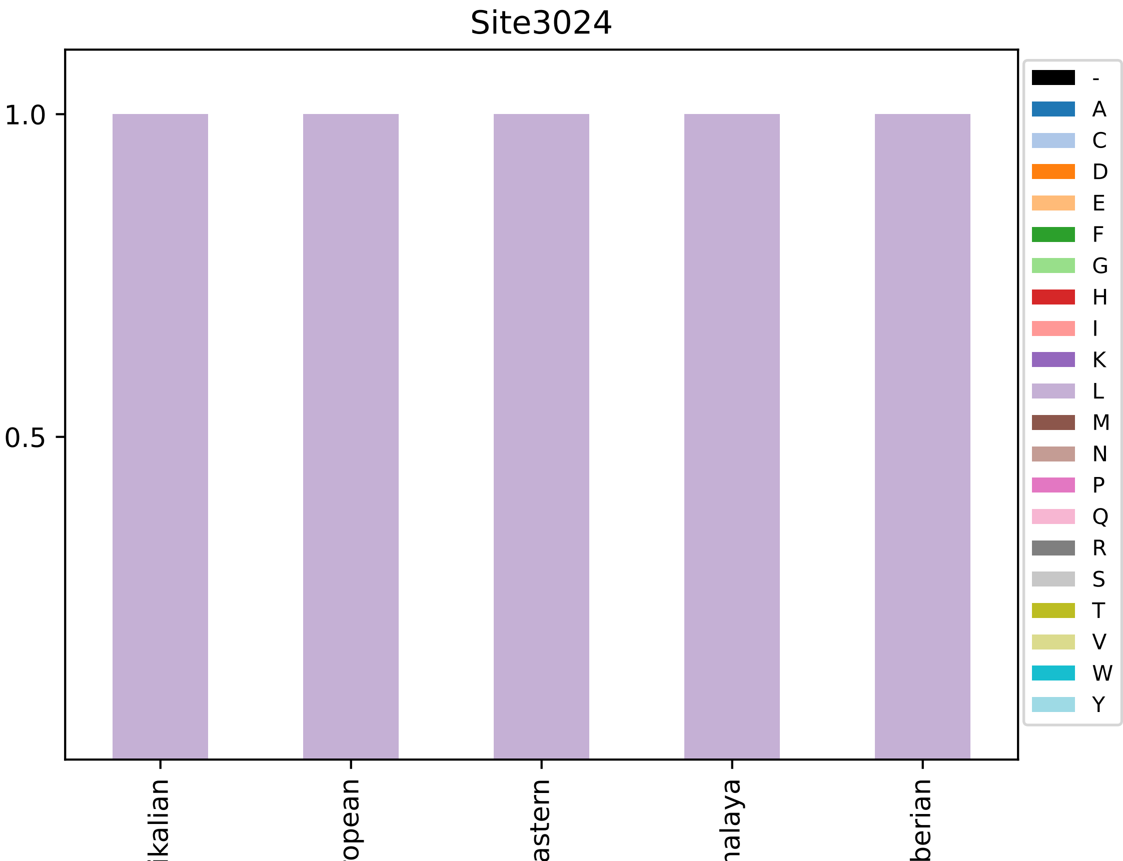

Site3024
1.0
0.5
-
A
C
D
E
F
G
H
I
K
L
M
N
P
Q
R
S
T
V
W
Y
ikalian
ropean
astern
nalaya
berian

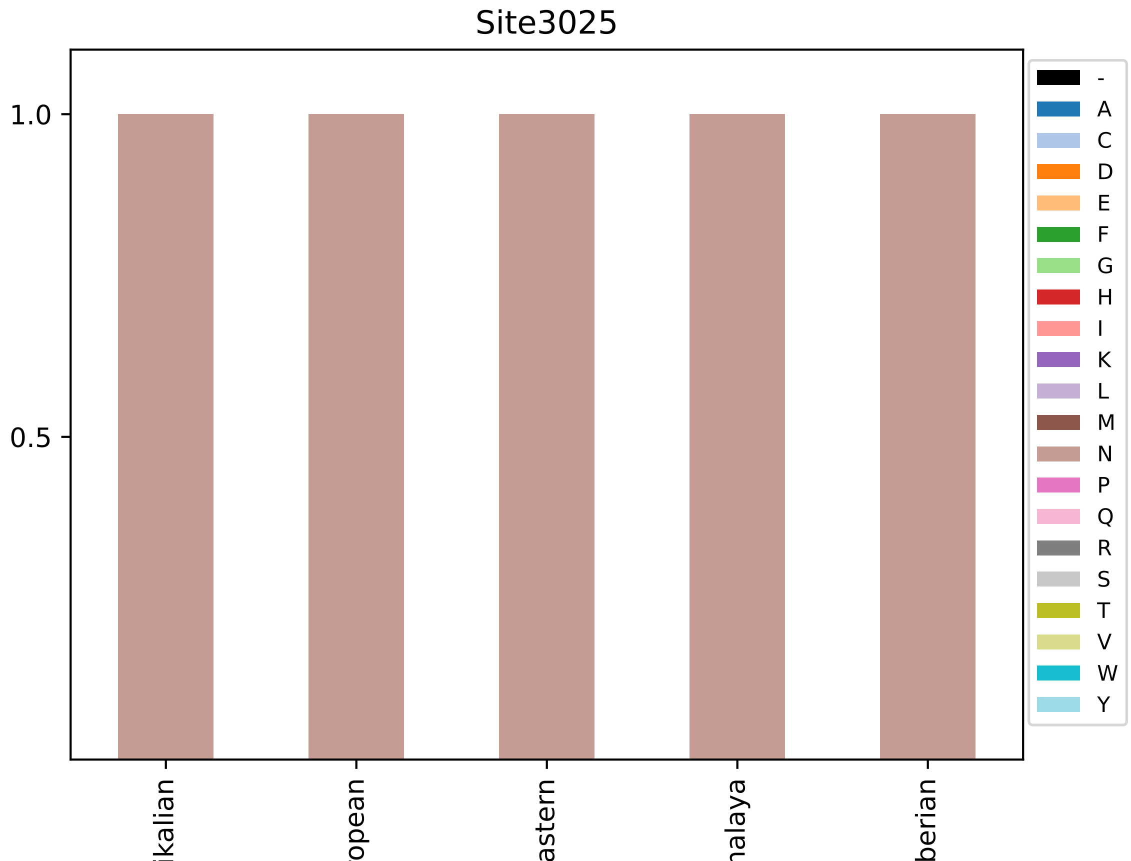

Site3025
1.0
0.5
-
A
C
D
E
F
G
H
I
K
L
M
N
P
Q
R
S
T
V
W
Y
ikalian
opean
astern
nalaya
berian

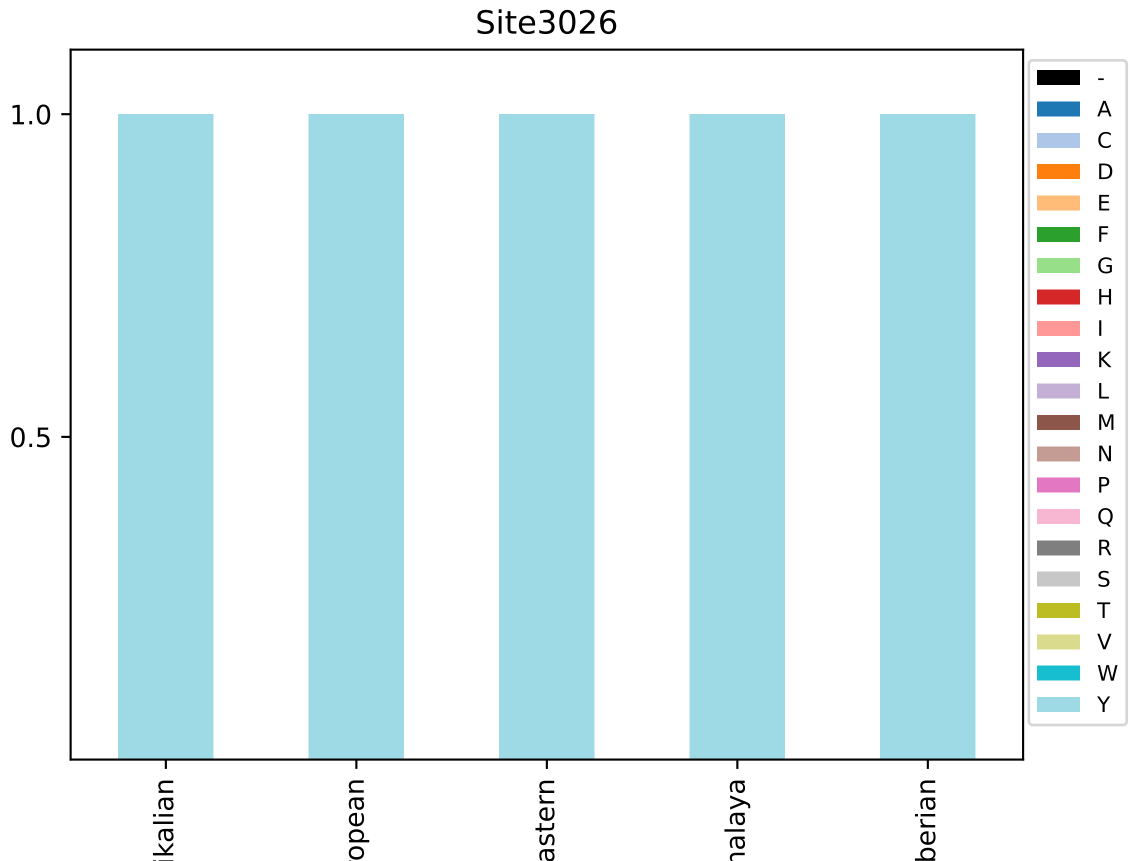

Site3026
1.0
0.5
-
A
C
D
E
F
G
H
I
K
L
M
N
P
Q
R
S
T
V
W
Y
ikalian
opean
astern
nalaya
berian

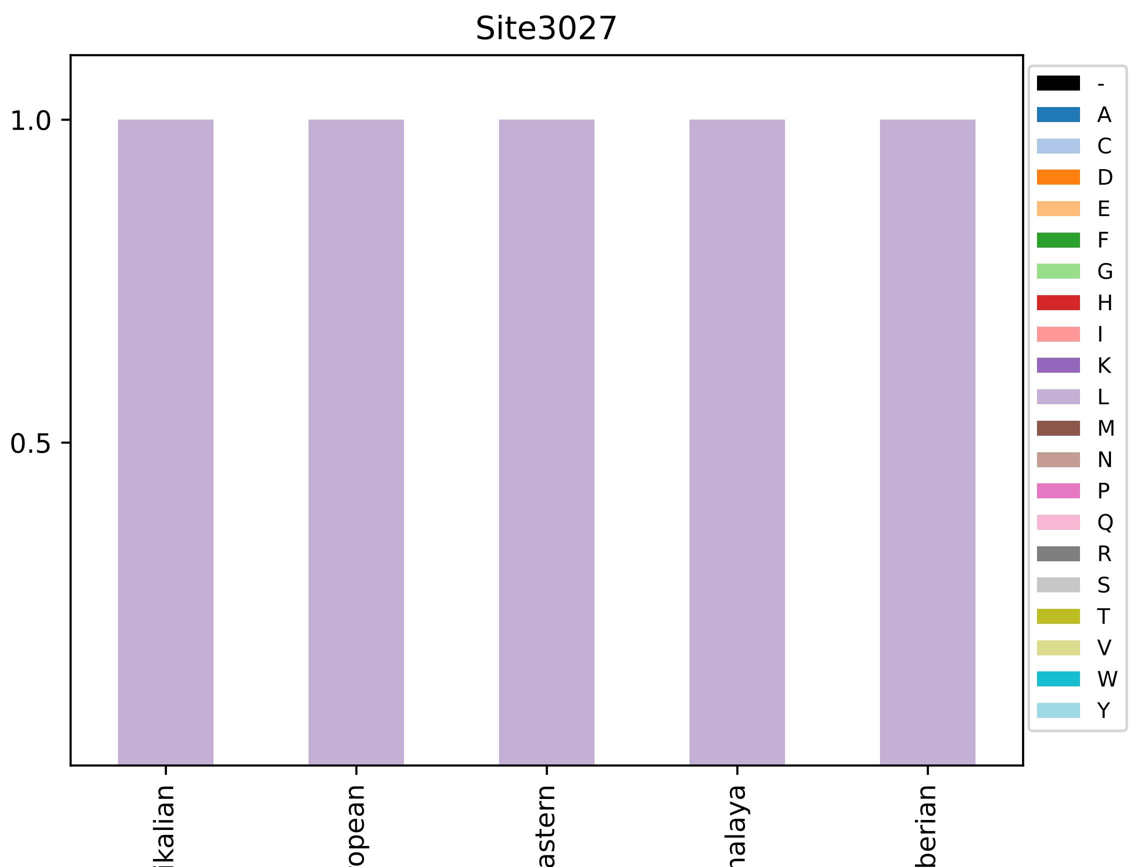
Site3027
1.0
0.5
ikalian
opean
astern
nalaya
berian
-
A
C
D
E
F
G
H
I
K
L
M
N
P
Q
R
S
T
V
W
Y

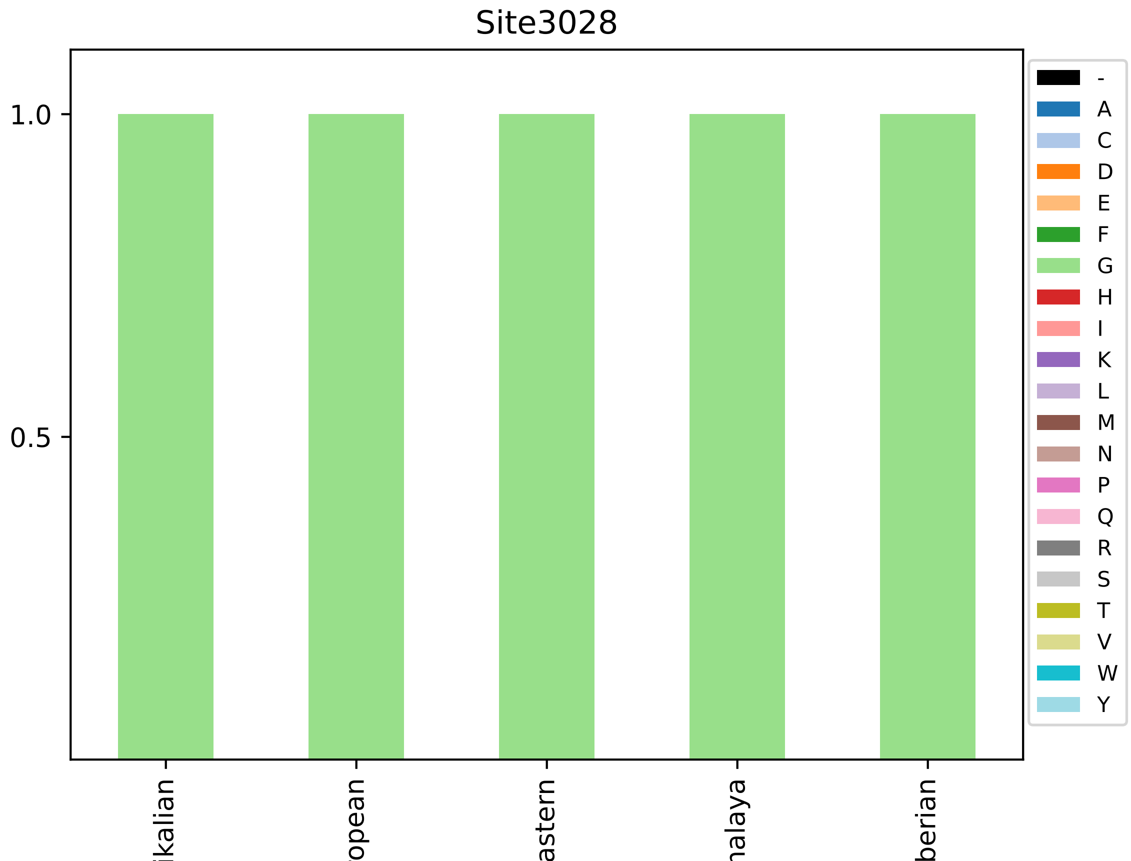

Site3028
1.0
0.5
-
A
C
D
E
F
G
H
I
K
L
M
N
P
Q
R
S
T
V
W
Y
ikalian
opean
astern
halaya
berian

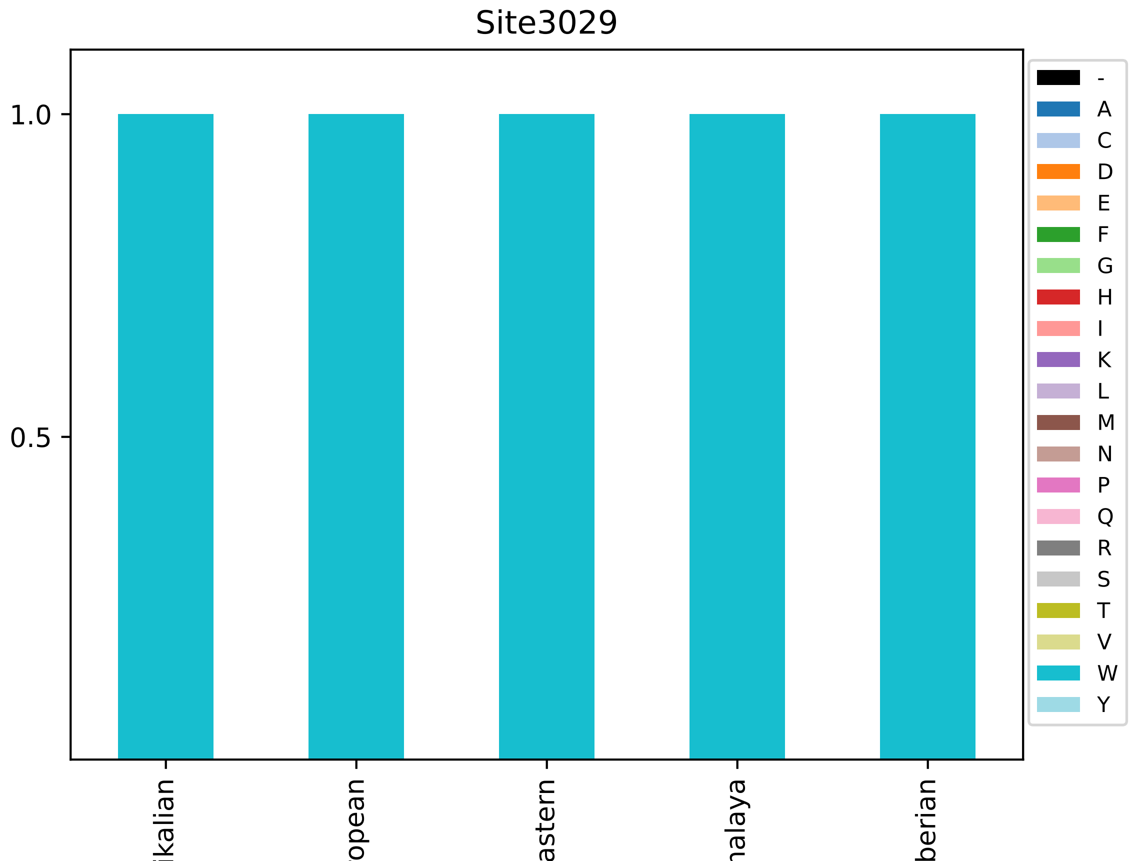
Site3029
1.0
0.5
-
A
C
D
E
F
G
H
I
K
L
M
N
P
Q
R
S
T
V
W
Y
ikalian
opean
astern
nalaya
berian

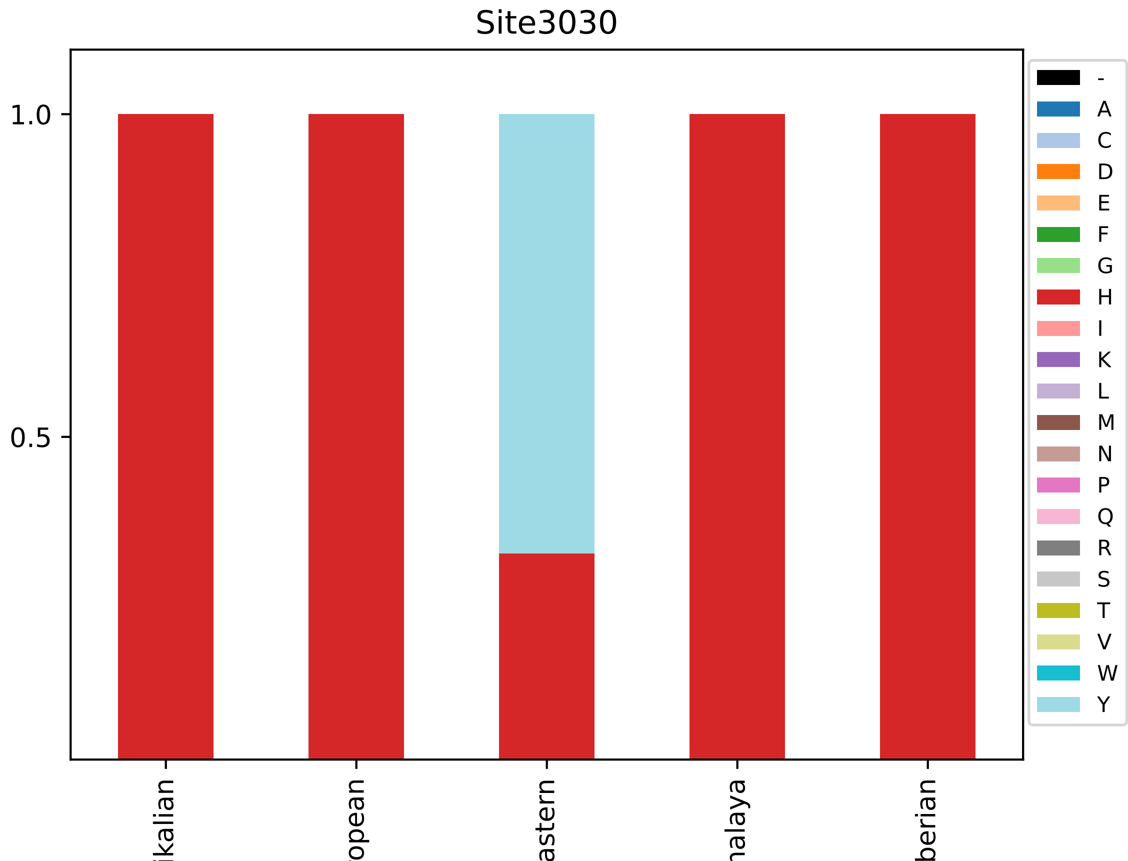

Site3030
1.0
0.5
-
A
C
D
E
F
G
H
I
K
L
M
N
P
Q
R
S
T
V
W
Y
ikalian
opean
astern
nalaya
berian

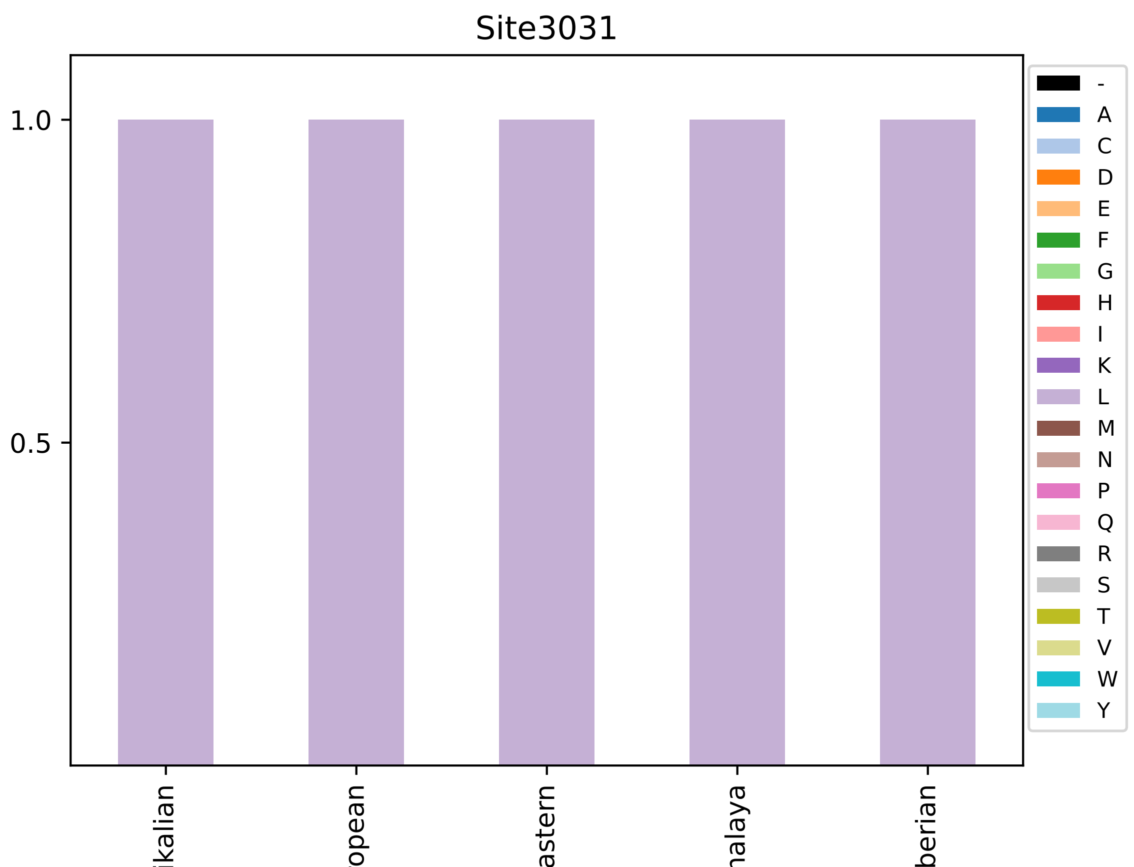

Site3031
1.0
0.5
ikalian
opean
astern
nalaya
berian
-
A
C
D
E
F
G
H
I
K
L
M
N
P
Q
R
S
T
V
W
Y

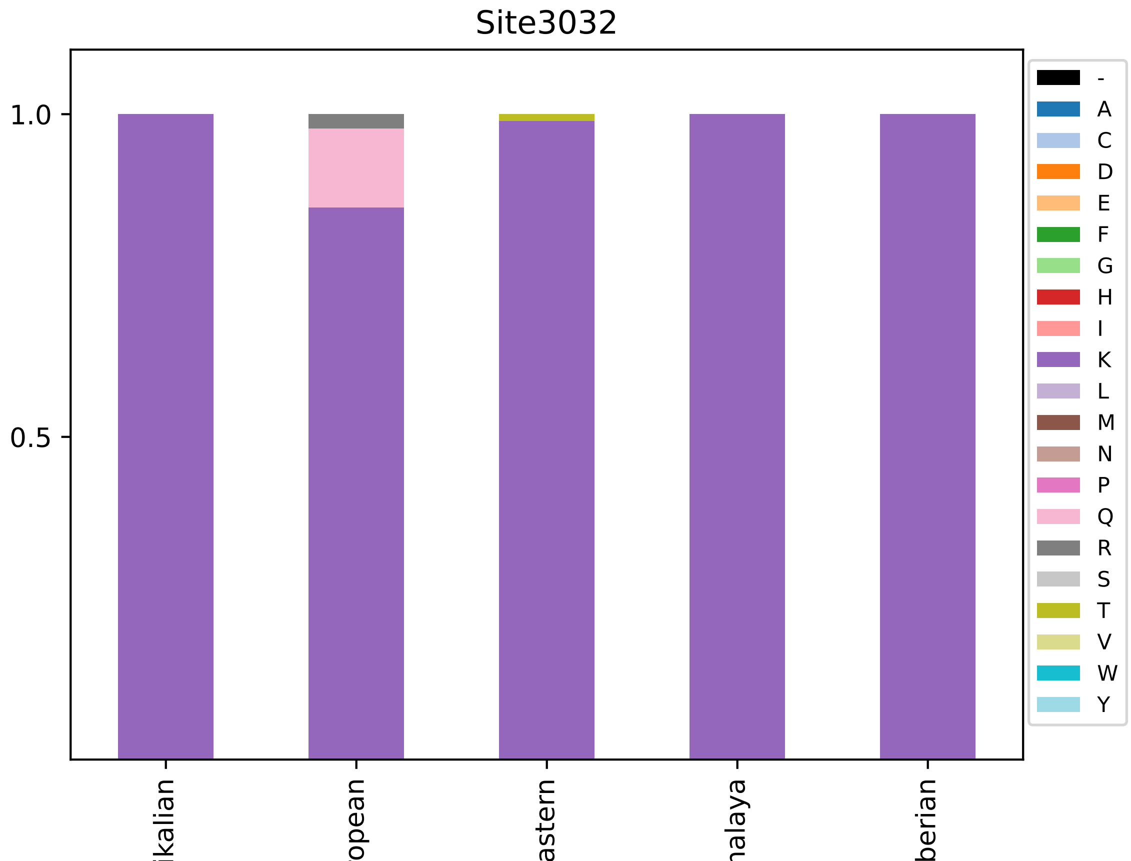
Site3032
1.0
0.5
-
A
C
D
E
F
G
H
I
K
L
M
N
P
Q
R
S
T
V
W
Y
ikalian
opean
astern
nalaya
berian

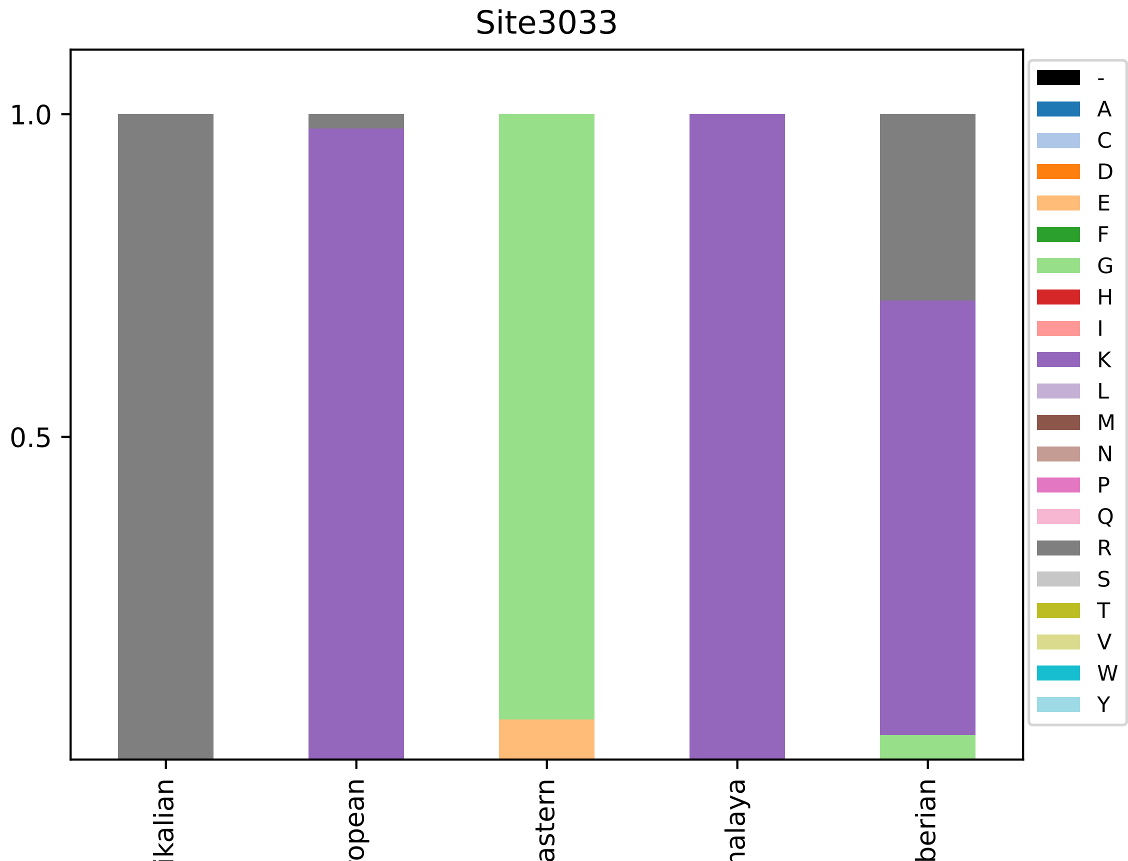
Site3033
1.0
0.5
-
A
C
D
E
F
G
H
I
K
L
M
N
P
Q
R
S
T
V
W
Y
ikalian
opean
astern
nalaya
berian

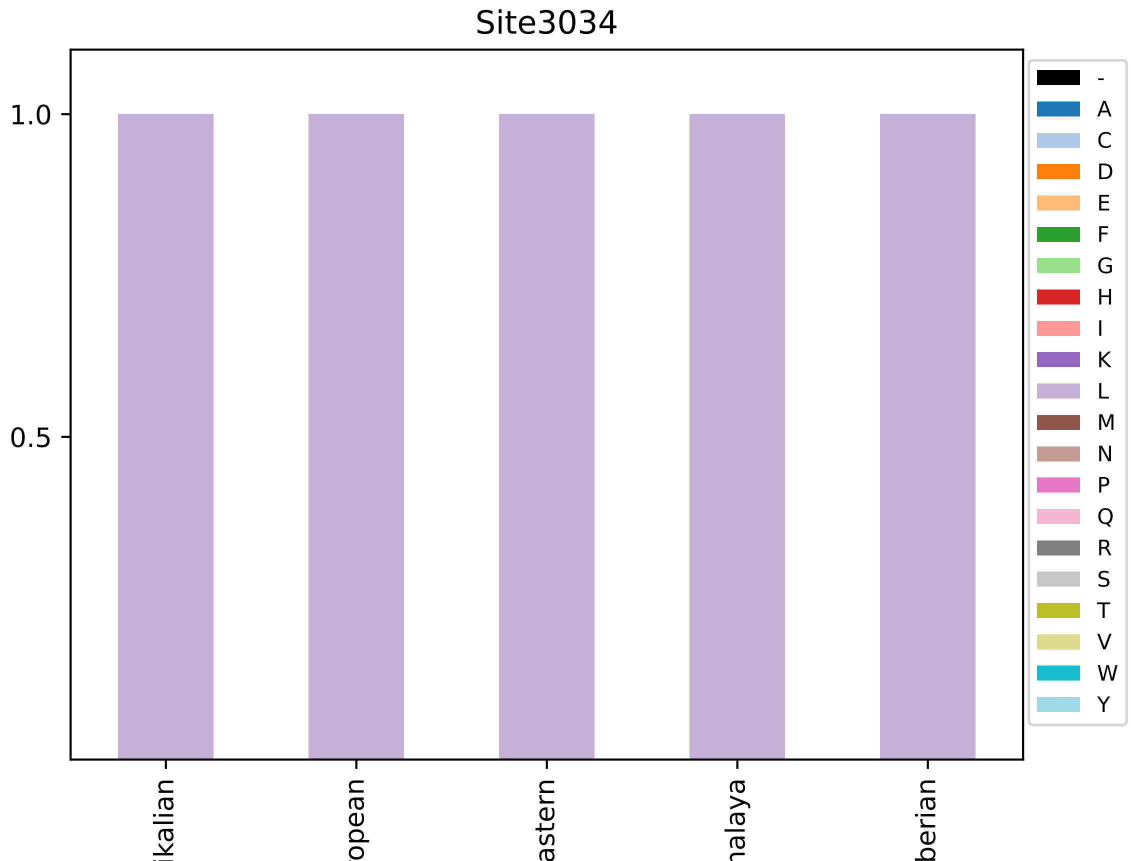
Site3034
1.0
0.5
-
A
C
D
E
F
G
H
I
K
L
M
N
P
Q
R
S
T
V
W
Y
ikalian
opean
astern
halaya
berian

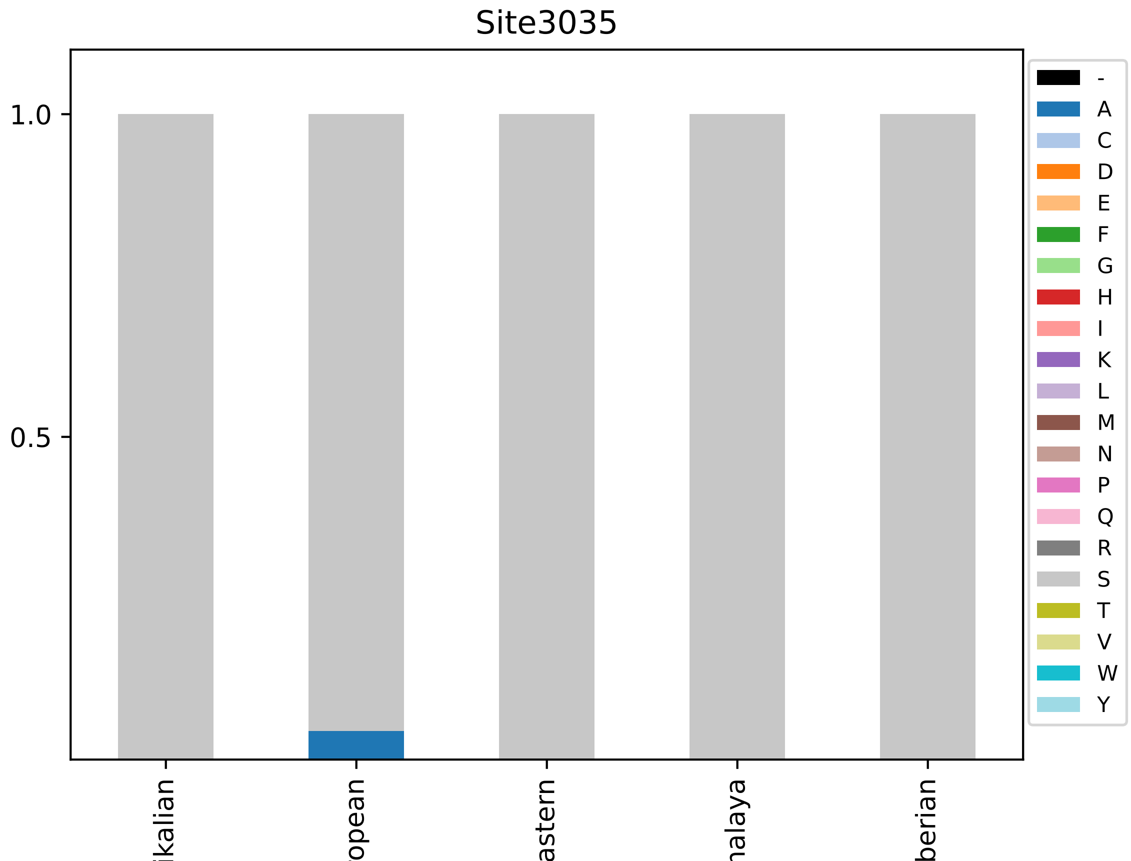
Site3035
1.0
0.5
-
A
C
D
E
F
G
H
I
K
L
M
N
P
Q
R
S
T
V
W
Y
ikalian
opean
astern
nalaya
berian

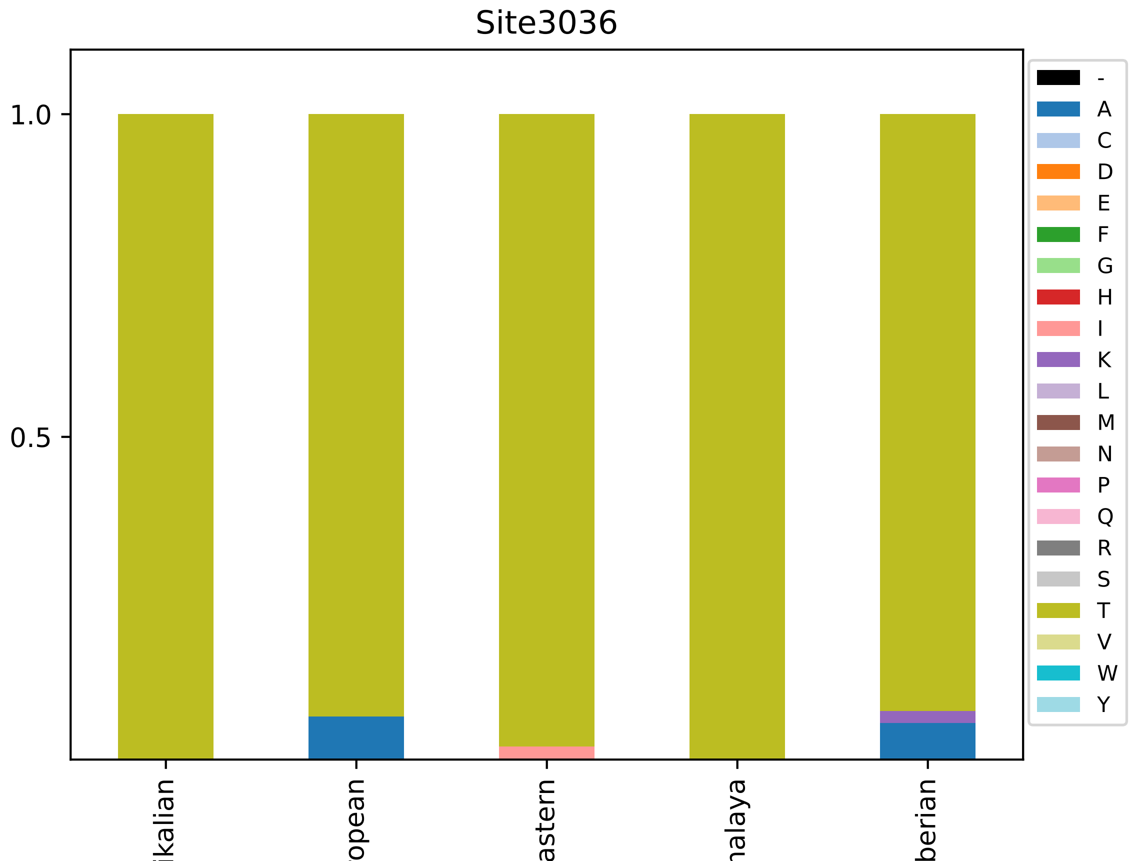

Site3036
1.0
0.5
ikalian
opean
astern
nalaya
berian
-
A
C
D
E
F
G
H
I
K
L
M
N
P
Q
R
S
T
V
W
Y

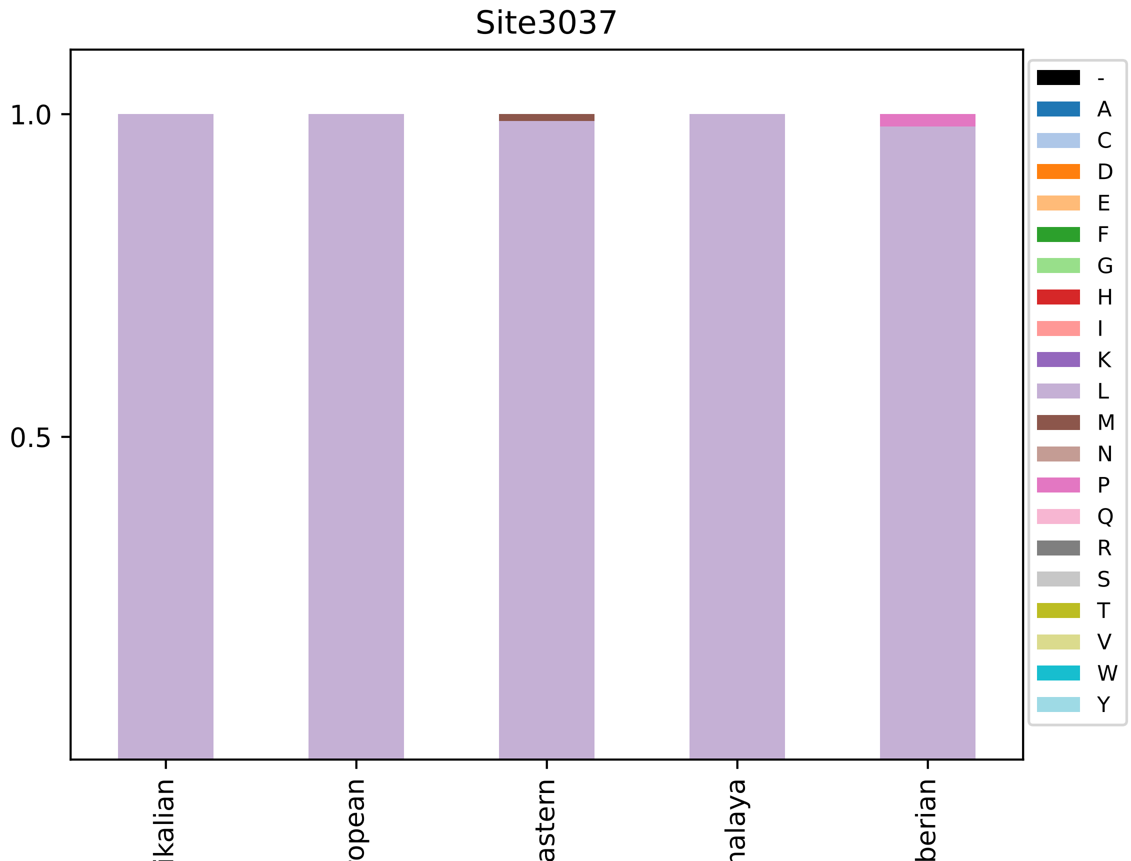
Site3037
1.0
0.5
ikalian
opean
astern
nalaya
berian
-
A
C
D
E
F
G
H
I
K
L
M
N
P
Q
R
S
T
V
W
Y

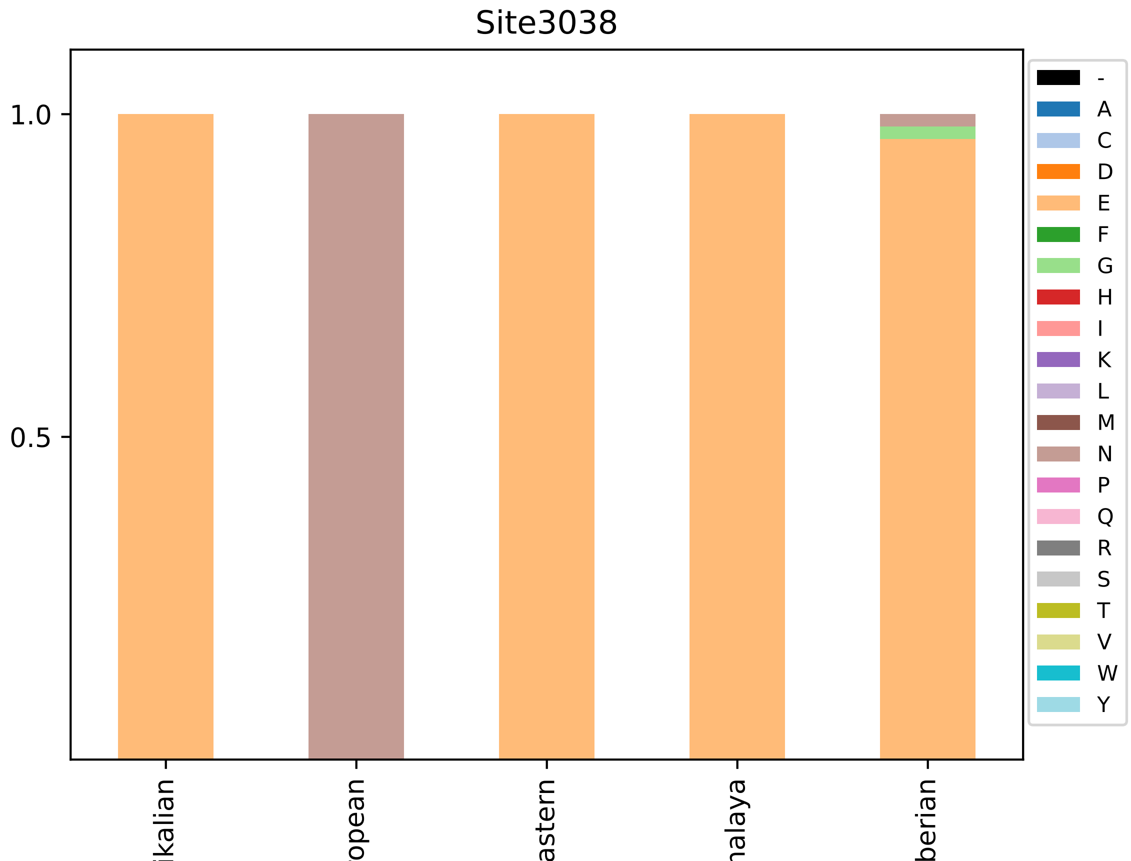
Site3038
1.0
0.5
-
A
C
D
E
F
G
H
I
K
L
M
N
P
Q
R
S
T
V
W
Y

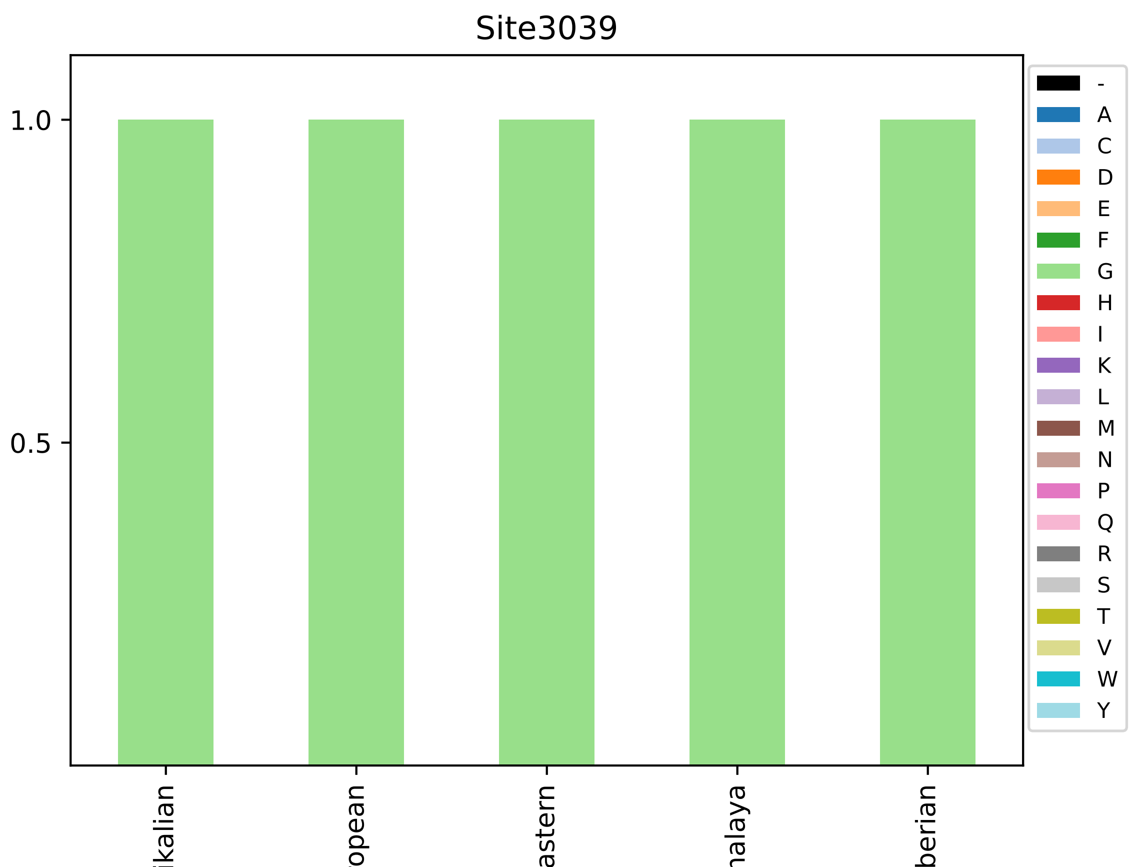

Site3039
1.0
0.5
-
A
C
D
E
F
G
H
I
K
L
M
N
P
Q
R
S
T
V
W
Y
ikalian
opean
astern
nalaya
berian

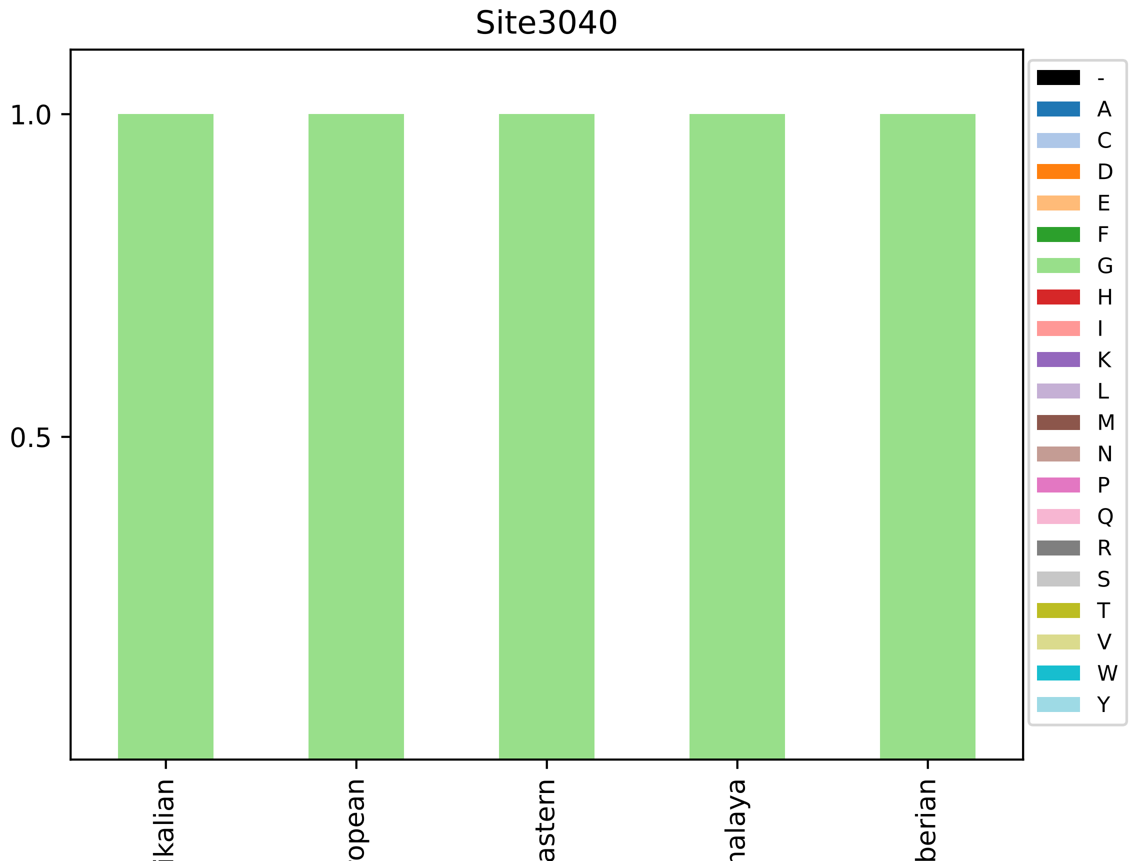
Site3040
1.0
0.5
-
A
C
D
E
F
G
H
I
K
L
M
N
P
Q
R
S
T
V
W
Y
ikalian
opean
astern
nalaya
berian

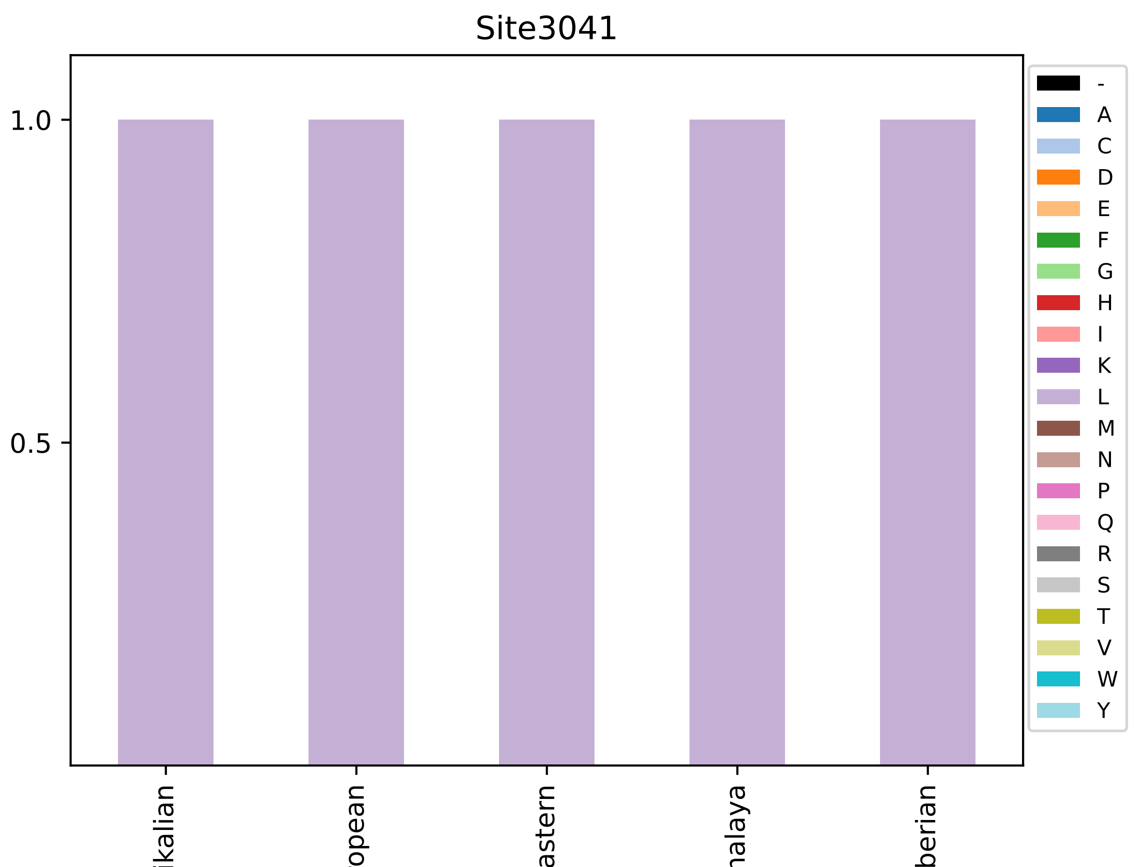

Site3041
1.0
0.5
-
A
C
D
E
F
G
H
I
K
L
M
N
P
Q
R
S
T
V
W
Y

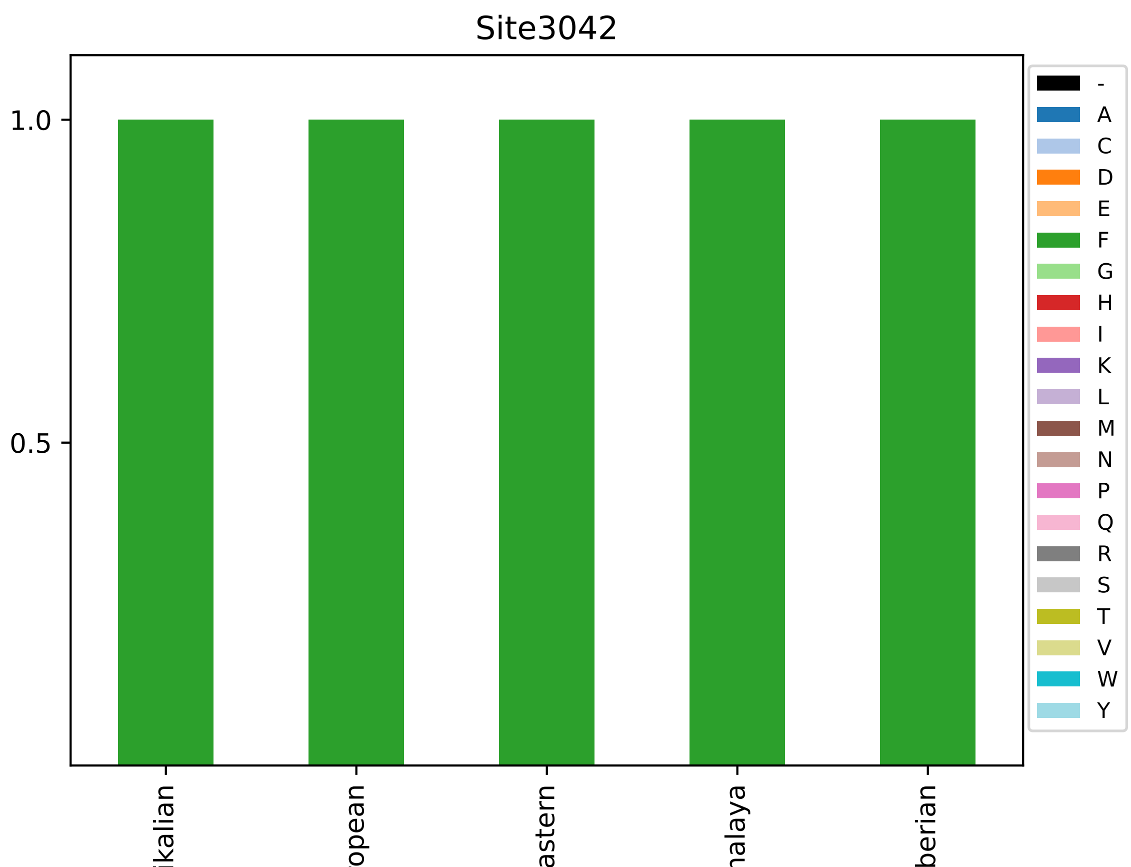

Site3042
1.0
0.5
-
A
C
D
E
F
G
H
I
K
L
M
N
P
Q
R
S
T
V
W
Y
ikalian
opean
astern
nalaya
berian

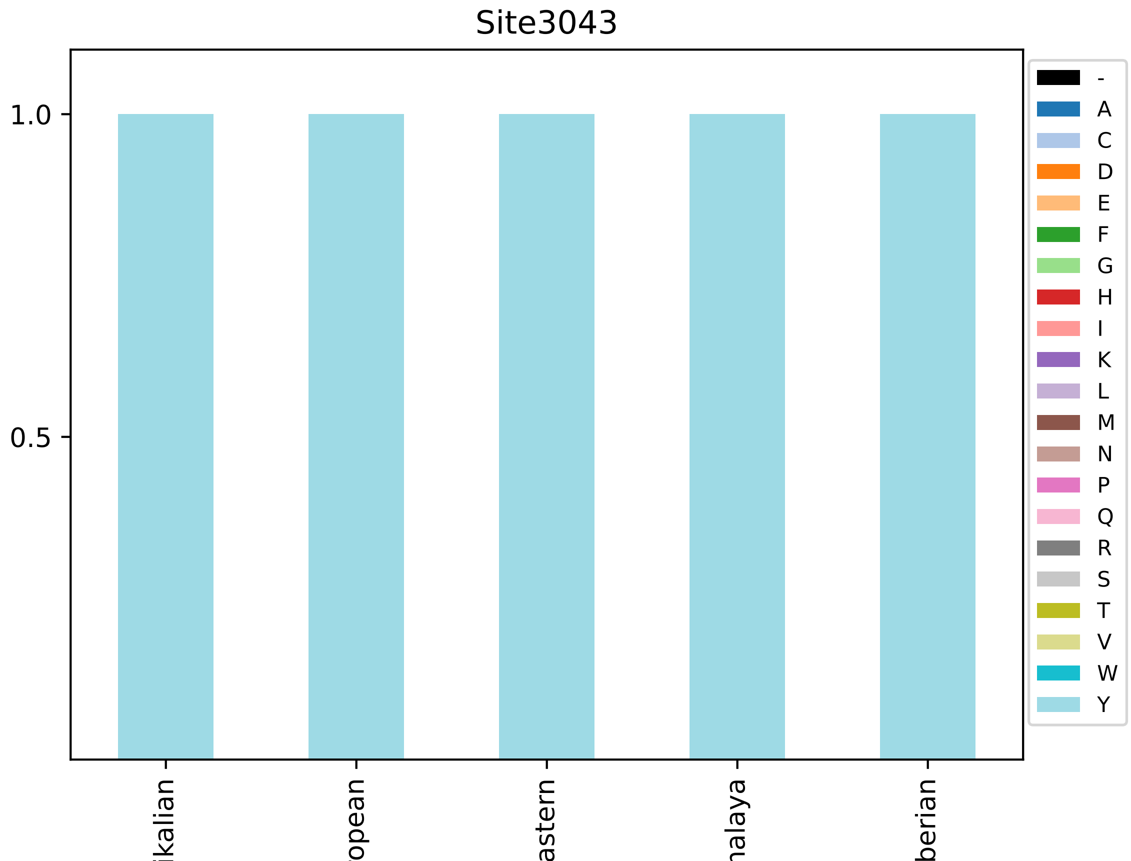

Site3043
1.0
0.5
-
A
C
D
E
F
G
H
I
K
L
M
N
P
Q
R
S
T
V
W
Y
ikalian
opean
astern
nalaya
berian

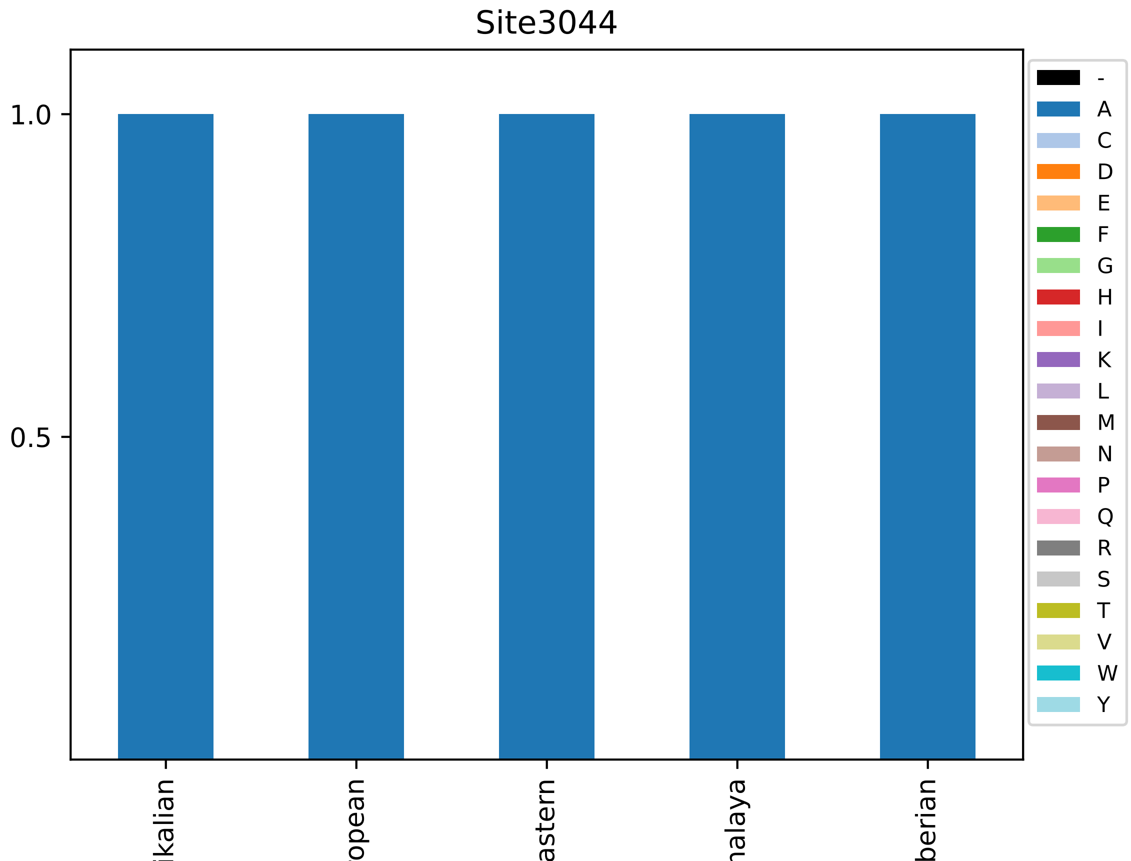

Site3044
1.0
0.5
-
A
C
D
E
F
G
H
I
K
L
M
N
P
Q
R
S
T
V
W
Y
ikalian
opean
astern
nalaya
berian

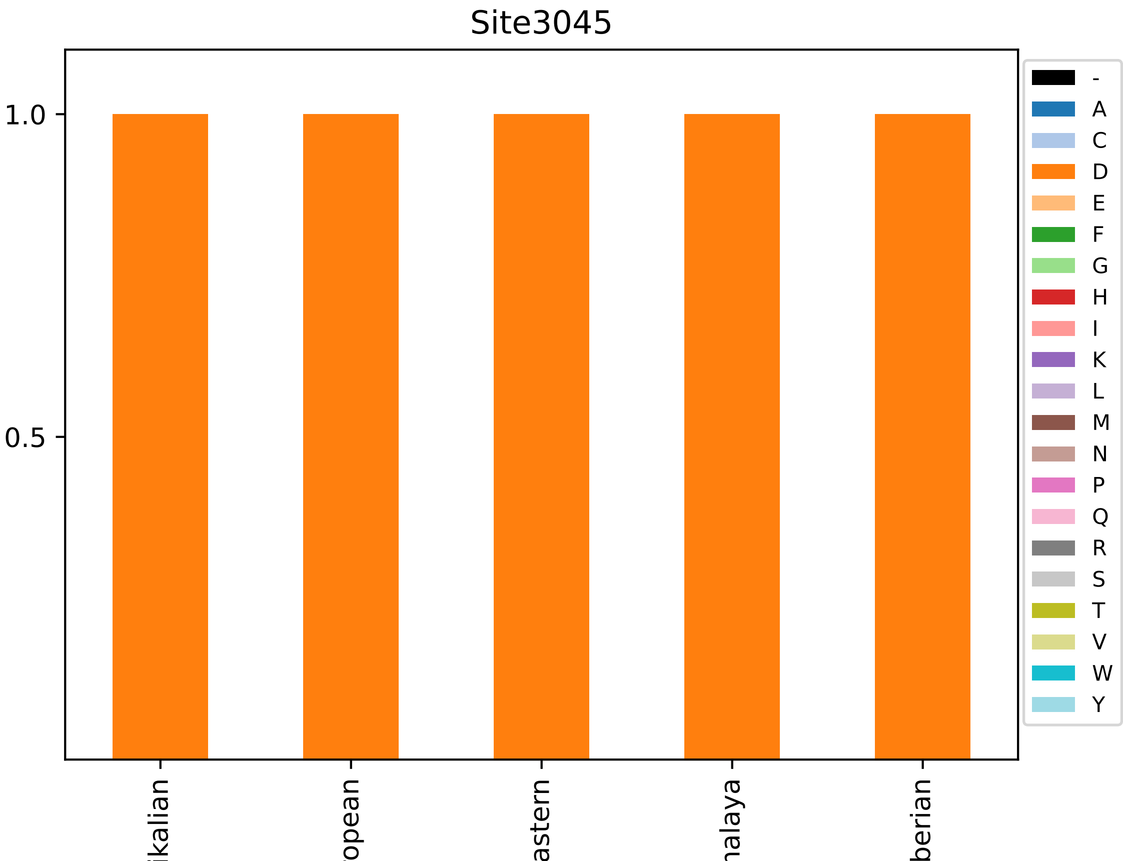

Site3045
1.0
0.5
-
A
C
D
E
F
G
H
I
K
L
M
N
P
Q
R
S
T
V
W
Y

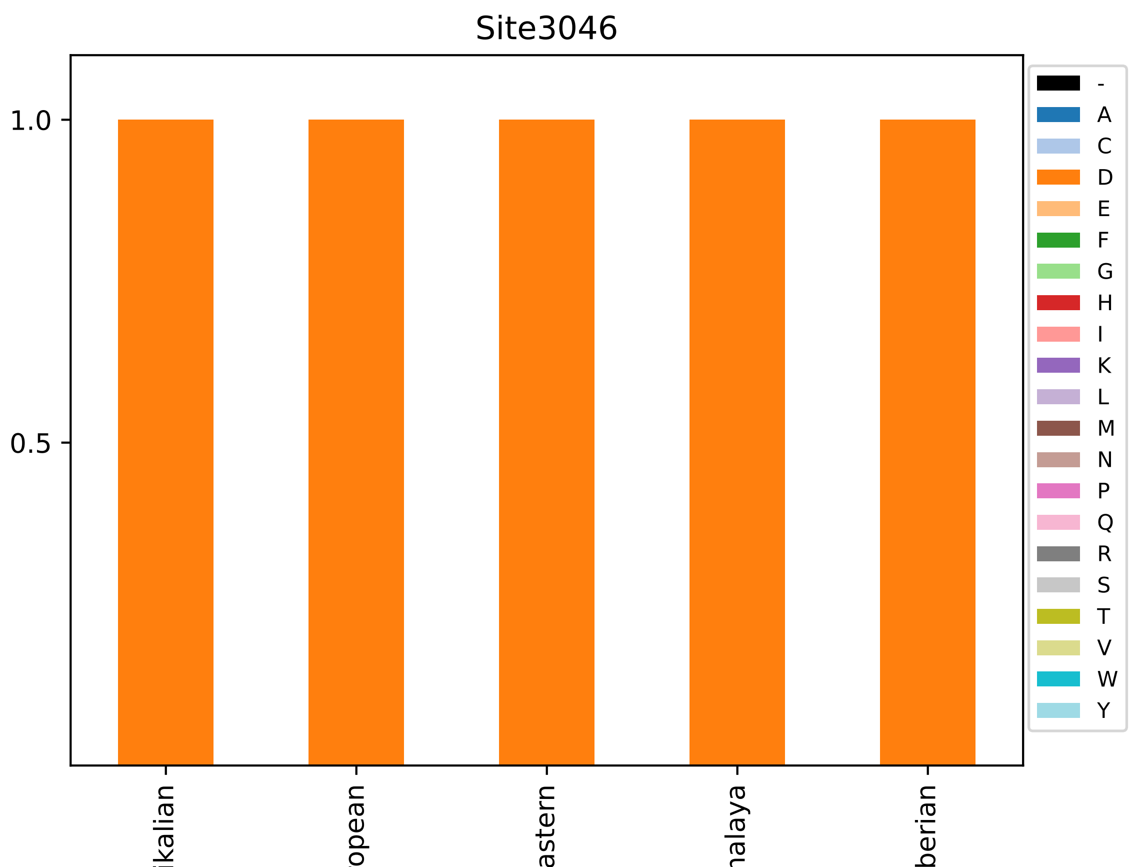
Site3046
1.0
0.5
-
A
C
D
E
F
G
H
I
K
L
M
N
P
Q
R
S
T
V
W
Y
ikalian
opean
astern
nalaya
berian

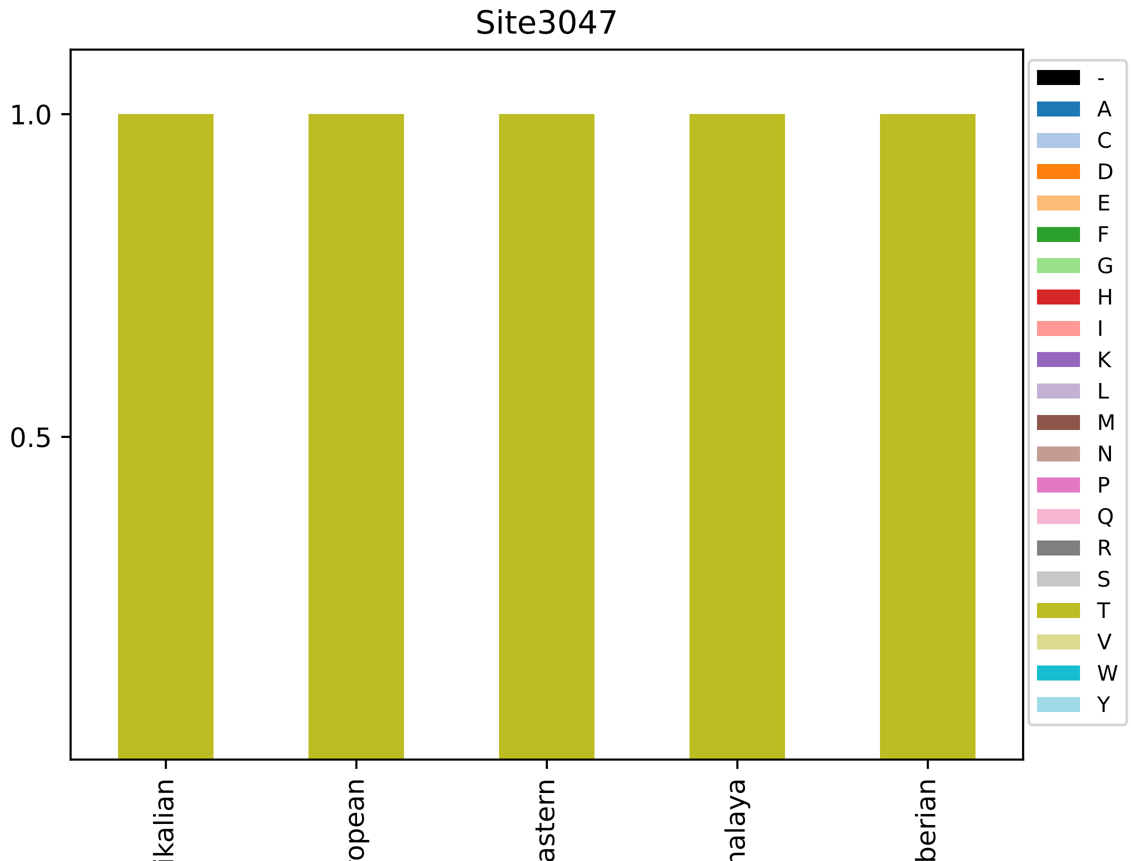
Site3047
1.0
0.5
ikalian
opean
astern
nalaya
berian
-
A
C
D
E
F
G
H
I
K
L
M
N
P
Q
R
S
T
V
W
Y

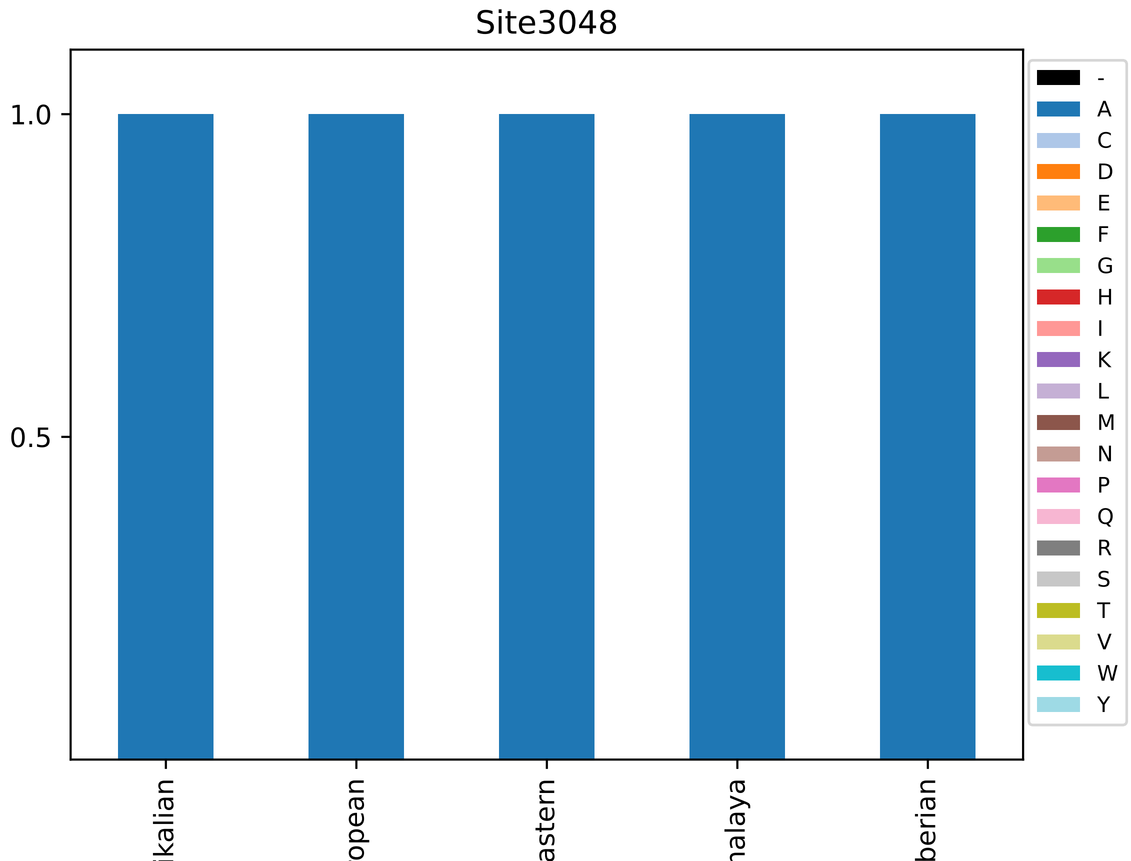

Site3048
1.0
0.5
ikalian
opean
astern
nalaya
berian
-
A
C
D
E
F
G
H
I
K
L
M
N
P
Q
R
S
T
V
W
Y

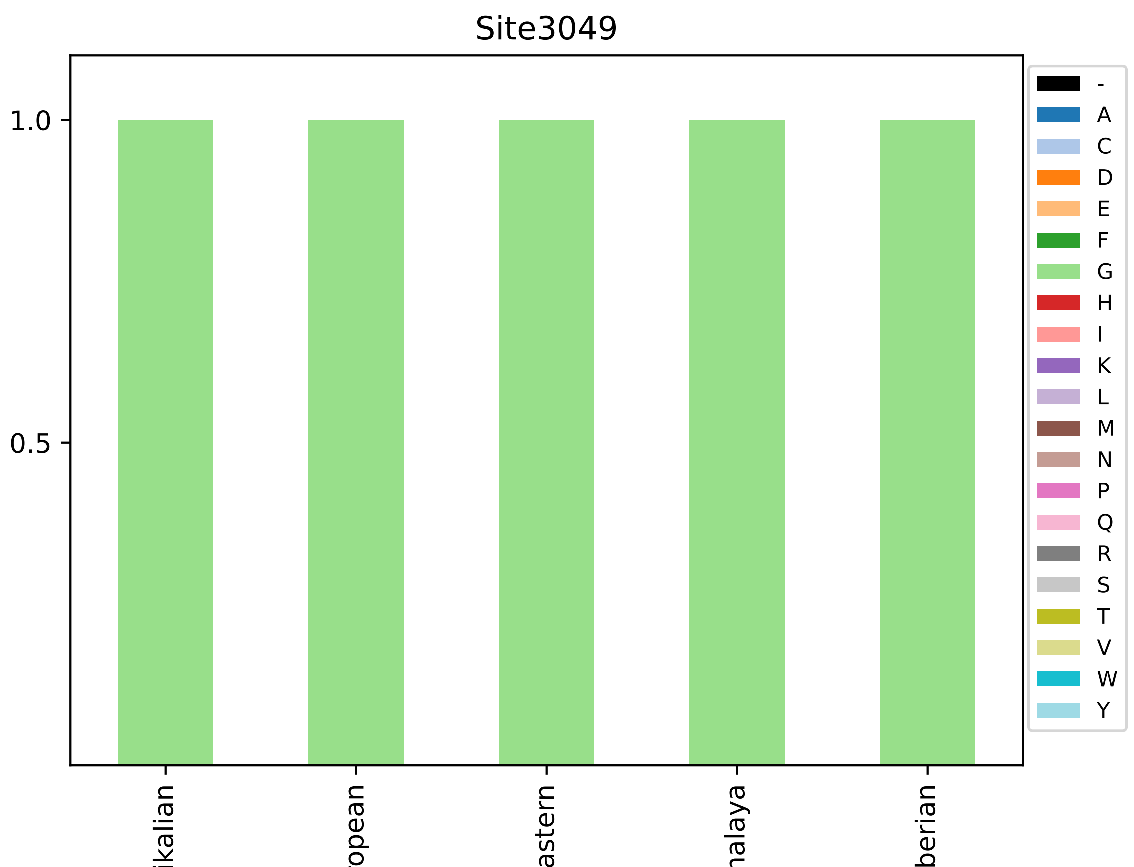
Site3049
1.0
0.5
-
A
C
D
E
F
G
H
I
K
L
M
N
P
Q
R
S
T
V
W
Y
ikalian
opean
astern
nalaya
berian

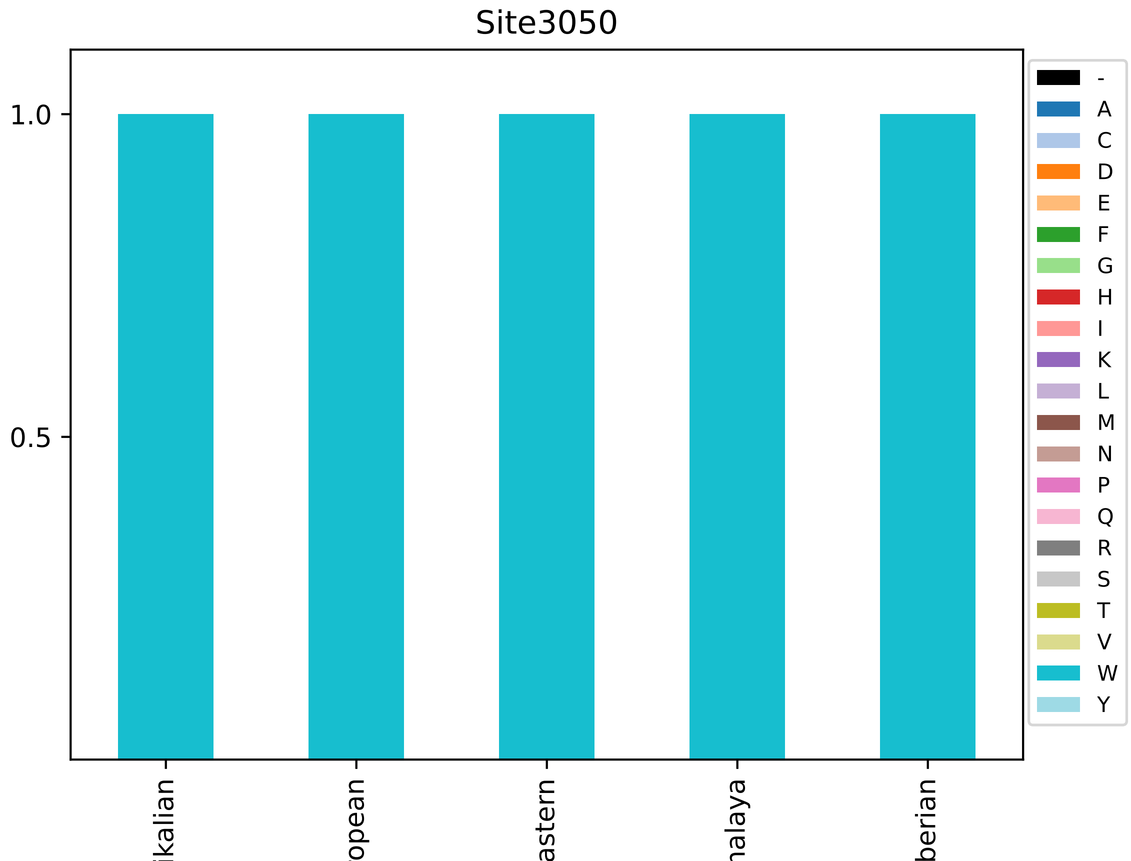
Site3050
1.0
0.5
-
A
C
D
E
F
G
H
I
K
L
M
N
P
Q
R
S
T
V
W
Y
ikalian
opean
astern
nalaya
berian

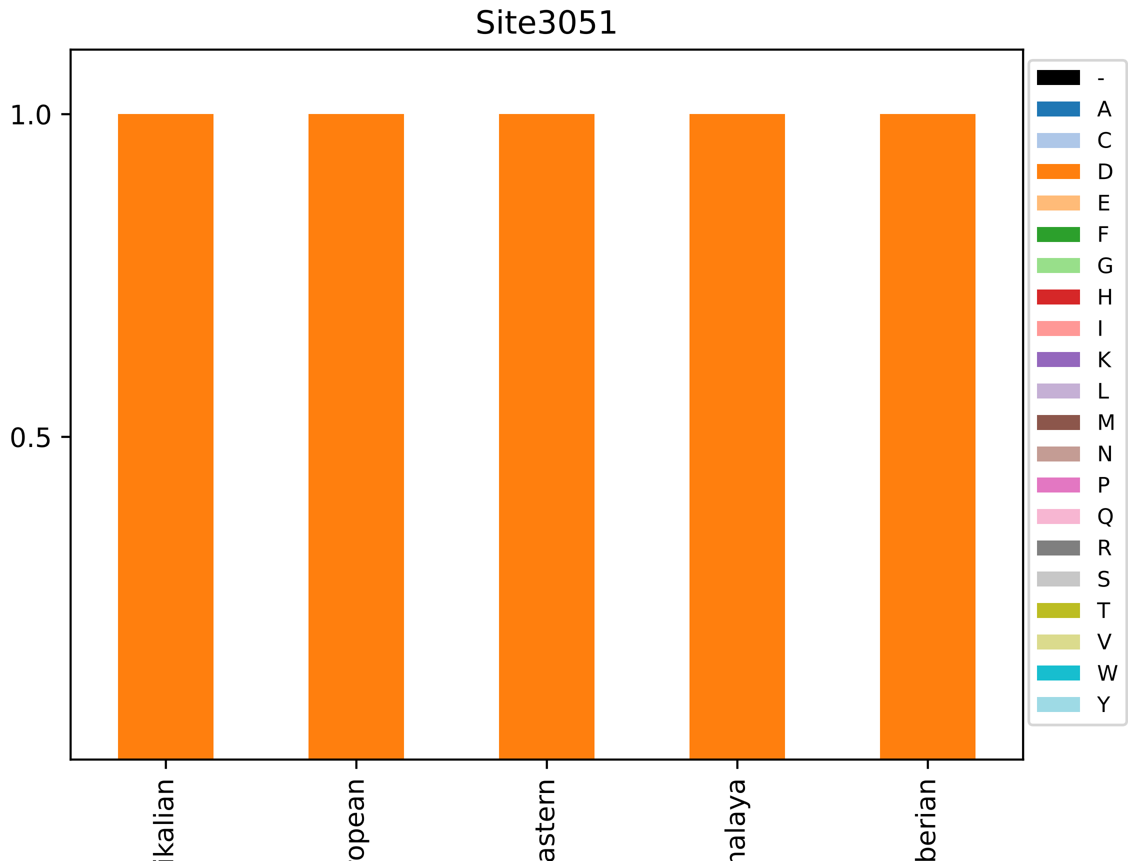
Site3051
1.0
0.5
ikalian
opean
astern
nalaya
berian
-
A
C
D
E
F
G
H
I
K
L
M
N
P
Q
R
S
T
V
W
Y

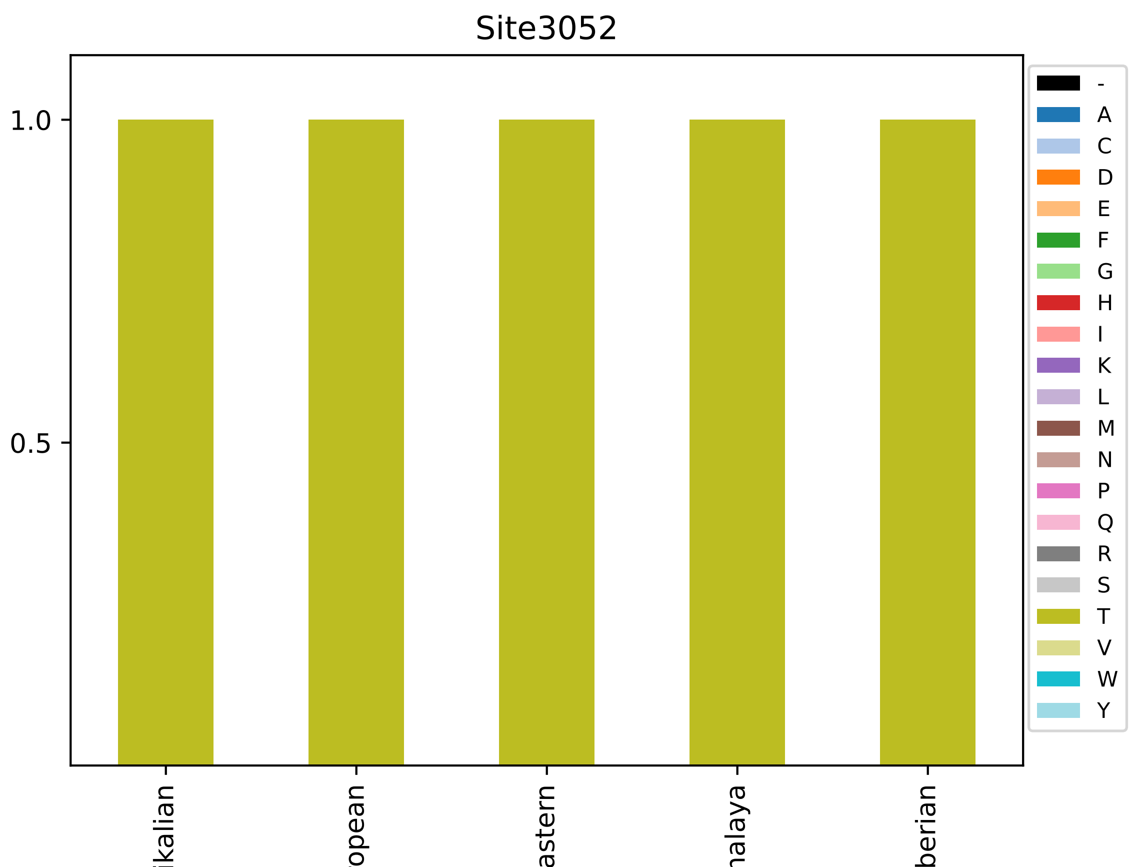
Site3052
1.0
0.5
ikalian
opean
astern
nalaya
berian
-
A
C
D
E
F
G
H
I
K
L
M
N
P
Q
R
S
T
V
W
Y

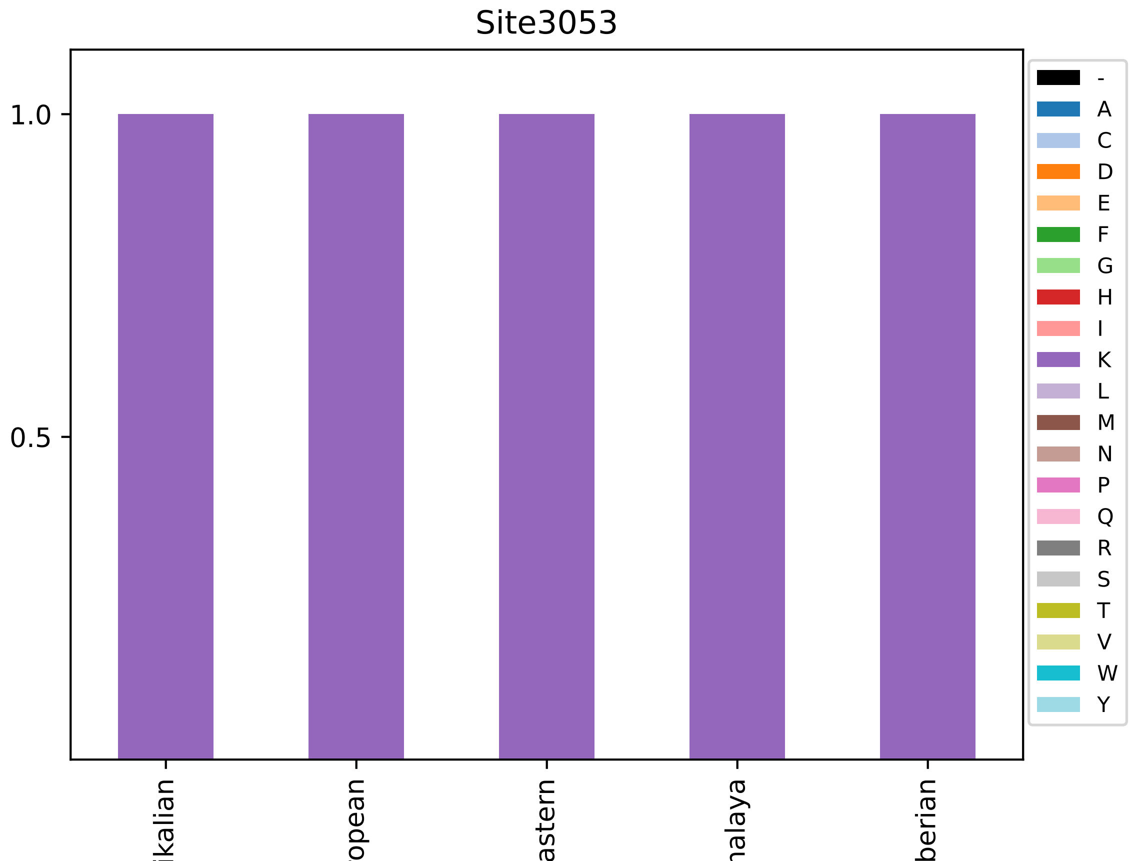

Site3053
1.0
0.5
-
A
C
D
E
F
G
H
I
K
L
M
N
P
Q
R
S
T
V
W
Y
ikalian
opean
astern
nalaya
berian

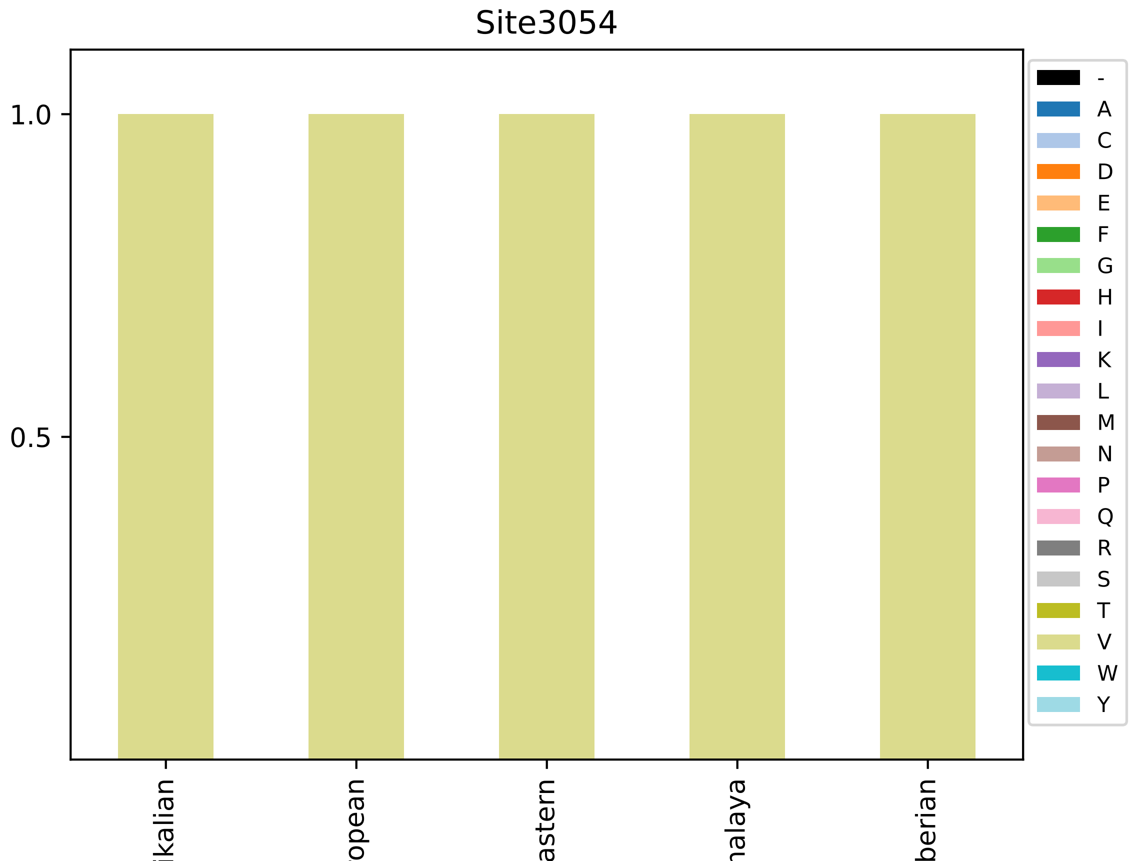
Site3054
1.0
0.5
-
A
C
D
E
F
G
H
I
K
L
M
N
P
Q
R
S
T
V
W
Y
ikalian
opean
astern
nalaya
berian

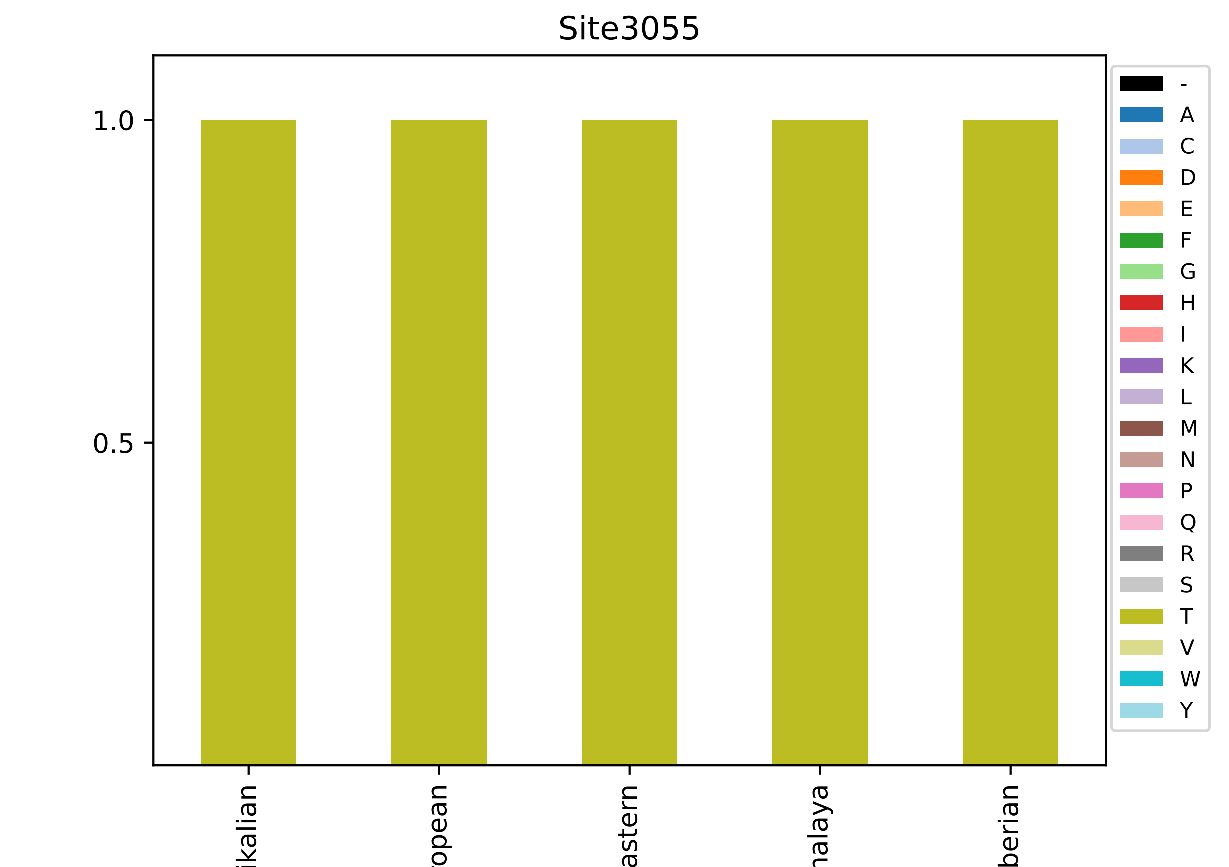

Site3055
1.0
0.5
-
A
C
D
E
F
G
H
I
K
L
M
N
P
Q
R
S
T
V
W
Y
ikalian
opean
astern
nalaya
berian

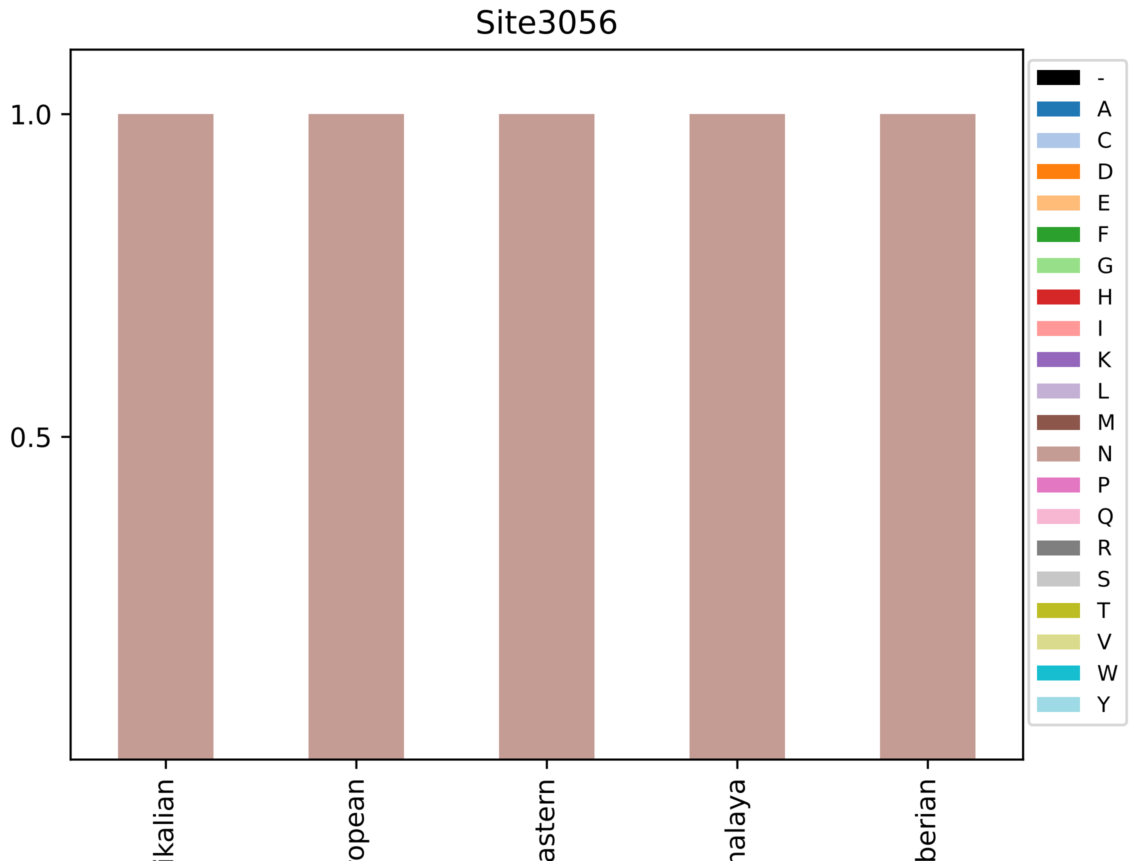

Site3056
1.0
0.5
-
A
C
D
E
F
G
H
I
K
L
M
N
P
Q
R
S
T
V
W
Y
ikalian
opean
astern
nalaya
berian

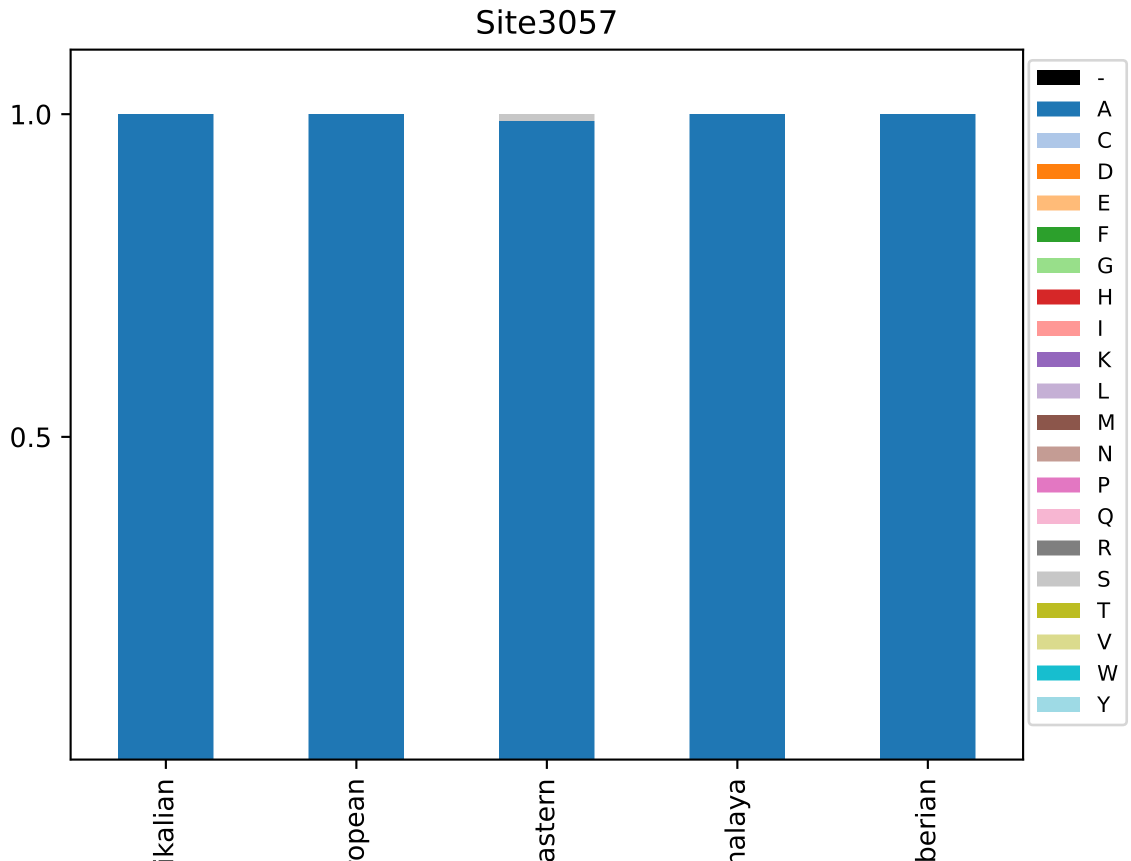
Site3057
1.0
0.5
-
A
C
D
E
F
G
H
I
K
L
M
N
P
Q
R
S
T
V
W
Y
ikalian
opean
astern
nalaya
berian

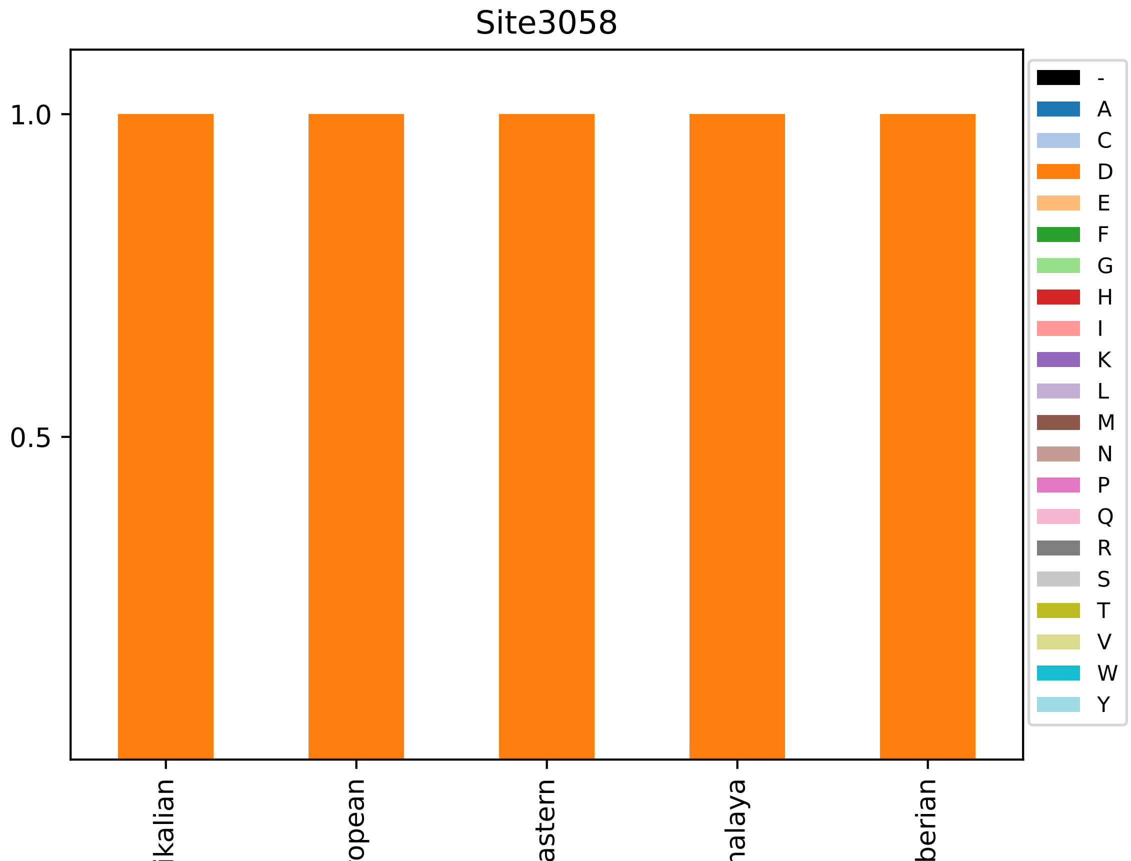
Site3058
1.0
0.5
-
A
C
D
E
F
G
H
I
K
L
M
N
P
Q
R
S
T
V
W
Y
ikalian
opean
astern
nalaya
berian

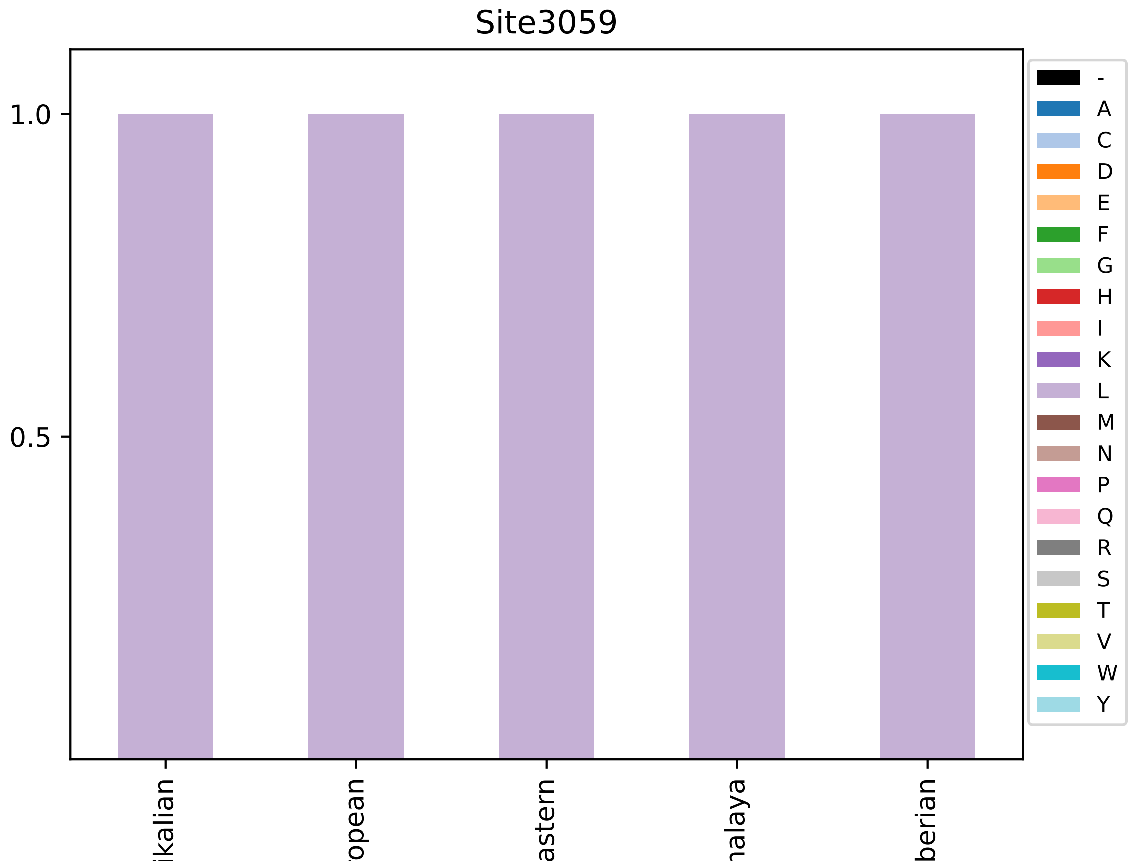

Site3059
1.0
0.5
-
A
C
D
E
F
G
H
I
K
L
M
N
P
Q
R
S
T
V
W
Y
ikalian
opean
astern
nalaya
berian

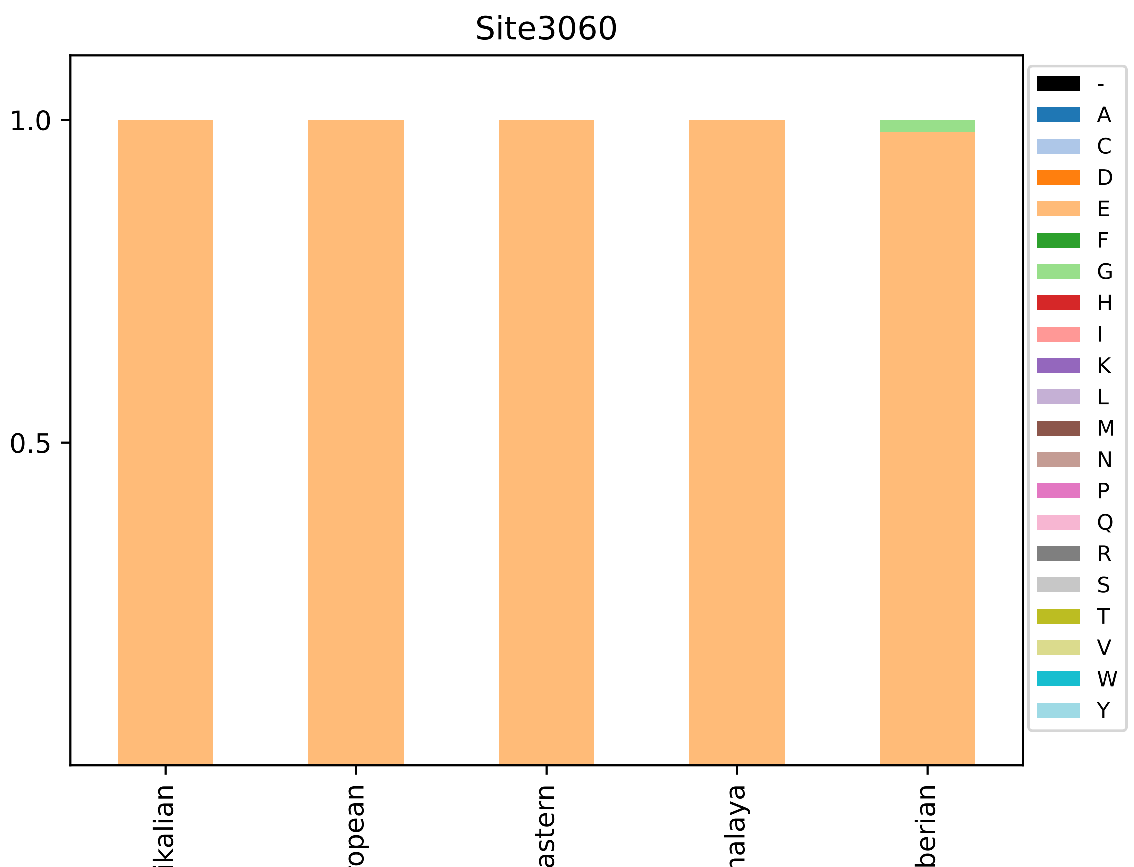
Site3060
1.0
0.5
-
A
C
D
E
F
G
H
I
K
L
M
N
P
Q
R
S
T
V
W
Y
ikalian
opean
astern
nalaya
berian

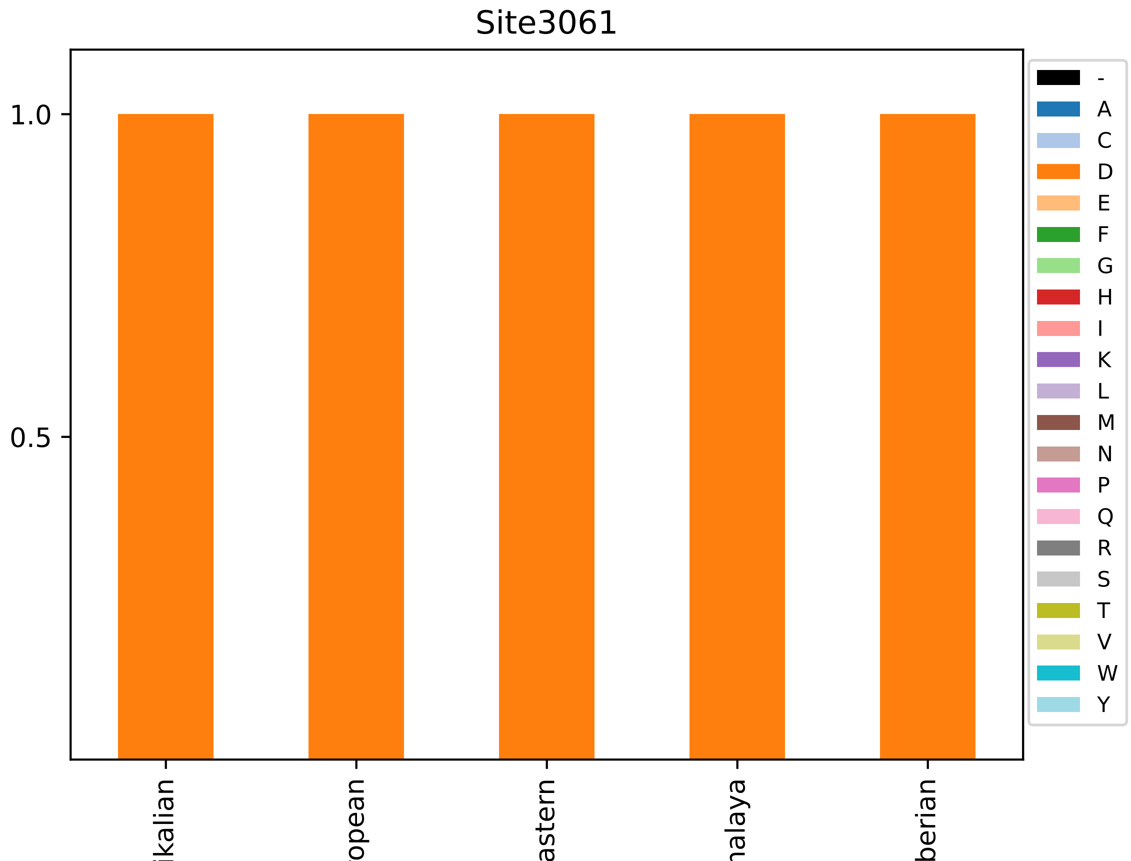
Site3061
1.0
0.5
-
A
C
D
E
F
G
H
I
K
L
M
N
P
Q
R
S
T
V
W
Y
ikalian
opean
astern
nalaya
berian

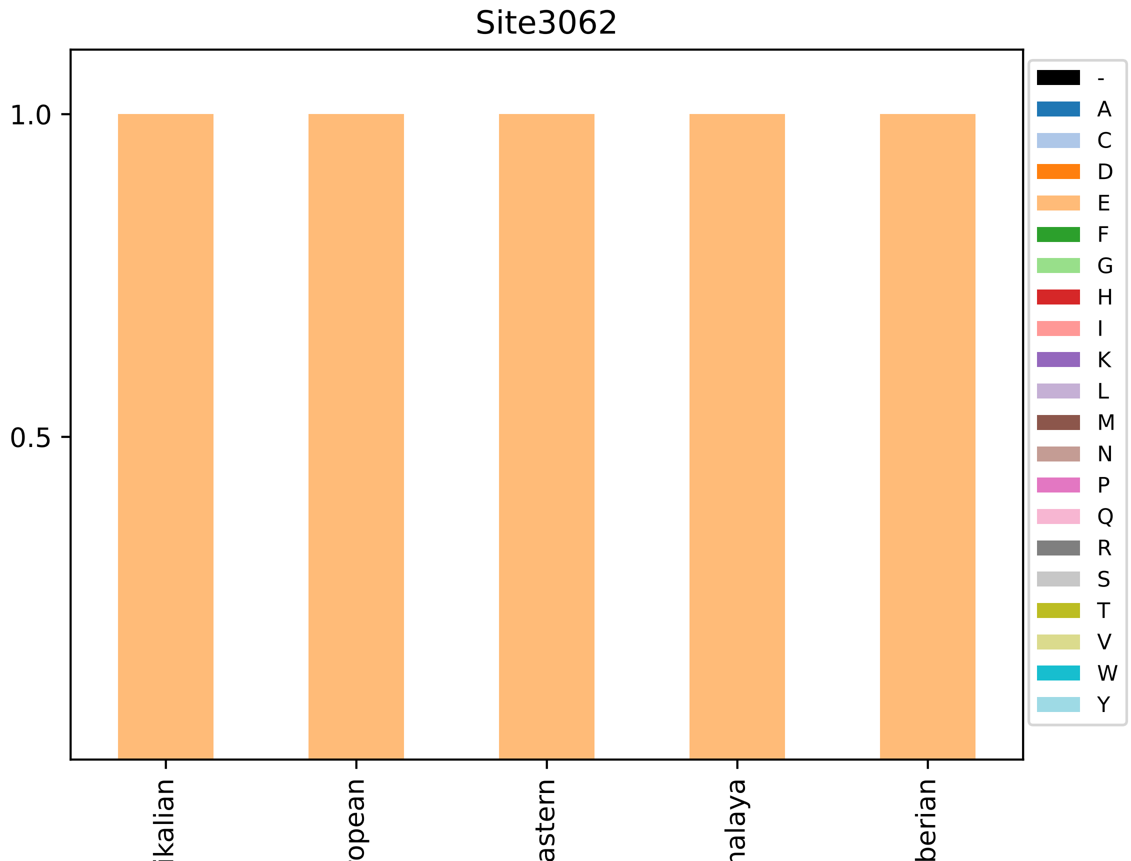
Site3062
1.0
0.5
-
A
C
D
E
F
G
H
I
K
L
M
N
P
Q
R
S
T
V
W
Y
ikalian
opean
astern
nalaya
berian

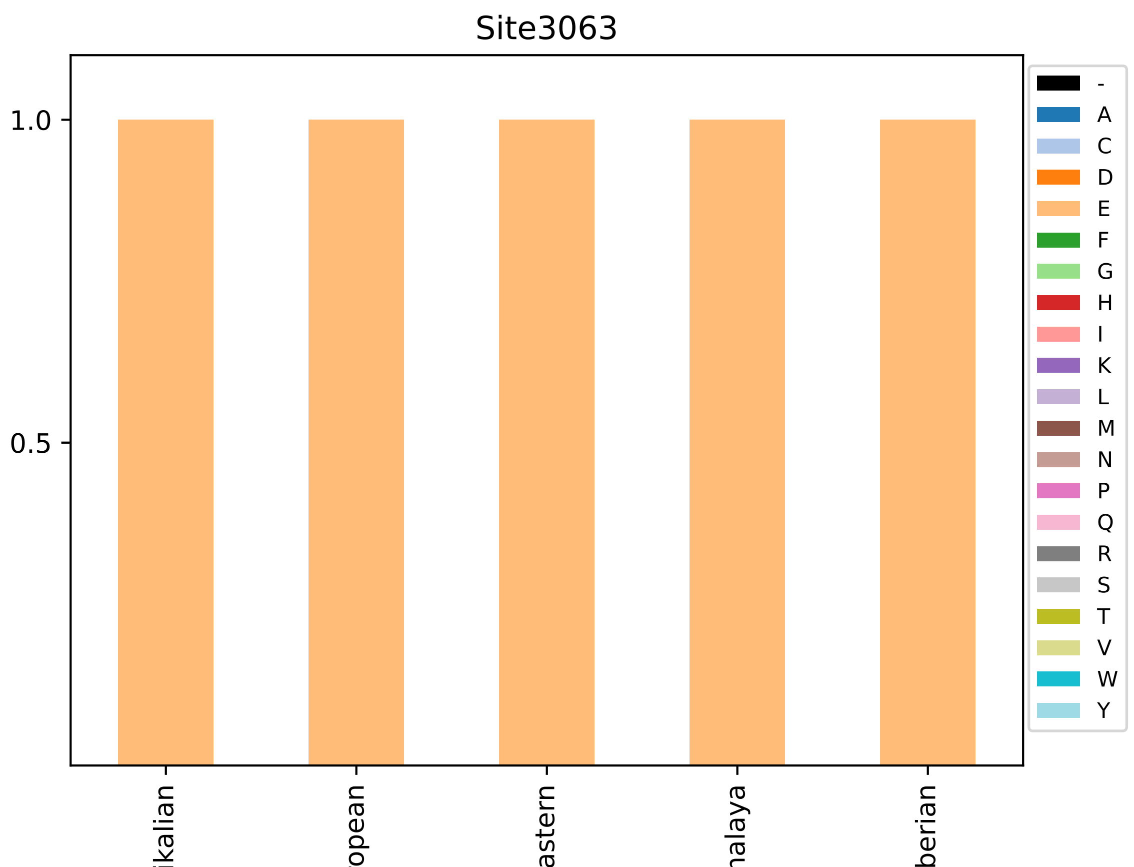

Site3063
1.0
0.5
-
A
C
D
E
F
G
H
I
K
L
M
N
P
Q
R
S
T
V
W
Y
ikalian
opean
astern
nalaya
berian

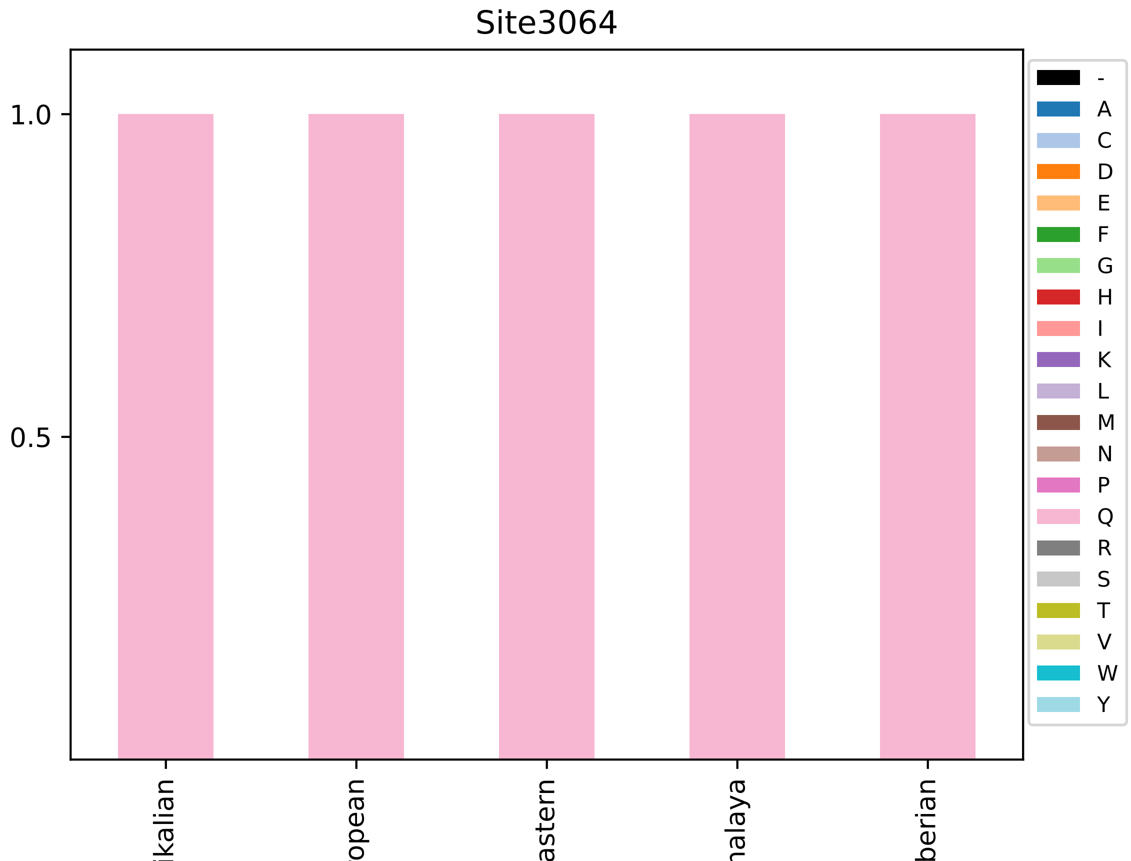

Site3064
1.0
0.5
-
A
C
D
E
F
G
H
I
K
L
M
N
P
Q
R
S
T
V
W
Y
ikalian
opean
astern
nalaya
berian

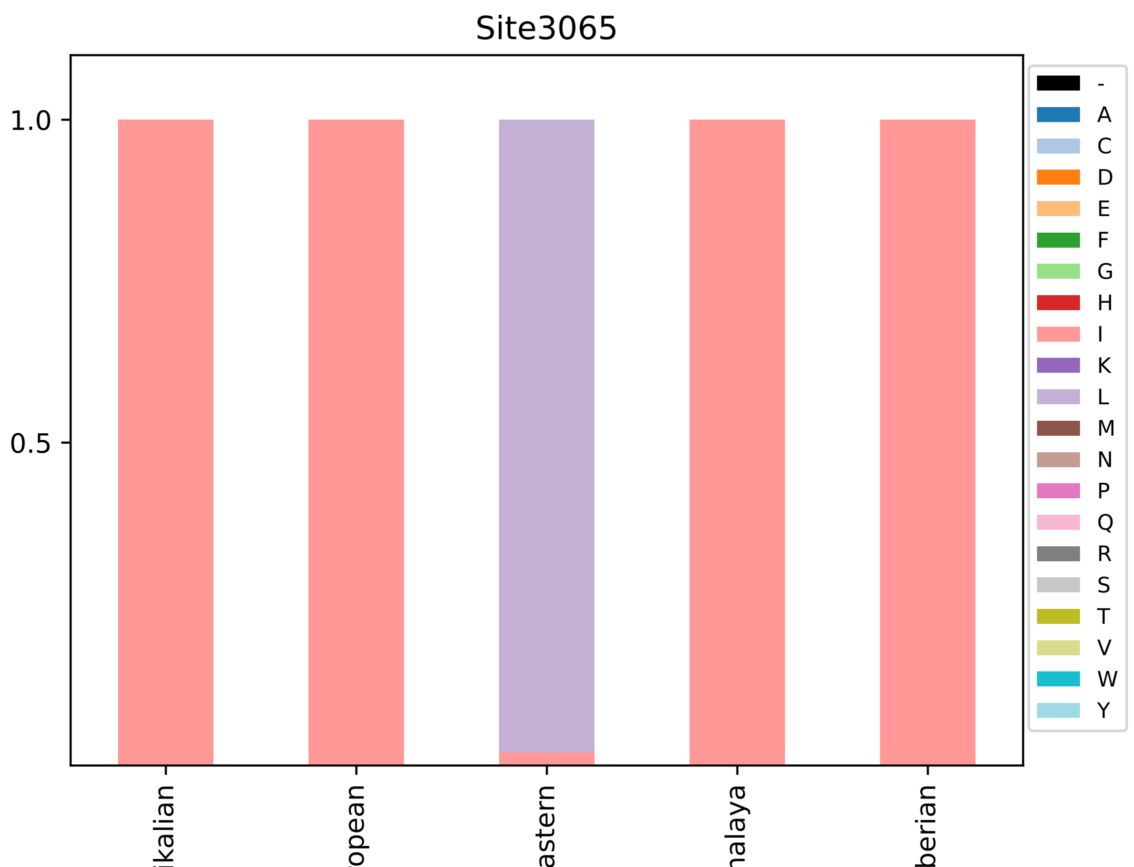

Site3065
1.0
0.5
-
A
C
D
E
F
G
H
I
K
L
M
N
P
Q
R
S
T
V
W
Y
ikalian
opean
astern
nalaya
berian

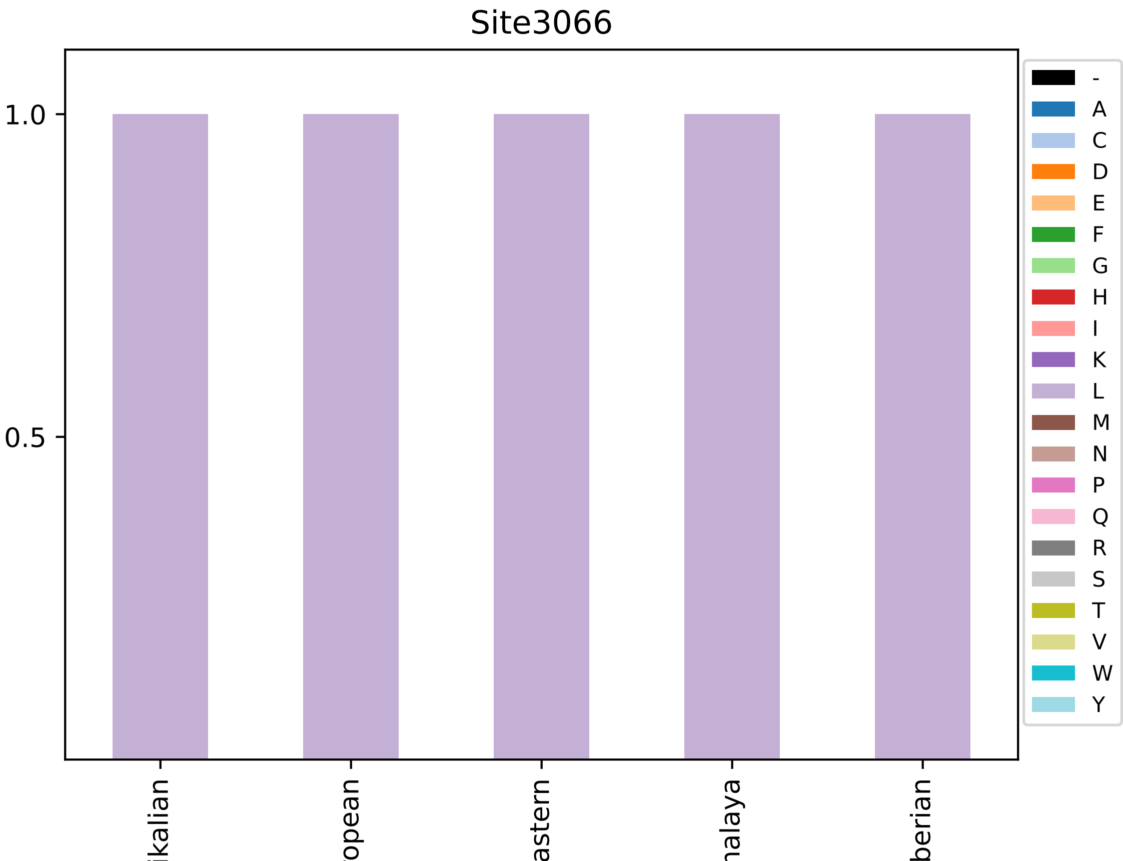
Site3066
1.0
0.5
-
A
C
D
E
F
G
H
I
K
L
M
N
P
Q
R
S
T
V
W
Y
ikalian
opean
astern
nalaya
berian

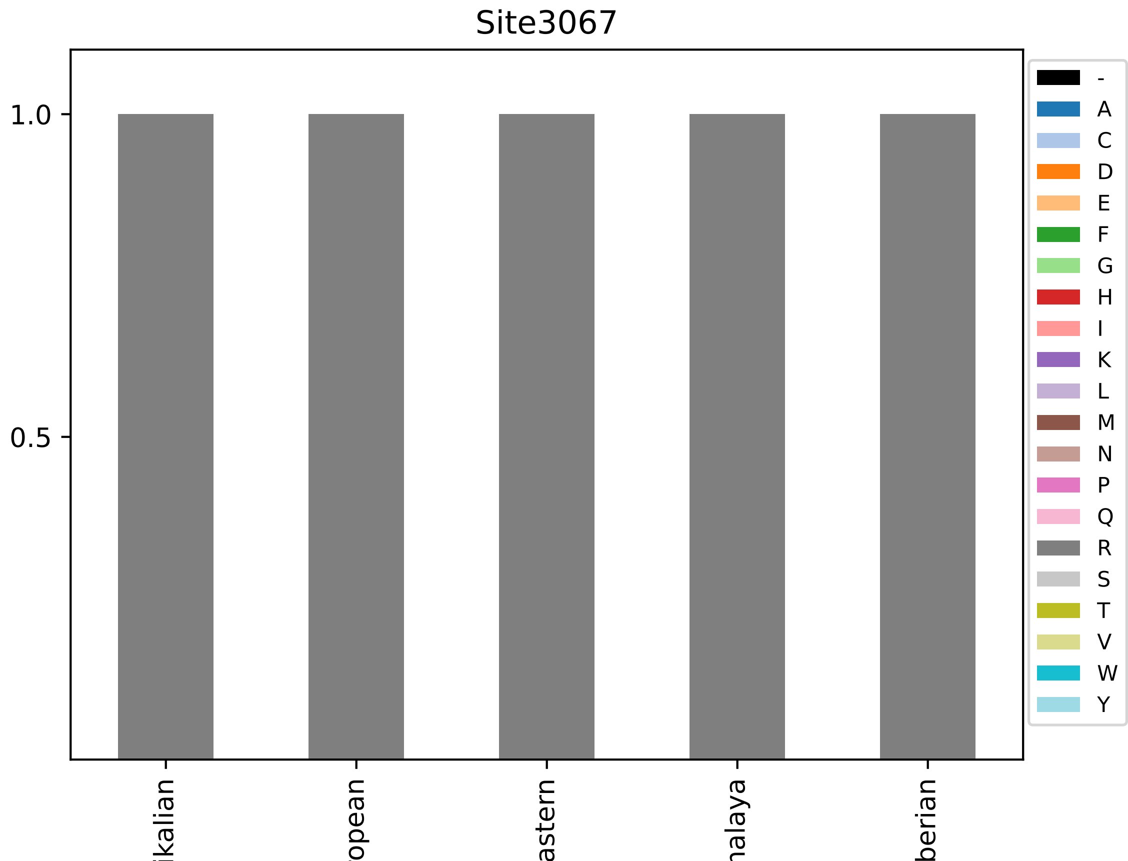
Site3067
1.0
0.5
-
A
C
D
E
F
G
H
I
K
L
M
N
P
Q
R
S
T
V
W
Y
ikalian
opean
astern
nalaya
berian

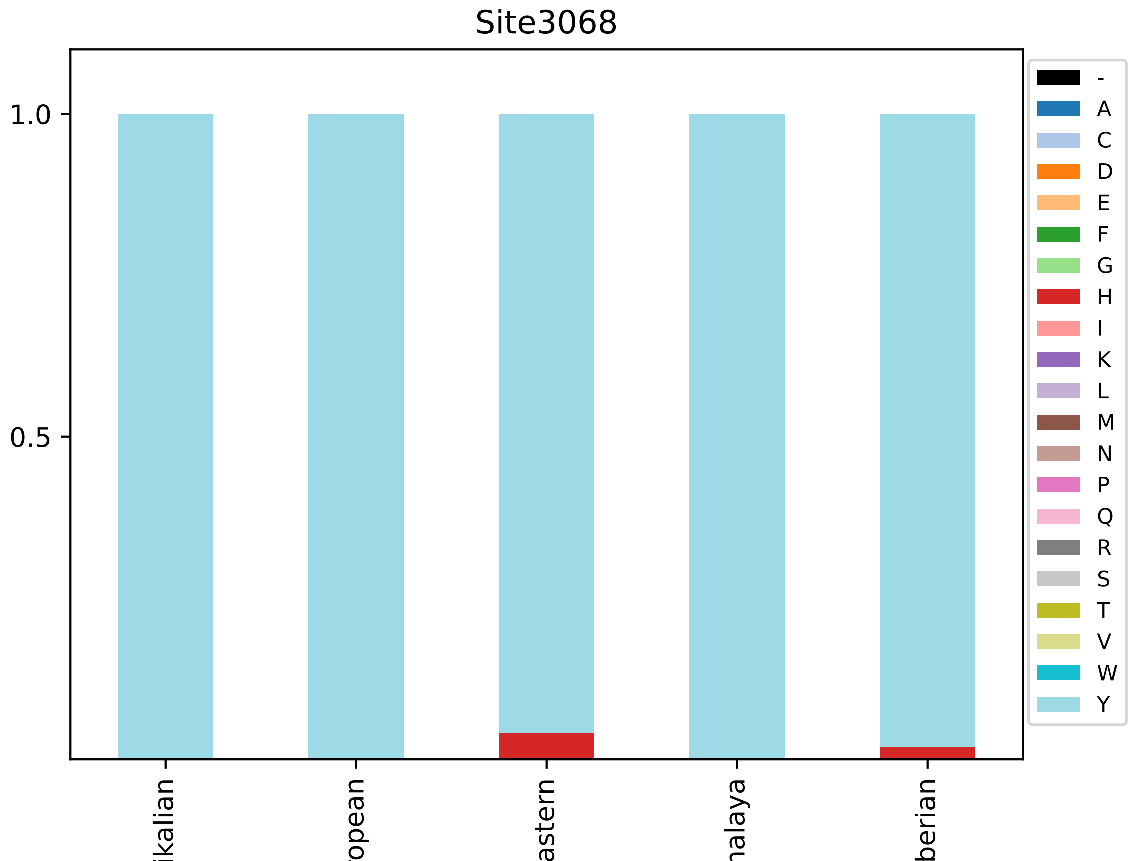

Site3068
1.0
0.5
-
A
C
D
E
F
G
H
I
K
L
M
N
P
Q
R
S
T
V
W
Y
ikalian
opean
astern
nalaya
berian

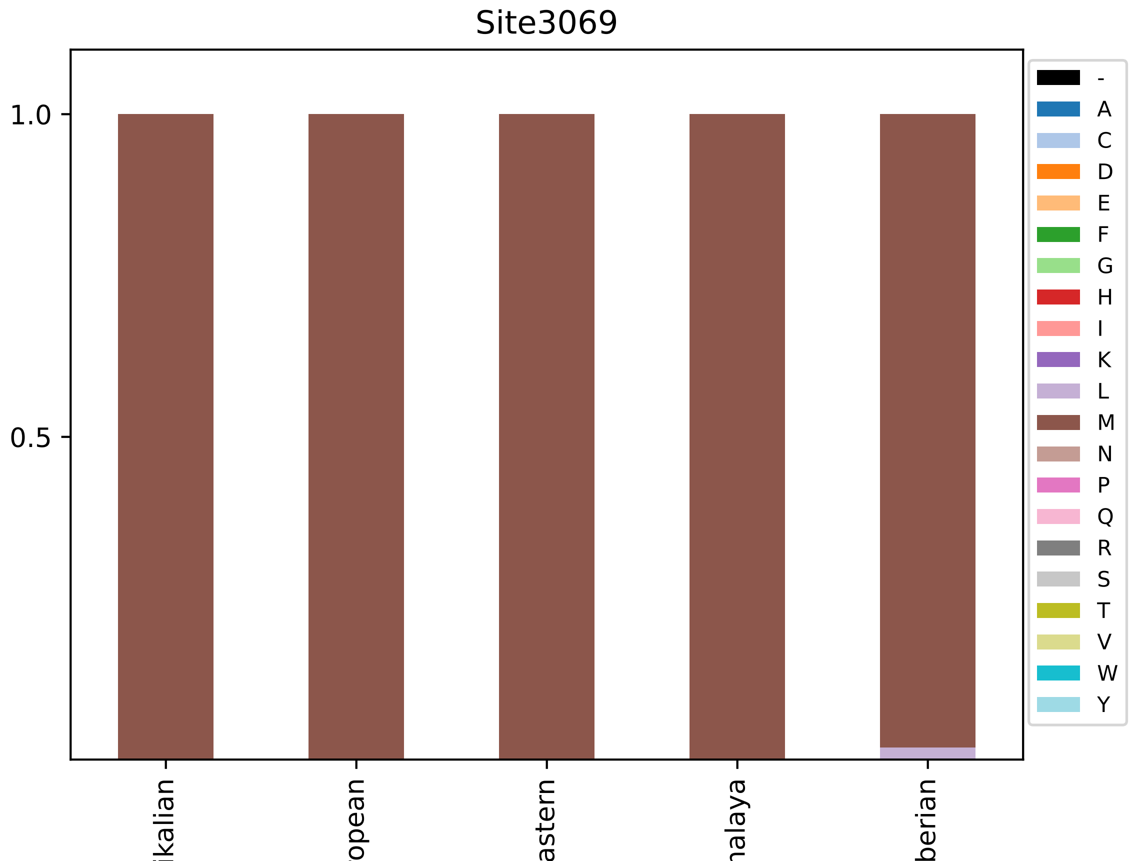
Site3069
1.0
0.5
-
A
C
D
E
F
G
H
I
K
L
M
N
P
Q
R
S
T
V
W
Y
ikalian
opean
astern
nalaya
berian

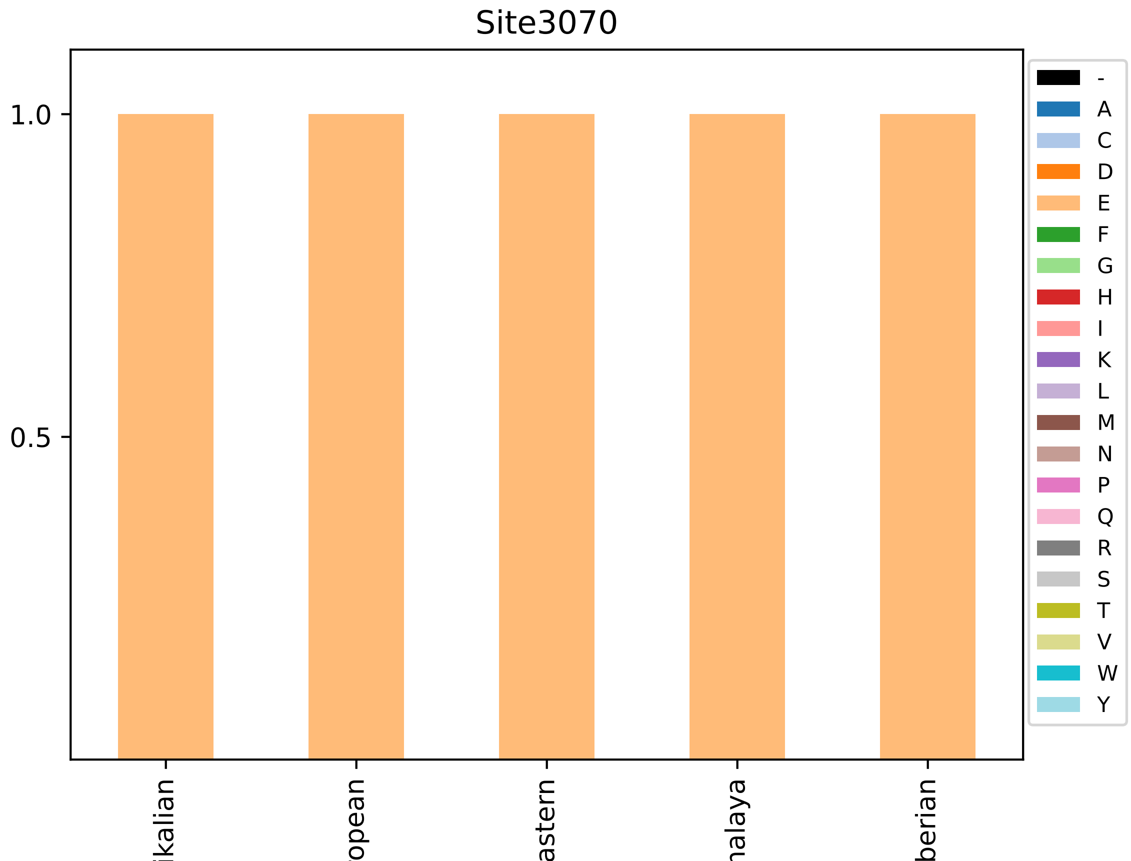
Site3070
1.0
0.5
-
A
C
D
E
F
G
H
I
K
L
M
N
P
Q
R
S
T
V
W
Y
ikalian
opean
astern
nalaya
berian

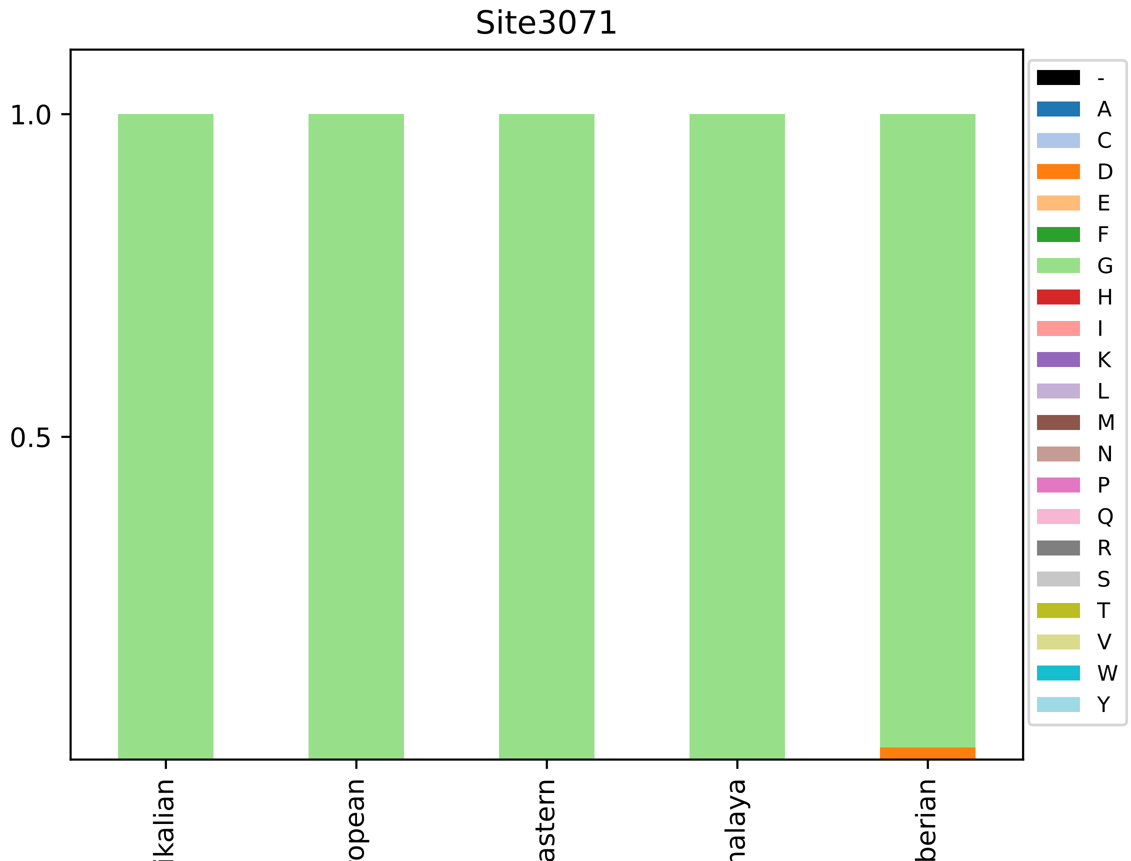
Site3071
1.0
0.5
ikalian
opean
astern
nalaya
berian
-
A
C
D
E
F
G
H
I
K
L
M
N
P
Q
R
S
T
V
W
Y

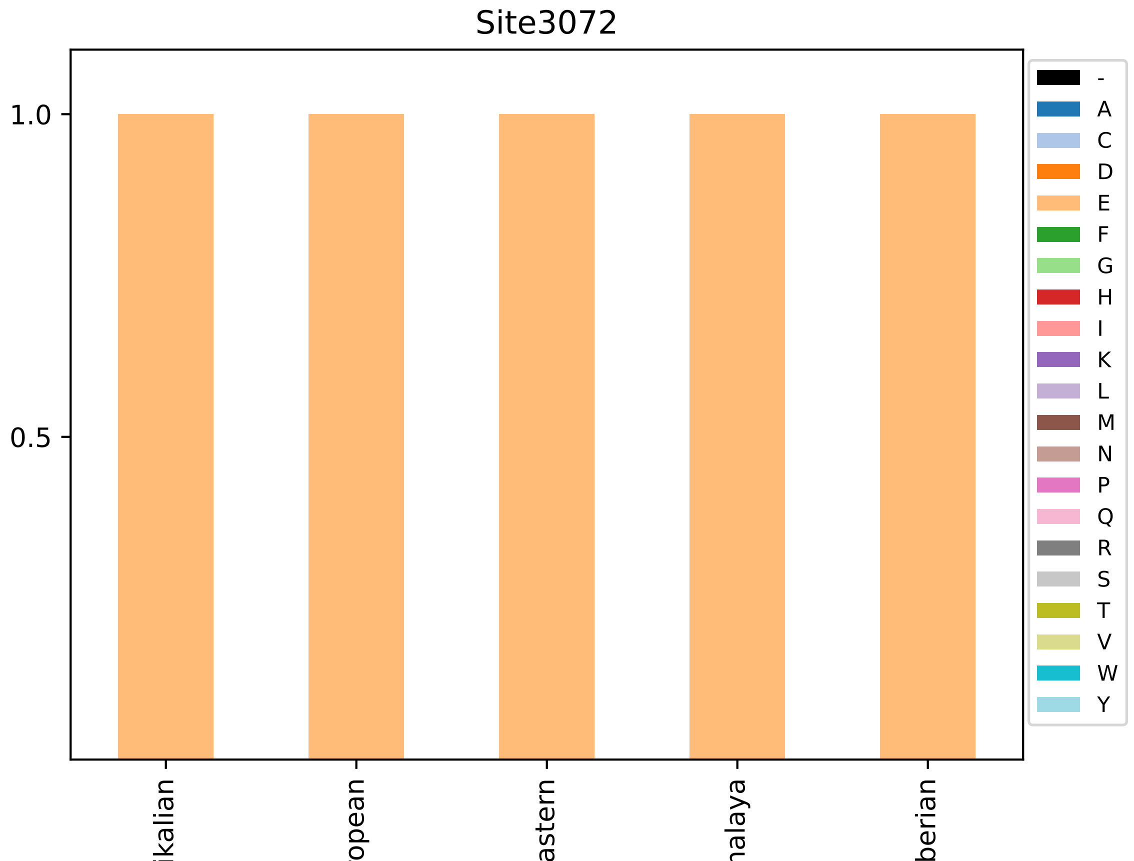

Site3072
1.0
0.5
ikalian
opean
astern
nalaya
berian
-
A
C
D
E
F
G
H
I
K
L
M
N
P
Q
R
S
T
V
W
Y

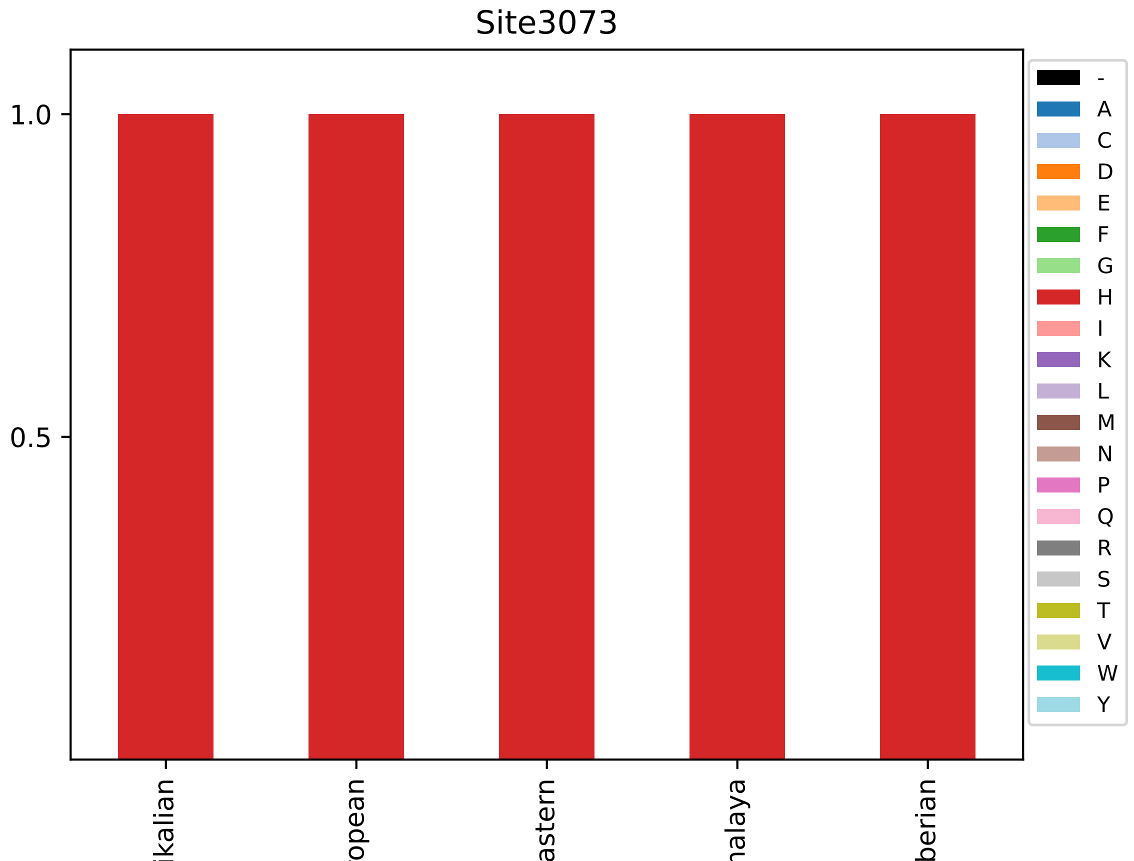

Site3073
1.0
0.5
-
A
C
D
E
F
G
H
I
K
L
M
N
P
Q
R
S
T
V
W
Y
ikalian
opean
astern
nalaya
berian

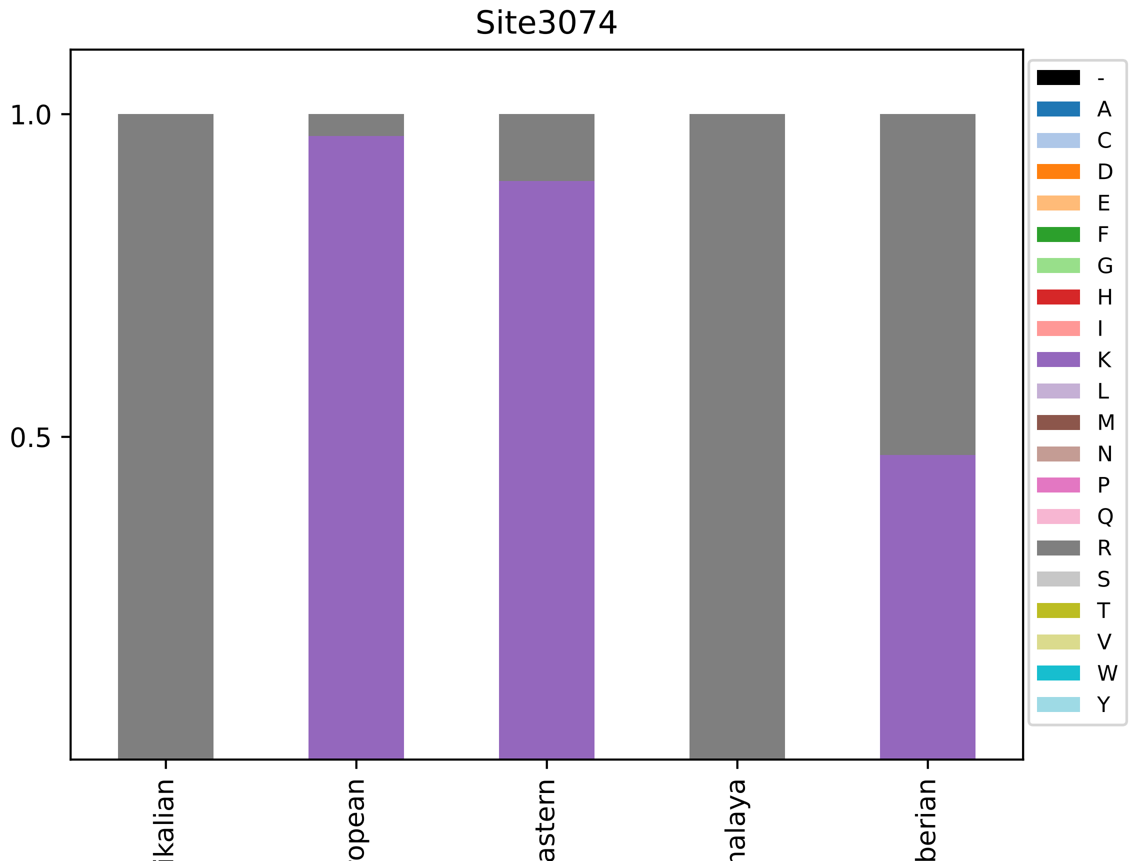

Site3074
1.0
0.5
-
A
C
D
E
F
G
H
I
K
L
M
N
P
Q
R
S
T
V
W
Y
ikalian
opean
astern
nalaya
berian

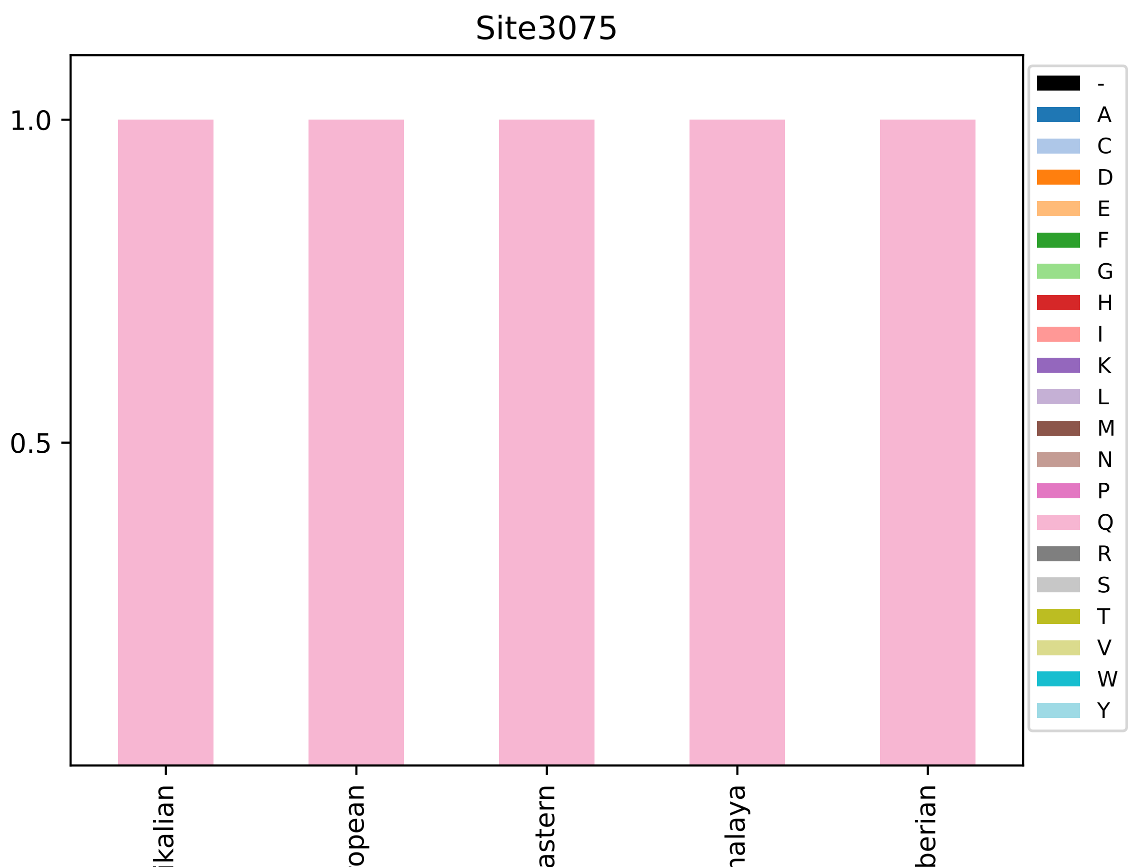

Site3075
1.0
0.5
-
A
C
D
E
F
G
H
I
K
L
M
N
P
Q
R
S
T
V
W
Y

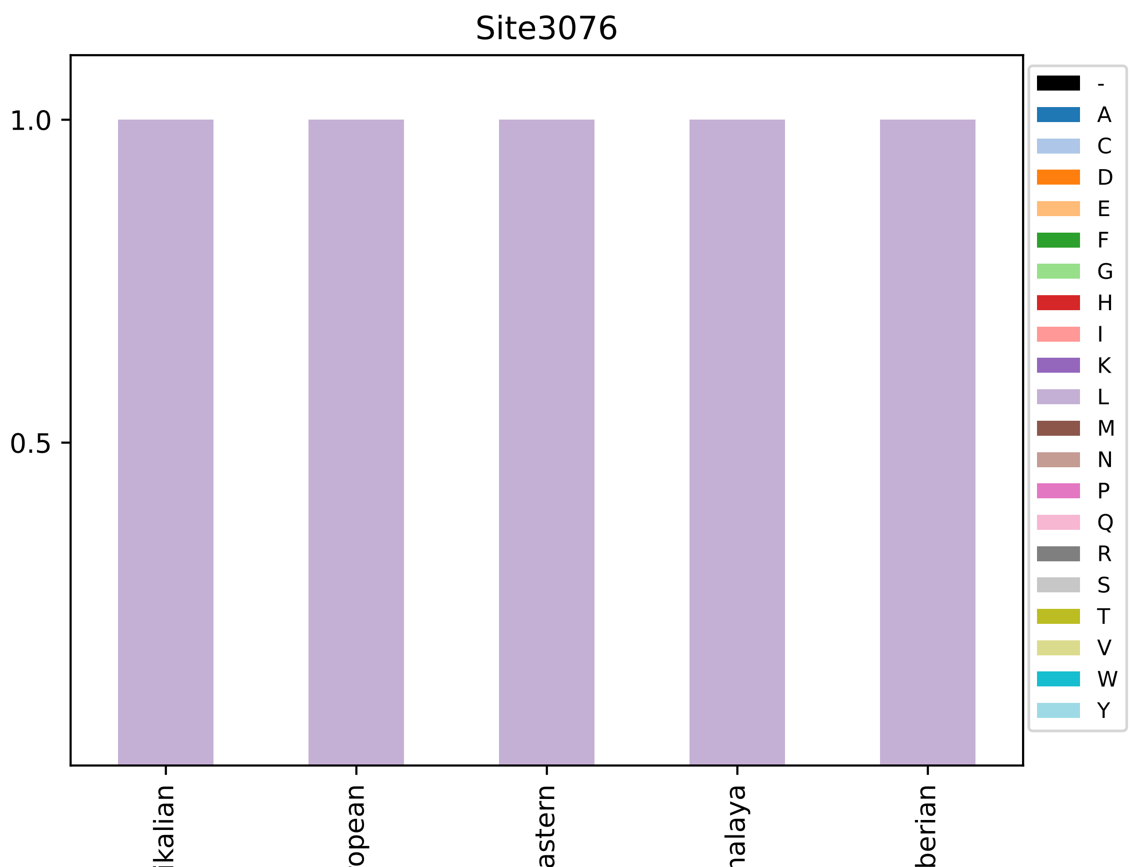

Site3076
1.0
0.5
ikalian
opean
astern
nalaya
berian
-
A
C
D
E
F
G
H
I
K
L
M
N
P
Q
R
S
T
V
W
Y

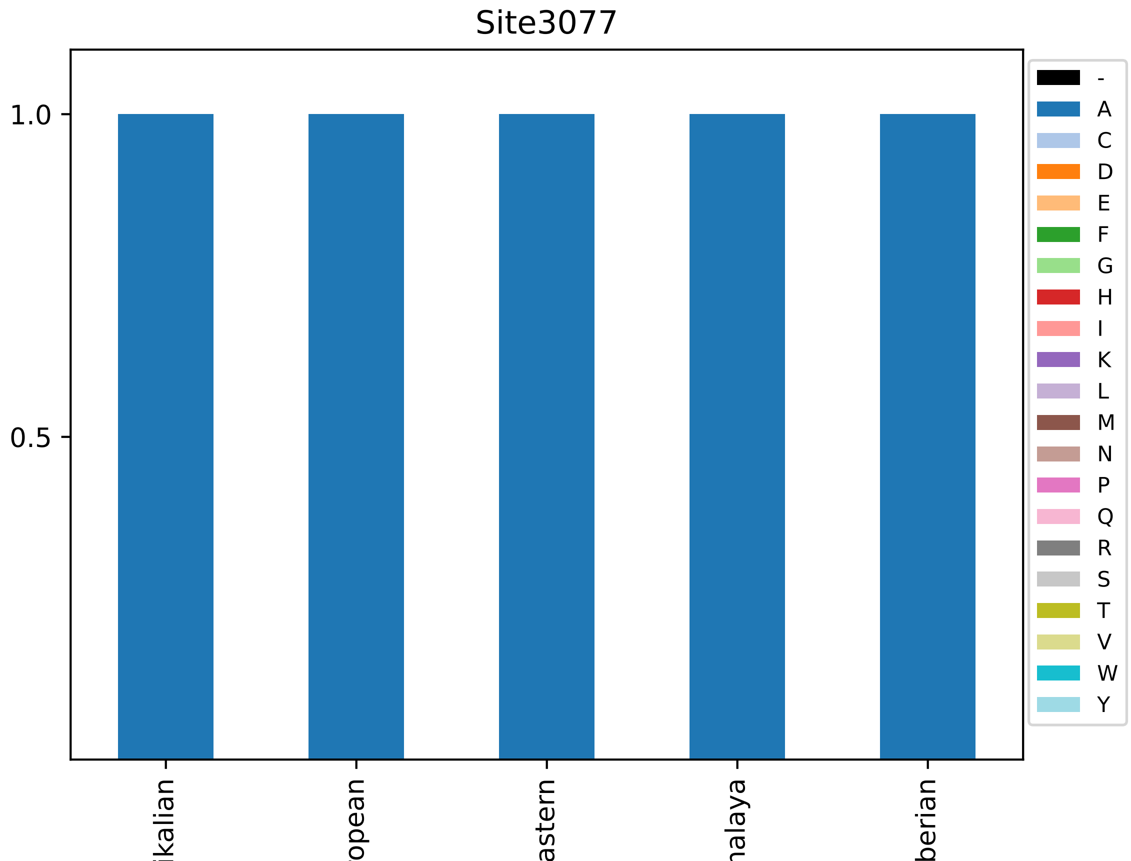

Site3077
1.0
0.5
ikalian
ropean
astern
nalaya
berian
-
A
C
D
E
F
G
H
I
K
L
M
N
P
Q
R
S
T
V
W
Y

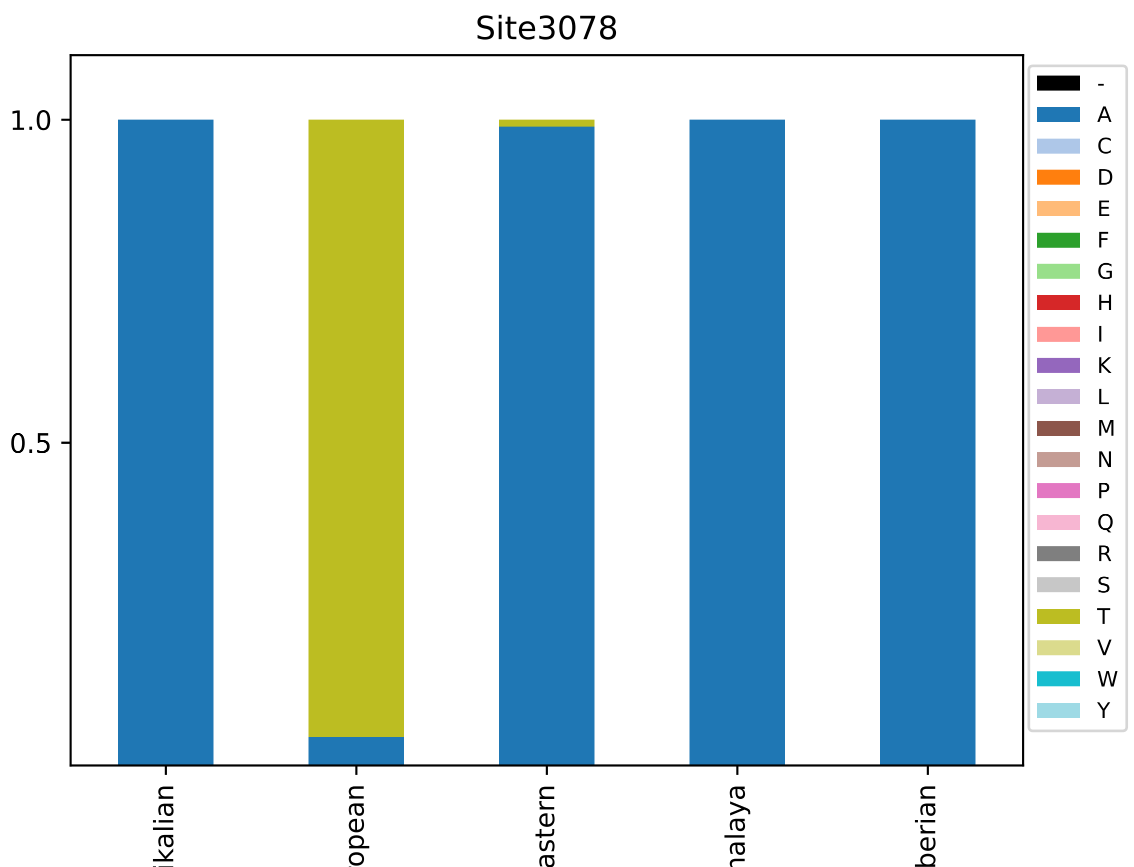
Site3078
1.0
0.5
-
A
C
D
E
F
G
H
I
K
L
M
N
P
Q
R
S
T
V
W
Y

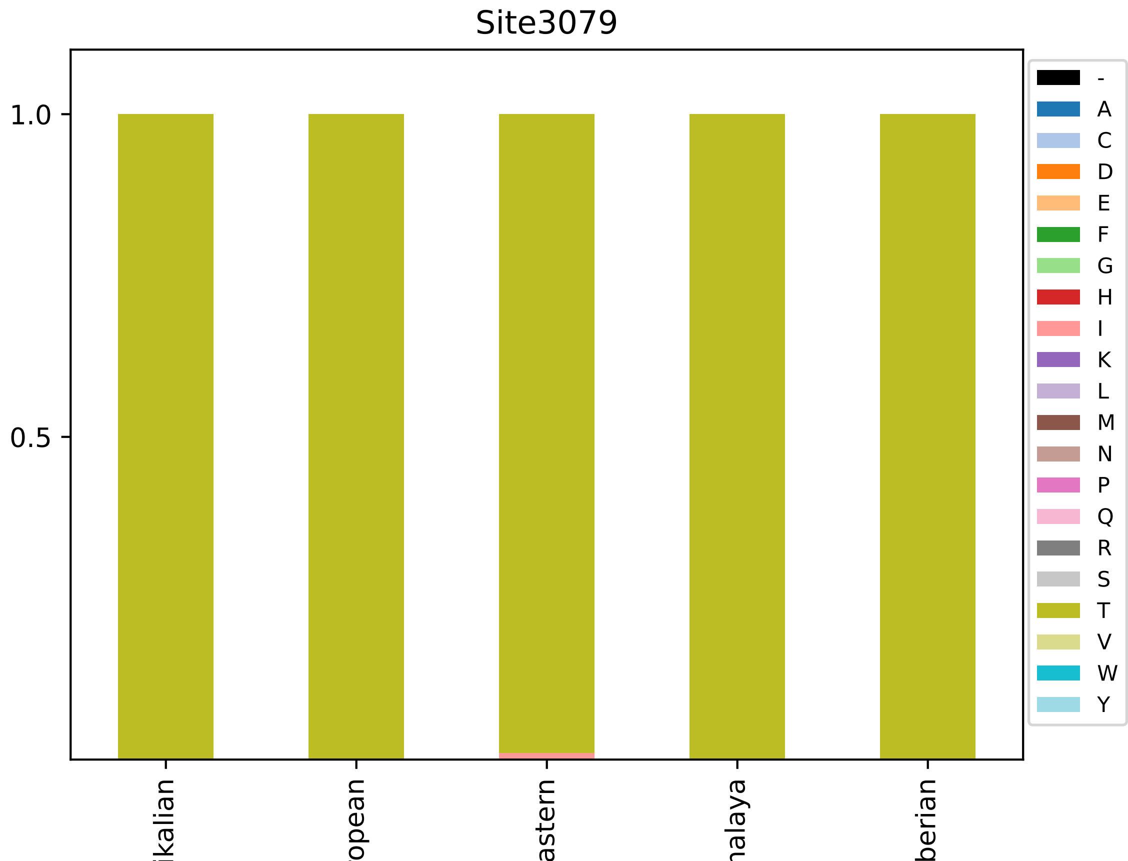
Site3079
1.0
0.5
-
A
C
D
E
F
G
H
I
K
L
M
N
P
Q
R
S
T
V
W
Y

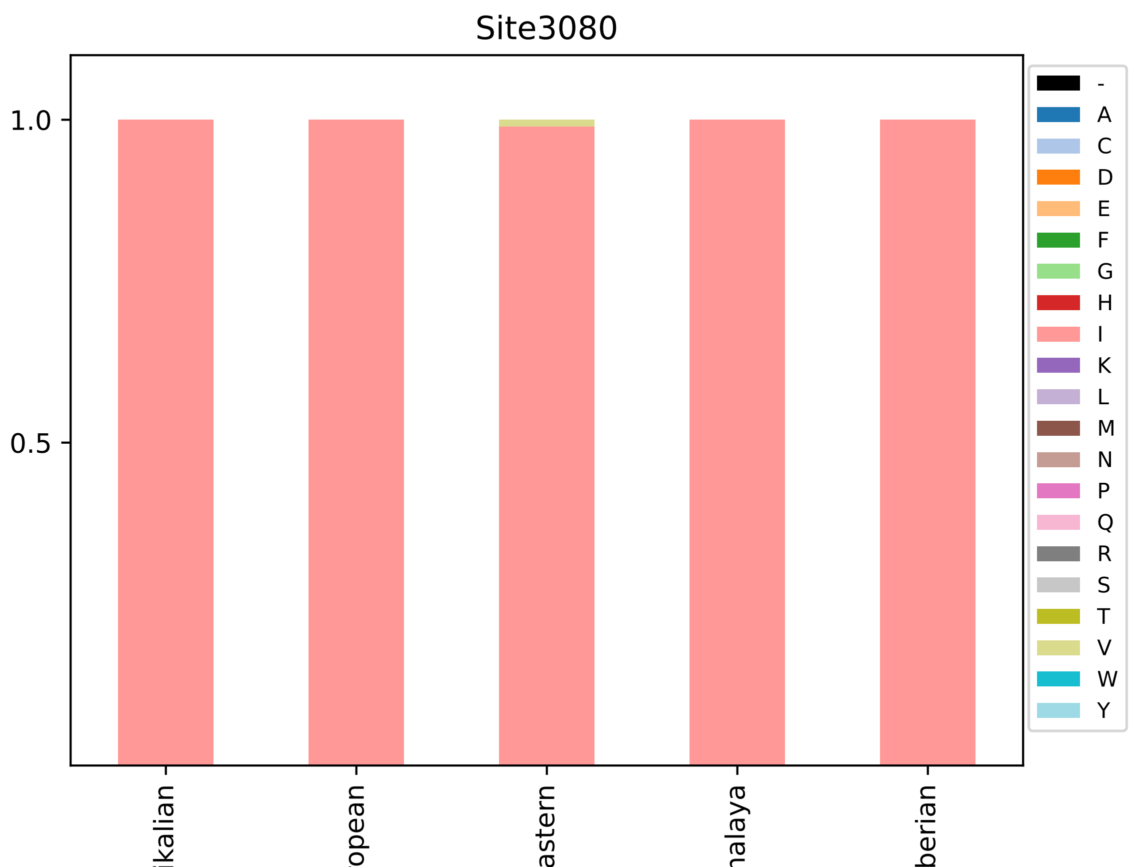

Site3080
1.0
0.5
-
A
C
D
E
F
G
H
I
K
L
M
N
P
Q
R
S
T
V
W
Y
ikalian
opean
astern
nalaya
berian

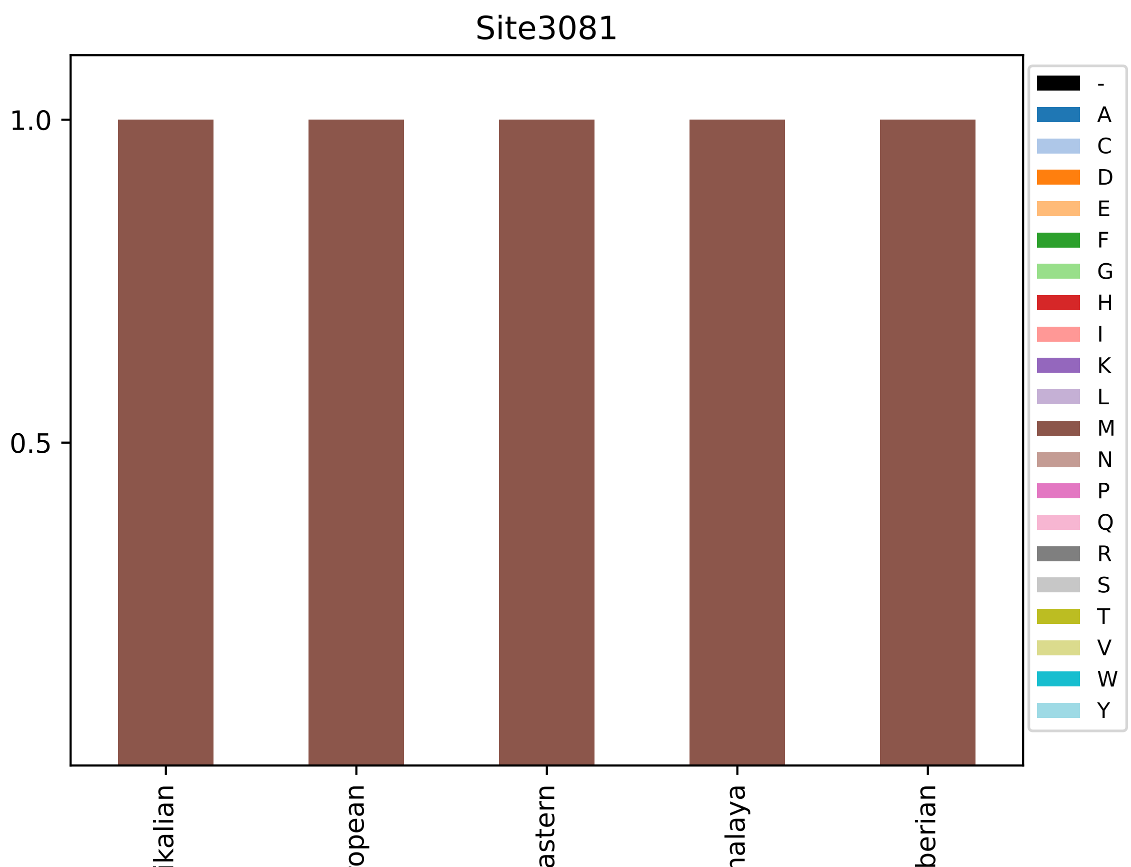
Site3081
1.0
0.5
ikalian
opean
astern
nalaya
berian
-
A
C
D
E
F
G
H
I
K
L
M
N
P
Q
R
S
T
V
W
Y

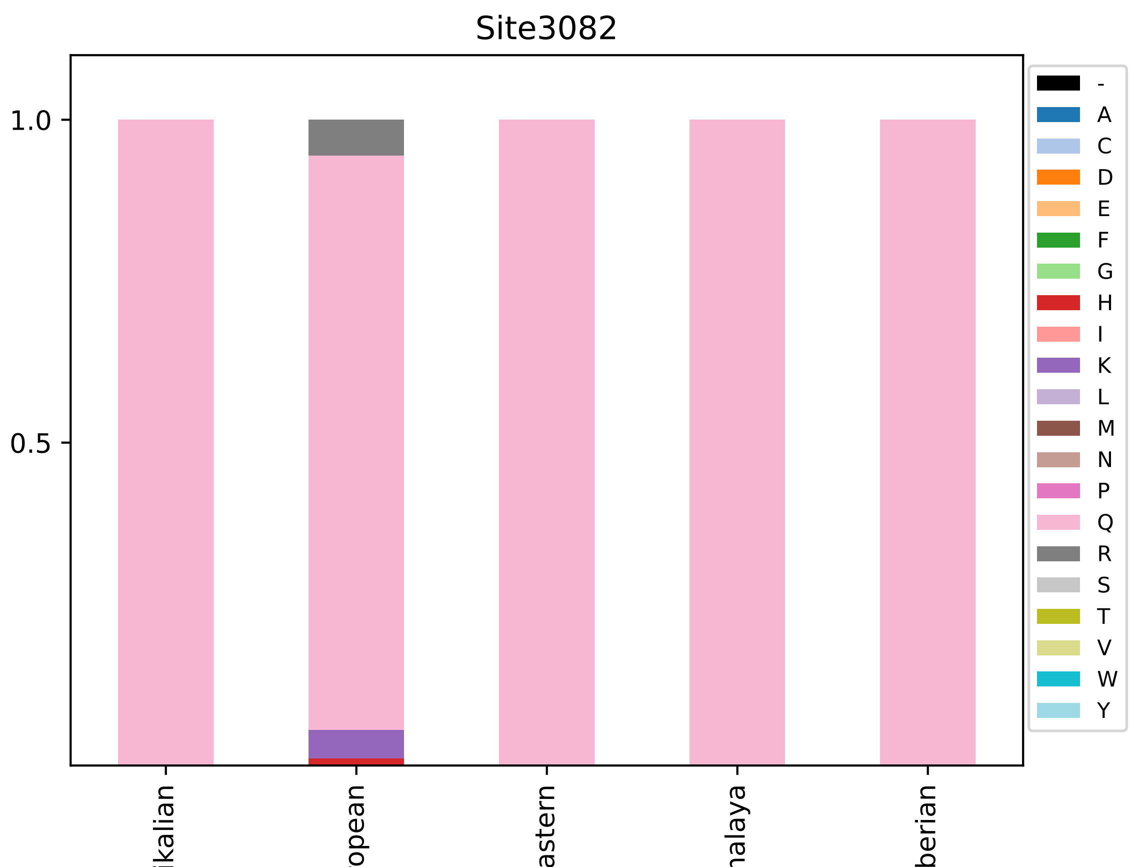
Site3082
1.0
0.5
-
A
C
D
E
F
G
H
I
K
L
M
N
P
Q
R
S
T
V
W
Y
ikalian
ropean
astern
nalaya
berian

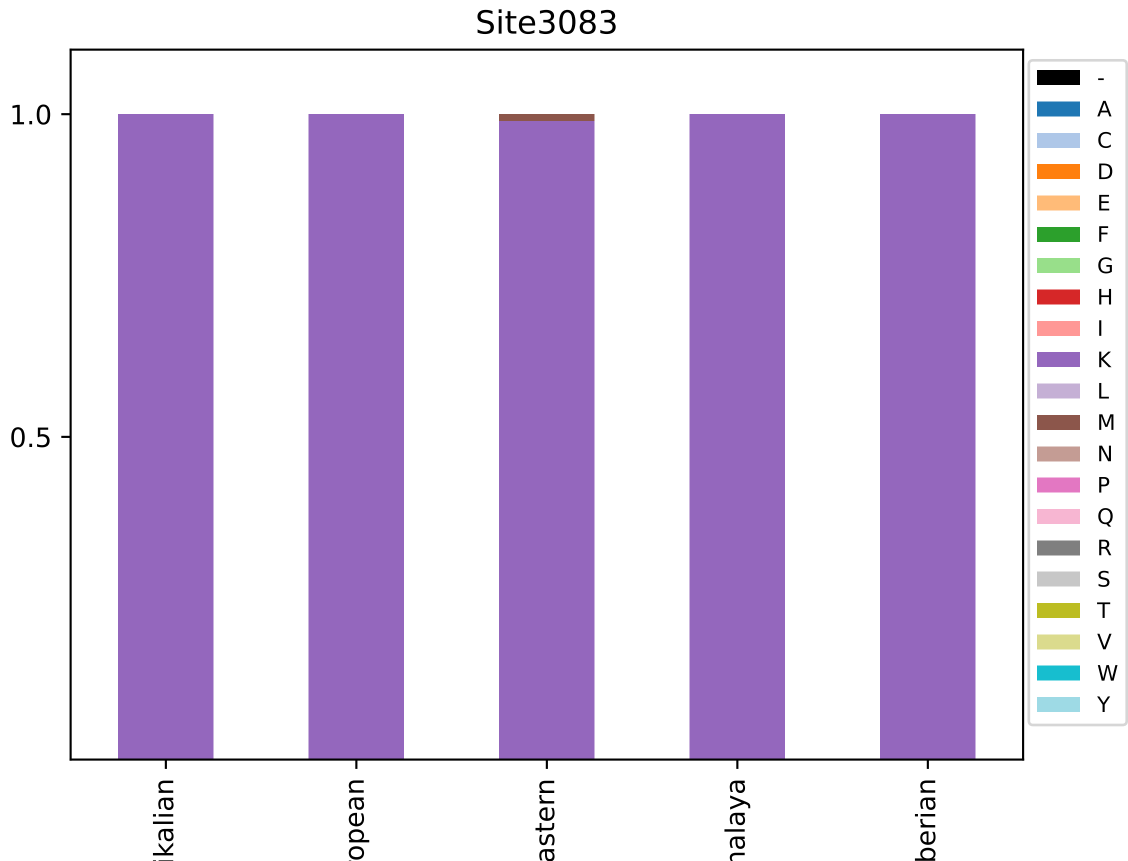
Site3083
1.0
0.5
-
A
C
D
E
F
G
H
I
K
L
M
N
P
Q
R
S
T
V
W
Y
ikalian
opean
astern
nalaya
berian

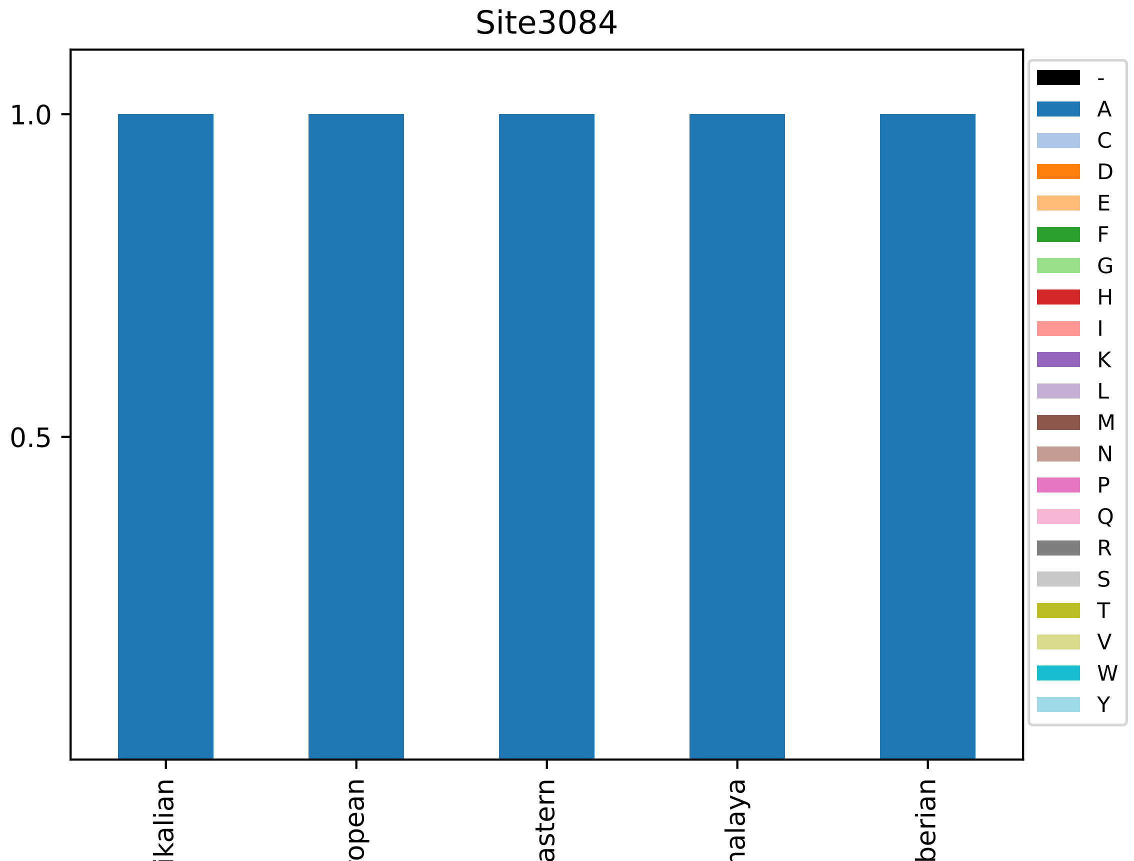

Site3084
1.0
0.5
-
A
C
D
E
F
G
H
I
K
L
M
N
P
Q
R
S
T
V
W
Y
ikalian
opean
astern
nalaya
berian

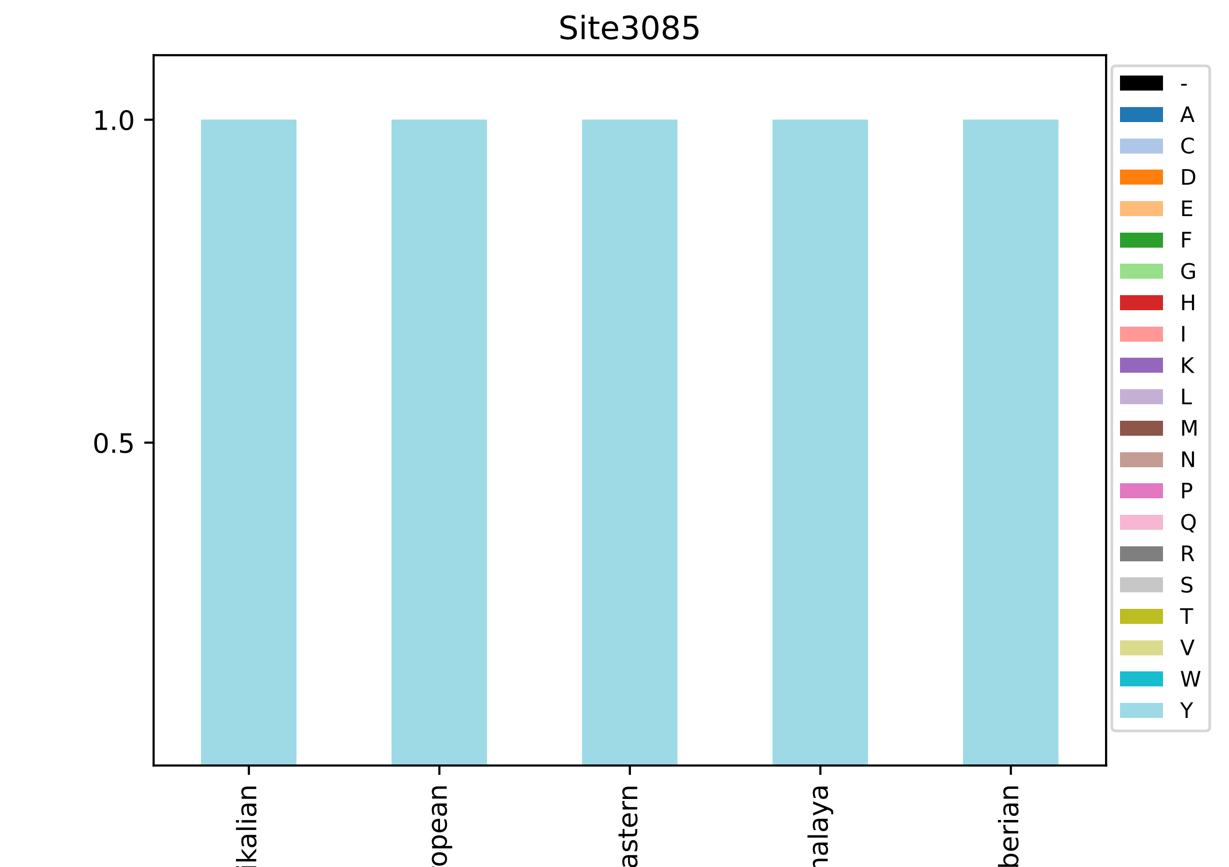
Site3085
1.0
0.5
-
A
C
D
E
F
G
H
I
K
L
M
N
P
Q
R
S
T
V
W
Y
ikalian
opean
astern
nalaya
berian

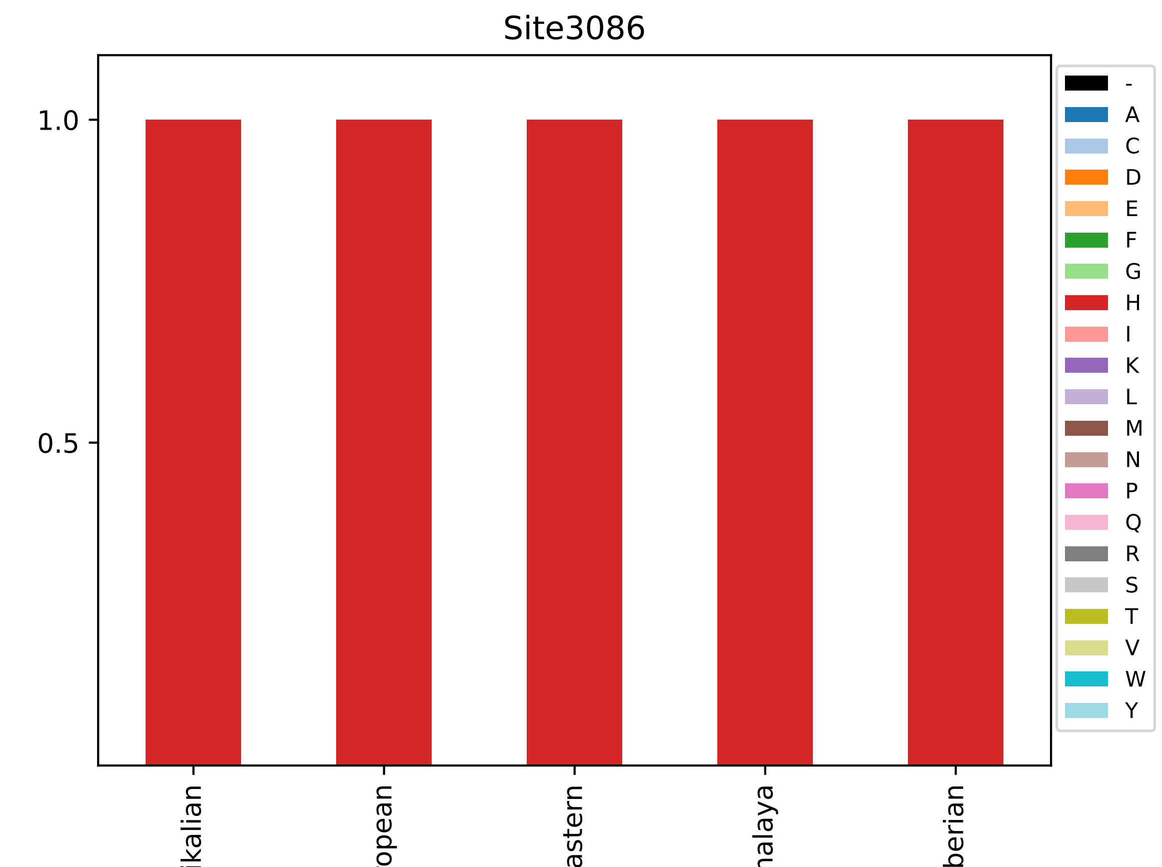

Site3086
1.0
0.5
-
A
C
D
E
F
G
H
I
K
L
M
N
P
Q
R
S
T
V
W
Y
ikalian
opean
astern
nalaya
berian

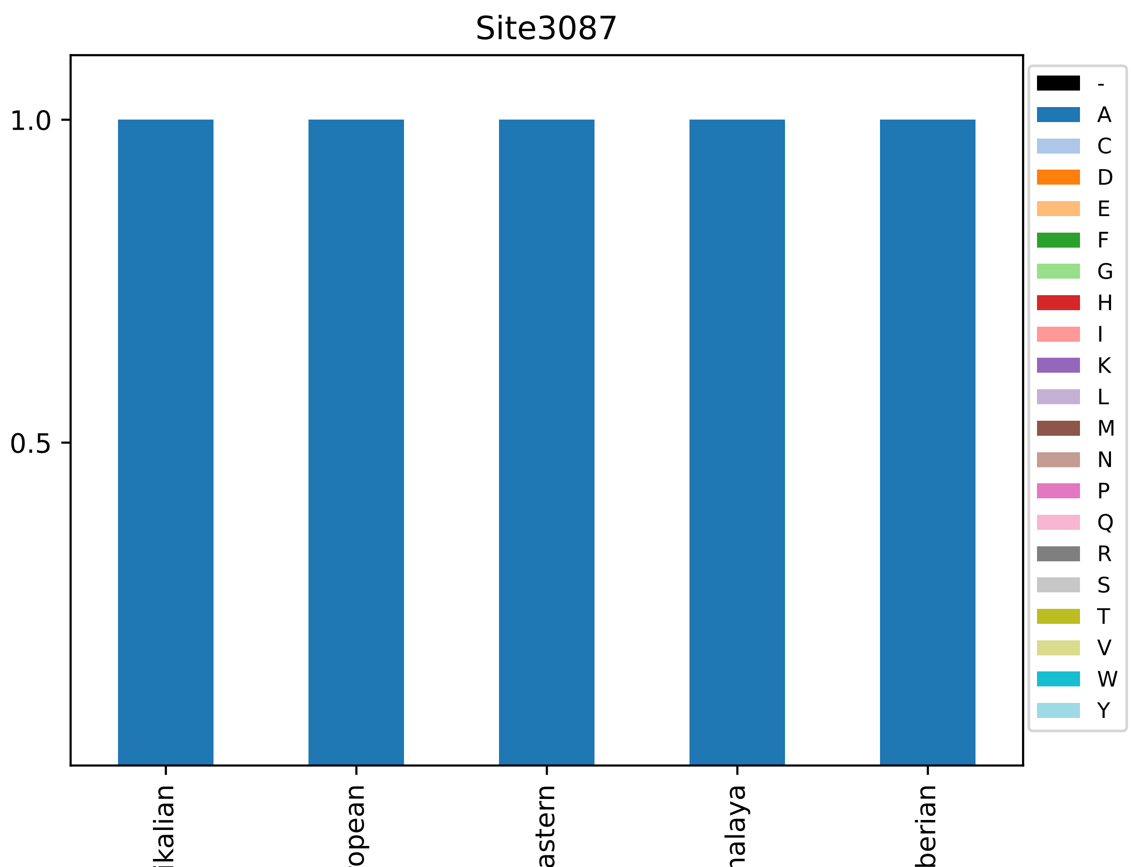
Site3087
1.0
0.5
-
A
C
D
E
F
G
H
I
K
L
M
N
P
Q
R
S
T
V
W
Y
ikalian
opean
astern
nalaya
berian

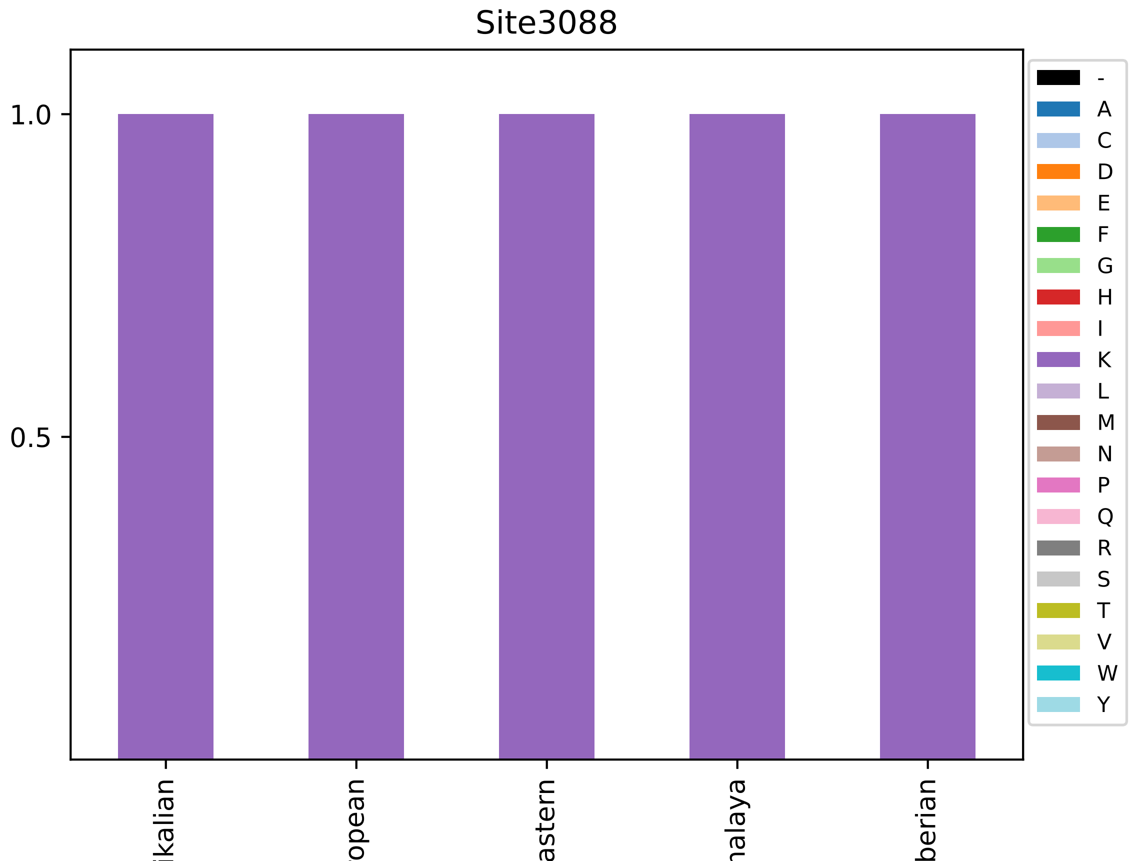

Site3088
1.0
0.5
-
A
C
D
E
F
G
H
I
K
L
M
N
P
Q
R
S
T
V
W
Y
ikalian
opean
astern
nalaya
berian

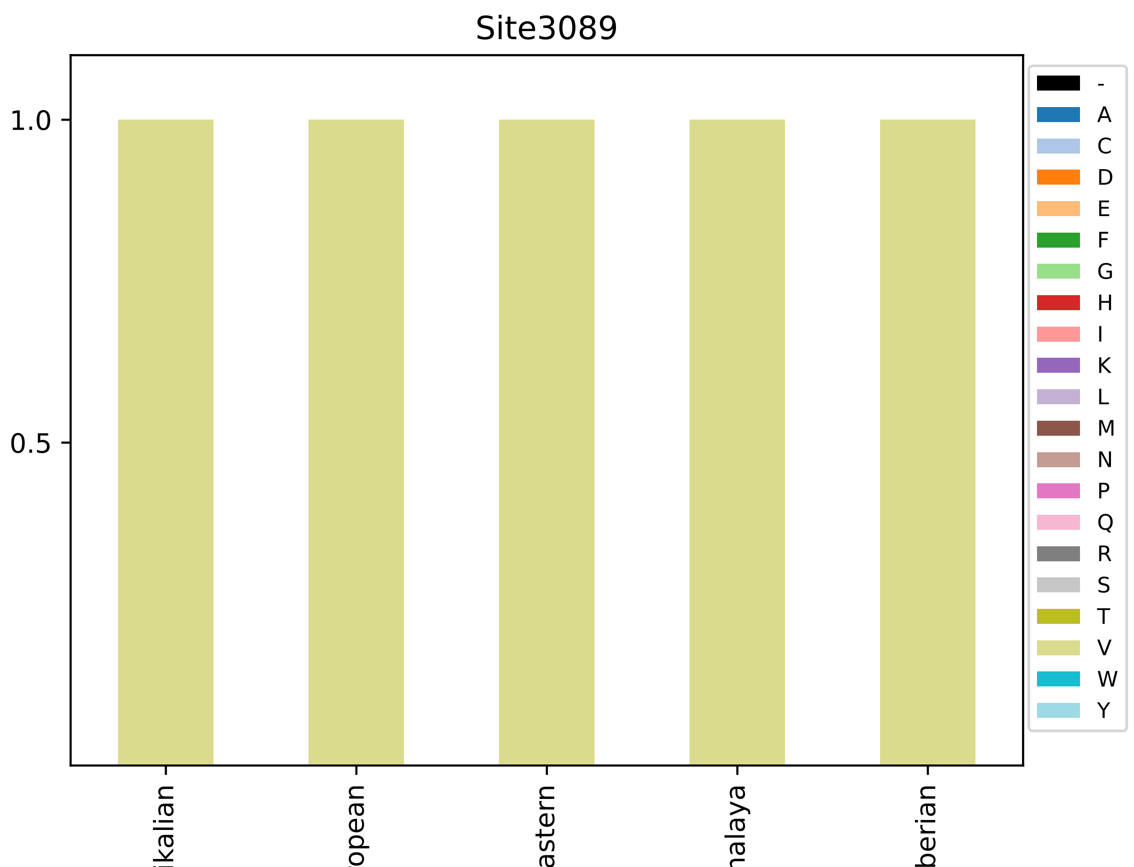

Site3089
1.0
0.5
-
A
C
D
E
F
G
H
I
K
L
M
N
P
Q
R
S
T
V
W
Y
ikalian
opean
astern
nalaya
berian

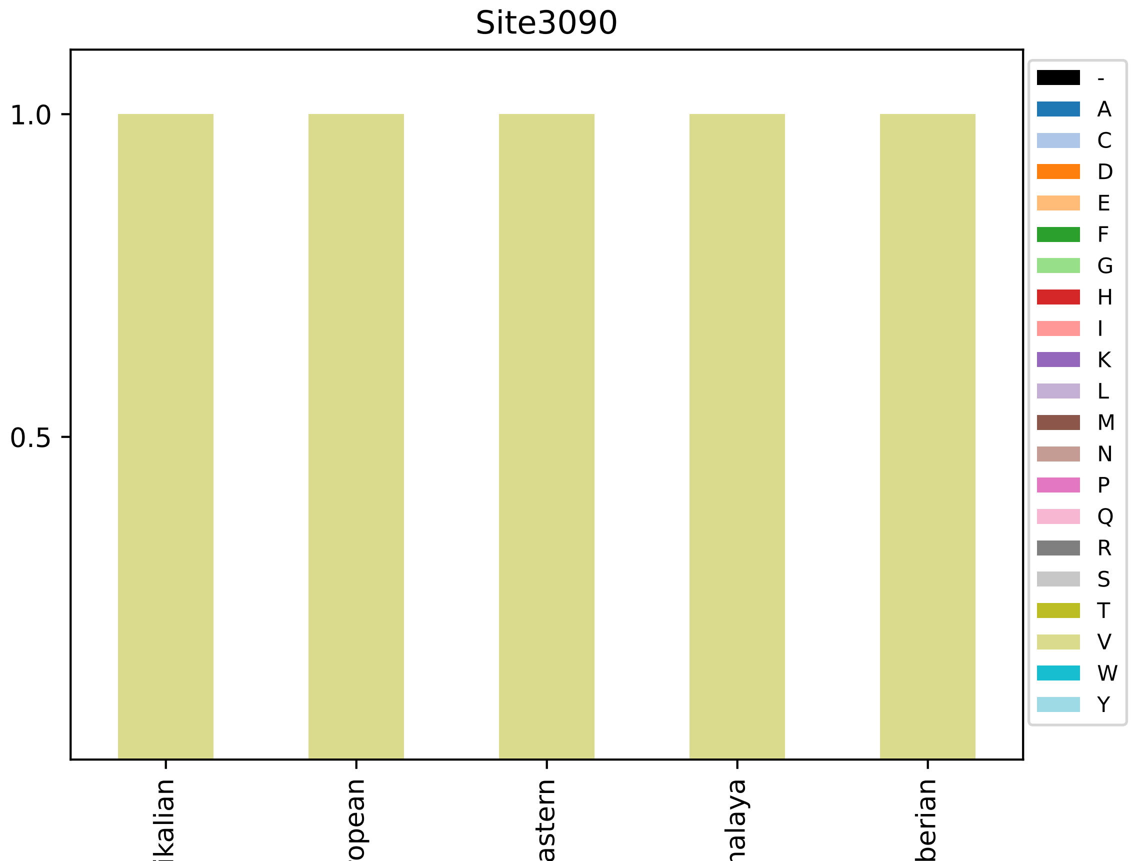
Site3090
1.0
0.5
-
A
C
D
E
F
G
H
I
K
L
M
N
P
Q
R
S
T
V
W
Y
ikalian
opean
astern
nalaya
berian

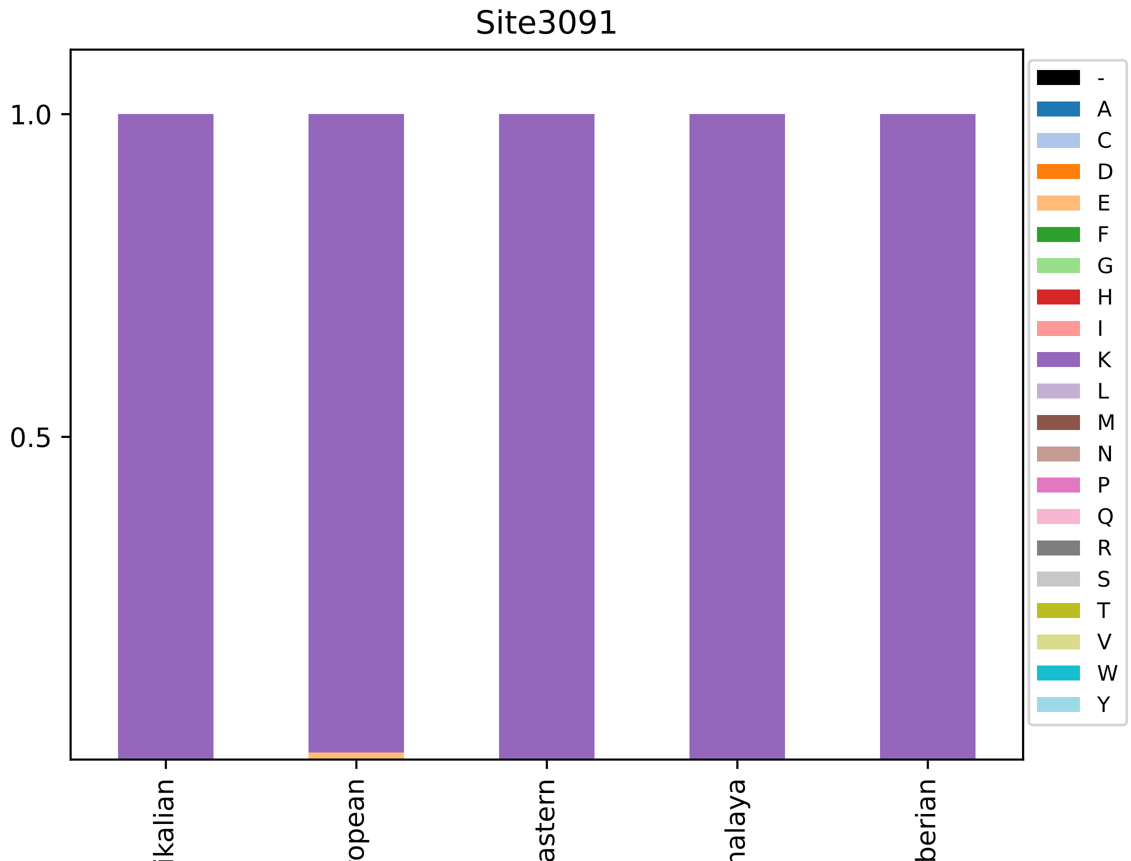
Site3091
1.0
0.5
-
A
C
D
E
F
G
H
I
K
L
M
N
P
Q
R
S
T
V
W
Y
ikalian
opean
astern
nalaya
berian

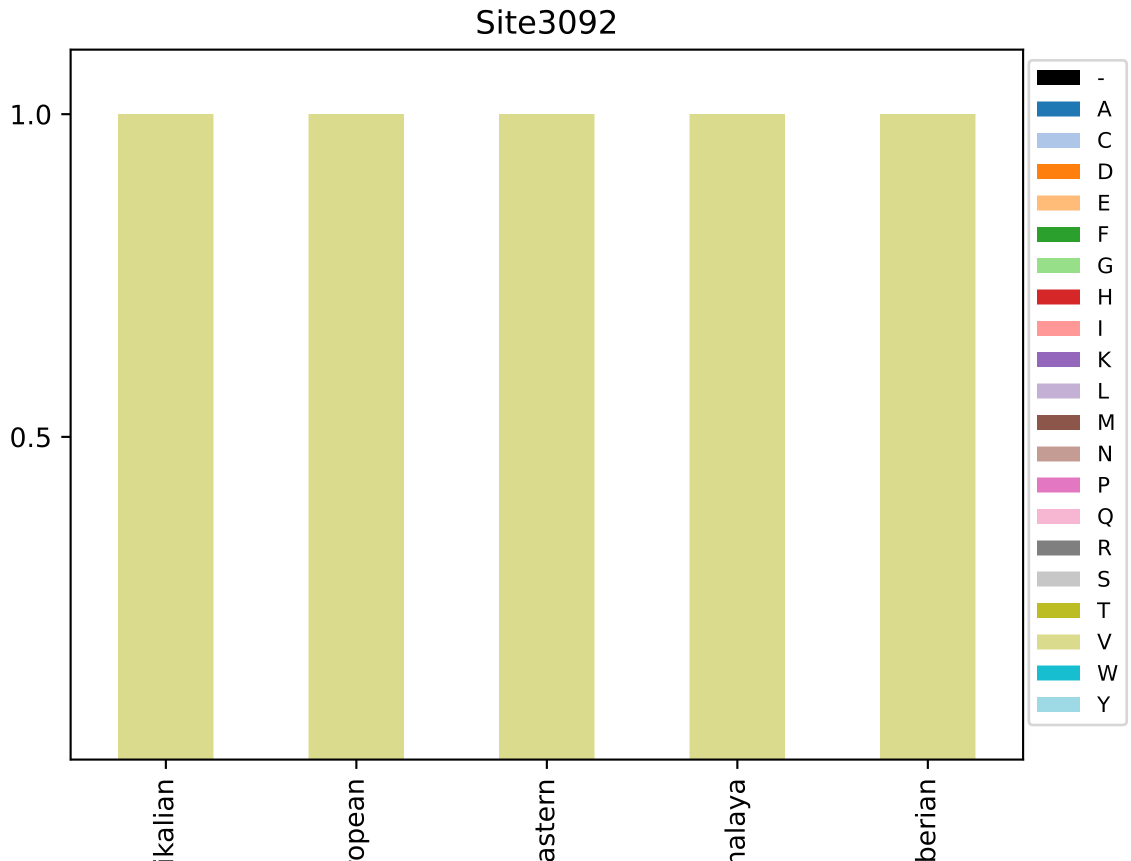
Site3092
1.0
0.5
-
A
C
D
E
F
G
H
I
K
L
M
N
P
Q
R
S
T
V
W
Y
ikalian
opean
astern
nalaya
berian

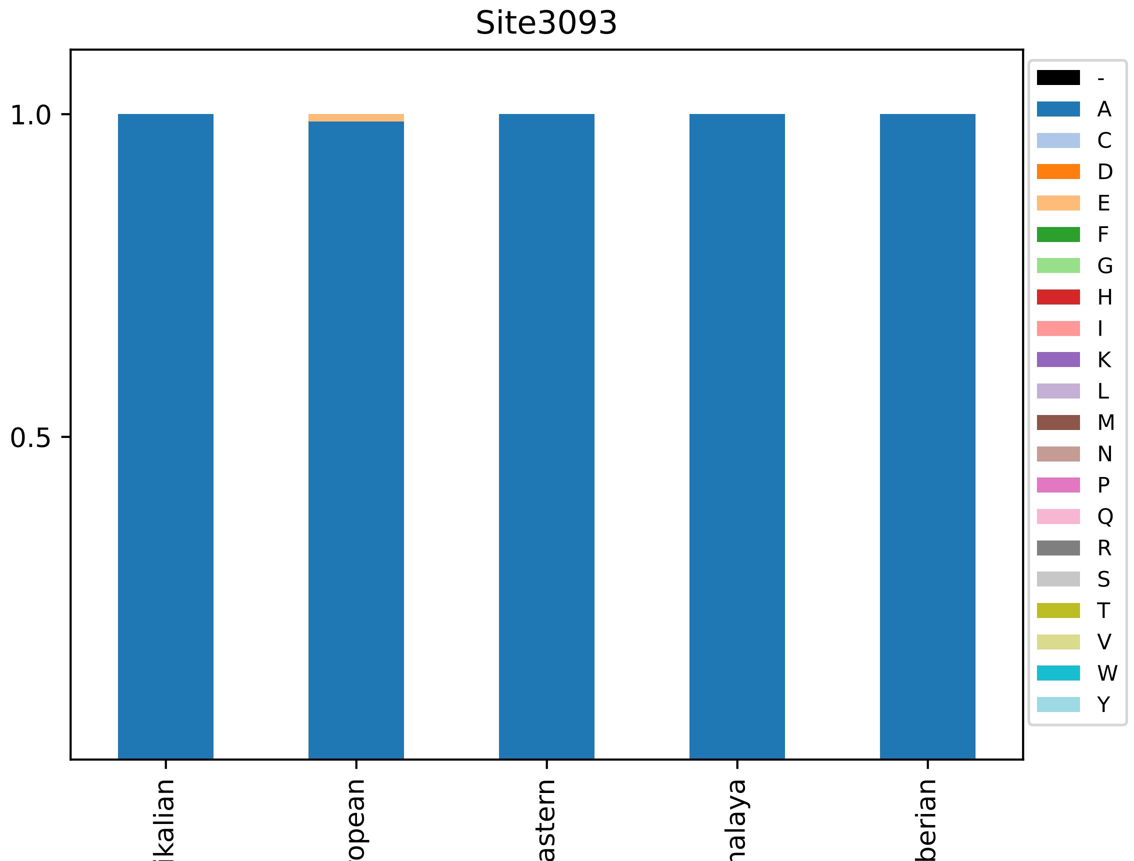
Site3093
1.0
0.5
-
A
C
D
E
F
G
H
I
K
L
M
N
P
Q
R
S
T
V
W
Y
ikalian
opean
astern
nalaya
berian

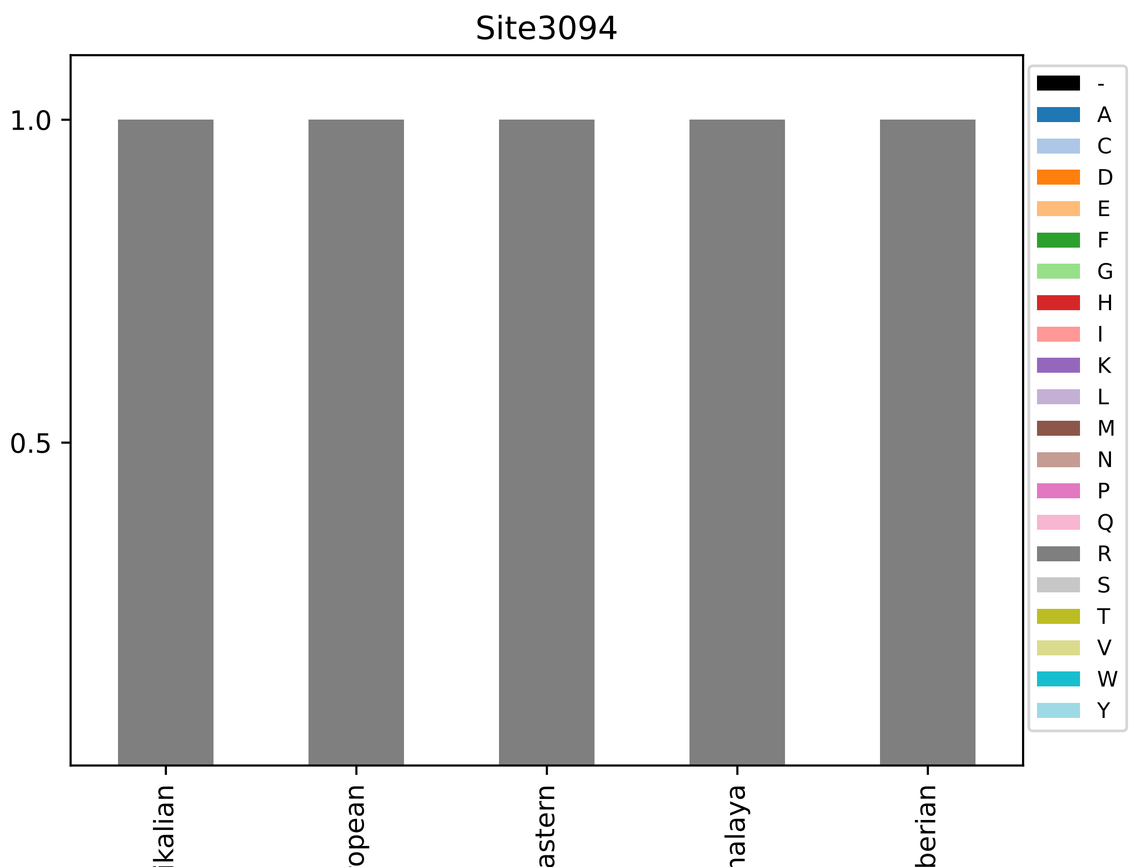
Site3094
1.0
0.5
-
A
C
D
E
F
G
H
I
K
L
M
N
P
Q
R
S
T
V
W
Y
ikalian
opean
astern
nalaya
berian

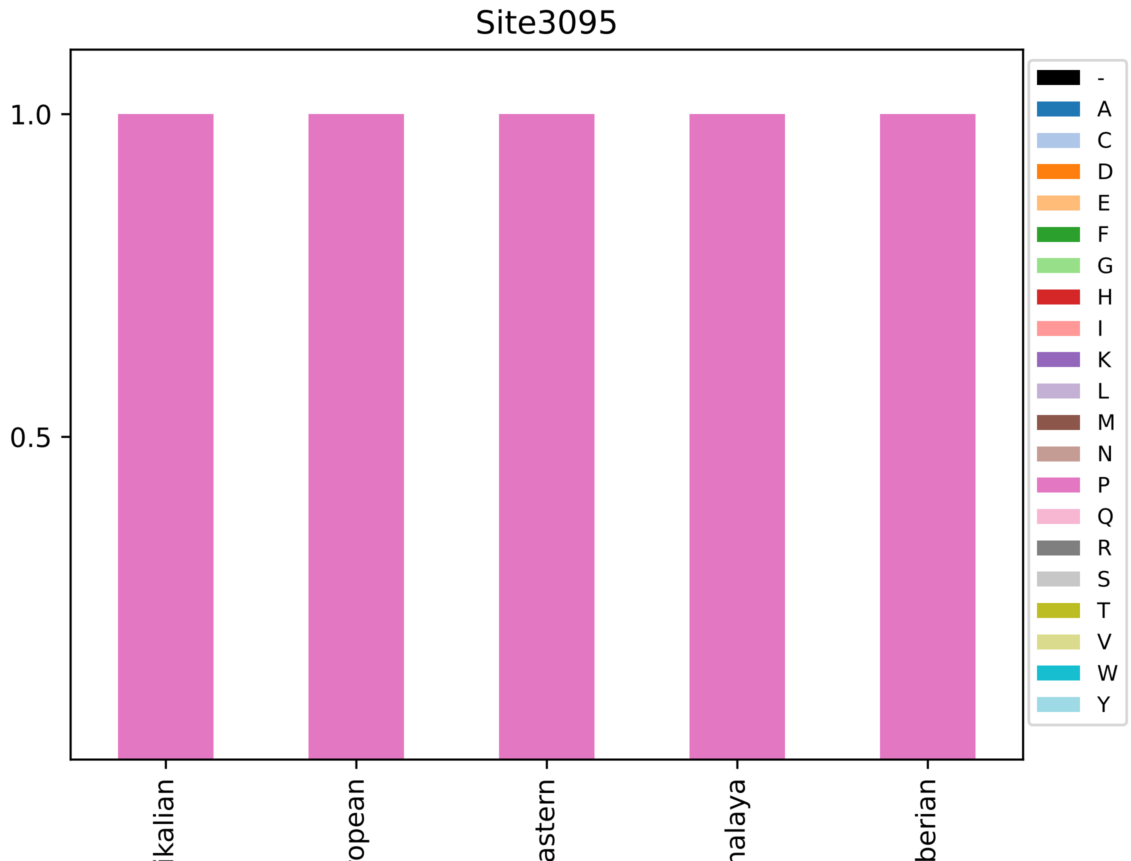

Site3095
1.0
0.5
-
A
C
D
E
F
G
H
I
K
L
M
N
P
Q
R
S
T
V
W
Y
ikalian
opean
astern
nalaya
berian

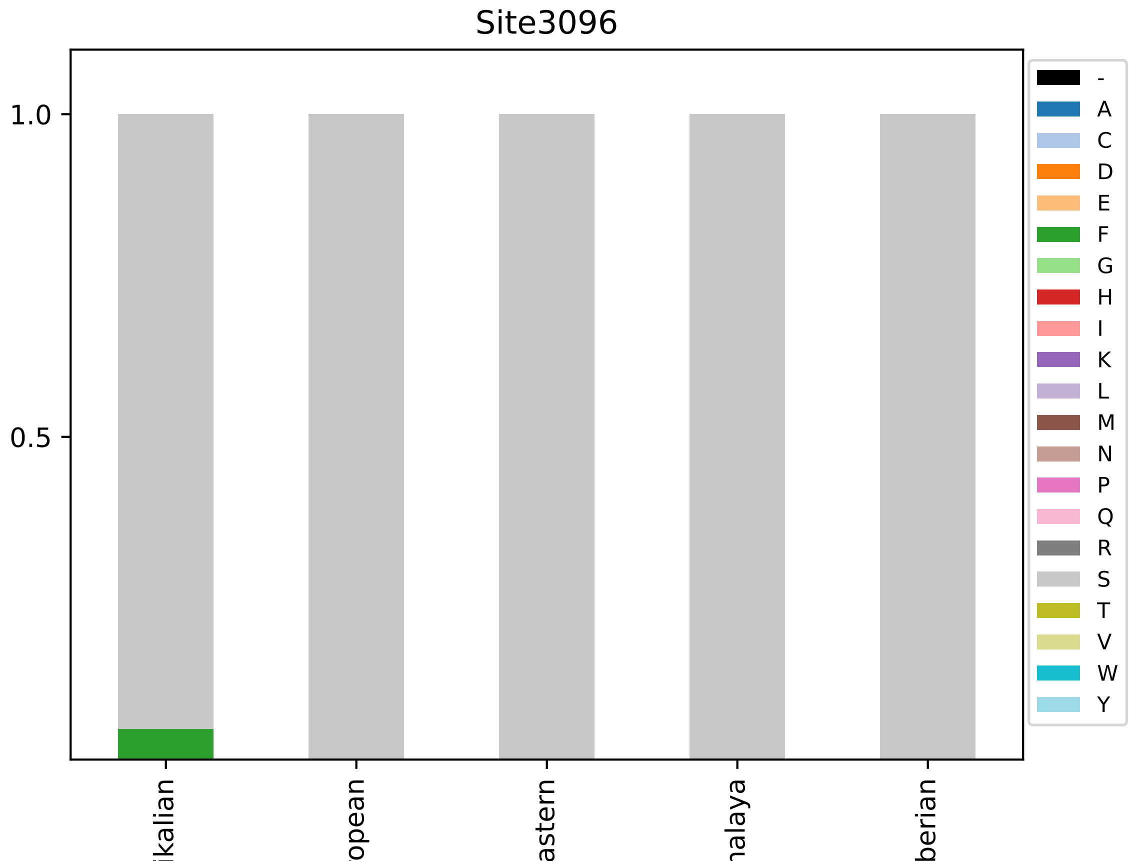

Site3096
1.0
0.5
-
A
C
D
E
F
G
H
I
K
L
M
N
P
Q
R
S
T
V
W
Y
ikalian
opean
astern
nalaya
berian

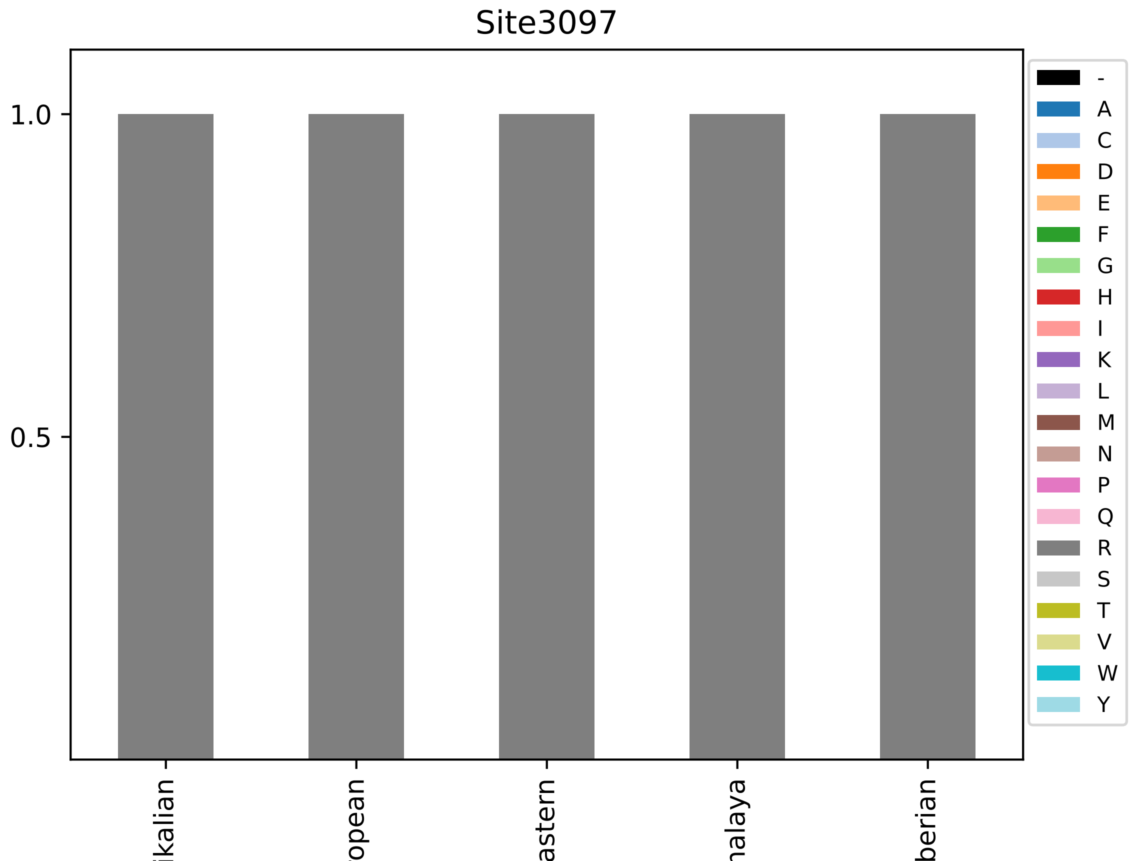

Site3097
1.0
0.5
-
A
C
D
E
F
G
H
I
K
L
M
N
P
Q
R
S
T
V
W
Y
ikalian
opean
astern
nalaya
berian

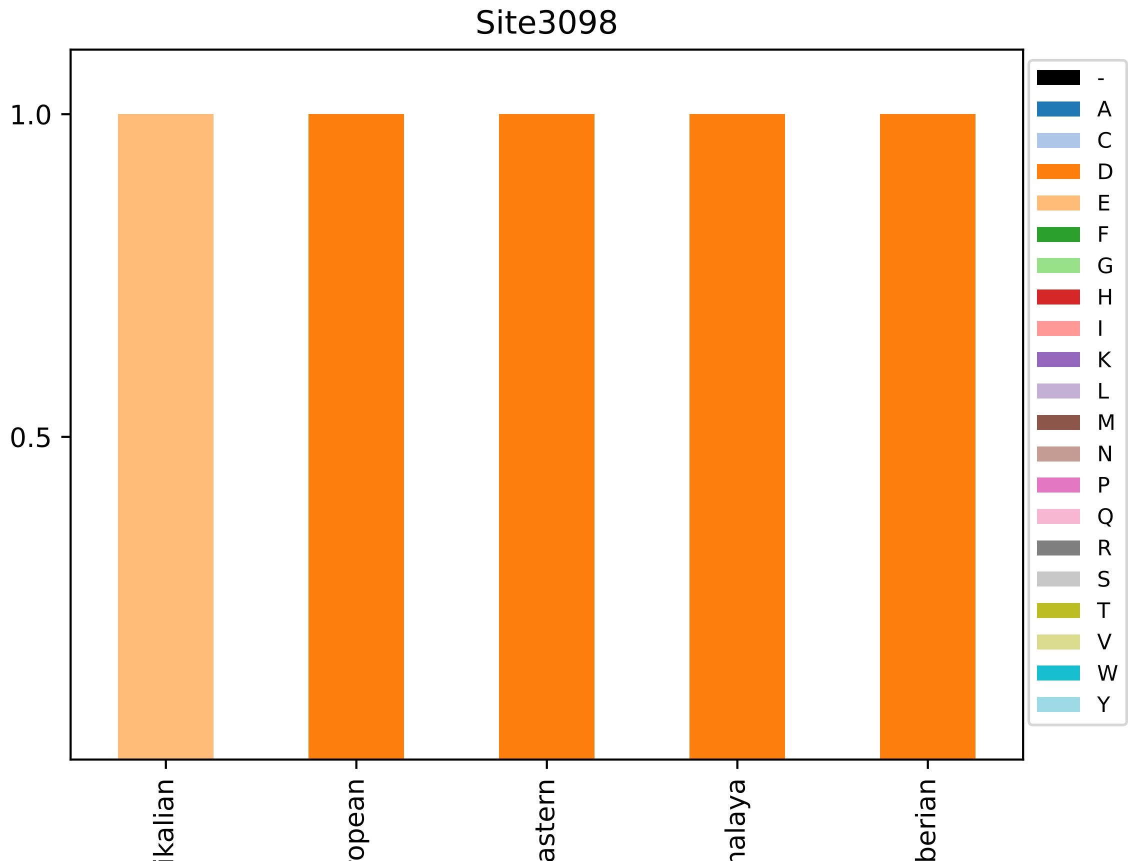
Site3098
1.0
0.5
ikalian
opean
astern
nalaya
berian
-
A
C
D
E
F
G
H
I
K
L
M
N
P
Q
R
S
T
V
W
Y

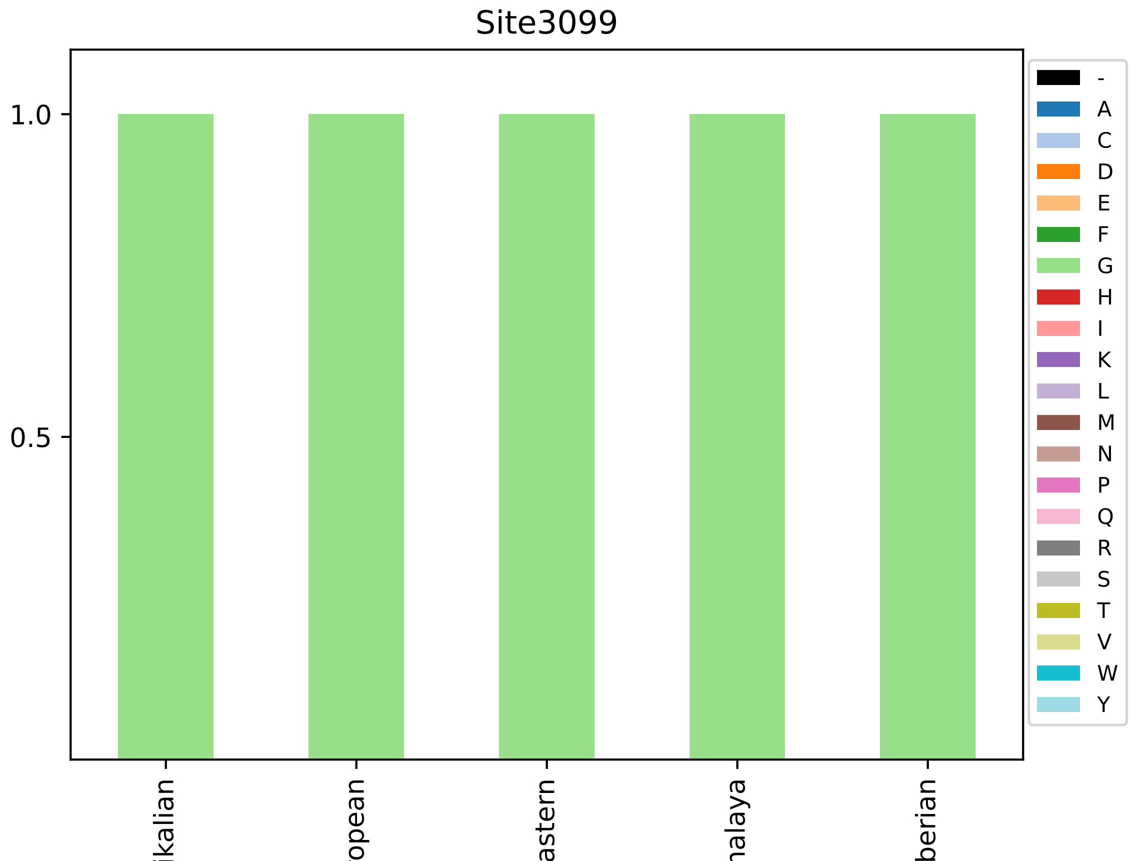

Site3099
1.0
0.5
-
A
C
D
E
F
G
H
I
K
L
M
N
P
Q
R
S
T
V
W
Y
ikalian
opean
astern
nalaya
berian

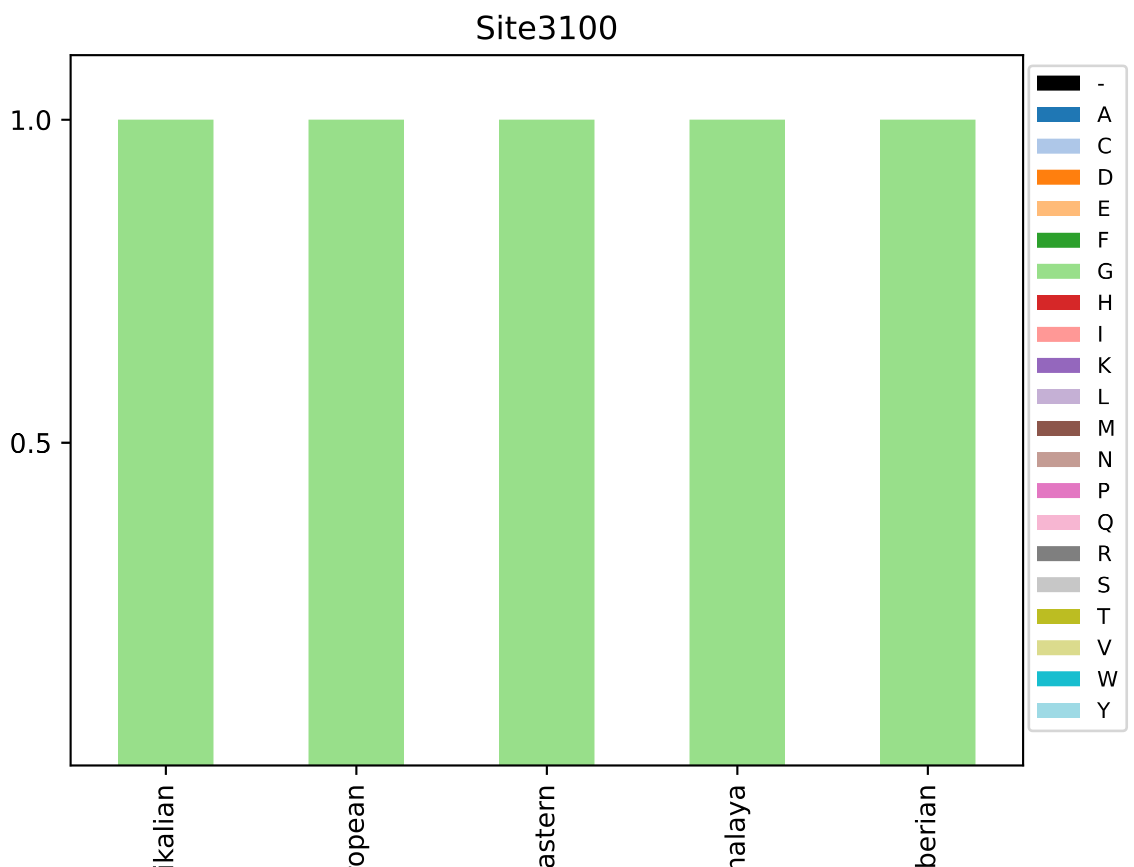
Site3100
1.0
0.5
-
A
C
D
E
F
G
H
I
K
L
M
N
P
Q
R
S
T
V
W
Y
ikalian
opean
astern
nalaya
berian

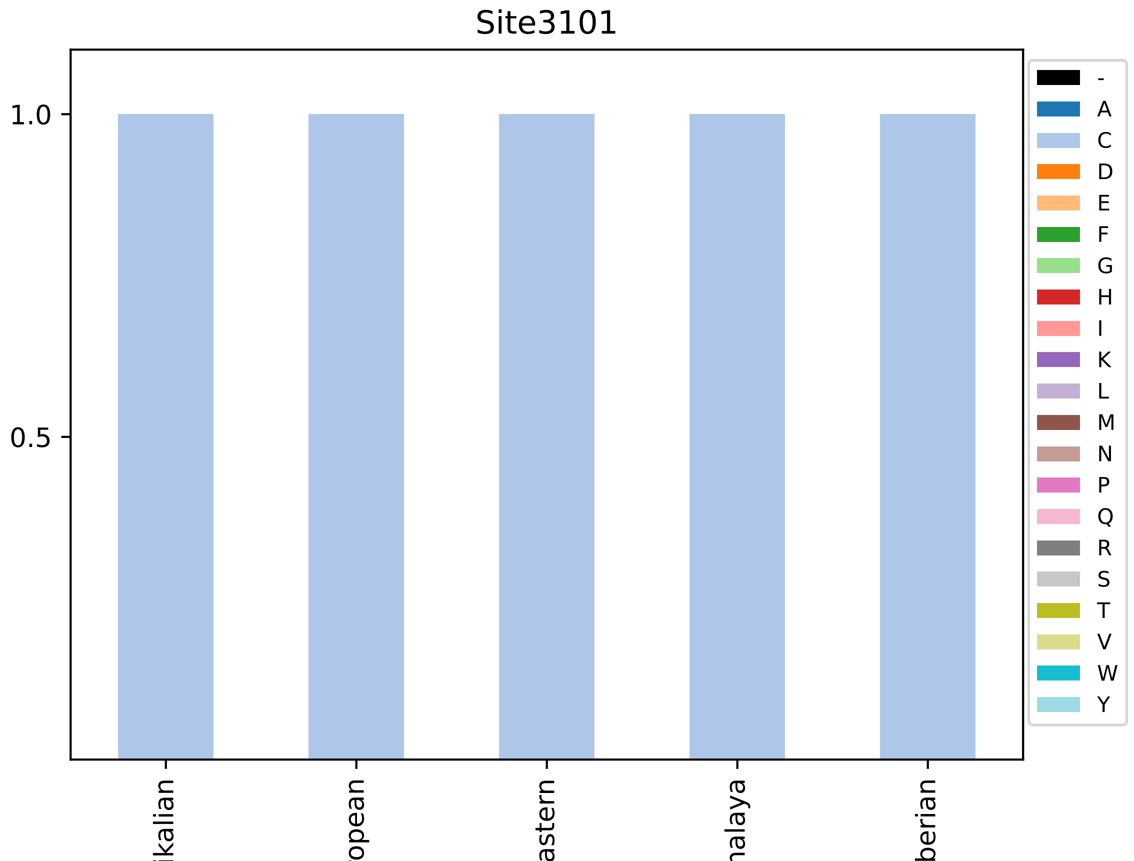

Site3101
1.0
0.5
-
A
C
D
E
F
G
H
I
K
L
M
N
P
Q
R
S
T
V
W
Y
ikalian
opean
astern
halaya
berian

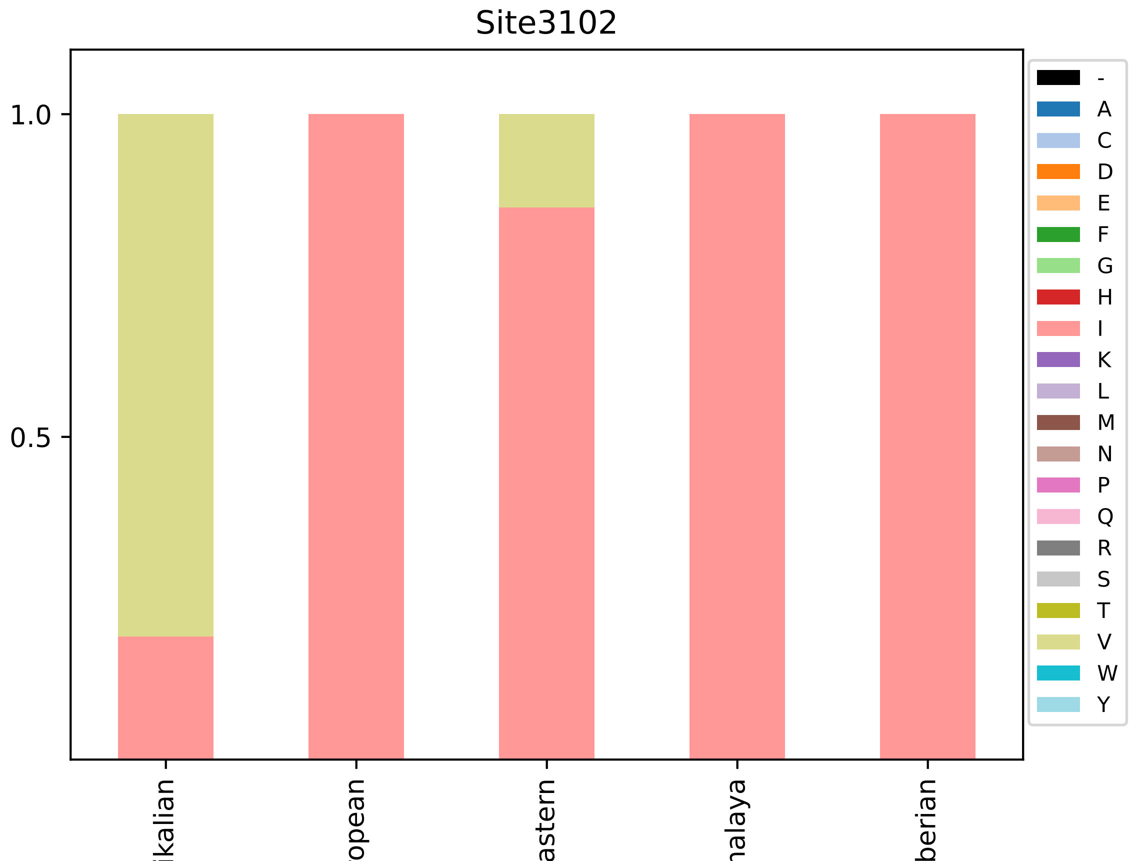
Site3102
1.0
0.5
-
A
C
D
E
F
G
H
I
K
L
M
N
P
Q
R
S
T
V
W
Y
ikalian
ropean
astern
nalaya
berian

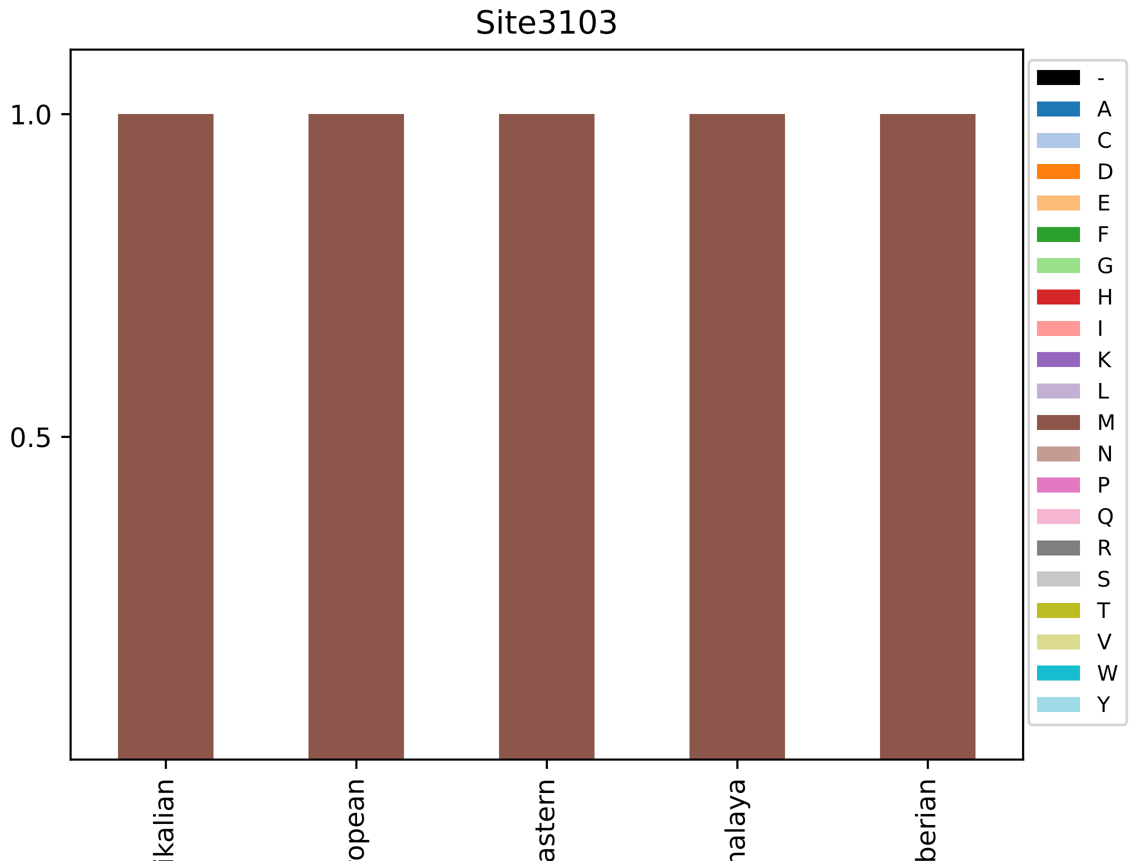

Site3103
1.0
0.5
-
A
C
D
E
F
G
H
I
K
L
M
N
P
Q
R
S
T
V
W
Y
ikalian
ropean
astern
nalaya
berian

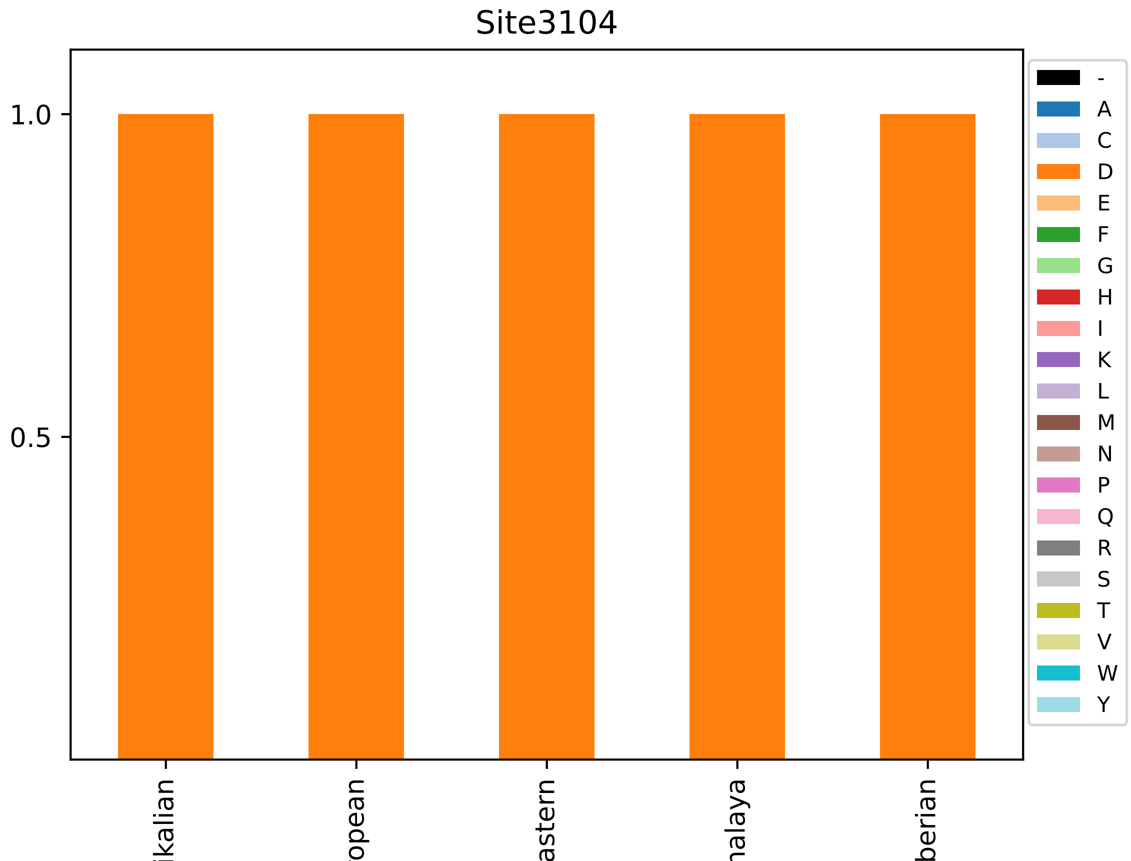
Site3104
1.0
0.5
-
A
C
D
E
F
G
H
I
K
L
M
N
P
Q
R
S
T
V
W
Y
ikalian
opean
astern
nalaya
berian

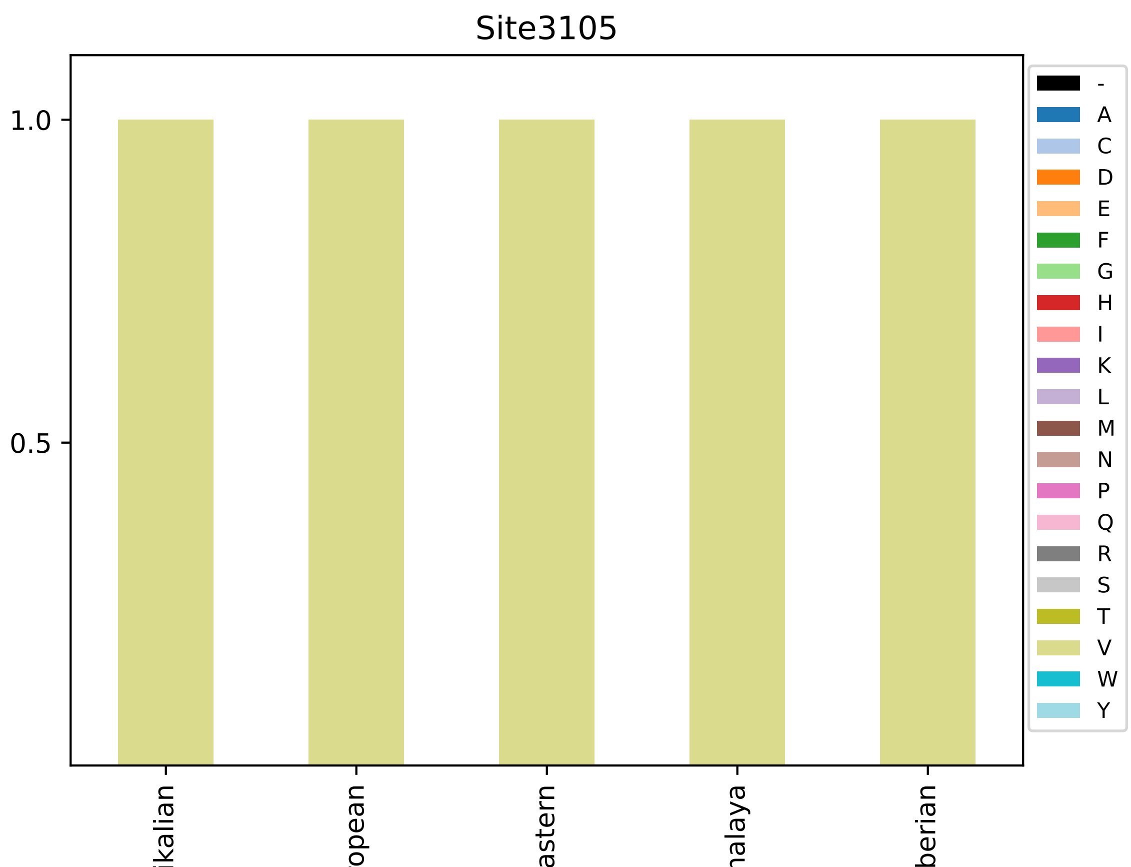

Site3105
1.0
0.5
ikalian
opean
astern
nalaya
berian
-
A
C
D
E
F
G
H
I
K
L
M
N
P
Q
R
S
T
V
W
Y

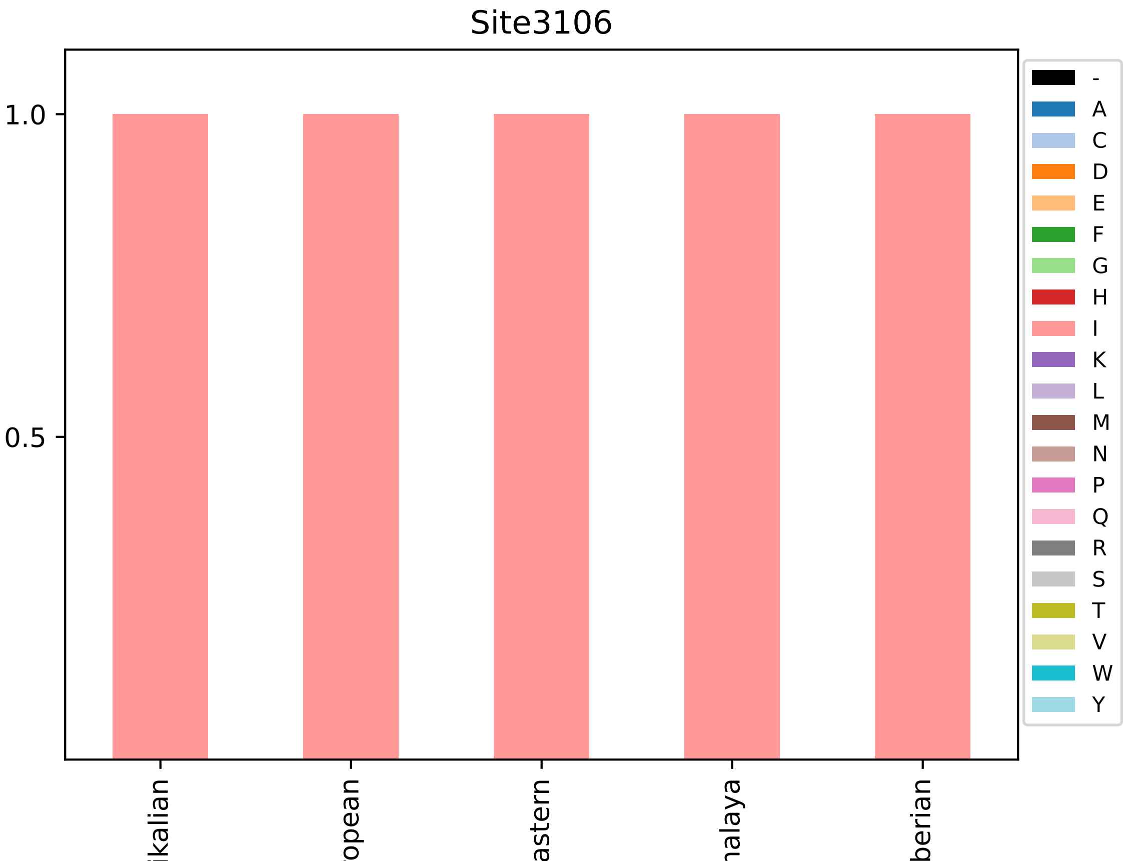

Site3106
1.0
0.5
-
A
C
D
E
F
G
H
I
K
L
M
N
P
Q
R
S
T
V
W
Y
ikalian
opean
astern
nalaya
berian

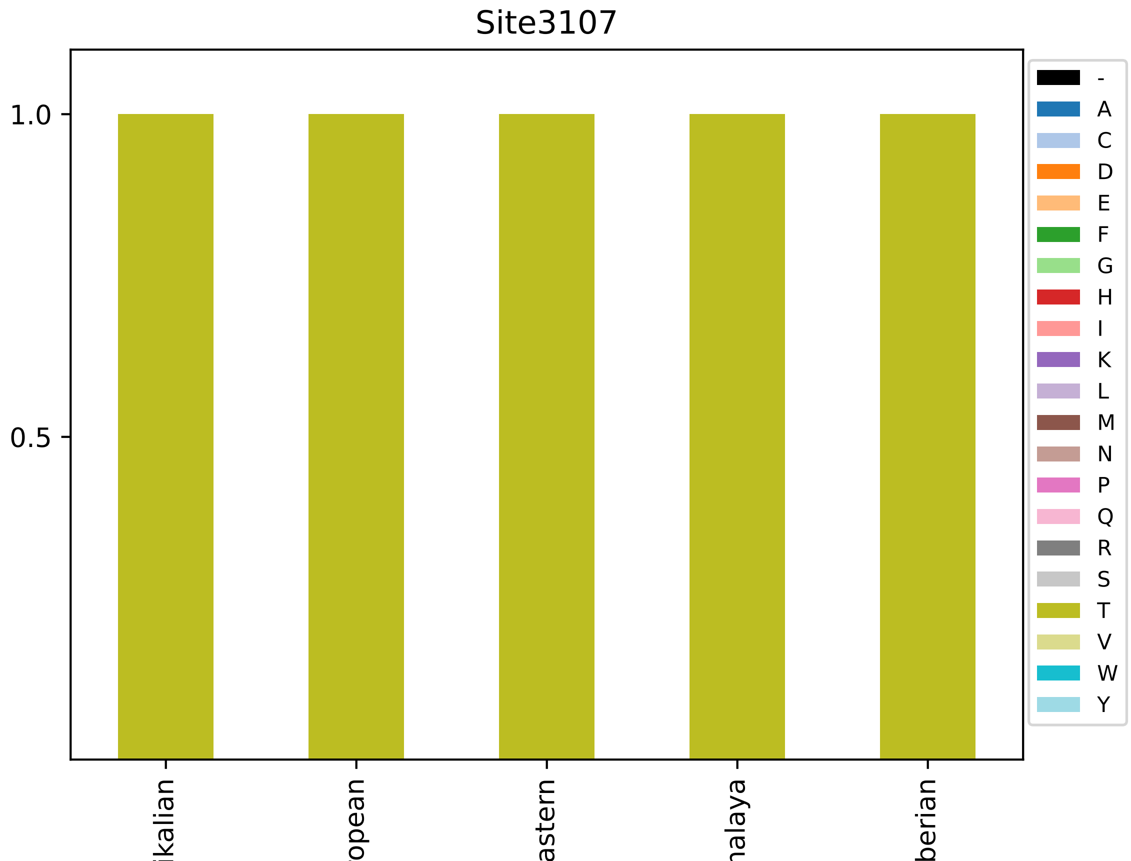

Site3107
1.0
0.5
-
A
C
D
E
F
G
H
I
K
L
M
N
P
Q
R
S
T
V
W
Y
ikalian
opean
astern
nalaya
berian

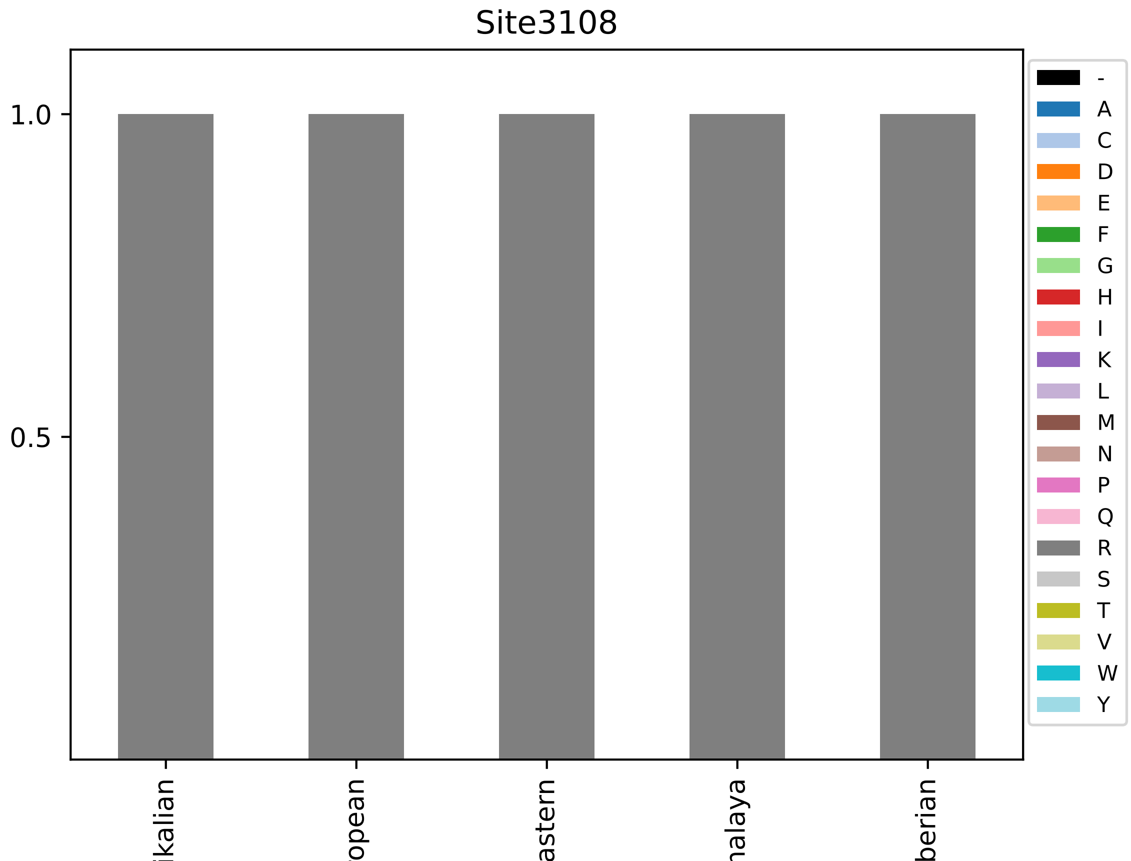

Site3108
1.0
0.5
-
A
C
D
E
F
G
H
I
K
L
M
N
P
Q
R
S
T
V
W
Y
ikalian
opean
astern
nalaya
berian

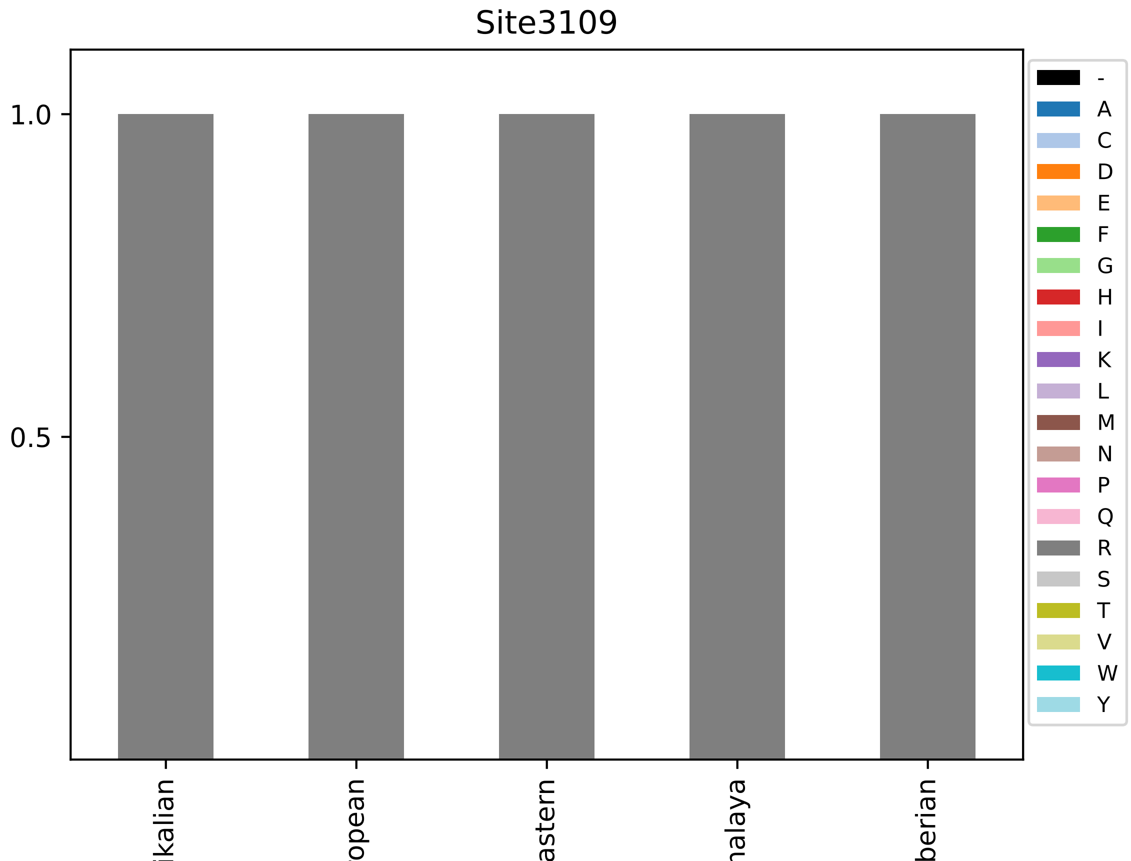
Site3109
1.0
0.5
ikalian
ropean
astern
nalaya
berian
-
A
C
D
E
F
G
H
I
K
L
M
N
P
Q
R
S
T
V
W
Y

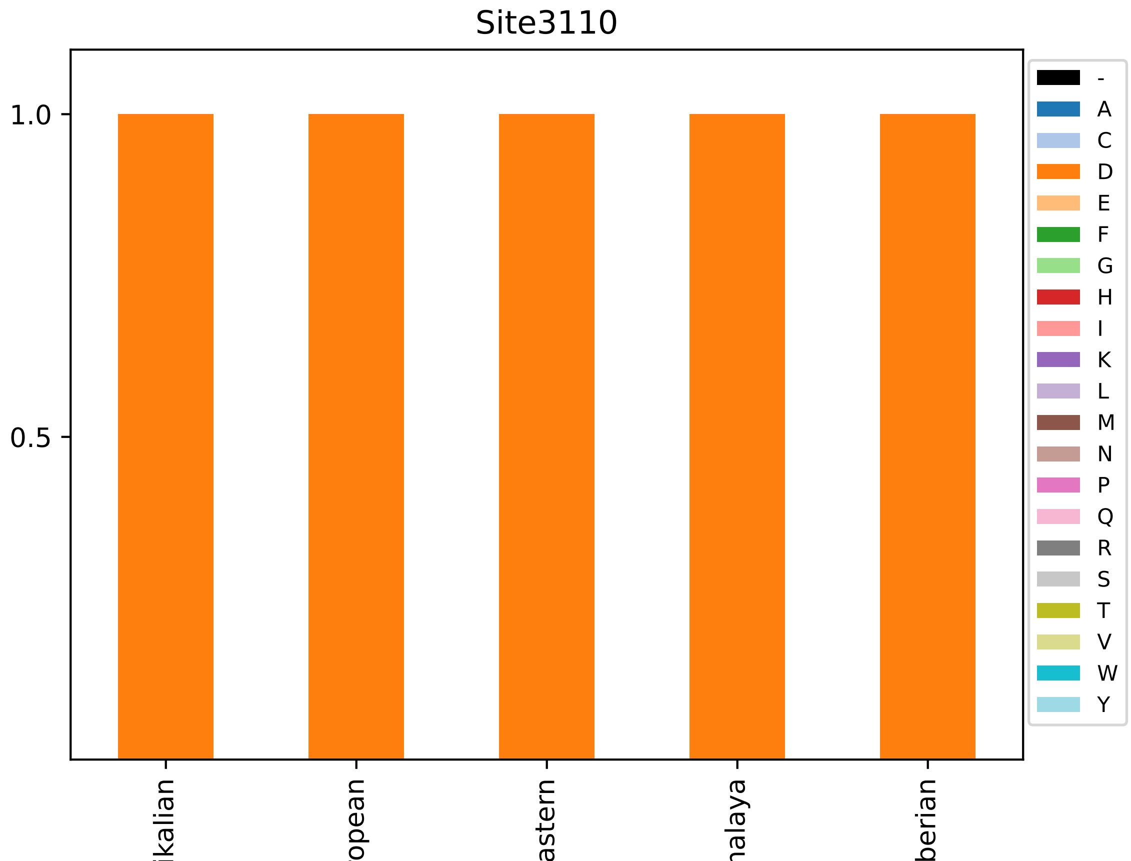

Site3110
1.0
0.5
ikalian
opean
astern
nalaya
berian
-
A
C
D
E
F
G
H
I
K
L
M
N
P
Q
R
S
T
V
W
Y

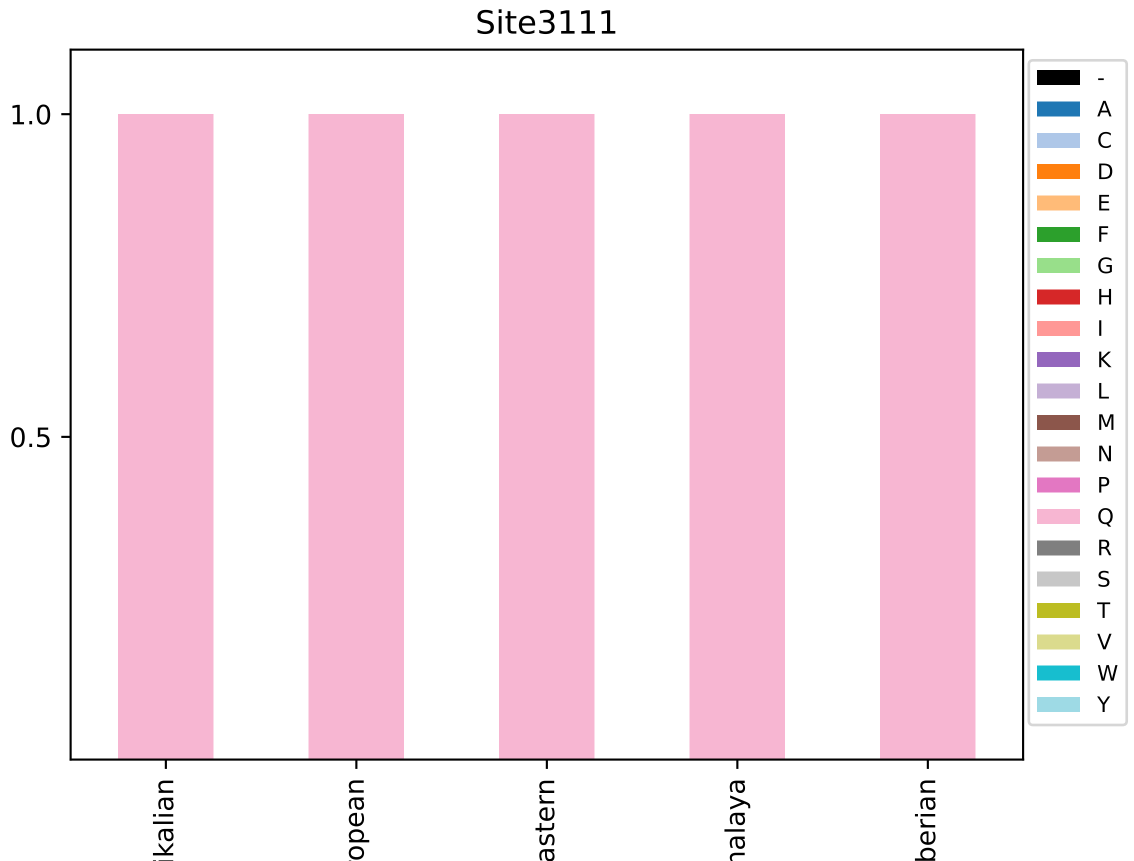
Site3111
1.0
0.5
-
A
C
D
E
F
G
H
I
K
L
M
N
P
Q
R
S
T
V
W
Y
ikalian
opean
astern
nalaya
berian

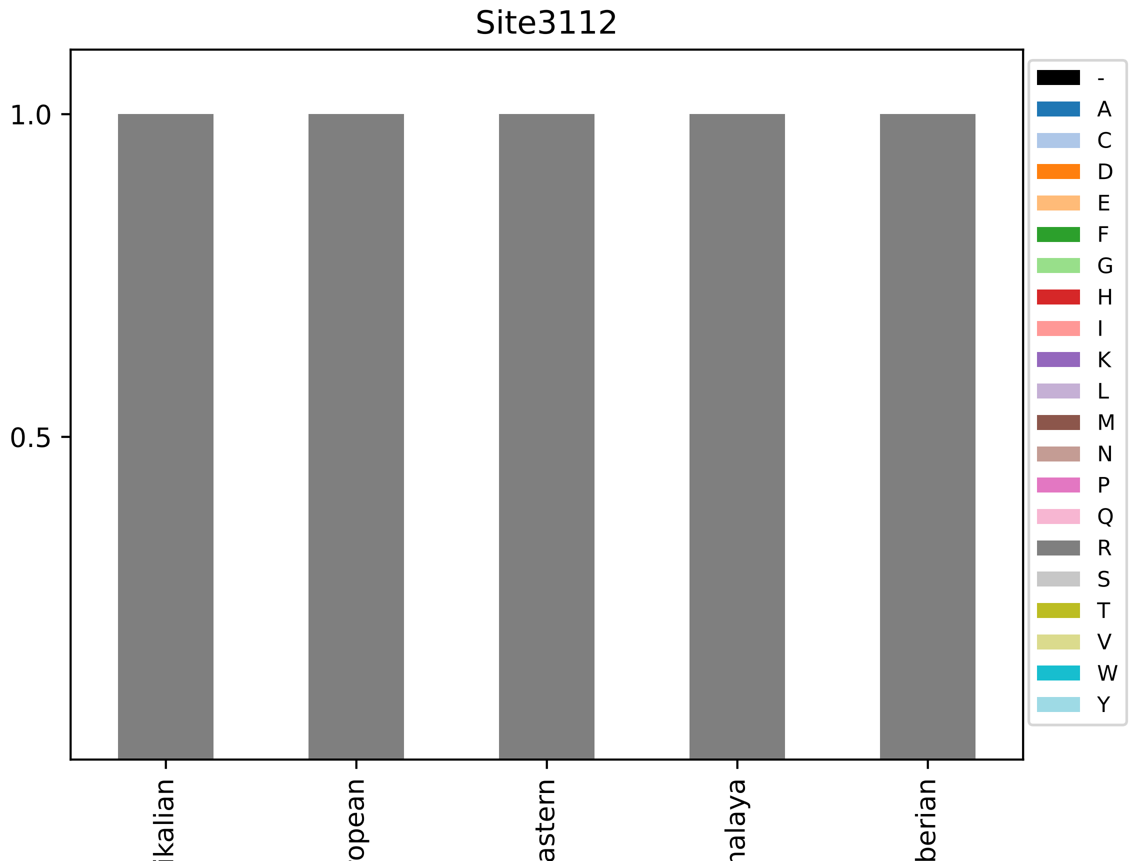

Site3112
1.0
0.5
-
A
C
D
E
F
G
H
I
K
L
M
N
P
Q
R
S
T
V
W
Y
ikalian
opean
astern
nalaya
berian

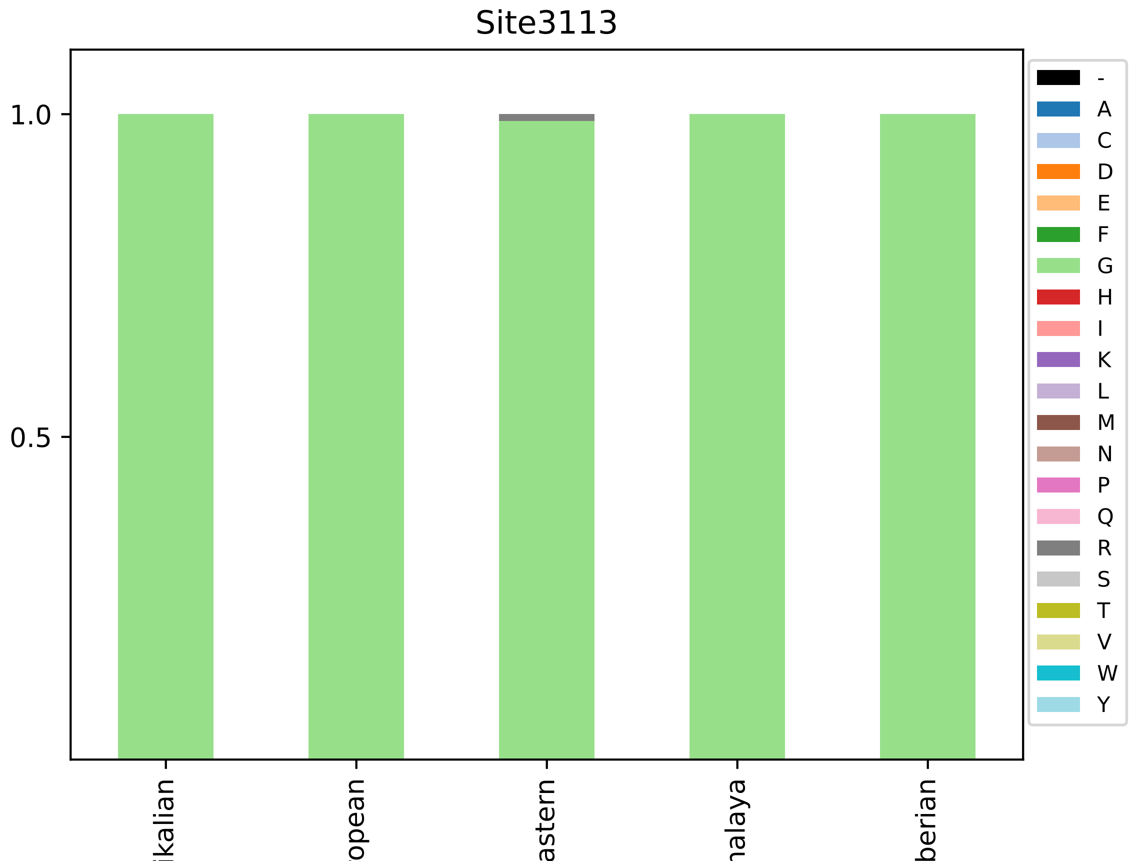

Site3113
1.0
0.5
-
A
C
D
E
F
G
H
I
K
L
M
N
P
Q
R
S
T
V
W
Y
ikalian
opean
astern
nalaya
berian

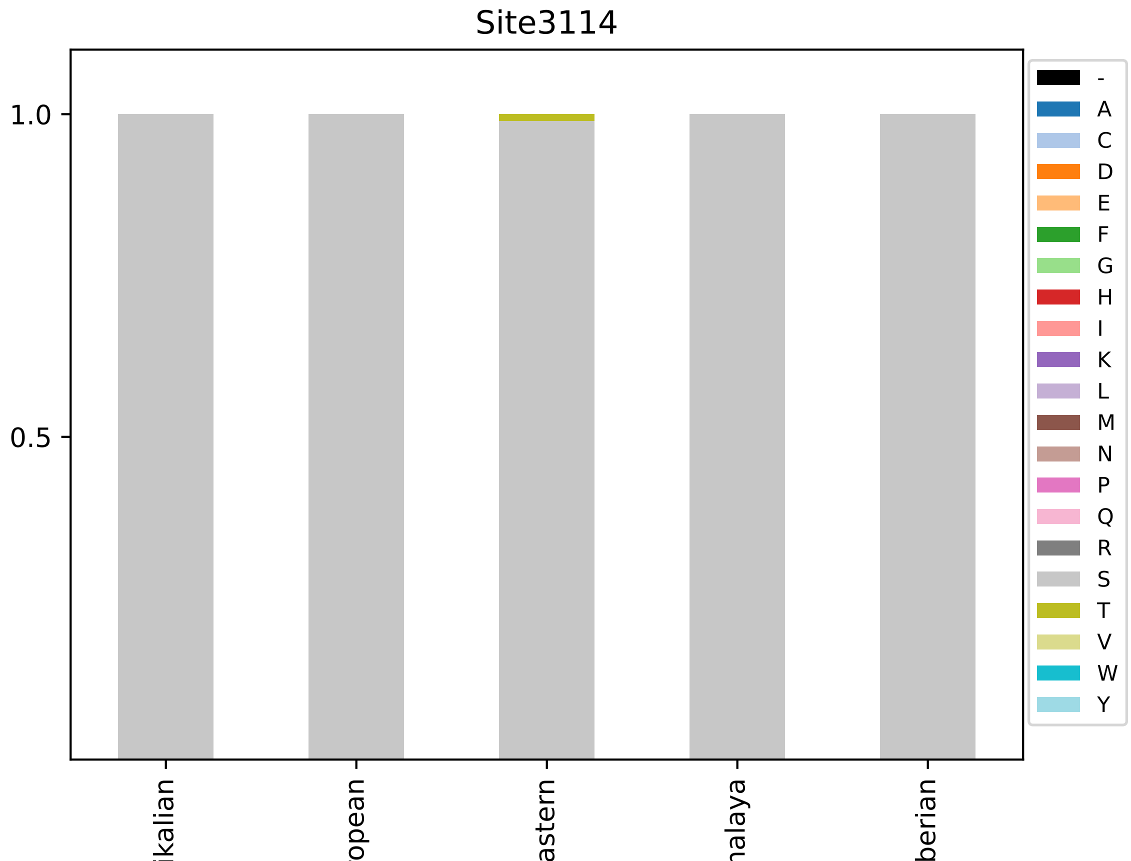
Site3114
1.0
0.5
-
A
C
D
E
F
G
H
I
K
L
M
N
P
Q
R
S
T
V
W
Y

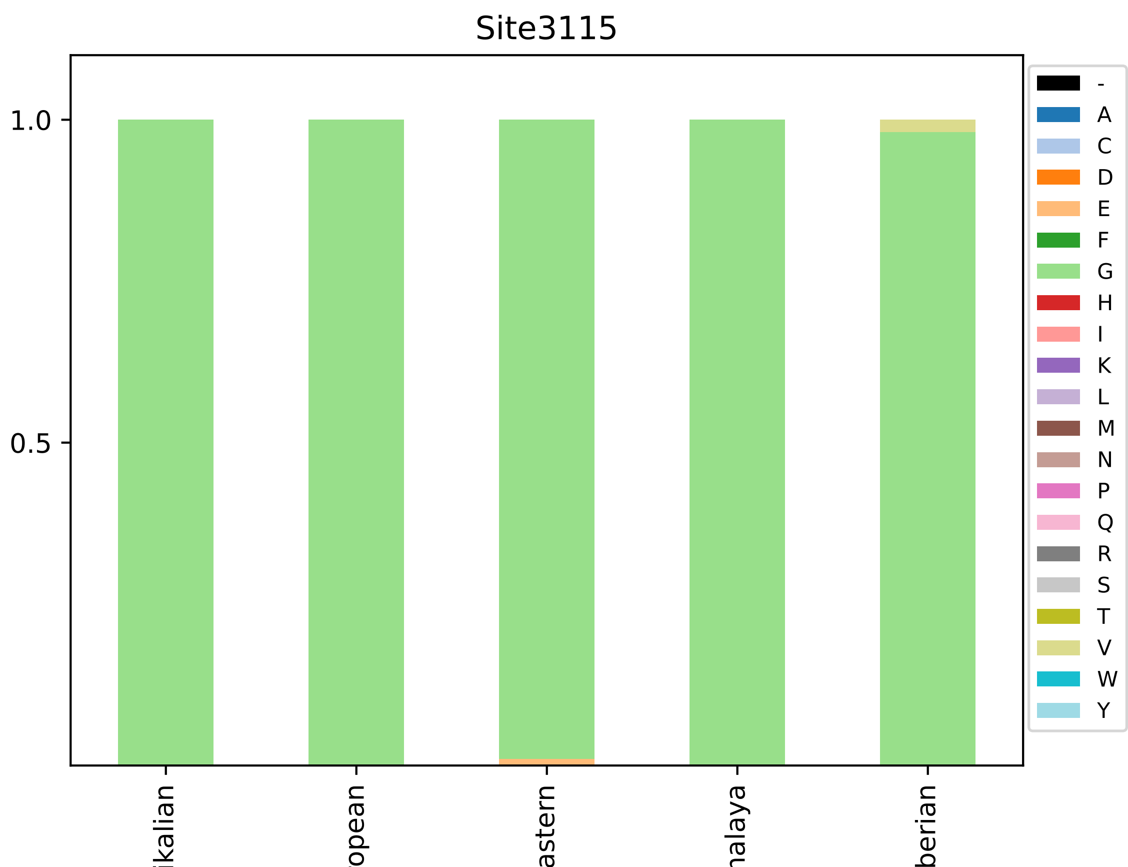
Site3115
1.0
0.5
ikalian
opean
astern
nalaya
berian
-
A
C
D
E
F
G
H
I
K
L
M
N
P
Q
R
S
T
V
W
Y

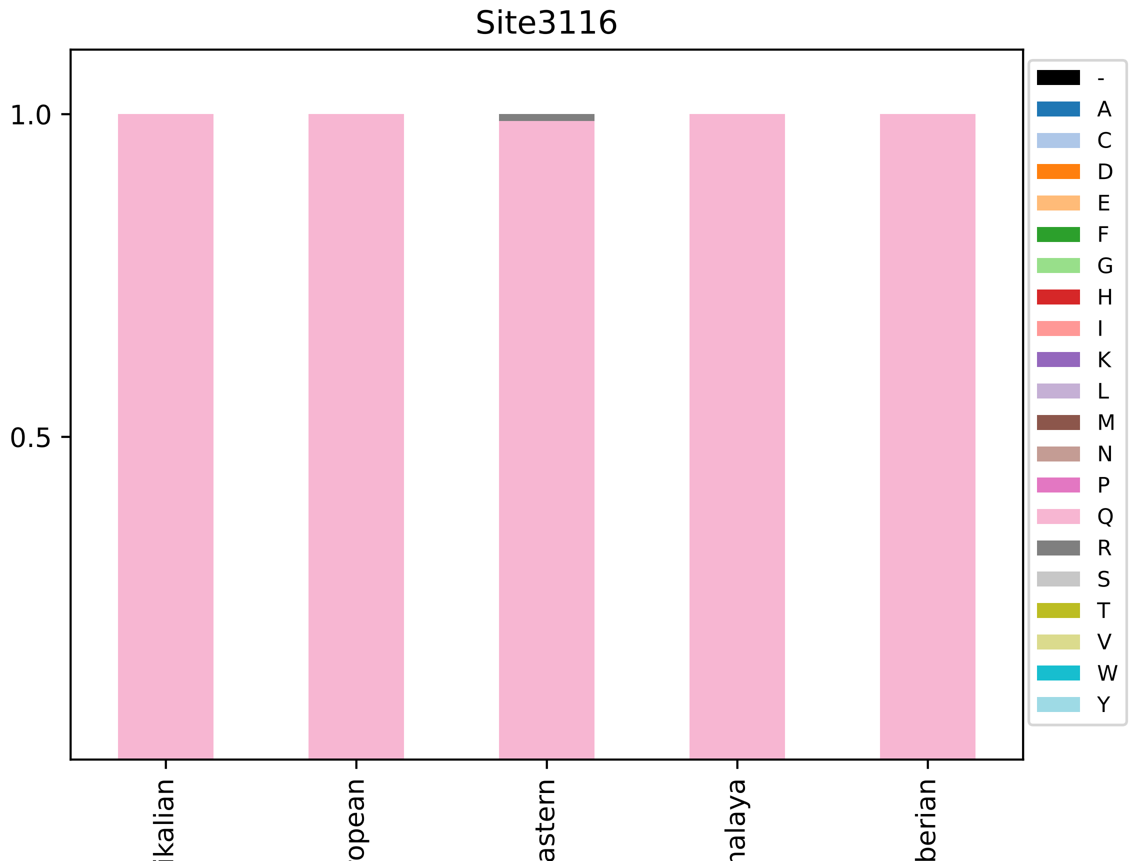
Site3116
1.0
0.5
-
A
C
D
E
F
G
H
I
K
L
M
N
P
Q
R
S
T
V
W
Y
ikalian
opean
astern
nalaya
berian

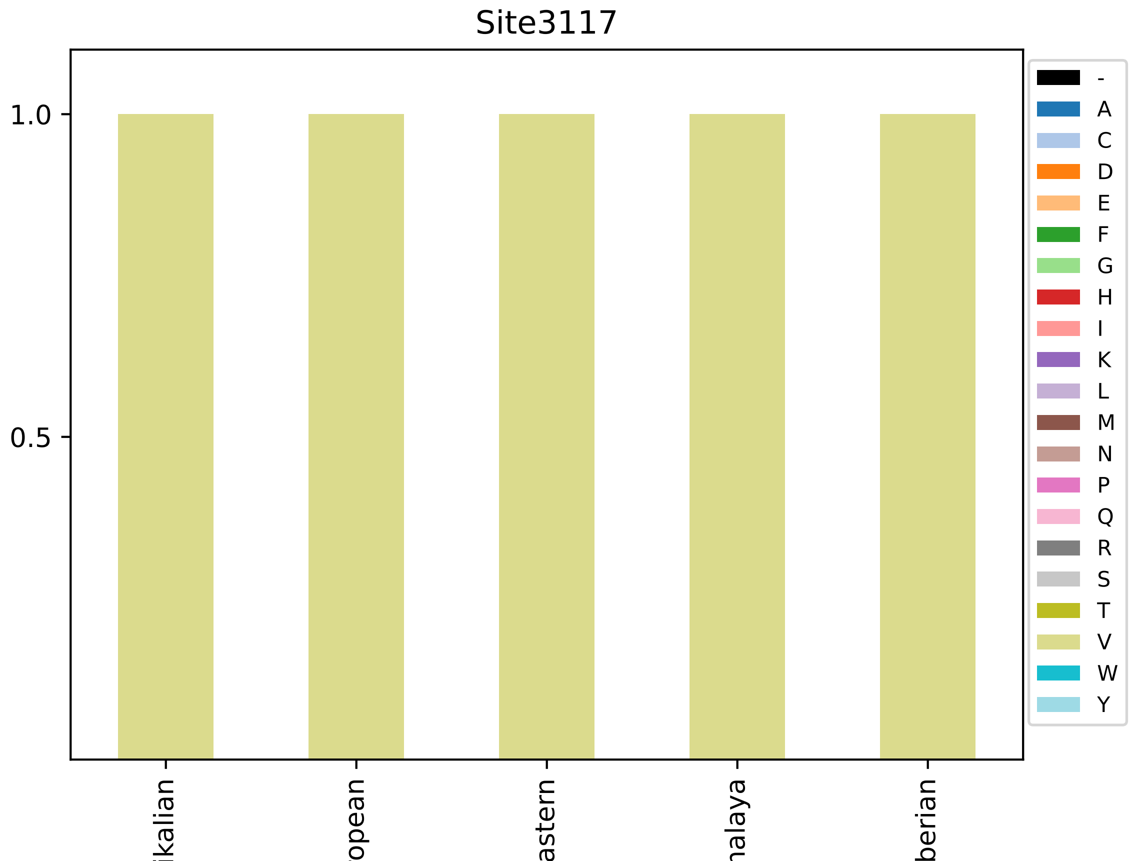

Site3117
1.0
0.5
-
A
C
D
E
F
G
H
I
K
L
M
N
P
Q
R
S
T
V
W
Y
ikalian
opean
astern
nalaya
berian

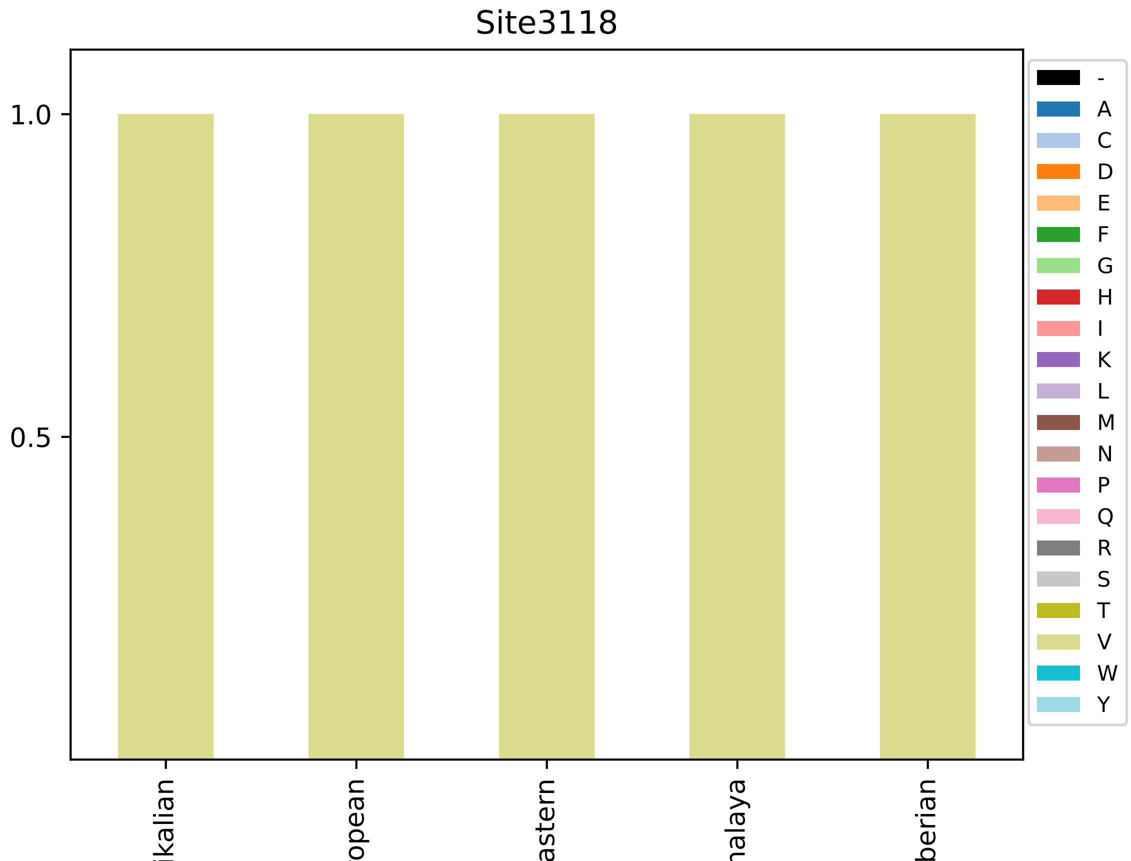
Site3118
1.0
0.5
ikalian
opean
astern
nalaya
berian
-
A
C
D
E
F
G
H
I
K
L
M
N
P
Q
R
S
T
V
W
Y

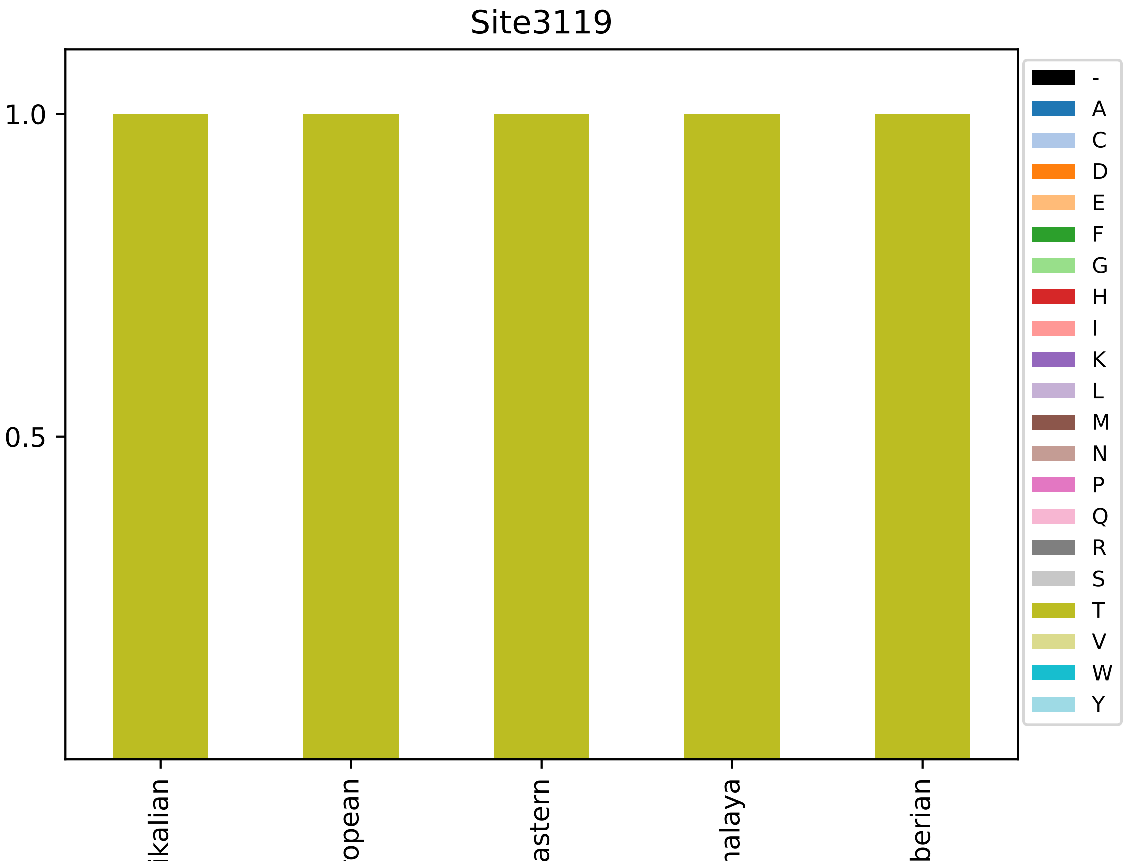
Site3119
1.0
0.5
-
A
C
D
E
F
G
H
I
K
L
M
N
P
Q
R
S
T
V
W
Y

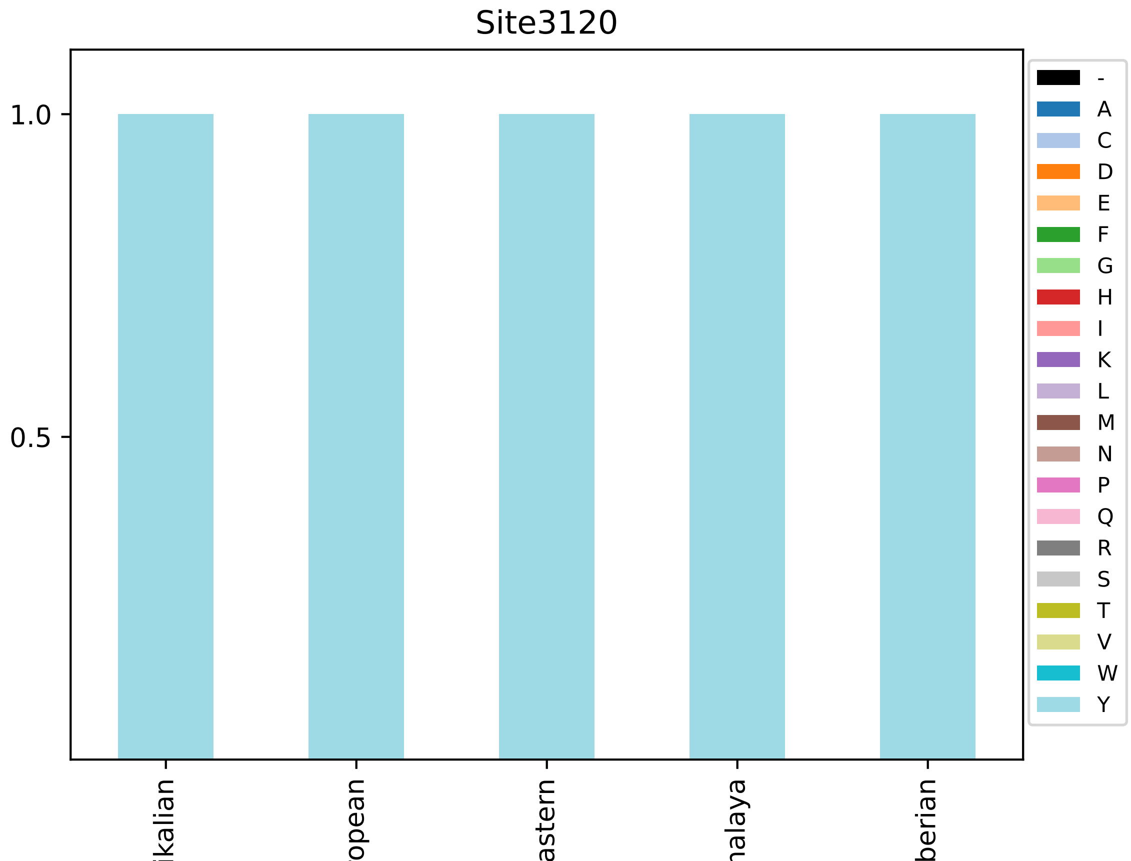
Site3120
1.0
0.5
-
A
C
D
E
F
G
H
I
K
L
M
N
P
Q
R
S
T
V
W
Y
ikalian
opean
astern
nalaya
berian

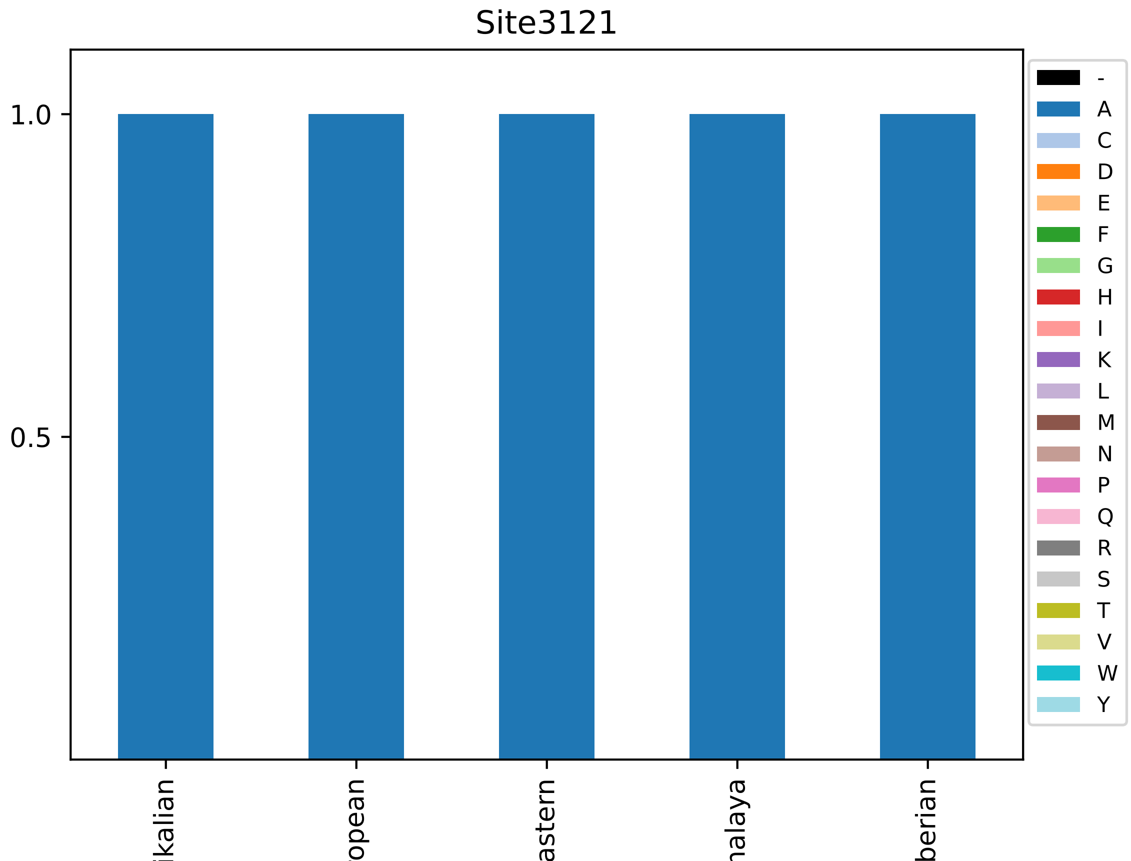
Site3121
1.0
0.5
-
A
C
D
E
F
G
H
I
K
L
M
N
P
Q
R
S
T
V
W
Y
ikalian
opean
astern
nalaya
berian

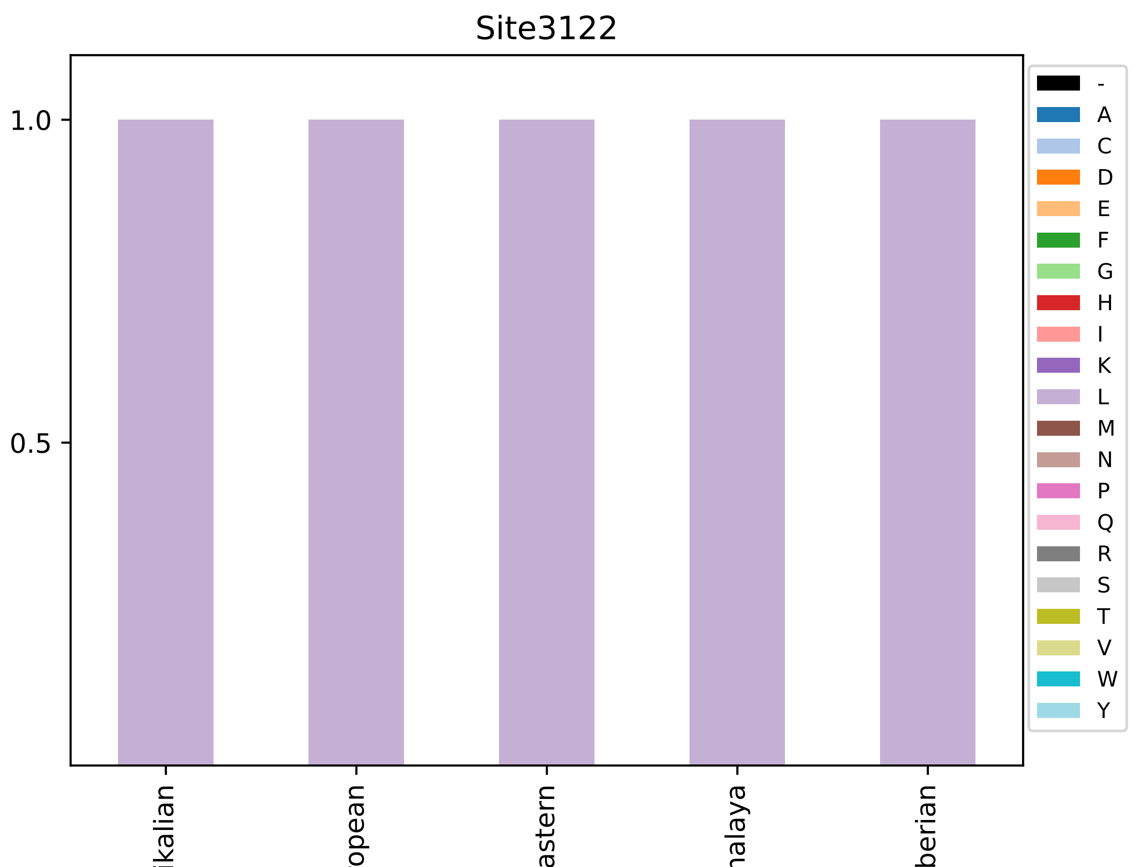

Site3122
1.0
0.5
-
A
C
D
E
F
G
H
I
K
L
M
N
P
Q
R
S
T
V
W
Y
ikalian
opean
astern
nalaya
berian

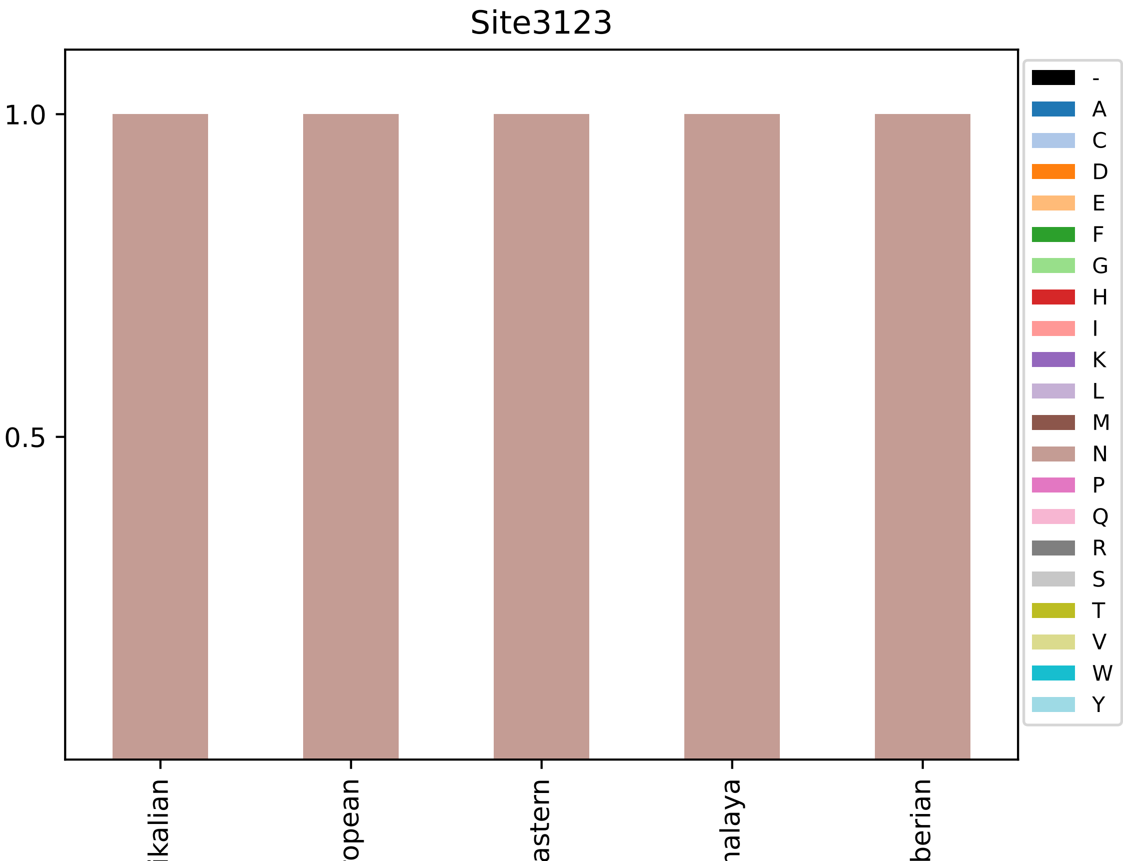
Site3123
1.0
0.5
-
A
C
D
E
F
G
H
I
K
L
M
N
P
Q
R
S
T
V
W
Y

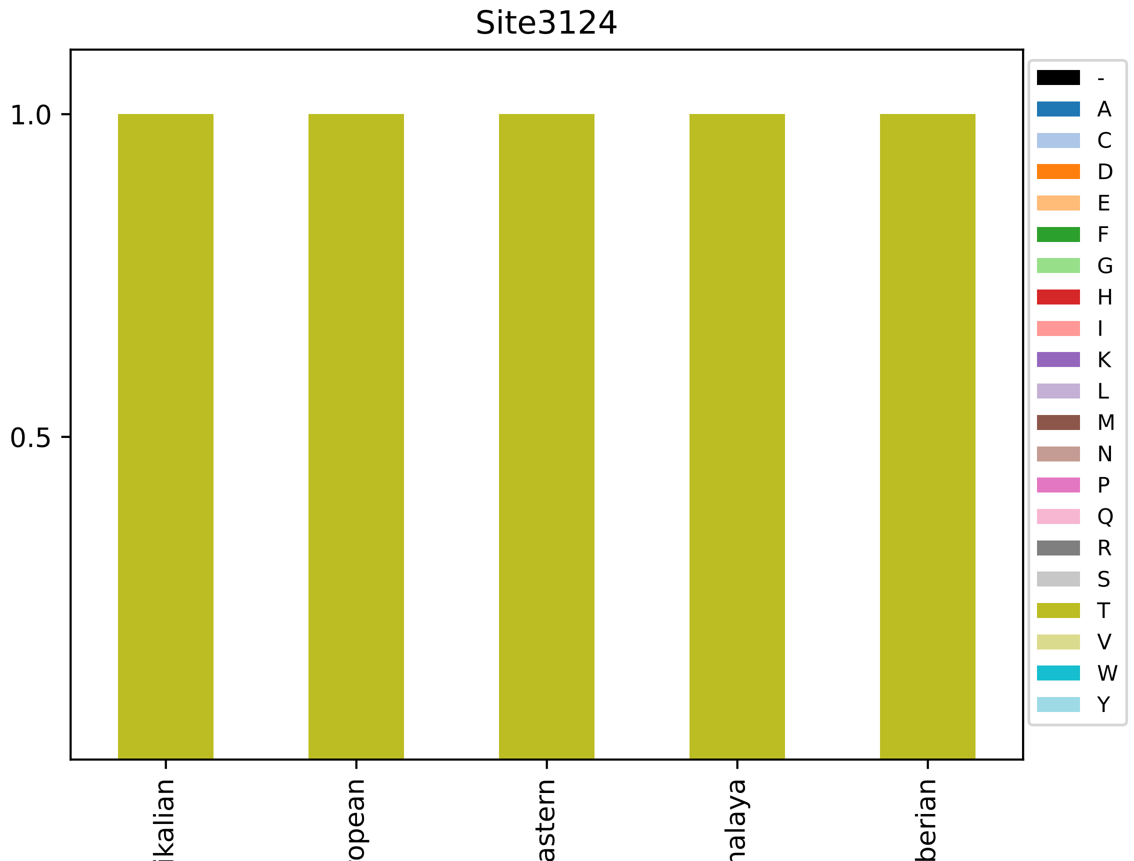

Site3124
1.0
0.5
-
A
C
D
E
F
G
H
I
K
L
M
N
P
Q
R
S
T
V
W
Y
ikalian
opean
astern
nalaya
berian

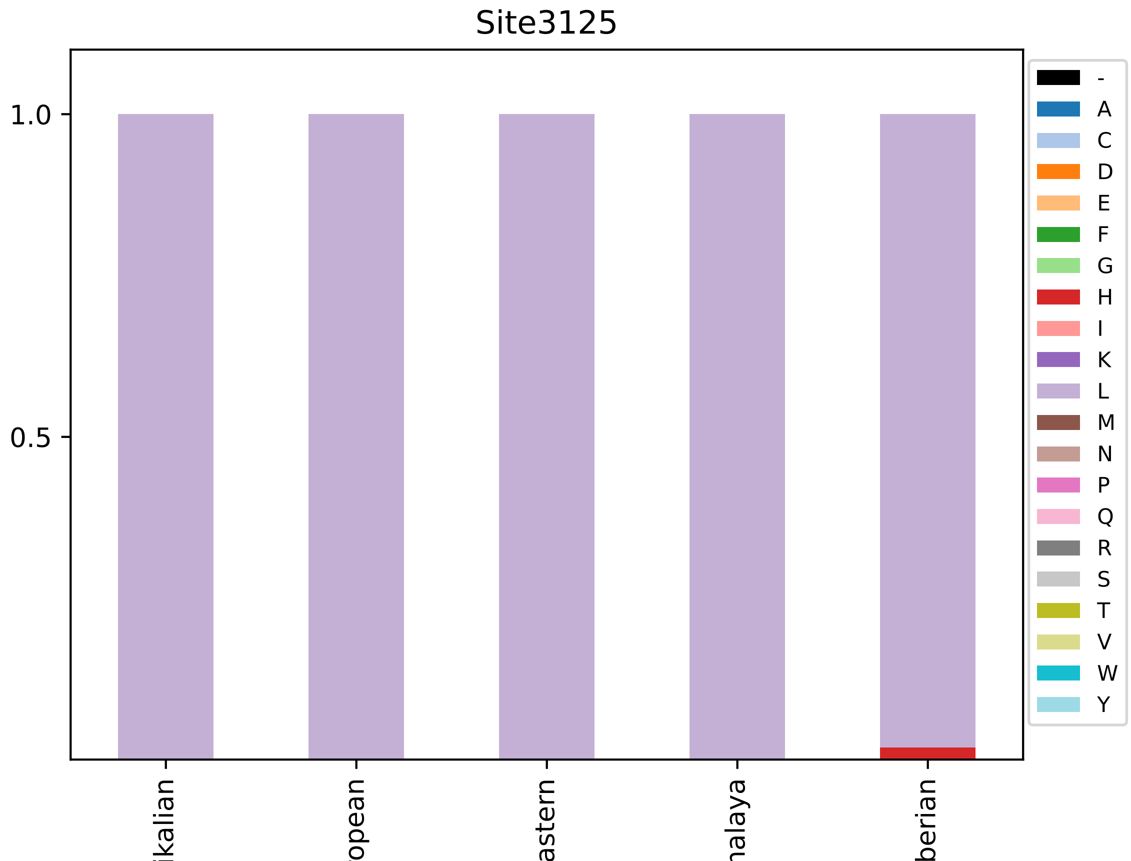
Site3125
1.0
0.5
-
A
C
D
E
F
G
H
I
K
L
M
N
P
Q
R
S
T
V
W
Y
ikalian
opean
astern
nalaya
berian

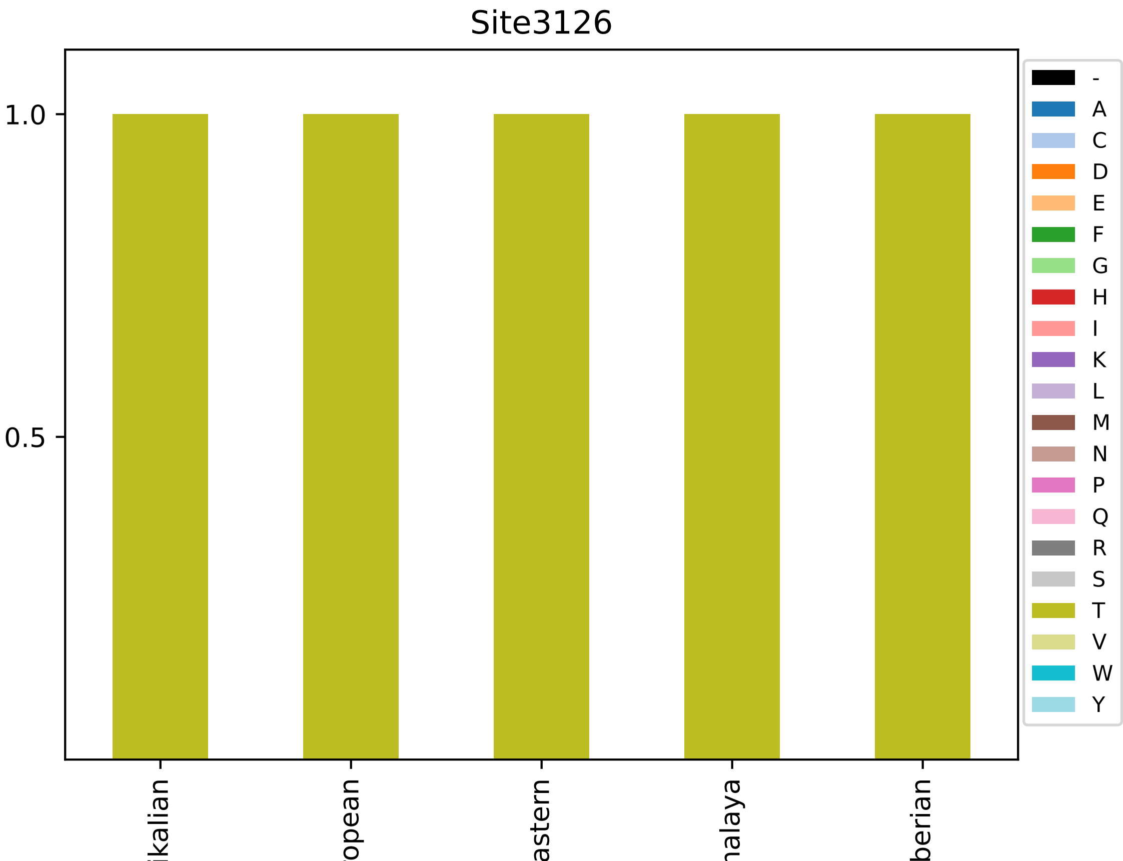
Site3126
1.0
0.5
-
A
C
D
E
F
G
H
I
K
L
M
N
P
Q
R
S
T
V
W
Y
ikalian
opean
astern
nalaya
berian

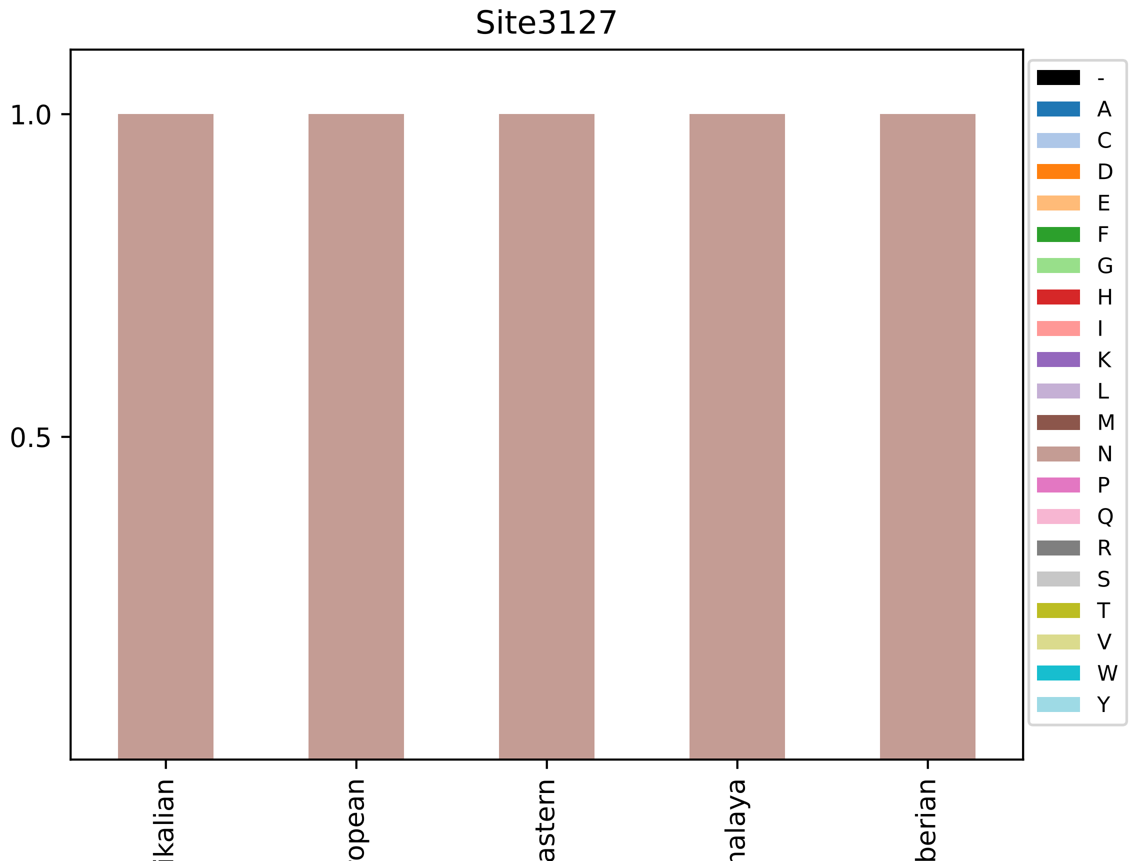
Site3127
1.0
0.5
-
A
C
D
E
F
G
H
I
K
L
M
N
P
Q
R
S
T
V
W
Y

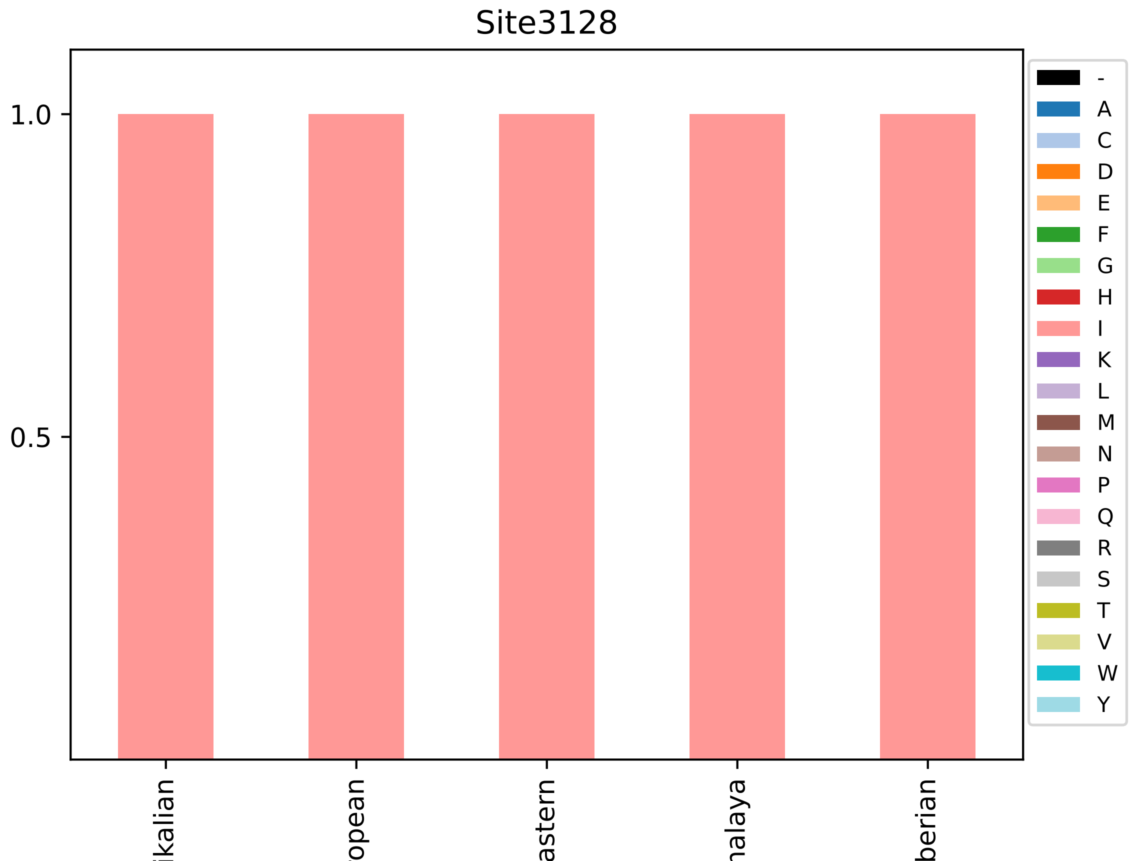

Site3128
1.0
0.5
ikalian
opean
astern
nalaya
berian
-
A
C
D
E
F
G
H
I
K
L
M
N
P
Q
R
S
T
V
W
Y

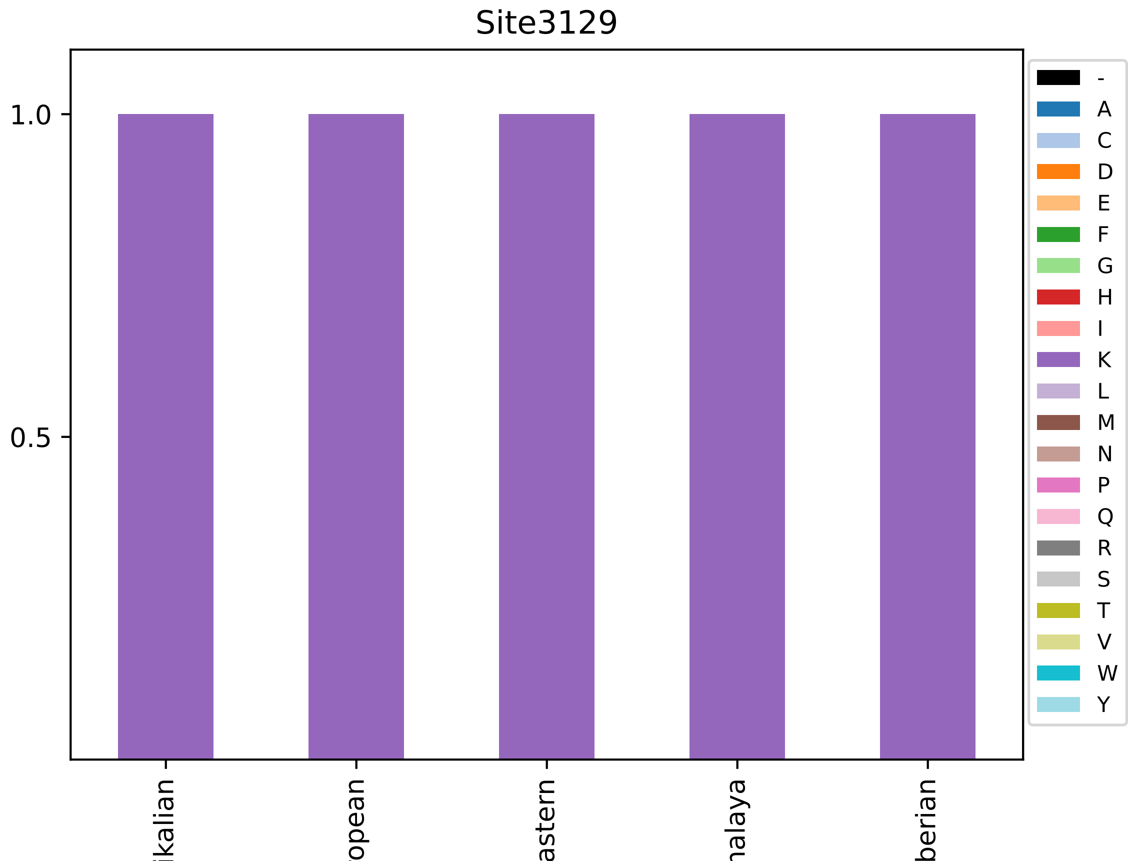
Site3129
1.0
0.5
ikalian
opean
astern
nalaya
berian
-
A
C
D
E
F
G
H
I
K
L
M
N
P
Q
R
S
T
V
W
Y

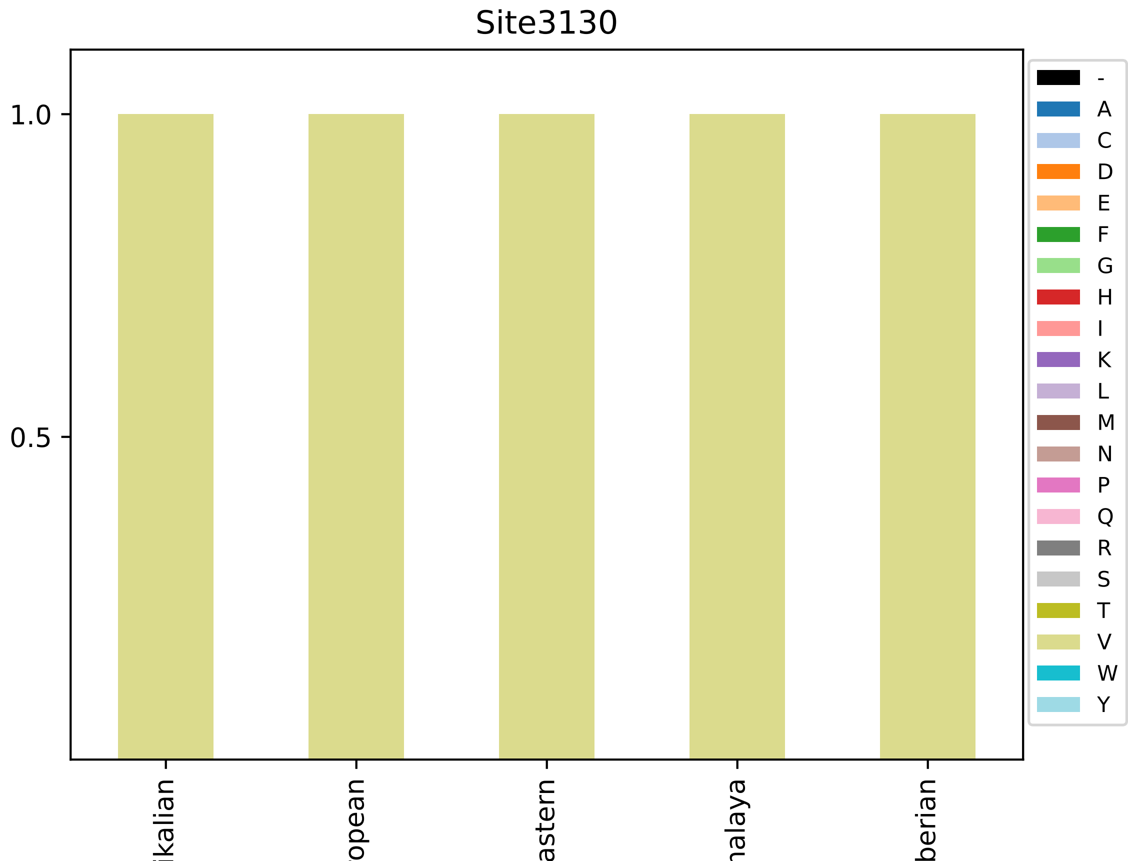

Site3130
1.0
0.5
-
A
C
D
E
F
G
H
I
K
L
M
N
P
Q
R
S
T
V
W
Y
ikalian
ropean
astern
nalaya
berian

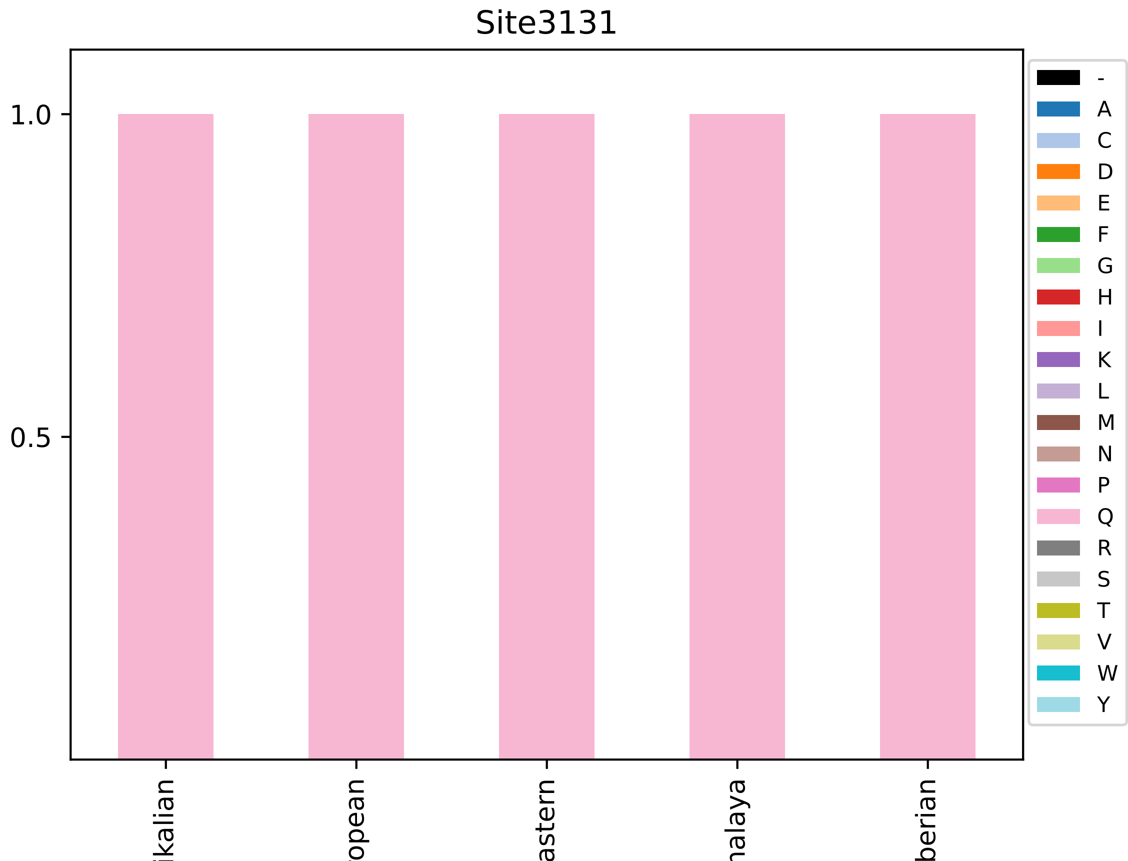
Site3131
1.0
0.5
-
A
C
D
E
F
G
H
I
K
L
M
N
P
Q
R
S
T
V
W
Y
ikalian
opean
astern
nalaya
berian

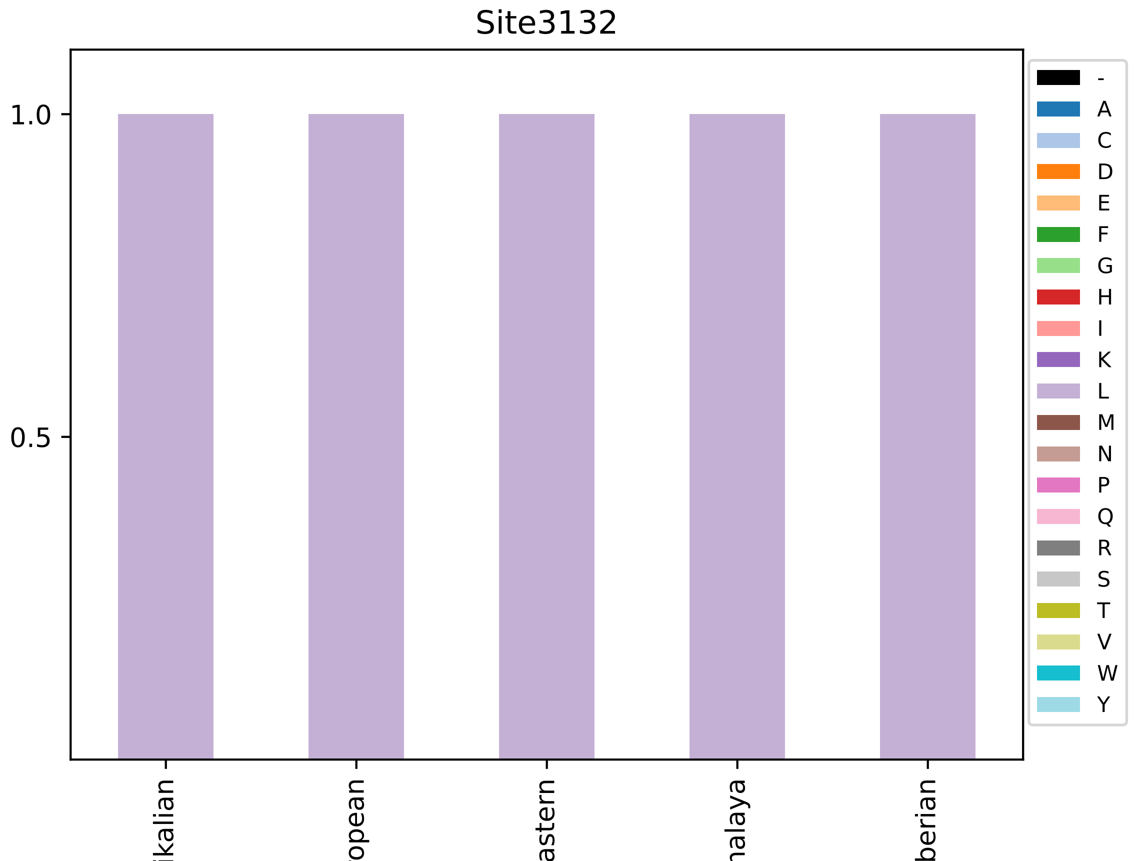
Site3132
1.0
0.5
-
A
C
D
E
F
G
H
I
K
L
M
N
P
Q
R
S
T
V
W
Y
ikalian
opean
astern
nalaya
berian

Site3133

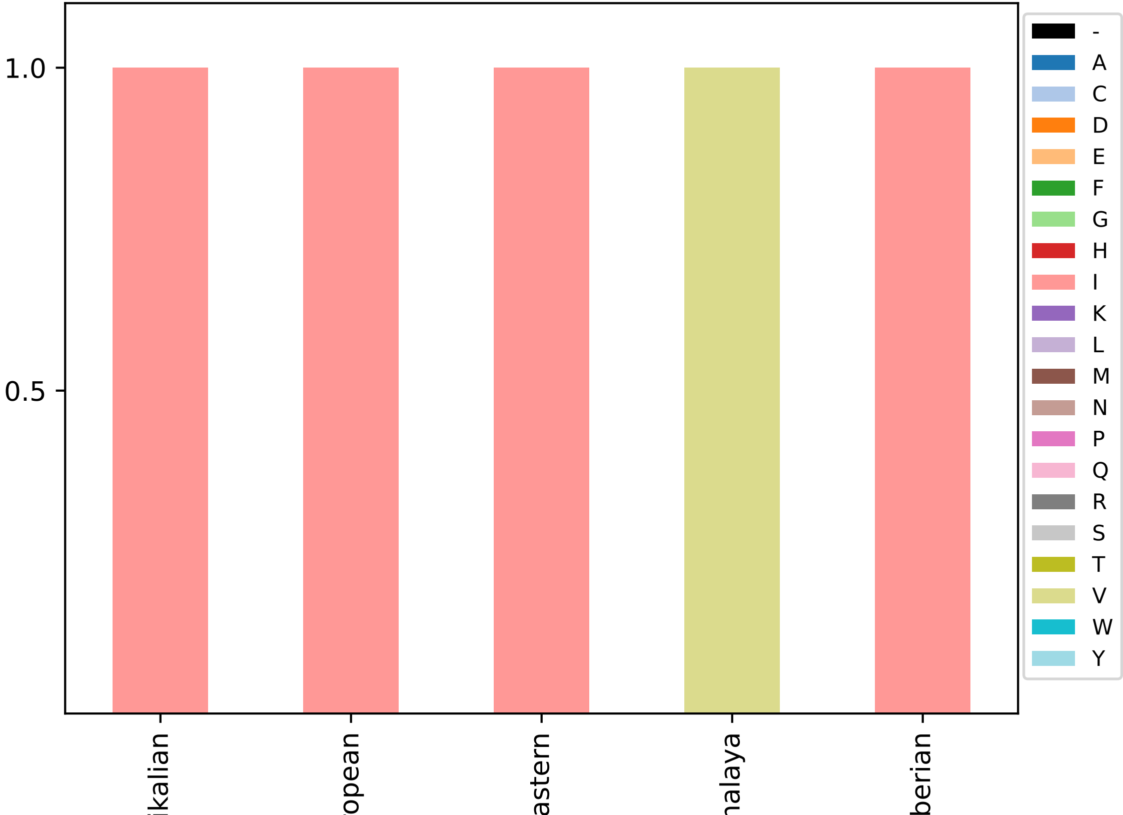

1.0
0.5
-
A
C
D
E
F
G
H
I
K
L
M
N
P
Q
R
S
T
V
W
Y
ikalian
opean
astern
nalaya
berian

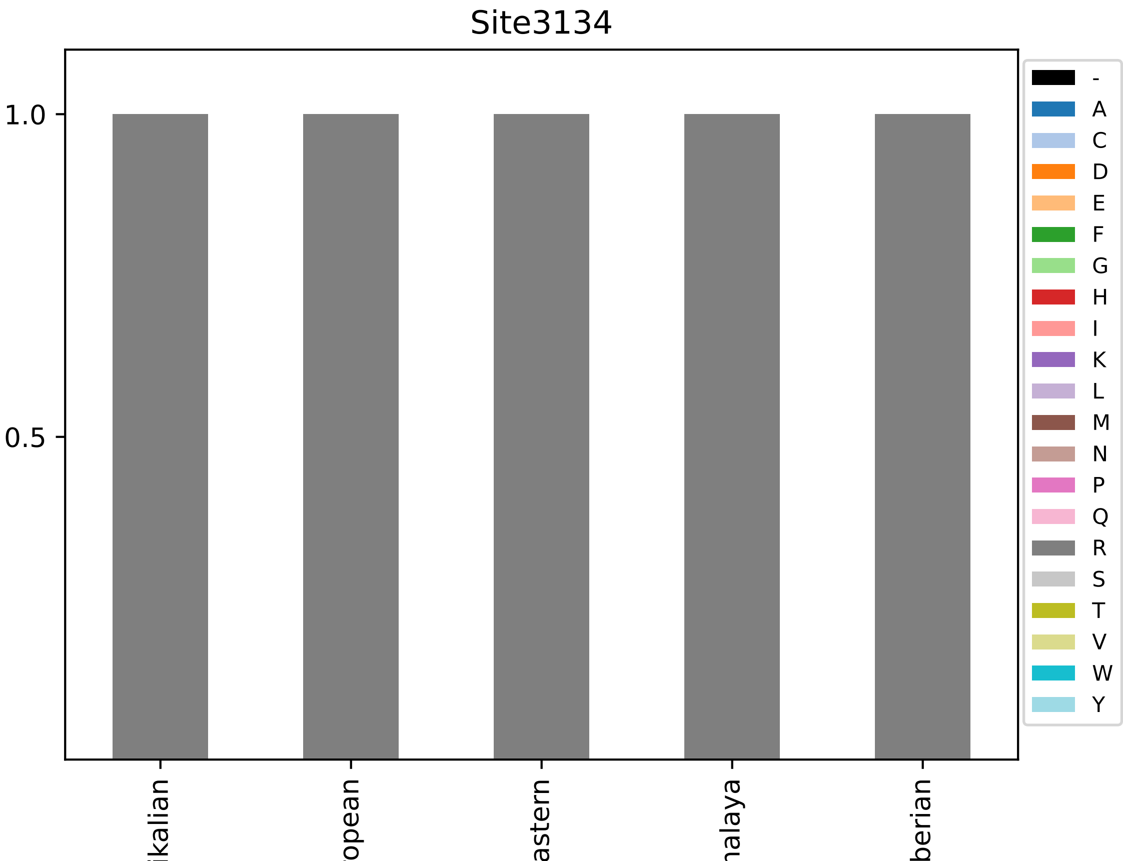

Site3134
1.0
0.5
ikalian
opean
astern
nalaya
berian
-
A
C
D
E
F
G
H
I
K
L
M
N
P
Q
R
S
T
V
W
Y

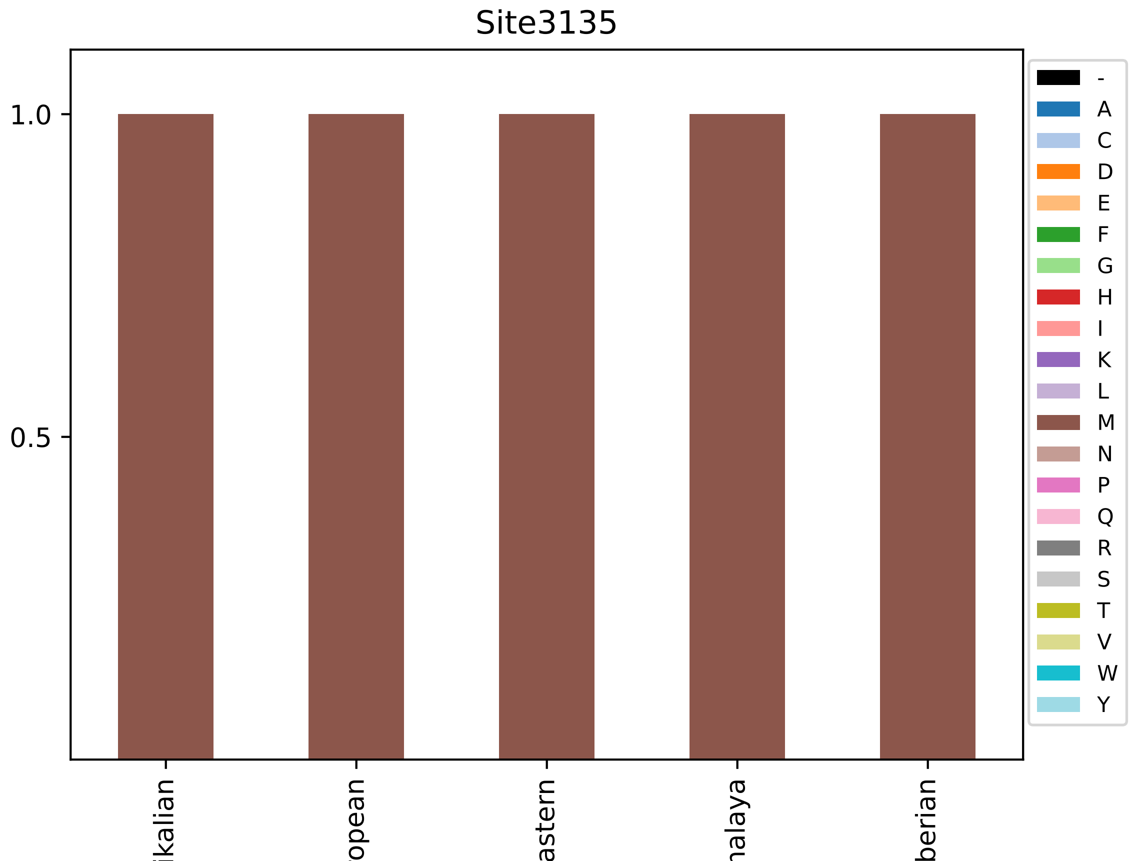
Site3135
1.0
0.5
-
A
C
D
E
F
G
H
I
K
L
M
N
P
Q
R
S
T
V
W
Y
ikalian
opean
astern
nalaya
berian

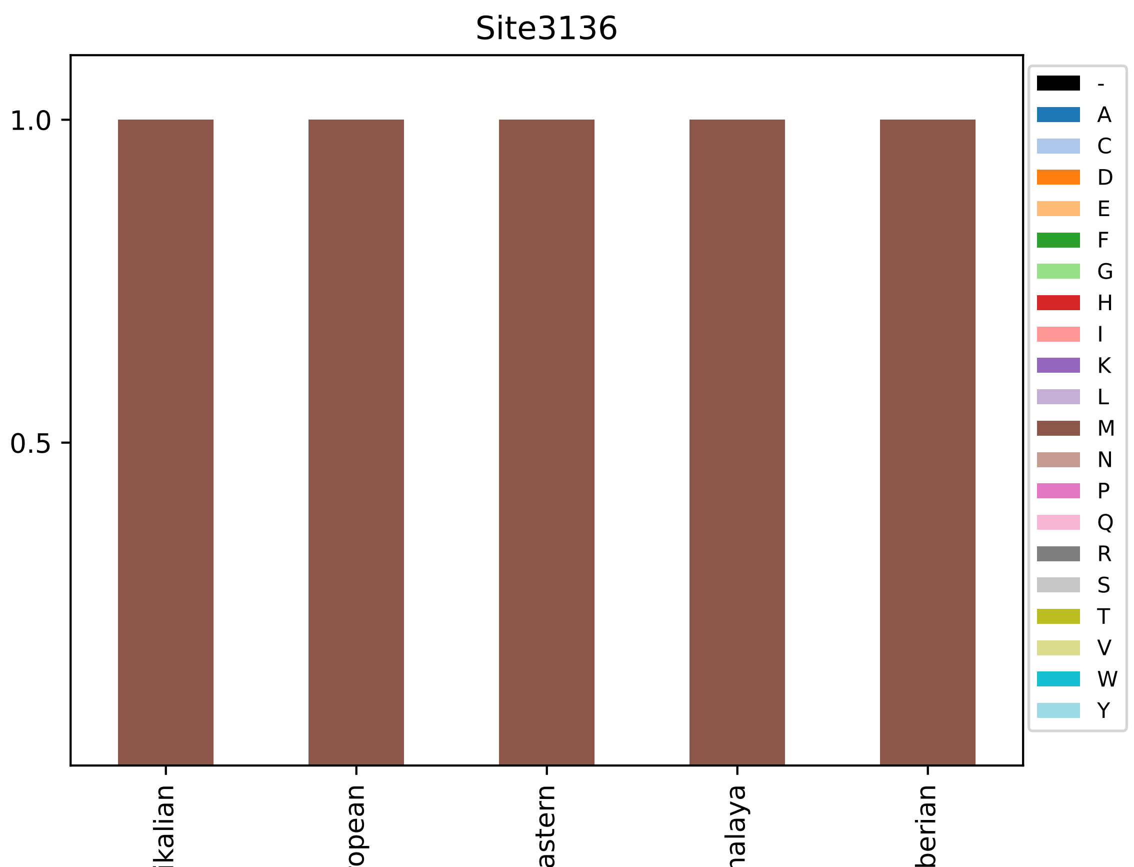
Site3136
1.0
0.5
-
A
C
D
E
F
G
H
I
K
L
M
N
P
Q
R
S
T
V
W
Y
ikalian
opean
astern
nalaya
berian

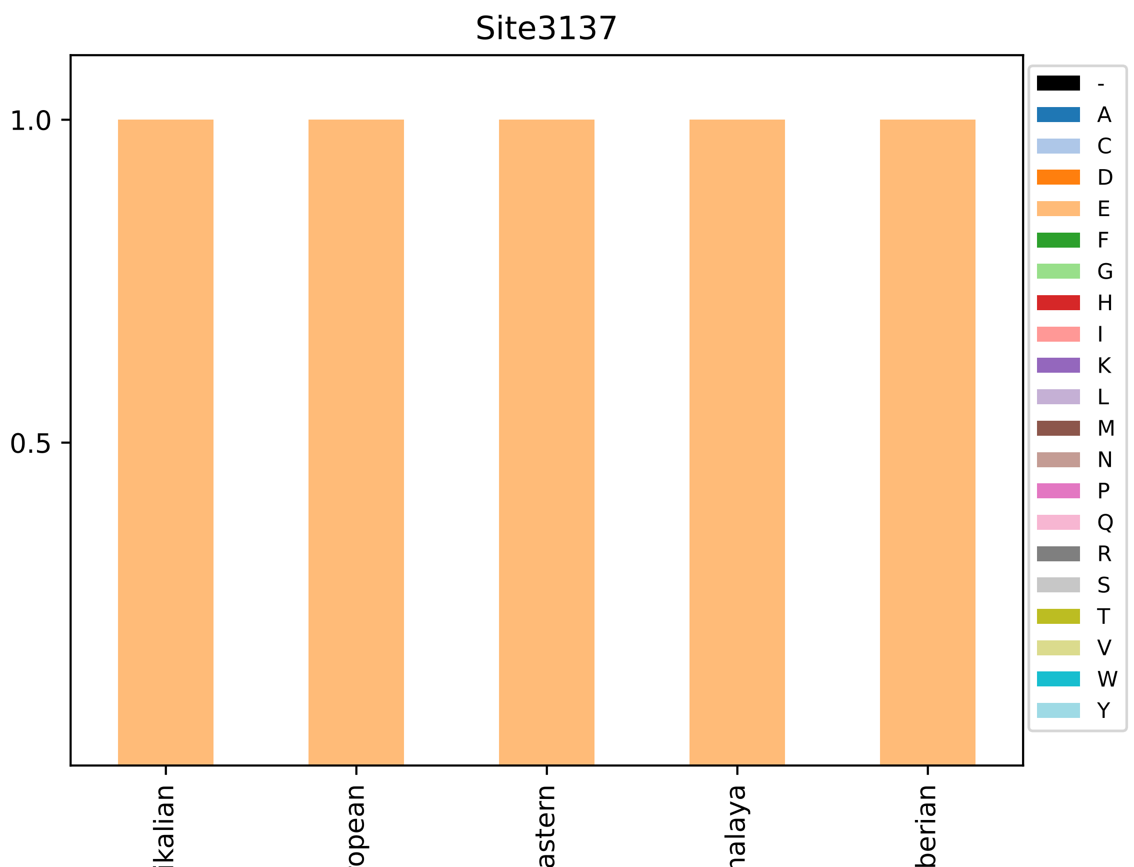
Site3137
1.0
0.5
ikalian
opean
astern
nalaya
berian
-
A
C
D
E
F
G
H
I
K
L
M
N
P
Q
R
S
T
V
W
Y

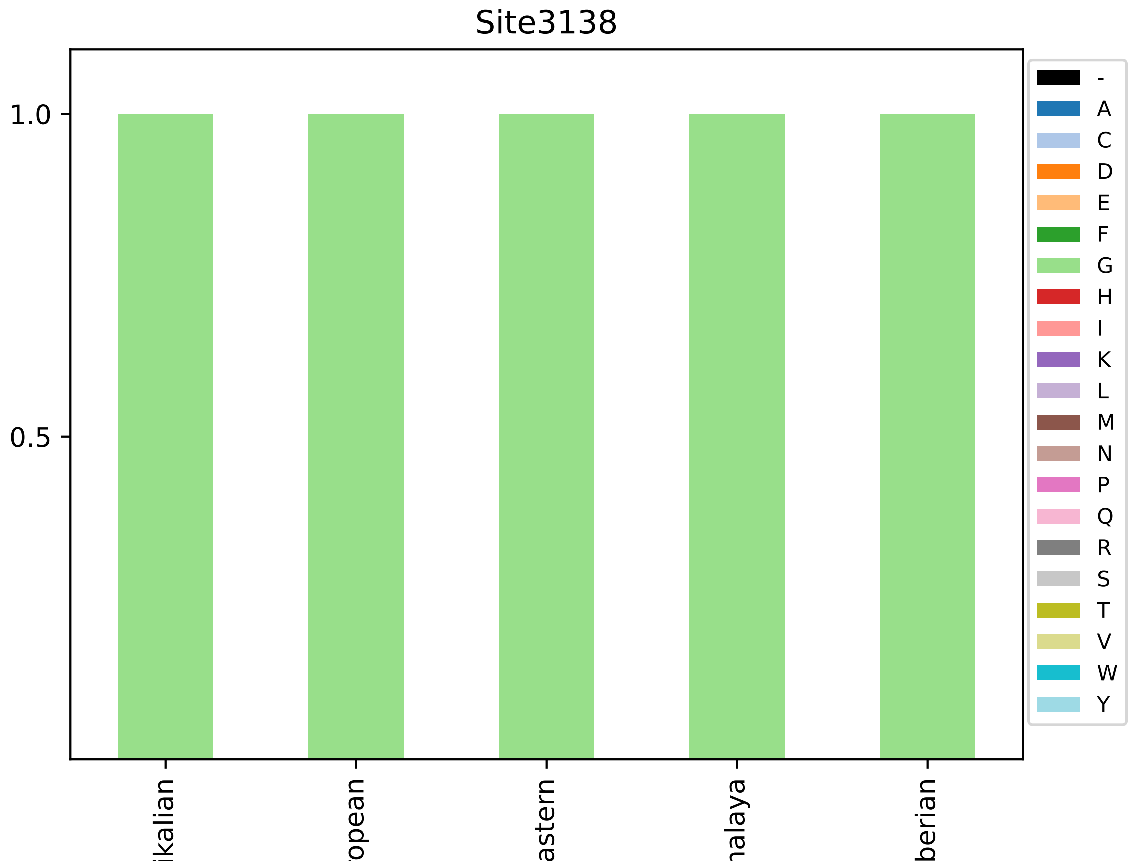

Site3138
1.0
0.5
-
A
C
D
E
F
G
H
I
K
L
M
N
P
Q
R
S
T
V
W
Y
ikalian
opean
astern
nalaya
berian

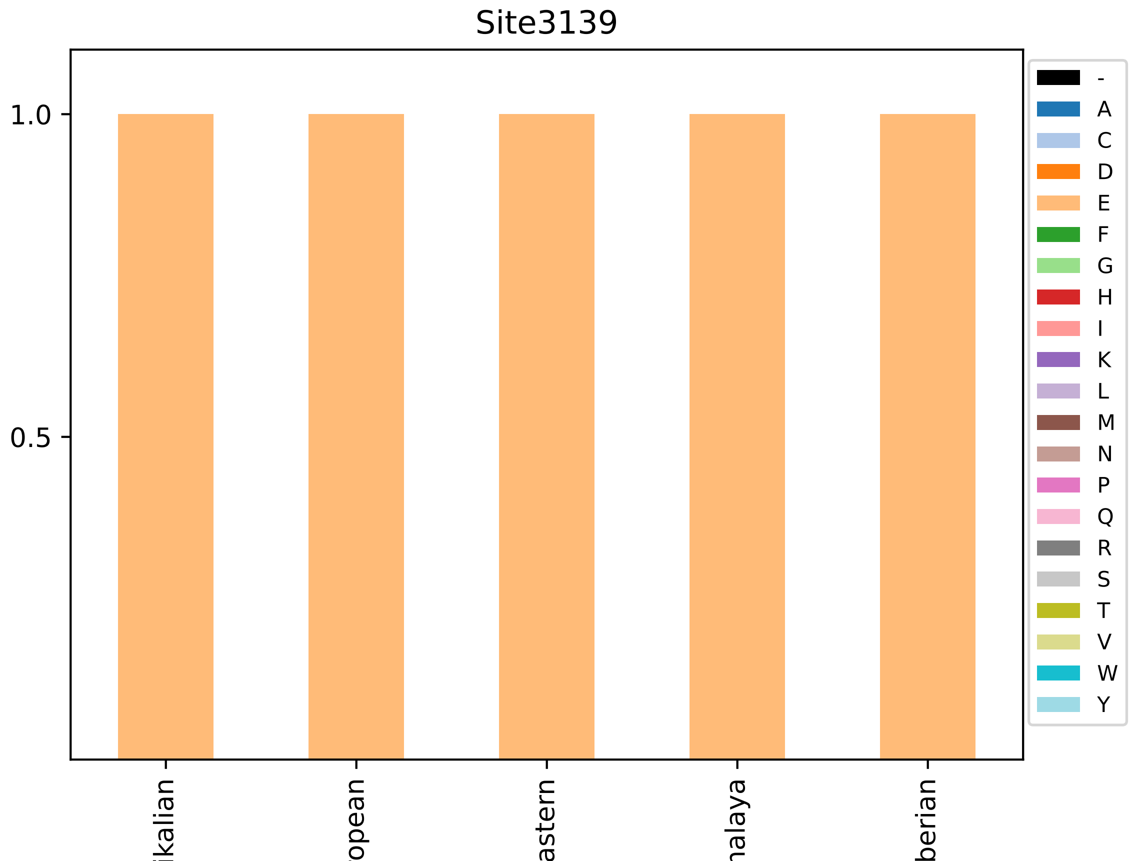
Site3139
1.0
0.5
-
A
C
D
E
F
G
H
I
K
L
M
N
P
Q
R
S
T
V
W
Y
ikalian
opean
astern
nalaya
berian

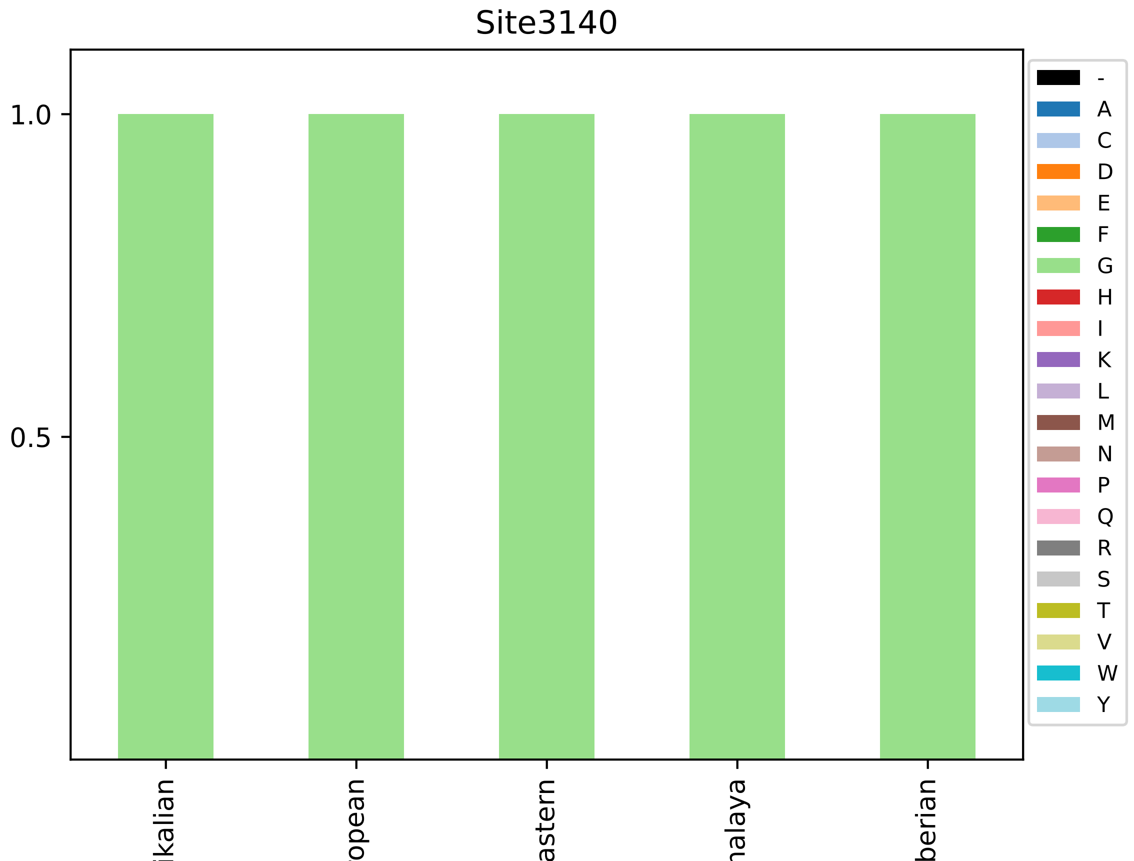

Site3140
1.0
0.5
-
A
C
D
E
F
G
H
I
K
L
M
N
P
Q
R
S
T
V
W
Y
ikalian
opean
astern
nalaya
berian

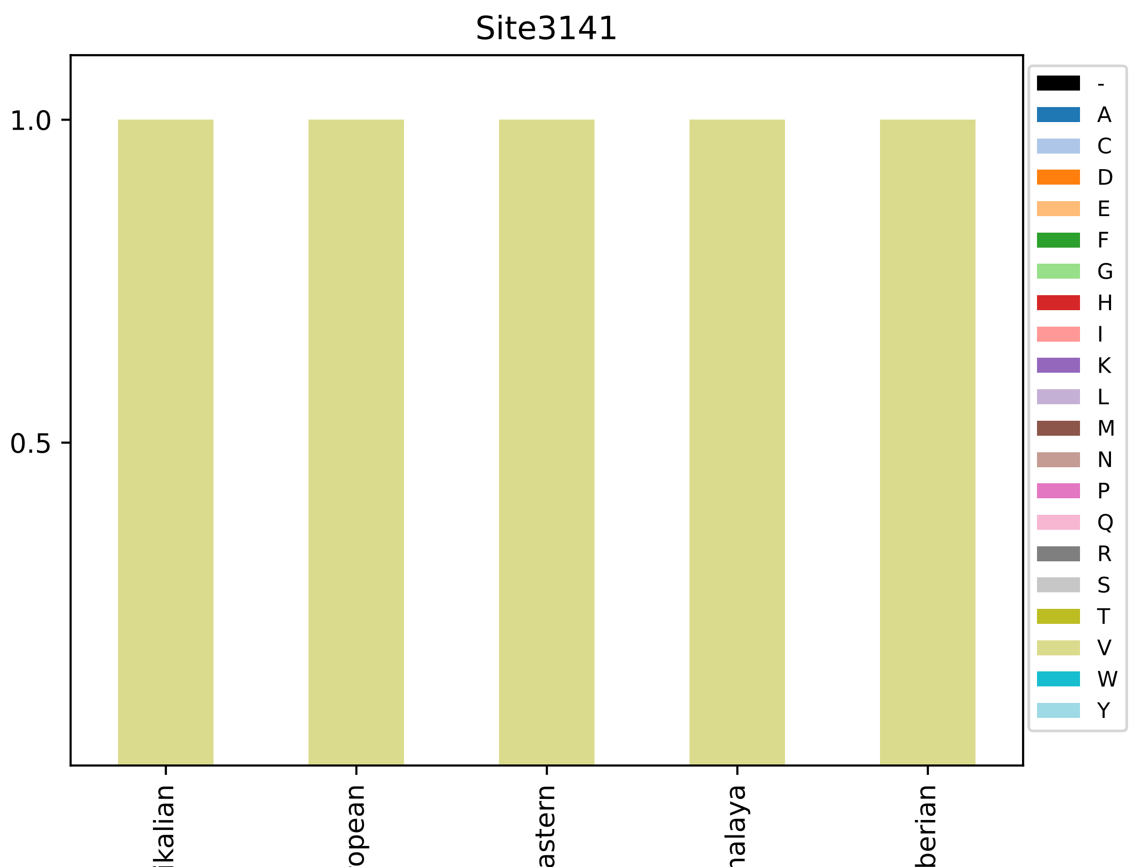

Site3141
1.0
0.5
ikalian
opean
astern
nalaya
berian
-
A
C
D
E
F
G
H
I
K
L
M
N
P
Q
R
S
T
V
W
Y

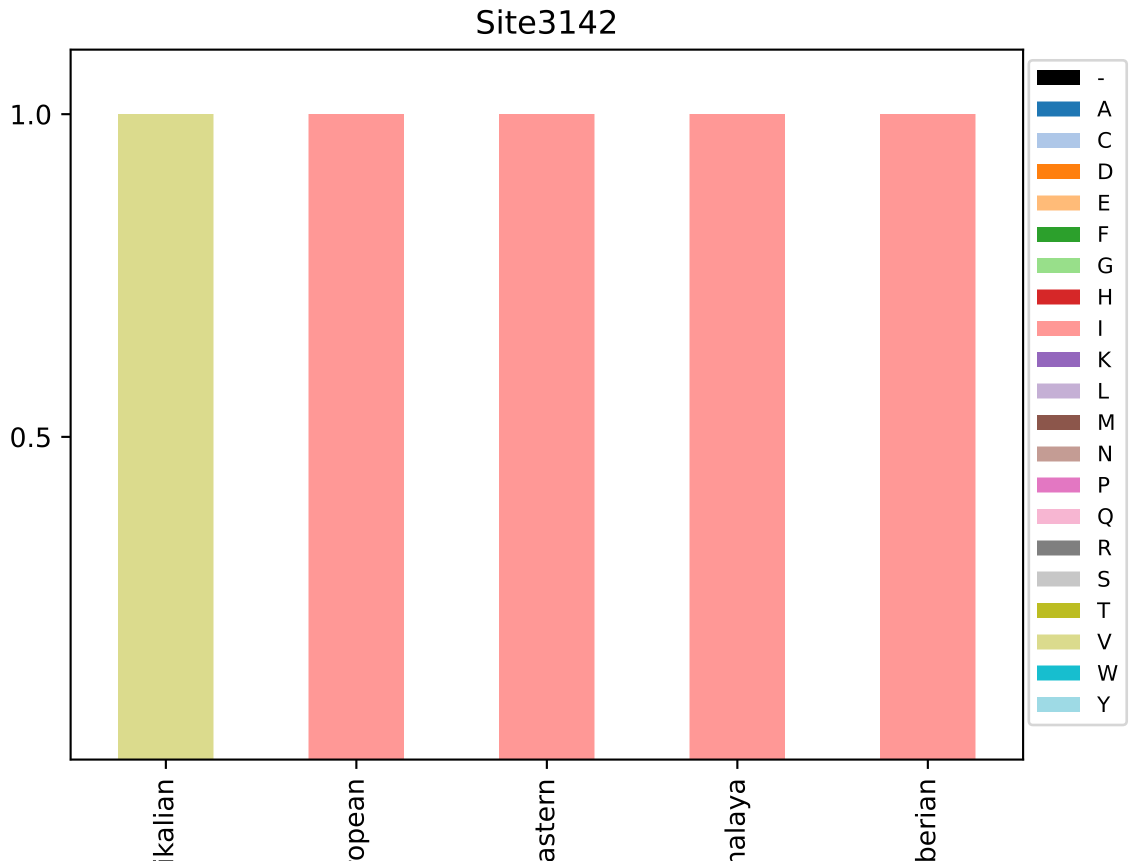
Site3142
1.0
0.5
-
A
C
D
E
F
G
H
I
K
L
M
N
P
Q
R
S
T
V
W
Y
ikalian
opean
astern
nalaya
berian

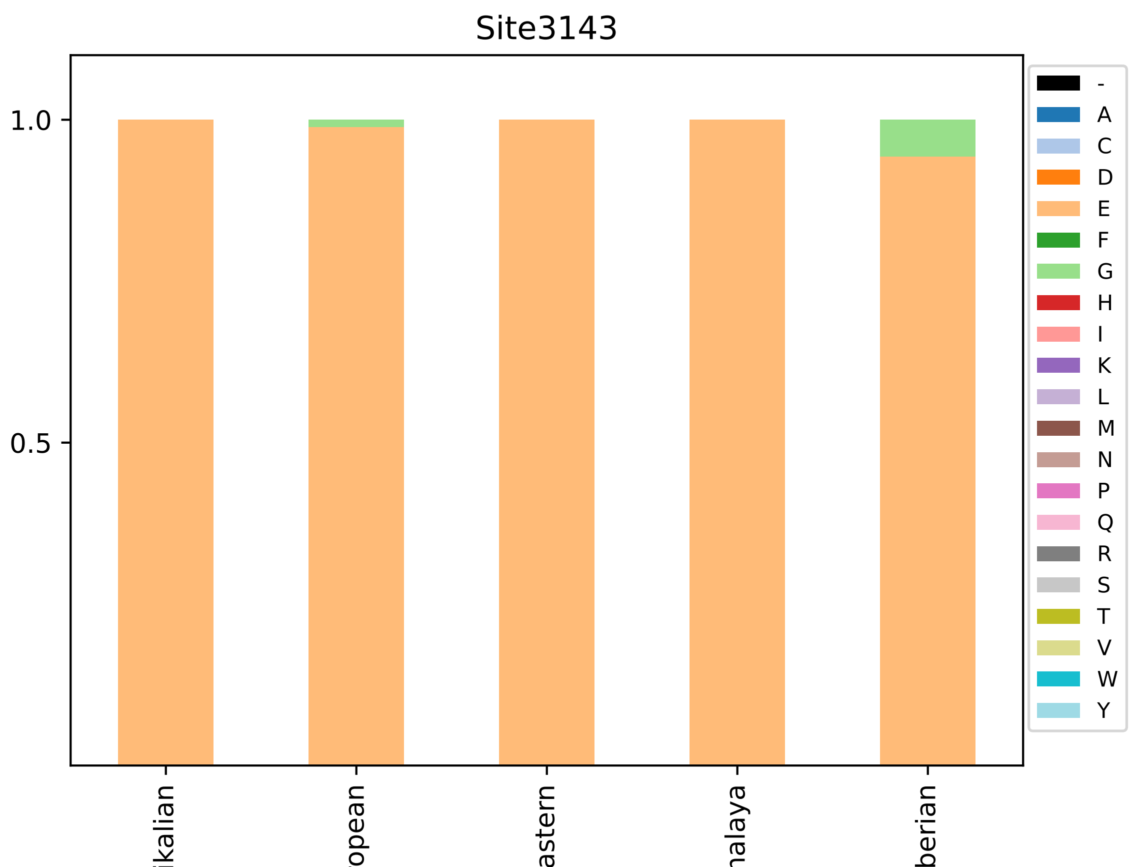
Site3143
1.0
0.5
-
A
C
D
E
F
G
H
I
K
L
M
N
P
Q
R
S
T
V
W
Y
ikalian
opean
astern
nalaya
berian

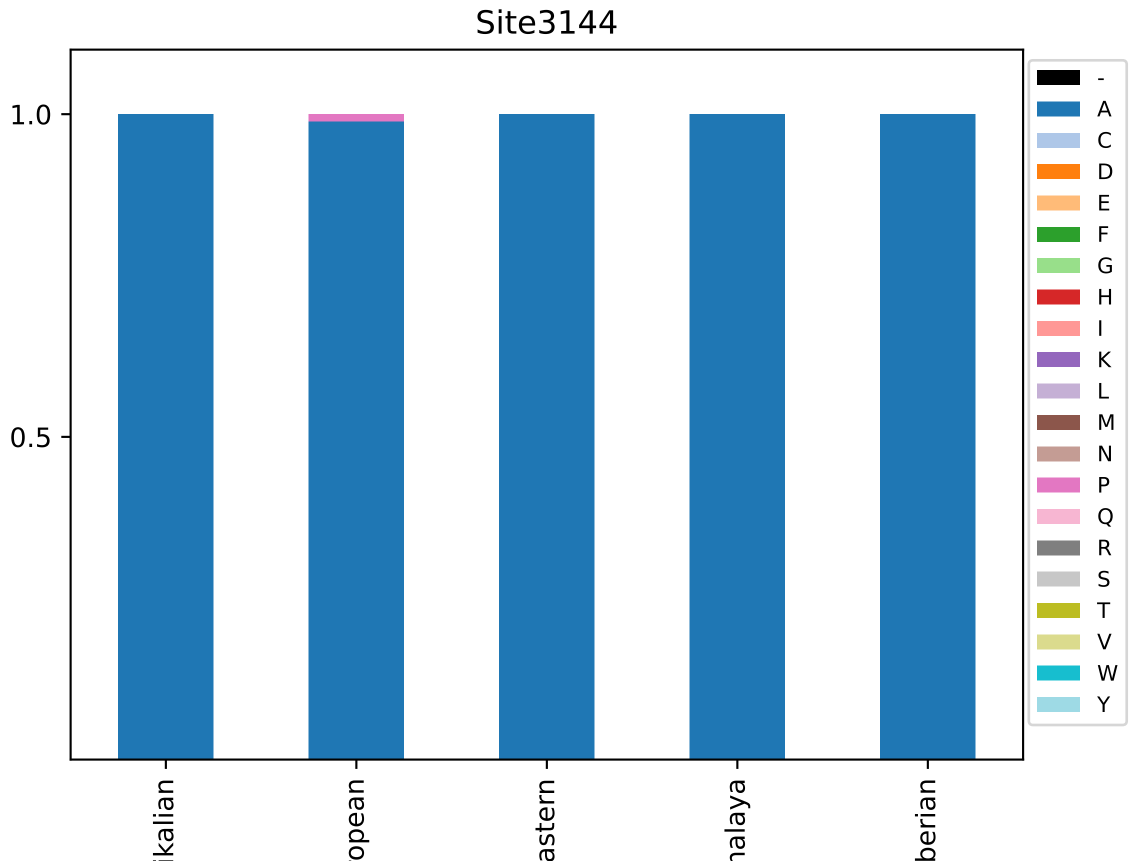

Site3144
1.0
0.5
-
A
C
D
E
F
G
H
I
K
L
M
N
P
Q
R
S
T
V
W
Y
ikalian
opean
astern
nalaya
berian

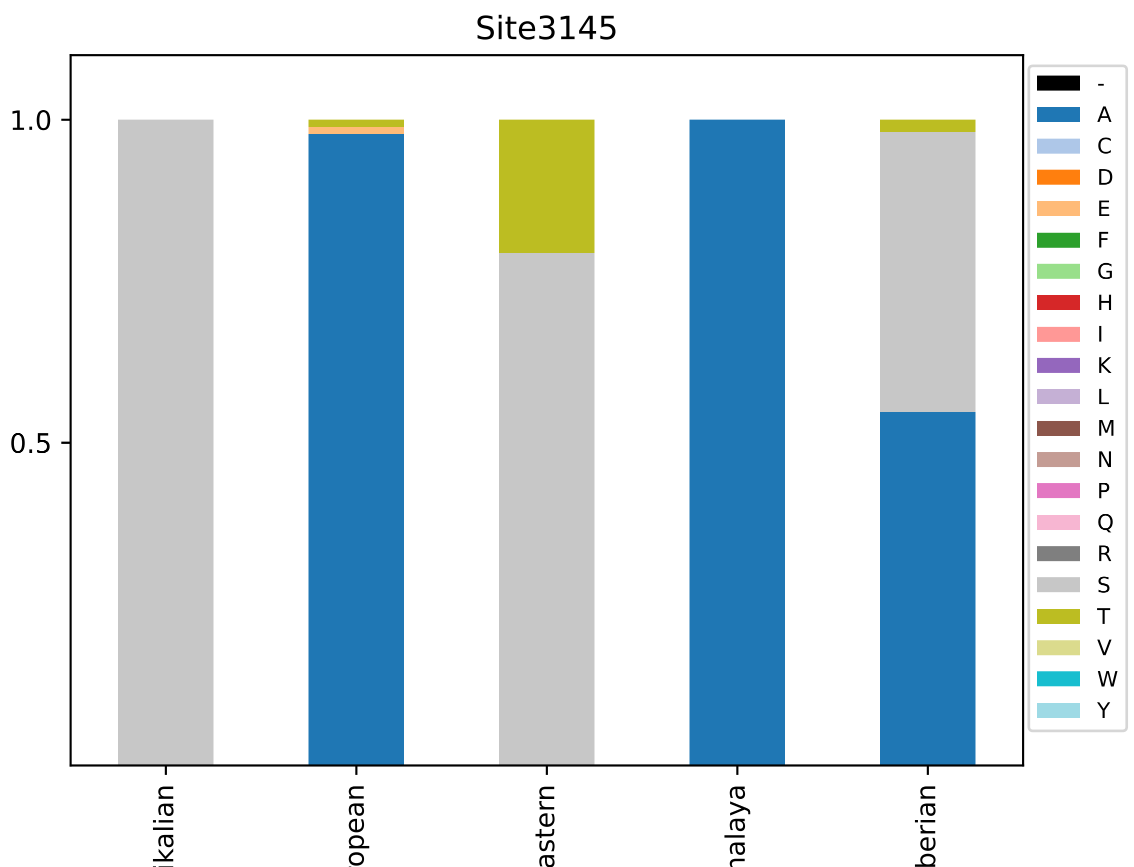

Site3145
1.0
0.5
ikalian
opean
astern
nalaya
berian
-
A
C
D
E
F
G
H
I
K
L
M
N
P
Q
R
S
T
V
W
Y

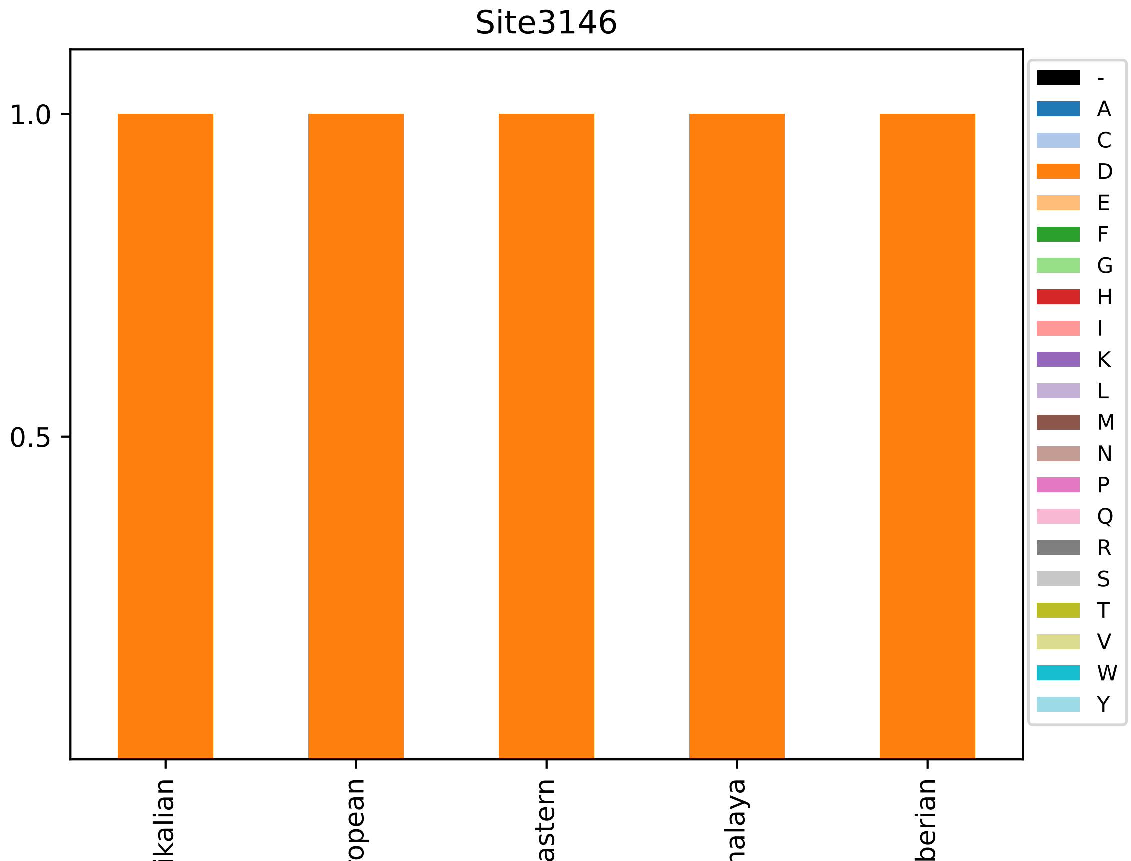
Site3146
1.0
0.5
-
A
C
D
E
F
G
H
I
K
L
M
N
P
Q
R
S
T
V
W
Y
ikalian
opean
astern
nalaya
berian

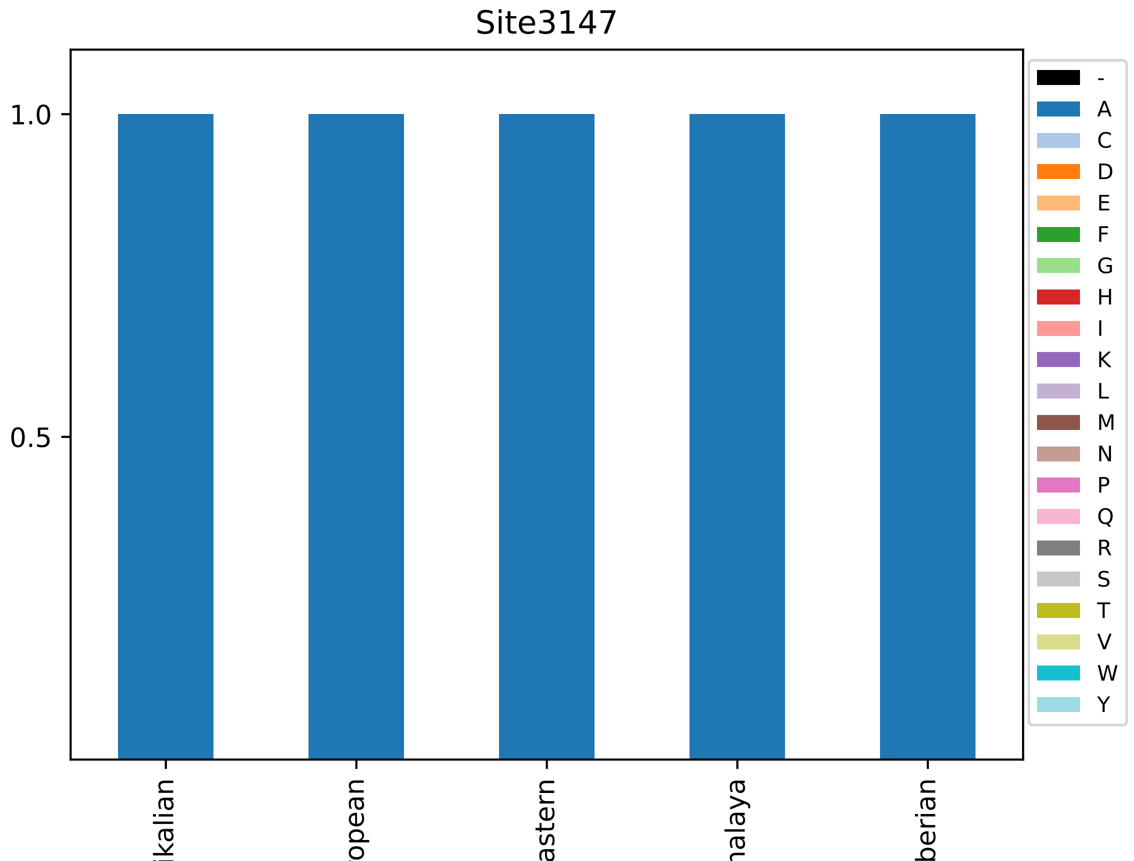

Site3147
1.0
0.5
-
A
C
D
E
F
G
H
I
K
L
M
N
P
Q
R
S
T
V
W
Y
ikalian
opean
astern
nalaya
berian

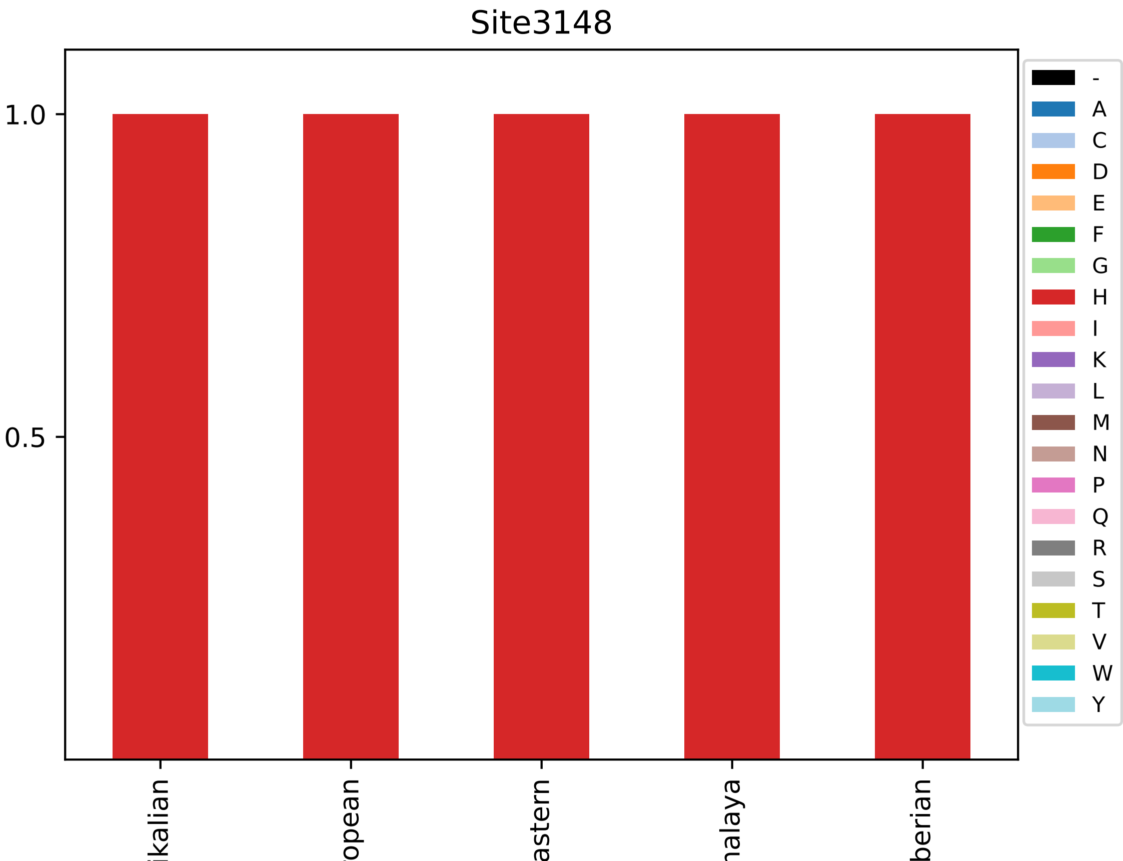

Site3148
1.0
0.5
-
A
C
D
E
F
G
H
I
K
L
M
N
P
Q
R
S
T
V
W
Y
ikalian
opean
astern
nalaya
berian

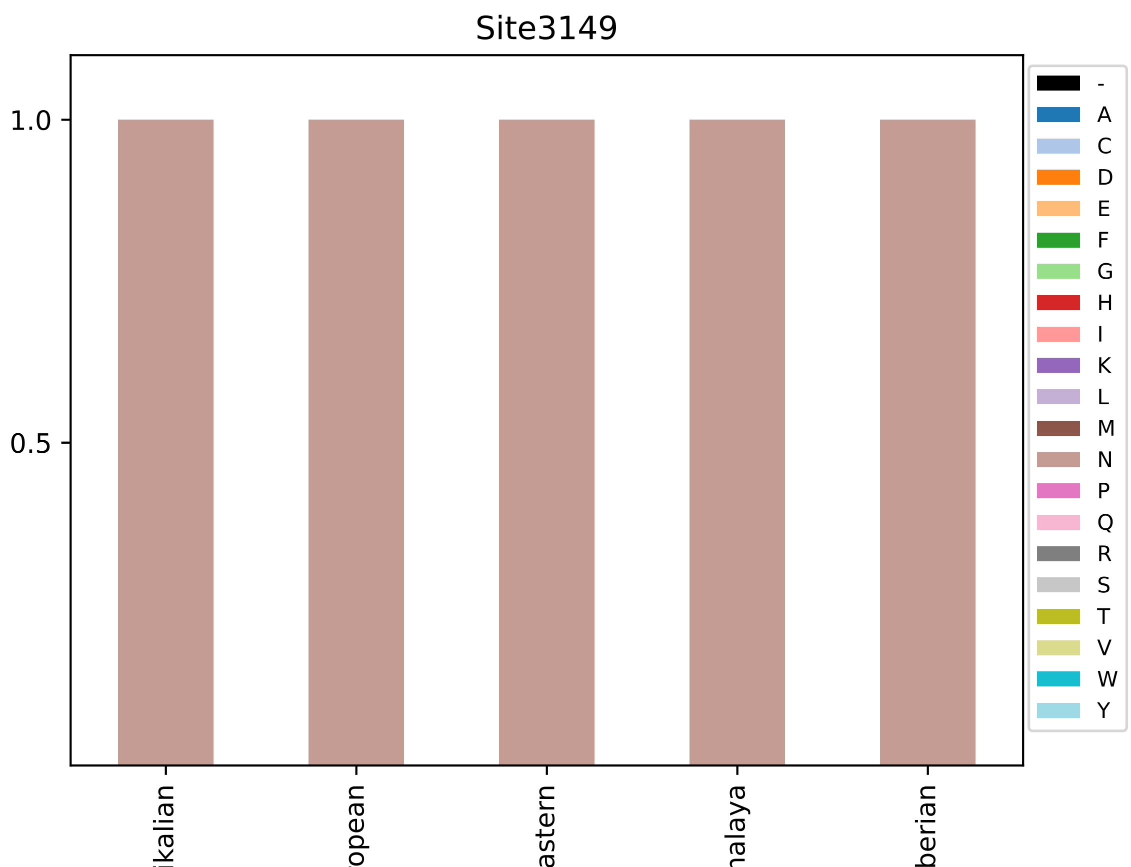
Site3149
1.0
0.5
-
A
C
D
E
F
G
H
I
K
L
M
N
P
Q
R
S
T
V
W
Y
ikalian
opean
astern
nalaya
berian

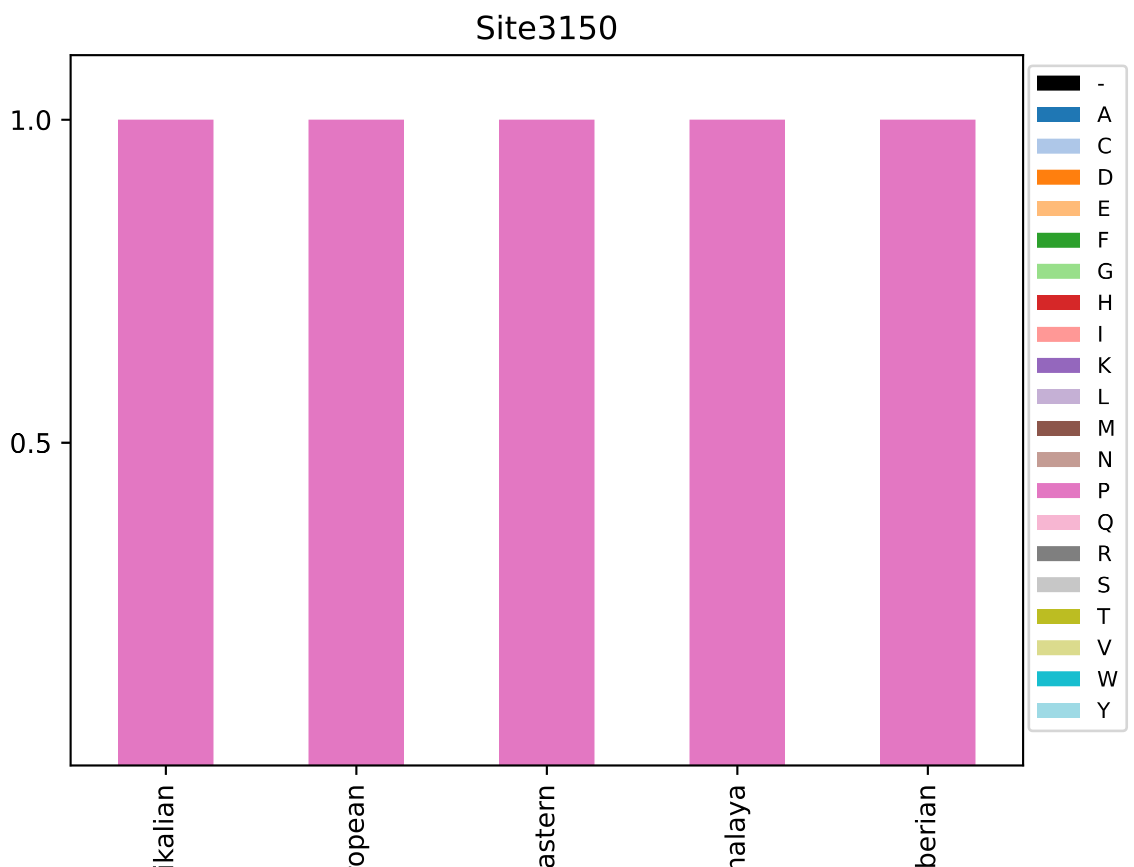
Site3150
1.0
0.5
-
A
C
D
E
F
G
H
I
K
L
M
N
P
Q
R
S
T
V
W
Y
ikalian
opean
astern
nalaya
berian

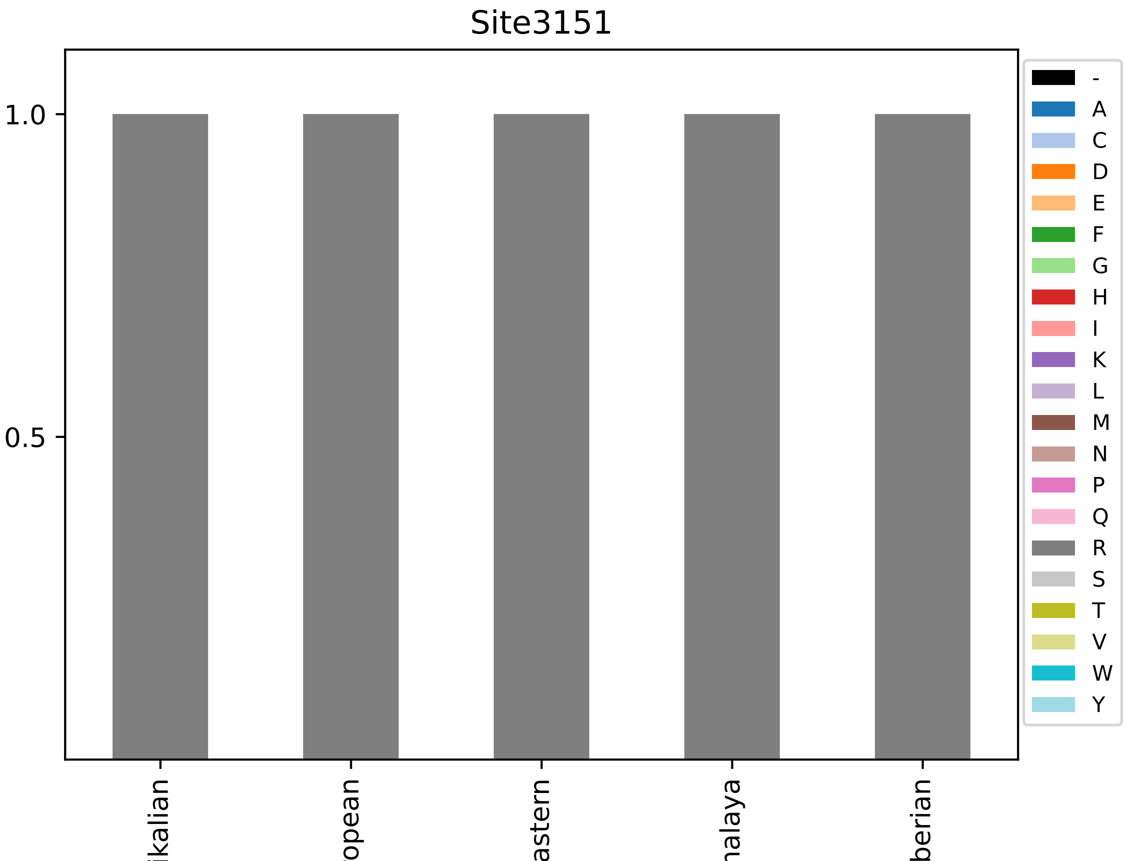
Site3151
1.0
0.5
-
A
C
D
E
F
G
H
I
K
L
M
N
P
Q
R
S
T
V
W
Y

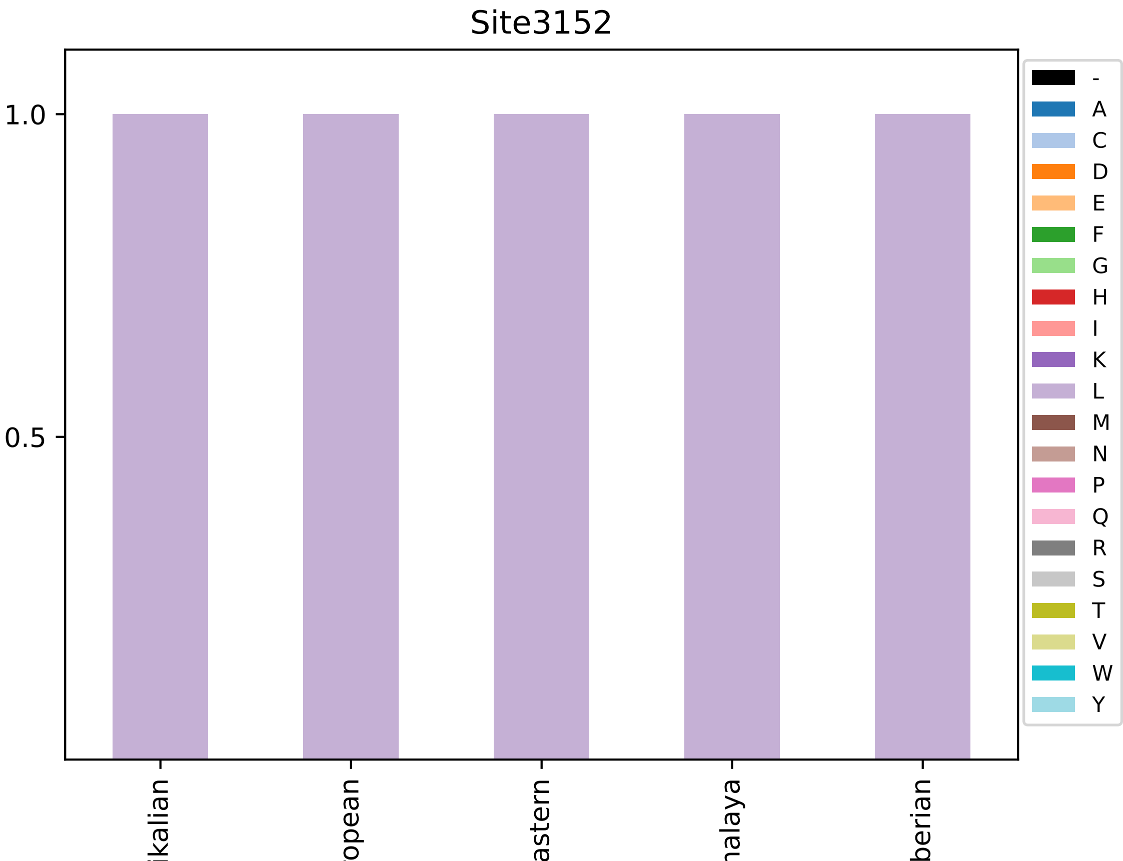

Site3152
1.0
0.5
-
A
C
D
E
F
G
H
I
K
L
M
N
P
Q
R
S
T
V
W
Y
ikalian
opean
astern
nalaya
berian

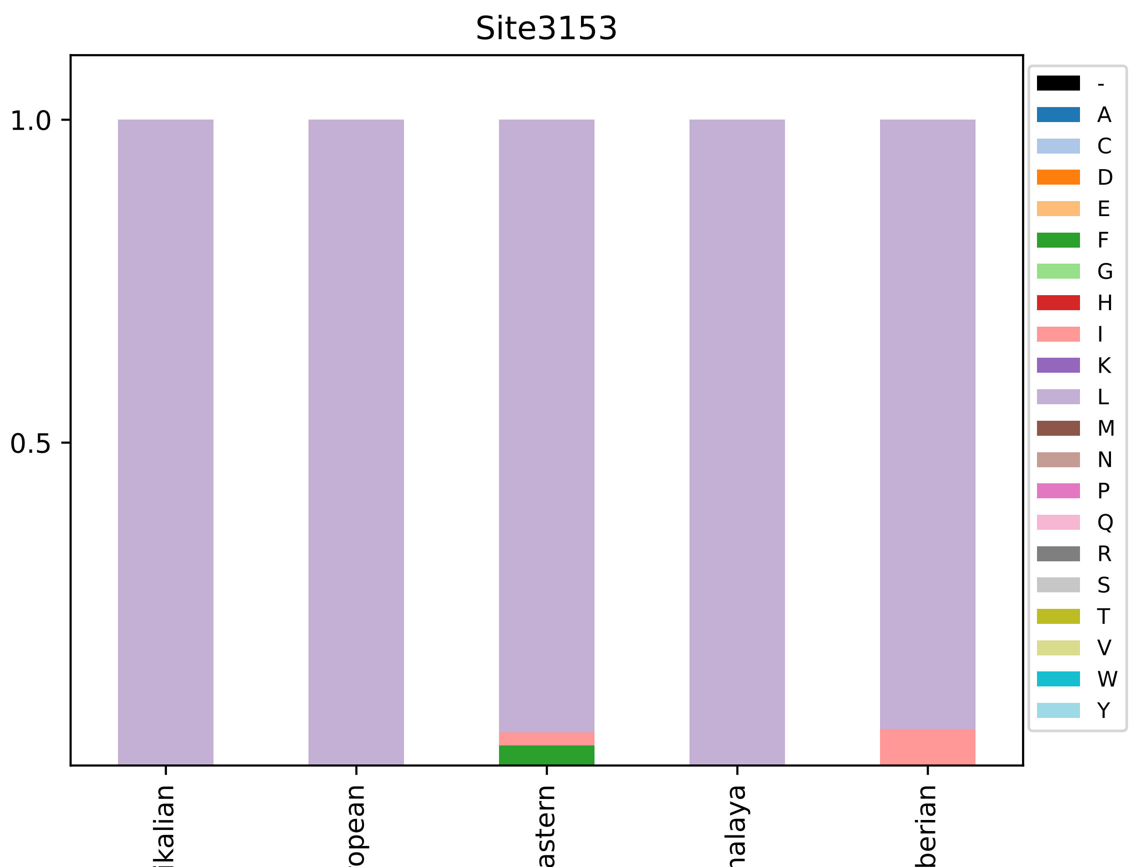
Site3153
1.0
0.5
-
A
C
D
E
F
G
H
I
K
L
M
N
P
Q
R
S
T
V
W
Y
ikalian
opean
astern
nalaya
berian

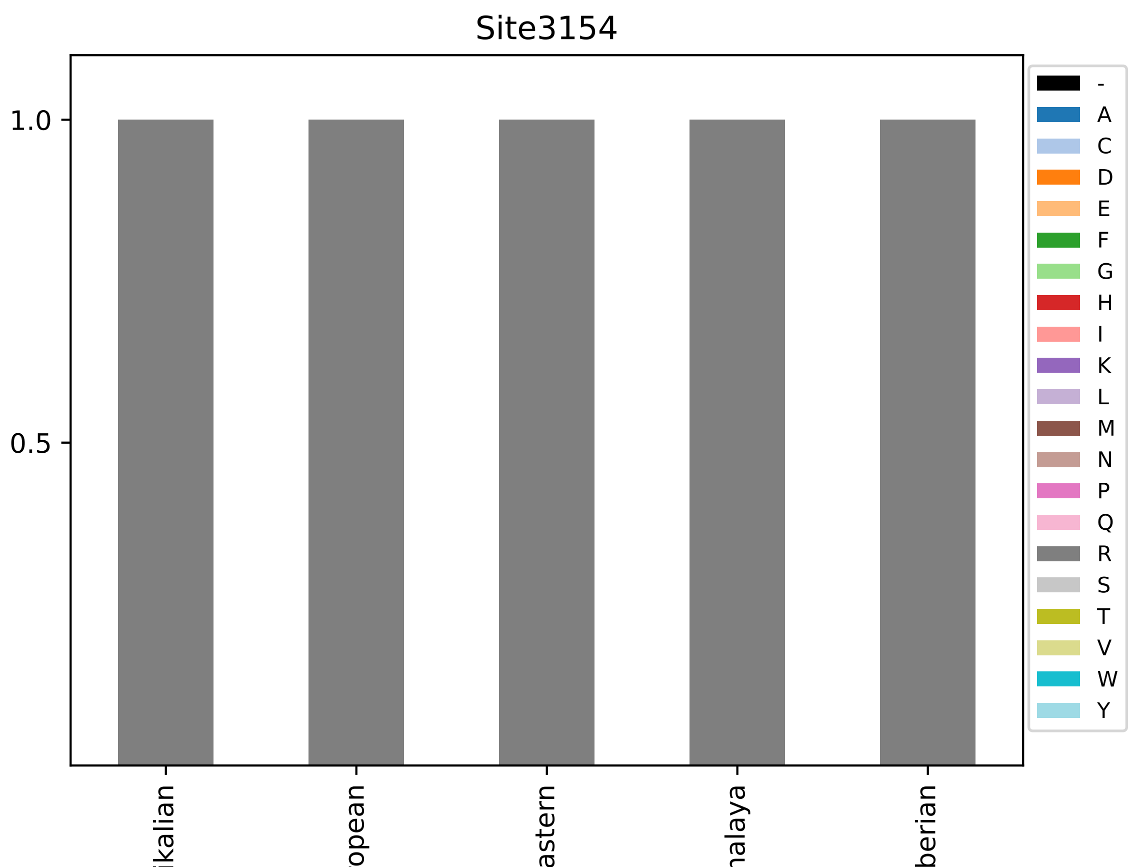
Site3154
1.0
0.5
-
A
C
D
E
F
G
H
I
K
L
M
N
P
Q
R
S
T
V
W
Y
ikalian
opean
astern
nalaya
berian

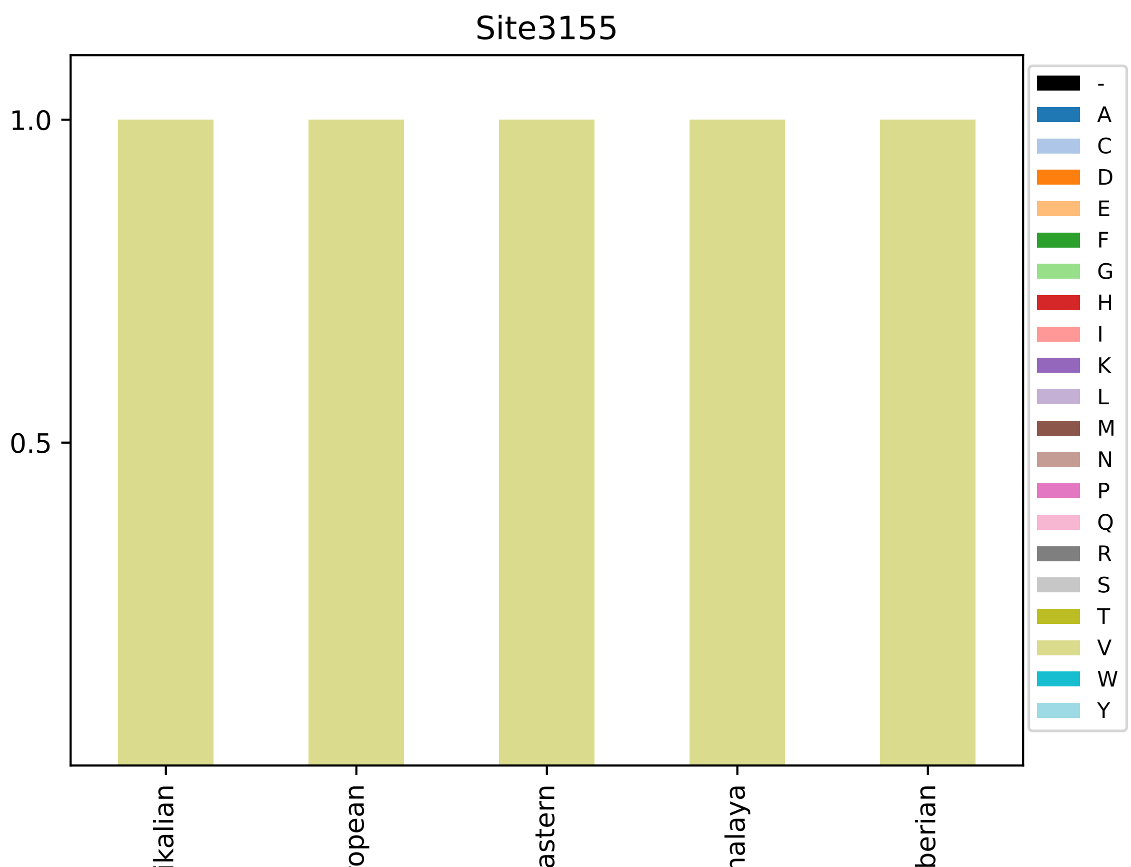
Site3155
1.0
0.5
-
A
C
D
E
F
G
H
I
K
L
M
N
P
Q
R
S
T
V
W
Y
ikalian
opean
astern
nalaya
berian

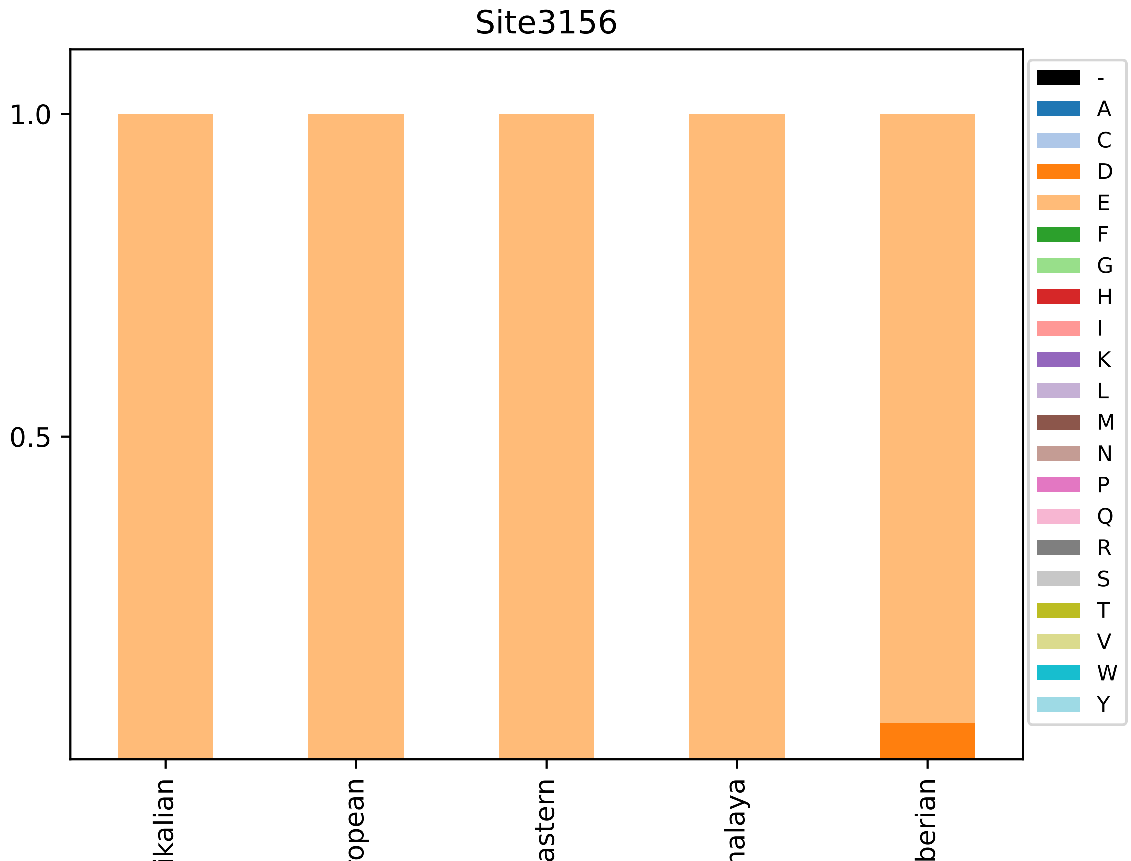
Site3156
1.0
0.5
-
A
C
D
E
F
G
H
I
K
L
M
N
P
Q
R
S
T
V
W
Y
ikalian
opean
astern
nalaya
berian

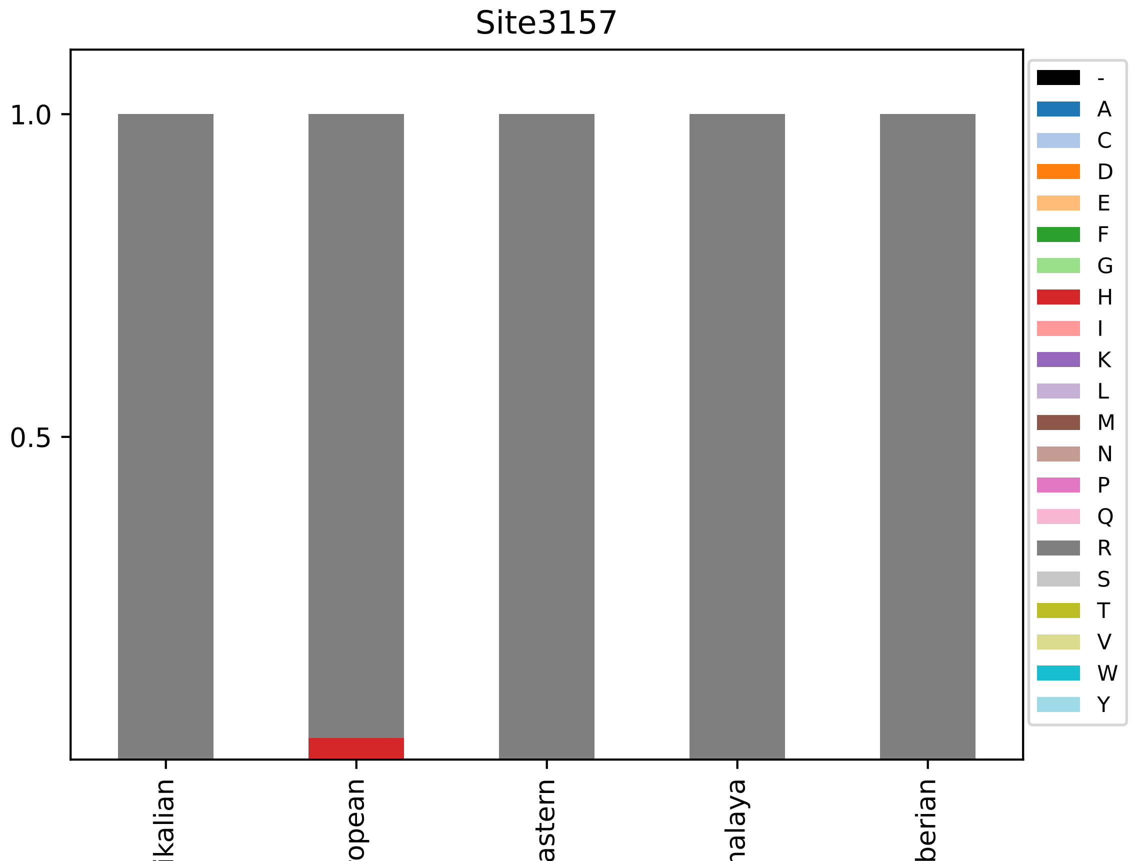
Site3157
1.0
0.5
-
A
C
D
E
F
G
H
I
K
L
M
N
P
Q
R
S
T
V
W
Y
ikalian
opean
astern
nalaya
berian

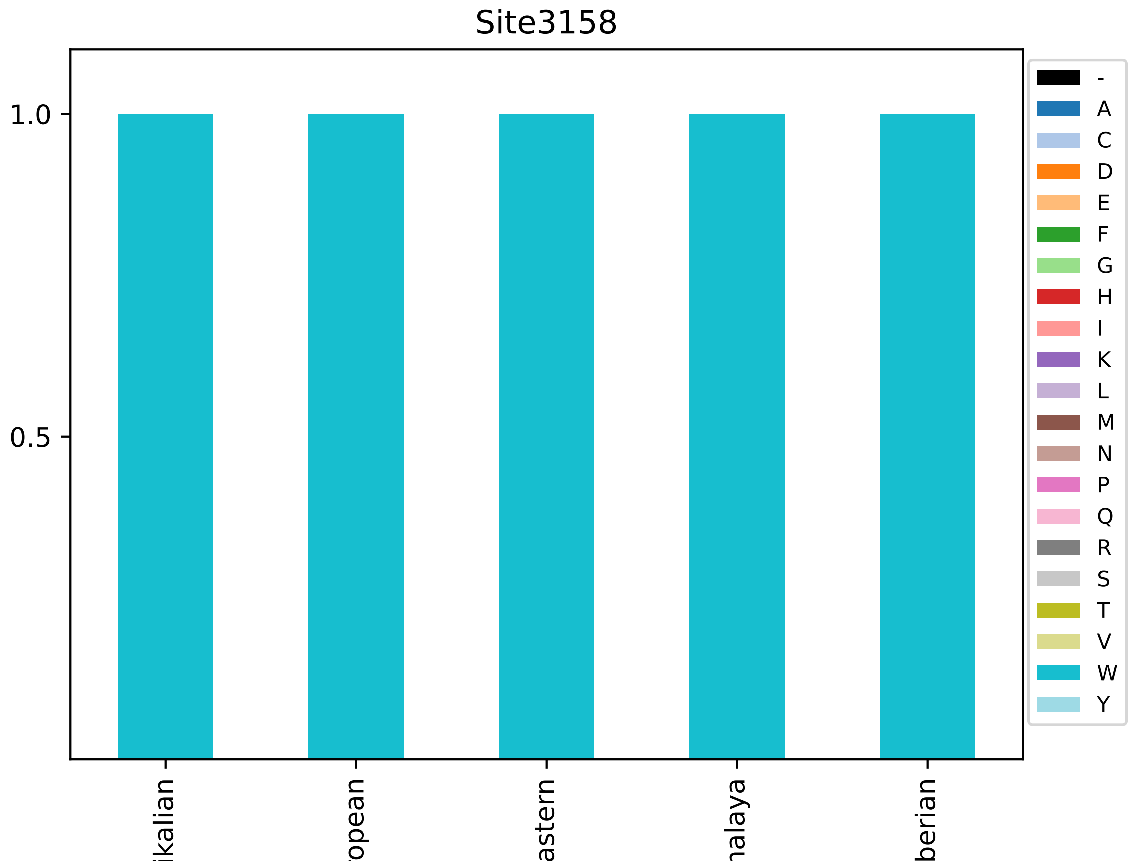

Site3158
1.0
0.5
ikalian
opean
astern
nalaya
berian
-
A
C
D
E
F
G
H
I
K
L
M
N
P
Q
R
S
T
V
W
Y

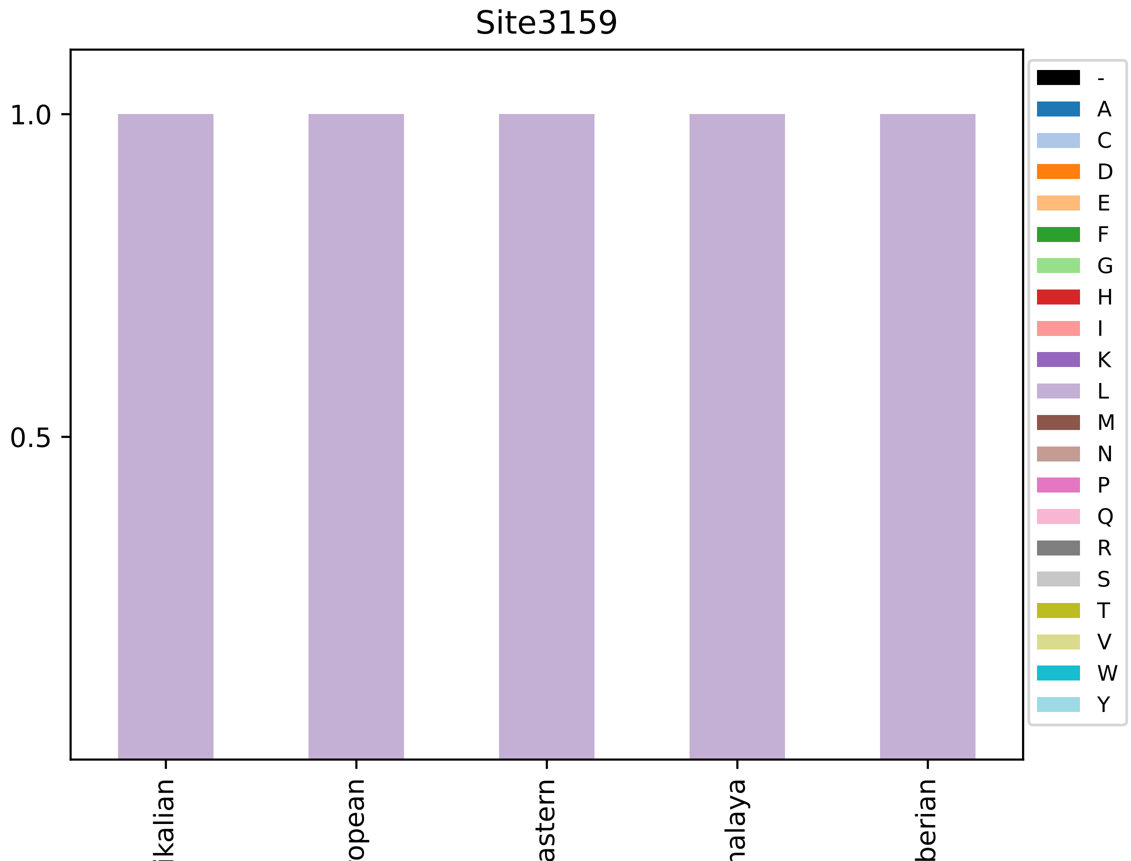

Site3159
1.0
0.5
-
A
C
D
E
F
G
H
I
K
L
M
N
P
Q
R
S
T
V
W
Y
ikalian
opean
astern
nalaya
berian

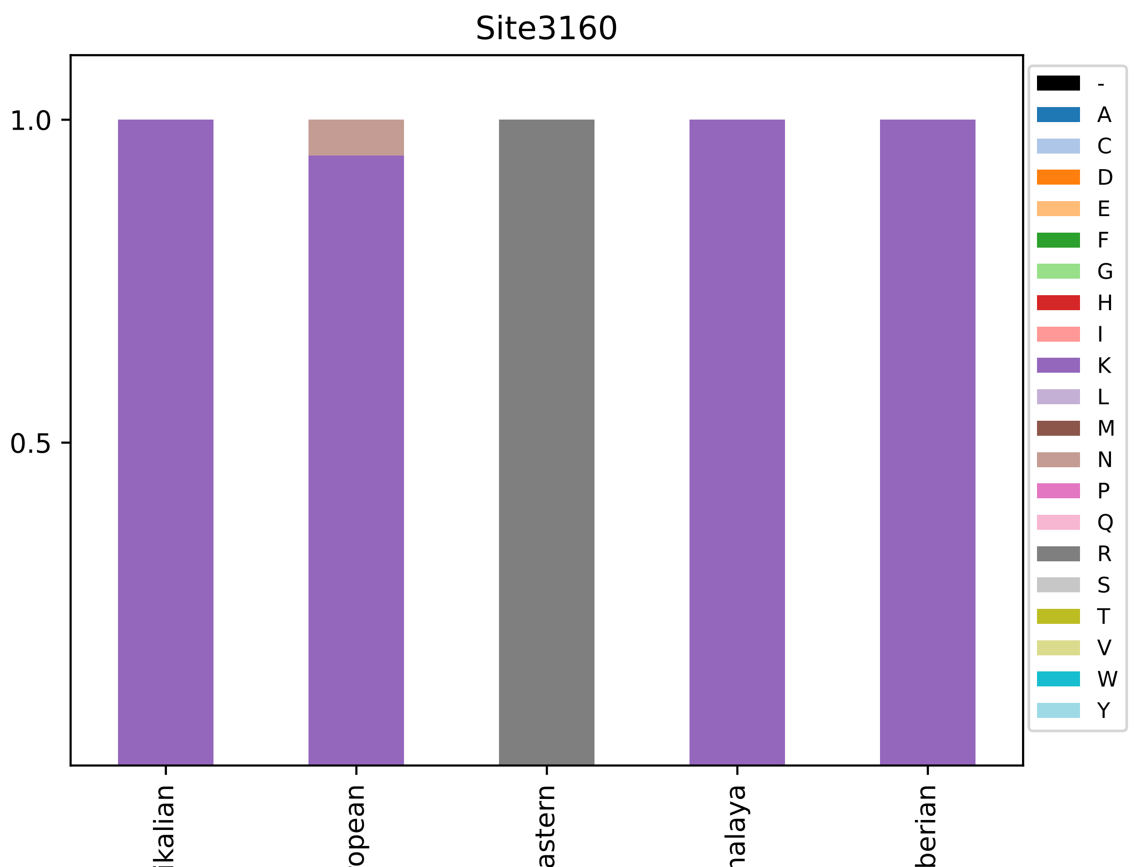

Site3160
1.0
0.5
-
A
C
D
E
F
G
H
I
K
L
M
N
P
Q
R
S
T
V
W
Y
ikalian
opean
astern
nalaya
berian

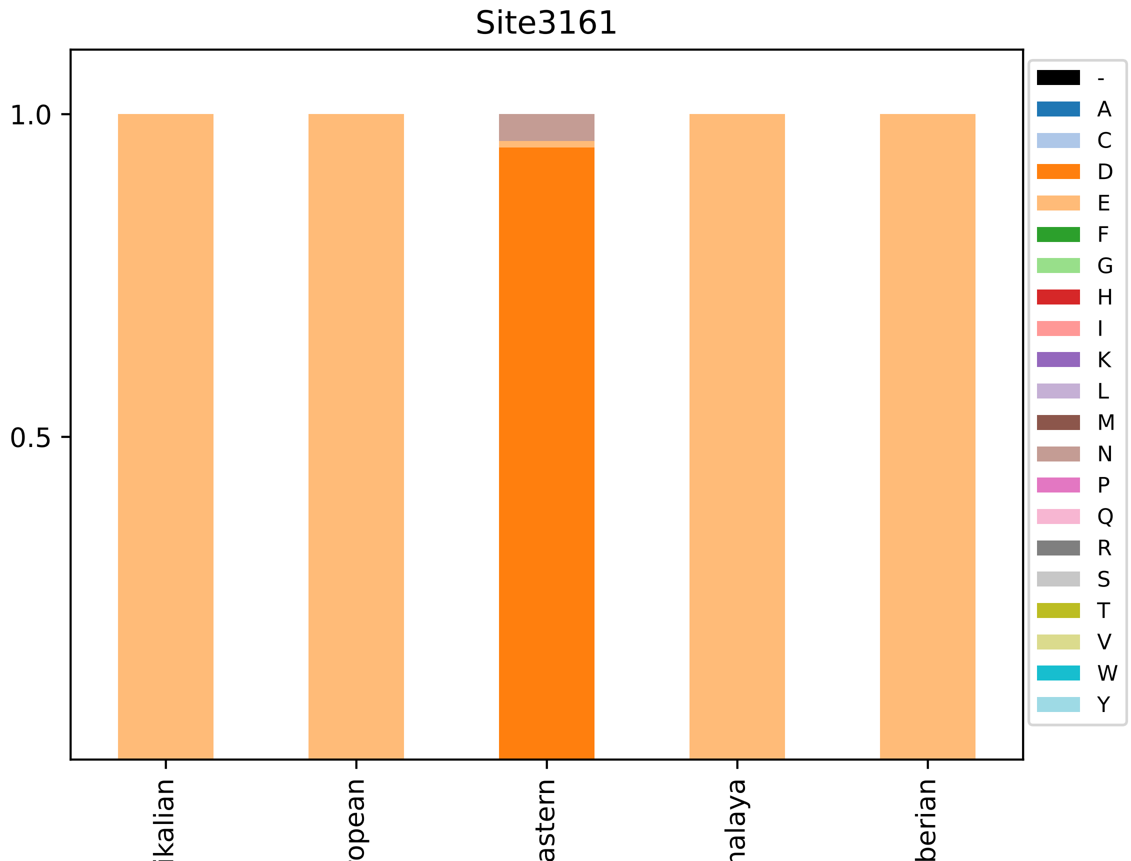
Site3161
1.0
0.5
-
A
C
D
E
F
G
H
I
K
L
M
N
P
Q
R
S
T
V
W
Y
ikalian
opean
astern
nalaya
berian

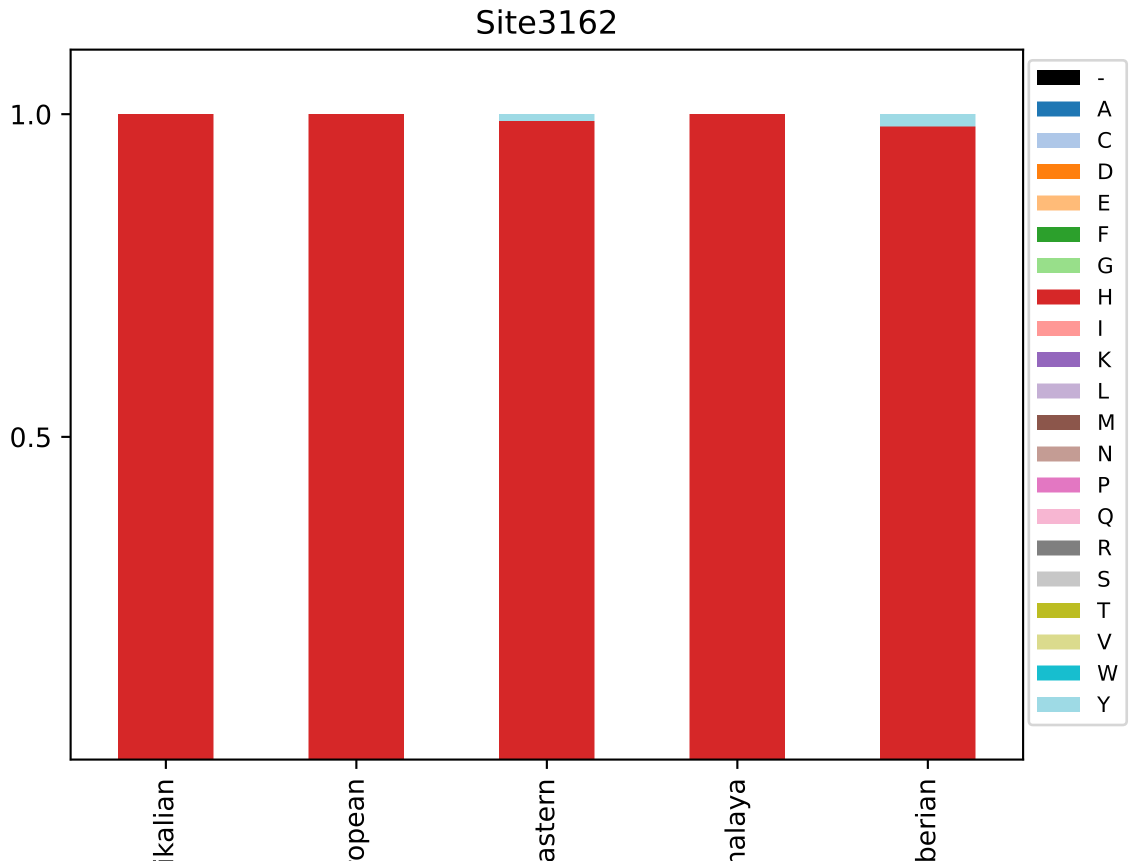
Site3162
1.0
0.5
-
A
C
D
E
F
G
H
I
K
L
M
N
P
Q
R
S
T
V
W
Y
ikalian
opean
astern
nalaya
berian

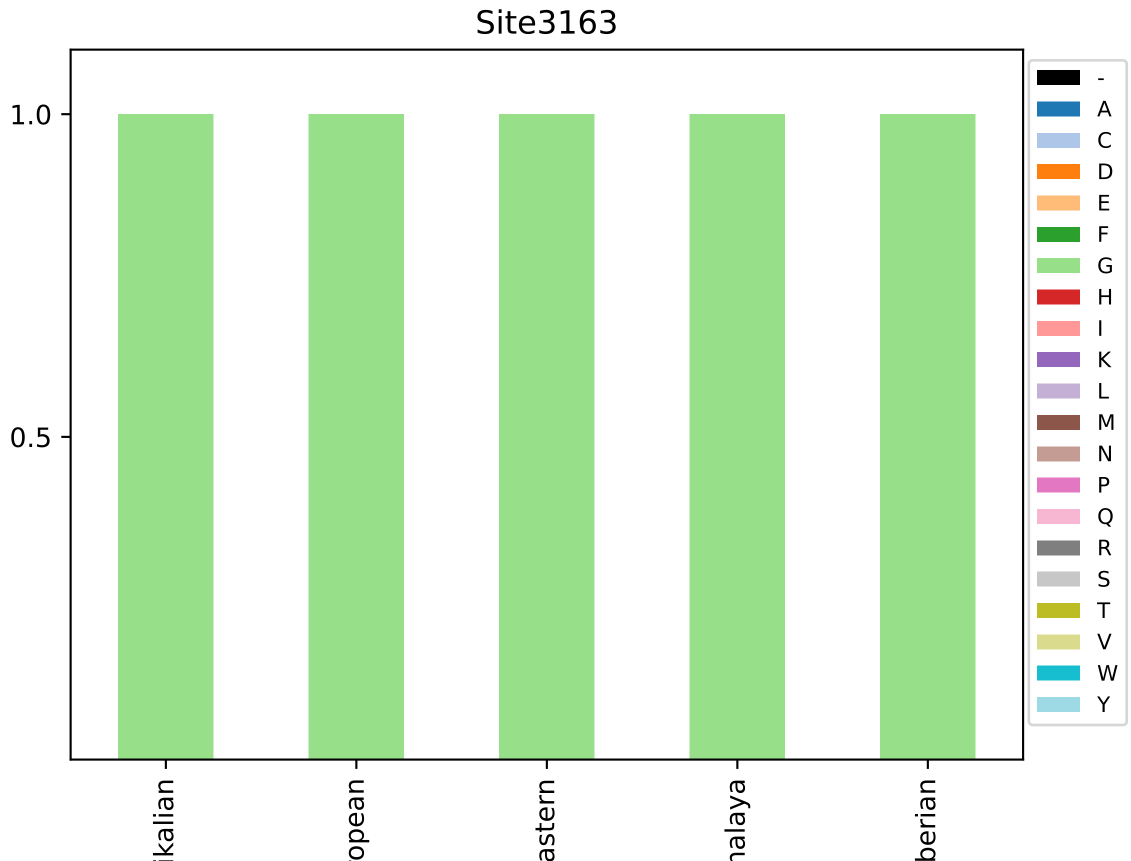
Site3163
1.0
0.5
-
A
C
D
E
F
G
H
I
K
L
M
N
P
Q
R
S
T
V
W
Y
ikalian
ropean
astern
nalaya
berian

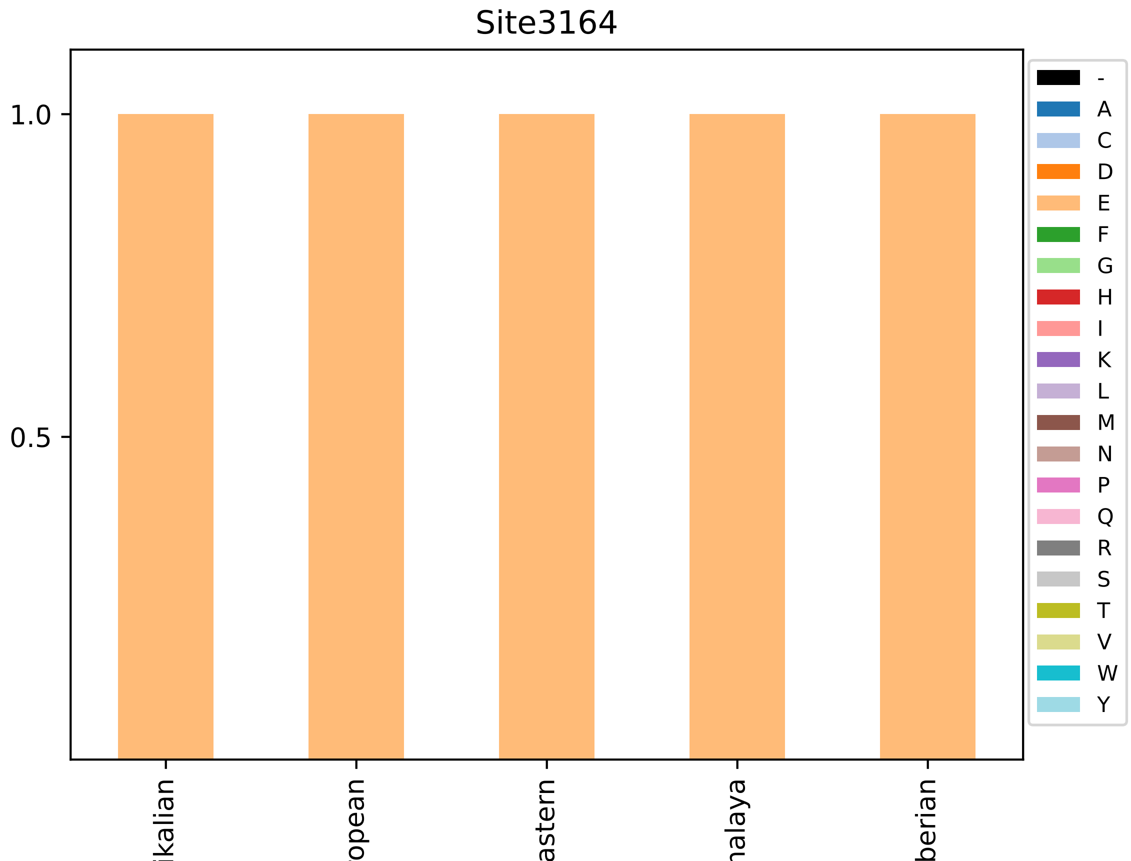

Site3164
1.0
0.5
ikalian
opean
astern
nalaya
berian
-
A
C
D
E
F
G
H
I
K
L
M
N
P
Q
R
S
T
V
W
Y

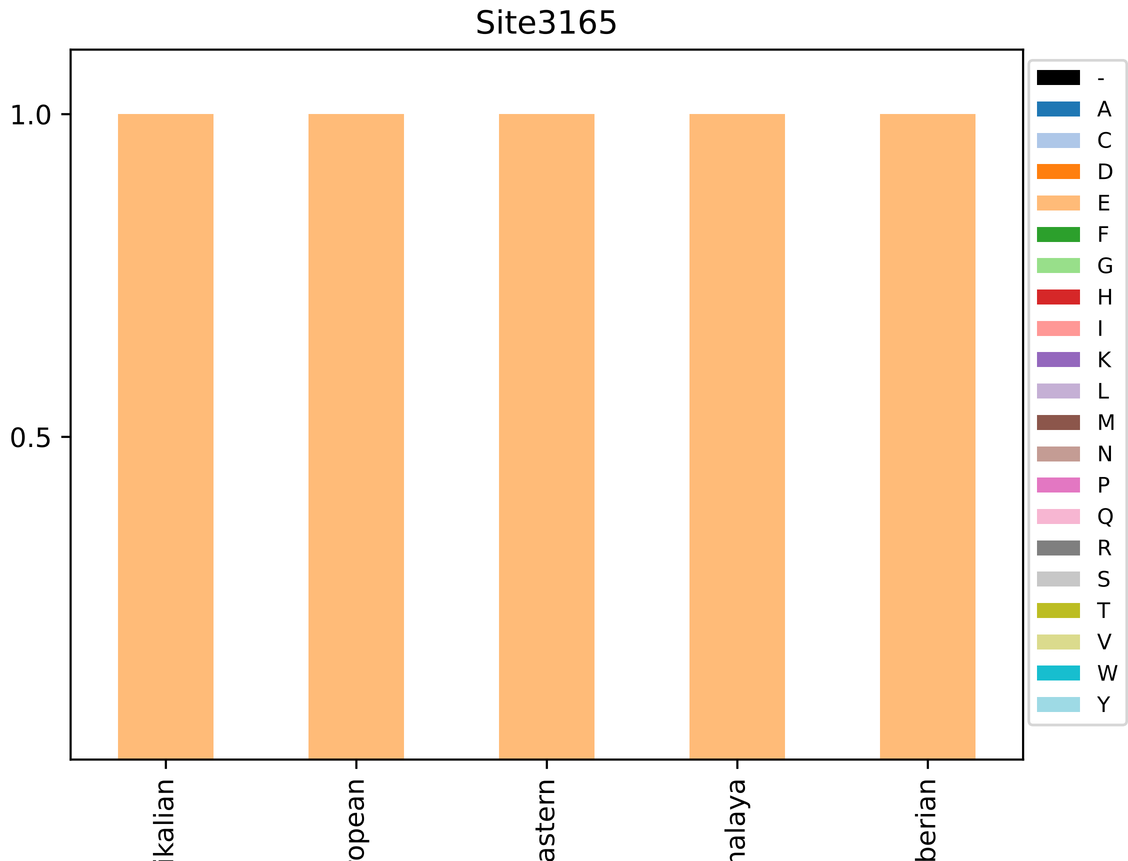

Site3165
1.0
0.5
ikalian
opean
astern
nalaya
berian
-
A
C
D
E
F
G
H
I
K
L
M
N
P
Q
R
S
T
V
W
Y

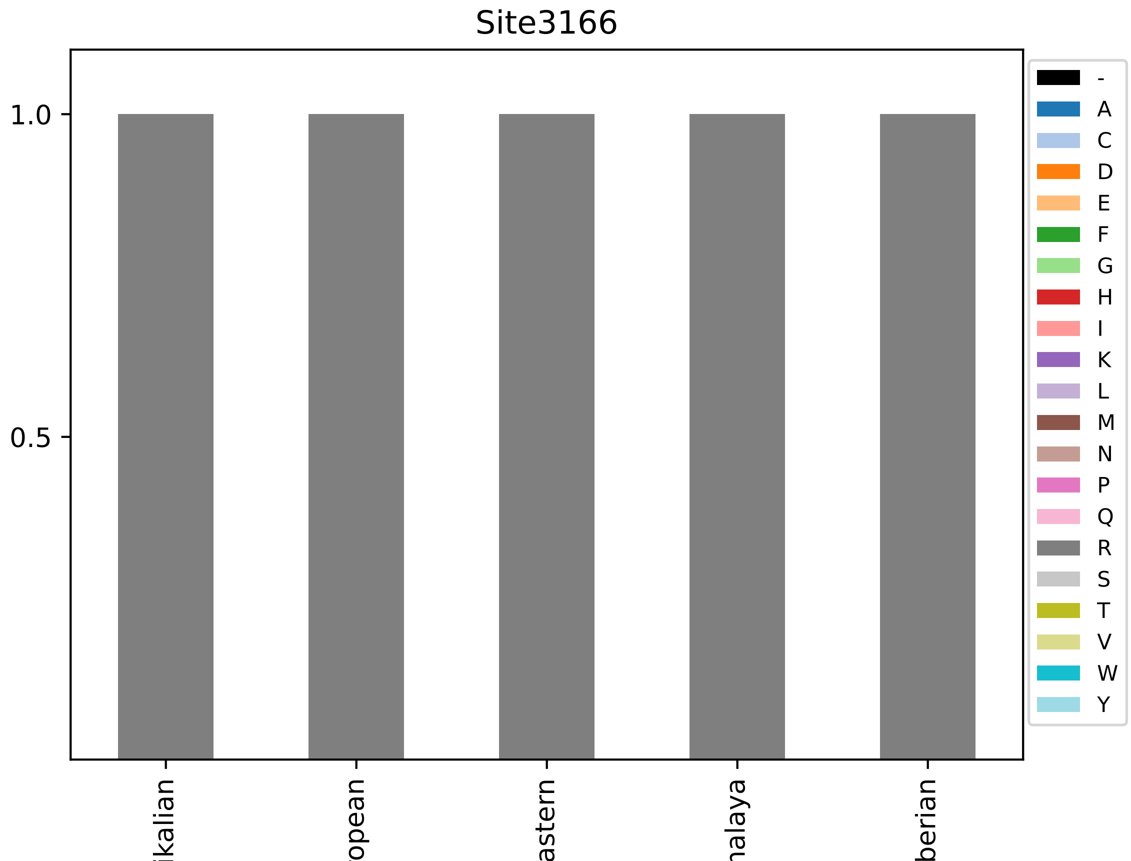

Site3166
1.0
0.5
-
A
C
D
E
F
G
H
I
K
L
M
N
P
Q
R
S
T
V
W
Y
ikalian
opean
astern
nalaya
berian

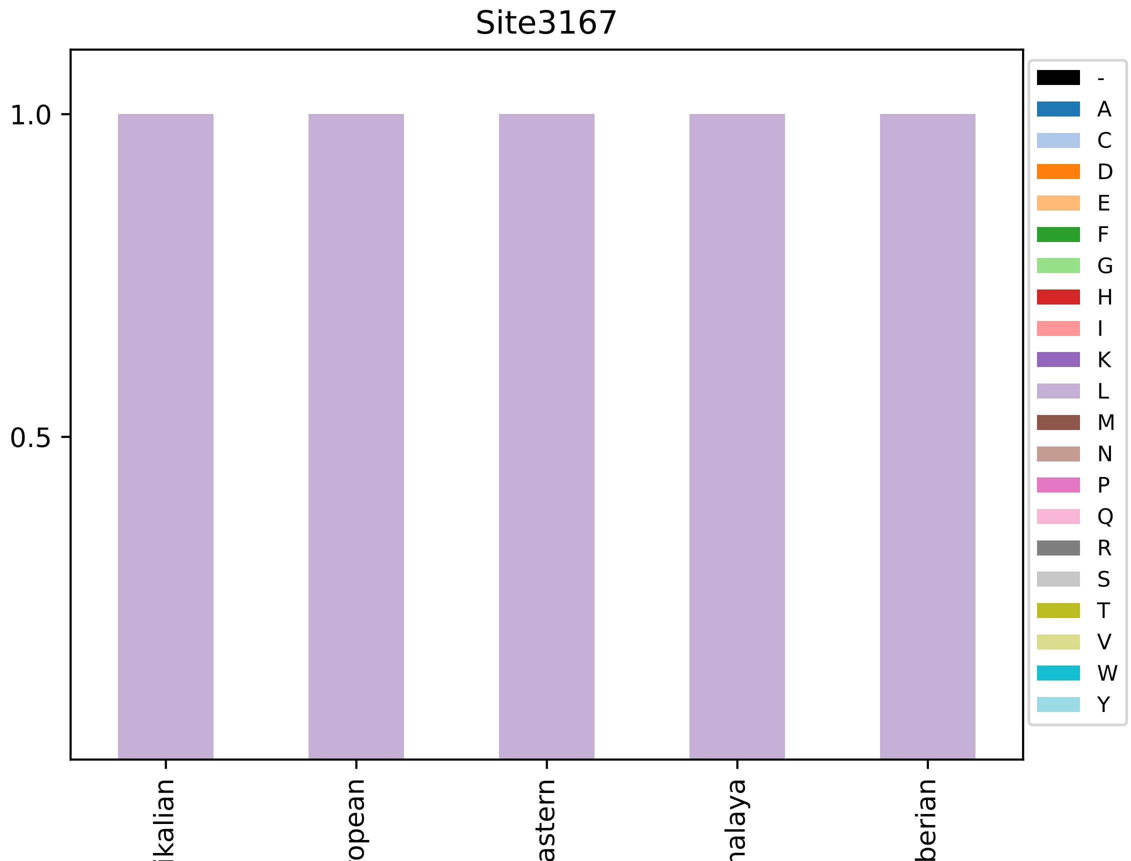
Site3167
1.0
0.5
-
A
C
D
E
F
G
H
I
K
L
M
N
P
Q
R
S
T
V
W
Y
ikalian
opean
astern
nalaya
berian

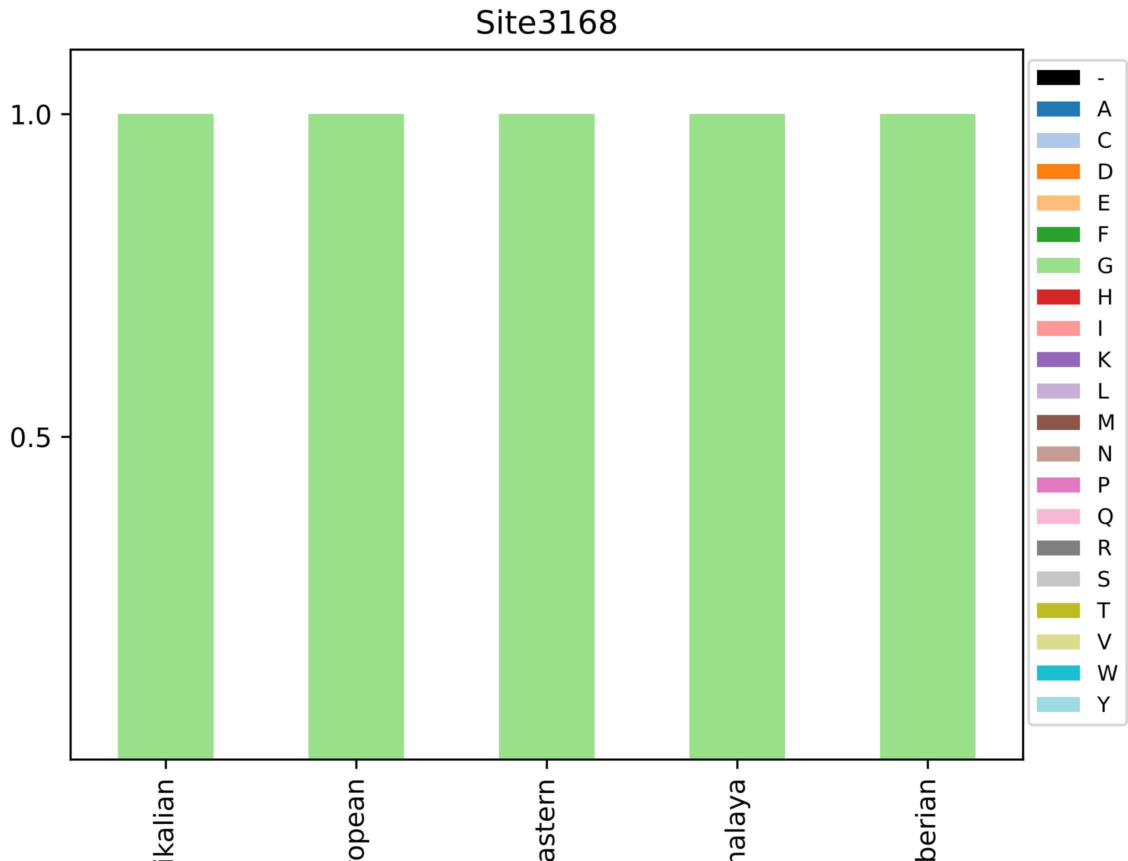

Site3168
1.0
0.5
-
A
C
D
E
F
G
H
I
K
L
M
N
P
Q
R
S
T
V
W
Y
ikalian
opean
astern
nalaya
berian

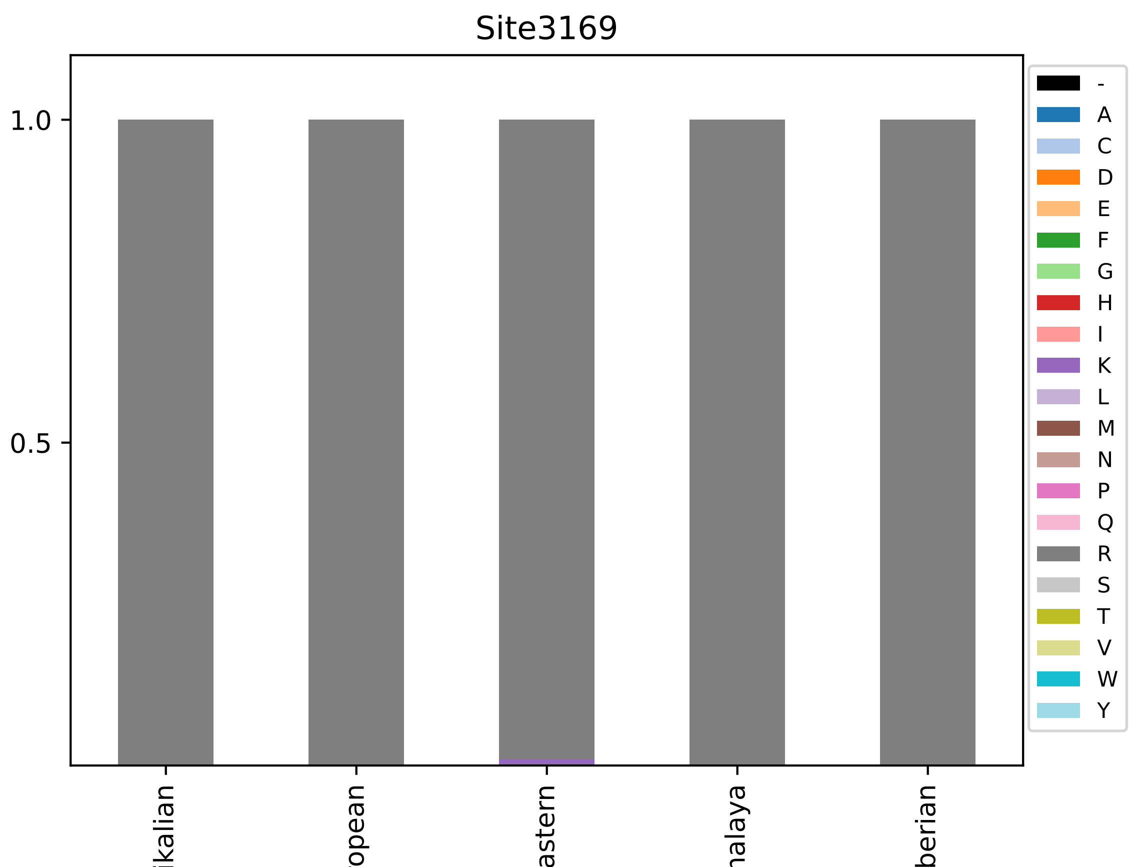

Site3169
1.0
0.5
-
A
C
D
E
F
G
H
I
K
L
M
N
P
Q
R
S
T
V
W
Y
ikalian
opean
astern
halaya
berian

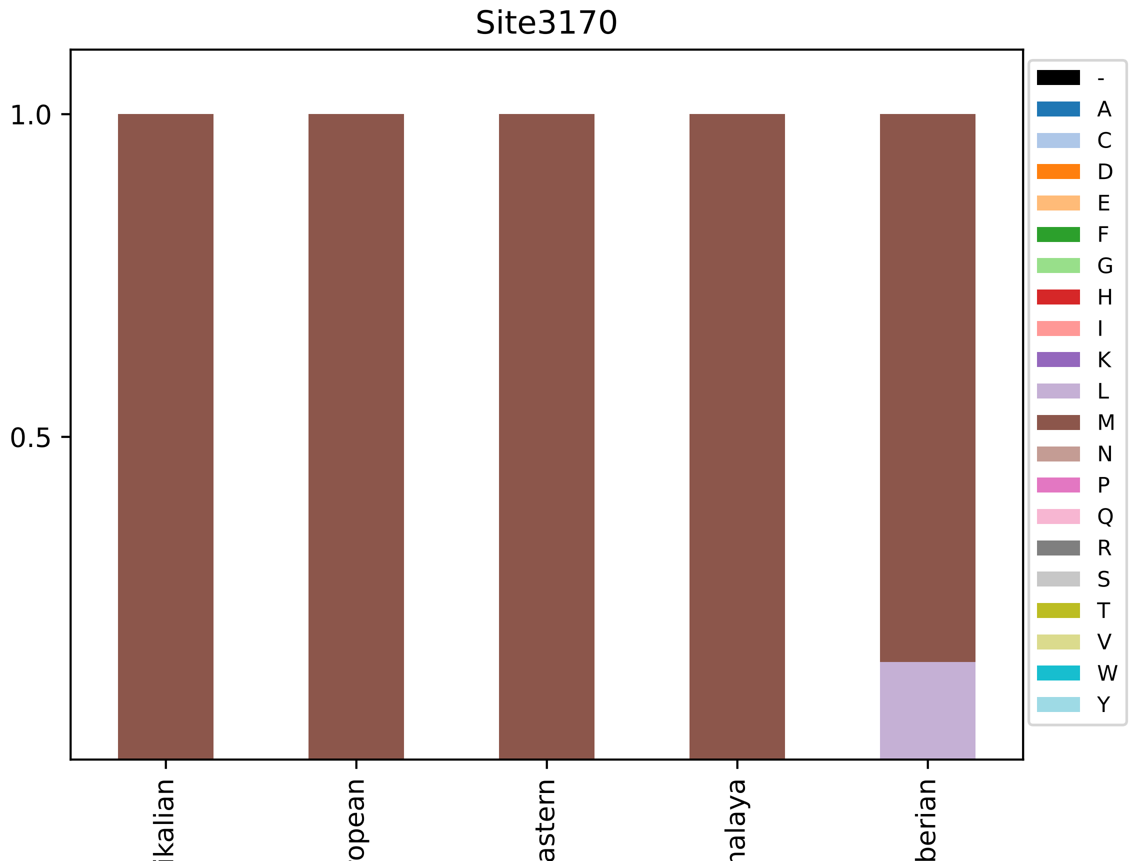

Site3170
1.0
0.5
ikalian
ropean
astern
nalaya
berian
-
A
C
D
E
F
G
H
I
K
L
M
N
P
Q
R
S
T
V
W
Y

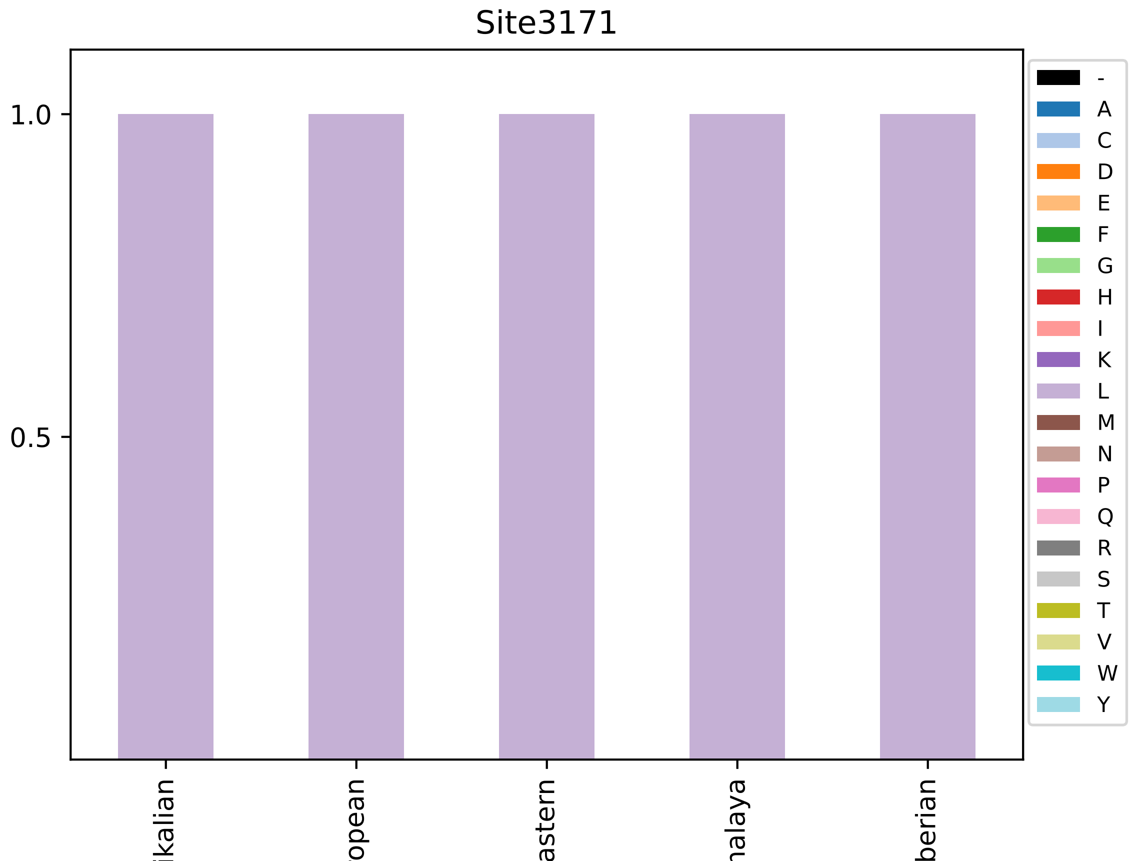
Site3171
1.0
0.5
-
A
C
D
E
F
G
H
I
K
L
M
N
P
Q
R
S
T
V
W
Y
ikalian
opean
astern
nalaya
berian

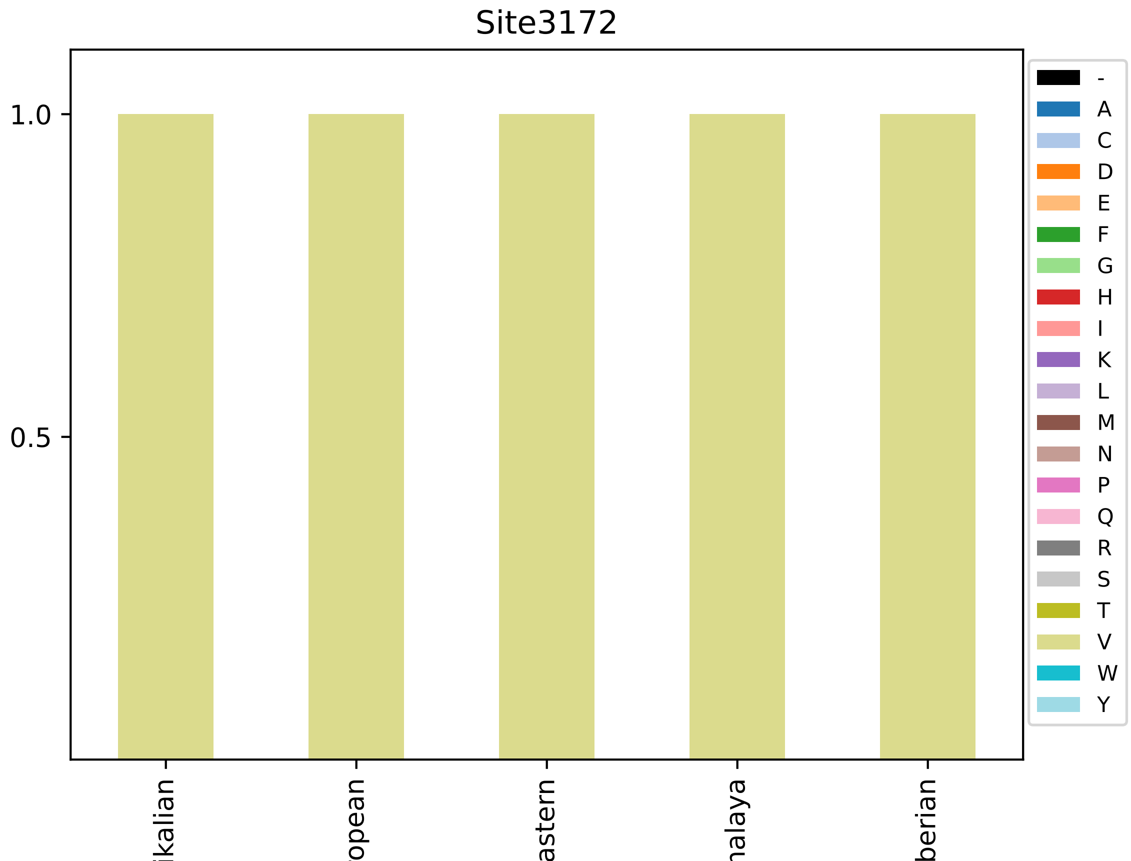
Site3172
1.0
0.5
-
A
C
D
E
F
G
H
I
K
L
M
N
P
Q
R
S
T
V
W
Y
ikalian
opean
astern
nalaya
berian

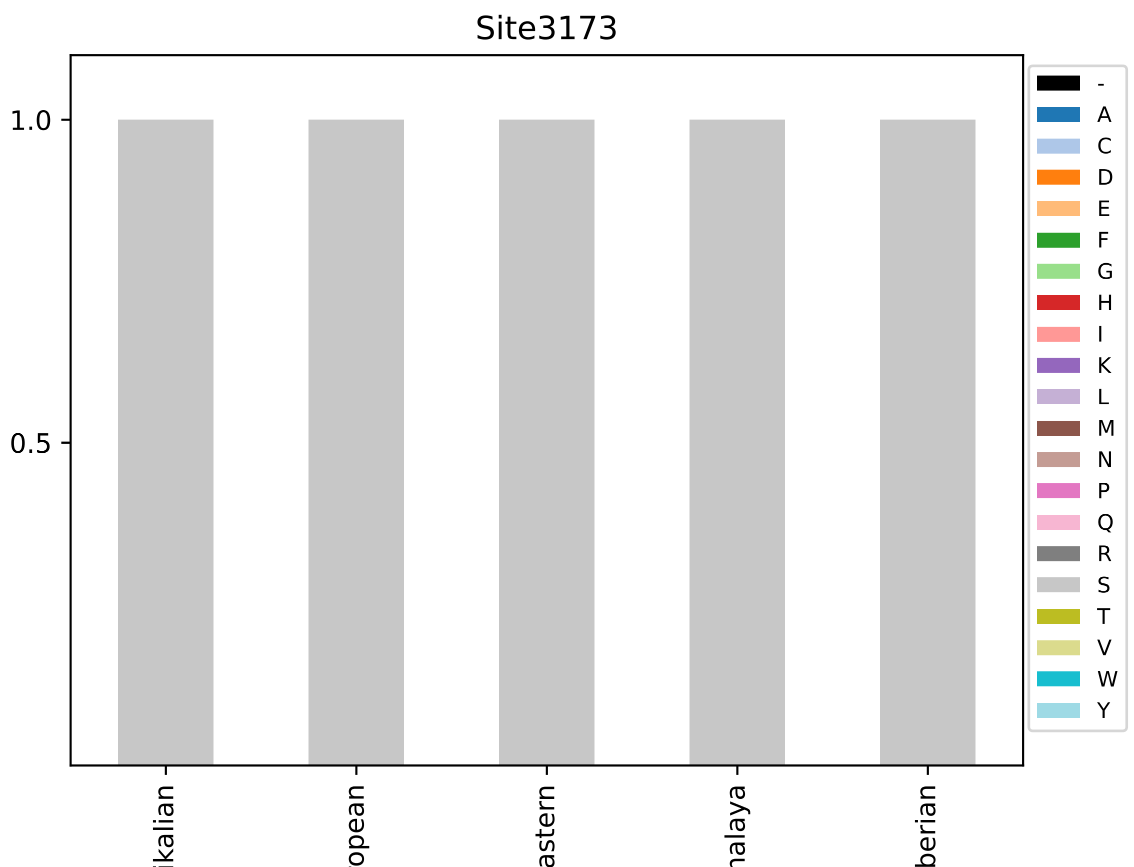
Site3173
1.0
0.5
-
A
C
D
E
F
G
H
I
K
L
M
N
P
Q
R
S
T
V
W
Y
ikalian
opean
astern
nalaya
berian

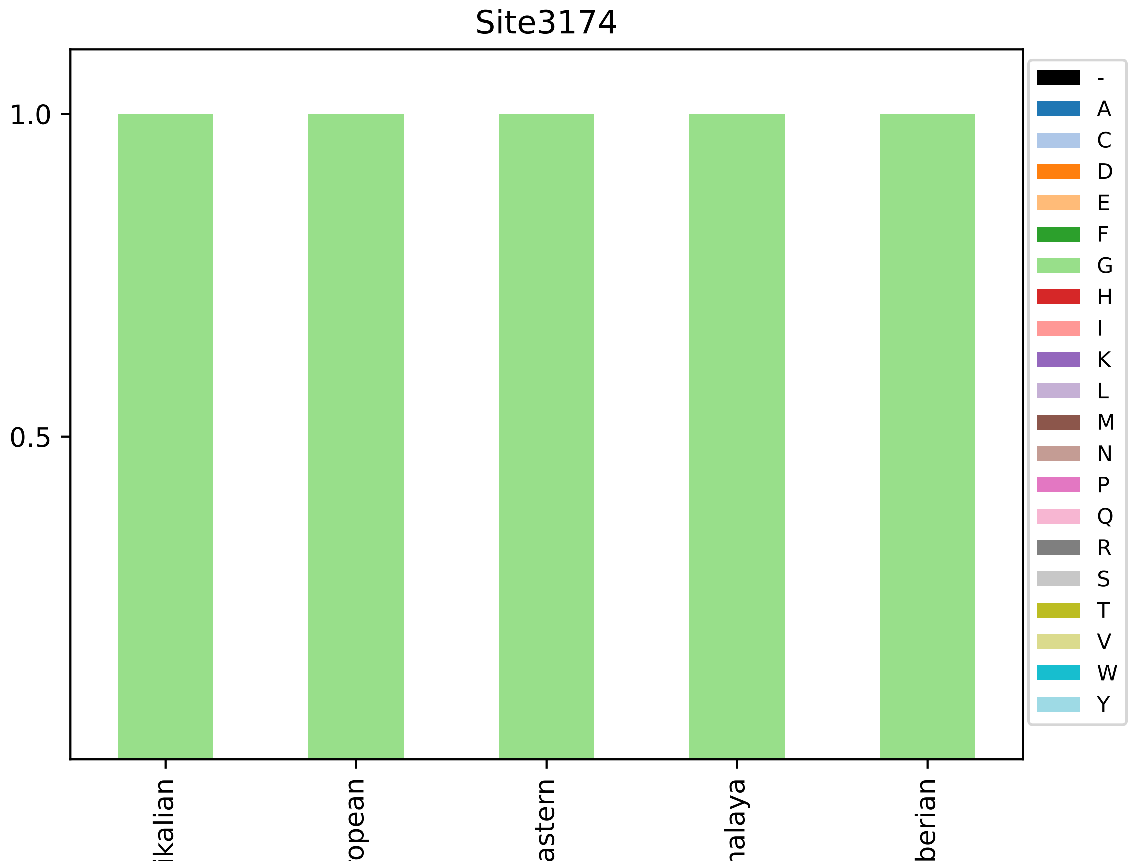
Site3174
1.0
0.5
-
A
C
D
E
F
G
H
I
K
L
M
N
P
Q
R
S
T
V
W
Y
ikalian
opean
astern
nalaya
berian

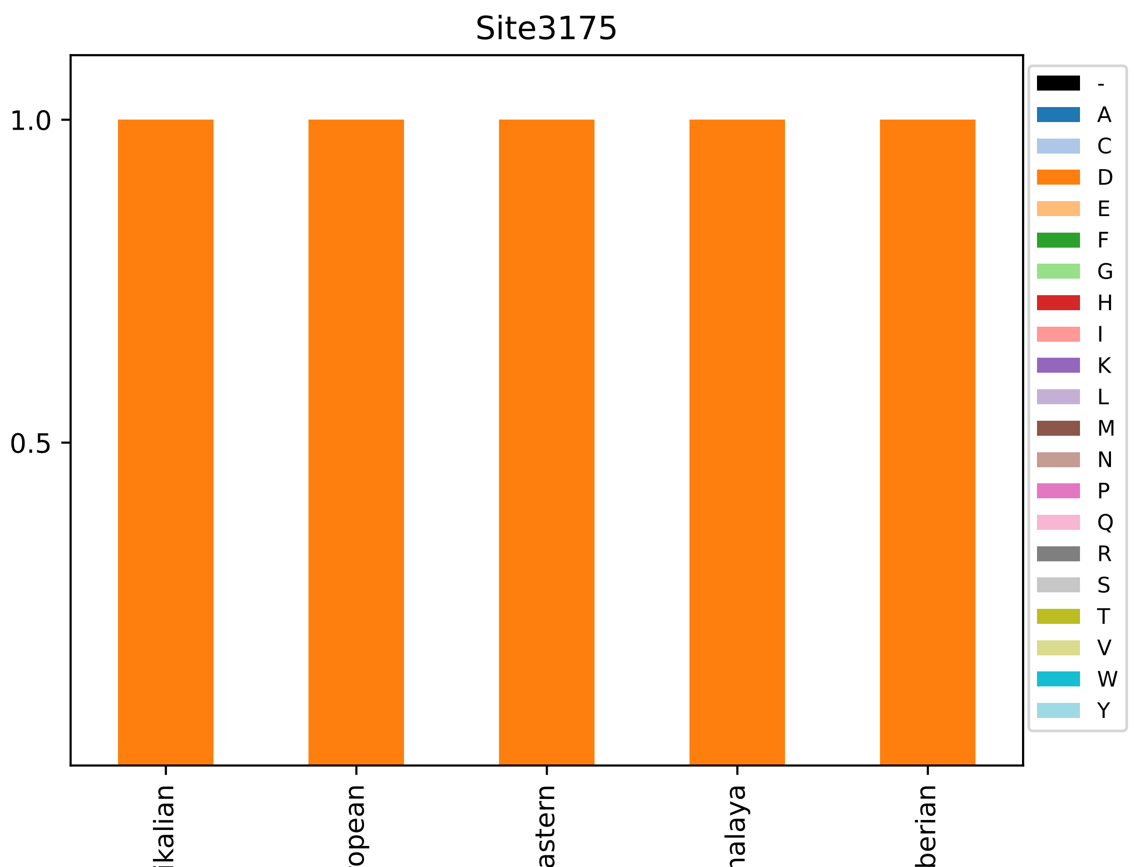

Site3175
1.0
0.5
-
A
C
D
E
F
G
H
I
K
L
M
N
P
Q
R
S
T
V
W
Y
ikalian
opean
astern
nalaya
berian

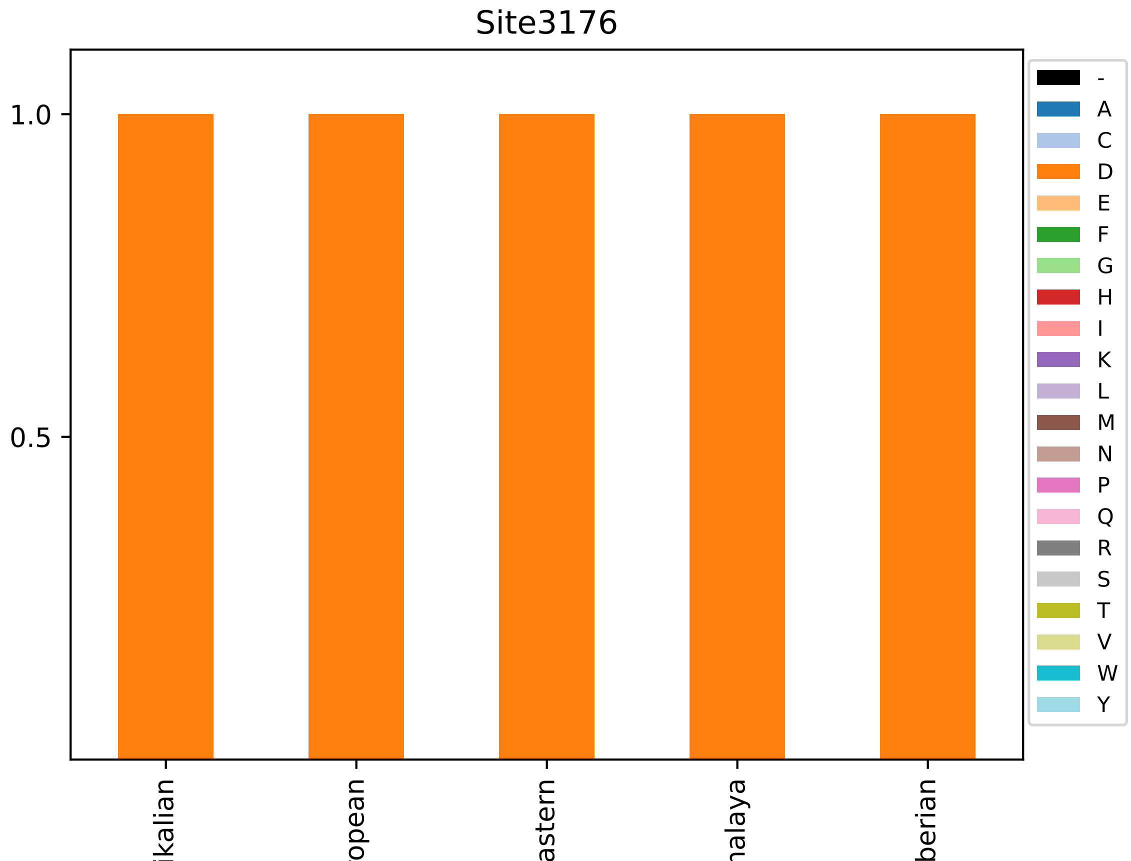

Site3176
1.0
0.5
ikalian
opean
astern
nalaya
berian
-
A
C
D
E
F
G
H
I
K
L
M
N
P
Q
R
S
T
V
W
Y

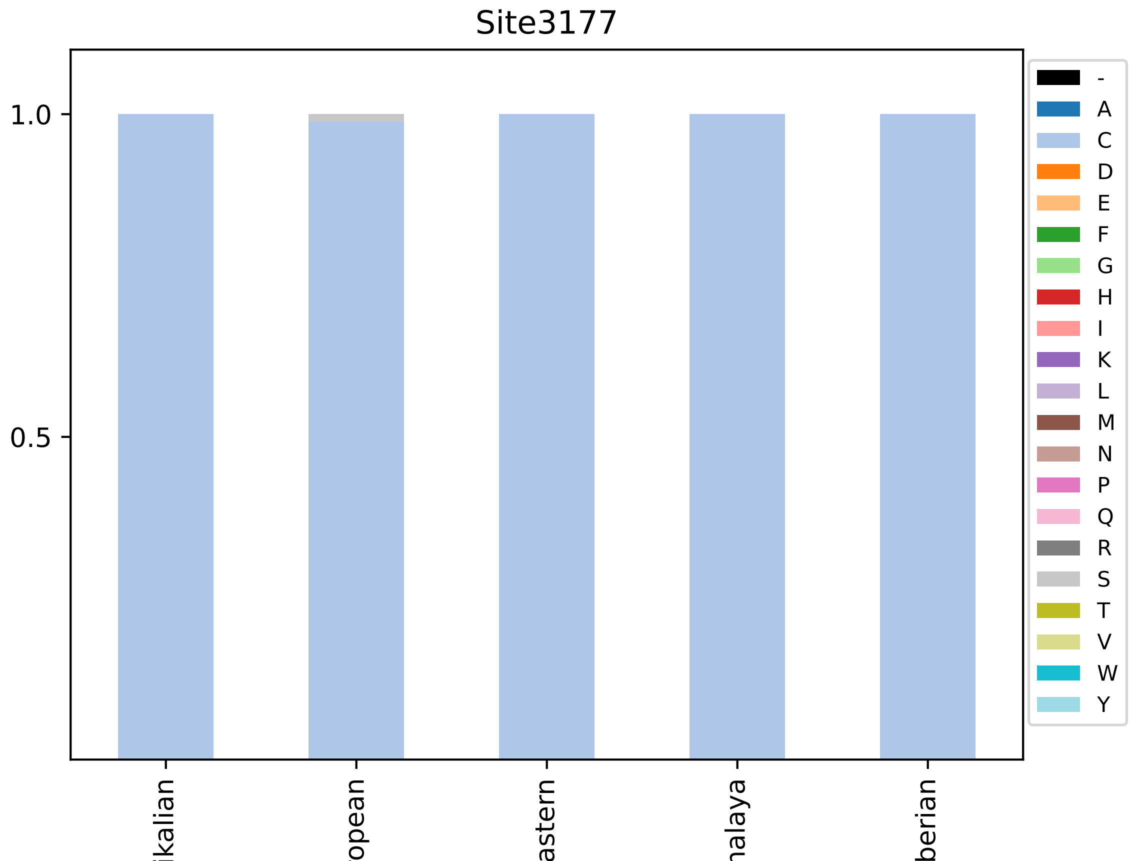
Site3177
1.0
0.5
-
A
C
D
E
F
G
H
I
K
L
M
N
P
Q
R
S
T
V
W
Y
ikalian
opean
astern
nalaya
berian

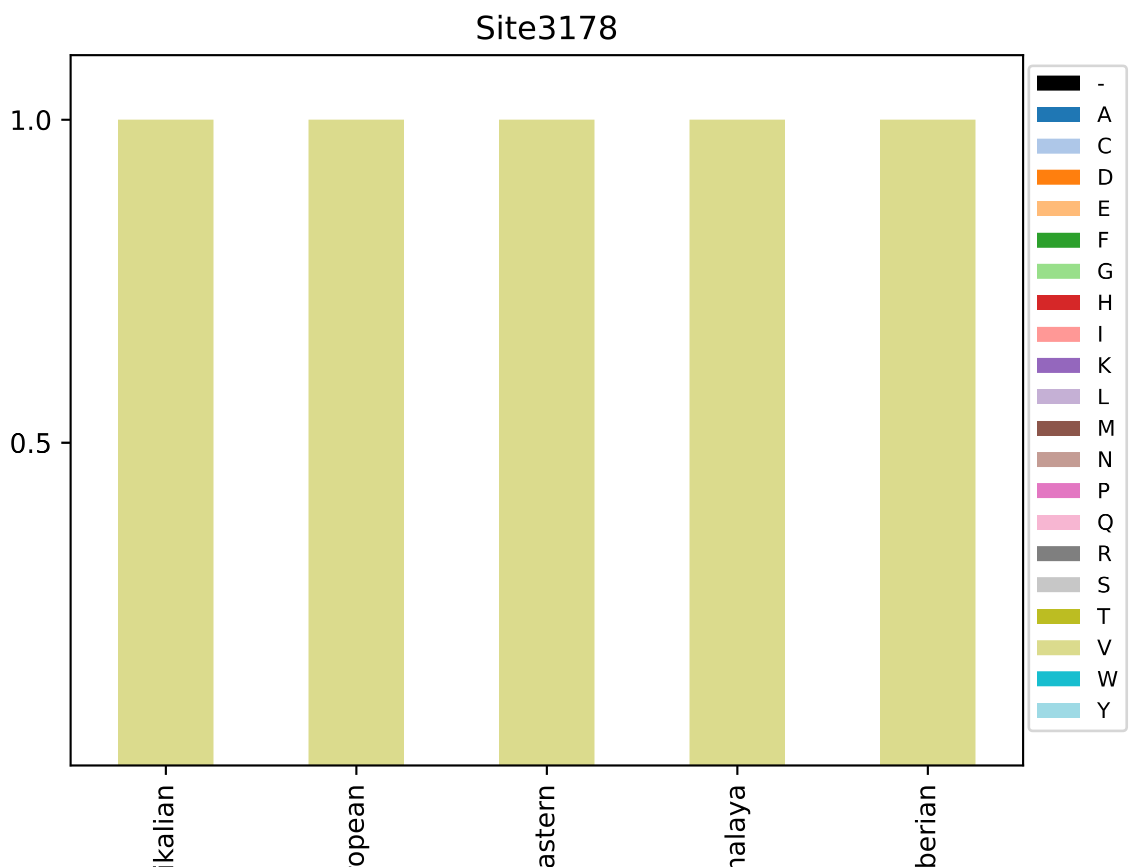
Site3178
1.0
0.5
-
A
C
D
E
F
G
H
I
K
L
M
N
P
Q
R
S
T
V
W
Y
ikalian
ropean
astern
nalaya
berian

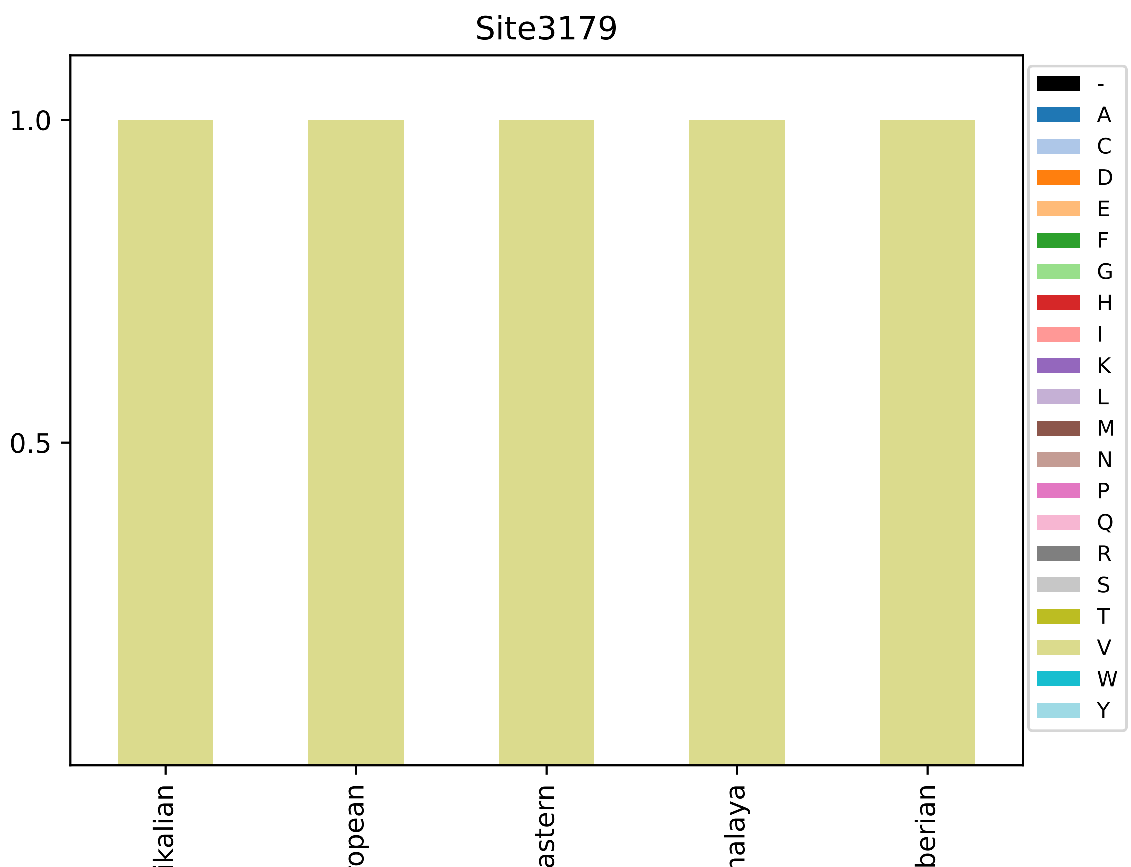
Site3179
1.0
0.5
-
A
C
D
E
F
G
H
I
K
L
M
N
P
Q
R
S
T
V
W
Y
ikalian
opean
astern
halaya
berian

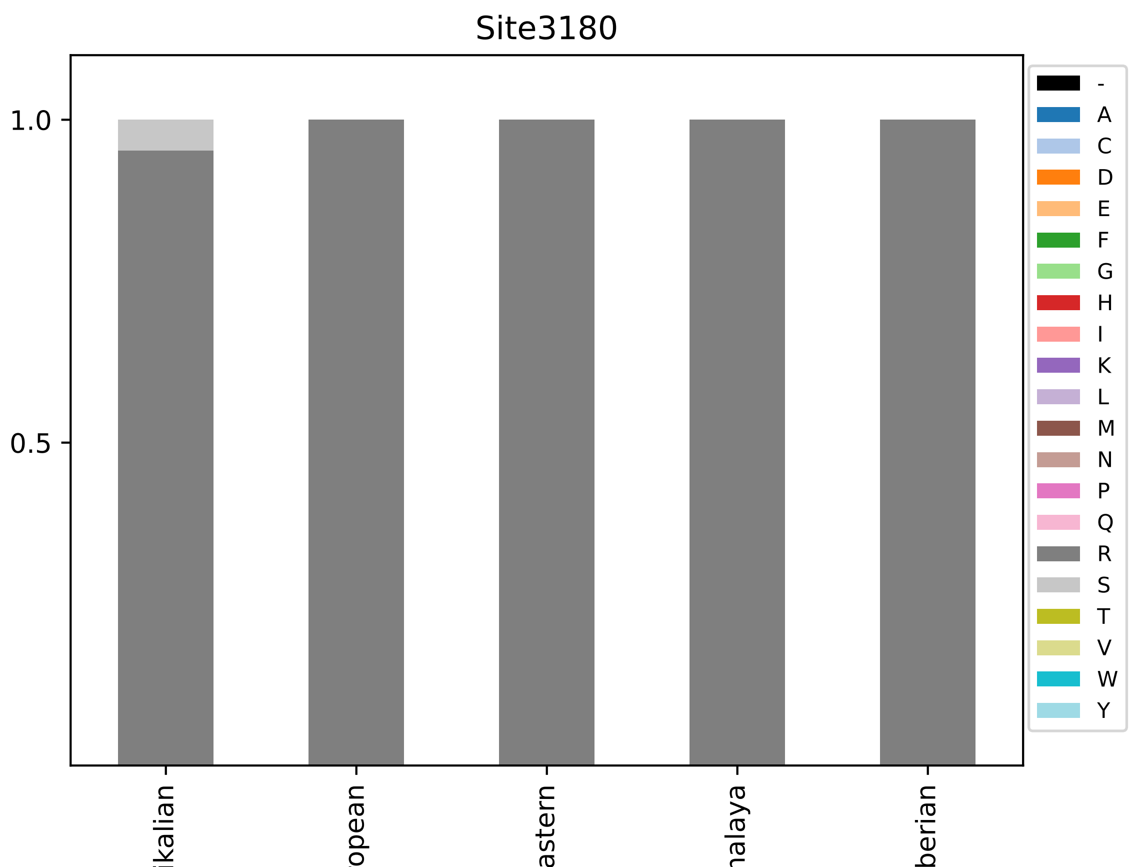
Site3180
1.0
0.5
ikalian
opean
astern
nalaya
berian
-
A
C
D
E
F
G
H
I
K
L
M
N
P
Q
R
S
T
V
W
Y

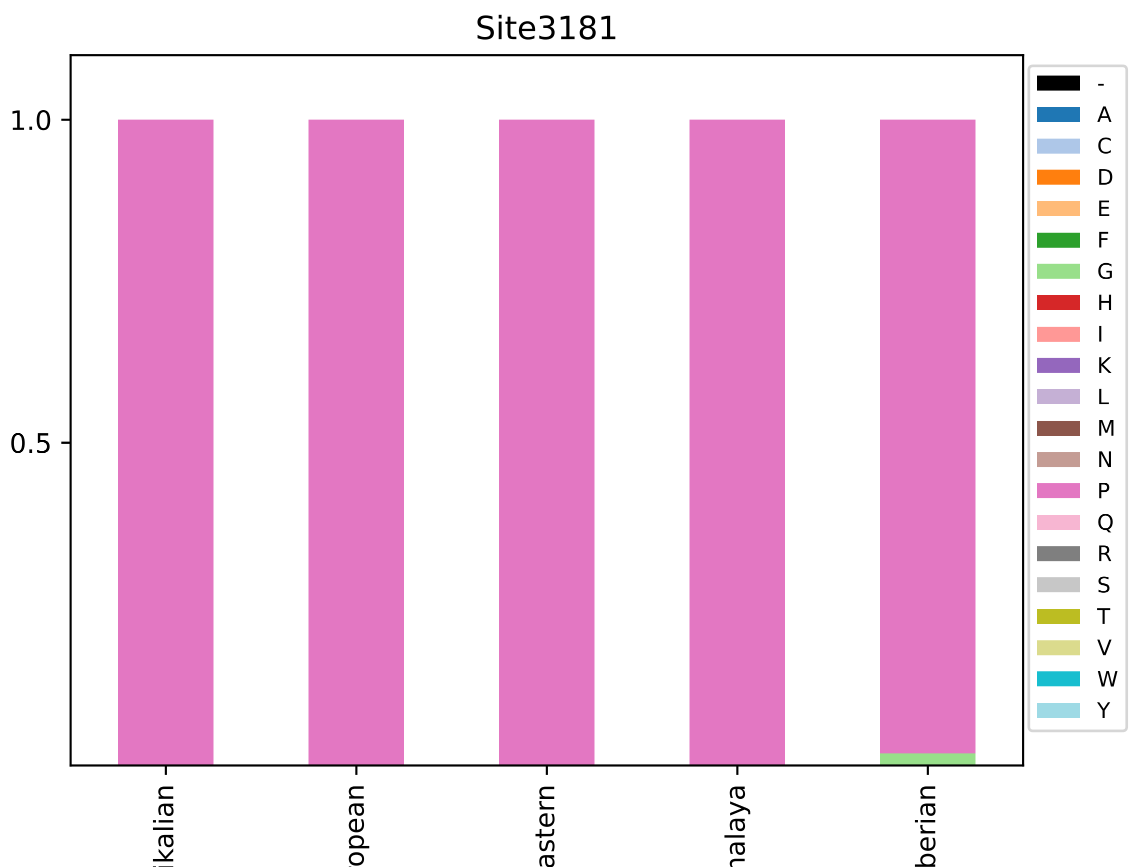

Site3181
1.0
0.5
-
A
C
D
E
F
G
H
I
K
L
M
N
P
Q
R
S
T
V
W
Y
ikalian
opean
astern
nalaya
berian

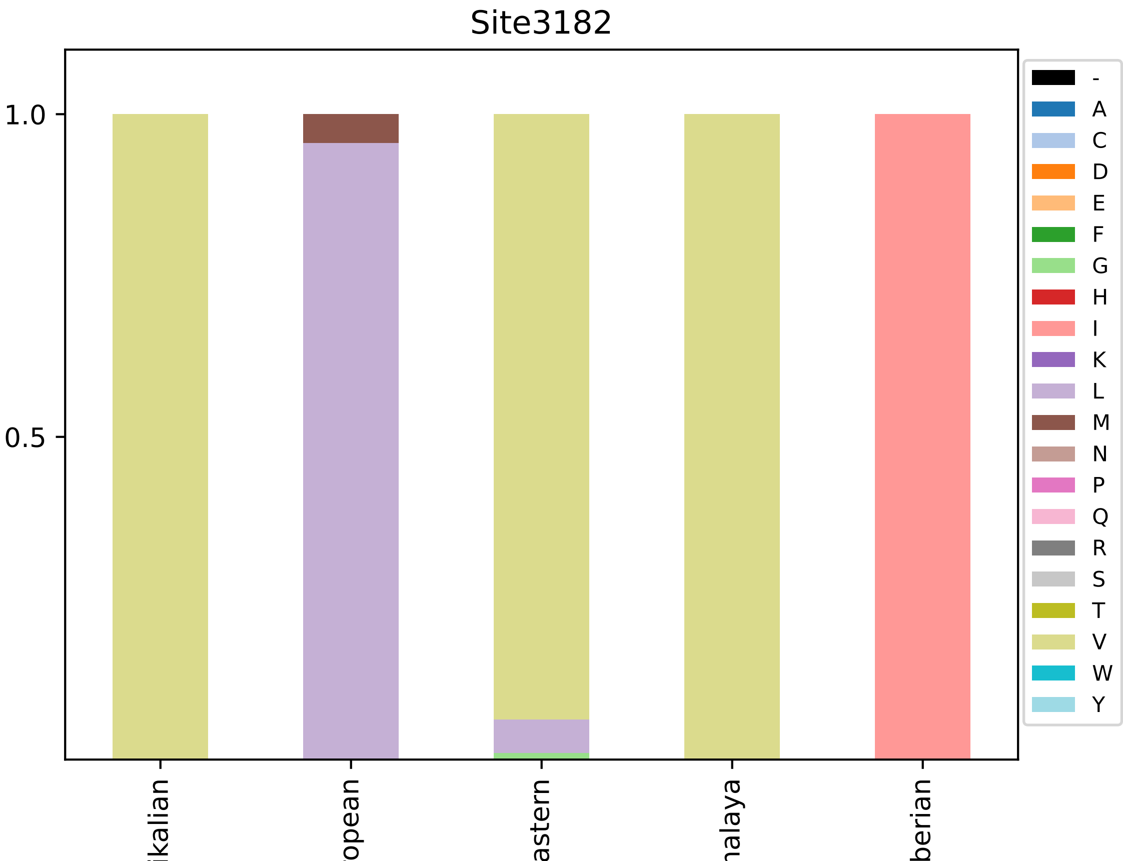
Site3182
1.0
0.5
-
A
C
D
E
F
G
H
I
K
L
M
N
P
Q
R
S
T
V
W
Y
ikalian
opean
astern
nalaya
berian

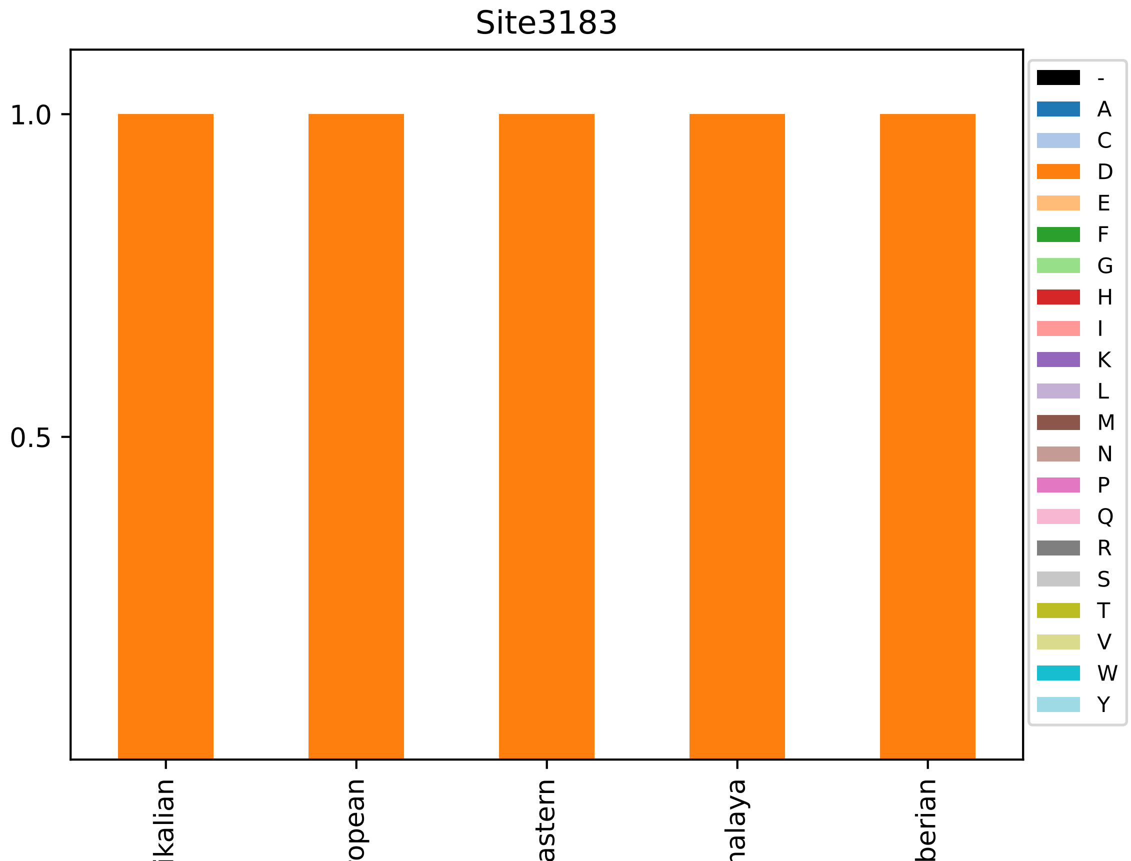

Site3183
1.0
0.5
-
A
C
D
E
F
G
H
I
K
L
M
N
P
Q
R
S
T
V
W
Y
ikalian
opean
astern
nalaya
berian

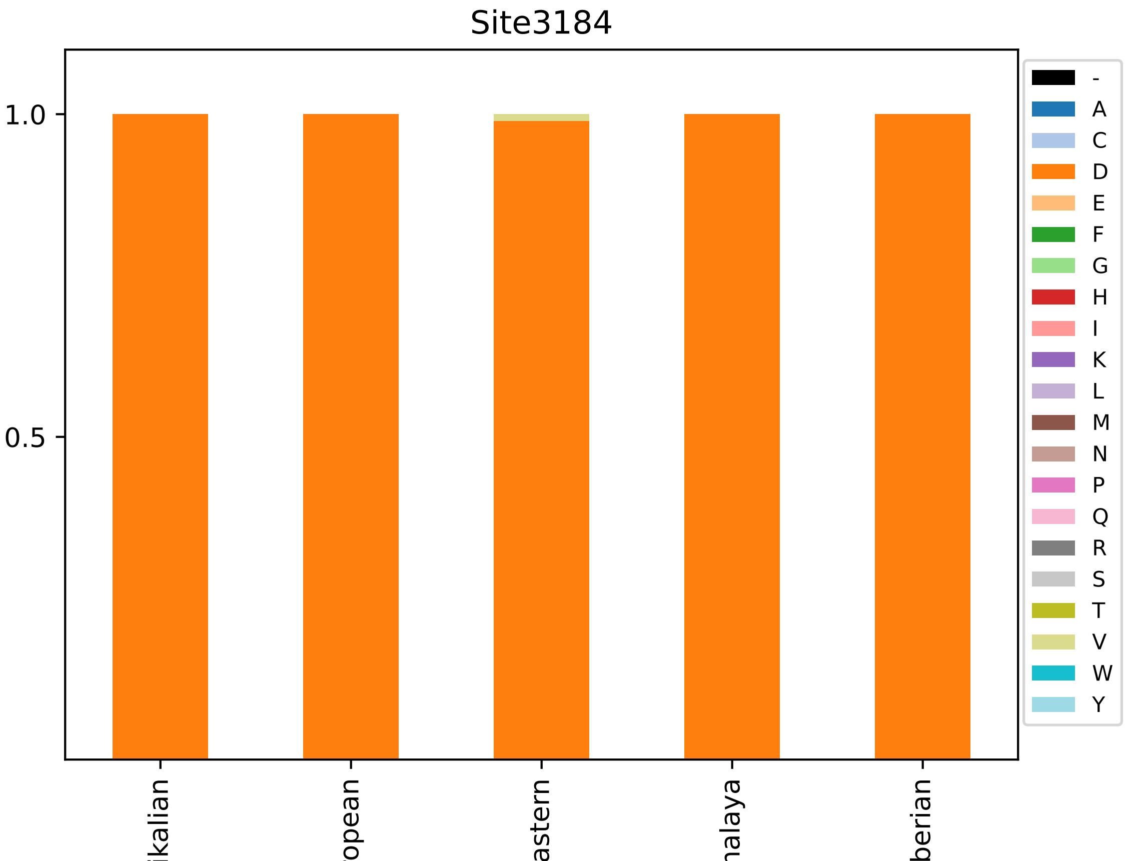
Site3184
1.0
0.5
-
A
C
D
E
F
G
H
I
K
L
M
N
P
Q
R
S
T
V
W
Y
ikalian
opean
astern
nalaya
berian

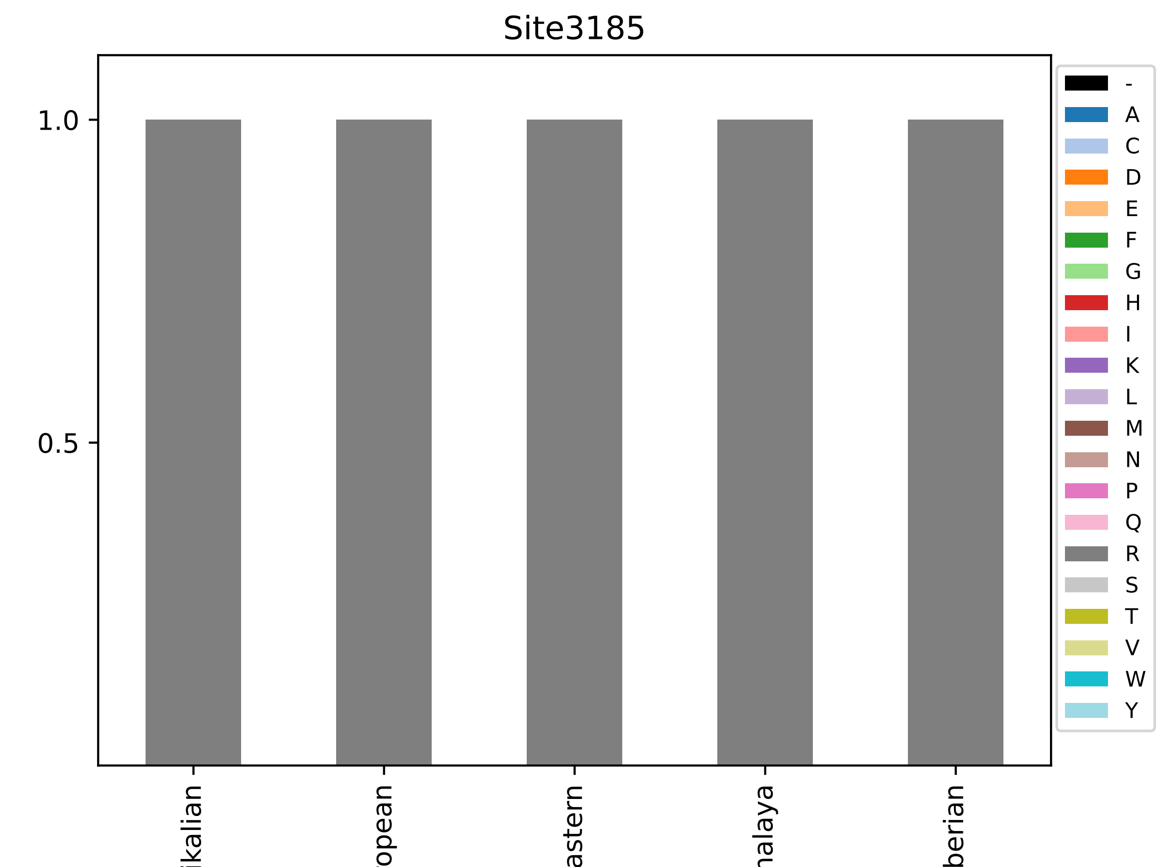
Site3185
1.0
0.5
ikalian
opean
astern
nalaya
berian
-
A
C
D
E
F
G
H
I
K
L
M
N
P
Q
R
S
T
V
W
Y

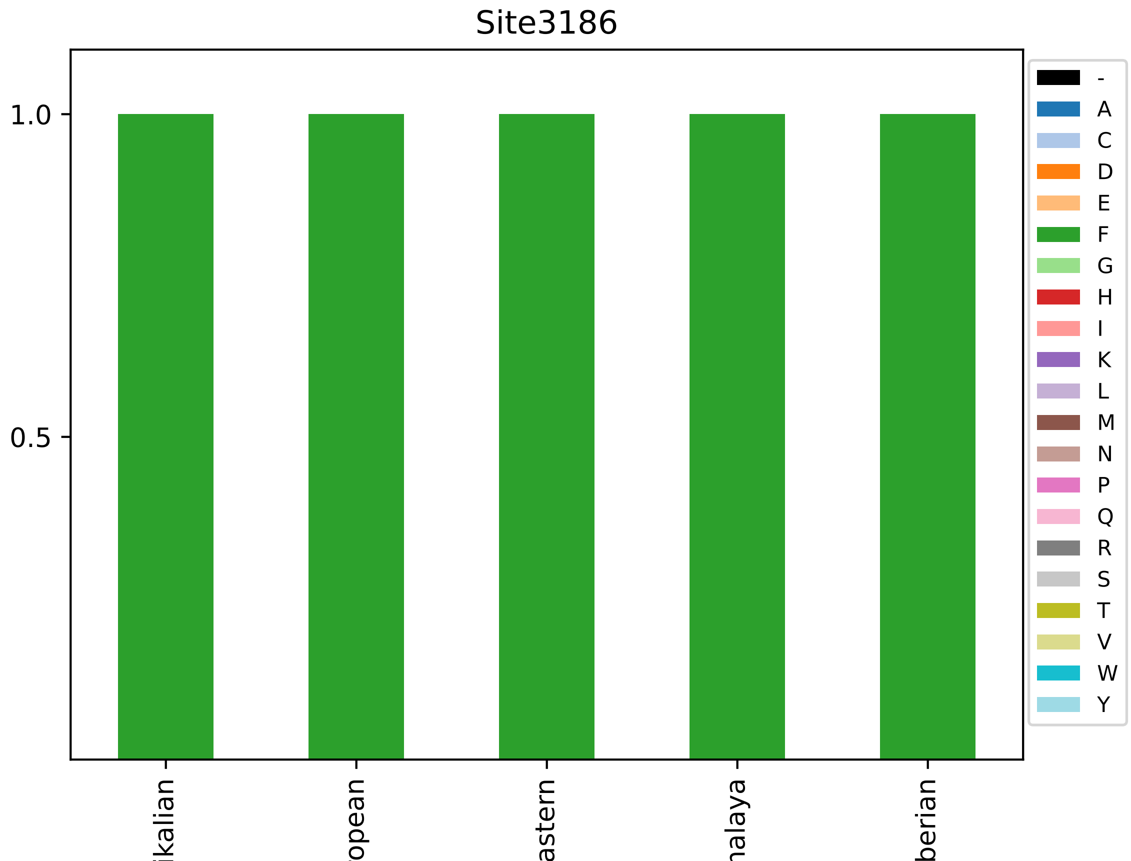
Site3186
1.0
0.5
-
A
C
D
E
F
G
H
I
K
L
M
N
P
Q
R
S
T
V
W
Y
ikalian
opean
astern
nalaya
berian

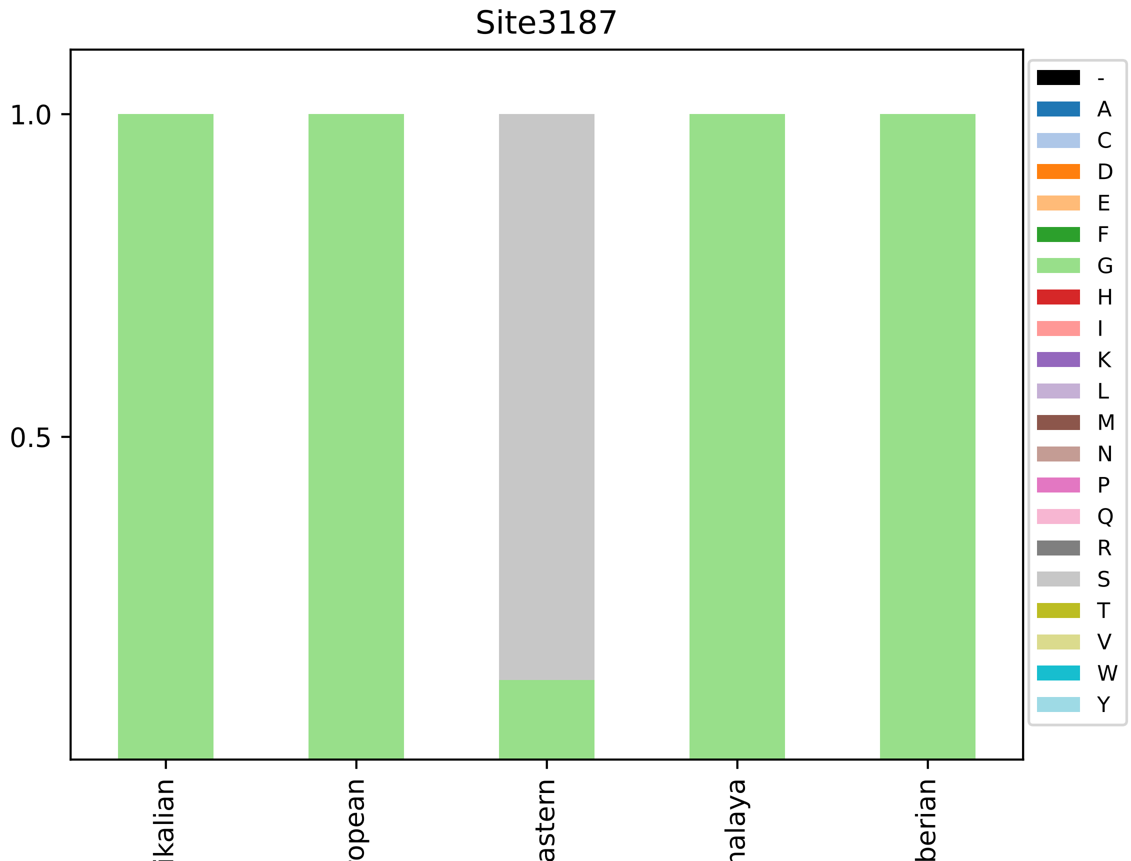

Site3187
1.0
0.5
ikalian
opean
astern
nalaya
berian
-
A
C
D
E
F
G
H
I
K
L
M
N
P
Q
R
S
T
V
W
Y

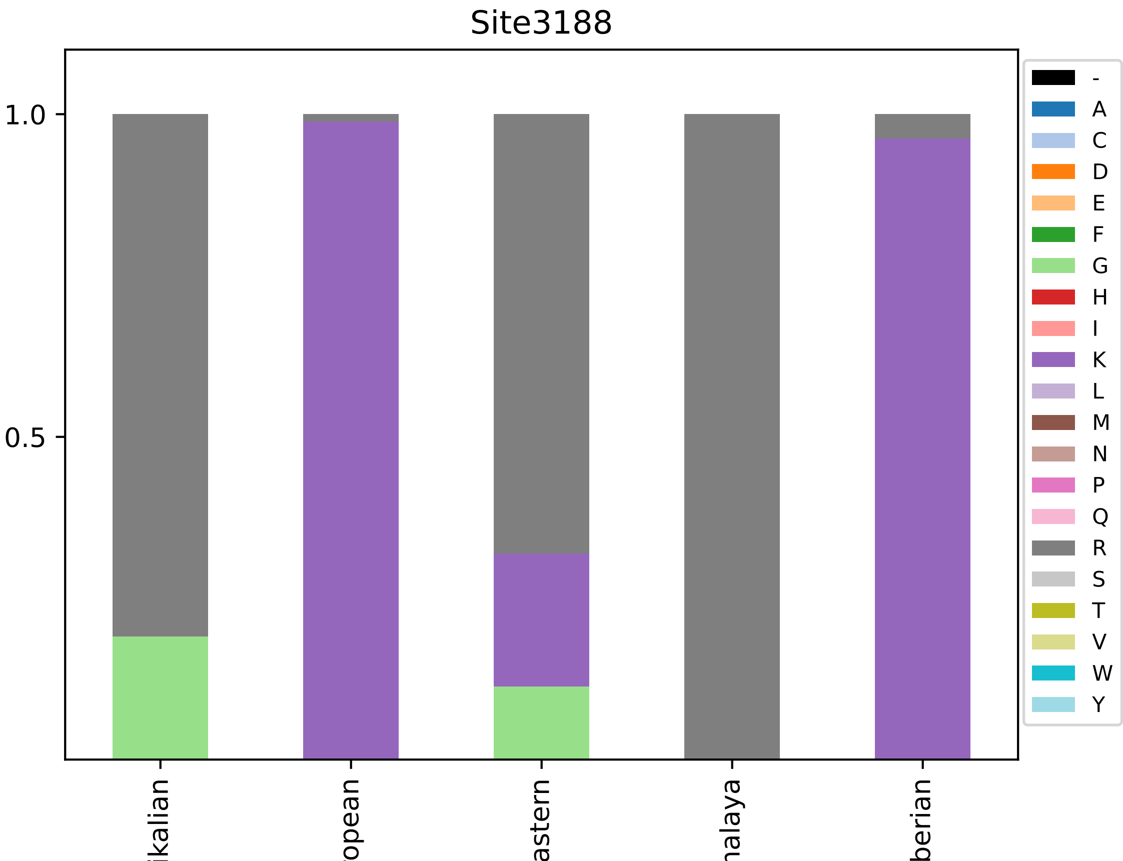
Site3188
1.0
0.5
-
A
C
D
E
F
G
H
I
K
L
M
N
P
Q
R
S
T
V
W
Y
ikalian
opean
astern
nalaya
berian

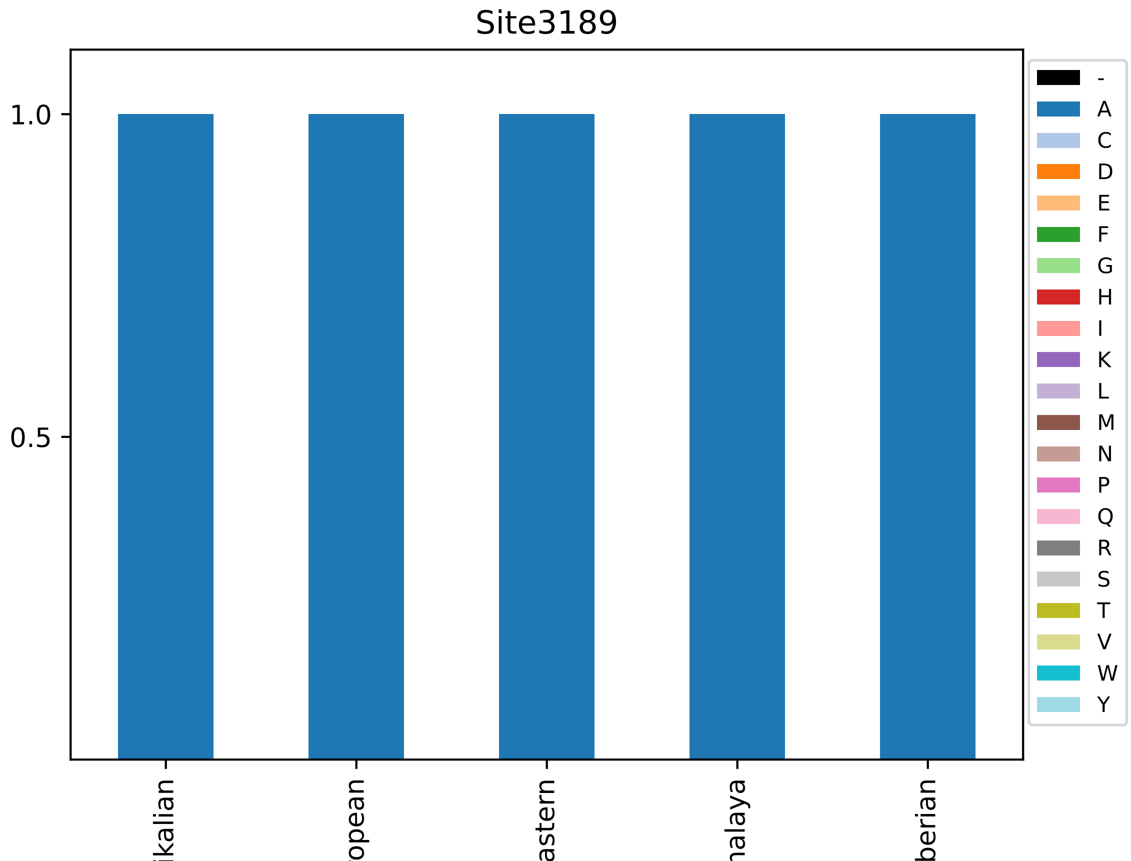
Site3189
1.0
0.5
-
A
C
D
E
F
G
H
I
K
L
M
N
P
Q
R
S
T
V
W
Y

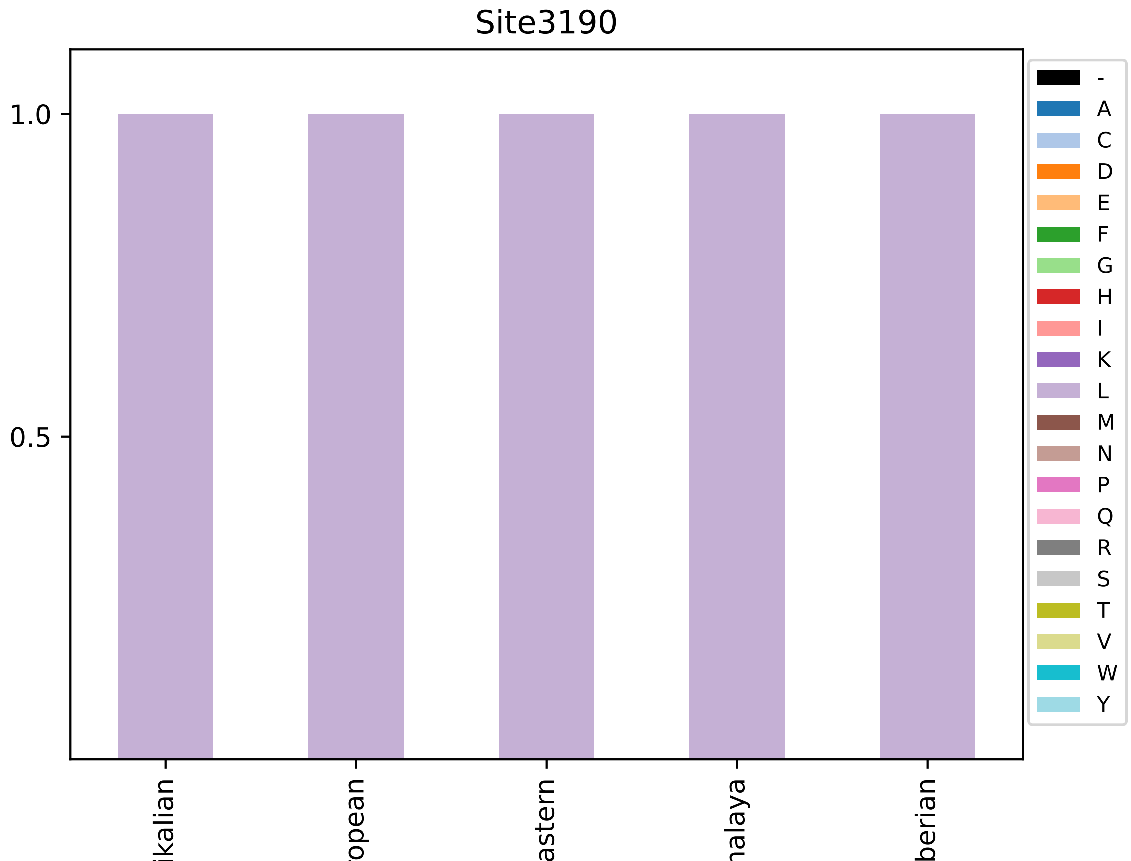
Site3190
1.0
0.5
ikalian
opean
astern
nalaya
berian
-
A
C
D
E
F
G
H
I
K
L
M
N
P
Q
R
S
T
V
W
Y

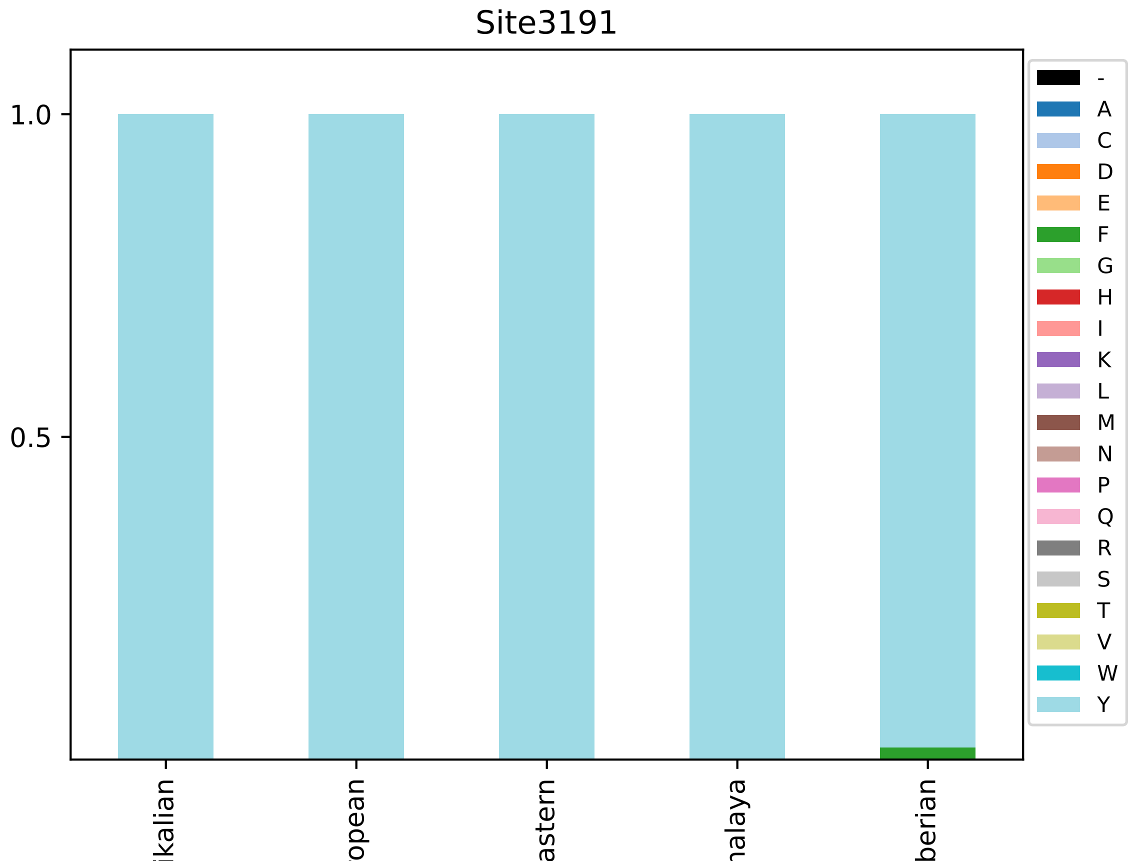

Site3191
1.0
0.5
ikalian
ropean
astern
nalaya
berian
-
A
C
D
E
F
G
H
I
K
L
M
N
P
Q
R
S
T
V
W
Y

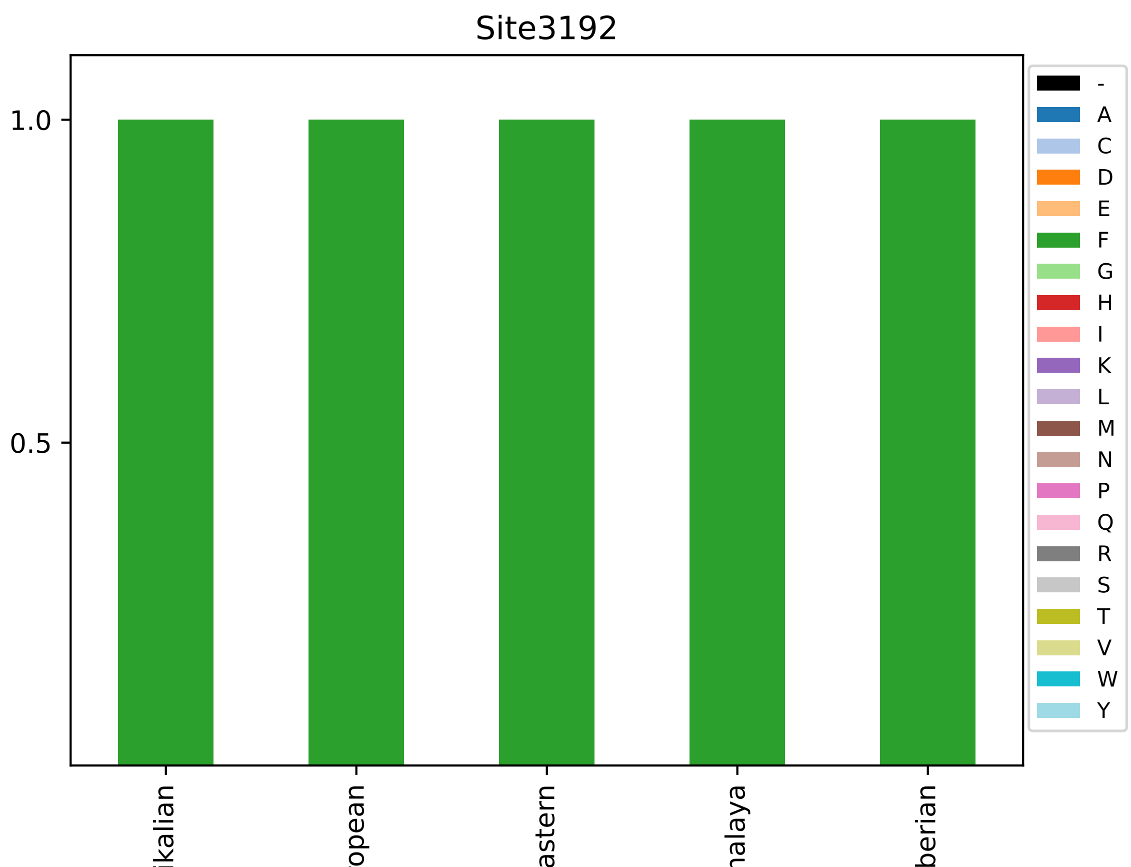
Site3192
1.0
0.5
ikalian
ropean
astern
nalaya
berian
-
A
C
D
E
F
G
H
I
K
L
M
N
P
Q
R
S
T
V
W
Y

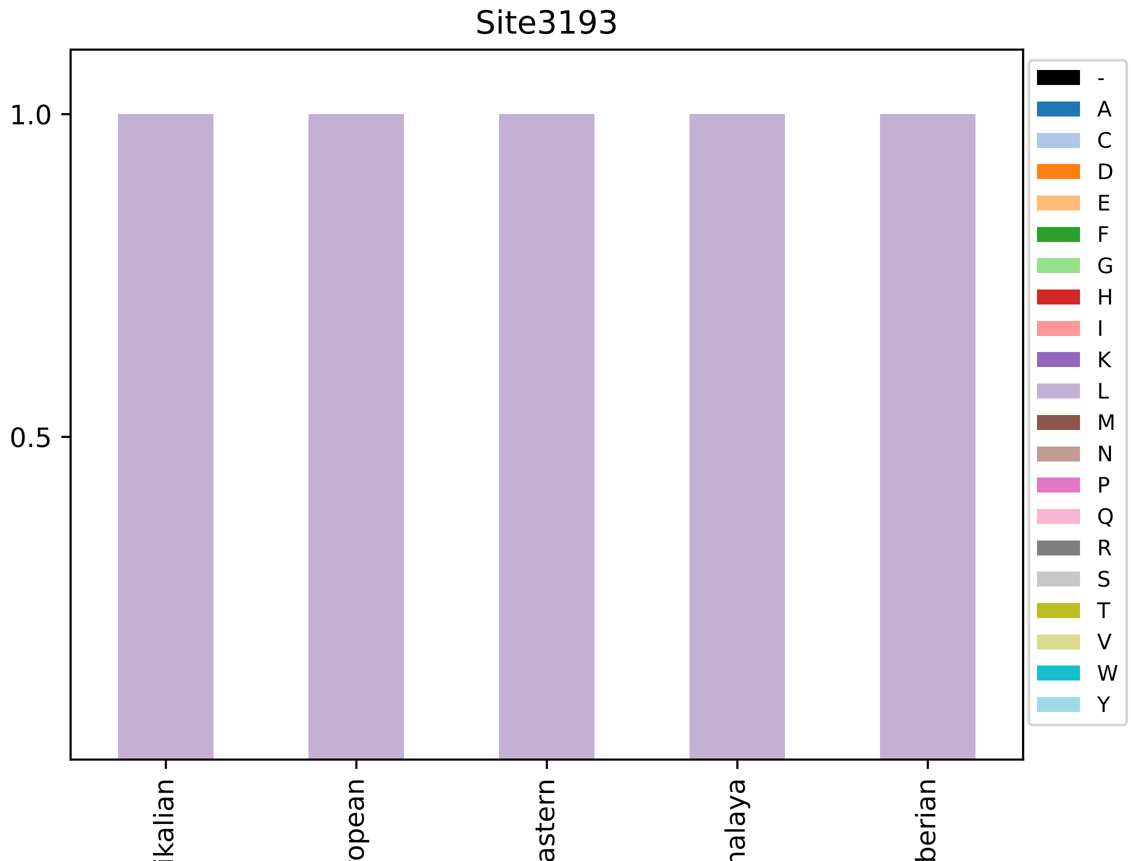
Site3193
1.0
0.5
-
A
C
D
E
F
G
H
I
K
L
M
N
P
Q
R
S
T
V
W
Y

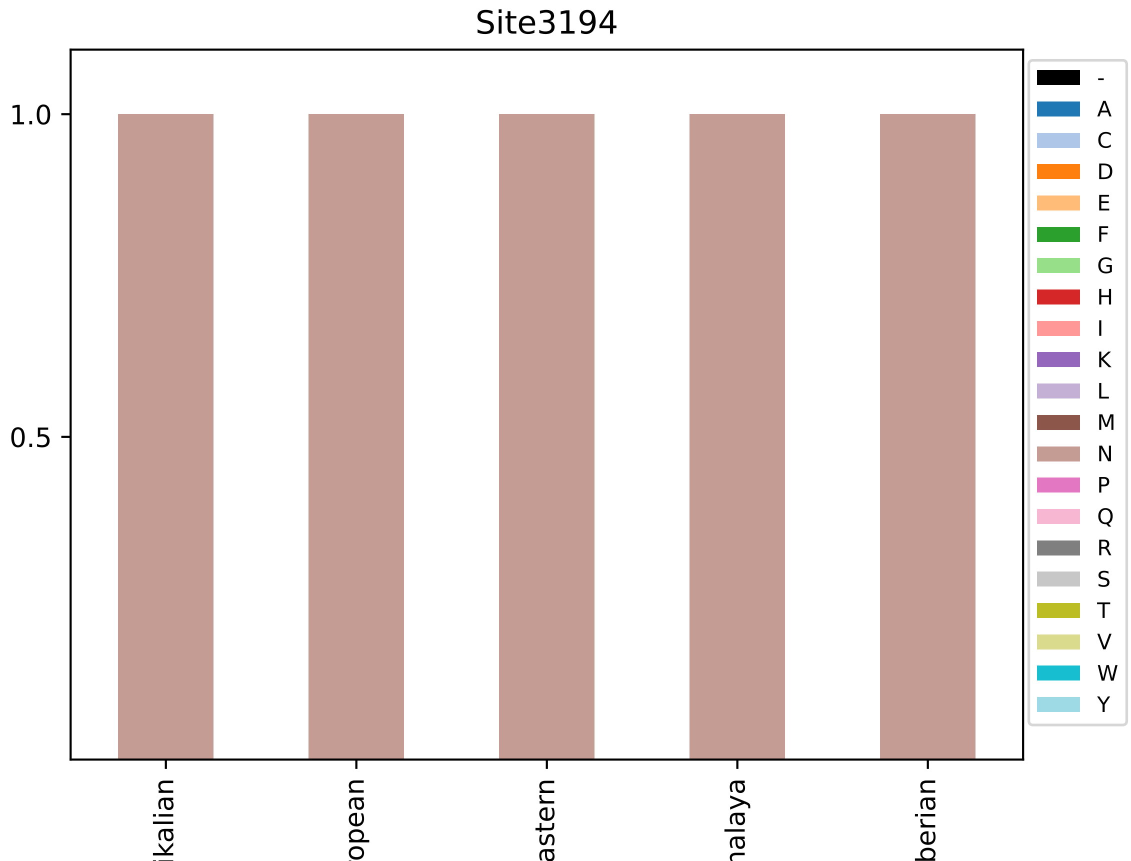
Site3194
1.0
0.5
-
A
C
D
E
F
G
H
I
K
L
M
N
P
Q
R
S
T
V
W
Y

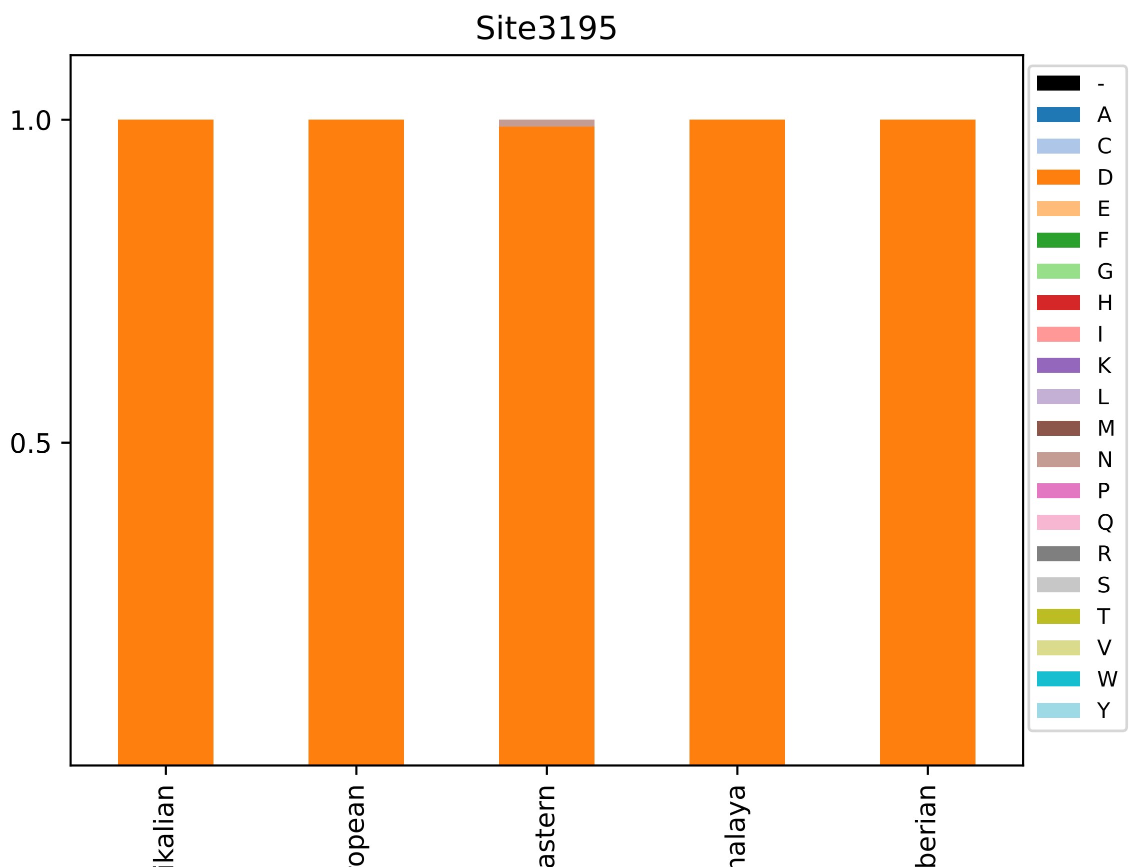

Site3195
1.0
0.5
-
A
C
D
E
F
G
H
I
K
L
M
N
P
Q
R
S
T
V
W
Y
ikalian
opean
astern
nalaya
berian

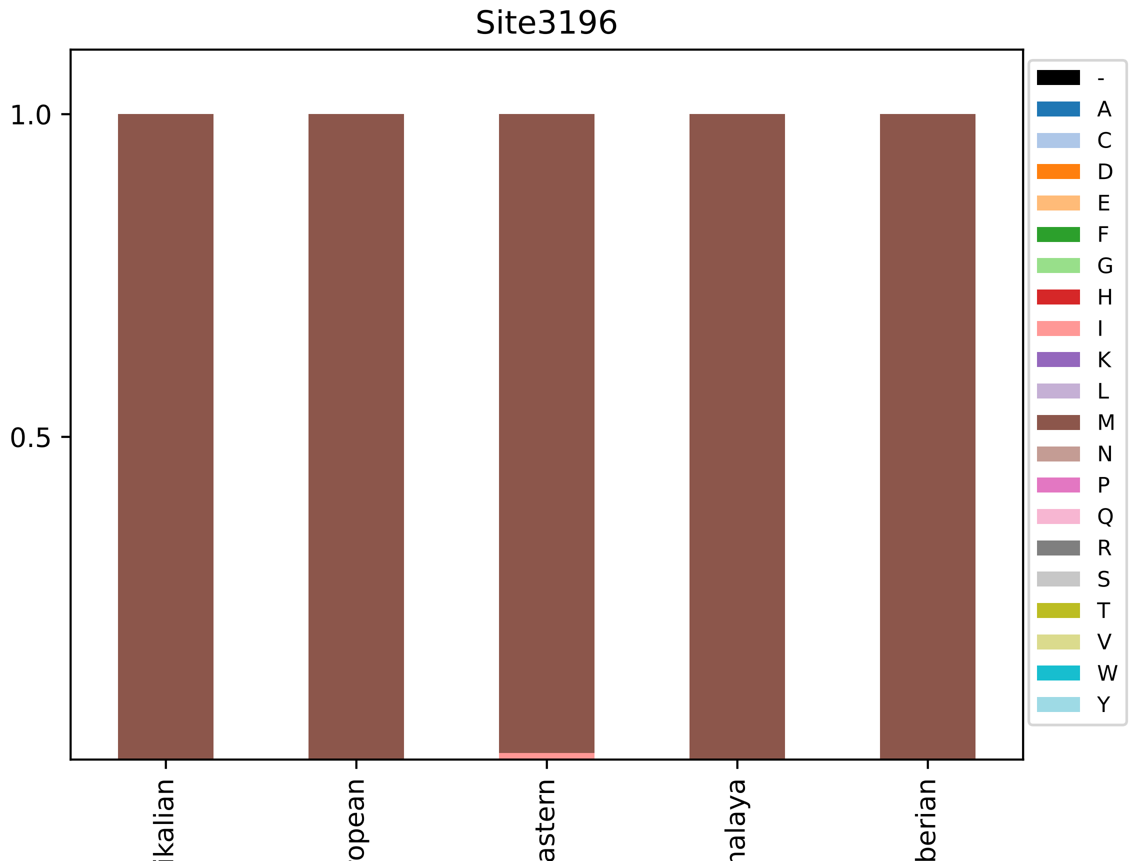

Site3196
1.0
0.5
ikalian
ropean
astern
nalaya
berian
-
A
C
D
E
F
G
H
I
K
L
M
N
P
Q
R
S
T
V
W
Y

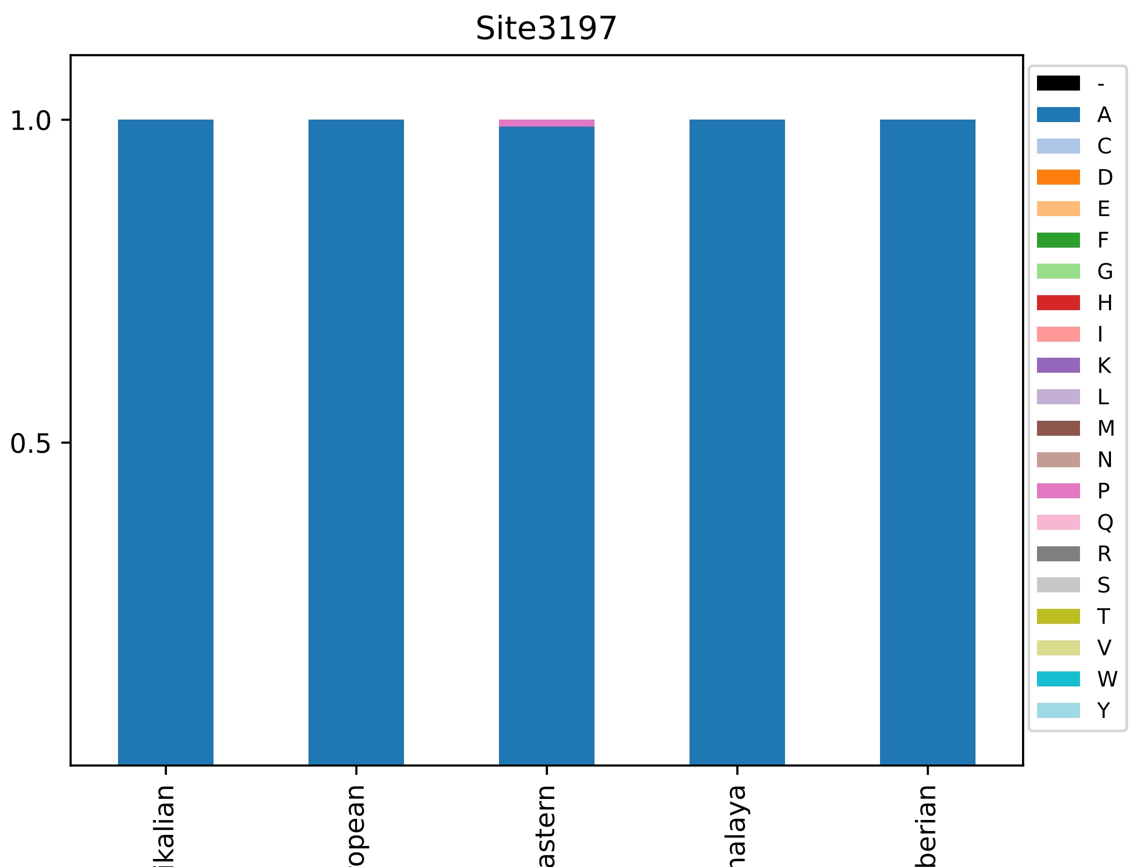
Site3197
1.0
0.5
-
A
C
D
E
F
G
H
I
K
L
M
N
P
Q
R
S
T
V
W
Y
ikalian
opean
astern
nalaya
berian

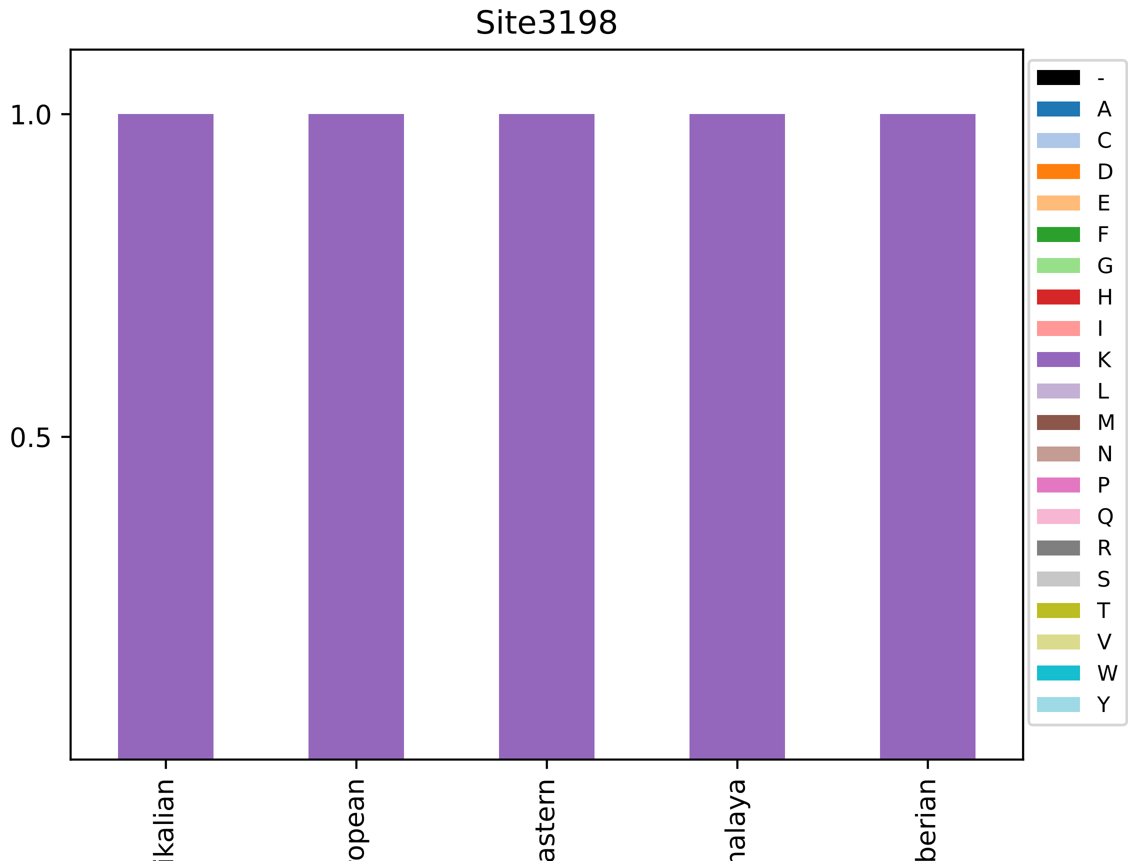
Site3198
1.0
0.5
-
A
C
D
E
F
G
H
I
K
L
M
N
P
Q
R
S
T
V
W
Y

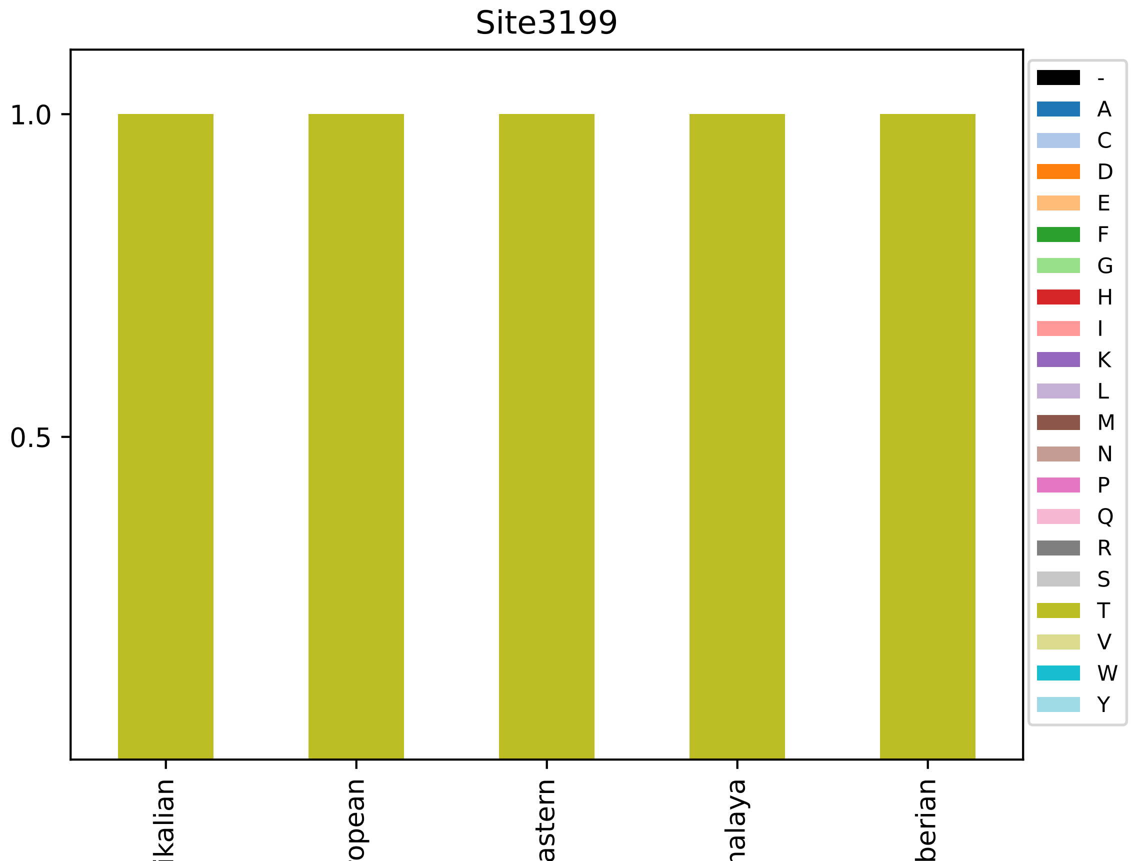

Site3199
1.0
0.5
-
A
C
D
E
F
G
H
I
K
L
M
N
P
Q
R
S
T
V
W
Y
ikalian
opean
astern
nalaya
berian

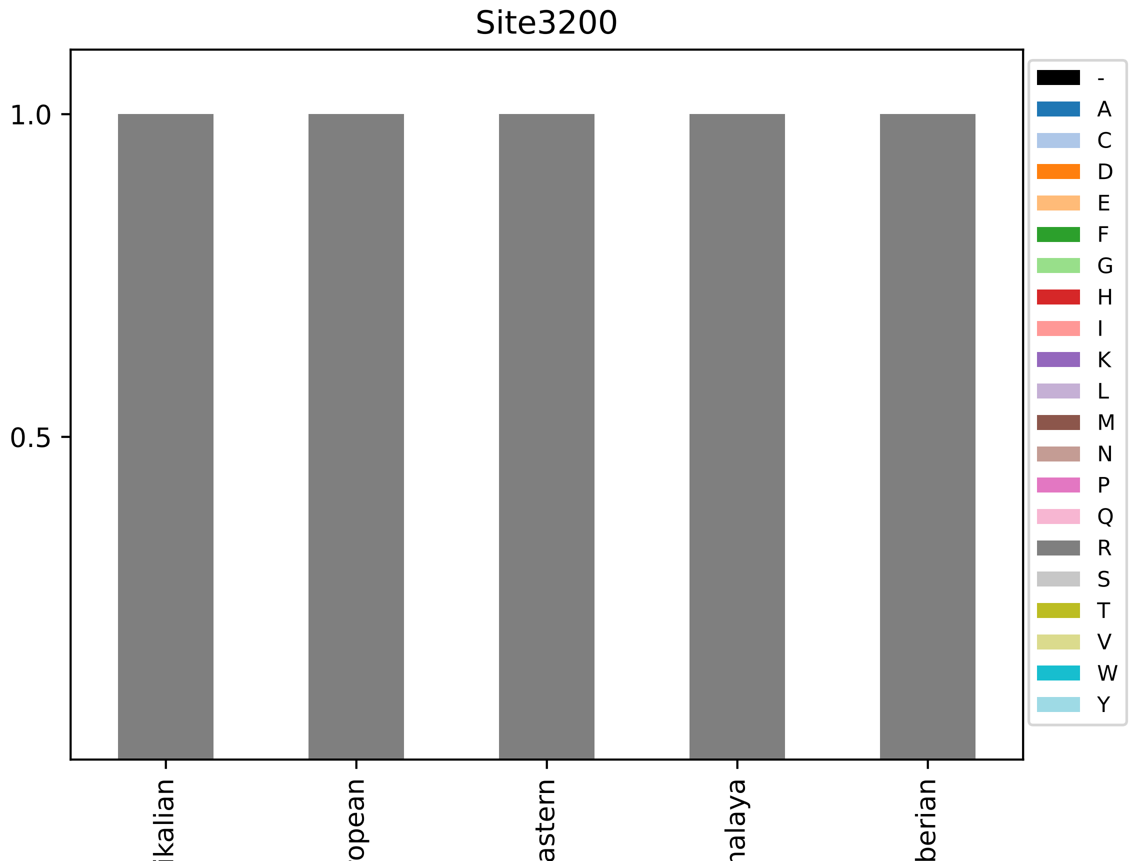

Site3200
1.0
0.5
ikalian
opean
astern
nalaya
berian
-
A
C
D
E
F
G
H
I
K
L
M
N
P
Q
R
S
T
V
W
Y

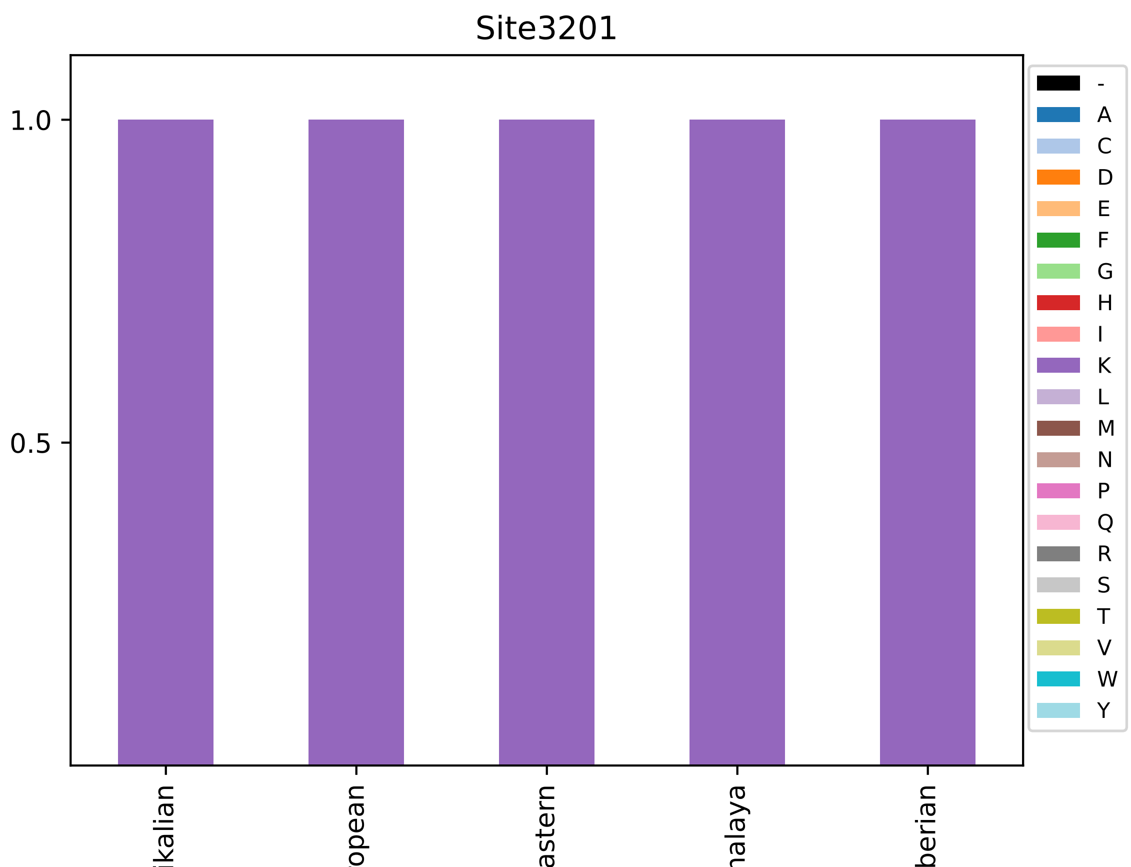
Site3201
1.0
0.5
-
A
C
D
E
F
G
H
I
K
L
M
N
P
Q
R
S
T
V
W
Y
ikalian
opean
astern
nalaya
berian

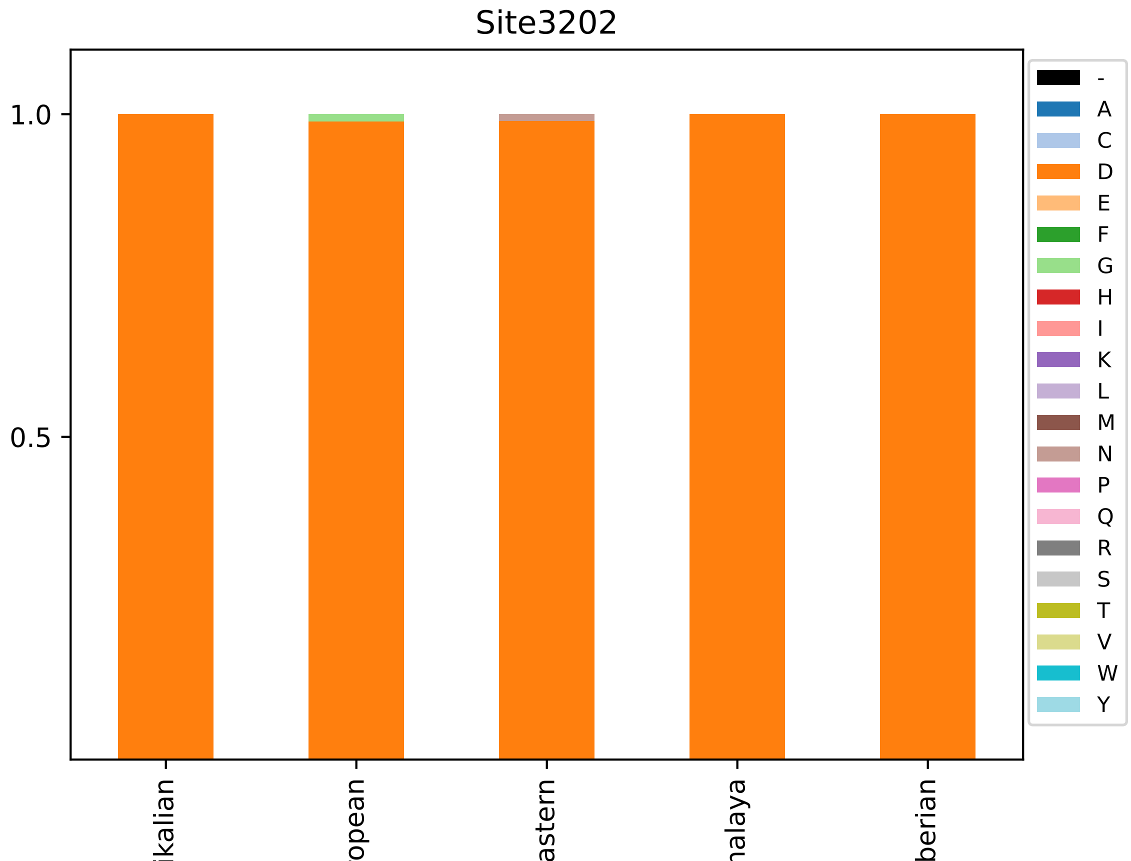
Site3202
1.0
0.5
-
A
C
D
E
F
G
H
I
K
L
M
N
P
Q
R
S
T
V
W
Y
ikalian
opean
astern
nalaya
berian

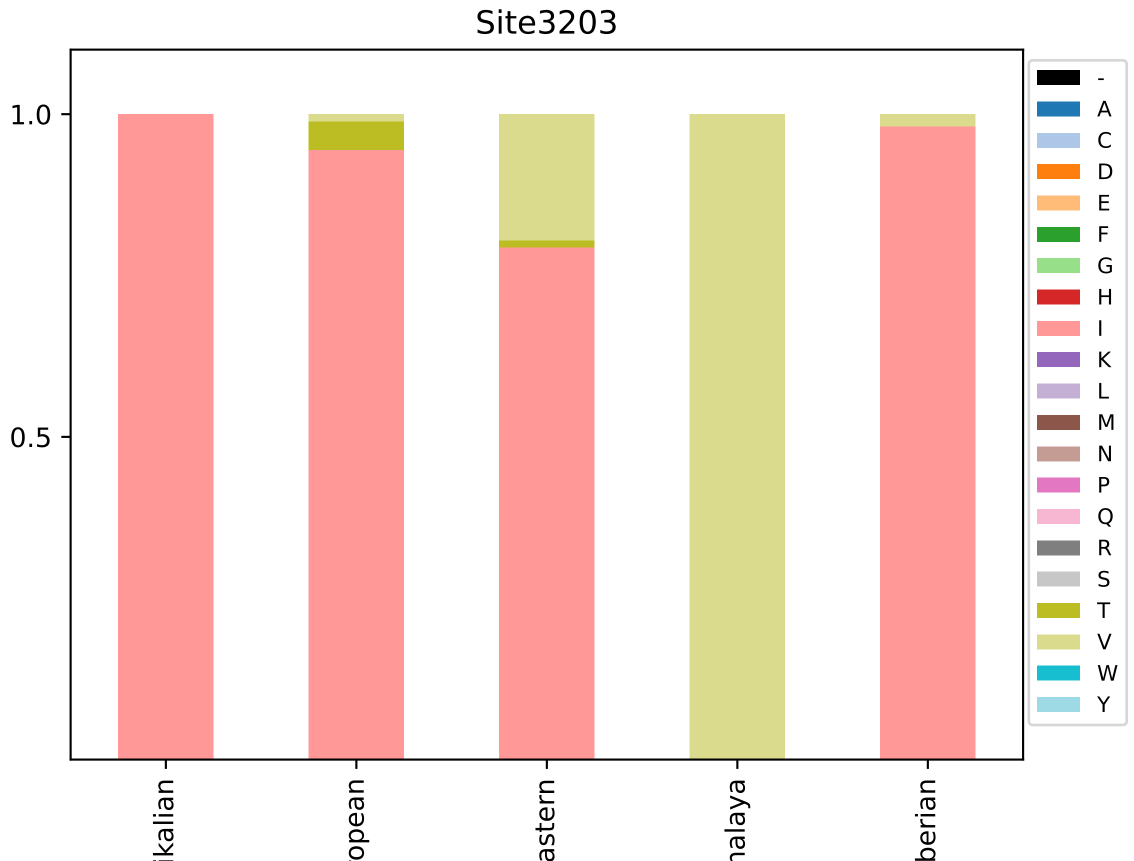

Site3203
1.0
0.5
ikalian
opean
astern
nalaya
berian
-
A
C
D
E
F
G
H
I
K
L
M
N
P
Q
R
S
T
V
W
Y

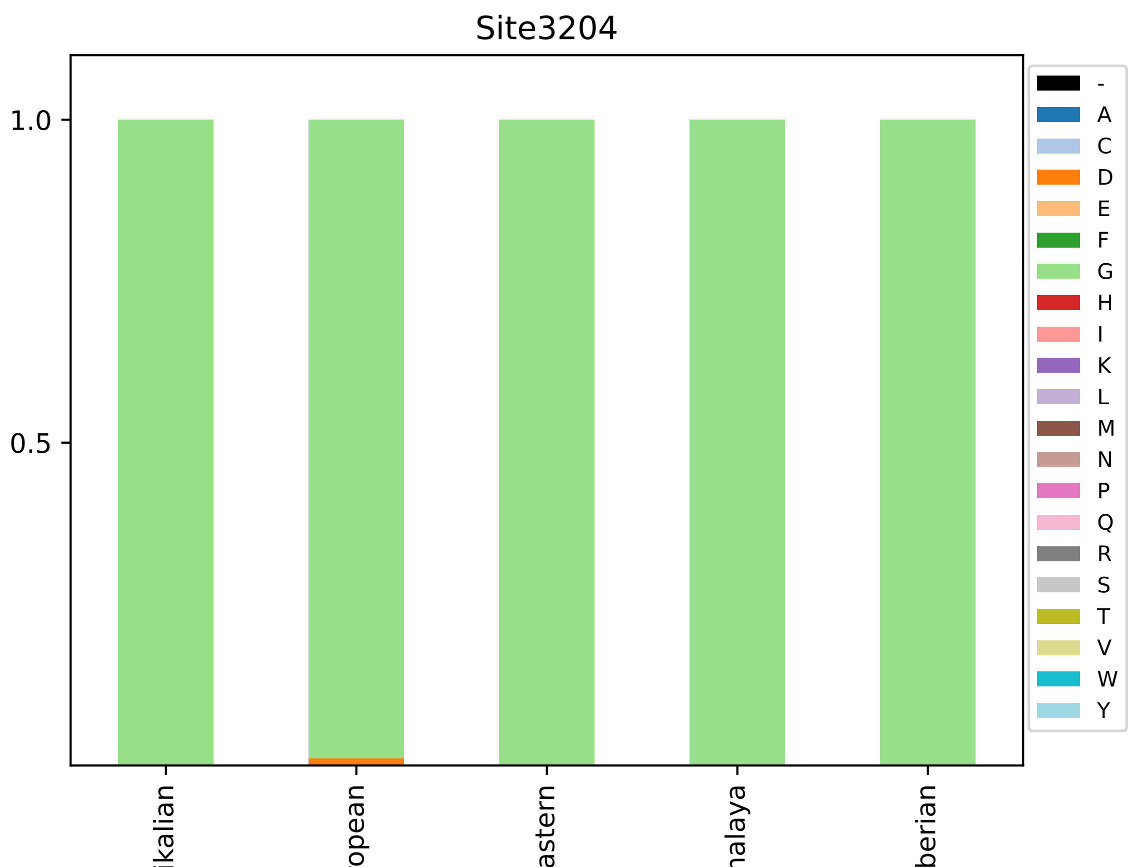
Site3204
1.0
0.5
-
A
C
D
E
F
G
H
I
K
L
M
N
P
Q
R
S
T
V
W
Y
ikalian
opean
astern
nalaya
berian

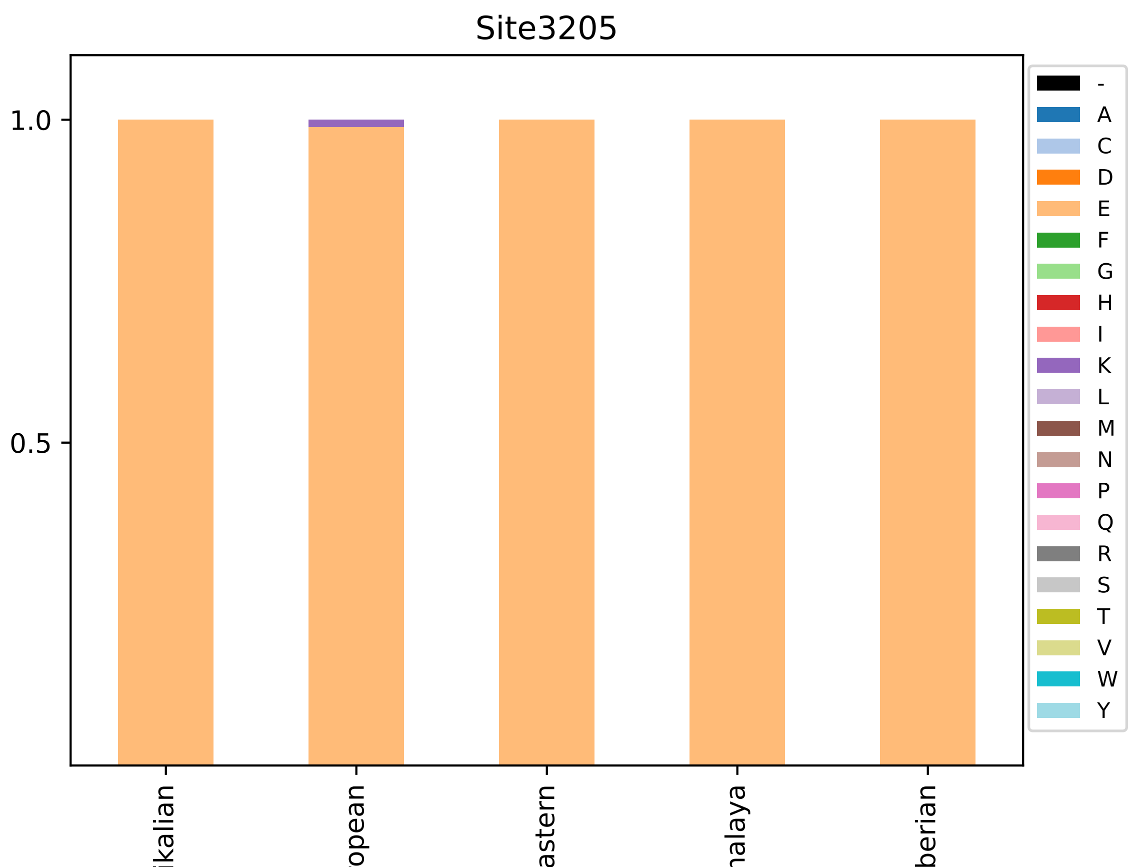
Site3205
1.0
0.5
-
A
C
D
E
F
G
H
I
K
L
M
N
P
Q
R
S
T
V
W
Y
ikalian
opean
astern
nalaya
berian

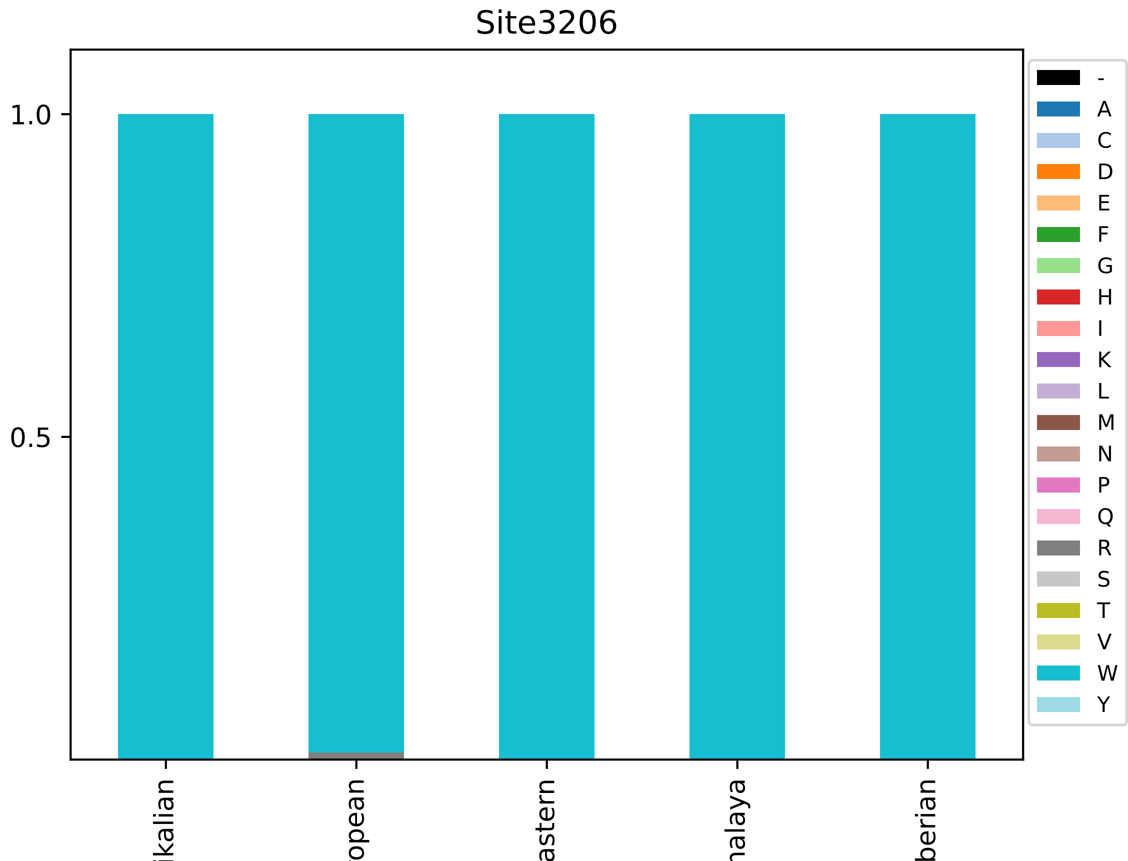

Site3206
1.0
0.5
-
A
C
D
E
F
G
H
I
K
L
M
N
P
Q
R
S
T
V
W
Y
ikalian
opean
astern
nalaya
berian

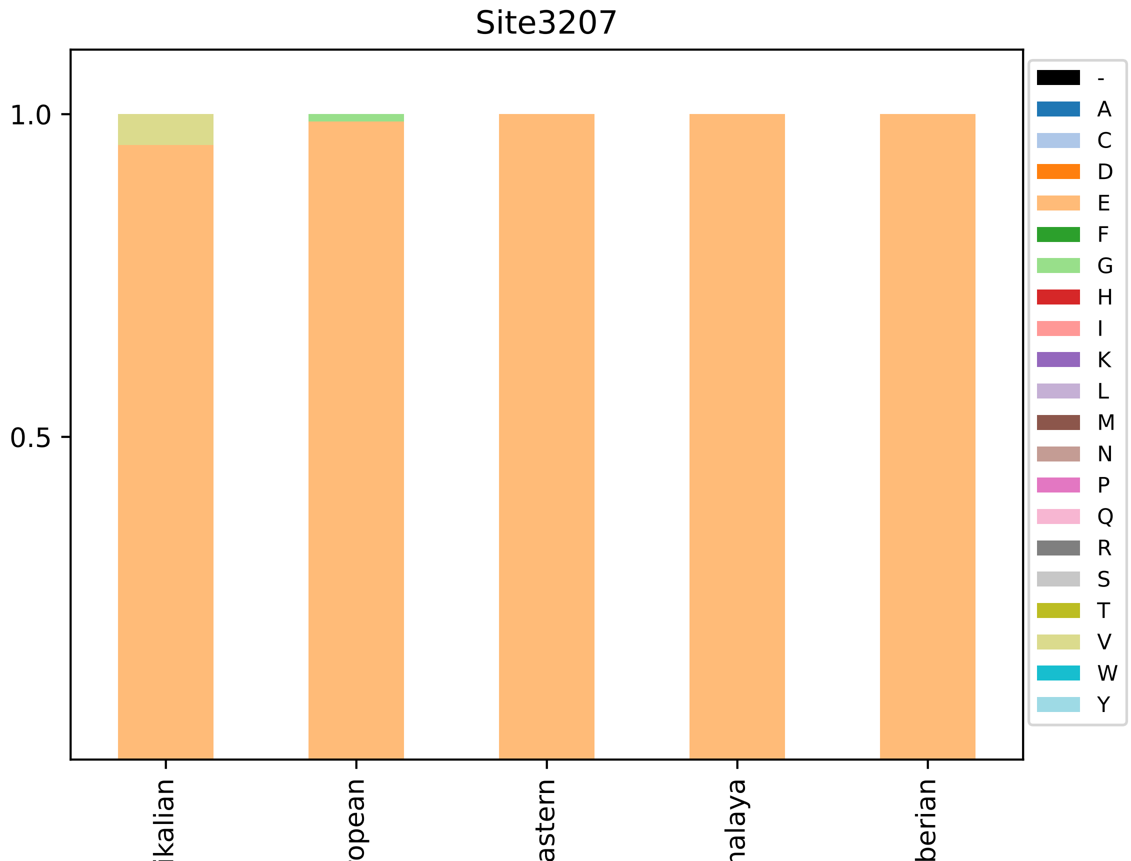
Site3207
1.0
0.5
-
A
C
D
E
F
G
H
I
K
L
M
N
P
Q
R
S
T
V
W
Y
ikalian
opean
astern
nalaya
berian

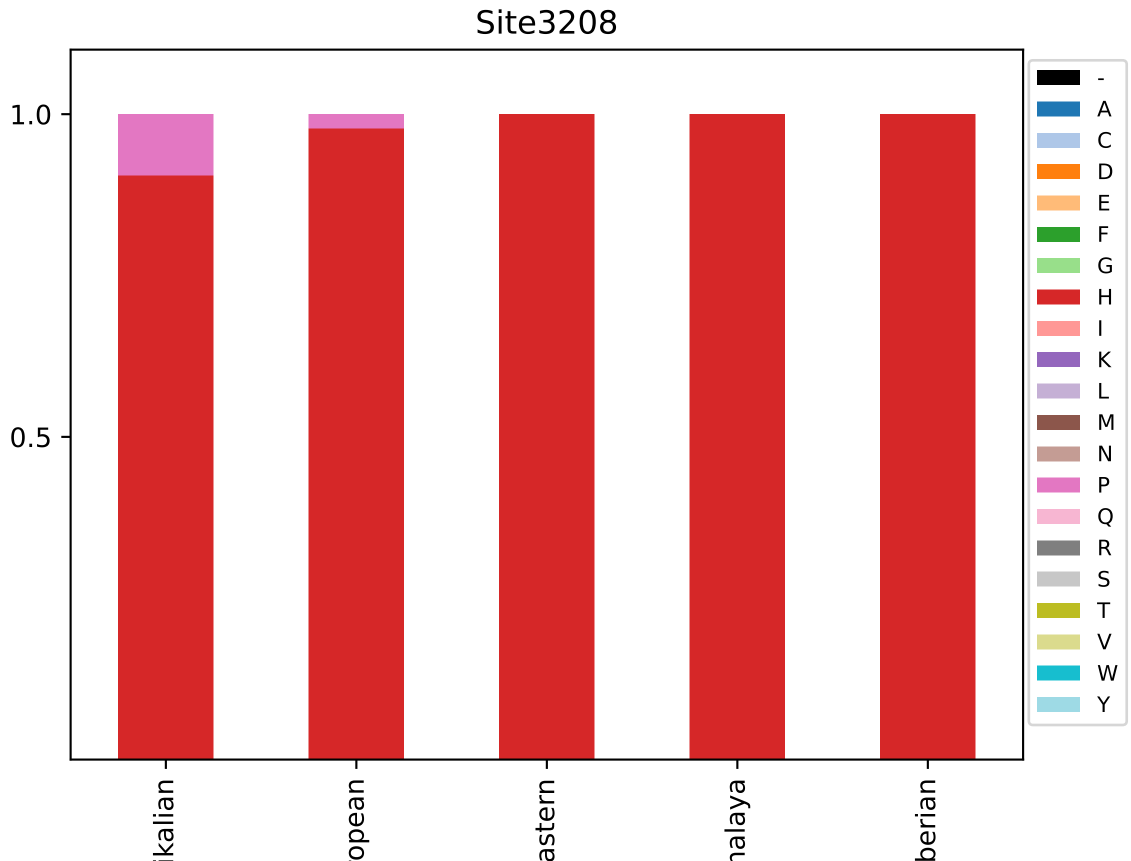
Site3208
1.0
0.5
-
A
C
D
E
F
G
H
I
K
L
M
N
P
Q
R
S
T
V
W
Y

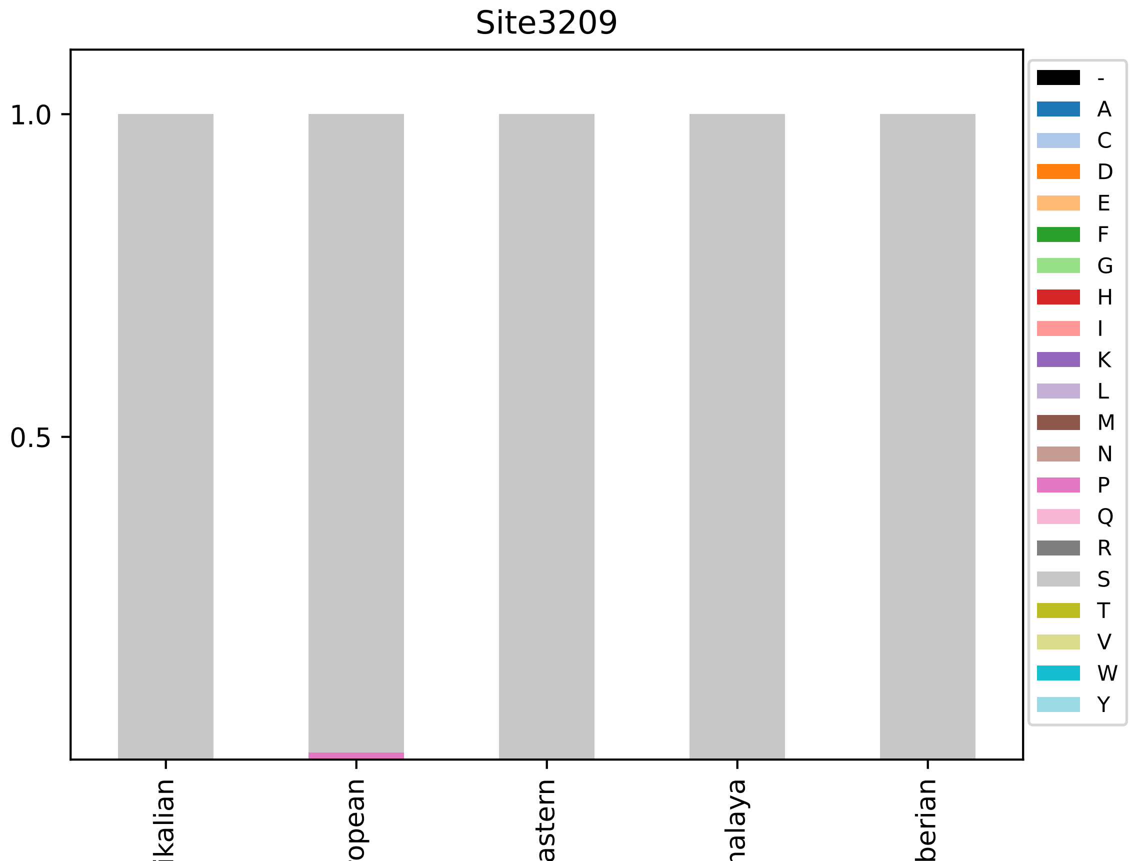

Site3209
1.0
0.5
-
A
C
D
E
F
G
H
I
K
L
M
N
P
Q
R
S
T
V
W
Y
ikalian
opean
astern
nalaya
berian

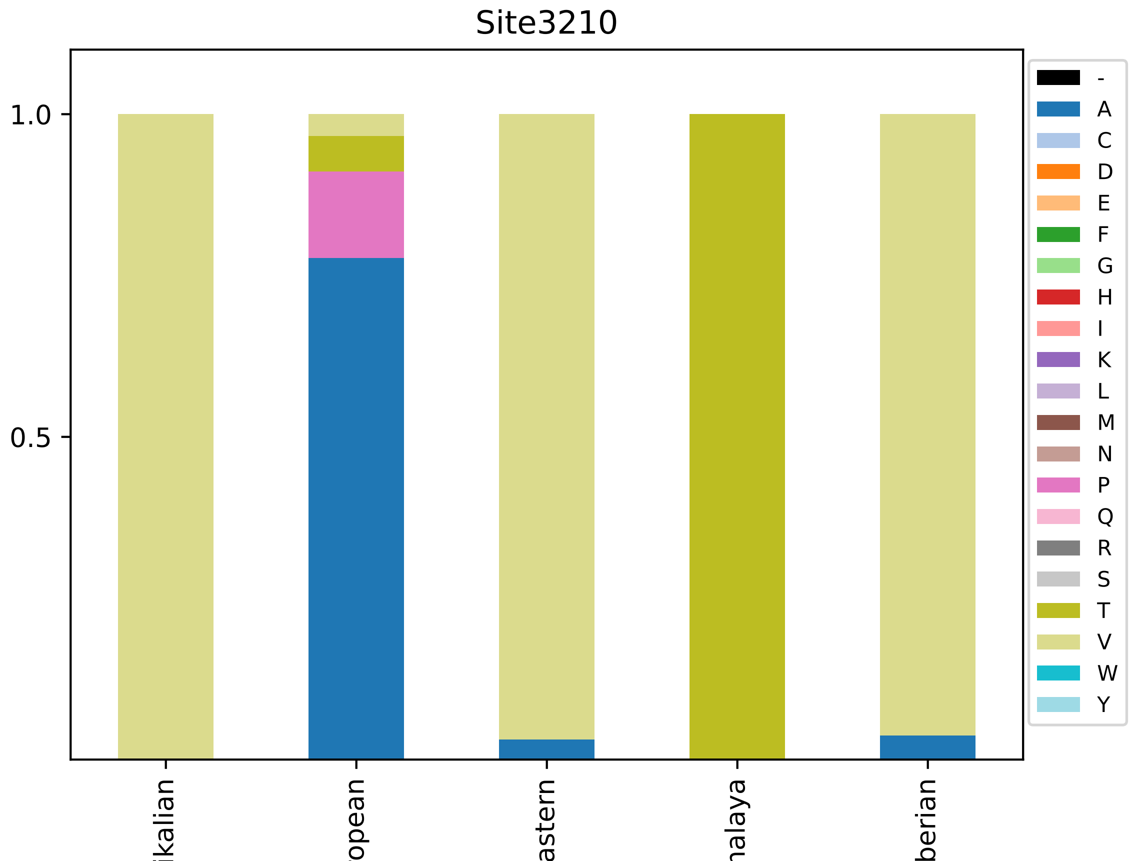
Site3210
1.0
0.5
-
A
C
D
E
F
G
H
I
K
L
M
N
P
Q
R
S
T
V
W
Y
ikalian
opean
astern
nalaya
berian

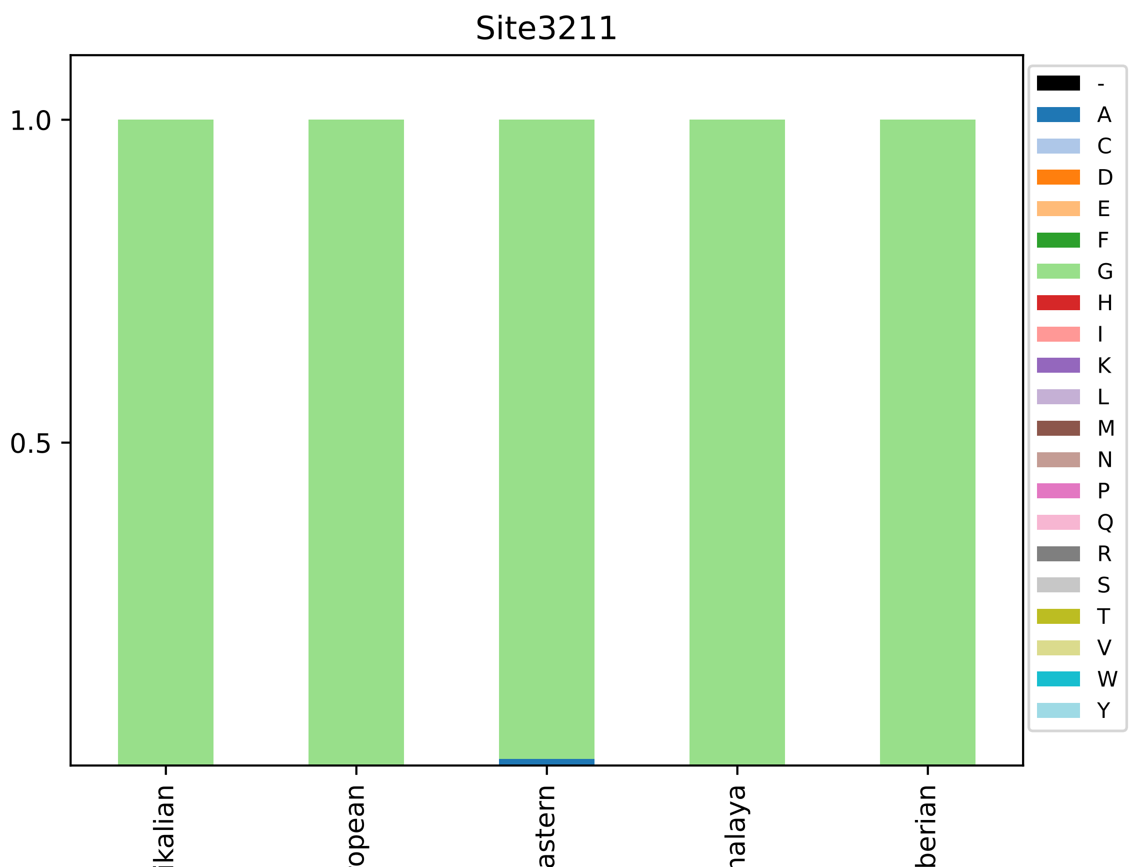

Site3211
1.0
0.5
-
A
C
D
E
F
G
H
I
K
L
M
N
P
Q
R
S
T
V
W
Y
ikalian
opean
astern
nalaya
berian

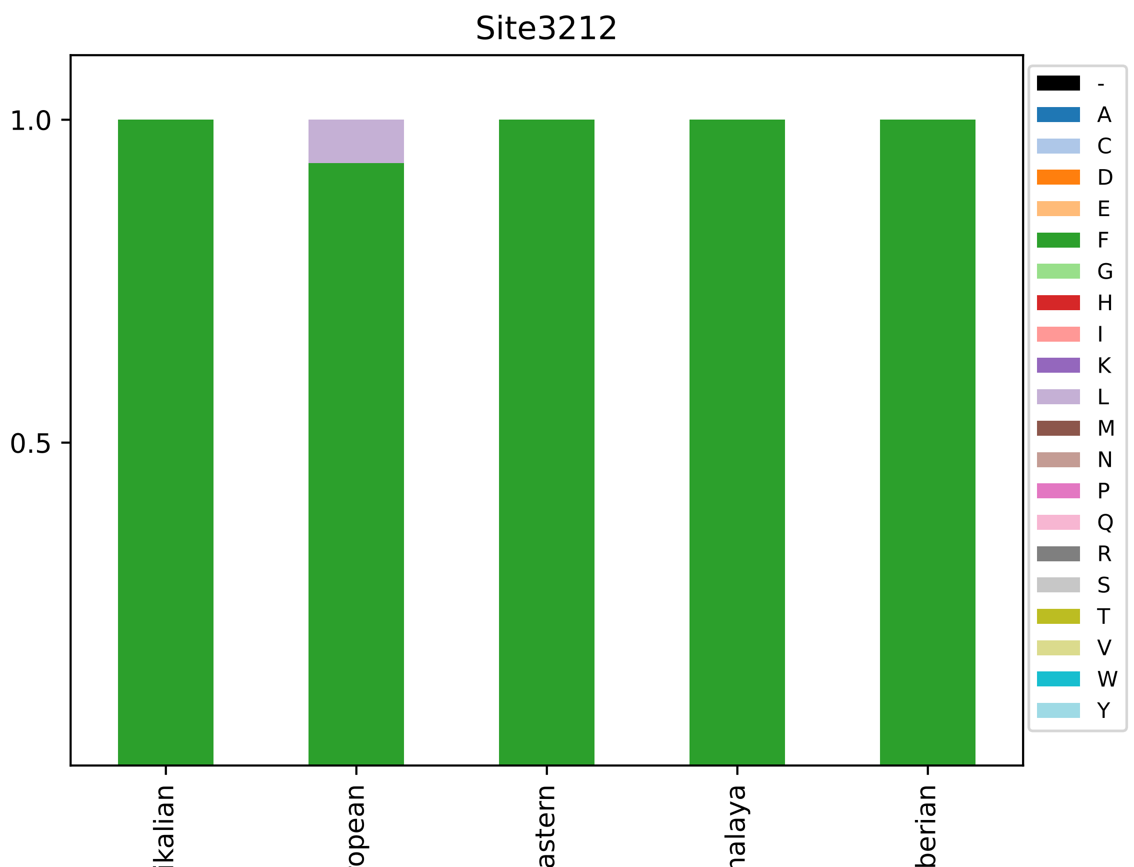

Site3212
1.0
0.5
ikalian
opean
astern
nalaya
berian
-
A
C
D
E
F
G
H
I
K
L
M
N
P
Q
R
S
T
V
W
Y

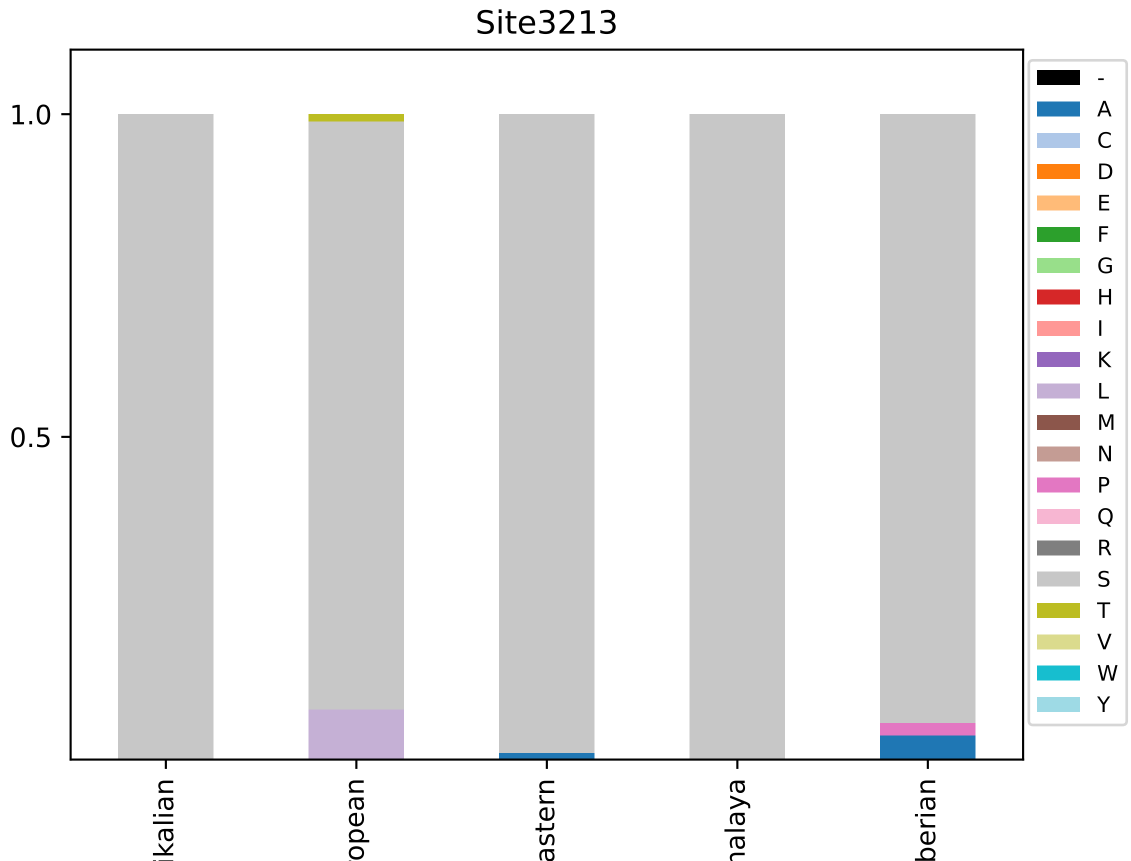

Site3213
1.0
0.5
-
A
C
D
E
F
G
H
I
K
L
M
N
P
Q
R
S
T
V
W
Y
ikalian
opean
astern
nalaya
berian

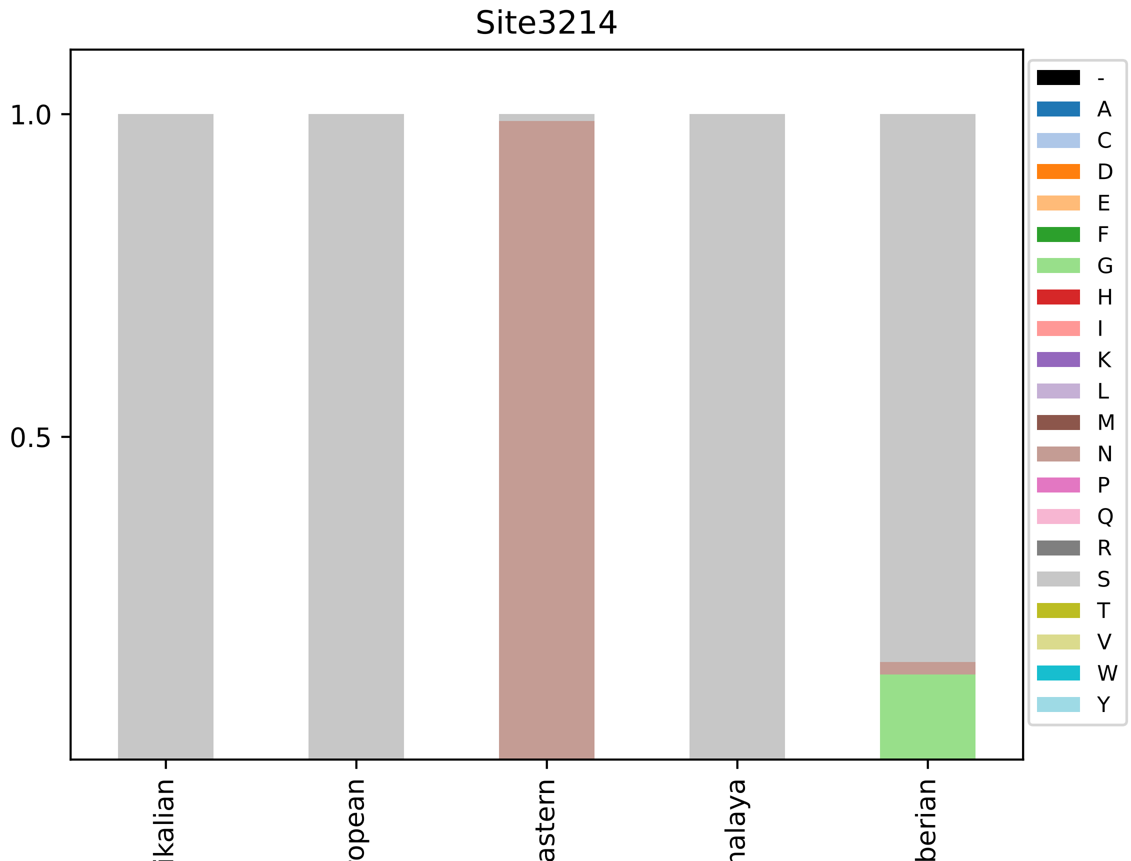

Site3214
1.0
0.5
-
A
C
D
E
F
G
H
I
K
L
M
N
P
Q
R
S
T
V
W
Y
ikalian
opean
astern
nalaya
berian

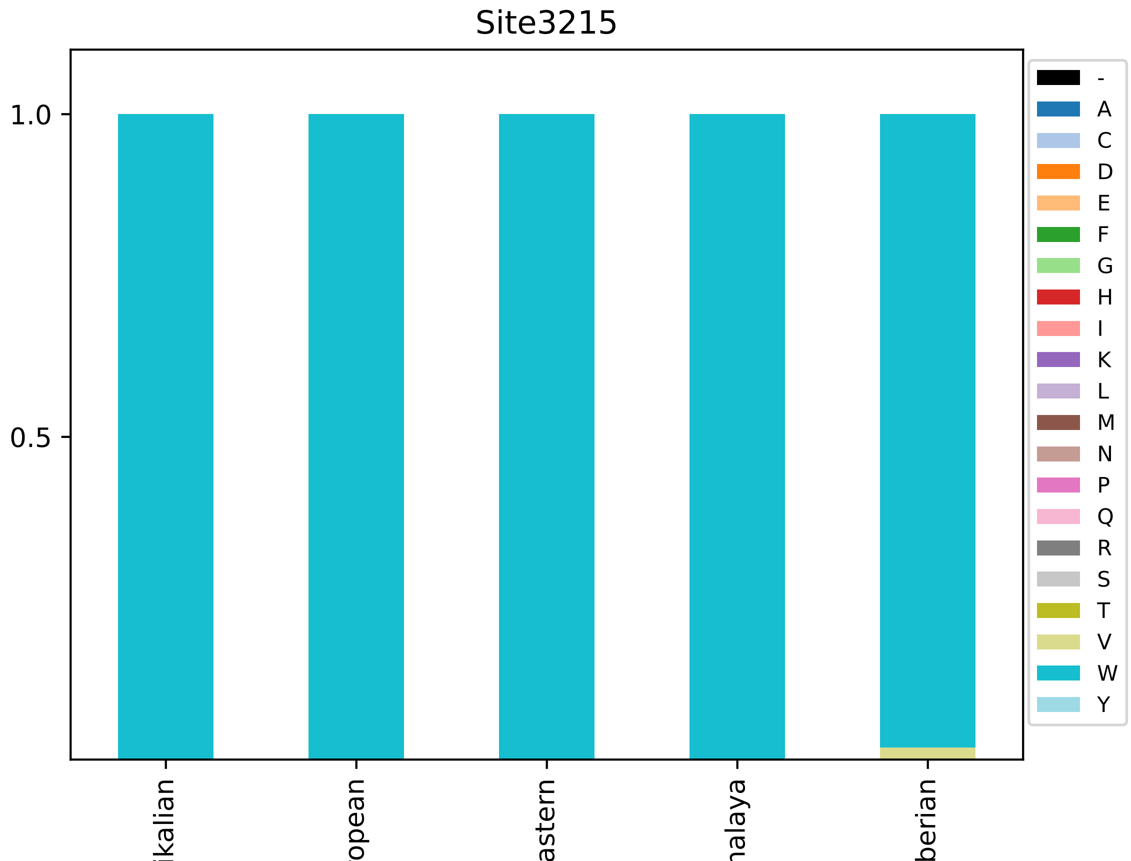

Site3215
1.0
0.5
-
A
C
D
E
F
G
H
I
K
L
M
N
P
Q
R
S
T
V
W
Y
ikalian
opean
astern
nalaya
berian

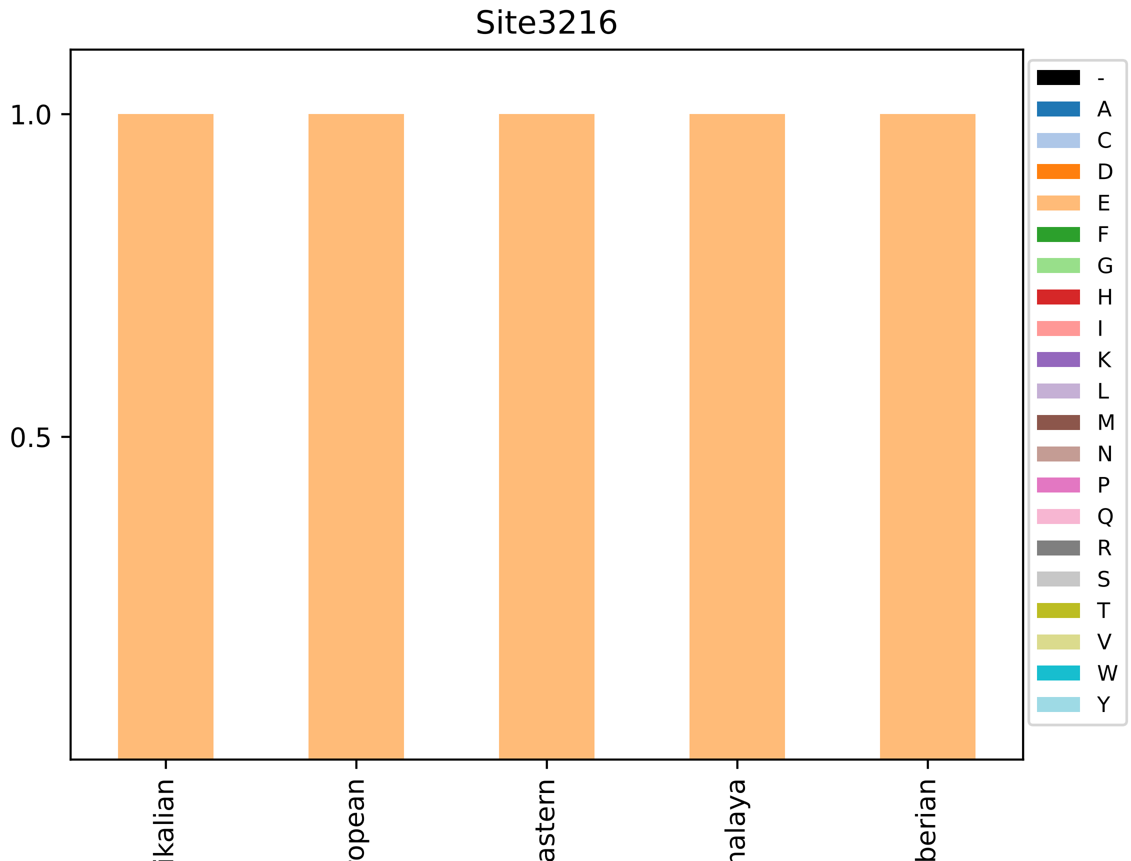
Site3216
1.0
0.5
ikalian
opean
astern
nalaya
berian
-
A
C
D
E
F
G
H
I
K
L
M
N
P
Q
R
S
T
V
W
Y

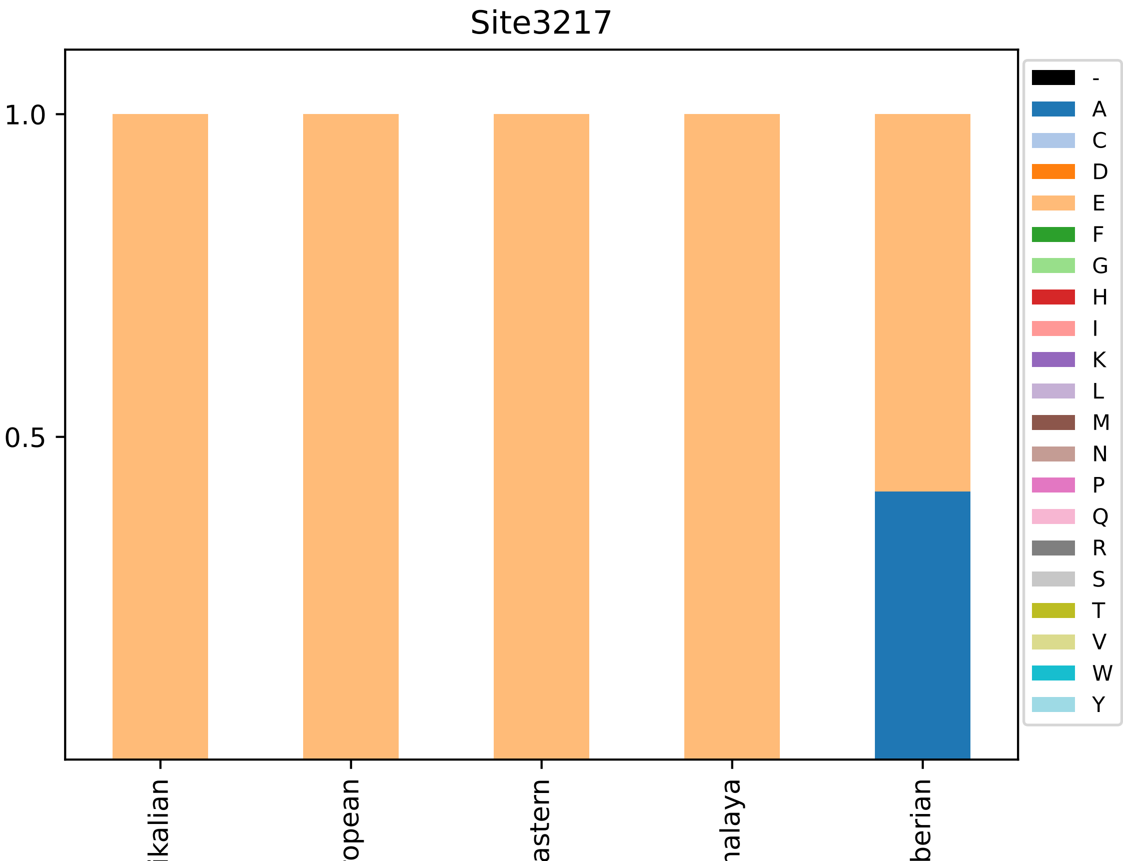

Site3217
1.0
0.5
-
A
C
D
E
F
G
H
I
K
L
M
N
P
Q
R
S
T
V
W
Y
ikalian
opean
astern
nalaya
berian

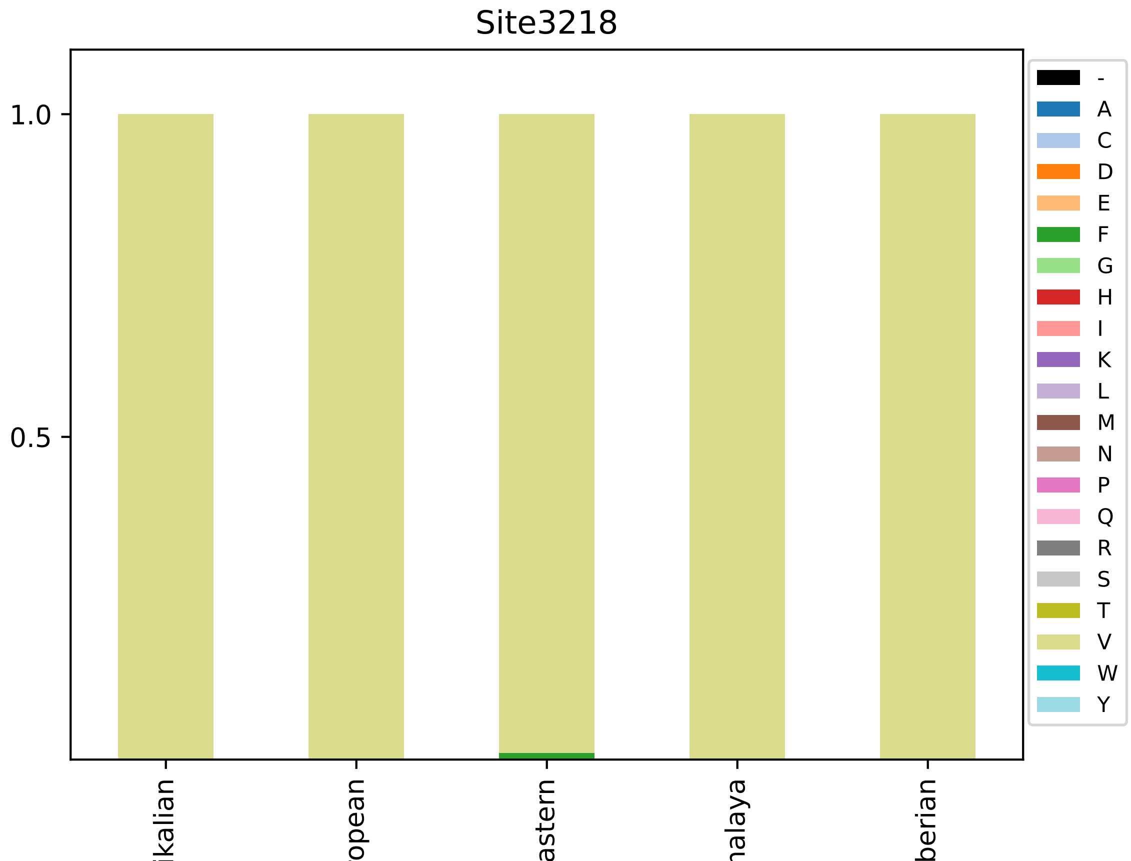

Site3218
1.0
0.5
-
A
C
D
E
F
G
H
I
K
L
M
N
P
Q
R
S
T
V
W
Y
ikalian
opean
astern
nalaya
berian

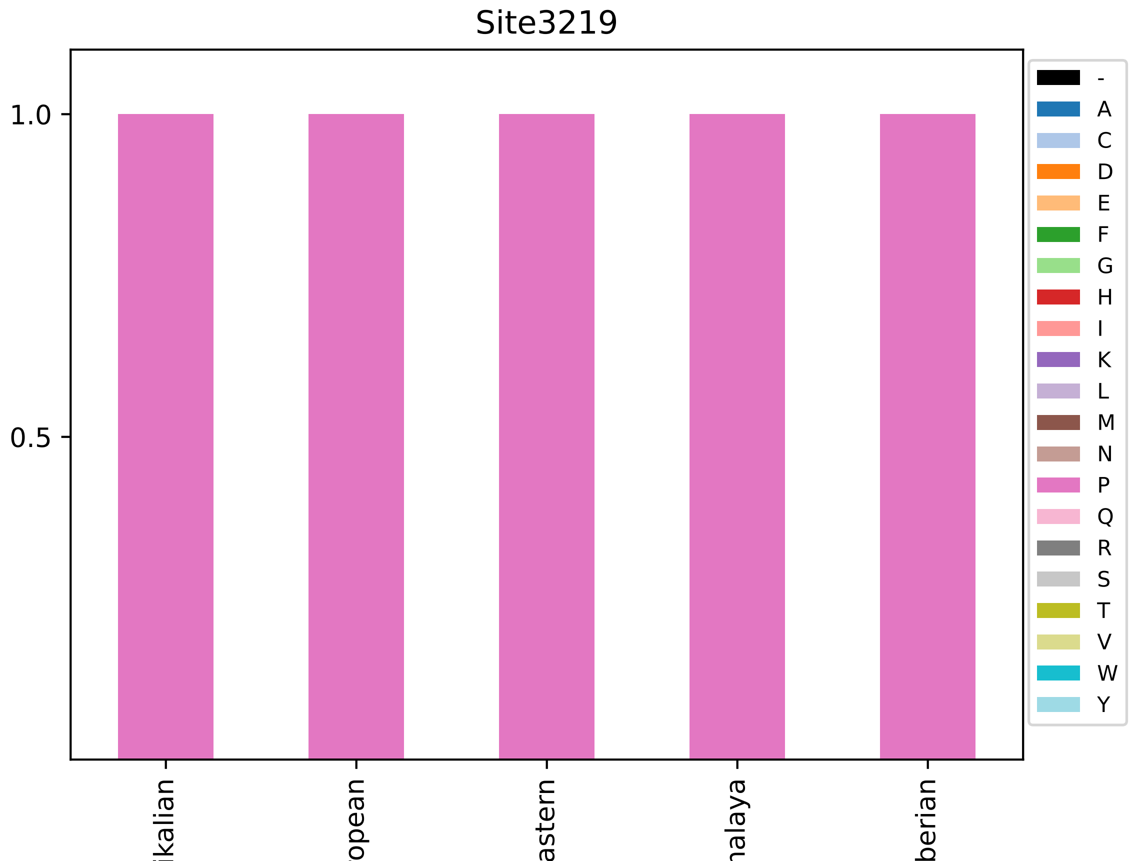
Site3219
1.0
0.5
-
A
C
D
E
F
G
H
I
K
L
M
N
P
Q
R
S
T
V
W
Y
ikalian
opean
astern
nalaya
berian

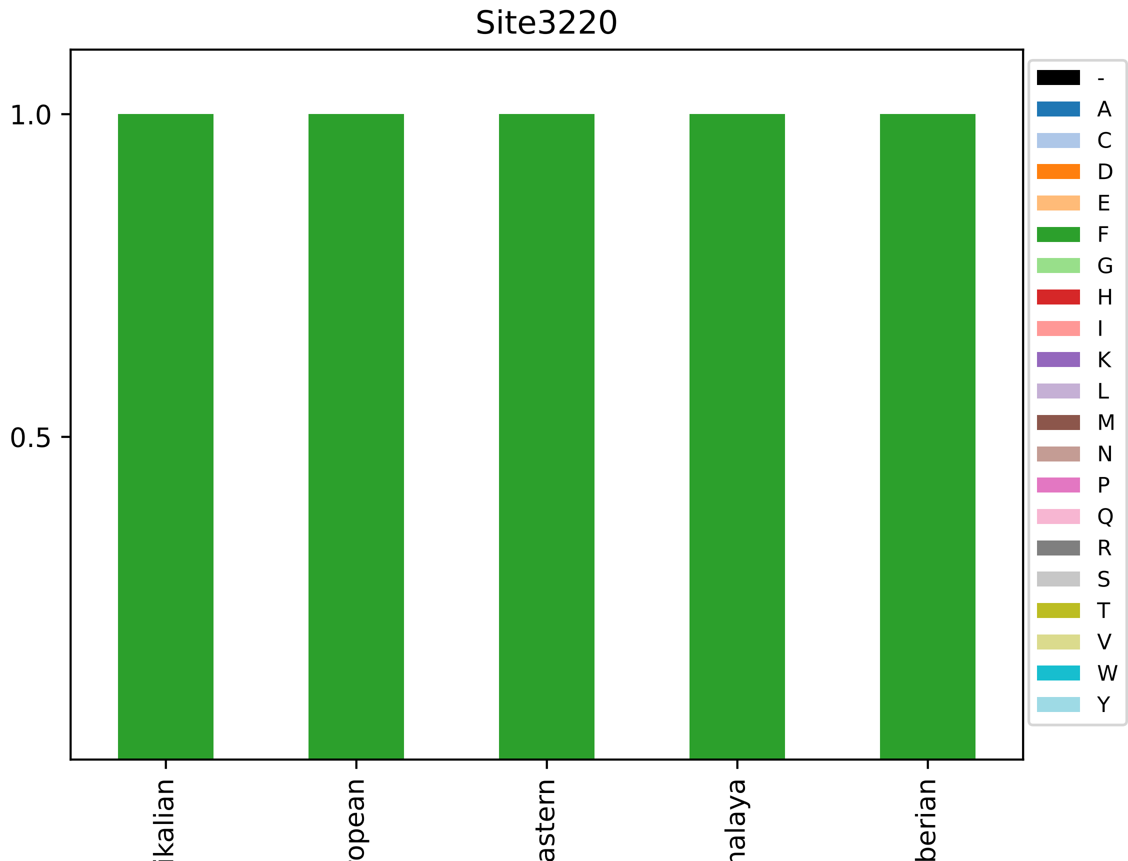

Site3220
1.0
0.5
-
A
C
D
E
F
G
H
I
K
L
M
N
P
Q
R
S
T
V
W
Y
ikalian
opean
astern
nalaya
berian

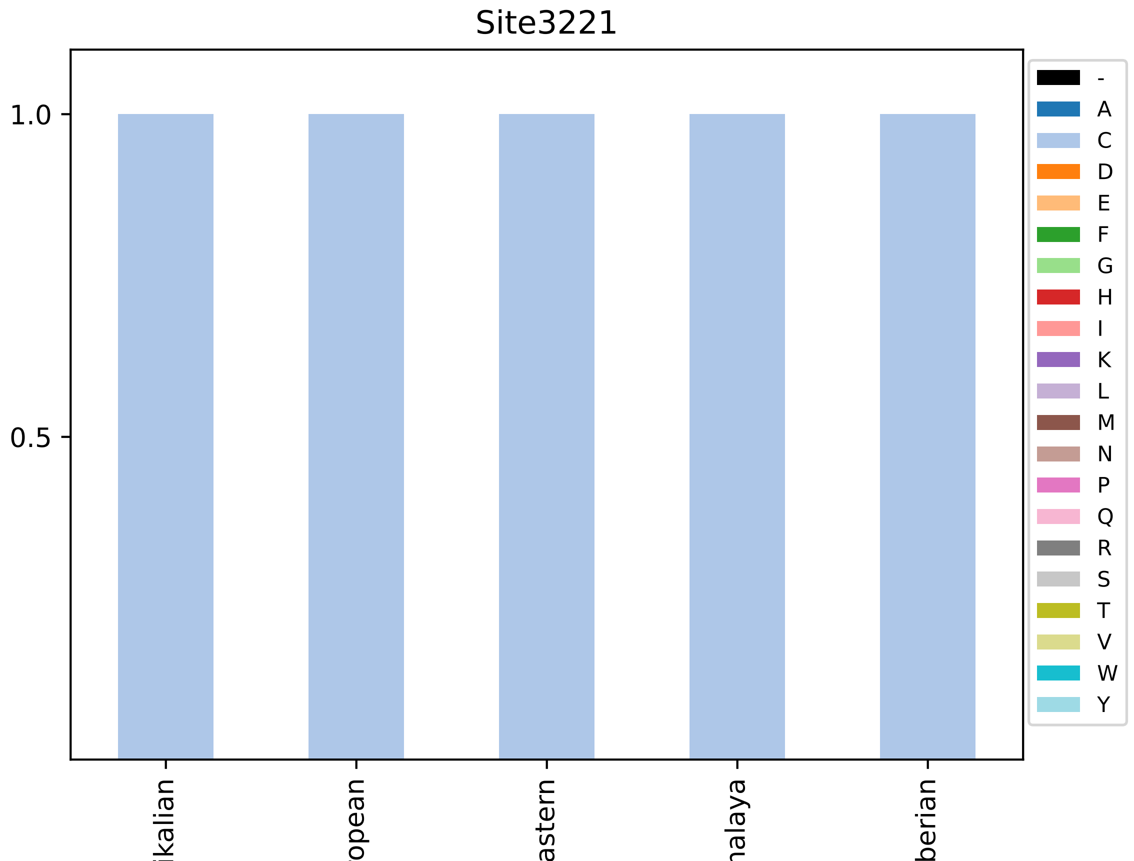
Site3221
1.0
0.5
ikalian
opean
astern
nalaya
berian
-
A
C
D
E
F
G
H
I
K
L
M
N
P
Q
R
S
T
V
W
Y

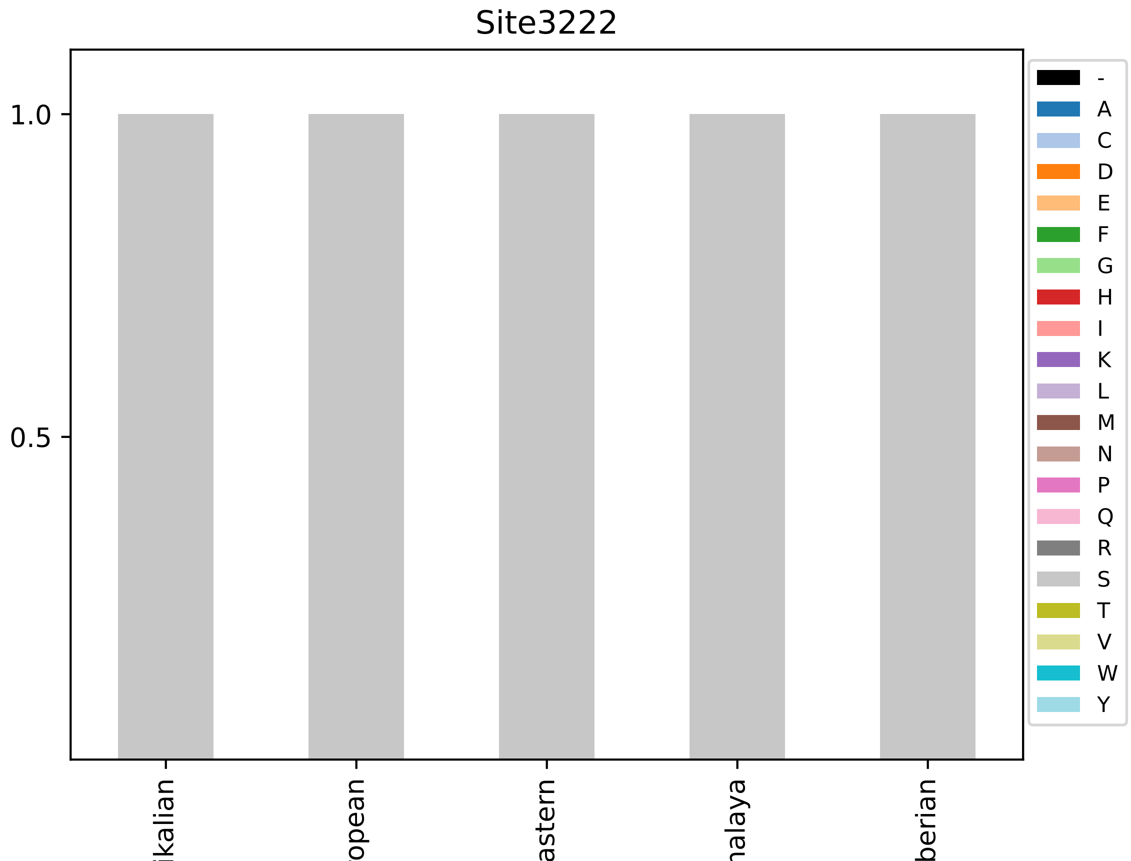

Site3222
1.0
0.5
-
A
C
D
E
F
G
H
I
K
L
M
N
P
Q
R
S
T
V
W
Y
ikalian
ropean
astern
nalaya
berian

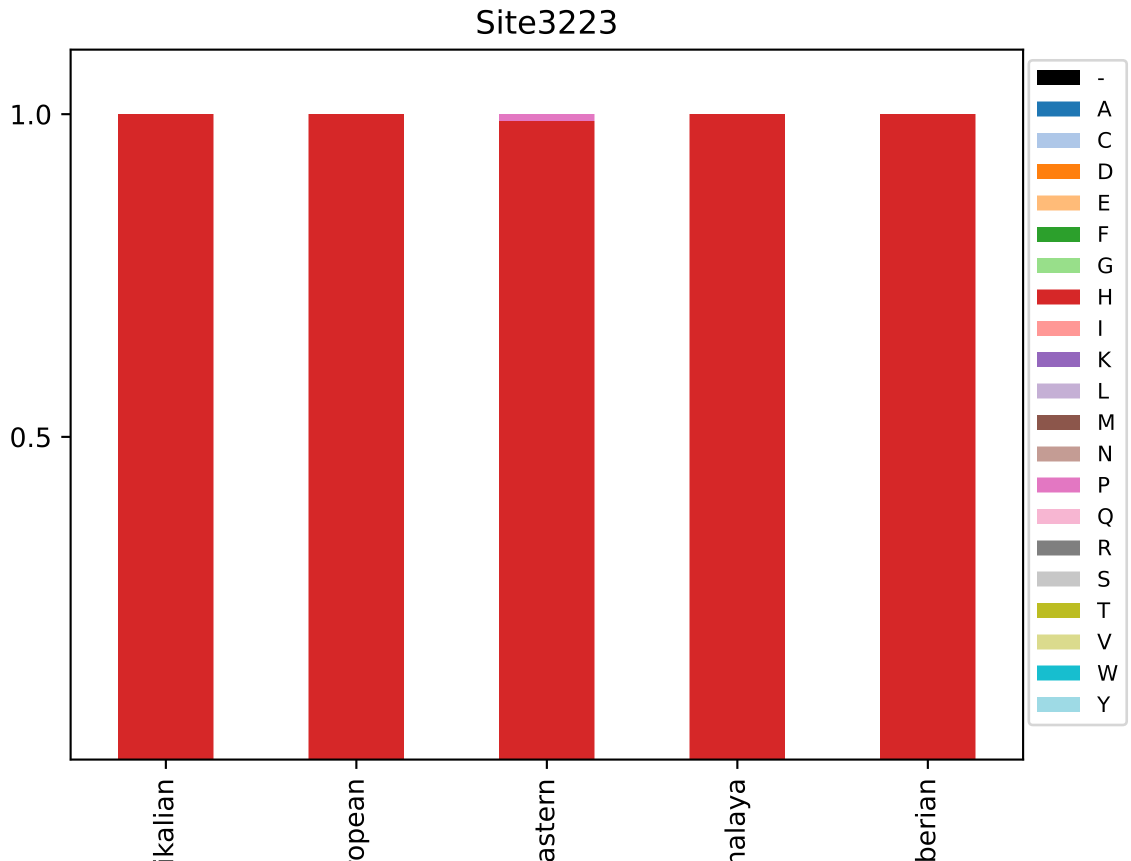
Site3223
1.0
0.5
-
A
C
D
E
F
G
H
I
K
L
M
N
P
Q
R
S
T
V
W
Y
ikalian
opean
astern
nalaya
berian

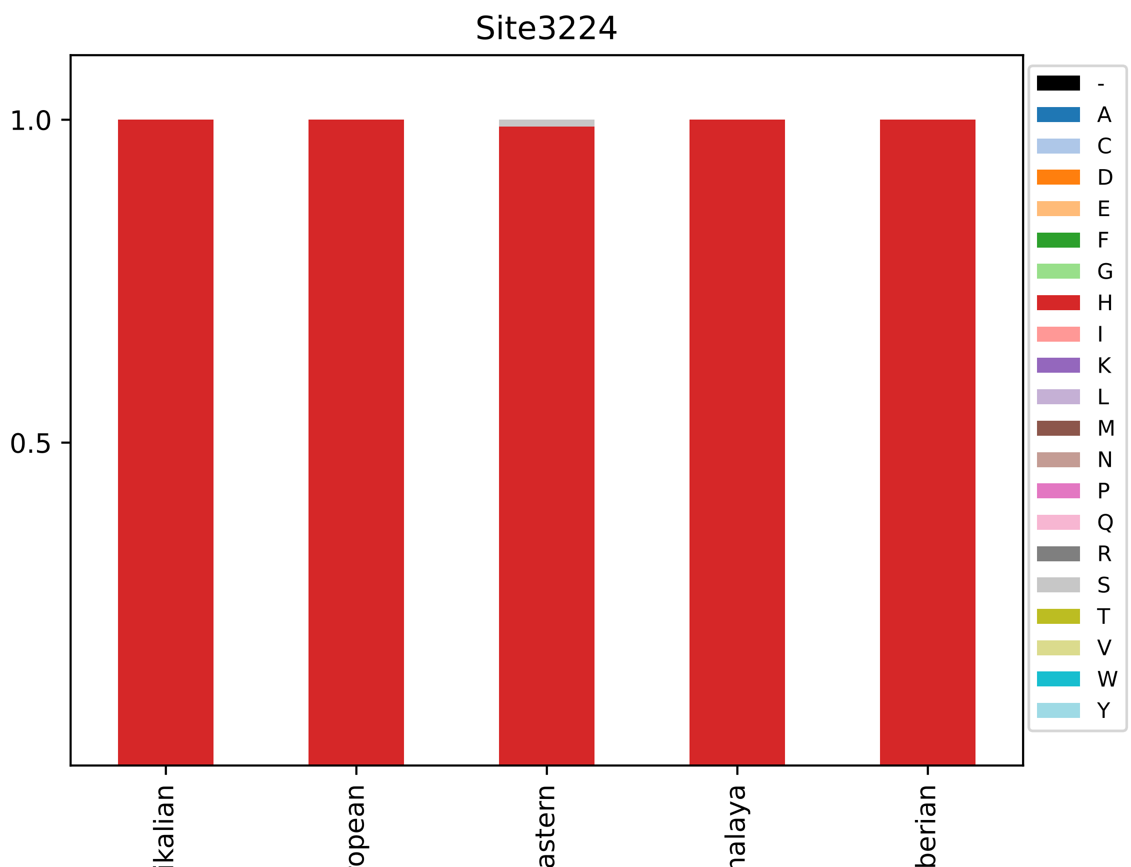
Site3224
1.0
0.5
-
A
C
D
E
F
G
H
I
K
L
M
N
P
Q
R
S
T
V
W
Y
ikalian
opean
astern
nalaya
berian

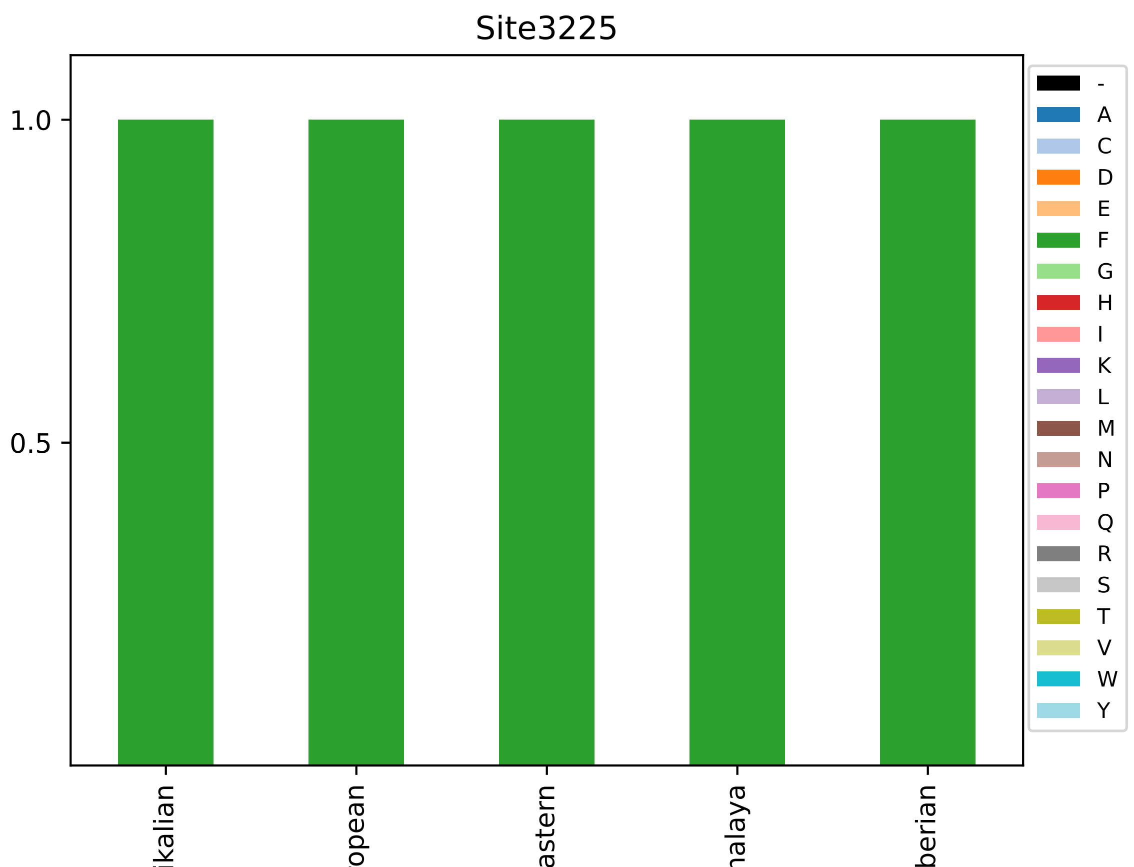
Site3225
1.0
0.5
-
A
C
D
E
F
G
H
I
K
L
M
N
P
Q
R
S
T
V
W
Y
ikalian
opean
astern
nalaya
berian

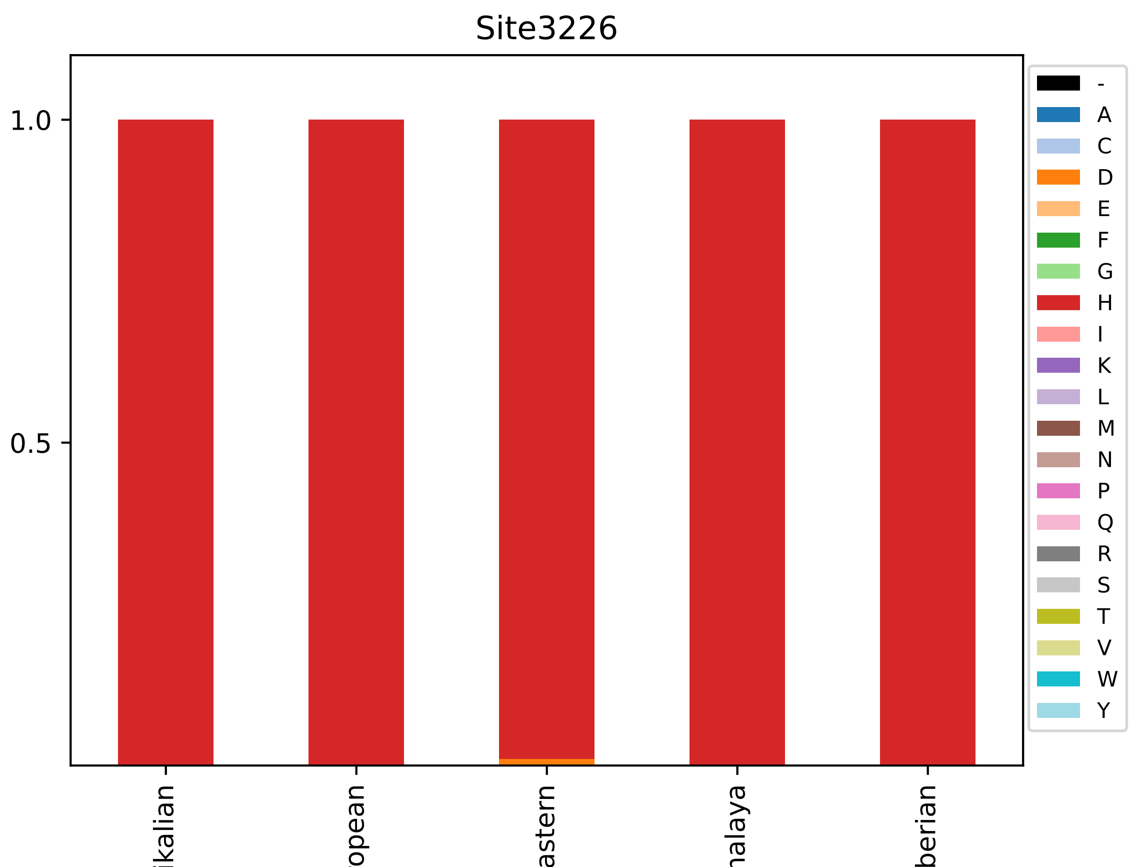
Site3226
1.0
0.5
-
A
C
D
E
F
G
H
I
K
L
M
N
P
Q
R
S
T
V
W
Y
ikalian
opean
astern
nalaya
berian

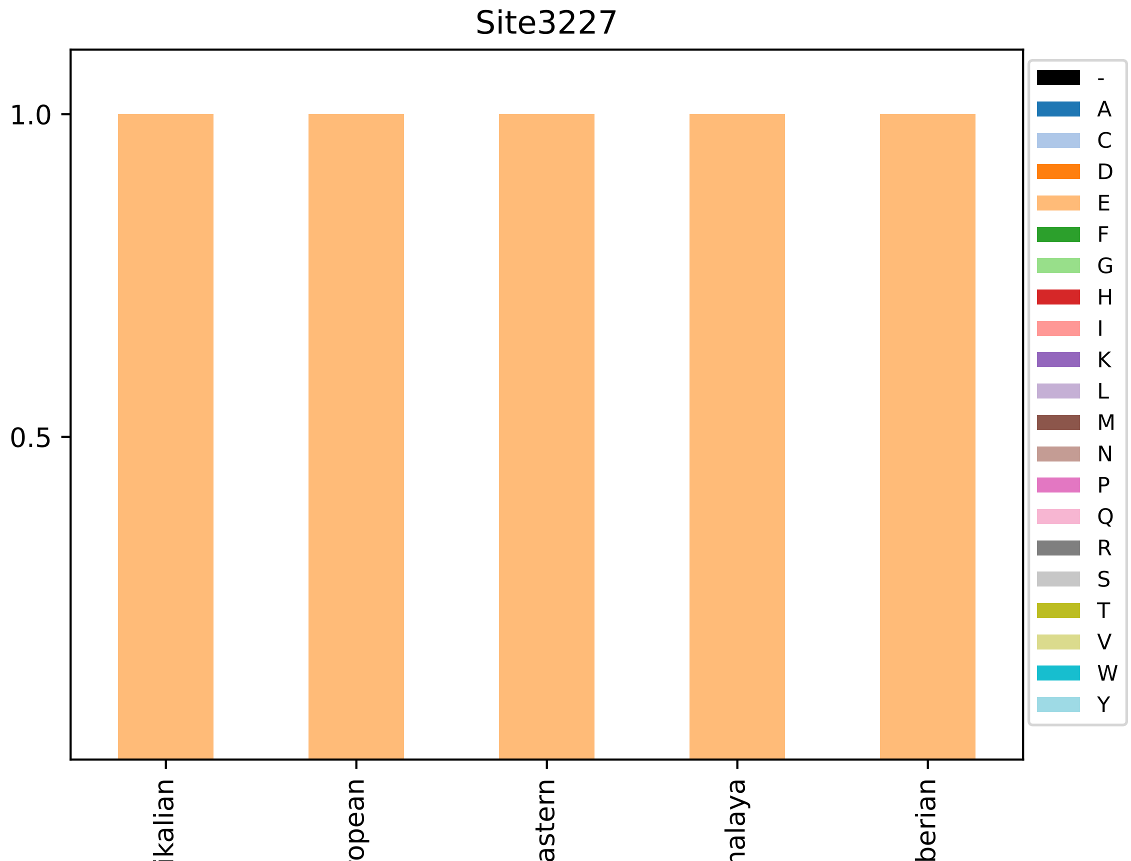
Site3227
1.0
0.5
-
A
C
D
E
F
G
H
I
K
L
M
N
P
Q
R
S
T
V
W
Y
ikalian
opean
astern
nalaya
berian

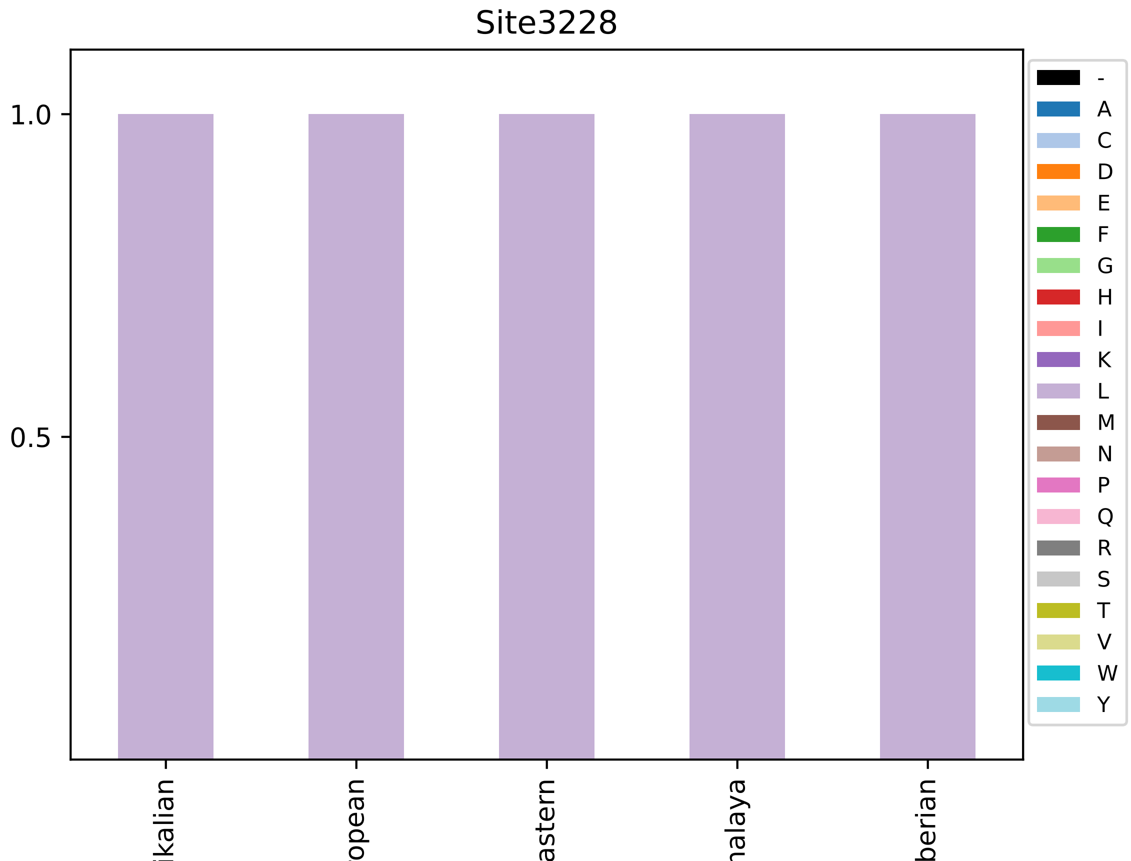
Site3228
1.0
0.5
-
A
C
D
E
F
G
H
I
K
L
M
N
P
Q
R
S
T
V
W
Y

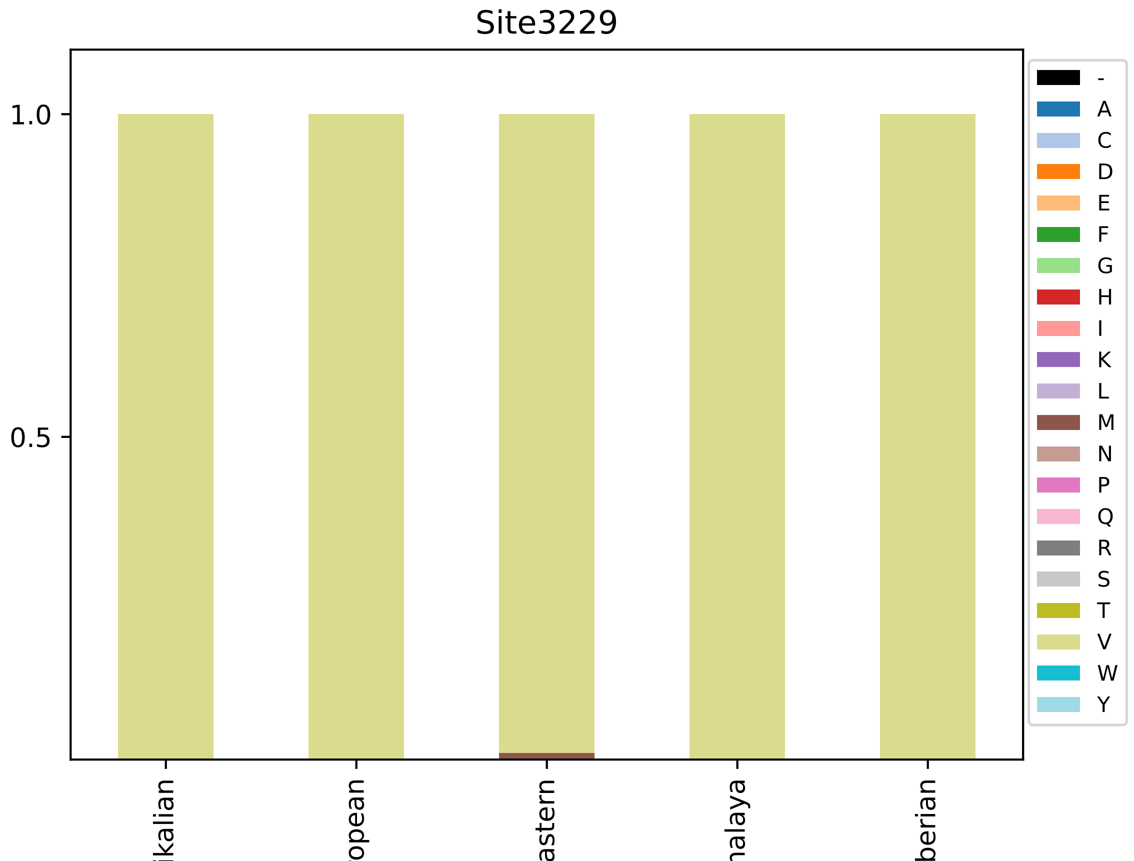
Site3229
1.0
0.5
ikalian
opean
astern
nalaya
berian
-
A
C
D
E
F
G
H
I
K
L
M
N
P
Q
R
S
T
V
W
Y

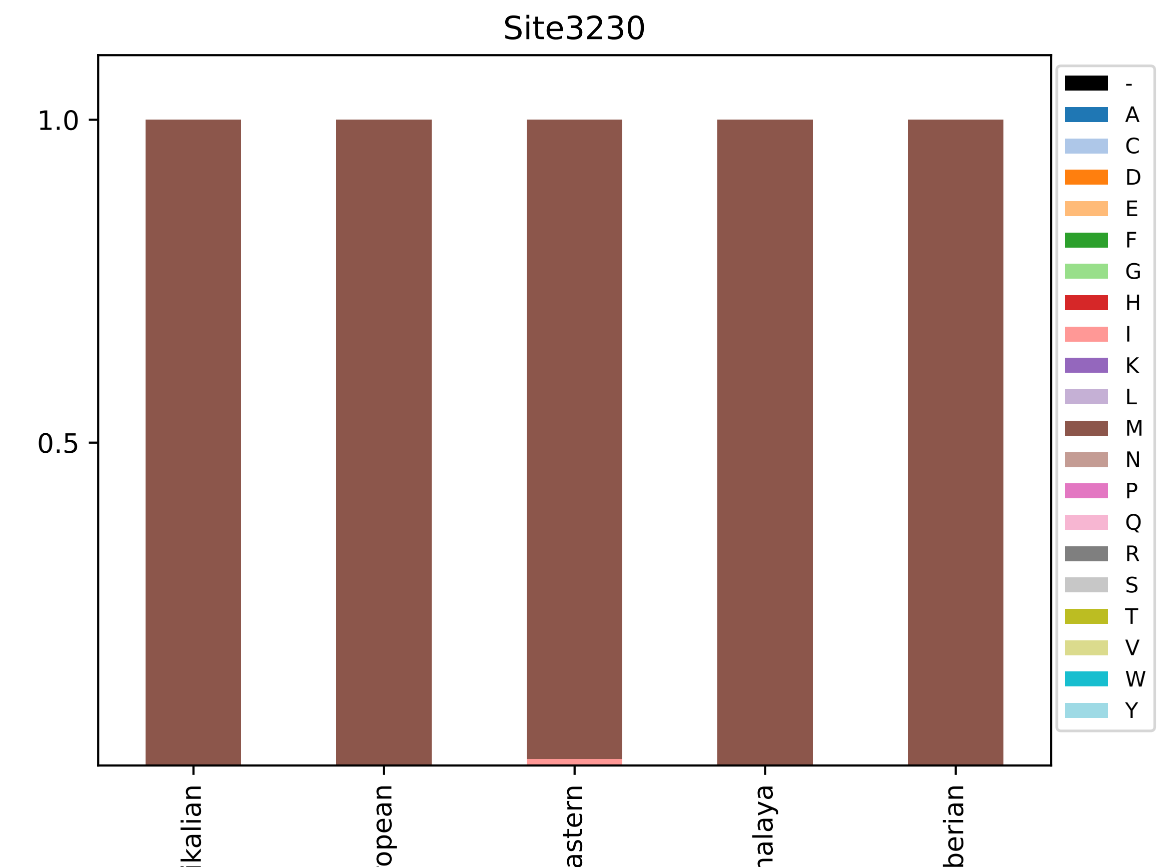

Site3230
1.0
0.5
ikalian
opean
astern
nalaya
berian
-
A
C
D
E
F
G
H
I
K
L
M
N
P
Q
R
S
T
V
W
Y

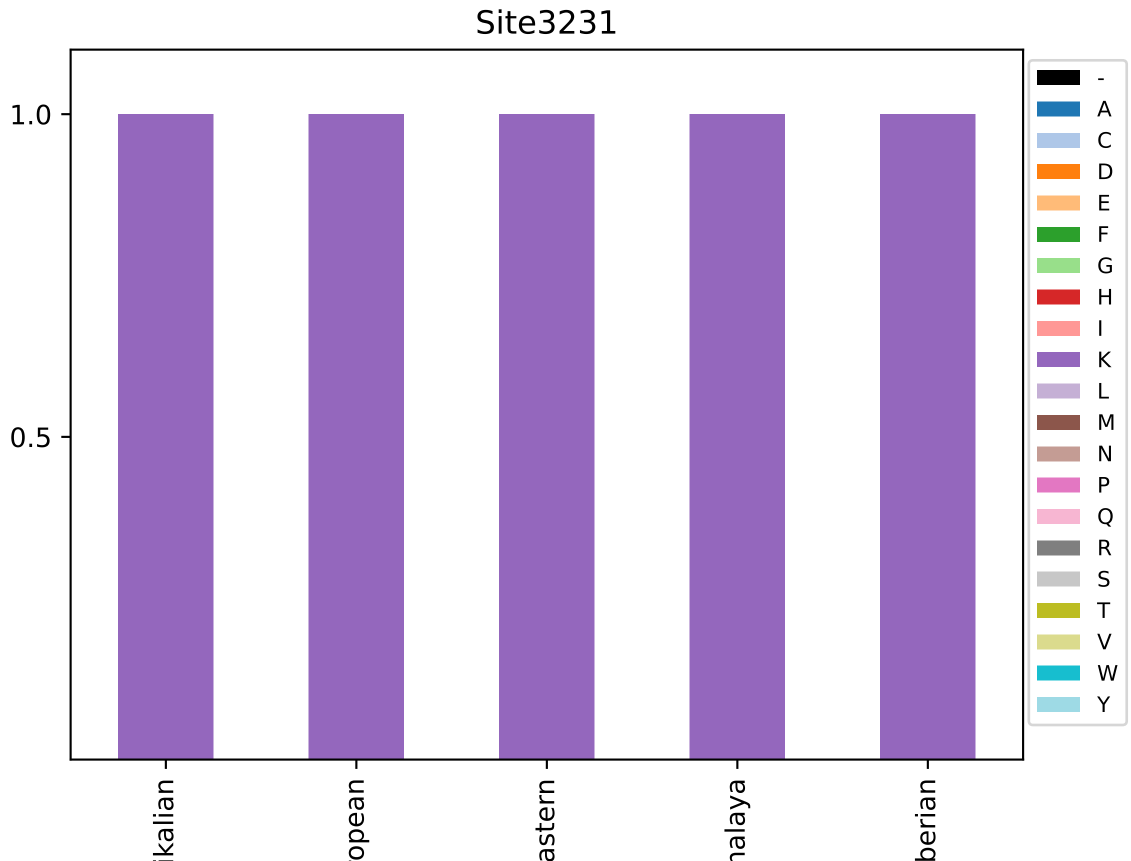

Site3231
1.0
0.5
-
A
C
D
E
F
G
H
I
K
L
M
N
P
Q
R
S
T
V
W
Y
ikalian
opean
astern
nalaya
berian

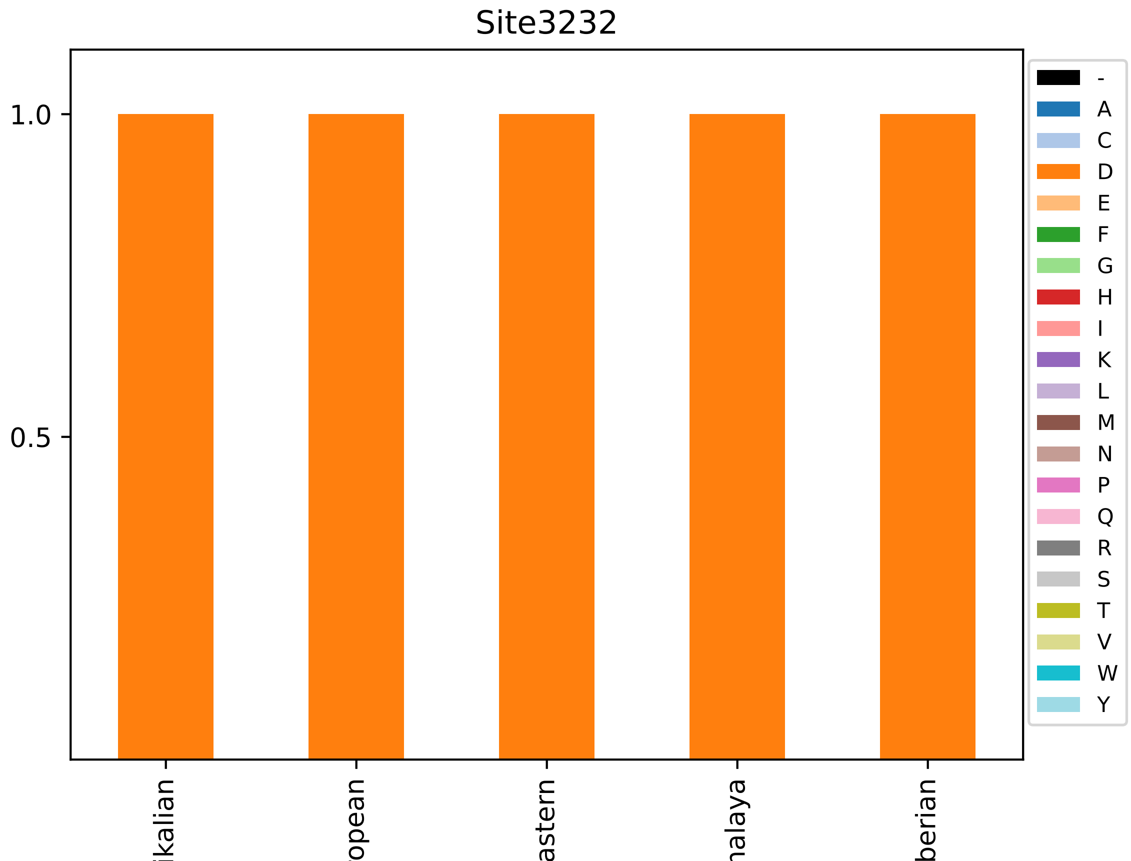
Site3232
1.0
0.5
-
A
C
D
E
F
G
H
I
K
L
M
N
P
Q
R
S
T
V
W
Y
ikalian
opean
astern
nalaya
berian

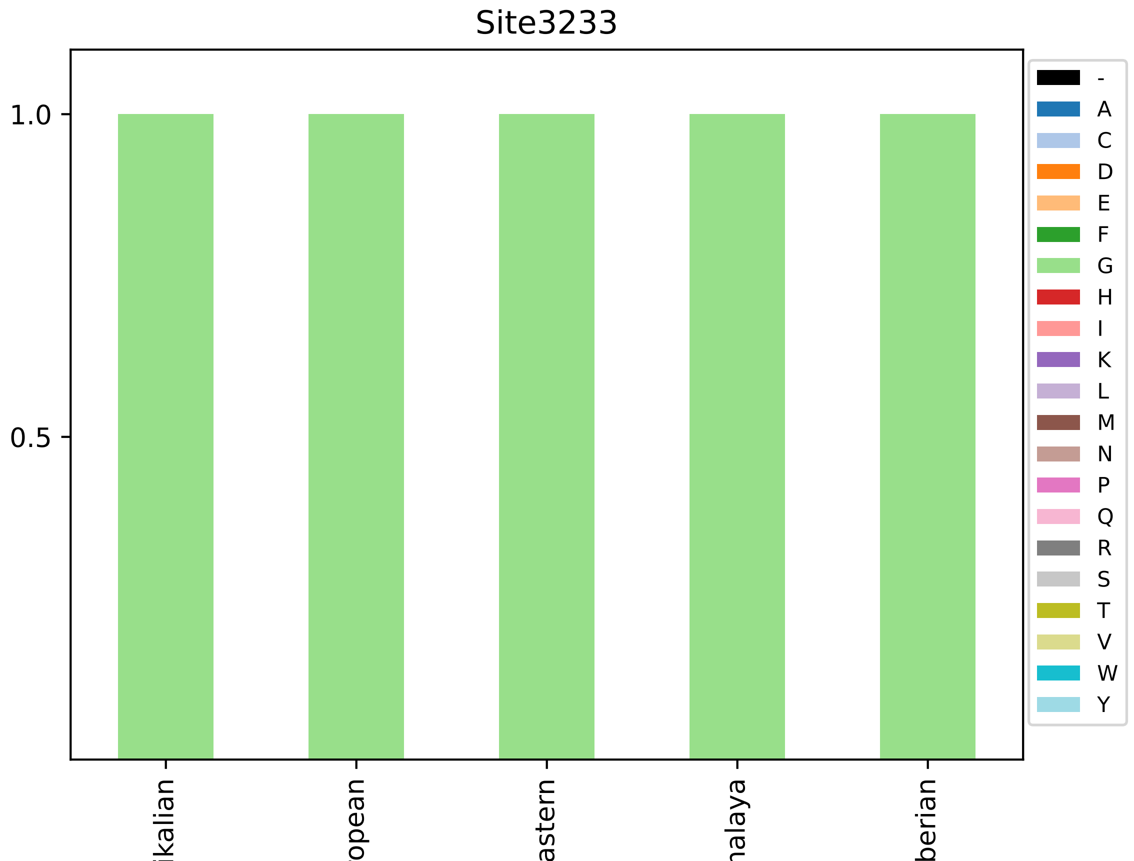

Site3233
1.0
0.5
-
A
C
D
E
F
G
H
I
K
L
M
N
P
Q
R
S
T
V
W
Y
ikalian
opean
astern
nalaya
berian

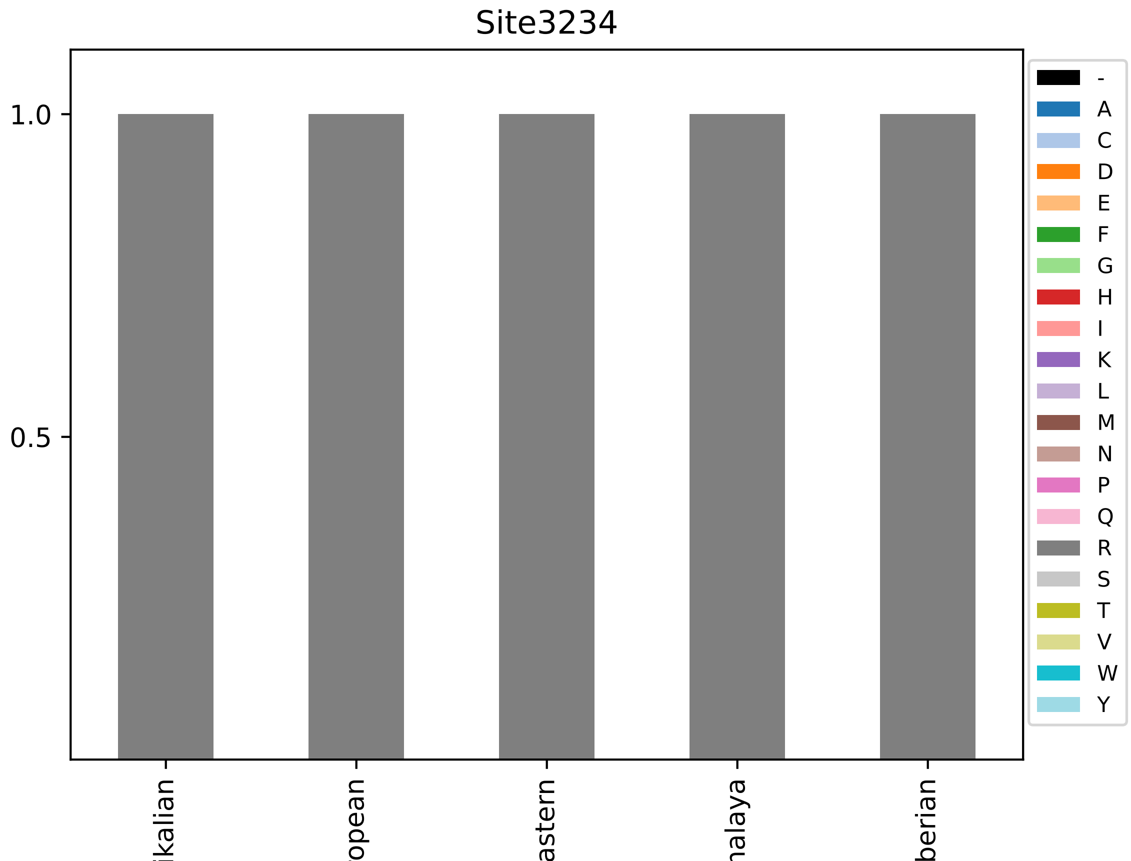
Site3234
1.0
0.5
-
A
C
D
E
F
G
H
I
K
L
M
N
P
Q
R
S
T
V
W
Y
ikalian
opean
astern
nalaya
berian

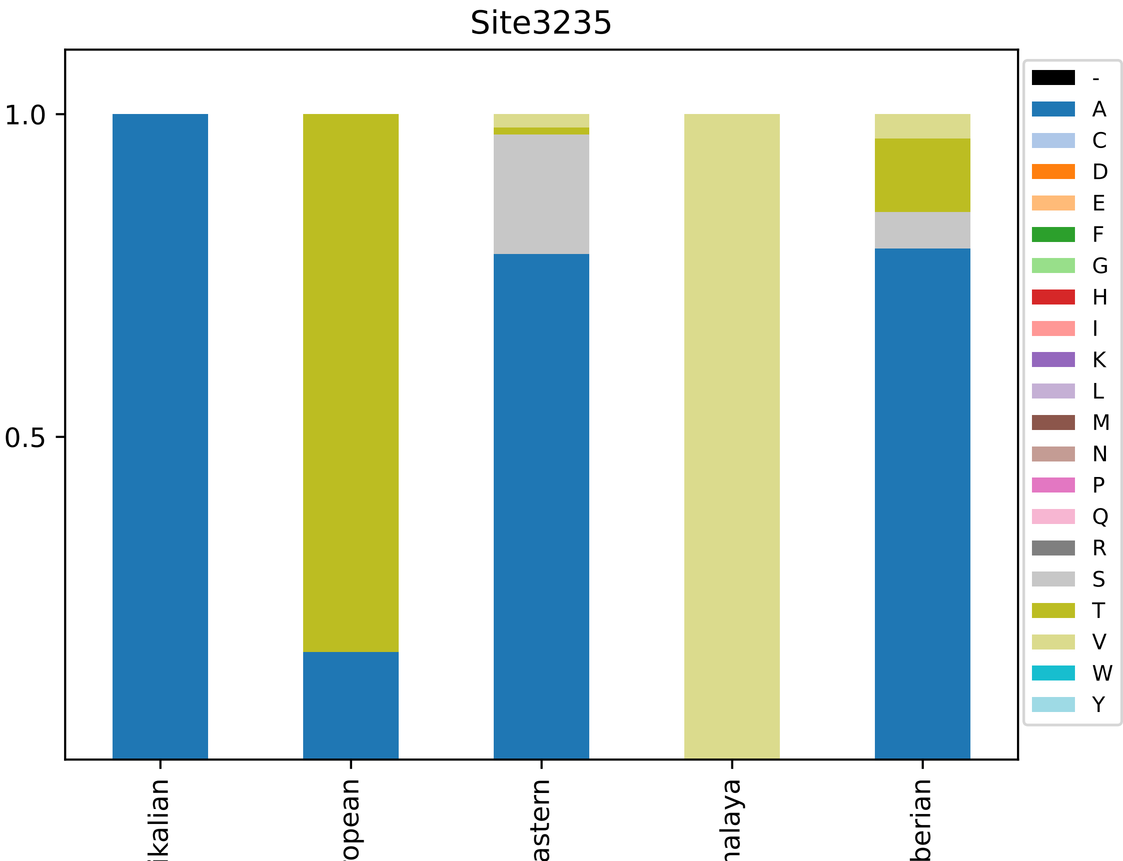

Site3235
1.0
0.5
-
A
C
D
E
F
G
H
I
K
L
M
N
P
Q
R
S
T
V
W
Y
ikalian
opean
astern
nalaya
berian

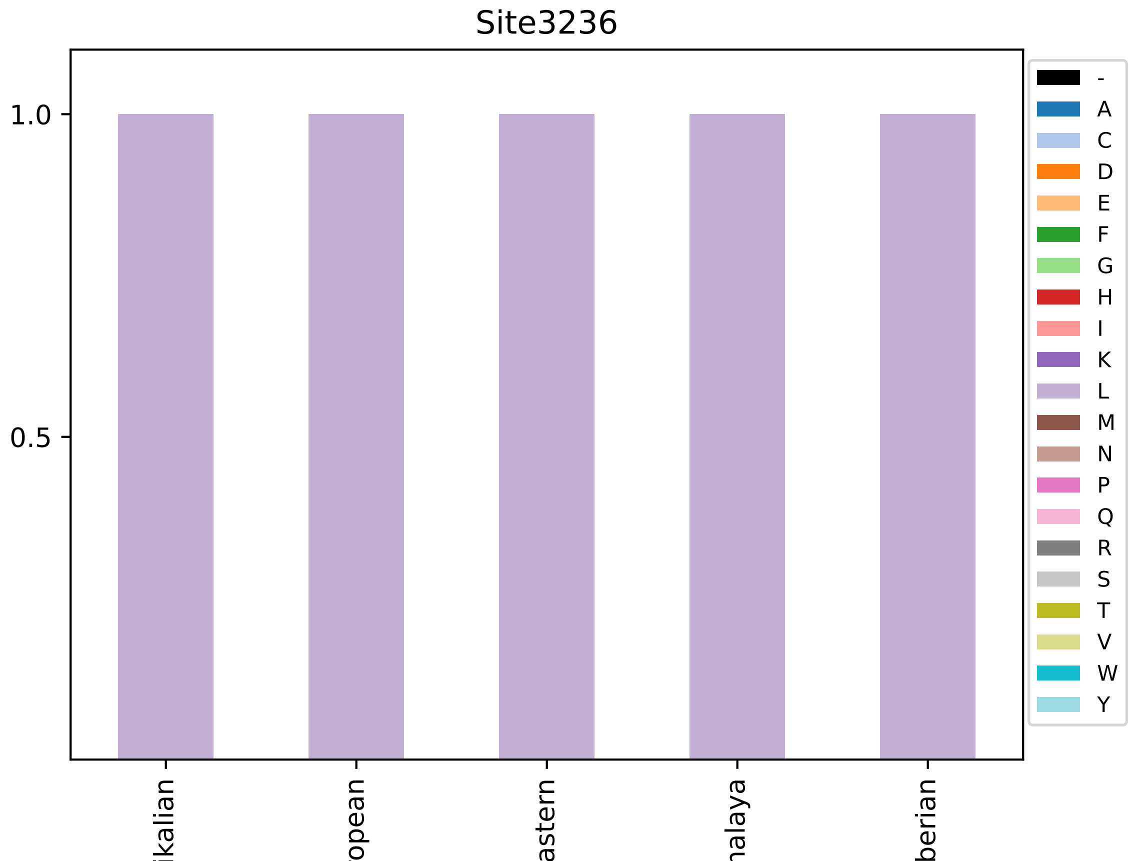

Site3236
1.0
0.5
-
A
C
D
E
F
G
H
I
K
L
M
N
P
Q
R
S
T
V
W
Y
ikalian
opean
astern
nalaya
berian

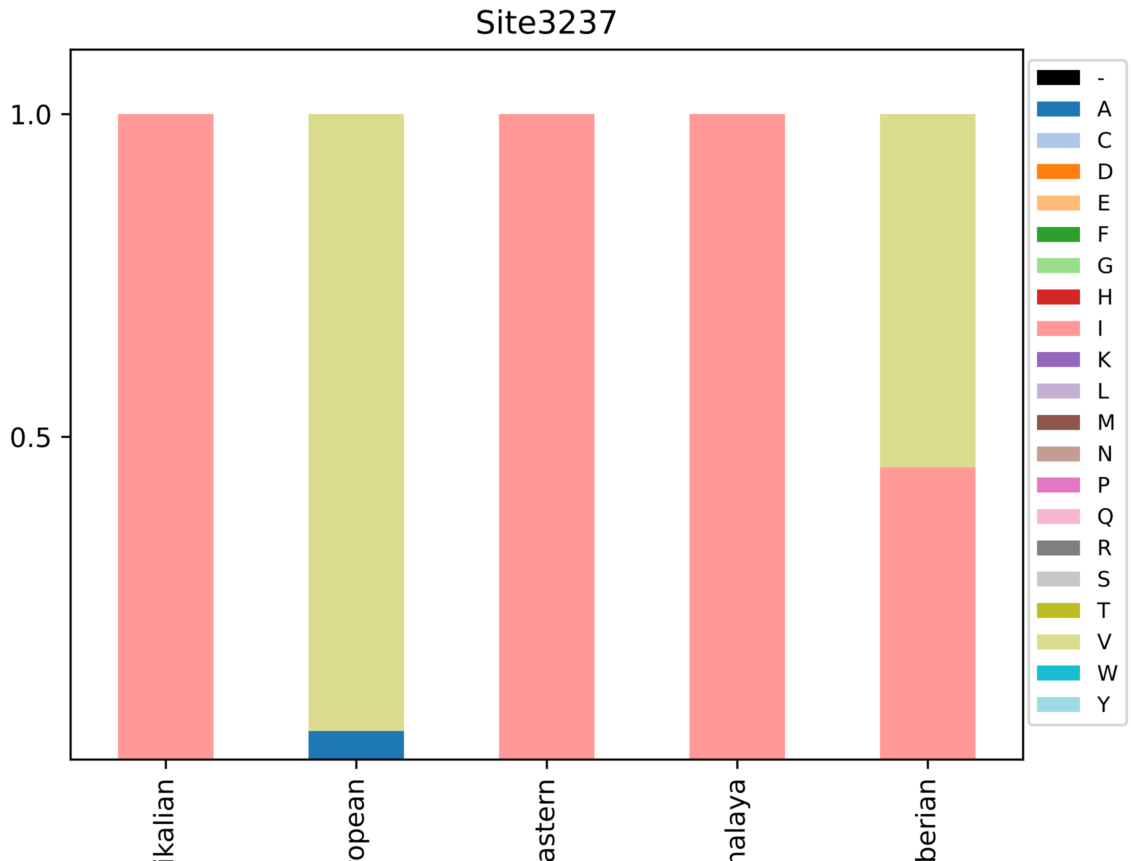
Site3237
1.0
0.5
-
A
C
D
E
F
G
H
I
K
L
M
N
P
Q
R
S
T
V
W
Y
ikalian
opean
astern
nalaya
berian

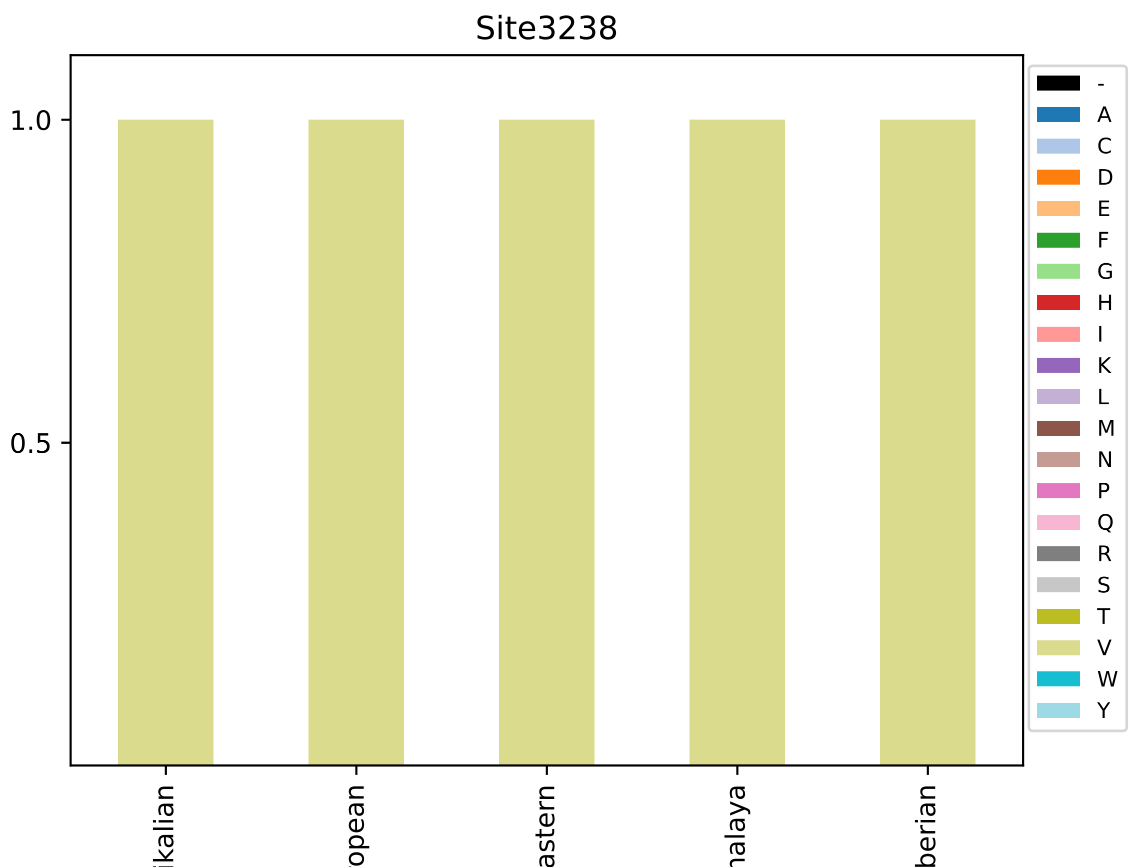

Site3238
1.0
0.5
-
A
C
D
E
F
G
H
I
K
L
M
N
P
Q
R
S
T
V
W
Y
ikalian
opean
astern
nalaya
berian

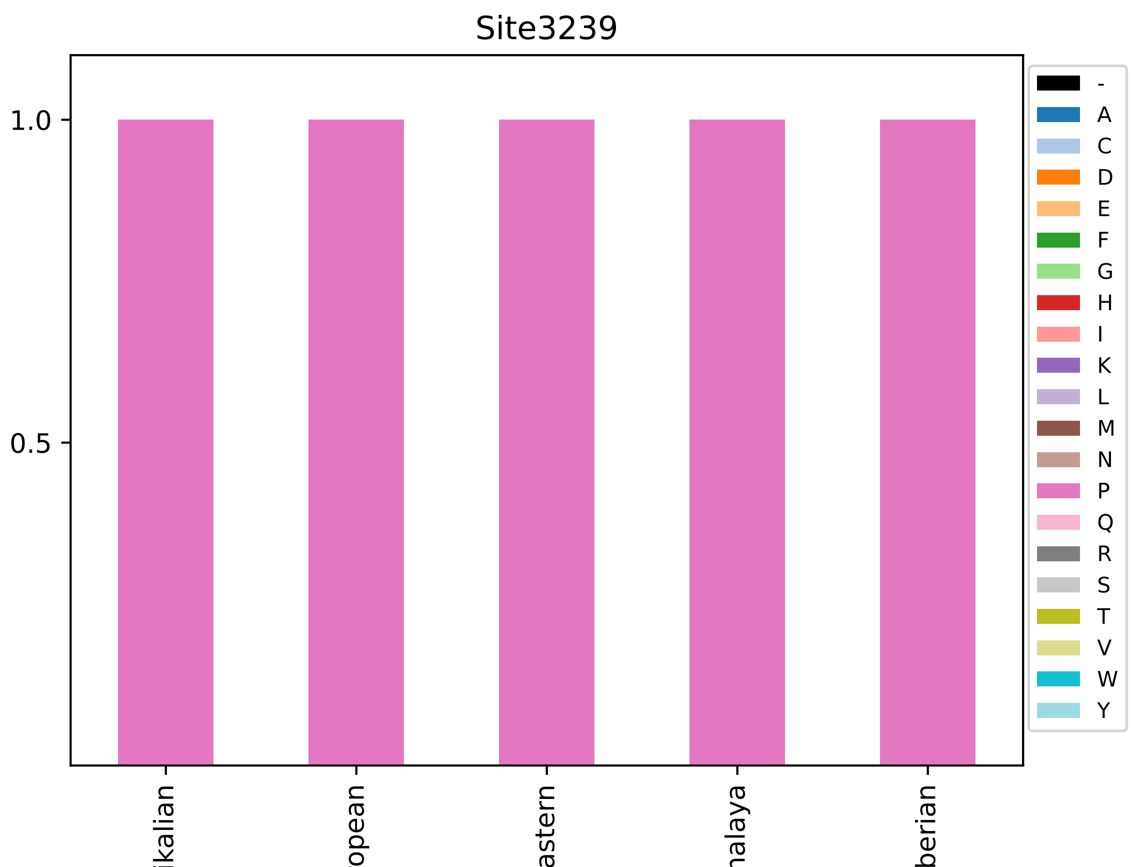

Site3239
1.0
0.5
-
A
C
D
E
F
G
H
I
K
L
M
N
P
Q
R
S
T
V
W
Y
ikalian
opean
astern
nalaya
berian

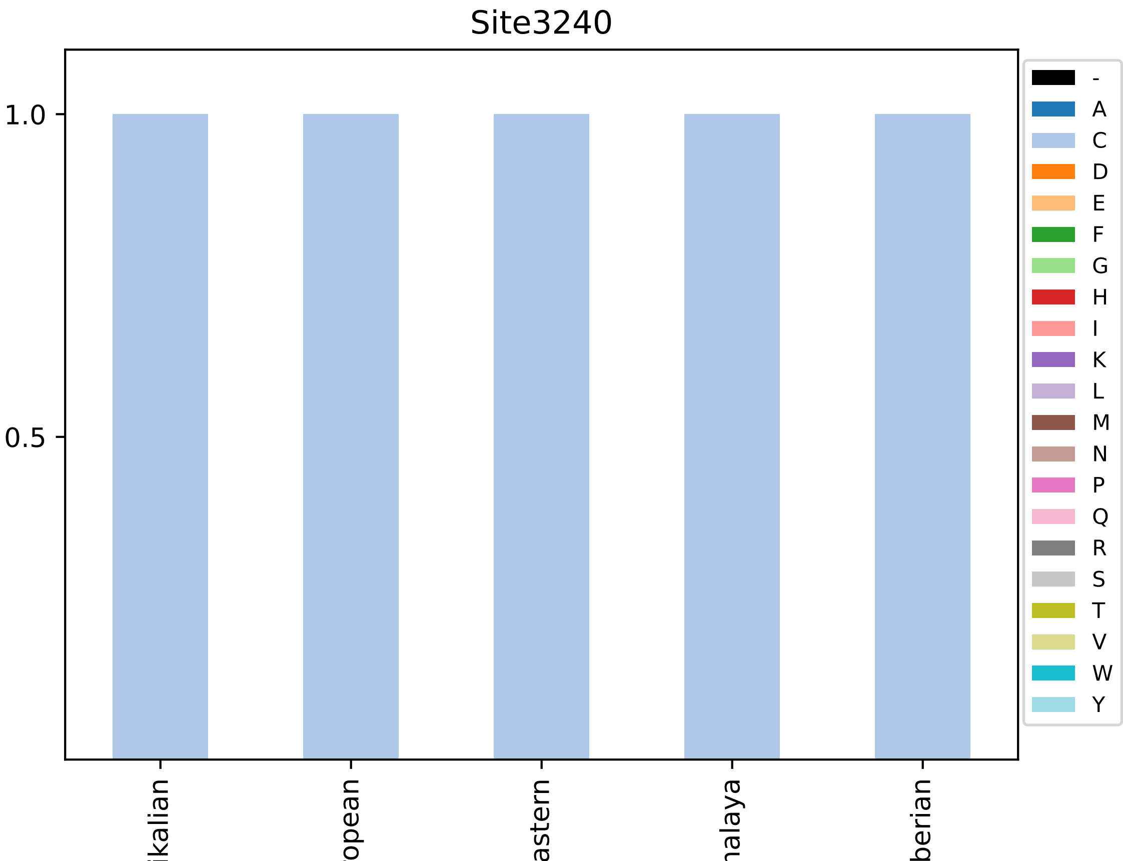
Site3240
1.0
0.5
-
A
C
D
E
F
G
H
I
K
L
M
N
P
Q
R
S
T
V
W
Y
ikalian
opean
astern
nalaya
berian

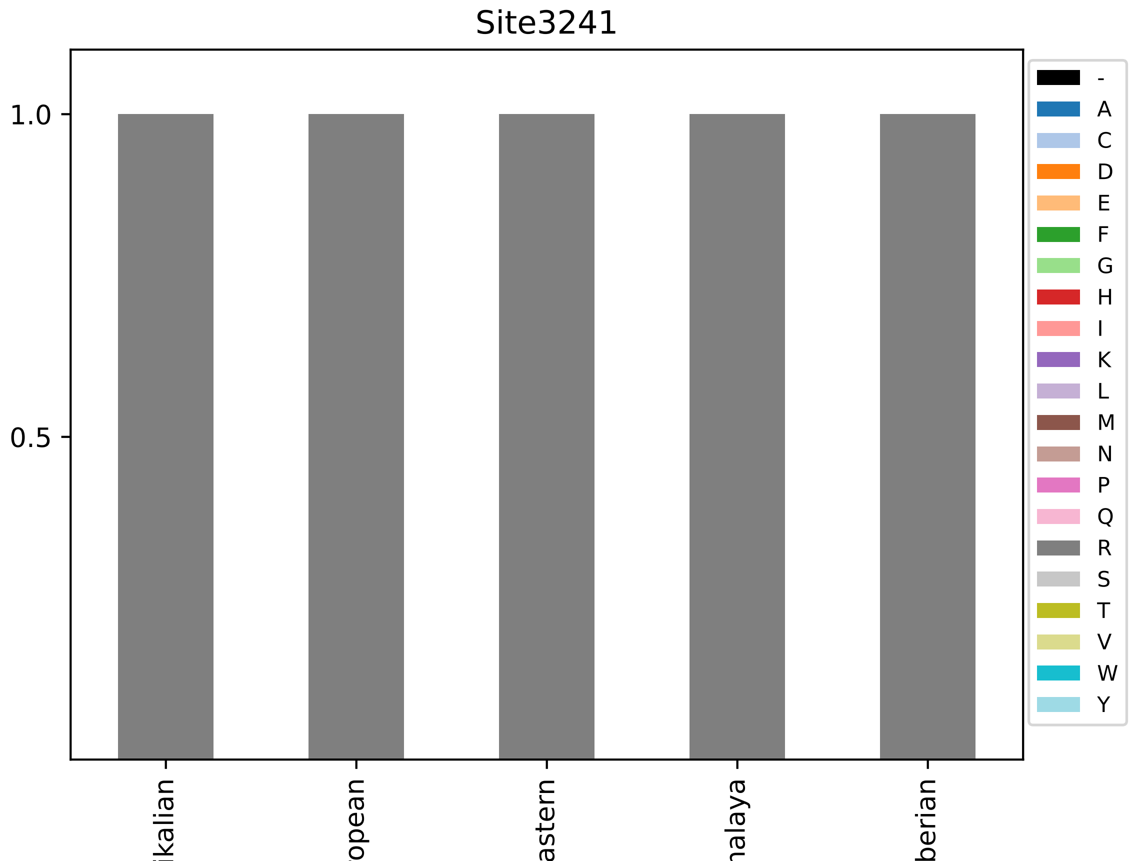
Site3241
1.0
0.5
-
A
C
D
E
F
G
H
I
K
L
M
N
P
Q
R
S
T
V
W
Y
ikalian
opean
astern
nalaya
berian

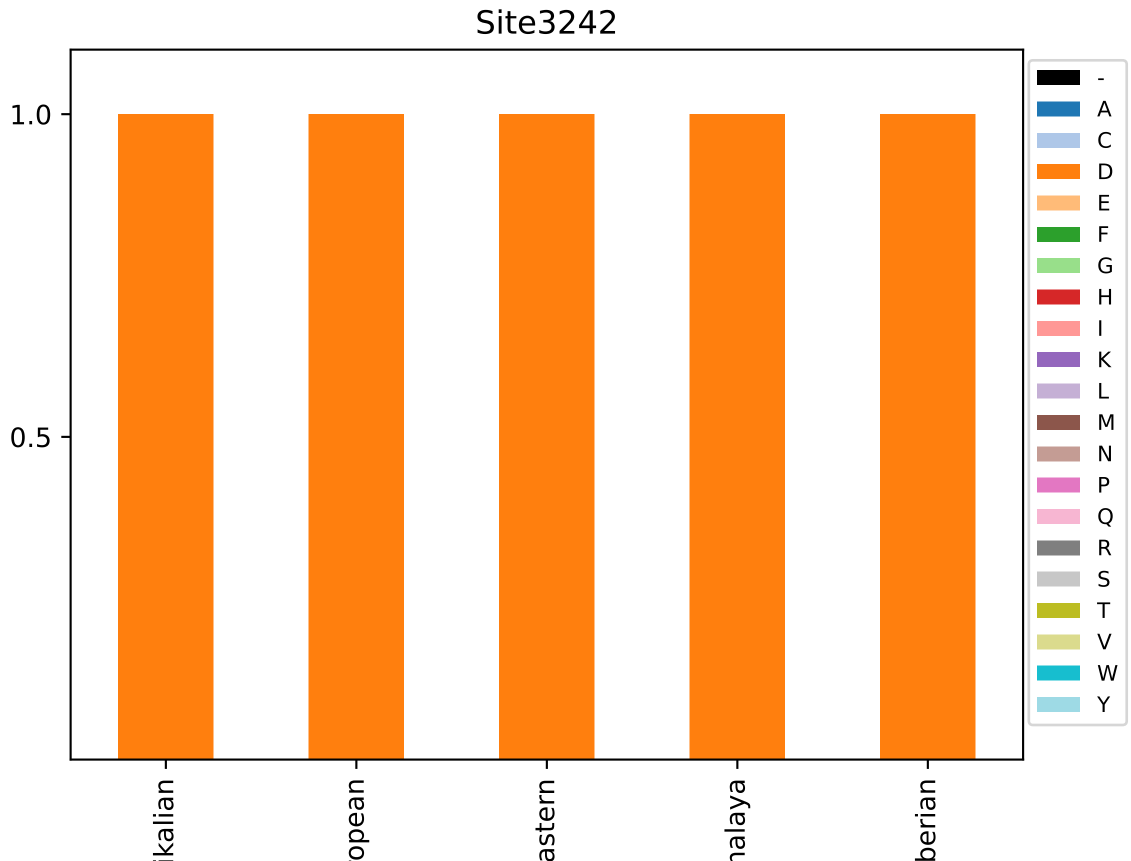
Site3242
1.0
0.5
-
A
C
D
E
F
G
H
I
K
L
M
N
P
Q
R
S
T
V
W
Y
ikalian
opean
astern
nalaya
berian

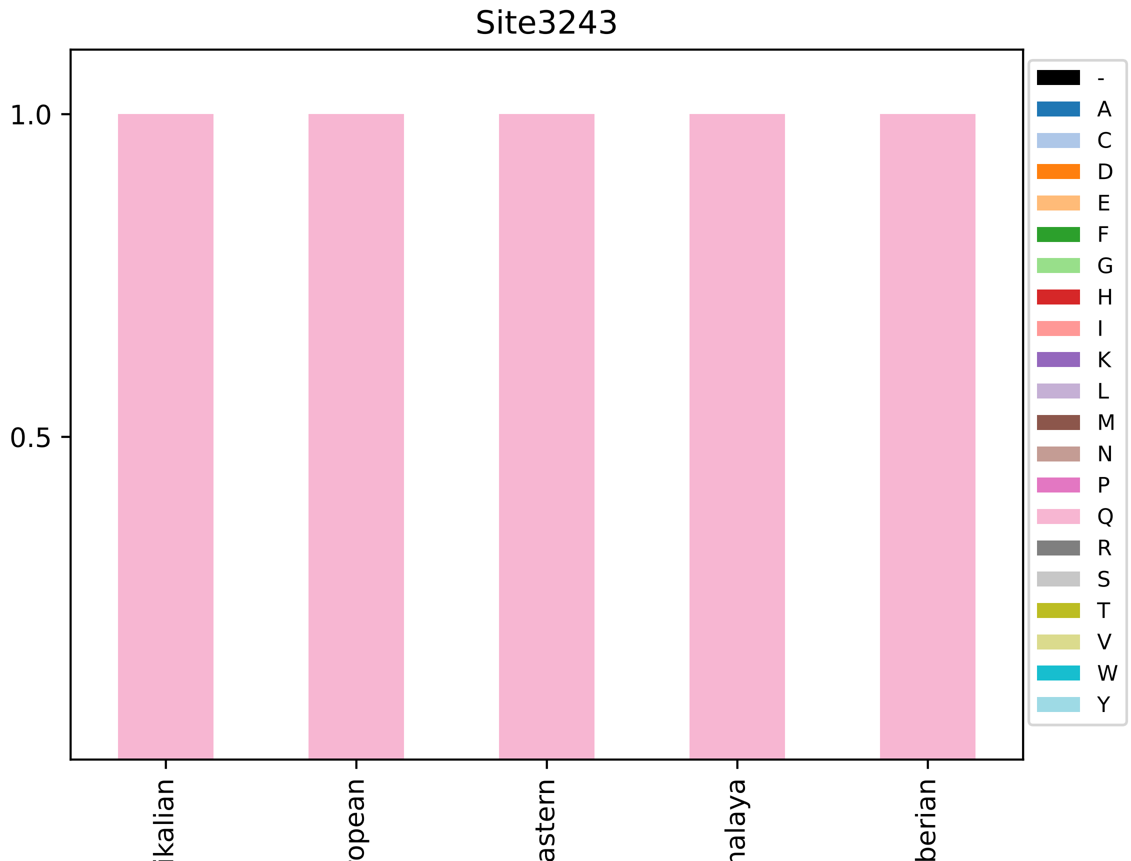
Site3243
1.0
0.5
-
A
C
D
E
F
G
H
I
K
L
M
N
P
Q
R
S
T
V
W
Y
ikalian
opean
astern
nalaya
berian

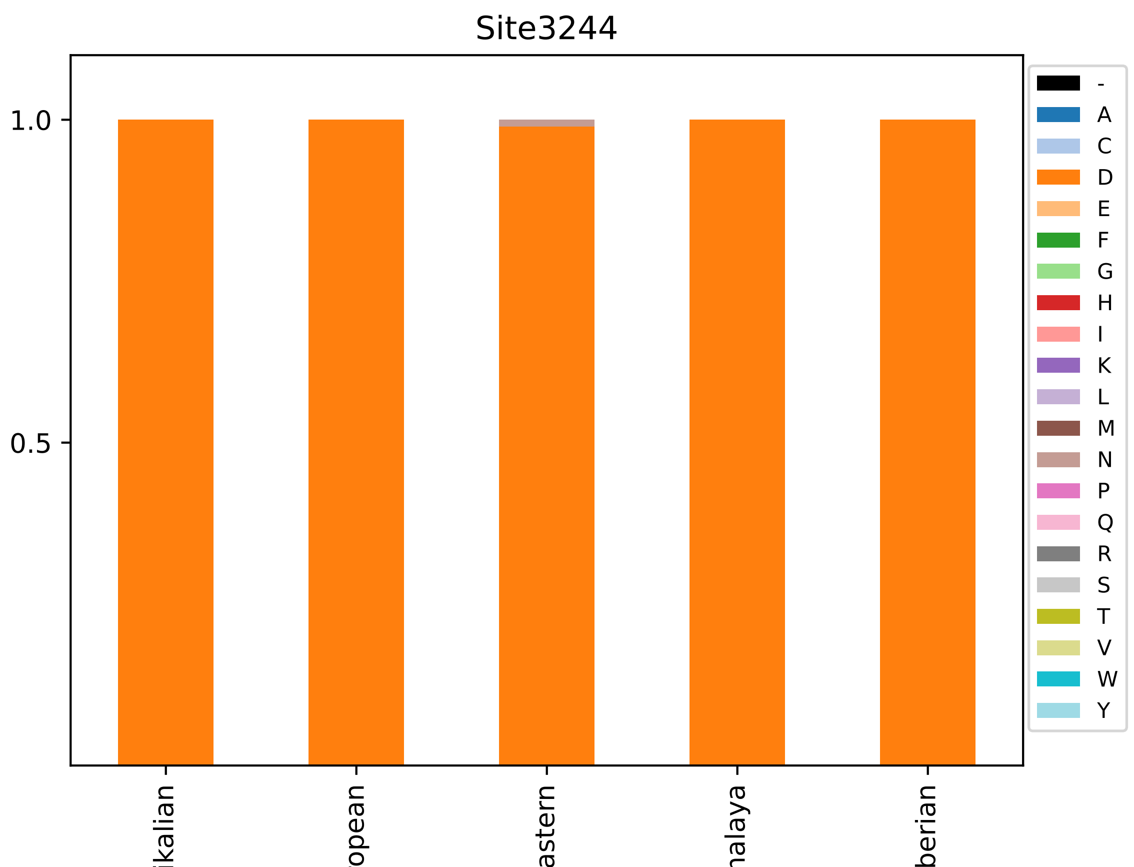

Site3244
1.0
0.5
-
A
C
D
E
F
G
H
I
K
L
M
N
P
Q
R
S
T
V
W
Y
ikalian
opean
astern
nalaya
berian

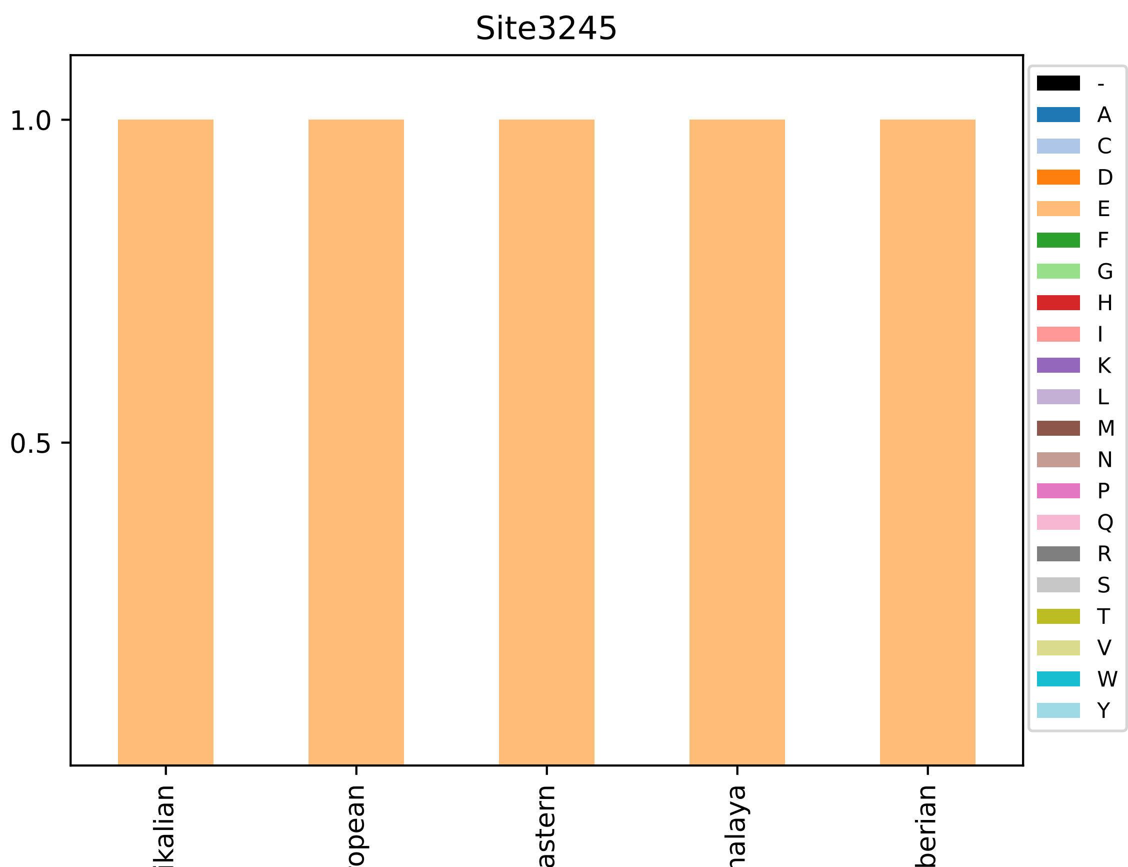
Site3245
1.0
0.5
-
A
C
D
E
F
G
H
I
K
L
M
N
P
Q
R
S
T
V
W
Y
ikalian
opean
astern
nalaya
berian

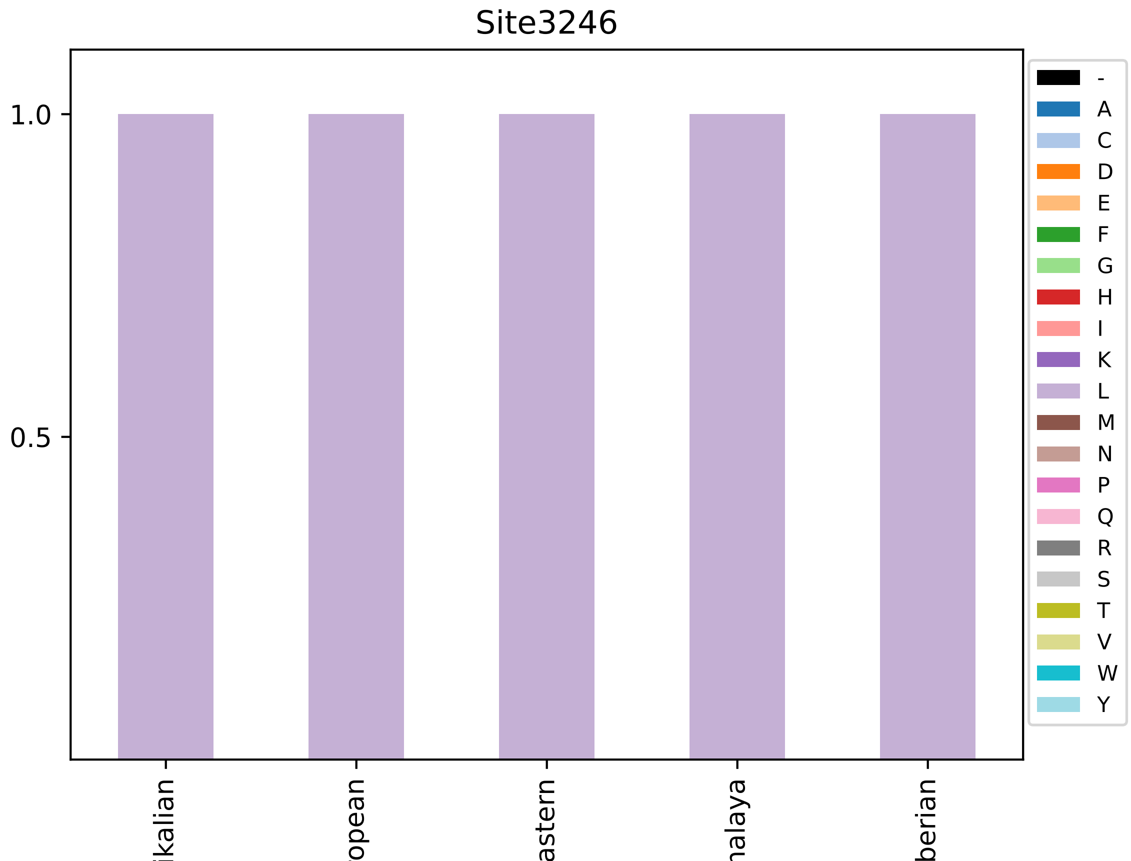
Site3246
1.0
0.5
ikalian
opean
astern
nalaya
berian
-
A
C
D
E
F
G
H
I
K
L
M
N
P
Q
R
S
T
V
W
Y

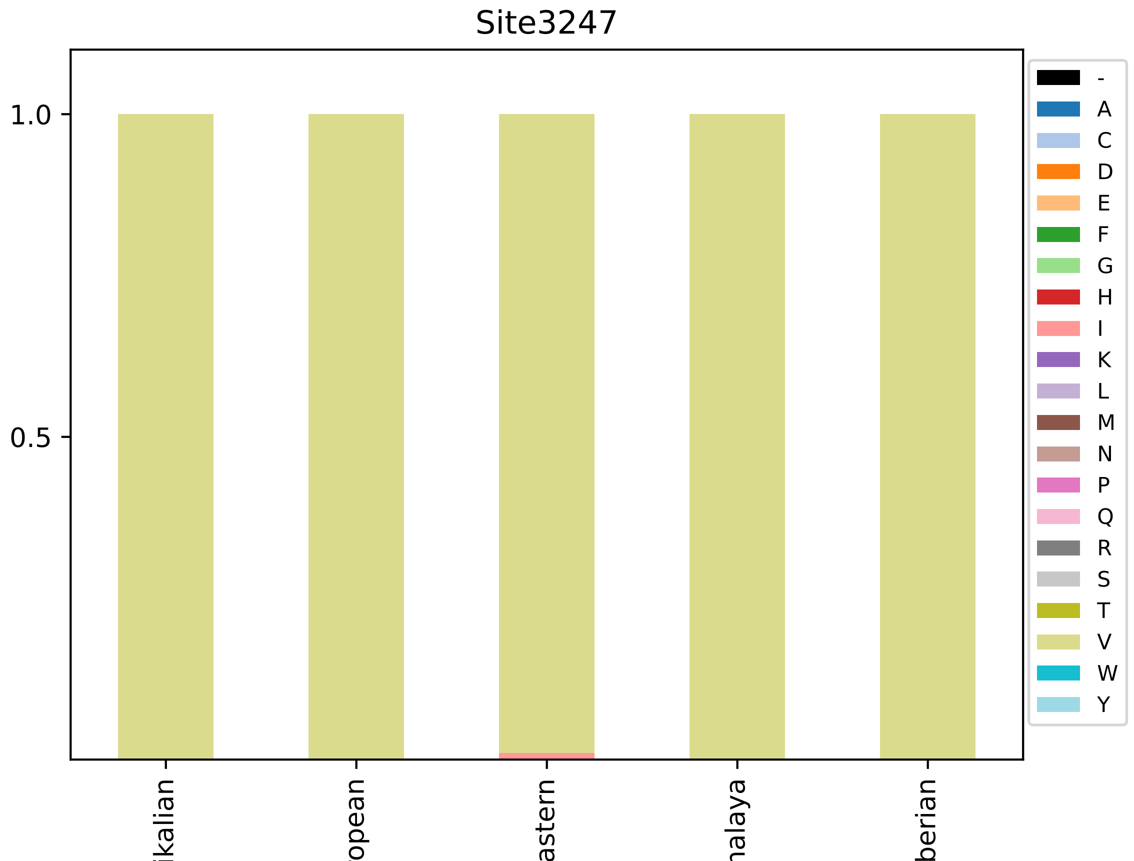
Site3247
1.0
0.5
-
A
C
D
E
F
G
H
I
K
L
M
N
P
Q
R
S
T
V
W
Y
ikalian
opean
astern
nalaya
berian

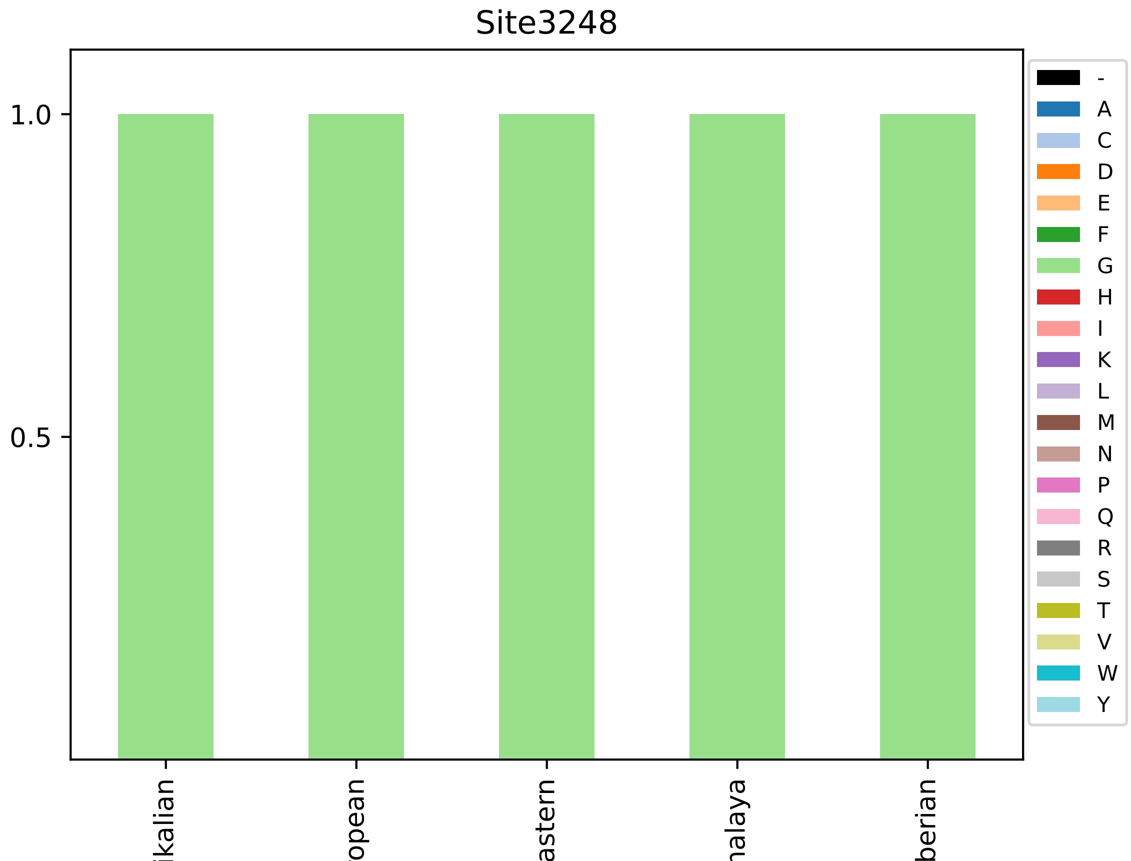

Site3248
1.0
0.5
-
A
C
D
E
F
G
H
I
K
L
M
N
P
Q
R
S
T
V
W
Y
ikalian
opean
astern
nalaya
berian

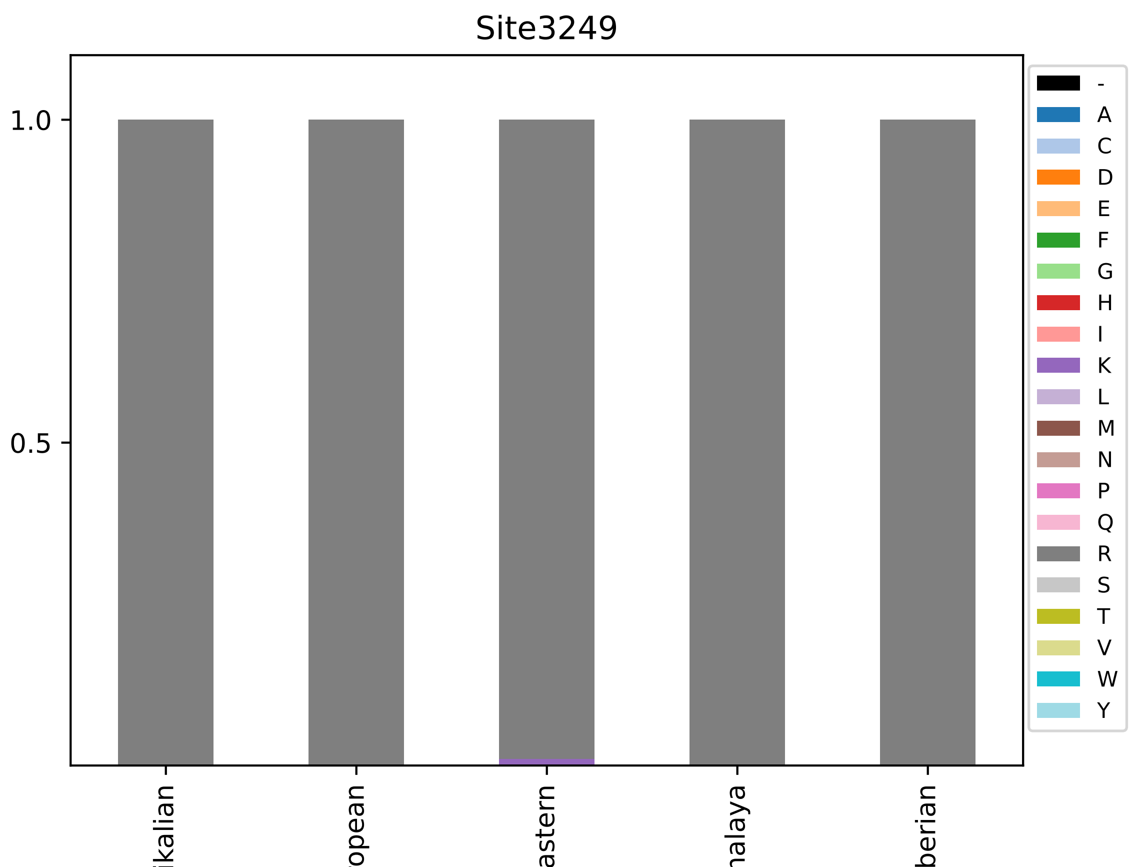

Site3249
1.0
0.5
-
A
C
D
E
F
G
H
I
K
L
M
N
P
Q
R
S
T
V
W
Y
ikalian
opean
astern
nalaya
berian

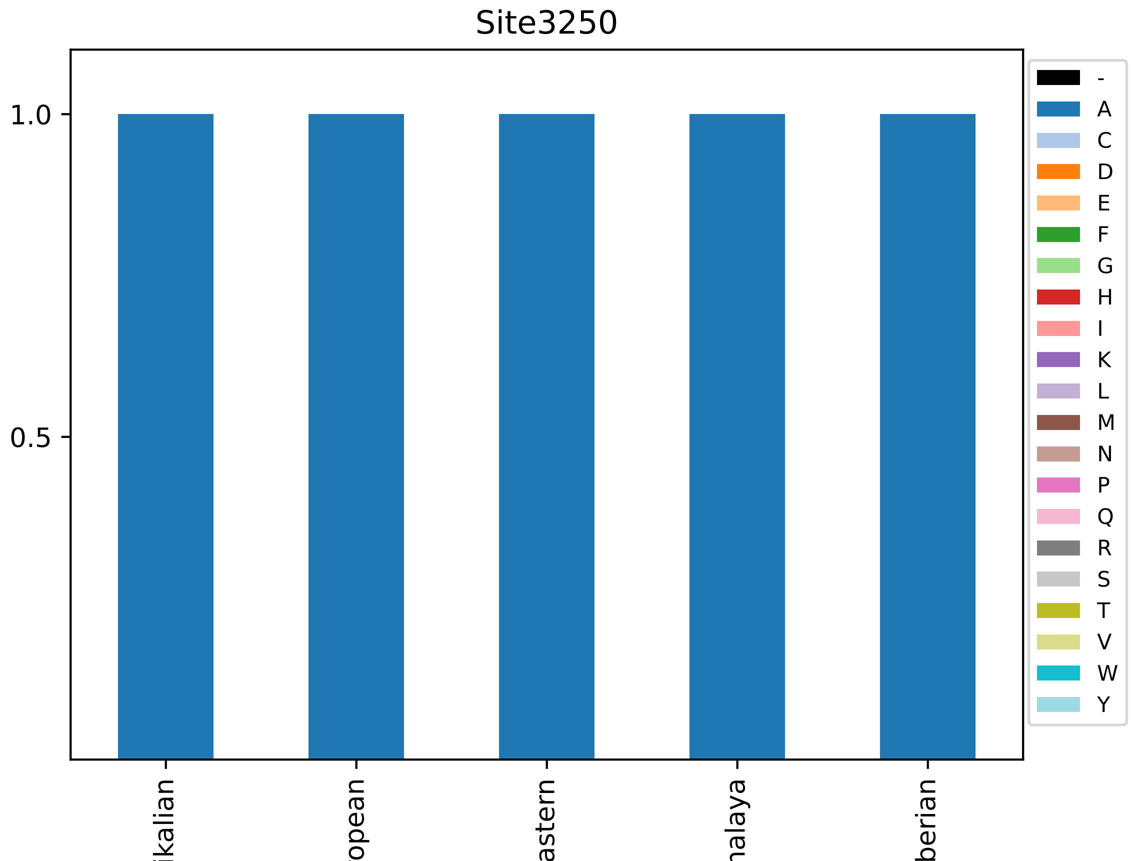

Site3250
1.0
0.5
-
A
C
D
E
F
G
H
I
K
L
M
N
P
Q
R
S
T
V
W
Y

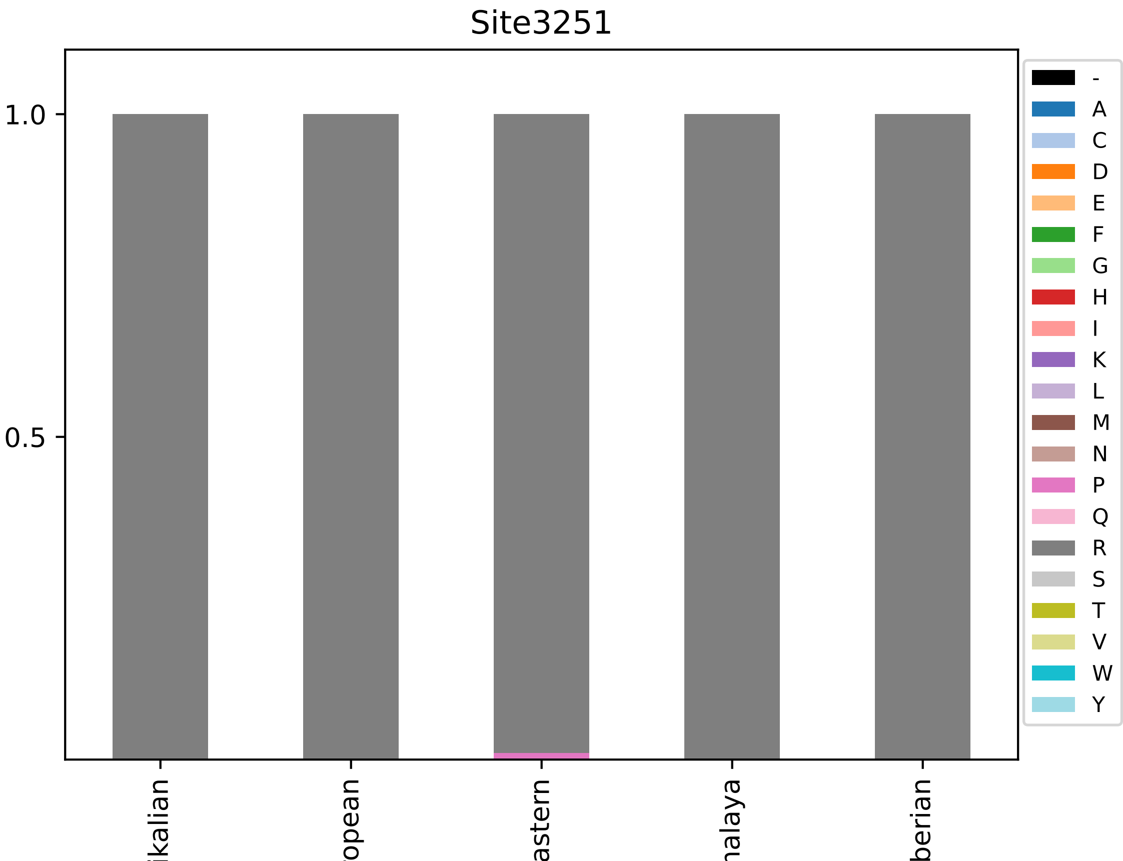

Site3251
1.0
0.5
ikalian
opean
astern
nalaya
berian
-
A
C
D
E
F
G
H
I
K
L
M
N
P
Q
R
S
T
V
W
Y

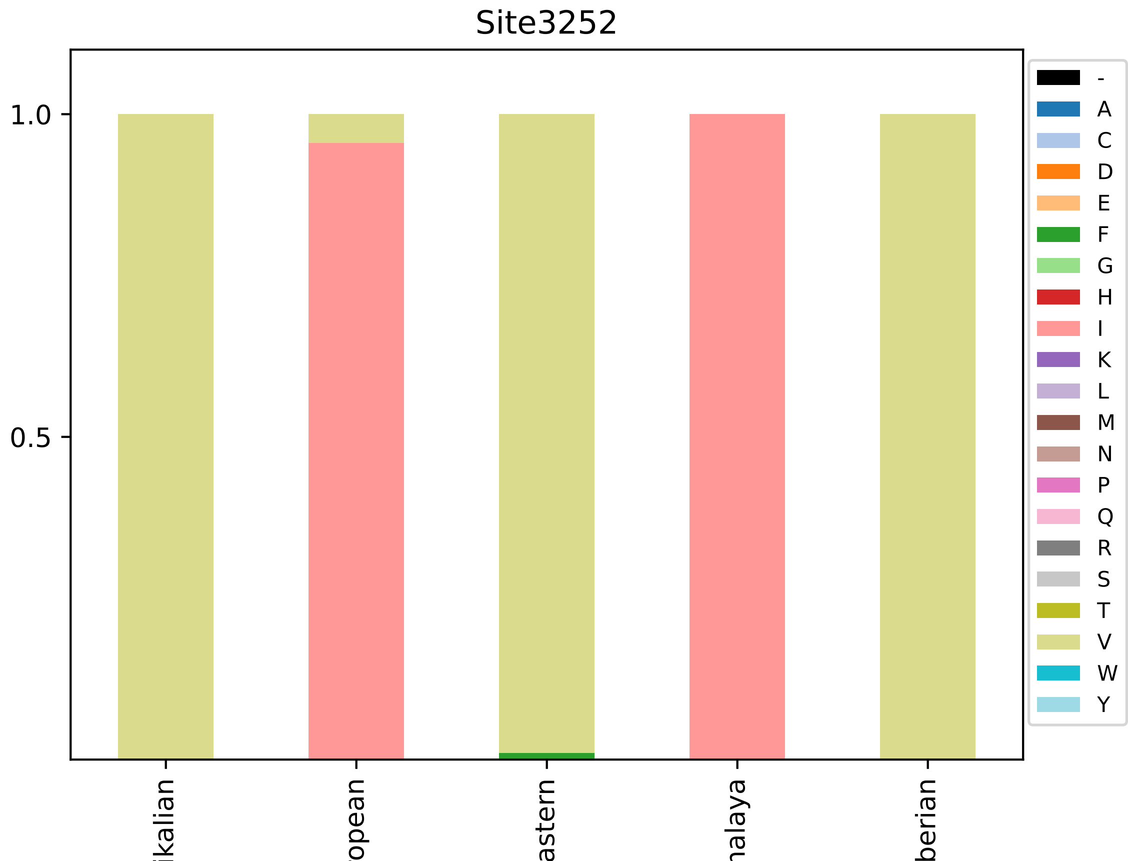
Site3252
1.0
0.5
-
A
C
D
E
F
G
H
I
K
L
M
N
P
Q
R
S
T
V
W
Y
ikalian
opean
astern
nalaya
berian

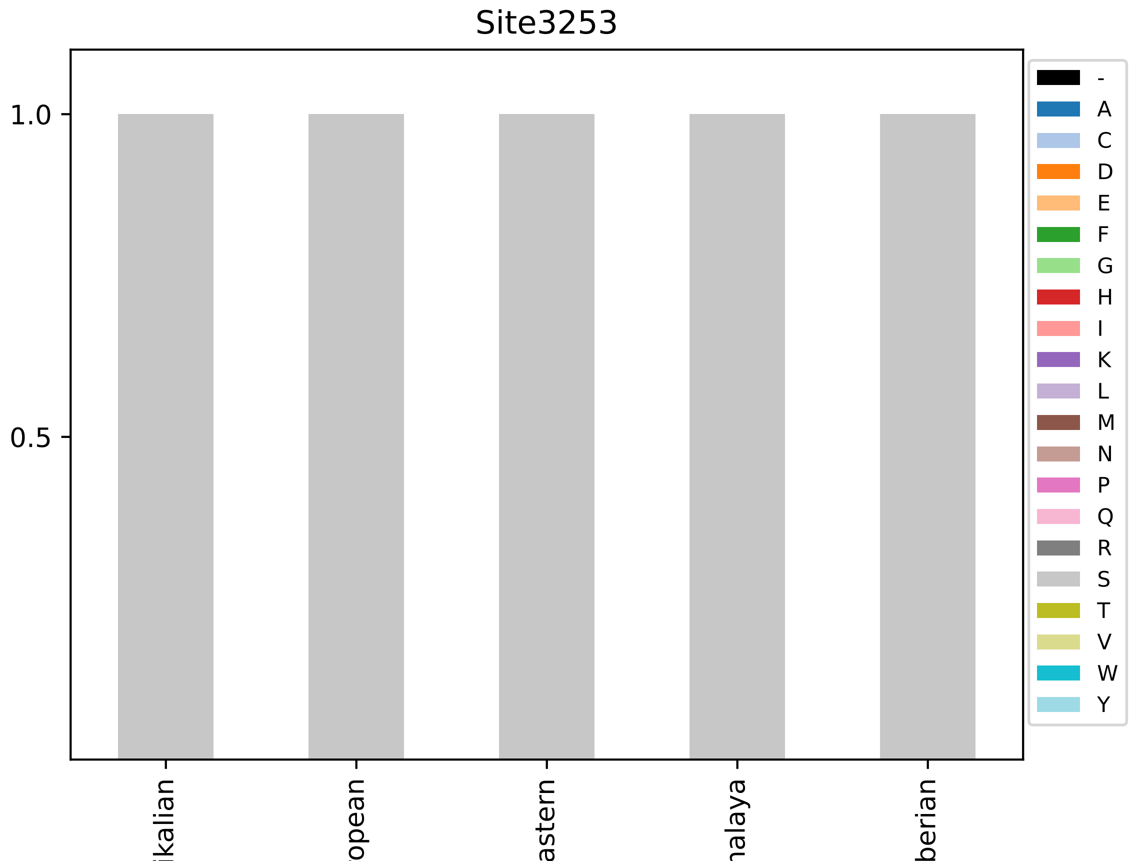

Site3253
1.0
0.5
-
A
C
D
E
F
G
H
I
K
L
M
N
P
Q
R
S
T
V
W
Y
ikalian
opean
astern
nalaya
berian

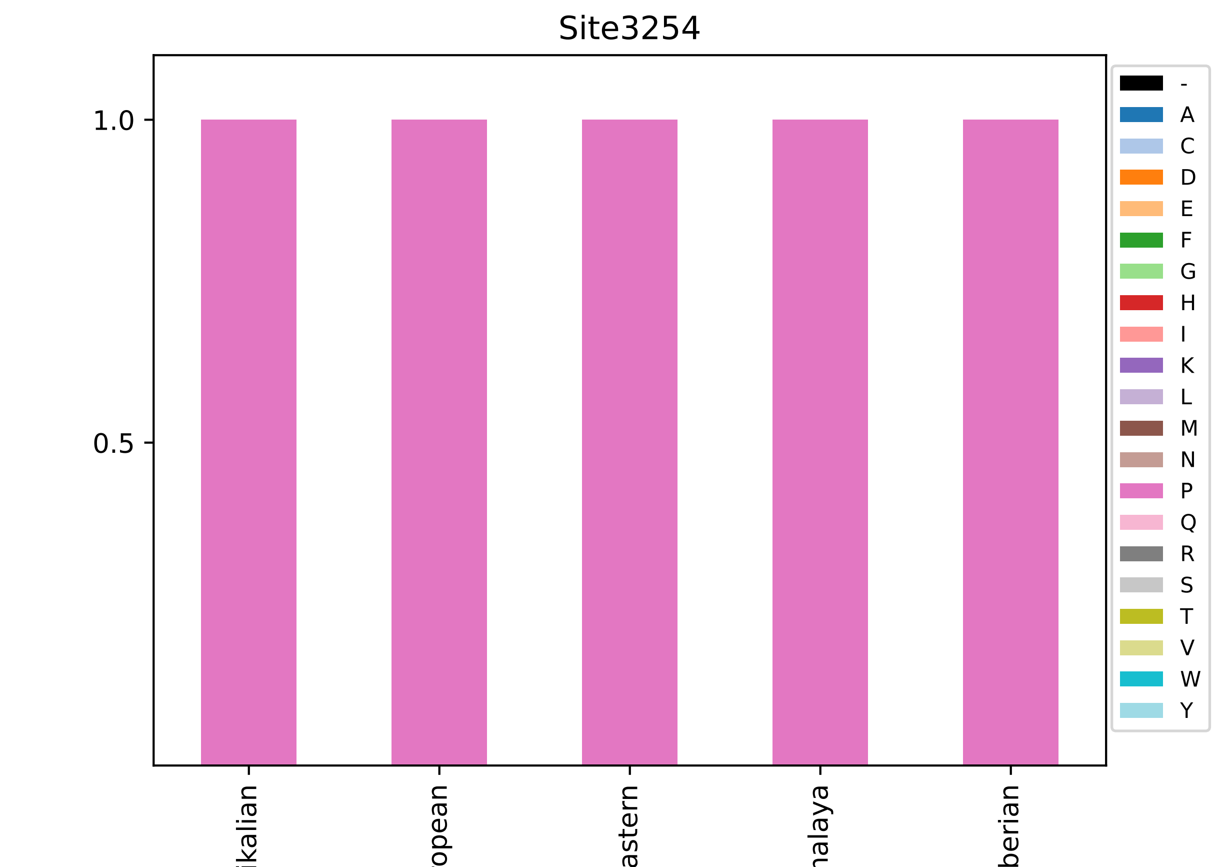
Site3254
1.0
0.5
-
A
C
D
E
F
G
H
I
K
L
M
N
P
Q
R
S
T
V
W
Y
ikalian
opean
astern
nalaya
berian

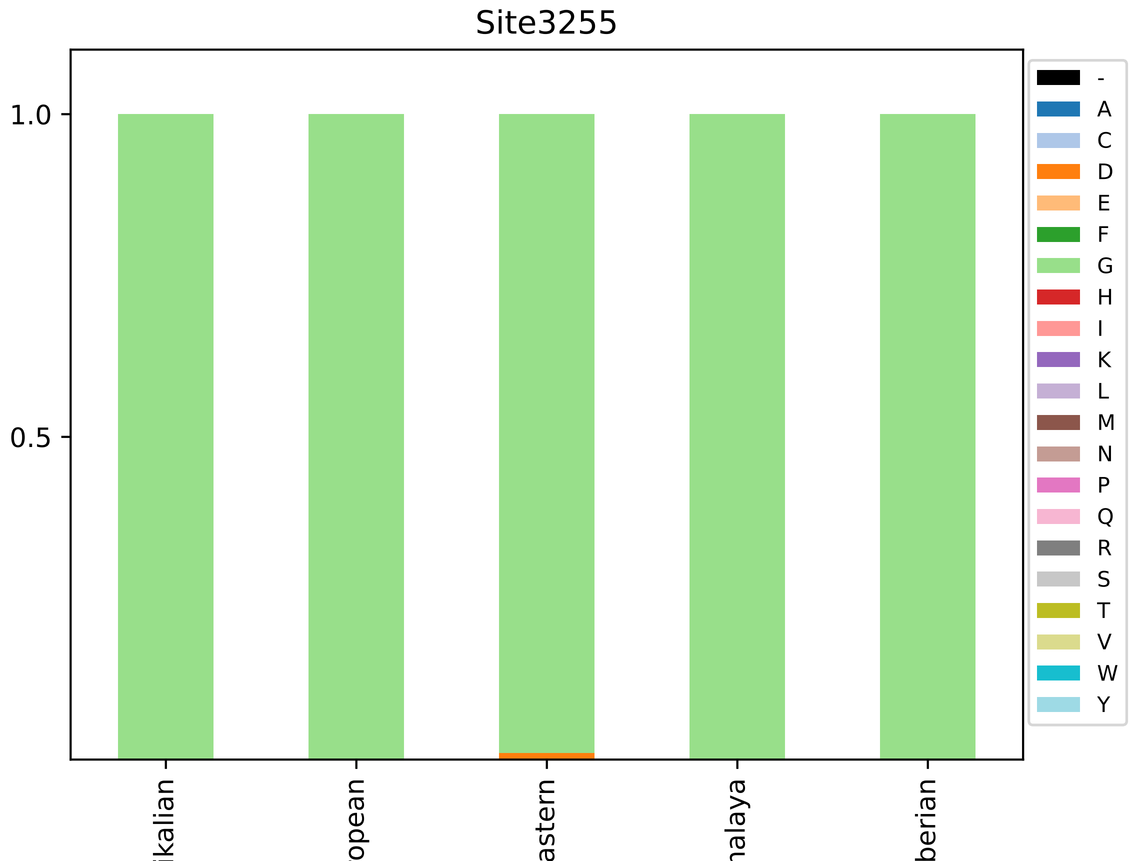

Site3255
1.0
0.5
-
A
C
D
E
F
G
H
I
K
L
M
N
P
Q
R
S
T
V
W
Y
ikalian
opean
astern
nalaya
berian

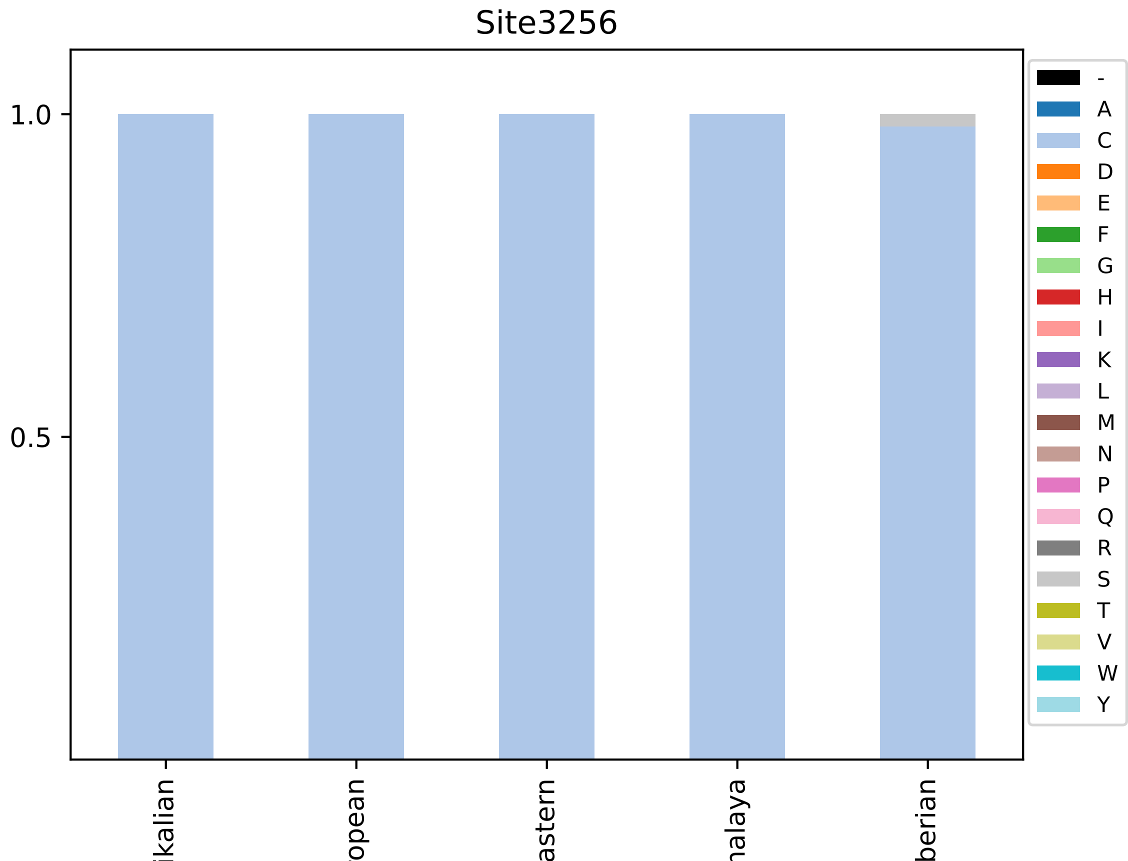
Site3256
1.0
0.5
-
A
C
D
E
F
G
H
I
K
L
M
N
P
Q
R
S
T
V
W
Y

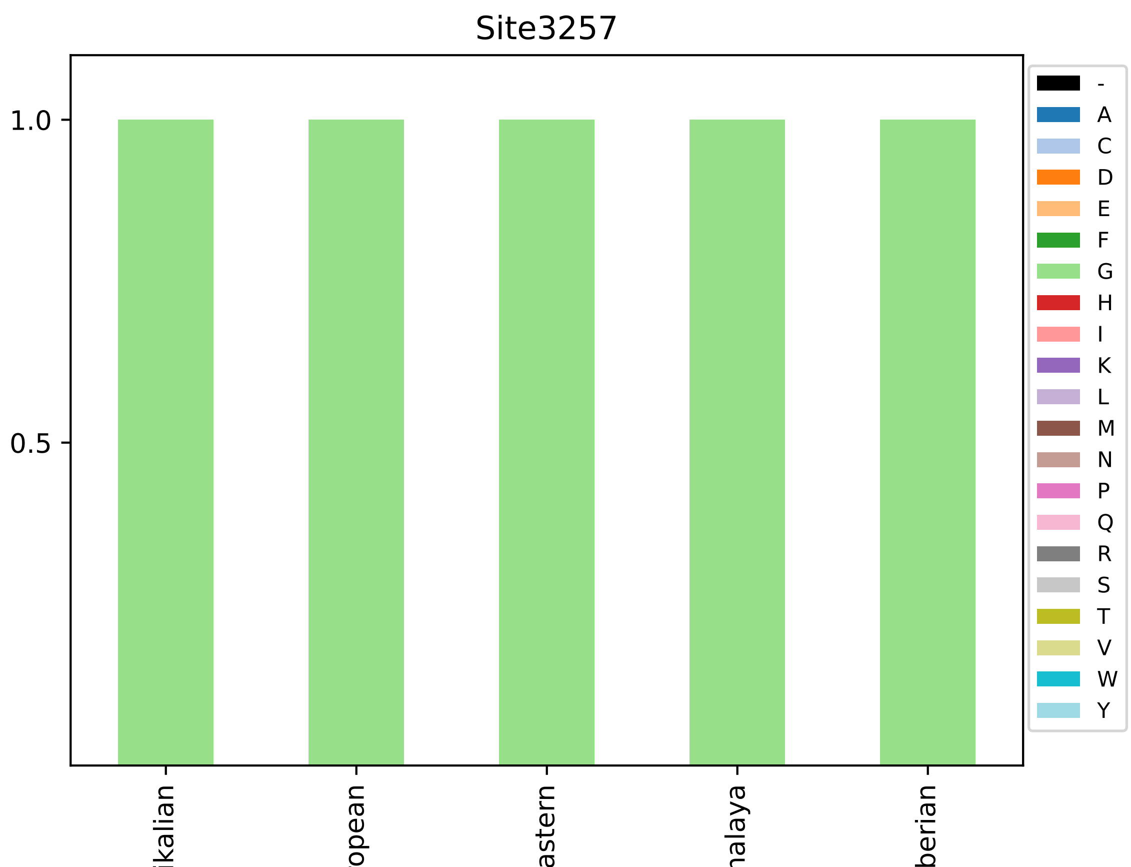

Site3257
1.0
0.5
-
A
C
D
E
F
G
H
I
K
L
M
N
P
Q
R
S
T
V
W
Y
ikalian
opean
astern
nalaya
berian

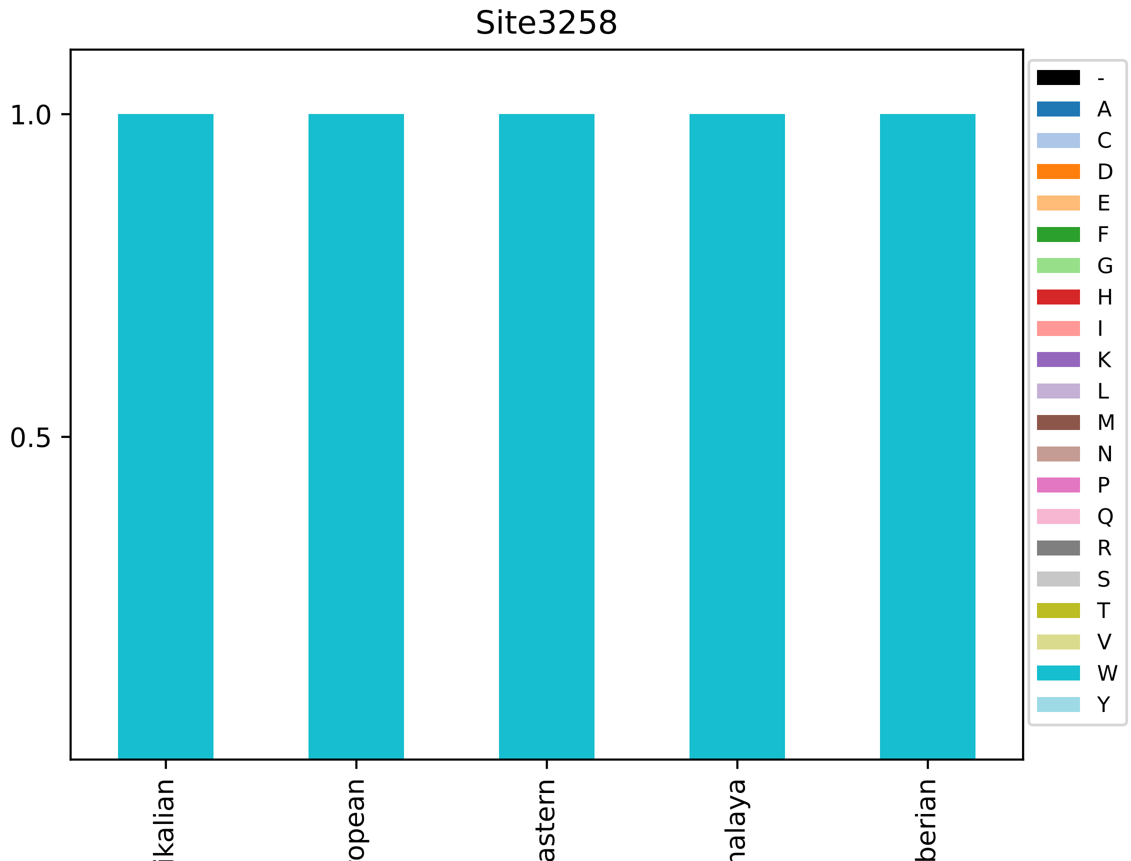
Site3258
1.0
0.5
-
A
C
D
E
F
G
H
I
K
L
M
N
P
Q
R
S
T
V
W
Y
ikalian
opean
astern
nalaya
berian

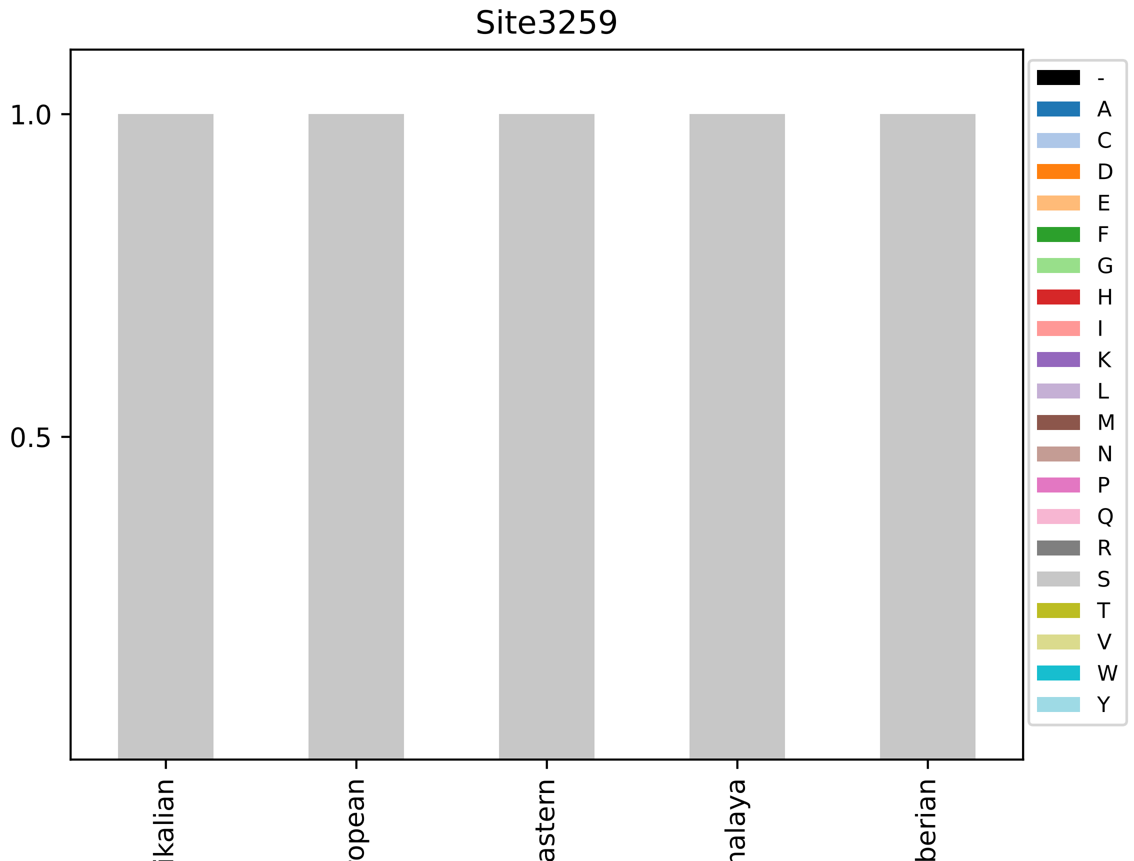
Site3259
1.0
0.5
-
A
C
D
E
F
G
H
I
K
L
M
N
P
Q
R
S
T
V
W
Y
ikalian
opean
astern
nalaya
berian

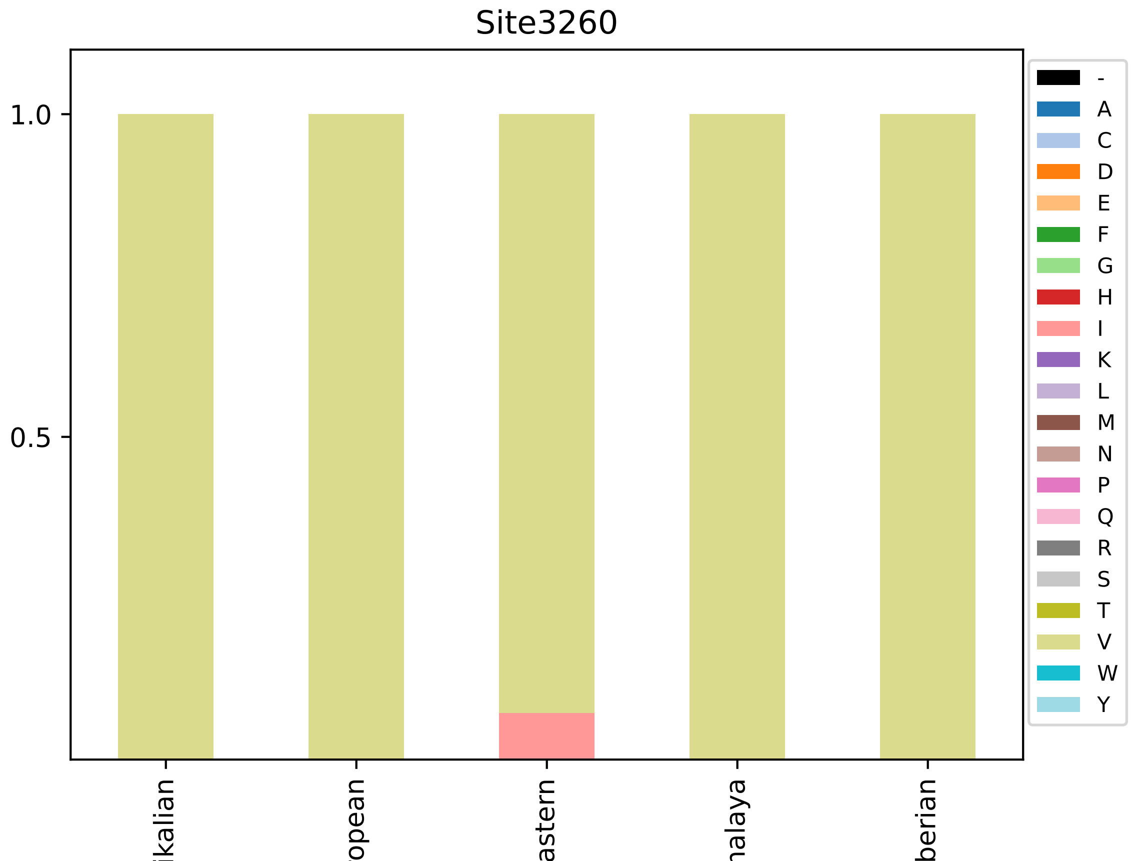

Site3260
1.0
0.5
-
A
C
D
E
F
G
H
I
K
L
M
N
P
Q
R
S
T
V
W
Y
ikalian
opean
astern
nalaya
berian

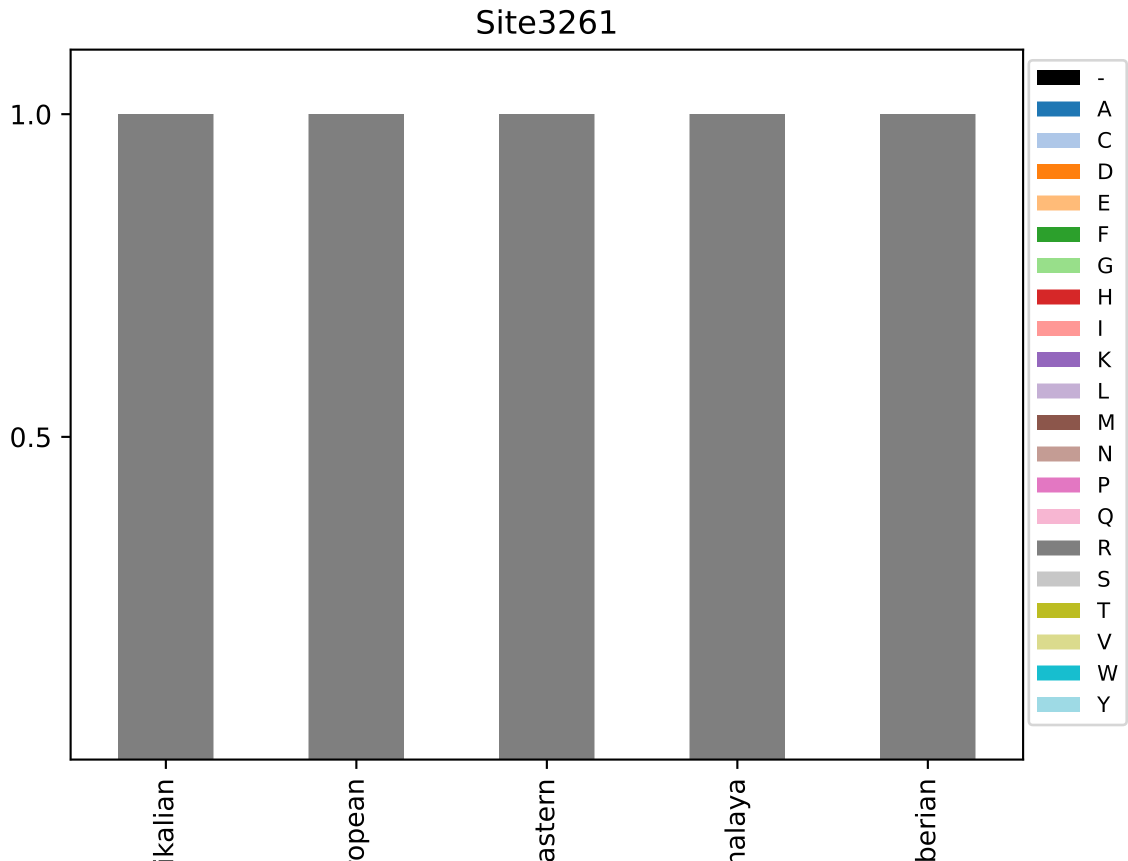

Site3261
1.0
0.5
-
A
C
D
E
F
G
H
I
K
L
M
N
P
Q
R
S
T
V
W
Y
ikalian
opean
astern
nalaya
berian

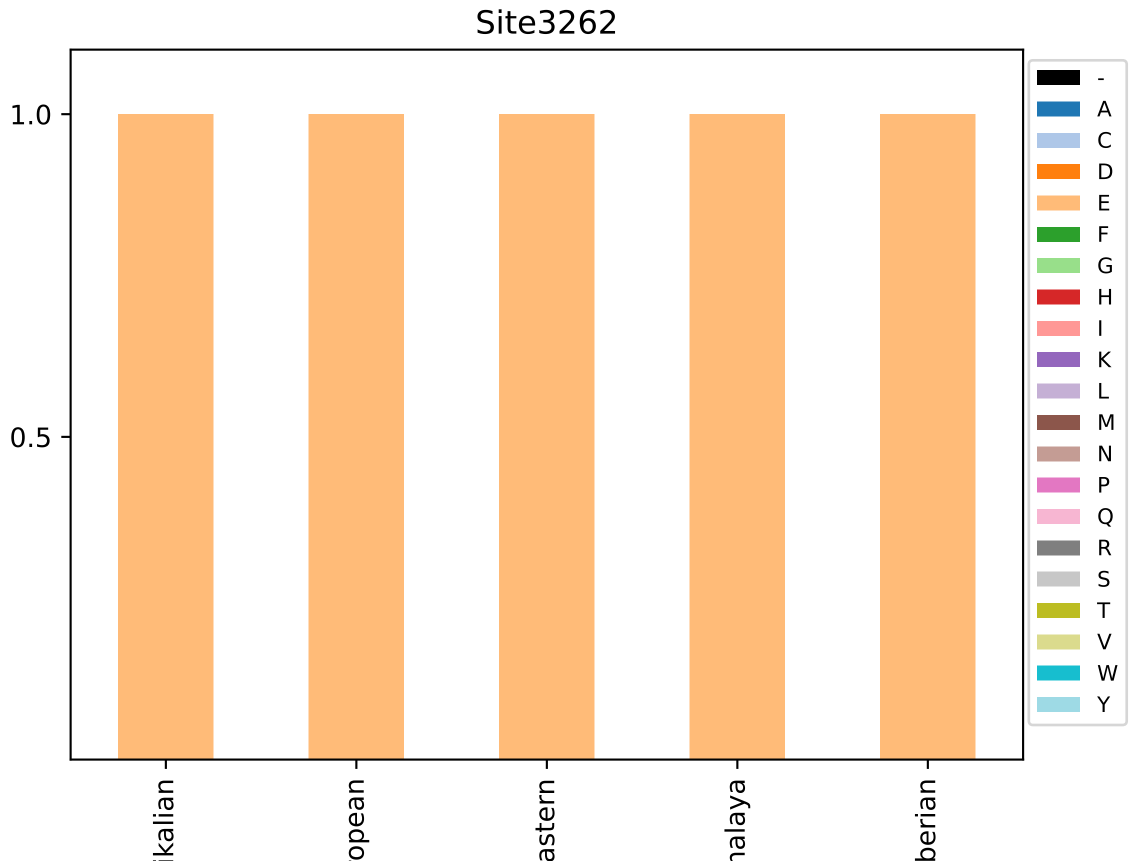
Site3262
1.0
0.5
ikalian
opean
astern
nalaya
berian
-
A
C
D
E
F
G
H
I
K
L
M
N
P
Q
R
S
T
V
W
Y

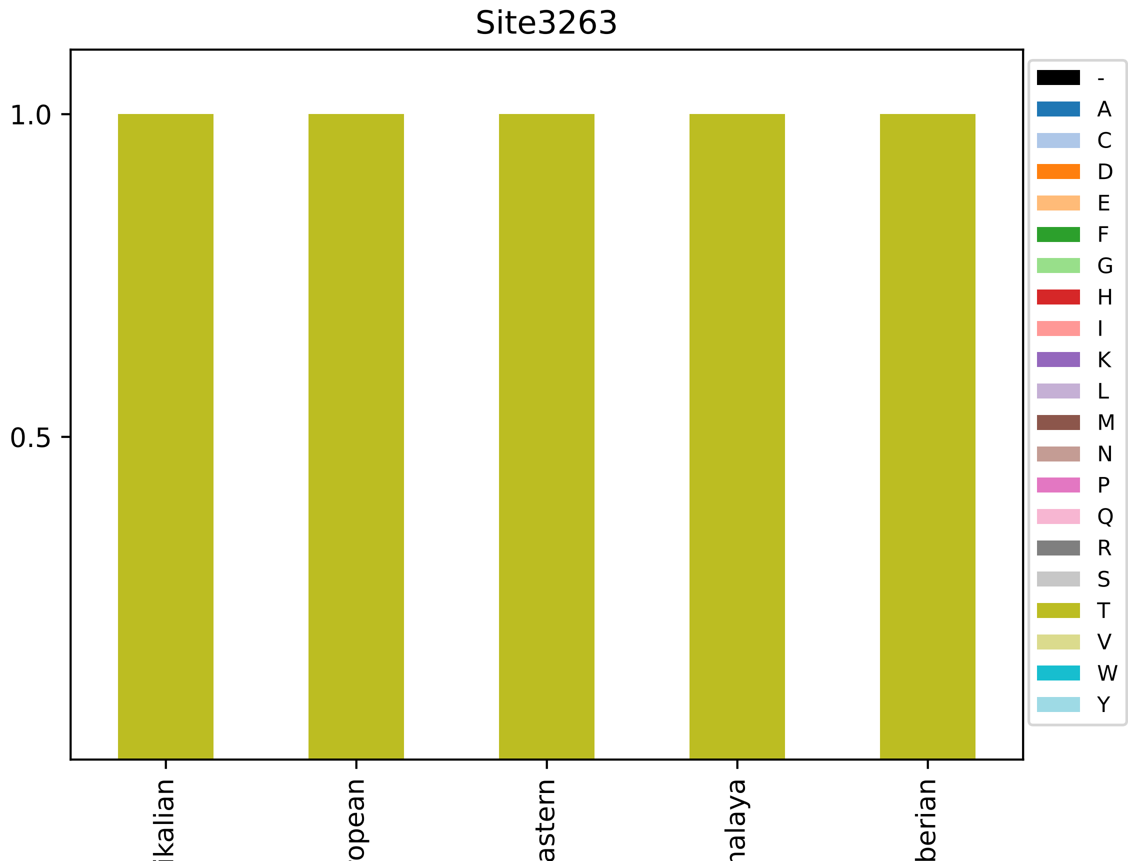
Site3263
1.0
0.5
-
A
C
D
E
F
G
H
I
K
L
M
N
P
Q
R
S
T
V
W
Y
ikalian
opean
astern
nalaya
berian

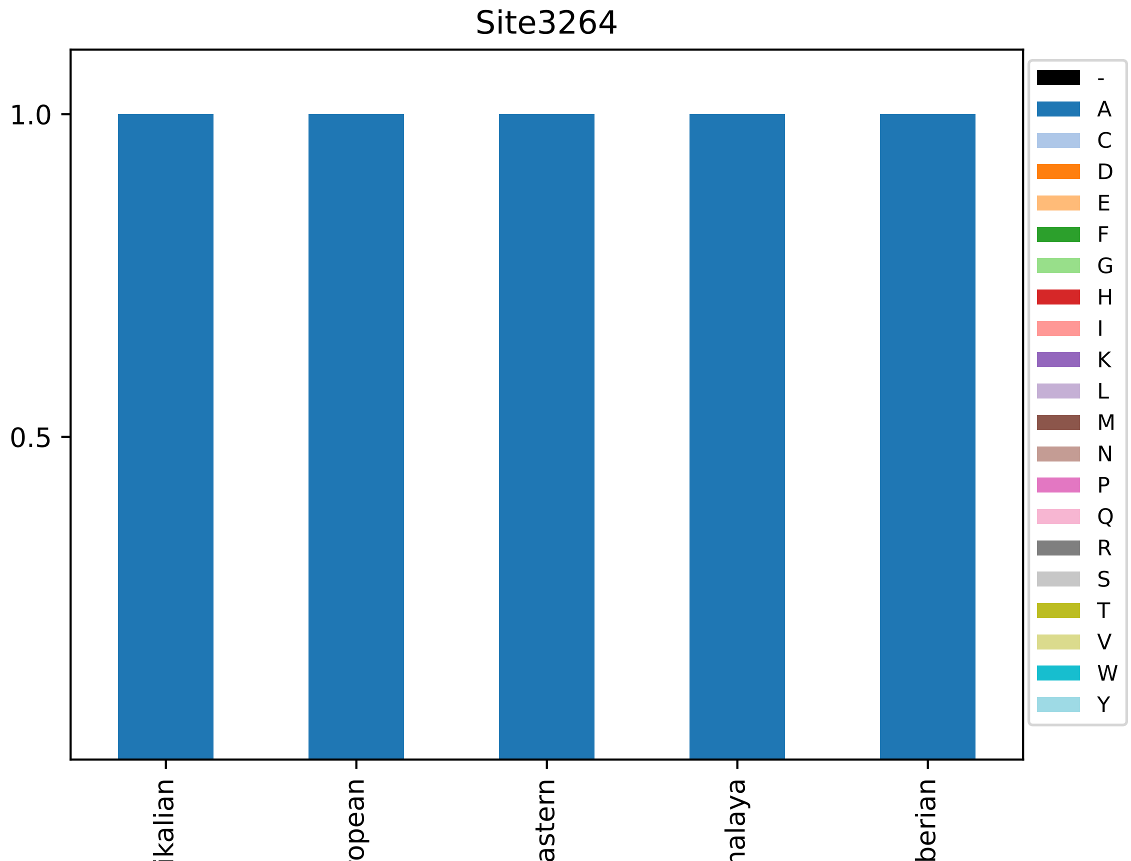

Site3264
1.0
0.5
-
A
C
D
E
F
G
H
I
K
L
M
N
P
Q
R
S
T
V
W
Y
ikalian
opean
astern
nalaya
berian

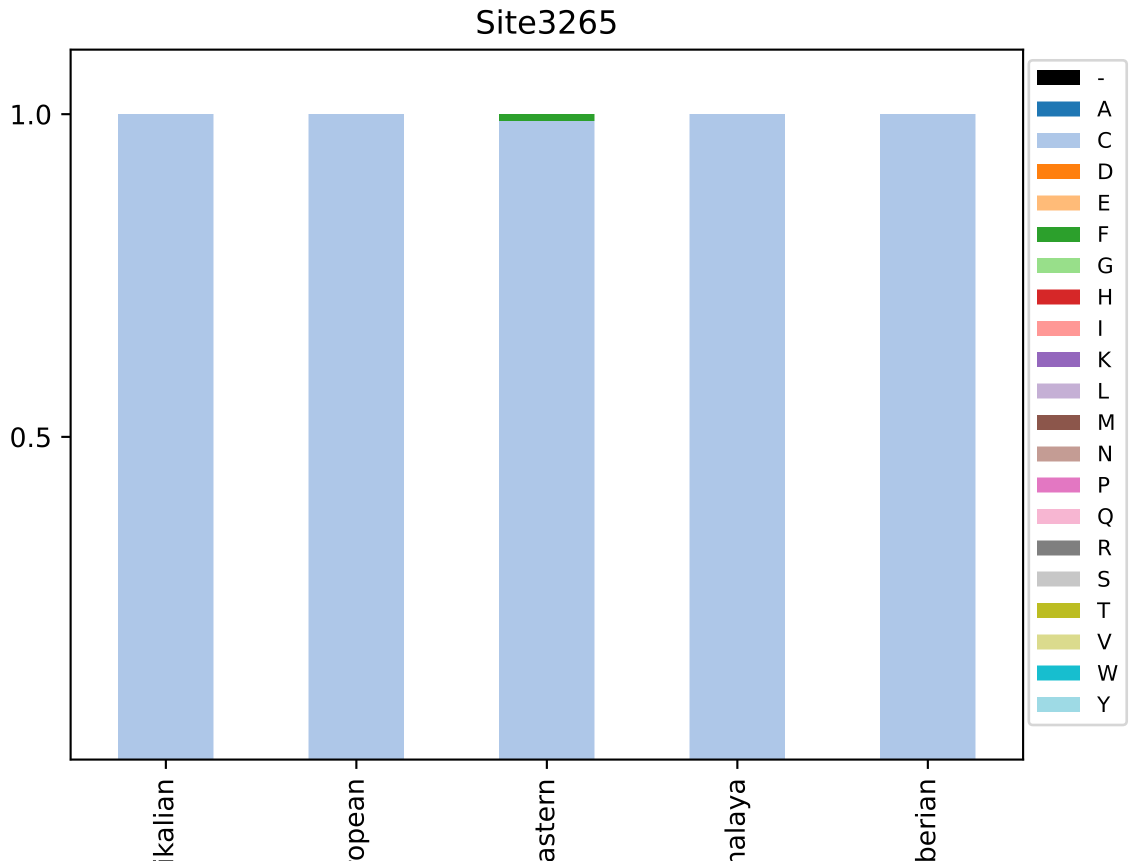
Site3265
1.0
0.5
-
A
C
D
E
F
G
H
I
K
L
M
N
P
Q
R
S
T
V
W
Y
ikalian
opean
astern
nalaya
berian

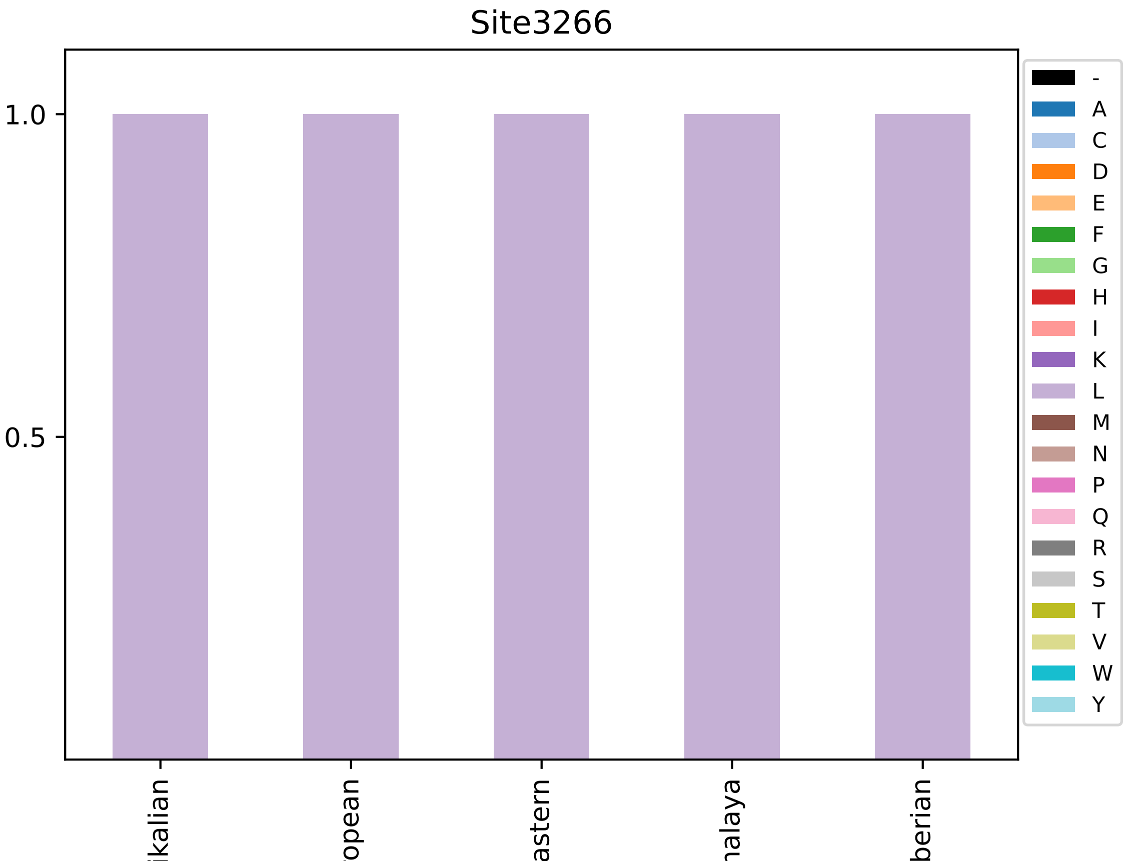
Site3266
1.0
0.5
-
A
C
D
E
F
G
H
I
K
L
M
N
P
Q
R
S
T
V
W
Y
ikalian
opean
astern
nalaya
berian

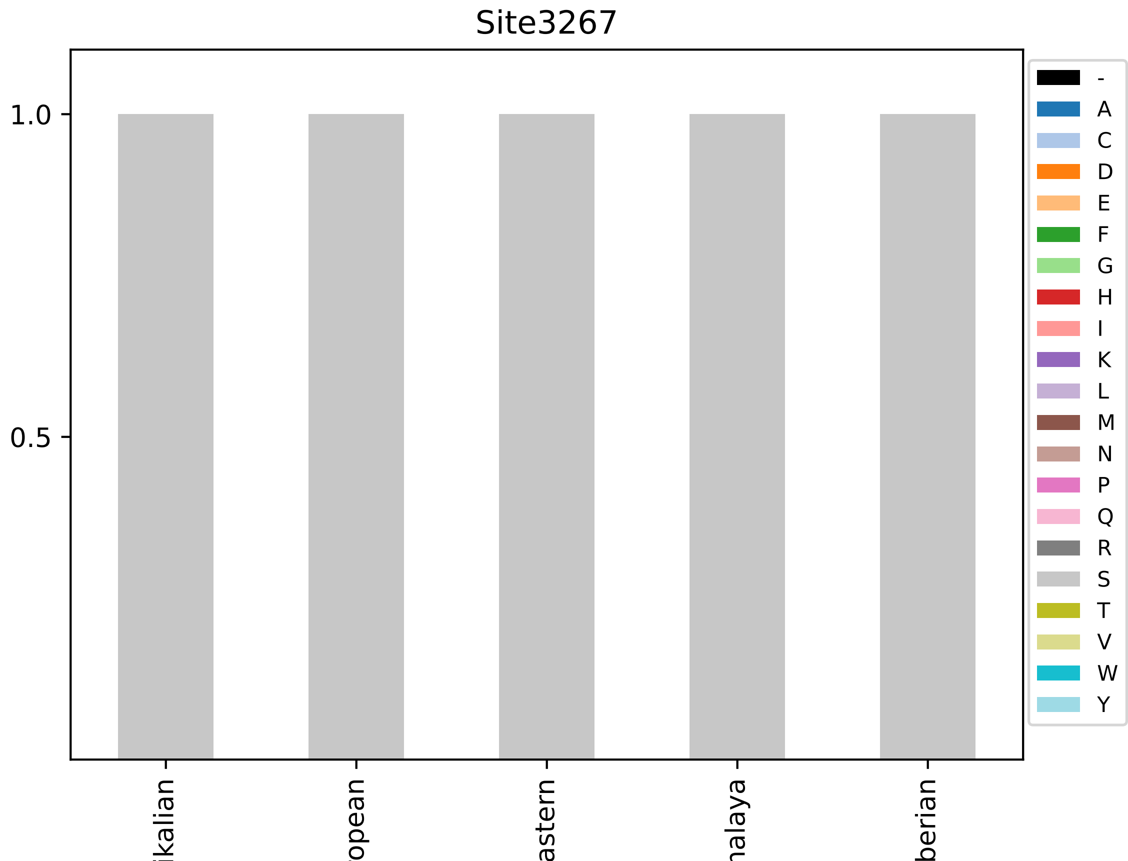

Site3267
1.0
0.5
ikalian
opean
astern
nalaya
berian
-
A
C
D
E
F
G
H
I
K
L
M
N
P
Q
R
S
T
V
W
Y

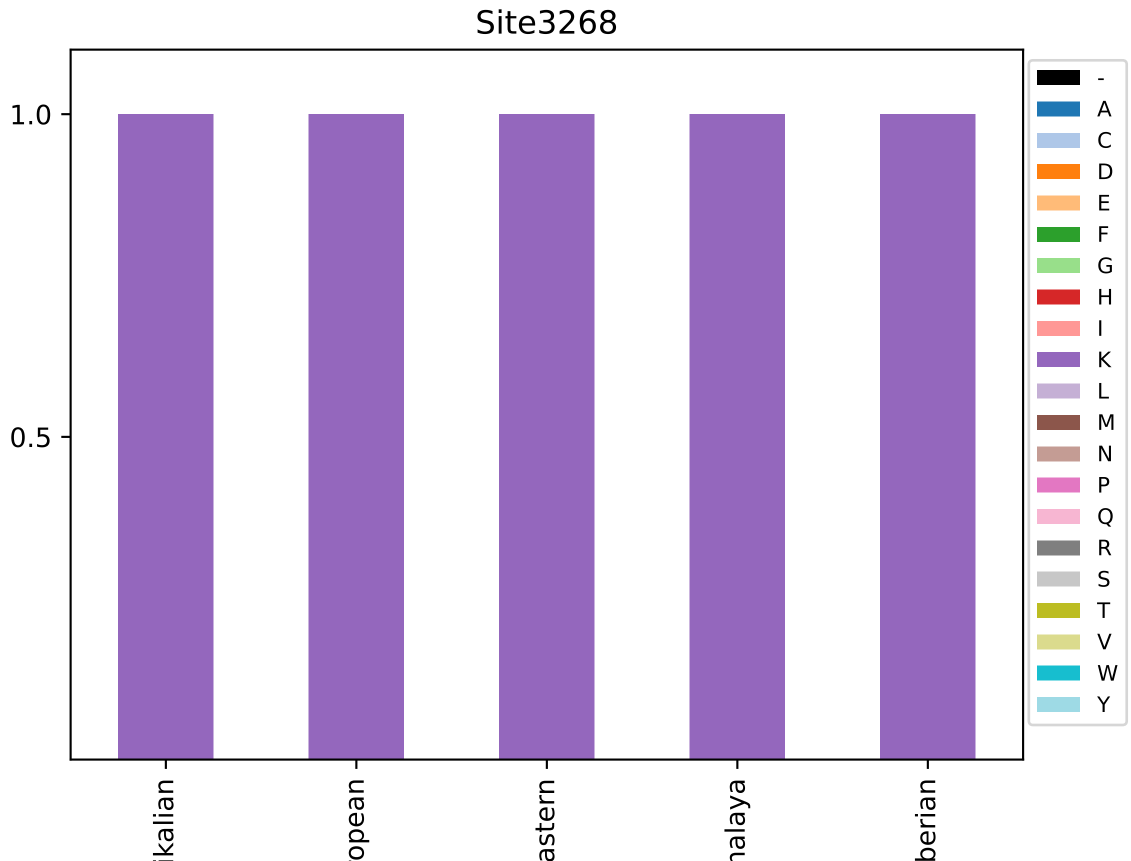

Site3268
1.0
0.5
-
A
C
D
E
F
G
H
I
K
L
M
N
P
Q
R
S
T
V
W
Y
ikalian
opean
astern
nalaya
berian

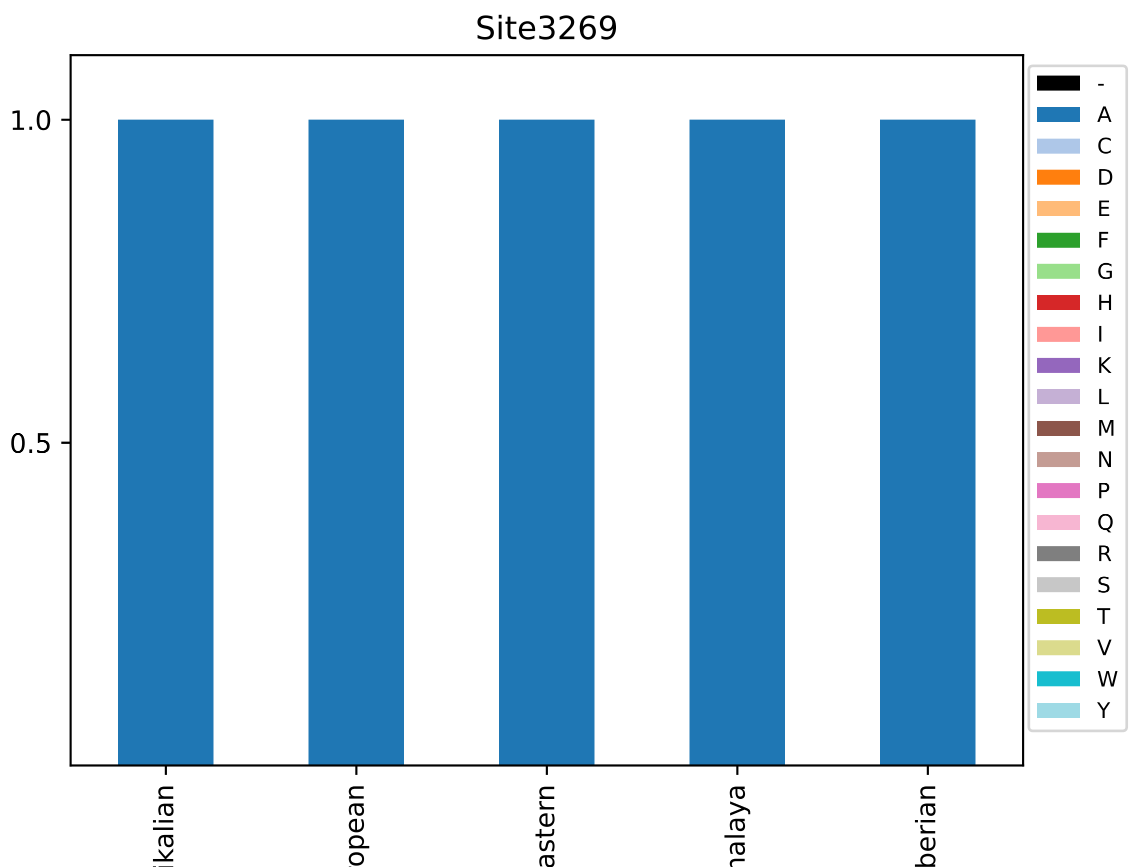
Site3269
1.0
0.5
ikalian
opean
astern
nalaya
berian
-
A
C
D
E
F
G
H
I
K
L
M
N
P
Q
R
S
T
V
W
Y

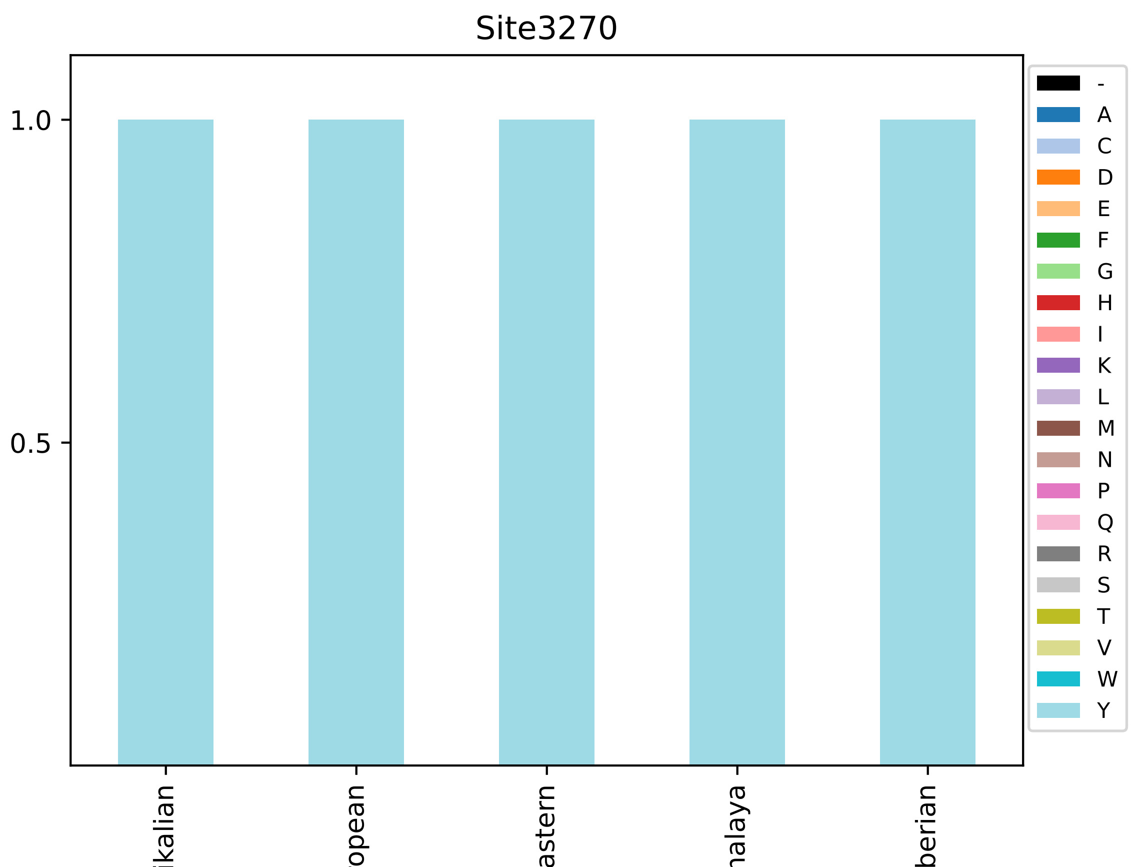
Site3270
1.0
0.5
-
A
C
D
E
F
G
H
I
K
L
M
N
P
Q
R
S
T
V
W
Y
ikalian
opean
astern
nalaya
berian

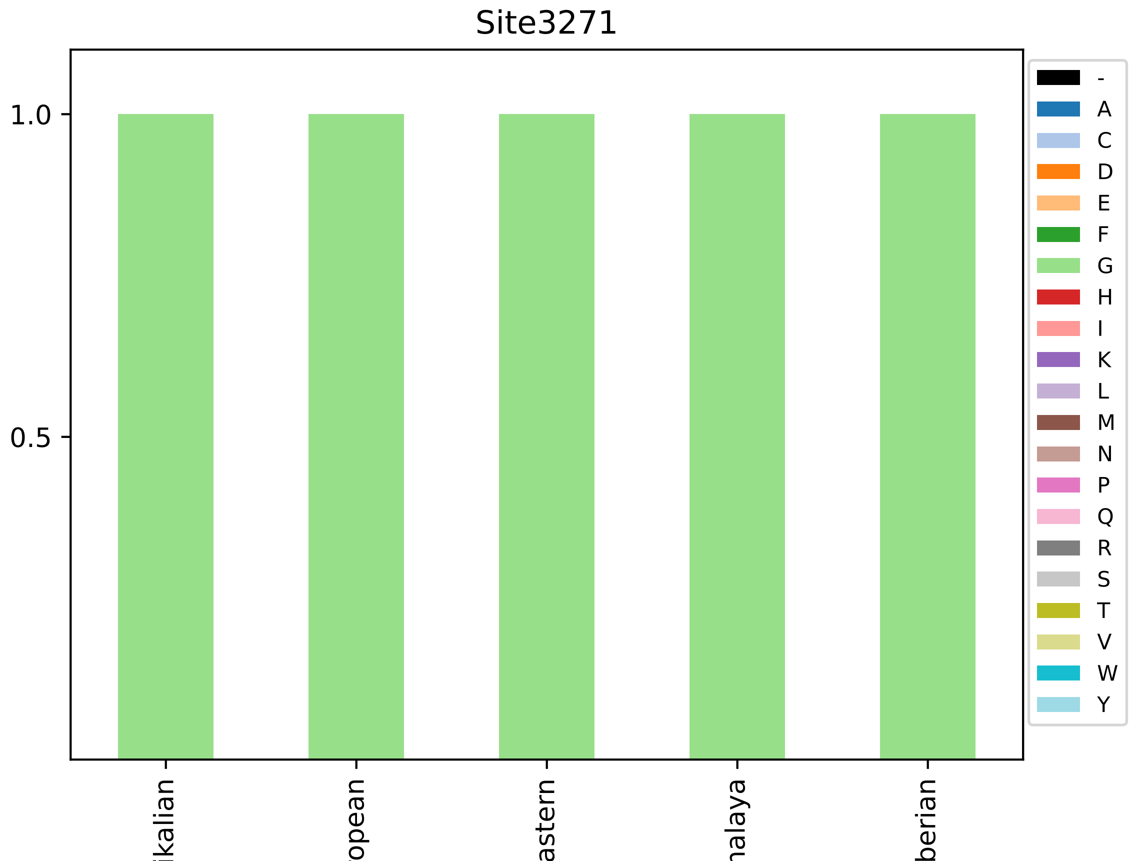
Site3271
1.0
0.5
-
A
C
D
E
F
G
H
I
K
L
M
N
P
Q
R
S
T
V
W
Y
ikalian
opean
astern
nalaya
berian

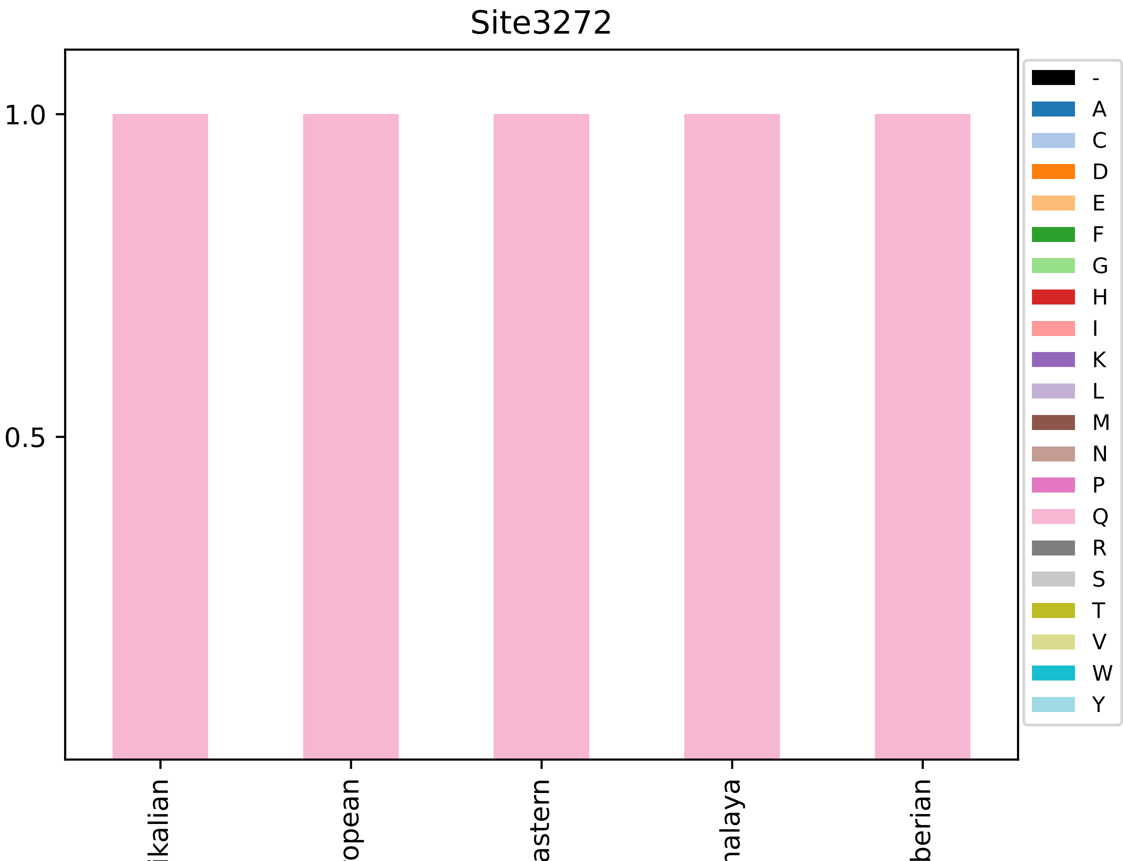

Site3272
1.0
0.5
-
A
C
D
E
F
G
H
I
K
L
M
N
P
Q
R
S
T
V
W
Y
ikalian
opean
astern
nalaya
berian

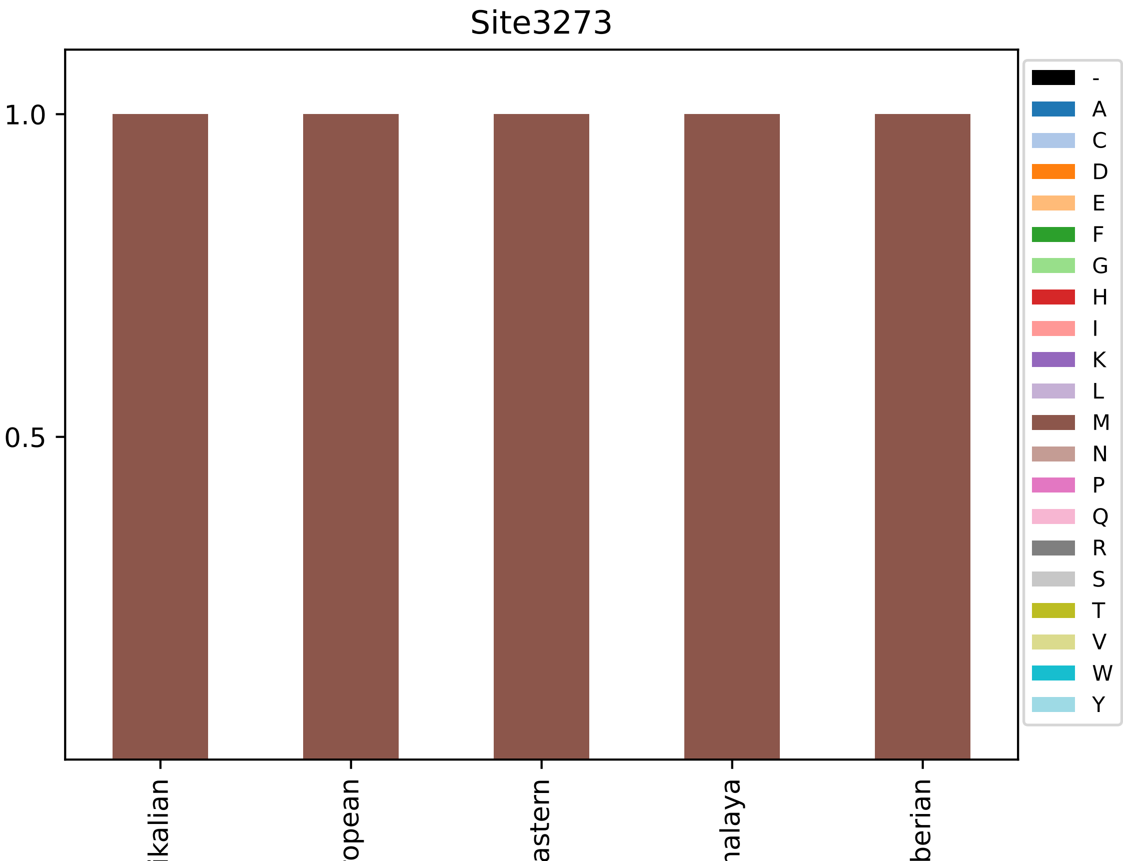

Site3273
1.0
0.5
-
A
C
D
E
F
G
H
I
K
L
M
N
P
Q
R
S
T
V
W
Y
ikalian
opean
astern
nalaya
berian

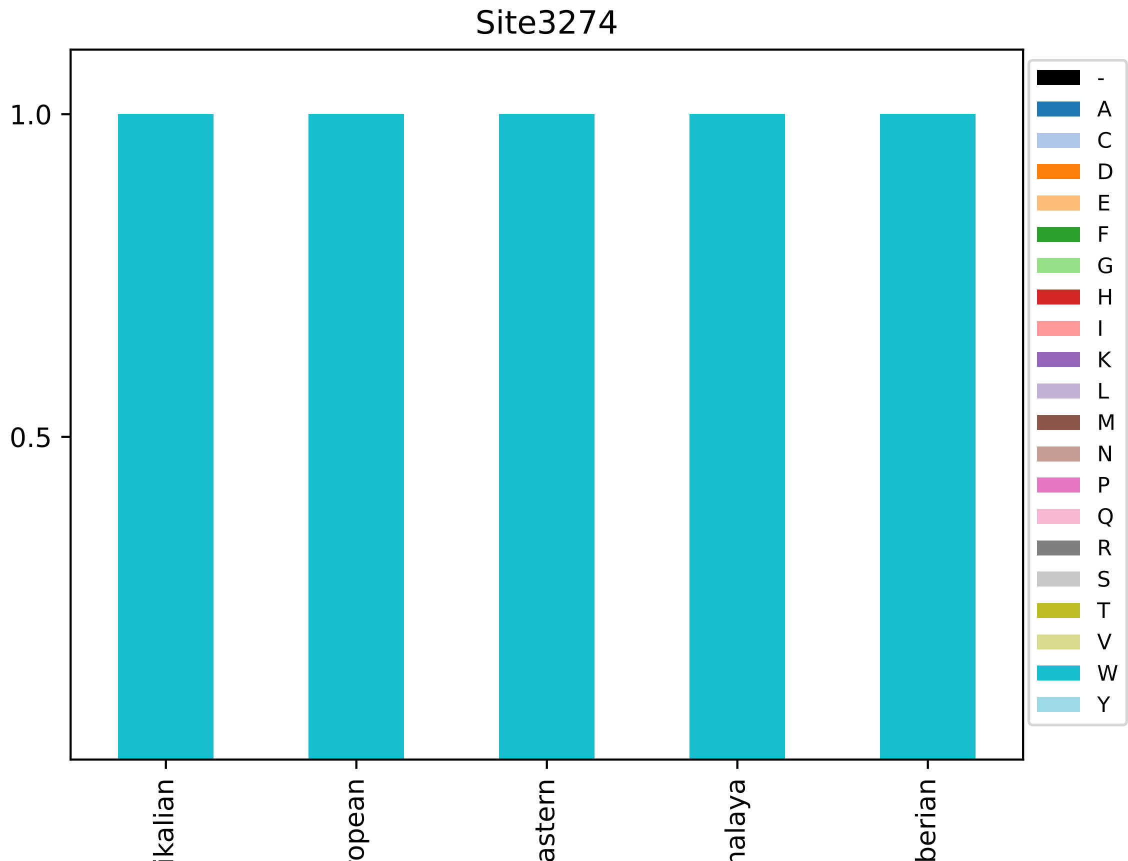

Site3274
1.0
0.5
-
A
C
D
E
F
G
H
I
K
L
M
N
P
Q
R
S
T
V
W
Y
ikalian
opean
astern
nalaya
berian

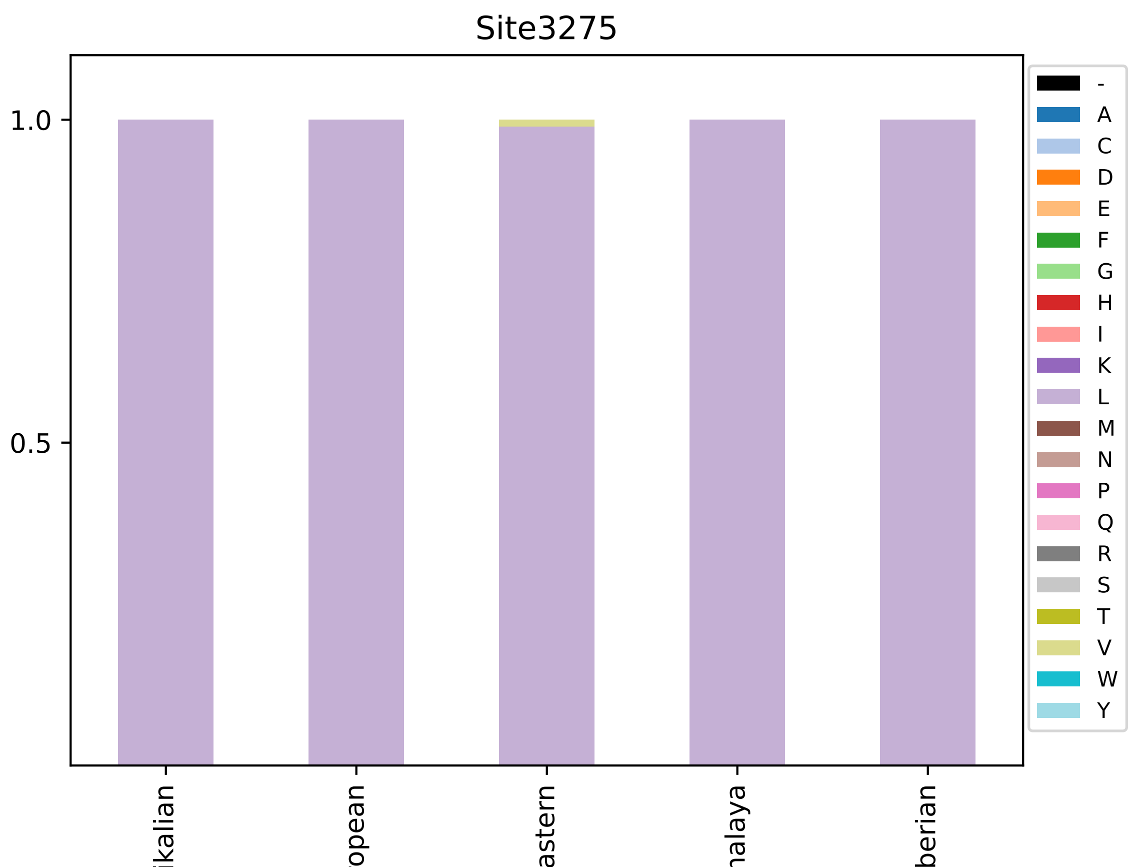

Site3275
1.0
0.5
ikalian
opean
astern
nalaya
berian
-
A
C
D
E
F
G
H
I
K
L
M
N
P
Q
R
S
T
V
W
Y

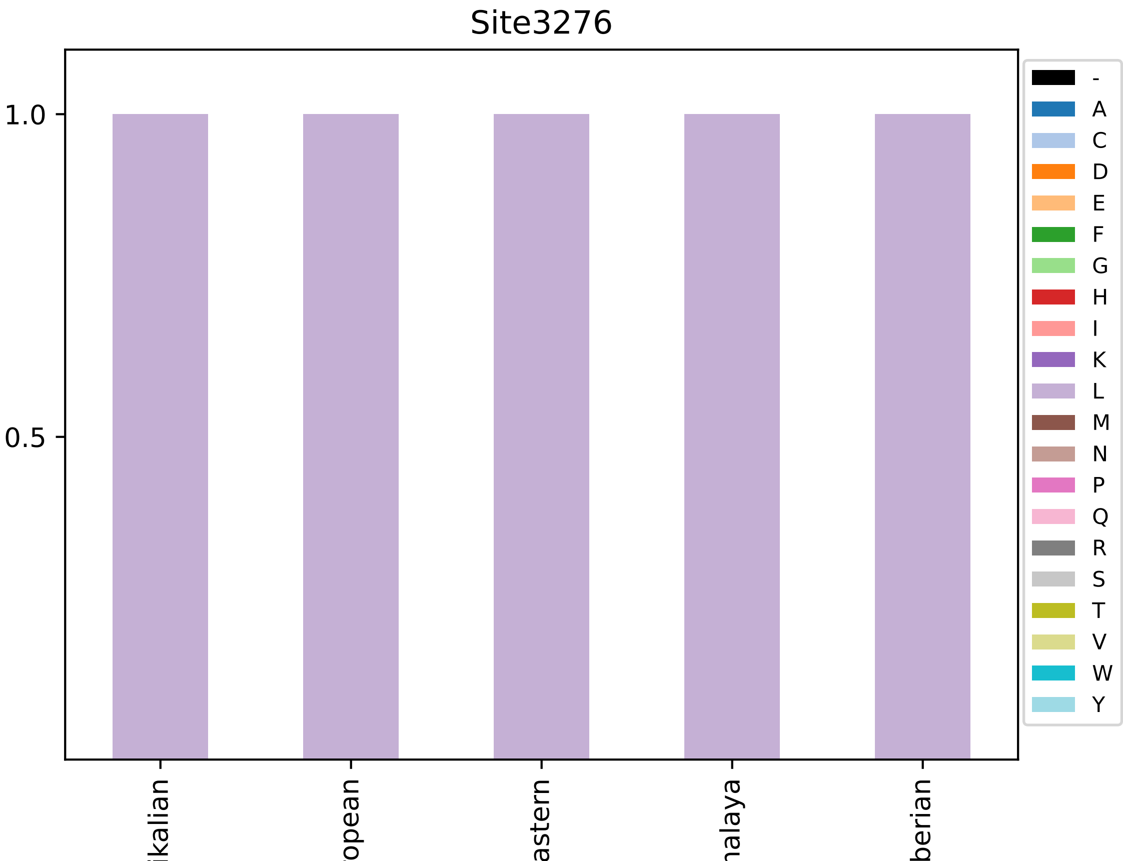

Site3276
1.0
0.5
-
A
C
D
E
F
G
H
I
K
L
M
N
P
Q
R
S
T
V
W
Y

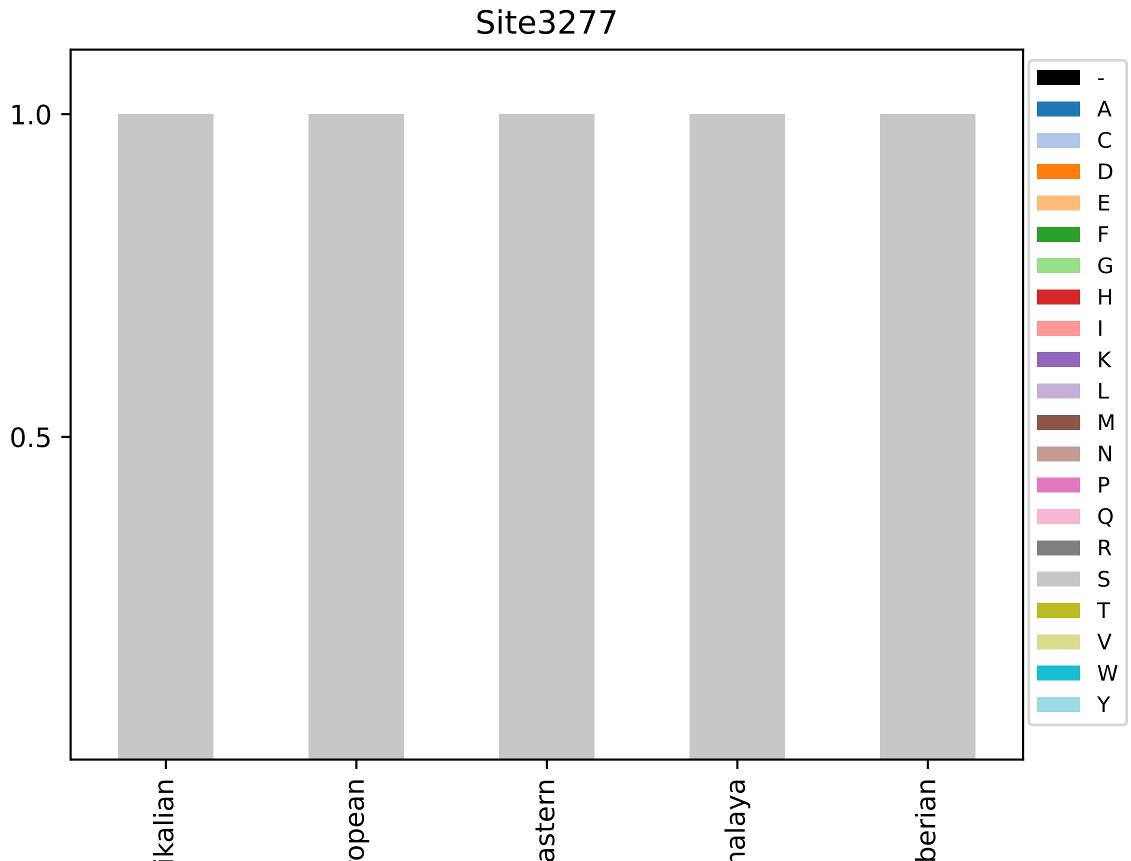
Site3277
1.0
0.5
ikalian
opean
astern
nalaya
berian
-
A
C
D
E
F
G
H
I
K
L
M
N
P
Q
R
S
T
V
W
Y

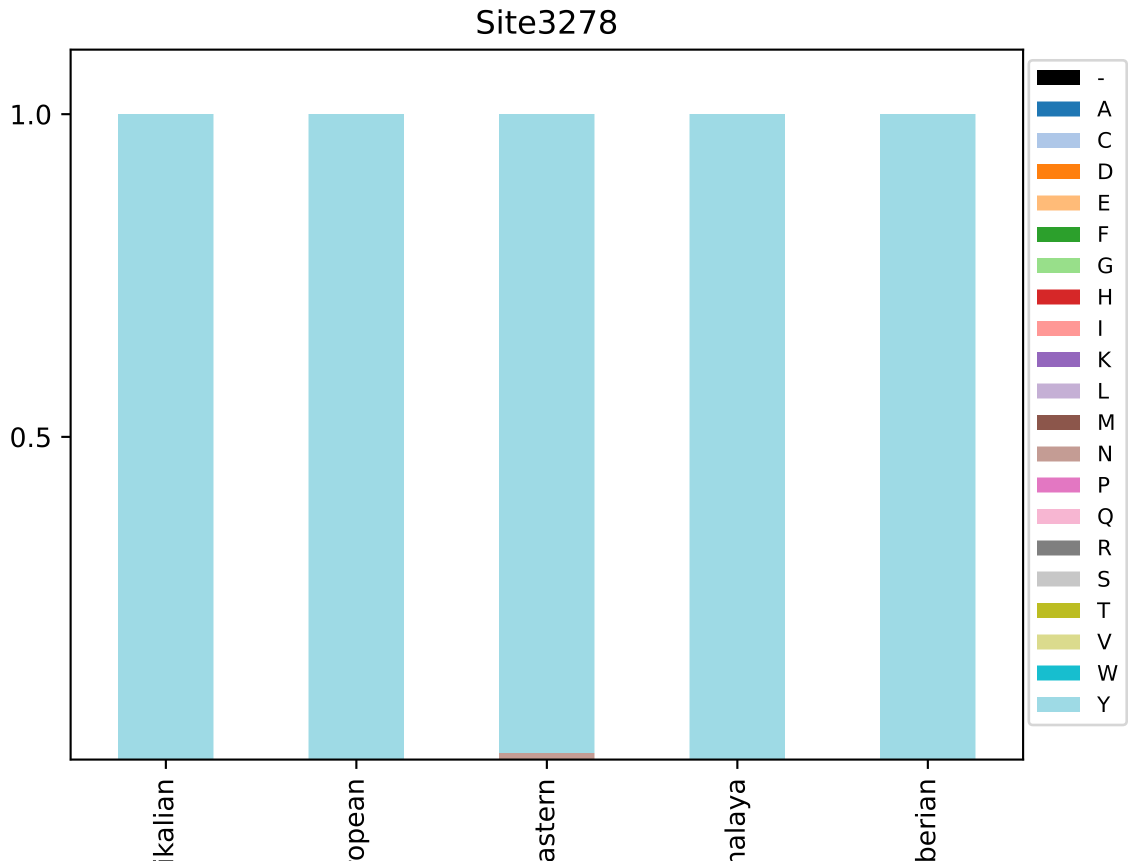
Site3278
1.0
0.5
-
A
C
D
E
F
G
H
I
K
L
M
N
P
Q
R
S
T
V
W
Y
ikalian
opean
astern
nalaya
berian

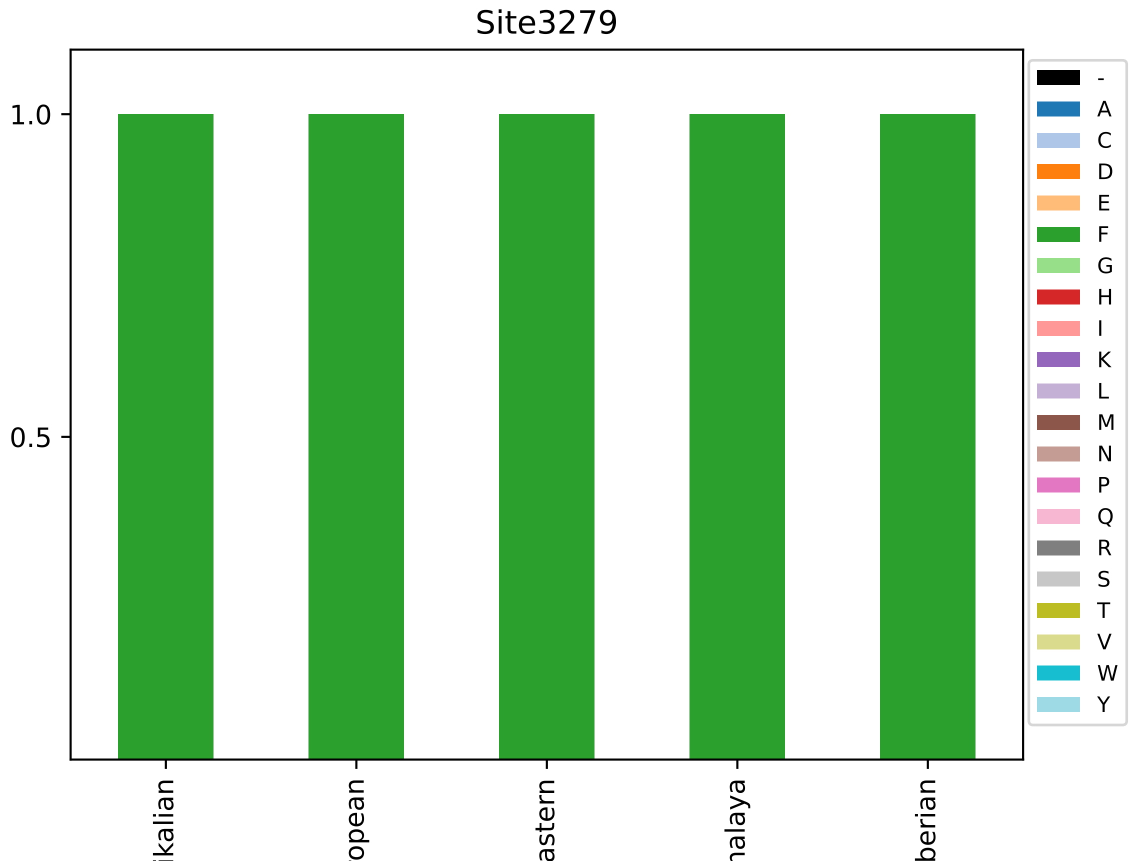

Site3279
1.0
0.5
ikalian
opean
astern
nalaya
berian
-
A
C
D
E
F
G
H
I
K
L
M
N
P
Q
R
S
T
V
W
Y

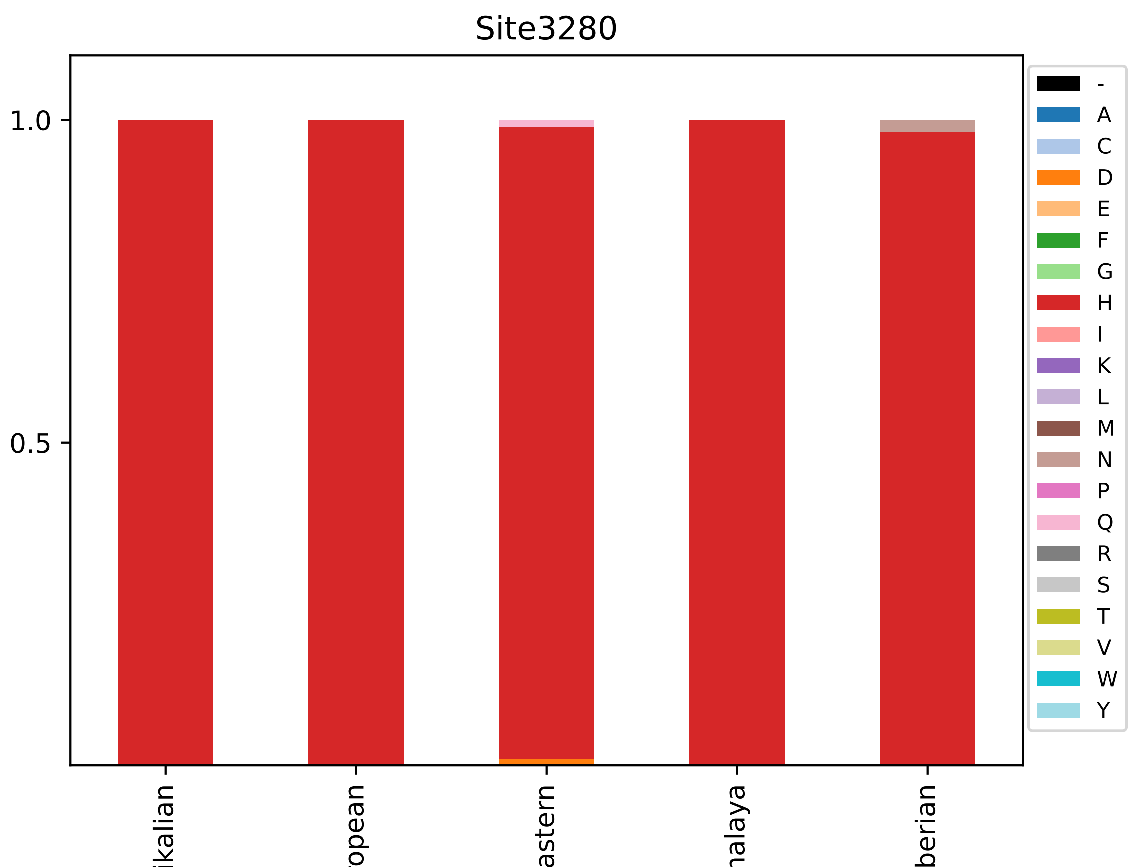

Site3280
1.0
0.5
ikalian
opean
astern
nalaya
berian
-
A
C
D
E
F
G
H
I
K
L
M
N
P
Q
R
S
T
V
W
Y

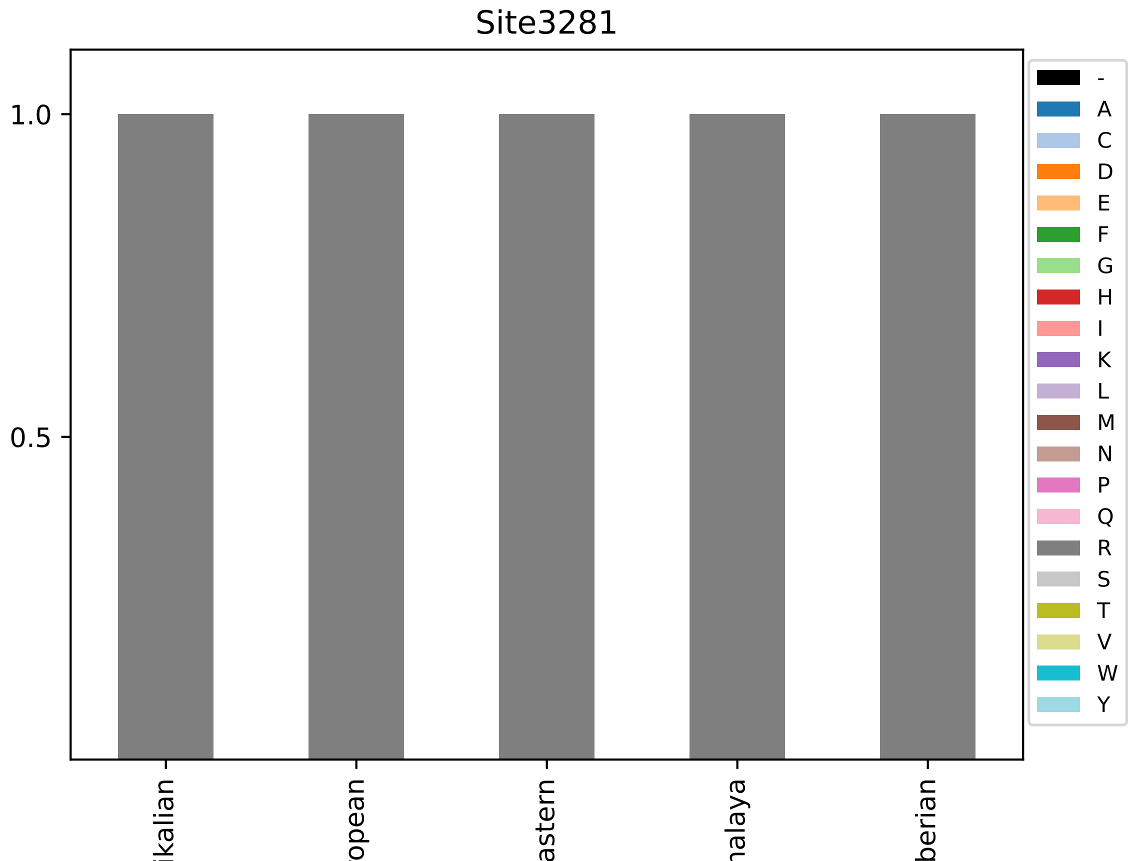
Site3281
1.0
0.5
-
A
C
D
E
F
G
H
I
K
L
M
N
P
Q
R
S
T
V
W
Y
ikalian
opean
astern
nalaya
berian

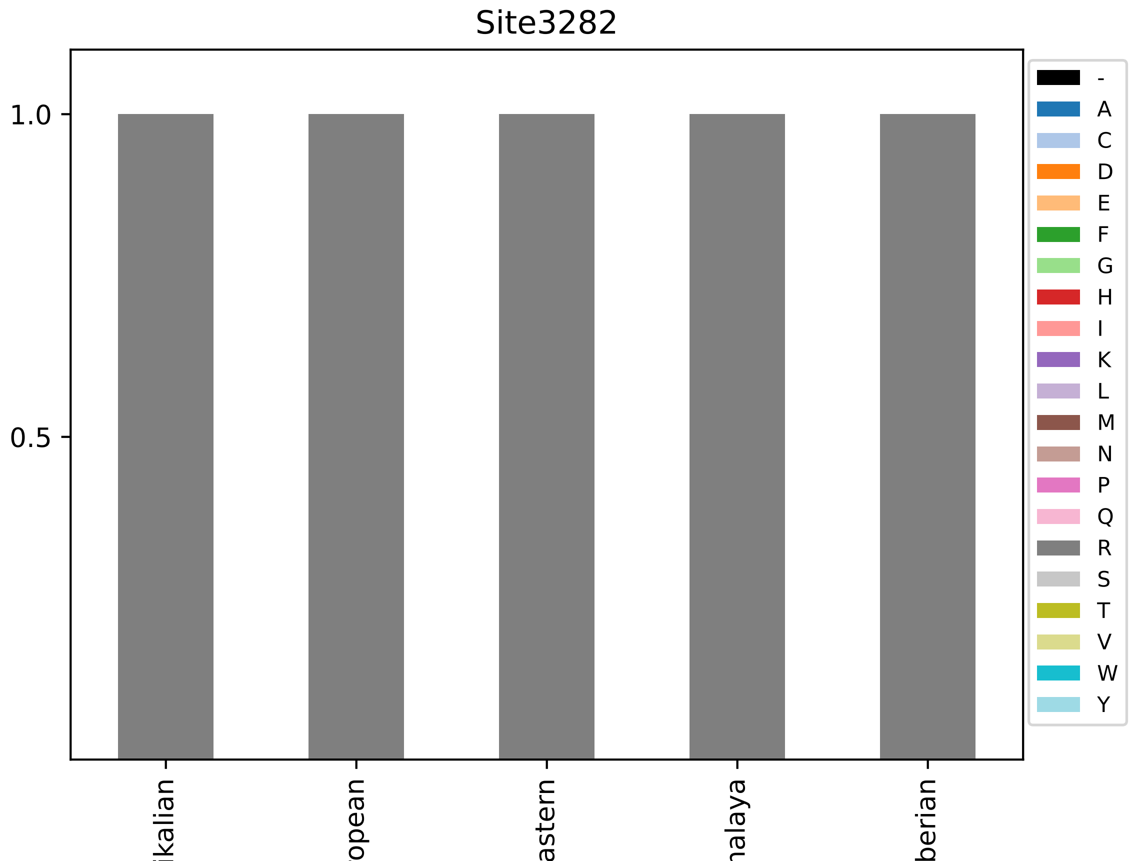
Site3282
1.0
0.5
-
A
C
D
E
F
G
H
I
K
L
M
N
P
Q
R
S
T
V
W
Y

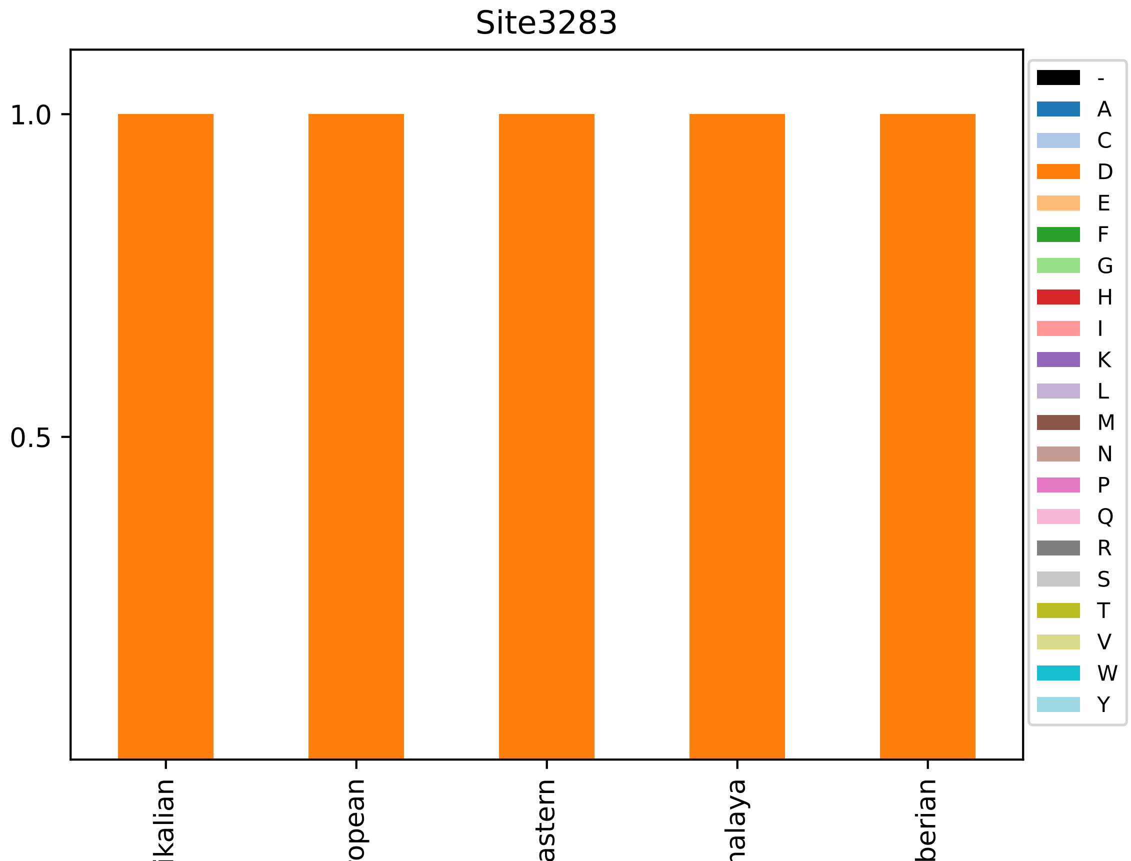
Site3283
1.0
0.5
-
A
C
D
E
F
G
H
I
K
L
M
N
P
Q
R
S
T
V
W
Y

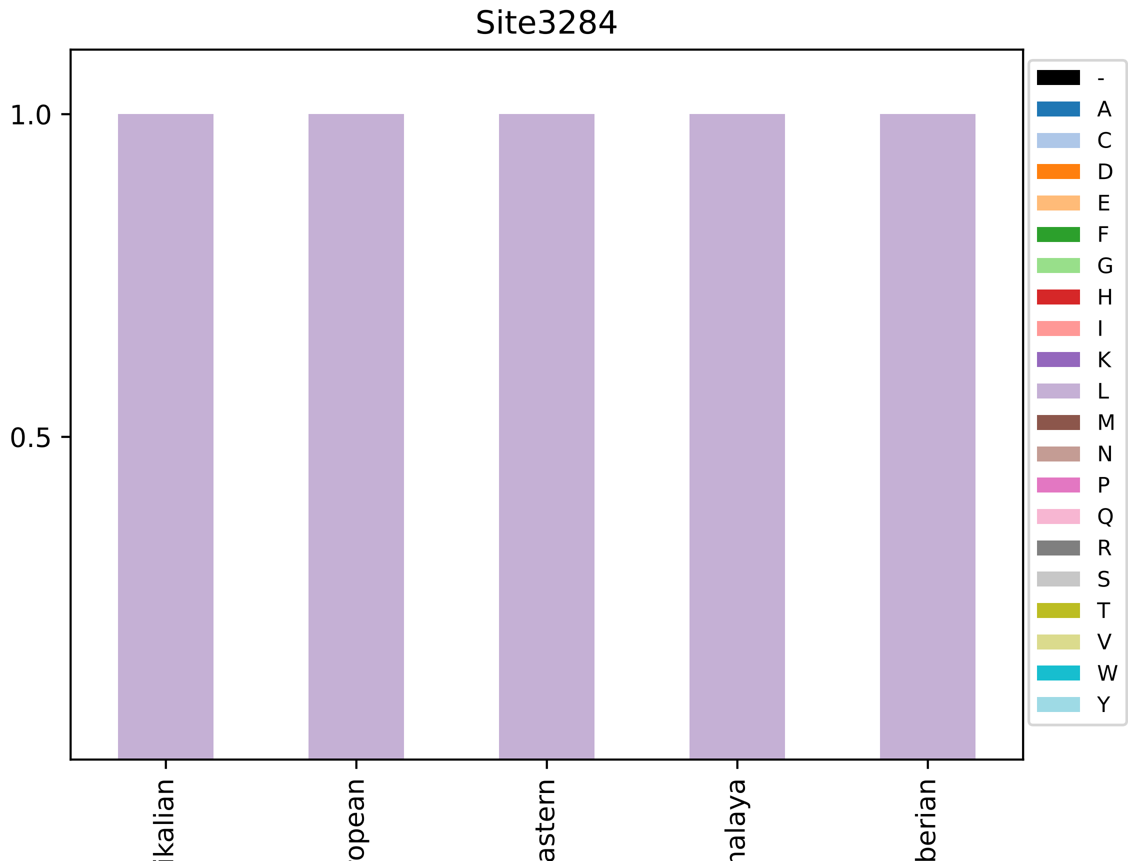
Site3284
1.0
0.5
-
A
C
D
E
F
G
H
I
K
L
M
N
P
Q
R
S
T
V
W
Y
ikalian
opean
astern
nalaya
berian

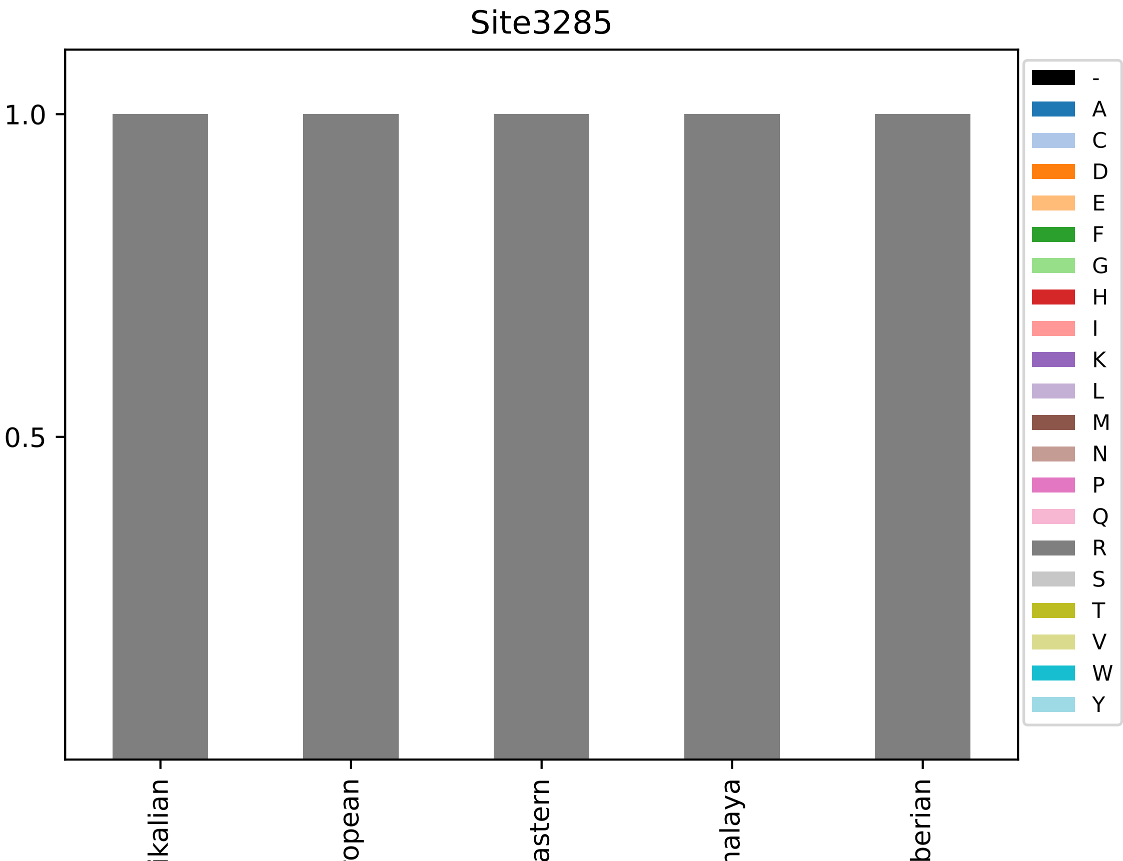
Site3285
1.0
0.5
ikalian
ropean
astern
nalaya
berian
-
A
C
D
E
F
G
H
I
K
L
M
N
P
Q
R
S
T
V
W
Y

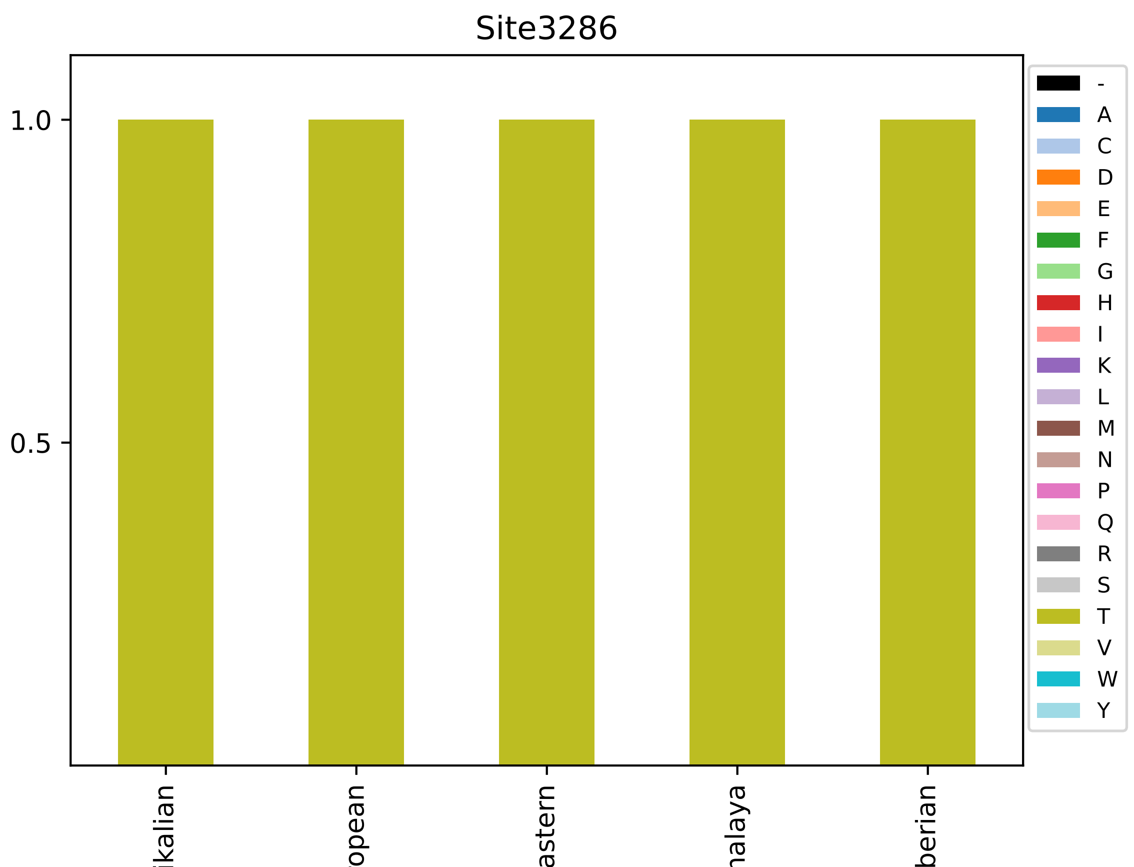

Site3286
1.0
0.5
-
A
C
D
E
F
G
H
I
K
L
M
N
P
Q
R
S
T
V
W
Y
ikalian
opean
astern
nalaya
berian

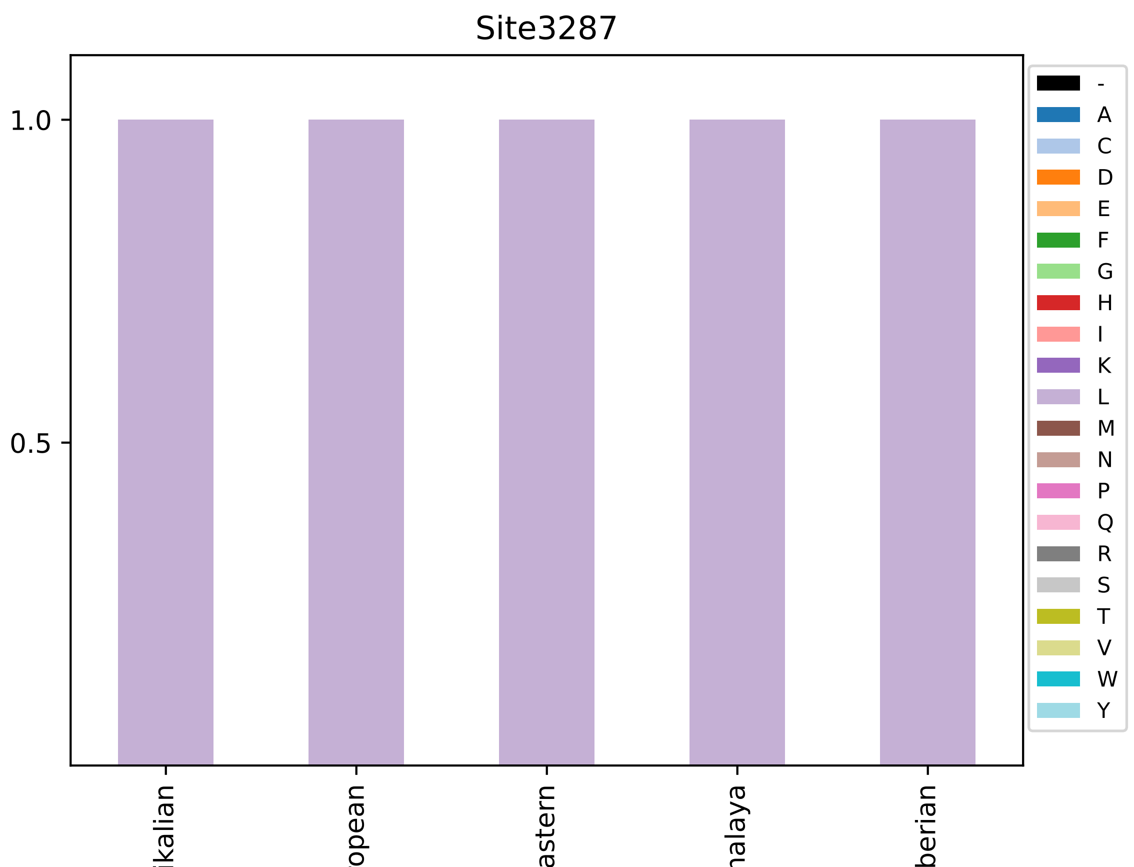
Site3287
1.0
0.5
-
A
C
D
E
F
G
H
I
K
L
M
N
P
Q
R
S
T
V
W
Y
ikalian
opean
astern
nalaya
berian

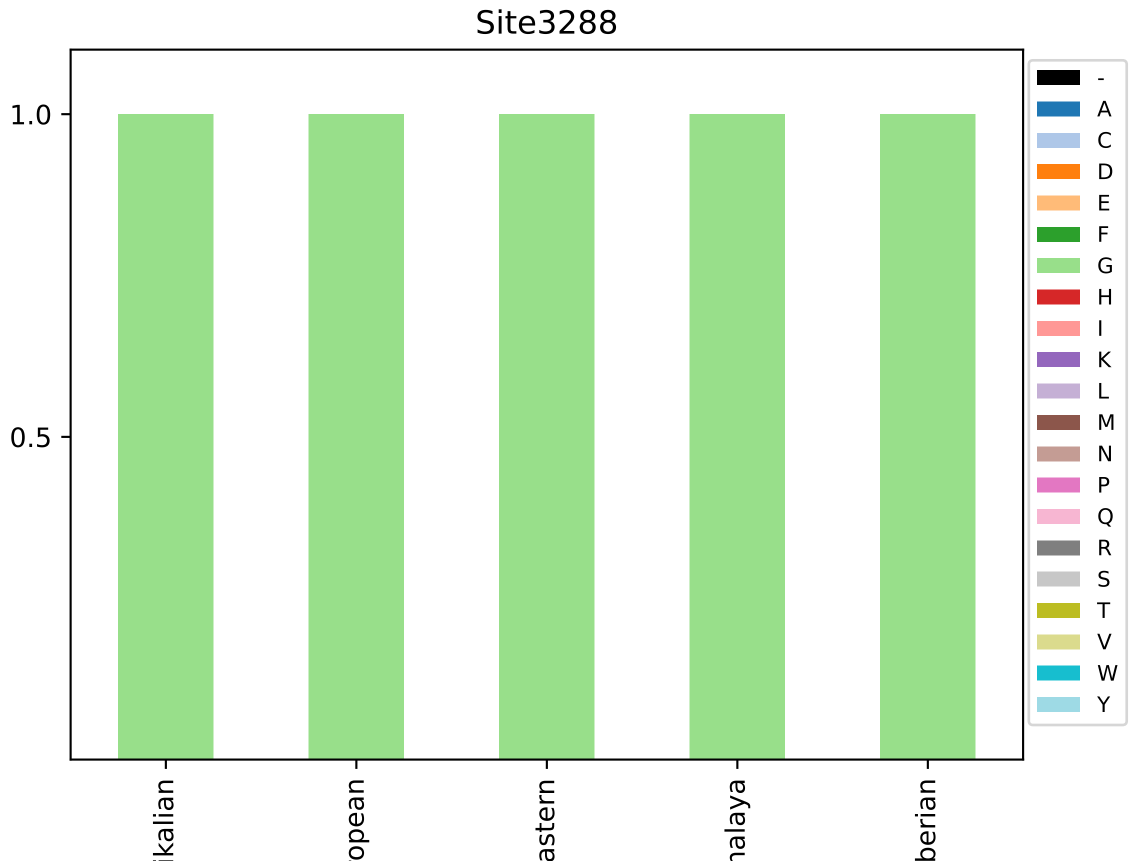

Site3288
1.0
0.5
-
A
C
D
E
F
G
H
I
K
L
M
N
P
Q
R
S
T
V
W
Y
ikalian
opean
astern
nalaya
berian

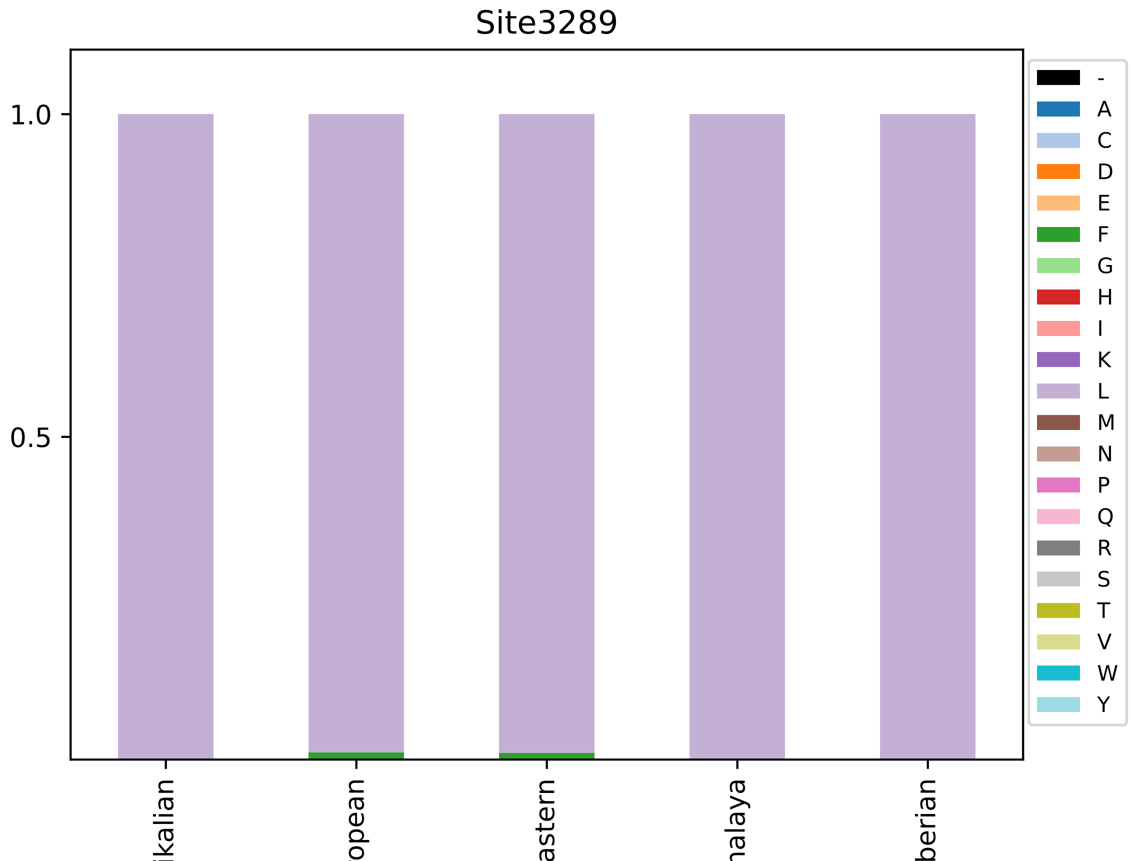
Site3289
1.0
0.5
-
A
C
D
E
F
G
H
I
K
L
M
N
P
Q
R
S
T
V
W
Y
ikalian
opean
astern
nalaya
berian

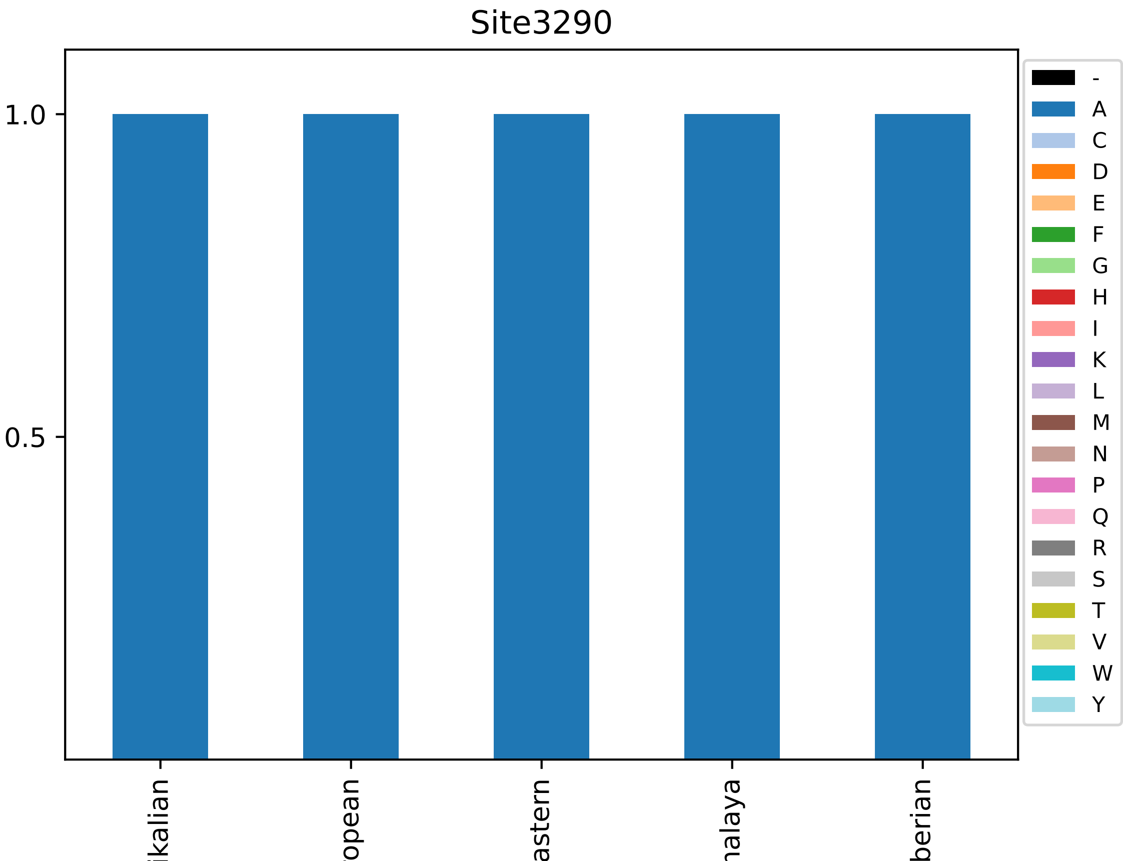

Site3290
1.0
0.5
-
A
C
D
E
F
G
H
I
K
L
M
N
P
Q
R
S
T
V
W
Y
ikalian
opean
astern
nalaya
berian

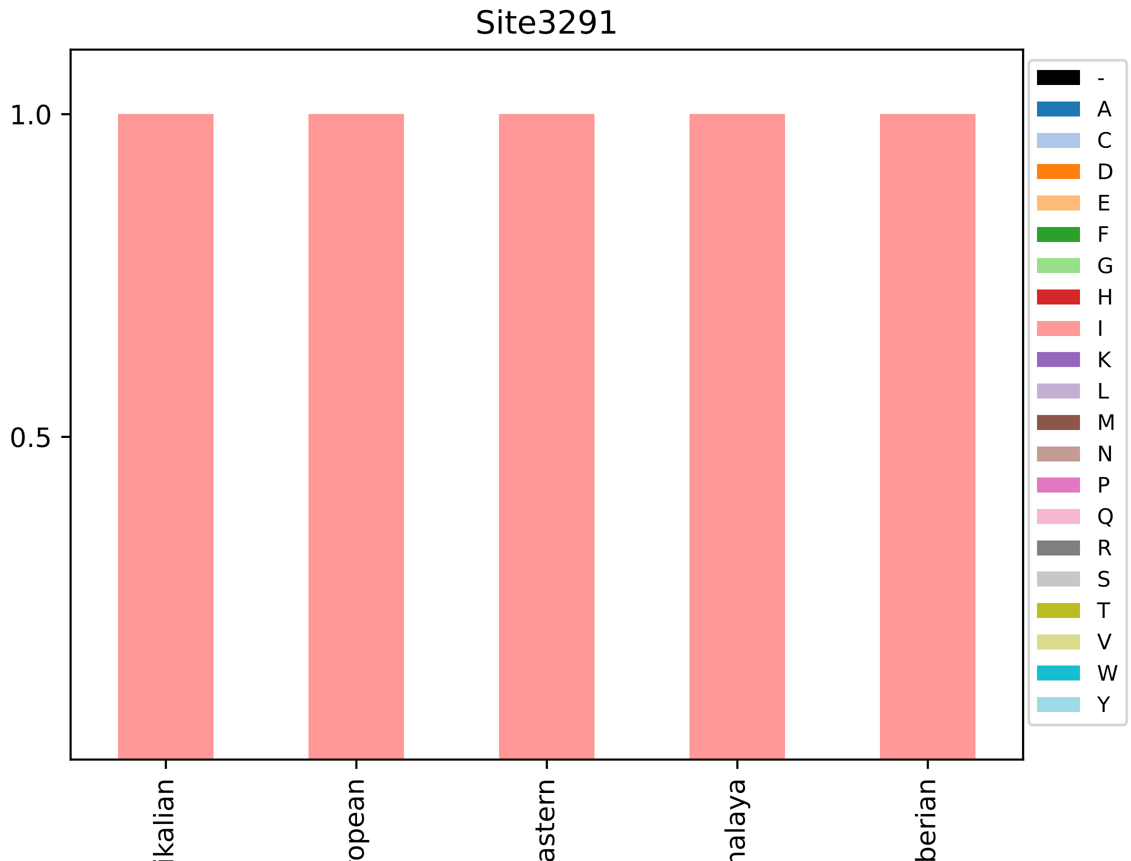
Site3291
1.0
0.5
-
A
C
D
E
F
G
H
I
K
L
M
N
P
Q
R
S
T
V
W
Y
ikalian
opean
astern
nalaya
berian

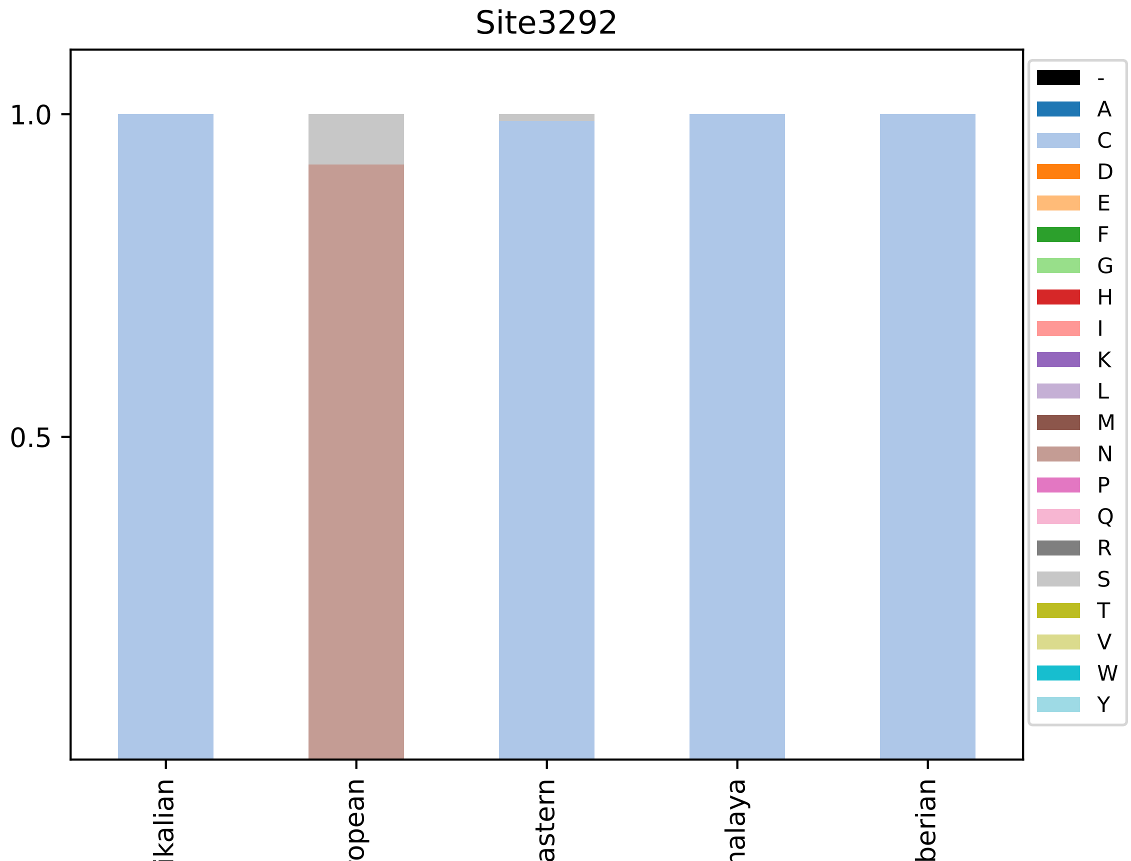
Site3292
1.0
0.5
-
A
C
D
E
F
G
H
I
K
L
M
N
P
Q
R
S
T
V
W
Y
ikalian
opean
astern
nalaya
berian

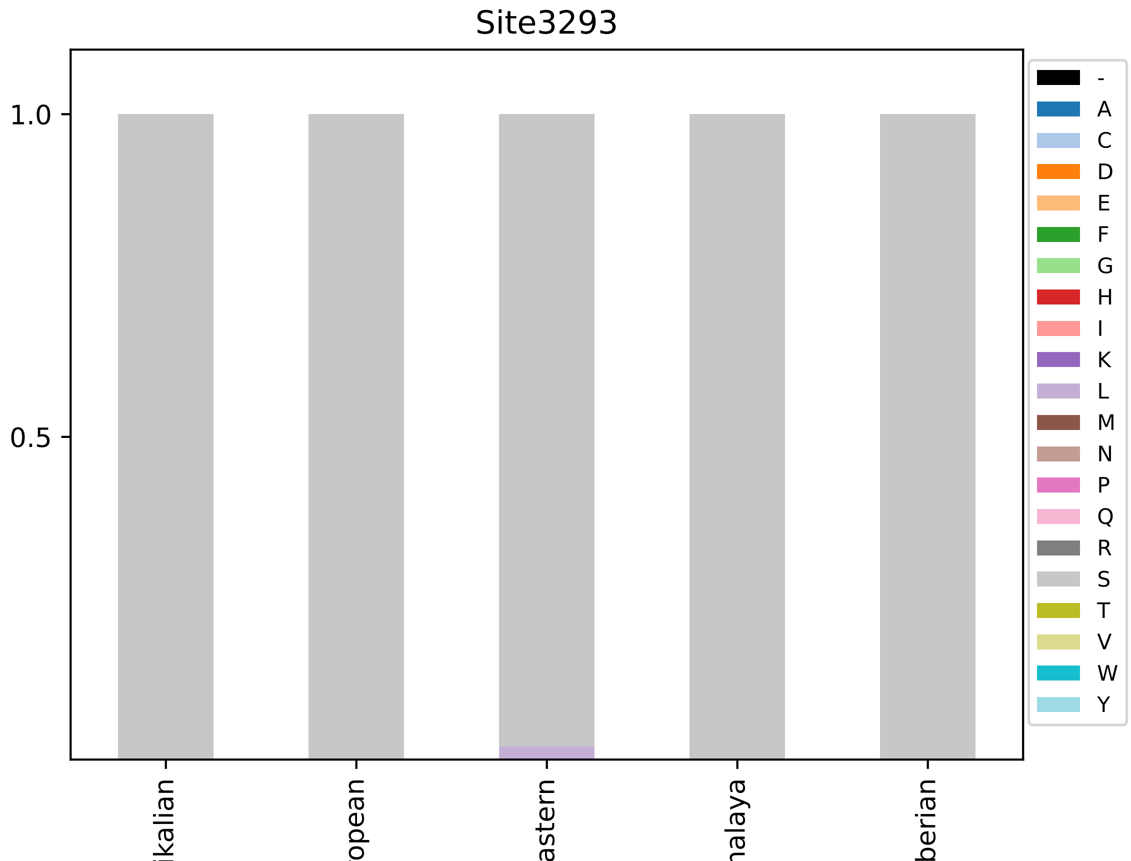

Site3293
1.0
0.5
-
A
C
D
E
F
G
H
I
K
L
M
N
P
Q
R
S
T
V
W
Y
ikalian
ropean
astern
nalaya
berian

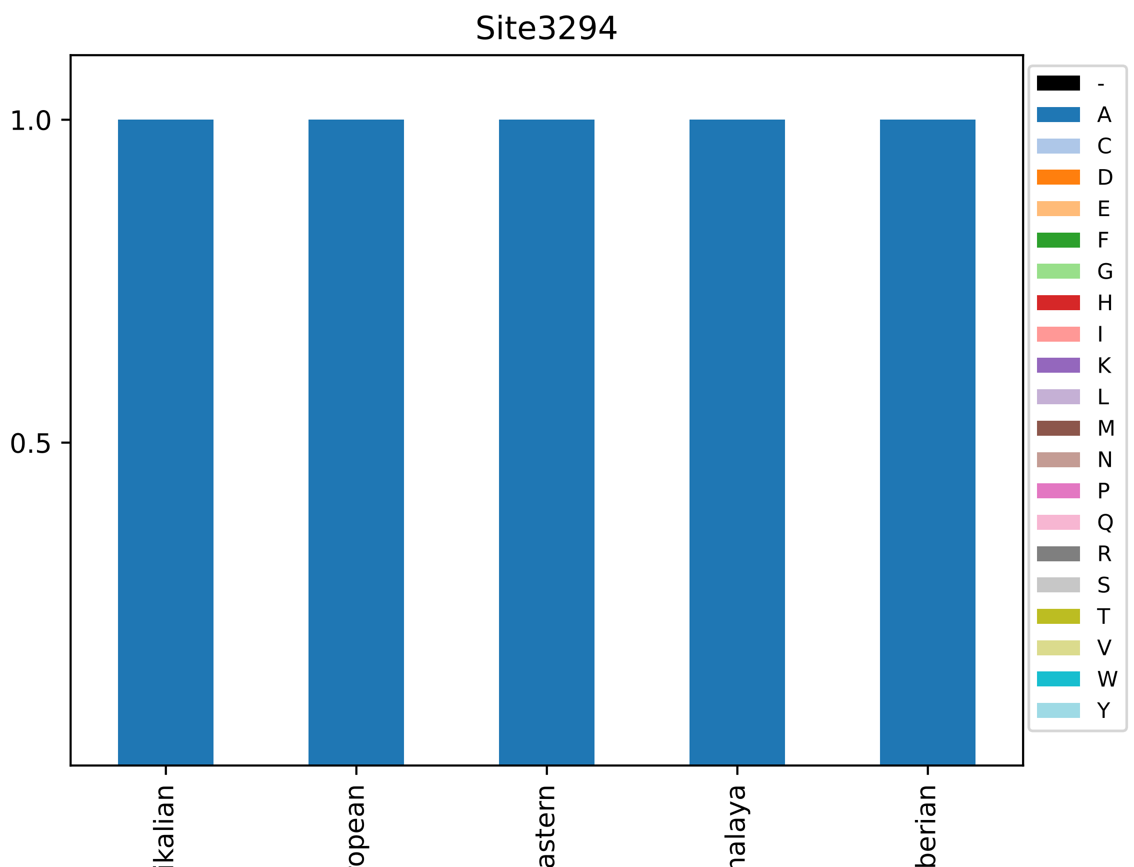
Site3294
1.0
0.5
-
A
C
D
E
F
G
H
I
K
L
M
N
P
Q
R
S
T
V
W
Y
ikalian
opean
astern
nalaya
berian

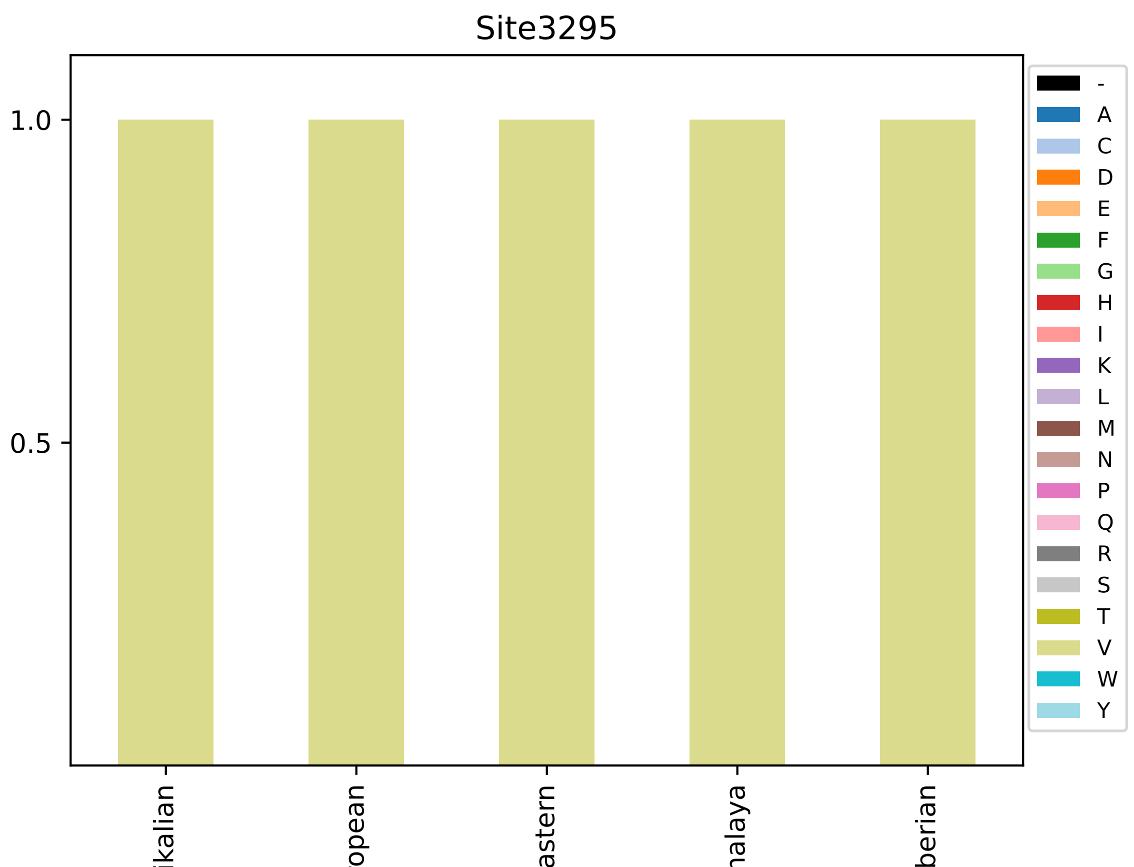
Site3295
1.0
0.5
-
A
C
D
E
F
G
H
I
K
L
M
N
P
Q
R
S
T
V
W
Y
ikalian
opean
astern
nalaya
berian

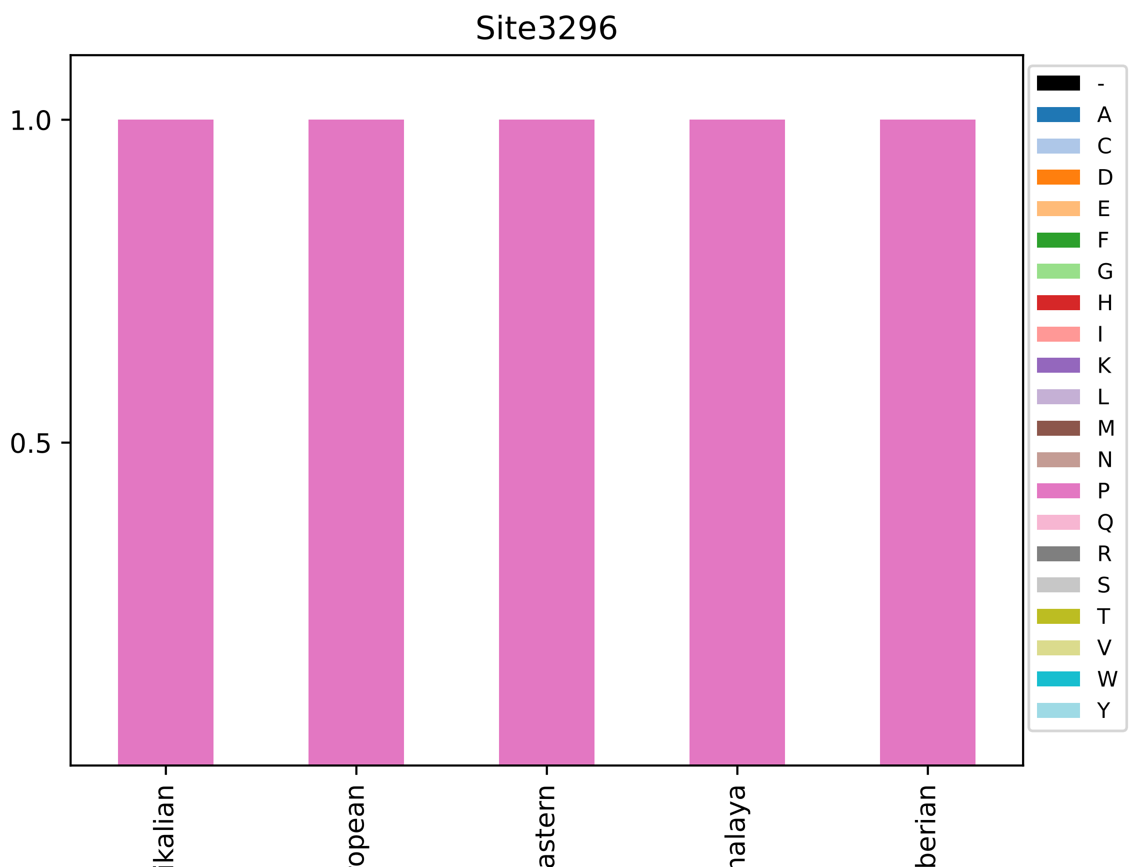
Site3296
1.0
0.5
-
A
C
D
E
F
G
H
I
K
L
M
N
P
Q
R
S
T
V
W
Y

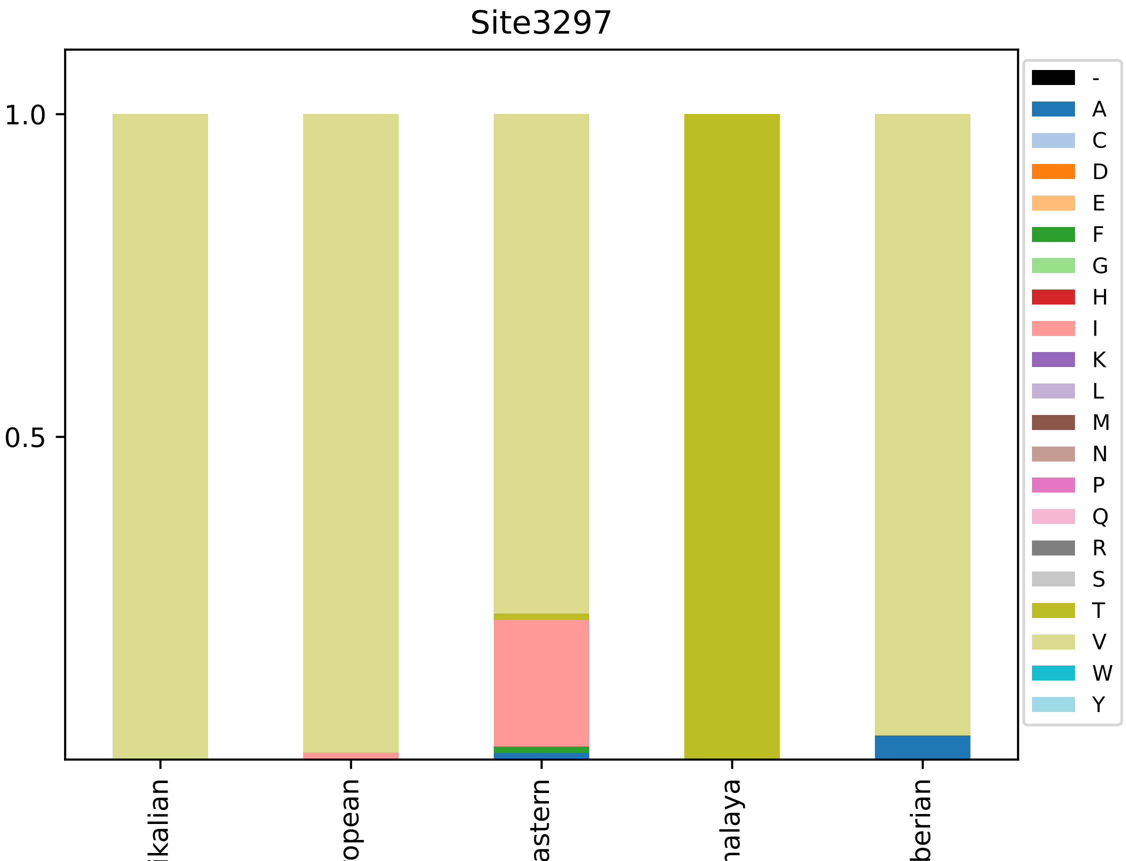
Site3297
1.0
0.5
-
A
C
D
E
F
G
H
I
K
L
M
N
P
Q
R
S
T
V
W
Y
ikalian
opean
astern
nalaya
berian

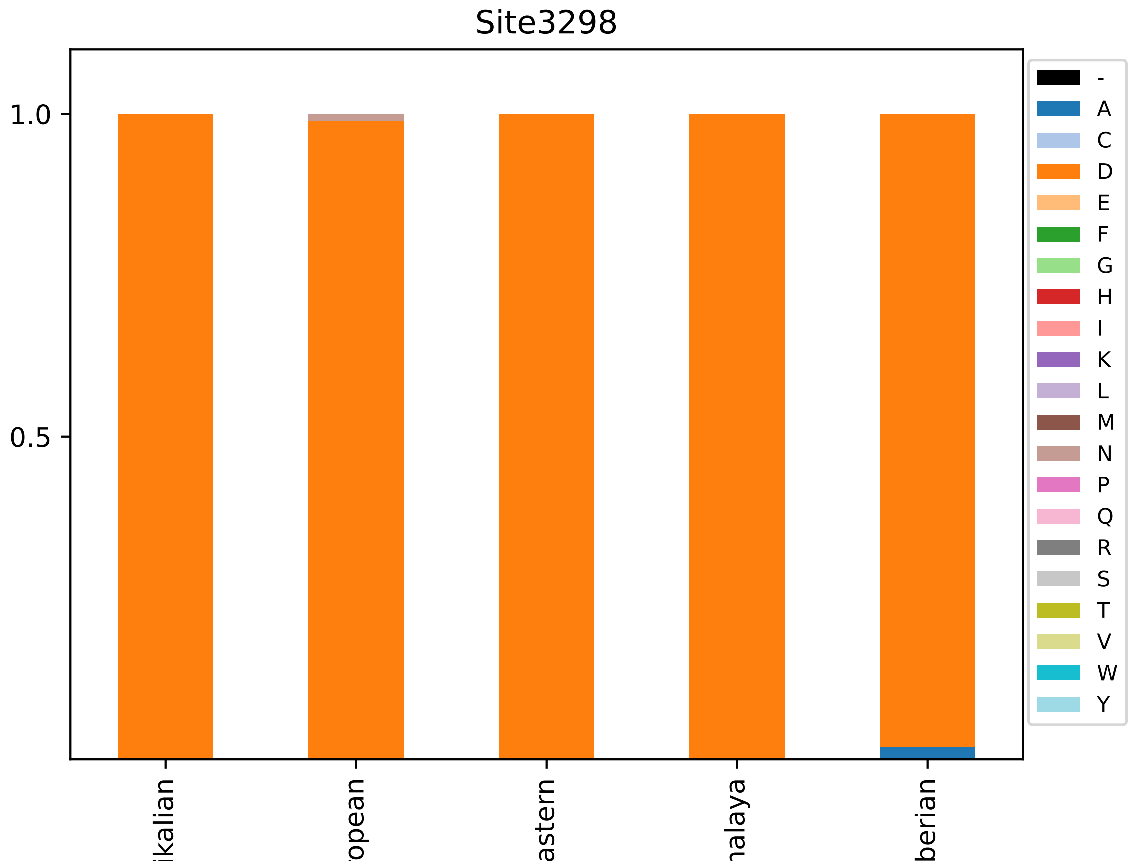

Site3298
1.0
0.5
-
A
C
D
E
F
G
H
I
K
L
M
N
P
Q
R
S
T
V
W
Y

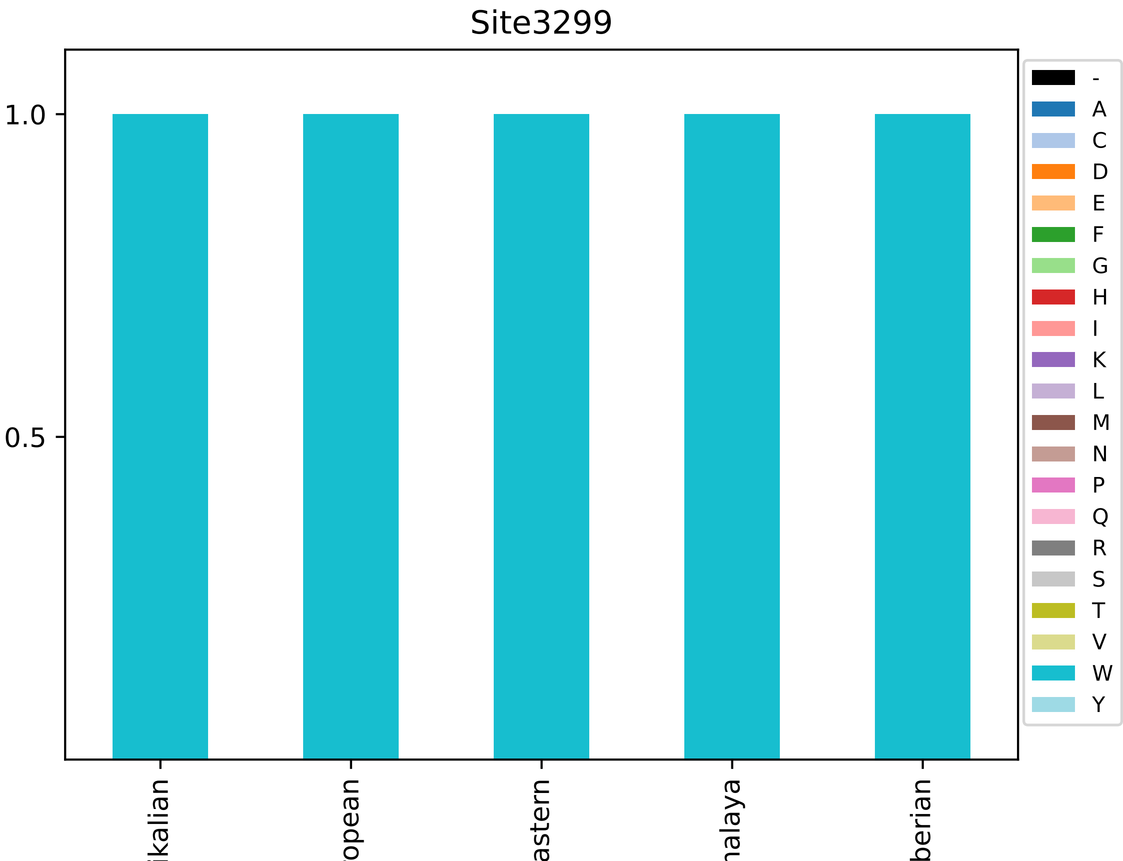

Site3299
1.0
0.5
-
A
C
D
E
F
G
H
I
K
L
M
N
P
Q
R
S
T
V
W
Y
ikalian
opean
astern
nalaya
berian

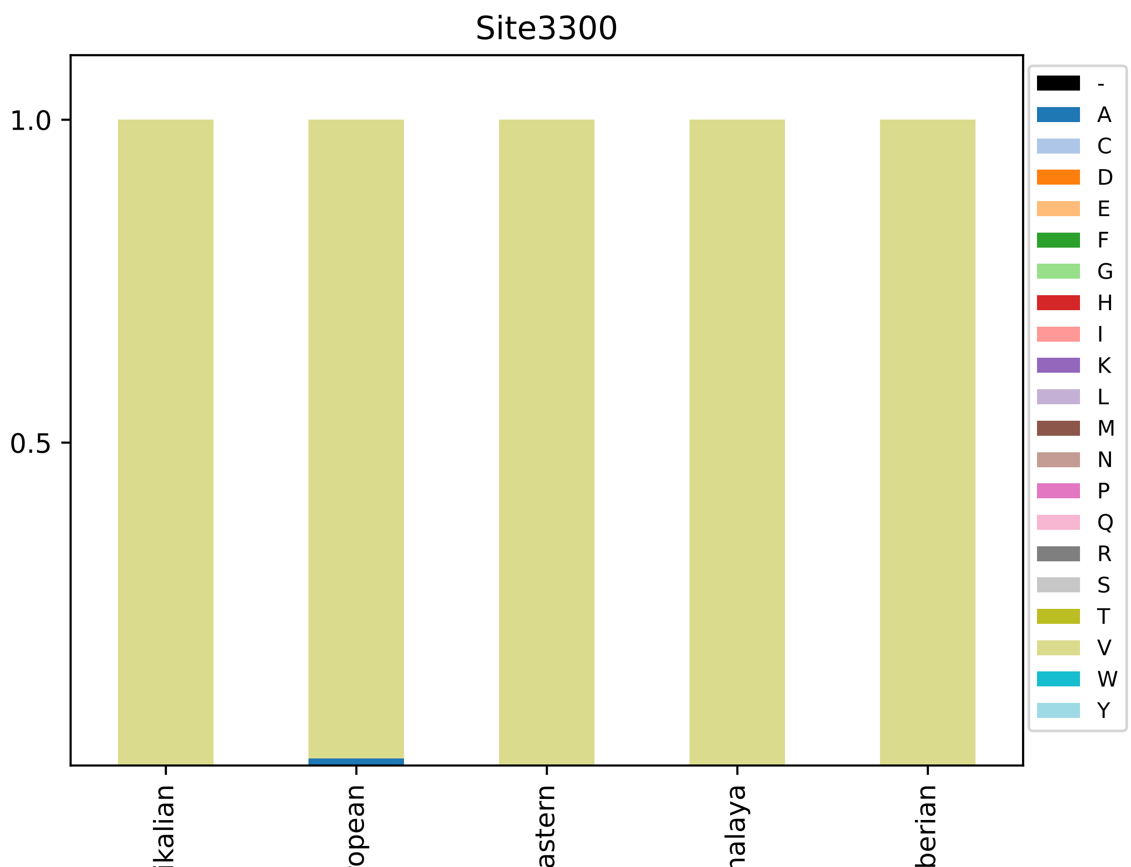
Site3300
1.0
0.5
ikalian
opean
astern
nalaya
berian
-
A
C
D
E
F
G
H
I
K
L
M
N
P
Q
R
S
T
V
W
Y

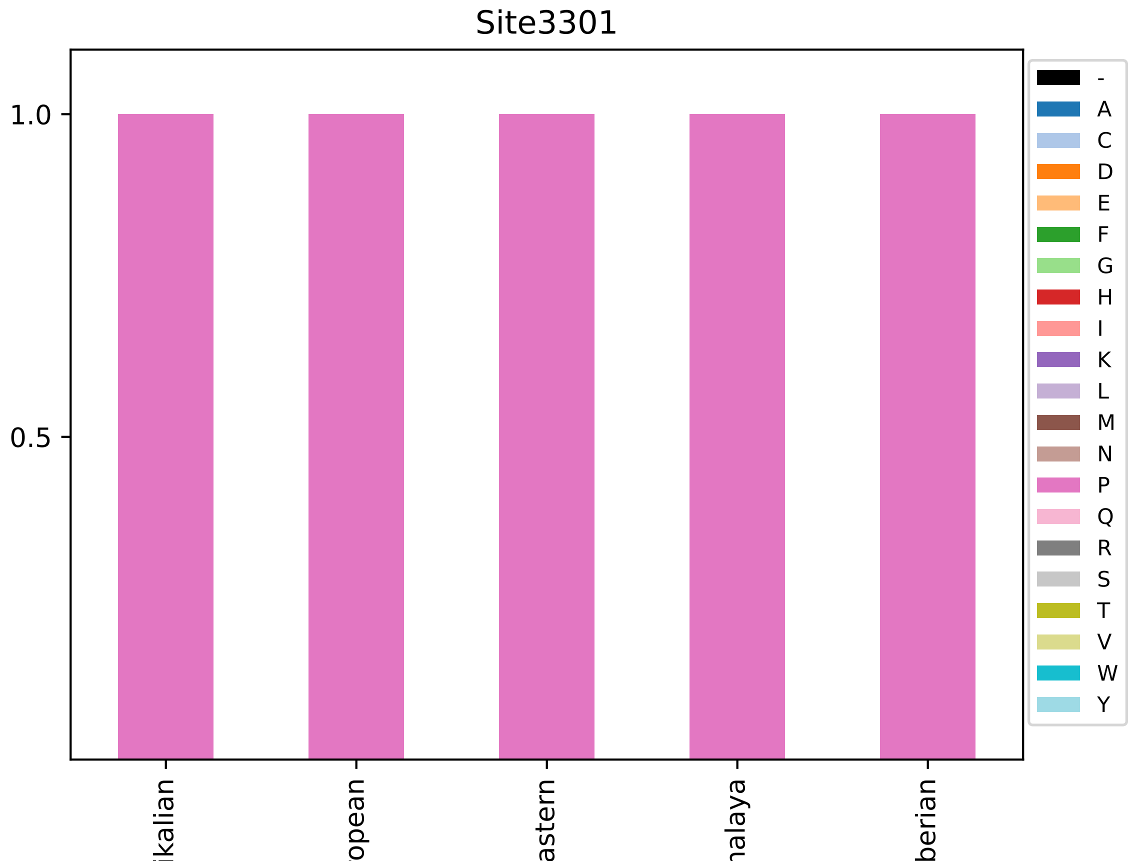
Site3301
1.0
0.5
-
A
C
D
E
F
G
H
I
K
L
M
N
P
Q
R
S
T
V
W
Y
ikalian
opean
astern
nalaya
berian

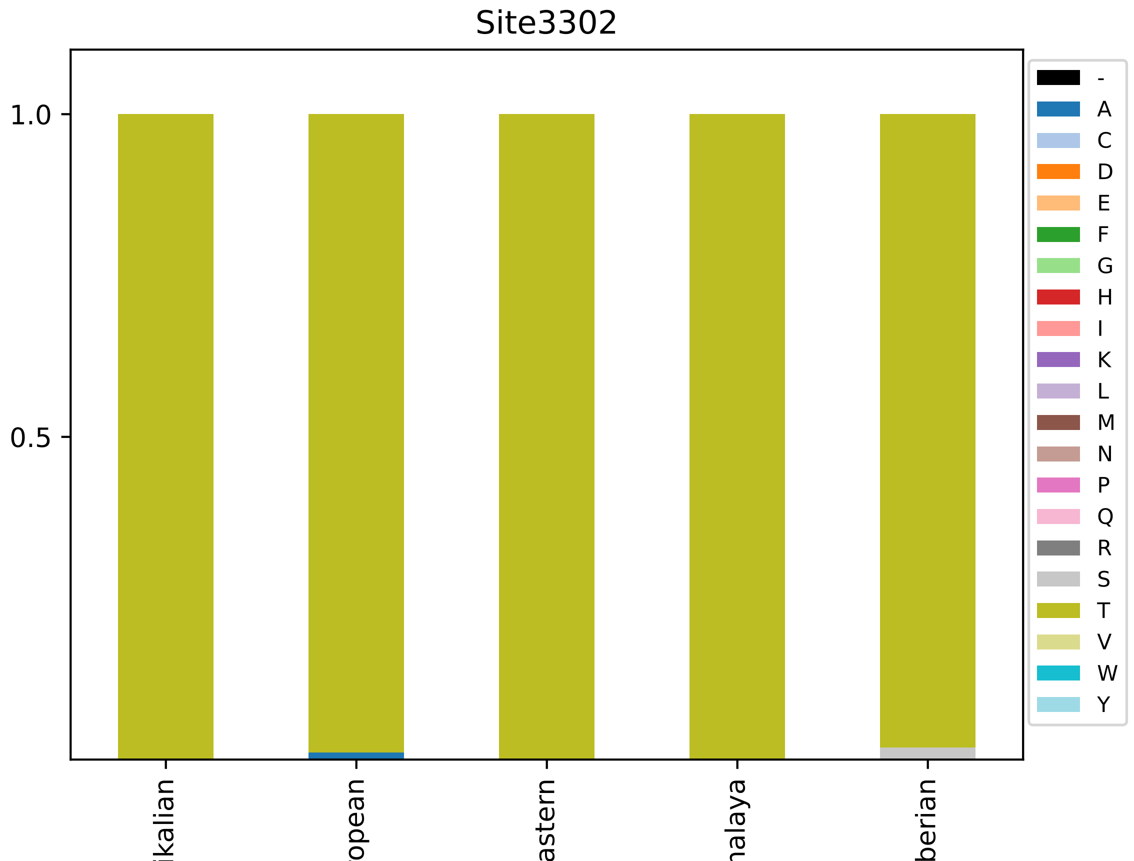

Site3302
1.0
0.5
-
A
C
D
E
F
G
H
I
K
L
M
N
P
Q
R
S
T
V
W
Y
ikalian
opean
astern
nalaya
berian

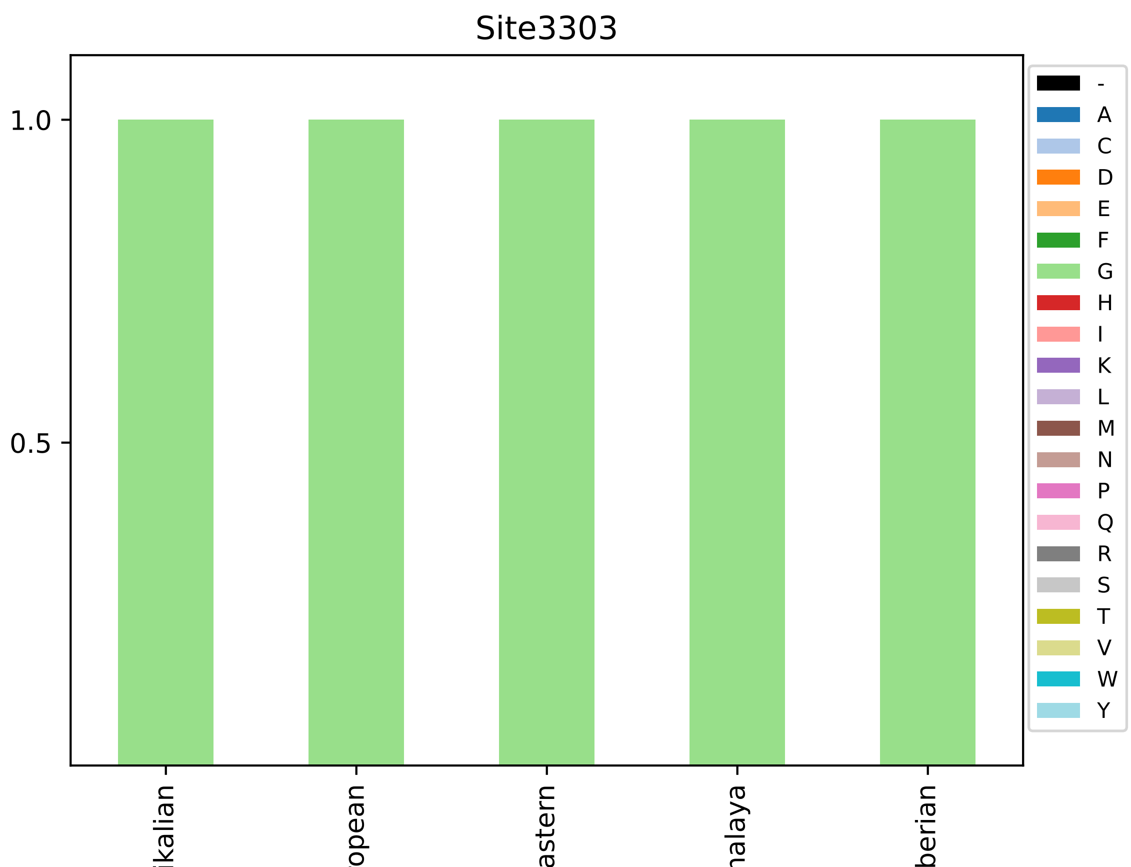

Site3303
1.0
0.5
-
A
C
D
E
F
G
H
I
K
L
M
N
P
Q
R
S
T
V
W
Y
ikalian
opean
astern
nalaya
berian

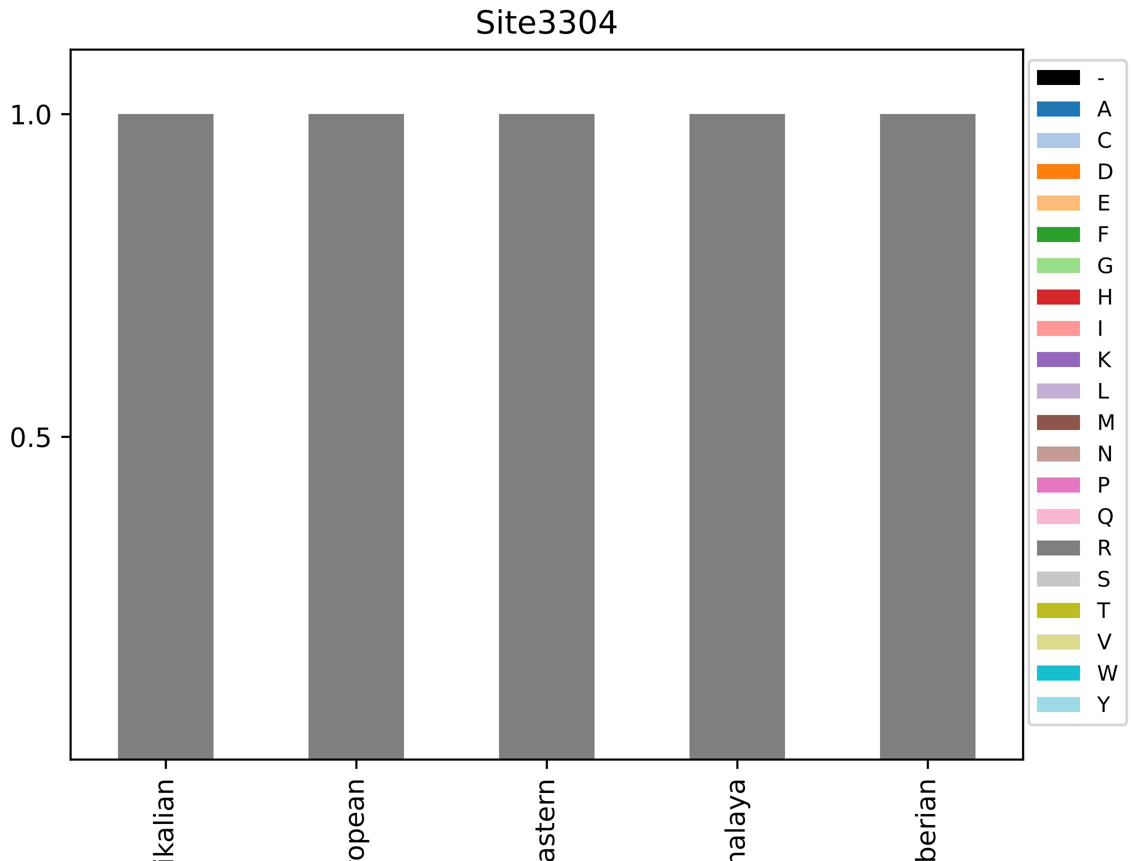
Site3304
1.0
0.5
ikalian
ropean
astern
halaya
berian
-
A
C
D
E
F
G
H
I
K
L
M
N
P
Q
R
S
T
V
W
Y

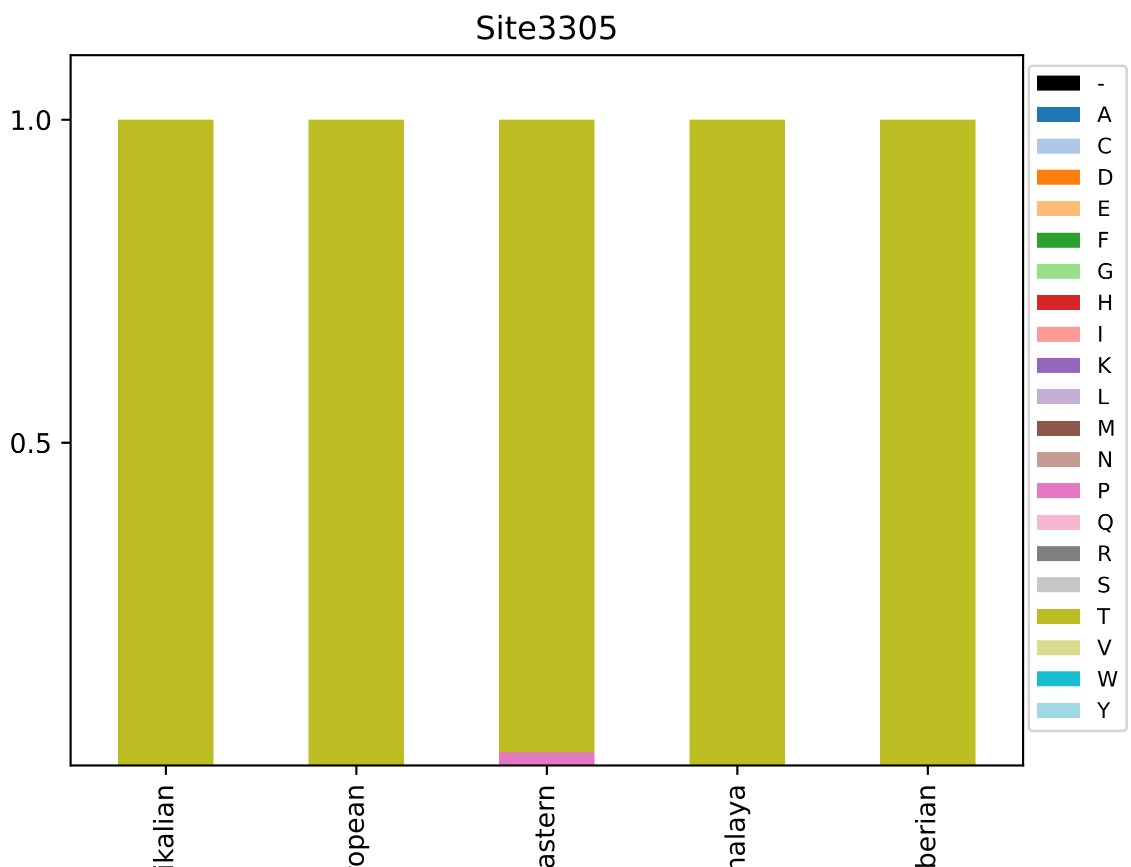
Site3305
1.0
0.5
-
A
C
D
E
F
G
H
I
K
L
M
N
P
Q
R
S
T
V
W
Y
ikalian
opean
astern
nalaya
berian

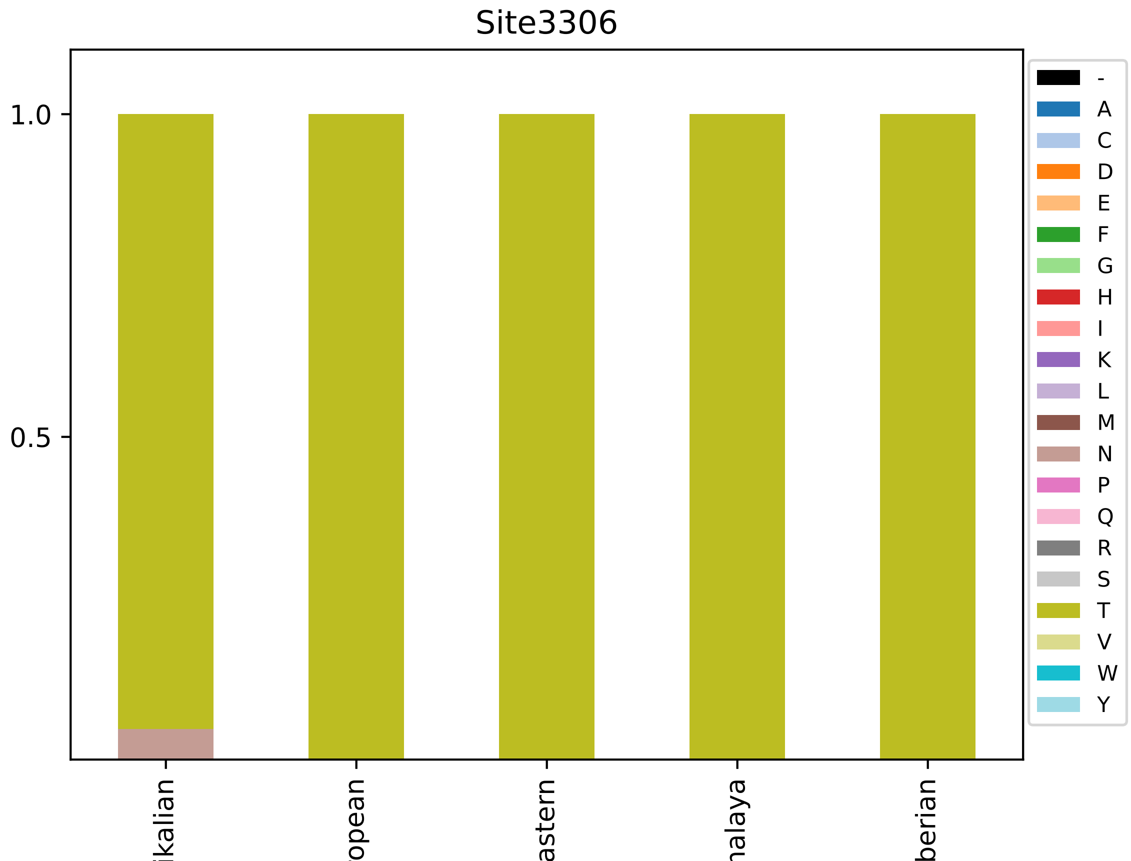

Site3306
1.0
0.5
-
A
C
D
E
F
G
H
I
K
L
M
N
P
Q
R
S
T
V
W
Y
ikalian
ropean
astern
nalaya
berian

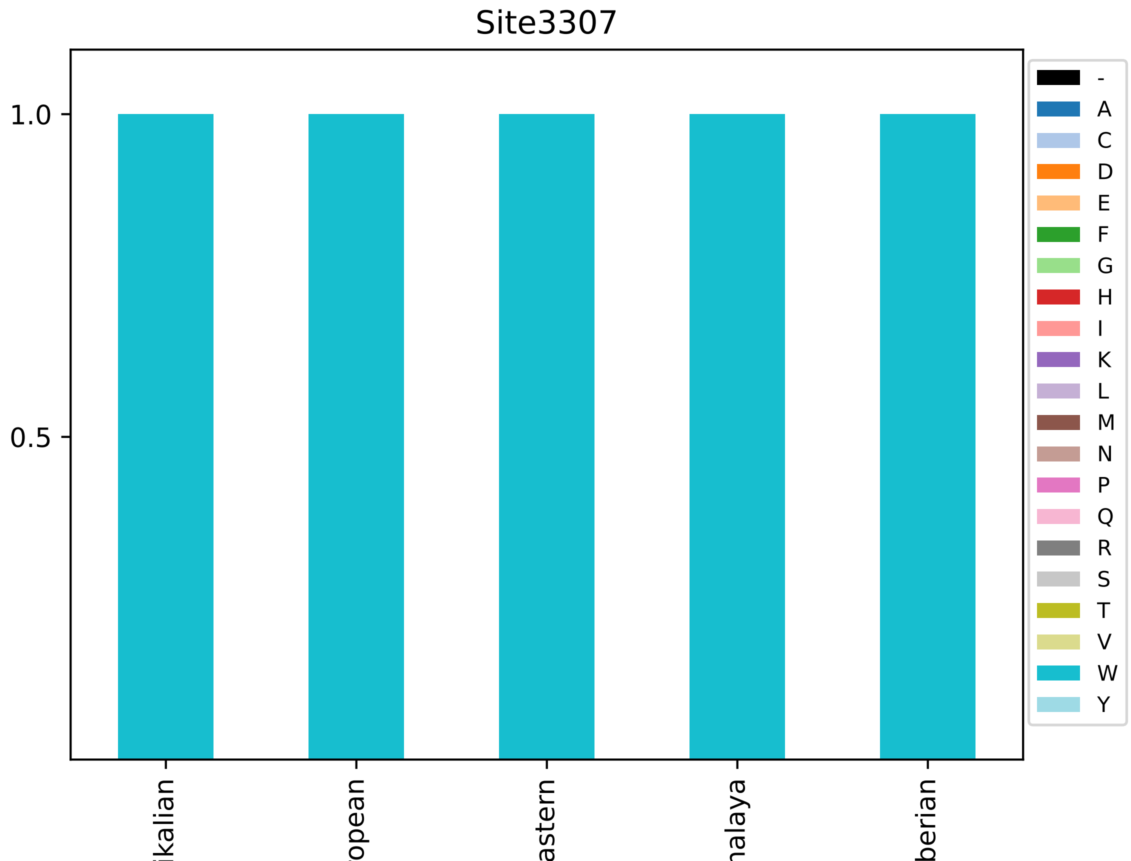

Site3307
1.0
0.5
-
A
C
D
E
F
G
H
I
K
L
M
N
P
Q
R
S
T
V
W
Y
ikalian
opean
astern
nalaya
berian

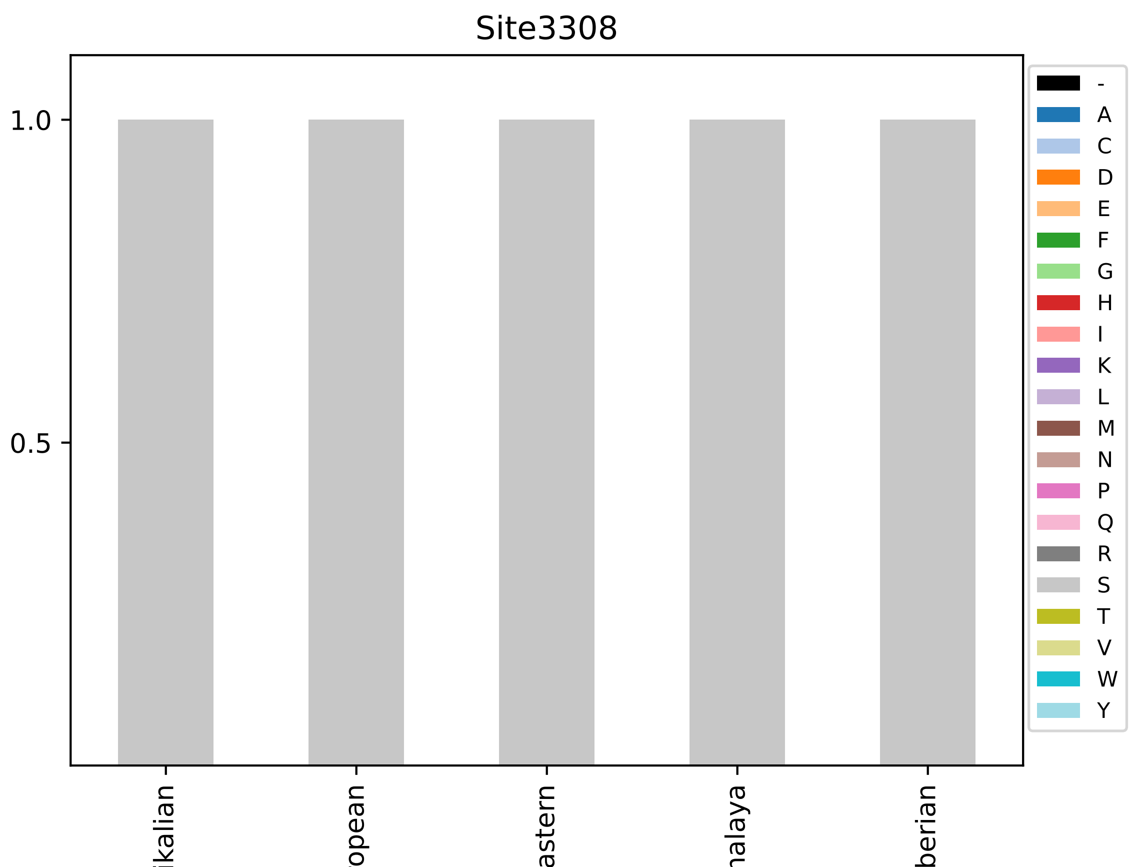
Site3308
1.0
0.5
-
A
C
D
E
F
G
H
I
K
L
M
N
P
Q
R
S
T
V
W
Y
ikalian
opean
astern
nalaya
berian

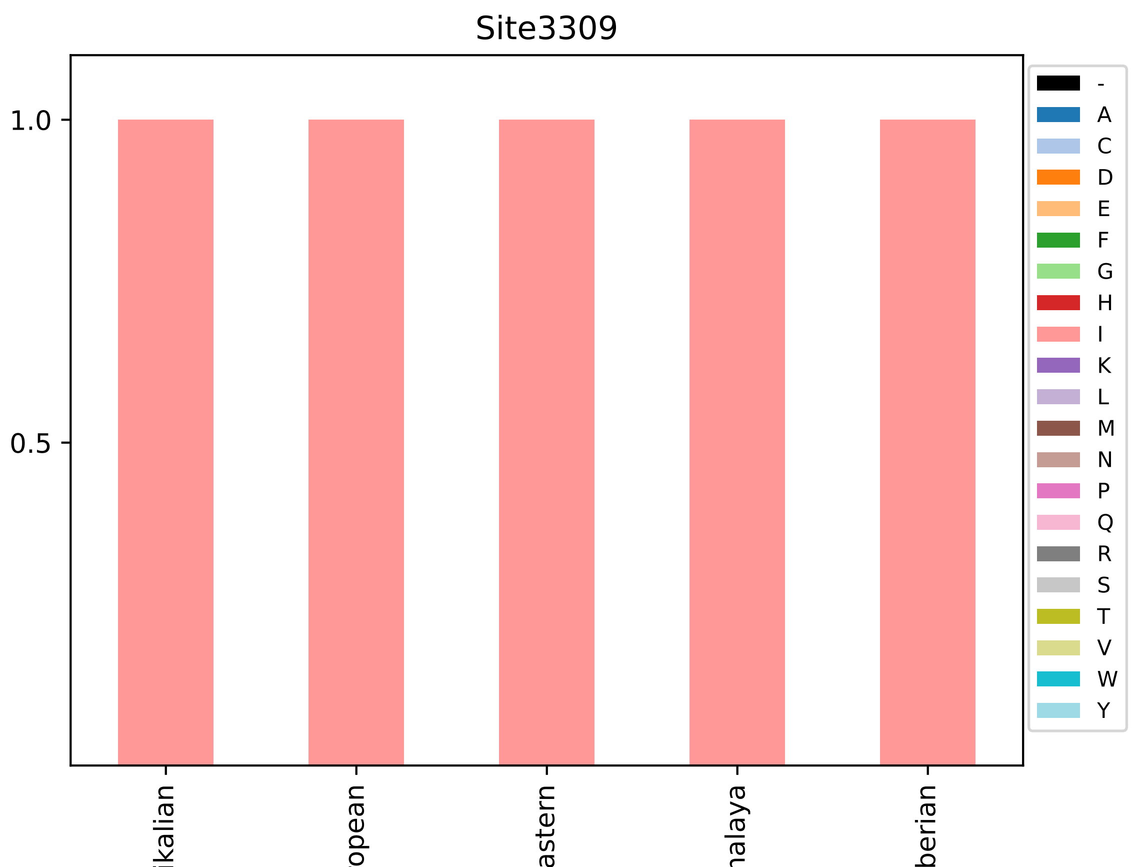

Site3309
1.0
0.5
ikalian
opean
astern
nalaya
berian
-
A
C
D
E
F
G
H
I
K
L
M
N
P
Q
R
S
T
V
W
Y

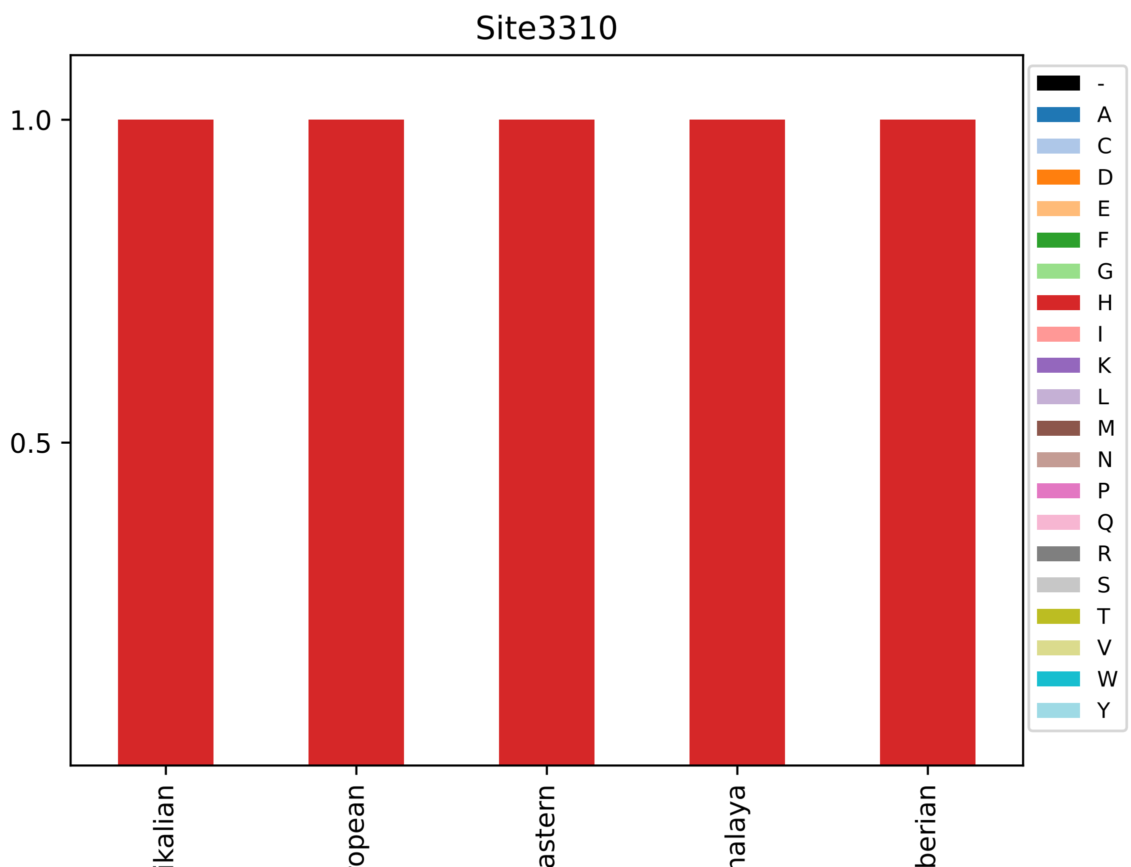
Site3310
1.0
0.5
-
A
C
D
E
F
G
H
I
K
L
M
N
P
Q
R
S
T
V
W
Y
ikalian
opean
astern
nalaya
berian

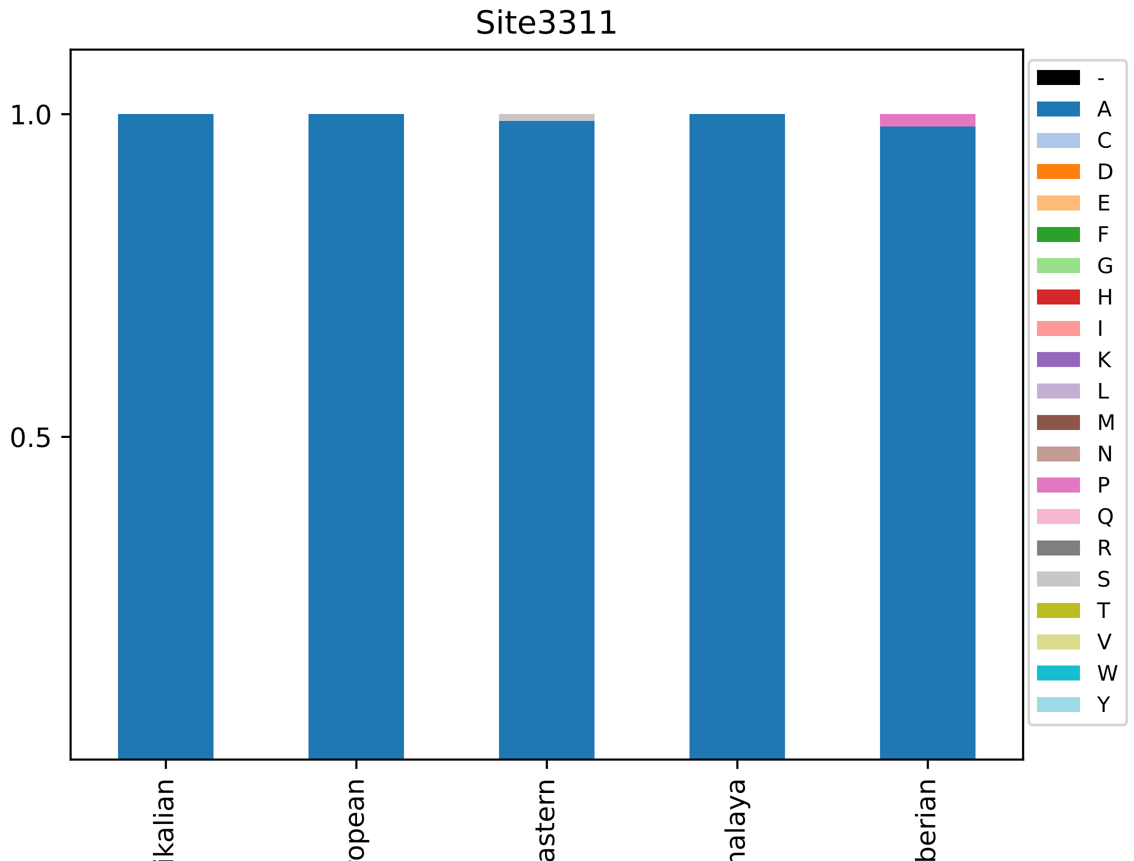

Site3311
1.0
0.5
-
A
C
D
E
F
G
H
I
K
L
M
N
P
Q
R
S
T
V
W
Y
ikalian
opean
astern
nalaya
berian

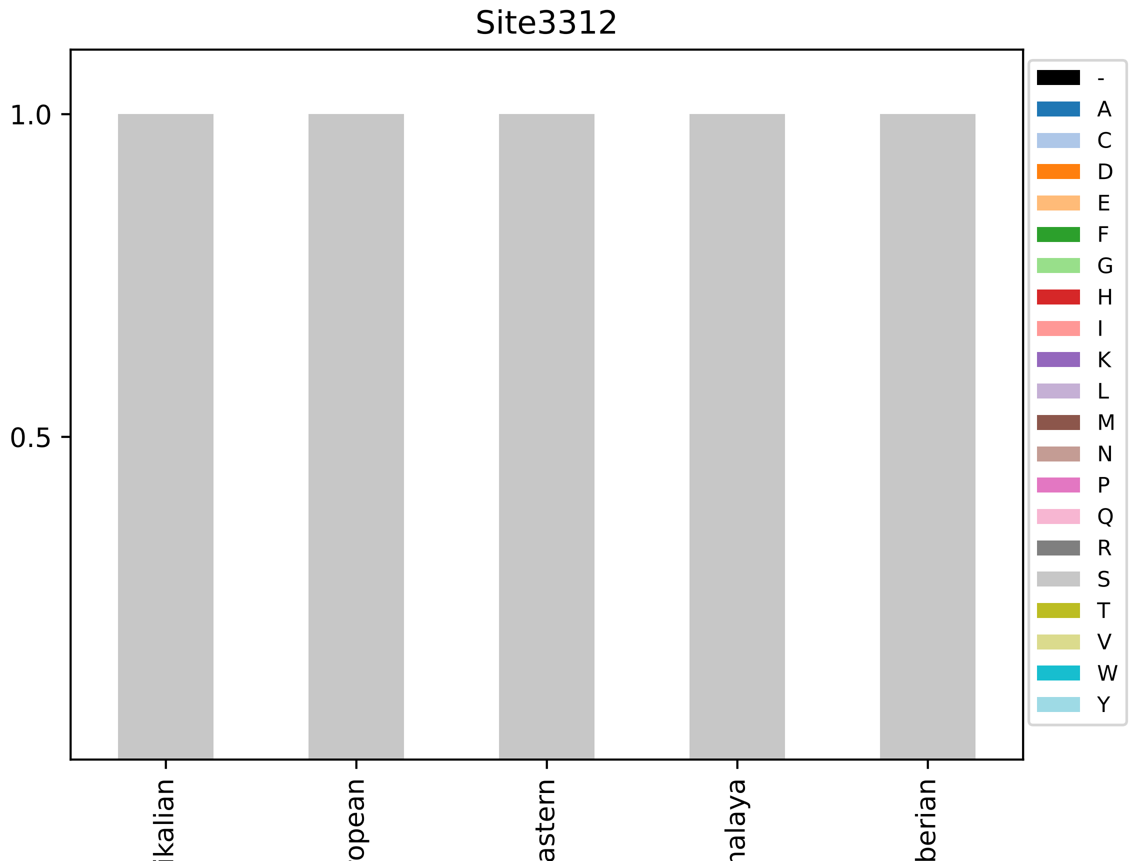
Site3312
1.0
0.5
-
A
C
D
E
F
G
H
I
K
L
M
N
P
Q
R
S
T
V
W
Y
ikalian
opean
astern
nalaya
berian

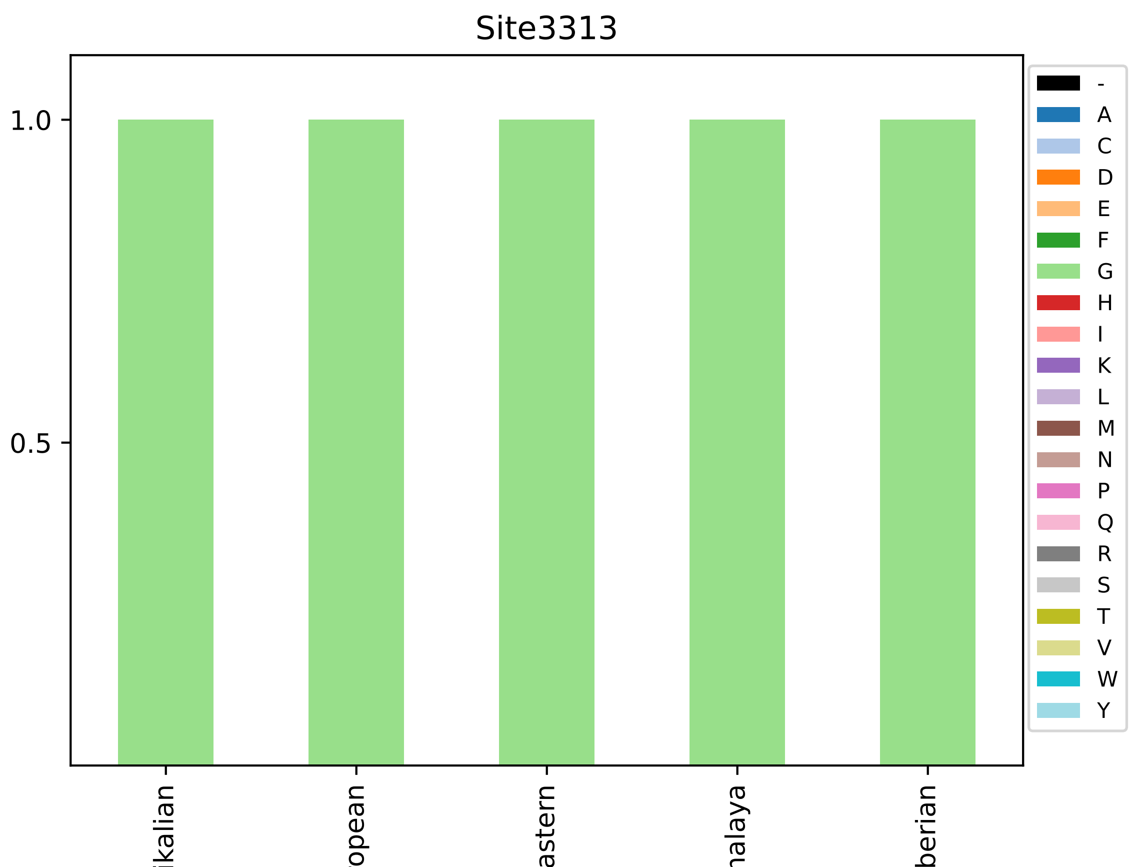

Site3313
1.0
0.5
-
A
C
D
E
F
G
H
I
K
L
M
N
P
Q
R
S
T
V
W
Y

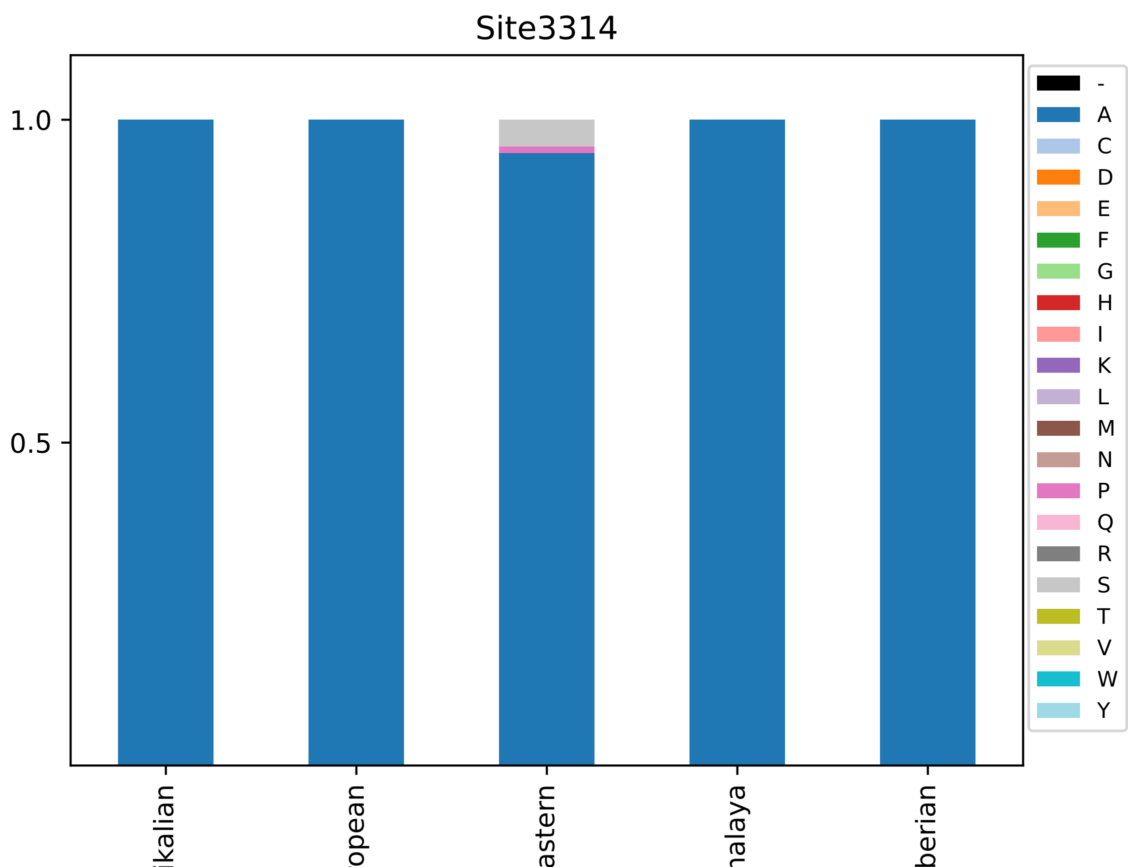

Site3314
1.0
0.5
-
A
C
D
E
F
G
H
I
K
L
M
N
P
Q
R
S
T
V
W
Y
ikalian
opean
astern
nalaya
berian

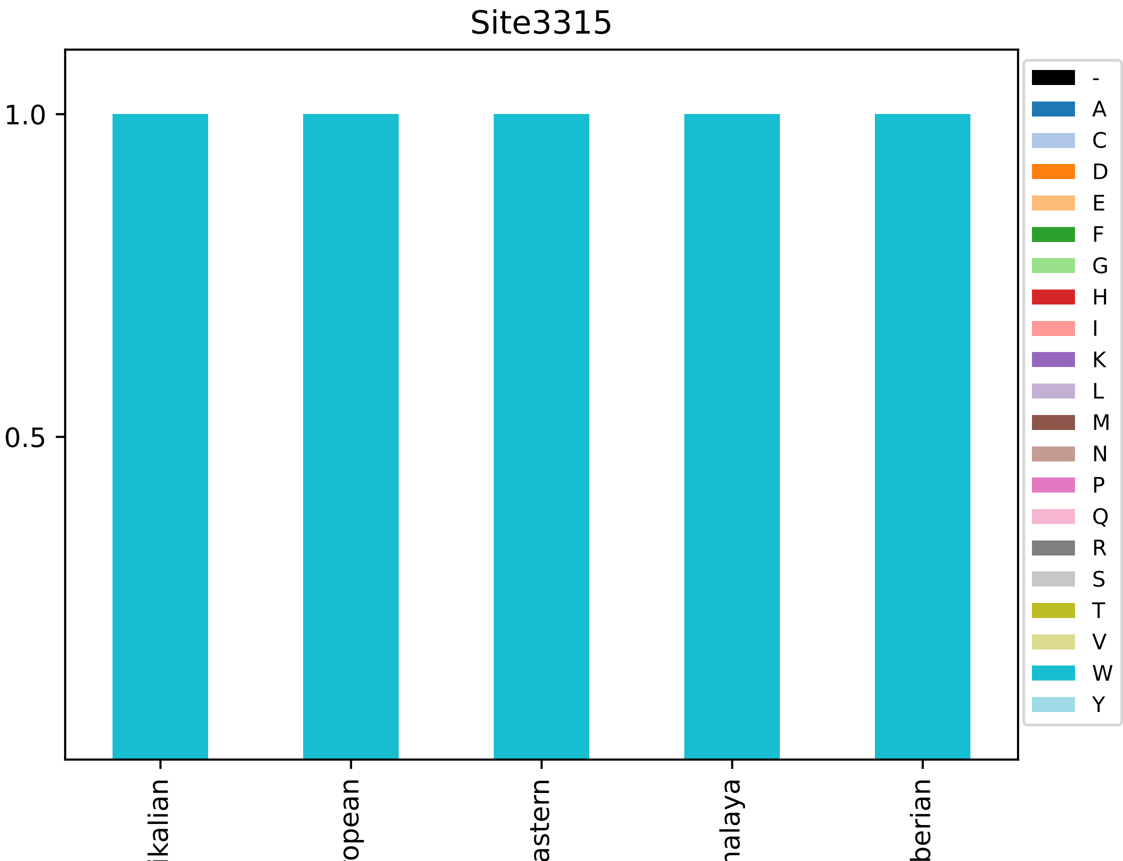
Site3315
1.0
0.5
-
A
C
D
E
F
G
H
I
K
L
M
N
P
Q
R
S
T
V
W
Y
ikalian
opean
astern
nalaya
berian

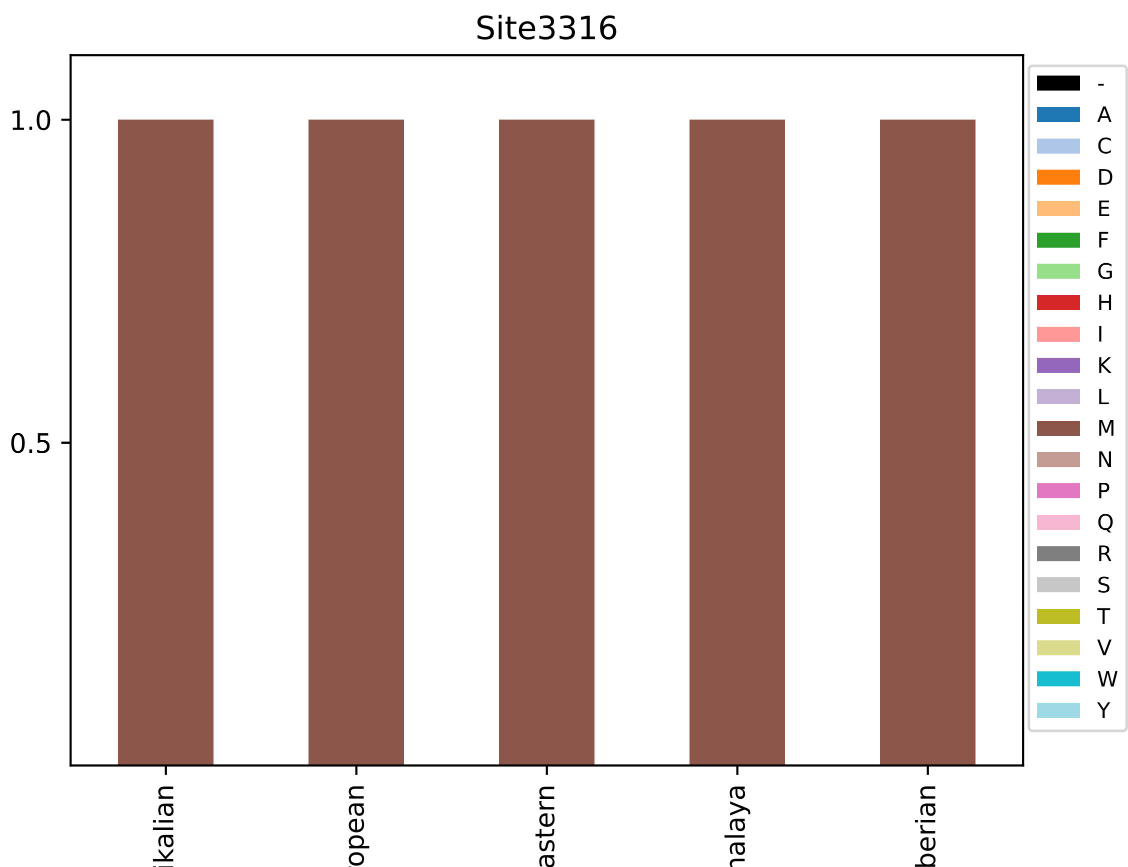

Site3316
1.0
0.5
-
A
C
D
E
F
G
H
I
K
L
M
N
P
Q
R
S
T
V
W
Y
ikalian
opean
astern
nalaya
berian

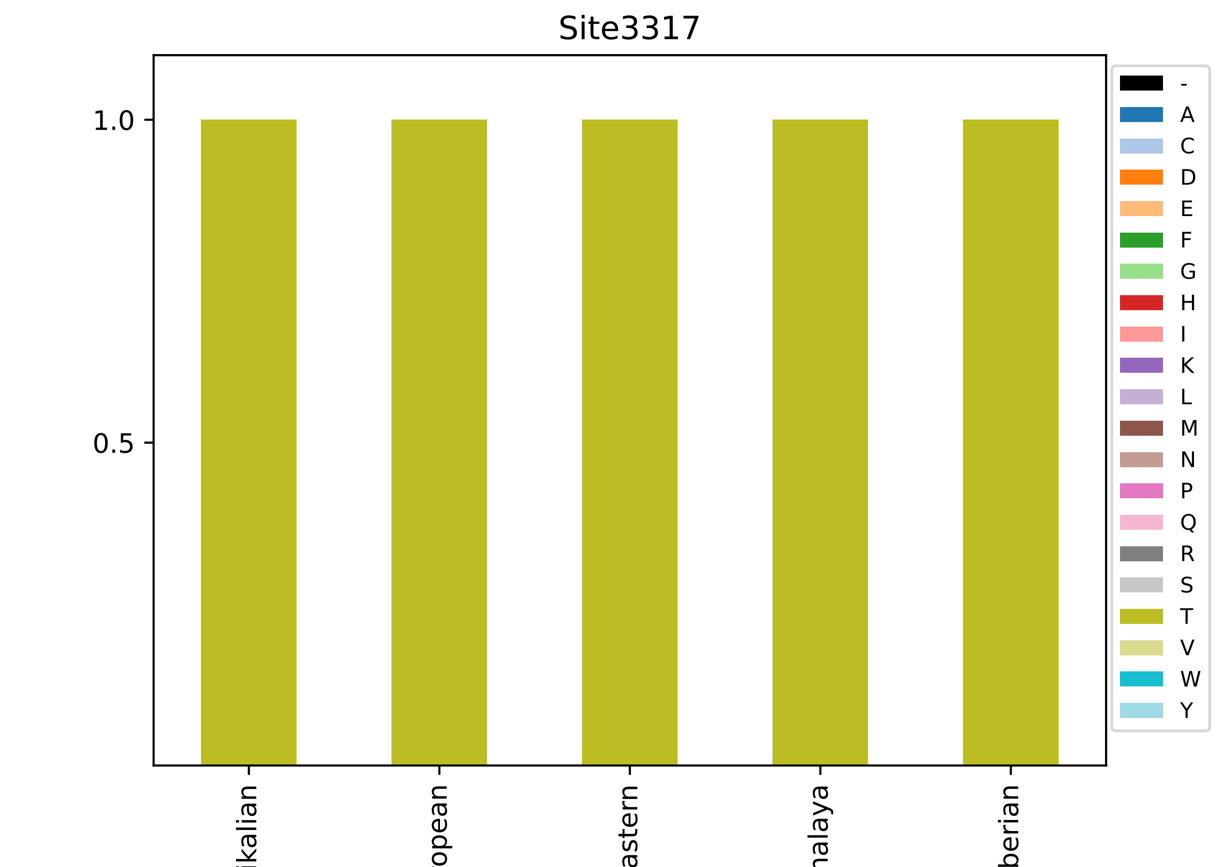

Site3317
1.0
0.5
-
A
C
D
E
F
G
H
I
K
L
M
N
P
Q
R
S
T
V
W
Y
ikalian
opean
astern
nalaya
berian

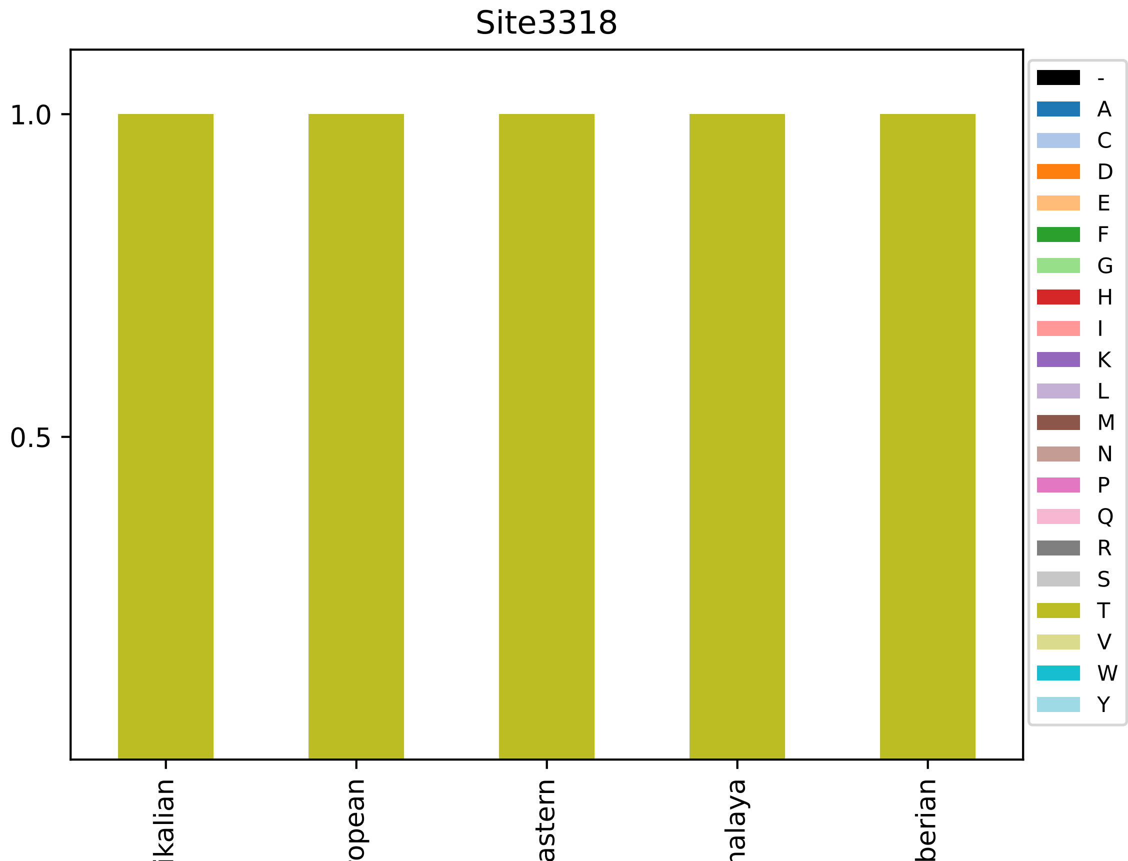

Site3318
1.0
0.5
-
A
C
D
E
F
G
H
I
K
L
M
N
P
Q
R
S
T
V
W
Y
ikalian
opean
astern
nalaya
berian

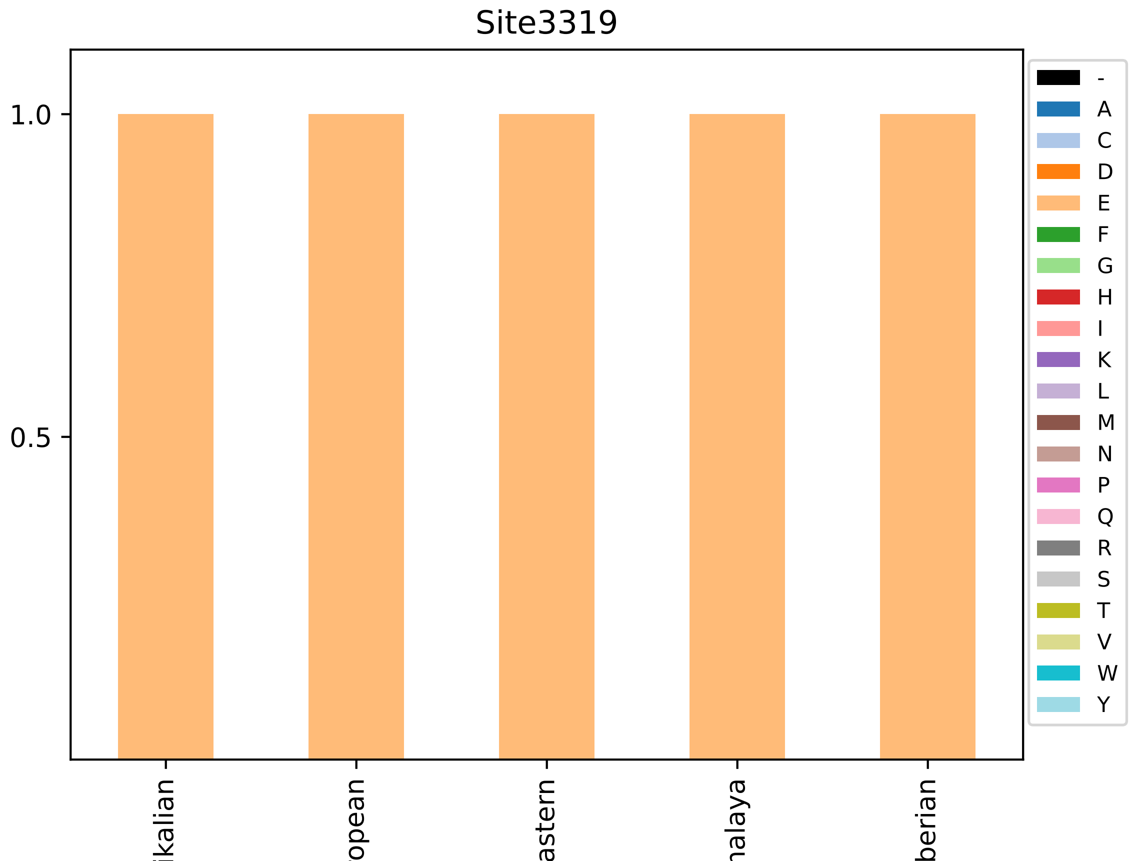

Site3319
1.0
0.5
-
A
C
D
E
F
G
H
I
K
L
M
N
P
Q
R
S
T
V
W
Y
ikalian
opean
astern
nalaya
berian

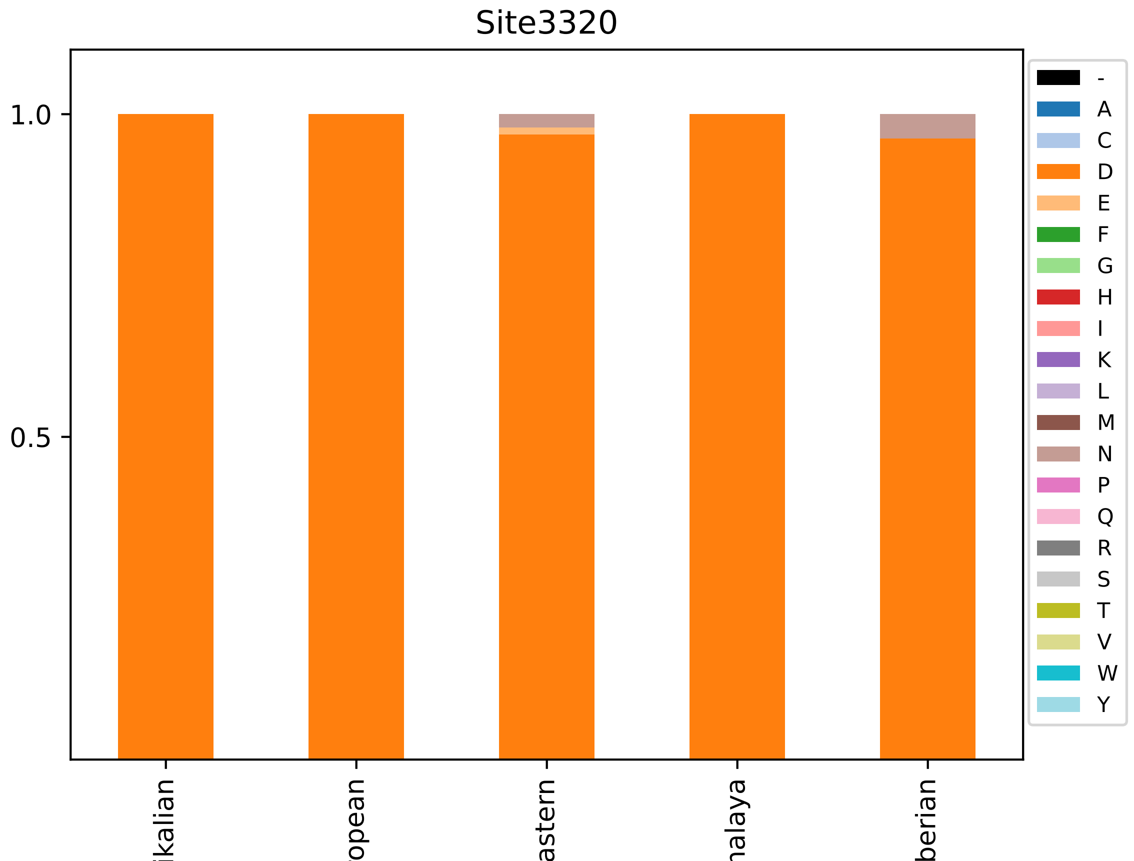

Site3320
1.0
0.5
-
A
C
D
E
F
G
H
I
K
L
M
N
P
Q
R
S
T
V
W
Y

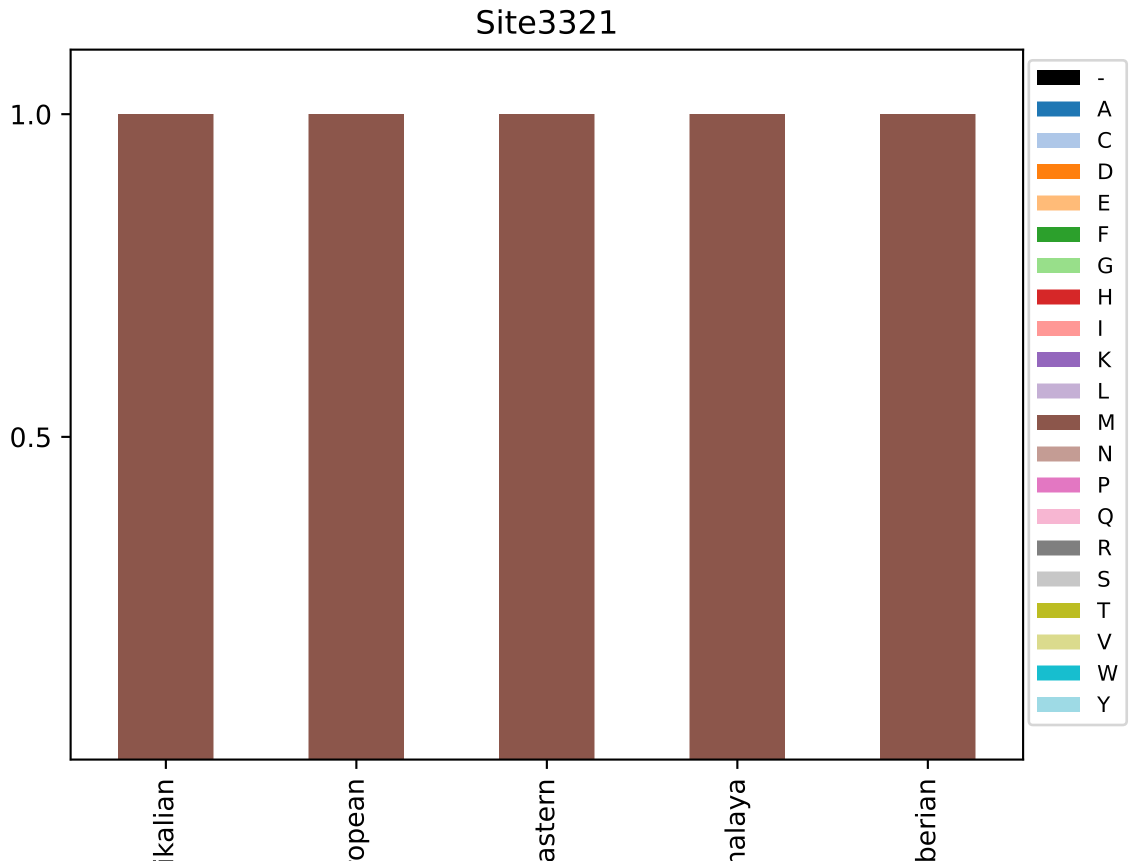
Site3321
1.0
0.5
-
A
C
D
E
F
G
H
I
K
L
M
N
P
Q
R
S
T
V
W
Y
ikalian
opean
astern
nalaya
berian

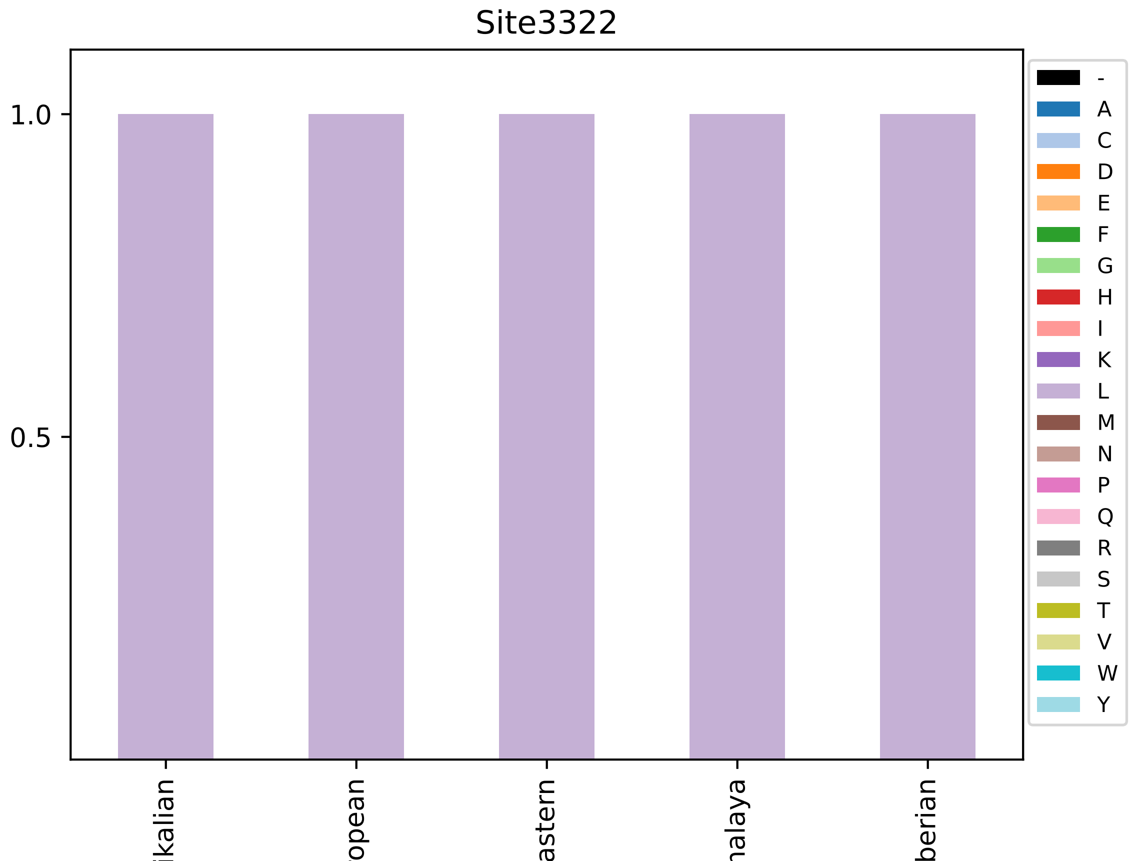
Site3322
1.0
0.5
-
A
C
D
E
F
G
H
I
K
L
M
N
P
Q
R
S
T
V
W
Y
ikalian
opean
astern
nalaya
berian

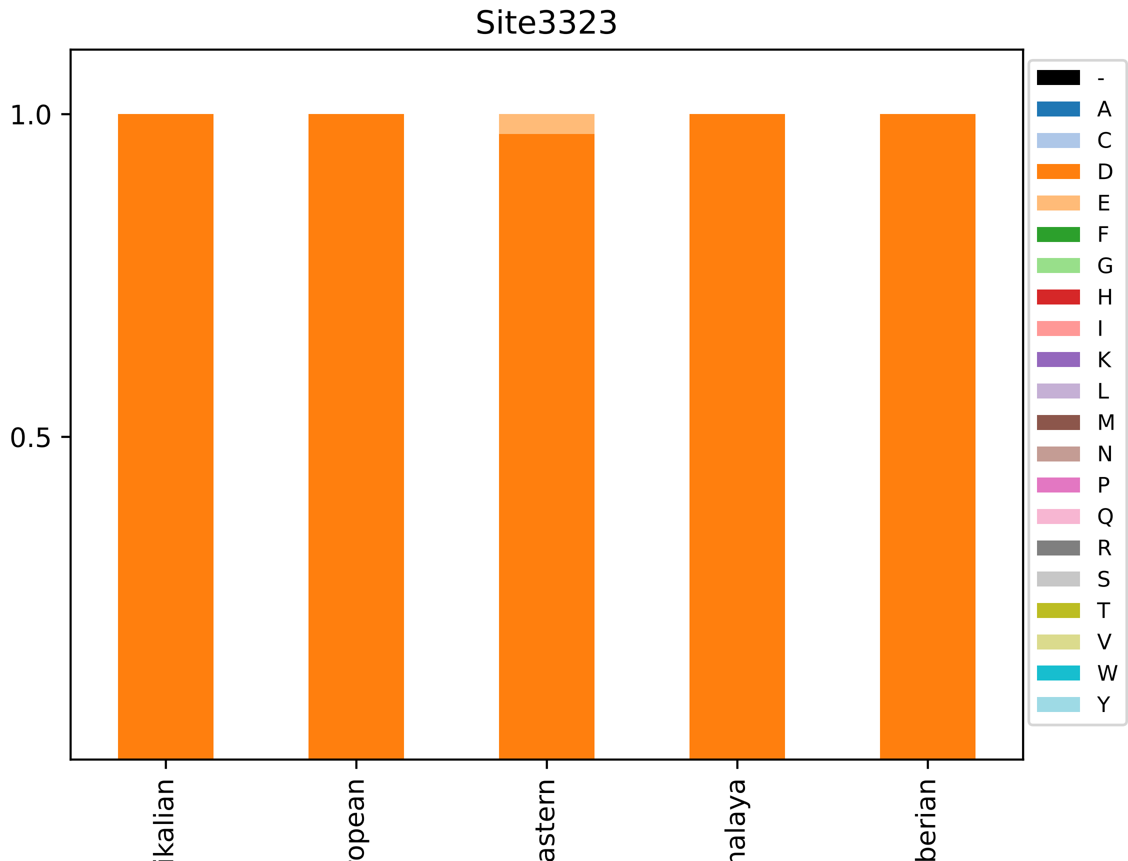

Site3323
1.0
0.5
-
A
C
D
E
F
G
H
I
K
L
M
N
P
Q
R
S
T
V
W
Y
ikalian
opean
astern
nalaya
berian

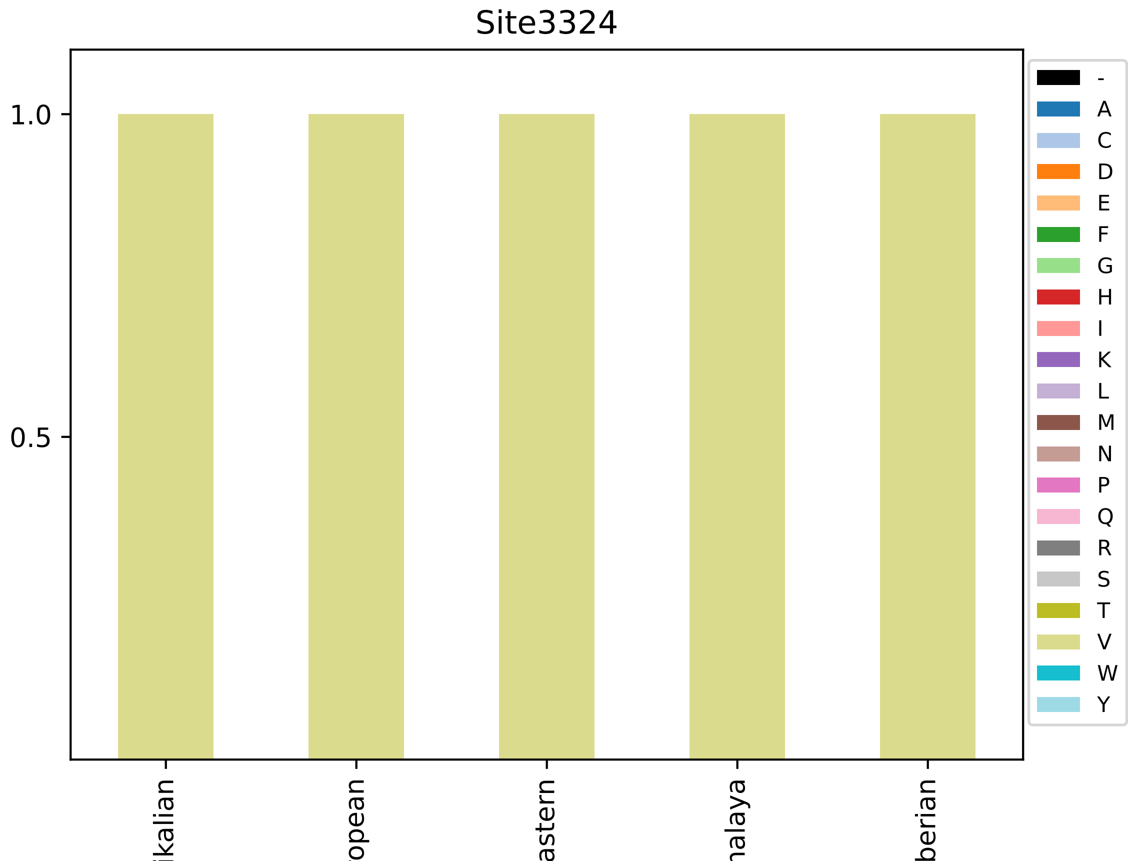
Site3324
1.0
0.5
-
A
C
D
E
F
G
H
I
K
L
M
N
P
Q
R
S
T
V
W
Y
ikalian
opean
astern
nalaya
berian

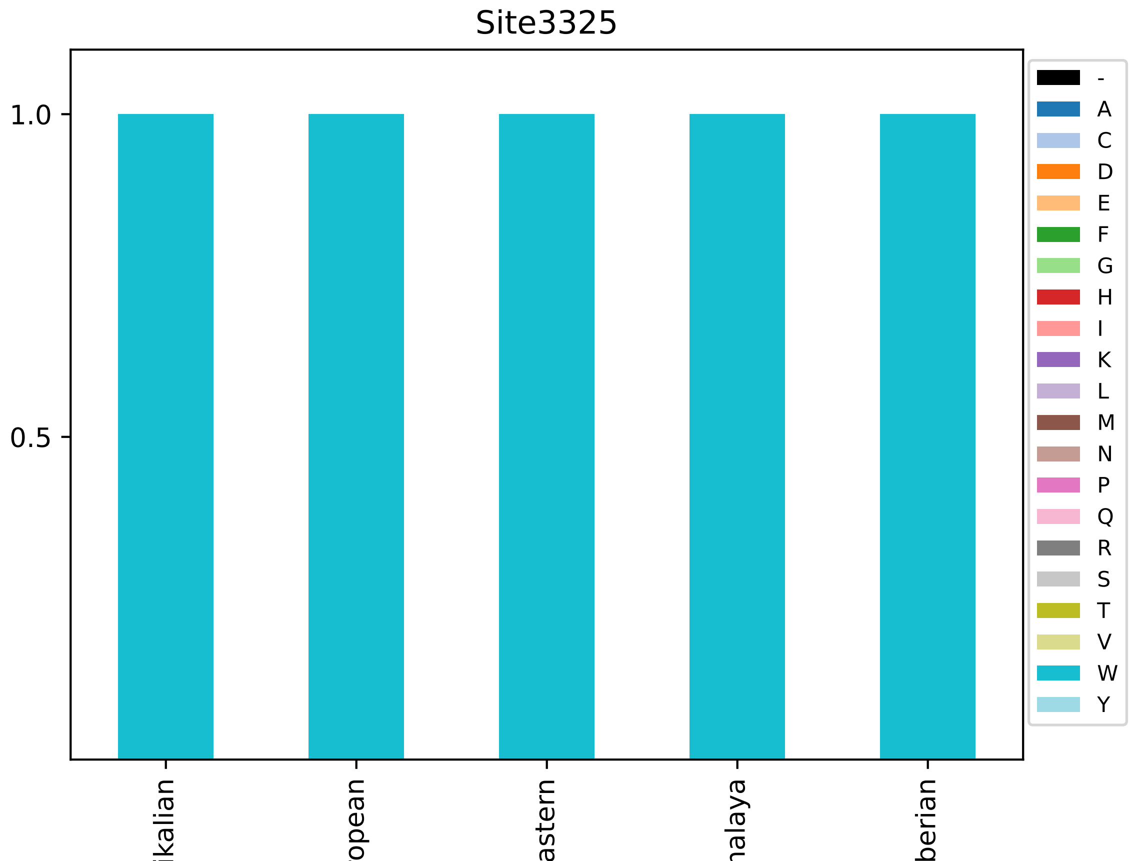

Site3325
1.0
0.5
ikalian
opean
astern
halaya
berian
-
A
C
D
E
F
G
H
I
K
L
M
N
P
Q
R
S
T
V
W
Y

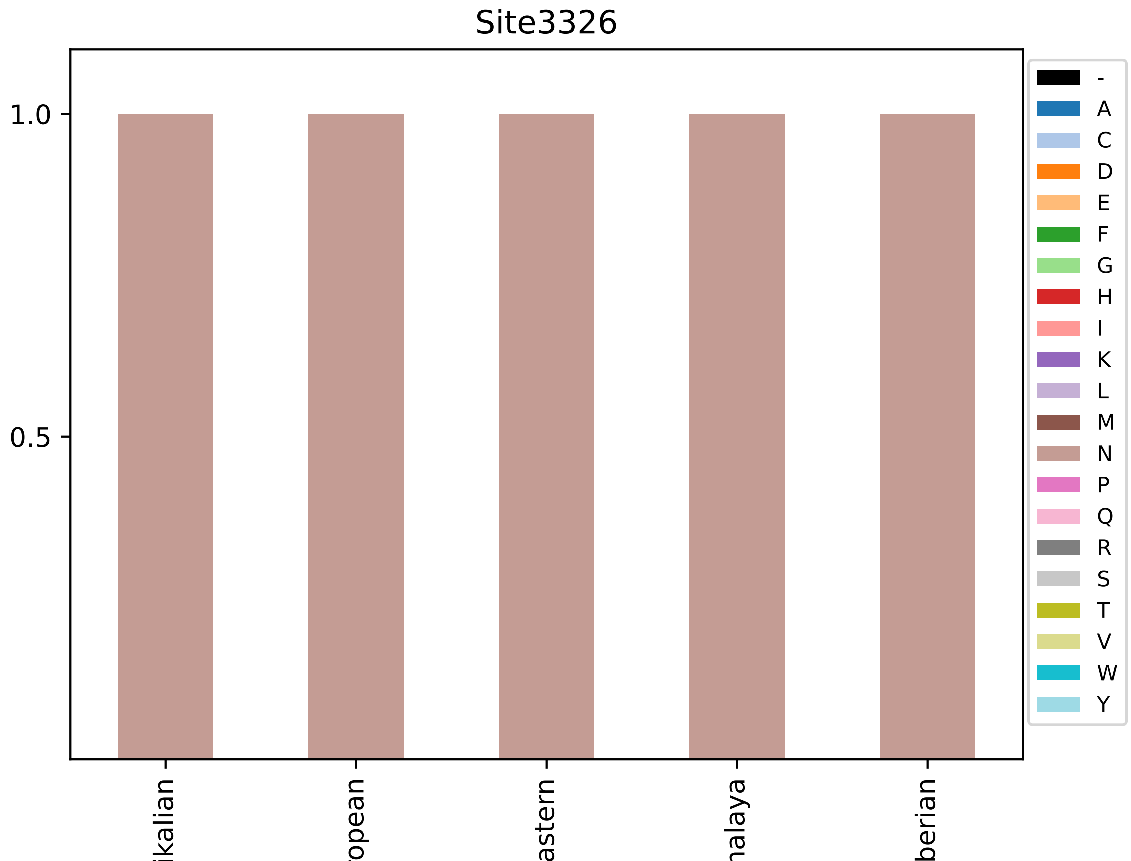
Site3326
1.0
0.5
-
A
C
D
E
F
G
H
I
K
L
M
N
P
Q
R
S
T
V
W
Y
ikalian
opean
astern
nalaya
berian

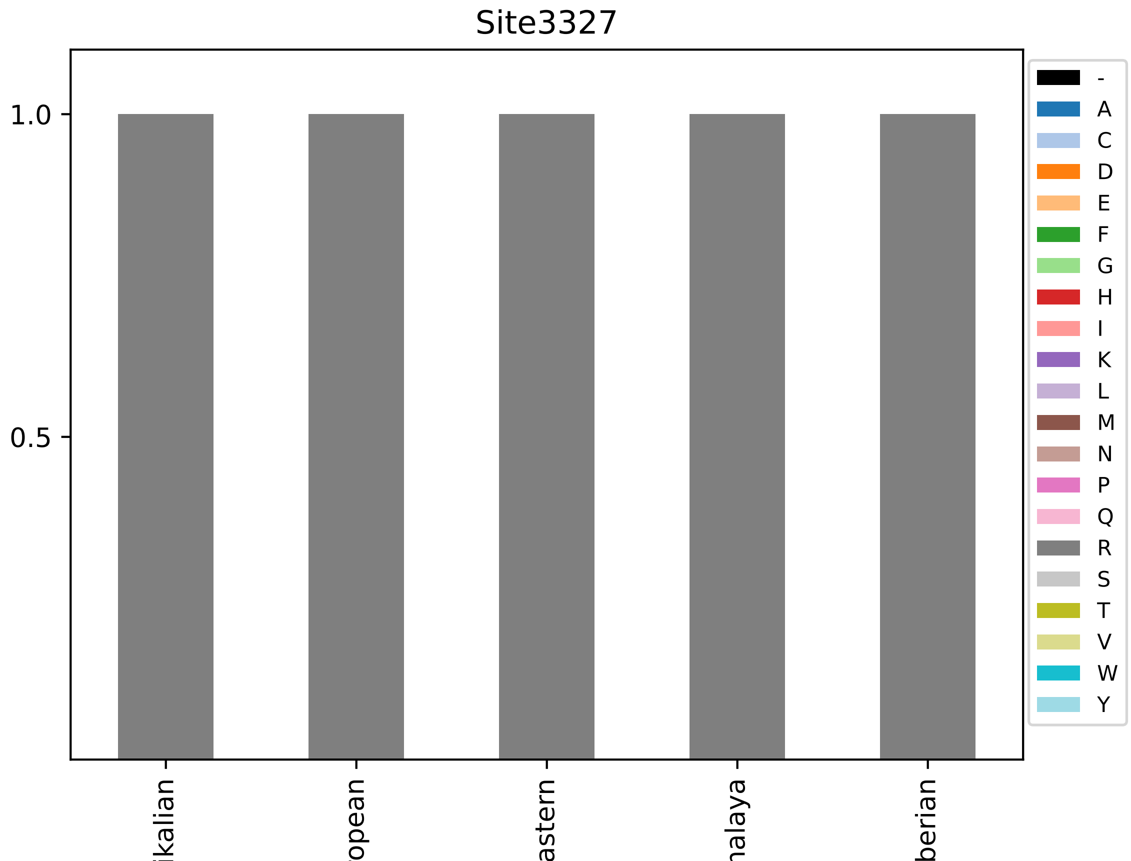

Site3327
1.0
0.5
-
A
C
D
E
F
G
H
I
K
L
M
N
P
Q
R
S
T
V
W
Y

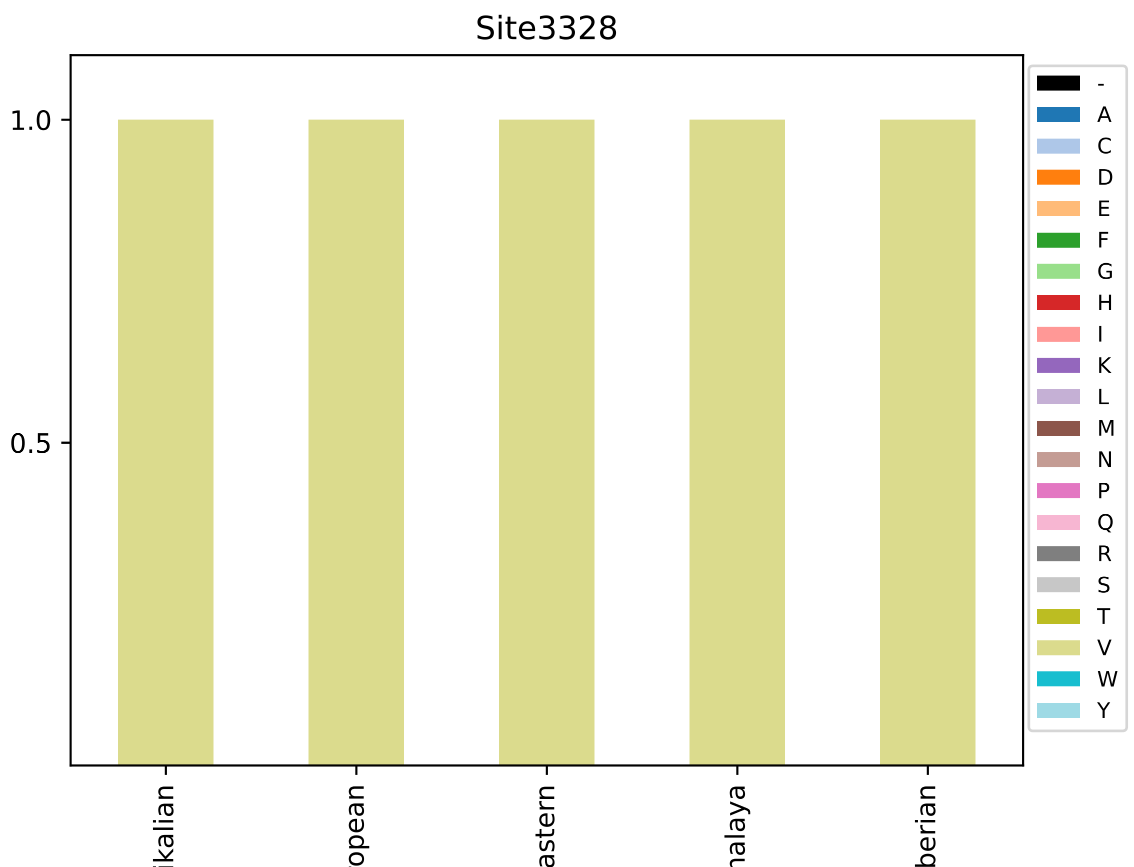
Site3328
1.0
0.5
-
A
C
D
E
F
G
H
I
K
L
M
N
P
Q
R
S
T
V
W
Y
ikalian
opean
astern
nalaya
berian

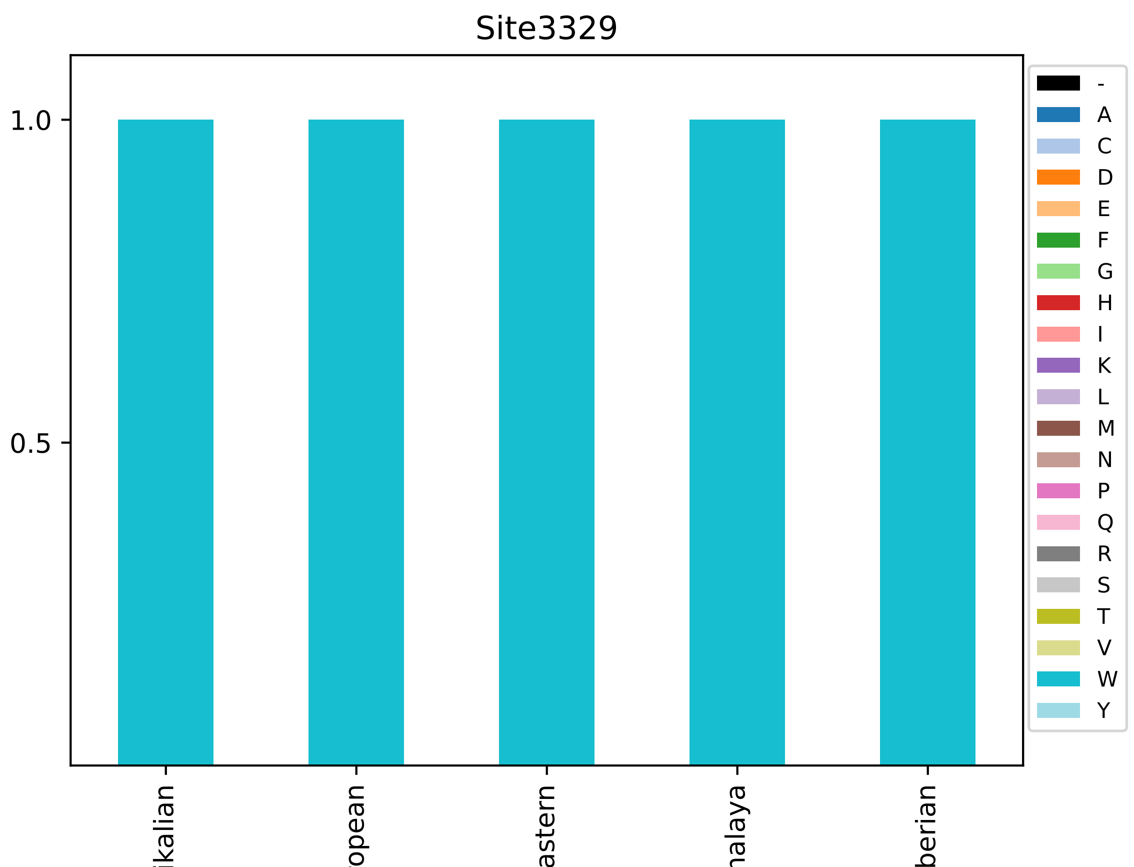

Site3329
1.0
0.5
ikalian
opean
astern
nalaya
berian
-
A
C
D
E
F
G
H
I
K
L
M
N
P
Q
R
S
T
V
W
Y

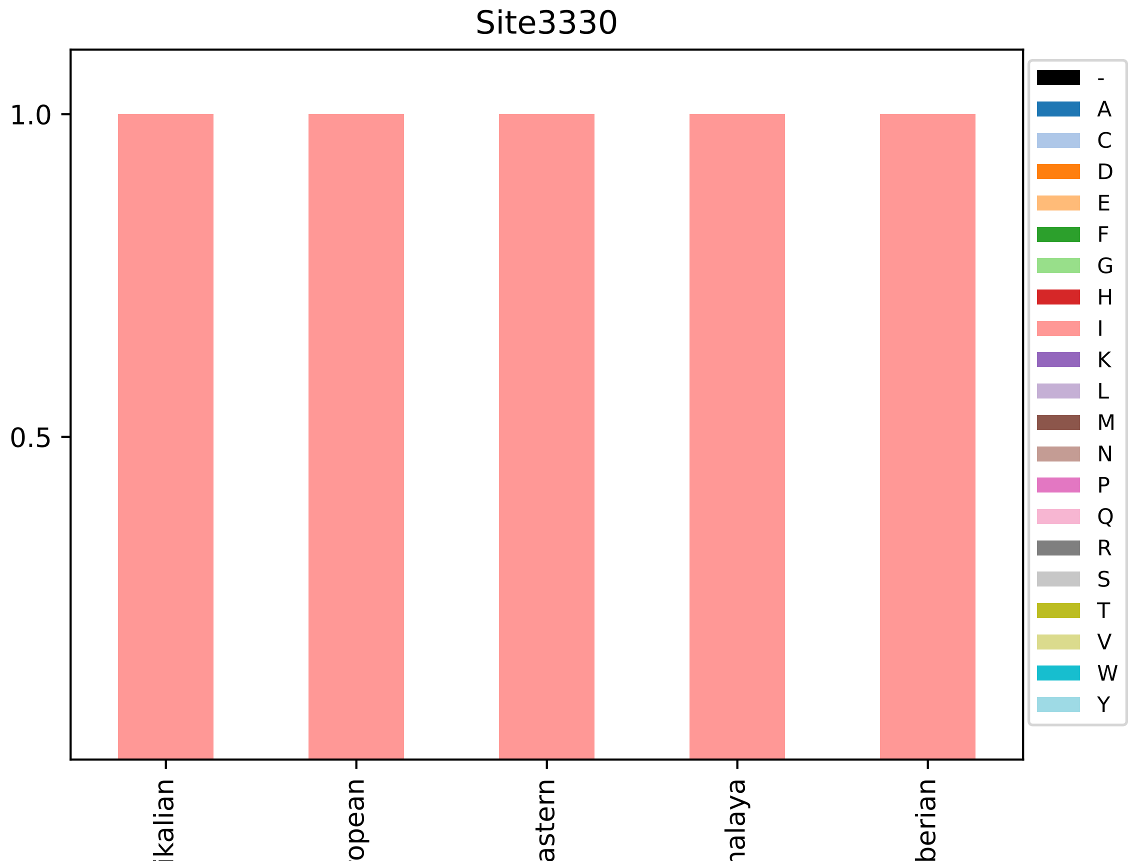

Site3330
1.0
0.5
ikalian
opean
astern
nalaya
berian
-
A
C
D
E
F
G
H
I
K
L
M
N
P
Q
R
S
T
V
W
Y

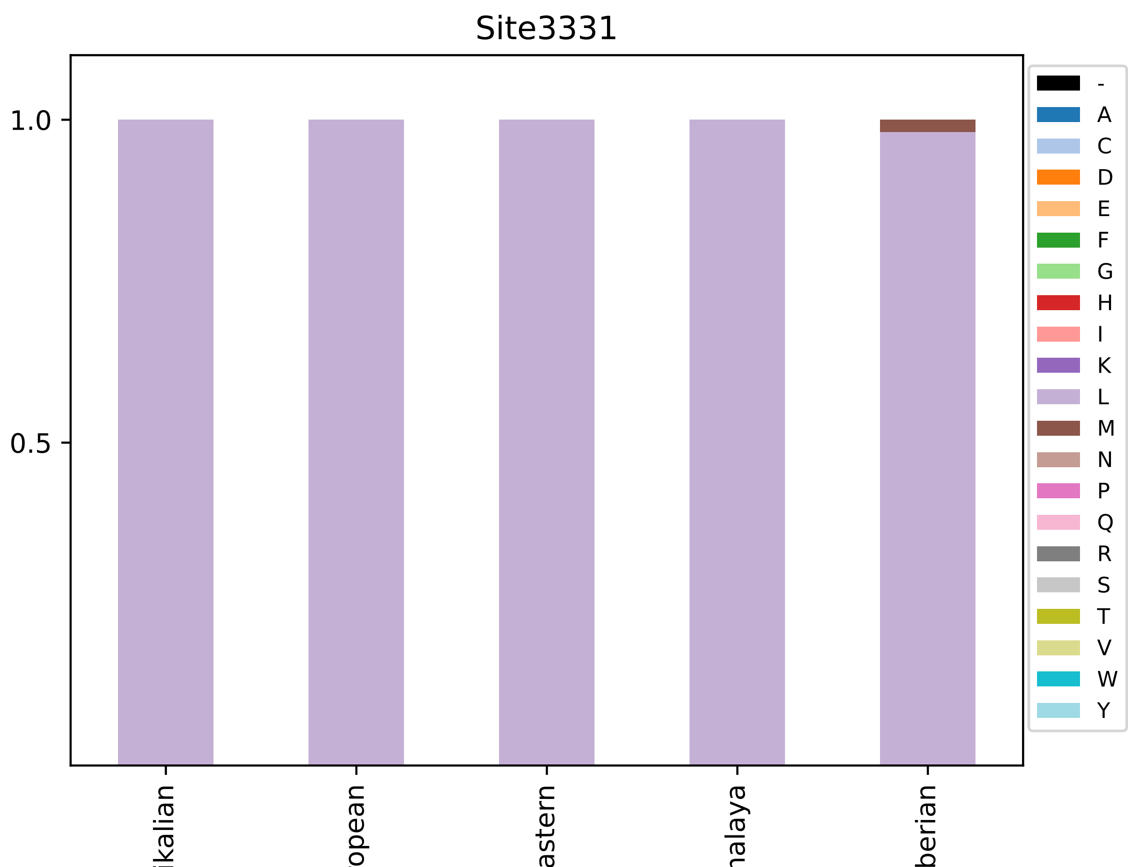

Site3331
1.0
0.5
-
A
C
D
E
F
G
H
I
K
L
M
N
P
Q
R
S
T
V
W
Y
ikalian
opean
astern
nalaya
berian

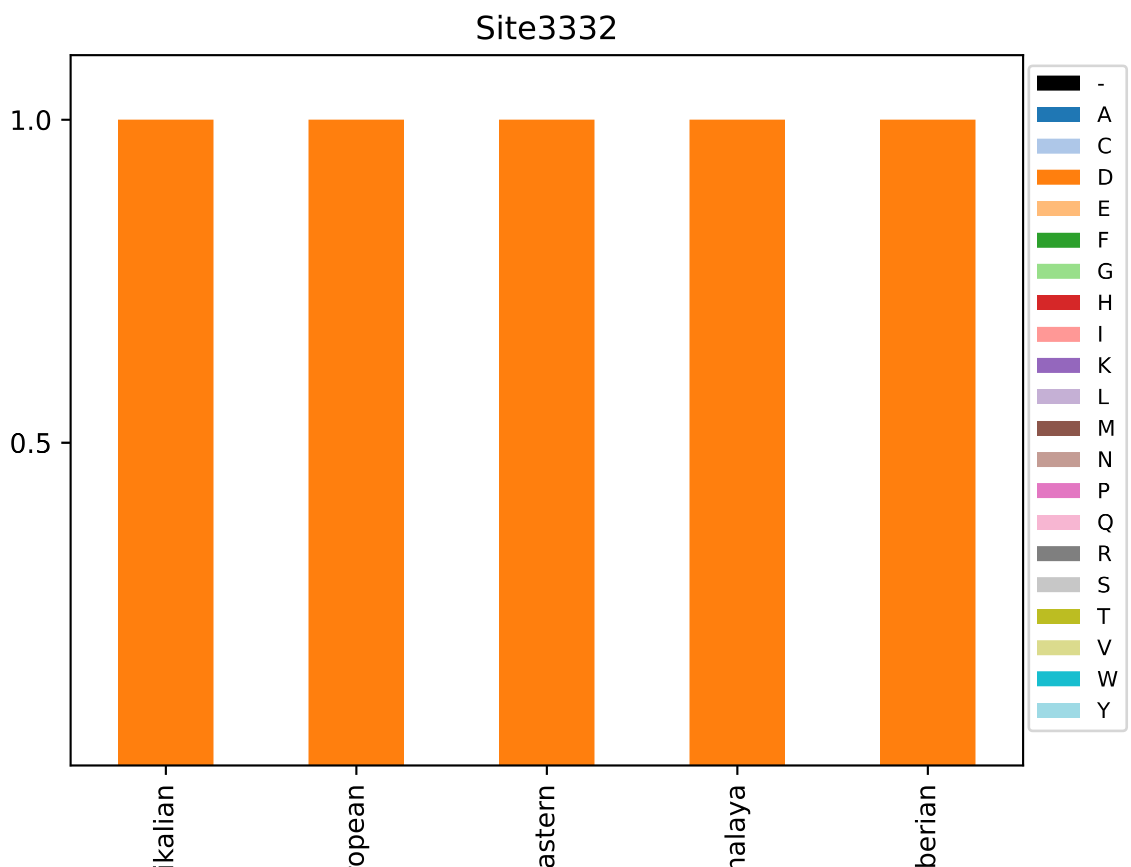
Site3332
1.0
0.5
ikalian
opean
astern
nalaya
berian
-
A
C
D
E
F
G
H
I
K
L
M
N
P
Q
R
S
T
V
W
Y

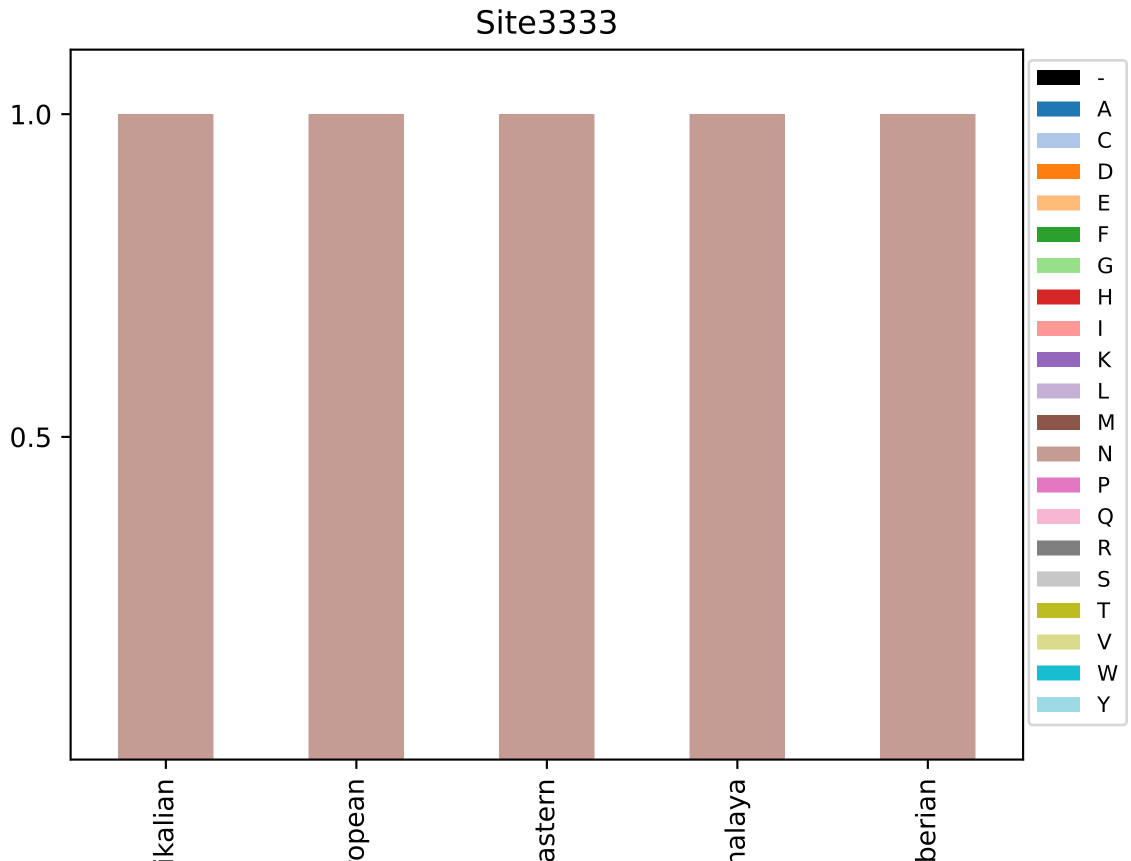
Site3333
1.0
0.5
-
A
C
D
E
F
G
H
I
K
L
M
N
P
Q
R
S
T
V
W
Y
ikalian
opean
astern
nalaya
berian

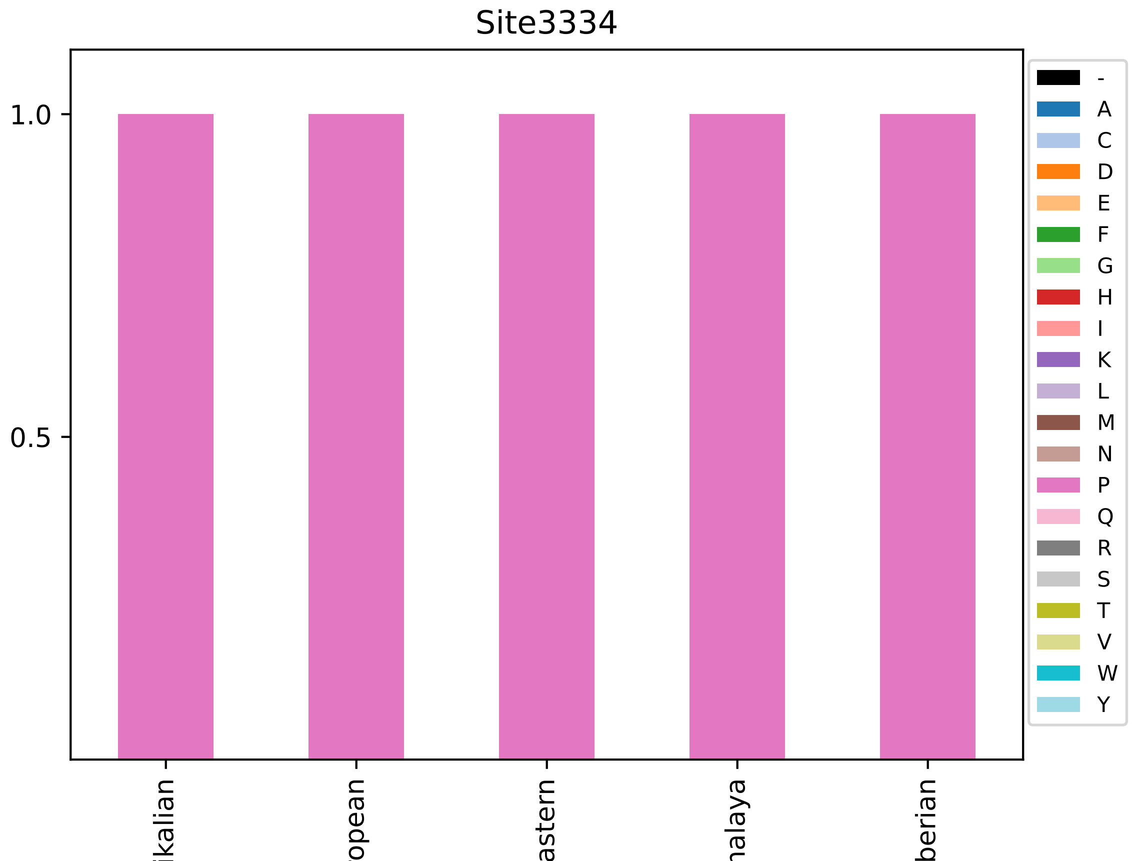
Site3334
1.0
0.5
ikalian
opean
astern
nalaya
berian
-
A
C
D
E
F
G
H
I
K
L
M
N
P
Q
R
S
T
V
W
Y

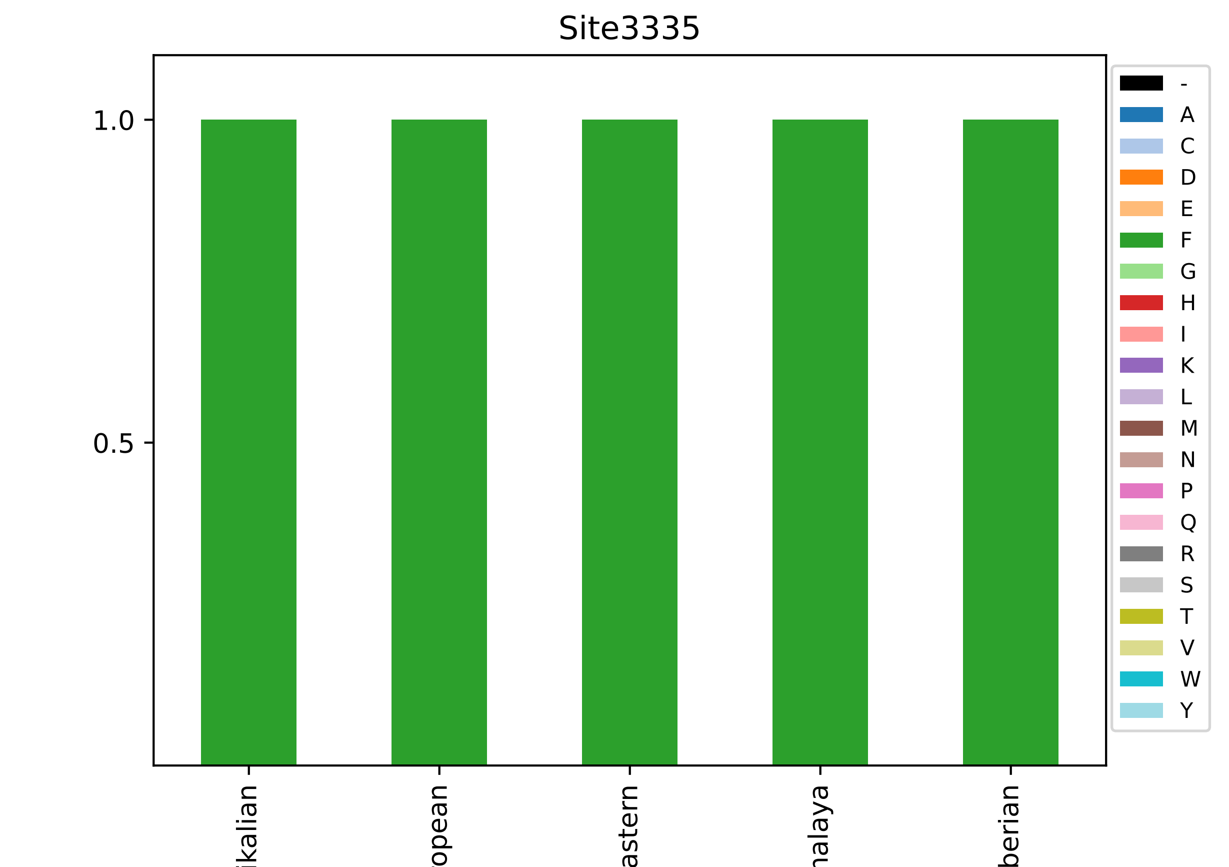

Site3335
1.0
0.5
-
A
C
D
E
F
G
H
I
K
L
M
N
P
Q
R
S
T
V
W
Y
ikalian
opean
astern
nalaya
berian

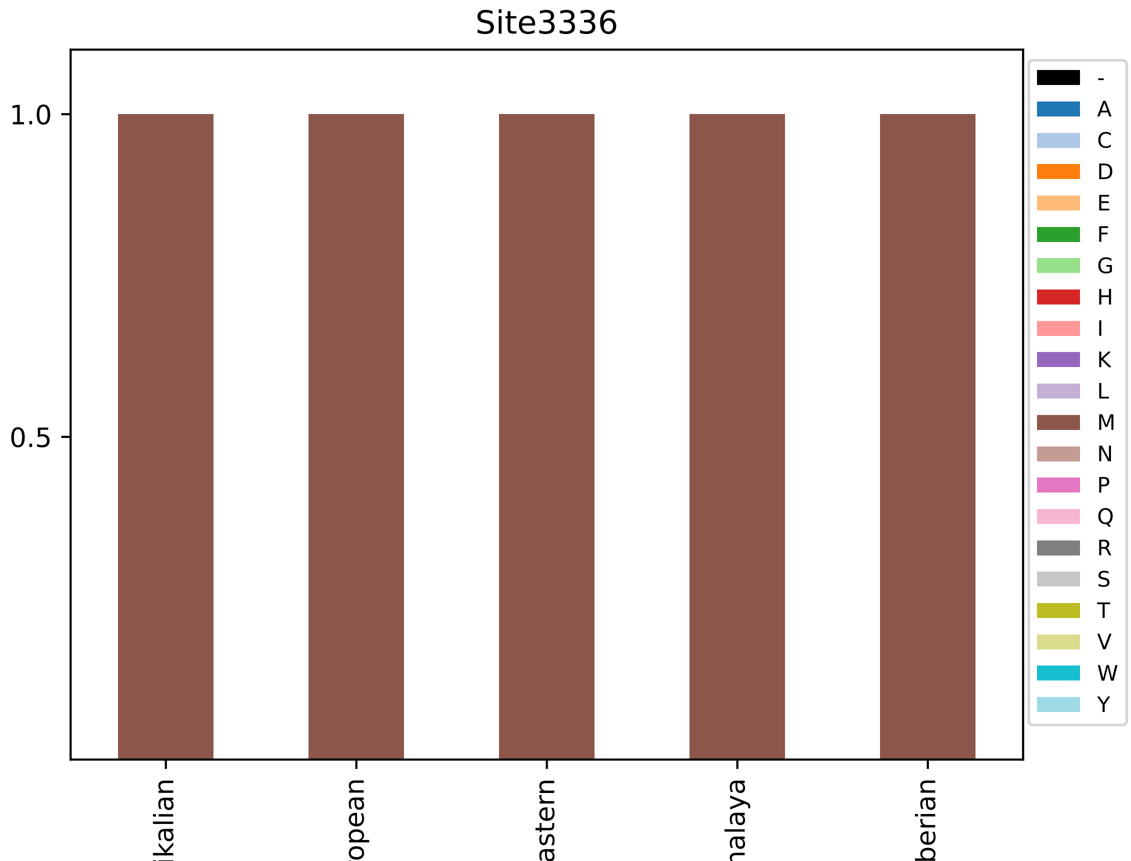

Site3336
1.0
0.5
-
A
C
D
E
F
G
H
I
K
L
M
N
P
Q
R
S
T
V
W
Y
ikalian
opean
astern
nalaya
berian

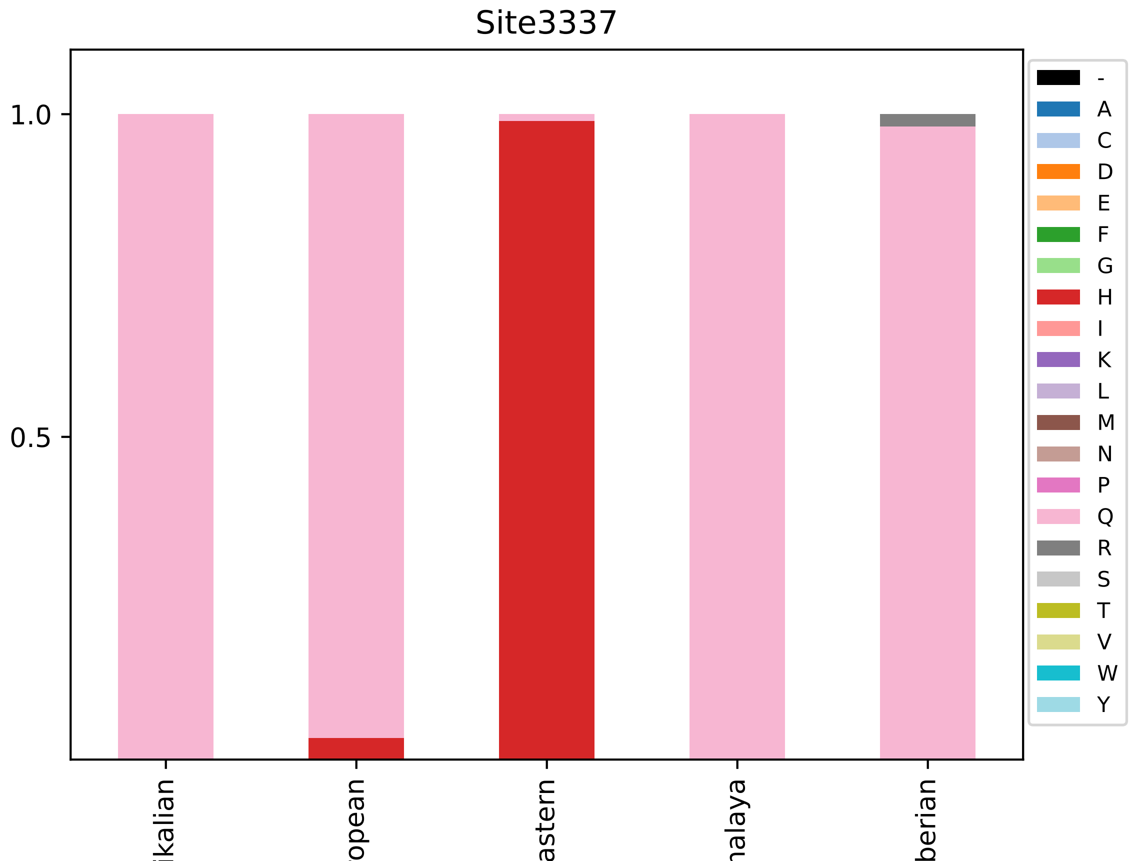

Site3337
1.0
0.5
ikalian
opean
astern
nalaya
berian
-
A
C
D
E
F
G
H
I
K
L
M
N
P
Q
R
S
T
V
W
Y

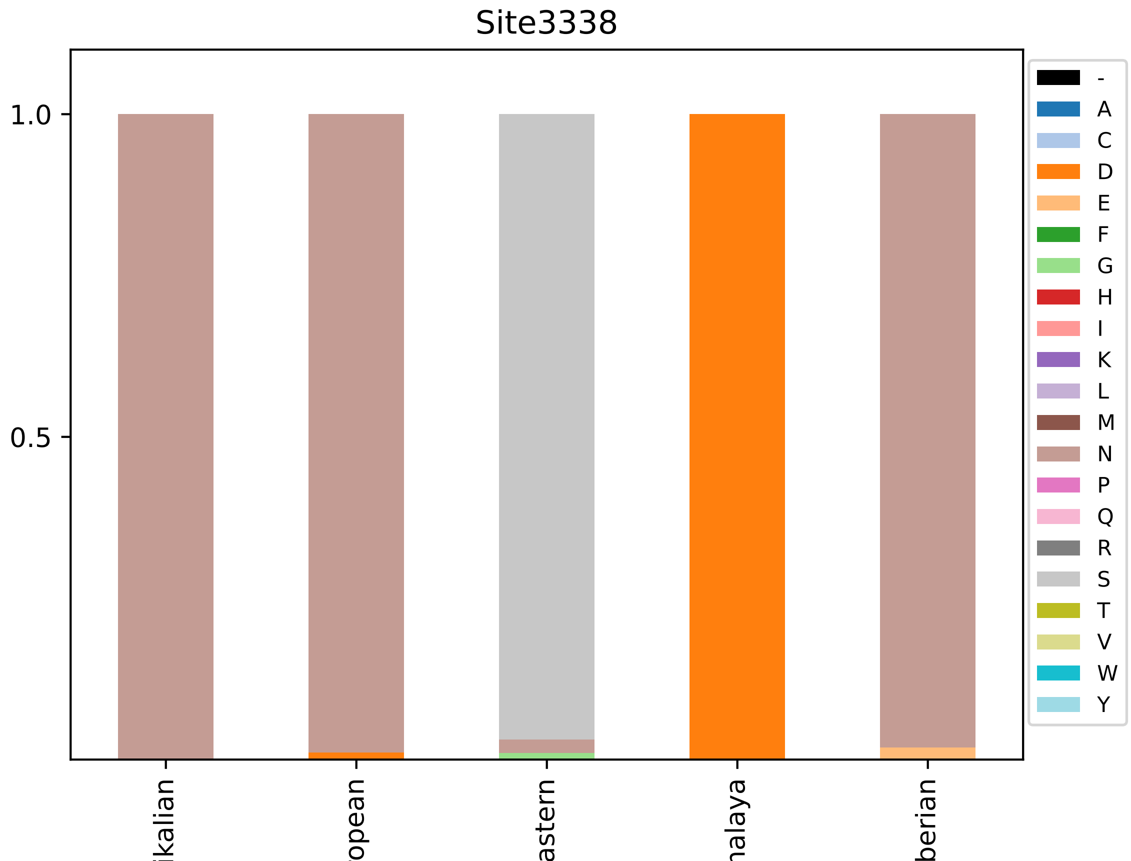

Site3338
1.0
0.5
-
A
C
D
E
F
G
H
I
K
L
M
N
P
Q
R
S
T
V
W
Y
ikalian
opean
astern
nalaya
berian

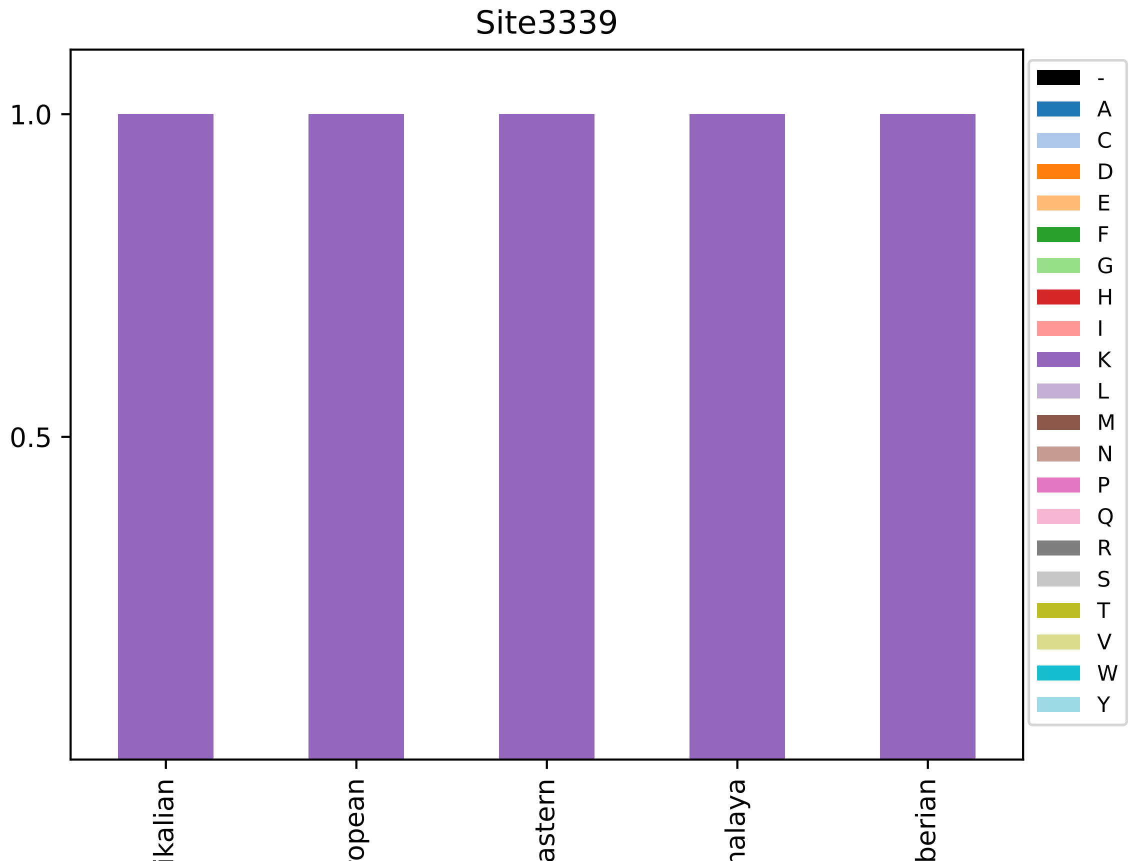

Site3339
1.0
0.5
-
A
C
D
E
F
G
H
I
K
L
M
N
P
Q
R
S
T
V
W
Y
ikalian
opean
astern
nalaya
berian

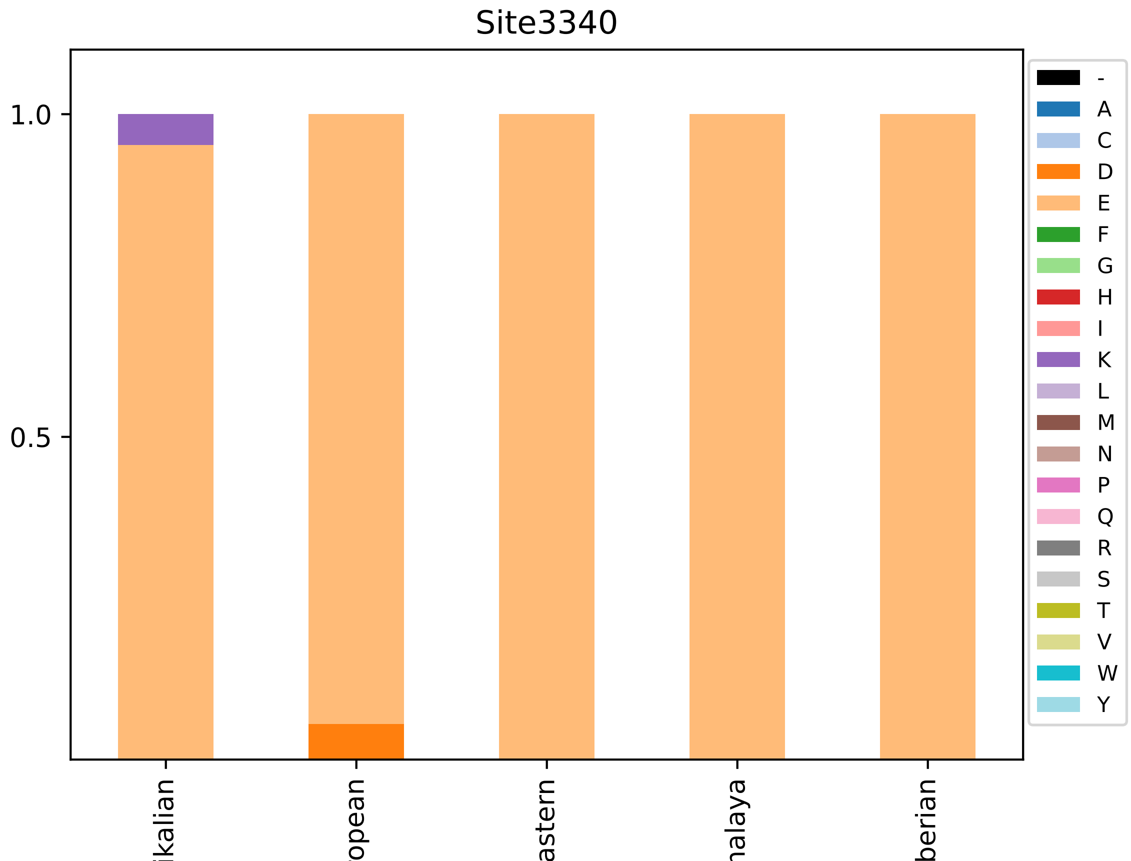

Site3340
1.0
0.5
-
A
C
D
E
F
G
H
I
K
L
M
N
P
Q
R
S
T
V
W
Y

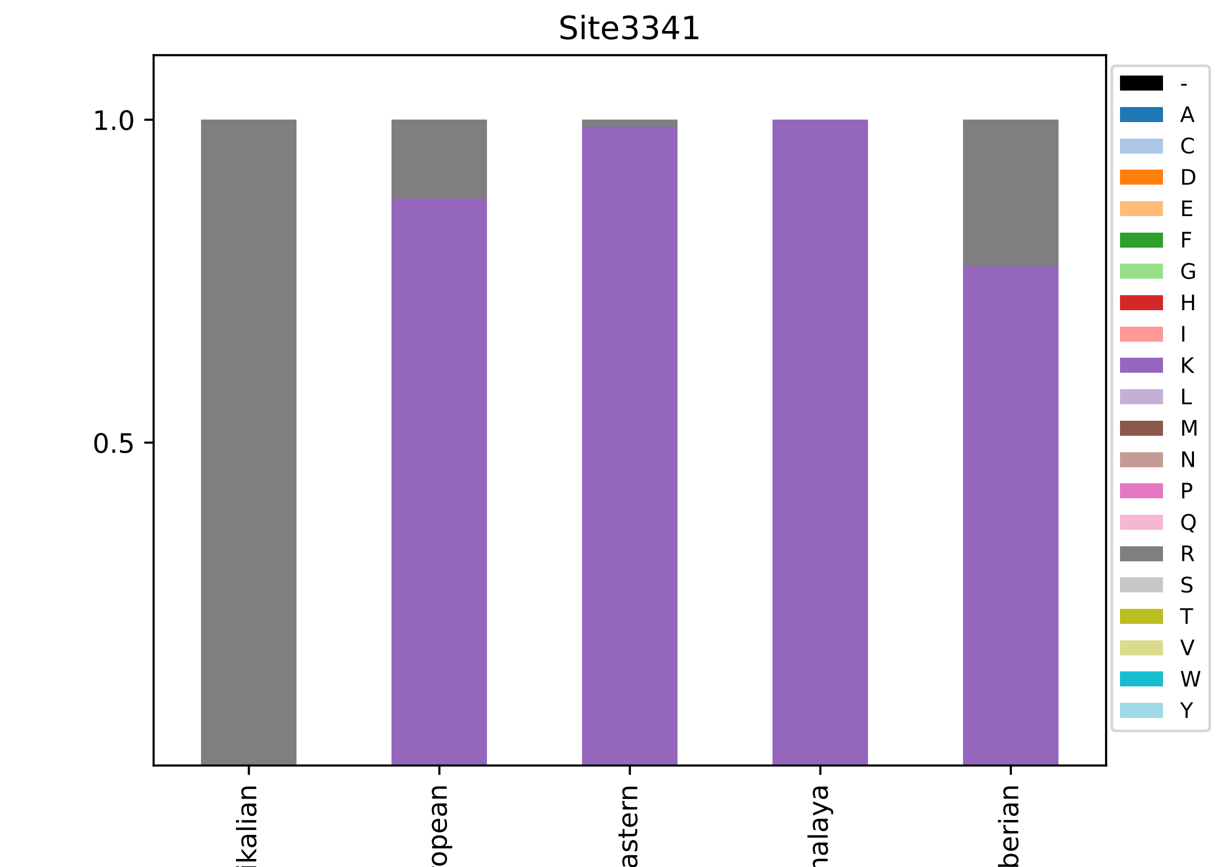
Site3341
1.0
0.5
-
A
C
D
E
F
G
H
I
K
L
M
N
P
Q
R
S
T
V
W
Y
ikalian
opean
astern
nalaya
berian

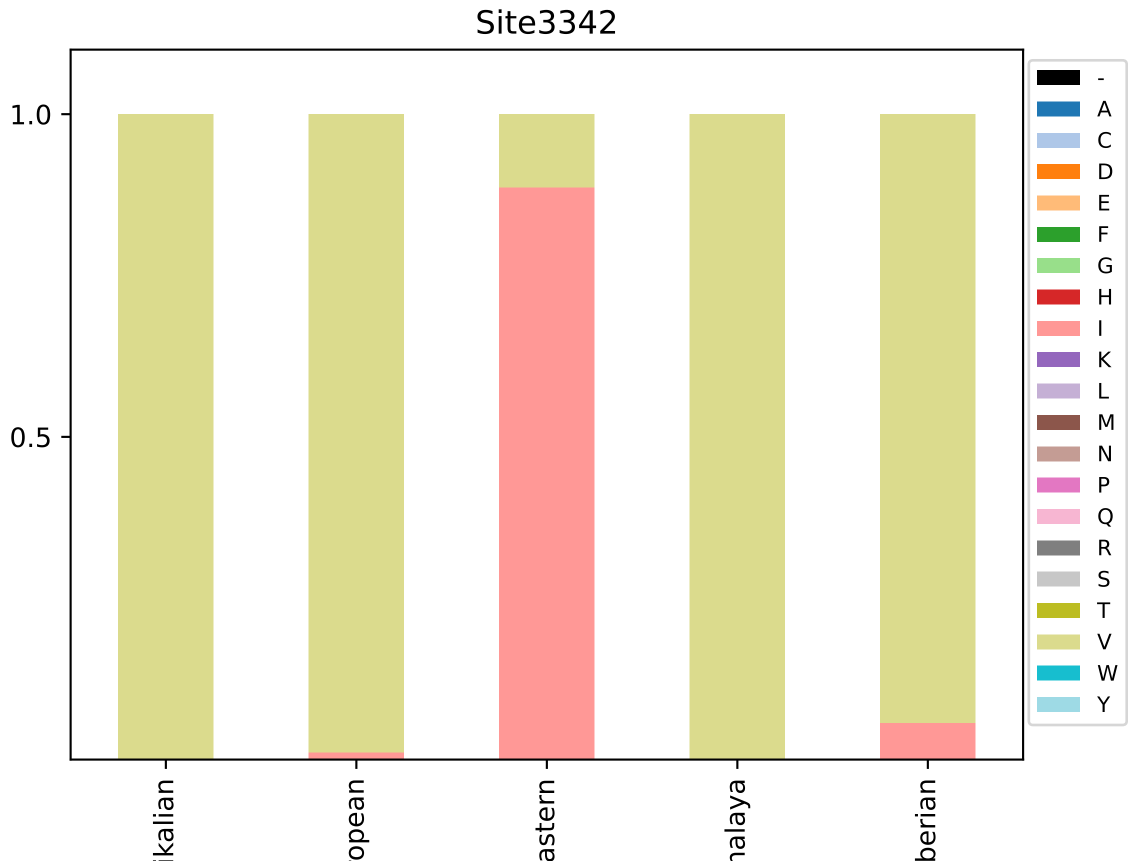

Site3342
1.0
0.5
-
A
C
D
E
F
G
H
I
K
L
M
N
P
Q
R
S
T
V
W
Y
ikalian
opean
astern
nalaya
berian

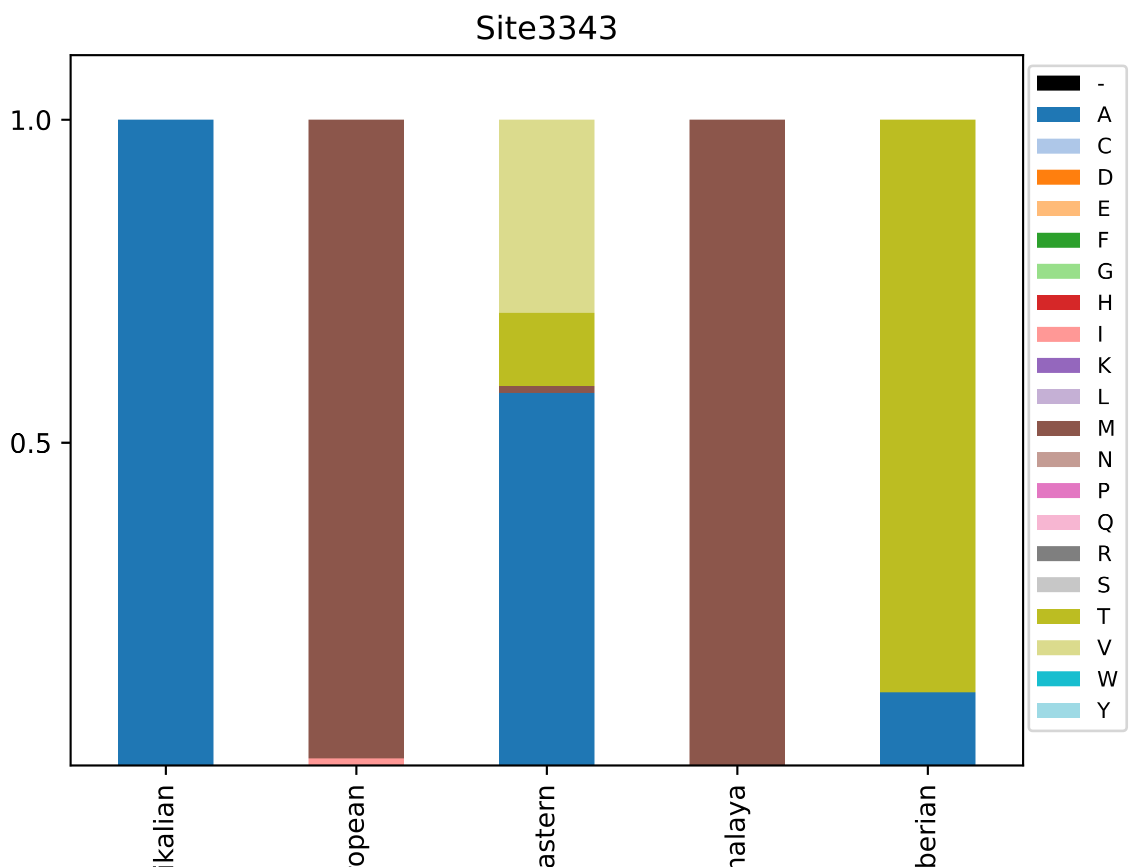
Site3343
1.0
0.5
ikalian
opean
astern
nalaya
berian
-
A
C
D
E
F
G
H
I
K
L
M
N
P
Q
R
S
T
V
W
Y

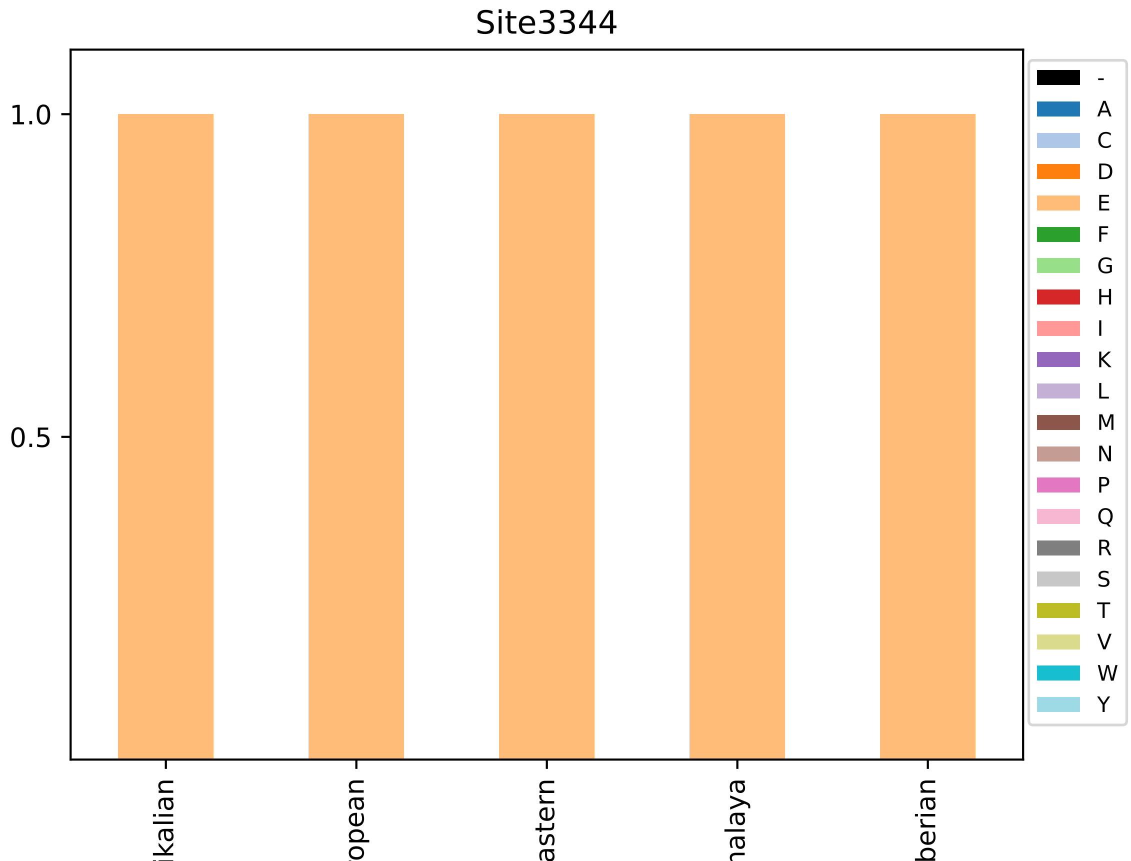
Site3344
1.0
0.5
-
A
C
D
E
F
G
H
I
K
L
M
N
P
Q
R
S
T
V
W
Y
ikalian
opean
astern
nalaya
berian

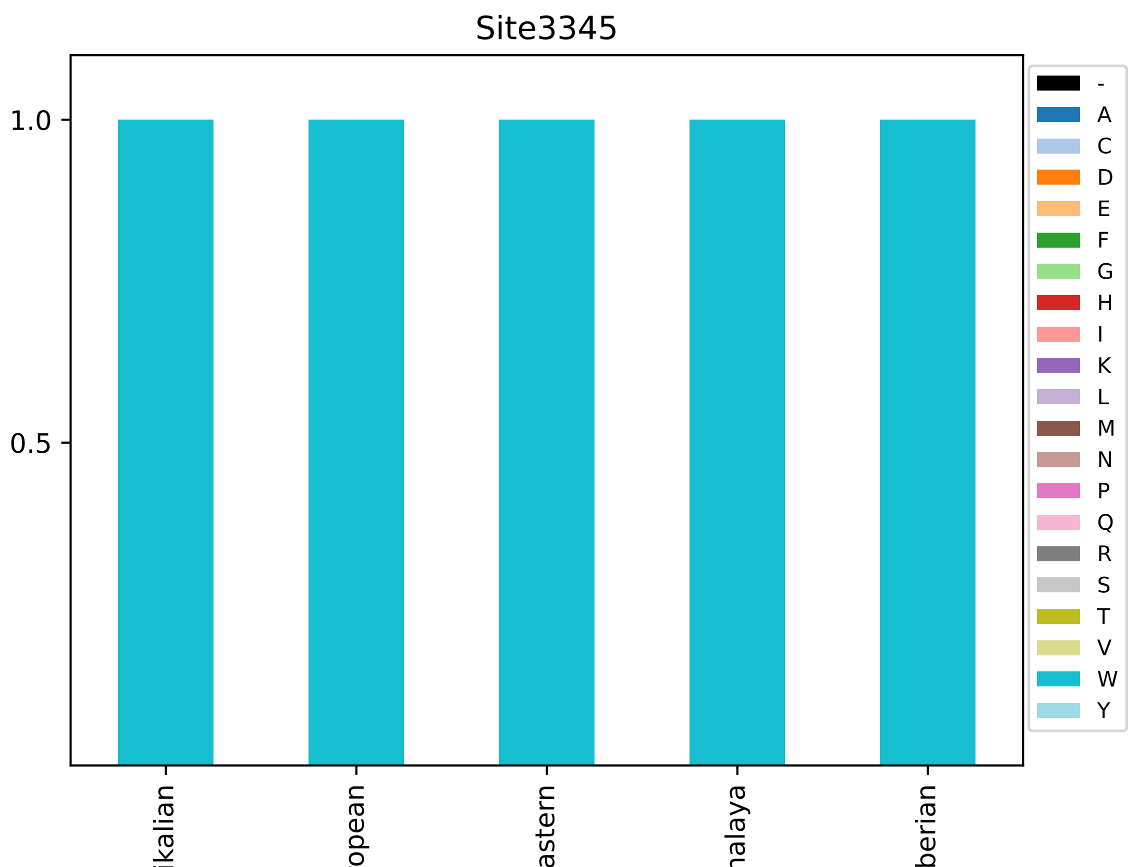
Site3345
1.0
0.5
-
A
C
D
E
F
G
H
I
K
L
M
N
P
Q
R
S
T
V
W
Y
ikalian
opean
astern
nalaya
berian

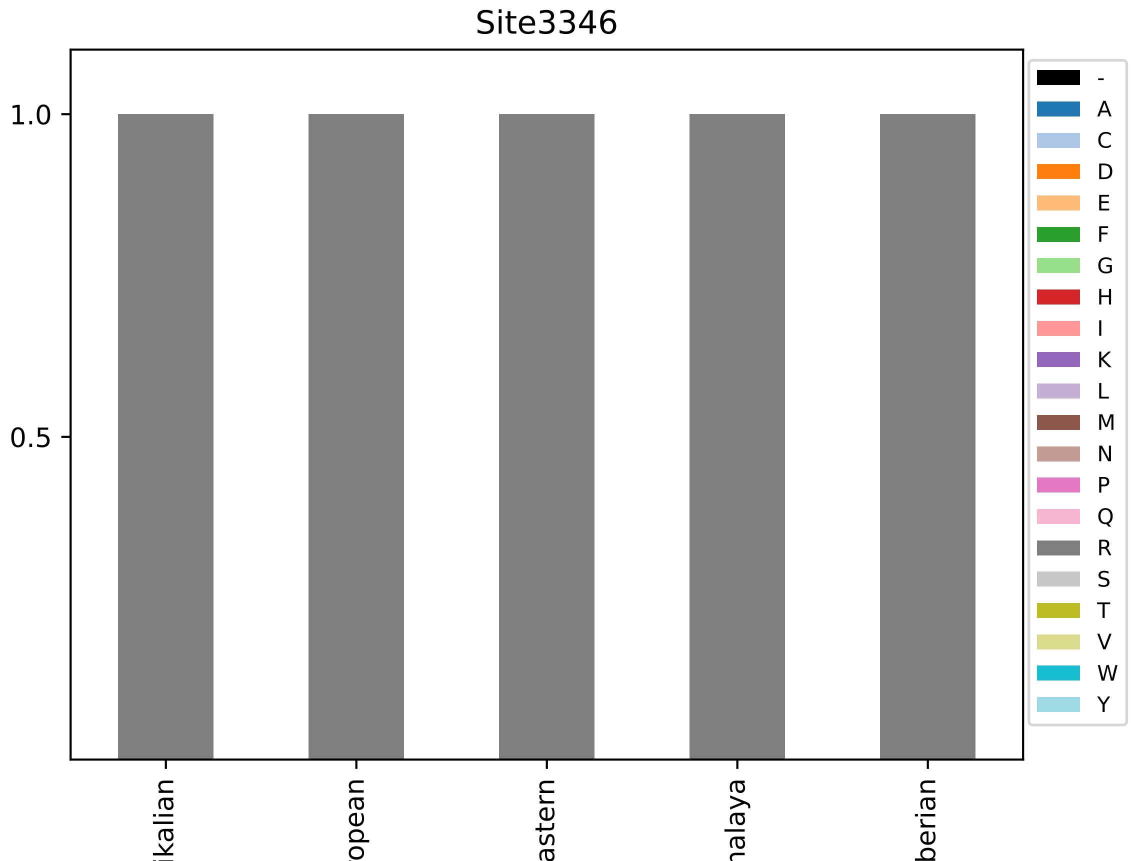
Site3346
1.0
0.5
-
A
C
D
E
F
G
H
I
K
L
M
N
P
Q
R
S
T
V
W
Y
ikalian
opean
astern
nalaya
berian

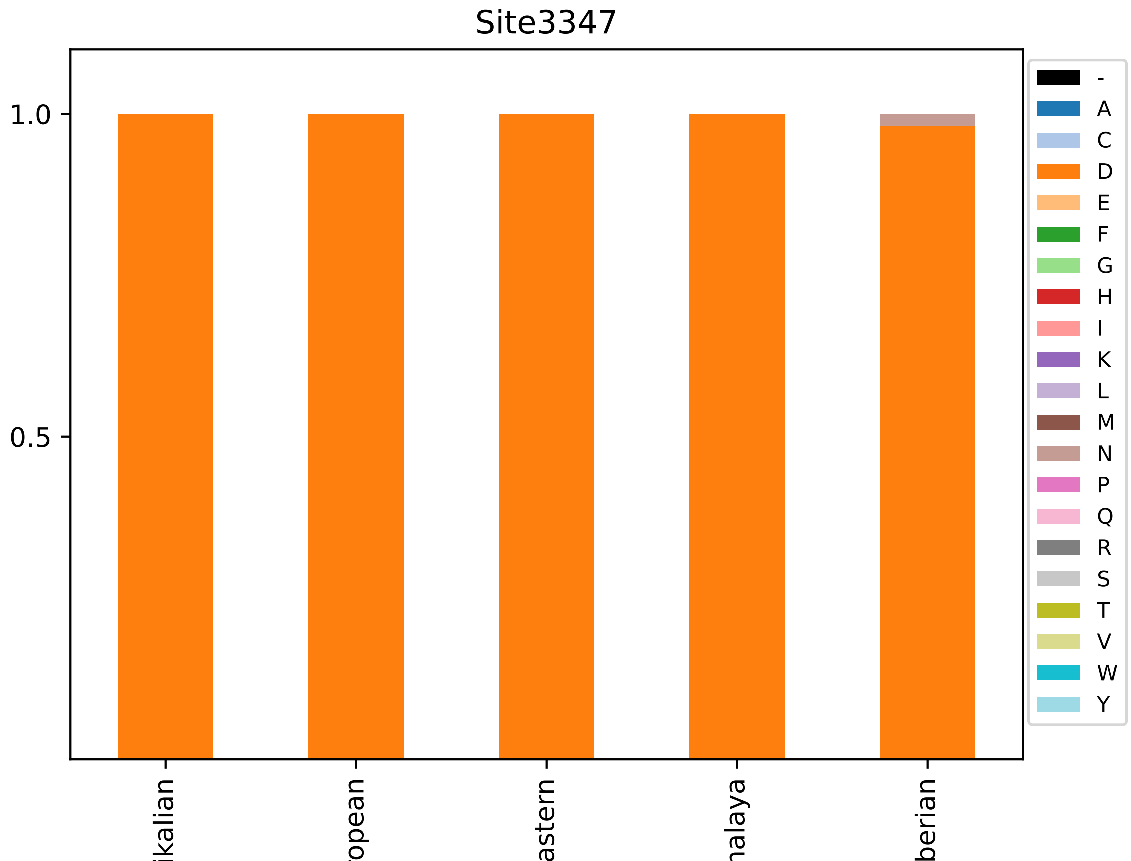
Site3347
1.0
0.5
ikalian
opean
astern
halaya
berian
-
A
C
D
E
F
G
H
I
K
L
M
N
P
Q
R
S
T
V
W
Y

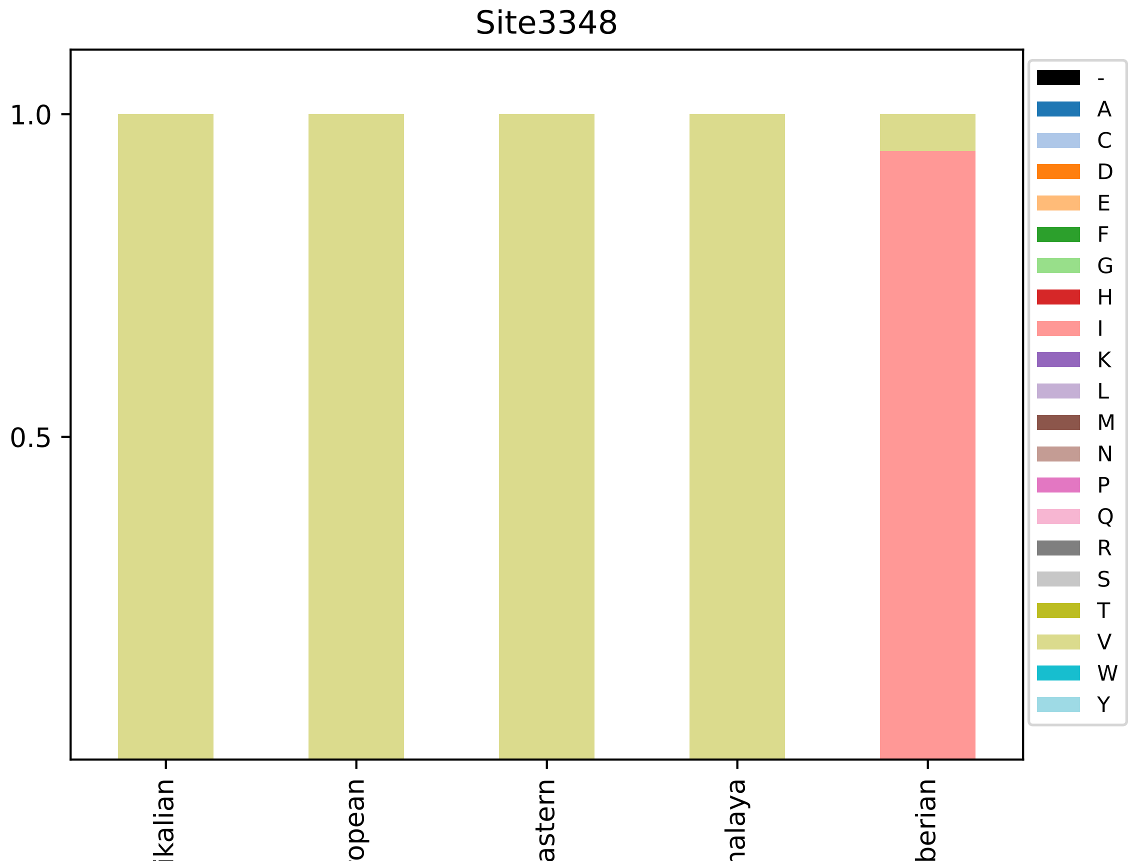
Site3348
1.0
0.5
-
A
C
D
E
F
G
H
I
K
L
M
N
P
Q
R
S
T
V
W
Y
ikalian
opean
astern
halaya
berian

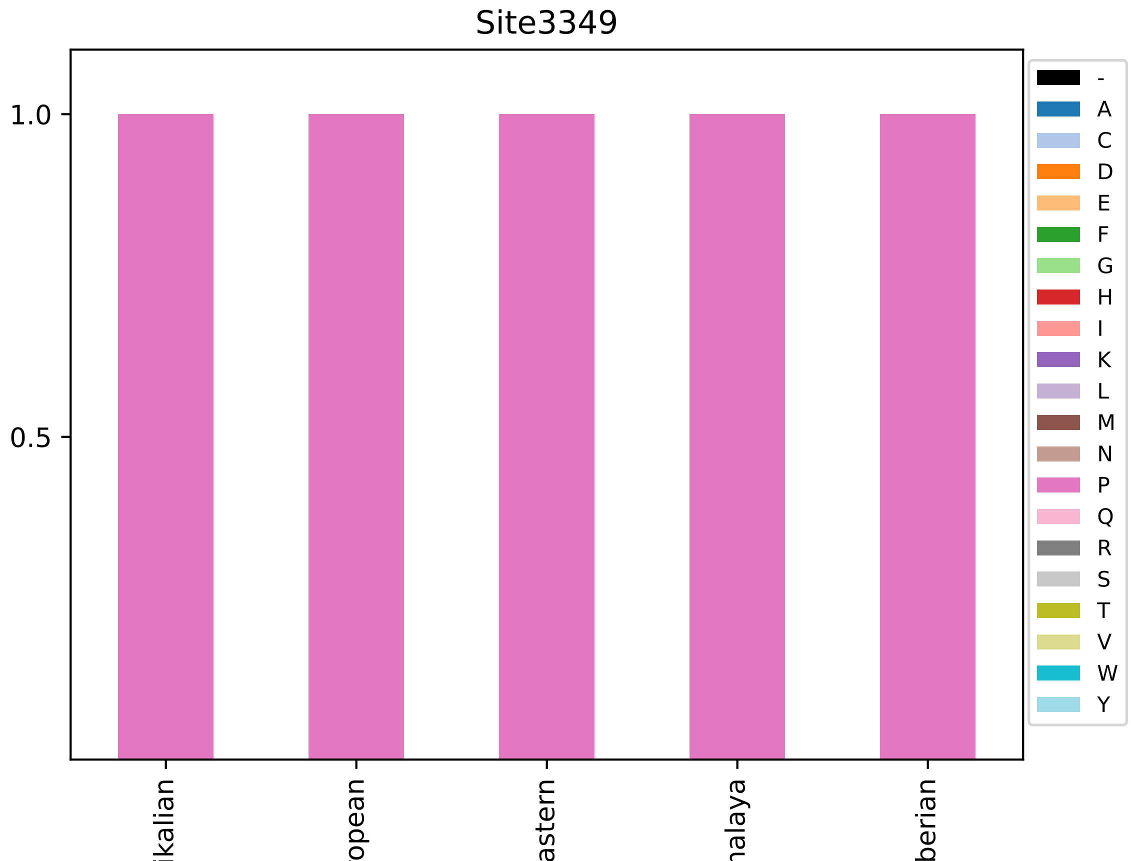
Site3349
1.0
0.5
-
A
C
D
E
F
G
H
I
K
L
M
N
P
Q
R
S
T
V
W
Y
ikalian
opean
astern
nalaya
berian

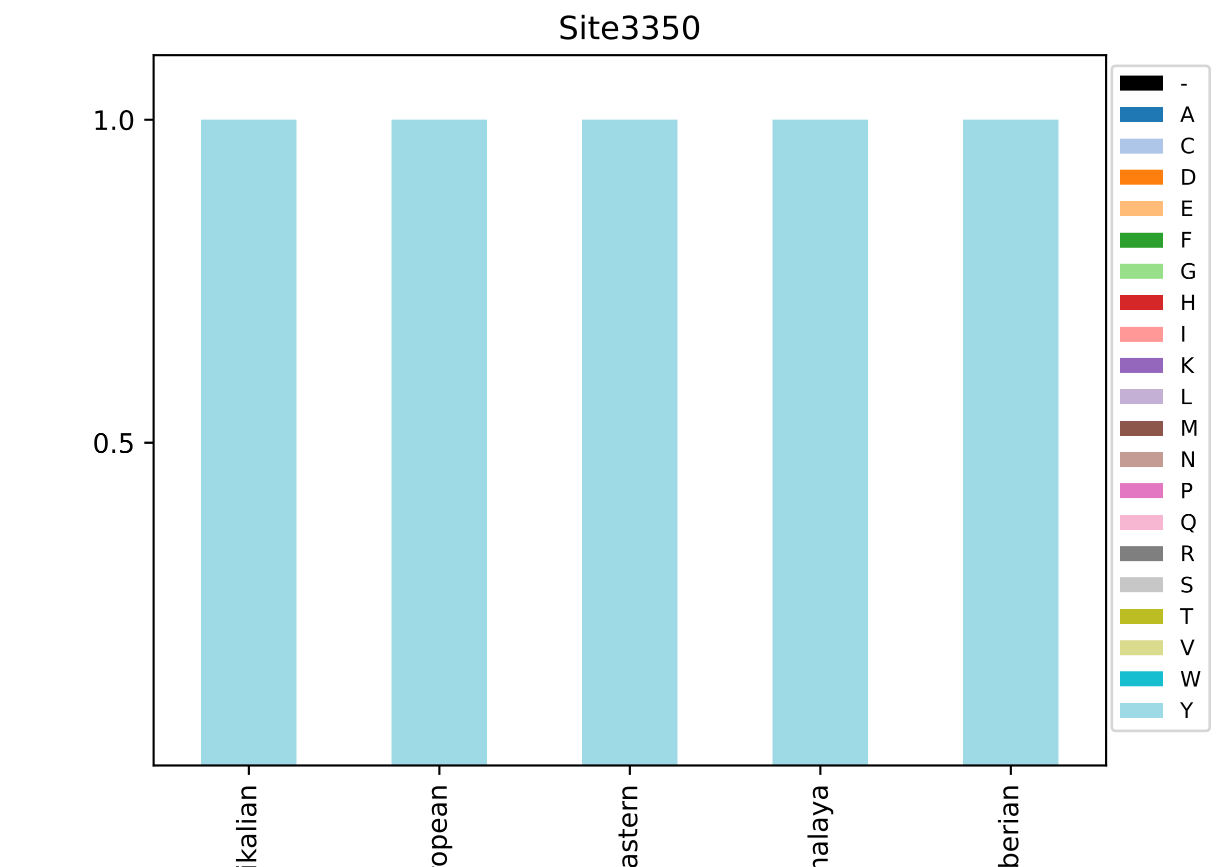

Site3350
1.0
0.5
-
A
C
D
E
F
G
H
I
K
L
M
N
P
Q
R
S
T
V
W
Y
ikalian
opean
astern
halaya
berian

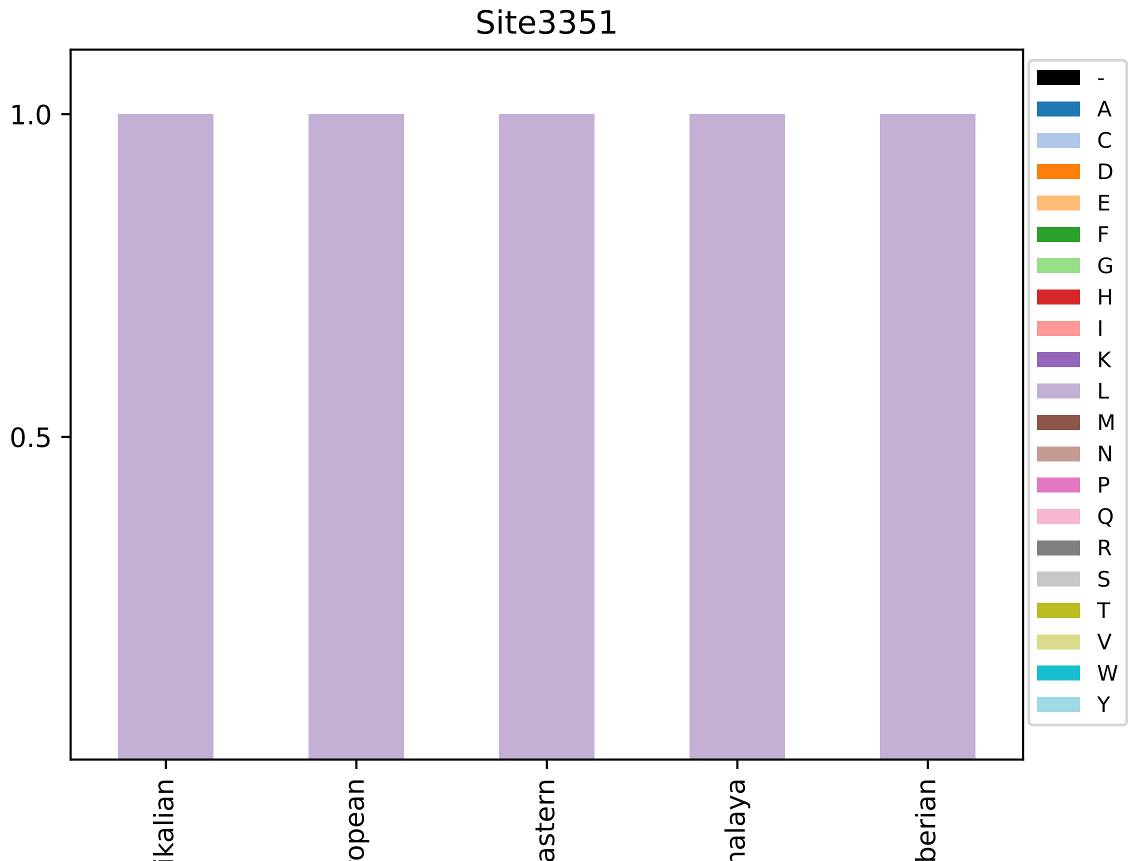

Site3351
1.0
0.5
-
A
C
D
E
F
G
H
I
K
L
M
N
P
Q
R
S
T
V
W
Y
ikalian
opean
astern
nalaya
berian

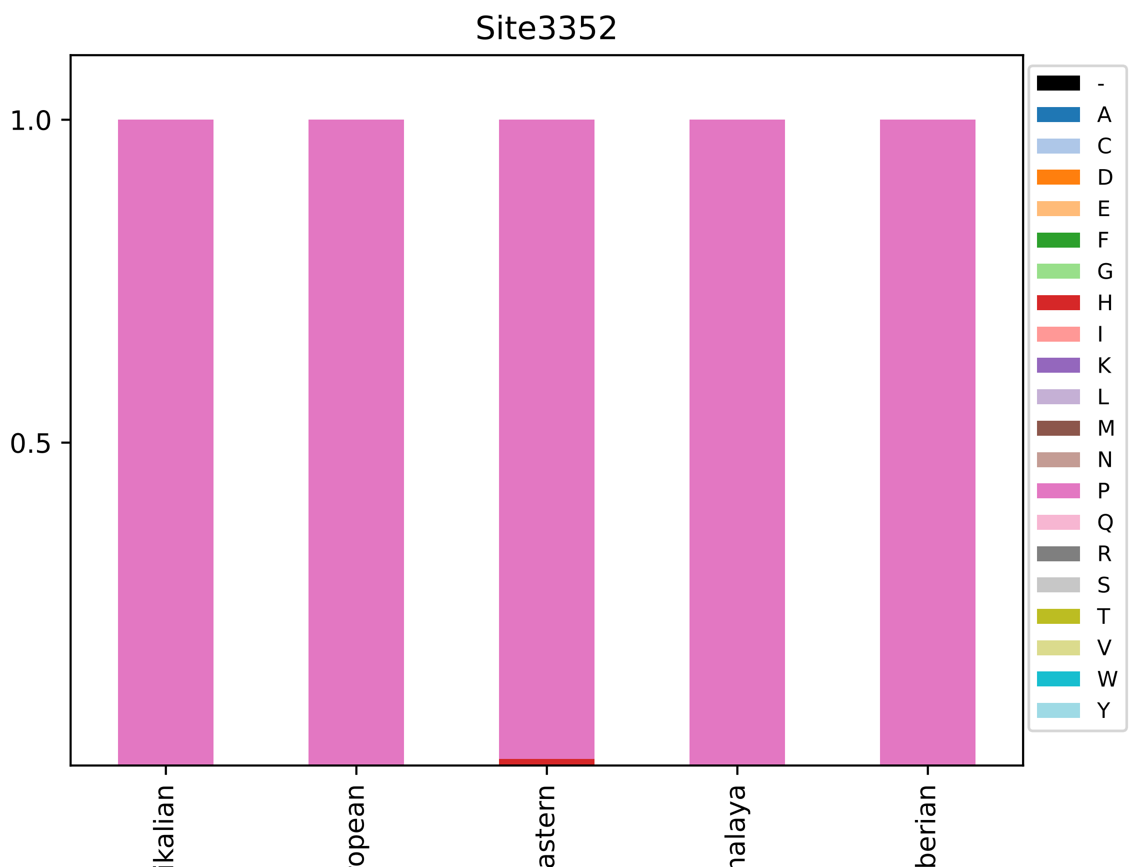
Site3352
1.0
0.5
-
A
C
D
E
F
G
H
I
K
L
M
N
P
Q
R
S
T
V
W
Y

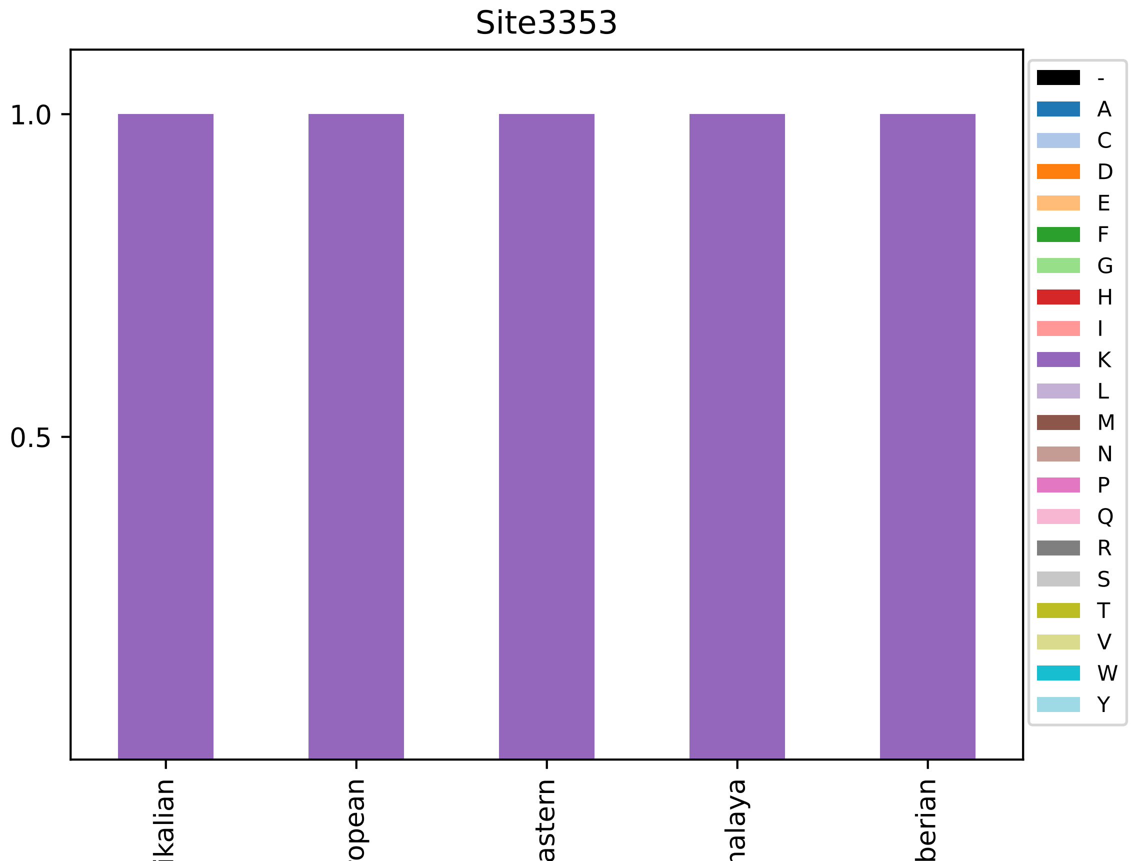
Site3353
1.0
0.5
-
A
C
D
E
F
G
H
I
K
L
M
N
P
Q
R
S
T
V
W
Y
ikalian
opean
astern
nalaya
berian

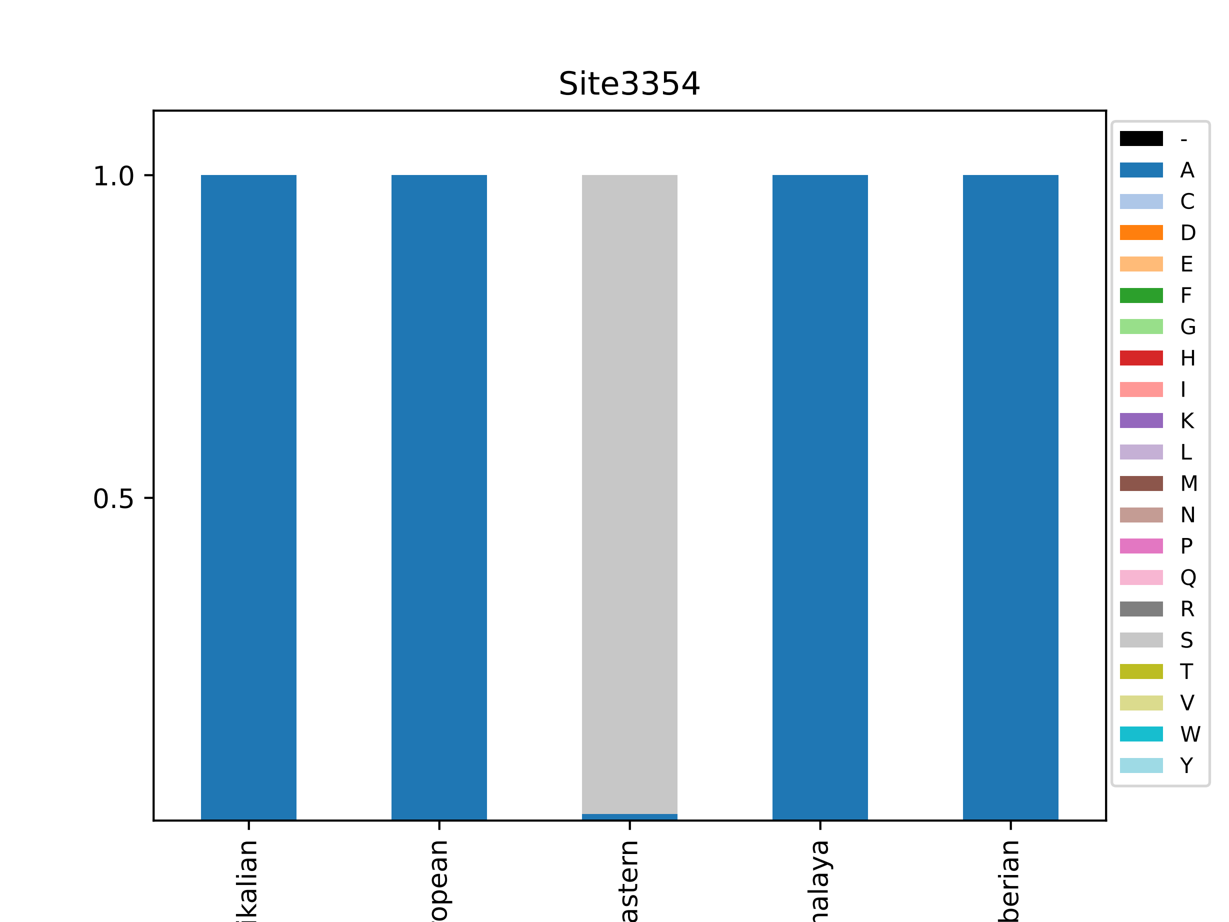
Site3354
1.0
0.5
-
A
C
D
E
F
G
H
I
K
L
M
N
P
Q
R
S
T
V
W
Y
ikalian
opean
astern
nalaya
berian

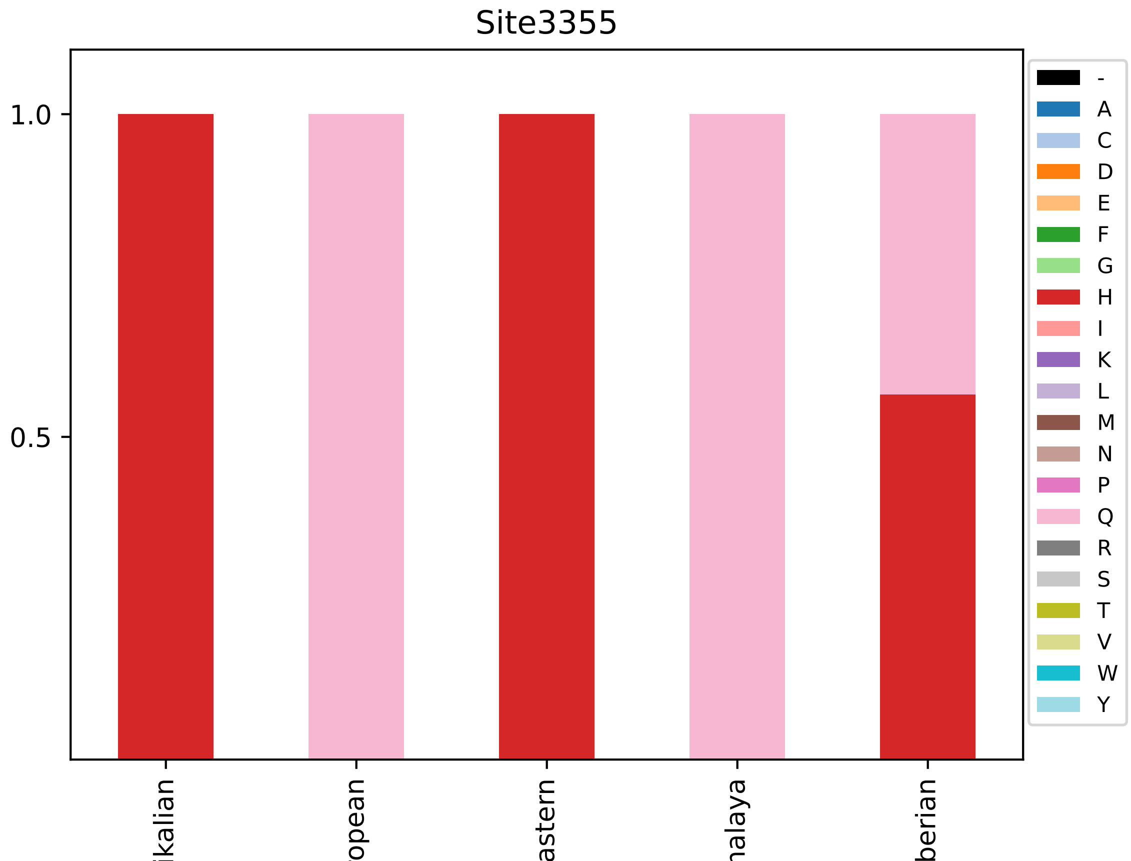

Site3355
1.0
0.5
-
A
C
D
E
F
G
H
I
K
L
M
N
P
Q
R
S
T
V
W
Y
ikalian
opean
astern
nalaya
berian

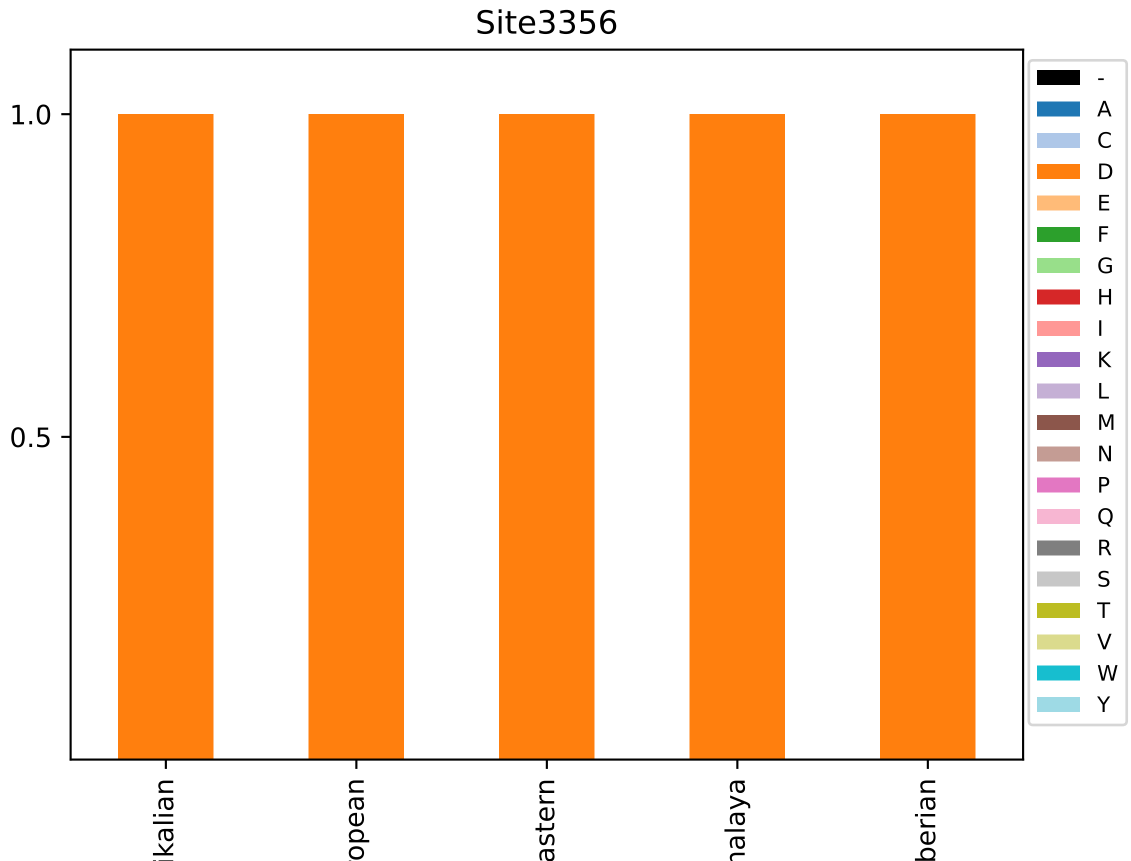
Site3356
1.0
0.5
-
A
C
D
E
F
G
H
I
K
L
M
N
P
Q
R
S
T
V
W
Y
ikalian
opean
astern
nalaya
berian

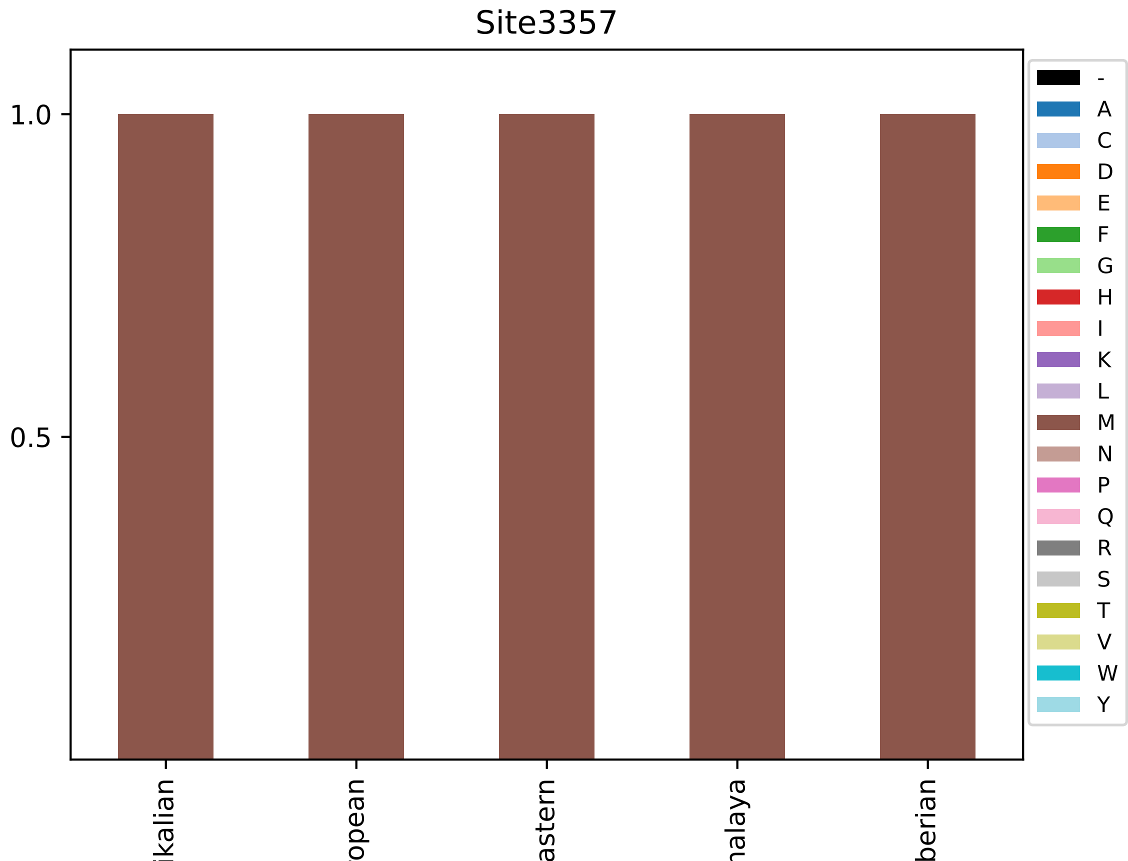

Site3357
1.0
0.5
-
A
C
D
E
F
G
H
I
K
L
M
N
P
Q
R
S
T
V
W
Y
ikalian
opean
astern
nalaya
berian

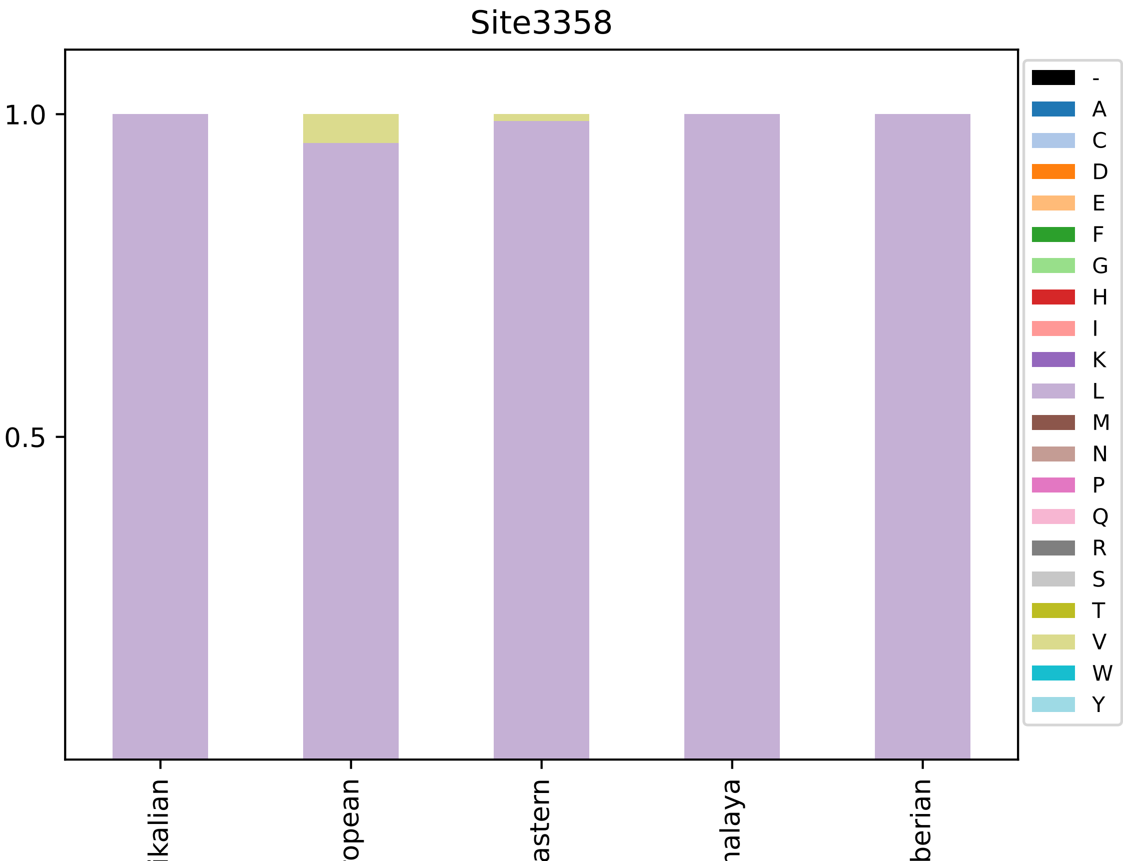

Site3358
1.0
0.5
-
A
C
D
E
F
G
H
I
K
L
M
N
P
Q
R
S
T
V
W
Y
ikalian
opean
astern
nalaya
berian

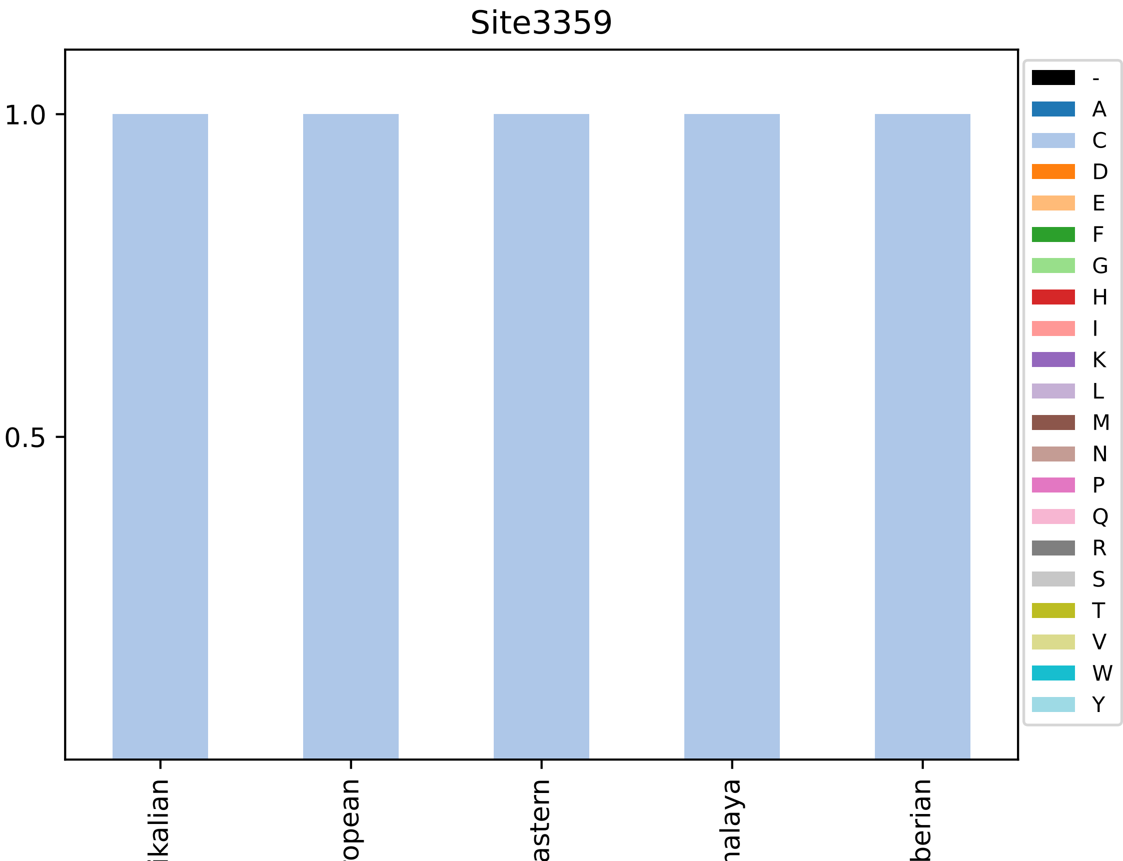

Site3359
1.0
0.5
-
A
C
D
E
F
G
H
I
K
L
M
N
P
Q
R
S
T
V
W
Y
ikalian
opean
astern
halaya
berian

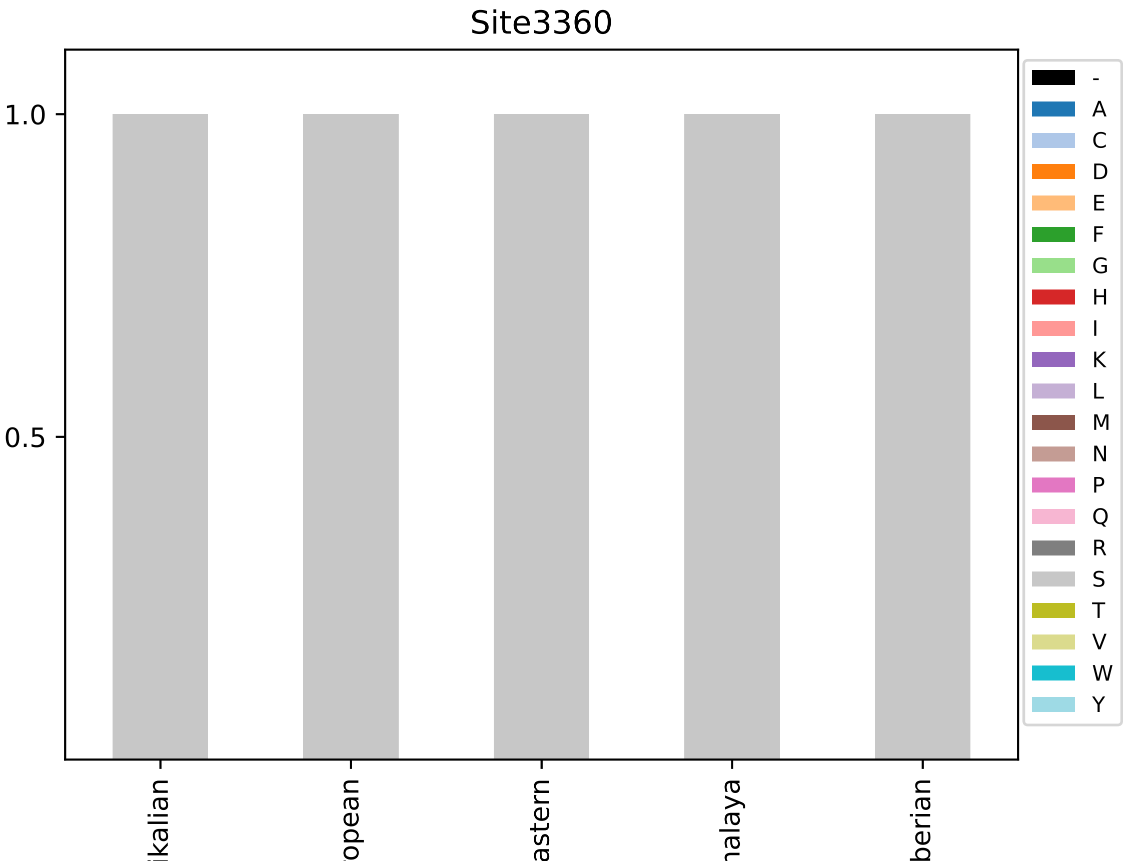
Site3360
1.0
0.5
-
A
C
D
E
F
G
H
I
K
L
M
N
P
Q
R
S
T
V
W
Y
ikalian
opean
astern
nalaya
berian

Site3361

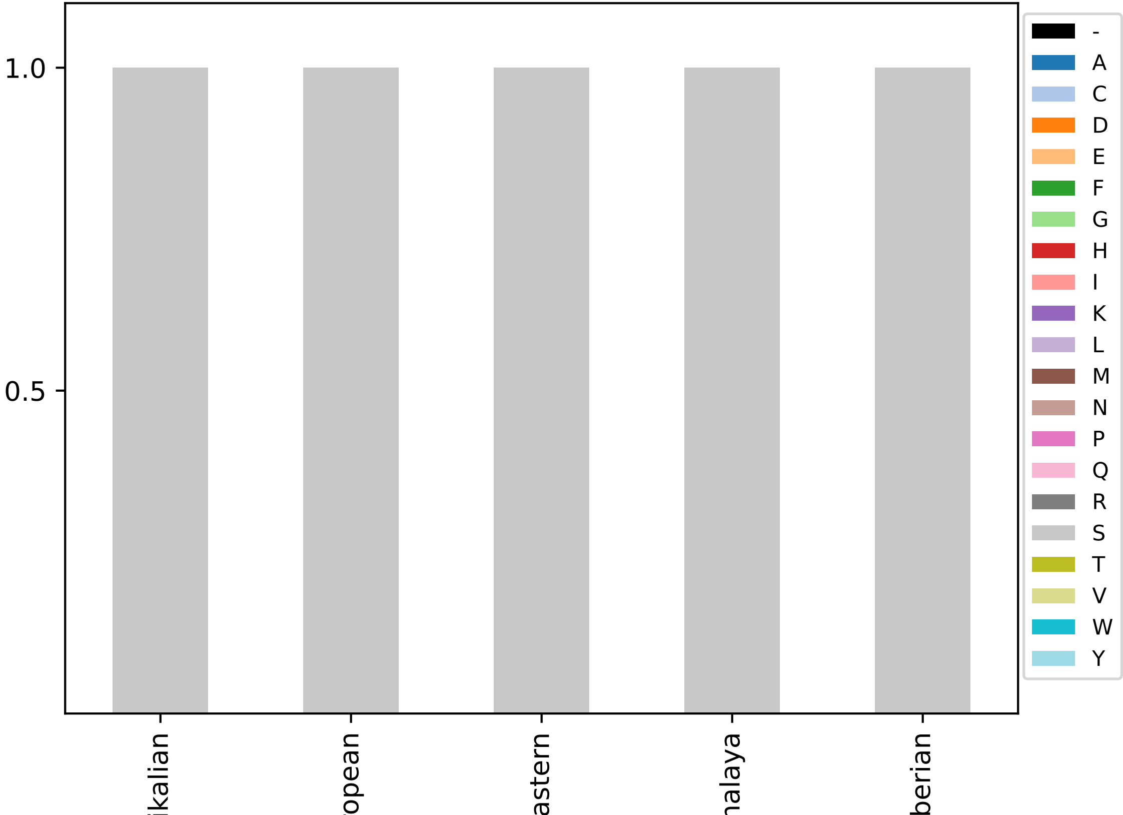

1.0
0.5
-
A
C
D
E
F
G
H
I
K
L
M
N
P
Q
R
S
T
V
W
Y
ikalian
opean
astern
nalaya
berian

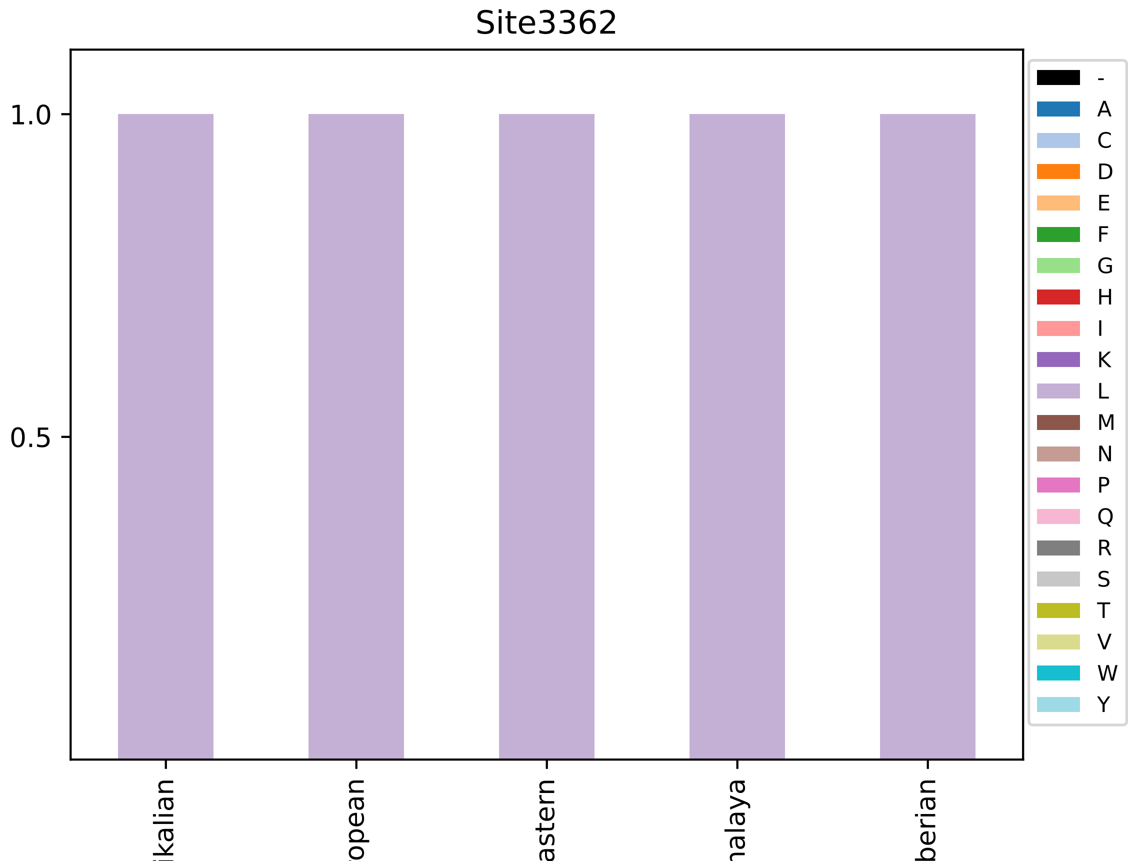

Site3362
1.0
0.5
-
A
C
D
E
F
G
H
I
K
L
M
N
P
Q
R
S
T
V
W
Y
ikalian
ropean
astern
nalaya
berian

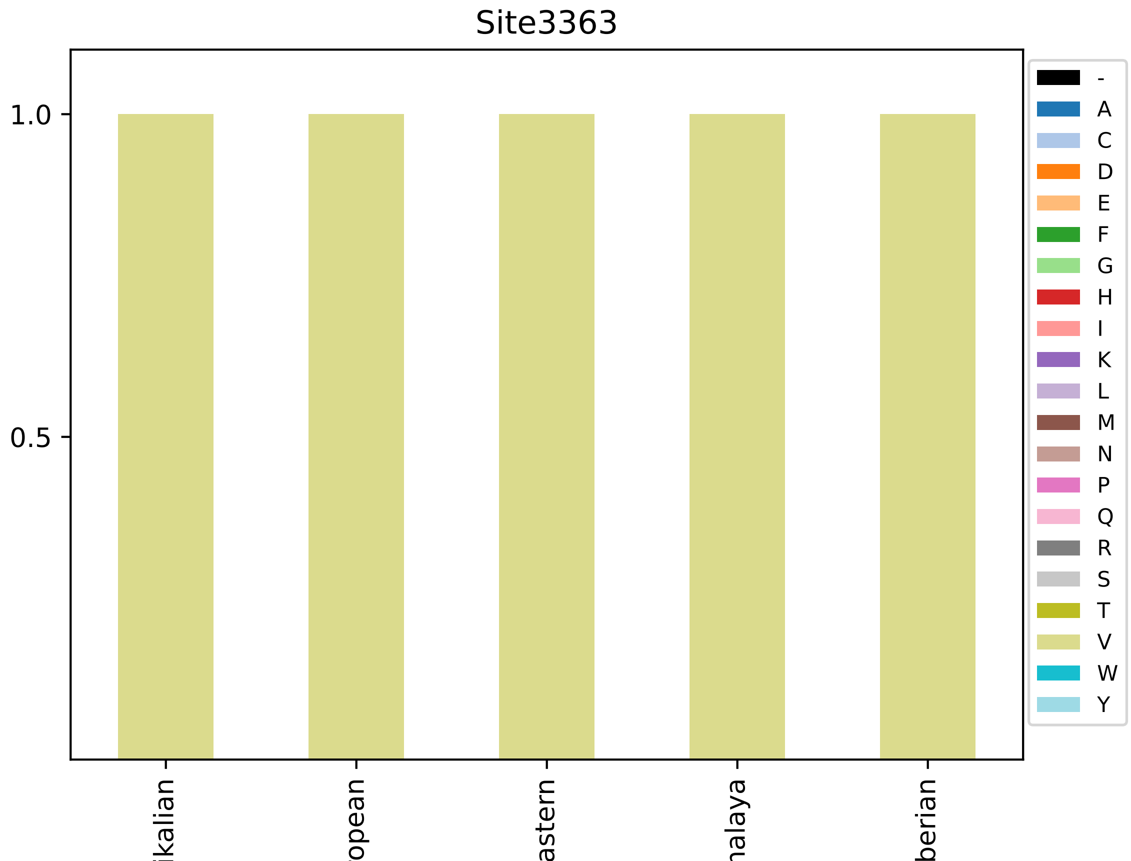

Site3363
1.0
0.5
ikalian
opean
astern
nalaya
berian
-
A
C
D
E
F
G
H
I
K
L
M
N
P
Q
R
S
T
V
W
Y

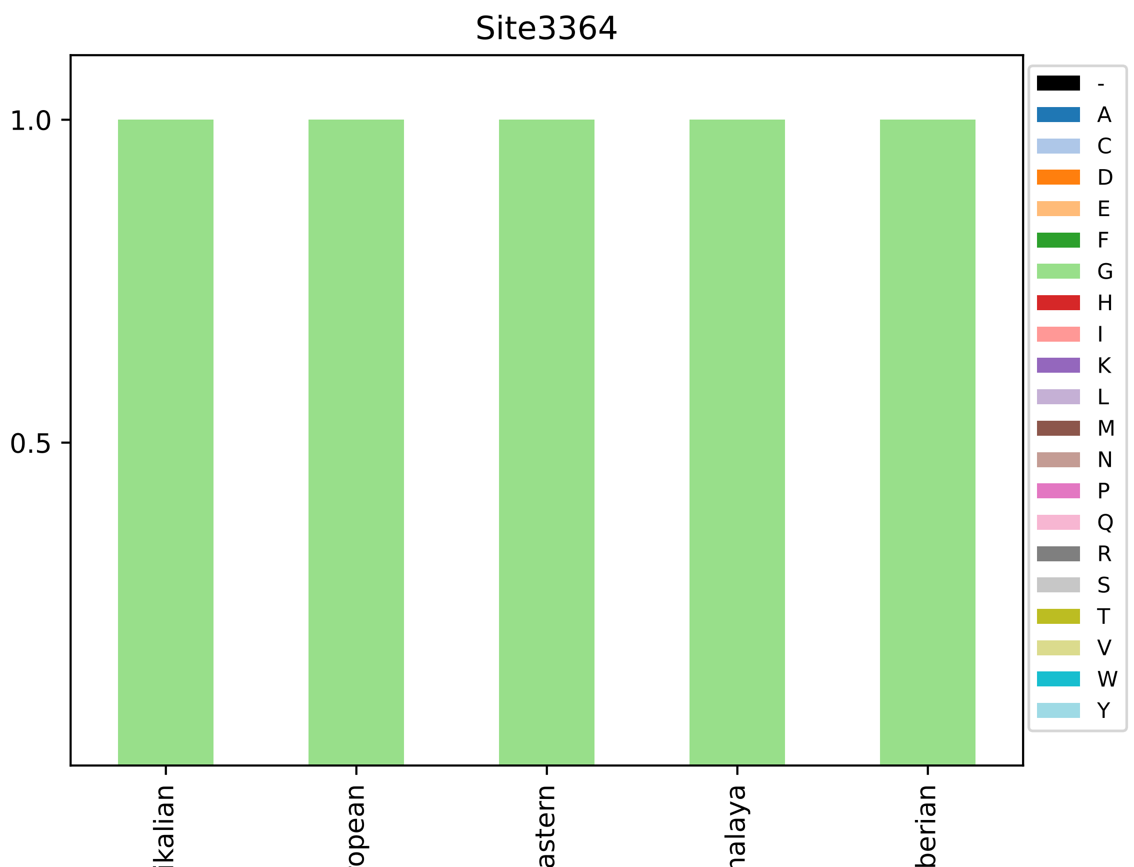

Site3364
1.0
0.5
-
A
C
D
E
F
G
H
I
K
L
M
N
P
Q
R
S
T
V
W
Y
ikalian
opean
astern
nalaya
berian

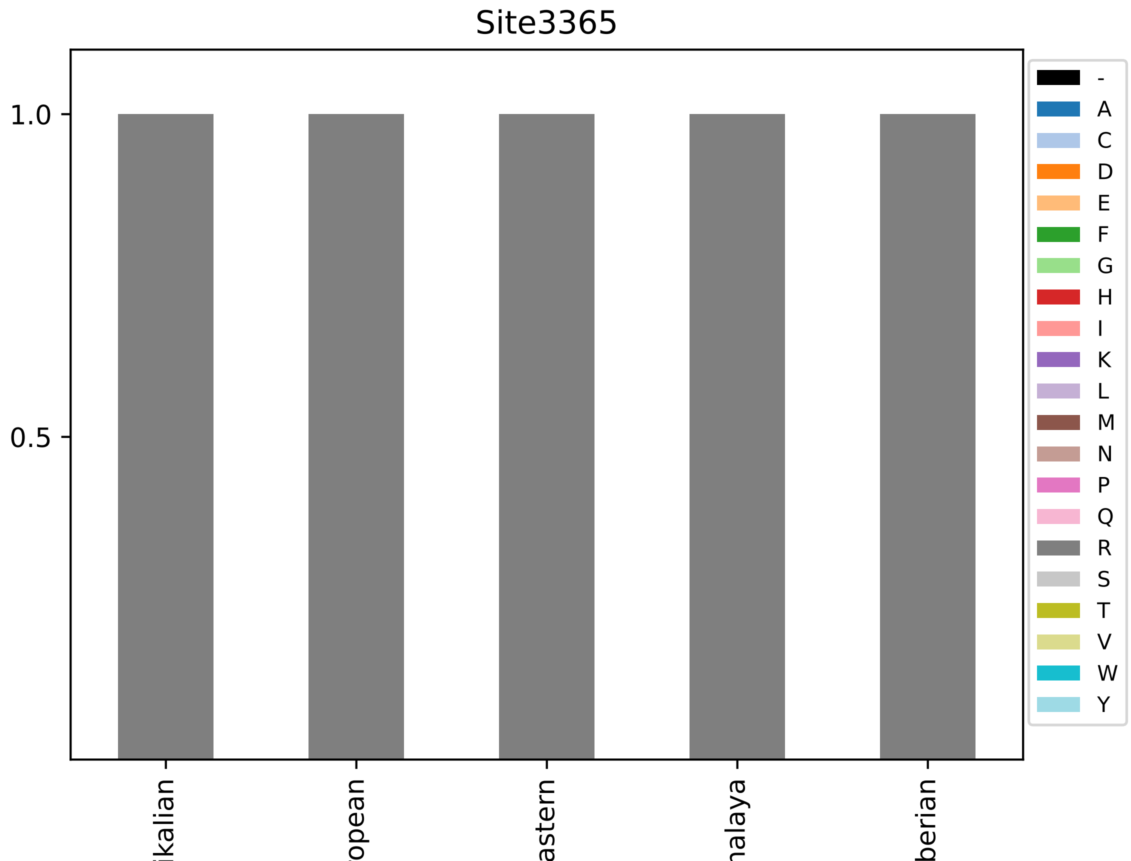
Site3365
1.0
0.5
-
A
C
D
E
F
G
H
I
K
L
M
N
P
Q
R
S
T
V
W
Y
ikalian
opean
astern
nalaya
berian

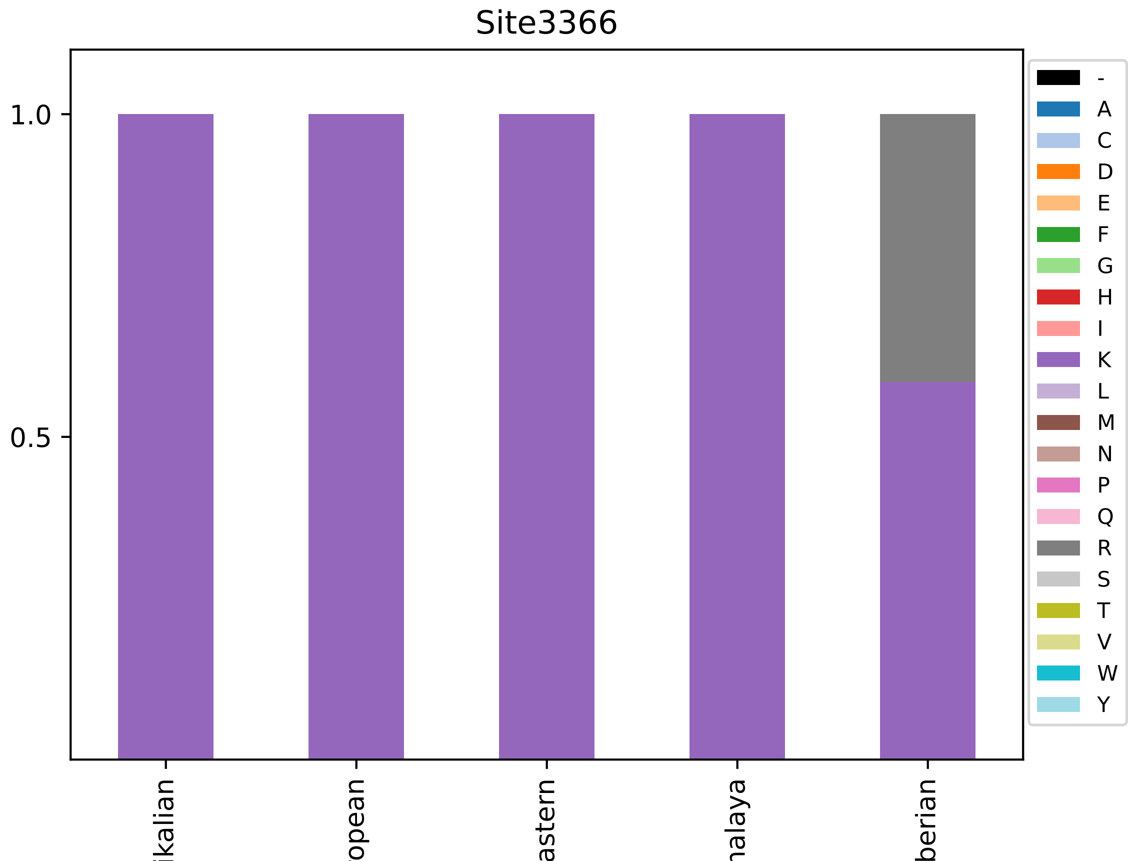

Site3366
1.0
0.5
ikalian
opean
astern
nalaya
berian
-
A
C
D
E
F
G
H
I
K
L
M
N
P
Q
R
S
T
V
W
Y

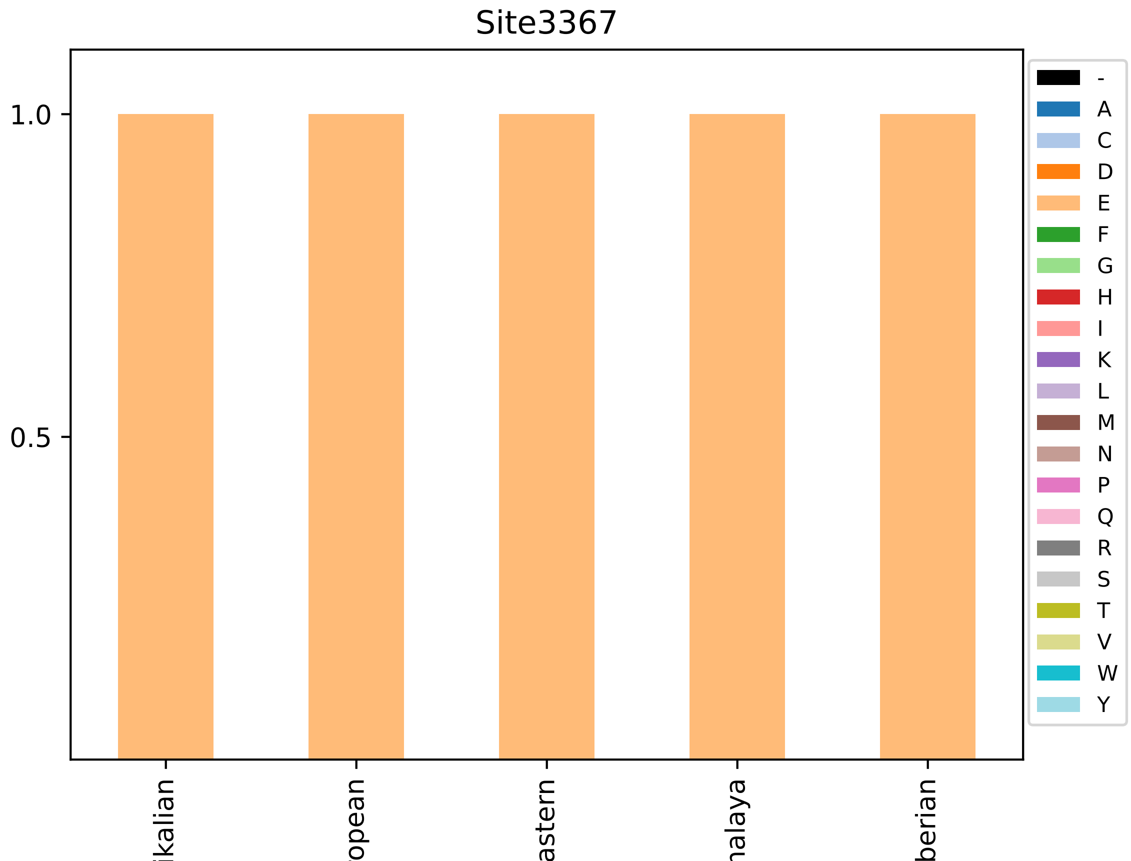

Site3367
1.0
0.5
ikalian
opean
astern
nalaya
berian
-
A
C
D
E
F
G
H
I
K
L
M
N
P
Q
R
S
T
V
W
Y

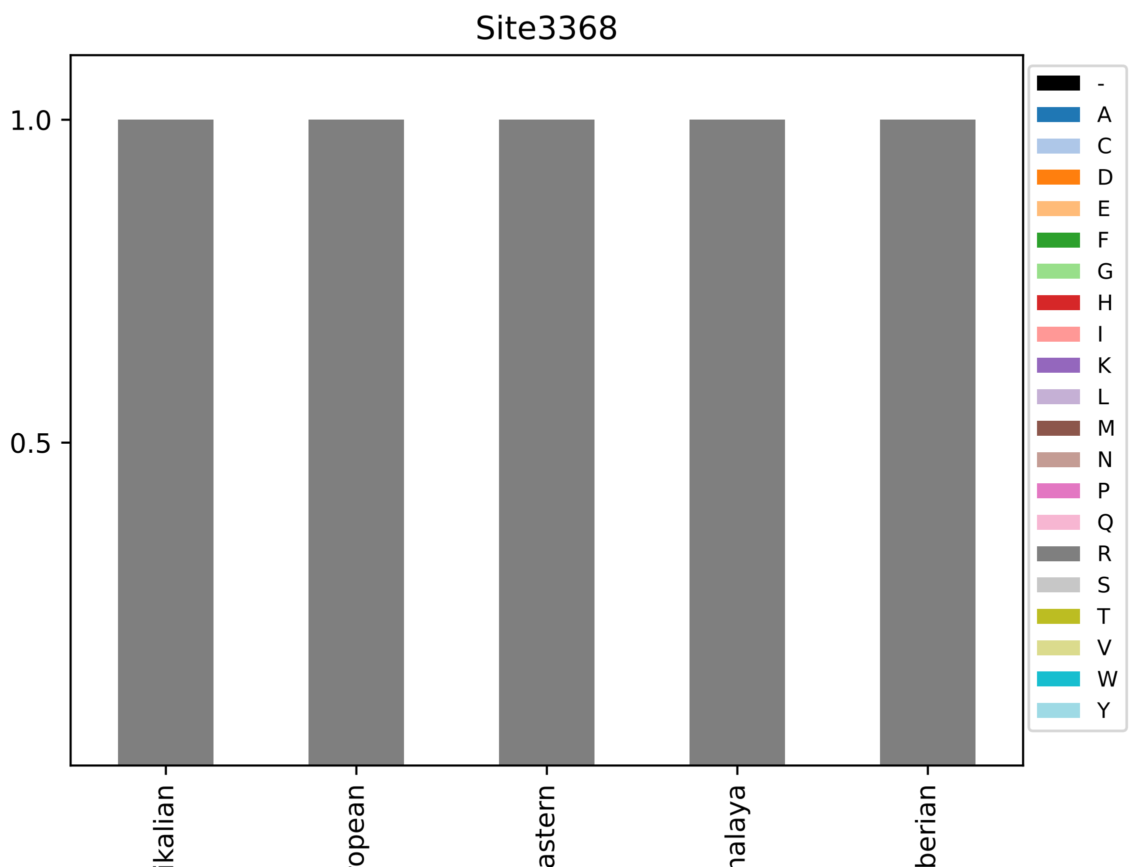

Site3368
1.0
0.5
-
A
C
D
E
F
G
H
I
K
L
M
N
P
Q
R
S
T
V
W
Y
ikalian
opean
astern
nalaya
berian

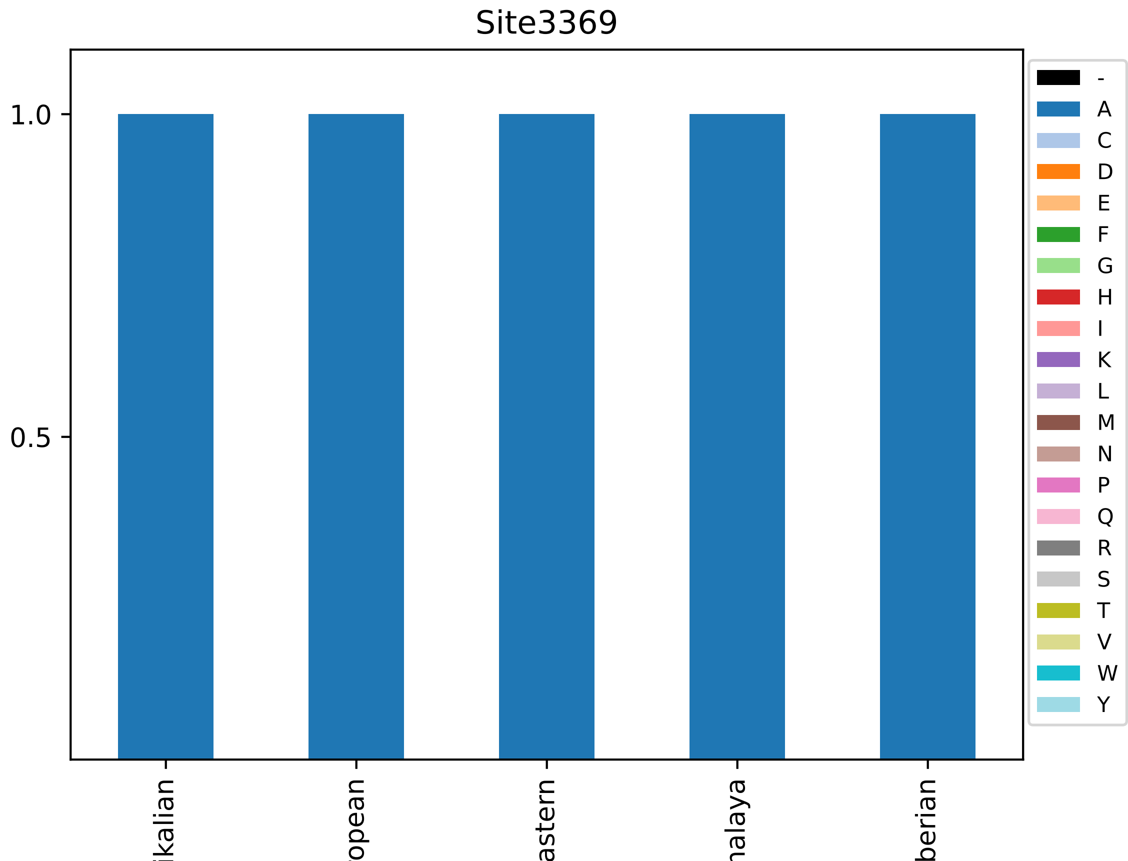
Site3369
1.0
0.5
ikalian
opean
astern
nalaya
berian
-
A
C
D
E
F
G
H
I
K
L
M
N
P
Q
R
S
T
V
W
Y

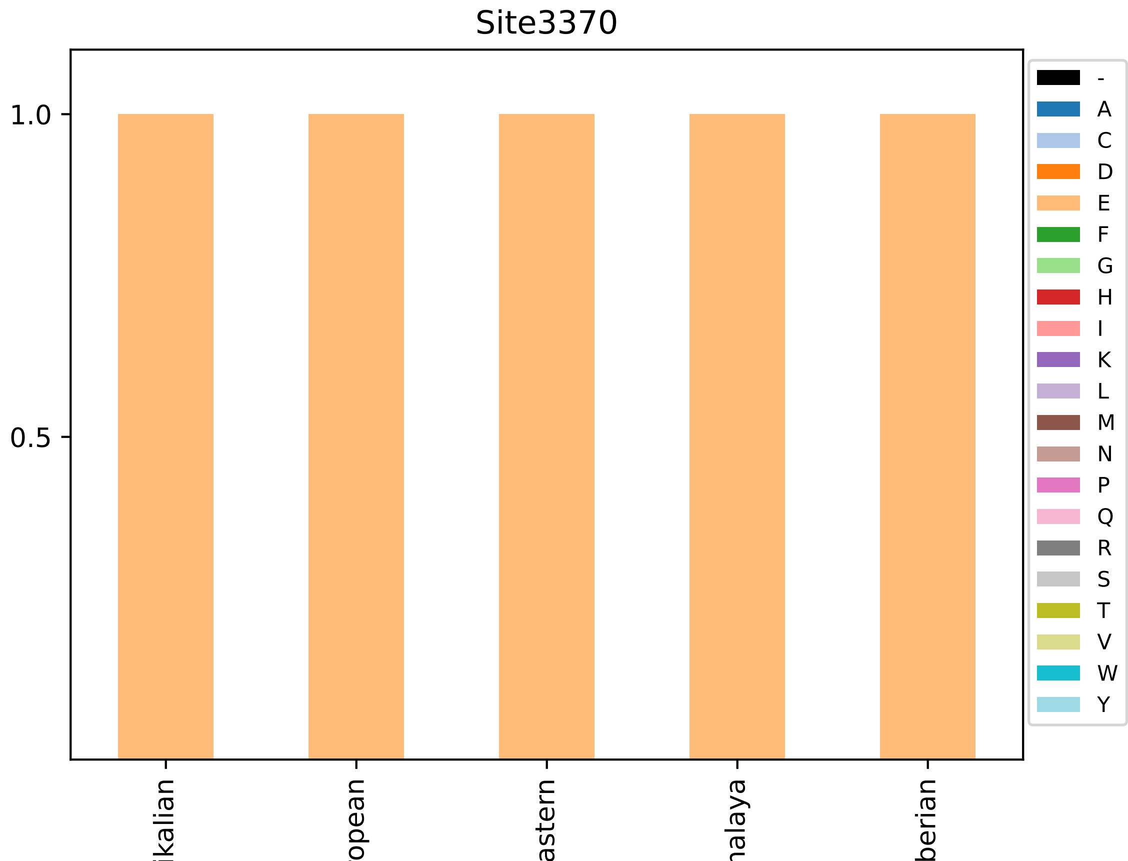
Site3370
1.0
0.5
-
A
C
D
E
F
G
H
I
K
L
M
N
P
Q
R
S
T
V
W
Y
ikalian
opean
astern
alaya
berian

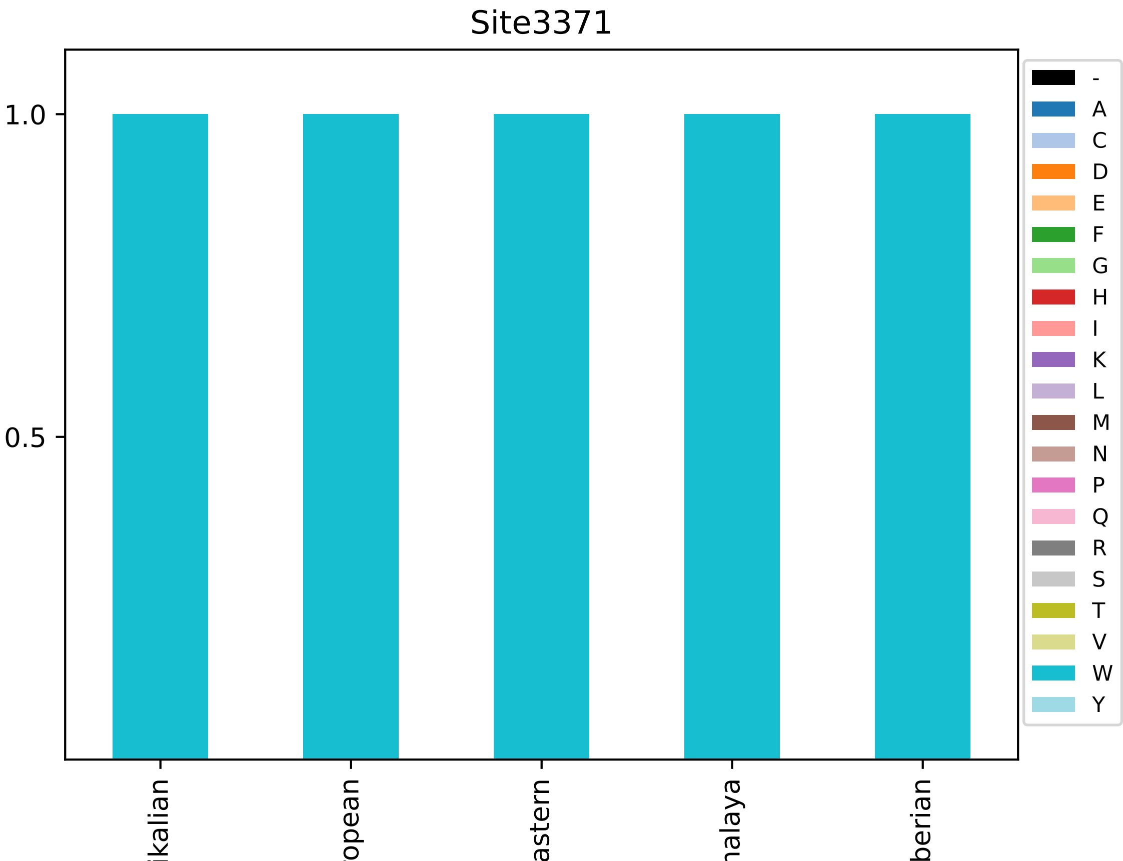
Site3371
1.0
0.5
-
A
C
D
E
F
G
H
I
K
L
M
N
P
Q
R
S
T
V
W
Y
ikalian
opean
astern
nalaya
berian

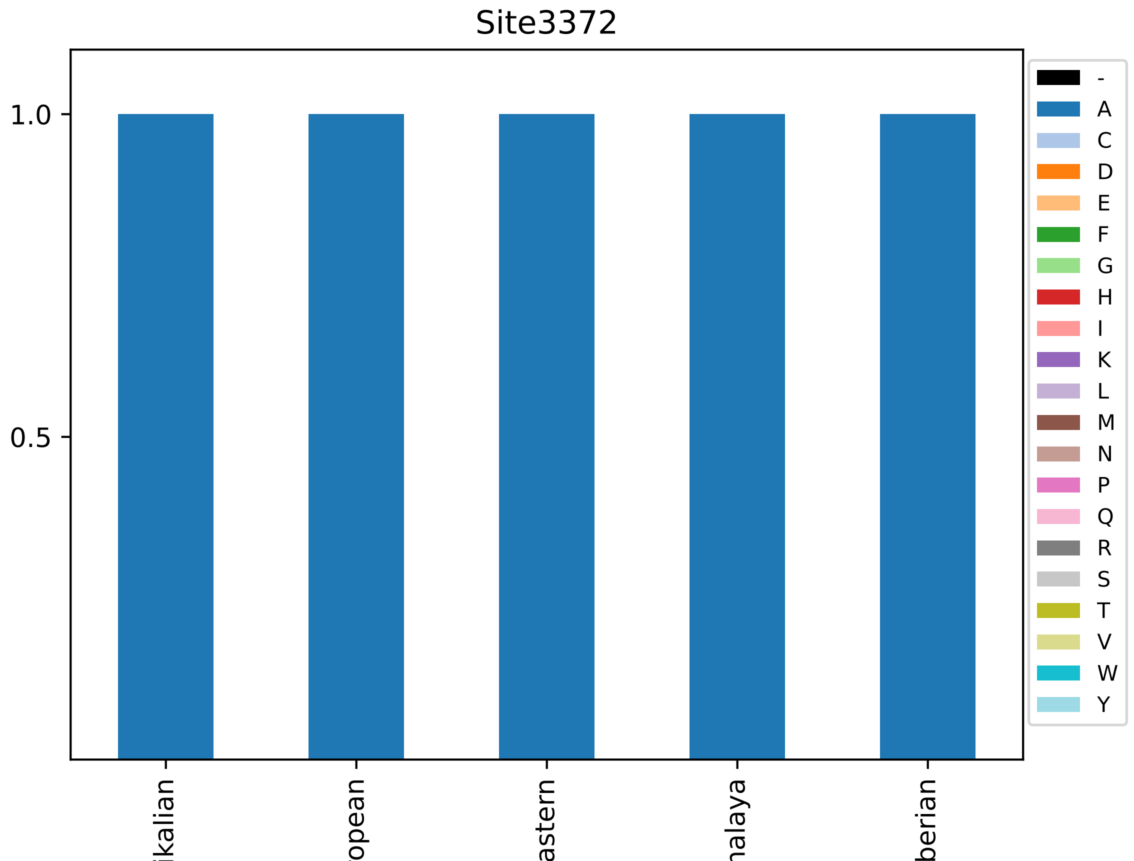
Site3372
1.0
0.5
-
A
C
D
E
F
G
H
I
K
L
M
N
P
Q
R
S
T
V
W
Y
ikalian
opean
astern
nalaya
berian

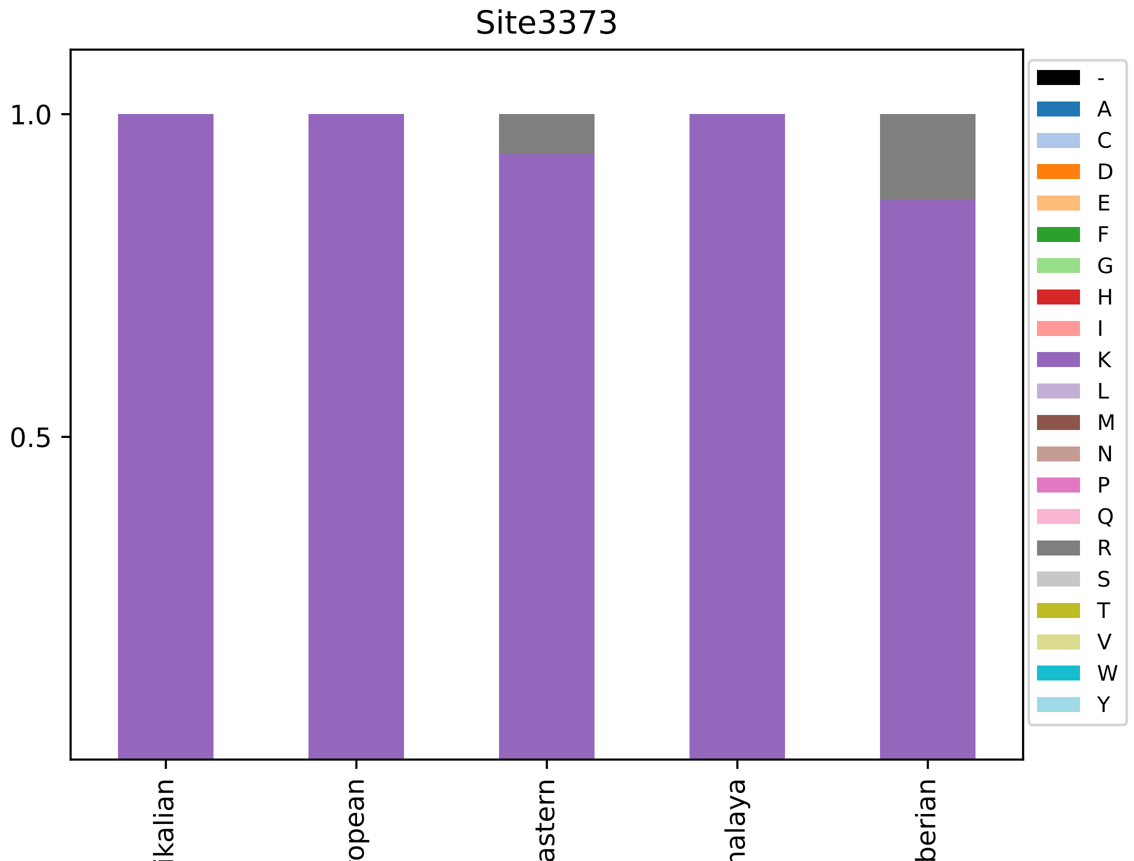

Site3373
1.0
0.5
-
A
C
D
E
F
G
H
I
K
L
M
N
P
Q
R
S
T
V
W
Y
ikalian
opean
astern
nalaya
berian

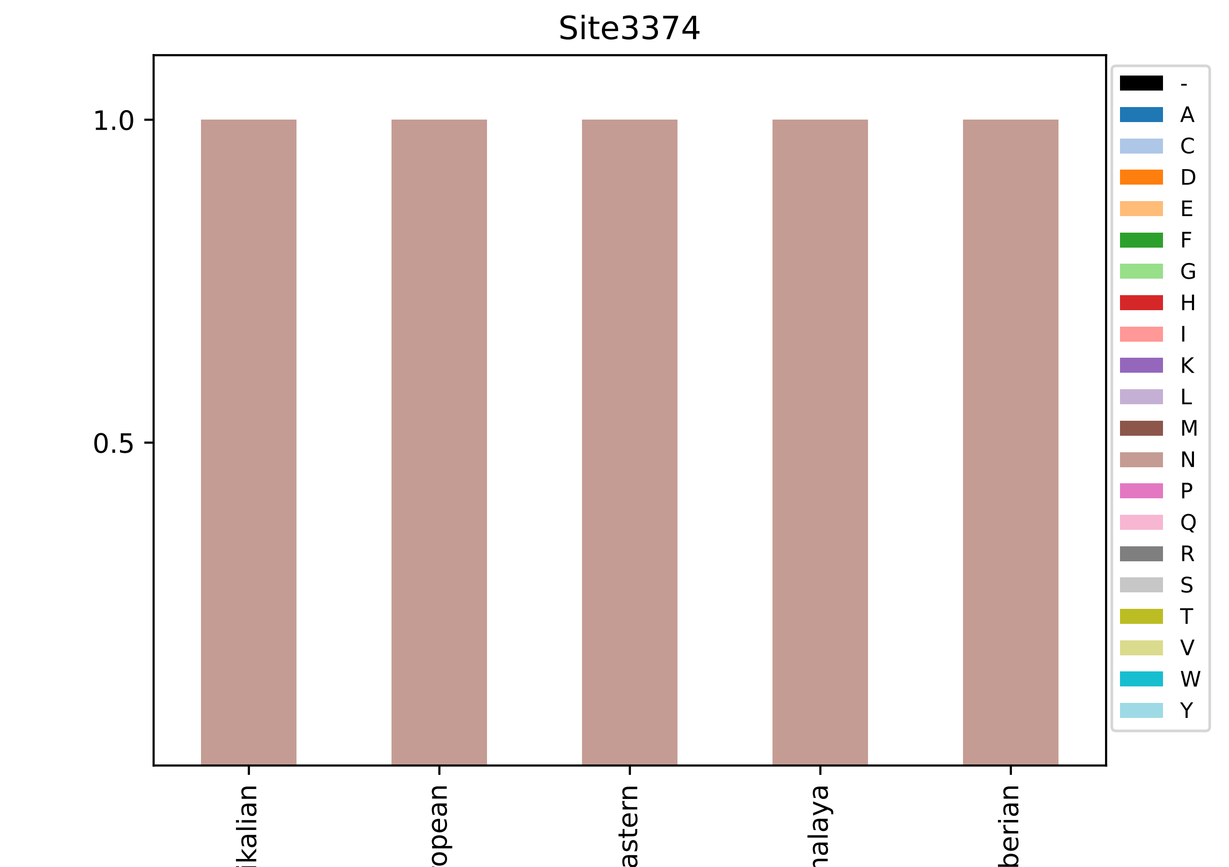

Site3374
1.0
0.5
-
A
C
D
E
F
G
H
I
K
L
M
N
P
Q
R
S
T
V
W
Y
ikalian
opean
astern
halaya
berian

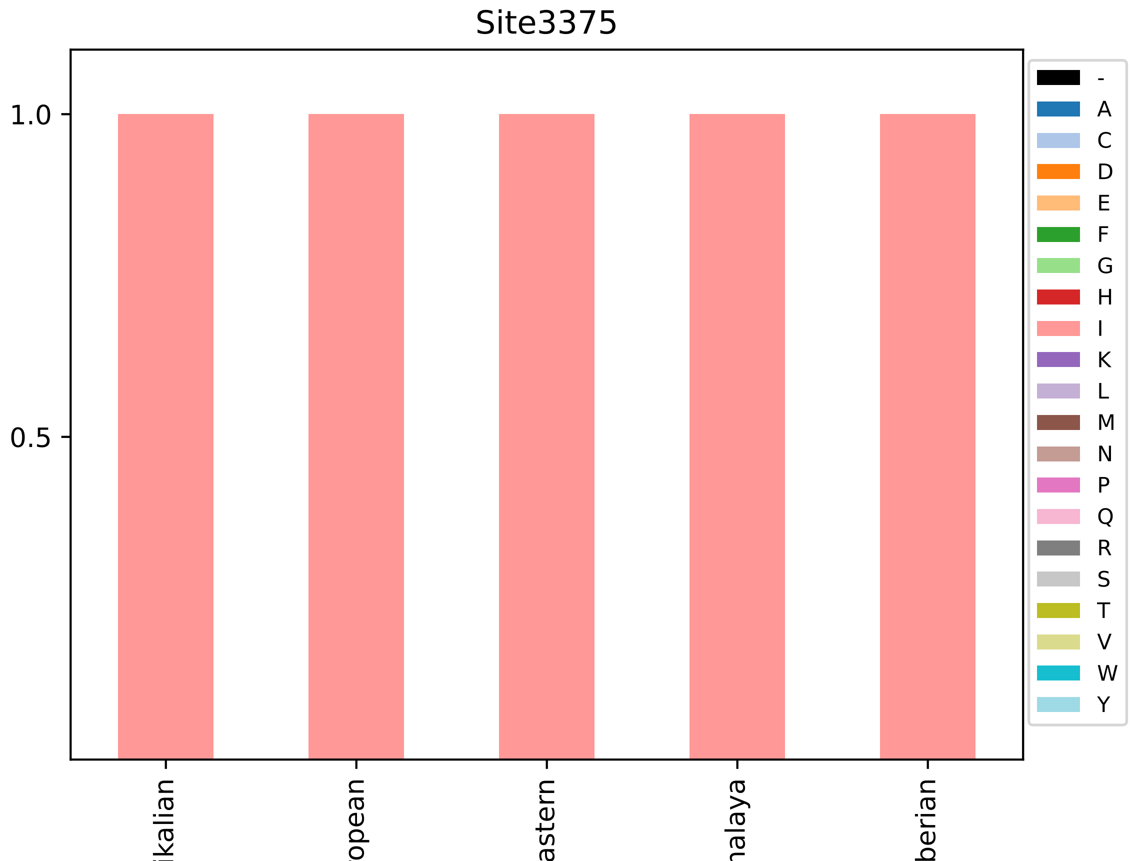
Site3375
1.0
0.5
-
A
C
D
E
F
G
H
I
K
L
M
N
P
Q
R
S
T
V
W
Y
ikalian
opean
astern
nalaya
berian

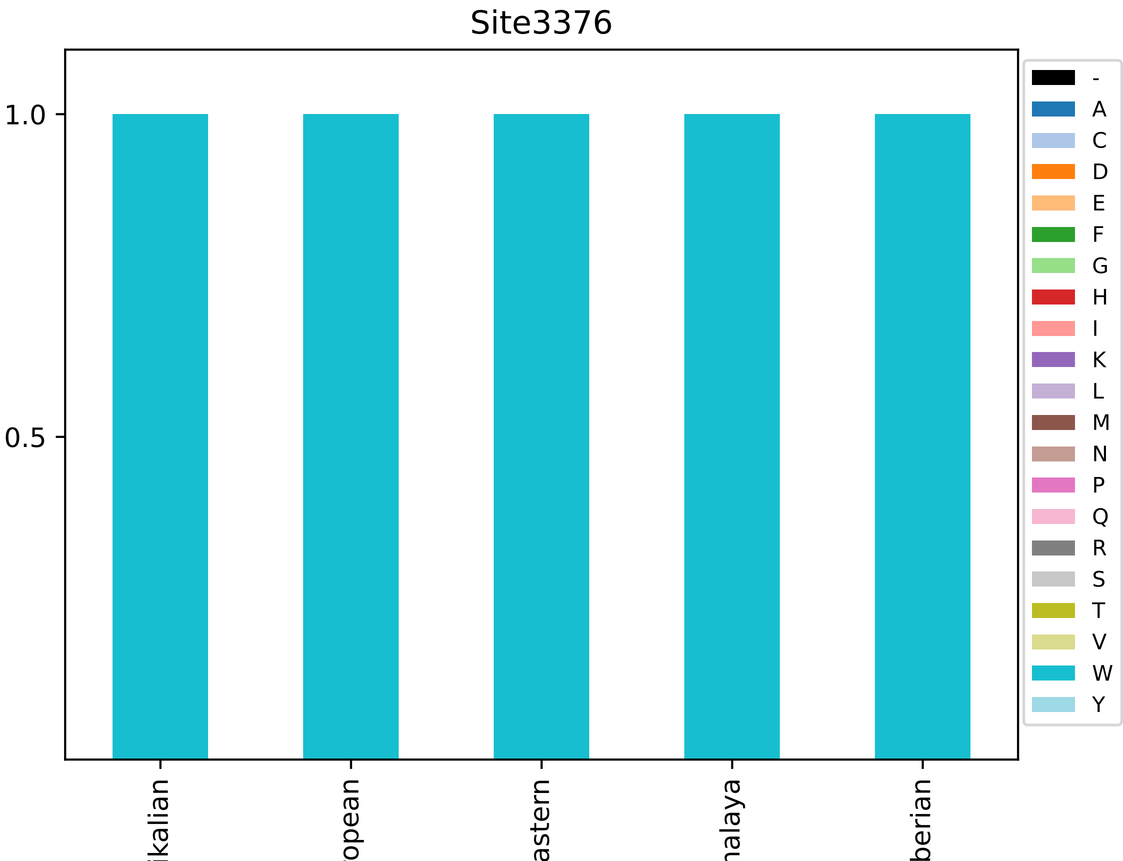

Site3376
1.0
0.5
-
A
C
D
E
F
G
H
I
K
L
M
N
P
Q
R
S
T
V
W
Y
ikalian
opean
astern
nalaya
berian

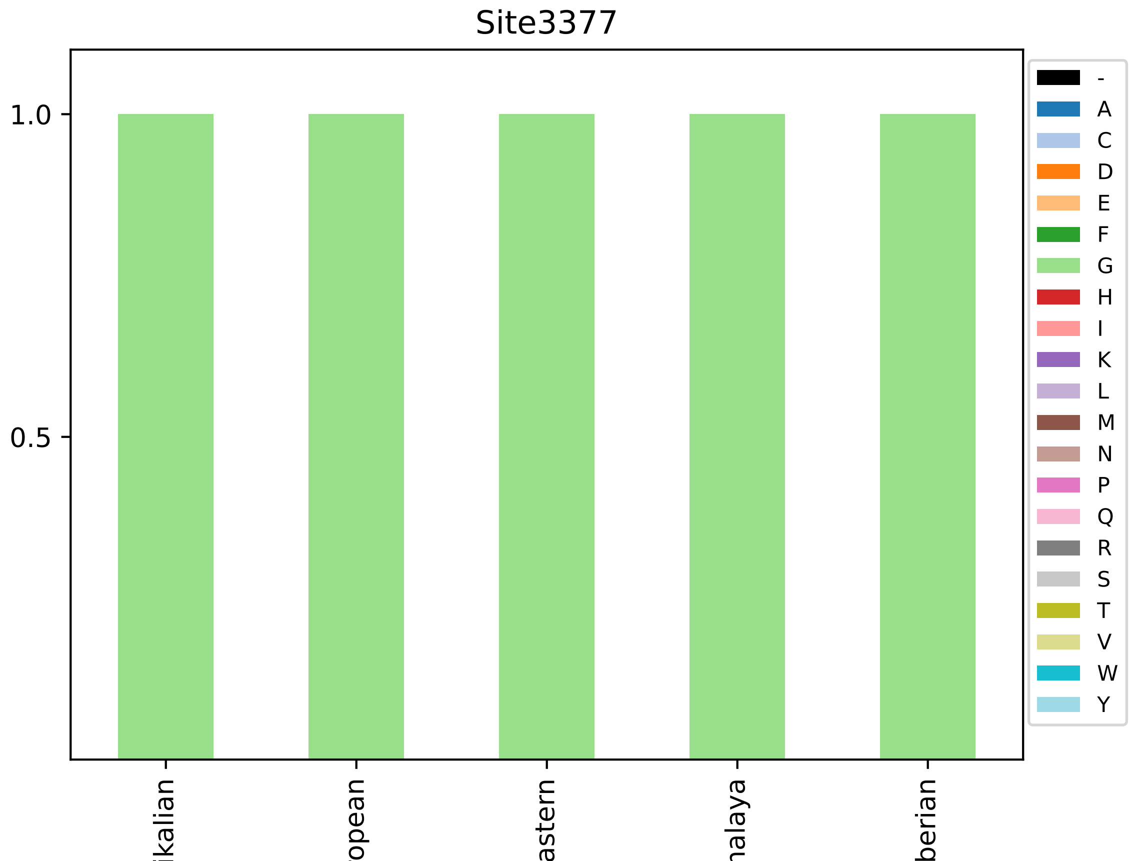

Site3377
1.0
0.5
-
A
C
D
E
F
G
H
I
K
L
M
N
P
Q
R
S
T
V
W
Y

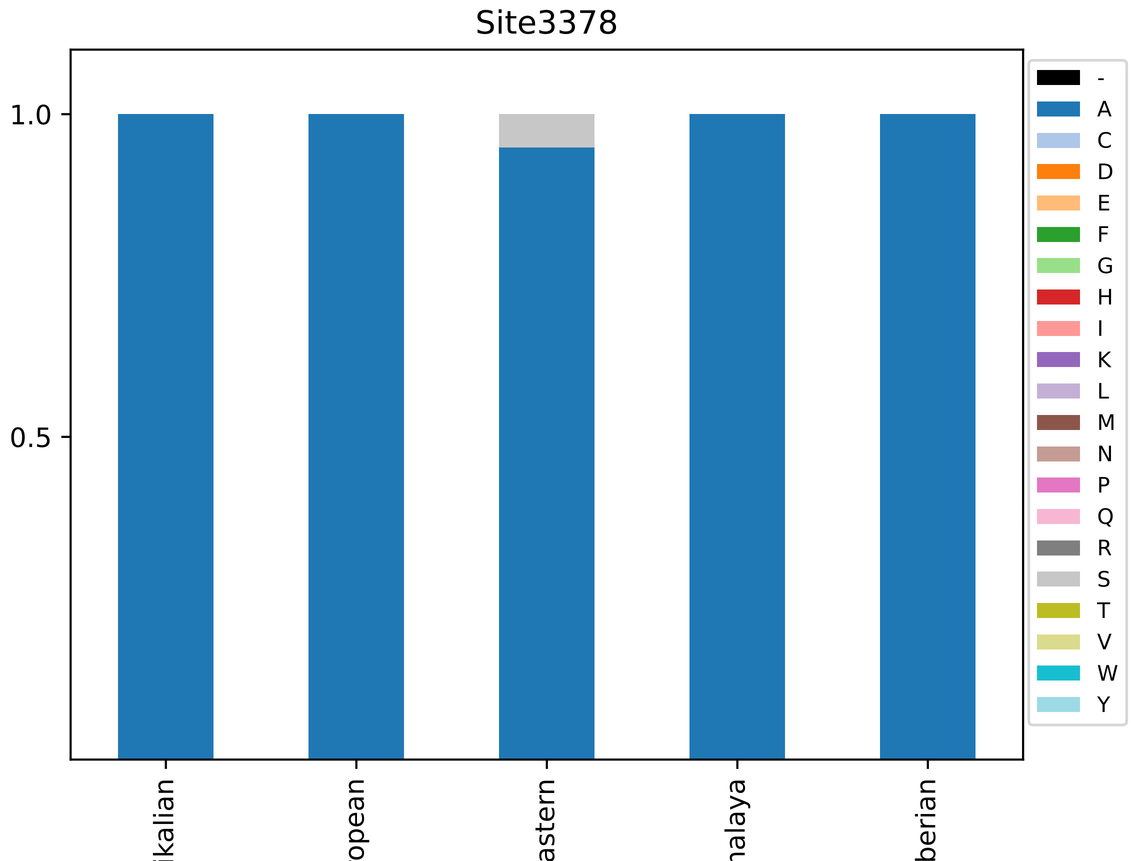
Site3378
1.0
0.5
-
A
C
D
E
F
G
H
I
K
L
M
N
P
Q
R
S
T
V
W
Y
ikalian
opean
astern
nalaya
berian

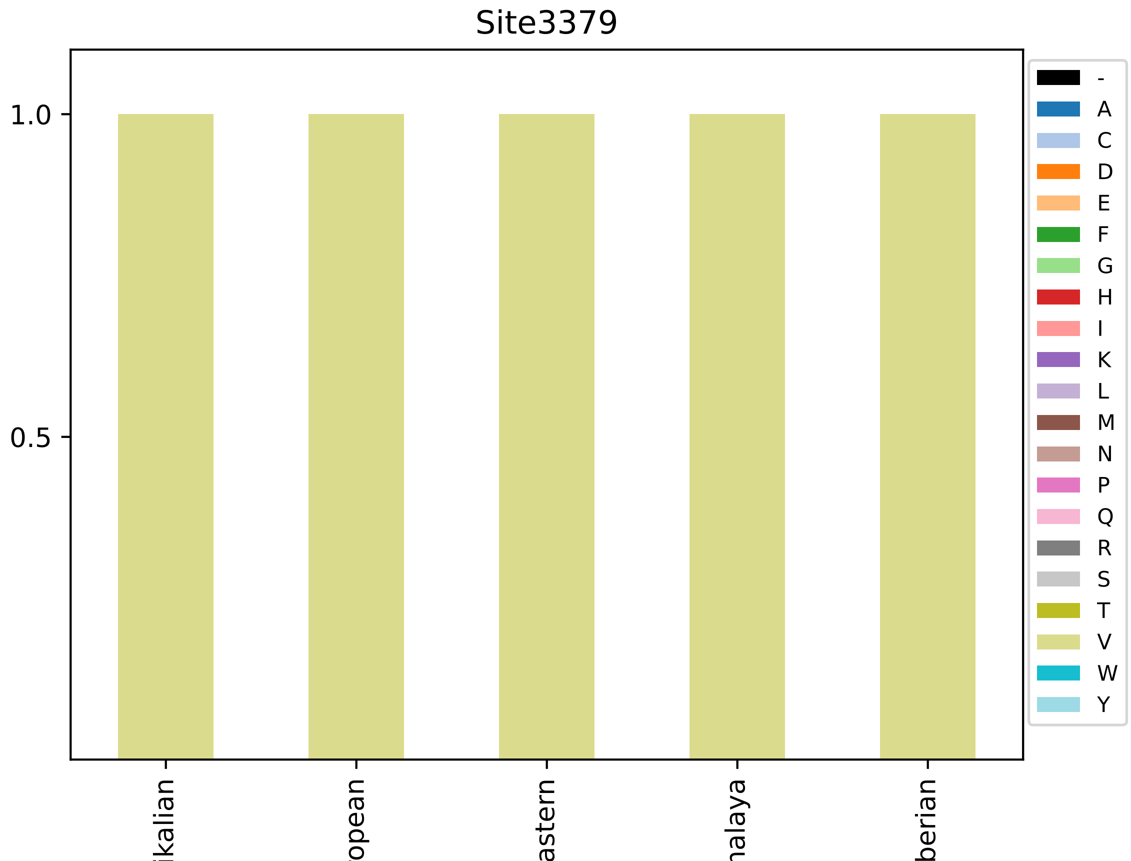
Site3379
1.0
0.5
ikalian
opean
astern
nalaya
berian
-
A
C
D
E
F
G
H
I
K
L
M
N
P
Q
R
S
T
V
W
Y

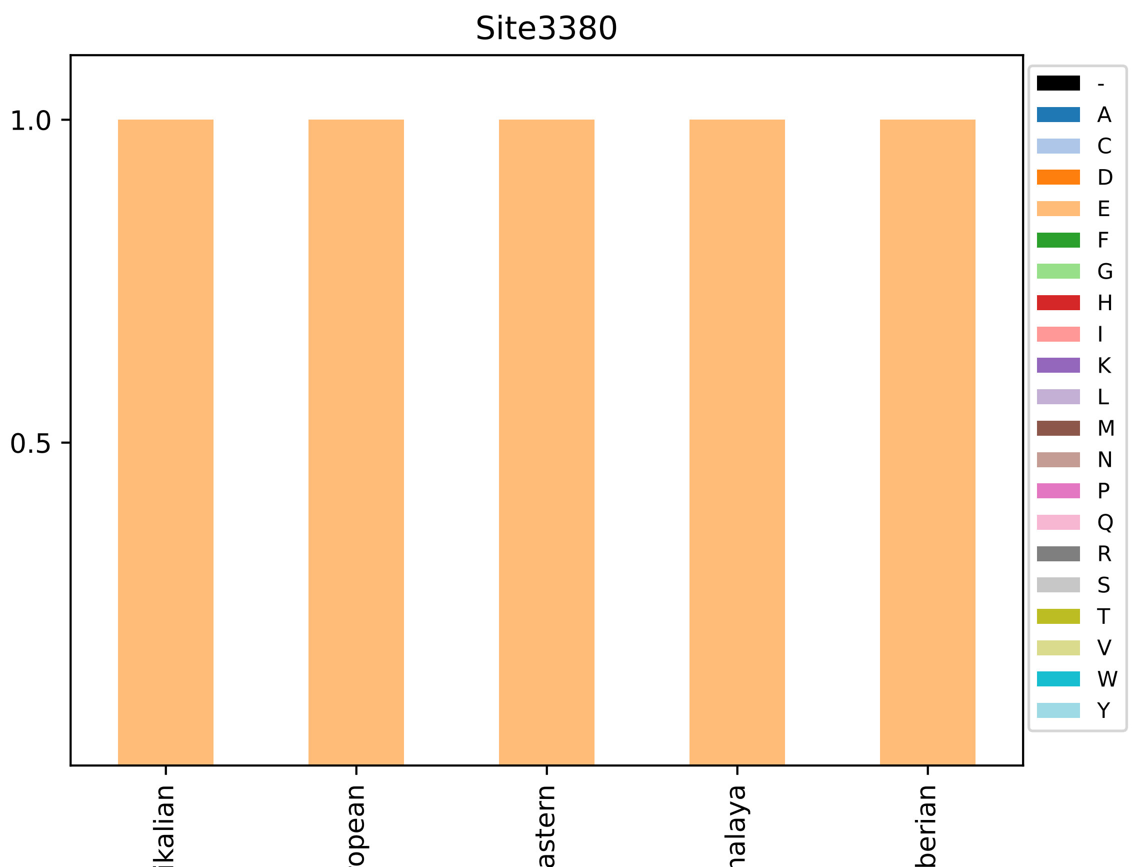

Site3380
1.0
0.5
-
A
C
D
E
F
G
H
I
K
L
M
N
P
Q
R
S
T
V
W
Y
ikalian
opean
astern
nalaya
berian

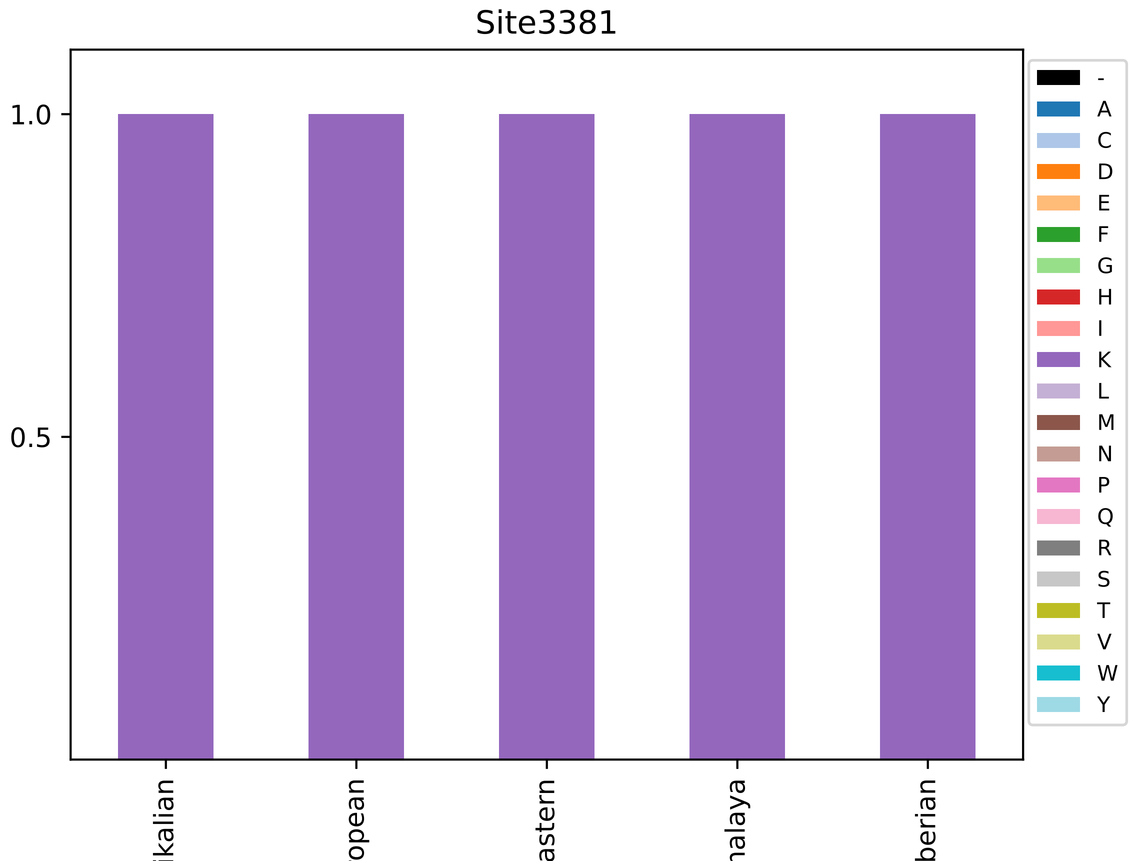
Site3381
1.0
0.5
ikalian
opean
astern
nalaya
berian
-
A
C
D
E
F
G
H
I
K
L
M
N
P
Q
R
S
T
V
W
Y

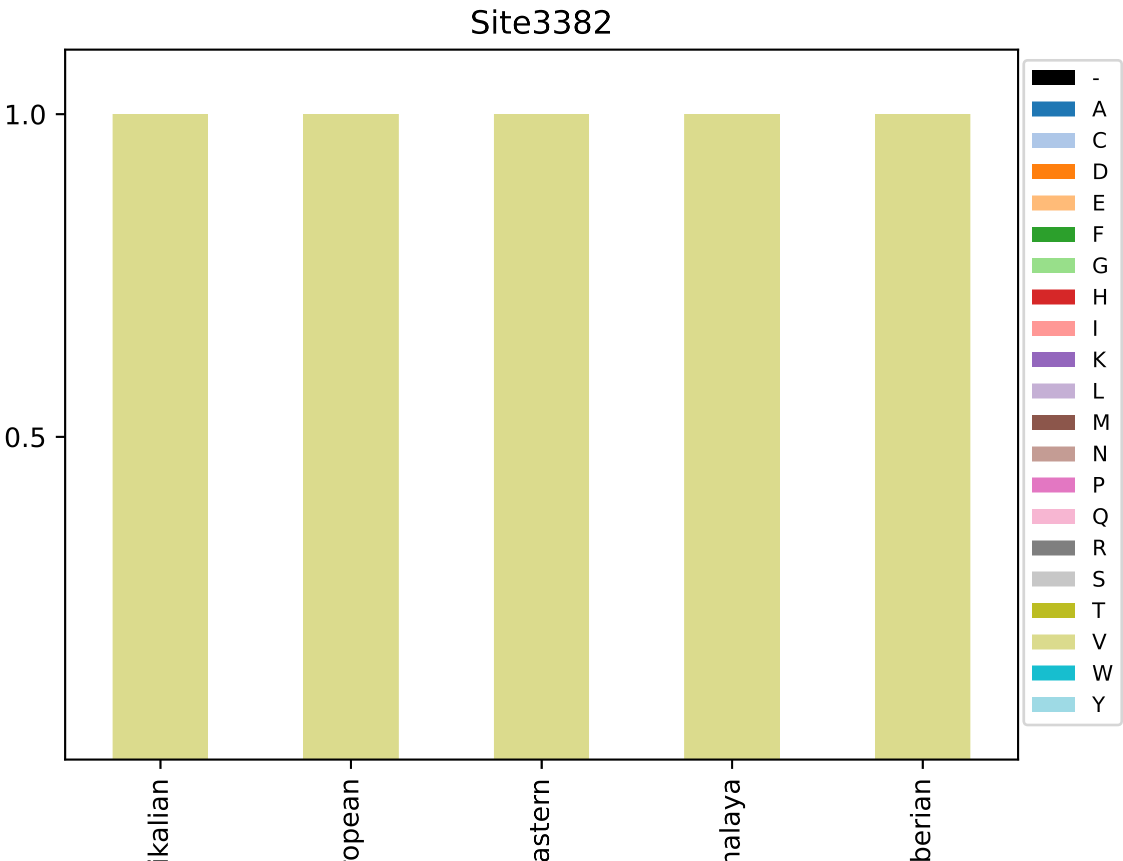

Site3382
1.0
0.5
-
A
C
D
E
F
G
H
I
K
L
M
N
P
Q
R
S
T
V
W
Y
ikalian
opean
astern
nalaya
berian

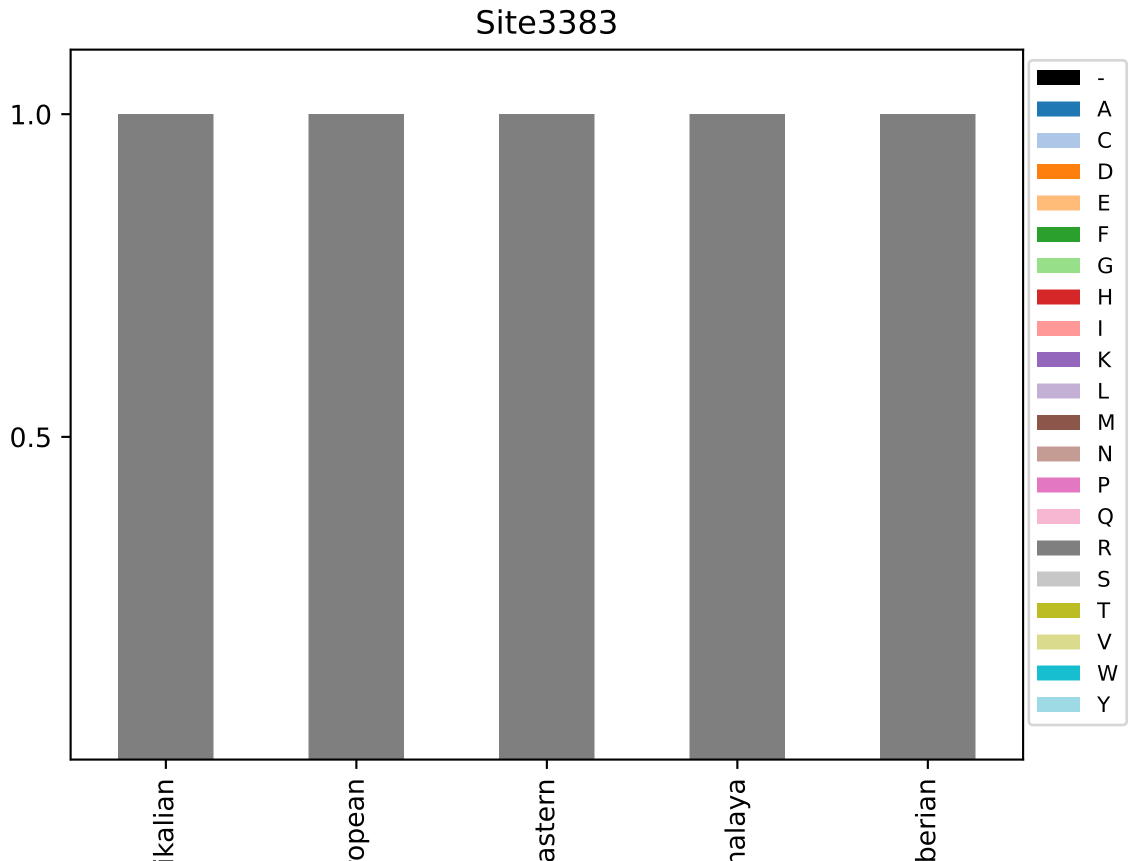
Site3383
1.0
0.5
-
A
C
D
E
F
G
H
I
K
L
M
N
P
Q
R
S
T
V
W
Y
ikalian
opean
astern
nalaya
berian

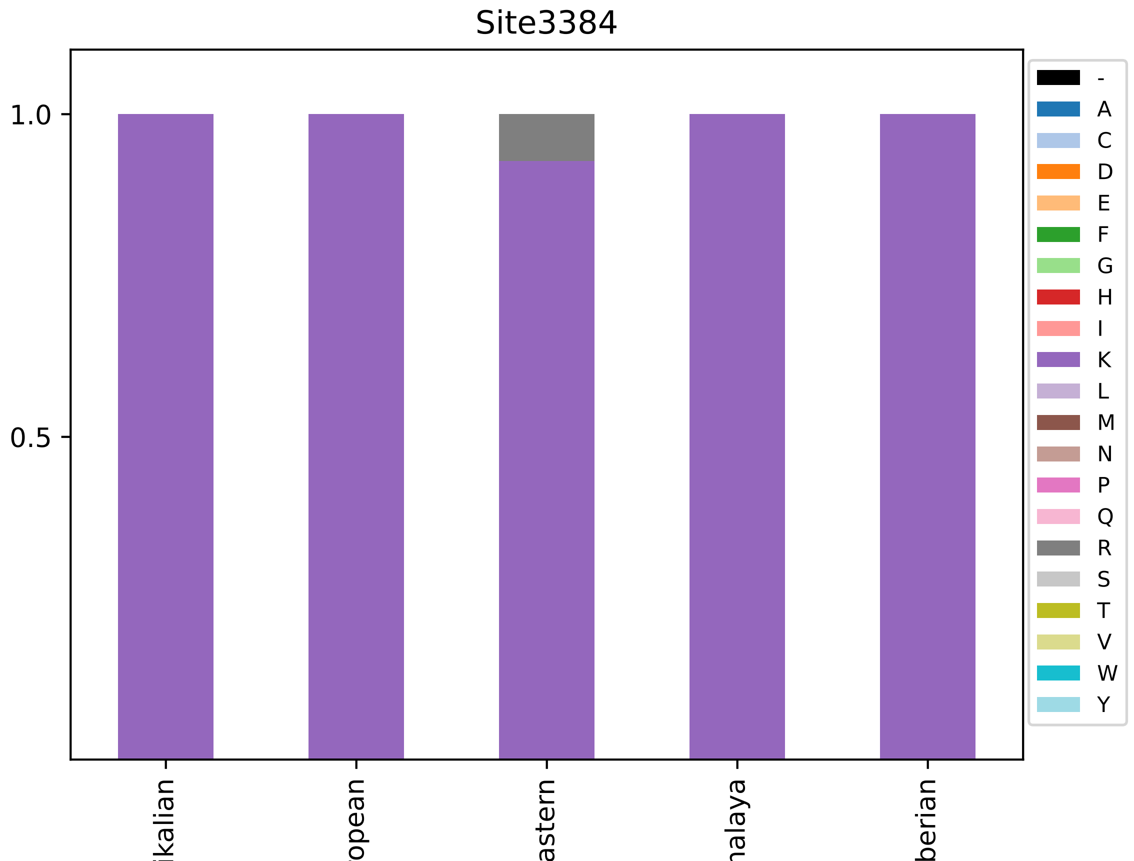

Site3384
1.0
0.5
ikalian
opean
astern
nalaya
berian
-
A
C
D
E
F
G
H
I
K
L
M
N
P
Q
R
S
T
V
W
Y

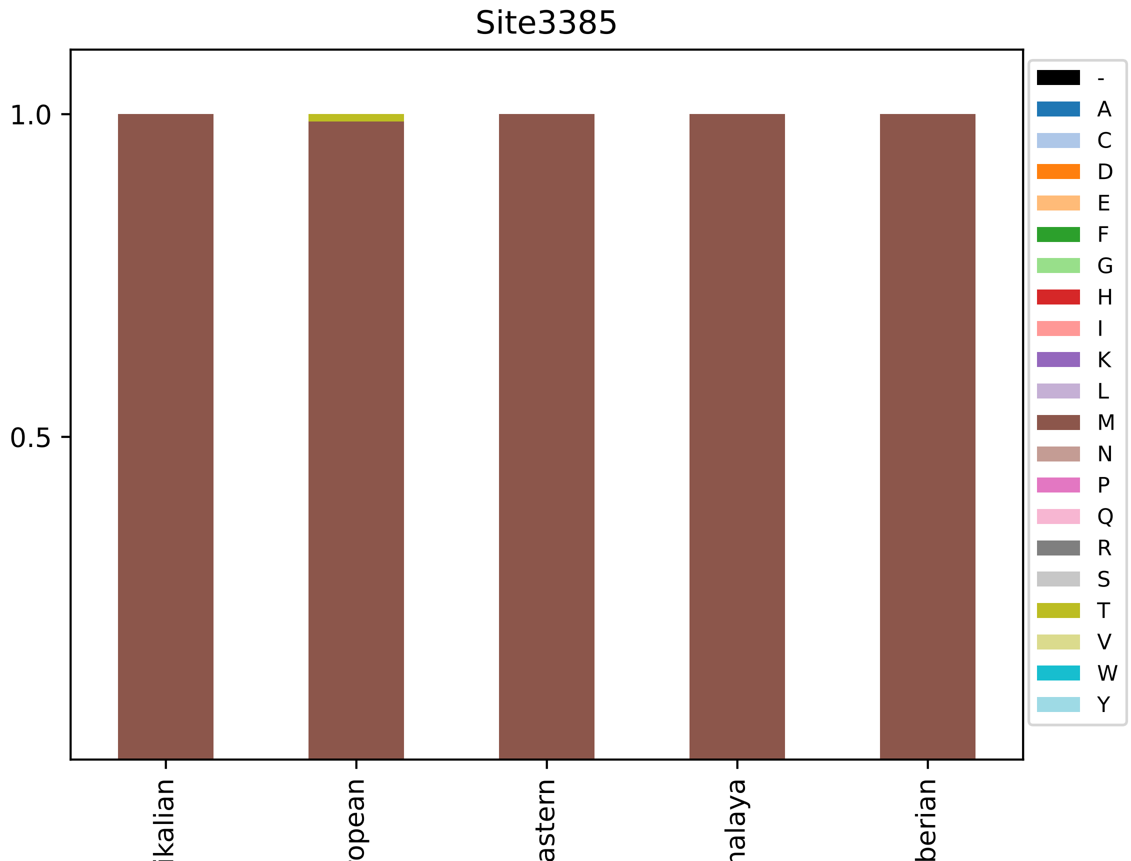
Site3385
1.0
0.5
-
A
C
D
E
F
G
H
I
K
L
M
N
P
Q
R
S
T
V
W
Y
ikalian
opean
astern
nalaya
berian

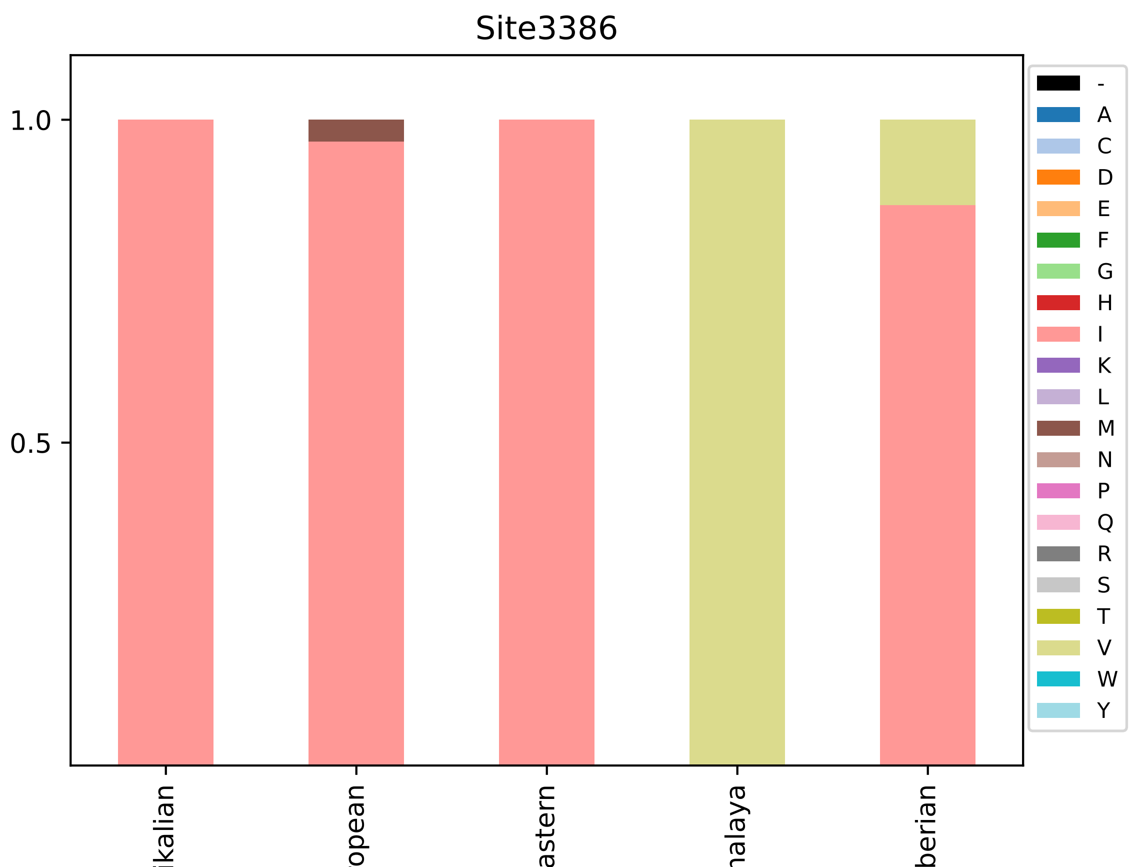

Site3386
1.0
0.5
ikalian
opean
astern
nalaya
berian
-
A
C
D
E
F
G
H
I
K
L
M
N
P
Q
R
S
T
V
W
Y

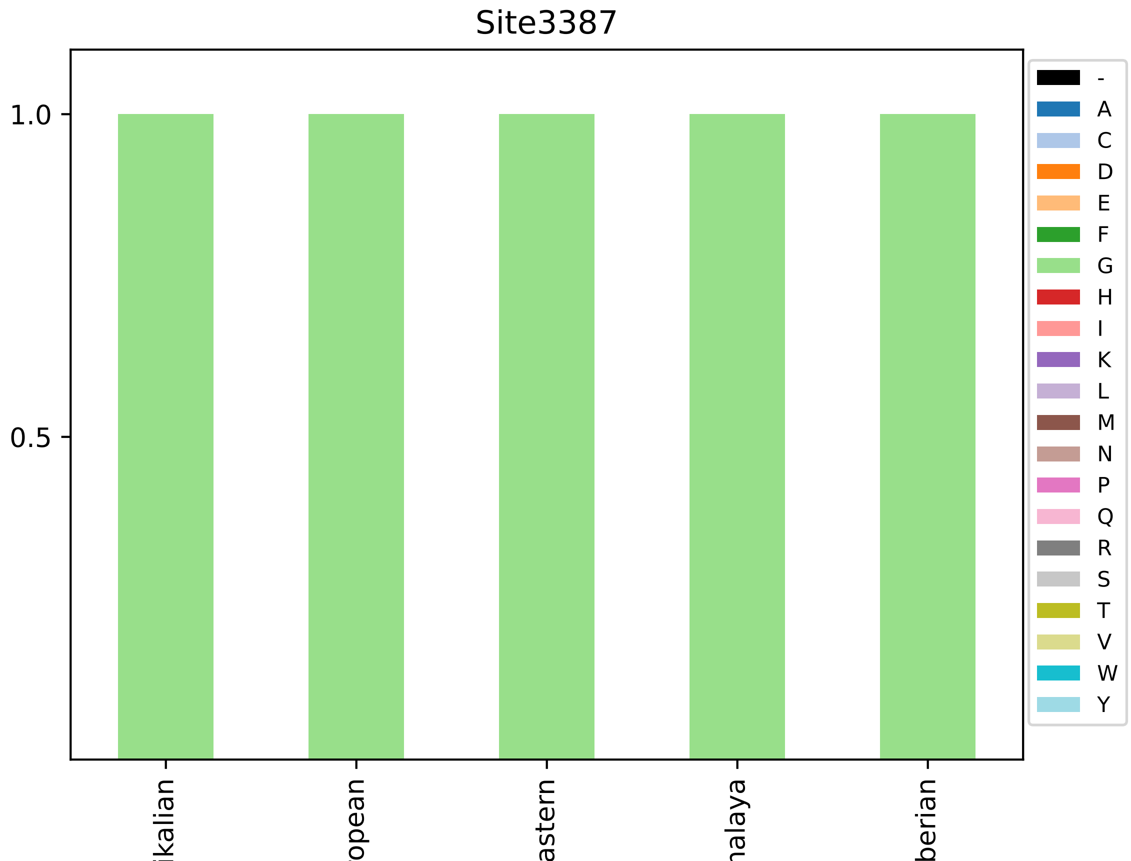

Site3387
1.0
0.5
-
A
C
D
E
F
G
H
I
K
L
M
N
P
Q
R
S
T
V
W
Y
ikalian
opean
astern
nalaya
berian

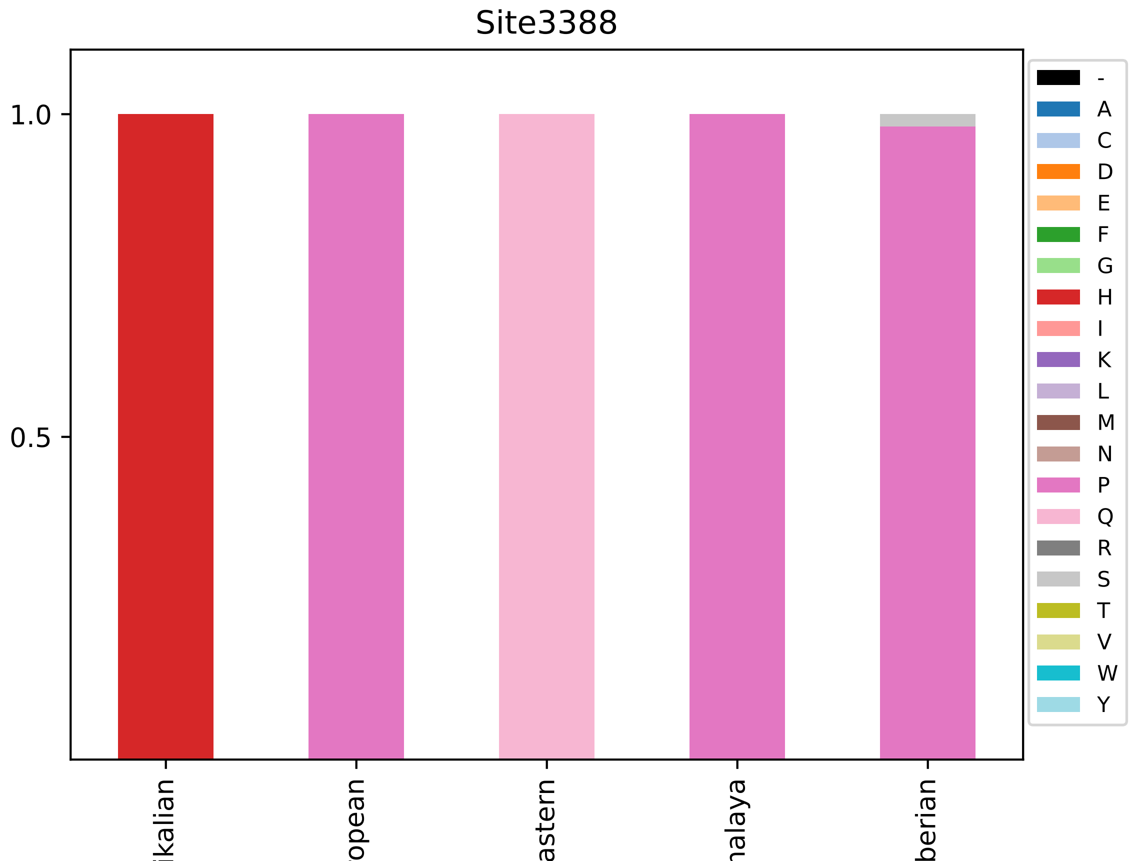

Site3388
1.0
0.5
ikalian
opean
astern
nalaya
berian
-
A
C
D
E
F
G
H
I
K
L
M
N
P
Q
R
S
T
V
W
Y

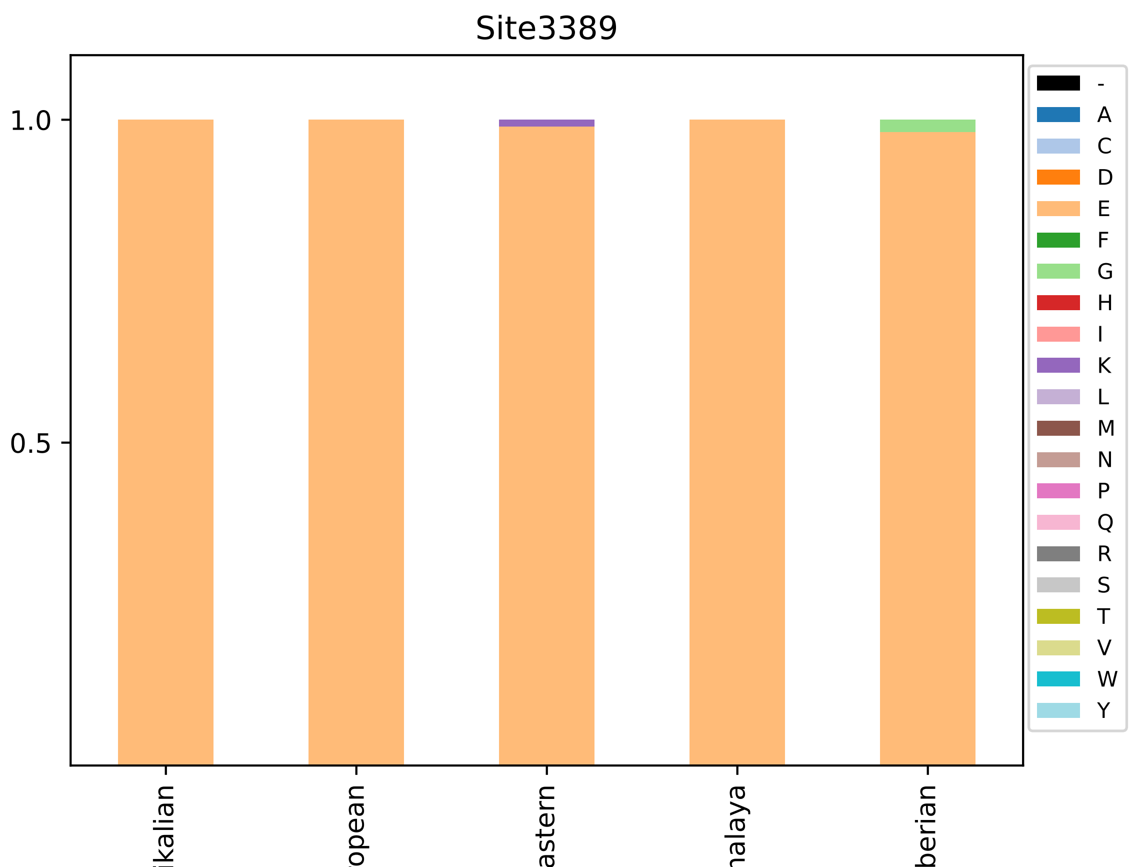

Site3389
1.0
0.5
-
A
C
D
E
F
G
H
I
K
L
M
N
P
Q
R
S
T
V
W
Y
ikalian
opean
astern
nalaya
berian

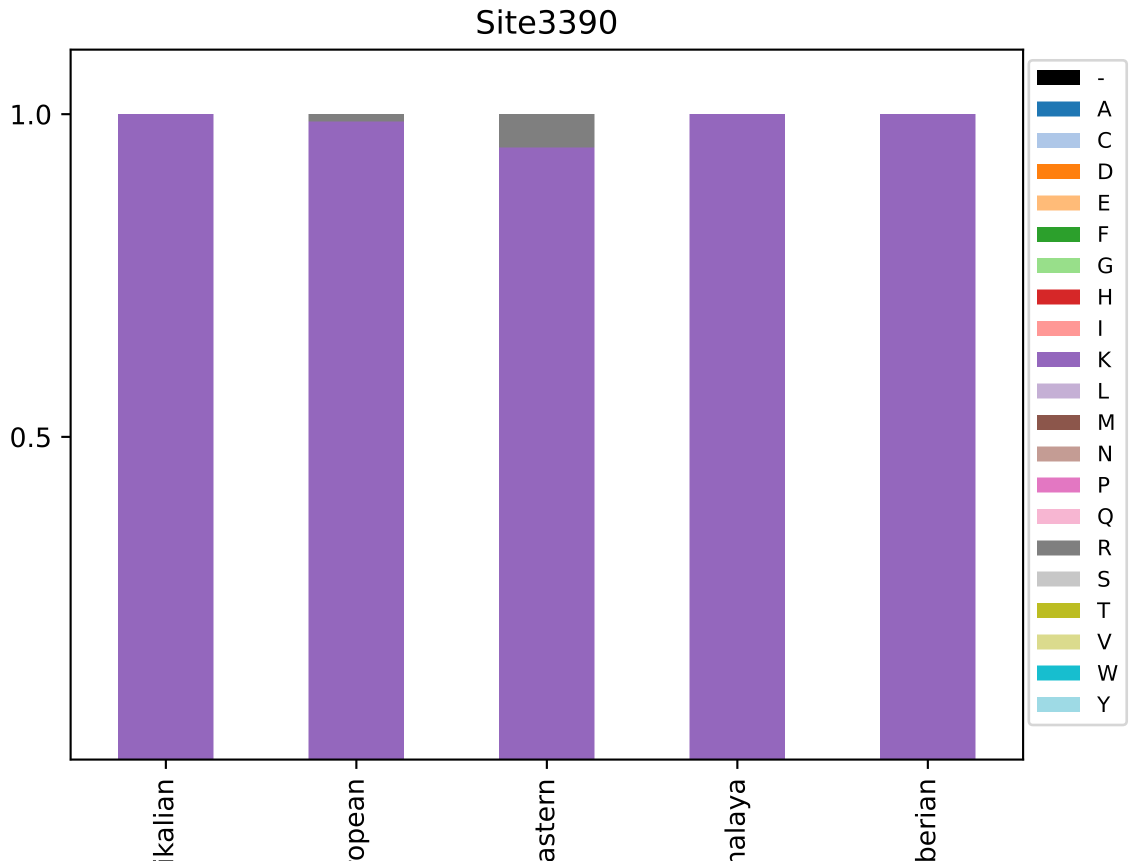
Site3390
1.0
0.5
ikalian
opean
astern
nalaya
berian
-
A
C
D
E
F
G
H
I
K
L
M
N
P
Q
R
S
T
V
W
Y

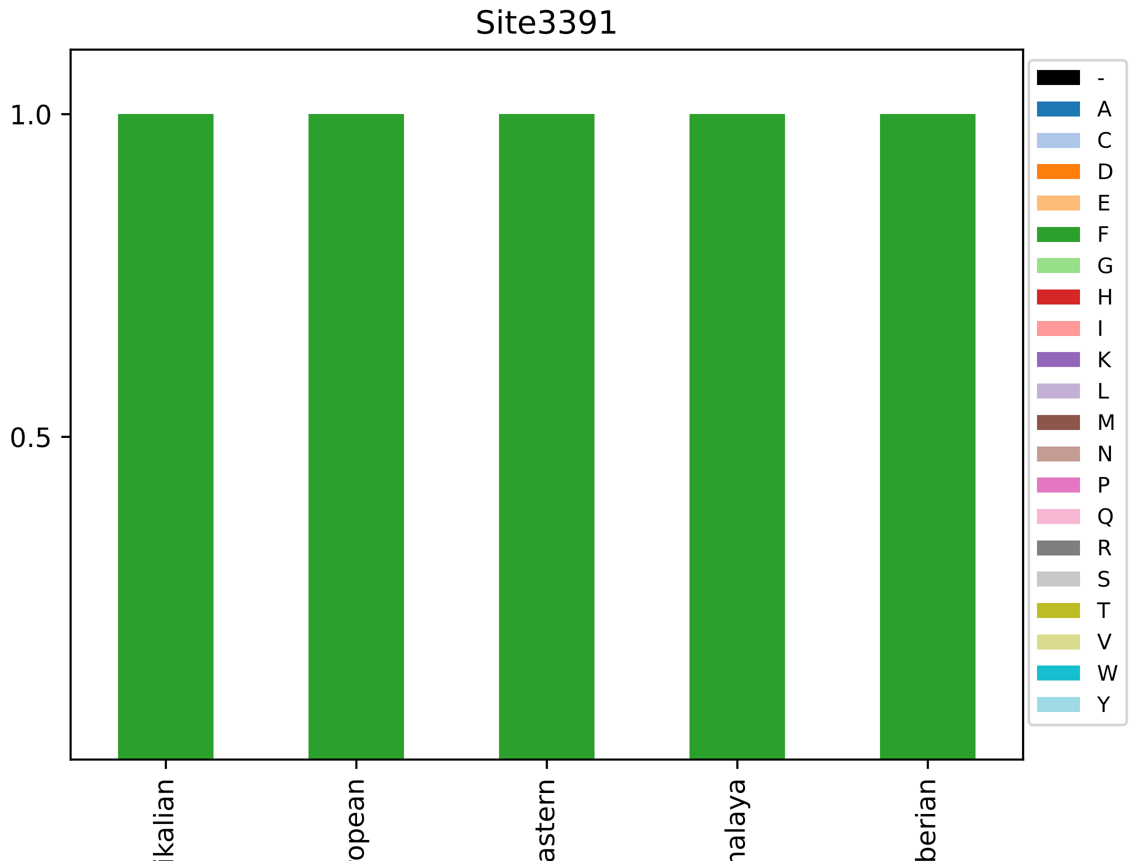
Site3391
1.0
0.5
-
A
C
D
E
F
G
H
I
K
L
M
N
P
Q
R
S
T
V
W
Y
ikalian
ropean
astern
nalaya
berian

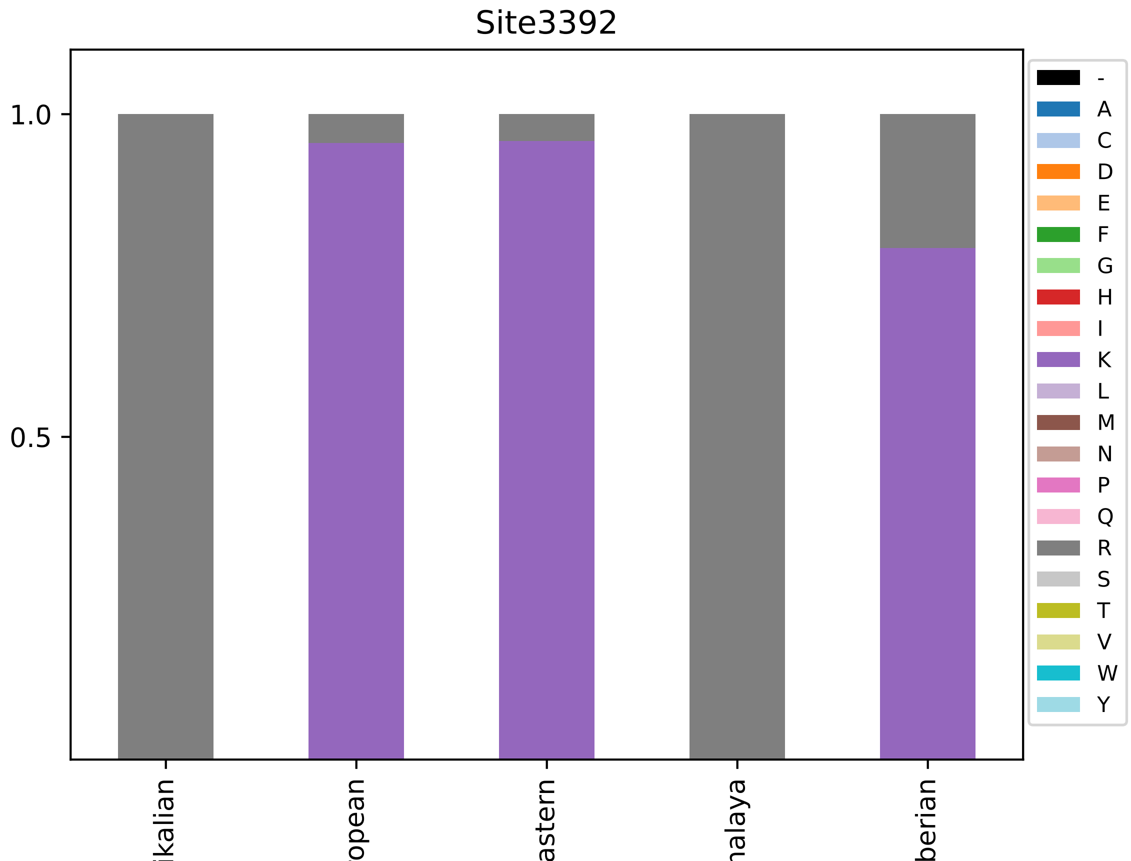
Site3392
1.0
0.5
-
A
C
D
E
F
G
H
I
K
L
M
N
P
Q
R
S
T
V
W
Y
ikalian
opean
astern
nalaya
berian

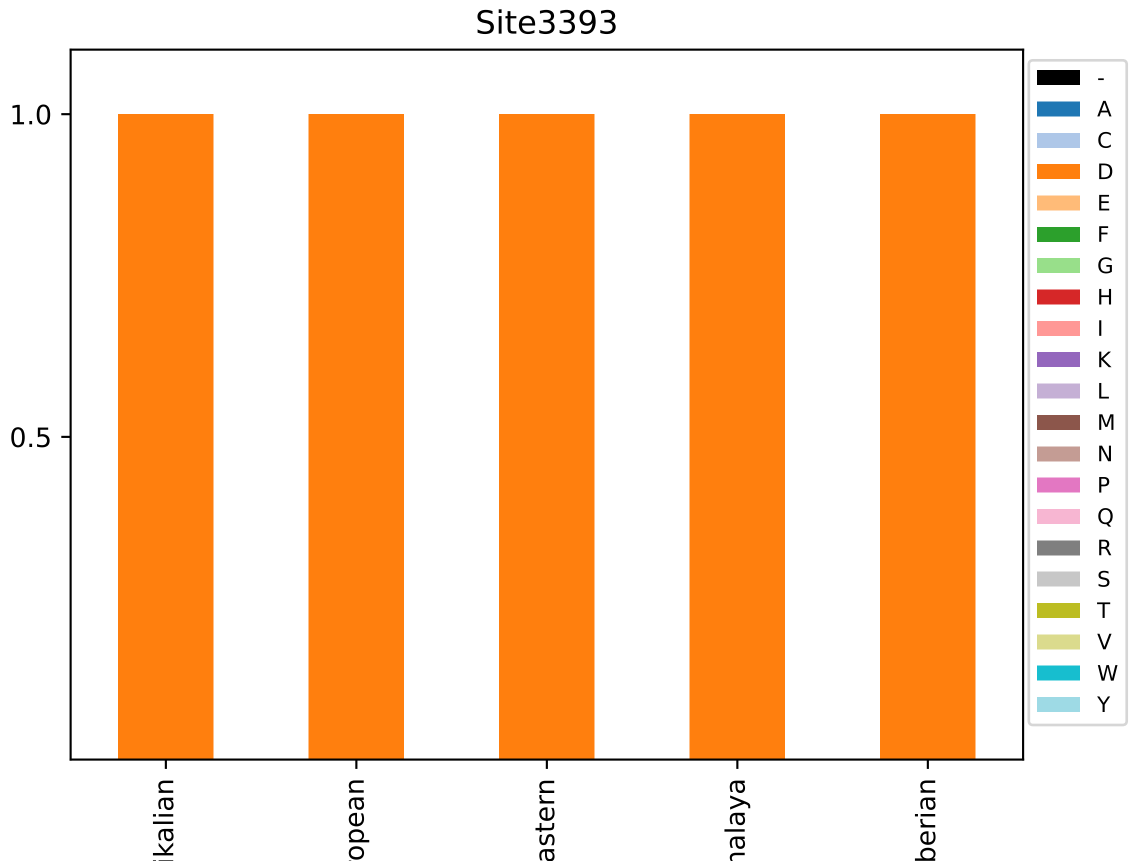
Site3393
1.0
0.5
-
A
C
D
E
F
G
H
I
K
L
M
N
P
Q
R
S
T
V
W
Y

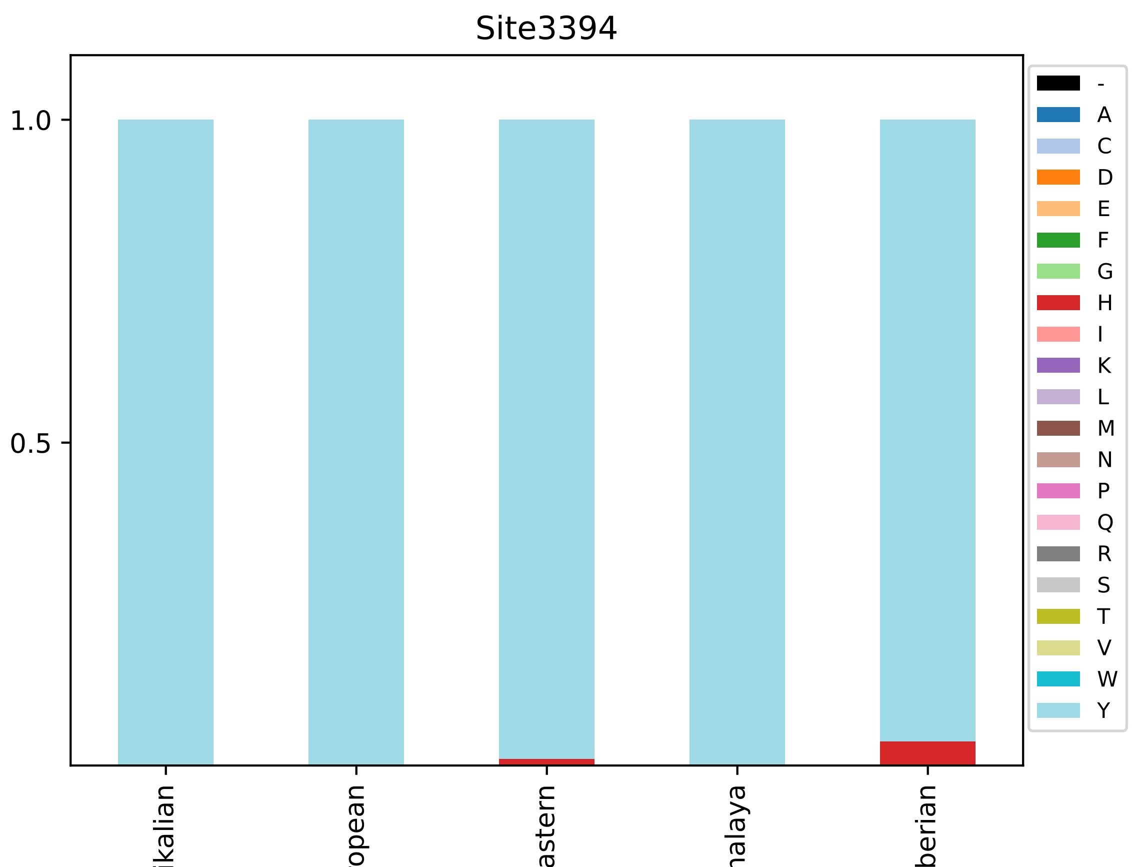

Site3394
1.0
0.5
-
A
C
D
E
F
G
H
I
K
L
M
N
P
Q
R
S
T
V
W
Y
ikalian
ropean
astern
nalaya
berian

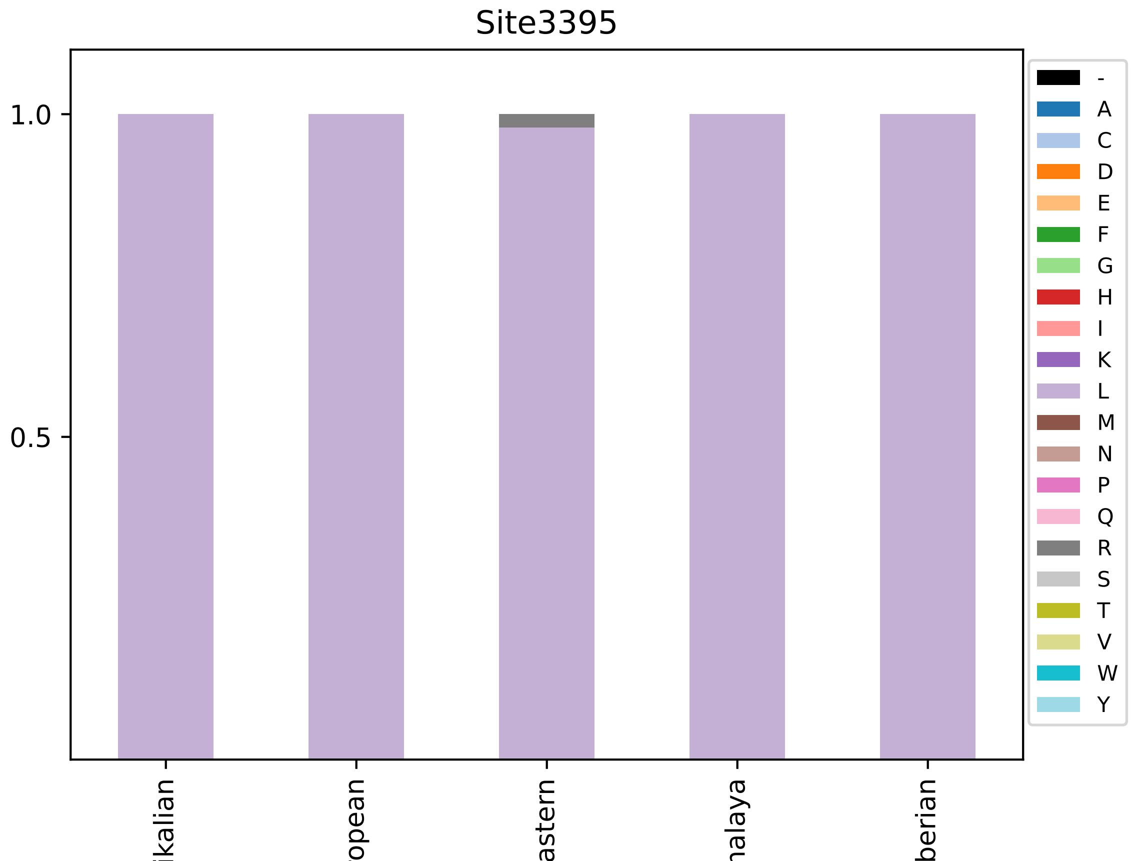
Site3395
1.0
0.5
-
A
C
D
E
F
G
H
I
K
L
M
N
P
Q
R
S
T
V
W
Y
ikalian
opean
astern
nalaya
berian

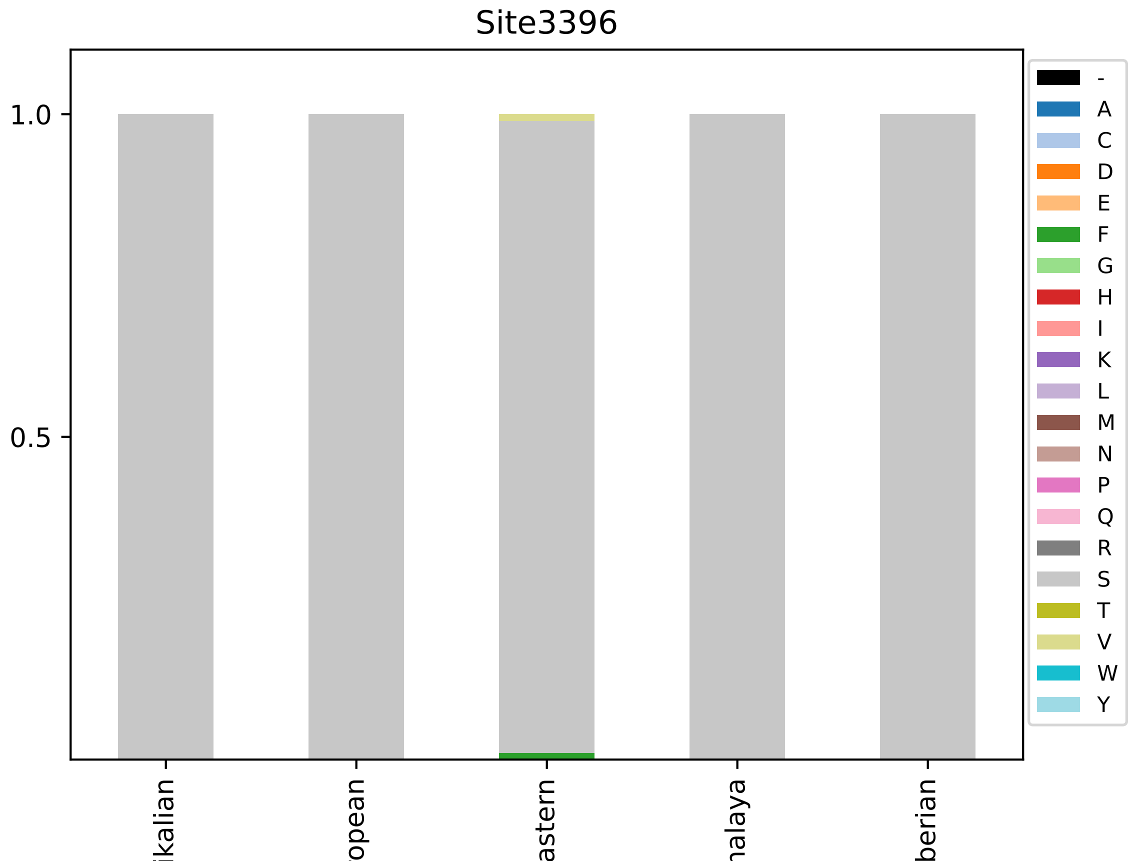

Site3396
1.0
0.5
ikalian
opean
astern
nalaya
berian
-
A
C
D
E
F
G
H
I
K
L
M
N
P
Q
R
S
T
V
W
Y

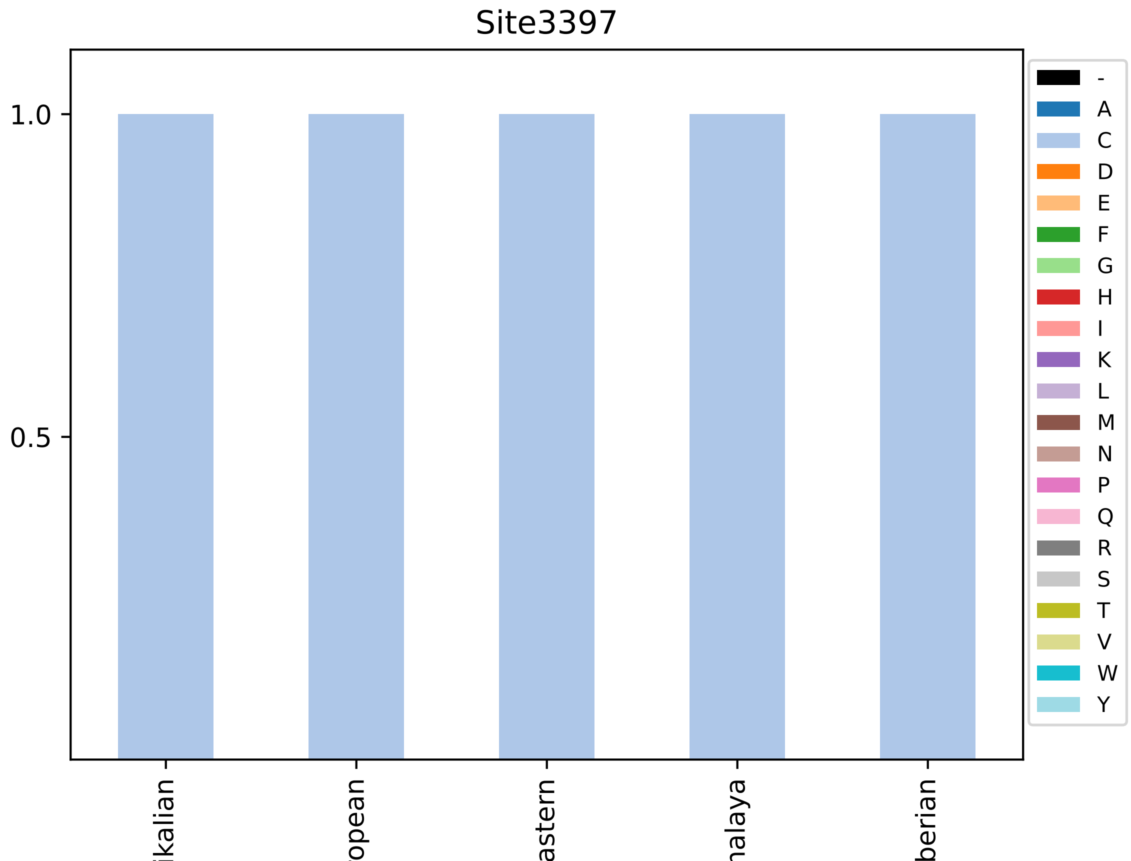
Site3397
1.0
0.5
-
A
C
D
E
F
G
H
I
K
L
M
N
P
Q
R
S
T
V
W
Y
ikalian
opean
astern
nalaya
berian

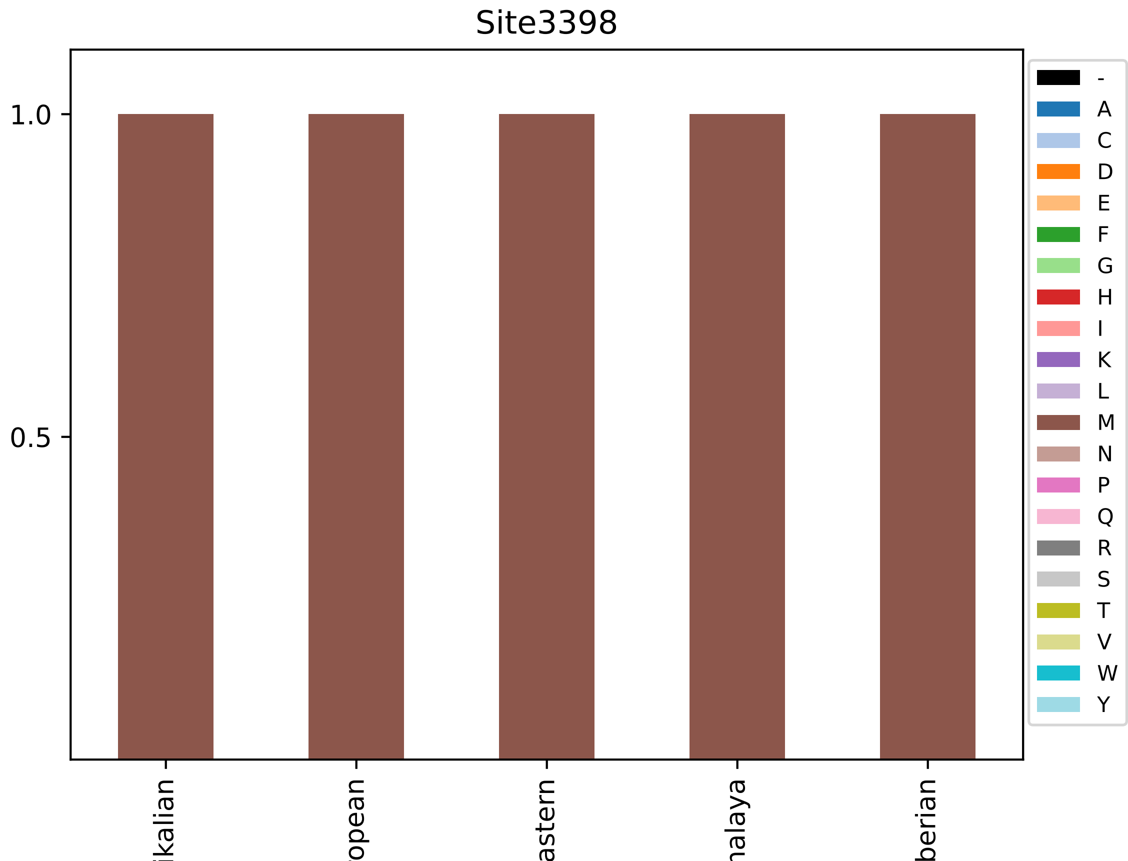
Site3398
1.0
0.5
ikalian
opean
astern
nalaya
berian
-
A
C
D
E
F
G
H
I
K
L
M
N
P
Q
R
S
T
V
W
Y

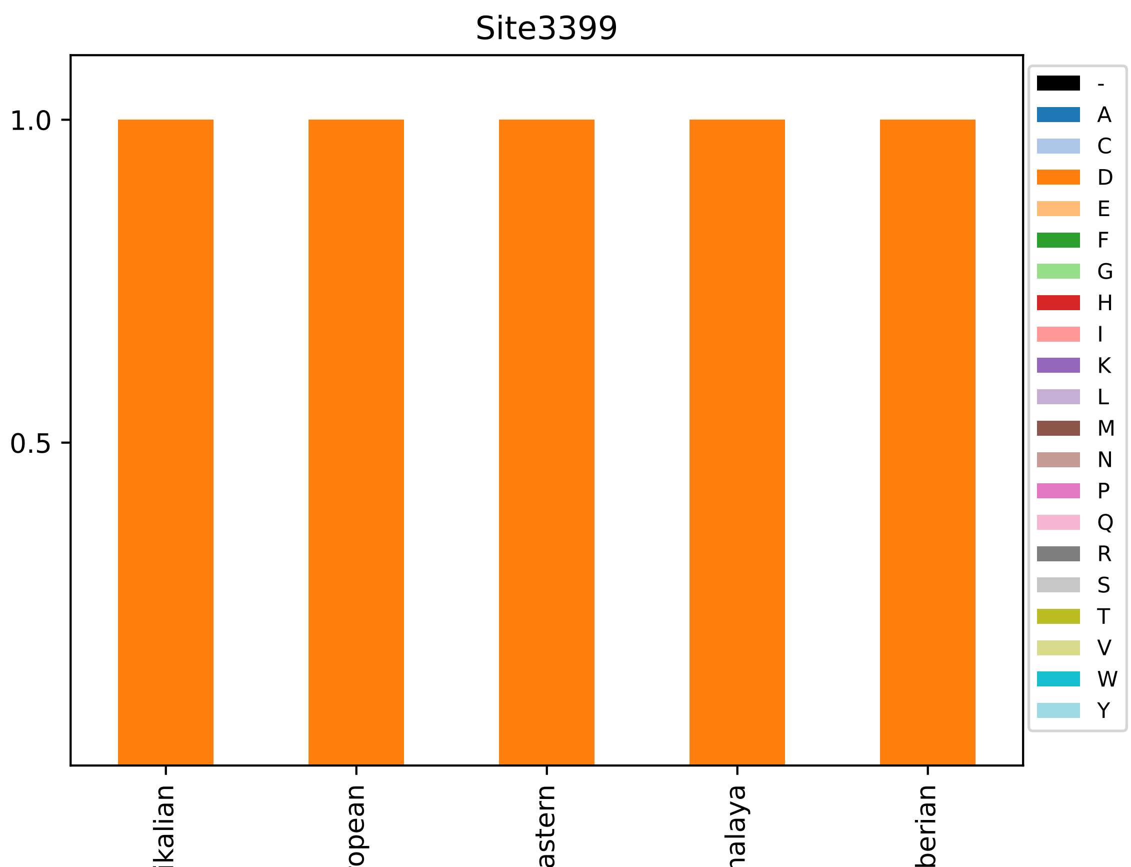

Site3399
1.0
0.5
-
A
C
D
E
F
G
H
I
K
L
M
N
P
Q
R
S
T
V
W
Y
ikalian
opean
astern
nalaya
berian

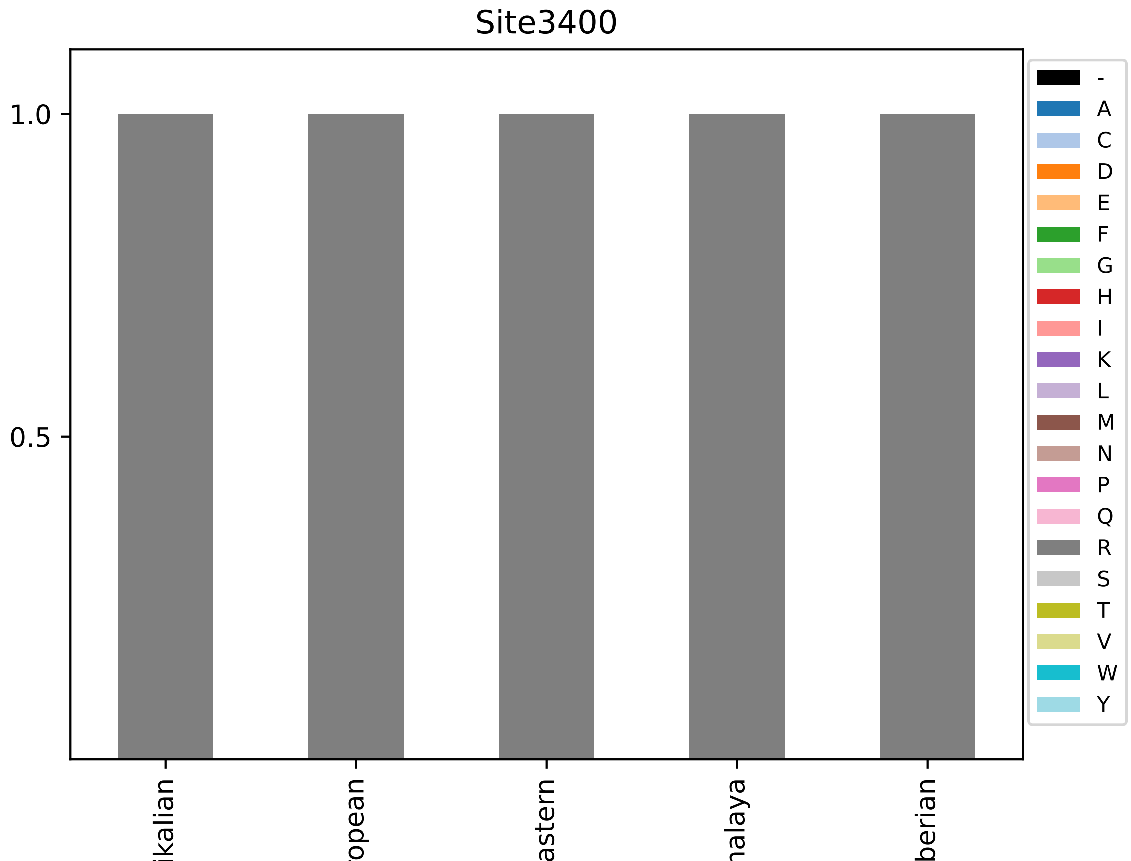
Site3400
1.0
0.5
-
A
C
D
E
F
G
H
I
K
L
M
N
P
Q
R
S
T
V
W
Y

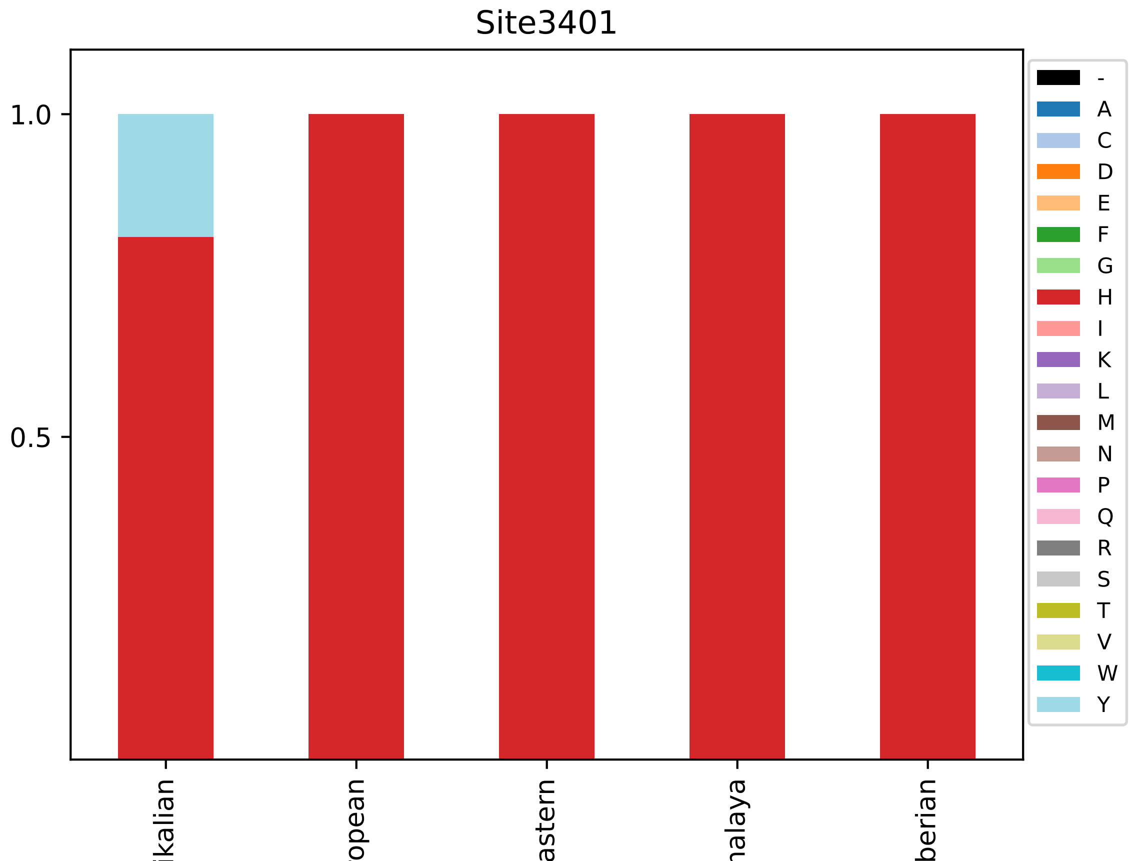

Site3401
1.0
0.5
ikalian
opean
astern
nalaya
berian
-
A
C
D
E
F
G
H
I
K
L
M
N
P
Q
R
S
T
V
W
Y

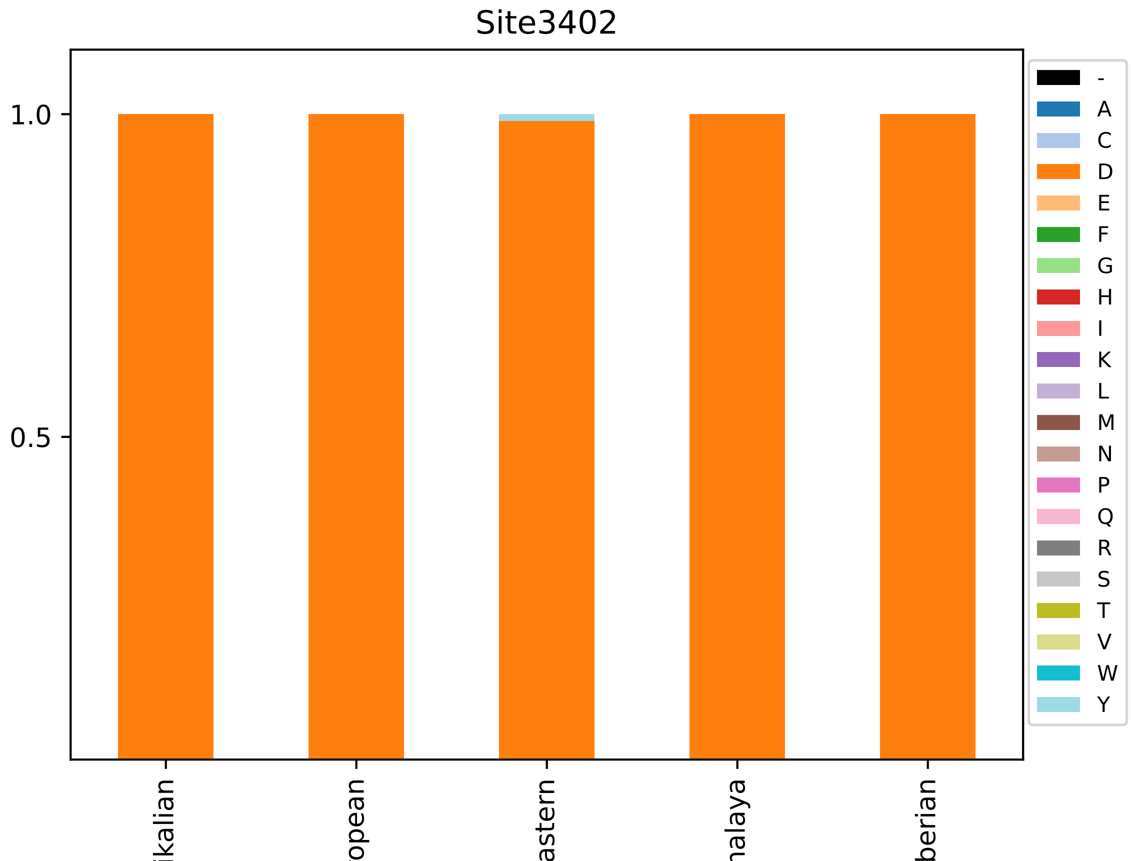

Site3402
1.0
0.5
-
A
C
D
E
F
G
H
I
K
L
M
N
P
Q
R
S
T
V
W
Y
ikalian
opean
astern
nalaya
berian

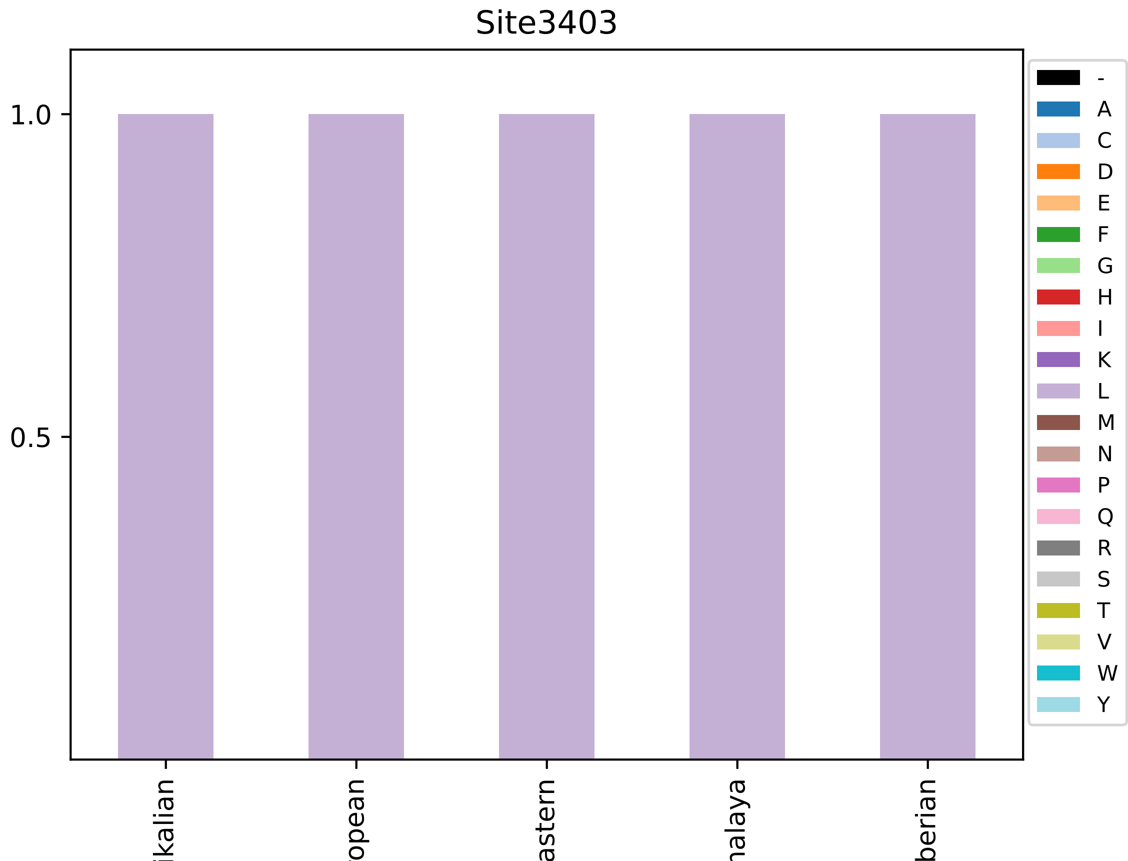

Site3403
1.0
0.5
-
A
C
D
E
F
G
H
I
K
L
M
N
P
Q
R
S
T
V
W
Y
ikalian
opean
astern
nalaya
berian

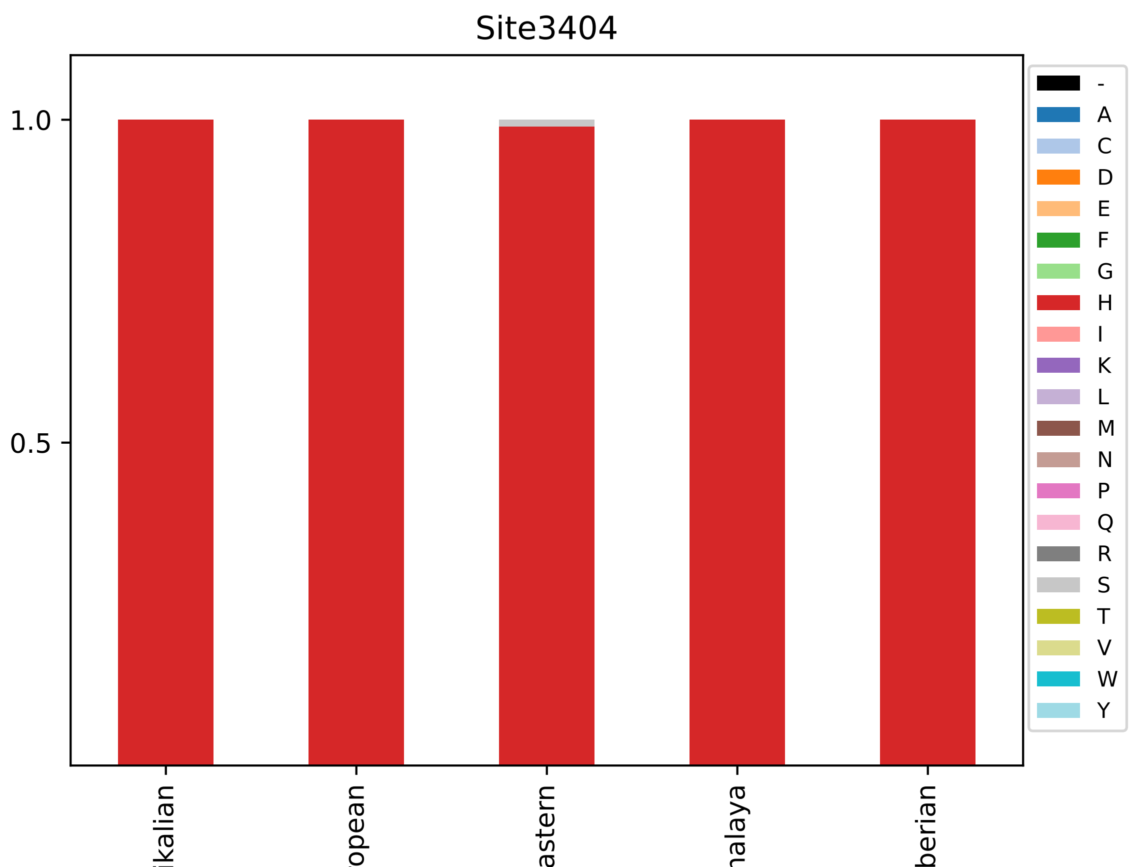

Site3404
1.0
0.5
-
A
C
D
E
F
G
H
I
K
L
M
N
P
Q
R
S
T
V
W
Y
ikalian
opean
astern
nalaya
berian

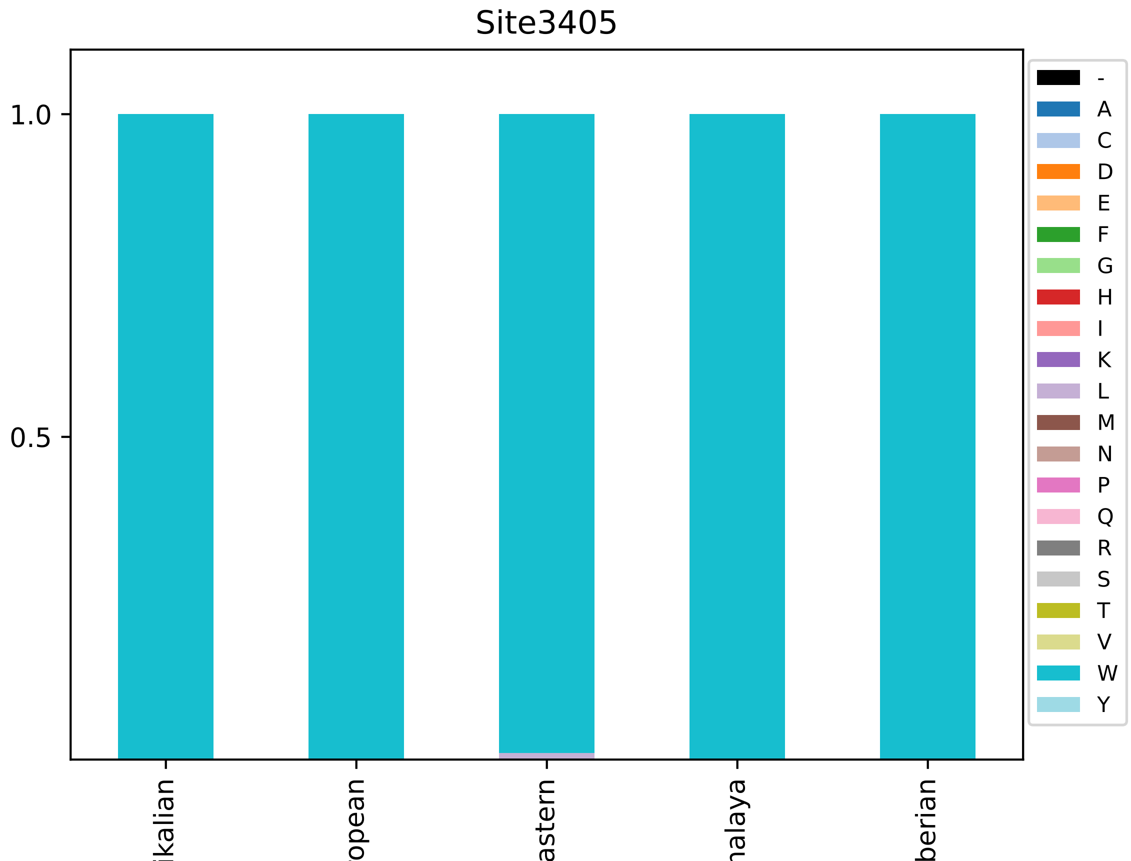
Site3405
1.0
0.5
-
A
C
D
E
F
G
H
I
K
L
M
N
P
Q
R
S
T
V
W
Y
ikalian
opean
astern
nalaya
berian

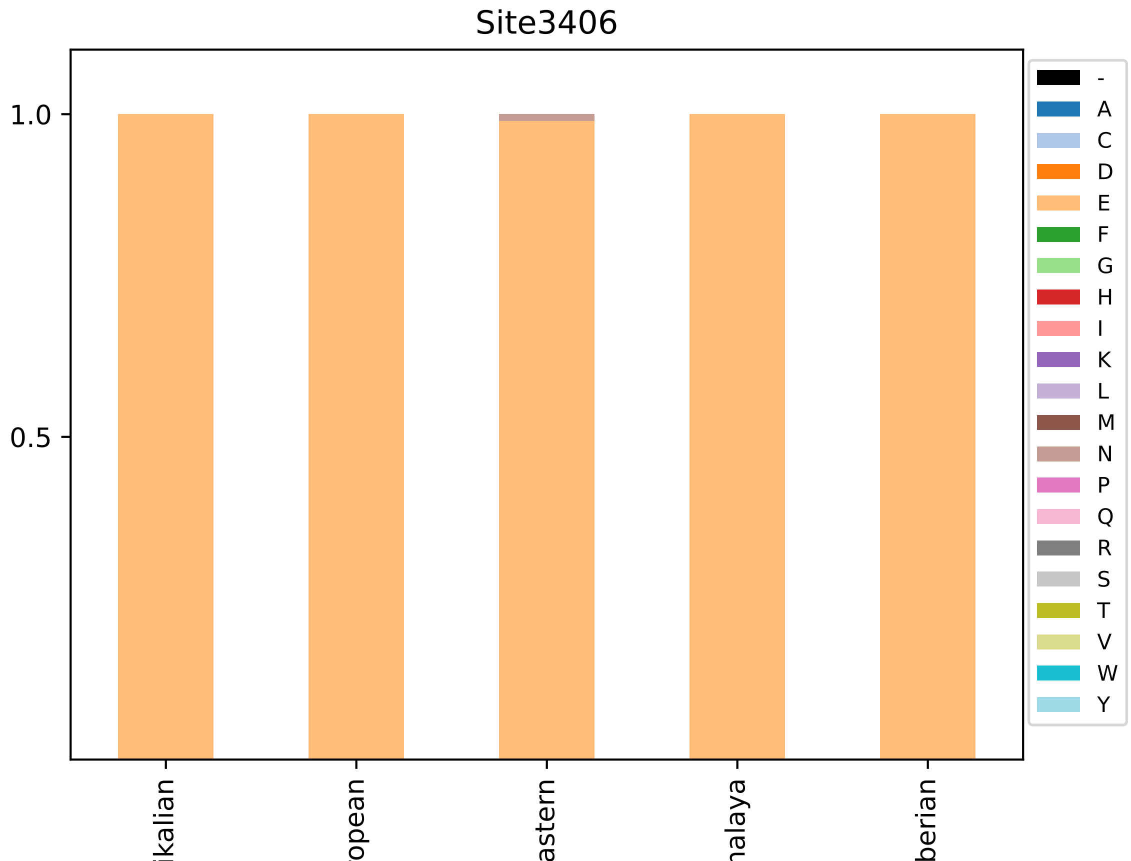

Site3406
1.0
0.5
ikalian
opean
astern
nalaya
berian
-
A
C
D
E
F
G
H
I
K
L
M
N
P
Q
R
S
T
V
W
Y

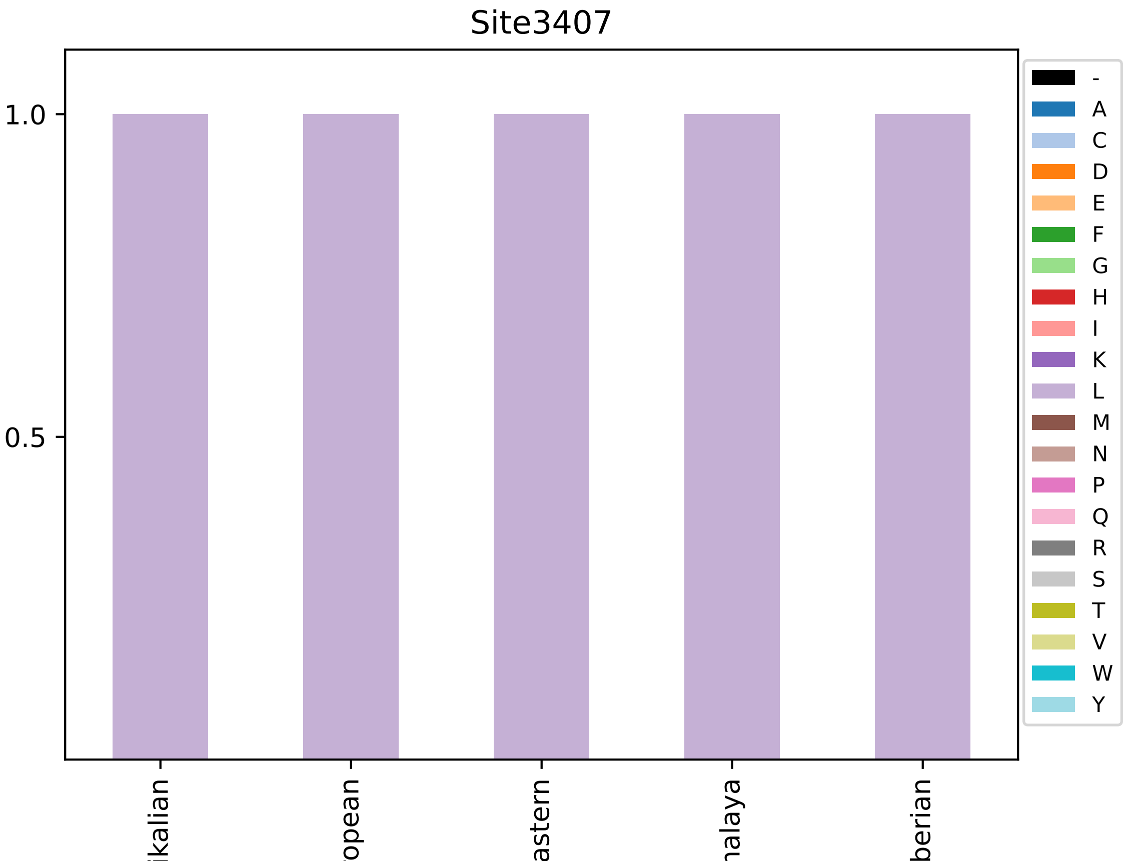
Site3407
1.0
0.5
-
A
C
D
E
F
G
H
I
K
L
M
N
P
Q
R
S
T
V
W
Y

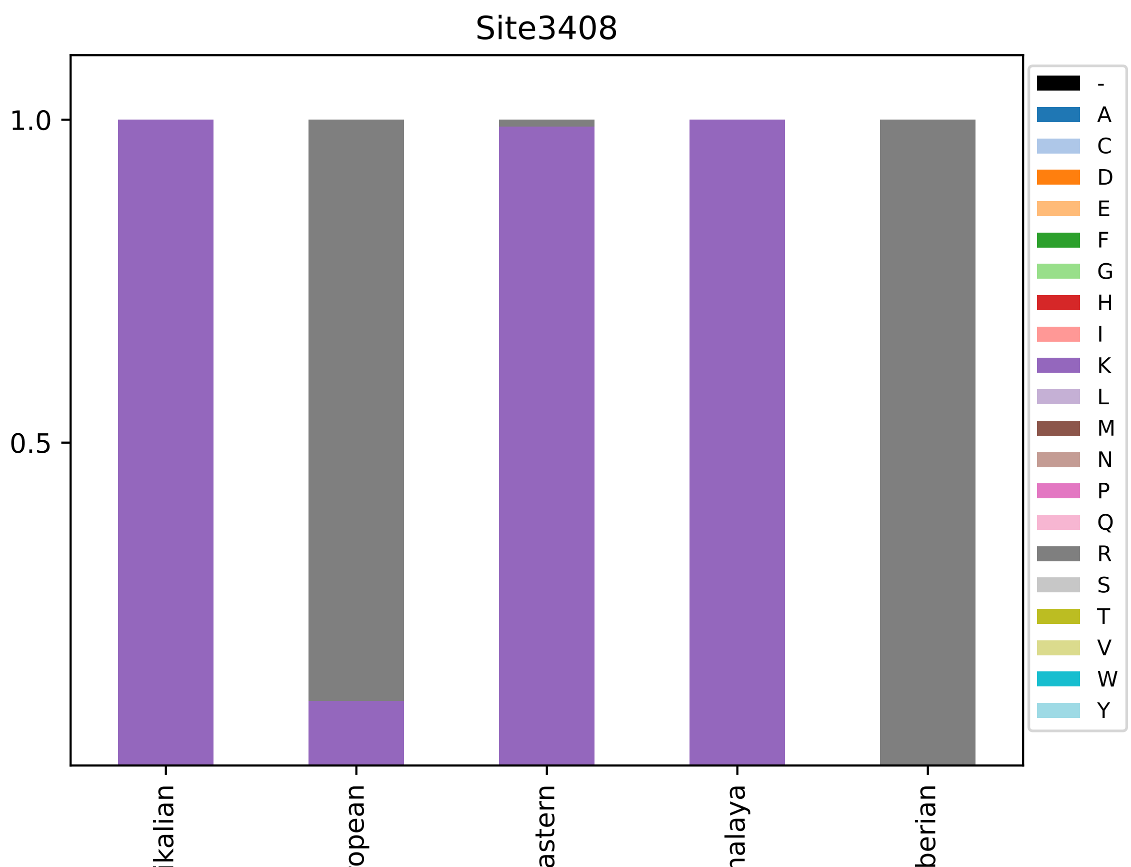

Site3408
1.0
0.5
-
A
C
D
E
F
G
H
I
K
L
M
N
P
Q
R
S
T
V
W
Y
ikalian
opean
astern
nalaya
berian

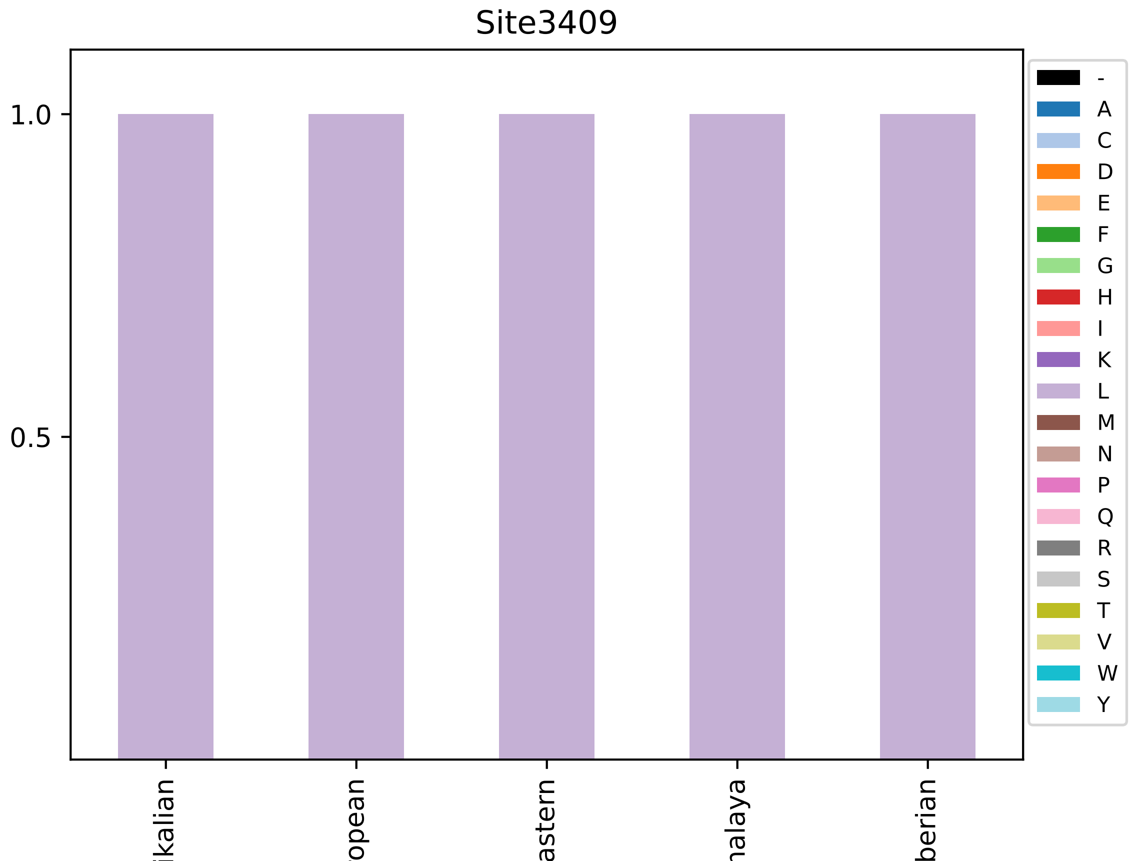
Site3409
1.0
0.5
-
A
C
D
E
F
G
H
I
K
L
M
N
P
Q
R
S
T
V
W
Y
ikalian
opean
astern
nalaya
berian

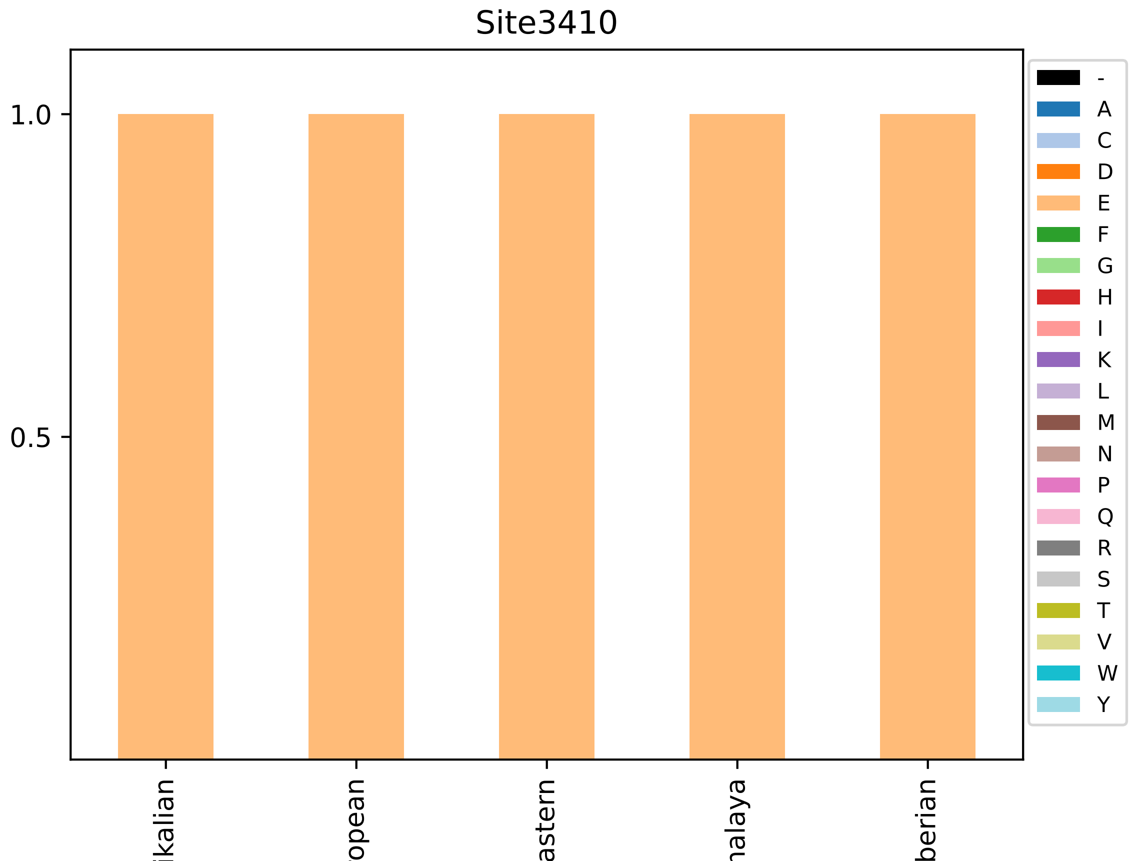

Site3410
1.0
0.5
-
A
C
D
E
F
G
H
I
K
L
M
N
P
Q
R
S
T
V
W
Y

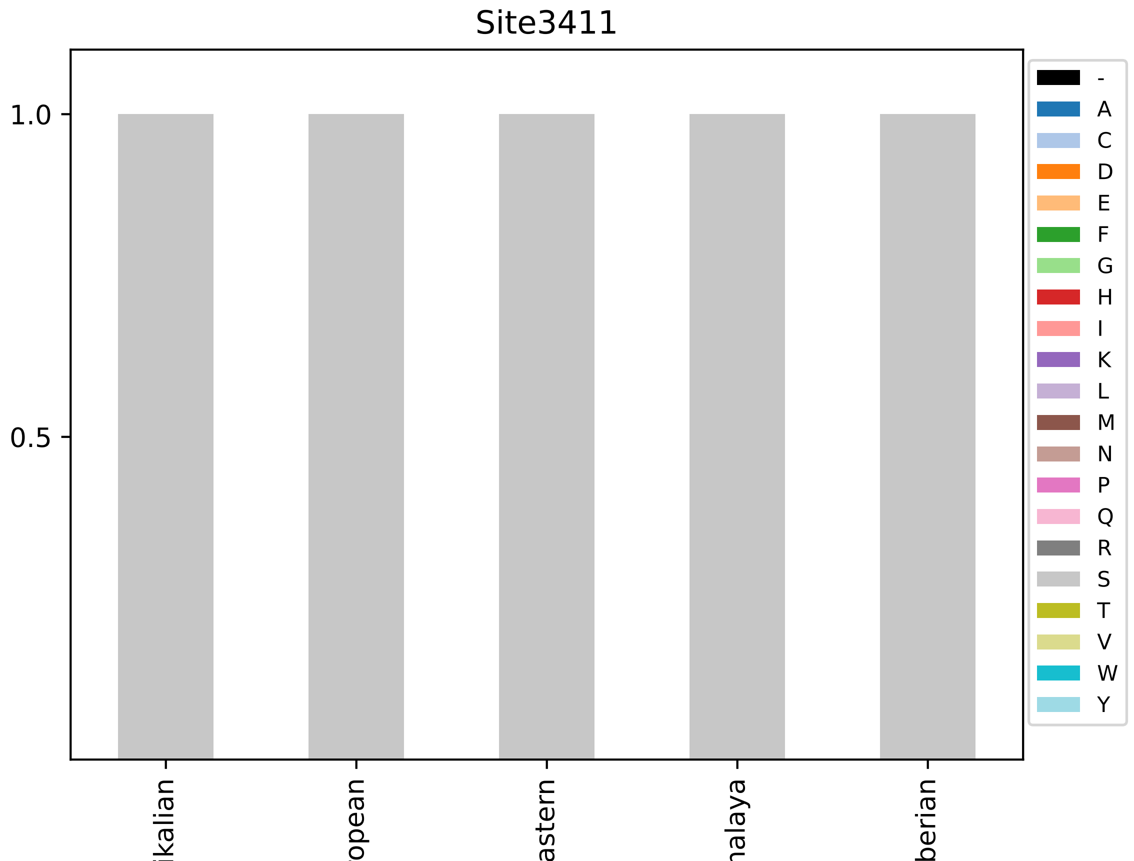
Site3411
1.0
0.5
-
A
C
D
E
F
G
H
I
K
L
M
N
P
Q
R
S
T
V
W
Y
ikalian
ropean
astern
nalaya
berian

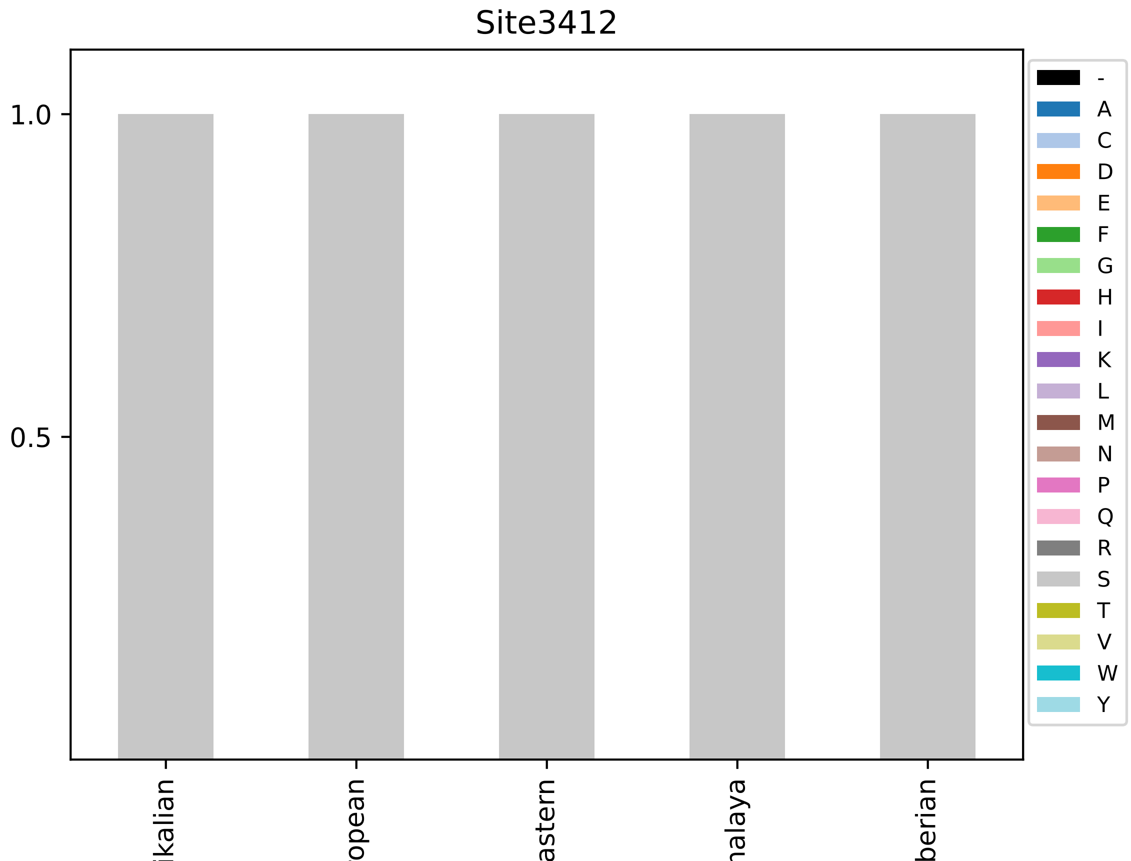

Site3412
1.0
0.5
-
A
C
D
E
F
G
H
I
K
L
M
N
P
Q
R
S
T
V
W
Y
ikalian
opean
astern
nalaya
berian

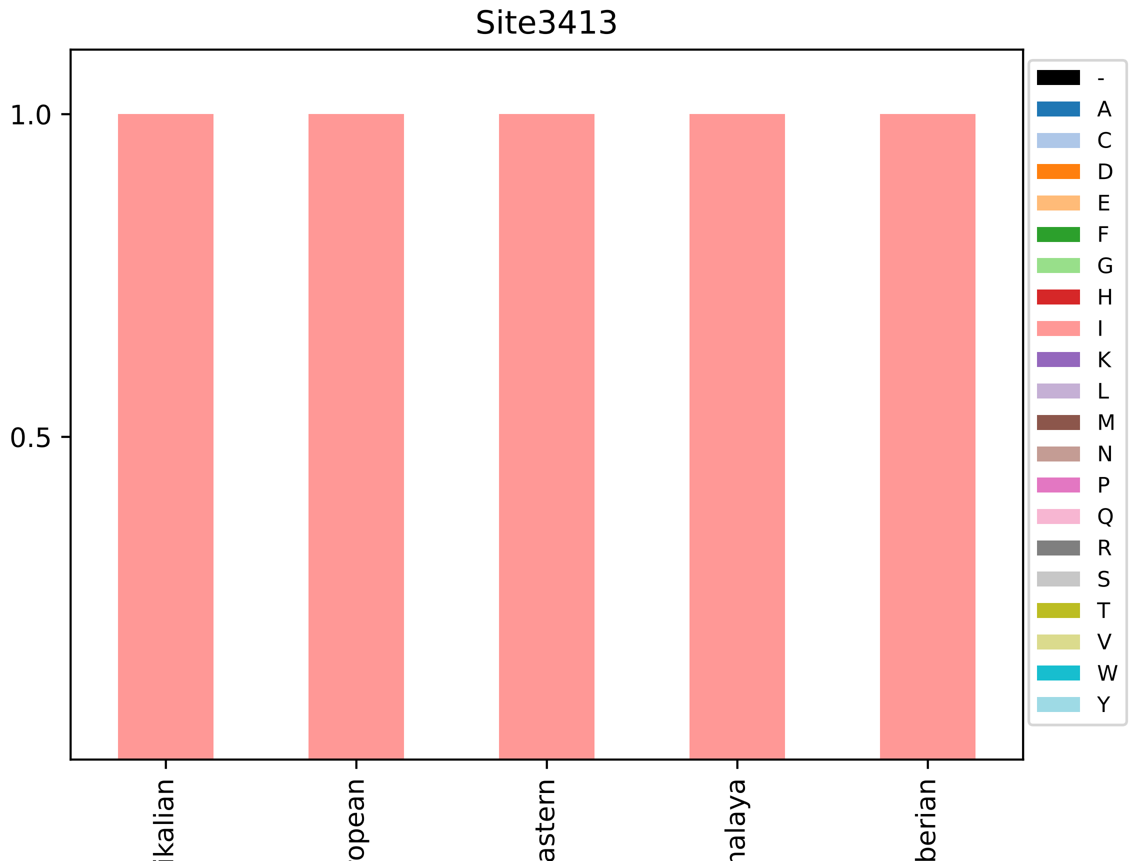

Site3413
1.0
0.5
-
A
C
D
E
F
G
H
I
K
L
M
N
P
Q
R
S
T
V
W
Y
ikalian
opean
astern
alaya
berian

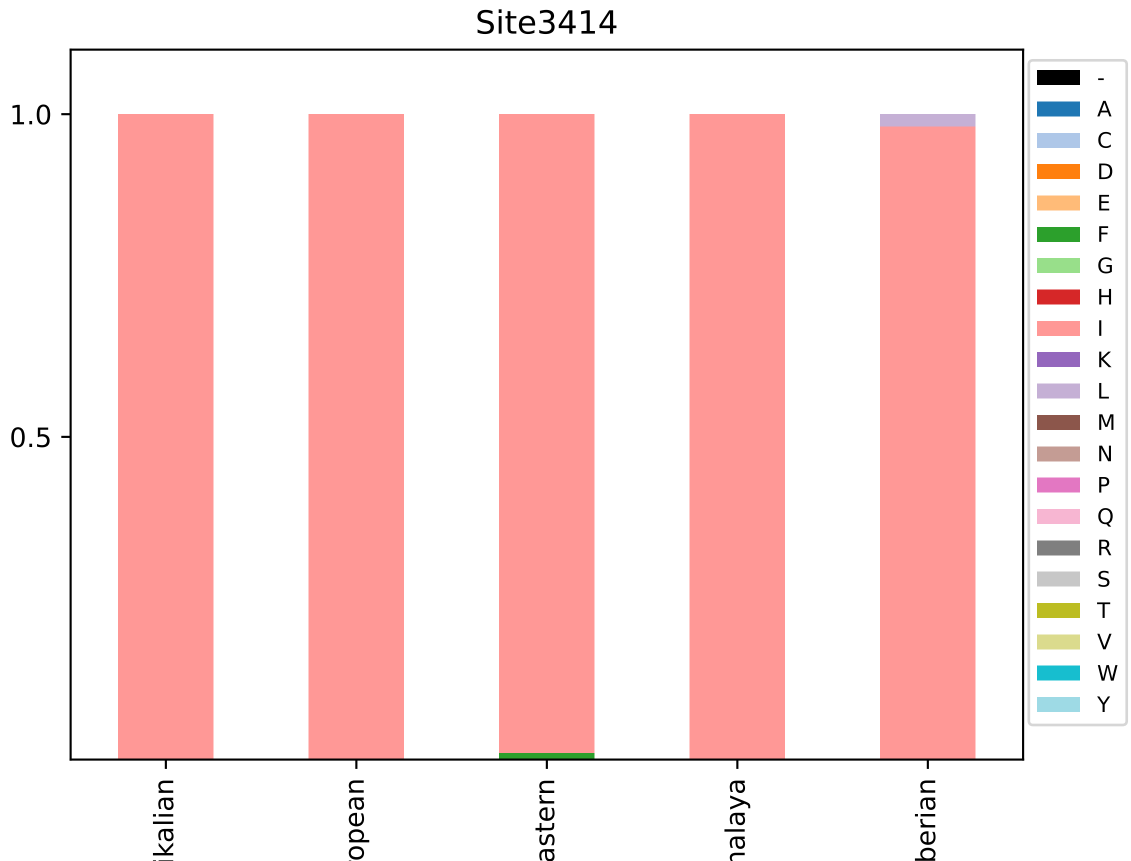
Site3414
1.0
0.5
-
A
C
D
E
F
G
H
I
K
L
M
N
P
Q
R
S
T
V
W
Y
ikalian
opean
astern
nalaya
berian

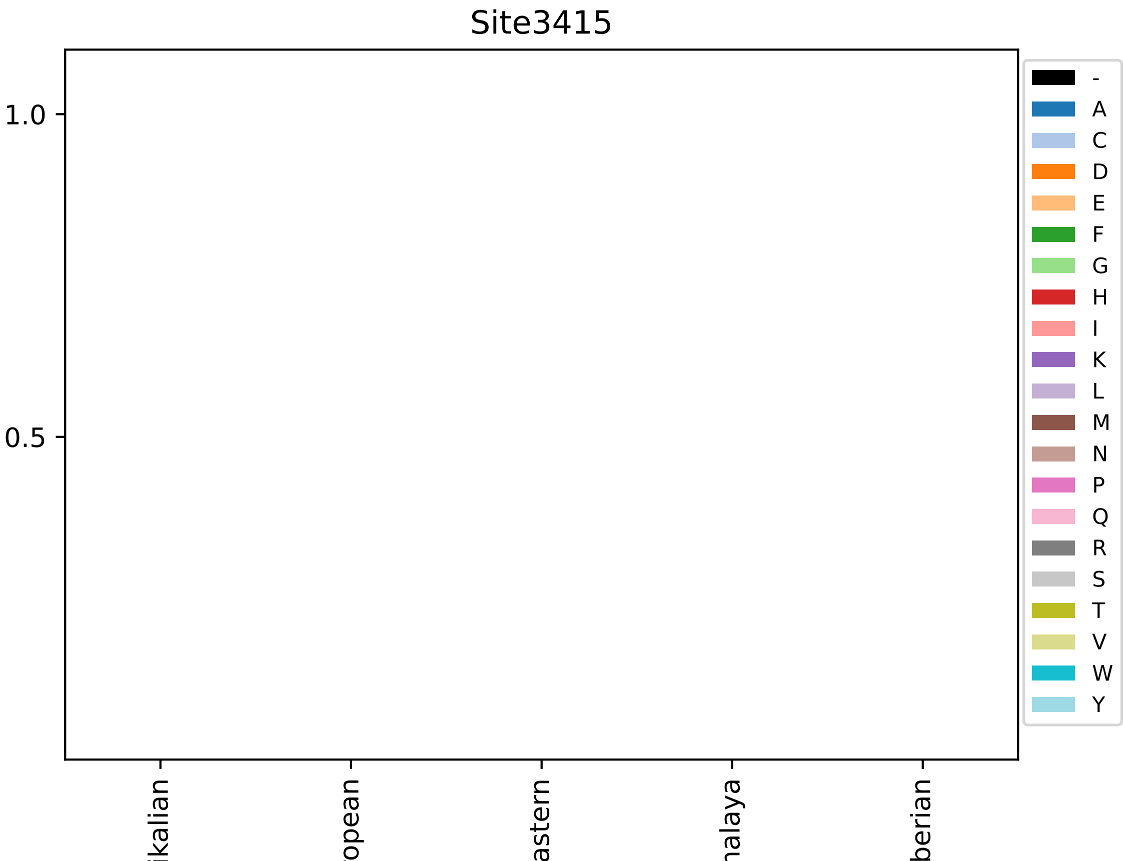

Site3415
1.0
0.5
-
A
C
D
E
F
G
H
I
K
L
M
N
P
Q
R
S
T
V
W
Y
