# Supplementary material for: Evolutionary Patterns and Genotype-Specific Amino Acid Mutations of Tick-Borne Encephalitis Virus
Source: Int J Mol Sci. 2025 Jan 23;26(3):954. doi: 10.3390/ijms26030954 (PMC11817229; doi:10.3390/ijms26030954)
Supplement: Supplementary file 1 [file ijms-26-00954-s001.zip › ijms-3423653-supplementary/Supplementary Data S2.zip!/host/host_aa_mutation.pdf]

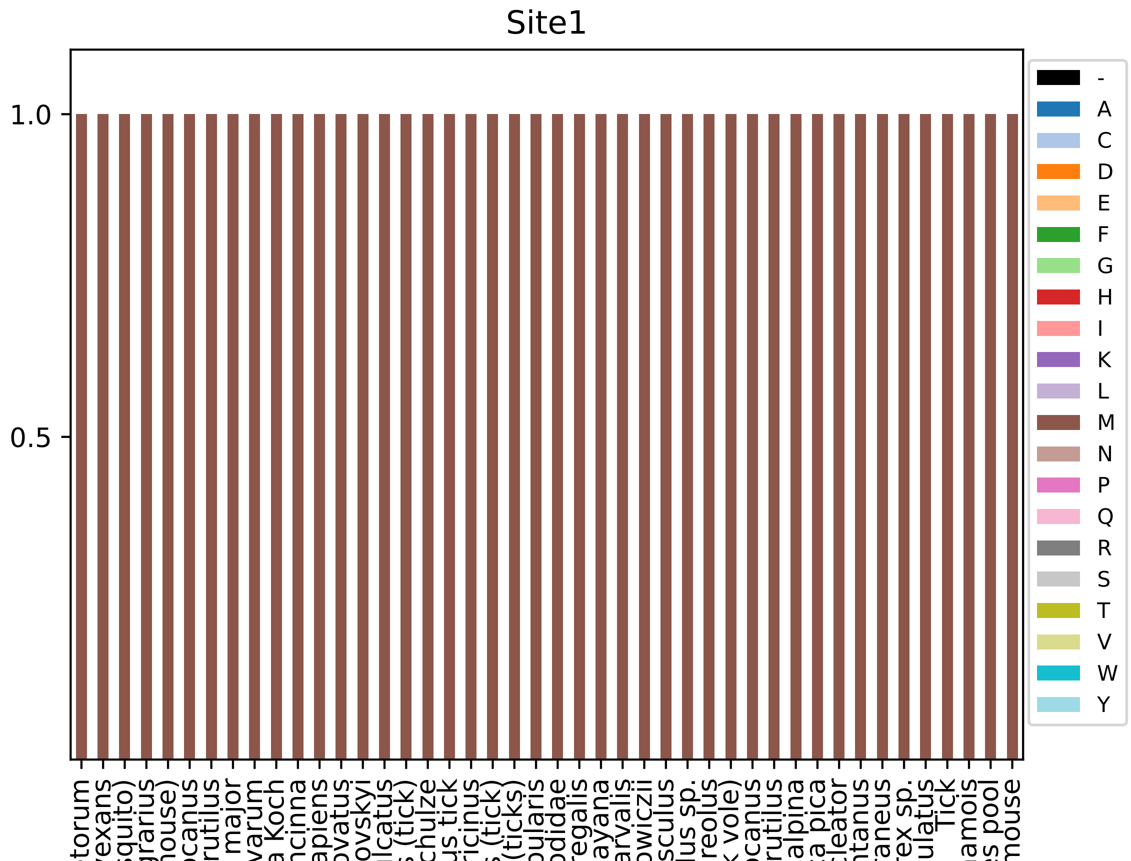
Site1
1.0
0.5
-
A
C
D
E
F
G
H
I
K
L
M
N
P
Q
R
S
T
V
W
Y

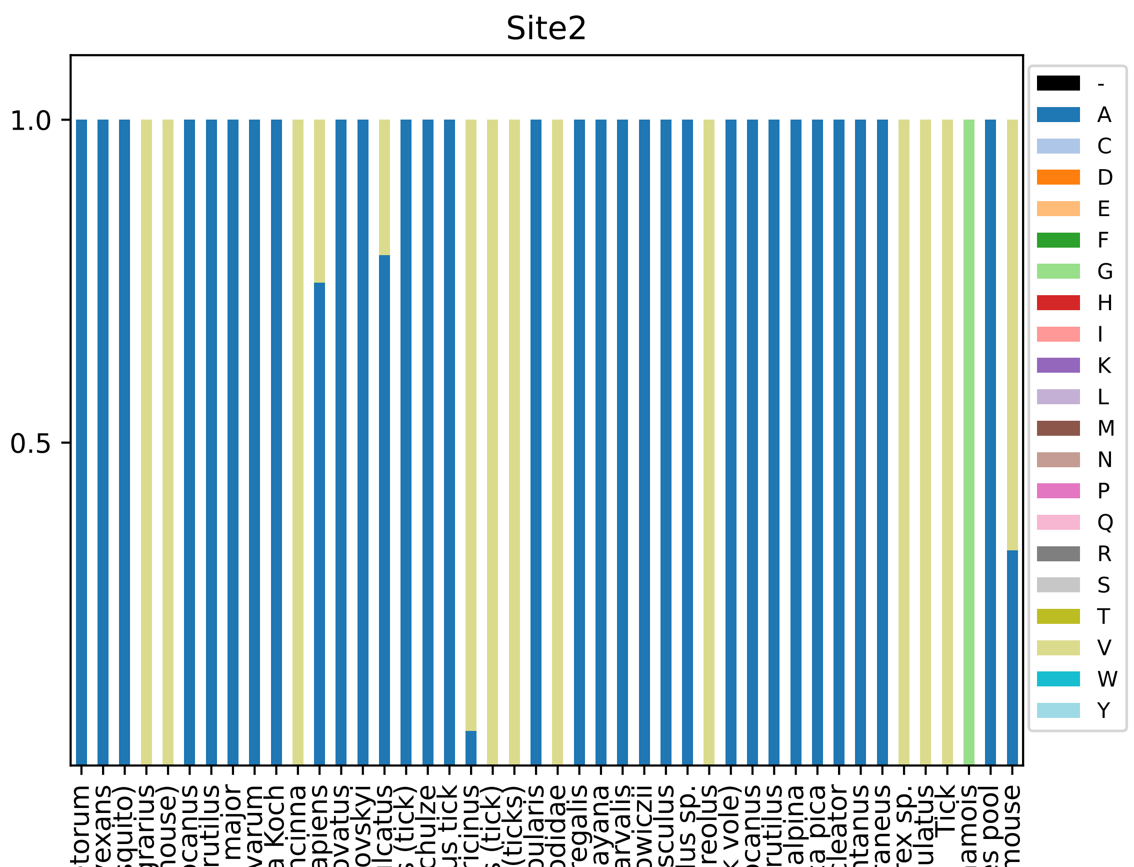

Site2
1.0
0.5
-
A
C
D
E
F
G
H
I
K
L
M
N
P
Q
R
S
T
V
W
Y

Site3

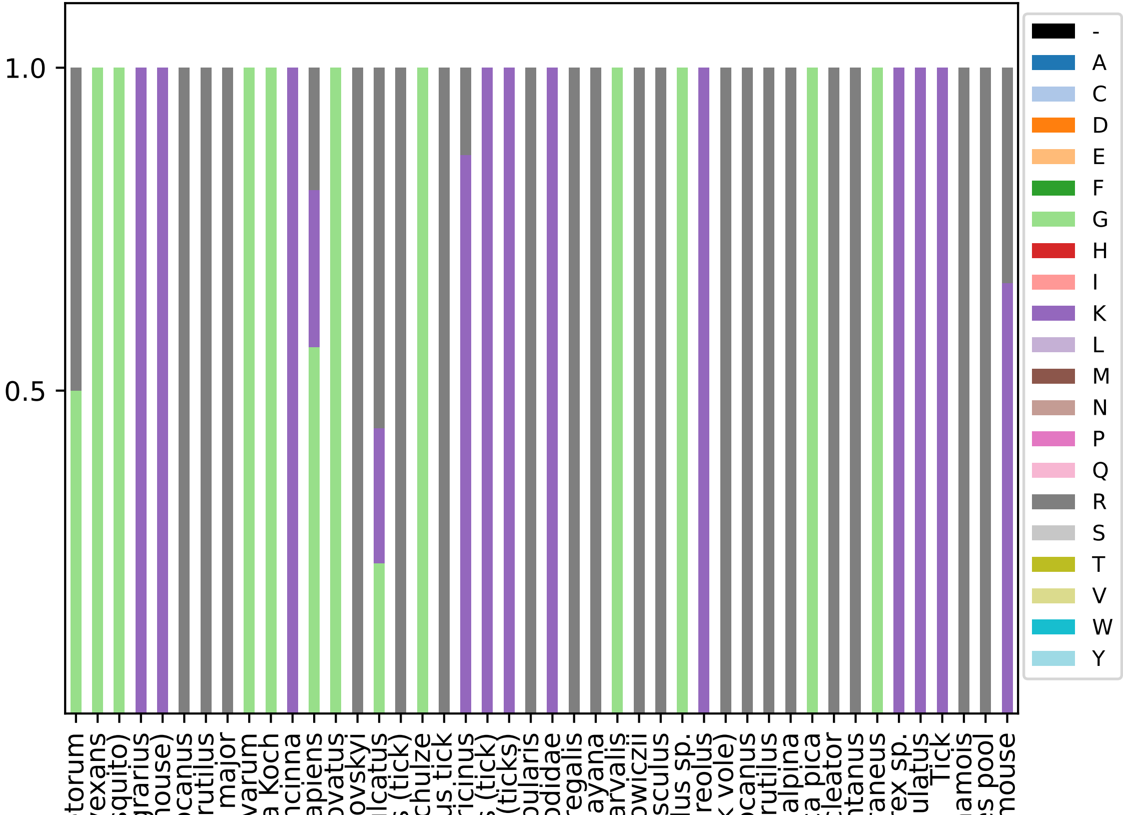

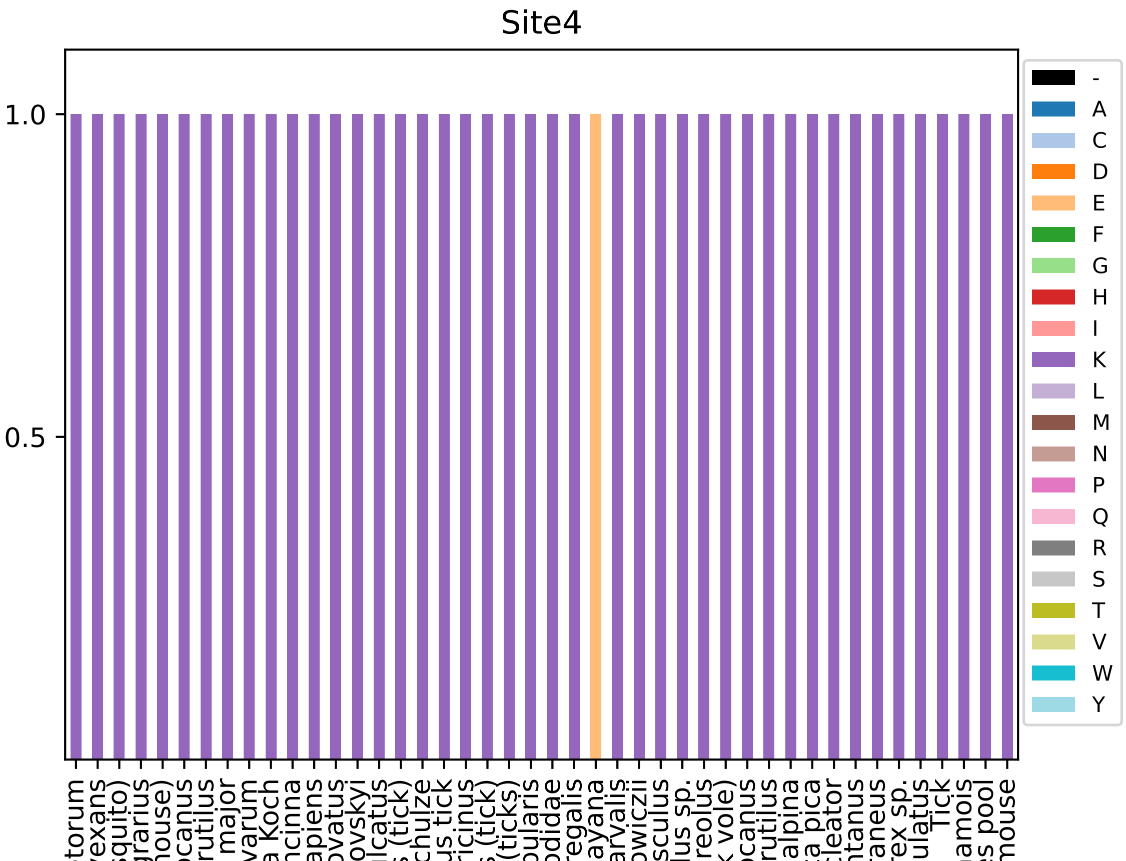
Site4
1.0
0.5
-
A
C
D
E
F
G
H
I
K
L
M
N
P
Q
R
S
T
V
W
Y

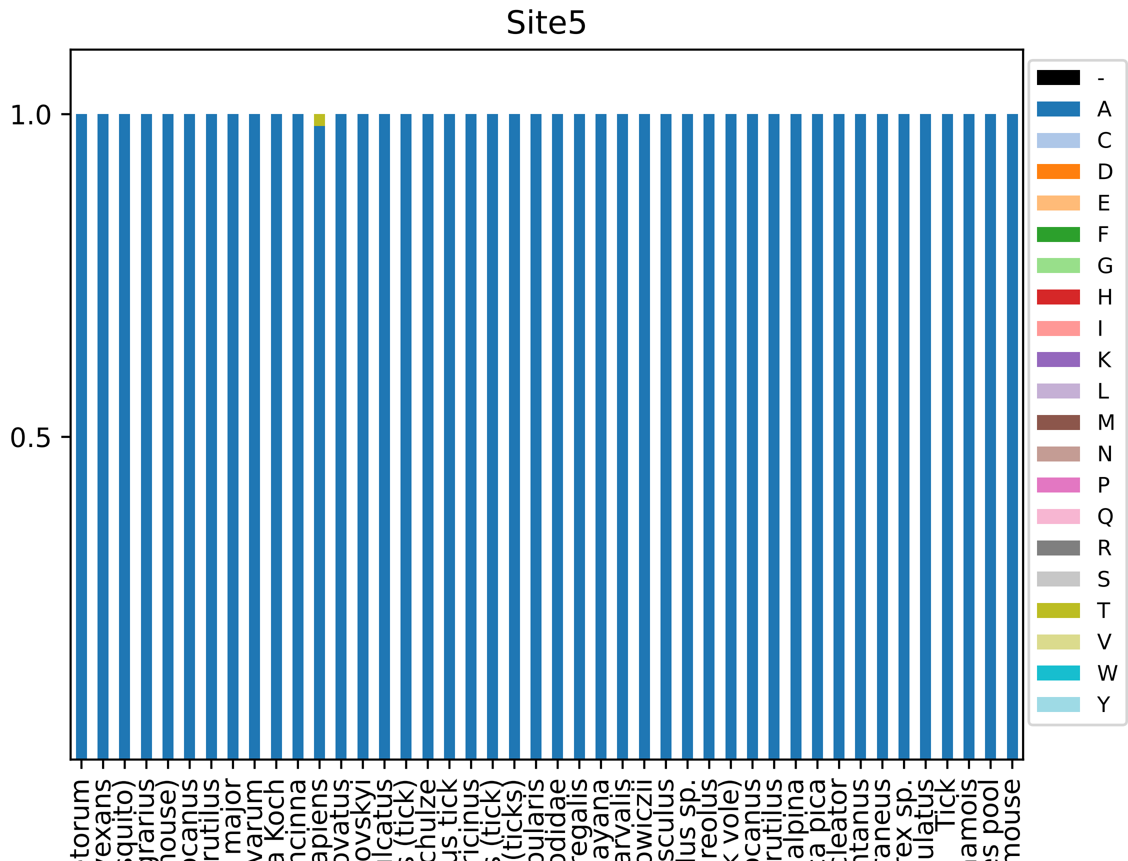
Site5
1.0
0.5
-
A
C
D
E
F
G
H
I
K
L
M
N
P
Q
R
S
T
V
W
Y

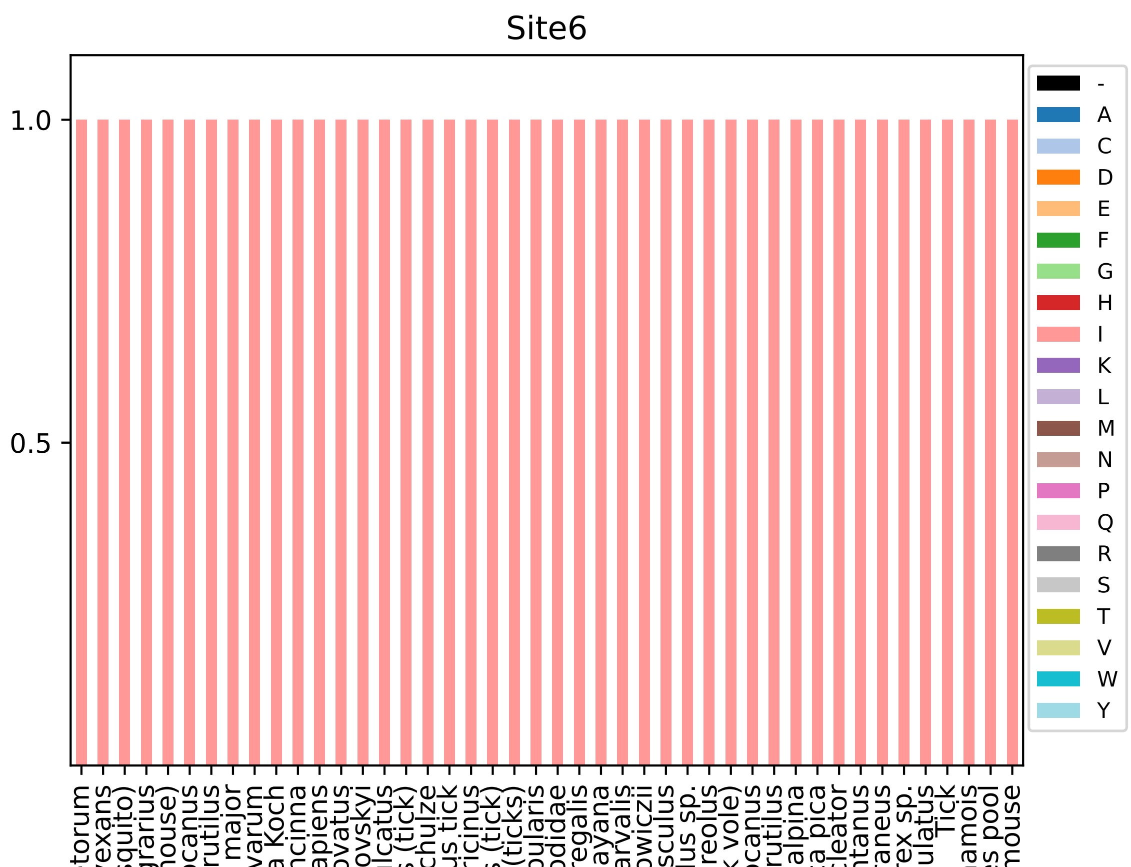
Site6
1.0
0.5
-
A
C
D
E
F
G
H
I
K
L
M
N
P
Q
R
S
T
V
W
Y

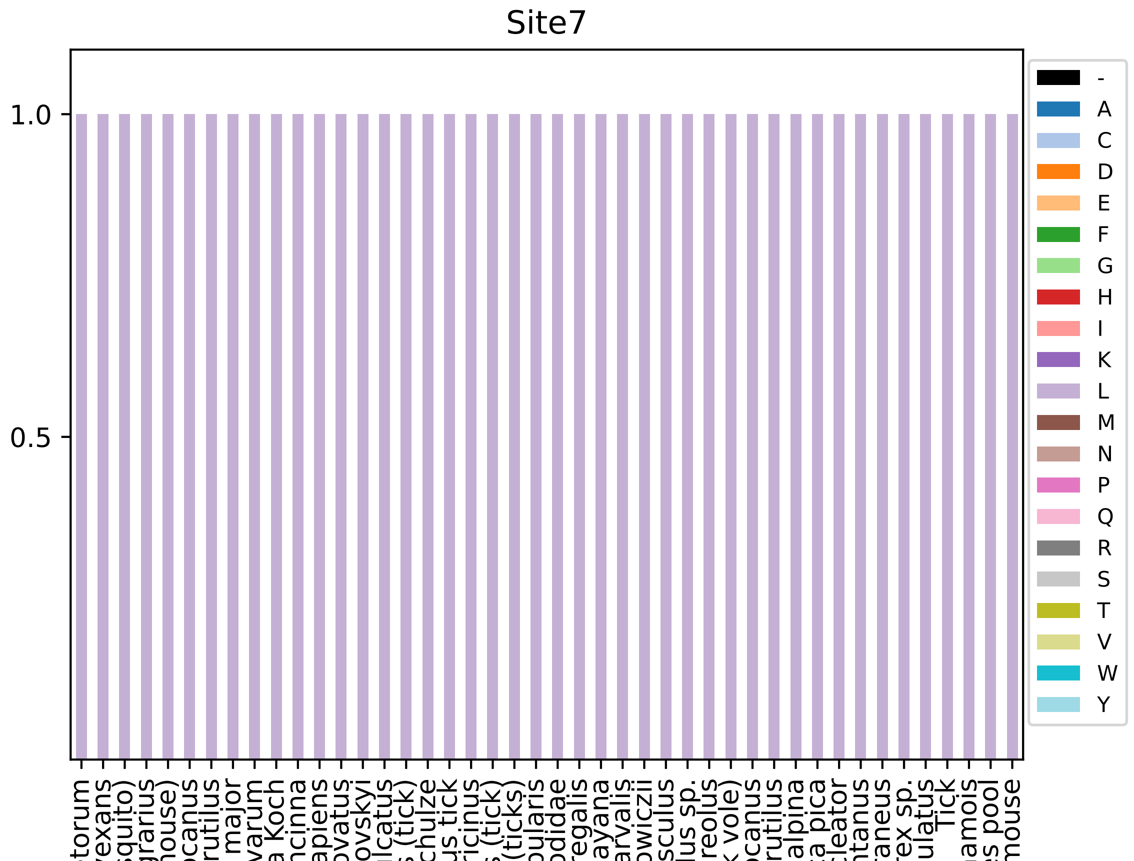
Site7
1.0
0.5
-
A
C
D
E
F
G
H
I
K
L
M
N
P
Q
R
S
T
V
W
Y

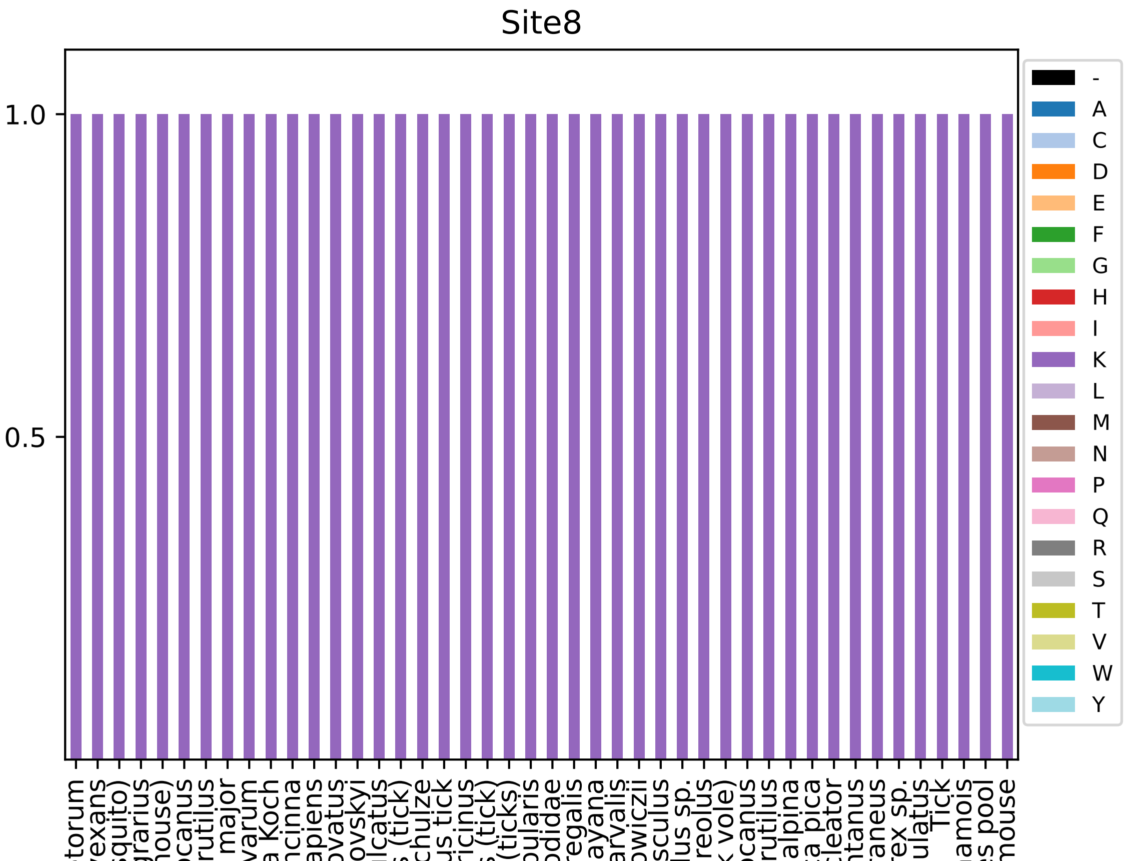
Site8
1.0
0.5
-
A
C
D
E
F
G
H
I
K
L
M
N
P
Q
R
S
T
V
W
Y

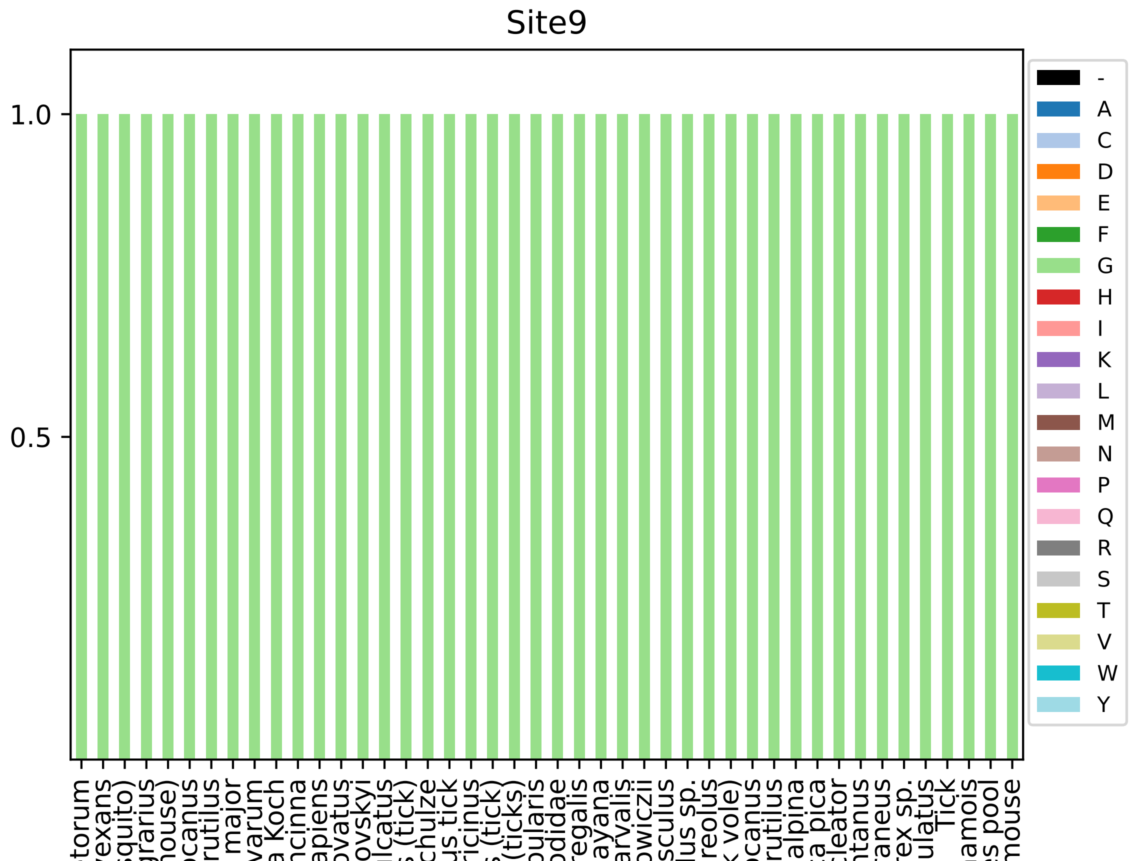

Site9
1.0
0.5
-
A
C
D
E
F
G
H
I
K
L
M
N
P
Q
R
S
T
V
W
Y

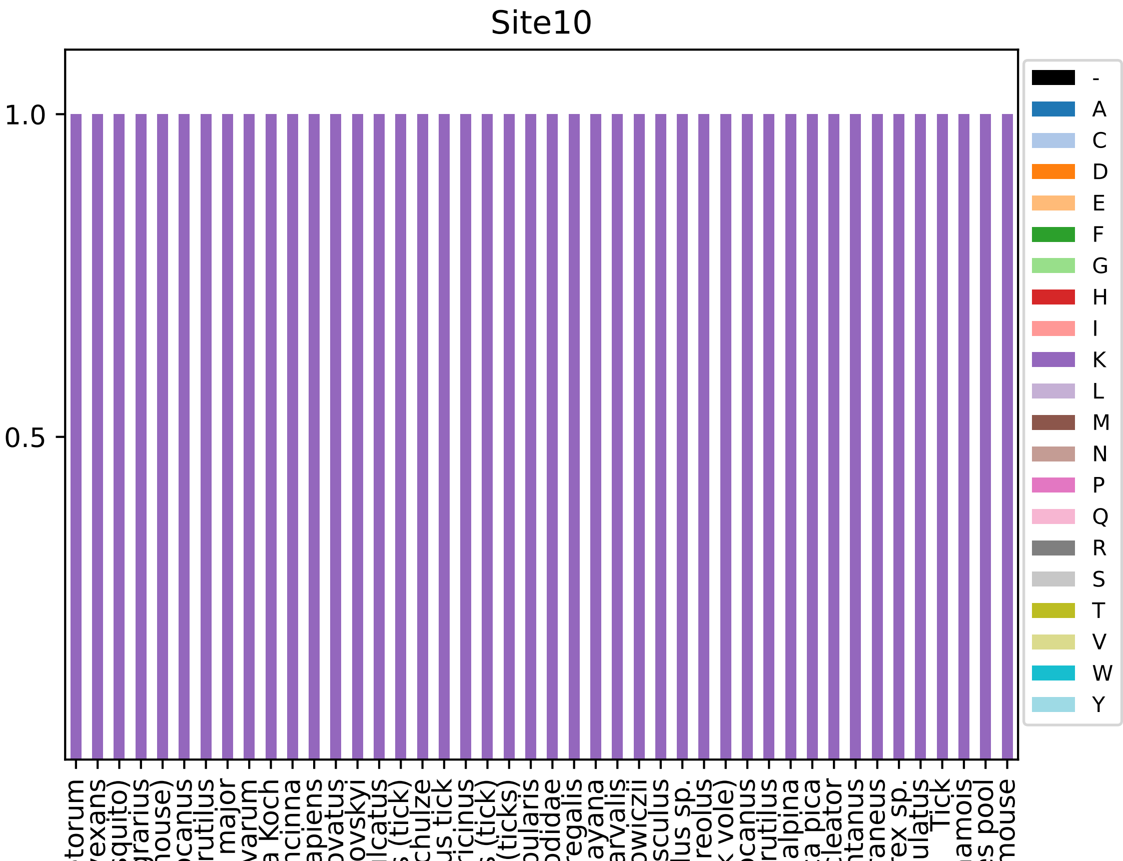
Site10
1.0
0.5
-
A
C
D
E
F
G
H
I
K
L
M
N
P
Q
R
S
T
V
W
Y

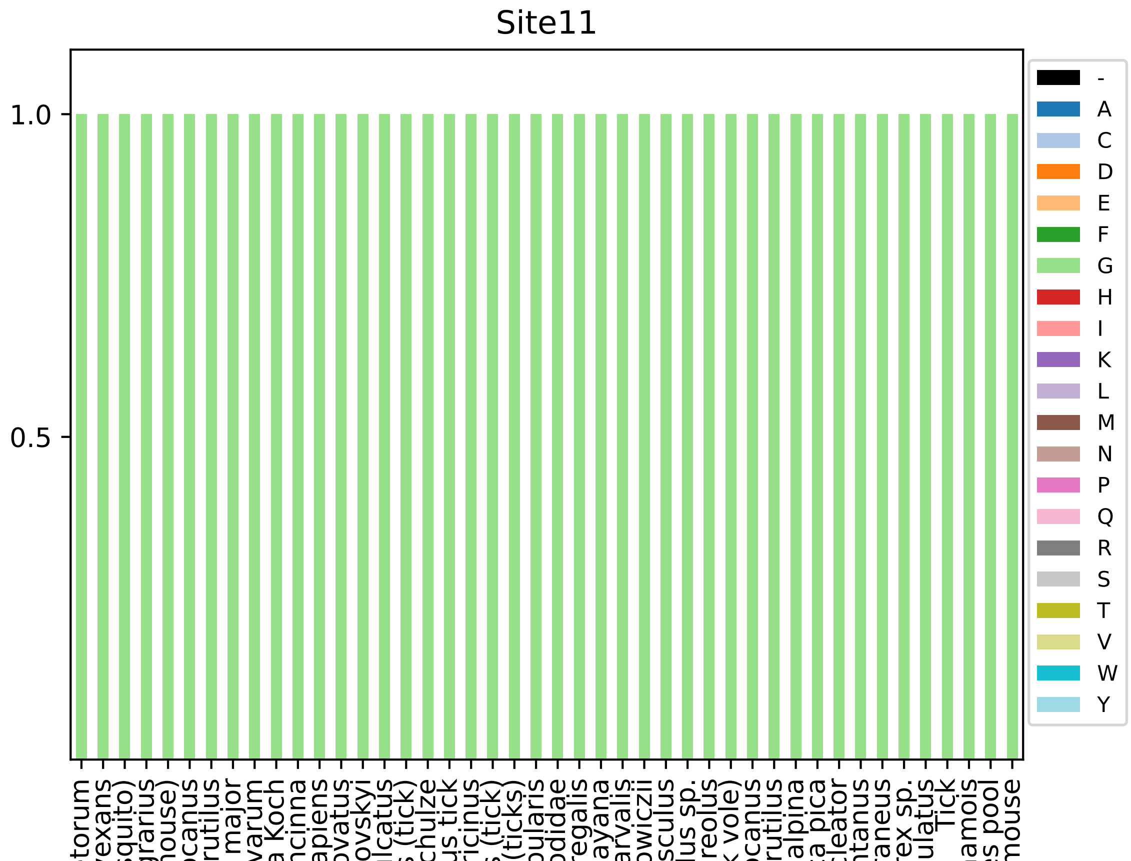
Site11
1.0
0.5
-
A
C
D
E
F
G
H
I
K
L
M
N
P
Q
R
S
T
V
W
Y

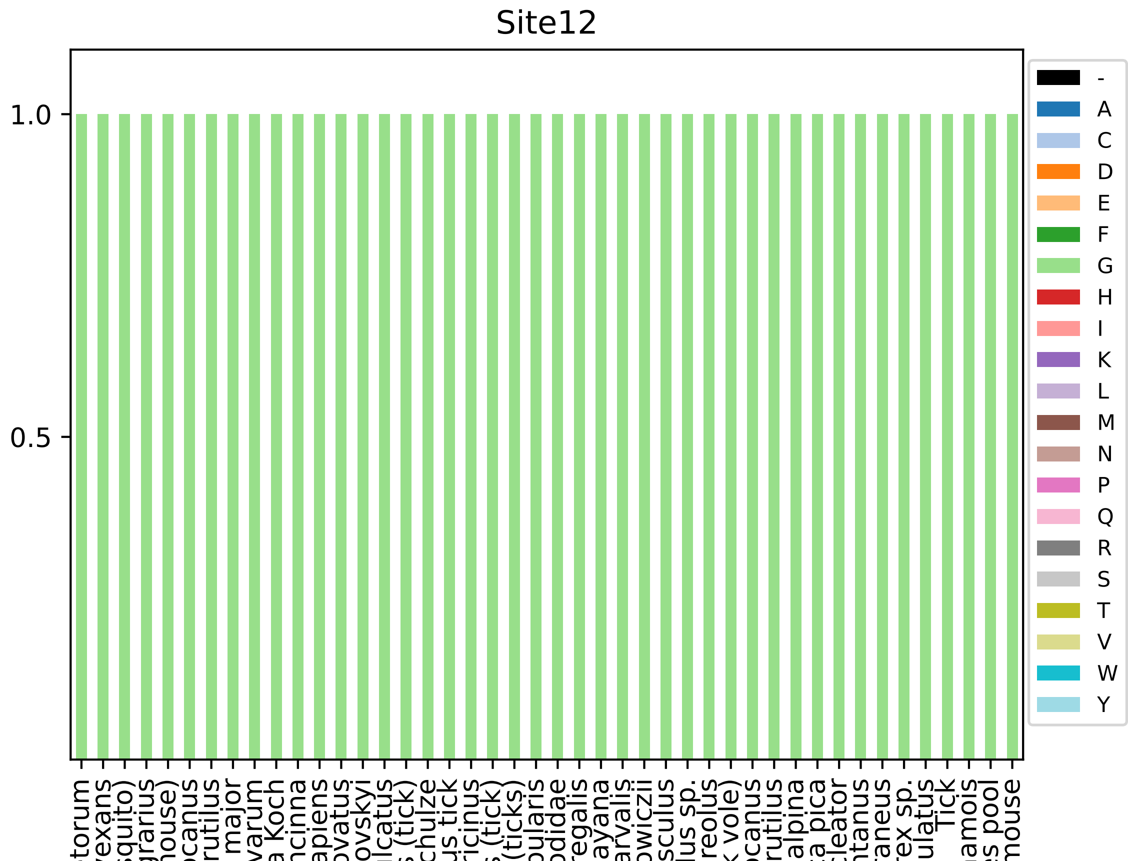
Site12
1.0
0.5
-
A
C
D
E
F
G
H
I
K
L
M
N
P
Q
R
S
T
V
W
Y

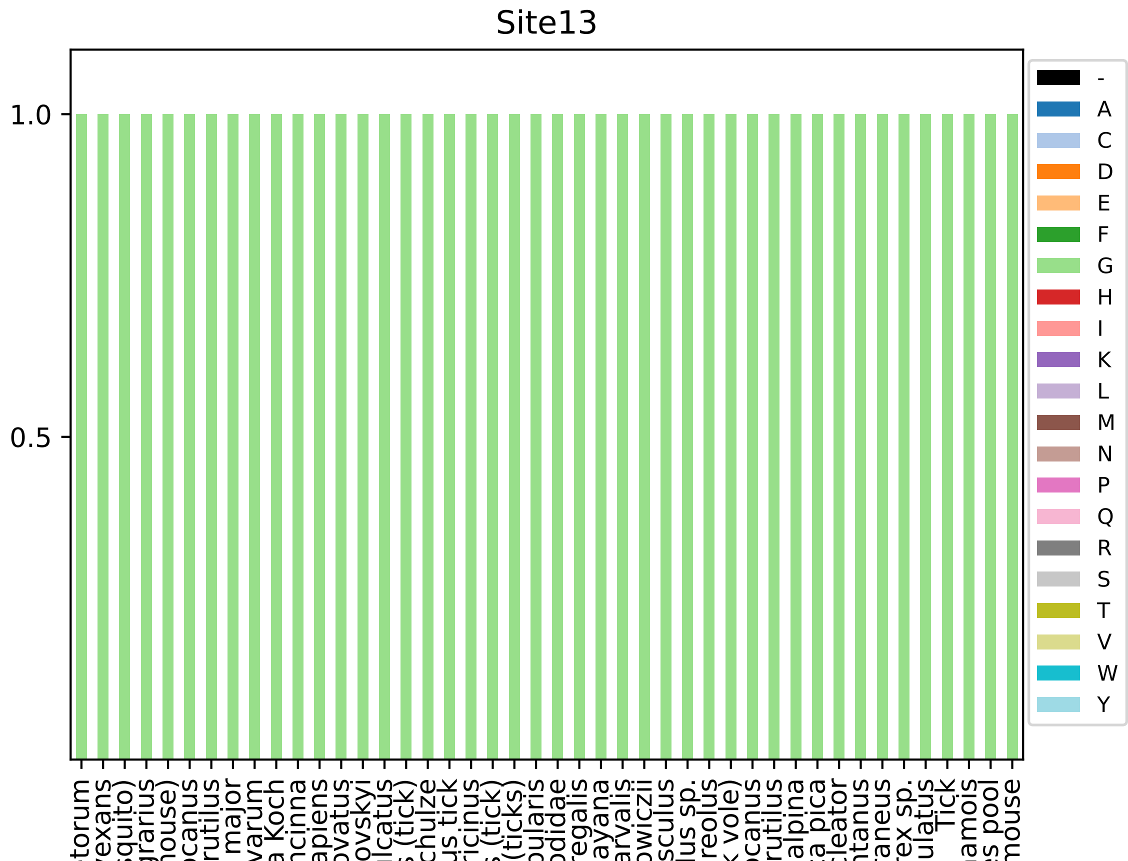
Site13
1.0
0.5
-
A
C
D
E
F
G
H
I
K
L
M
N
P
Q
R
S
T
V
W
Y

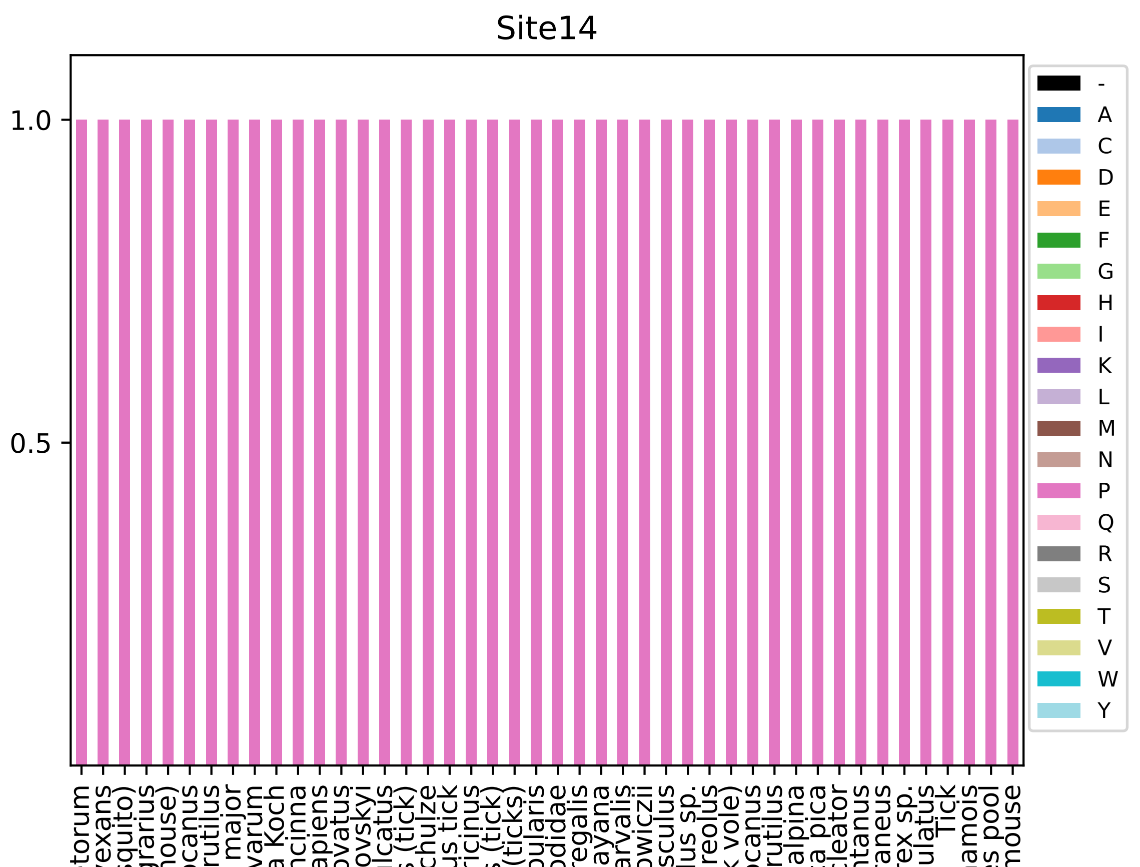
Site14
1.0
0.5
-
A
C
D
E
F
G
H
I
K
L
M
N
P
Q
R
S
T
V
W
Y

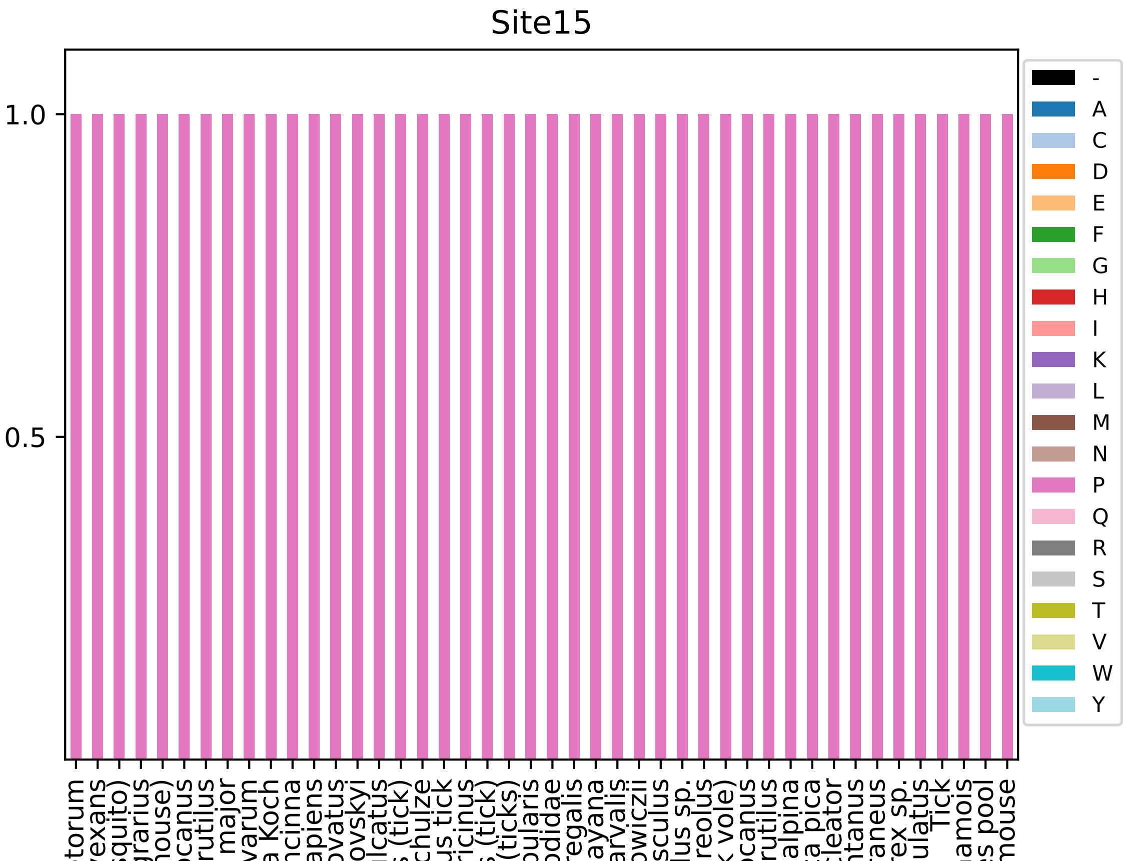
Site15
1.0
0.5
-
A
C
D
E
F
G
H
I
K
L
M
N
P
Q
R
S
T
V
W
Y

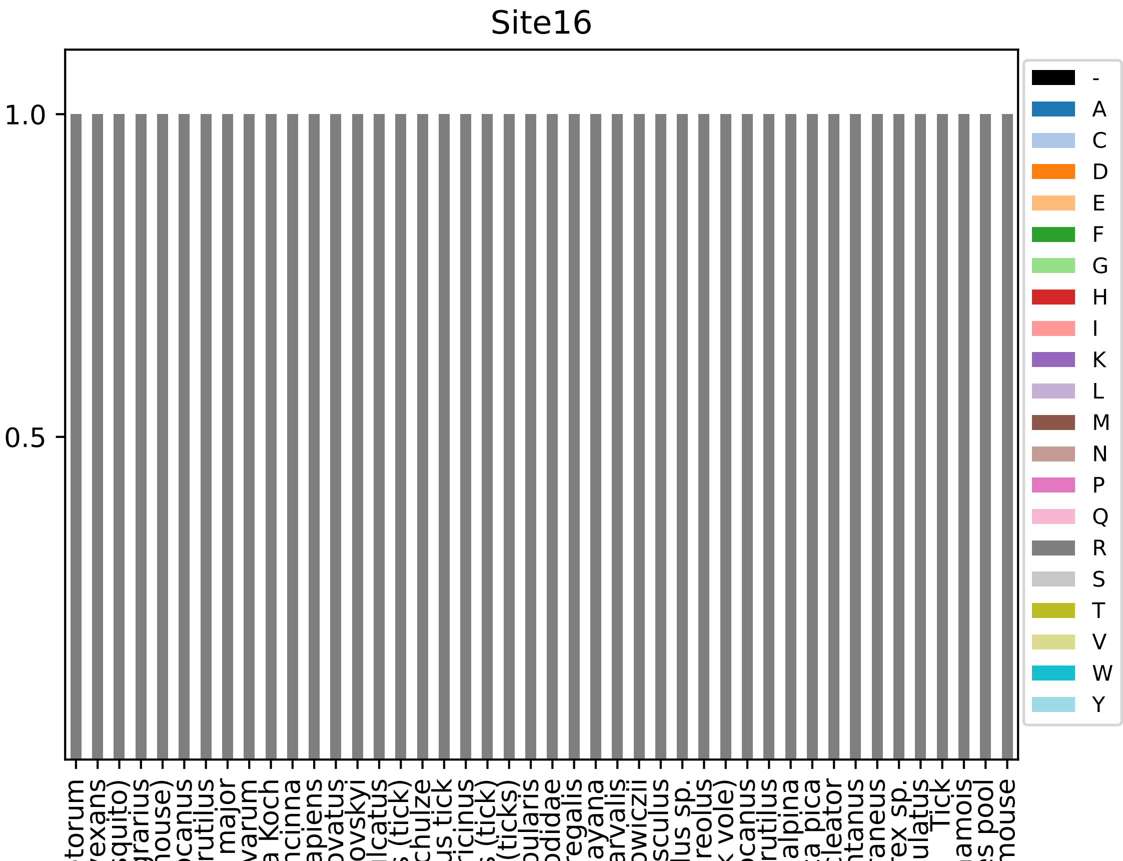

Site16
1.0
0.5
-
A
C
D
E
F
G
H
I
K
L
M
N
P
Q
R
S
T
V
W
Y

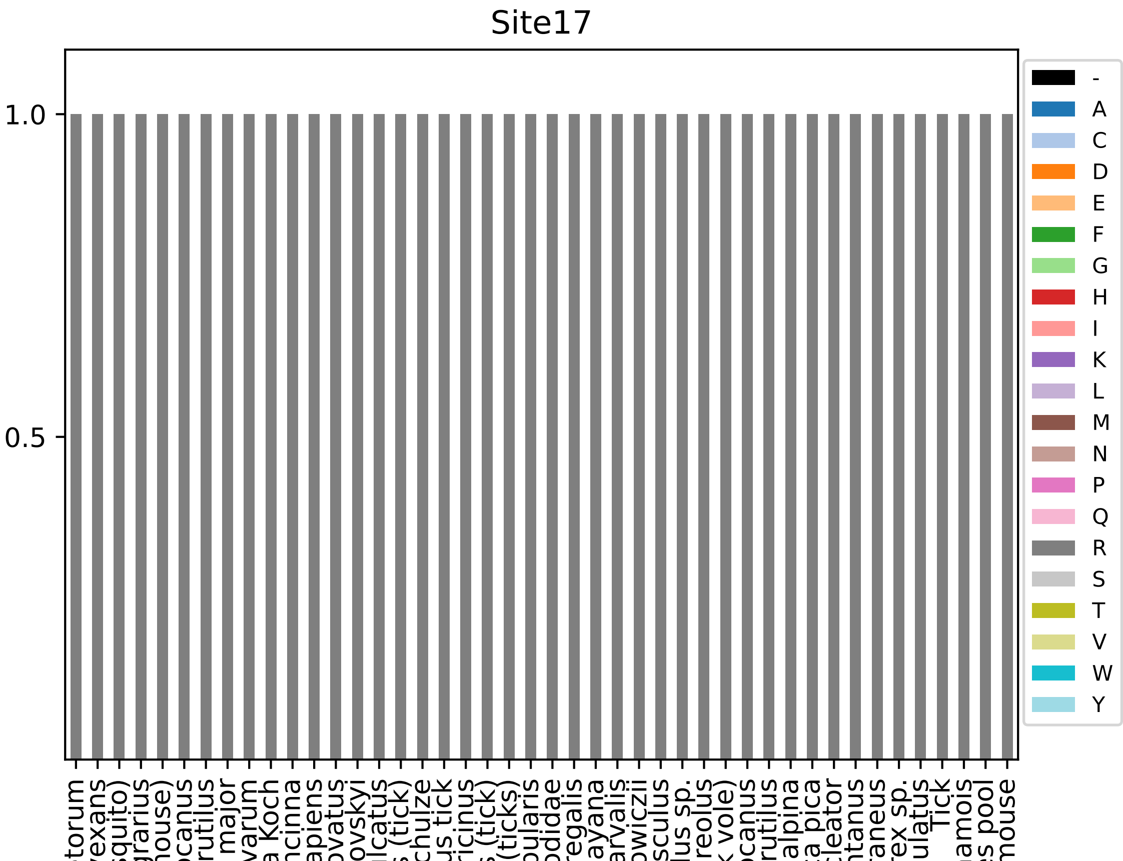
Site17
1.0
0.5
-
A
C
D
E
F
G
H
I
K
L
M
N
P
Q
R
S
T
V
W
Y

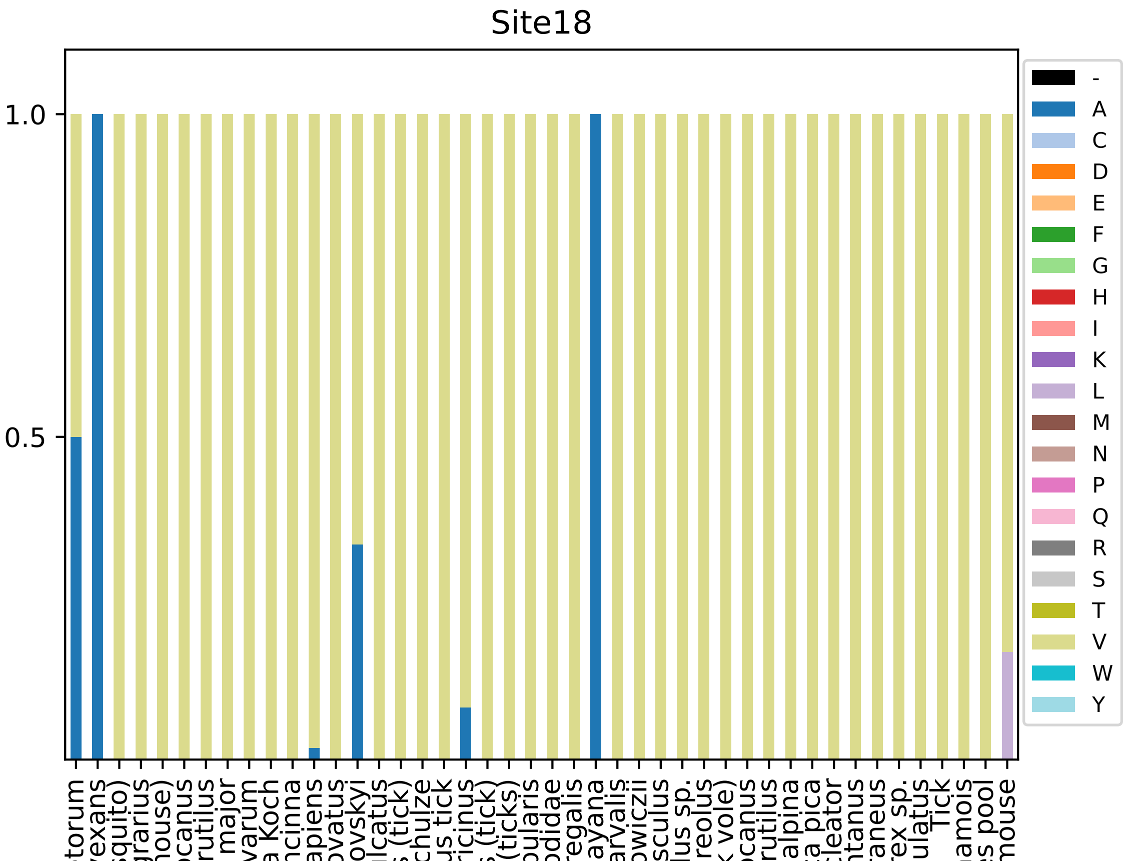
Site18
1.0
0.5
-
A
C
D
E
F
G
H
I
K
L
M
N
P
Q
R
S
T
V
W
Y

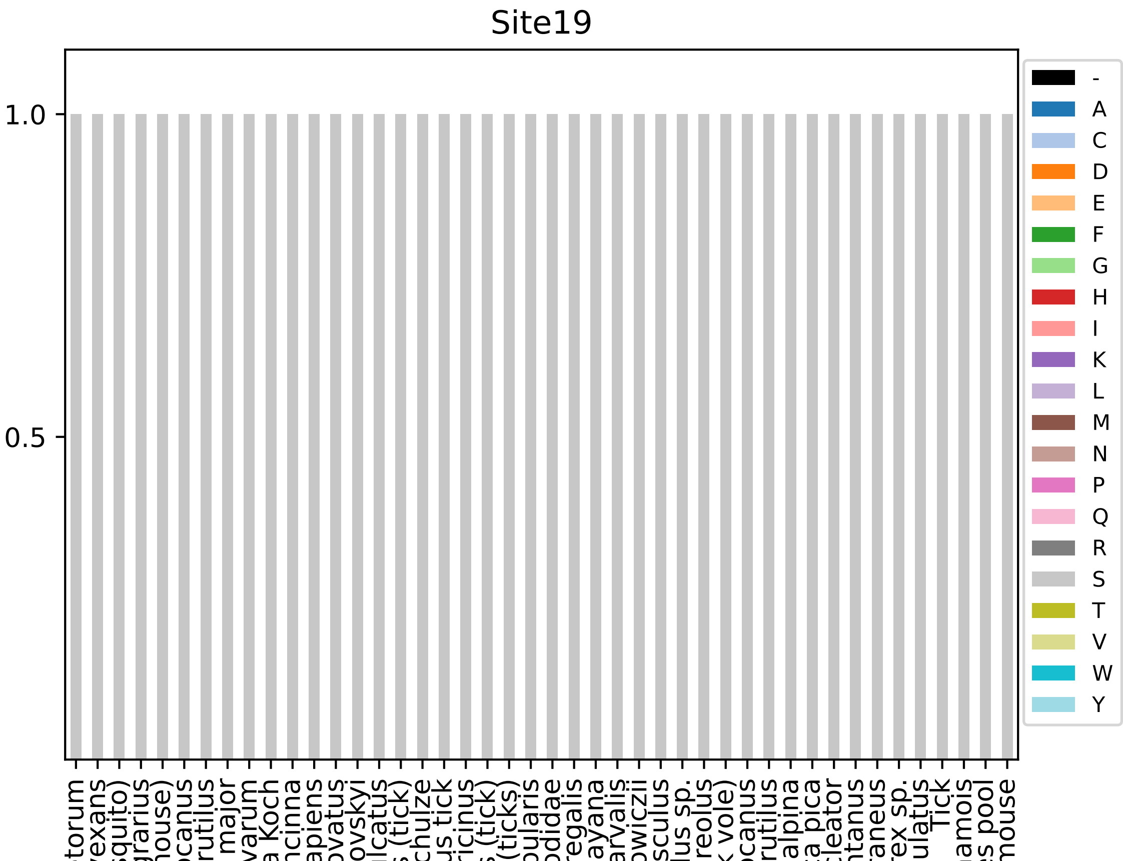
Site19
1.0
0.5
-
A
C
D
E
F
G
H
I
K
L
M
N
P
Q
R
S
T
V
W
Y

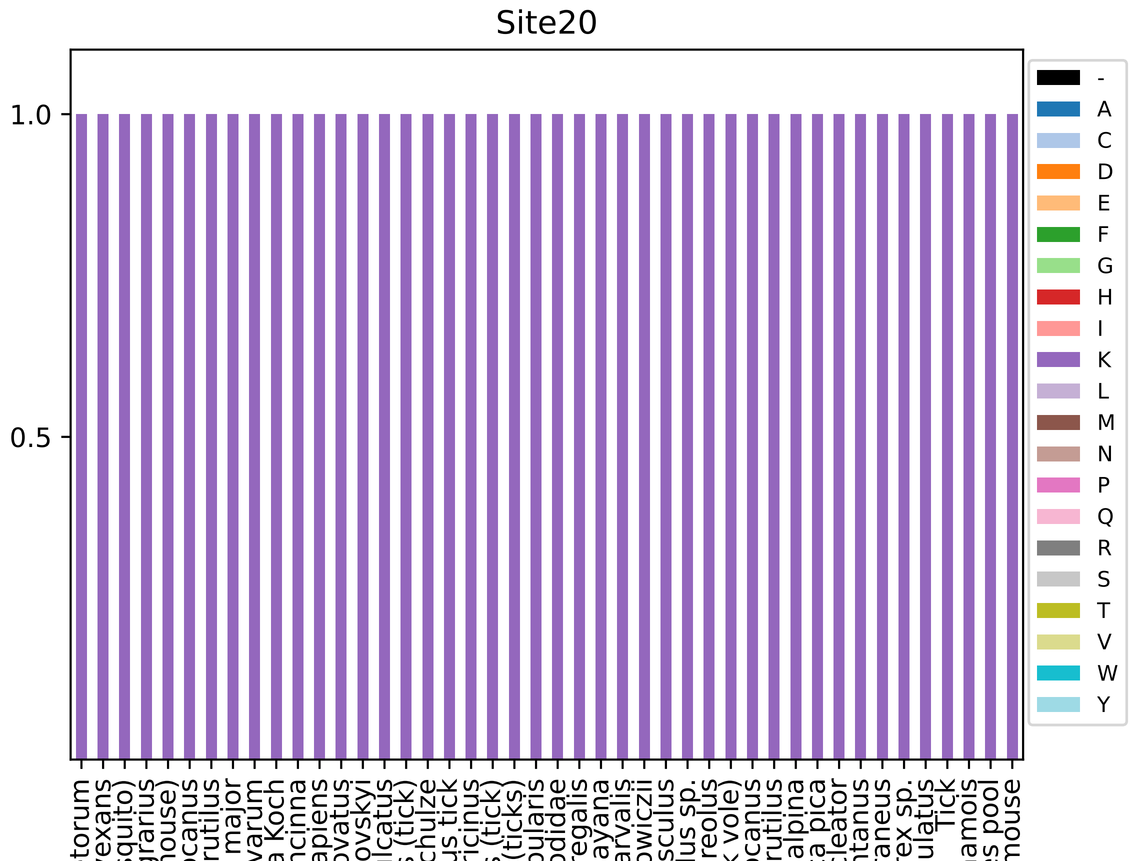
Site20
1.0
0.5
-
A
C
D
E
F
G
H
I
K
L
M
N
P
Q
R
S
T
V
W
Y
Tick

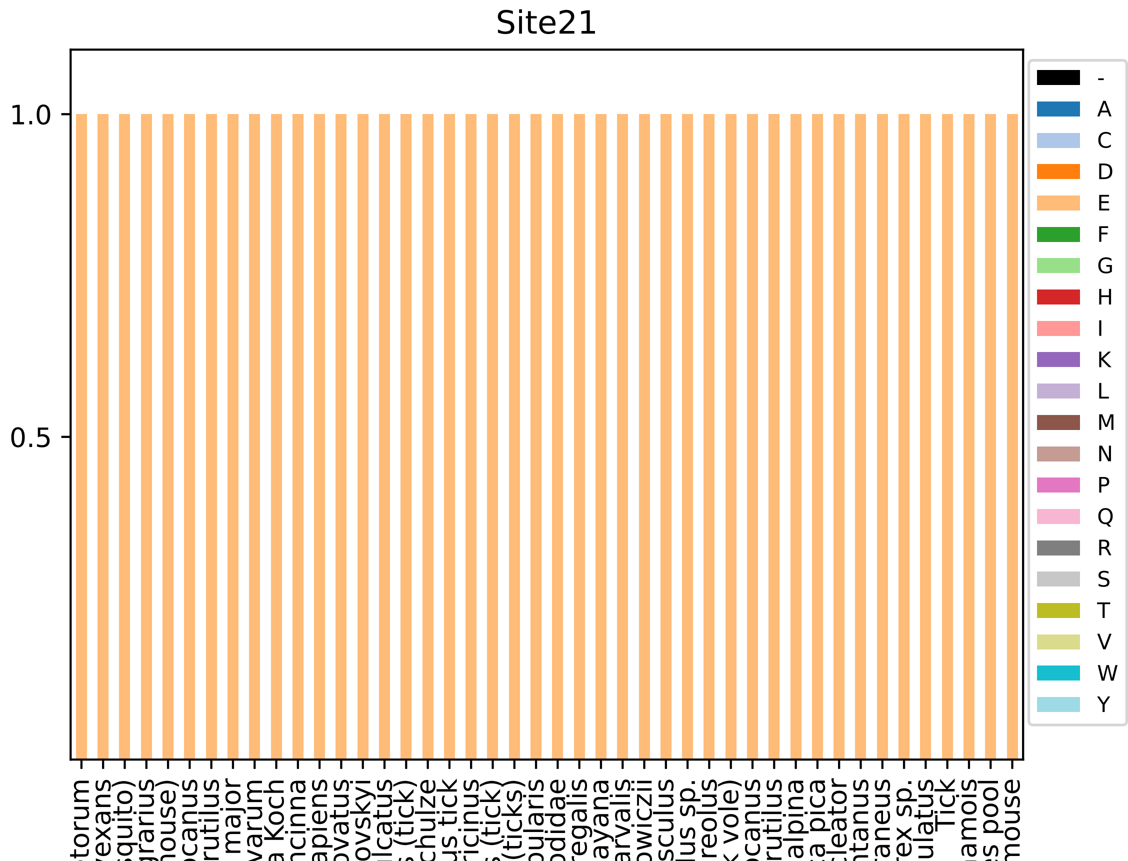
Site21
1.0
0.5
-
A
C
D
E
F
G
H
I
K
L
M
N
P
Q
R
S
T
V
W
Y

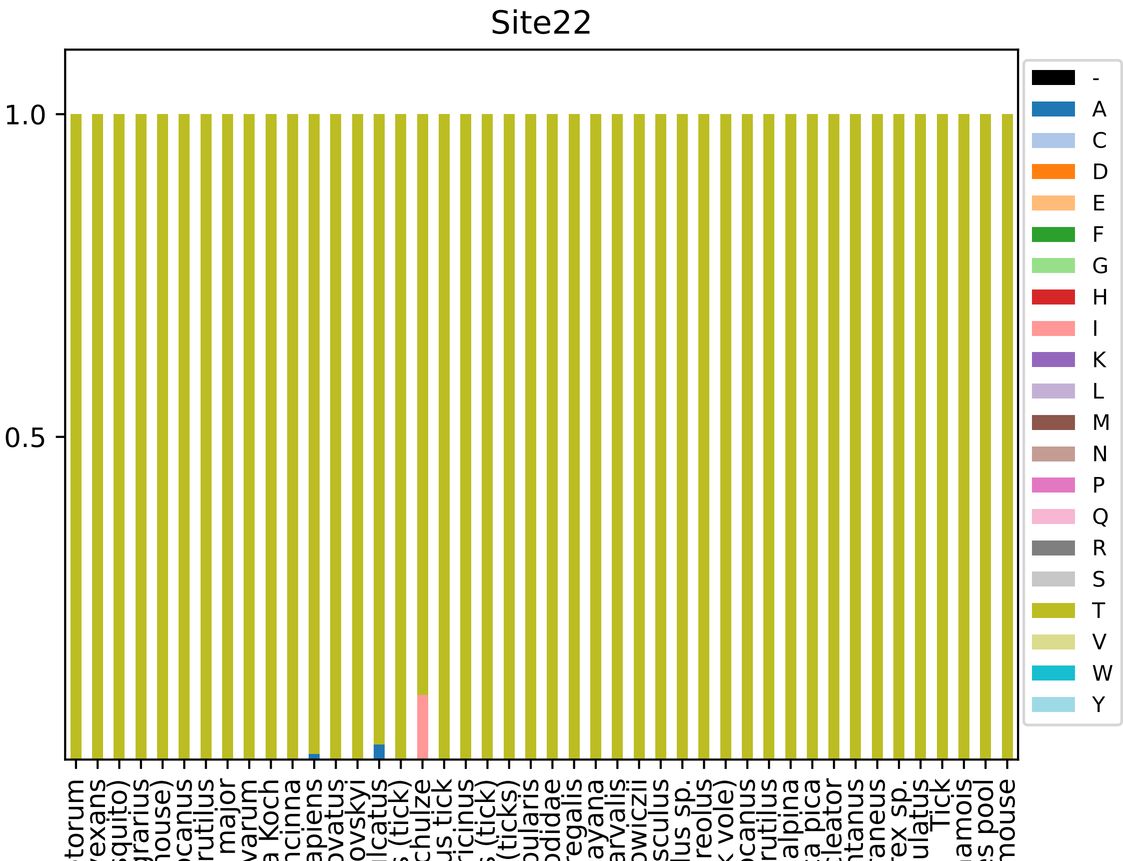
Site22
1.0
0.5
-
A
C
D
E
F
G
H
I
K
L
M
N
P
Q
R
S
T
V
W
Y
Tick

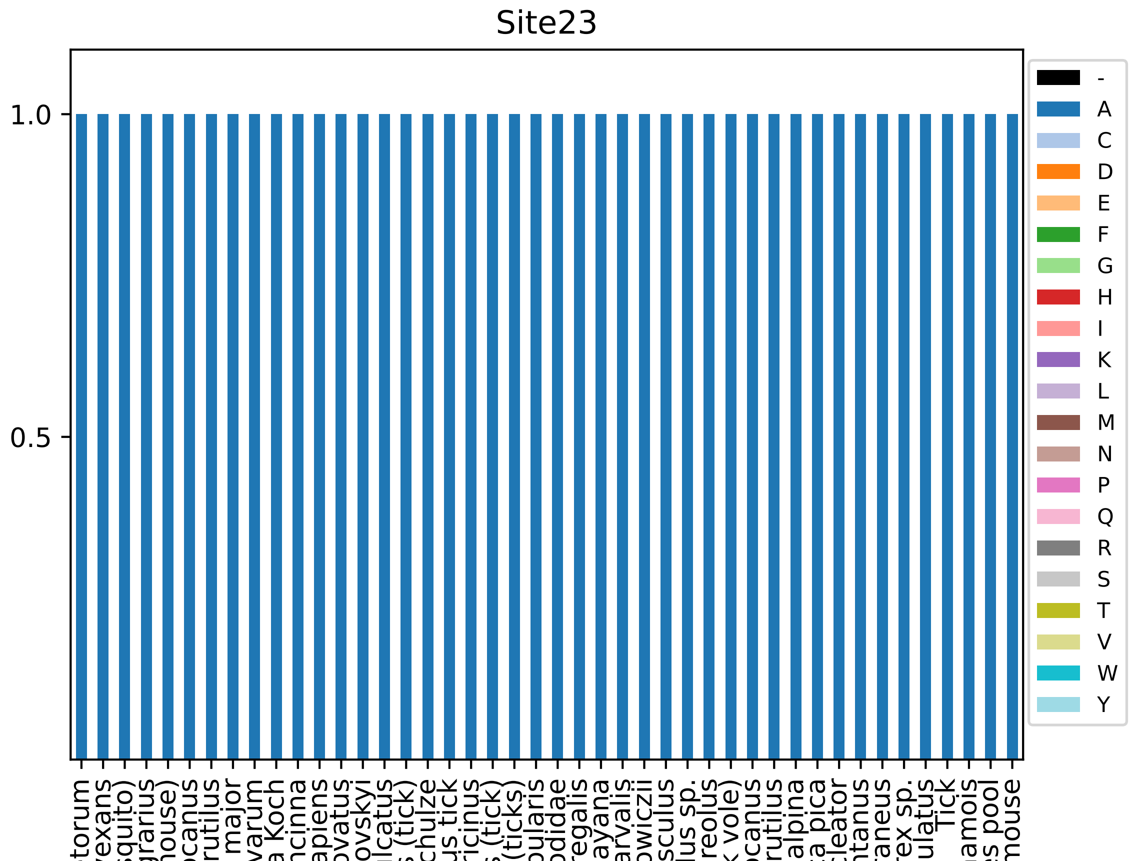
Site23
1.0
0.5
-
A
C
D
E
F
G
H
I
K
L
M
N
P
Q
R
S
T
V
W
Y

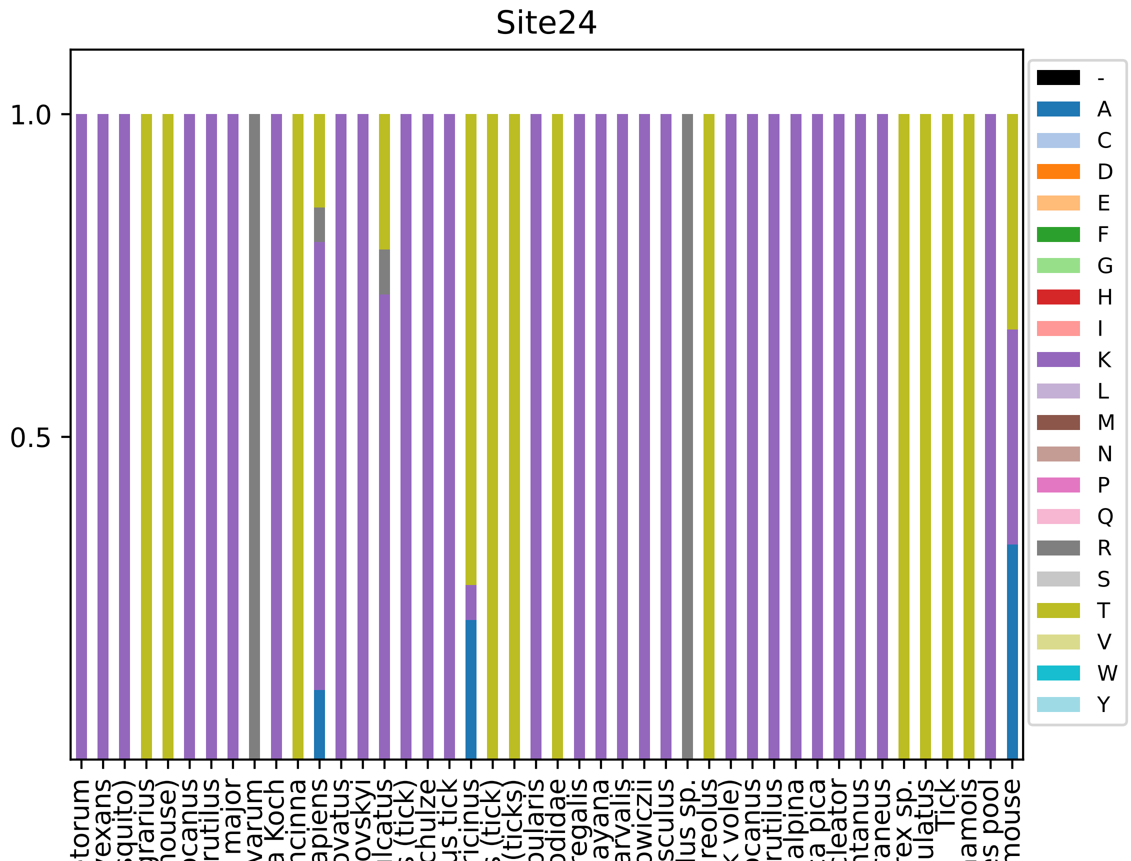
Site24
1.0
0.5
-
A
C
D
E
F
G
H
I
K
L
M
N
P
Q
R
S
T
V
W
Y

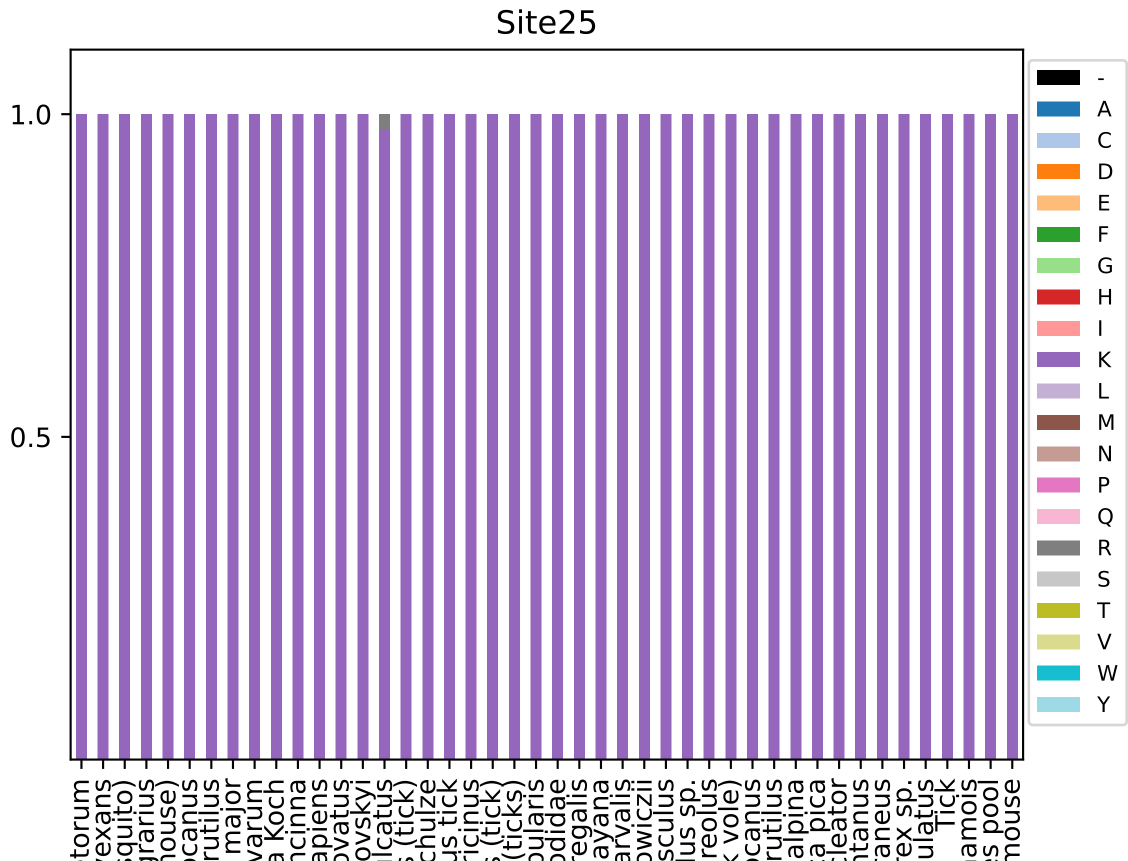

Site25
1.0
0.5
-
A
C
D
E
F
G
H
I
K
L
M
N
P
Q
R
S
T
V
W
Y

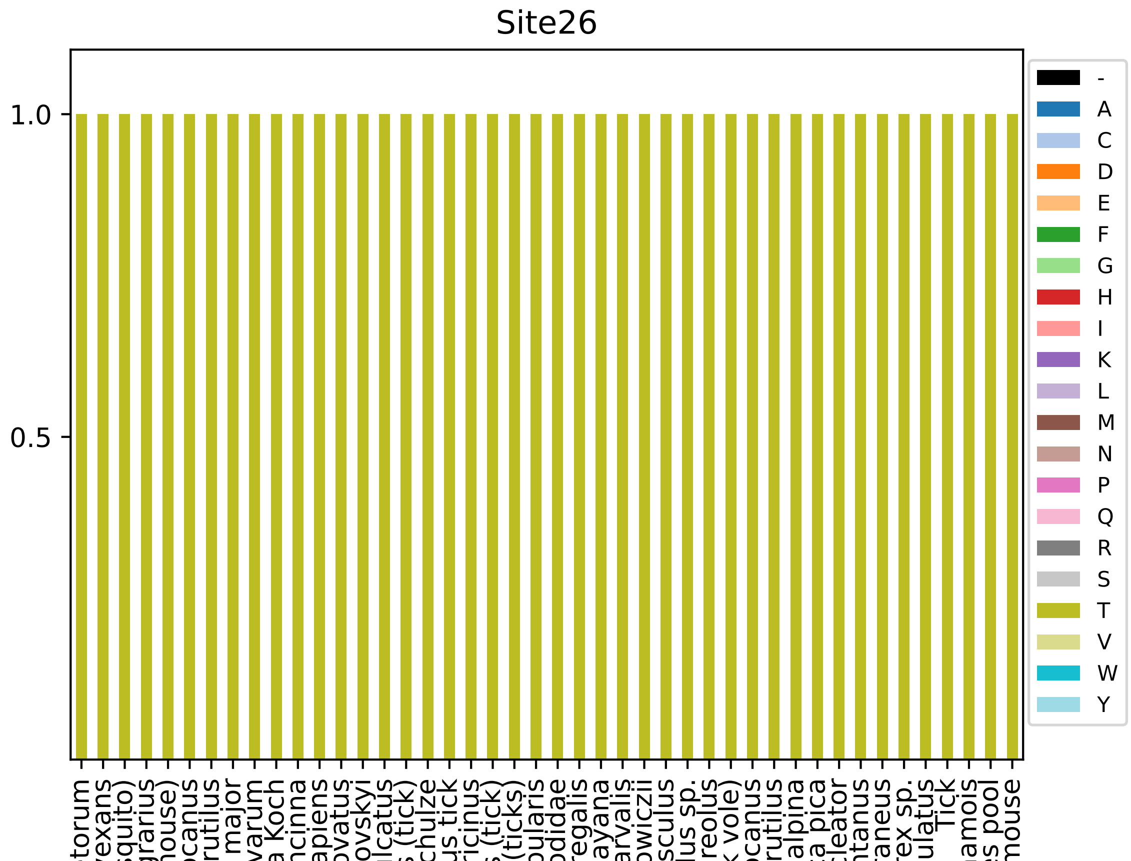
Site26
1.0
0.5
-
A
C
D
E
F
G
H
I
K
L
M
N
P
Q
R
S
T
V
W
Y

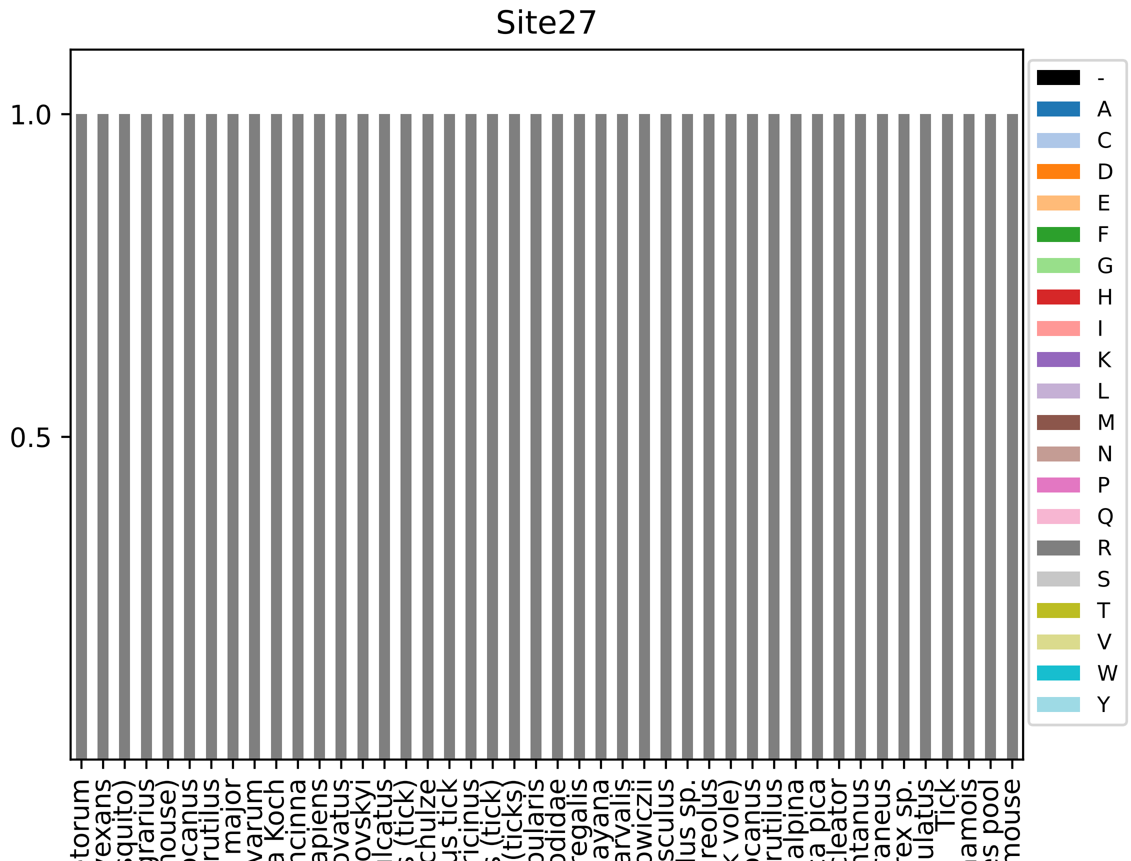
Site27
1.0
0.5
-
A
C
D
E
F
G
H
I
K
L
M
N
P
Q
R
S
T
V
W
Y

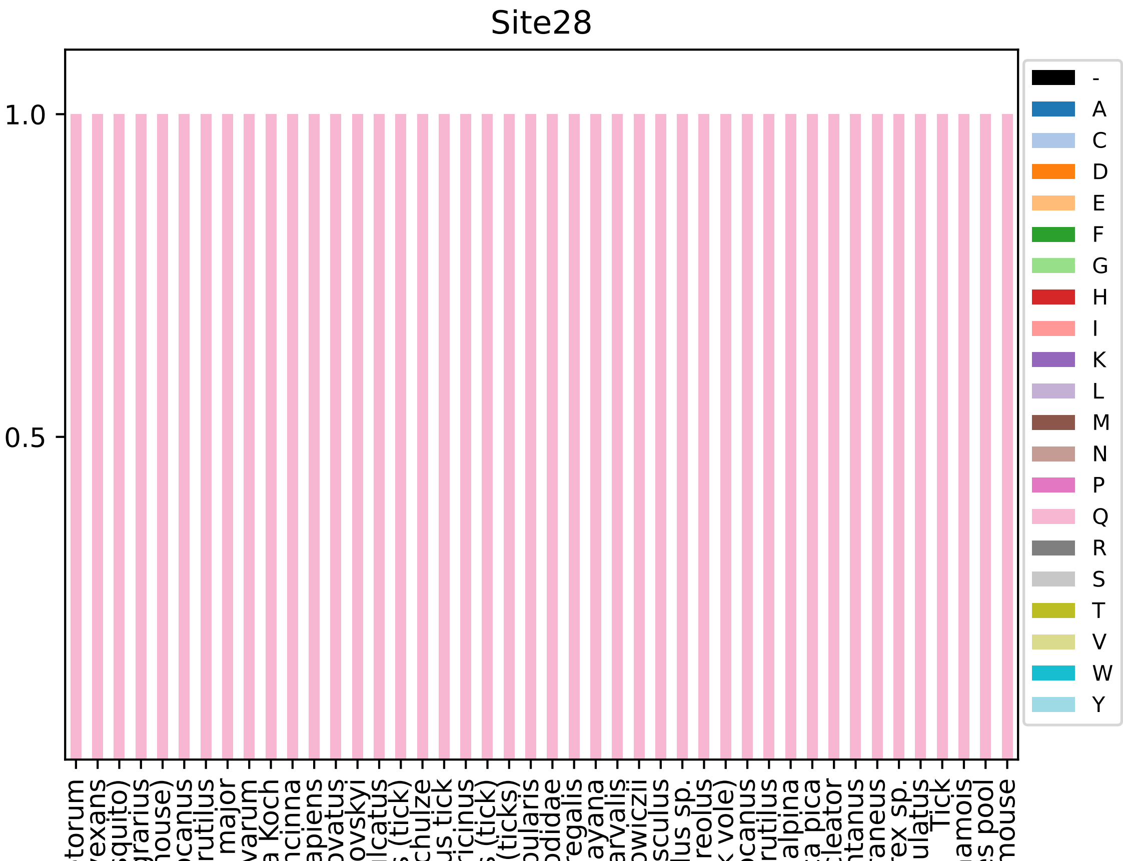
Site28
1.0
0.5
-
A
C
D
E
F
G
H
I
K
L
M
N
P
Q
R
S
T
V
W
Y

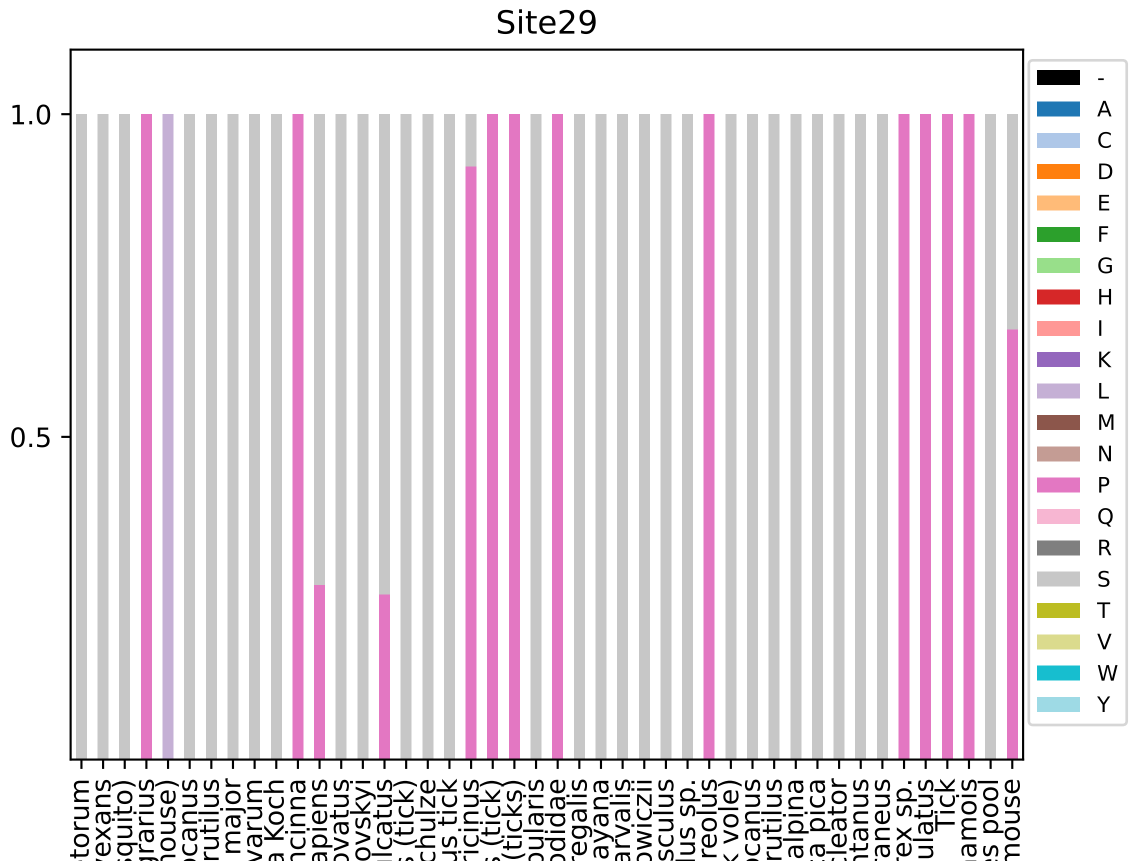
Site29
1.0
0.5
-
A
C
D
E
F
G
H
I
K
L
M
N
P
Q
R
S
T
V
W
Y

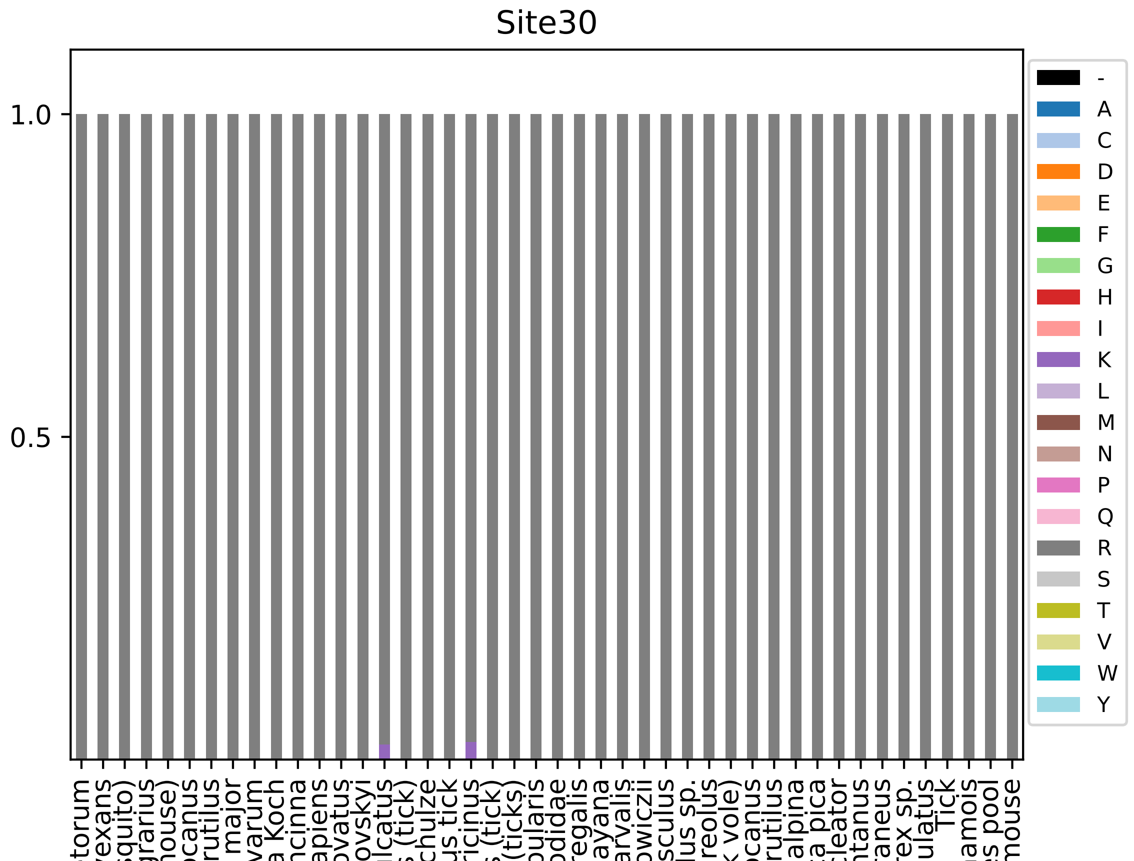

Site30
1.0
0.5
-
A
C
D
E
F
G
H
I
K
L
M
N
P
Q
R
S
T
V
W
Y

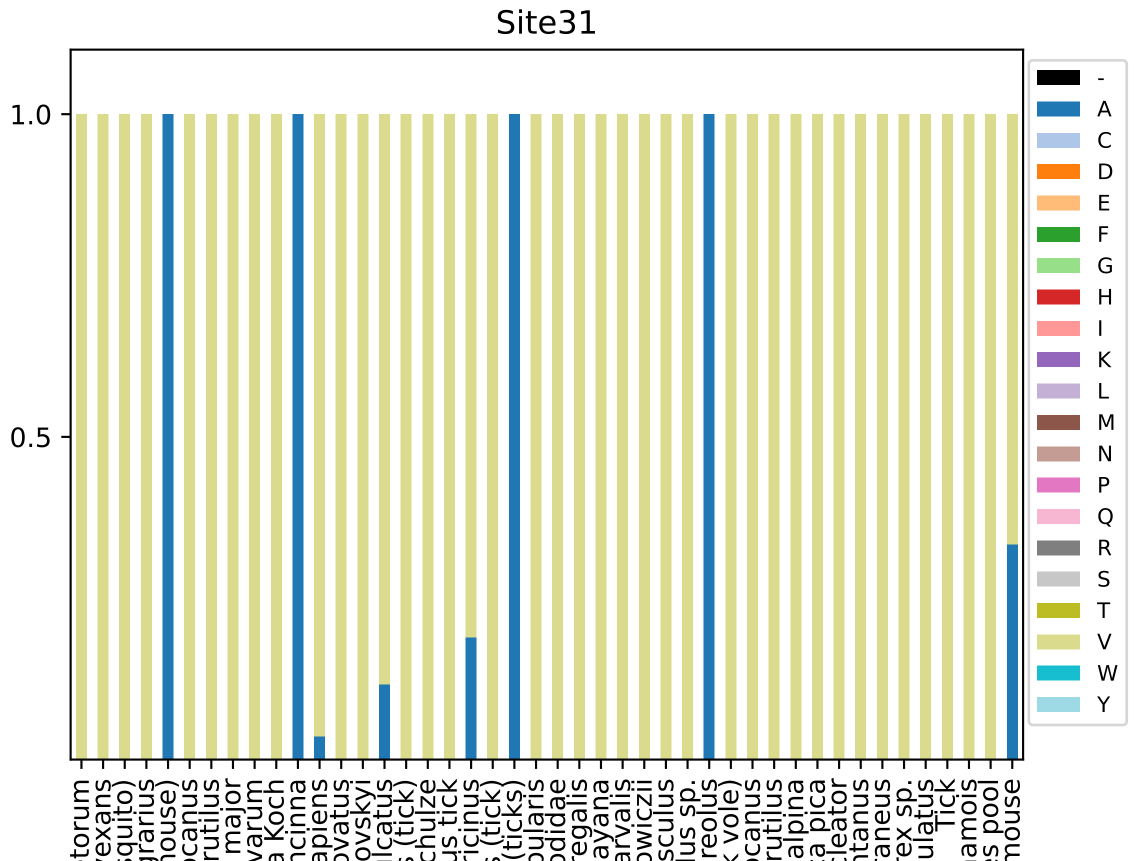

Site31
1.0
0.5
-
A
C
D
E
F
G
H
I
K
L
M
N
P
Q
R
S
T
V
W
Y

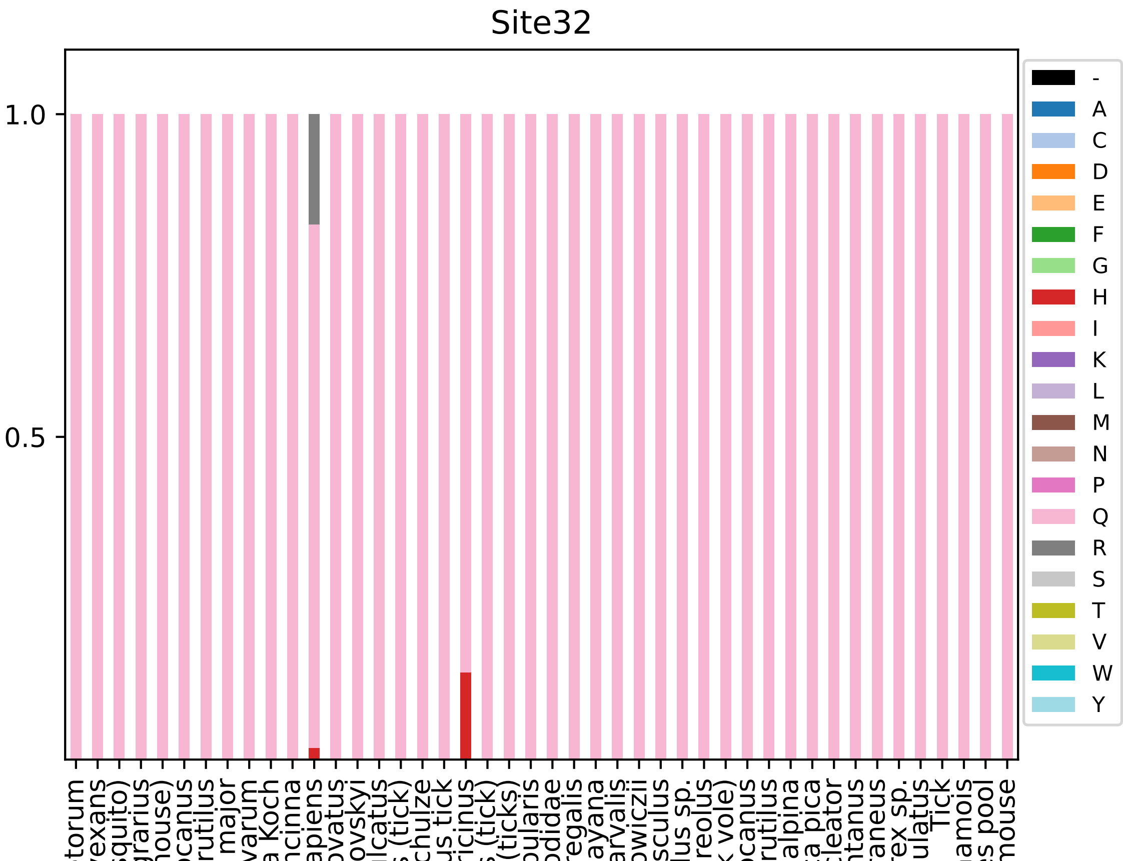
Site32
1.0
0.5
-
A
C
D
E
F
G
H
I
K
L
M
N
P
Q
R
S
T
V
W
Y

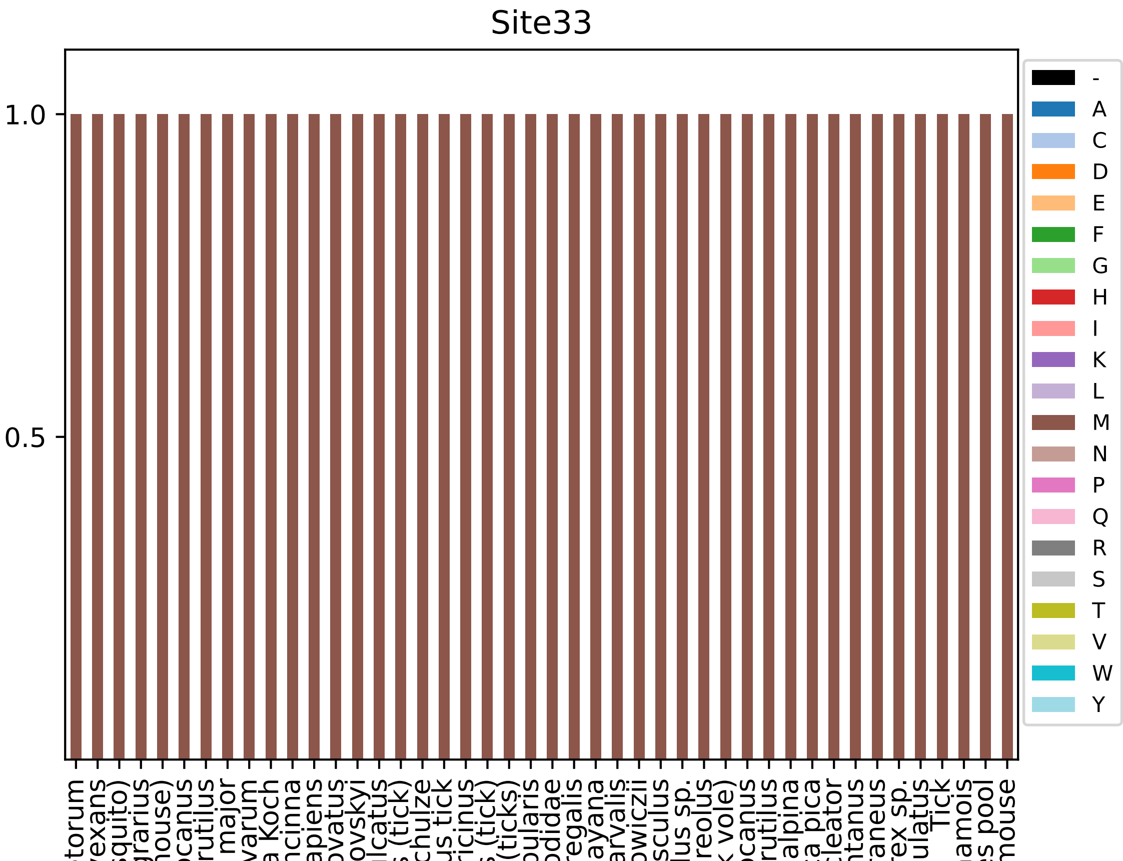
Site33
1.0
0.5
-
A
C
D
E
F
G
H
I
K
L
M
N
P
Q
R
S
T
V
W
Y

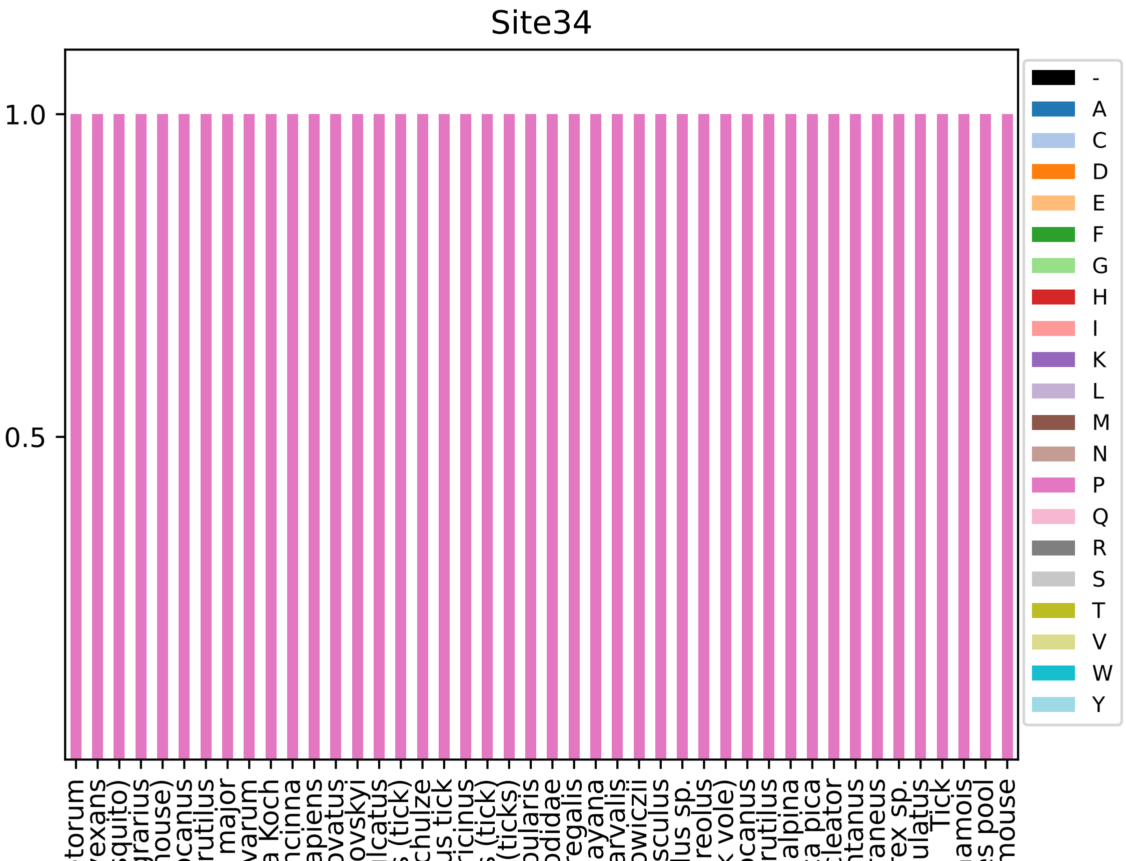

Site34
1.0
0.5
-
A
C
D
E
F
G
H
I
K
L
M
N
P
Q
R
S
T
V
W
Y

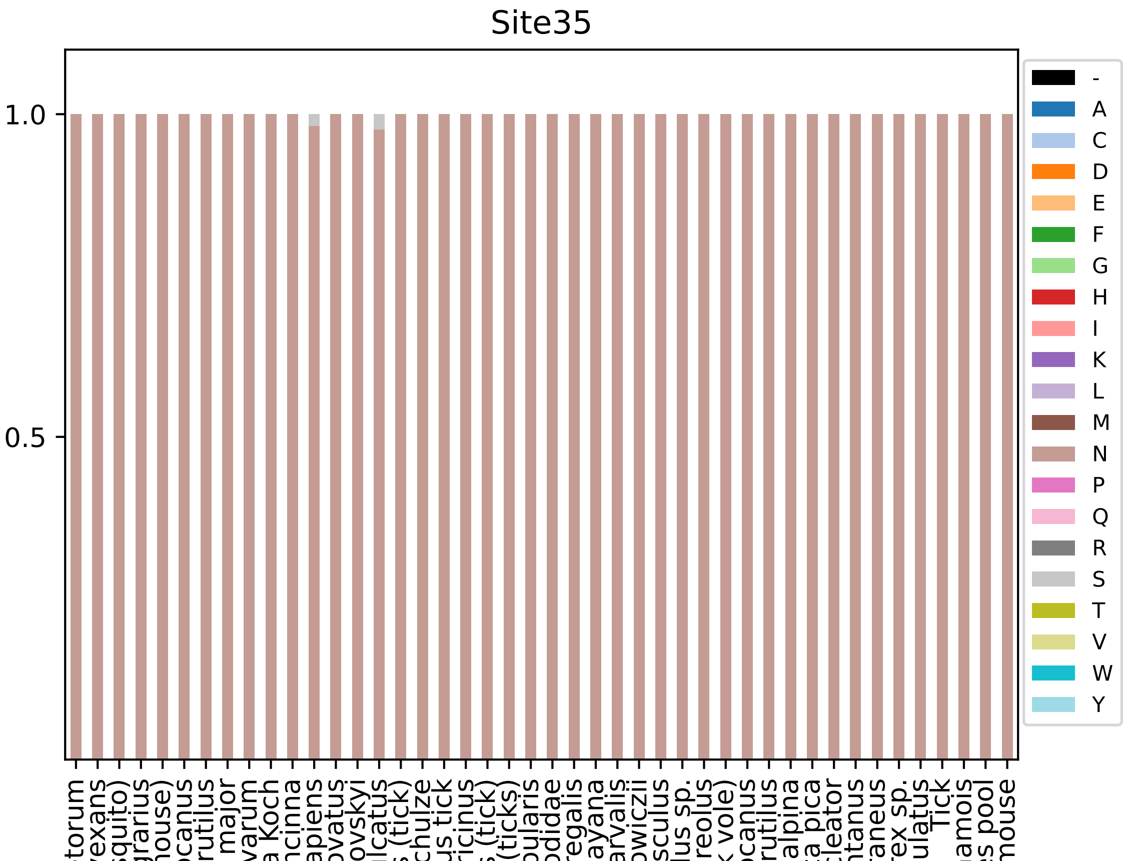
Site35
1.0
0.5
-
A
C
D
E
F
G
H
I
K
L
M
N
P
Q
R
S
T
V
W
Y

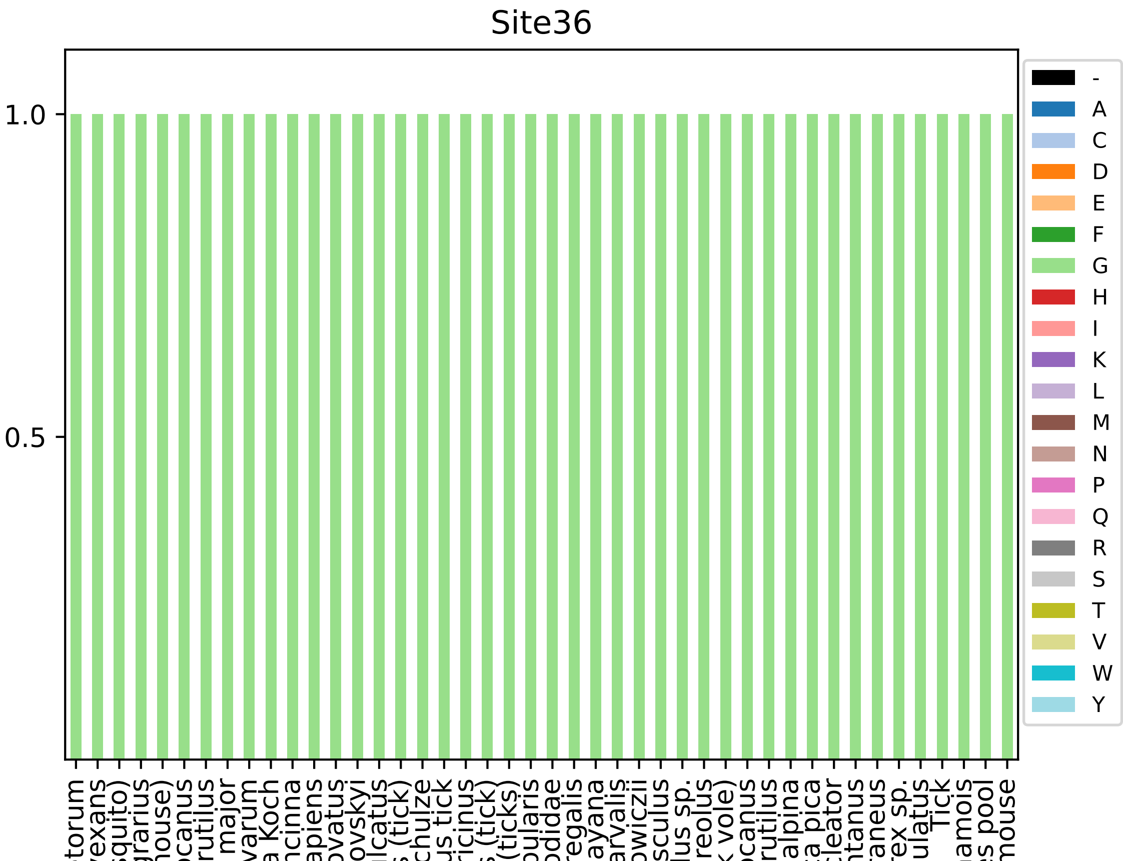
Site36
1.0
0.5
-
A
C
D
E
F
G
H
I
K
L
M
N
P
Q
R
S
T
V
W
Y

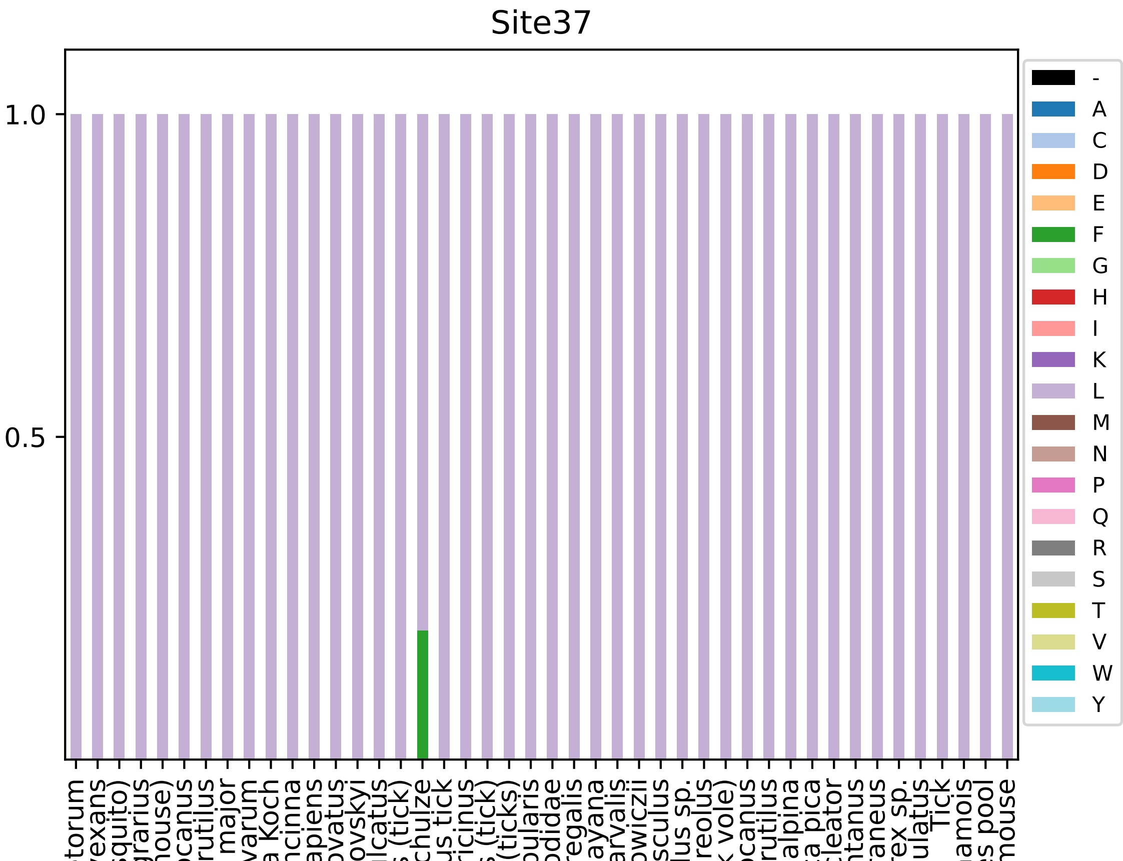
Site37
1.0
0.5
-
A
C
D
E
F
G
H
I
K
L
M
N
P
Q
R
S
T
V
W
Y

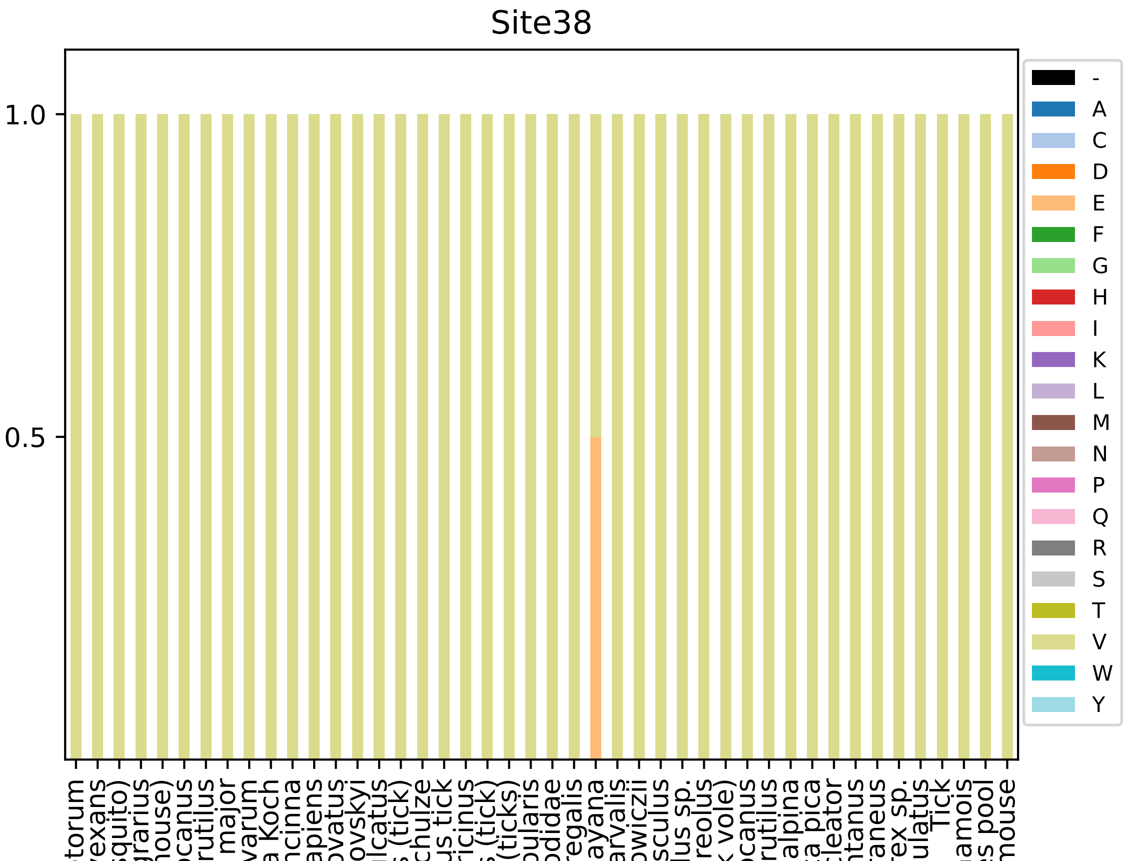
Site38
1.0
0.5
-
A
C
D
E
F
G
H
I
K
L
M
N
P
Q
R
S
T
V
W
Y

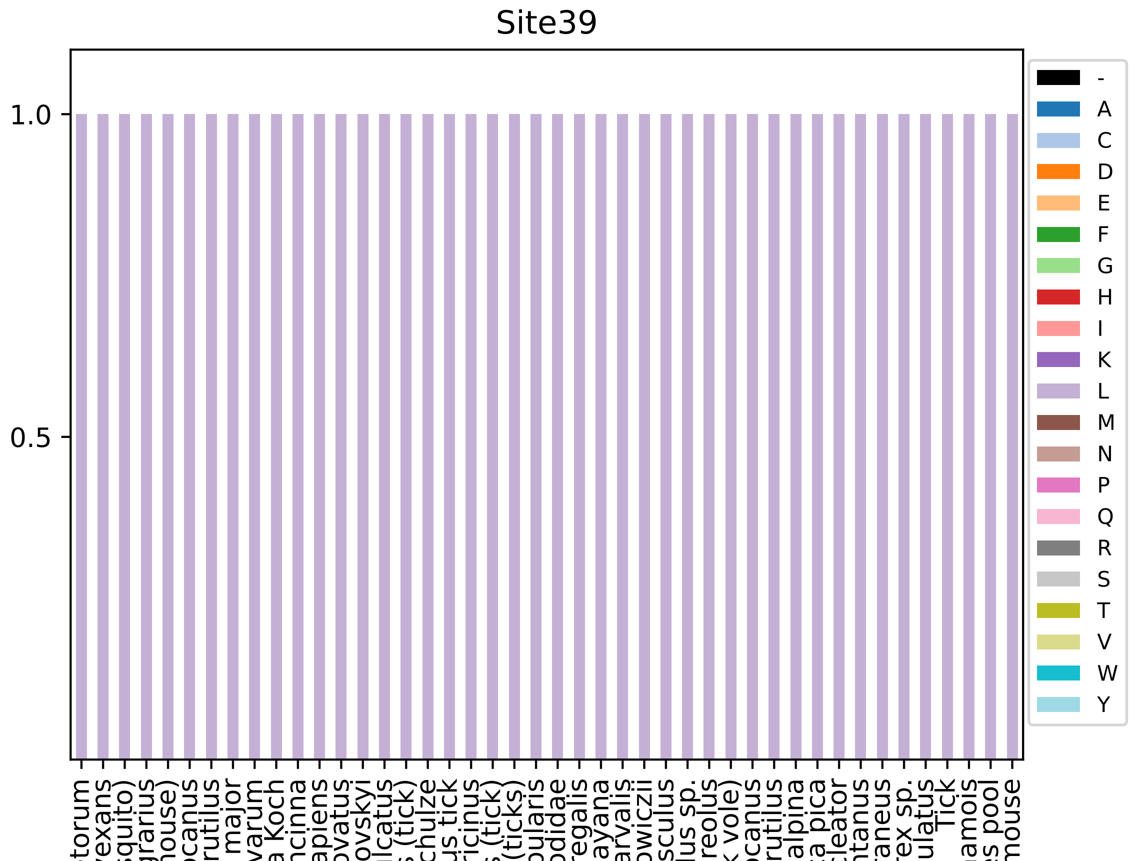
Site39
1.0
0.5
-
A
C
D
E
F
G
H
I
K
L
M
N
P
Q
R
S
T
V
W
Y

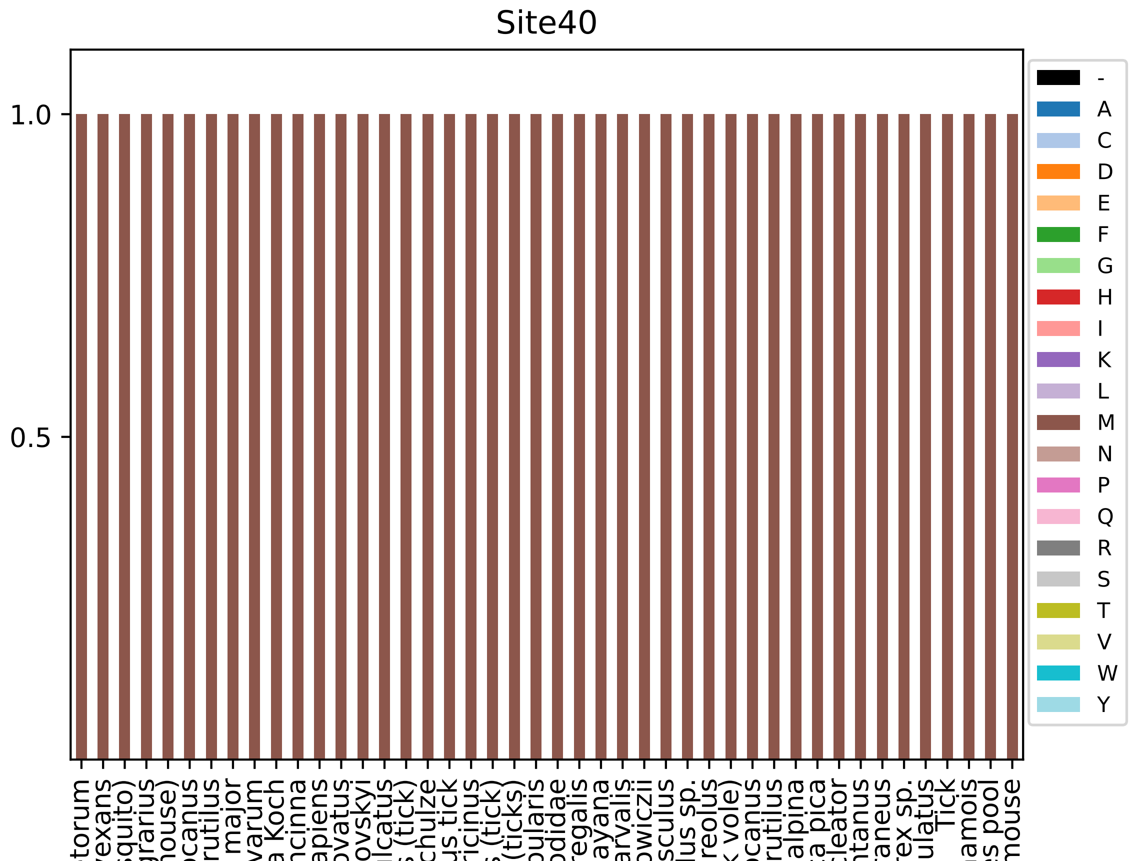
Site40
1.0
0.5
-
A
C
D
E
F
G
H
I
K
L
M
N
P
Q
R
S
T
V
W
Y

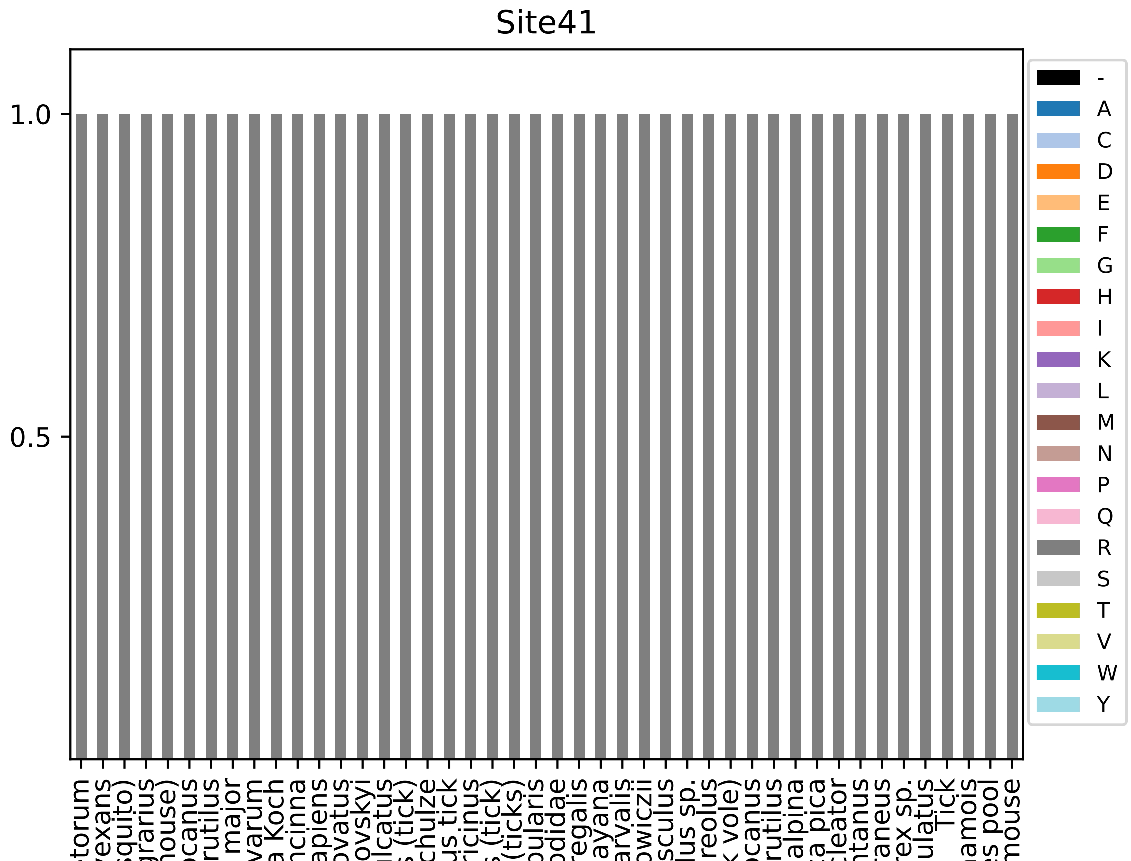
Site41
1.0
0.5
-
A
C
D
E
F
G
H
I
K
L
M
N
P
Q
R
S
T
V
W
Y

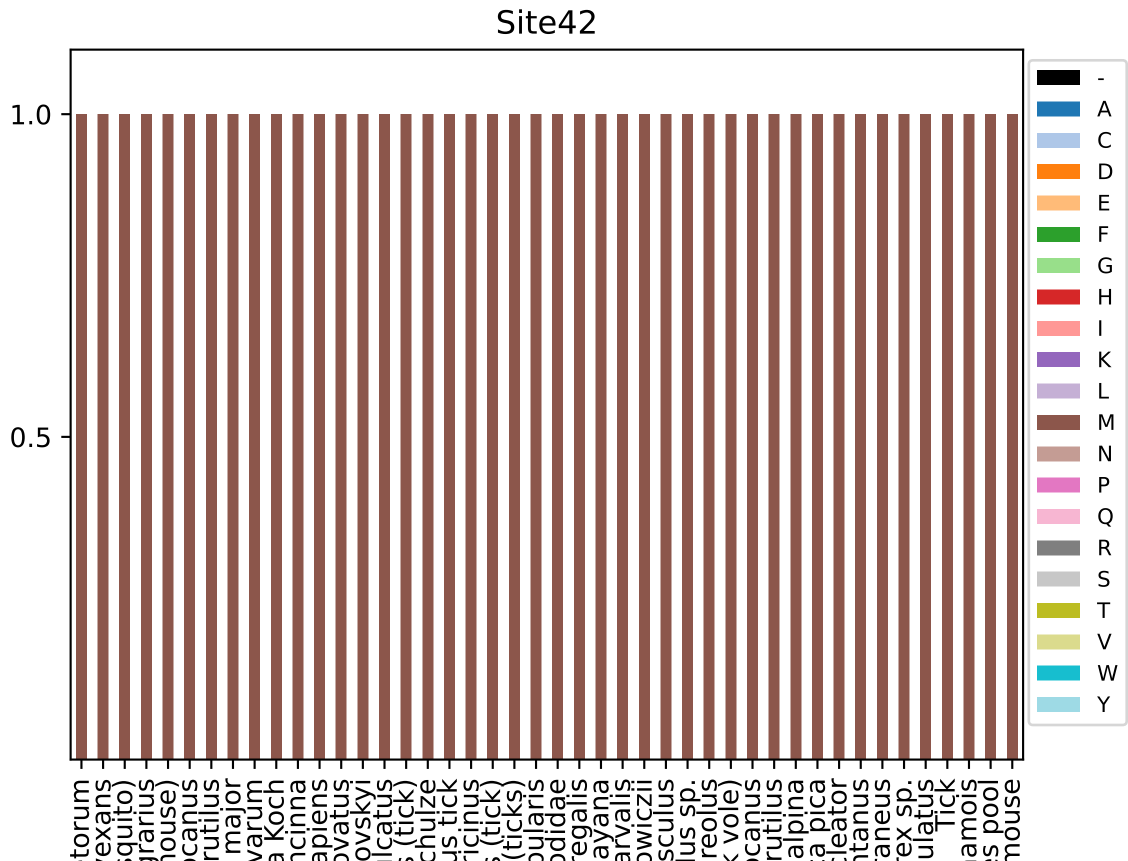
Site42
1.0
0.5
-
A
C
D
E
F
G
H
I
K
L
M
N
P
Q
R
S
T
V
W
Y

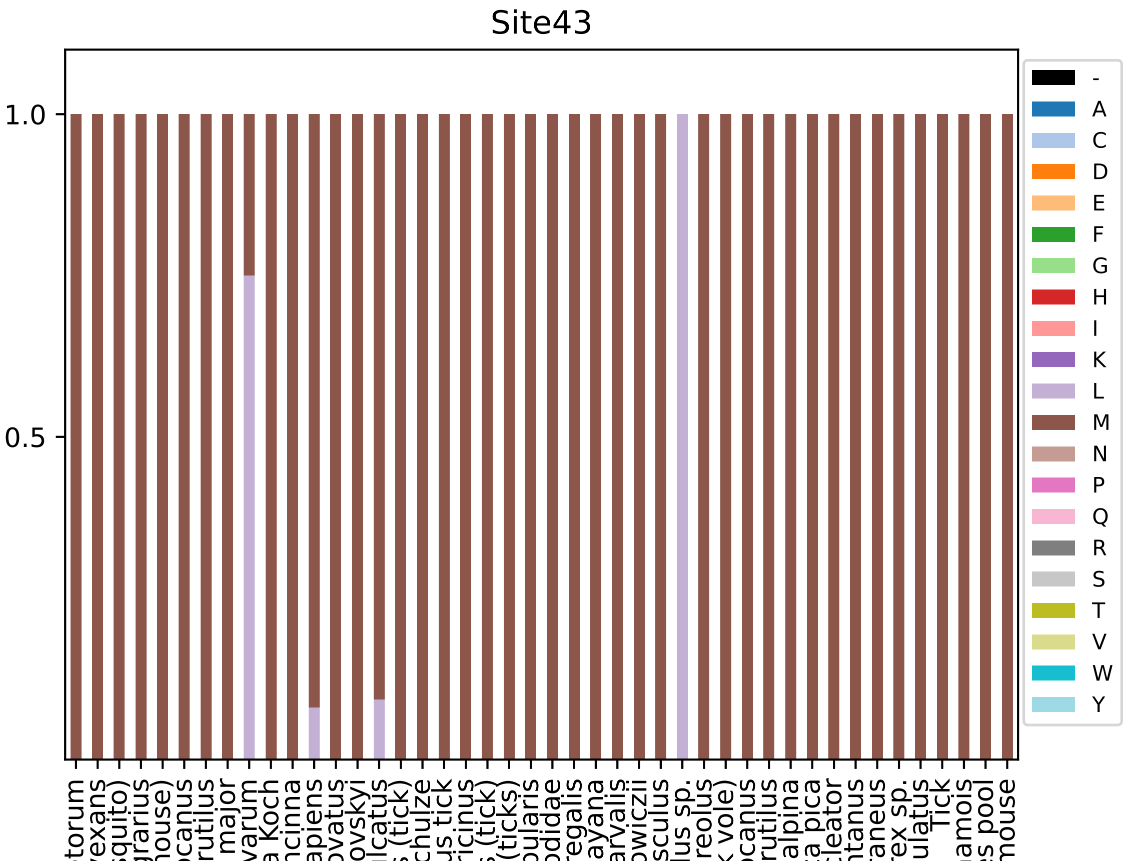

Site43
1.0
0.5
-
A
C
D
E
F
G
H
I
K
L
M
N
P
Q
R
S
T
V
W
Y

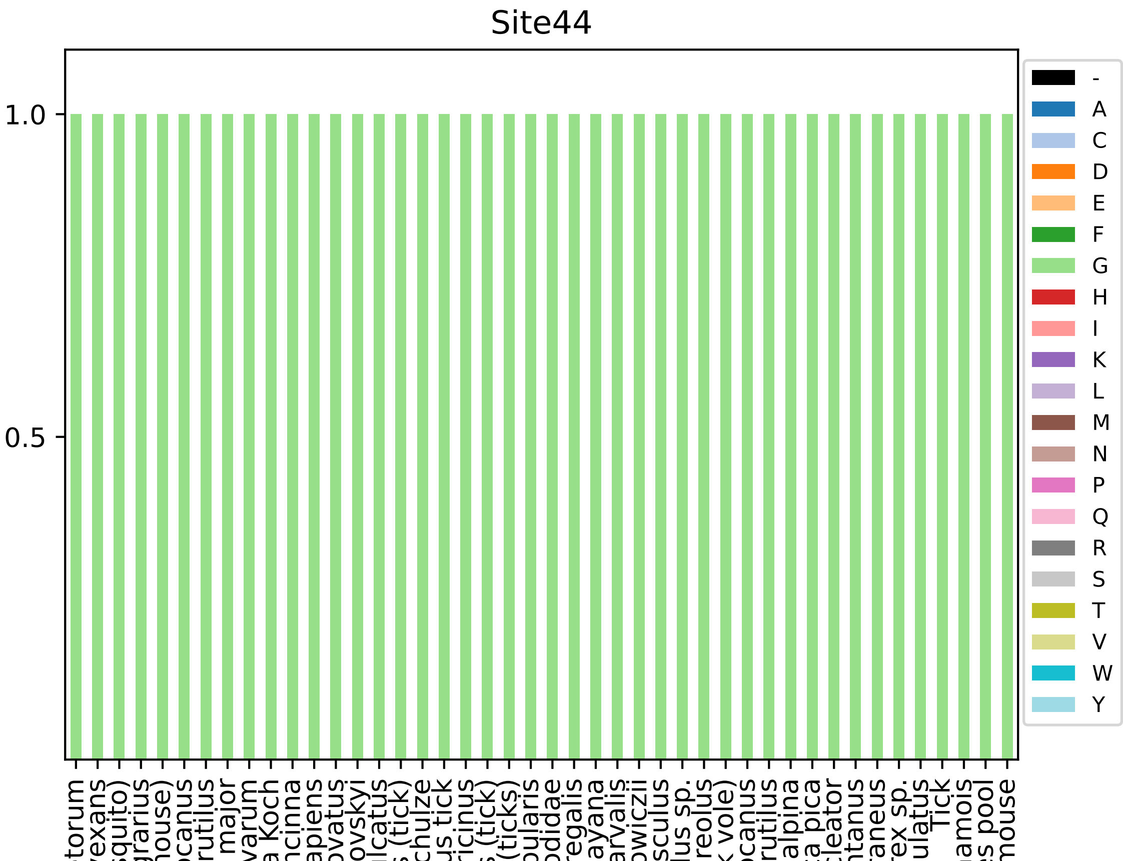
Site44
1.0
0.5
-
A
C
D
E
F
G
H
I
K
L
M
N
P
Q
R
S
T
V
W
Y

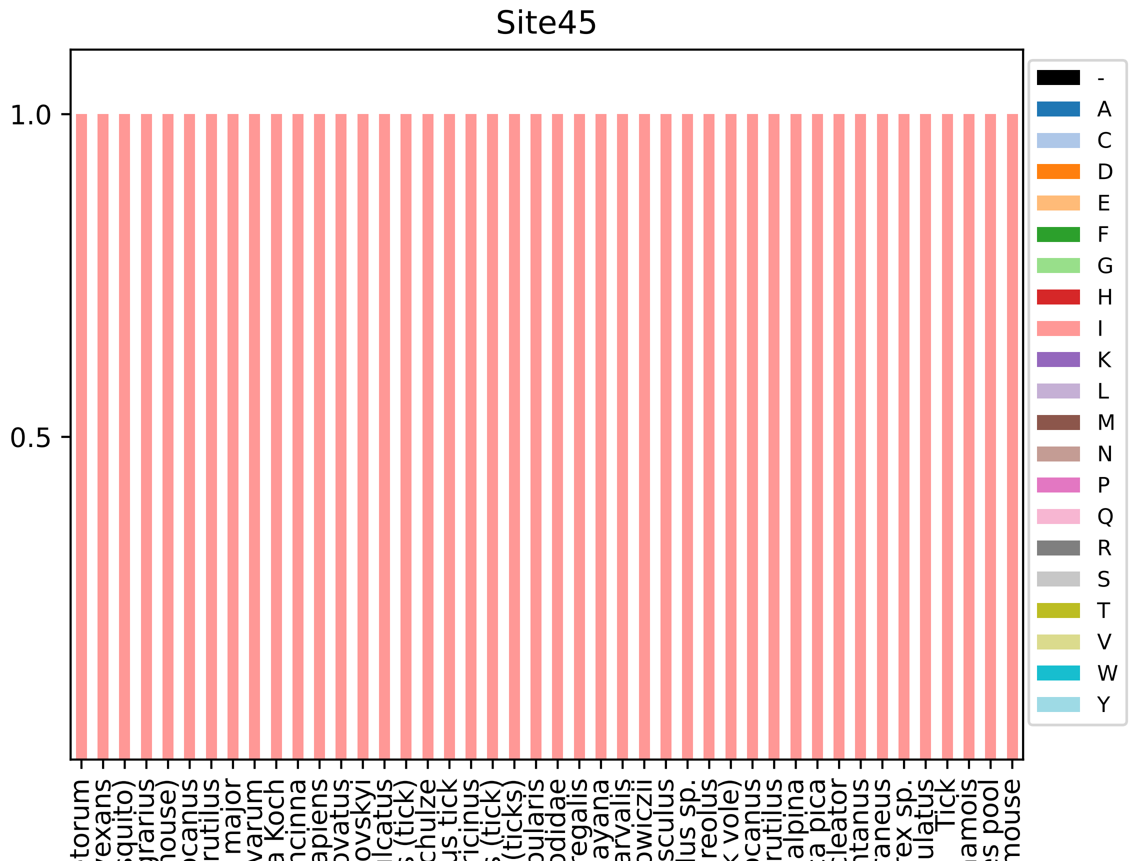
Site45
1.0
0.5
-
A
C
D
E
F
G
H
I
K
L
M
N
P
Q
R
S
T
V
W
Y

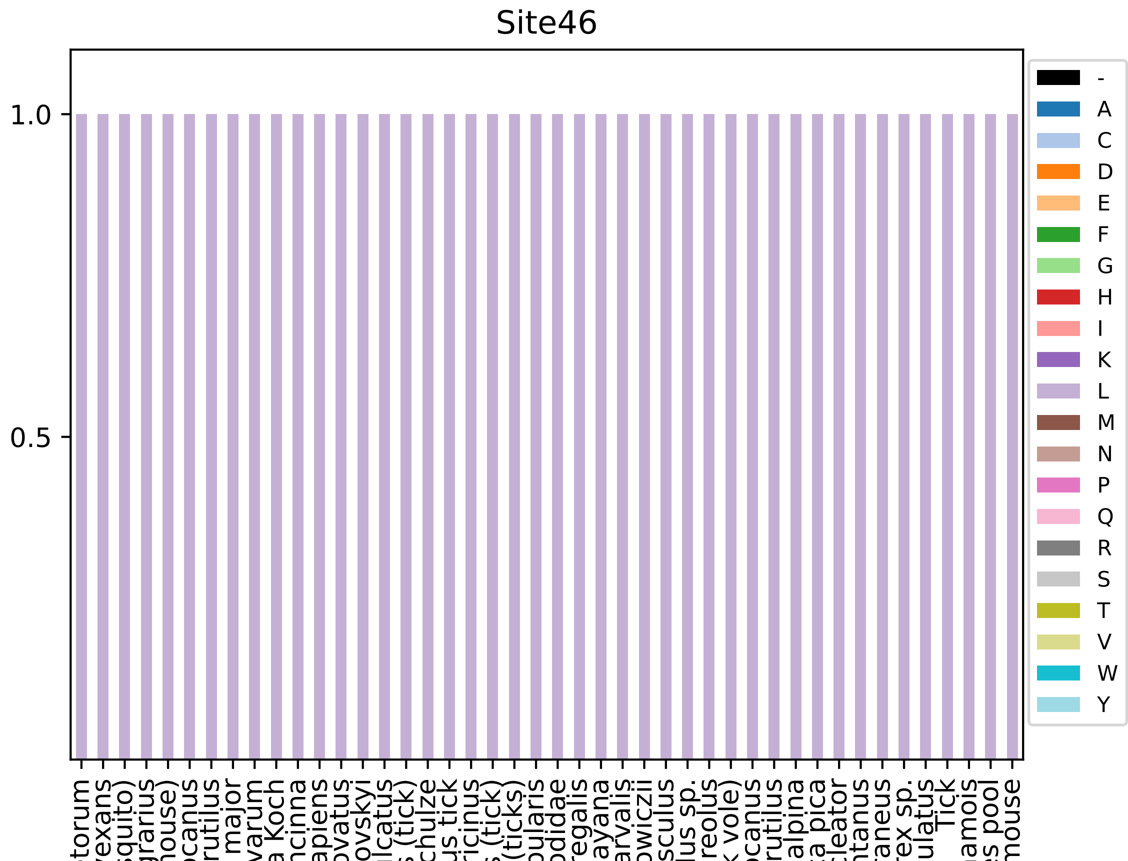
Site46
1.0
0.5
-
A
C
D
E
F
G
H
I
K
L
M
N
P
Q
R
S
T
V
W
Y

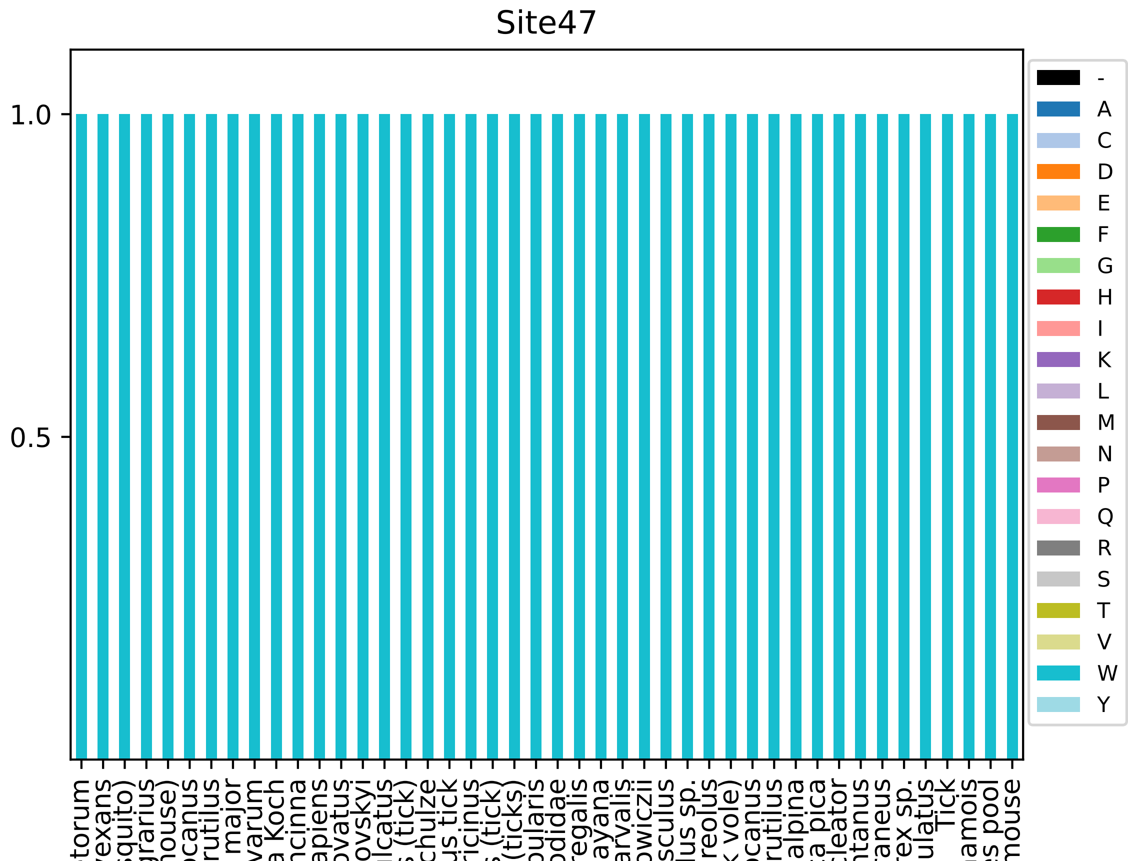
Site47
1.0
0.5
-
A
C
D
E
F
G
H
I
K
L
M
N
P
Q
R
S
T
V
W
Y

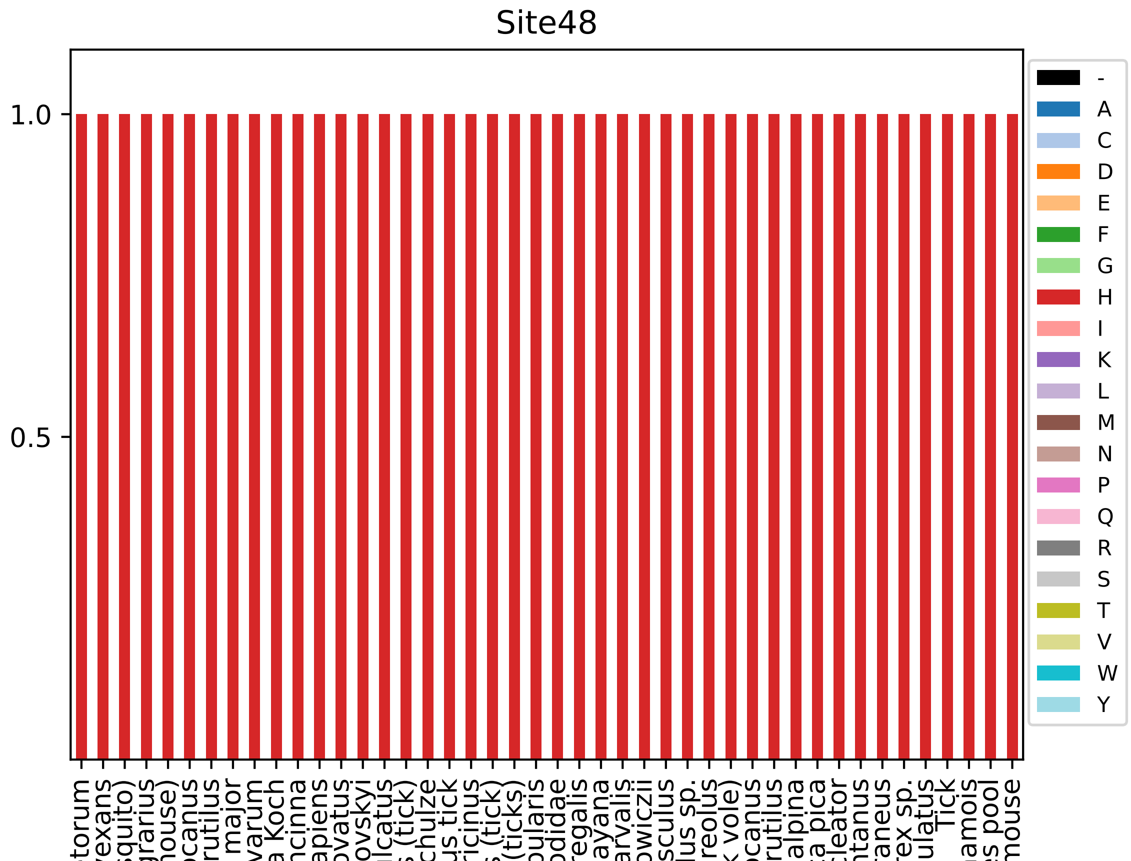
Site48
1.0
0.5
-
A
C
D
E
F
G
H
I
K
L
M
N
P
Q
R
S
T
V
W
Y

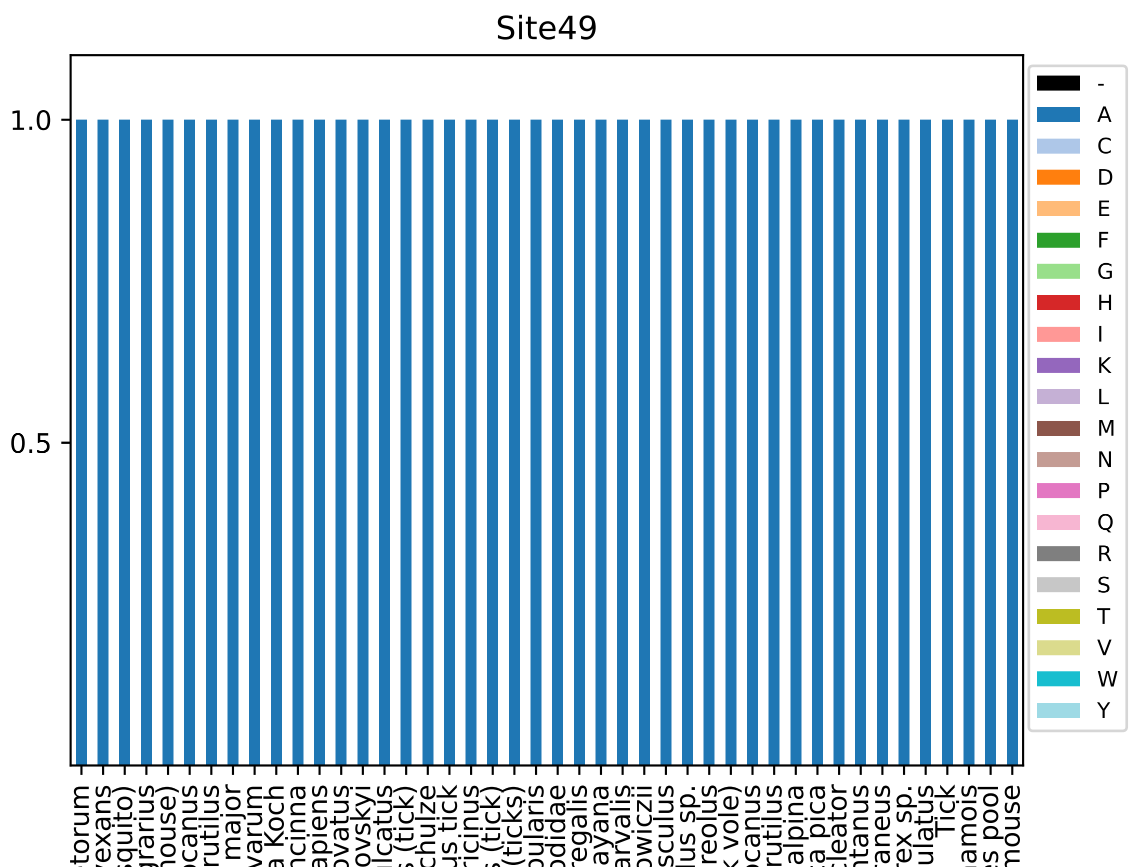

Site49
1.0
0.5
-
A
C
D
E
F
G
H
I
K
L
M
N
P
Q
R
S
T
V
W
Y

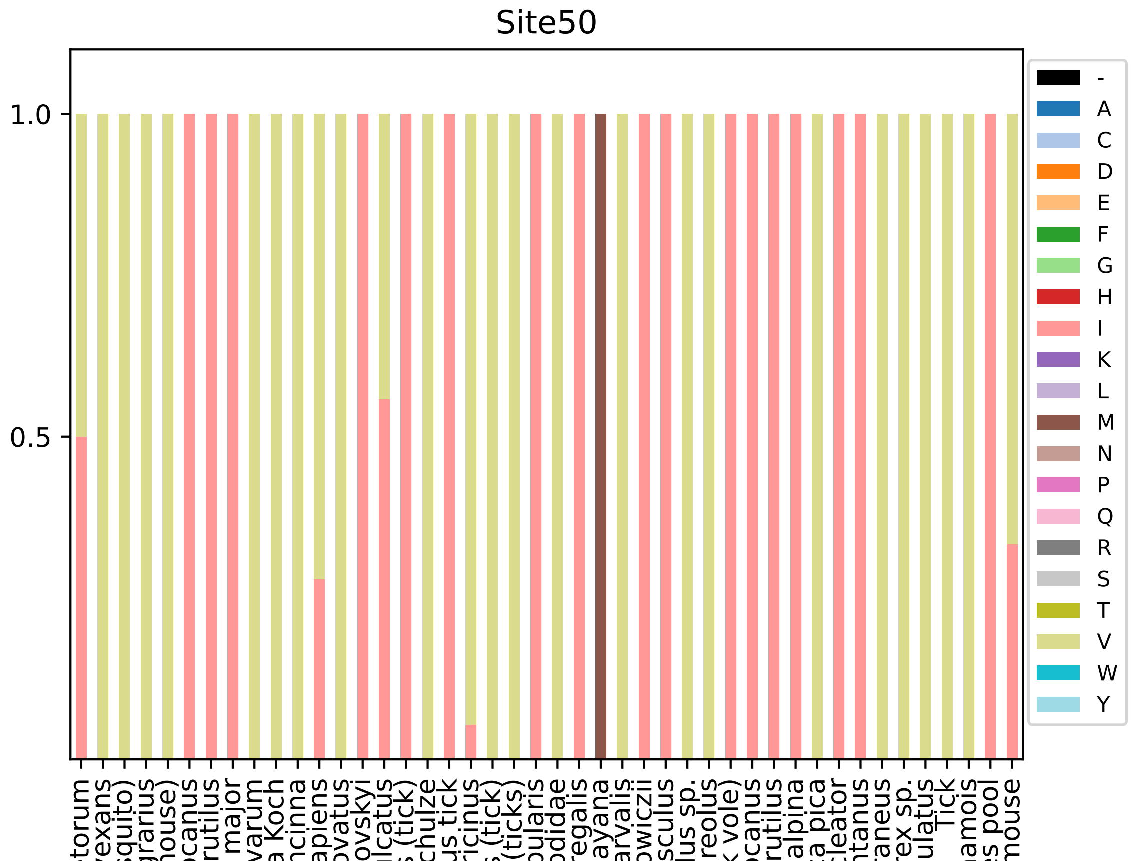
Site50
1.0
0.5
-
A
C
D
E
F
G
H
I
K
L
M
N
P
Q
R
S
T
V
W
Y

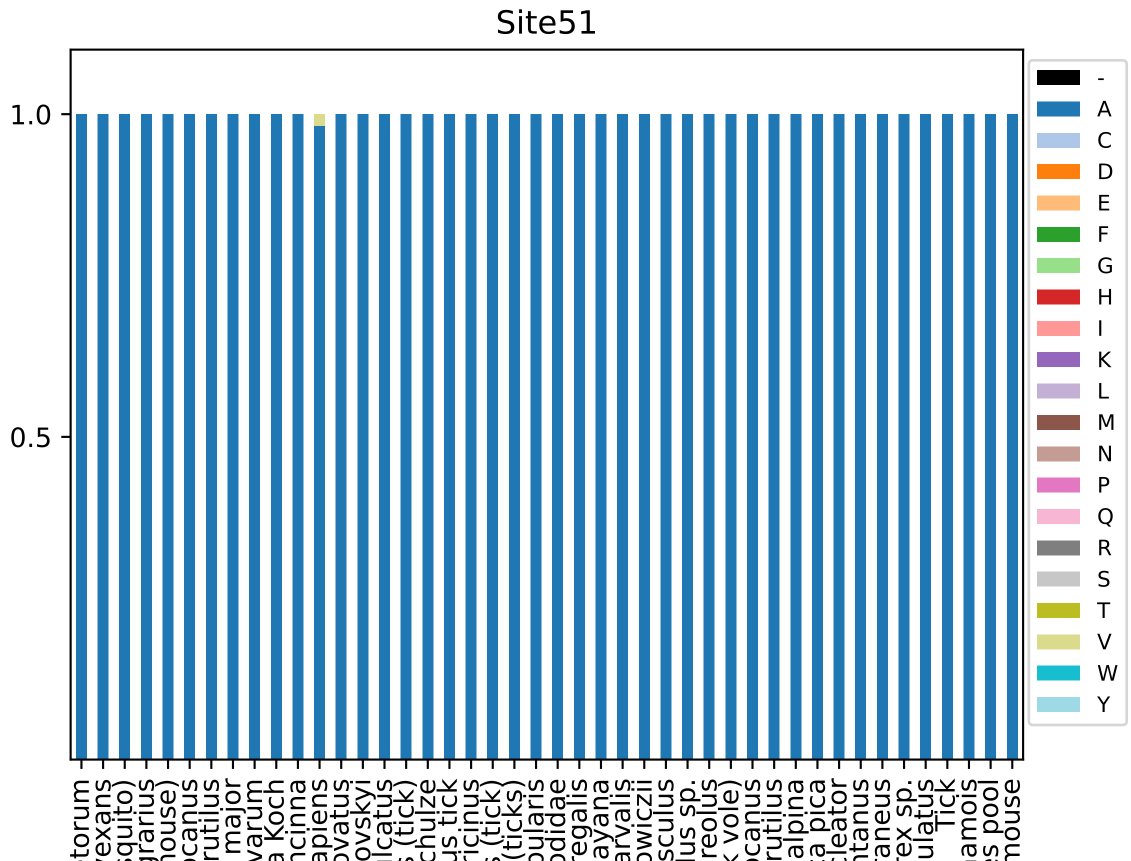
Site51
1.0
0.5
-
A
C
D
E
F
G
H
I
K
L
M
N
P
Q
R
S
T
V
W
Y

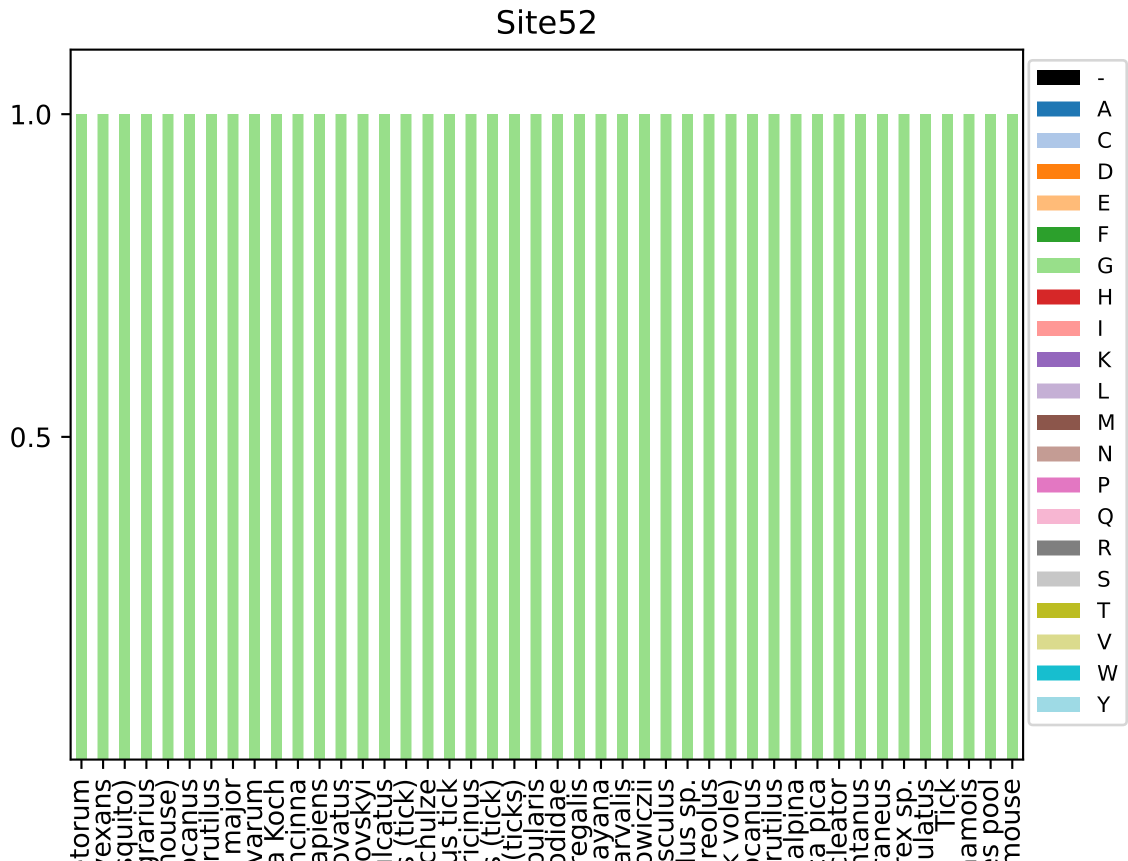
Site52
1.0
0.5
-
A
C
D
E
F
G
H
I
K
L
M
N
P
Q
R
S
T
V
W
Y

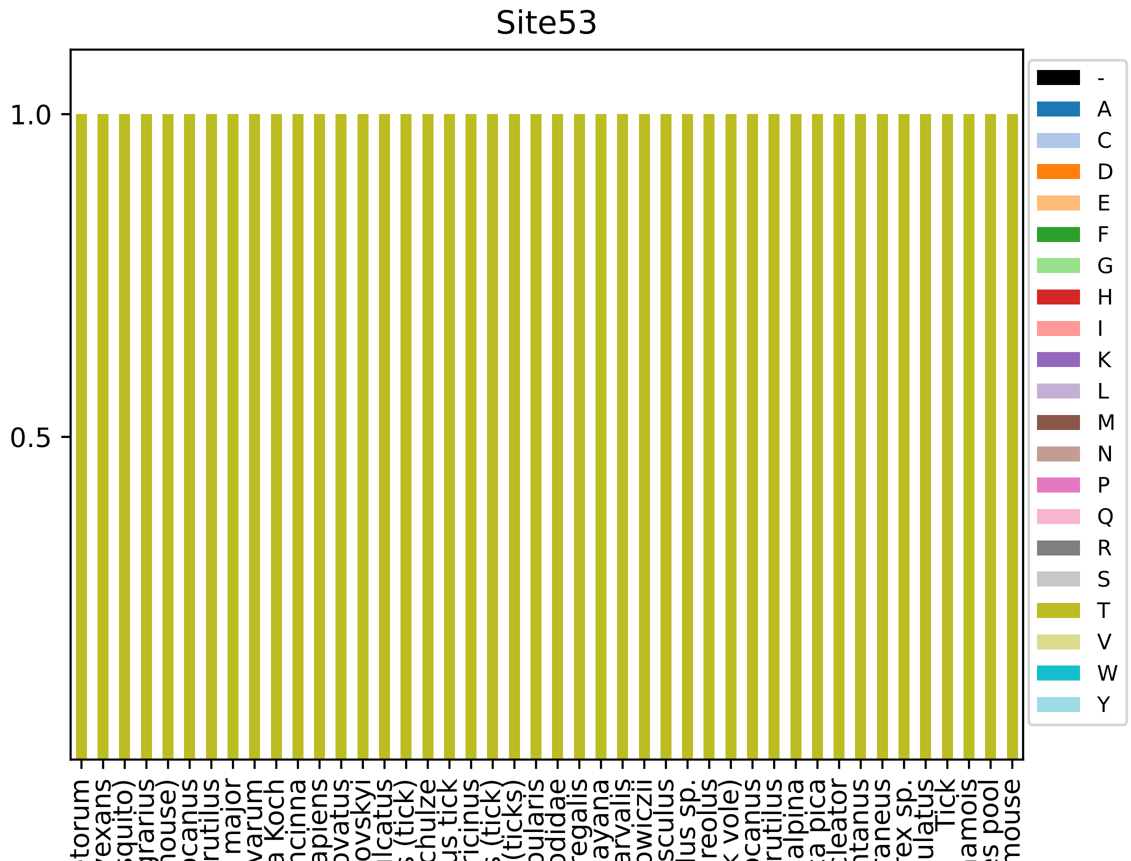
Site53
1.0
0.5
-
A
C
D
E
F
G
H
I
K
L
M
N
P
Q
R
S
T
V
W
Y

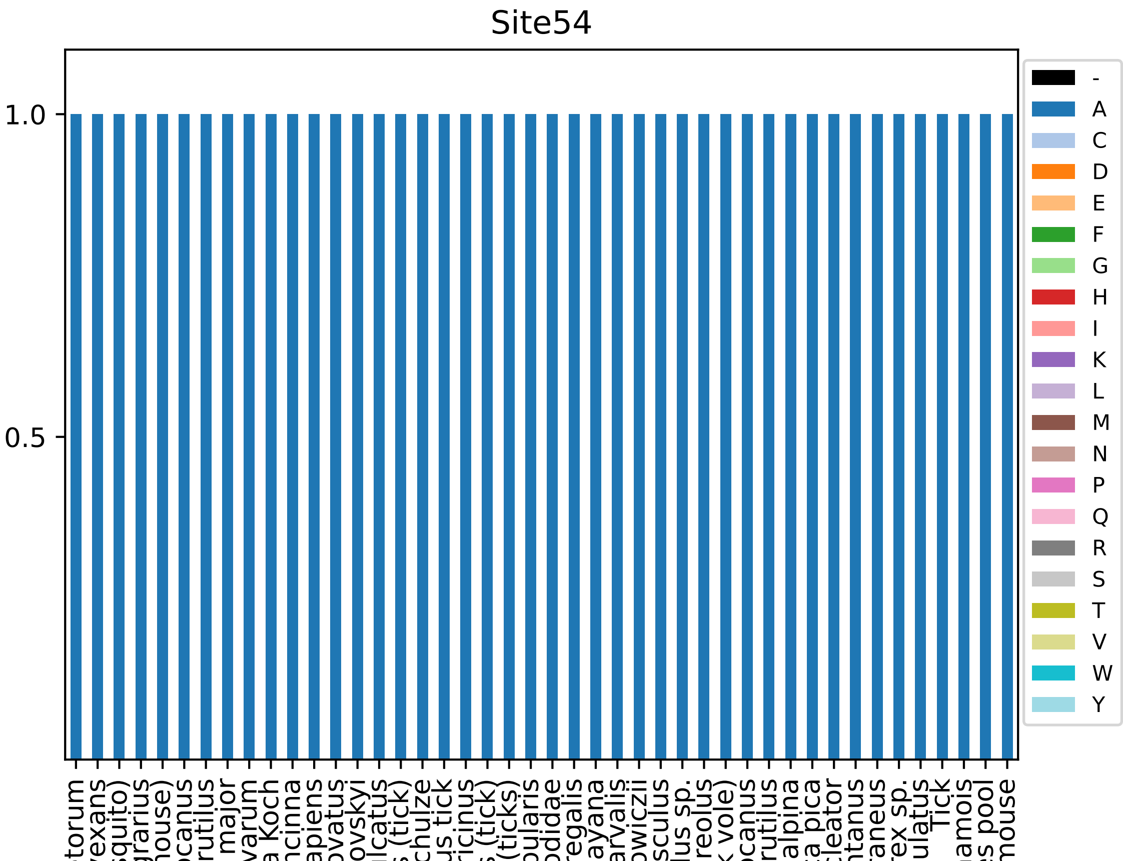
Site54
1.0
0.5
-
A
C
D
E
F
G
H
I
K
L
M
N
P
Q
R
S
T
V
W
Y

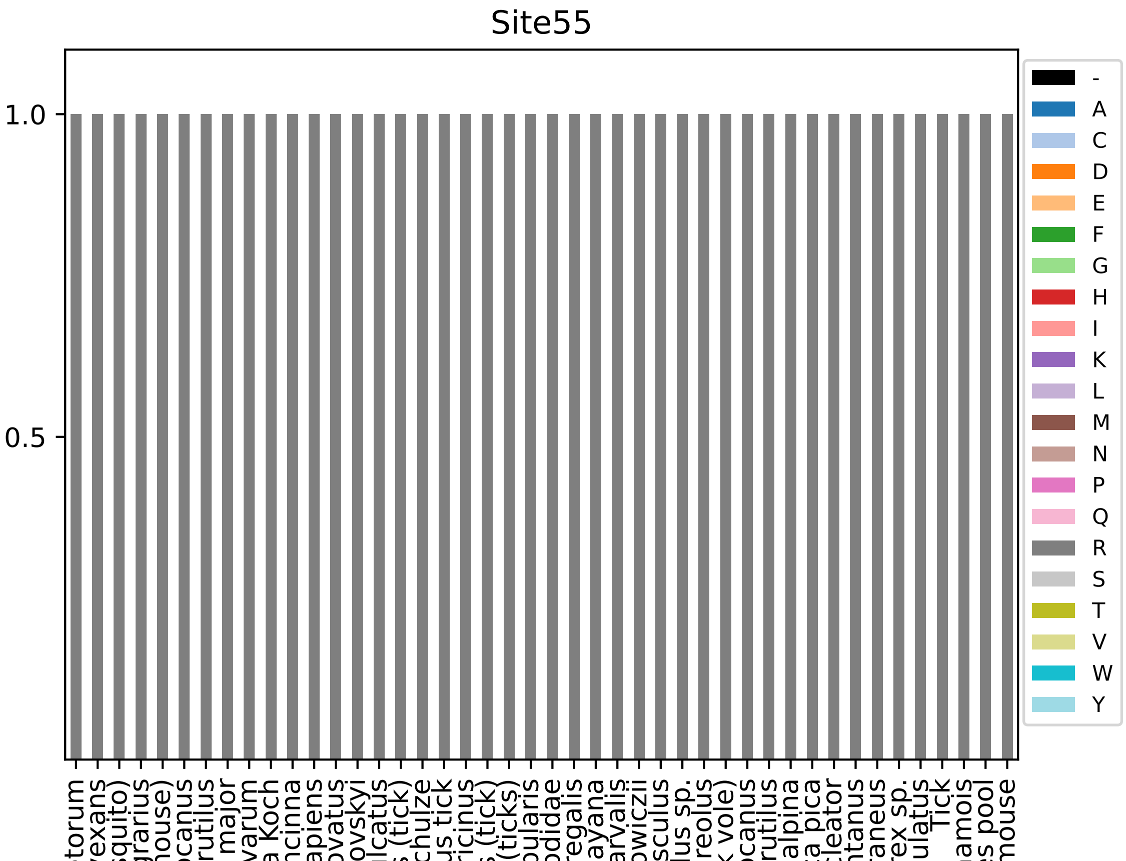
Site55
1.0
0.5
-
A
C
D
E
F
G
H
I
K
L
M
N
P
Q
R
S
T
V
W
Y

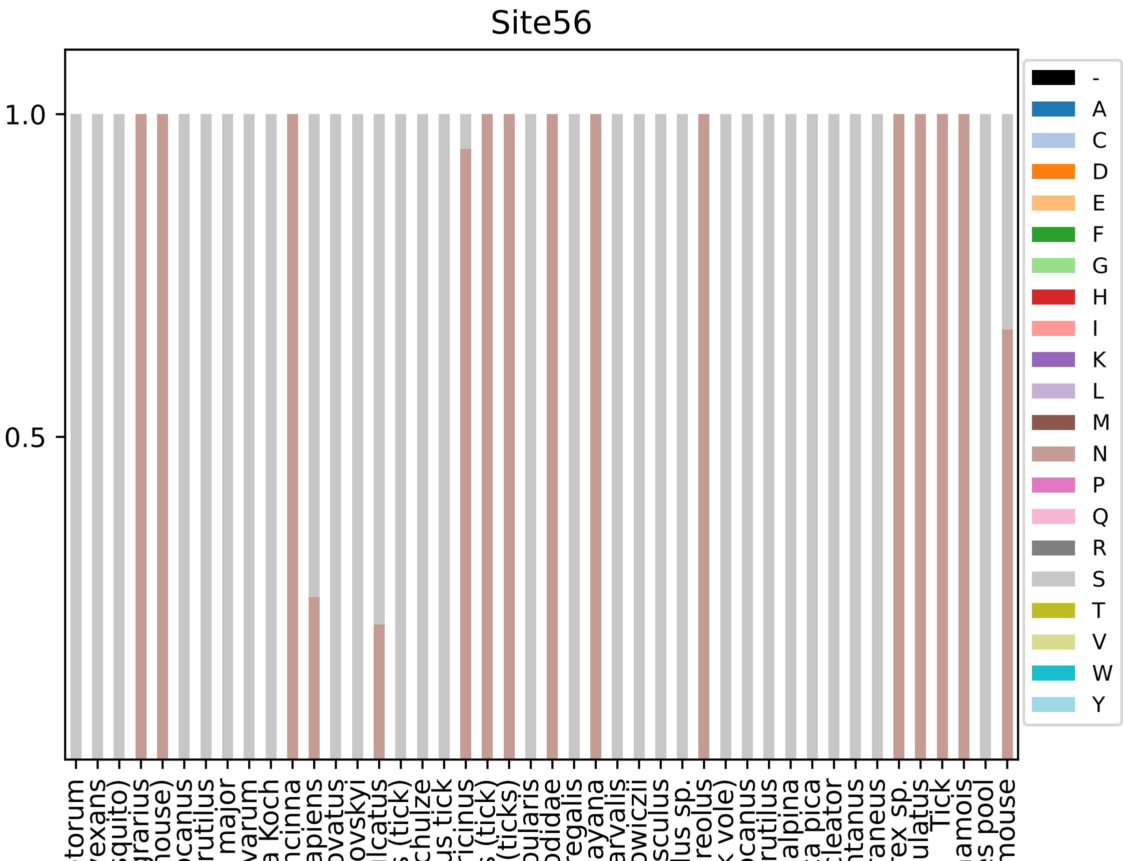
Site56
1.0
0.5
-
A
C
D
E
F
G
H
I
K
L
M
N
P
Q
R
S
T
V
W
Y

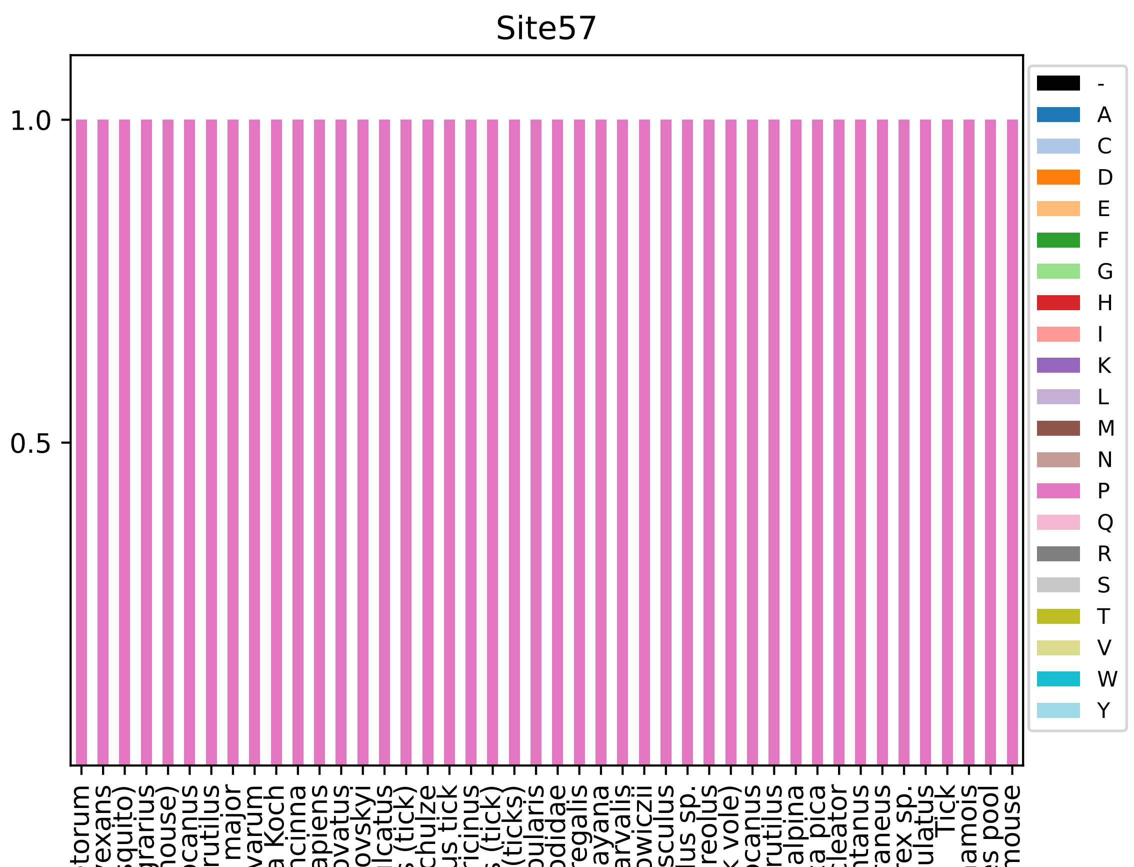

Site57
1.0
0.5
-
A
C
D
E
F
G
H
I
K
L
M
N
P
Q
R
S
T
V
W
Y

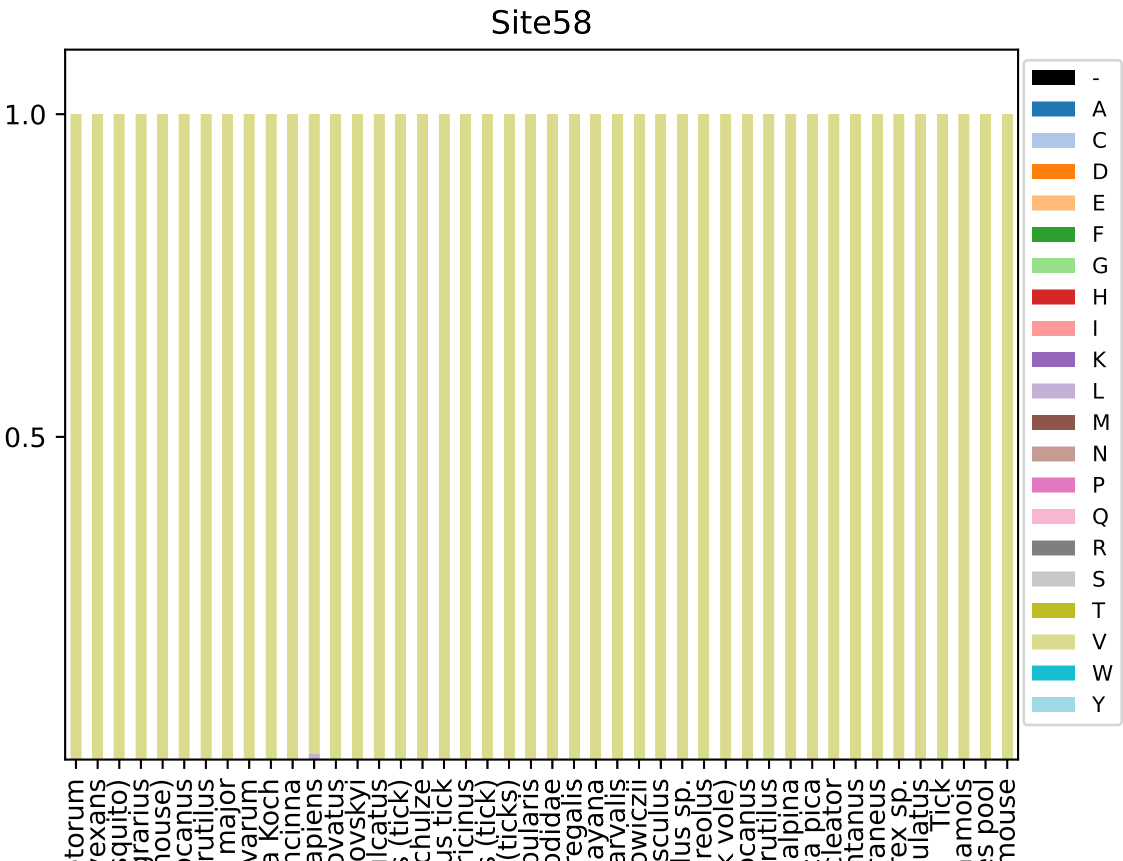
Site58
1.0
0.5
-
A
C
D
E
F
G
H
I
K
L
M
N
P
Q
R
S
T
V
W
Y

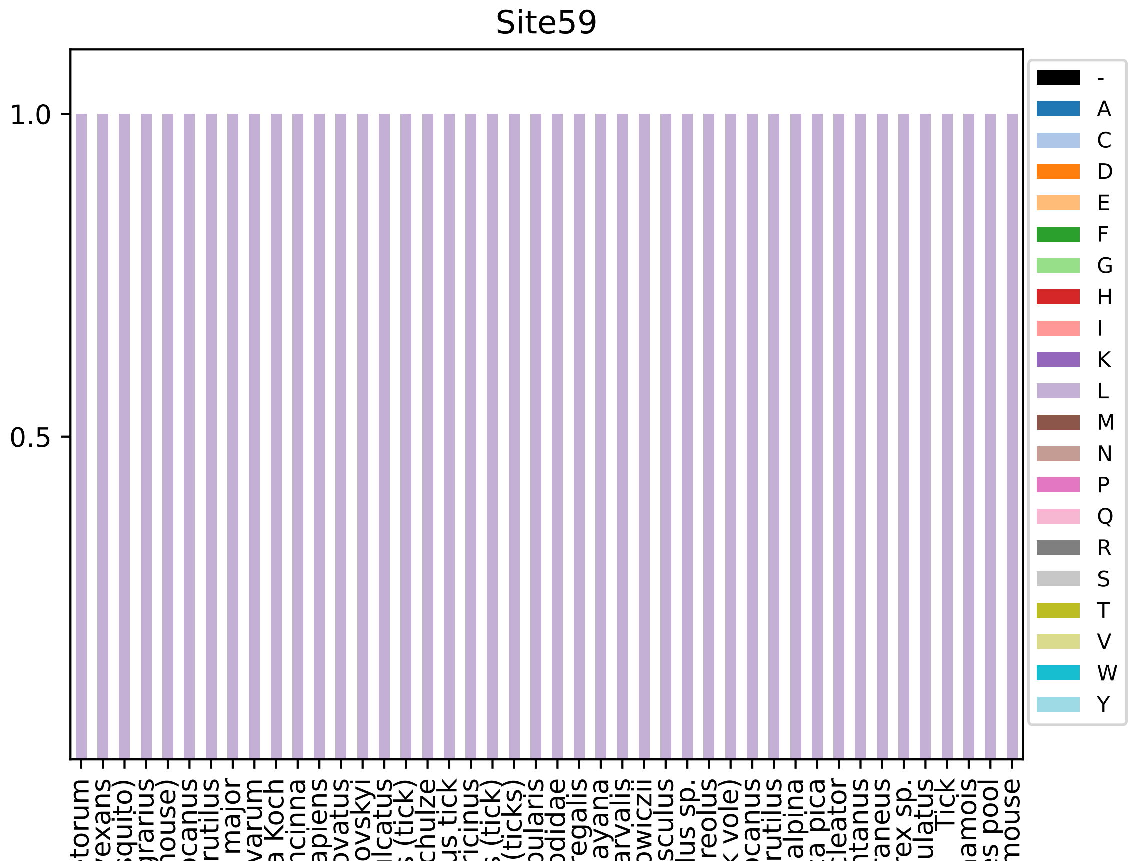
Site59
1.0
0.5
-
A
C
D
E
F
G
H
I
K
L
M
N
P
Q
R
S
T
V
W
Y

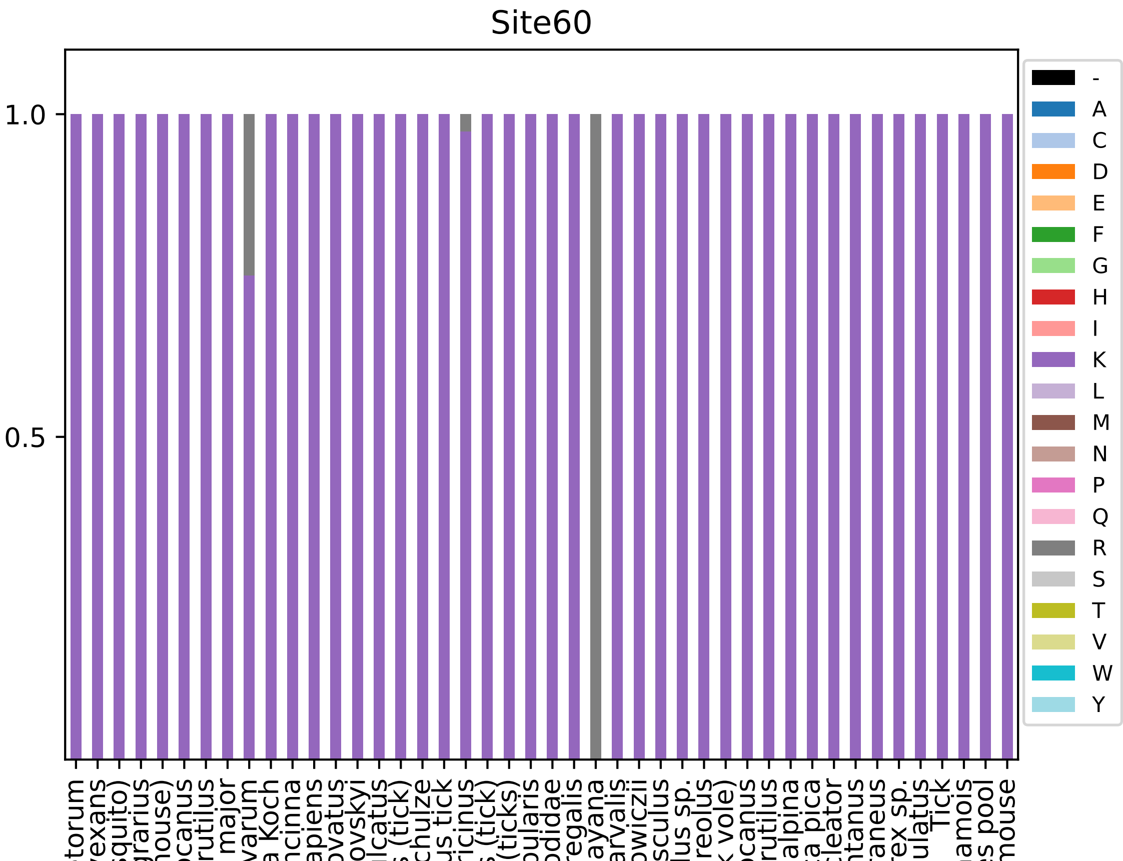
Site60
1.0
0.5
-
A
C
D
E
F
G
H
I
K
L
M
N
P
Q
R
S
T
V
W
Y

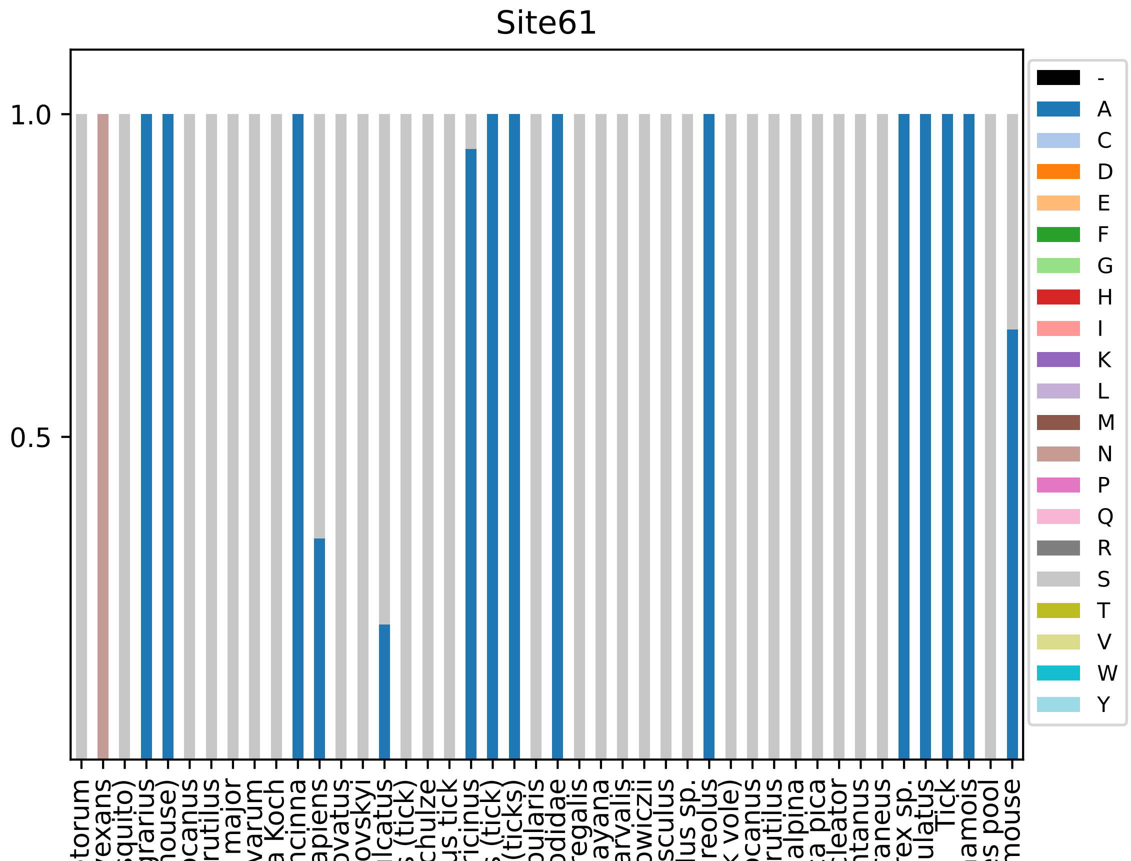

Site61
1.0
0.5
-
A
C
D
E
F
G
H
I
K
L
M
N
P
Q
R
S
T
V
W
Y

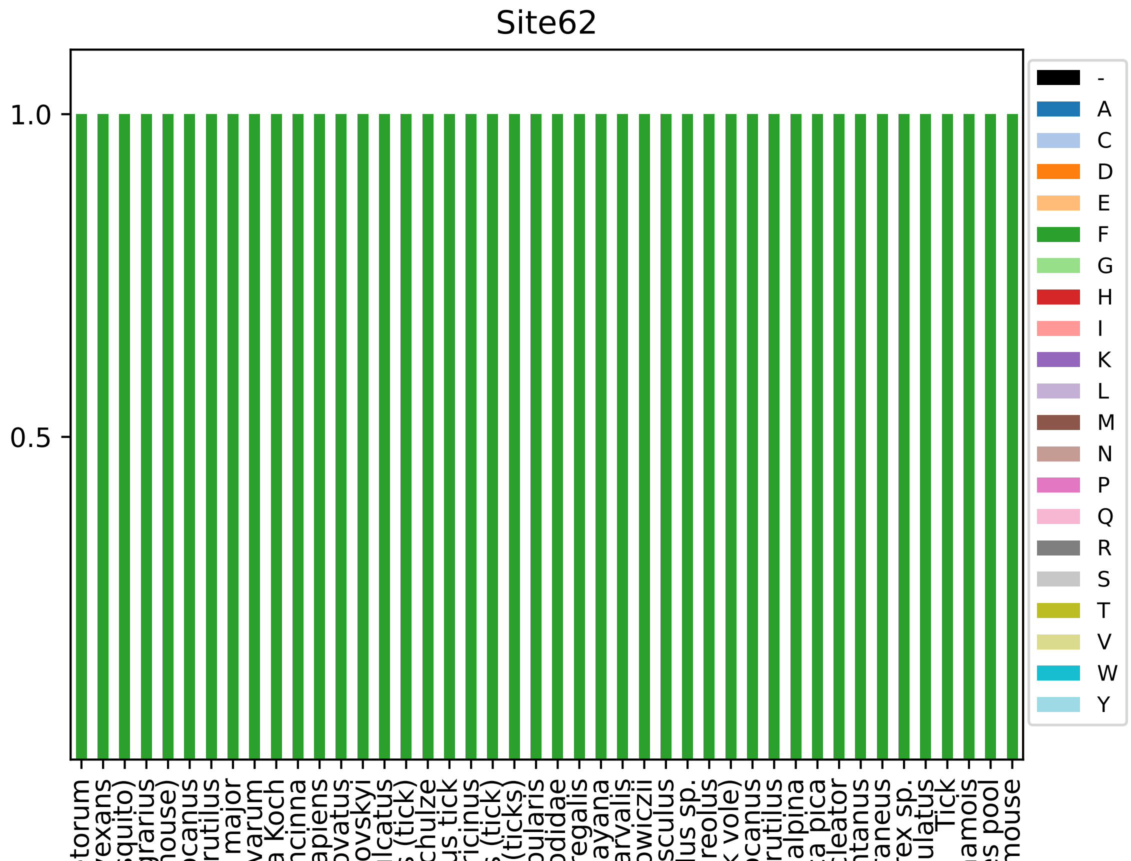
Site62
1.0
0.5
-
A
C
D
E
F
G
H
I
K
L
M
N
P
Q
R
S
T
V
W
Y
Tick

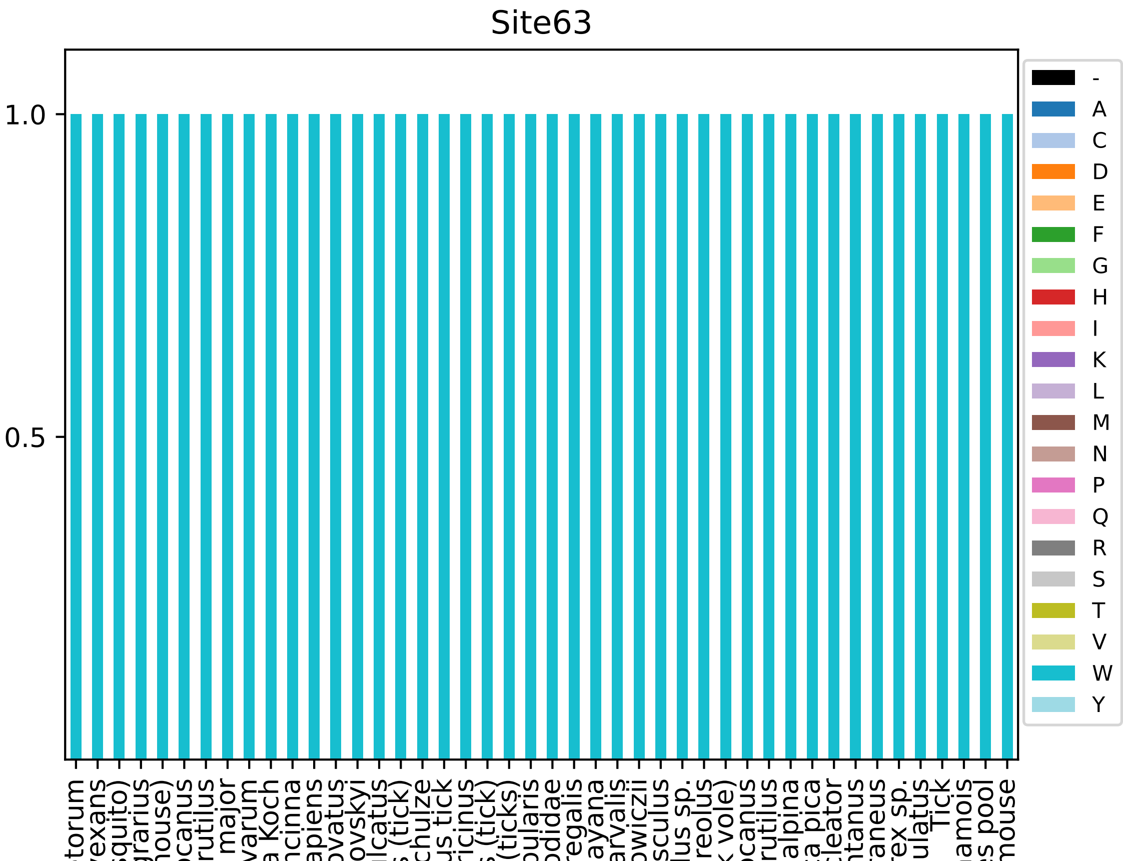
Site63
1.0
0.5
-
A
C
D
E
F
G
H
I
K
L
M
N
P
Q
R
S
T
V
W
Y

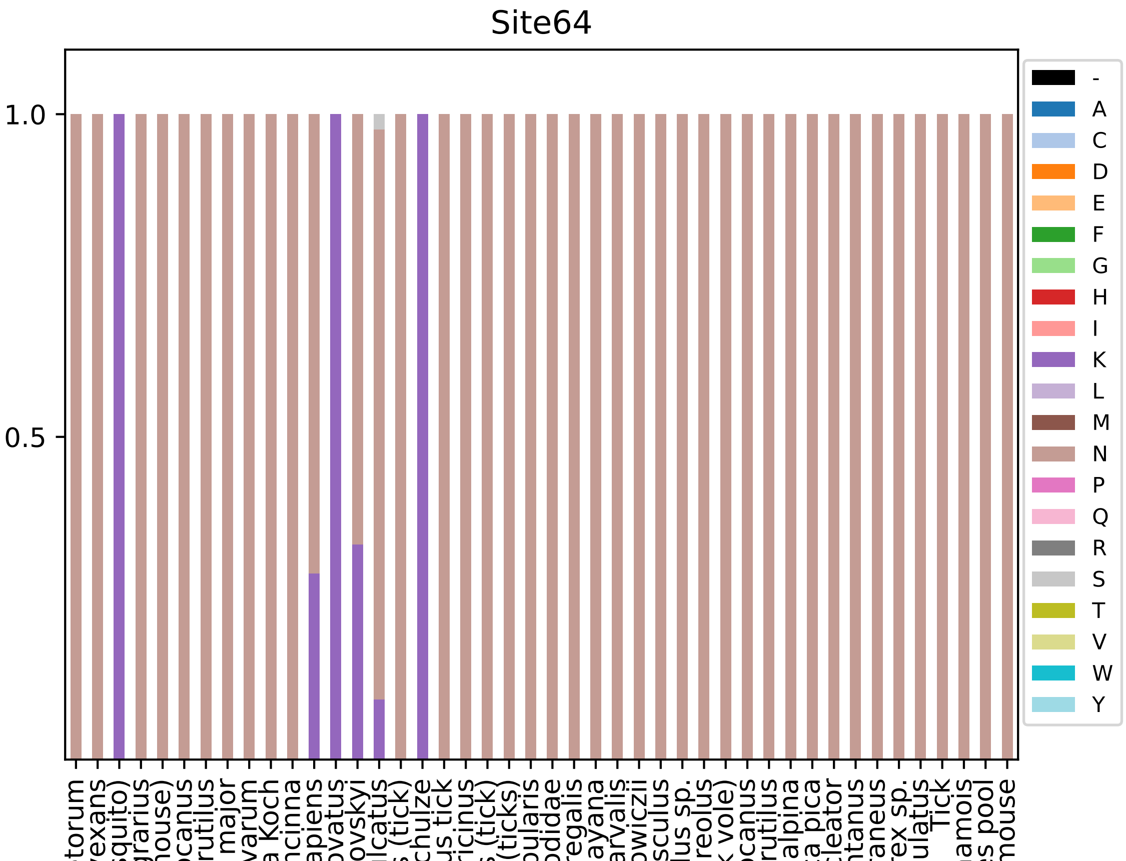
Site64
1.0
0.5
-
A
C
D
E
F
G
H
I
K
L
M
N
P
Q
R
S
T
V
W
Y

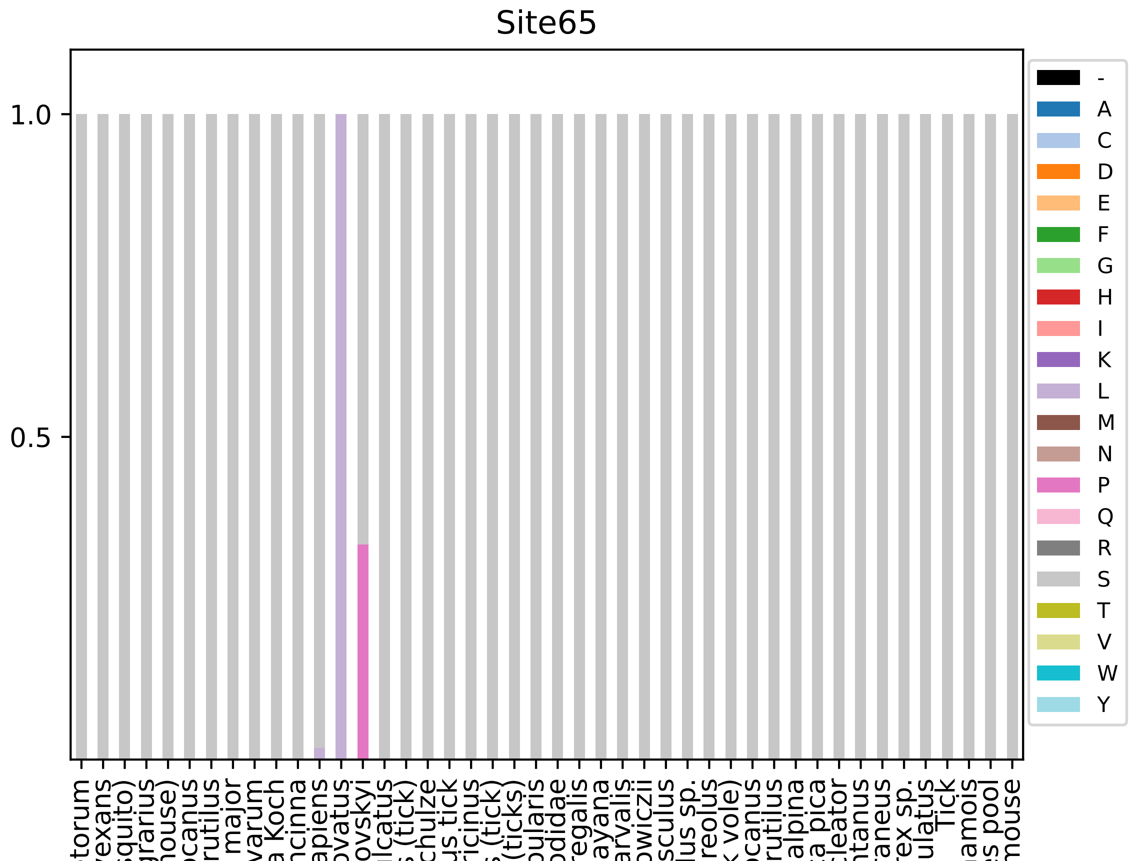
Site65
1.0
0.5
-
A
C
D
E
F
G
H
I
K
L
M
N
P
Q
R
S
T
V
W
Y

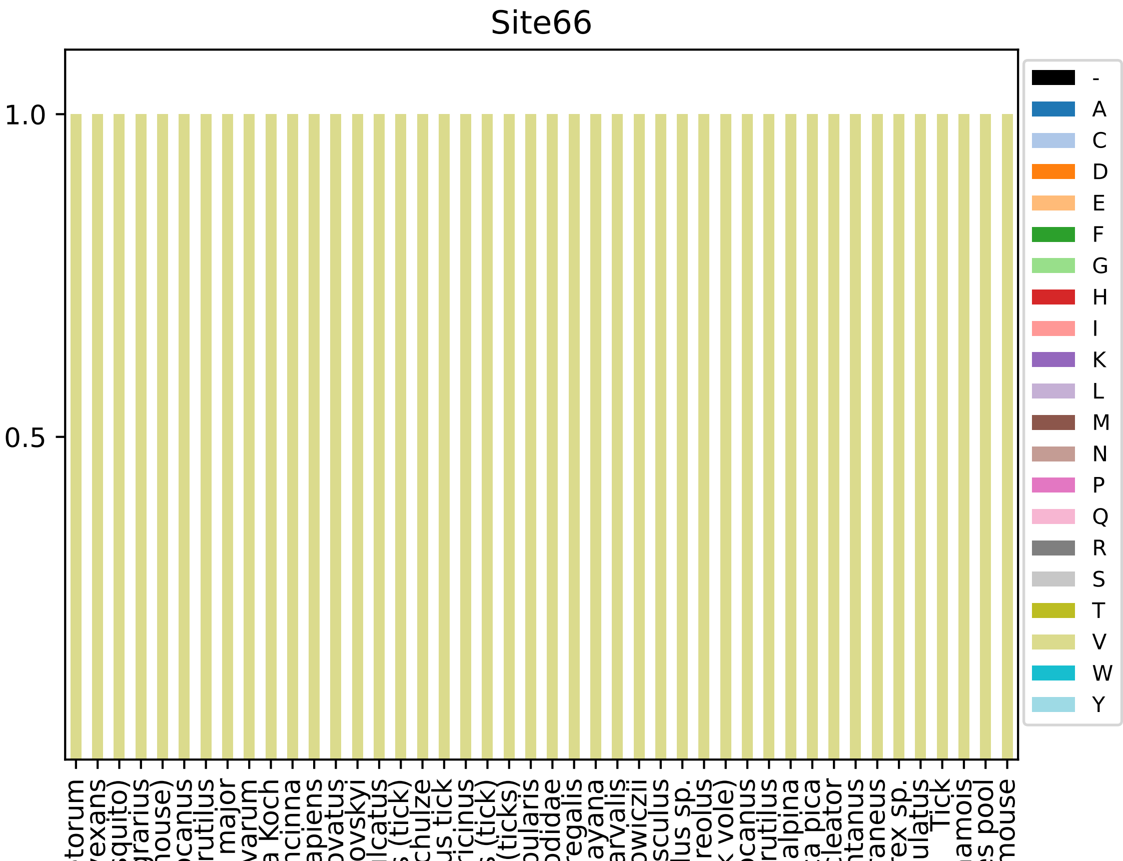
Site66
1.0
0.5
-
A
C
D
E
F
G
H
I
K
L
M
N
P
Q
R
S
T
V
W
Y

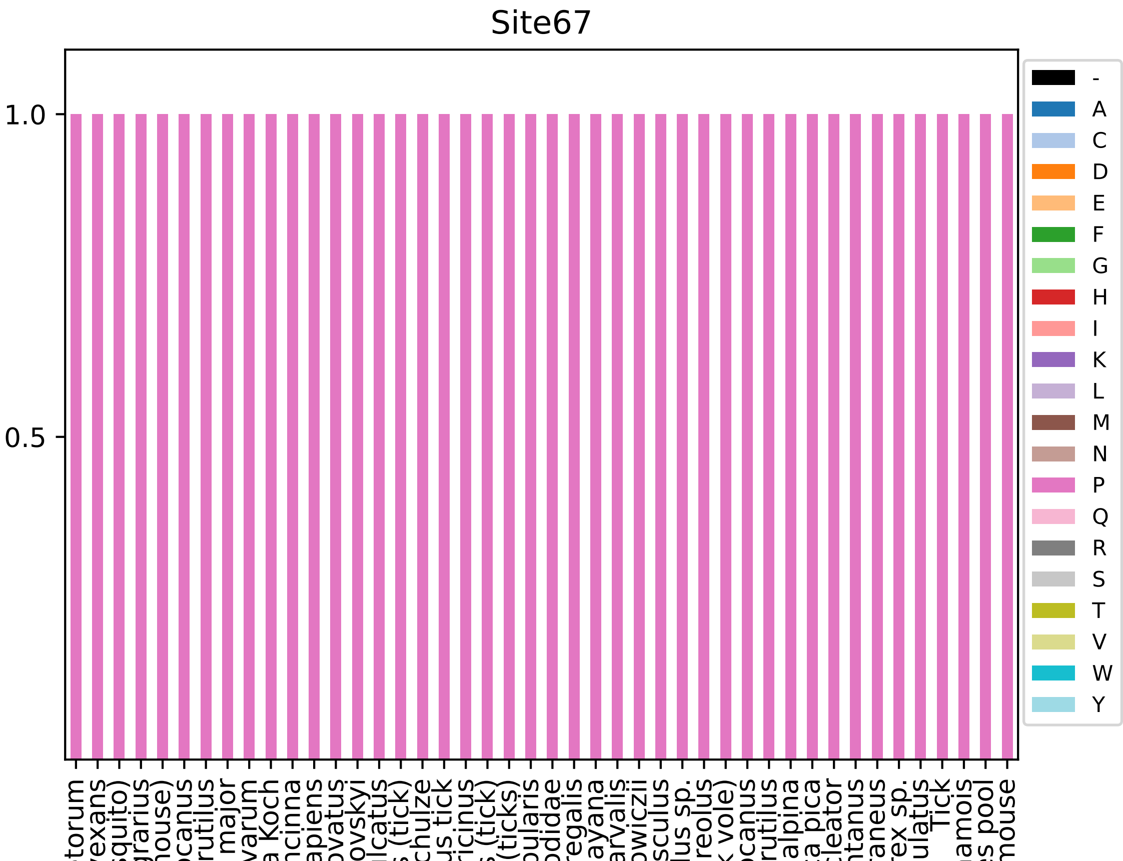

Site67
1.0
0.5
-
A
C
D
E
F
G
H
I
K
L
M
N
P
Q
R
S
T
V
W
Y

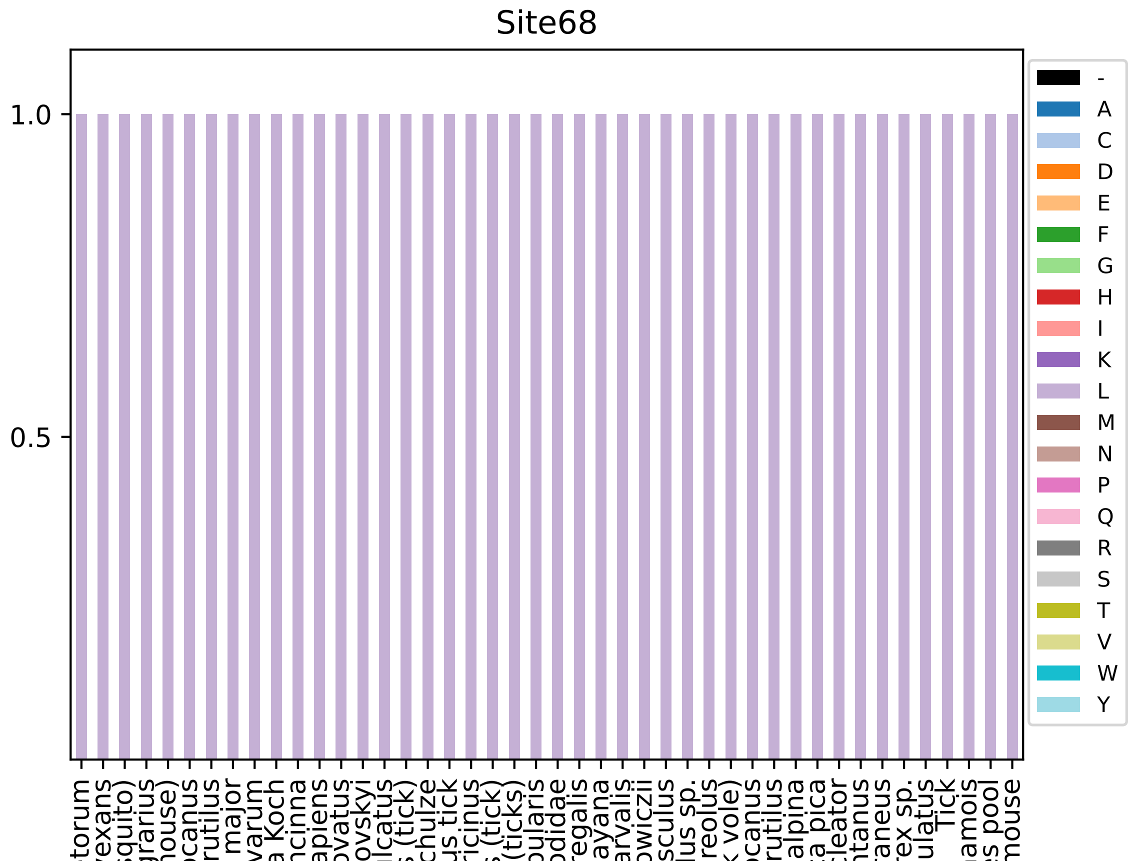
Site68
1.0
0.5
-
A
C
D
E
F
G
H
I
K
L
M
N
P
Q
R
S
T
V
W
Y

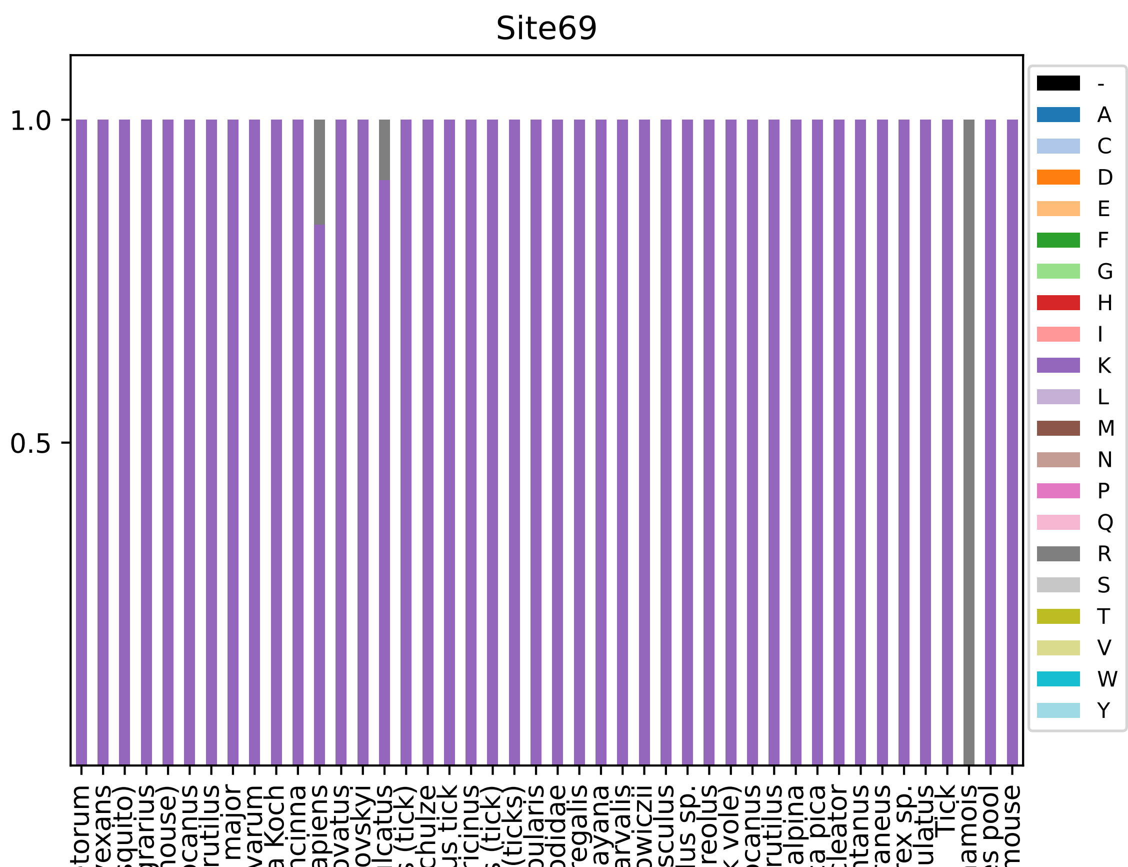

Site69
1.0
0.5
-
A
C
D
E
F
G
H
I
K
L
M
N
P
Q
R
S
T
V
W
Y
Tick

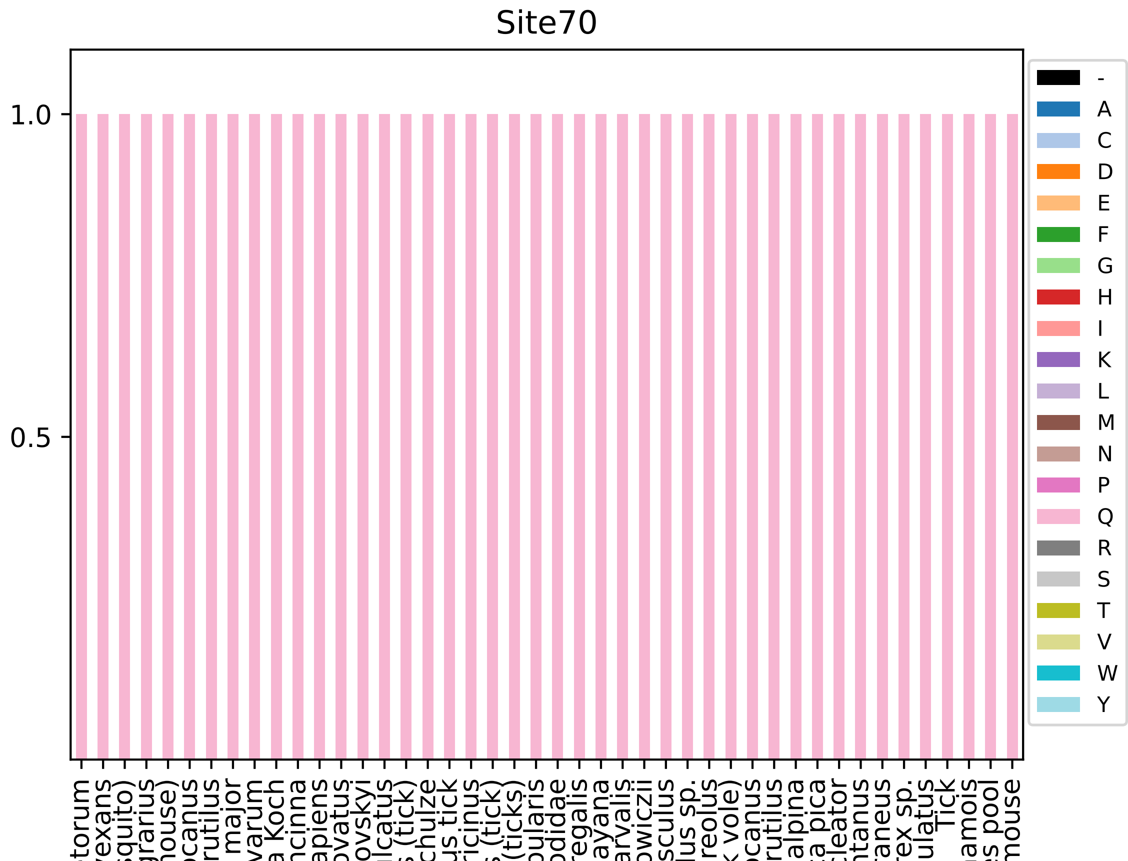
Site70
1.0
0.5
-
A
C
D
E
F
G
H
I
K
L
M
N
P
Q
R
S
T
V
W
Y
Tick

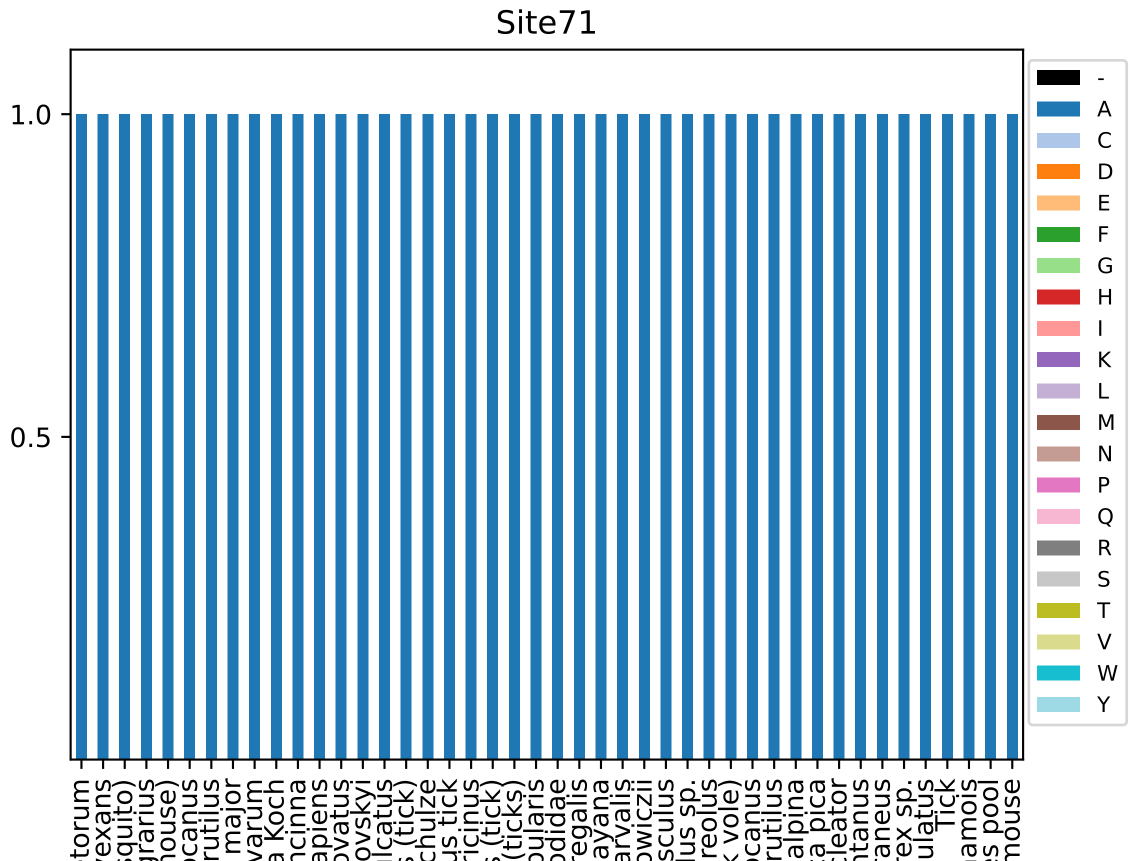

Site71
1.0
0.5
-
A
C
D
E
F
G
H
I
K
L
M
N
P
Q
R
S
T
V
W
Y

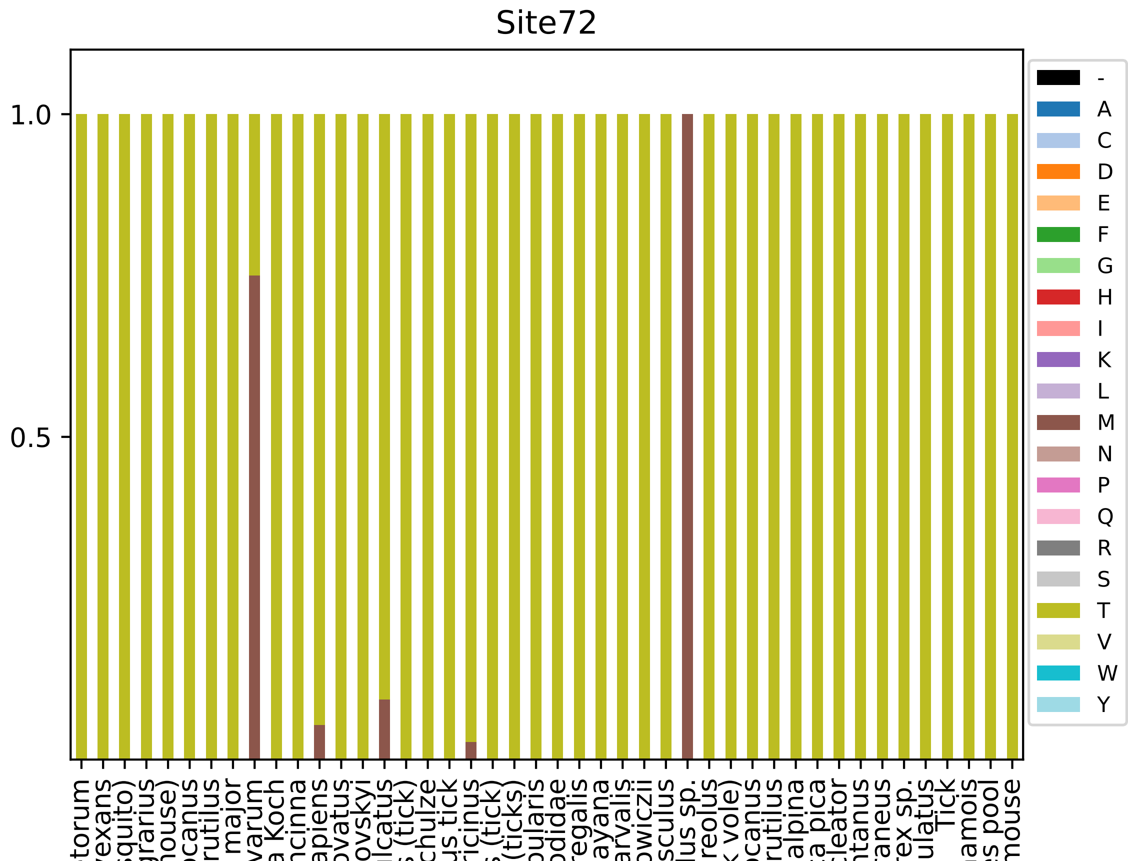

Site72
1.0
0.5
-
A
C
D
E
F
G
H
I
K
L
M
N
P
Q
R
S
T
V
W
Y

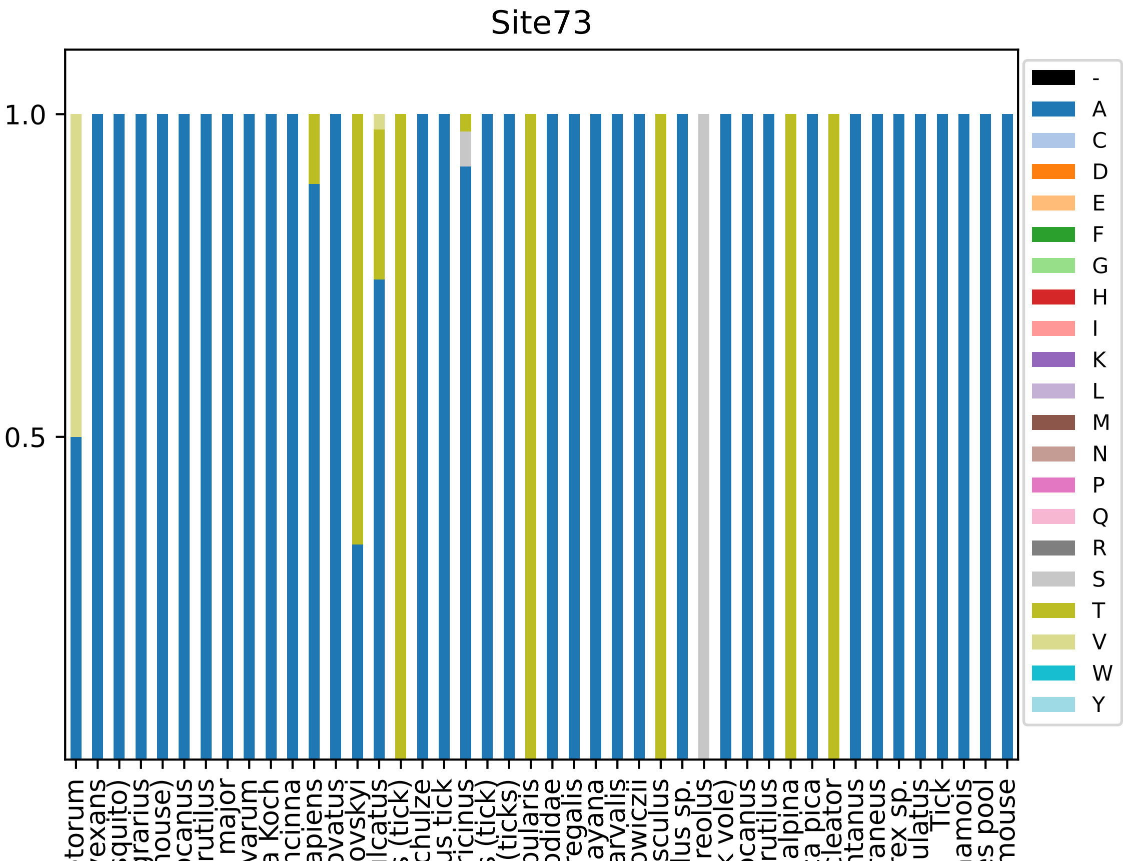
Site73
1.0
0.5
-
A
C
D
E
F
G
H
I
K
L
M
N
P
Q
R
S
T
V
W
Y

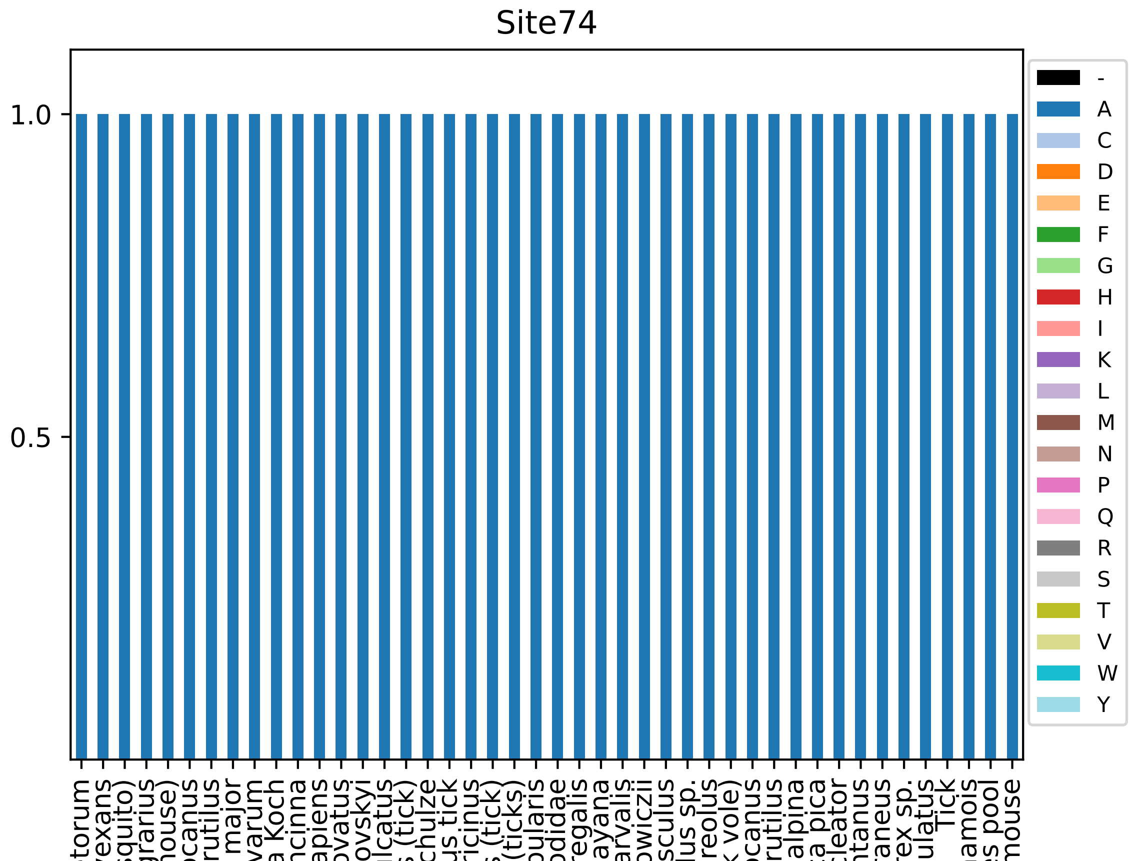
Site74
1.0
0.5
-
A
C
D
E
F
G
H
I
K
L
M
N
P
Q
R
S
T
V
W
Y

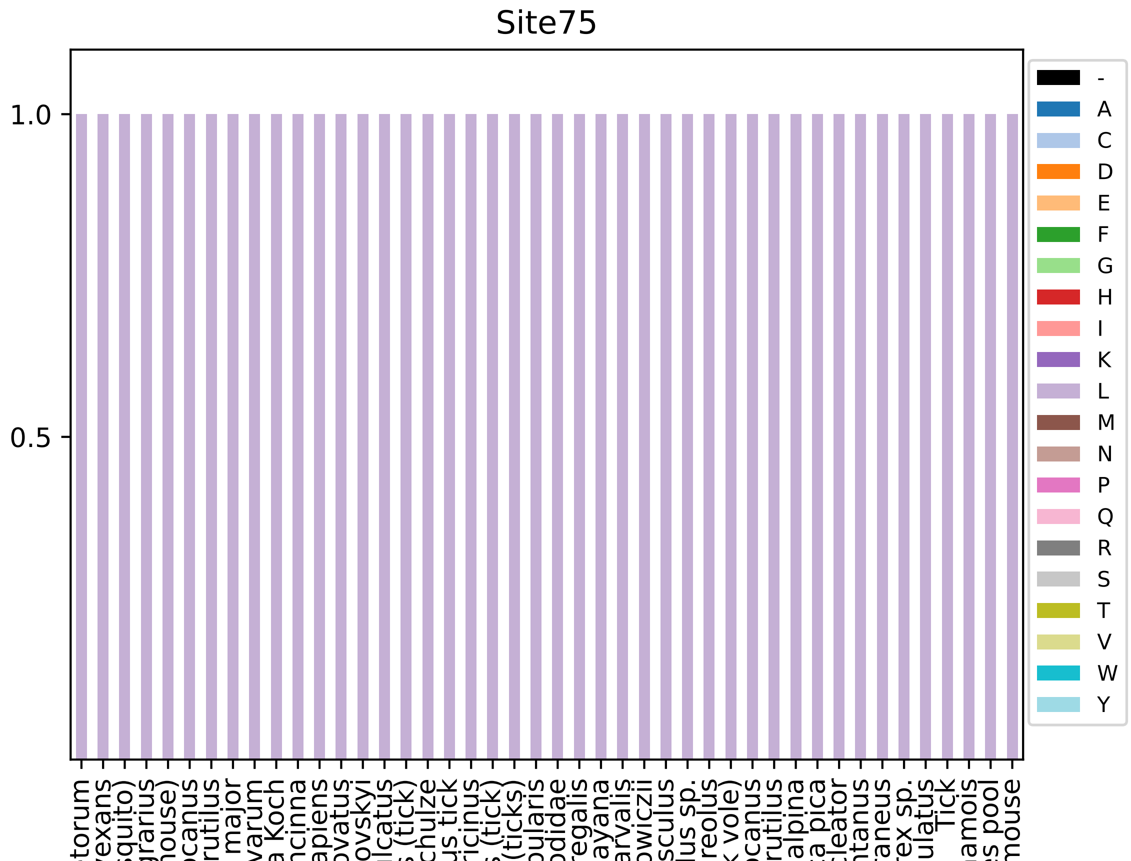

Site75
1.0
0.5
-
A
C
D
E
F
G
H
I
K
L
M
N
P
Q
R
S
T
V
W
Y

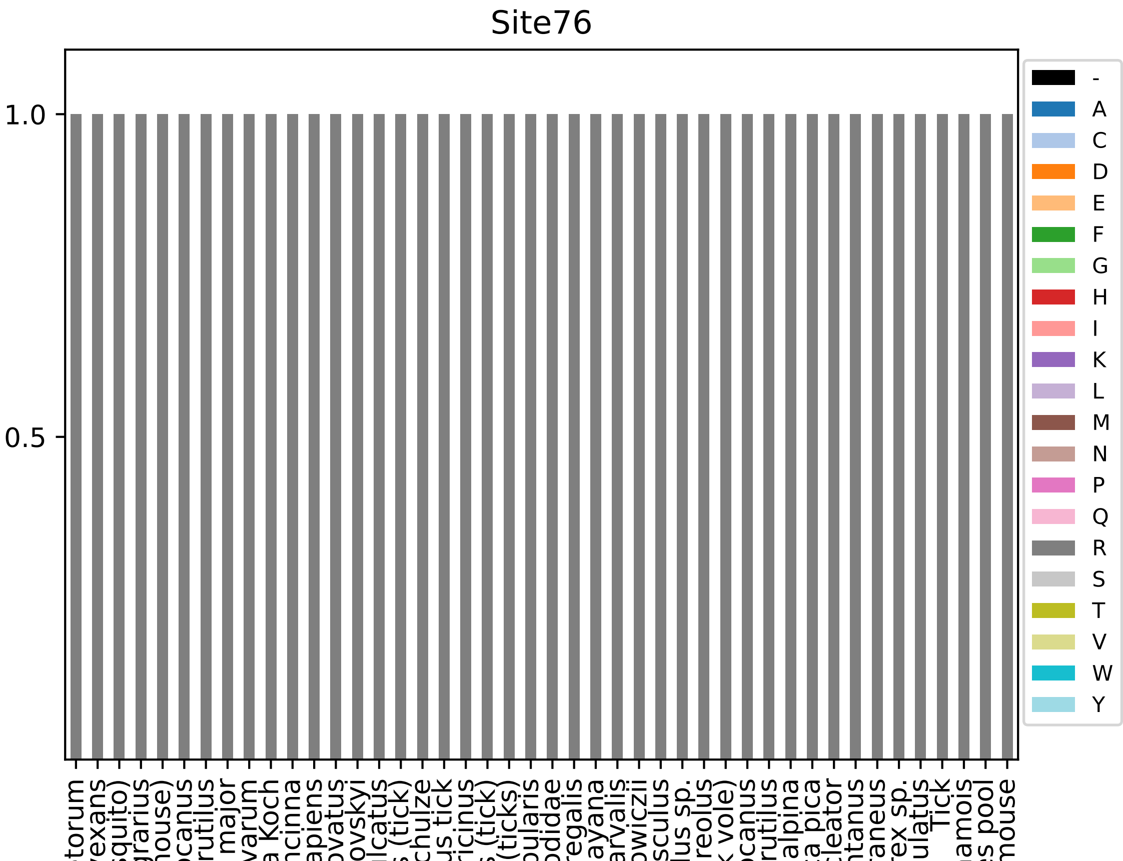

Site76
1.0
0.5
-
A
C
D
E
F
G
H
I
K
L
M
N
P
Q
R
S
T
V
W
Y

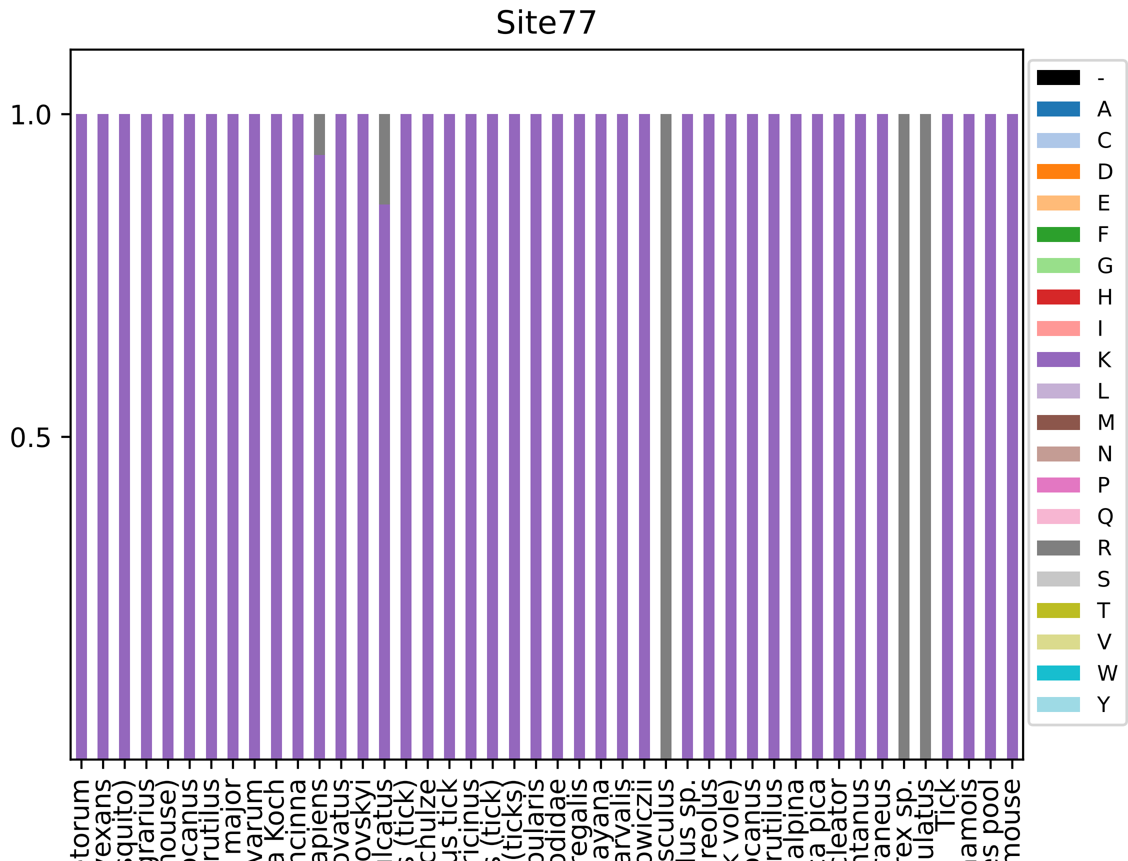
Site77
1.0
0.5
-
A
C
D
E
F
G
H
I
K
L
M
N
P
Q
R
S
T
V
W
Y

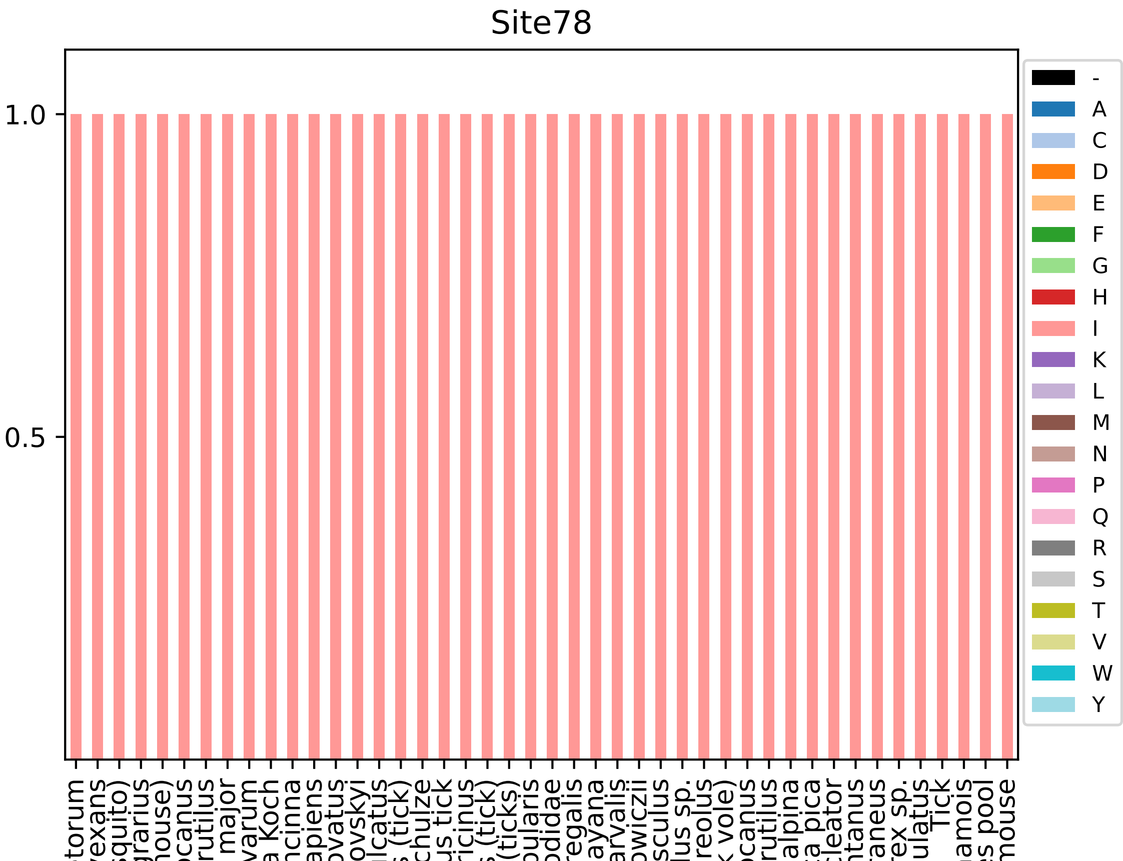

Site78
1.0
0.5
-
A
C
D
E
F
G
H
I
K
L
M
N
P
Q
R
S
T
V
W
Y
Tick

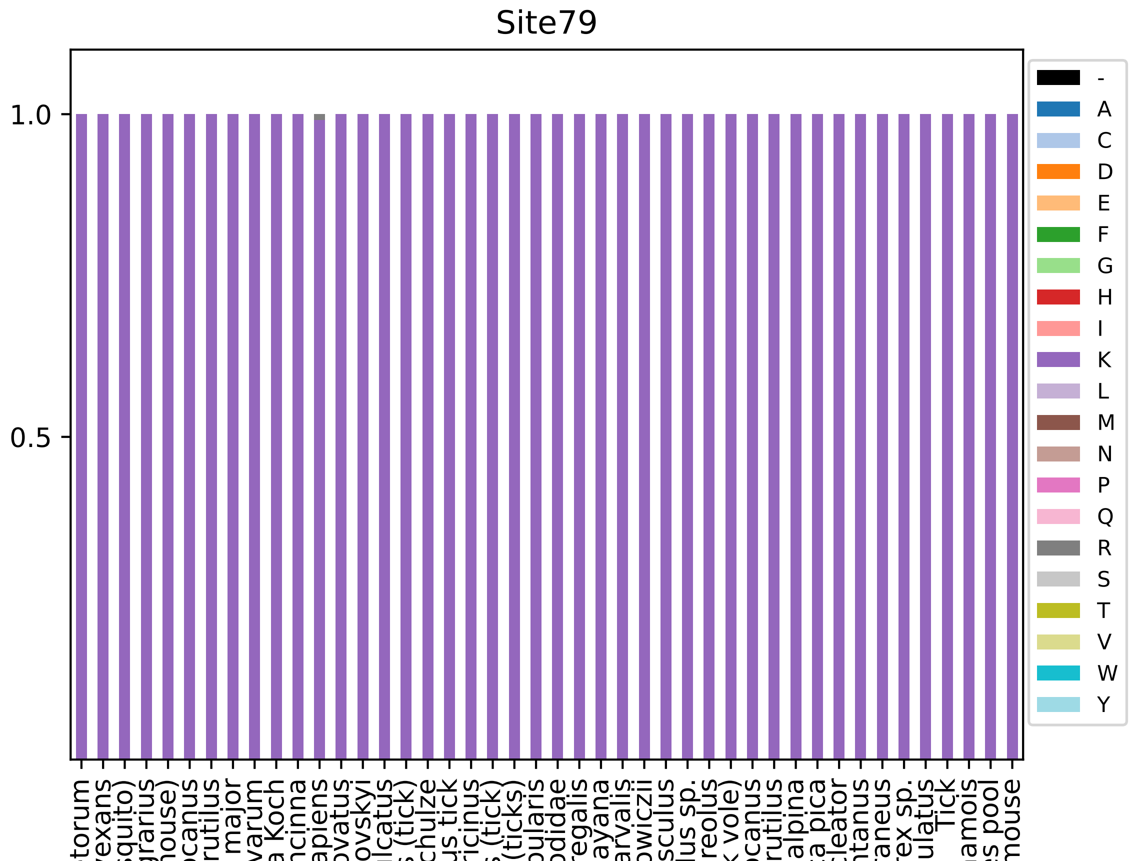
Site79
1.0
0.5
-
A
C
D
E
F
G
H
I
K
L
M
N
P
Q
R
S
T
V
W
Y

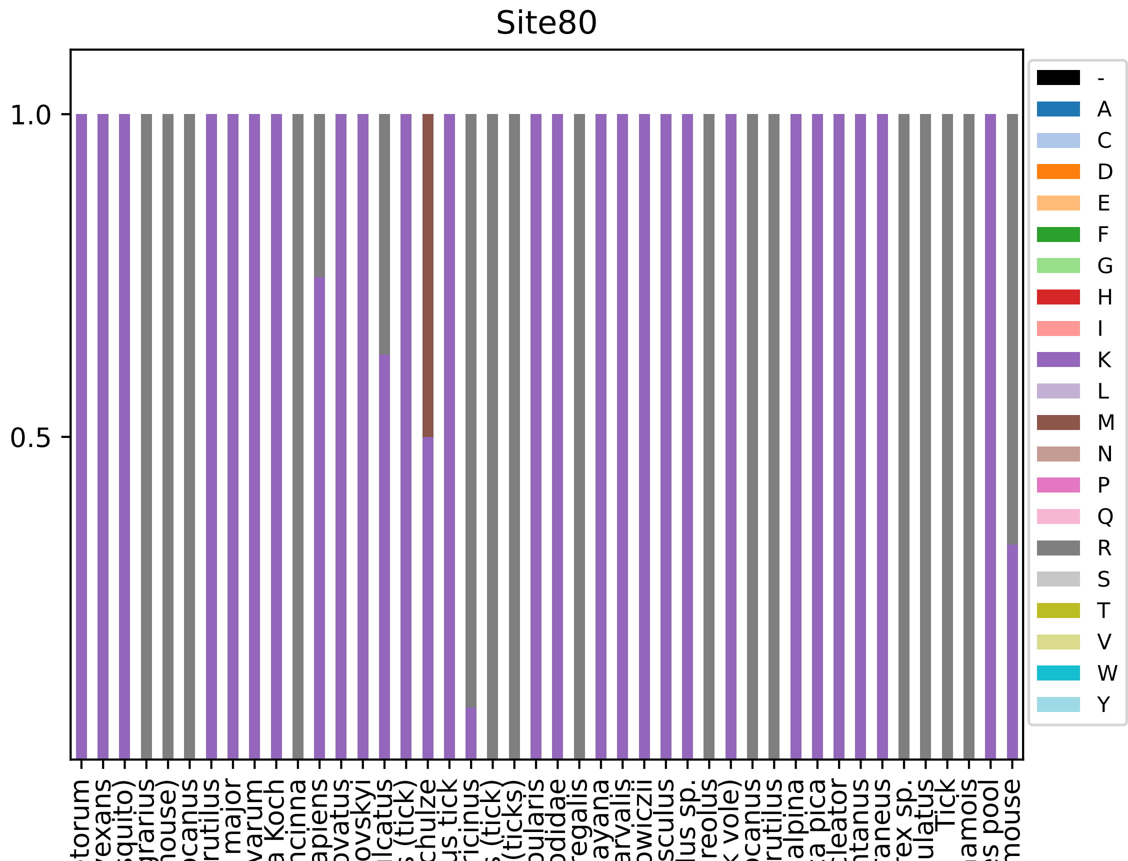
Site80
1.0
0.5
-
A
C
D
E
F
G
H
I
K
L
M
N
P
Q
R
S
T
V
W
Y
Tick

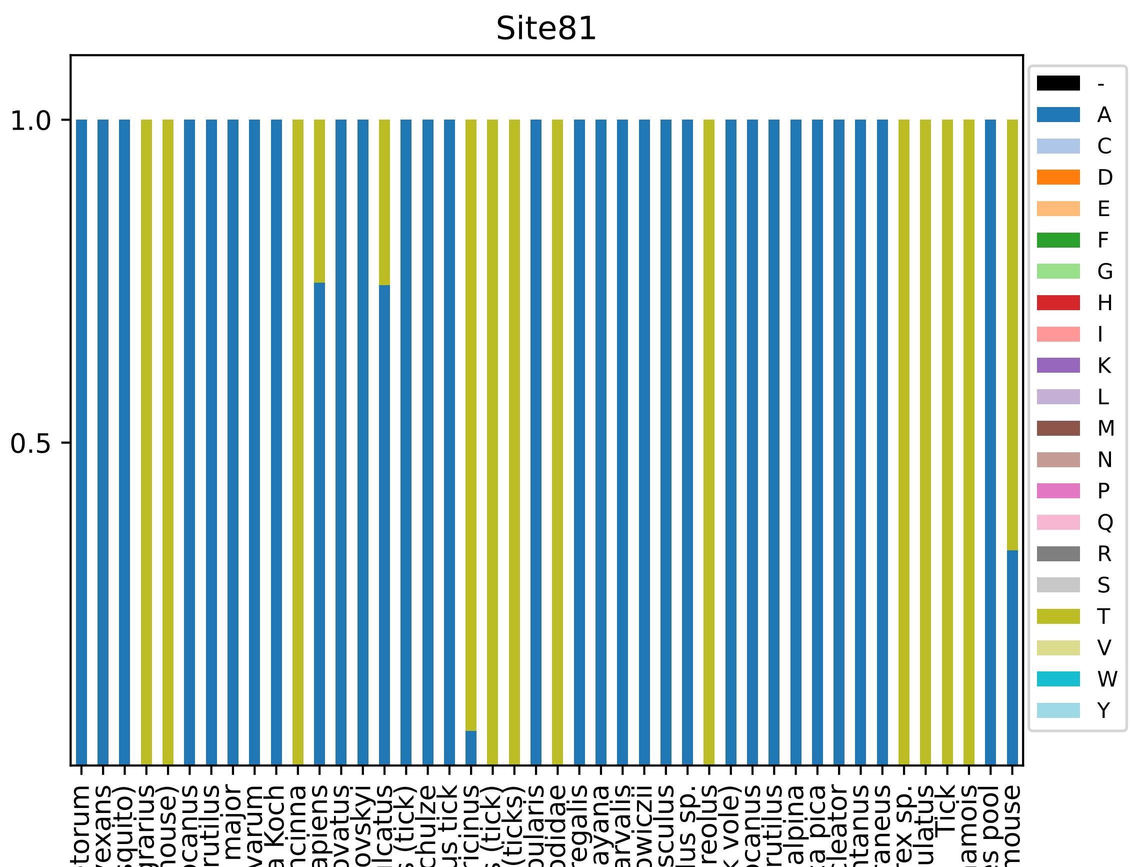
Site81
1.0
0.5
-
A
C
D
E
F
G
H
I
K
L
M
N
P
Q
R
S
T
V
W
Y

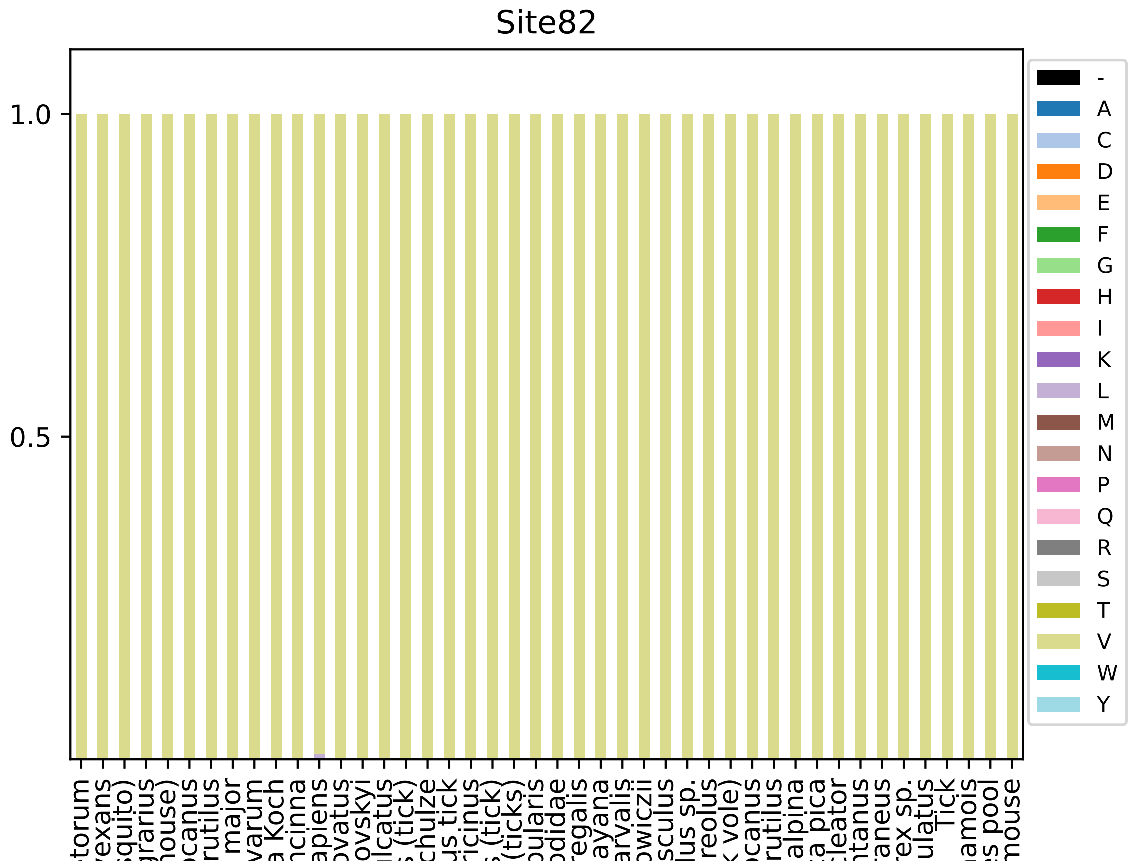
Site82
1.0
0.5
-
A
C
D
E
F
G
H
I
K
L
M
N
P
Q
R
S
T
V
W
Y

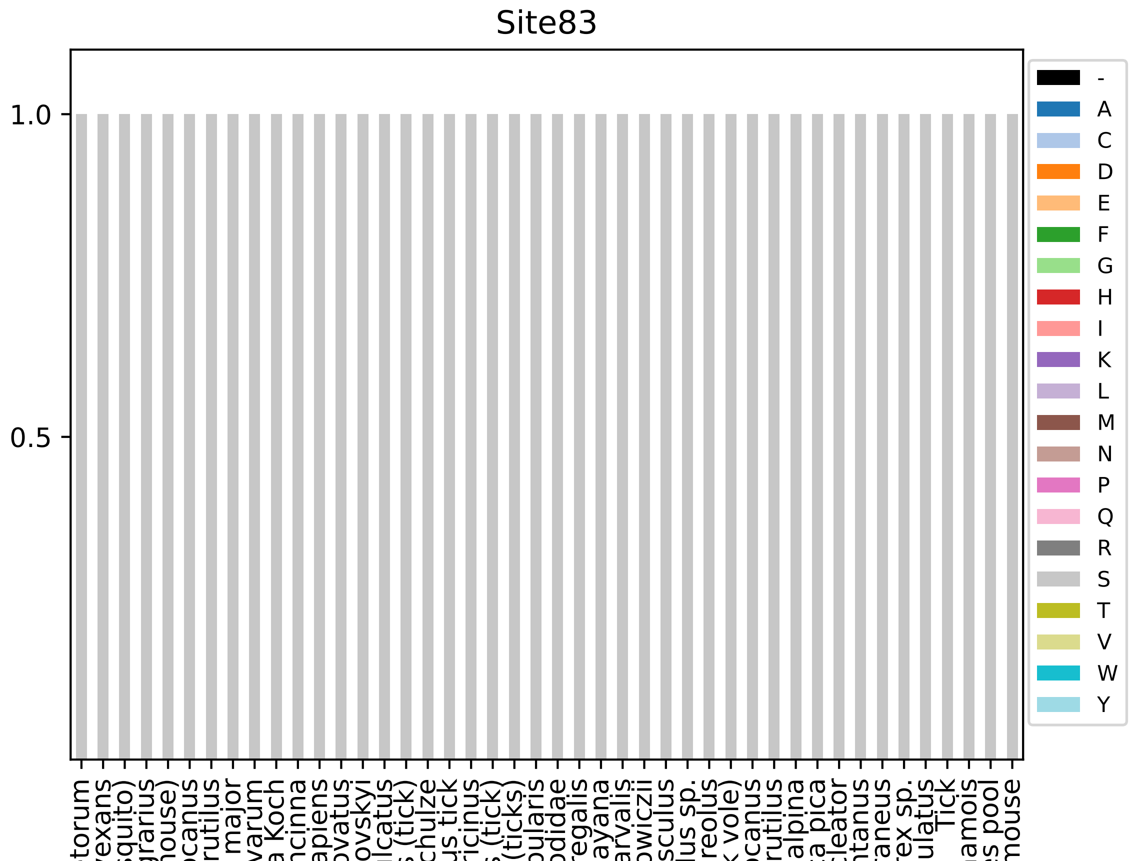
Site83
1.0
0.5
-
A
C
D
E
F
G
H
I
K
L
M
N
P
Q
R
S
T
V
W
Y

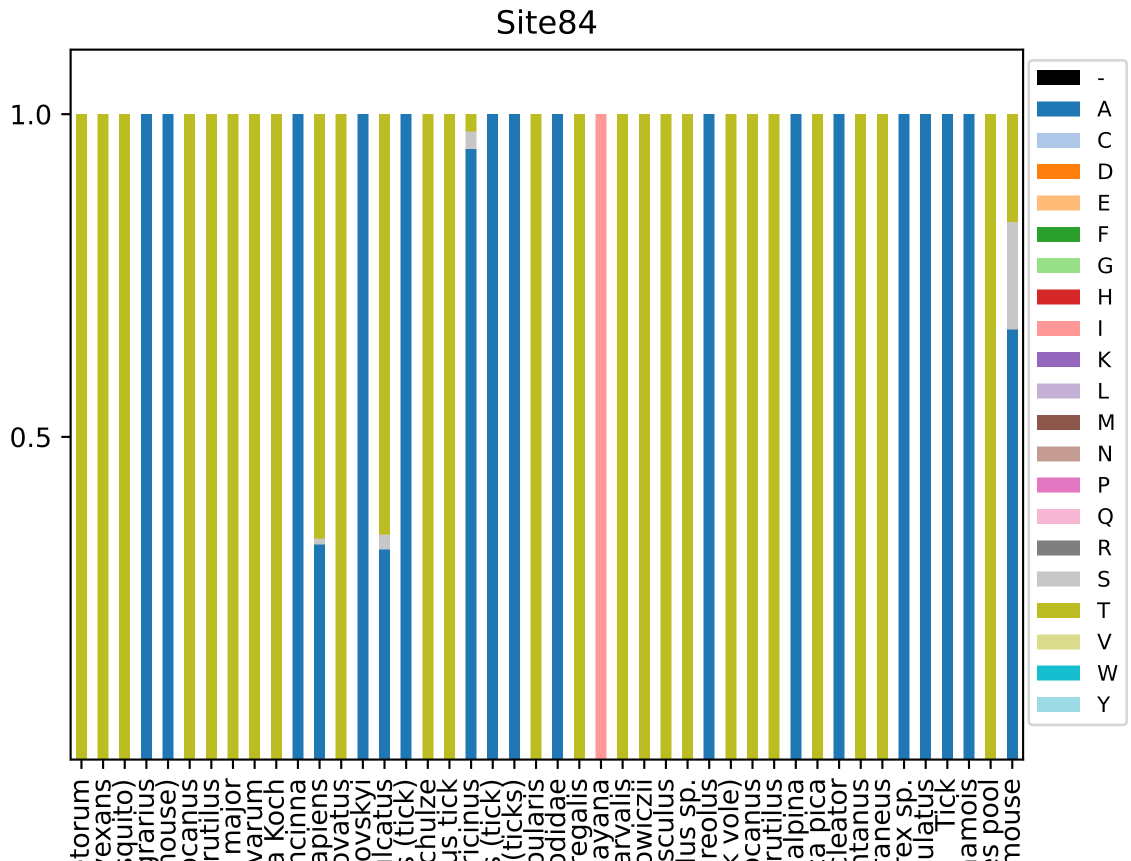
Site84
1.0
0.5
-
A
C
D
E
F
G
H
I
K
L
M
N
P
Q
R
S
T
V
W
Y

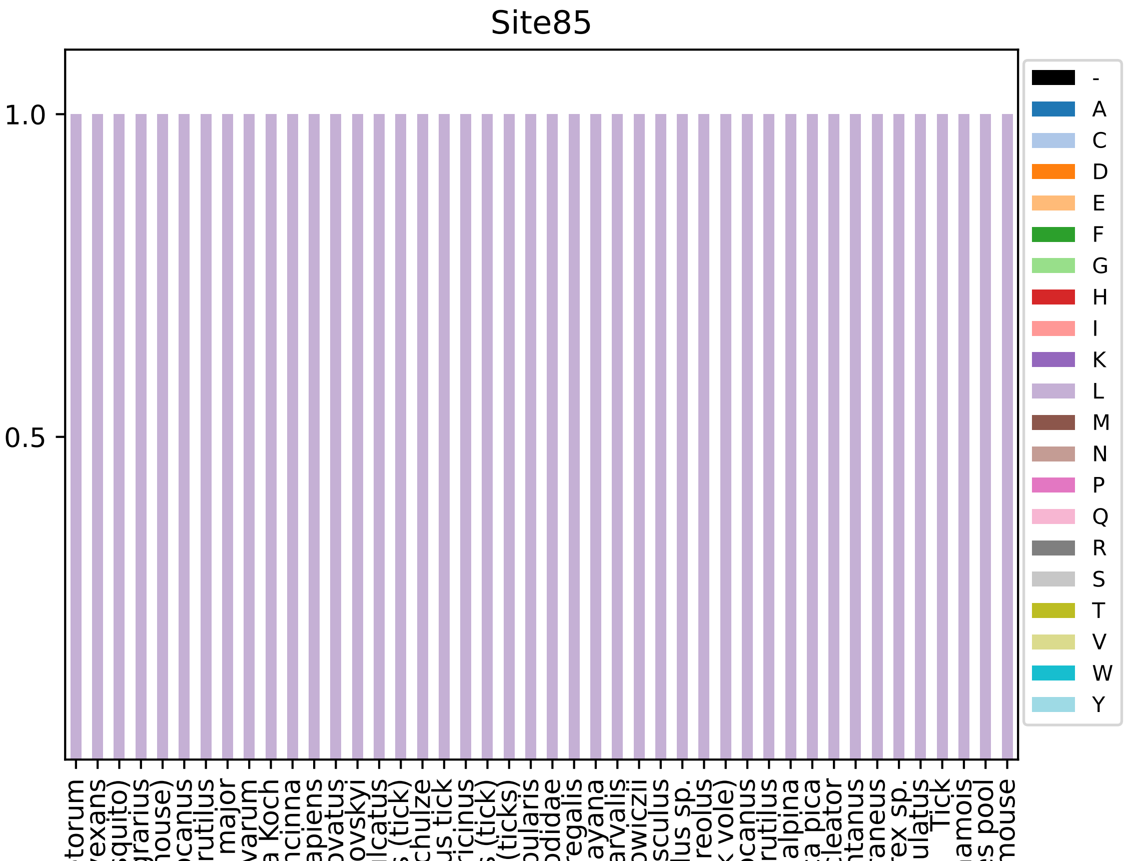
Site85
1.0
0.5
-
A
C
D
E
F
G
H
I
K
L
M
N
P
Q
R
S
T
V
W
Y

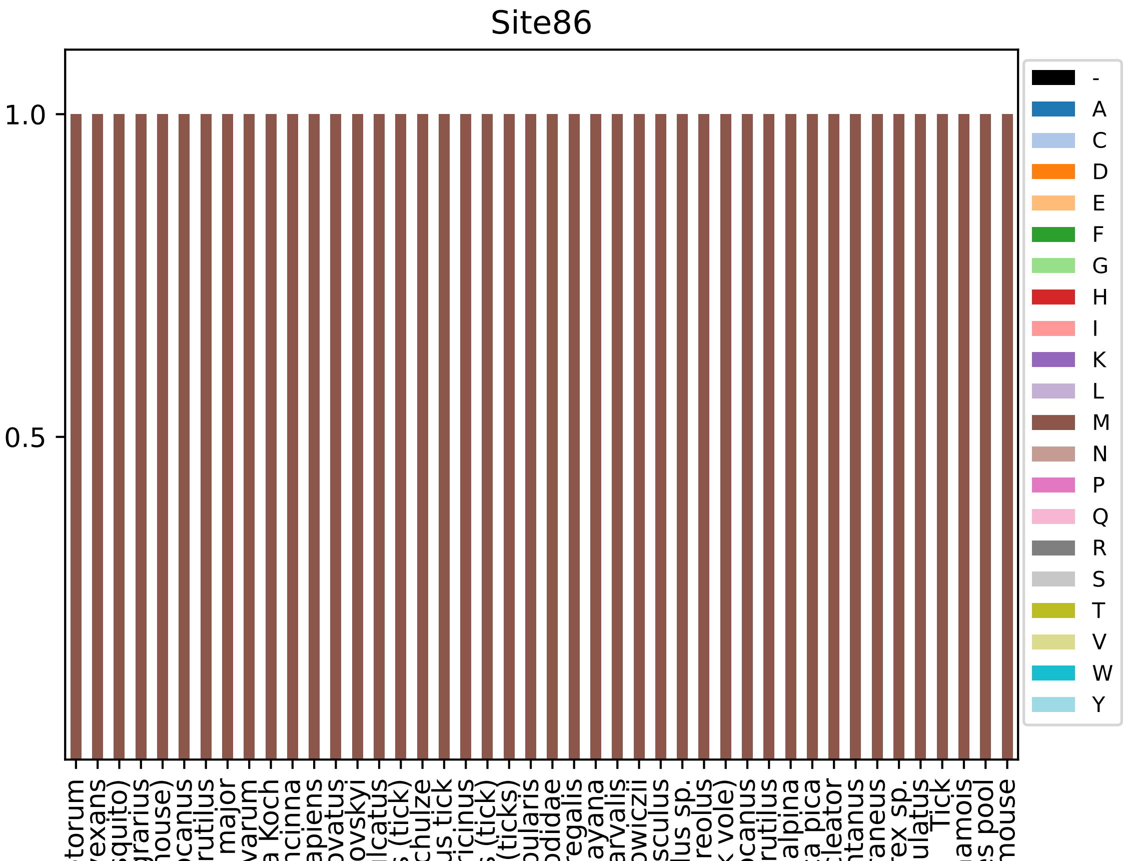
Site86
1.0
0.5
-
A
C
D
E
F
G
H
I
K
L
M
N
P
Q
R
S
T
V
W
Y

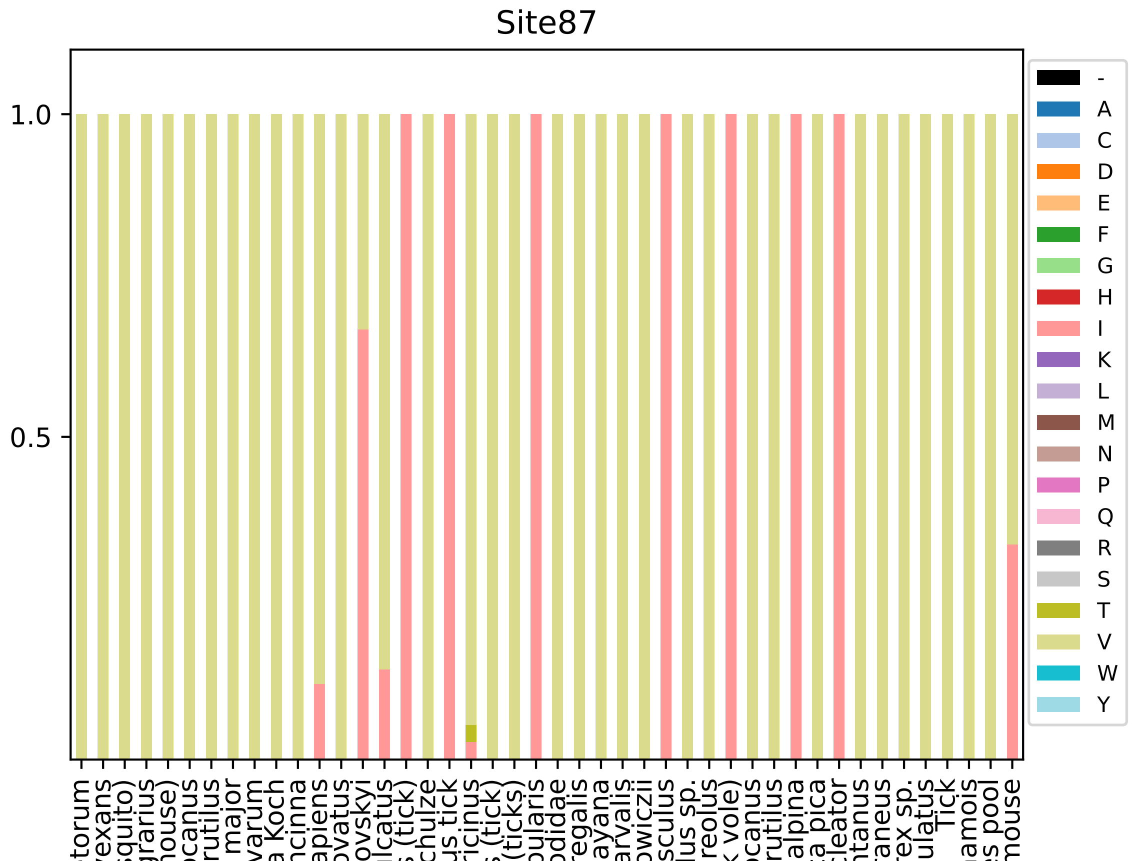
Site87
1.0
0.5
-
A
C
D
E
F
G
H
I
K
L
M
N
P
Q
R
S
T
V
W
Y

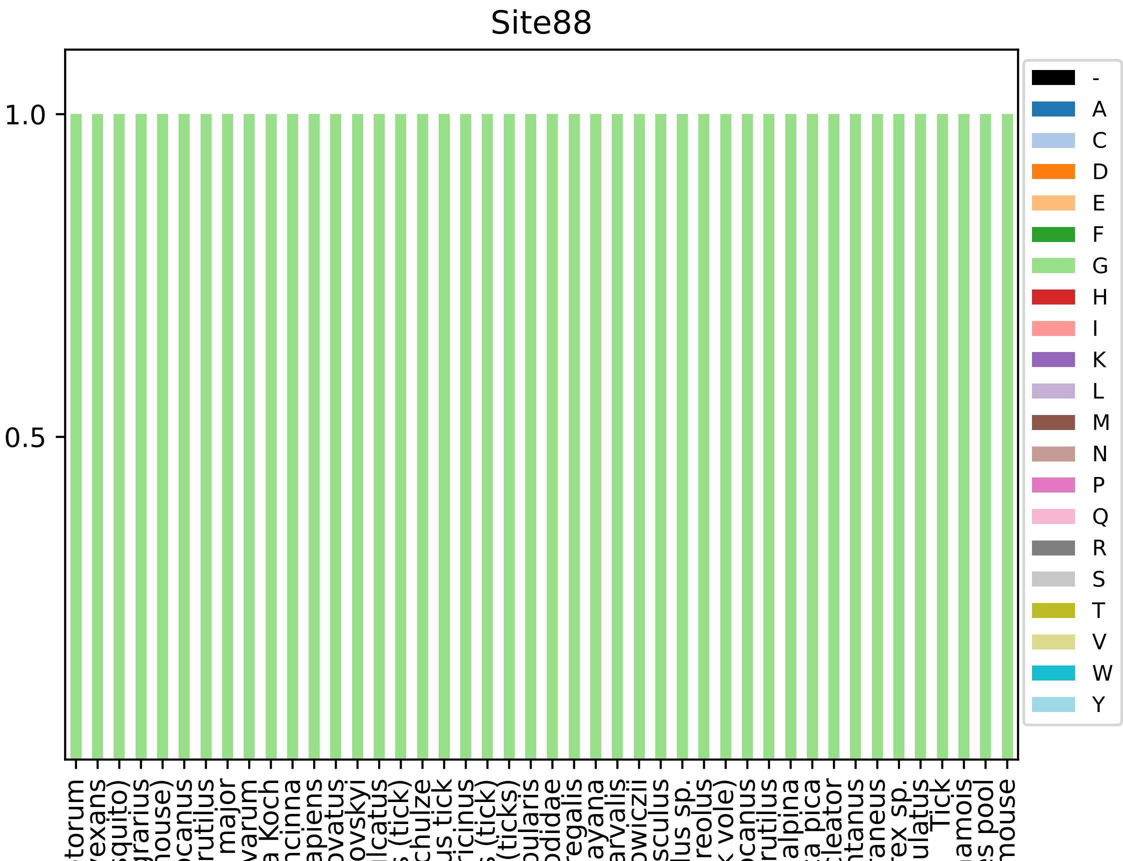

Site88
1.0
0.5
-
A
C
D
E
F
G
H
I
K
L
M
N
P
Q
R
S
T
V
W
Y

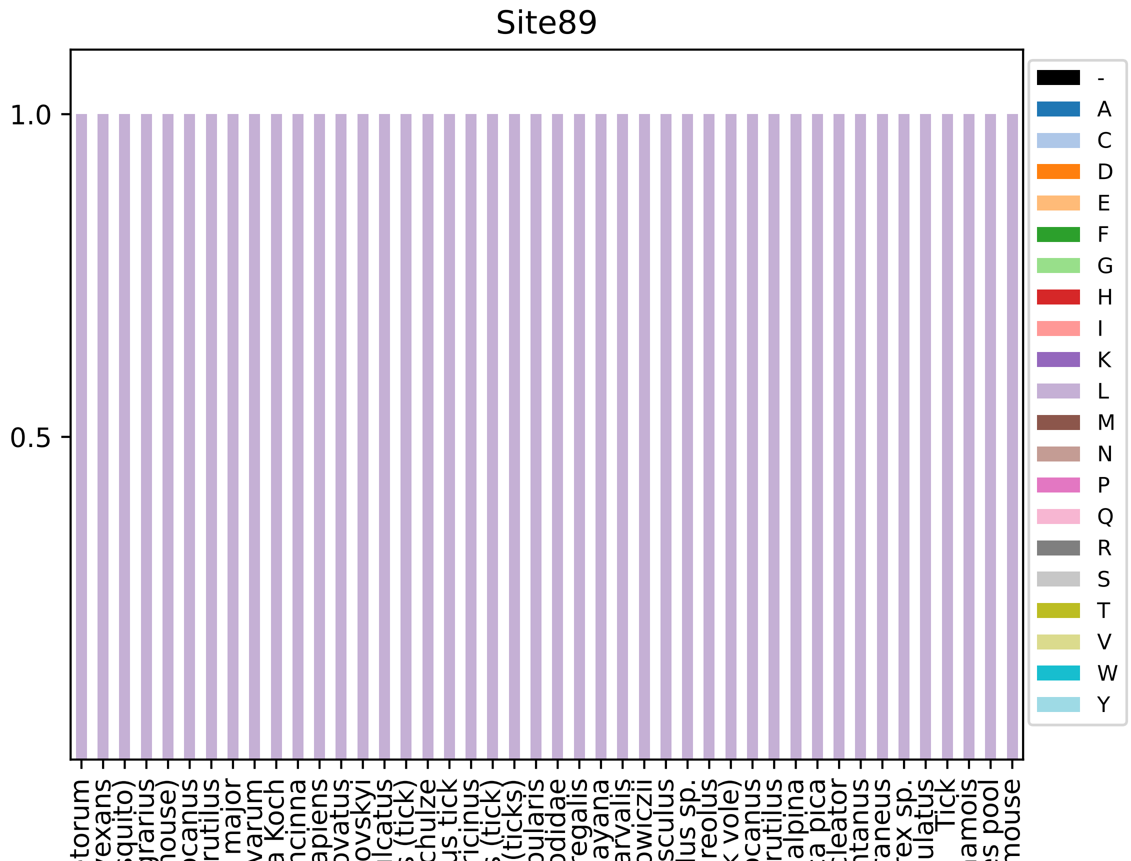
Site89
1.0
0.5
-
A
C
D
E
F
G
H
I
K
L
M
N
P
Q
R
S
T
V
W
Y

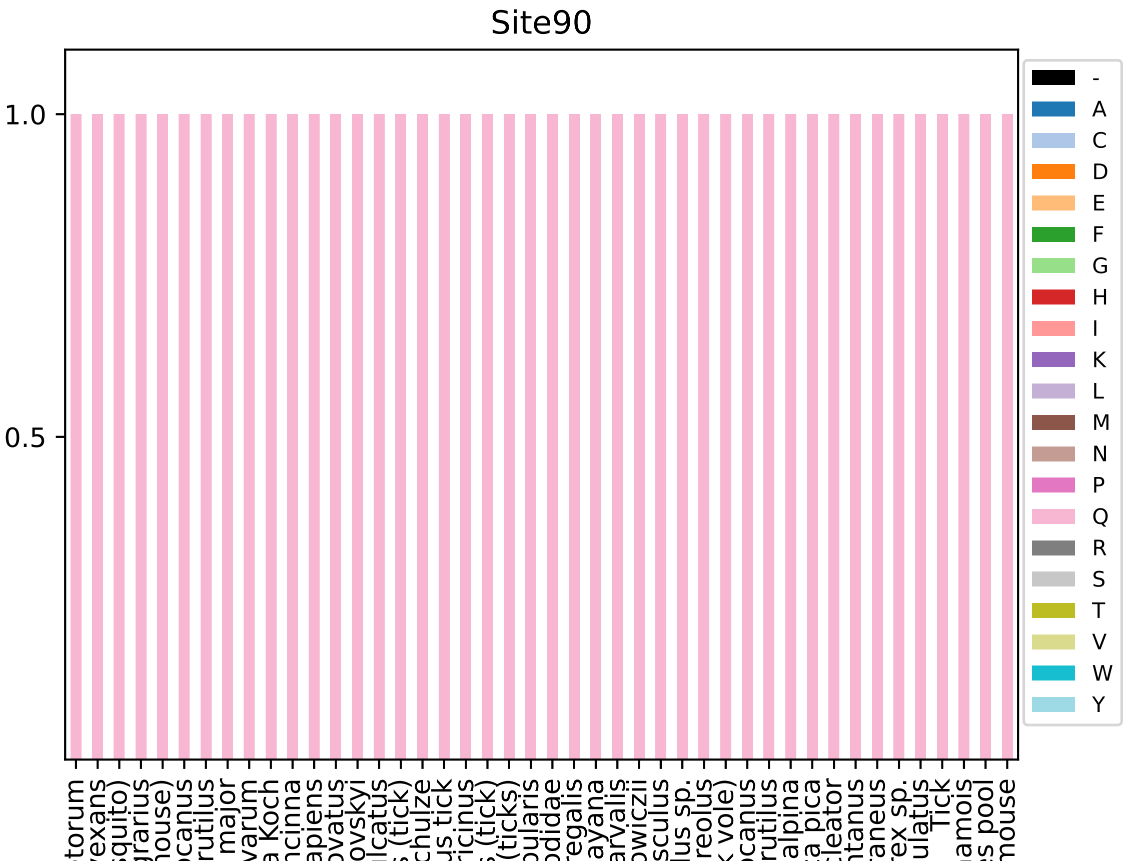
Site90
1.0
0.5
-
A
C
D
E
F
G
H
I
K
L
M
N
P
Q
R
S
T
V
W
Y

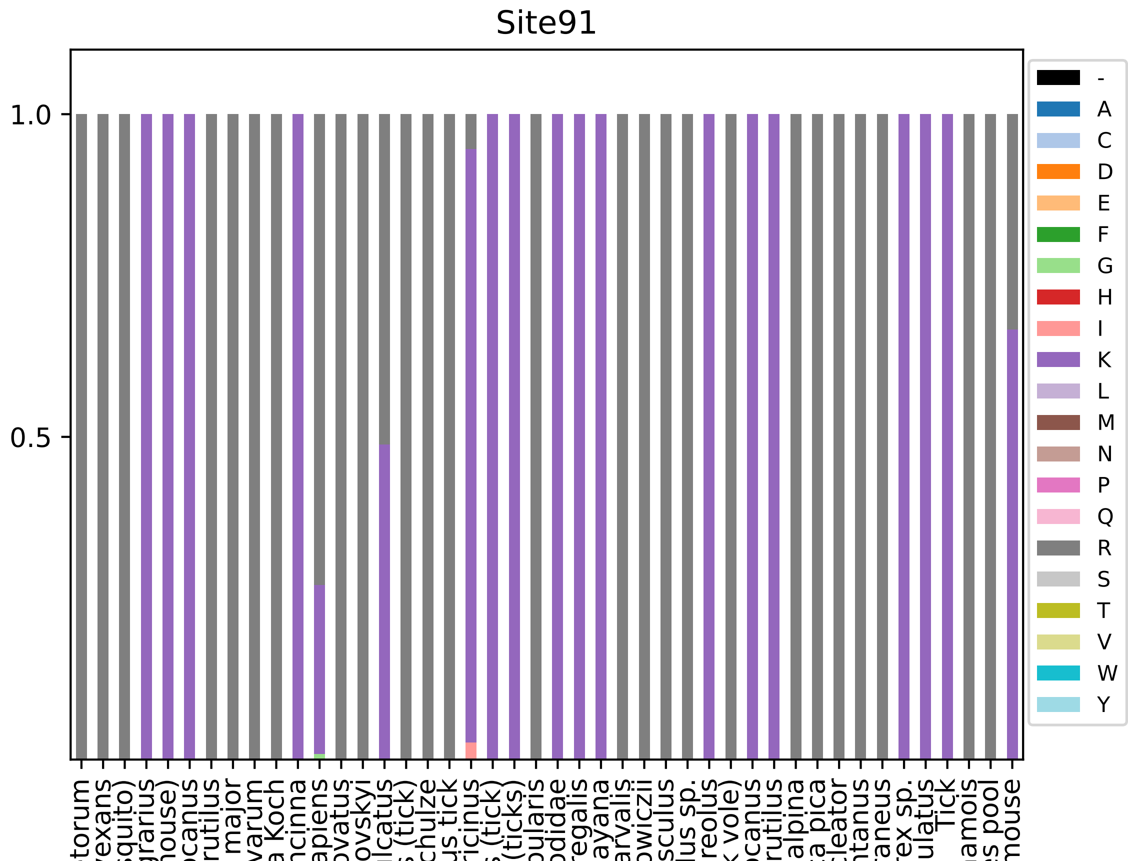
Site91
1.0
0.5
-
A
C
D
E
F
G
H
I
K
L
M
N
P
Q
R
S
T
V
W
Y

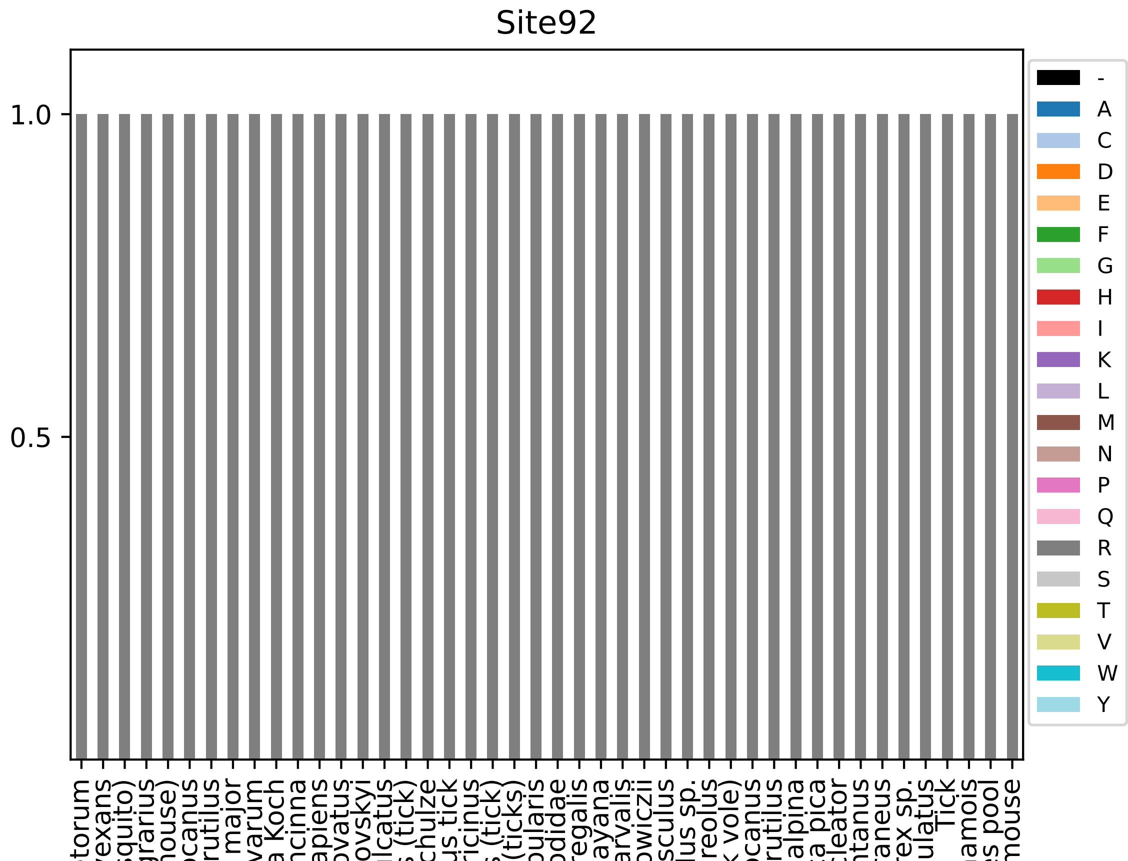

Site92
1.0
0.5
-
A
C
D
E
F
G
H
I
K
L
M
N
P
Q
R
S
T
V
W
Y

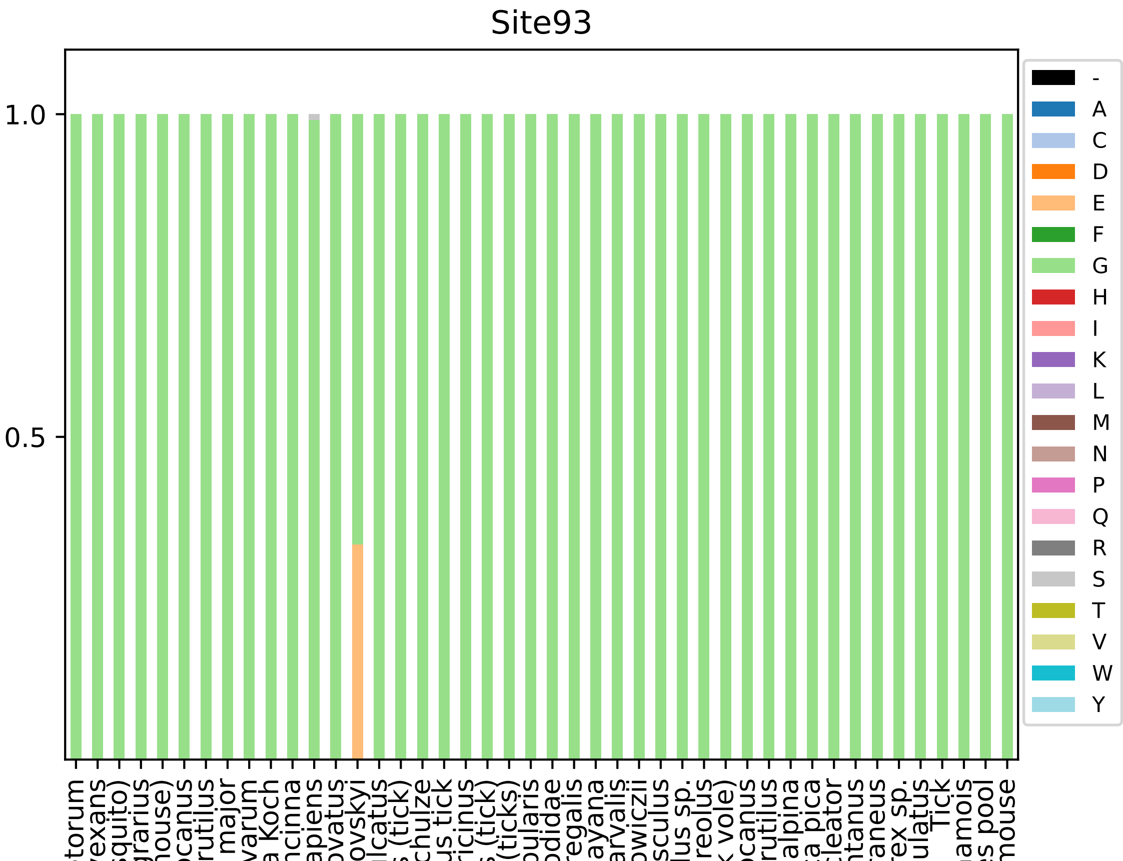

Site93
1.0
0.5
-
A
C
D
E
F
G
H
I
K
L
M
N
P
Q
R
S
T
V
W
Y

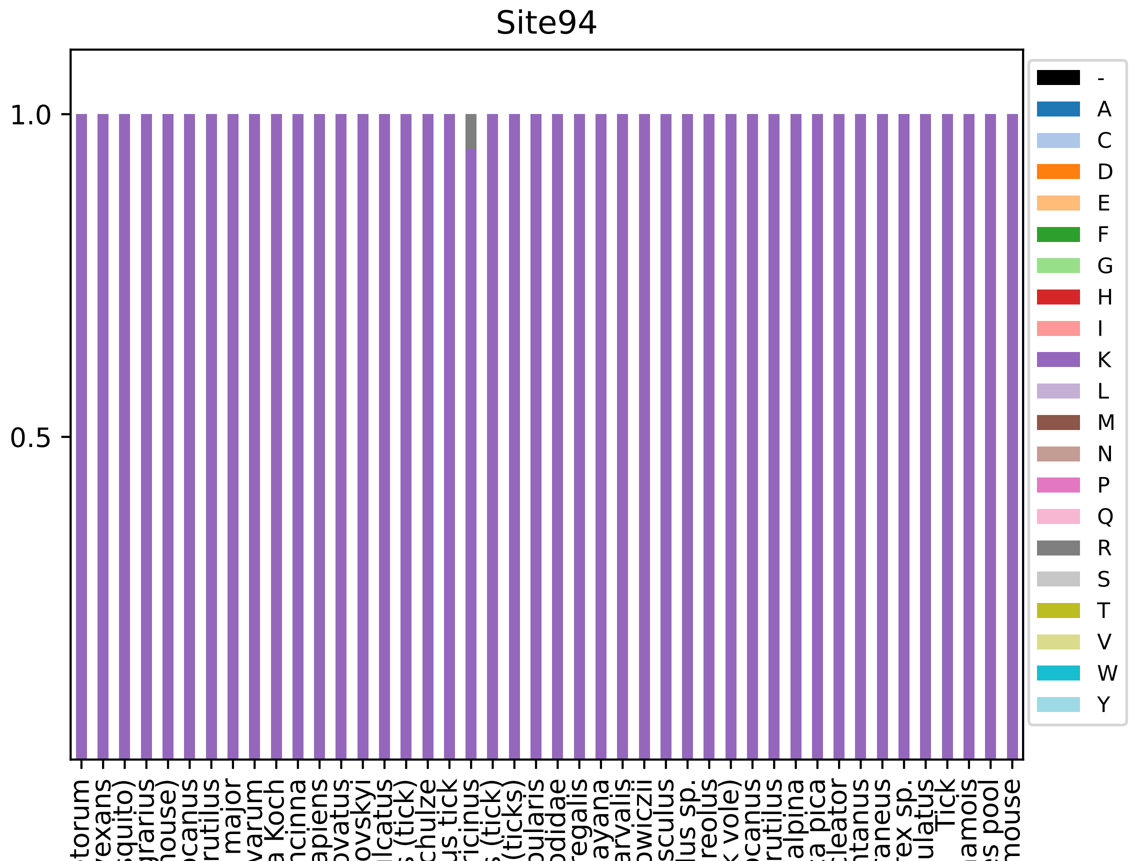
Site94
1.0
0.5
-
A
C
D
E
F
G
H
I
K
L
M
N
P
Q
R
S
T
V
W
Y

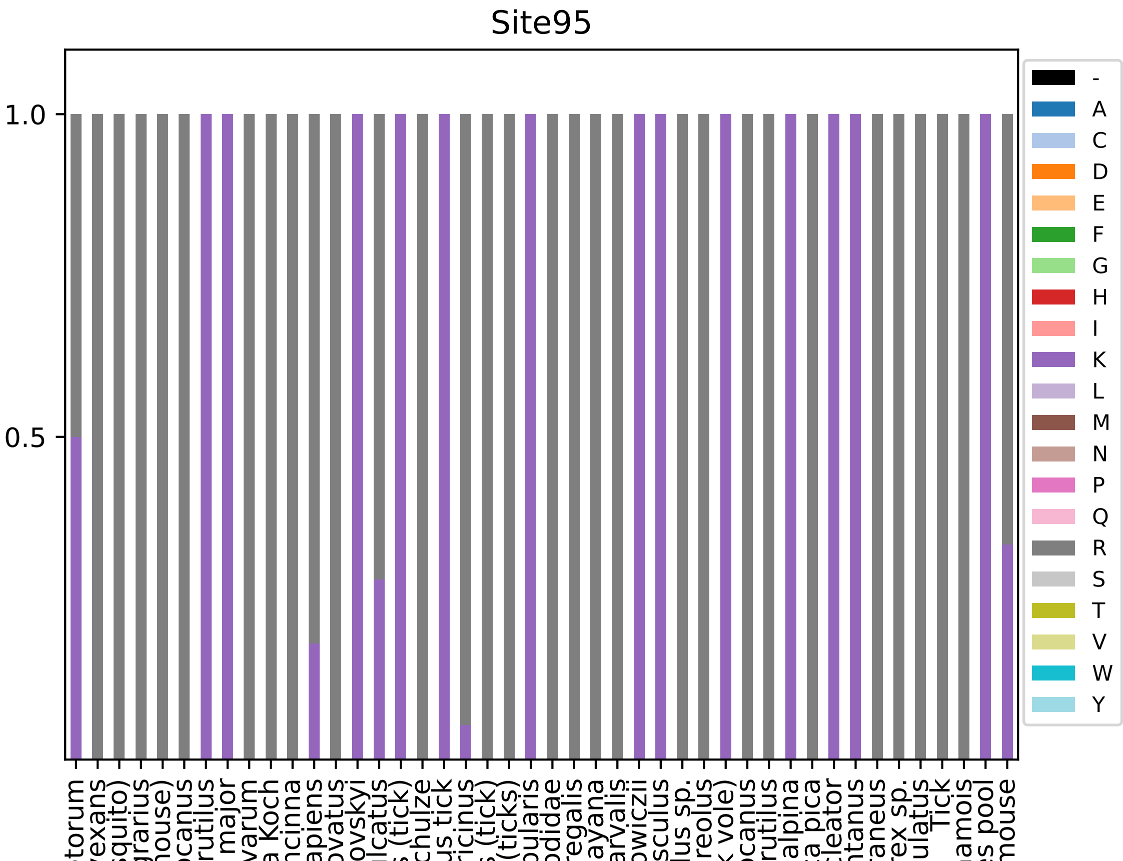
Site95
1.0
0.5
-
A
C
D
E
F
G
H
I
K
L
M
N
P
Q
R
S
T
V
W
Y

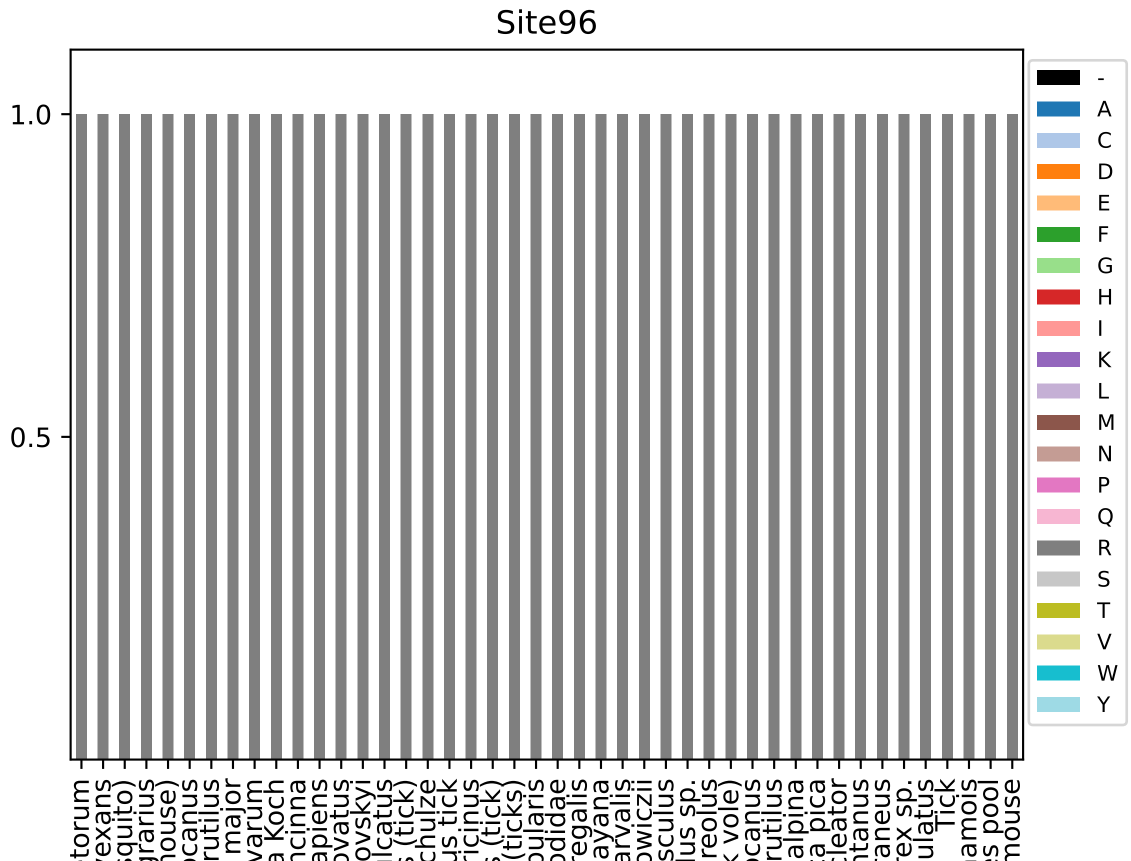

Site96
1.0
0.5
-
A
C
D
E
F
G
H
I
K
L
M
N
P
Q
R
S
T
V
W
Y

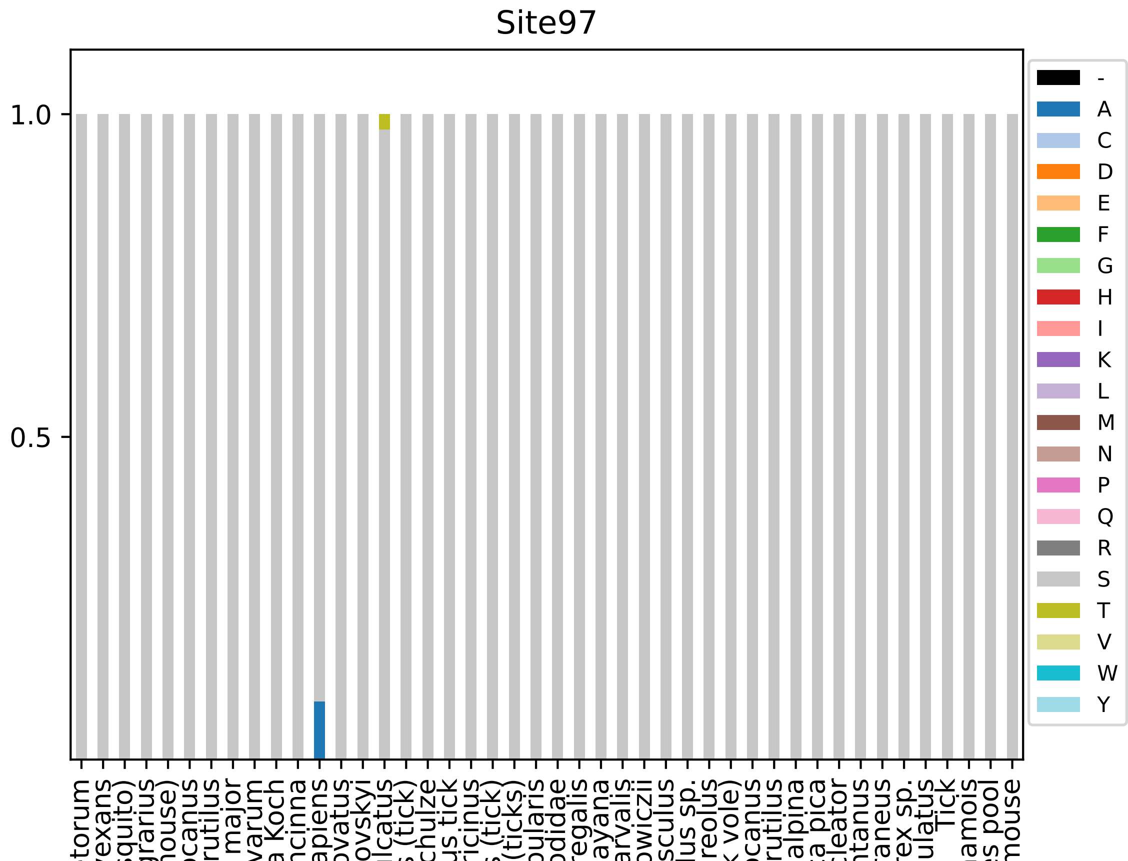
Site97
1.0
0.5
-
A
C
D
E
F
G
H
I
K
L
M
N
P
Q
R
S
T
V
W
Y

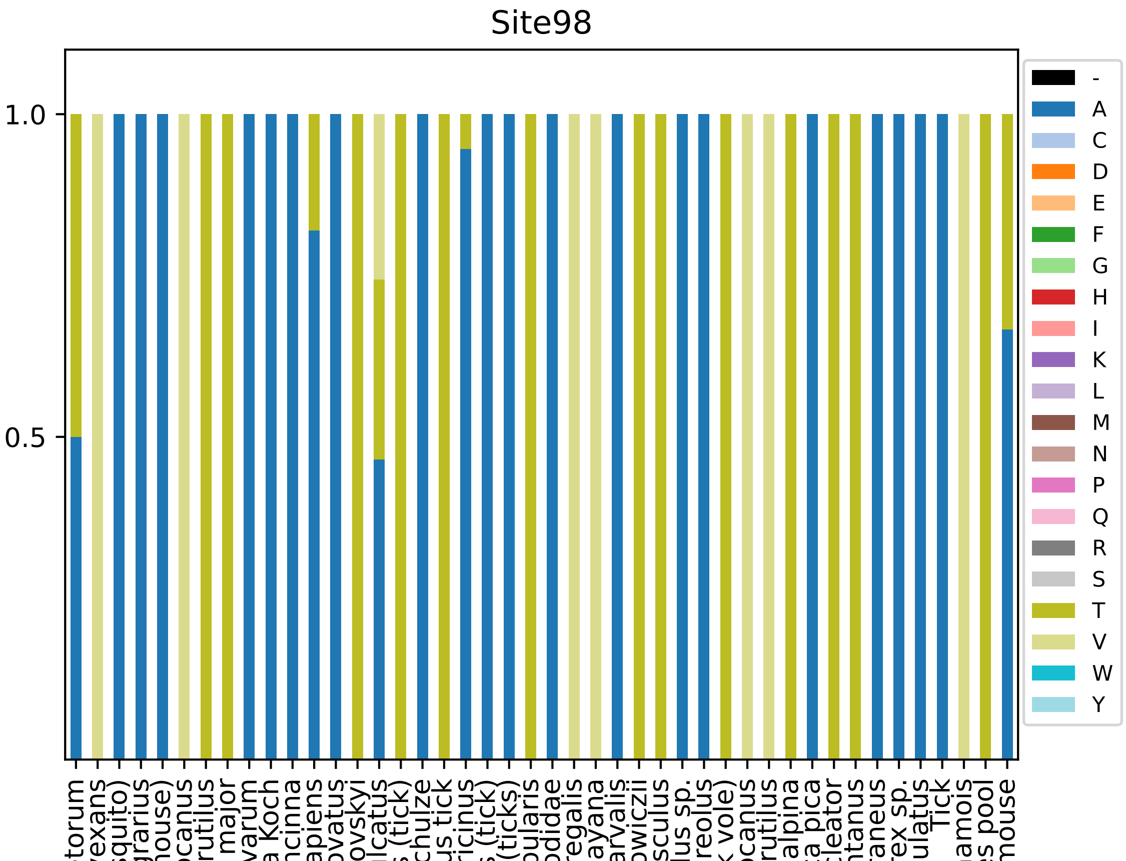

Site98
1.0
0.5
-
A
C
D
E
F
G
H
I
K
L
M
N
P
Q
R
S
T
V
W
Y

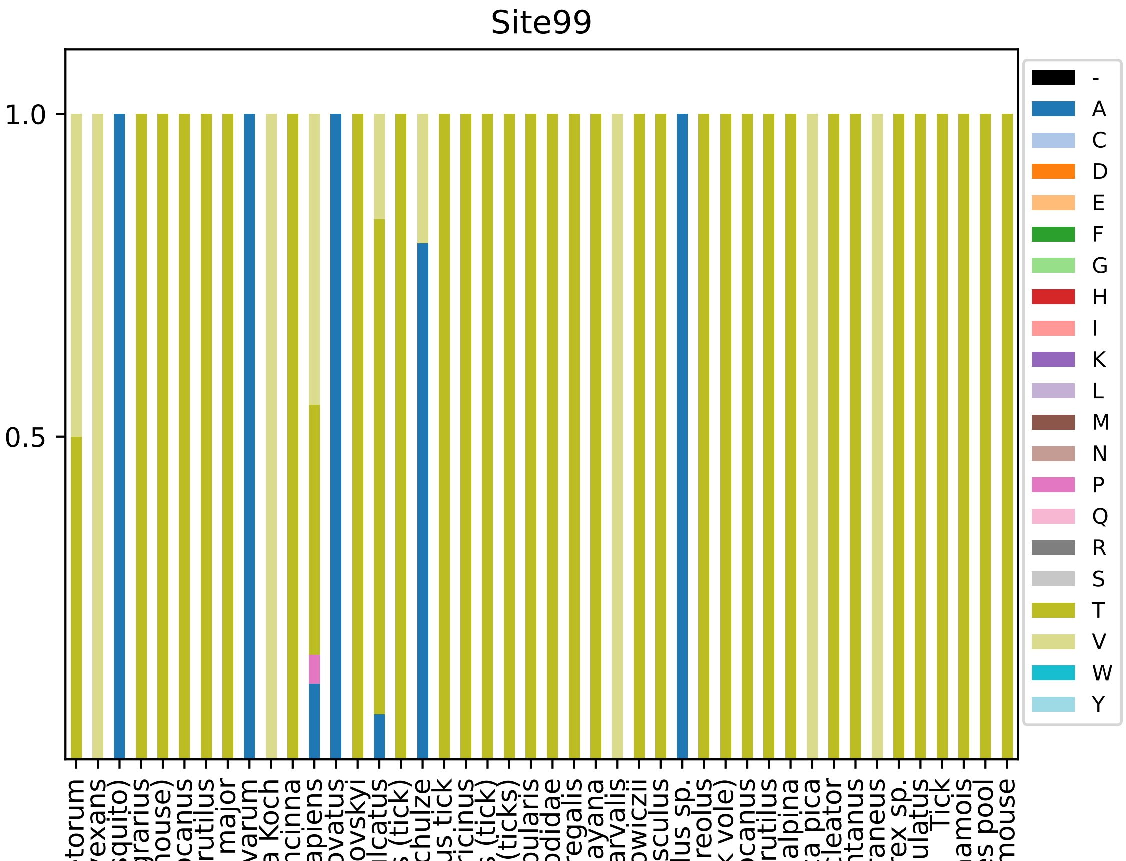
Site99
1.0
0.5
-
A
C
D
E
F
G
H
I
K
L
M
N
P
Q
R
S
T
V
W
Y

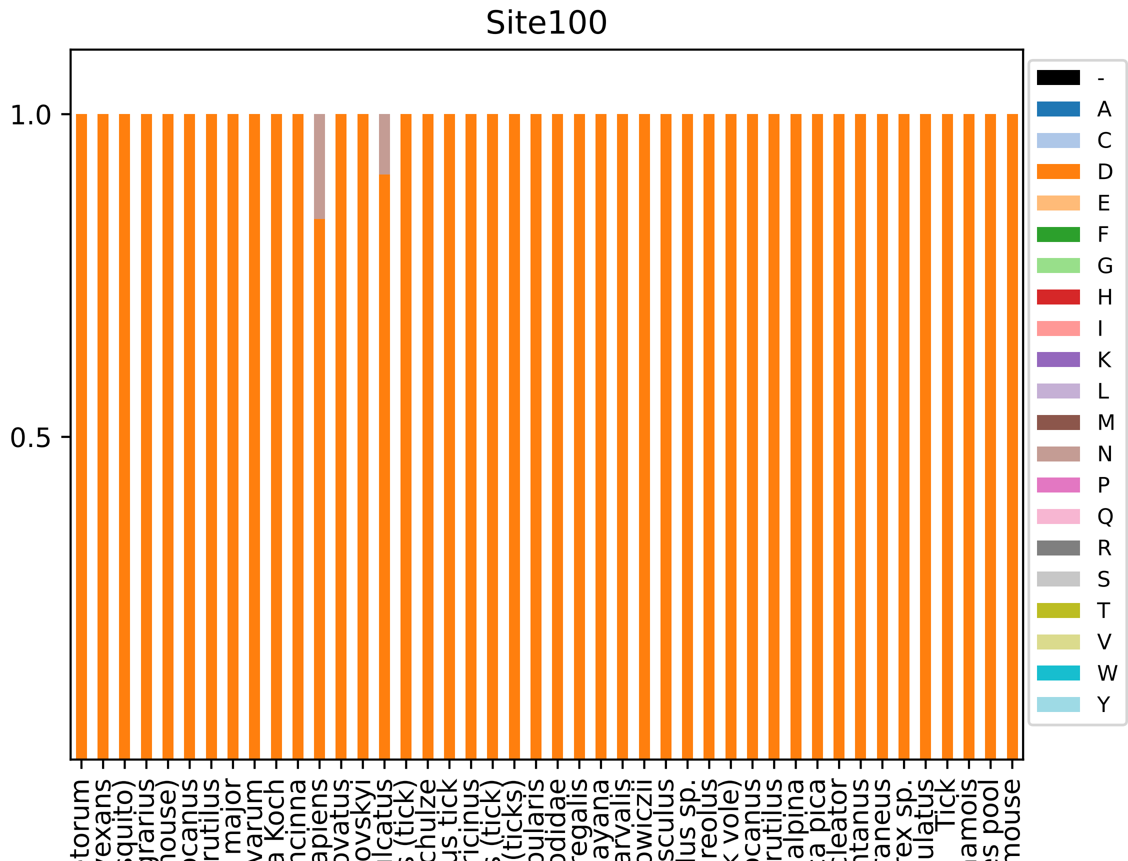

Site100
1.0
0.5
-
A
C
D
E
F
G
H
I
K
L
M
N
P
Q
R
S
T
V
W
Y

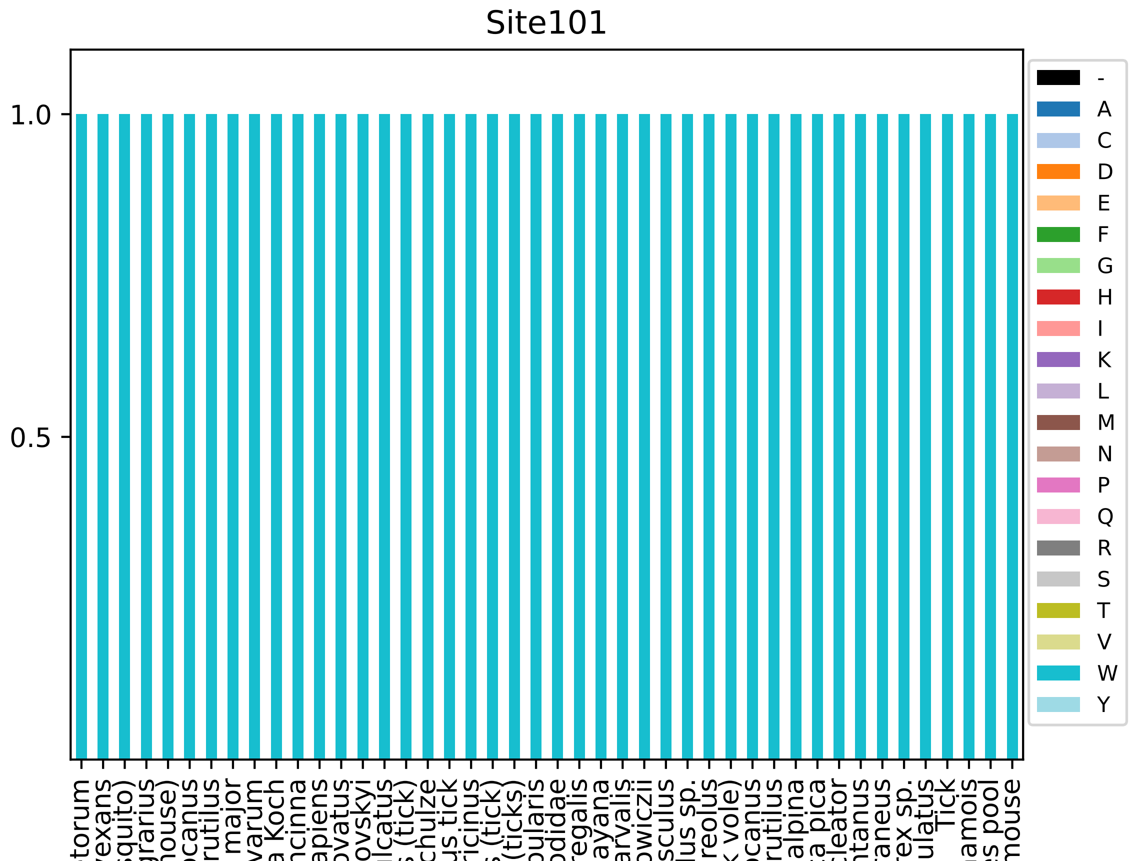
Site101
1.0
0.5
-
A
C
D
E
F
G
H
I
K
L
M
N
P
Q
R
S
T
V
W
Y

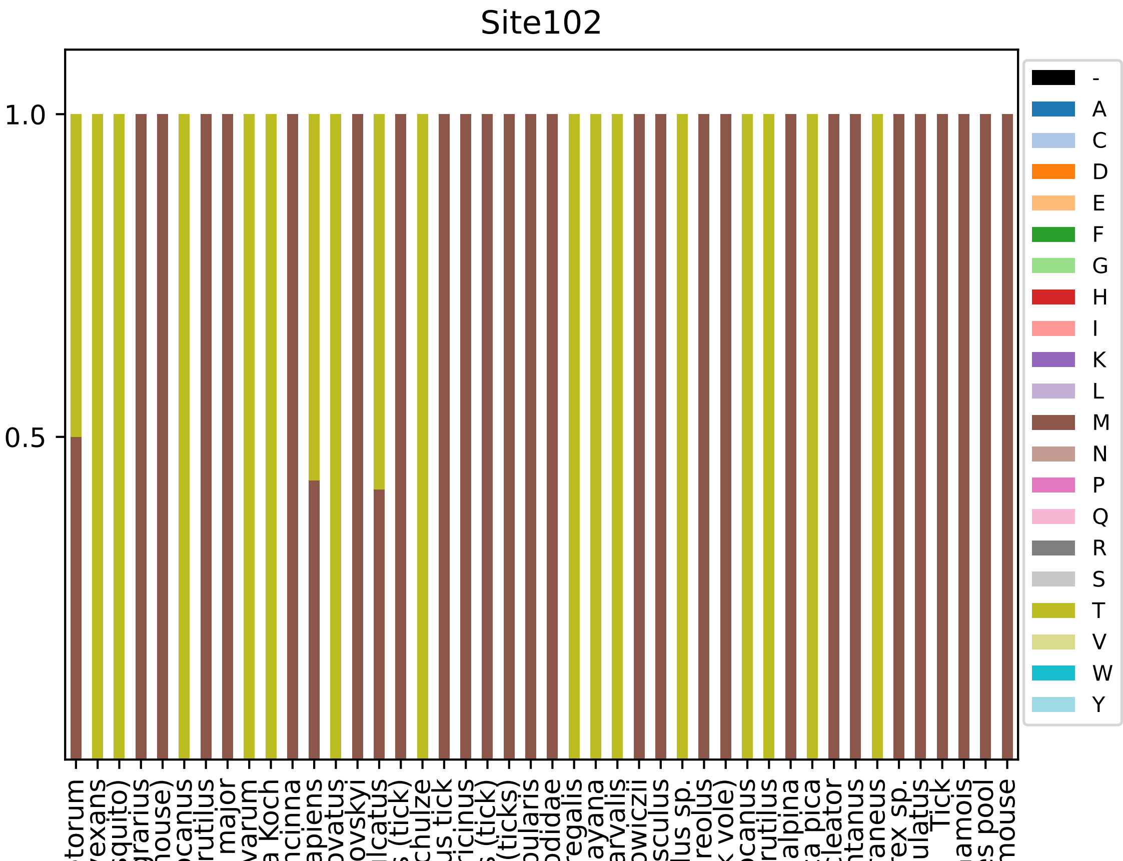
Site102
1.0
0.5
-
A
C
D
E
F
G
H
I
K
L
M
N
P
Q
R
S
T
V
W
Y

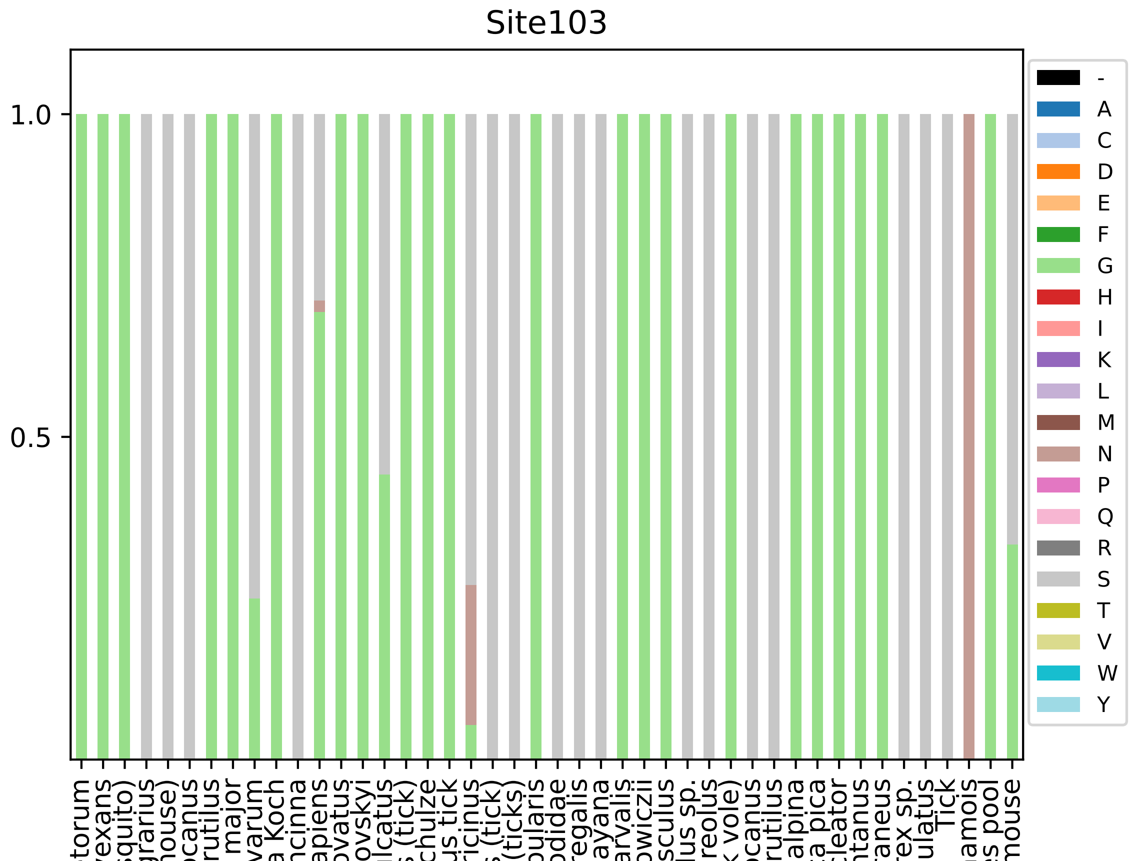
Site103
1.0
0.5
-
A
C
D
E
F
G
H
I
K
L
M
N
P
Q
R
S
T
V
W
Y

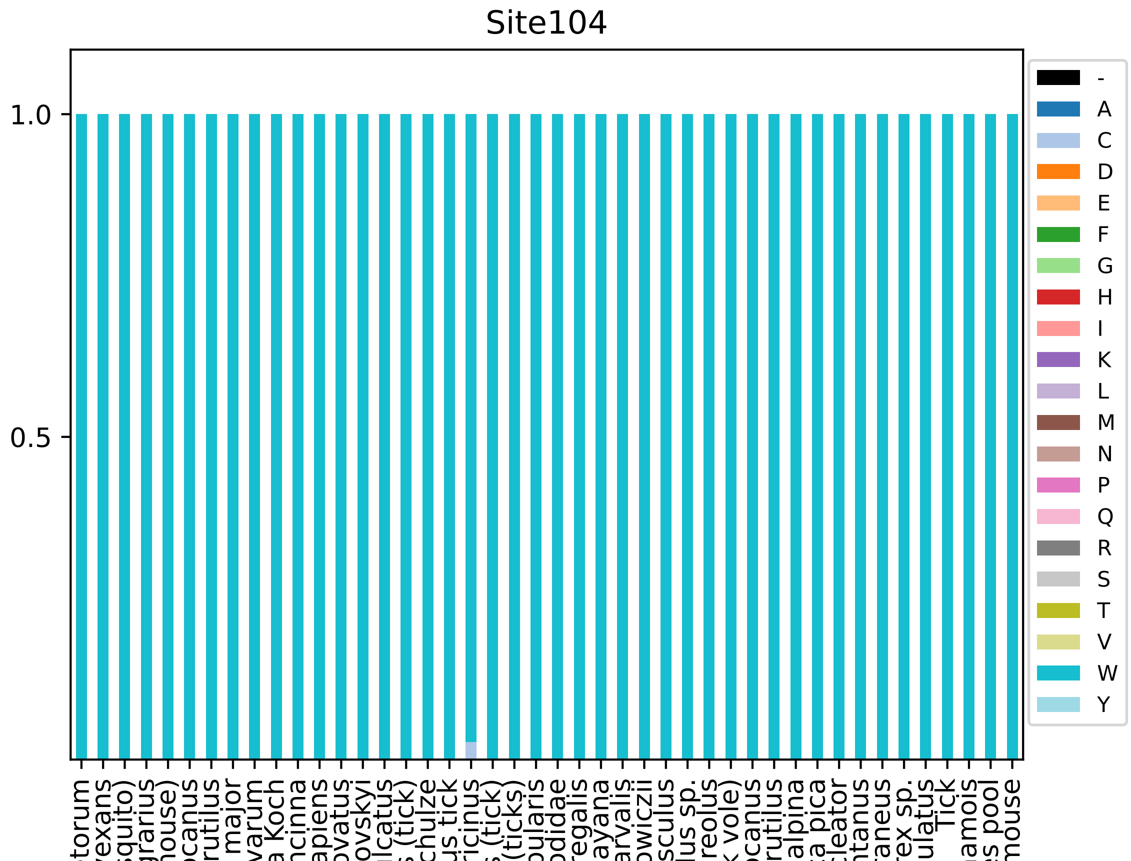
Site104
1.0
0.5
-
A
C
D
E
F
G
H
I
K
L
M
N
P
Q
R
S
T
V
W
Y

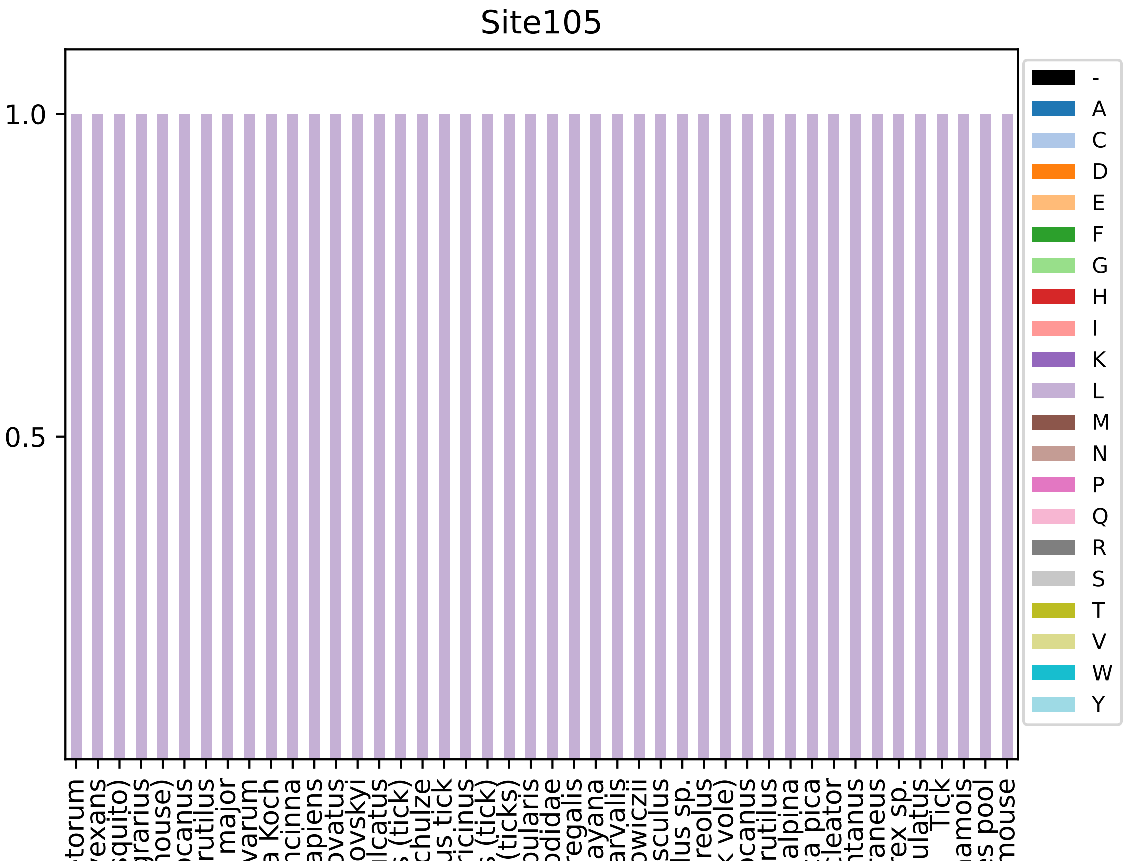
Site105
1.0
0.5
-
A
C
D
E
F
G
H
I
K
L
M
N
P
Q
R
S
T
V
W
Y

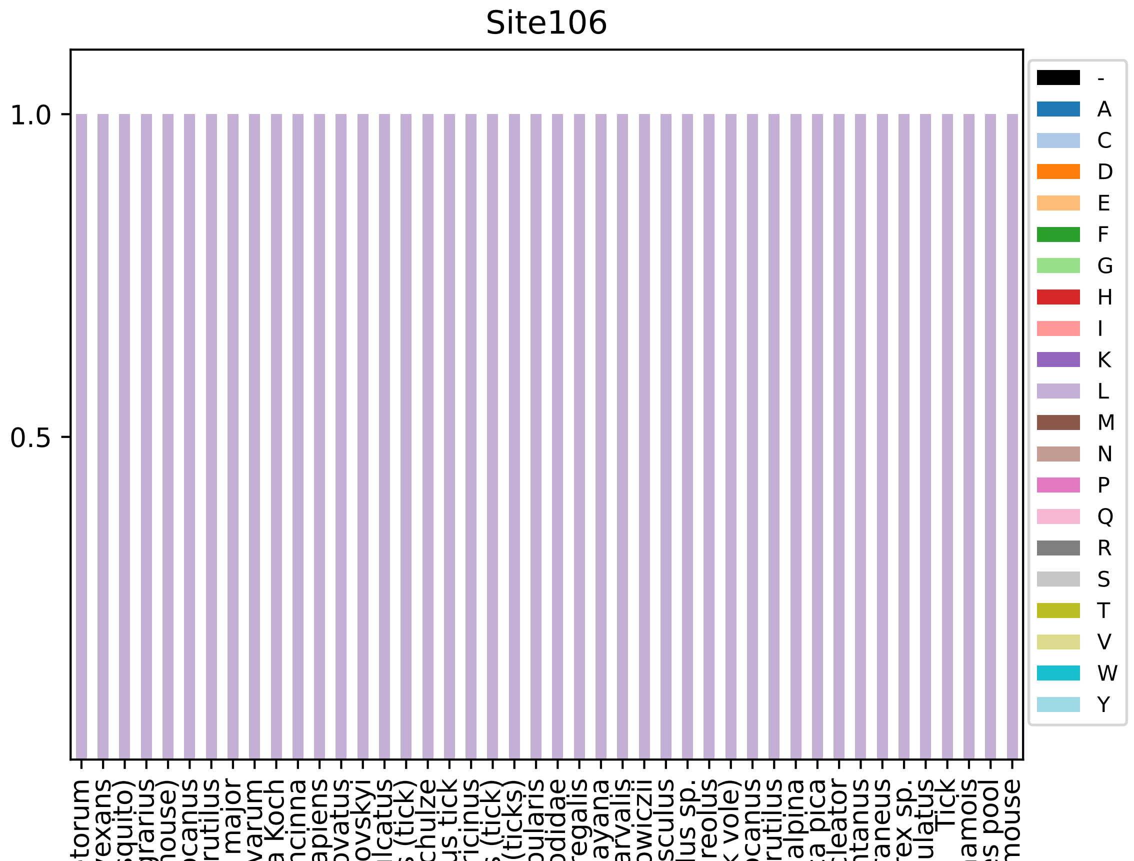
Site106
1.0
0.5
-
A
C
D
E
F
G
H
I
K
L
M
N
P
Q
R
S
T
V
W
Y

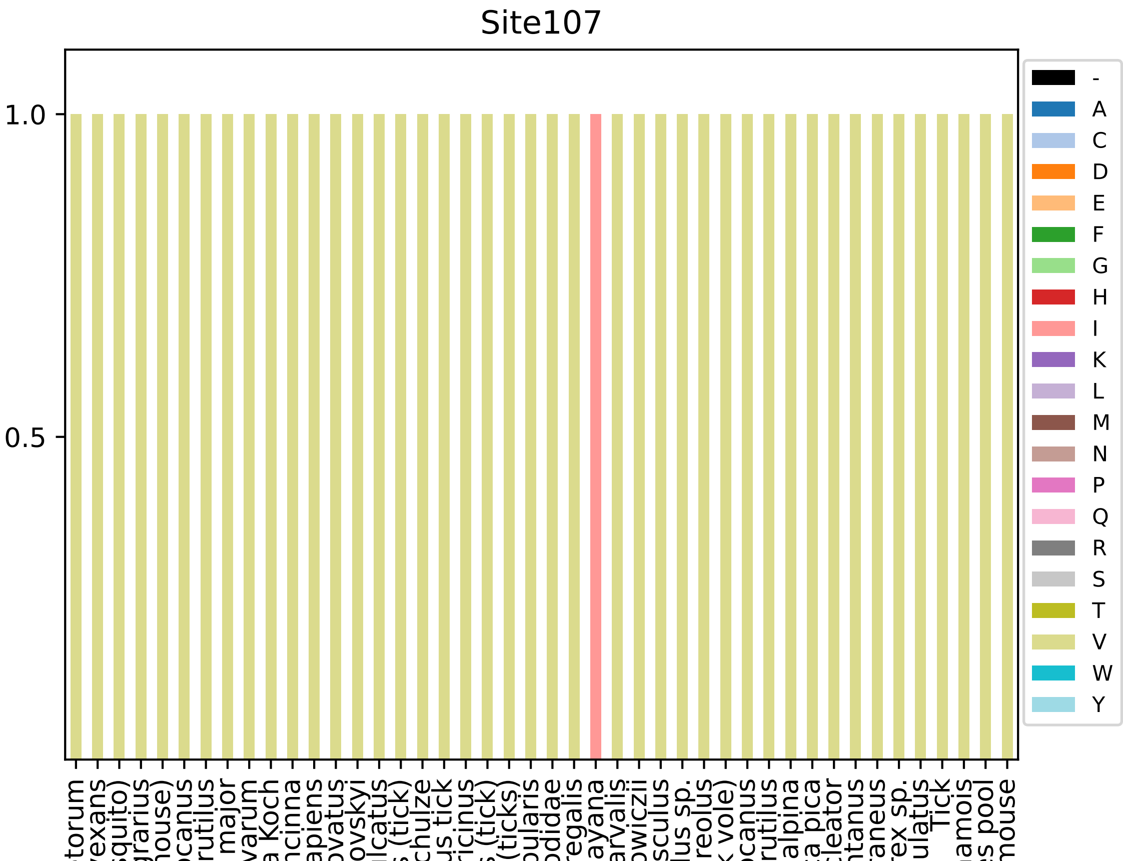

Site107
1.0
0.5
-
A
C
D
E
F
G
H
I
K
L
M
N
P
Q
R
S
T
V
W
Y
torum
vexans
squito)
garius
nouse)
ocanus
rutilus
major
varum
a Koch
ncinna
apiens
ovatus
ovskyi
ulcatus
s (tick)
chulze
us tick
ricinus
s (tick)
(ticks)
pularis
odidae
regalis
ayana
arvalis
owiczii
sculus
lus sp.
reolus
k vole)
ocanus
rutilus
alpina
a pica
leator
ntanus
aneus
ex sp.
ulatus
Tick
amois
es pool
mouse

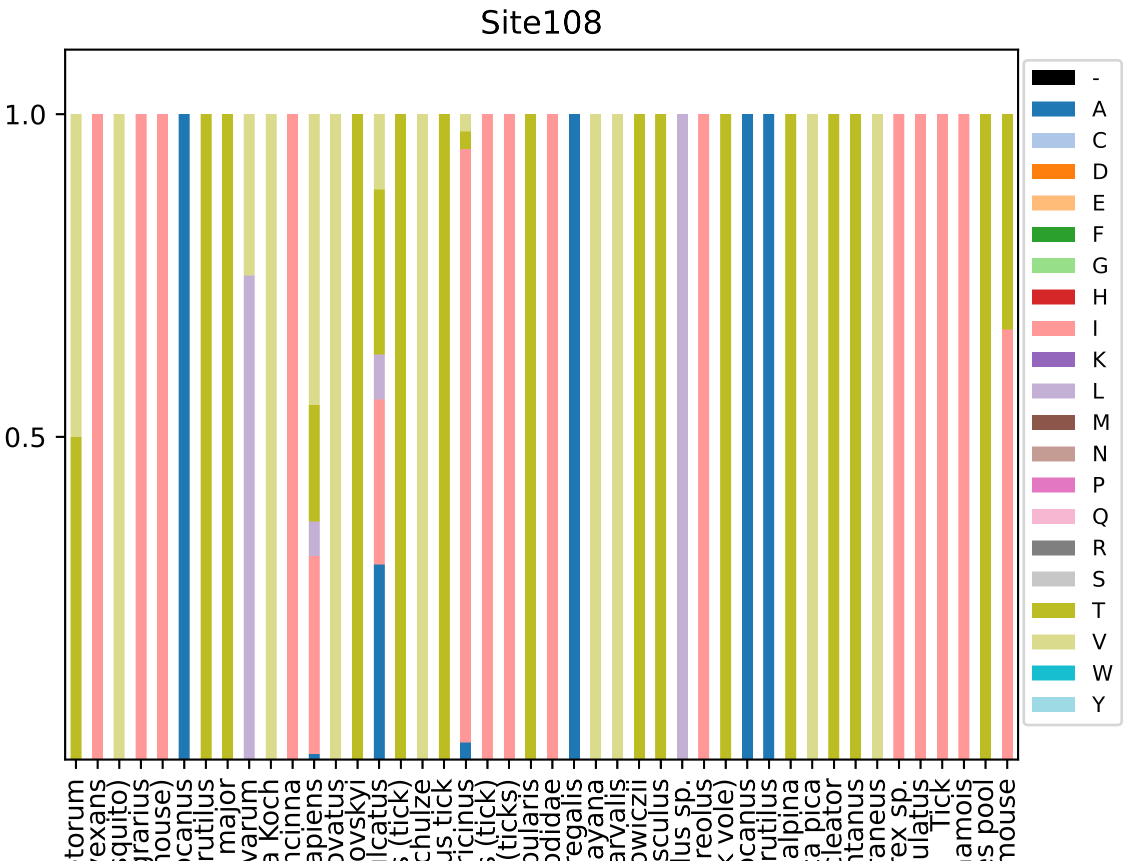

Site108
1.0
0.5
-
A
C
D
E
F
G
H
I
K
L
M
N
P
Q
R
S
T
V
W
Y

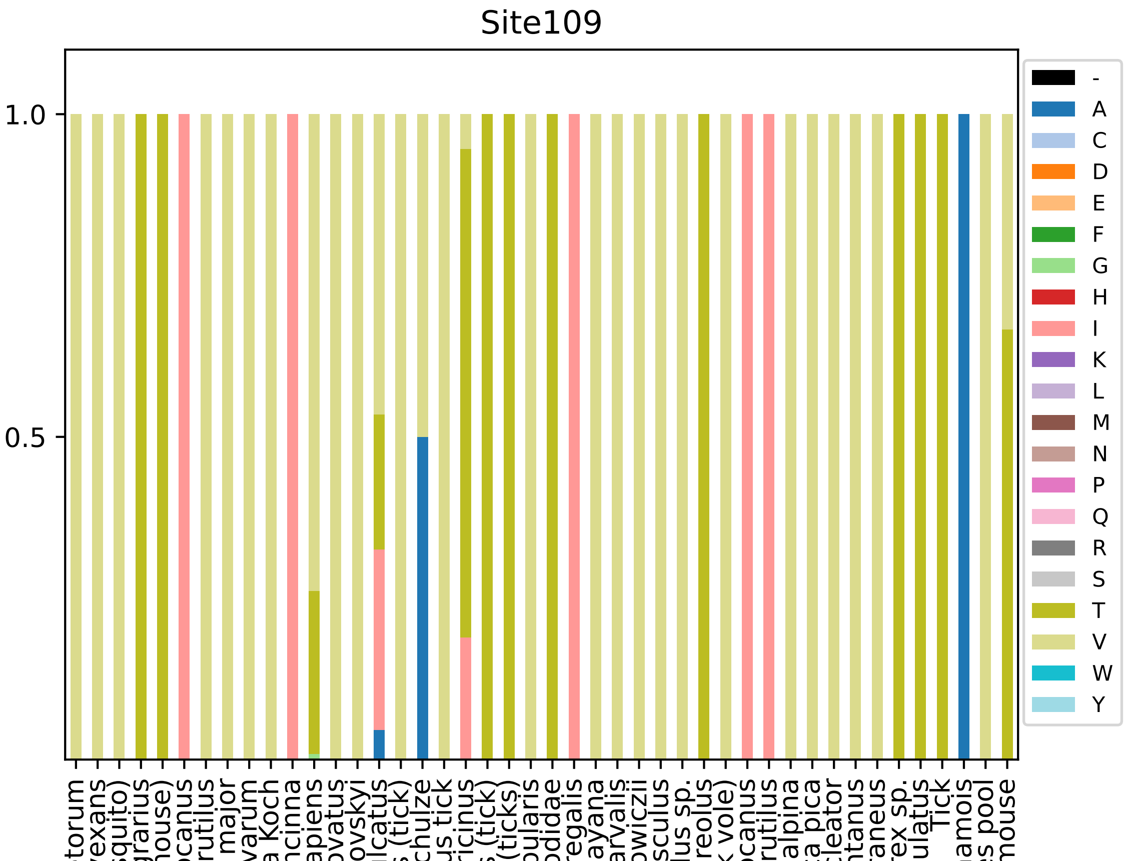

Site109
1.0
0.5
-
A
C
D
E
F
G
H
I
K
L
M
N
P
Q
R
S
T
V
W
Y

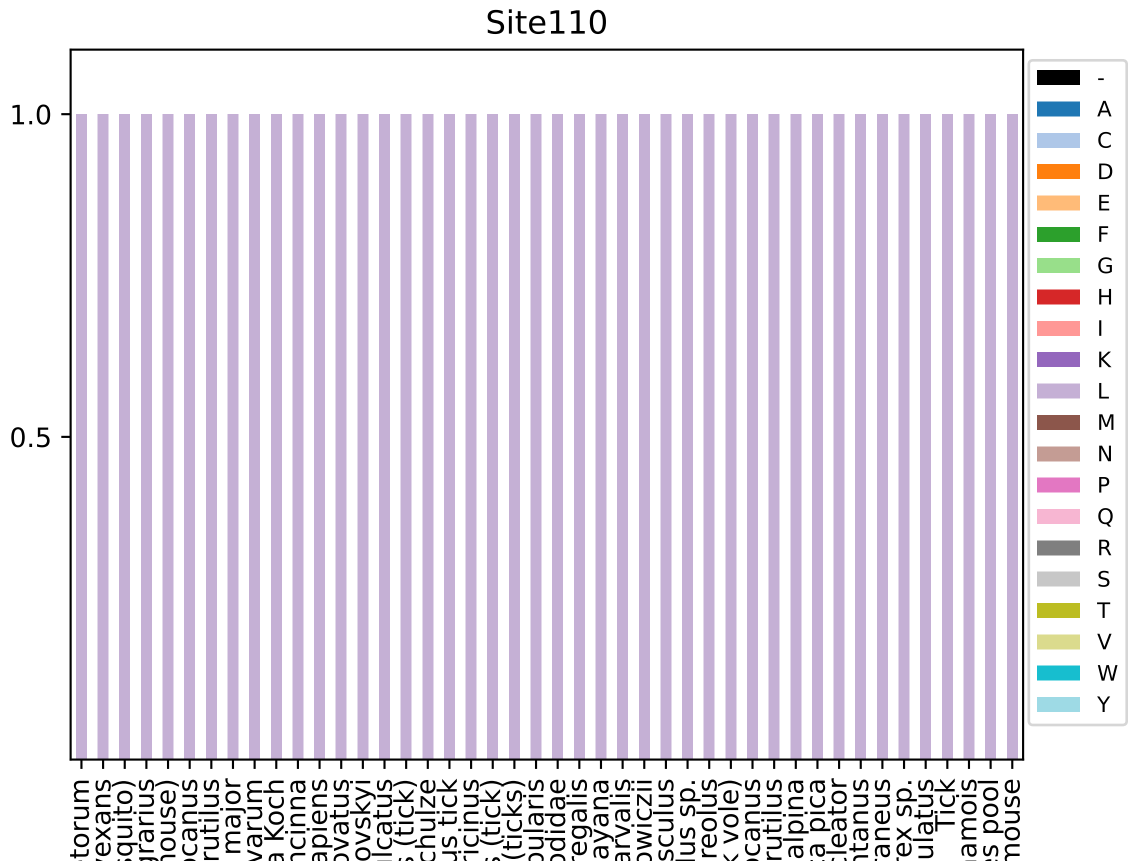
Site110
1.0
0.5
-
A
C
D
E
F
G
H
I
K
L
M
N
P
Q
R
S
T
V
W
Y

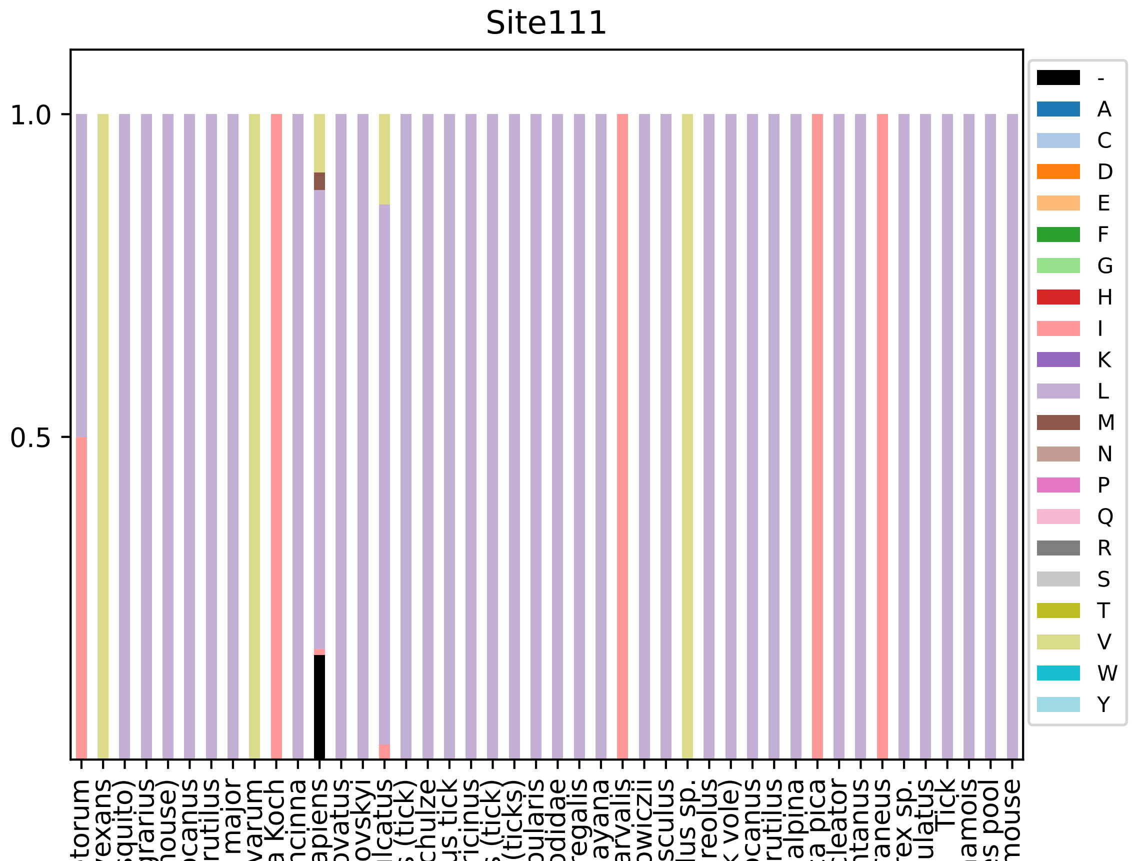
Site111
1.0
0.5
-
A
C
D
E
F
G
H
I
K
L
M
N
P
Q
R
S
T
V
W
Y

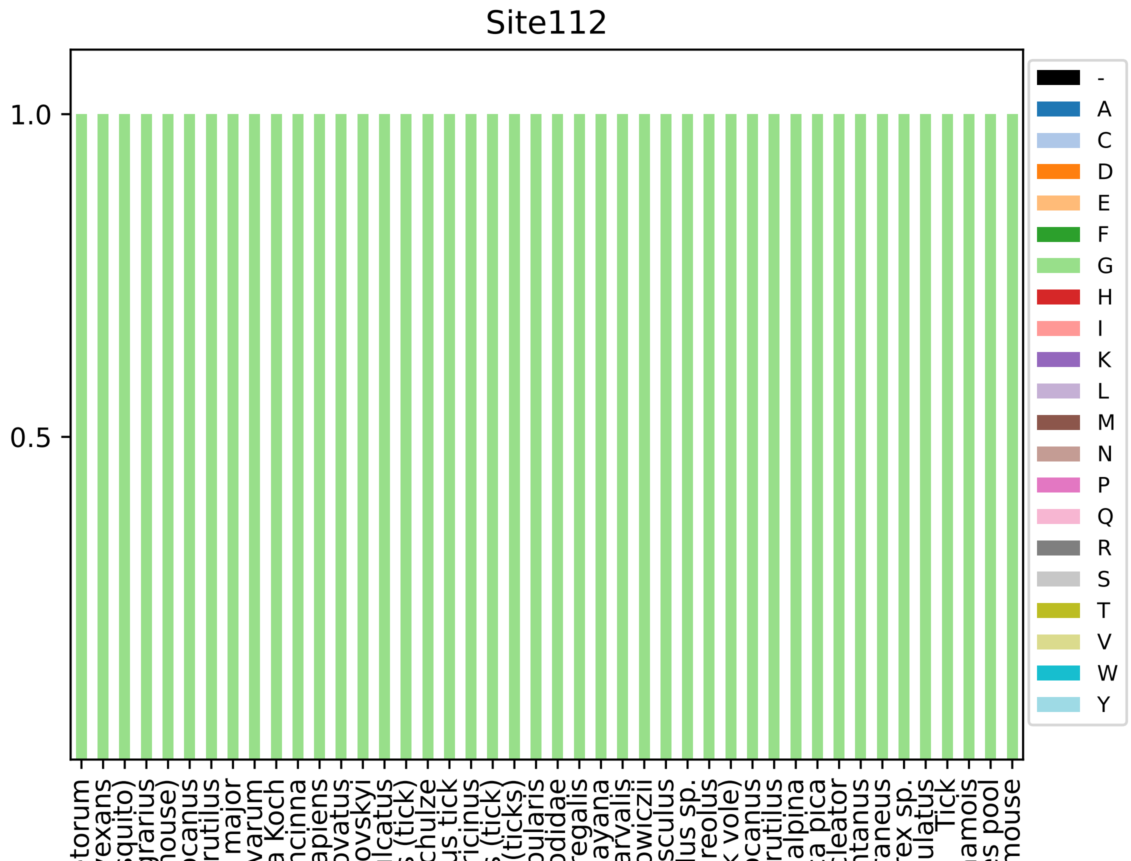
Site112
1.0
0.5
-
A
C
D
E
F
G
H
I
K
L
M
N
P
Q
R
S
T
V
W
Y

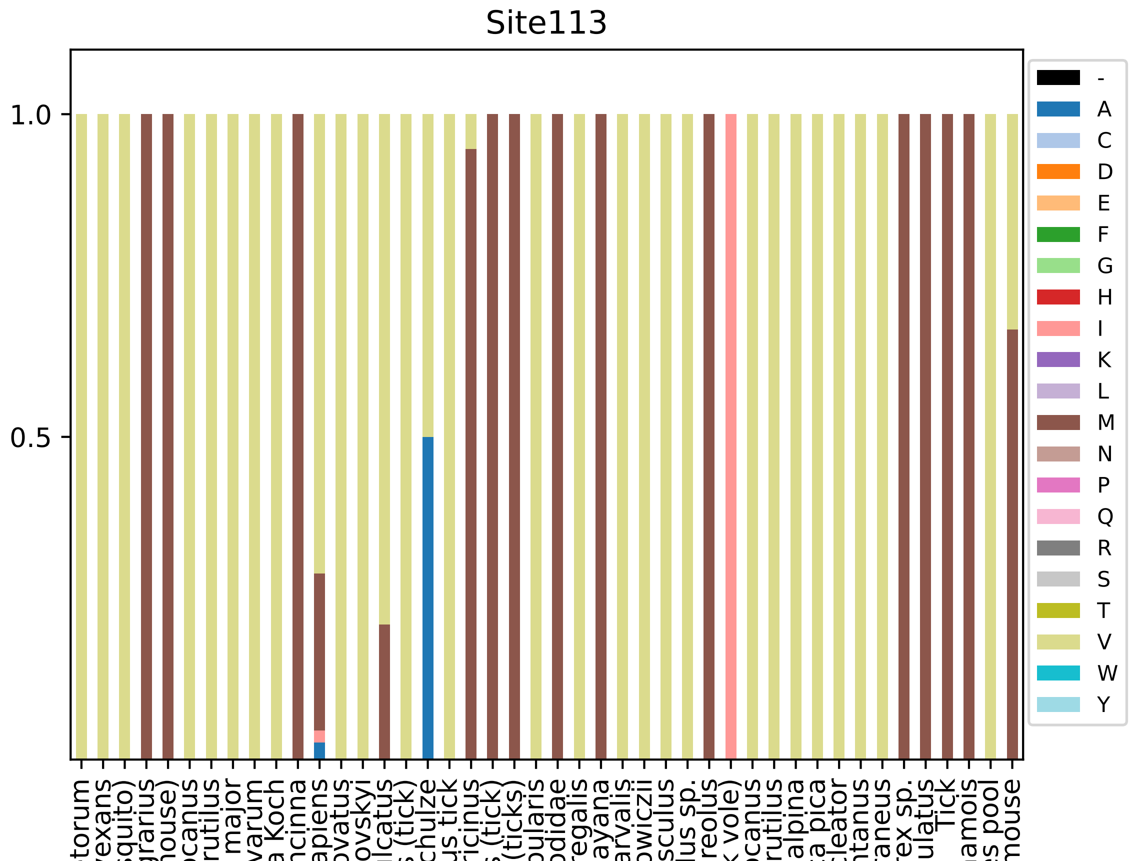

Site113
1.0
0.5
-
A
C
D
E
F
G
H
I
K
L
M
N
P
Q
R
S
T
V
W
Y

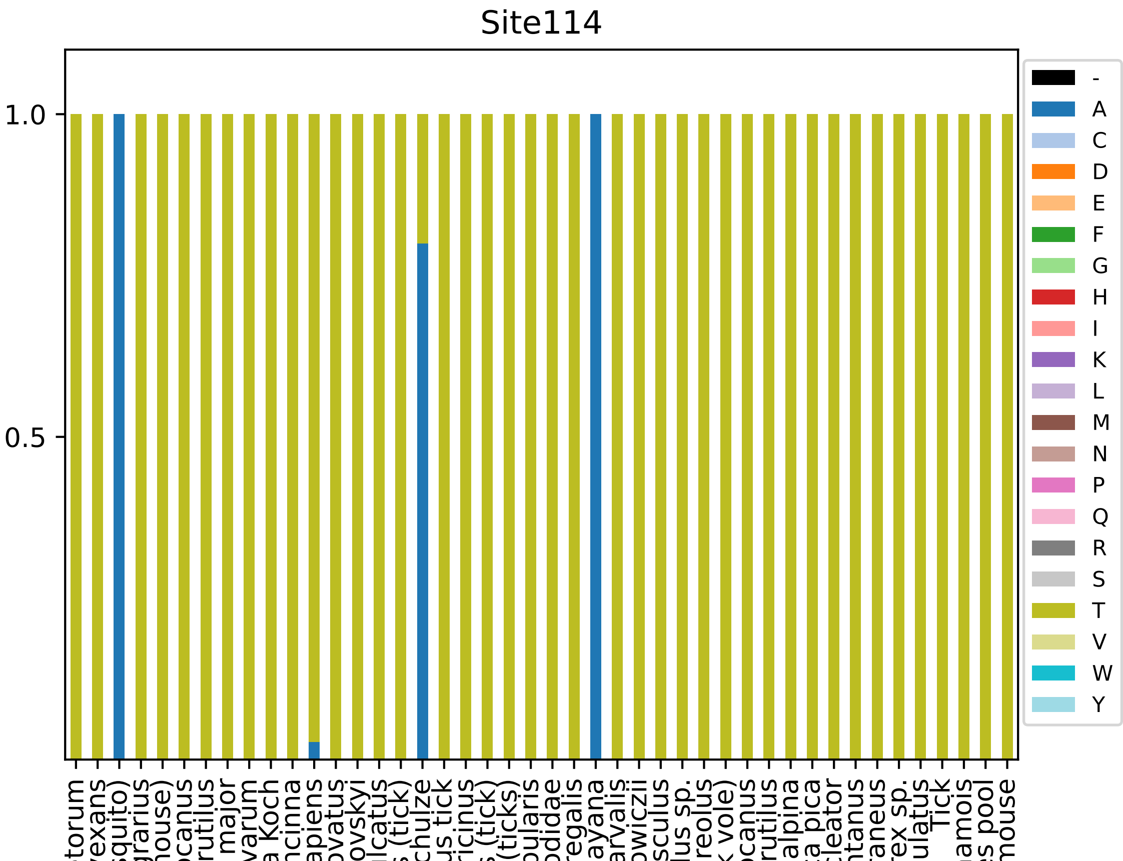
Site114
1.0
0.5
-
A
C
D
E
F
G
H
I
K
L
M
N
P
Q
R
S
T
V
W
Y

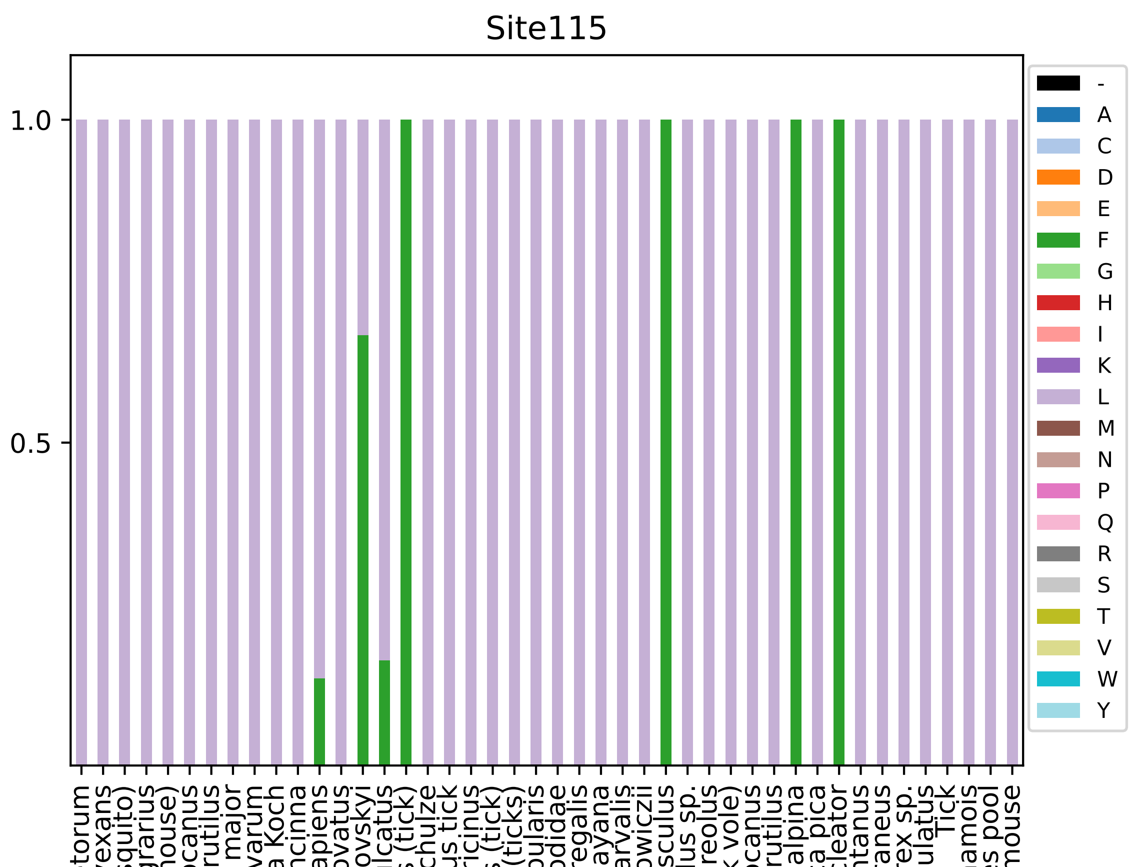
Site115
1.0
0.5
-
A
C
D
E
F
G
H
I
K
L
M
N
P
Q
R
S
T
V
W
Y

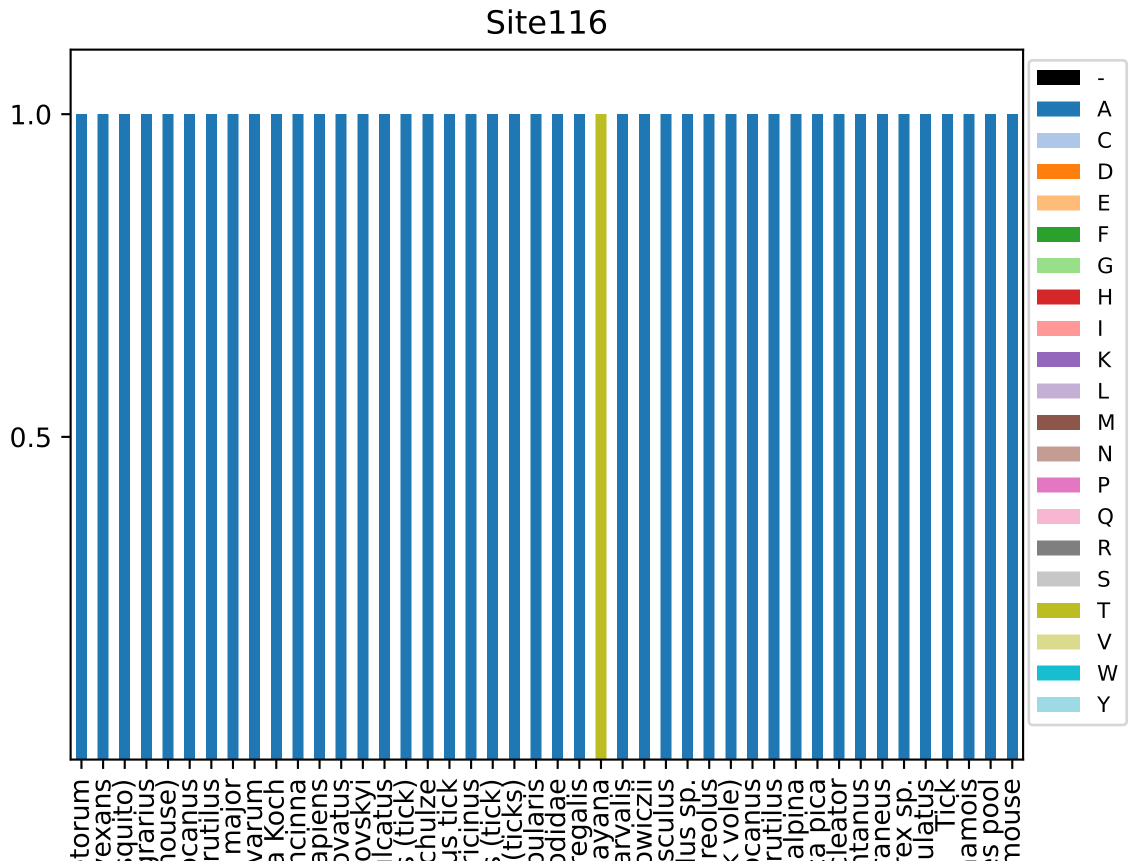

Site116
1.0
0.5
-
A
C
D
E
F
G
H
I
K
L
M
N
P
Q
R
S
T
V
W
Y

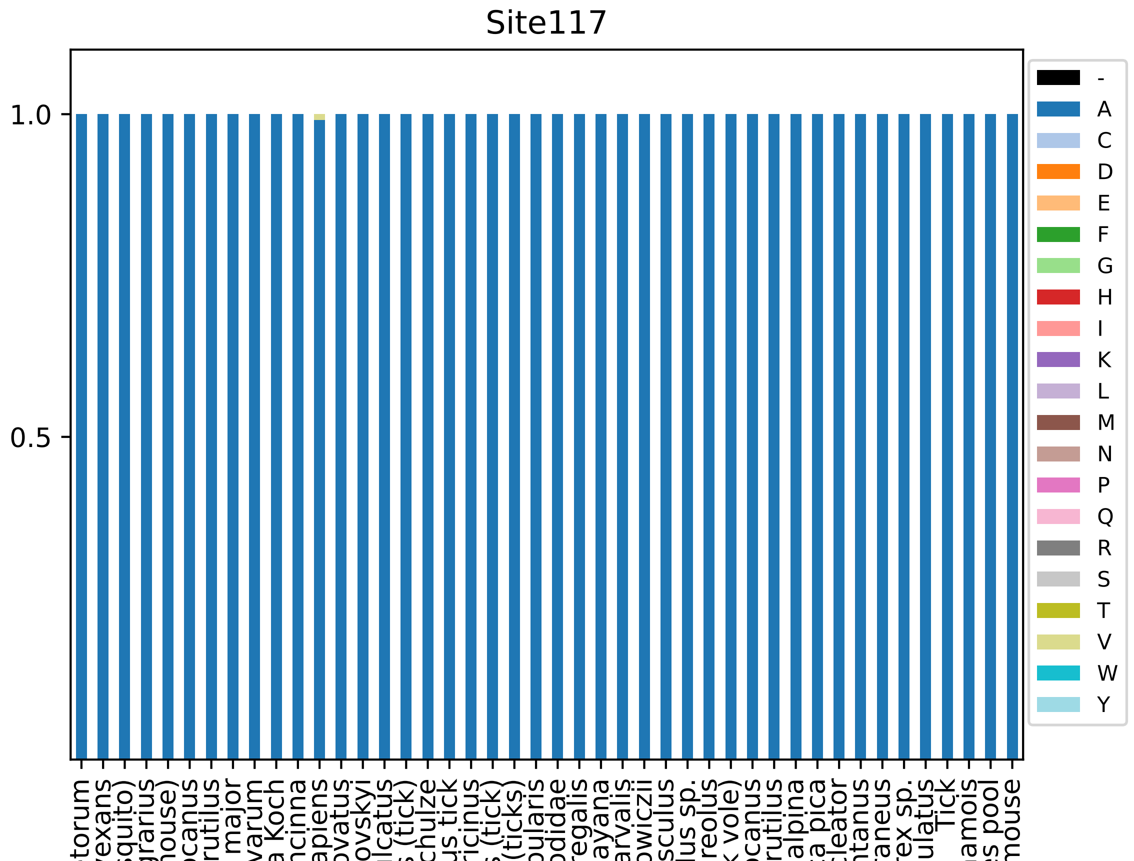

Site117
1.0
0.5
-
A
C
D
E
F
G
H
I
K
L
M
N
P
Q
R
S
T
V
W
Y

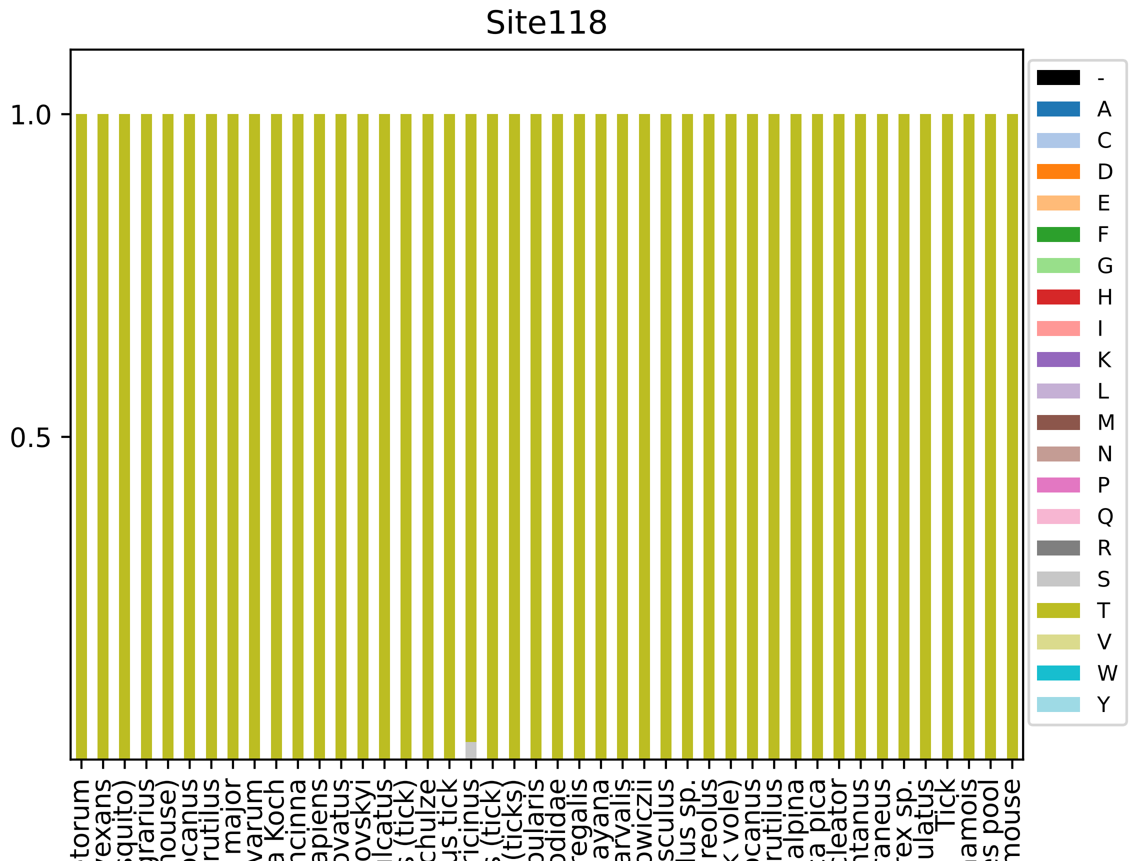

Site118
1.0
0.5
-
A
C
D
E
F
G
H
I
K
L
M
N
P
Q
R
S
T
V
W
Y

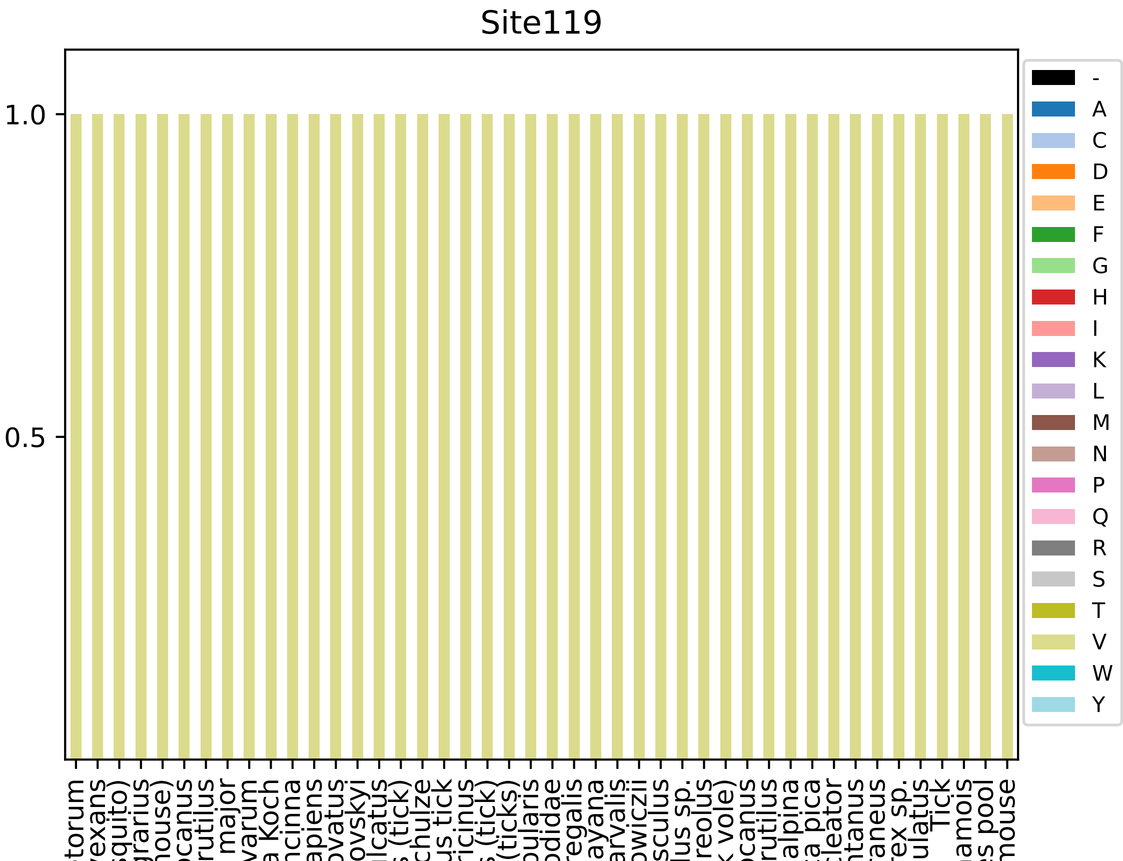
Site119
1.0
0.5
-
A
C
D
E
F
G
H
I
K
L
M
N
P
Q
R
S
T
V
W
Y

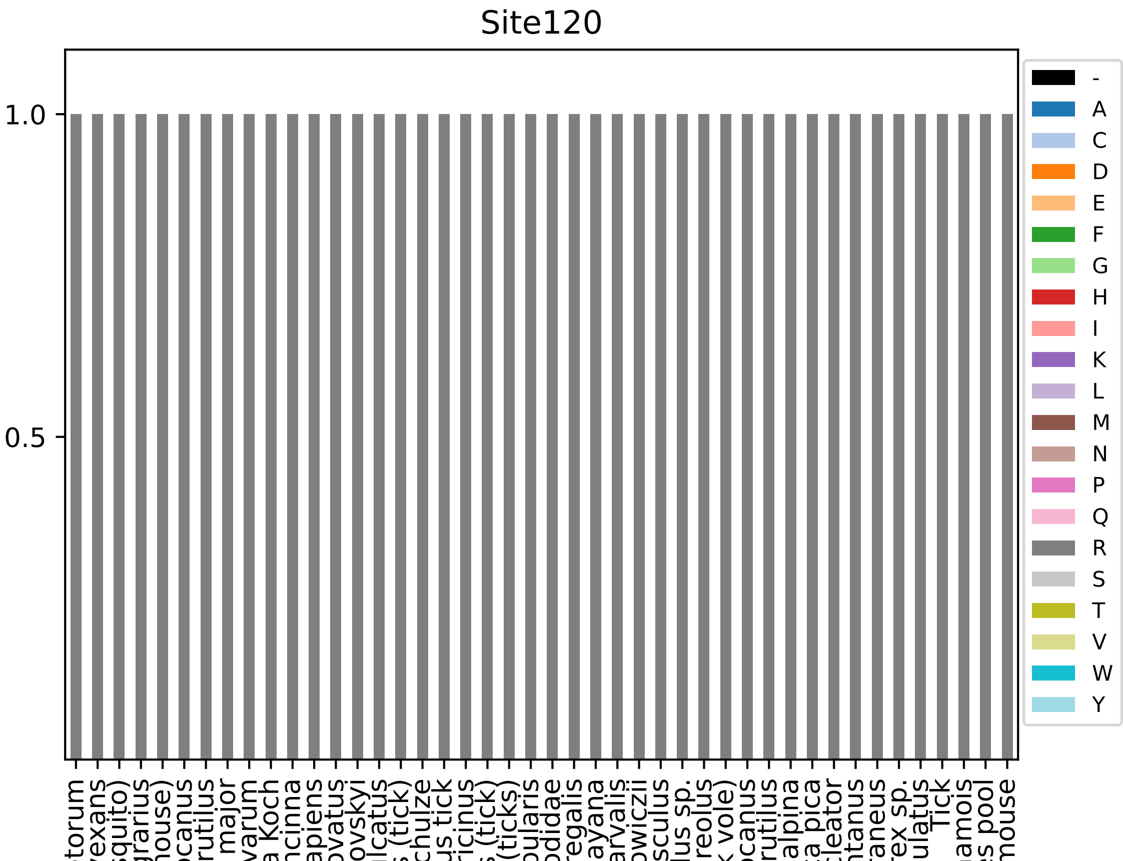

Site120
1.0
0.5
-
A
C
D
E
F
G
H
I
K
L
M
N
P
Q
R
S
T
V
W
Y

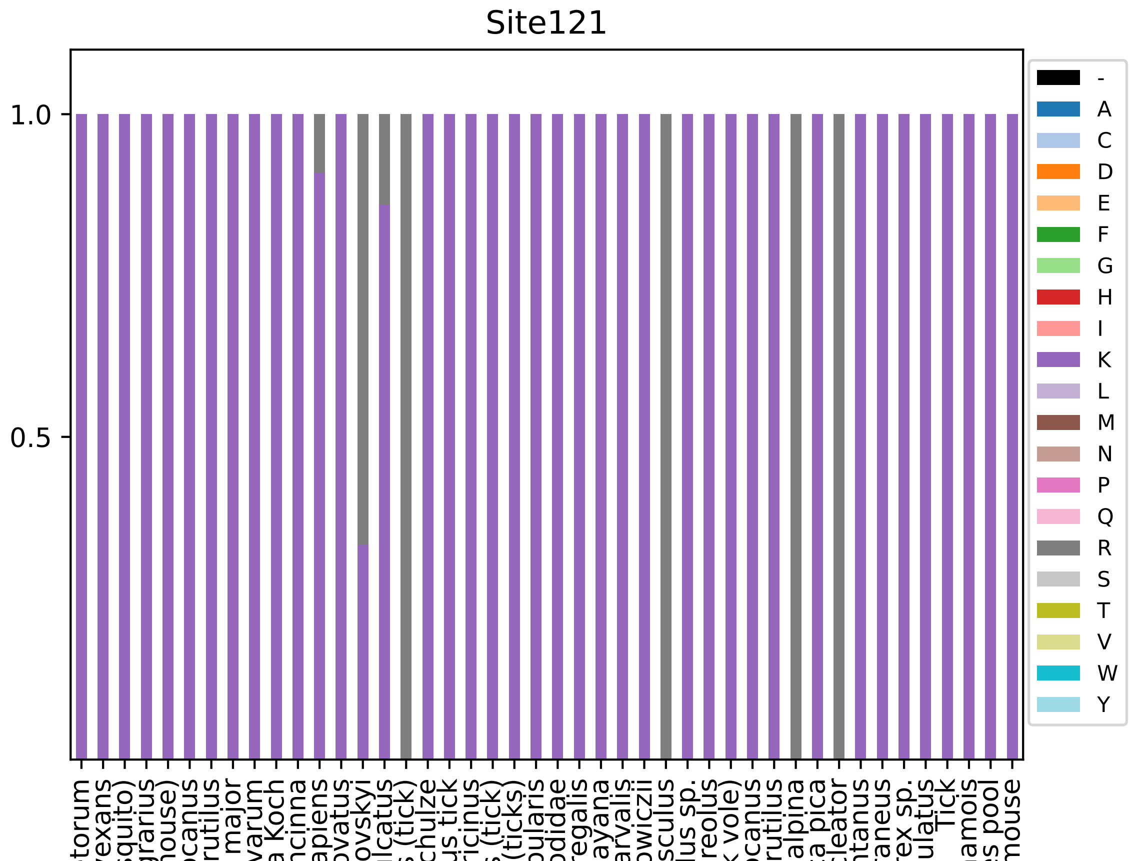
Site121
1.0
0.5
-
A
C
D
E
F
G
H
I
K
L
M
N
P
Q
R
S
T
V
W
Y

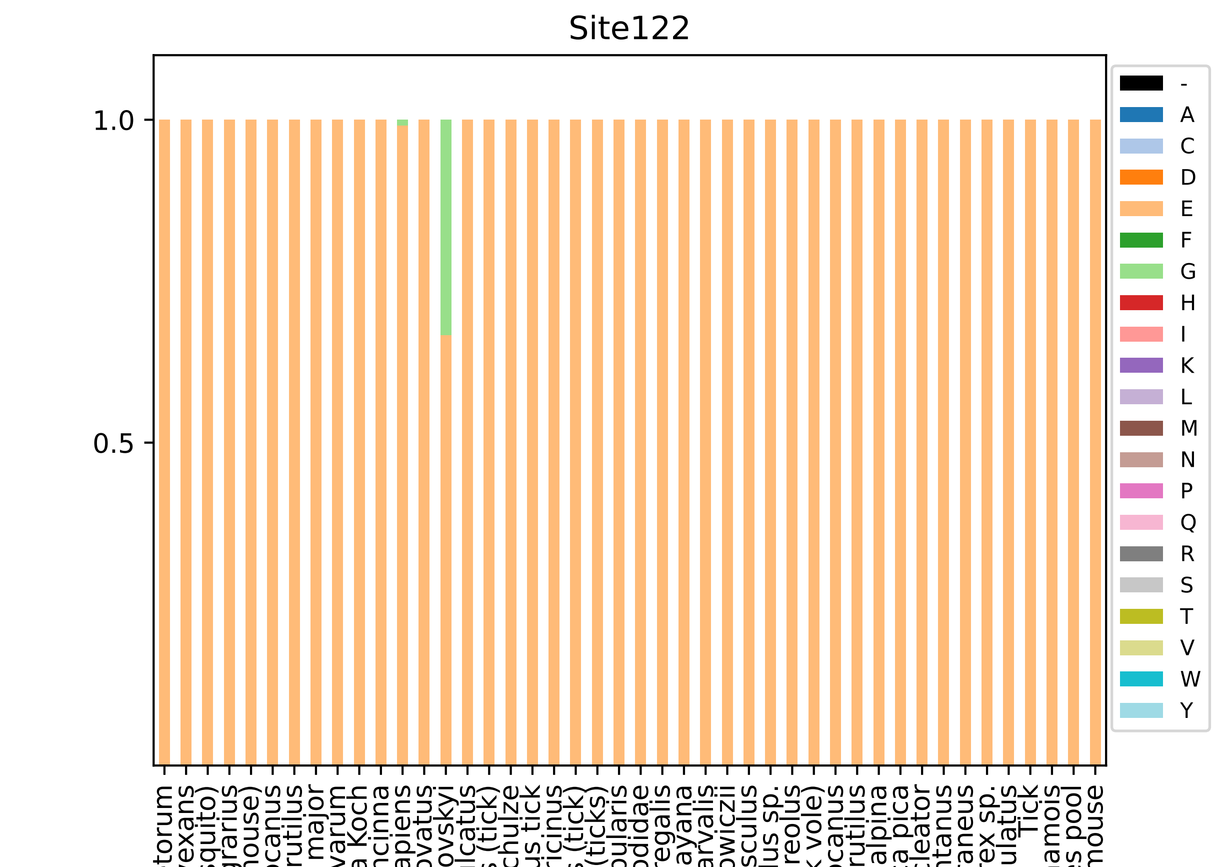

Site122
1.0
0.5
-
A
C
D
E
F
G
H
I
K
L
M
N
P
Q
R
S
T
V
W
Y

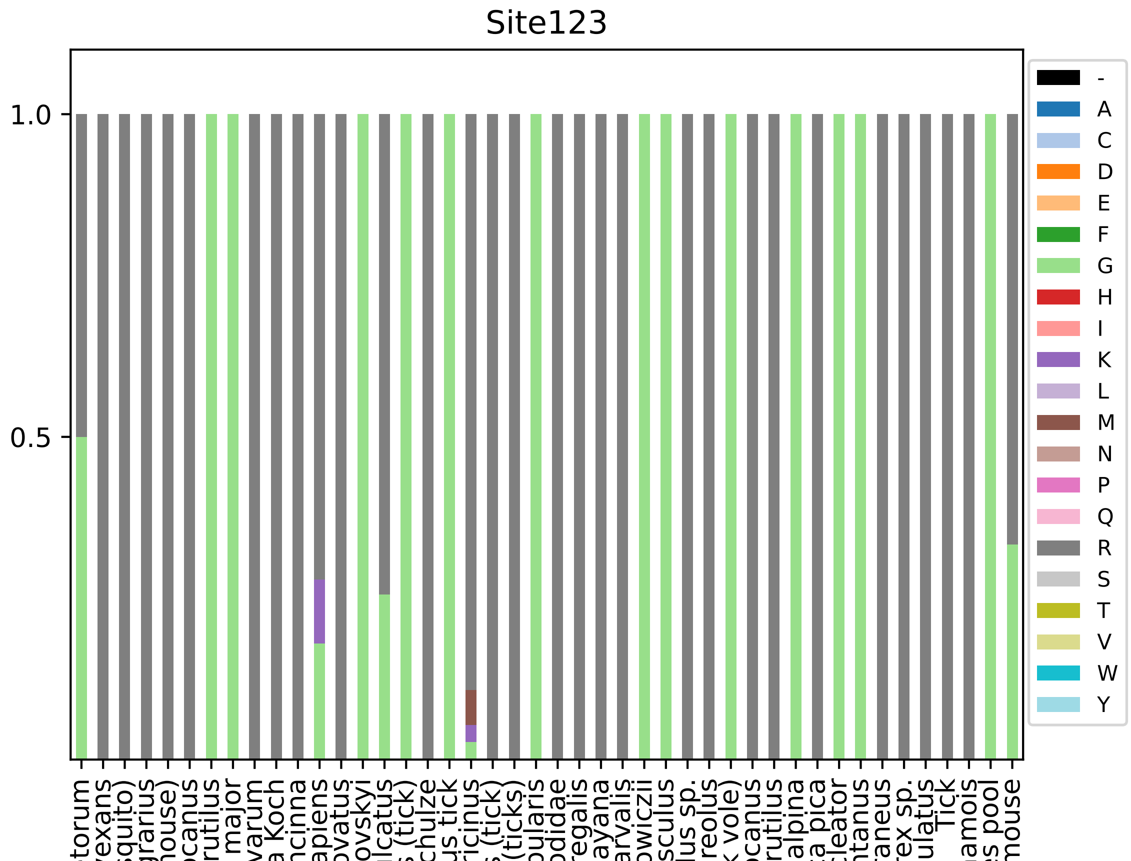

Site123
1.0
0.5
-
A
C
D
E
F
G
H
I
K
L
M
N
P
Q
R
S
T
V
W
Y

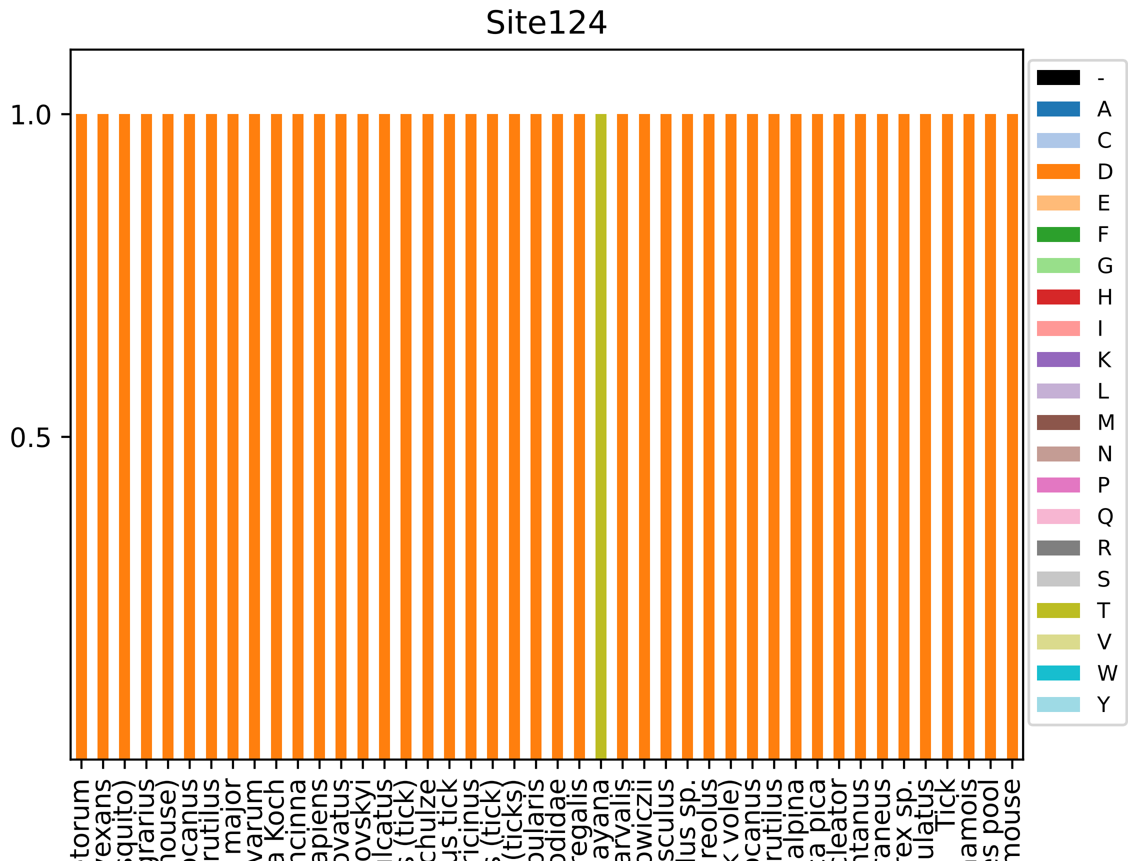
Site124
1.0
0.5
-
A
C
D
E
F
G
H
I
K
L
M
N
P
Q
R
S
T
V
W
Y

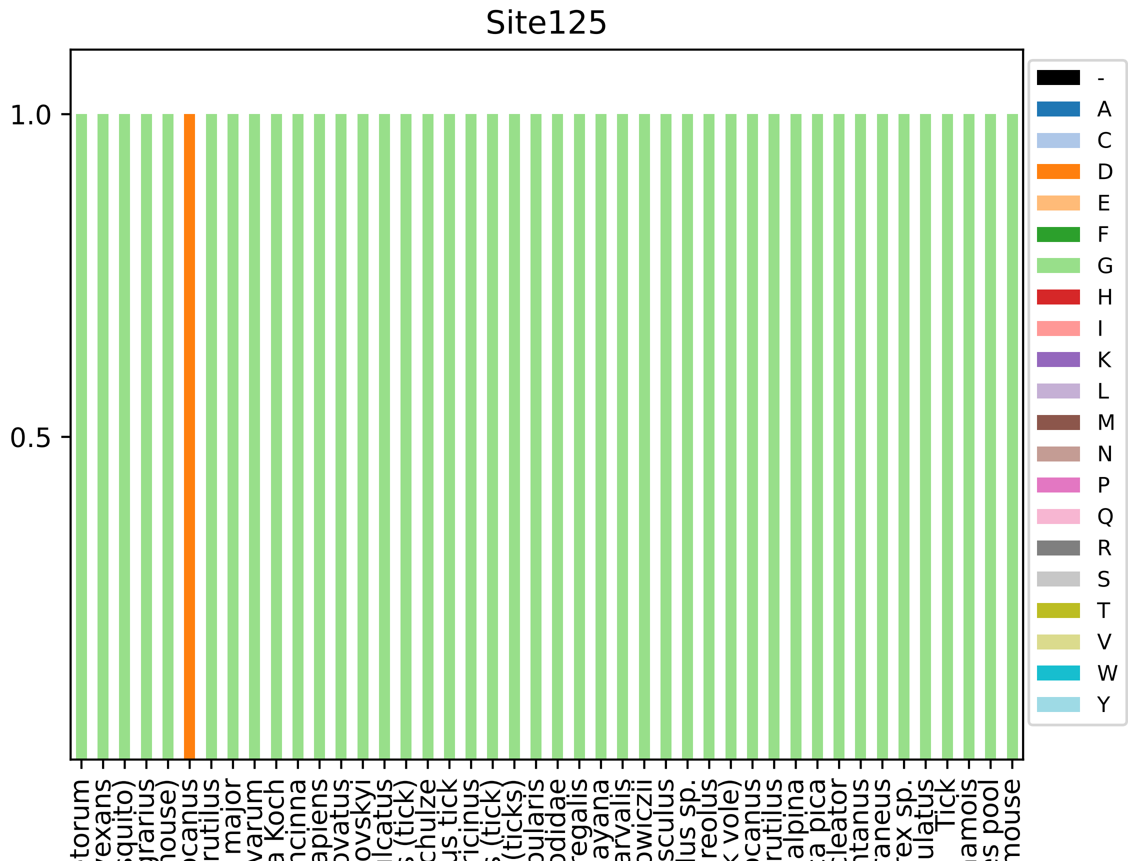
Site125
1.0
0.5
-
A
C
D
E
F
G
H
I
K
L
M
N
P
Q
R
S
T
V
W
Y
torum
vexans
squito)
grarius
nouse)
ocanus
rutilus
major
varum
a Koch
ncinna
apiens
ovatus
ovskyi
ulcatus
s (tick)
chulze
us tick
ricinus
s (ticks)
s (ticks)
pularis
podidae
regalis
ayana
arvalis
owiczii
sculus
lus sp.
reolus
k vole)
ocanus
rutilus
alpina
ca pica
cleator
ntanus
raneus
rex sp.
ulatus
Tick
amois
es pool
mouse

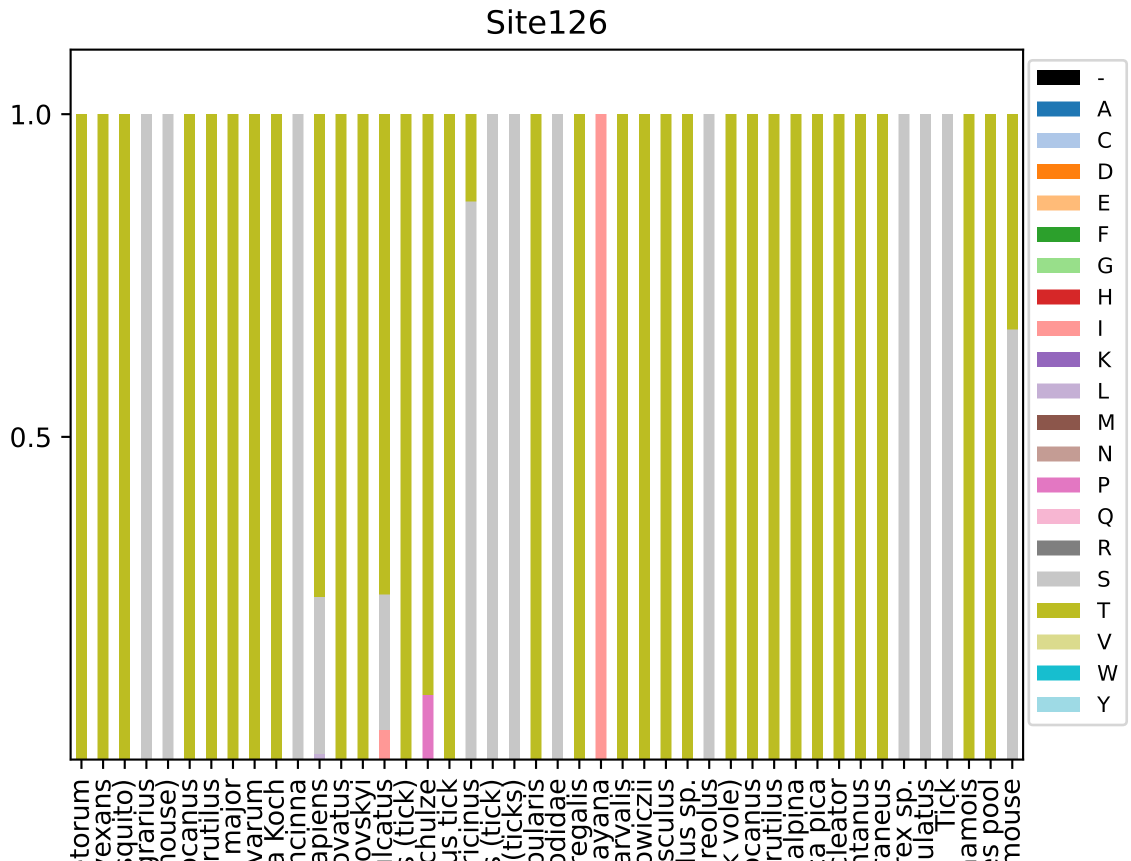

Site126
1.0
0.5
-
A
C
D
E
F
G
H
I
K
L
M
N
P
Q
R
S
T
V
W
Y

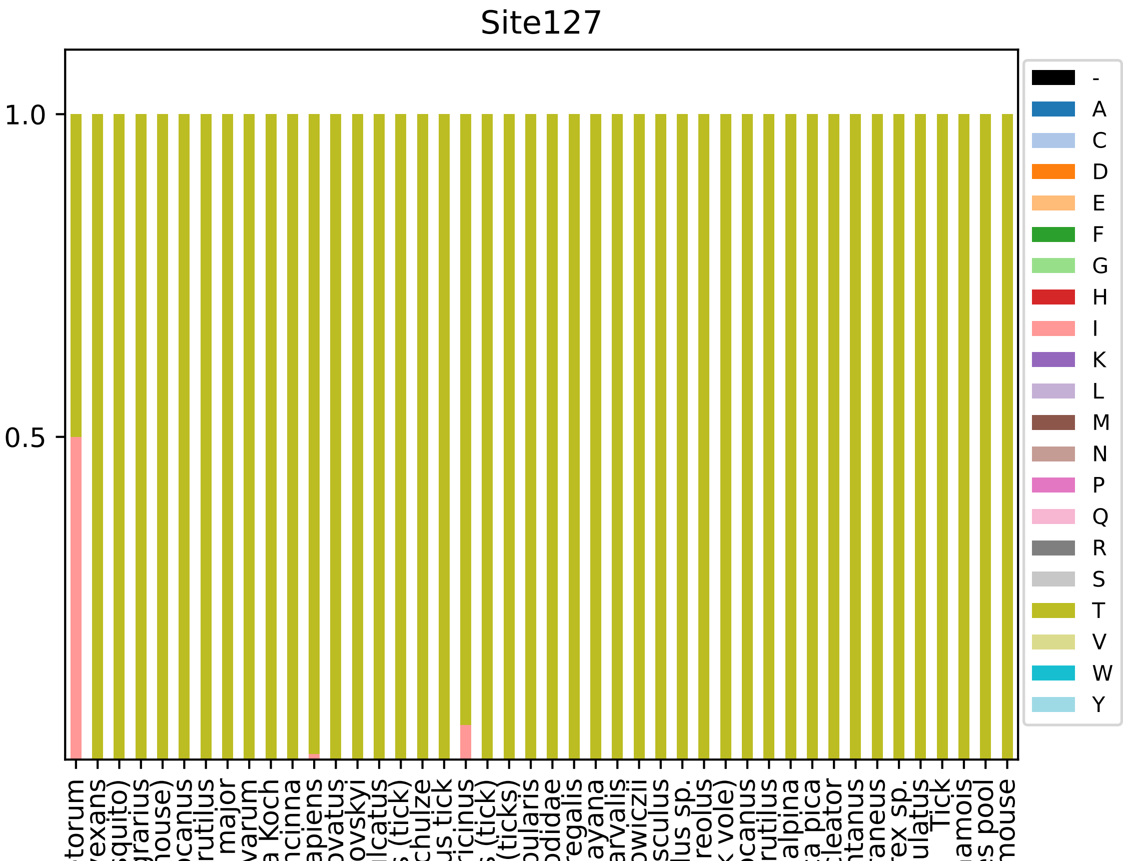

Site127
1.0
0.5
-
A
C
D
E
F
G
H
I
K
L
M
N
P
Q
R
S
T
V
W
Y

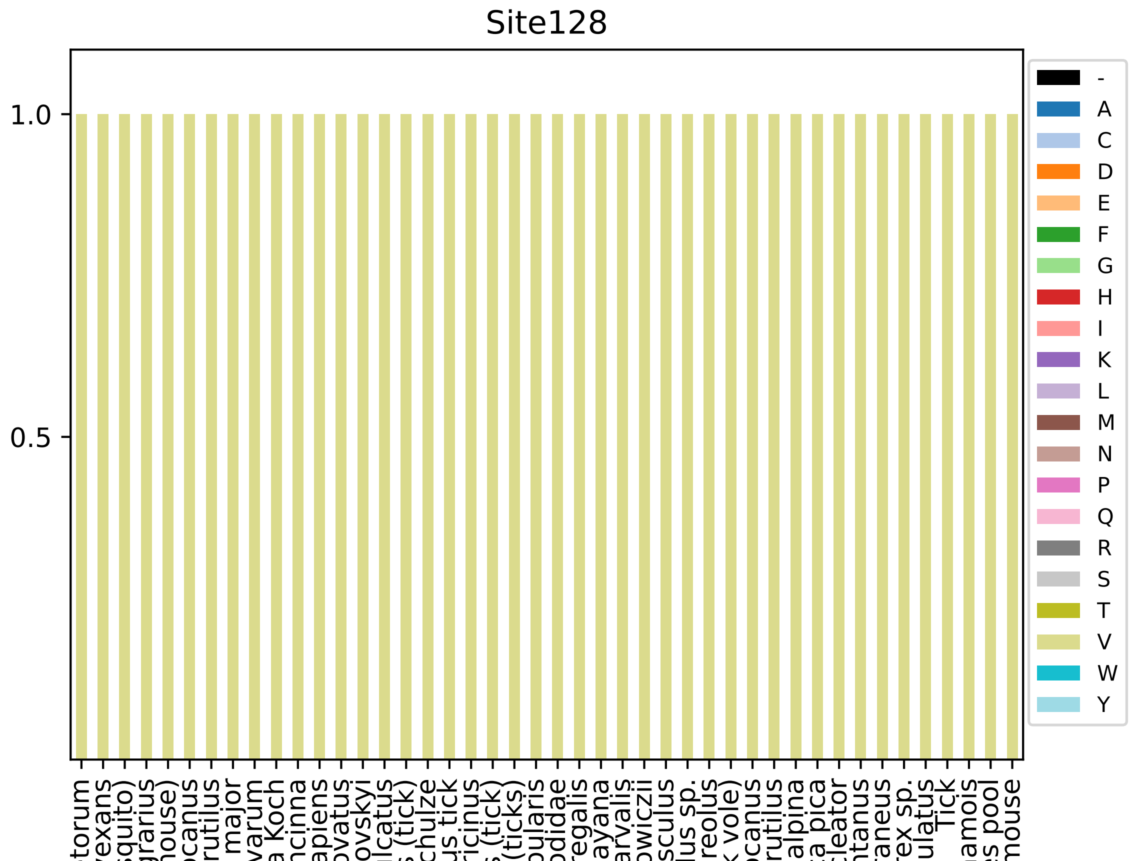

Site128
1.0
0.5
-
A
C
D
E
F
G
H
I
K
L
M
N
P
Q
R
S
T
V
W
Y

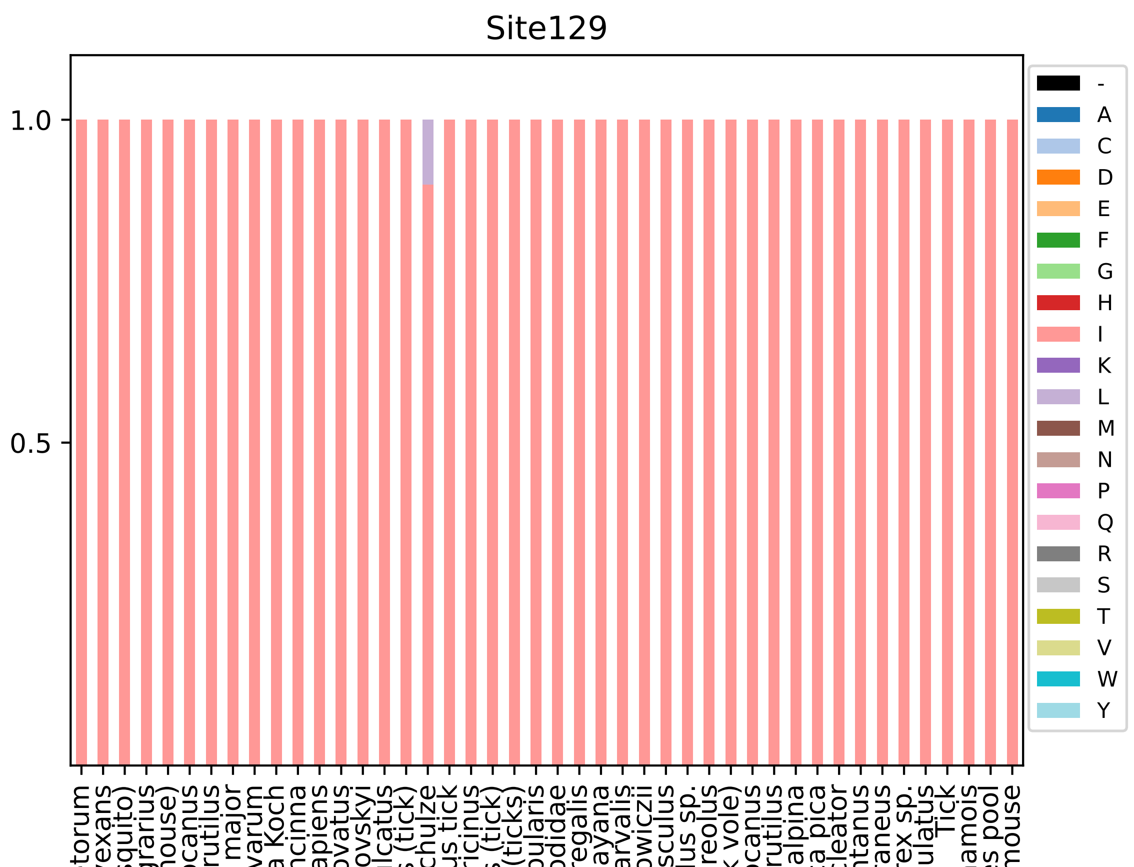

Site129
1.0
0.5
-
A
C
D
E
F
G
H
I
K
L
M
N
P
Q
R
S
T
V
W
Y

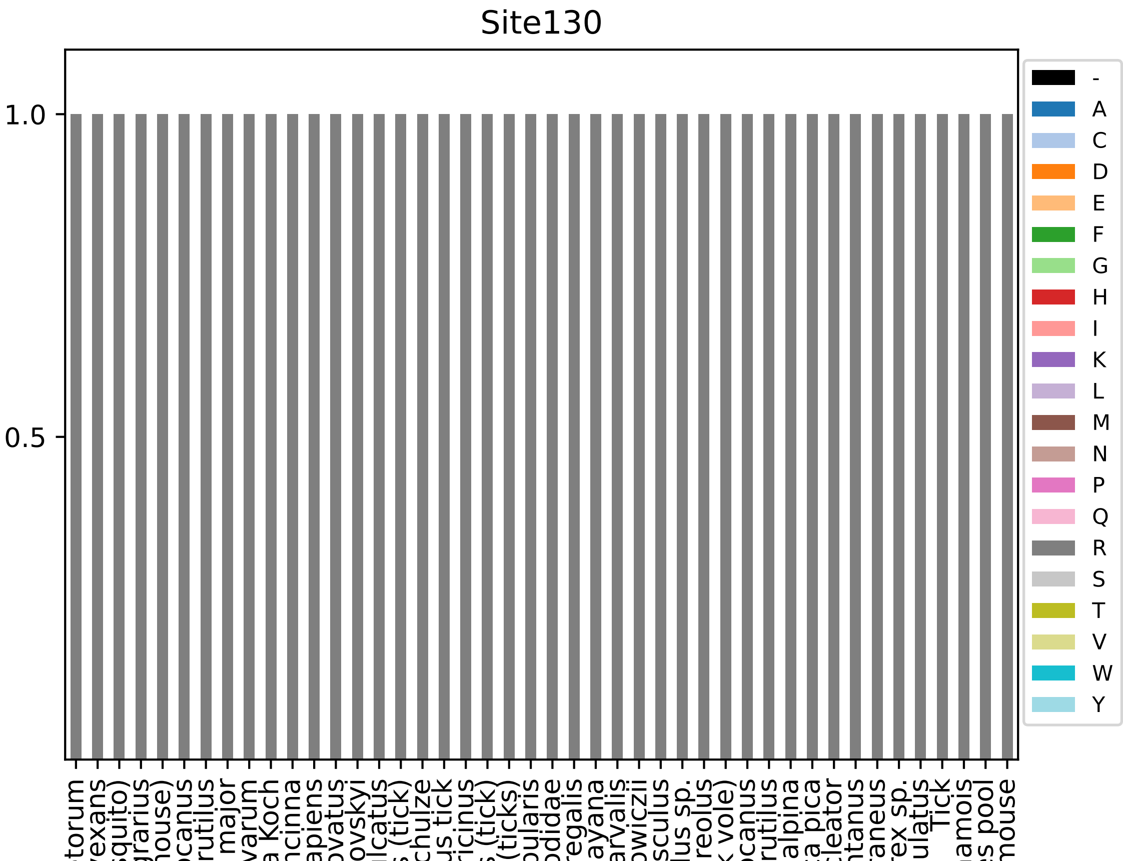
Site130
1.0
0.5
-
A
C
D
E
F
G
H
I
K
L
M
N
P
Q
R
S
T
V
W
Y

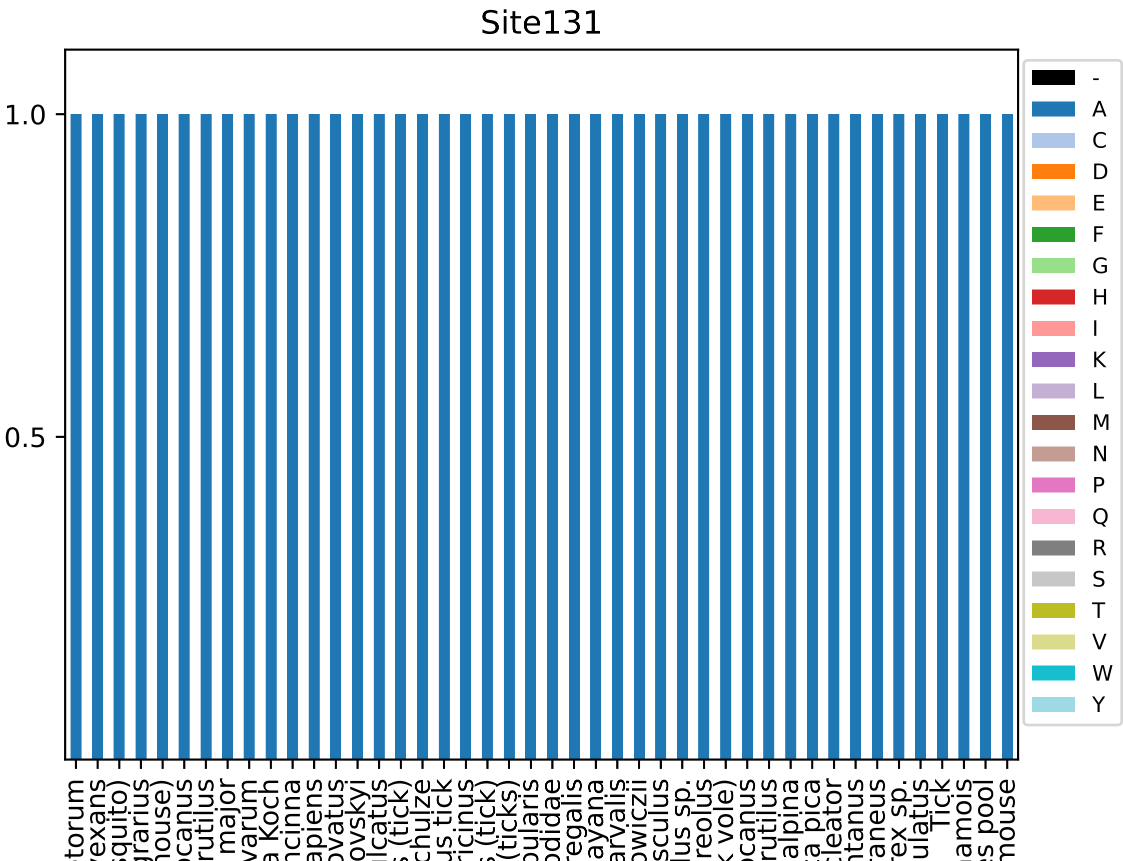
Site131
1.0
0.5
-
A
C
D
E
F
G
H
I
K
L
M
N
P
Q
R
S
T
V
W
Y

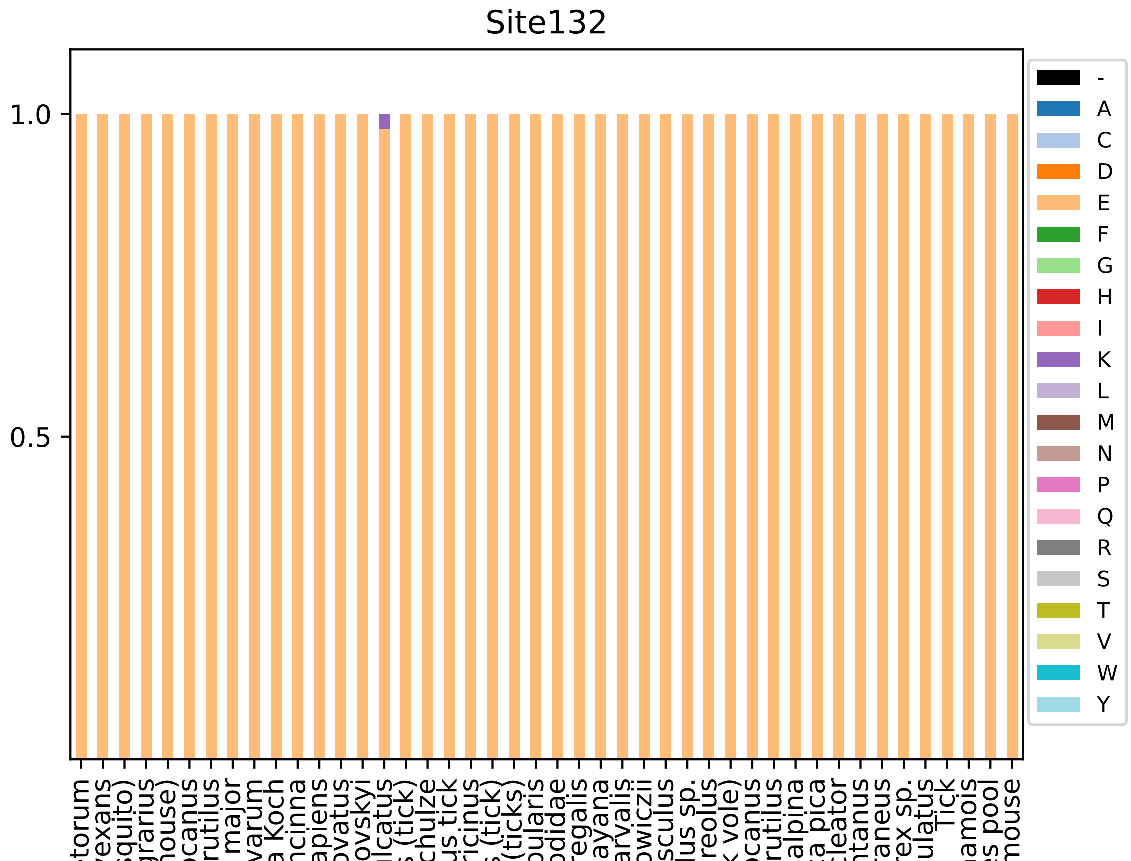
Site132
1.0
0.5
-
A
C
D
E
F
G
H
I
K
L
M
N
P
Q
R
S
T
V
W
Y

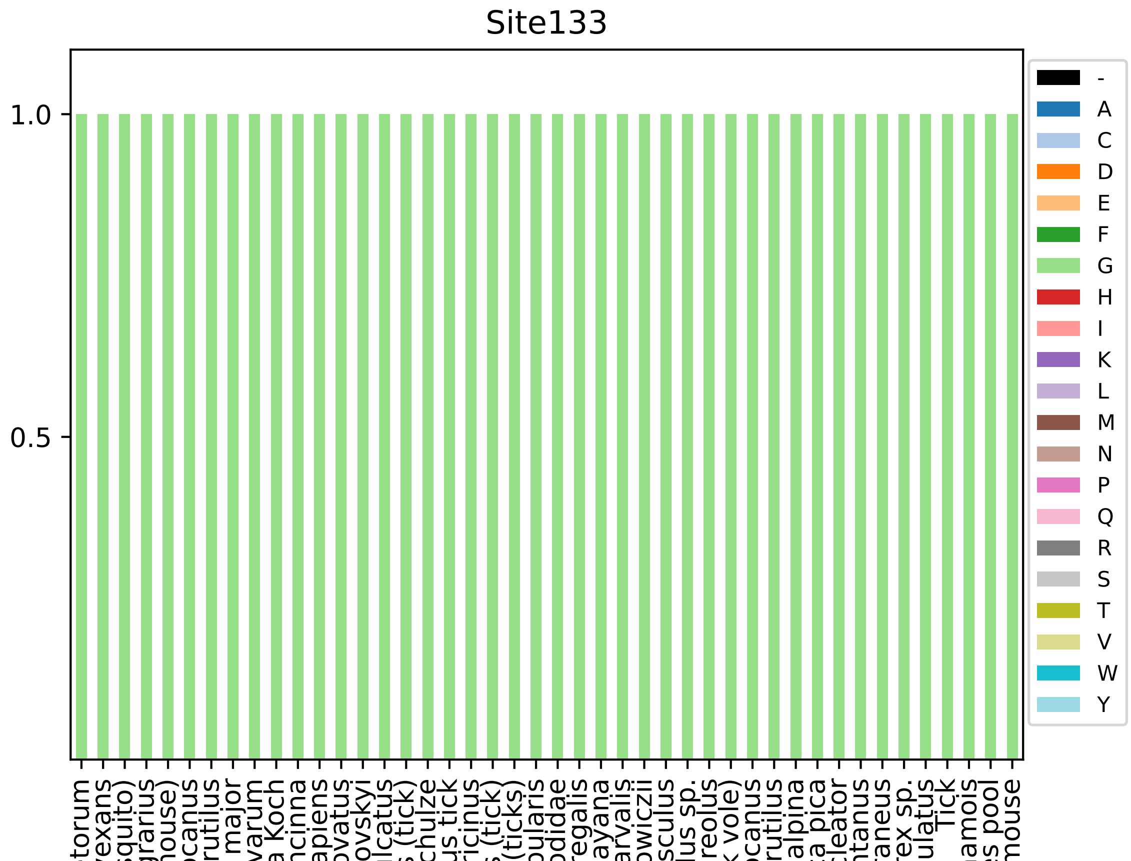

Site133
1.0
0.5
-
A
C
D
E
F
G
H
I
K
L
M
N
P
Q
R
S
T
V
W
Y

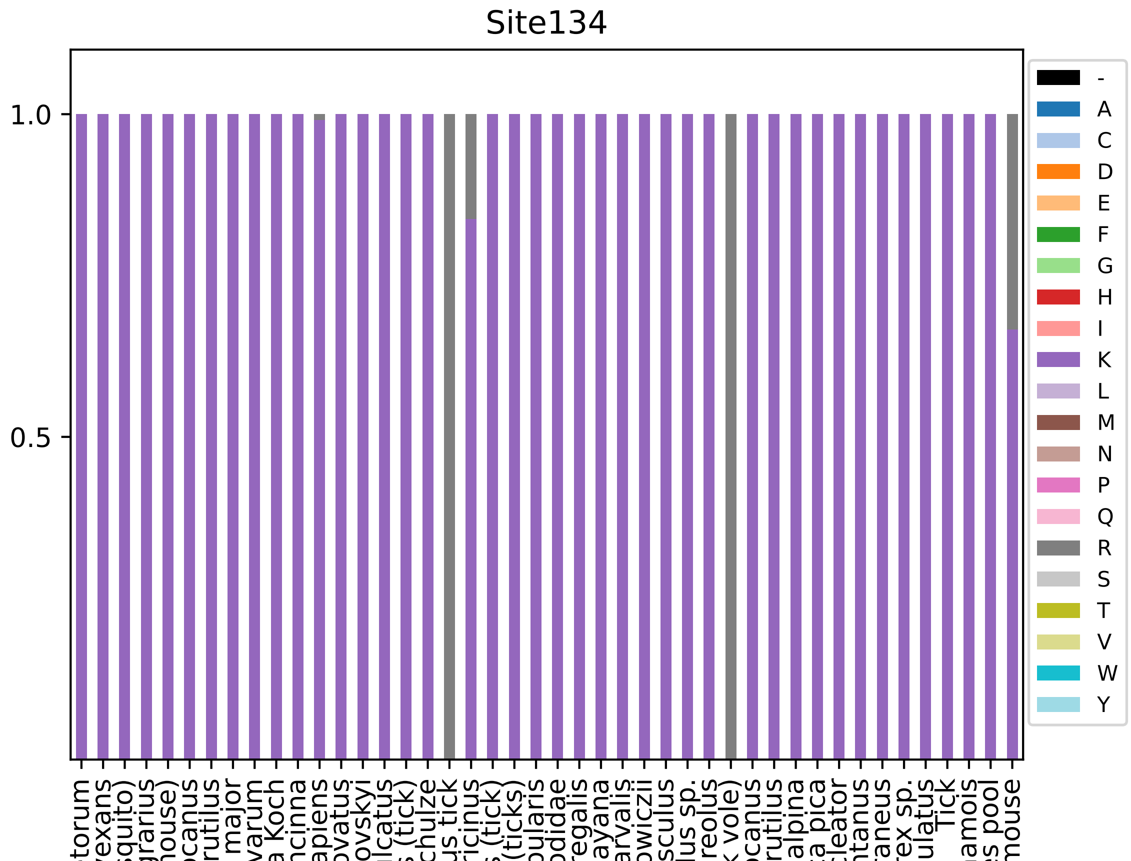
Site134
1.0
0.5
-
A
C
D
E
F
G
H
I
K
L
M
N
P
Q
R
S
T
V
W
Y

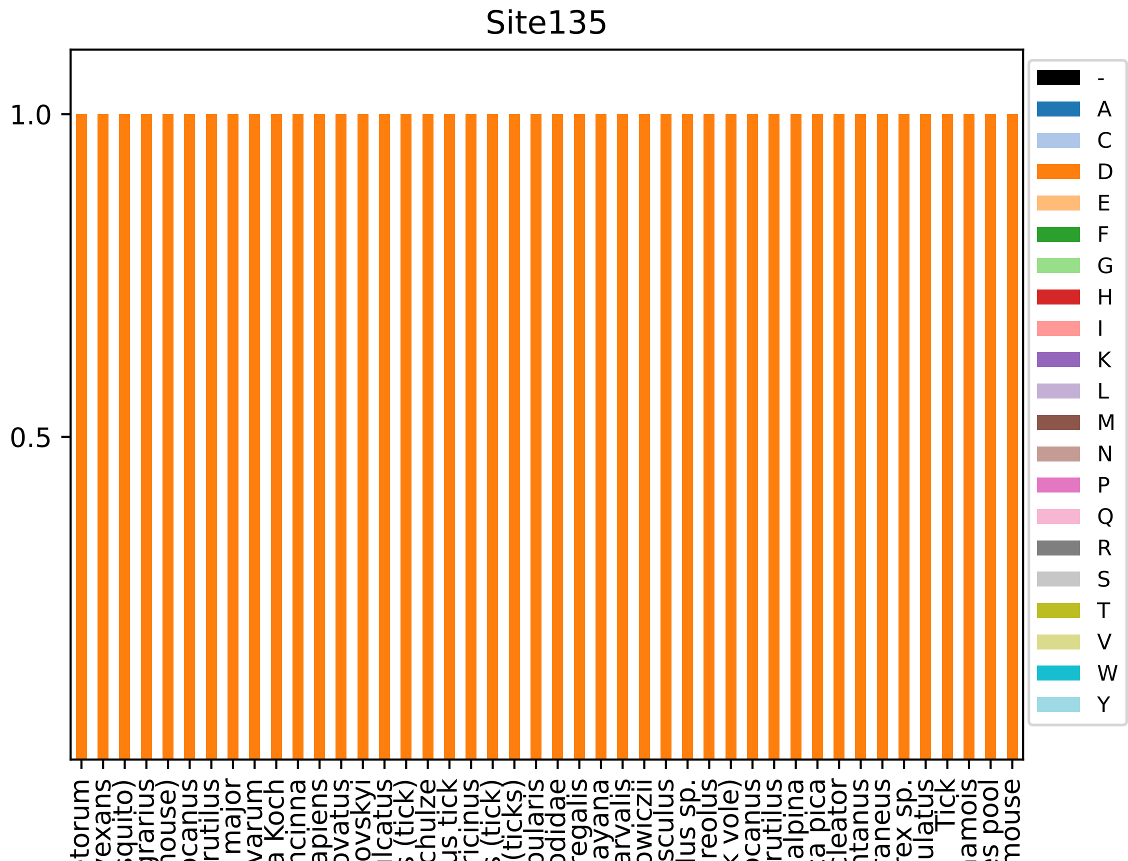
Site135
1.0
0.5
-
A
C
D
E
F
G
H
I
K
L
M
N
P
Q
R
S
T
V
W
Y

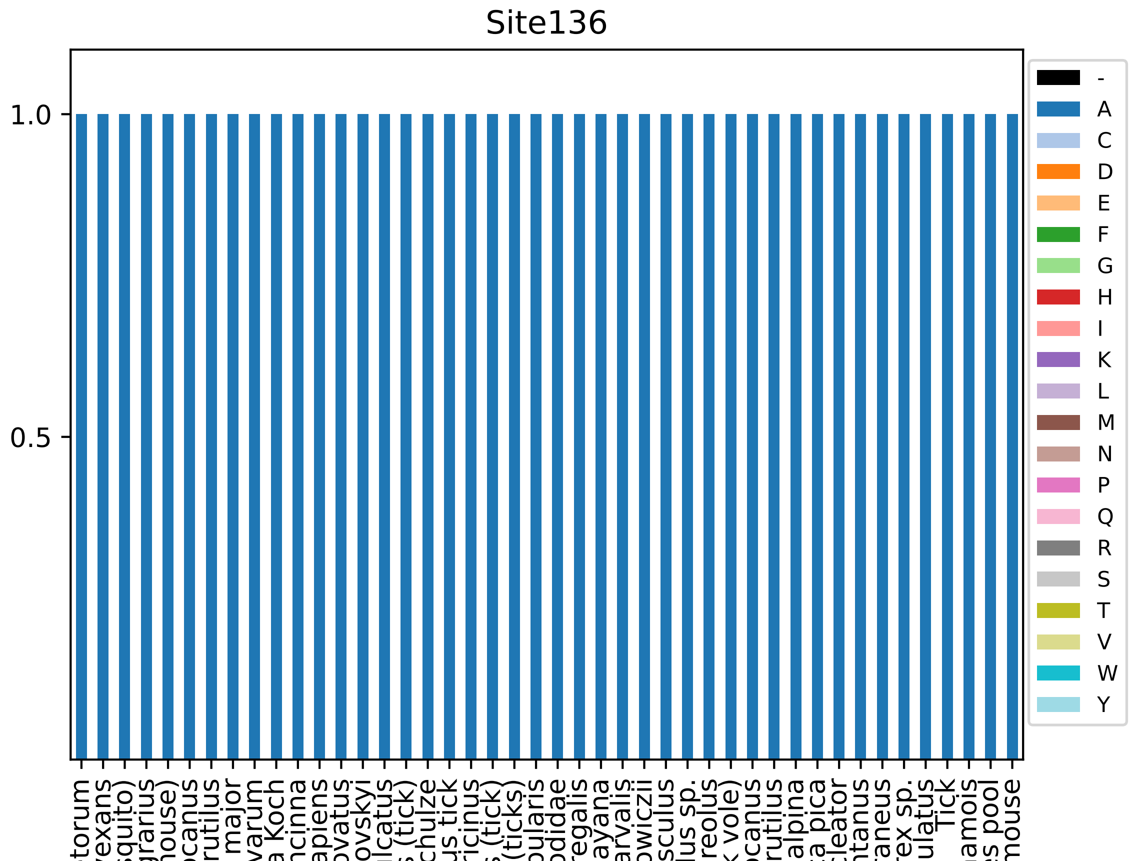
Site136
1.0
0.5
-
A
C
D
E
F
G
H
I
K
L
M
N
P
Q
R
S
T
V
W
Y

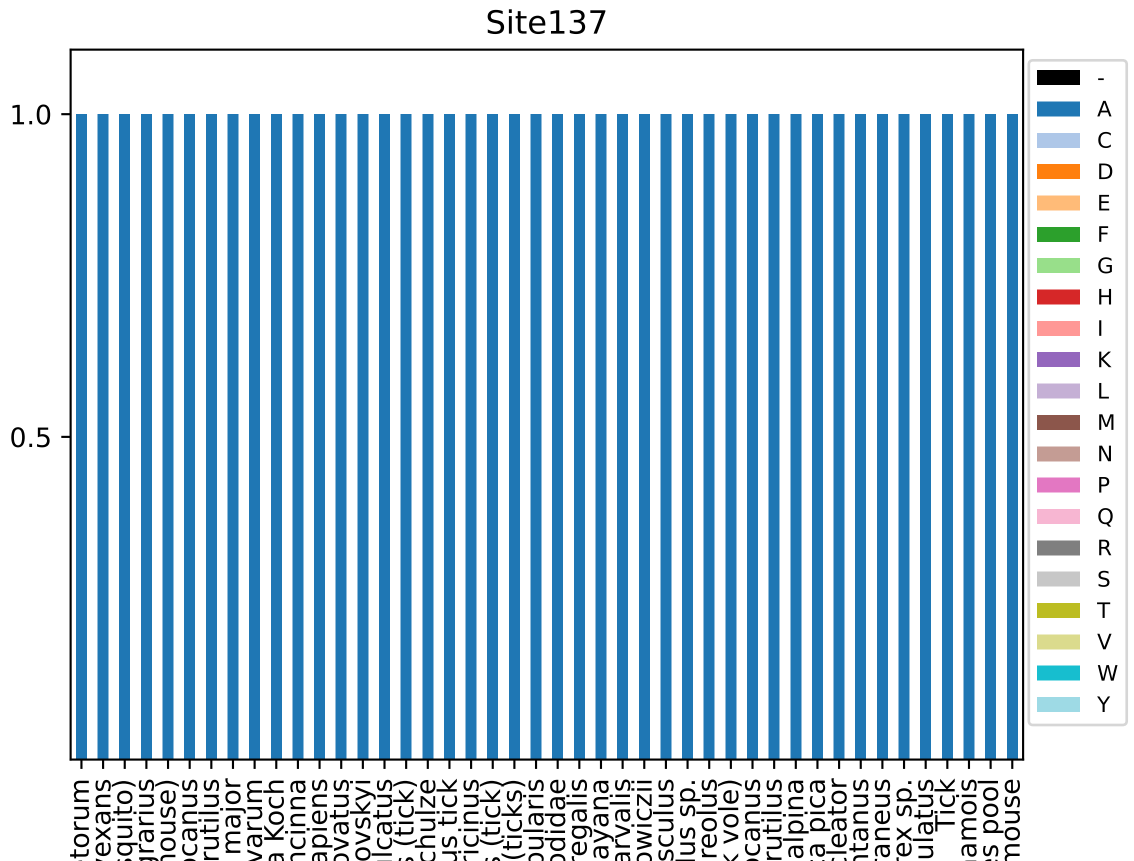
Site137
1.0
0.5
-
A
C
D
E
F
G
H
I
K
L
M
N
P
Q
R
S
T
V
W
Y

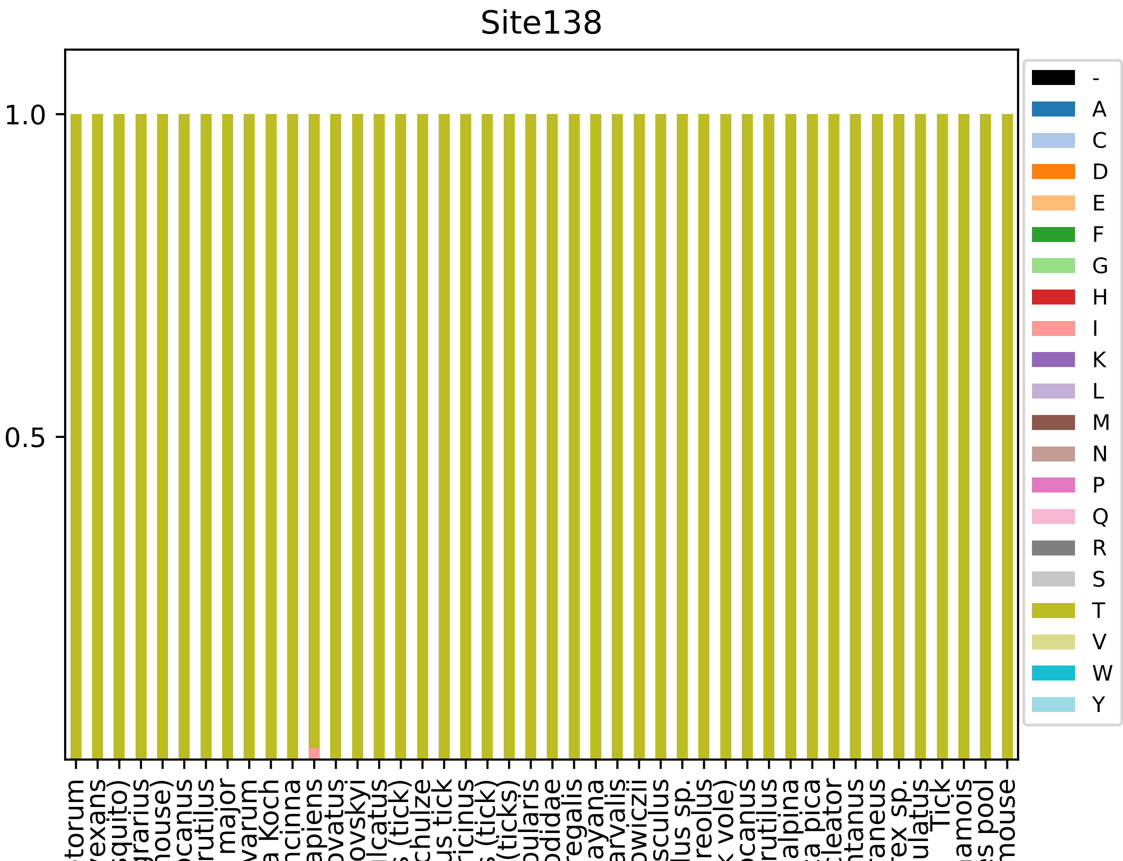
Site138
1.0
0.5
-
A
C
D
E
F
G
H
I
K
L
M
N
P
Q
R
S
T
V
W
Y

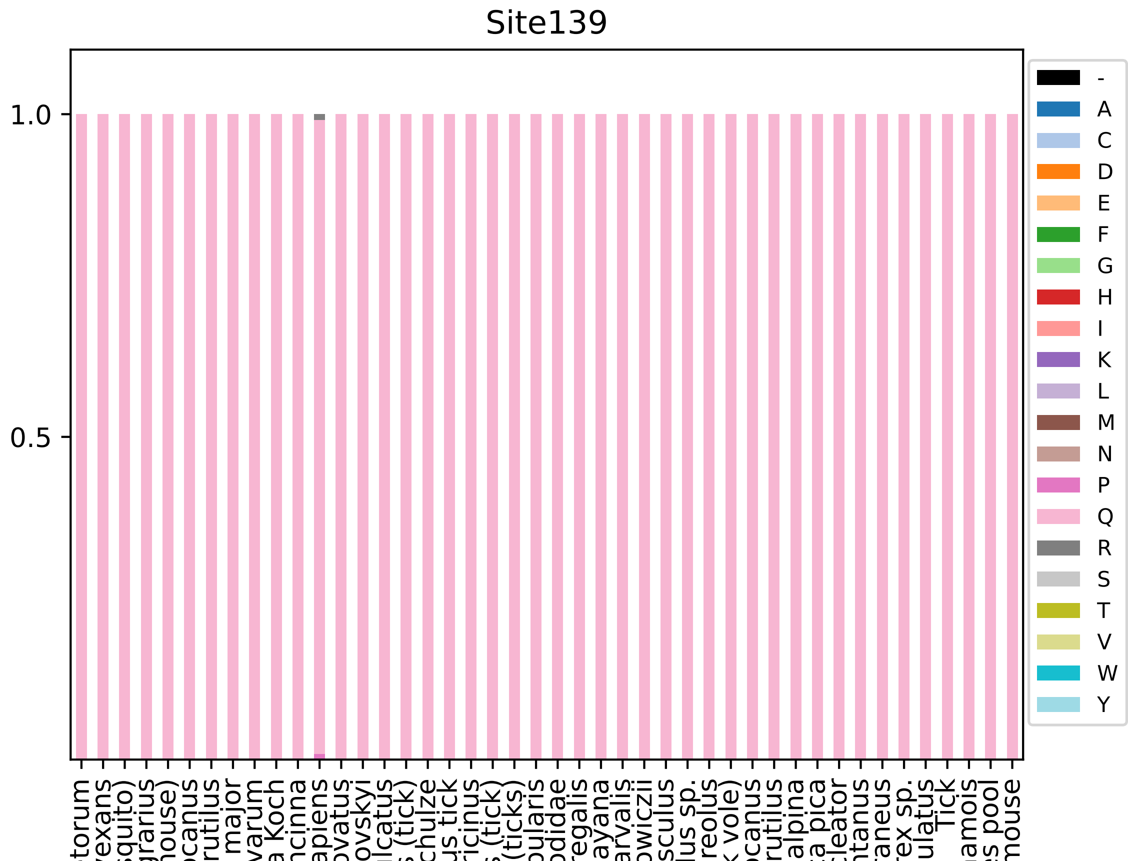
Site139
1.0
0.5
-
A
C
D
E
F
G
H
I
K
L
M
N
P
Q
R
S
T
V
W
Y

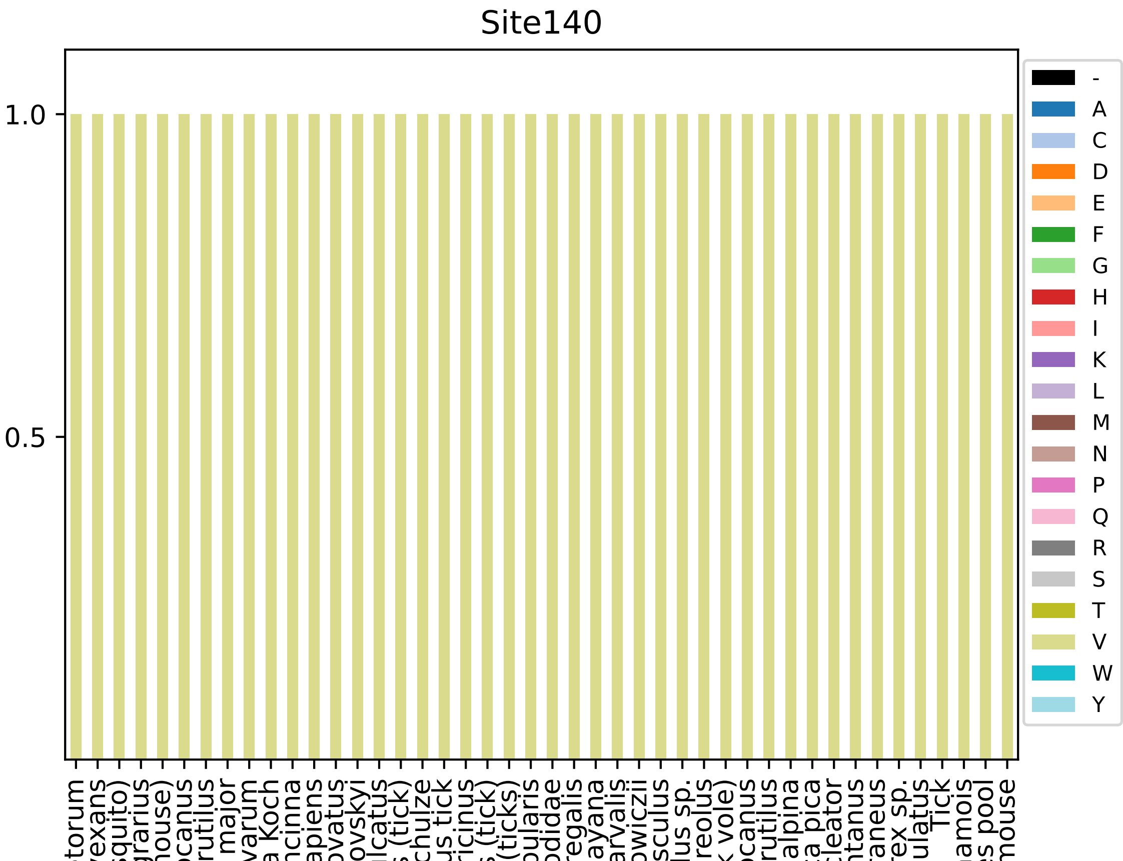
Site140
1.0
0.5
-
A
C
D
E
F
G
H
I
K
L
M
N
P
Q
R
S
T
V
W
Y

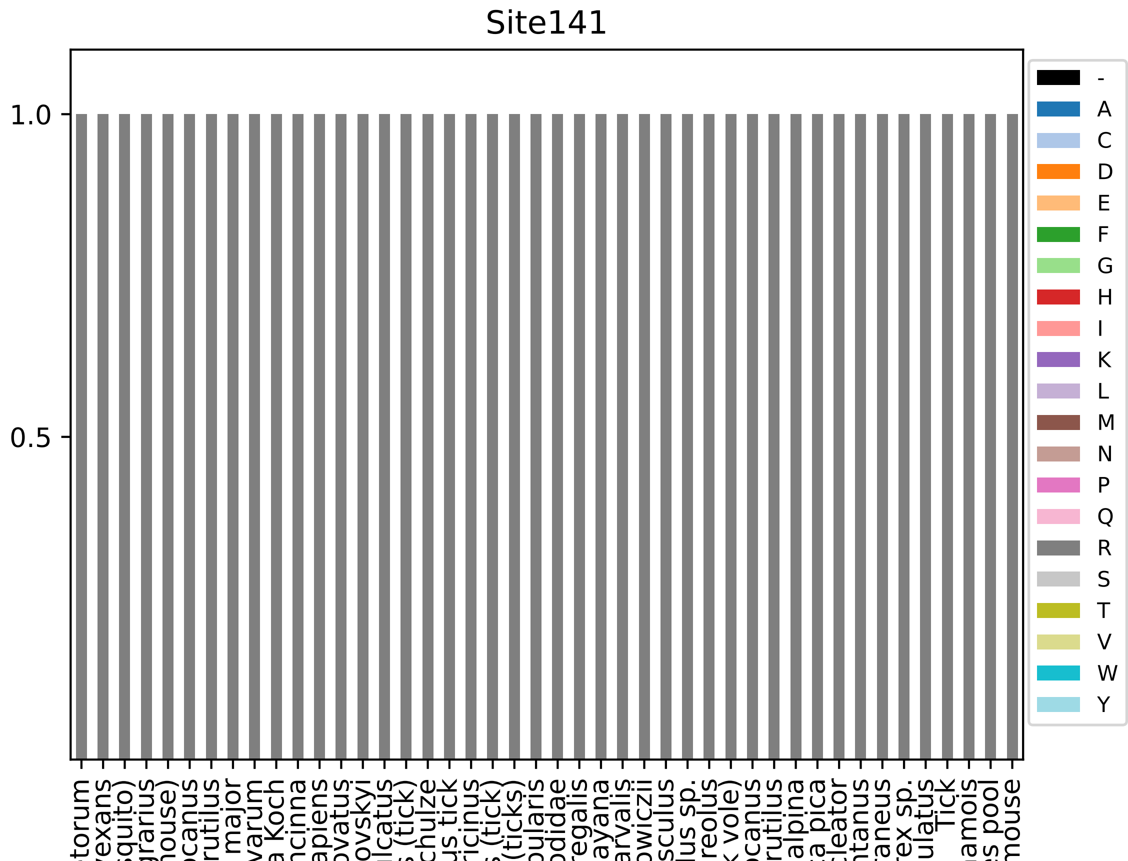
Site141
1.0
0.5
-
A
C
D
E
F
G
H
I
K
L
M
N
P
Q
R
S
T
V
W
Y

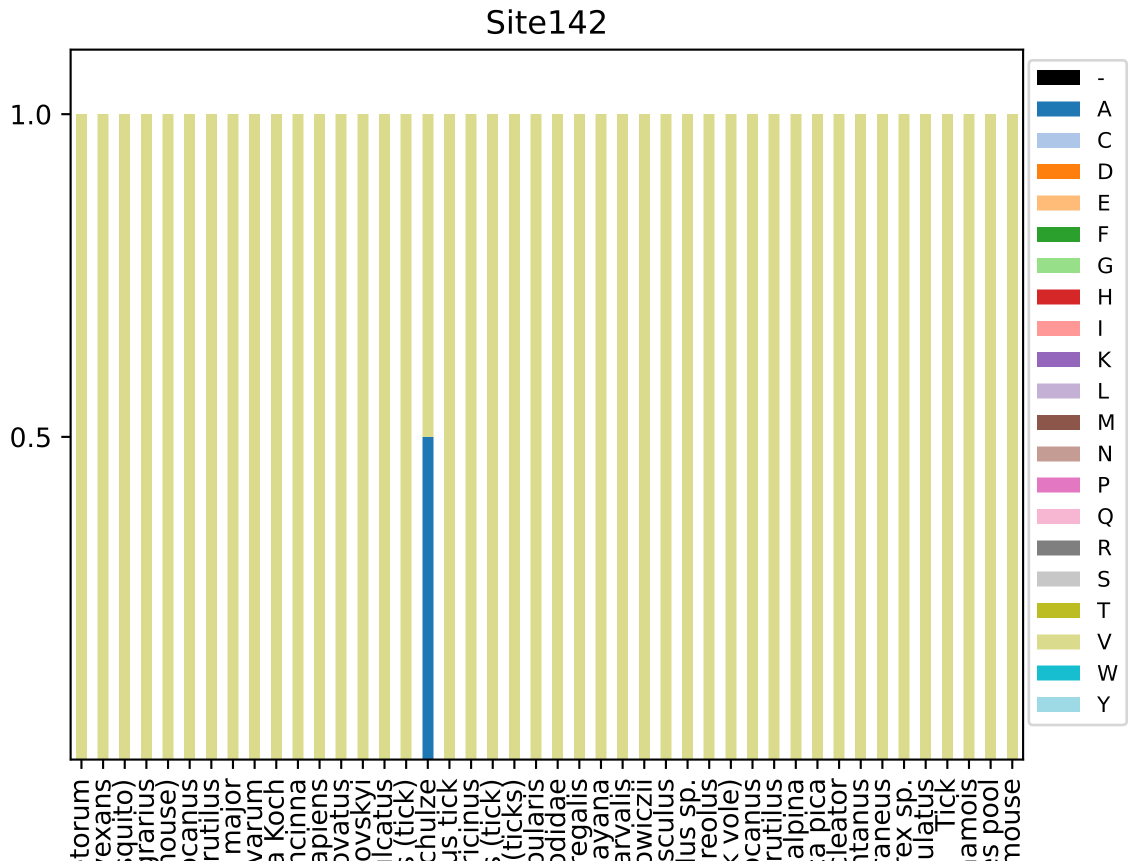
Site142
1.0
0.5
-
A
C
D
E
F
G
H
I
K
L
M
N
P
Q
R
S
T
V
W
Y

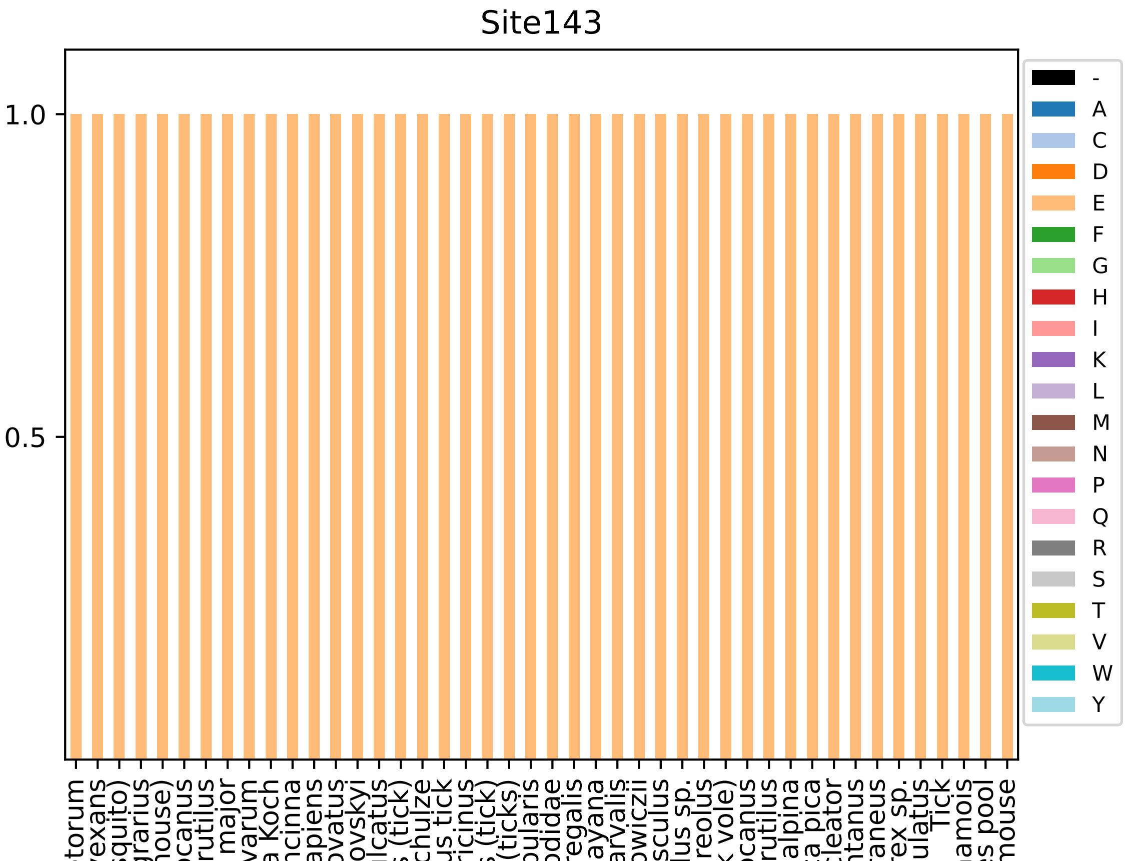

Site143
1.0
0.5
-
A
C
D
E
F
G
H
I
K
L
M
N
P
Q
R
S
T
V
W
Y

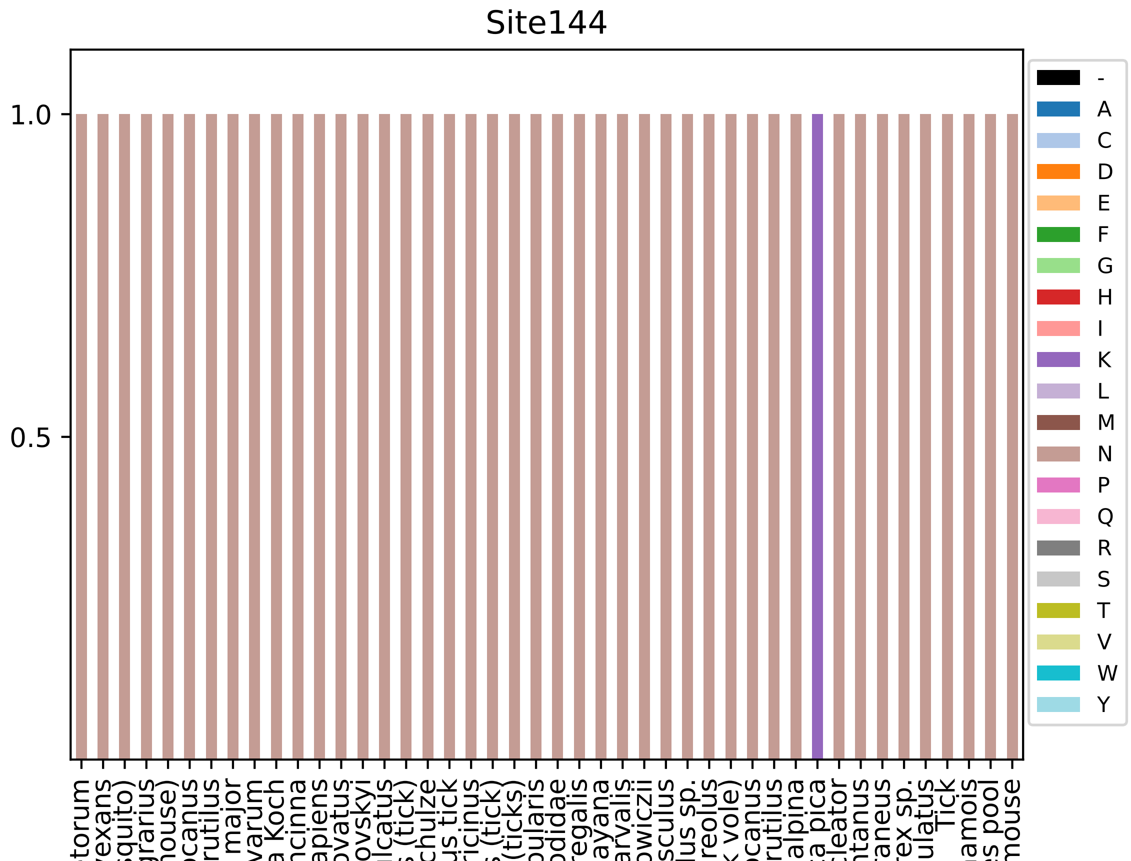

Site144
1.0
0.5
-
A
C
D
E
F
G
H
I
K
L
M
N
P
Q
R
S
T
V
W
Y

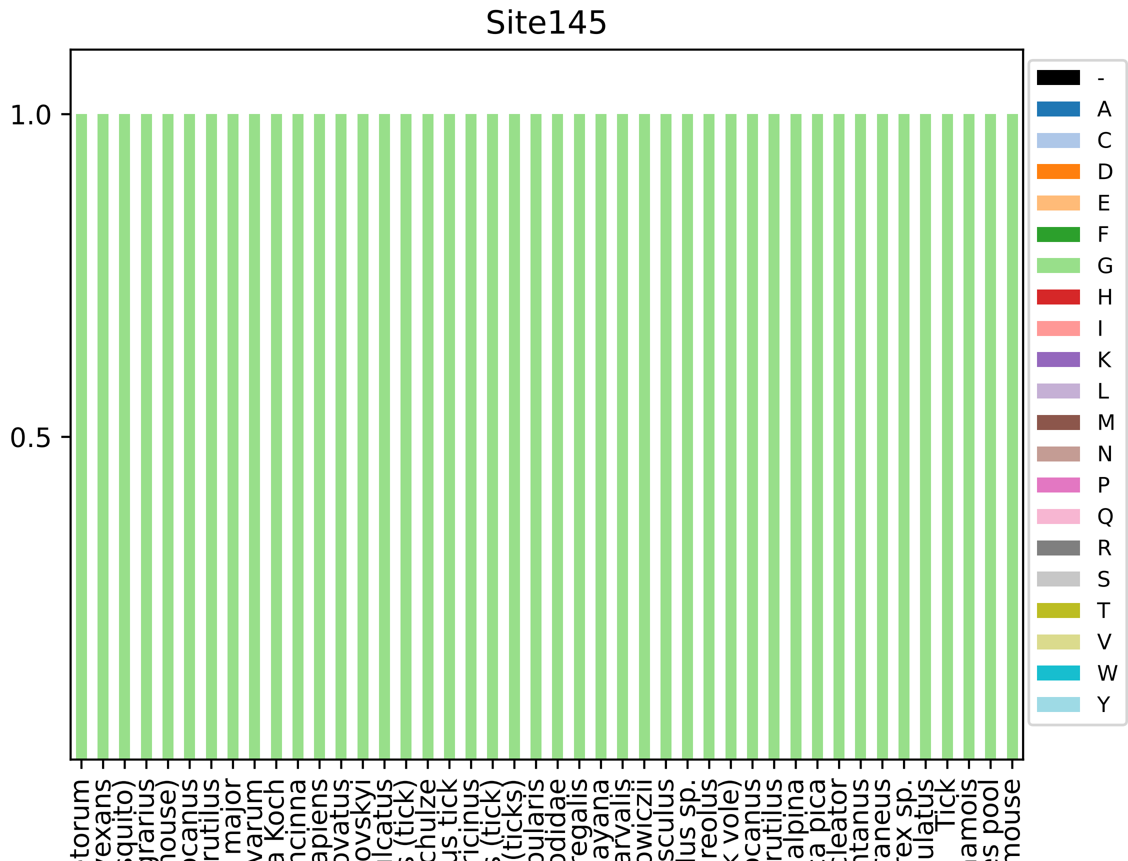
Site145
1.0
0.5
-
A
C
D
E
F
G
H
I
K
L
M
N
P
Q
R
S
T
V
W
Y

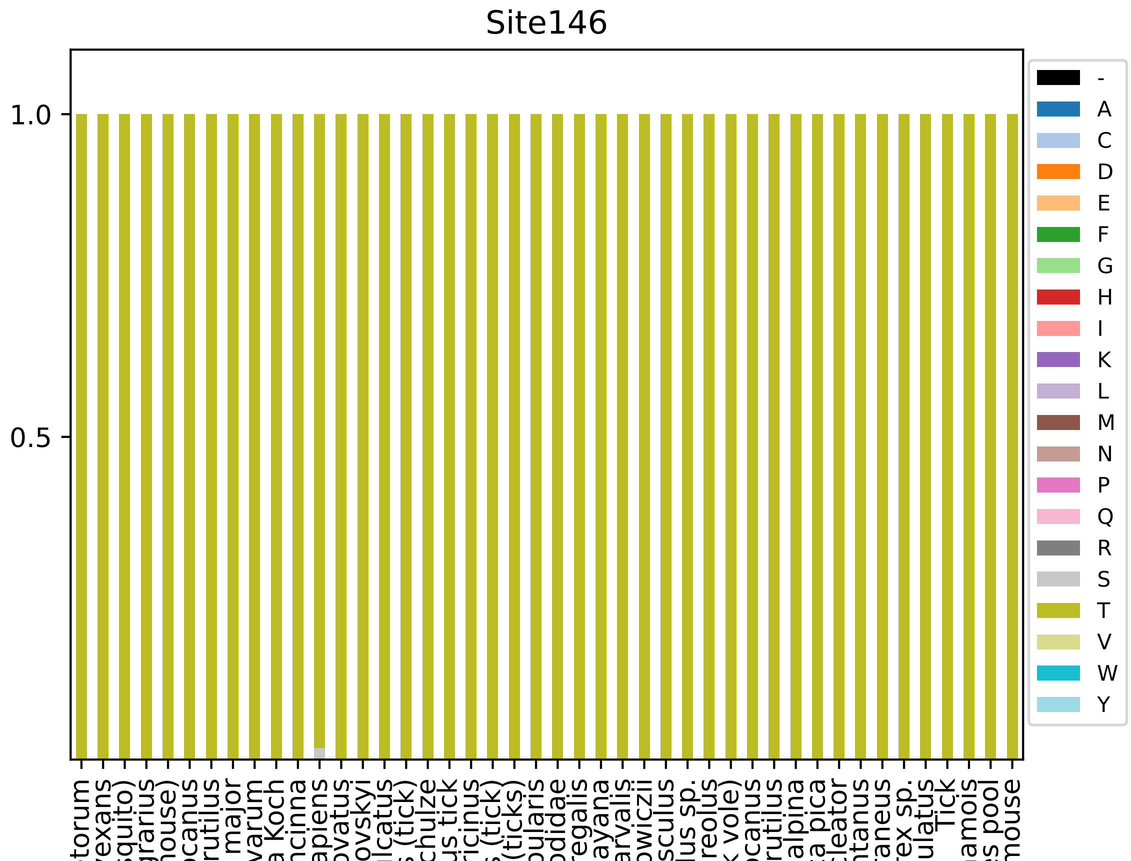

Site146
1.0
0.5
-
A
C
D
E
F
G
H
I
K
L
M
N
P
Q
R
S
T
V
W
Y

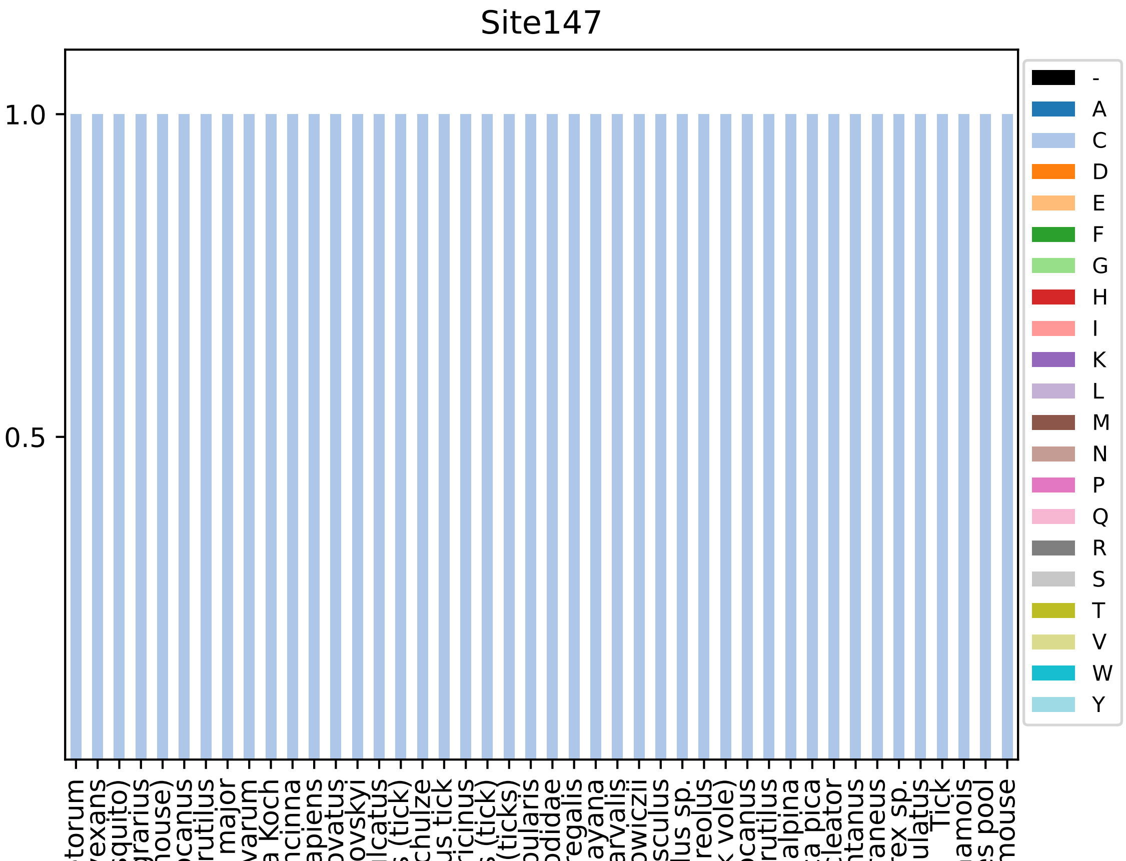
Site147
1.0
0.5
-
A
C
D
E
F
G
H
I
K
L
M
N
P
Q
R
S
T
V
W
Y

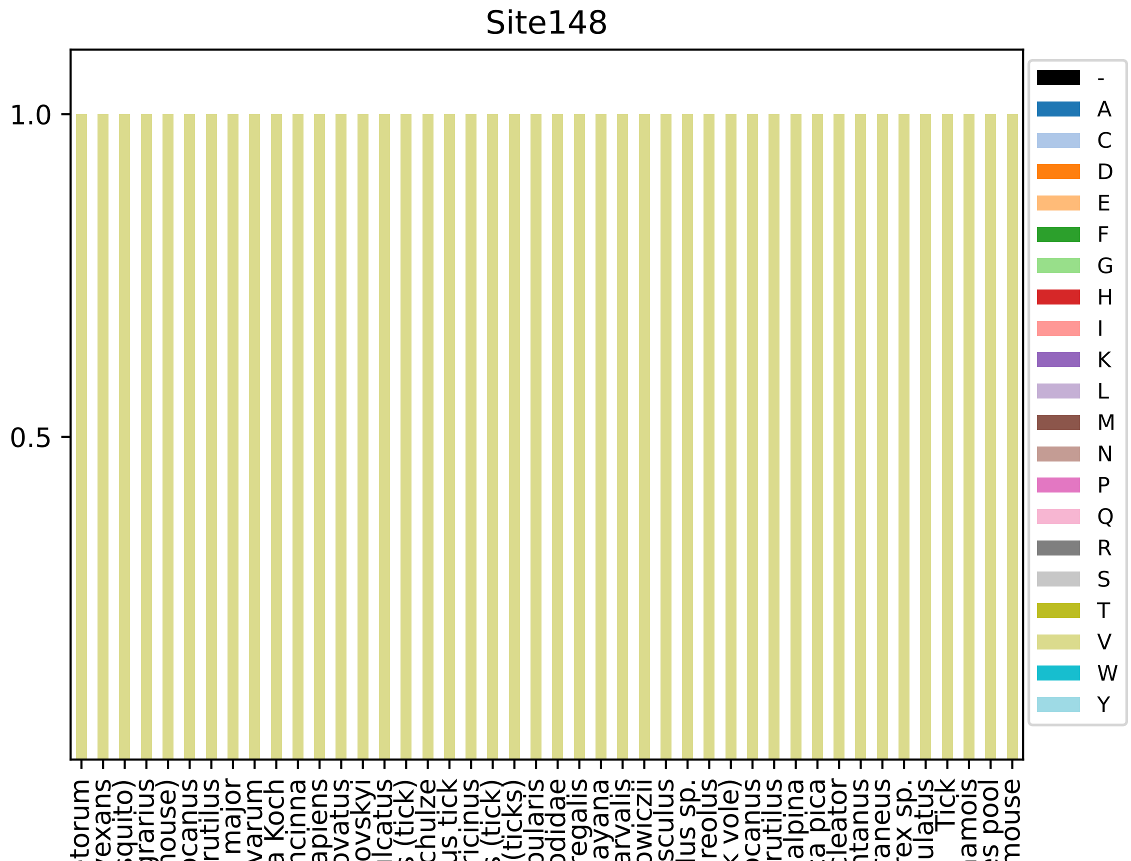
Site148
1.0
0.5
-
A
C
D
E
F
G
H
I
K
L
M
N
P
Q
R
S
T
V
W
Y

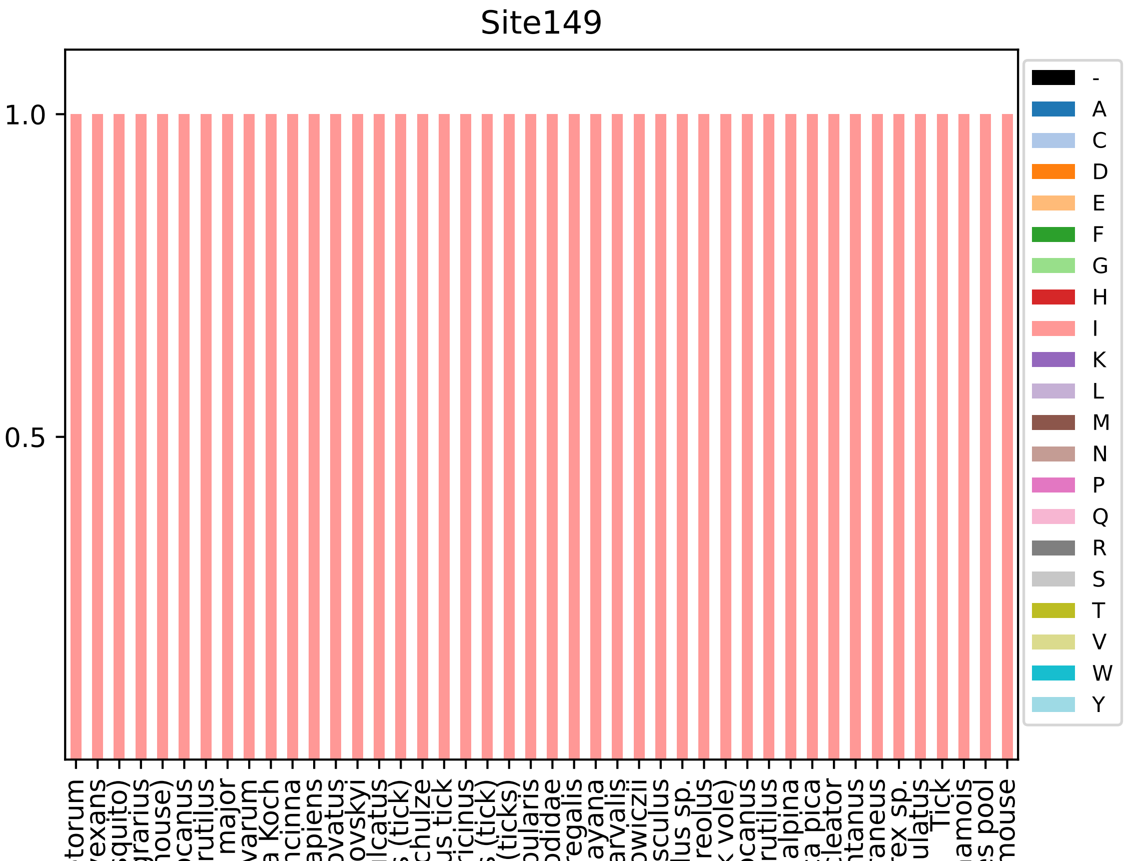
Site149
1.0
0.5
-
A
C
D
E
F
G
H
I
K
L
M
N
P
Q
R
S
T
V
W
Y

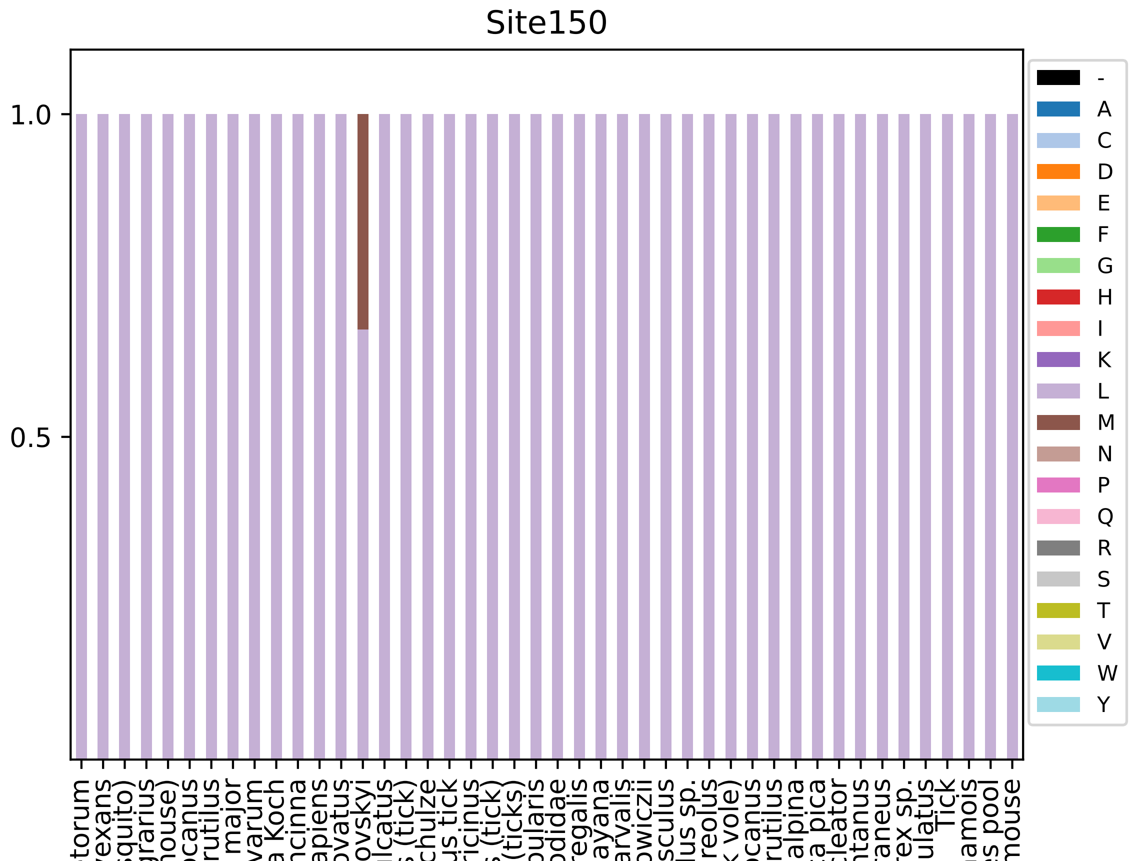

Site150
1.0
0.5
-
A
C
D
E
F
G
H
I
K
L
M
N
P
Q
R
S
T
V
W
Y

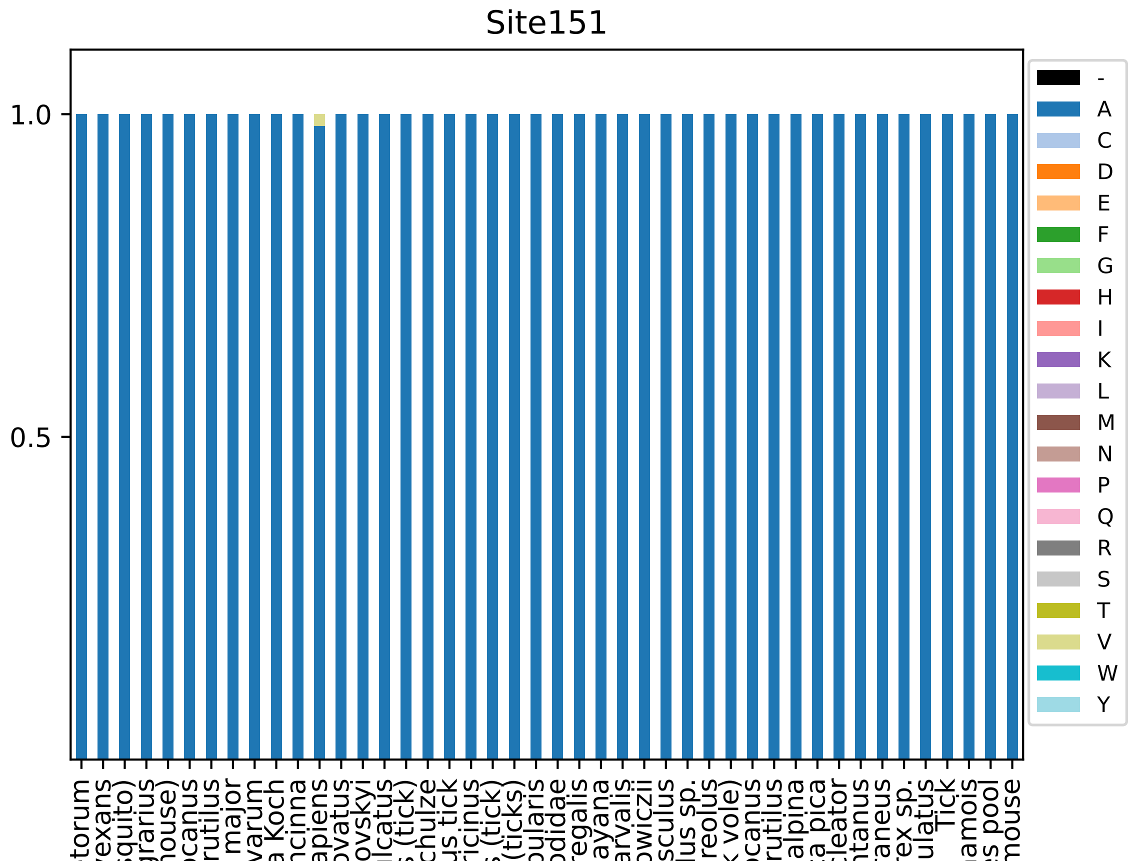
Site151
1.0
0.5
-
A
C
D
E
F
G
H
I
K
L
M
N
P
Q
R
S
T
V
W
Y
Tick

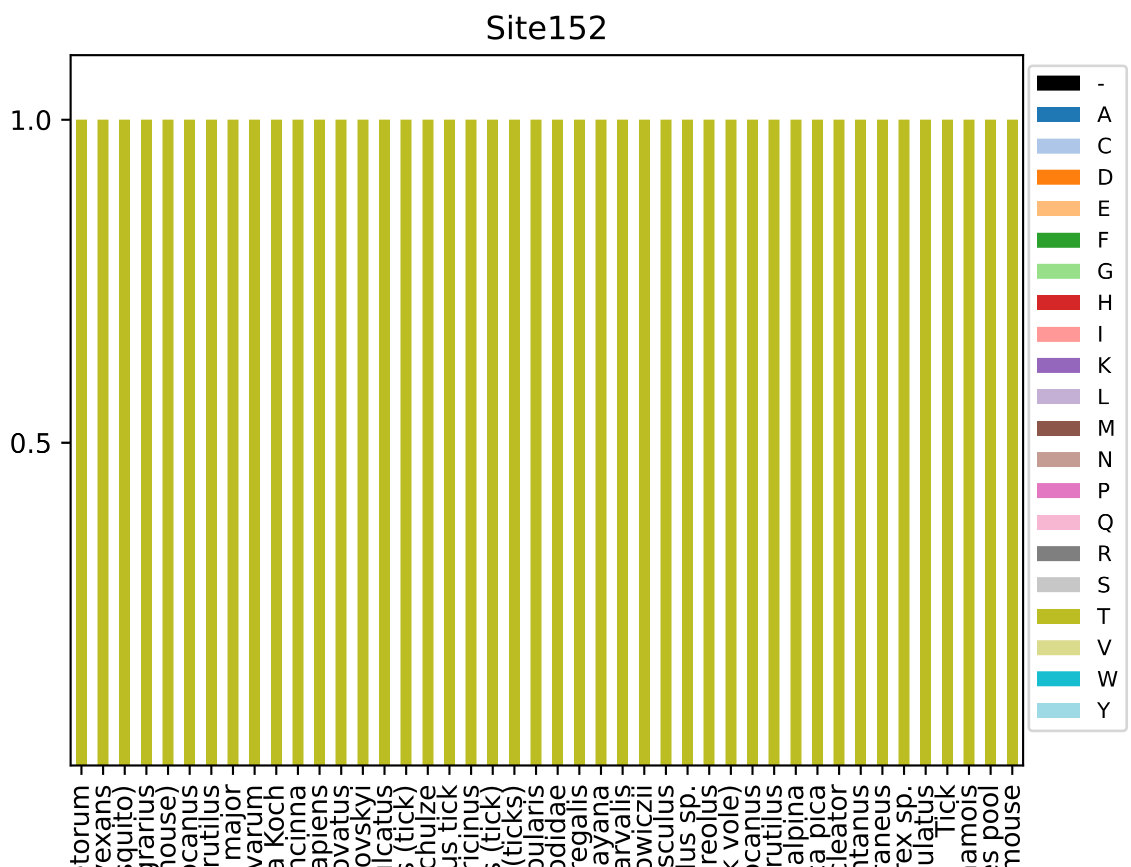

Site152
1.0
0.5
-
A
C
D
E
F
G
H
I
K
L
M
N
P
Q
R
S
T
V
W
Y

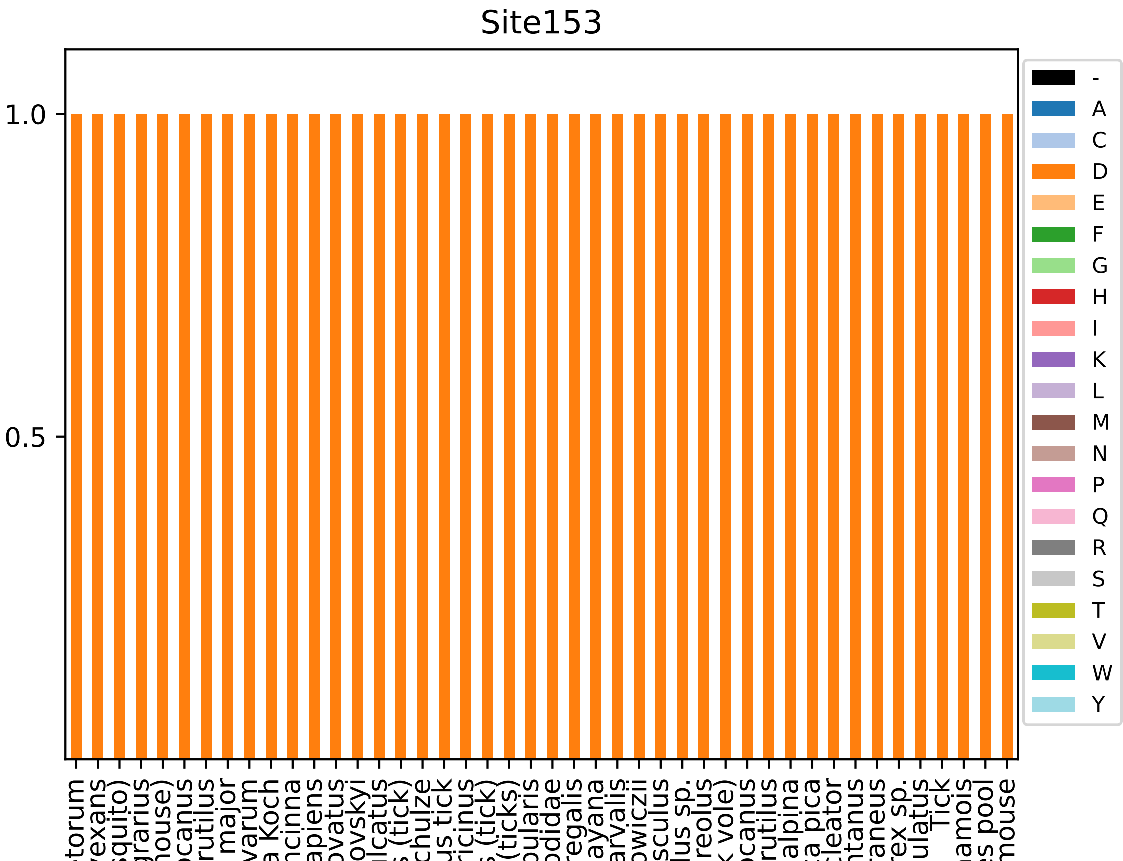
Site153
1.0
0.5
-
A
C
D
E
F
G
H
I
K
L
M
N
P
Q
R
S
T
V
W
Y

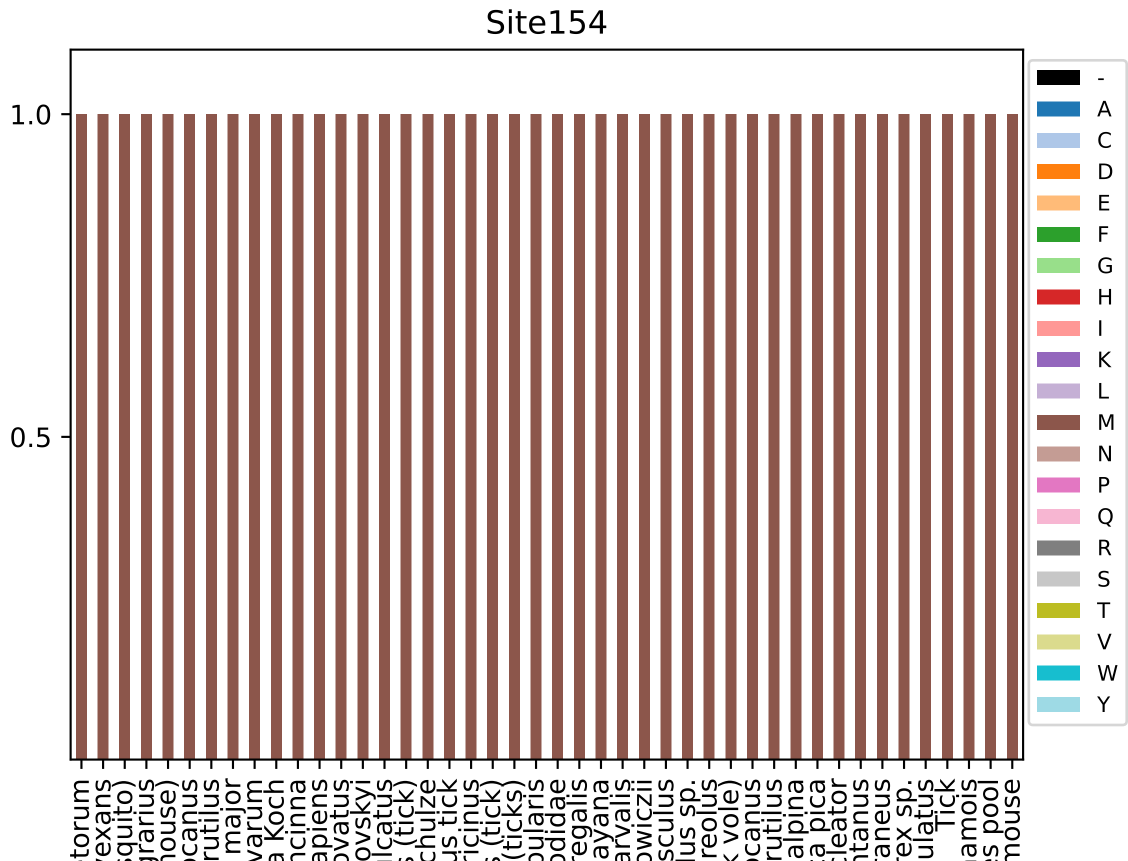
Site154
1.0
0.5
-
A
C
D
E
F
G
H
I
K
L
M
N
P
Q
R
S
T
V
W
Y

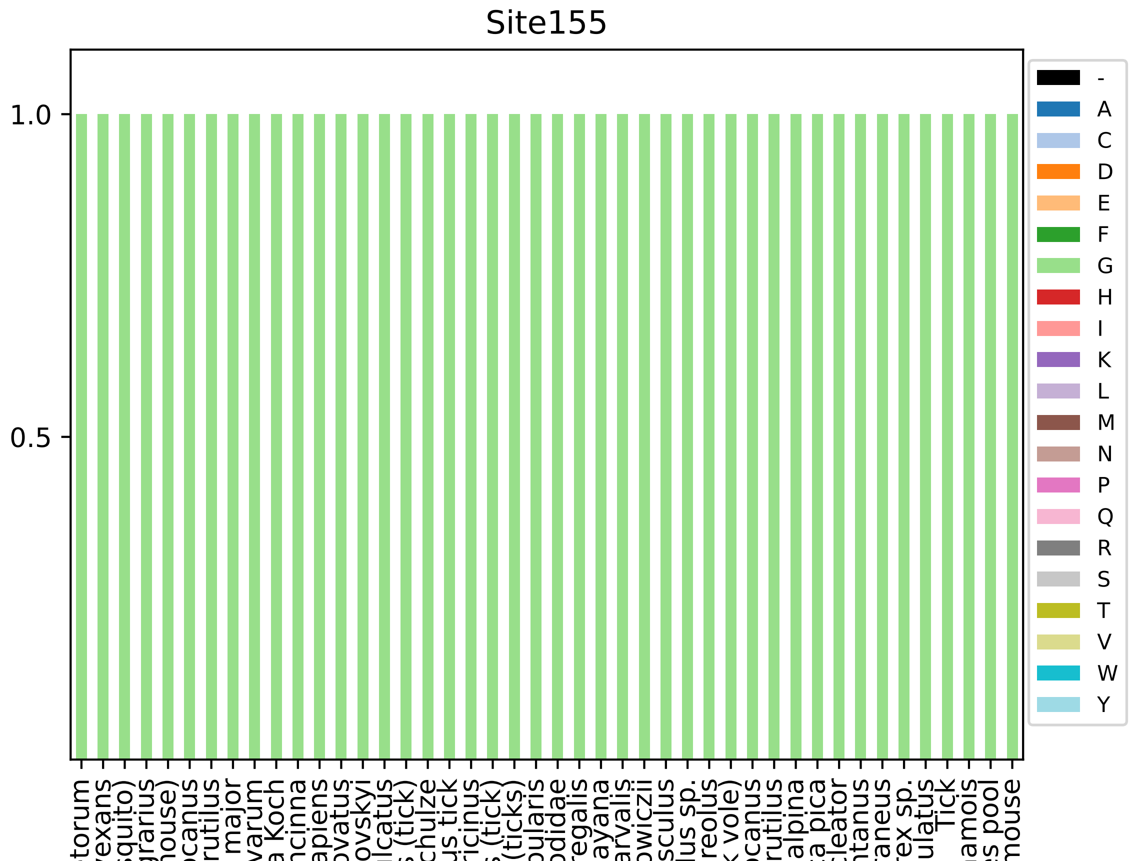
Site155
1.0
0.5
-
A
C
D
E
F
G
H
I
K
L
M
N
P
Q
R
S
T
V
W
Y

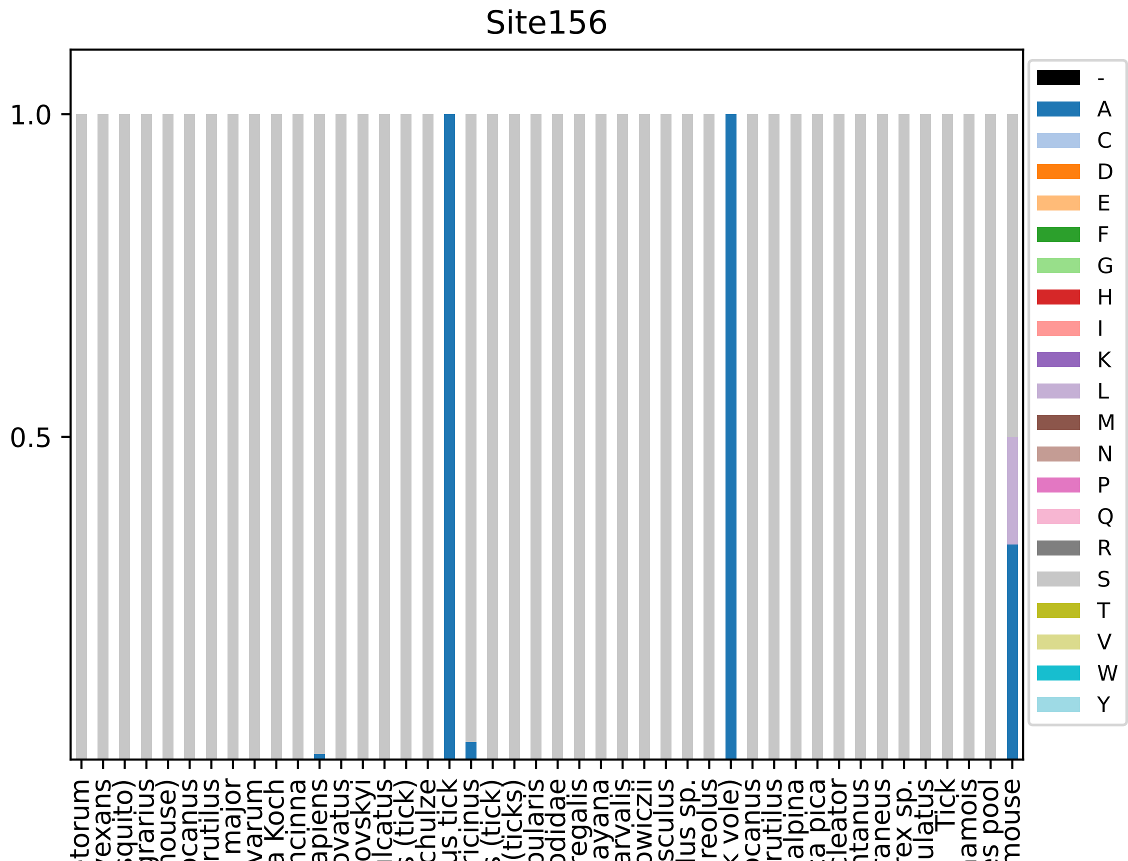
Site156
1.0
0.5
-
A
C
D
E
F
G
H
I
K
L
M
N
P
Q
R
S
T
V
W
Y

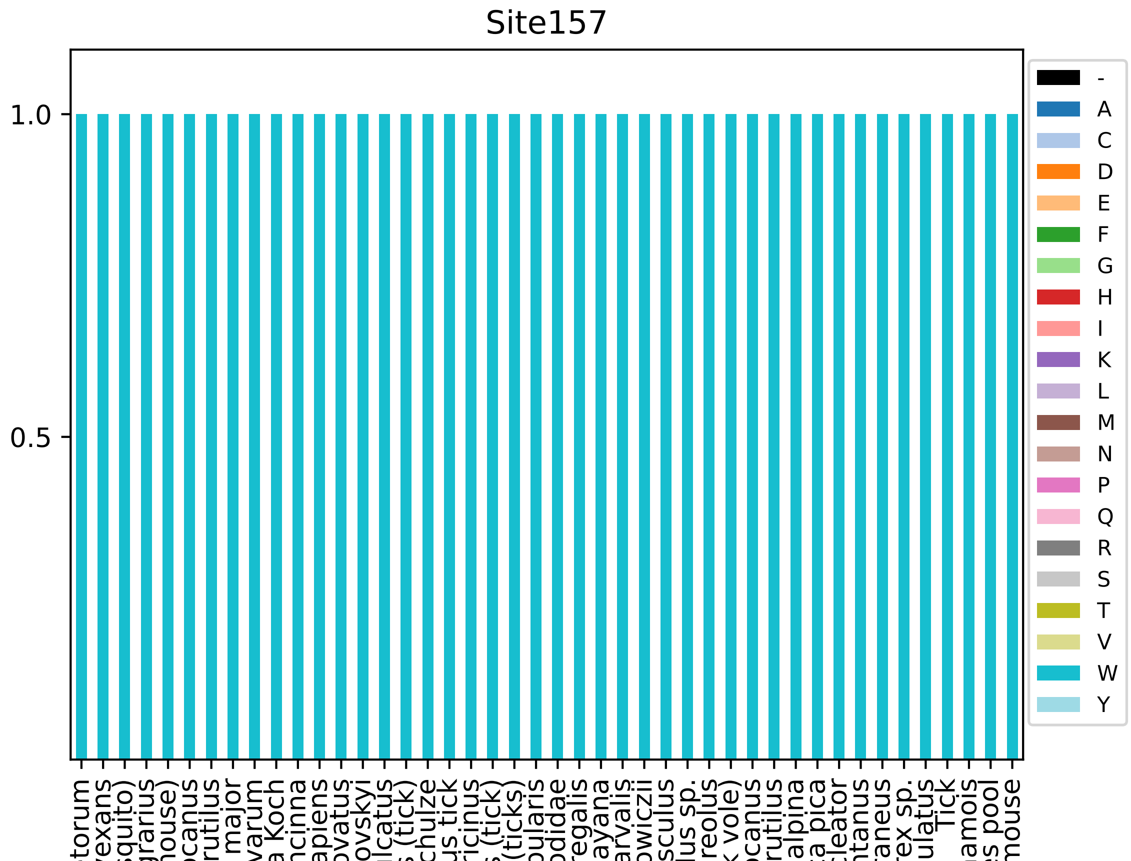
Site157
1.0
0.5
-
A
C
D
E
F
G
H
I
K
L
M
N
P
Q
R
S
T
V
W
Y

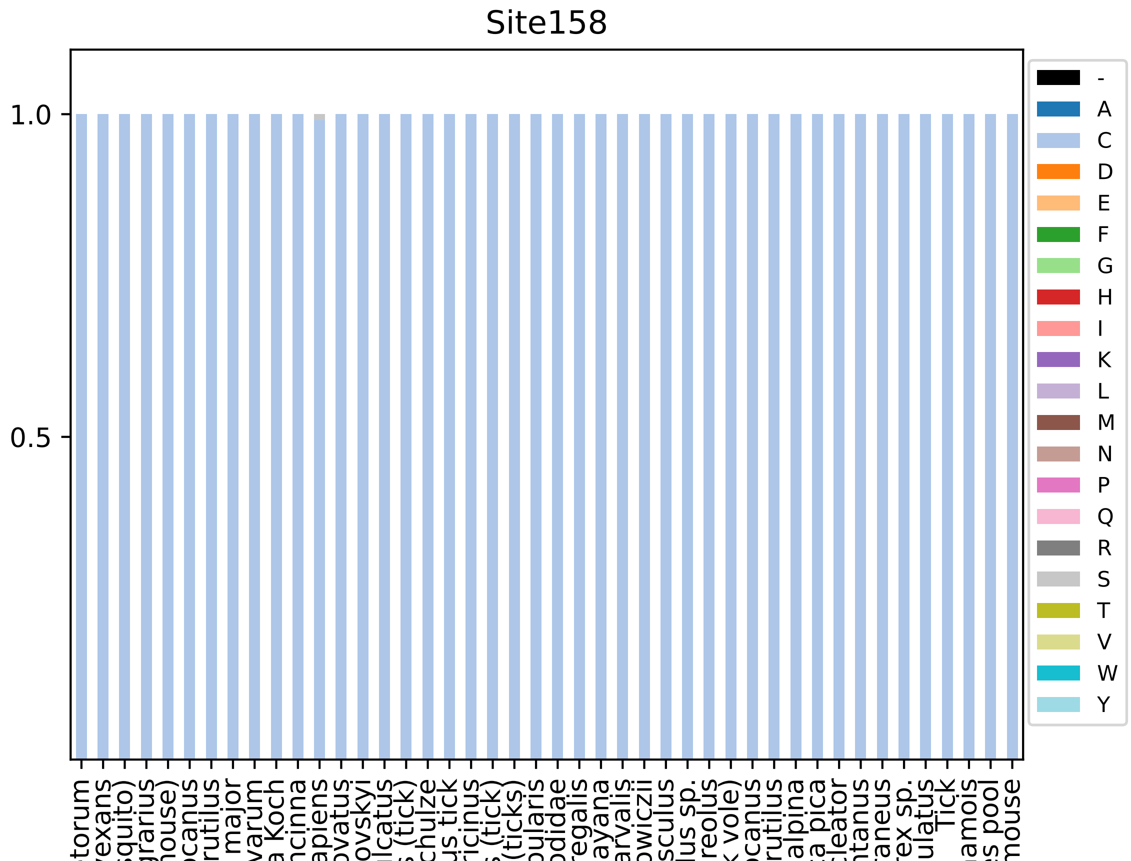

Site158
1.0
0.5
-
A
C
D
E
F
G
H
I
K
L
M
N
P
Q
R
S
T
V
W
Y

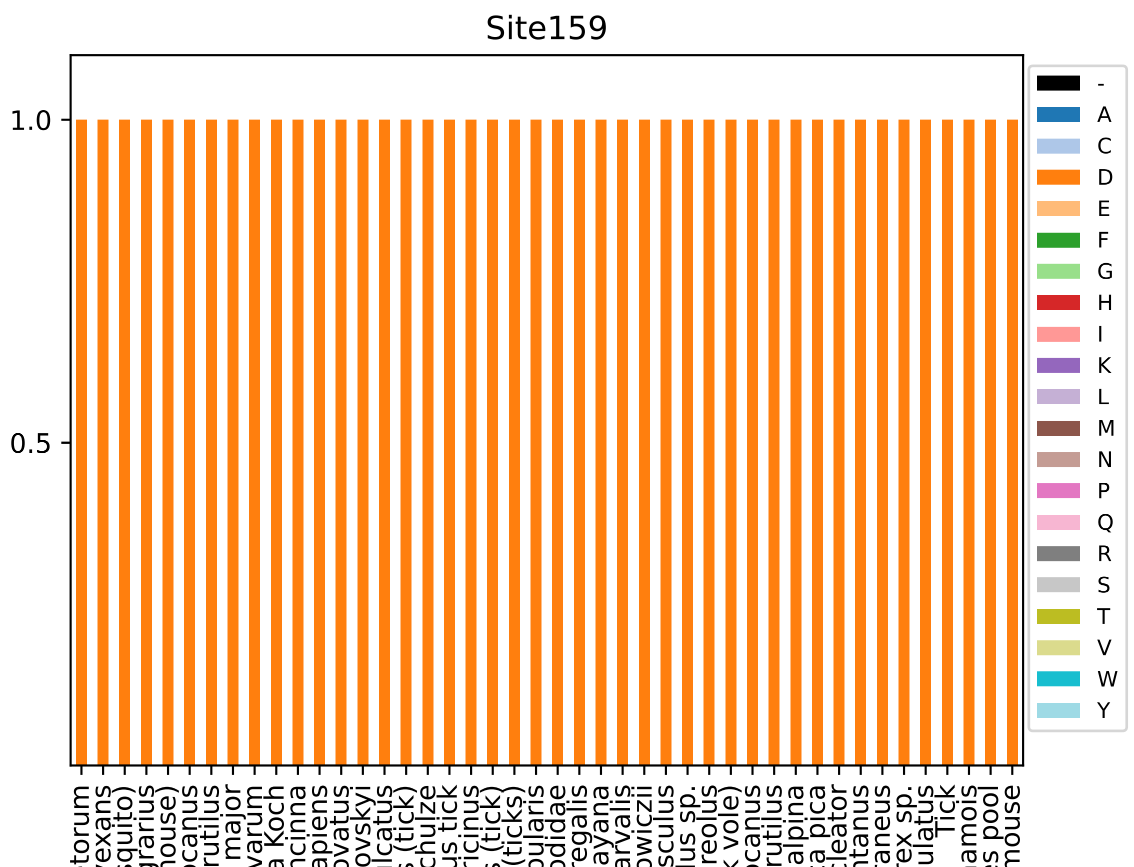
Site159
1.0
0.5
-
A
C
D
E
F
G
H
I
K
L
M
N
P
Q
R
S
T
V
W
Y

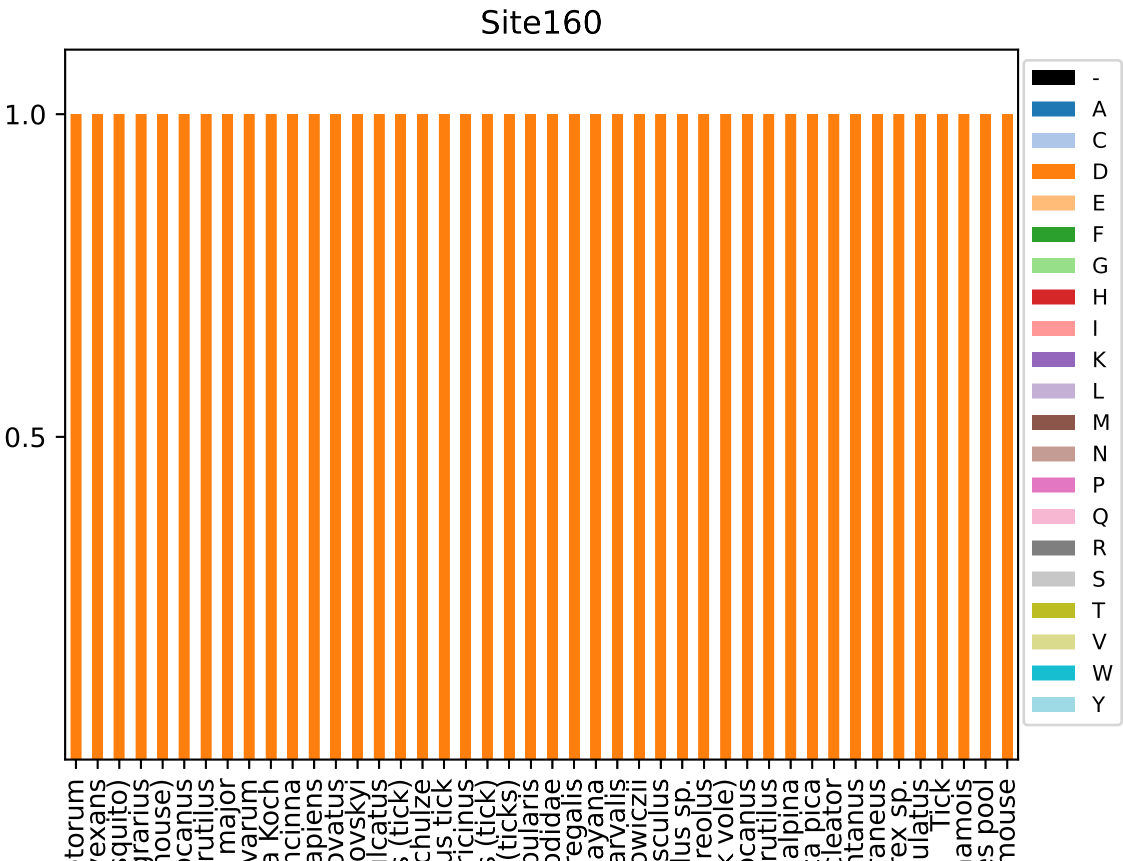

Site160
1.0
0.5
-
A
C
D
E
F
G
H
I
K
L
M
N
P
Q
R
S
T
V
W
Y

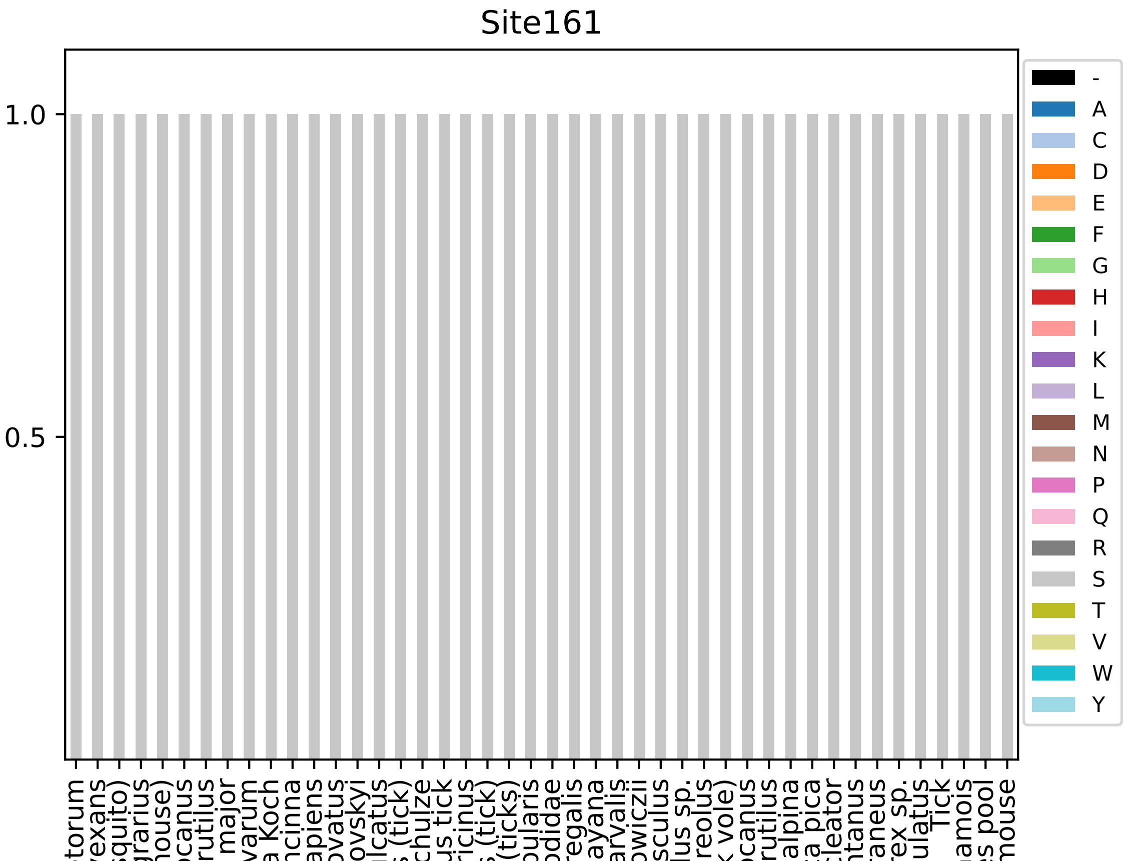
Site161
1.0
0.5
-
A
C
D
E
F
G
H
I
K
L
M
N
P
Q
R
S
T
V
W
Y

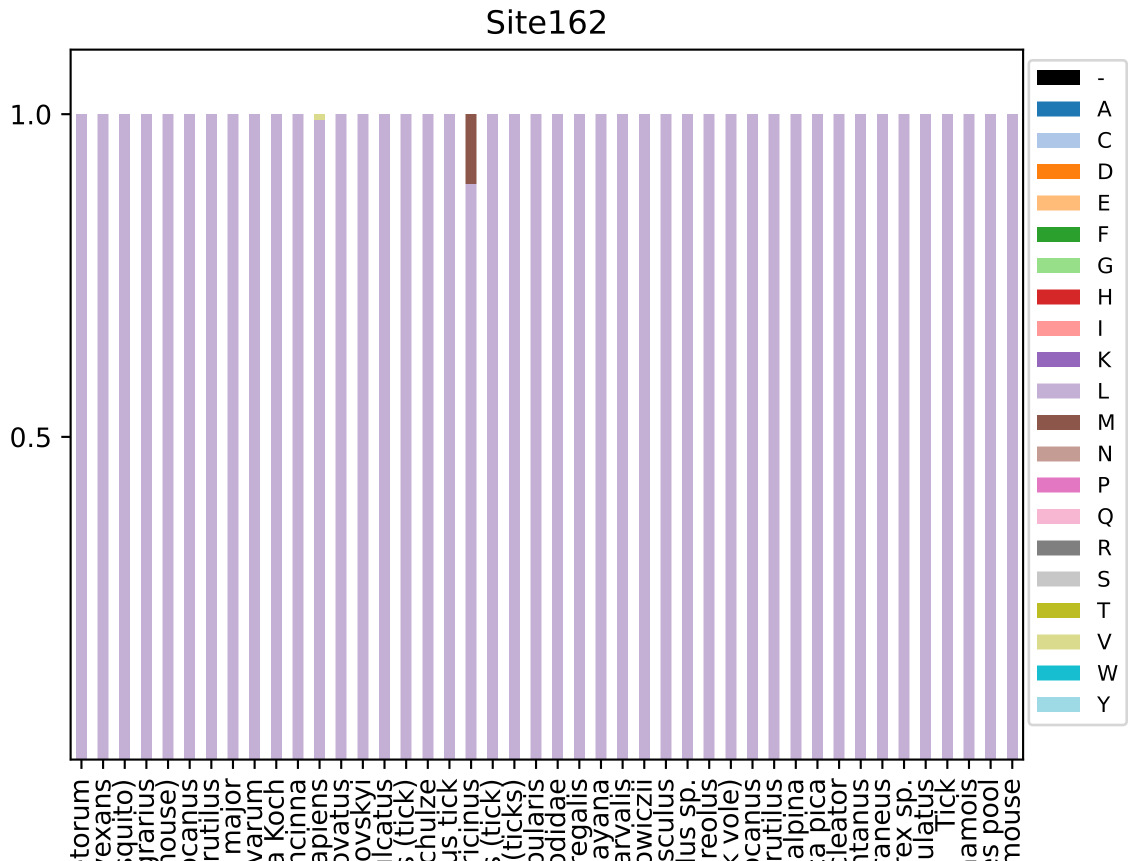
Site162
1.0
0.5
-
A
C
D
E
F
G
H
I
K
L
M
N
P
Q
R
S
T
V
W
Y

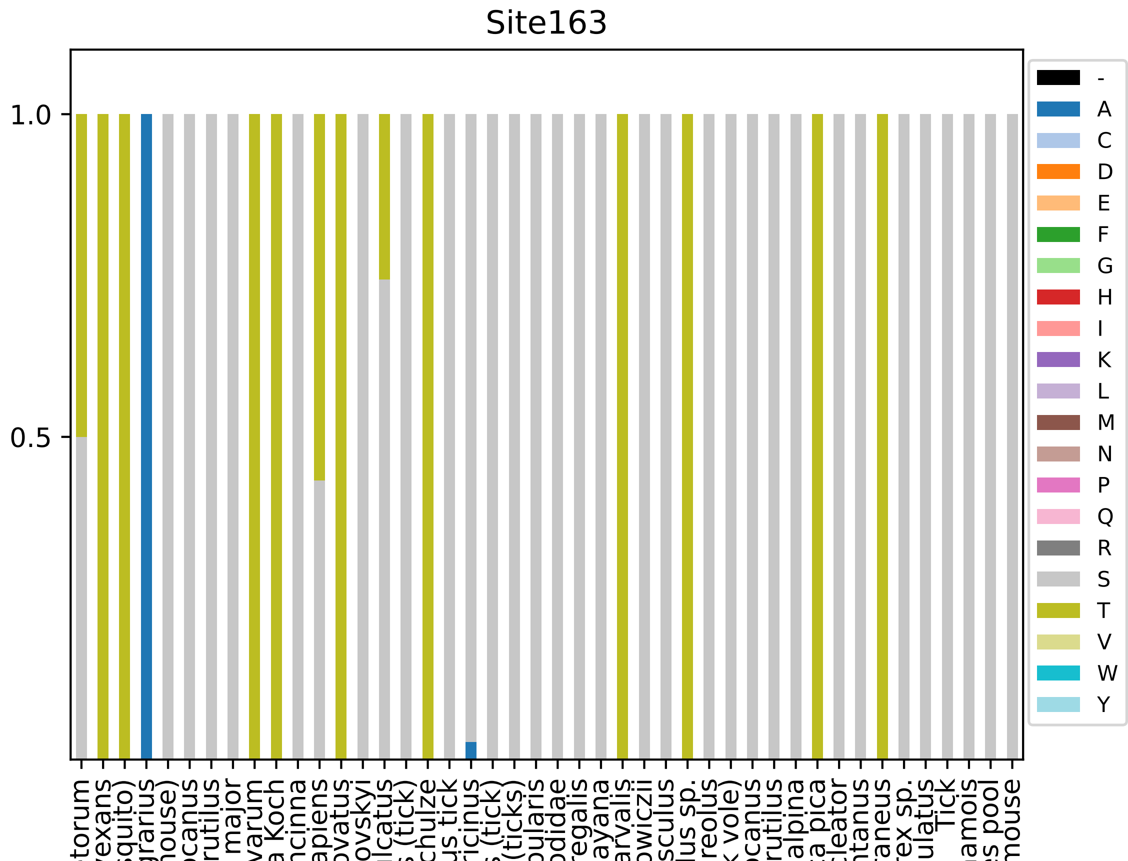
Site163
1.0
0.5
-
A
C
D
E
F
G
H
I
K
L
M
N
P
Q
R
S
T
V
W
Y
Tick

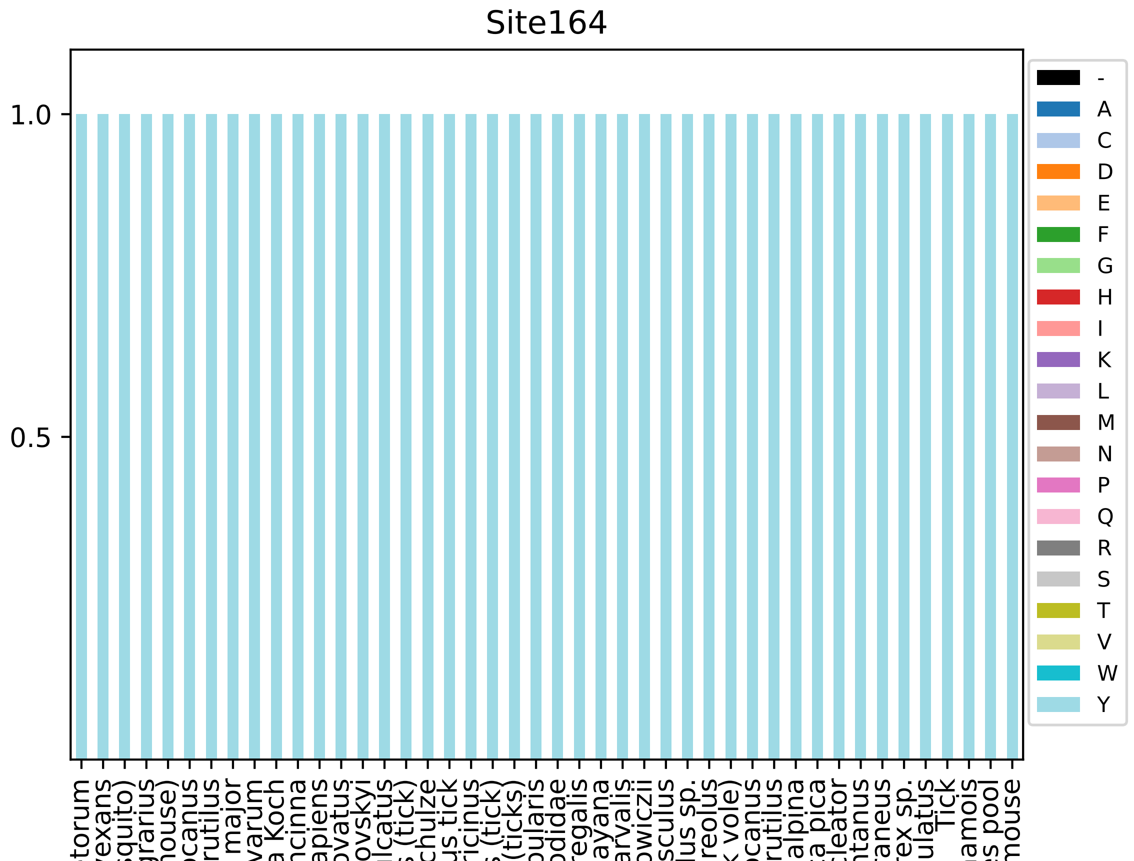

Site164
1.0
0.5
-
A
C
D
E
F
G
H
I
K
L
M
N
P
Q
R
S
T
V
W
Y

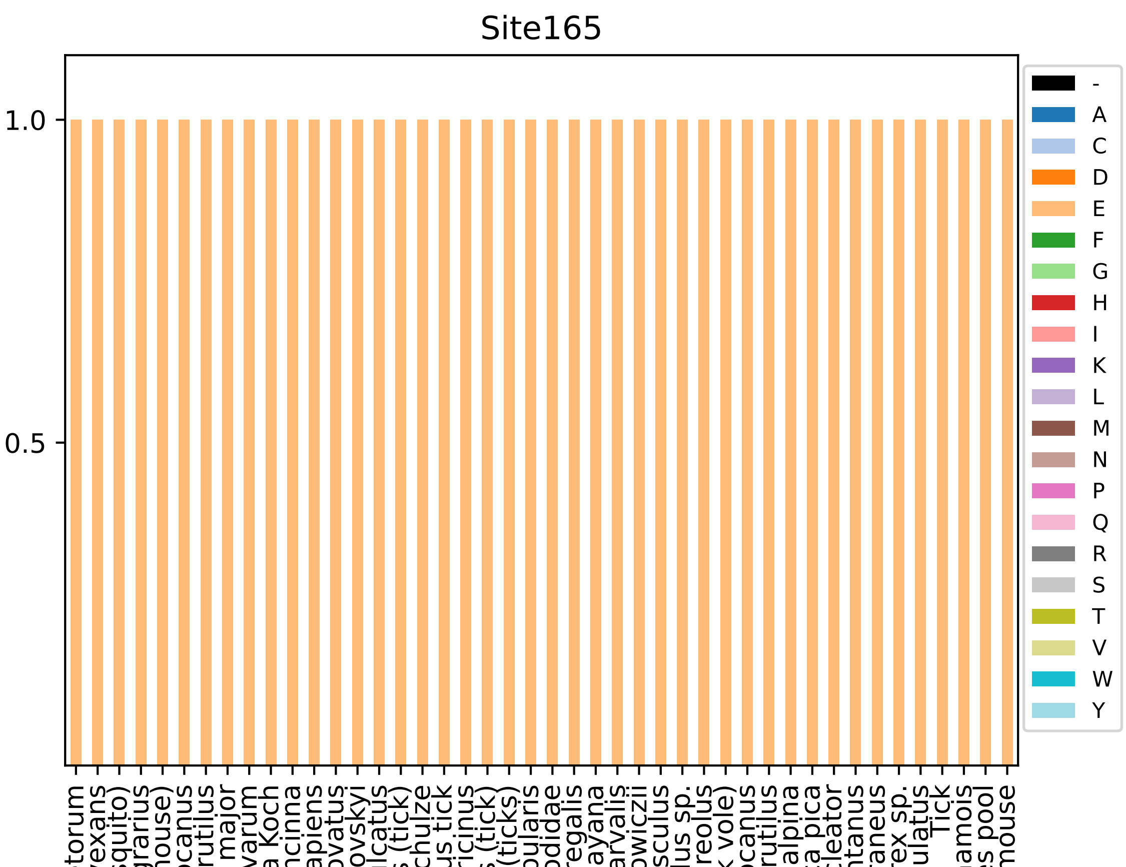
Site165
1.0
0.5
-
A
C
D
E
F
G
H
I
K
L
M
N
P
Q
R
S
T
V
W
Y

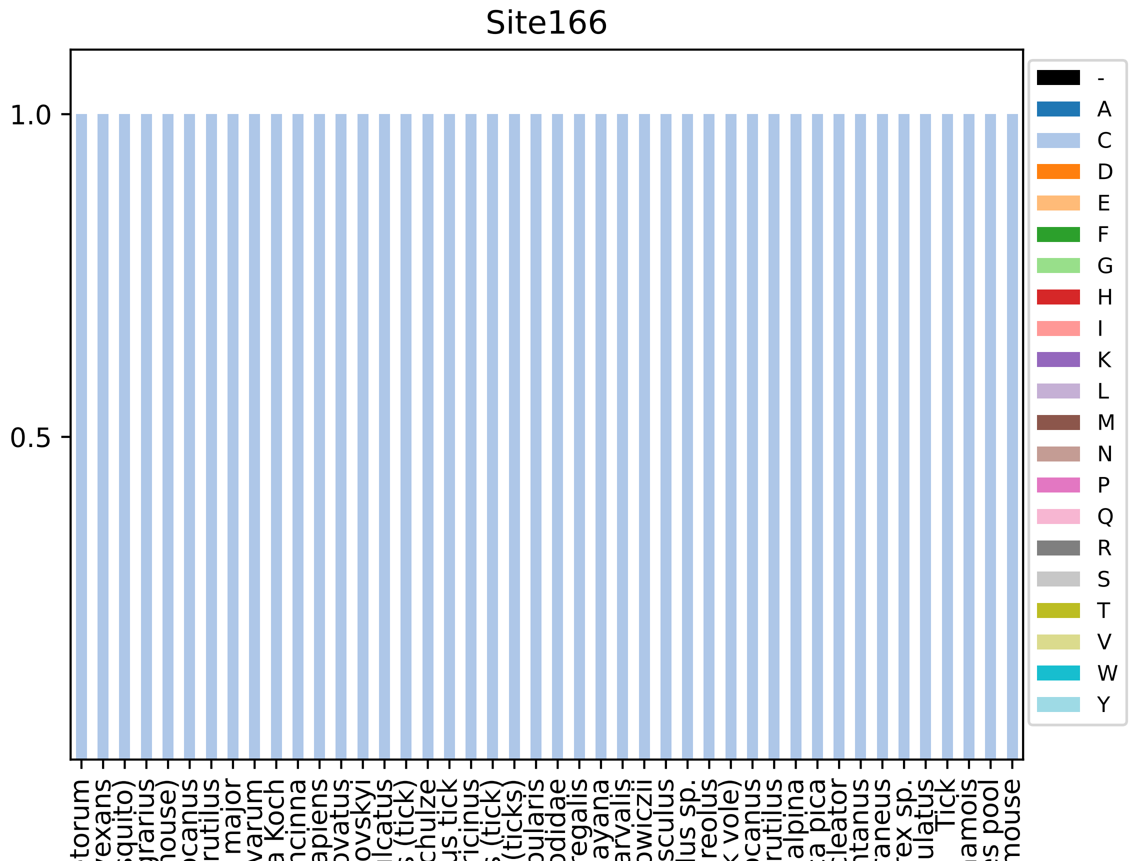
Site166
1.0
0.5
-
A
C
D
E
F
G
H
I
K
L
M
N
P
Q
R
S
T
V
W
Y

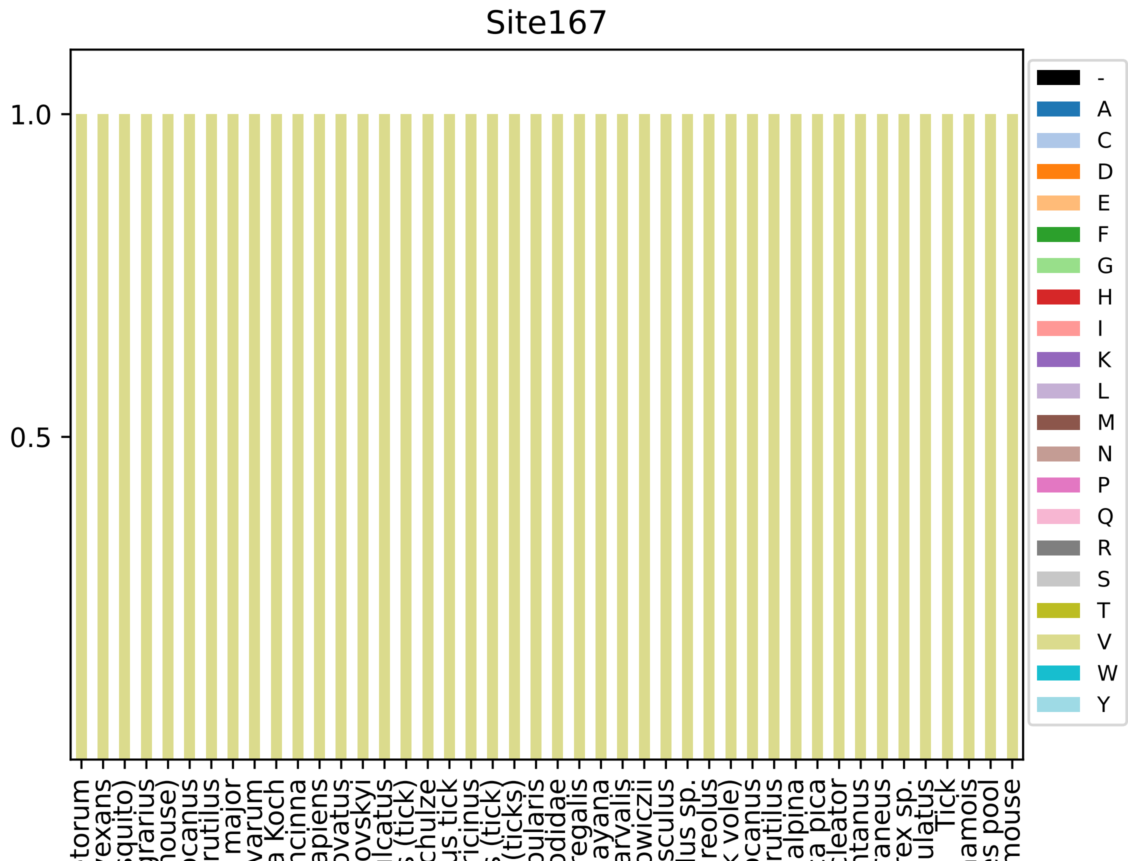
Site167
1.0
0.5
-
A
C
D
E
F
G
H
I
K
L
M
N
P
Q
R
S
T
V
W
Y

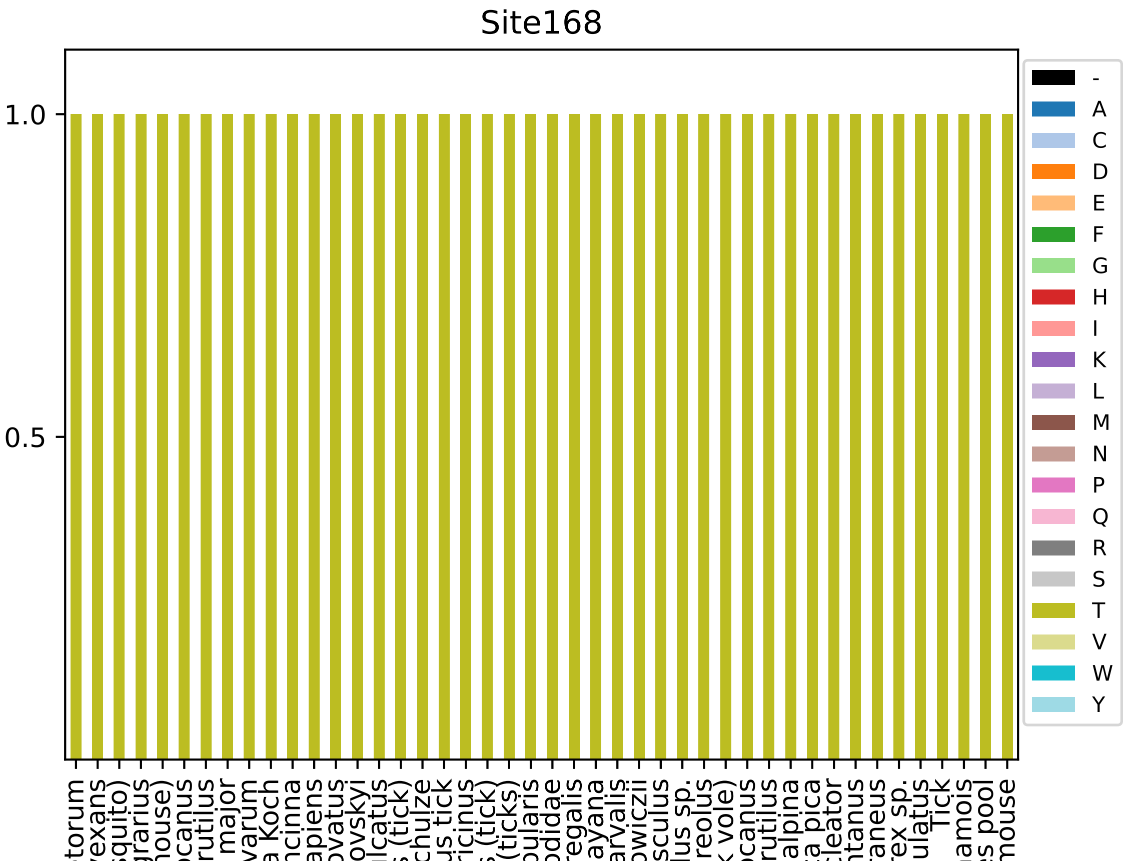
Site168
1.0
0.5
-
A
C
D
E
F
G
H
I
K
L
M
N
P
Q
R
S
T
V
W
Y

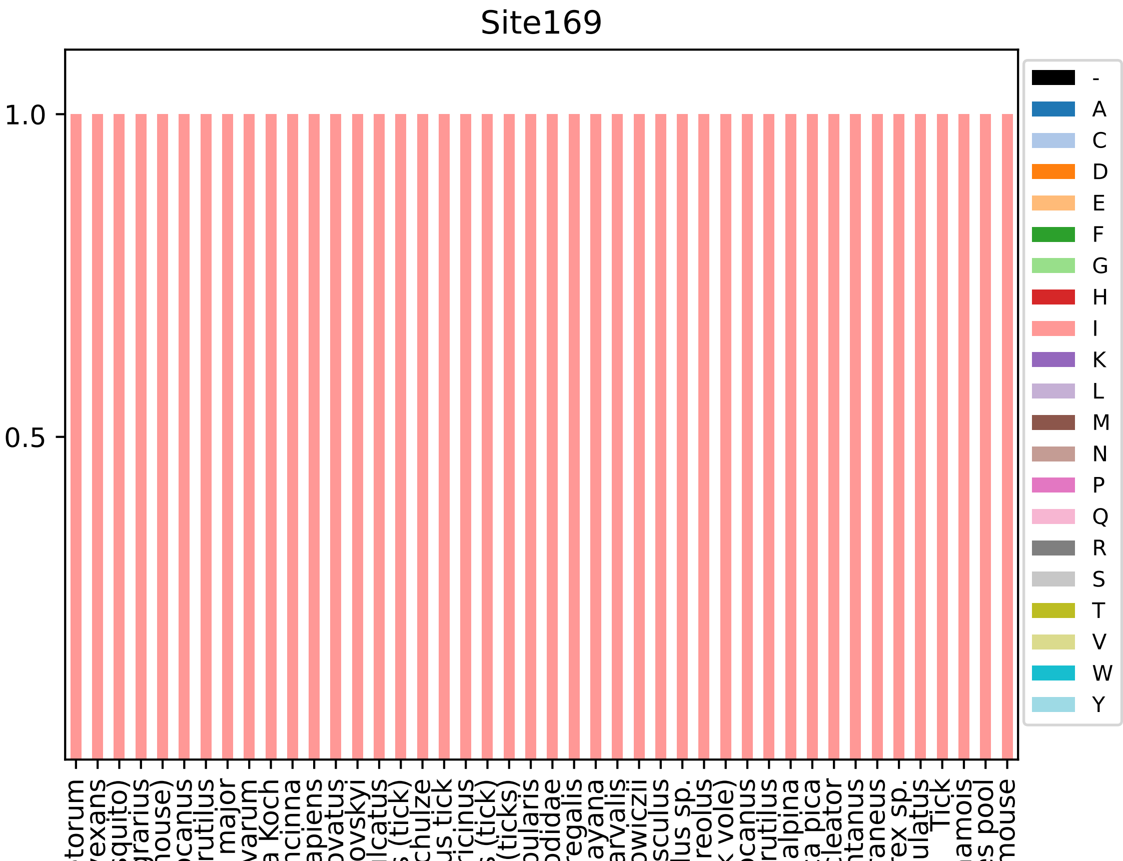

Site169
1.0
0.5
-
A
C
D
E
F
G
H
I
K
L
M
N
P
Q
R
S
T
V
W
Y

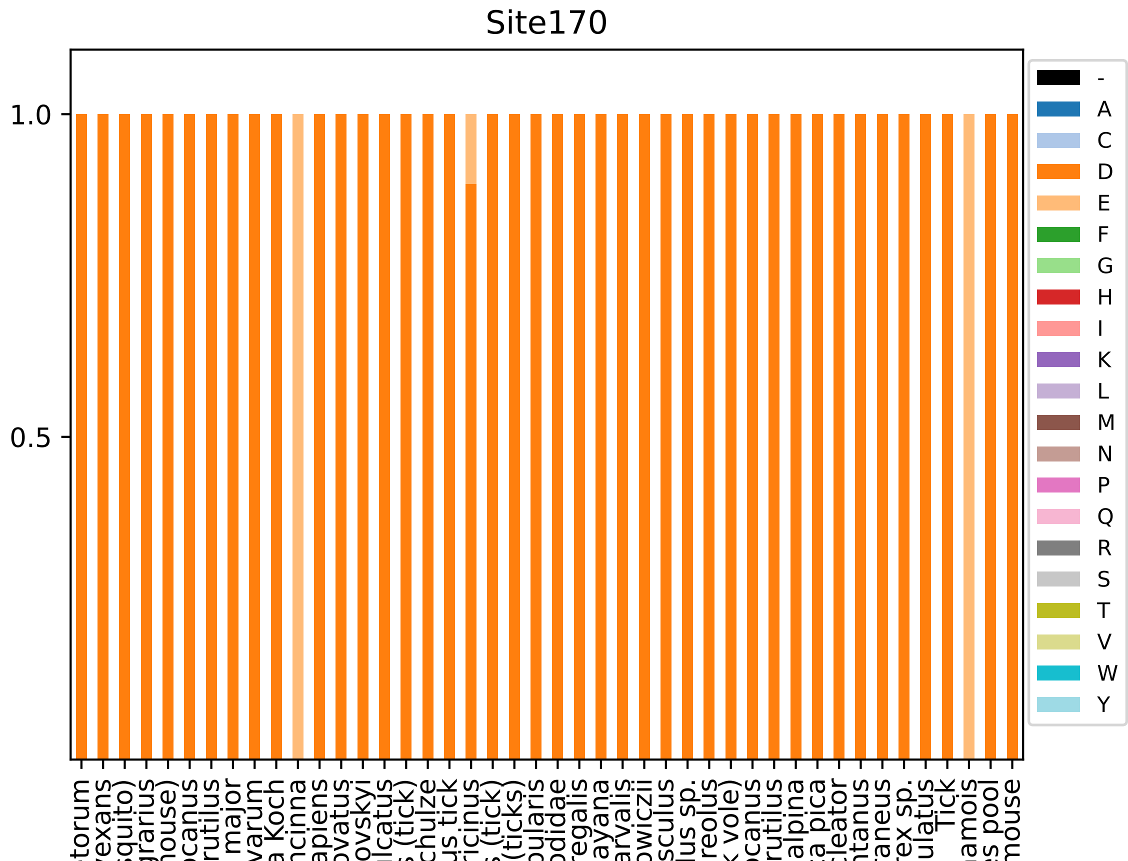

Site170
1.0
0.5
-
A
C
D
E
F
G
H
I
K
L
M
N
P
Q
R
S
T
V
W
Y

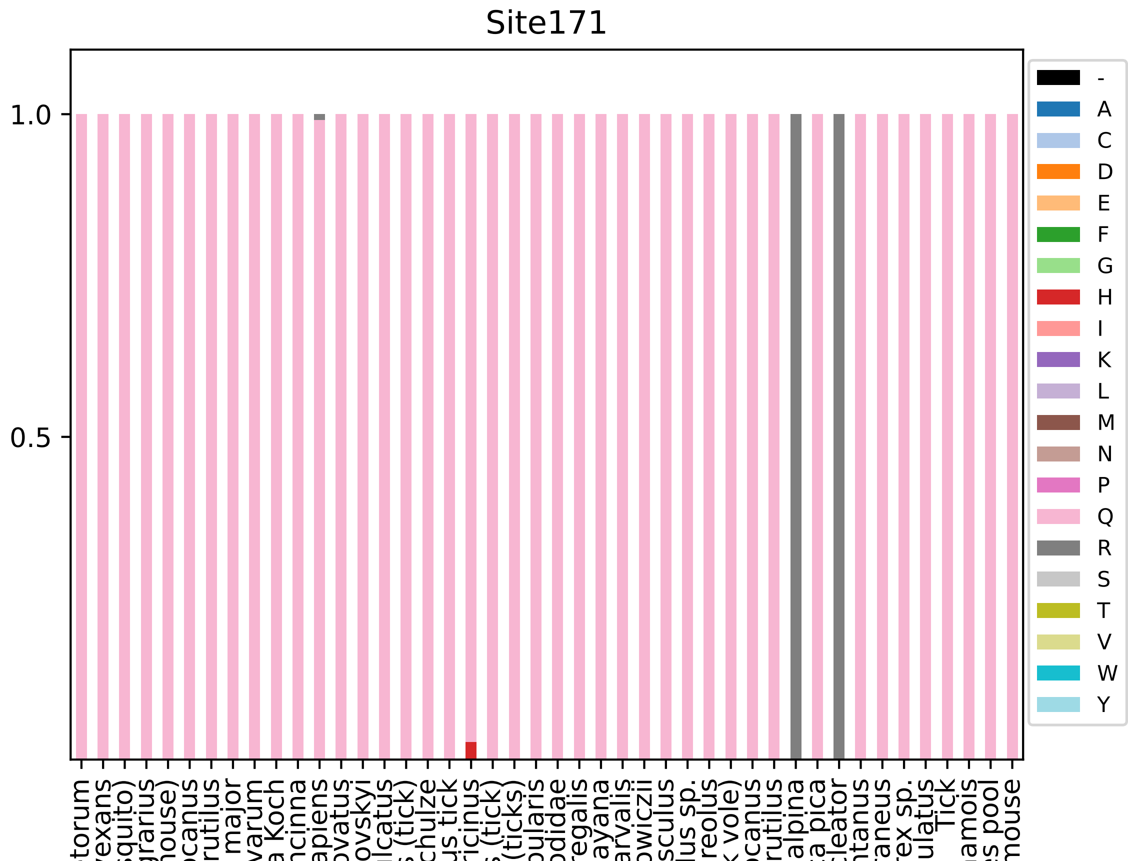
Site171
1.0
0.5
-
A
C
D
E
F
G
H
I
K
L
M
N
P
Q
R
S
T
V
W
Y

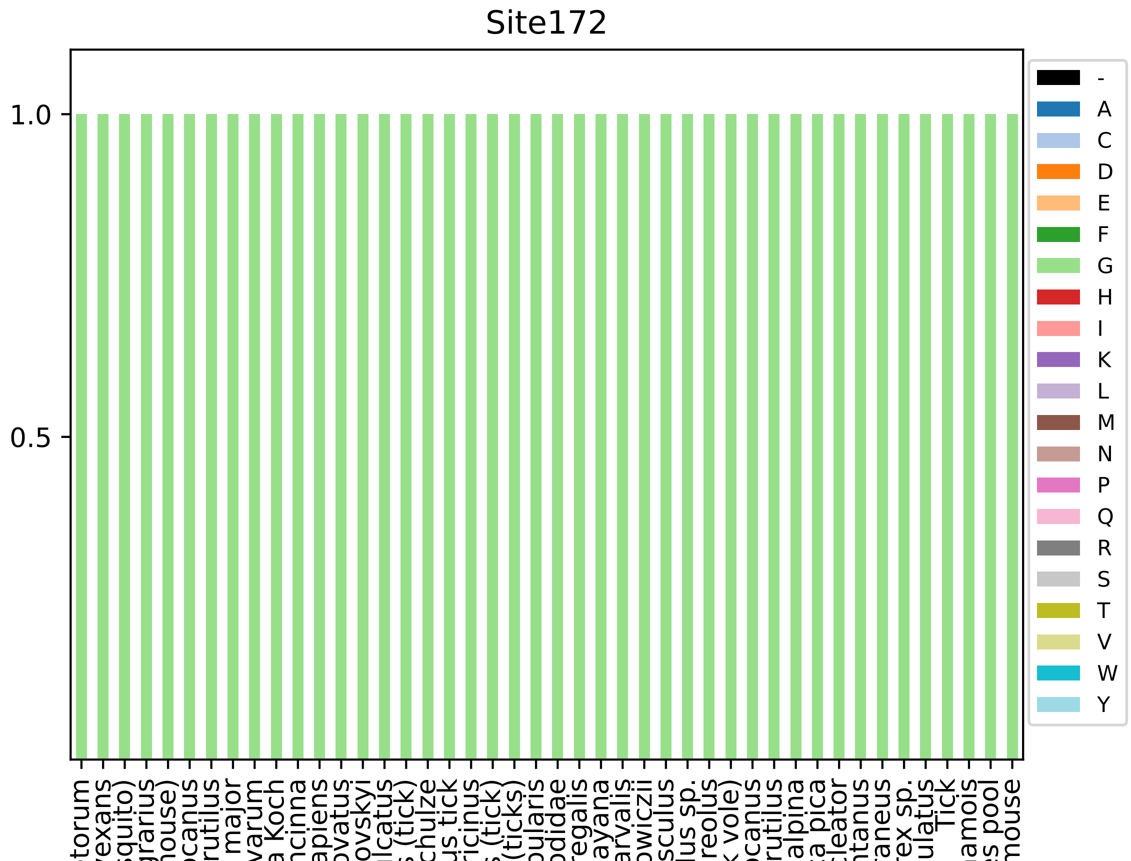
Site172
1.0
0.5
-
A
C
D
E
F
G
H
I
K
L
M
N
P
Q
R
S
T
V
W
Y
Tick

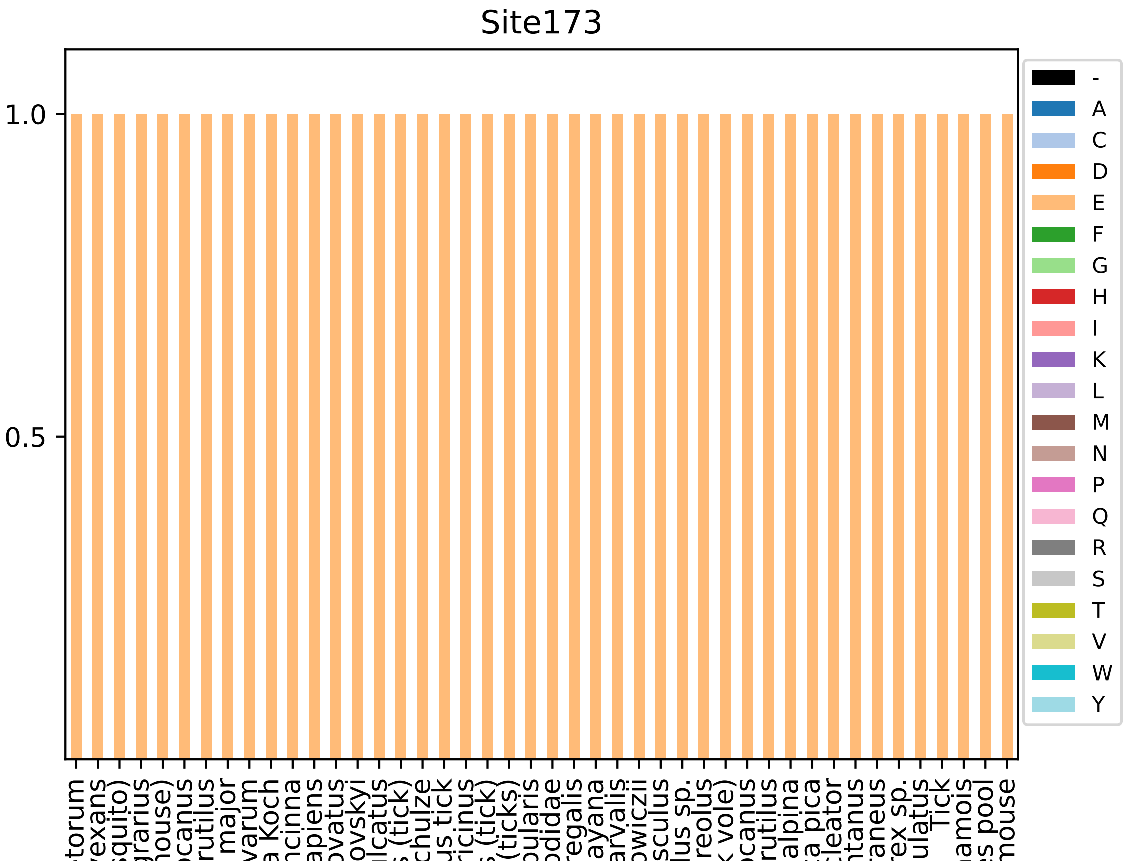
Site173
1.0
0.5
-
A
C
D
E
F
G
H
I
K
L
M
N
P
Q
R
S
T
V
W
Y

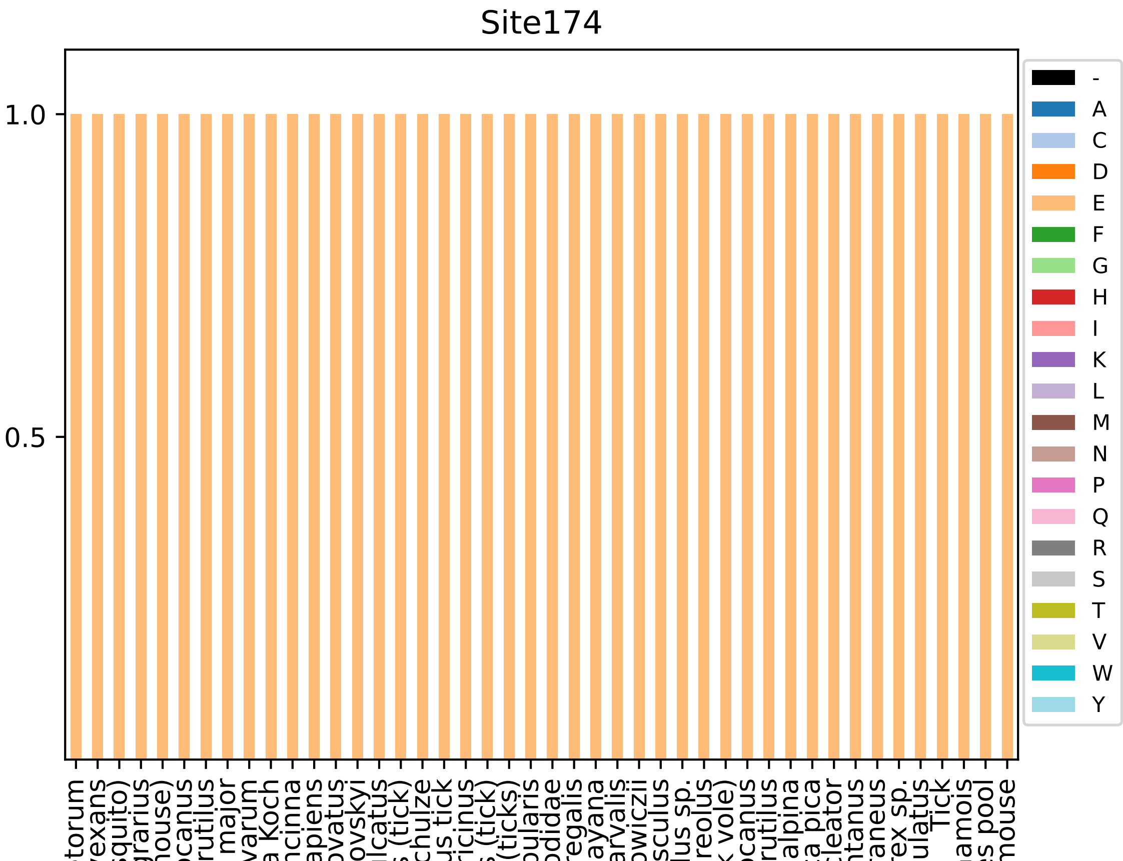
Site174
1.0
0.5
-
A
C
D
E
F
G
H
I
K
L
M
N
P
Q
R
S
T
V
W
Y

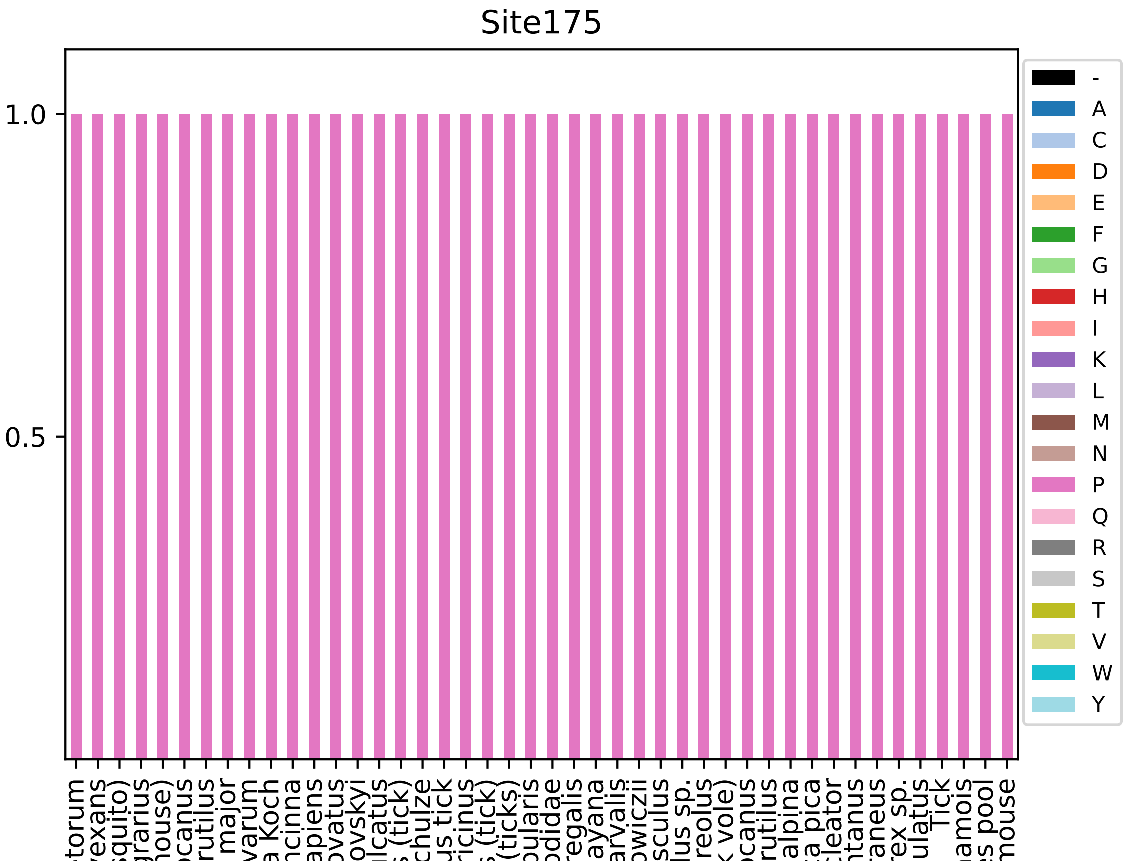

Site176

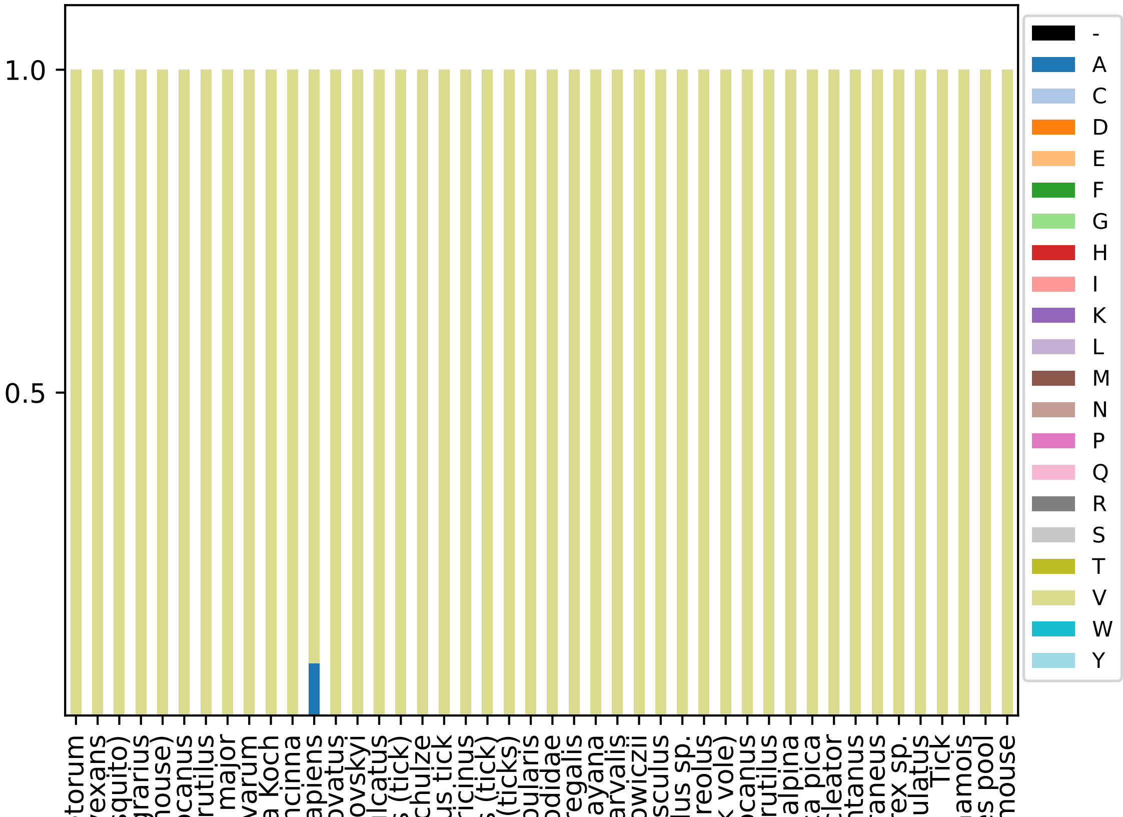

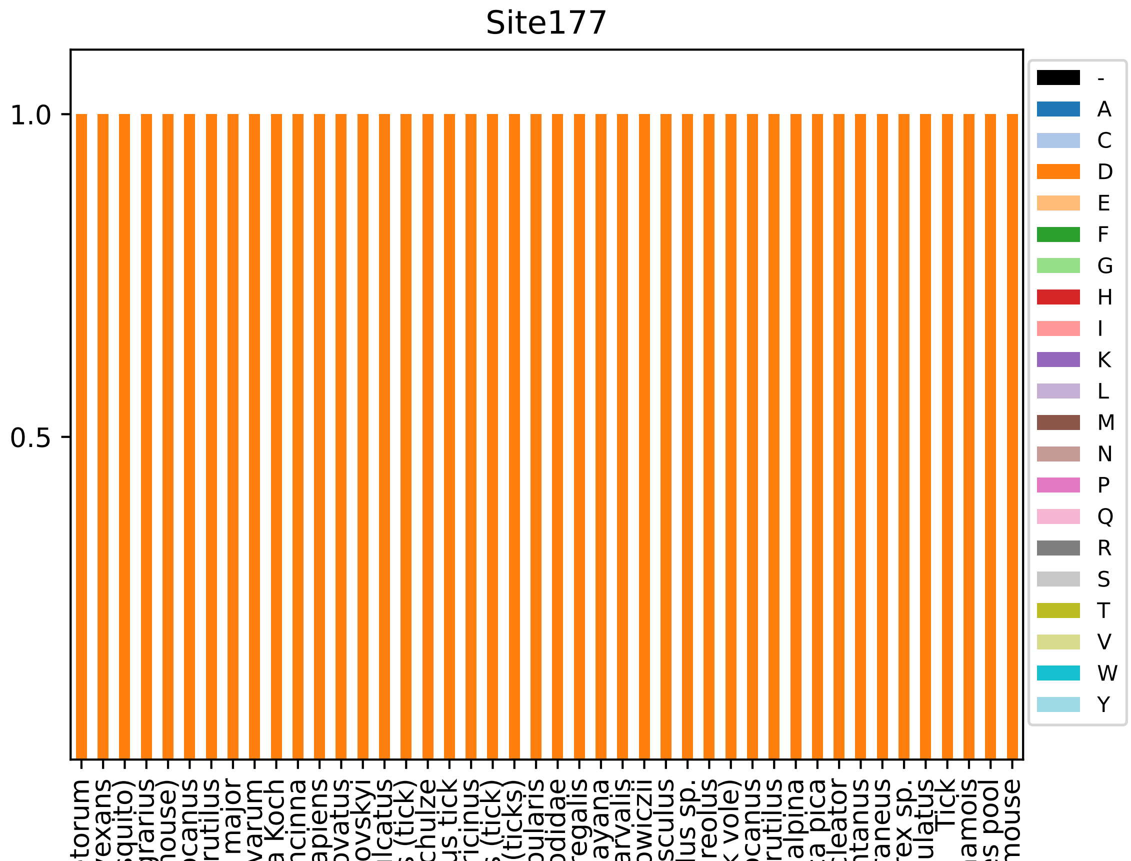
Site177
1.0
0.5
-
A
C
D
E
F
G
H
I
K
L
M
N
P
Q
R
S
T
V
W
Y

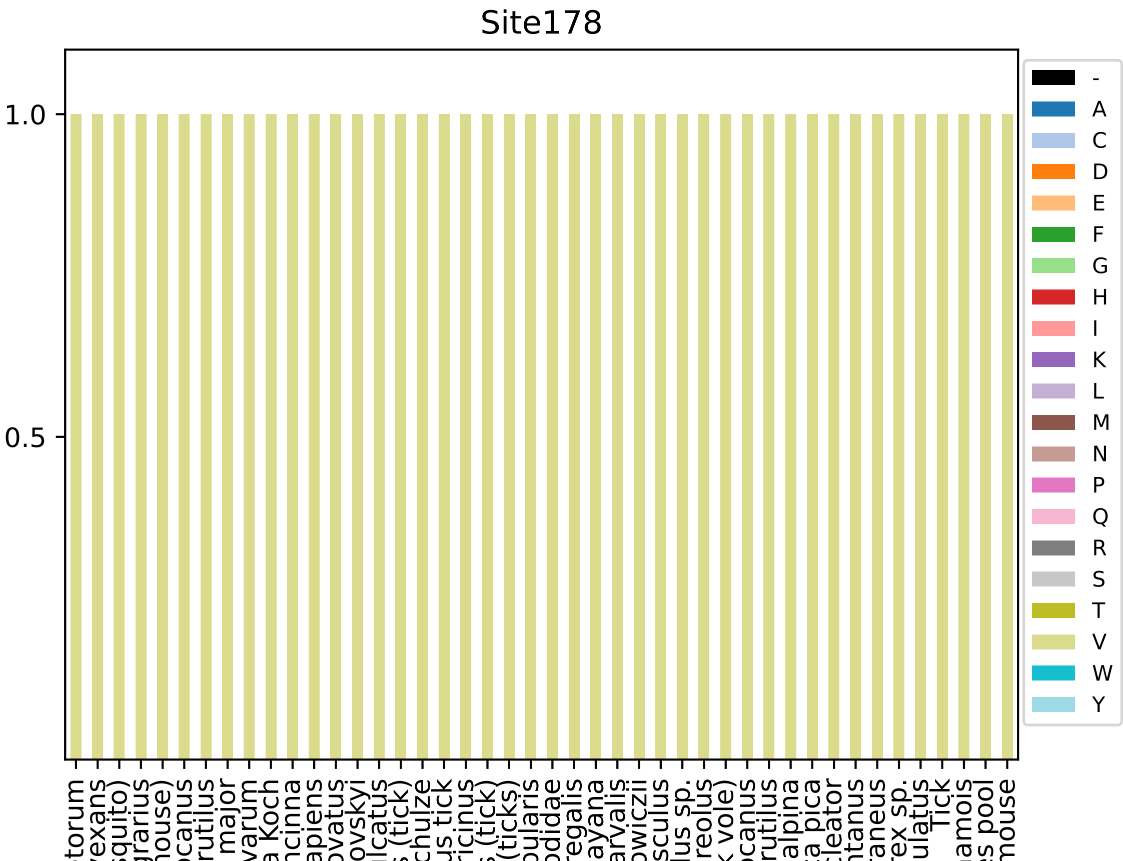
Site178
1.0
0.5
-
A
C
D
E
F
G
H
I
K
L
M
N
P
Q
R
S
T
V
W
Y

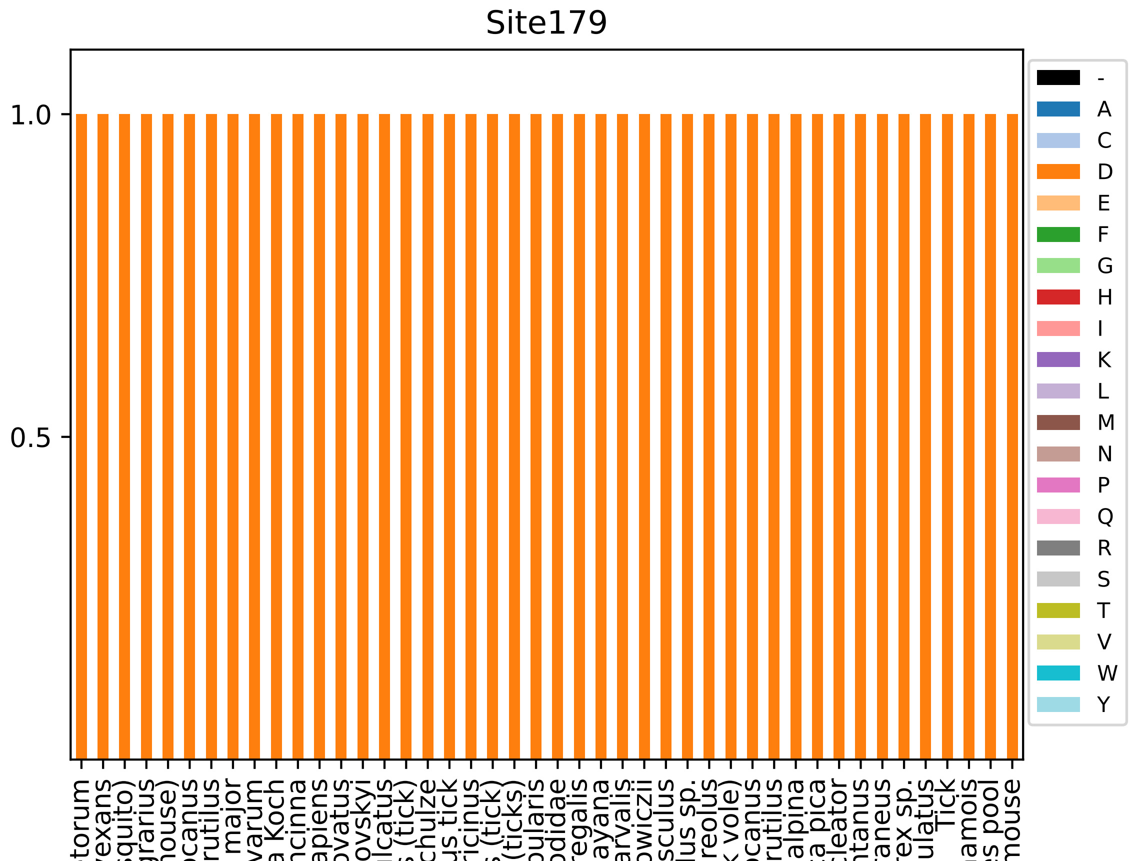
Site179
1.0
0.5
-
A
C
D
E
F
G
H
I
K
L
M
N
P
Q
R
S
T
V
W
Y

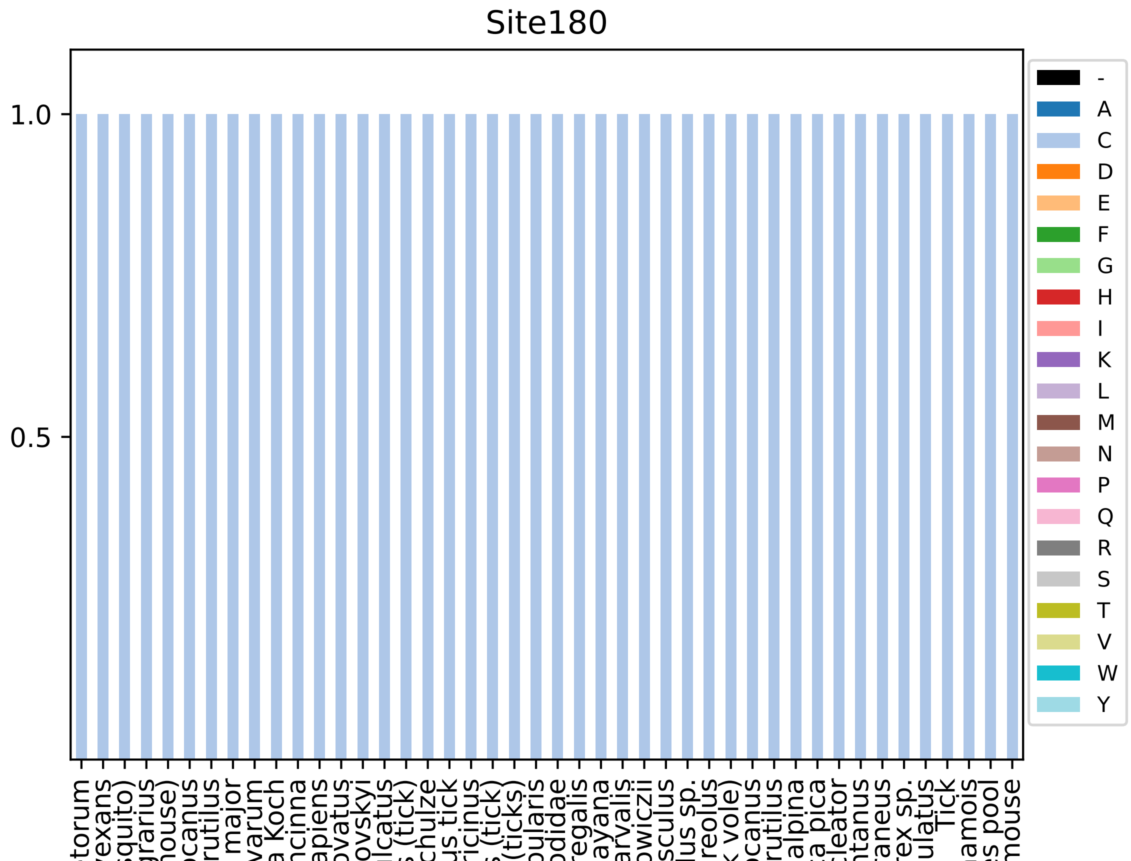
Site180
1.0
0.5
-
A
C
D
E
F
G
H
I
K
L
M
N
P
Q
R
S
T
V
W
Y

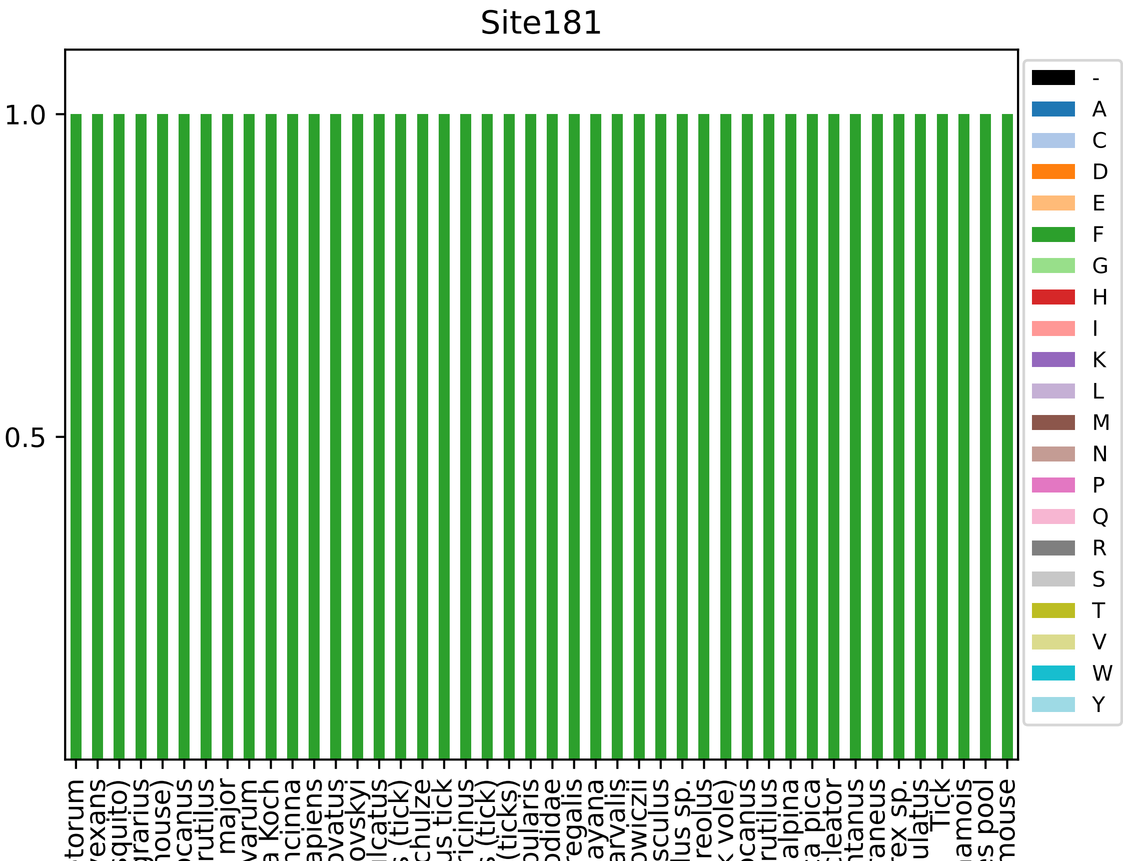
Site181
1.0
0.5
-
A
C
D
E
F
G
H
I
K
L
M
N
P
Q
R
S
T
V
W
Y

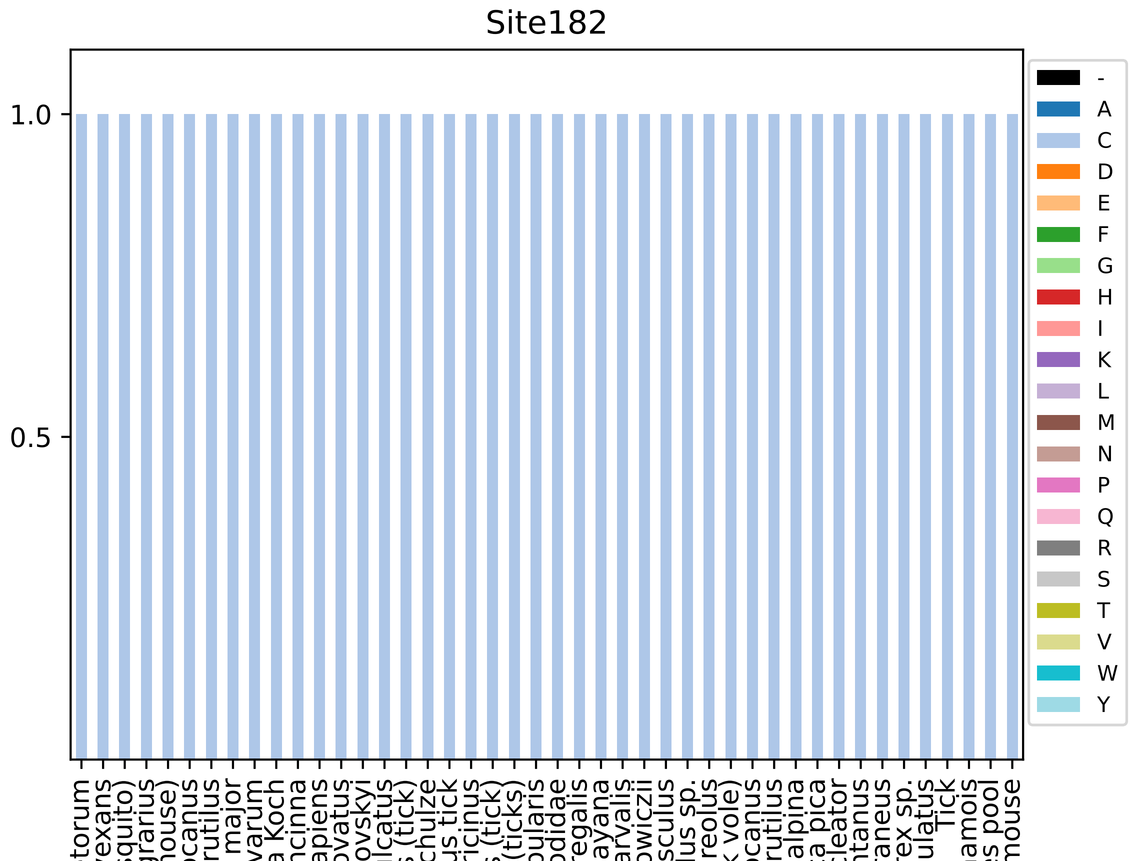

Site182
1.0
0.5
-
A
C
D
E
F
G
H
I
K
L
M
N
P
Q
R
S
T
V
W
Y

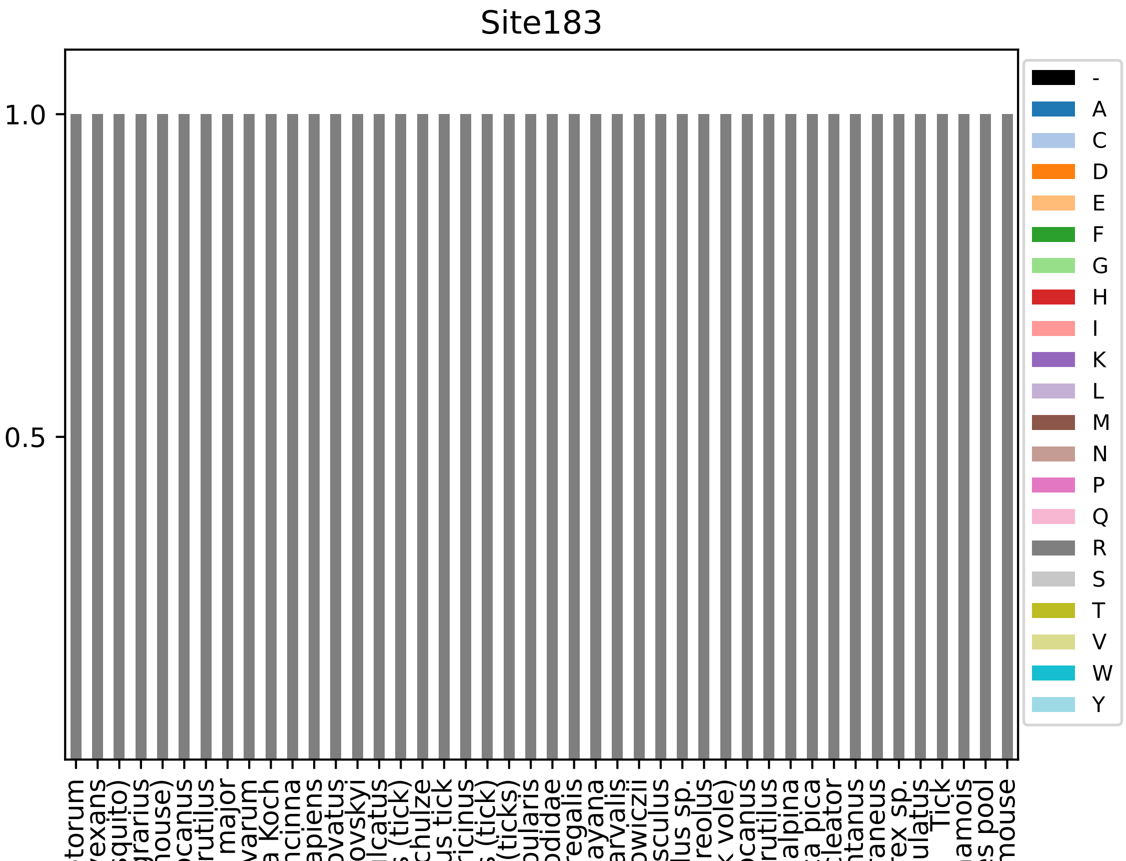
Site183
1.0
0.5
-
A
C
D
E
F
G
H
I
K
L
M
N
P
Q
R
S
T
V
W
Y

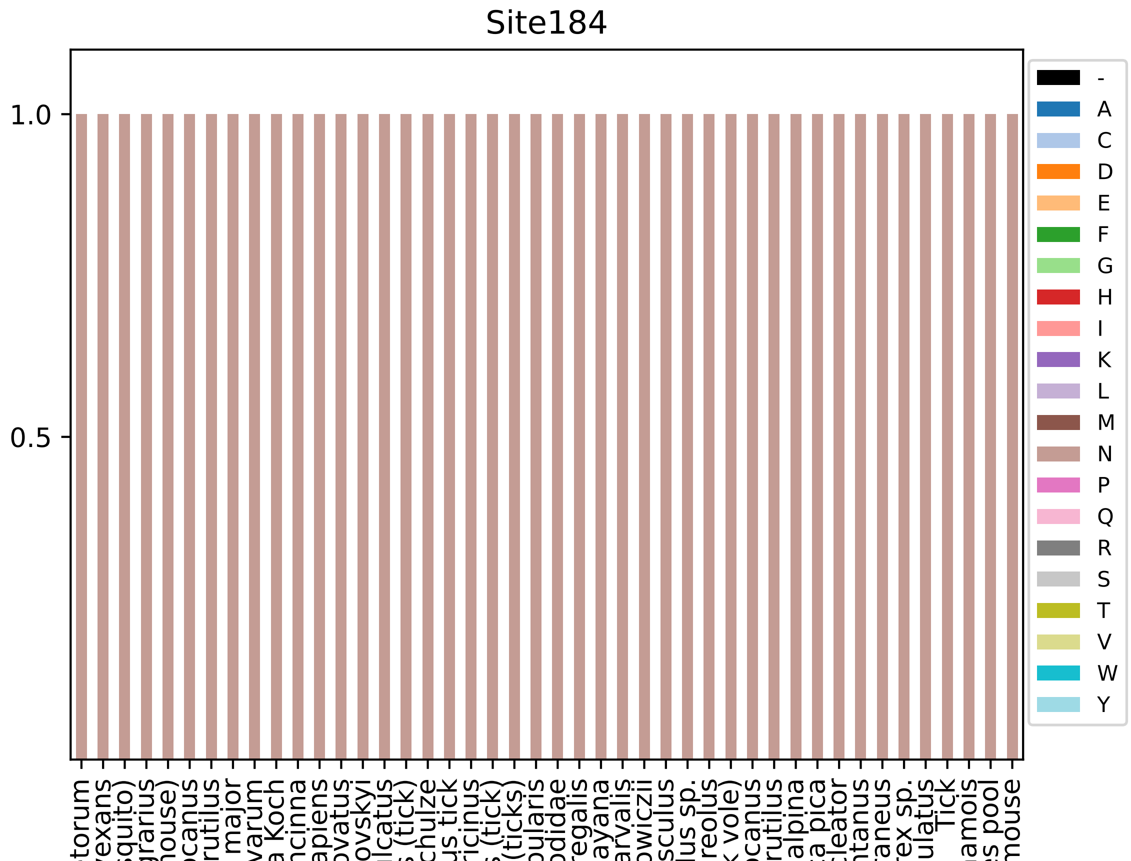
Site184
1.0
0.5
-
A
C
D
E
F
G
H
I
K
L
M
N
P
Q
R
S
T
V
W
Y

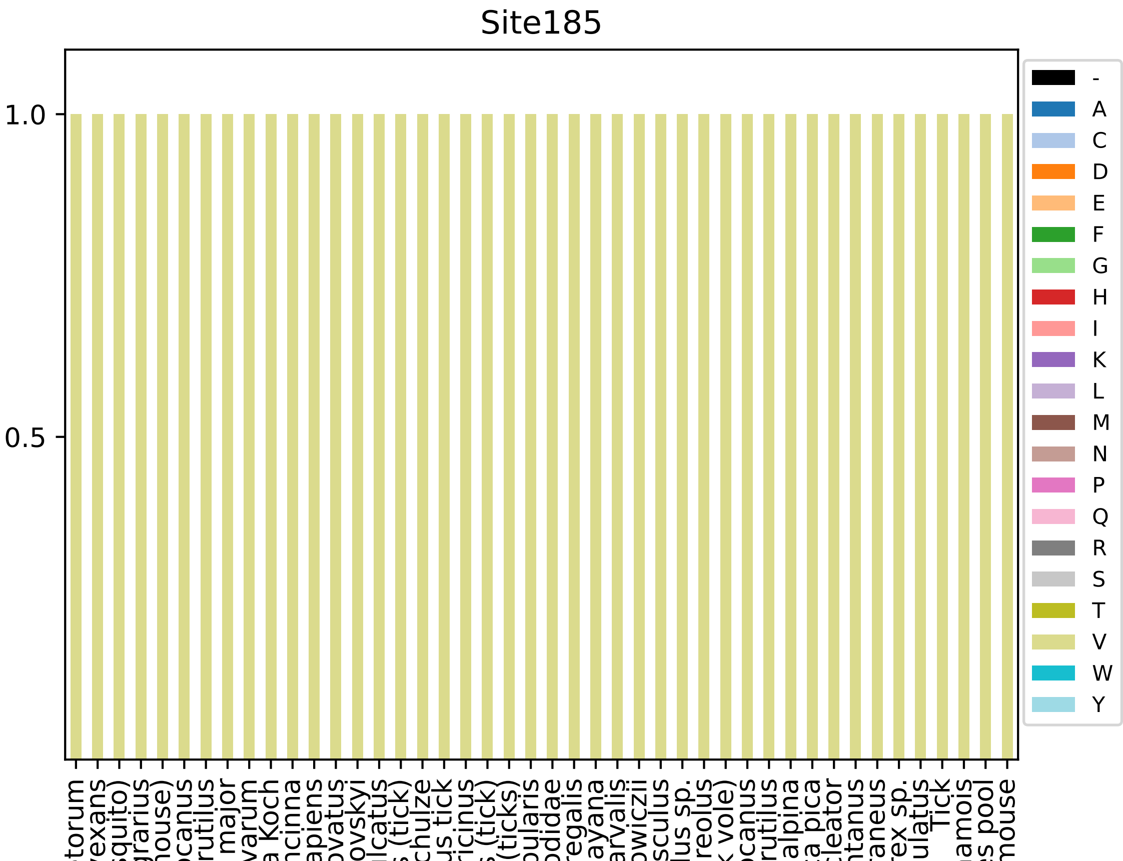
Site185
1.0
0.5
-
A
C
D
E
F
G
H
I
K
L
M
N
P
Q
R
S
T
V
W
Y

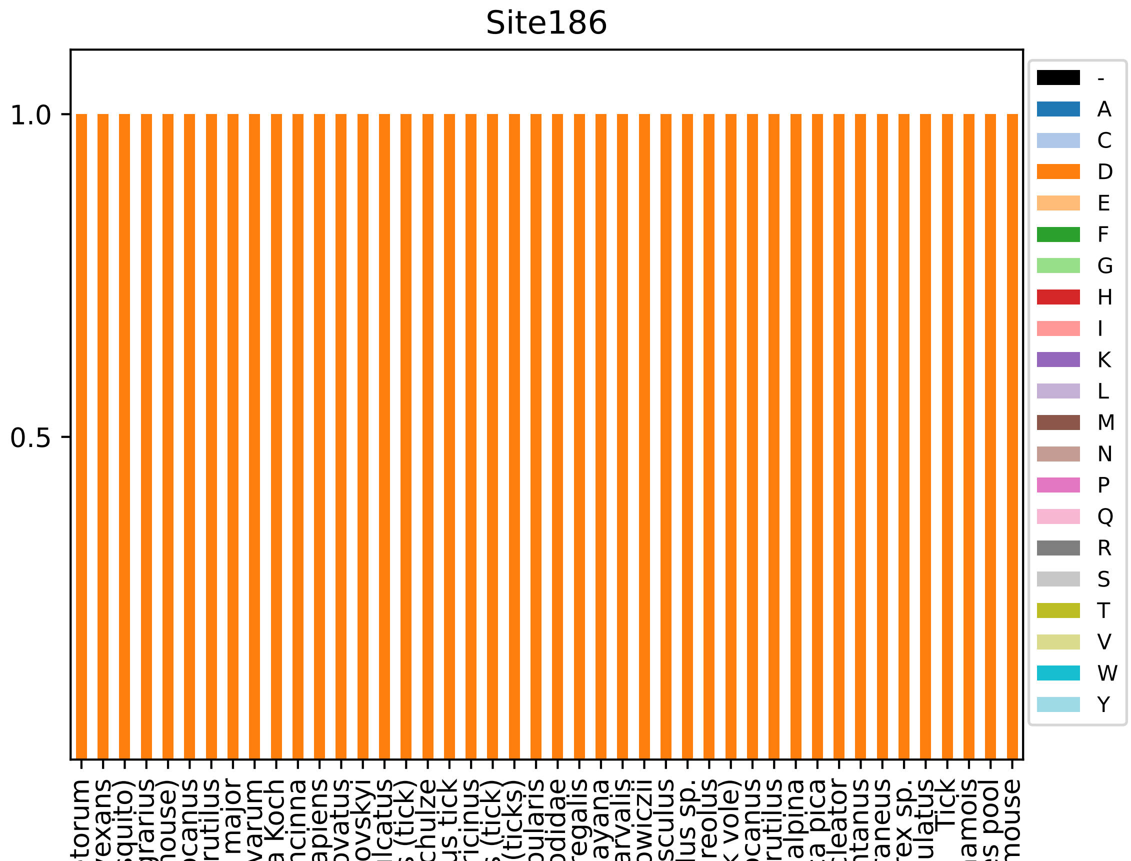
Site186
1.0
0.5
-
A
C
D
E
F
G
H
I
K
L
M
N
P
Q
R
S
T
V
W
Y

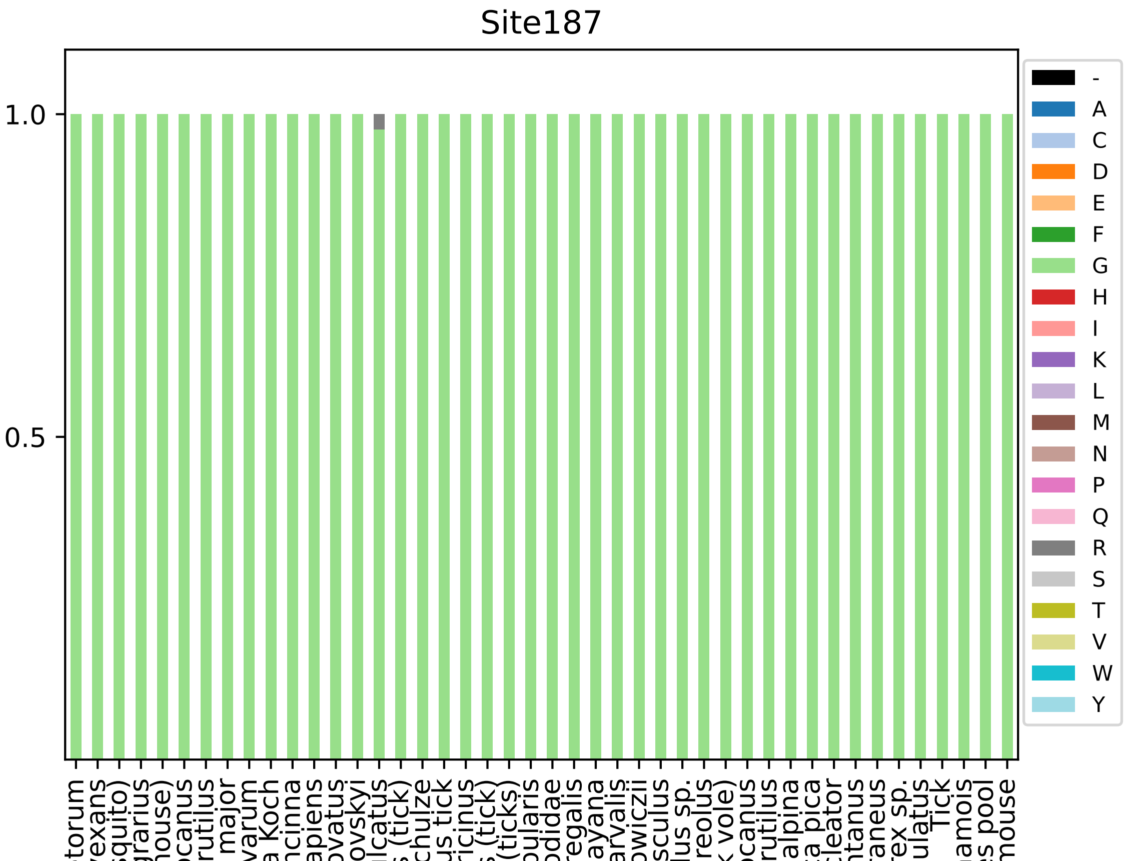
Site187
1.0
0.5
-
A
C
D
E
F
G
H
I
K
L
M
N
P
Q
R
S
T
V
W
Y
Tick

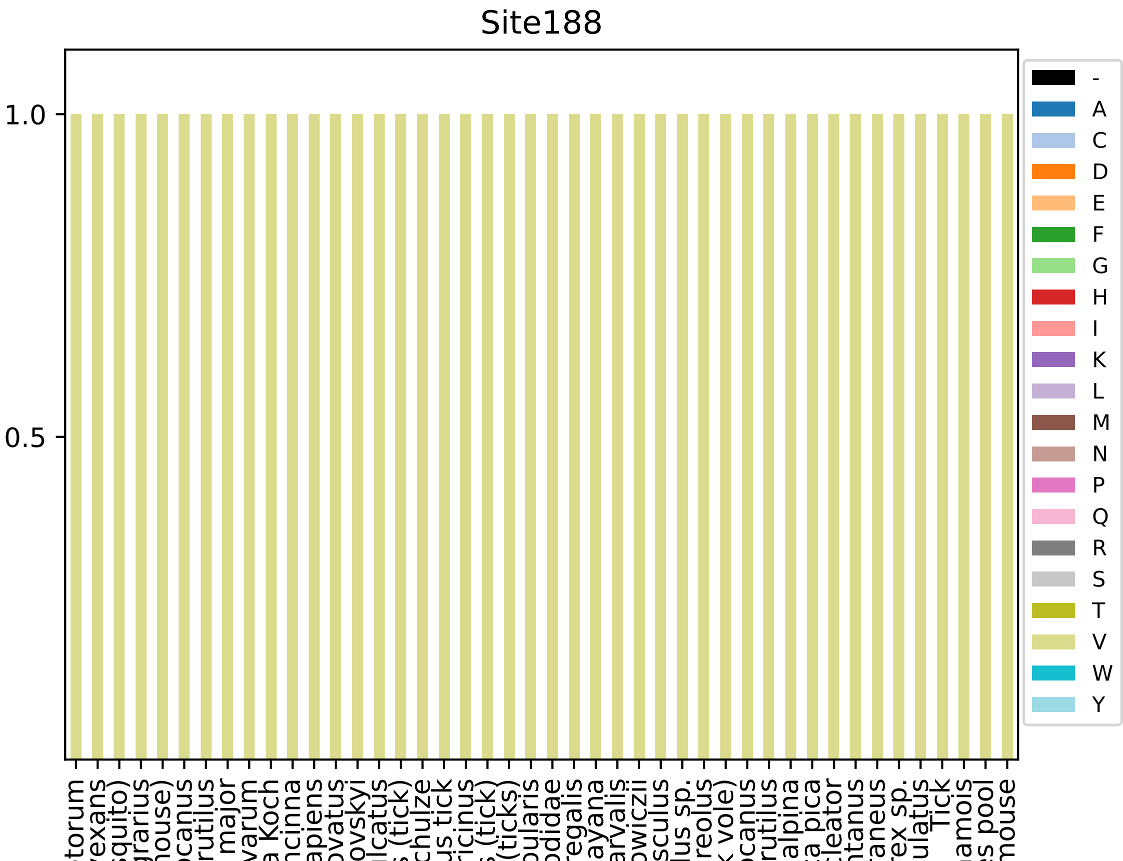

Site188
1.0
0.5
-
A
C
D
E
F
G
H
I
K
L
M
N
P
Q
R
S
T
V
W
Y

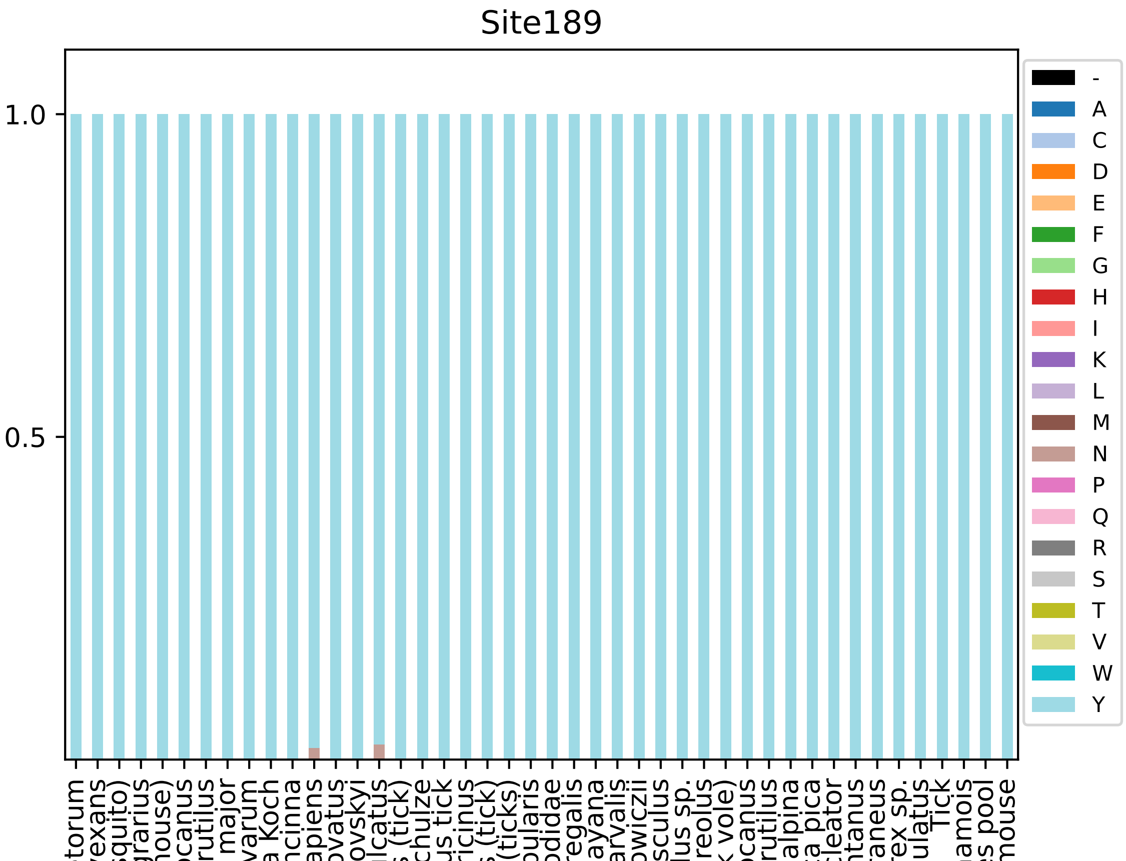

Site189
1.0
0.5
-
A
C
D
E
F
G
H
I
K
L
M
N
P
Q
R
S
T
V
W
Y

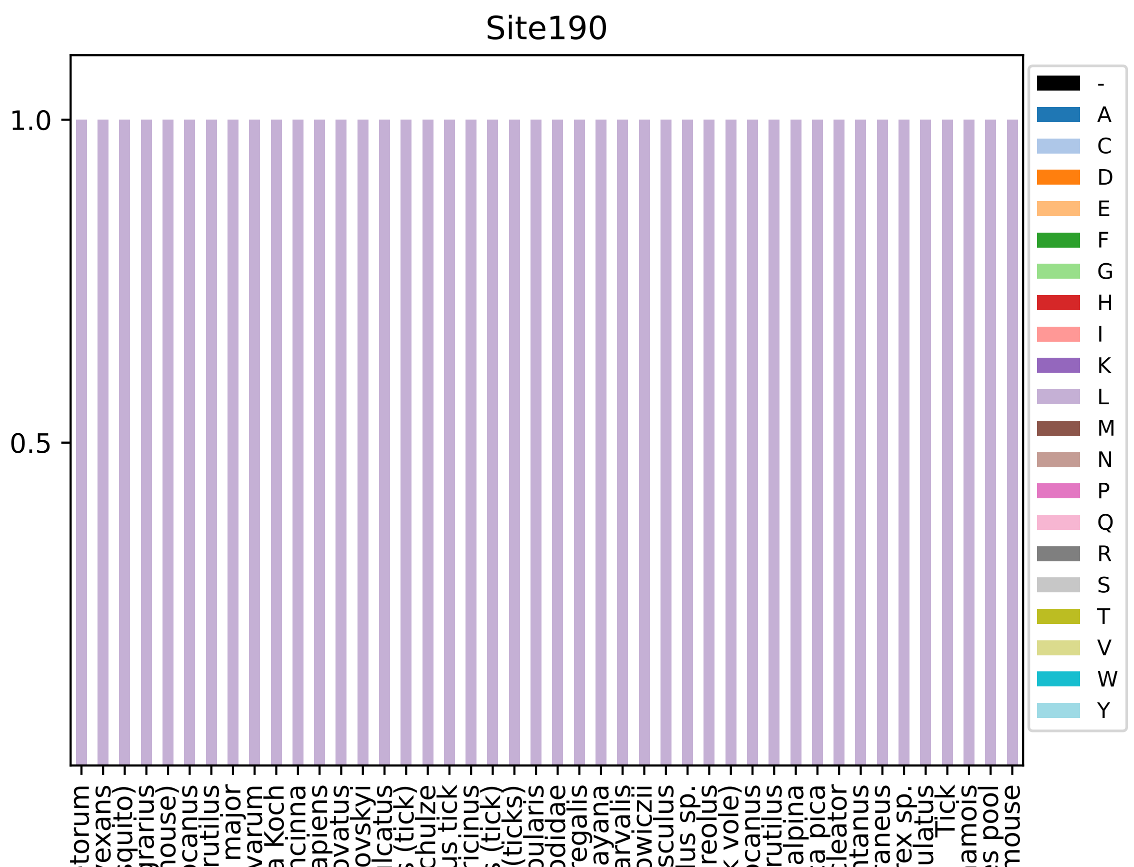
Site190
1.0
0.5
-
A
C
D
E
F
G
H
I
K
L
M
N
P
Q
R
S
T
V
W
Y

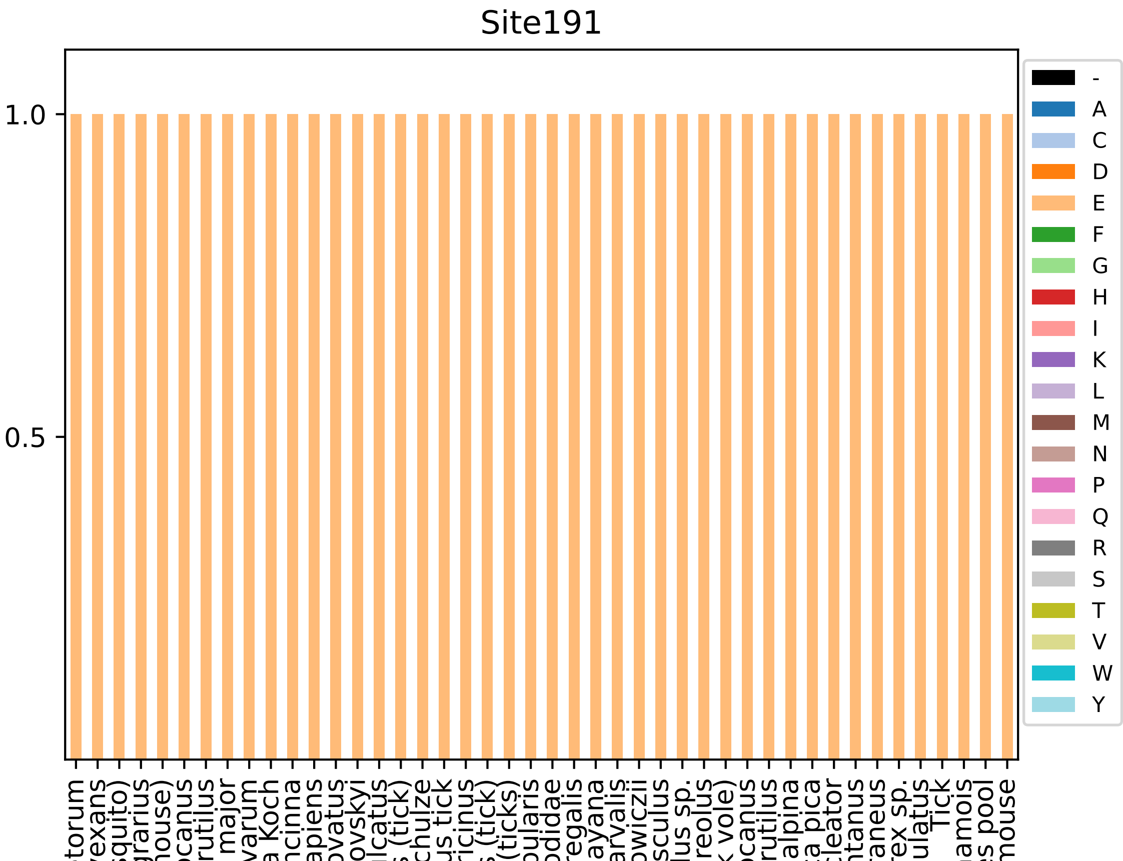
Site191
1.0
0.5
-
A
C
D
E
F
G
H
I
K
L
M
N
P
Q
R
S
T
V
W
Y

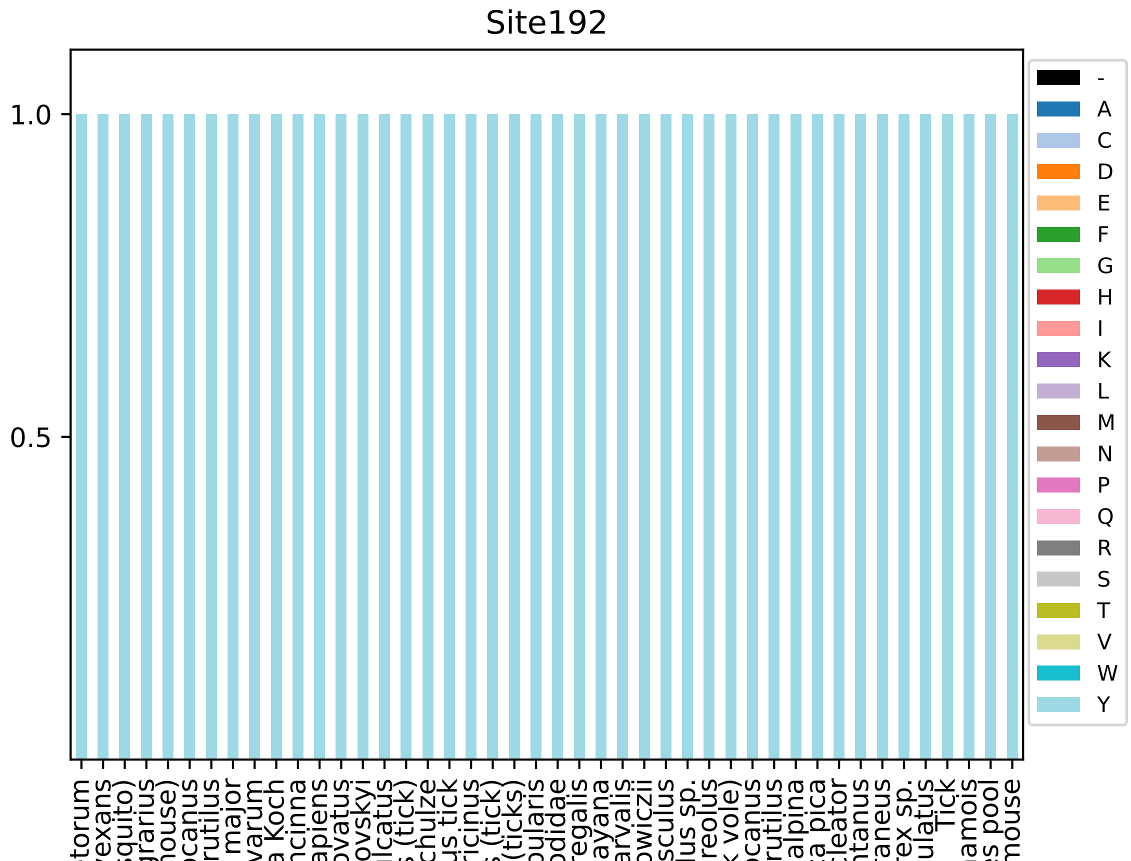

Site192
1.0
0.5
-
A
C
D
E
F
G
H
I
K
L
M
N
P
Q
R
S
T
V
W
Y

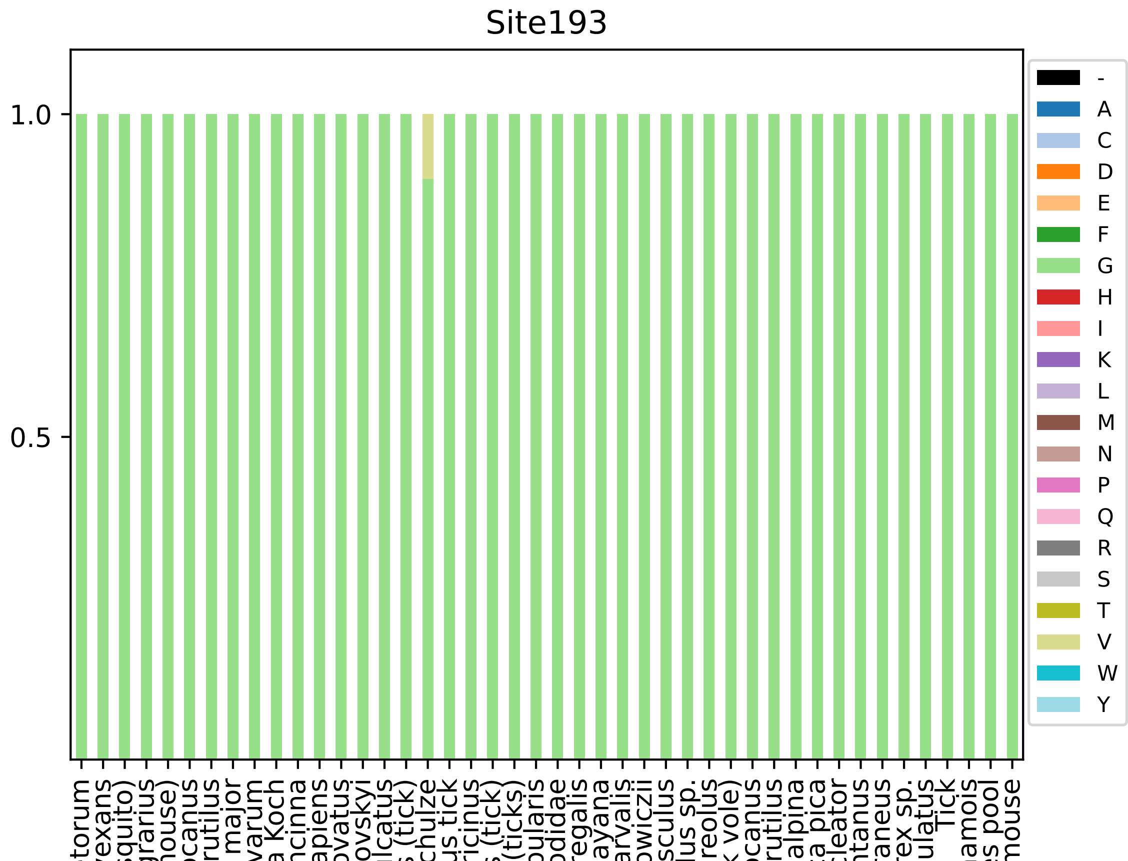
Site193
1.0
0.5
-
A
C
D
E
F
G
H
I
K
L
M
N
P
Q
R
S
T
V
W
Y

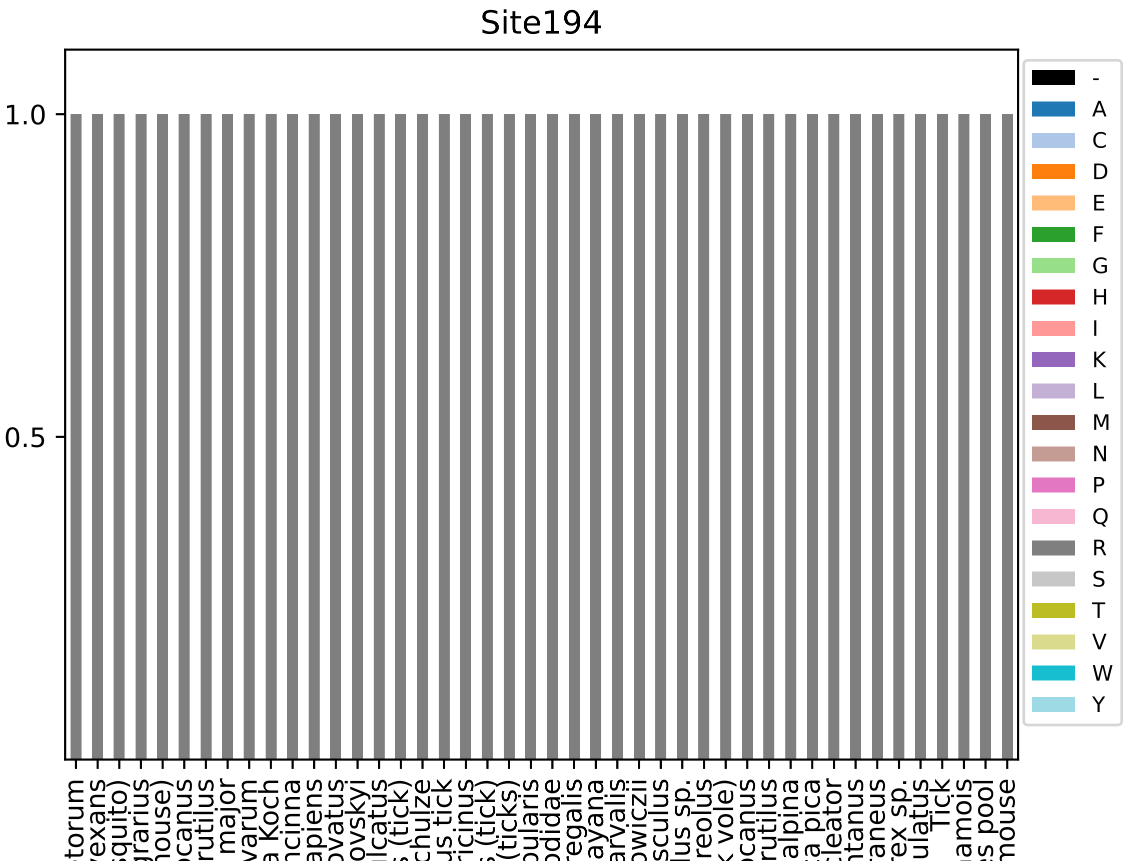

Site194
1.0
0.5
-
A
C
D
E
F
G
H
I
K
L
M
N
P
Q
R
S
T
V
W
Y

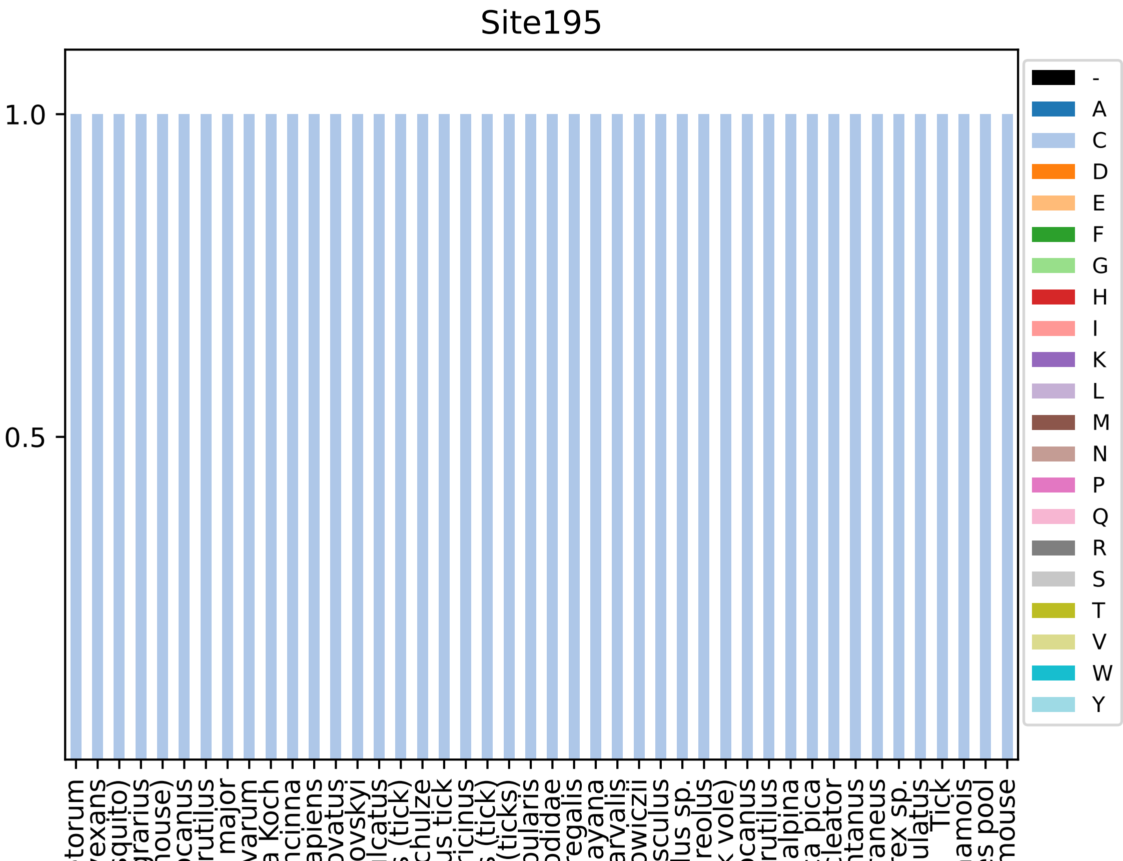
Site195
1.0
0.5
-
A
C
D
E
F
G
H
I
K
L
M
N
P
Q
R
S
T
V
W
Y

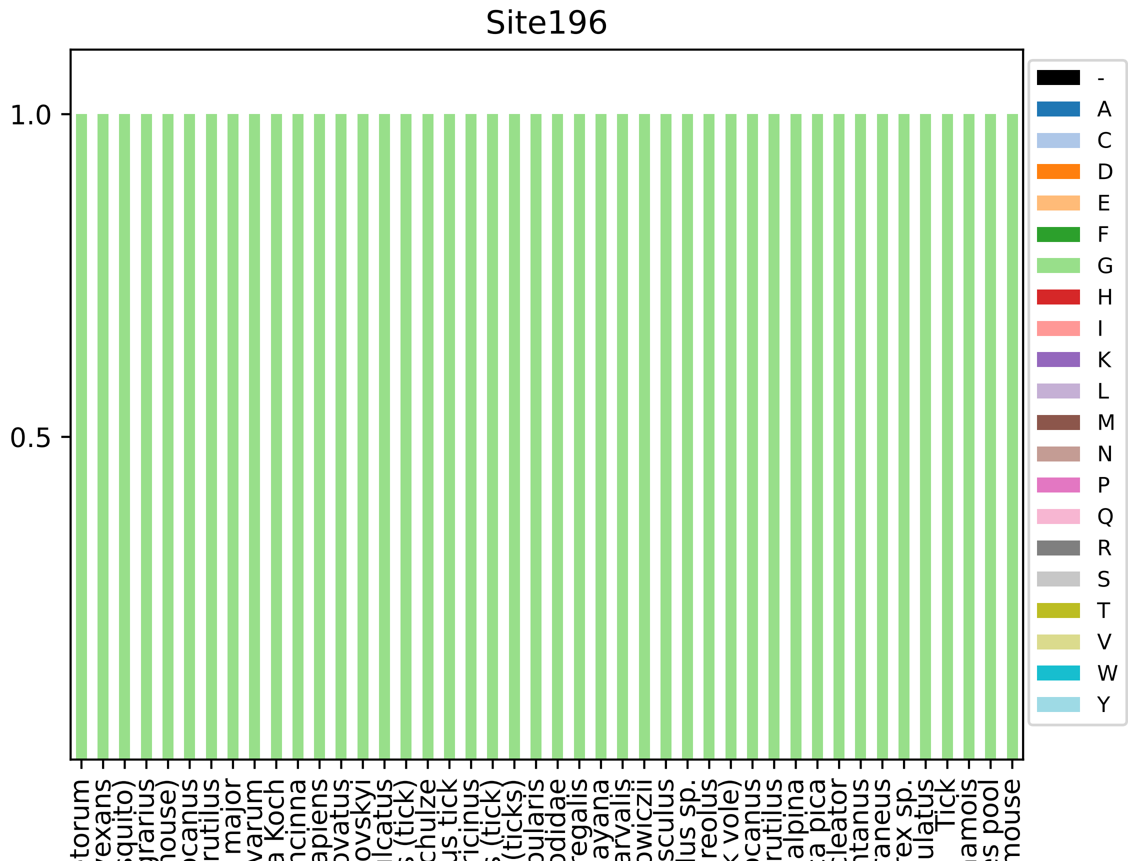

Site196
1.0
0.5
-
A
C
D
E
F
G
H
I
K
L
M
N
P
Q
R
S
T
V
W
Y

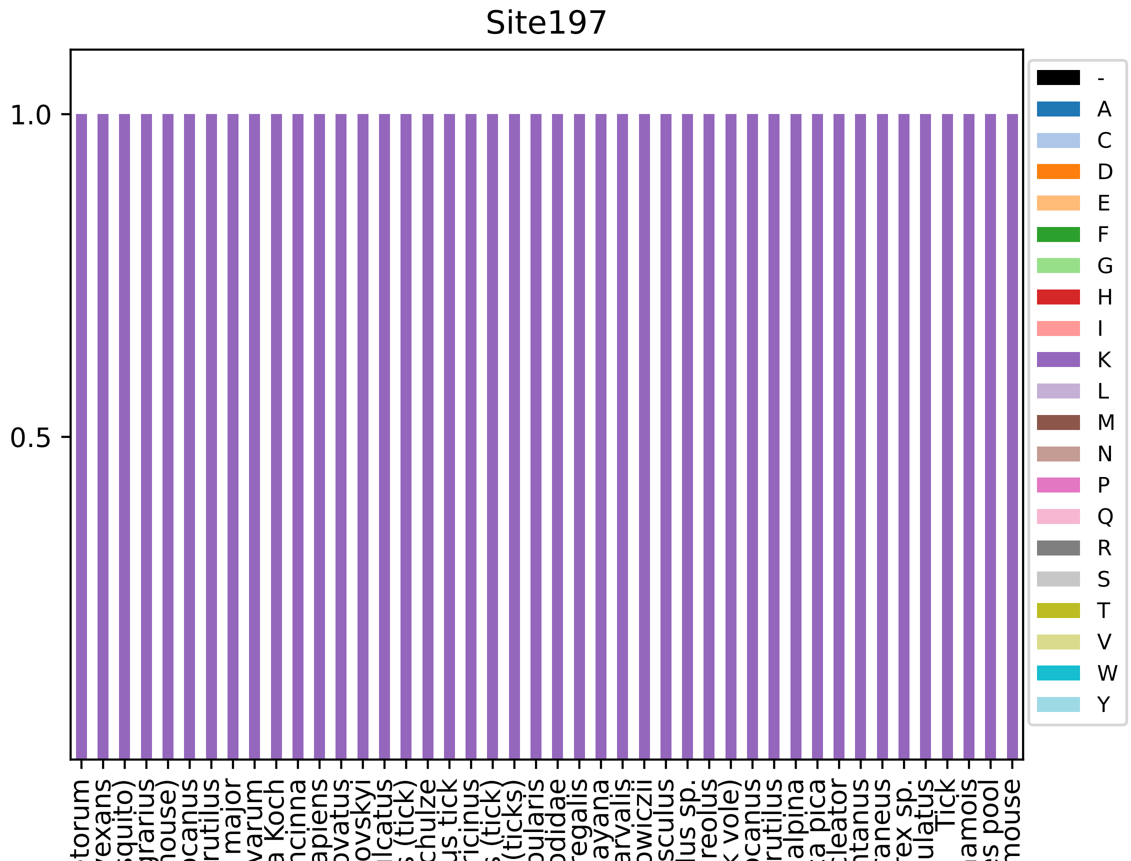
Site197
1.0
0.5
-
A
C
D
E
F
G
H
I
K
L
M
N
P
Q
R
S
T
V
W
Y

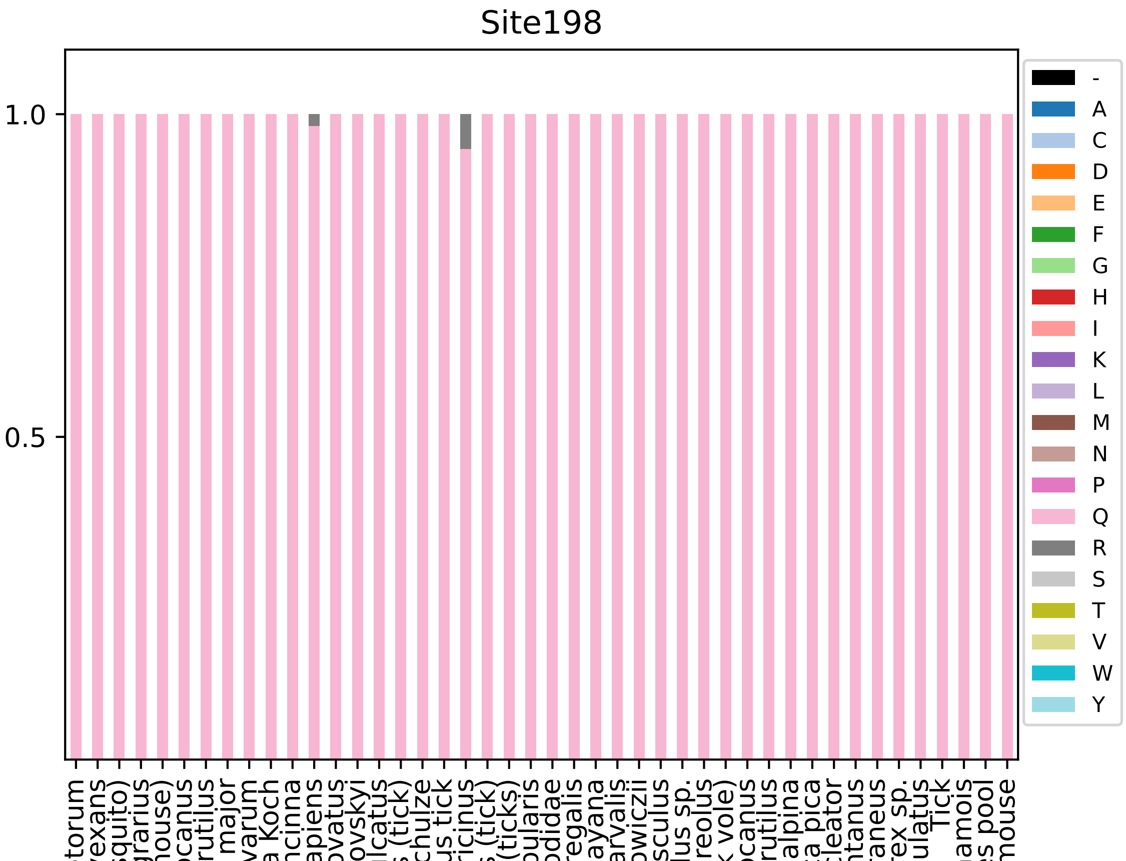
Site198
1.0
0.5
-
A
C
D
E
F
G
H
I
K
L
M
N
P
Q
R
S
T
V
W
Y

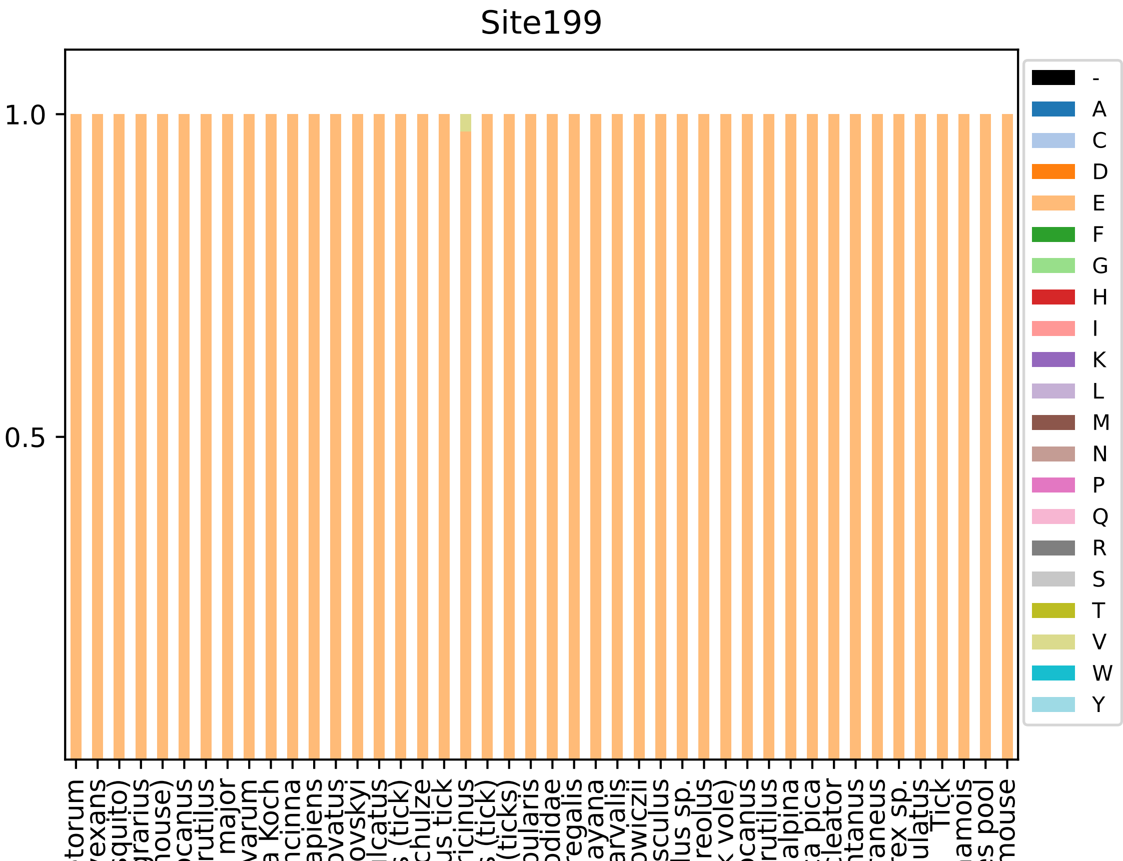

Site199
1.0
0.5
-
A
C
D
E
F
G
H
I
K
L
M
N
P
Q
R
S
T
V
W
Y
Tick

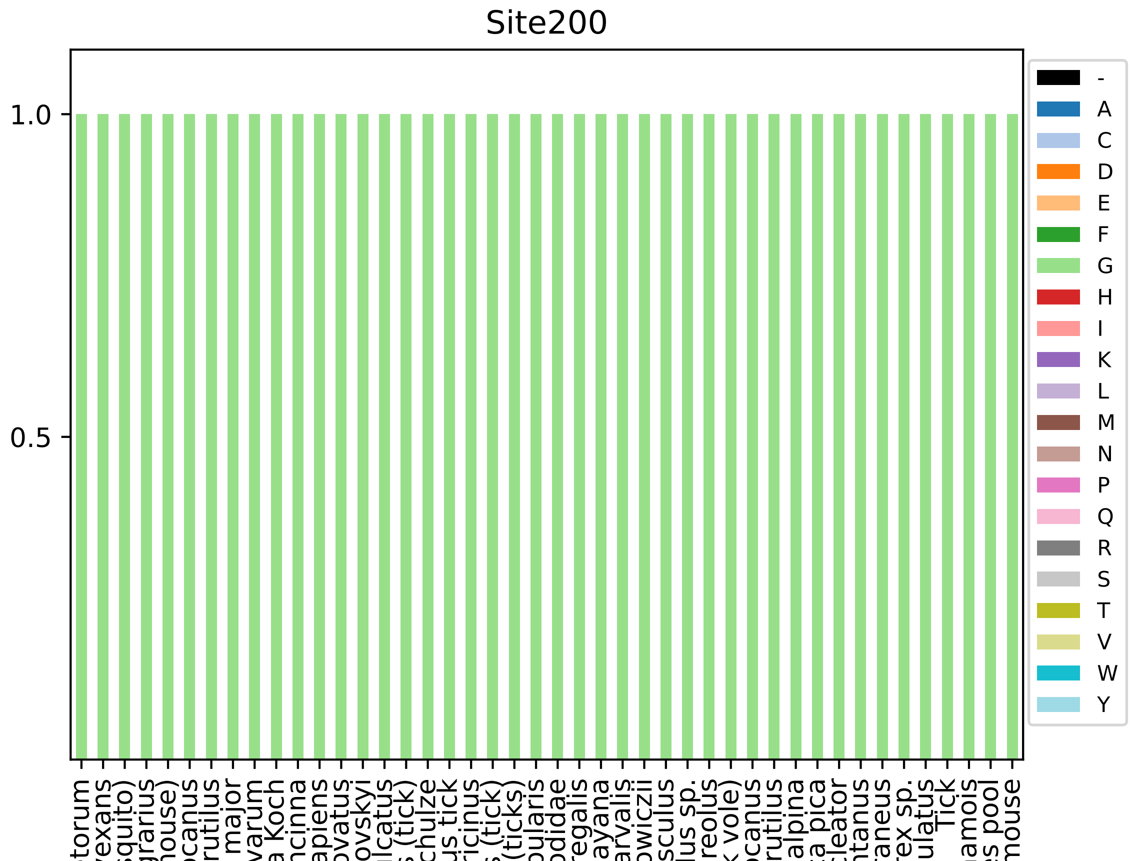
Site200
1.0
0.5
-
A
C
D
E
F
G
H
I
K
L
M
N
P
Q
R
S
T
V
W
Y

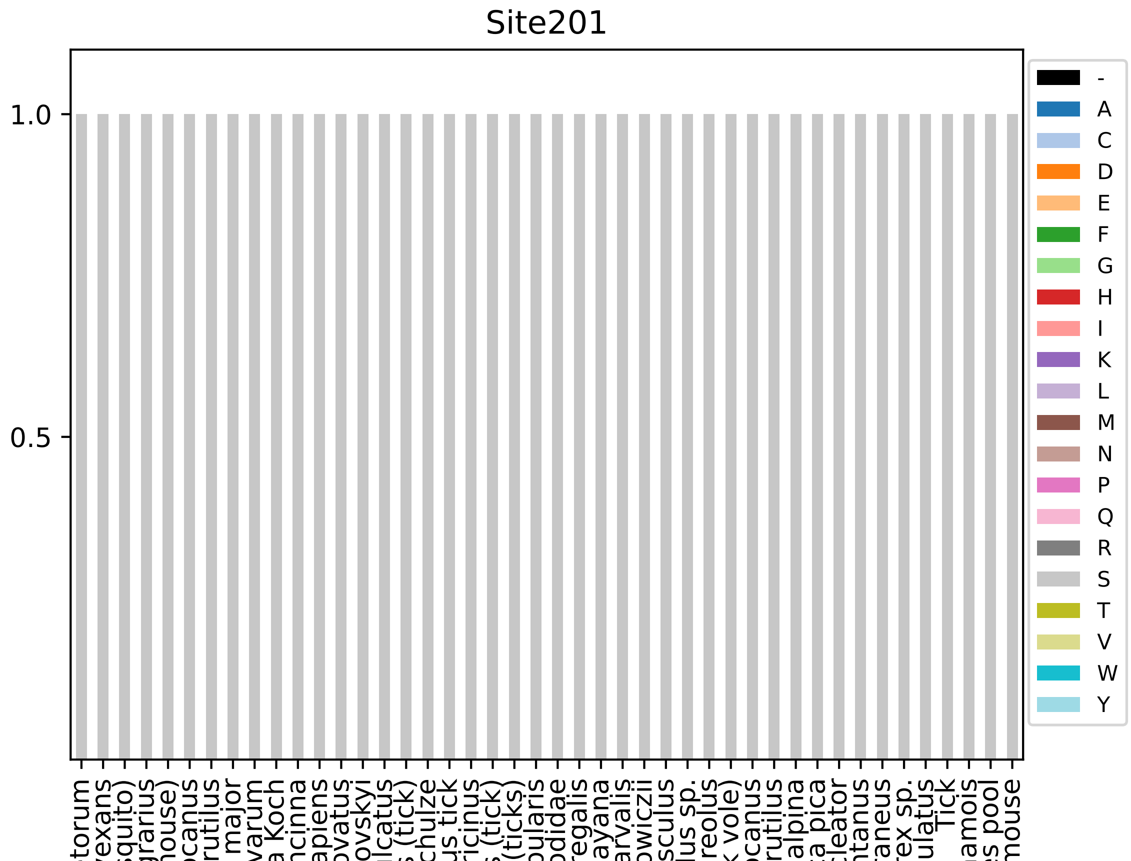
Site201
1.0
0.5
-
A
C
D
E
F
G
H
I
K
L
M
N
P
Q
R
S
T
V
W
Y

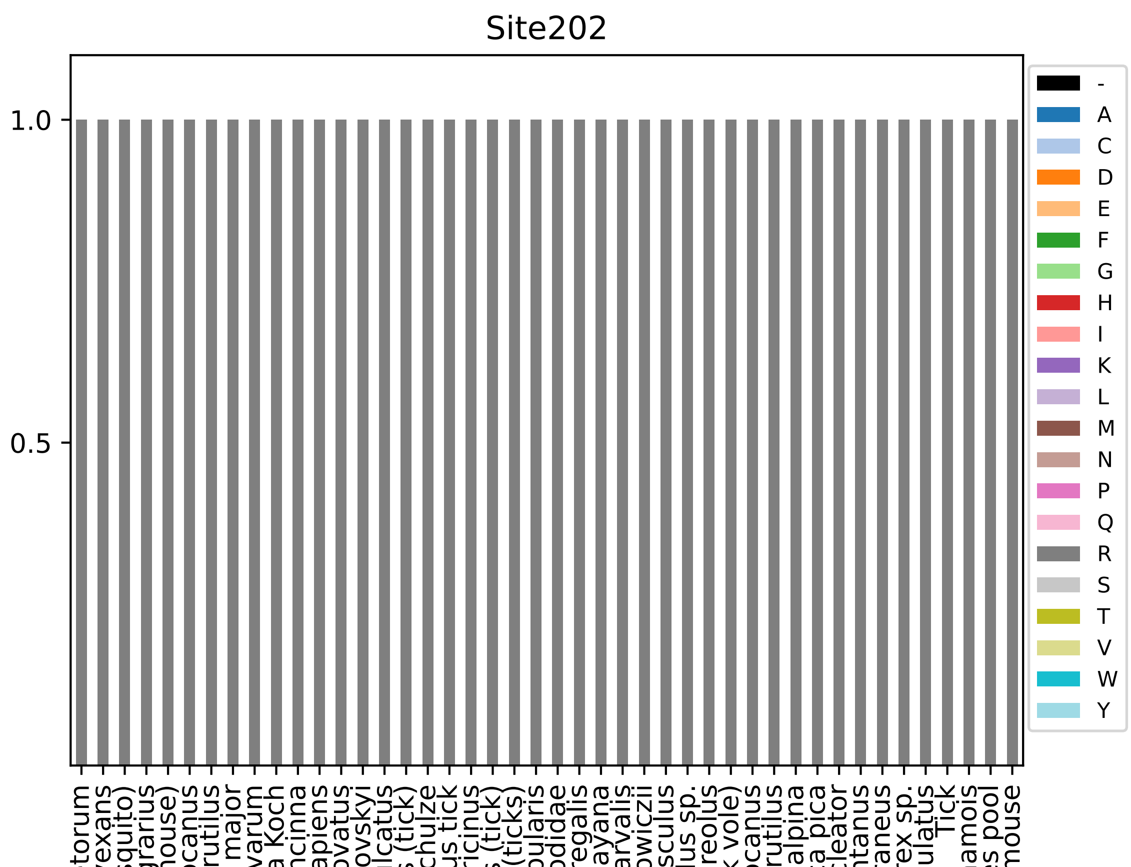
Site202
1.0
0.5
-
A
C
D
E
F
G
H
I
K
L
M
N
P
Q
R
S
T
V
W
Y

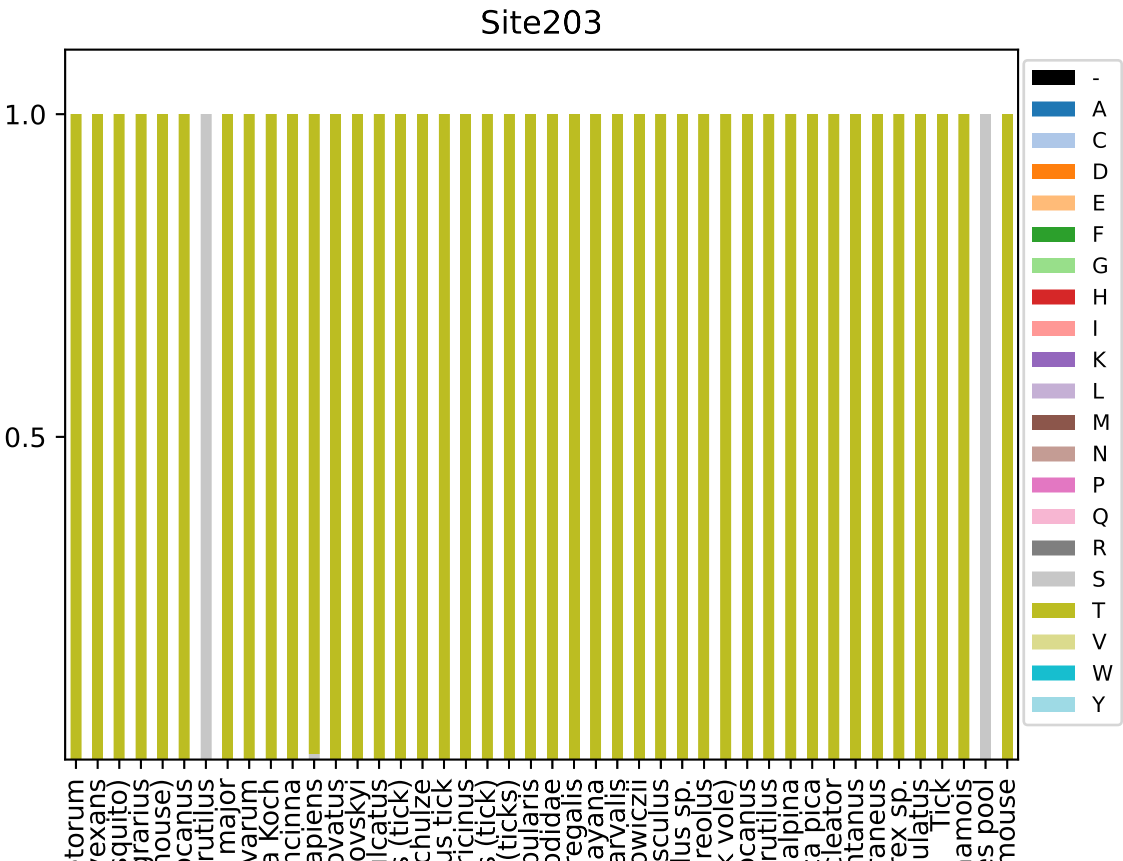
Site203
1.0
0.5
-
A
C
D
E
F
G
H
I
K
L
M
N
P
Q
R
S
T
V
W
Y

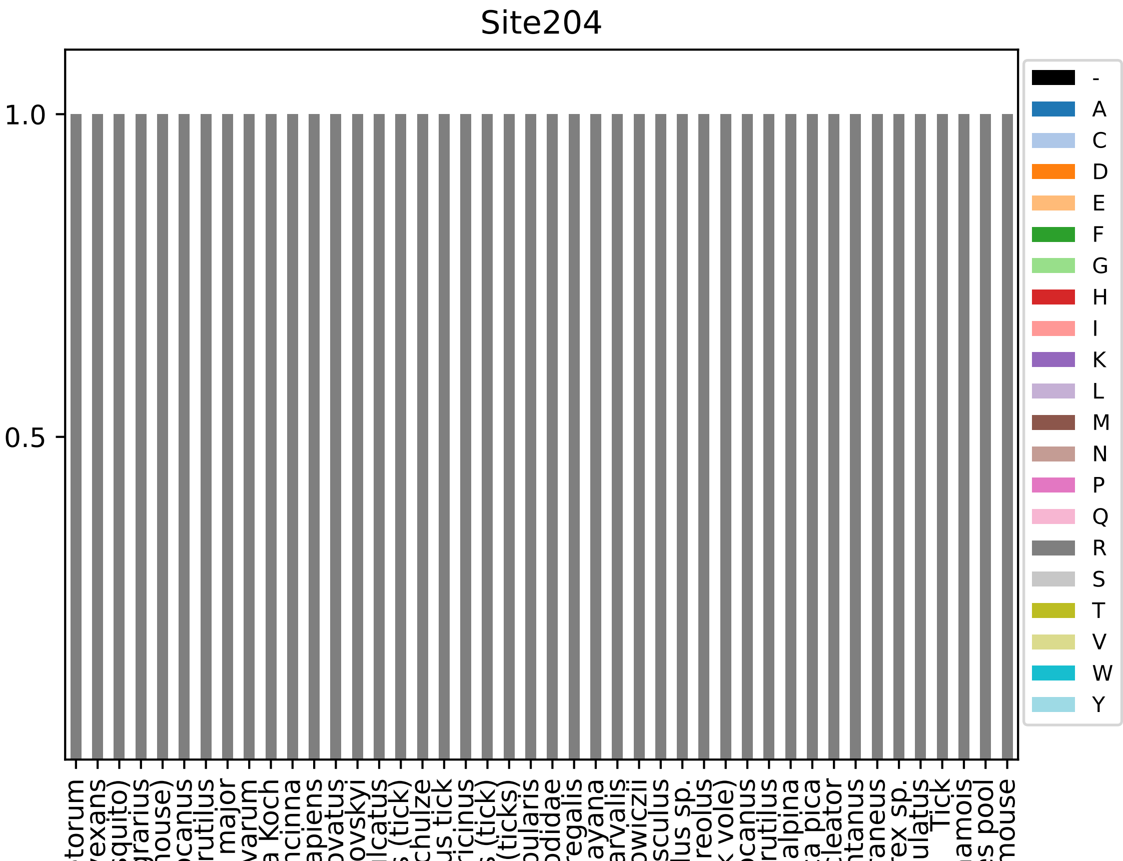
Site204
1.0
0.5
-
A
C
D
E
F
G
H
I
K
L
M
N
P
Q
R
S
T
V
W
Y

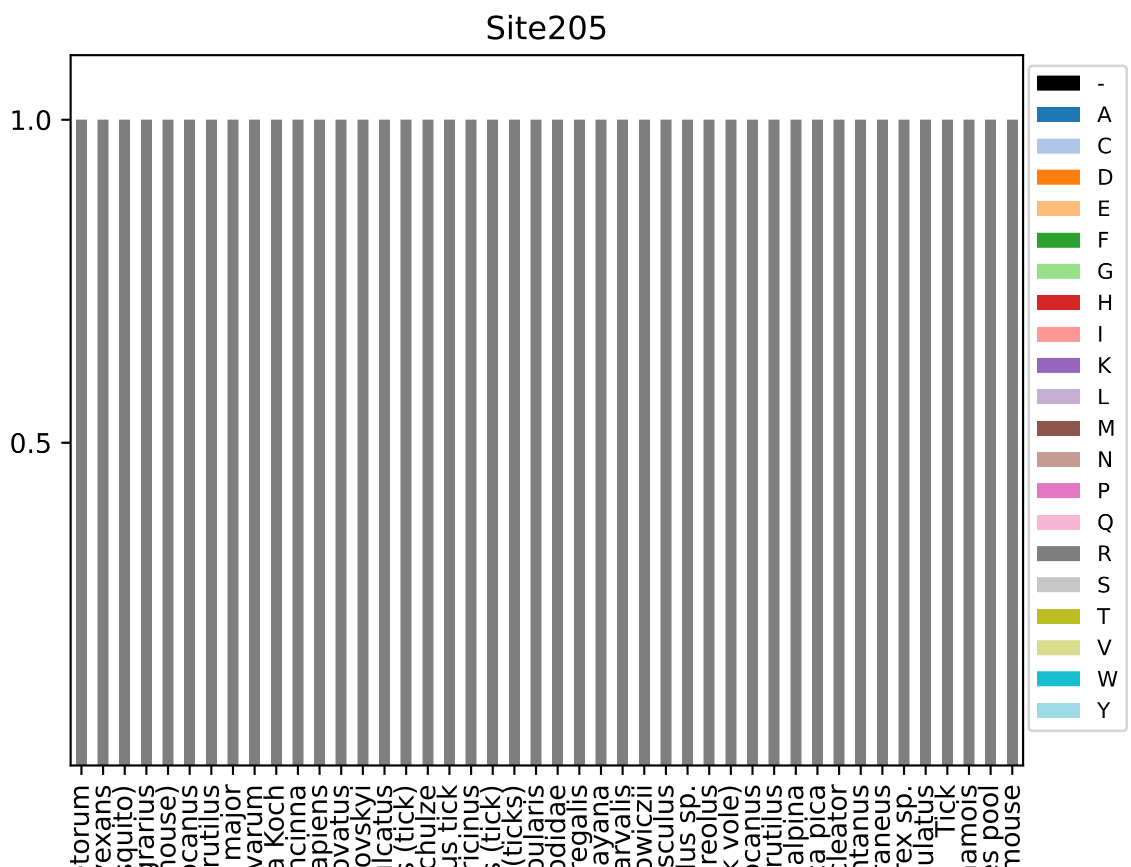
Site205
1.0
0.5
-
A
C
D
E
F
G
H
I
K
L
M
N
P
Q
R
S
T
V
W
Y

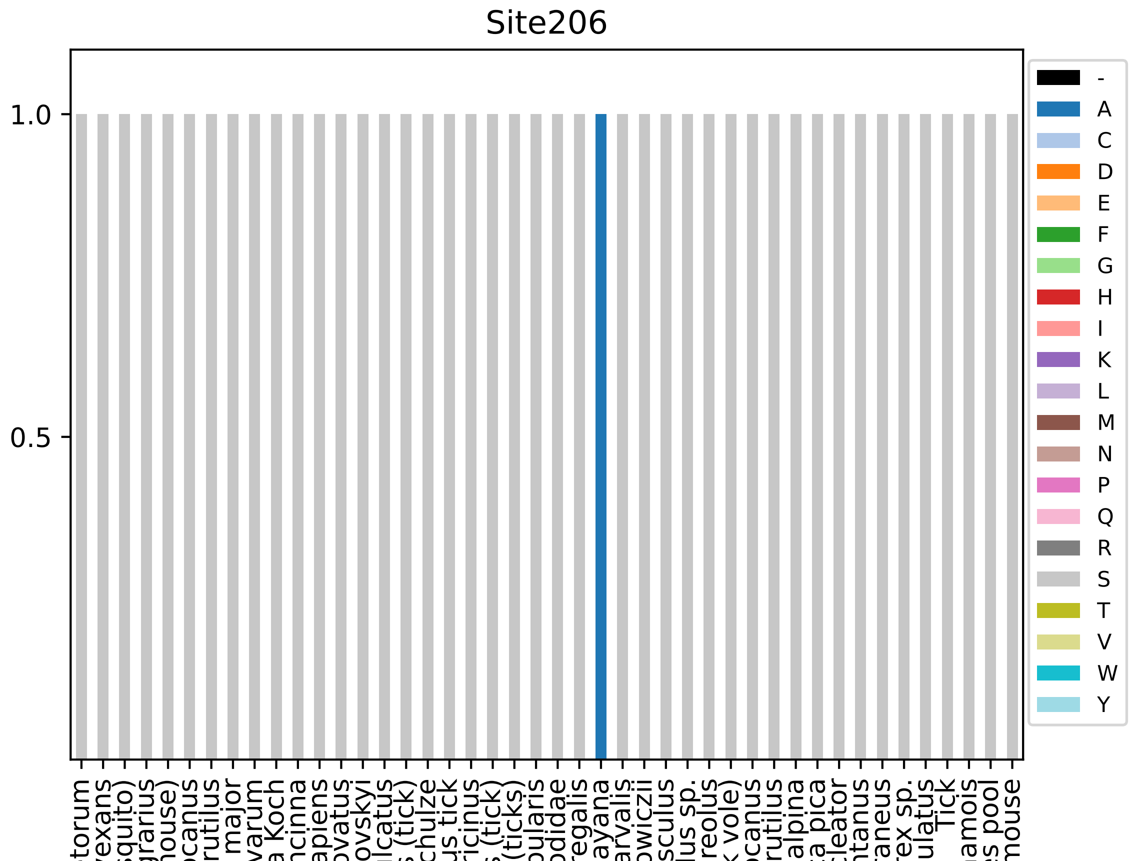
Site206
1.0
0.5
-
A
C
D
E
F
G
H
I
K
L
M
N
P
Q
R
S
T
V
W
Y

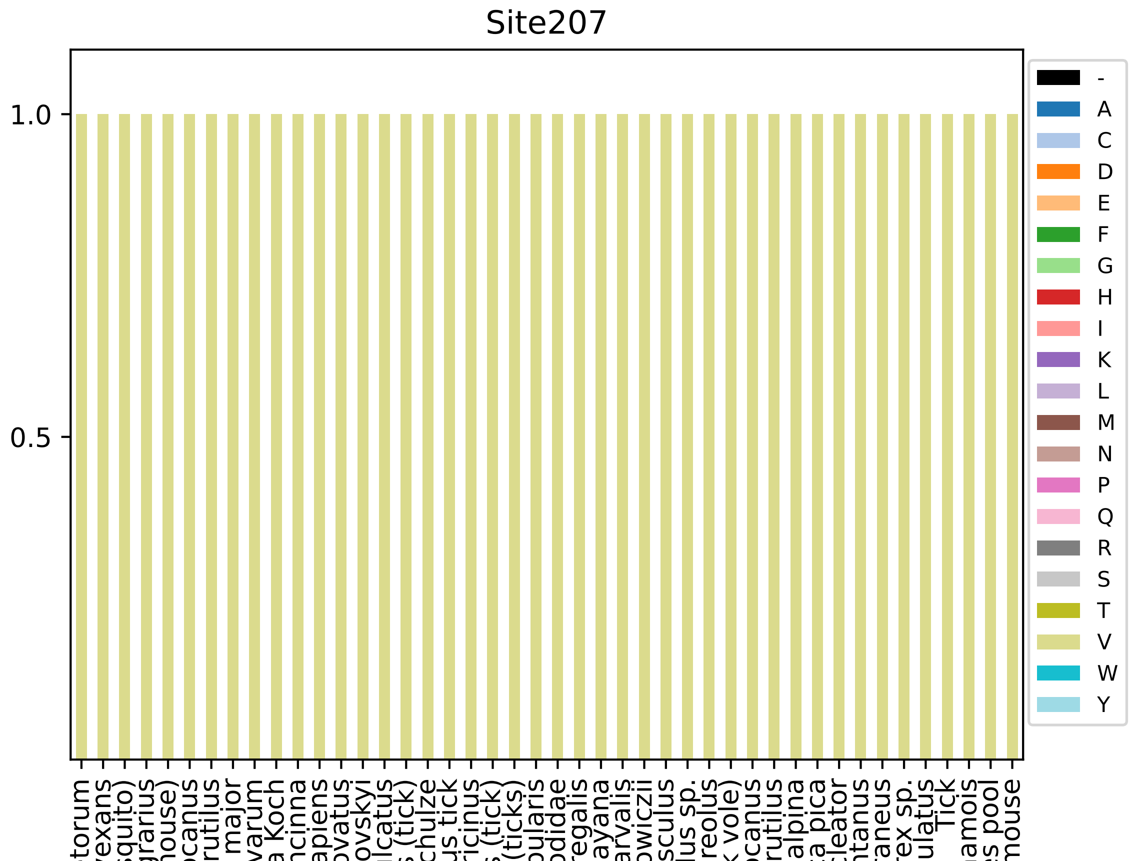

Site207
1.0
0.5
-
A
C
D
E
F
G
H
I
K
L
M
N
P
Q
R
S
T
V
W
Y

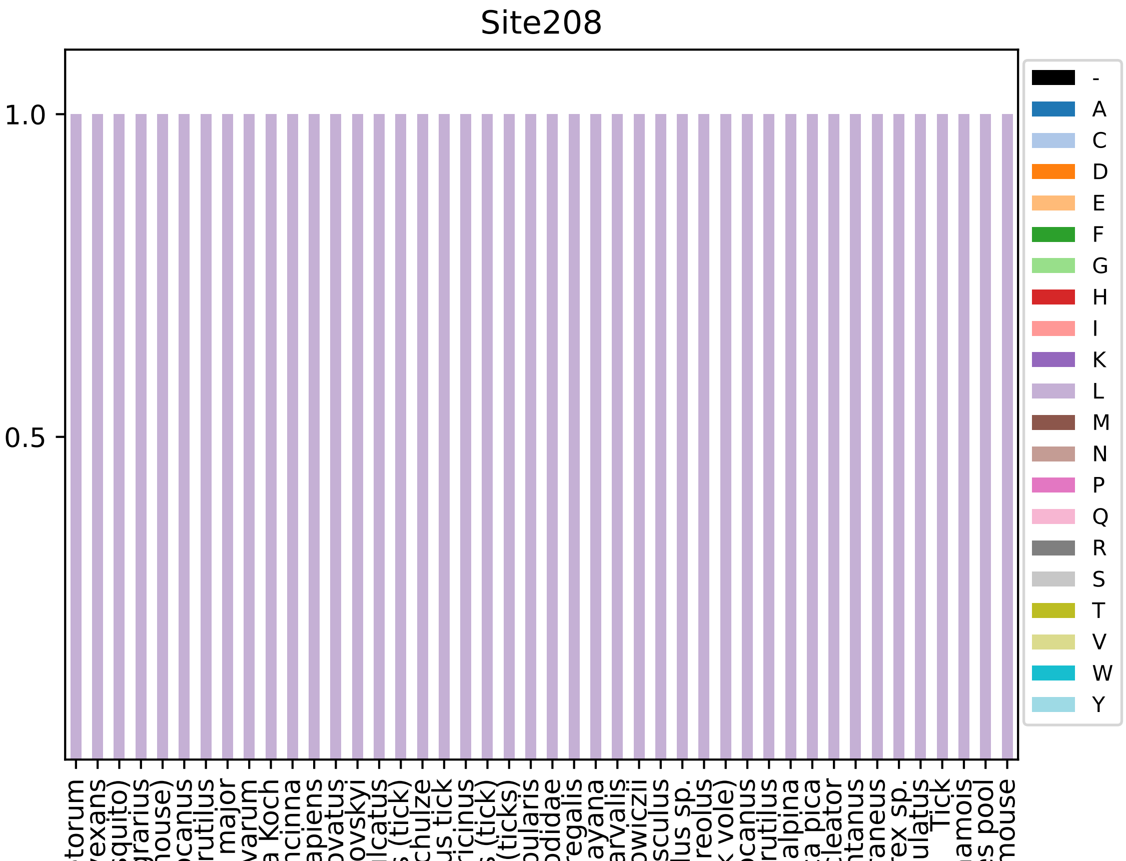
Site208
1.0
0.5
-
A
C
D
E
F
G
H
I
K
L
M
N
P
Q
R
S
T
V
W
Y

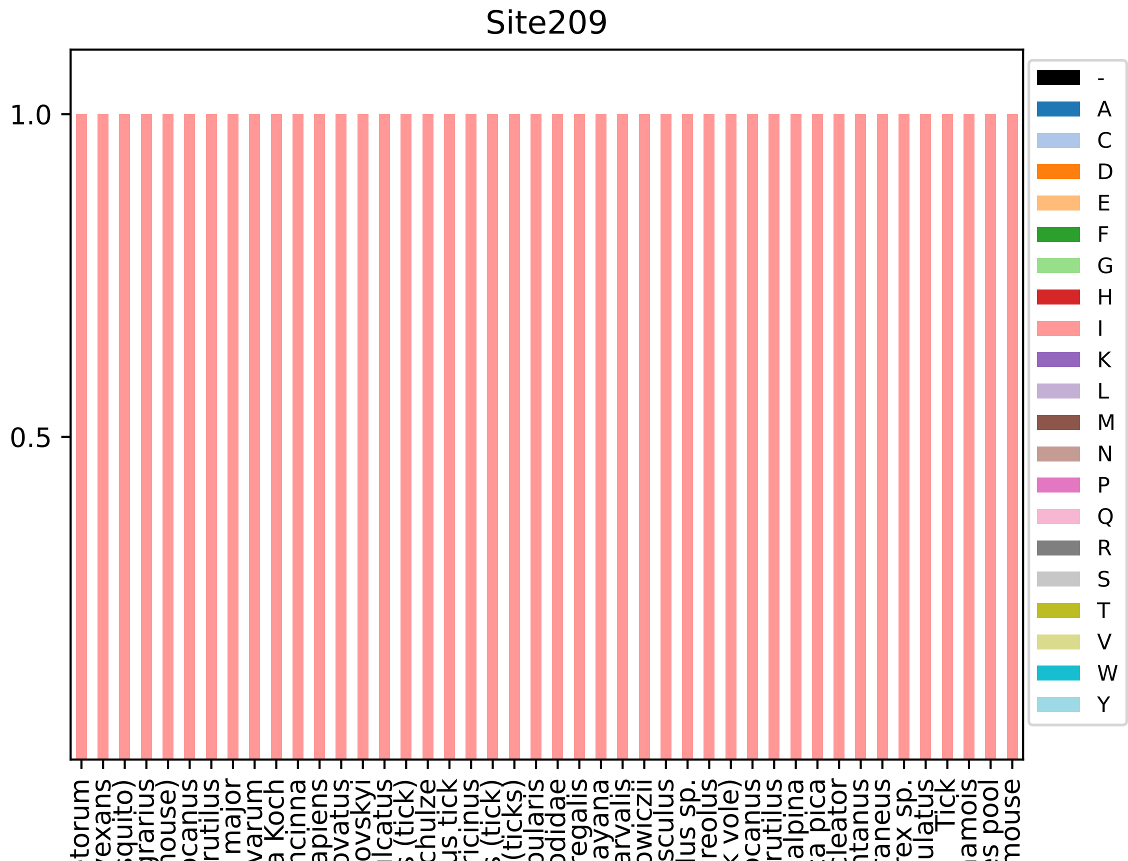

Site209
1.0
0.5
-
A
C
D
E
F
G
H
I
K
L
M
N
P
Q
R
S
T
V
W
Y

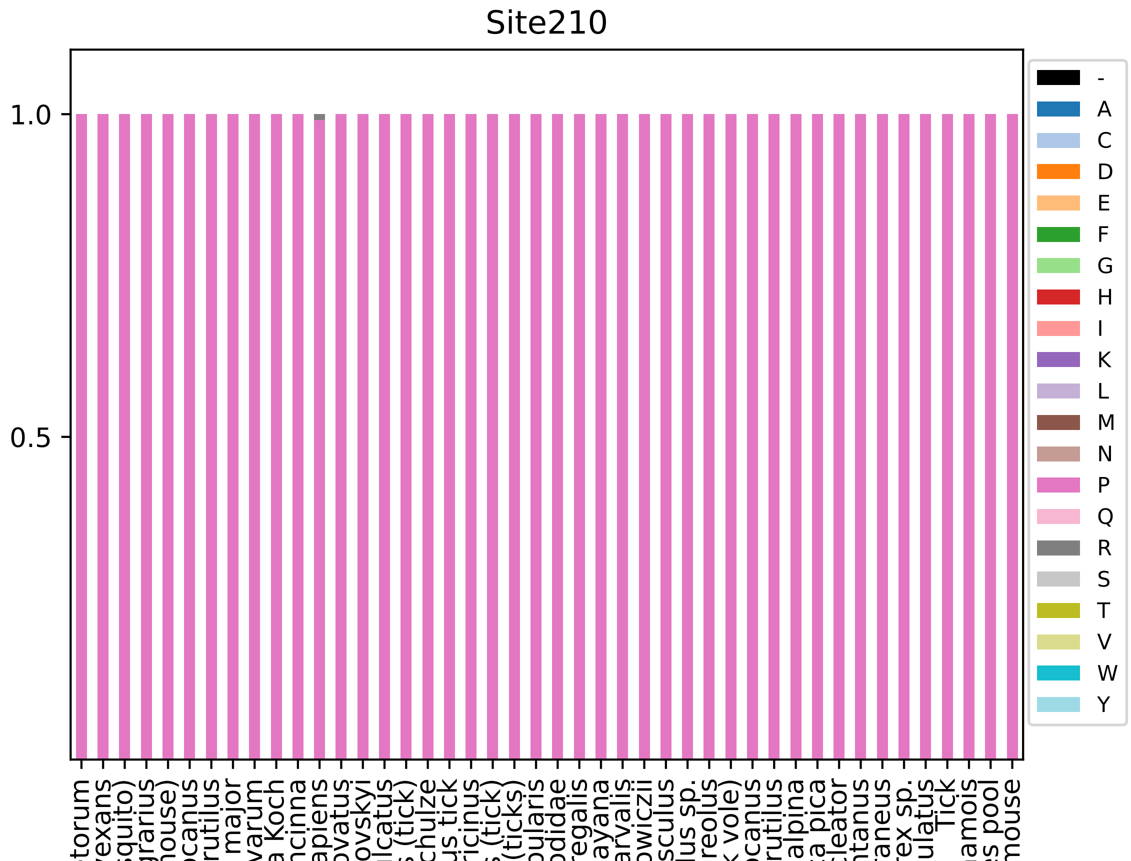

Site210
1.0
0.5
-
A
C
D
E
F
G
H
I
K
L
M
N
P
Q
R
S
T
V
W
Y

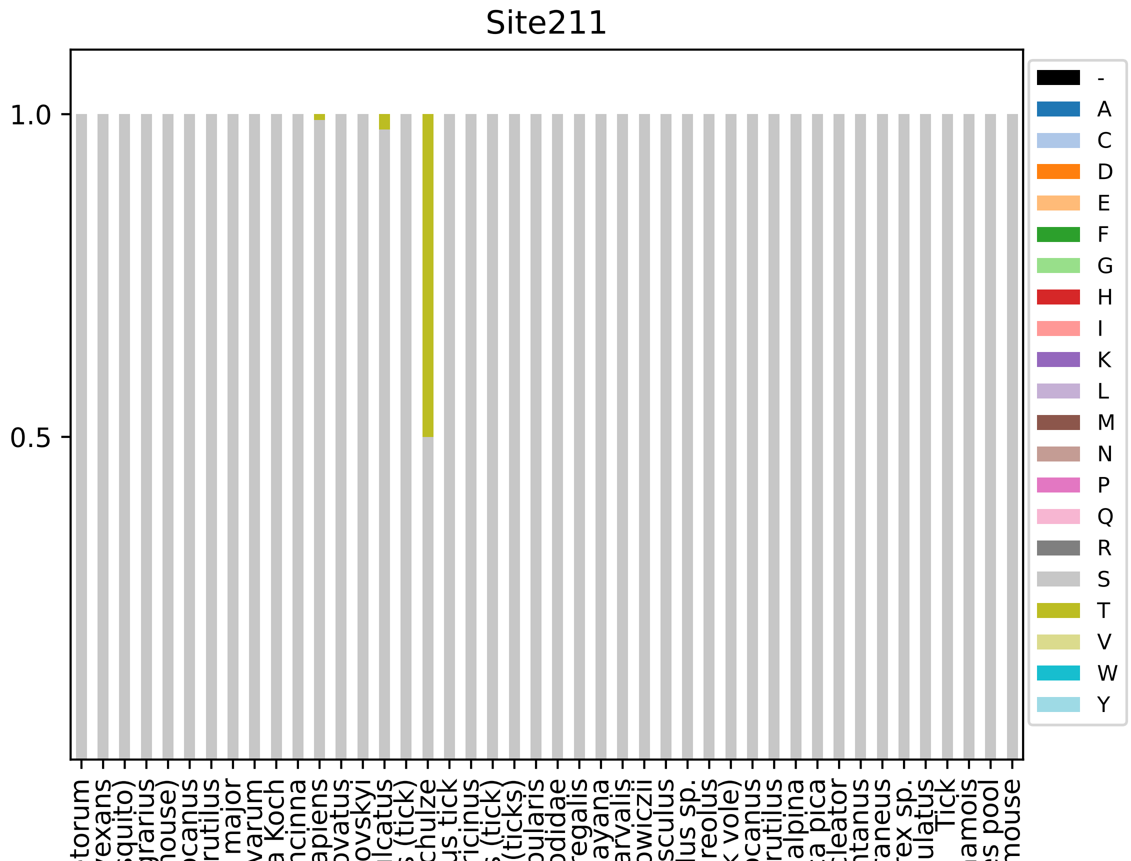

Site211
1.0
0.5
-
A
C
D
E
F
G
H
I
K
L
M
N
P
Q
R
S
T
V
W
Y

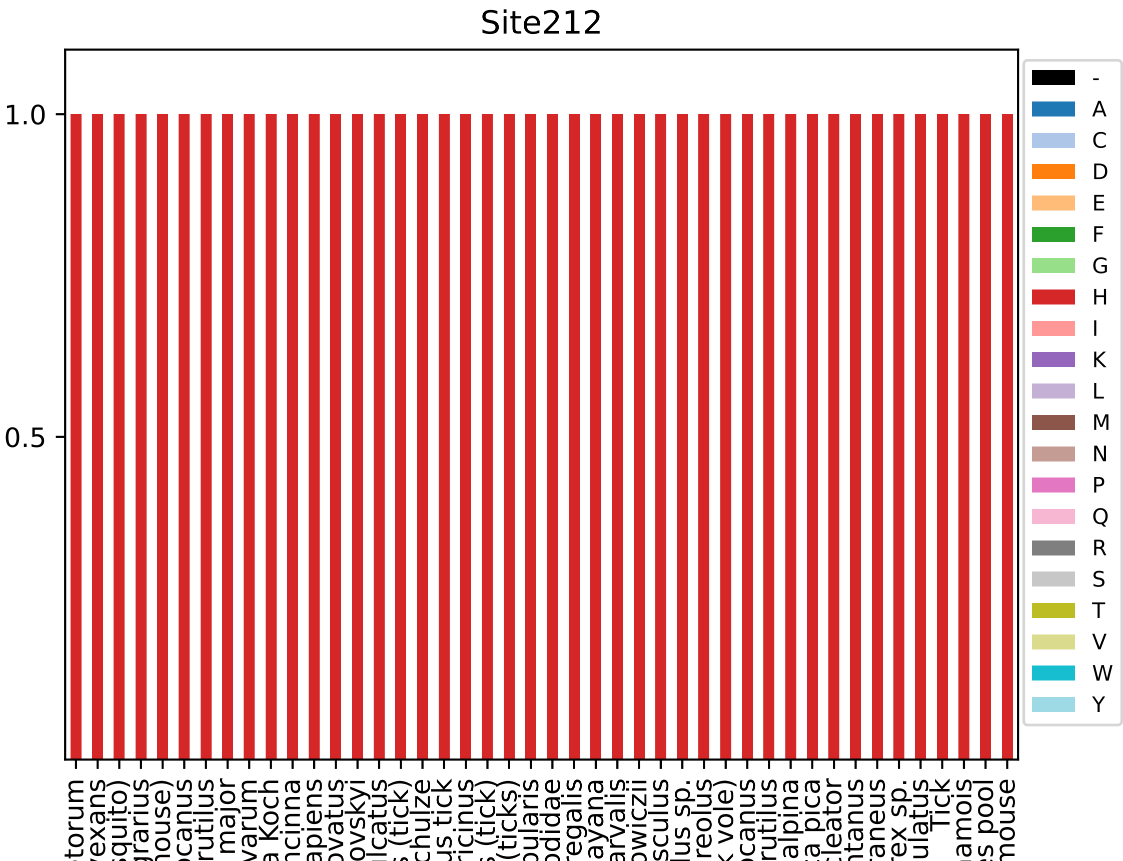
Site212
1.0
0.5
-
A
C
D
E
F
G
H
I
K
L
M
N
P
Q
R
S
T
V
W
Y

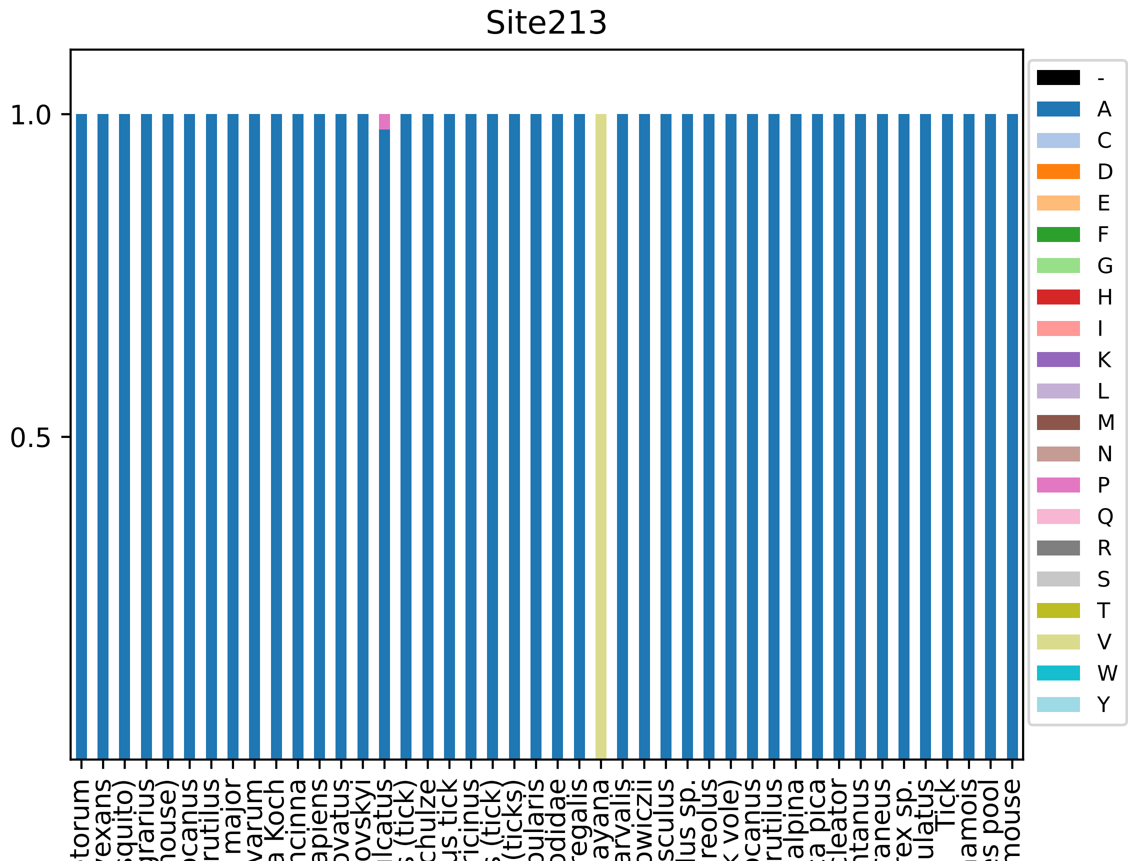
Site213
1.0
0.5
-
A
C
D
E
F
G
H
I
K
L
M
N
P
Q
R
S
T
V
W
Y
Tick

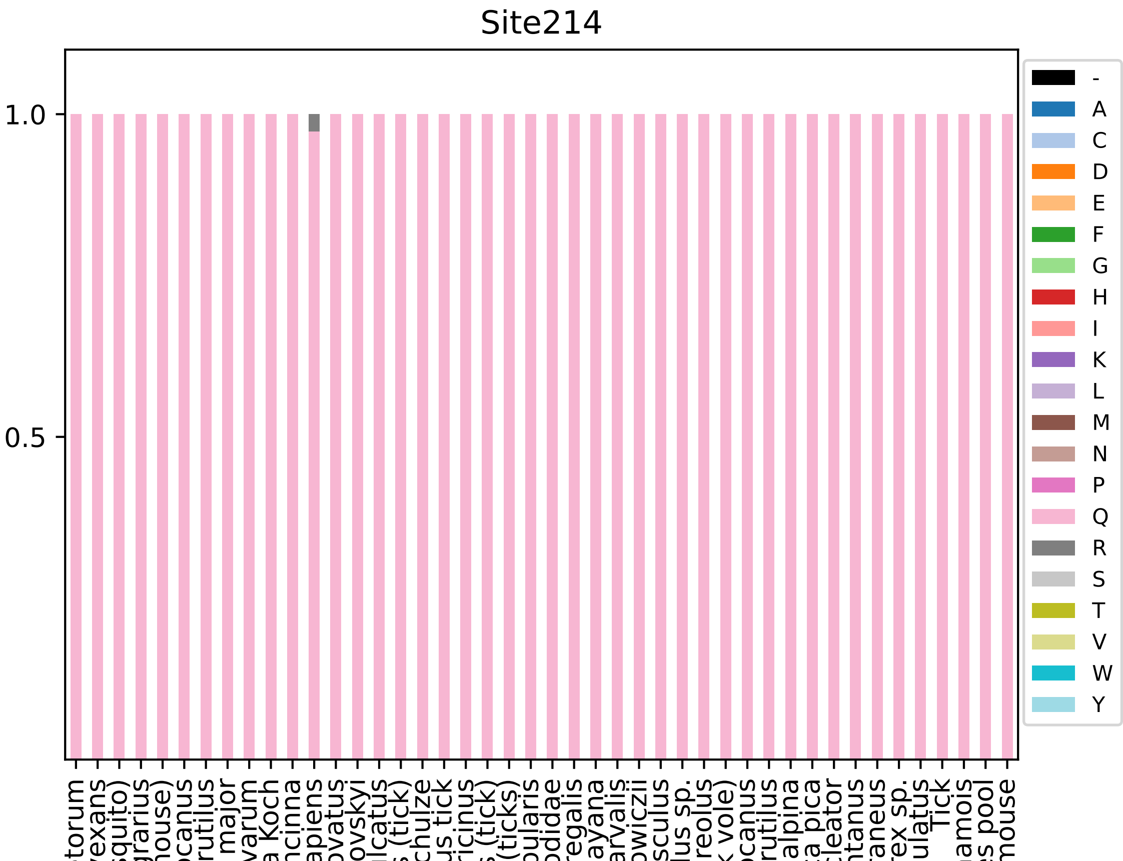

Site214
1.0
0.5
-
A
C
D
E
F
G
H
I
K
L
M
N
P
Q
R
S
T
V
W
Y

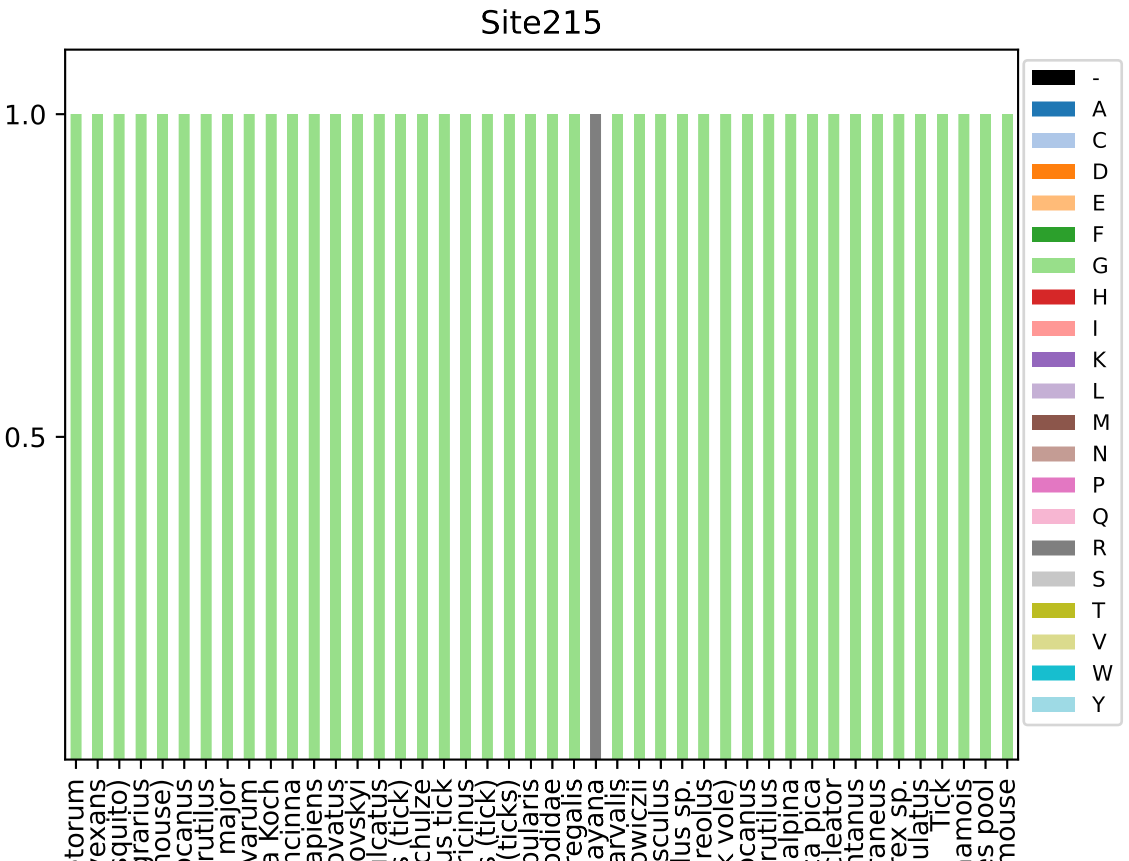
Site215
1.0
0.5
-
A
C
D
E
F
G
H
I
K
L
M
N
P
Q
R
S
T
V
W
Y

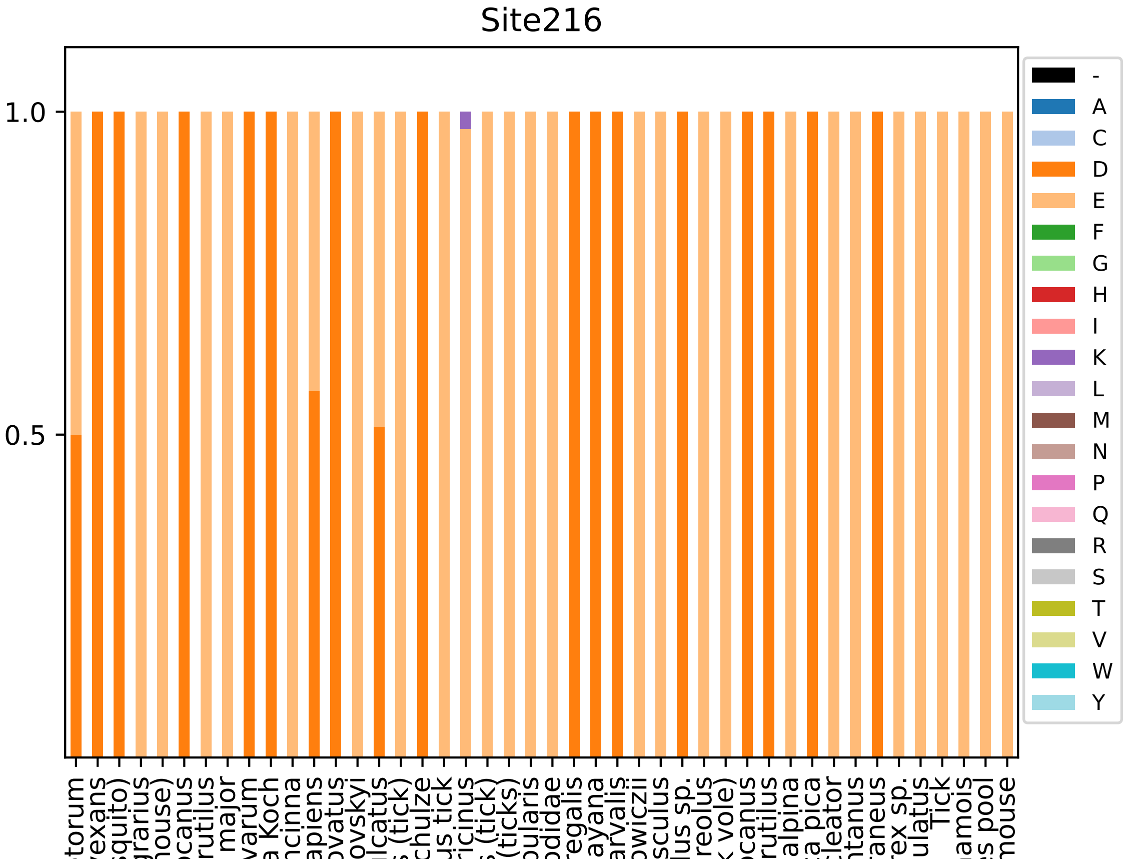
Site216
1.0
0.5
-
A
C
D
E
F
G
H
I
K
L
M
N
P
Q
R
S
T
V
W
Y
Tick

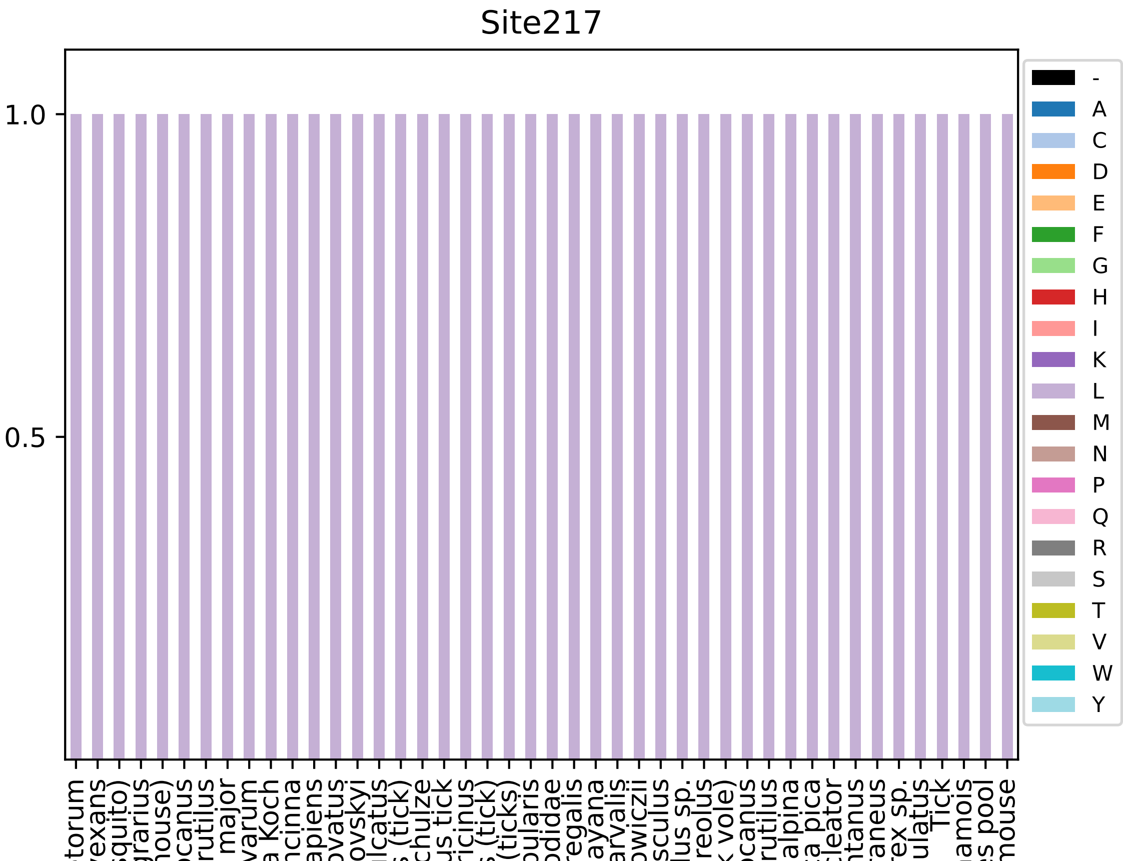
Site217
1.0
0.5
-
A
C
D
E
F
G
H
I
K
L
M
N
P
Q
R
S
T
V
W
Y

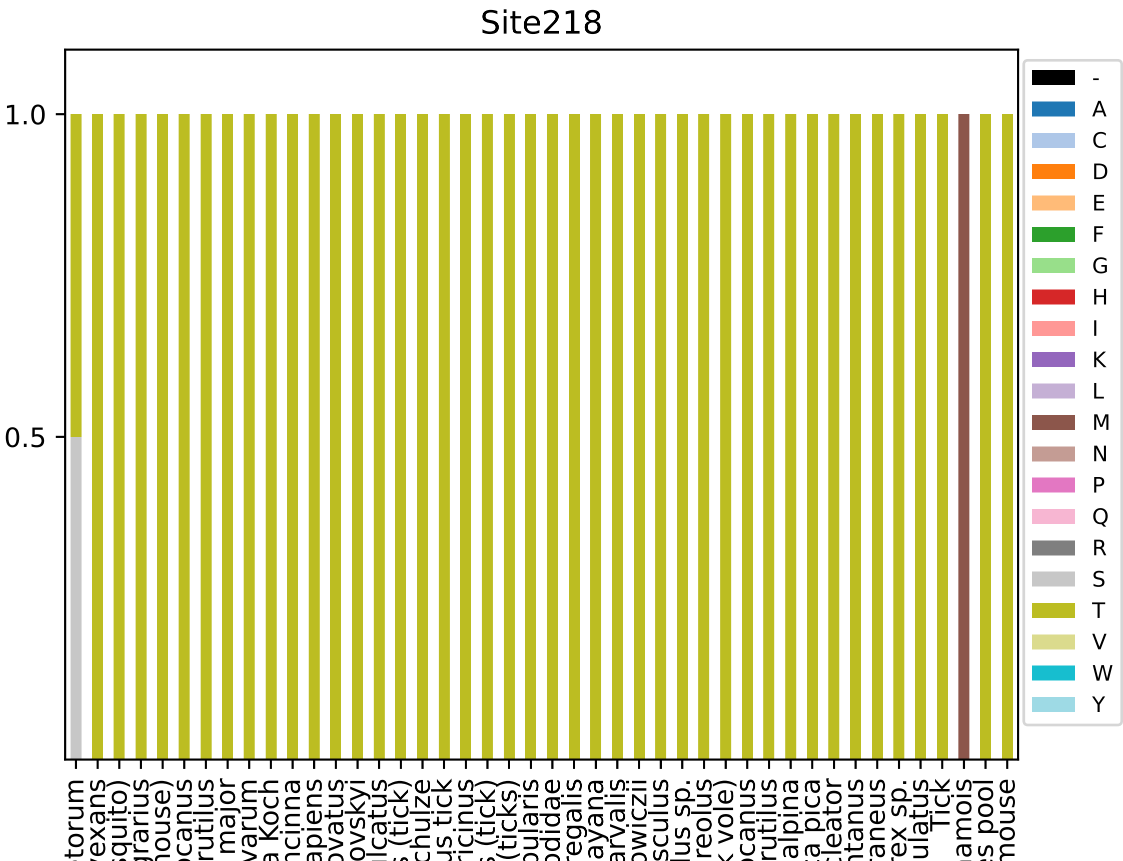
Site218
1.0
0.5
-
A
C
D
E
F
G
H
I
K
L
M
N
P
Q
R
S
T
V
W
Y
Tick

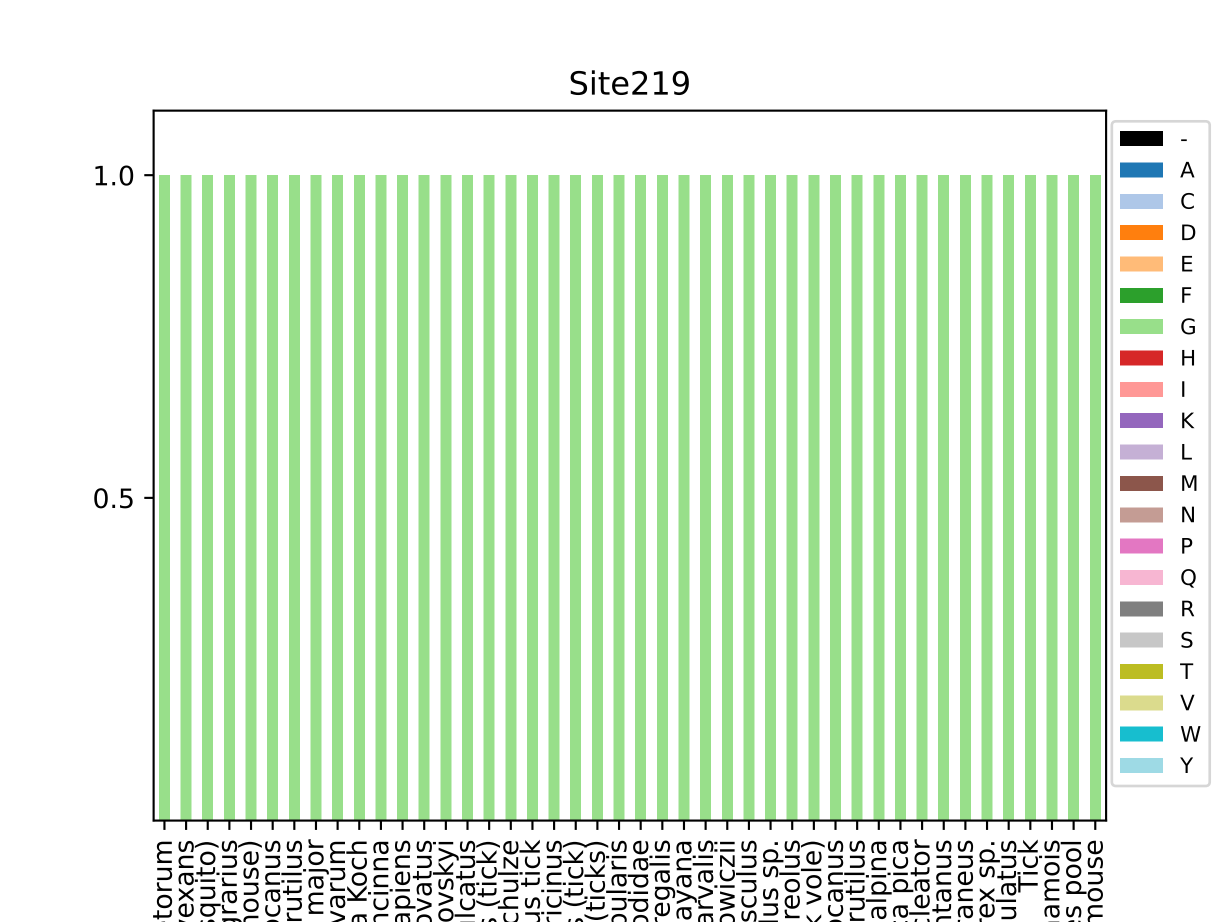
Site219
1.0
0.5
-
A
C
D
E
F
G
H
I
K
L
M
N
P
Q
R
S
T
V
W
Y

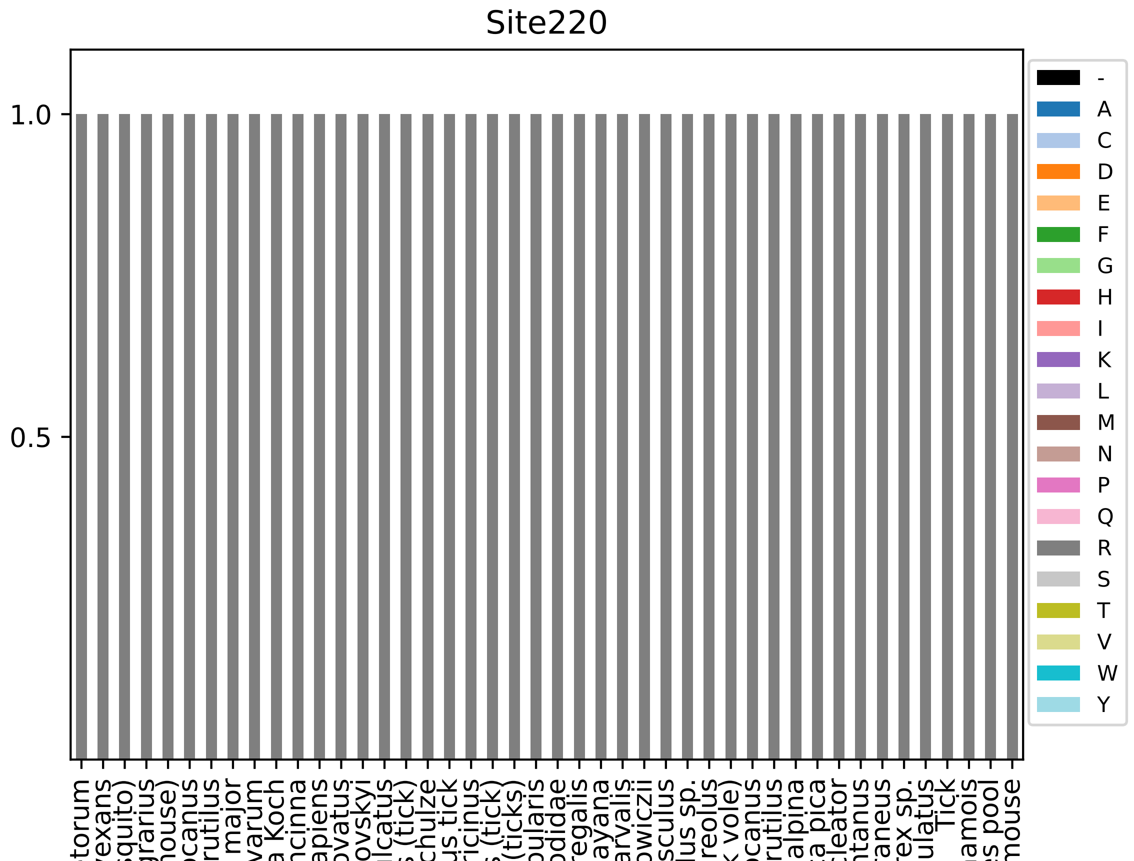

Site220
1.0
0.5
-
A
C
D
E
F
G
H
I
K
L
M
N
P
Q
R
S
T
V
W
Y

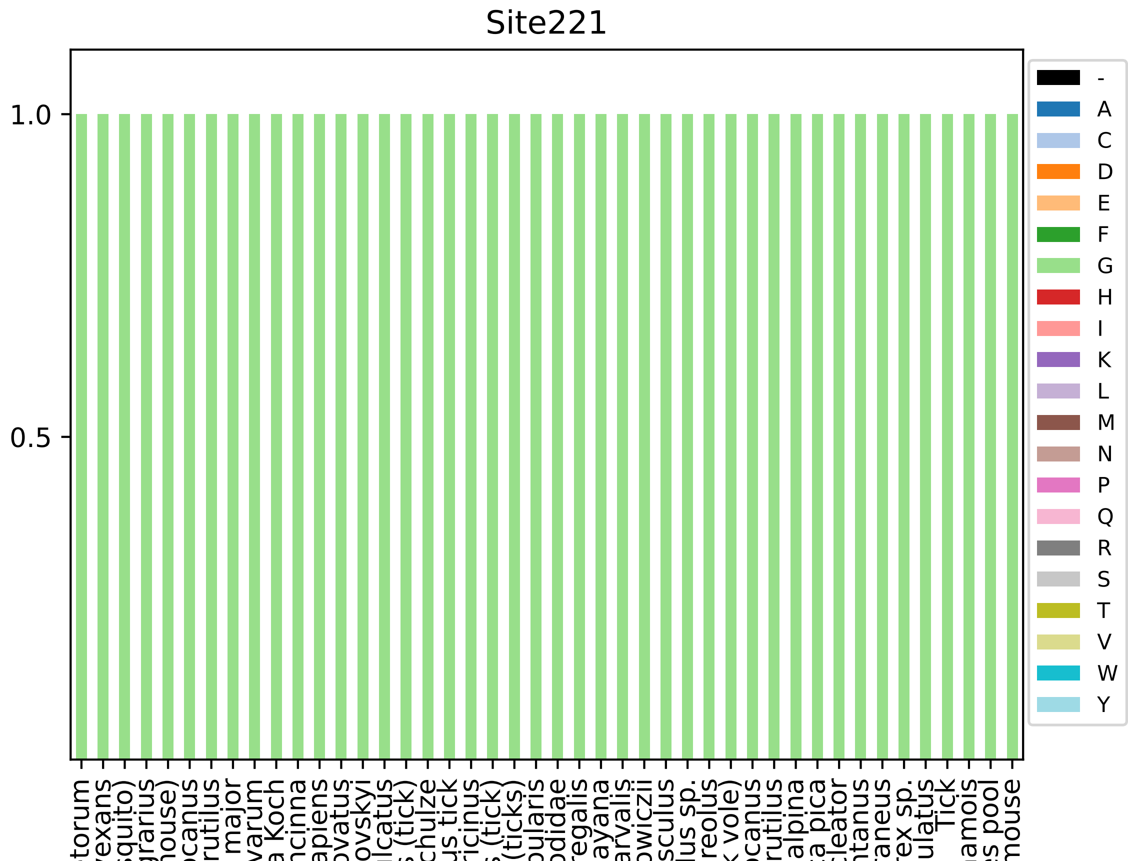
Site221
1.0
0.5
-
A
C
D
E
F
G
H
I
K
L
M
N
P
Q
R
S
T
V
W
Y

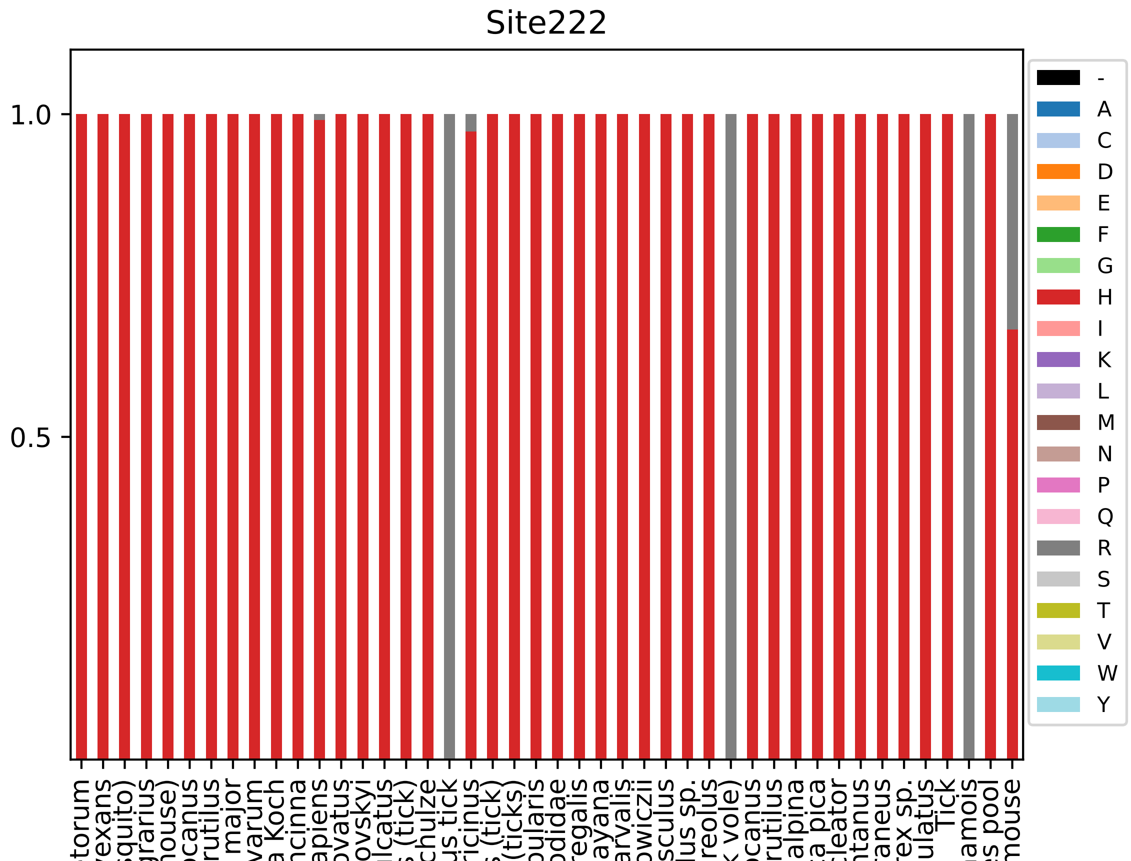
Site222
1.0
0.5
-
A
C
D
E
F
G
H
I
K
L
M
N
P
Q
R
S
T
V
W
Y

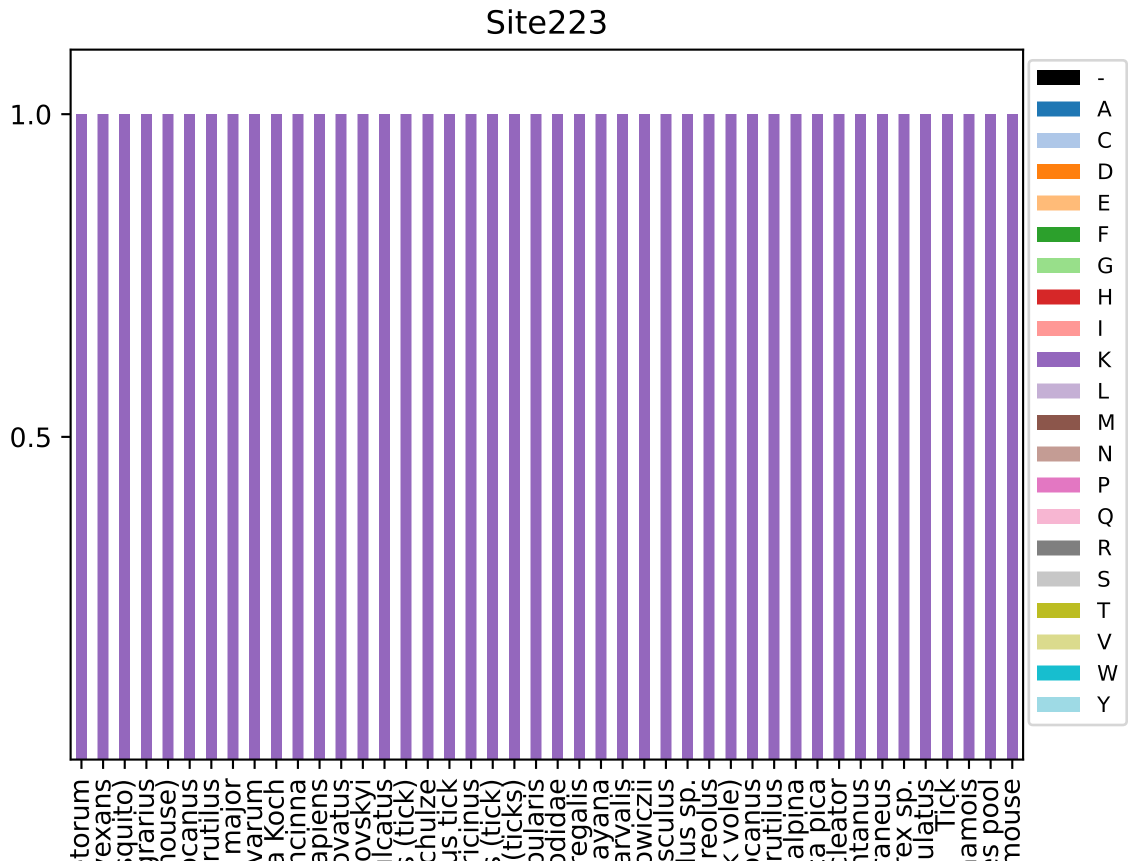
Site223
1.0
0.5
-
A
C
D
E
F
G
H
I
K
L
M
N
P
Q
R
S
T
V
W
Y

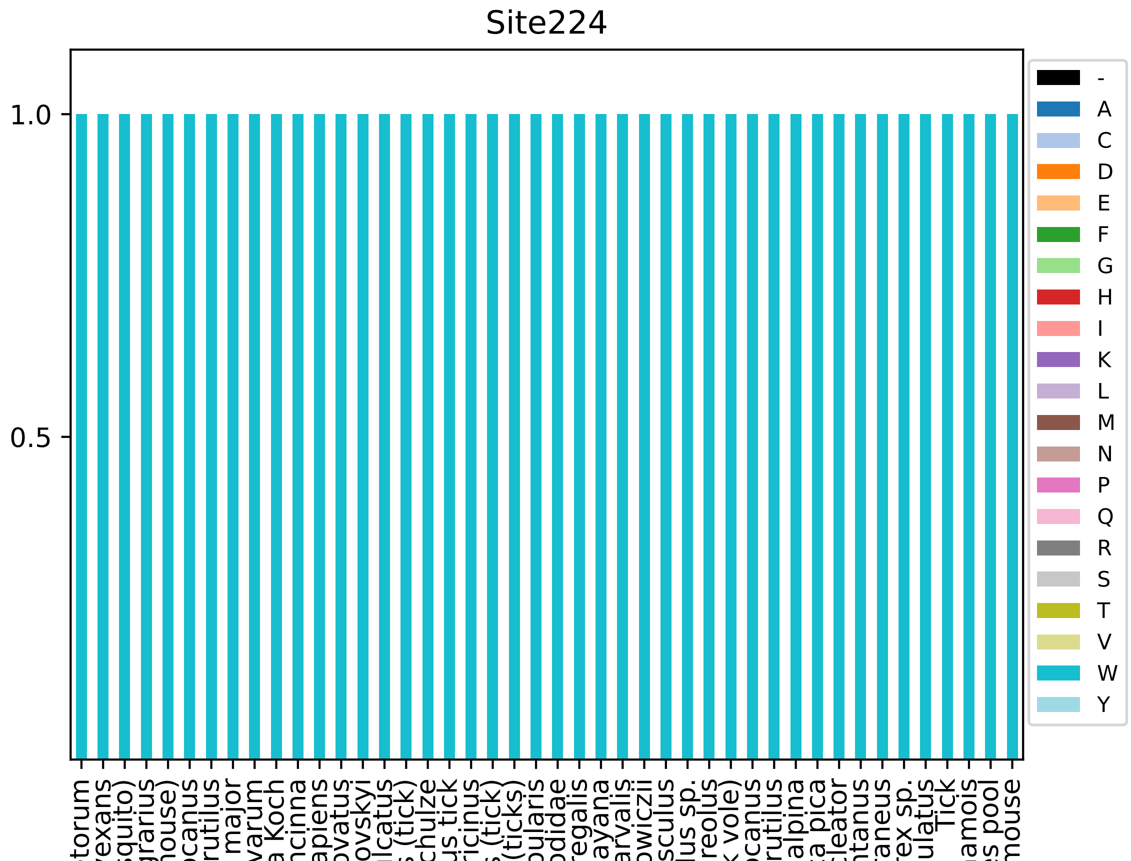
Site224
1.0
0.5
-
A
C
D
E
F
G
H
I
K
L
M
N
P
Q
R
S
T
V
W
Y

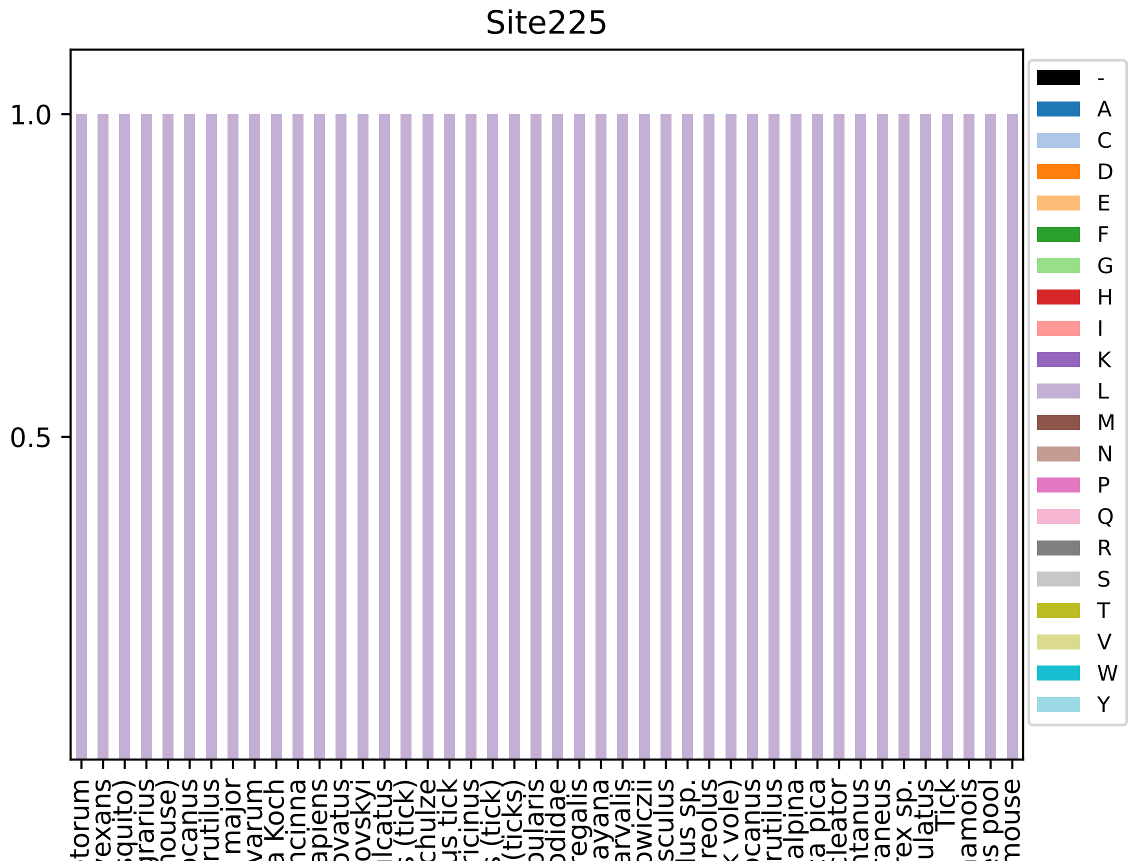
Site225
1.0
0.5
-
A
C
D
E
F
G
H
I
K
L
M
N
P
Q
R
S
T
V
W
Y

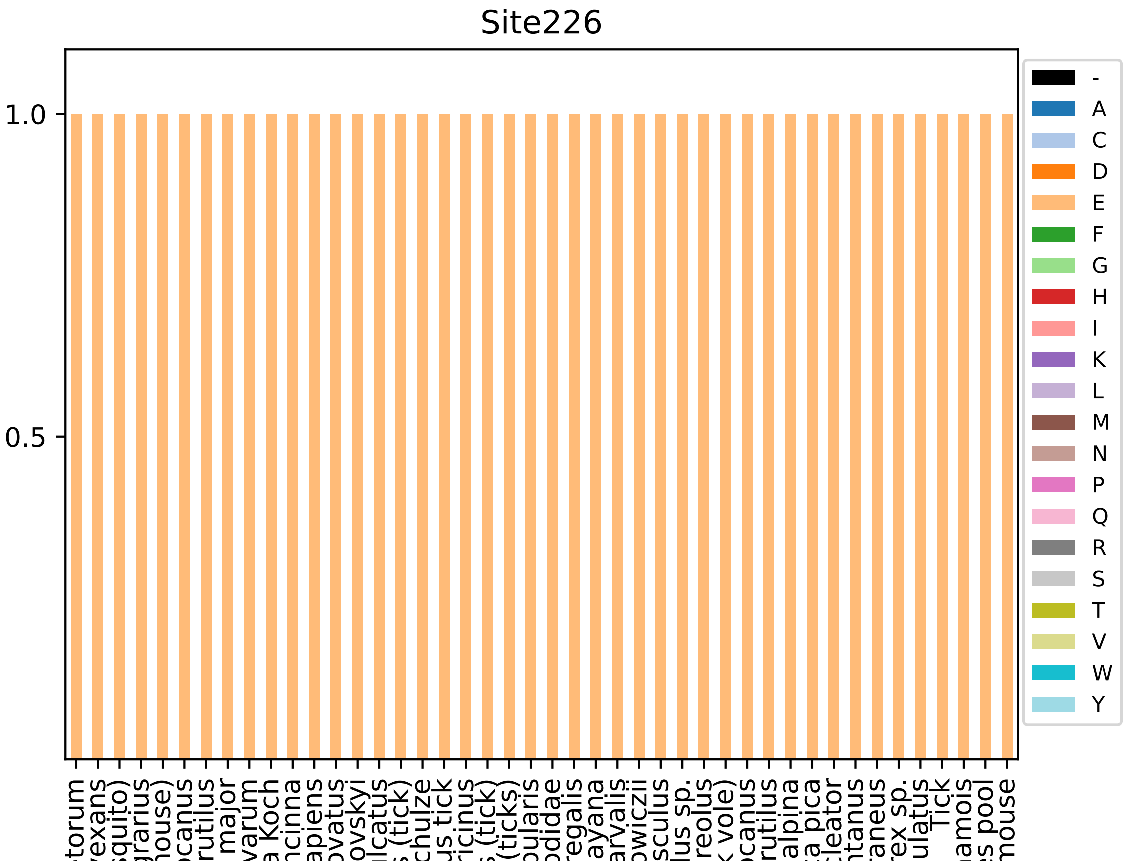

Site226
1.0
0.5
-
A
C
D
E
F
G
H
I
K
L
M
N
P
Q
R
S
T
V
W
Y

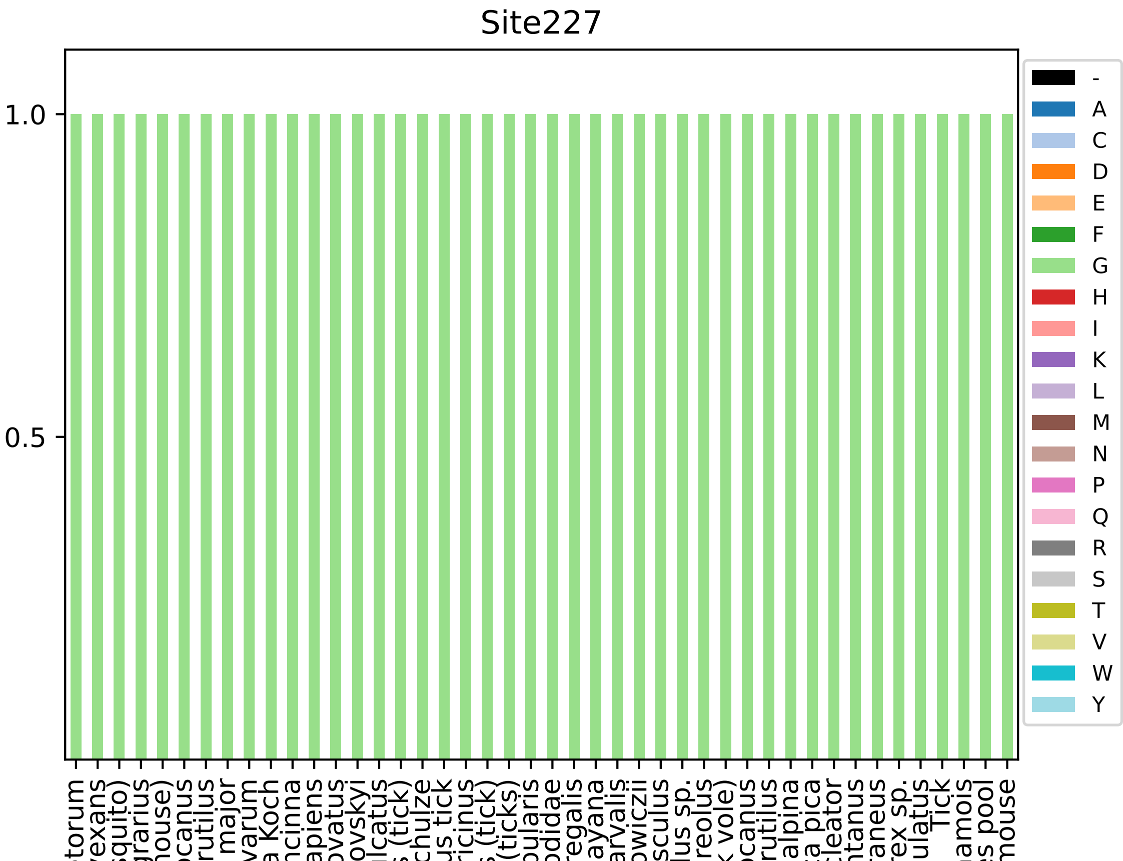

Site227
1.0
0.5
-
A
C
D
E
F
G
H
I
K
L
M
N
P
Q
R
S
T
V
W
Y

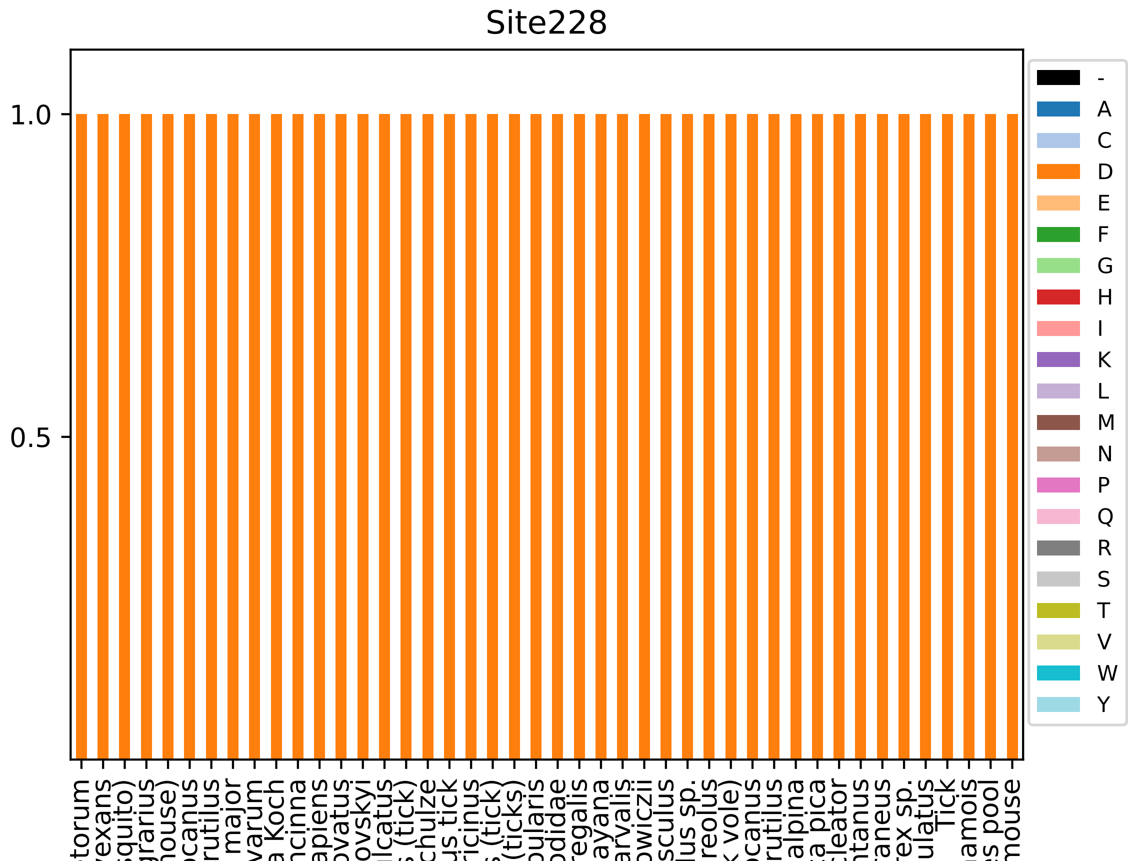
Site228
1.0
0.5
-
A
C
D
E
F
G
H
I
K
L
M
N
P
Q
R
S
T
V
W
Y

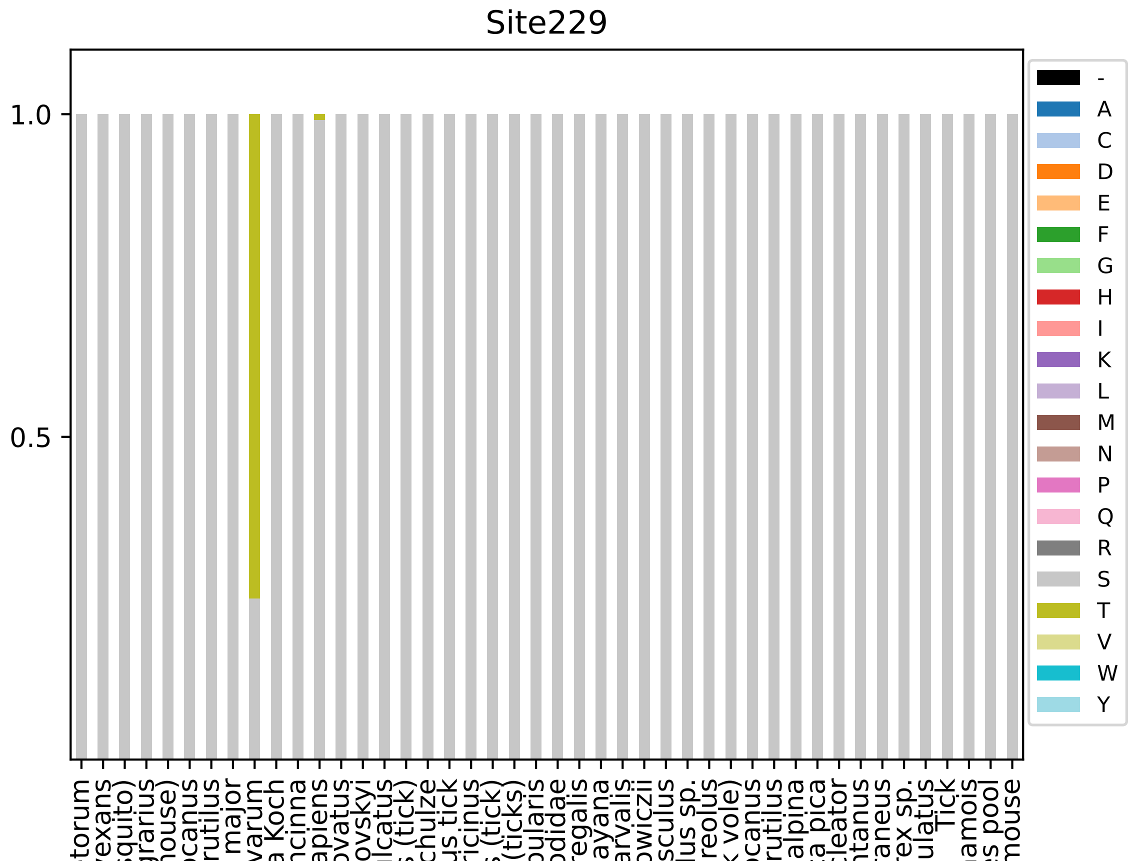
Site229
1.0
0.5
-
A
C
D
E
F
G
H
I
K
L
M
N
P
Q
R
S
T
V
W
Y

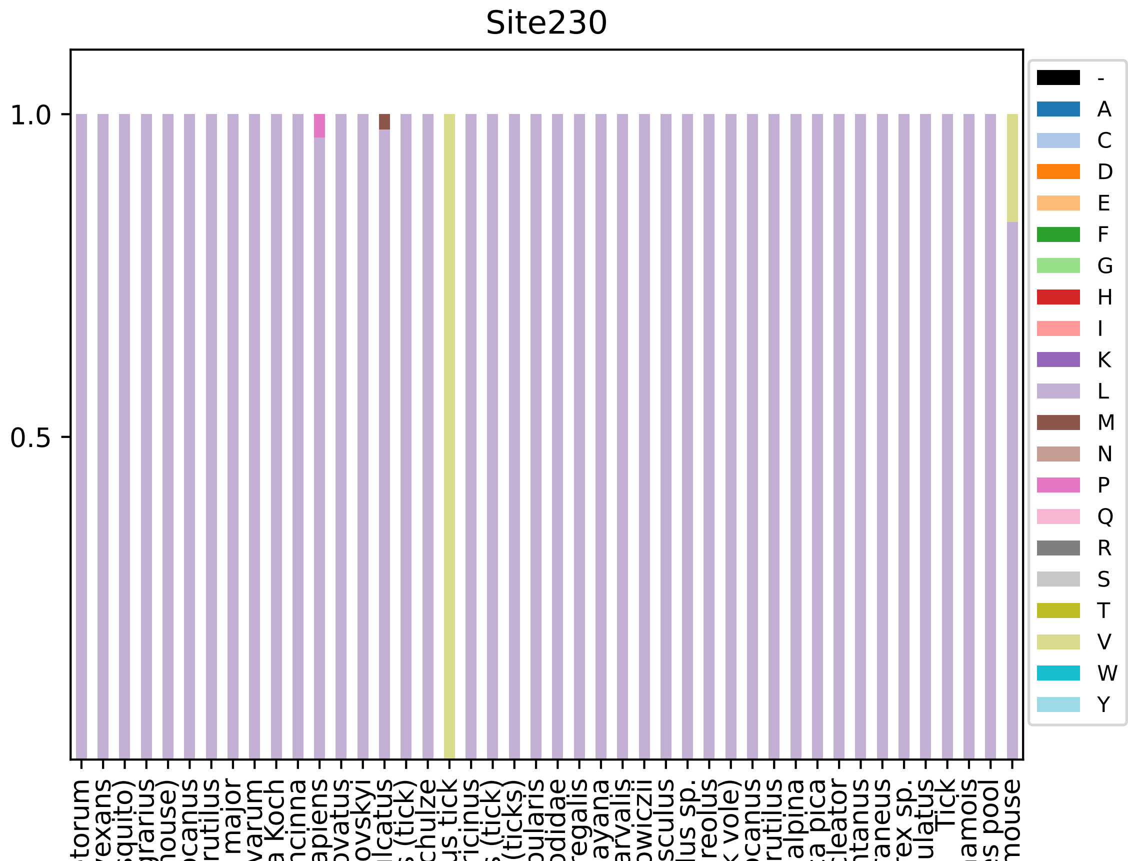
Site230
1.0
0.5
-
A
C
D
E
F
G
H
I
K
L
M
N
P
Q
R
S
T
V
W
Y

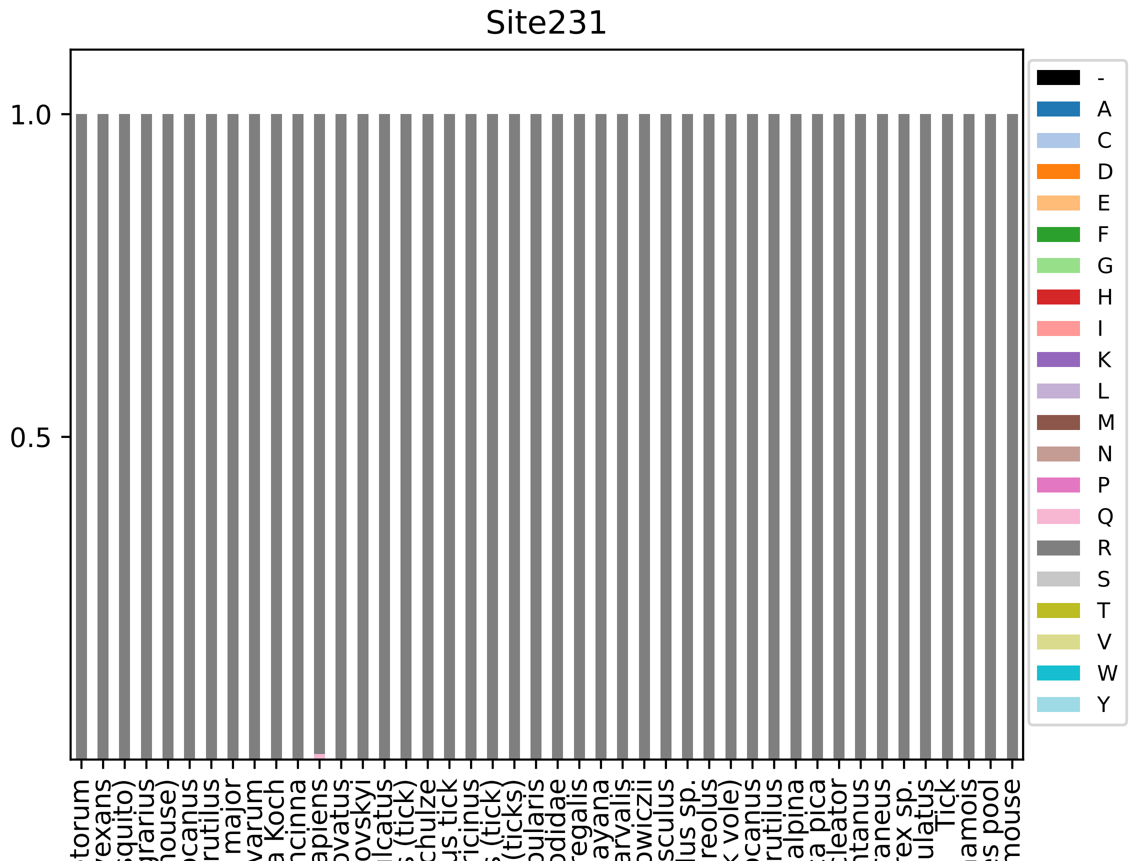
Site231
1.0
0.5
-
A
C
D
E
F
G
H
I
K
L
M
N
P
Q
R
S
T
V
W
Y

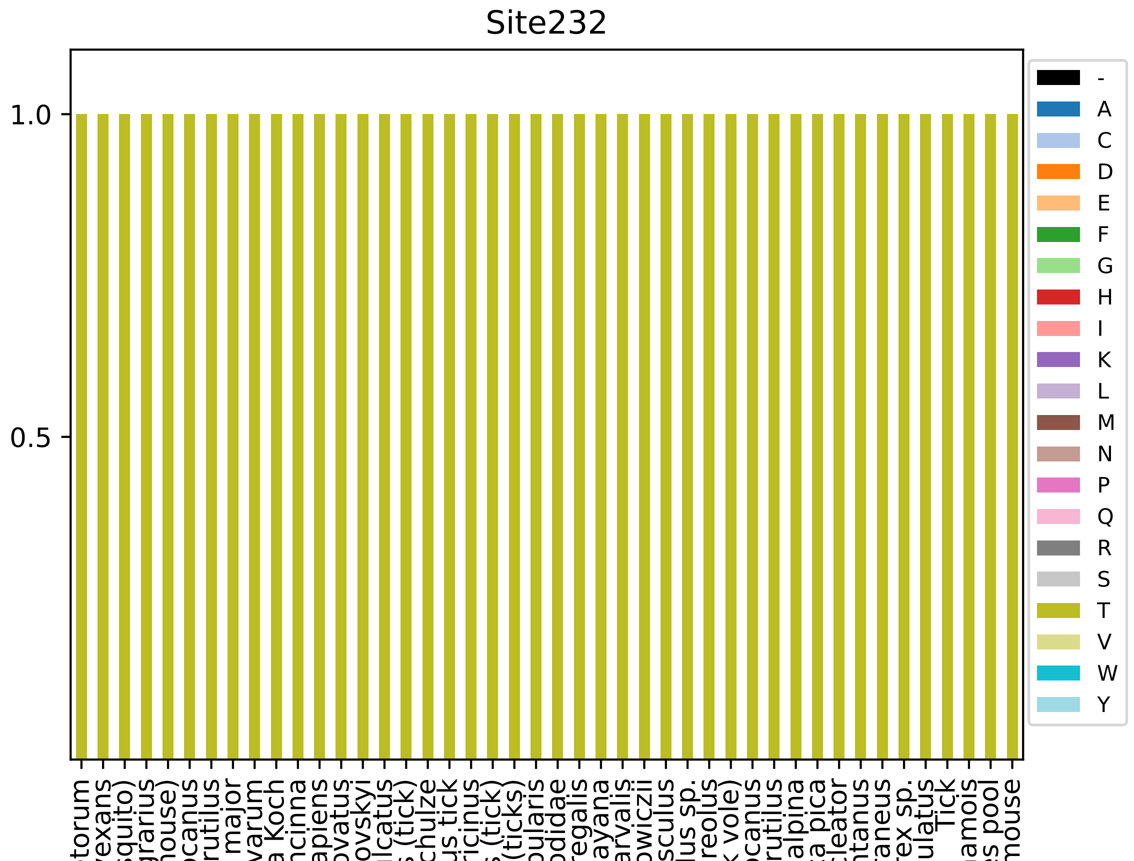
Site232
1.0
0.5
-
A
C
D
E
F
G
H
I
K
L
M
N
P
Q
R
S
T
V
W
Y
Tick

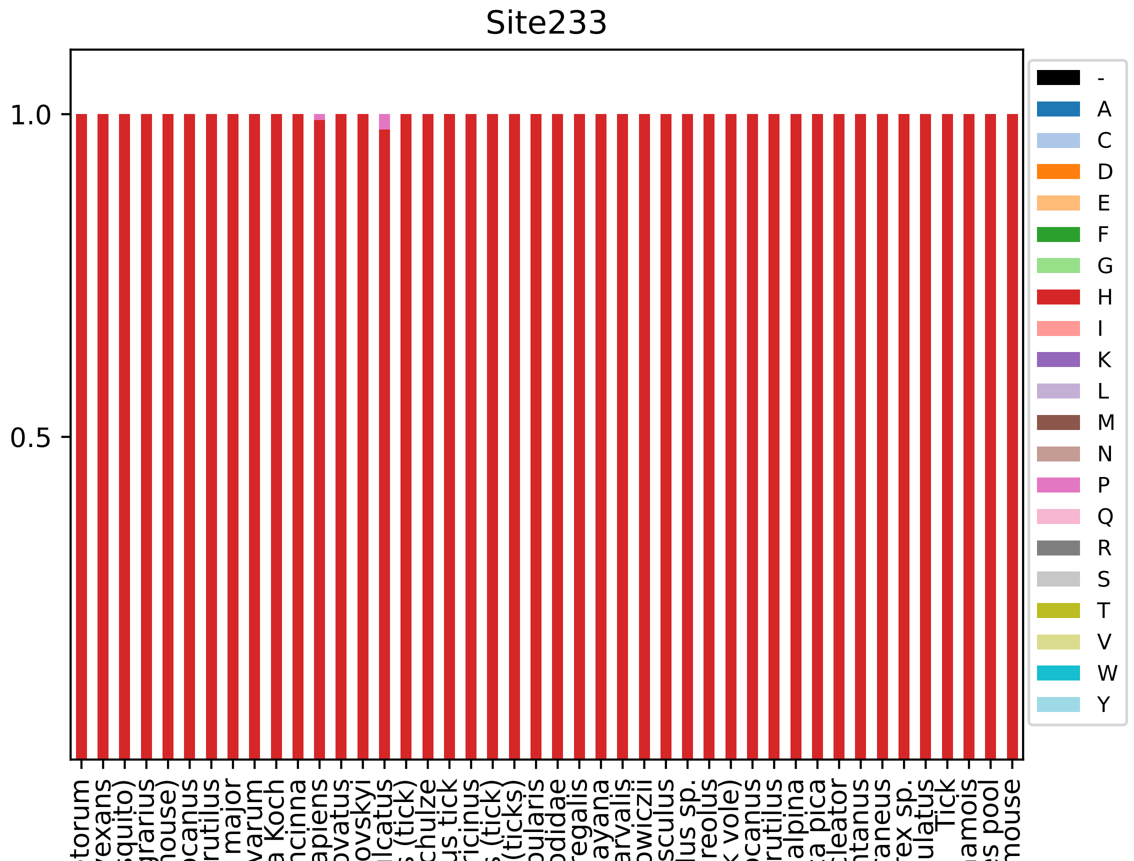
Site233
1.0
0.5
-
A
C
D
E
F
G
H
I
K
L
M
N
P
Q
R
S
T
V
W
Y

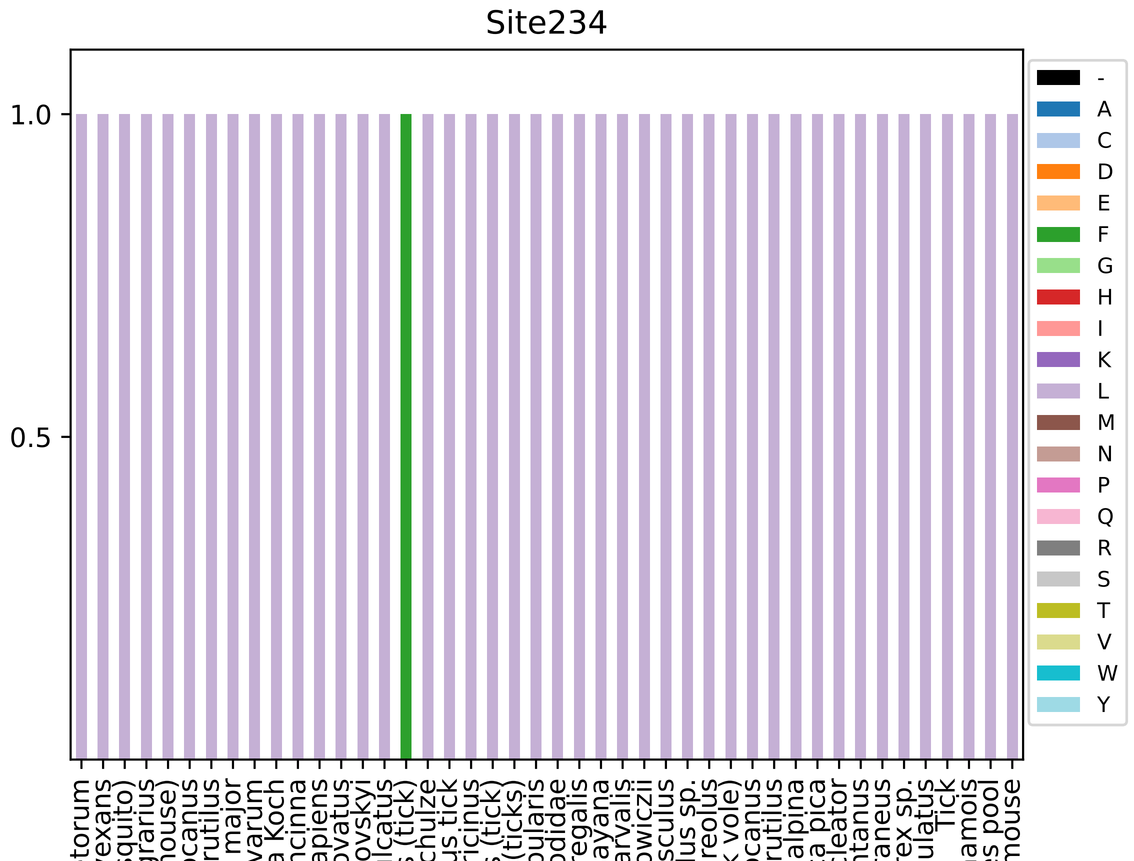

Site234
1.0
0.5
-
A
C
D
E
F
G
H
I
K
L
M
N
P
Q
R
S
T
V
W
Y

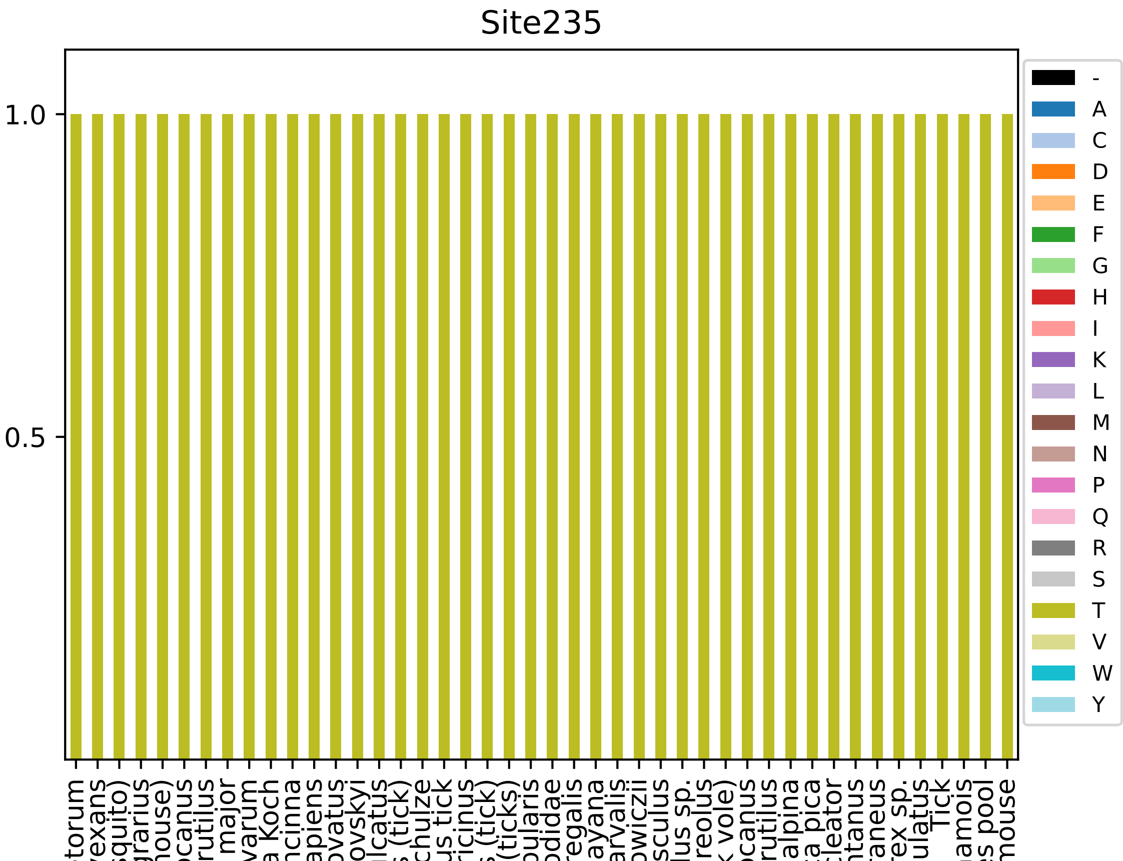

Site235
1.0
0.5
-
A
C
D
E
F
G
H
I
K
L
M
N
P
Q
R
S
T
V
W
Y

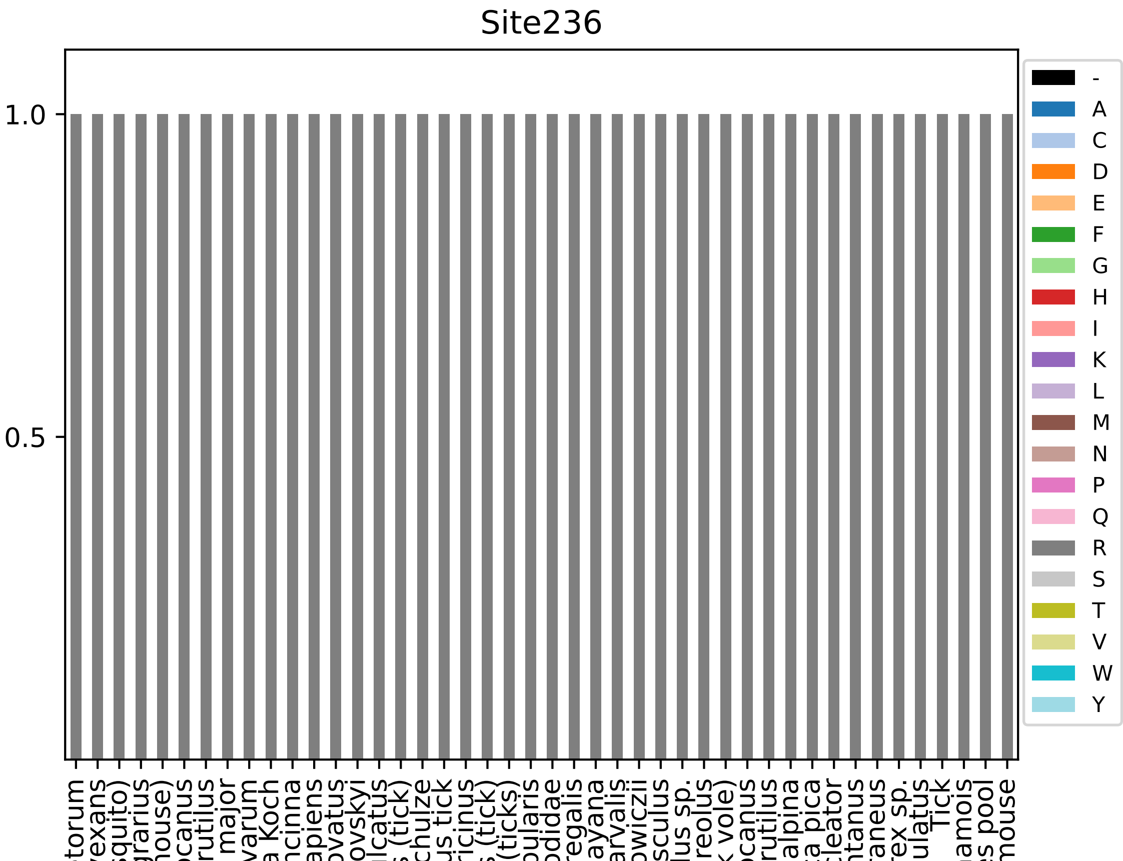
Site236
1.0
0.5
-
A
C
D
E
F
G
H
I
K
L
M
N
P
Q
R
S
T
V
W
Y

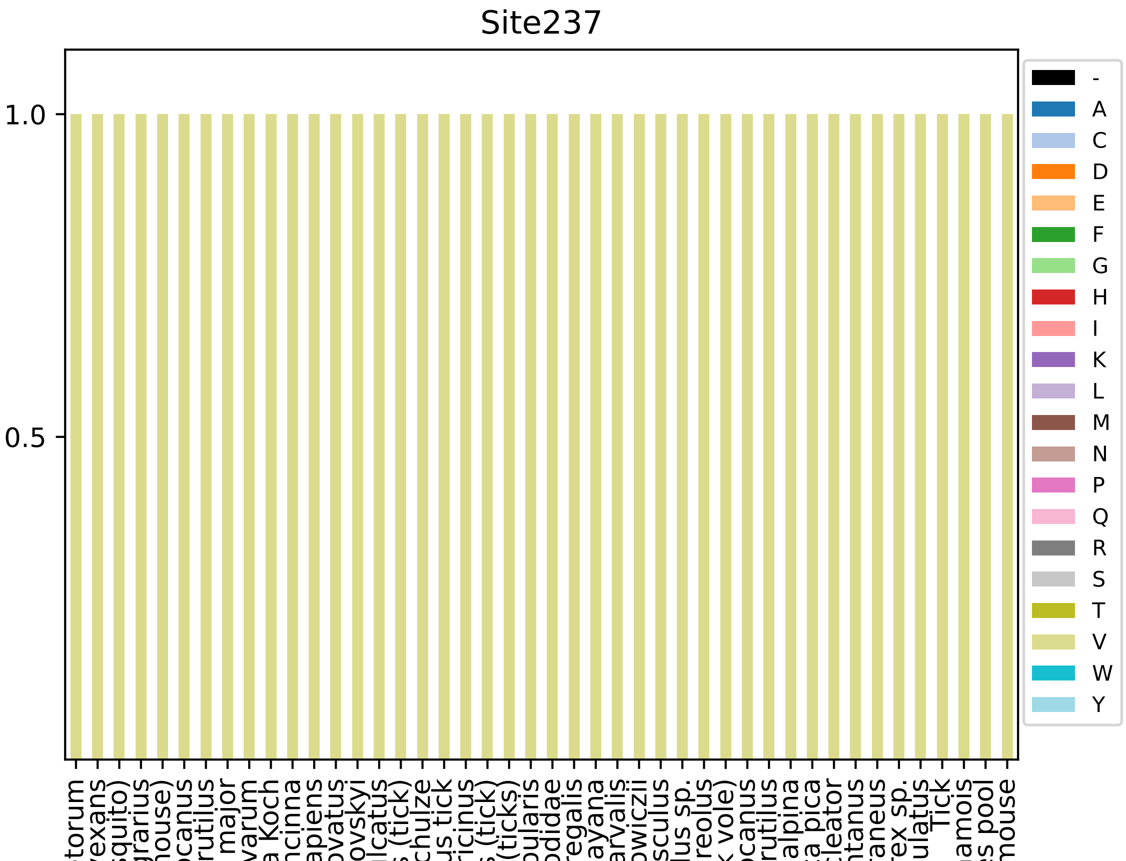

Site237
1.0
0.5
-
A
C
D
E
F
G
H
I
K
L
M
N
P
Q
R
S
T
V
W
Y

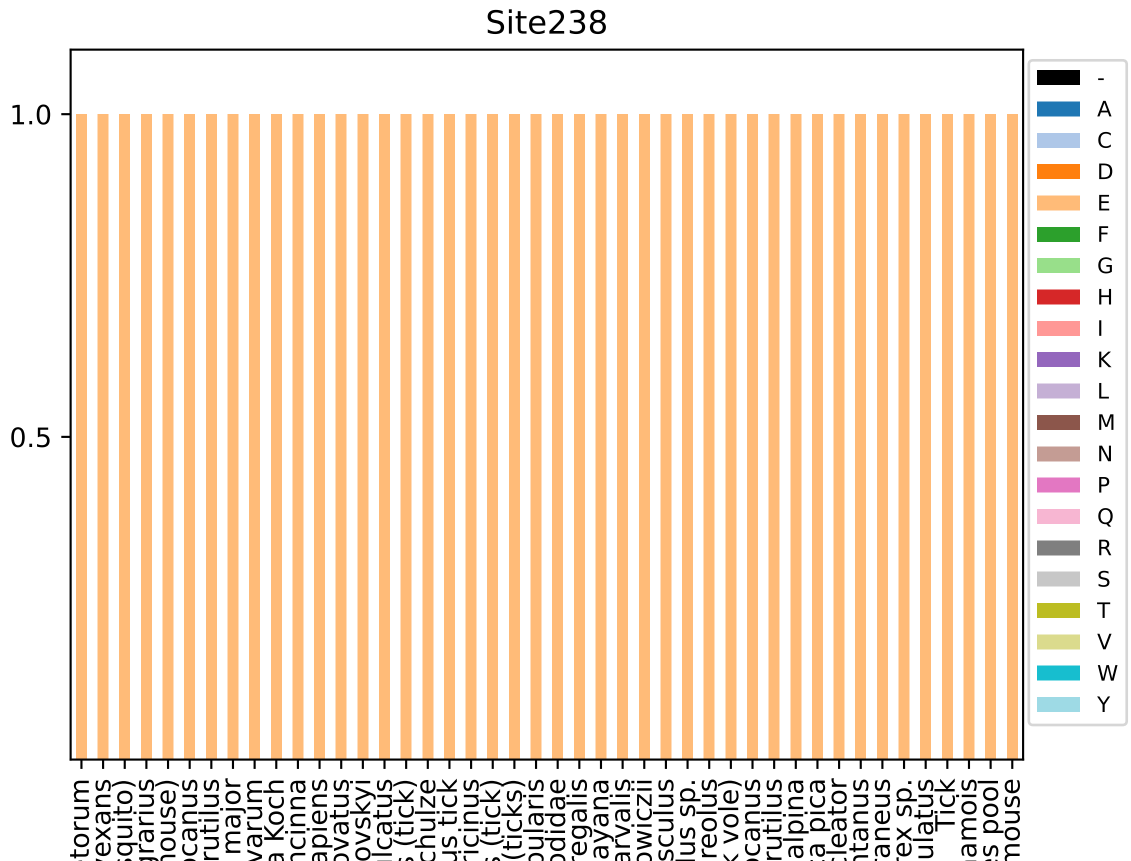
Site238
1.0
0.5
-
A
C
D
E
F
G
H
I
K
L
M
N
P
Q
R
S
T
V
W
Y

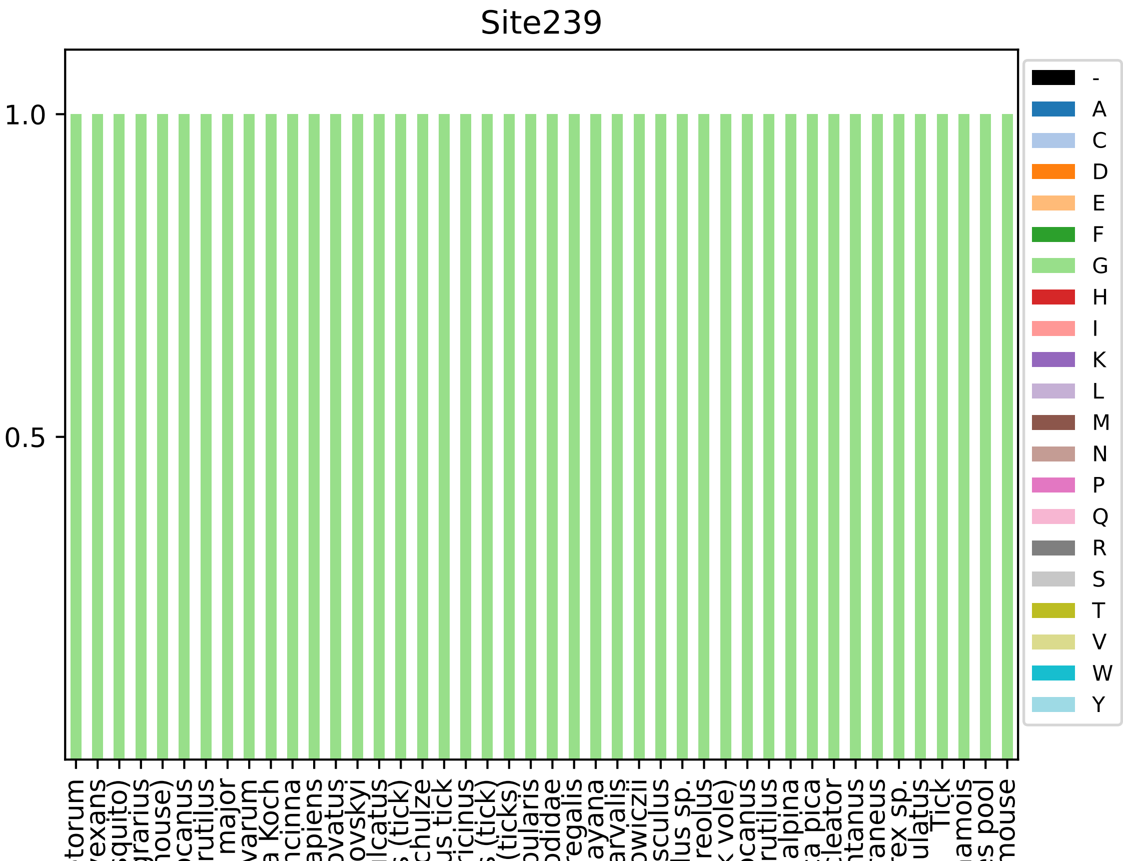

Site239
1.0
0.5
-
A
C
D
E
F
G
H
I
K
L
M
N
P
Q
R
S
T
V
W
Y

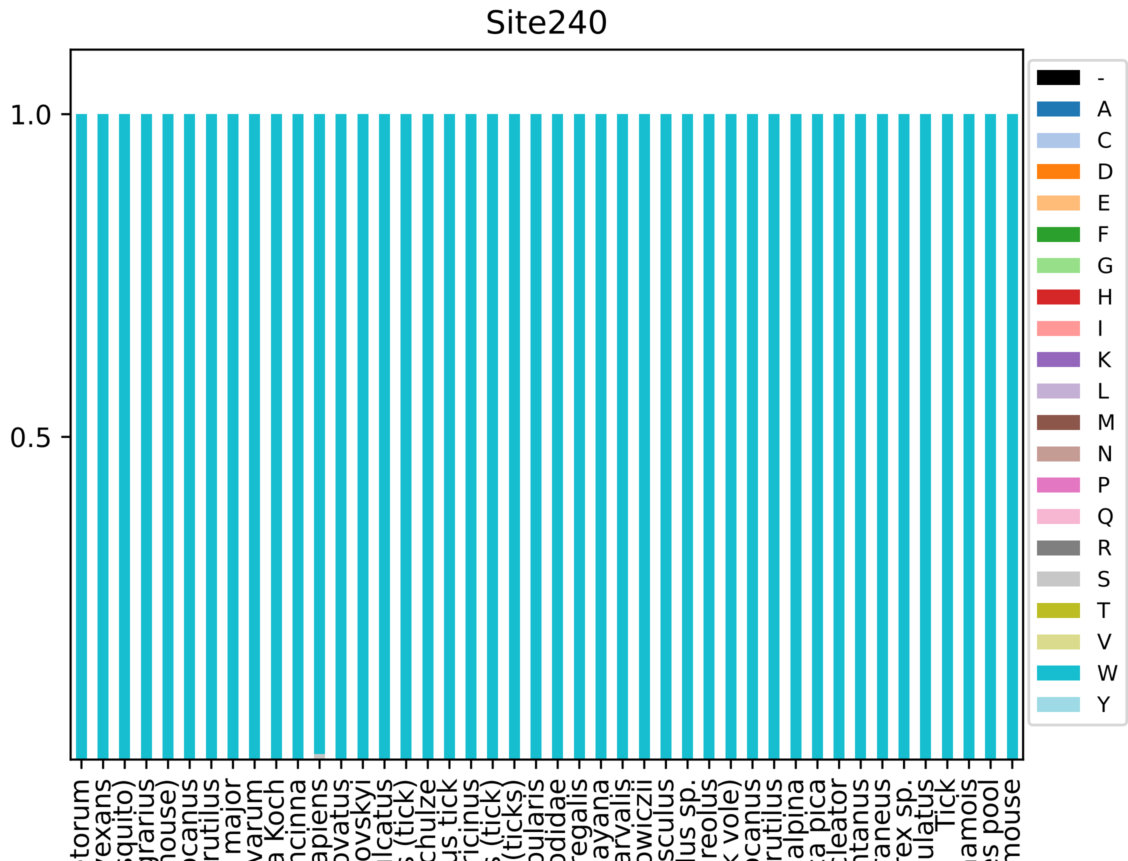
Site240
1.0
0.5
-
A
C
D
E
F
G
H
I
K
L
M
N
P
Q
R
S
T
V
W
Y

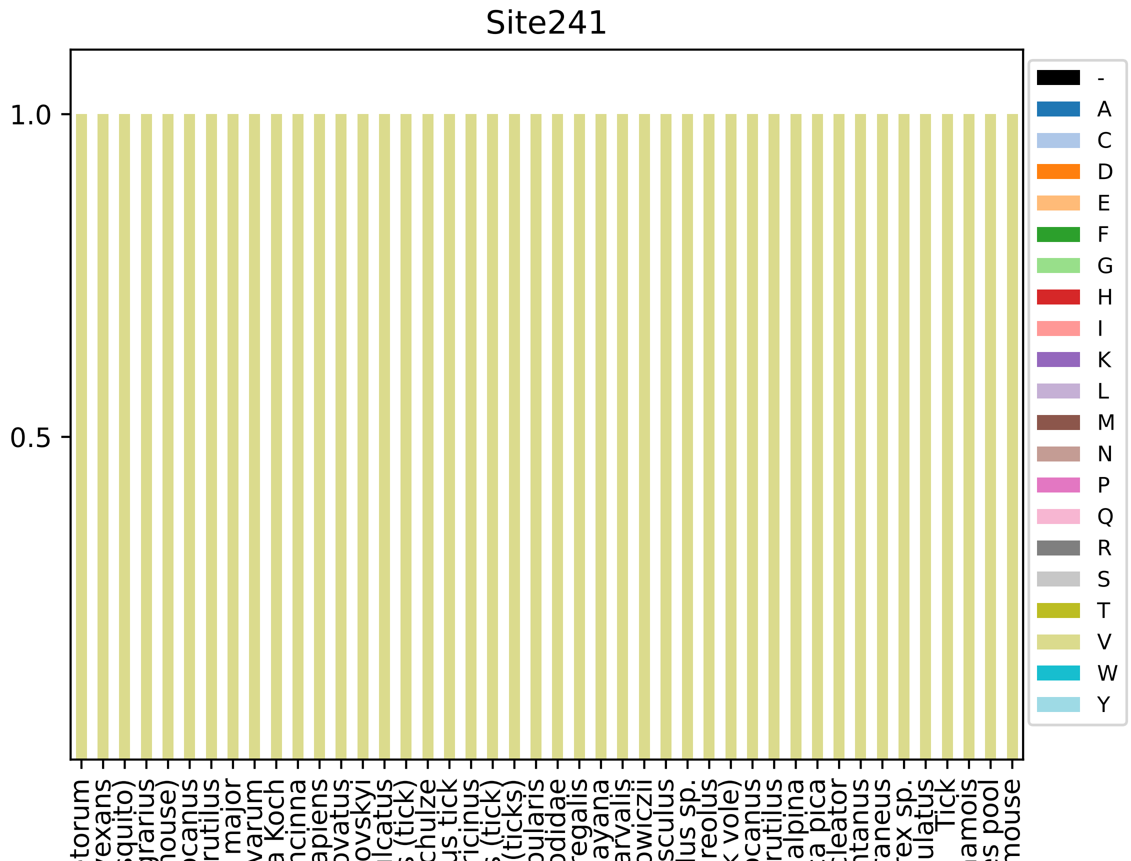
Site241
1.0
0.5
-
A
C
D
E
F
G
H
I
K
L
M
N
P
Q
R
S
T
V
W
Y

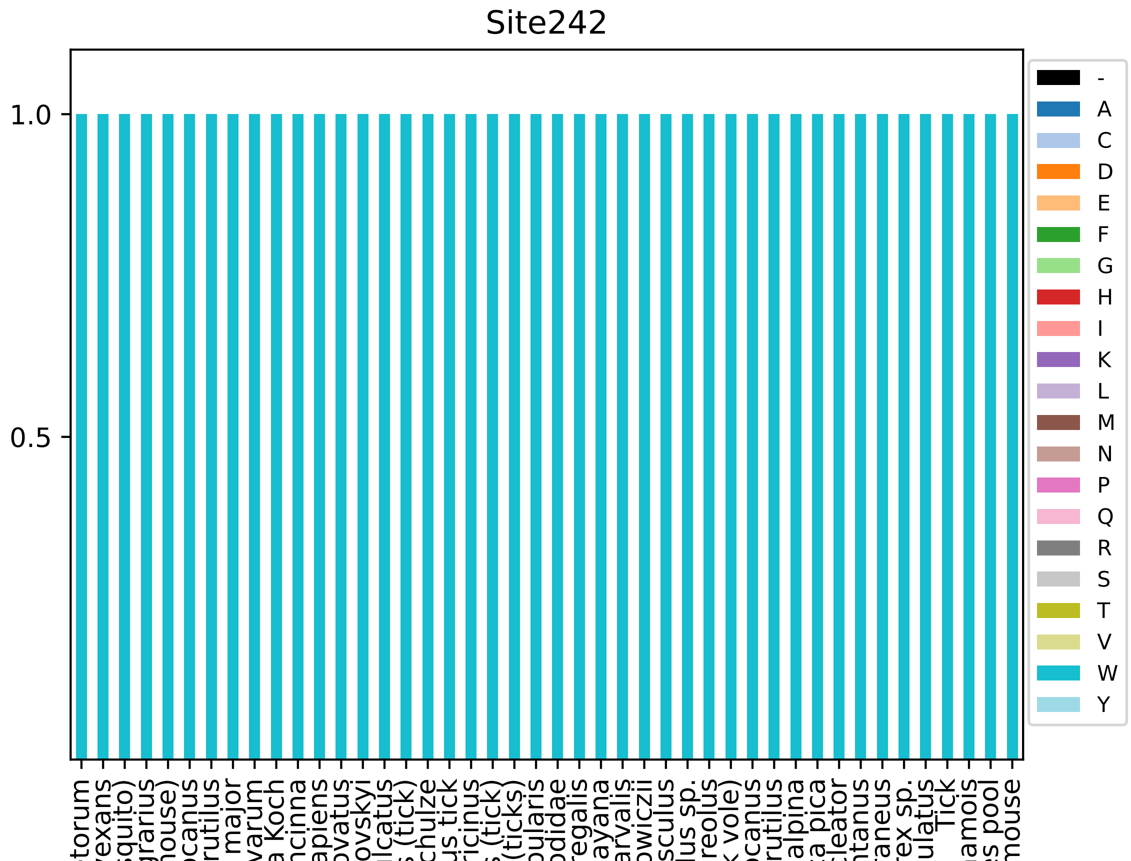
Site242
1.0
0.5
-
A
C
D
E
F
G
H
I
K
L
M
N
P
Q
R
S
T
V
W
Y

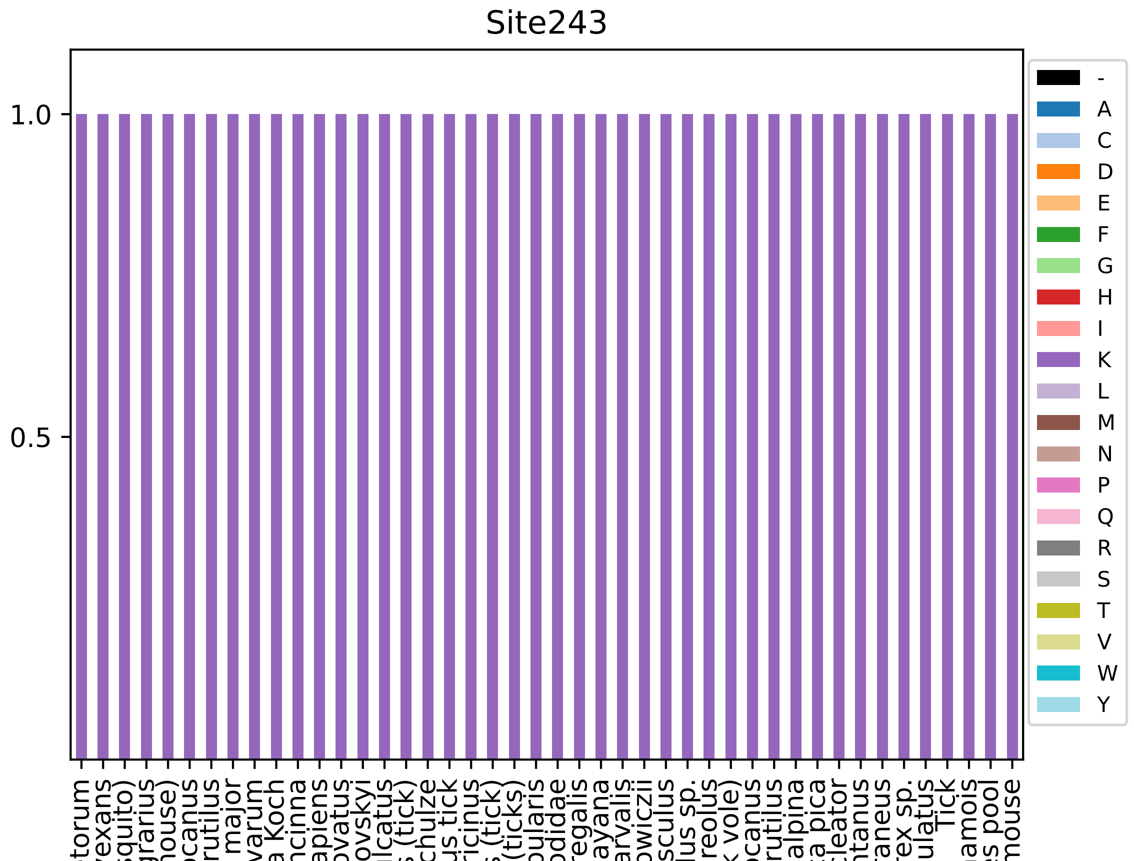

Site243
1.0
0.5
-
A
C
D
E
F
G
H
I
K
L
M
N
P
Q
R
S
T
V
W
Y

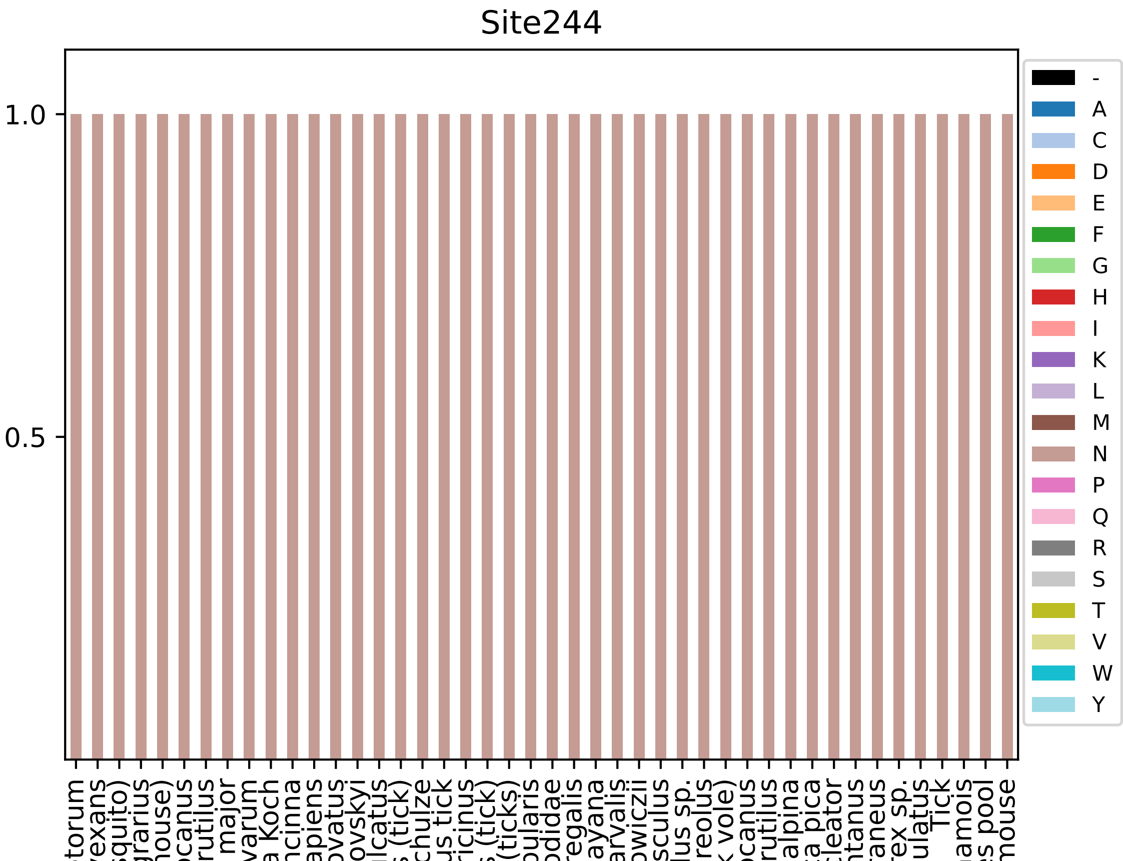
Site244
1.0
0.5
-
A
C
D
E
F
G
H
I
K
L
M
N
P
Q
R
S
T
V
W
Y

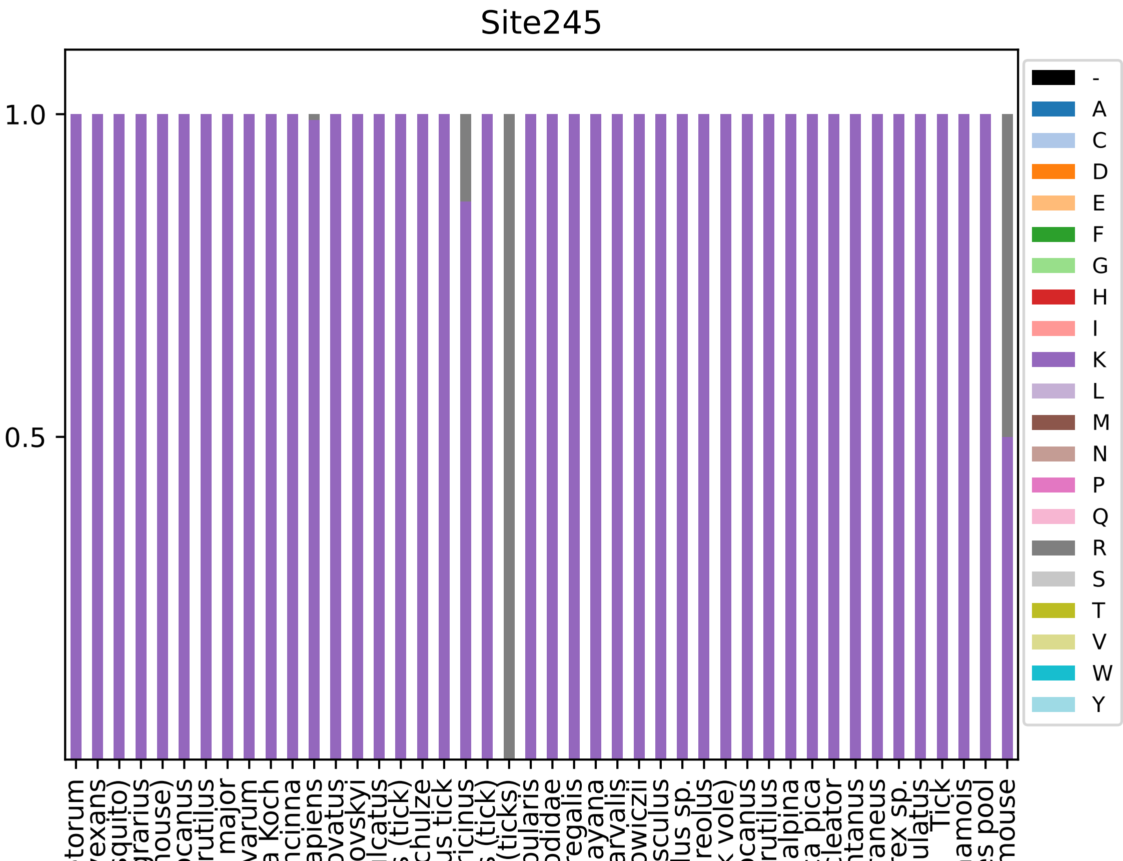
Site245
1.0
0.5
-
A
C
D
E
F
G
H
I
K
L
M
N
P
Q
R
S
T
V
W
Y

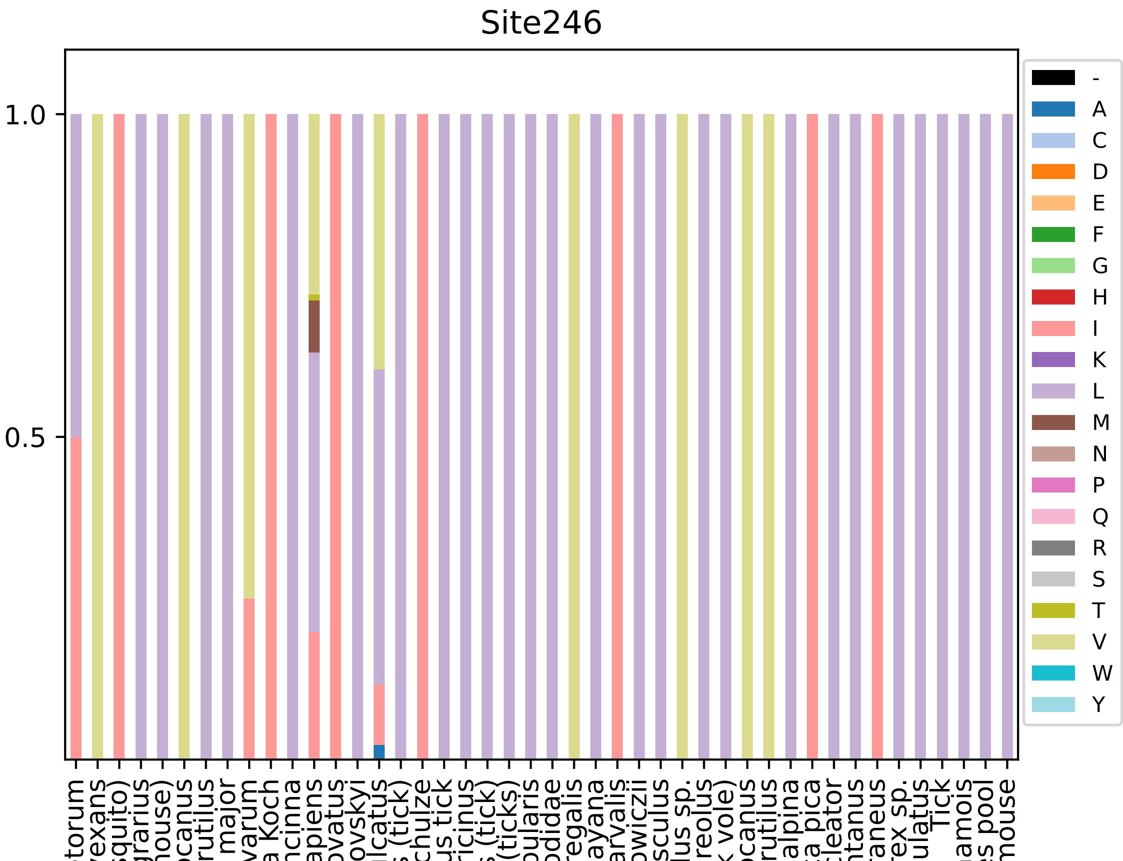

Site246
1.0
0.5
-
A
C
D
E
F
G
H
I
K
L
M
N
P
Q
R
S
T
V
W
Y

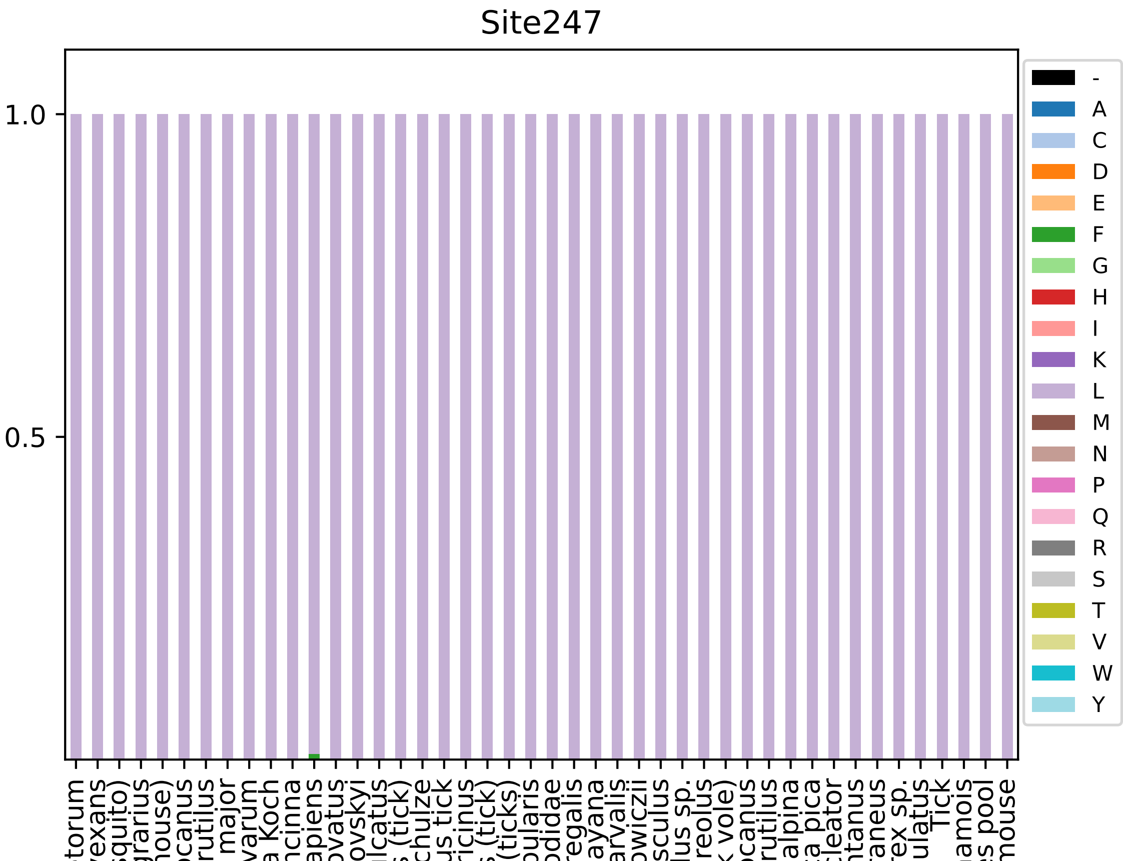
Site247
1.0
0.5
-
A
C
D
E
F
G
H
I
K
L
M
N
P
Q
R
S
T
V
W
Y

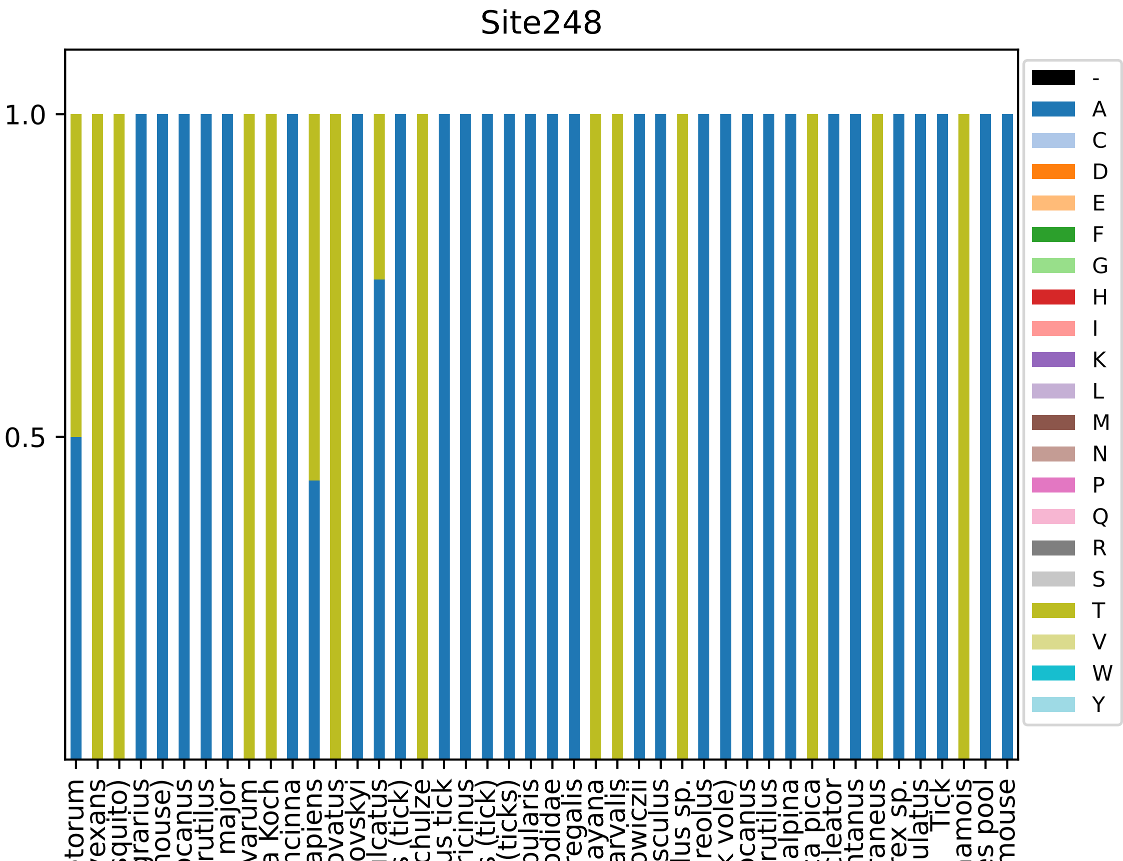
Site248
1.0
0.5
-
A
C
D
E
F
G
H
I
K
L
M
N
P
Q
R
S
T
V
W
Y

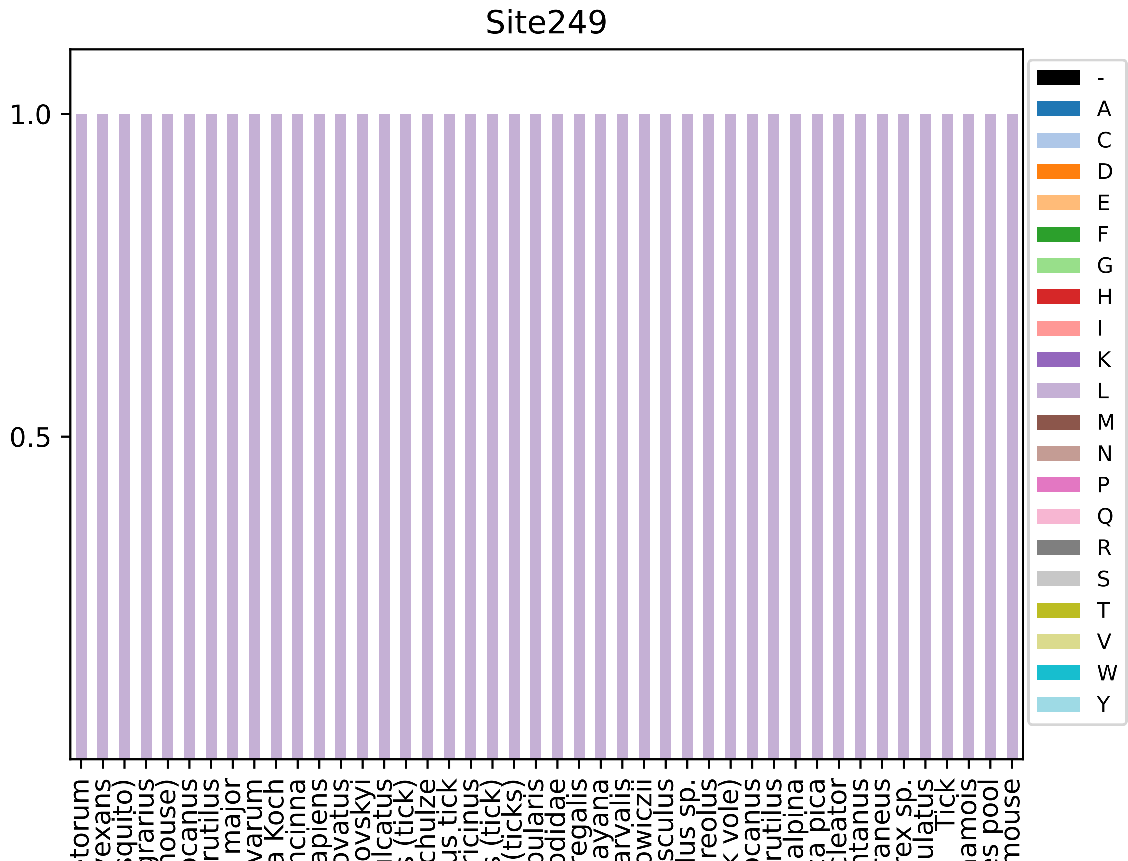
Site249
1.0
0.5
-
A
C
D
E
F
G
H
I
K
L
M
N
P
Q
R
S
T
V
W
Y

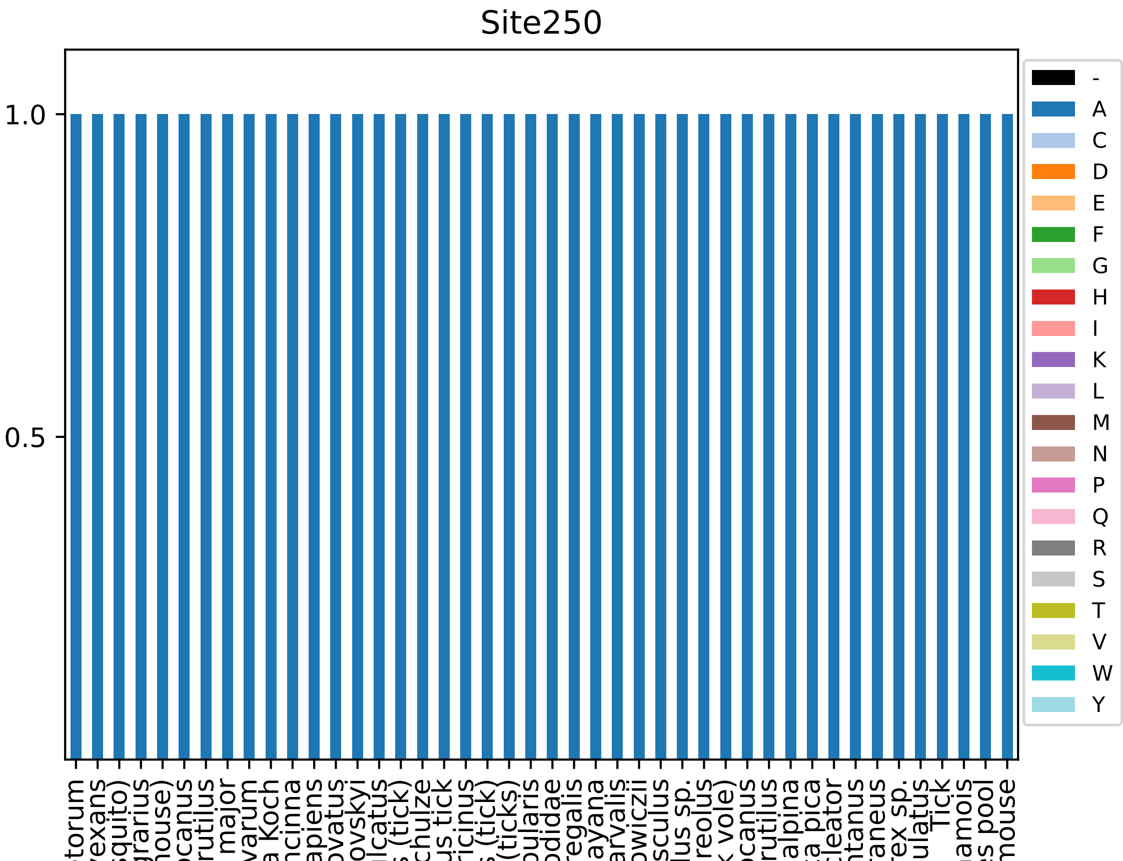

Site250
1.0
0.5
-
A
C
D
E
F
G
H
I
K
L
M
N
P
Q
R
S
T
V
W
Y

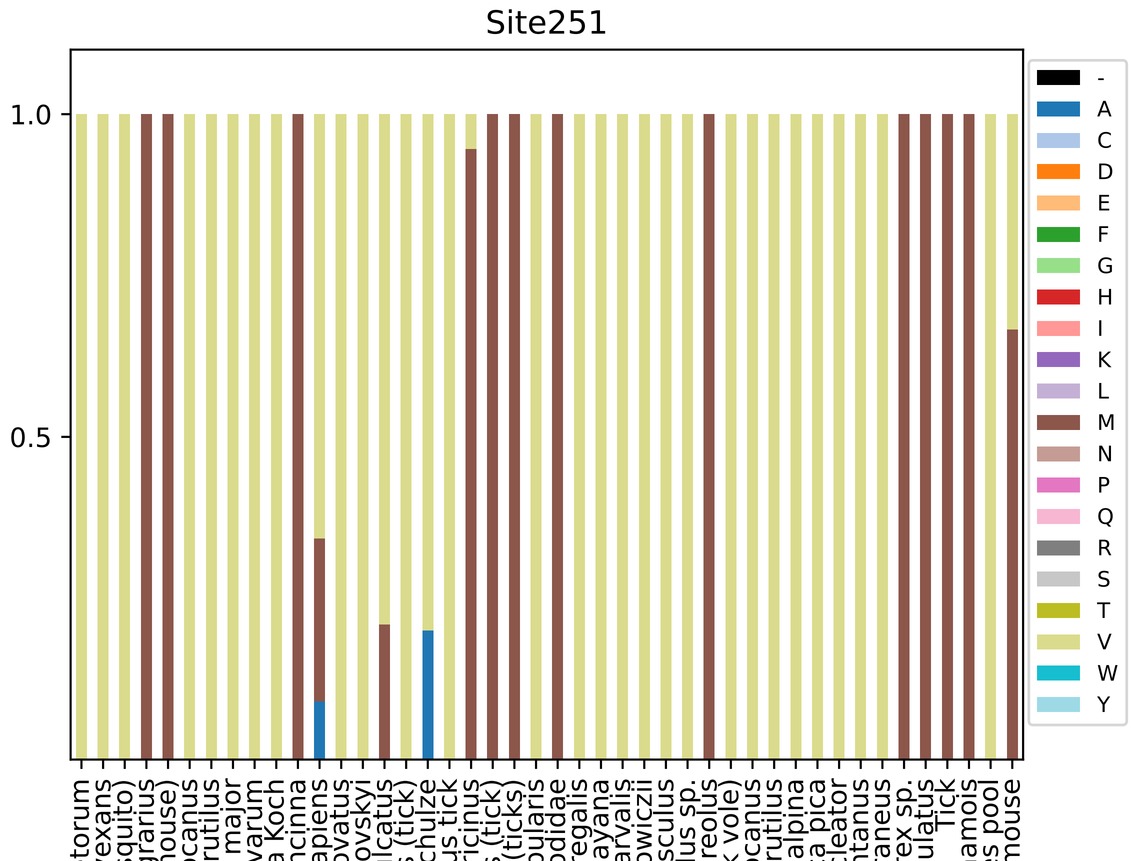

Site251
1.0
0.5
-
A
C
D
E
F
G
H
I
K
L
M
N
P
Q
R
S
T
V
W
Y
Tick

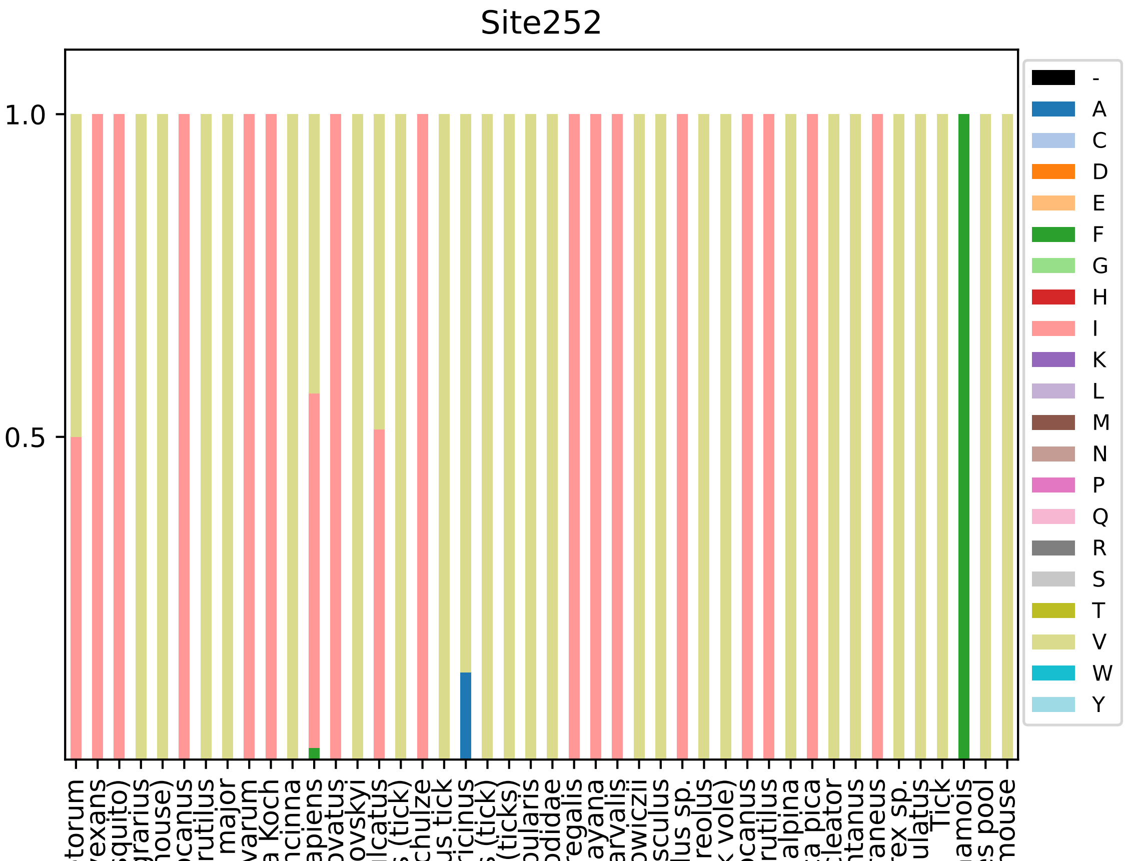
Site252
1.0
0.5
-
A
C
D
E
F
G
H
I
K
L
M
N
P
Q
R
S
T
V
W
Y

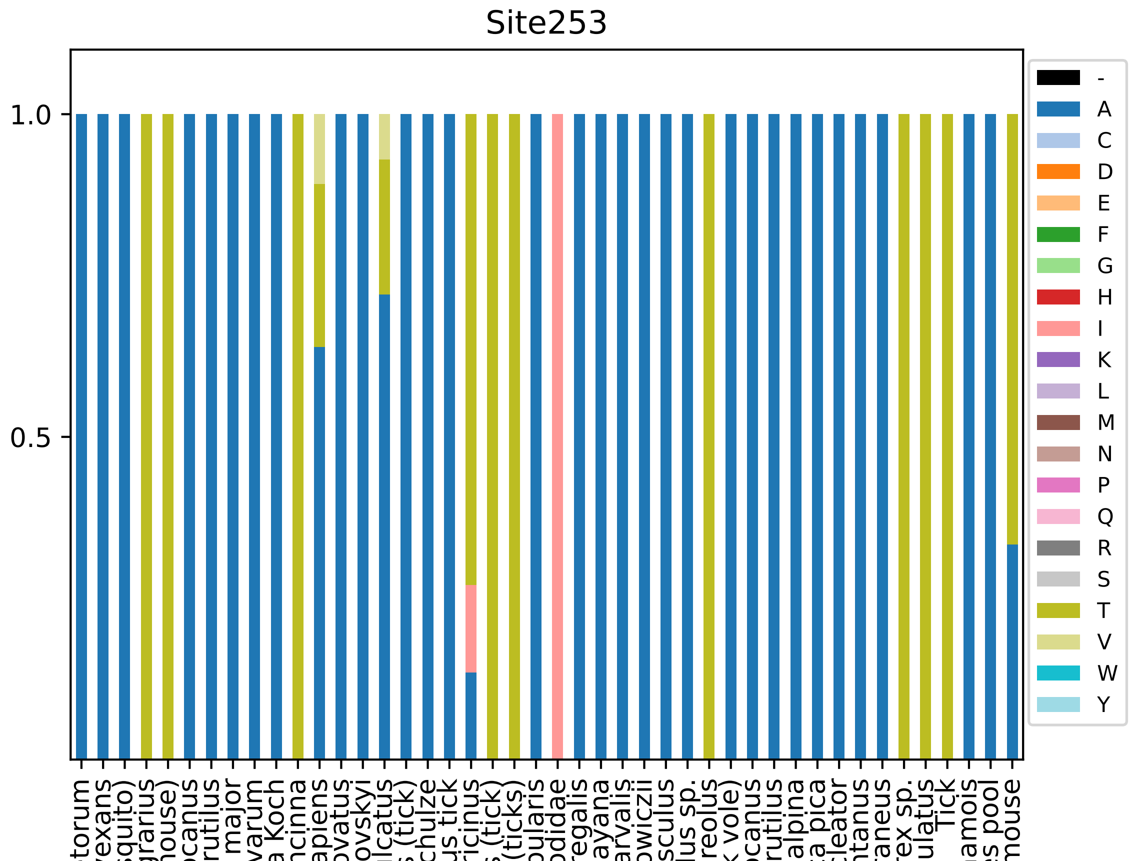
Site253
1.0
0.5
-
A
C
D
E
F
G
H
I
K
L
M
N
P
Q
R
S
T
V
W
Y

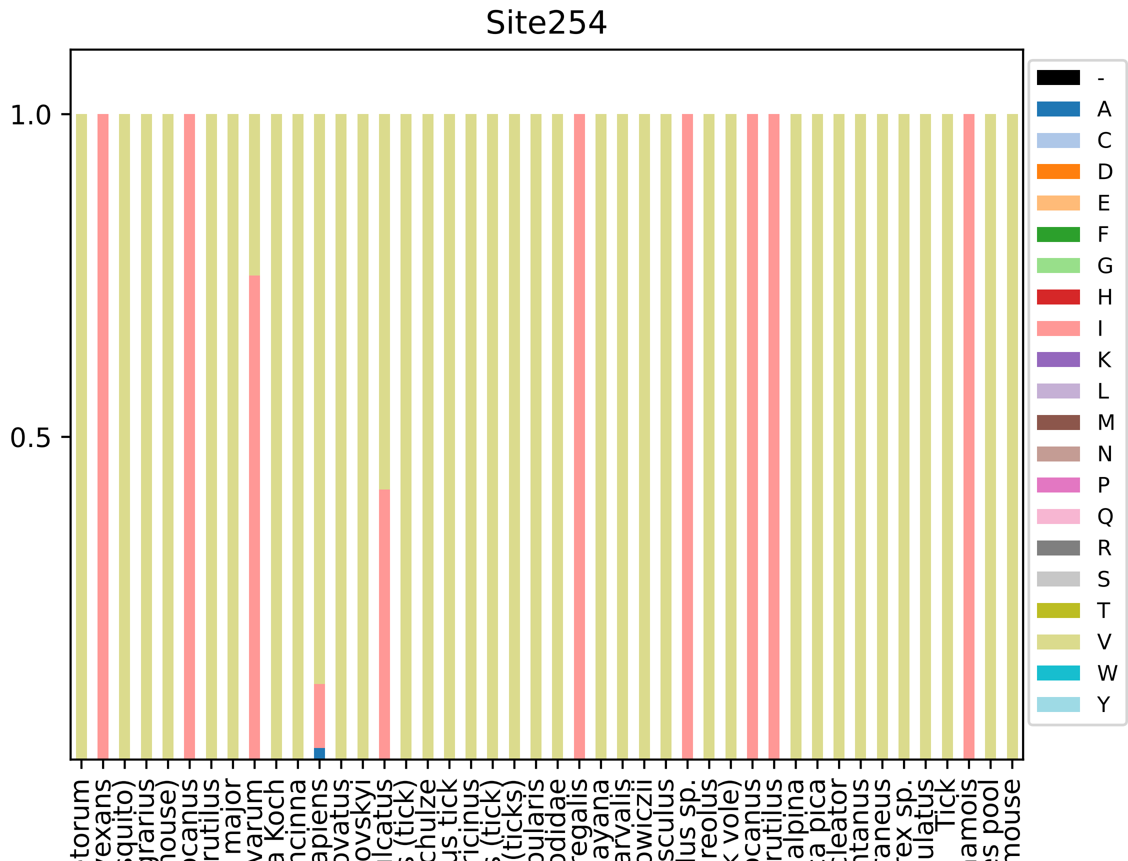

Site254
1.0
0.5
-
A
C
D
E
F
G
H
I
K
L
M
N
P
Q
R
S
T
V
W
Y

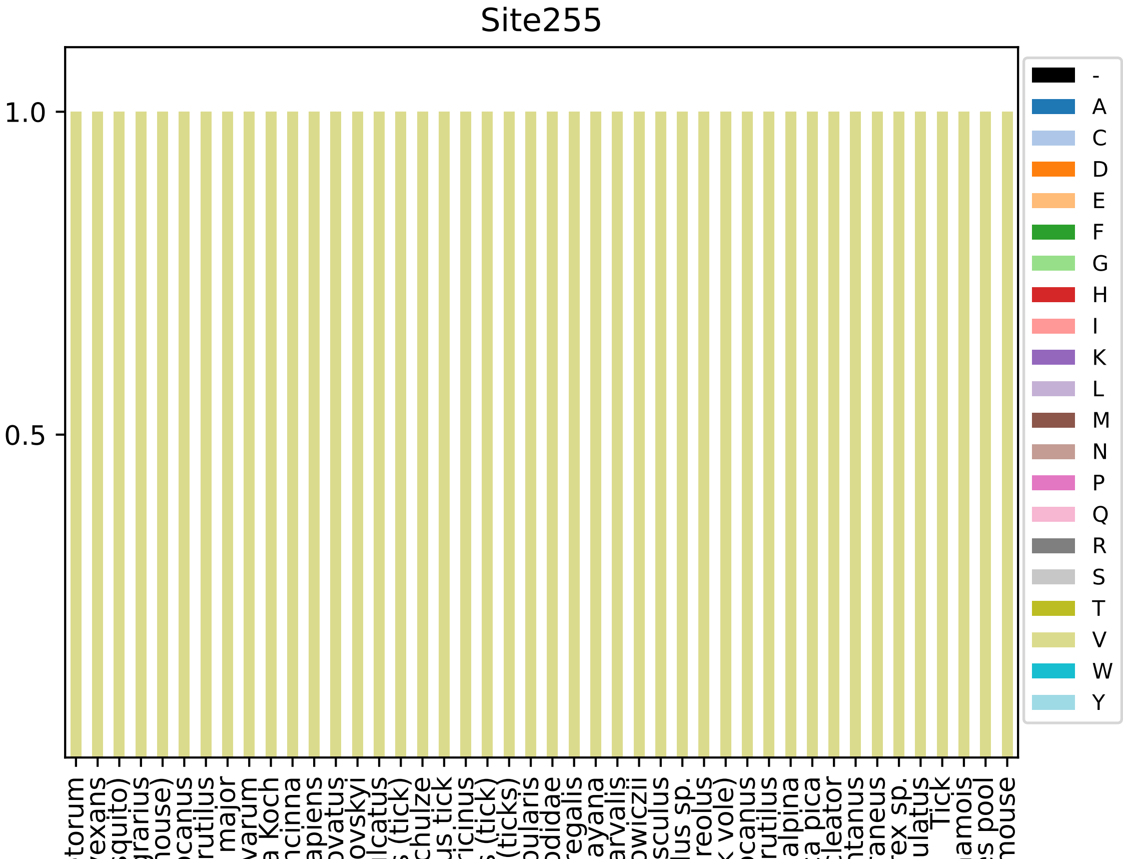
Site255
1.0
0.5
-
A
C
D
E
F
G
H
I
K
L
M
N
P
Q
R
S
T
V
W
Y

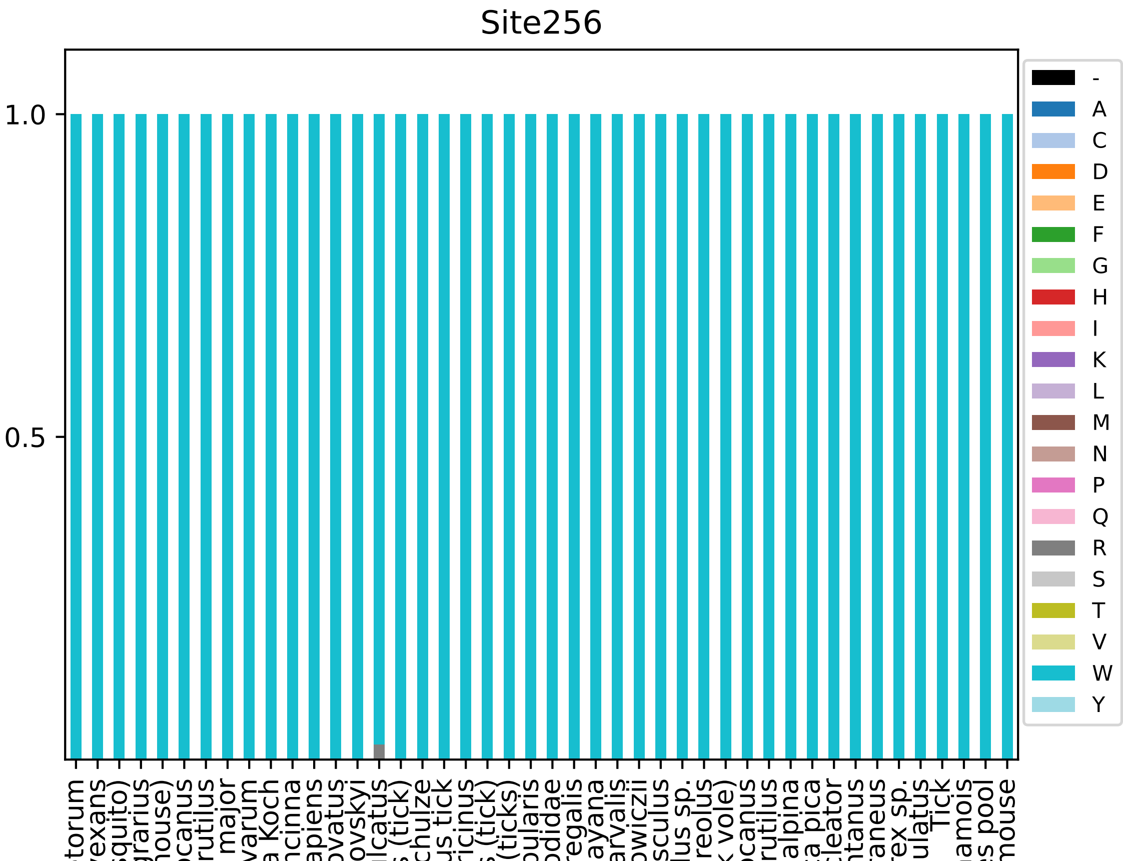
Site256
1.0
0.5
-
A
C
D
E
F
G
H
I
K
L
M
N
P
Q
R
S
T
V
W
Y

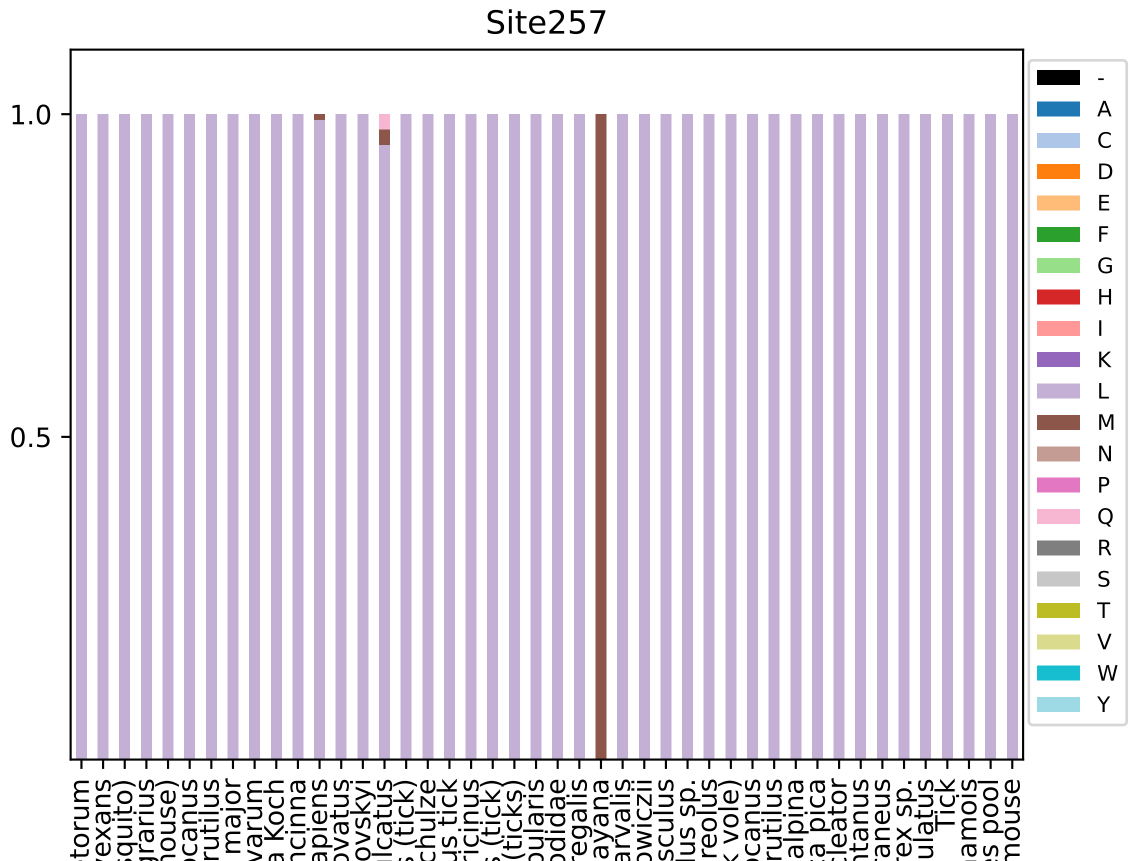

Site257
1.0
0.5
-
A
C
D
E
F
G
H
I
K
L
M
N
P
Q
R
S
T
V
W
Y

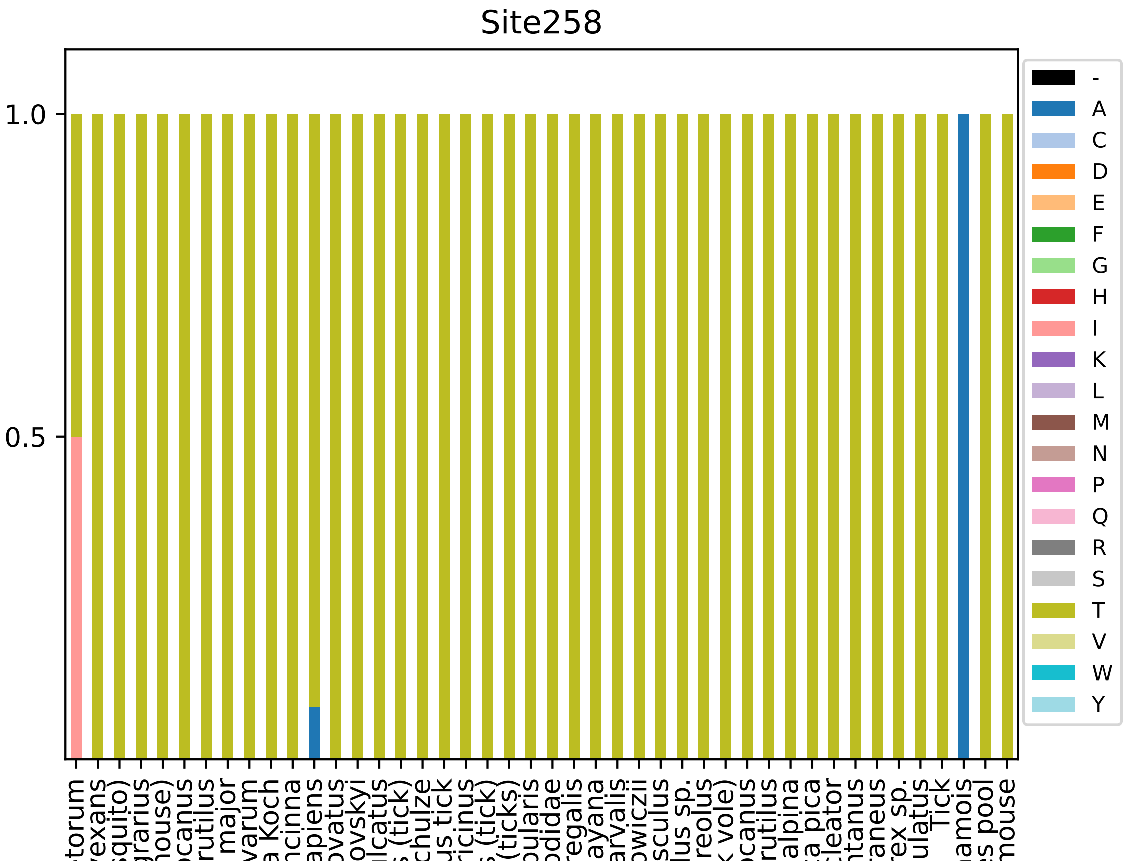

Site258
1.0
0.5
-
A
C
D
E
F
G
H
I
K
L
M
N
P
Q
R
S
T
V
W
Y

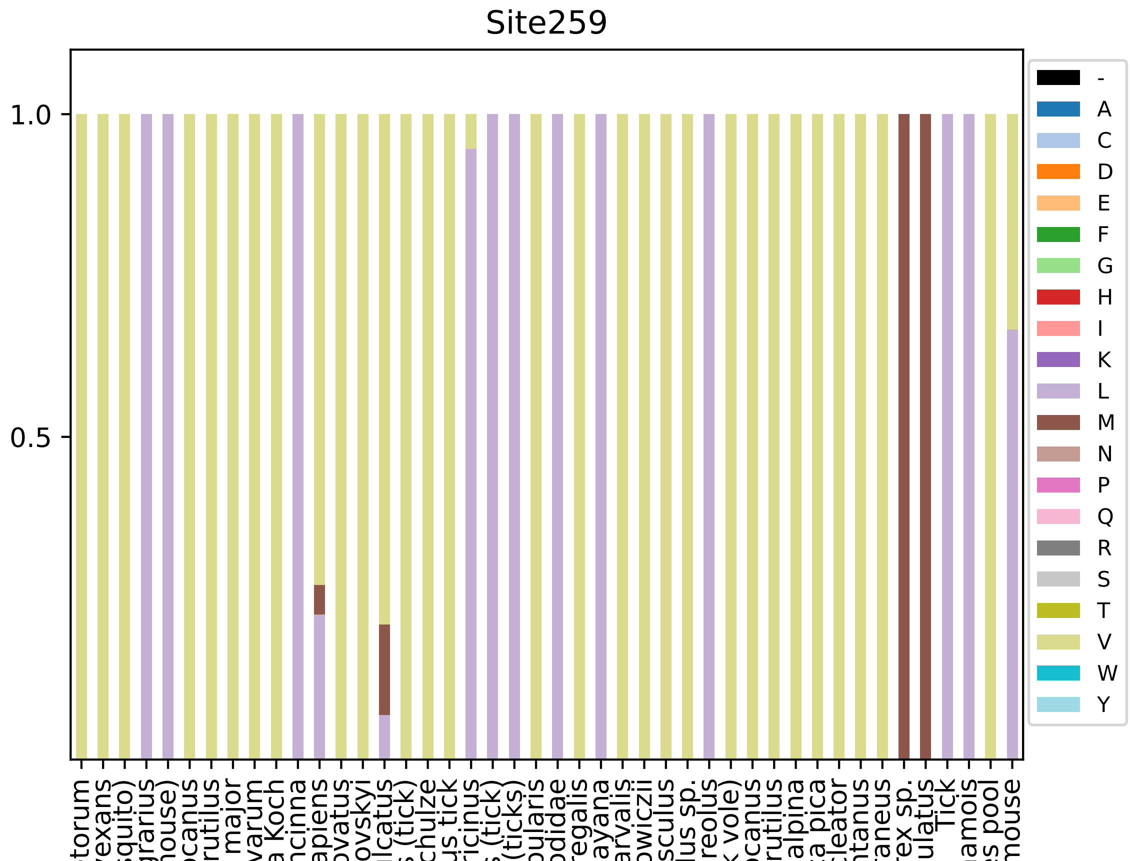
Site259
1.0
0.5
-
A
C
D
E
F
G
H
I
K
L
M
N
P
Q
R
S
T
V
W
Y

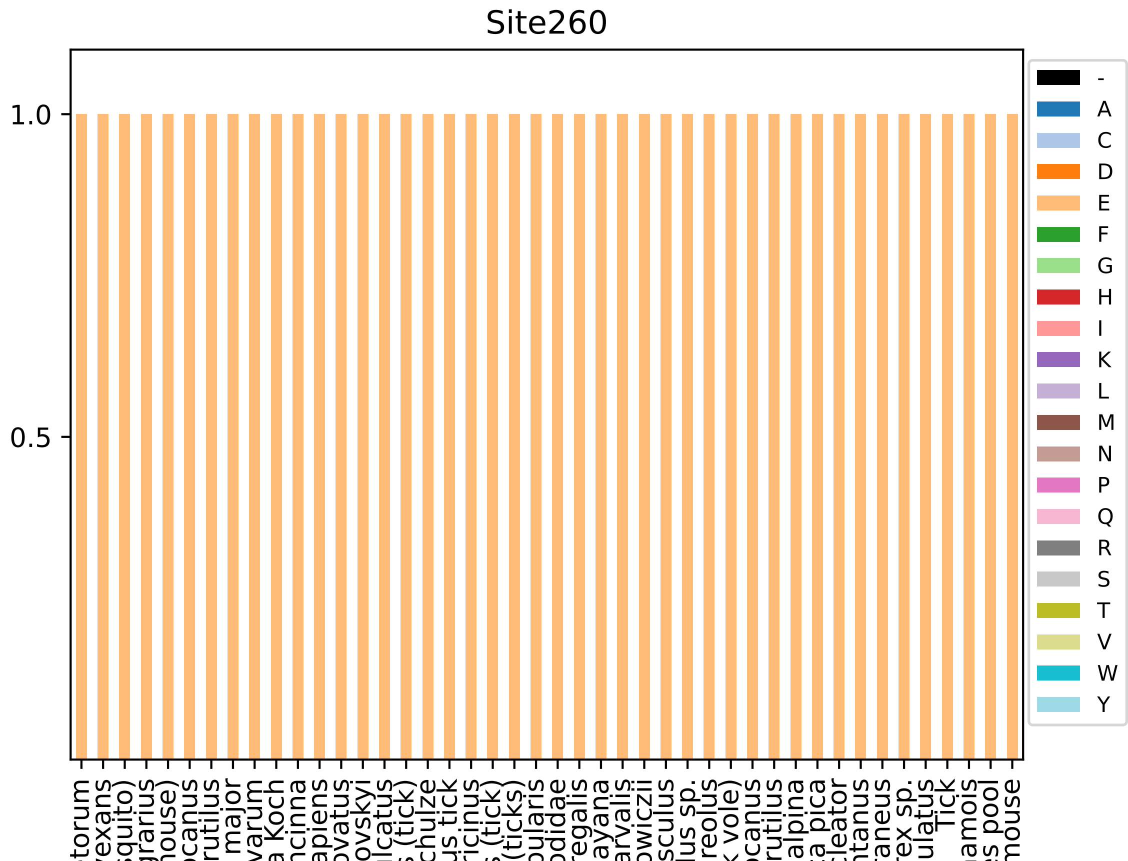
Site260
1.0
0.5
-
A
C
D
E
F
G
H
I
K
L
M
N
P
Q
R
S
T
V
W
Y

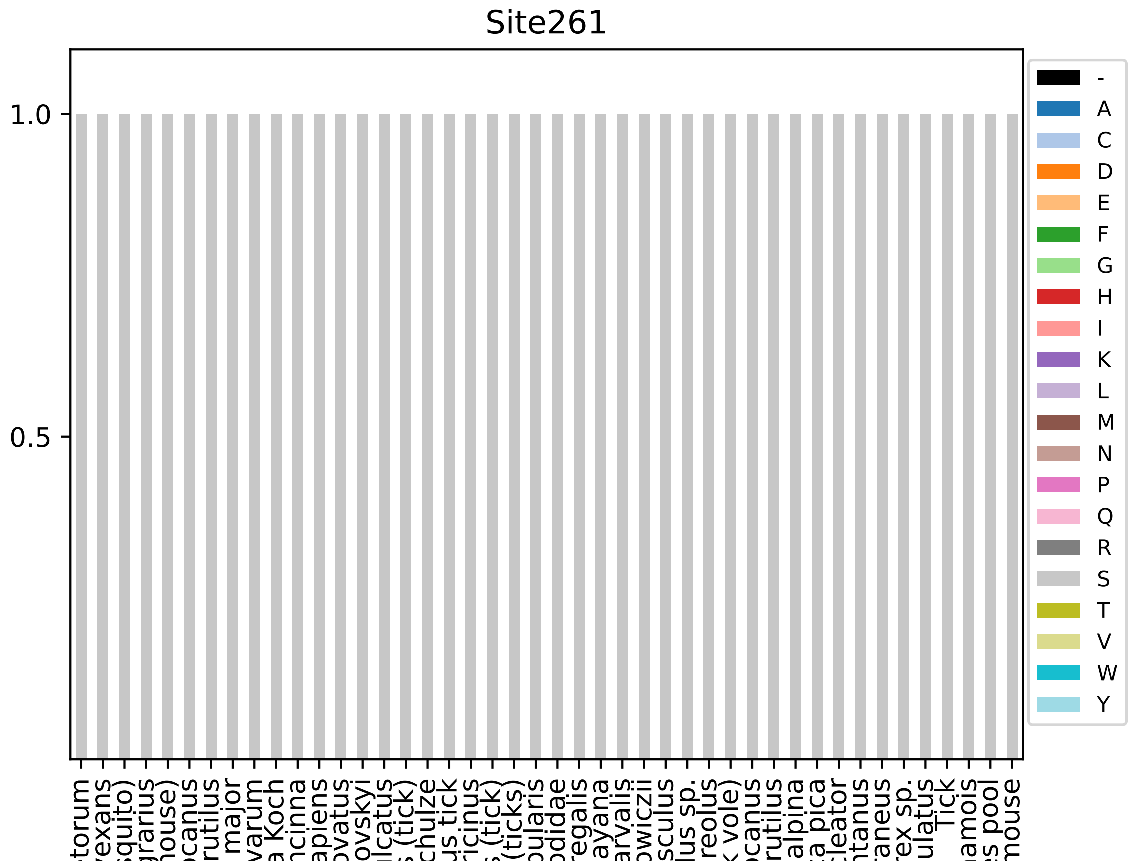
Site261
1.0
0.5
-
A
C
D
E
F
G
H
I
K
L
M
N
P
Q
R
S
T
V
W
Y

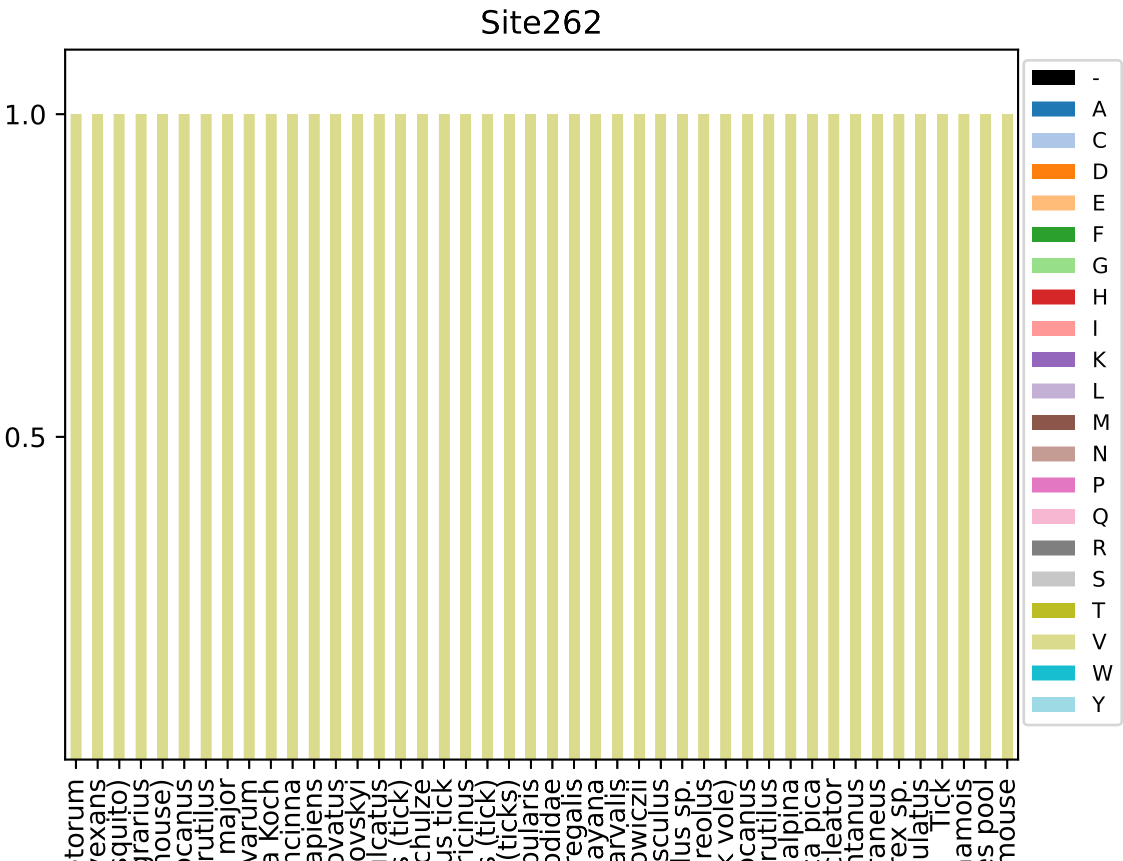
Site262
1.0
0.5
-
A
C
D
E
F
G
H
I
K
L
M
N
P
Q
R
S
T
V
W
Y

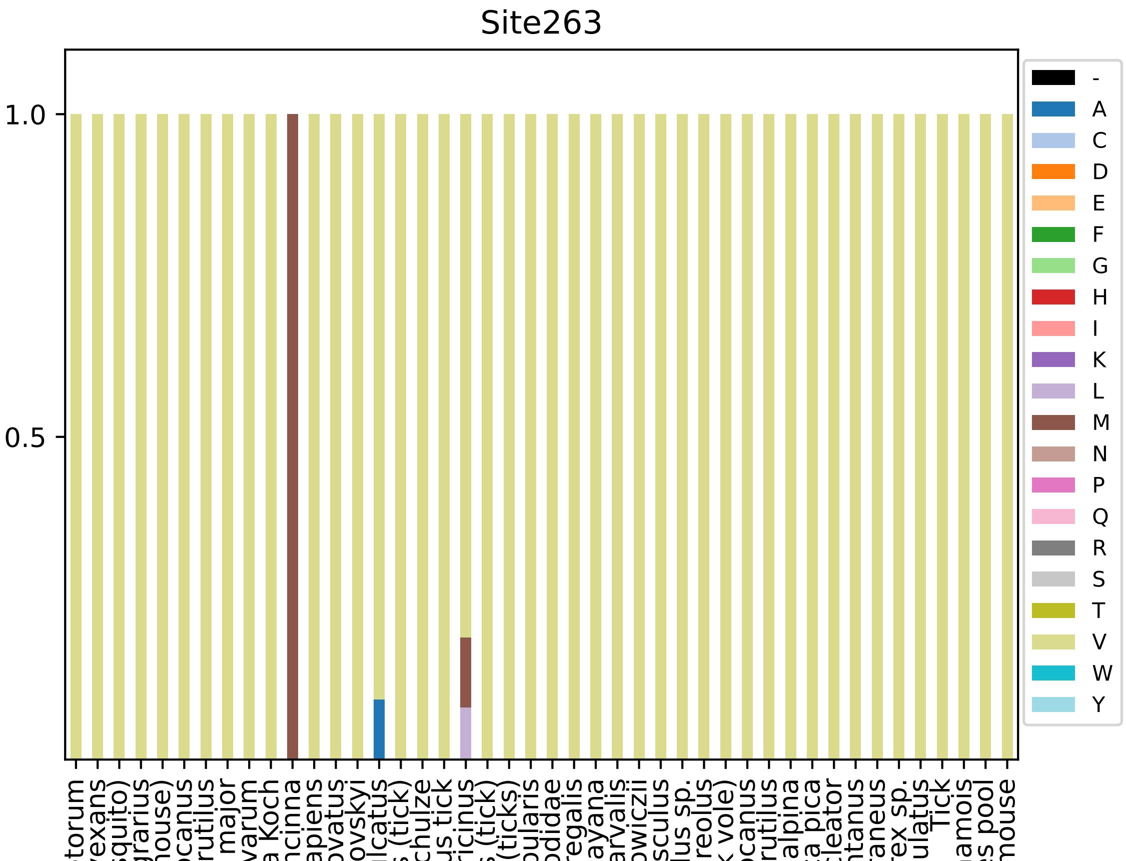

Site263
1.0
0.5
-
A
C
D
E
F
G
H
I
K
L
M
N
P
Q
R
S
T
V
W
Y

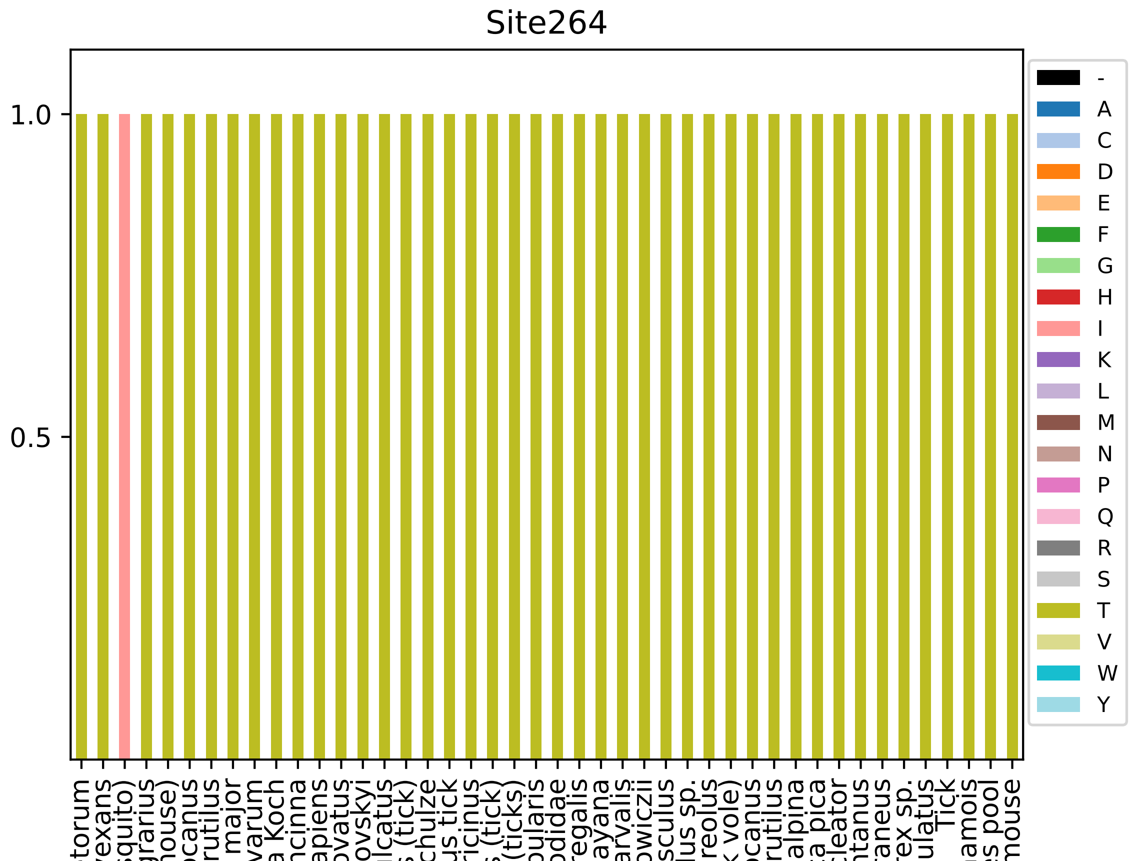
Site264
1.0
0.5
-
A
C
D
E
F
G
H
I
K
L
M
N
P
Q
R
S
T
V
W
Y

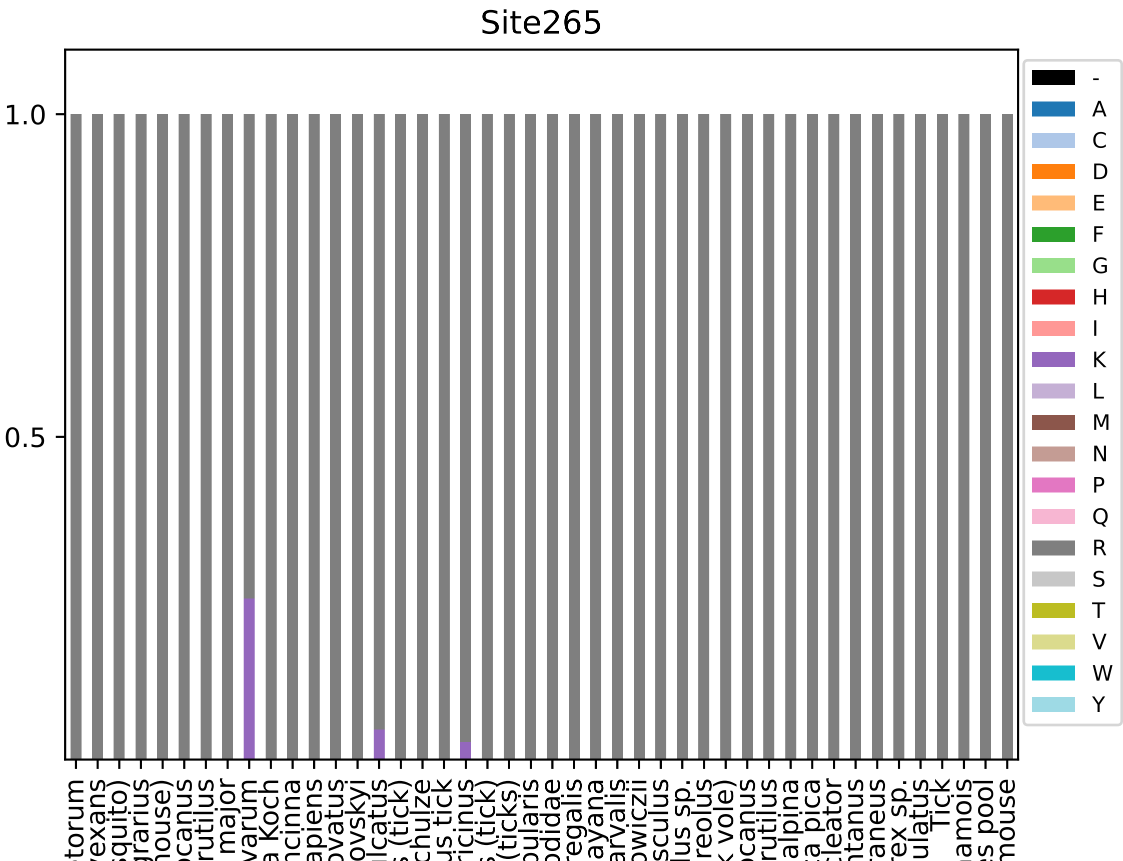
Site265
1.0
0.5
-
A
C
D
E
F
G
H
I
K
L
M
N
P
Q
R
S
T
V
W
Y

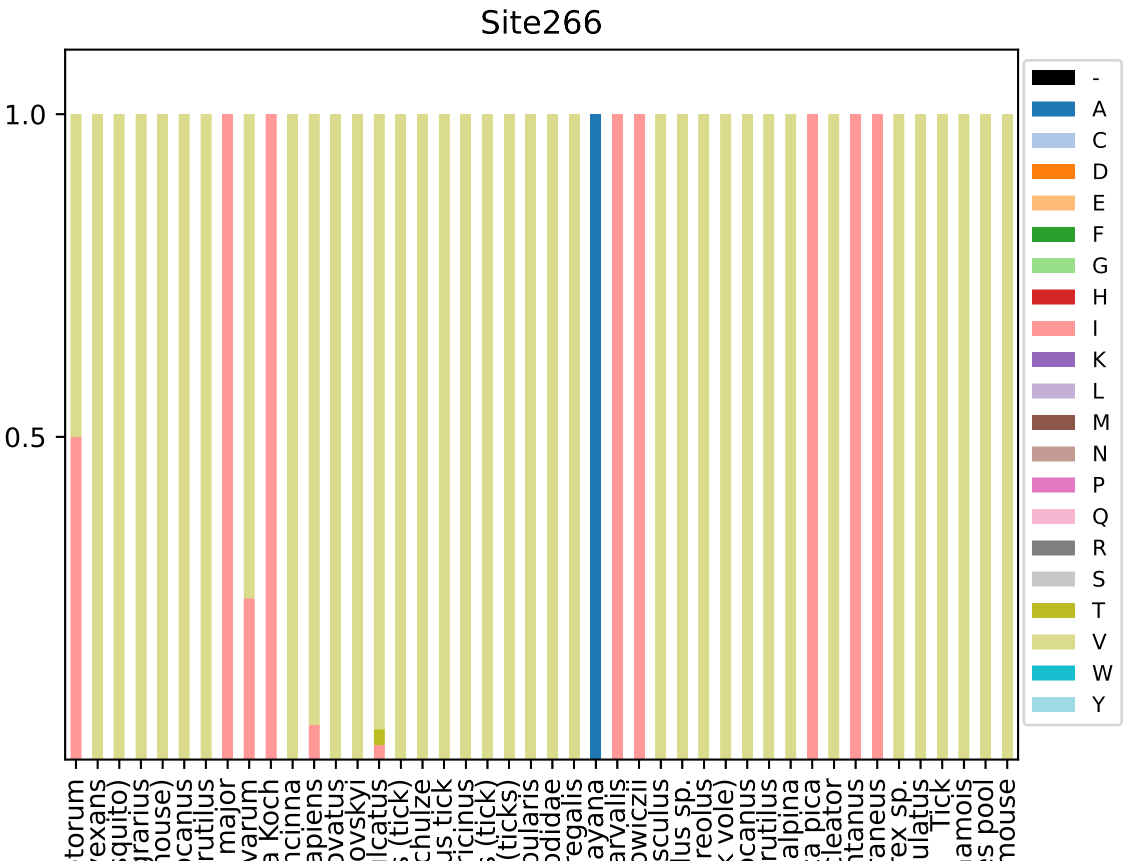
Site266
1.0
0.5
-
A
C
D
E
F
G
H
I
K
L
M
N
P
Q
R
S
T
V
W
Y

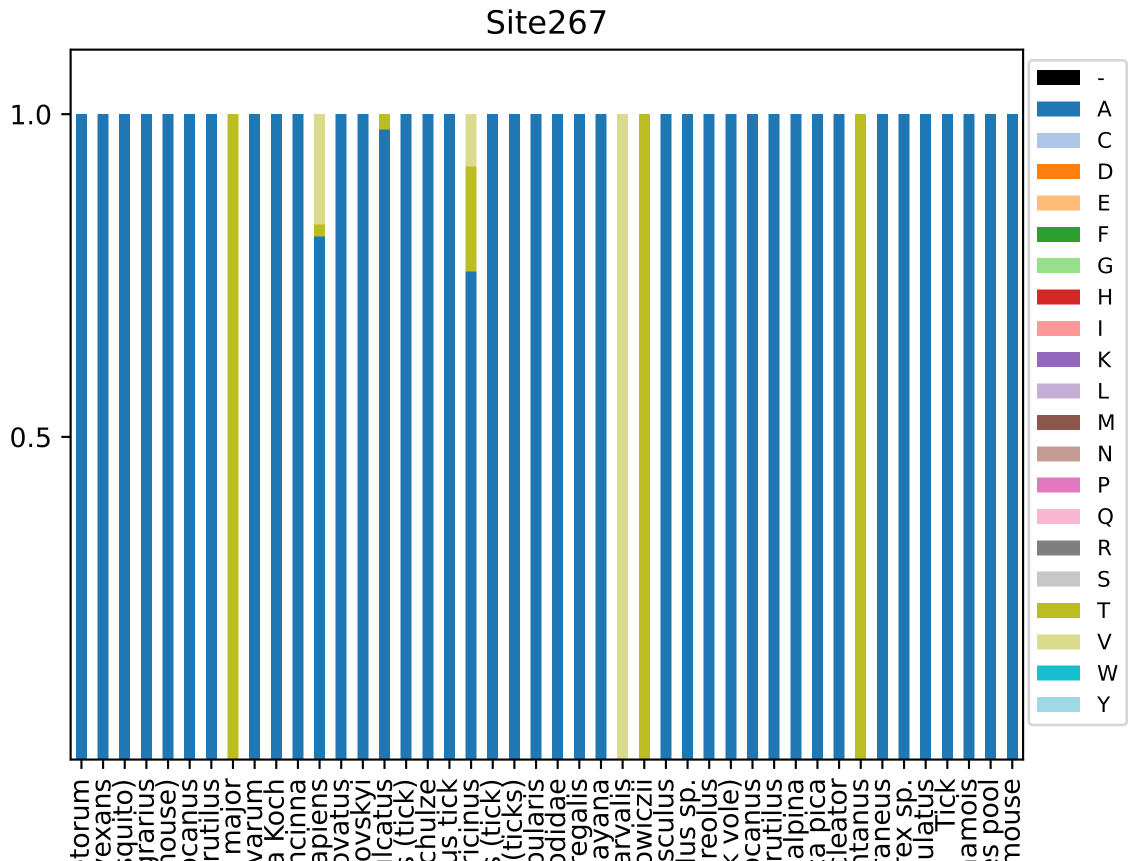
Site267
1.0
0.5
-
A
C
D
E
F
G
H
I
K
L
M
N
P
Q
R
S
T
V
W
Y
Tick

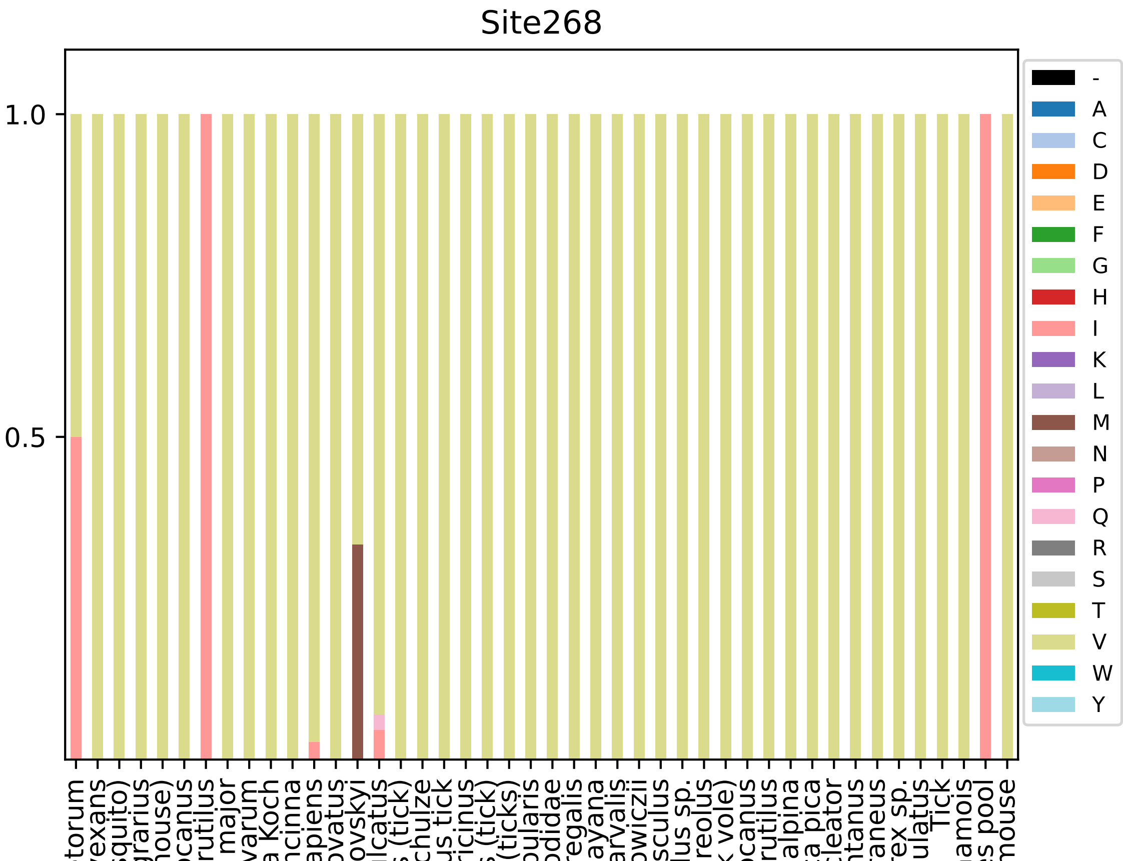

Site268
1.0
0.5
-
A
C
D
E
F
G
H
I
K
L
M
N
P
Q
R
S
T
V
W
Y

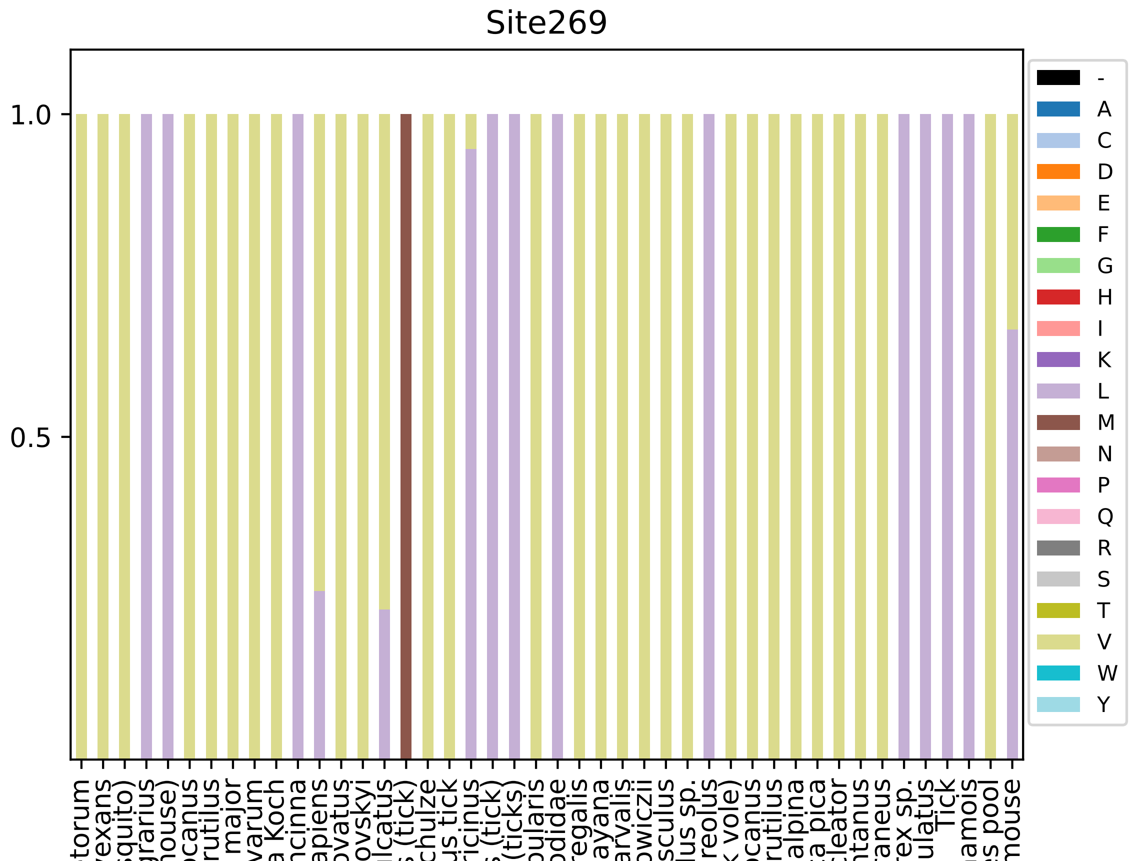

Site269
1.0
0.5
-
A
C
D
E
F
G
H
I
K
L
M
N
P
Q
R
S
T
V
W
Y

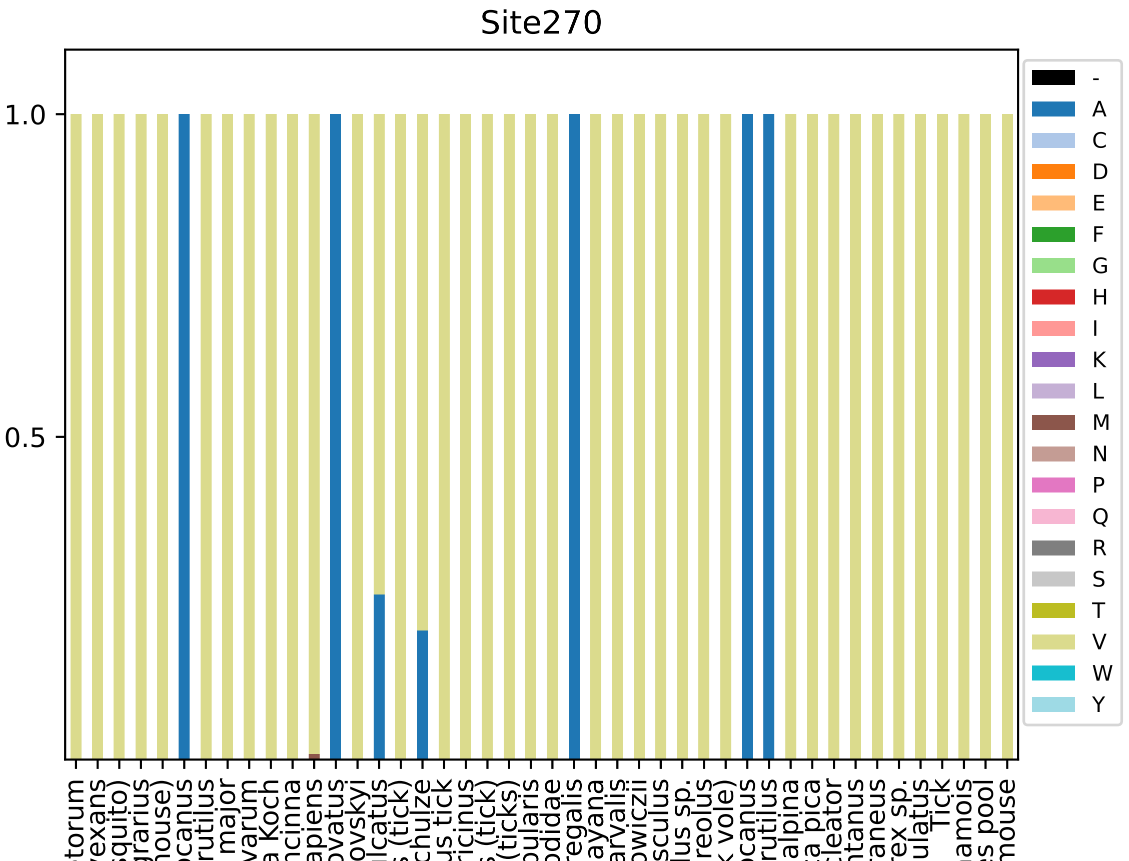
Site270
1.0
0.5
-
A
C
D
E
F
G
H
I
K
L
M
N
P
Q
R
S
T
V
W
Y

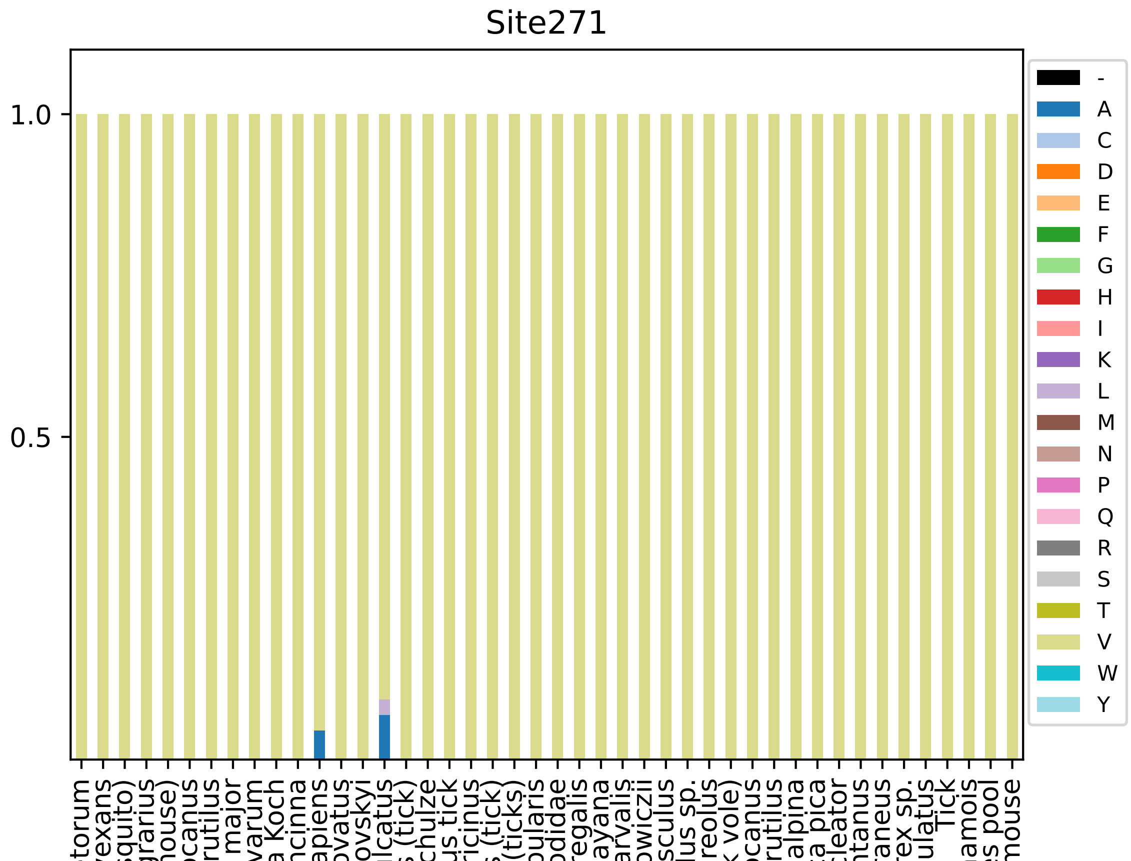

Site271
1.0
0.5
-
A
C
D
E
F
G
H
I
K
L
M
N
P
Q
R
S
T
V
W
Y

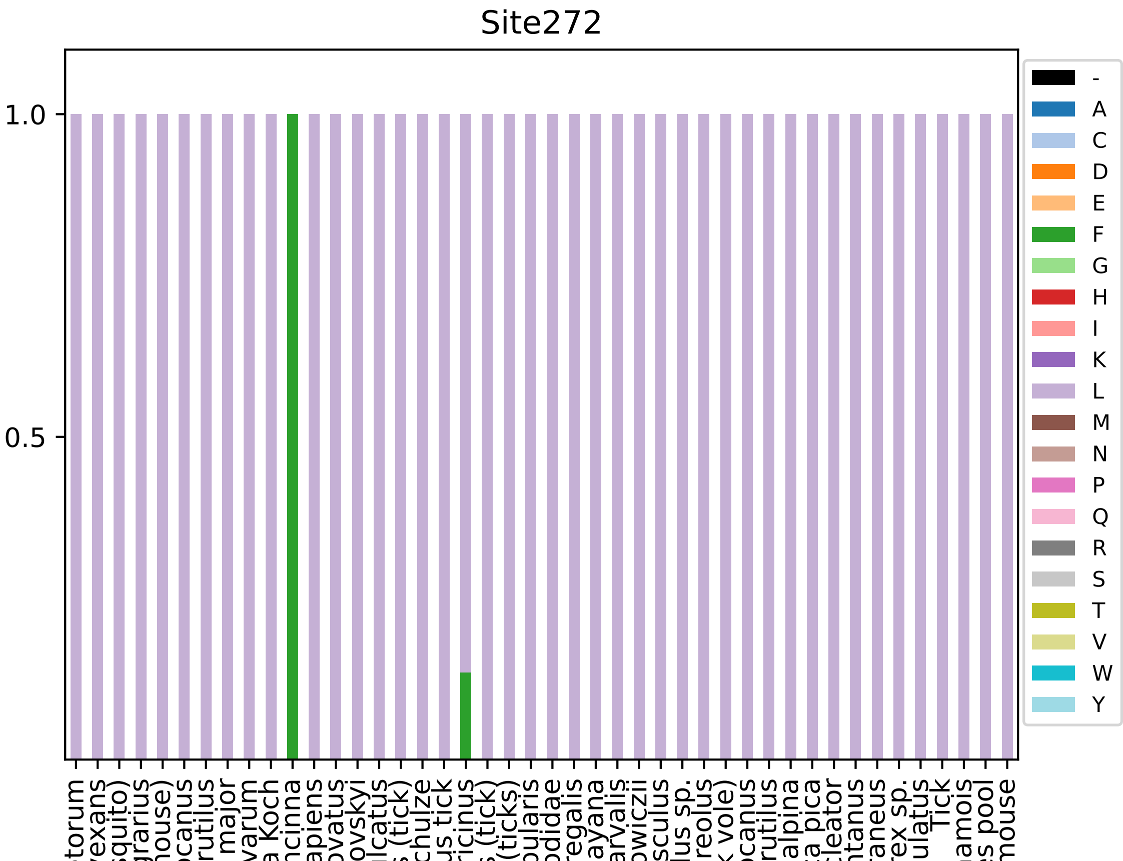
Site272
1.0
0.5
-
A
C
D
E
F
G
H
I
K
L
M
N
P
Q
R
S
T
V
W
Y

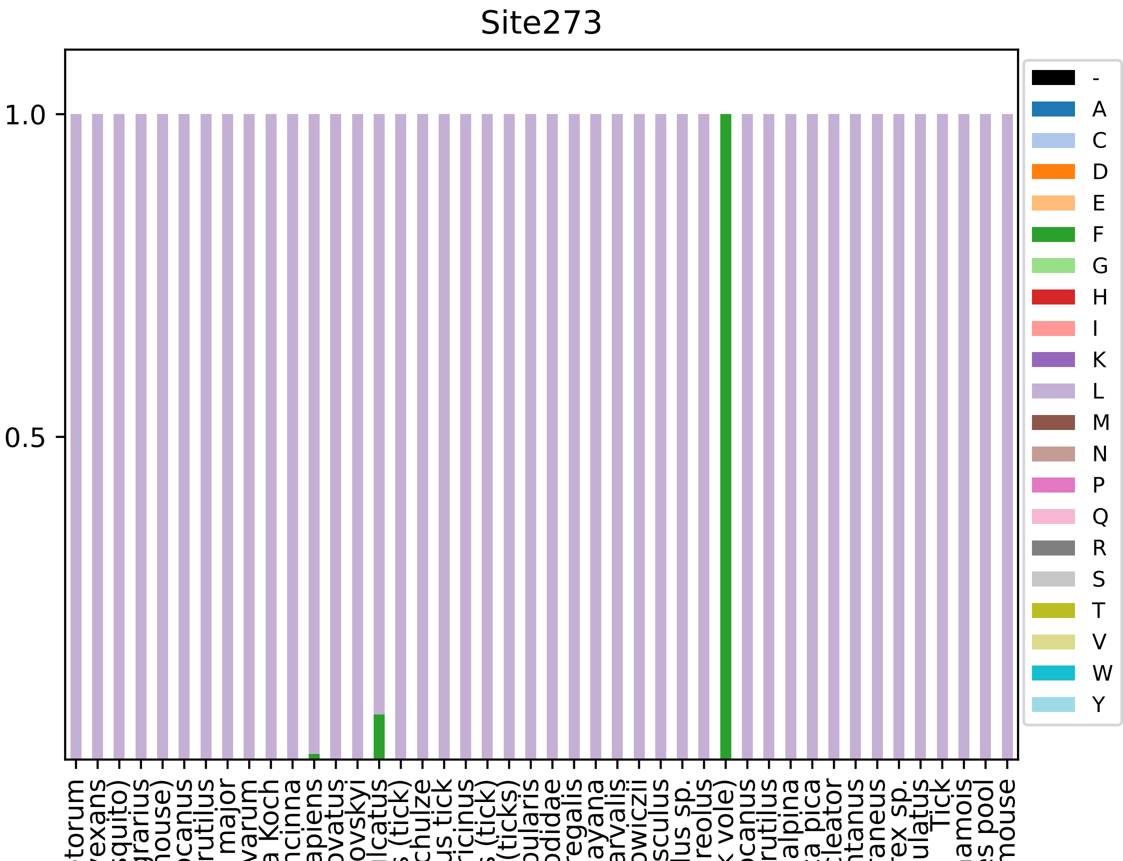
Site273
1.0
0.5
-
A
C
D
E
F
G
H
I
K
L
M
N
P
Q
R
S
T
V
W
Y

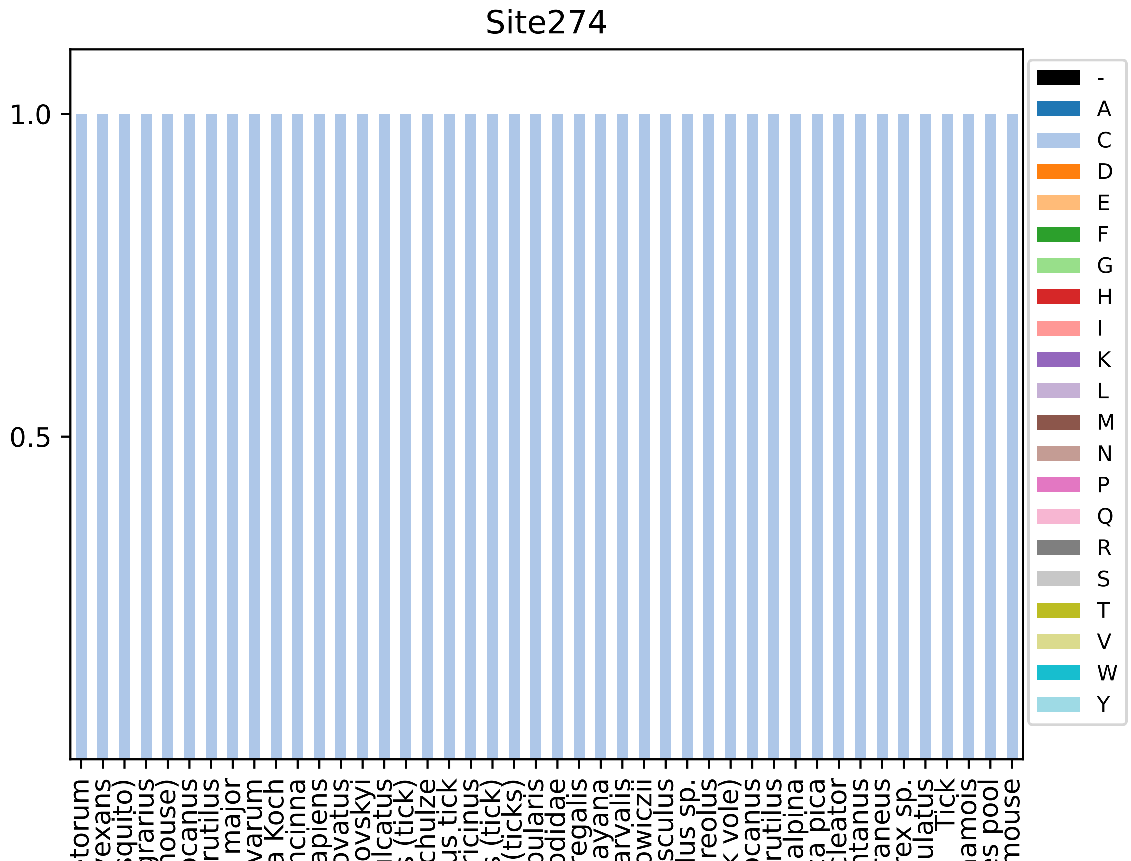

Site274
1.0
0.5
-
A
C
D
E
F
G
H
I
K
L
M
N
P
Q
R
S
T
V
W
Y

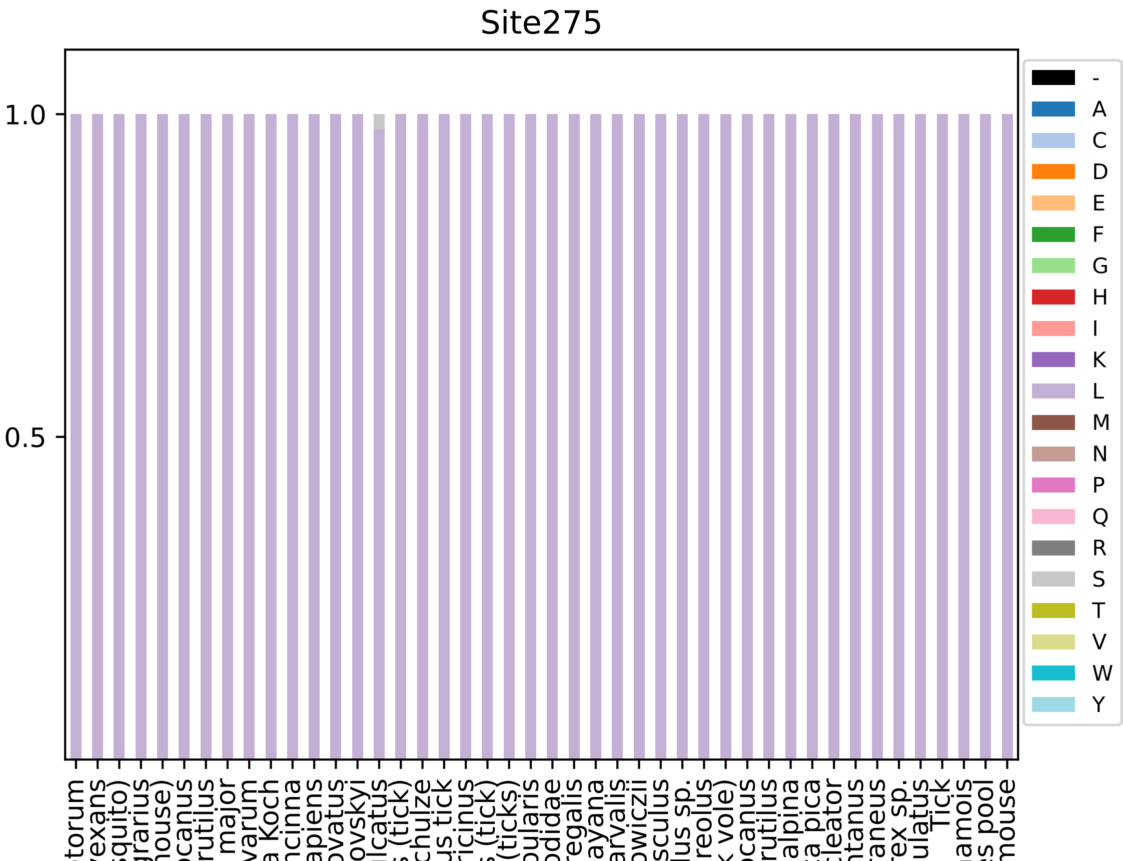
Site275
1.0
0.5
-
A
C
D
E
F
G
H
I
K
L
M
N
P
Q
R
S
T
V
W
Y

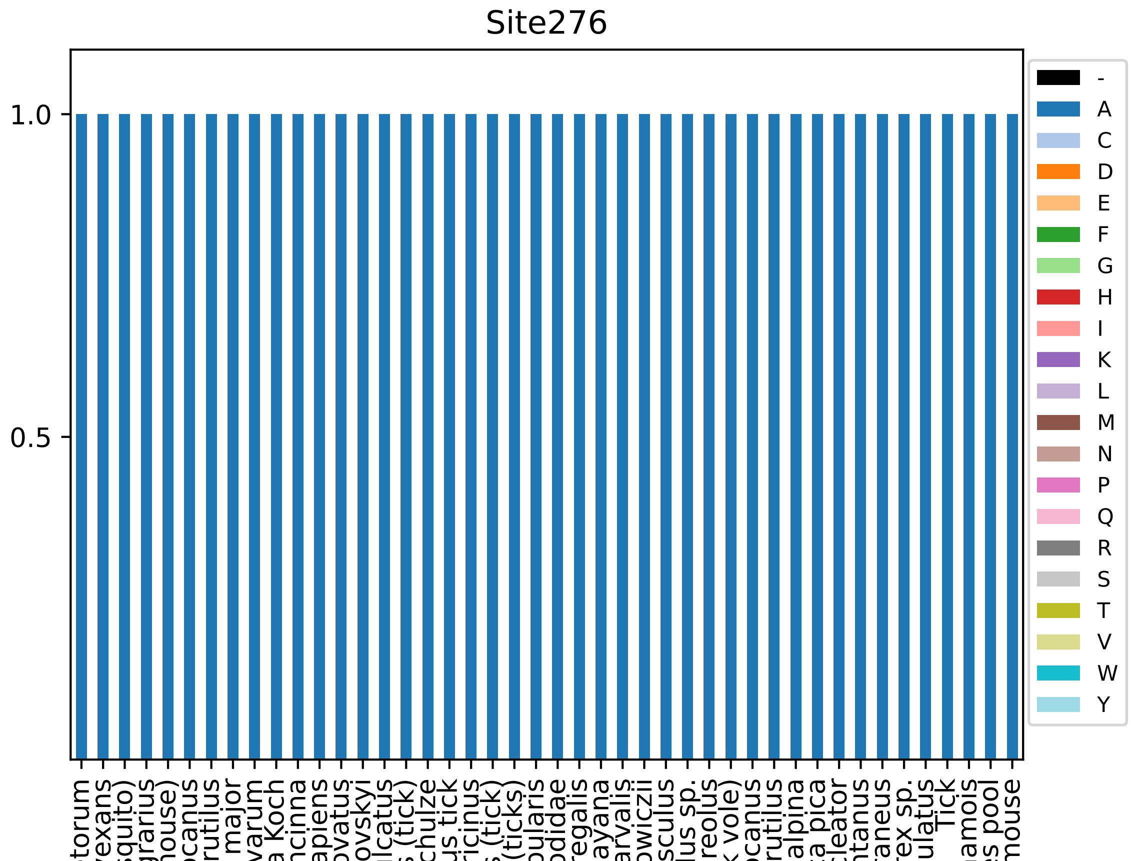
Site276
1.0
0.5
-
A
C
D
E
F
G
H
I
K
L
M
N
P
Q
R
S
T
V
W
Y

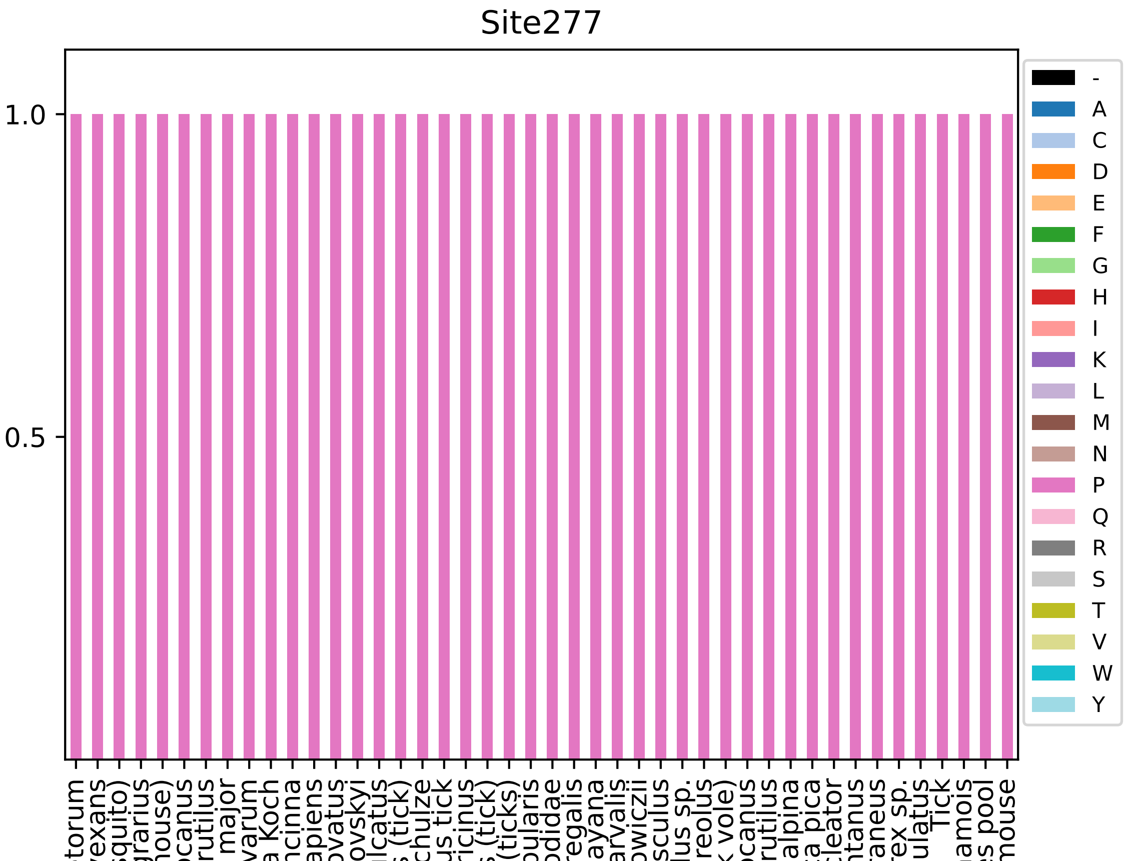

Site277
1.0
0.5
-
A
C
D
E
F
G
H
I
K
L
M
N
P
Q
R
S
T
V
W
Y

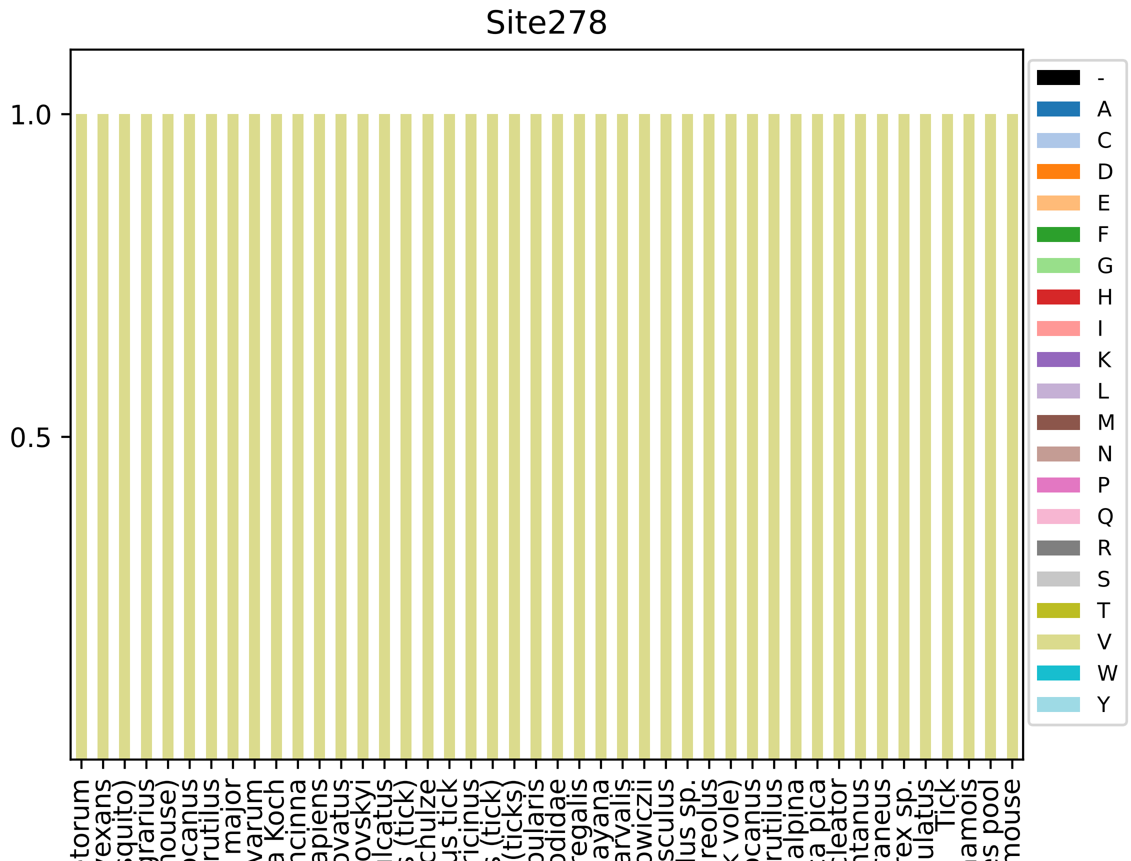
Site278
1.0
0.5
-
A
C
D
E
F
G
H
I
K
L
M
N
P
Q
R
S
T
V
W
Y

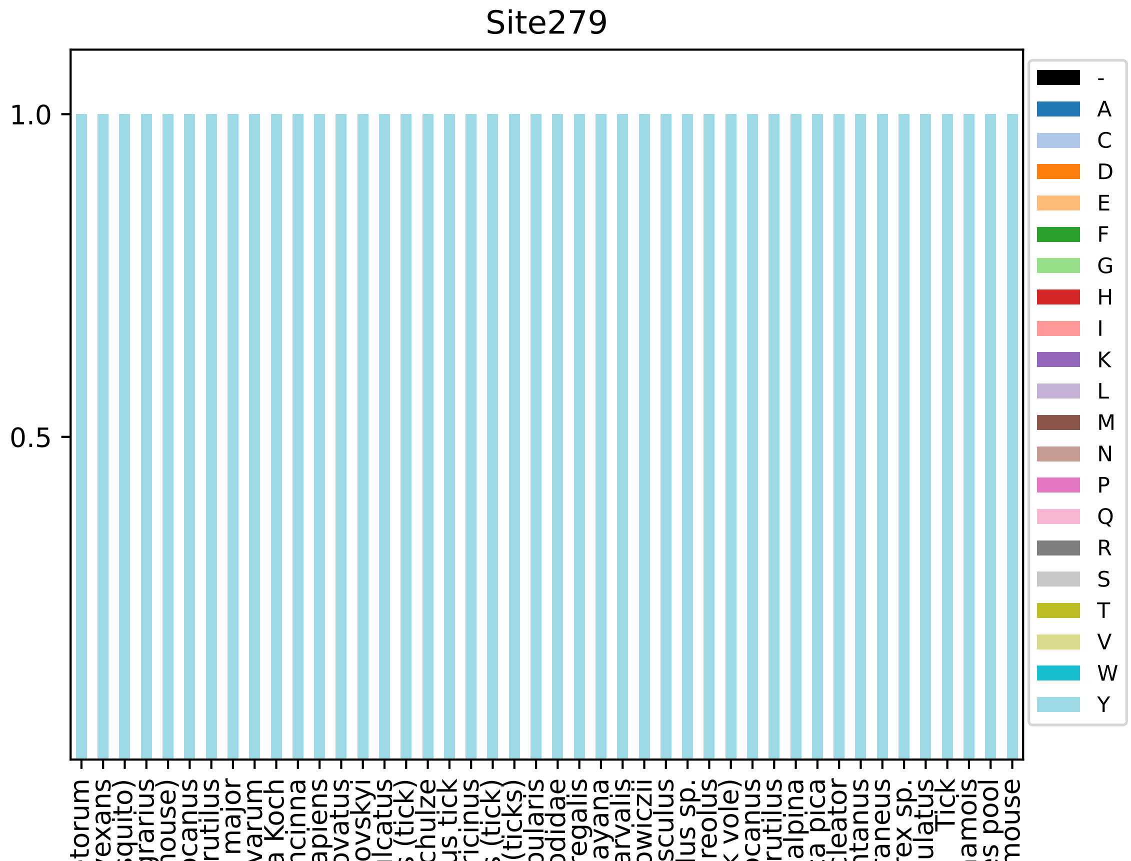
Site279
1.0
0.5
-
A
C
D
E
F
G
H
I
K
L
M
N
P
Q
R
S
T
V
W
Y

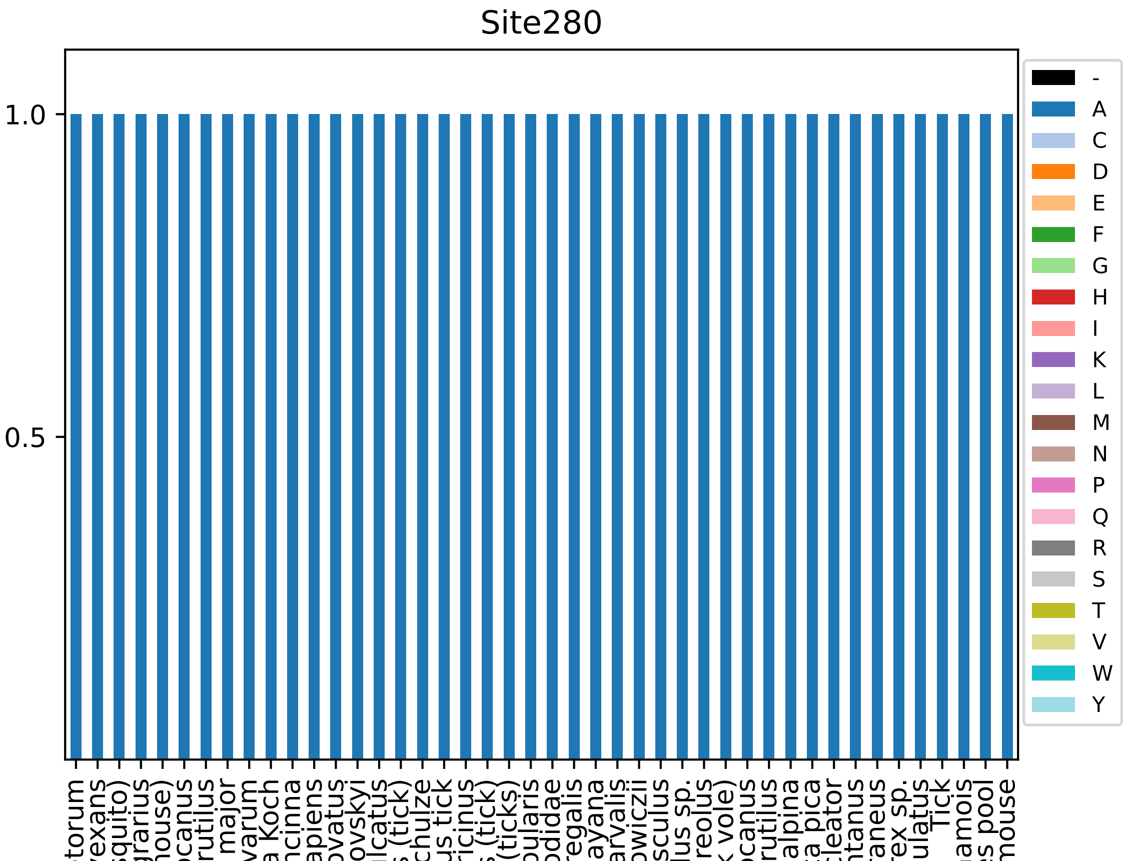
Site280
1.0
0.5
-
A
C
D
E
F
G
H
I
K
L
M
N
P
Q
R
S
T
V
W
Y

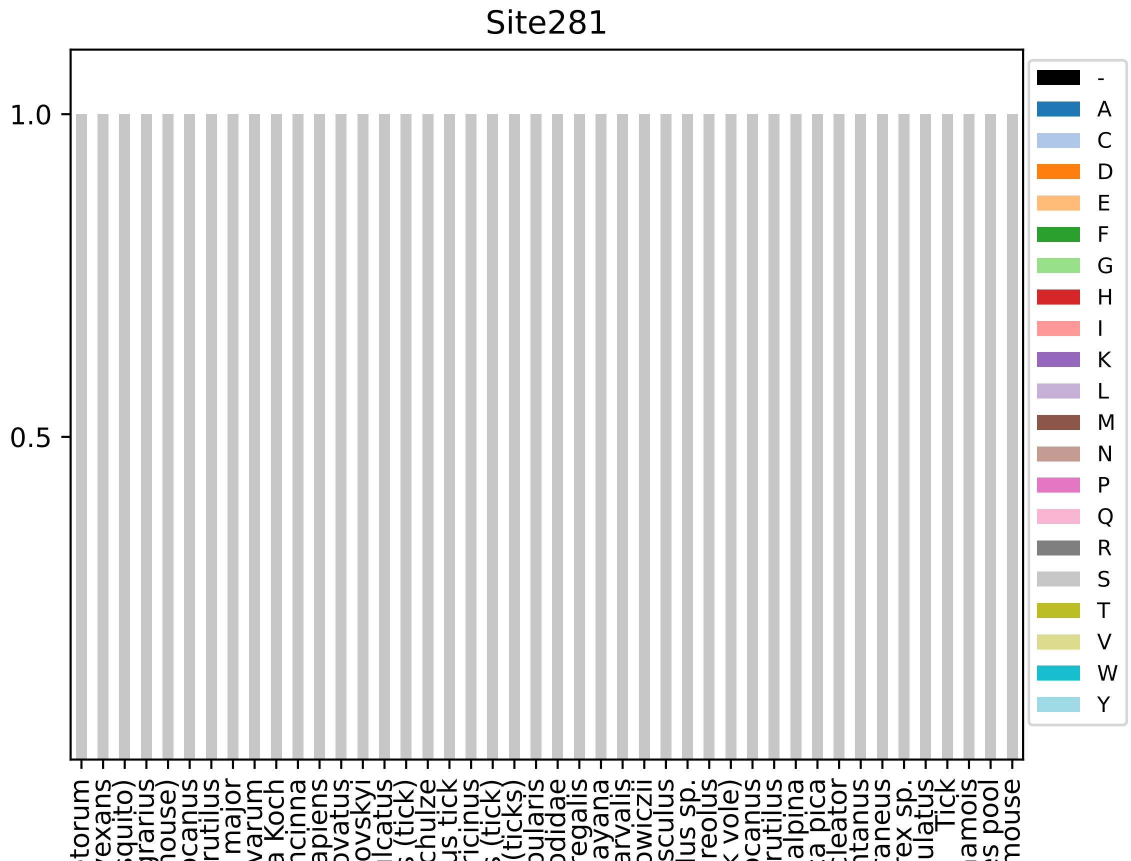
Site281
1.0
0.5
-
A
C
D
E
F
G
H
I
K
L
M
N
P
Q
R
S
T
V
W
Y

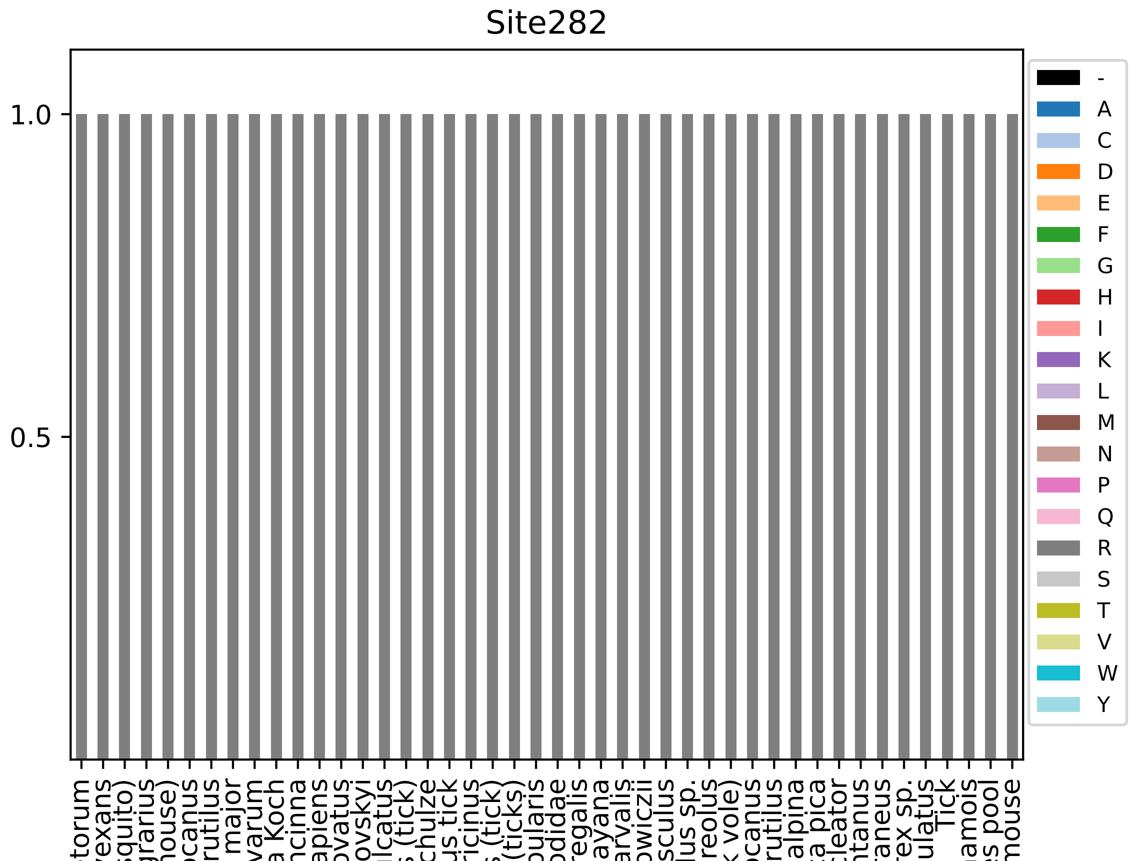
Site282
1.0
0.5
-
A
C
D
E
F
G
H
I
K
L
M
N
P
Q
R
S
T
V
W
Y

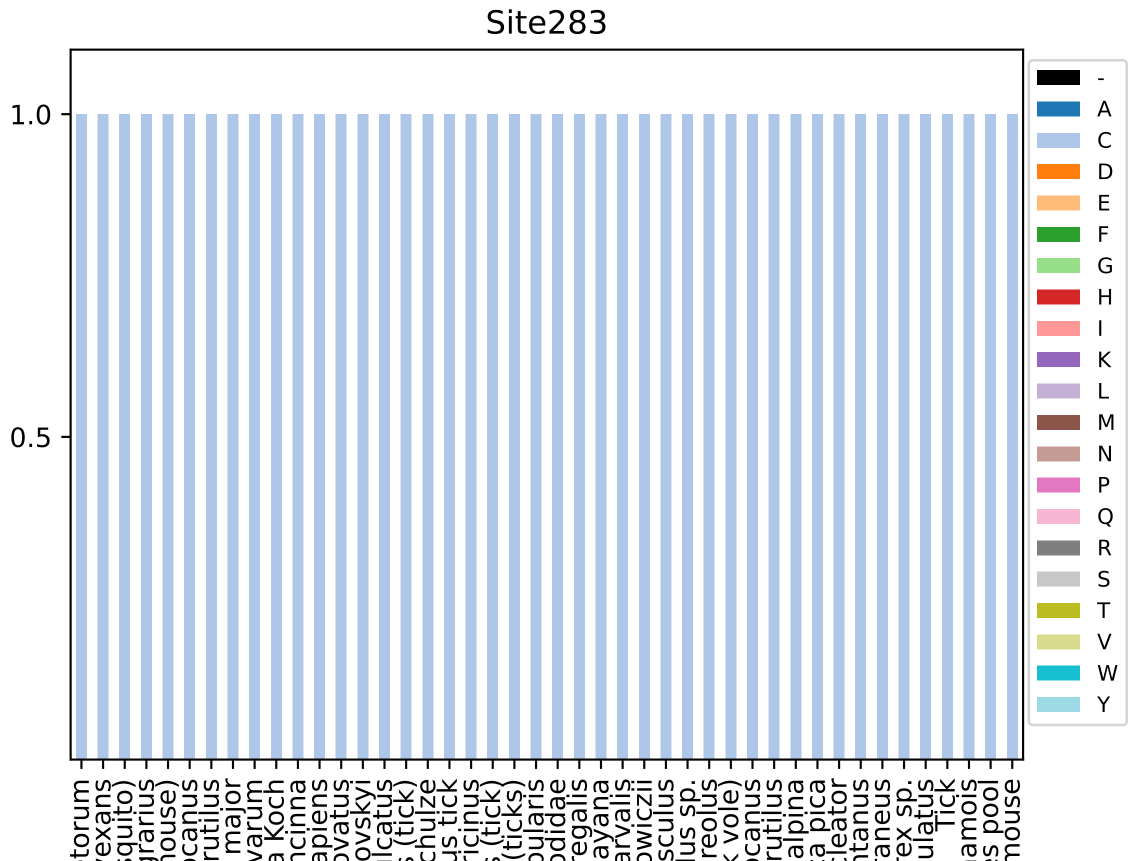

Site283
1.0
0.5
-
A
C
D
E
F
G
H
I
K
L
M
N
P
Q
R
S
T
V
W
Y

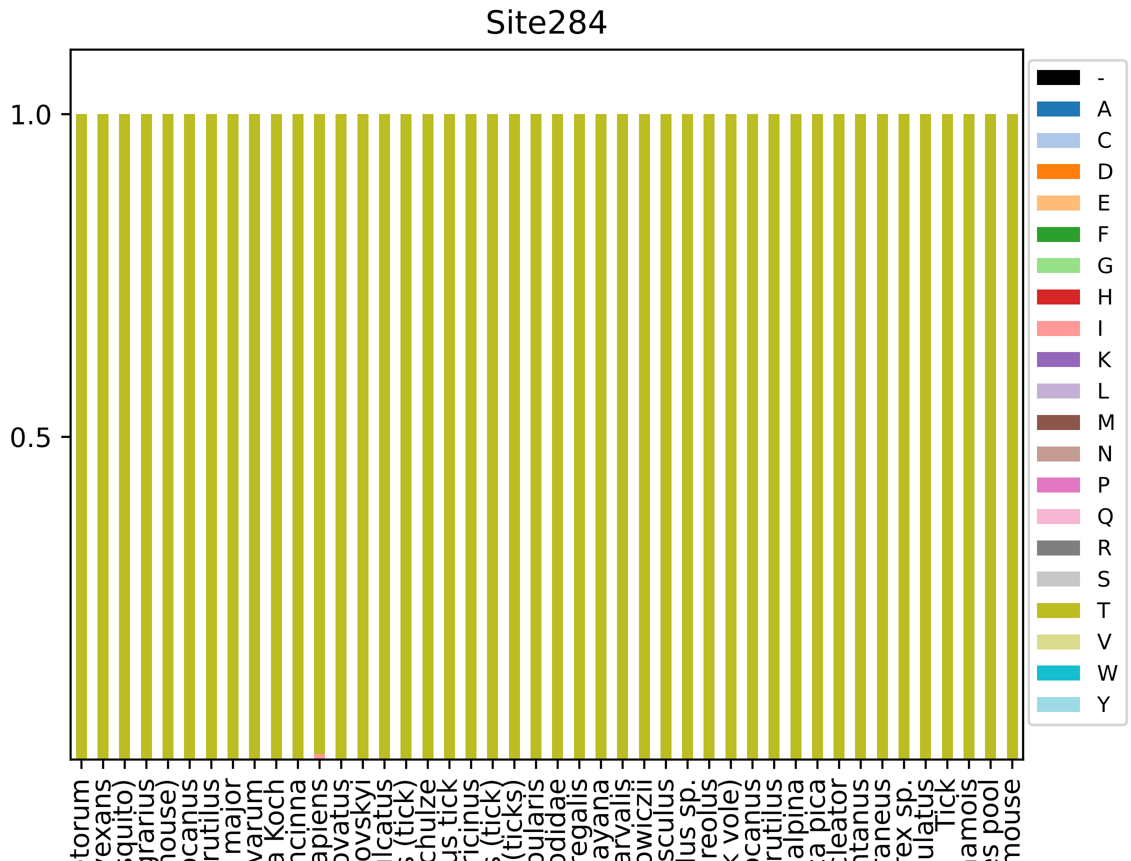
Site284
1.0
0.5
-
A
C
D
E
F
G
H
I
K
L
M
N
P
Q
R
S
T
V
W
Y

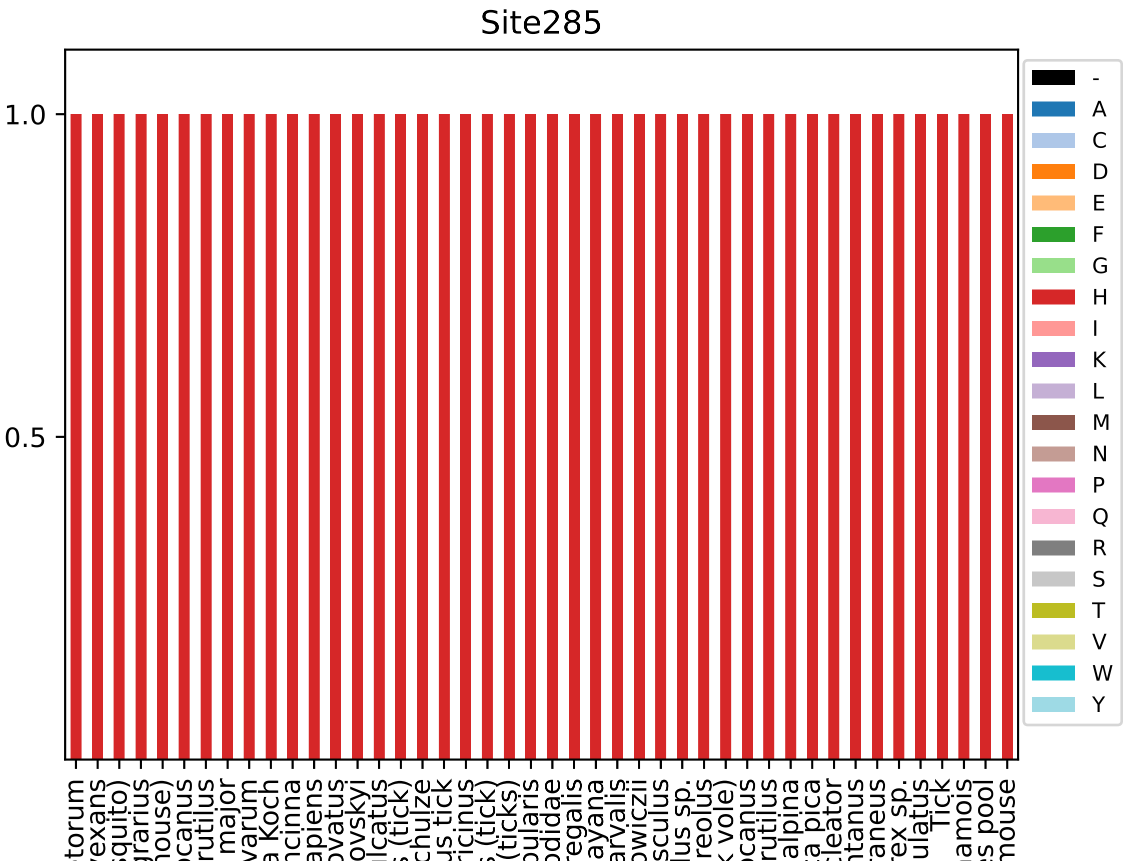
Site285
1.0
0.5
-
A
C
D
E
F
G
H
I
K
L
M
N
P
Q
R
S
T
V
W
Y

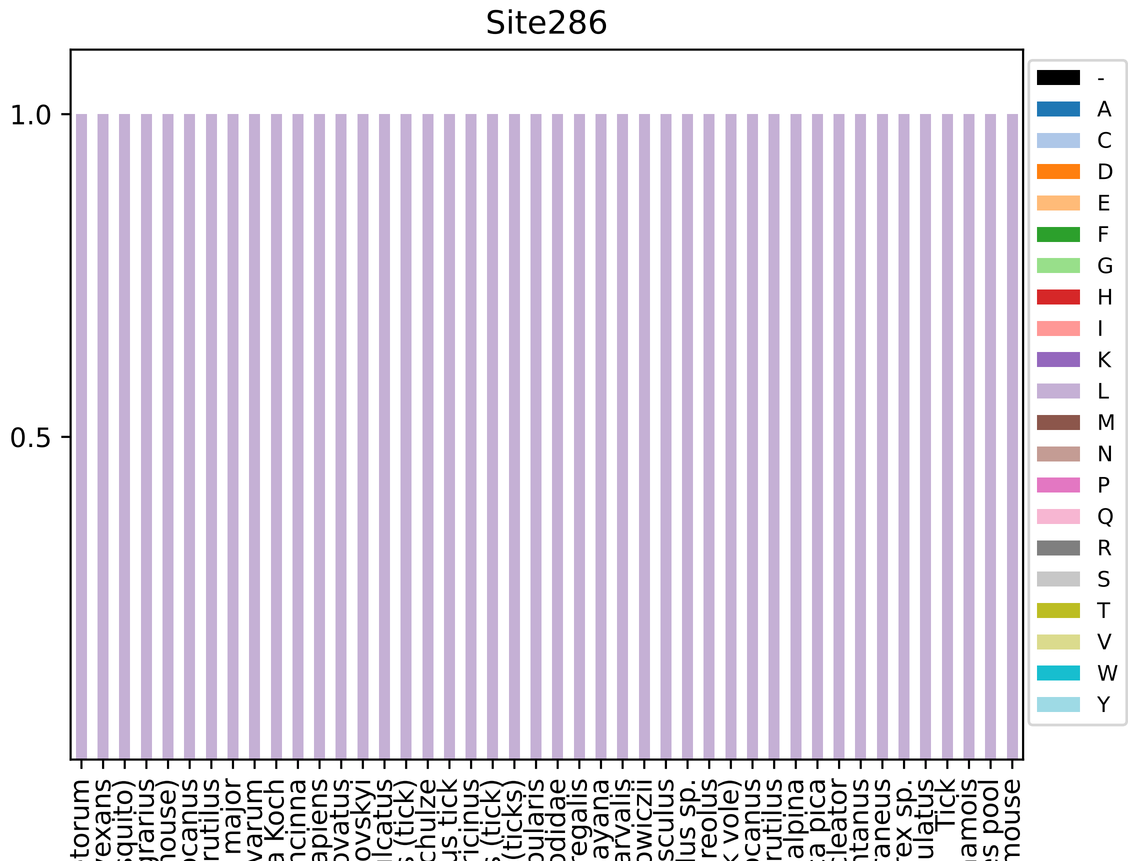

Site286
1.0
0.5
-
A
C
D
E
F
G
H
I
K
L
M
N
P
Q
R
S
T
V
W
Y

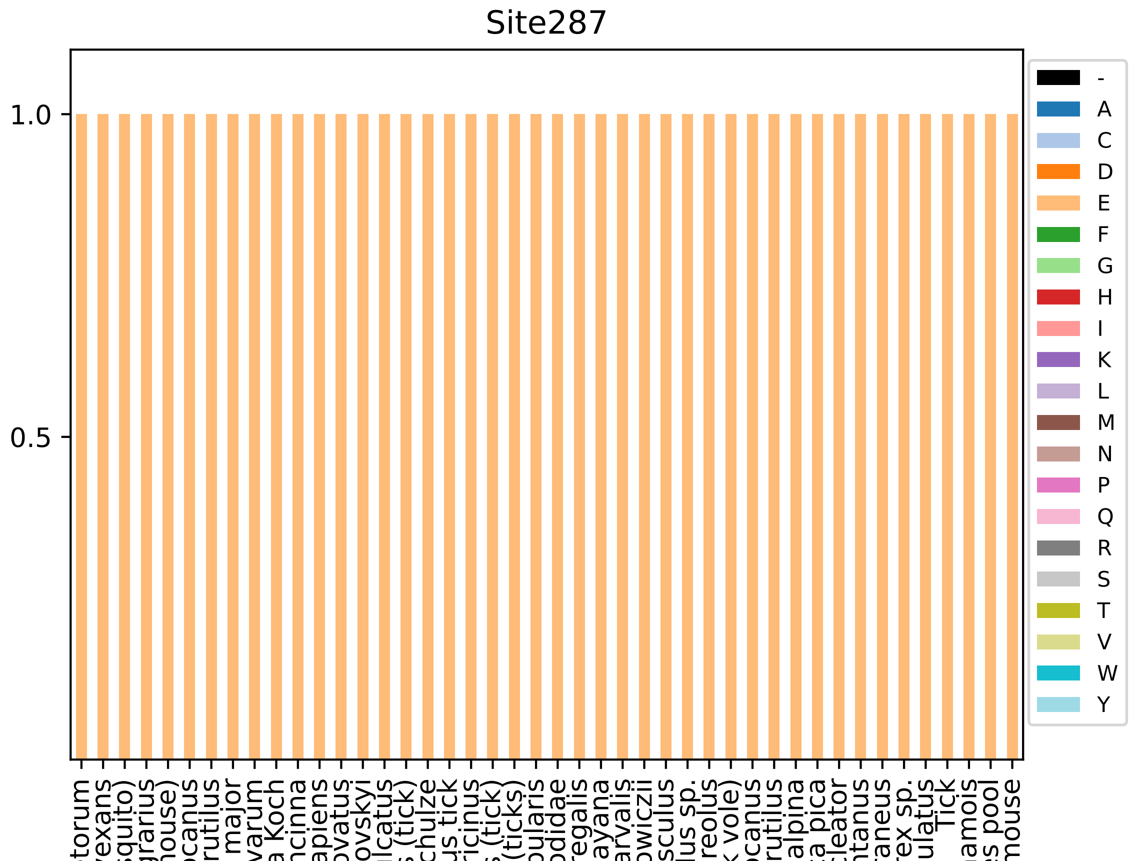
Site287
1.0
0.5
-
A
C
D
E
F
G
H
I
K
L
M
N
P
Q
R
S
T
V
W
Y

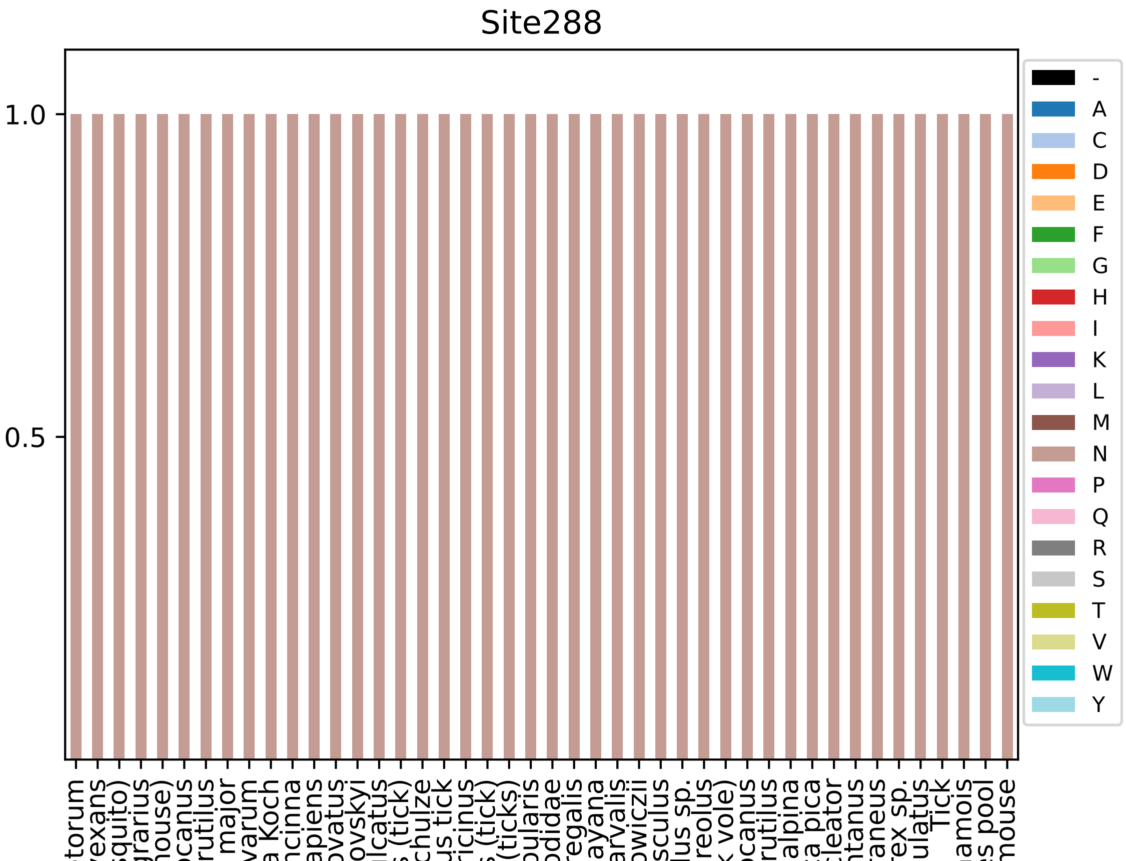

Site288
1.0
0.5
-
A
C
D
E
F
G
H
I
K
L
M
N
P
Q
R
S
T
V
W
Y

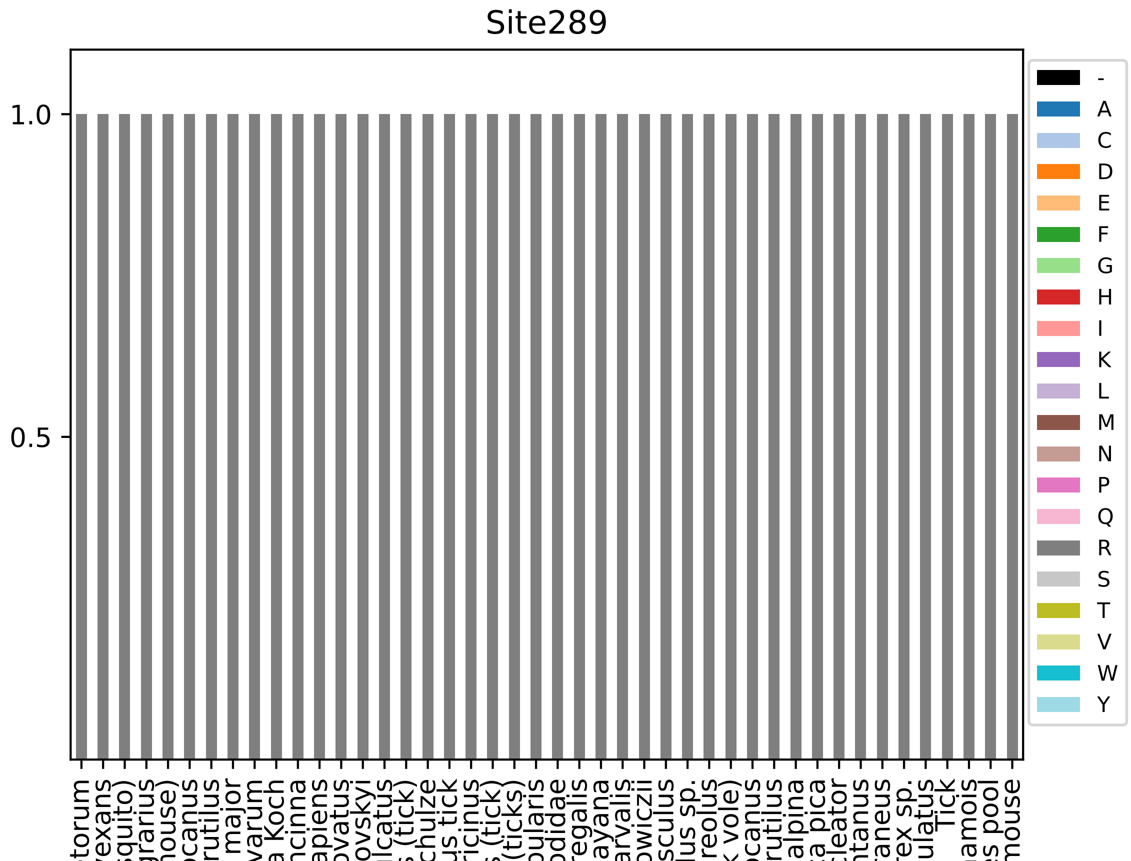
Site289
1.0
0.5
-
A
C
D
E
F
G
H
I
K
L
M
N
P
Q
R
S
T
V
W
Y

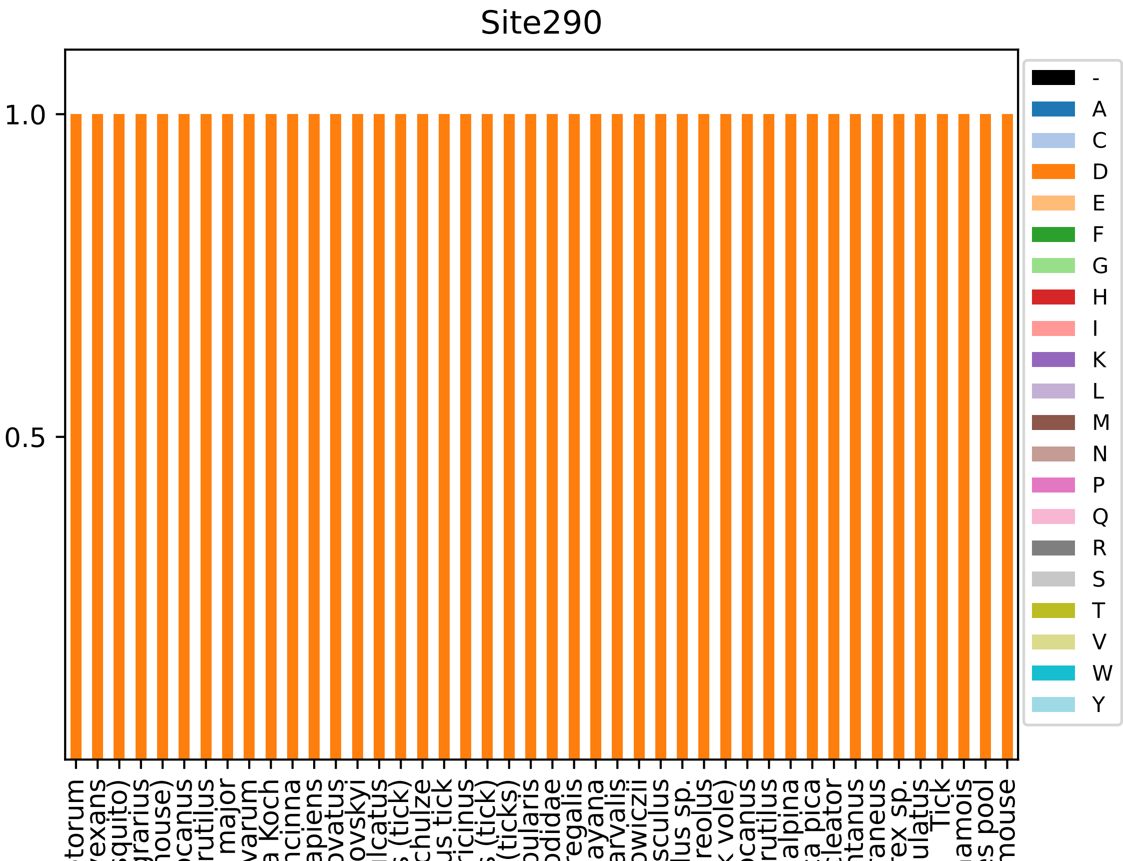
Site290
1.0
0.5
-
A
C
D
E
F
G
H
I
K
L
M
N
P
Q
R
S
T
V
W
Y

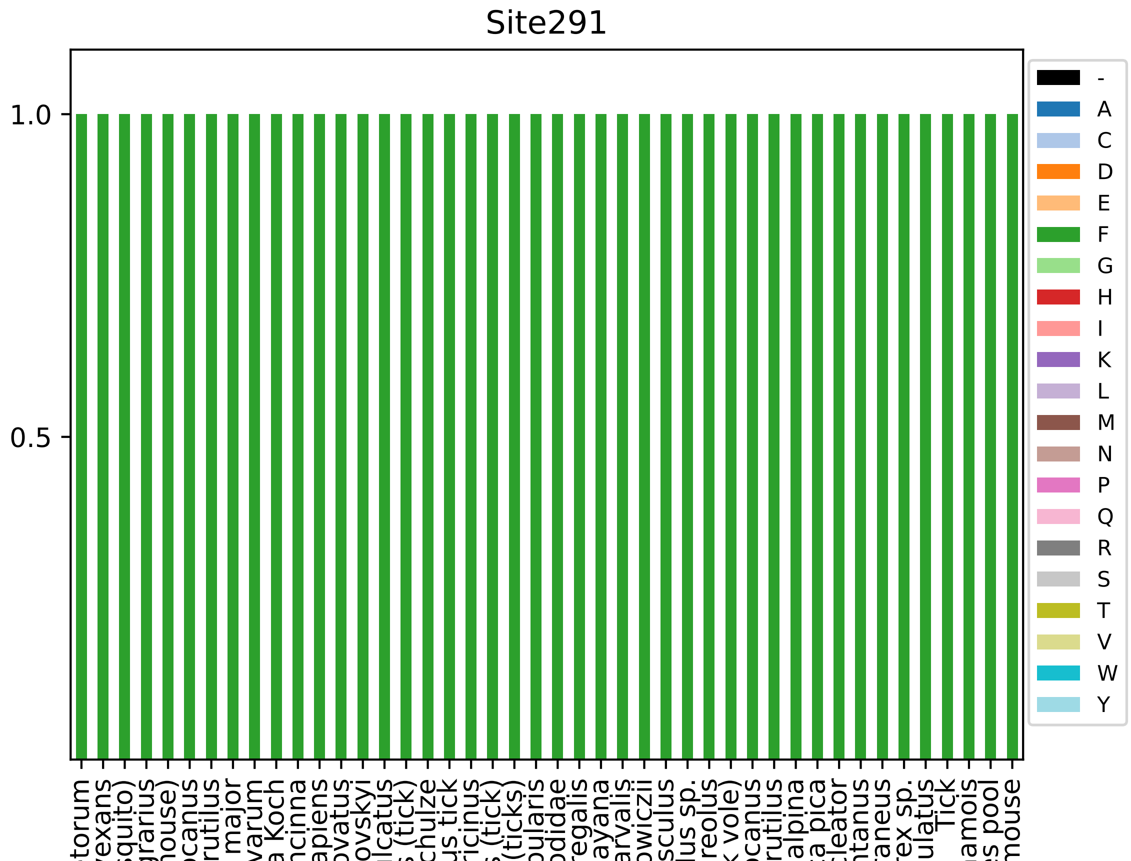
Site291
1.0
0.5
-
A
C
D
E
F
G
H
I
K
L
M
N
P
Q
R
S
T
V
W
Y

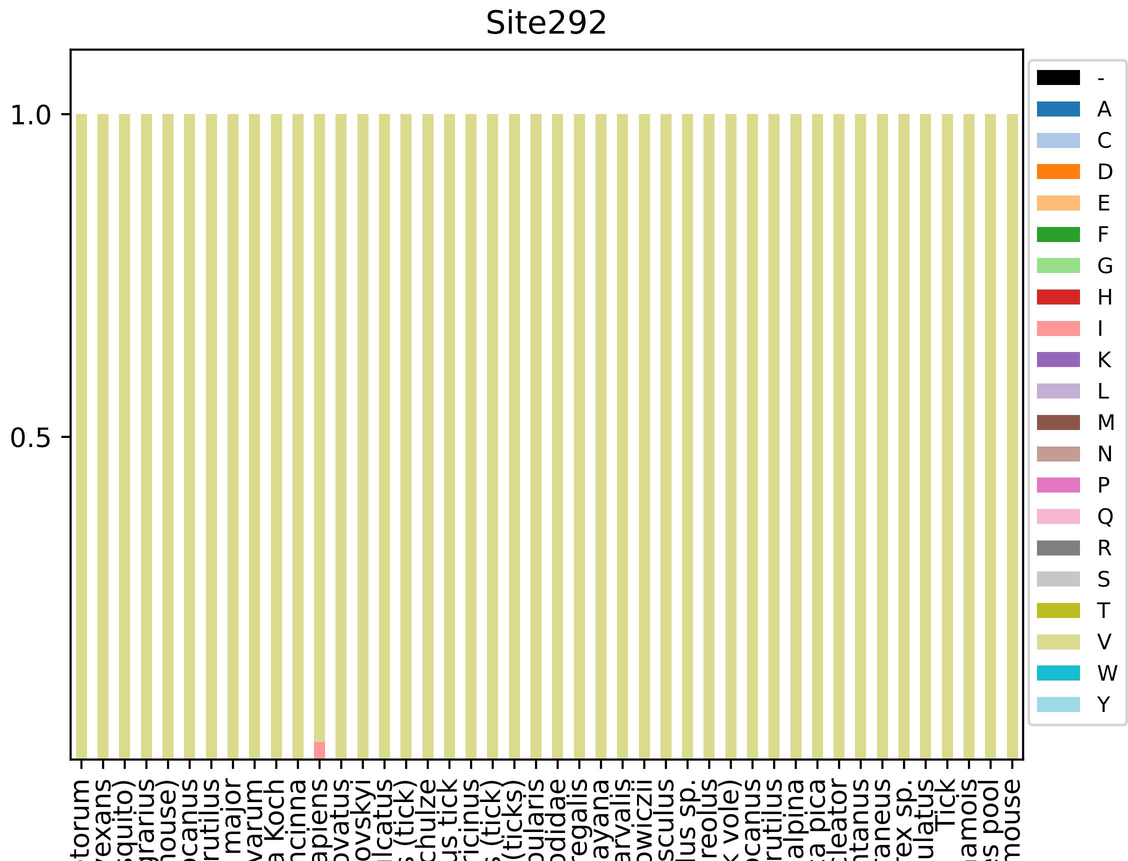

Site292
1.0
0.5
-
A
C
D
E
F
G
H
I
K
L
M
N
P
Q
R
S
T
V
W
Y

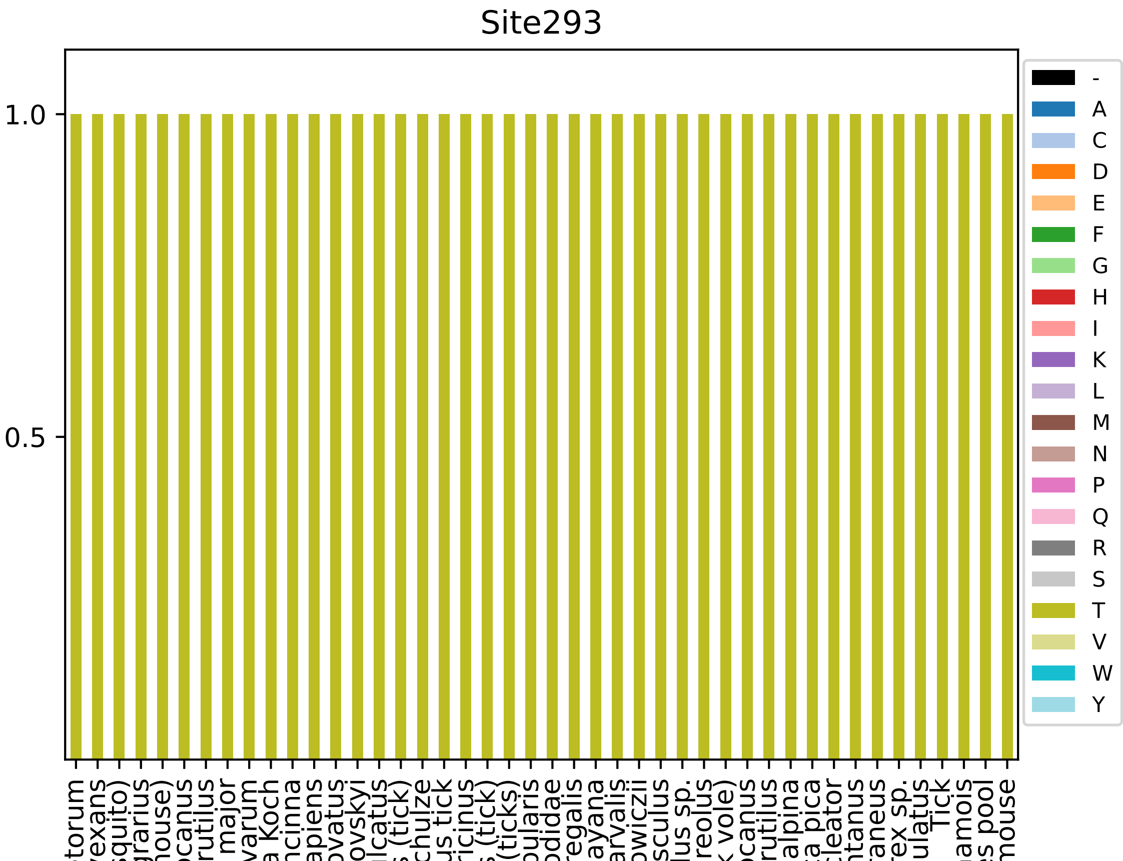
Site293
1.0
0.5
-
A
C
D
E
F
G
H
I
K
L
M
N
P
Q
R
S
T
V
W
Y

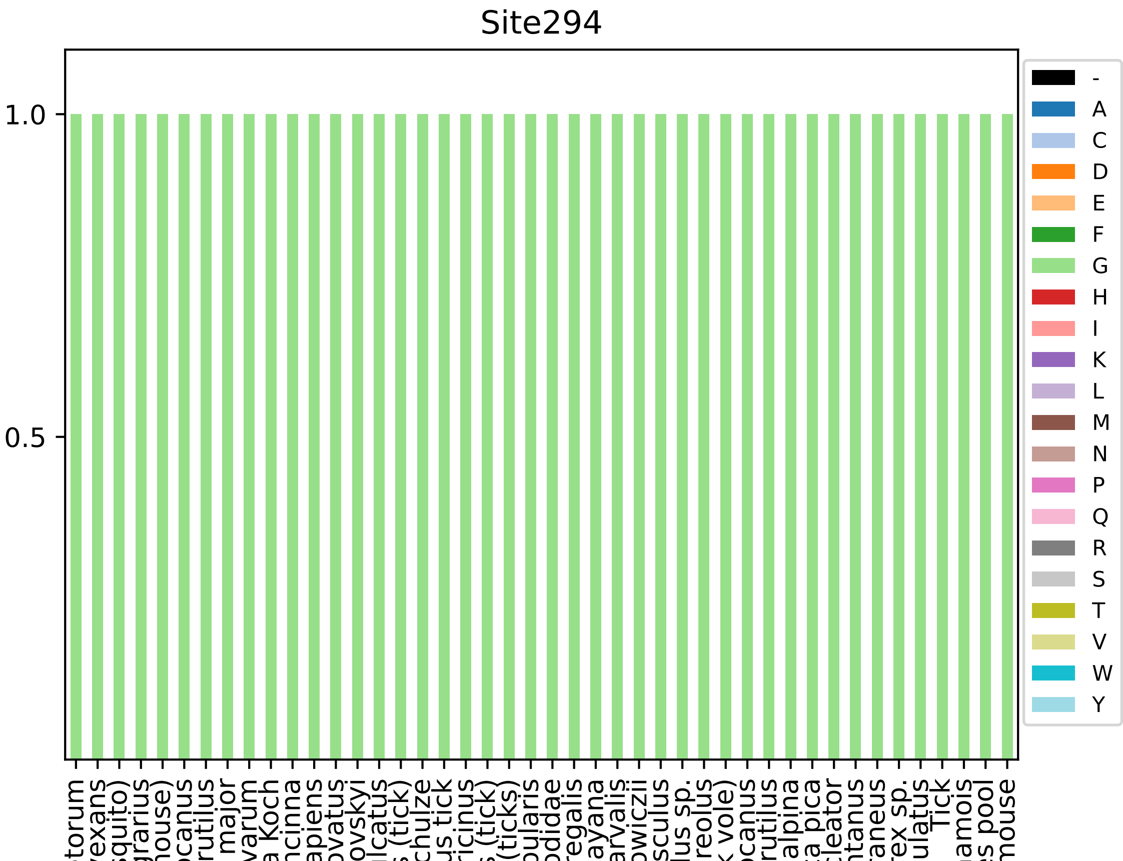
Site294
1.0
0.5
-
A
C
D
E
F
G
H
I
K
L
M
N
P
Q
R
S
T
V
W
Y

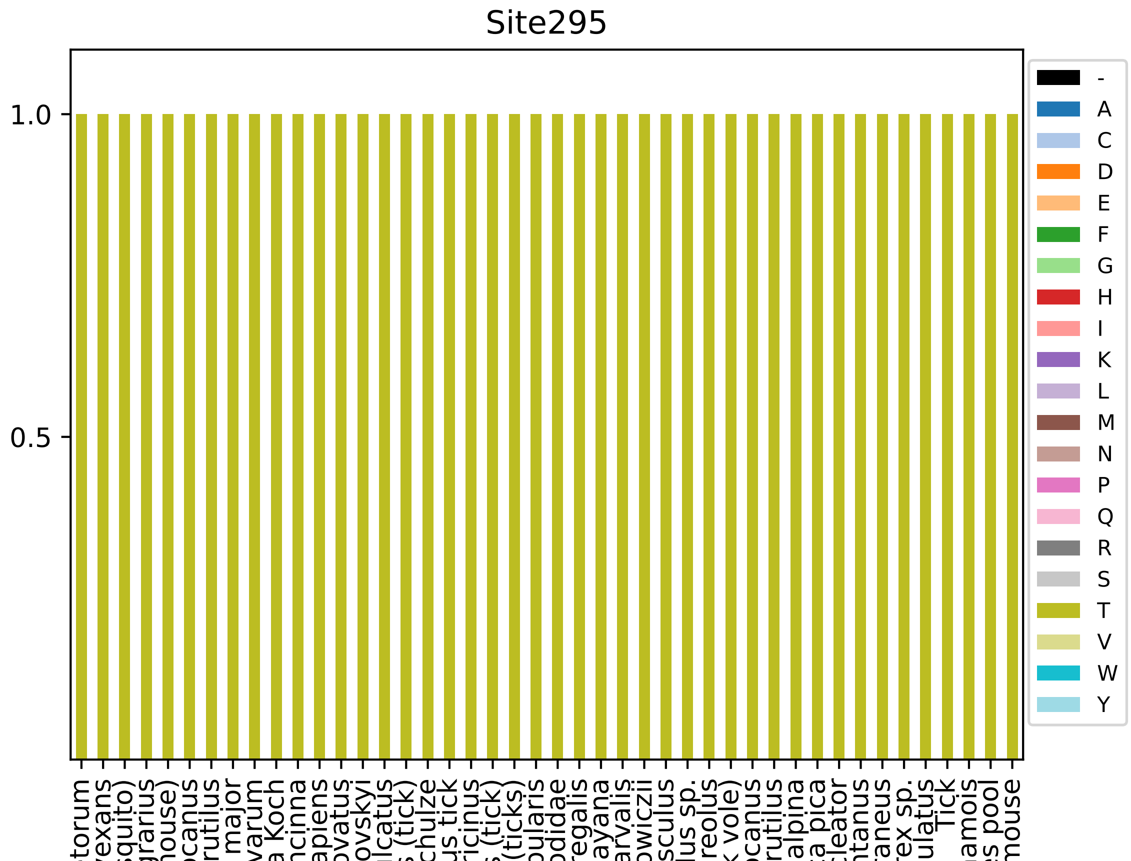
Site295
1.0
0.5
-
A
C
D
E
F
G
H
I
K
L
M
N
P
Q
R
S
T
V
W
Y

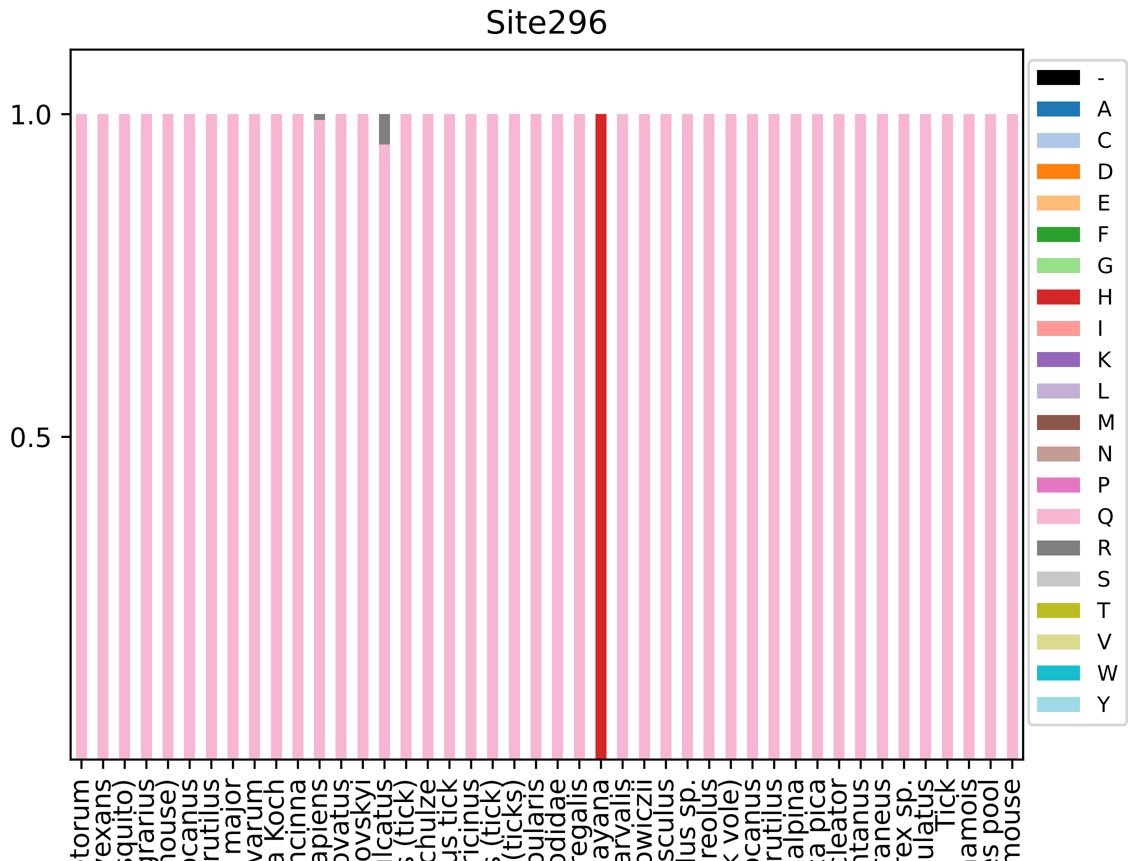

Site296
1.0
0.5
-
A
C
D
E
F
G
H
I
K
L
M
N
P
Q
R
S
T
V
W
Y

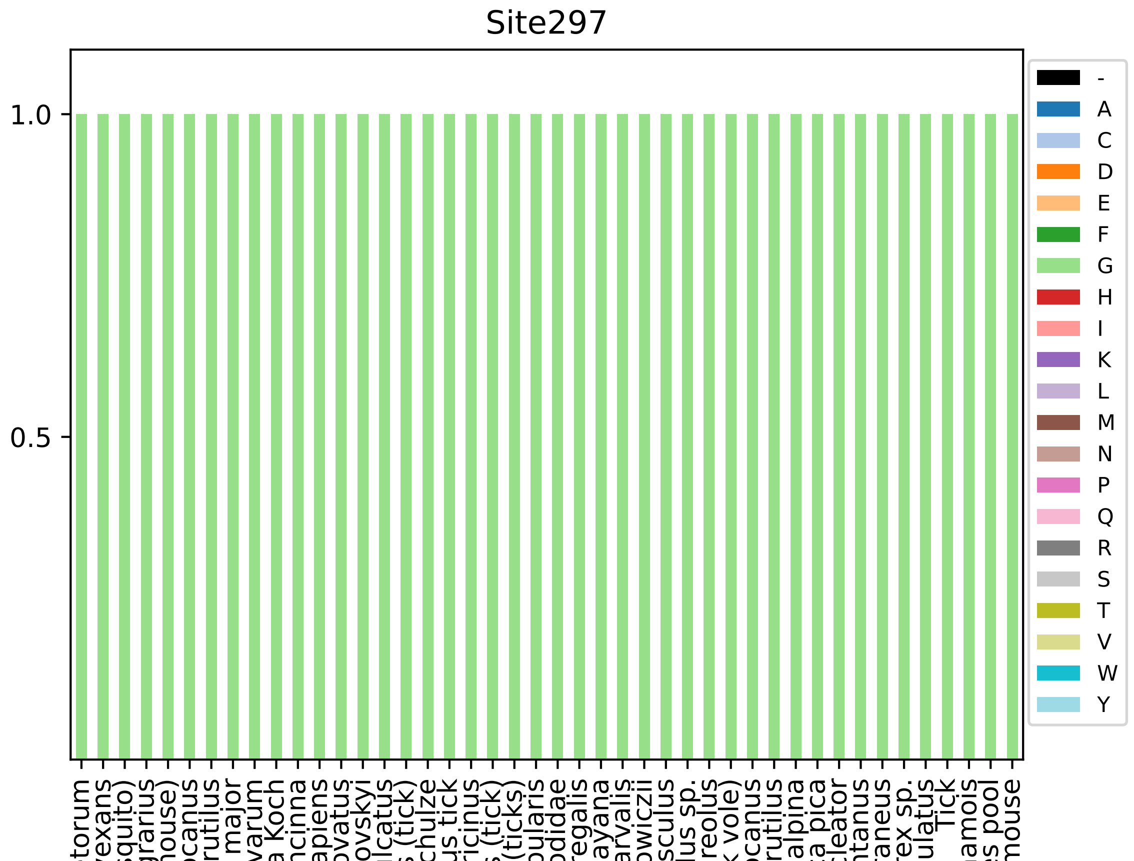
Site297
1.0
0.5
-
A
C
D
E
F
G
H
I
K
L
M
N
P
Q
R
S
T
V
W
Y

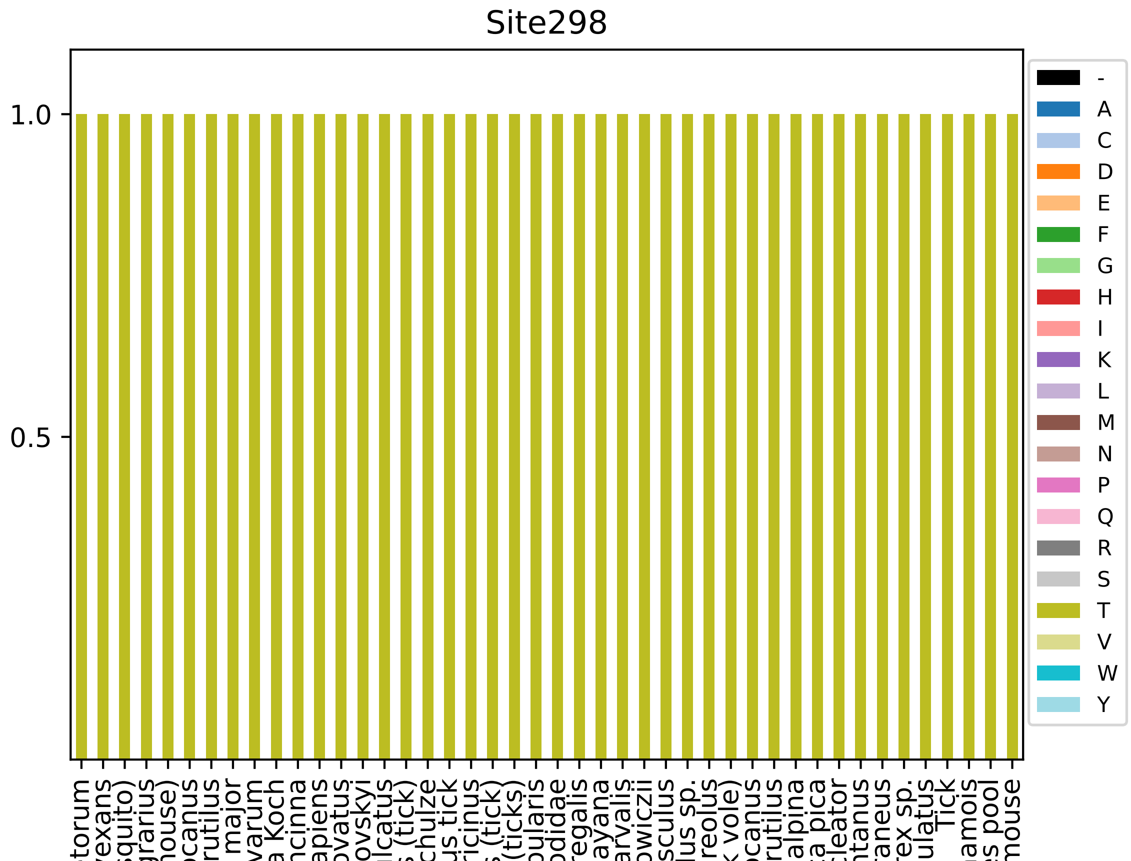
Site298
1.0
0.5
-
A
C
D
E
F
G
H
I
K
L
M
N
P
Q
R
S
T
V
W
Y
Tick

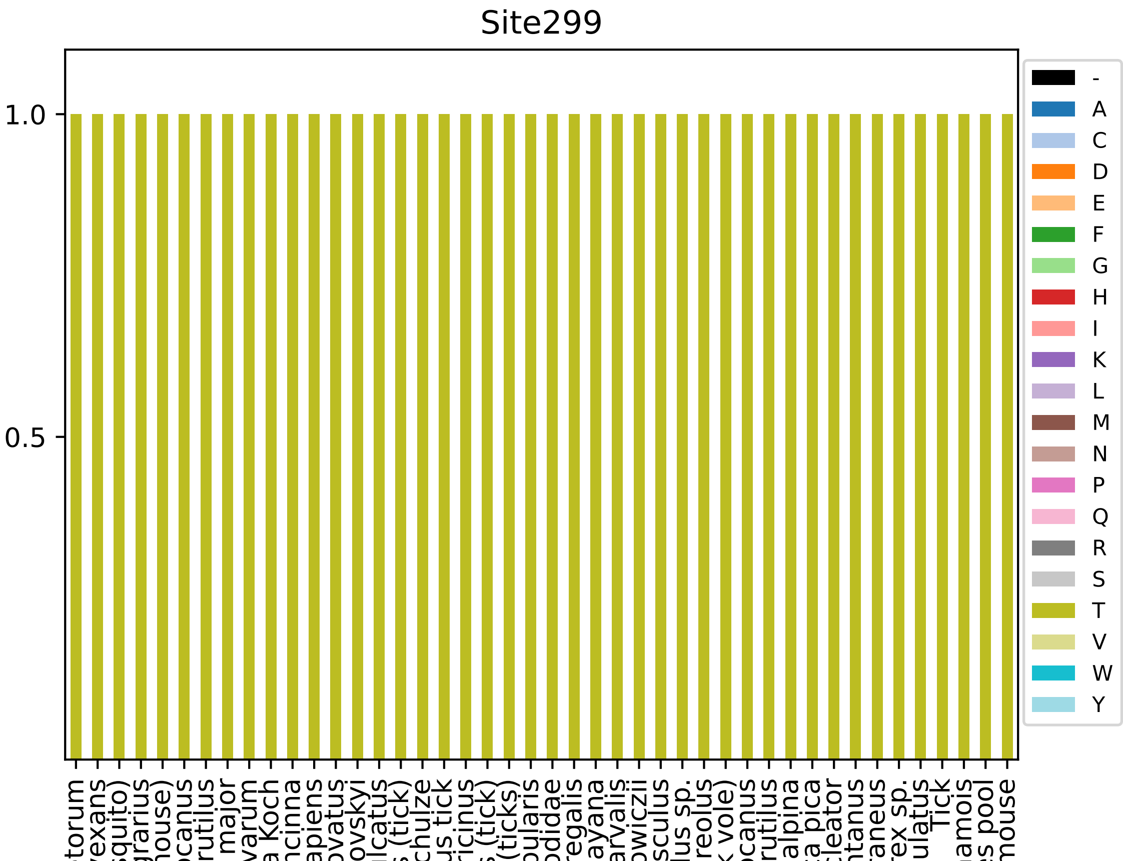
Site299
1.0
0.5
-
A
C
D
E
F
G
H
I
K
L
M
N
P
Q
R
S
T
V
W
Y

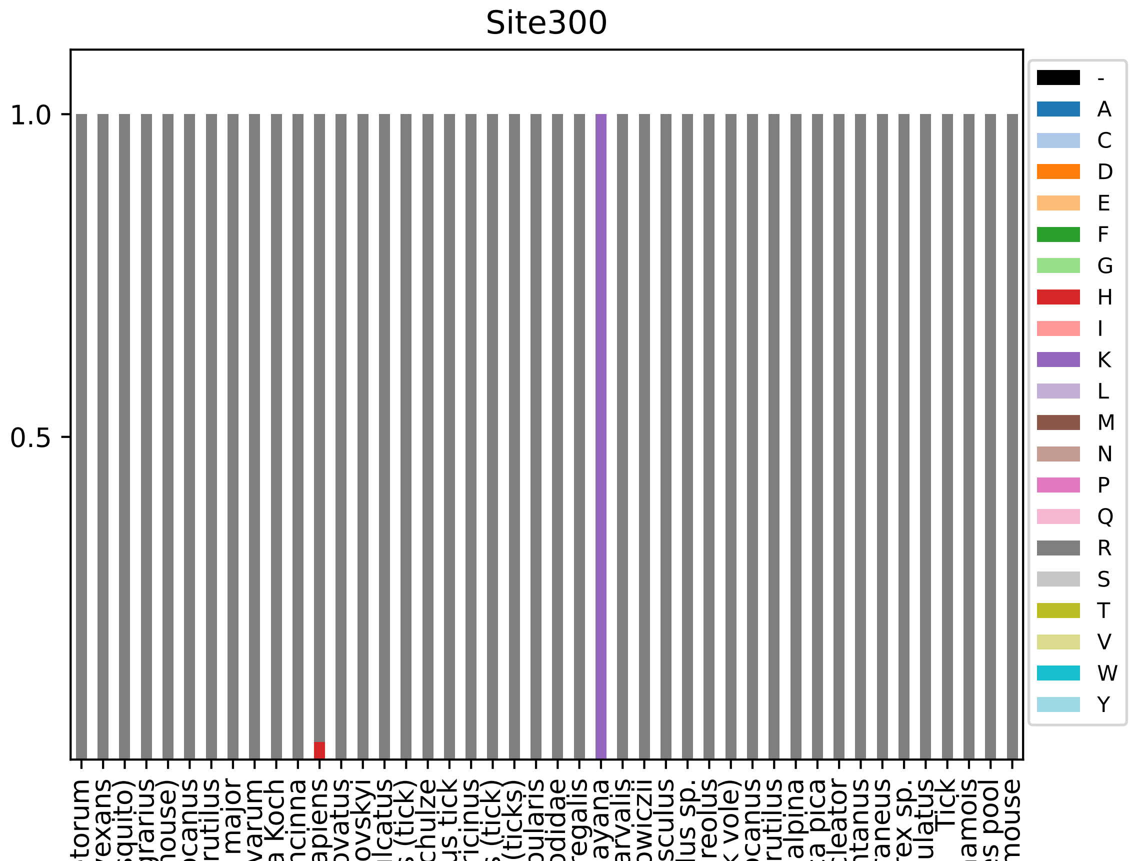
Site300
1.0
0.5
-
A
C
D
E
F
G
H
I
K
L
M
N
P
Q
R
S
T
V
W
Y

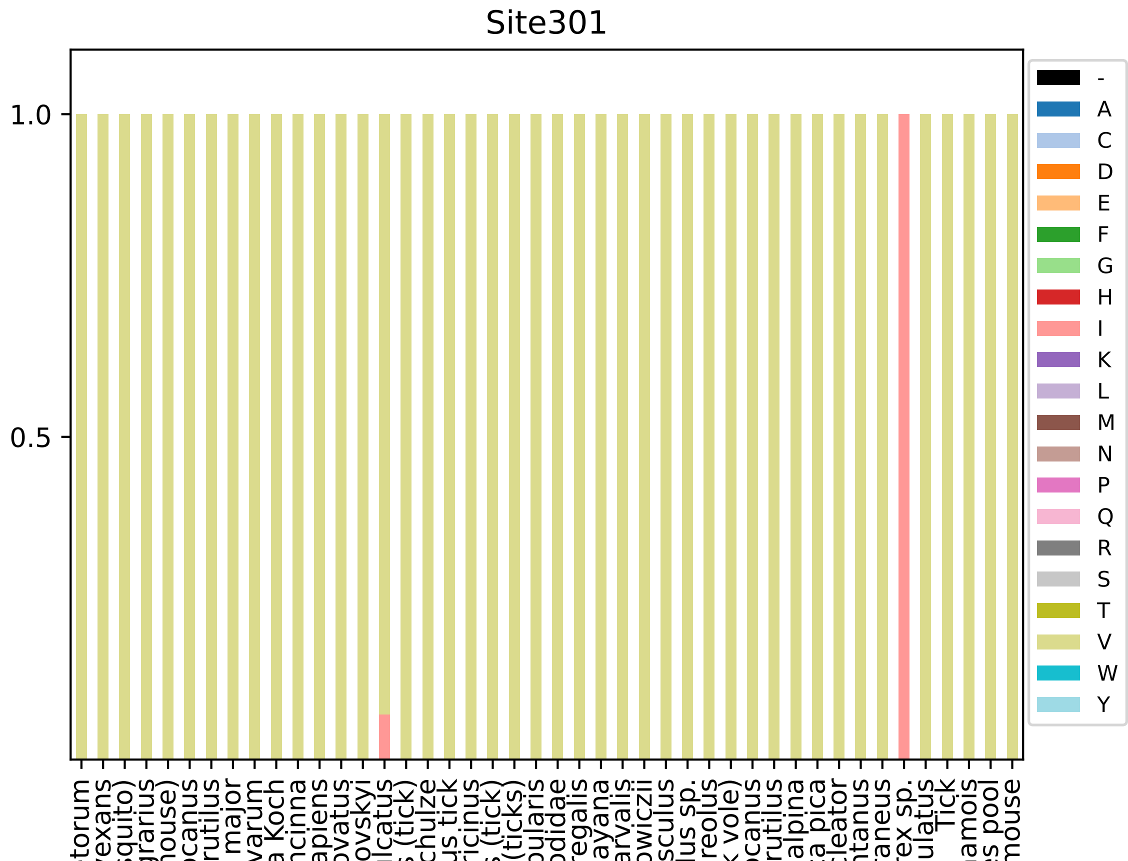
Site301
1.0
0.5
-
A
C
D
E
F
G
H
I
K
L
M
N
P
Q
R
S
T
V
W
Y

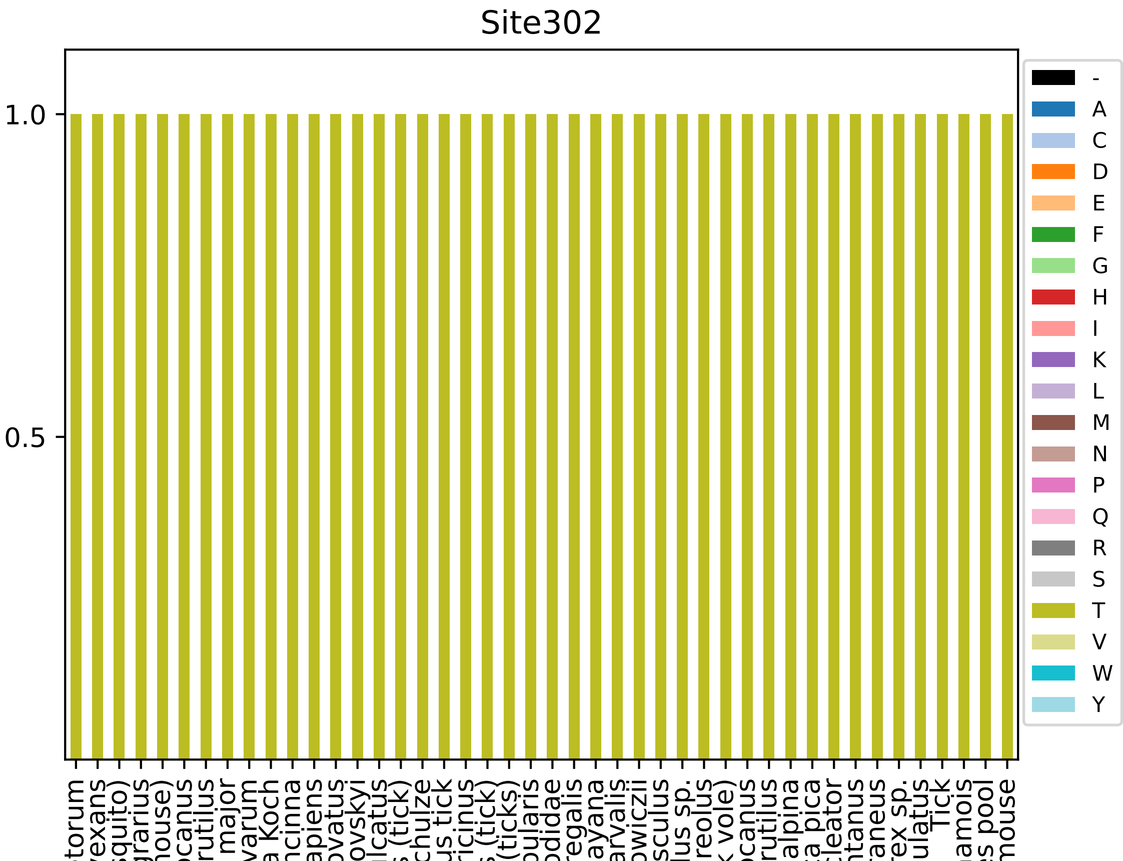
Site302
1.0
0.5
-
A
C
D
E
F
G
H
I
K
L
M
N
P
Q
R
S
T
V
W
Y

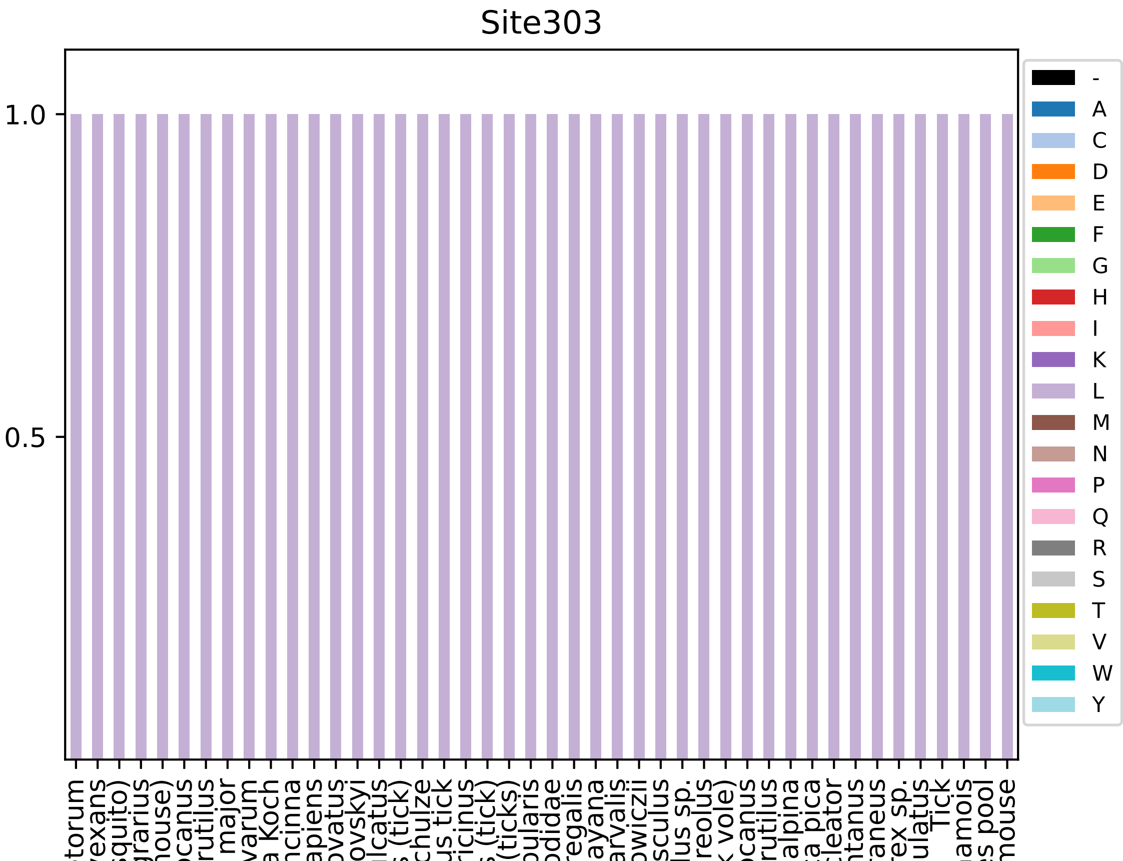
Site303
1.0
0.5
-
A
C
D
E
F
G
H
I
K
L
M
N
P
Q
R
S
T
V
W
Y
torum
vexans
squito)
grarius
nouse)
ocanus
rutilus
major
varum
a Koch
ncinna
apiens
ovatus
ovskyi
ulcatus
s (tick)
chulze
us tick
ricinus
s (tick)
(ticks)
pularis
odidae
regalis
ayana
arvalis
owiczii
isculus
lus sp.
reolus
k vole)
ocanus
rutilus
alpina
a pica
cleator
ntanus
raneus
rex sp.
ulatus
Tick
amois
es pool
mouse

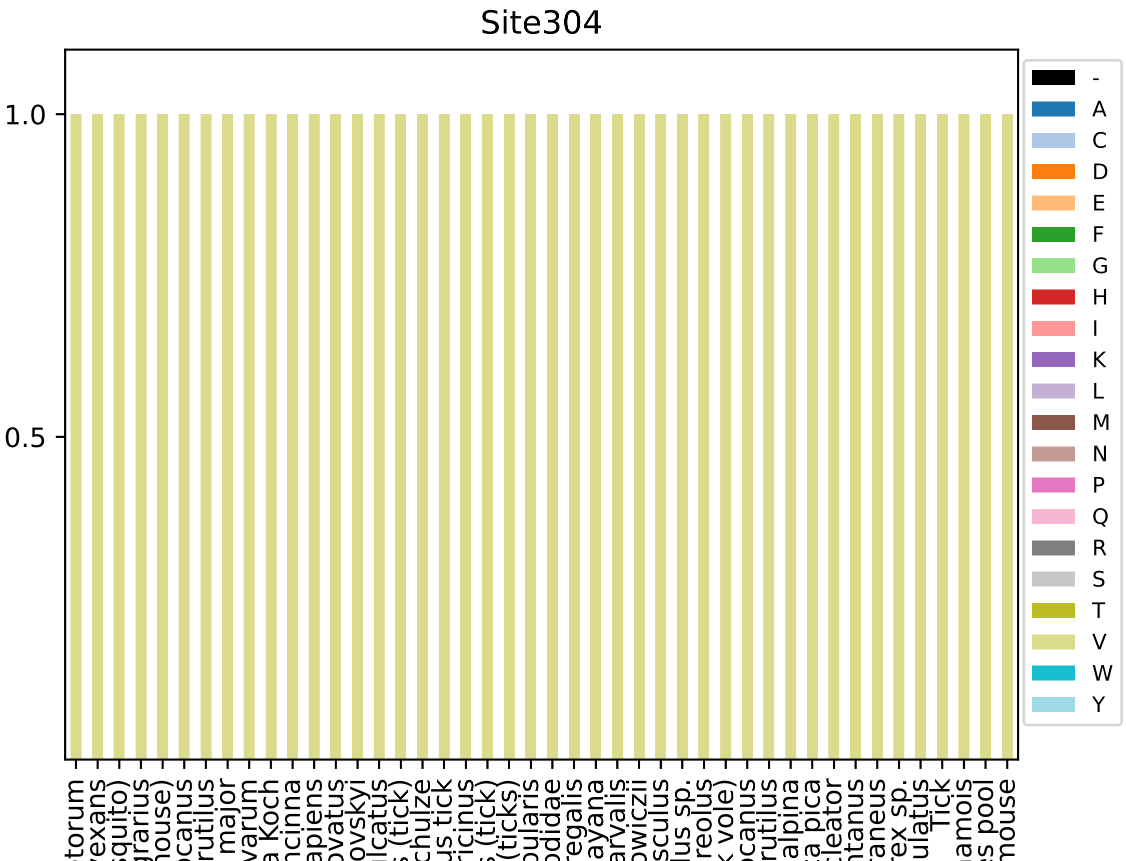
Site304
1.0
0.5
-
A
C
D
E
F
G
H
I
K
L
M
N
P
Q
R
S
T
V
W
Y

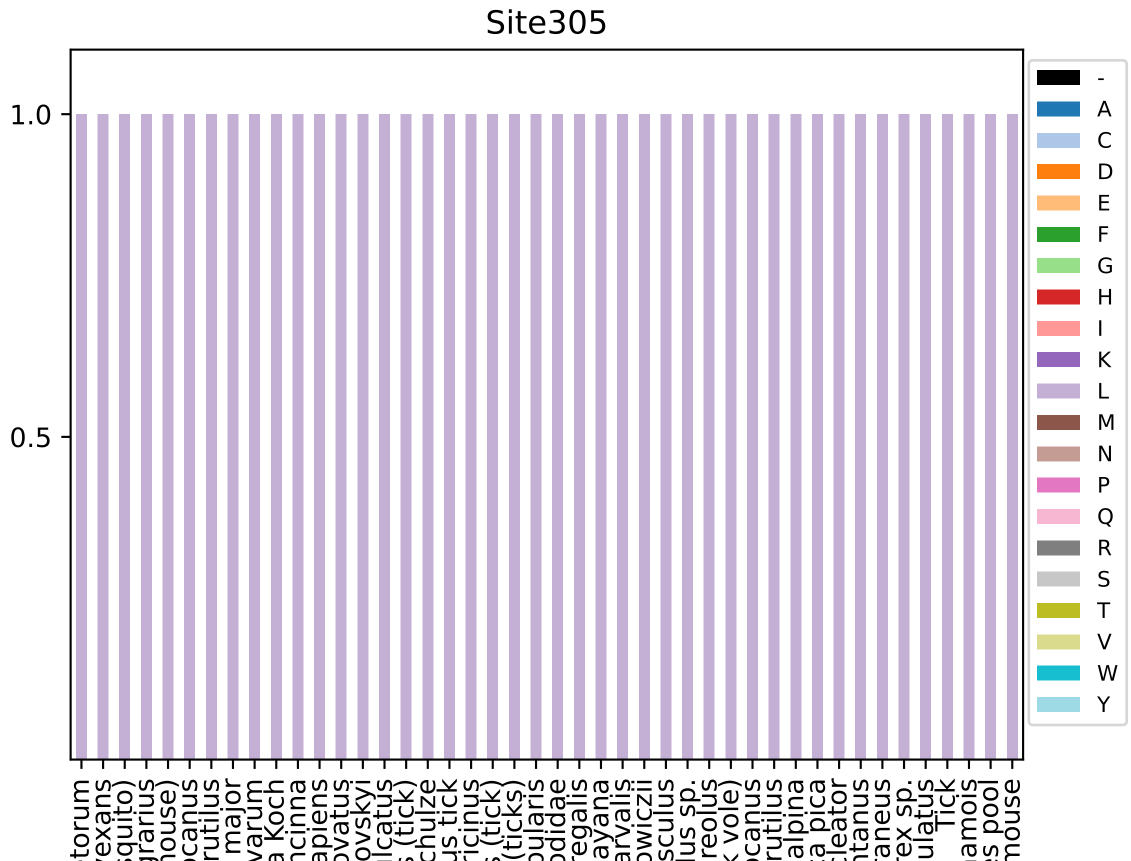

Site305
1.0
0.5
-
A
C
D
E
F
G
H
I
K
L
M
N
P
Q
R
S
T
V
W
Y

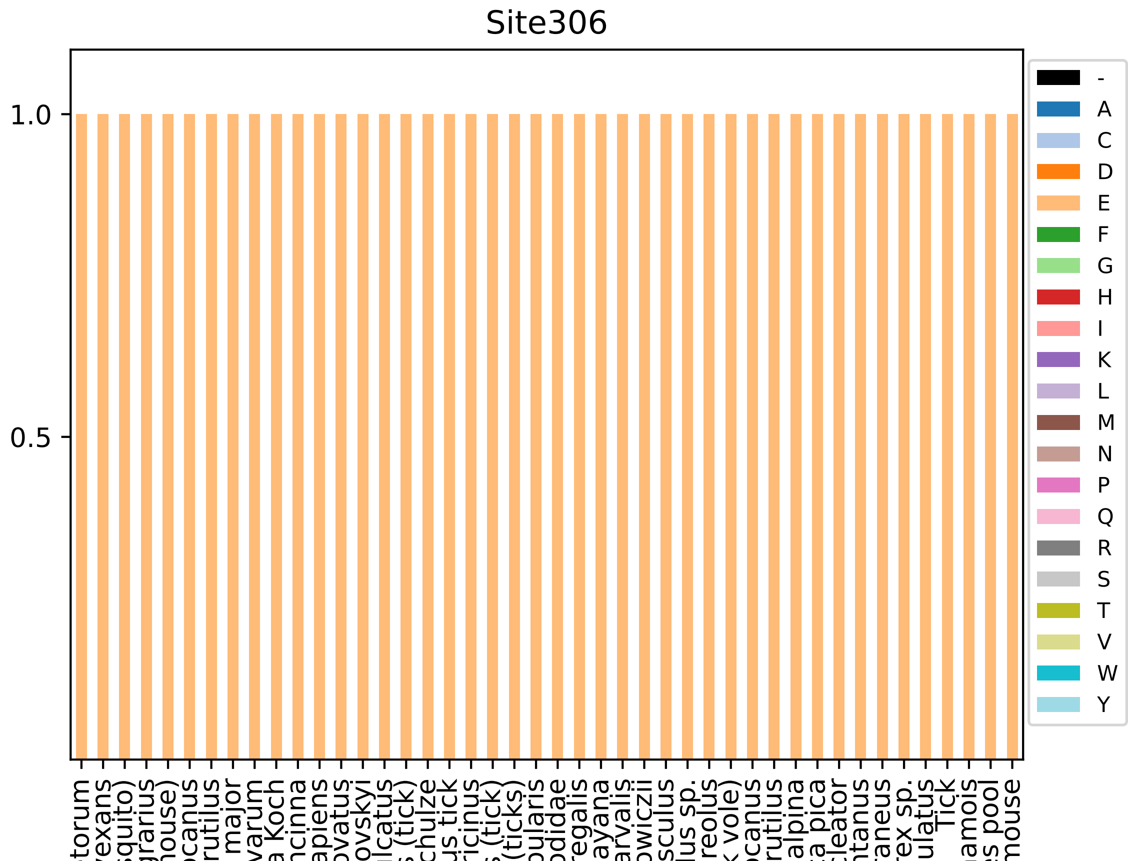
Site306
1.0
0.5
-
A
C
D
E
F
G
H
I
K
L
M
N
P
Q
R
S
T
V
W
Y

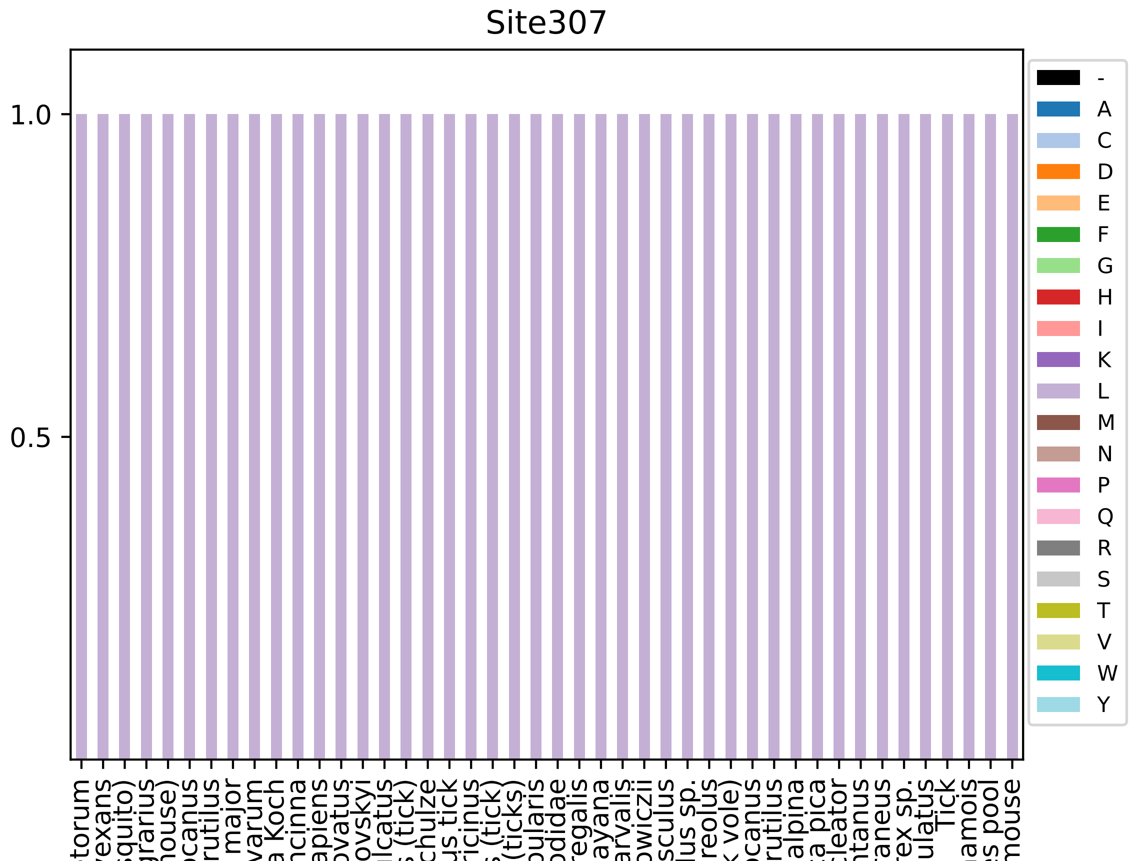
Site307
1.0
0.5
-
A
C
D
E
F
G
H
I
K
L
M
N
P
Q
R
S
T
V
W
Y

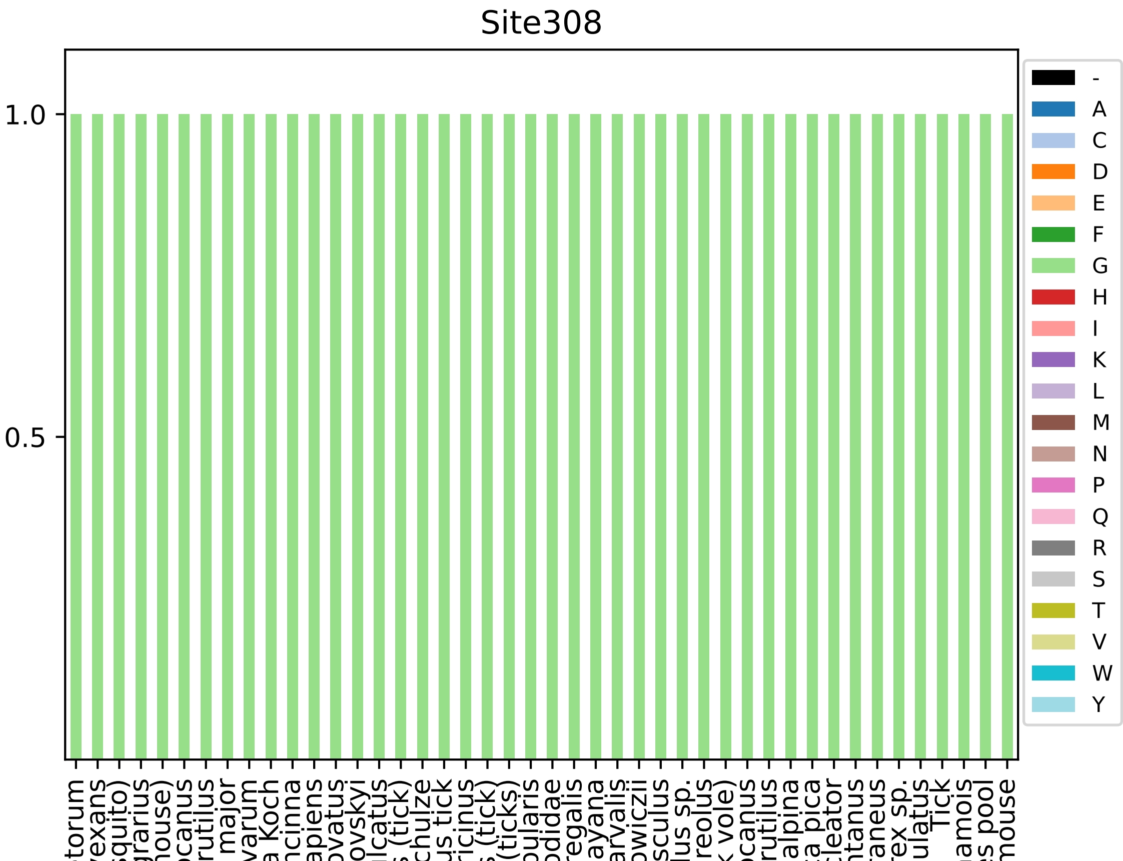
Site308
1.0
0.5
-
A
C
D
E
F
G
H
I
K
L
M
N
P
Q
R
S
T
V
W
Y

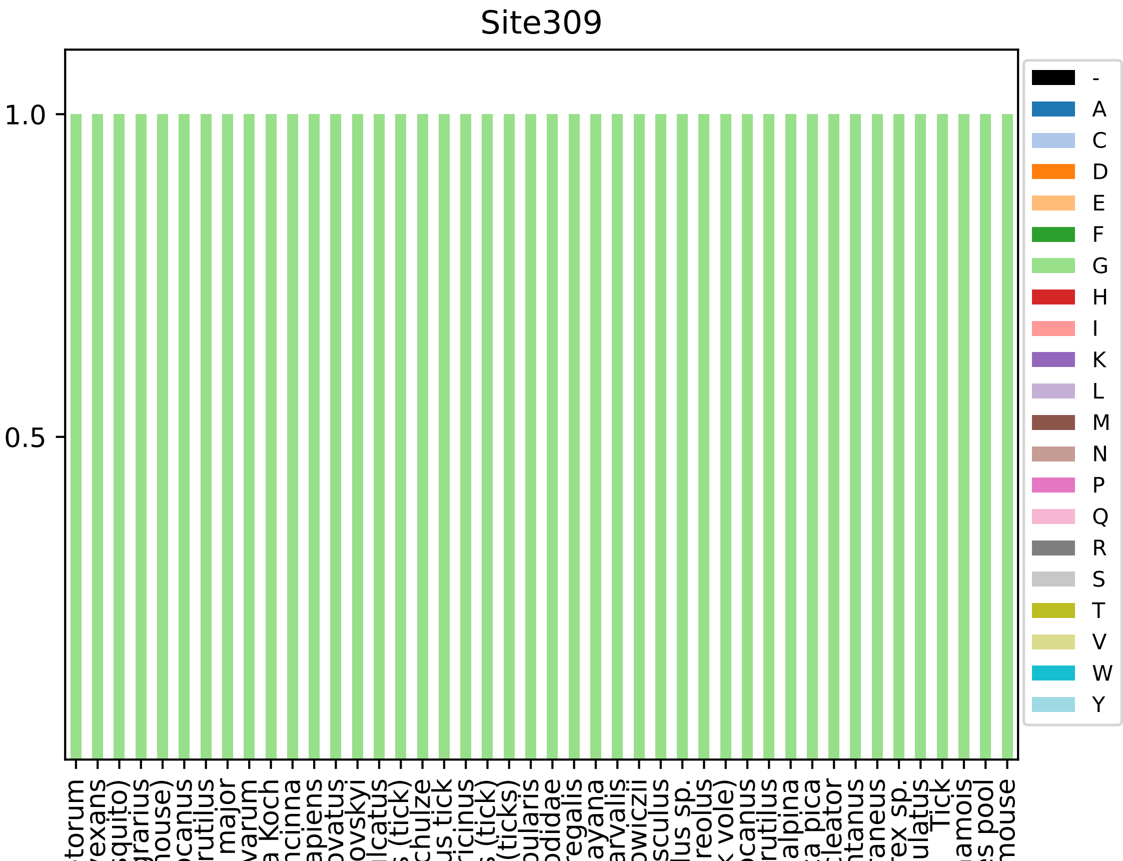
Site309
1.0
0.5
-
A
C
D
E
F
G
H
I
K
L
M
N
P
Q
R
S
T
V
W
Y

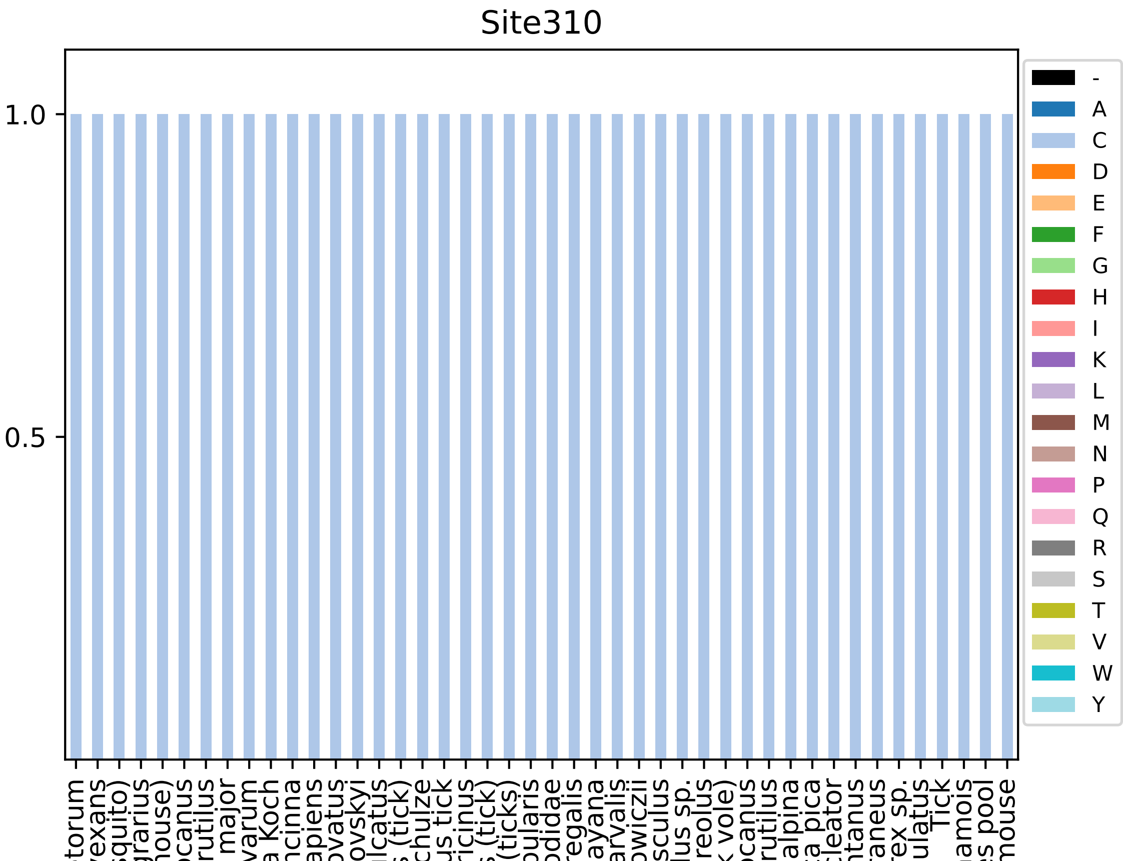
Site310
1.0
0.5
-
A
C
D
E
F
G
H
I
K
L
M
N
P
Q
R
S
T
V
W
Y

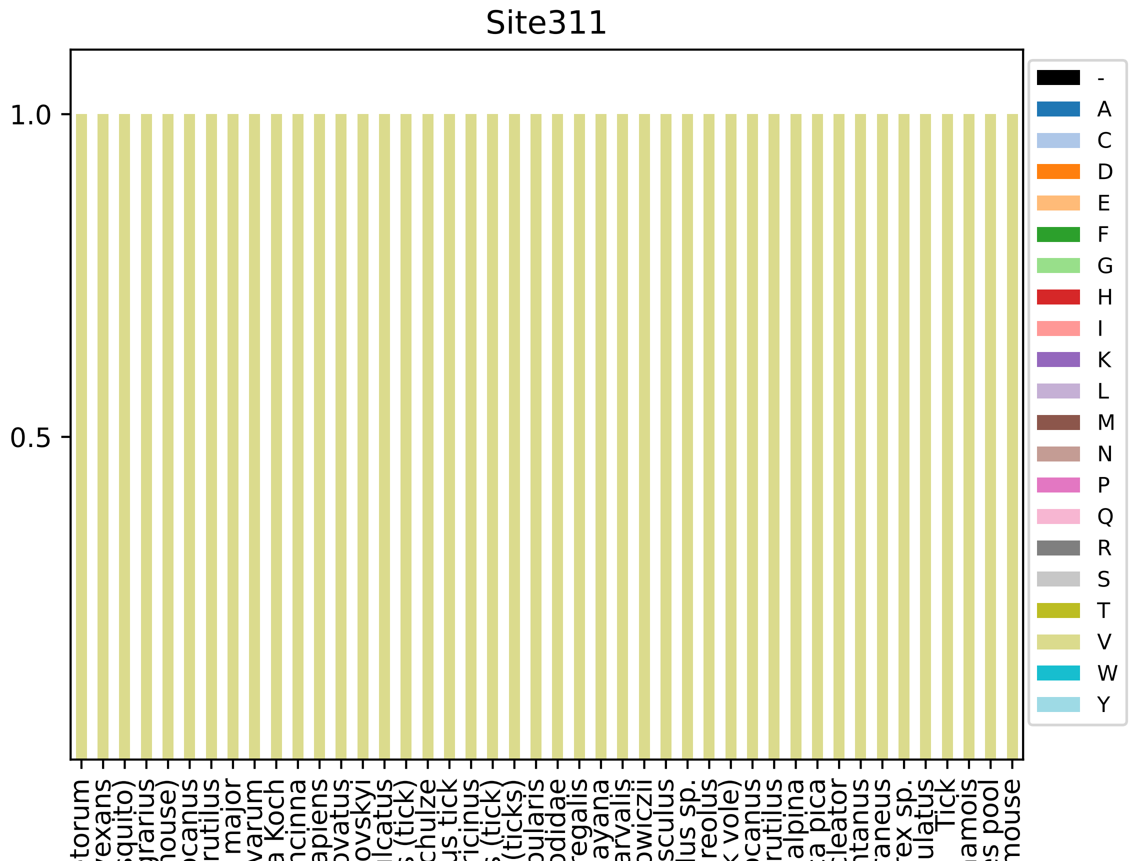
Site311
1.0
0.5
-
A
C
D
E
F
G
H
I
K
L
M
N
P
Q
R
S
T
V
W
Y

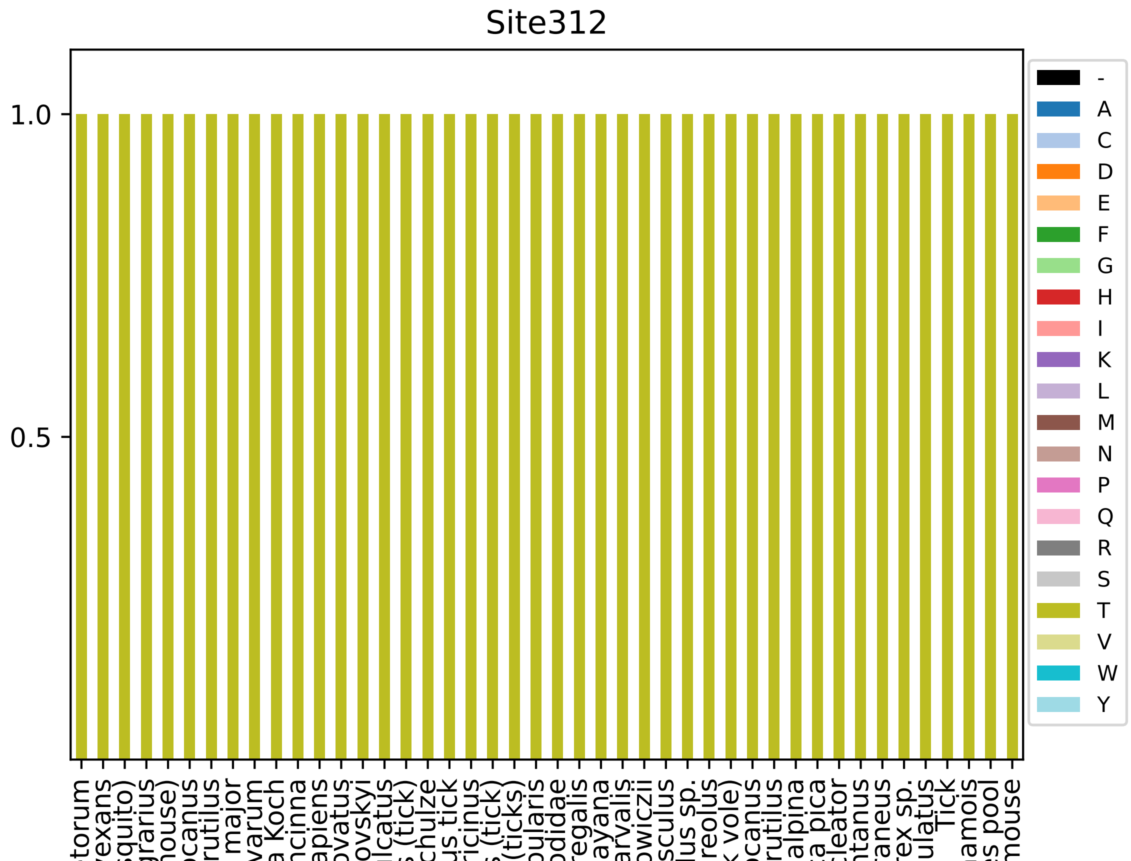

Site312
1.0
0.5
-
A
C
D
E
F
G
H
I
K
L
M
N
P
Q
R
S
T
V
W
Y

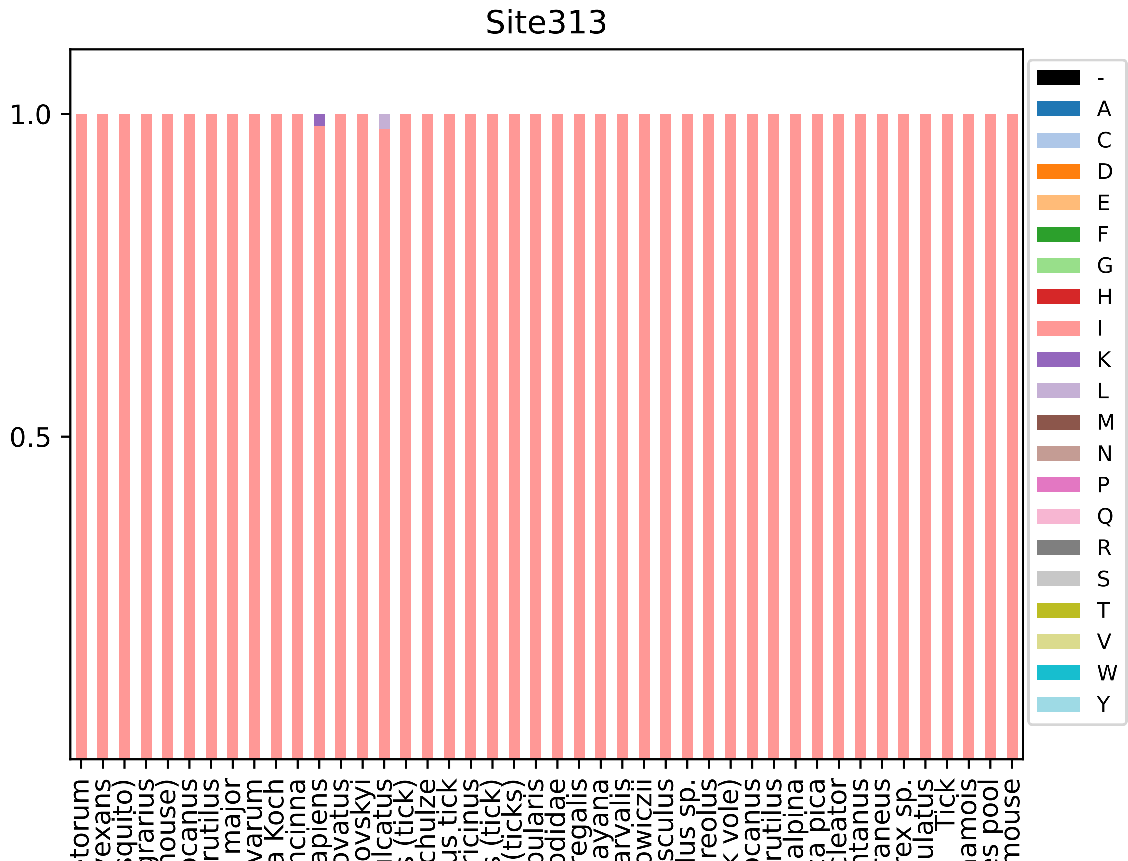
Site313
1.0
0.5
-
A
C
D
E
F
G
H
I
K
L
M
N
P
Q
R
S
T
V
W
Y

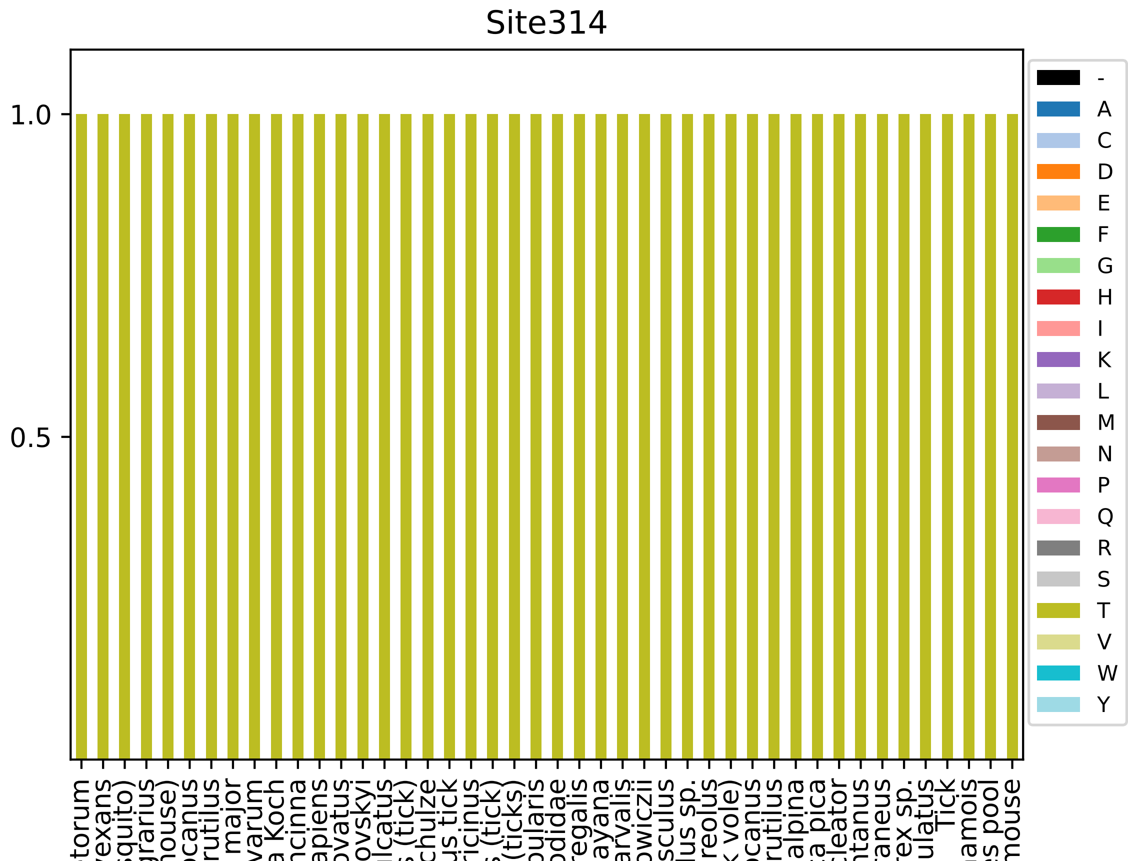
Site314
1.0
0.5
-
A
C
D
E
F
G
H
I
K
L
M
N
P
Q
R
S
T
V
W
Y

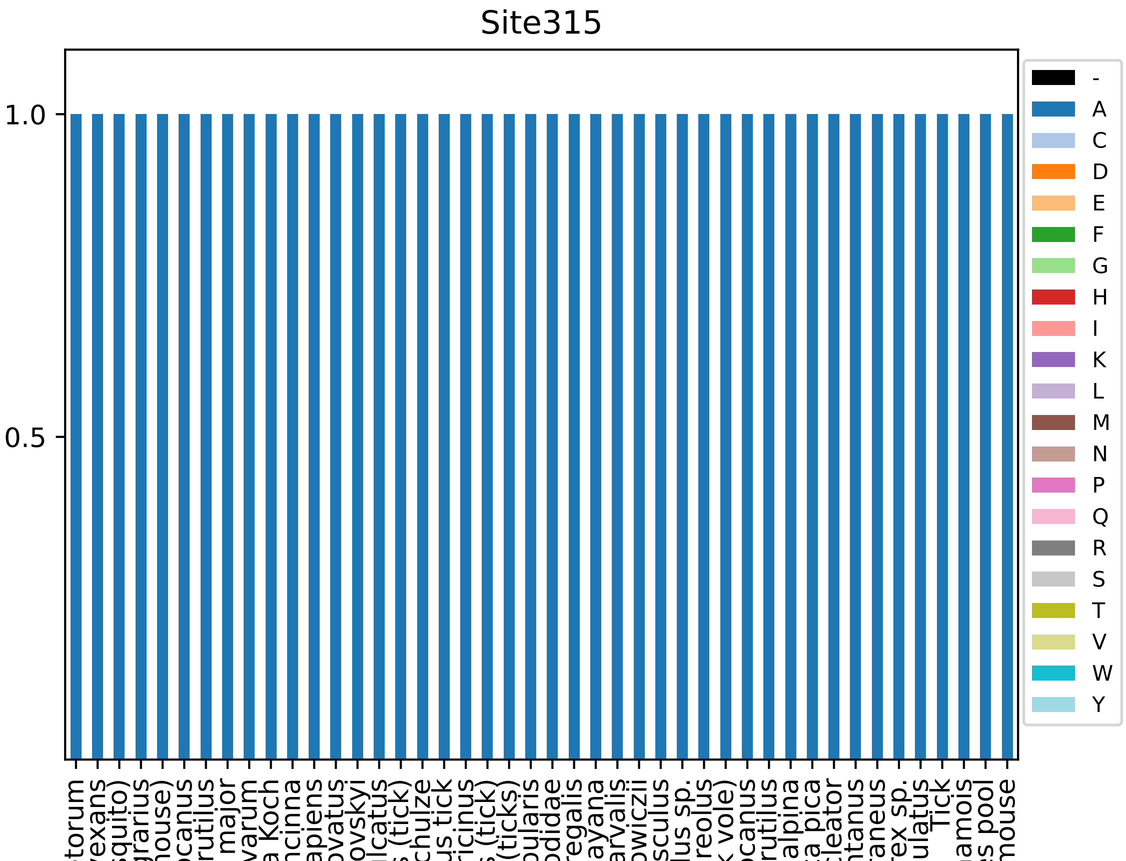
Site315
1.0
0.5
-
A
C
D
E
F
G
H
I
K
L
M
N
P
Q
R
S
T
V
W
Y

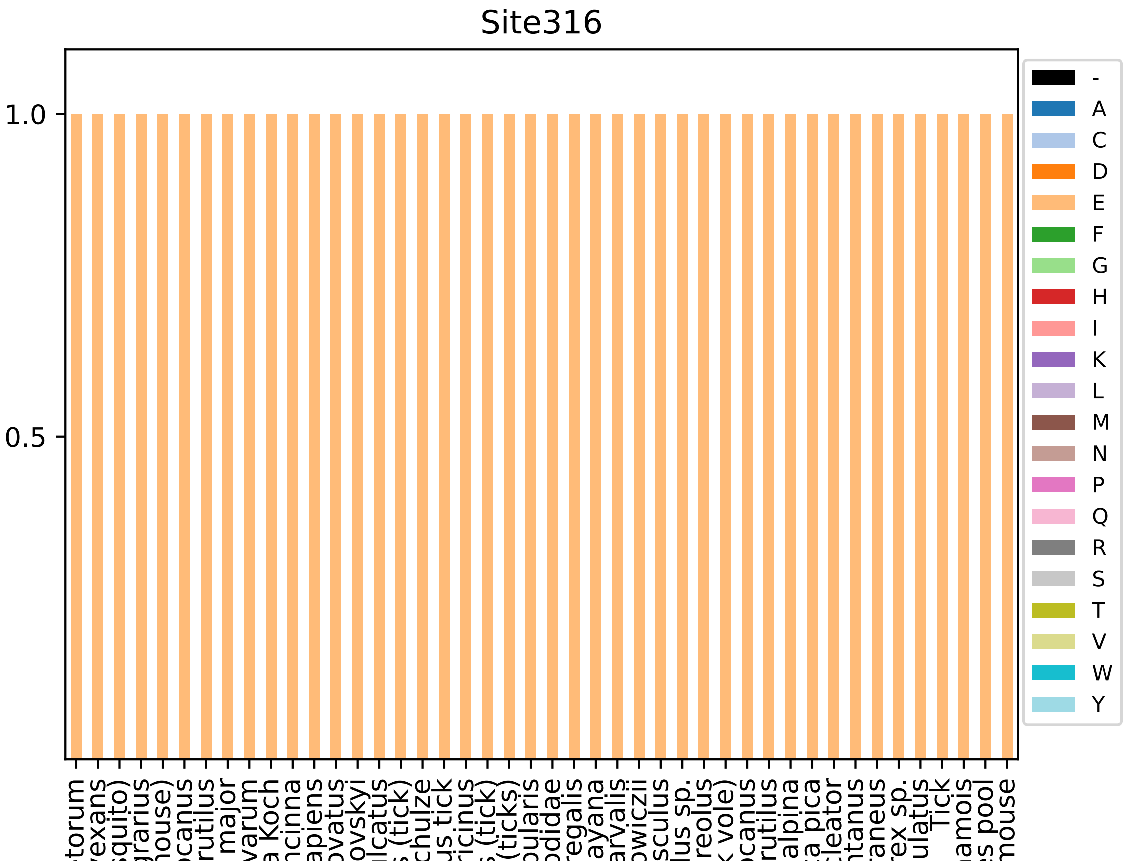
Site316
1.0
0.5
-
A
C
D
E
F
G
H
I
K
L
M
N
P
Q
R
S
T
V
W
Y

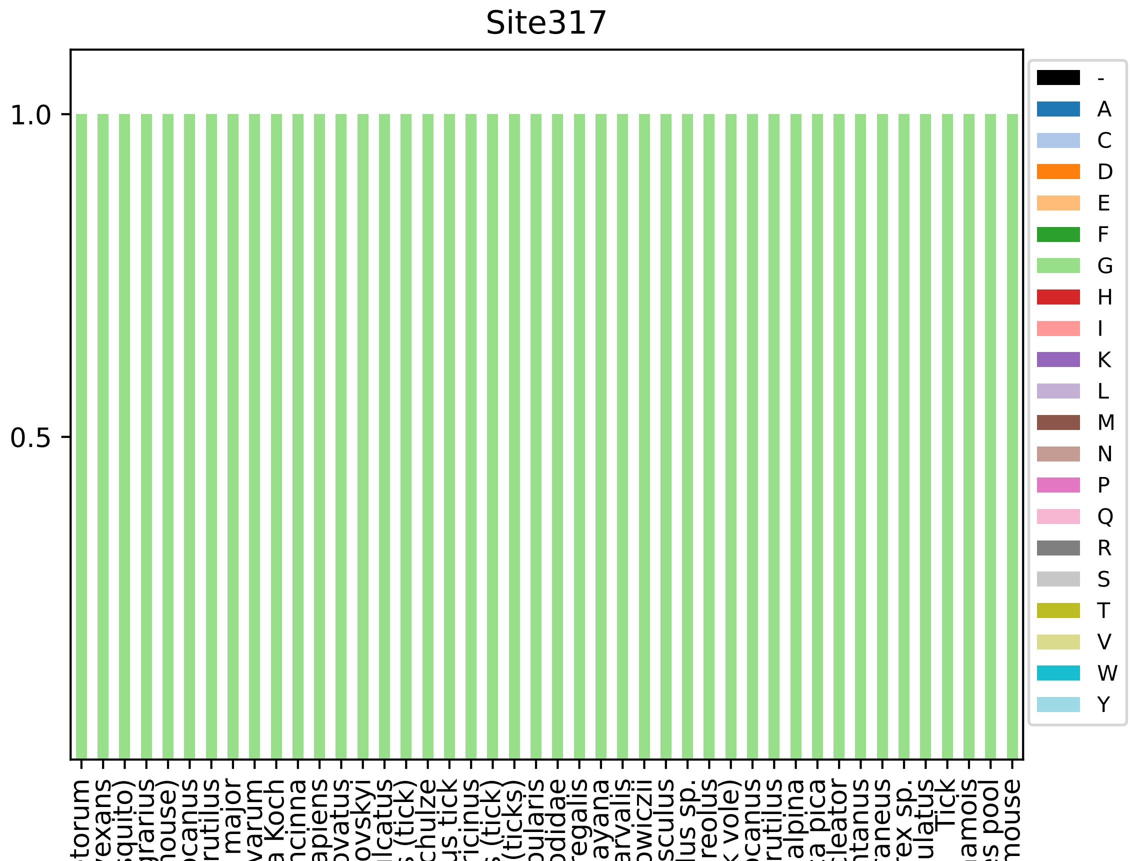
Site317
1.0
0.5
-
A
C
D
E
F
G
H
I
K
L
M
N
P
Q
R
S
T
V
W
Y

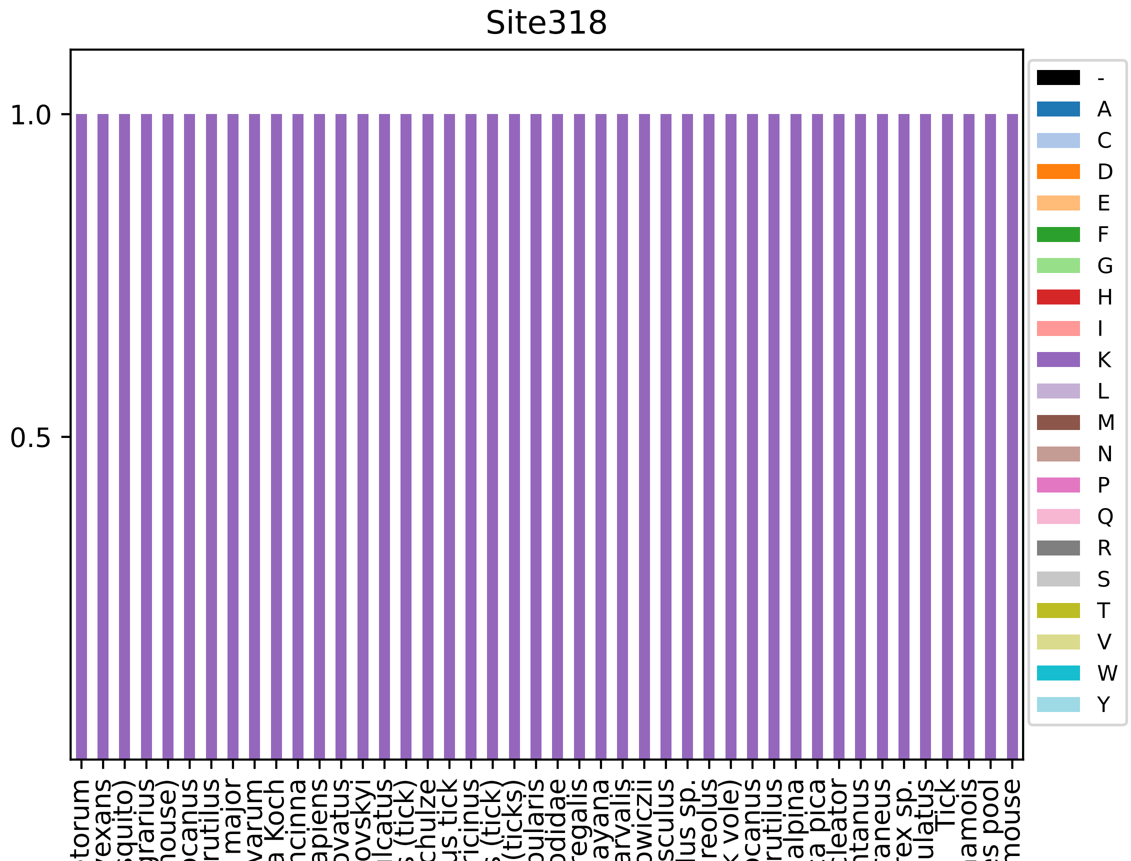

Site318
1.0
0.5
-
A
C
D
E
F
G
H
I
K
L
M
N
P
Q
R
S
T
V
W
Y

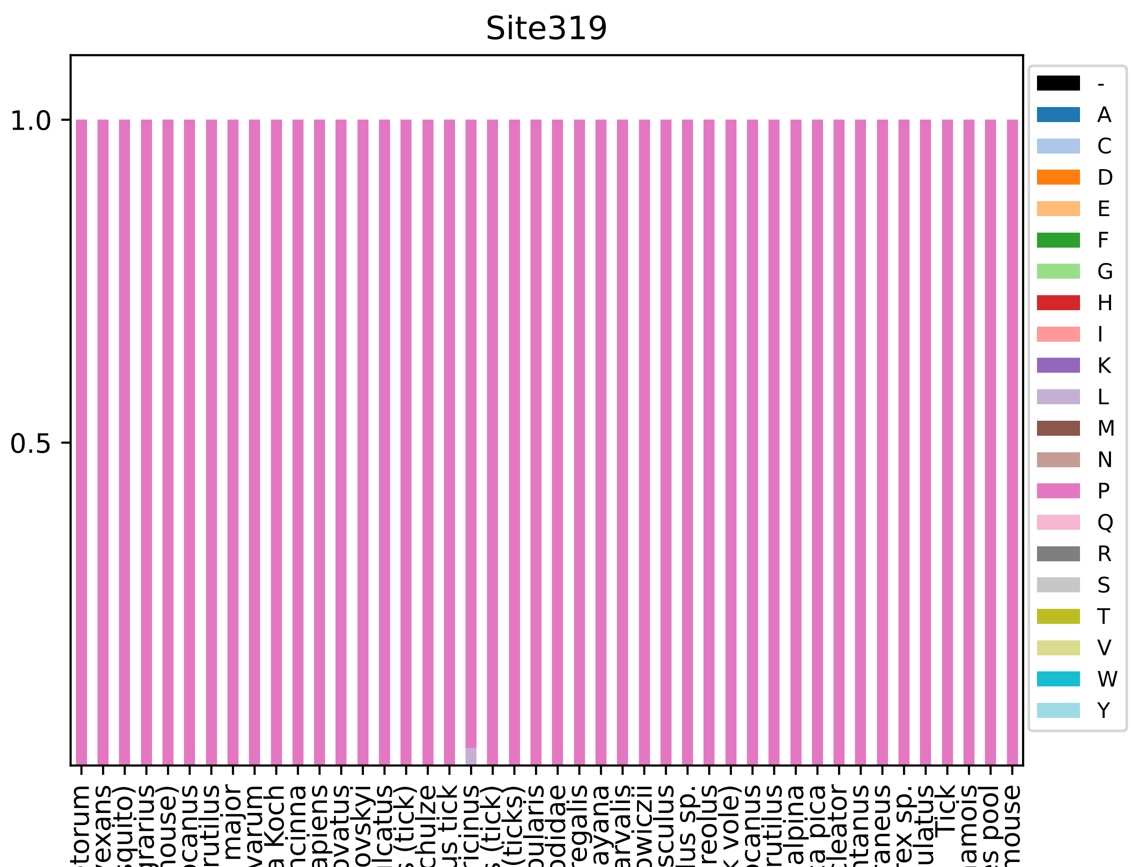
Site319
1.0
0.5
-
A
C
D
E
F
G
H
I
K
L
M
N
P
Q
R
S
T
V
W
Y

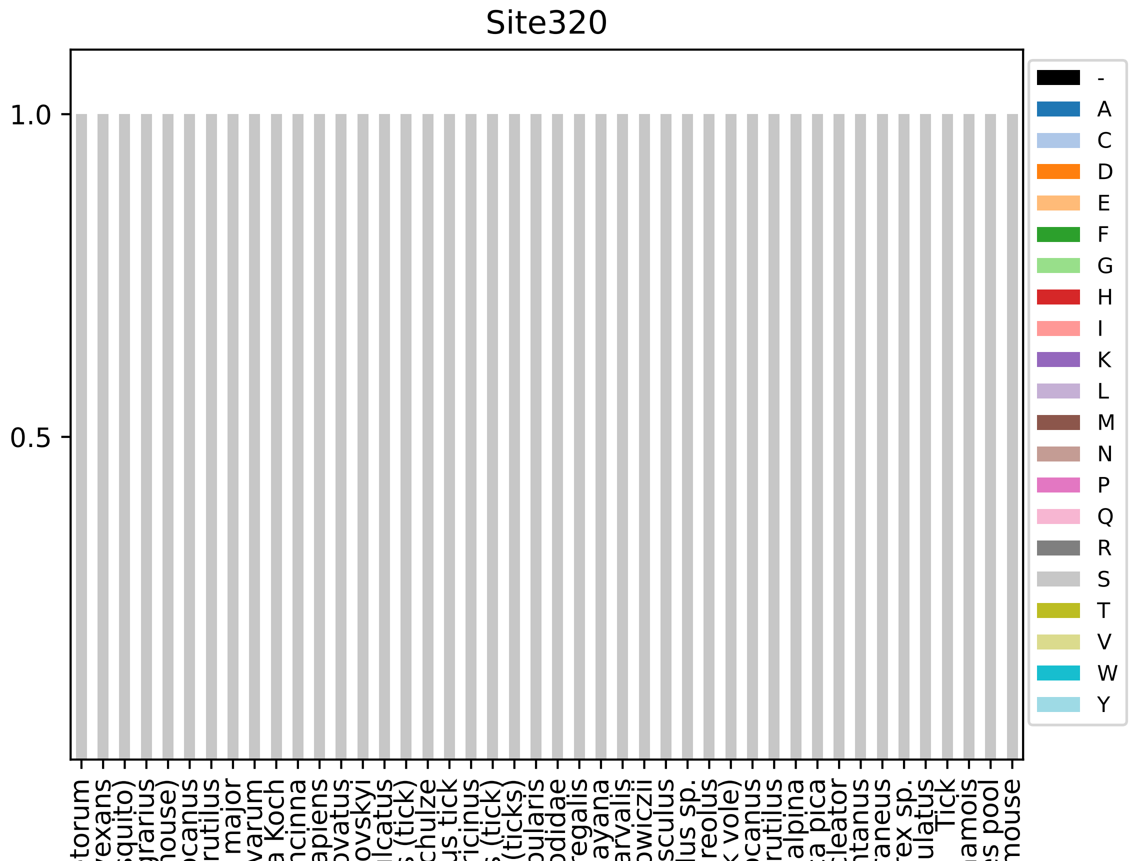

Site320
1.0
0.5
-
A
C
D
E
F
G
H
I
K
L
M
N
P
Q
R
S
T
V
W
Y

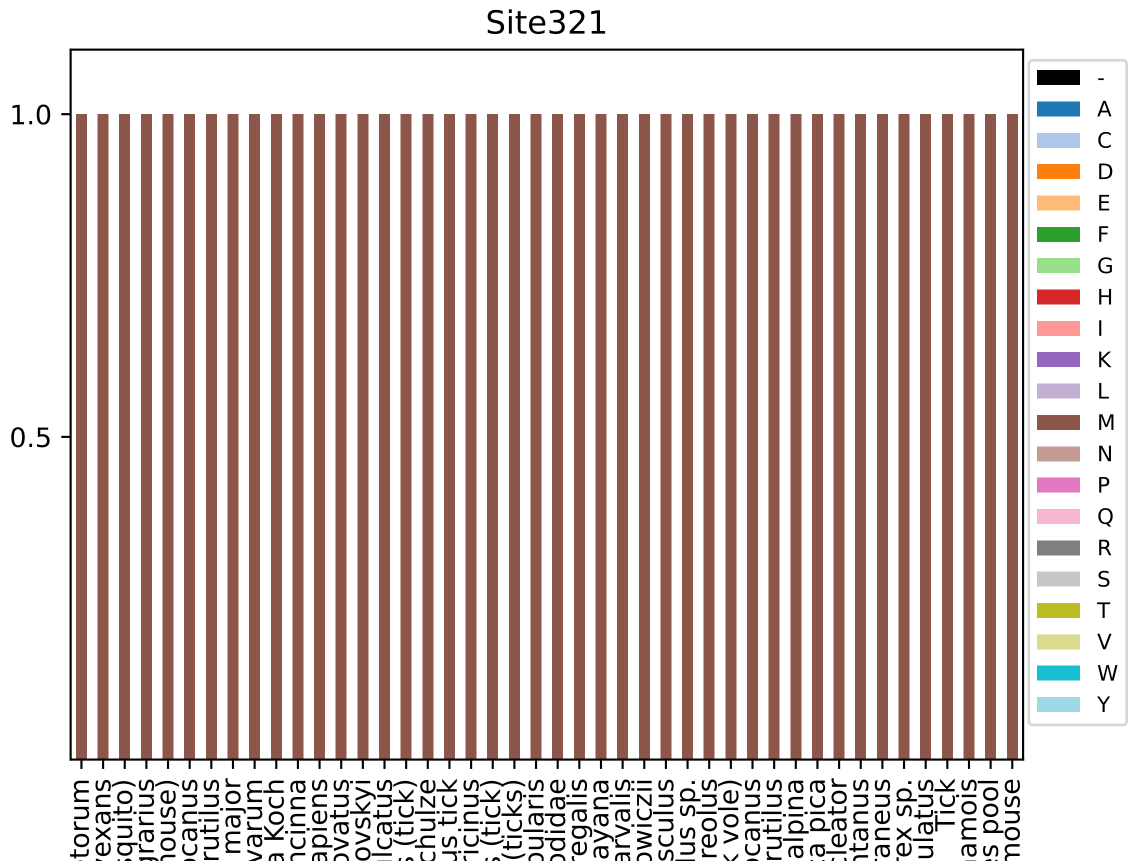
Site321
1.0
0.5
-
A
C
D
E
F
G
H
I
K
L
M
N
P
Q
R
S
T
V
W
Y
Tick

Site322

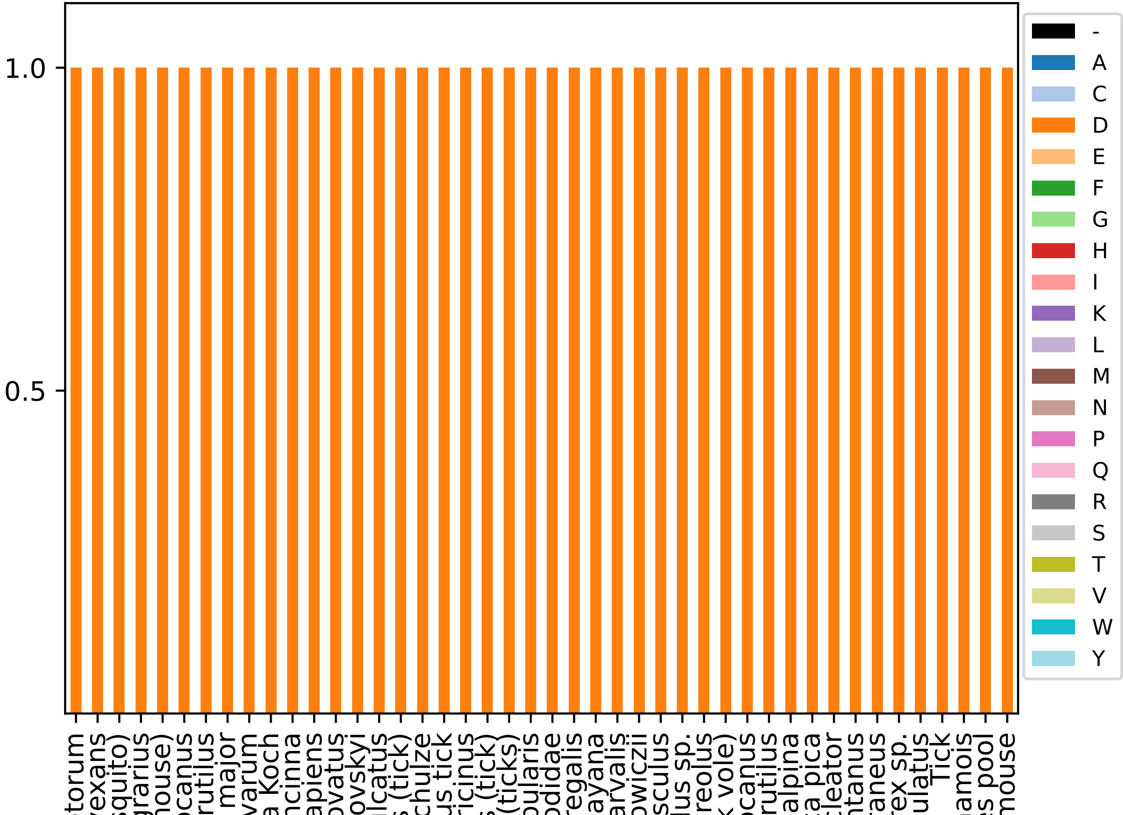

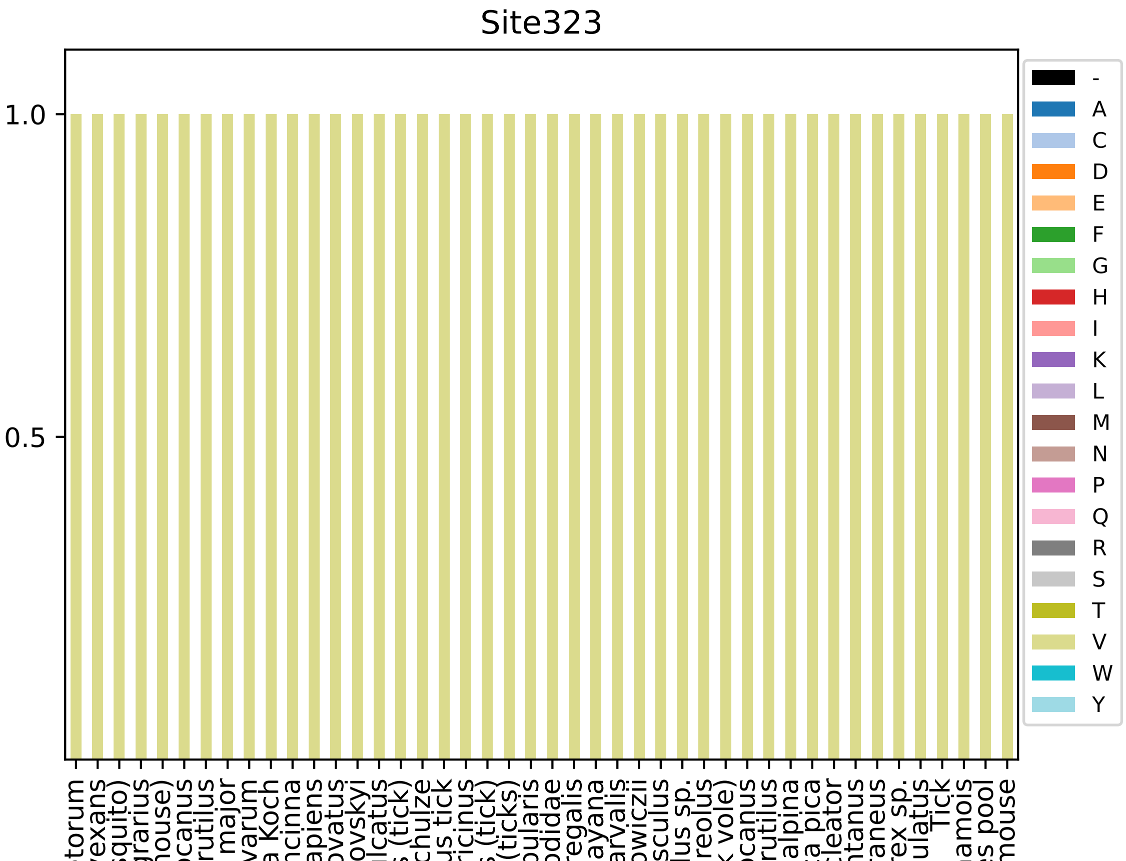

Site323
1.0
0.5
-
A
C
D
E
F
G
H
I
K
L
M
N
P
Q
R
S
T
V
W
Y

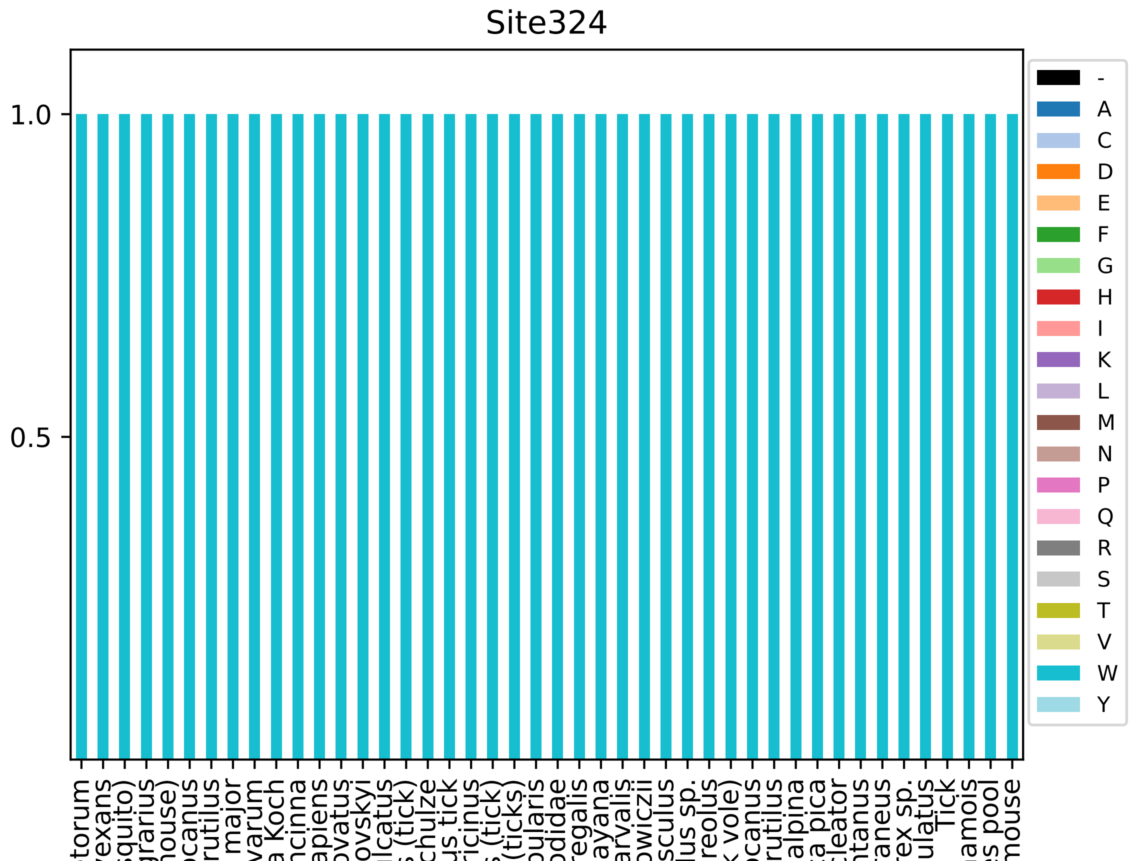

Site324
1.0
0.5
-
A
C
D
E
F
G
H
I
K
L
M
N
P
Q
R
S
T
V
W
Y

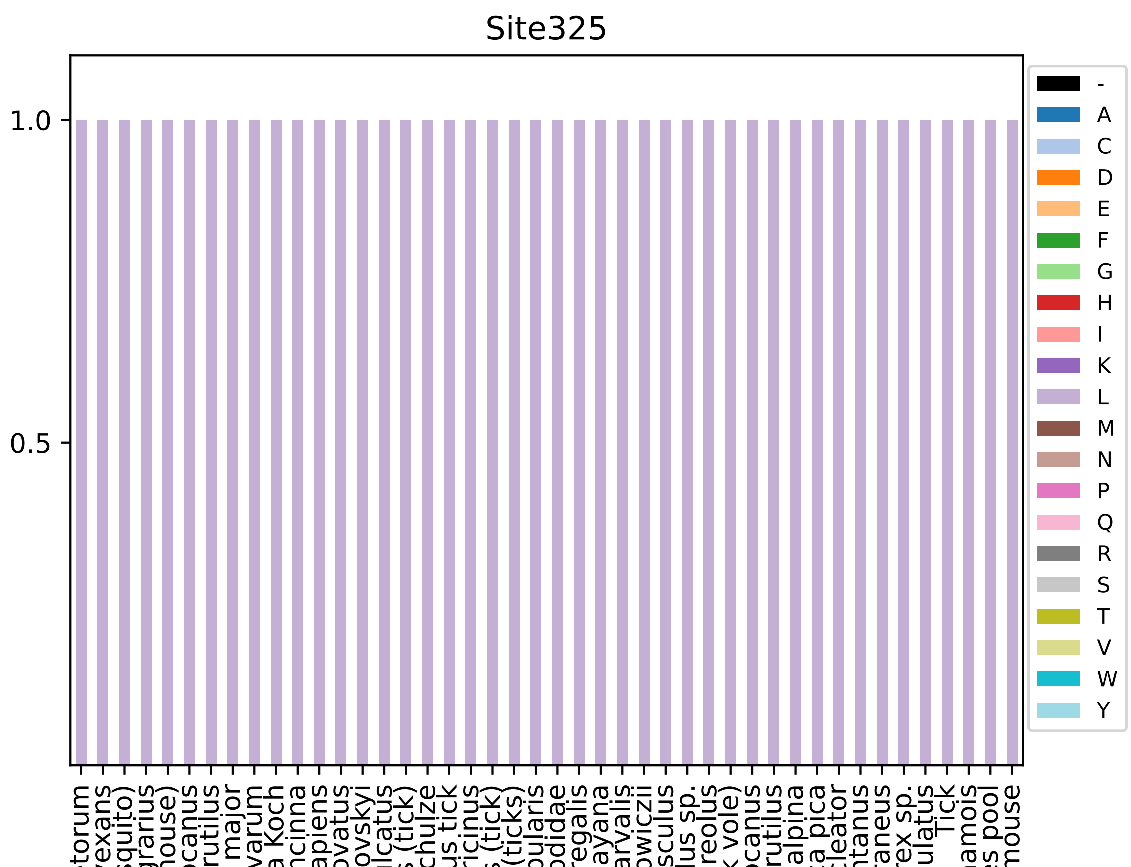
Site325
1.0
0.5
-
A
C
D
E
F
G
H
I
K
L
M
N
P
Q
R
S
T
V
W
Y

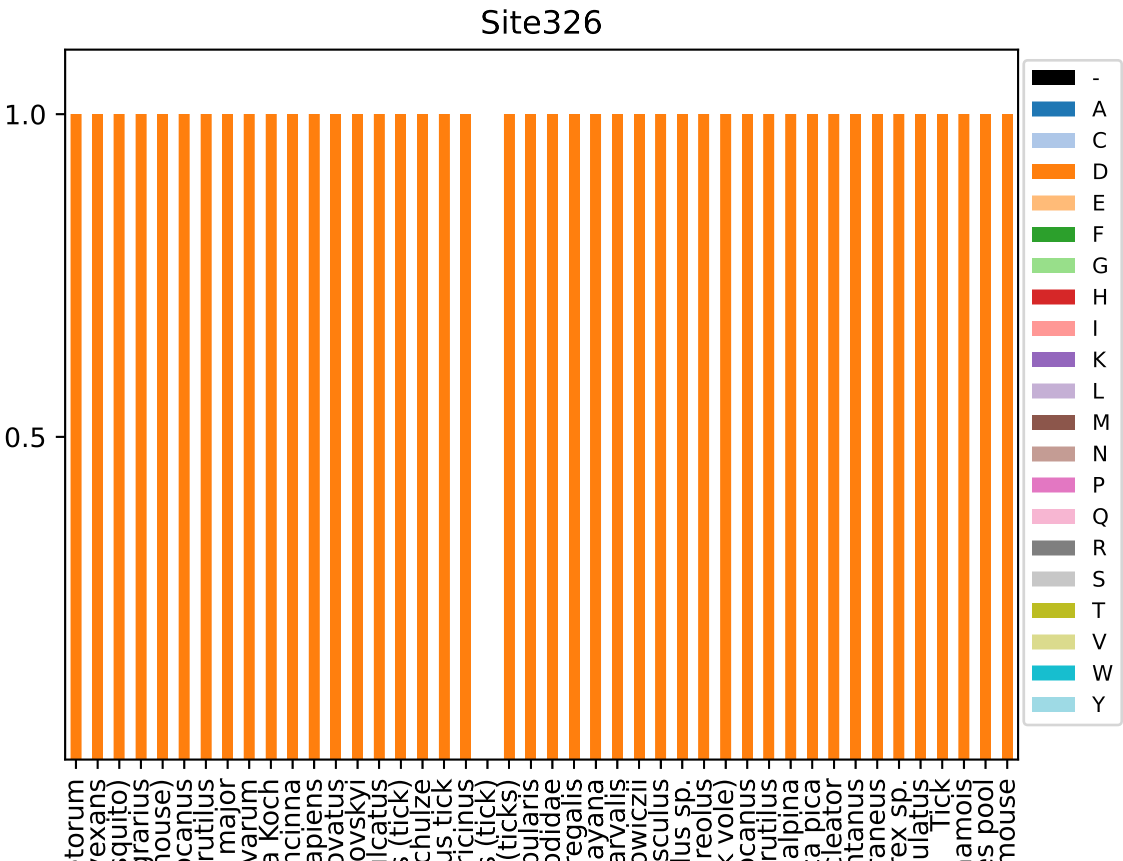
Site326
1.0
0.5
-
A
C
D
E
F
G
H
I
K
L
M
N
P
Q
R
S
T
V
W
Y

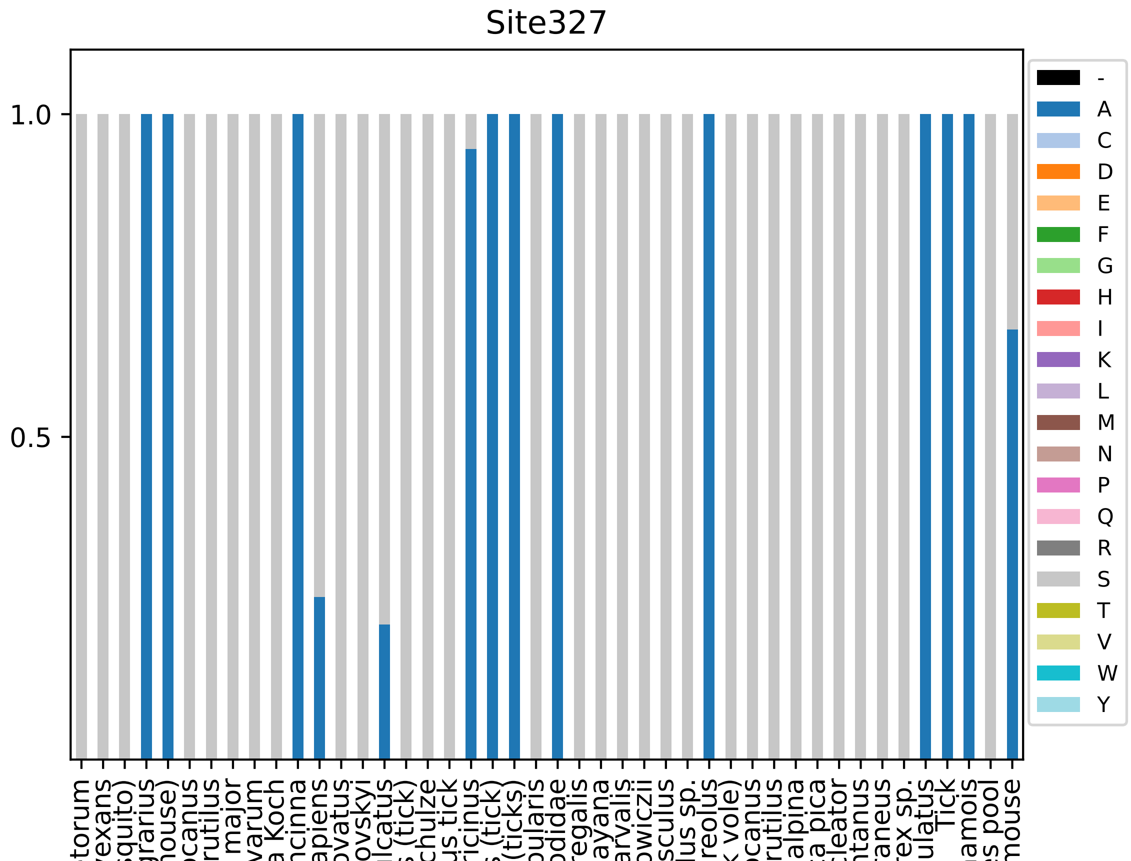
Site327
1.0
0.5
-
A
C
D
E
F
G
H
I
K
L
M
N
P
Q
R
S
T
V
W
Y

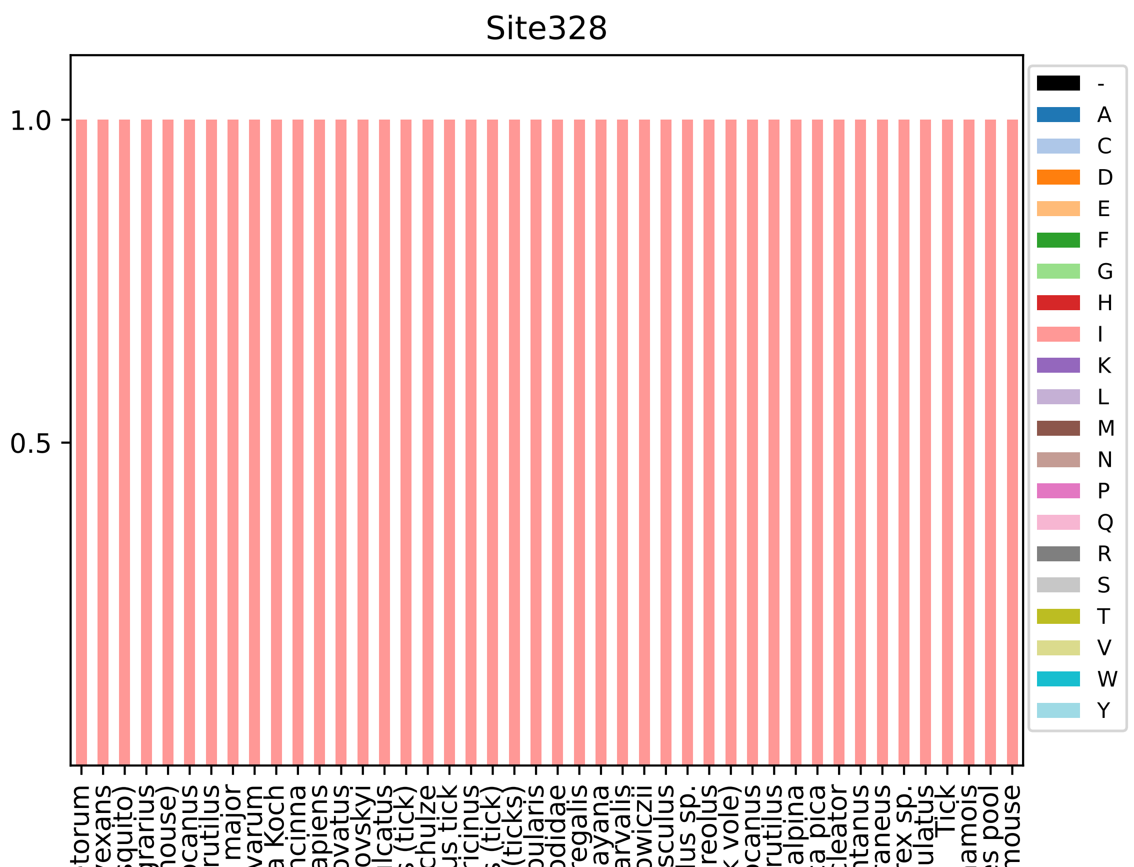

Site328
1.0
0.5
-
A
C
D
E
F
G
H
I
K
L
M
N
P
Q
R
S
T
V
W
Y

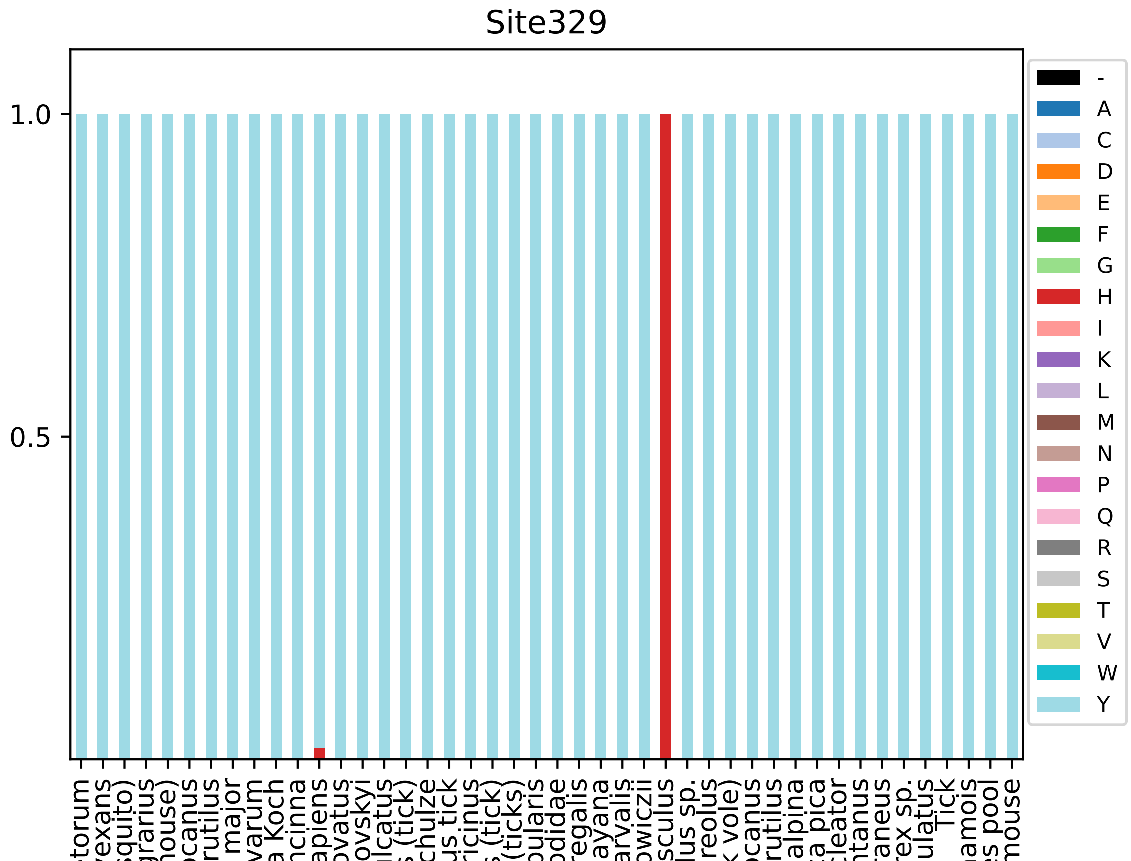

Site329
1.0
0.5
-
A
C
D
E
F
G
H
I
K
L
M
N
P
Q
R
S
T
V
W
Y

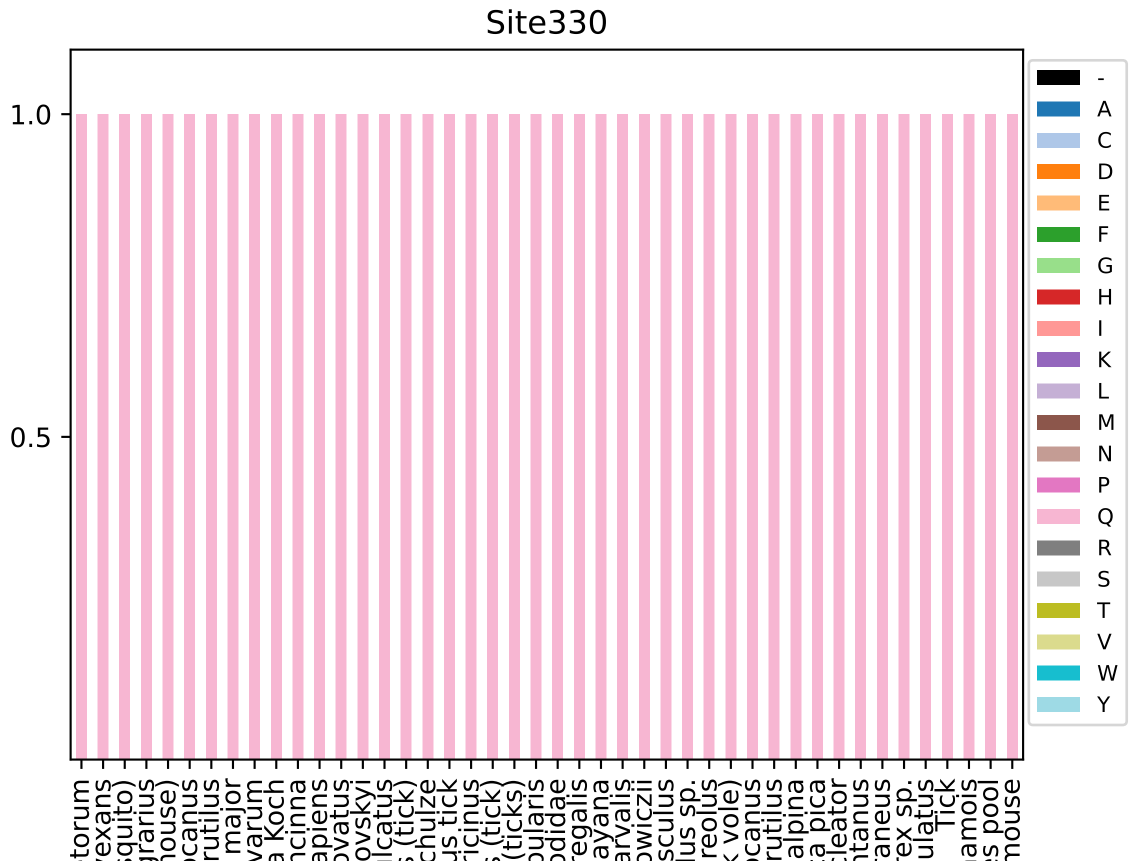
Site330
1.0
0.5
-
A
C
D
E
F
G
H
I
K
L
M
N
P
Q
R
S
T
V
W
Y

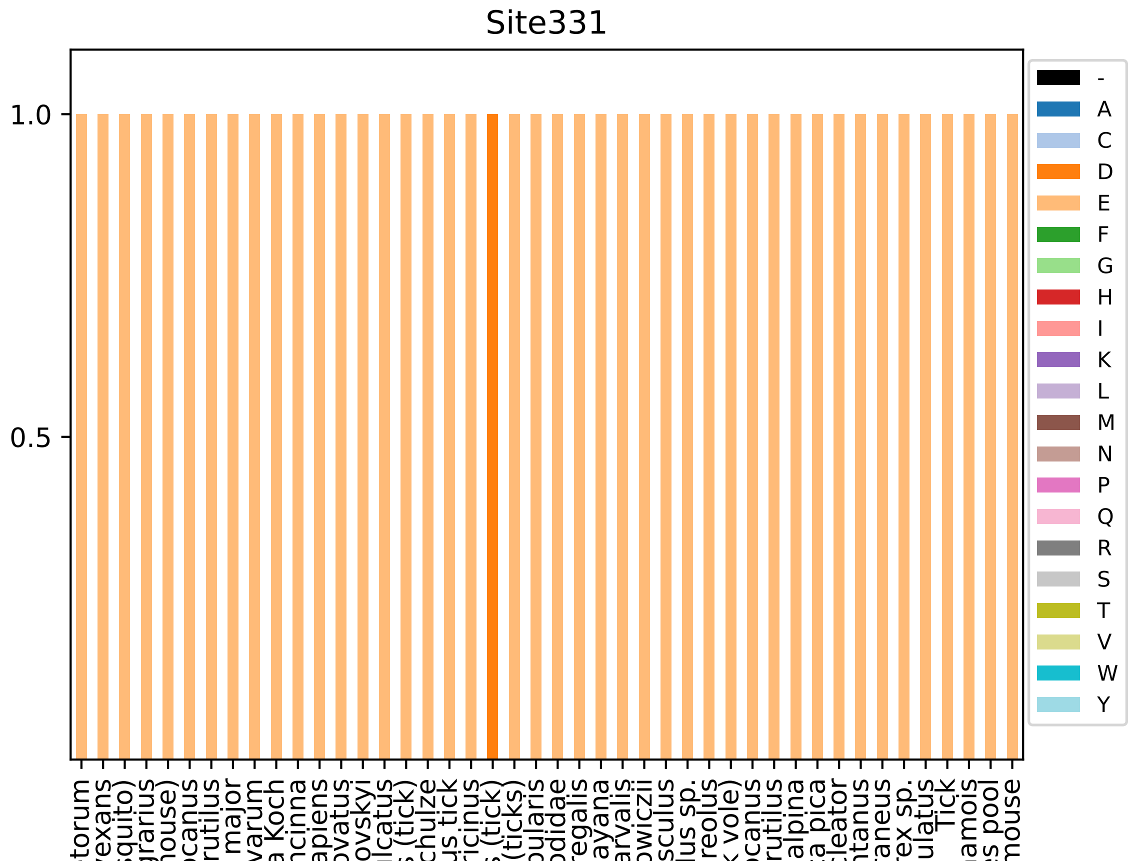
Site331
1.0
0.5
-
A
C
D
E
F
G
H
I
K
L
M
N
P
Q
R
S
T
V
W
Y

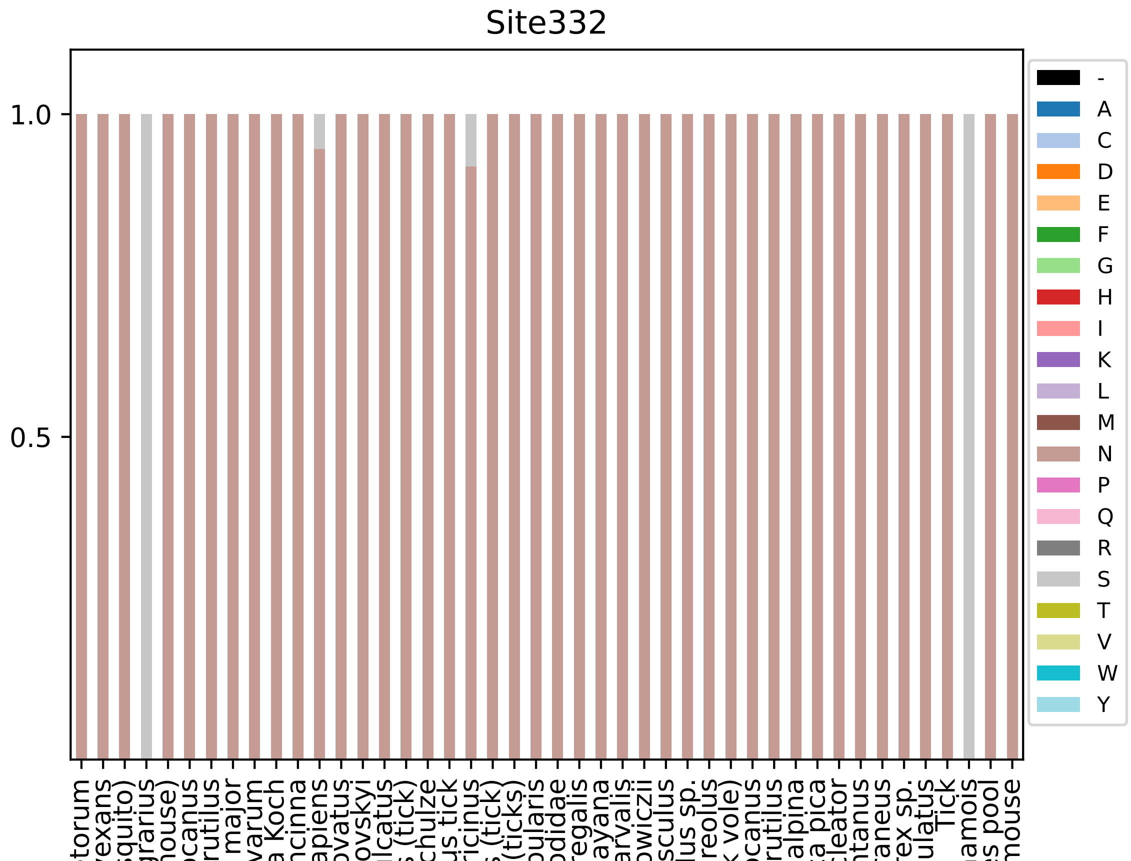

Site332
1.0
0.5
-
A
C
D
E
F
G
H
I
K
L
M
N
P
Q
R
S
T
V
W
Y

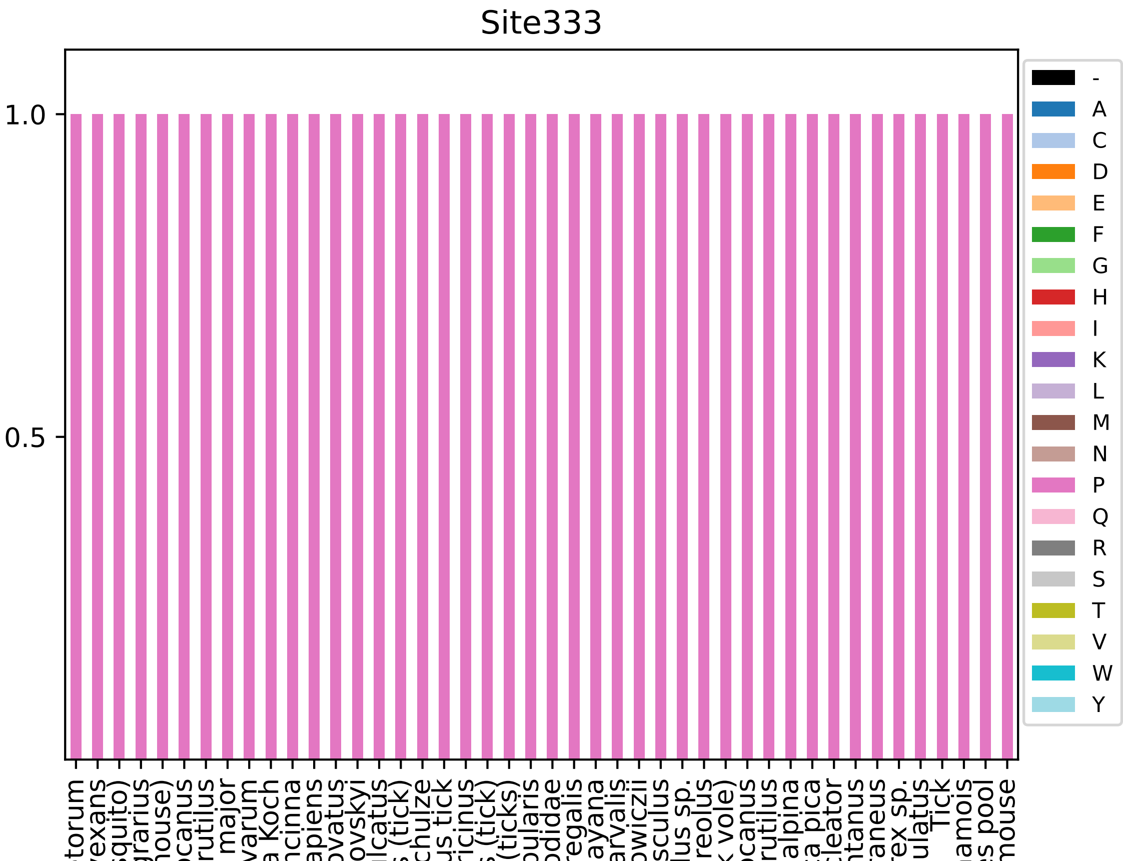

Site333
1.0
0.5
-
A
C
D
E
F
G
H
I
K
L
M
N
P
Q
R
S
T
V
W
Y

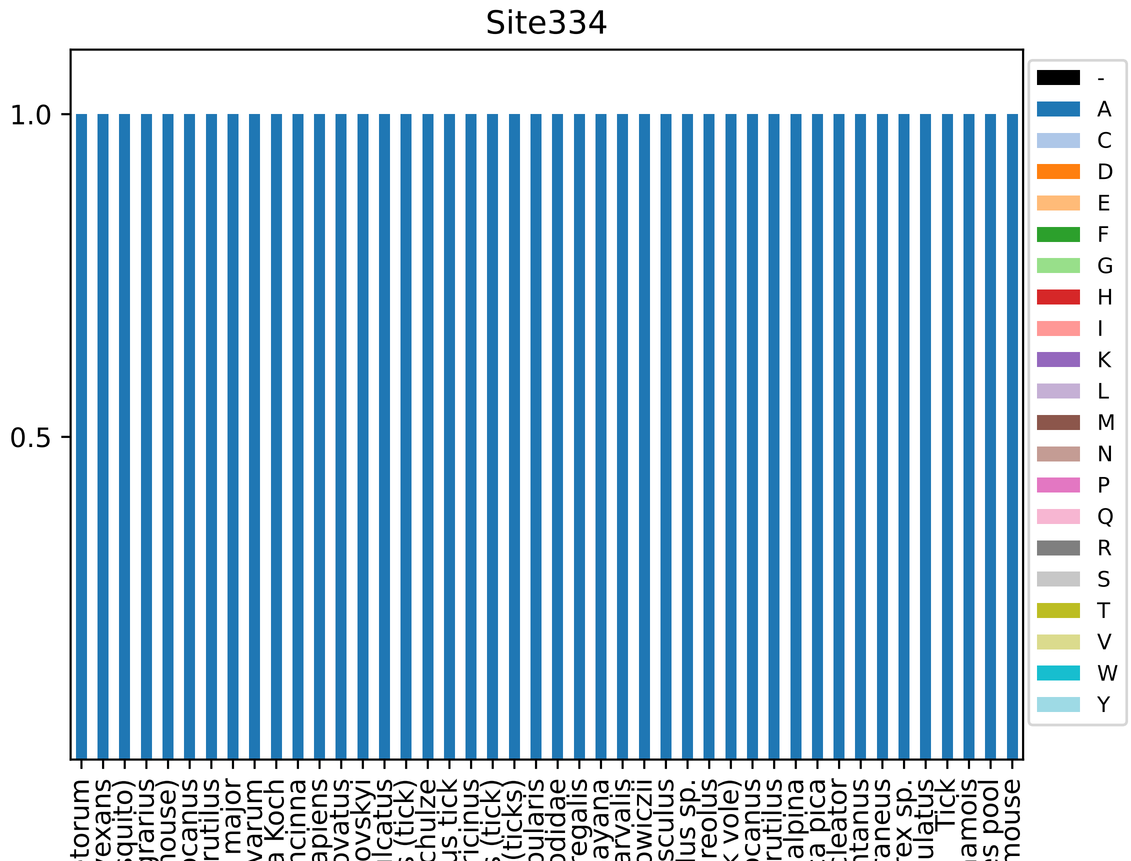
Site334
1.0
0.5
-
A
C
D
E
F
G
H
I
K
L
M
N
P
Q
R
S
T
V
W
Y
Tick

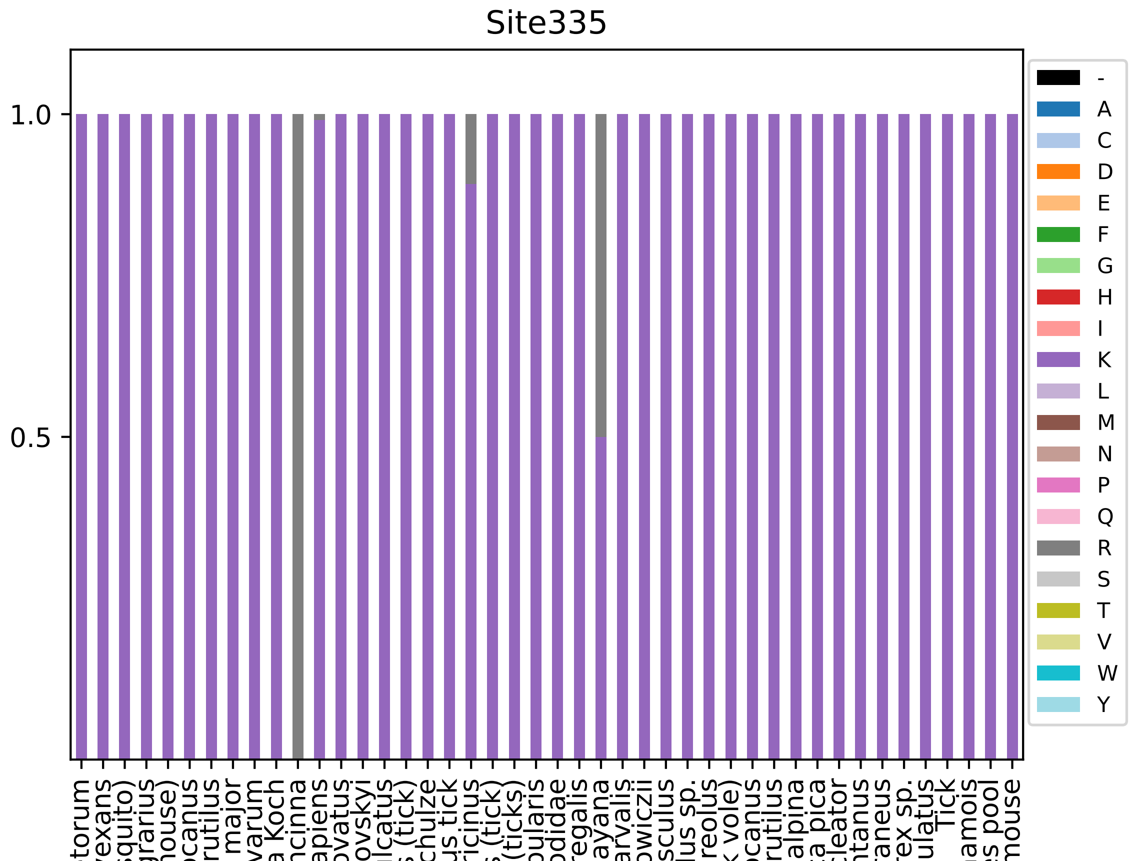
Site335
1.0
0.5
-
A
C
D
E
F
G
H
I
K
L
M
N
P
Q
R
S
T
V
W
Y

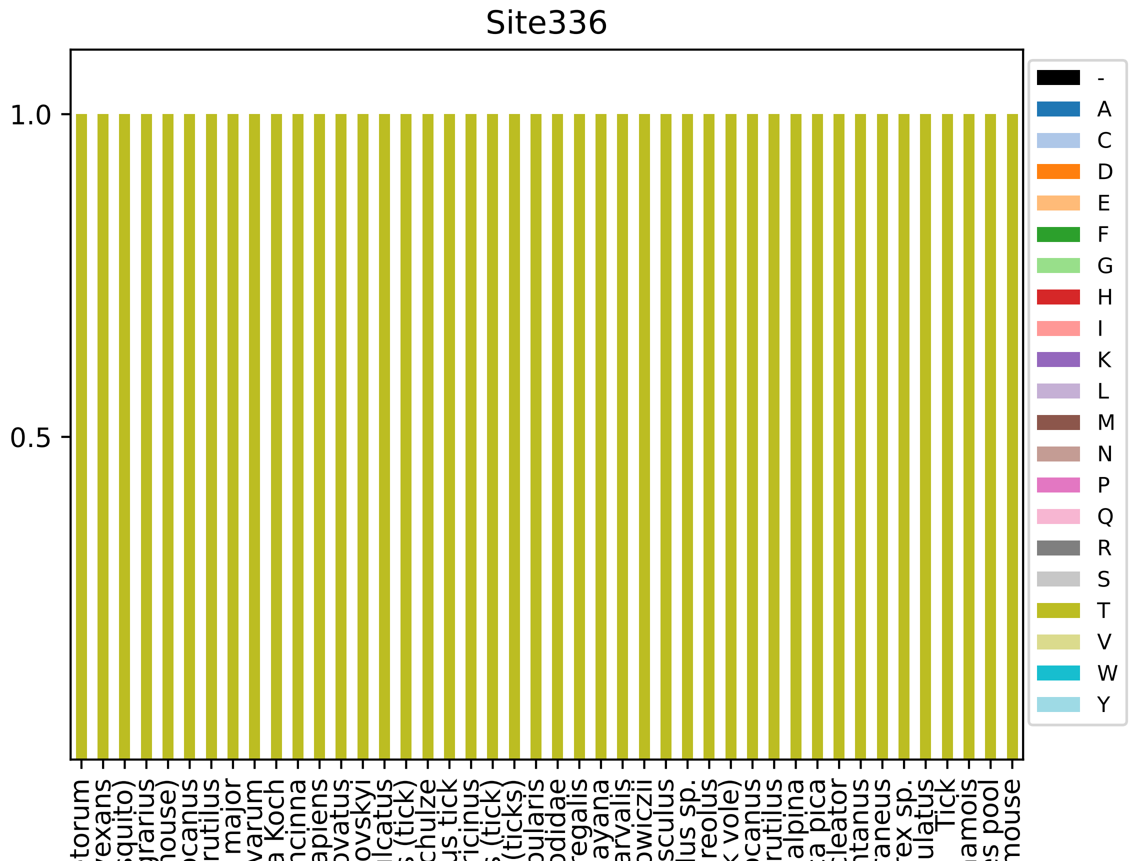

Site336
1.0
0.5
-
A
C
D
E
F
G
H
I
K
L
M
N
P
Q
R
S
T
V
W
Y

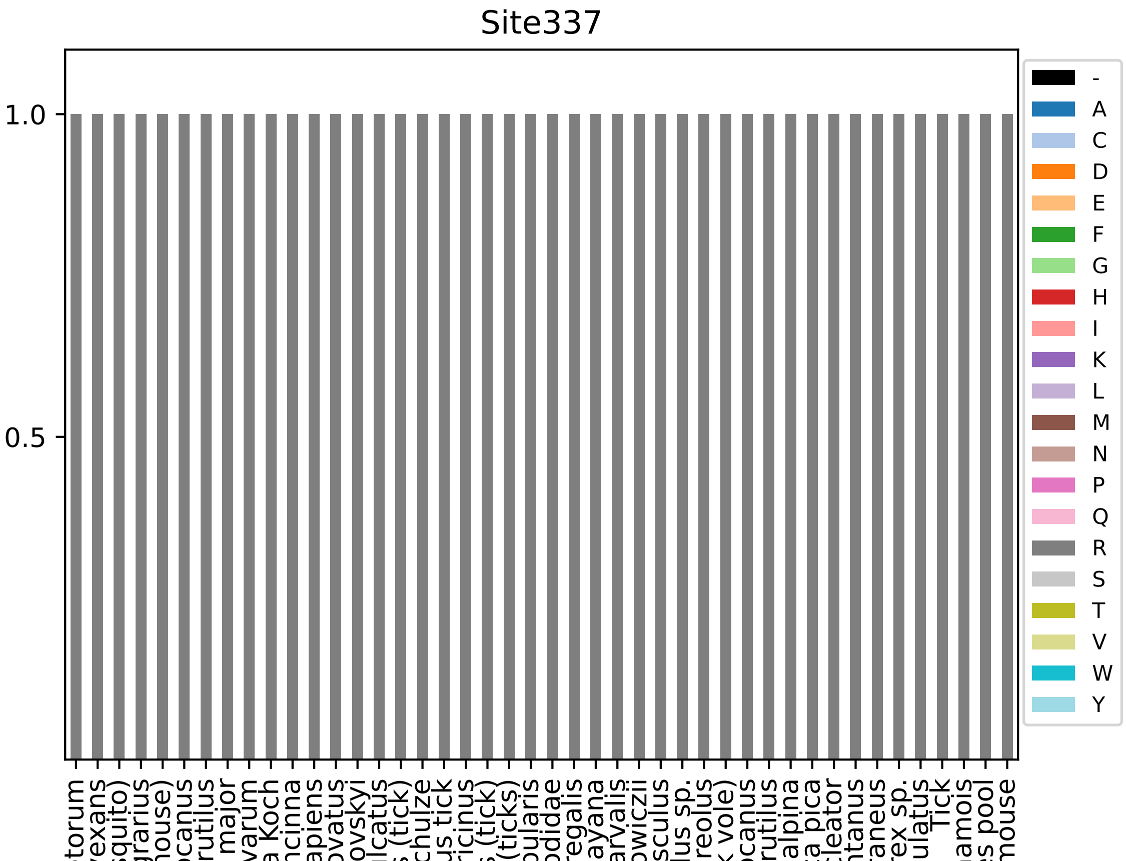
Site337
1.0
0.5
-
A
C
D
E
F
G
H
I
K
L
M
N
P
Q
R
S
T
V
W
Y

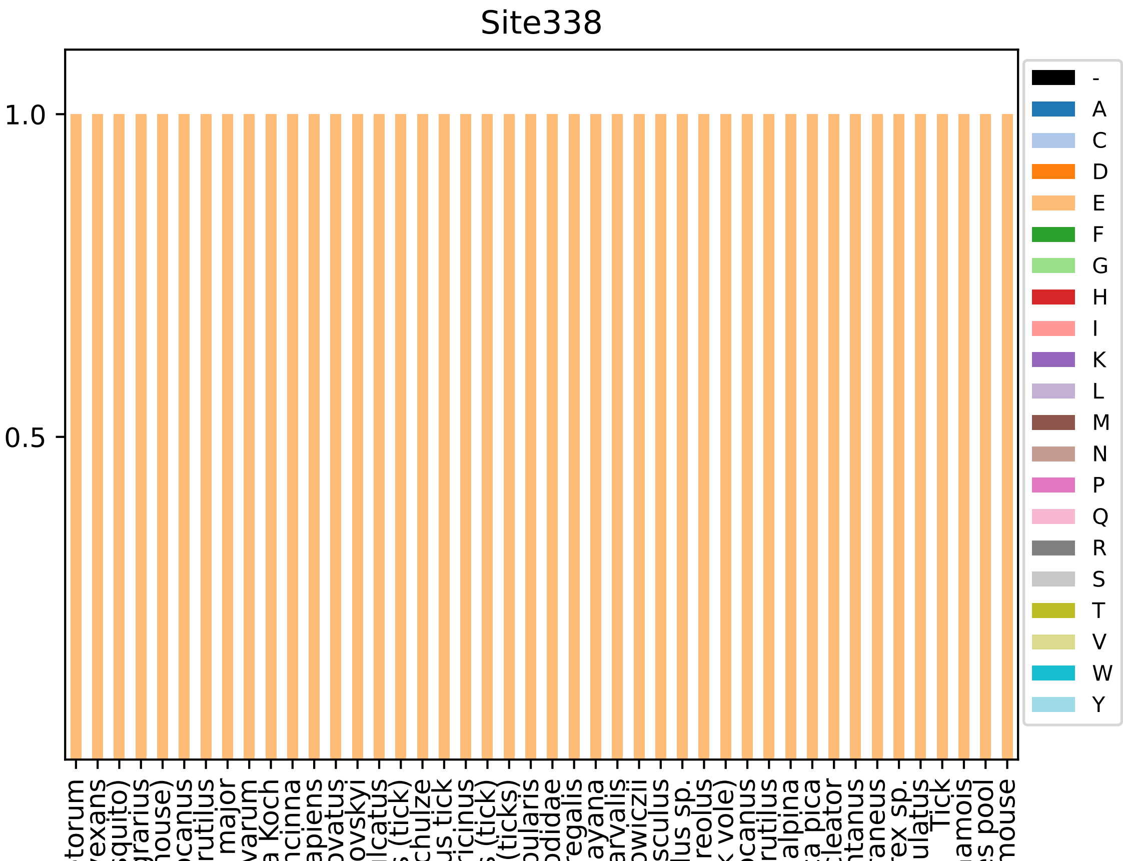

Site338
1.0
0.5
-
A
C
D
E
F
G
H
I
K
L
M
N
P
Q
R
S
T
V
W
Y

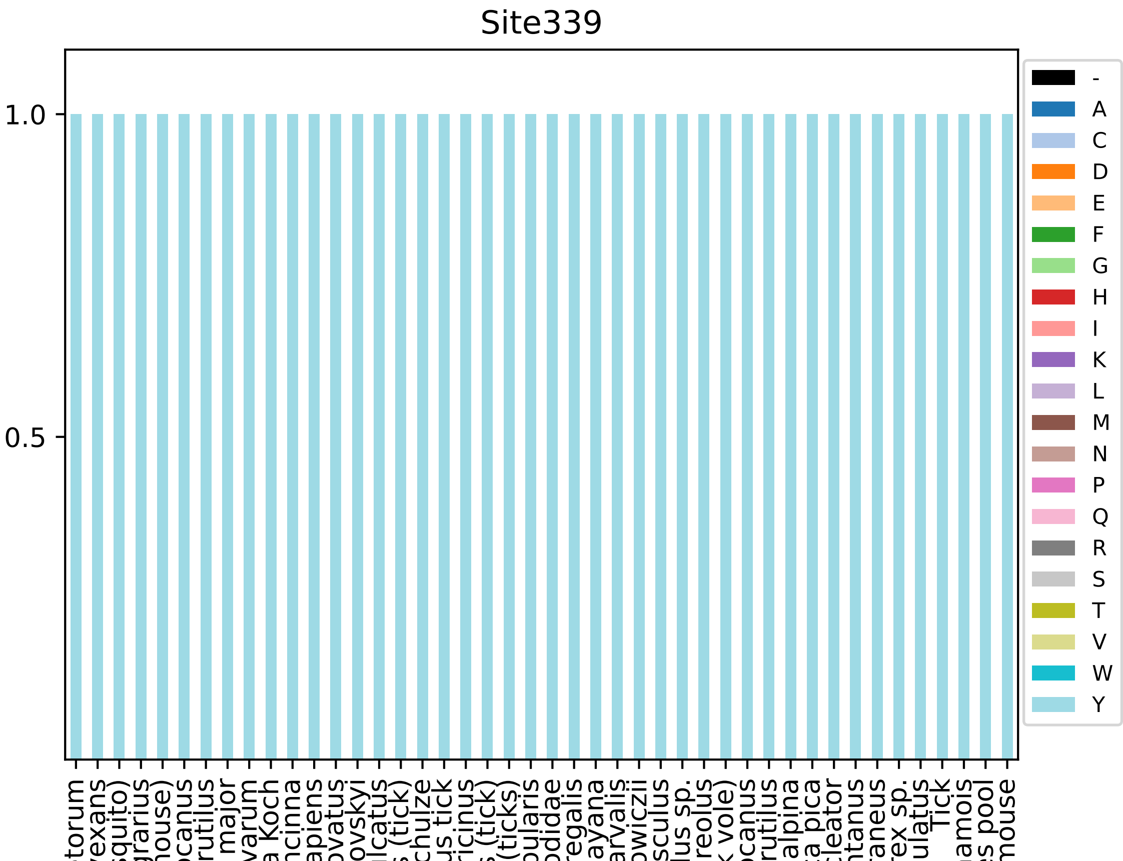
Site339
1.0
0.5
-
A
C
D
E
F
G
H
I
K
L
M
N
P
Q
R
S
T
V
W
Y

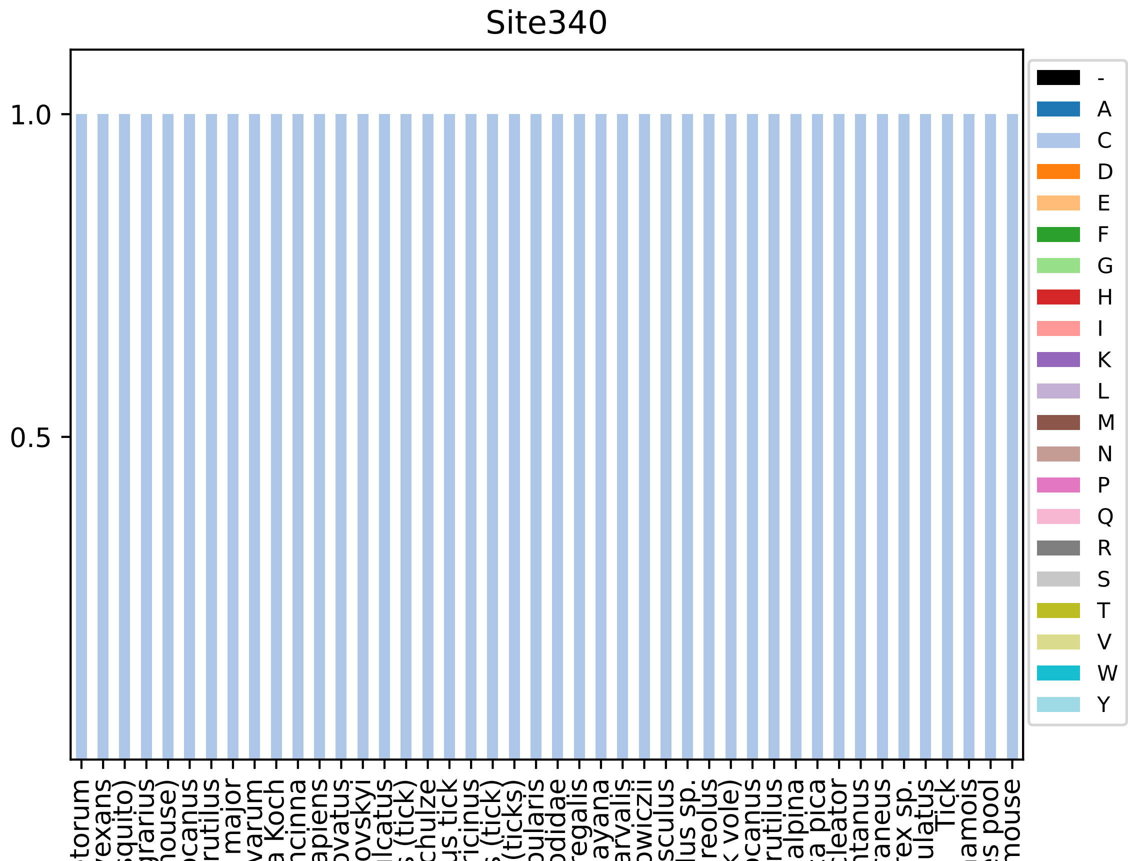

Site340
1.0
0.5
-
A
C
D
E
F
G
H
I
K
L
M
N
P
Q
R
S
T
V
W
Y

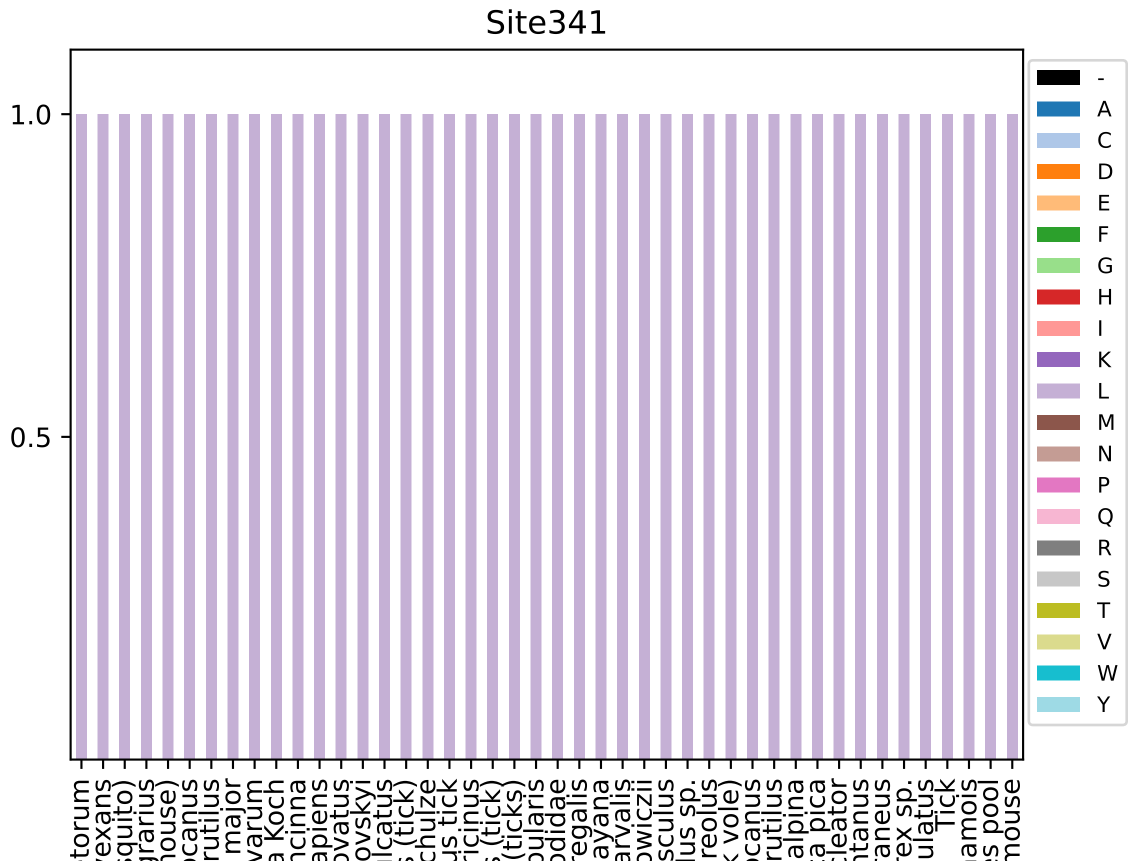
Site341
1.0
0.5
-
A
C
D
E
F
G
H
I
K
L
M
N
P
Q
R
S
T
V
W
Y

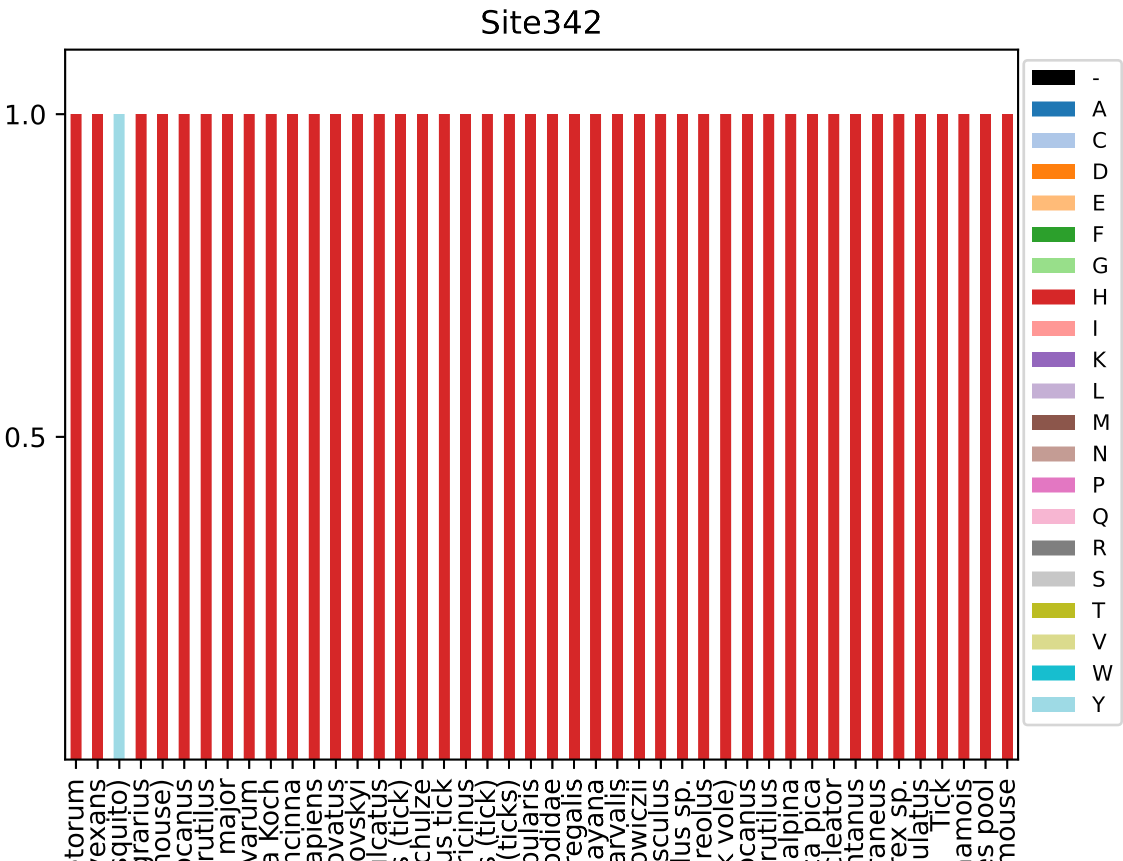

Site342
1.0
0.5
-
A
C
D
E
F
G
H
I
K
L
M
N
P
Q
R
S
T
V
W
Y

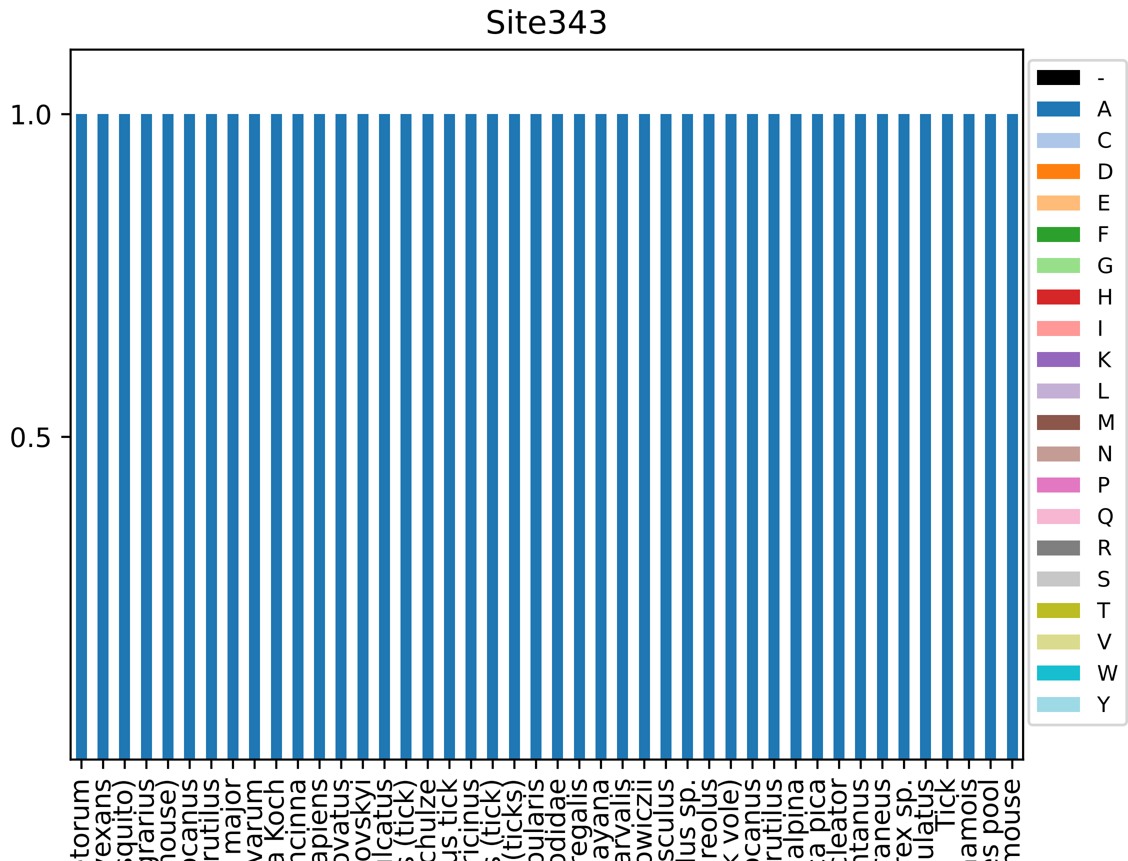

Site343
1.0
0.5
-
A
C
D
E
F
G
H
I
K
L
M
N
P
Q
R
S
T
V
W
Y

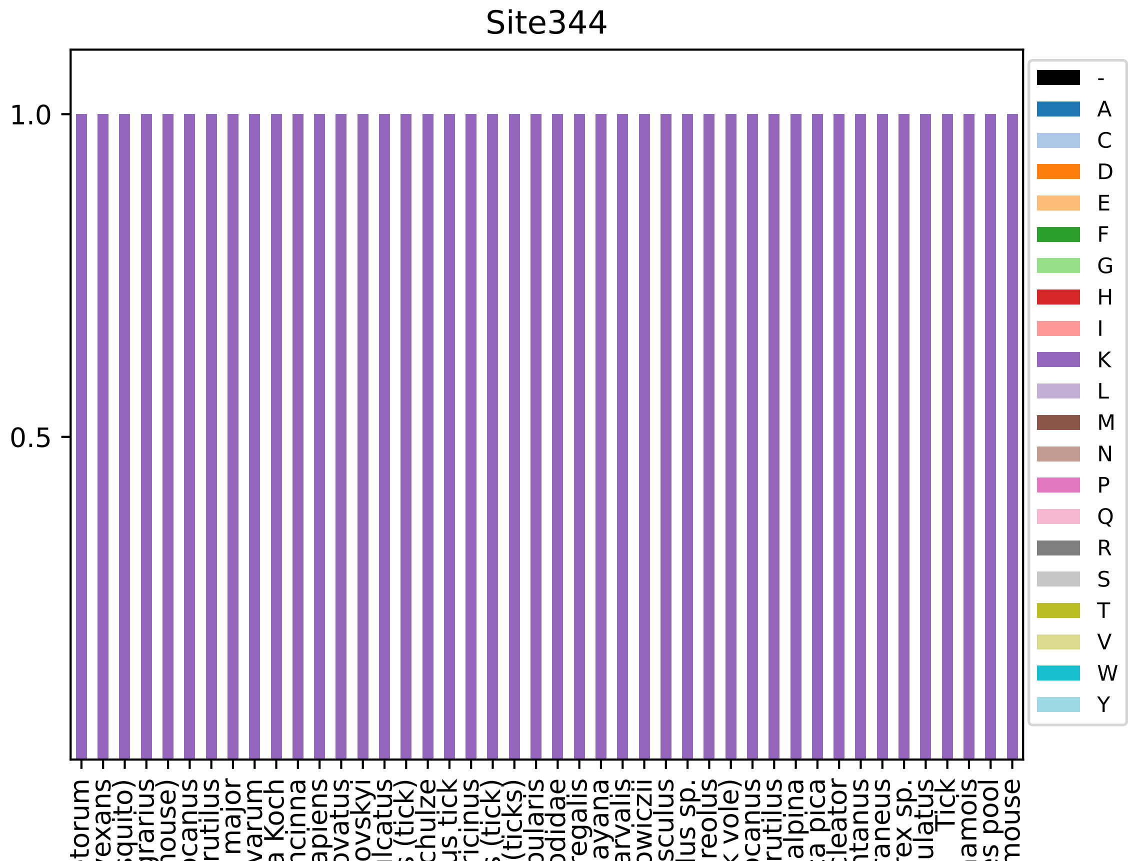
Site344
1.0
0.5
-
A
C
D
E
F
G
H
I
K
L
M
N
P
Q
R
S
T
V
W
Y

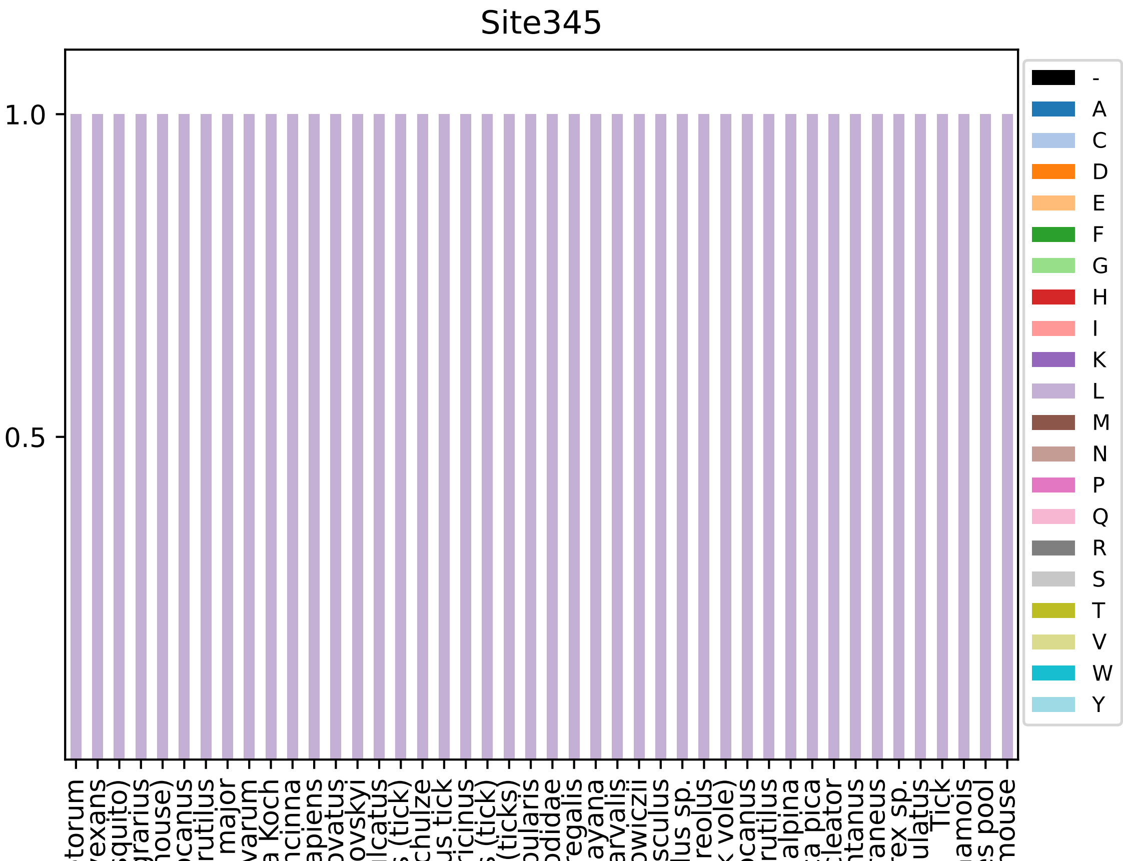
Site345
1.0
0.5
-
A
C
D
E
F
G
H
I
K
L
M
N
P
Q
R
S
T
V
W
Y

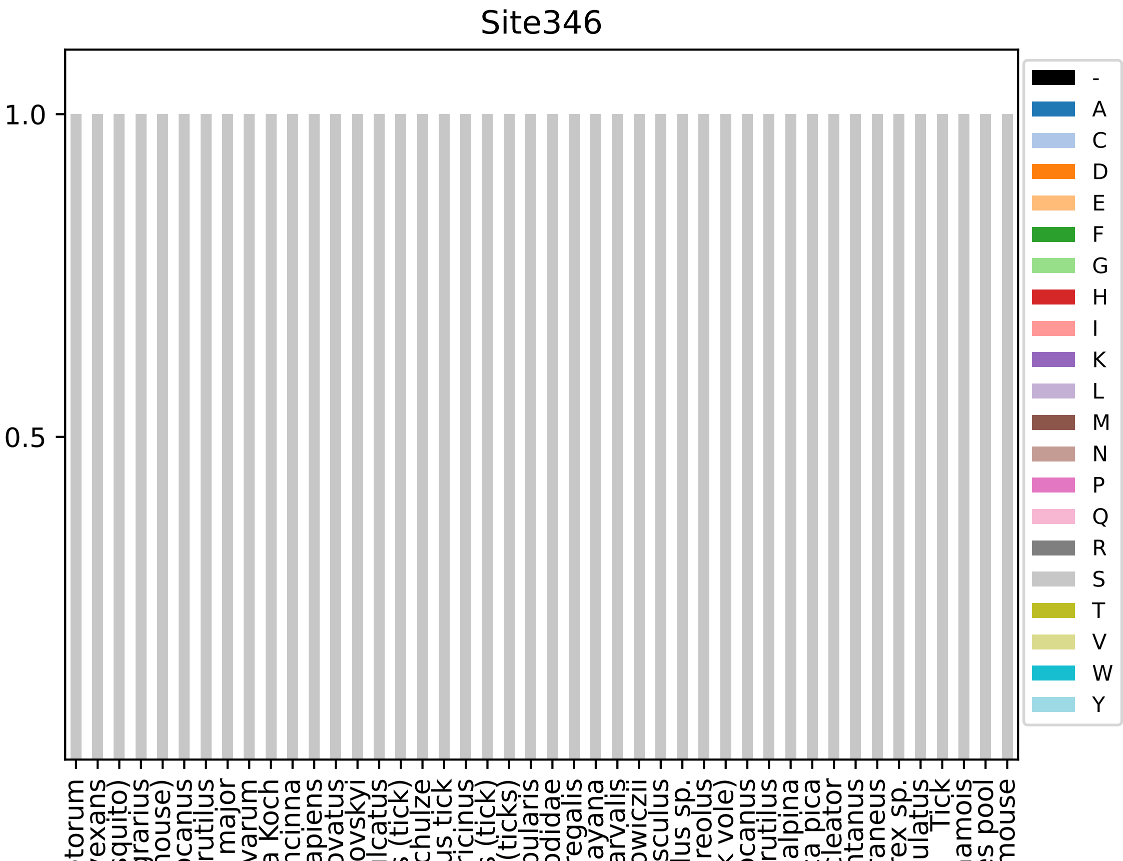
Site346
1.0
0.5
-
A
C
D
E
F
G
H
I
K
L
M
N
P
Q
R
S
T
V
W
Y

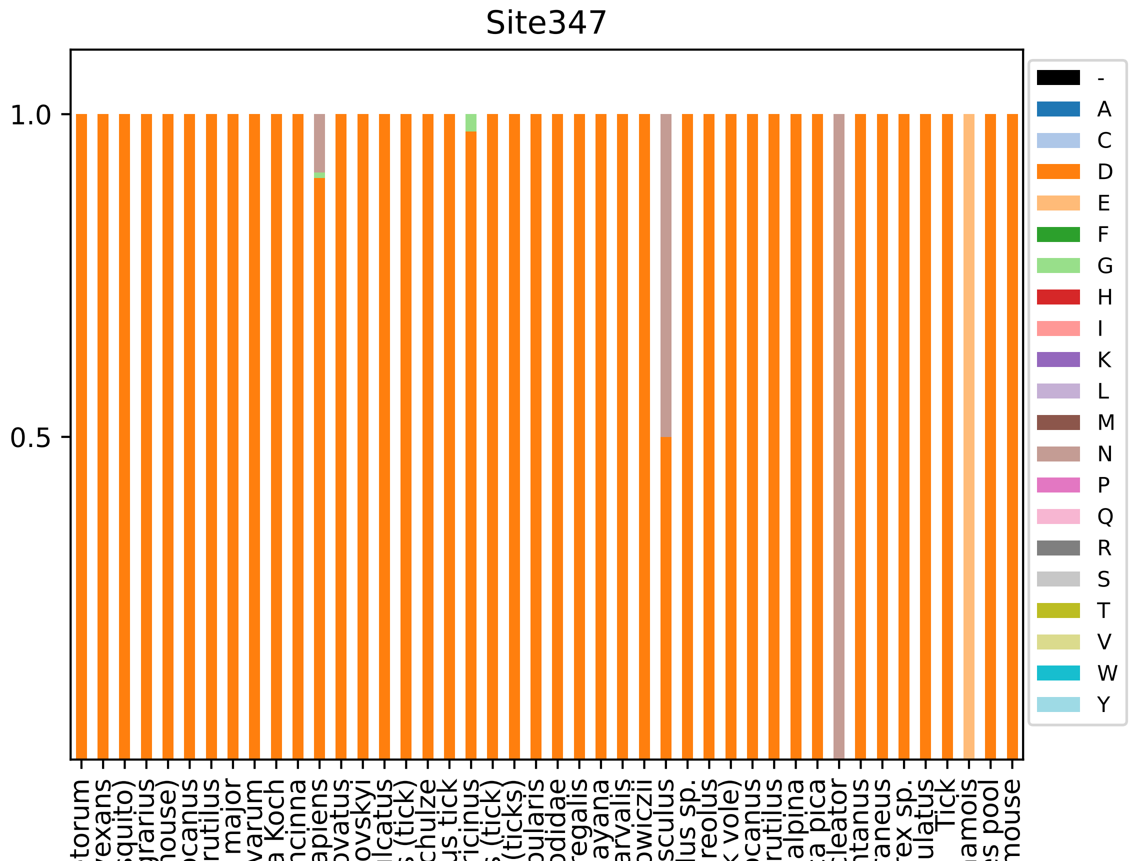
Site347
1.0
0.5
-
A
C
D
E
F
G
H
I
K
L
M
N
P
Q
R
S
T
V
W
Y

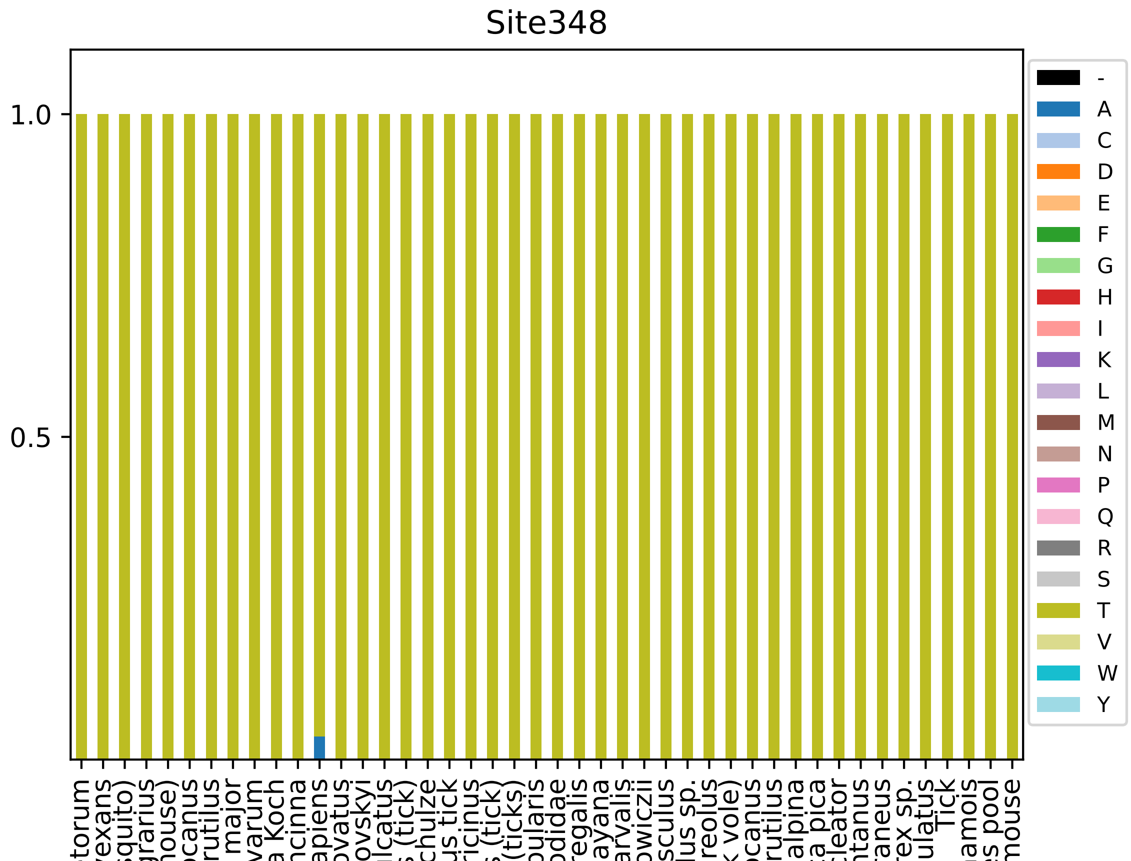
Site348
1.0
0.5
-
A
C
D
E
F
G
H
I
K
L
M
N
P
Q
R
S
T
V
W
Y

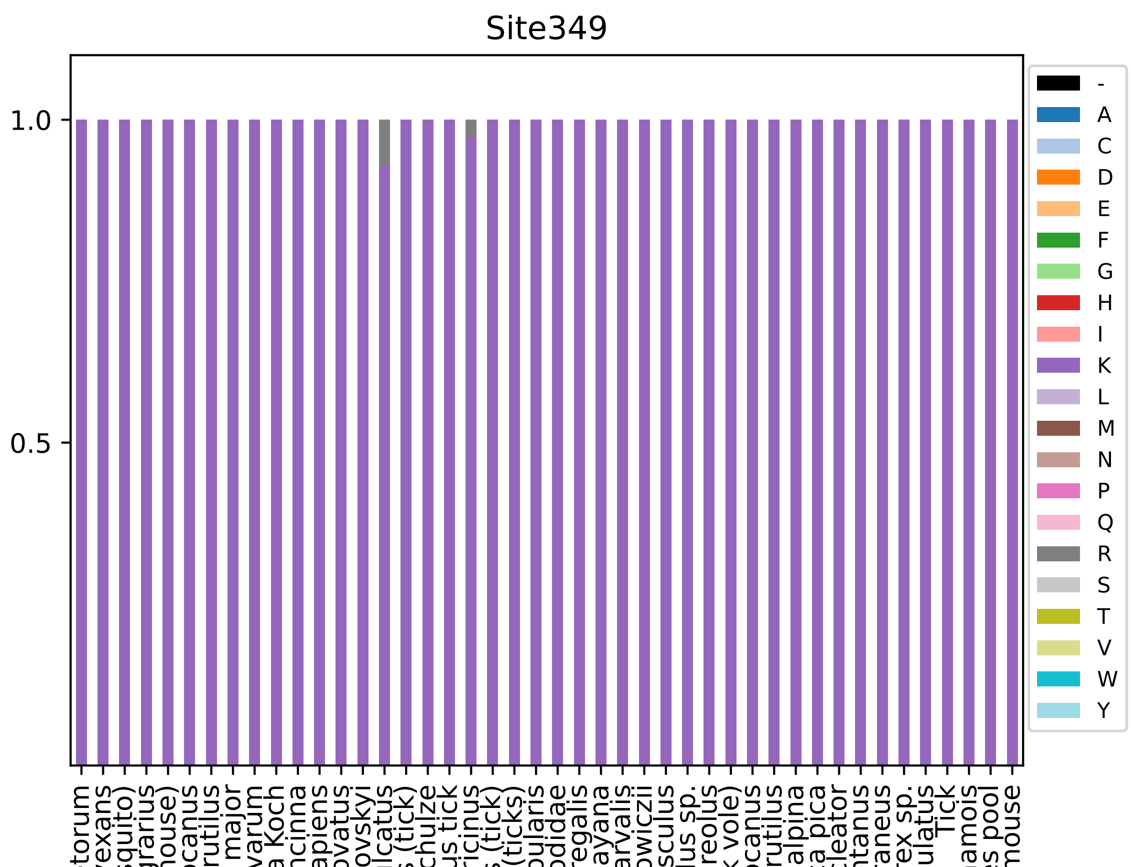

Site349
1.0
0.5
-
A
C
D
E
F
G
H
I
K
L
M
N
P
Q
R
S
T
V
W
Y

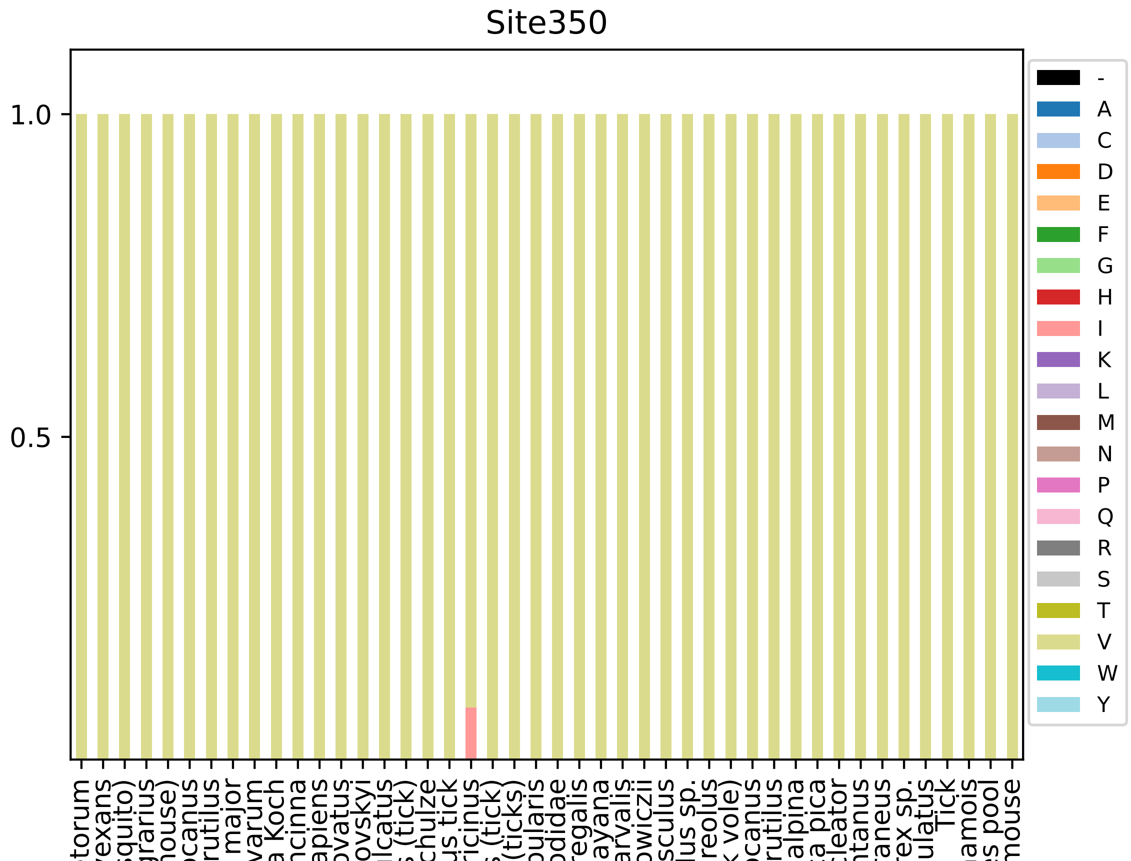
Site350
1.0
0.5
-
A
C
D
E
F
G
H
I
K
L
M
N
P
Q
R
S
T
V
W
Y

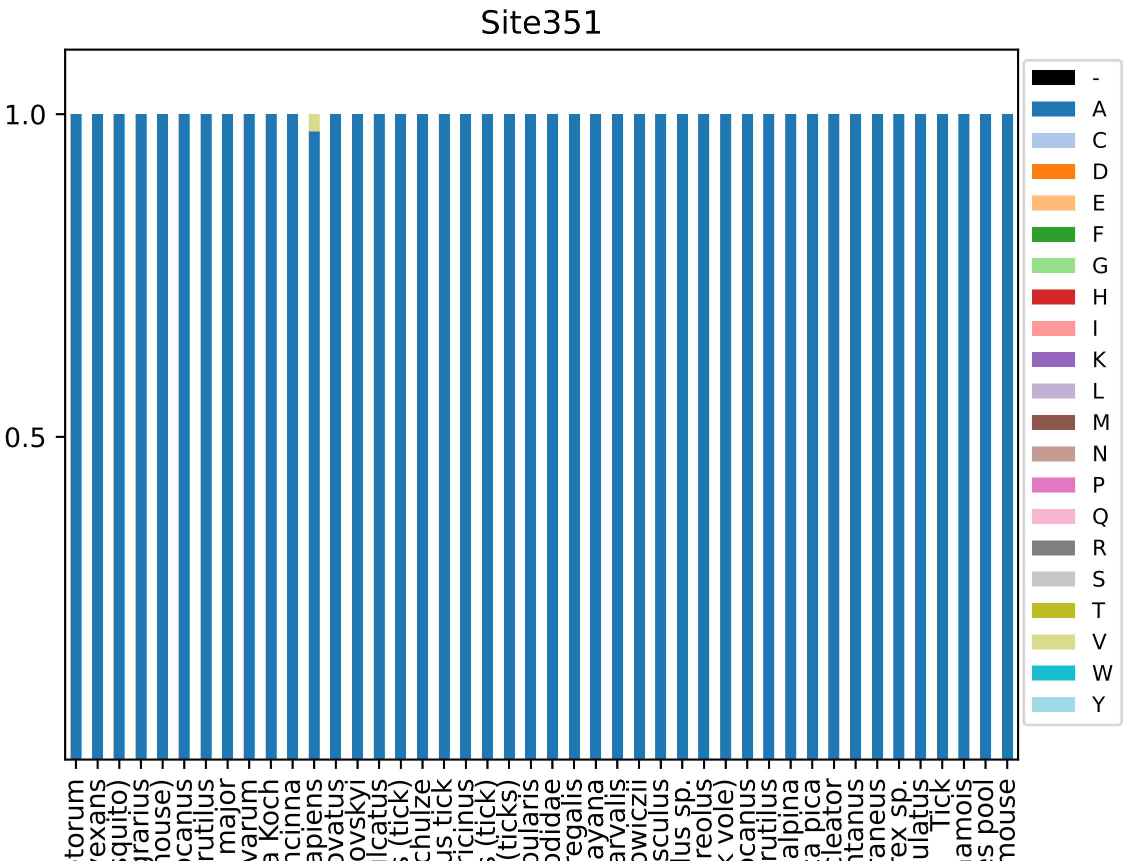
Site351
1.0
0.5
-
A
C
D
E
F
G
H
I
K
L
M
N
P
Q
R
S
T
V
W
Y

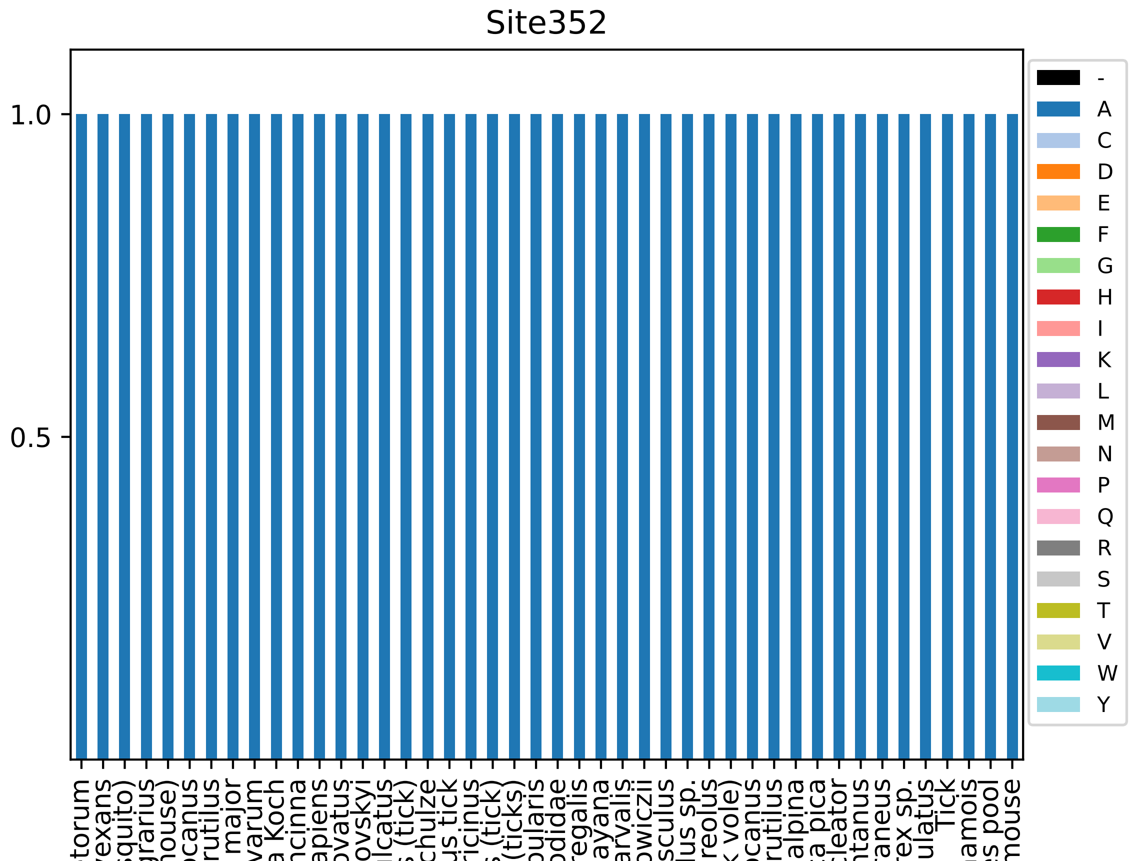
Site352
1.0
0.5
-
A
C
D
E
F
G
H
I
K
L
M
N
P
Q
R
S
T
V
W
Y

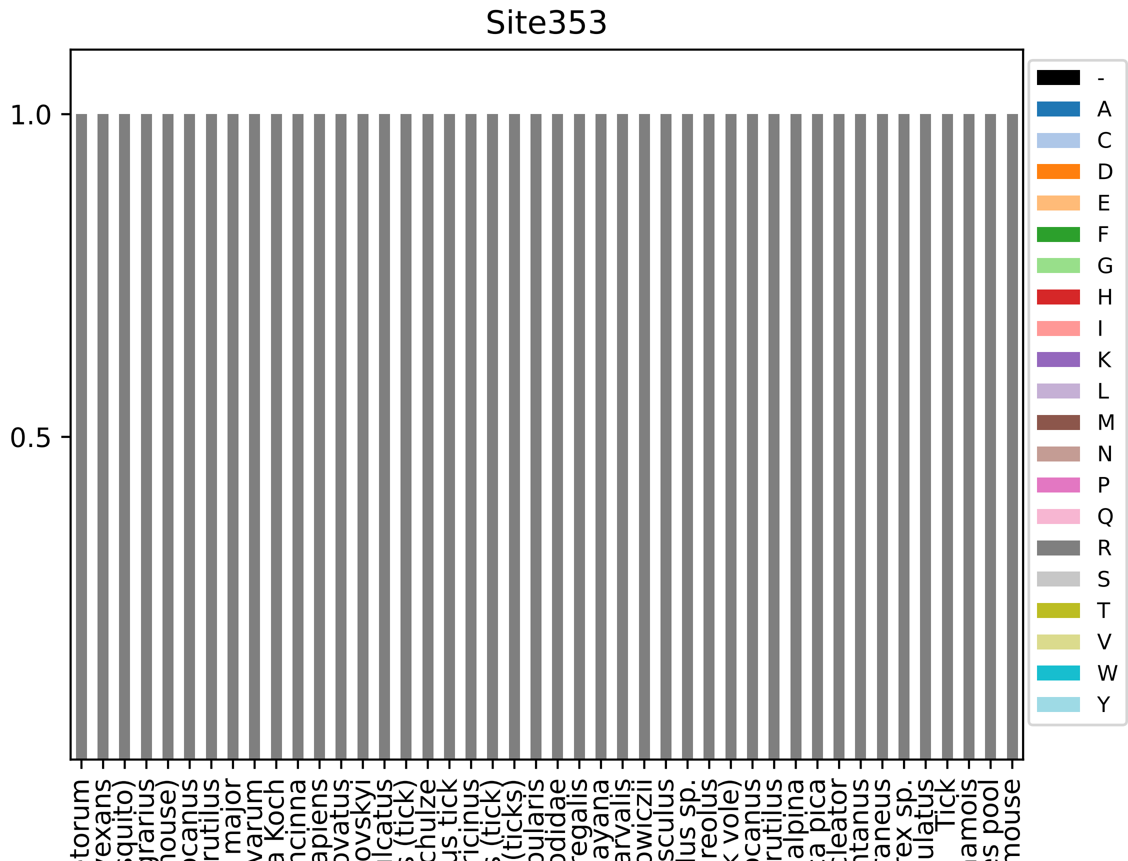
Site353
1.0
0.5
-
A
C
D
E
F
G
H
I
K
L
M
N
P
Q
R
S
T
V
W
Y

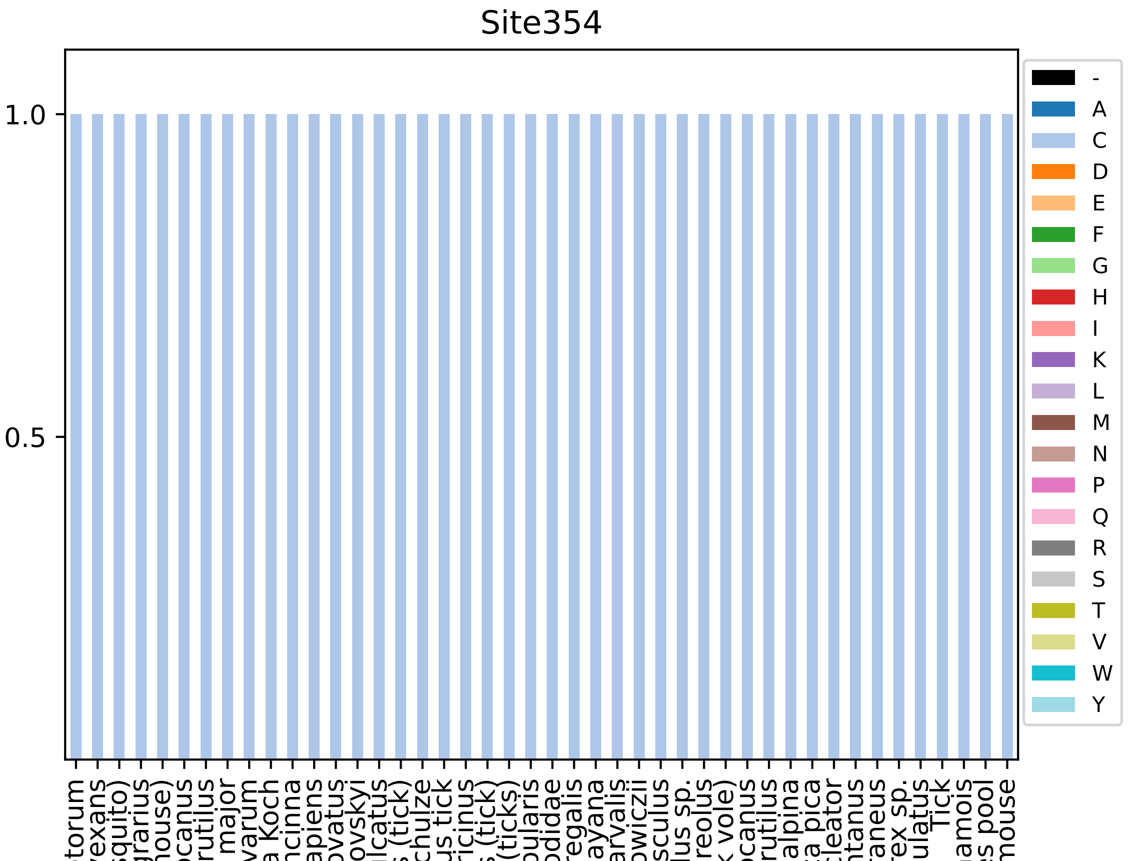

Site354
1.0
0.5
-
A
C
D
E
F
G
H
I
K
L
M
N
P
Q
R
S
T
V
W
Y

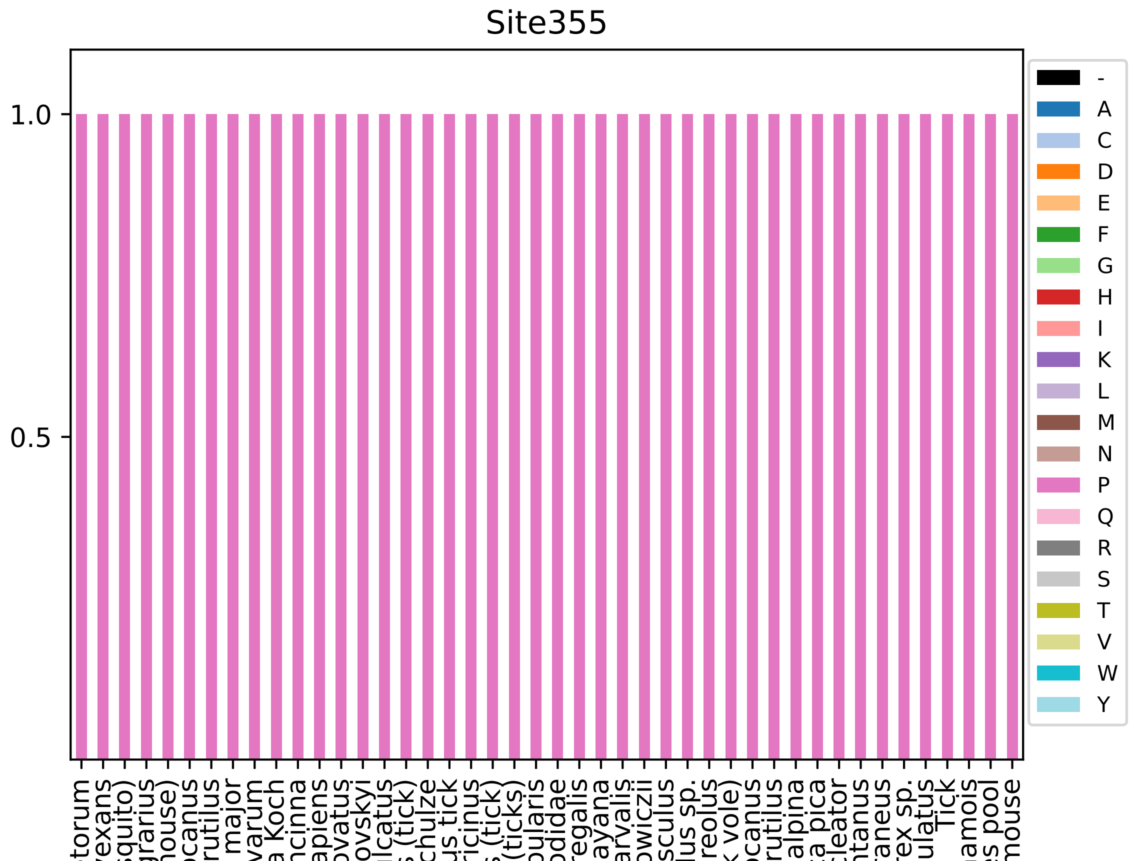
Site355
1.0
0.5
-
A
C
D
E
F
G
H
I
K
L
M
N
P
Q
R
S
T
V
W
Y

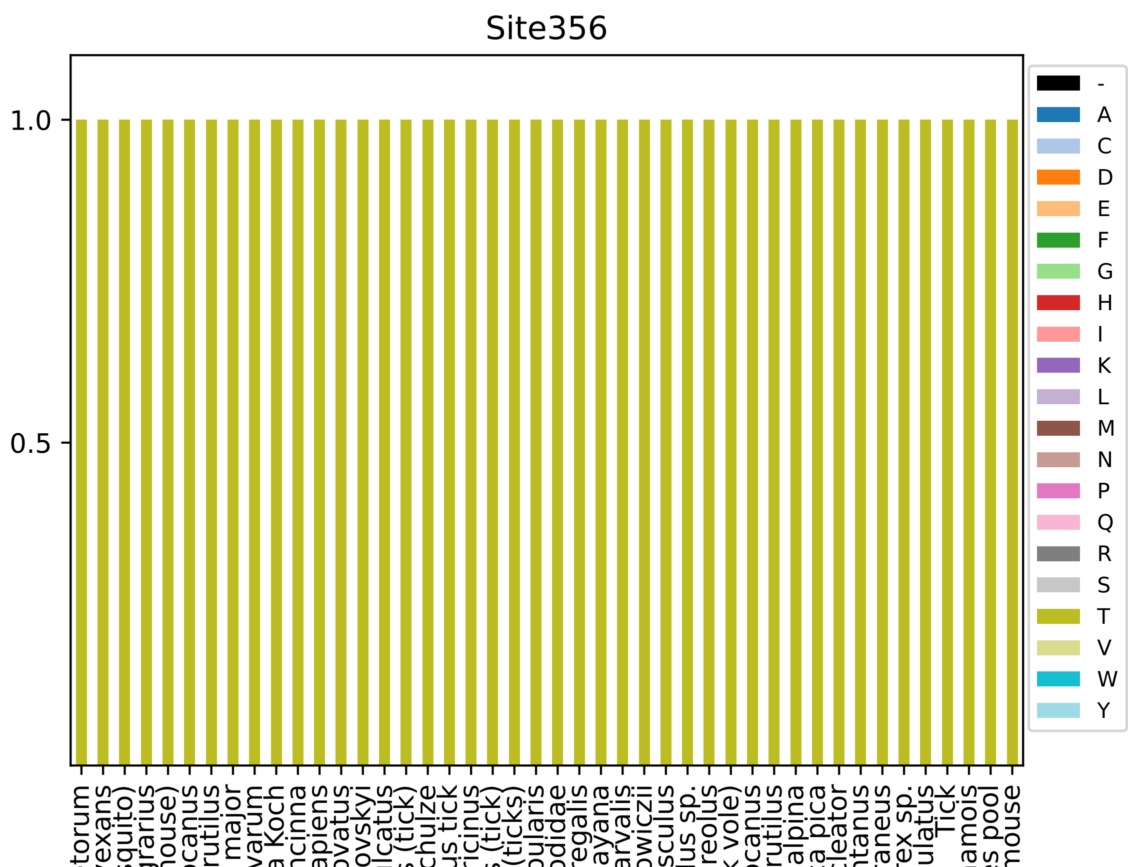
Site356
1.0
0.5
-
A
C
D
E
F
G
H
I
K
L
M
N
P
Q
R
S
T
V
W
Y

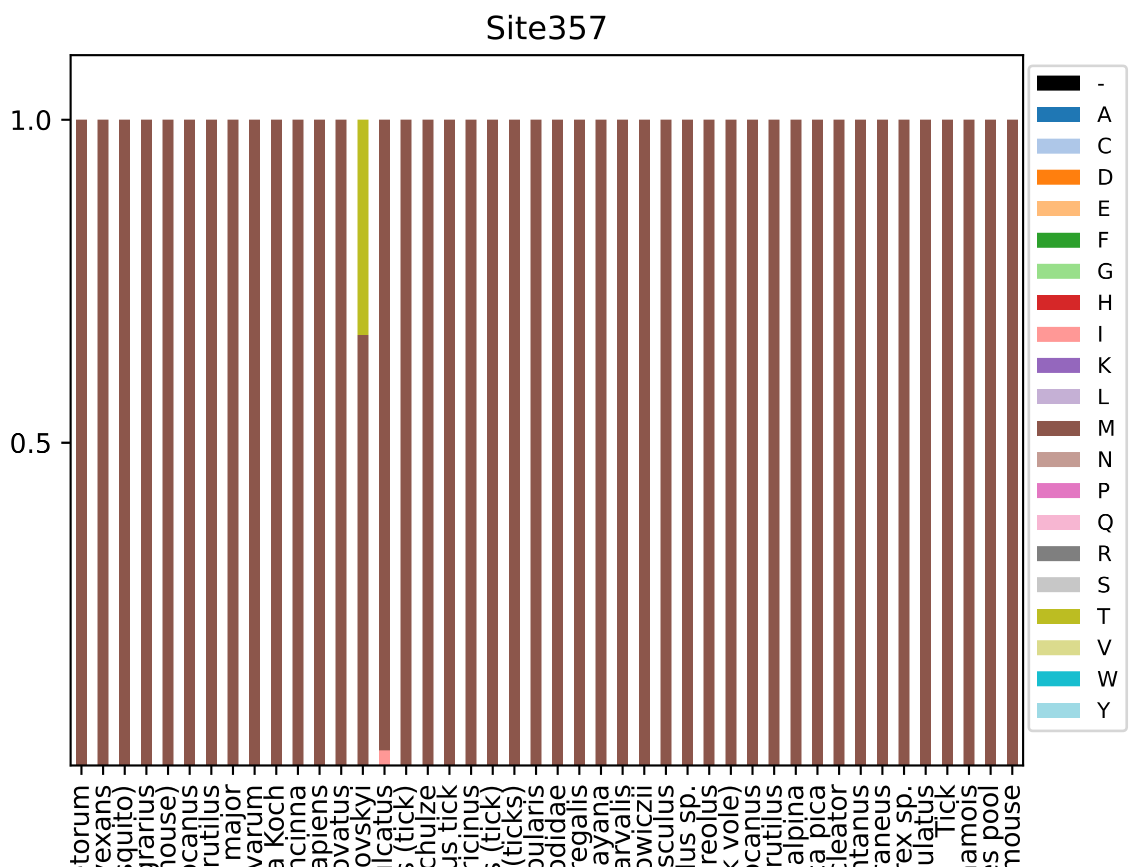
Site357
1.0
0.5
-
A
C
D
E
F
G
H
I
K
L
M
N
P
Q
R
S
T
V
W
Y

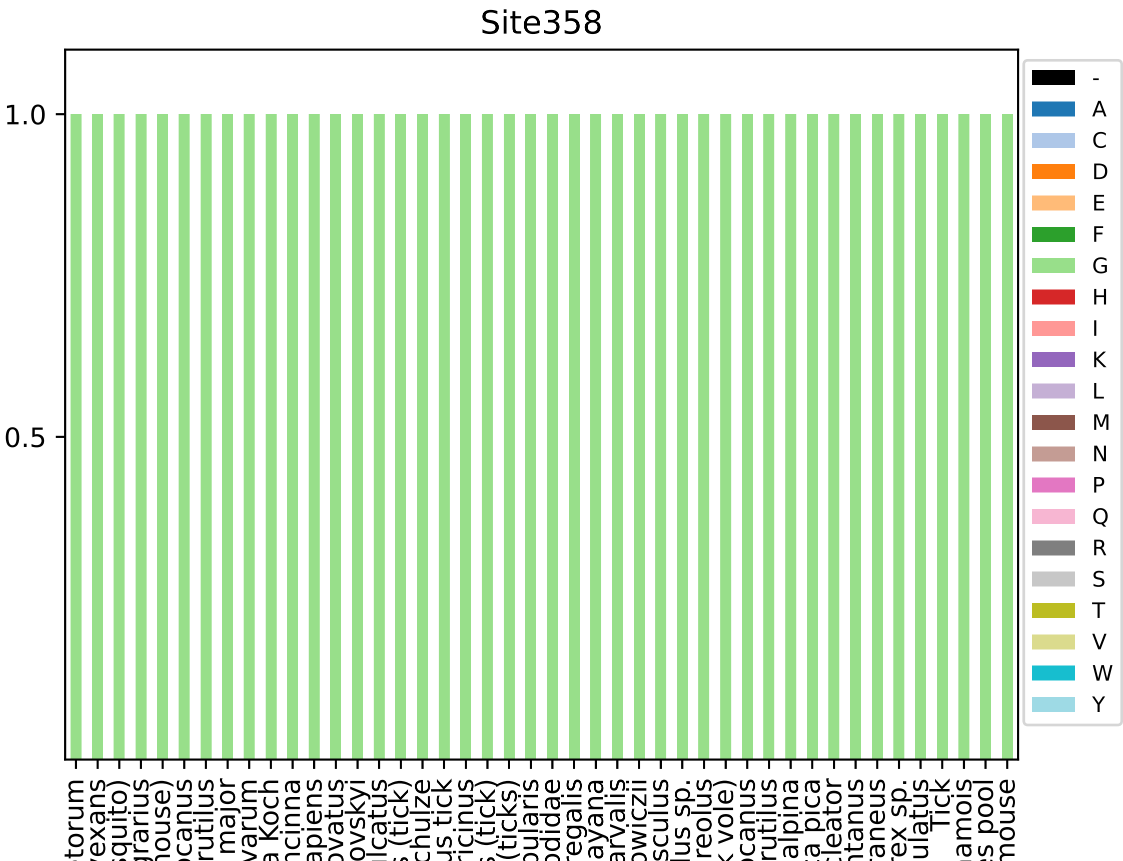
Site358
1.0
0.5
-
A
C
D
E
F
G
H
I
K
L
M
N
P
Q
R
S
T
V
W
Y

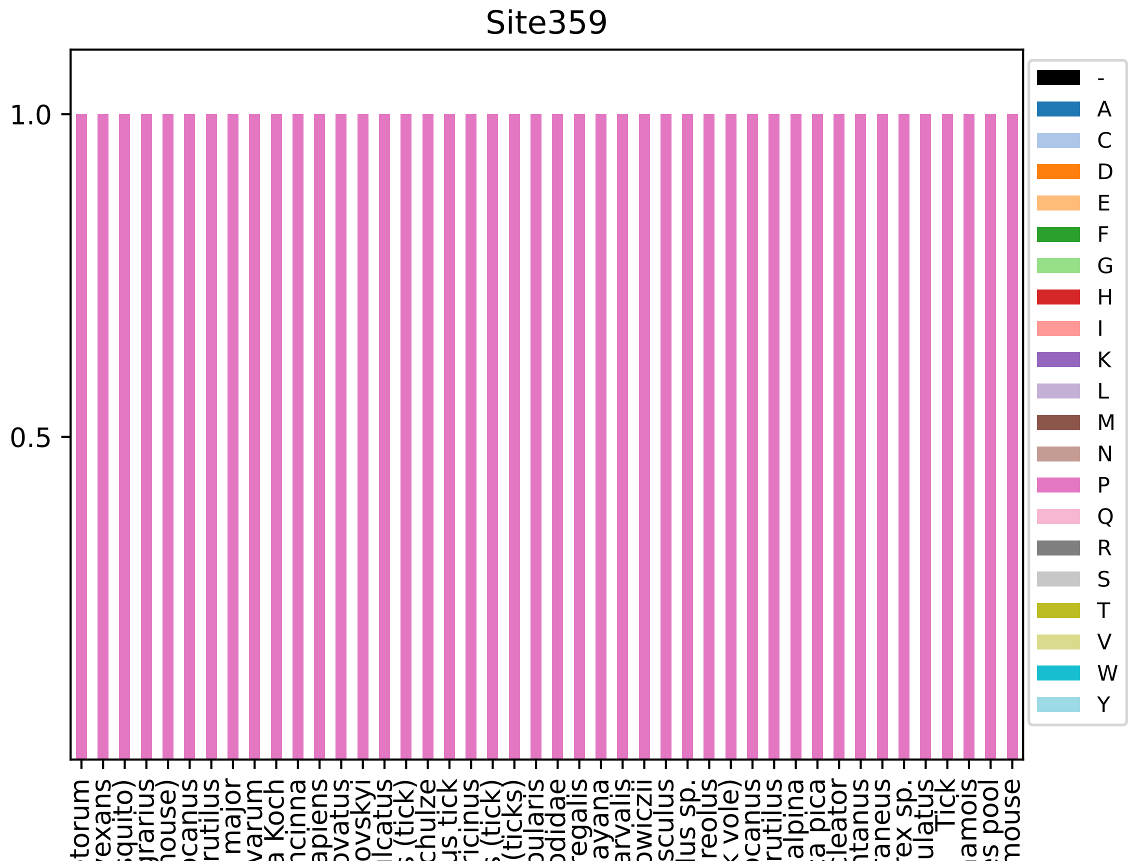
Site359
1.0
0.5
-
A
C
D
E
F
G
H
I
K
L
M
N
P
Q
R
S
T
V
W
Y

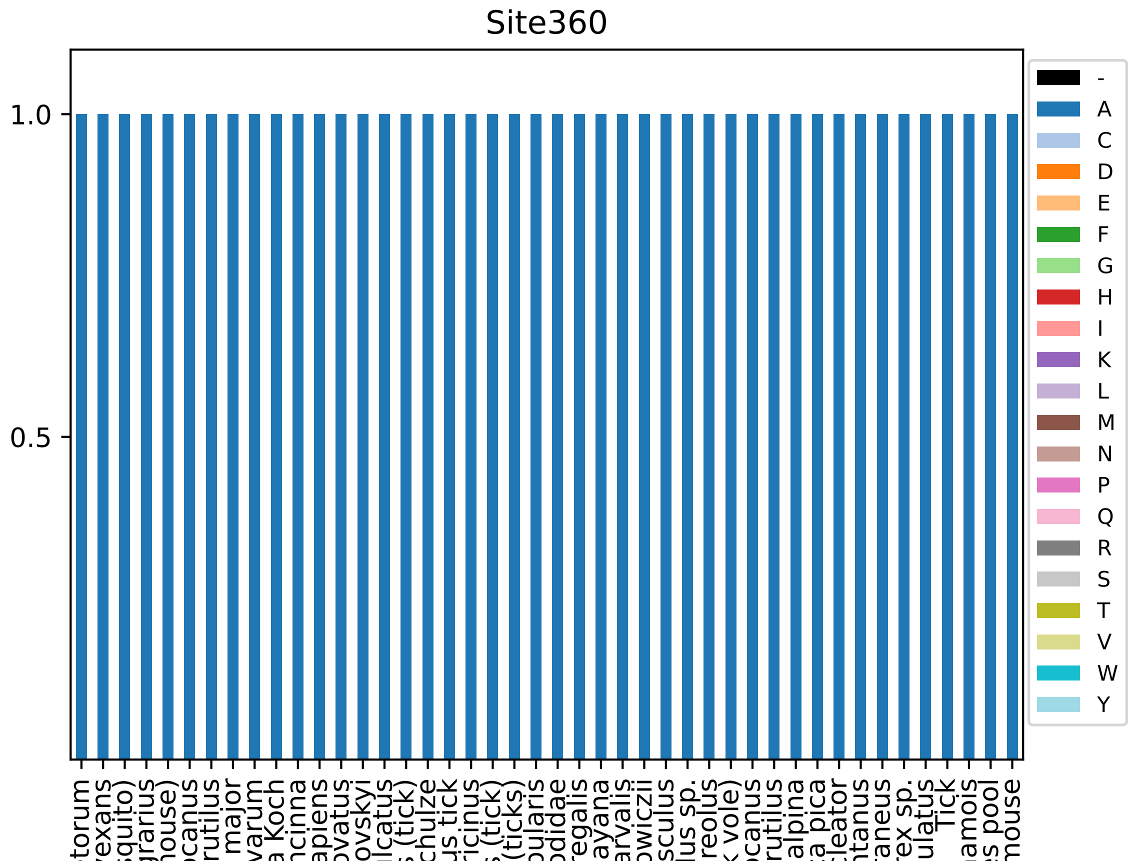
Site360
1.0
0.5
-
A
C
D
E
F
G
H
I
K
L
M
N
P
Q
R
S
T
V
W
Y

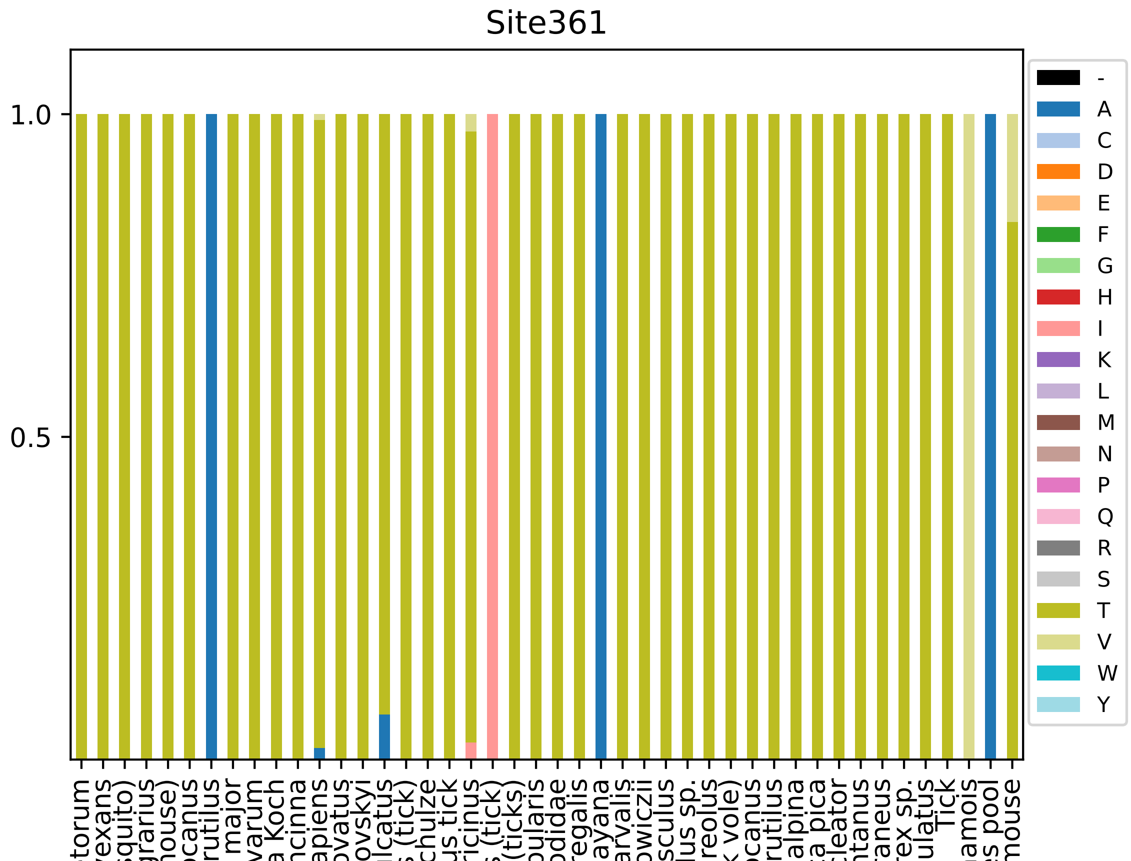
Site361
1.0
0.5
-
A
C
D
E
F
G
H
I
K
L
M
N
P
Q
R
S
T
V
W
Y
Tick

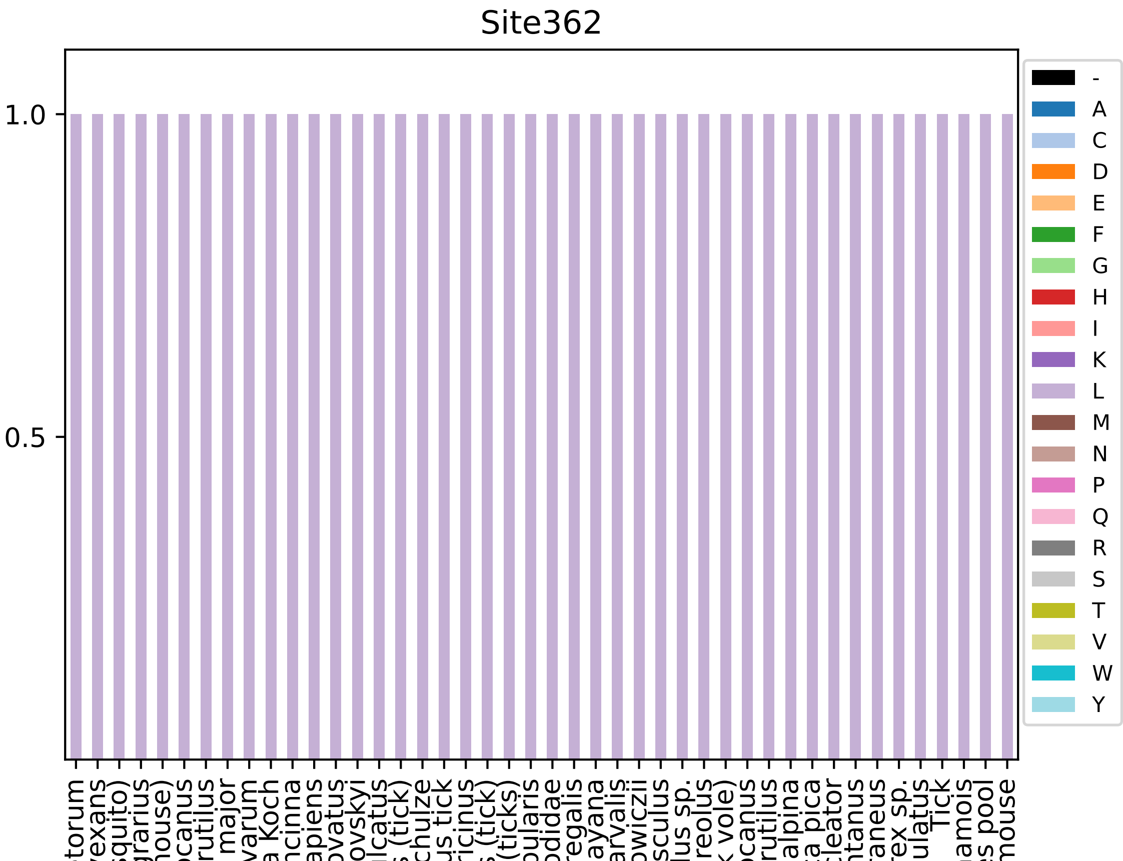
Site362
1.0
0.5
-
A
C
D
E
F
G
H
I
K
L
M
N
P
Q
R
S
T
V
W
Y

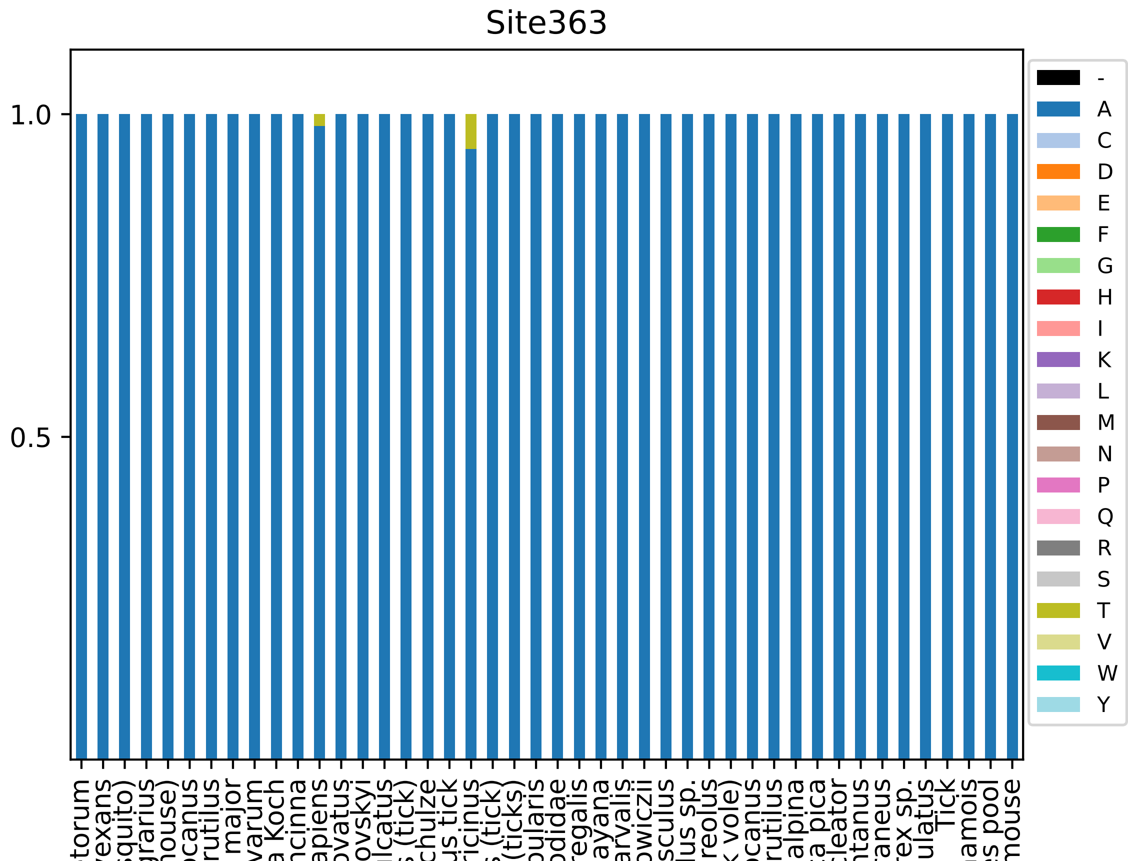
Site363
1.0
0.5
-
A
C
D
E
F
G
H
I
K
L
M
N
P
Q
R
S
T
V
W
Y
Tick

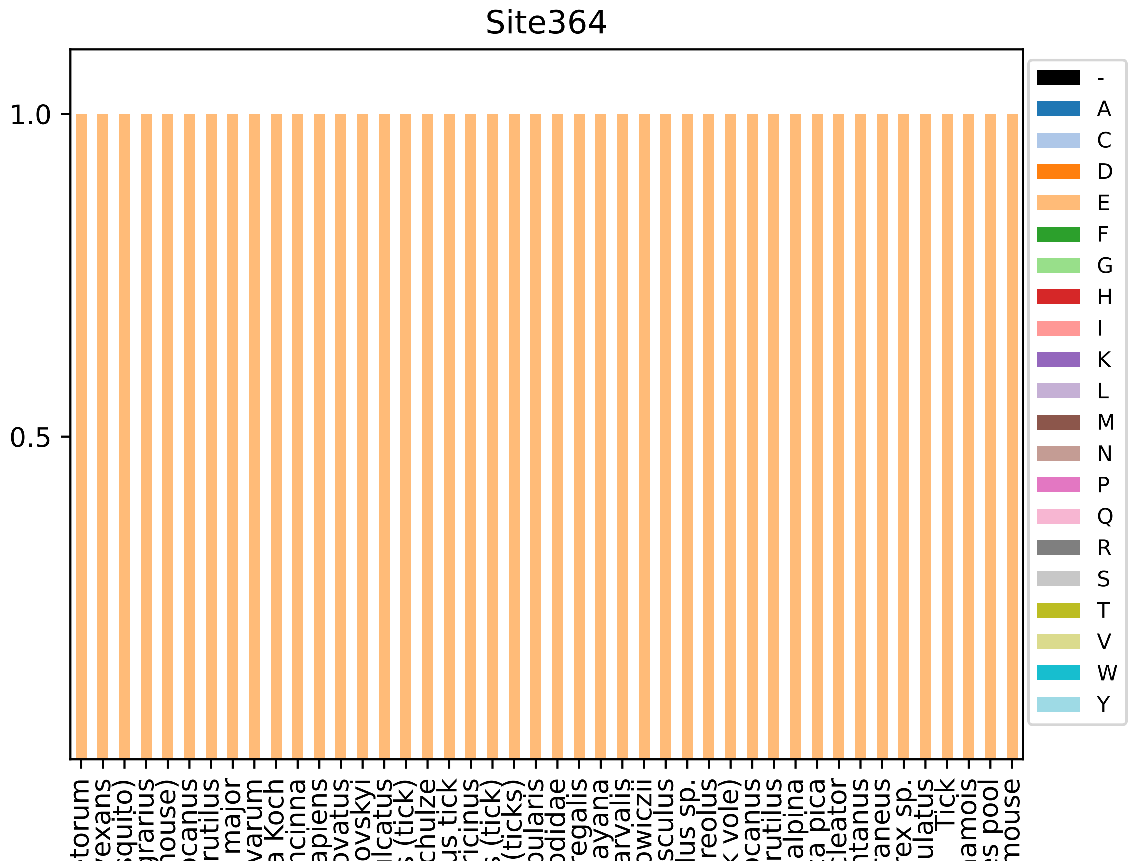
Site364
1.0
0.5
-
A
C
D
E
F
G
H
I
K
L
M
N
P
Q
R
S
T
V
W
Y

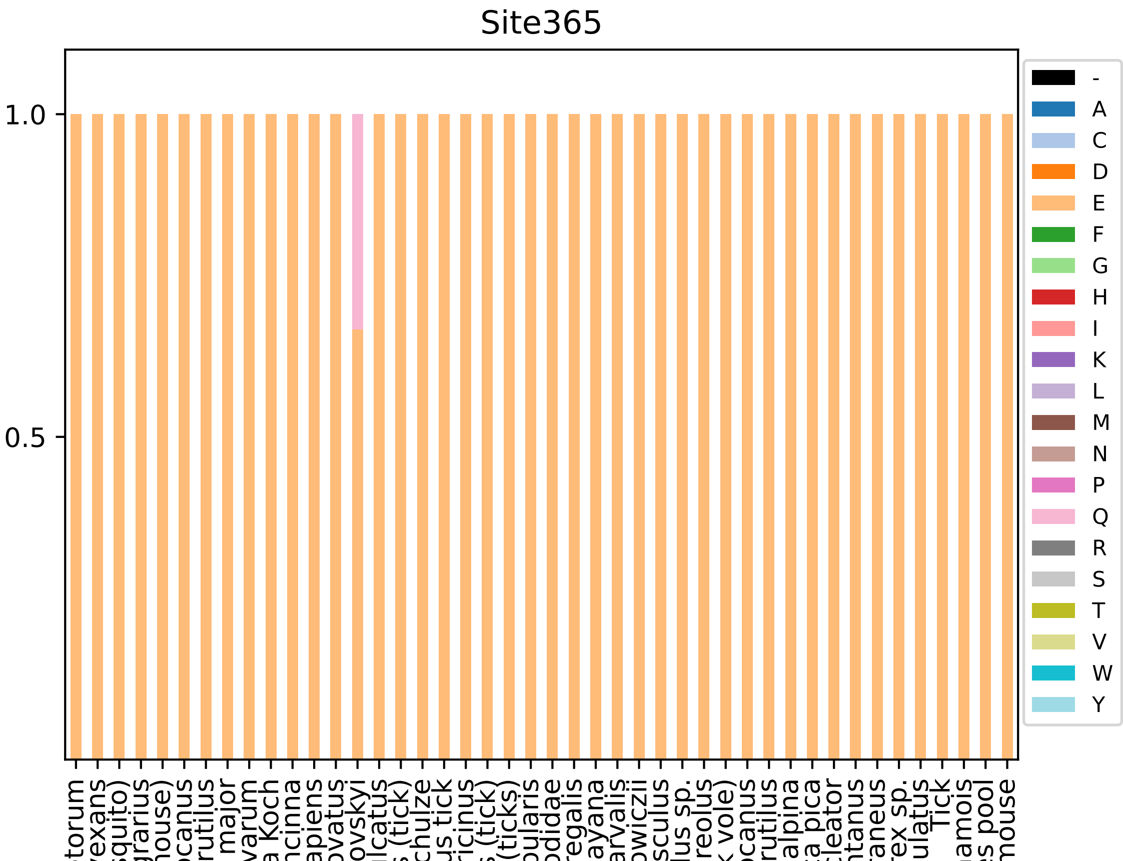
Site365
1.0
0.5
-
A
C
D
E
F
G
H
I
K
L
M
N
P
Q
R
S
T
V
W
Y

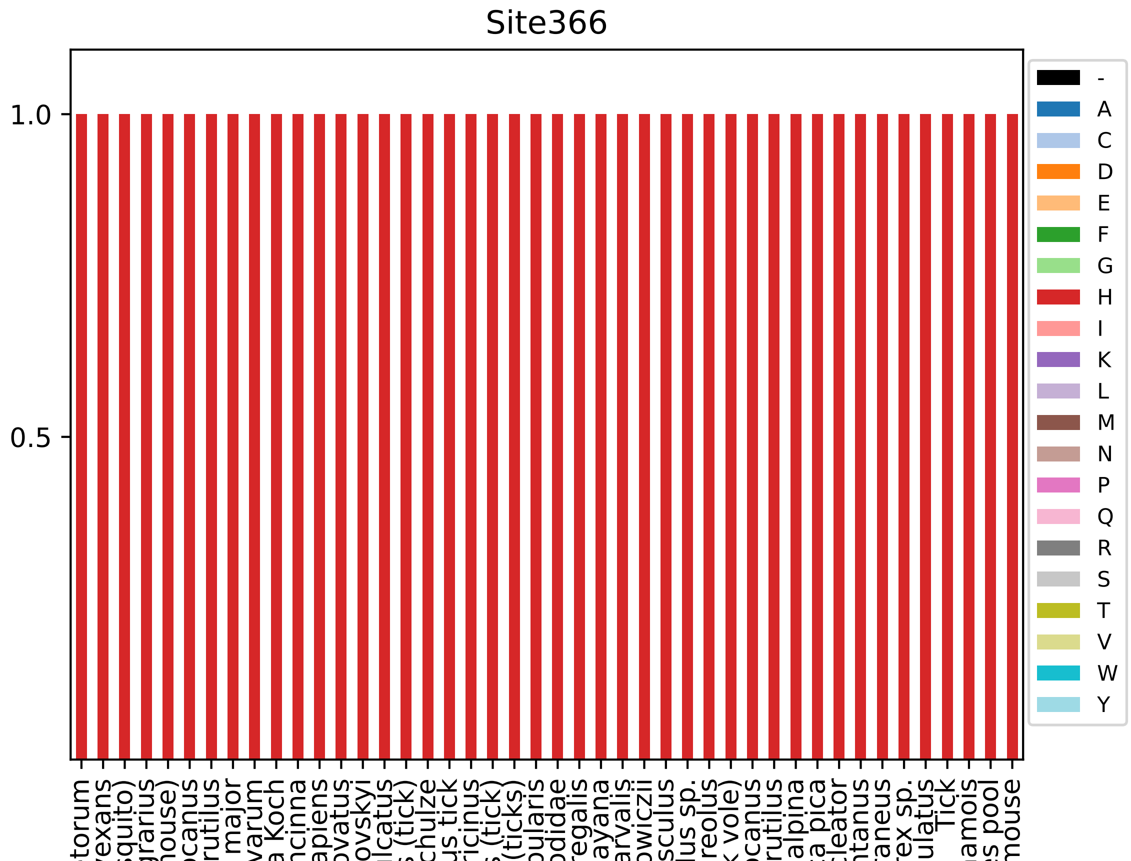
Site366
1.0
0.5
-
A
C
D
E
F
G
H
I
K
L
M
N
P
Q
R
S
T
V
W
Y

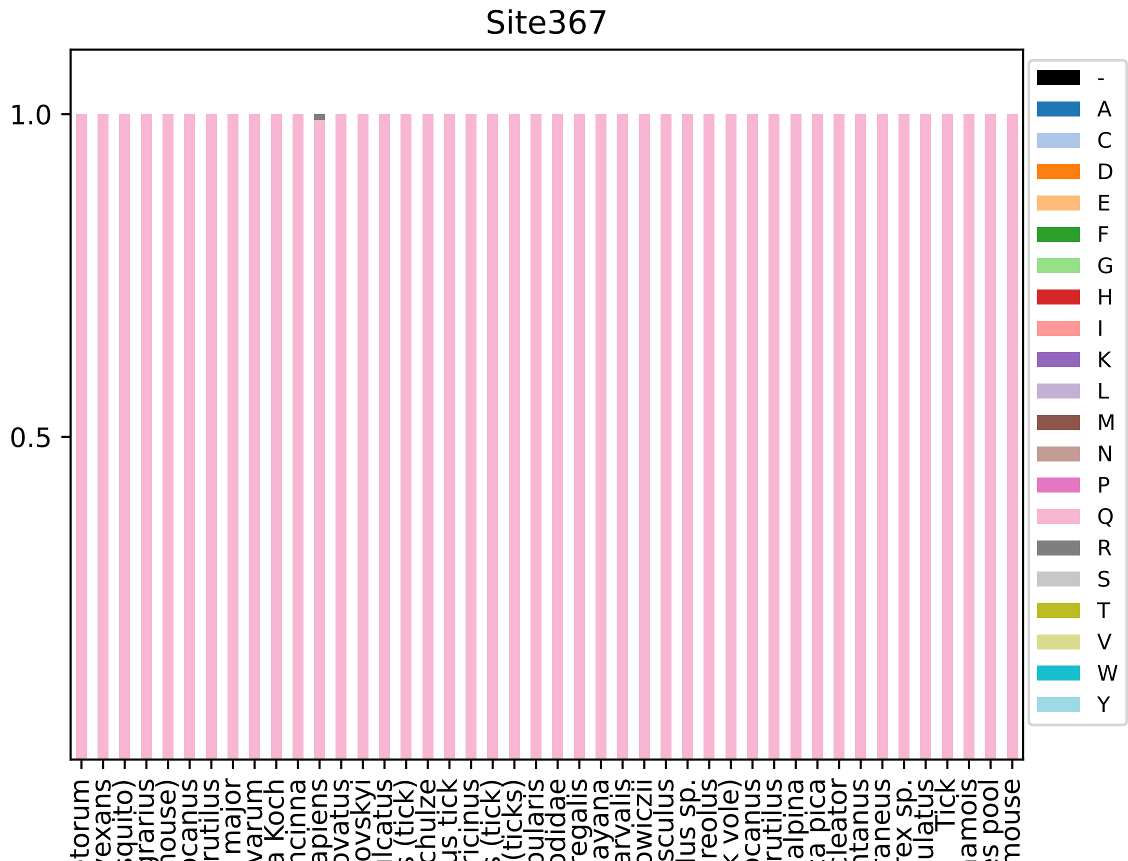

Site367
1.0
0.5
-
A
C
D
E
F
G
H
I
K
L
M
N
P
Q
R
S
T
V
W
Y

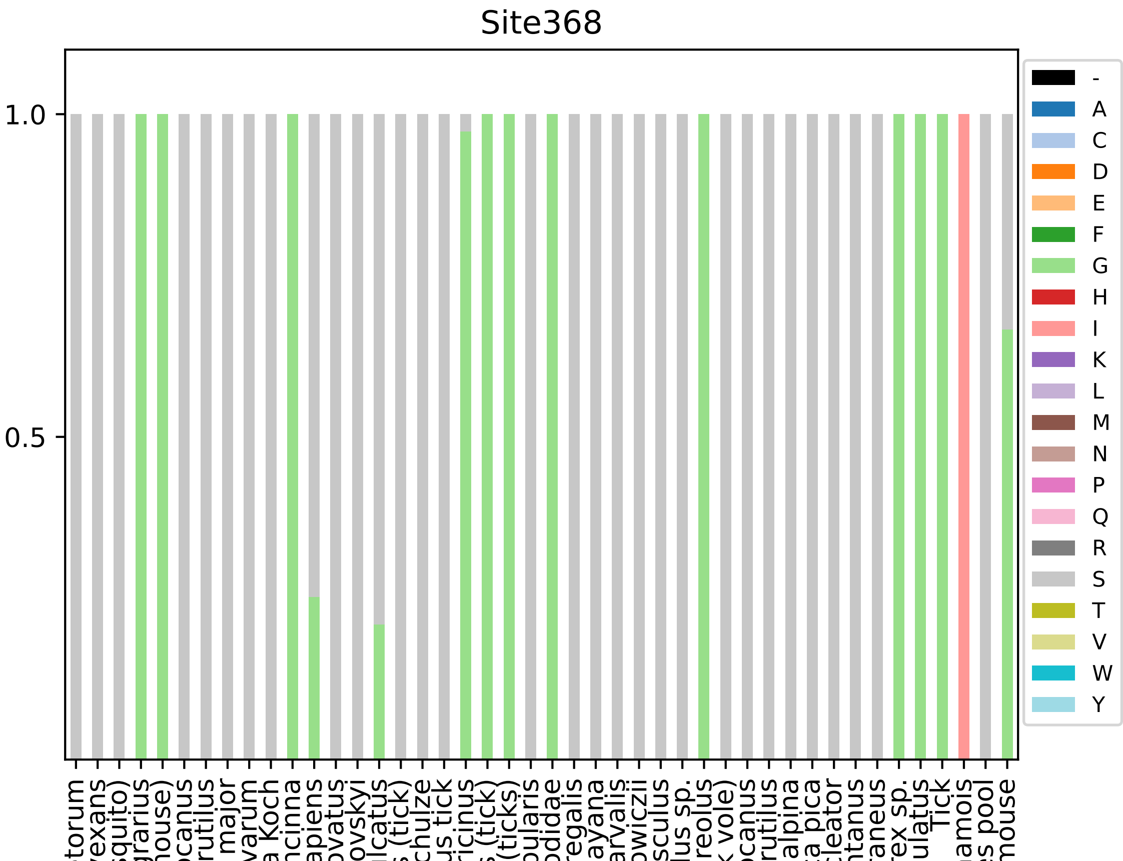

Site368
1.0
0.5
-
A
C
D
E
F
G
H
I
K
L
M
N
P
Q
R
S
T
V
W
Y
Tick

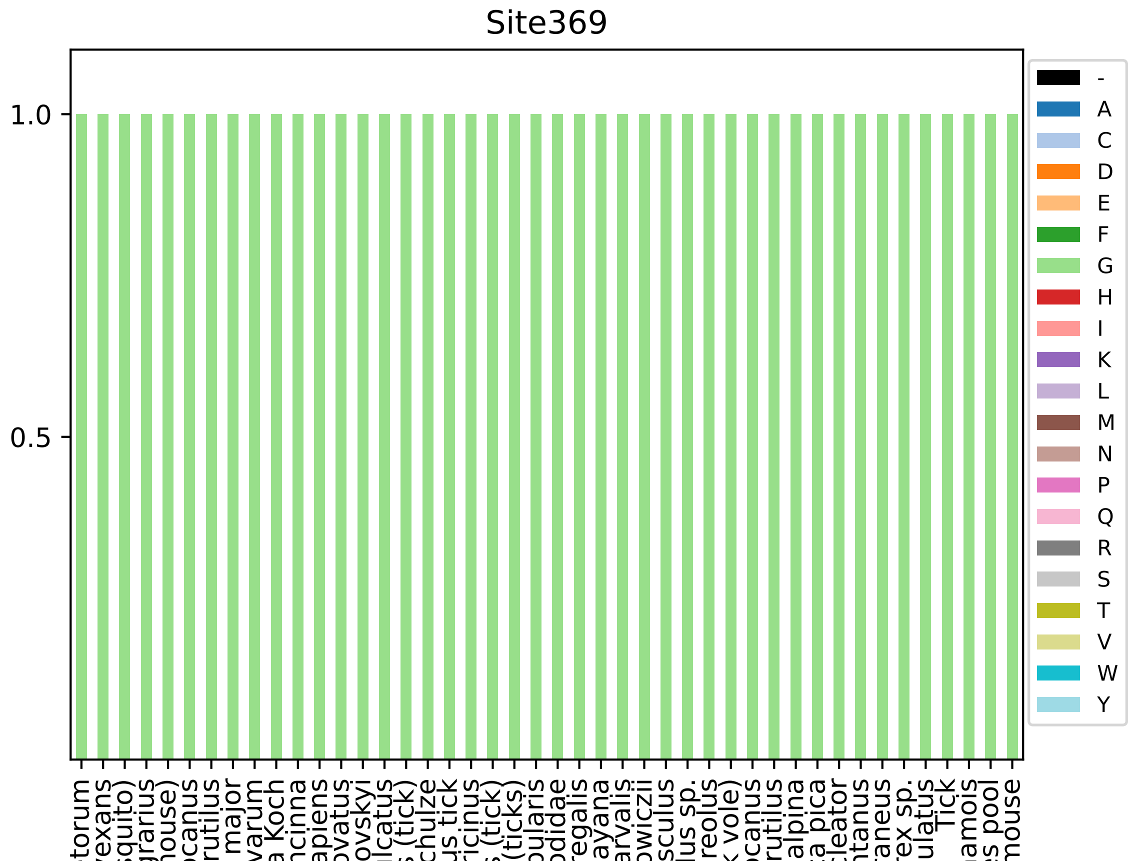
Site369
1.0
0.5
-
A
C
D
E
F
G
H
I
K
L
M
N
P
Q
R
S
T
V
W
Y

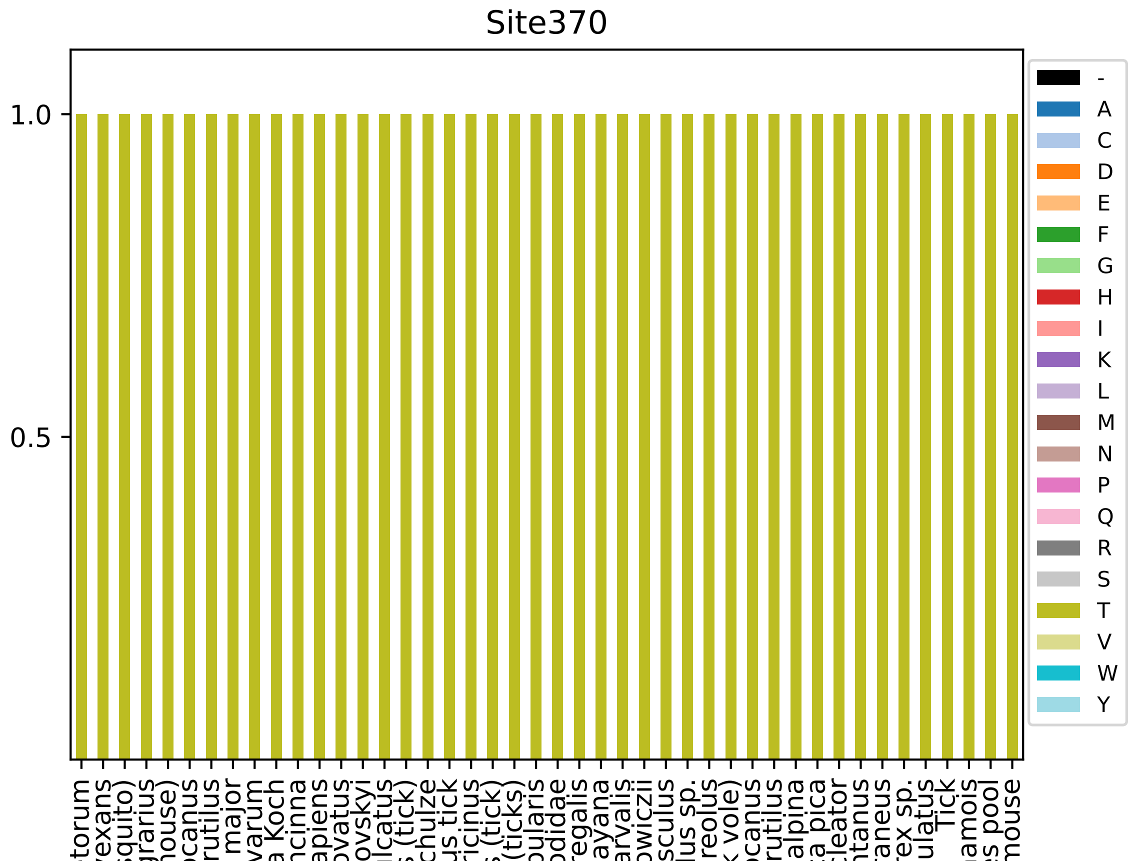
Site370
1.0
0.5
-
A
C
D
E
F
G
H
I
K
L
M
N
P
Q
R
S
T
V
W
Y

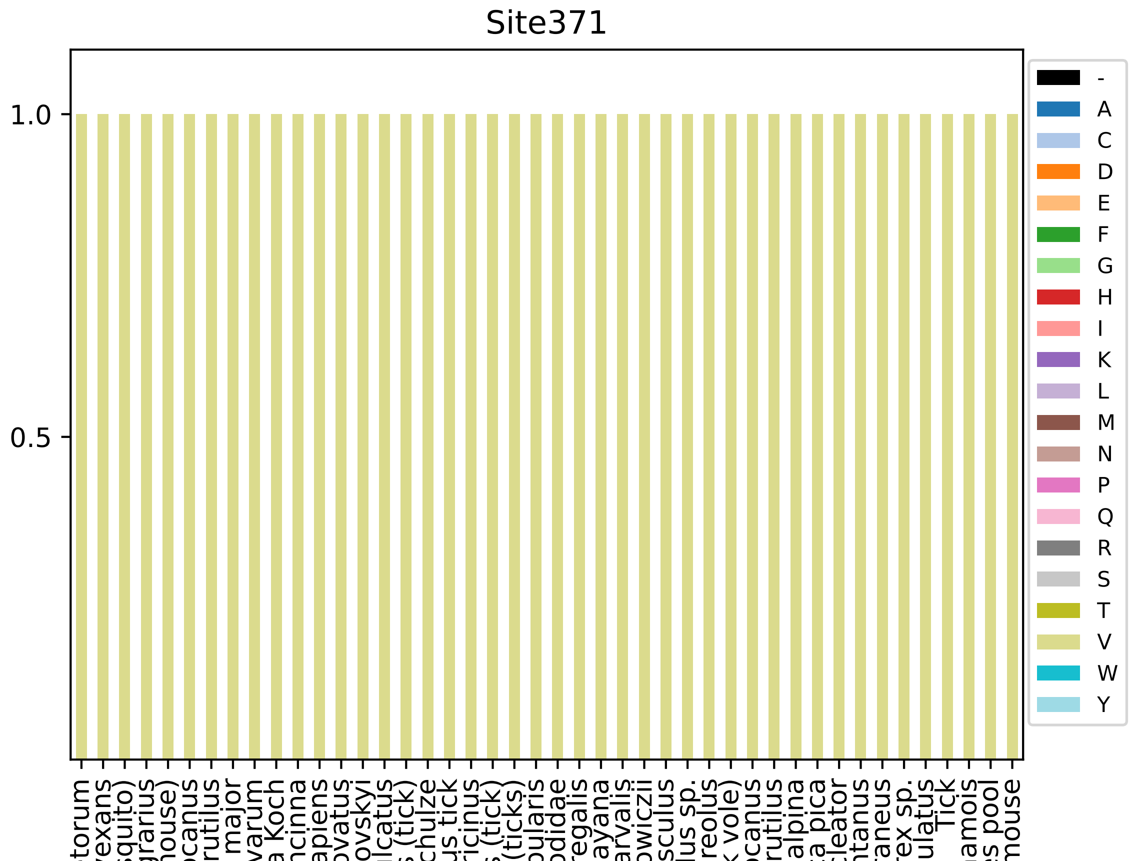
Site371
1.0
0.5
-
A
C
D
E
F
G
H
I
K
L
M
N
P
Q
R
S
T
V
W
Y

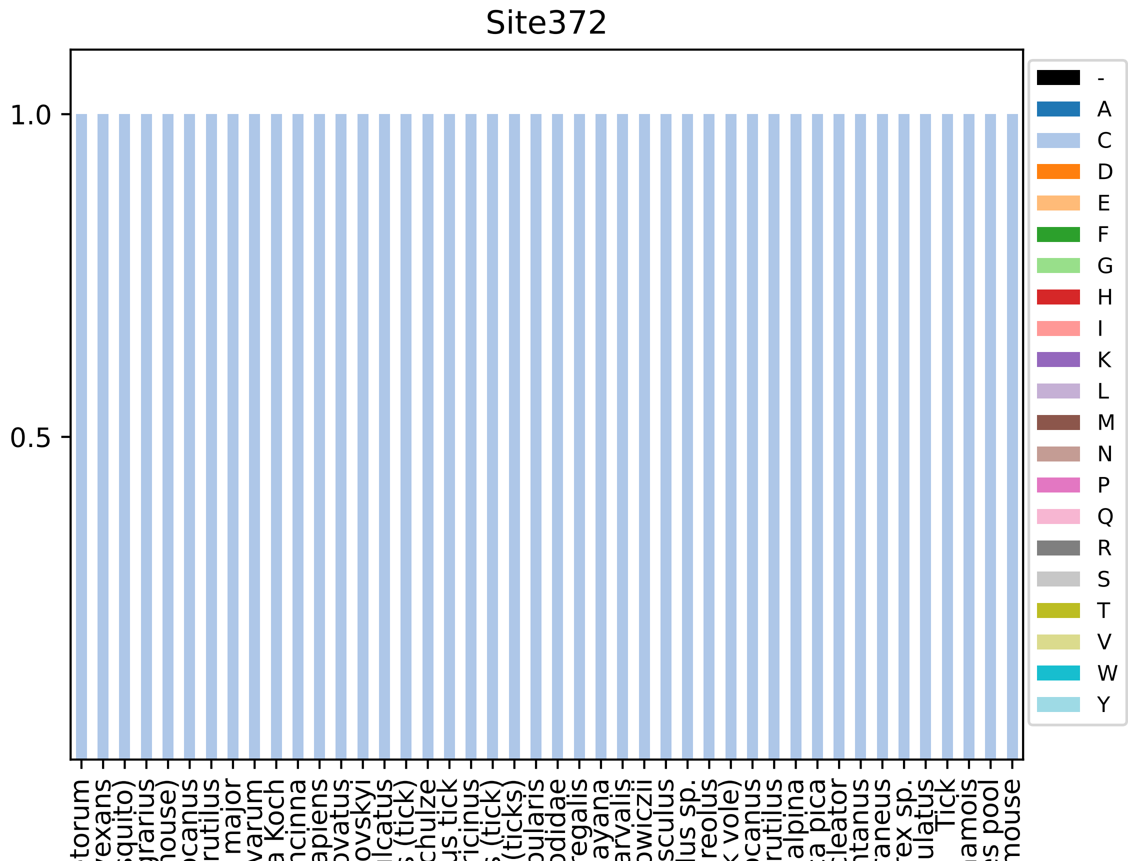
Site372
1.0
0.5
-
A
C
D
E
F
G
H
I
K
L
M
N
P
Q
R
S
T
V
W
Y

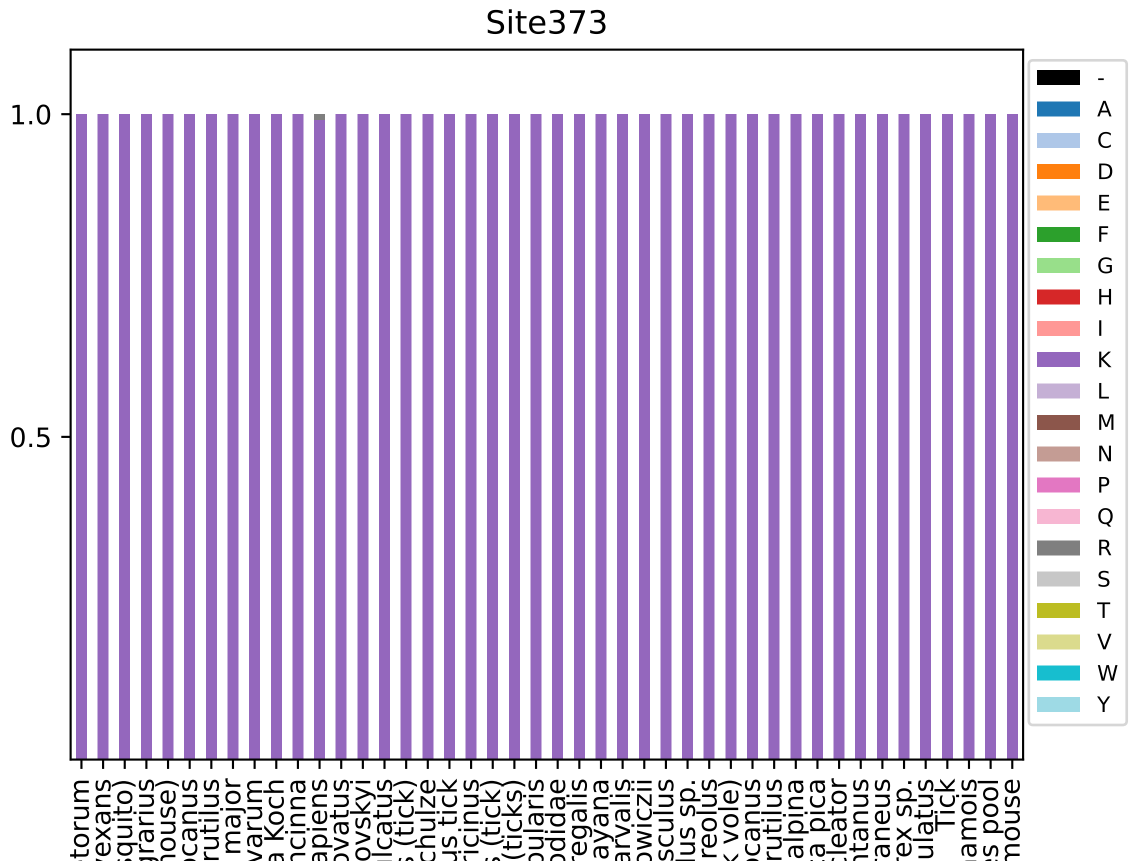

Site373
1.0
0.5
-
A
C
D
E
F
G
H
I
K
L
M
N
P
Q
R
S
T
V
W
Y

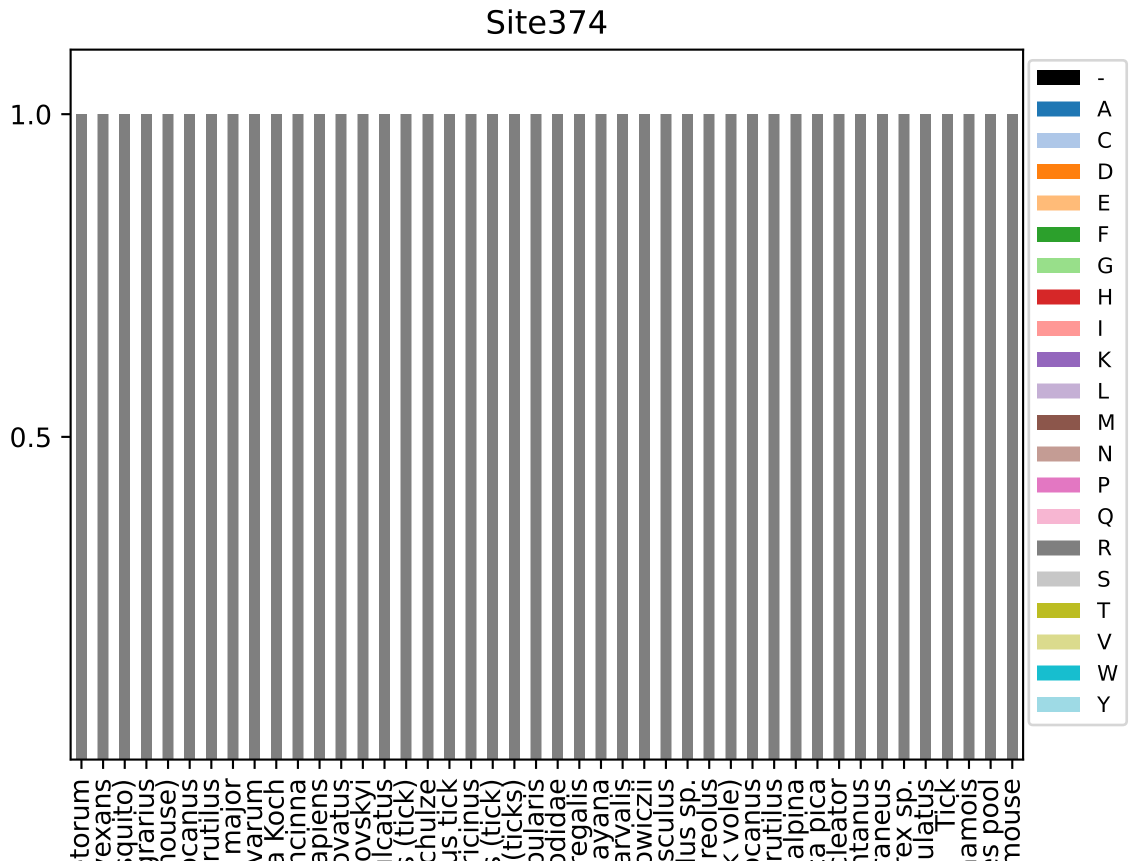
Site374
1.0
0.5
-
A
C
D
E
F
G
H
I
K
L
M
N
P
Q
R
S
T
V
W
Y

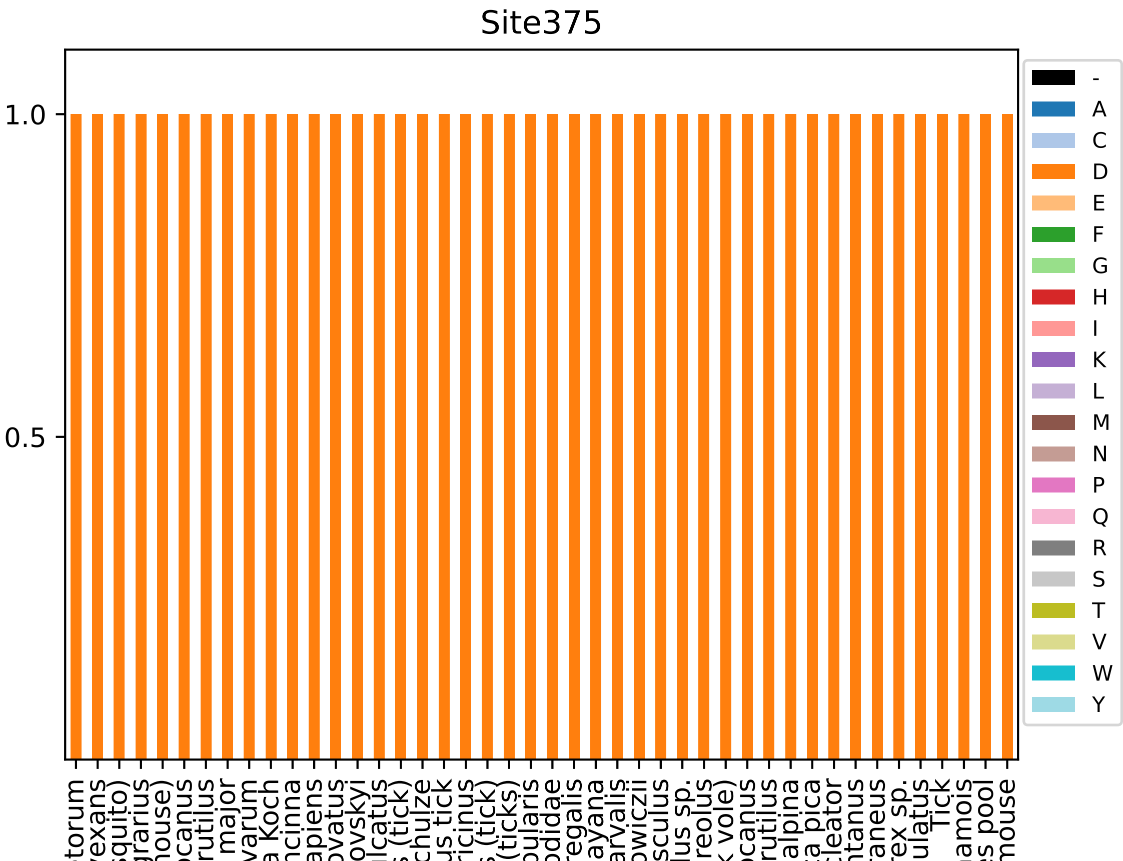

Site375
1.0
0.5
-
A
C
D
E
F
G
H
I
K
L
M
N
P
Q
R
S
T
V
W
Y

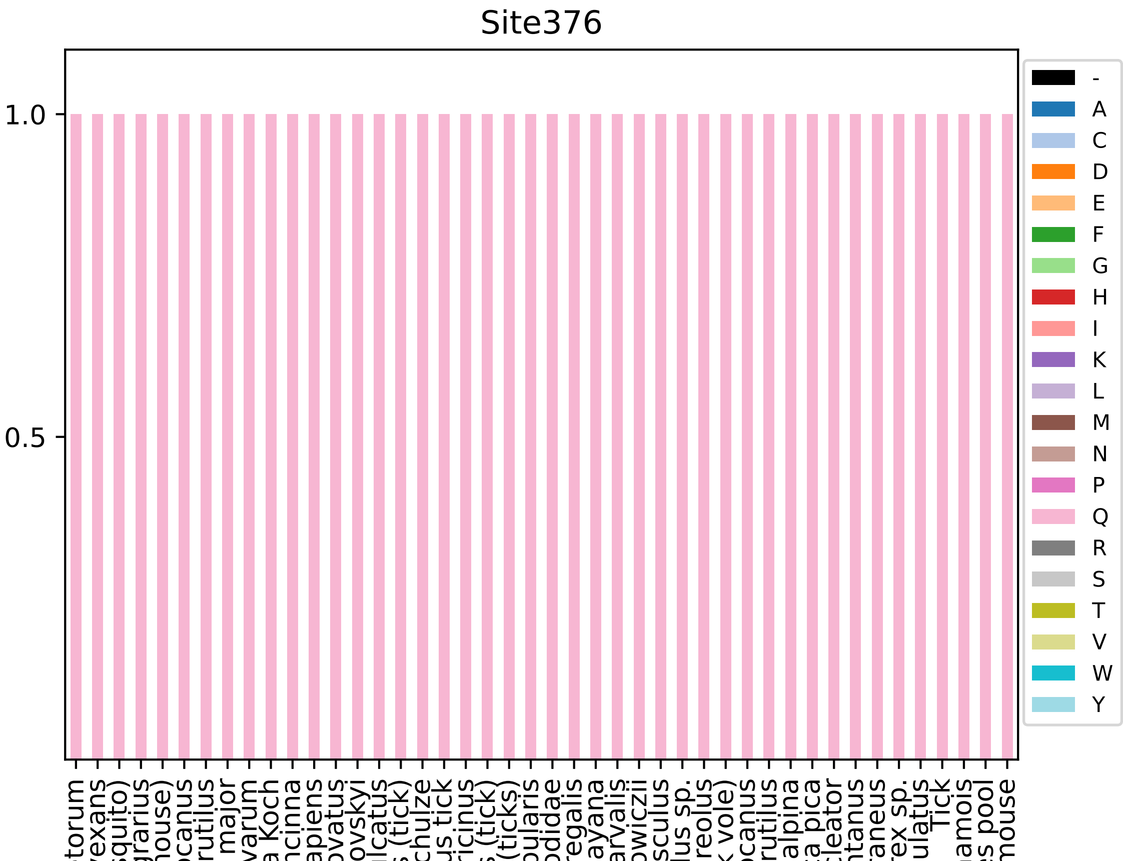

Site376
1.0
0.5
-
A
C
D
E
F
G
H
I
K
L
M
N
P
Q
R
S
T
V
W
Y

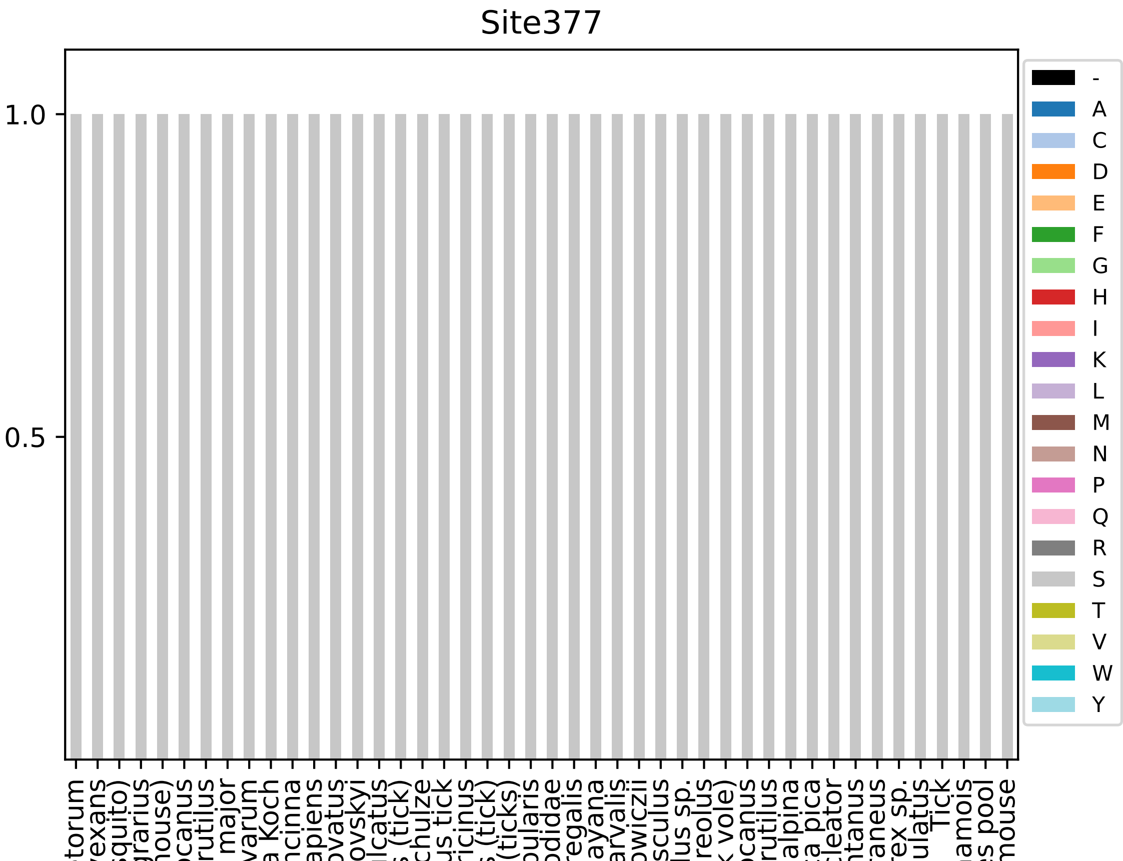

Site377
1.0
0.5
-
A
C
D
E
F
G
H
I
K
L
M
N
P
Q
R
S
T
V
W
Y

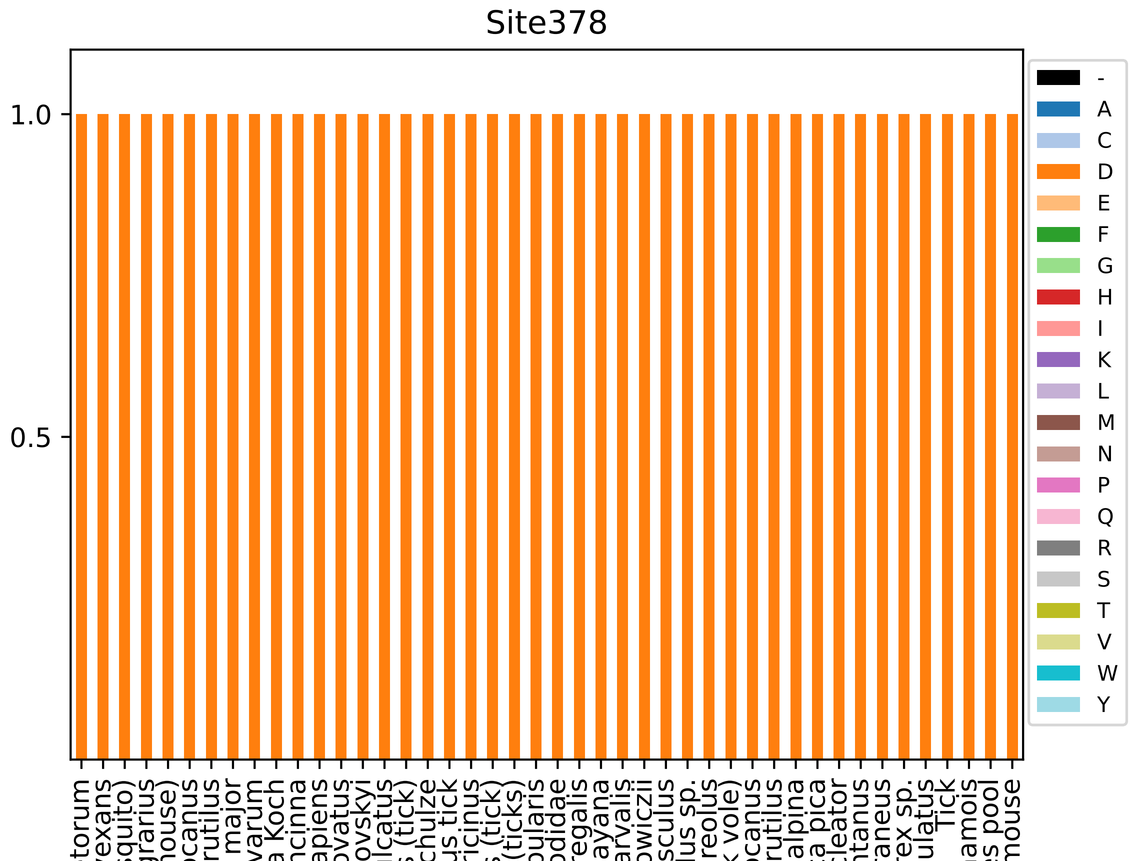
Site378
1.0
0.5
-
A
C
D
E
F
G
H
I
K
L
M
N
P
Q
R
S
T
V
W
Y
Tick

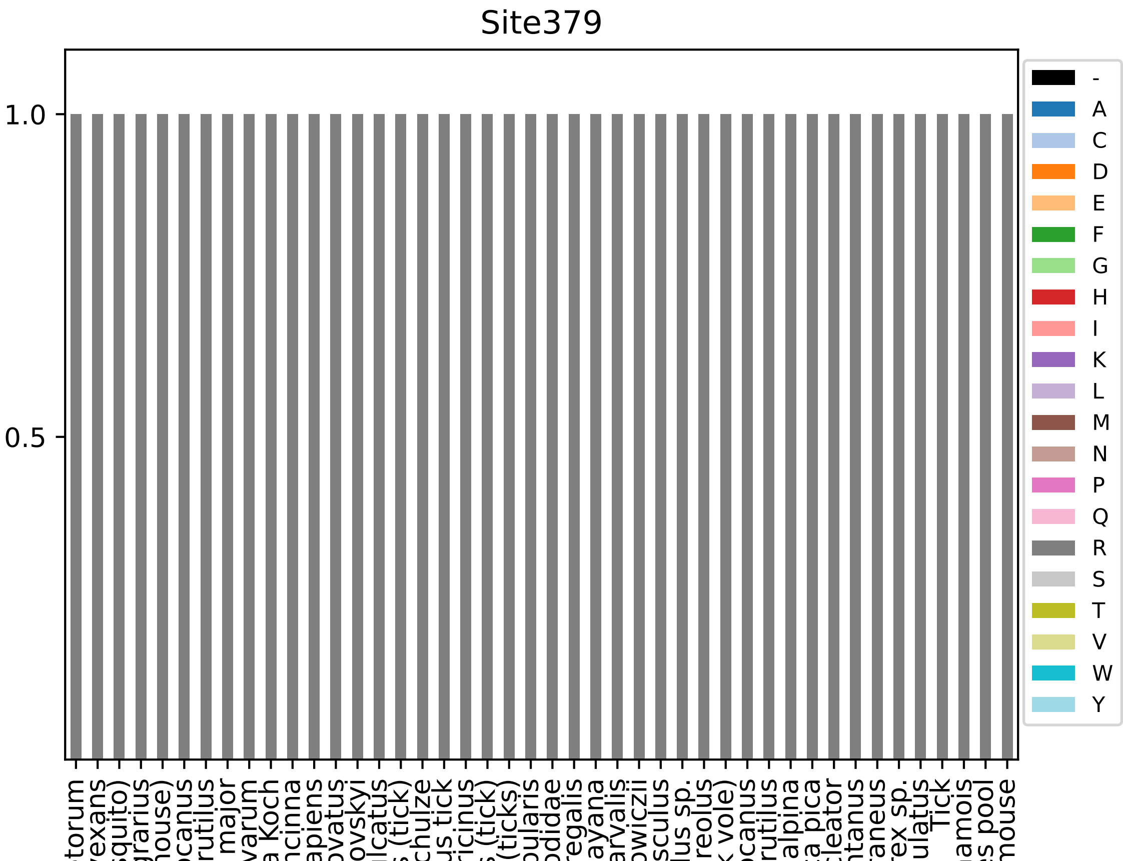
Site379
1.0
0.5
-
A
C
D
E
F
G
H
I
K
L
M
N
P
Q
R
S
T
V
W
Y

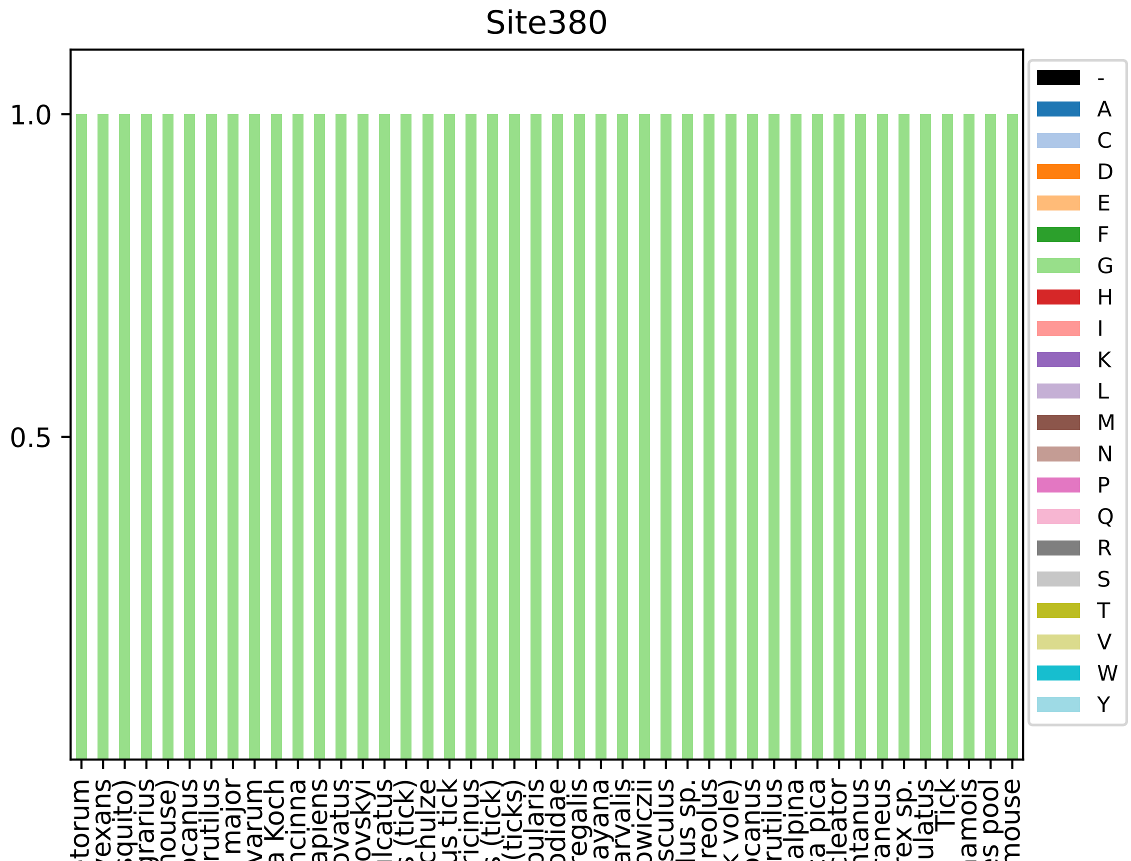
Site380
1.0
0.5
-
A
C
D
E
F
G
H
I
K
L
M
N
P
Q
R
S
T
V
W
Y

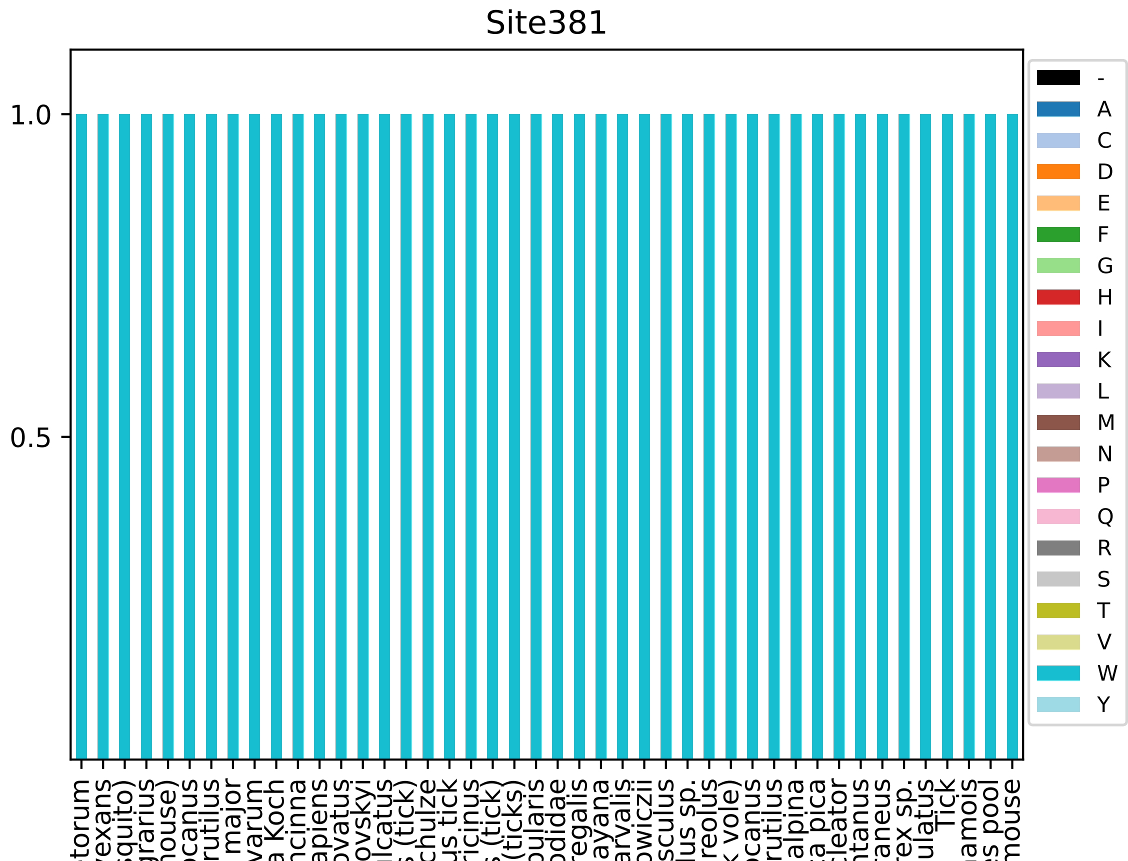

Site381
1.0
0.5
-
A
C
D
E
F
G
H
I
K
L
M
N
P
Q
R
S
T
V
W
Y

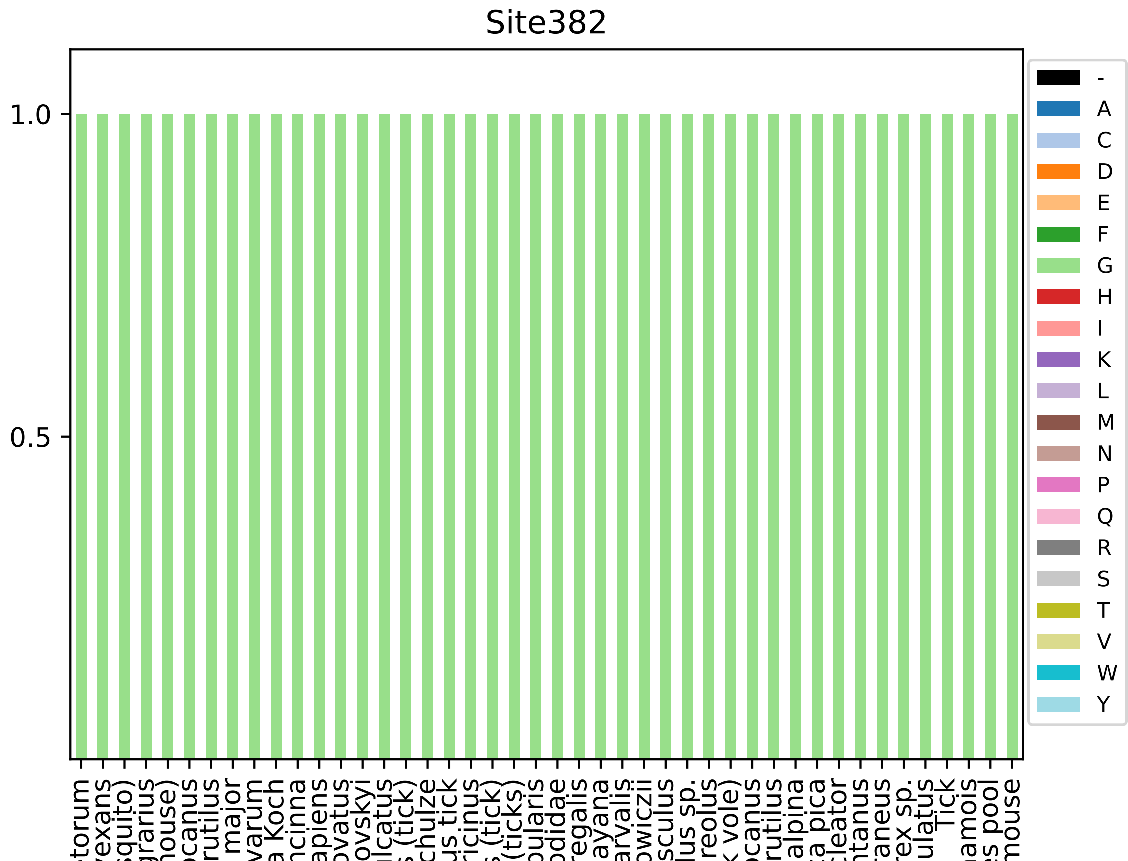
Site382
1.0
0.5
-
A
C
D
E
F
G
H
I
K
L
M
N
P
Q
R
S
T
V
W
Y

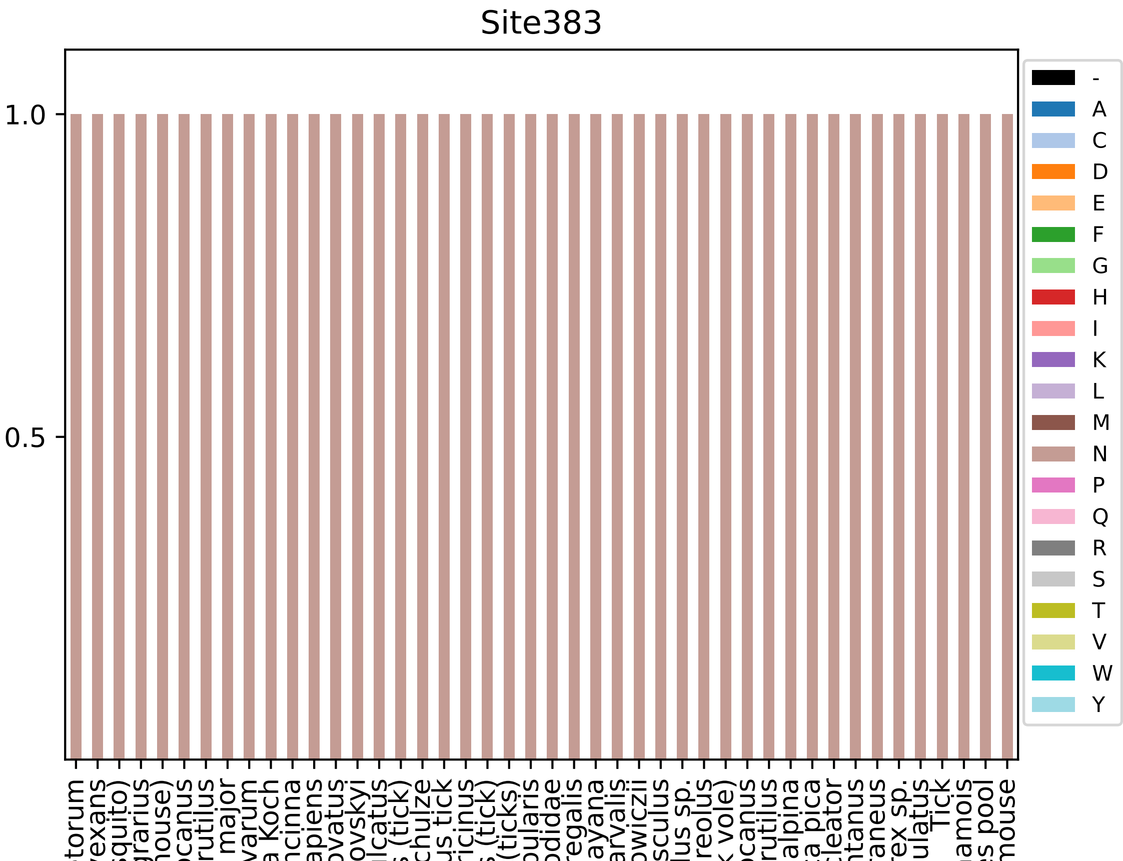

Site383
1.0
0.5
-
A
C
D
E
F
G
H
I
K
L
M
N
P
Q
R
S
T
V
W
Y

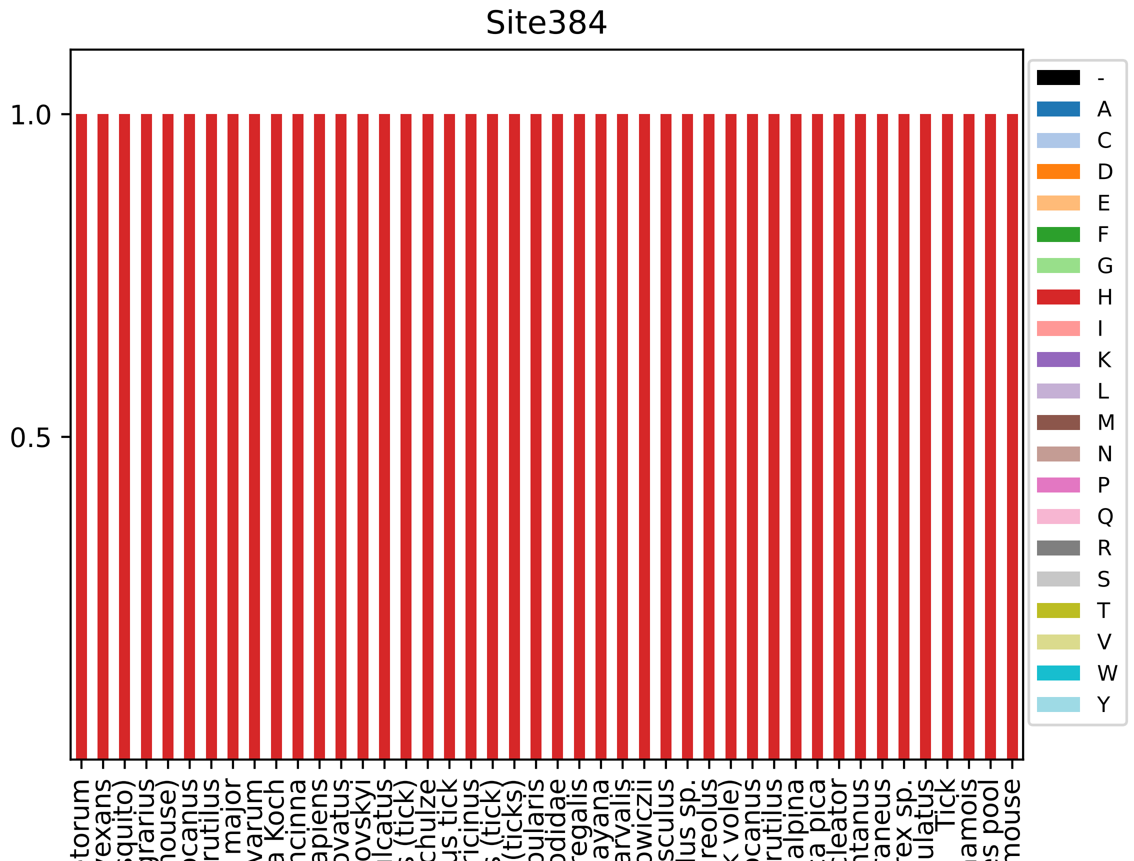
Site384
1.0
0.5
-
A
C
D
E
F
G
H
I
K
L
M
N
P
Q
R
S
T
V
W
Y

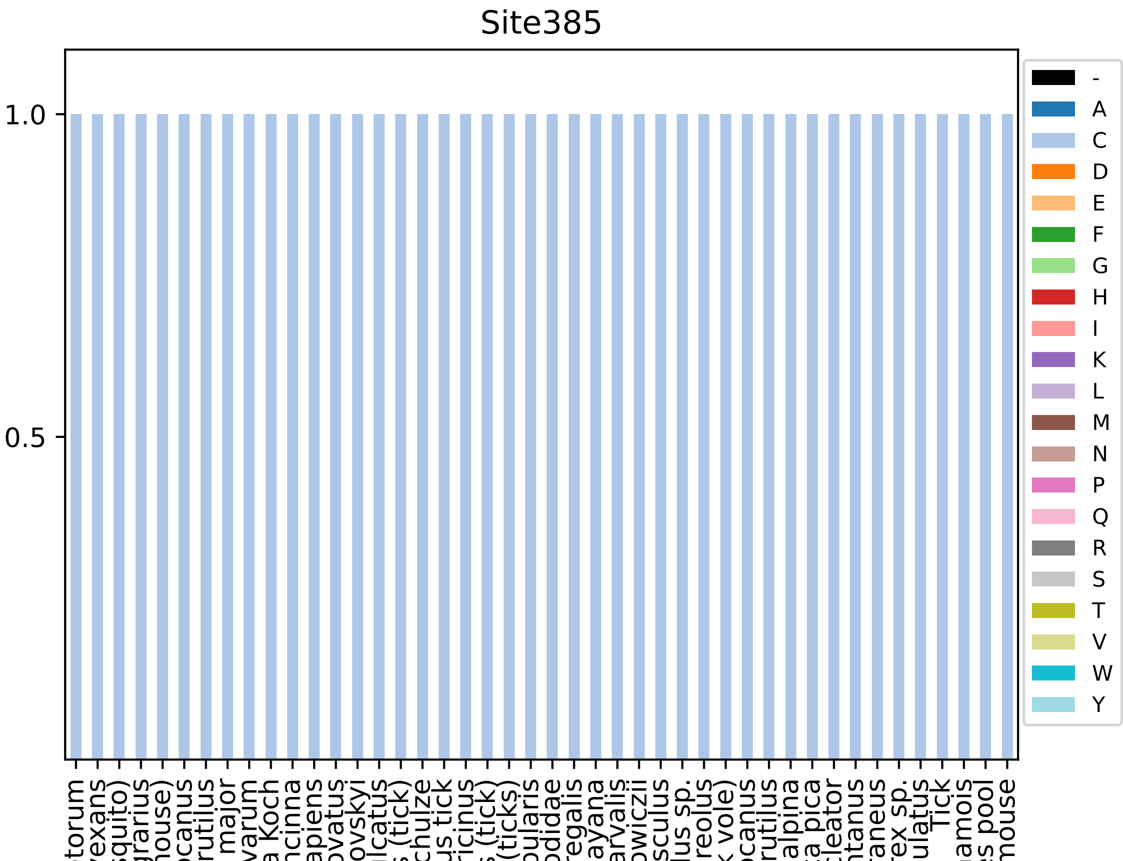
Site385
1.0
0.5
-
A
C
D
E
F
G
H
I
K
L
M
N
P
Q
R
S
T
V
W
Y

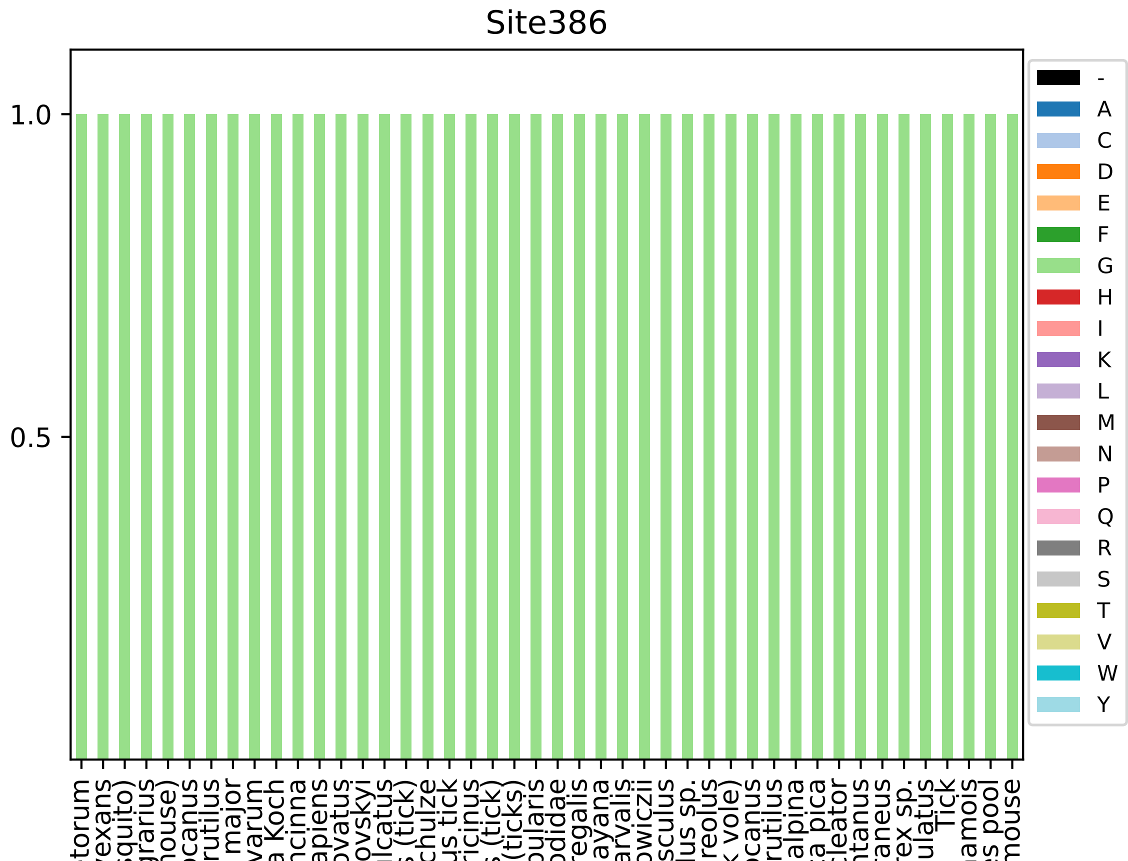
Site386
1.0
0.5
-
A
C
D
E
F
G
H
I
K
L
M
N
P
Q
R
S
T
V
W
Y

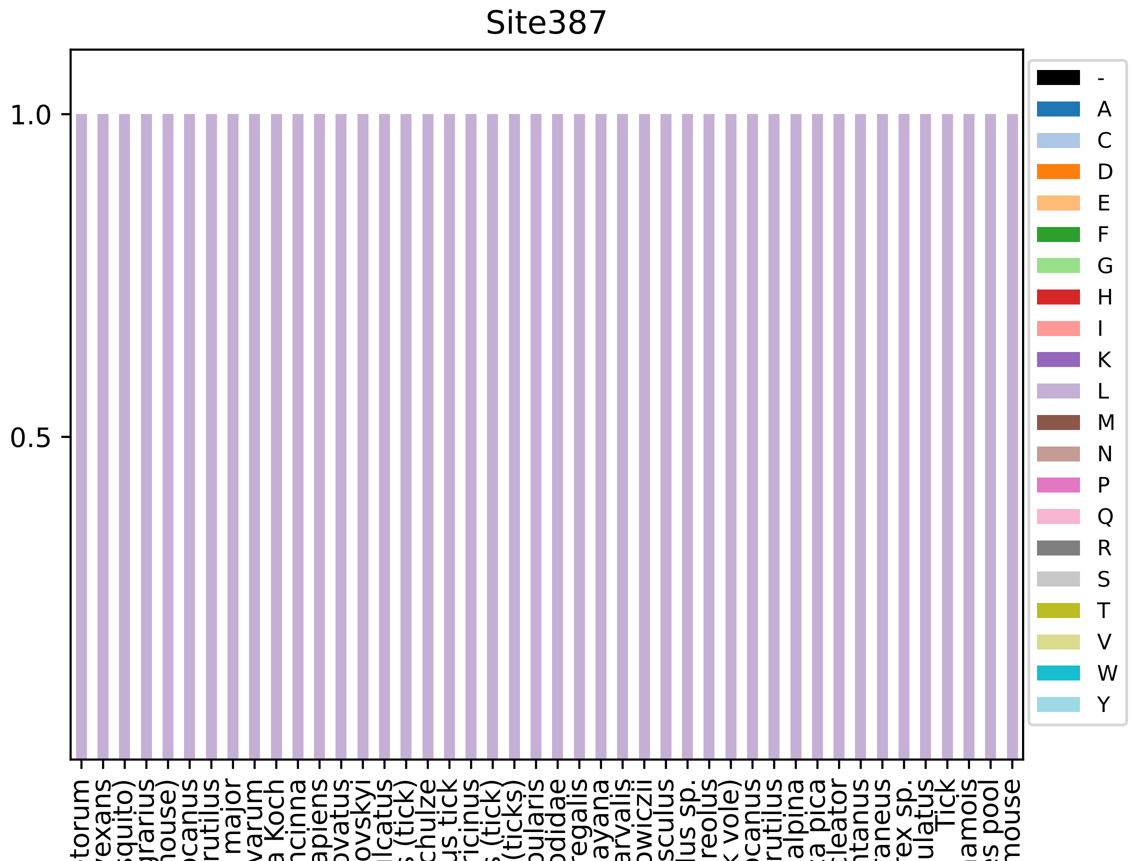
Site387
1.0
0.5
-
A
C
D
E
F
G
H
I
K
L
M
N
P
Q
R
S
T
V
W
Y

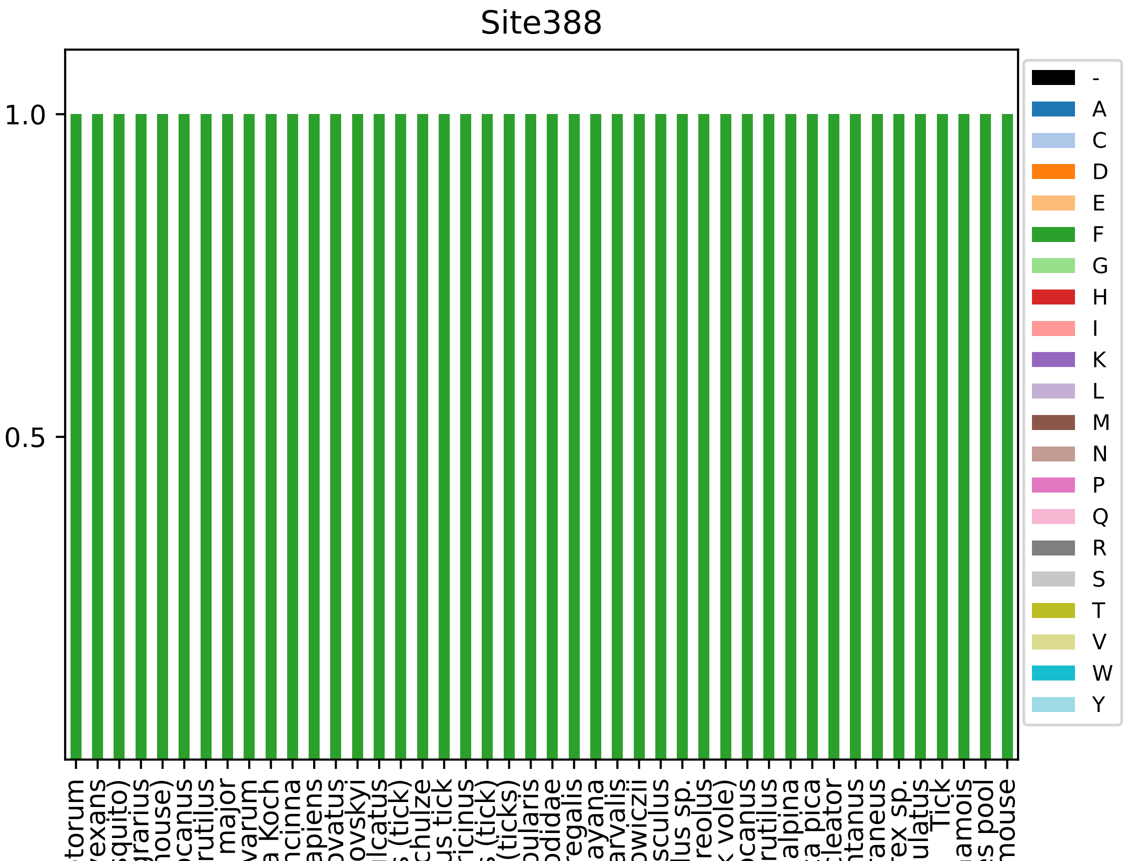
Site388
1.0
0.5
-
A
C
D
E
F
G
H
I
K
L
M
N
P
Q
R
S
T
V
W
Y

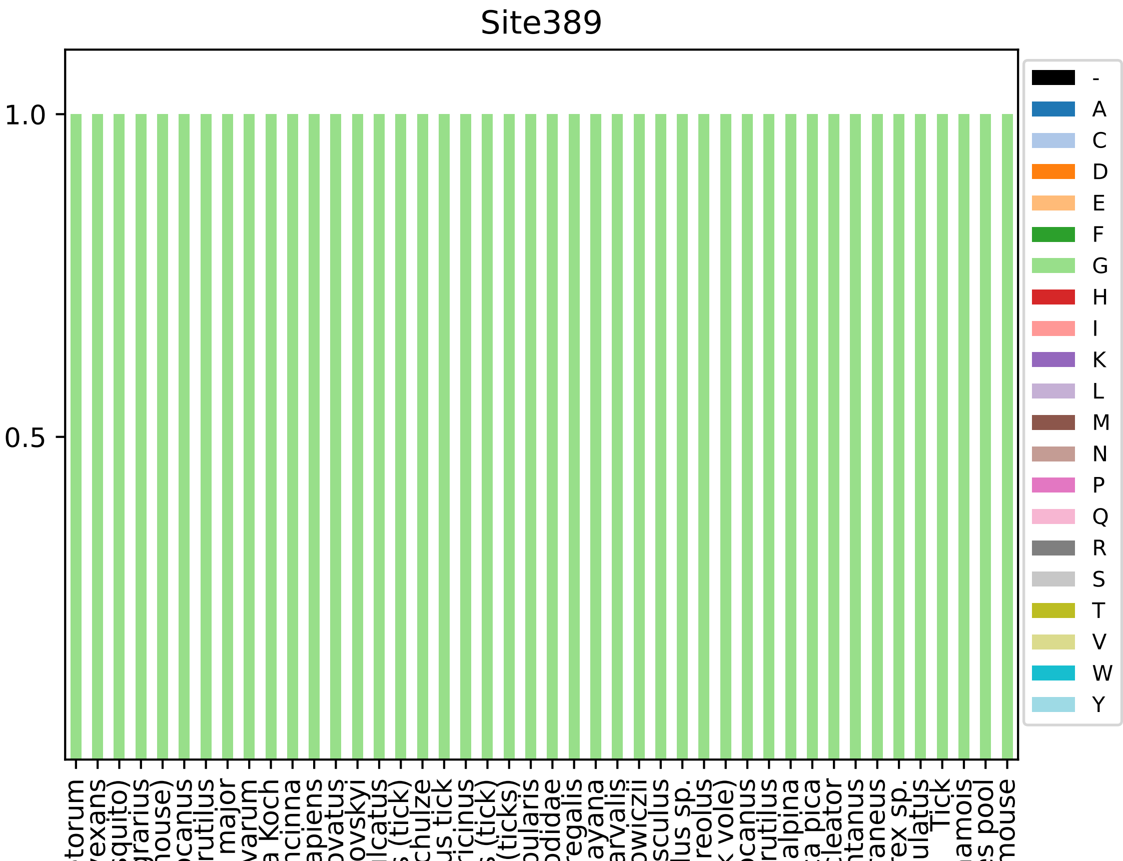

Site389
1.0
0.5
-
A
C
D
E
F
G
H
I
K
L
M
N
P
Q
R
S
T
V
W
Y

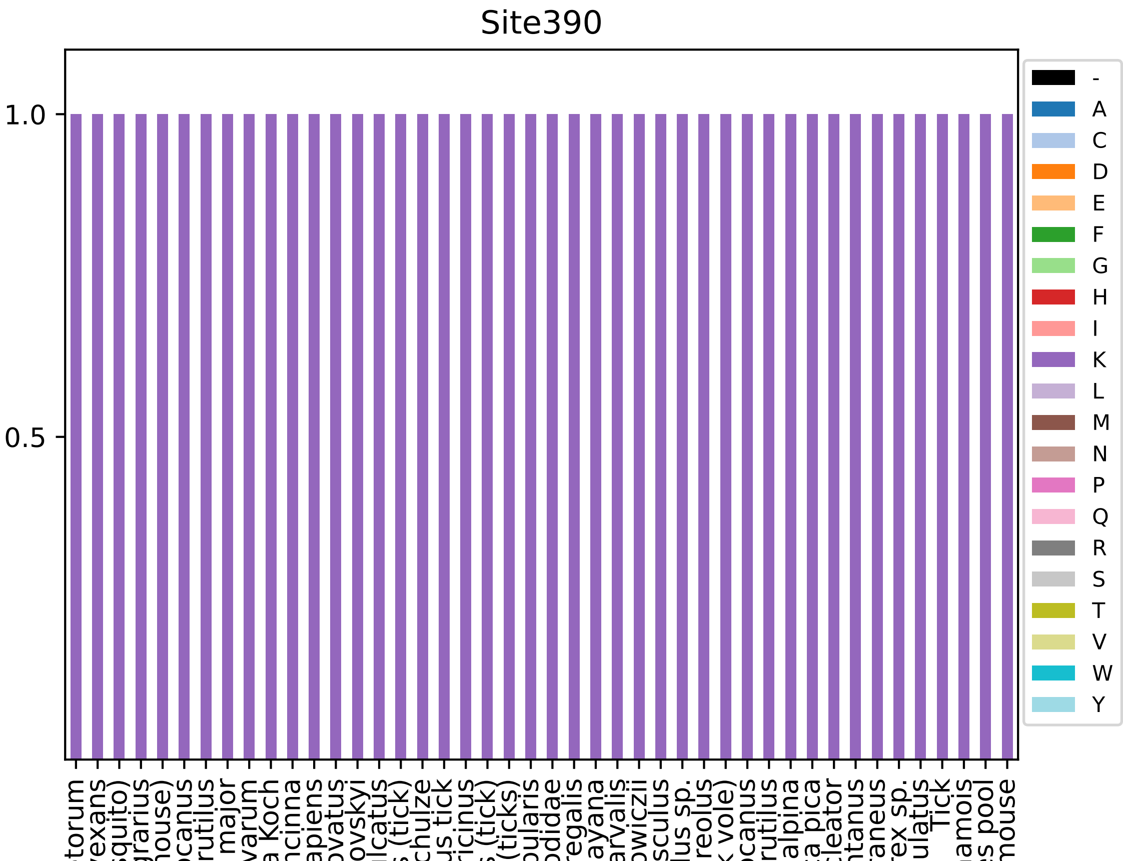

Site390
1.0
0.5
-
A
C
D
E
F
G
H
I
K
L
M
N
P
Q
R
S
T
V
W
Y
Tick

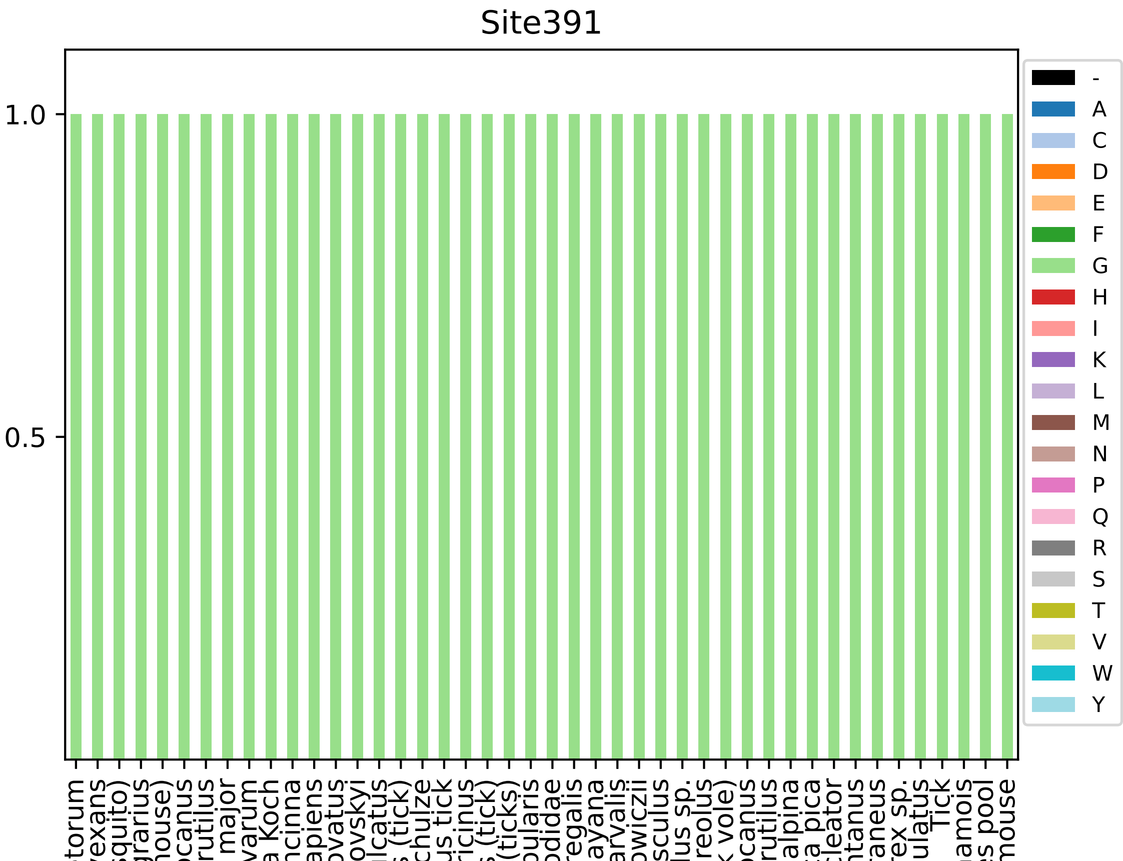
Site391
1.0
0.5
-
A
C
D
E
F
G
H
I
K
L
M
N
P
Q
R
S
T
V
W
Y

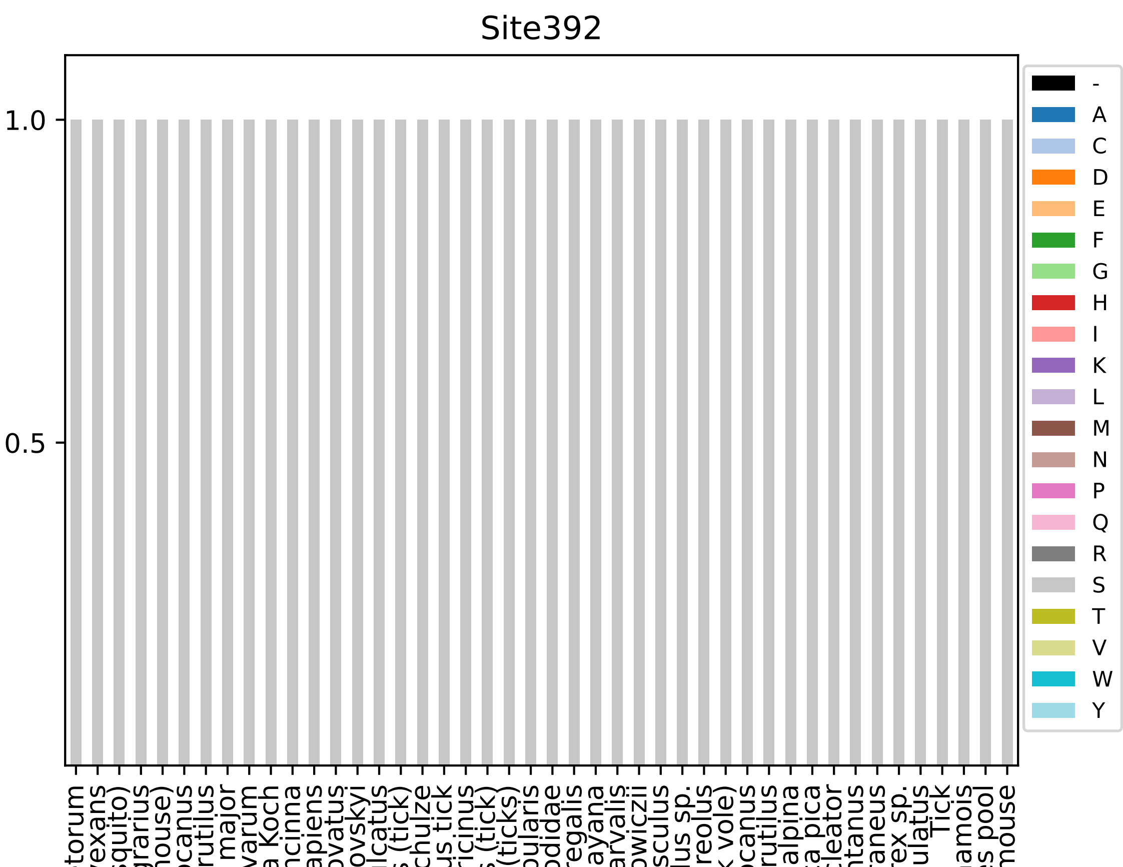

Site392
1.0
0.5
-
A
C
D
E
F
G
H
I
K
L
M
N
P
Q
R
S
T
V
W
Y

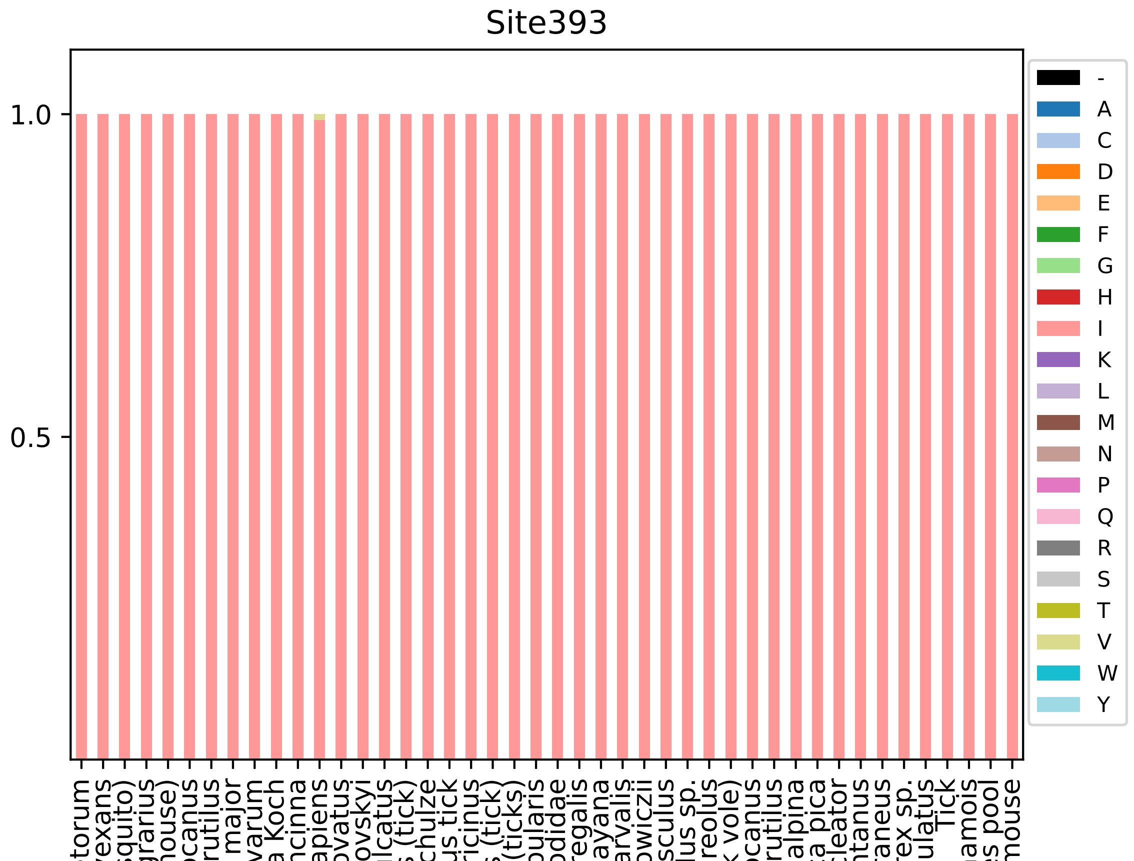

Site393
1.0
0.5
-
A
C
D
E
F
G
H
I
K
L
M
N
P
Q
R
S
T
V
W
Y

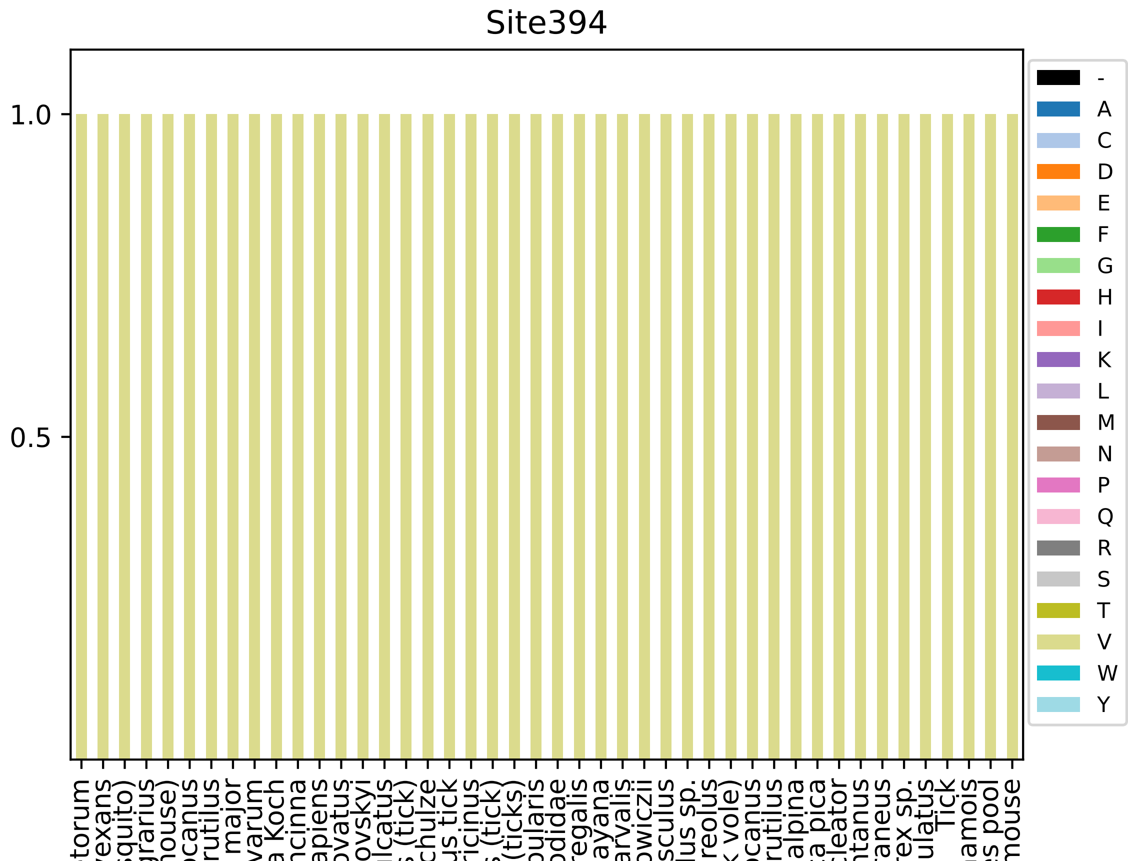
Site394
1.0
0.5
-
A
C
D
E
F
G
H
I
K
L
M
N
P
Q
R
S
T
V
W
Y

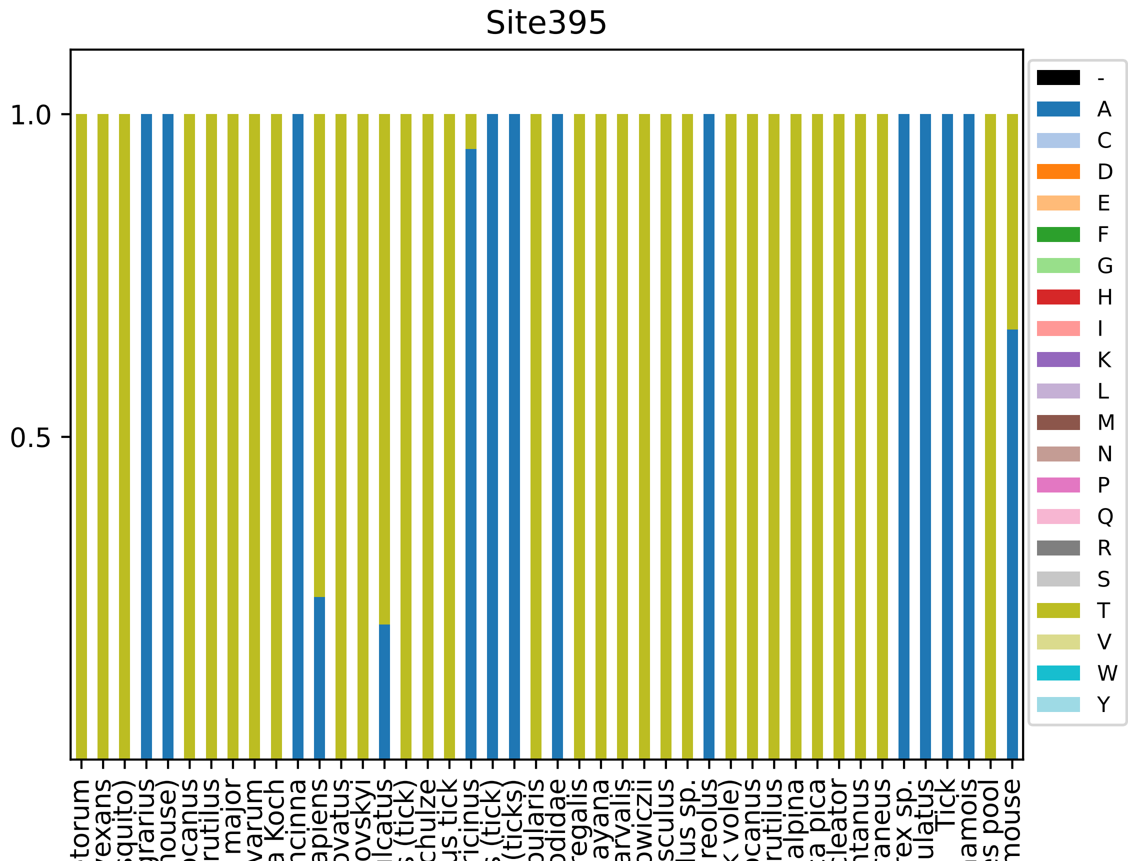
Site395
1.0
0.5
-
A
C
D
E
F
G
H
I
K
L
M
N
P
Q
R
S
T
V
W
Y

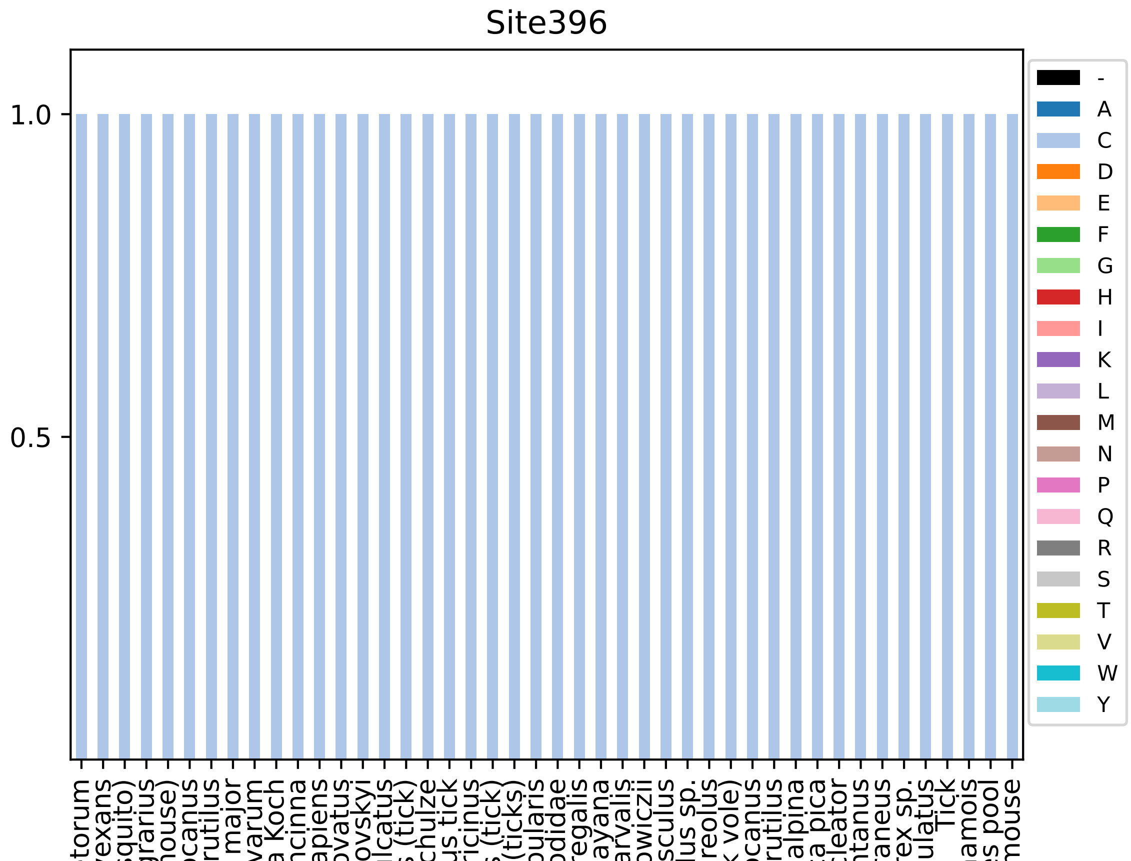
Site396
1.0
0.5
-
A
C
D
E
F
G
H
I
K
L
M
N
P
Q
R
S
T
V
W
Y

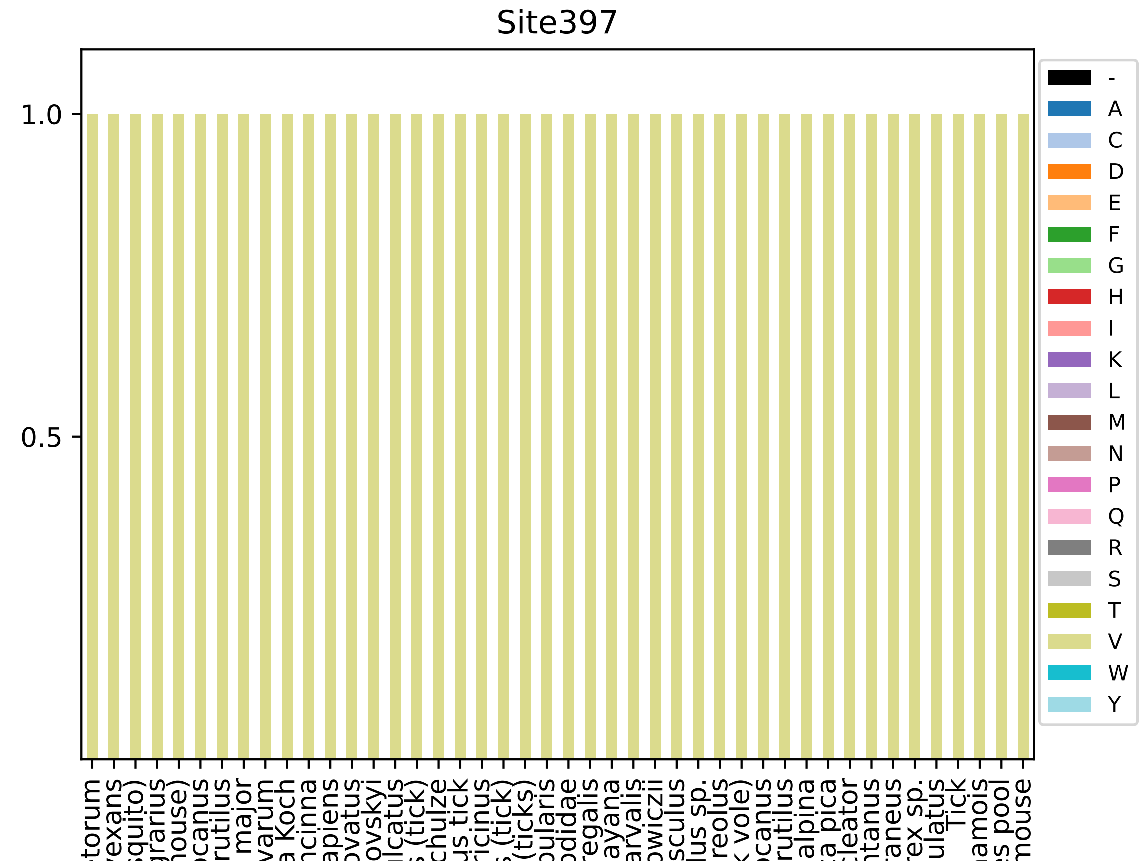
Site397
1.0
0.5
-
A
C
D
E
F
G
H
I
K
L
M
N
P
Q
R
S
T
V
W
Y

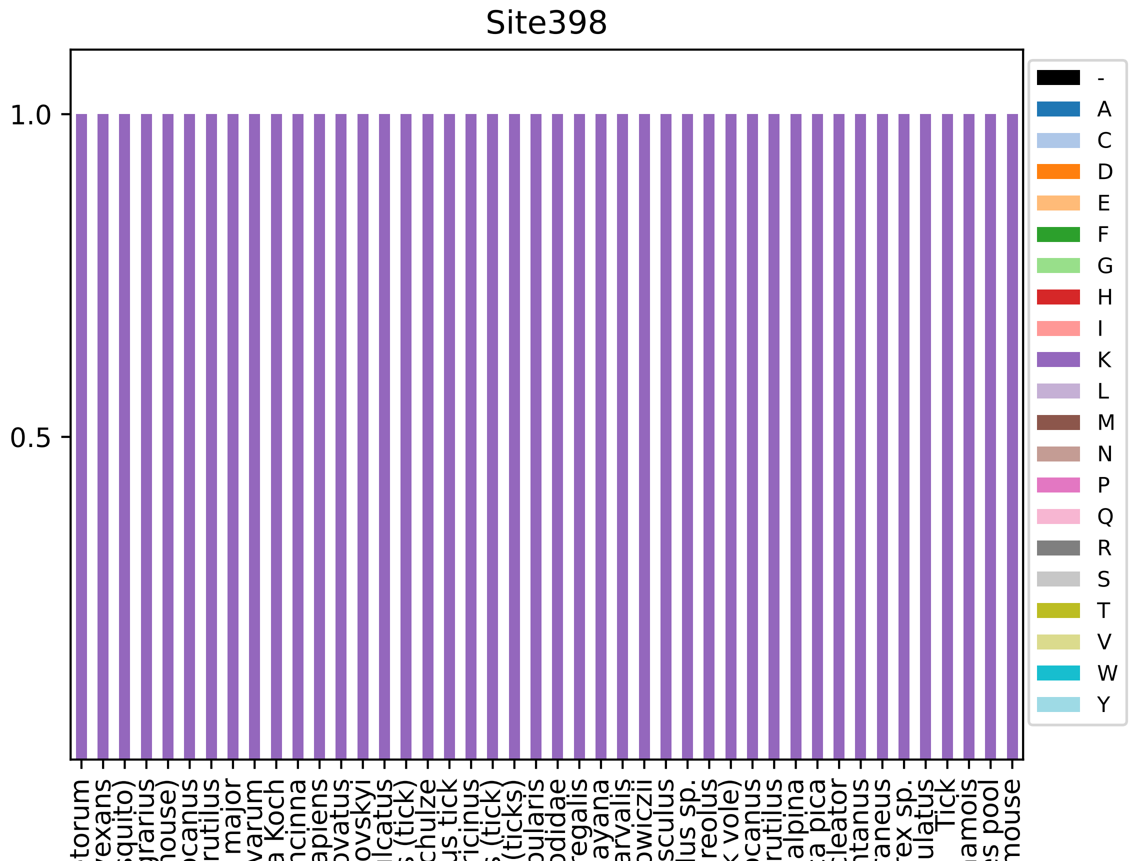
Site398
1.0
0.5
-
A
C
D
E
F
G
H
I
K
L
M
N
P
Q
R
S
T
V
W
Y

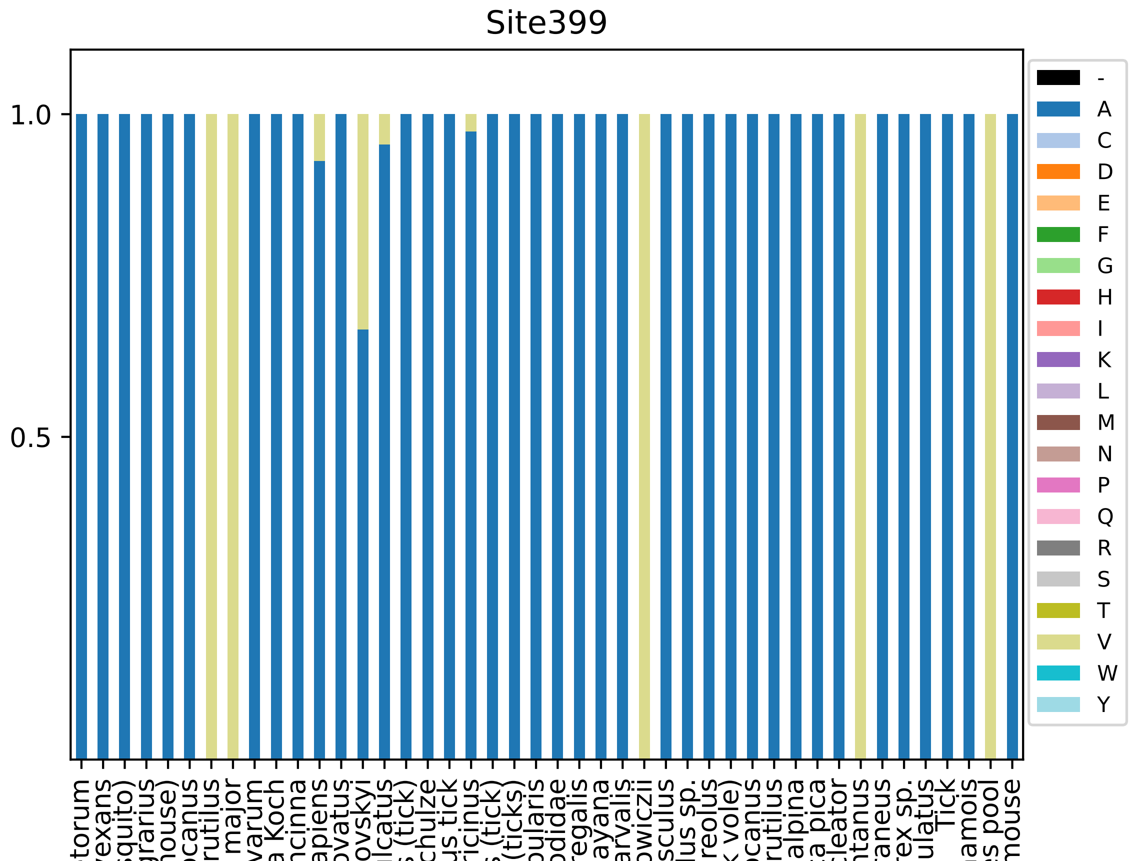
Site399
1.0
0.5
-
A
C
D
E
F
G
H
I
K
L
M
N
P
Q
R
S
T
V
W
Y

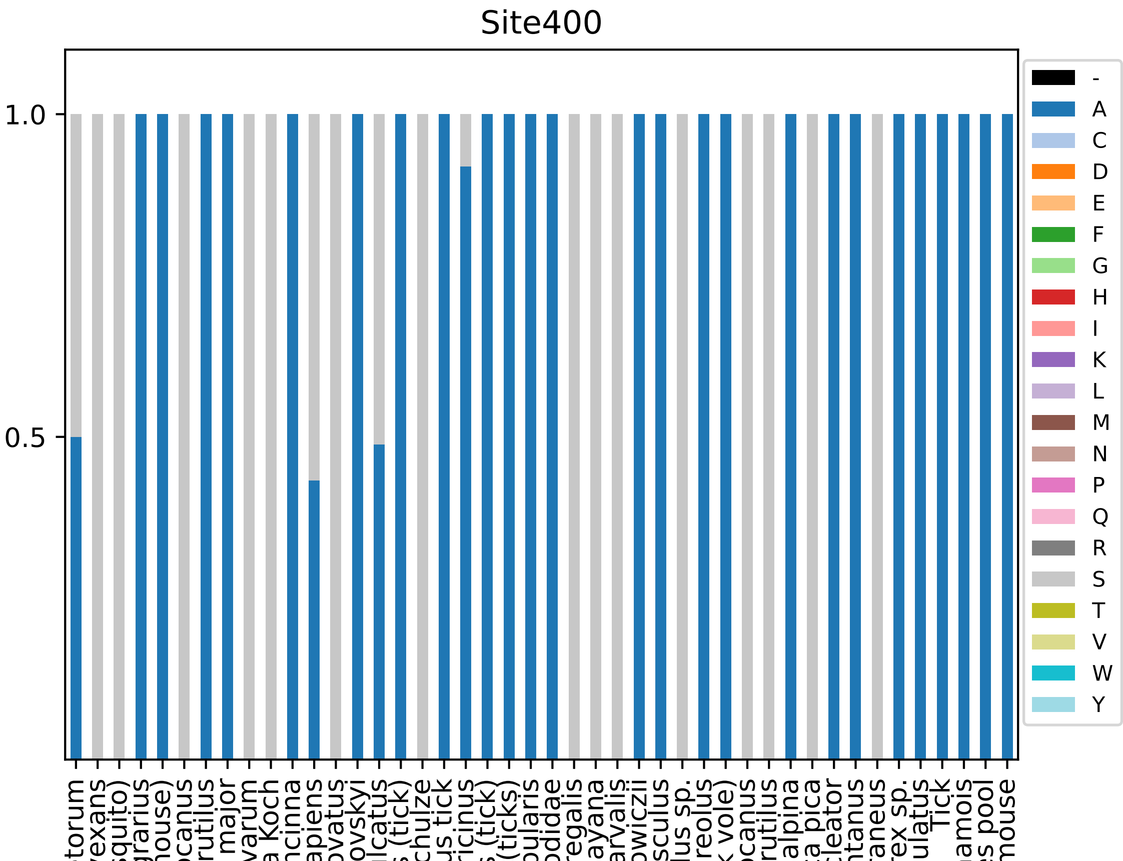
Site400
1.0
0.5
-
A
C
D
E
F
G
H
I
K
L
M
N
P
Q
R
S
T
V
W
Y

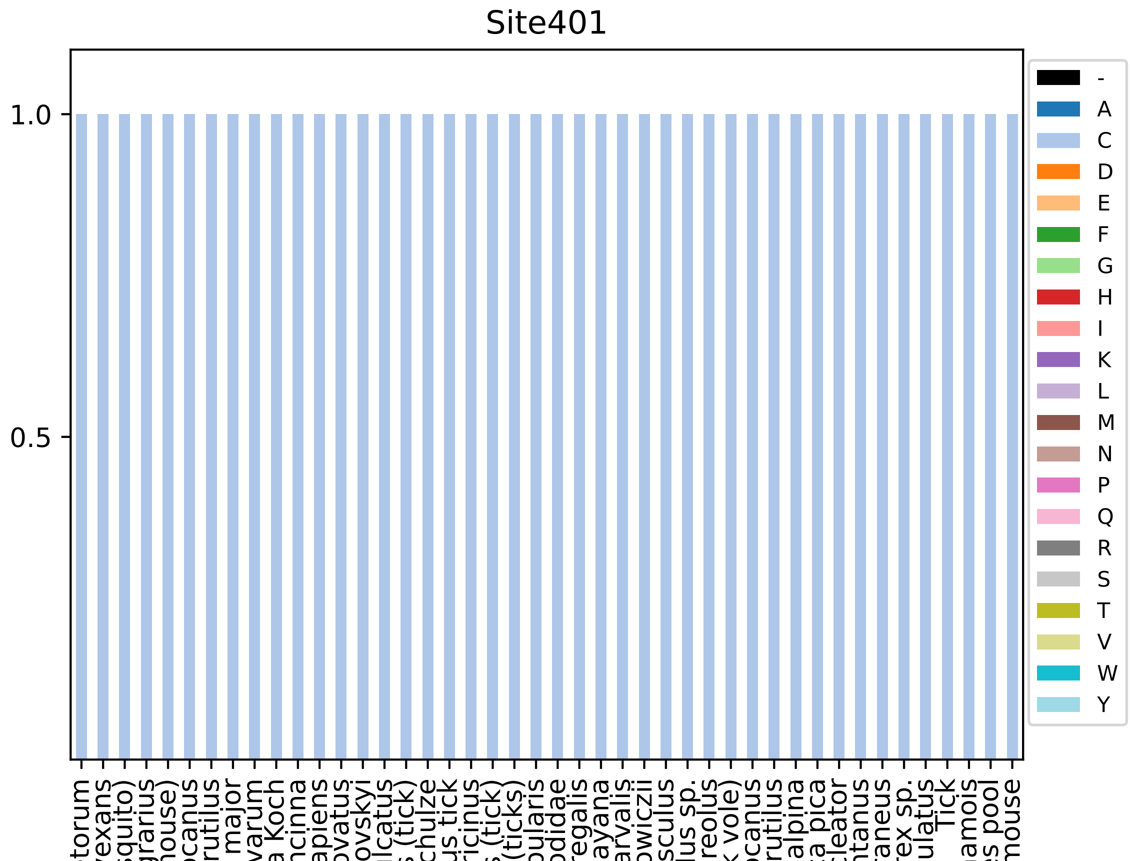
Site401
1.0
0.5
-
A
C
D
E
F
G
H
I
K
L
M
N
P
Q
R
S
T
V
W
Y

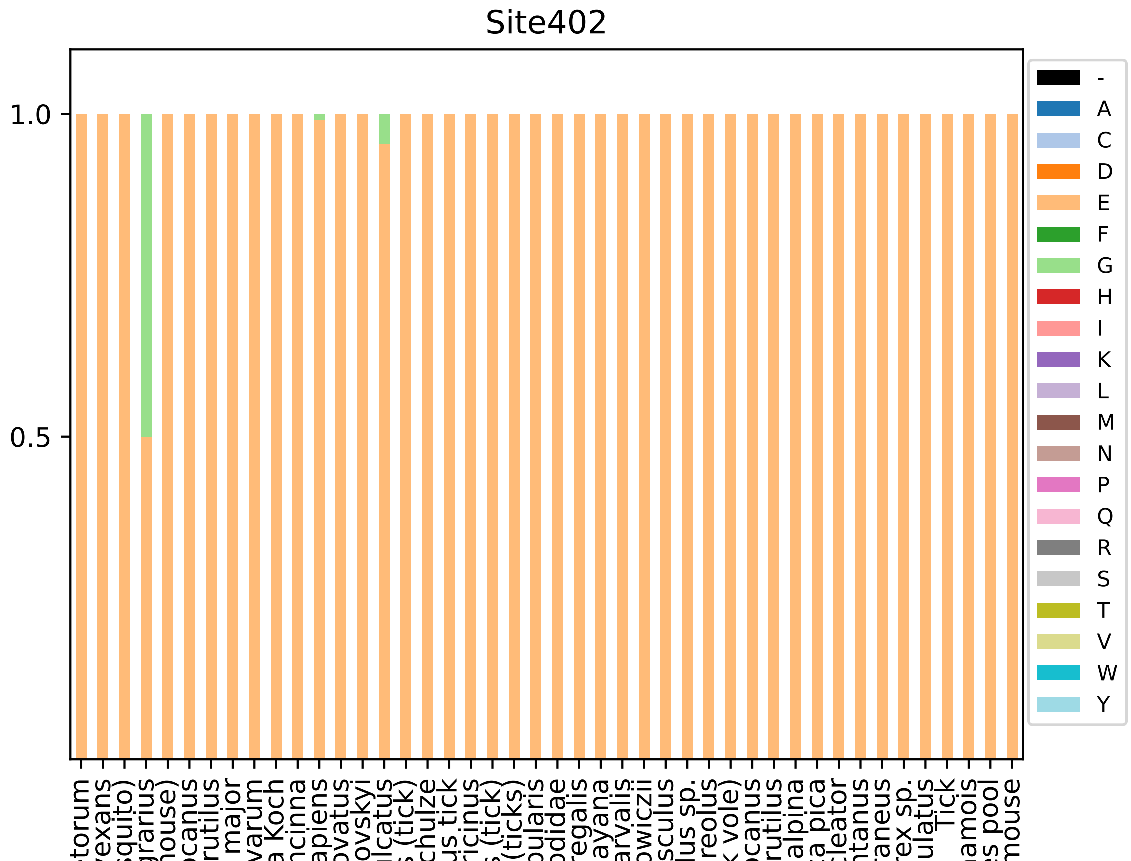

Site402
1.0
0.5
-
A
C
D
E
F
G
H
I
K
L
M
N
P
Q
R
S
T
V
W
Y

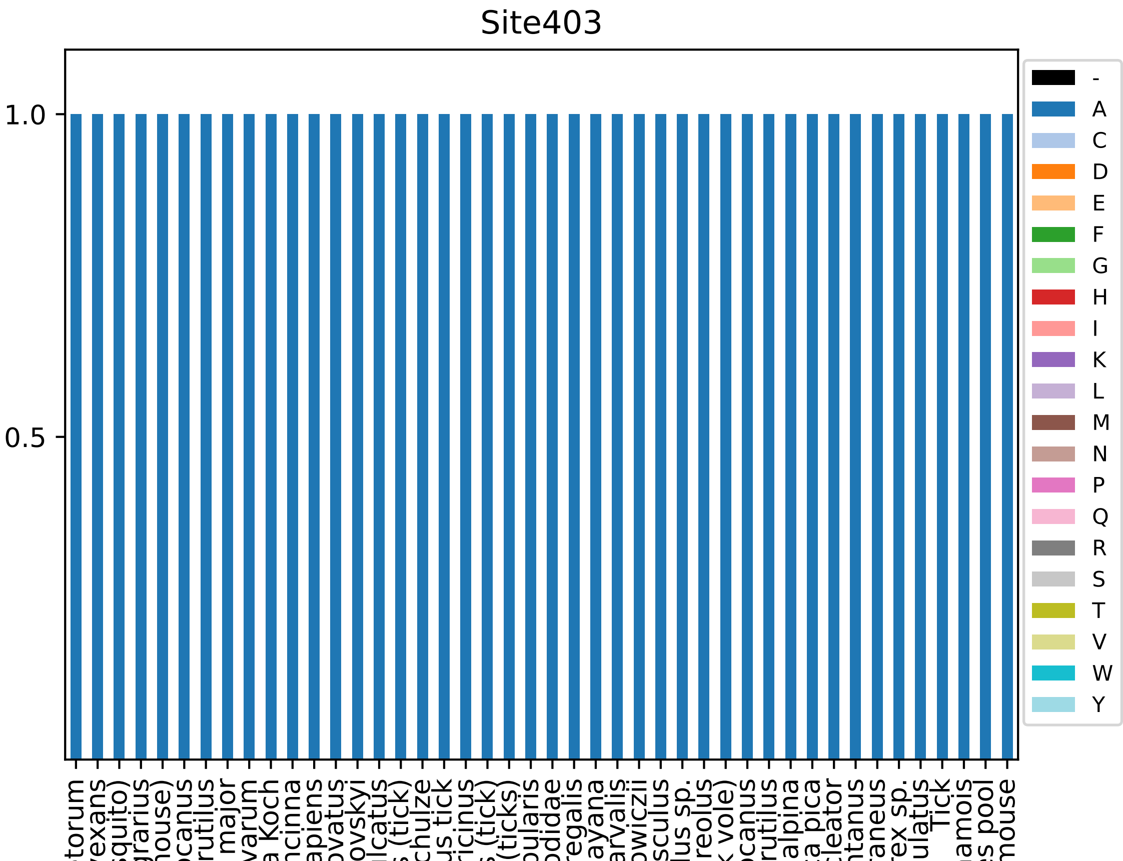
Site403
1.0
0.5
-
A
C
D
E
F
G
H
I
K
L
M
N
P
Q
R
S
T
V
W
Y

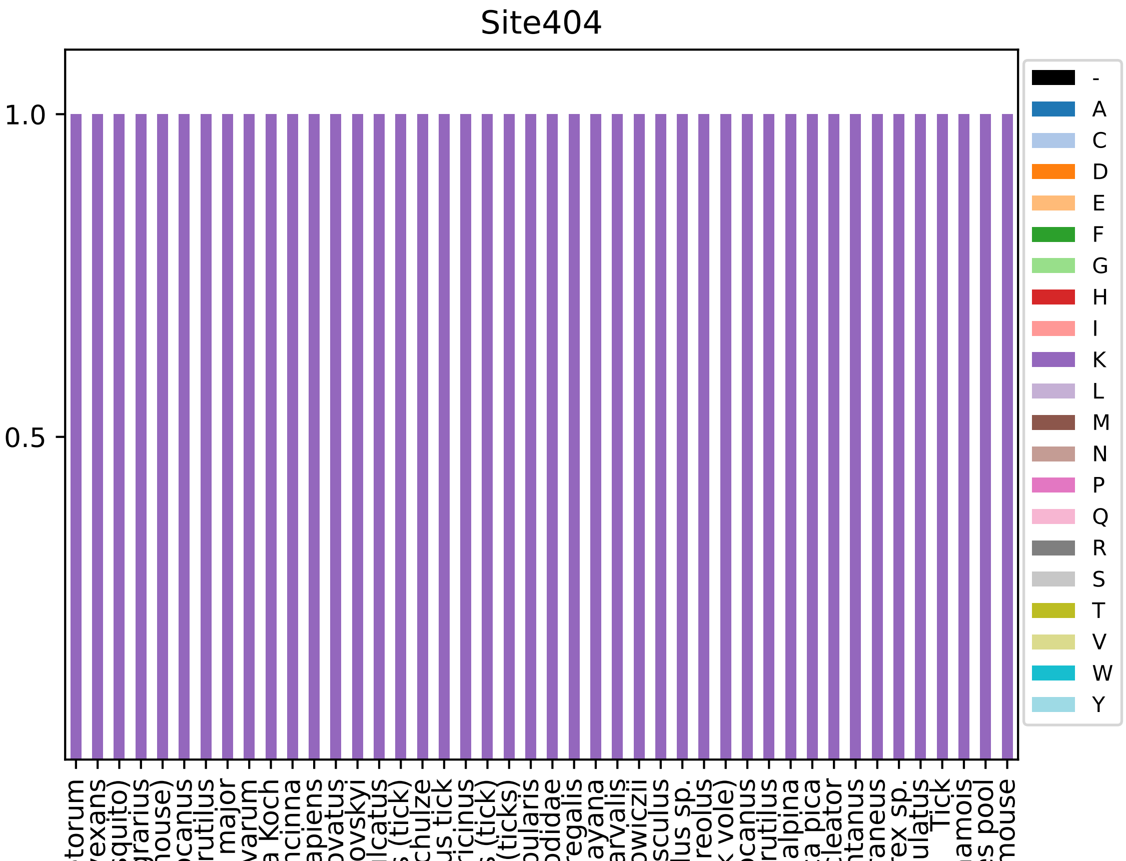
Site404
1.0
0.5
-
A
C
D
E
F
G
H
I
K
L
M
N
P
Q
R
S
T
V
W
Y
torum
vexans
squito)
garius
mouse)
canus
rutilus
major
varum
a Koch
ncinna
apiens
ovatus
ovskyi
lcatus
s (tick)
chulze
us tick
ricinus
s (ticks)
(ticks)
pularis
odidae
regalis
ayana
arvalis
owiczii
sculus
lus sp.
reolus
k vole)
canus
rutilus
alpina
a pica
leator
ntanus
raneus
rex sp.
ulatus
Tick
amois
es pool
mouse

Site405

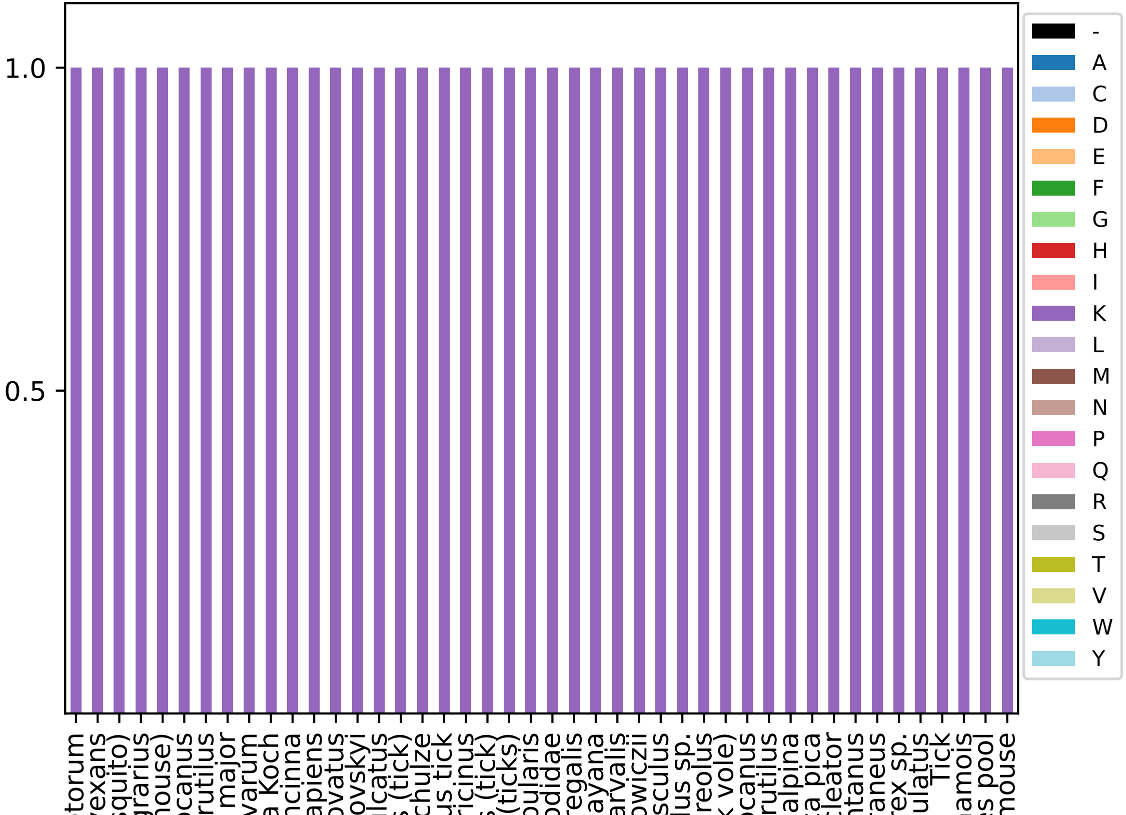

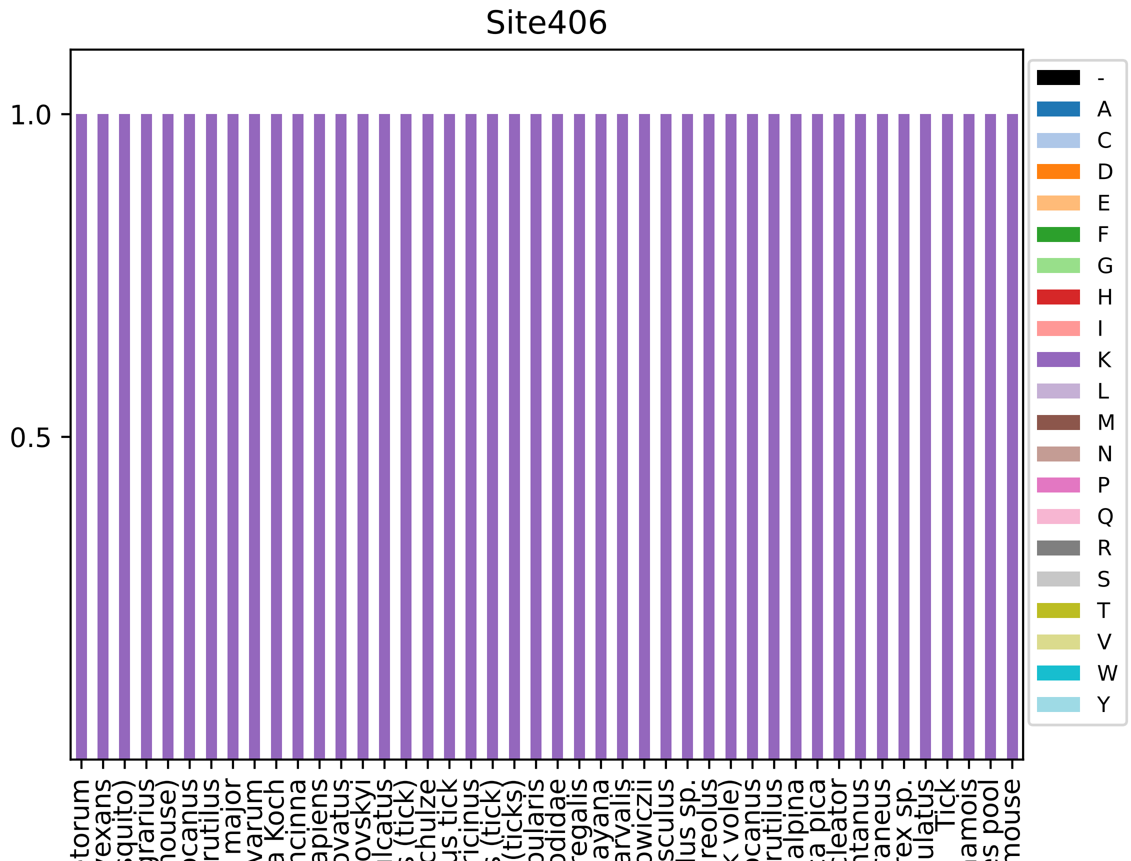

Site406
1.0
0.5
-
A
C
D
E
F
G
H
I
K
L
M
N
P
Q
R
S
T
V
W
Y

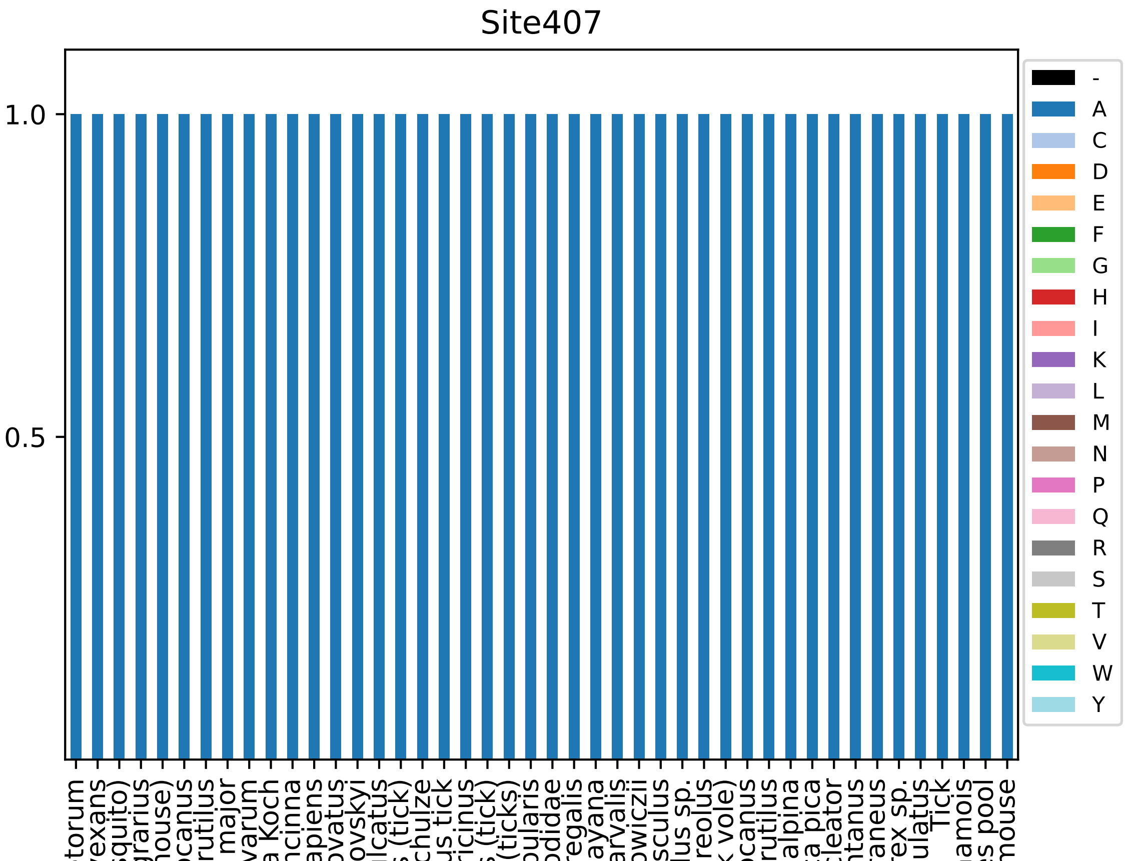
Site407
1.0
0.5
-
A
C
D
E
F
G
H
I
K
L
M
N
P
Q
R
S
T
V
W
Y

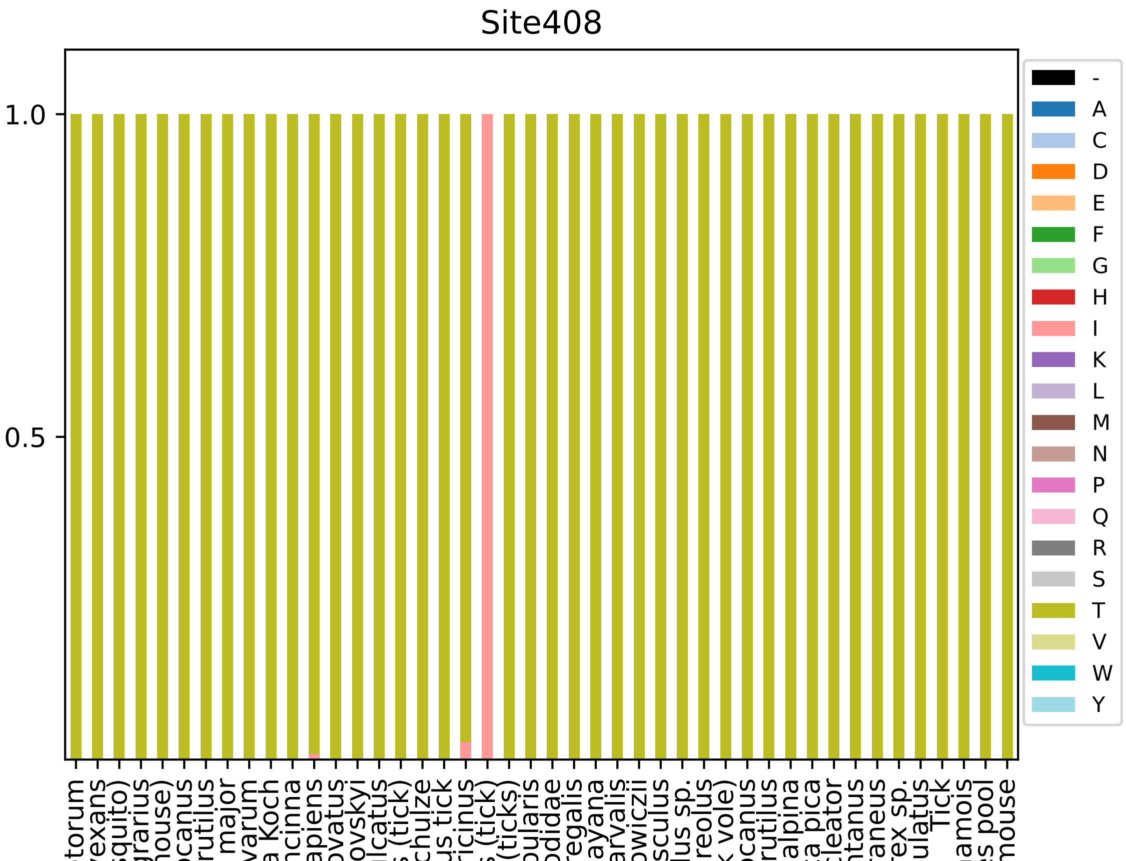
Site408
1.0
0.5
-
A
C
D
E
F
G
H
I
K
L
M
N
P
Q
R
S
T
V
W
Y

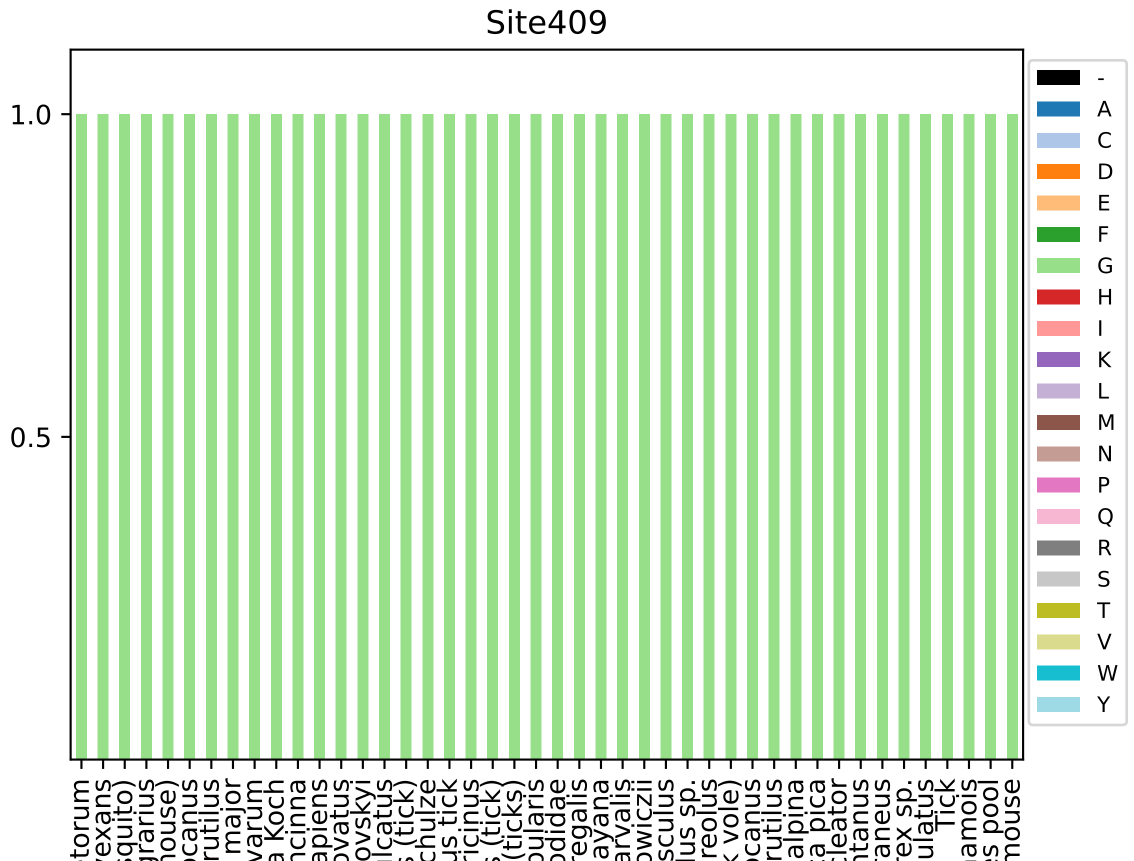
Site409
1.0
0.5
-
A
C
D
E
F
G
H
I
K
L
M
N
P
Q
R
S
T
V
W
Y

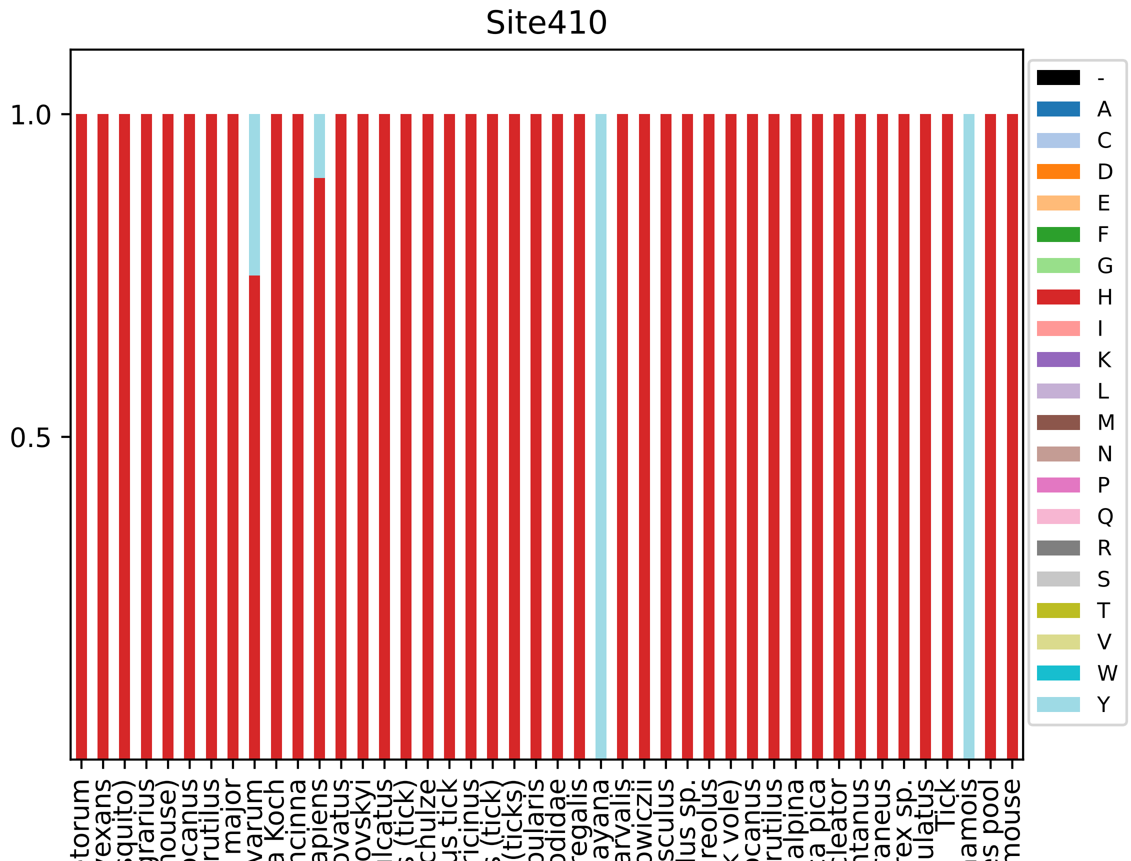
Site410
1.0
0.5
-
A
C
D
E
F
G
H
I
K
L
M
N
P
Q
R
S
T
V
W
Y

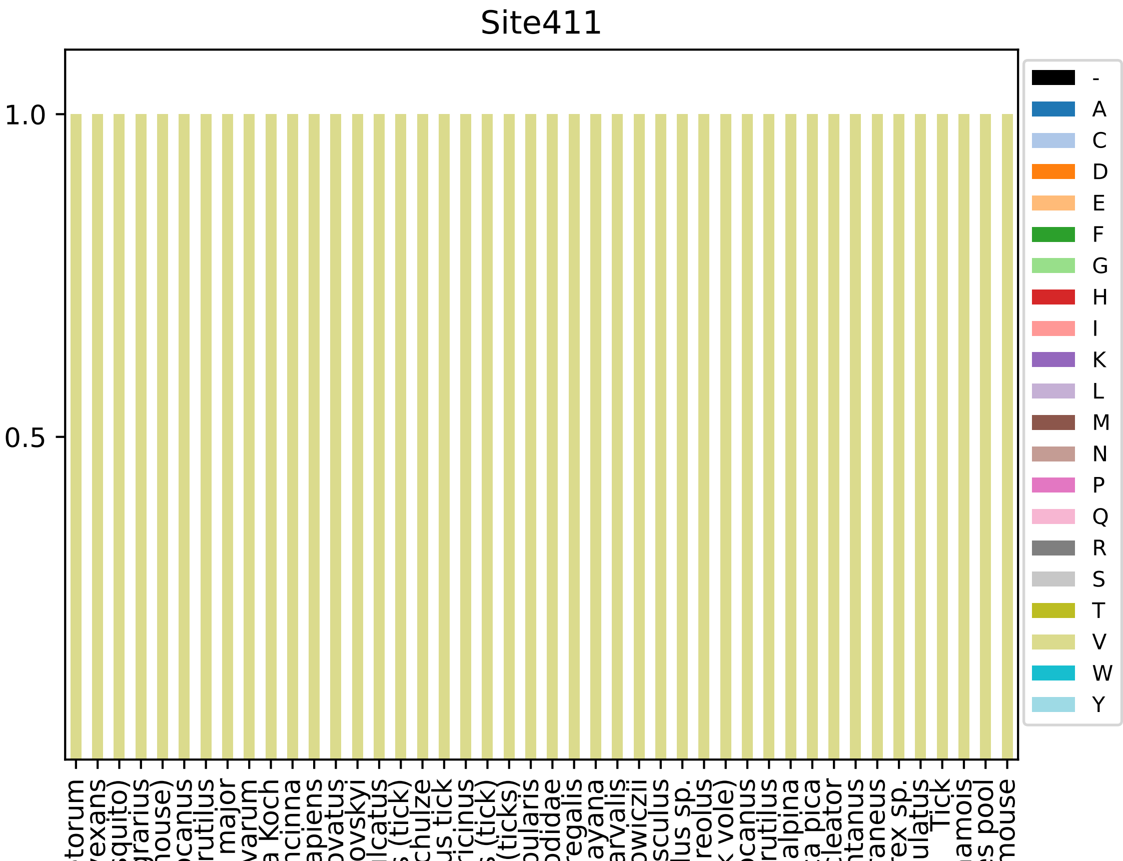
Site411
1.0
0.5
-
A
C
D
E
F
G
H
I
K
L
M
N
P
Q
R
S
T
V
W
Y
torum
vexans
squito)
grarius
nouse)
ocanus
rutilus
major
varum
a Koch
ncinna
apiens
ovatus
ovskyi
lcatus
s (tick)
chulze
us tick
ricinus
s (tick)
(ticks)
pularis
podidae
regalis
ayana
arvalis
owiczii
sculus
lus sp.
reolus
k vole)
ocanus
rutilus
alpina
a pica
cleator
ntanus
raneus
rex sp.
ulatus
Tick
amois
es pool
mouse

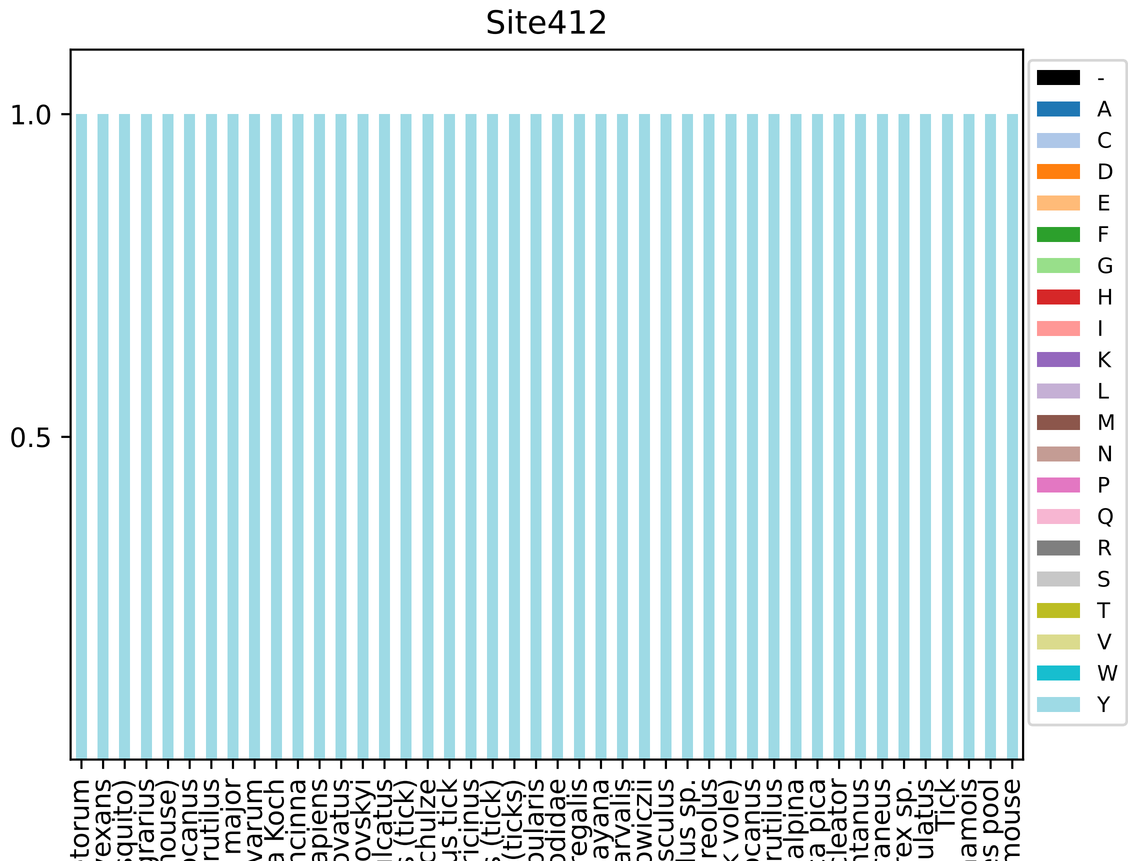

Site412
1.0
0.5
-
A
C
D
E
F
G
H
I
K
L
M
N
P
Q
R
S
T
V
W
Y

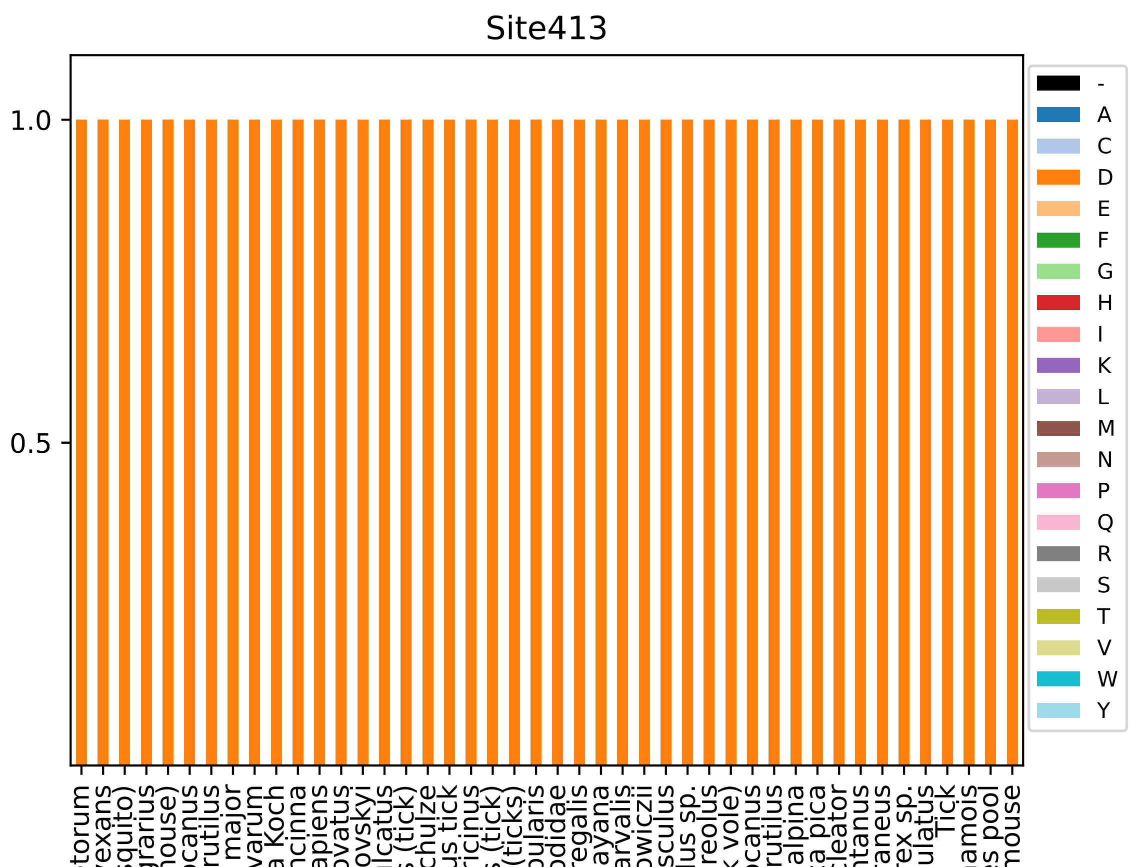
Site413
1.0
0.5
-
A
C
D
E
F
G
H
I
K
L
M
N
P
Q
R
S
T
V
W
Y

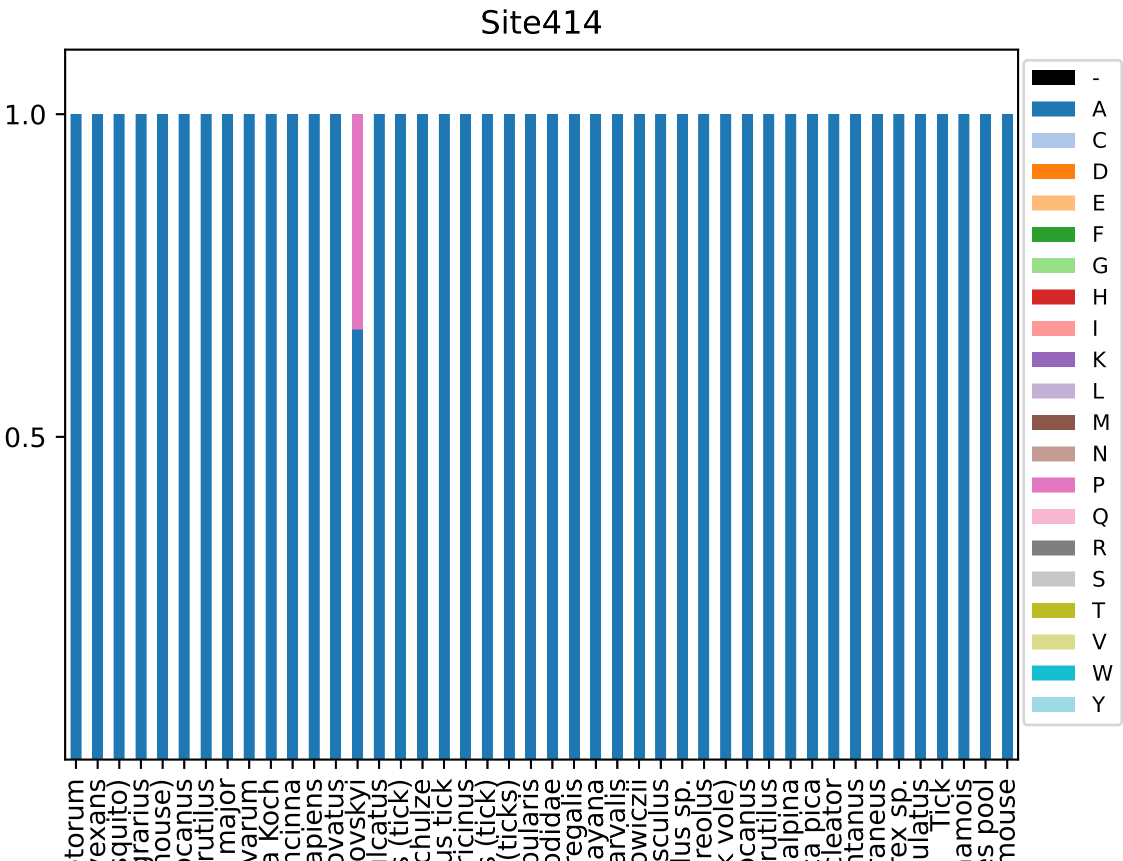

Site414
1.0
0.5
-
A
C
D
E
F
G
H
I
K
L
M
N
P
Q
R
S
T
V
W
Y

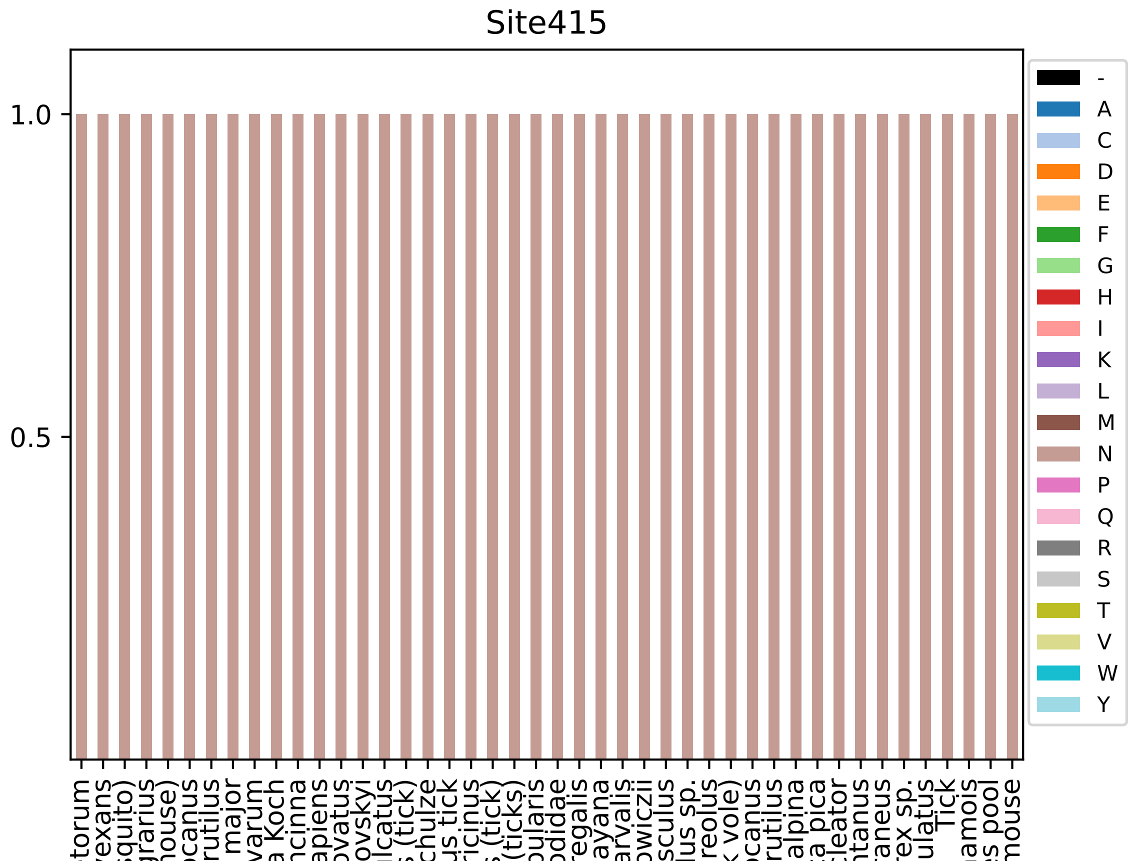
Site415
1.0
0.5
-
A
C
D
E
F
G
H
I
K
L
M
N
P
Q
R
S
T
V
W
Y

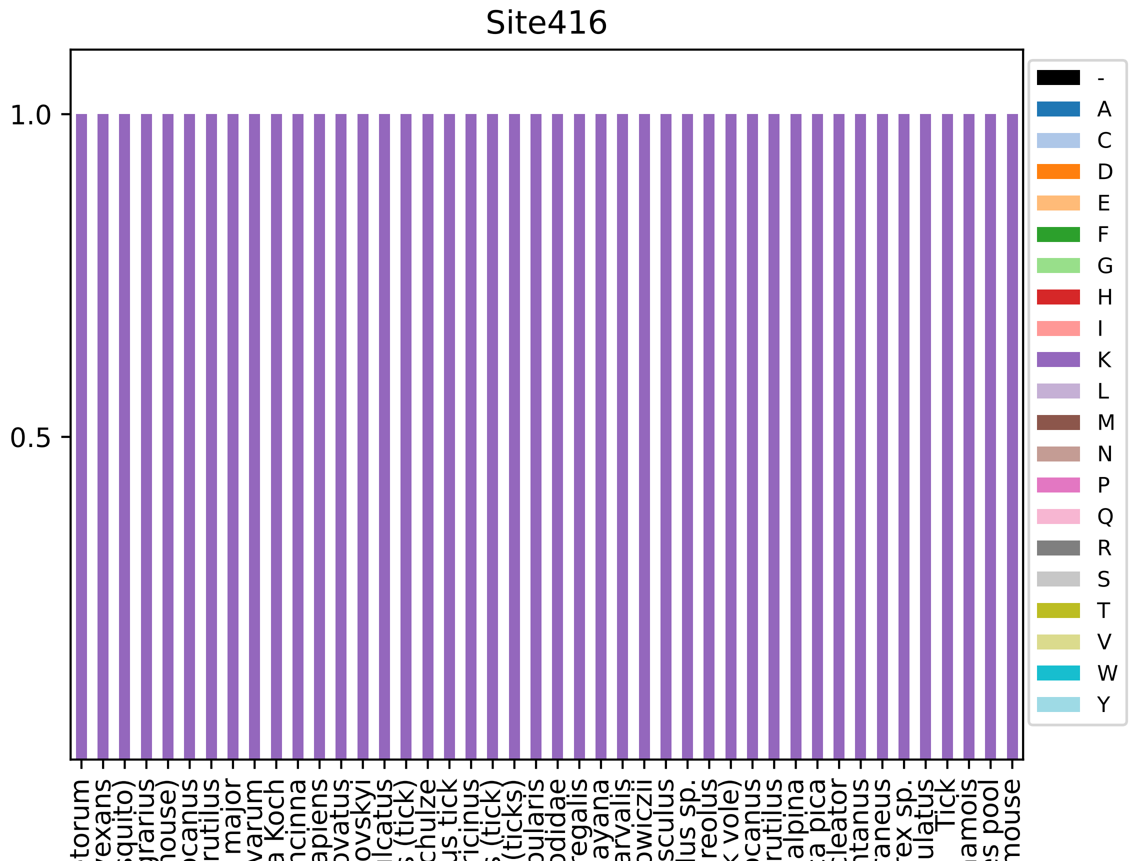
Site416
1.0
0.5
-
A
C
D
E
F
G
H
I
K
L
M
N
P
Q
R
S
T
V
W
Y
Tick

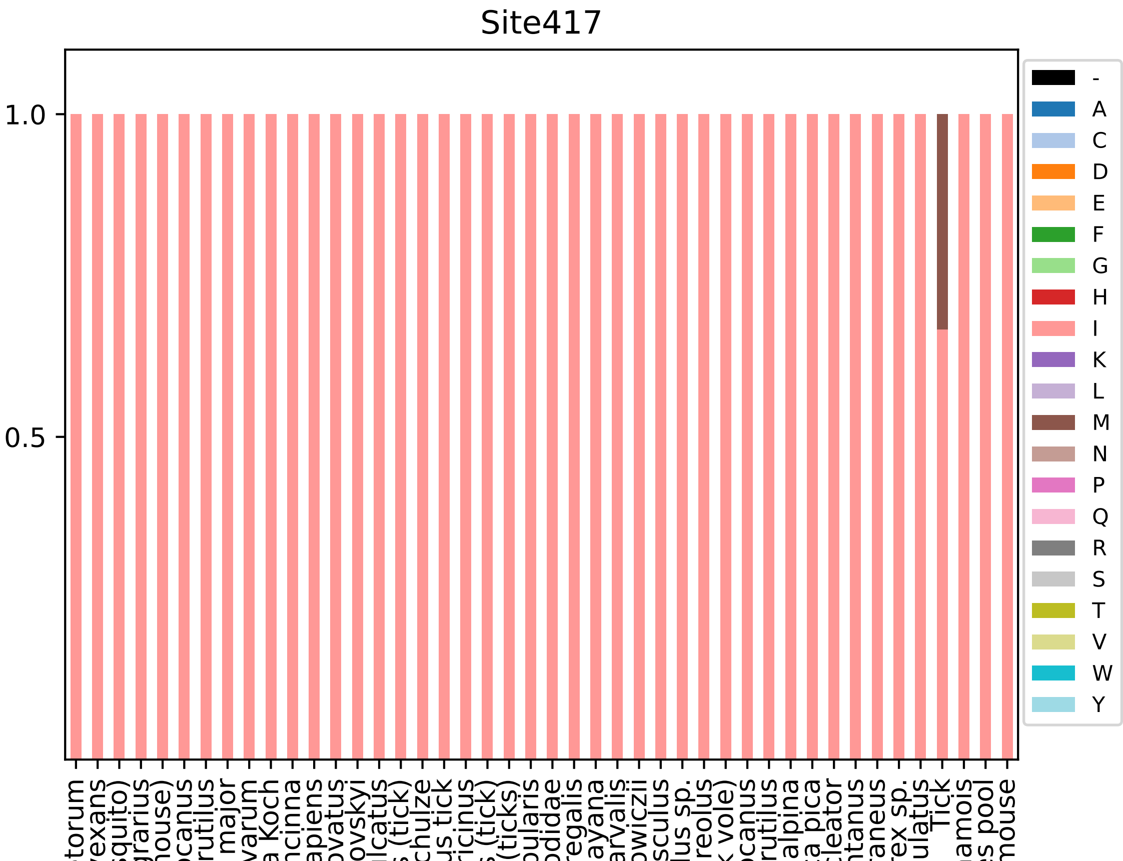
Site417
1.0
0.5
-
A
C
D
E
F
G
H
I
K
L
M
N
P
Q
R
S
T
V
W
Y
Tick

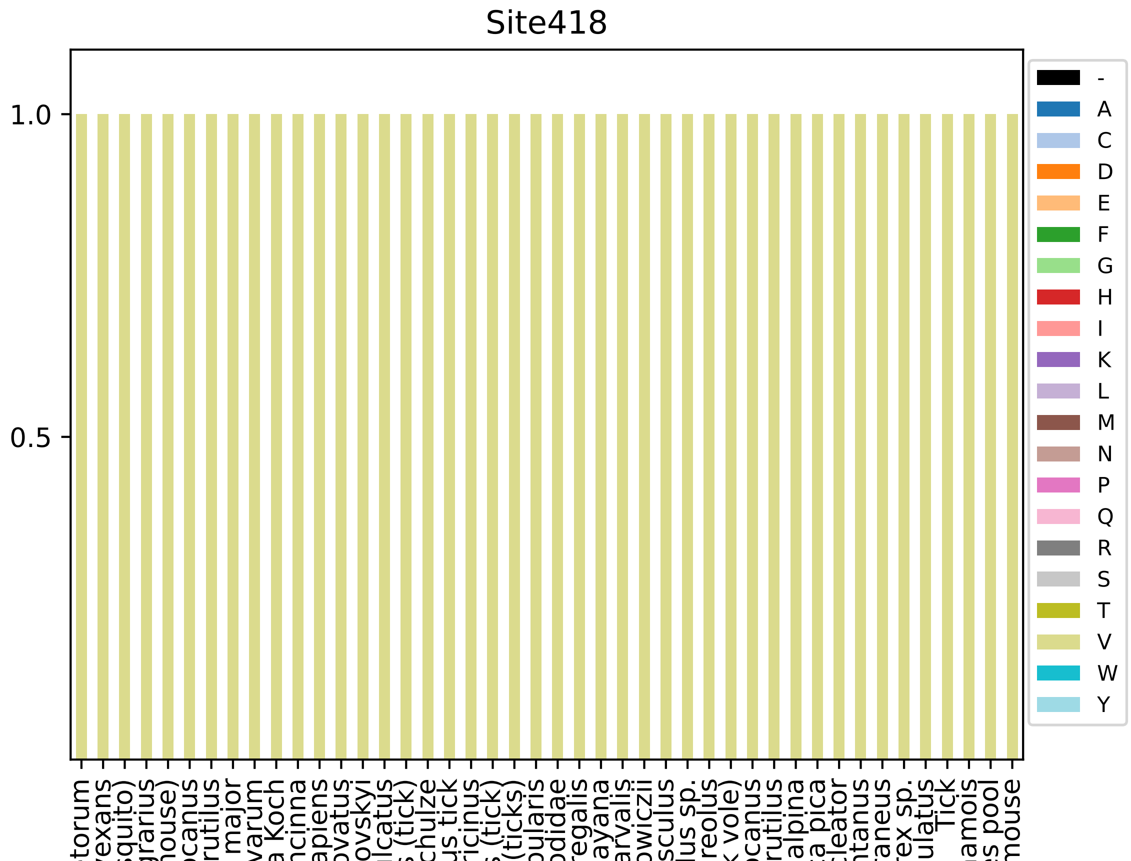

Site418
1.0
0.5
-
A
C
D
E
F
G
H
I
K
L
M
N
P
Q
R
S
T
V
W
Y

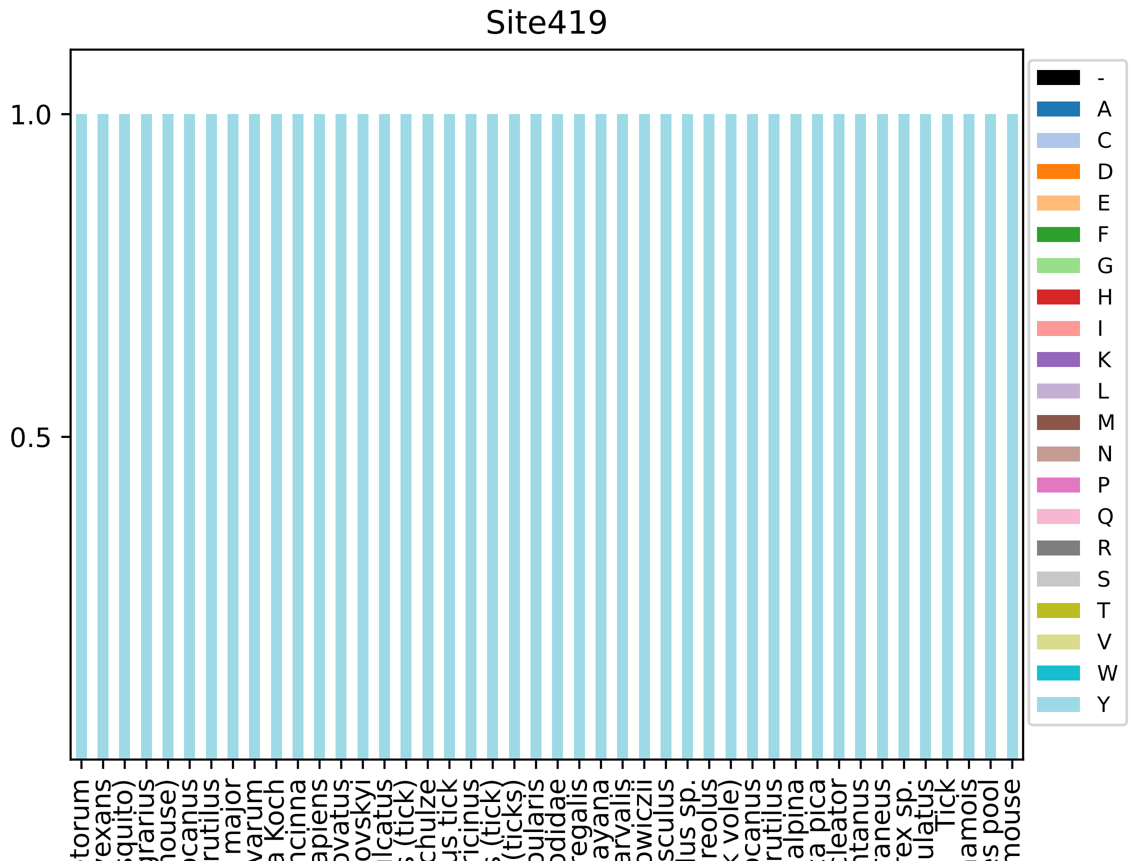
Site419
1.0
0.5
-
A
C
D
E
F
G
H
I
K
L
M
N
P
Q
R
S
T
V
W
Y

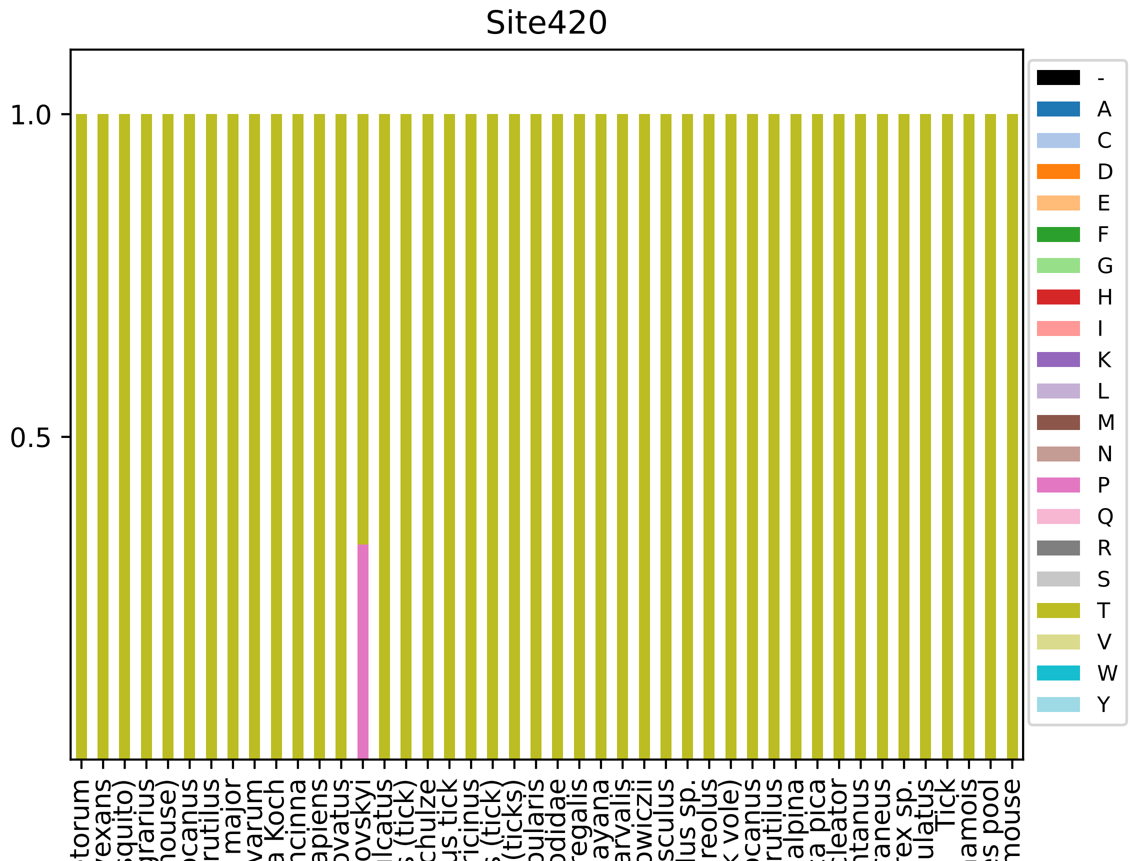
Site420
1.0
0.5
-
A
C
D
E
F
G
H
I
K
L
M
N
P
Q
R
S
T
V
W
Y

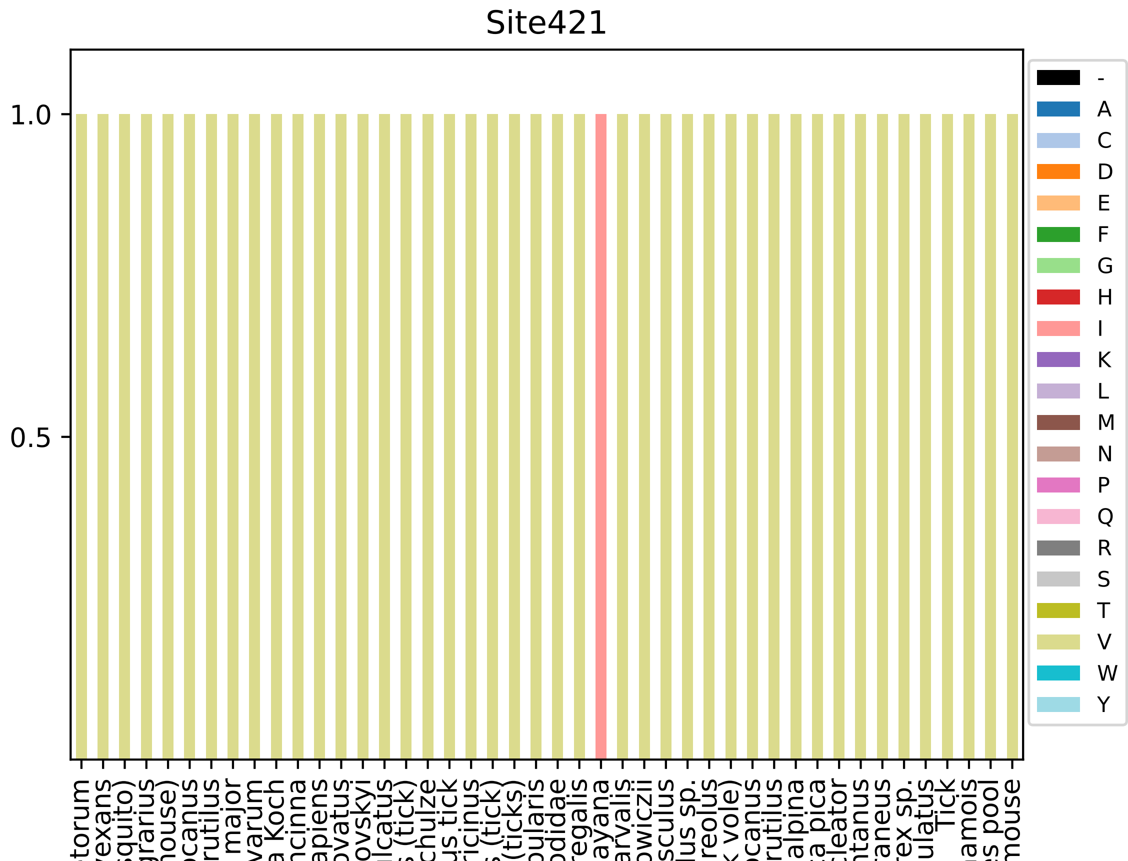

Site421
1.0
0.5
-
A
C
D
E
F
G
H
I
K
L
M
N
P
Q
R
S
T
V
W
Y

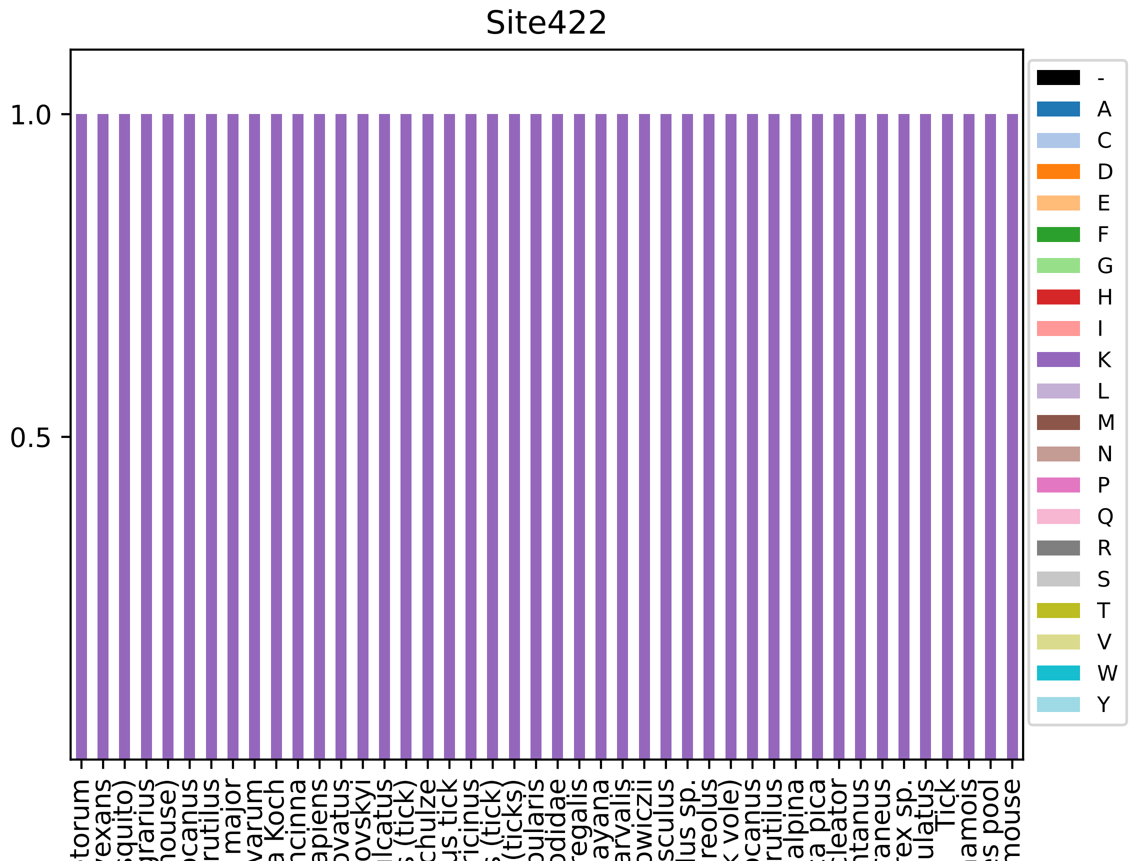
Site422
1.0
0.5
-
A
C
D
E
F
G
H
I
K
L
M
N
P
Q
R
S
T
V
W
Y

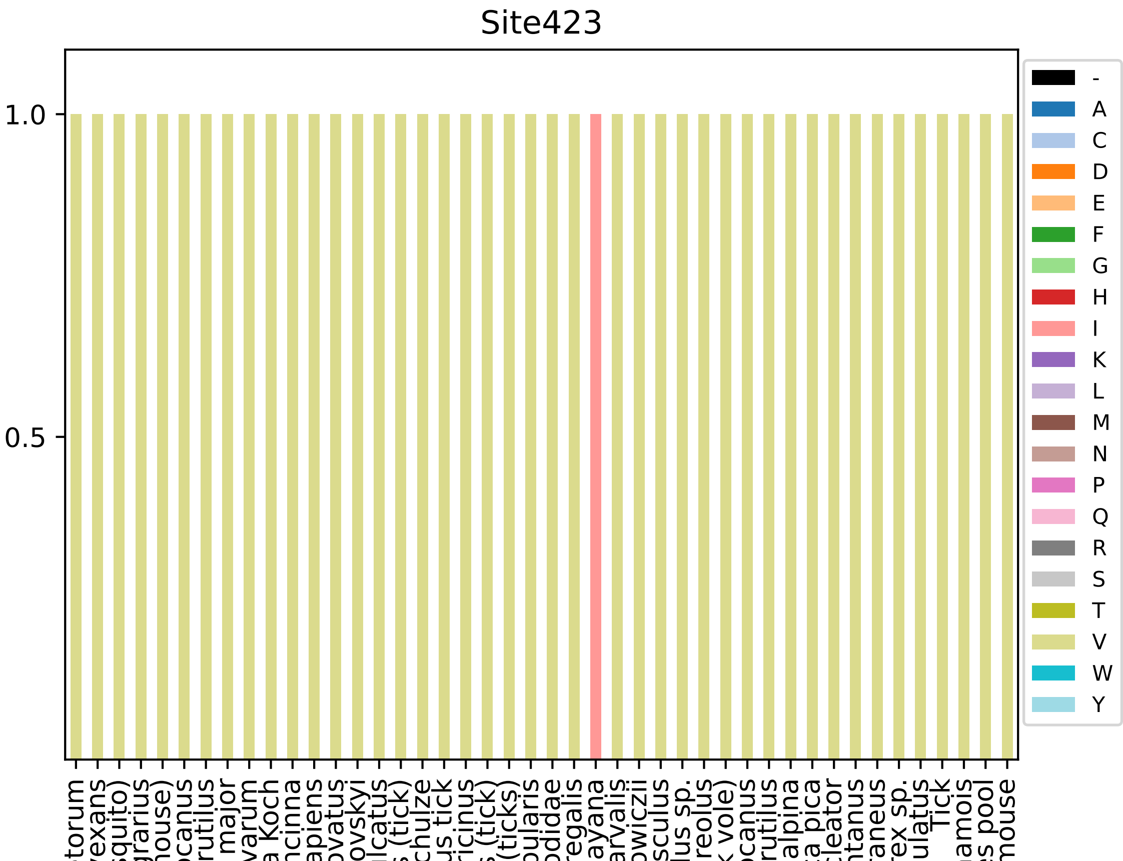
Site423
1.0
0.5
-
A
C
D
E
F
G
H
I
K
L
M
N
P
Q
R
S
T
V
W
Y

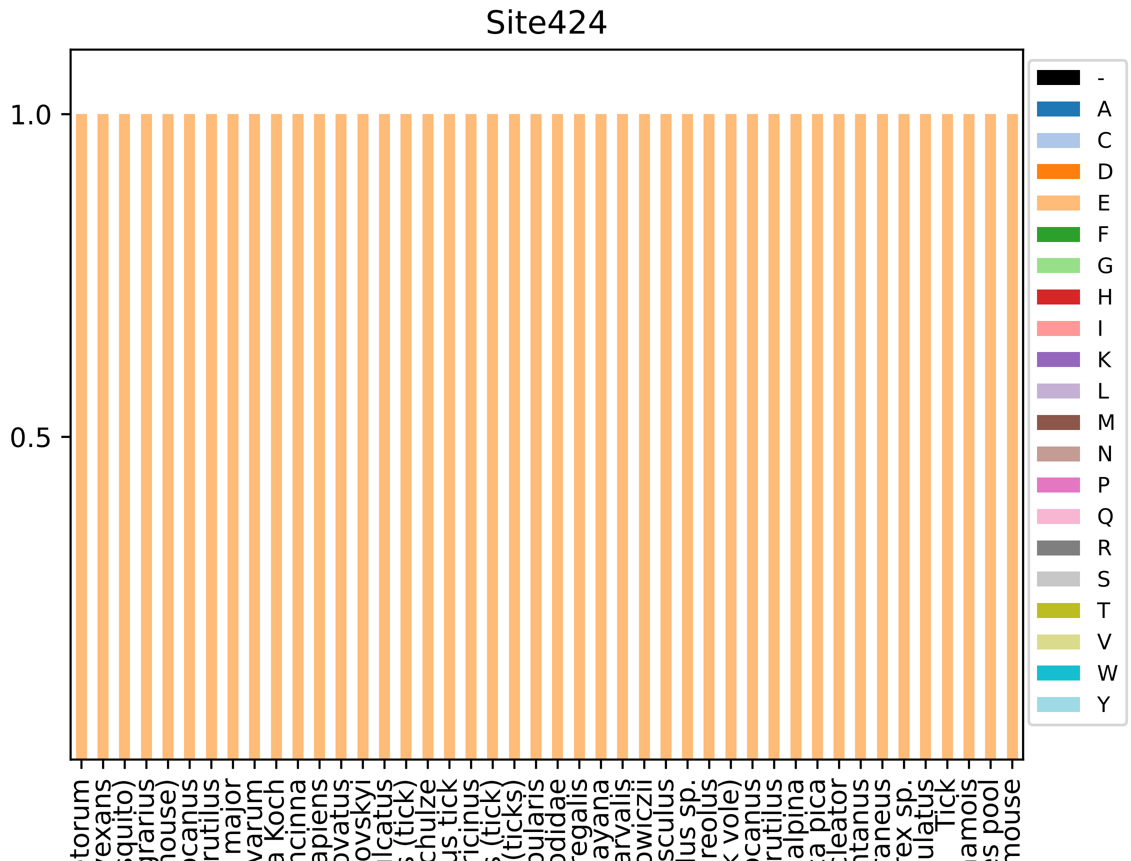
Site424
1.0
0.5
-
A
C
D
E
F
G
H
I
K
L
M
N
P
Q
R
S
T
V
W
Y

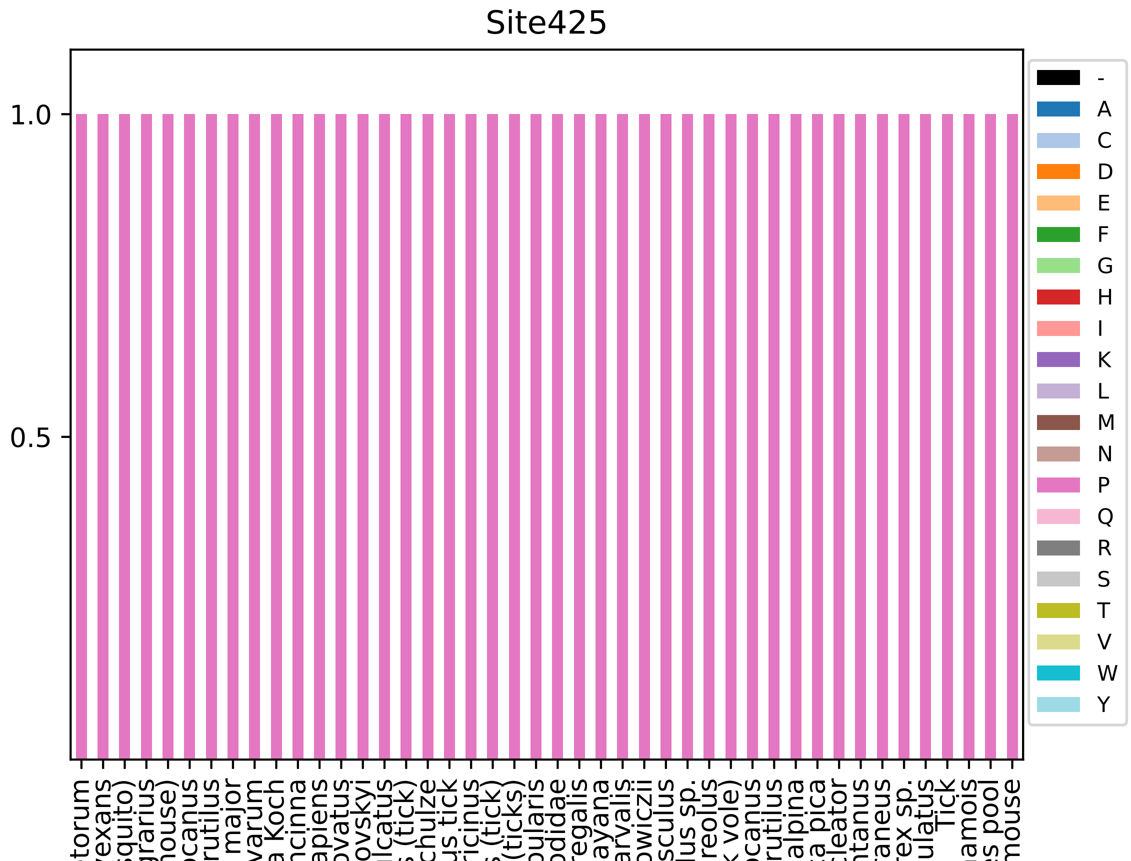

Site425
1.0
0.5
-
A
C
D
E
F
G
H
I
K
L
M
N
P
Q
R
S
T
V
W
Y

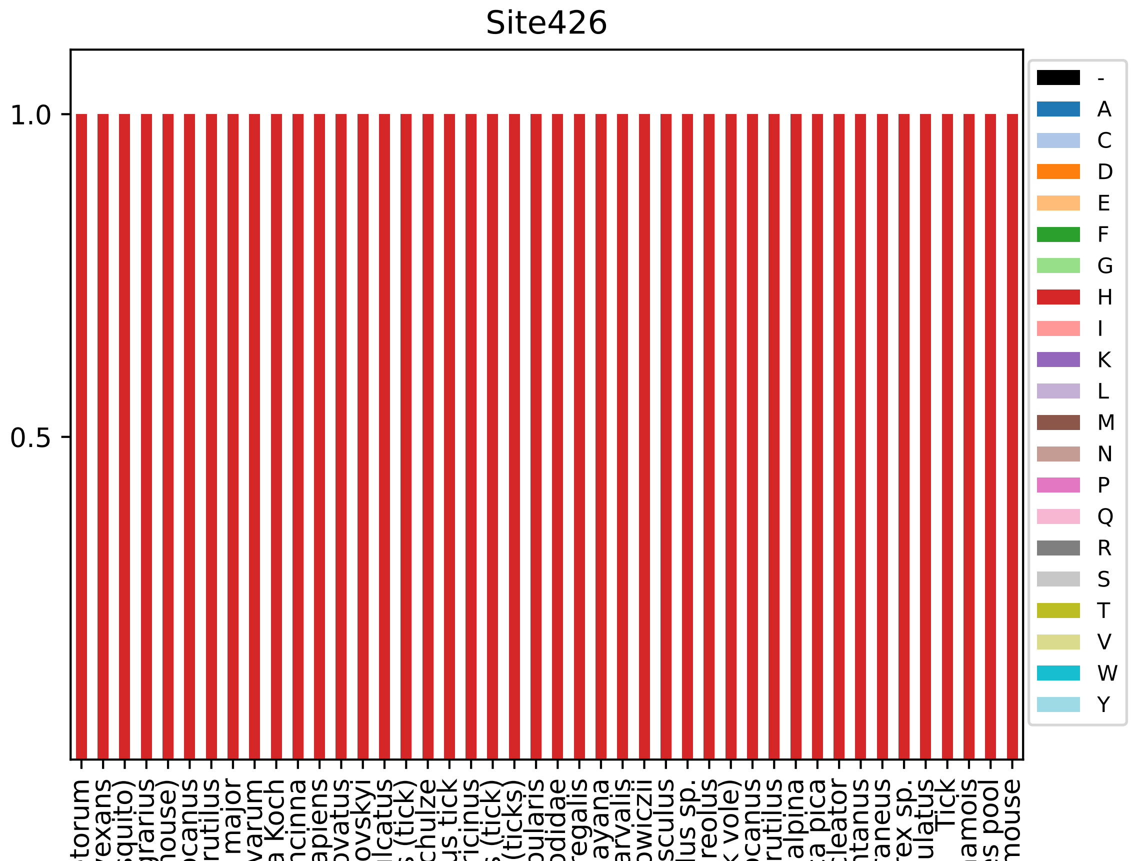
Site426
1.0
0.5
-
A
C
D
E
F
G
H
I
K
L
M
N
P
Q
R
S
T
V
W
Y

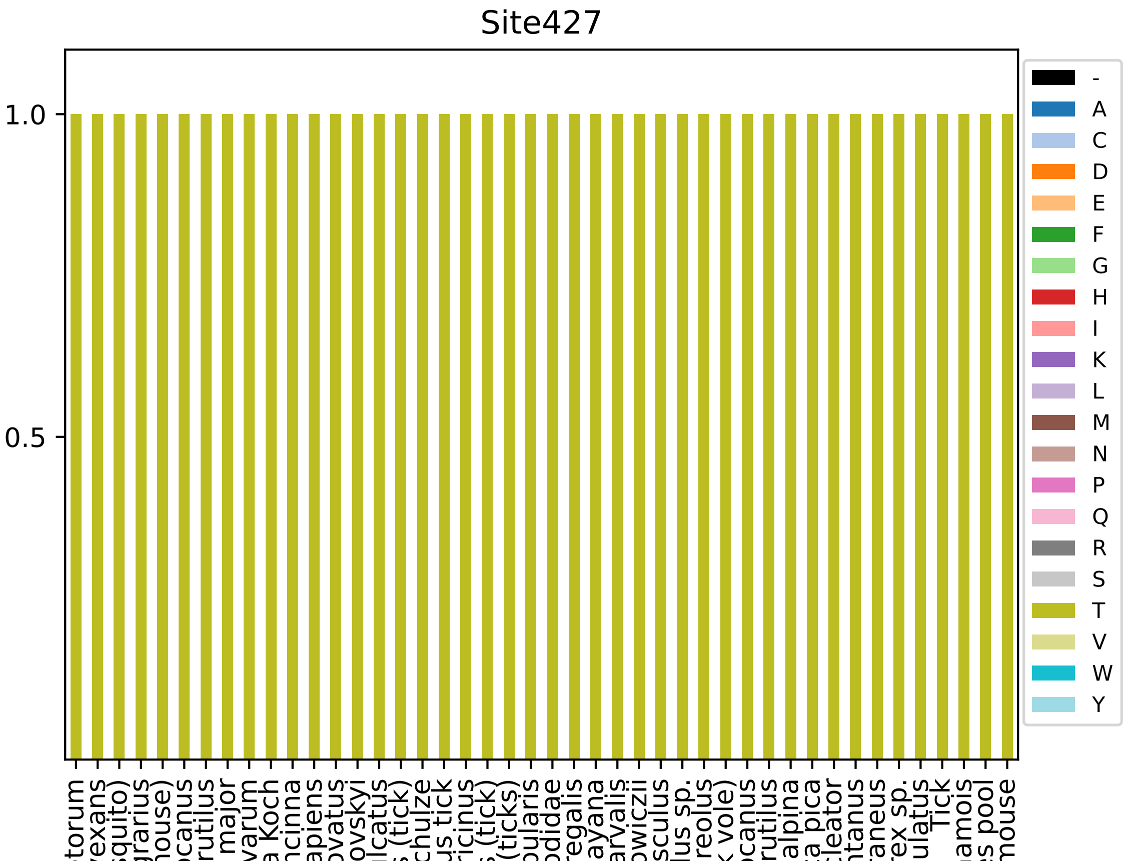
Site427
1.0
0.5
-
A
C
D
E
F
G
H
I
K
L
M
N
P
Q
R
S
T
V
W
Y

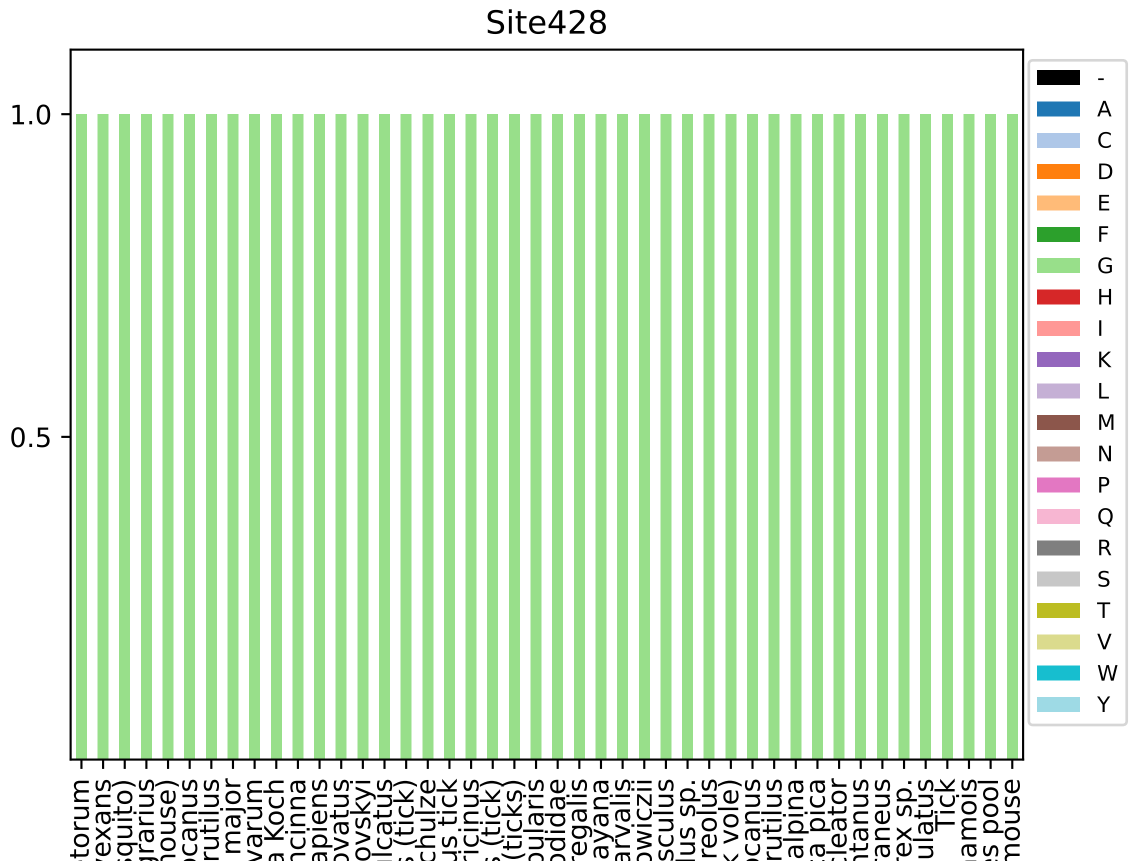
Site428
1.0
0.5
-
A
C
D
E
F
G
H
I
K
L
M
N
P
Q
R
S
T
V
W
Y

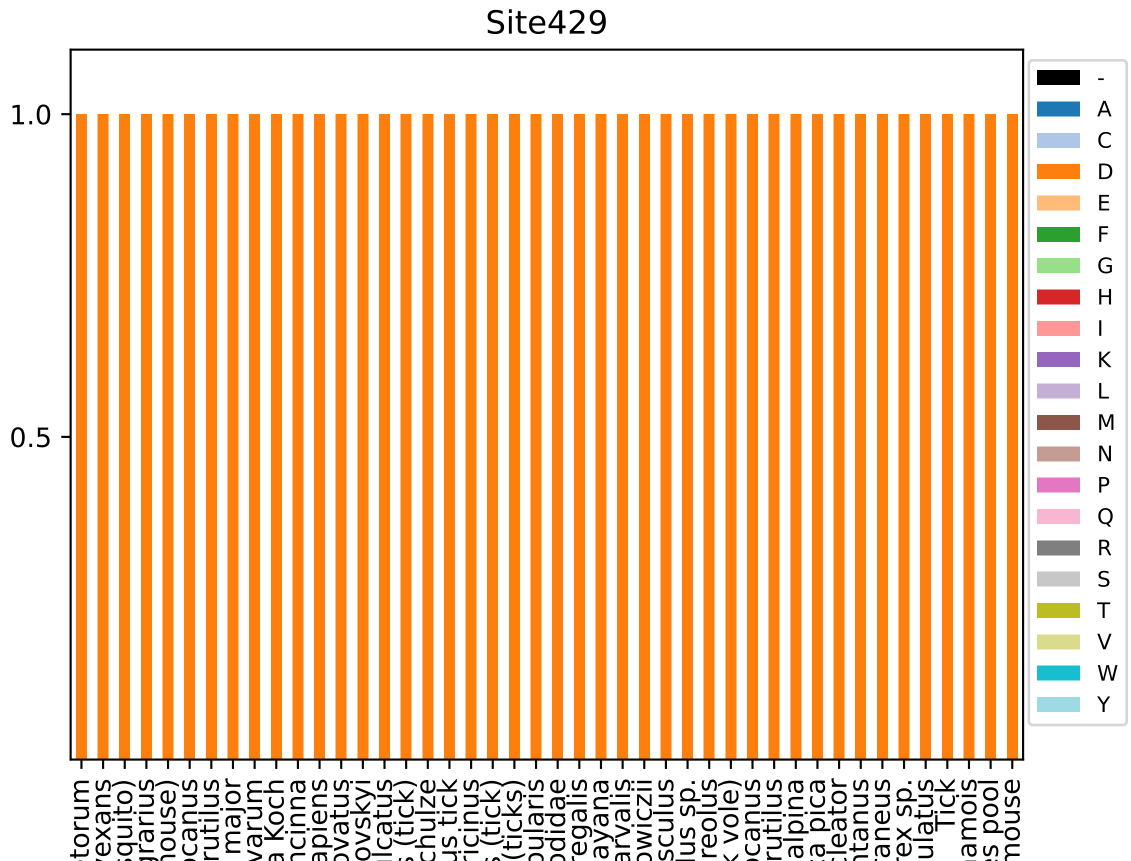
Site429
1.0
0.5
-
A
C
D
E
F
G
H
I
K
L
M
N
P
Q
R
S
T
V
W
Y

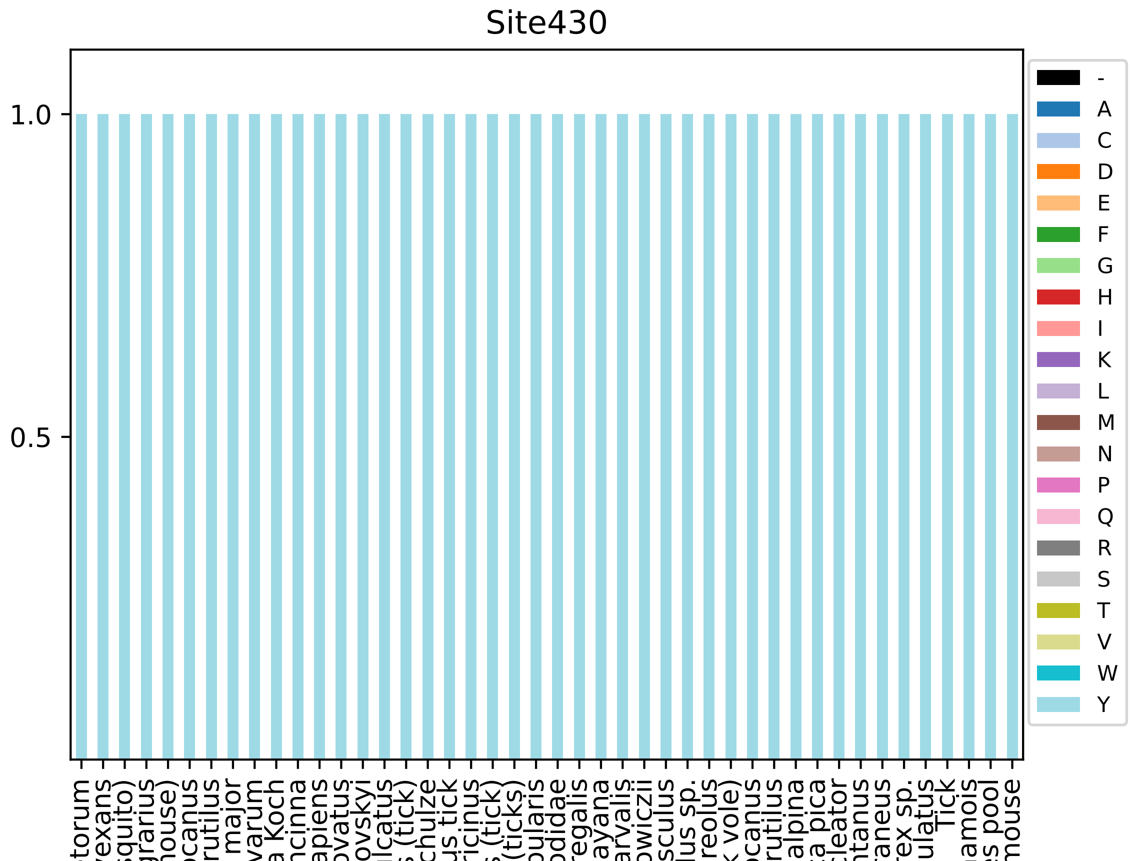
Site430
1.0
0.5
-
A
C
D
E
F
G
H
I
K
L
M
N
P
Q
R
S
T
V
W
Y

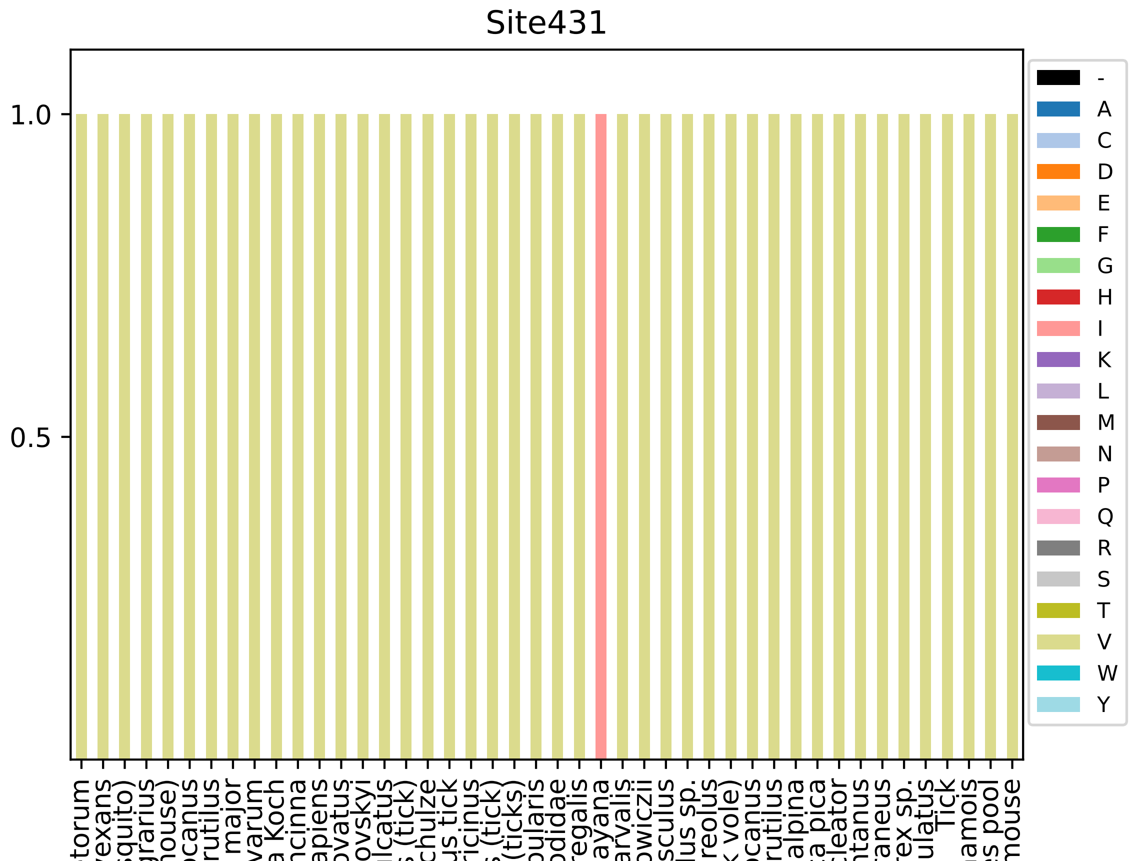
Site431
1.0
0.5
-
A
C
D
E
F
G
H
I
K
L
M
N
P
Q
R
S
T
V
W
Y

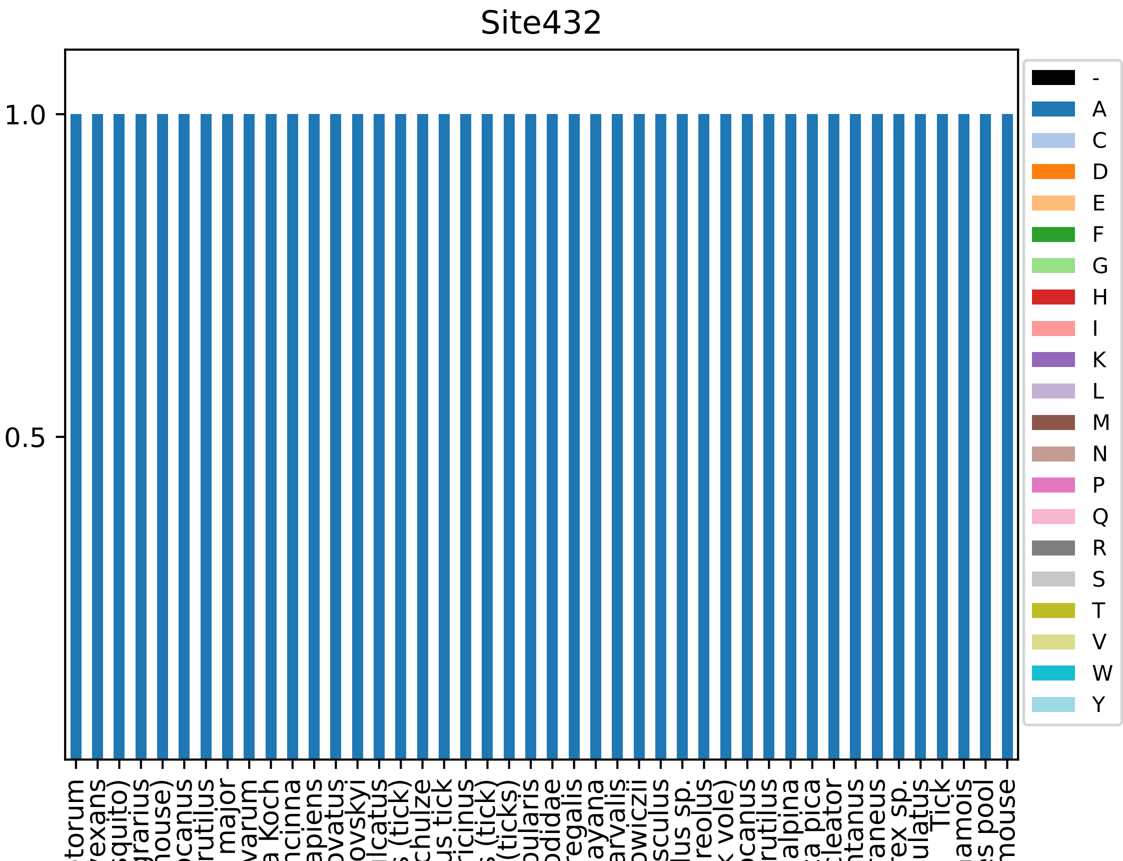
Site432
1.0
0.5
-
A
C
D
E
F
G
H
I
K
L
M
N
P
Q
R
S
T
V
W
Y

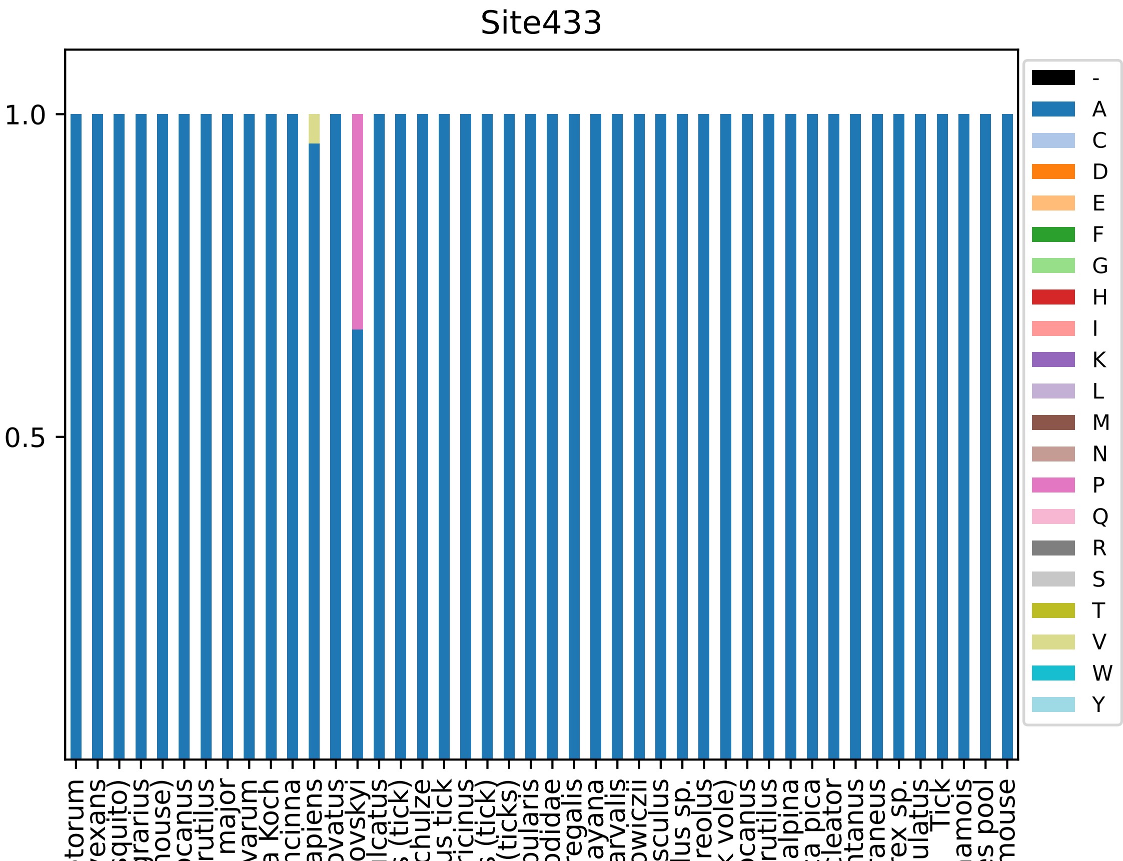
Site433
1.0
0.5
-
A
C
D
E
F
G
H
I
K
L
M
N
P
Q
R
S
T
V
W
Y

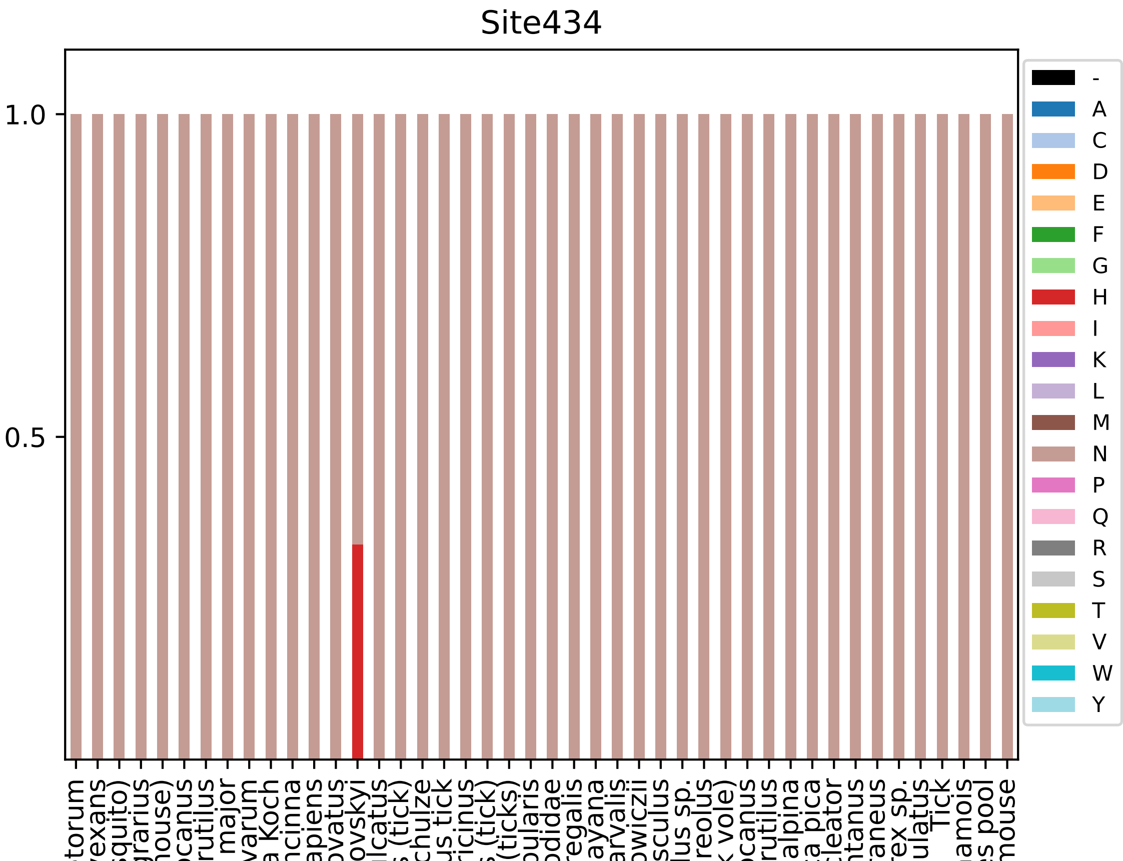

Site434
1.0
0.5
-
A
C
D
E
F
G
H
I
K
L
M
N
P
Q
R
S
T
V
W
Y

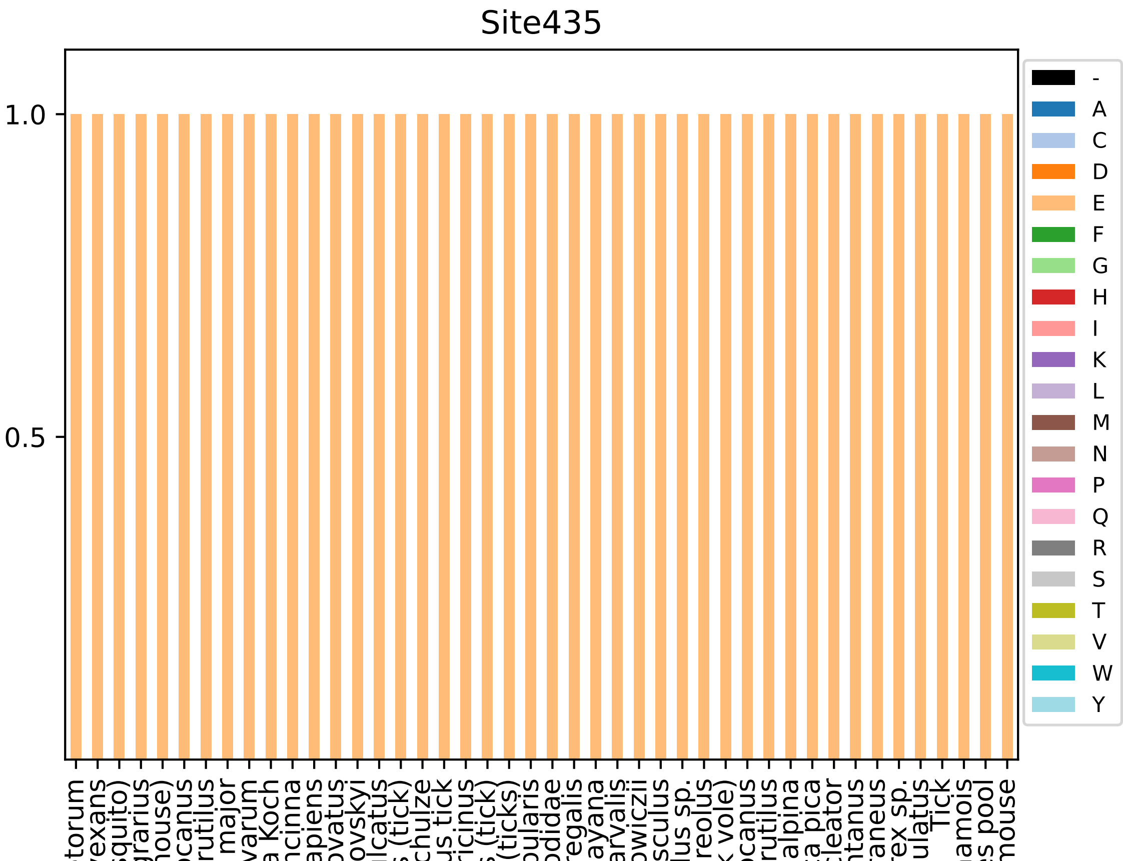
Site435
1.0
0.5
-
A
C
D
E
F
G
H
I
K
L
M
N
P
Q
R
S
T
V
W
Y

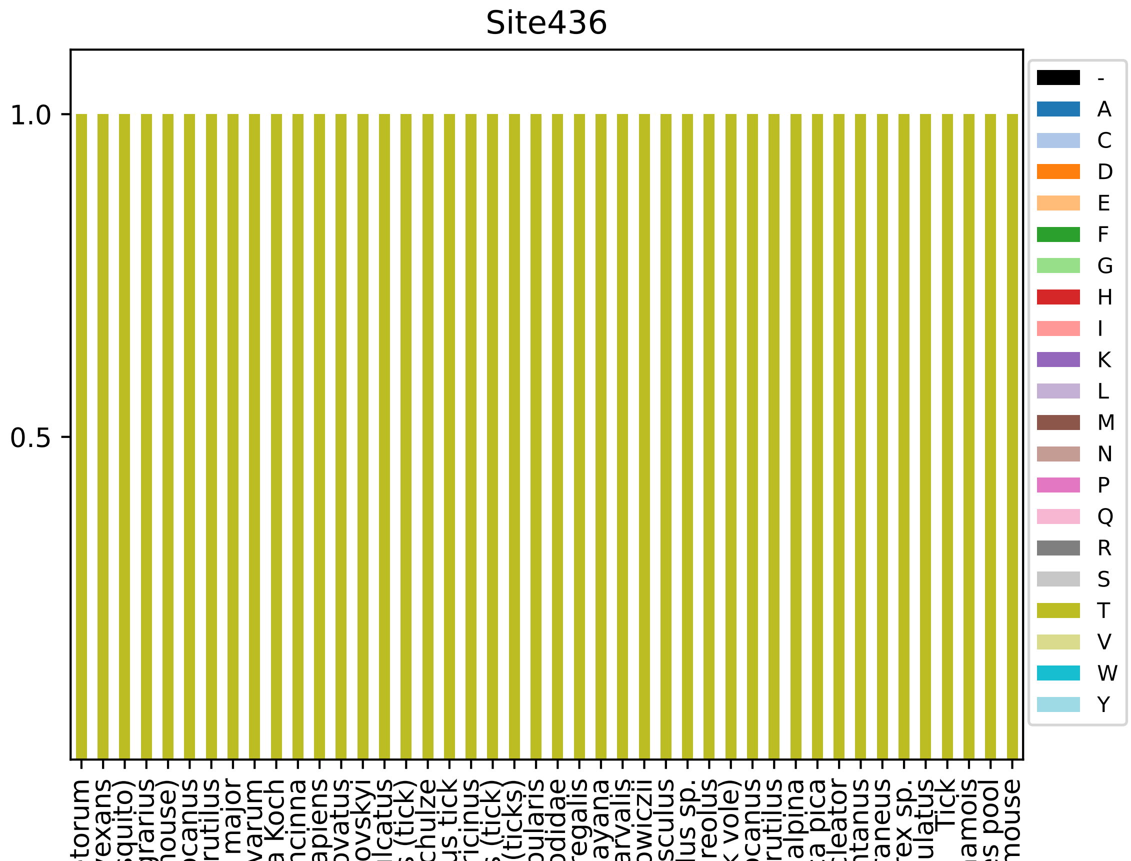

Site436
1.0
0.5
-
A
C
D
E
F
G
H
I
K
L
M
N
P
Q
R
S
T
V
W
Y
Tick

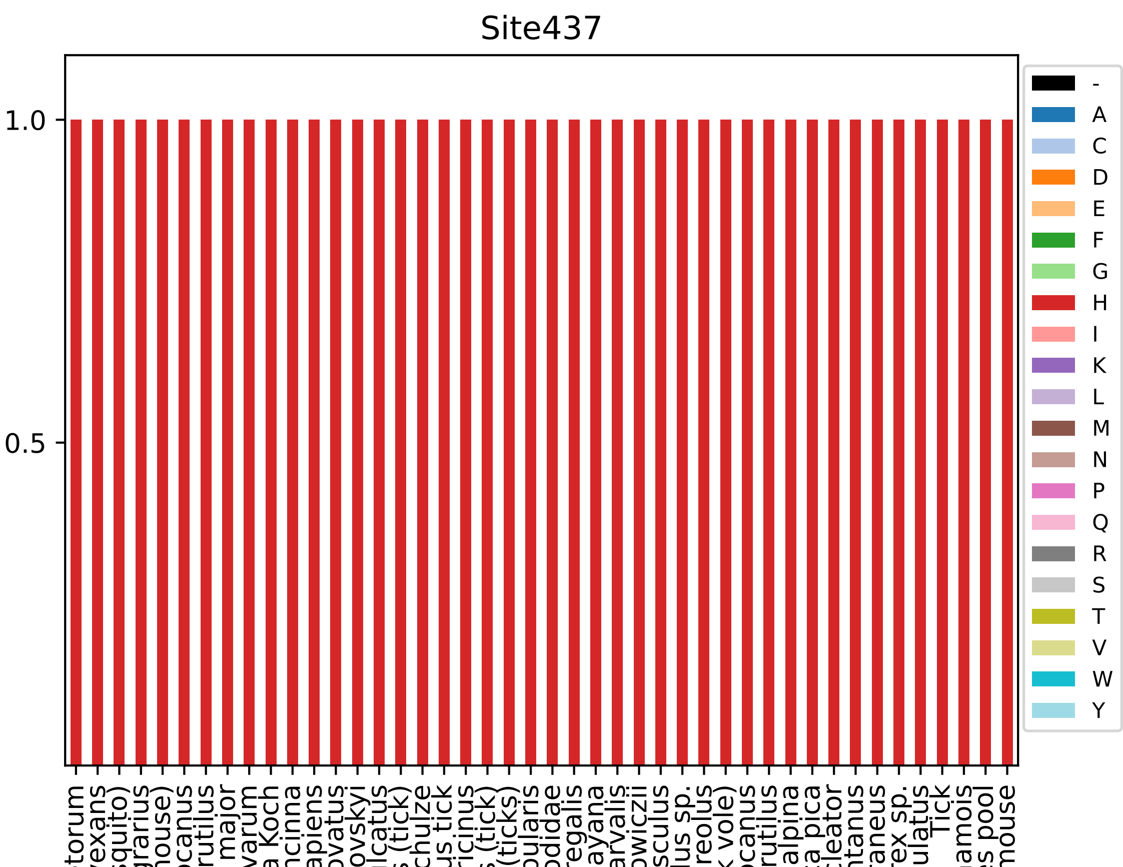
Site437
1.0
0.5
-
A
C
D
E
F
G
H
I
K
L
M
N
P
Q
R
S
T
V
W
Y

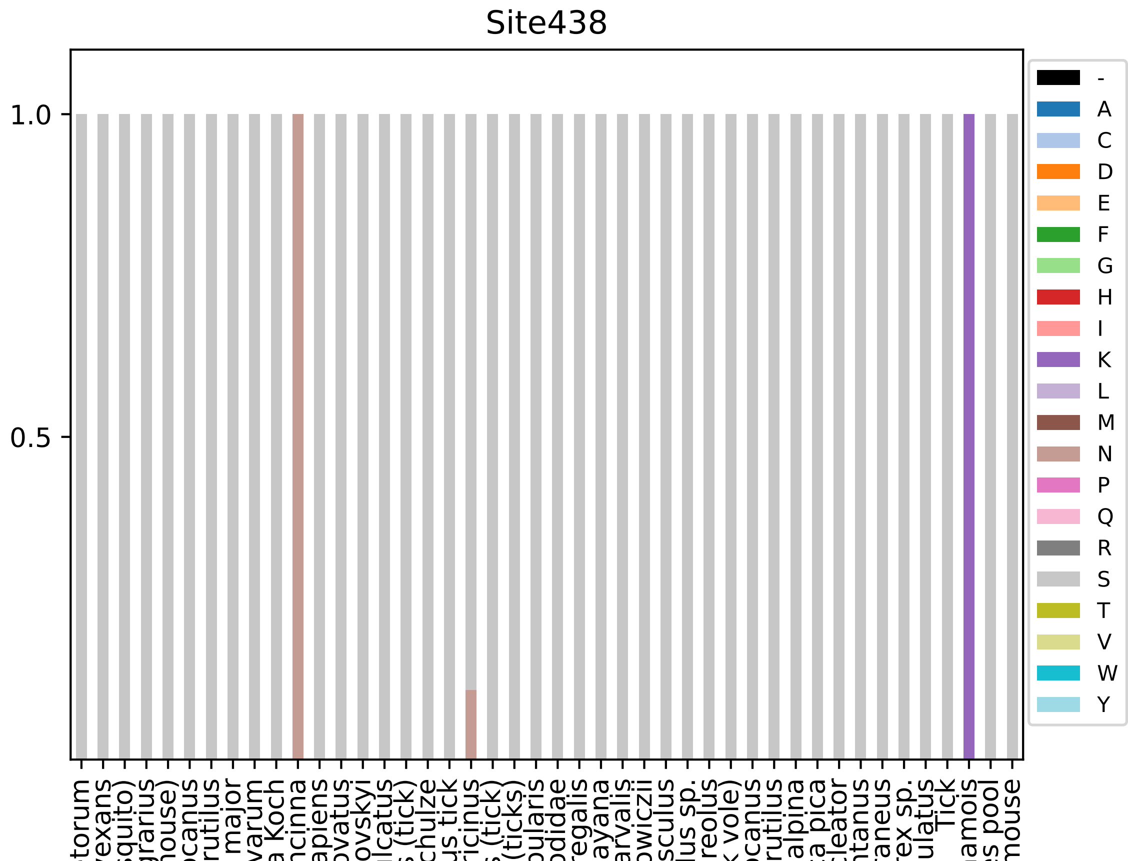

Site438
1.0
0.5
-
A
C
D
E
F
G
H
I
K
L
M
N
P
Q
R
S
T
V
W
Y

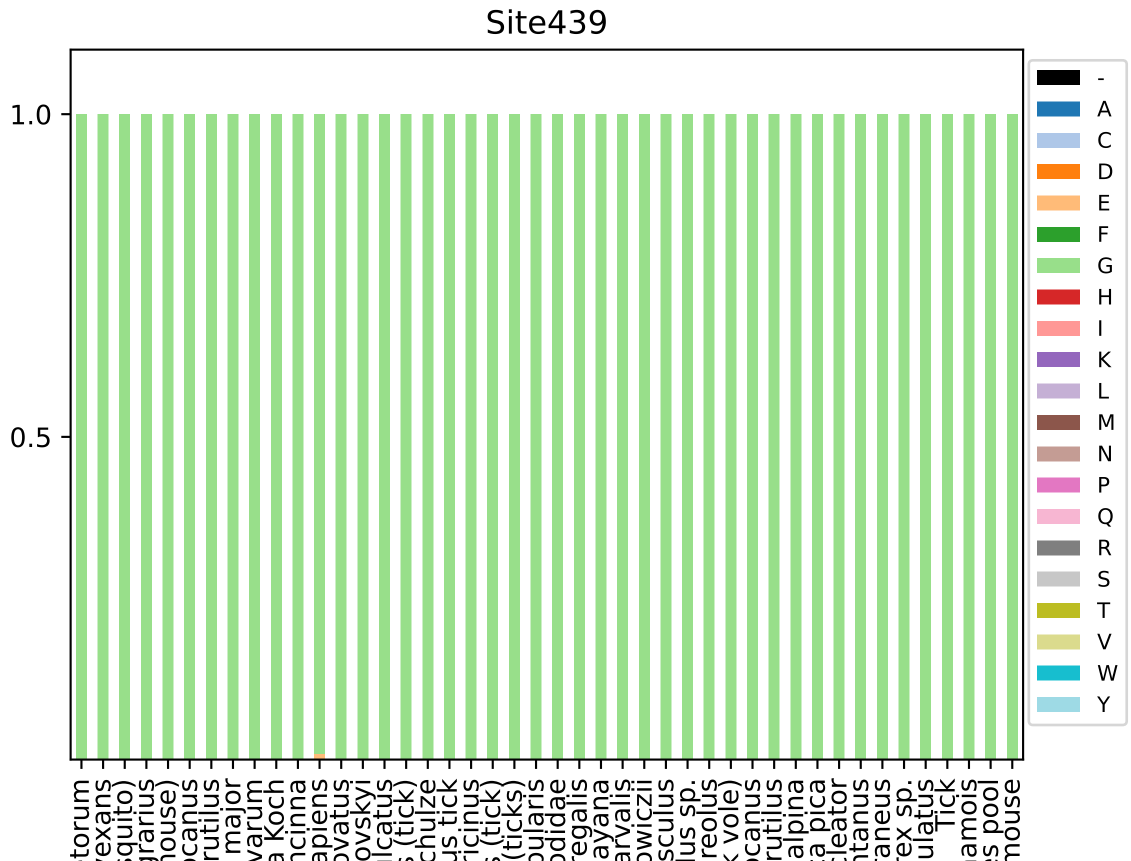

Site439
1.0
0.5
-
A
C
D
E
F
G
H
I
K
L
M
N
P
Q
R
S
T
V
W
Y

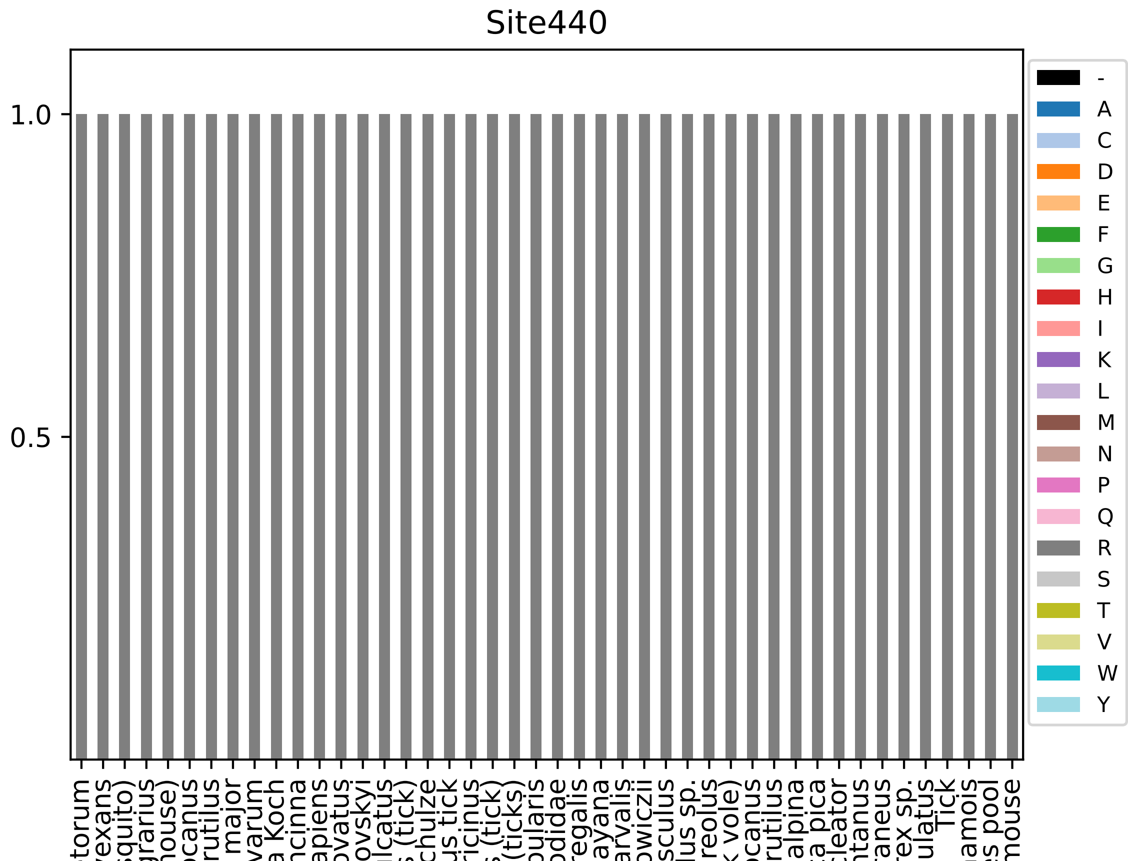
Site440
1.0
0.5
-
A
C
D
E
F
G
H
I
K
L
M
N
P
Q
R
S
T
V
W
Y
Tick

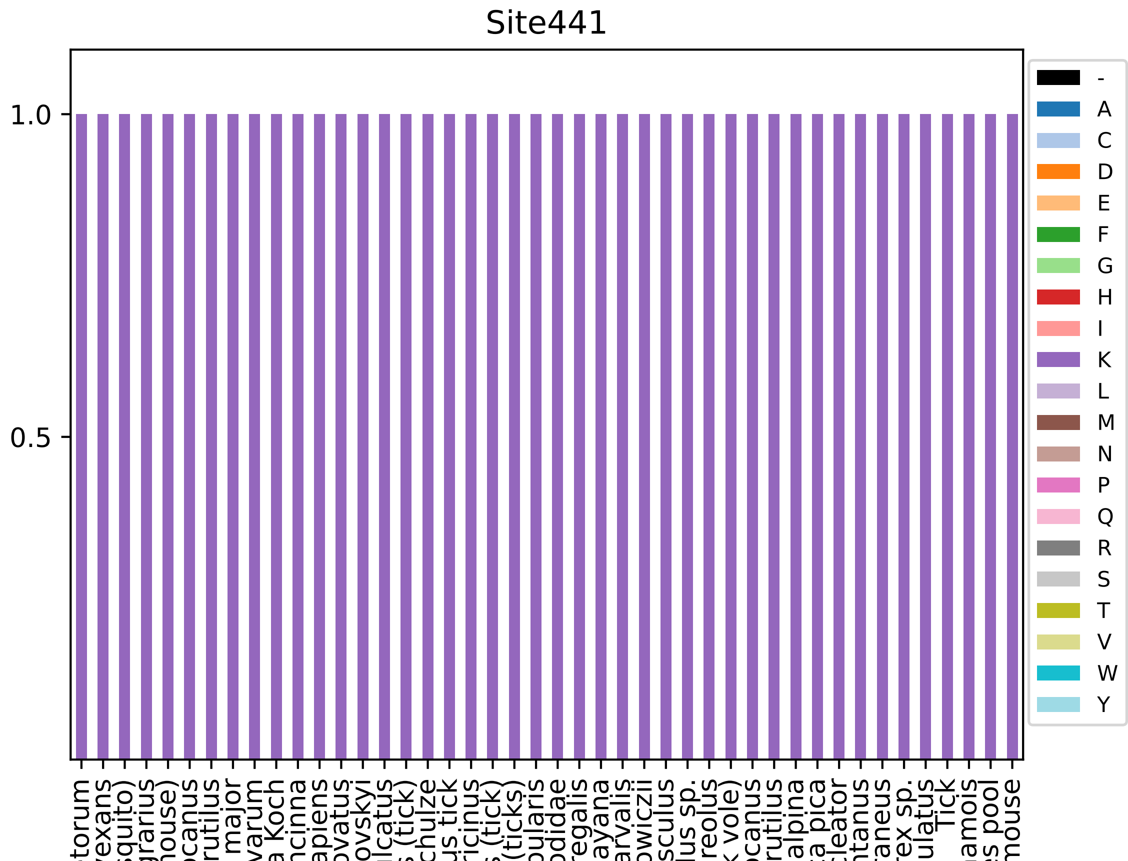
Site441
1.0
0.5
-
A
C
D
E
F
G
H
I
K
L
M
N
P
Q
R
S
T
V
W
Y
Tick

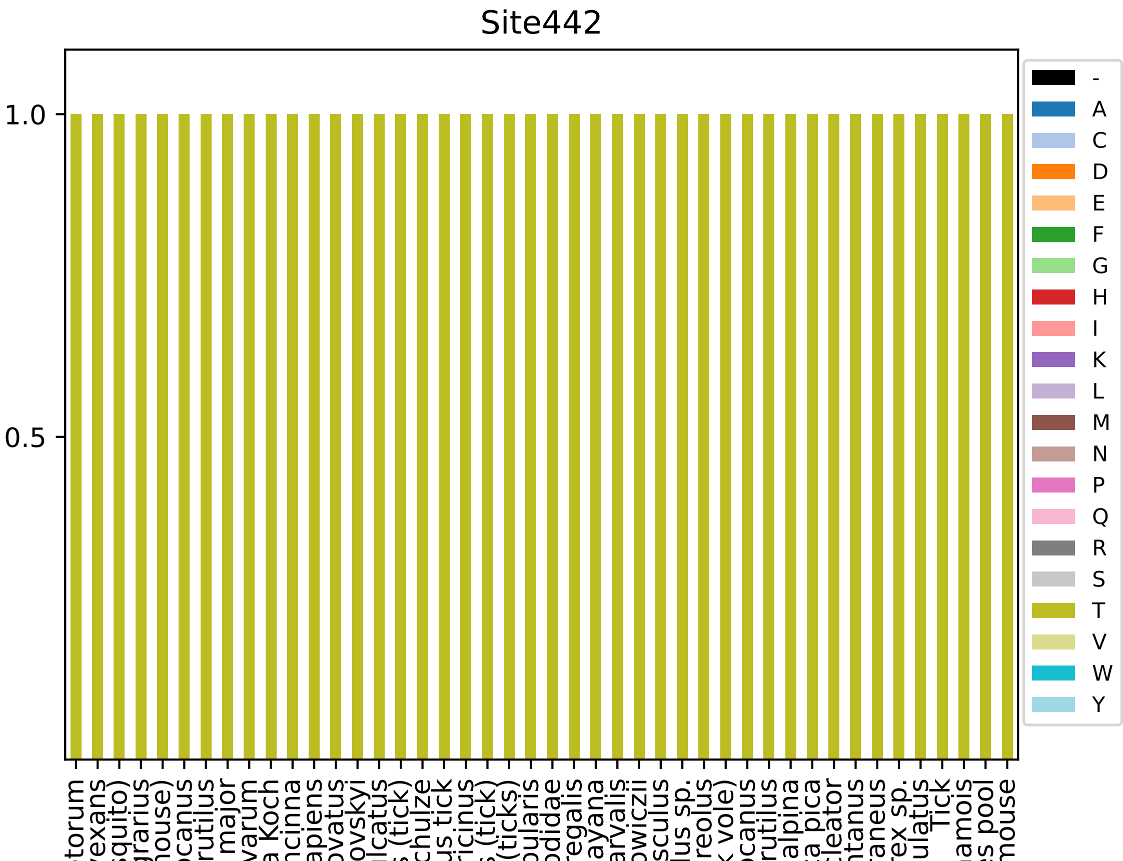
Site442
1.0
0.5
-
A
C
D
E
F
G
H
I
K
L
M
N
P
Q
R
S
T
V
W
Y

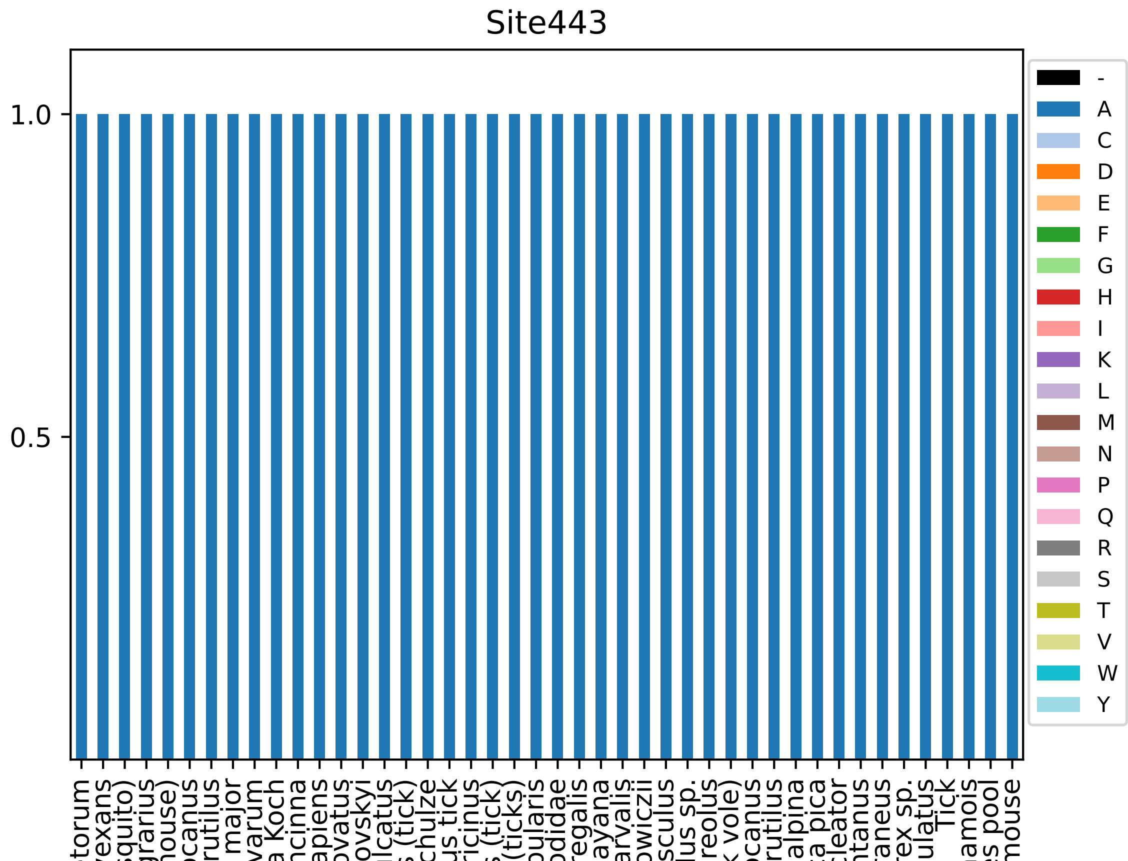

Site443
1.0
0.5
-
A
C
D
E
F
G
H
I
K
L
M
N
P
Q
R
S
T
V
W
Y

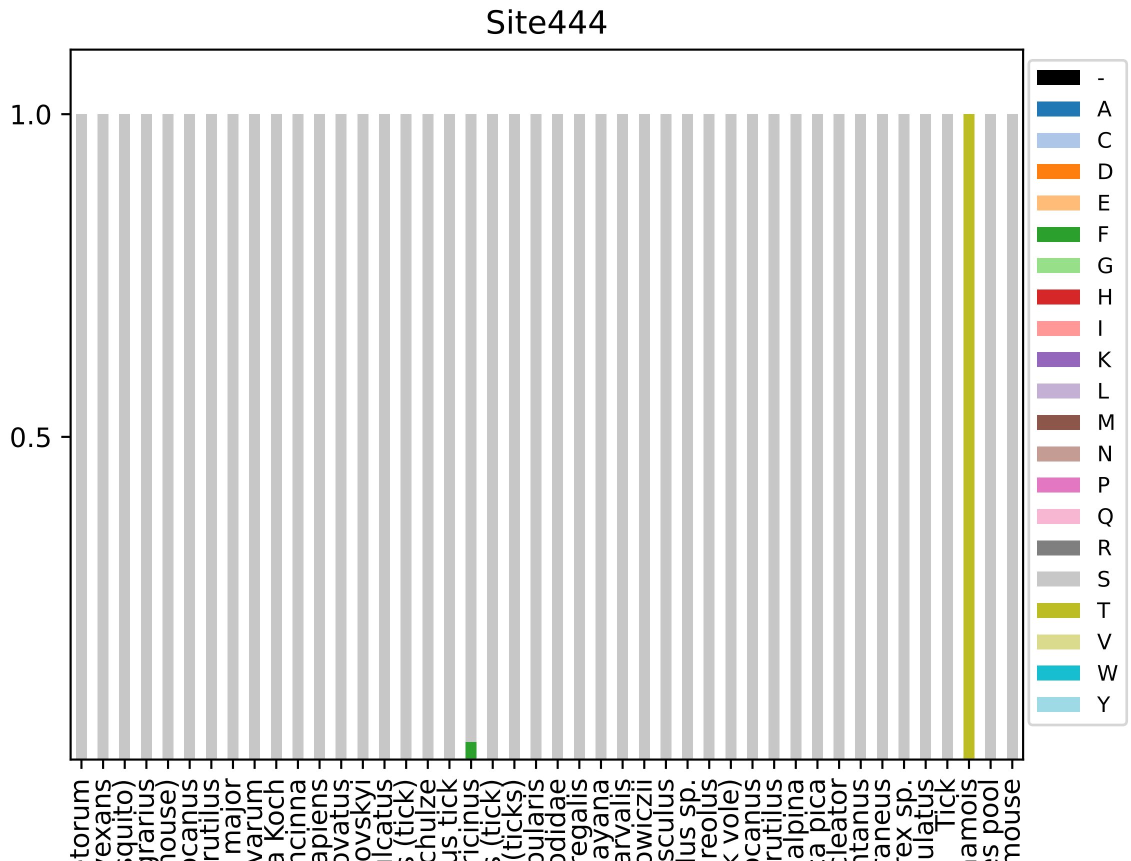
Site444
1.0
0.5
-
A
C
D
E
F
G
H
I
K
L
M
N
P
Q
R
S
T
V
W
Y

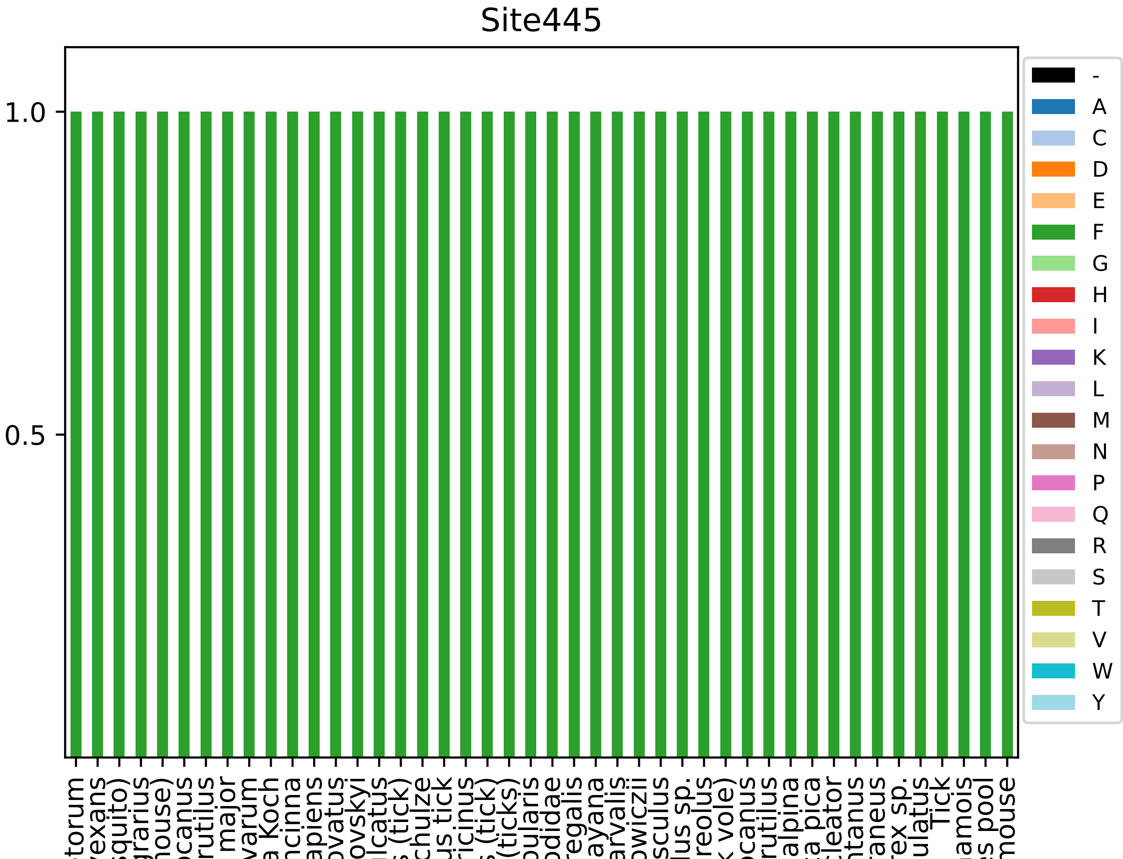
Site445
1.0
0.5
-
A
C
D
E
F
G
H
I
K
L
M
N
P
Q
R
S
T
V
W
Y

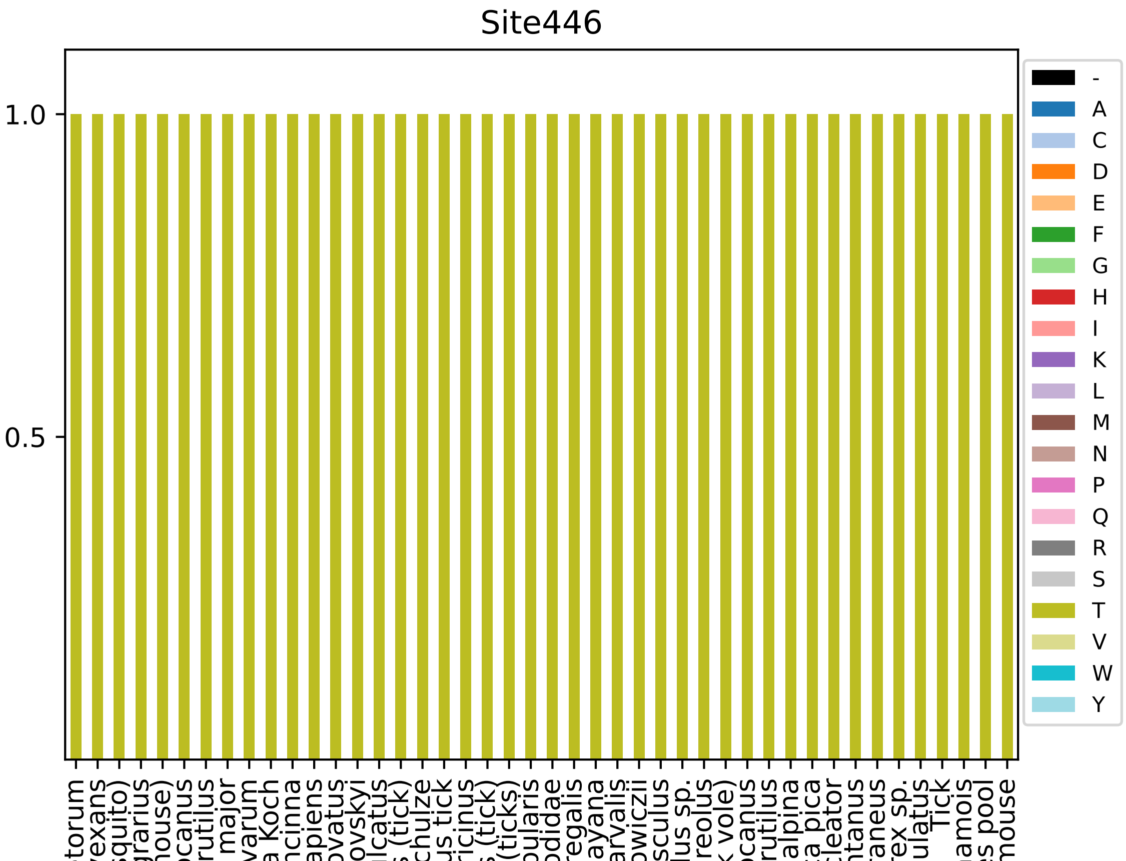
Site446
1.0
0.5
-
A
C
D
E
F
G
H
I
K
L
M
N
P
Q
R
S
T
V
W
Y

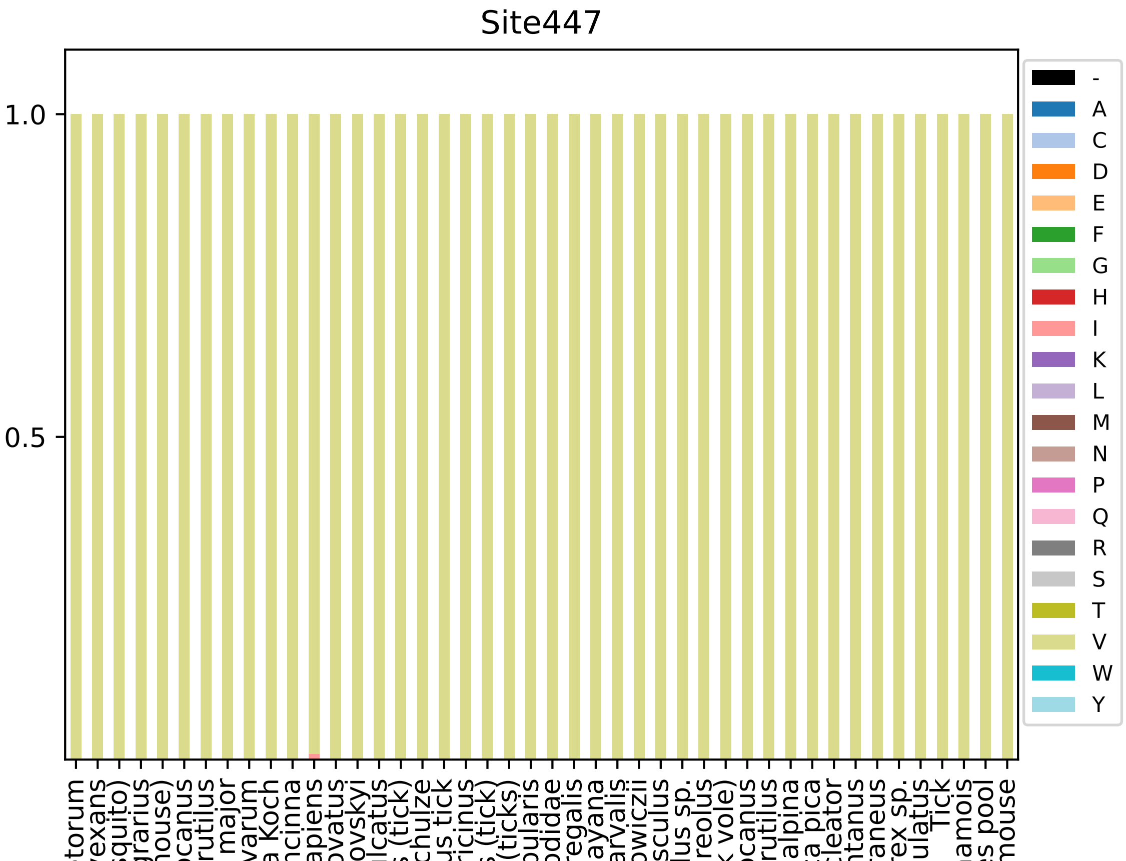
Site447
1.0
0.5
-
A
C
D
E
F
G
H
I
K
L
M
N
P
Q
R
S
T
V
W
Y

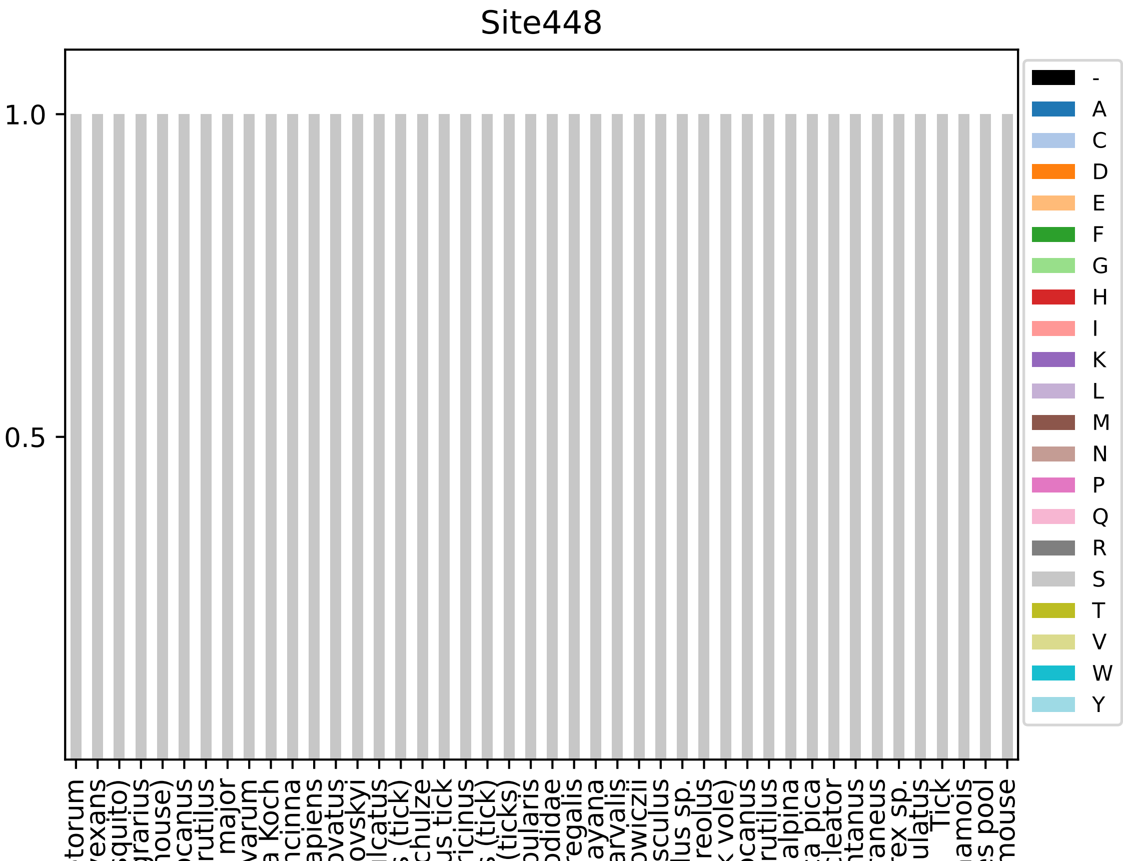
Site448
1.0
0.5
-
A
C
D
E
F
G
H
I
K
L
M
N
P
Q
R
S
T
V
W
Y

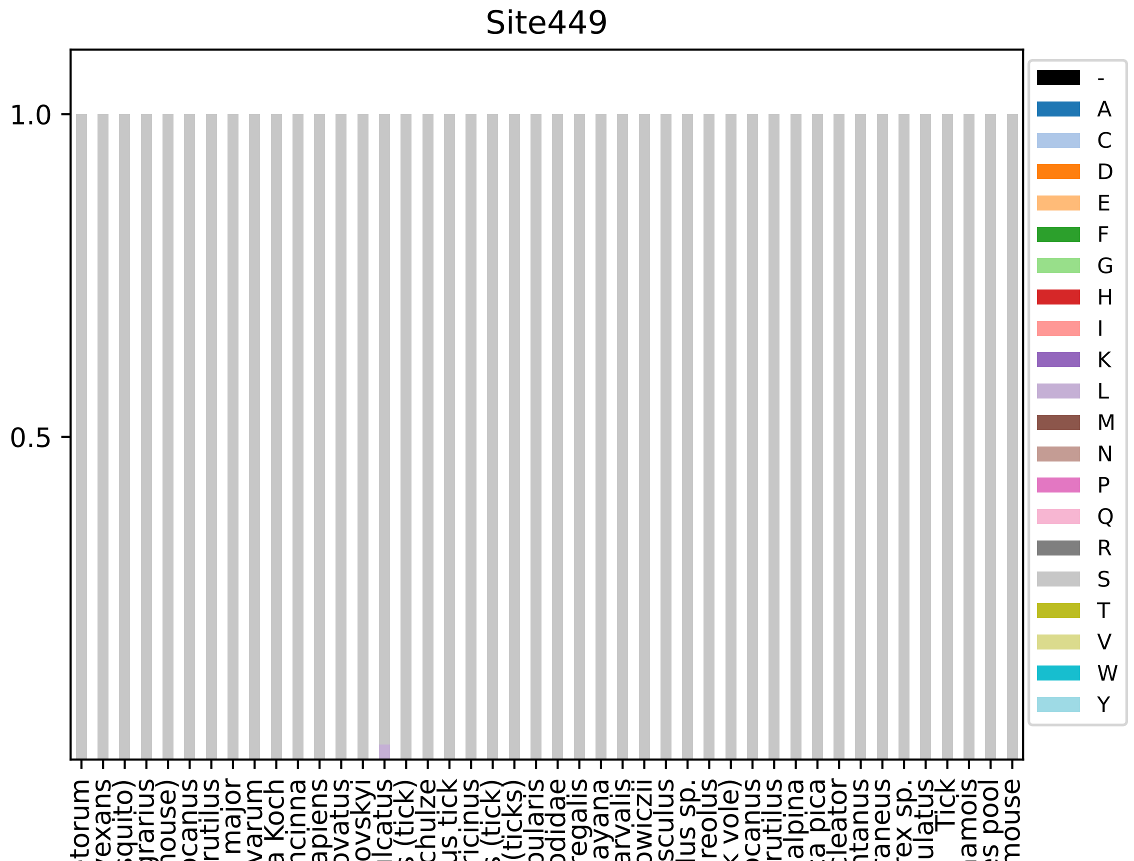

Site449
1.0
0.5
-
A
C
D
E
F
G
H
I
K
L
M
N
P
Q
R
S
T
V
W
Y

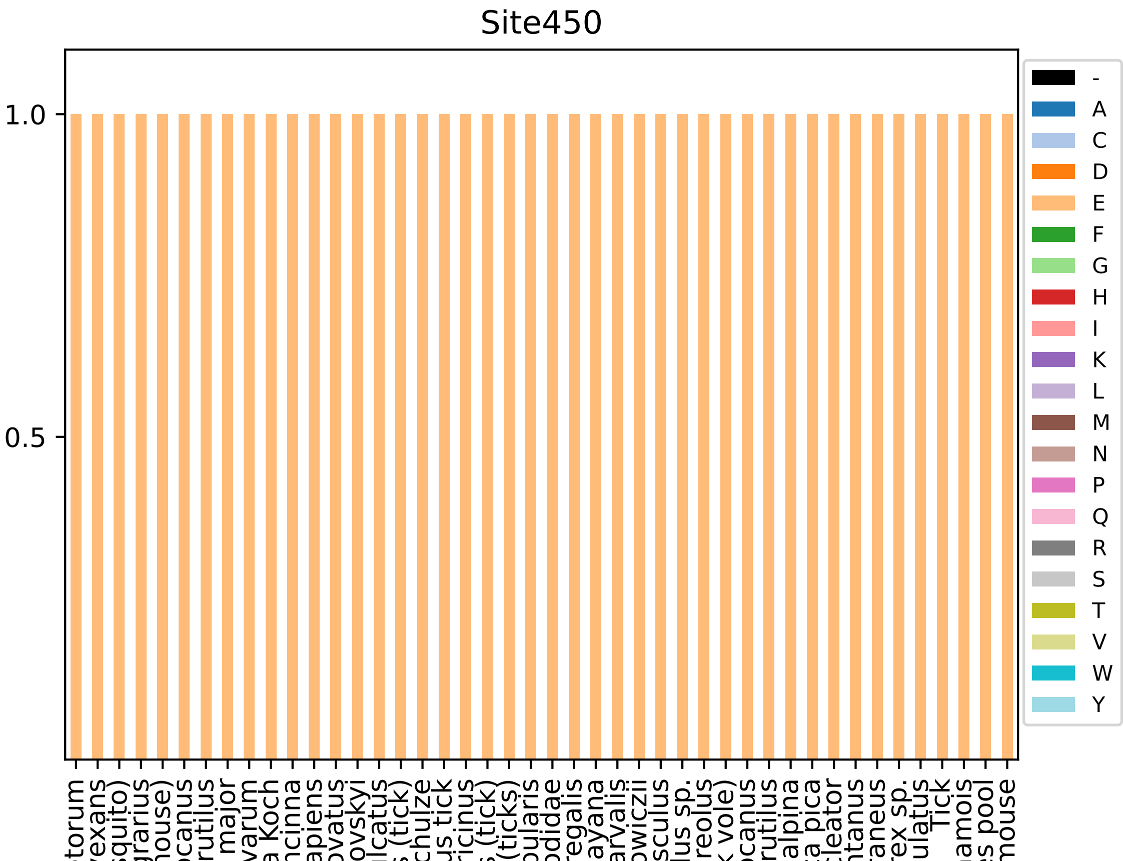

Site450
1.0
0.5
-
A
C
D
E
F
G
H
I
K
L
M
N
P
Q
R
S
T
V
W
Y

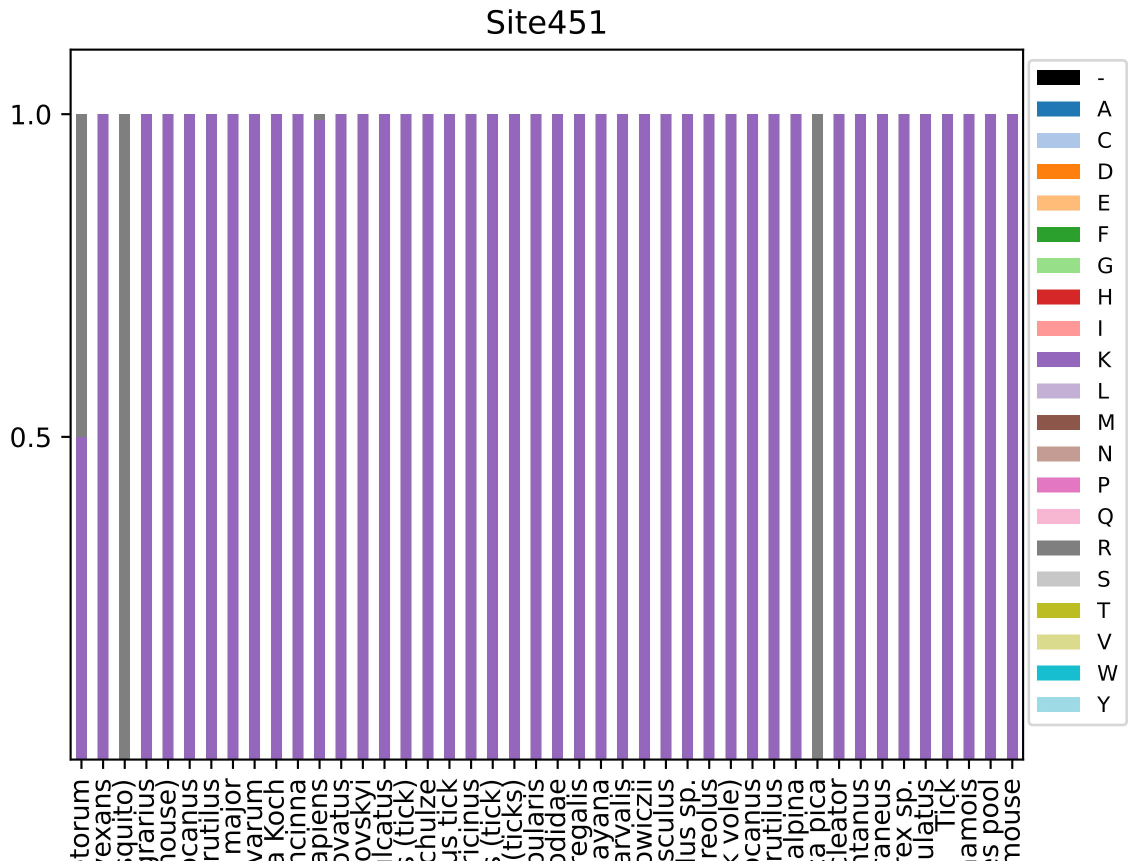
Site451
1.0
0.5
-
A
C
D
E
F
G
H
I
K
L
M
N
P
Q
R
S
T
V
W
Y

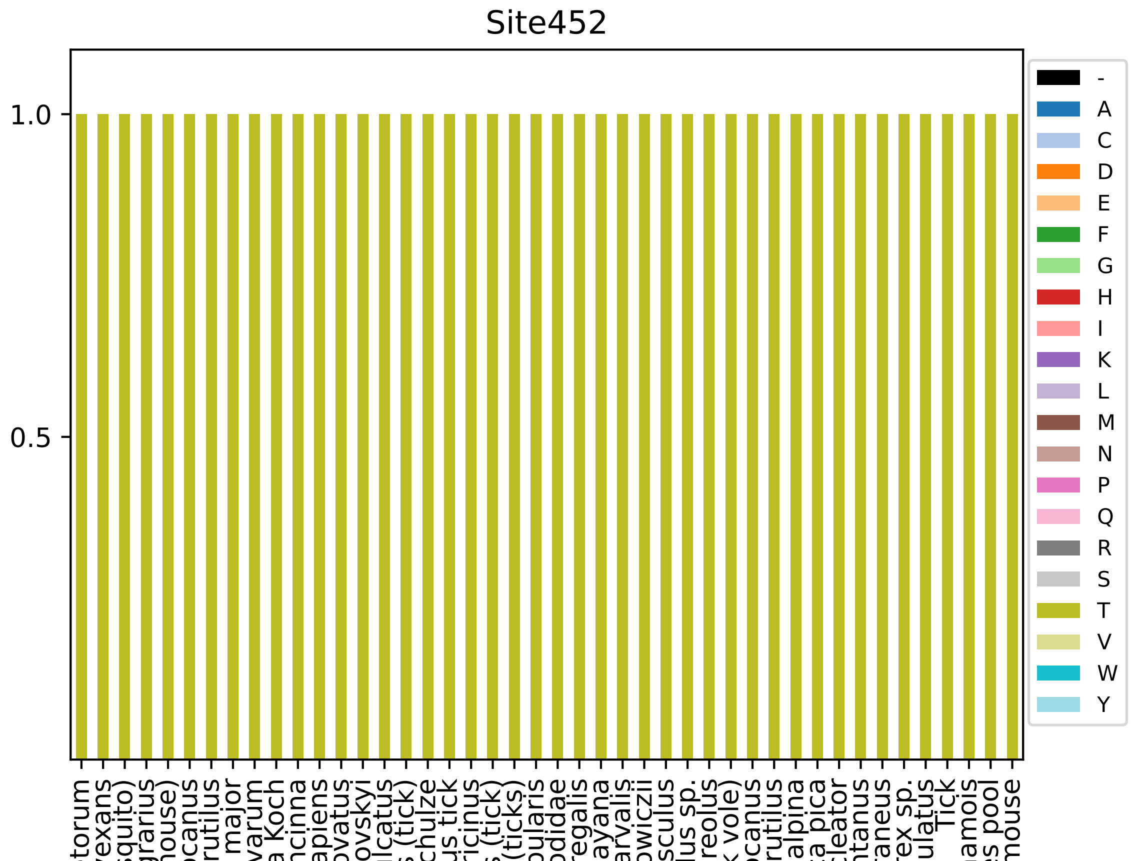
Site452
1.0
0.5
-
A
C
D
E
F
G
H
I
K
L
M
N
P
Q
R
S
T
V
W
Y

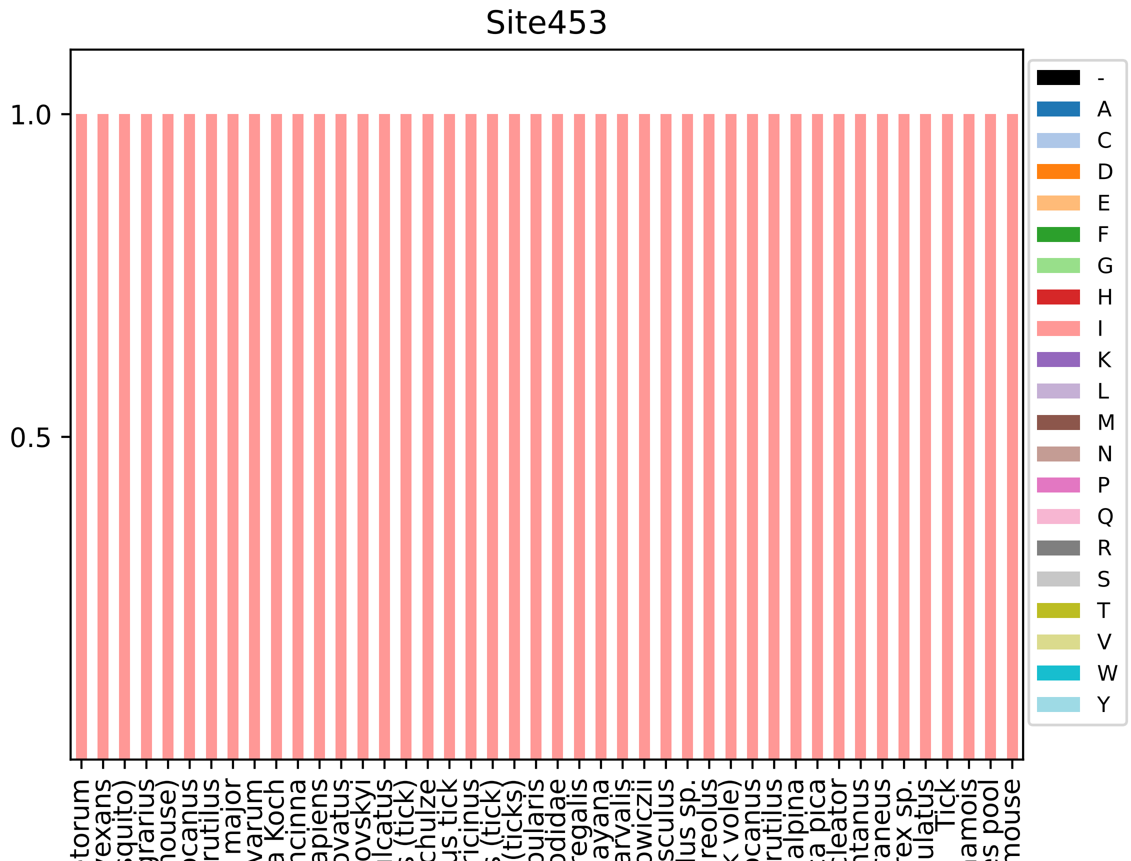
Site453
1.0
0.5
-
A
C
D
E
F
G
H
I
K
L
M
N
P
Q
R
S
T
V
W
Y

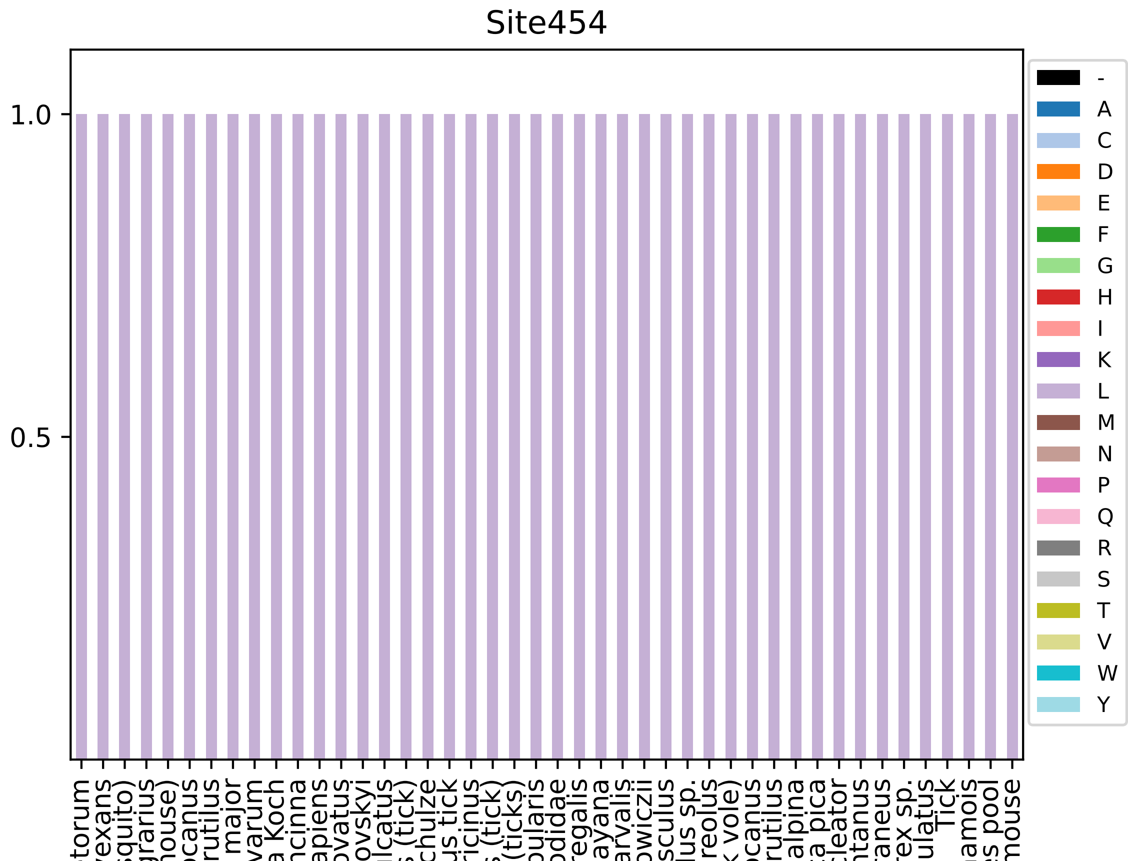

Site454
1.0
0.5
-
A
C
D
E
F
G
H
I
K
L
M
N
P
Q
R
S
T
V
W
Y

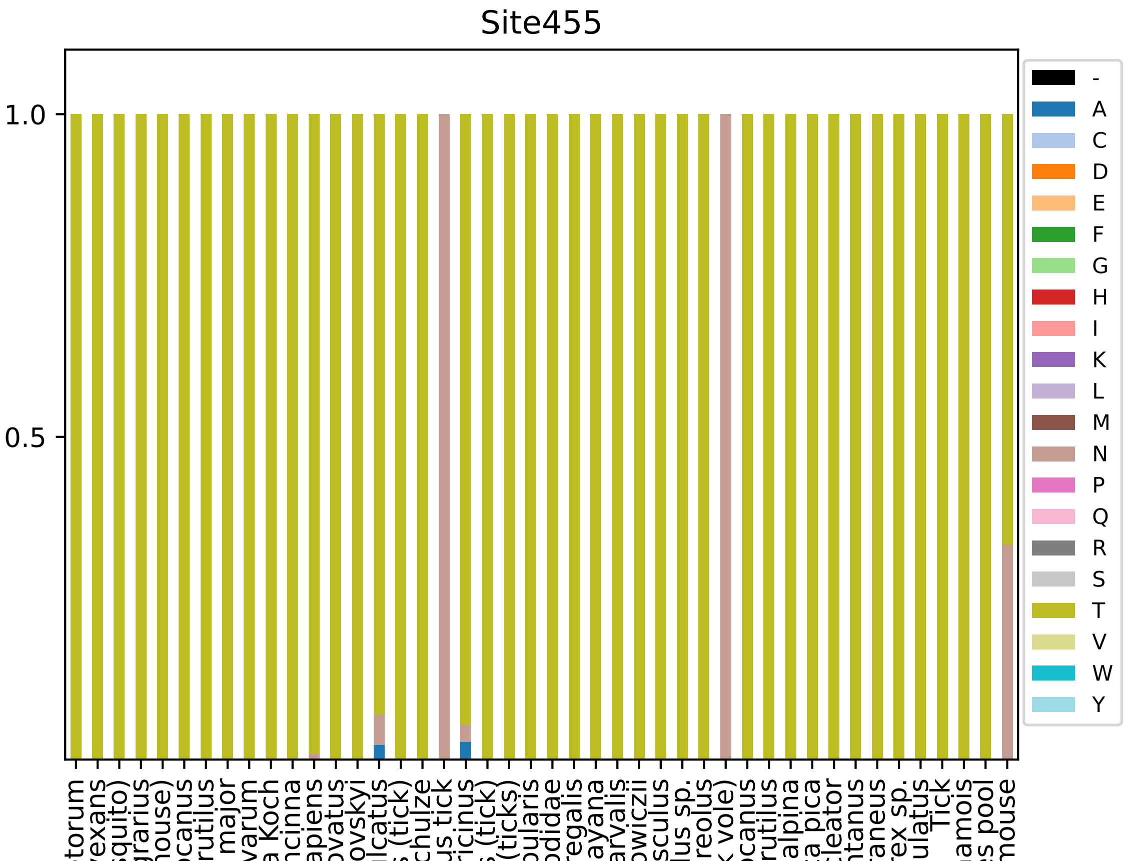

Site455
1.0
0.5
-
A
C
D
E
F
G
H
I
K
L
M
N
P
Q
R
S
T
V
W
Y

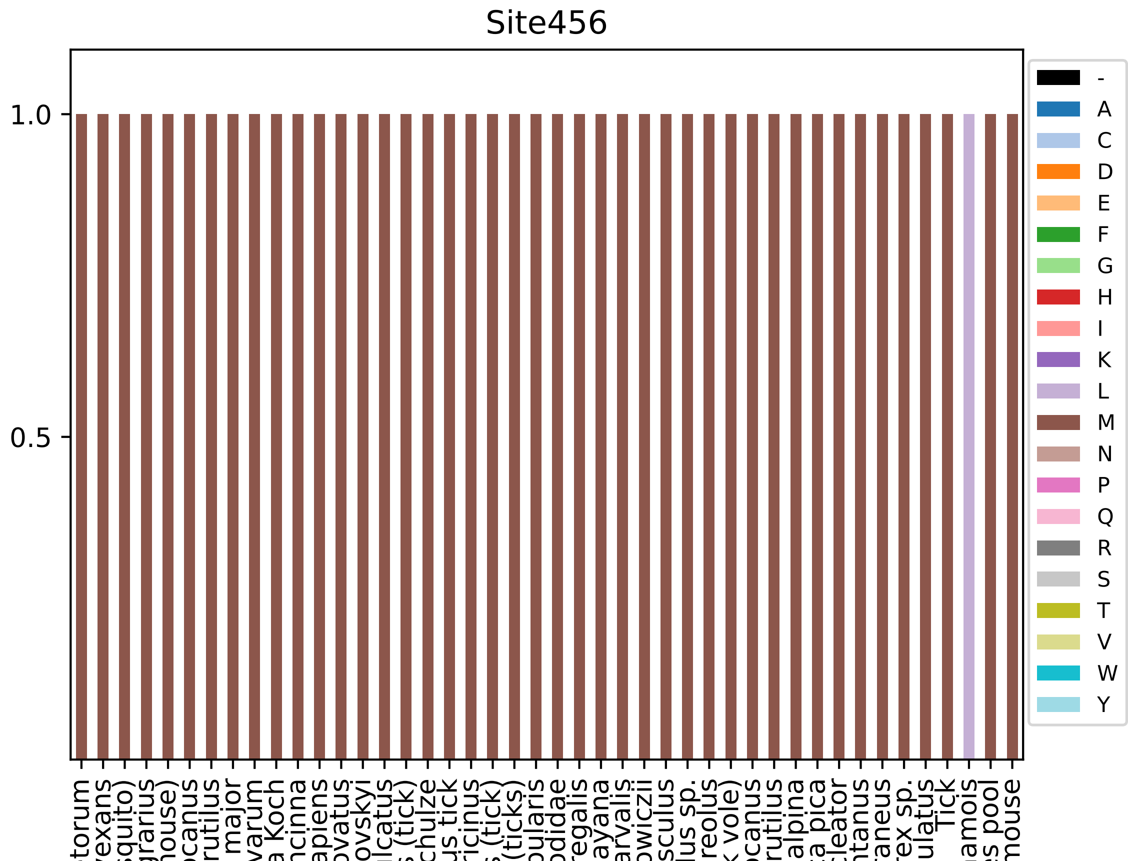
Site456
1.0
0.5
-
A
C
D
E
F
G
H
I
K
L
M
N
P
Q
R
S
T
V
W
Y
Tick

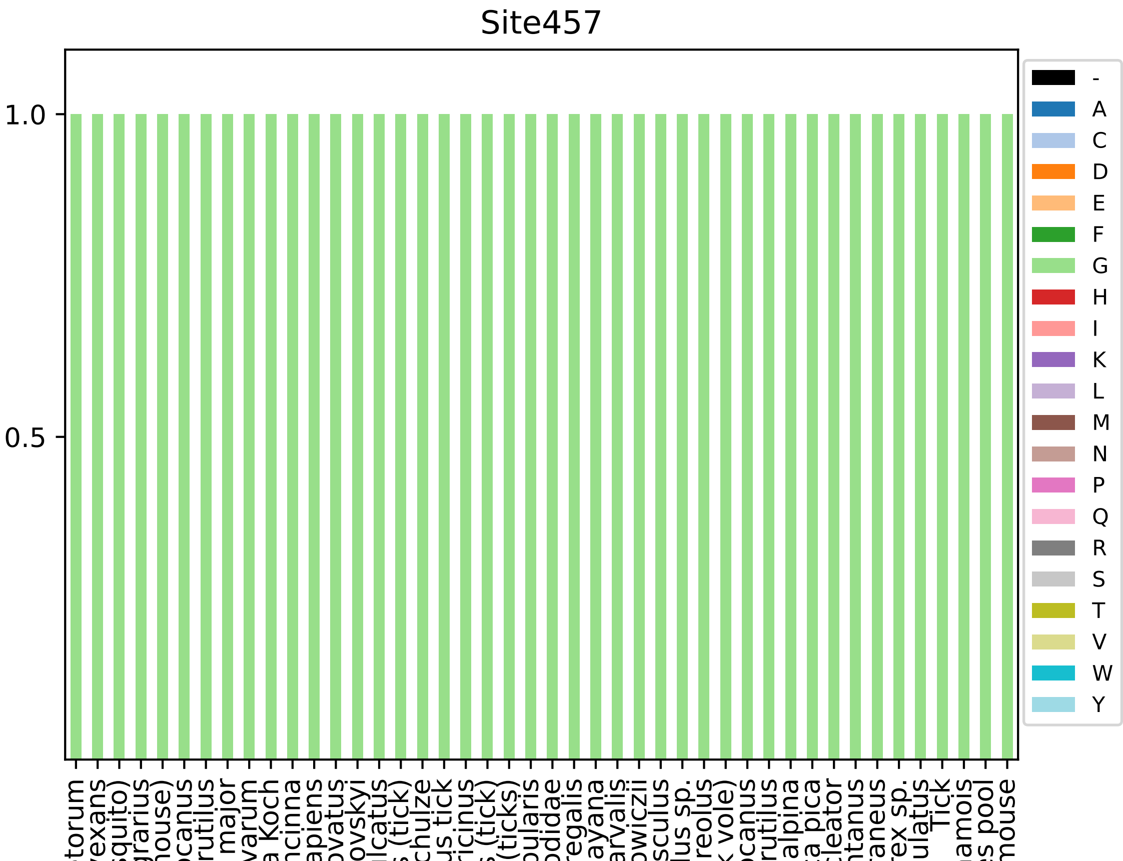

Site457
1.0
0.5
-
A
C
D
E
F
G
H
I
K
L
M
N
P
Q
R
S
T
V
W
Y

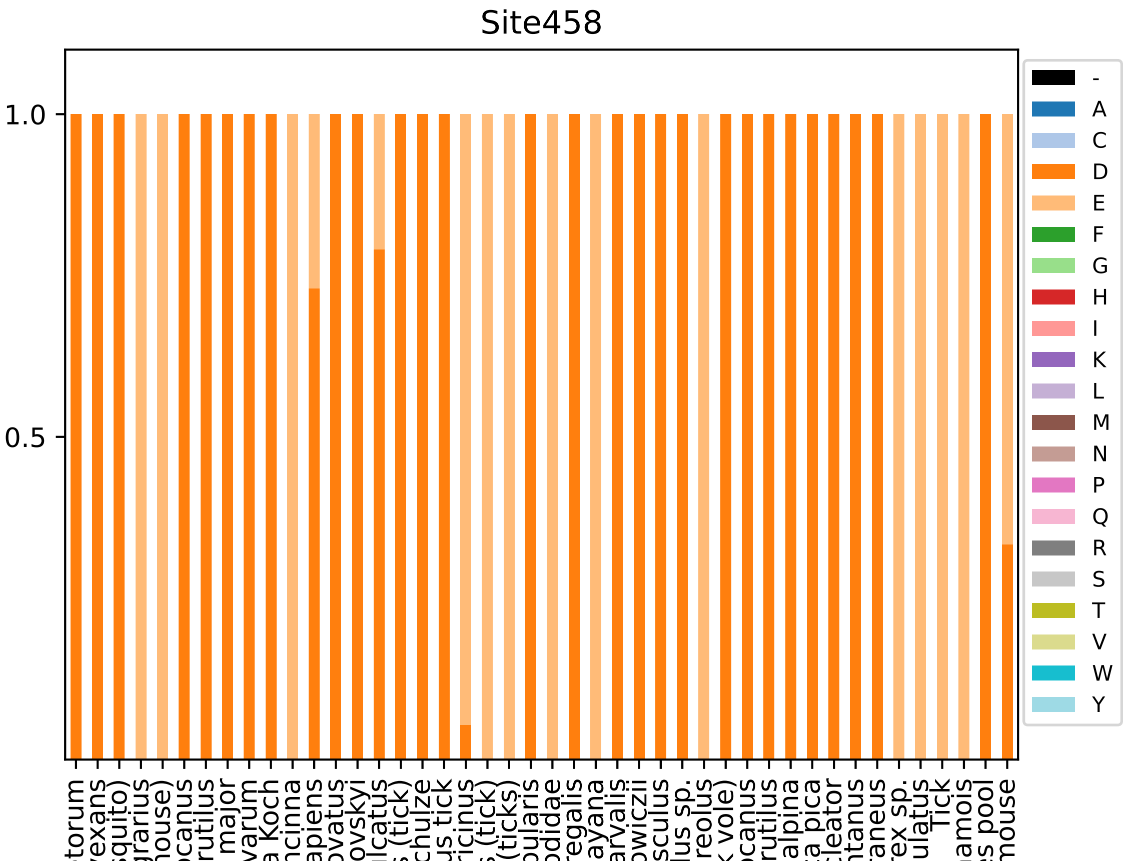
Site458
1.0
0.5
-
A
C
D
E
F
G
H
I
K
L
M
N
P
Q
R
S
T
V
W
Y

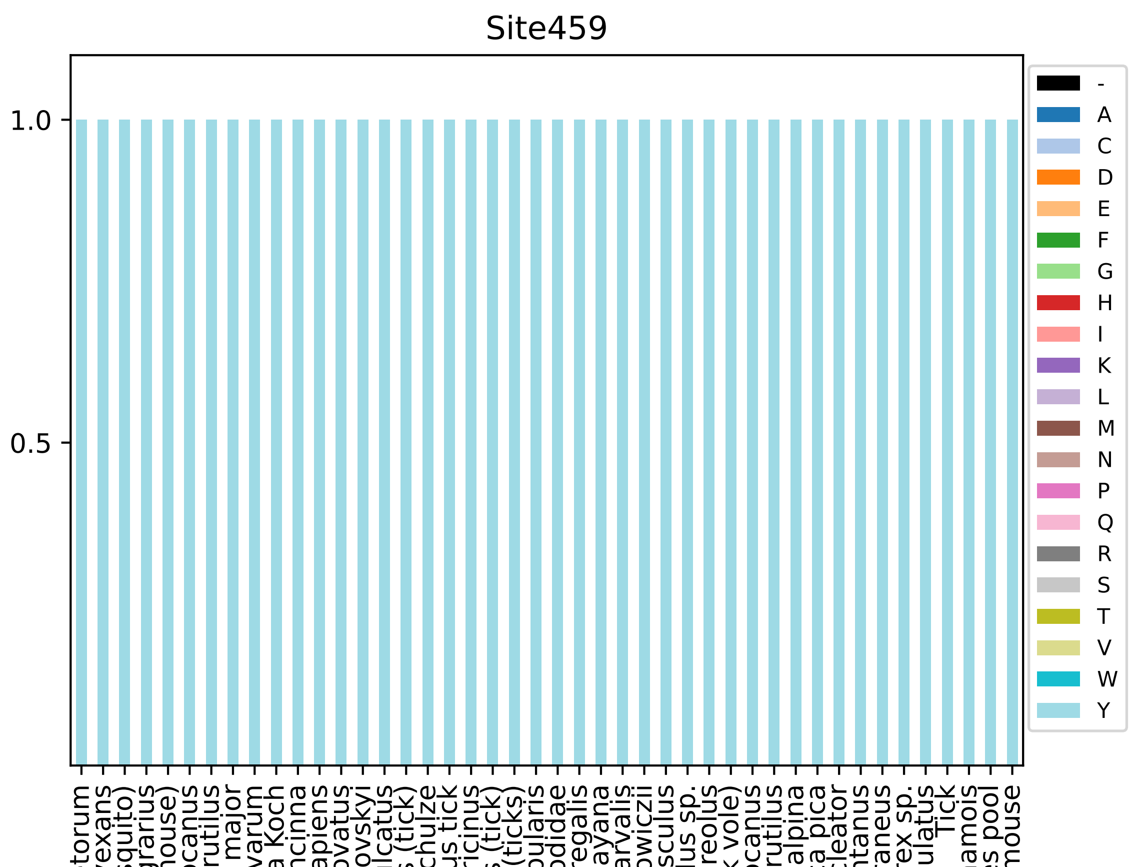

Site459
1.0
0.5
-
A
C
D
E
F
G
H
I
K
L
M
N
P
Q
R
S
T
V
W
Y

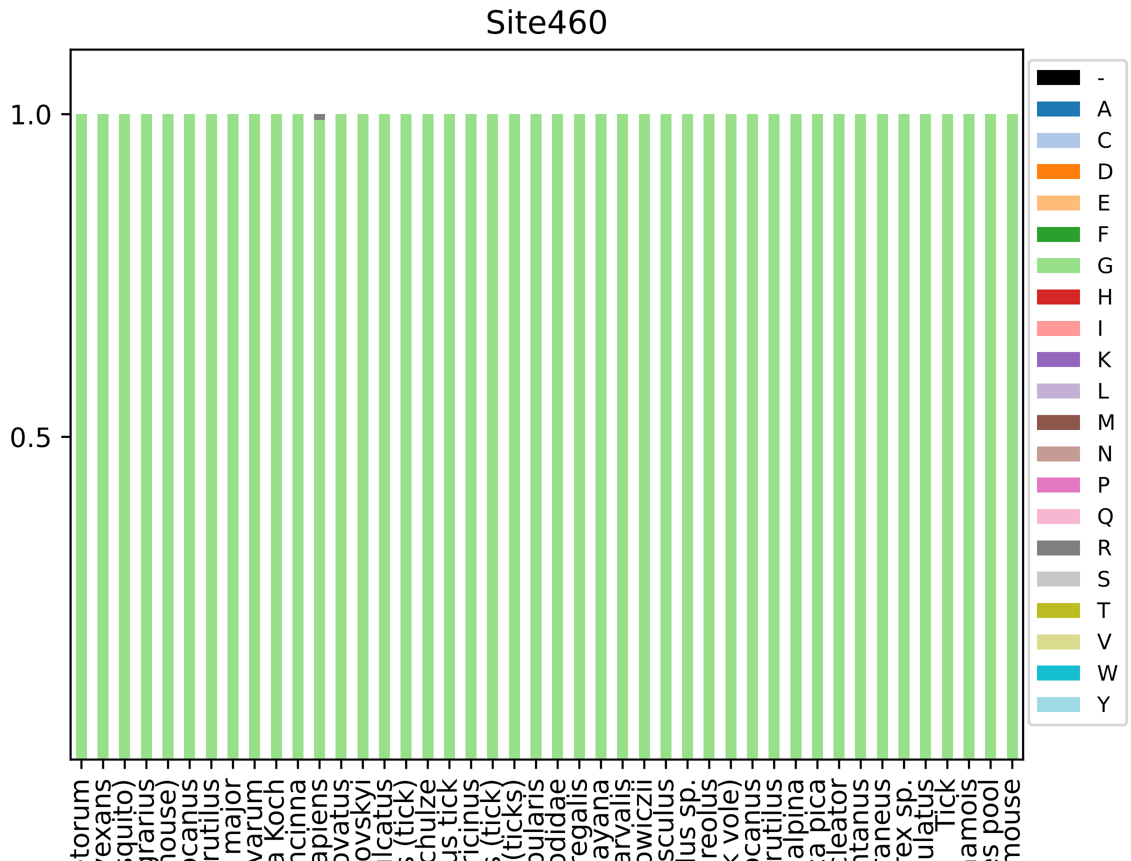
Site460
1.0
0.5
-
A
C
D
E
F
G
H
I
K
L
M
N
P
Q
R
S
T
V
W
Y

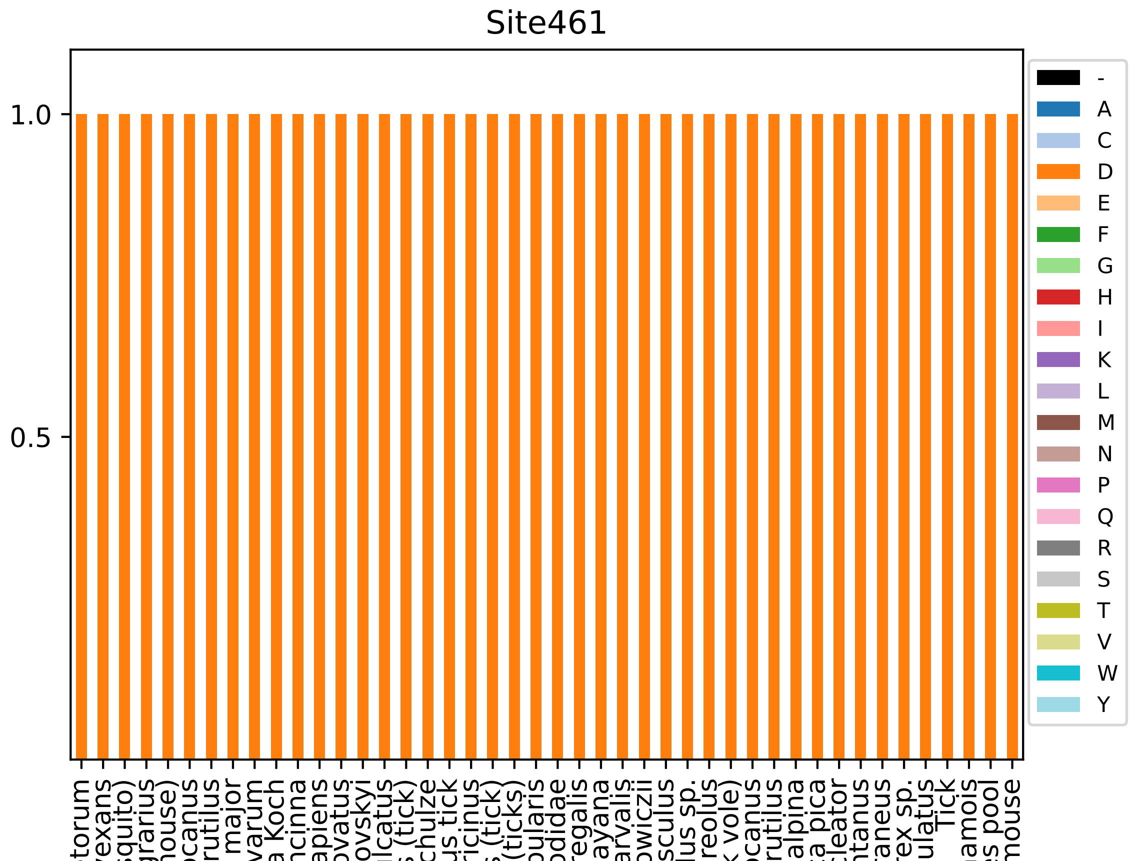
Site461
1.0
0.5
-
A
C
D
E
F
G
H
I
K
L
M
N
P
Q
R
S
T
V
W
Y

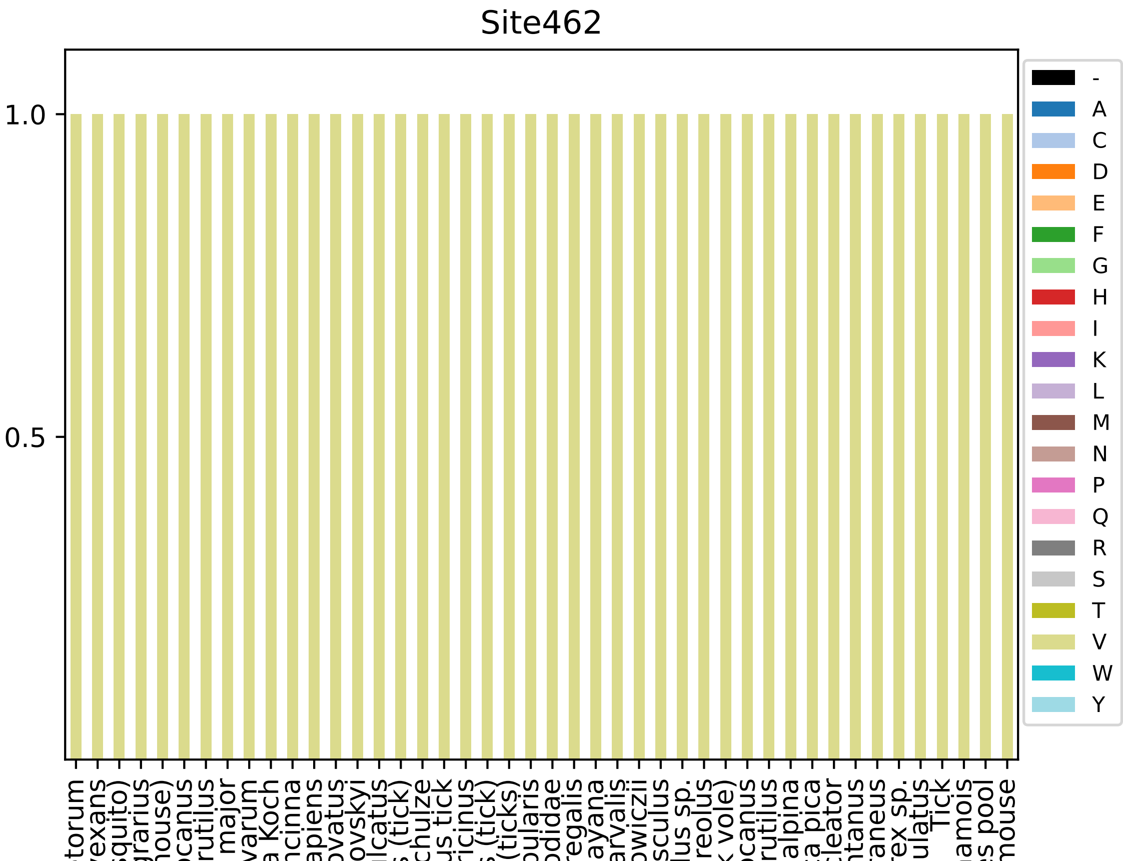
Site462
1.0
0.5
-
A
C
D
E
F
G
H
I
K
L
M
N
P
Q
R
S
T
V
W
Y
torum
vexans
squito)
grarius
mouse)
ocanus
rutilus
major
varum
a Koch
ncinna
apiens
ovatus
ovskyi
ulcatus
s (tick)
chulze
us tick
ricinus
s (tick)
(ticks)
pularis
podidae
regalis
ayana
arvalis
owiczii
sculus
lus sp.
reolus
k vole)
ocanus
rutilus
alpina
a pica
leator
ntanus
raneus
rex sp.
ulatus
Tick
amois
es pool
mouse

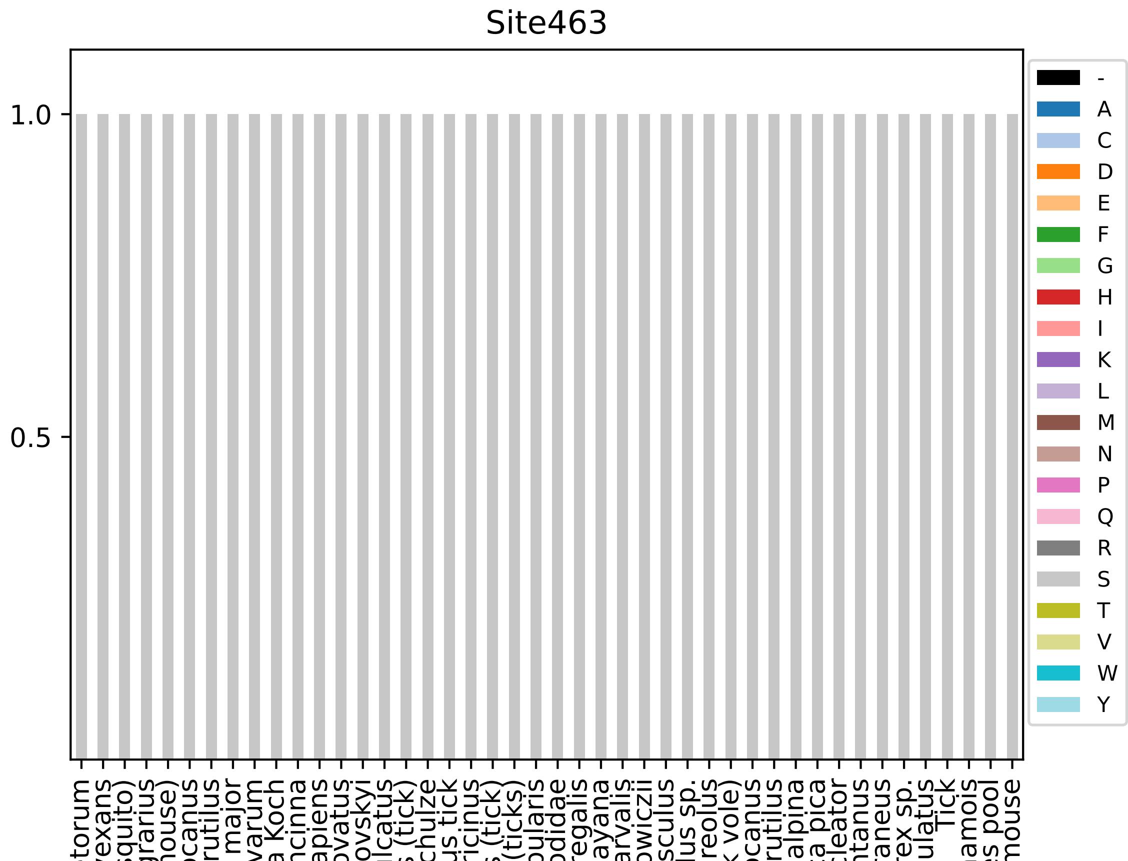

Site463
1.0
0.5
-
A
C
D
E
F
G
H
I
K
L
M
N
P
Q
R
S
T
V
W
Y

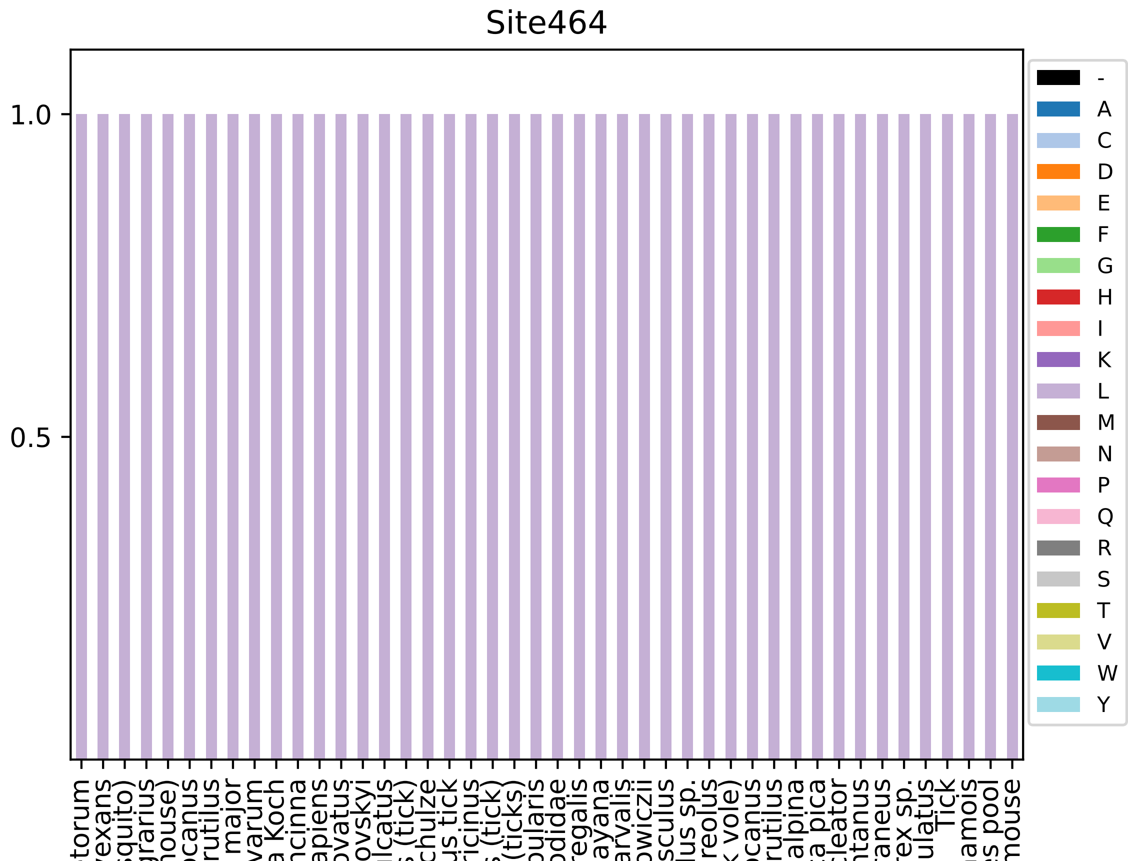
Site464
1.0
0.5
-
A
C
D
E
F
G
H
I
K
L
M
N
P
Q
R
S
T
V
W
Y

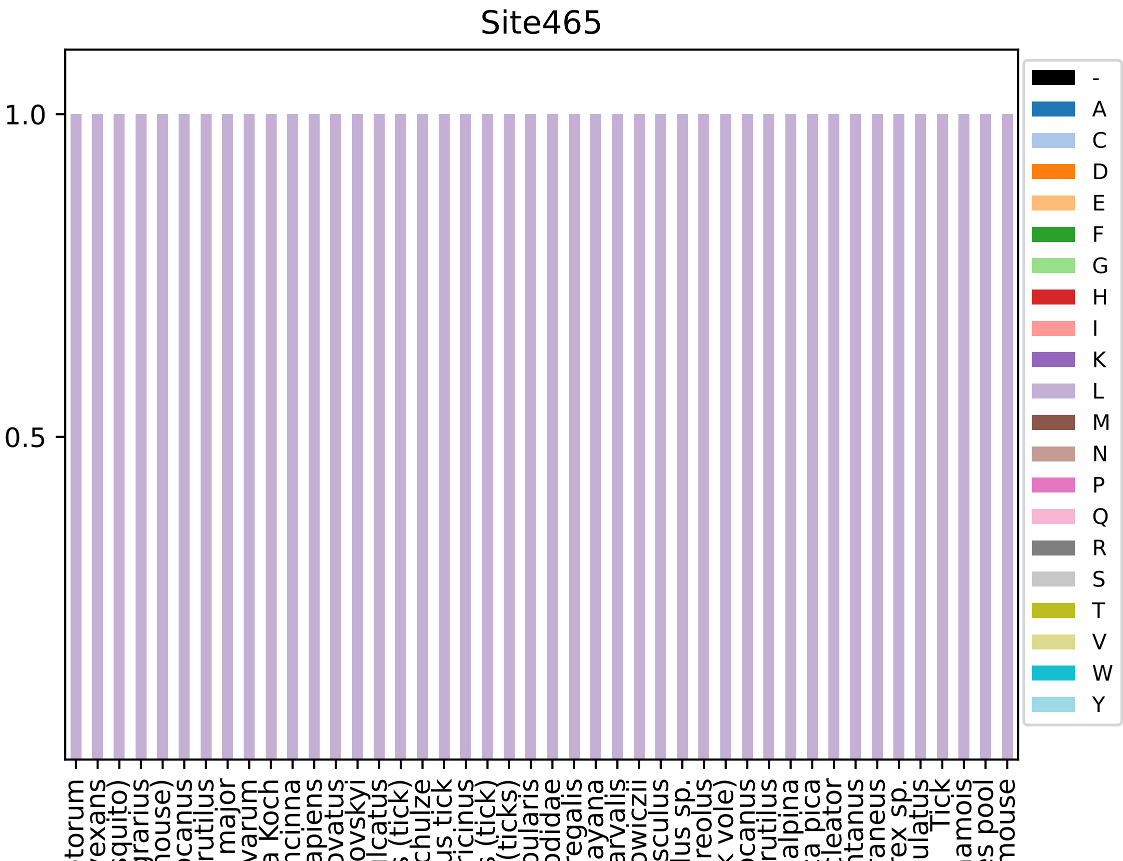
Site465
1.0
0.5
-
A
C
D
E
F
G
H
I
K
L
M
N
P
Q
R
S
T
V
W
Y

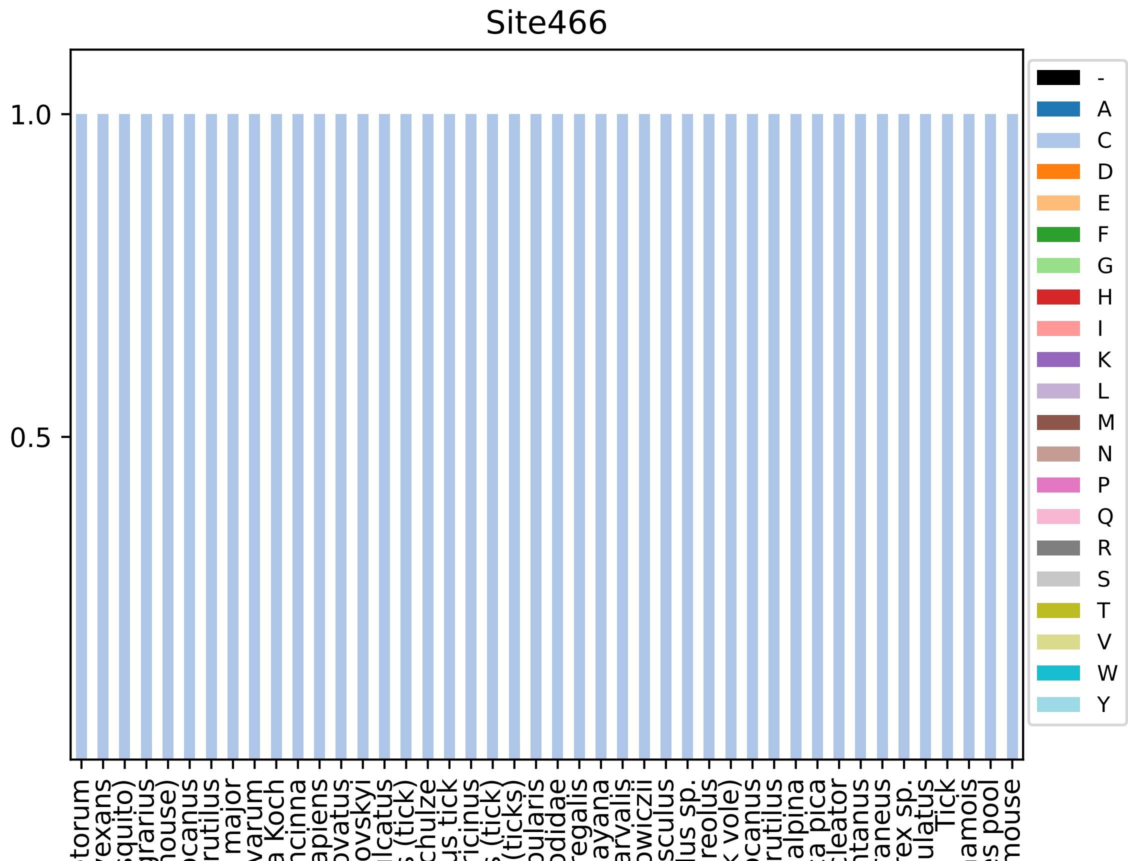
Site466
1.0
0.5
-
A
C
D
E
F
G
H
I
K
L
M
N
P
Q
R
S
T
V
W
Y

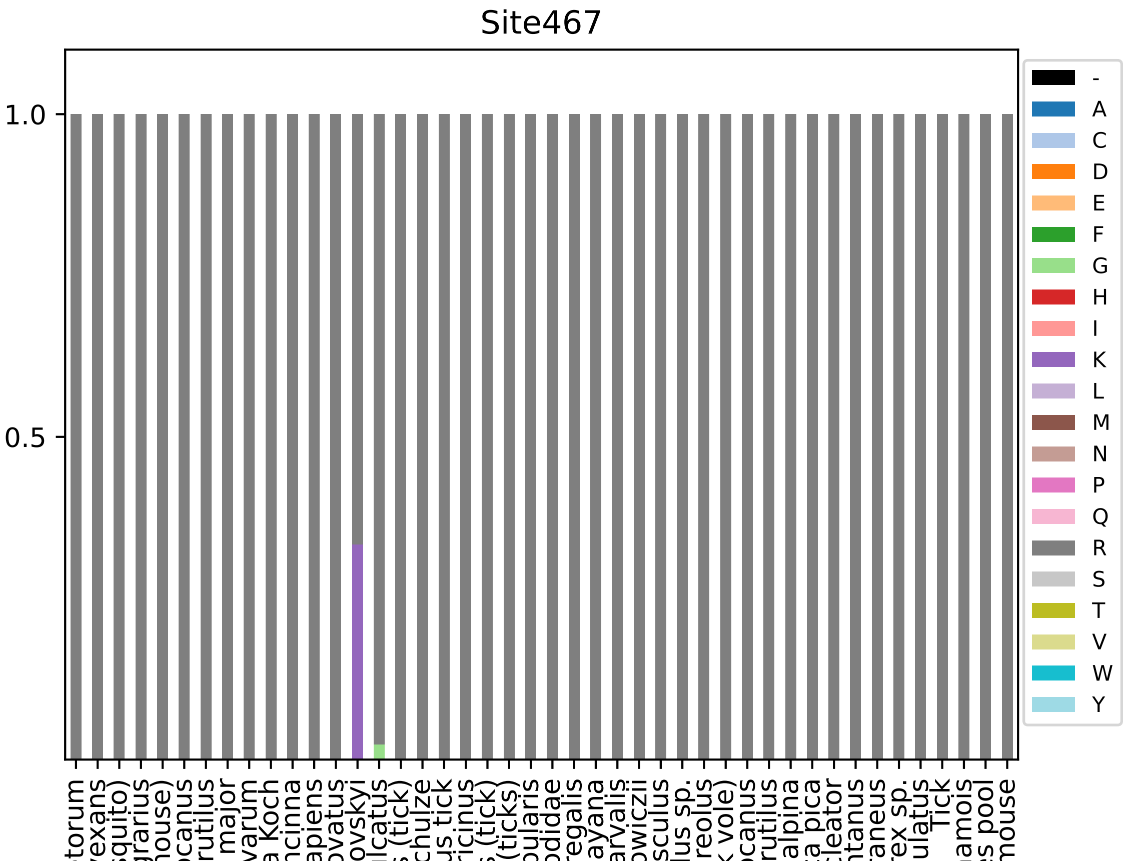
Site467
1.0
0.5
-
A
C
D
E
F
G
H
I
K
L
M
N
P
Q
R
S
T
V
W
Y

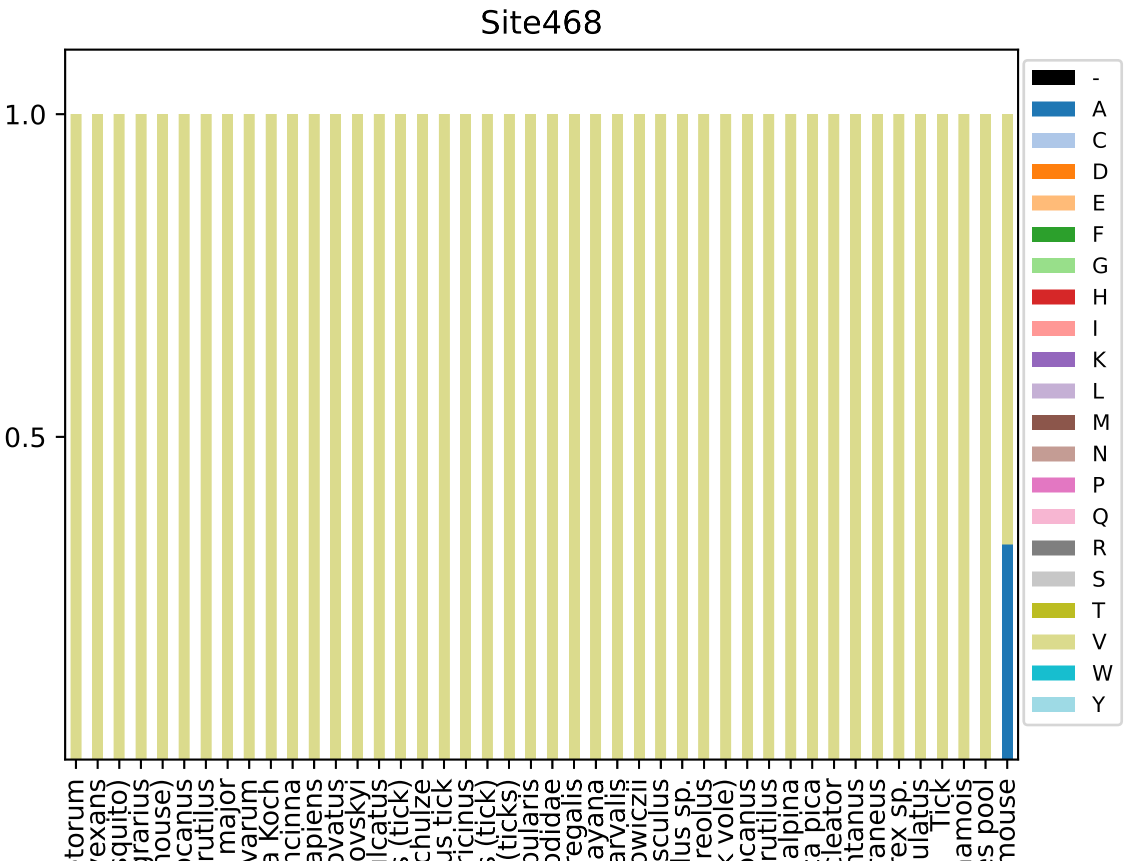

Site468
1.0
0.5
-
A
C
D
E
F
G
H
I
K
L
M
N
P
Q
R
S
T
V
W
Y

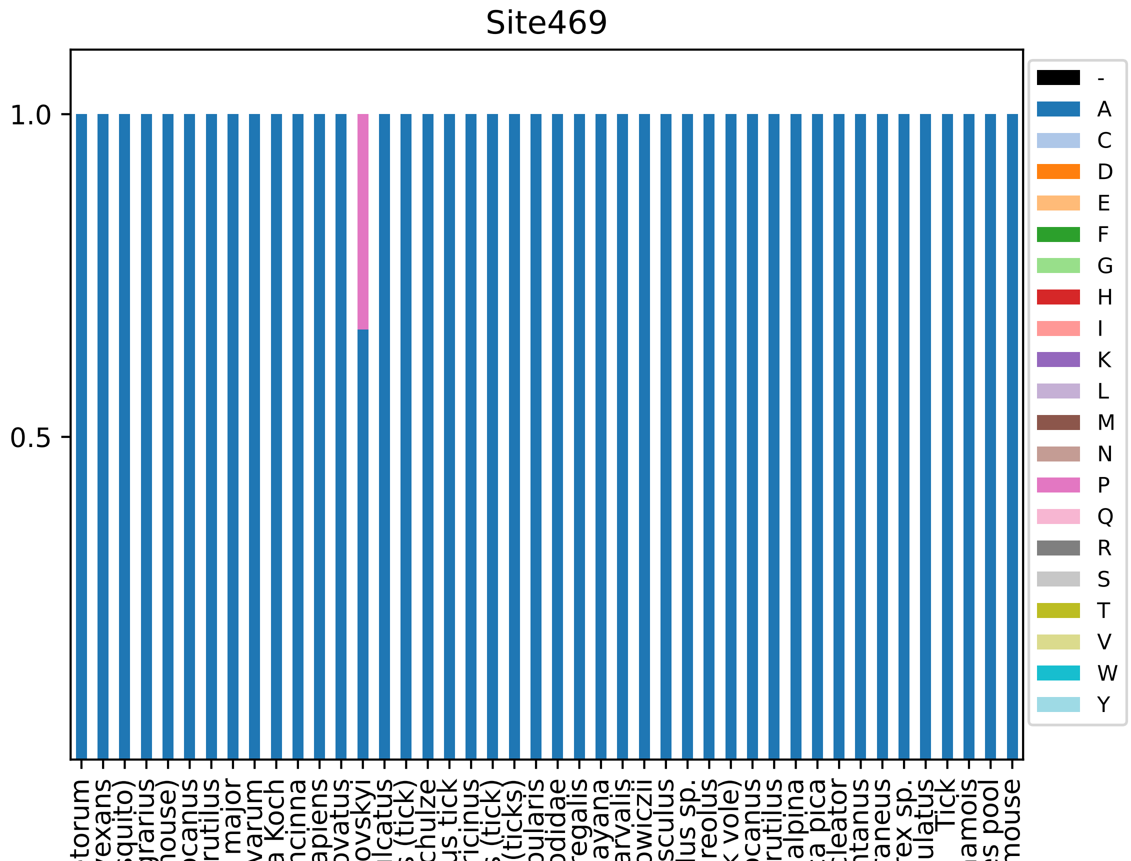
Site469
1.0
0.5
-
A
C
D
E
F
G
H
I
K
L
M
N
P
Q
R
S
T
V
W
Y

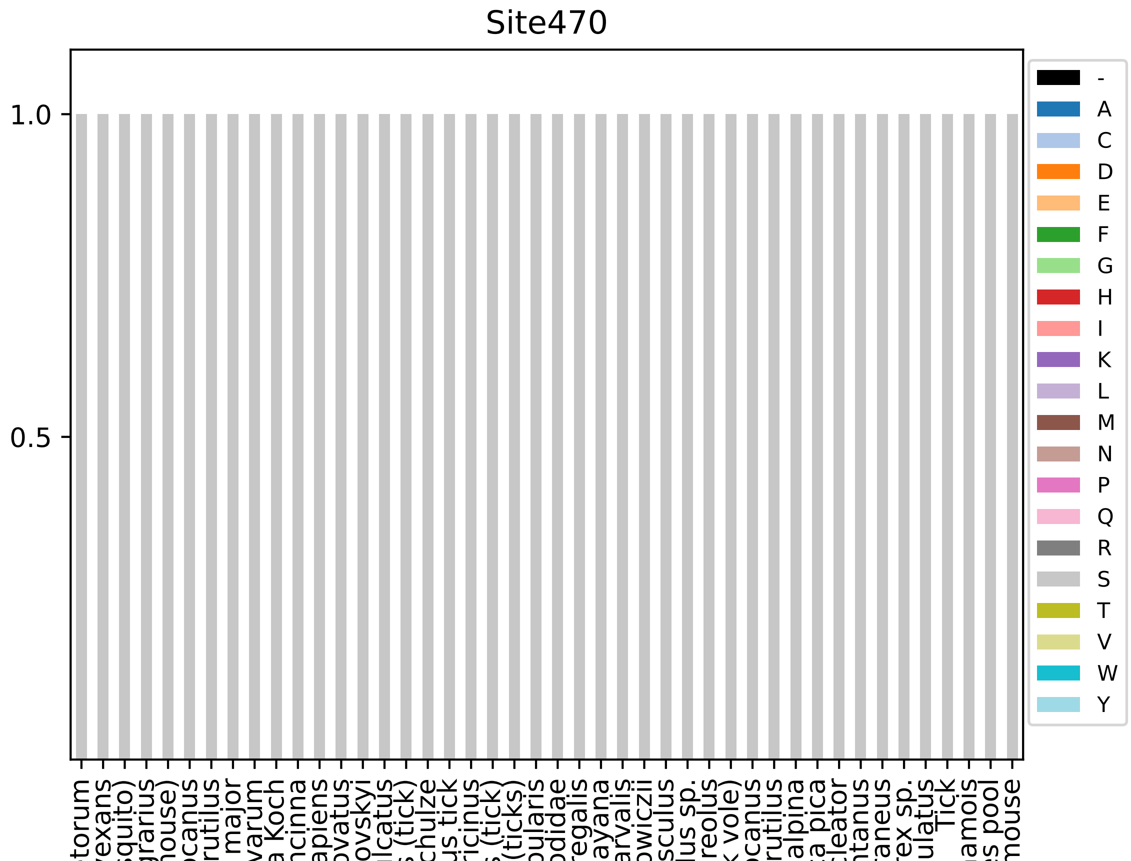
Site470
1.0
0.5
-
A
C
D
E
F
G
H
I
K
L
M
N
P
Q
R
S
T
V
W
Y

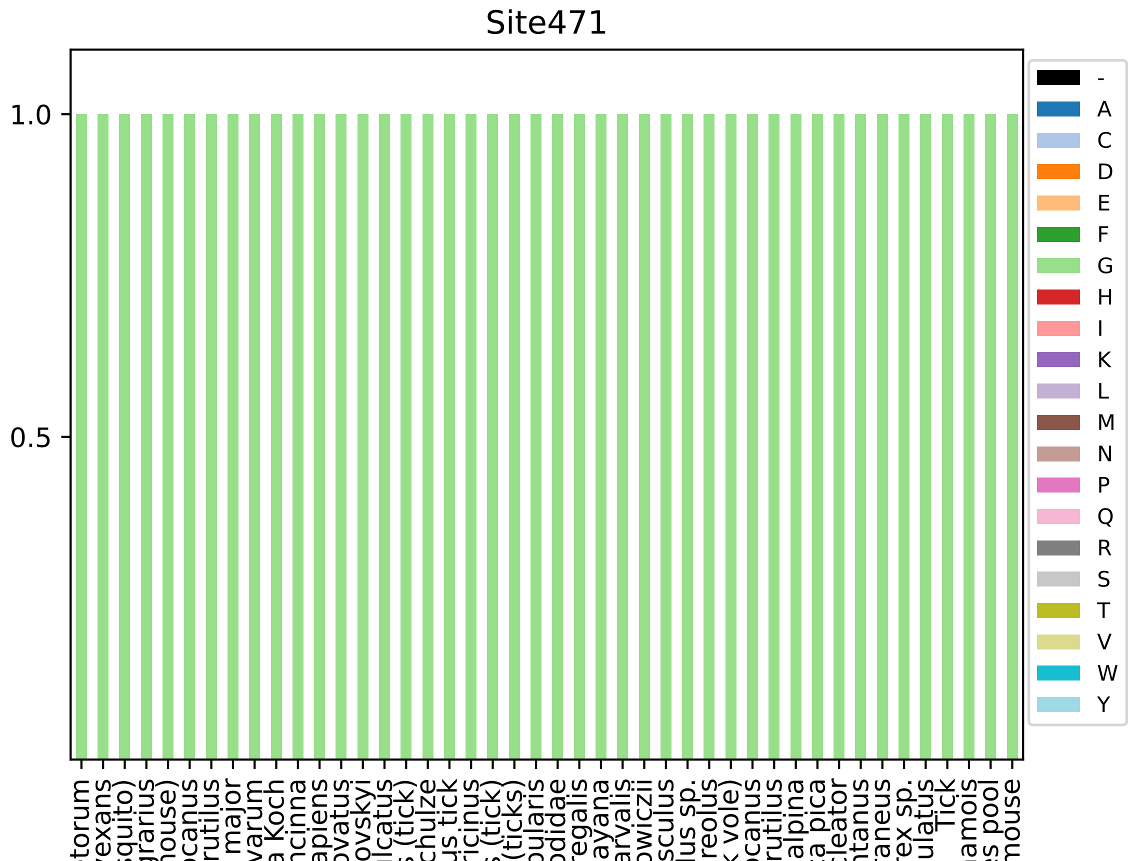

Site471
1.0
0.5
-
A
C
D
E
F
G
H
I
K
L
M
N
P
Q
R
S
T
V
W
Y

Site472

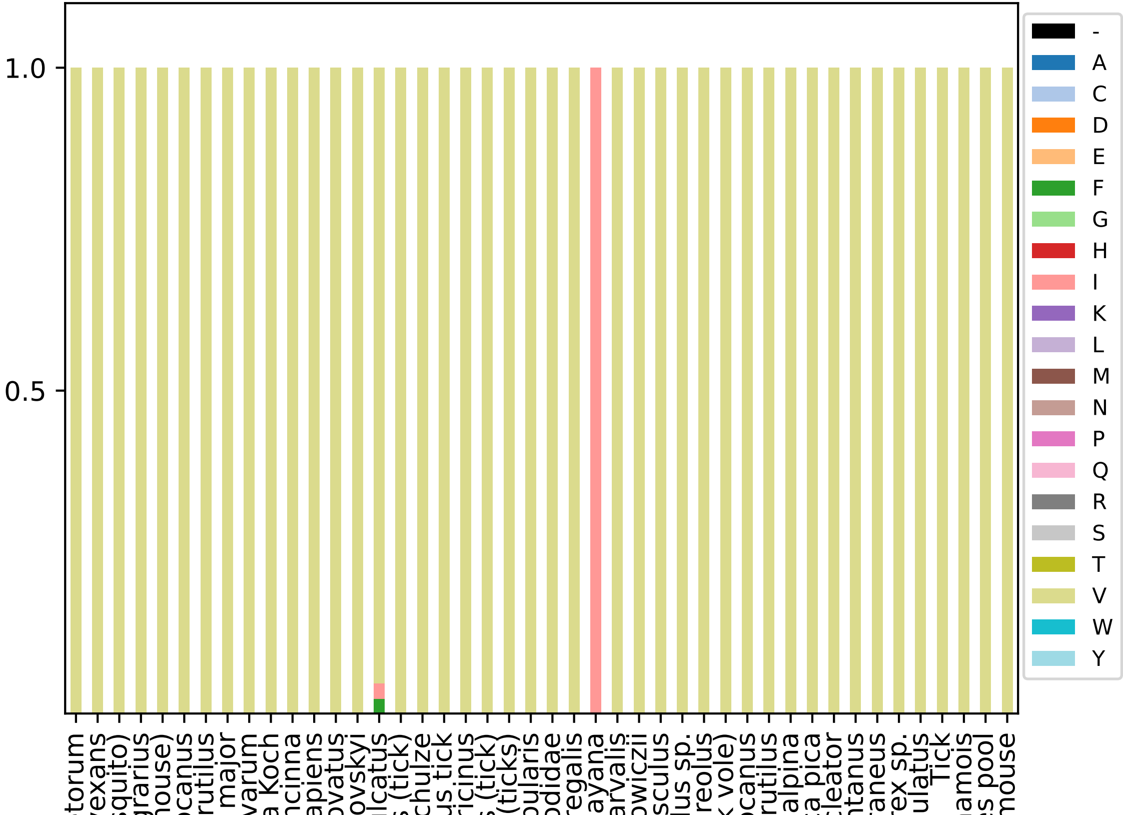

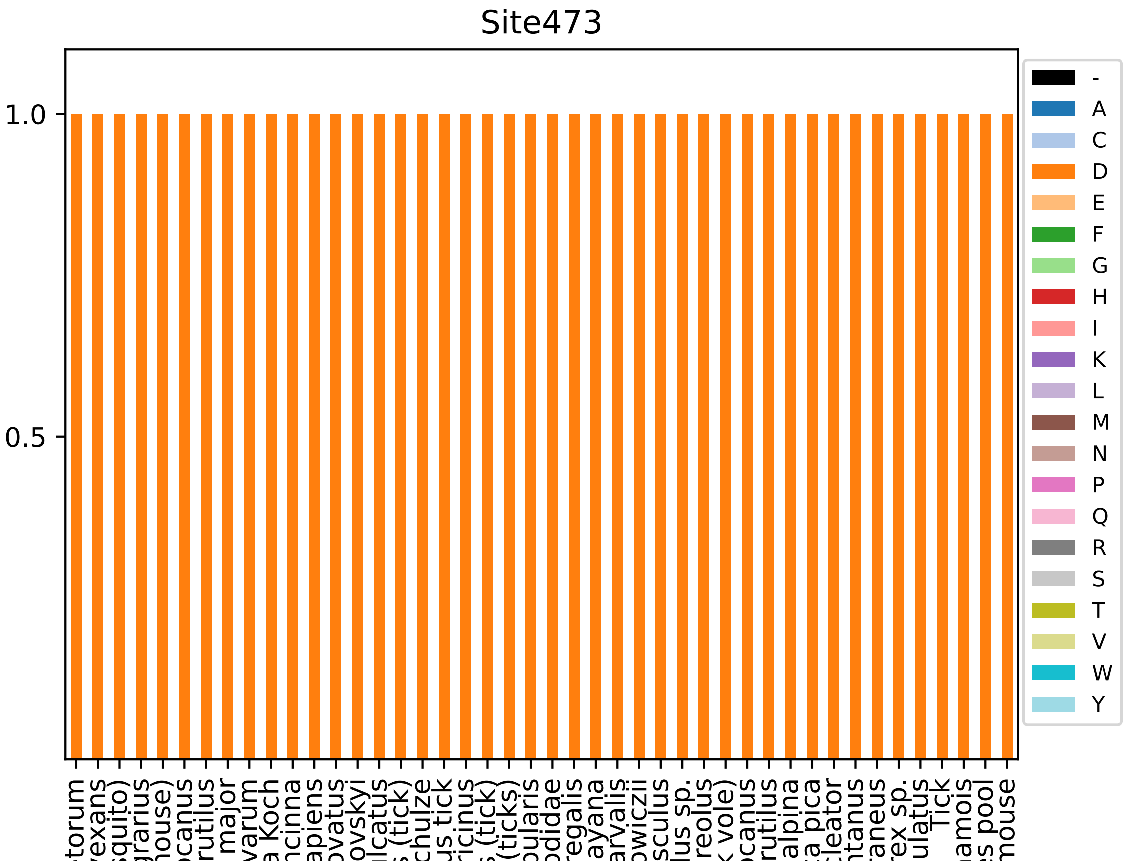
Site473
1.0
0.5
-
A
C
D
E
F
G
H
I
K
L
M
N
P
Q
R
S
T
V
W
Y

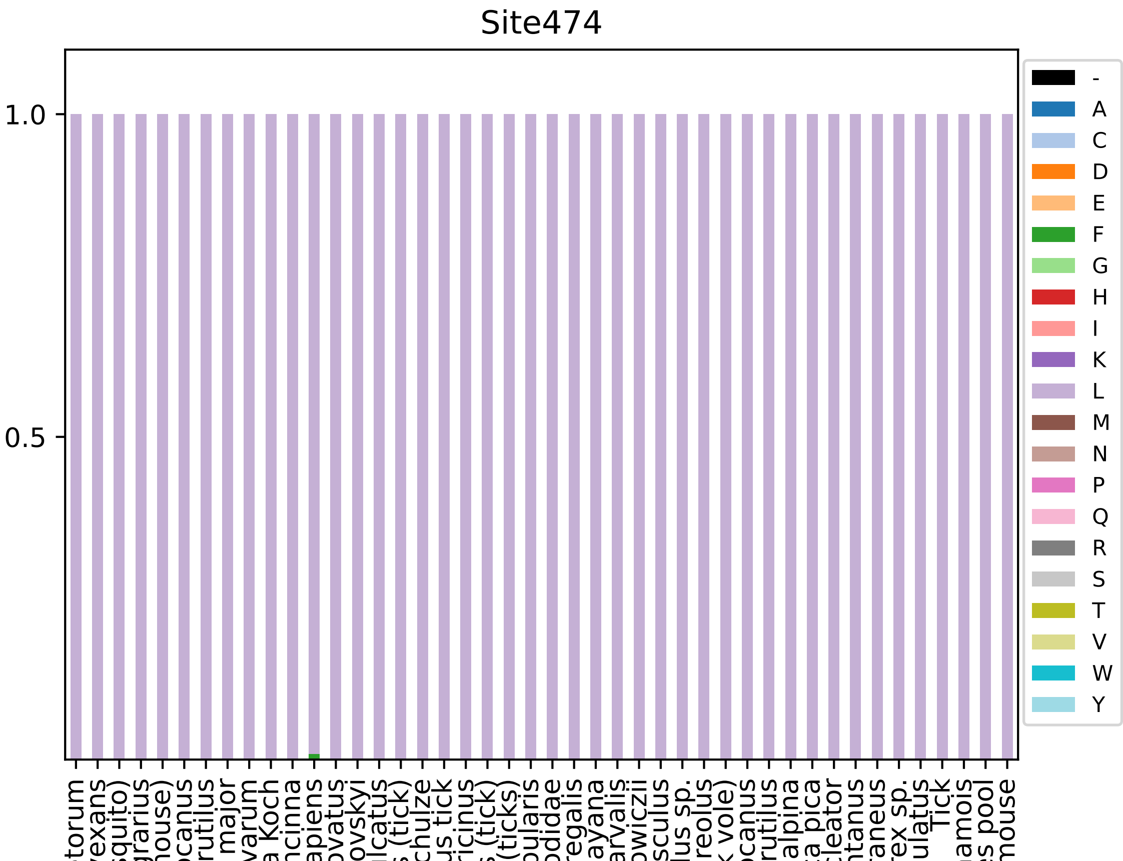

Site474
1.0
0.5
-
A
C
D
E
F
G
H
I
K
L
M
N
P
Q
R
S
T
V
W
Y

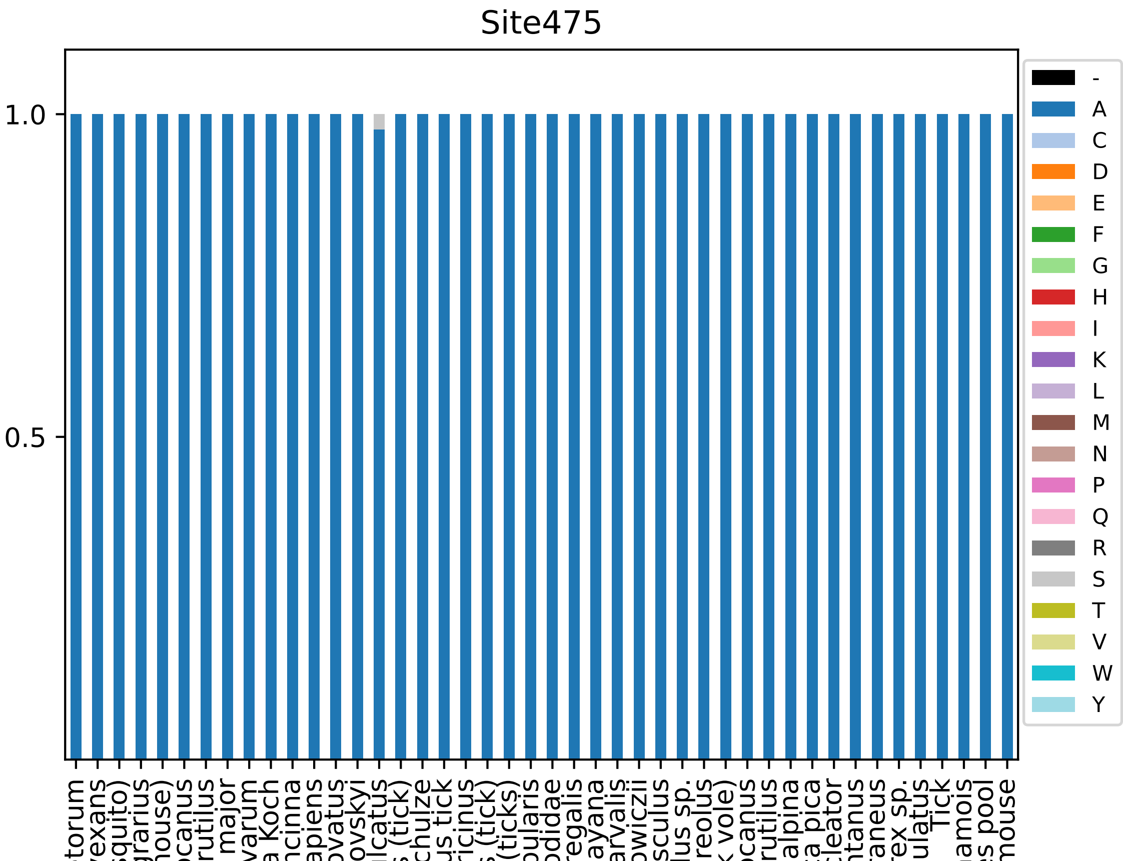

Site475
1.0
0.5
-
A
C
D
E
F
G
H
I
K
L
M
N
P
Q
R
S
T
V
W
Y

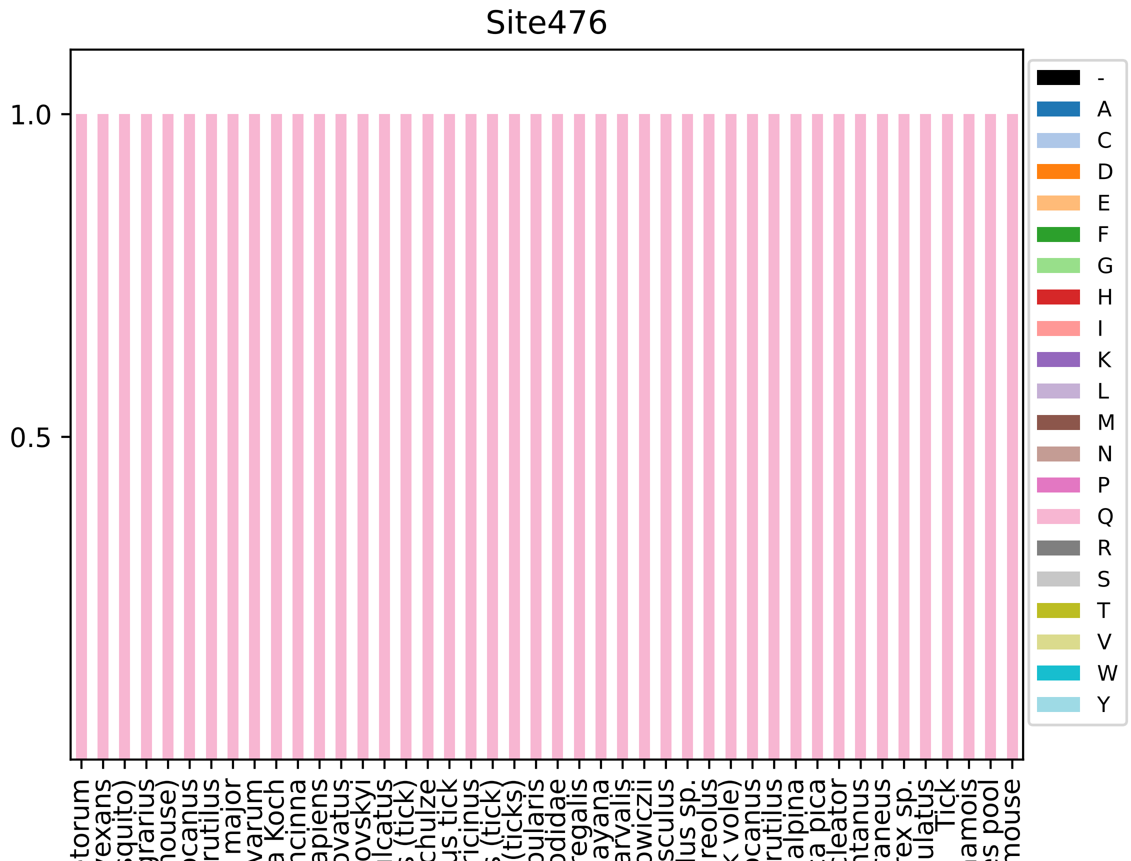

Site476
1.0
0.5
-
A
C
D
E
F
G
H
I
K
L
M
N
P
Q
R
S
T
V
W
Y

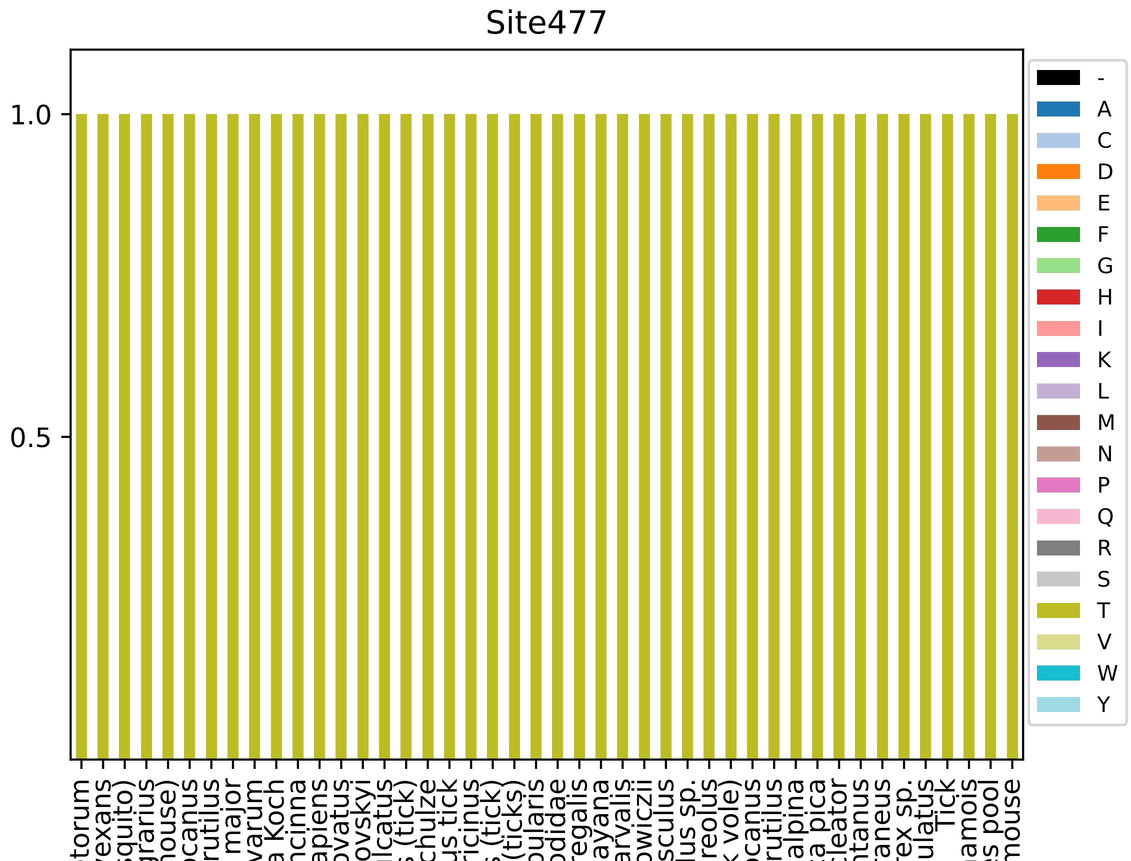
Site477
1.0
0.5
-
A
C
D
E
F
G
H
I
K
L
M
N
P
Q
R
S
T
V
W
Y

Site478

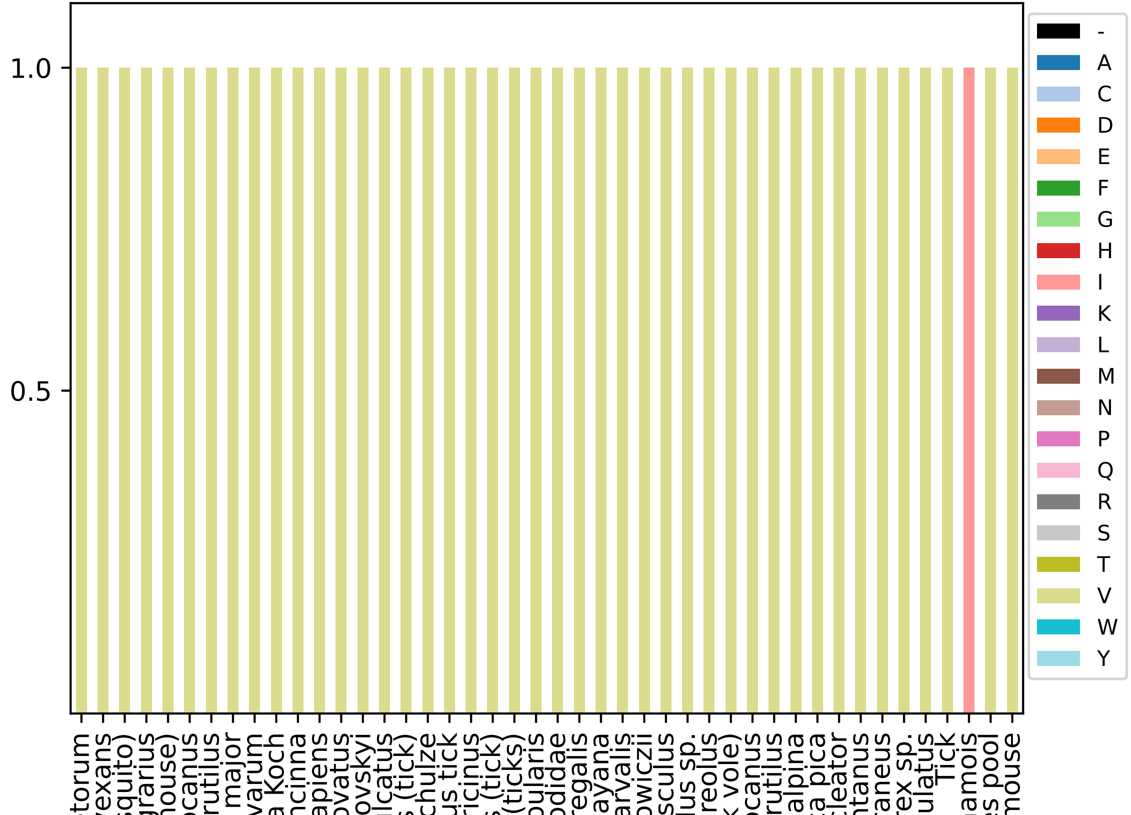

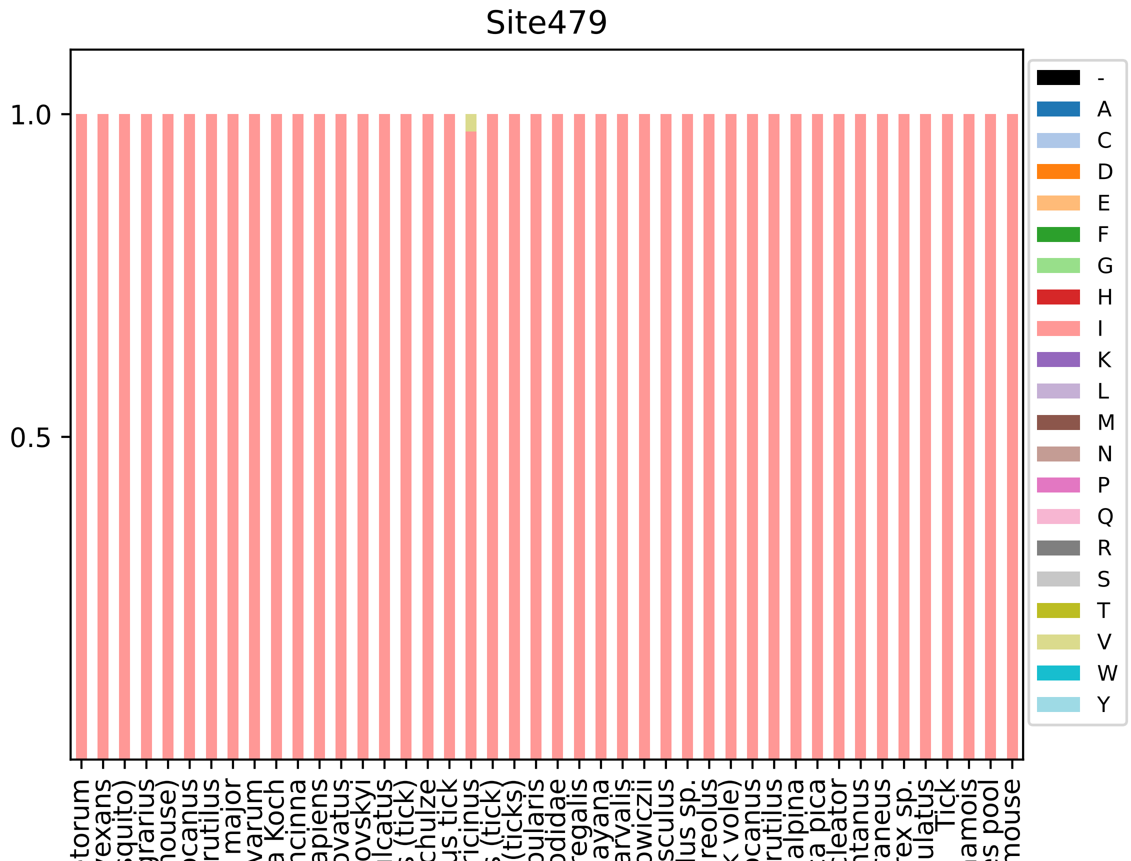
Site479
1.0
0.5
-
A
C
D
E
F
G
H
I
K
L
M
N
P
Q
R
S
T
V
W
Y

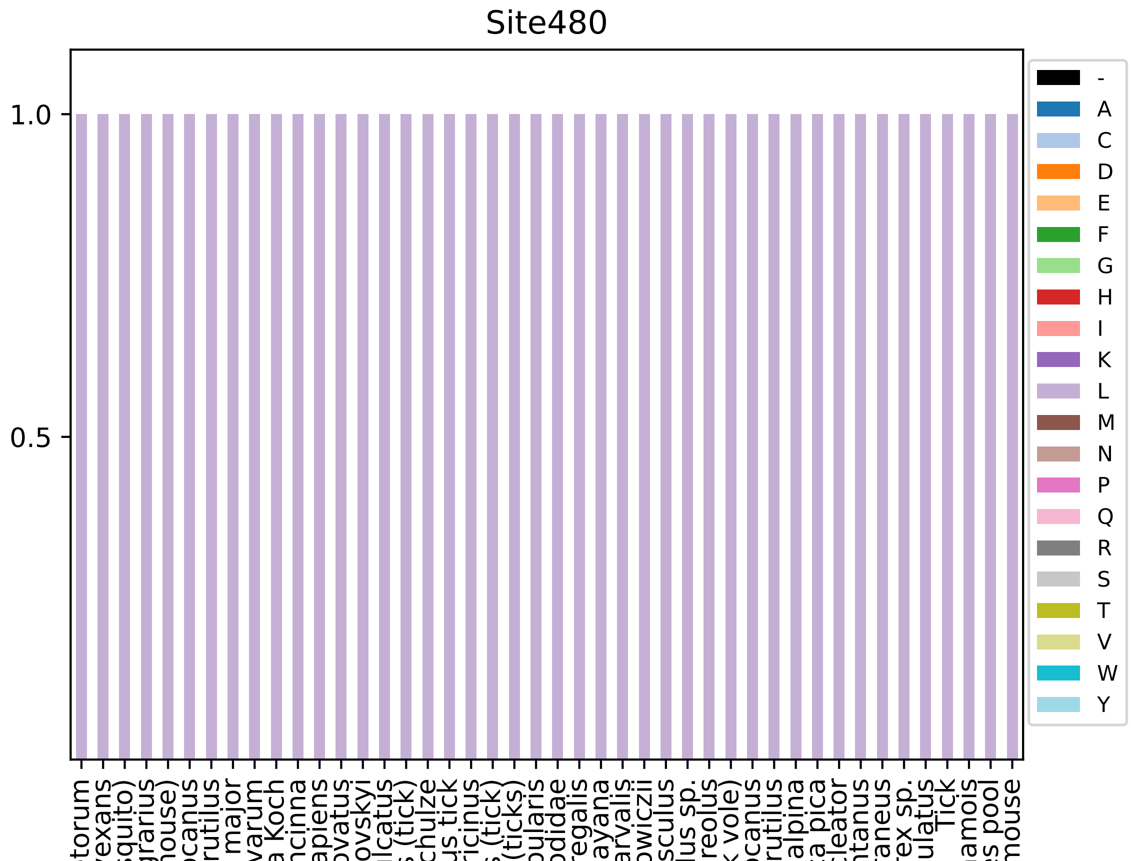

Site480
1.0
0.5
-
A
C
D
E
F
G
H
I
K
L
M
N
P
Q
R
S
T
V
W
Y
Tick

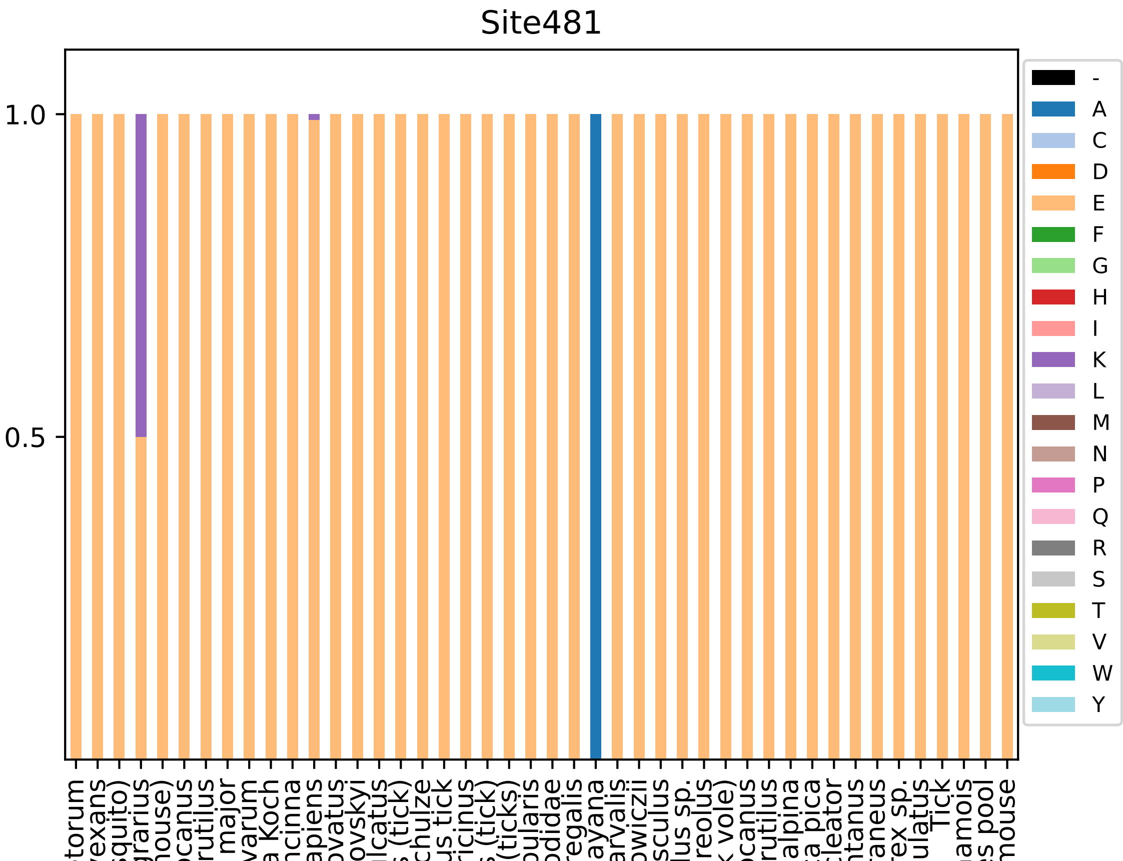
Site481
1.0
0.5
-
A
C
D
E
F
G
H
I
K
L
M
N
P
Q
R
S
T
V
W
Y

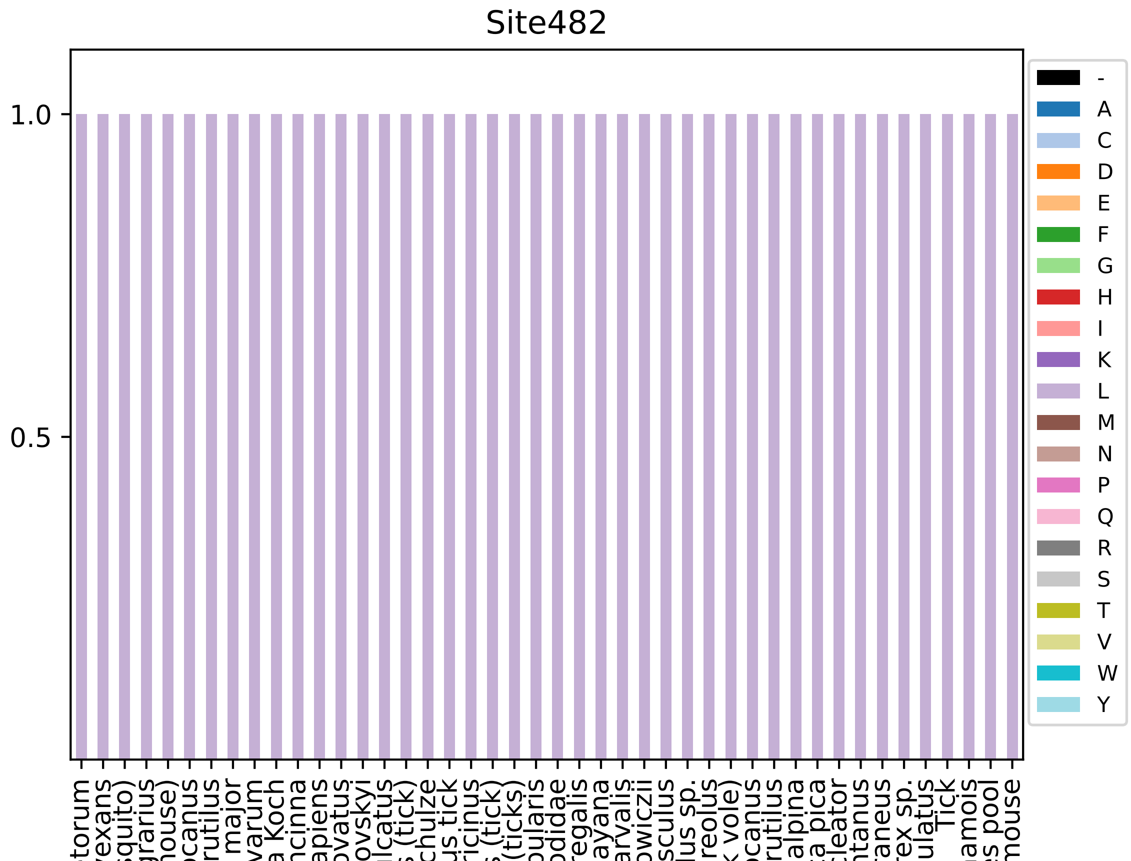
Site482
1.0
0.5
-
A
C
D
E
F
G
H
I
K
L
M
N
P
Q
R
S
T
V
W
Y

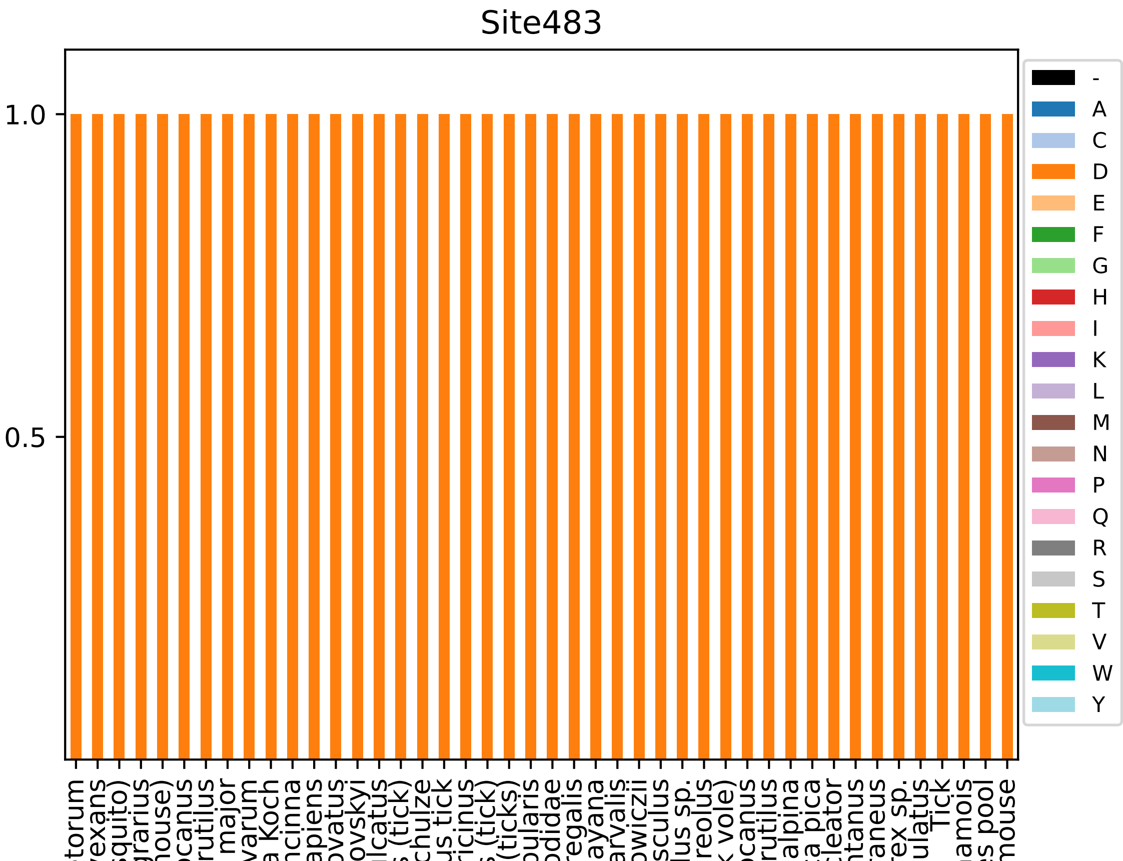
Site483
1.0
0.5
-
A
C
D
E
F
G
H
I
K
L
M
N
P
Q
R
S
T
V
W
Y

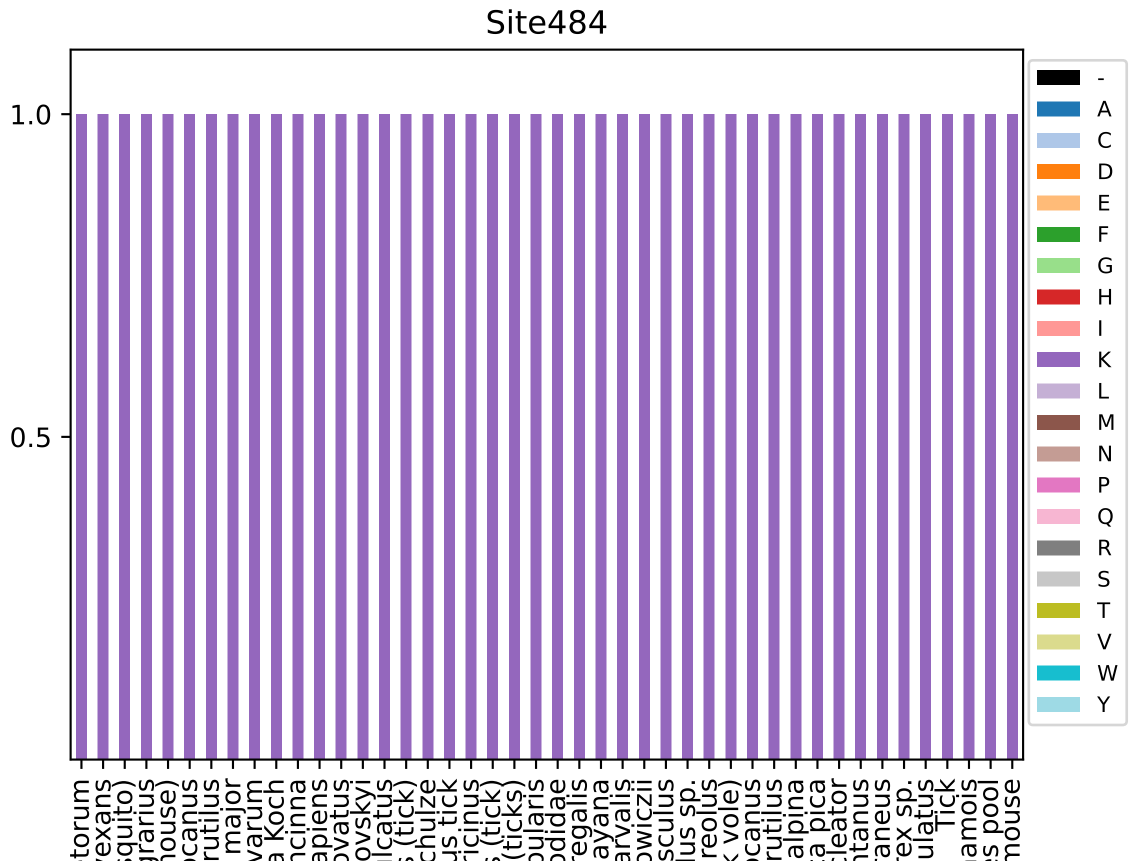
Site484
1.0
0.5
-
A
C
D
E
F
G
H
I
K
L
M
N
P
Q
R
S
T
V
W
Y

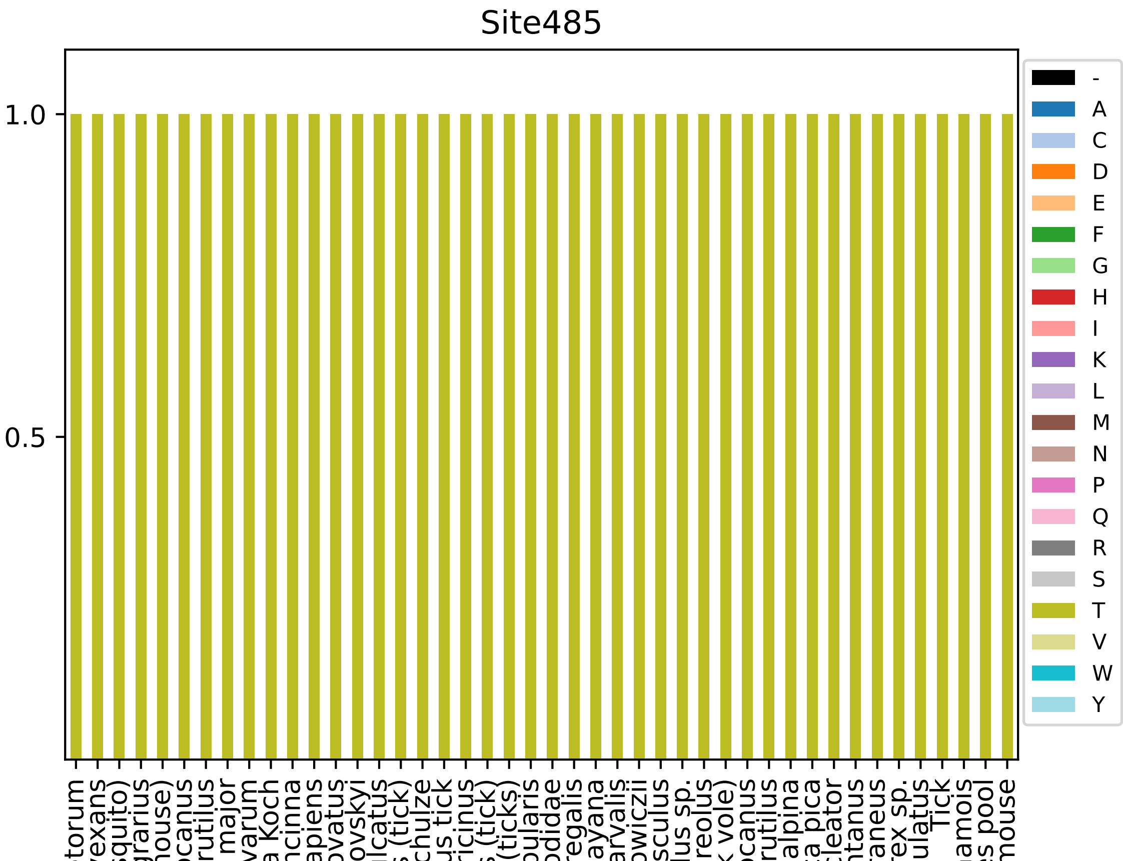
Site485
1.0
0.5
-
A
C
D
E
F
G
H
I
K
L
M
N
P
Q
R
S
T
V
W
Y

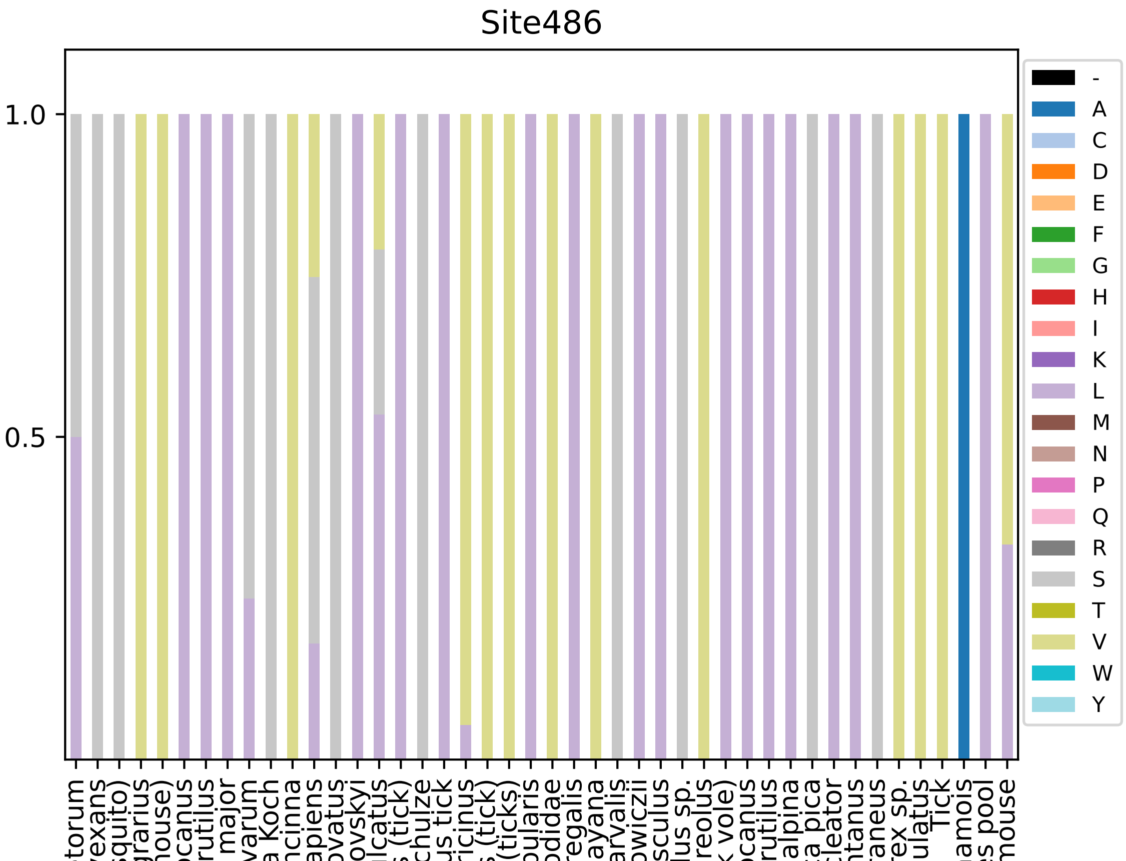
Site486
1.0
0.5
-
A
C
D
E
F
G
H
I
K
L
M
N
P
Q
R
S
T
V
W
Y

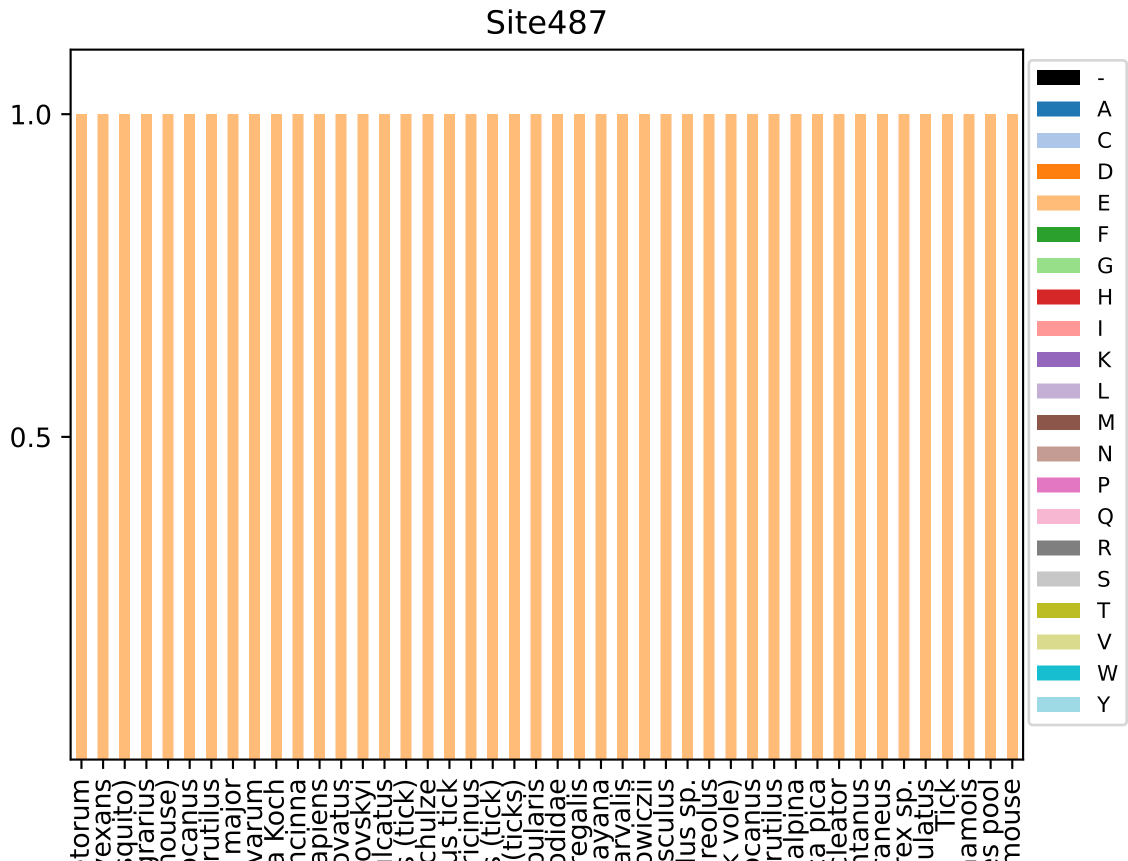

Site487
1.0
0.5
-
A
C
D
E
F
G
H
I
K
L
M
N
P
Q
R
S
T
V
W
Y

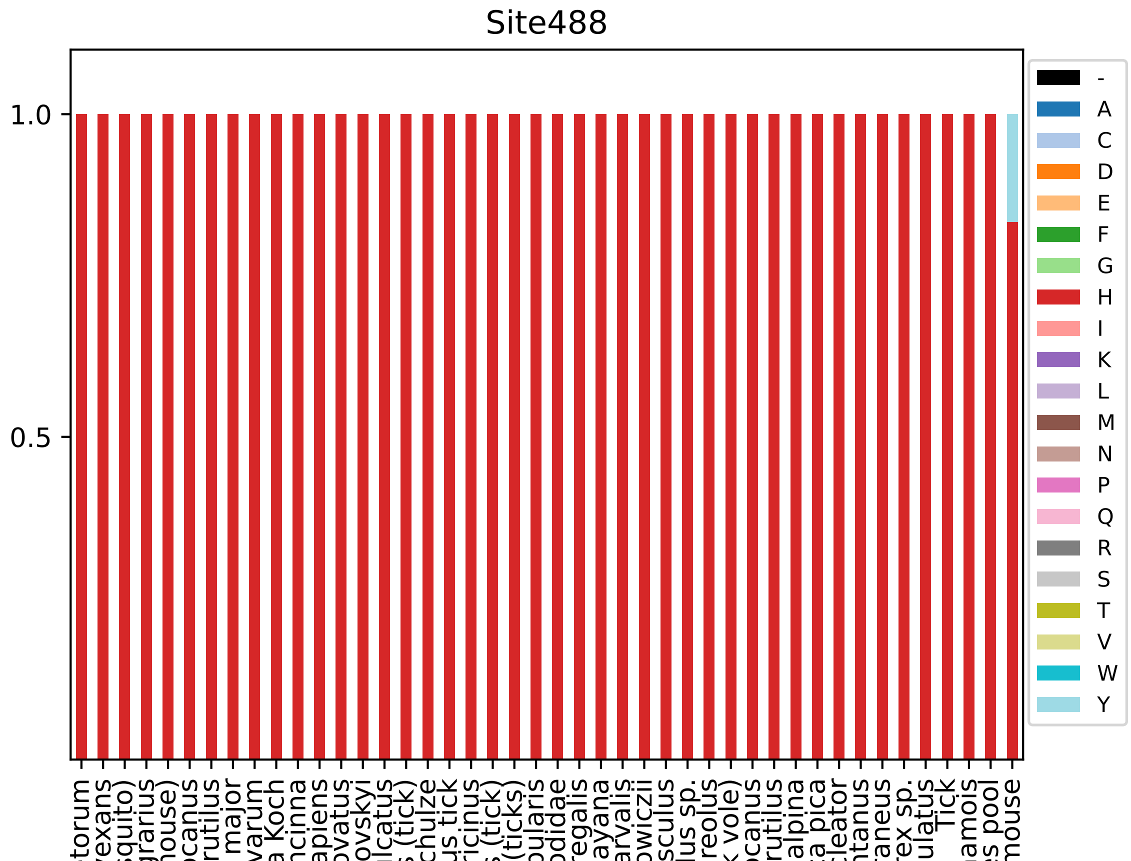
Site488
1.0
0.5
-
A
C
D
E
F
G
H
I
K
L
M
N
P
Q
R
S
T
V
W
Y

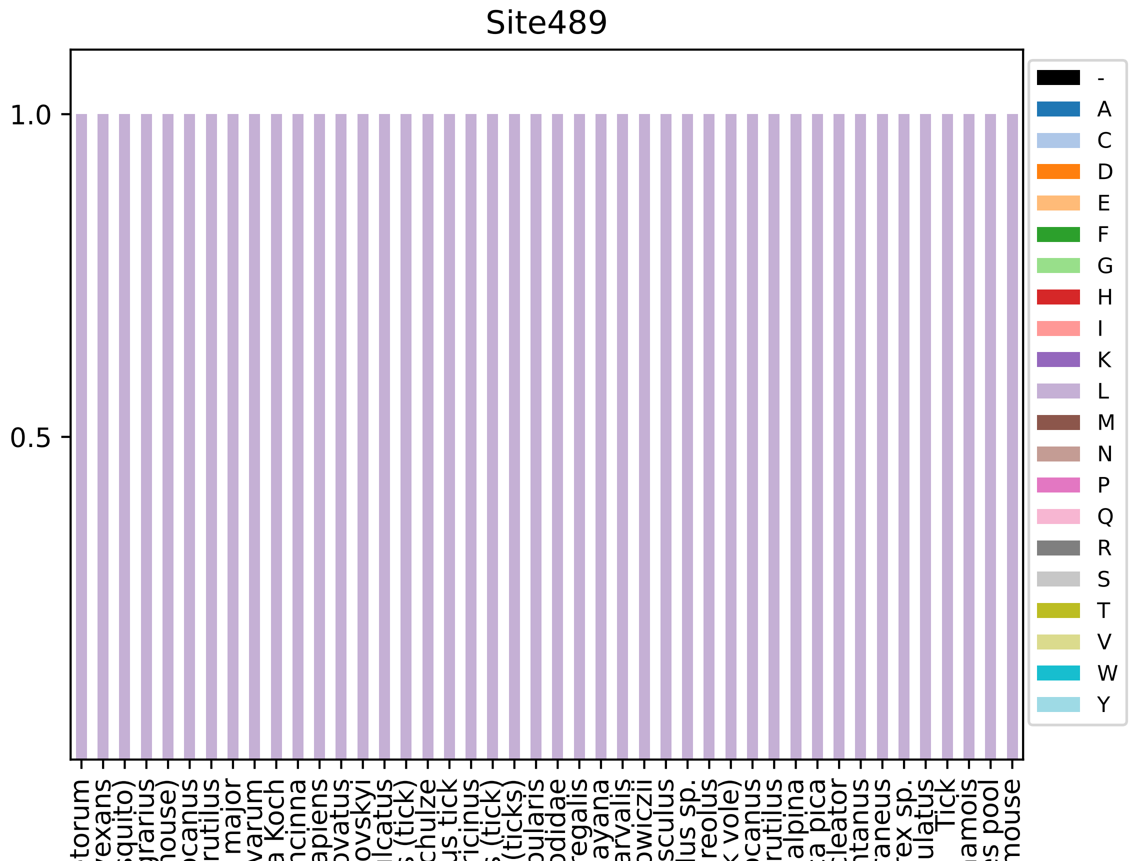
Site489
1.0
0.5
-
A
C
D
E
F
G
H
I
K
L
M
N
P
Q
R
S
T
V
W
Y
Tick

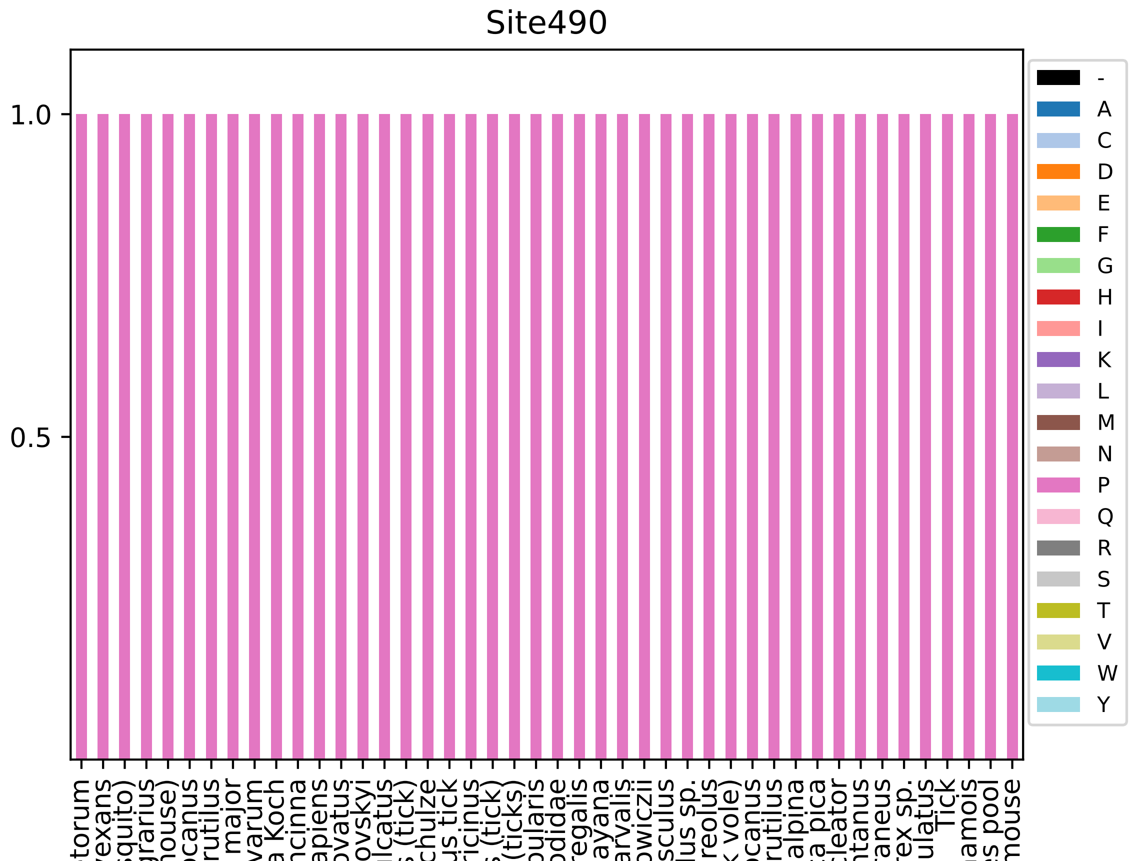

Site490
1.0
0.5
-
A
C
D
E
F
G
H
I
K
L
M
N
P
Q
R
S
T
V
W
Y

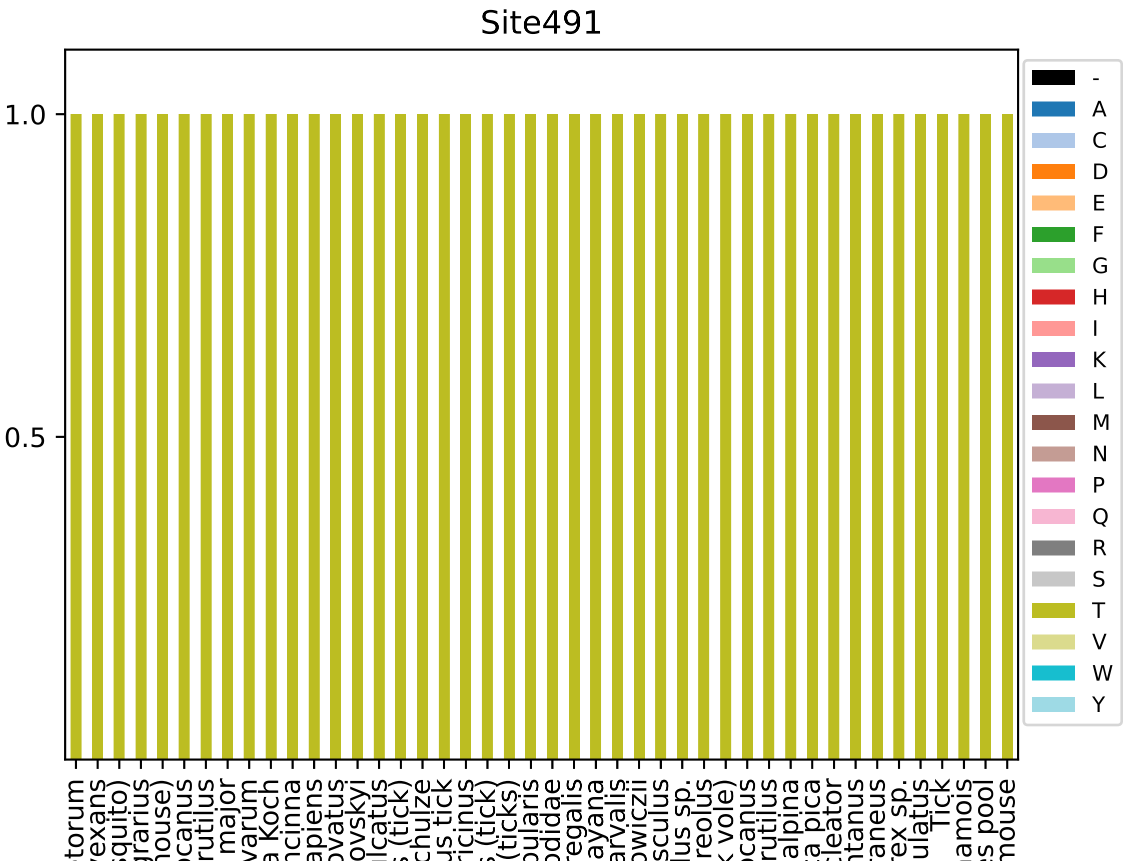
Site491
1.0
0.5
-
A
C
D
E
F
G
H
I
K
L
M
N
P
Q
R
S
T
V
W
Y

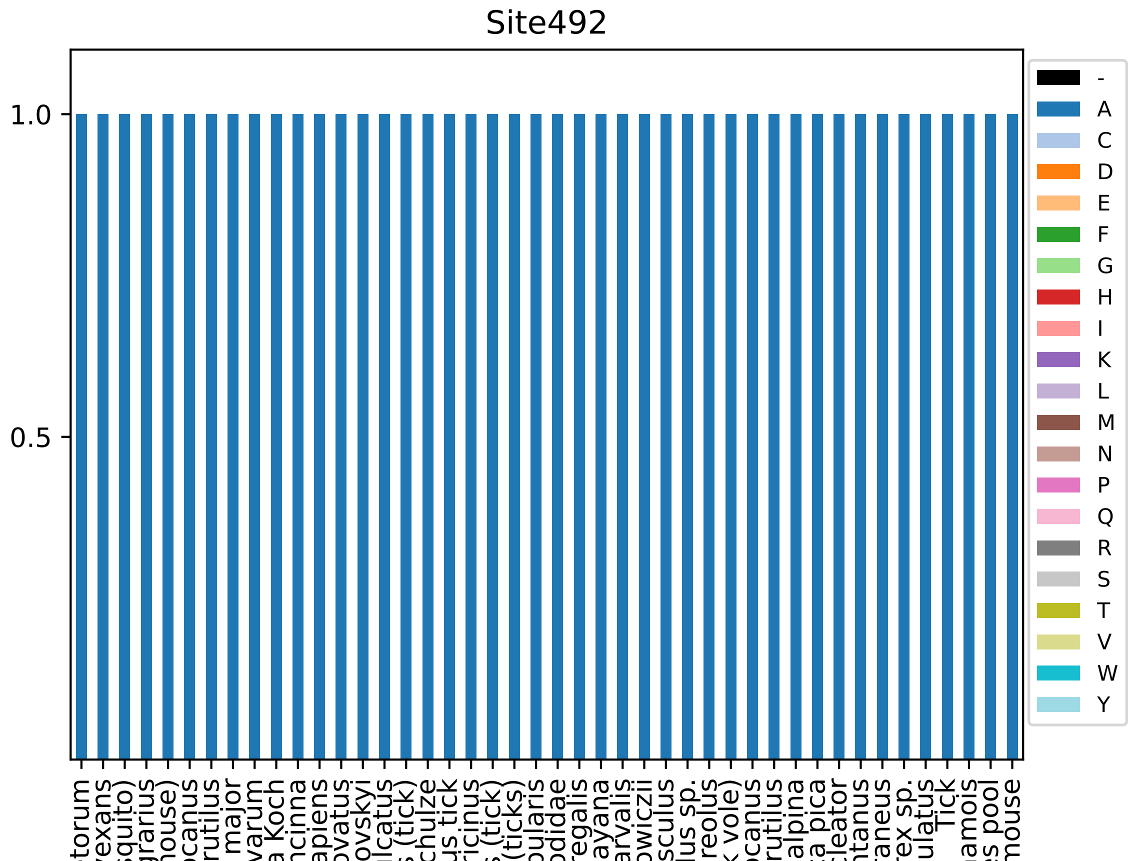
Site492
1.0
0.5
-
A
C
D
E
F
G
H
I
K
L
M
N
P
Q
R
S
T
V
W
Y

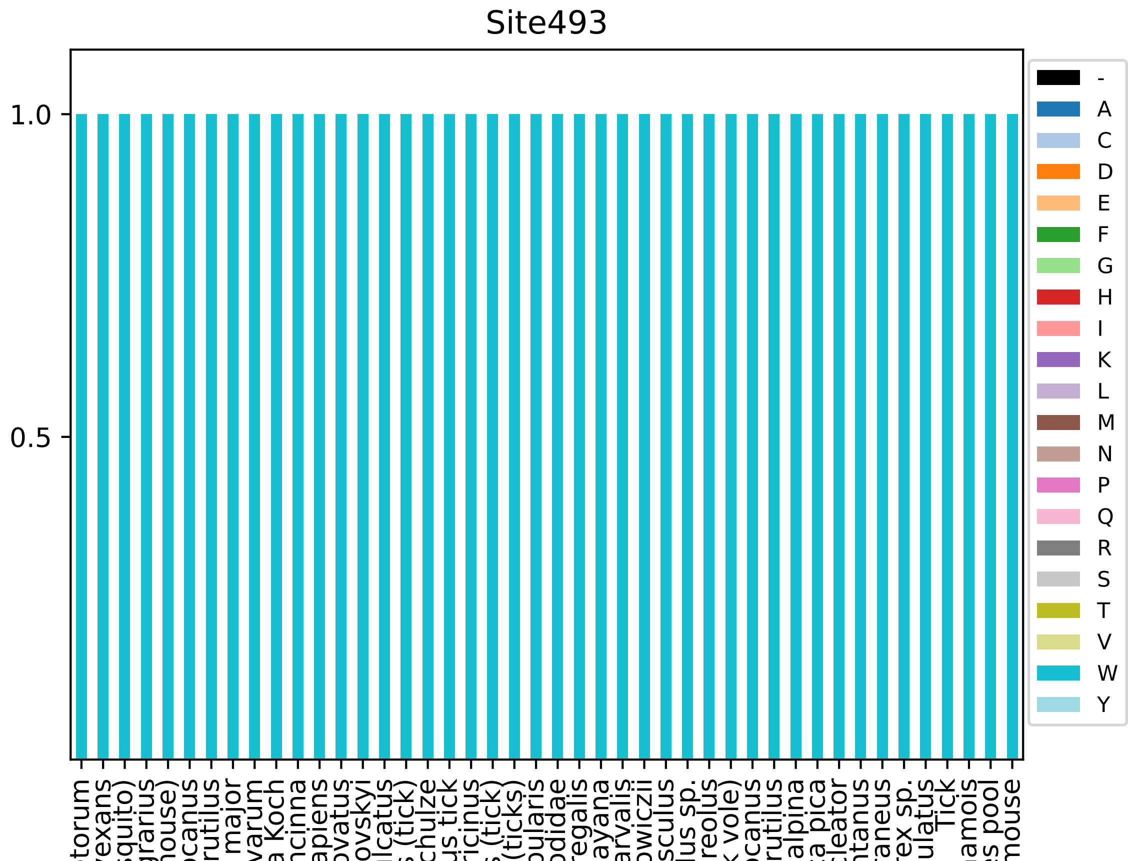
Site493
1.0
0.5
-
A
C
D
E
F
G
H
I
K
L
M
N
P
Q
R
S
T
V
W
Y

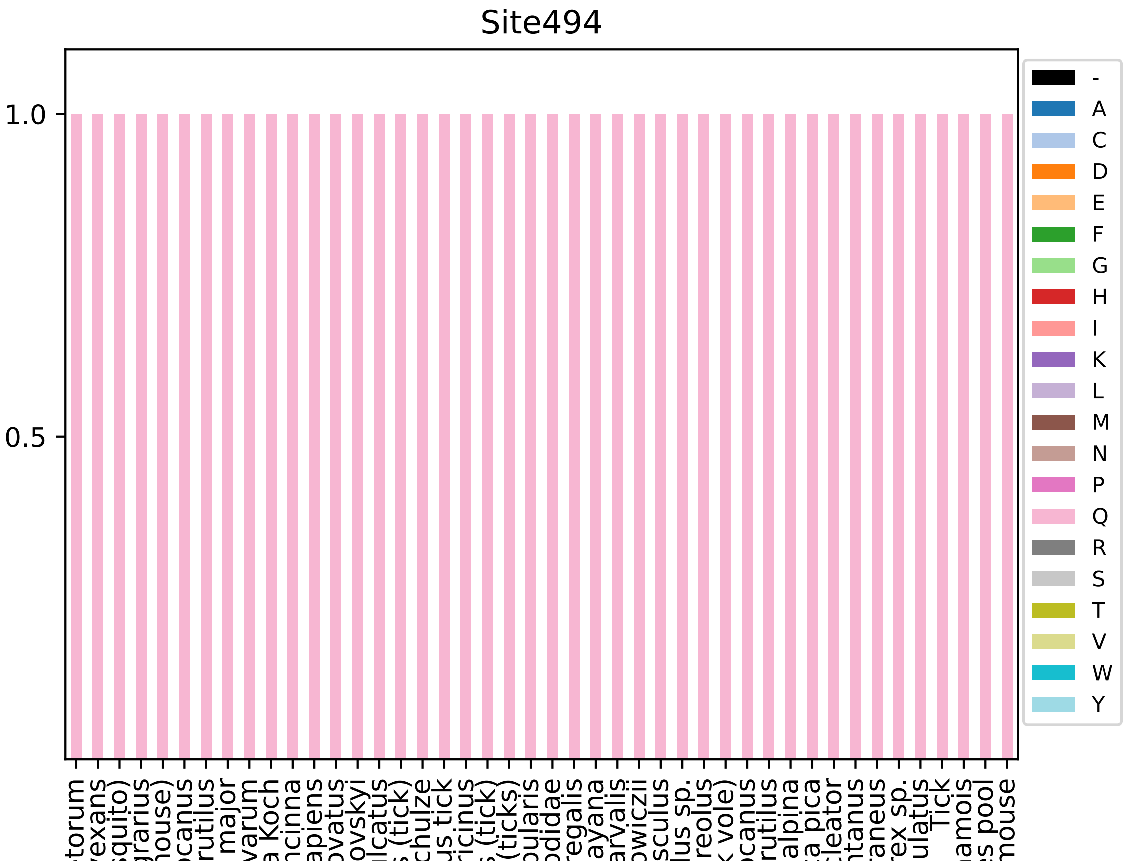

Site494
1.0
0.5
-
A
C
D
E
F
G
H
I
K
L
M
N
P
Q
R
S
T
V
W
Y

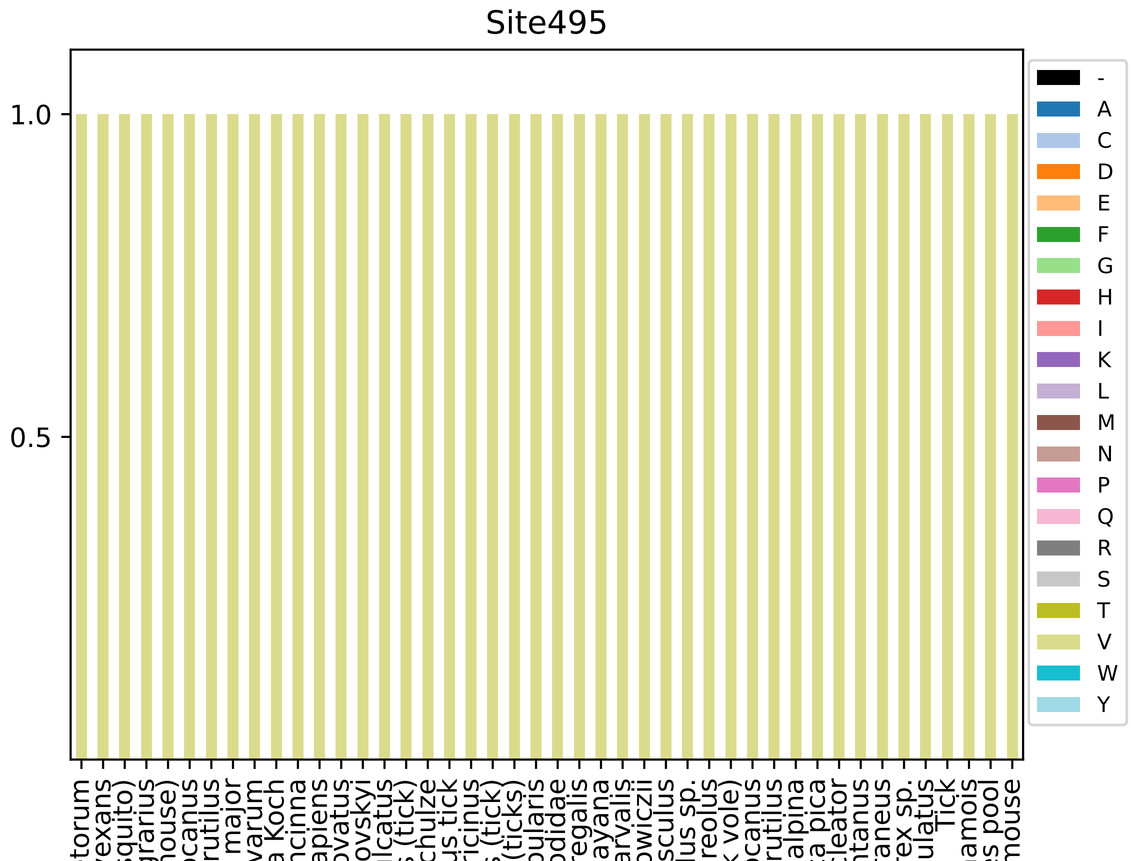
Site495
1.0
0.5
-
A
C
D
E
F
G
H
I
K
L
M
N
P
Q
R
S
T
V
W
Y

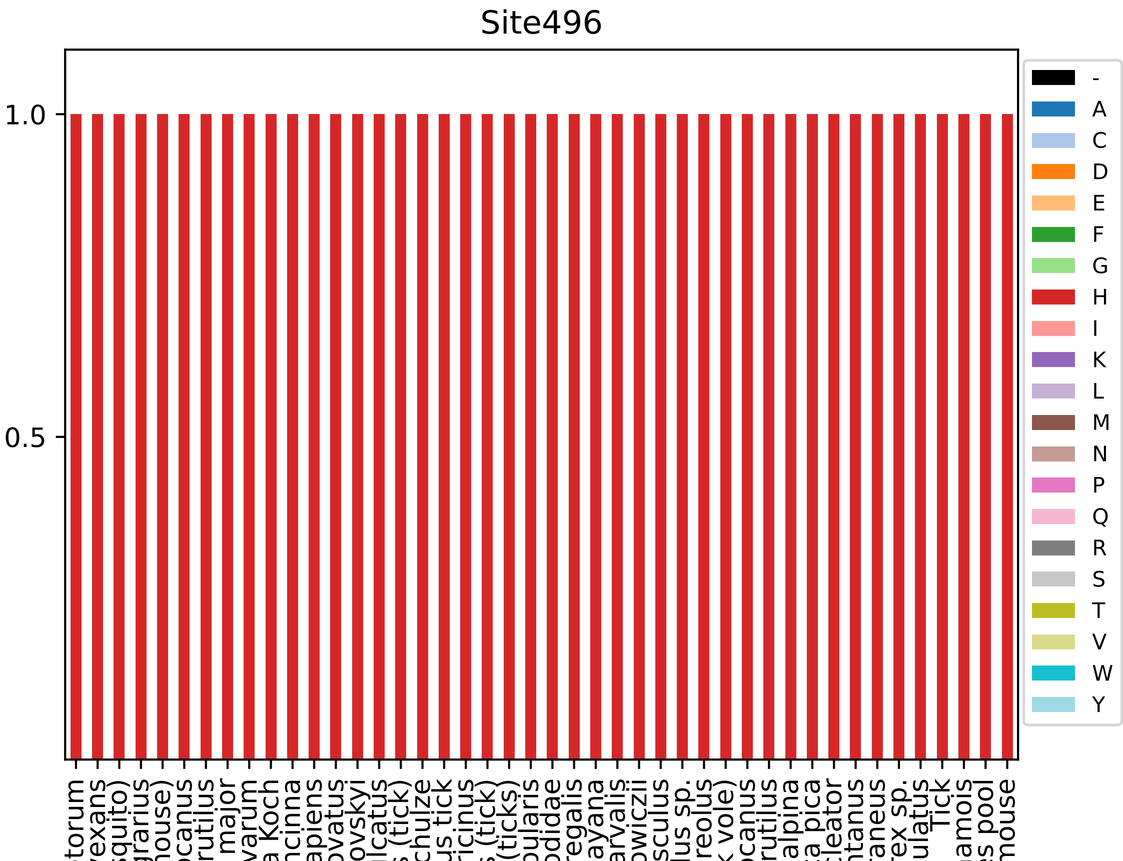
Site496
1.0
0.5
-
A
C
D
E
F
G
H
I
K
L
M
N
P
Q
R
S
T
V
W
Y
Tick

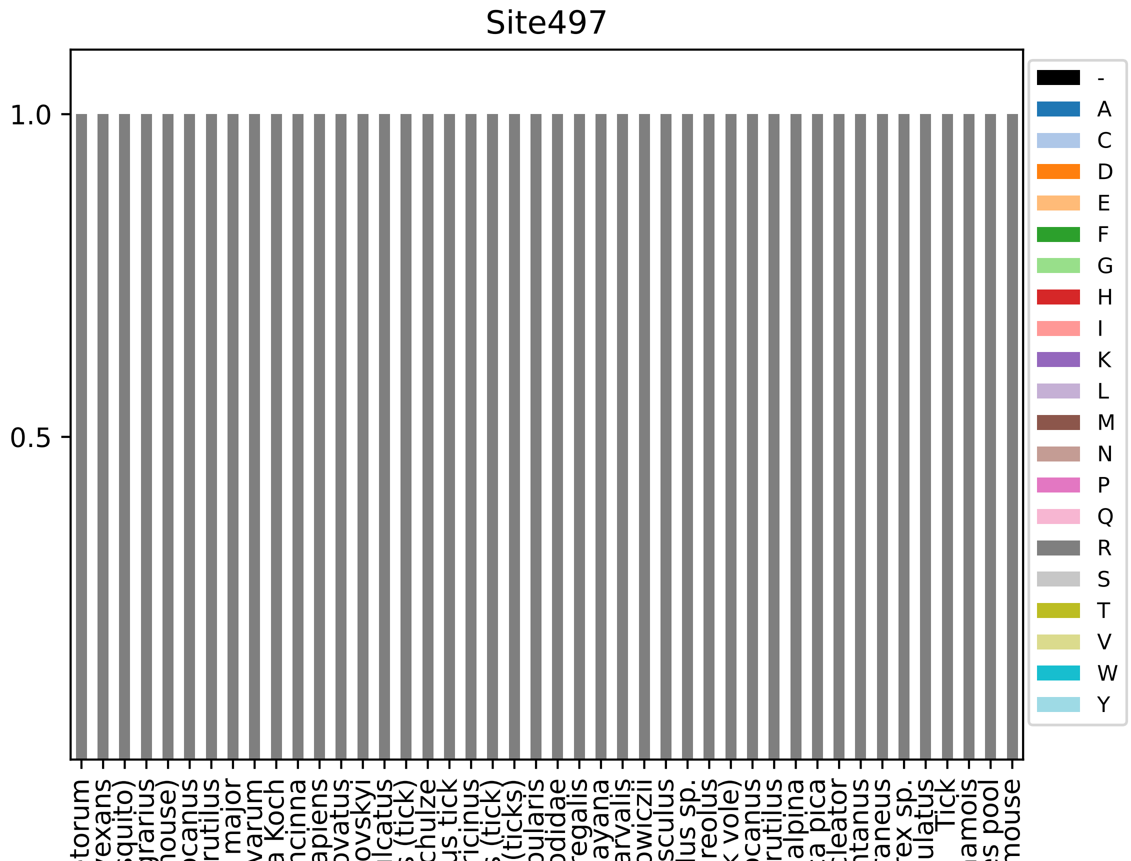
Site497
1.0
0.5
-
A
C
D
E
F
G
H
I
K
L
M
N
P
Q
R
S
T
V
W
Y

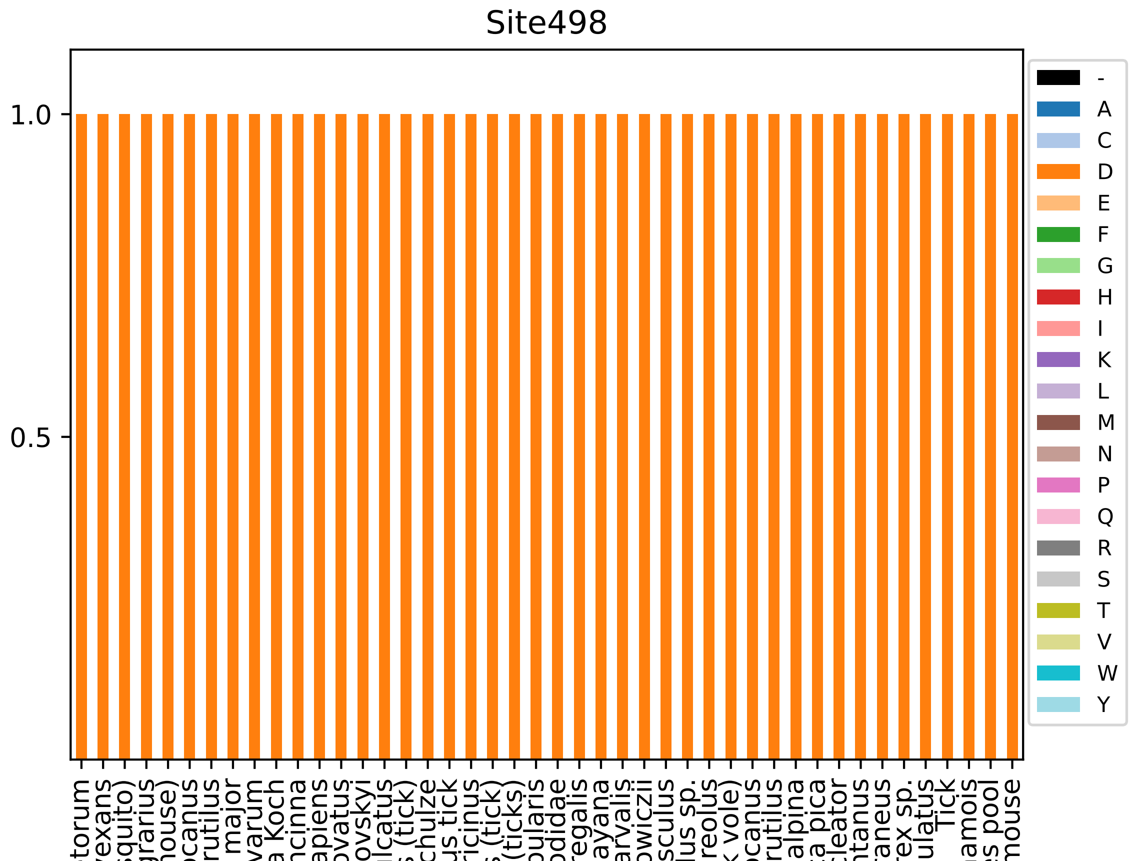

Site498
1.0
0.5
-
A
C
D
E
F
G
H
I
K
L
M
N
P
Q
R
S
T
V
W
Y

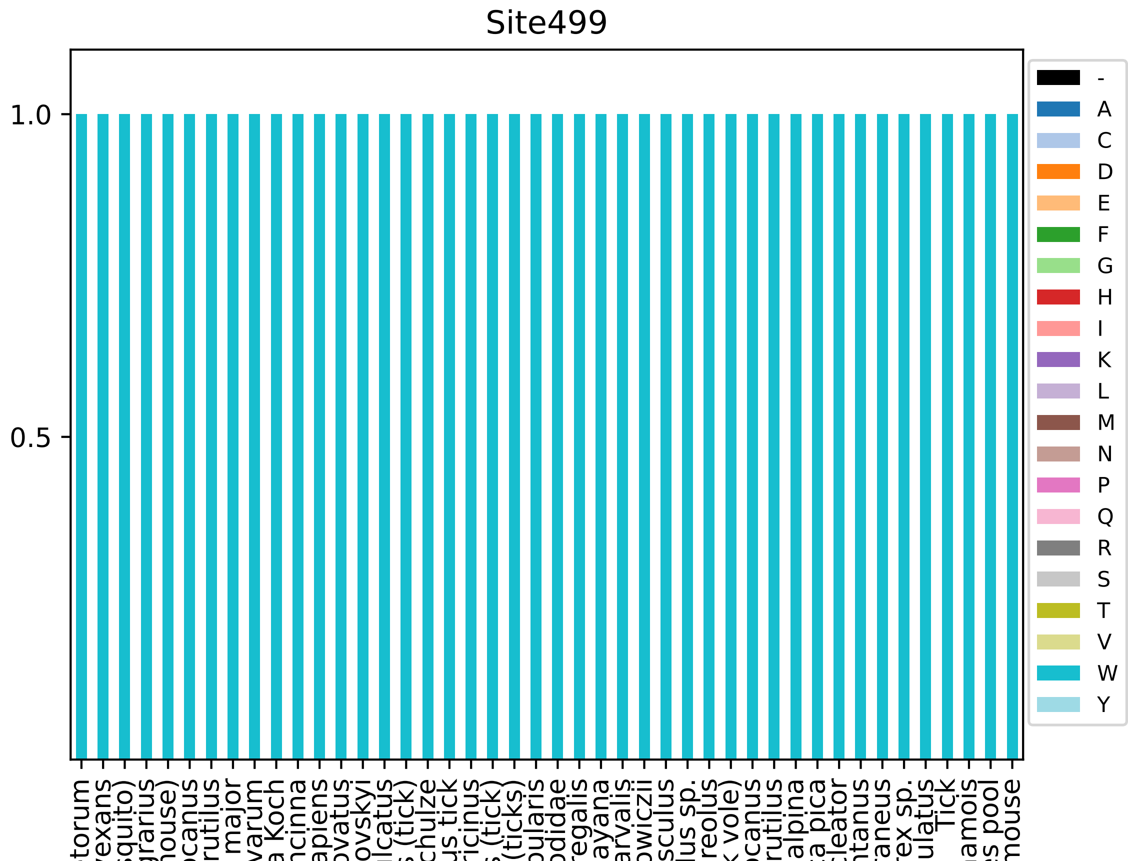
Site499
1.0
0.5
-
A
C
D
E
F
G
H
I
K
L
M
N
P
Q
R
S
T
V
W
Y

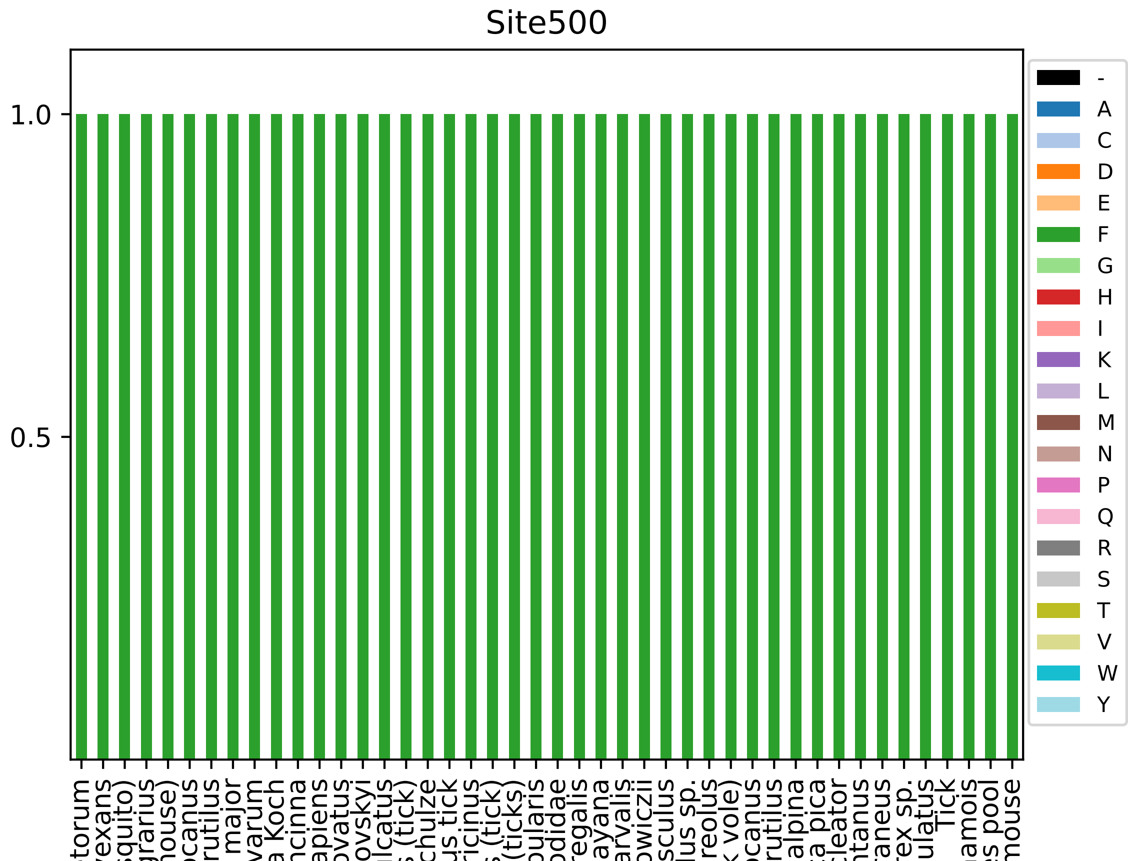
Site500
1.0
0.5
-
A
C
D
E
F
G
H
I
K
L
M
N
P
Q
R
S
T
V
W
Y

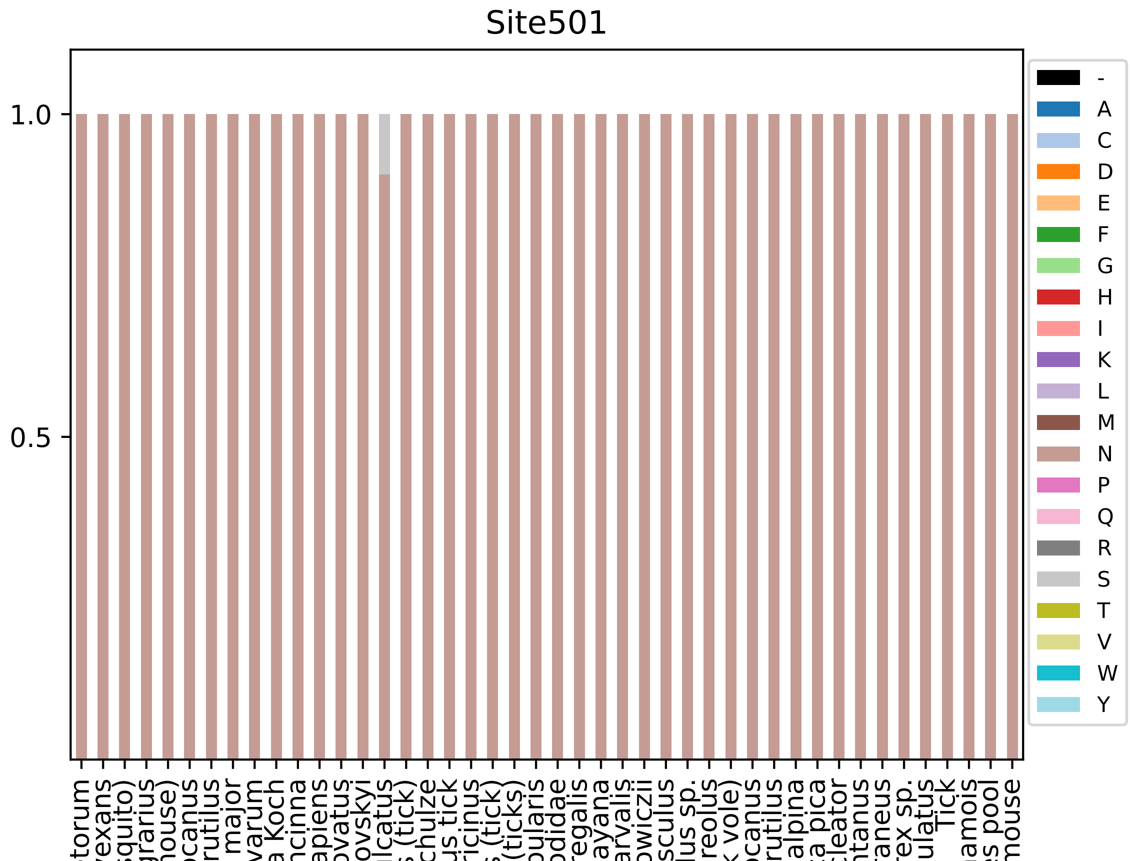
Site501
1.0
0.5
-
A
C
D
E
F
G
H
I
K
L
M
N
P
Q
R
S
T
V
W
Y

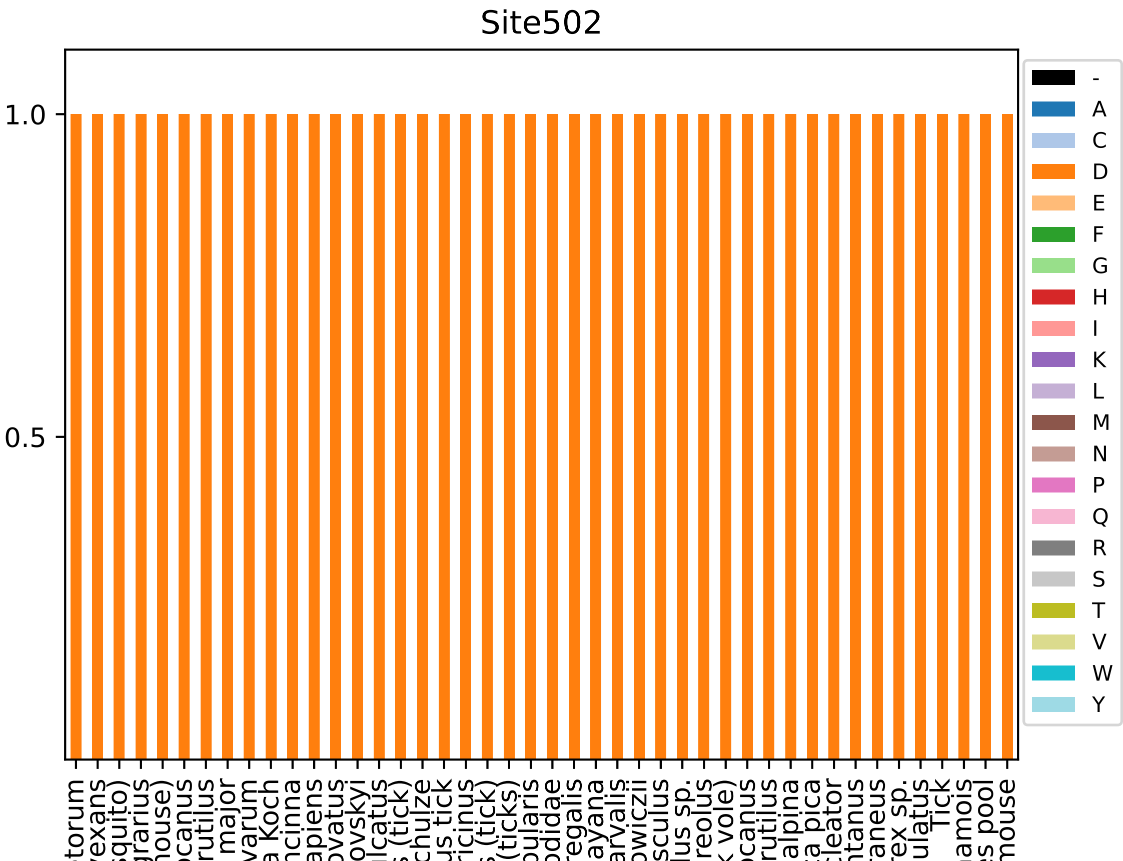
Site502
1.0
0.5
-
A
C
D
E
F
G
H
I
K
L
M
N
P
Q
R
S
T
V
W
Y

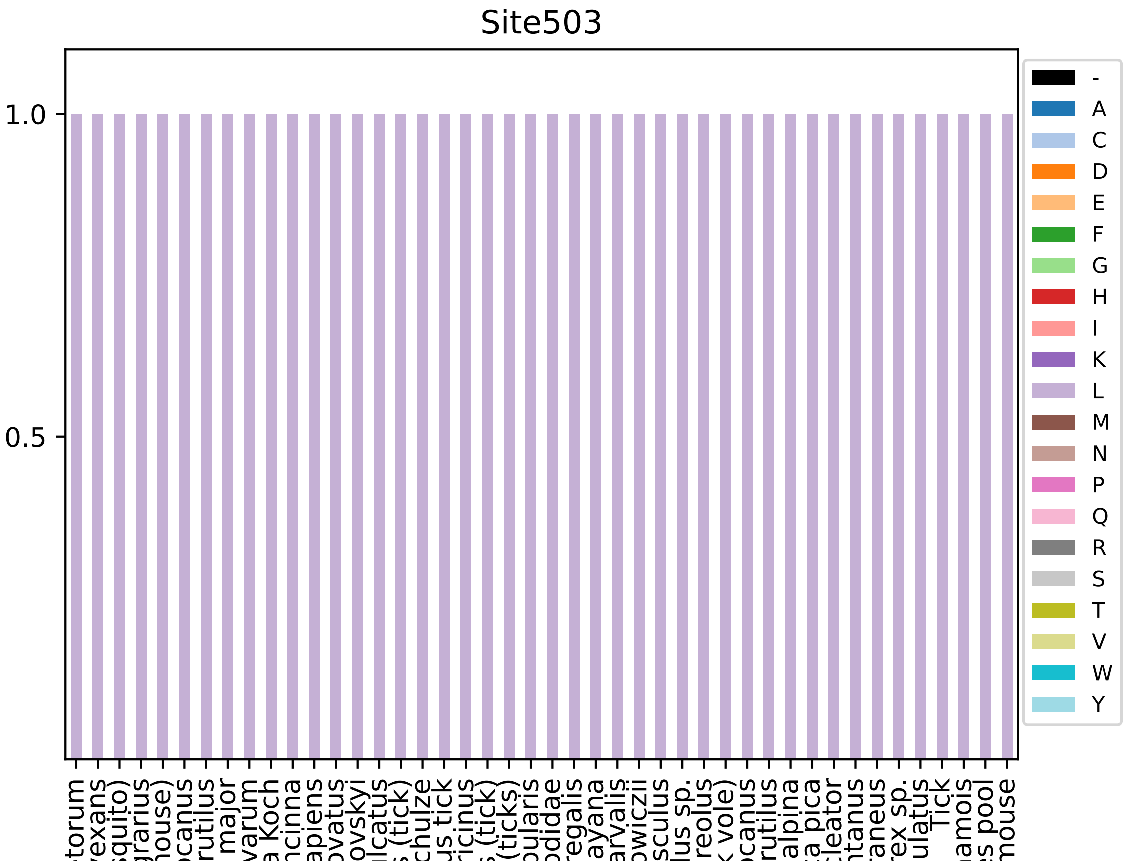
Site503
1.0
0.5
-
A
C
D
E
F
G
H
I
K
L
M
N
P
Q
R
S
T
V
W
Y

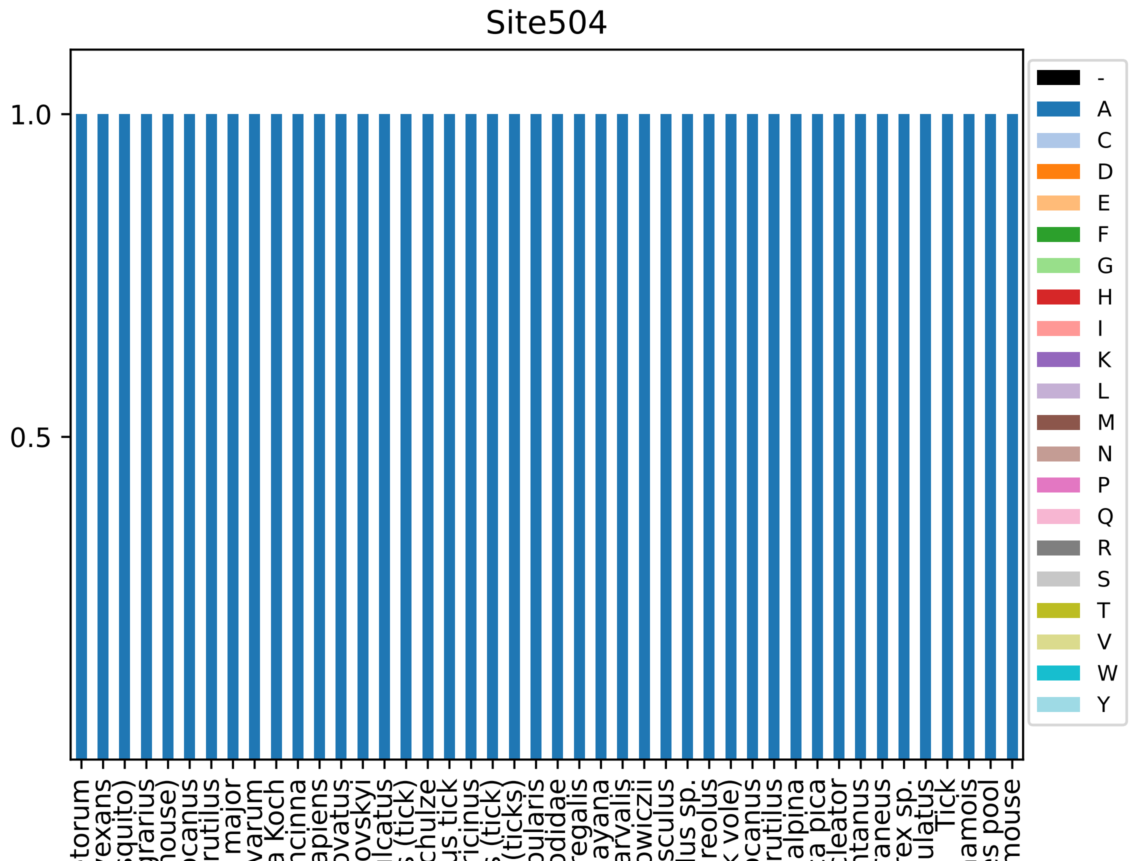

Site504
1.0
0.5
-
A
C
D
E
F
G
H
I
K
L
M
N
P
Q
R
S
T
V
W
Y

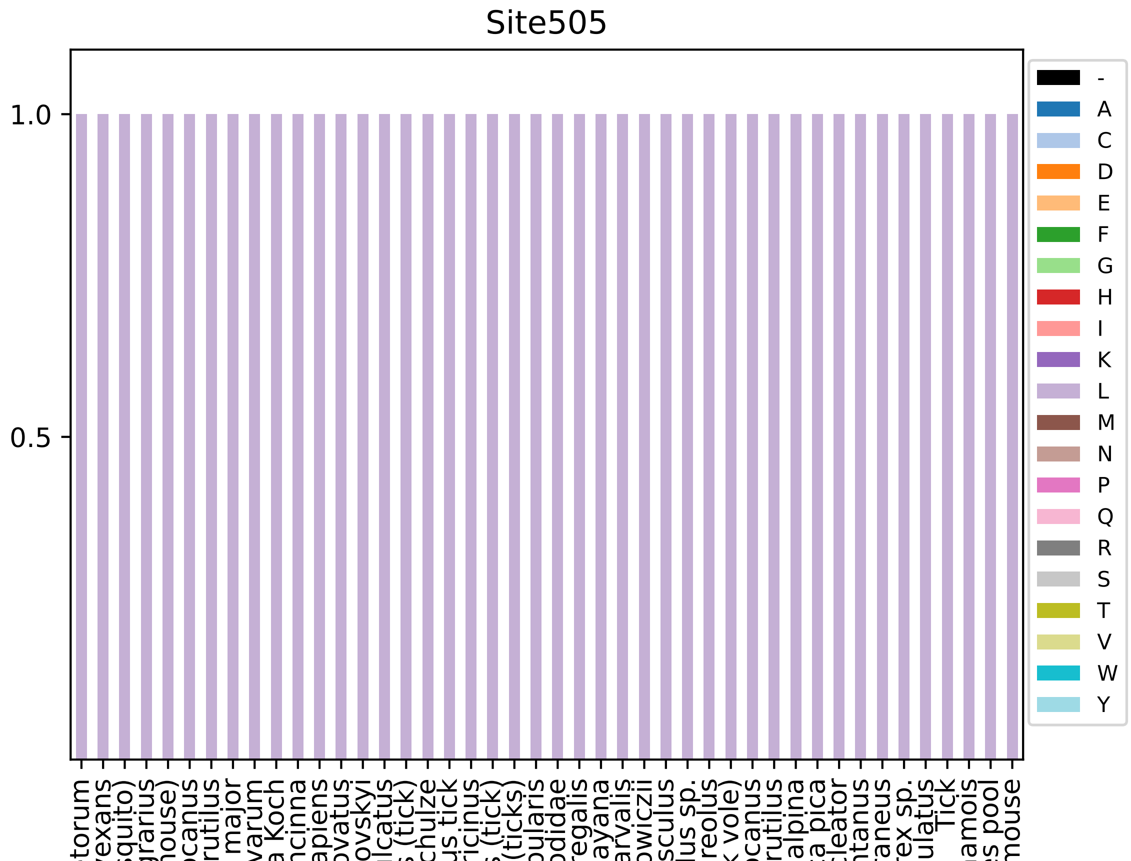
Site505
1.0
0.5
-
A
C
D
E
F
G
H
I
K
L
M
N
P
Q
R
S
T
V
W
Y

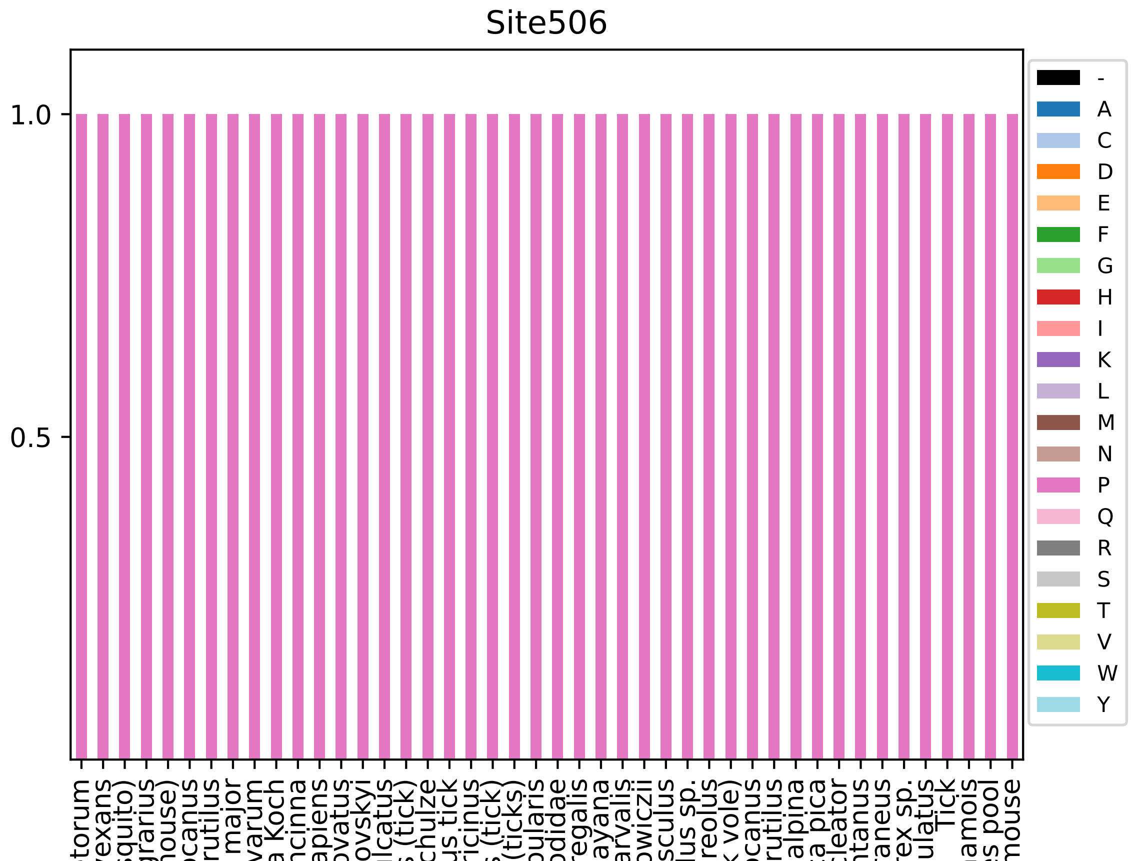
Site506
1.0
0.5
-
A
C
D
E
F
G
H
I
K
L
M
N
P
Q
R
S
T
V
W
Y

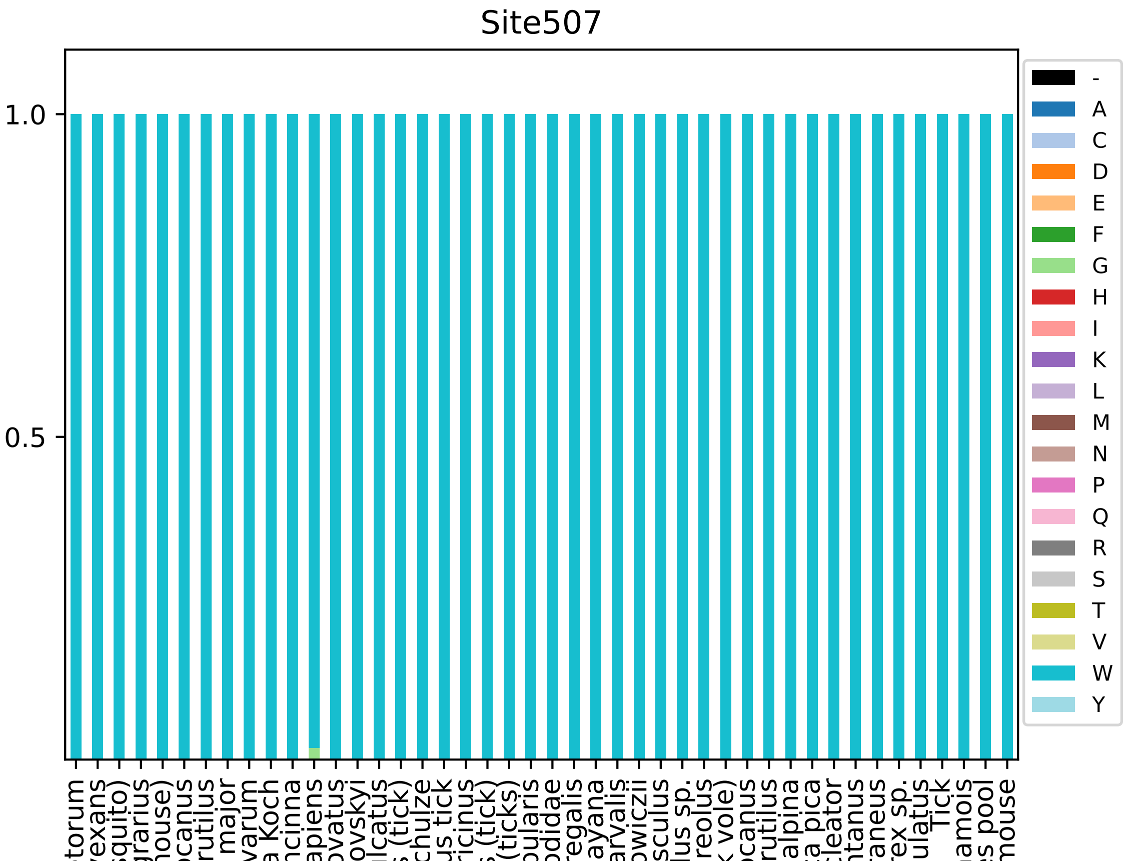
Site507
1.0
0.5
-
A
C
D
E
F
G
H
I
K
L
M
N
P
Q
R
S
T
V
W
Y

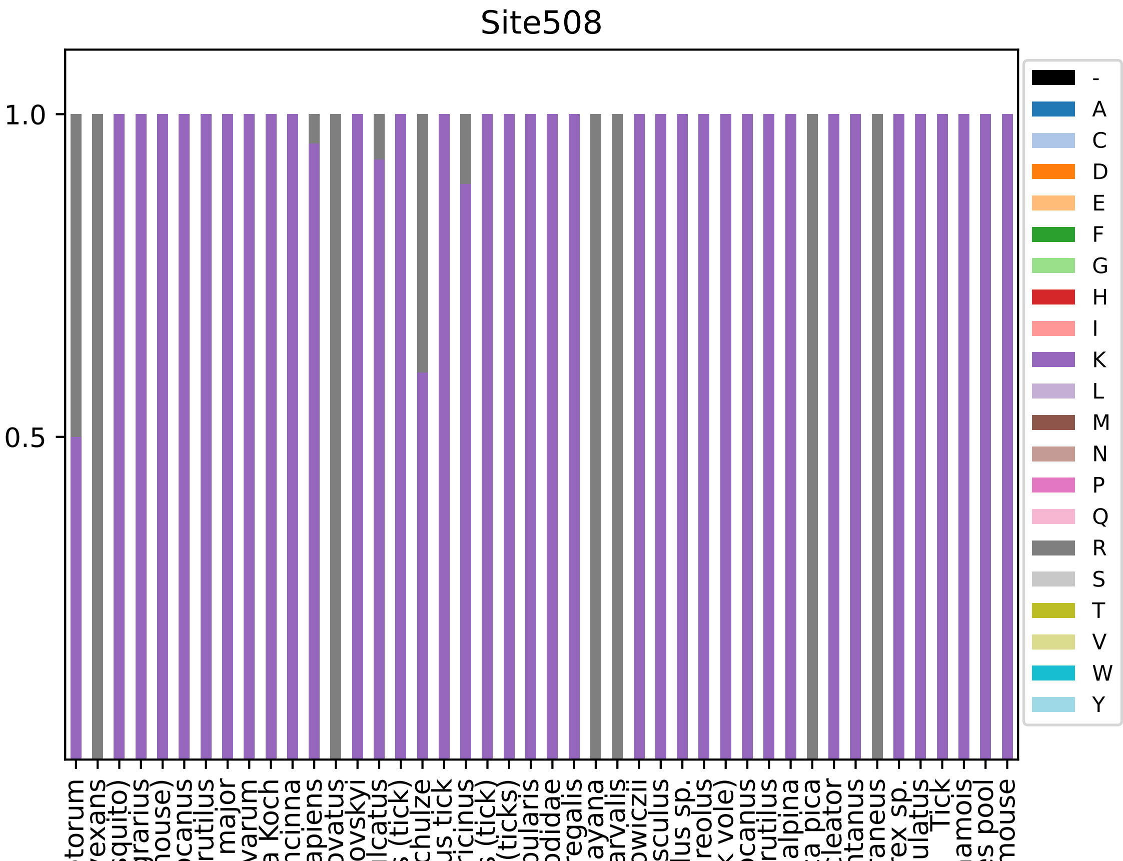
Site508
1.0
0.5
-
A
C
D
E
F
G
H
I
K
L
M
N
P
Q
R
S
T
V
W
Y

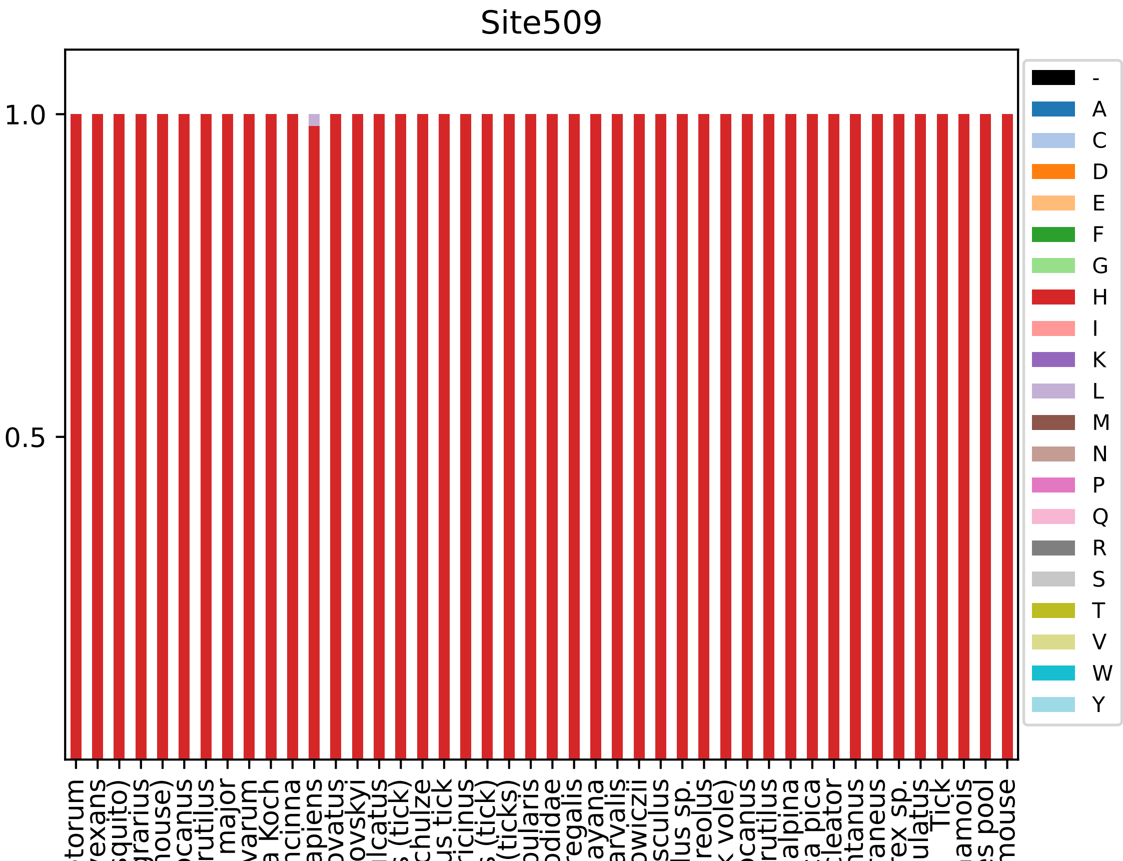
Site509
1.0
0.5
-
A
C
D
E
F
G
H
I
K
L
M
N
P
Q
R
S
T
V
W
Y

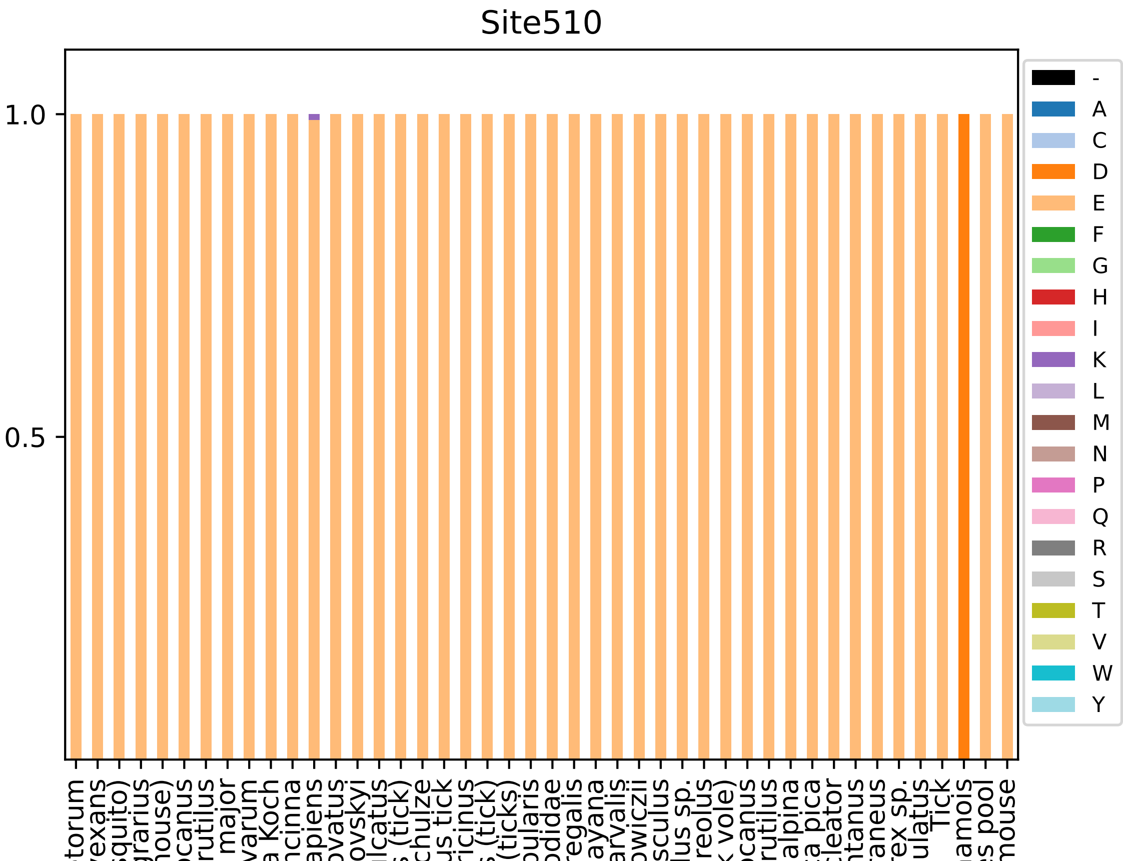
Site510
1.0
0.5
-
A
C
D
E
F
G
H
I
K
L
M
N
P
Q
R
S
T
V
W
Y

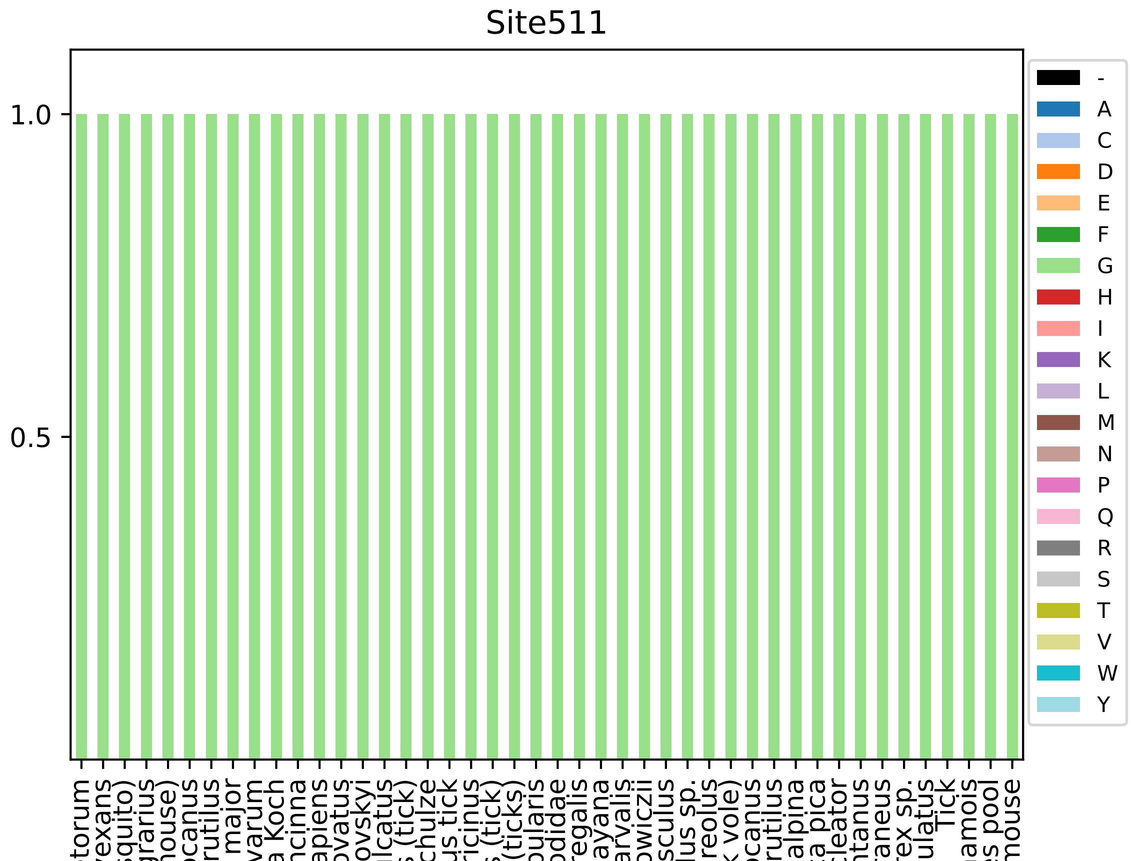

Site511
1.0
0.5
-
A
C
D
E
F
G
H
I
K
L
M
N
P
Q
R
S
T
V
W
Y

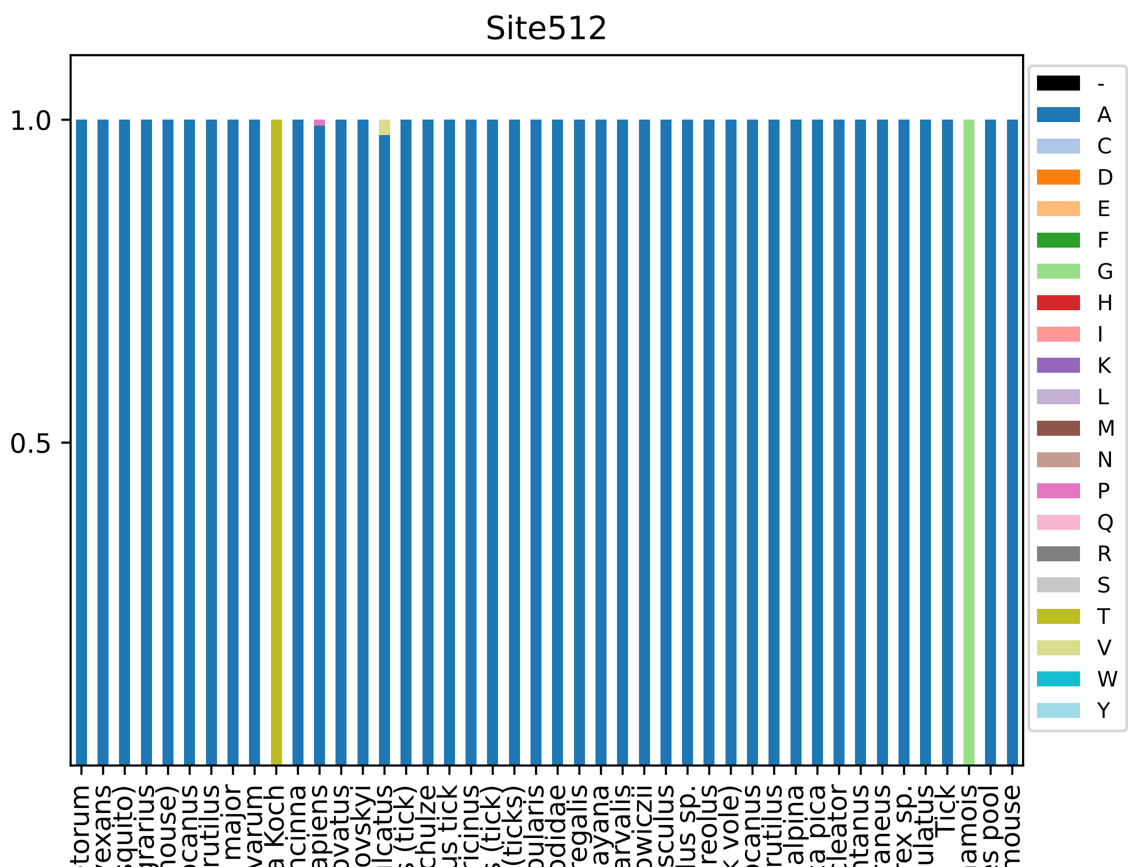

Site512
1.0
0.5
-
A
C
D
E
F
G
H
I
K
L
M
N
P
Q
R
S
T
V
W
Y

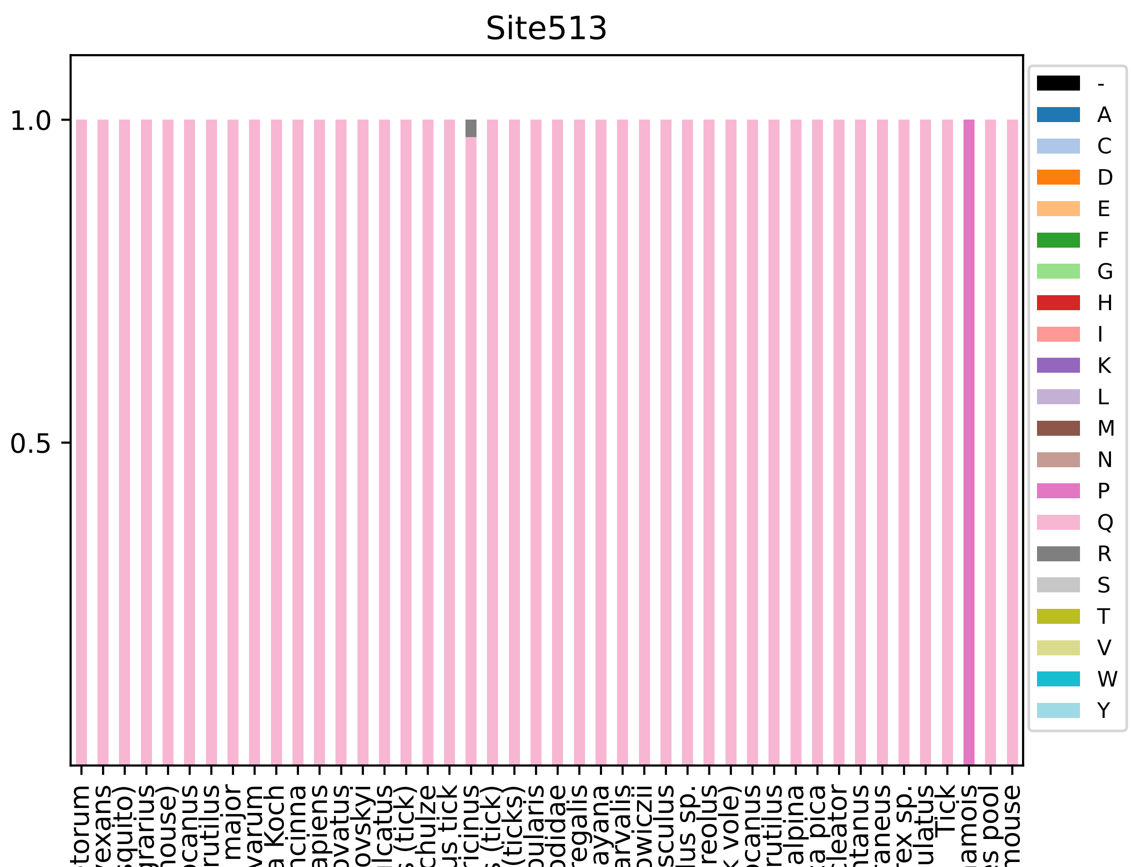
Site513
1.0
0.5
-
A
C
D
E
F
G
H
I
K
L
M
N
P
Q
R
S
T
V
W
Y
Tick

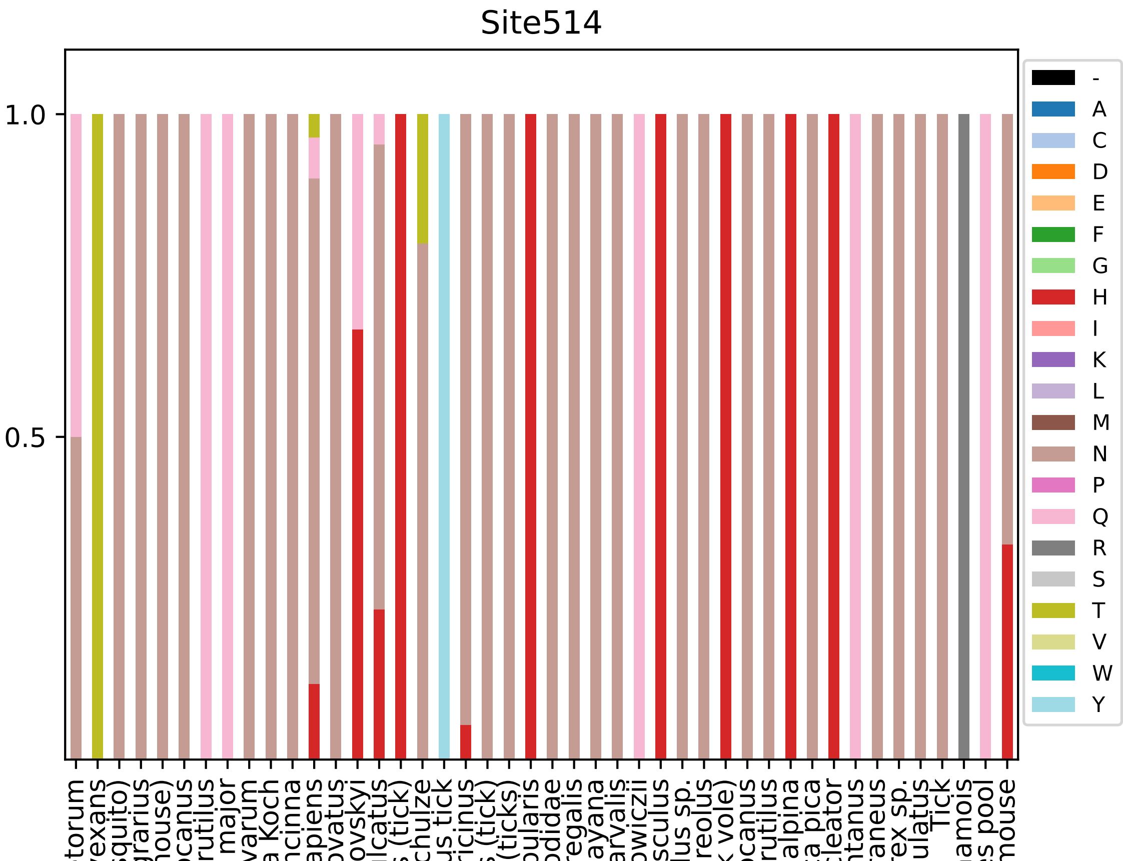

Site514
1.0
0.5
-
A
C
D
E
F
G
H
I
K
L
M
N
P
Q
R
S
T
V
W
Y

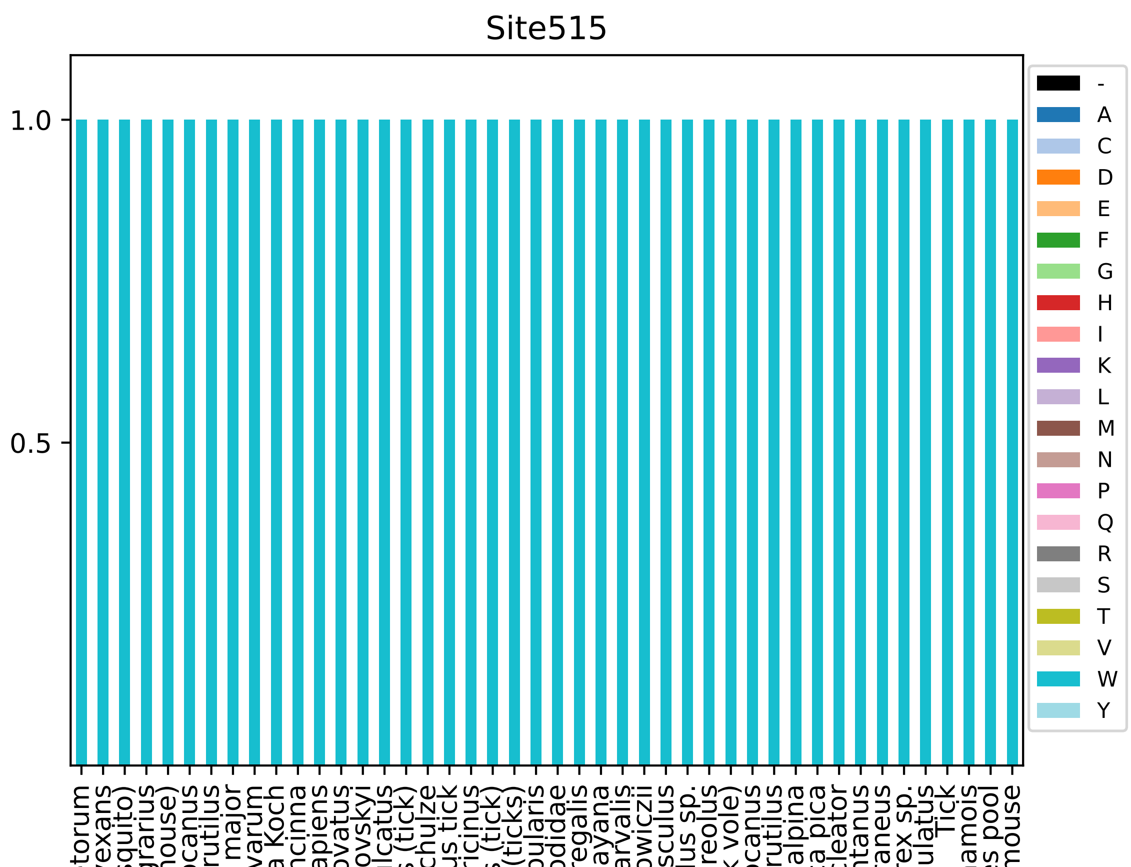
Site515
1.0
0.5
-
A
C
D
E
F
G
H
I
K
L
M
N
P
Q
R
S
T
V
W
Y

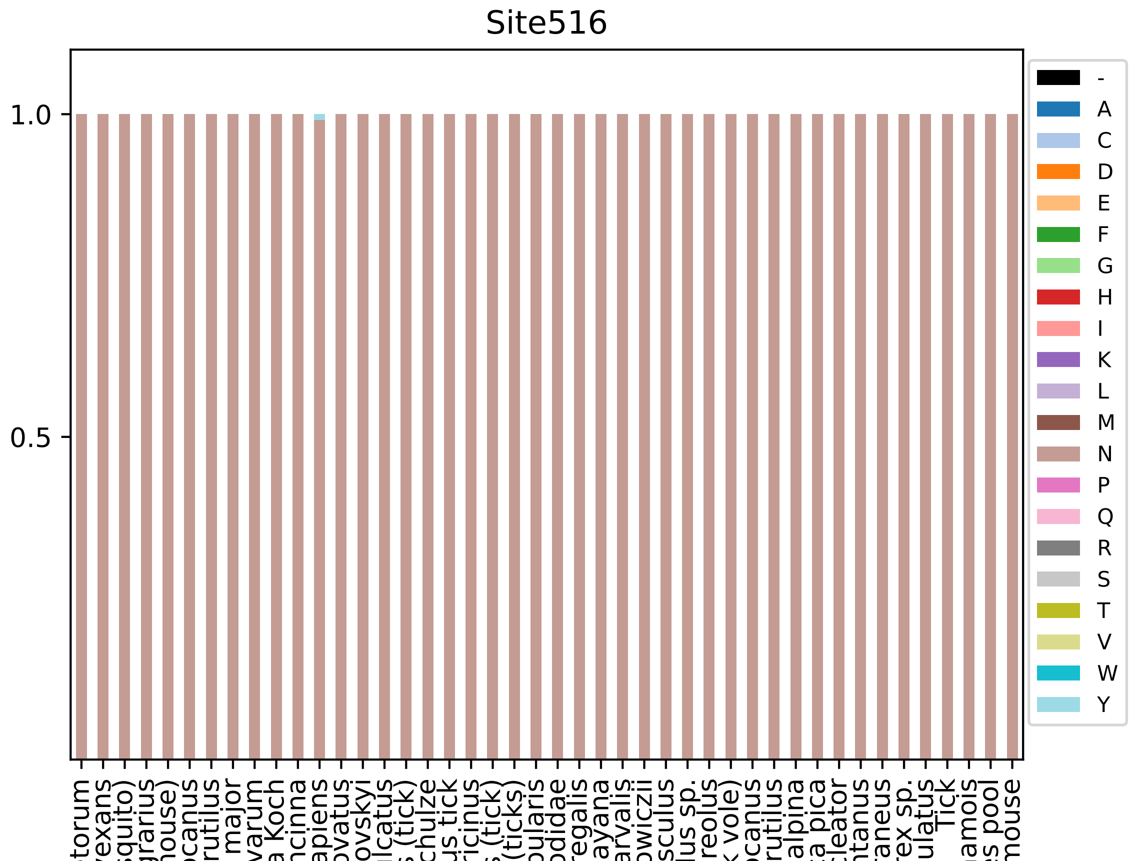
Site516
1.0
0.5
-
A
C
D
E
F
G
H
I
K
L
M
N
P
Q
R
S
T
V
W
Y

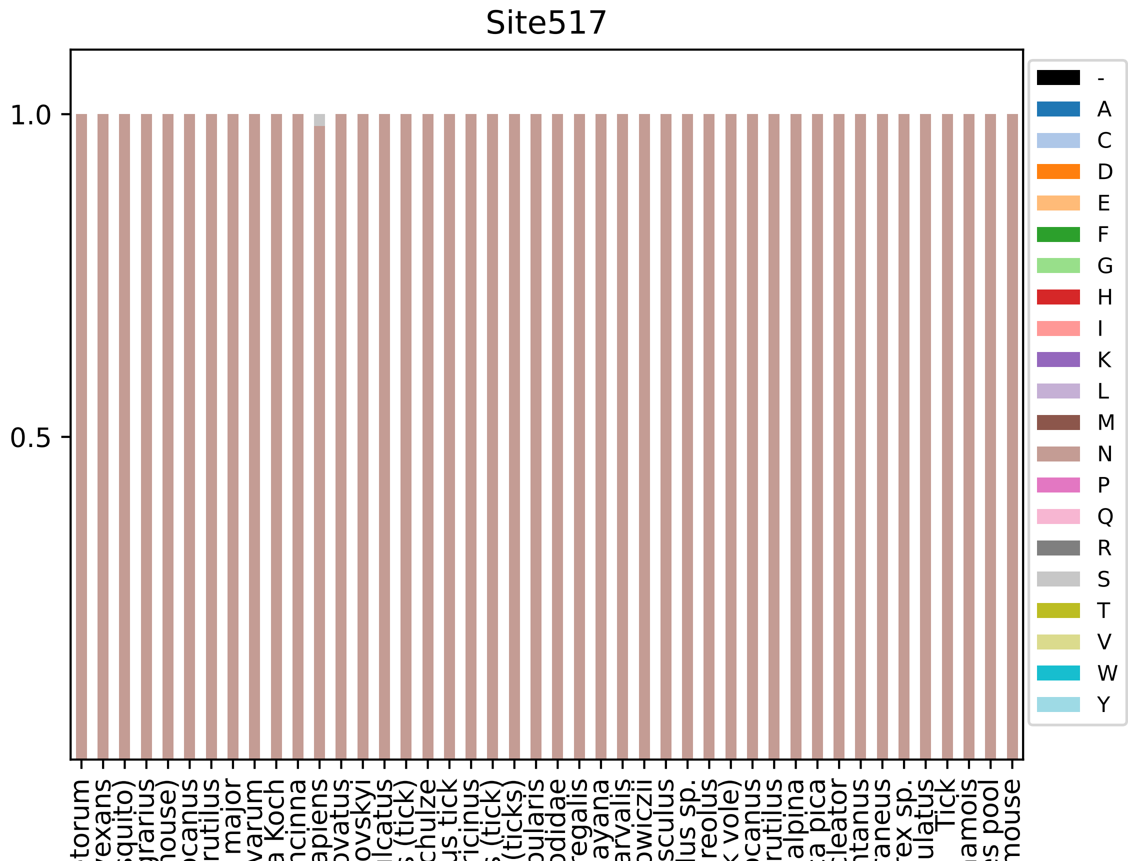

Site517
1.0
0.5
-
A
C
D
E
F
G
H
I
K
L
M
N
P
Q
R
S
T
V
W
Y

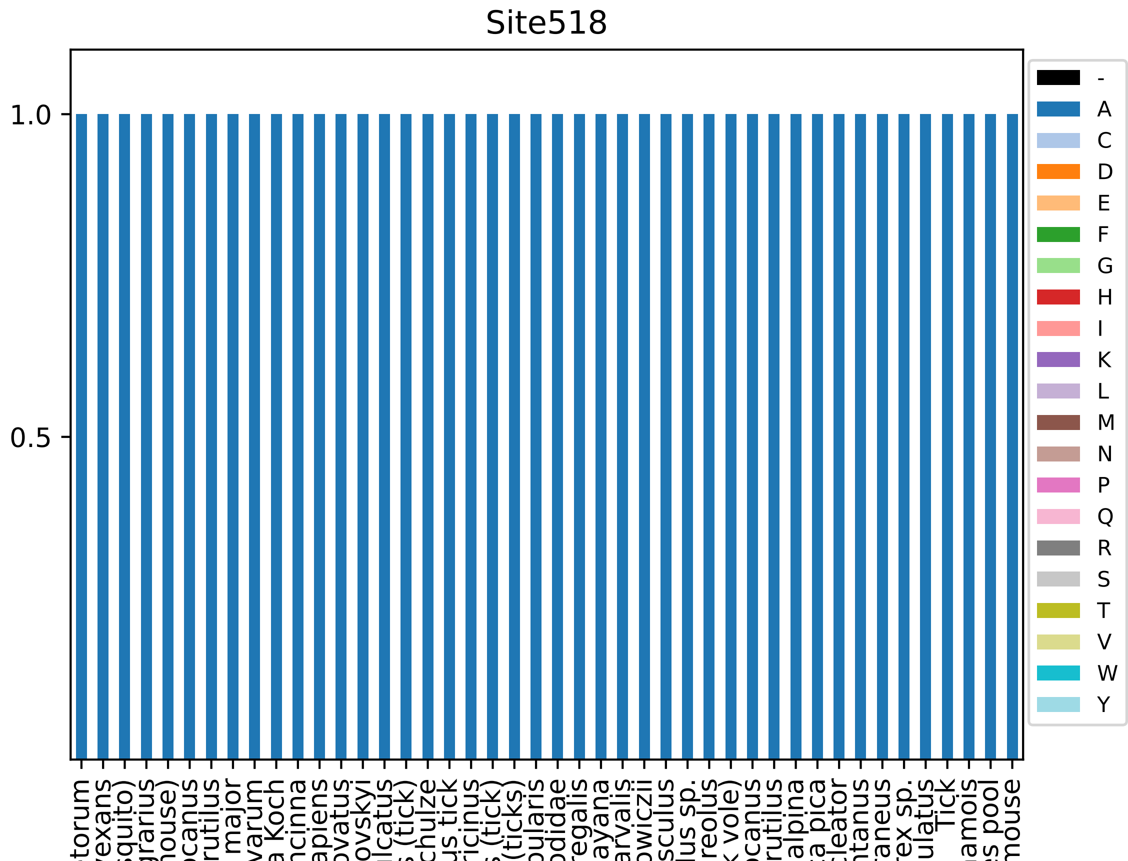
Site518
1.0
0.5
-
A
C
D
E
F
G
H
I
K
L
M
N
P
Q
R
S
T
V
W
Y

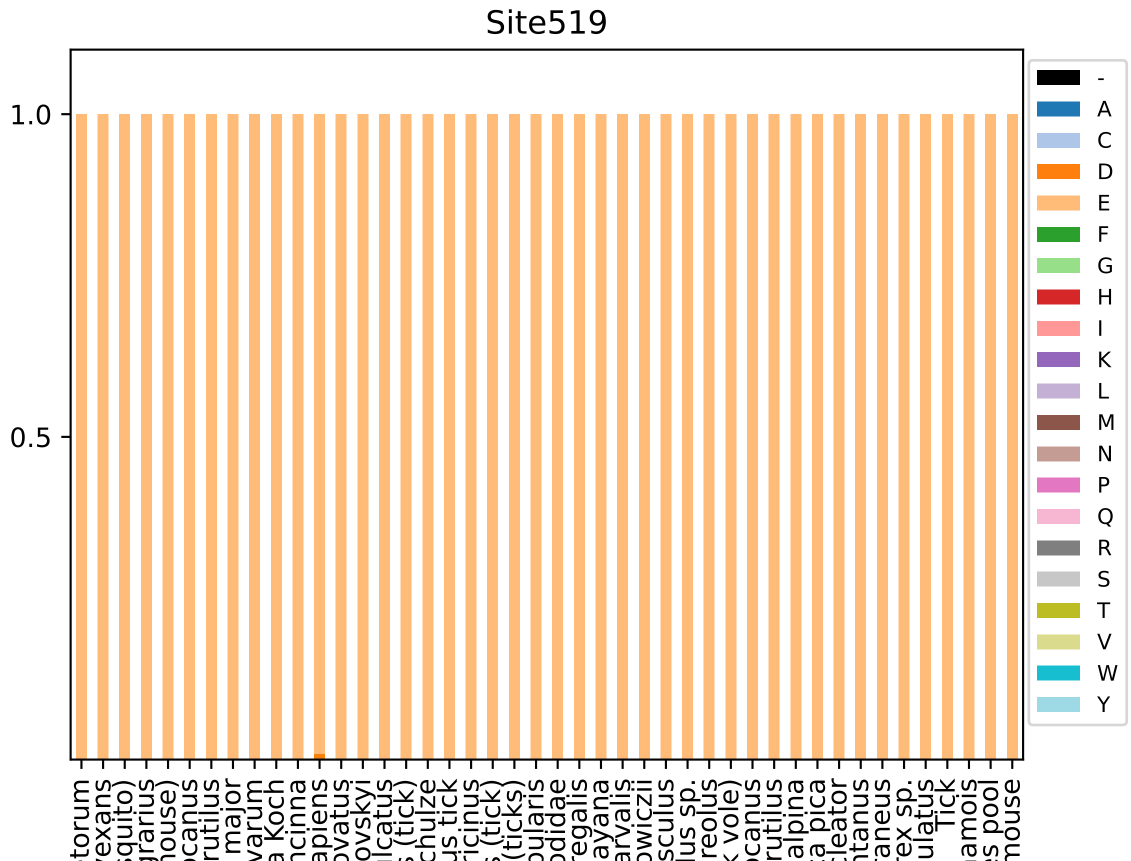

Site519
1.0
0.5
-
A
C
D
E
F
G
H
I
K
L
M
N
P
Q
R
S
T
V
W
Y

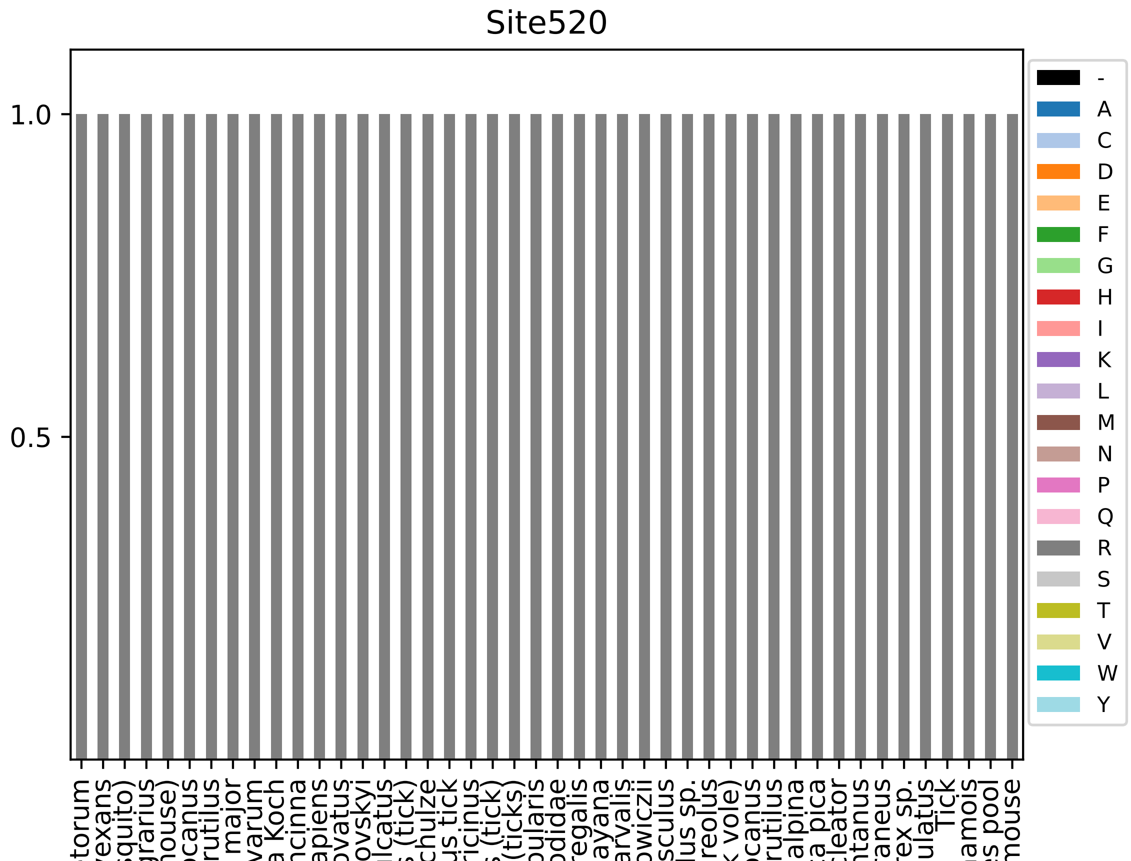

Site520
1.0
0.5
-
A
C
D
E
F
G
H
I
K
L
M
N
P
Q
R
S
T
V
W
Y

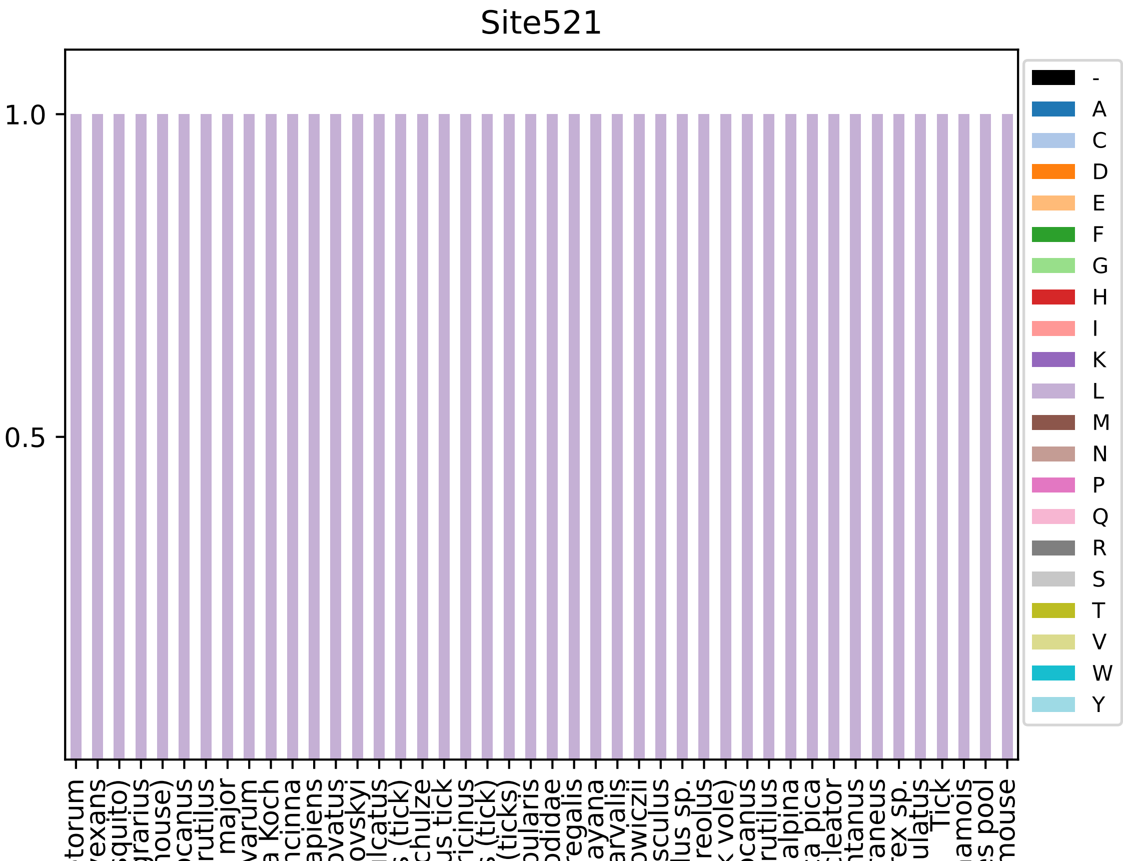
Site521
1.0
0.5
-
A
C
D
E
F
G
H
I
K
L
M
N
P
Q
R
S
T
V
W
Y

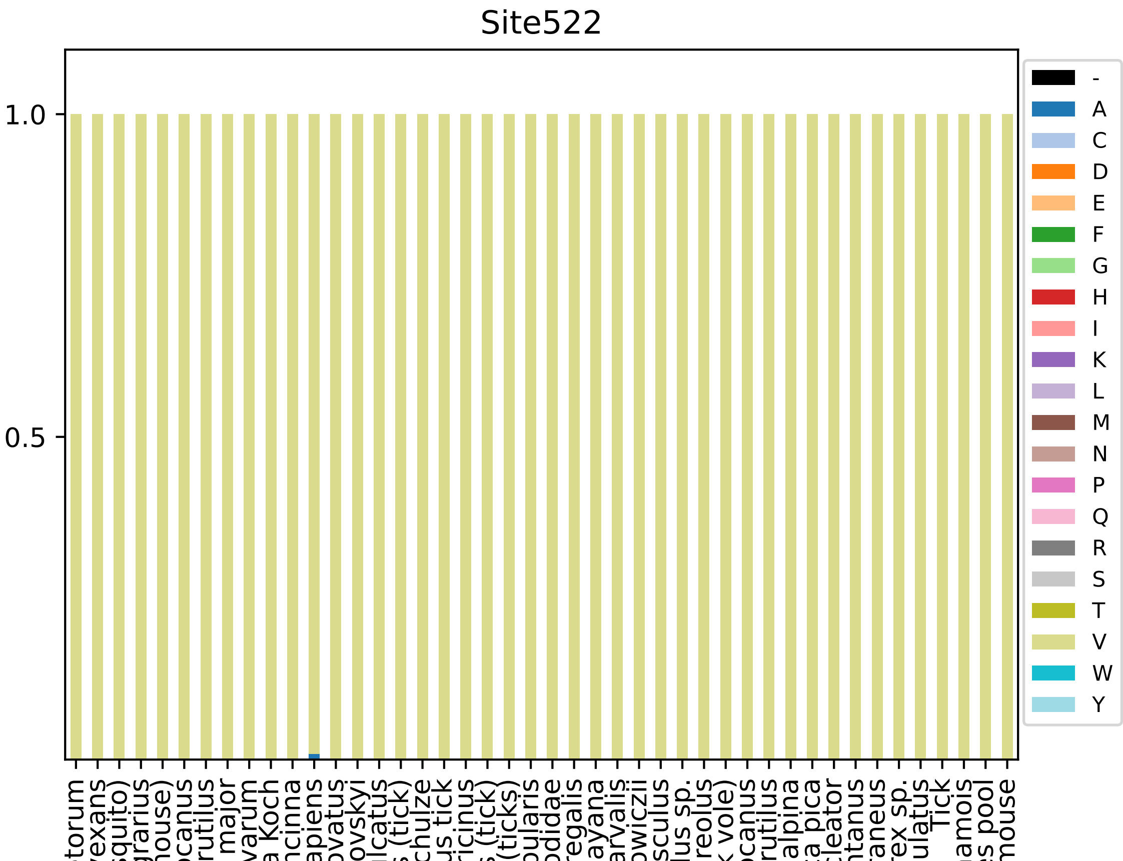
Site522
1.0
0.5
-
A
C
D
E
F
G
H
I
K
L
M
N
P
Q
R
S
T
V
W
Y

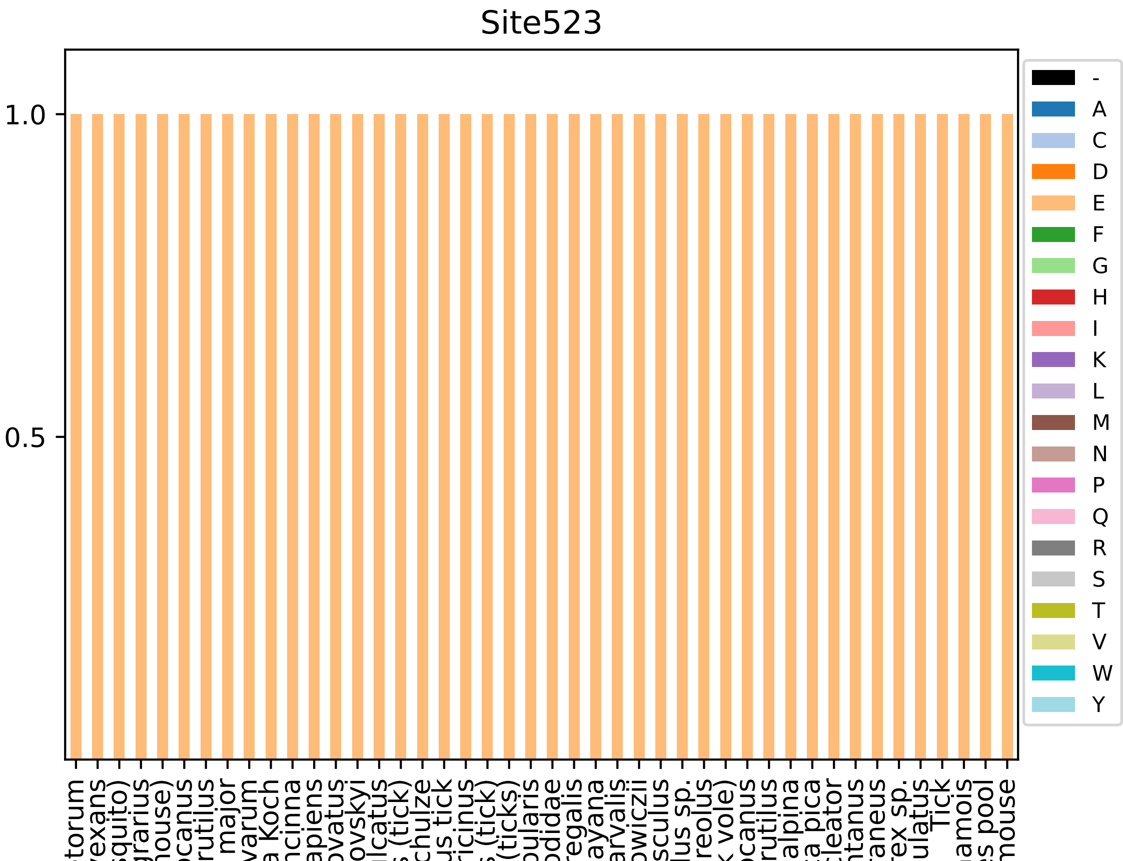
Site523
1.0
0.5
-
A
C
D
E
F
G
H
I
K
L
M
N
P
Q
R
S
T
V
W
Y

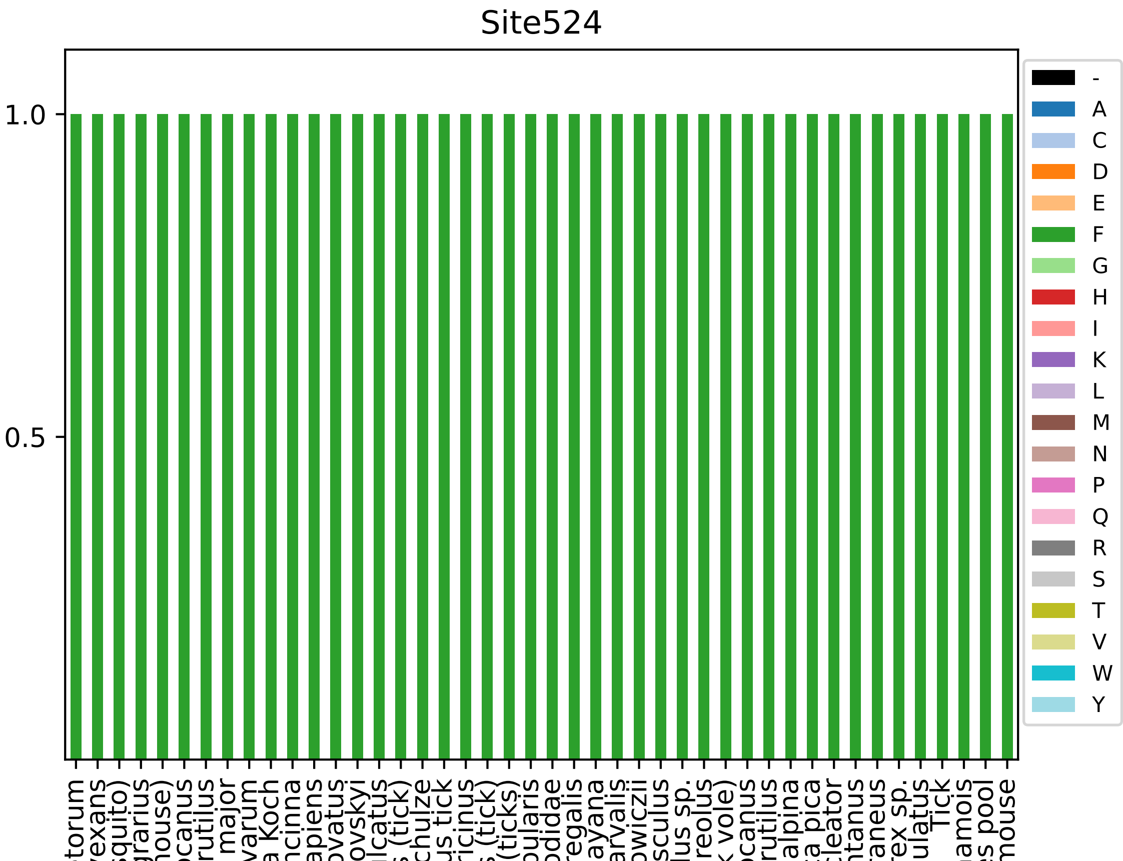
Site524
1.0
0.5
-
A
C
D
E
F
G
H
I
K
L
M
N
P
Q
R
S
T
V
W
Y

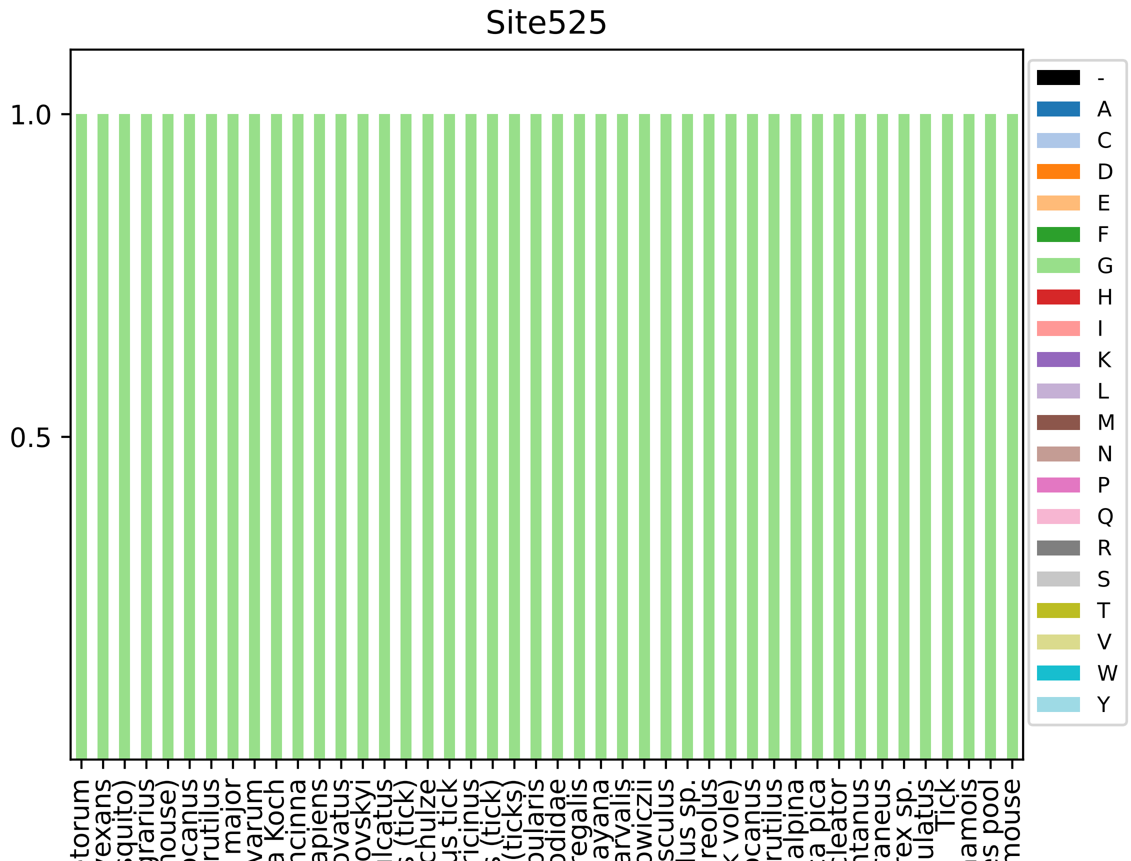
Site525
1.0
0.5
-
A
C
D
E
F
G
H
I
K
L
M
N
P
Q
R
S
T
V
W
Y

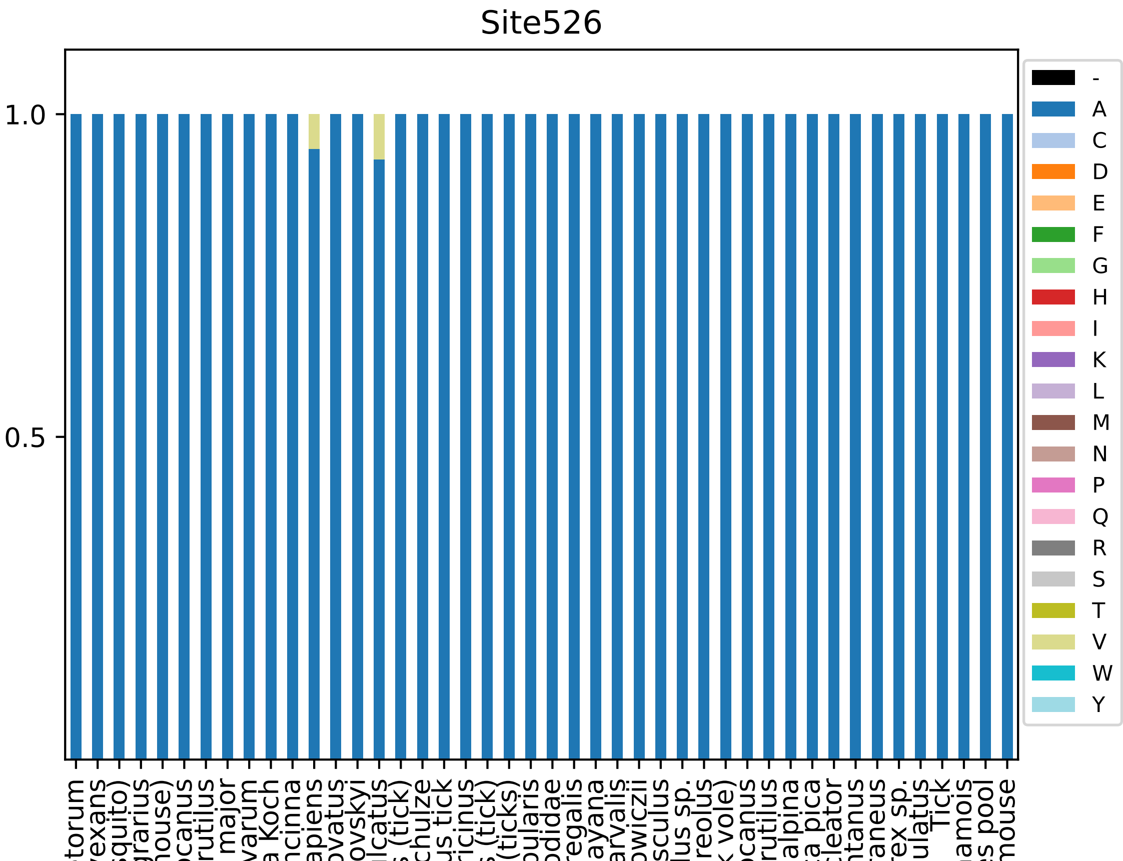

Site526
1.0
0.5
-
A
C
D
E
F
G
H
I
K
L
M
N
P
Q
R
S
T
V
W
Y

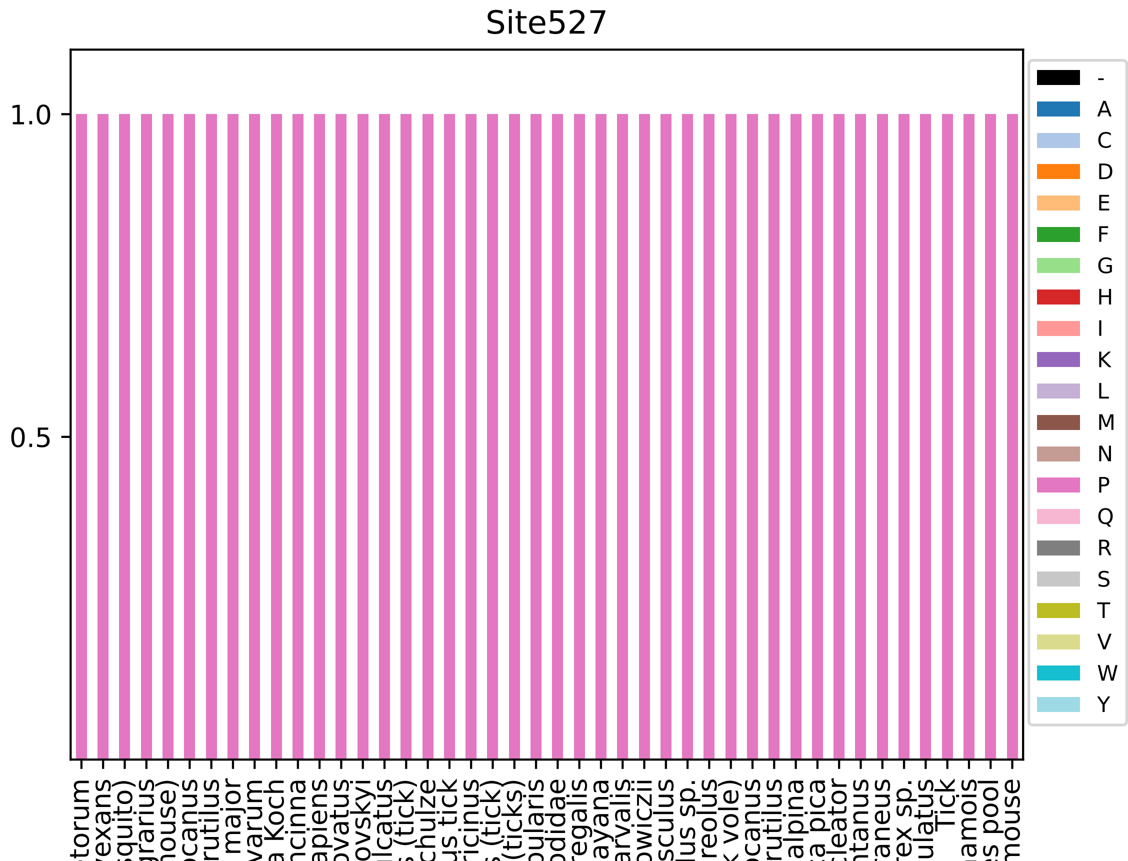

Site527
1.0
0.5
-
A
C
D
E
F
G
H
I
K
L
M
N
P
Q
R
S
T
V
W
Y

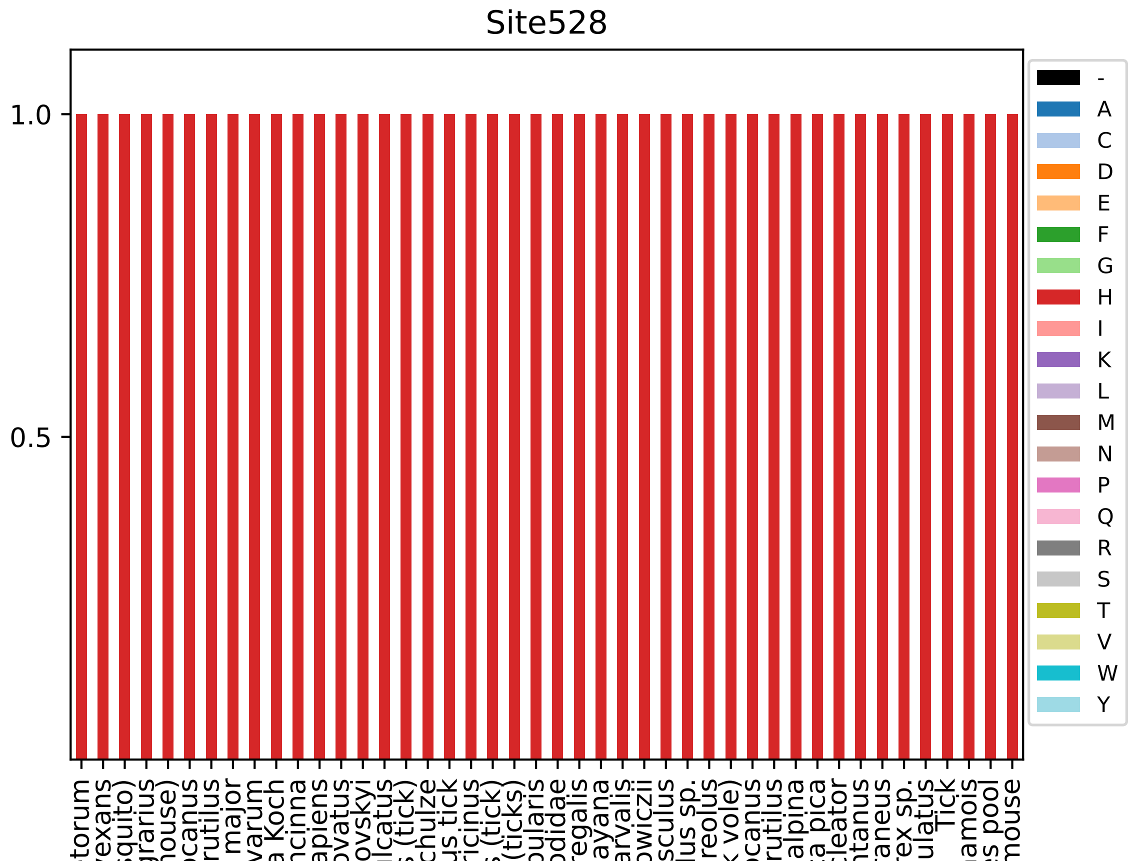
Site528
1.0
0.5
-
A
C
D
E
F
G
H
I
K
L
M
N
P
Q
R
S
T
V
W
Y

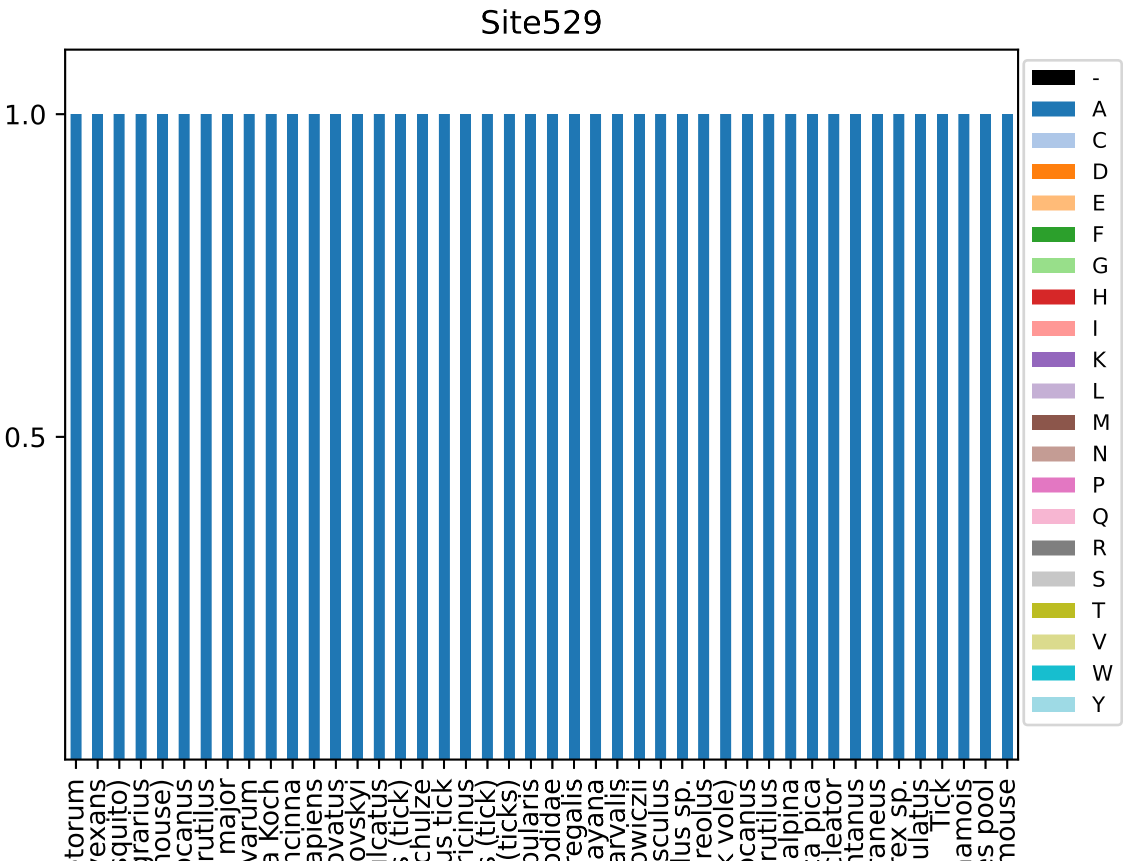
Site529
1.0
0.5
-
A
C
D
E
F
G
H
I
K
L
M
N
P
Q
R
S
T
V
W
Y

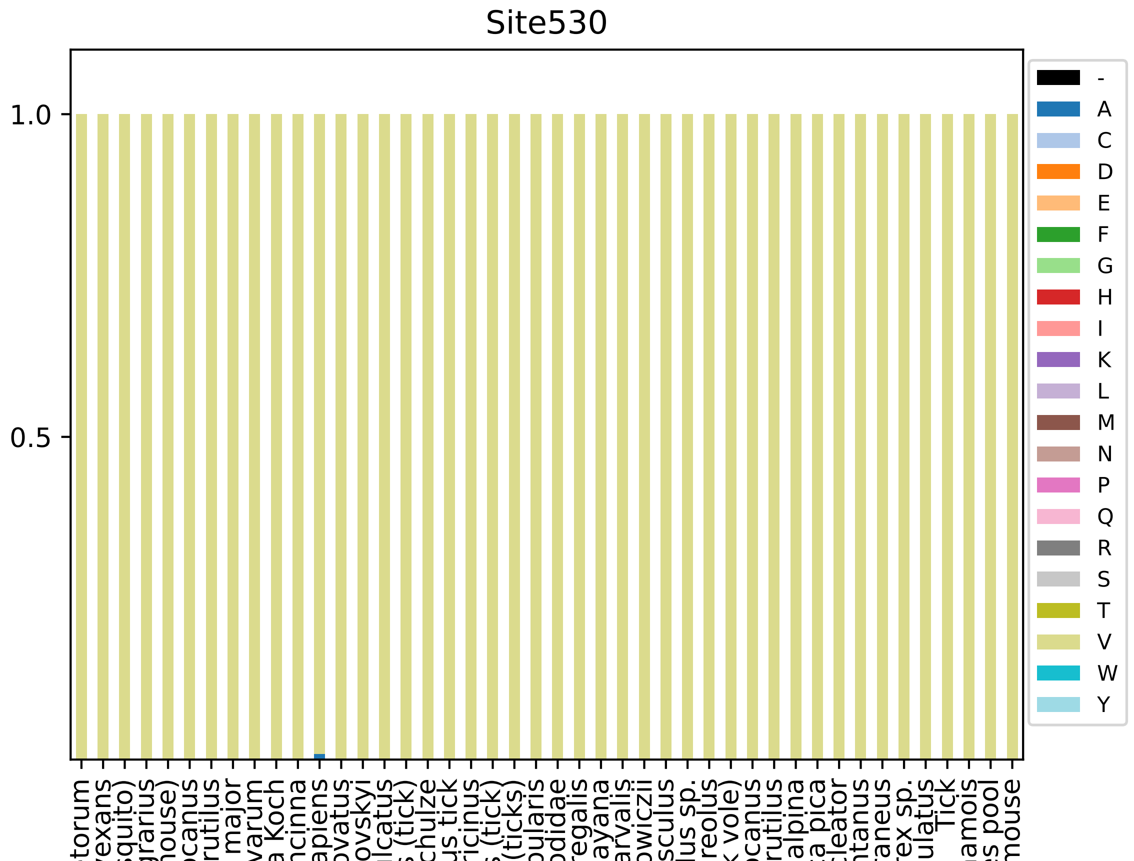
Site530
1.0
0.5
-
A
C
D
E
F
G
H
I
K
L
M
N
P
Q
R
S
T
V
W
Y

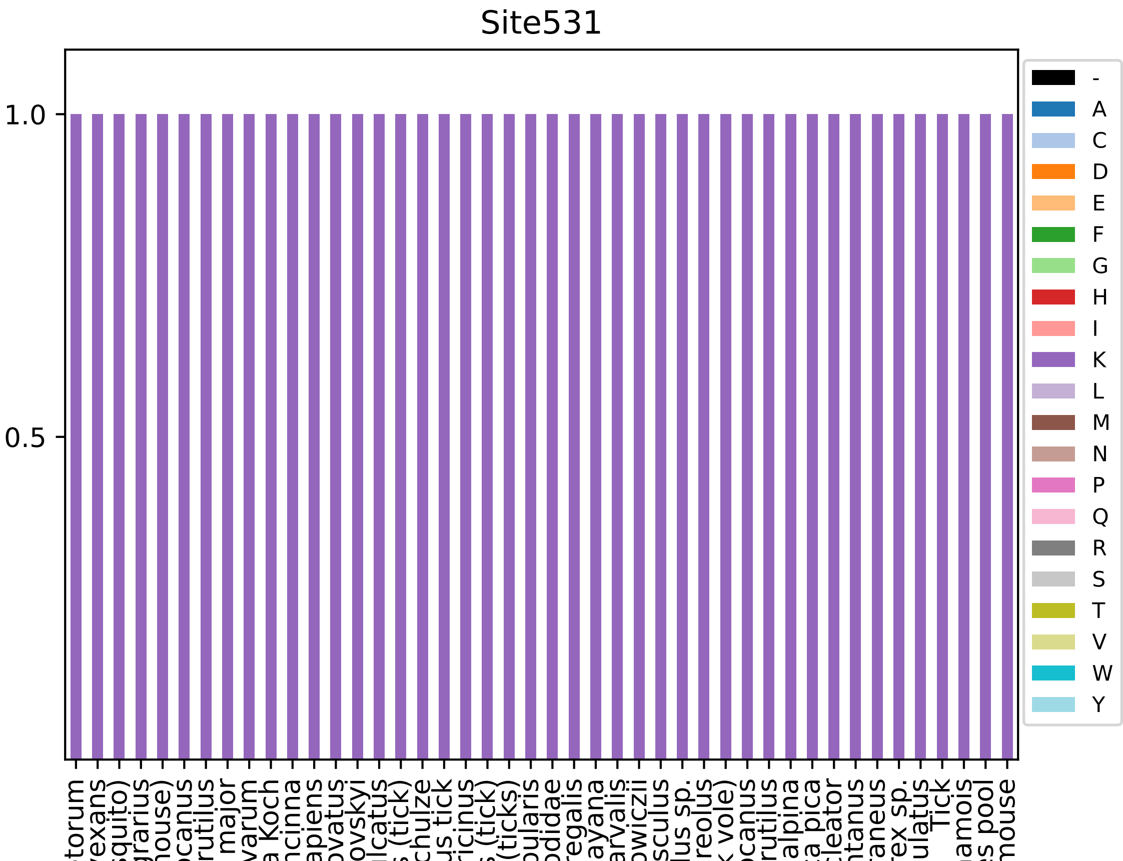
Site531
1.0
0.5
-
A
C
D
E
F
G
H
I
K
L
M
N
P
Q
R
S
T
V
W
Y

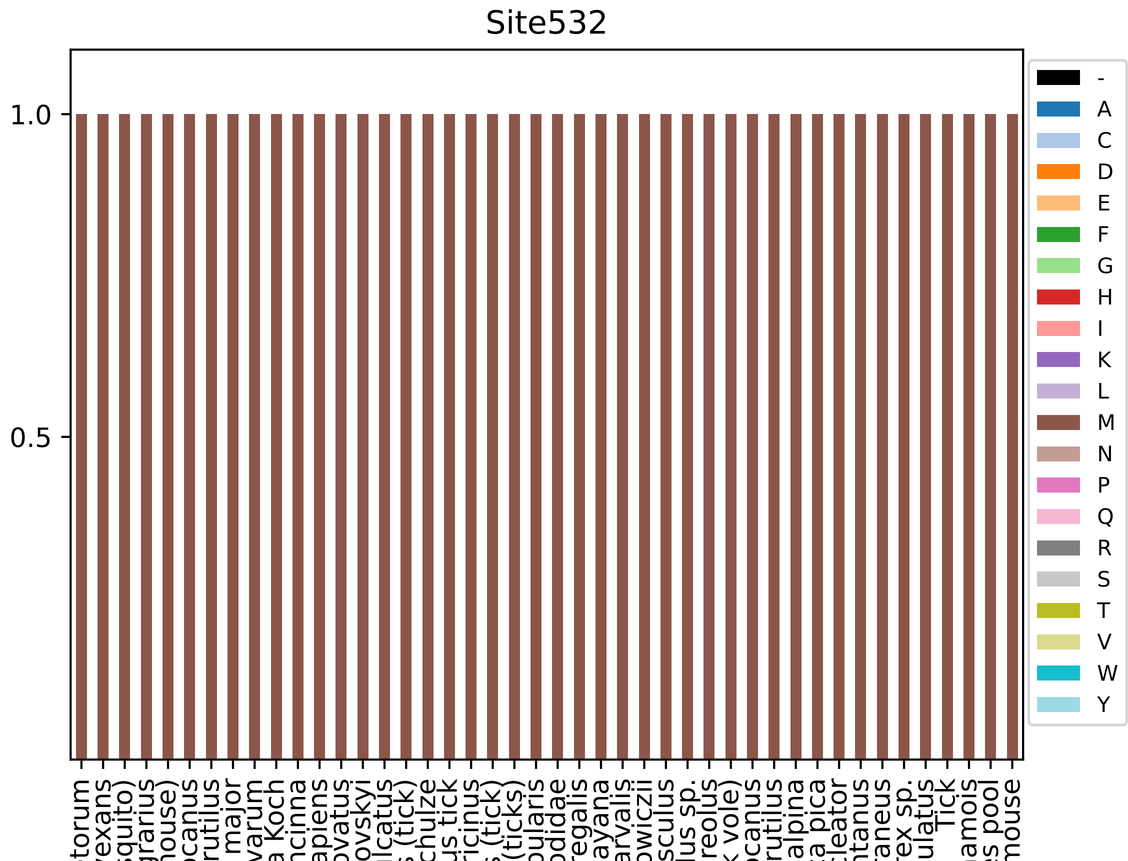
Site532
1.0
0.5
-
A
C
D
E
F
G
H
I
K
L
M
N
P
Q
R
S
T
V
W
Y

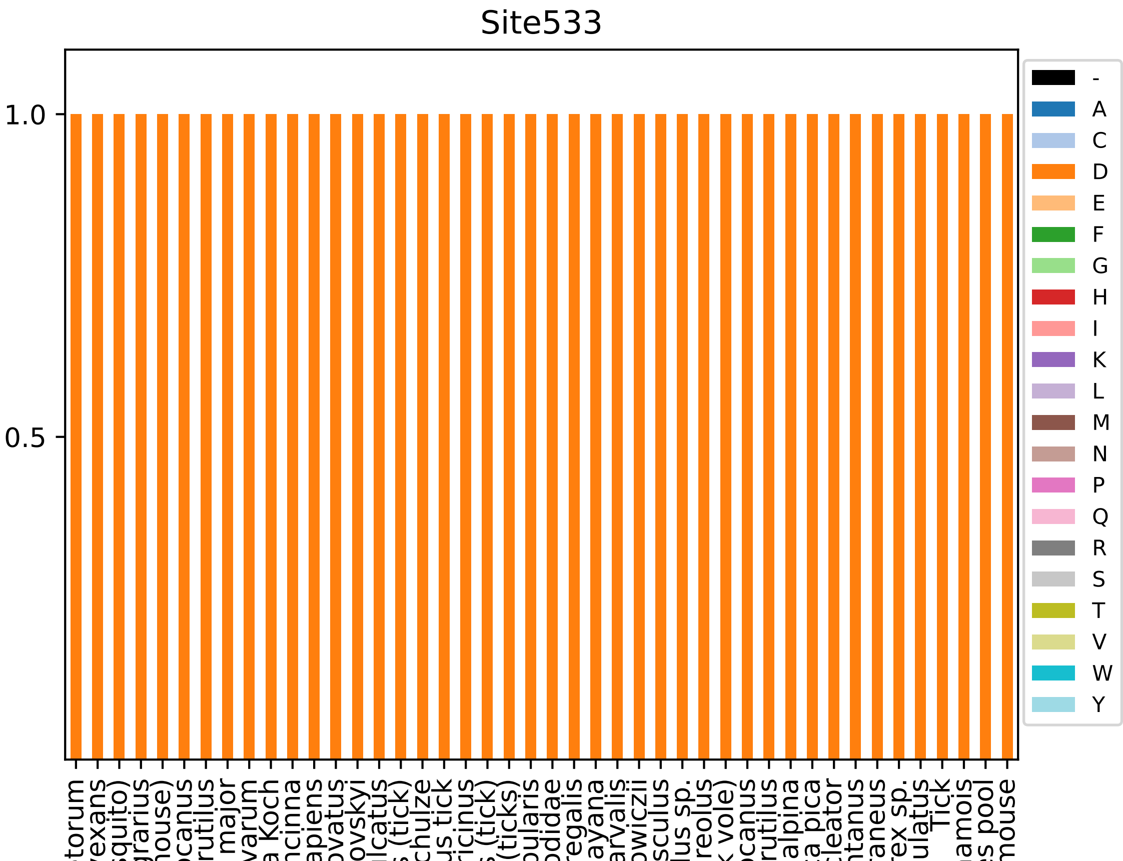

Site533
1.0
0.5
-
A
C
D
E
F
G
H
I
K
L
M
N
P
Q
R
S
T
V
W
Y

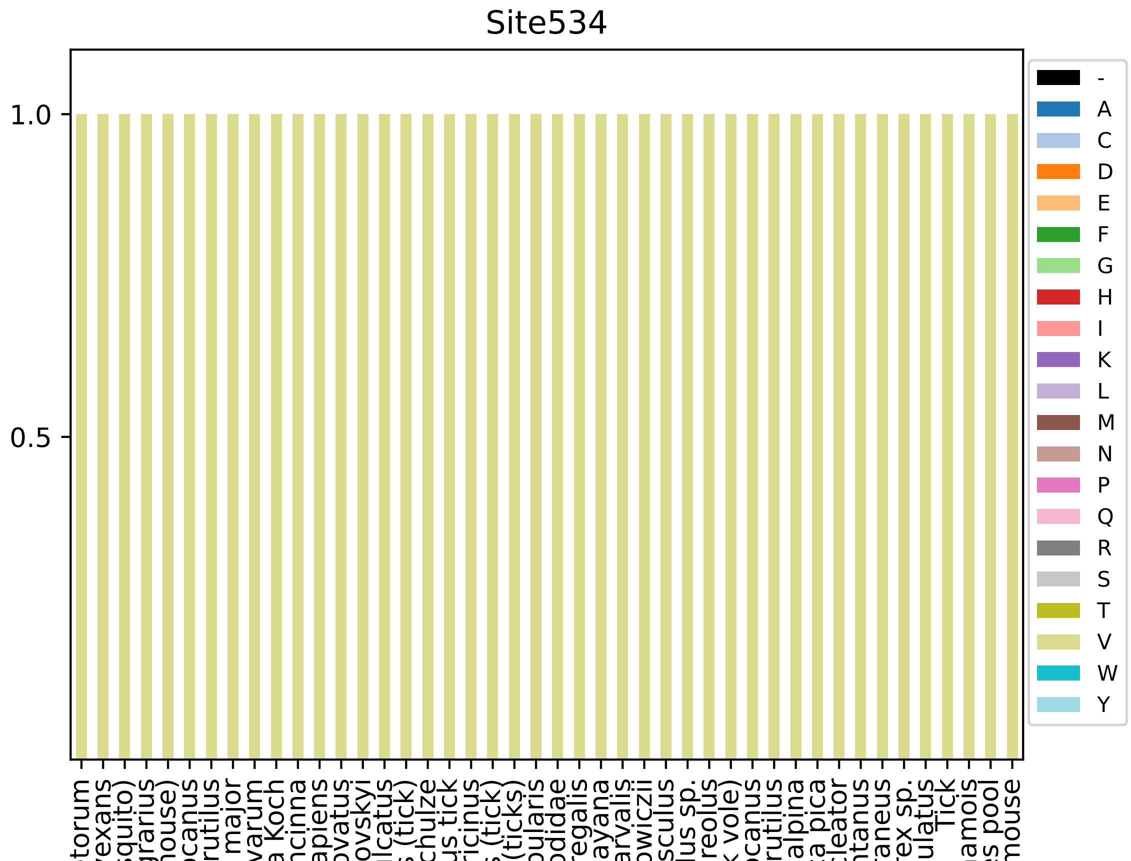

Site534
1.0
0.5
-
A
C
D
E
F
G
H
I
K
L
M
N
P
Q
R
S
T
V
W
Y

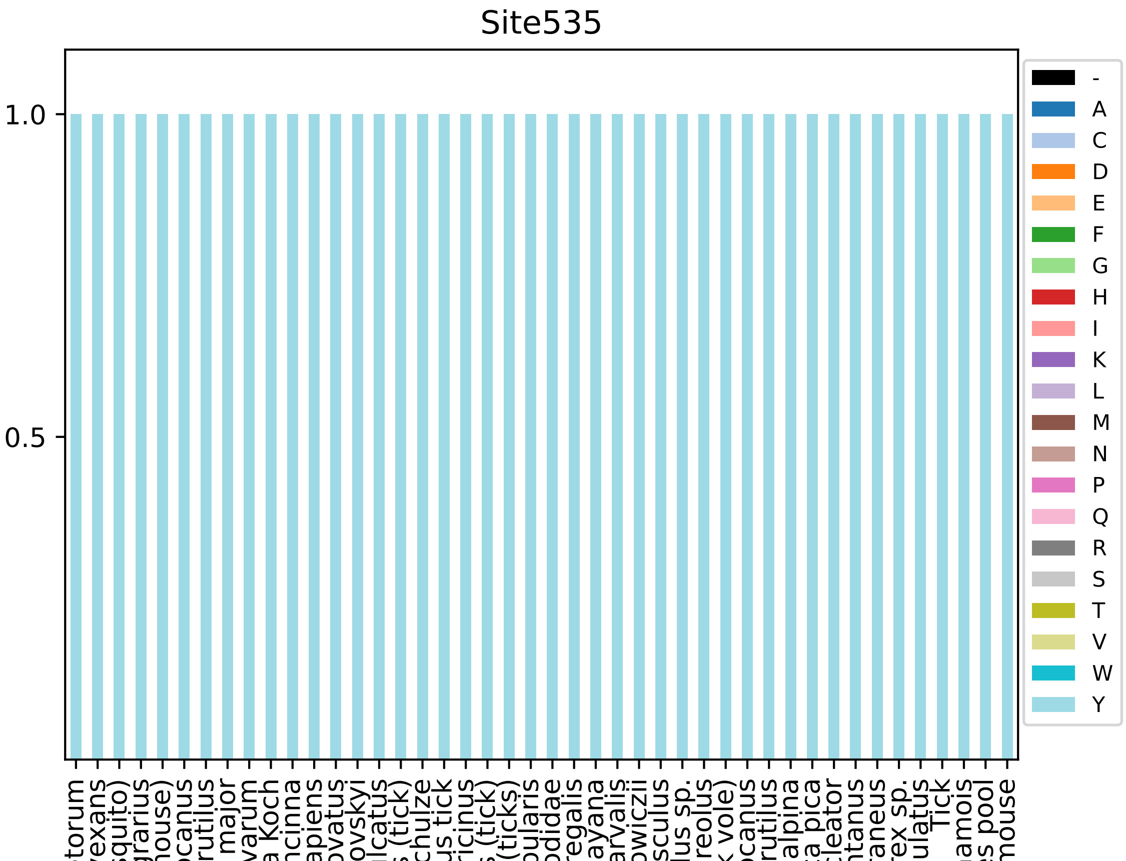
Site535
1.0
0.5
-
A
C
D
E
F
G
H
I
K
L
M
N
P
Q
R
S
T
V
W
Y

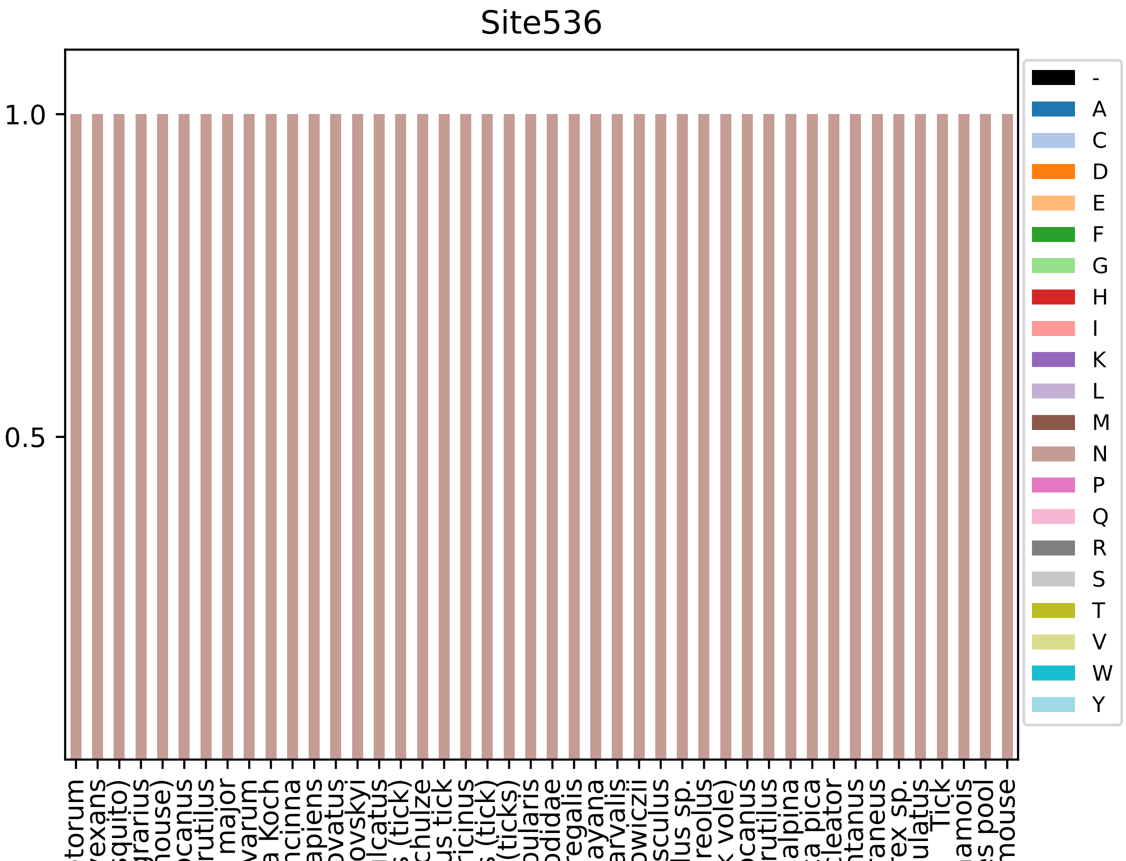
Site536
1.0
0.5
-
A
C
D
E
F
G
H
I
K
L
M
N
P
Q
R
S
T
V
W
Y

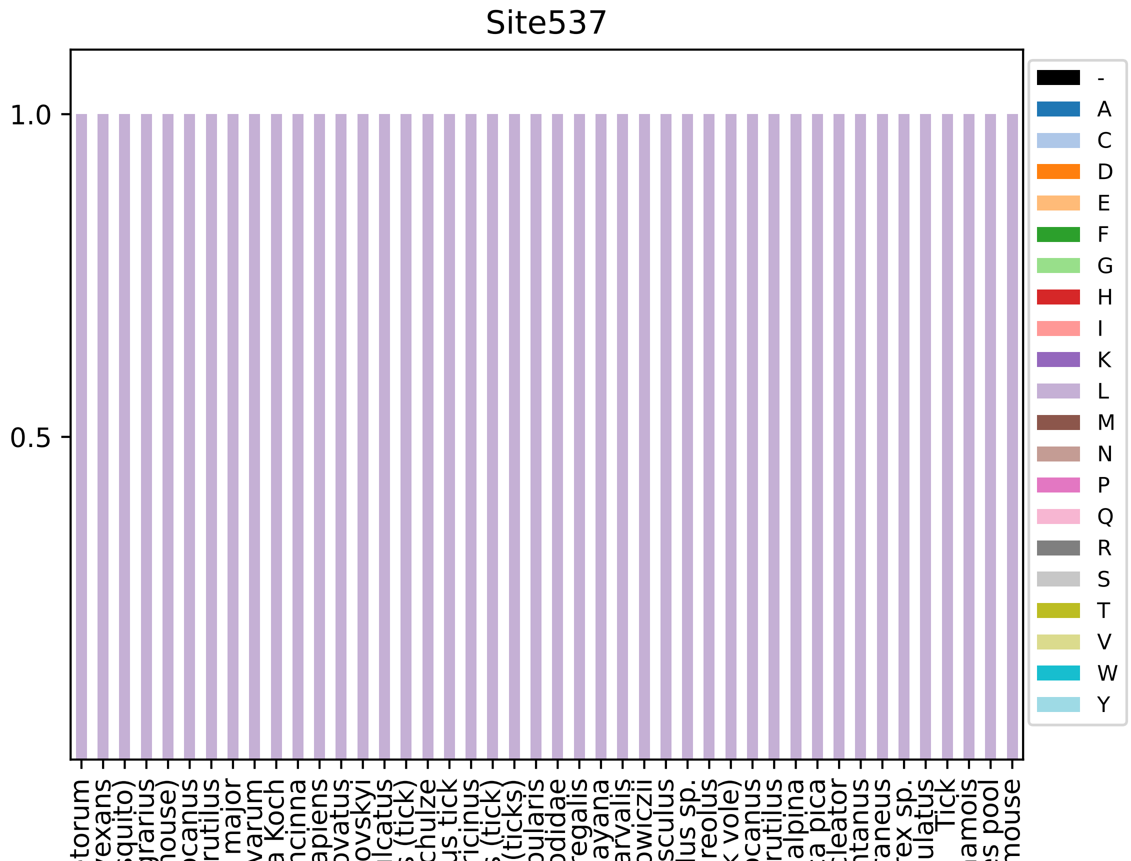
Site537
1.0
0.5
-
A
C
D
E
F
G
H
I
K
L
M
N
P
Q
R
S
T
V
W
Y

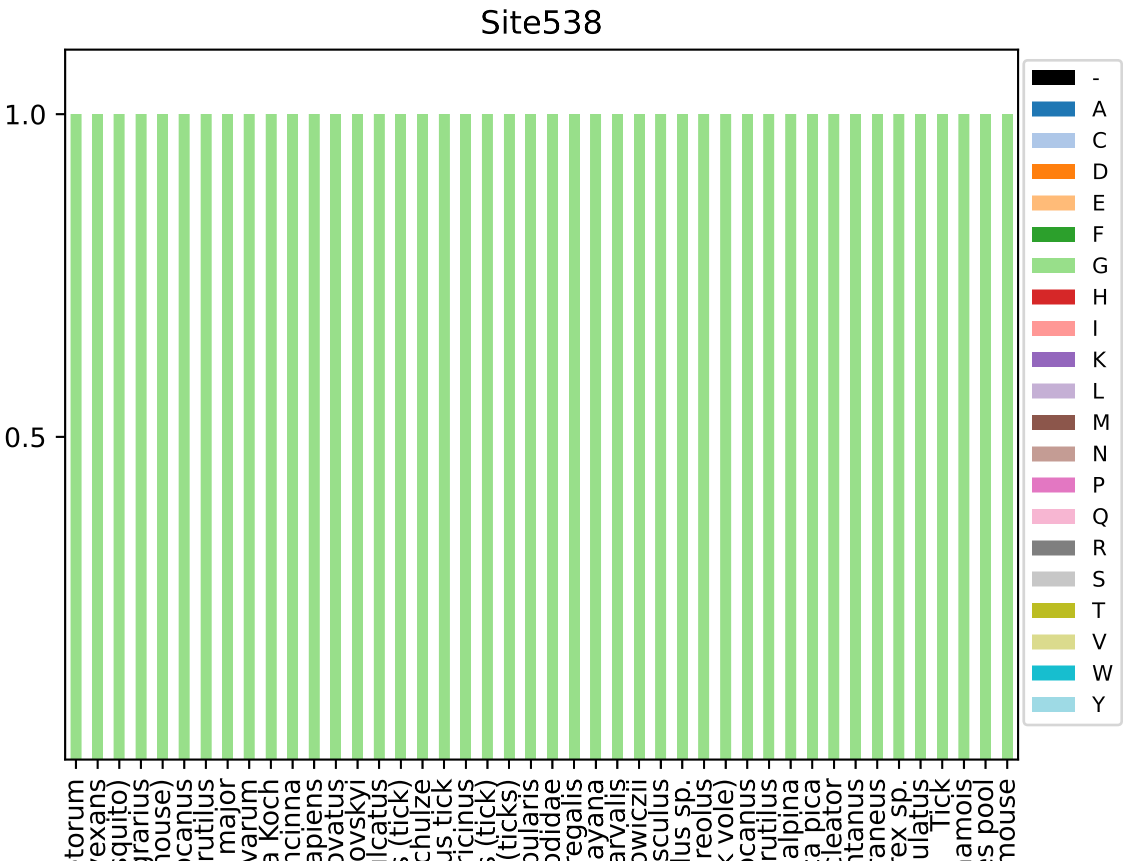
Site538
1.0
0.5
-
A
C
D
E
F
G
H
I
K
L
M
N
P
Q
R
S
T
V
W
Y

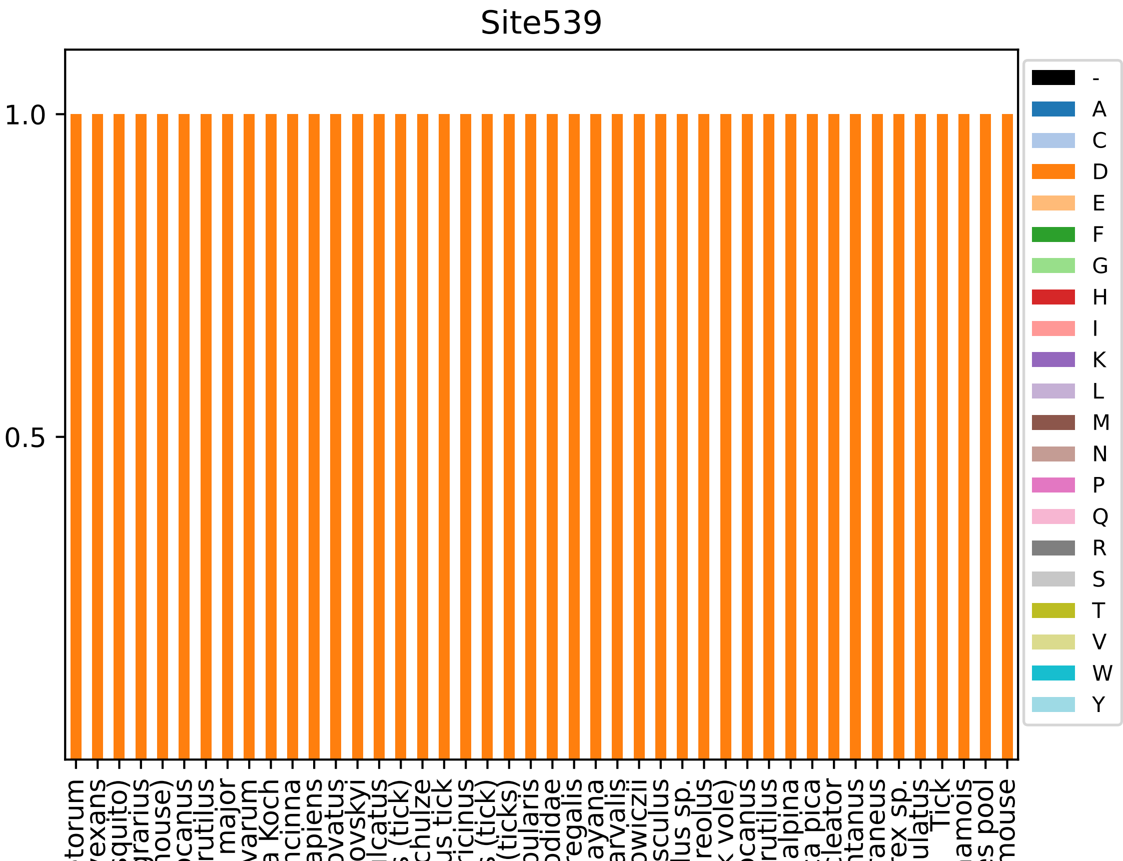
Site539
1.0
0.5
-
A
C
D
E
F
G
H
I
K
L
M
N
P
Q
R
S
T
V
W
Y
torum
vexans
squito)
grarius
house)
ocanus
rutilus
major
varum
a Koch
ncinna
apiens
ovatus
ovskyi
ulcatus
s (tick)
chulze
us tick
ricinus
s (tick)
(ticks)
pularis
odidae
regalis
ayana
arvalis
owiczii
sculus
lus sp.
reolus
x vole)
ocanus
rutilus
alpina
a pica
cleator
ntanus
raneus
rex sp.
ulatus
Tick
amois
es pool
mouse

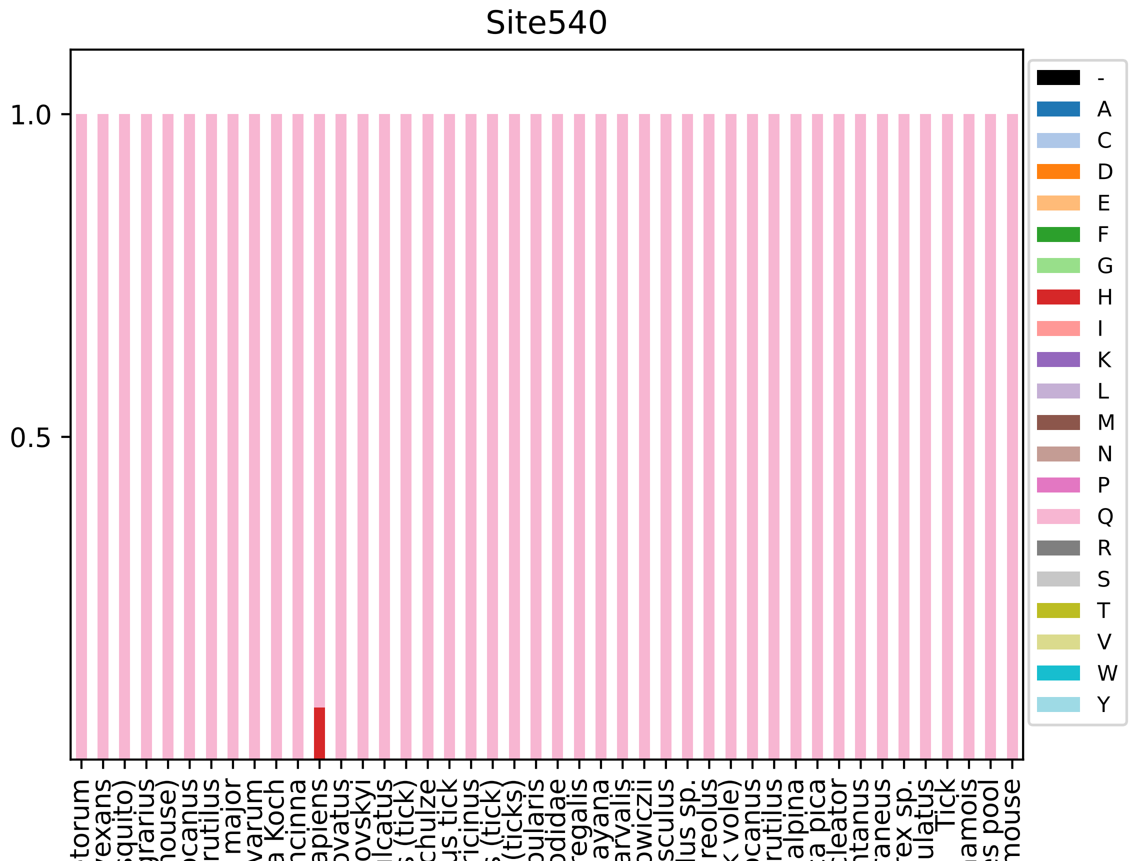
Site540
1.0
0.5
-
A
C
D
E
F
G
H
I
K
L
M
N
P
Q
R
S
T
V
W
Y

Site541

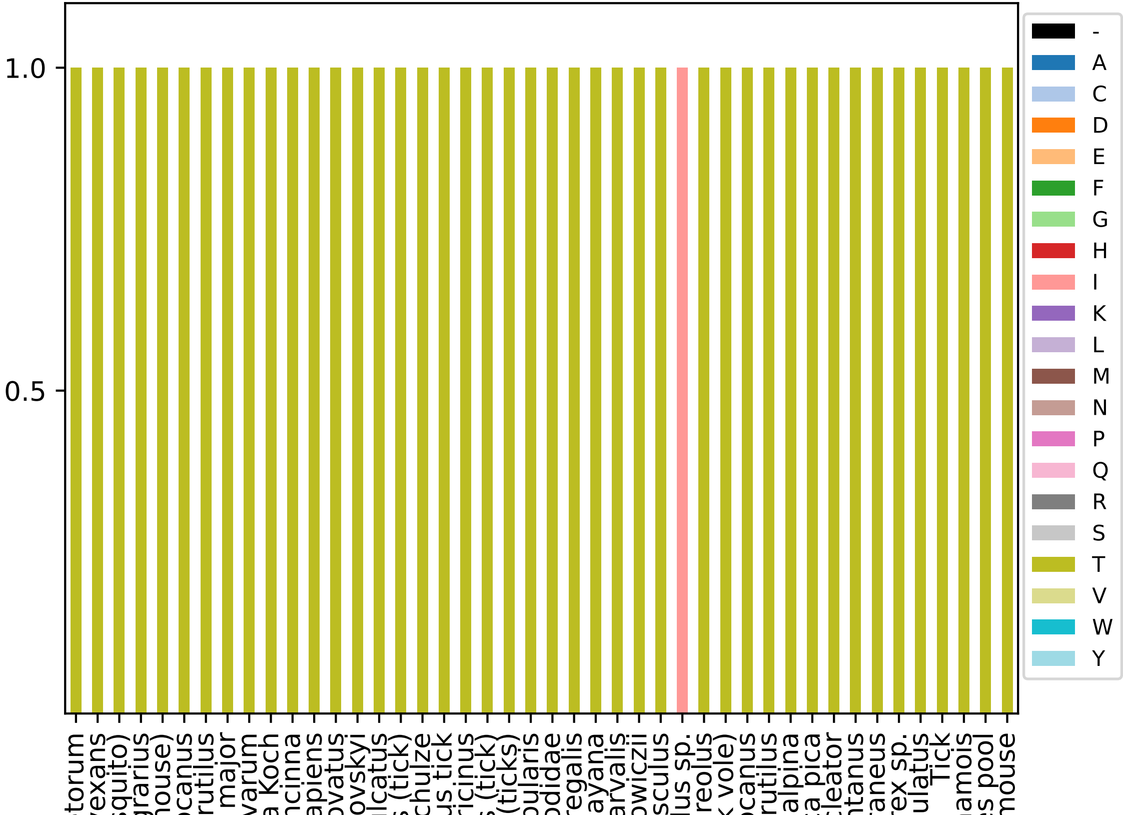

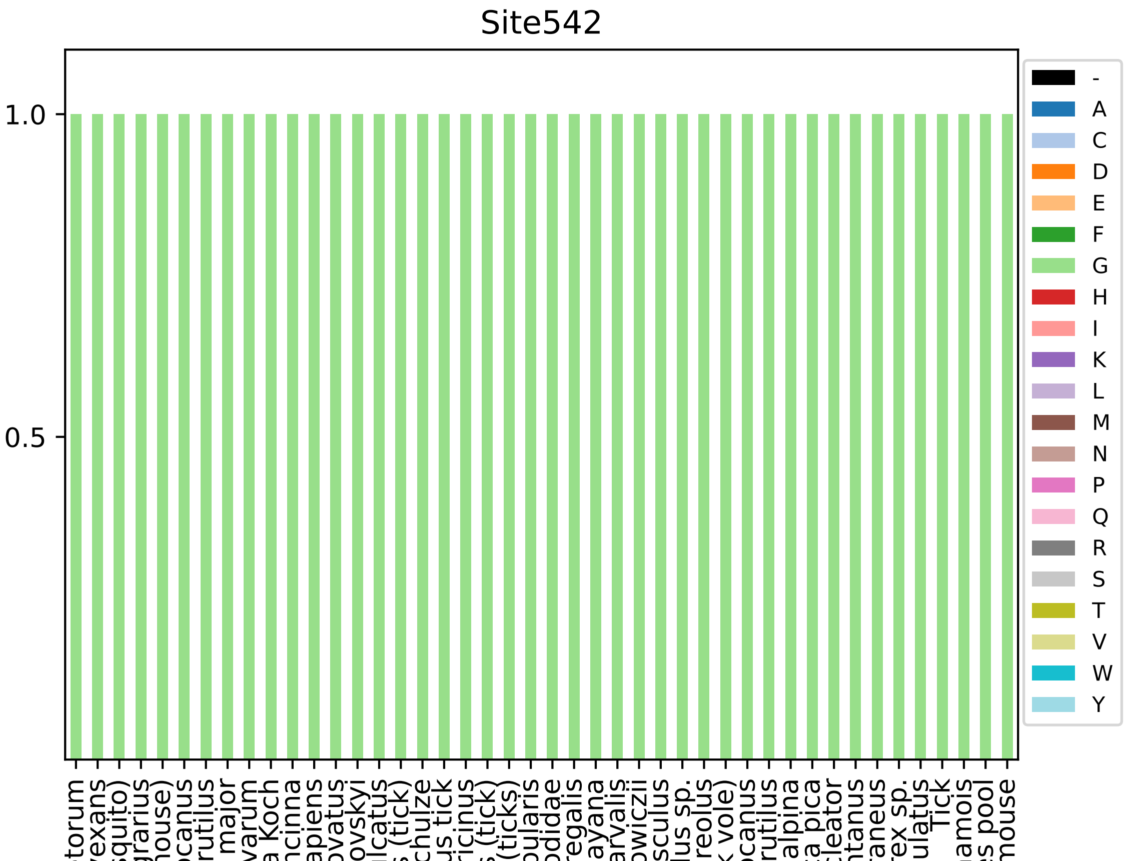
Site542
1.0
0.5
-
A
C
D
E
F
G
H
I
K
L
M
N
P
Q
R
S
T
V
W
Y

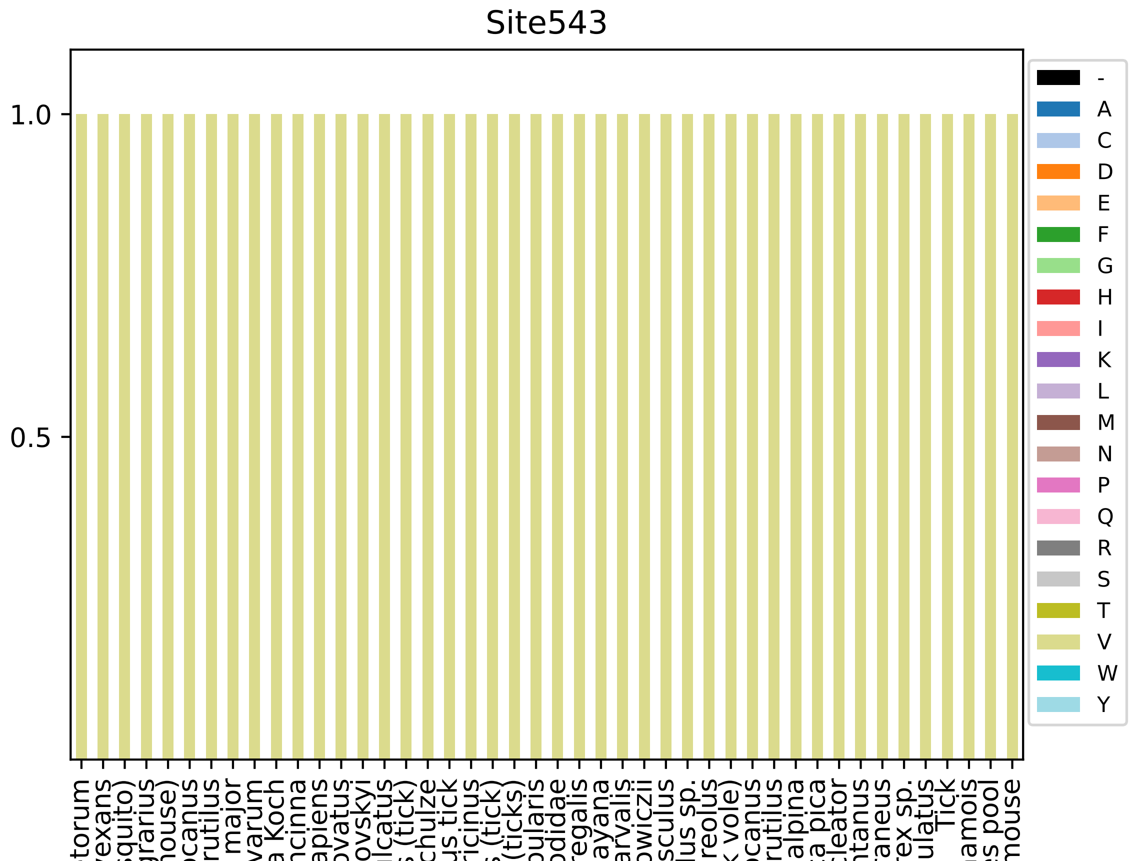
Site543
1.0
0.5
-
A
C
D
E
F
G
H
I
K
L
M
N
P
Q
R
S
T
V
W
Y

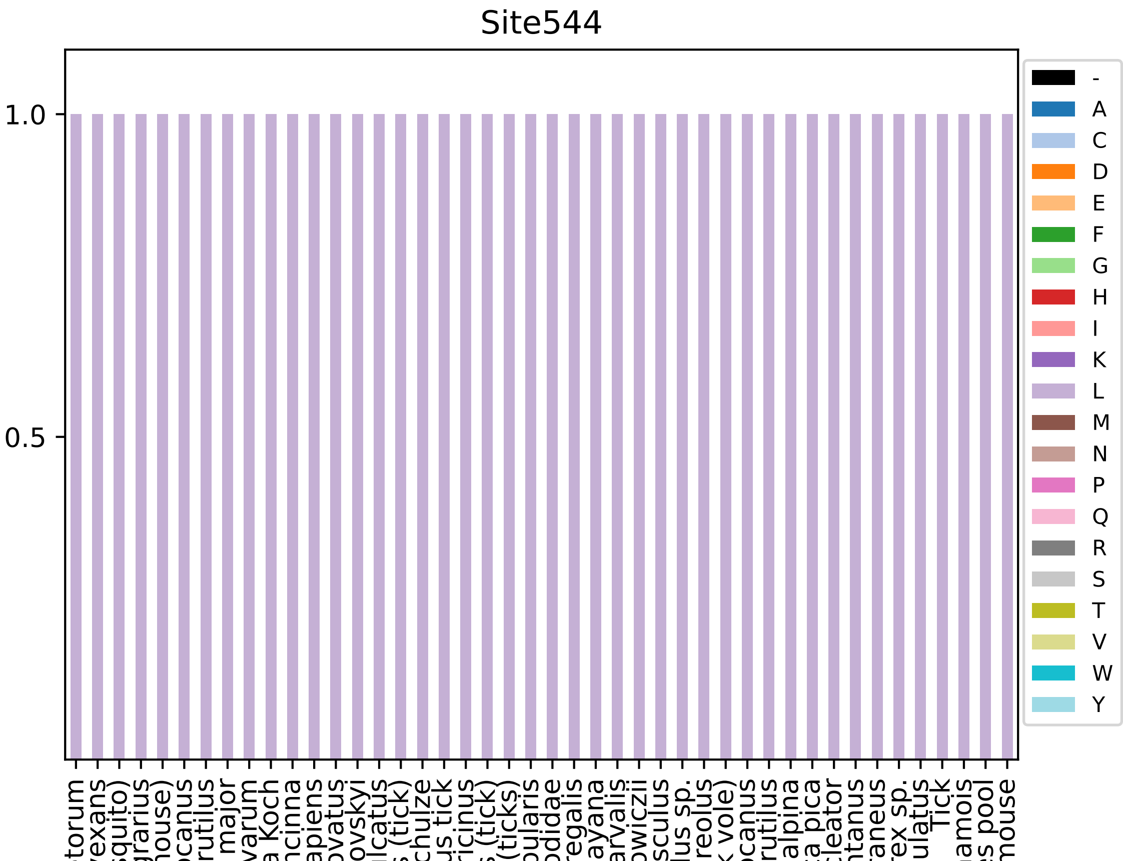

Site544
1.0
0.5
-
A
C
D
E
F
G
H
I
K
L
M
N
P
Q
R
S
T
V
W
Y

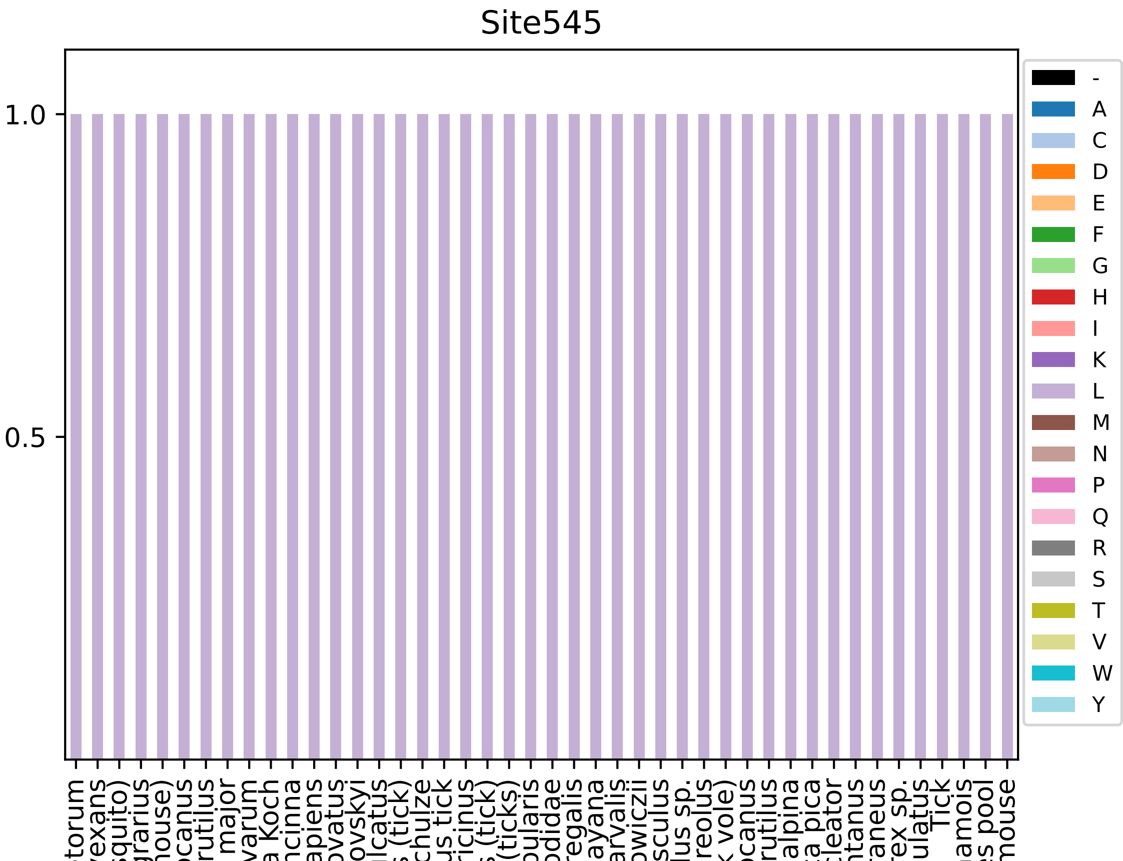
Site545
1.0
0.5
-
A
C
D
E
F
G
H
I
K
L
M
N
P
Q
R
S
T
V
W
Y

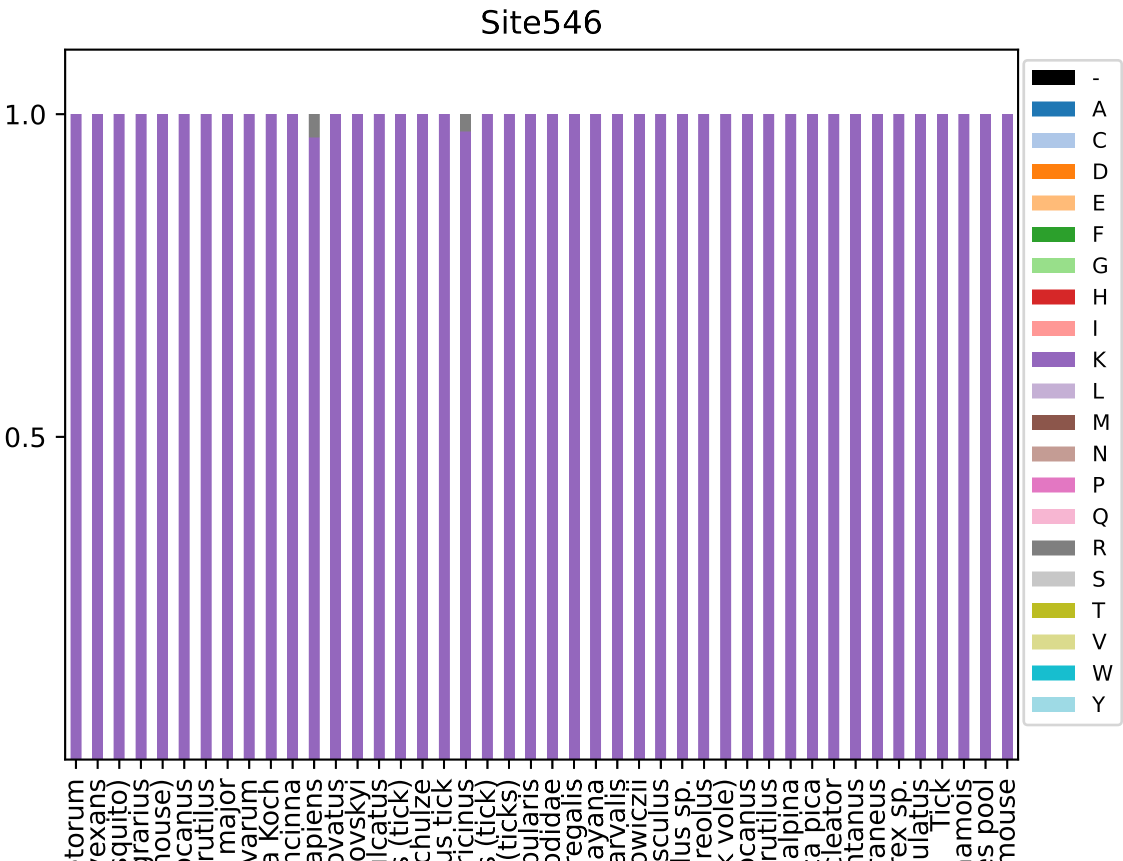
Site546
1.0
0.5
-
A
C
D
E
F
G
H
I
K
L
M
N
P
Q
R
S
T
V
W
Y

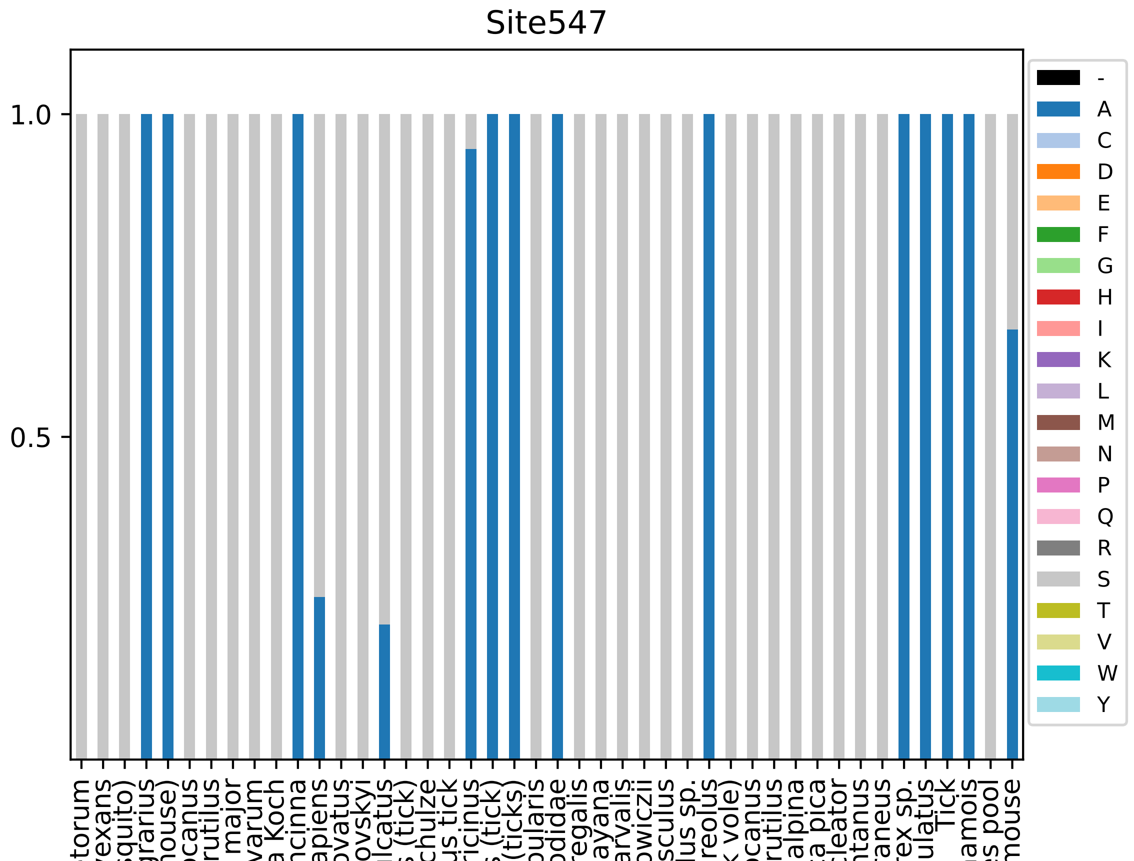

Site547
1.0
0.5
-
A
C
D
E
F
G
H
I
K
L
M
N
P
Q
R
S
T
V
W
Y
Tick

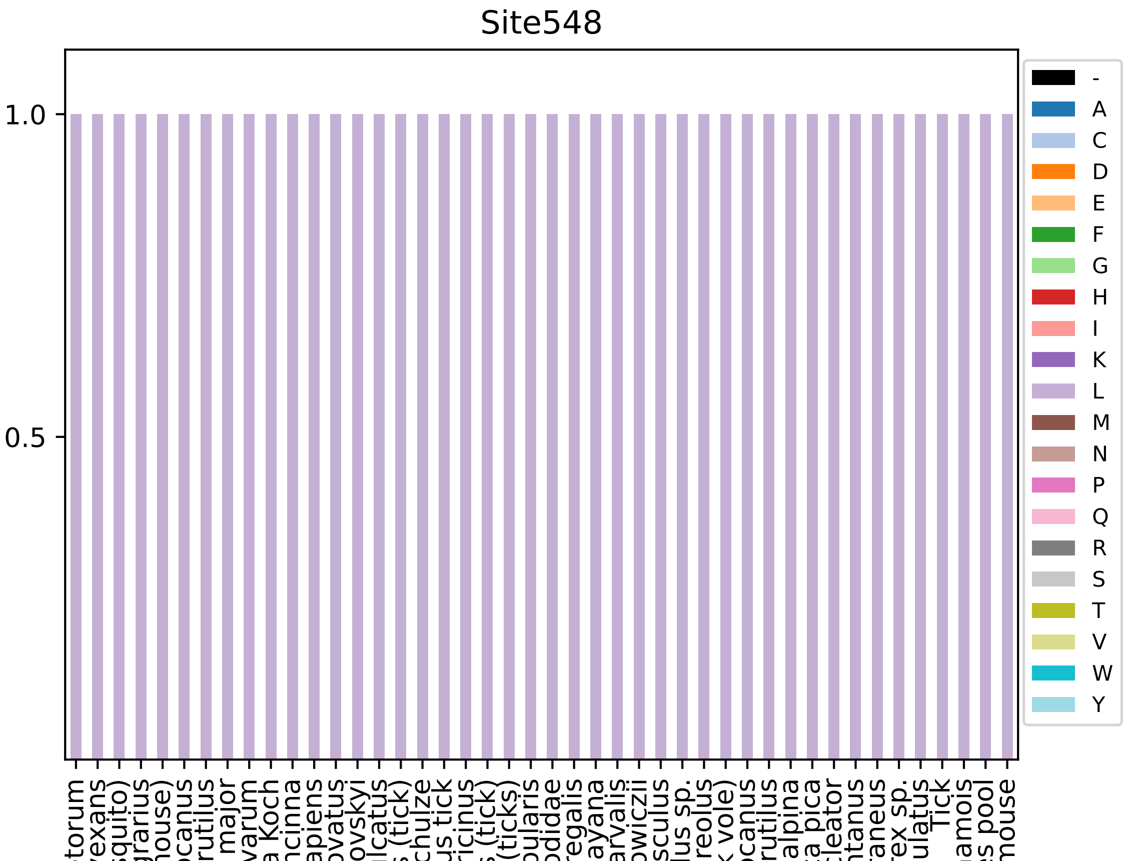

Site548
1.0
0.5
-
A
C
D
E
F
G
H
I
K
L
M
N
P
Q
R
S
T
V
W
Y
torum
vexans
squito)
grarius
mouse)
ocanus
rutilus
major
varum
a Koch
ncinna
apiens
ovatus
ovskyi
lcatus
(tick)
chulze
us tick
ricinus
(tick)
(ticks)
pularis
podidae
regalis
ayana
arvalis
owiczii
sculus
lus sp.
reolus
k vole)
ocanus
rutilus
alpina
a pica
leator
ntanus
aneus
ex sp.
ulatus
Tick
amois
es pool
mouse

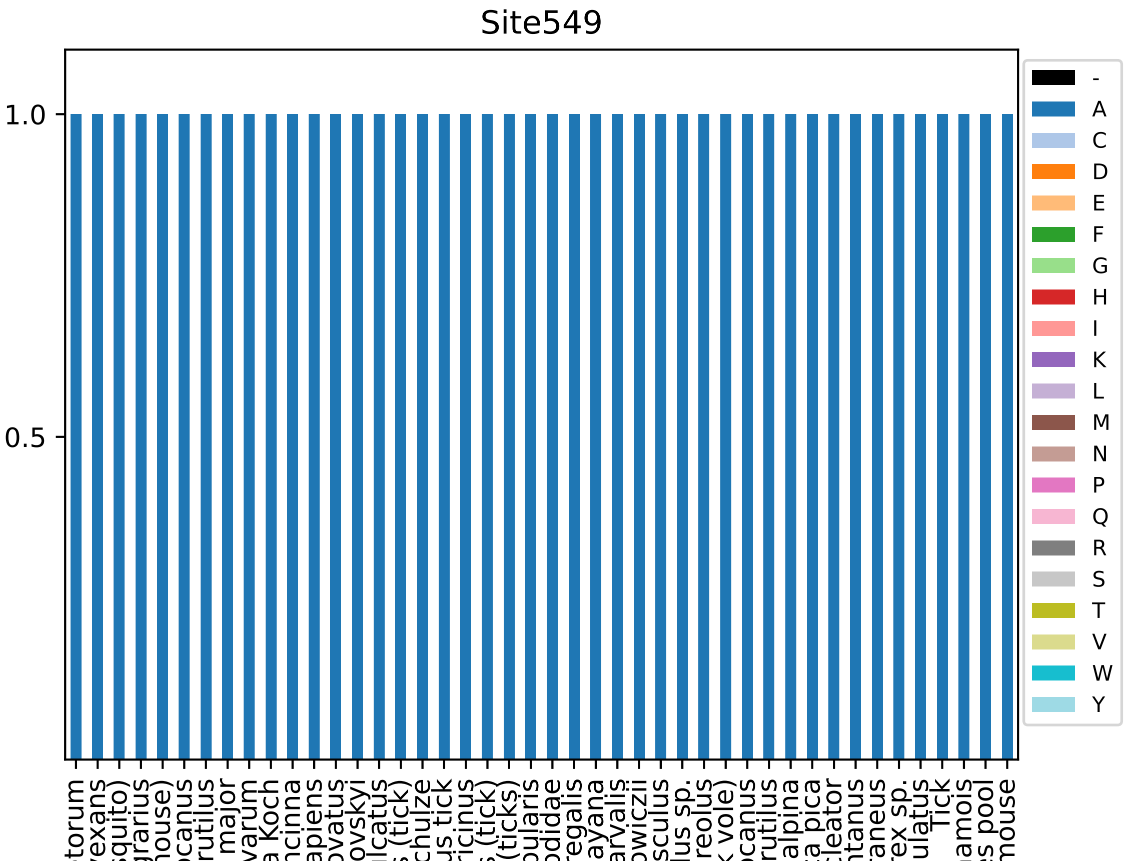

Site549
1.0
0.5
-
A
C
D
E
F
G
H
I
K
L
M
N
P
Q
R
S
T
V
W
Y

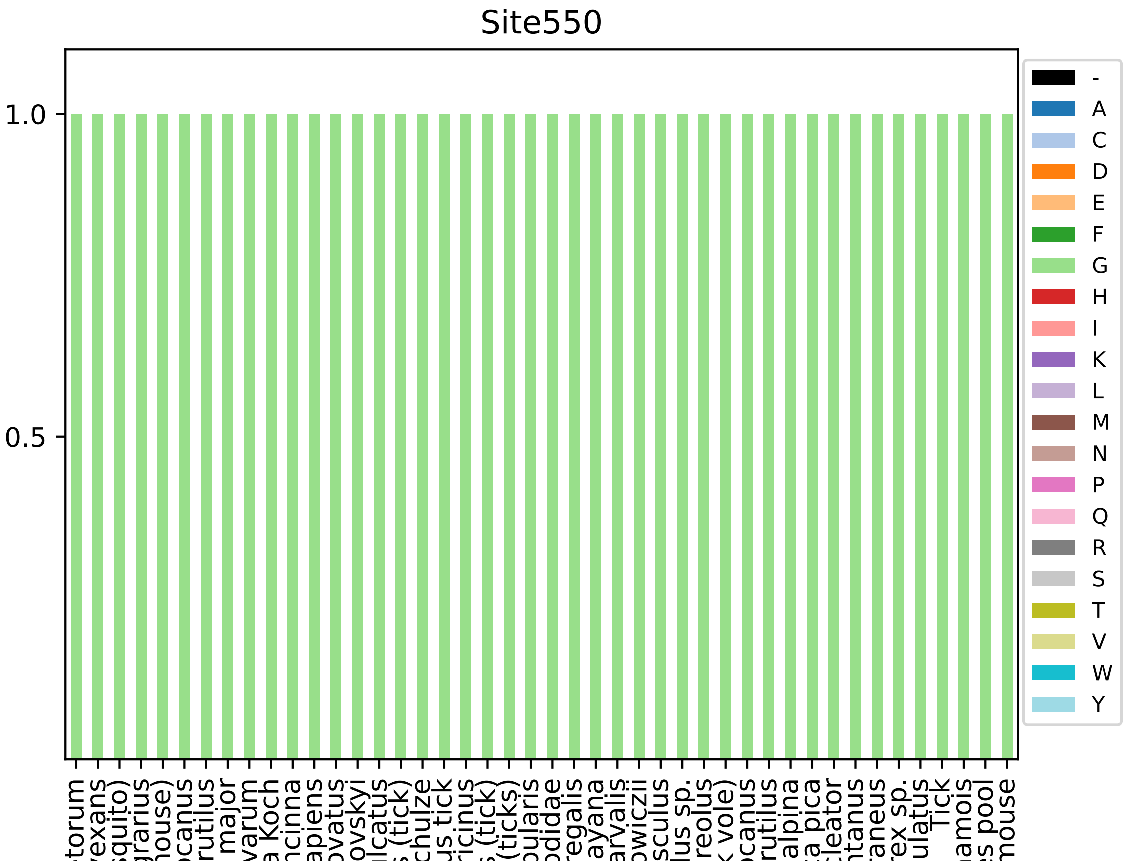
Site550
1.0
0.5
-
A
C
D
E
F
G
H
I
K
L
M
N
P
Q
R
S
T
V
W
Y

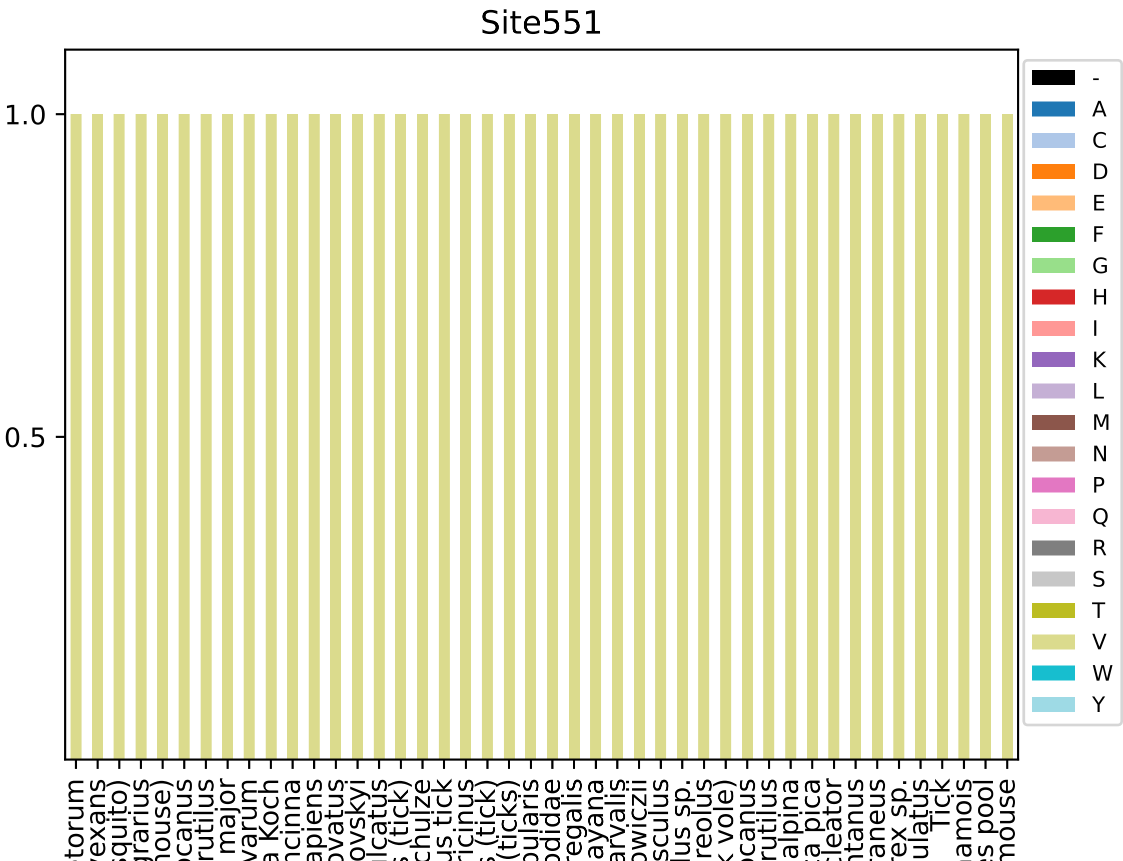
Site551
1.0
0.5
-
A
C
D
E
F
G
H
I
K
L
M
N
P
Q
R
S
T
V
W
Y

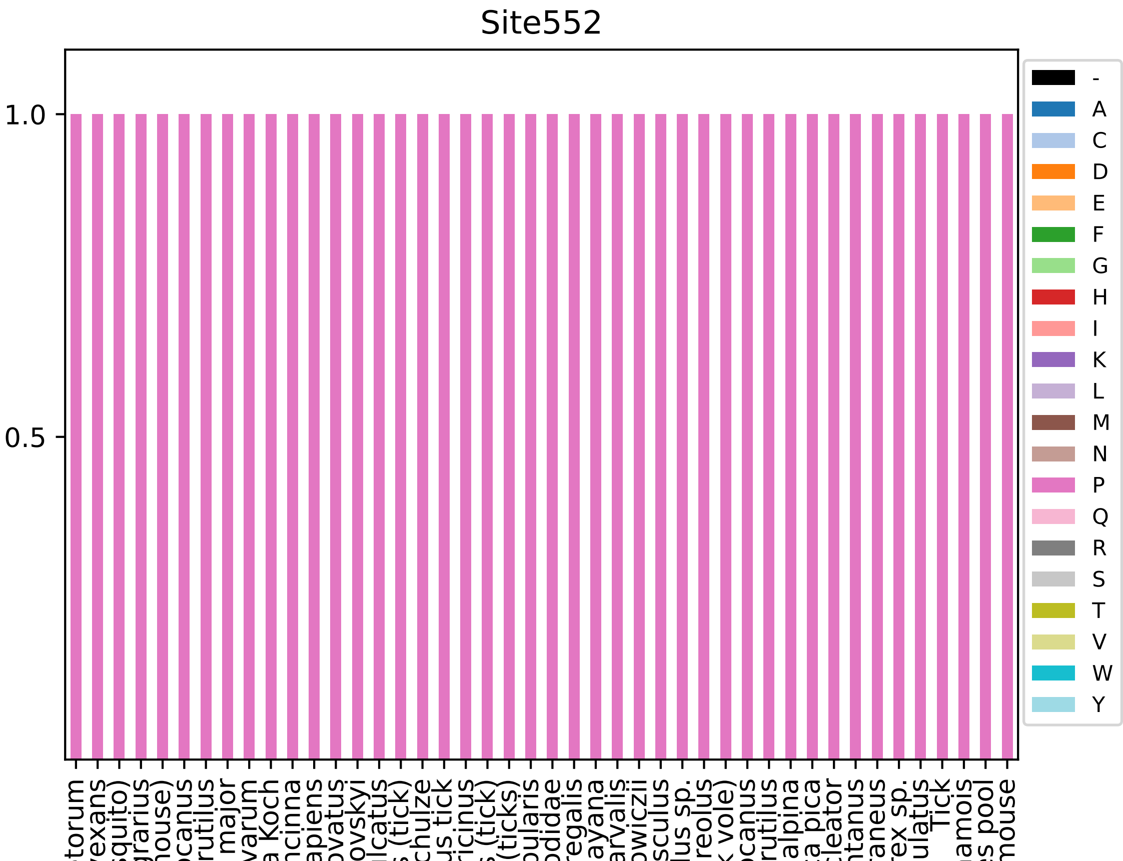
Site552
1.0
0.5
-
A
C
D
E
F
G
H
I
K
L
M
N
P
Q
R
S
T
V
W
Y

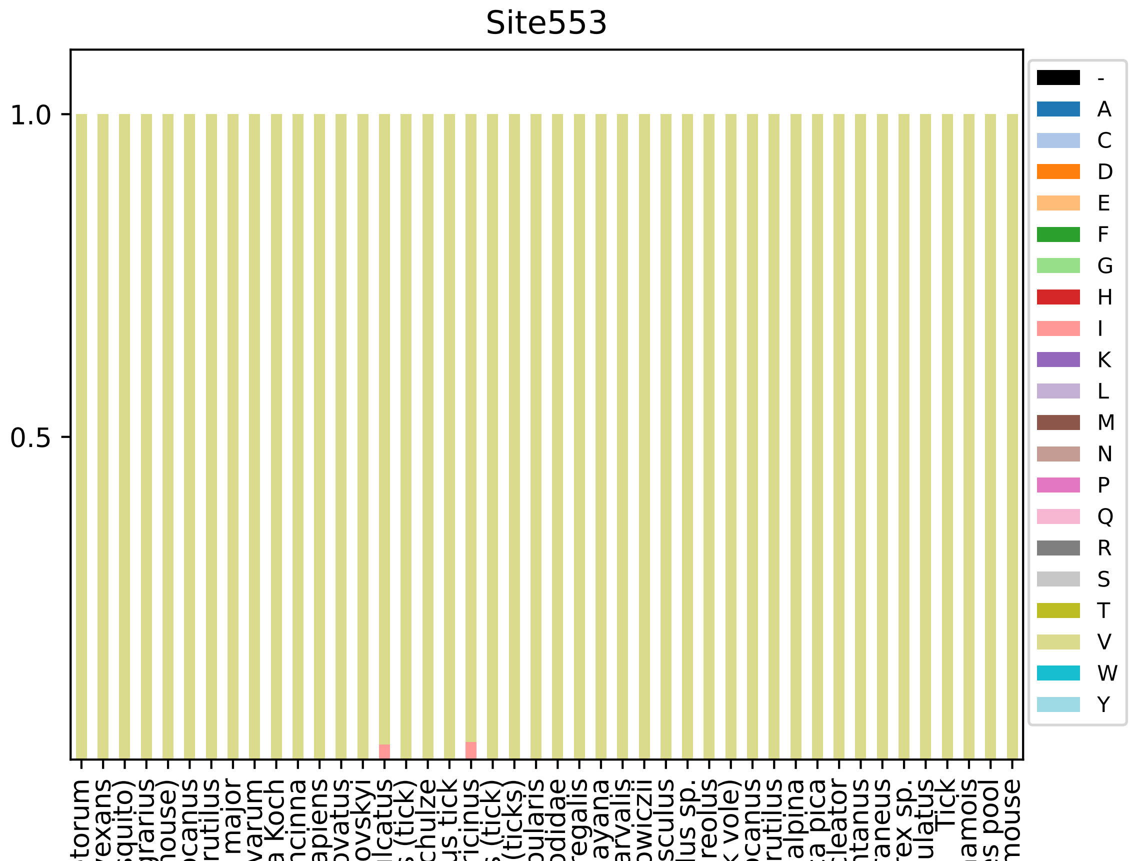
Site553
1.0
0.5
-
A
C
D
E
F
G
H
I
K
L
M
N
P
Q
R
S
T
V
W
Y

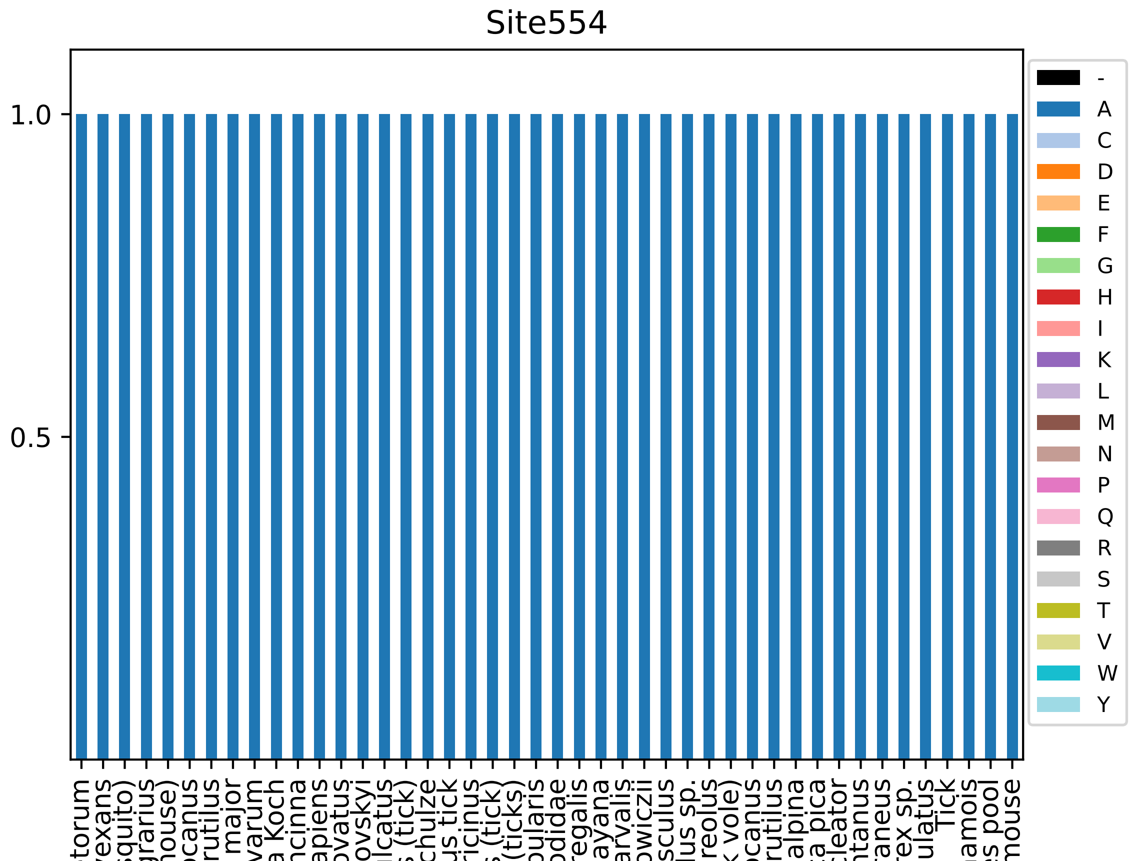
Site554
1.0
0.5
-
A
C
D
E
F
G
H
I
K
L
M
N
P
Q
R
S
T
V
W
Y

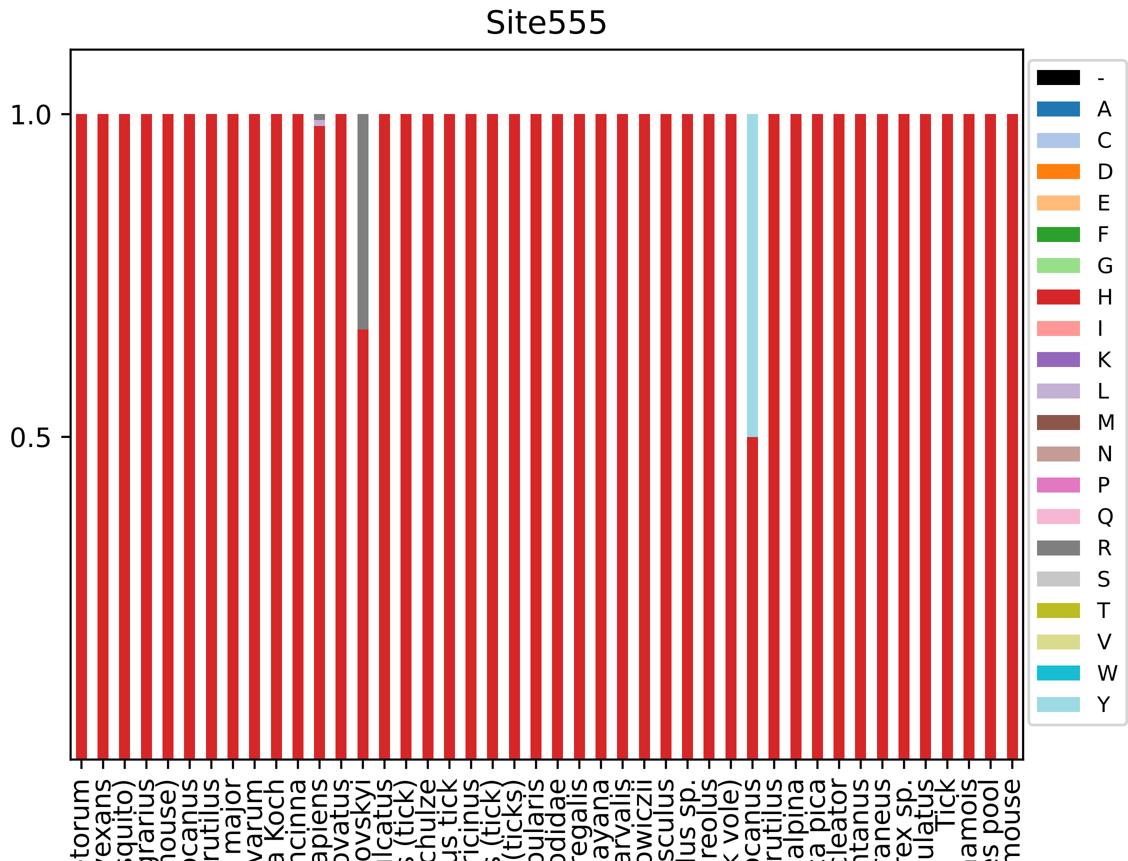
Site555
1.0
0.5
-
A
C
D
E
F
G
H
I
K
L
M
N
P
Q
R
S
T
V
W
Y

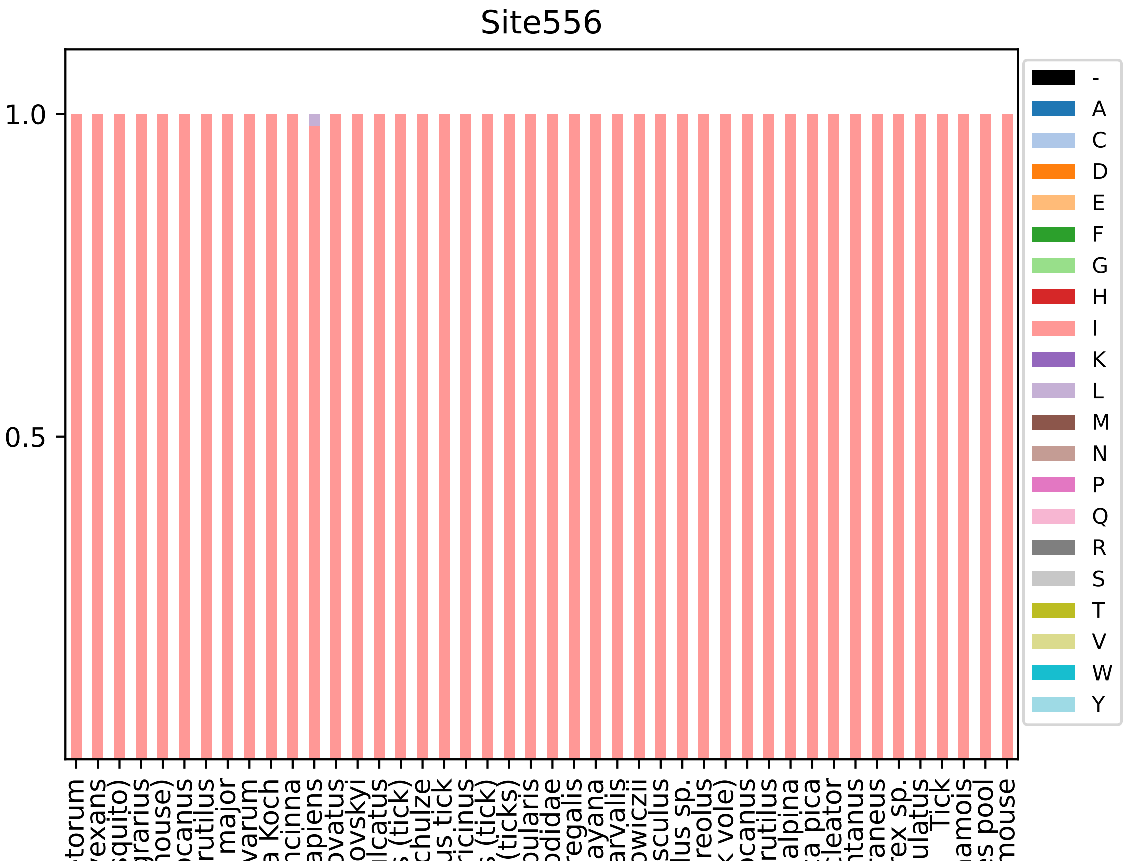

Site556
1.0
0.5
-
A
C
D
E
F
G
H
I
K
L
M
N
P
Q
R
S
T
V
W
Y

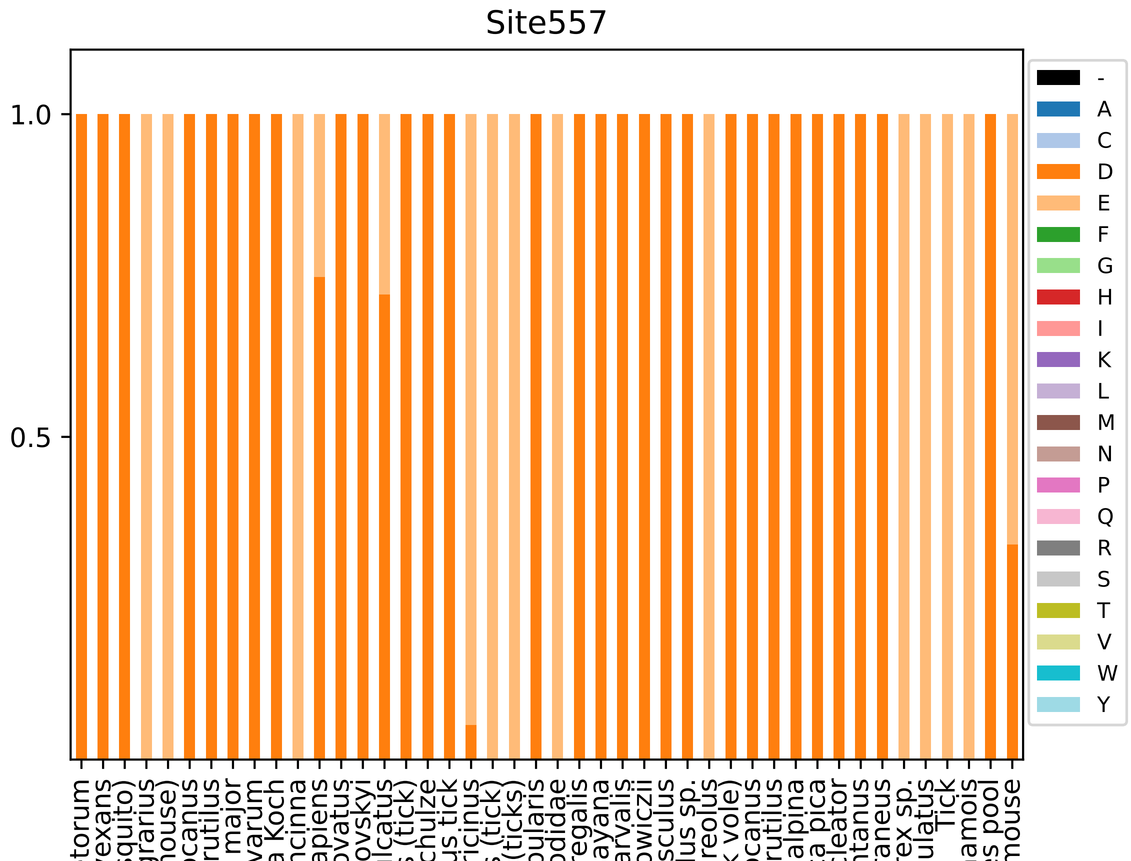
Site557
1.0
0.5
-
A
C
D
E
F
G
H
I
K
L
M
N
P
Q
R
S
T
V
W
Y

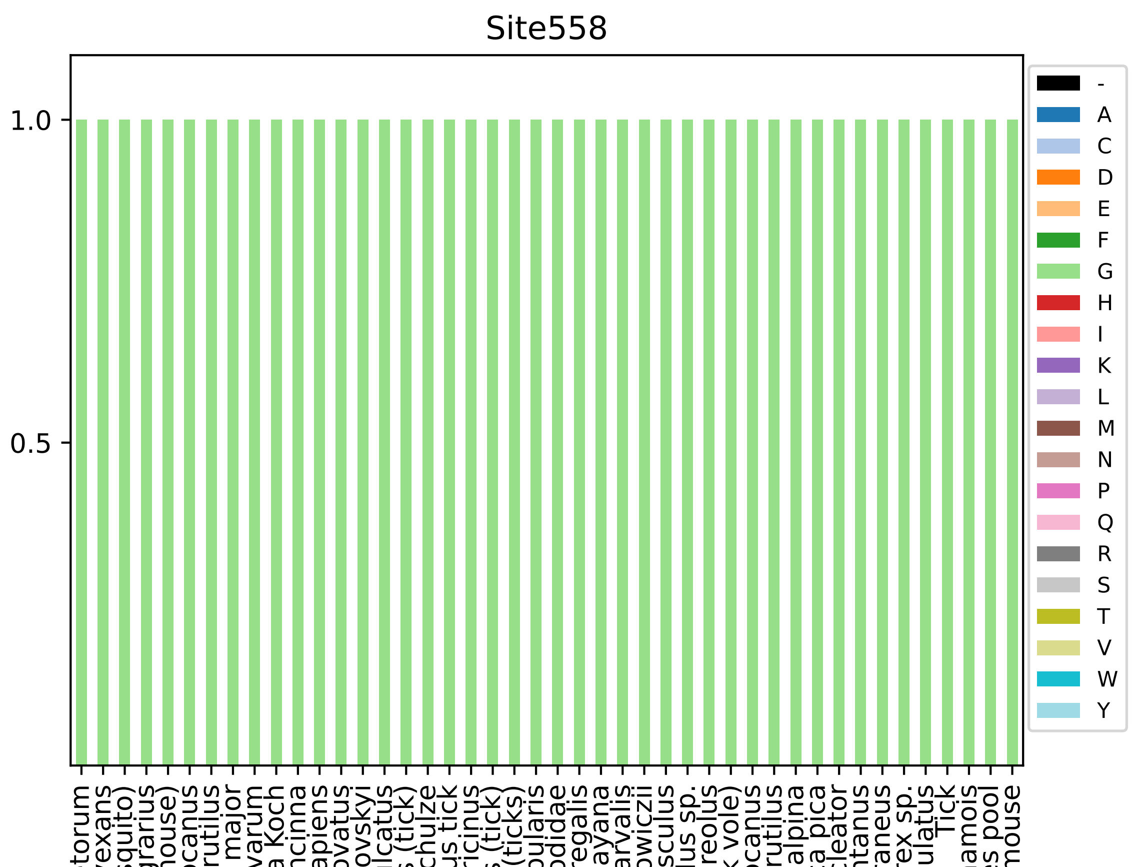
Site558
1.0
0.5
-
A
C
D
E
F
G
H
I
K
L
M
N
P
Q
R
S
T
V
W
Y

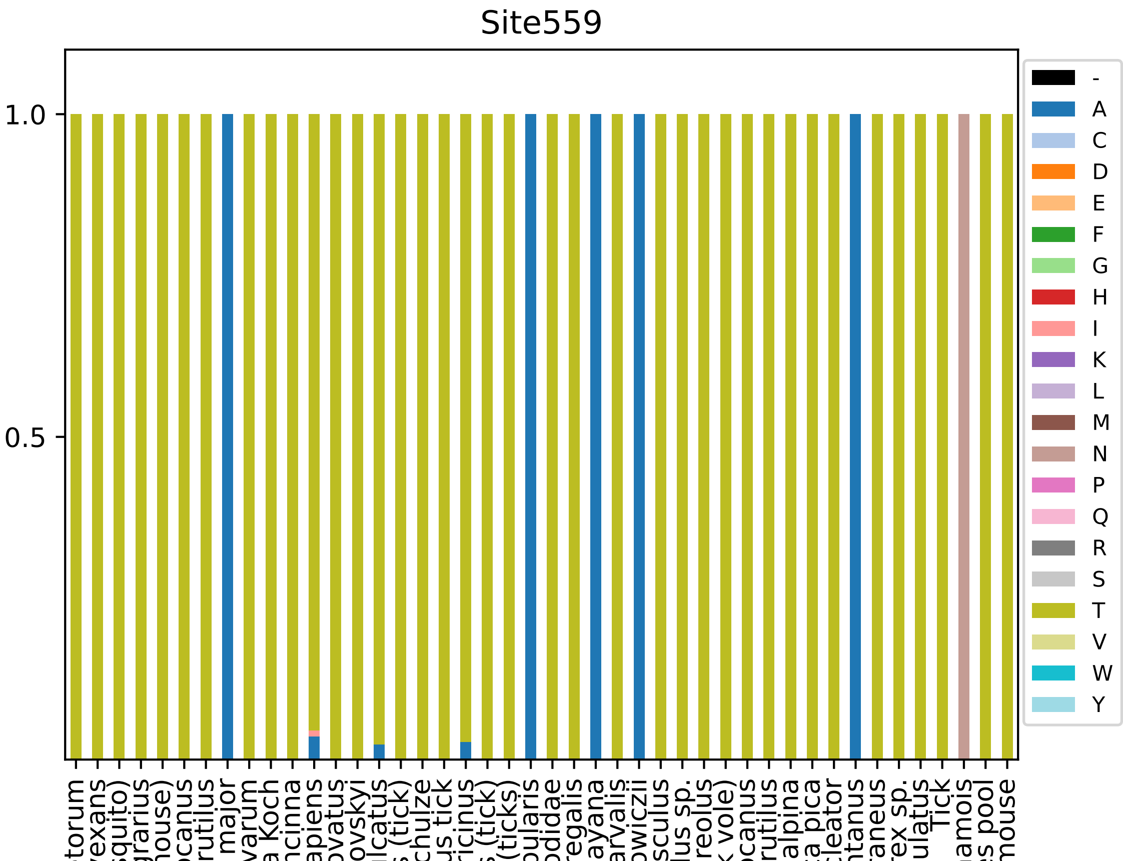
Site559
1.0
0.5
-
A
C
D
E
F
G
H
I
K
L
M
N
P
Q
R
S
T
V
W
Y

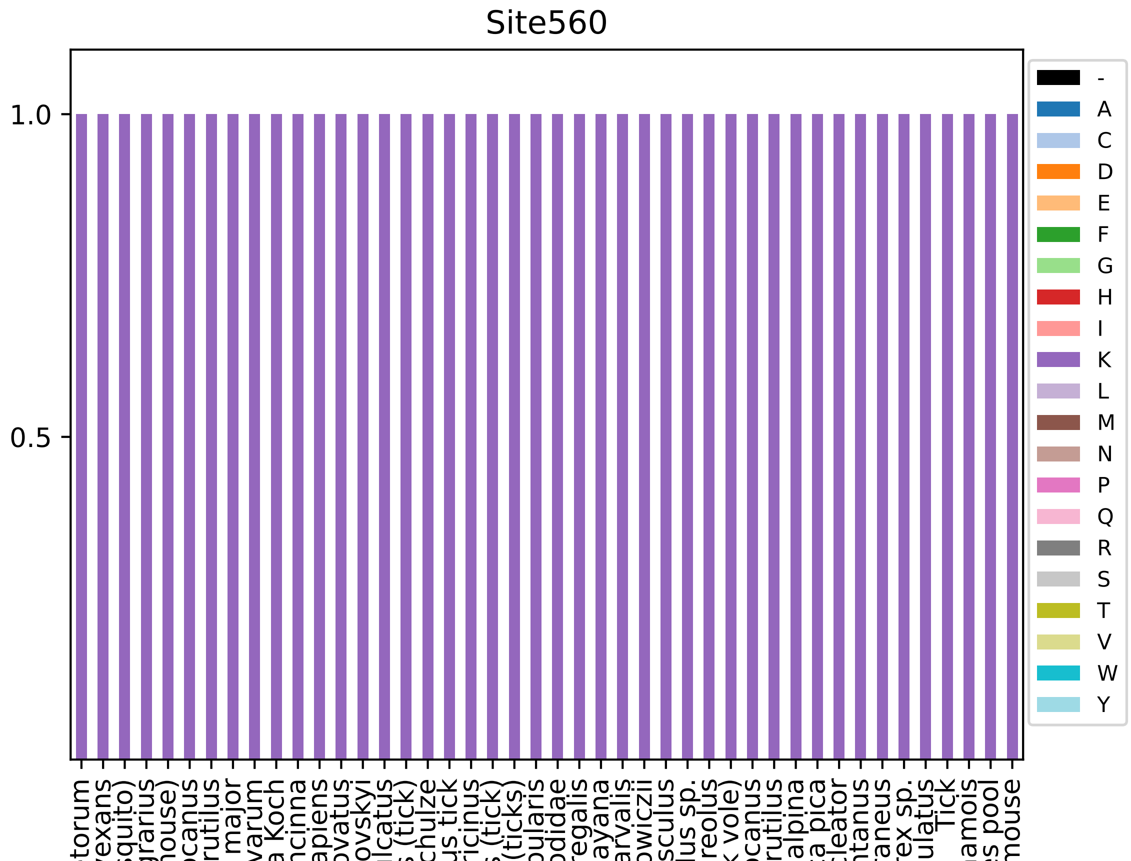
Site560
1.0
0.5
-
A
C
D
E
F
G
H
I
K
L
M
N
P
Q
R
S
T
V
W
Y

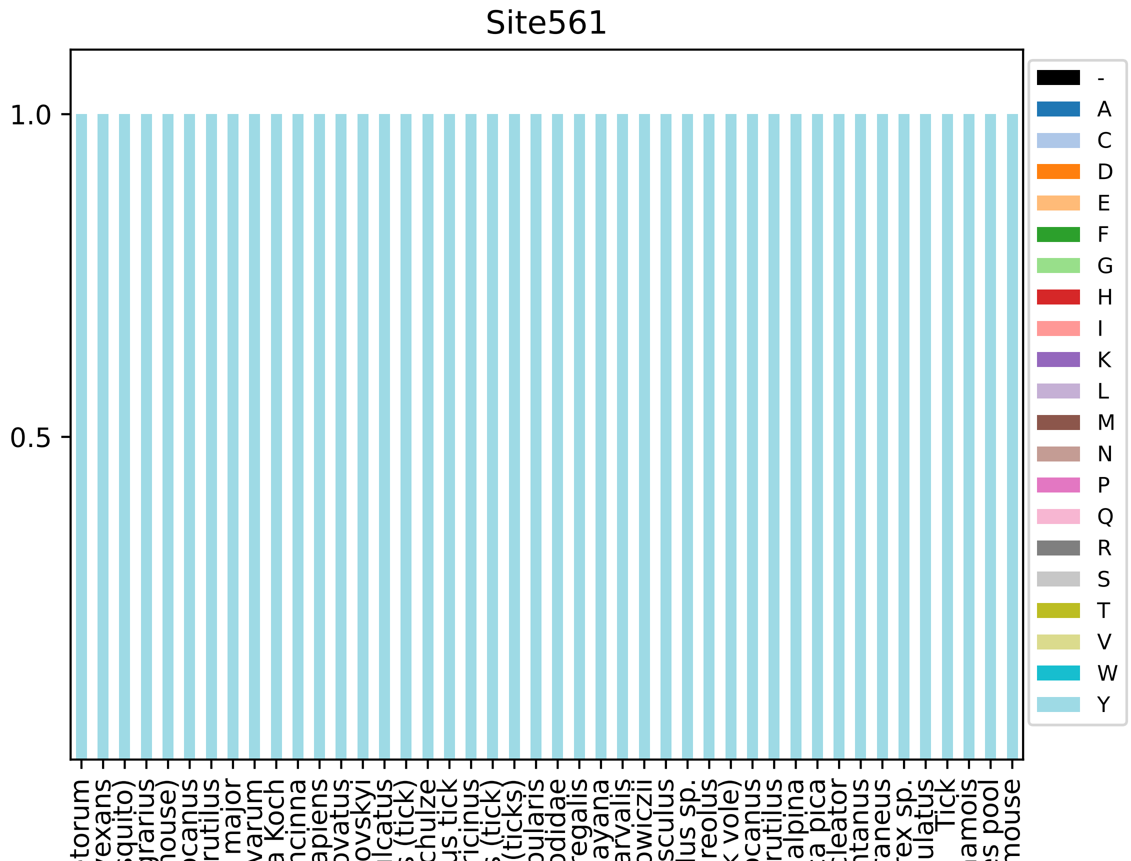
Site561
1.0
0.5
-
A
C
D
E
F
G
H
I
K
L
M
N
P
Q
R
S
T
V
W
Y

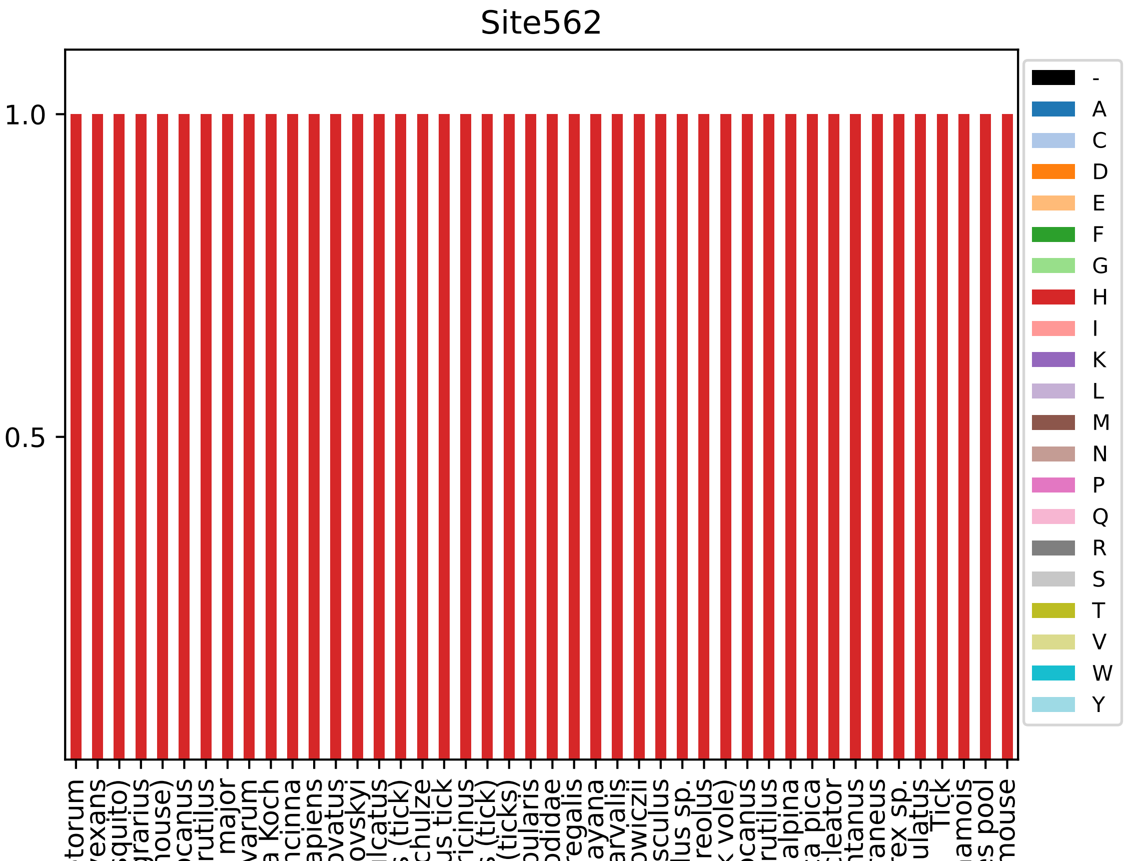

Site562
1.0
0.5
-
A
C
D
E
F
G
H
I
K
L
M
N
P
Q
R
S
T
V
W
Y

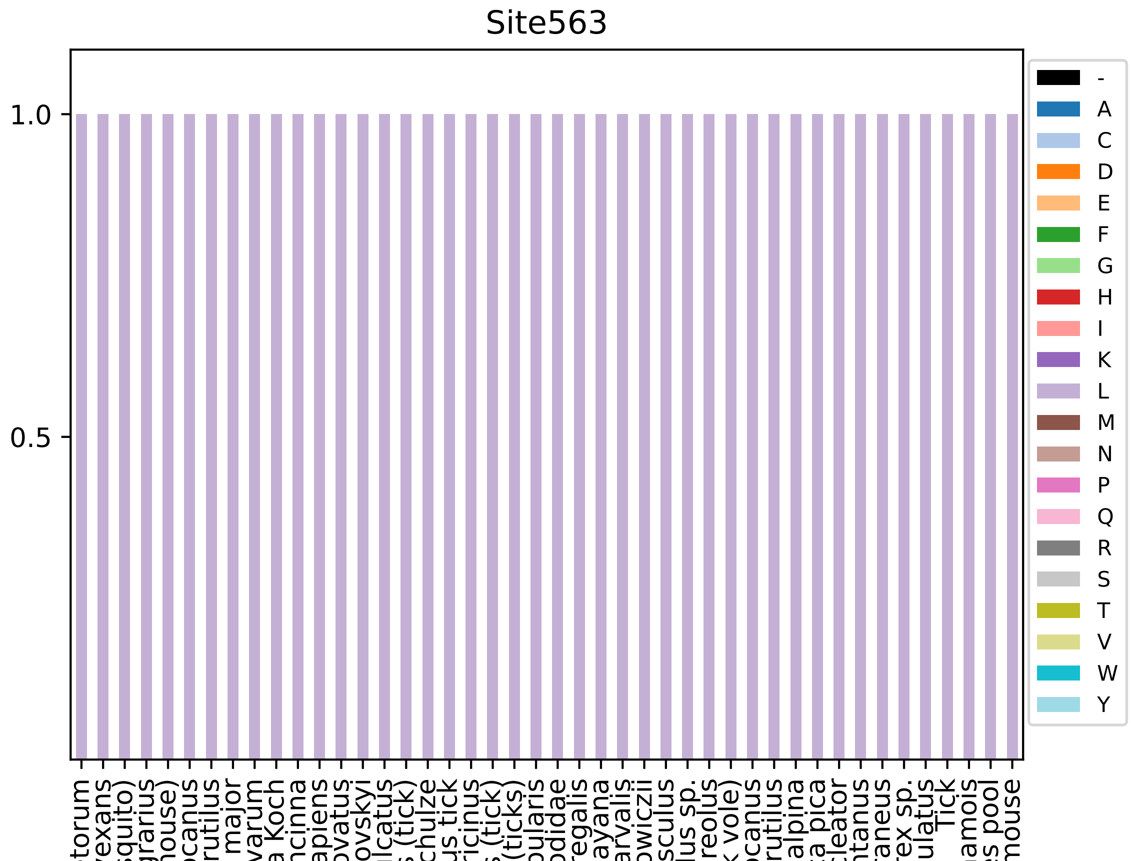
Site563
1.0
0.5
-
A
C
D
E
F
G
H
I
K
L
M
N
P
Q
R
S
T
V
W
Y

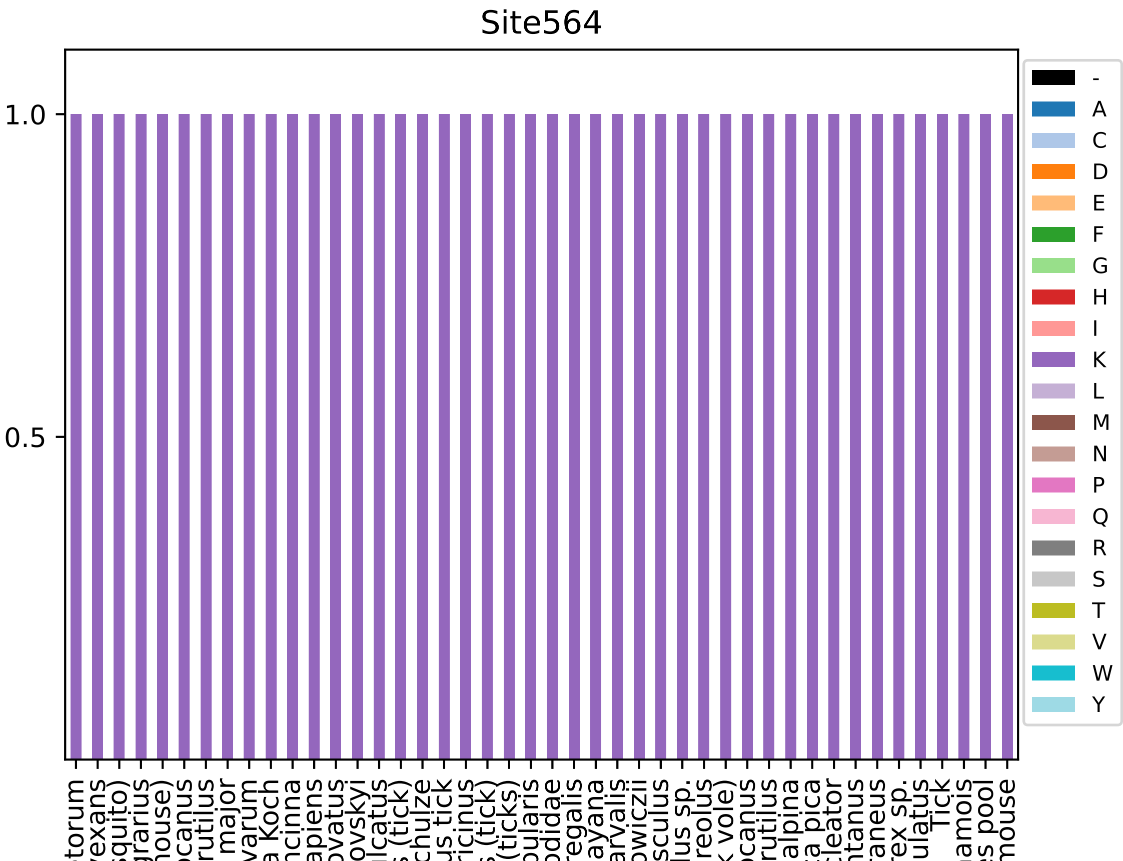
Site564
1.0
0.5
-
A
C
D
E
F
G
H
I
K
L
M
N
P
Q
R
S
T
V
W
Y

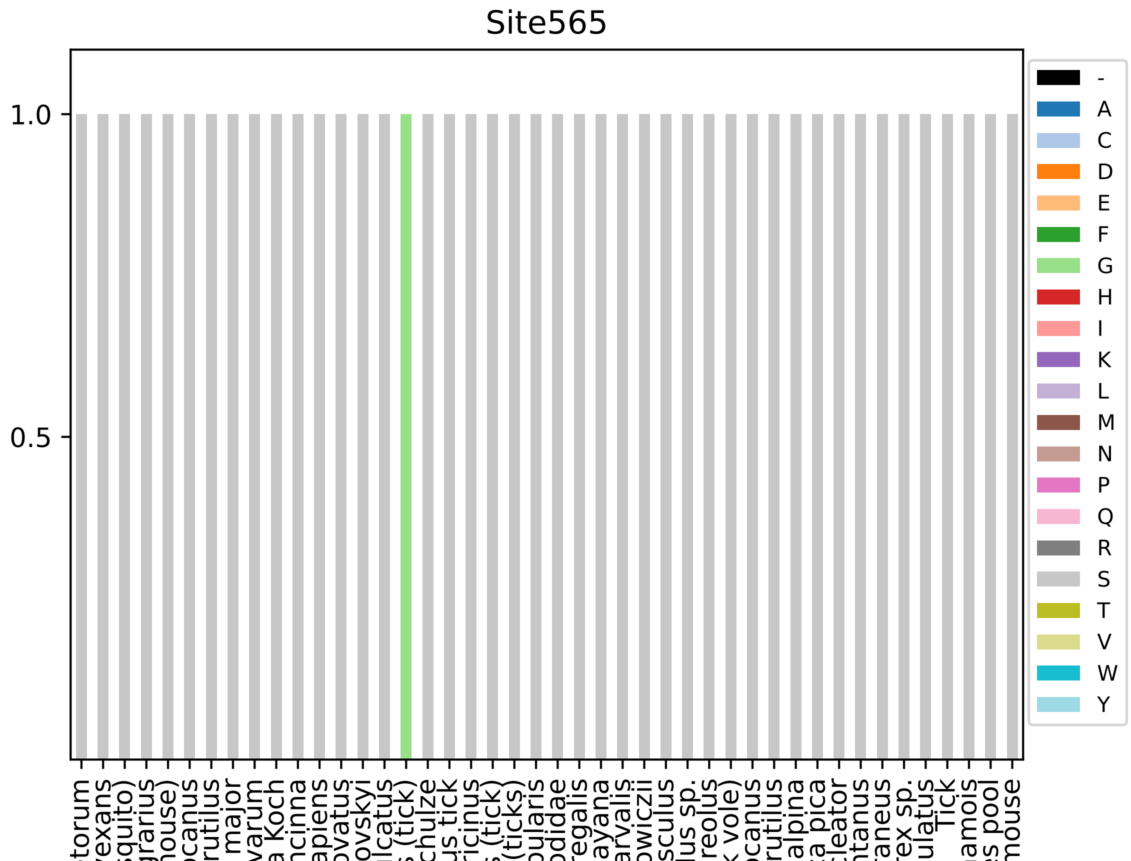
Site565
1.0
0.5
-
A
C
D
E
F
G
H
I
K
L
M
N
P
Q
R
S
T
V
W
Y

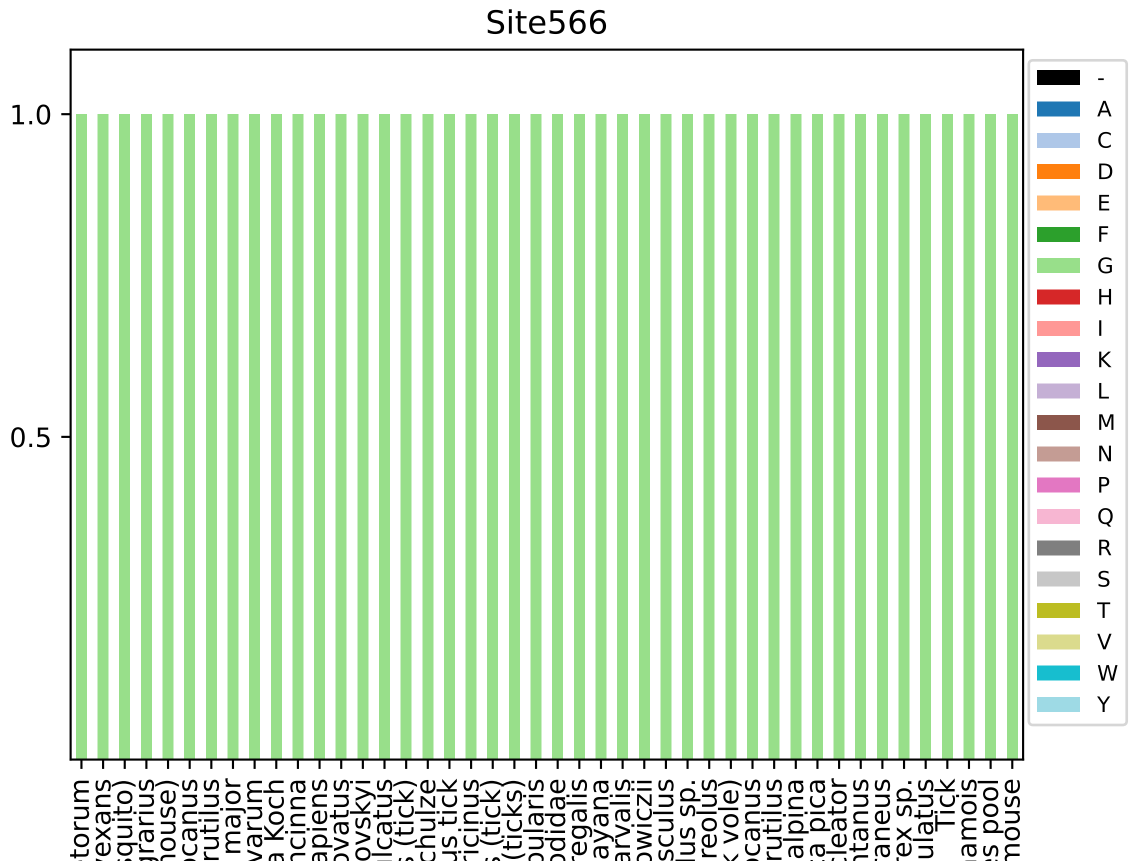
Site566
1.0
0.5
-
A
C
D
E
F
G
H
I
K
L
M
N
P
Q
R
S
T
V
W
Y

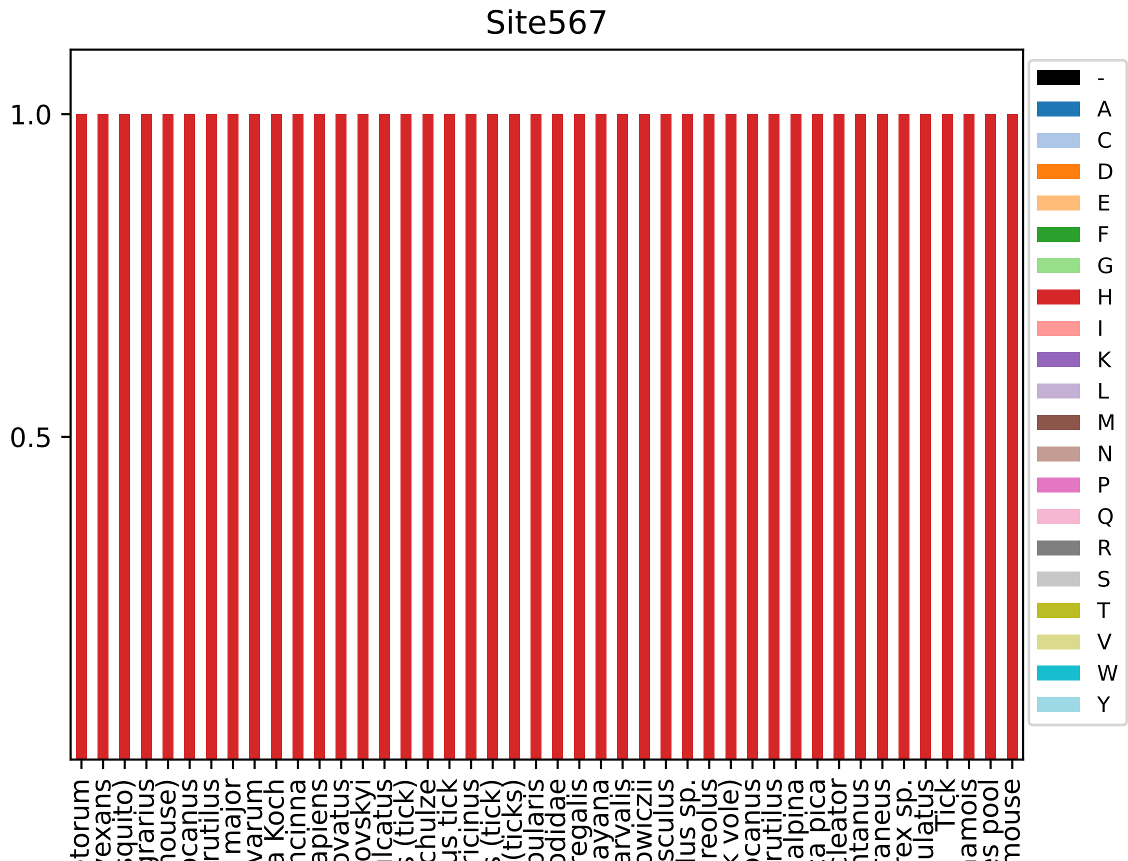
Site567
1.0
0.5
-
A
C
D
E
F
G
H
I
K
L
M
N
P
Q
R
S
T
V
W
Y

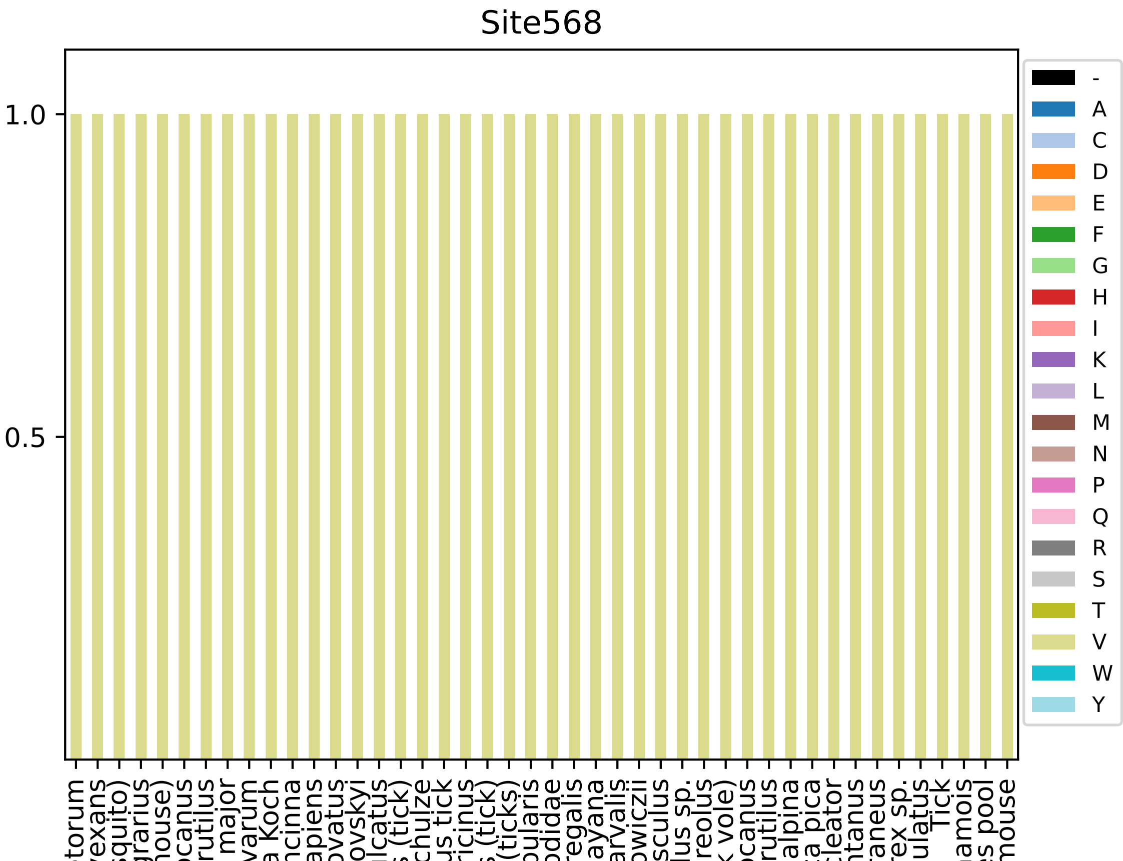
Site568
1.0
0.5
-
A
C
D
E
F
G
H
I
K
L
M
N
P
Q
R
S
T
V
W
Y

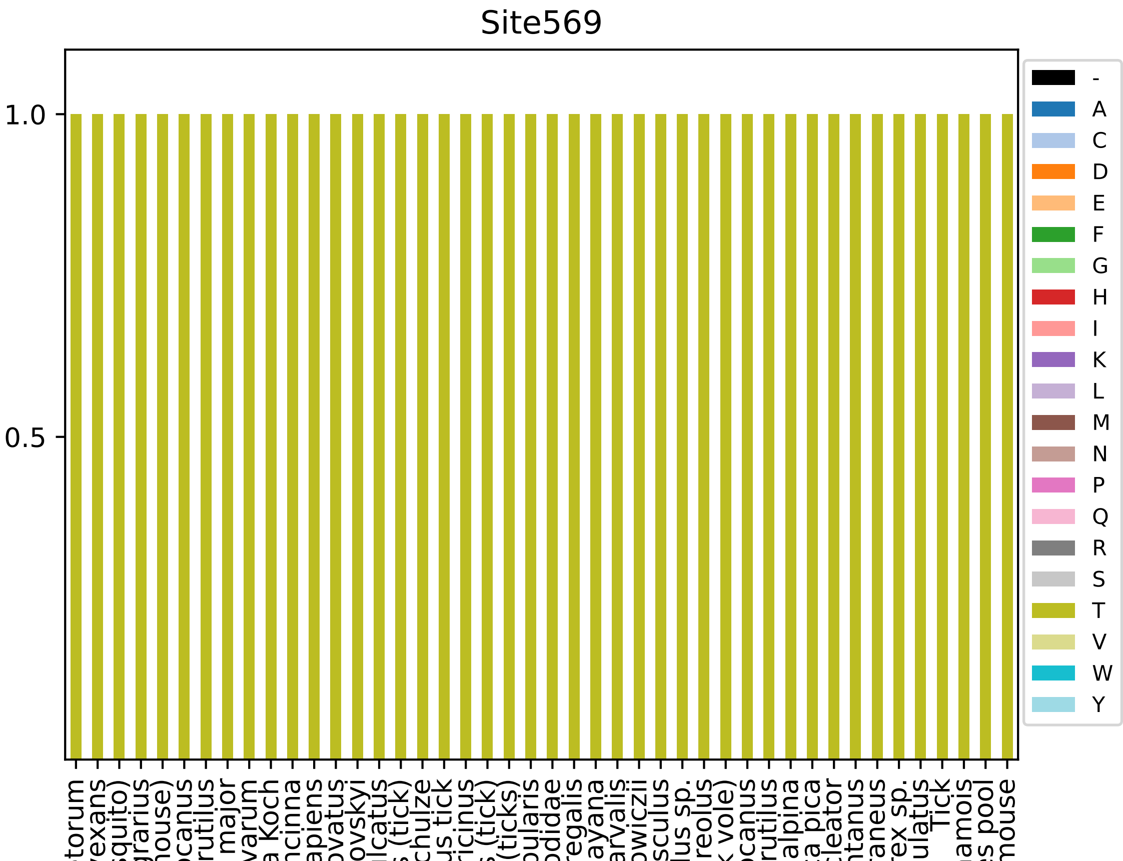
Site569
1.0
0.5
-
A
C
D
E
F
G
H
I
K
L
M
N
P
Q
R
S
T
V
W
Y

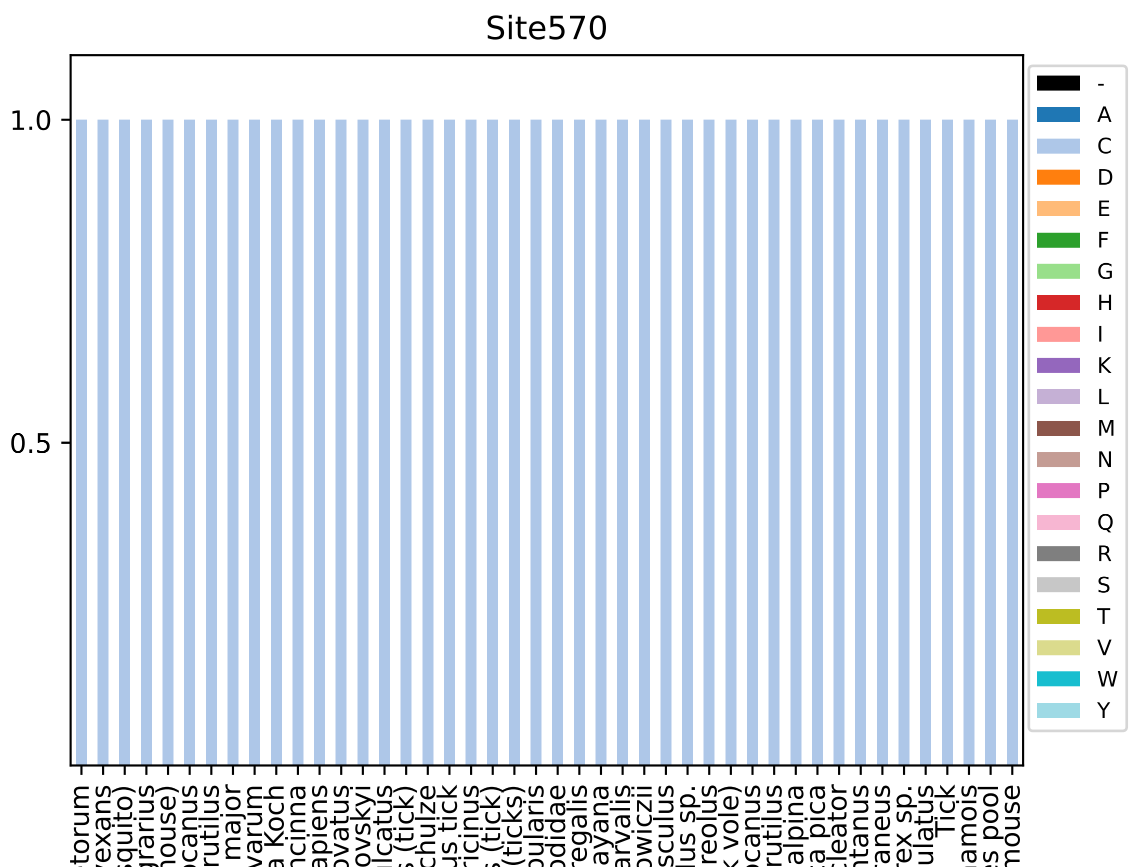
Site570
1.0
0.5
-
A
C
D
E
F
G
H
I
K
L
M
N
P
Q
R
S
T
V
W
Y

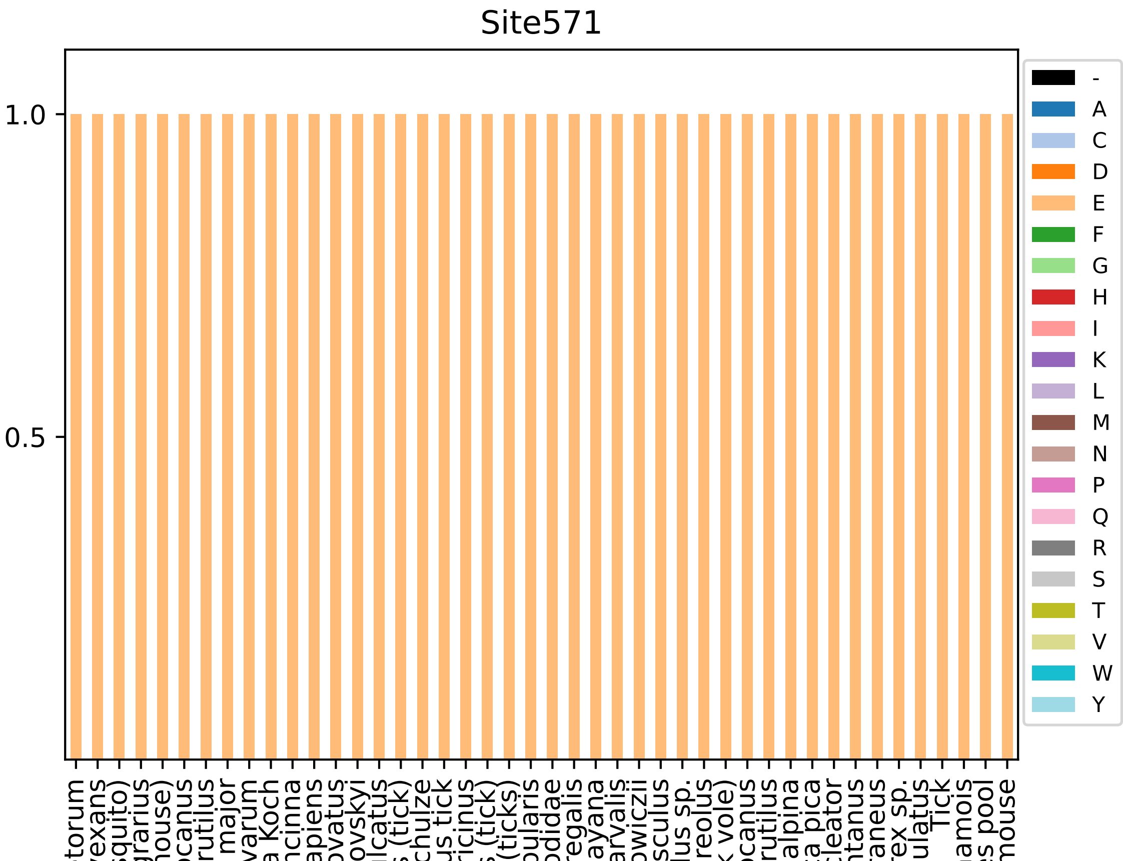
Site571
1.0
0.5
-
A
C
D
E
F
G
H
I
K
L
M
N
P
Q
R
S
T
V
W
Y

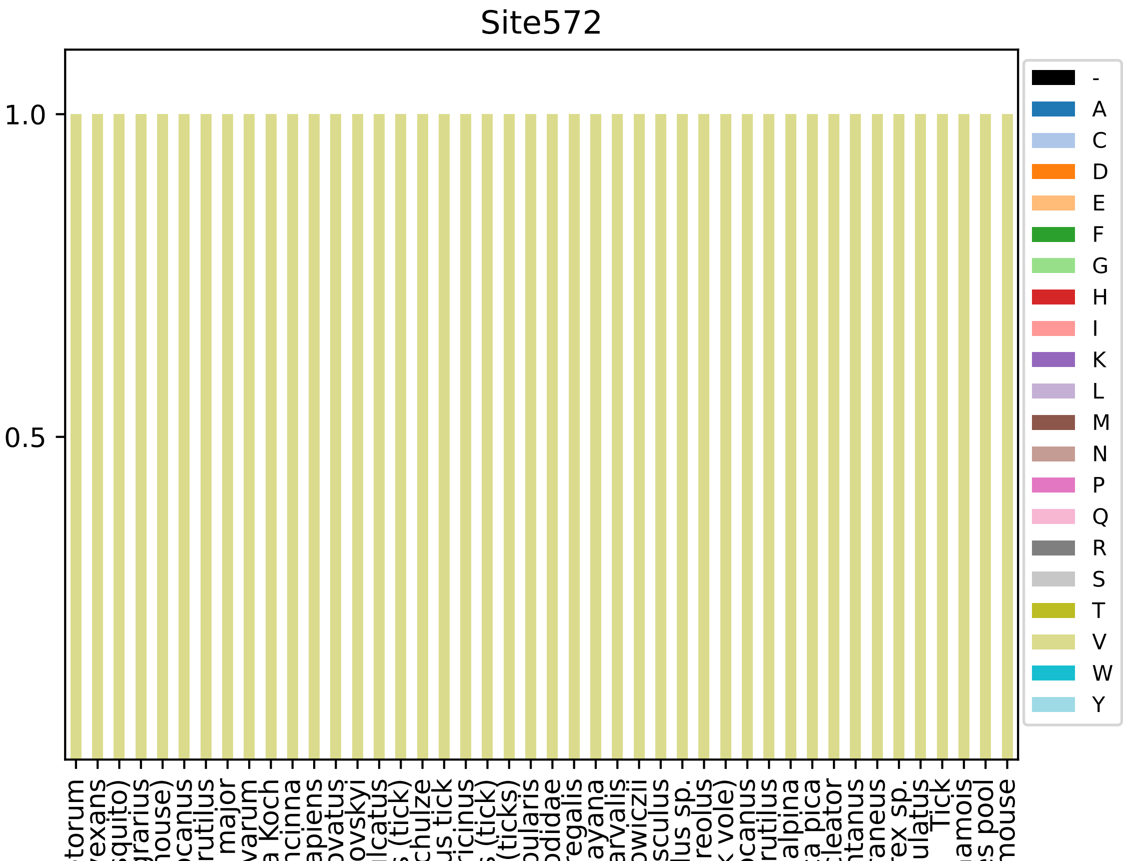
Site572
1.0
0.5
-
A
C
D
E
F
G
H
I
K
L
M
N
P
Q
R
S
T
V
W
Y

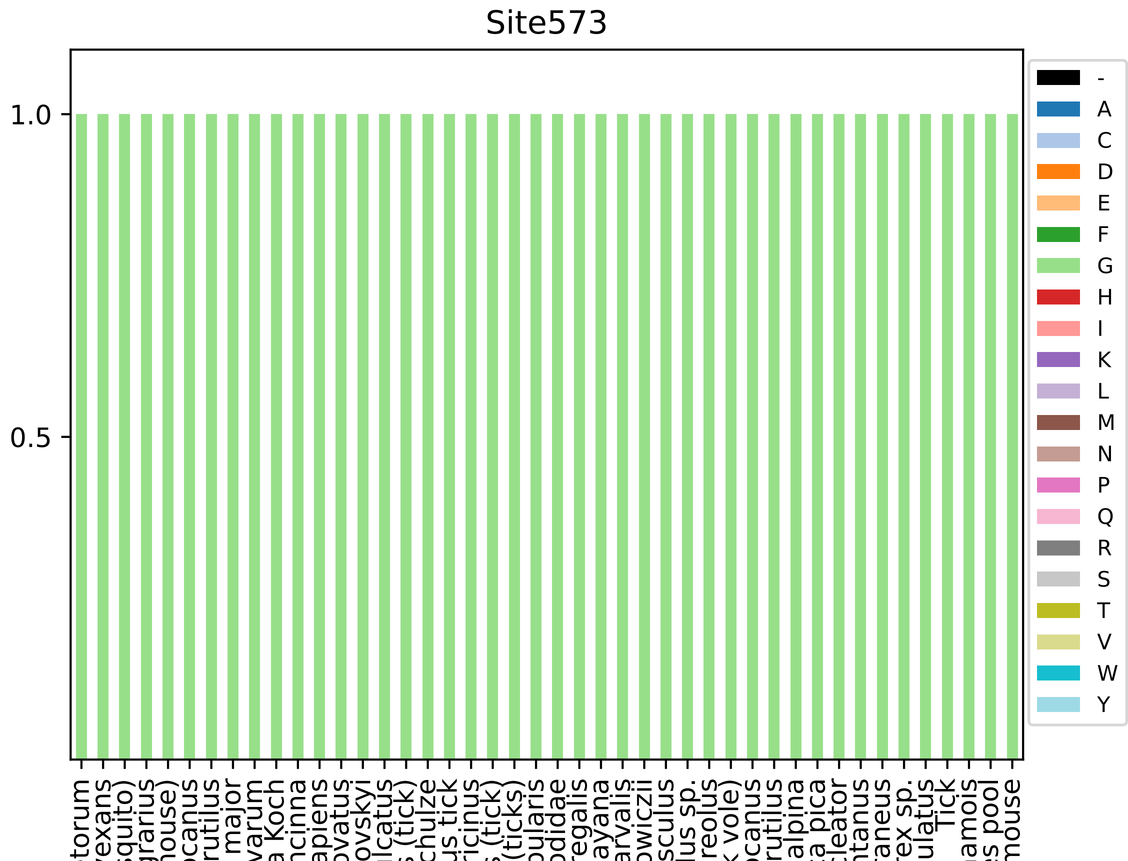

Site573
1.0
0.5
-
A
C
D
E
F
G
H
I
K
L
M
N
P
Q
R
S
T
V
W
Y

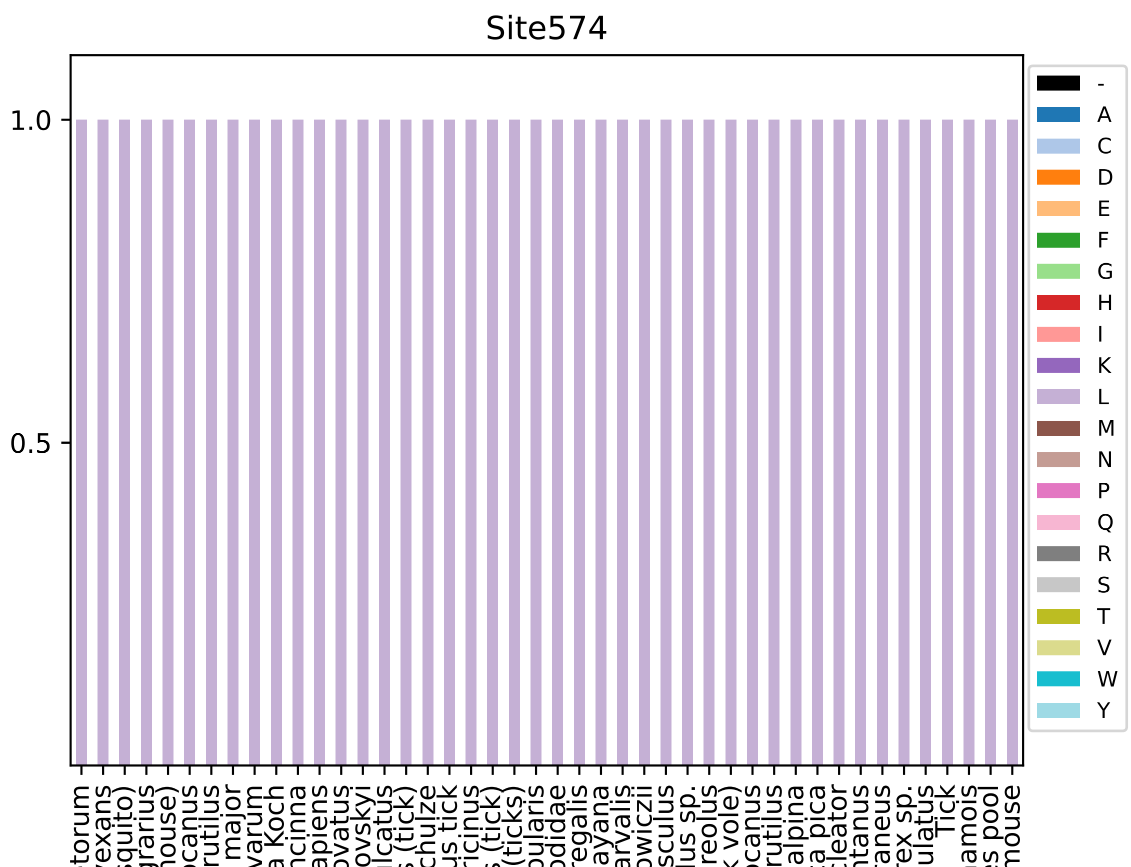

Site574
1.0
0.5
-
A
C
D
E
F
G
H
I
K
L
M
N
P
Q
R
S
T
V
W
Y

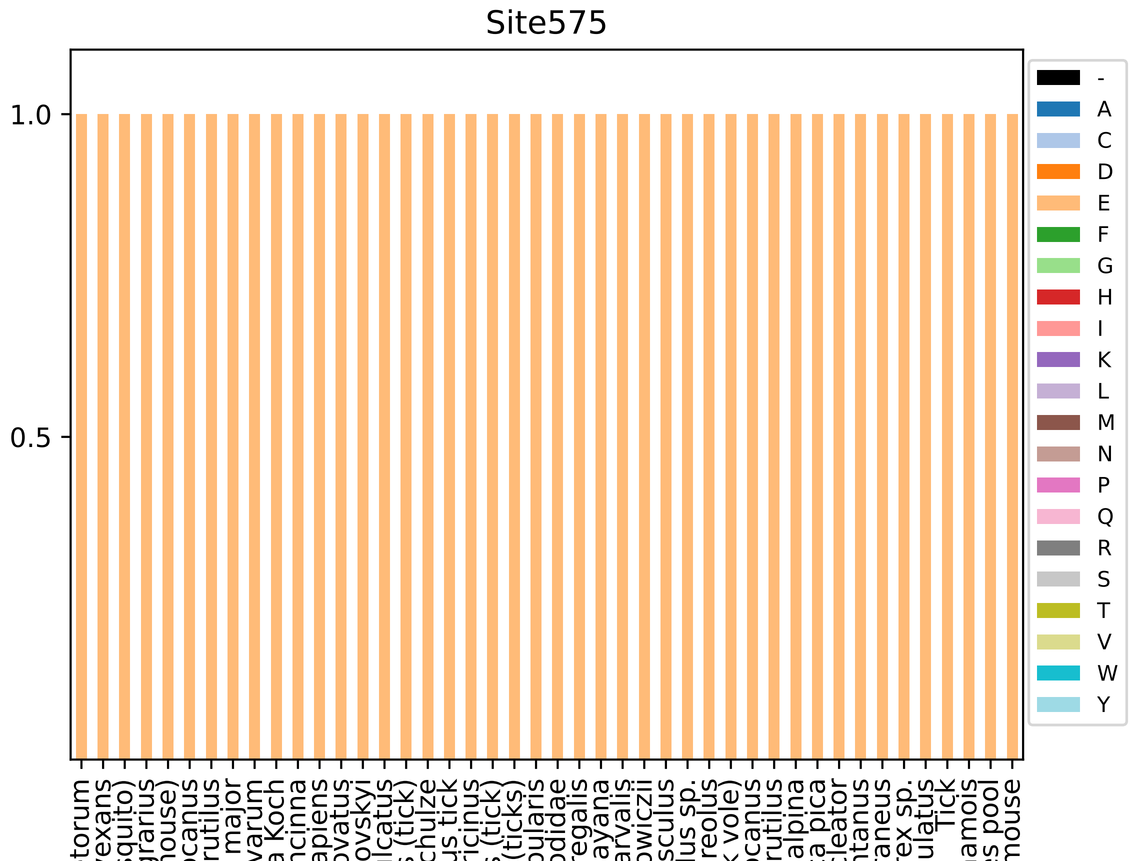
Site575
1.0
0.5
-
A
C
D
E
F
G
H
I
K
L
M
N
P
Q
R
S
T
V
W
Y

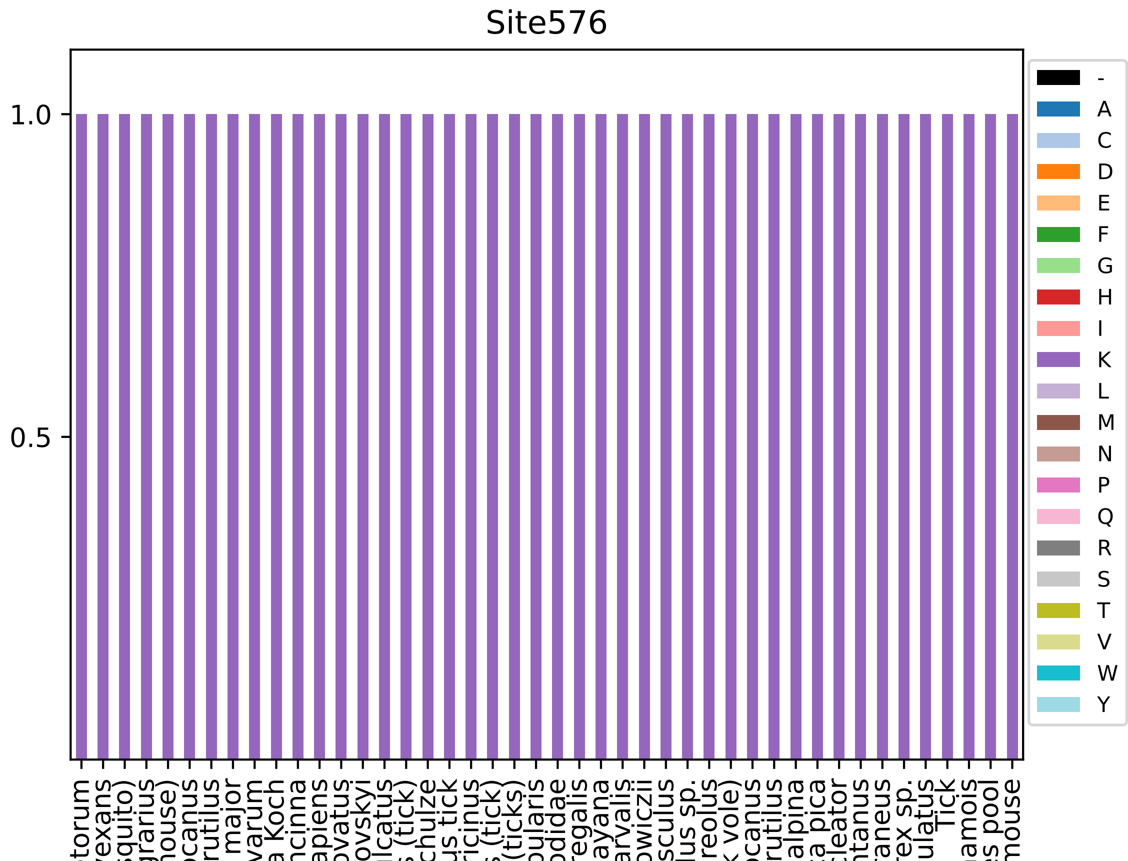

Site576
1.0
0.5
-
A
C
D
E
F
G
H
I
K
L
M
N
P
Q
R
S
T
V
W
Y

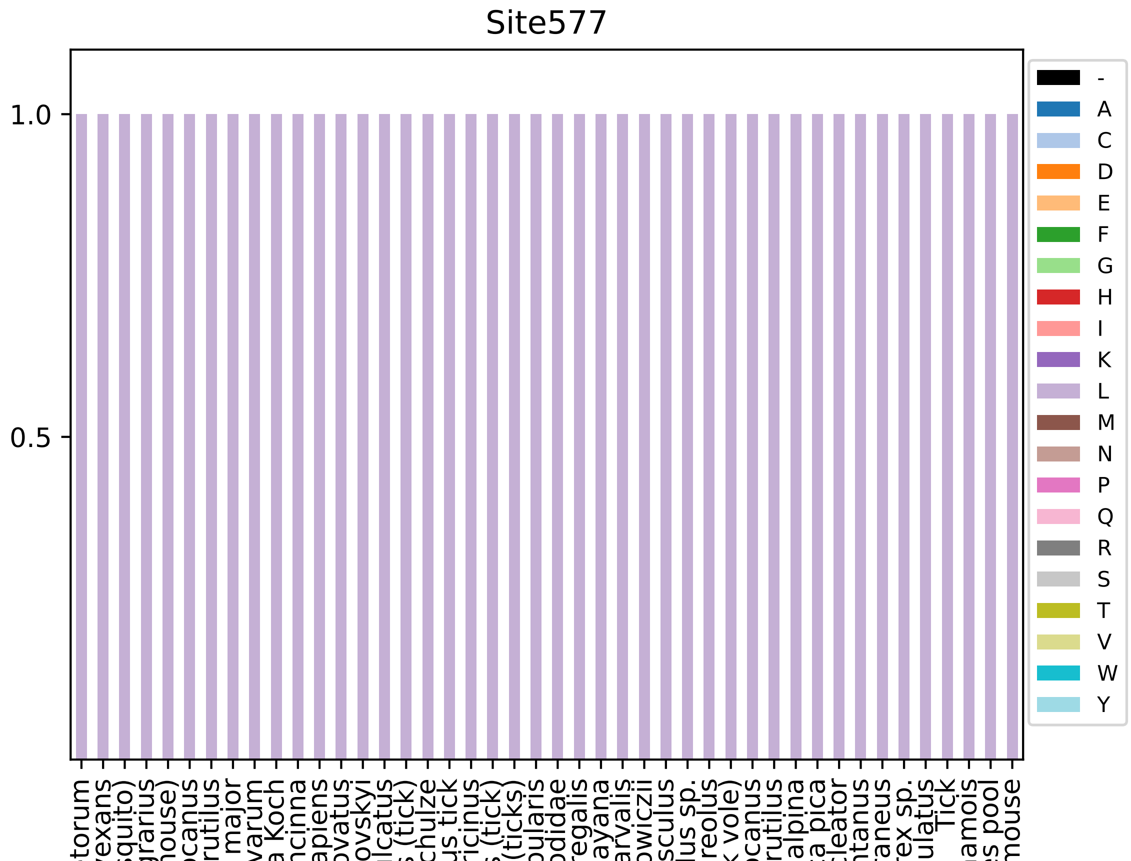
Site577
1.0
0.5
-
A
C
D
E
F
G
H
I
K
L
M
N
P
Q
R
S
T
V
W
Y

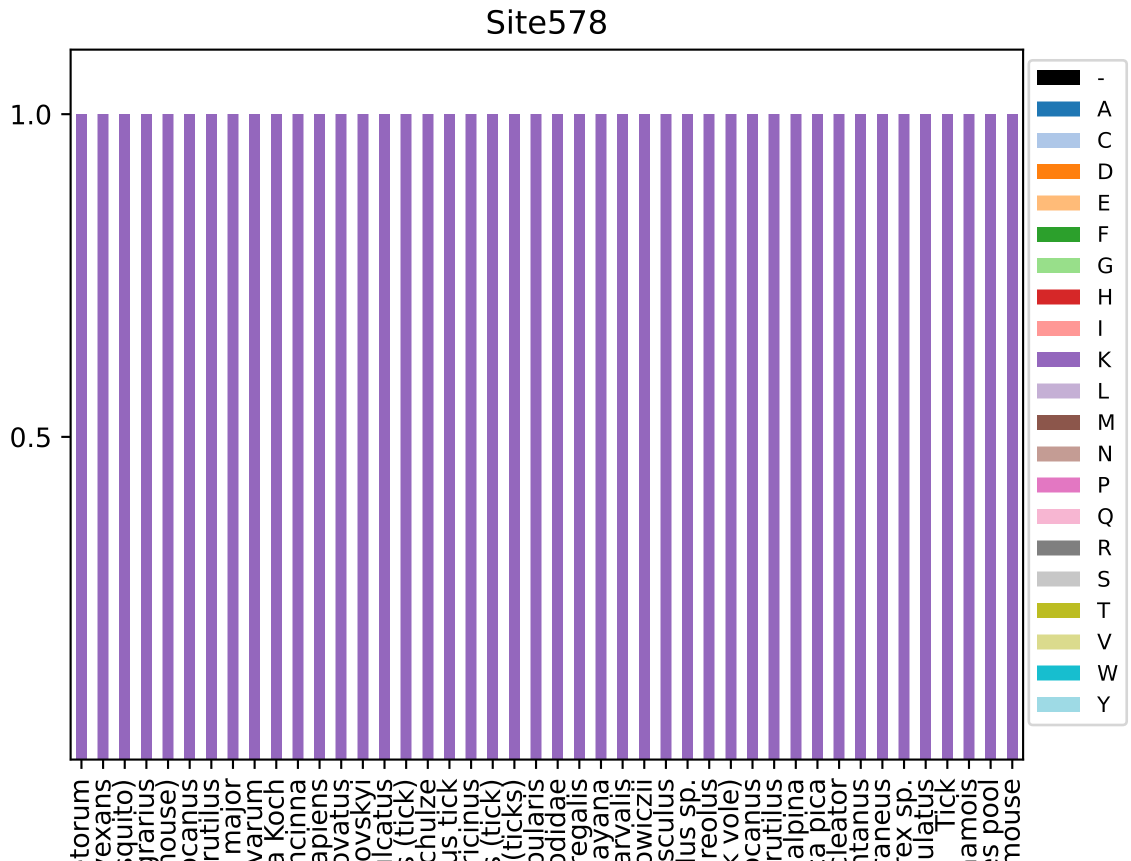

Site578
1.0
0.5
-
A
C
D
E
F
G
H
I
K
L
M
N
P
Q
R
S
T
V
W
Y
Tick

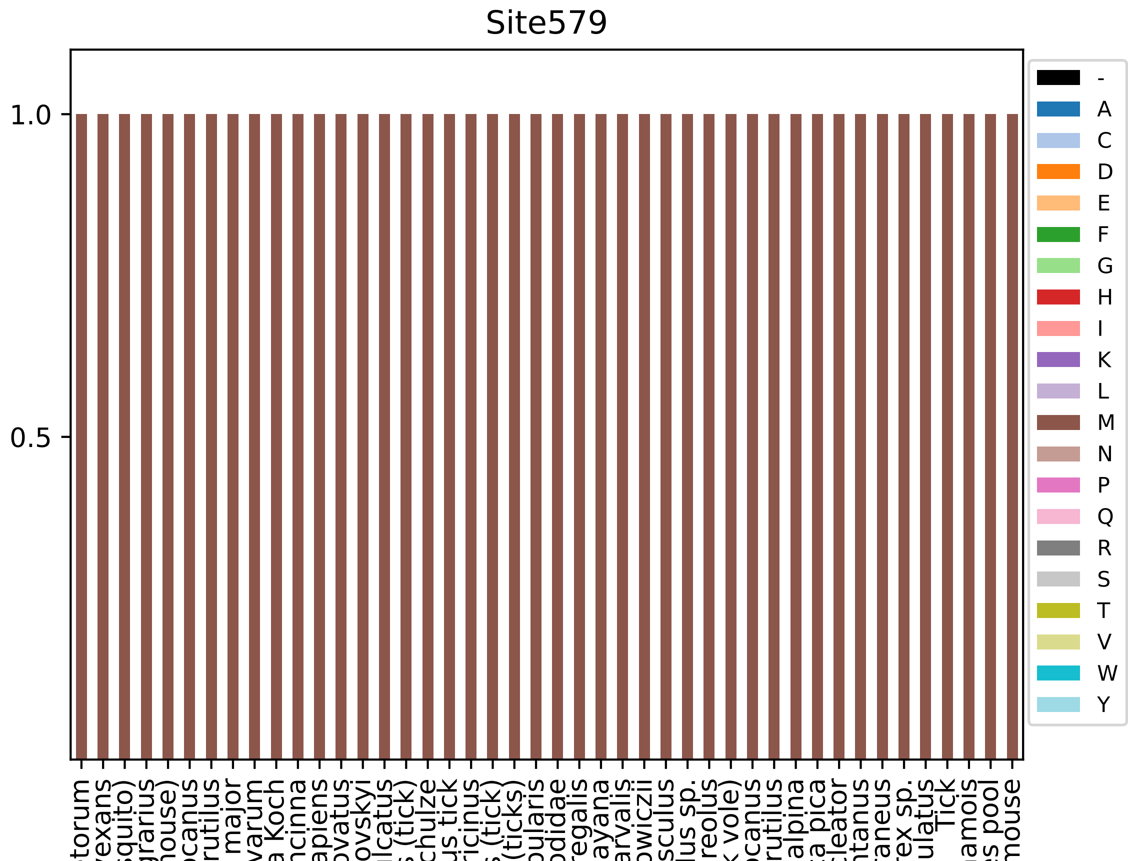
Site579
1.0
0.5
-
A
C
D
E
F
G
H
I
K
L
M
N
P
Q
R
S
T
V
W
Y

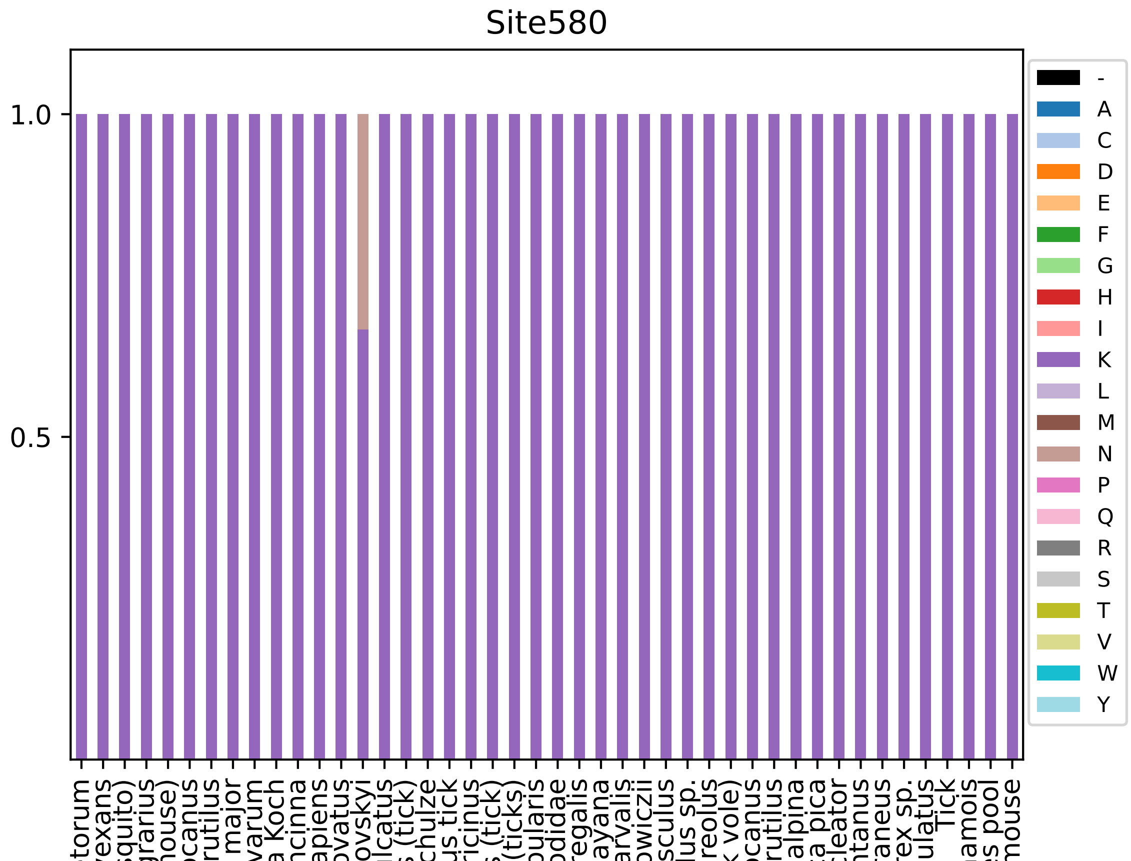
Site580
1.0
0.5
-
A
C
D
E
F
G
H
I
K
L
M
N
P
Q
R
S
T
V
W
Y
Tick

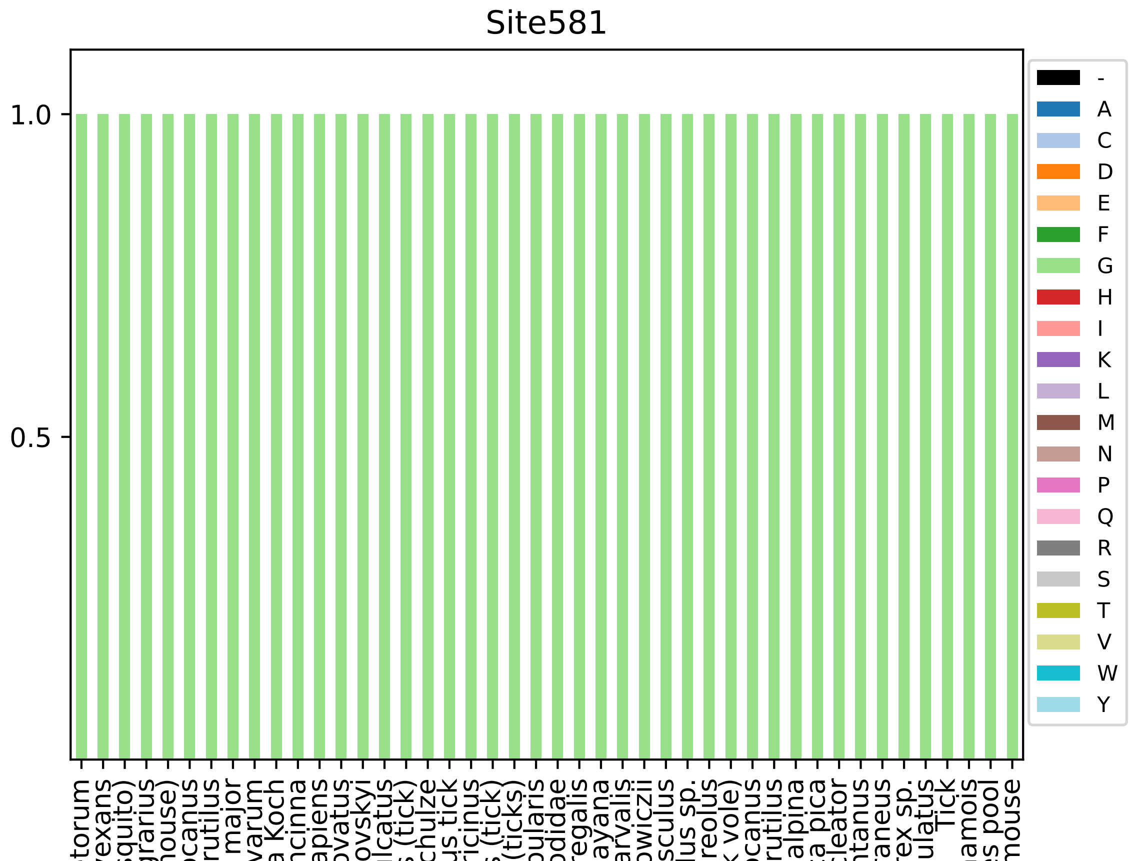
Site581
1.0
0.5
-
A
C
D
E
F
G
H
I
K
L
M
N
P
Q
R
S
T
V
W
Y

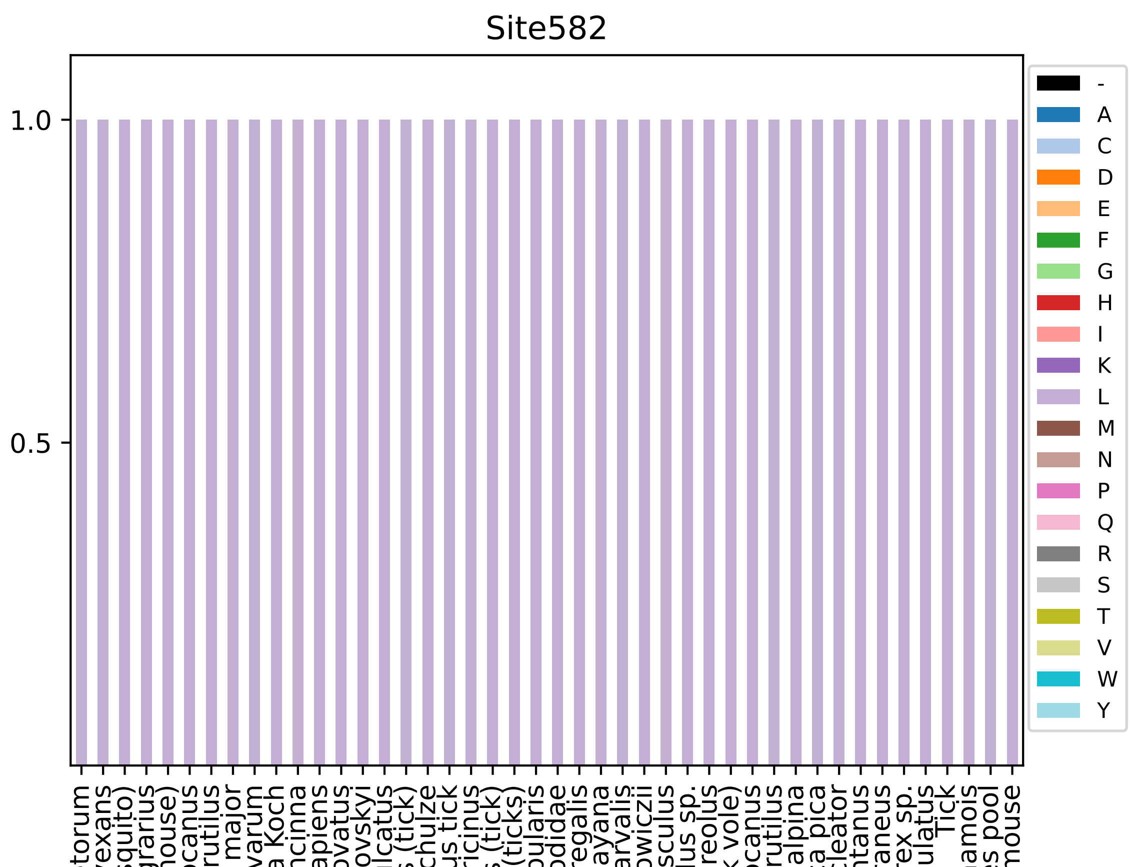
Site582
1.0
0.5
-
A
C
D
E
F
G
H
I
K
L
M
N
P
Q
R
S
T
V
W
Y
torum
vexans
squito)
grarius
nouse)
ocanus
rutilus
major
varum
a Koch
ncinna
apiens
ovatus
ovskyi
ulcatus
s (tick)
chulze
us tick
ricinus
s (tick)
(ticks)
pularis
podidae
regalis
ayana
arvalis
owiczii
sculus
lus sp.
reolus
k vole)
ocanus
rutilus
alpina
a pica
leator
ntanus
raneus
rex sp.
ulatus
Tick
amois
es pool
mouse

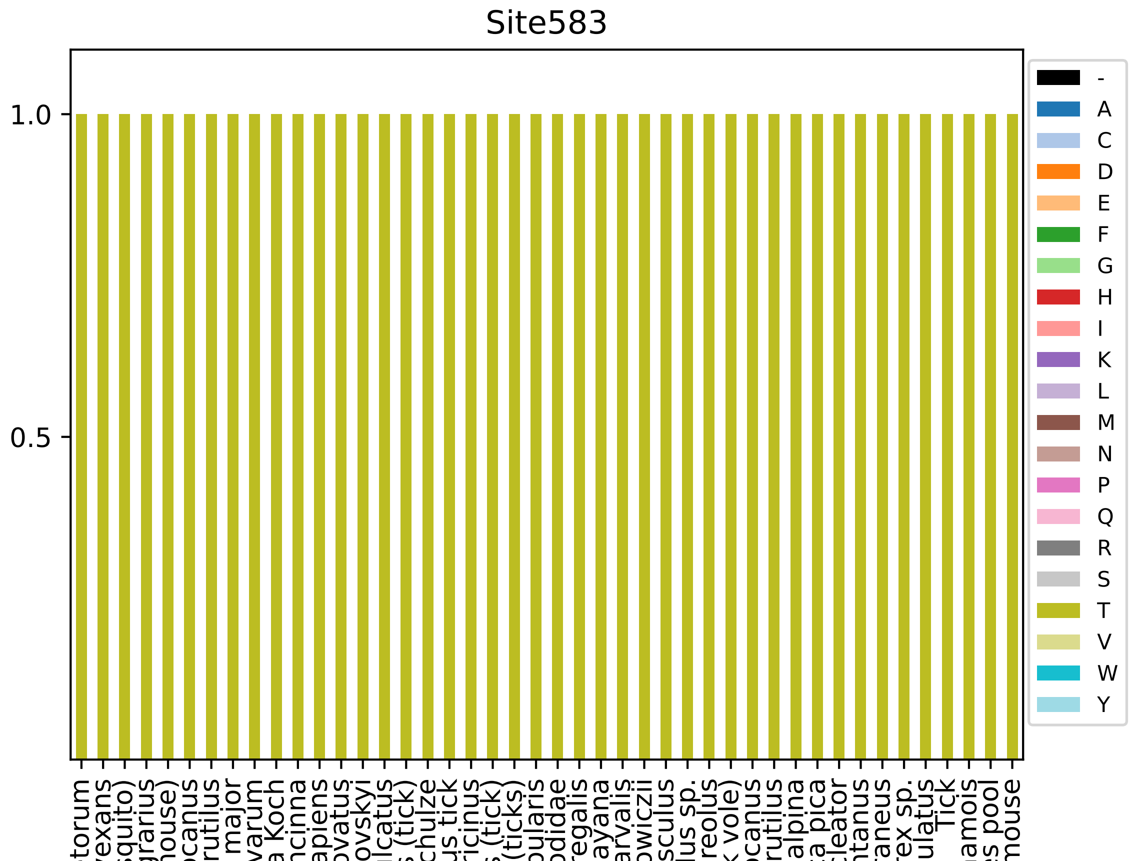
Site583
1.0
0.5
-
A
C
D
E
F
G
H
I
K
L
M
N
P
Q
R
S
T
V
W
Y

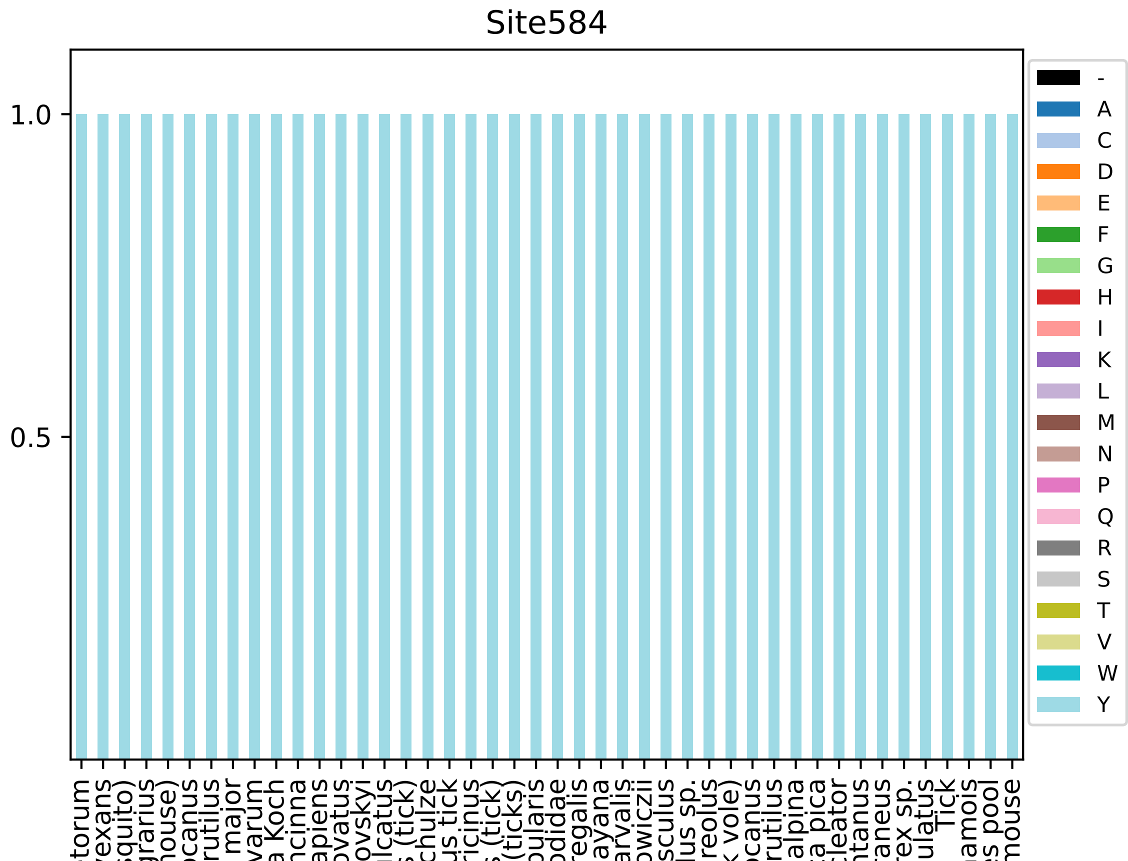

Site584
1.0
0.5
-
A
C
D
E
F
G
H
I
K
L
M
N
P
Q
R
S
T
V
W
Y

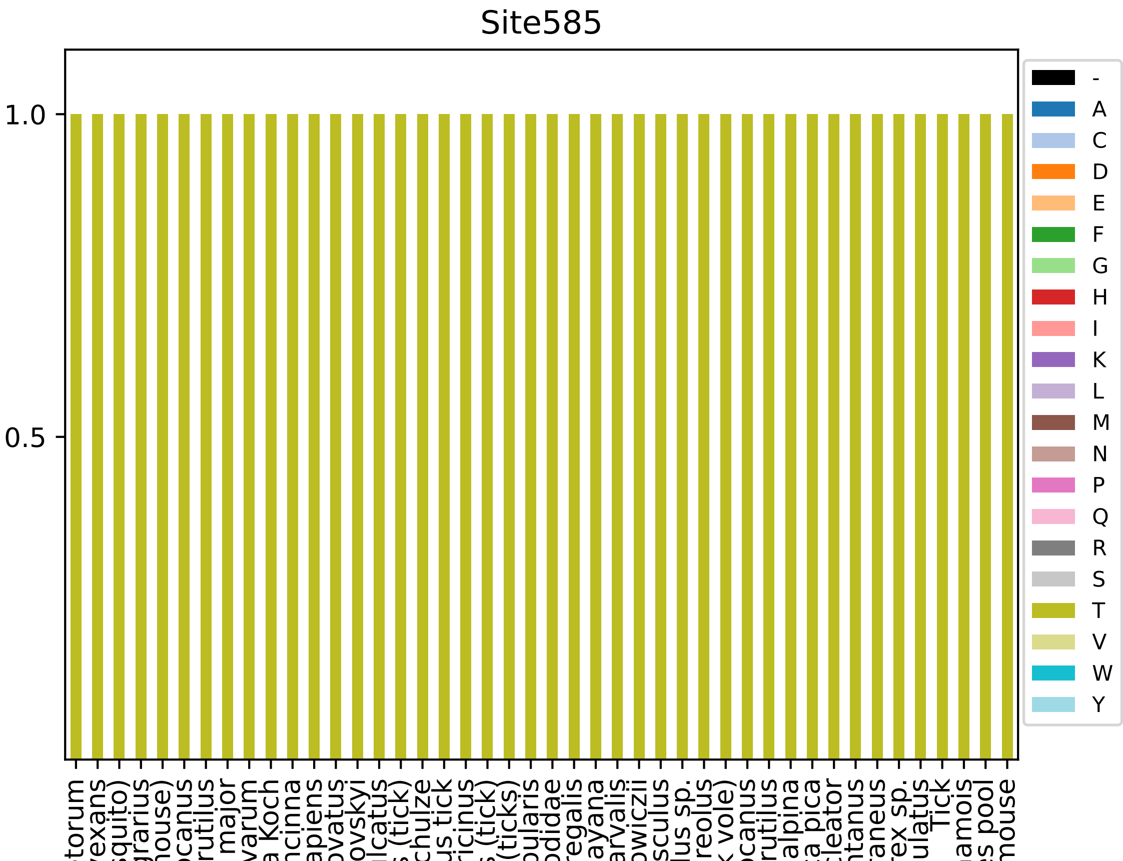
Site585
1.0
0.5
-
A
C
D
E
F
G
H
I
K
L
M
N
P
Q
R
S
T
V
W
Y

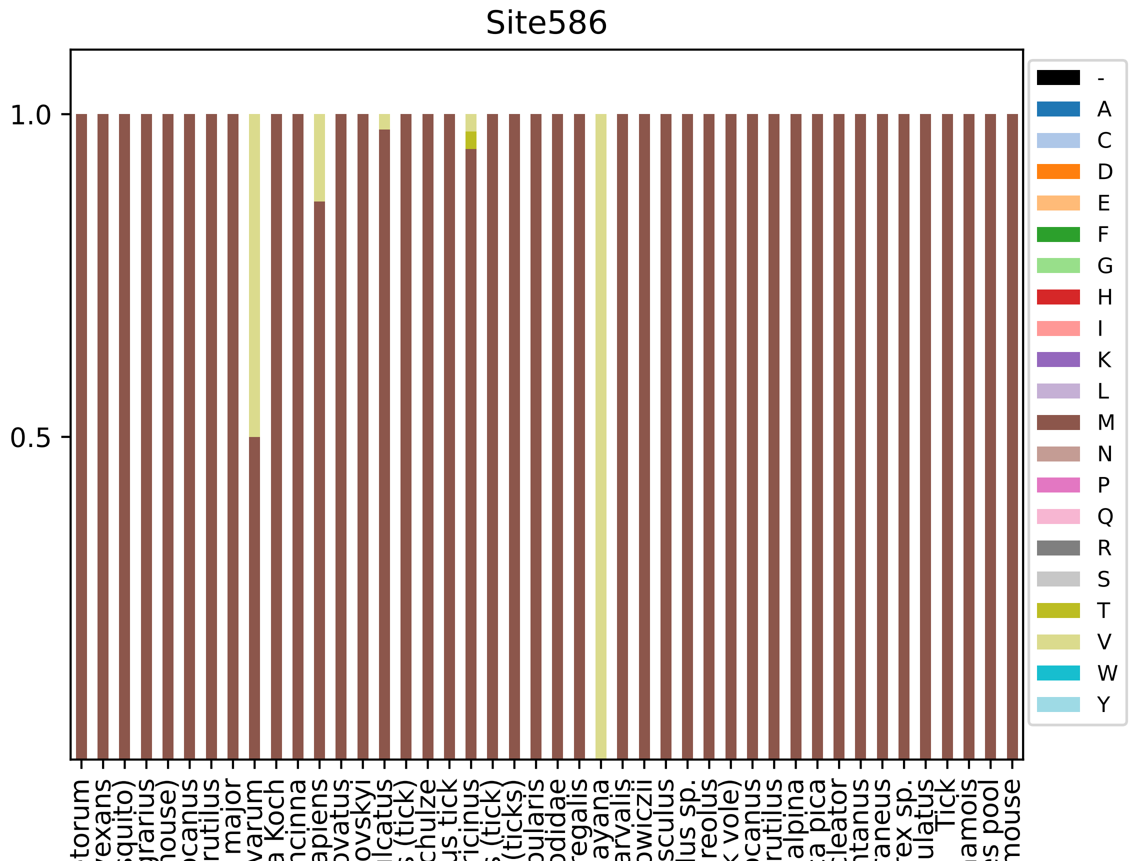

Site586
1.0
0.5
-
A
C
D
E
F
G
H
I
K
L
M
N
P
Q
R
S
T
V
W
Y

Site587

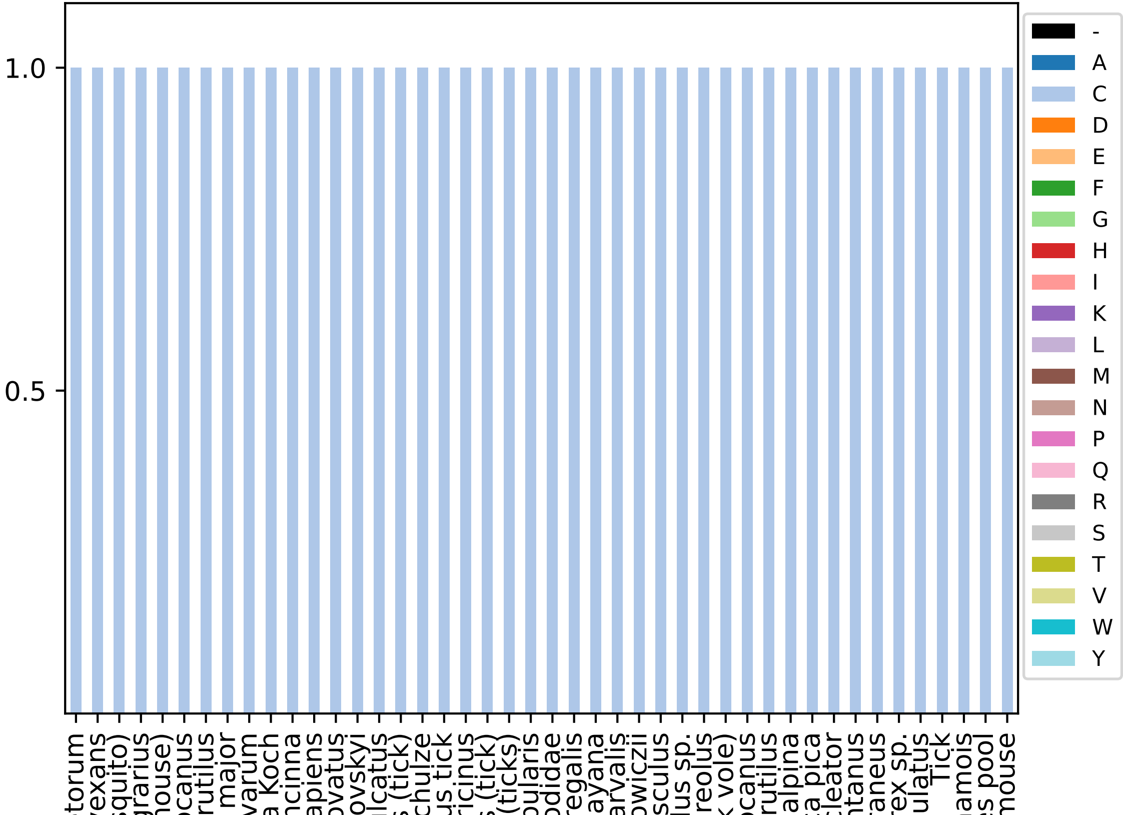

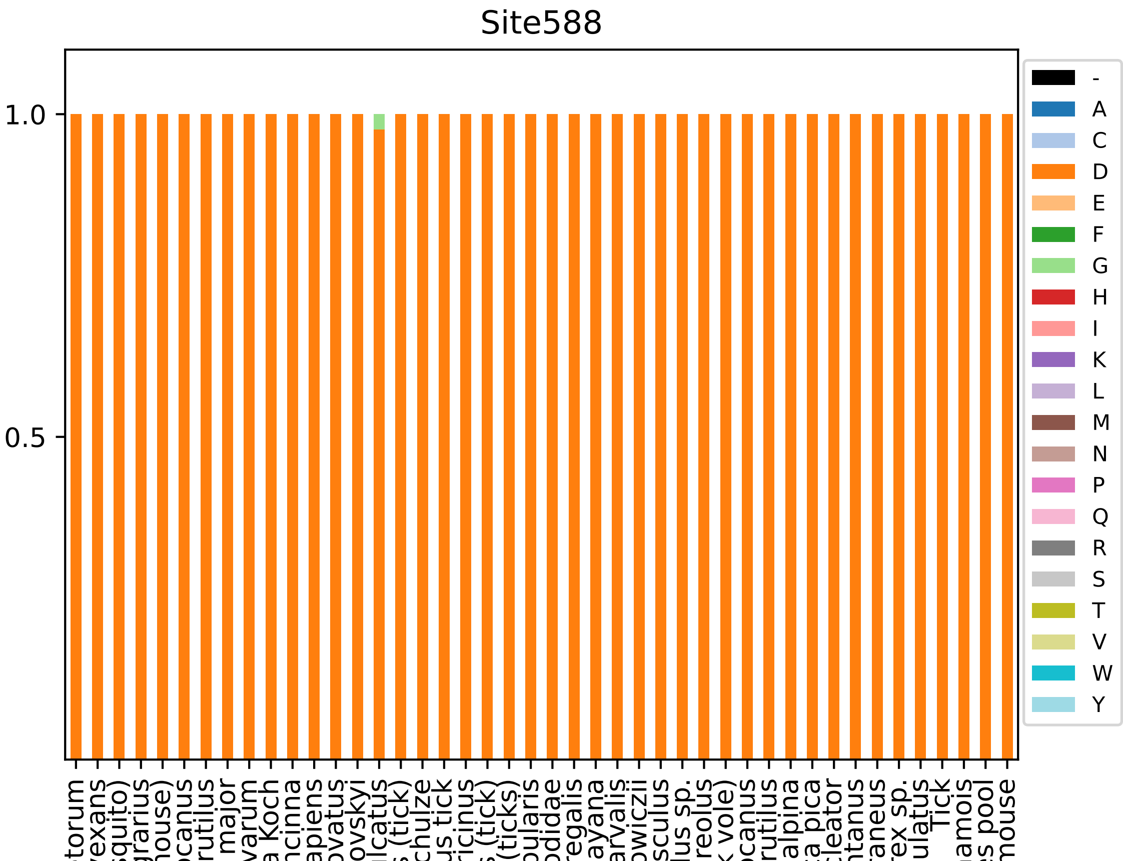

Site588
1.0
0.5
-
A
C
D
E
F
G
H
I
K
L
M
N
P
Q
R
S
T
V
W
Y

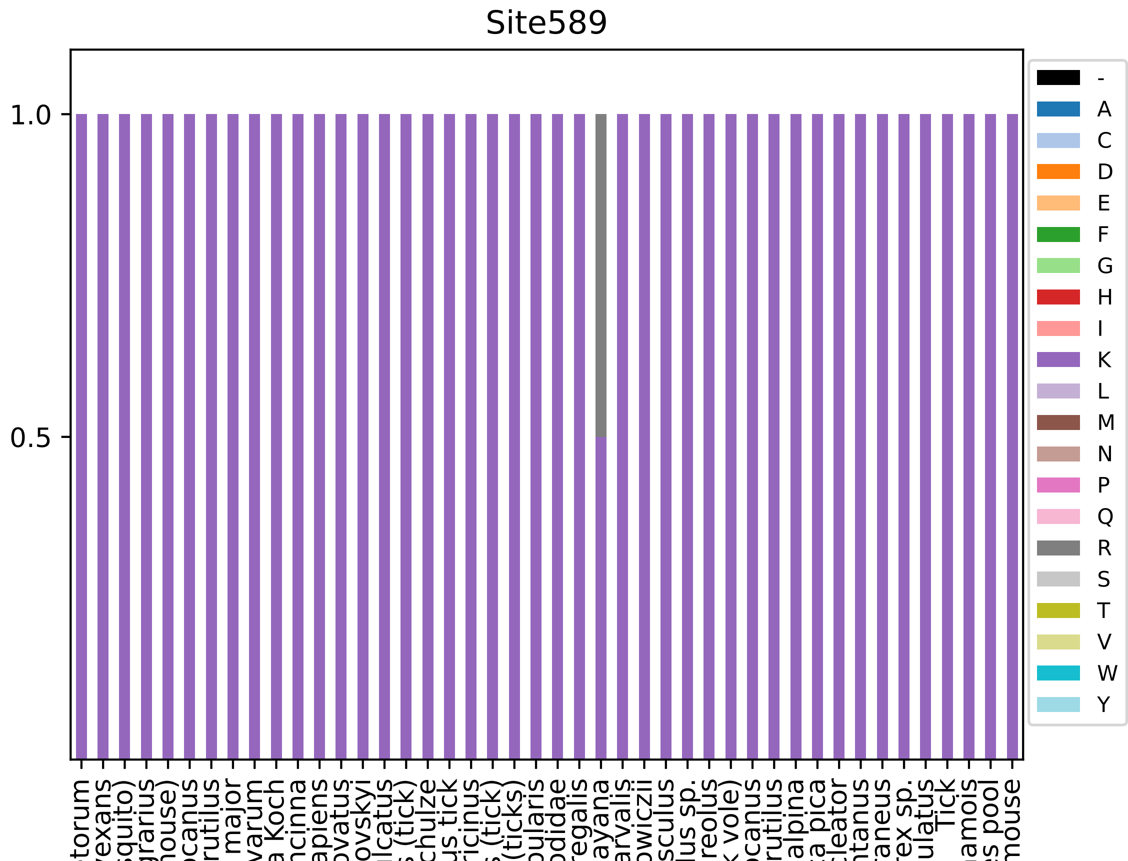
Site589
1.0
0.5
-
A
C
D
E
F
G
H
I
K
L
M
N
P
Q
R
S
T
V
W
Y

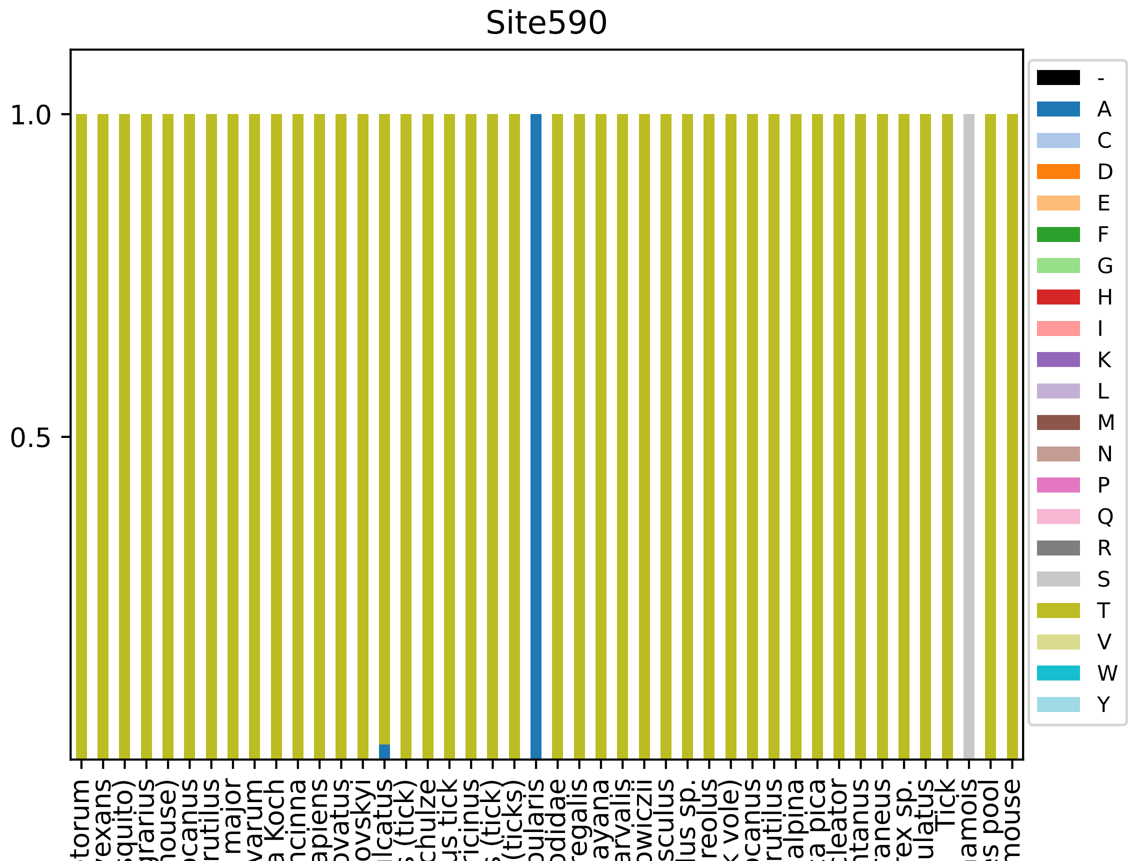
Site590
1.0
0.5
-
A
C
D
E
F
G
H
I
K
L
M
N
P
Q
R
S
T
V
W
Y

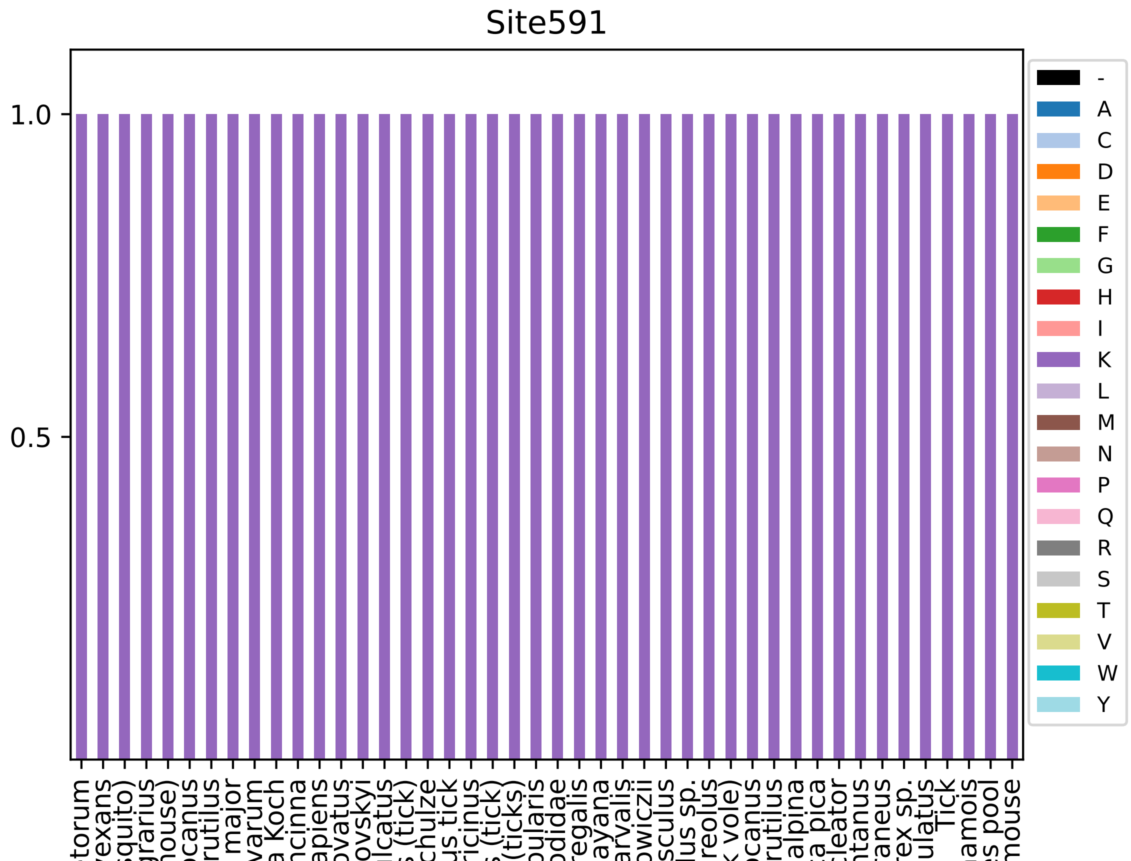
Site591
1.0
0.5
-
A
C
D
E
F
G
H
I
K
L
M
N
P
Q
R
S
T
V
W
Y

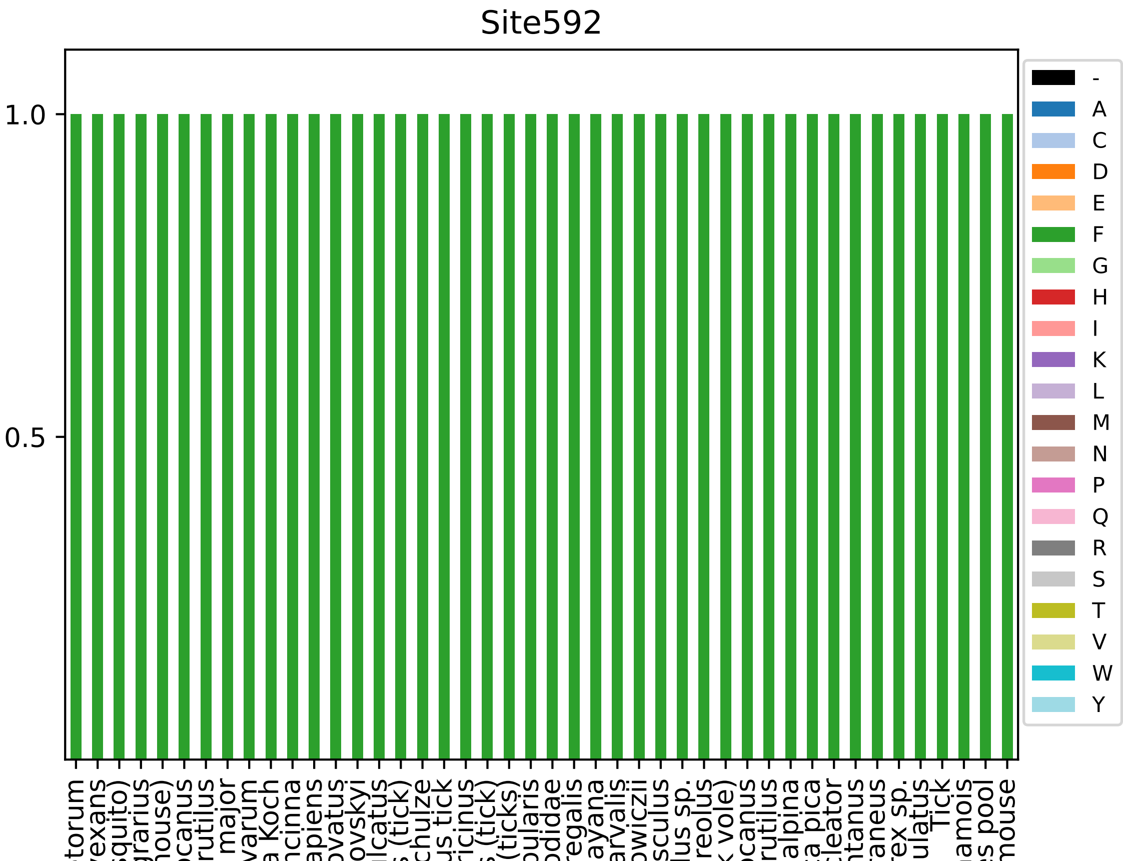

Site592
1.0
0.5
-
A
C
D
E
F
G
H
I
K
L
M
N
P
Q
R
S
T
V
W
Y

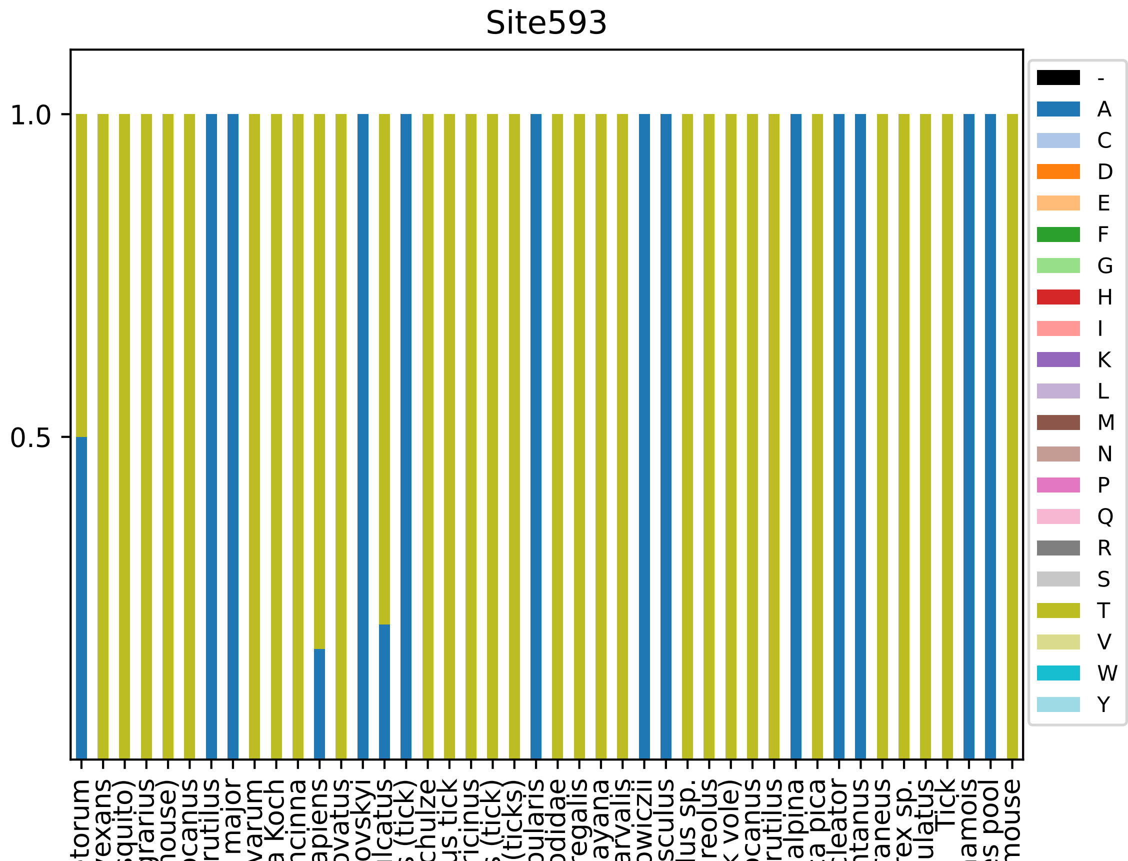
Site593
1.0
0.5
-
A
C
D
E
F
G
H
I
K
L
M
N
P
Q
R
S
T
V
W
Y

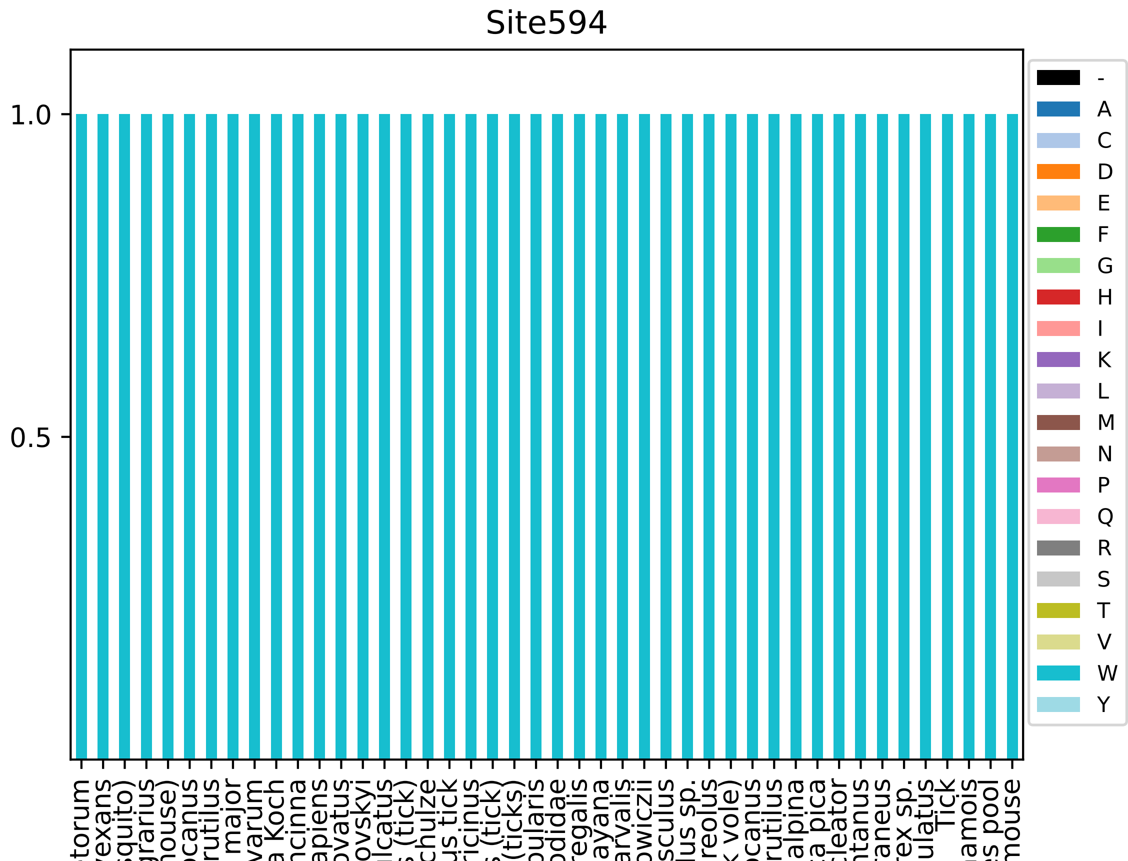
Site594
1.0
0.5
-
A
C
D
E
F
G
H
I
K
L
M
N
P
Q
R
S
T
V
W
Y

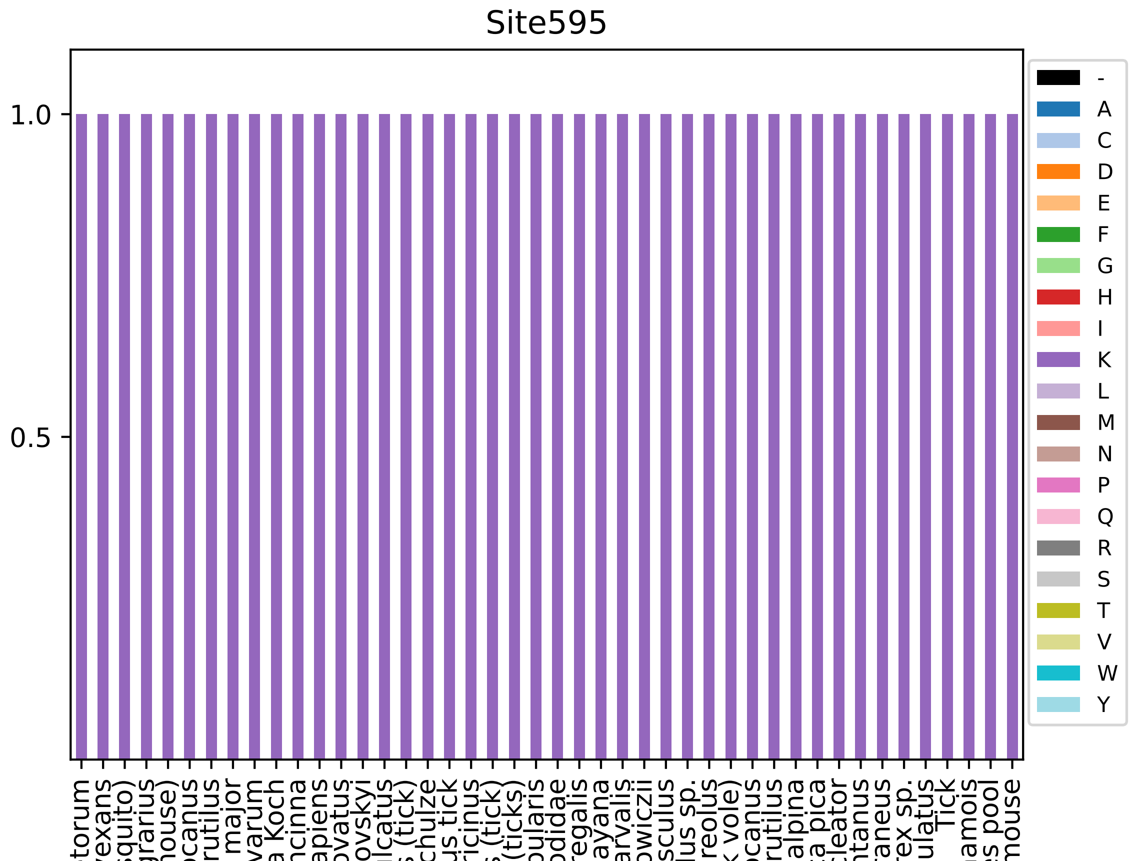
Site595
1.0
0.5
-
A
C
D
E
F
G
H
I
K
L
M
N
P
Q
R
S
T
V
W
Y

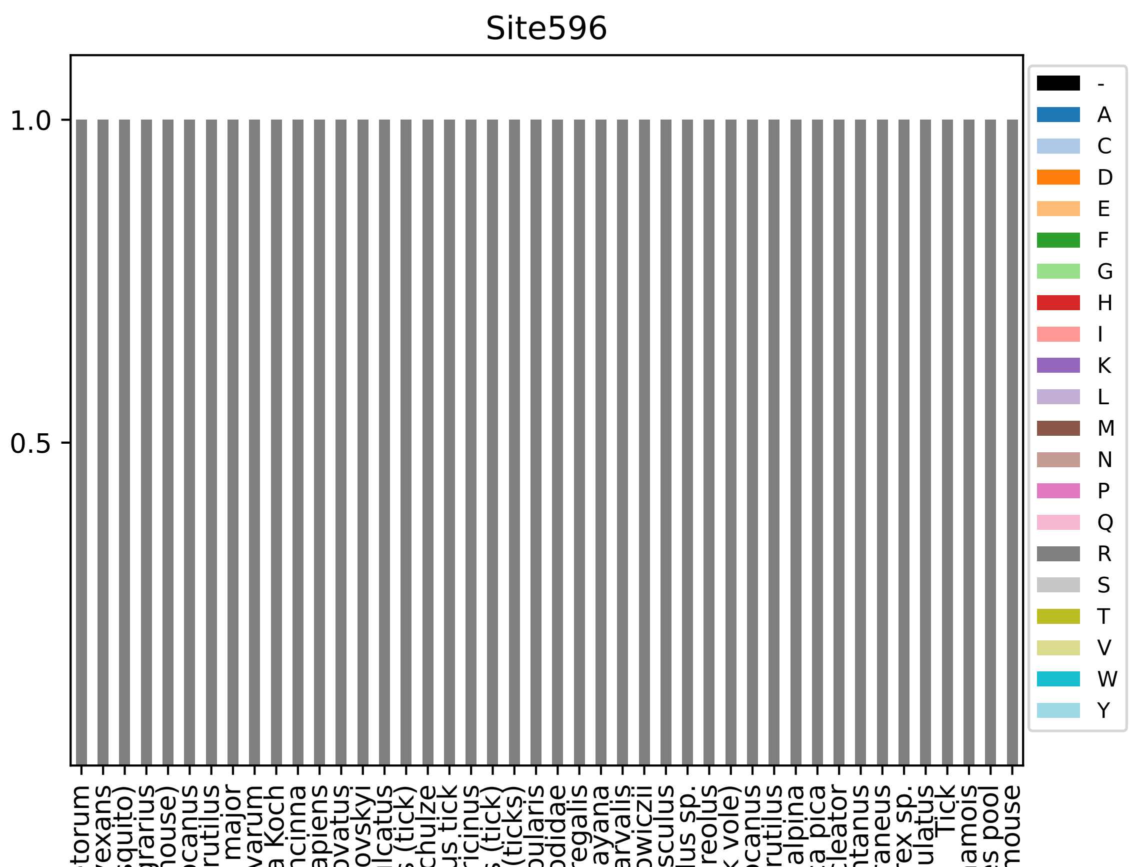

Site596
1.0
0.5
-
A
C
D
E
F
G
H
I
K
L
M
N
P
Q
R
S
T
V
W
Y

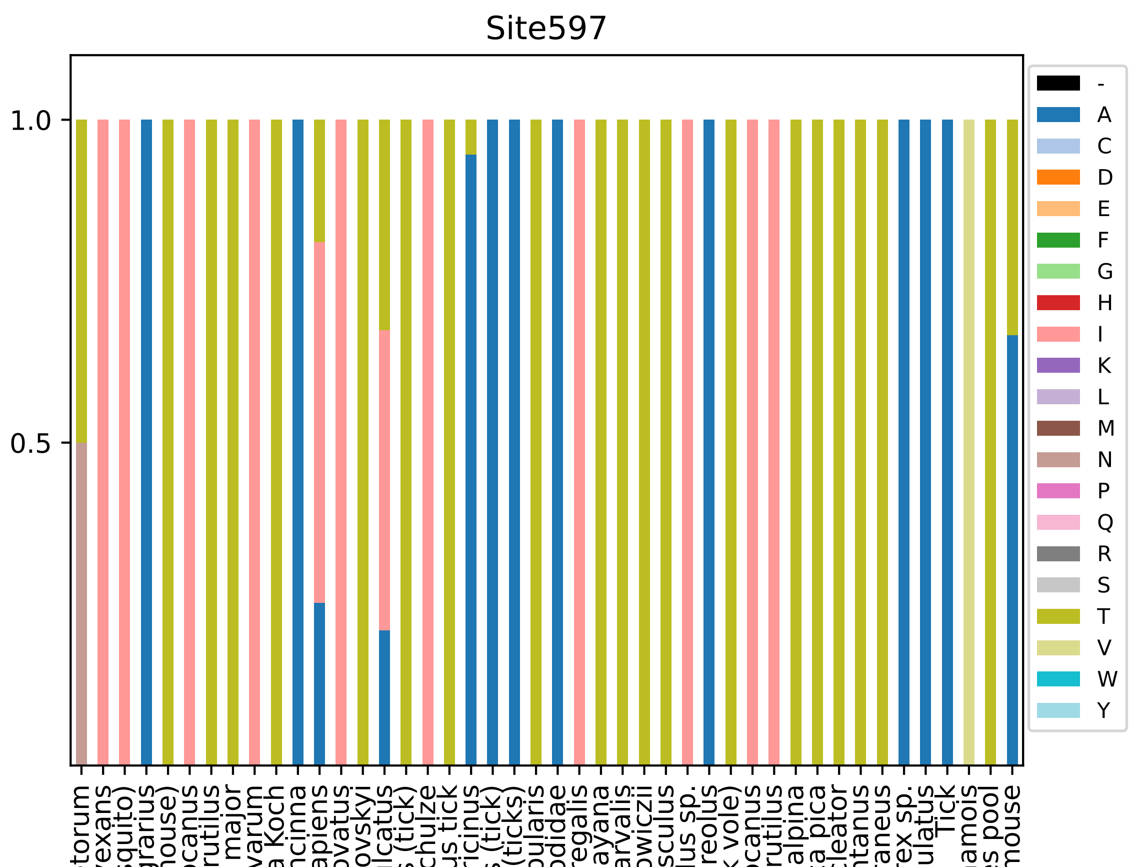
Site597
1.0
0.5
-
A
C
D
E
F
G
H
I
K
L
M
N
P
Q
R
S
T
V
W
Y

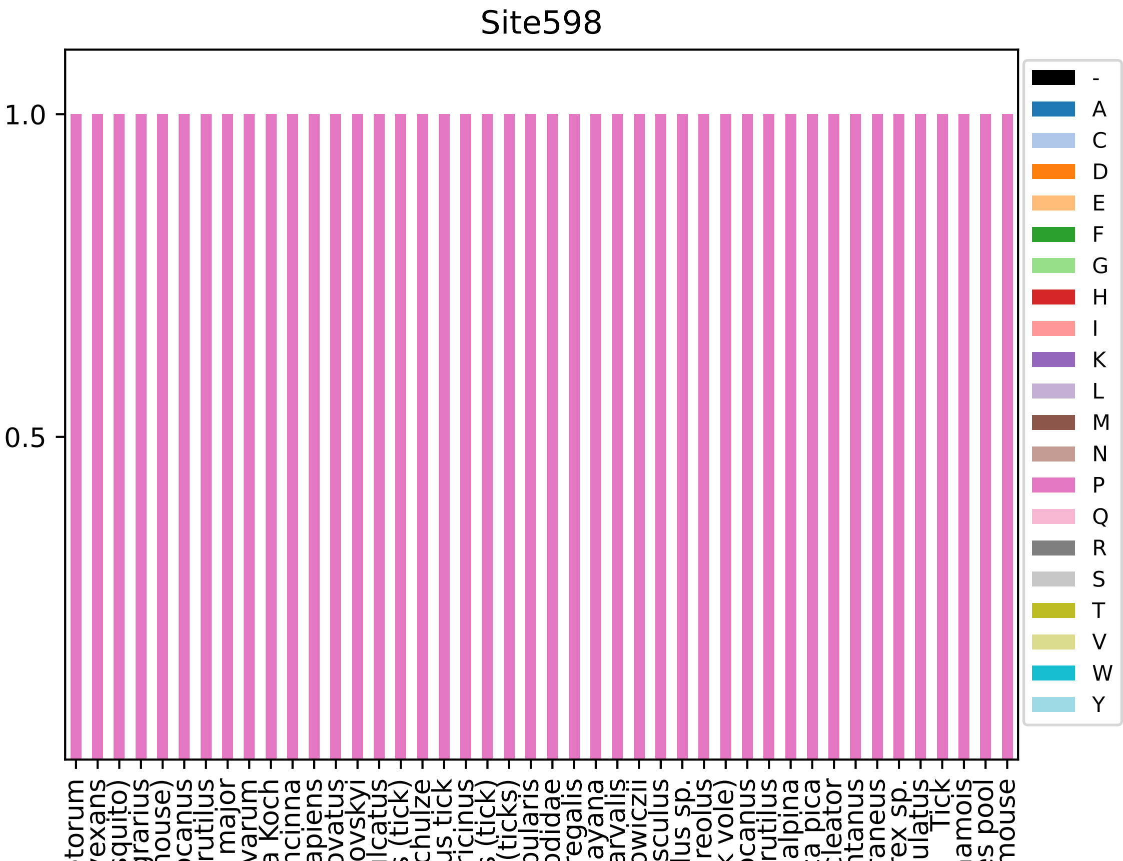
Site598
1.0
0.5
-
A
C
D
E
F
G
H
I
K
L
M
N
P
Q
R
S
T
V
W
Y

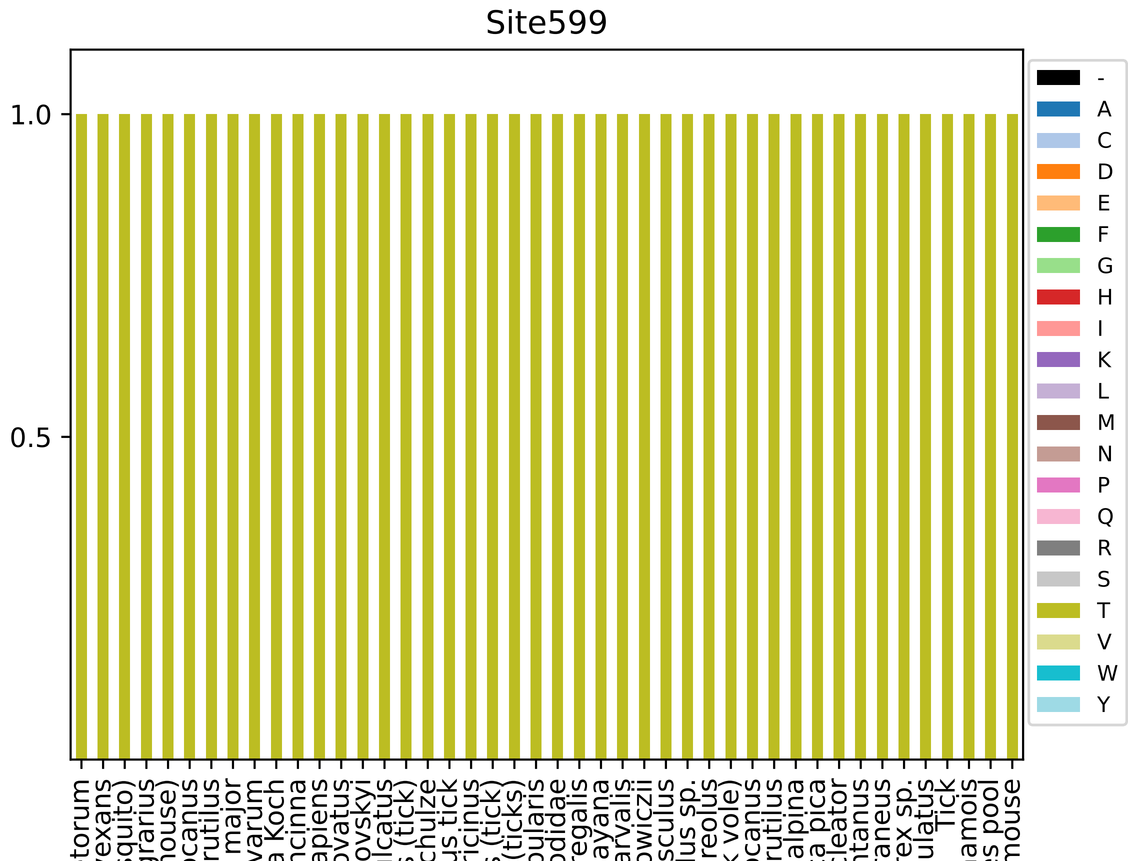
Site599
1.0
0.5
-
A
C
D
E
F
G
H
I
K
L
M
N
P
Q
R
S
T
V
W
Y

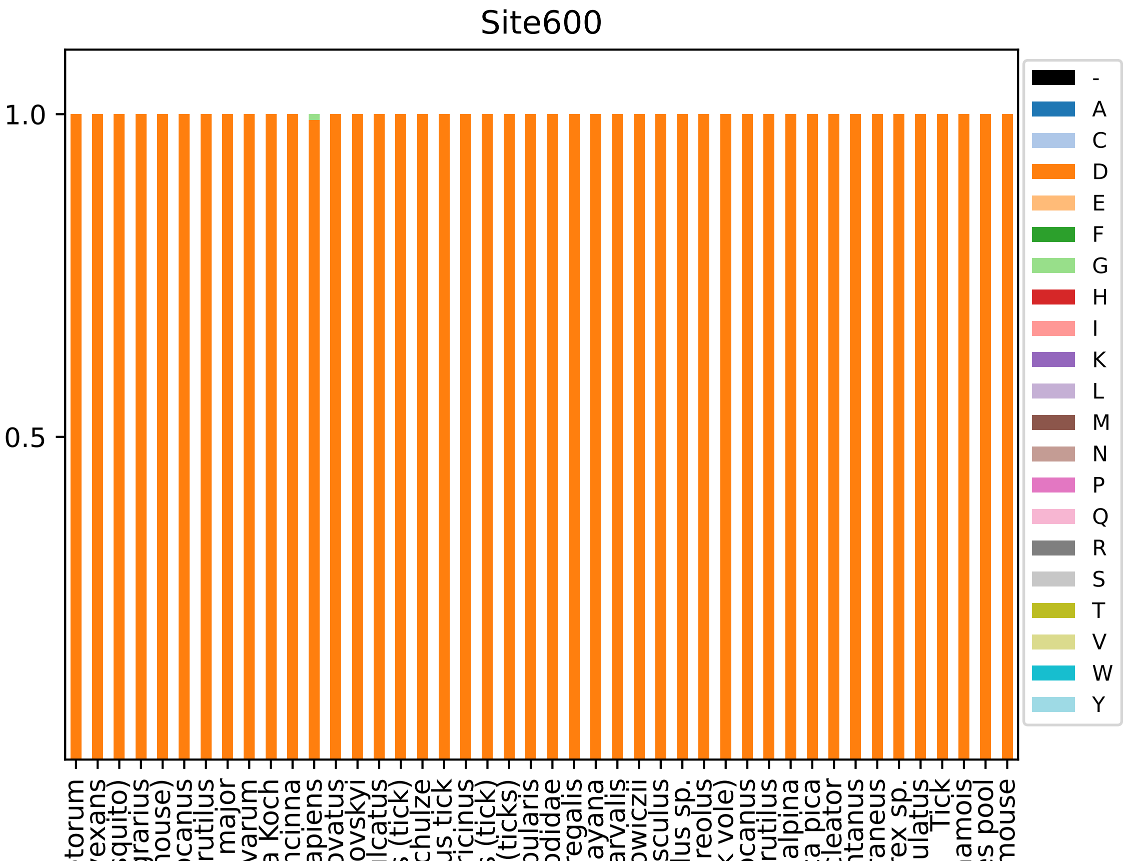
Site600
1.0
0.5
-
A
C
D
E
F
G
H
I
K
L
M
N
P
Q
R
S
T
V
W
Y

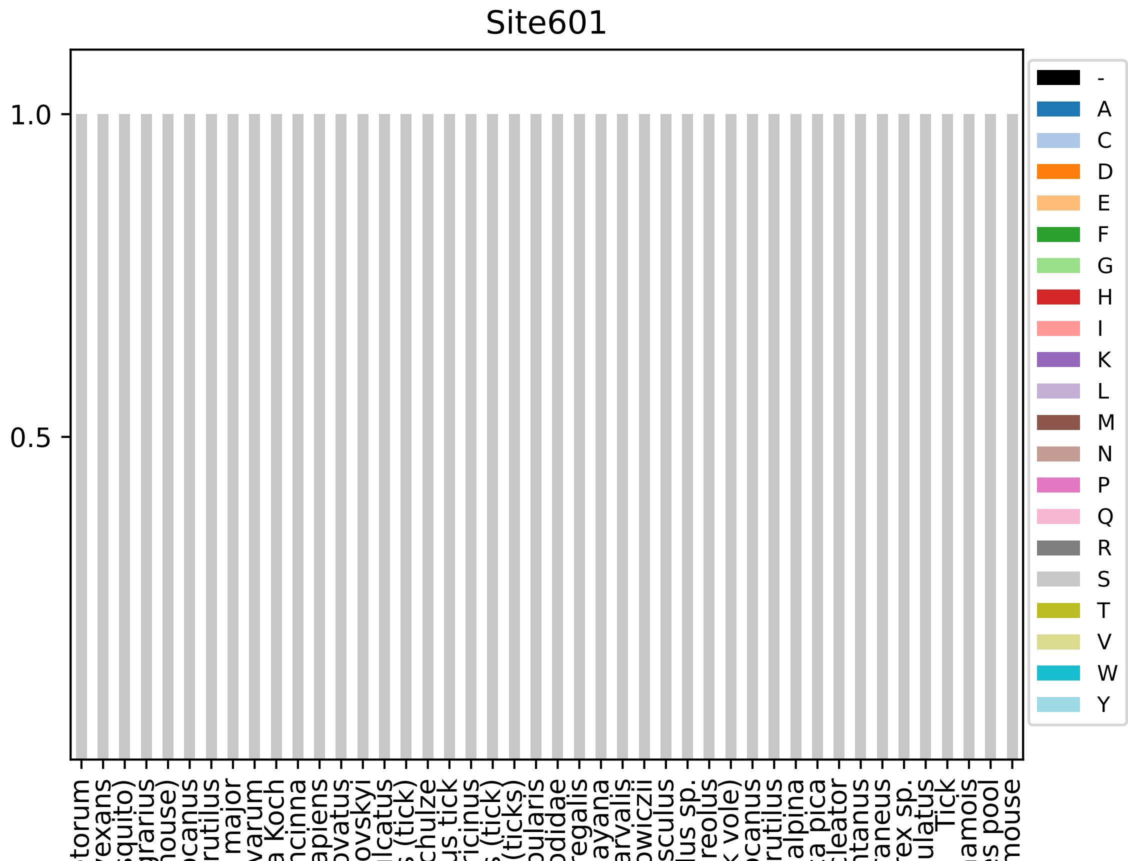

Site601
1.0
0.5
-
A
C
D
E
F
G
H
I
K
L
M
N
P
Q
R
S
T
V
W
Y

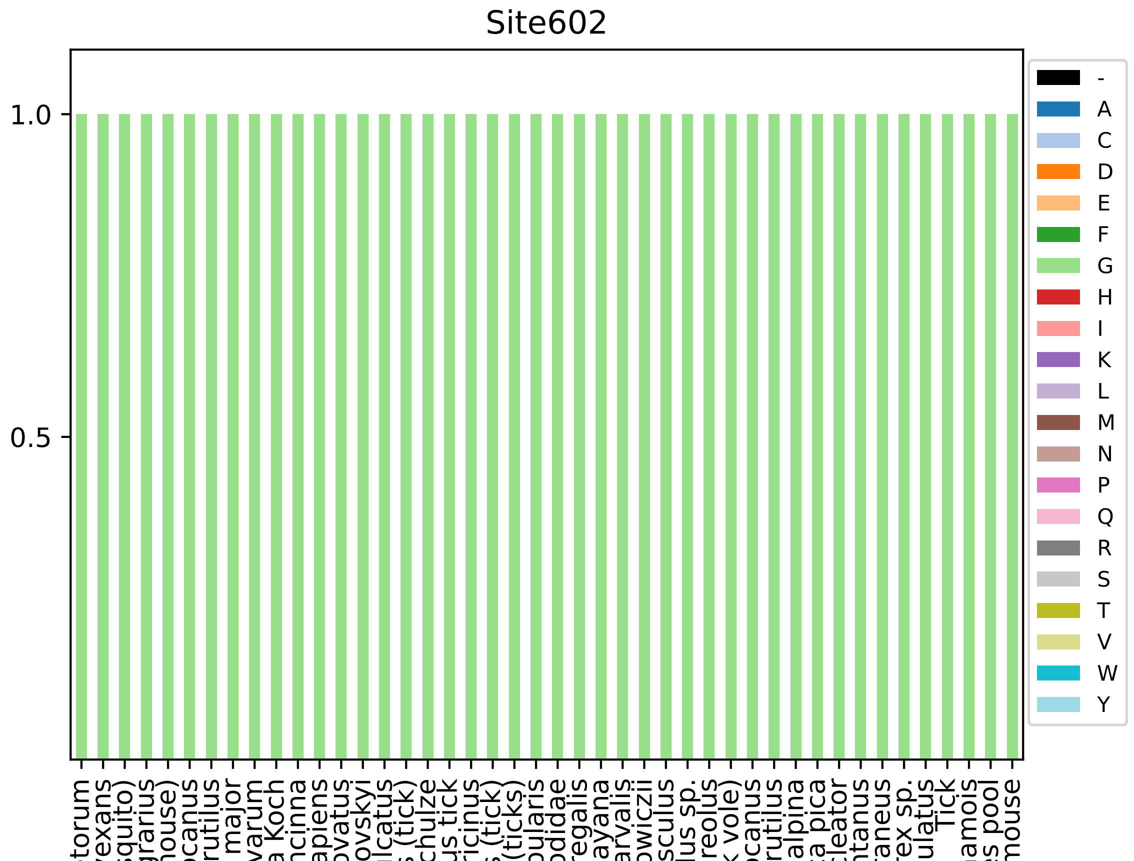
Site602
1.0
0.5
-
A
C
D
E
F
G
H
I
K
L
M
N
P
Q
R
S
T
V
W
Y

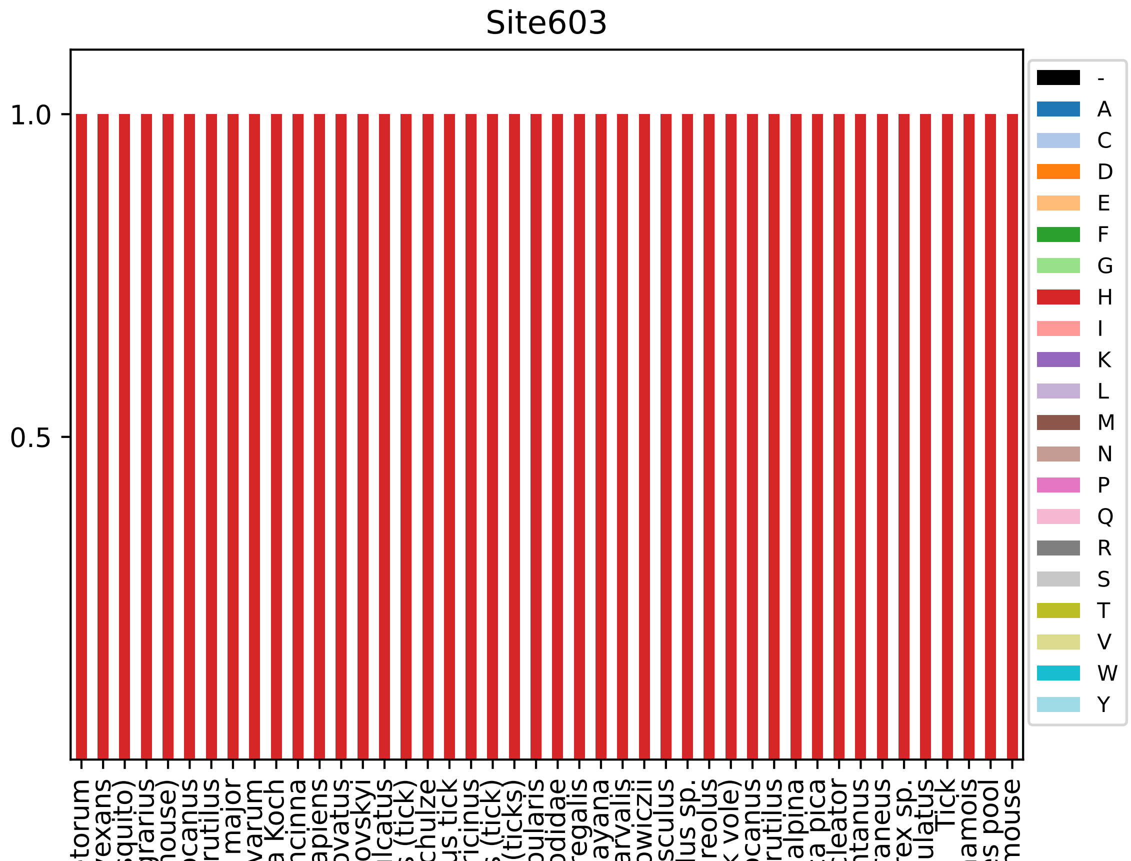
Site603
1.0
0.5
-
A
C
D
E
F
G
H
I
K
L
M
N
P
Q
R
S
T
V
W
Y

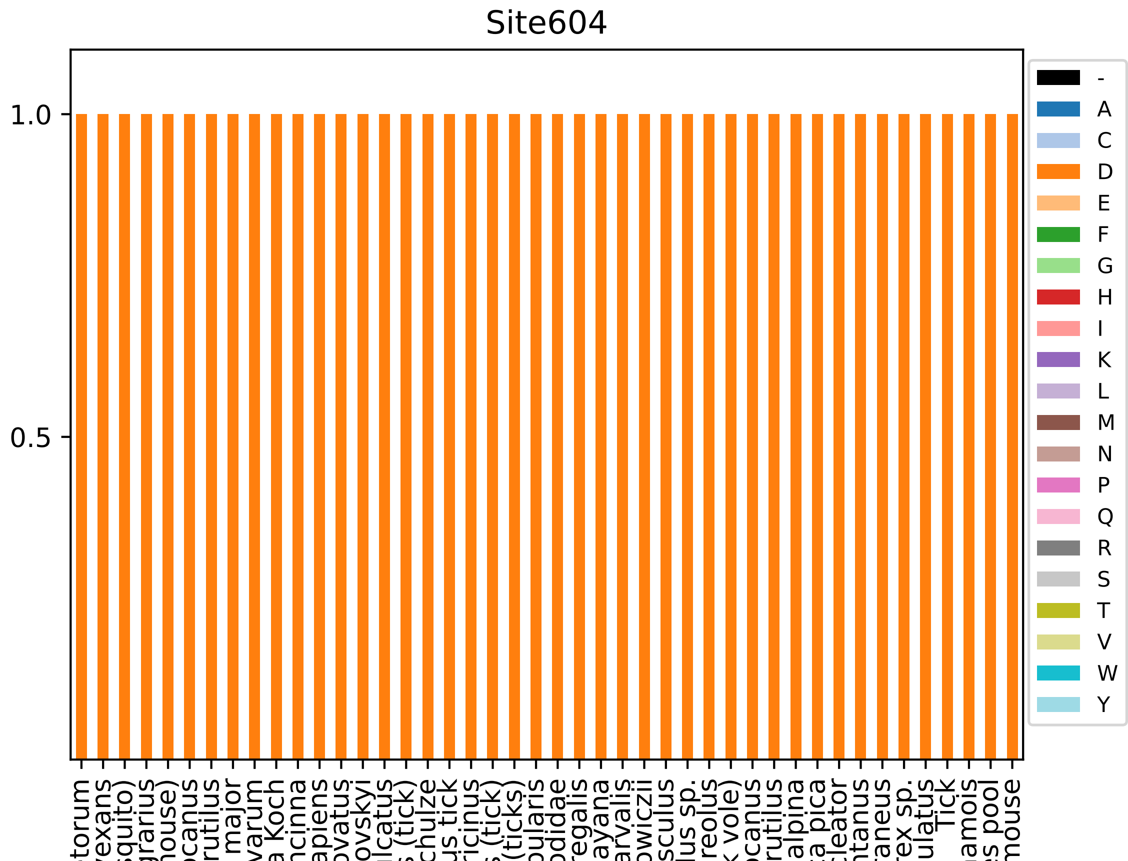
Site604
1.0
0.5
-
A
C
D
E
F
G
H
I
K
L
M
N
P
Q
R
S
T
V
W
Y

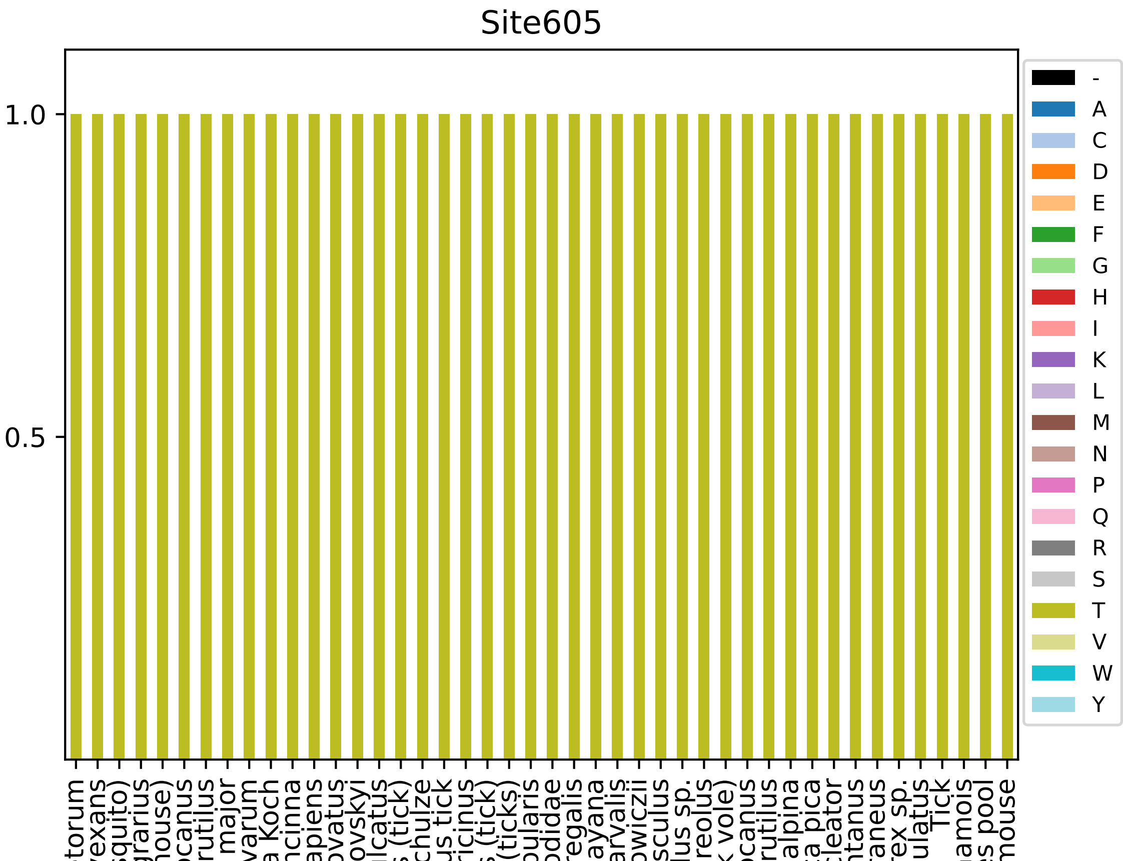

Site605
1.0
0.5
-
A
C
D
E
F
G
H
I
K
L
M
N
P
Q
R
S
T
V
W
Y

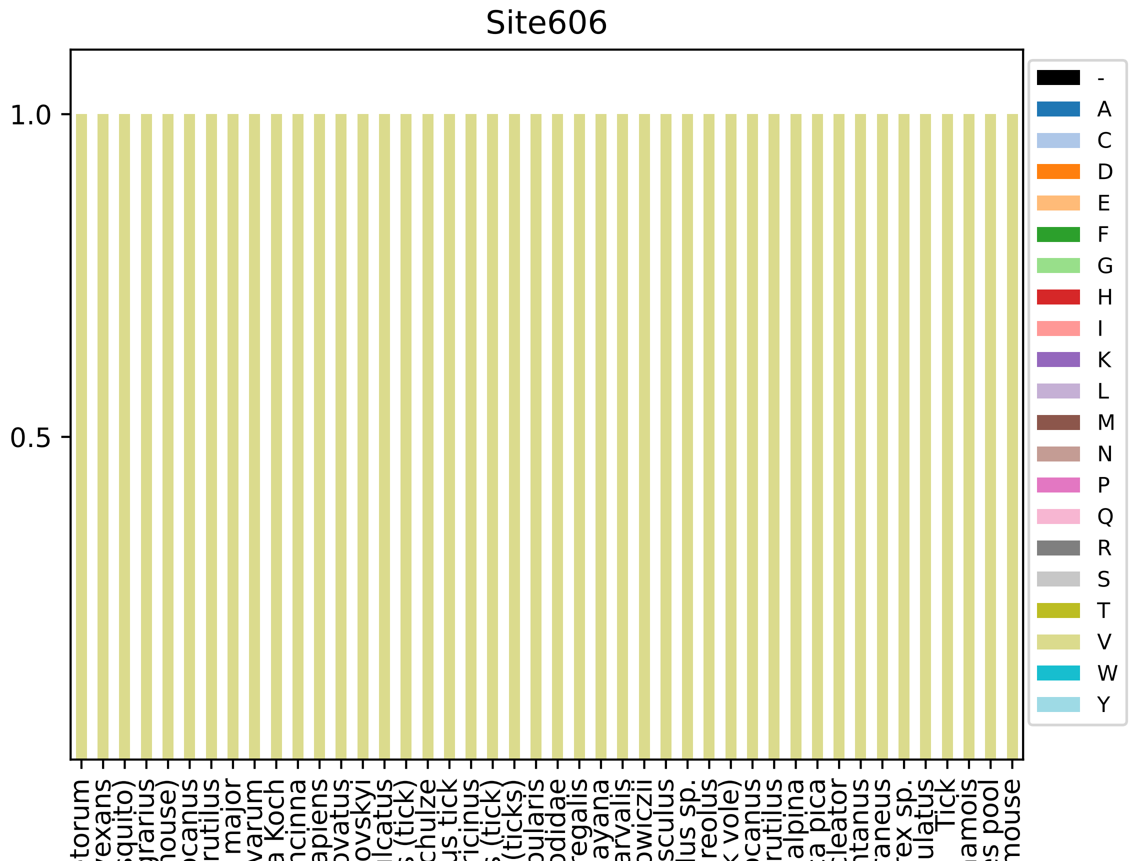
Site606
1.0
0.5
-
A
C
D
E
F
G
H
I
K
L
M
N
P
Q
R
S
T
V
W
Y

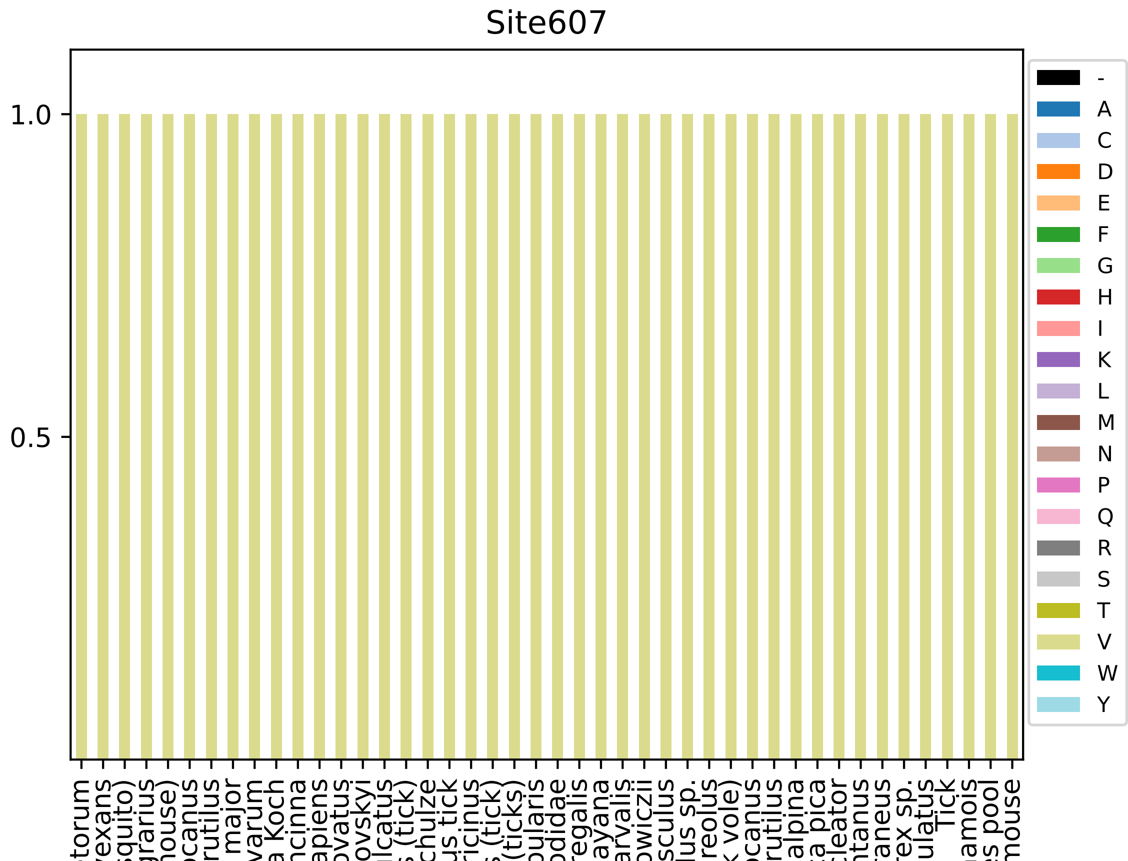

Site607
1.0
0.5
-
A
C
D
E
F
G
H
I
K
L
M
N
P
Q
R
S
T
V
W
Y

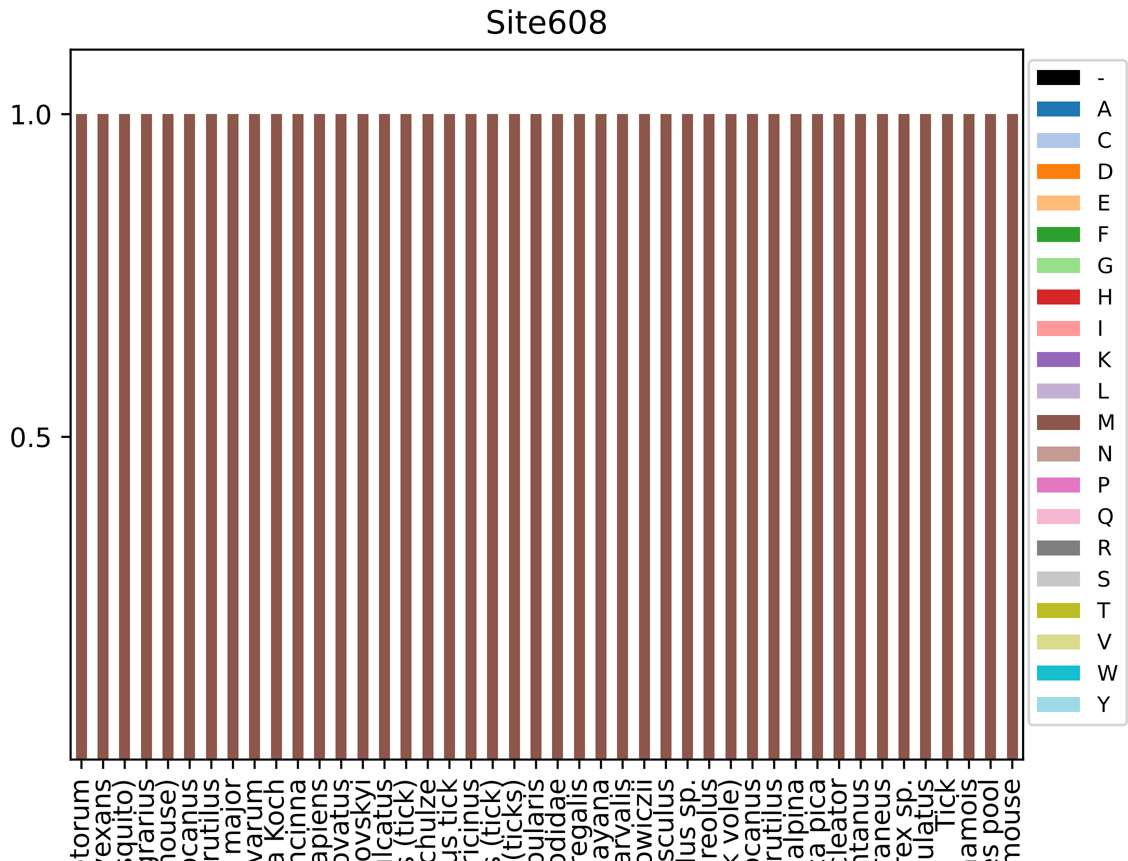
Site608
1.0
0.5
-
A
C
D
E
F
G
H
I
K
L
M
N
P
Q
R
S
T
V
W
Y

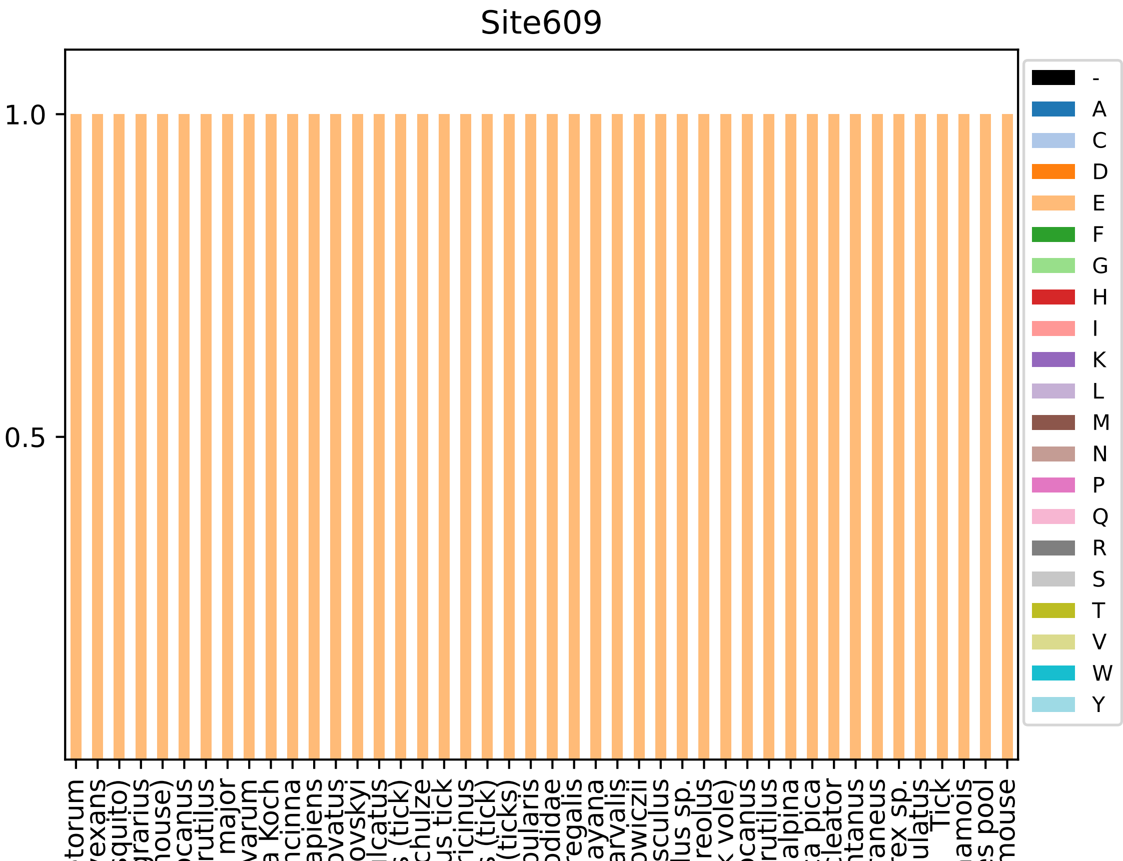
Site609
1.0
0.5
-
A
C
D
E
F
G
H
I
K
L
M
N
P
Q
R
S
T
V
W
Y

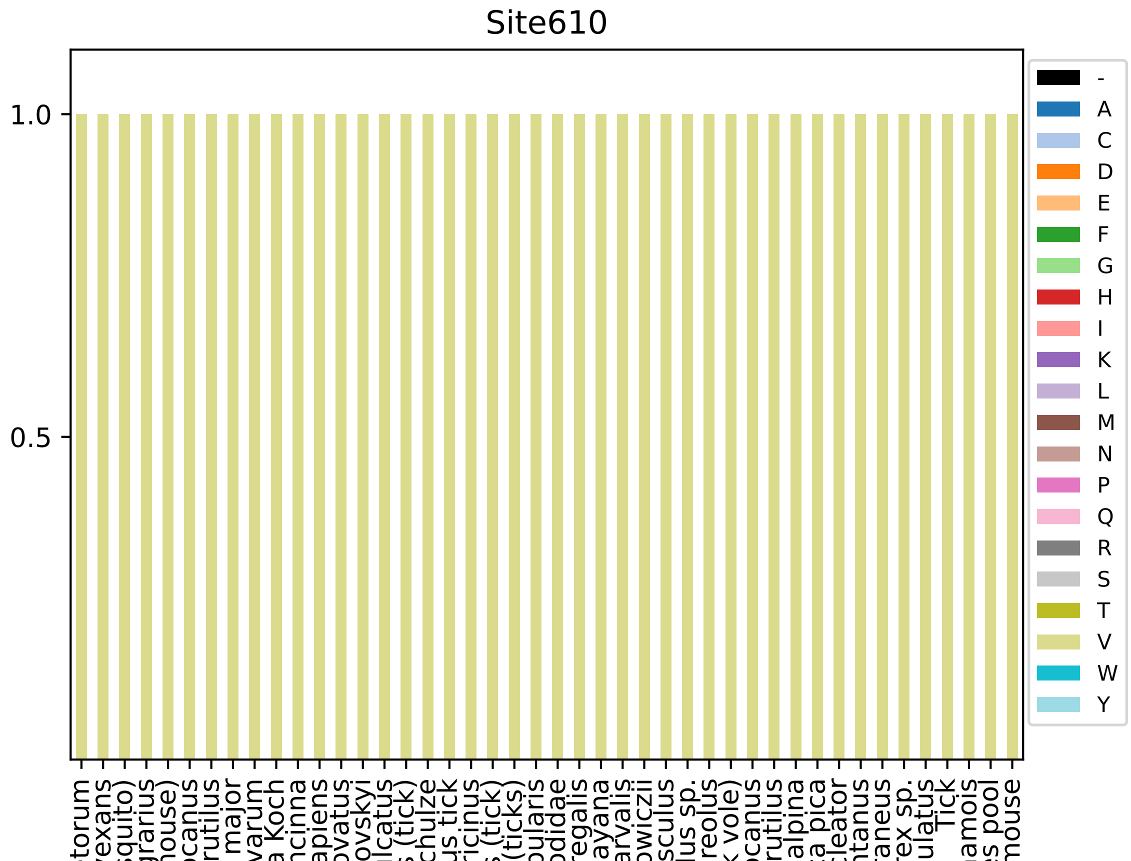

Site610
1.0
0.5
-
A
C
D
E
F
G
H
I
K
L
M
N
P
Q
R
S
T
V
W
Y

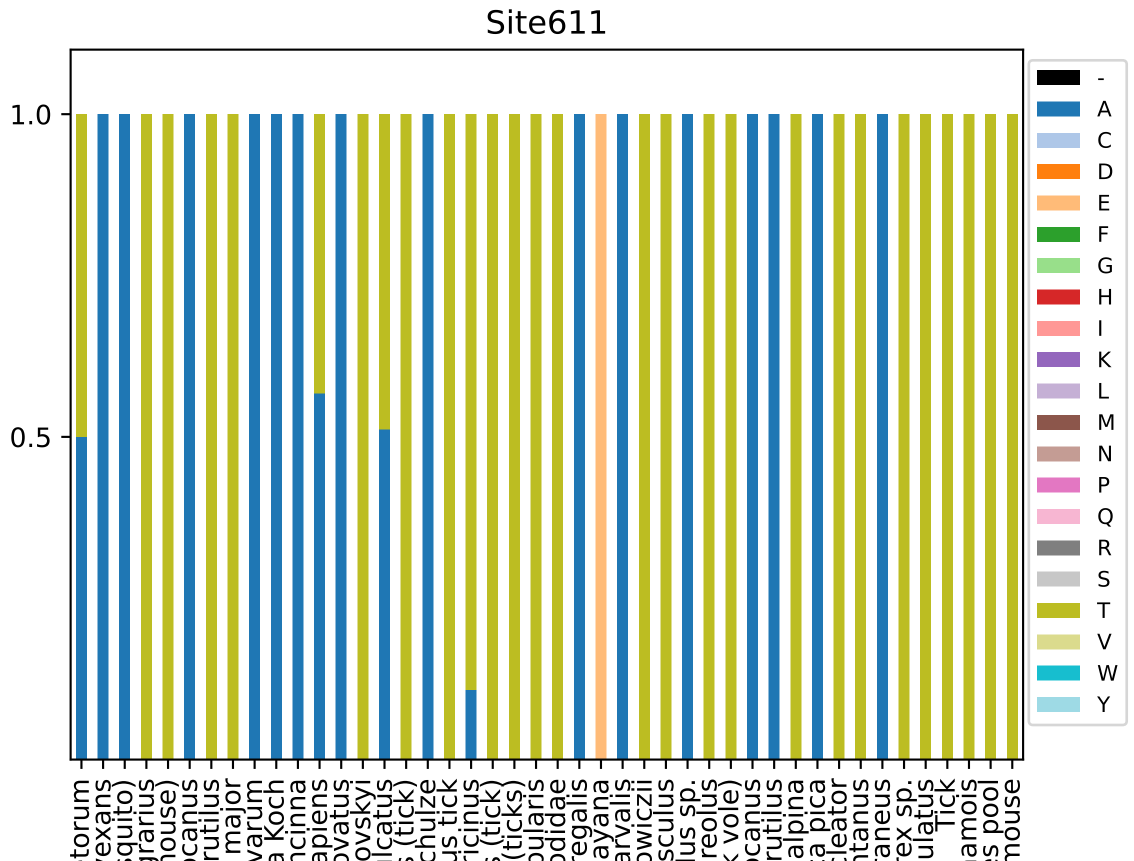

Site611
1.0
0.5
-
A
C
D
E
F
G
H
I
K
L
M
N
P
Q
R
S
T
V
W
Y

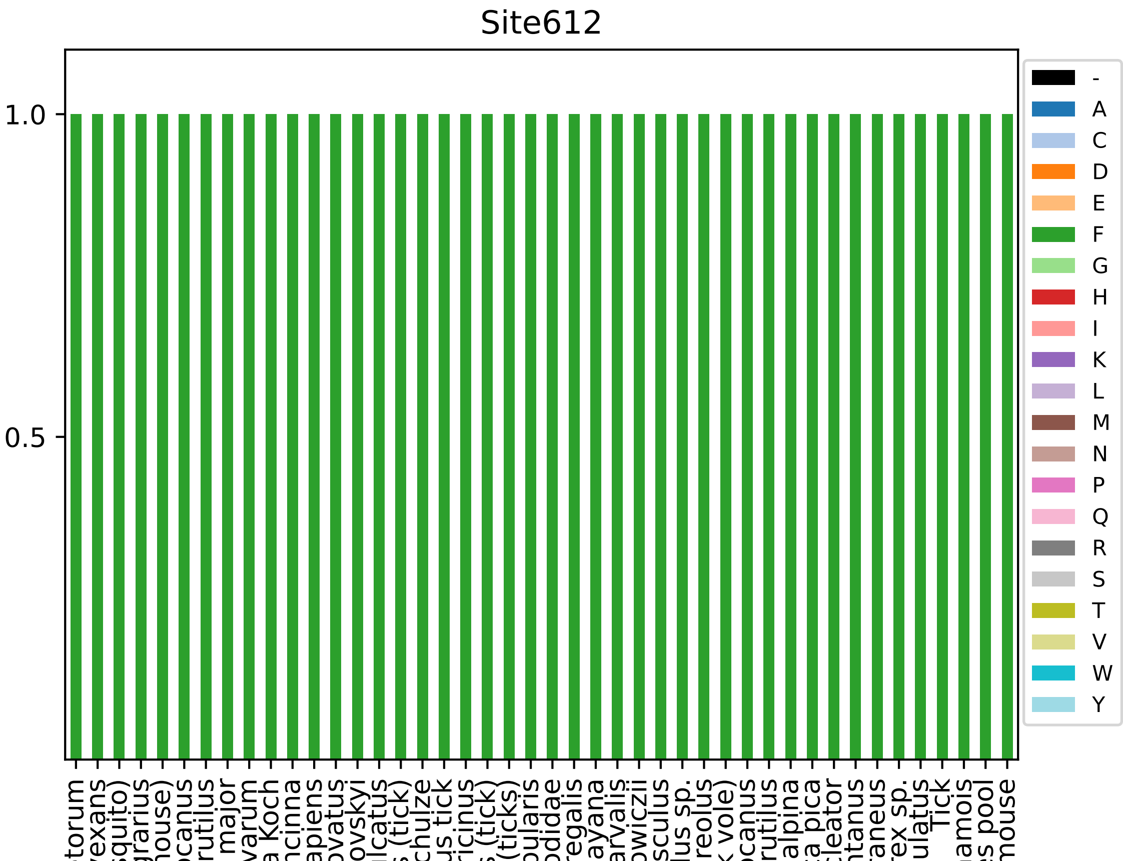

Site612
1.0
0.5
-
A
C
D
E
F
G
H
I
K
L
M
N
P
Q
R
S
T
V
W
Y

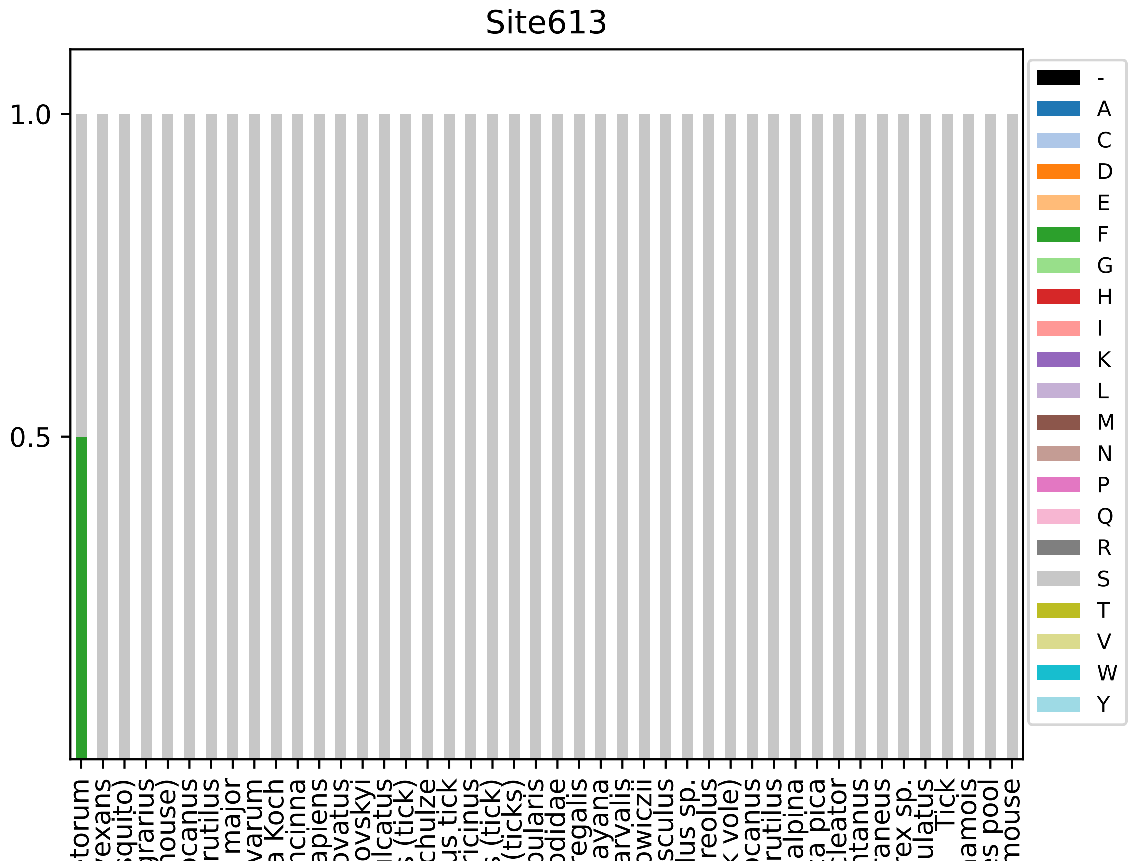

Site613
1.0
0.5
-
A
C
D
E
F
G
H
I
K
L
M
N
P
Q
R
S
T
V
W
Y

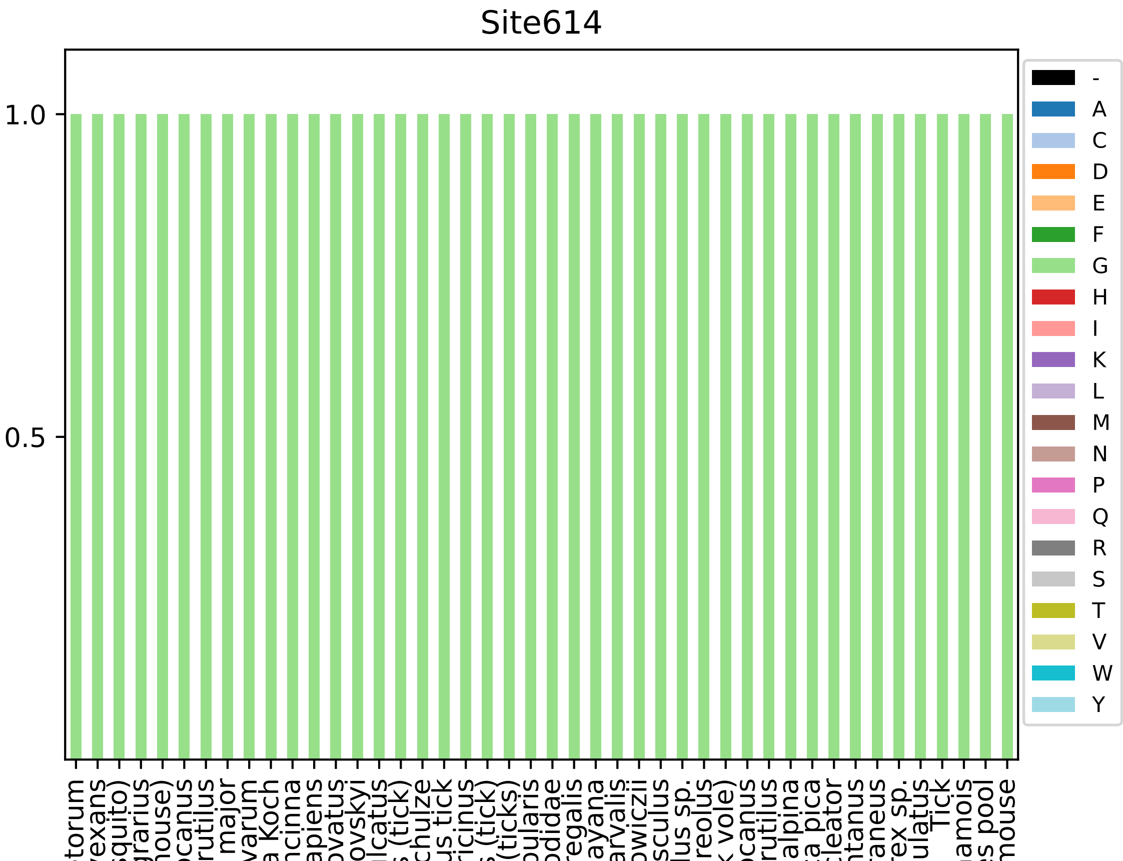
Site614
1.0
0.5
-
A
C
D
E
F
G
H
I
K
L
M
N
P
Q
R
S
T
V
W
Y

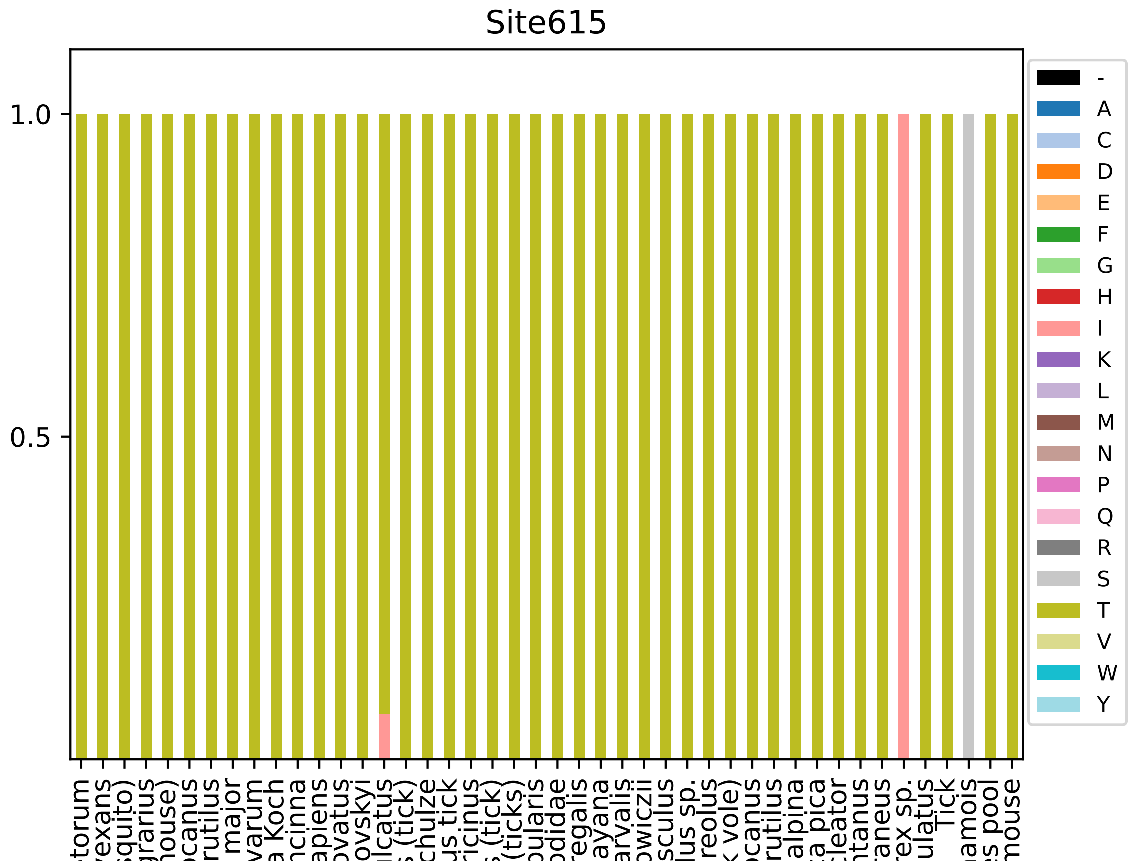
Site615
1.0
0.5
-
A
C
D
E
F
G
H
I
K
L
M
N
P
Q
R
S
T
V
W
Y

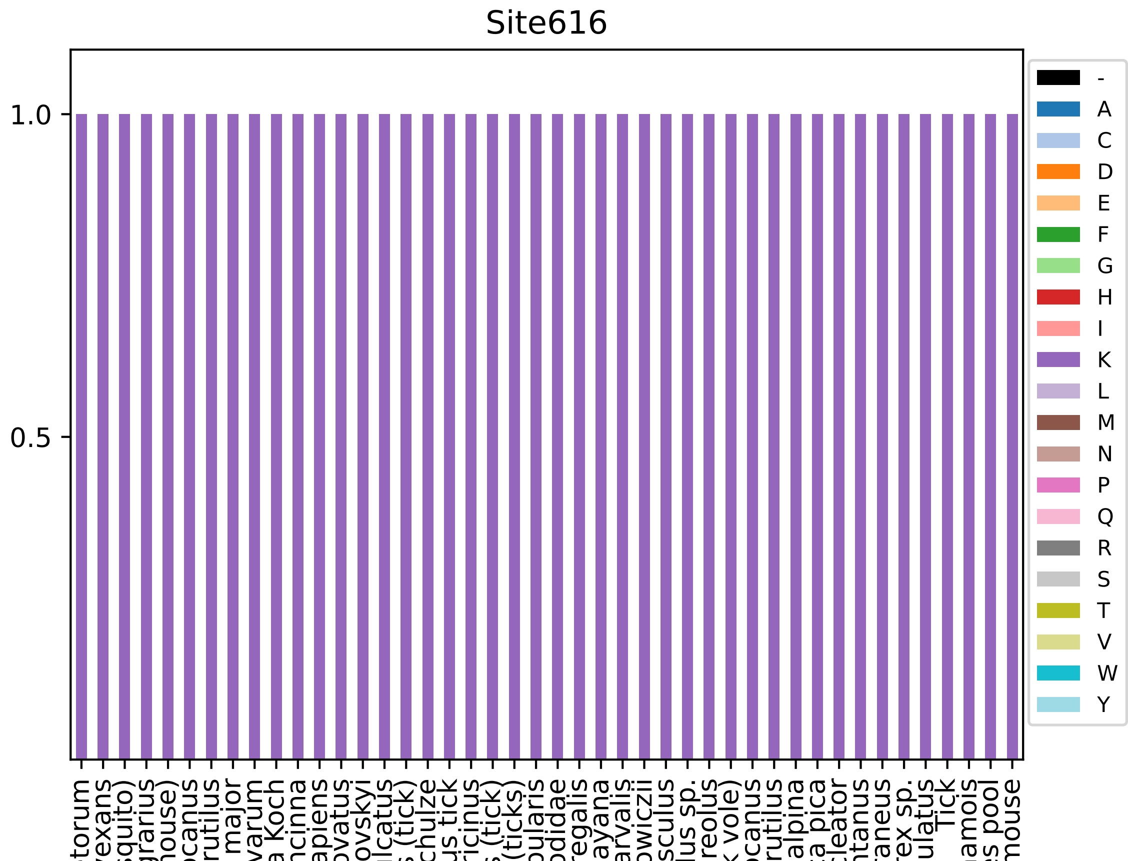
Site616
1.0
0.5
-
A
C
D
E
F
G
H
I
K
L
M
N
P
Q
R
S
T
V
W
Y

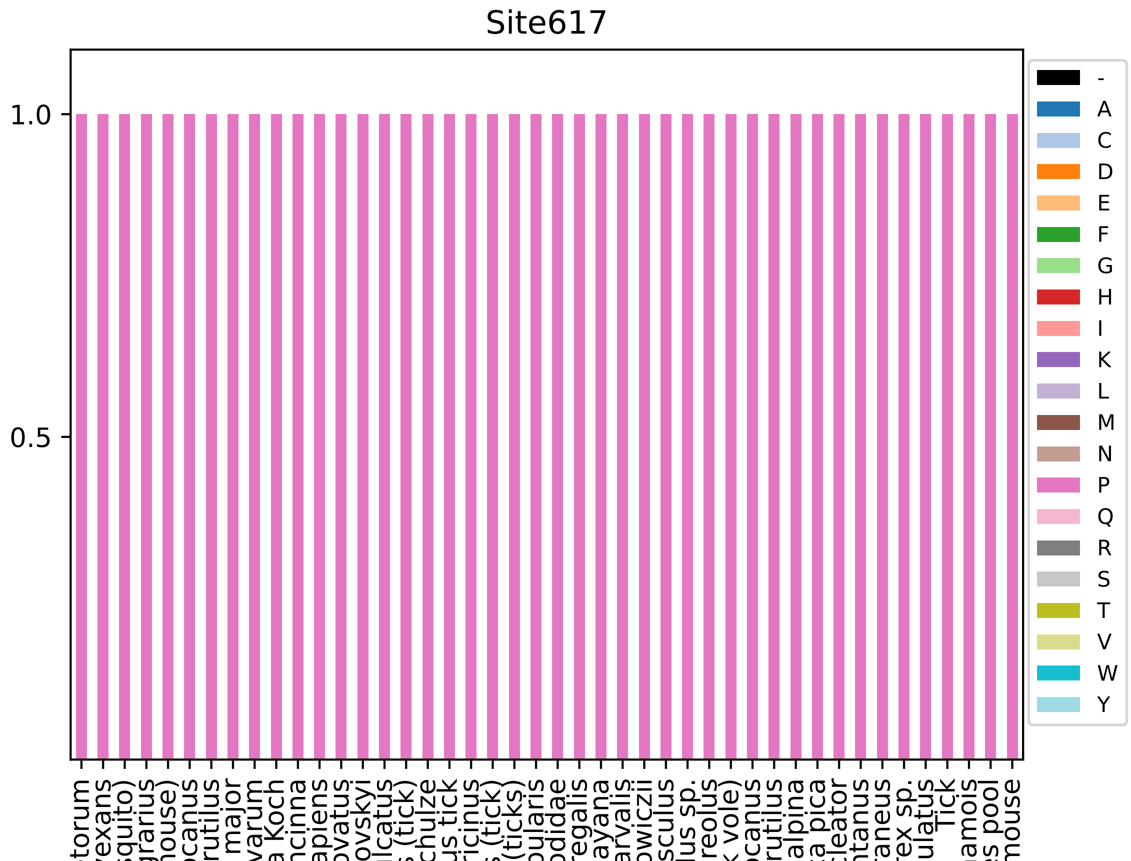
Site617
1.0
0.5
-
A
C
D
E
F
G
H
I
K
L
M
N
P
Q
R
S
T
V
W
Y

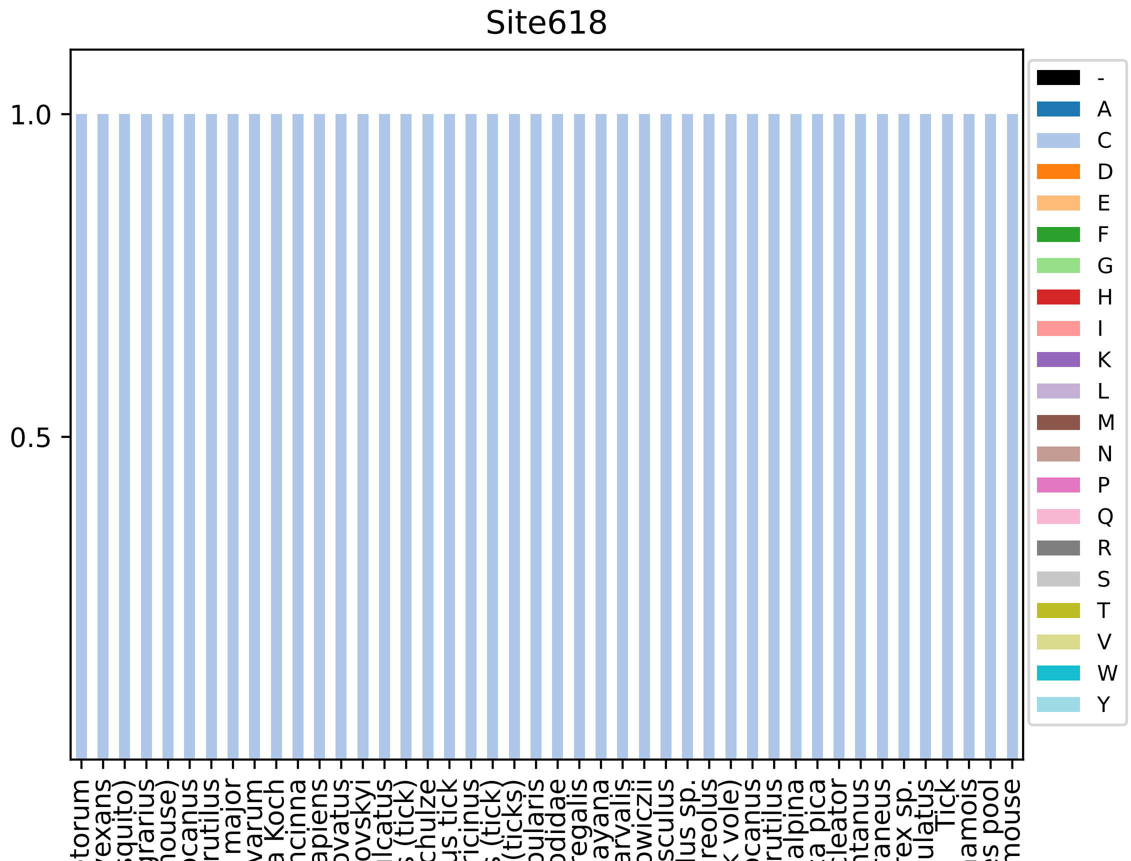
Site618
1.0
0.5
-
A
C
D
E
F
G
H
I
K
L
M
N
P
Q
R
S
T
V
W
Y

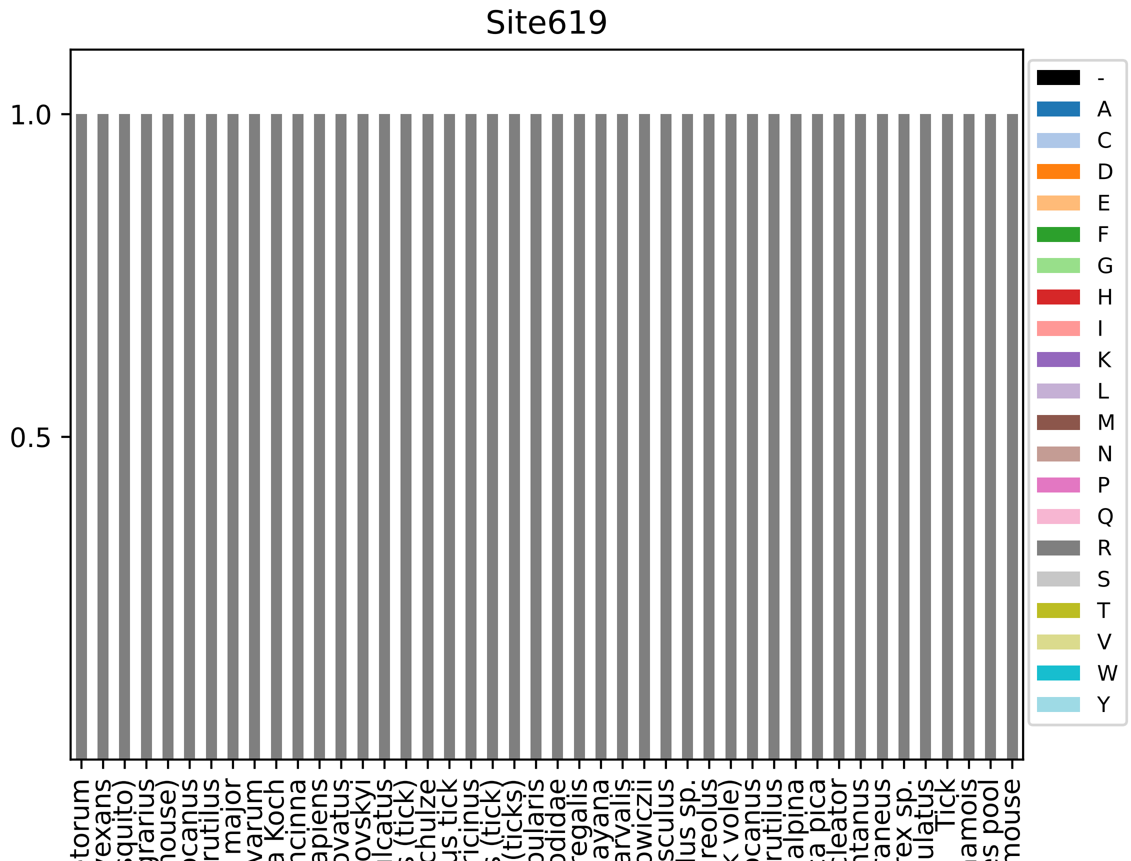
Site619
1.0
0.5
-
A
C
D
E
F
G
H
I
K
L
M
N
P
Q
R
S
T
V
W
Y

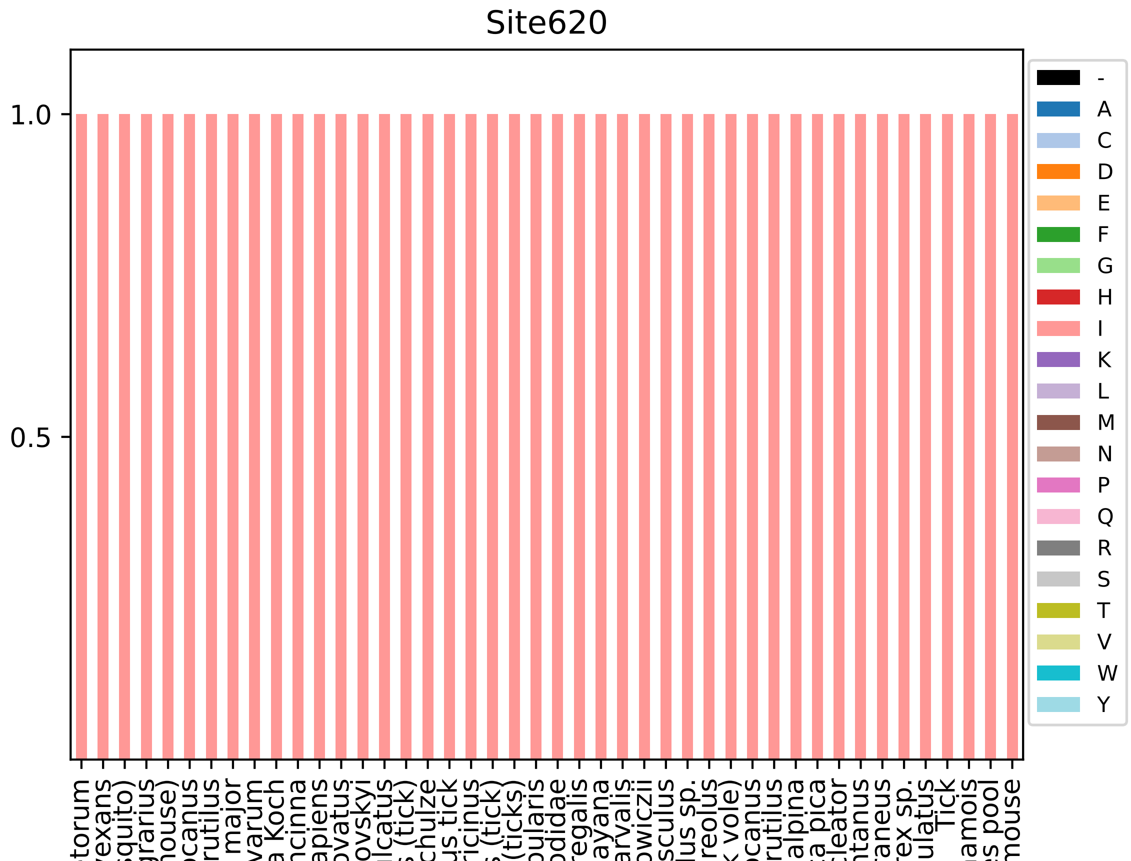
Site620
1.0
0.5
-
A
C
D
E
F
G
H
I
K
L
M
N
P
Q
R
S
T
V
W
Y

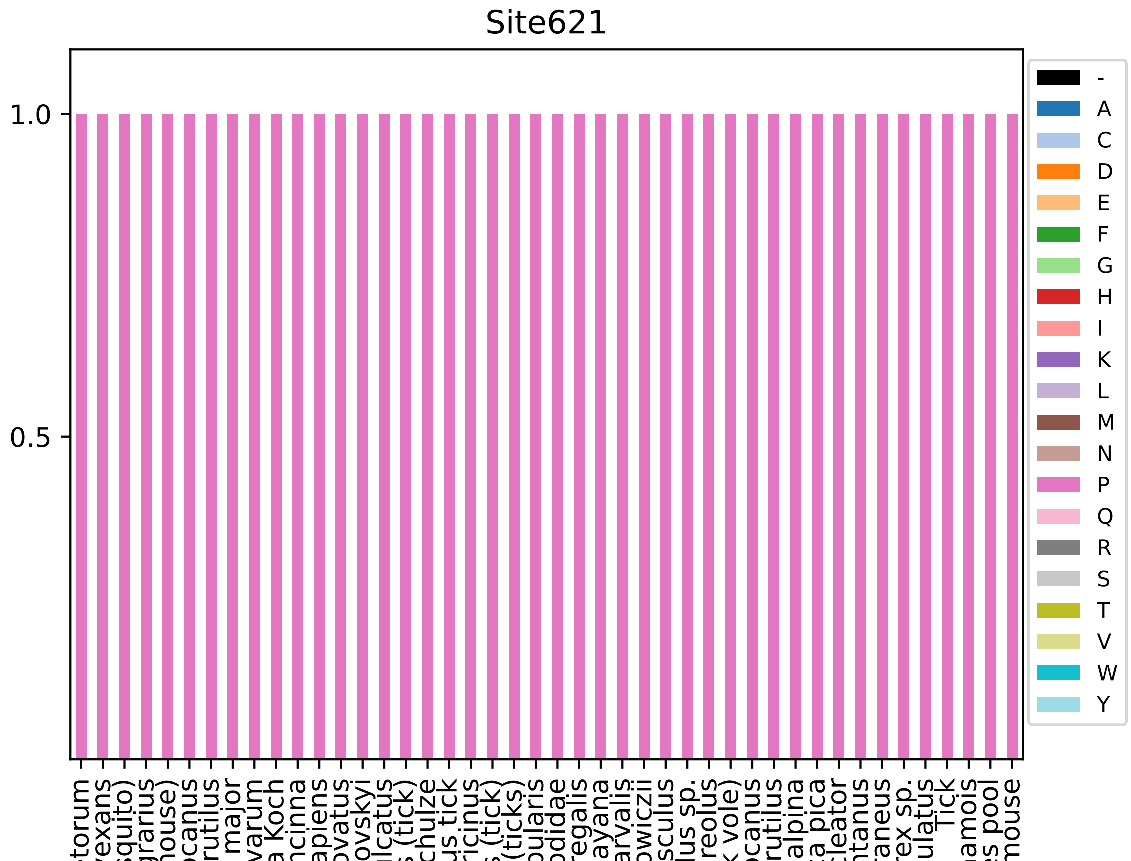
Site621
1.0
0.5
-
A
C
D
E
F
G
H
I
K
L
M
N
P
Q
R
S
T
V
W
Y

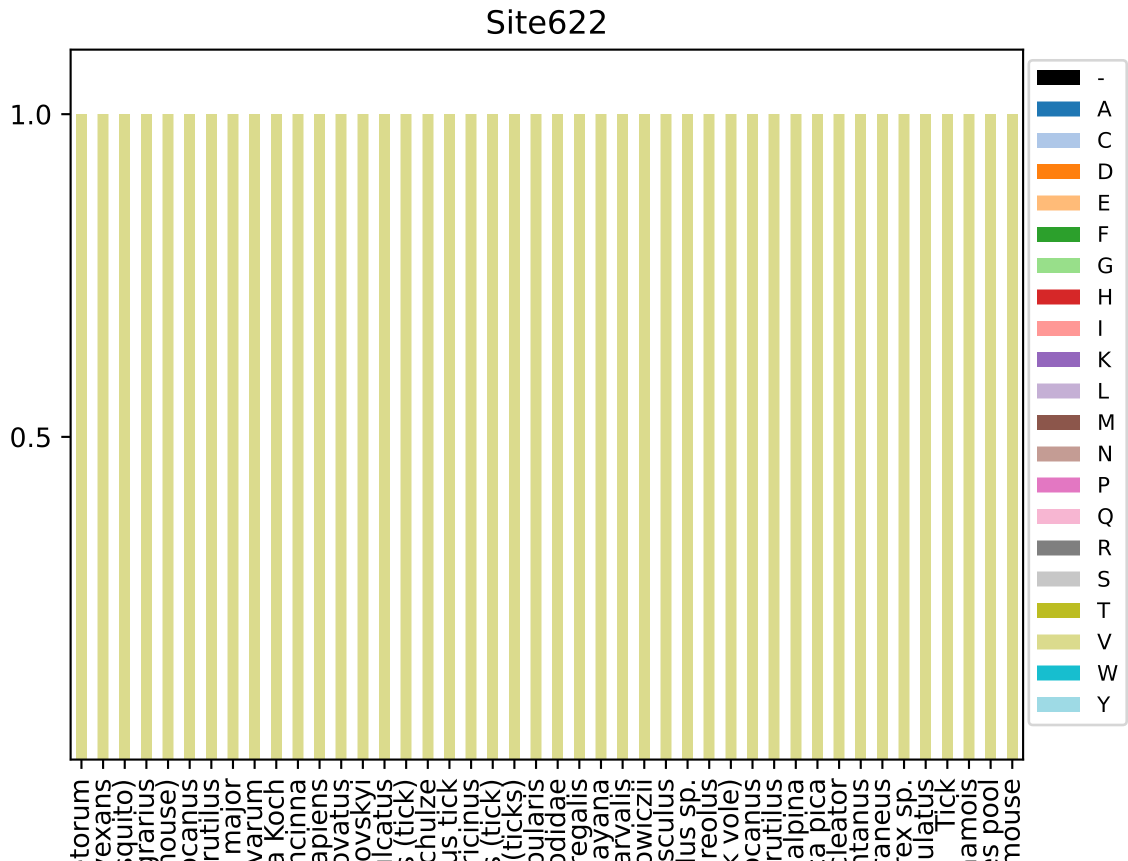
Site622
1.0
0.5
-
A
C
D
E
F
G
H
I
K
L
M
N
P
Q
R
S
T
V
W
Y

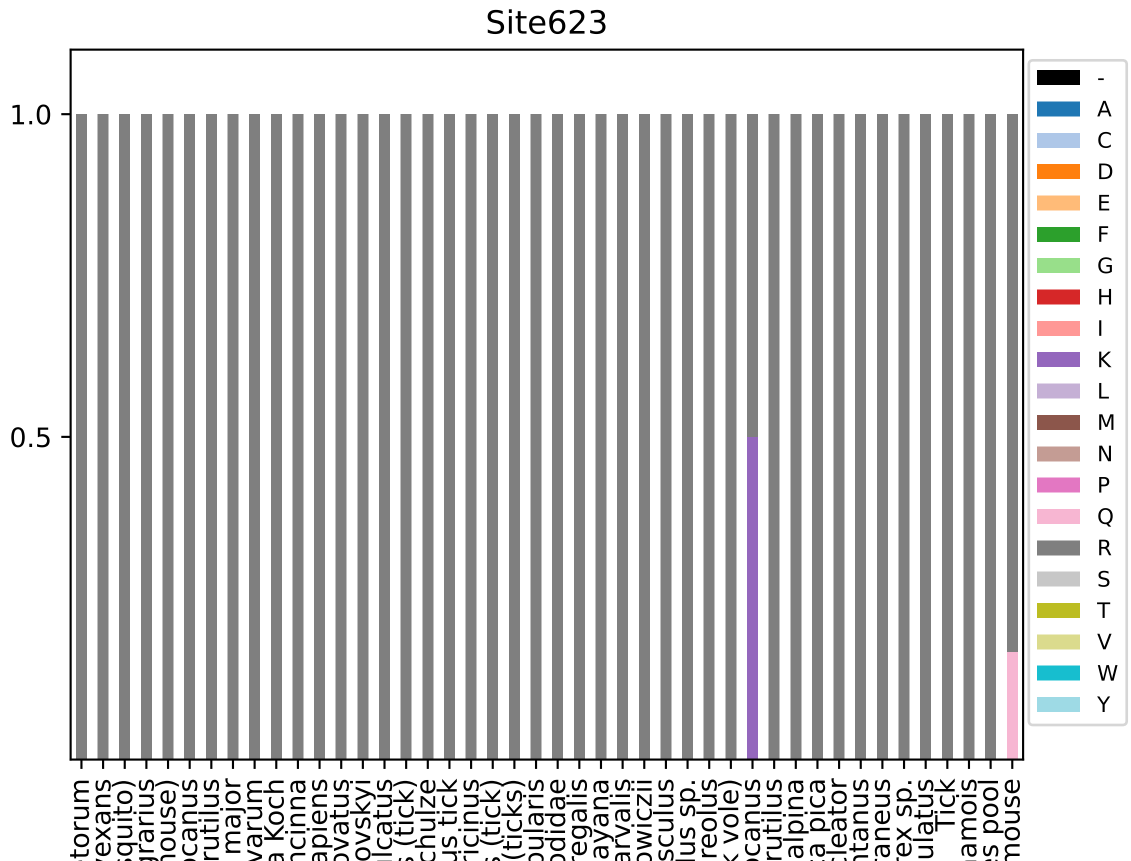

Site623
1.0
0.5
-
A
C
D
E
F
G
H
I
K
L
M
N
P
Q
R
S
T
V
W
Y

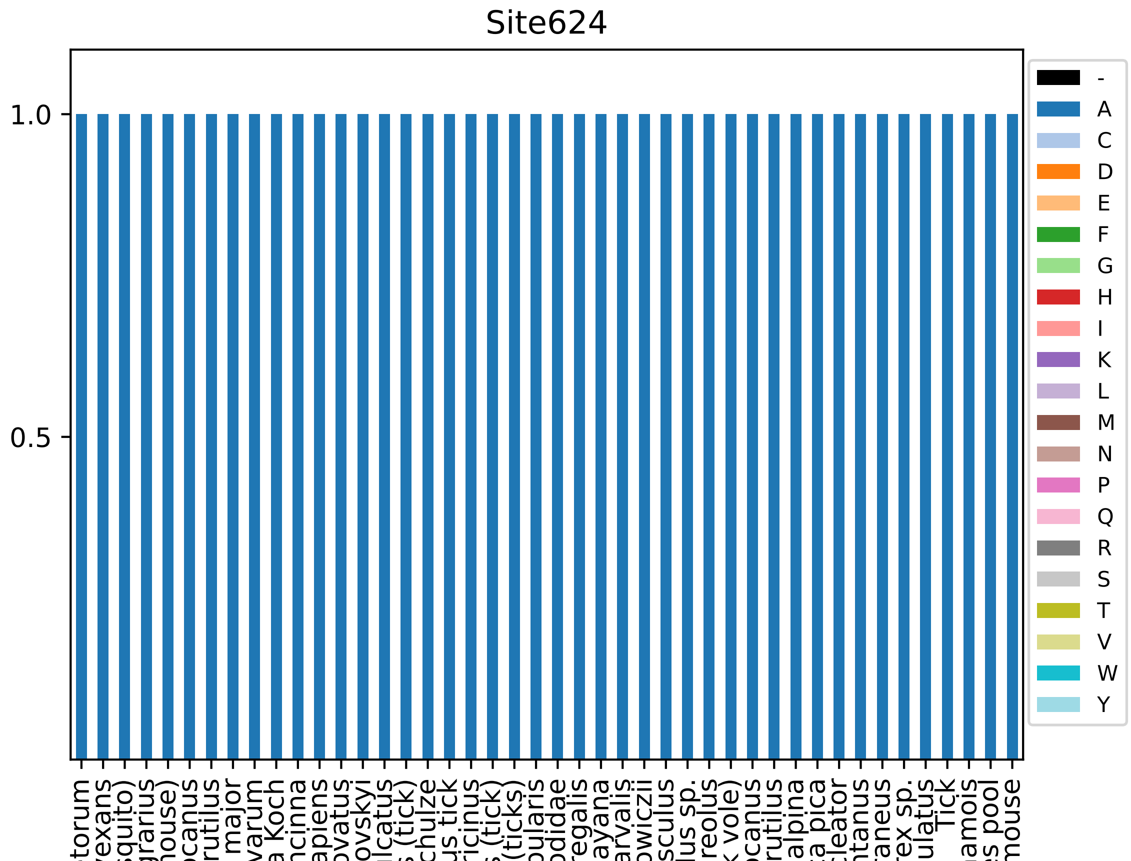
Site624
1.0
0.5
-
A
C
D
E
F
G
H
I
K
L
M
N
P
Q
R
S
T
V
W
Y

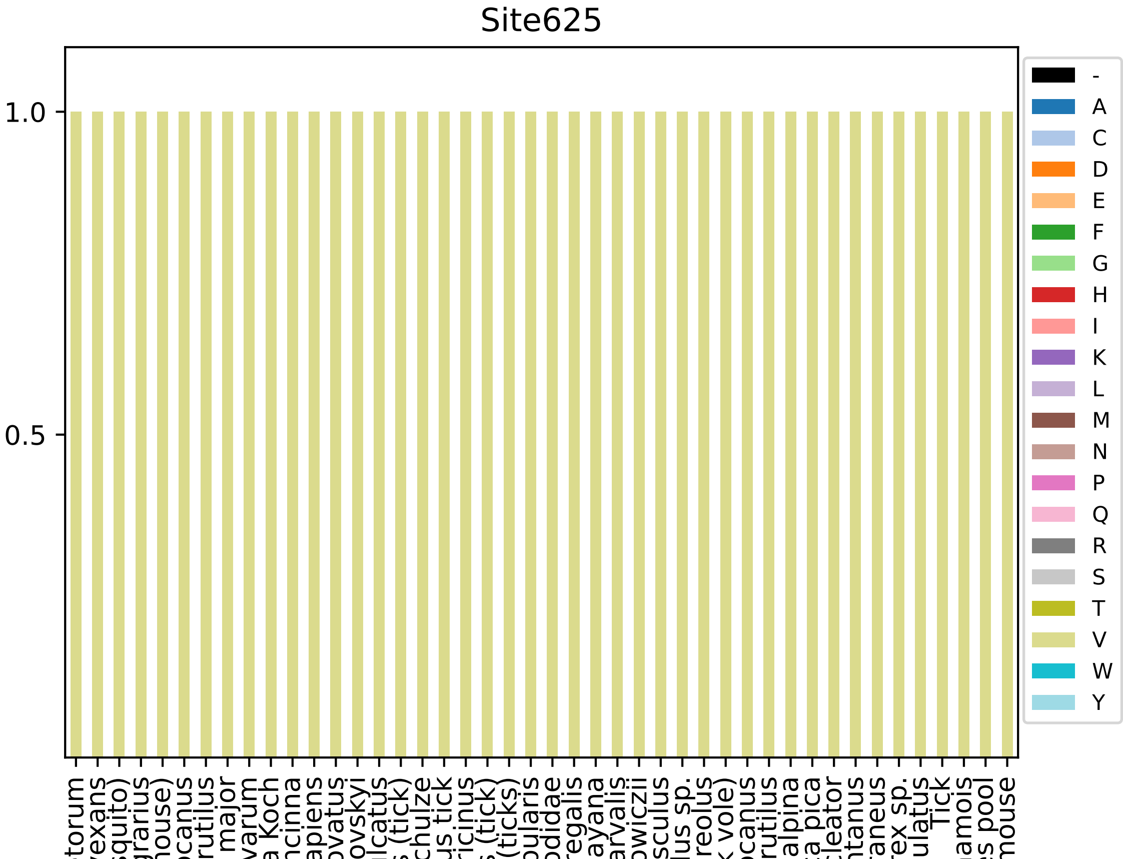
Site625
1.0
0.5
-
A
C
D
E
F
G
H
I
K
L
M
N
P
Q
R
S
T
V
W
Y

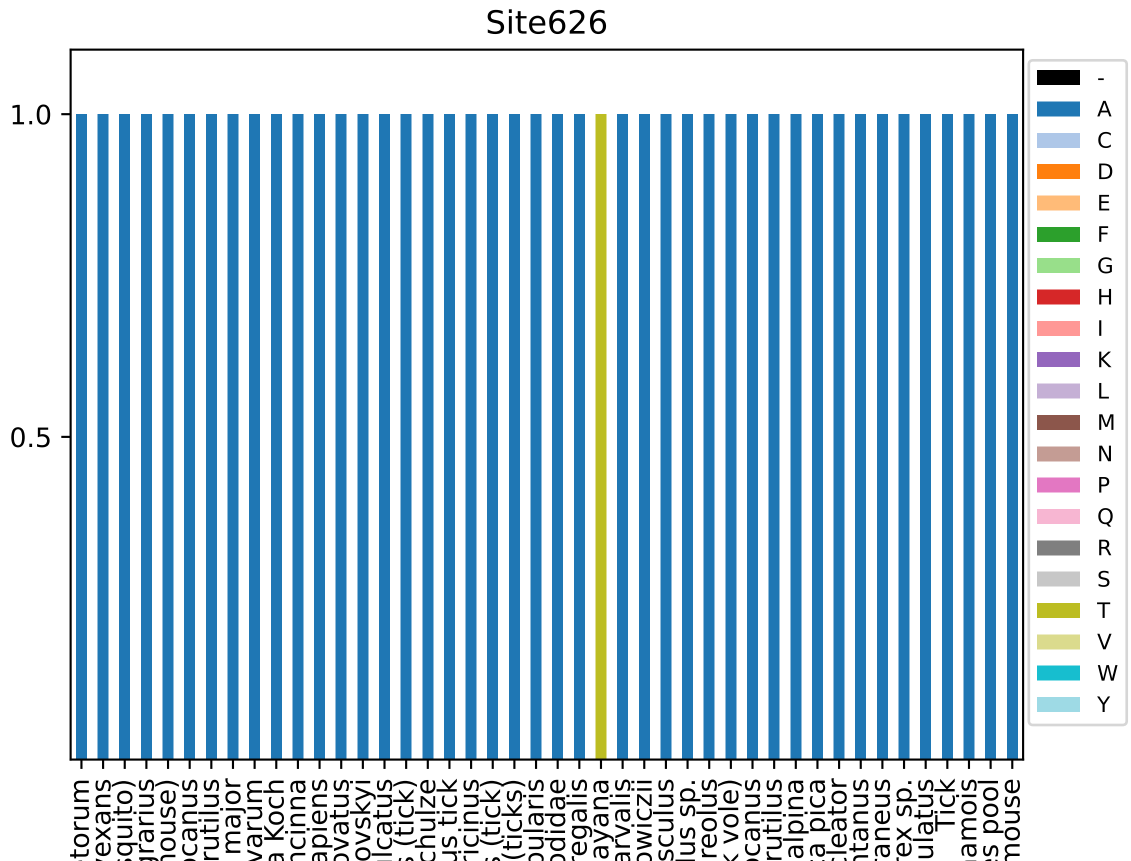
Site626
1.0
0.5
-
A
C
D
E
F
G
H
I
K
L
M
N
P
Q
R
S
T
V
W
Y

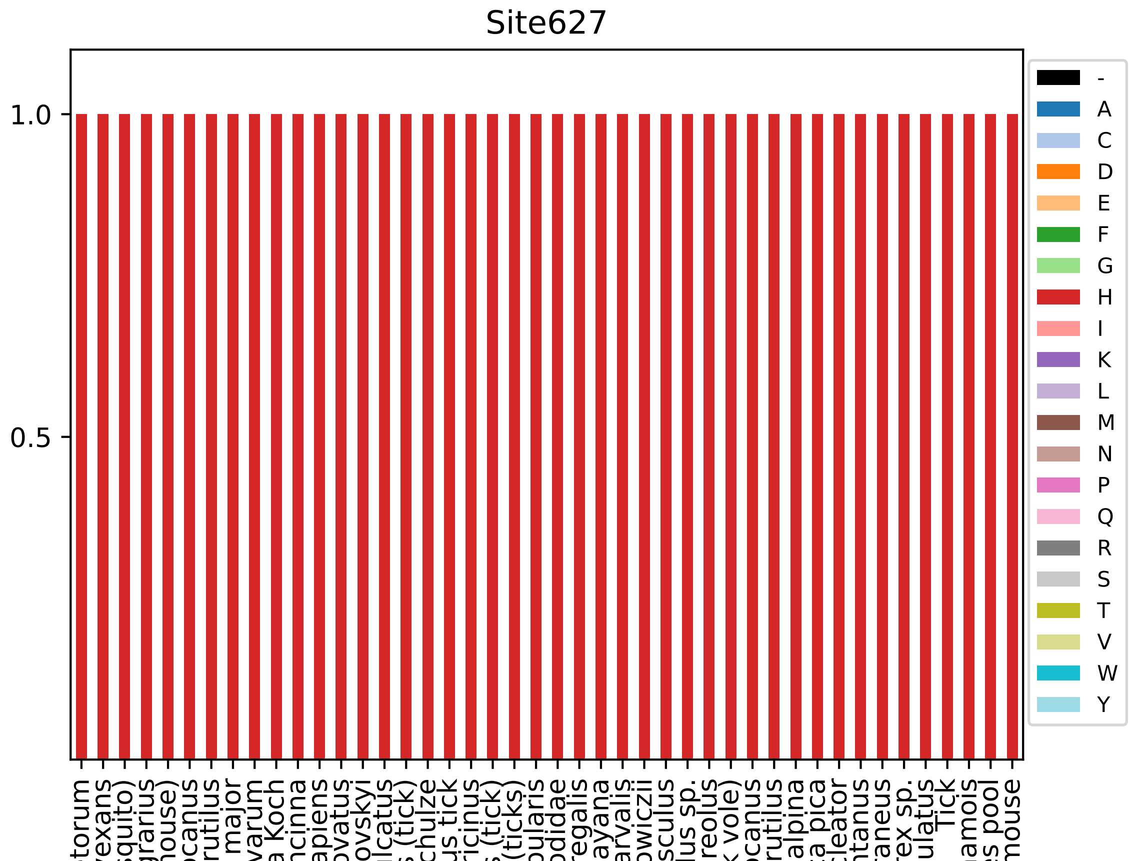
Site627
1.0
0.5
-
A
C
D
E
F
G
H
I
K
L
M
N
P
Q
R
S
T
V
W
Y

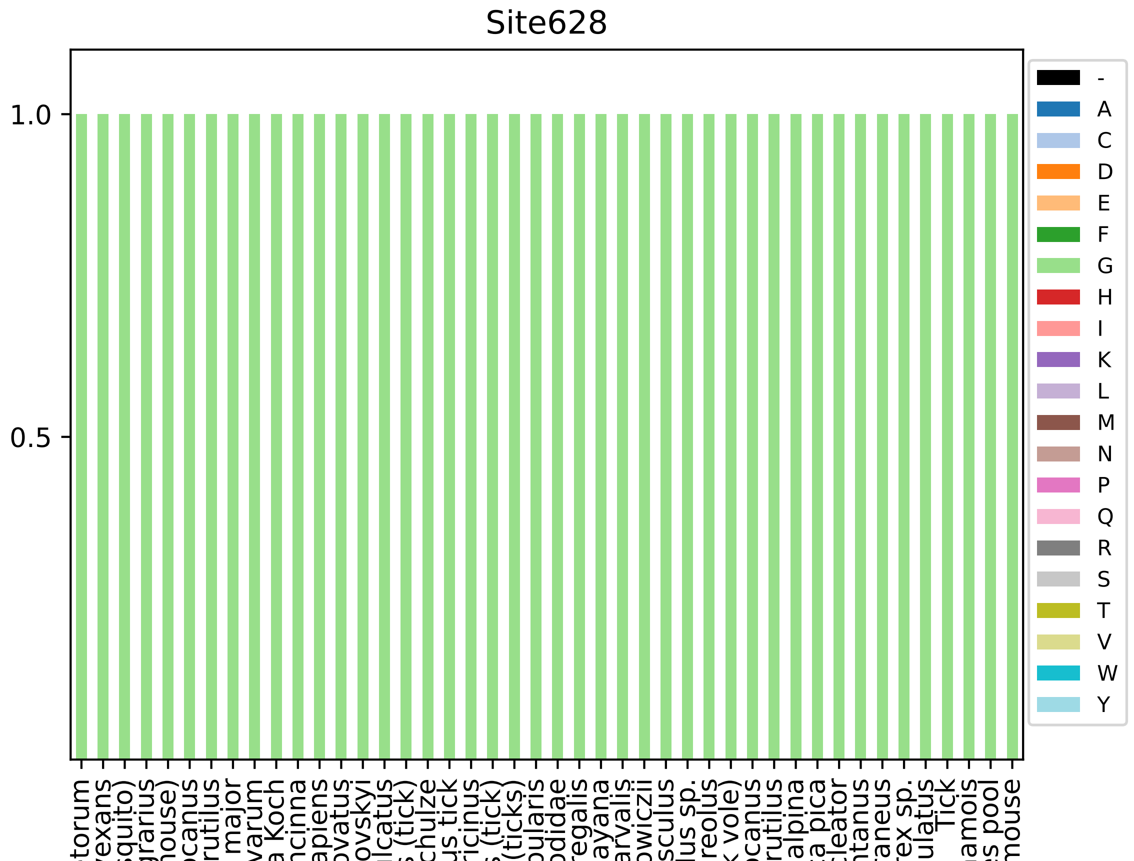
Site628
1.0
0.5
-
A
C
D
E
F
G
H
I
K
L
M
N
P
Q
R
S
T
V
W
Y

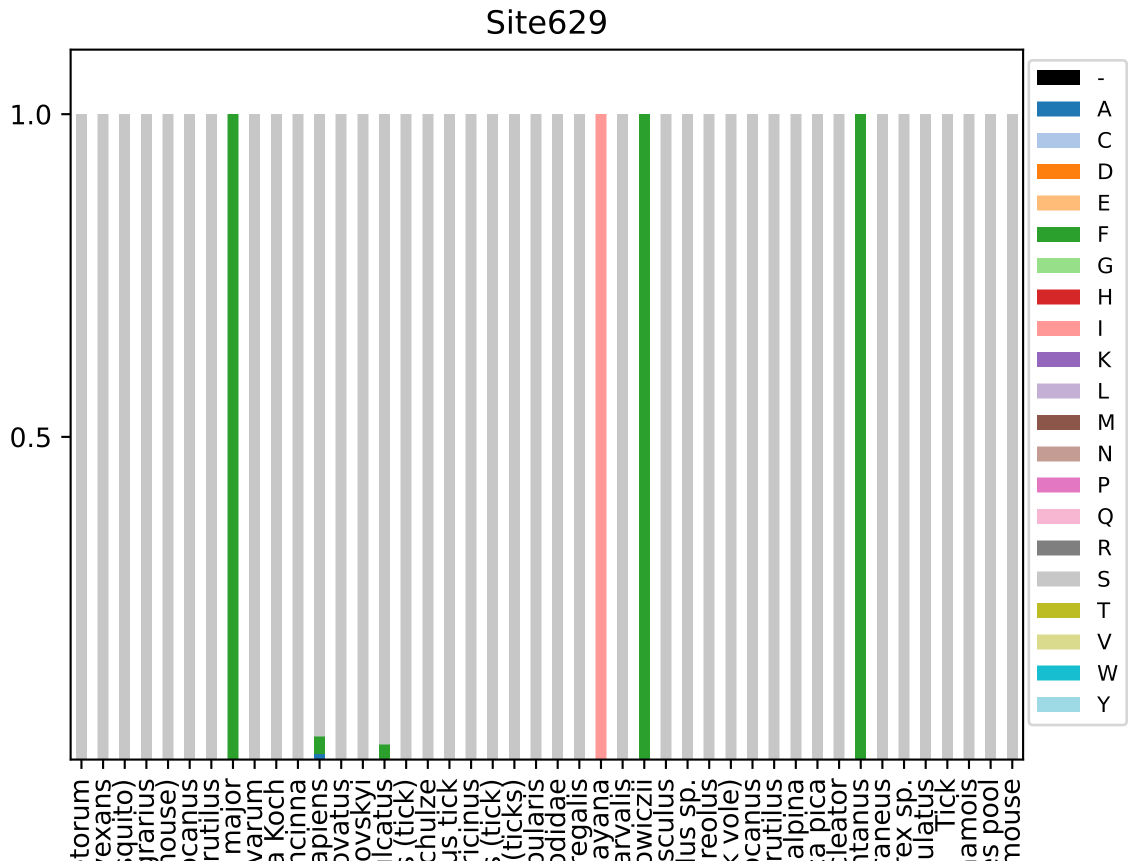

Site629
1.0
0.5
-
A
C
D
E
F
G
H
I
K
L
M
N
P
Q
R
S
T
V
W
Y

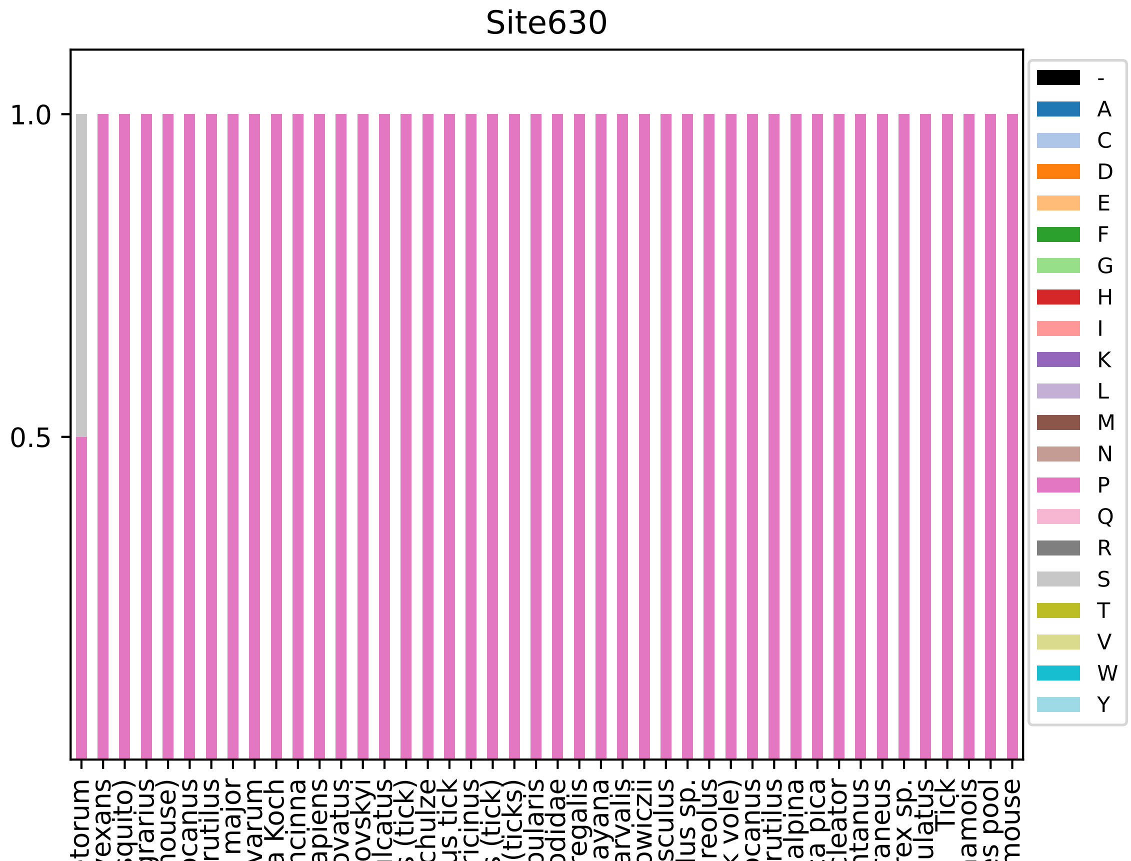
Site630
1.0
0.5
-
A
C
D
E
F
G
H
I
K
L
M
N
P
Q
R
S
T
V
W
Y

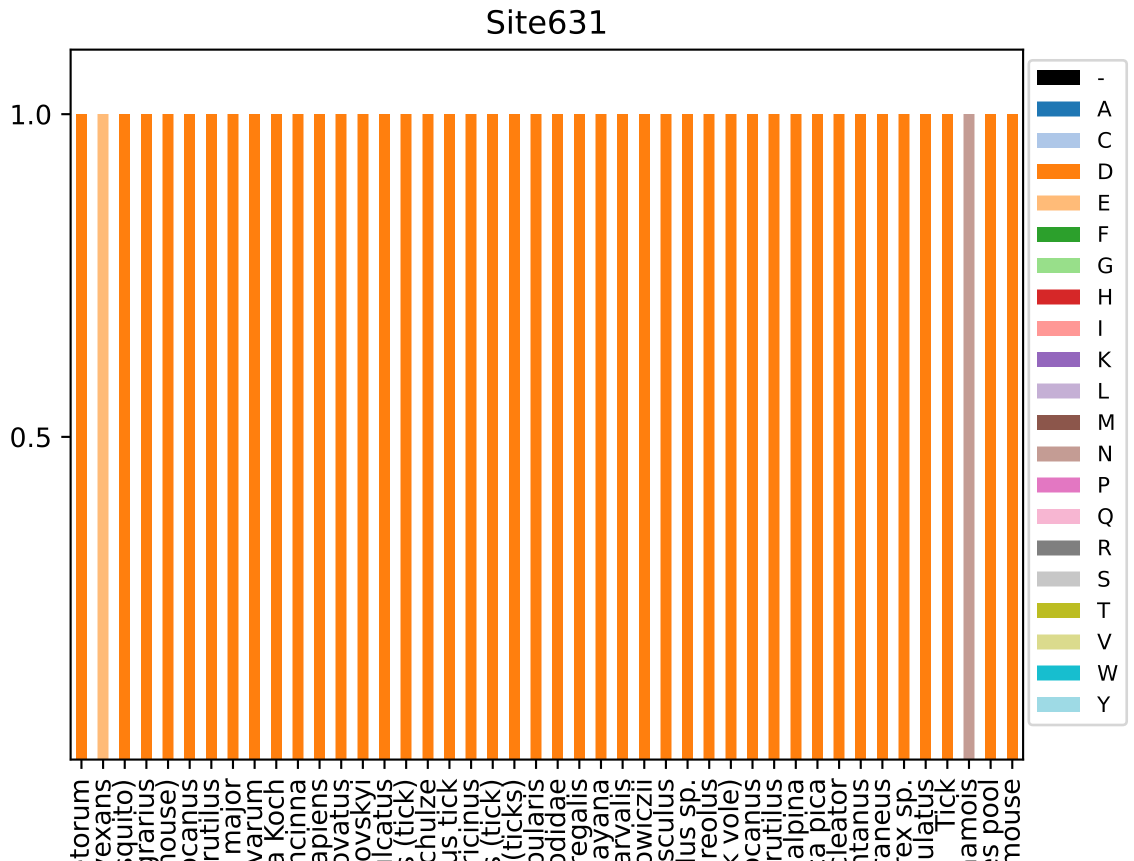
Site631
1.0
0.5
-
A
C
D
E
F
G
H
I
K
L
M
N
P
Q
R
S
T
V
W
Y

Site632

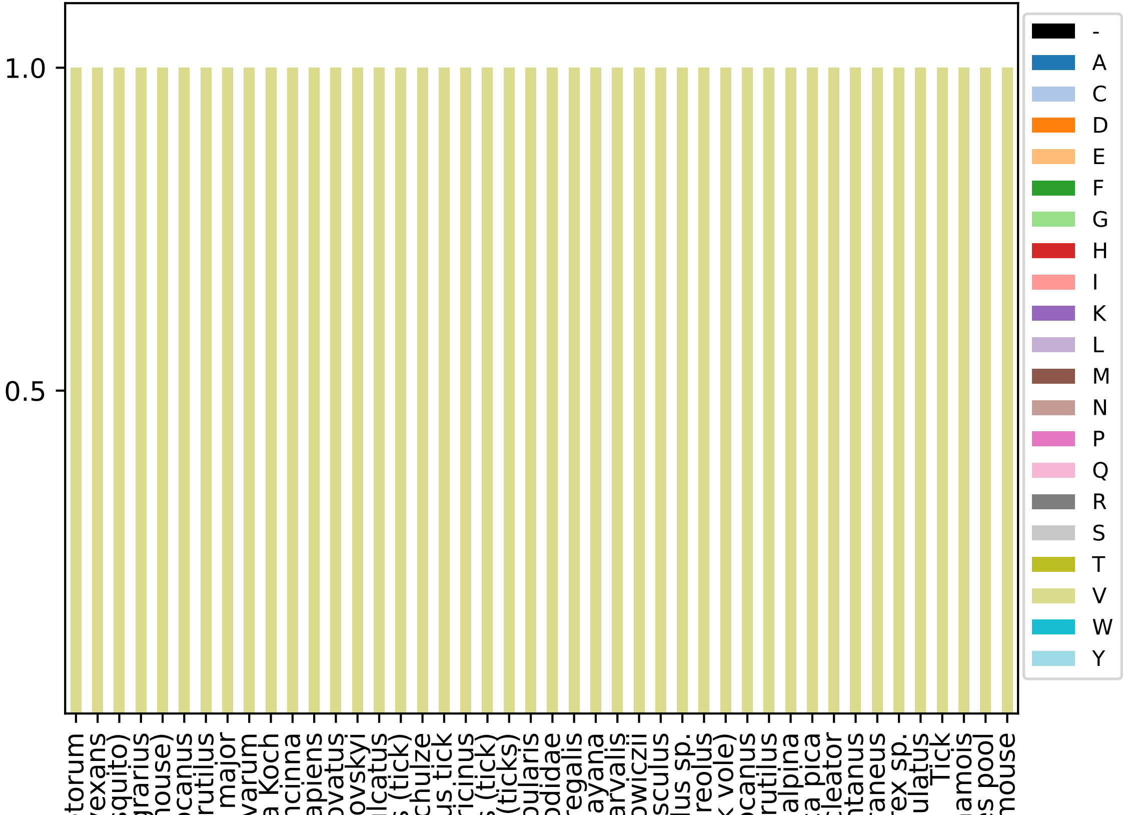

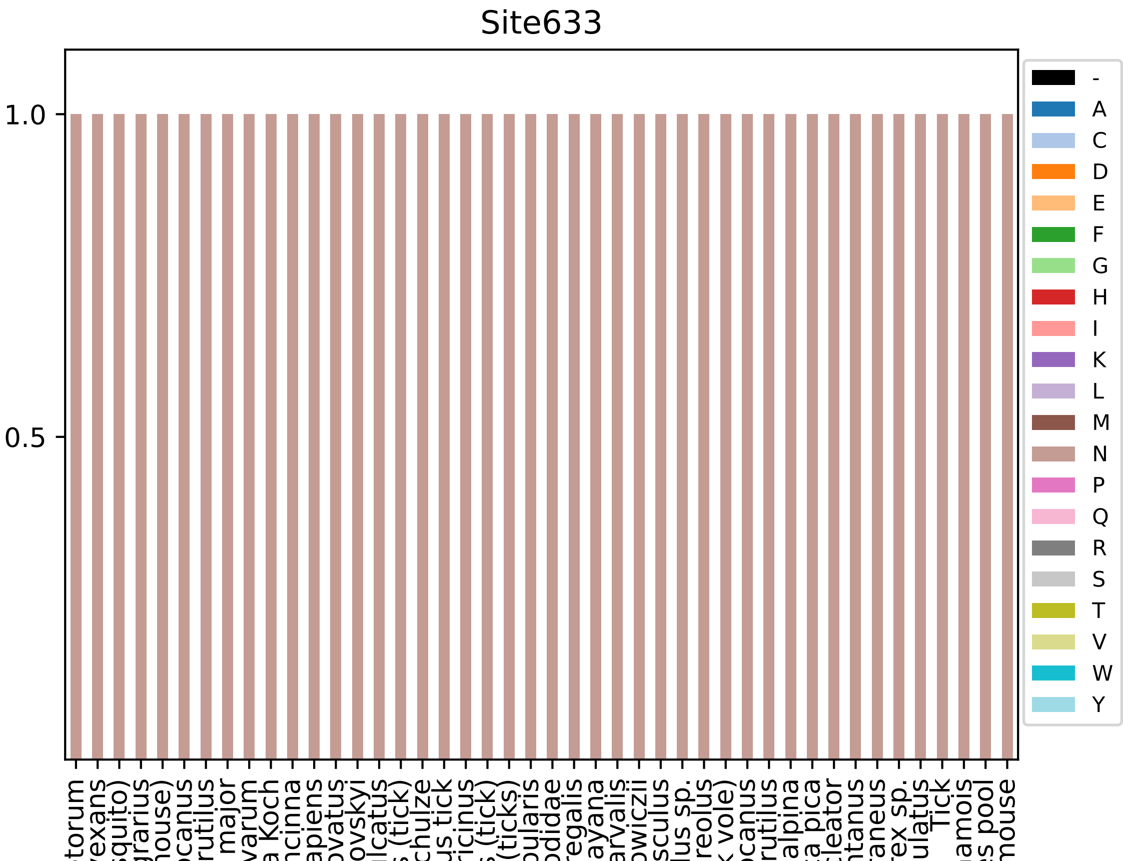
Site633
1.0
0.5
-
A
C
D
E
F
G
H
I
K
L
M
N
P
Q
R
S
T
V
W
Y

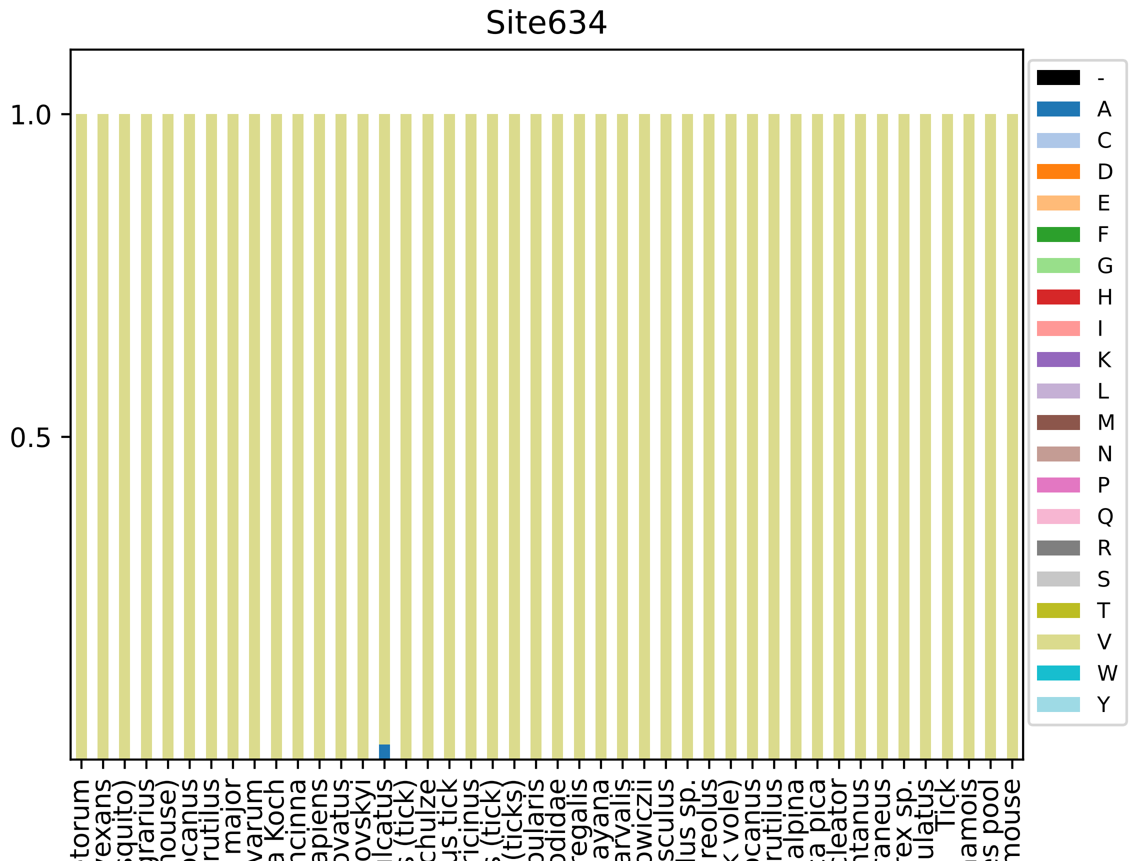
Site634
1.0
0.5
-
A
C
D
E
F
G
H
I
K
L
M
N
P
Q
R
S
T
V
W
Y

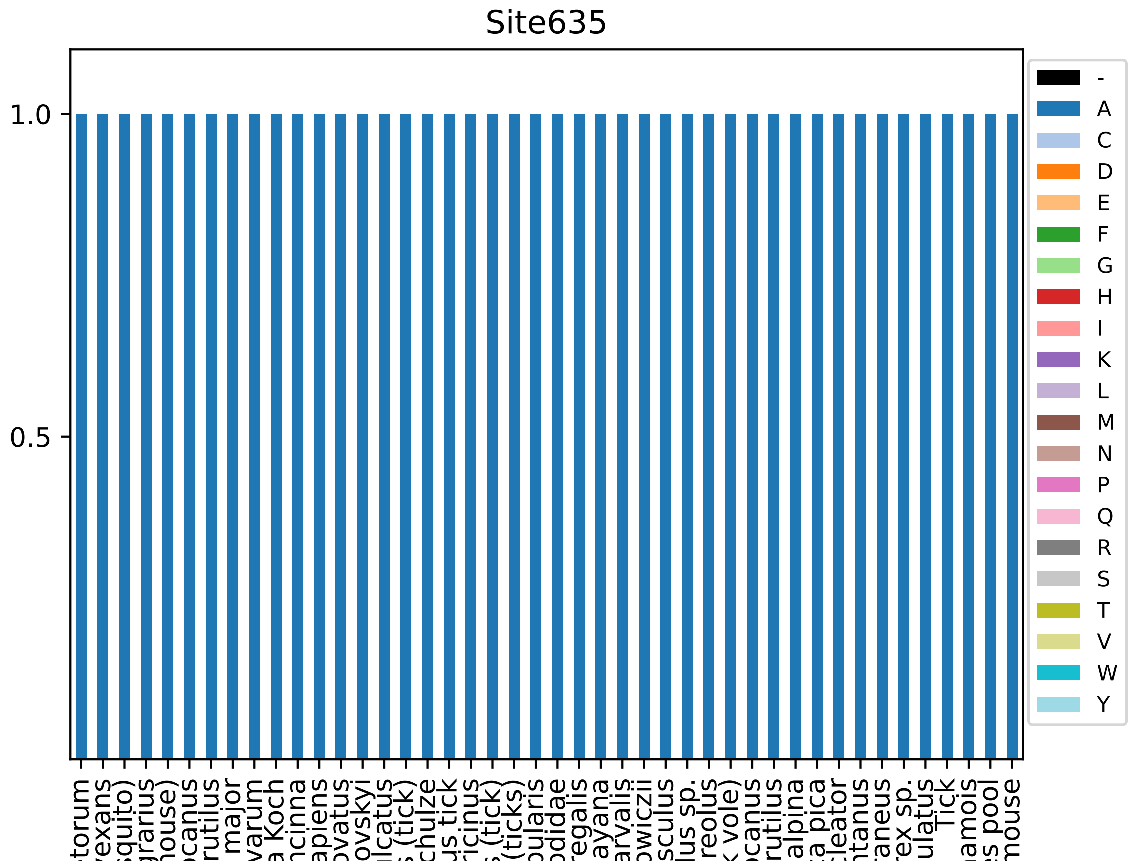
Site635
1.0
0.5
-
A
C
D
E
F
G
H
I
K
L
M
N
P
Q
R
S
T
V
W
Y

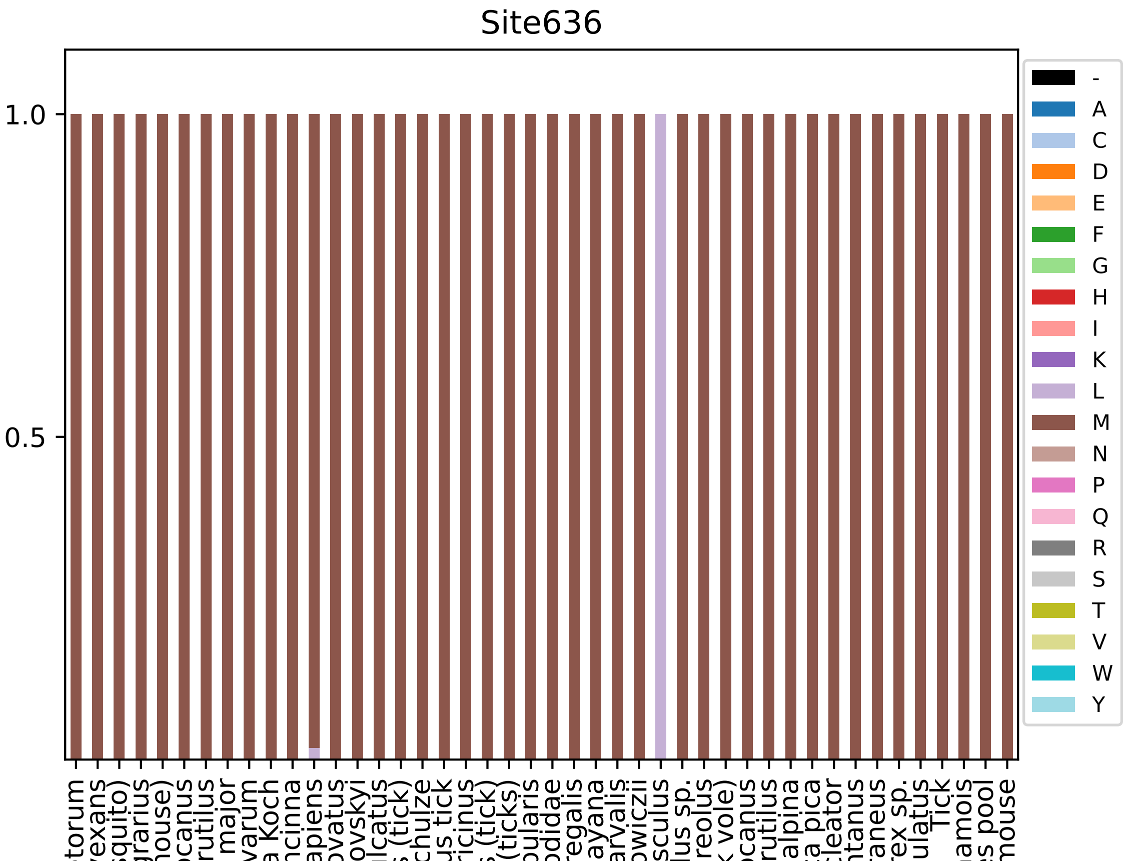

Site636
1.0
0.5
-
A
C
D
E
F
G
H
I
K
L
M
N
P
Q
R
S
T
V
W
Y

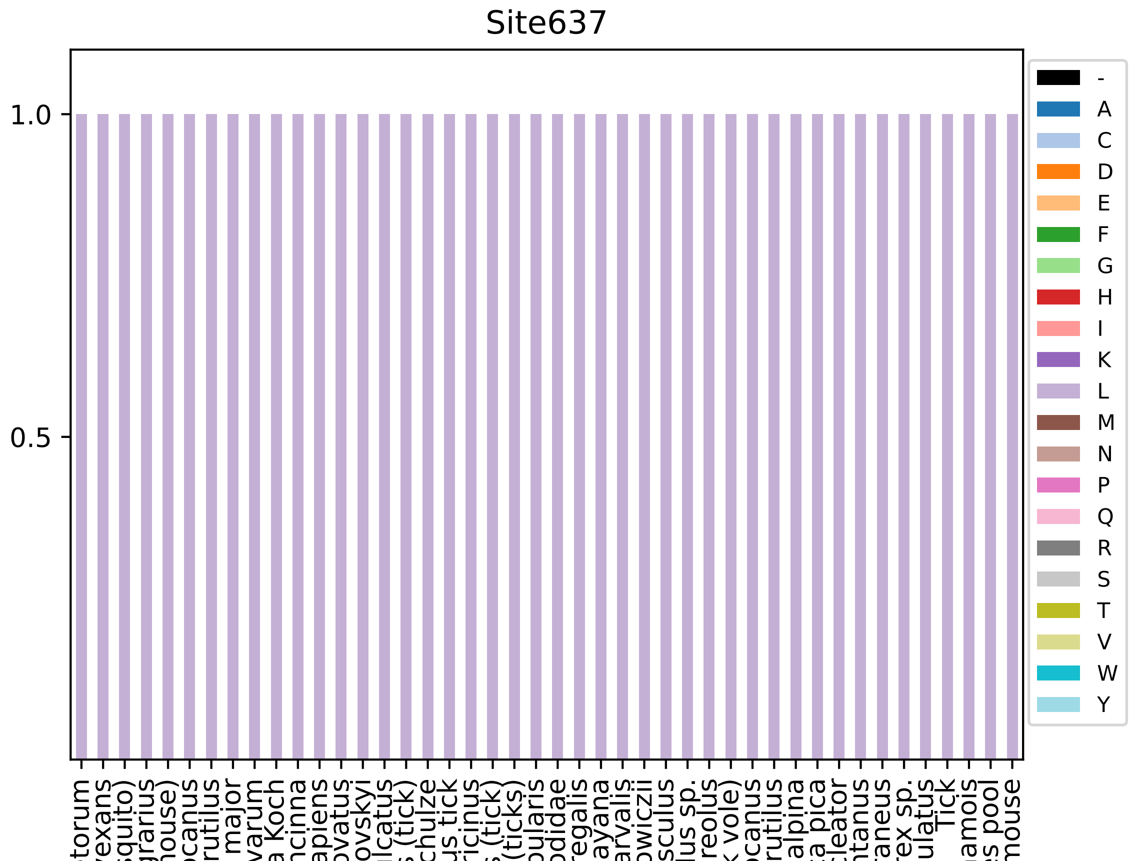
Site637
1.0
0.5
-
A
C
D
E
F
G
H
I
K
L
M
N
P
Q
R
S
T
V
W
Y

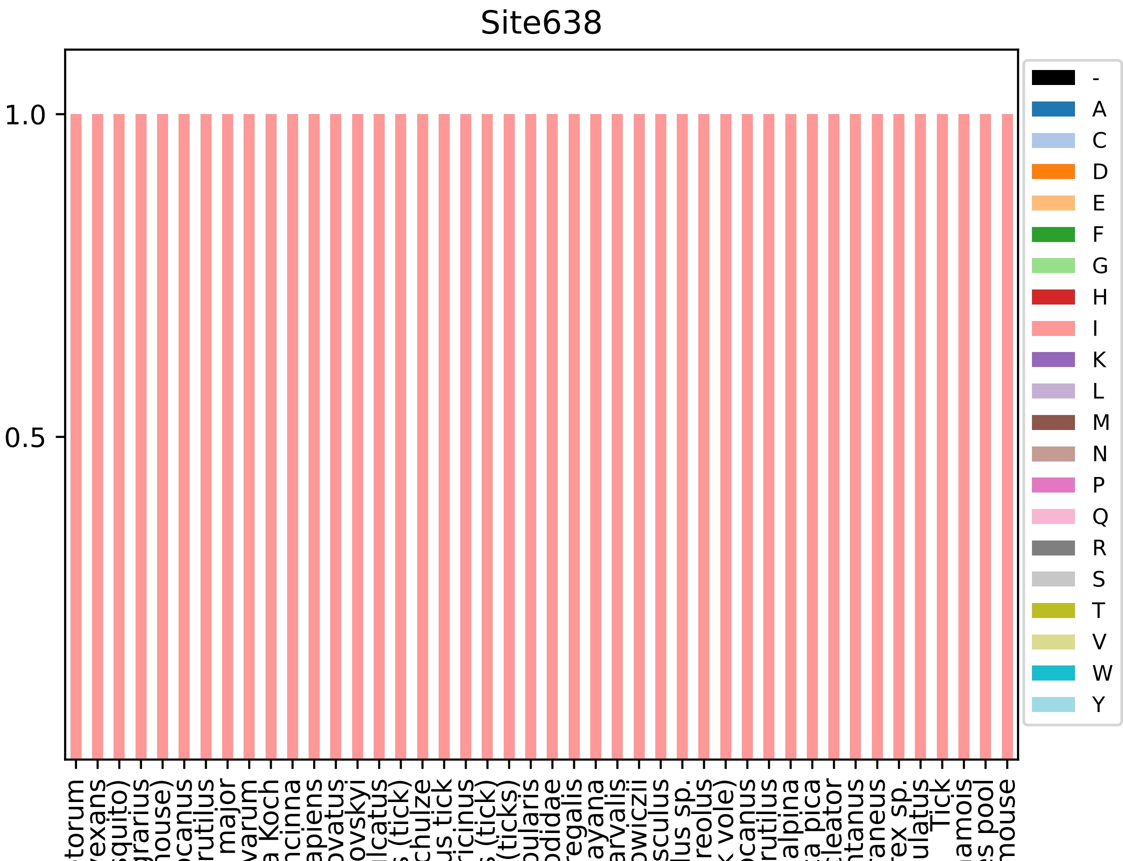
Site638
1.0
0.5
-
A
C
D
E
F
G
H
I
K
L
M
N
P
Q
R
S
T
V
W
Y

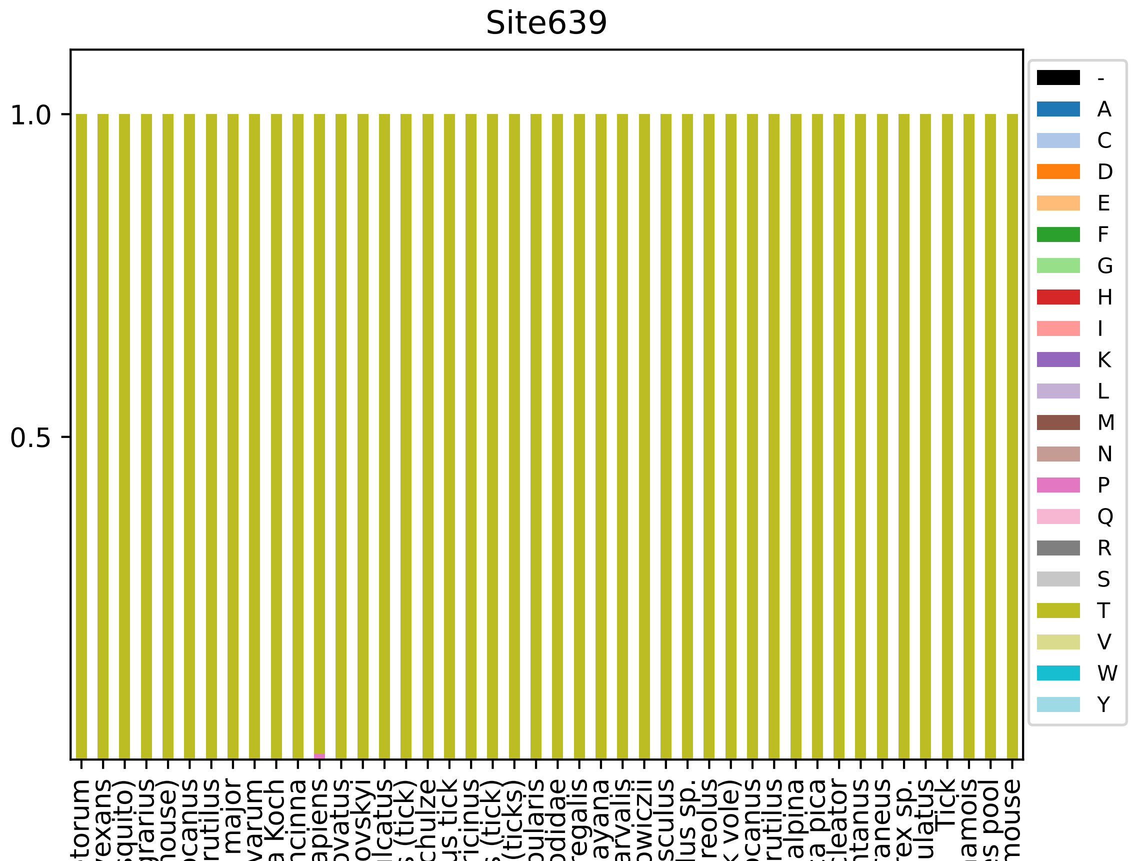
Site639
1.0
0.5
-
A
C
D
E
F
G
H
I
K
L
M
N
P
Q
R
S
T
V
W
Y

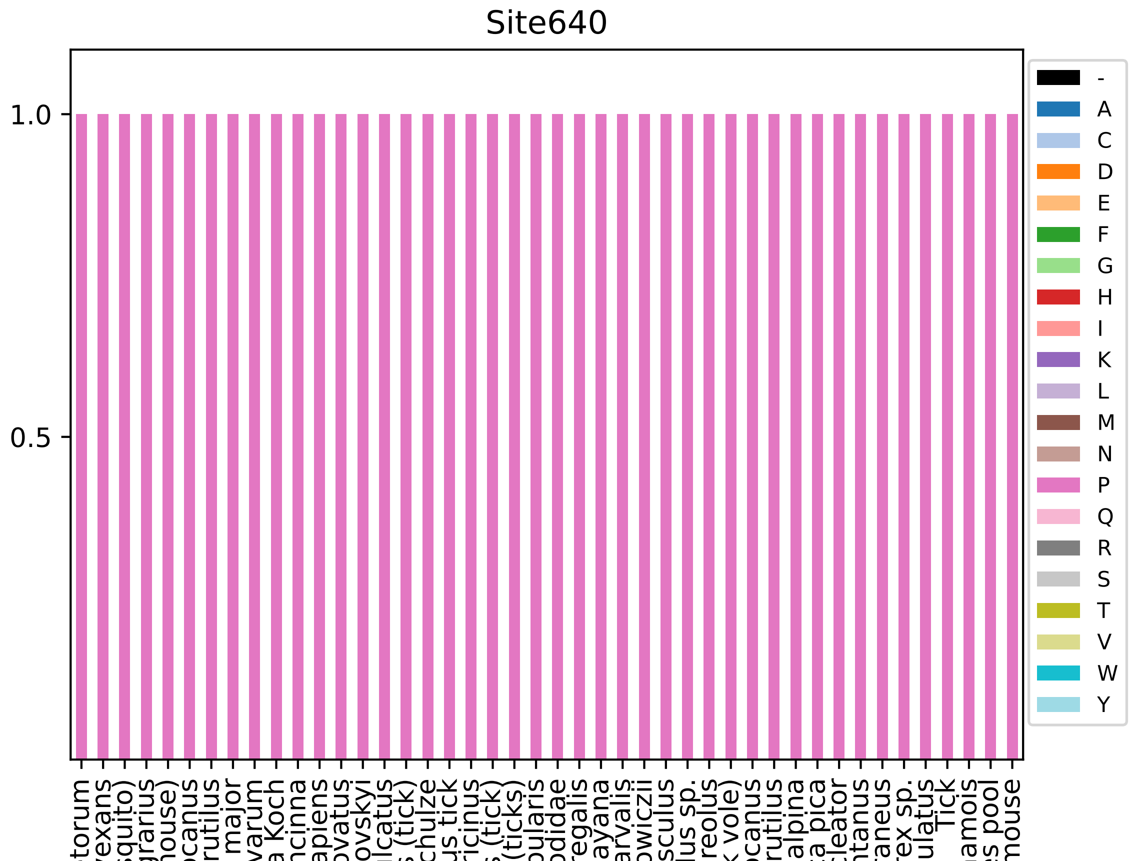

Site640
1.0
0.5
-
A
C
D
E
F
G
H
I
K
L
M
N
P
Q
R
S
T
V
W
Y

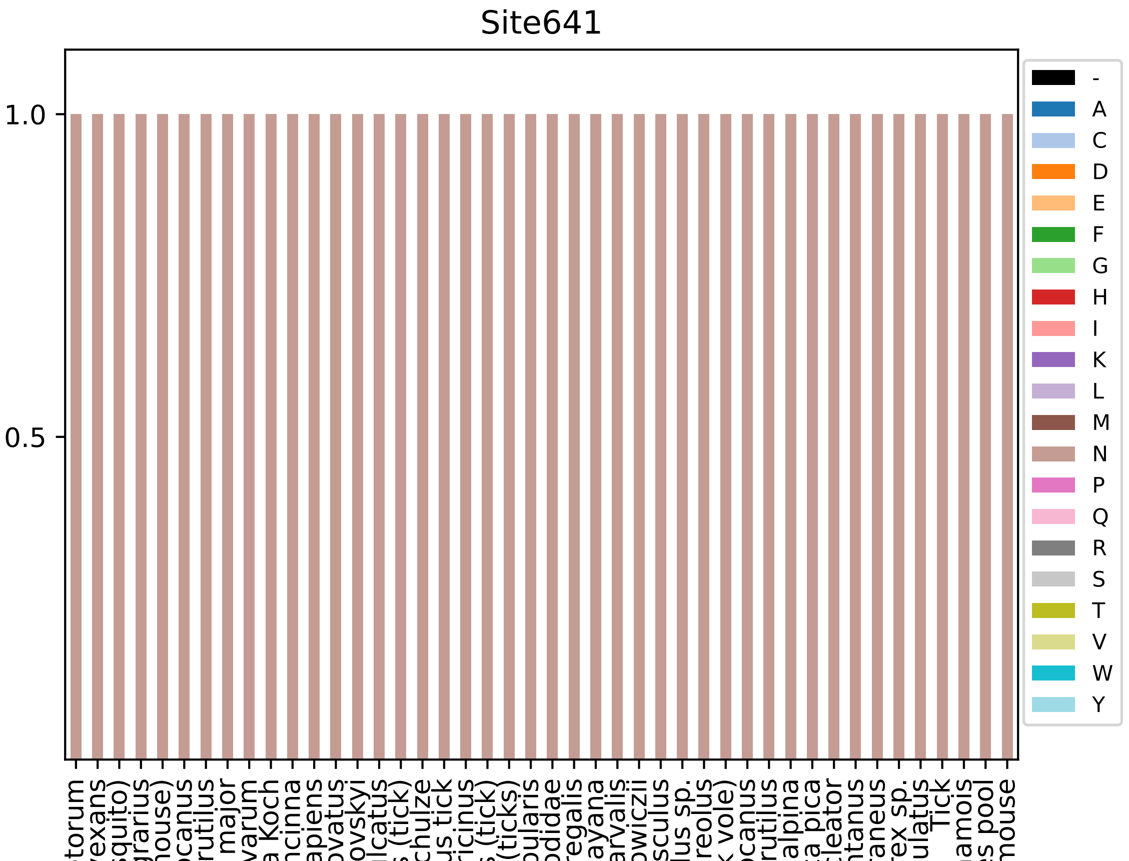
Site641
1.0
0.5
-
A
C
D
E
F
G
H
I
K
L
M
N
P
Q
R
S
T
V
W
Y

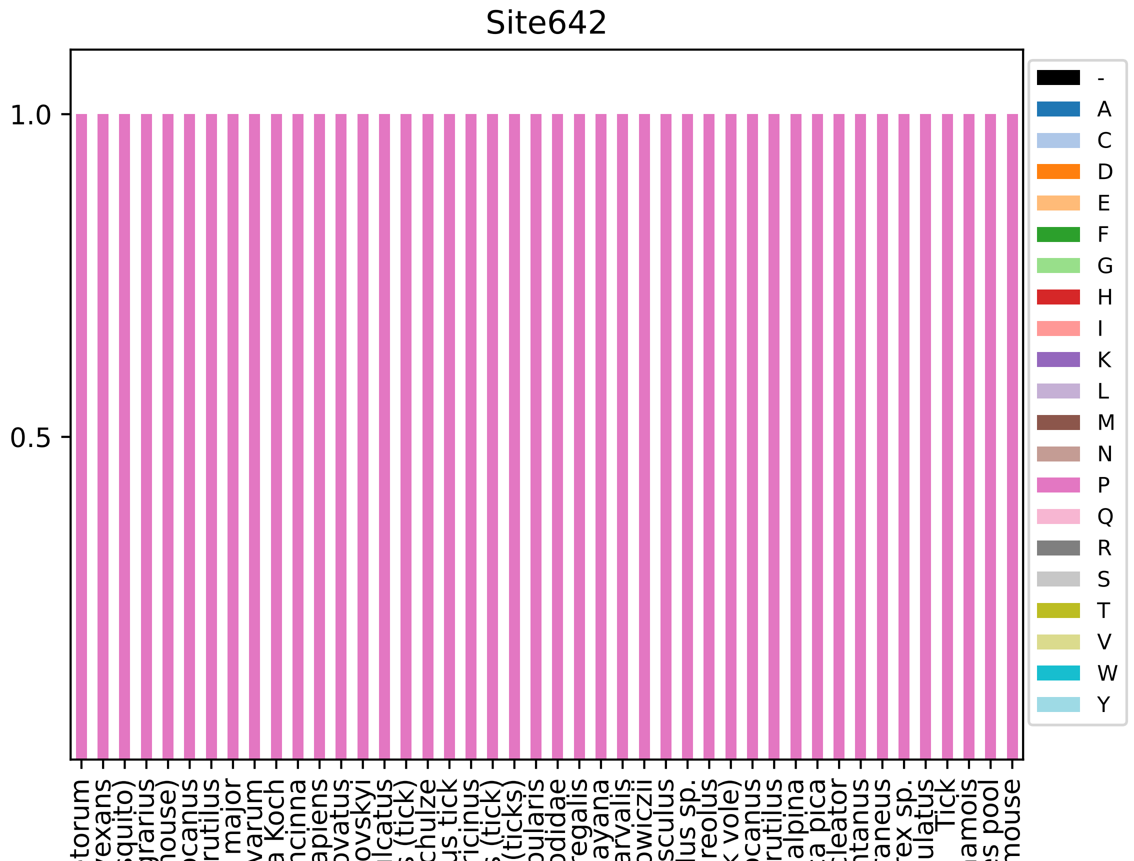

Site642
1.0
0.5
-
A
C
D
E
F
G
H
I
K
L
M
N
P
Q
R
S
T
V
W
Y

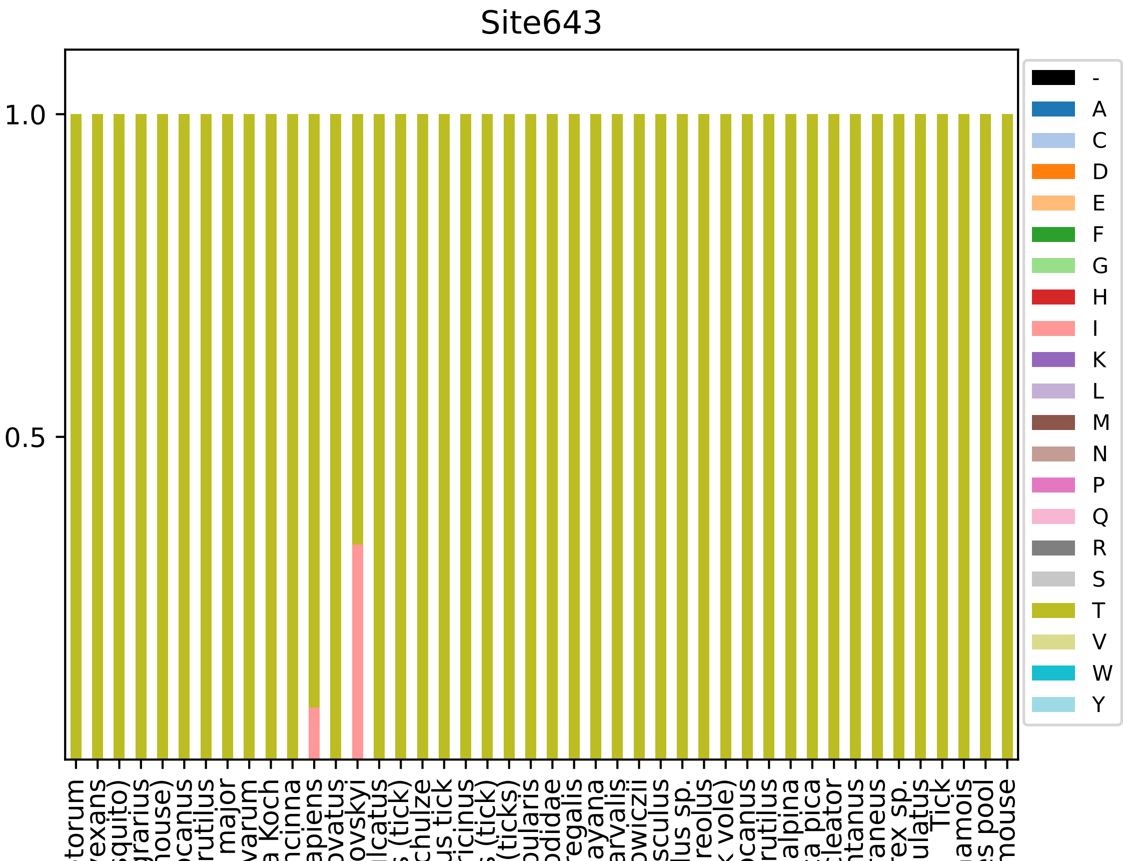
Site643
1.0
0.5
-
A
C
D
E
F
G
H
I
K
L
M
N
P
Q
R
S
T
V
W
Y

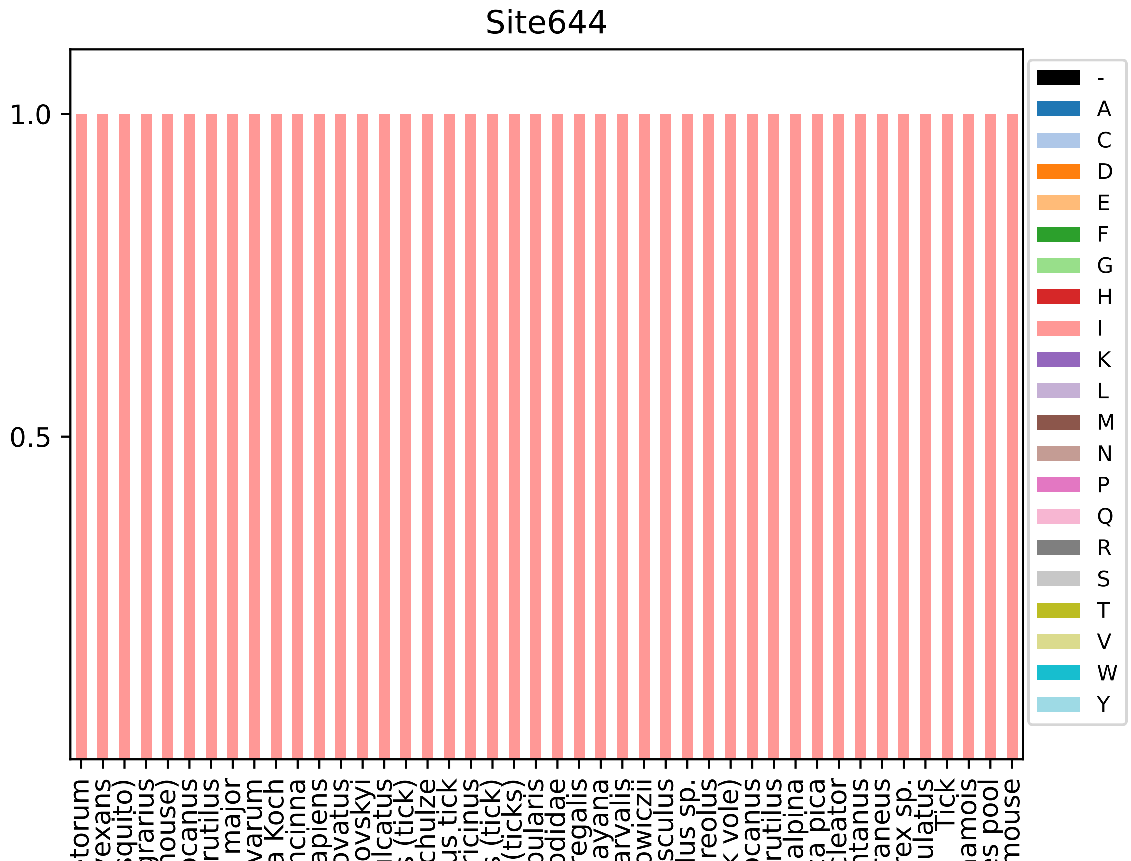
Site644
1.0
0.5
-
A
C
D
E
F
G
H
I
K
L
M
N
P
Q
R
S
T
V
W
Y

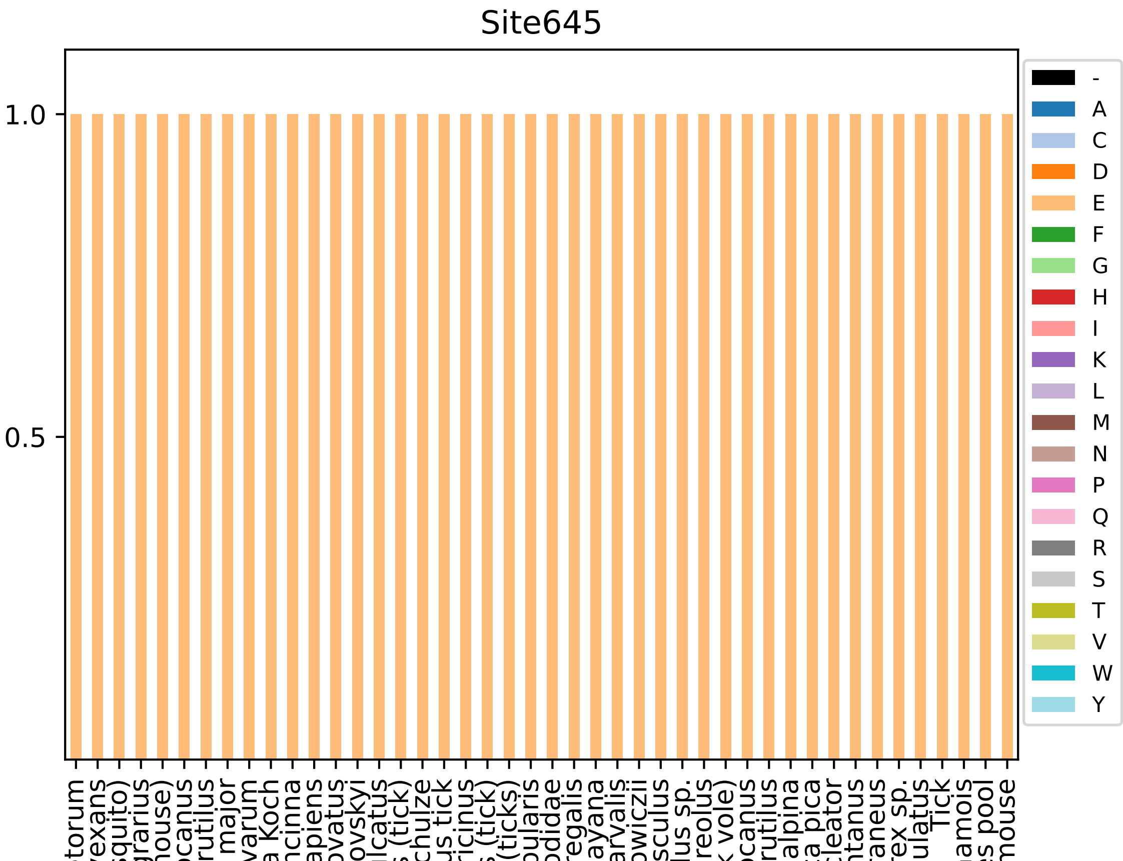

Site645
1.0
0.5
-
A
C
D
E
F
G
H
I
K
L
M
N
P
Q
R
S
T
V
W
Y

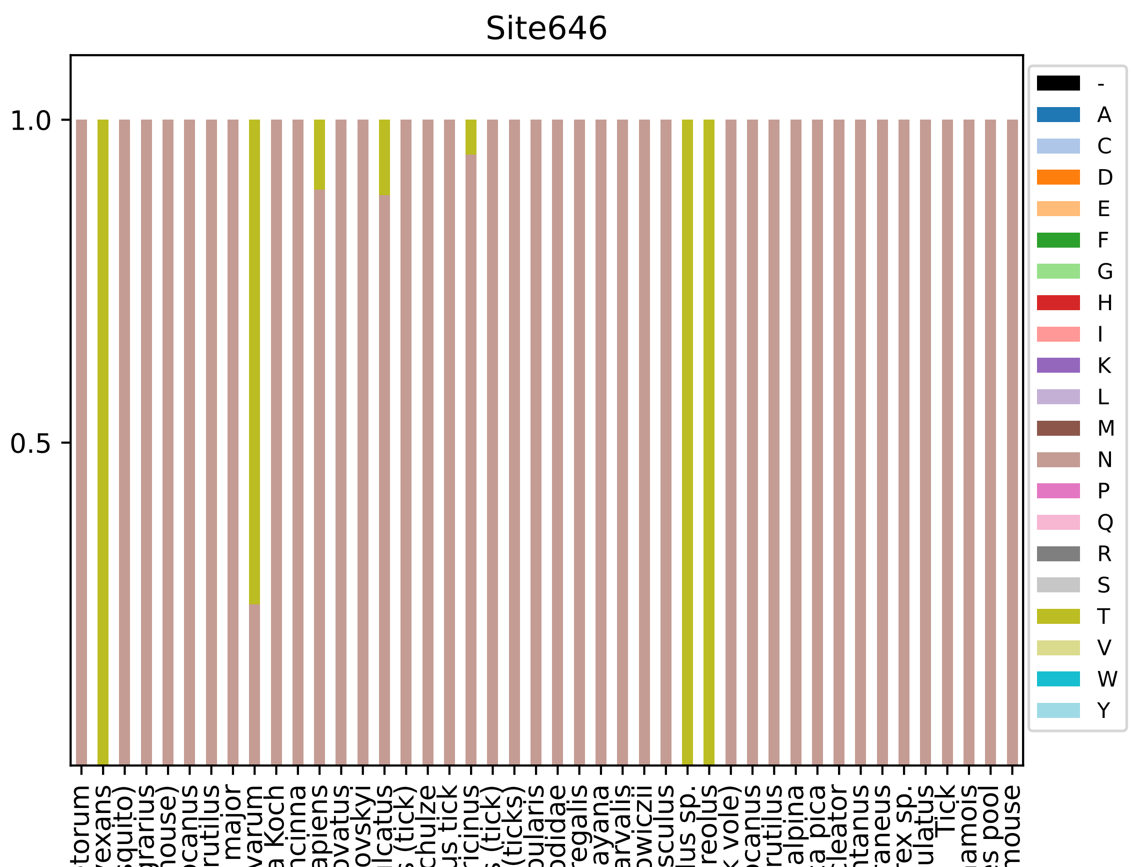

Site646
1.0
0.5
-
A
C
D
E
F
G
H
I
K
L
M
N
P
Q
R
S
T
V
W
Y

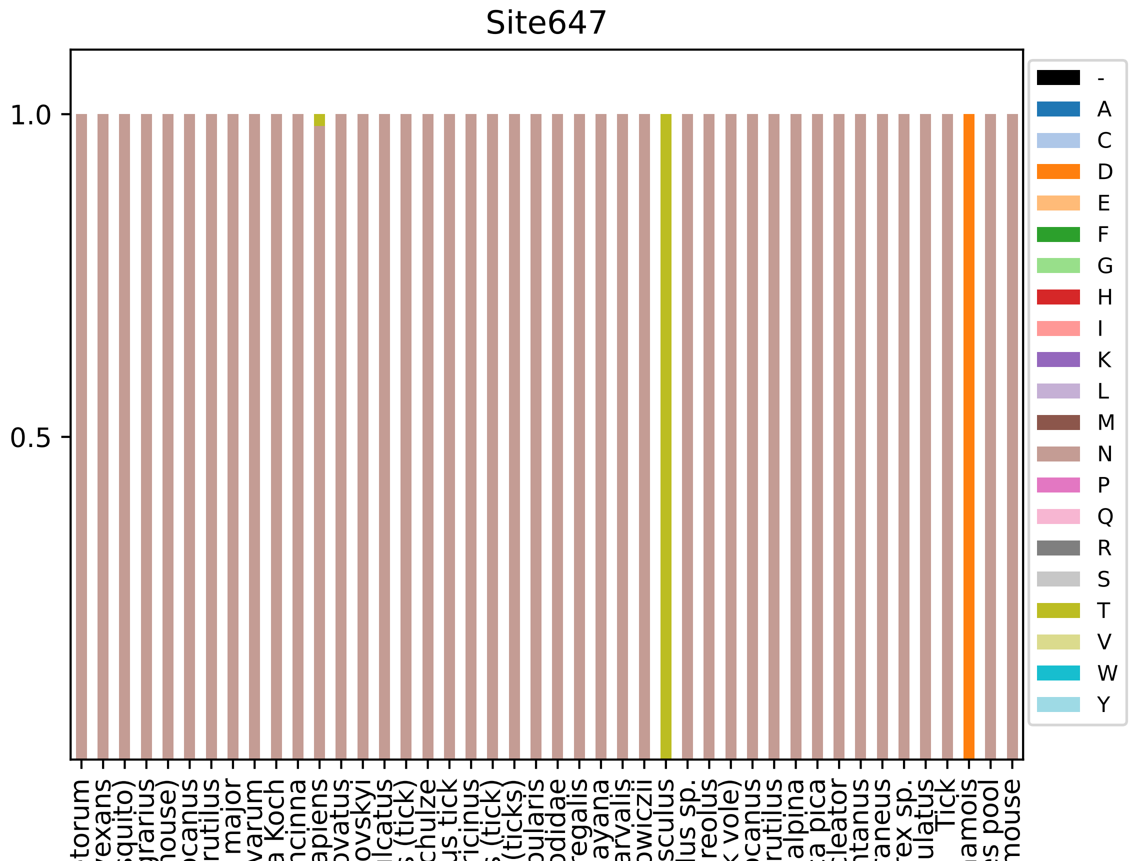

Site647
1.0
0.5
-
A
C
D
E
F
G
H
I
K
L
M
N
P
Q
R
S
T
V
W
Y

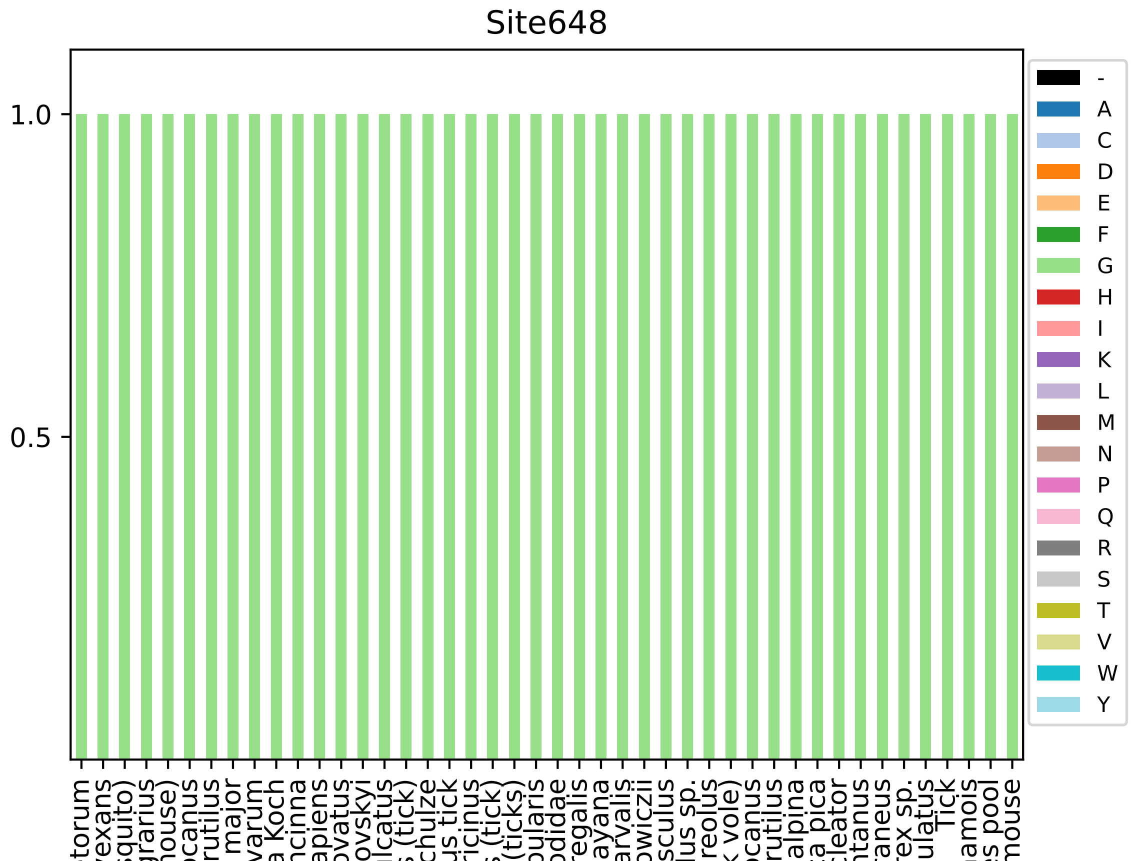
Site648
1.0
0.5
-
A
C
D
E
F
G
H
I
K
L
M
N
P
Q
R
S
T
V
W
Y

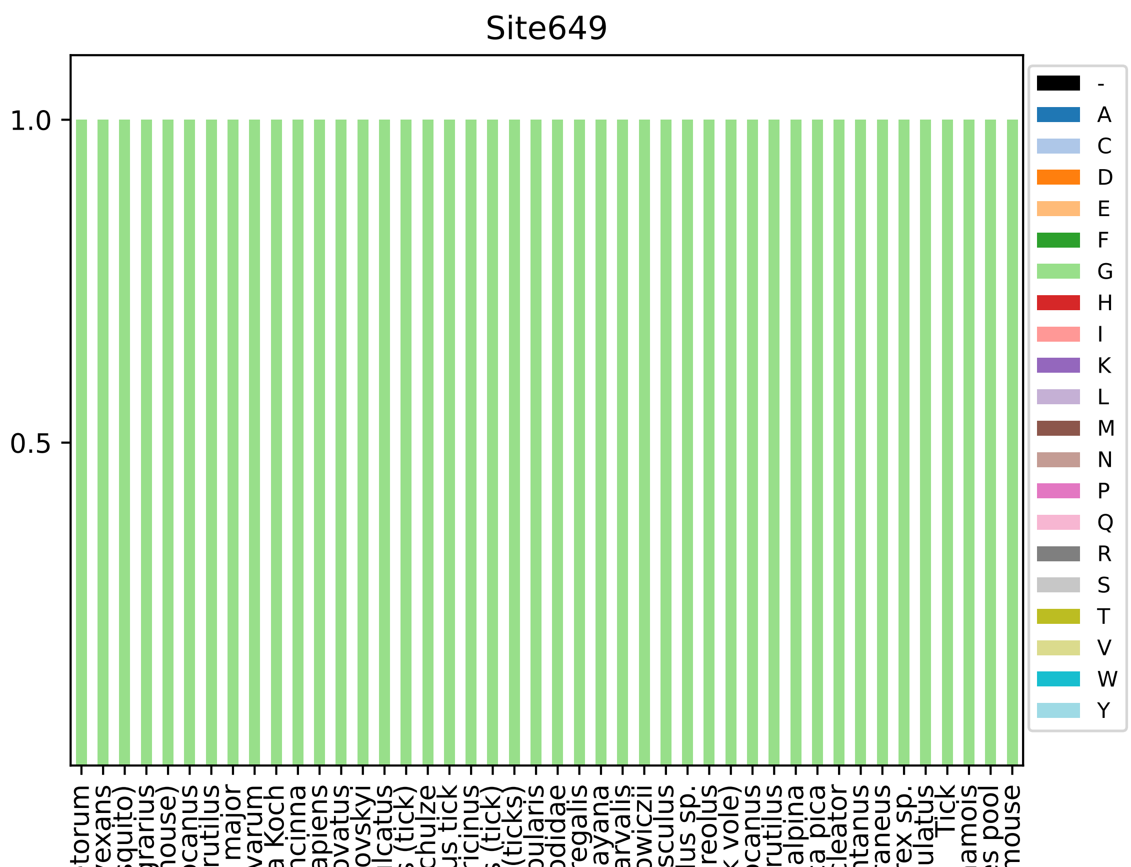
Site649
1.0
0.5
-
A
C
D
E
F
G
H
I
K
L
M
N
P
Q
R
S
T
V
W
Y

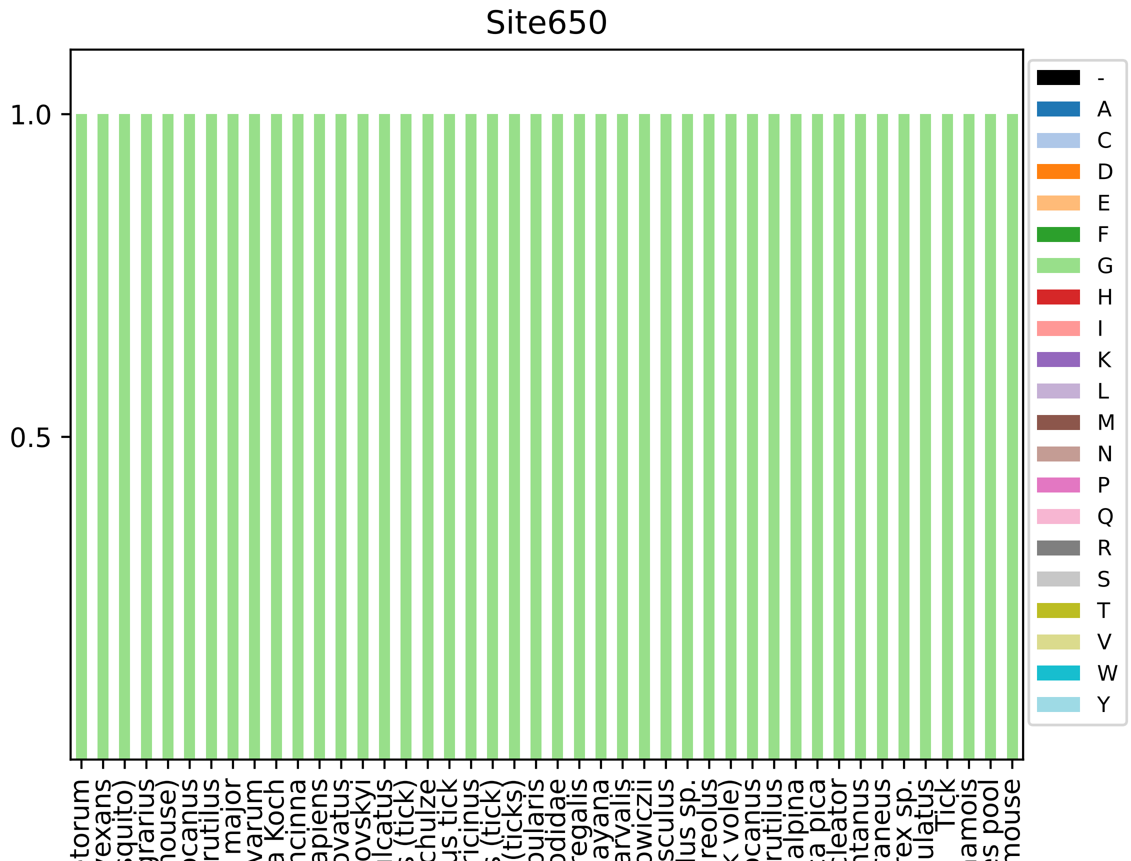
Site650
1.0
0.5
-
A
C
D
E
F
G
H
I
K
L
M
N
P
Q
R
S
T
V
W
Y

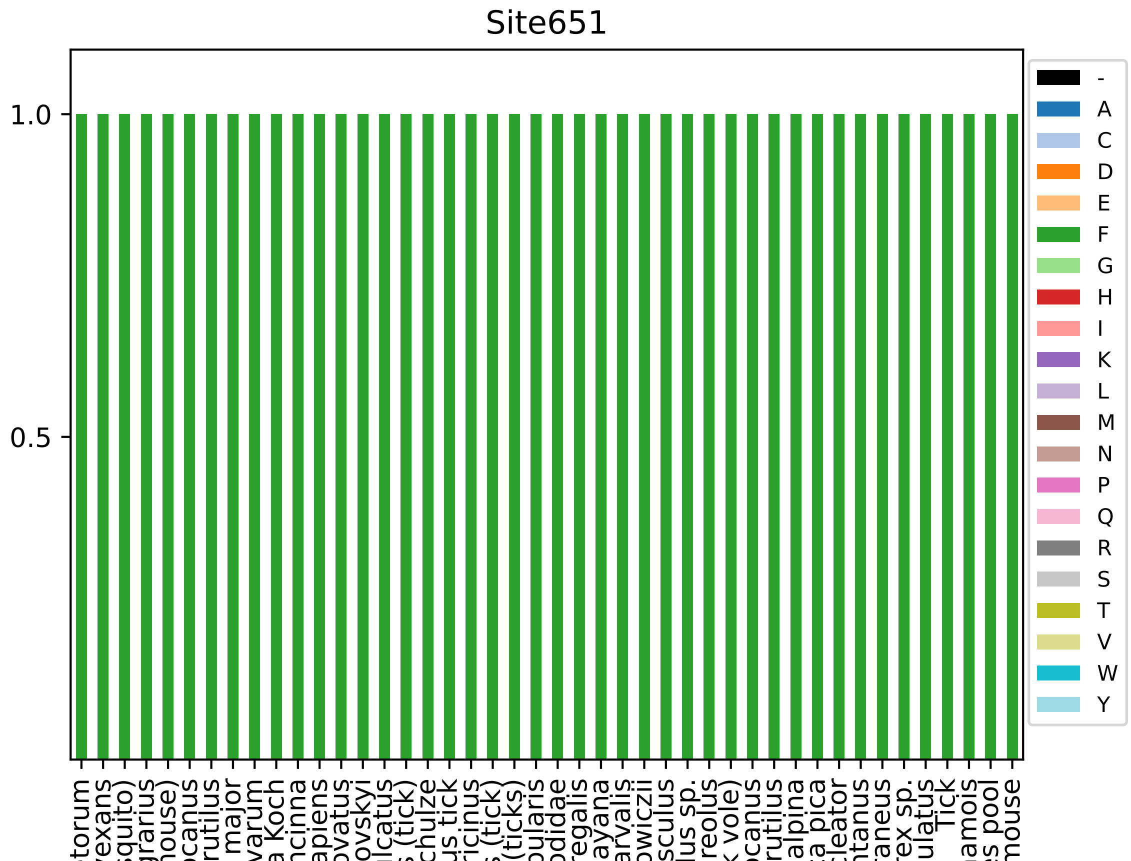
Site651
1.0
0.5
-
A
C
D
E
F
G
H
I
K
L
M
N
P
Q
R
S
T
V
W
Y

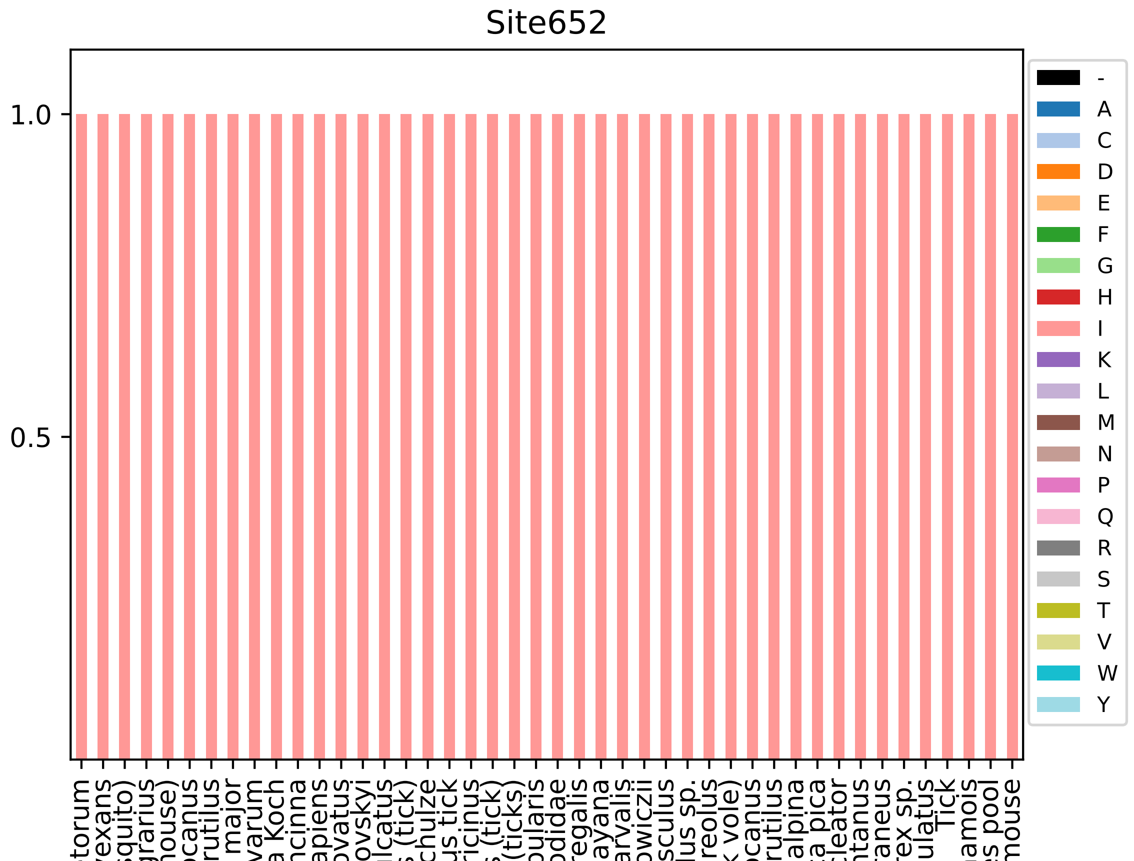

Site652
1.0
0.5
-
A
C
D
E
F
G
H
I
K
L
M
N
P
Q
R
S
T
V
W
Y

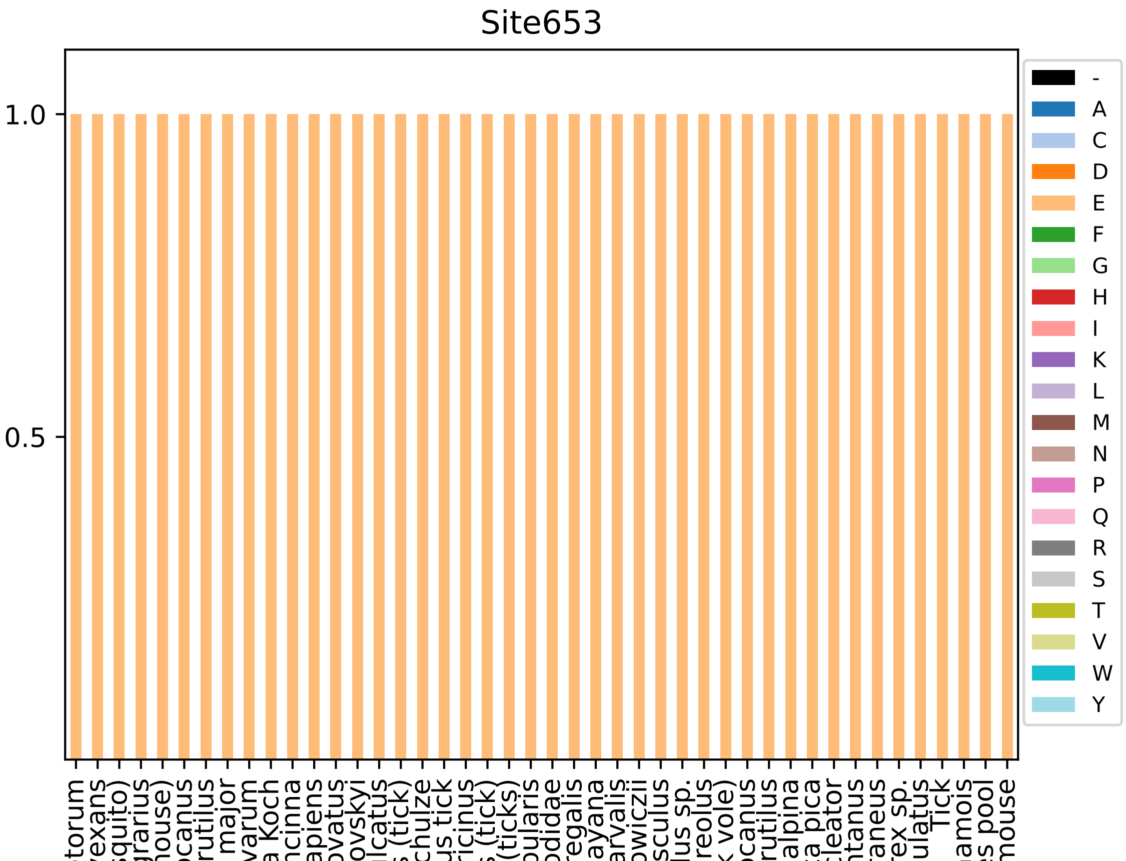
Site653
1.0
0.5
-
A
C
D
E
F
G
H
I
K
L
M
N
P
Q
R
S
T
V
W
Y

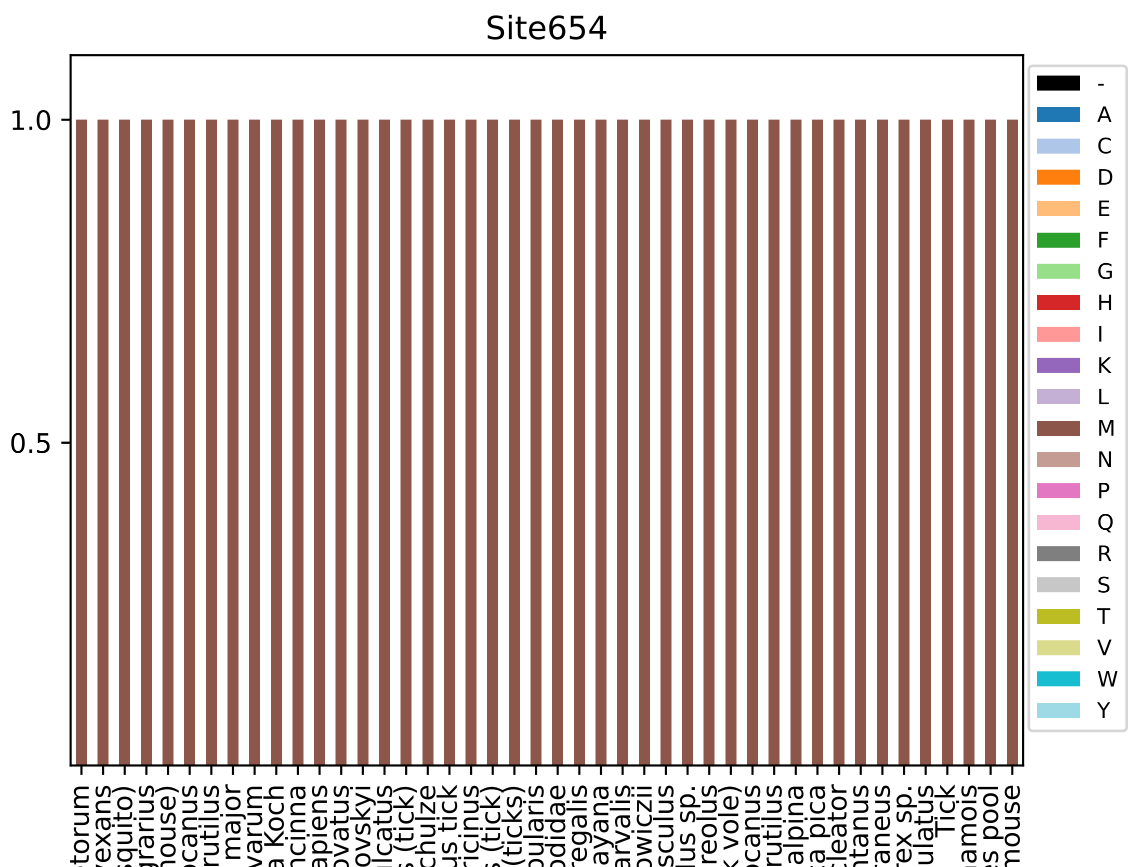
Site654
1.0
0.5
-
A
C
D
E
F
G
H
I
K
L
M
N
P
Q
R
S
T
V
W
Y
Tick

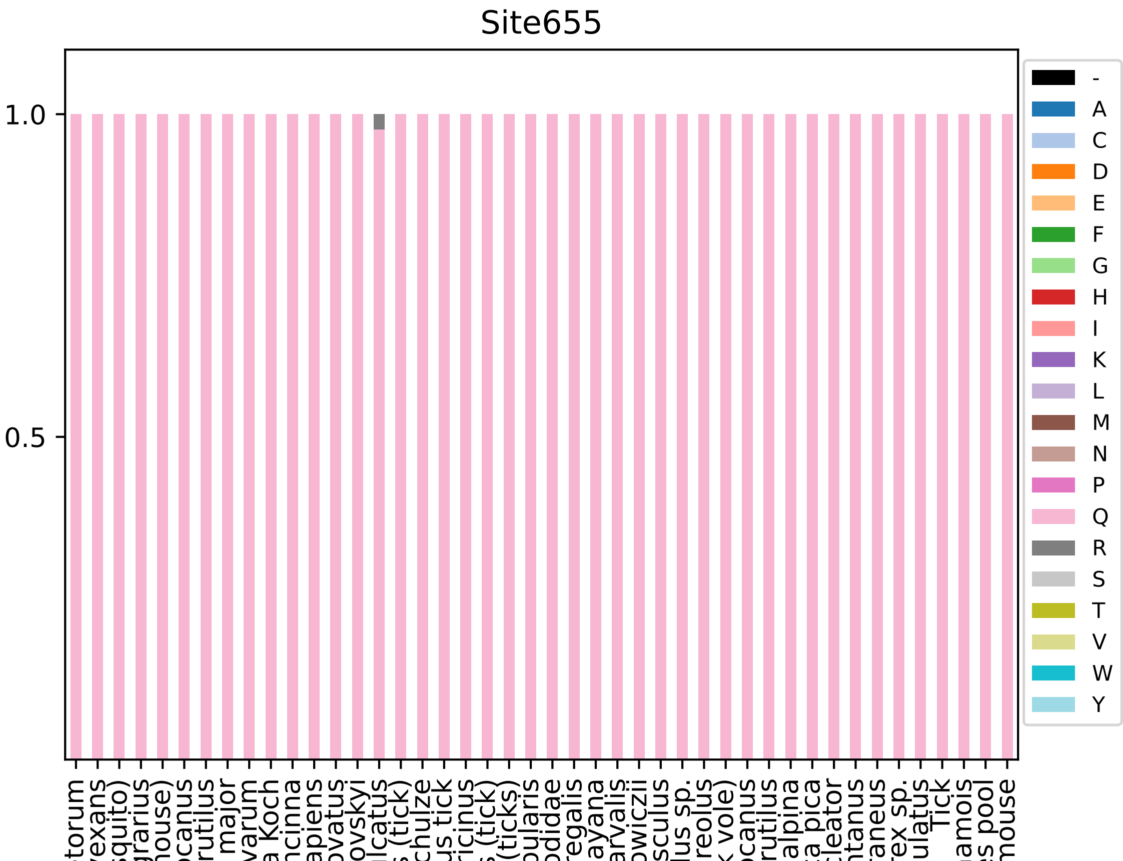
Site655
1.0
0.5
-
A
C
D
E
F
G
H
I
K
L
M
N
P
Q
R
S
T
V
W
Y

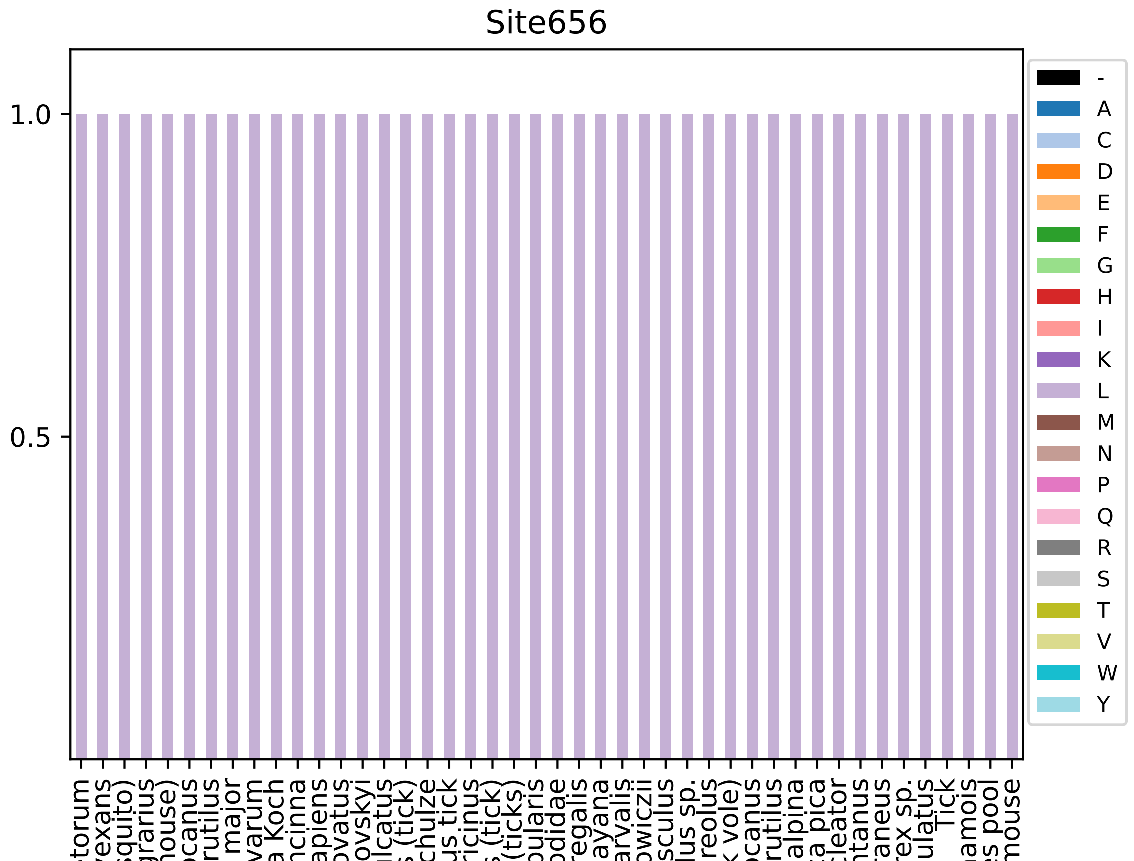

Site656
1.0
0.5
-
A
C
D
E
F
G
H
I
K
L
M
N
P
Q
R
S
T
V
W
Y

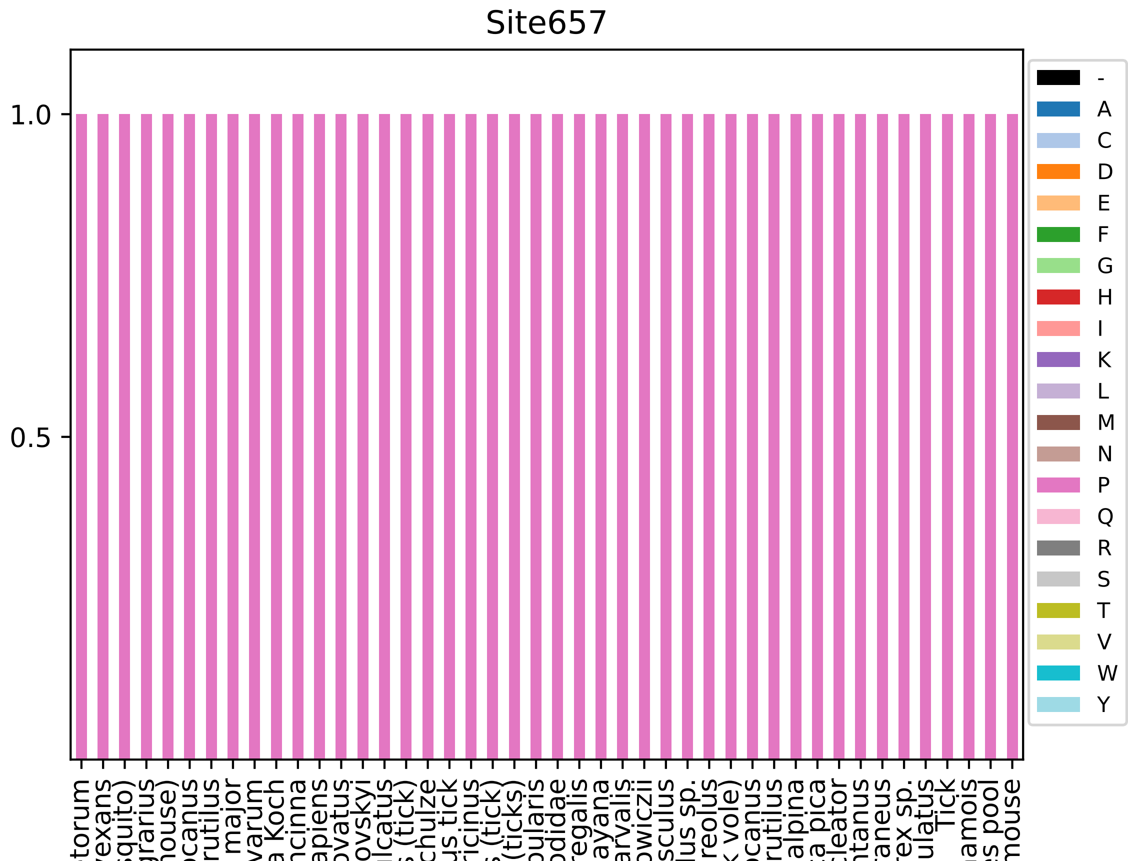
Site657
1.0
0.5
-
A
C
D
E
F
G
H
I
K
L
M
N
P
Q
R
S
T
V
W
Y

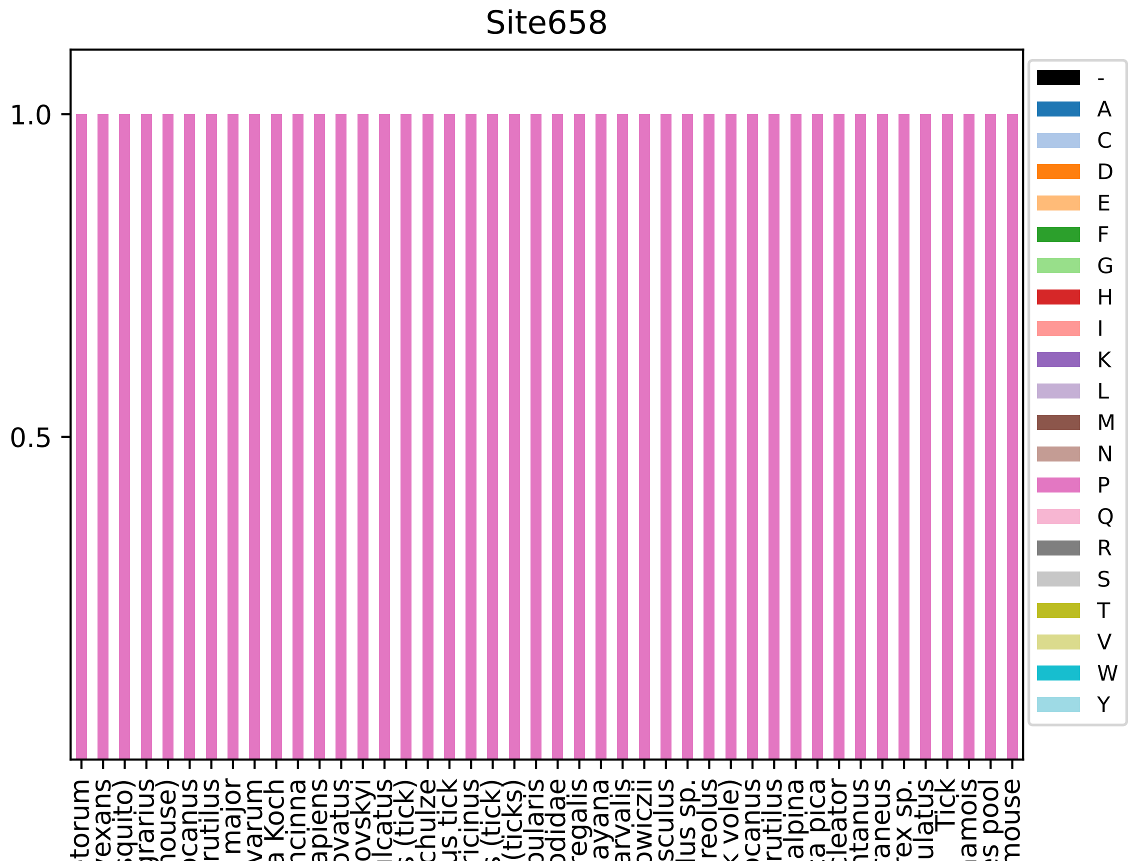
Site658
1.0
0.5
-
A
C
D
E
F
G
H
I
K
L
M
N
P
Q
R
S
T
V
W
Y

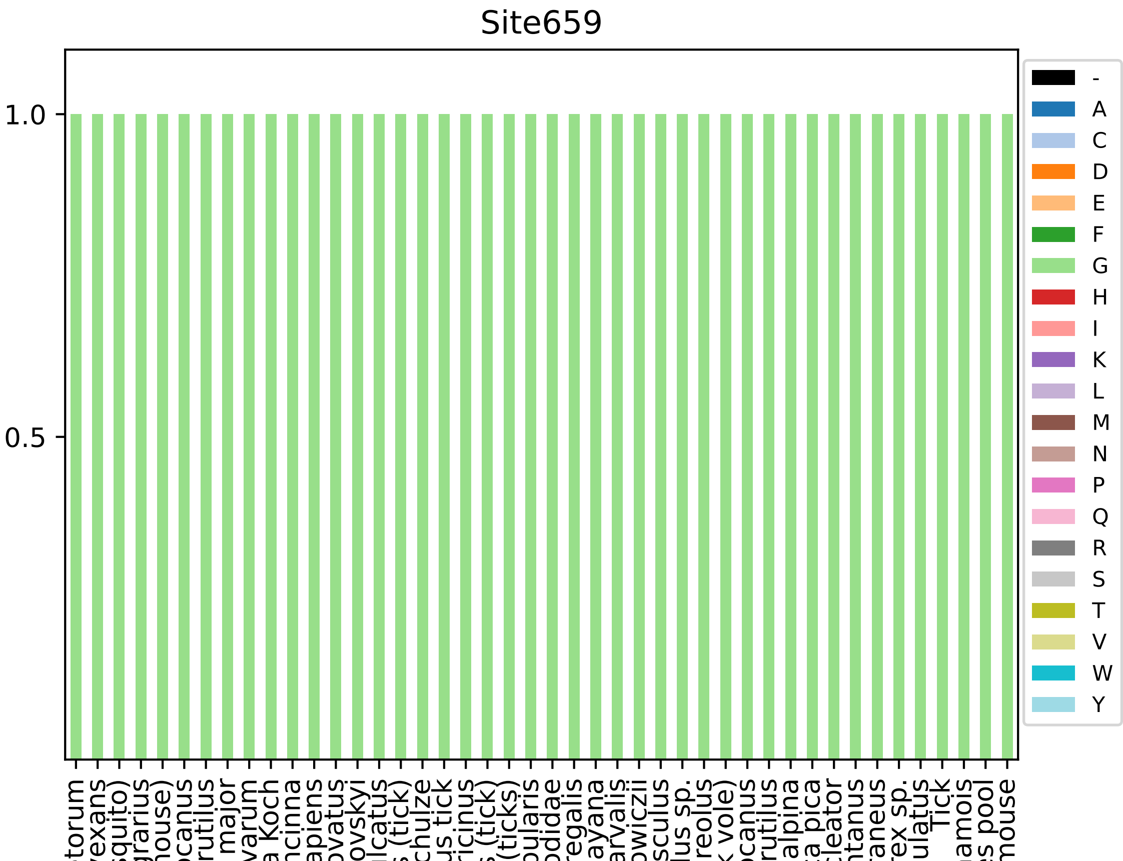

Site659
1.0
0.5
-
A
C
D
E
F
G
H
I
K
L
M
N
P
Q
R
S
T
V
W
Y

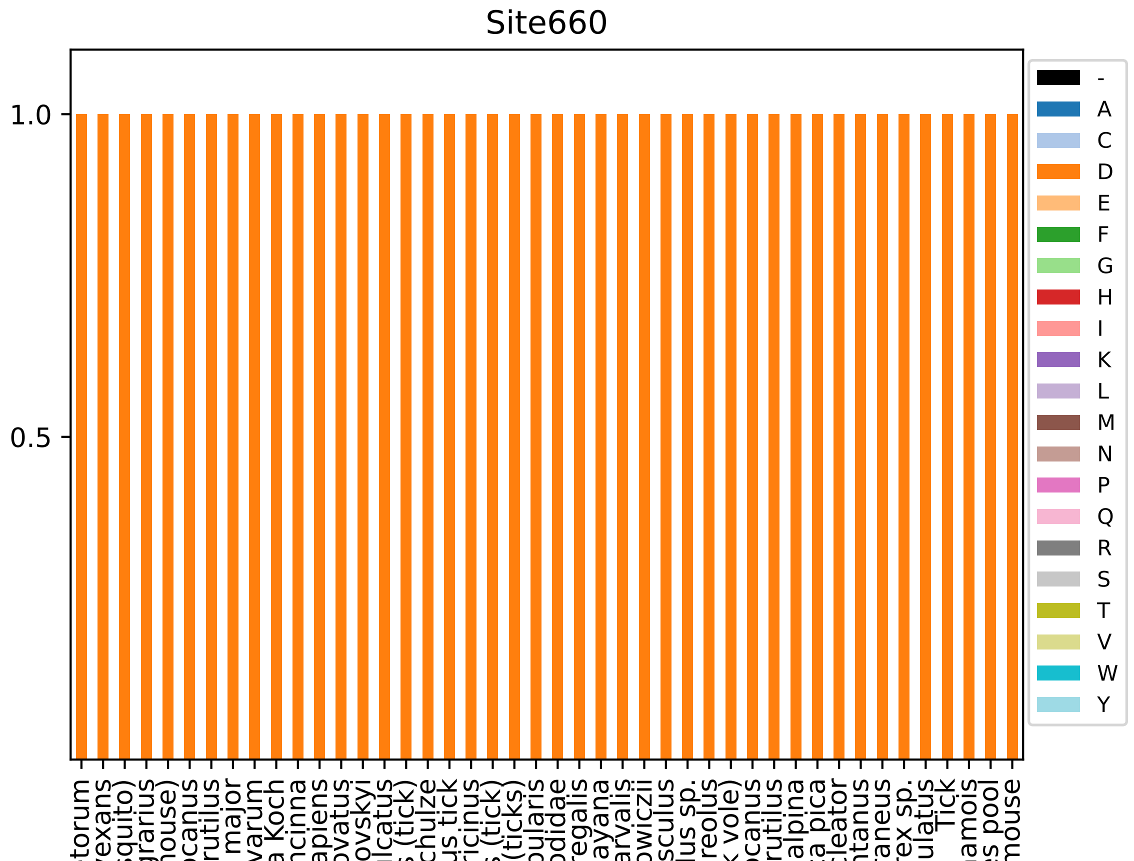

Site660
1.0
0.5
-
A
C
D
E
F
G
H
I
K
L
M
N
P
Q
R
S
T
V
W
Y

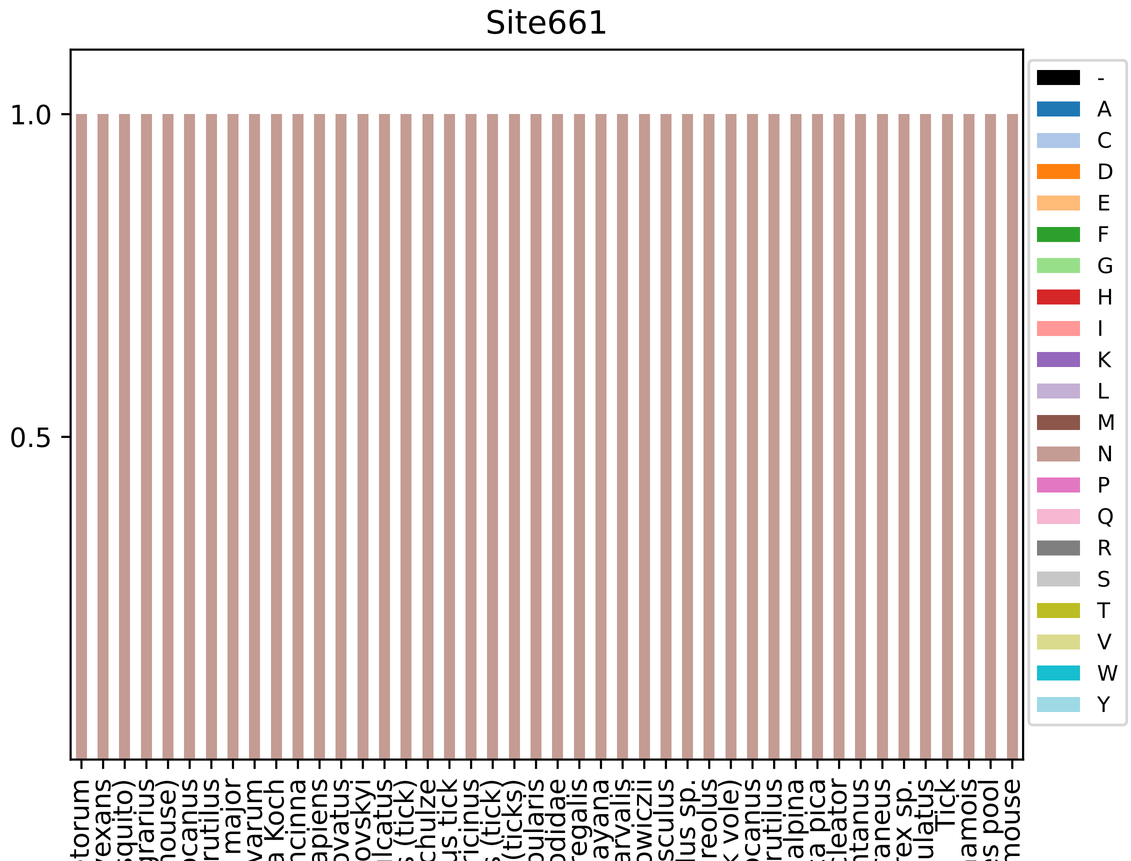
Site661
1.0
0.5
-
A
C
D
E
F
G
H
I
K
L
M
N
P
Q
R
S
T
V
W
Y

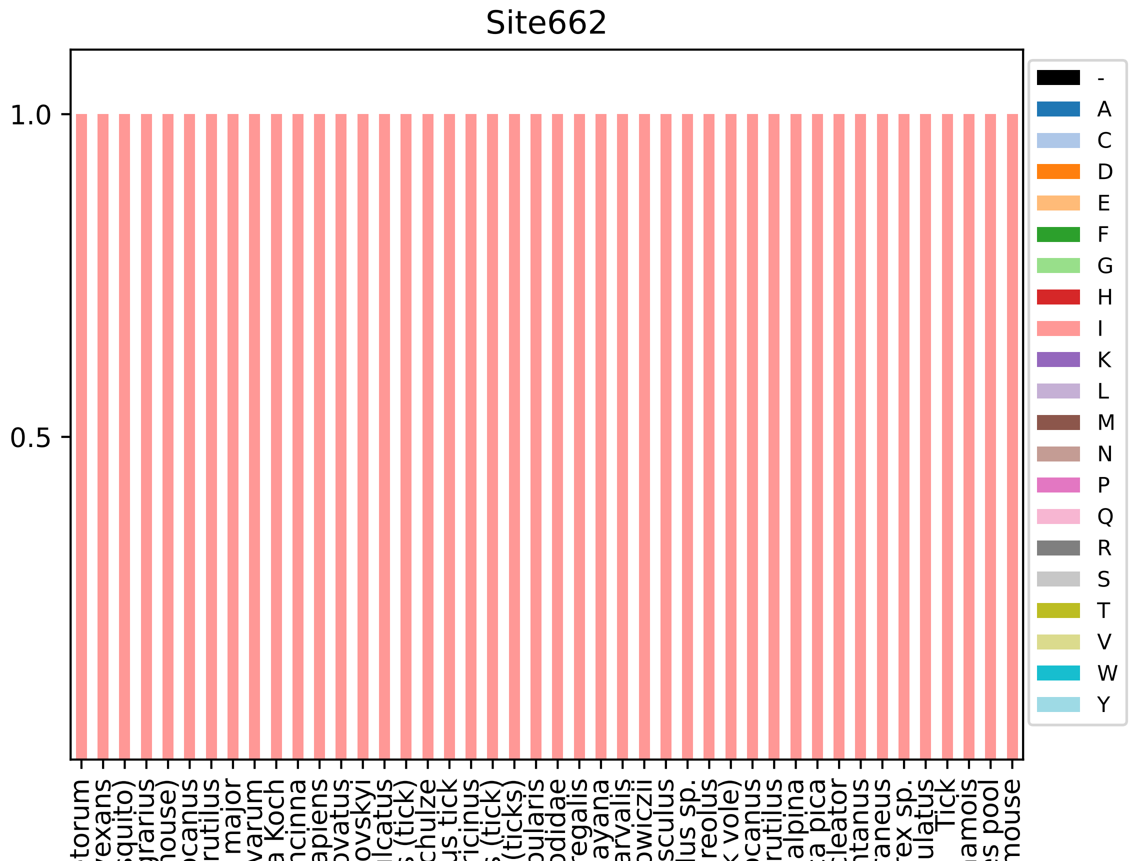

Site662
1.0
0.5
-
A
C
D
E
F
G
H
I
K
L
M
N
P
Q
R
S
T
V
W
Y

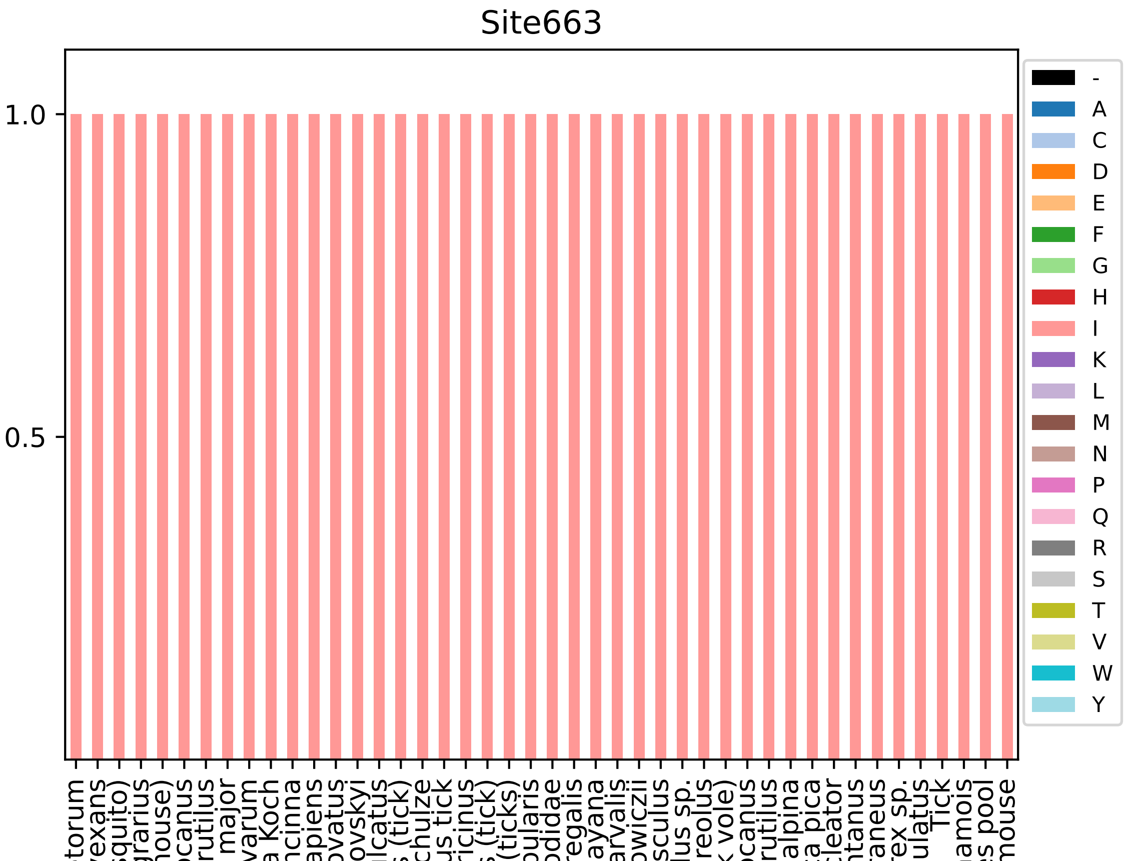
Site663
1.0
0.5
-
A
C
D
E
F
G
H
I
K
L
M
N
P
Q
R
S
T
V
W
Y

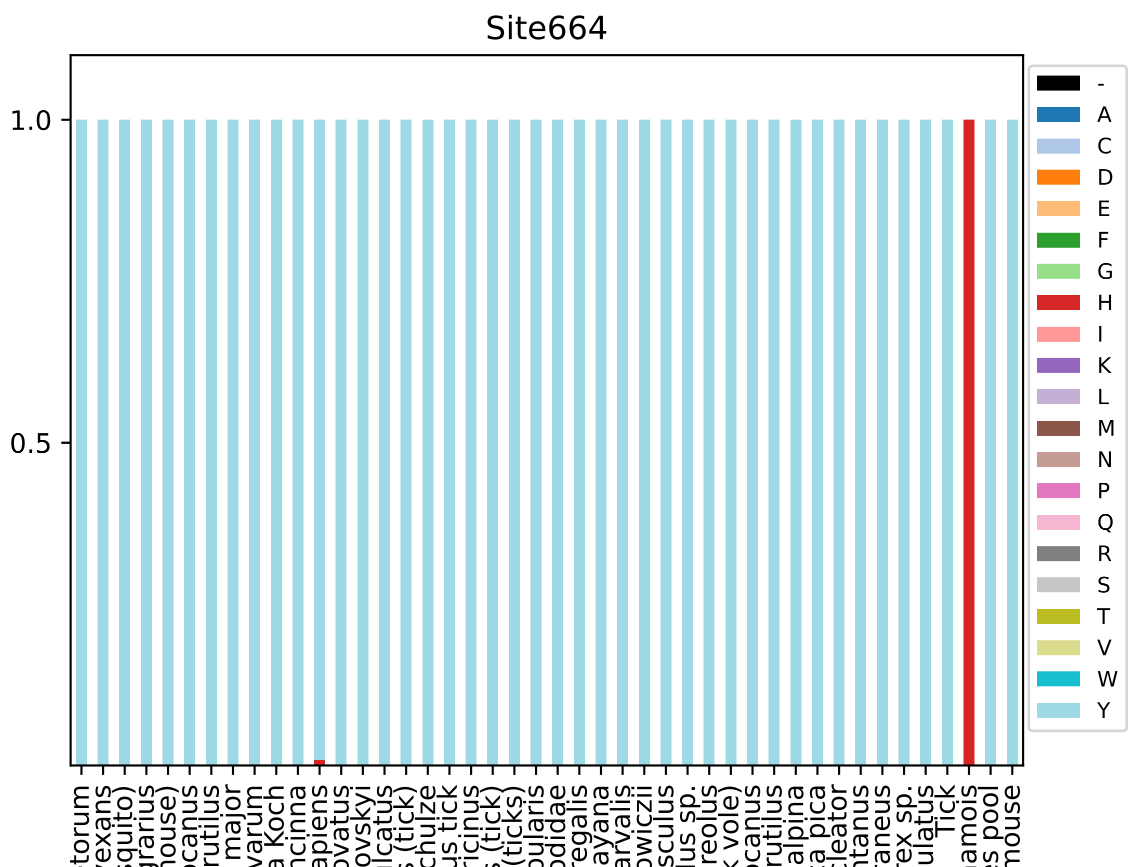
Site664
1.0
0.5
-
A
C
D
E
F
G
H
I
K
L
M
N
P
Q
R
S
T
V
W
Y
torum
vexans
squito)
garius
nouse)
ocanus
rutilus
major
varum
a Koch
ncinna
apiens
ovatus
ovskyi
ulcatus
s (tick)
chulze
us tick
ricinus
s (tick)
(ticks)
pularis
podidae
regalis
ayana
arvalis
owiczii
sculus
lus sp.
reolus
k vole)
ocanus
rutilus
alpina
a pica
cleator
ntanus
raneus
rex sp.
ulatus
Tick
amois
es pool
mouse

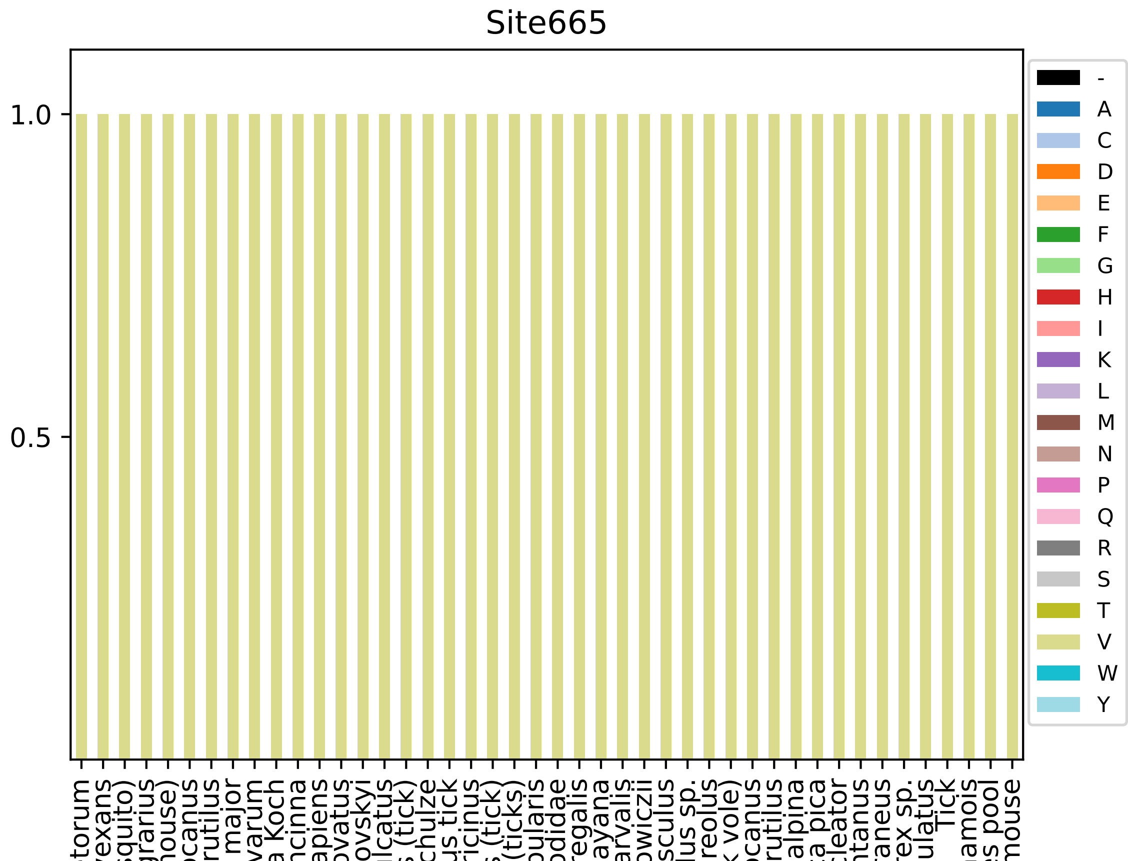

Site665
1.0
0.5
-
A
C
D
E
F
G
H
I
K
L
M
N
P
Q
R
S
T
V
W
Y

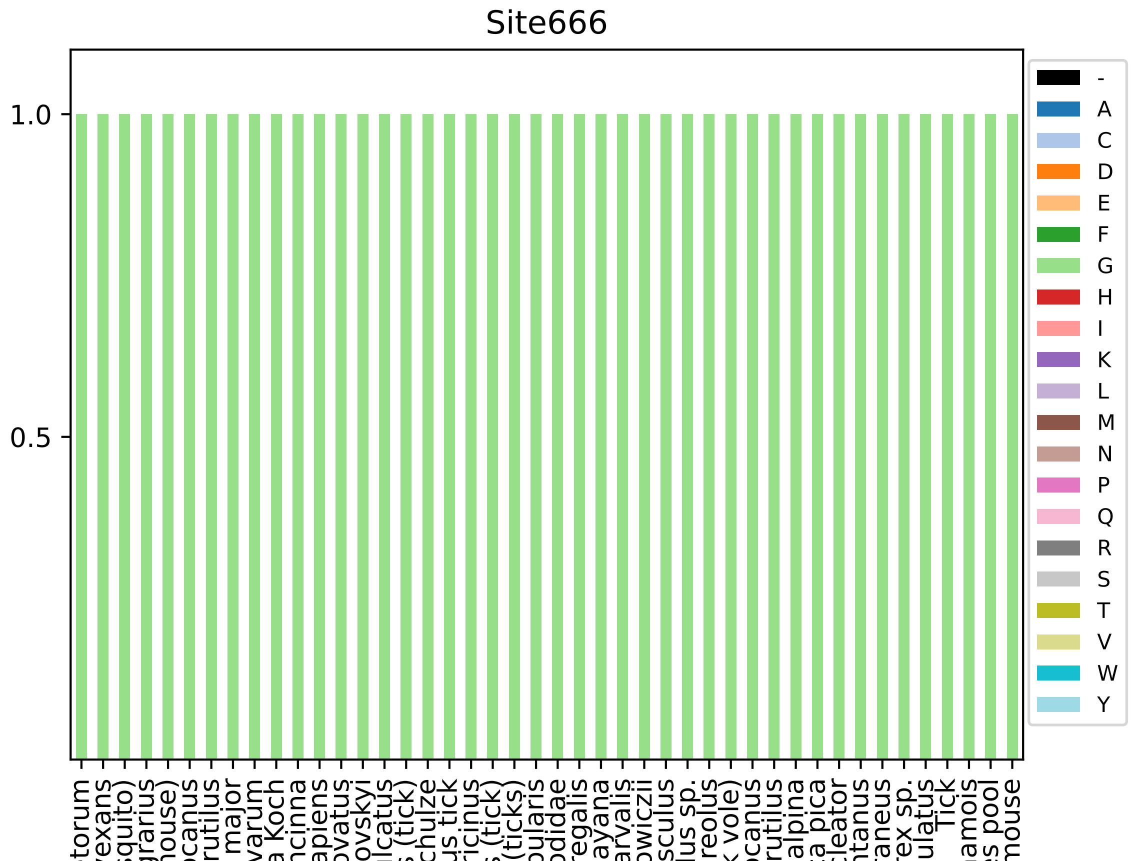
Site666
1.0
0.5
-
A
C
D
E
F
G
H
I
K
L
M
N
P
Q
R
S
T
V
W
Y

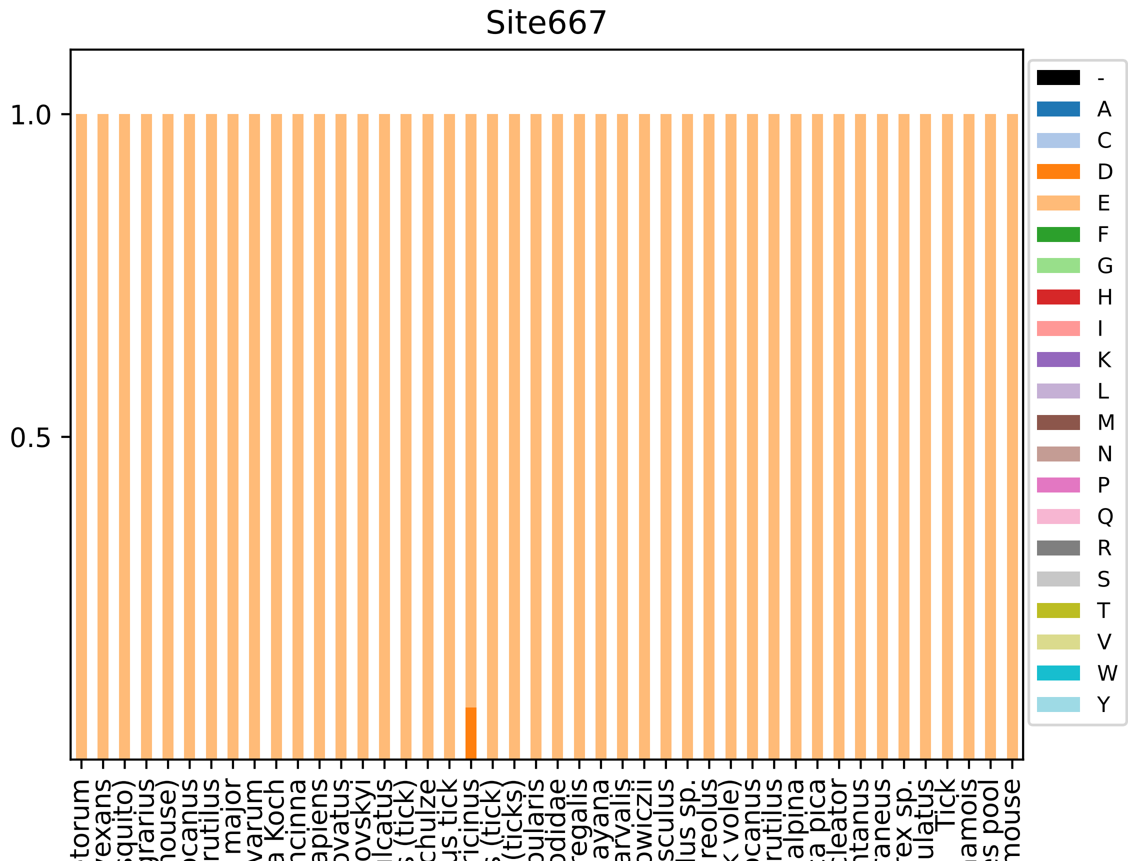
Site667
1.0
0.5
-
A
C
D
E
F
G
H
I
K
L
M
N
P
Q
R
S
T
V
W
Y

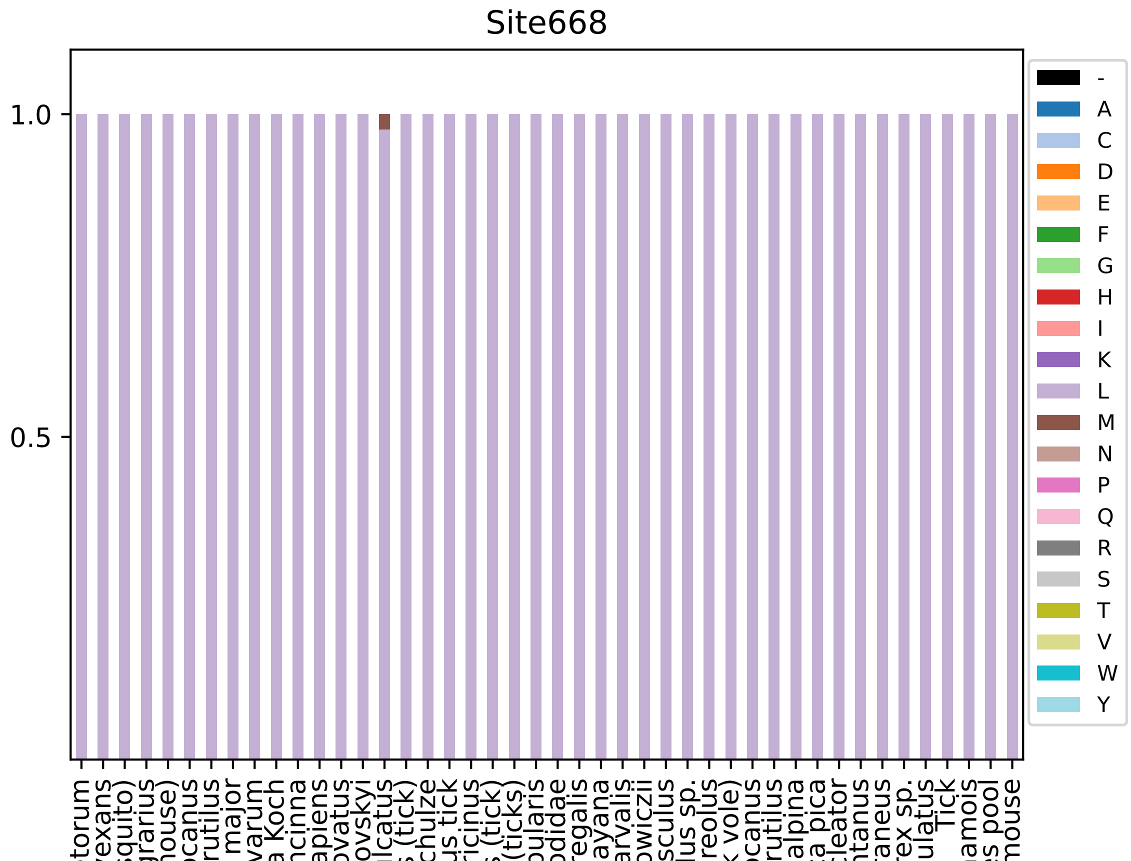
Site668
1.0
0.5
-
A
C
D
E
F
G
H
I
K
L
M
N
P
Q
R
S
T
V
W
Y

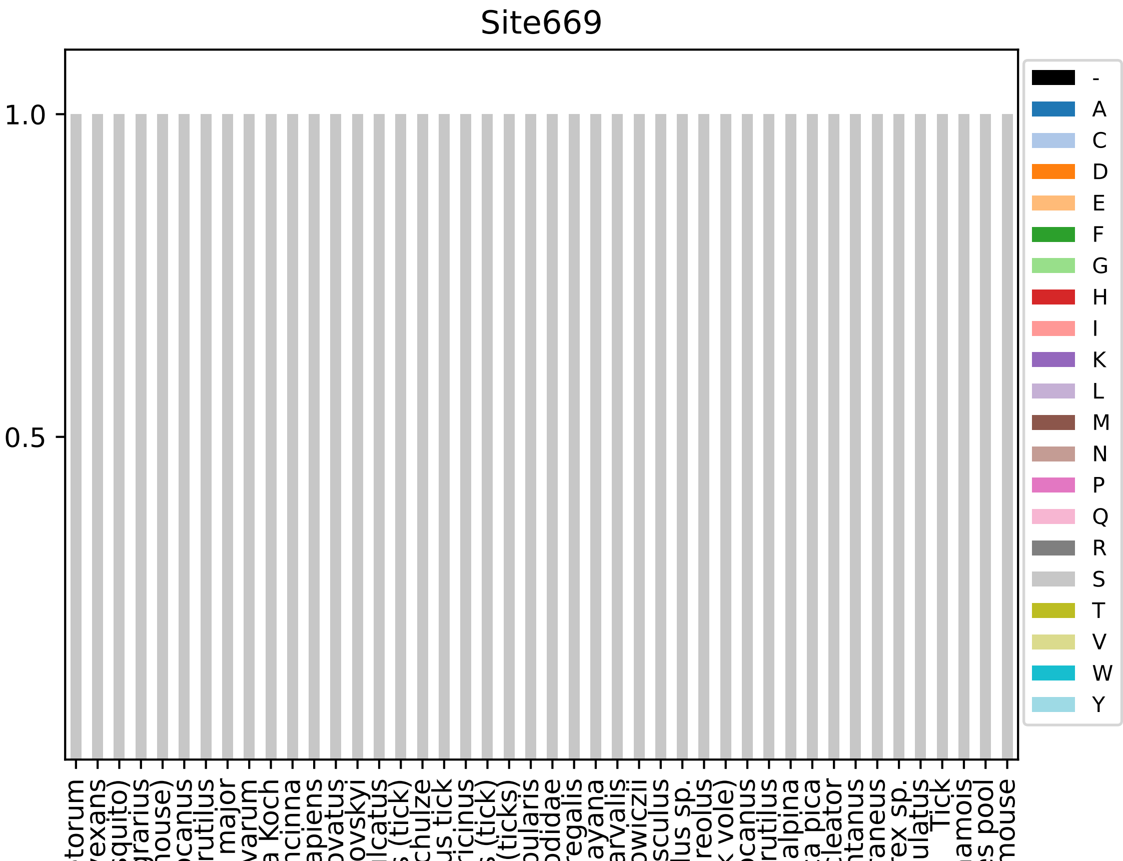
Site669
1.0
0.5
-
A
C
D
E
F
G
H
I
K
L
M
N
P
Q
R
S
T
V
W
Y

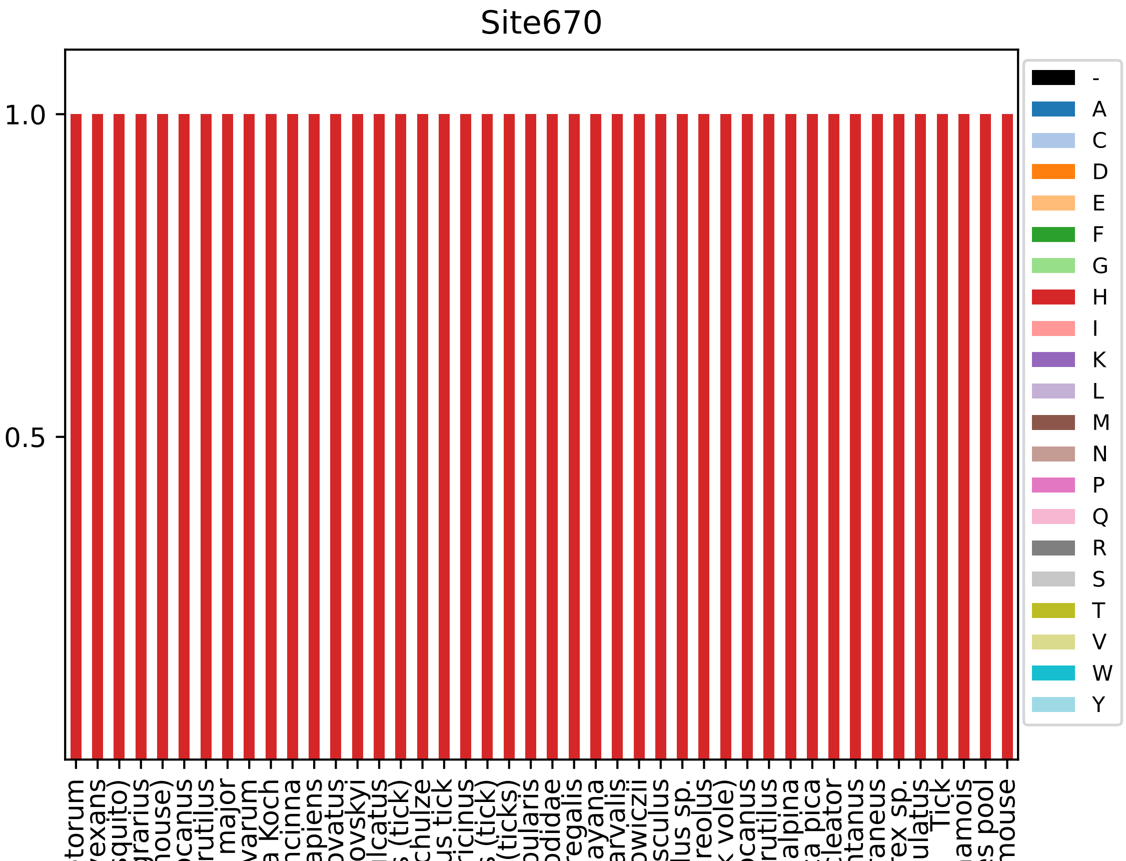
Site670
1.0
0.5
-
A
C
D
E
F
G
H
I
K
L
M
N
P
Q
R
S
T
V
W
Y

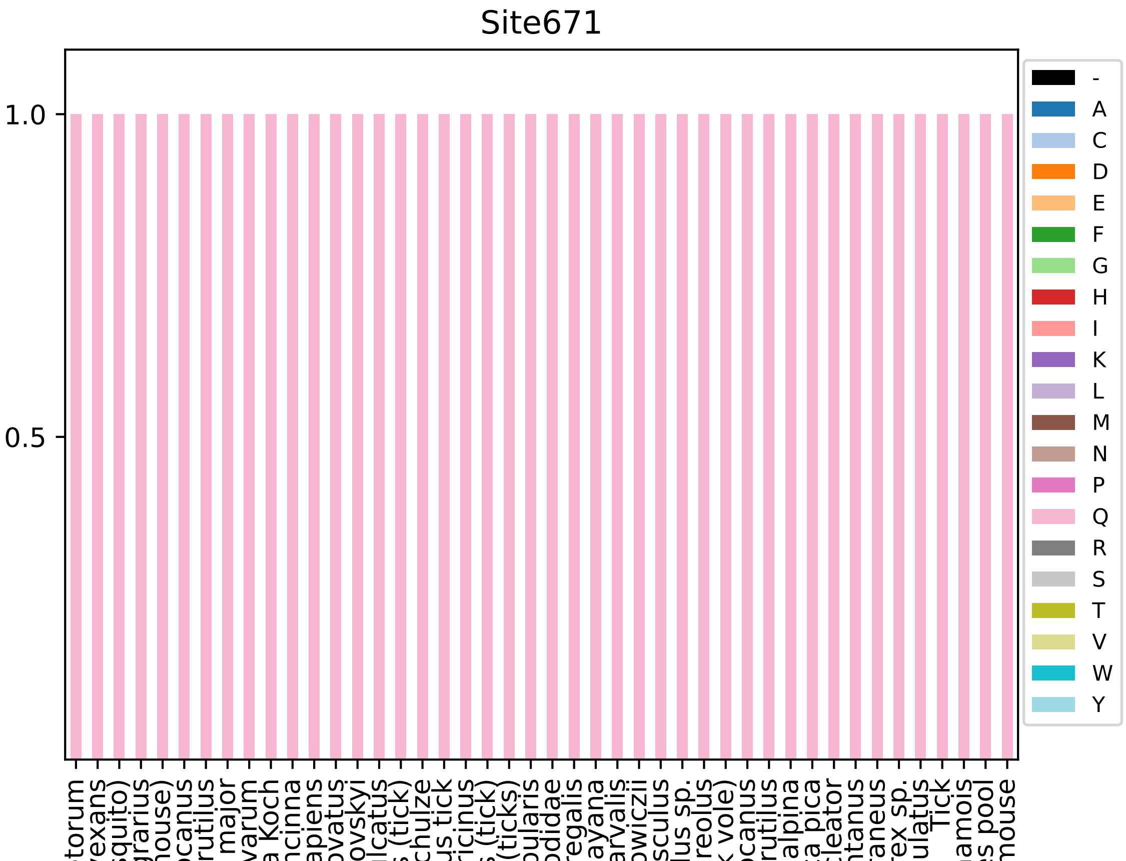

Site671
1.0
0.5
-
A
C
D
E
F
G
H
I
K
L
M
N
P
Q
R
S
T
V
W
Y

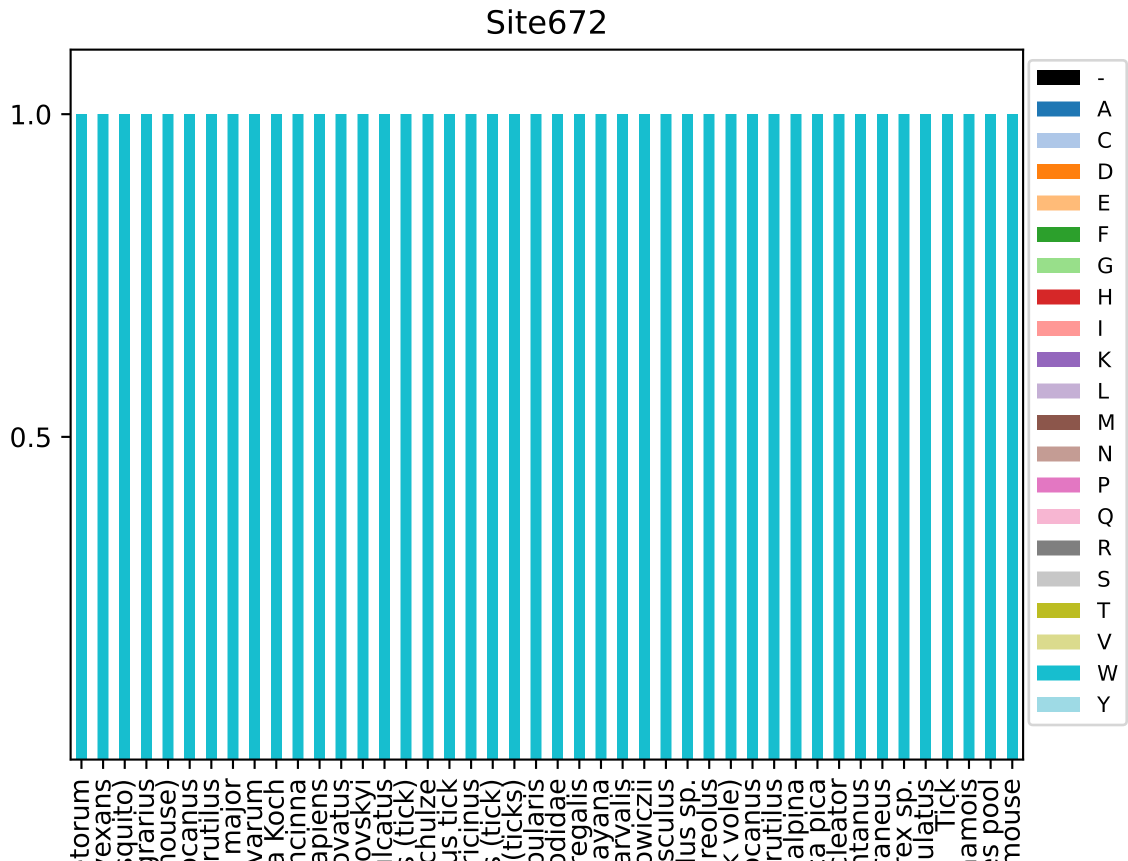
Site672
1.0
0.5
-
A
C
D
E
F
G
H
I
K
L
M
N
P
Q
R
S
T
V
W
Y

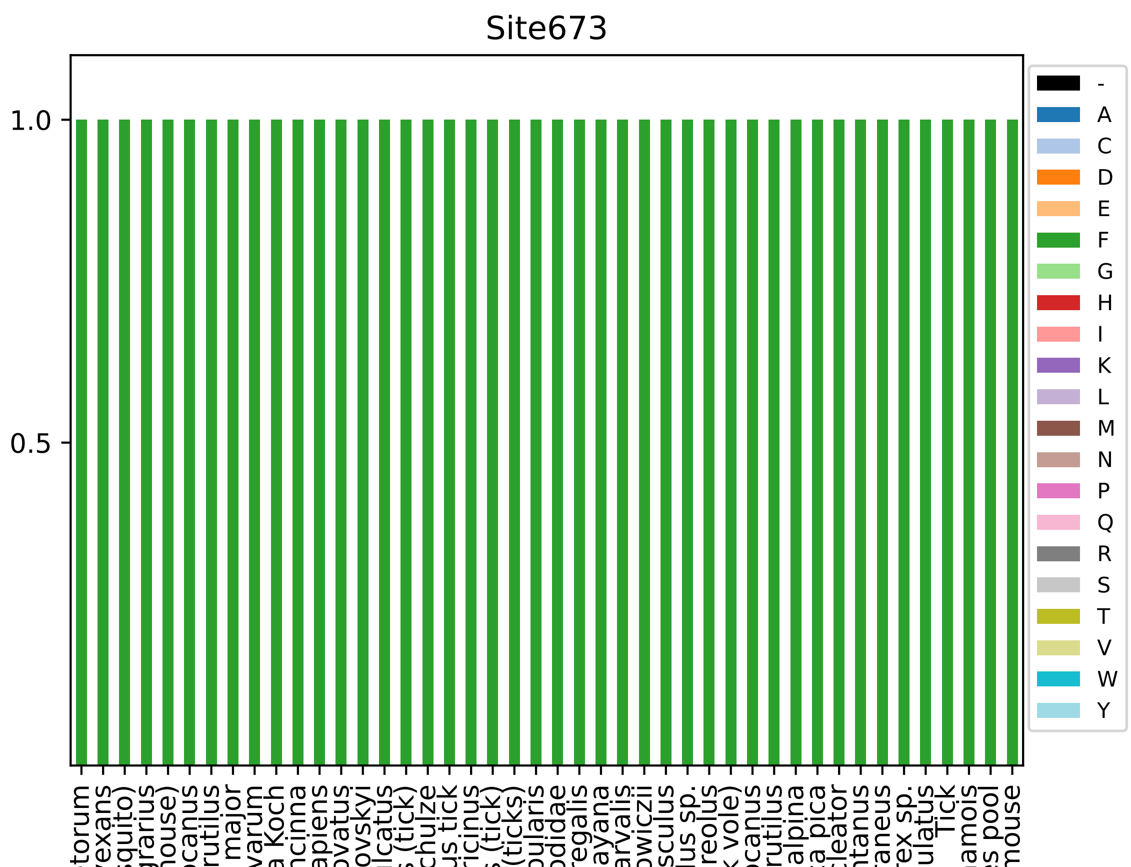
Site673
1.0
0.5
-
A
C
D
E
F
G
H
I
K
L
M
N
P
Q
R
S
T
V
W
Y

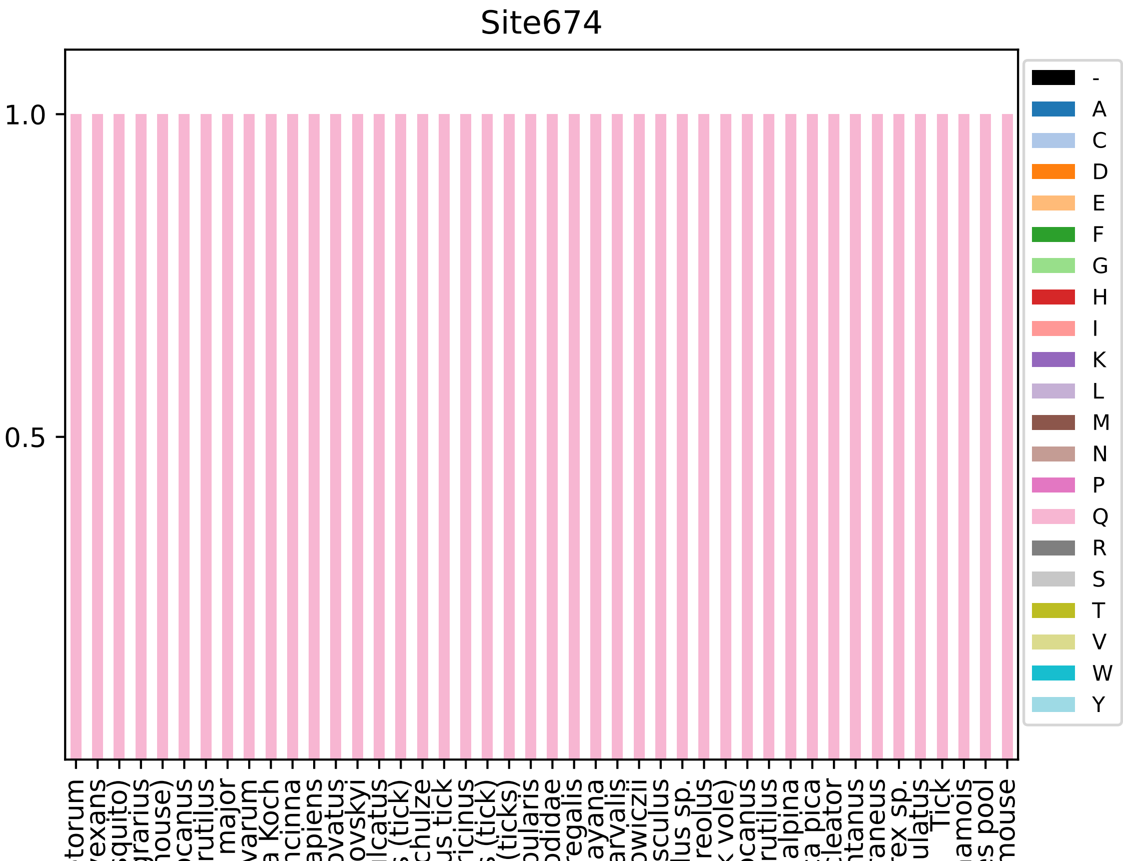
Site674
1.0
0.5
-
A
C
D
E
F
G
H
I
K
L
M
N
P
Q
R
S
T
V
W
Y

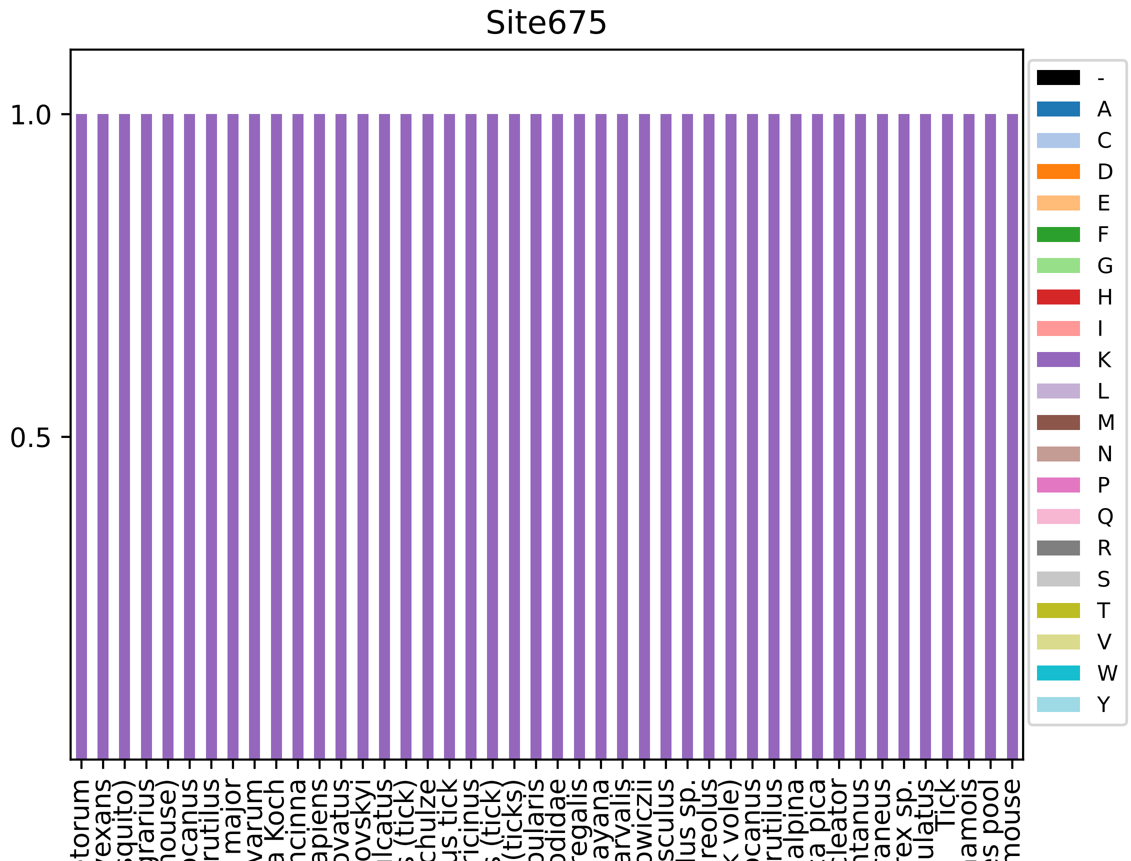
Site675
1.0
0.5
-
A
C
D
E
F
G
H
I
K
L
M
N
P
Q
R
S
T
V
W
Y

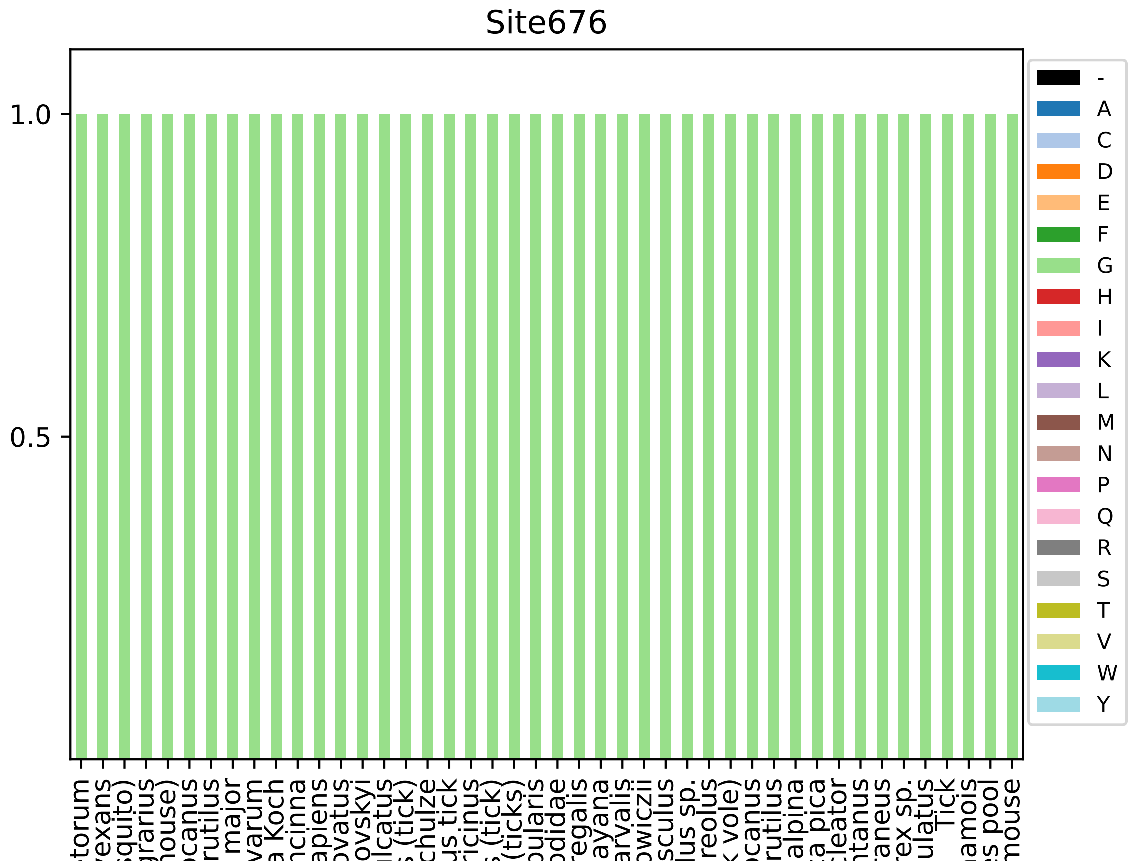

Site676
1.0
0.5
-
A
C
D
E
F
G
H
I
K
L
M
N
P
Q
R
S
T
V
W
Y

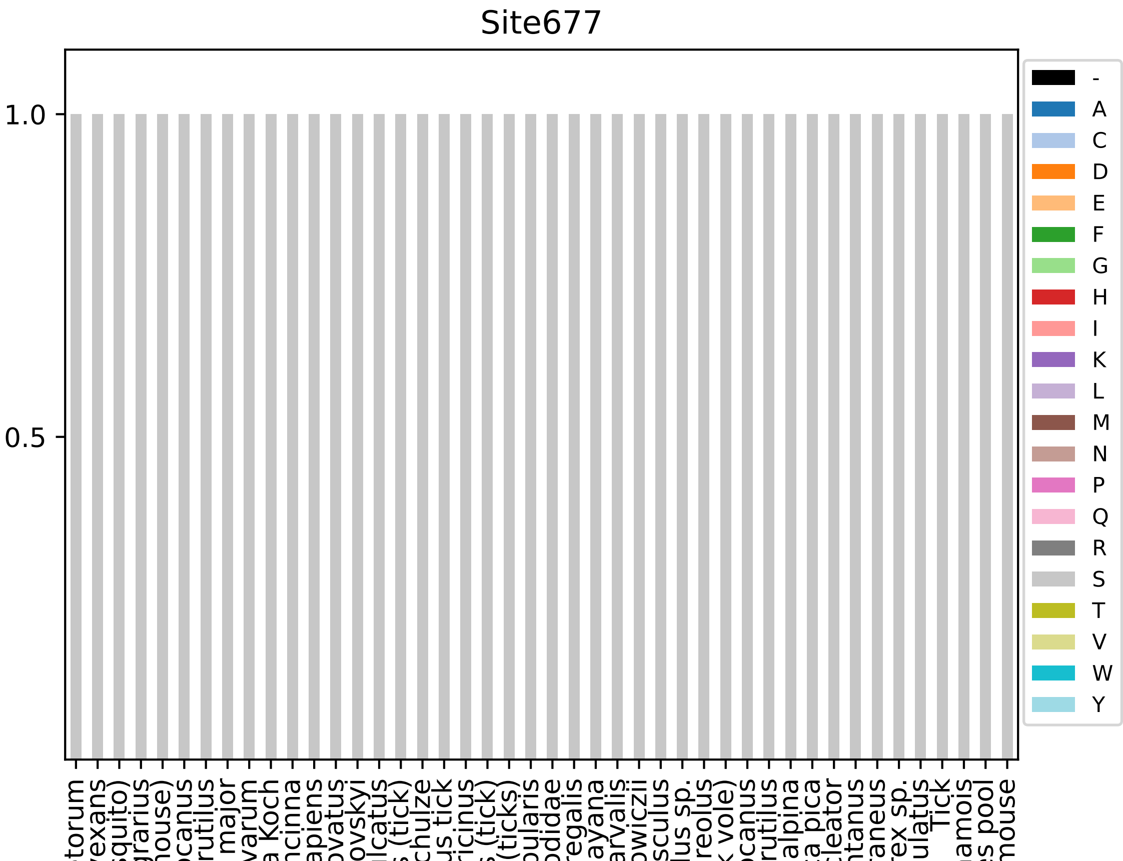

Site677
1.0
0.5
-
A
C
D
E
F
G
H
I
K
L
M
N
P
Q
R
S
T
V
W
Y

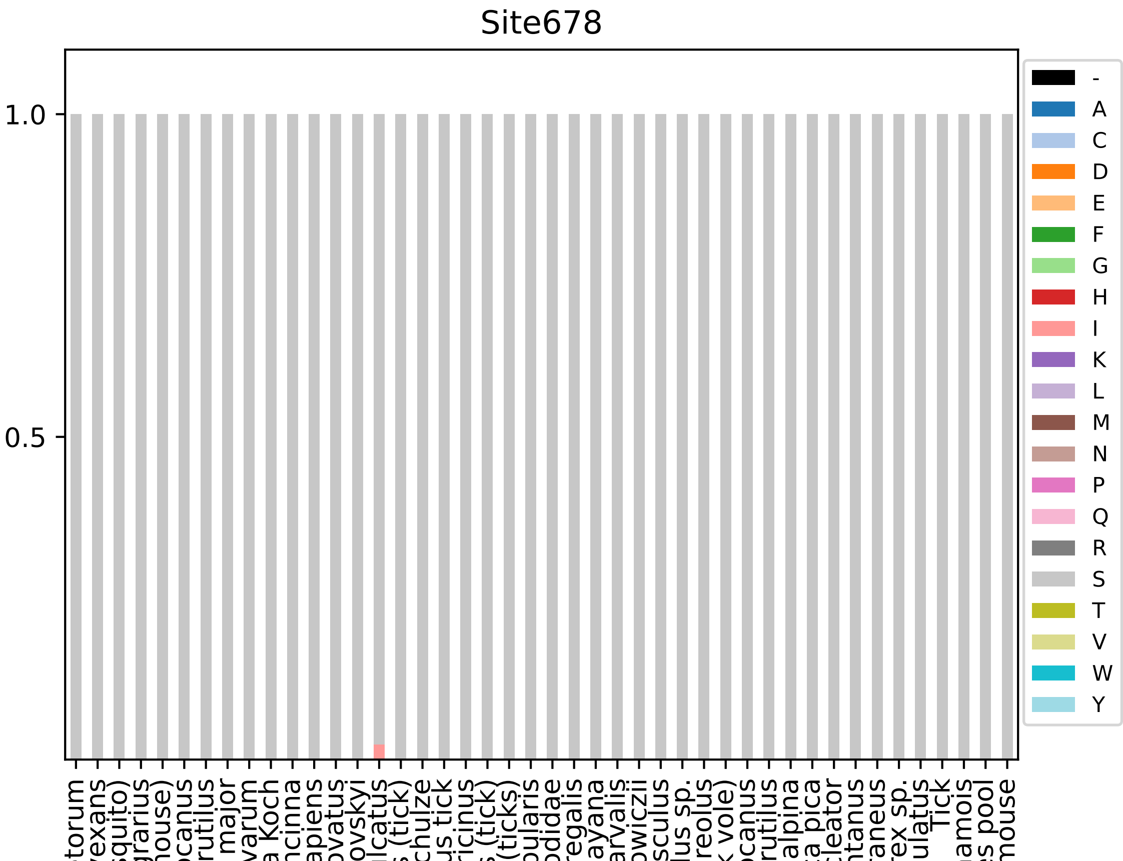
Site678
1.0
0.5
-
A
C
D
E
F
G
H
I
K
L
M
N
P
Q
R
S
T
V
W
Y

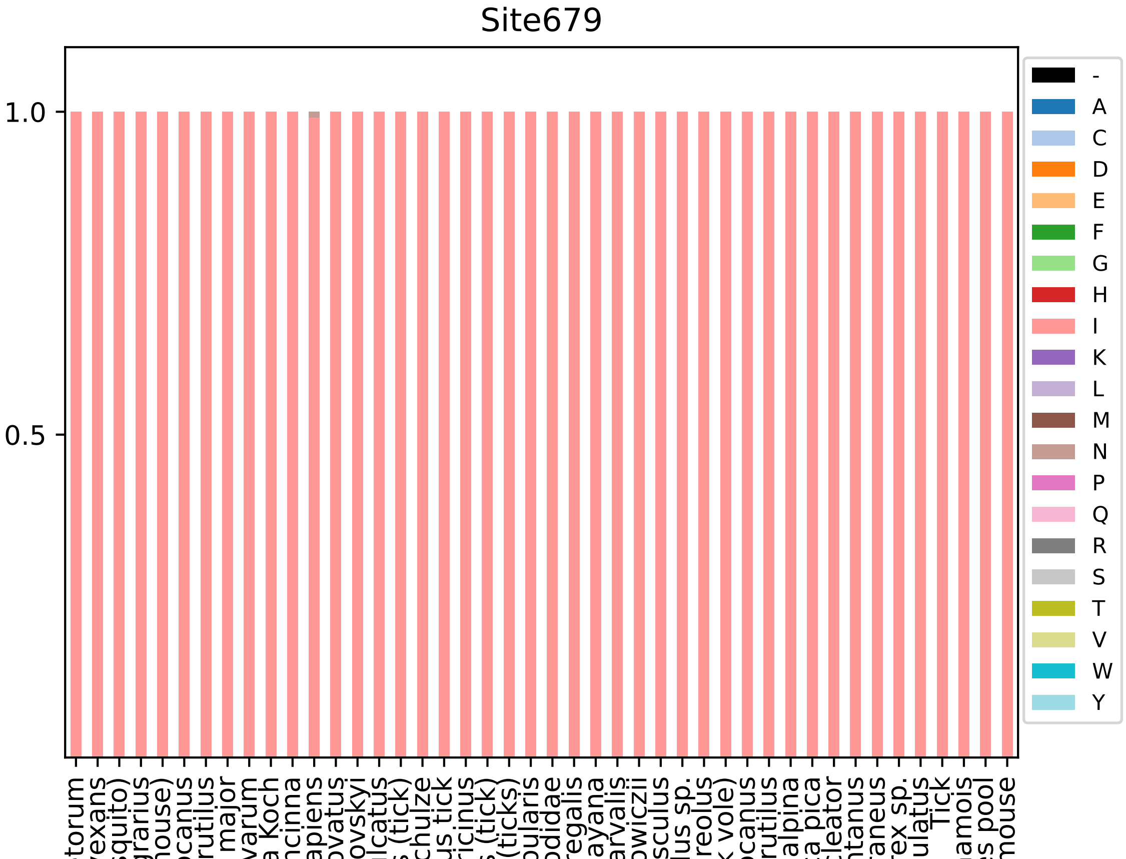
Site679
1.0
0.5
-
A
C
D
E
F
G
H
I
K
L
M
N
P
Q
R
S
T
V
W
Y

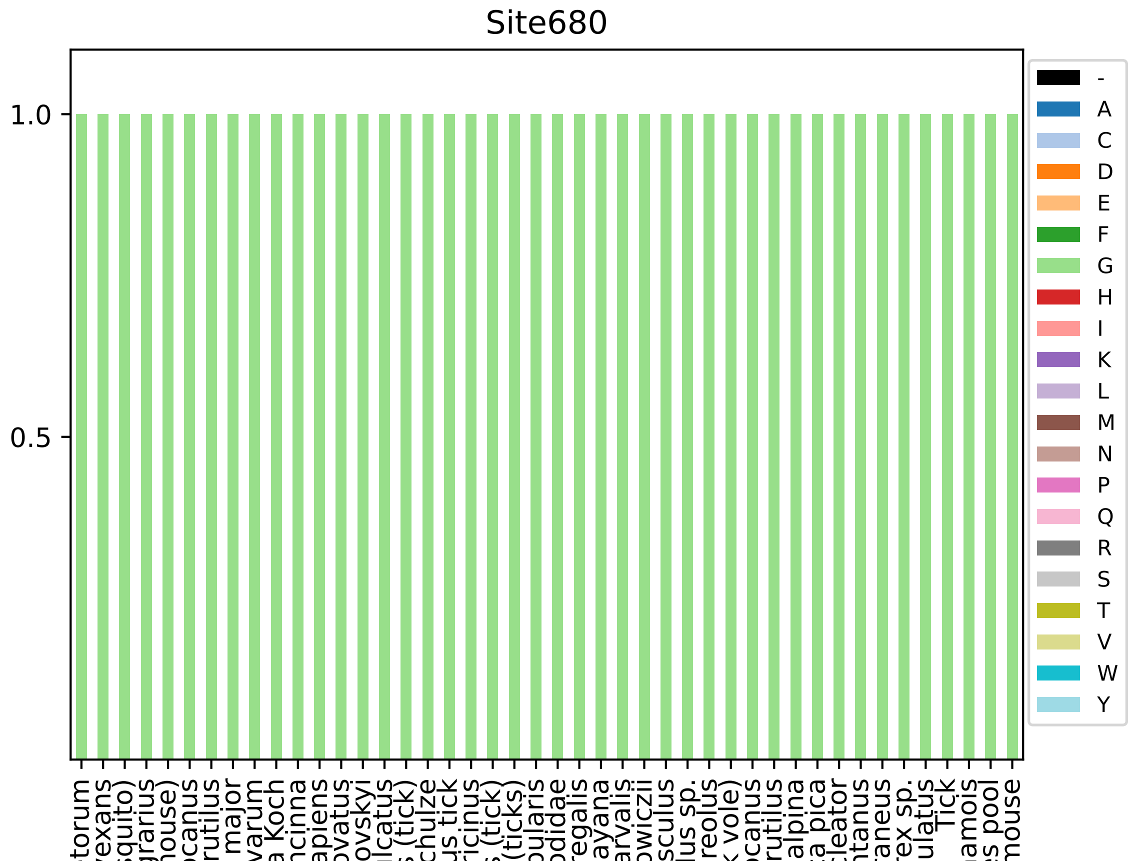

Site680
1.0
0.5
-
A
C
D
E
F
G
H
I
K
L
M
N
P
Q
R
S
T
V
W
Y

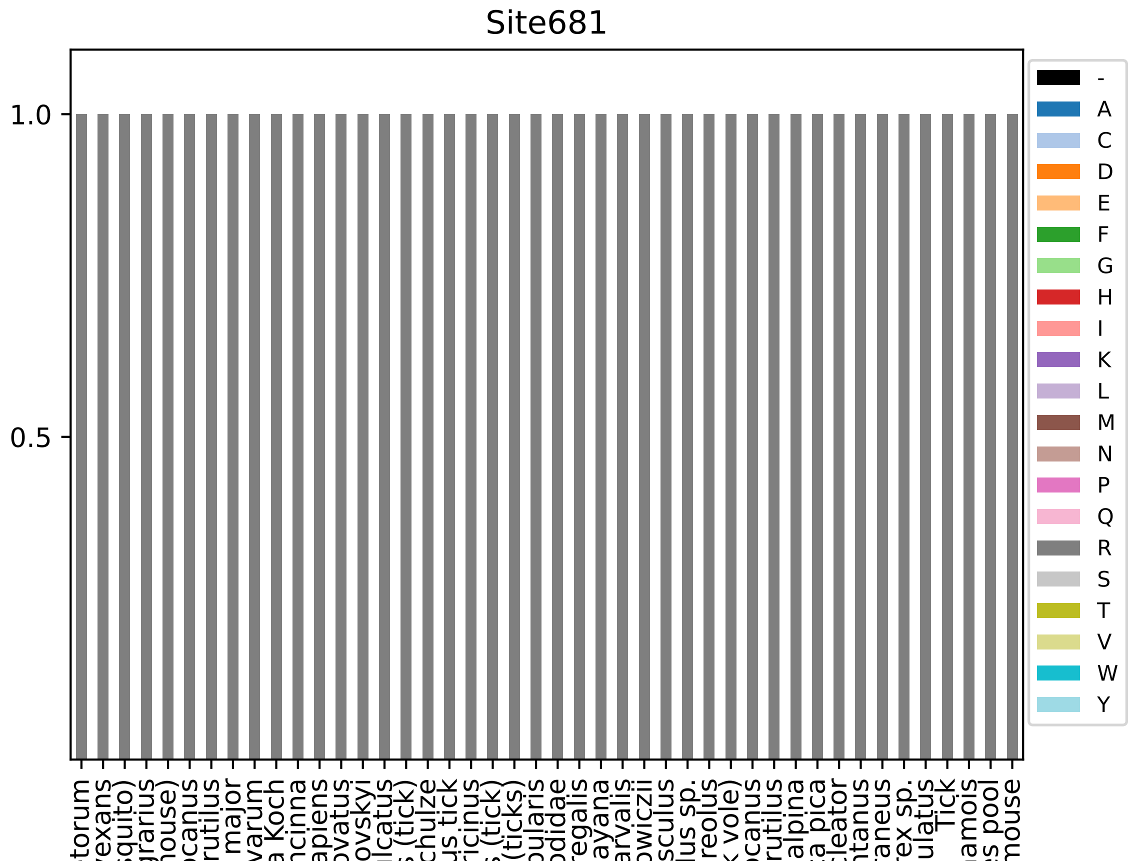
Site681
1.0
0.5
-
A
C
D
E
F
G
H
I
K
L
M
N
P
Q
R
S
T
V
W
Y

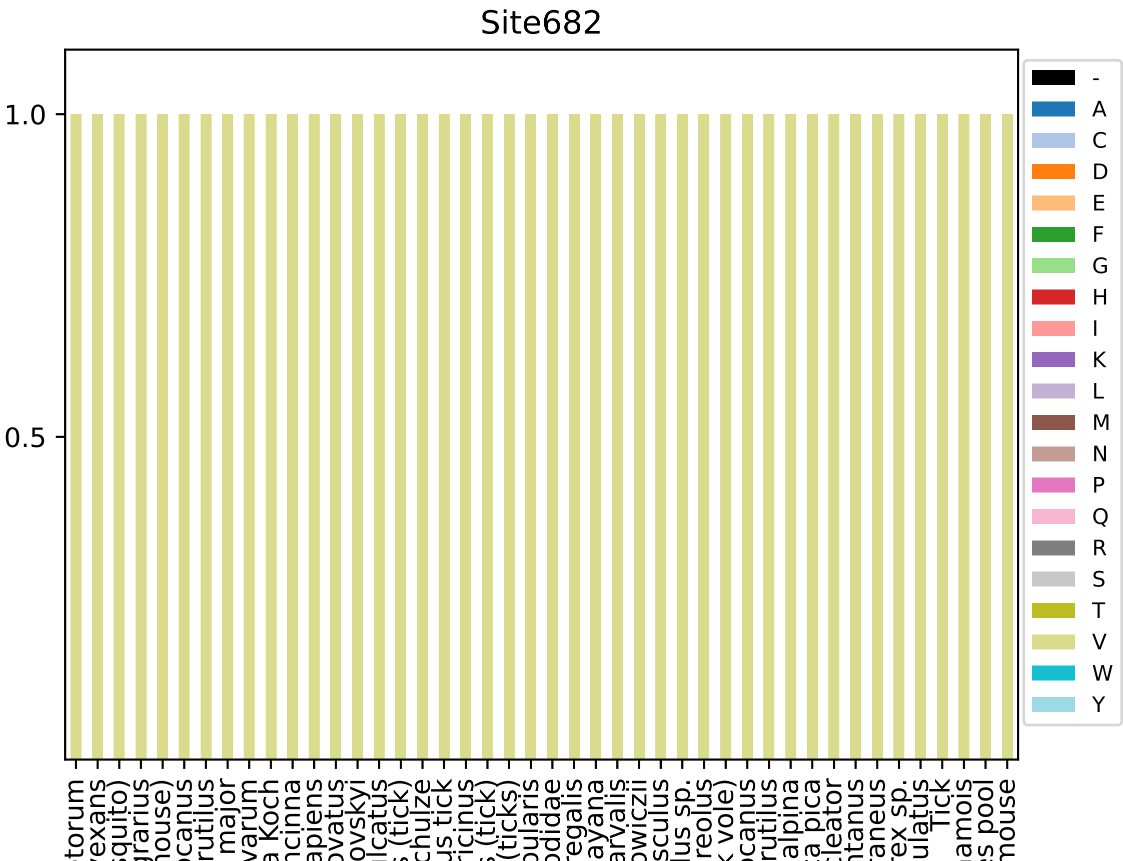
Site682
1.0
0.5
-
A
C
D
E
F
G
H
I
K
L
M
N
P
Q
R
S
T
V
W
Y

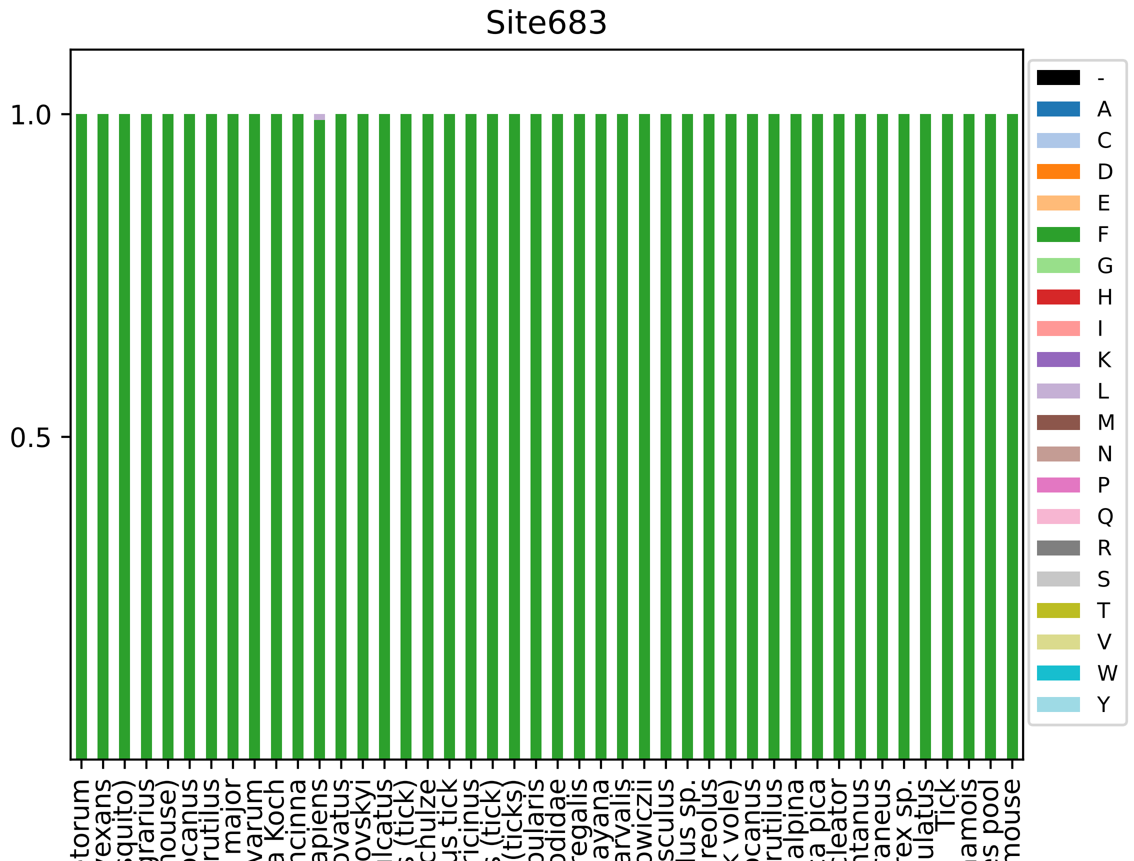
Site683
1.0
0.5
-
A
C
D
E
F
G
H
I
K
L
M
N
P
Q
R
S
T
V
W
Y

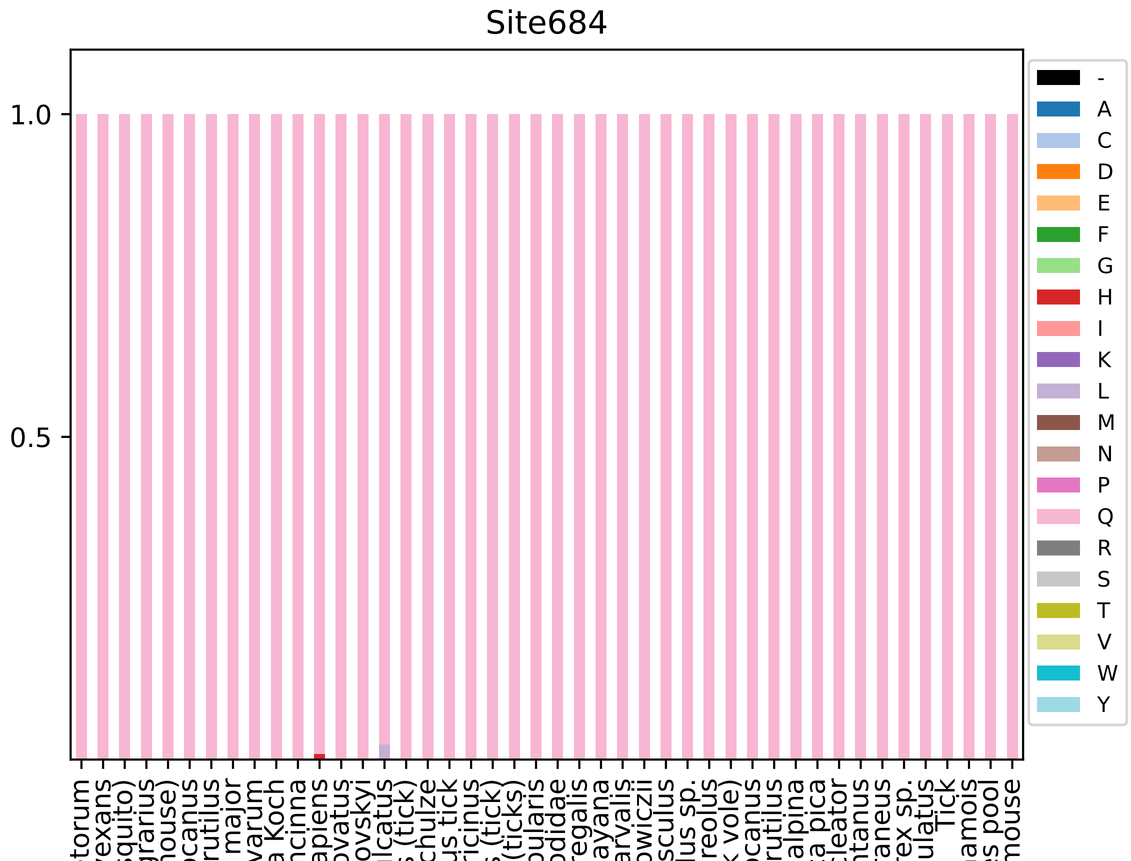

Site684
1.0
0.5
-
A
C
D
E
F
G
H
I
K
L
M
N
P
Q
R
S
T
V
W
Y

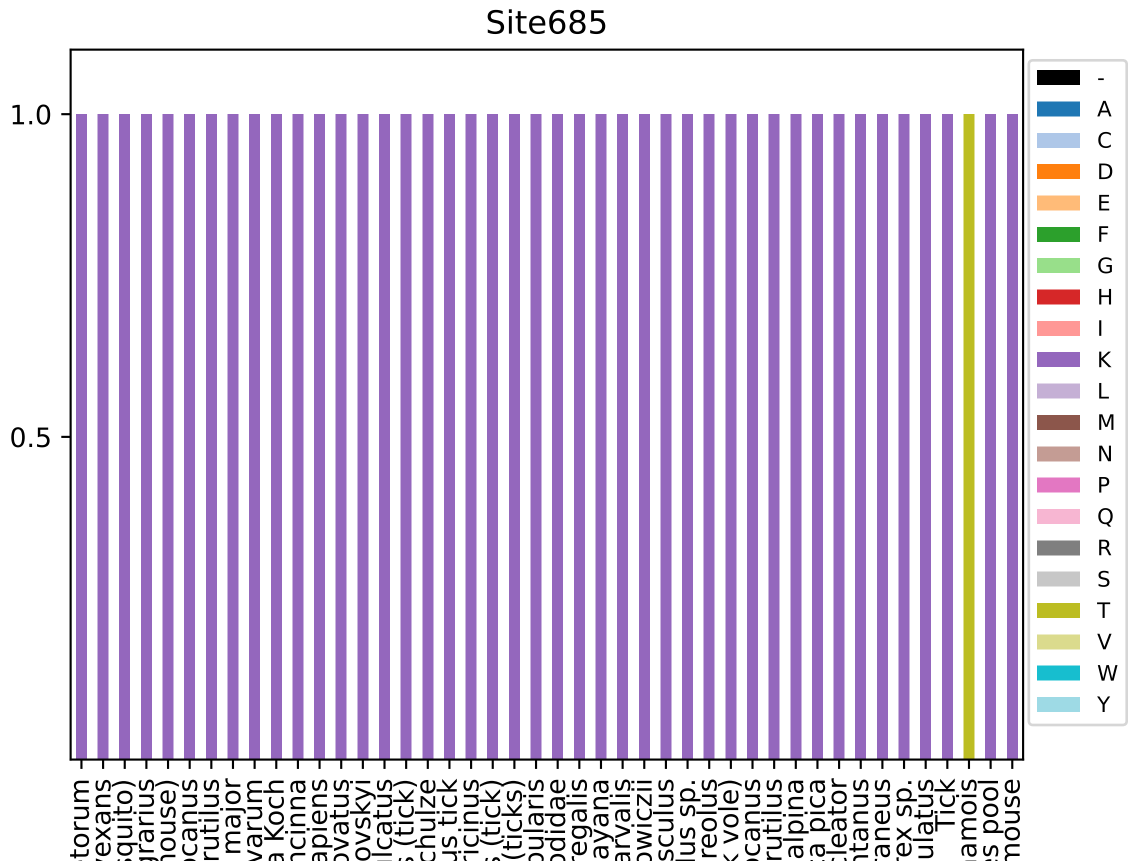
Site685
1.0
0.5
-
A
C
D
E
F
G
H
I
K
L
M
N
P
Q
R
S
T
V
W
Y

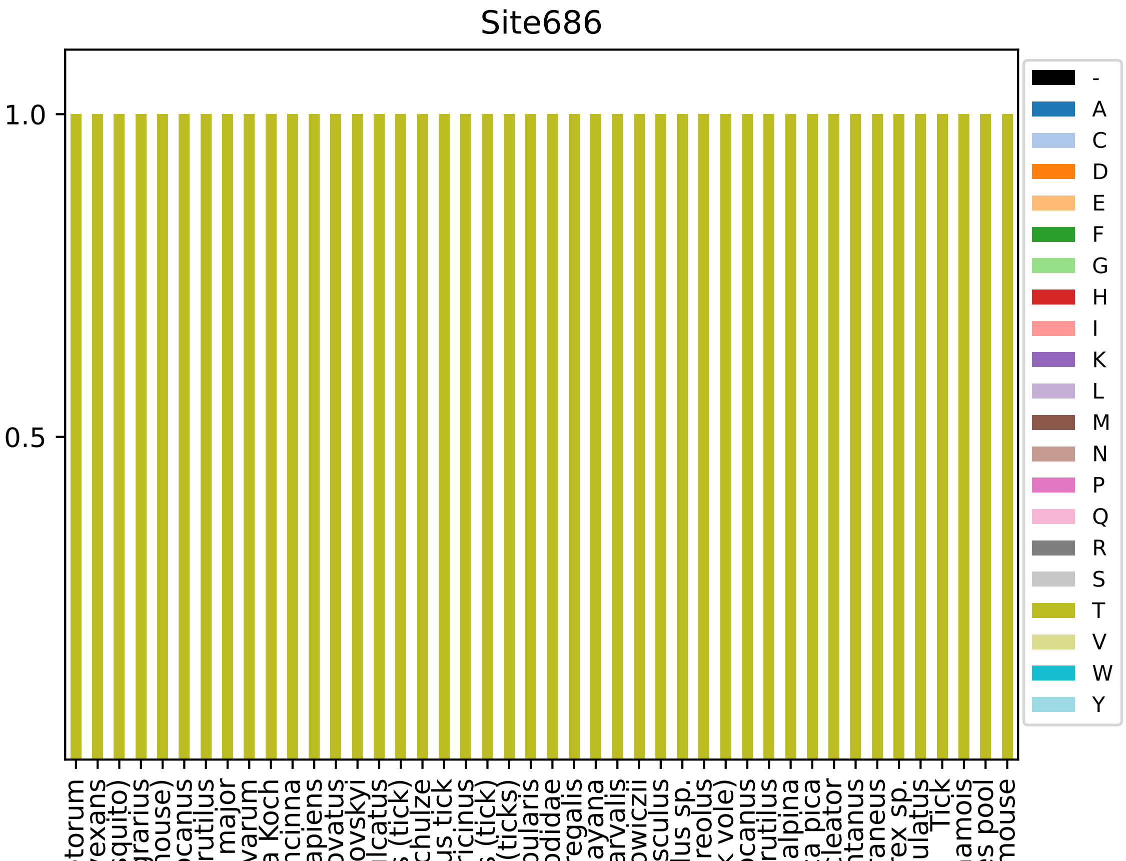

Site686
1.0
0.5
-
A
C
D
E
F
G
H
I
K
L
M
N
P
Q
R
S
T
V
W
Y

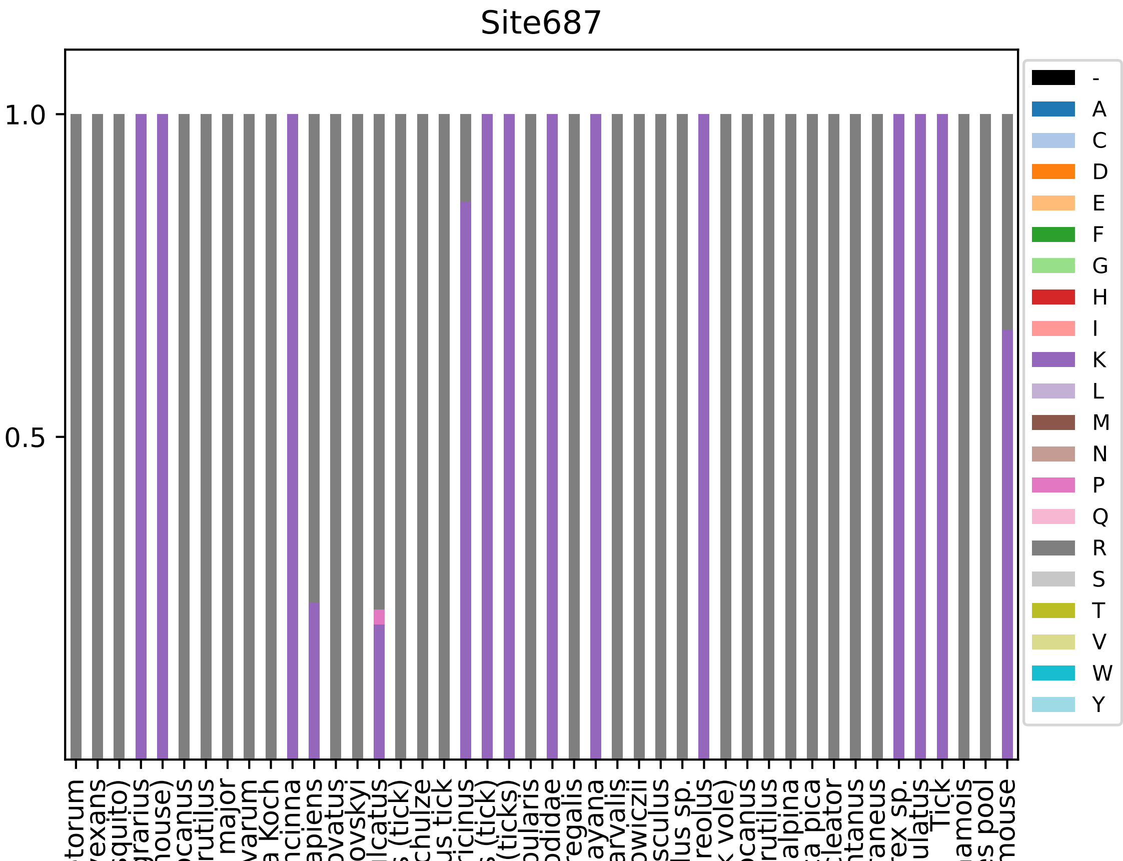

Site687
1.0
0.5
-
A
C
D
E
F
G
H
I
K
L
M
N
P
Q
R
S
T
V
W
Y

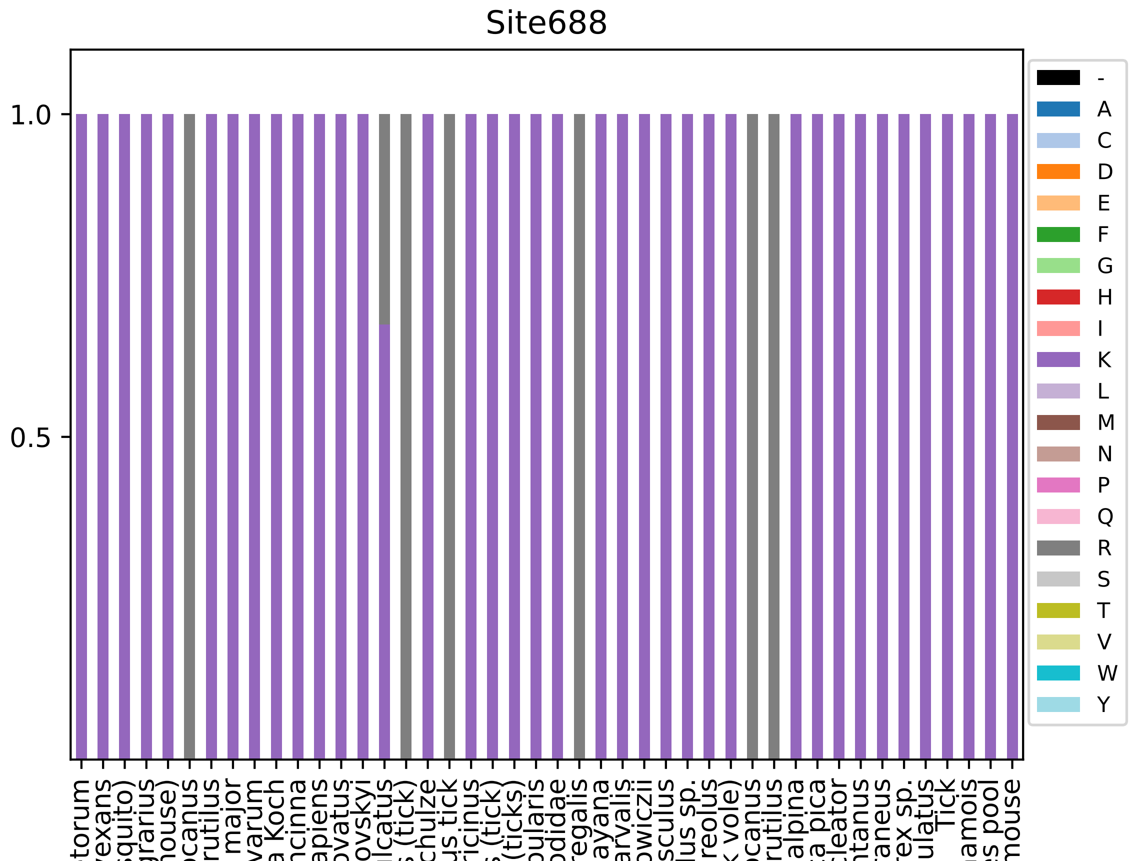

Site688
1.0
0.5
-
A
C
D
E
F
G
H
I
K
L
M
N
P
Q
R
S
T
V
W
Y

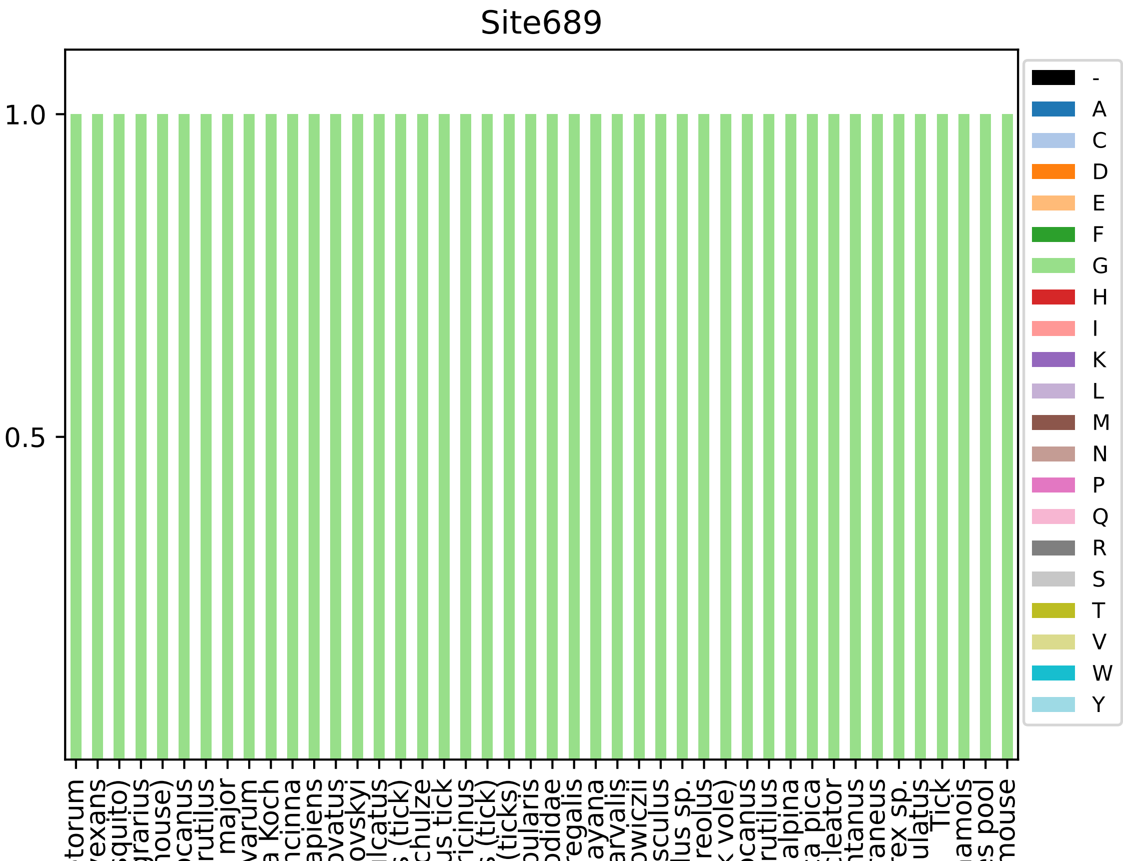

Site689
1.0
0.5
-
A
C
D
E
F
G
H
I
K
L
M
N
P
Q
R
S
T
V
W
Y

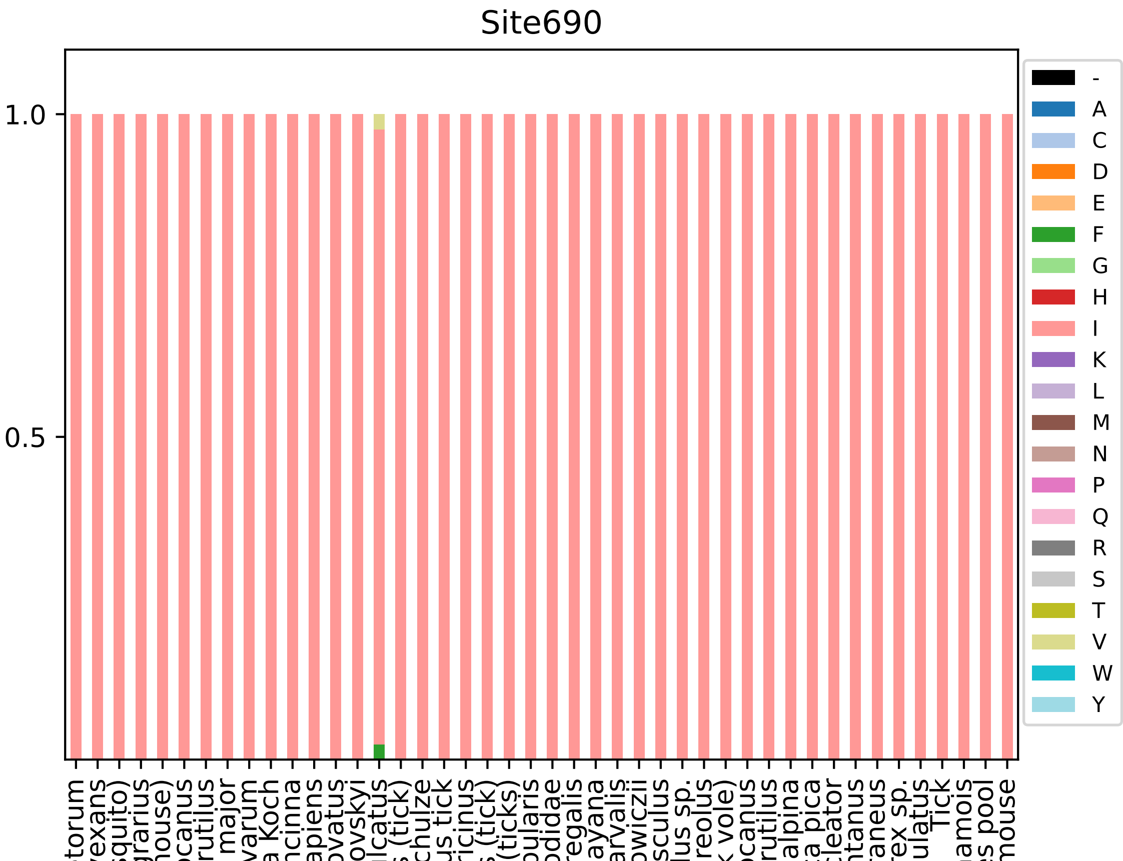

Site690
1.0
0.5
-
A
C
D
E
F
G
H
I
K
L
M
N
P
Q
R
S
T
V
W
Y

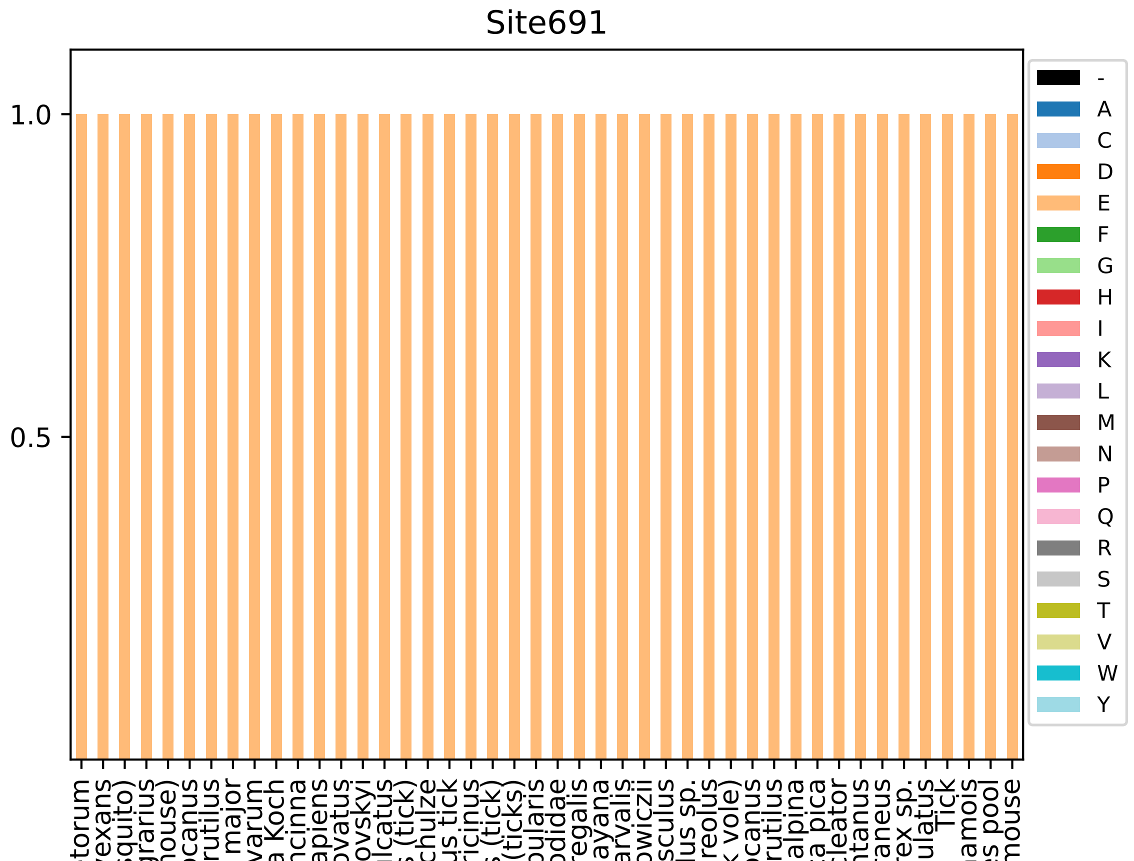

Site691
1.0
0.5
-
A
C
D
E
F
G
H
I
K
L
M
N
P
Q
R
S
T
V
W
Y

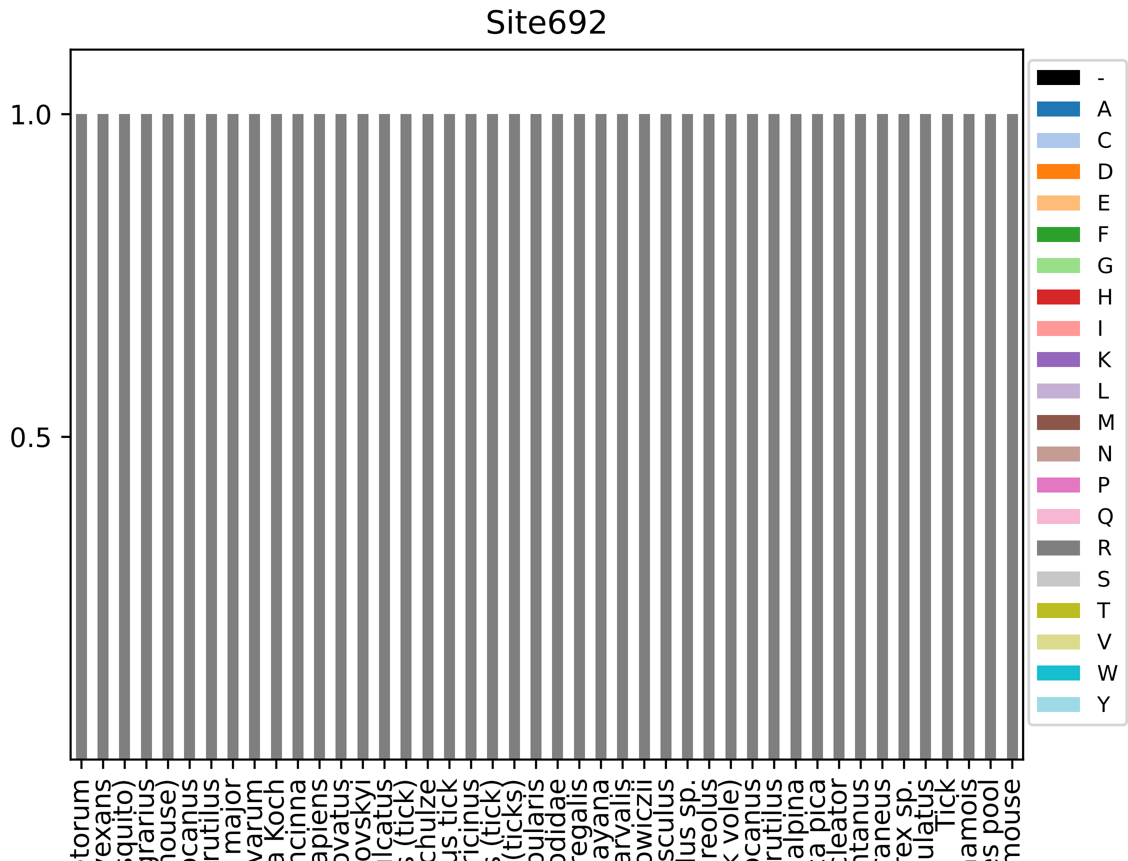
Site692
1.0
0.5
-
A
C
D
E
F
G
H
I
K
L
M
N
P
Q
R
S
T
V
W
Y

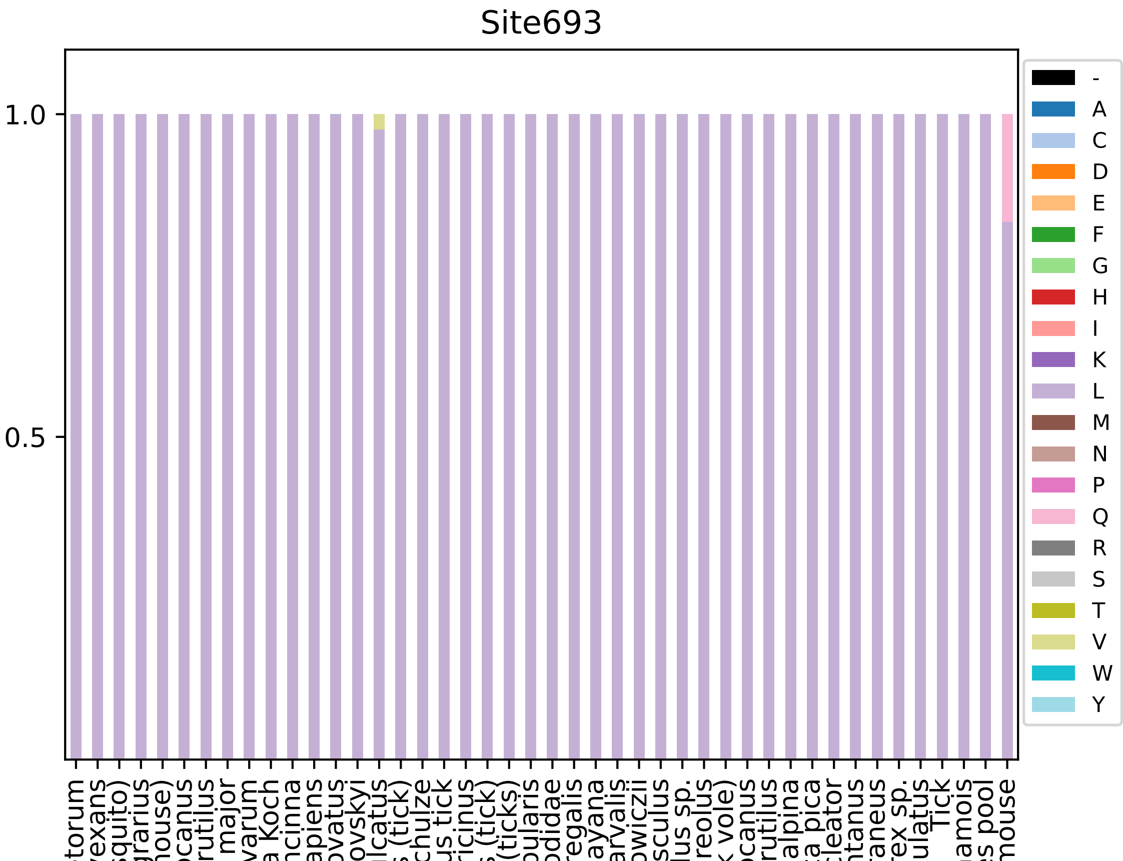
Site693
1.0
0.5
-
A
C
D
E
F
G
H
I
K
L
M
N
P
Q
R
S
T
V
W
Y
Tick

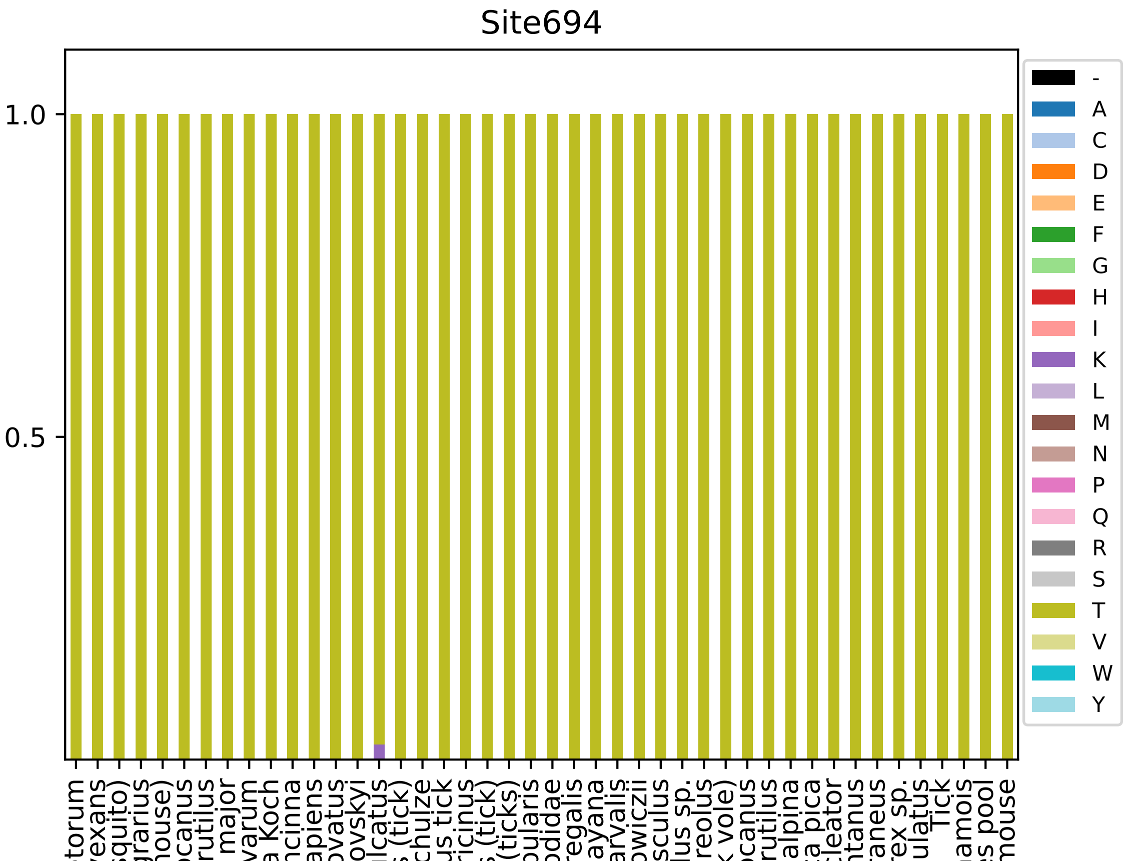
Site694
1.0
0.5
-
A
C
D
E
F
G
H
I
K
L
M
N
P
Q
R
S
T
V
W
Y

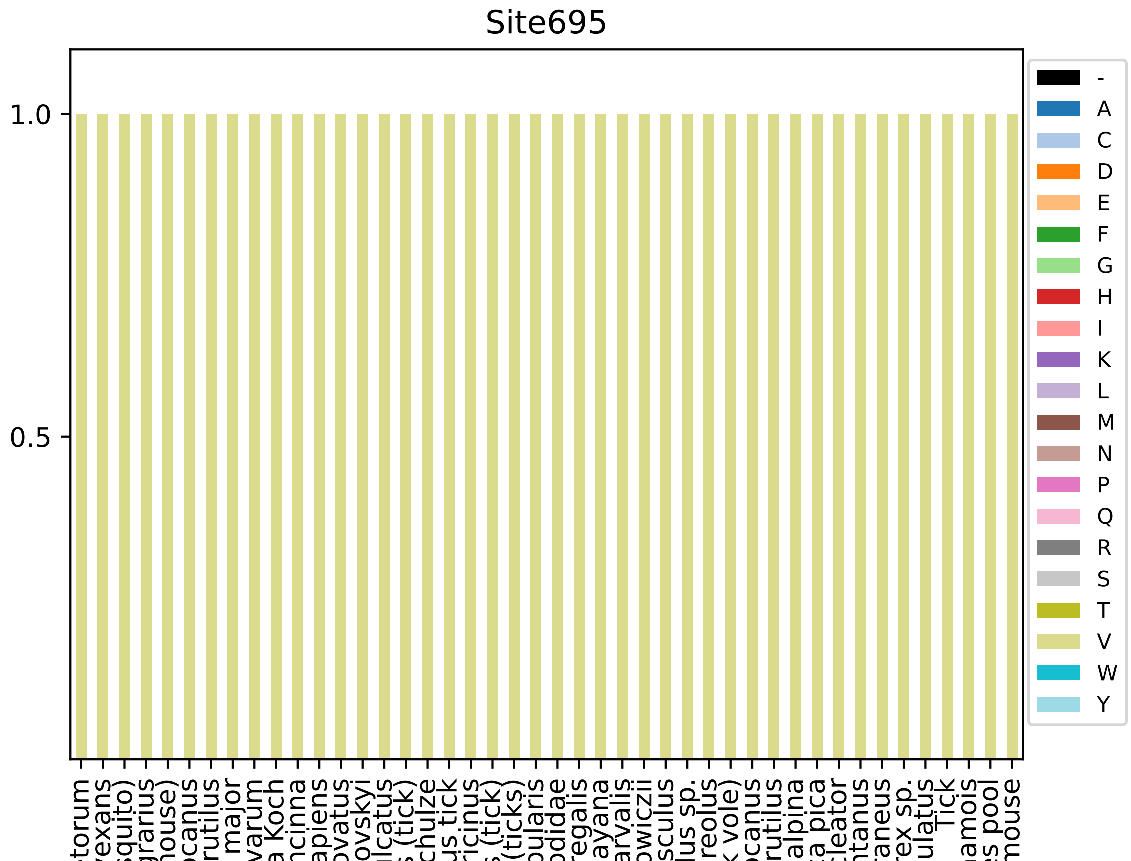
Site695
1.0
0.5
-
A
C
D
E
F
G
H
I
K
L
M
N
P
Q
R
S
T
V
W
Y

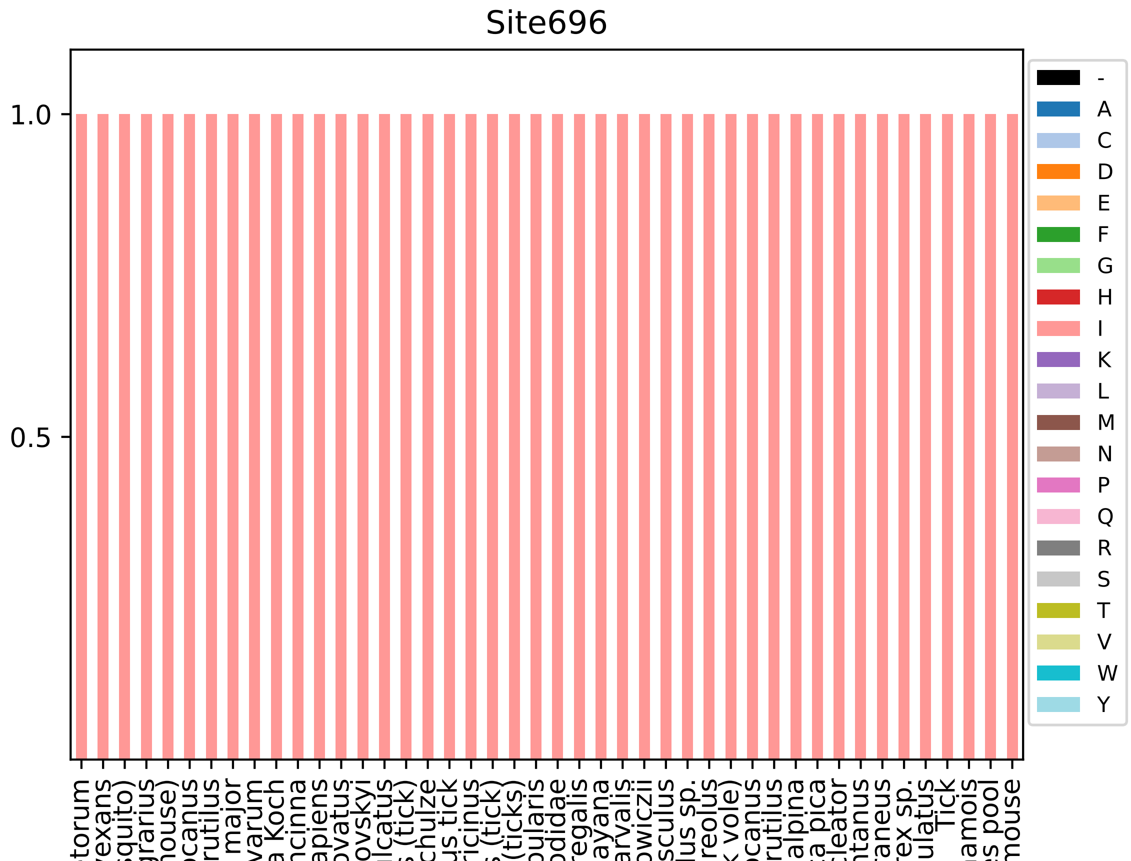
Site696
1.0
0.5
-
A
C
D
E
F
G
H
I
K
L
M
N
P
Q
R
S
T
V
W
Y

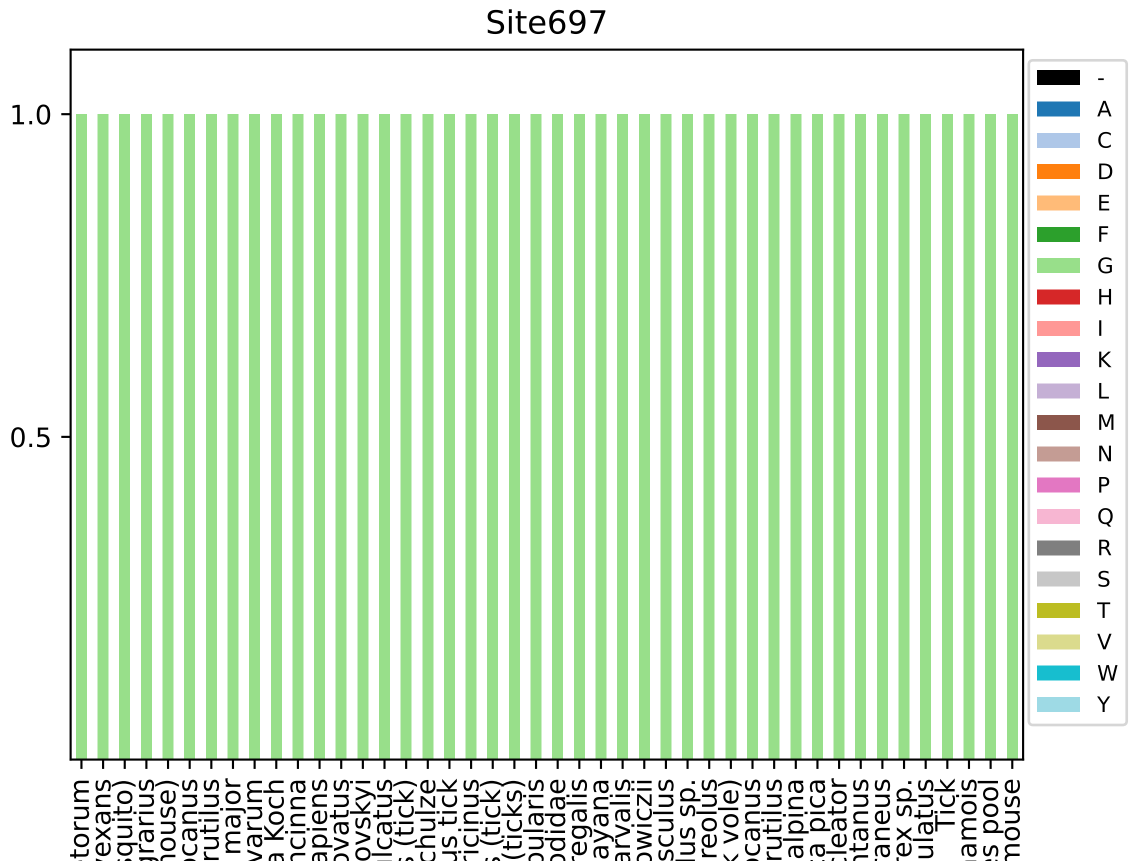

Site697
1.0
0.5
-
A
C
D
E
F
G
H
I
K
L
M
N
P
Q
R
S
T
V
W
Y

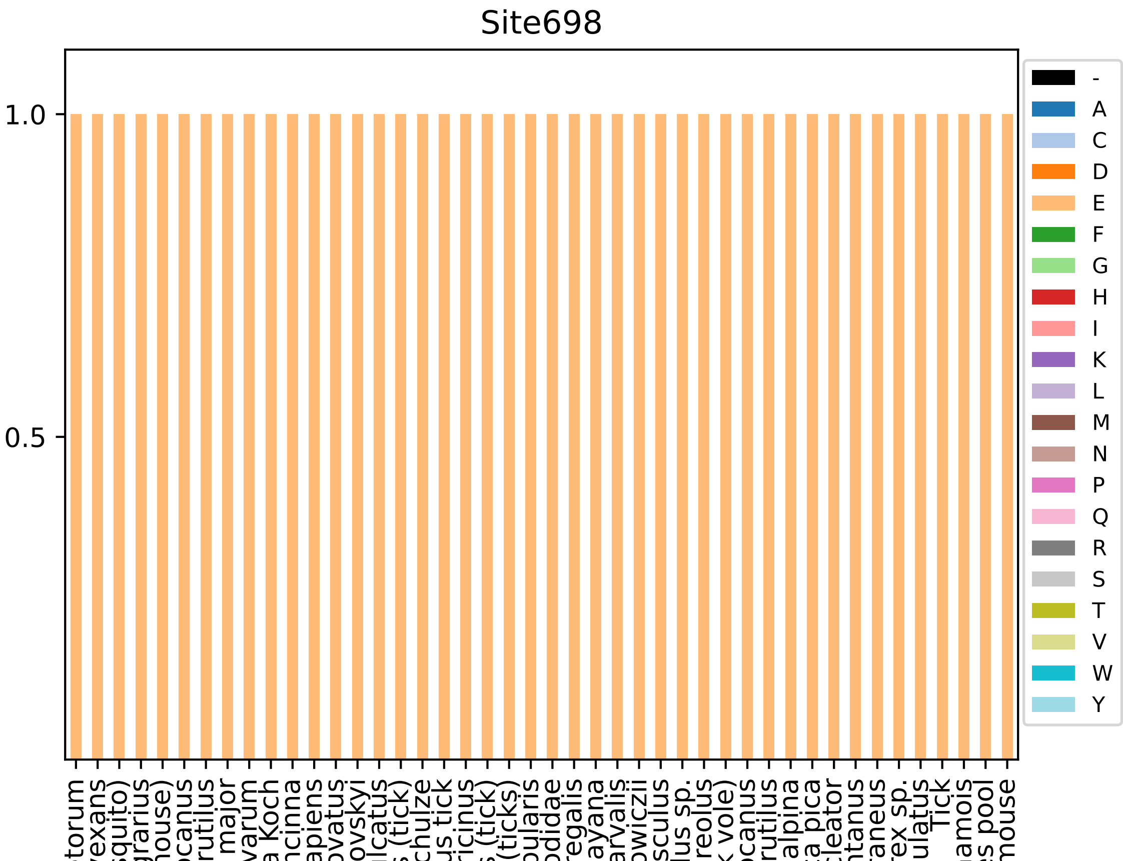

Site698
1.0
0.5
-
A
C
D
E
F
G
H
I
K
L
M
N
P
Q
R
S
T
V
W
Y

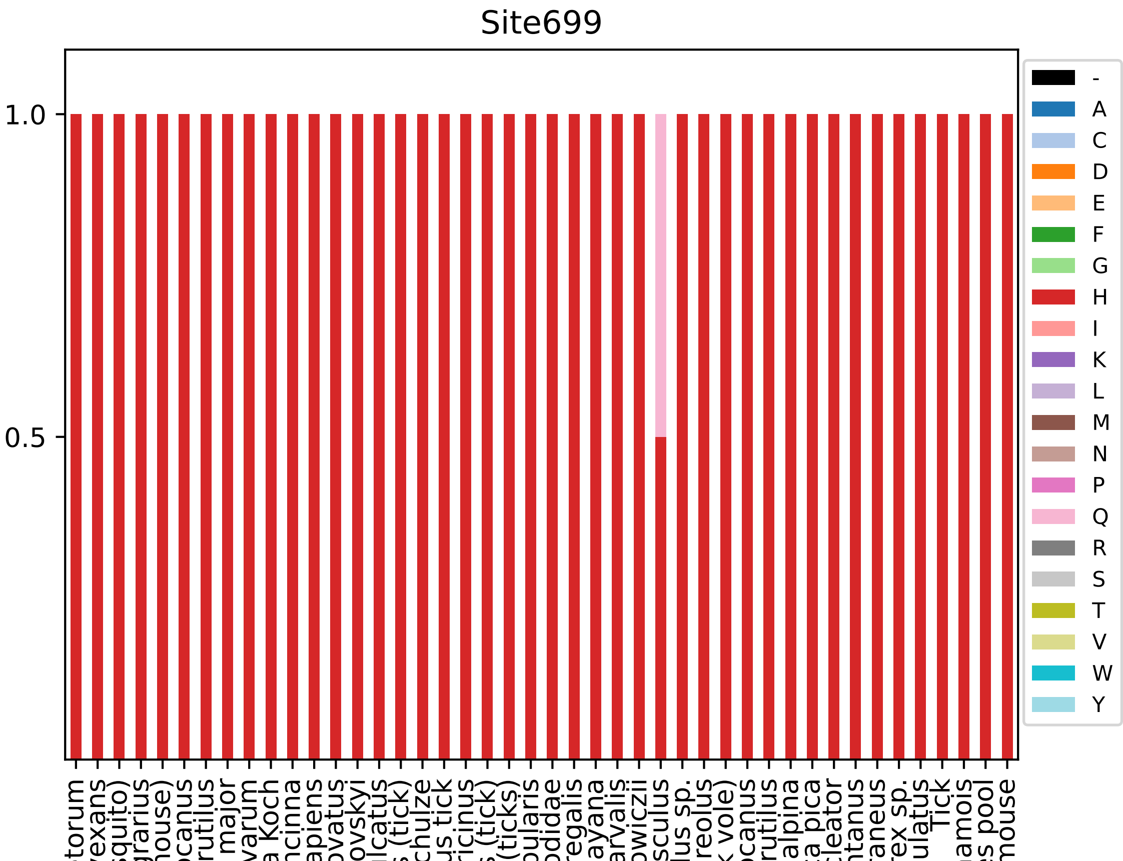
Site699
1.0
0.5
-
A
C
D
E
F
G
H
I
K
L
M
N
P
Q
R
S
T
V
W
Y

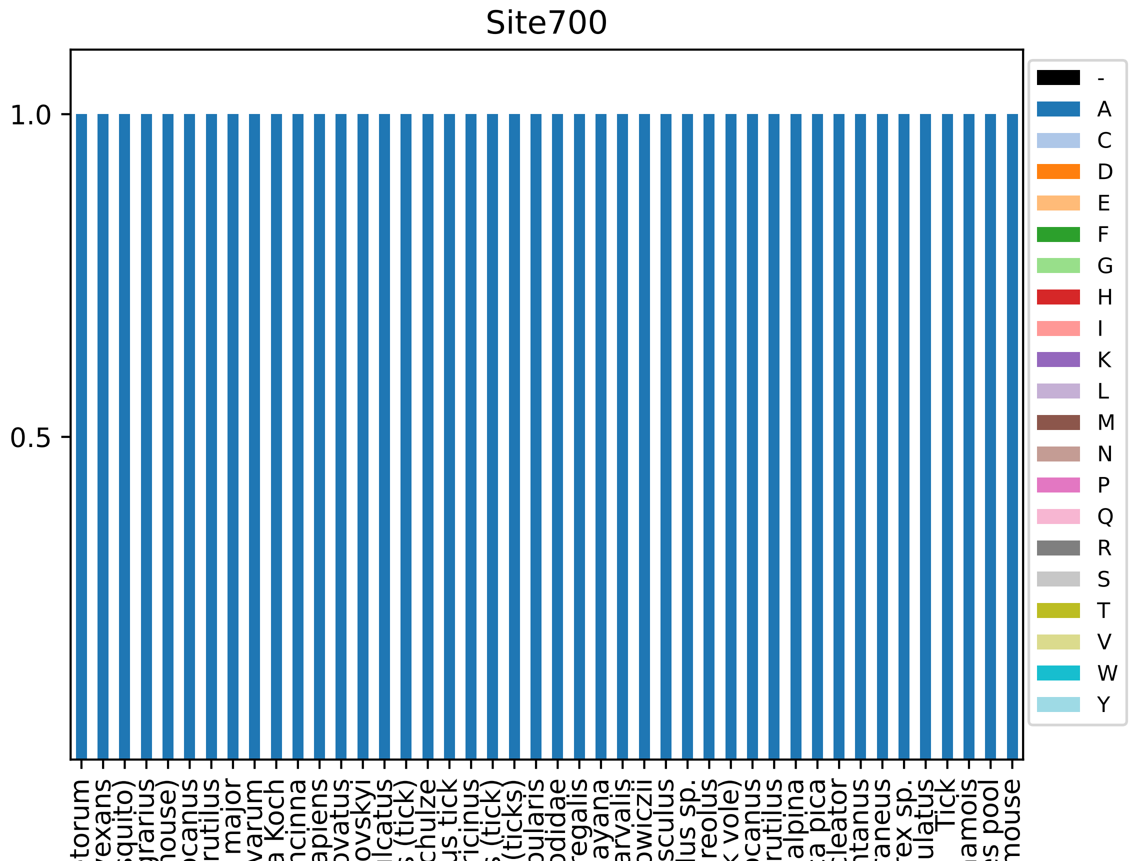

Site700
1.0
0.5
-
A
C
D
E
F
G
H
I
K
L
M
N
P
Q
R
S
T
V
W
Y

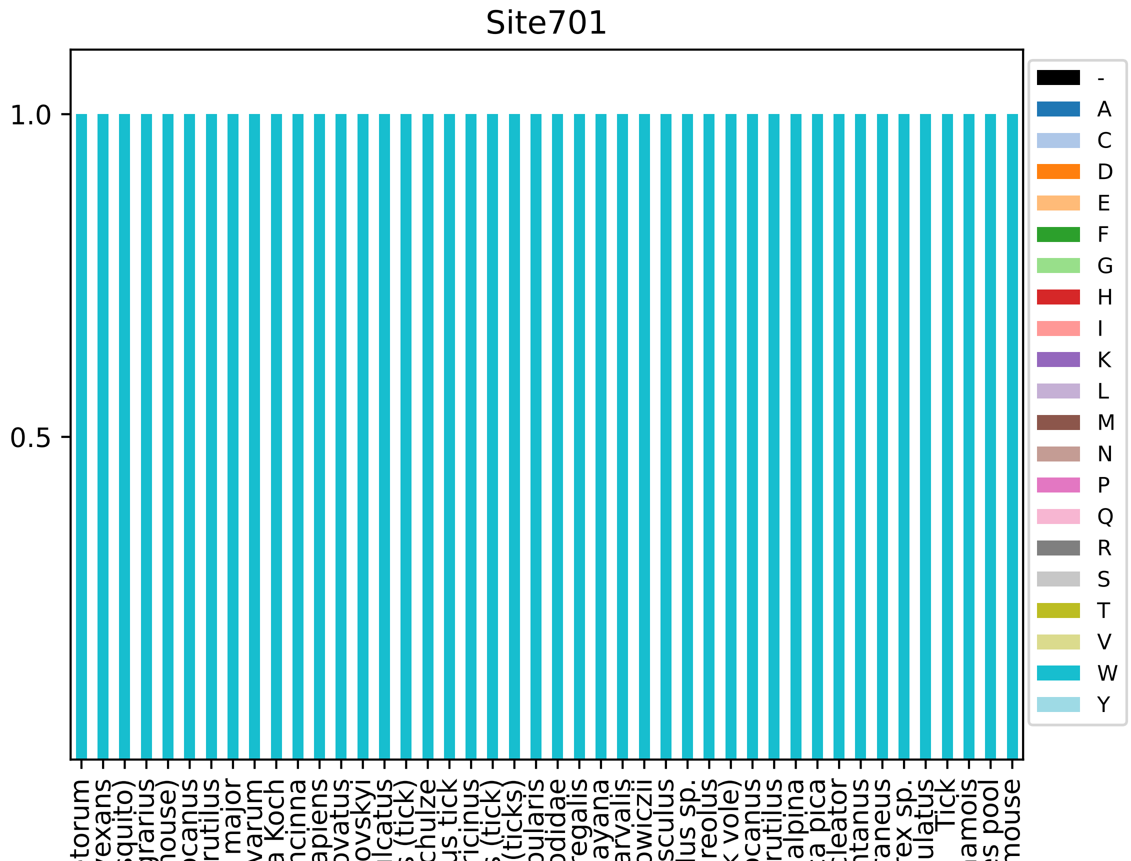

Site701
1.0
0.5
-
A
C
D
E
F
G
H
I
K
L
M
N
P
Q
R
S
T
V
W
Y
torum
vexans
squito)
garius
nouse)
ocanus
rutilus
major
varum
a Koch
ncinna
apiens
ovatus
ovskyi
ulcatus
s (tick)
chulze
us tick
ricinus
s (tick)
(ticks)
pularis
podidae
regalis
ayana
arvalis
owiczii
sculus
lus sp.
reolus
x vole)
ocanus
rutilus
alpina
a pica
cleator
ntanus
raneus
rex sp.
ulatus
Tick
amois
es pool
mouse

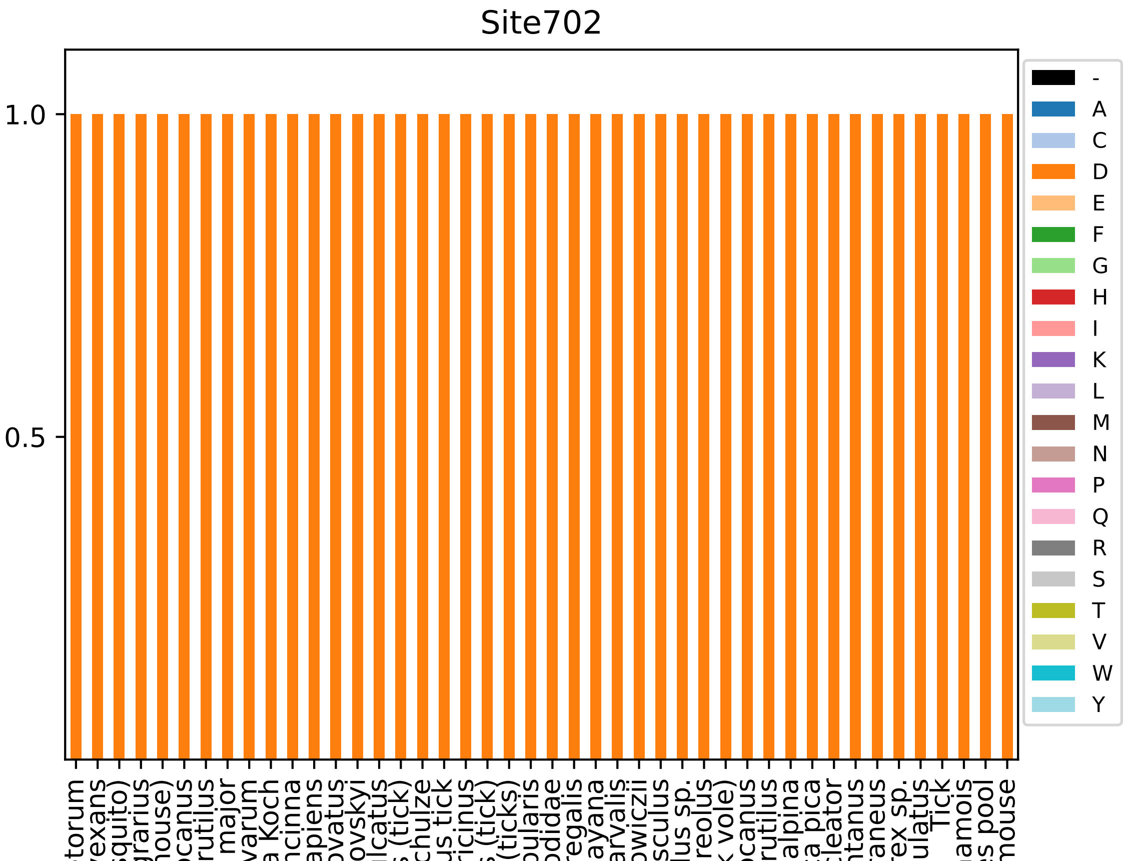

Site702
1.0
0.5
-
A
C
D
E
F
G
H
I
K
L
M
N
P
Q
R
S
T
V
W
Y

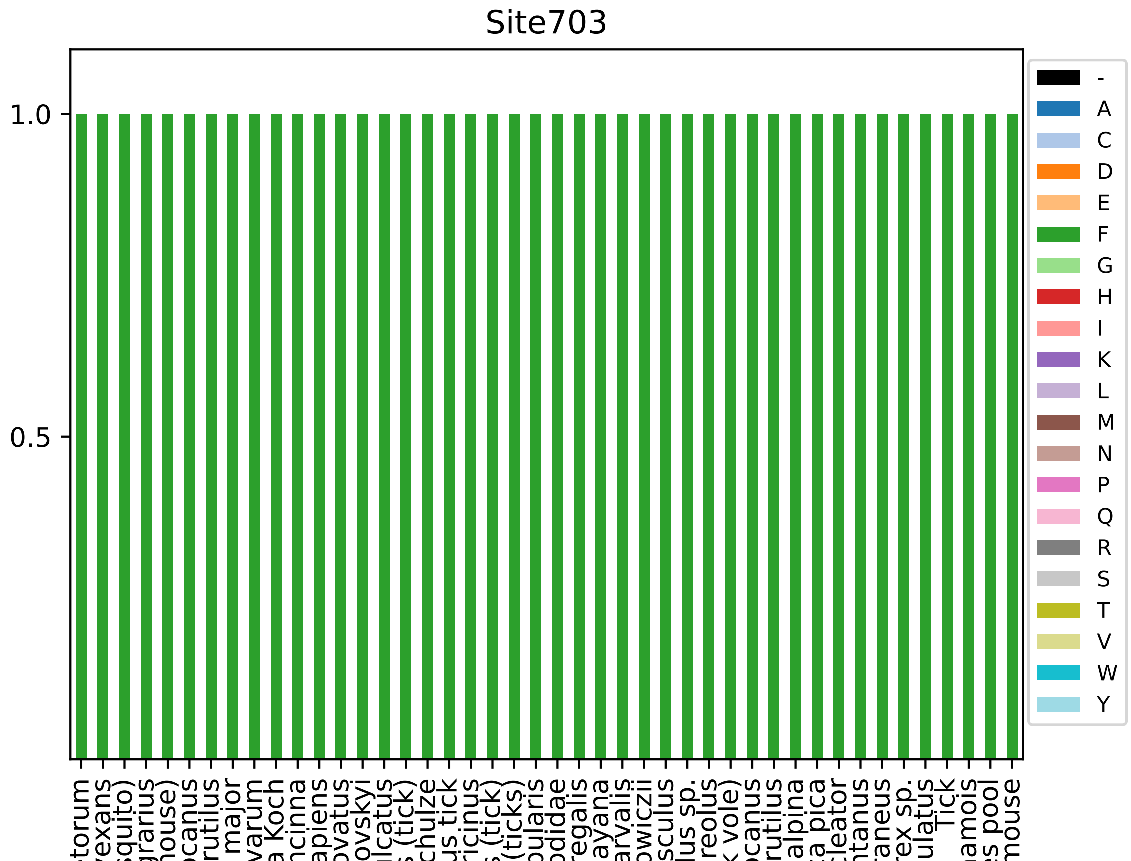
Site703
1.0
0.5
-
A
C
D
E
F
G
H
I
K
L
M
N
P
Q
R
S
T
V
W
Y
torum
vexans
squito)
grarius
mouse)
ocanus
rutilus
major
varum
a Koch
ncinna
apiens
ovatus
ovskyi
ulcatus
s (tick)
chulze
us tick
ricinus
s (tick)
(ticks)
pularis
odidae
regalis
ayana
arvalis
owiczii
sculus
lus sp.
reolus
x vole)
ocanus
rutilus
alpina
a pica
cleator
ntanus
raneus
rex sp.
ulatus
Tick
amois
es pool
mouse

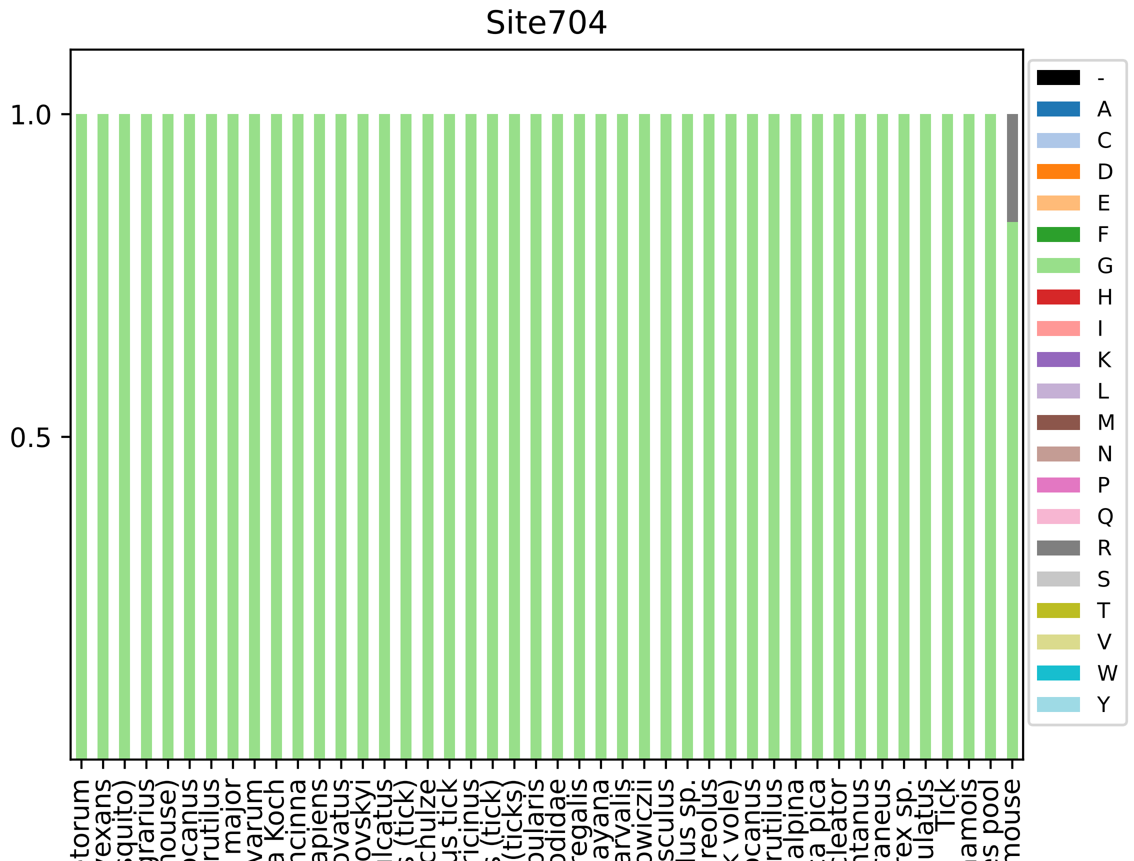
Site704
1.0
0.5
-
A
C
D
E
F
G
H
I
K
L
M
N
P
Q
R
S
T
V
W
Y

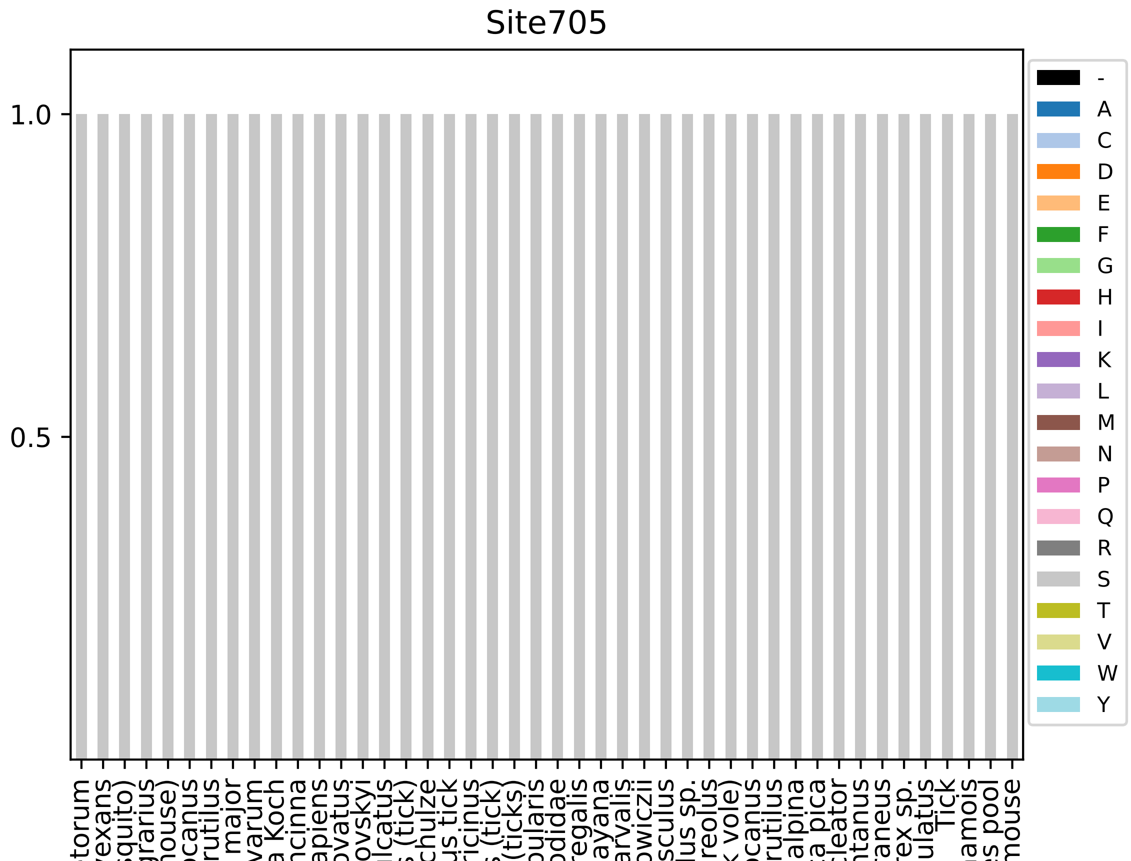

Site705
1.0
0.5
-
A
C
D
E
F
G
H
I
K
L
M
N
P
Q
R
S
T
V
W
Y

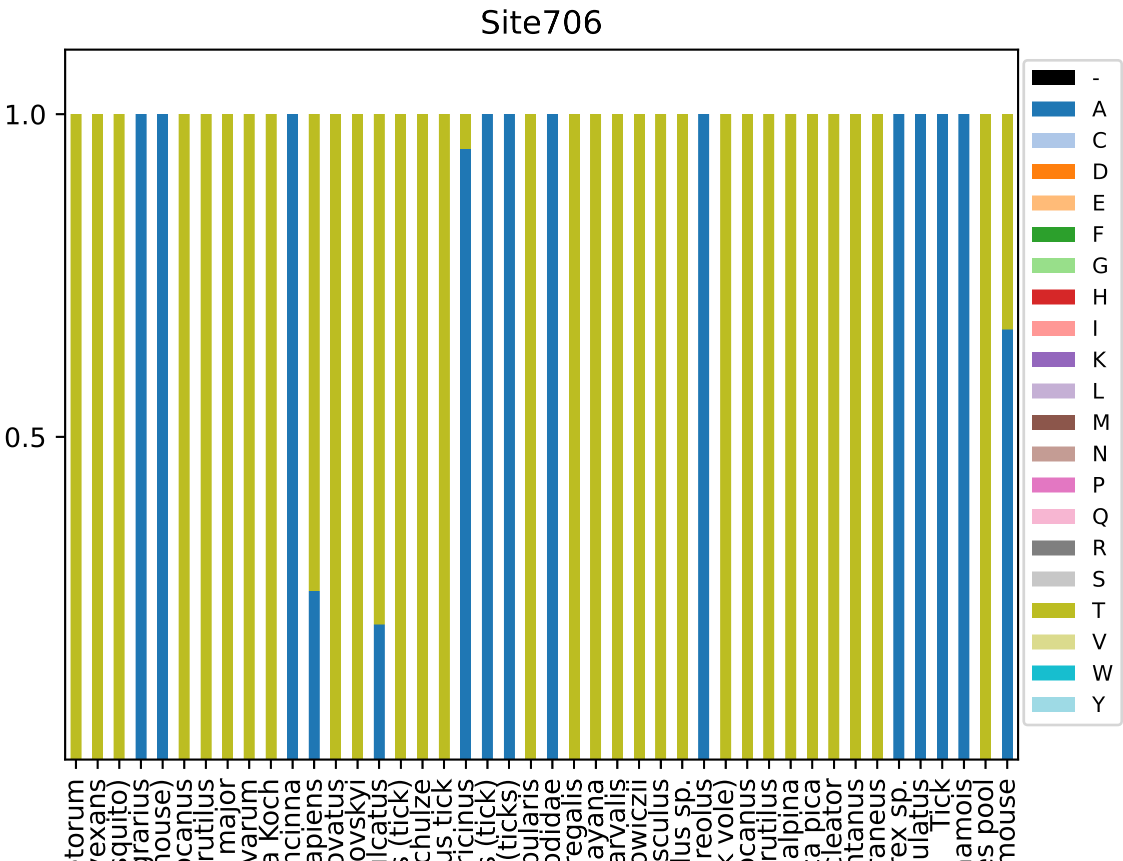
Site706
1.0
0.5
-
A
C
D
E
F
G
H
I
K
L
M
N
P
Q
R
S
T
V
W
Y
Tick

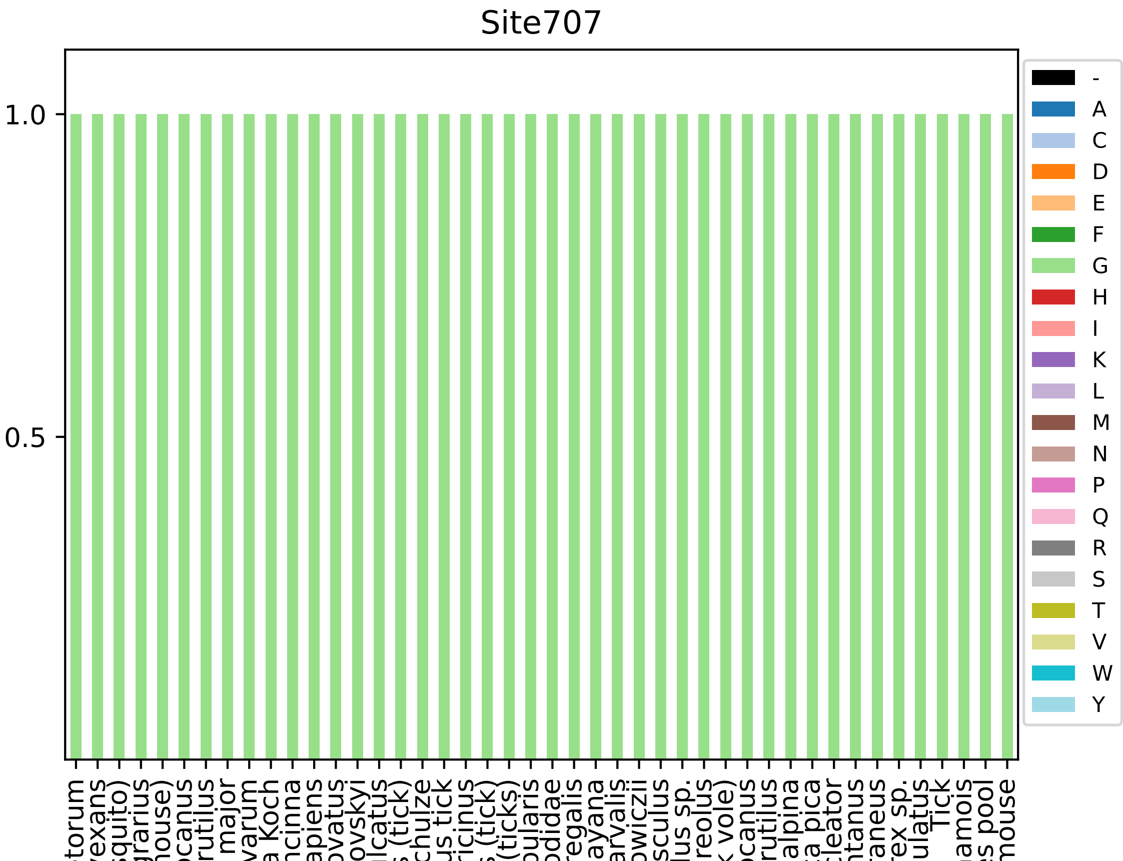

Site707
1.0
0.5
-
A
C
D
E
F
G
H
I
K
L
M
N
P
Q
R
S
T
V
W
Y

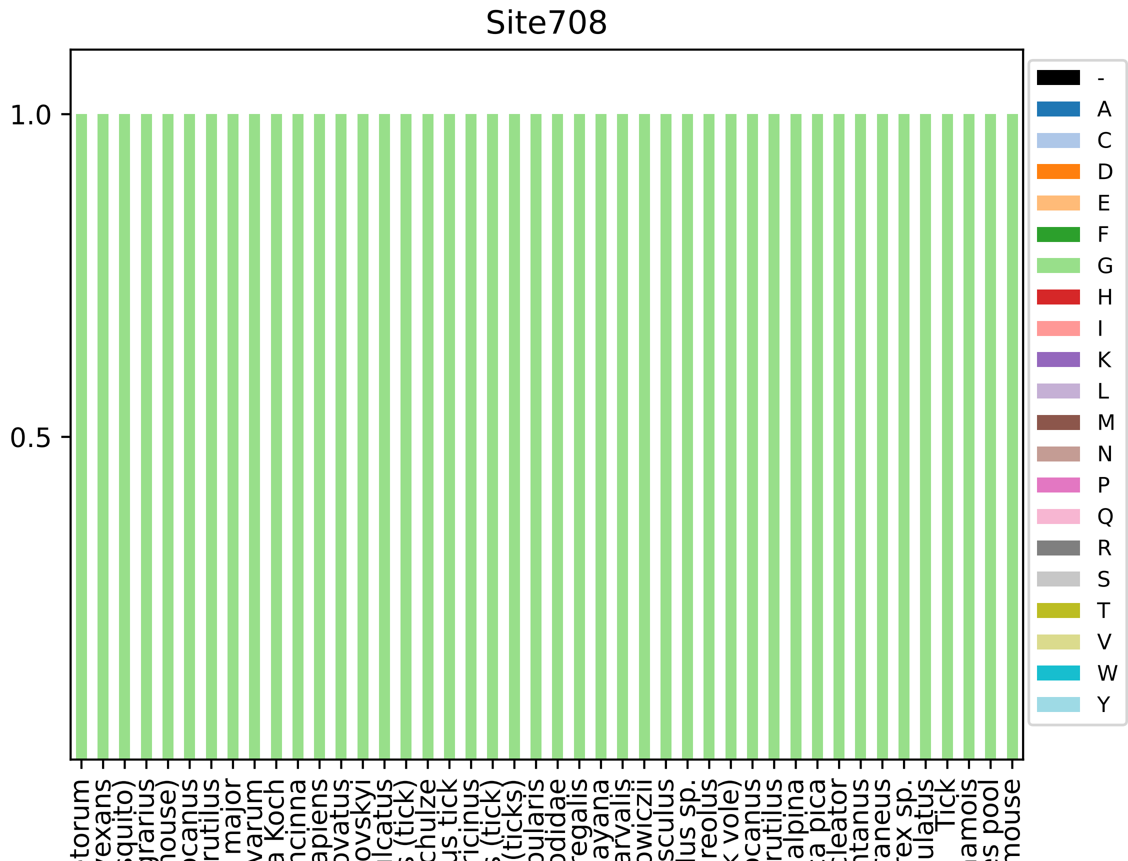
Site708
1.0
0.5
-
A
C
D
E
F
G
H
I
K
L
M
N
P
Q
R
S
T
V
W
Y

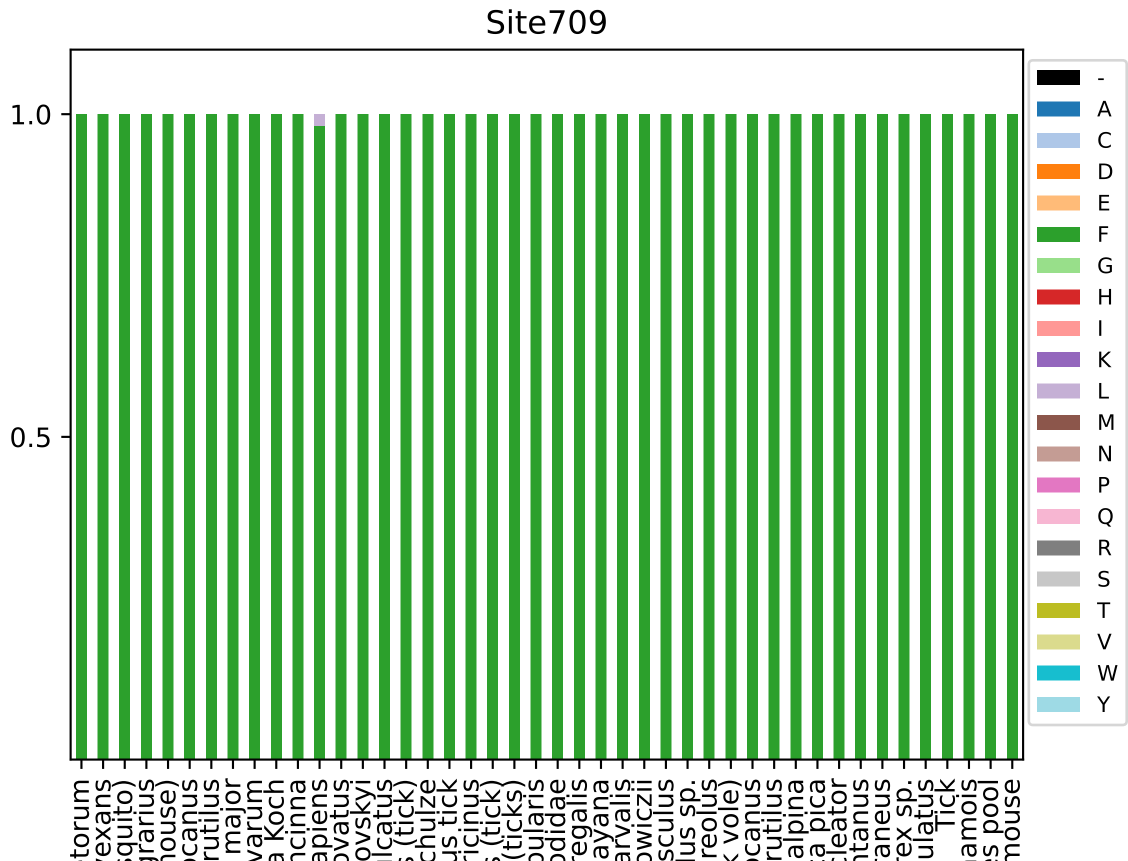

Site709
1.0
0.5
-
A
C
D
E
F
G
H
I
K
L
M
N
P
Q
R
S
T
V
W
Y

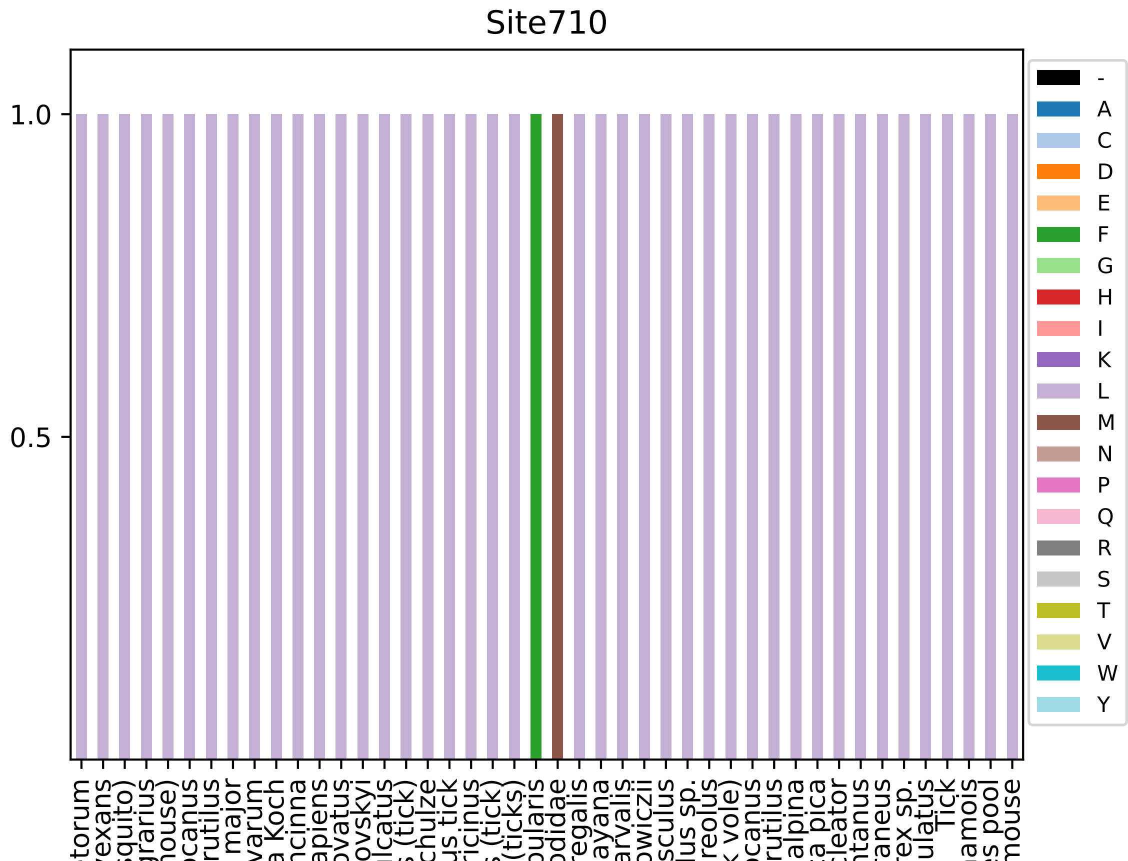
Site710
1.0
0.5
-
A
C
D
E
F
G
H
I
K
L
M
N
P
Q
R
S
T
V
W
Y
torum
vexans
squito)
grarius
house)
ocanus
rutilus
major
varum
a Koch
ncinna
apiens
ovatus
ovskyi
lcatus
s (tick)
chulze
us tick
ricinus
s (ticks)
oularis
odidae
regalis
ayana
arvalis
owiczii
sculus
lus sp.
reolus
k vole)
ocanus
rutilus
alpina
a pica
leator
ntanus
raneus
rex sp.
ulatus
Tick
amois
es pool
mouse

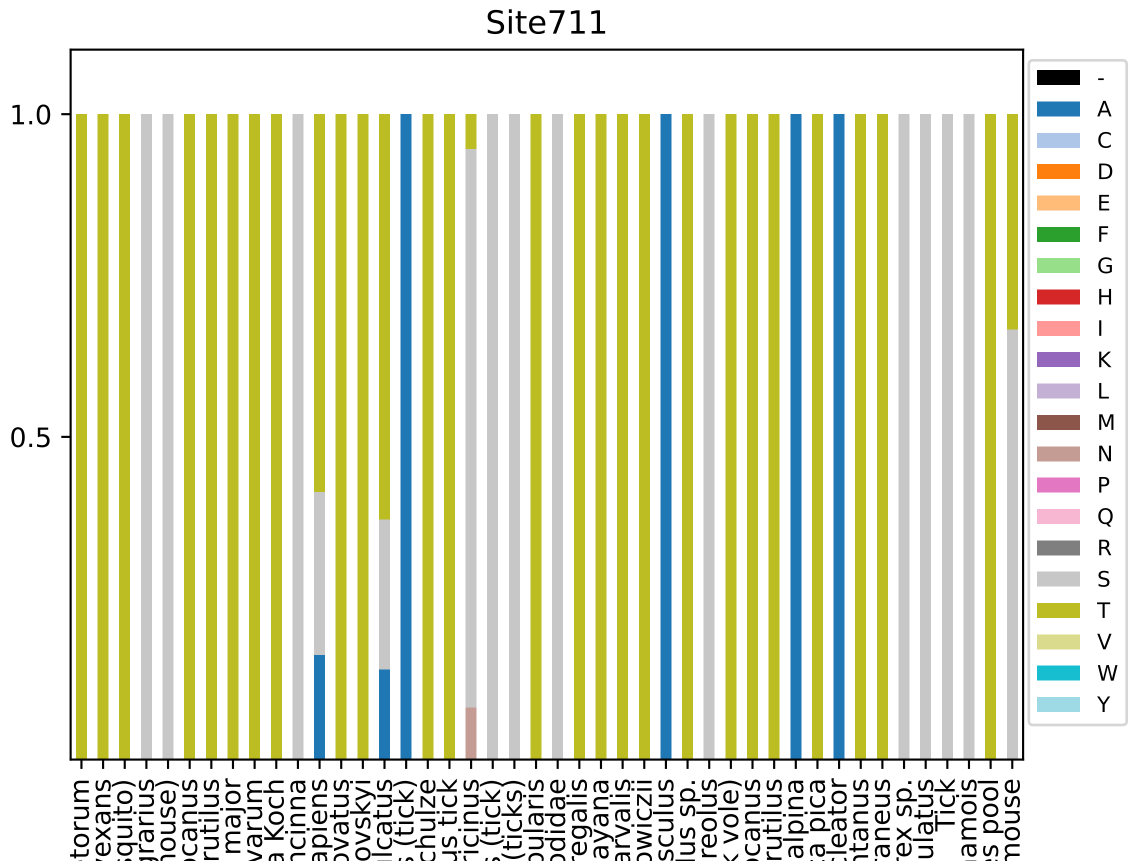
Site711
1.0
0.5
-
A
C
D
E
F
G
H
I
K
L
M
N
P
Q
R
S
T
V
W
Y

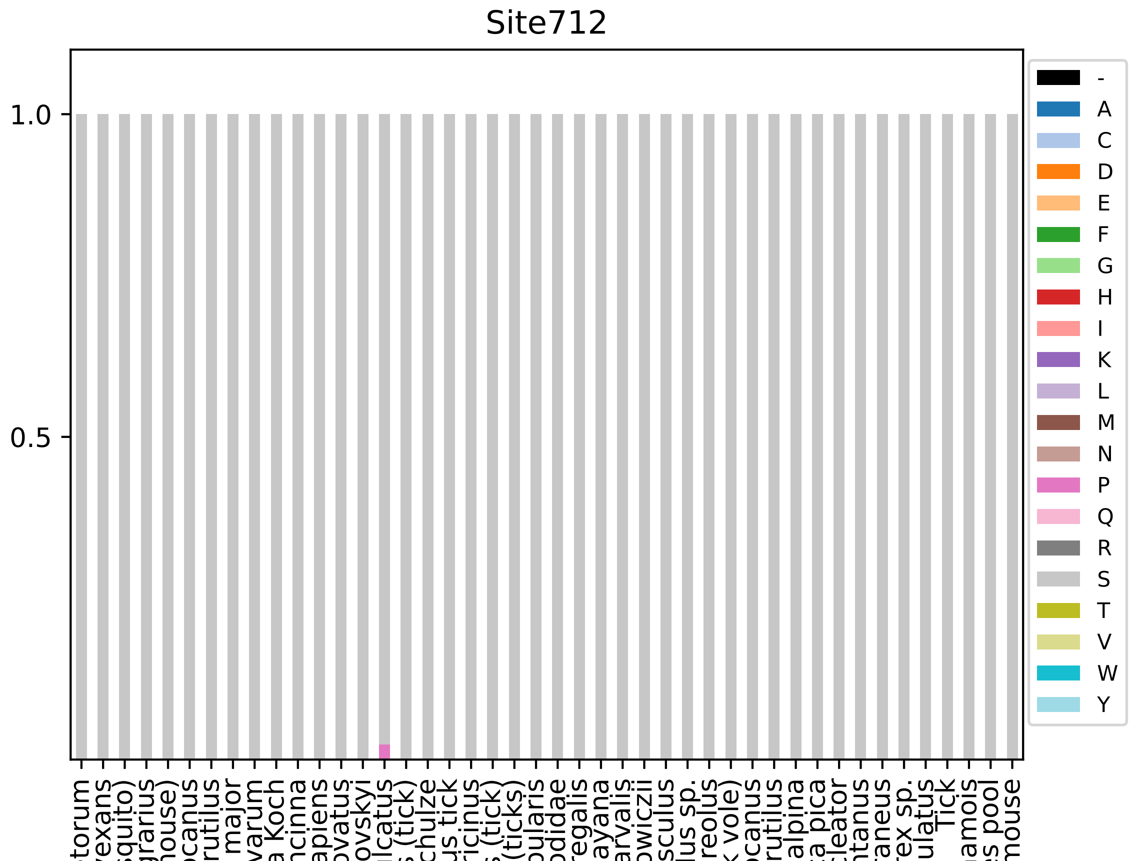
Site712
1.0
0.5
-
A
C
D
E
F
G
H
I
K
L
M
N
P
Q
R
S
T
V
W
Y

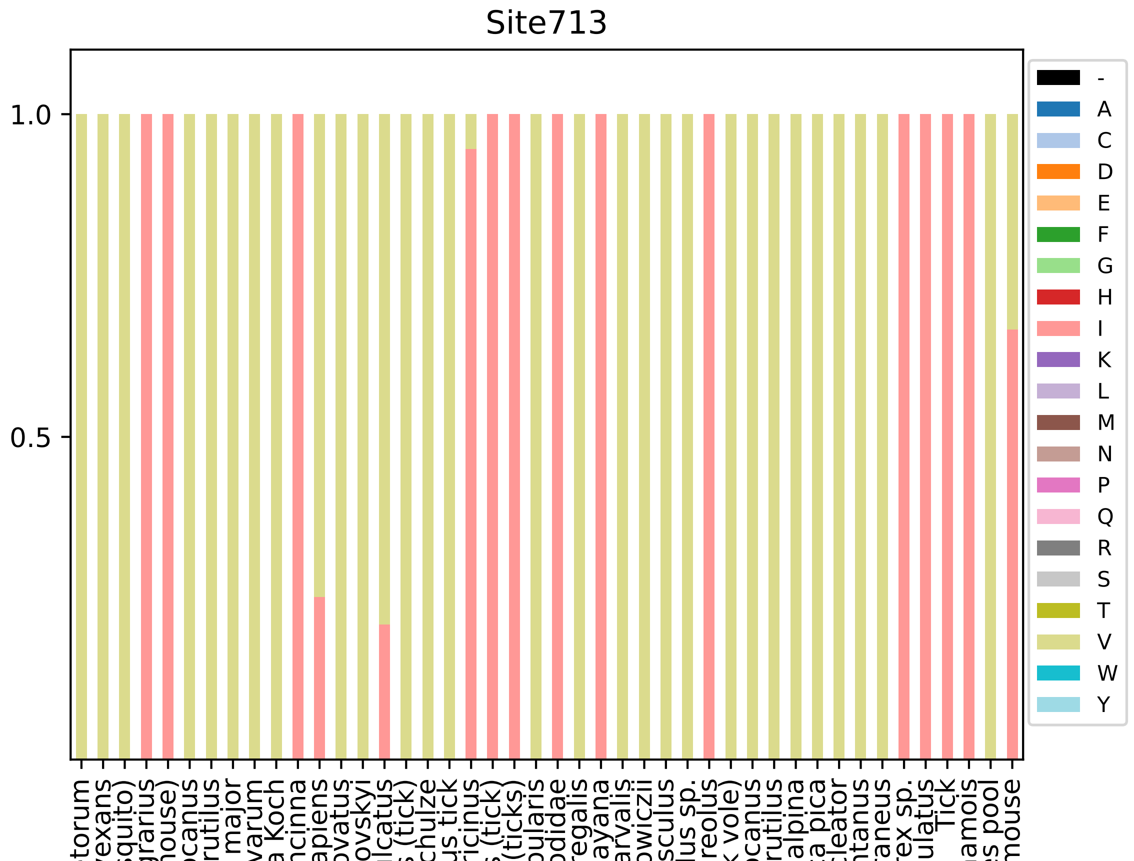
Site713
1.0
0.5
-
A
C
D
E
F
G
H
I
K
L
M
N
P
Q
R
S
T
V
W
Y
torum
vexans
squito)
grarius
house)
ocanus
rutilus
major
varum
a Koch
ncinna
apiens
ovatus
ovskyi
lcatus
s (tick)
chulze
us tick
ricinus
s (tick)
(ticks)
pularis
podidae
regalis
ayana
arvalis
owiczii
sculus
lus sp.
reolus
k vole)
ocanus
rutilus
alpina
a pica
cleator
ntanus
raneus
rex sp.
ulatus
Tick
amois
es pool
mouse

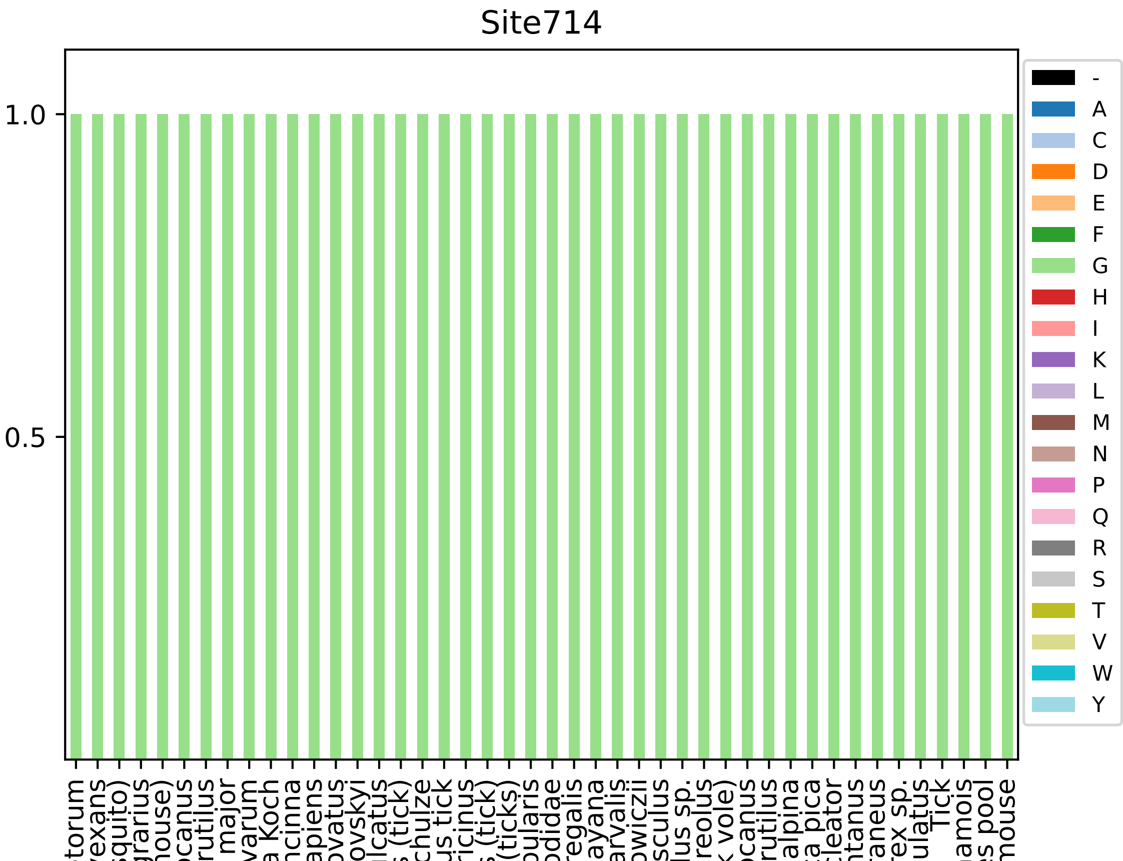

Site714
1.0
0.5
-
A
C
D
E
F
G
H
I
K
L
M
N
P
Q
R
S
T
V
W
Y

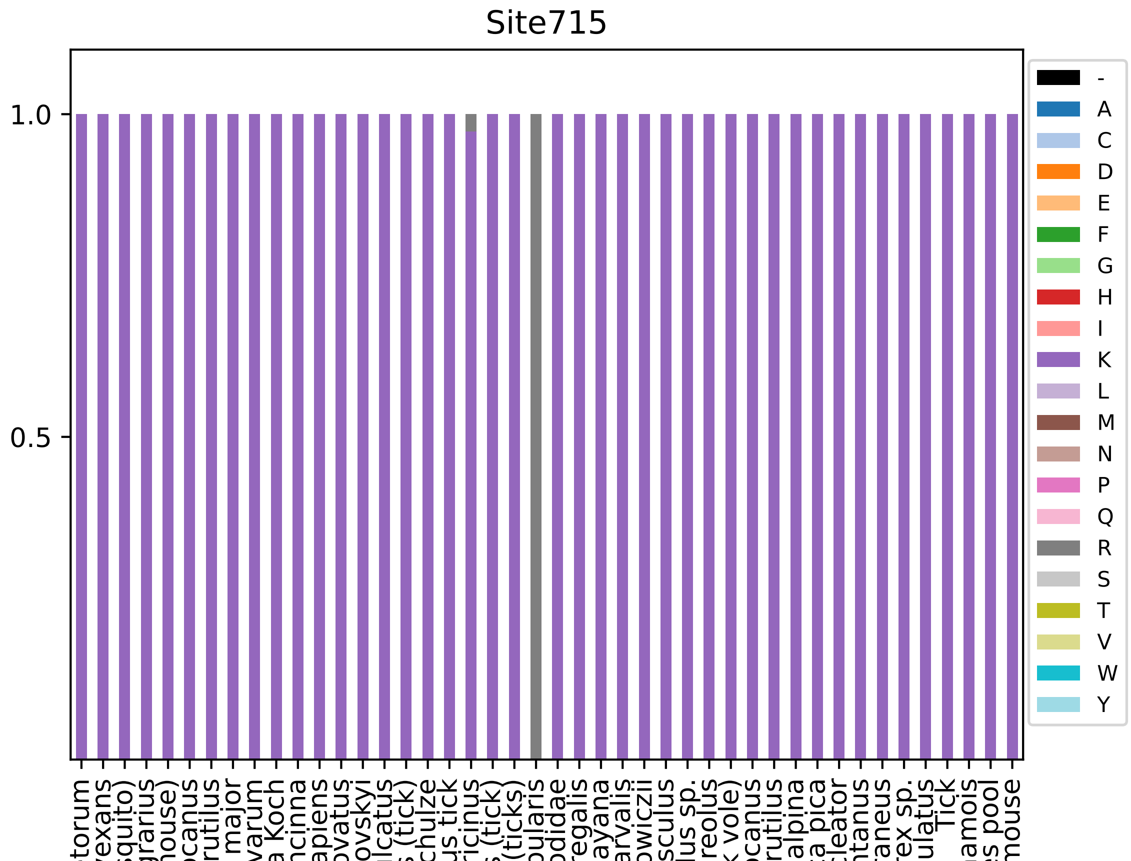
Site715
1.0
0.5
-
A
C
D
E
F
G
H
I
K
L
M
N
P
Q
R
S
T
V
W
Y
Tick

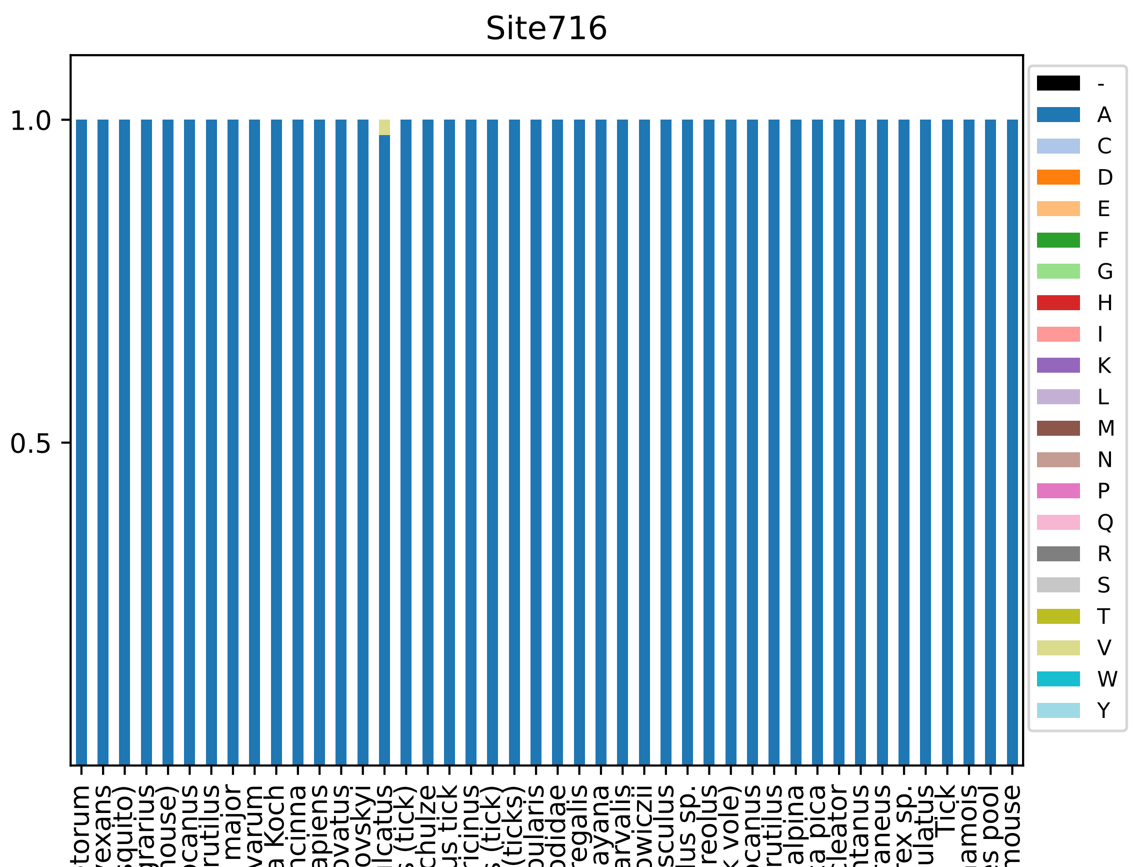
Site716
1.0
0.5
-
A
C
D
E
F
G
H
I
K
L
M
N
P
Q
R
S
T
V
W
Y

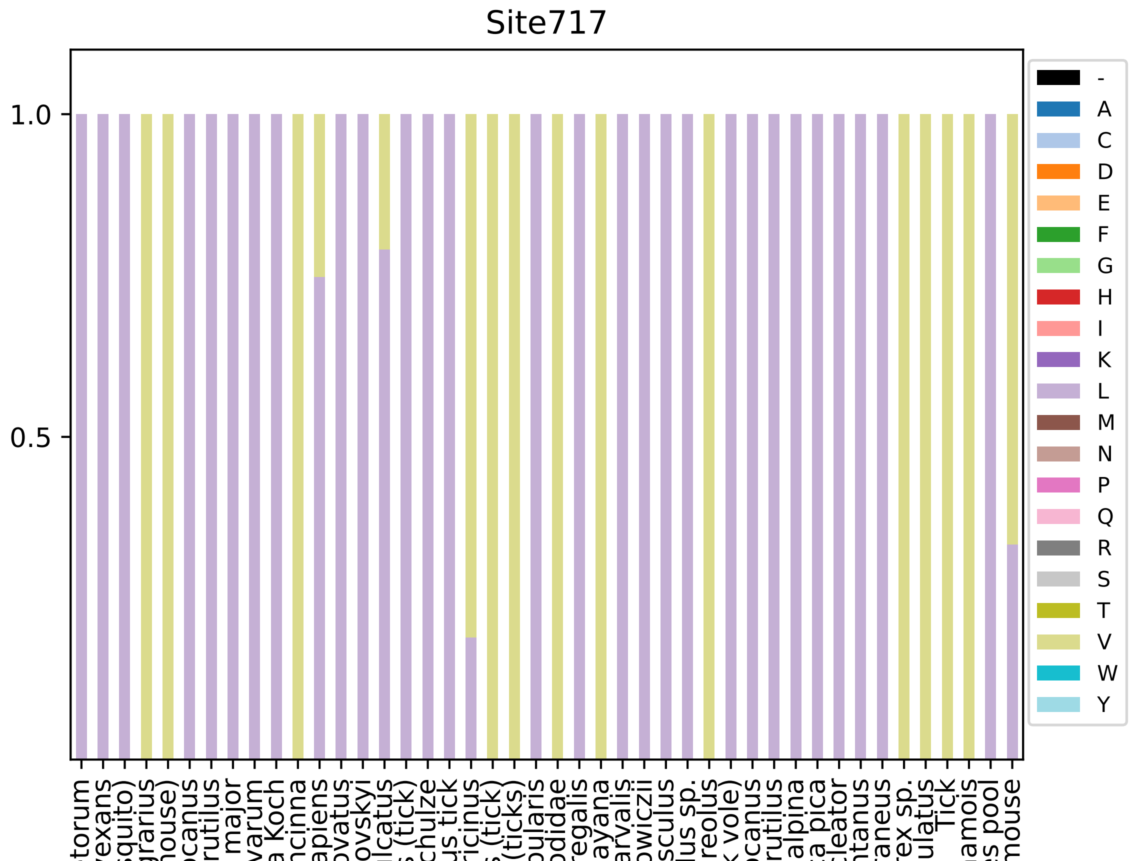
Site717
1.0
0.5
-
A
C
D
E
F
G
H
I
K
L
M
N
P
Q
R
S
T
V
W
Y

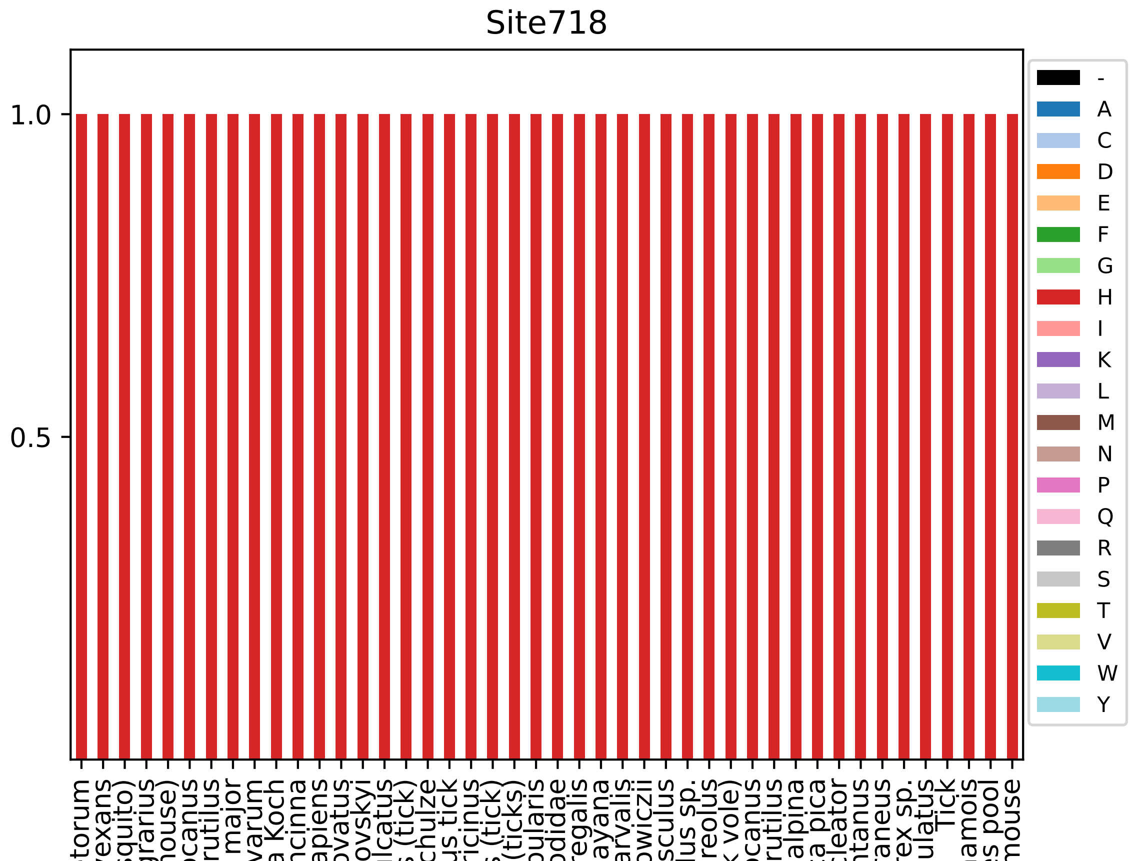
Site718
1.0
0.5
-
A
C
D
E
F
G
H
I
K
L
M
N
P
Q
R
S
T
V
W
Y

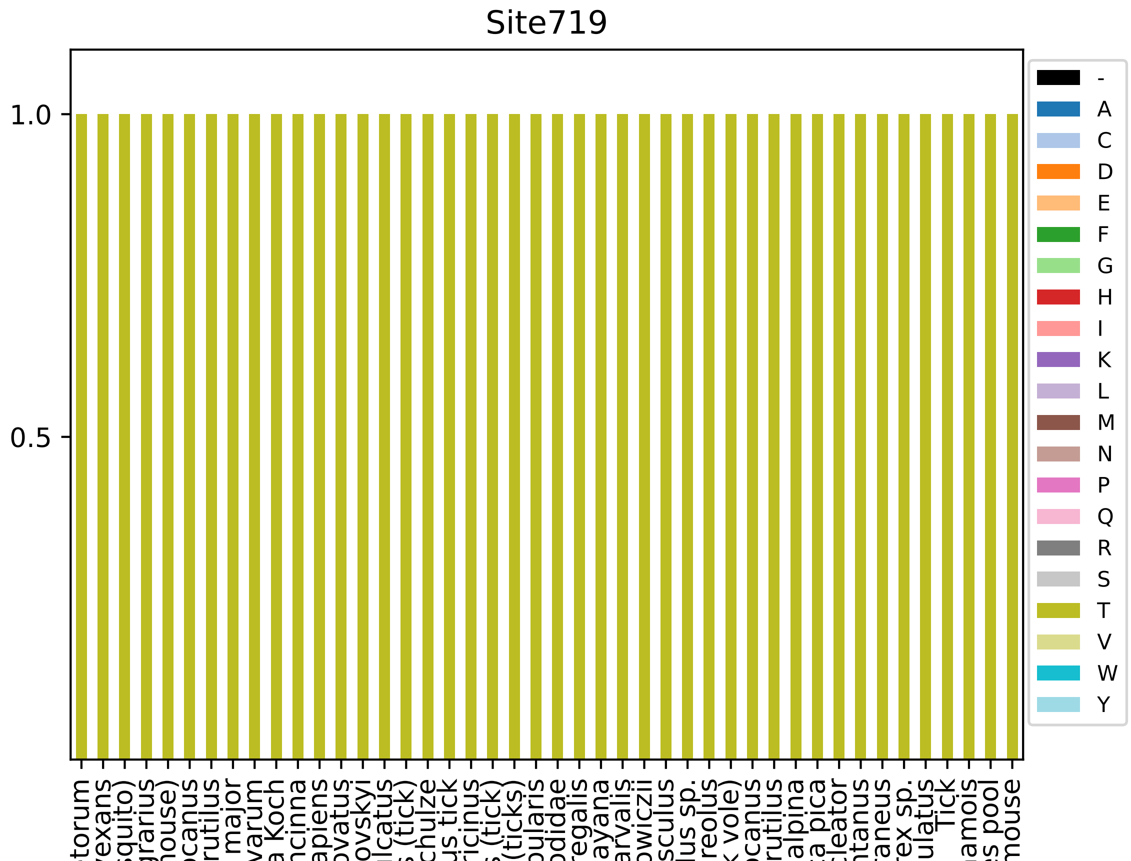
Site719
1.0
0.5
-
A
C
D
E
F
G
H
I
K
L
M
N
P
Q
R
S
T
V
W
Y

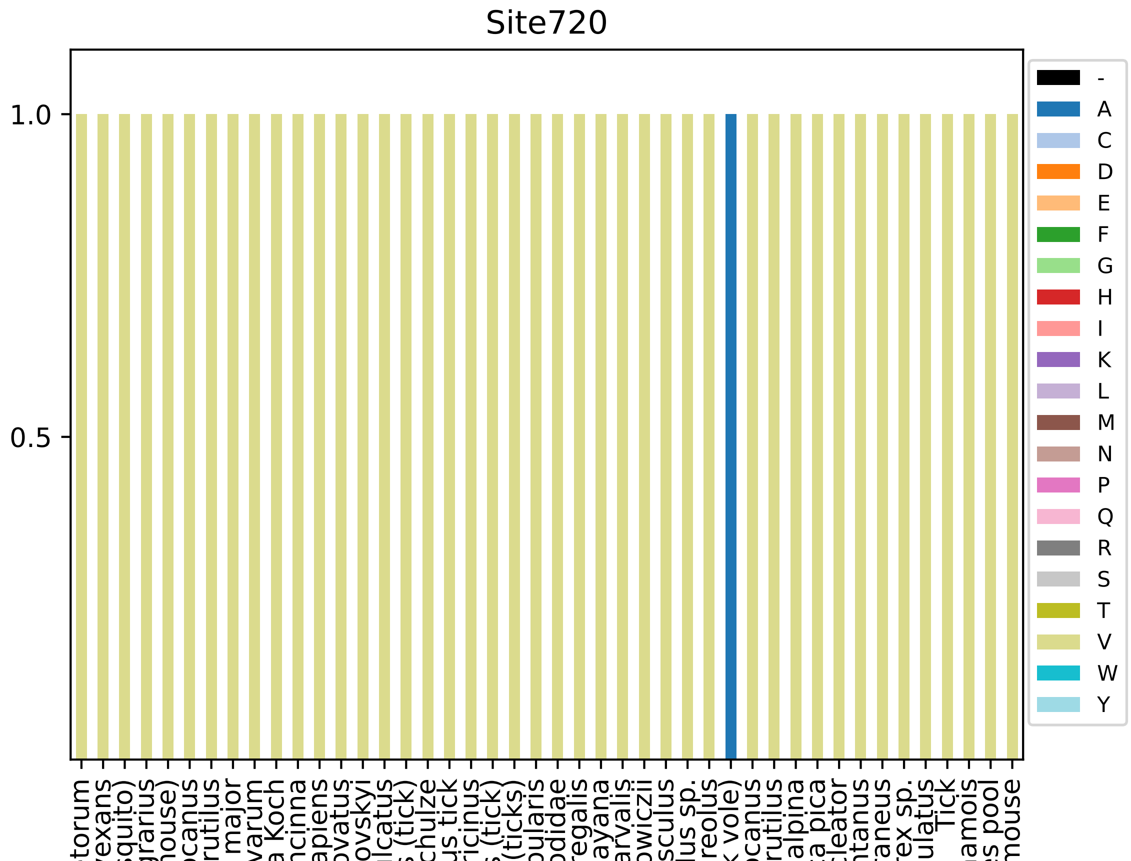
Site720
1.0
0.5
-
A
C
D
E
F
G
H
I
K
L
M
N
P
Q
R
S
T
V
W
Y

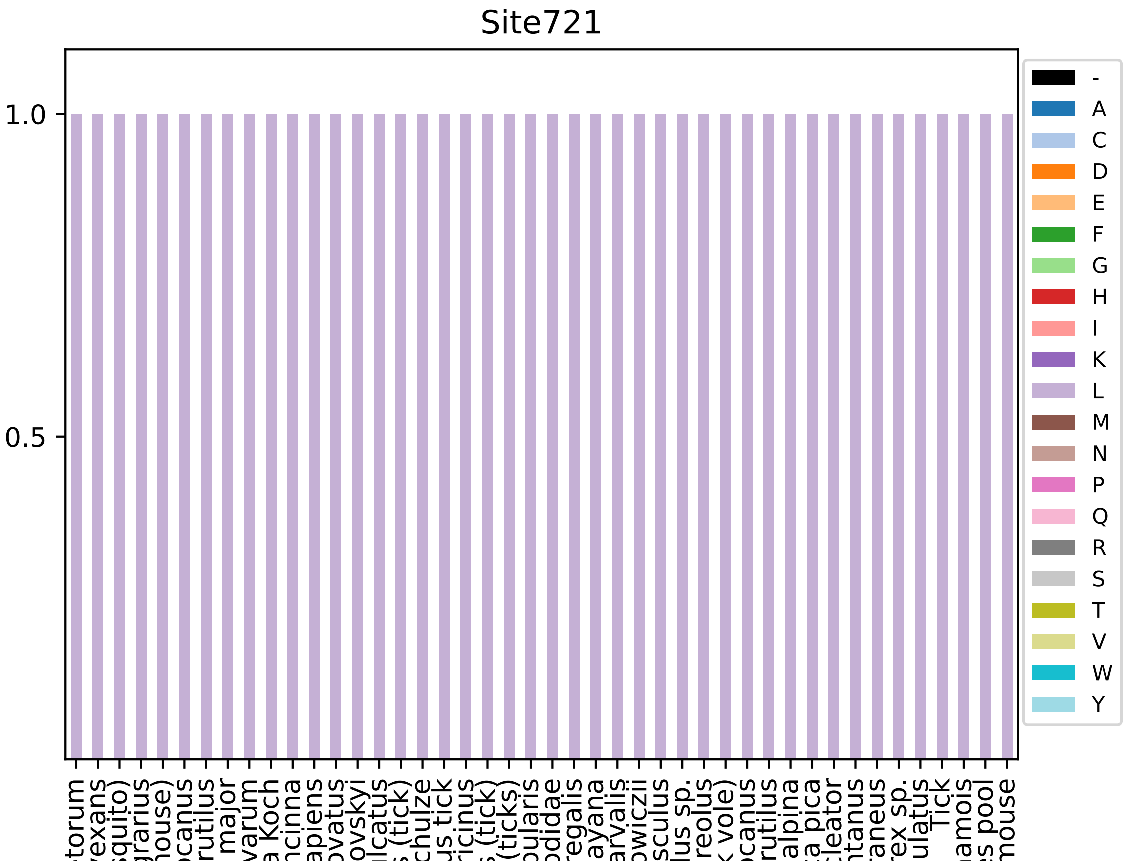

Site721
1.0
0.5
-
A
C
D
E
F
G
H
I
K
L
M
N
P
Q
R
S
T
V
W
Y

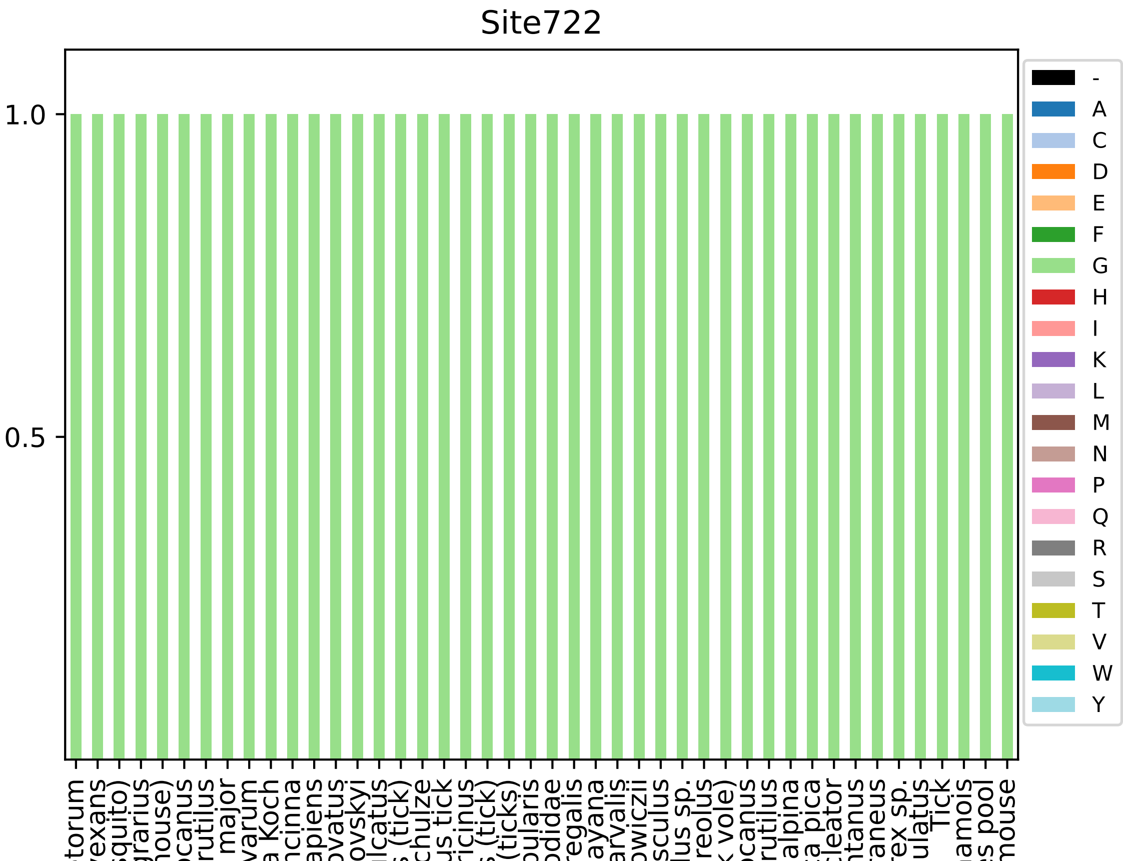
Site722
1.0
0.5
-
A
C
D
E
F
G
H
I
K
L
M
N
P
Q
R
S
T
V
W
Y

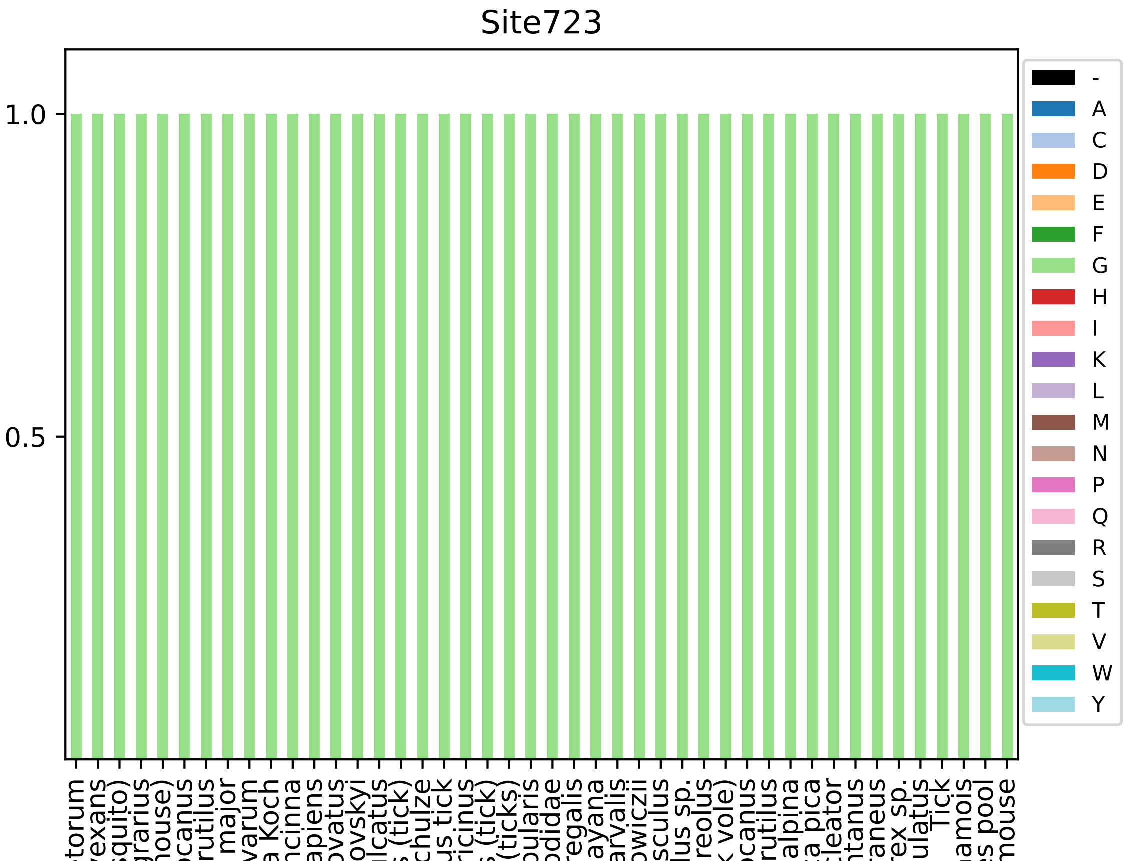
Site723
1.0
0.5
-
A
C
D
E
F
G
H
I
K
L
M
N
P
Q
R
S
T
V
W
Y

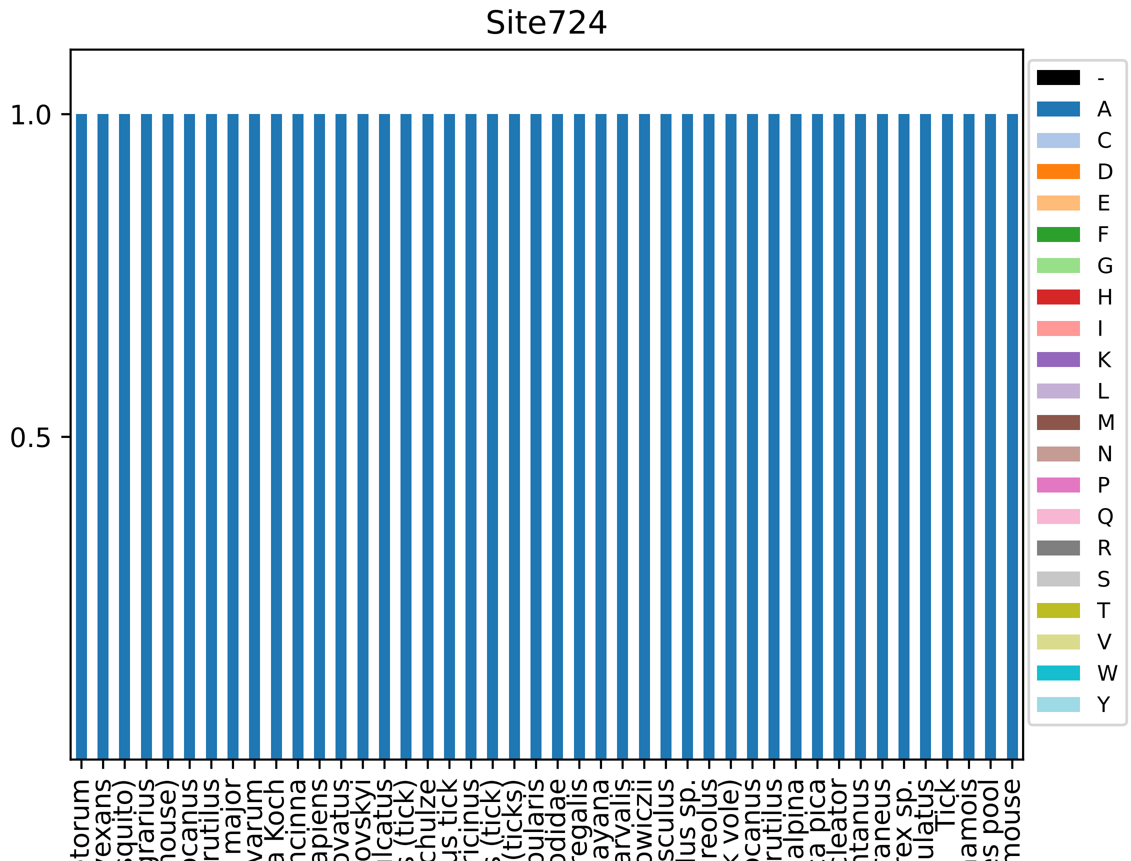

Site724
1.0
0.5
-
A
C
D
E
F
G
H
I
K
L
M
N
P
Q
R
S
T
V
W
Y

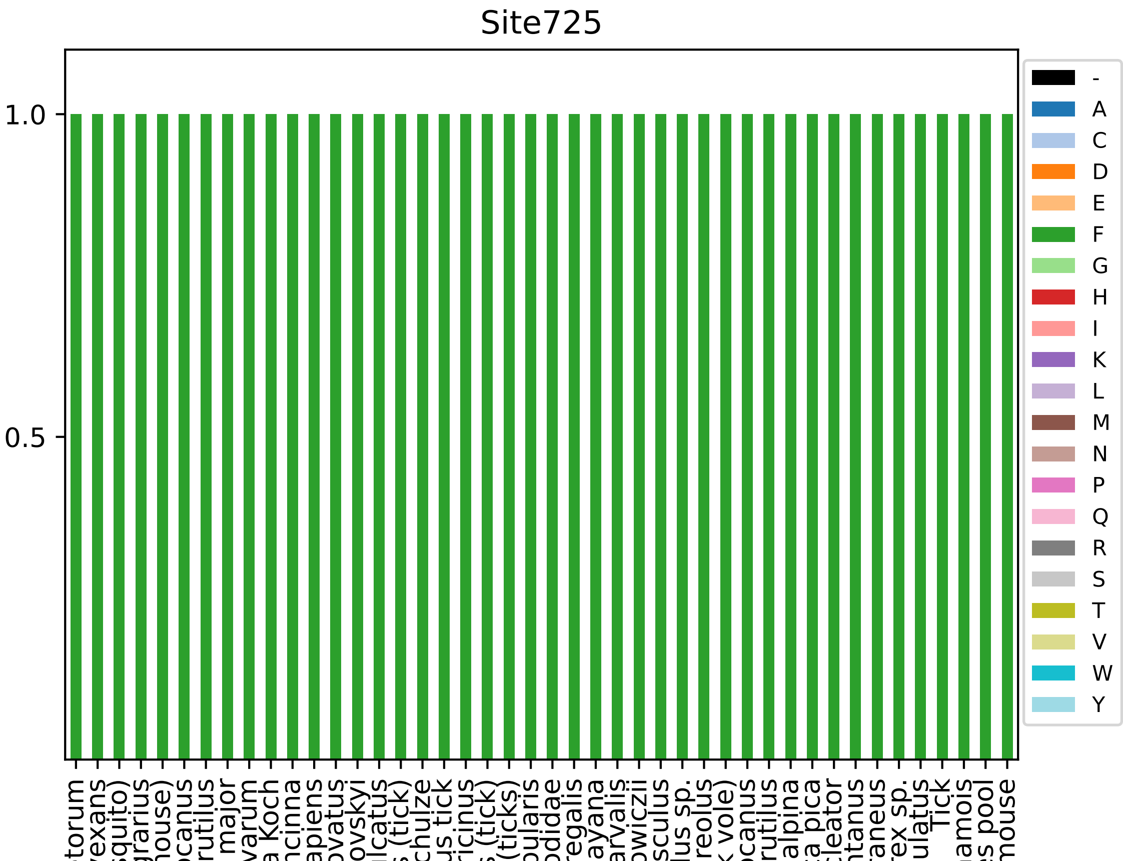
Site725
1.0
0.5
-
A
C
D
E
F
G
H
I
K
L
M
N
P
Q
R
S
T
V
W
Y

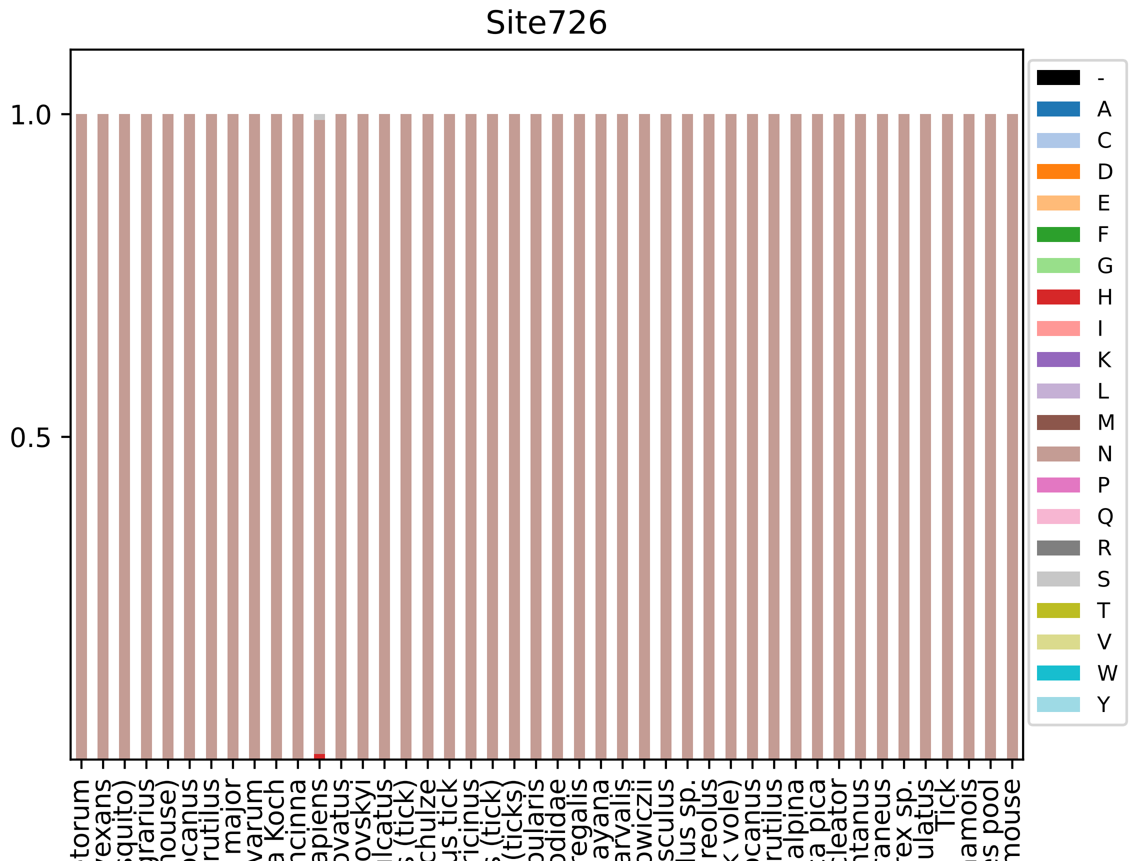
Site726
1.0
0.5
-
A
C
D
E
F
G
H
I
K
L
M
N
P
Q
R
S
T
V
W
Y

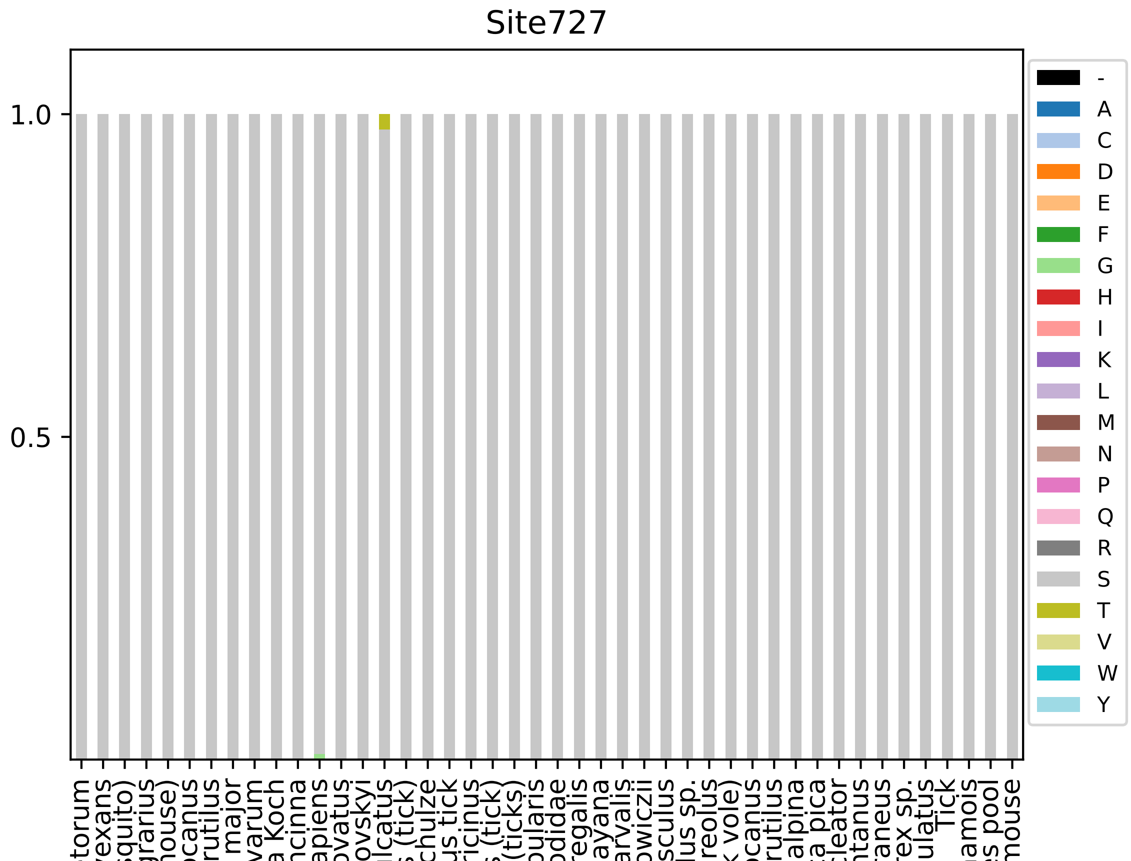
Site727
1.0
0.5
-
A
C
D
E
F
G
H
I
K
L
M
N
P
Q
R
S
T
V
W
Y

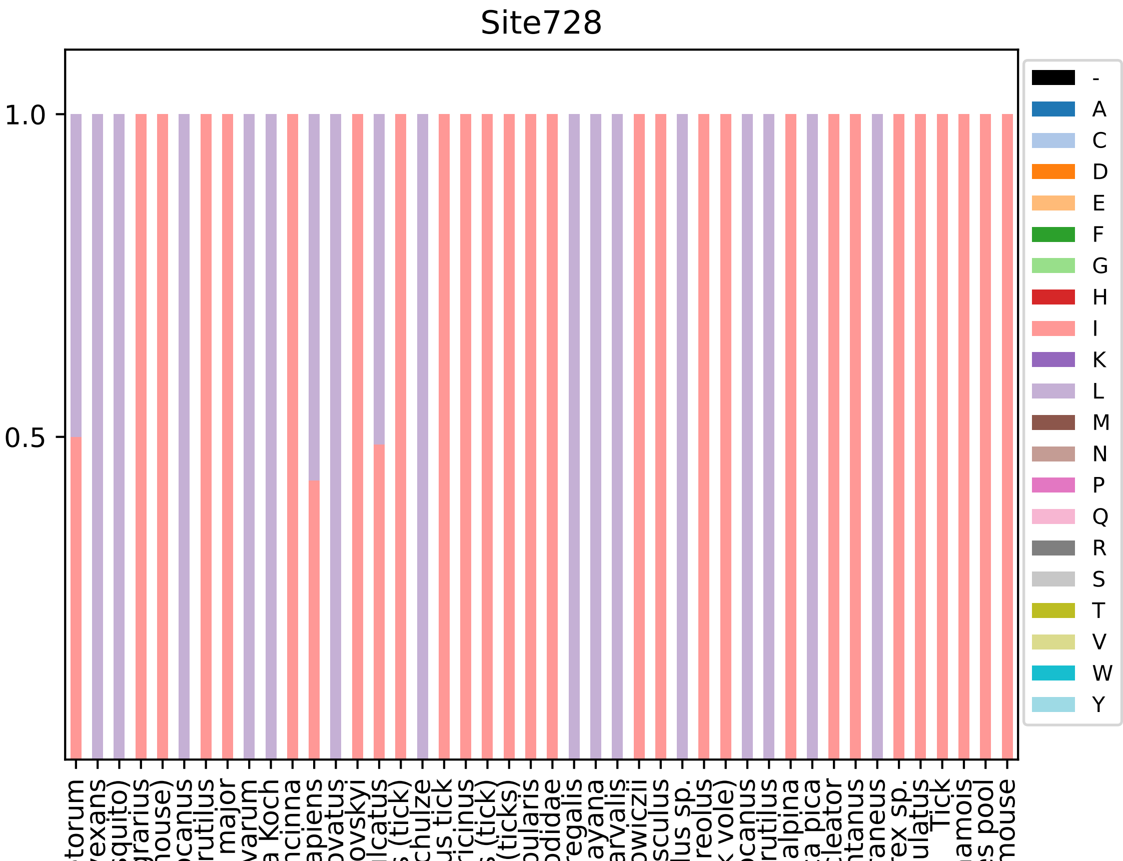
Site728
1.0
0.5
-
A
C
D
E
F
G
H
I
K
L
M
N
P
Q
R
S
T
V
W
Y

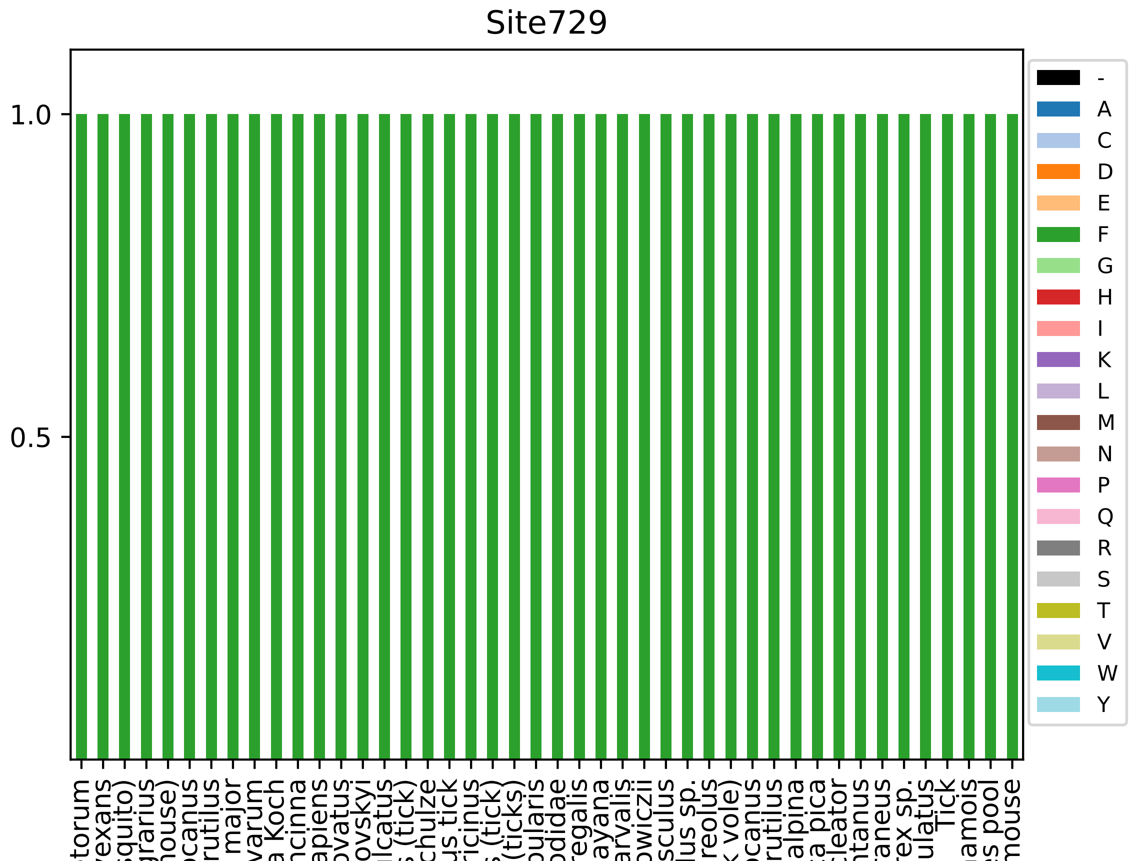
Site729
1.0
0.5
-
A
C
D
E
F
G
H
I
K
L
M
N
P
Q
R
S
T
V
W
Y

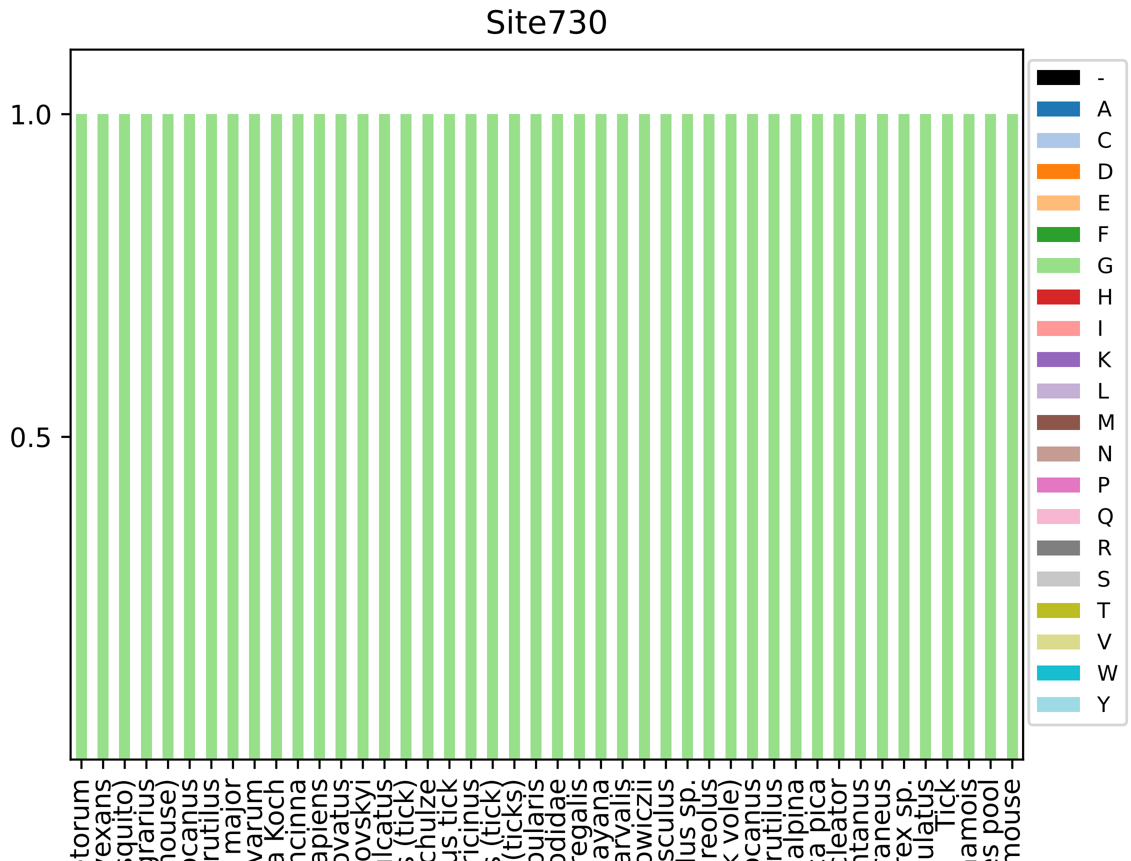
Site730
1.0
0.5
-
A
C
D
E
F
G
H
I
K
L
M
N
P
Q
R
S
T
V
W
Y

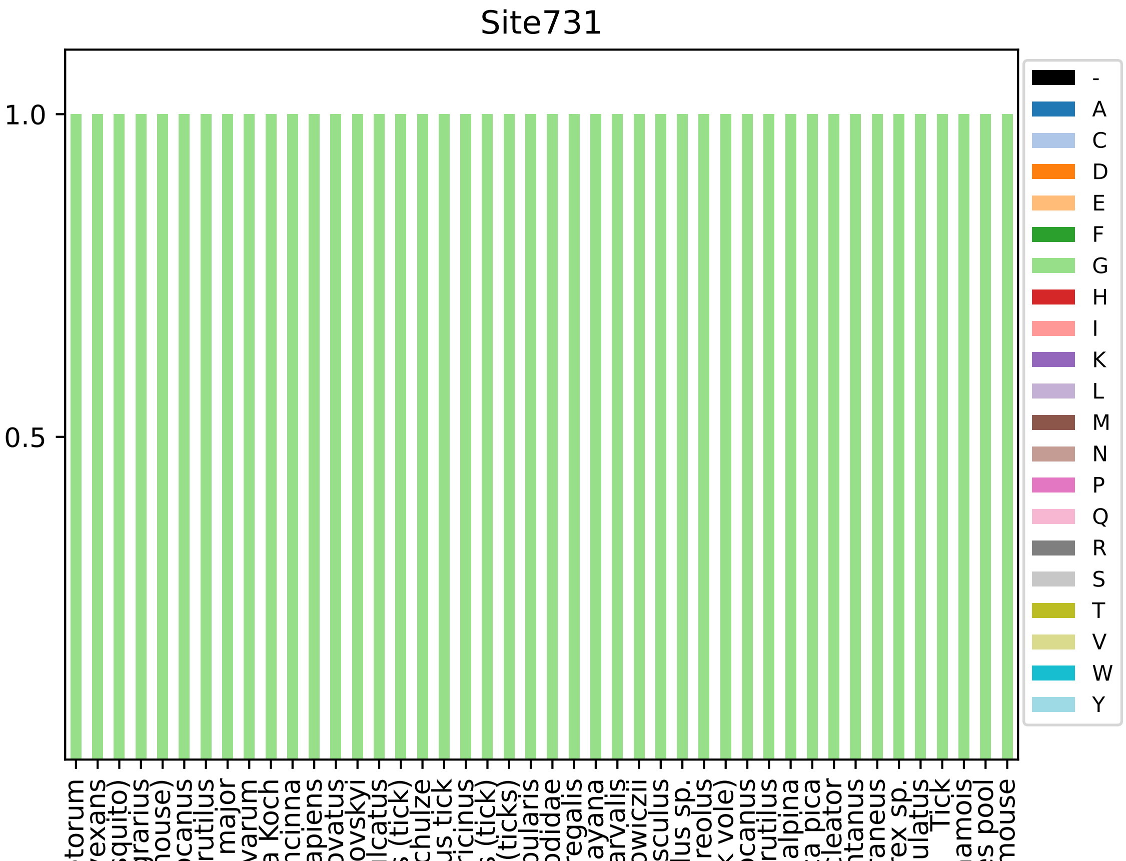

Site731
1.0
0.5
-
A
C
D
E
F
G
H
I
K
L
M
N
P
Q
R
S
T
V
W
Y

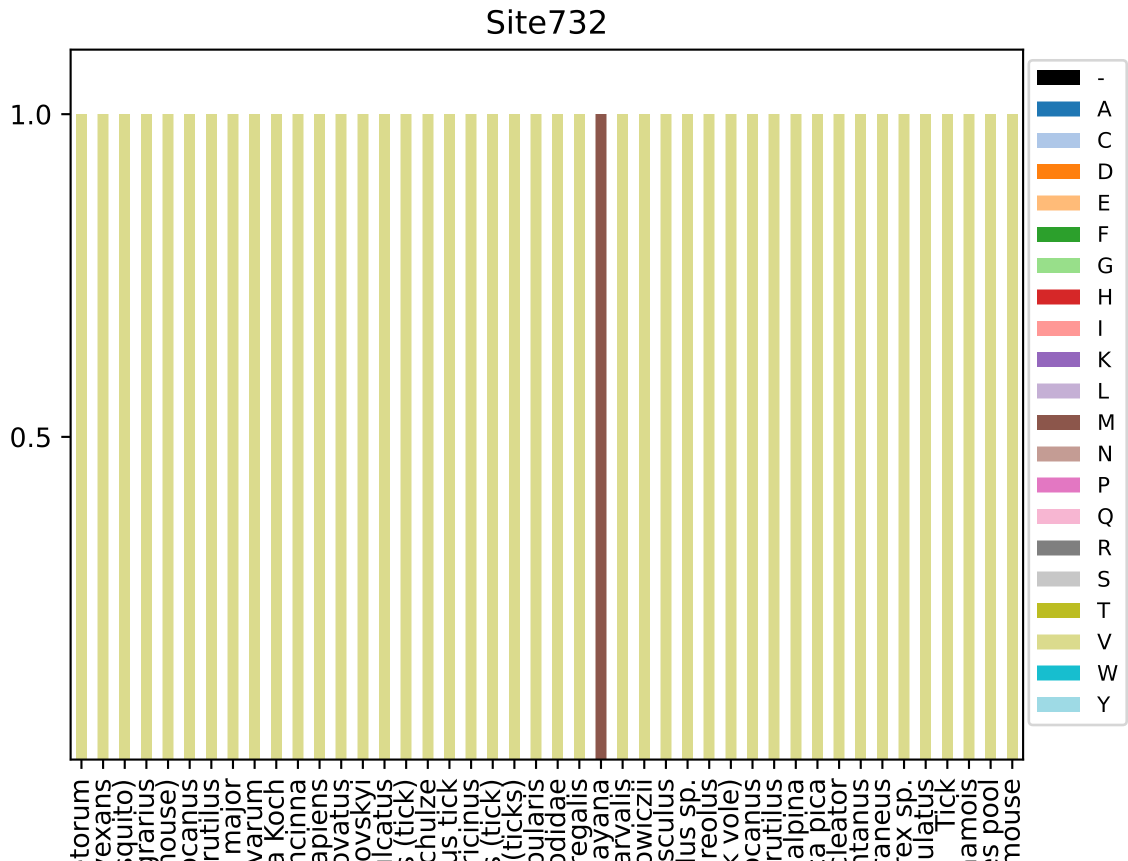
Site732
1.0
0.5
-
A
C
D
E
F
G
H
I
K
L
M
N
P
Q
R
S
T
V
W
Y
torum
vexans
squito)
grarius
nouse)
ocanus
rutilus
major
varum
a Koch
ncinna
apiens
ovatus
ovskyi
ulcatus
s (tick)
chulze
us tick
ricinus
s (ticks)
pularis
odidae
regalis
ayana
arvalis
owiczii
sculus
lus sp.
reolus
k vole)
ocanus
rutilus
alpina
a pica
leator
ntanus
aneus
rex sp.
ulatus
Tick
amois
es pool
mouse

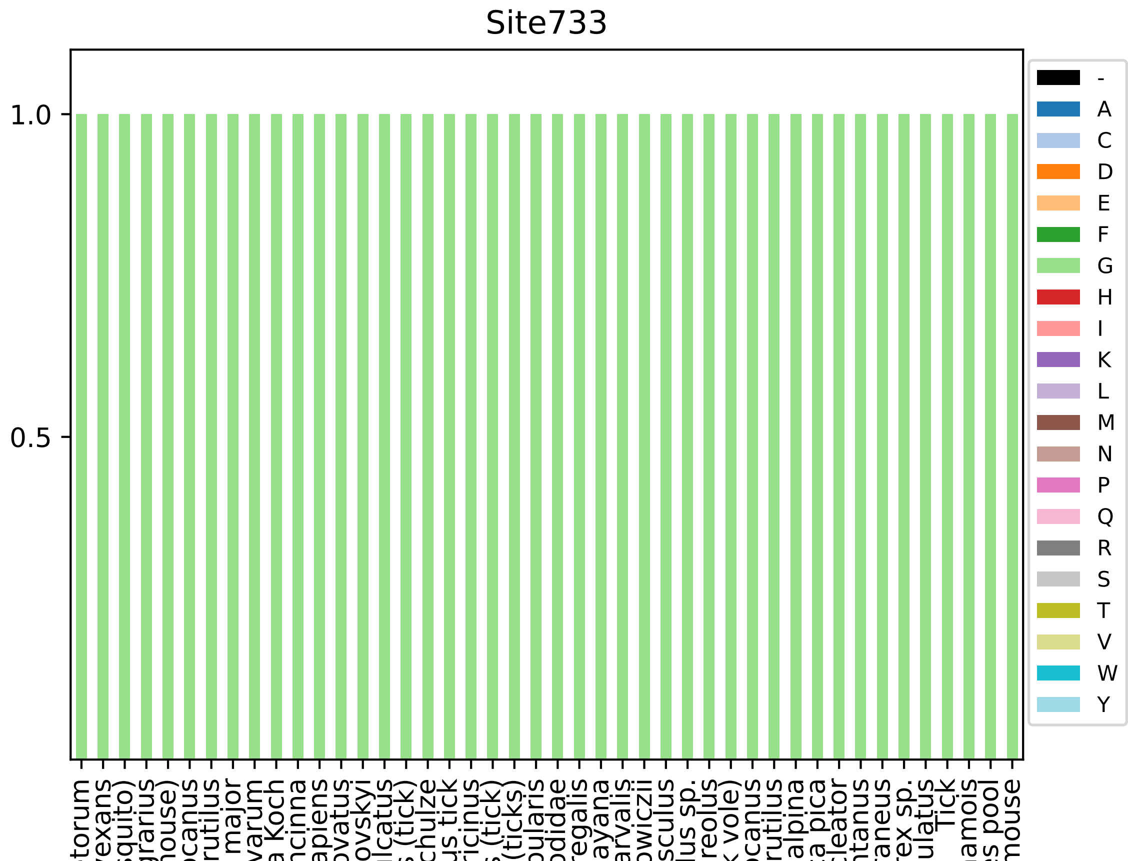
Site733
1.0
0.5
-
A
C
D
E
F
G
H
I
K
L
M
N
P
Q
R
S
T
V
W
Y

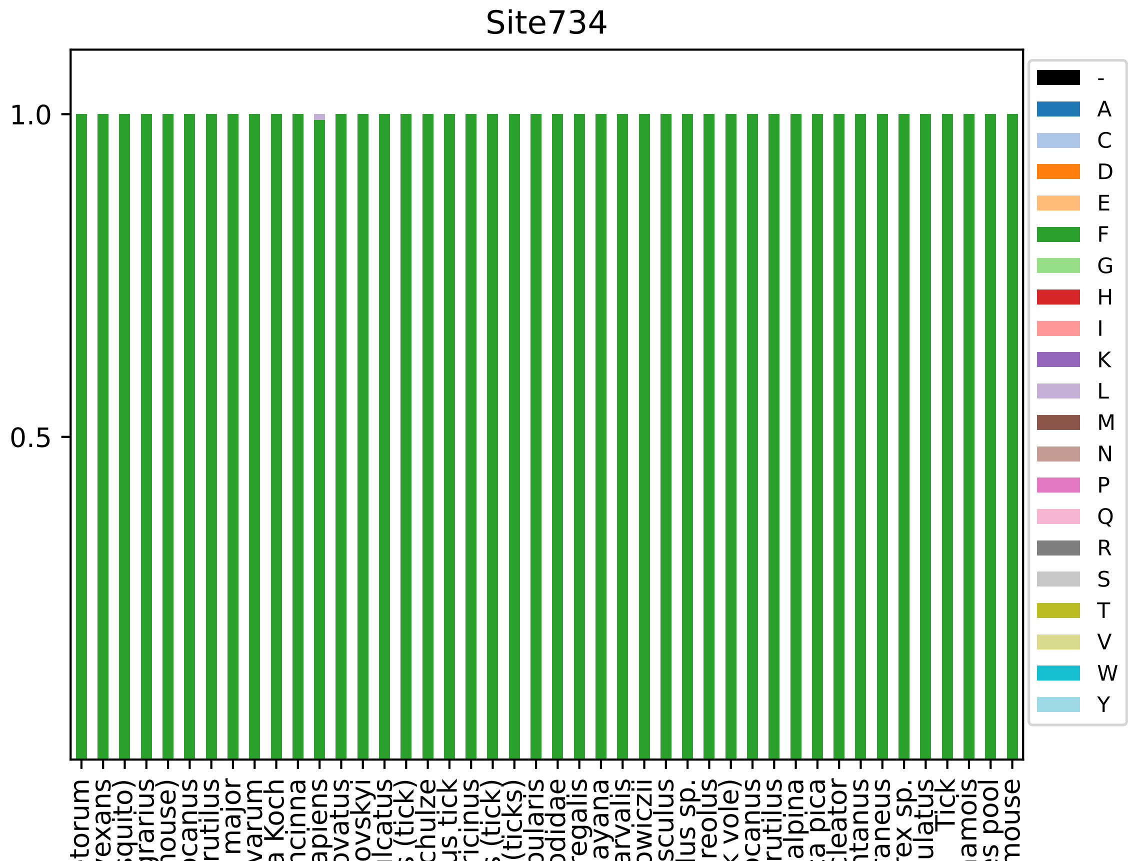
Site734
1.0
0.5
-
A
C
D
E
F
G
H
I
K
L
M
N
P
Q
R
S
T
V
W
Y

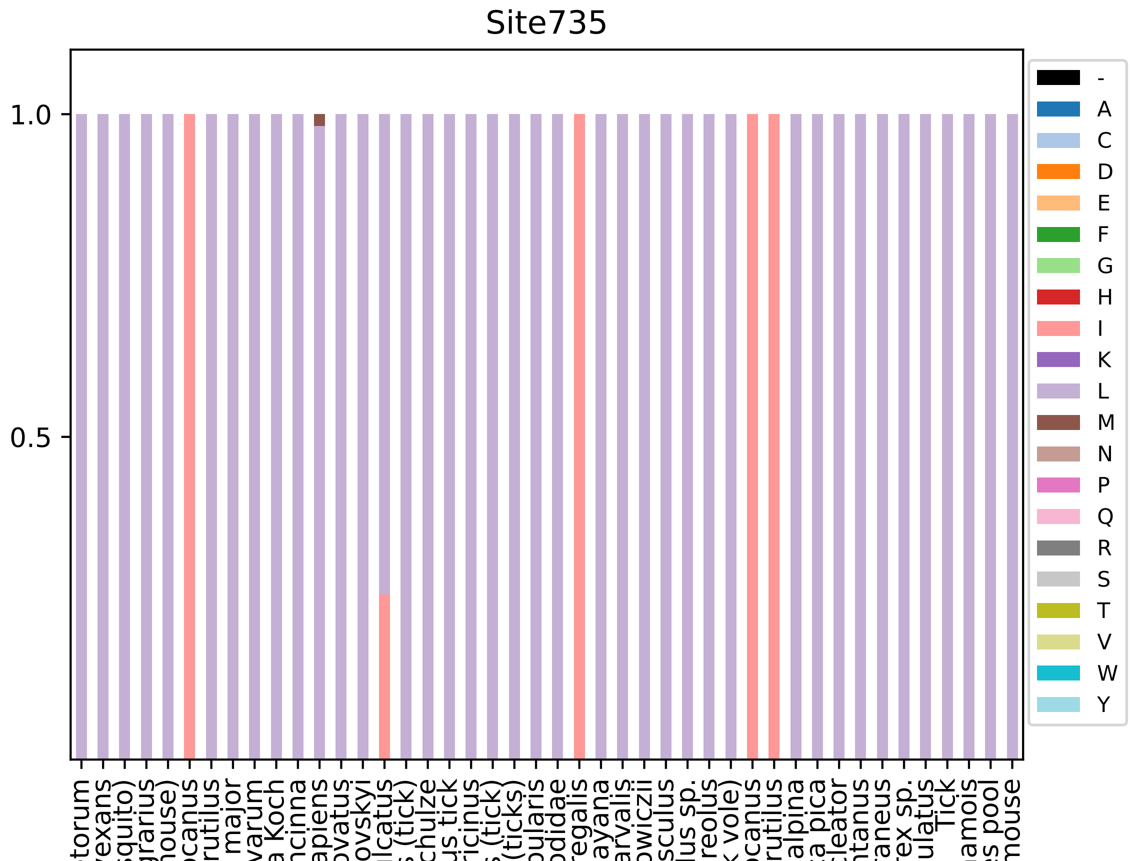
Site735
1.0
0.5
-
A
C
D
E
F
G
H
I
K
L
M
N
P
Q
R
S
T
V
W
Y

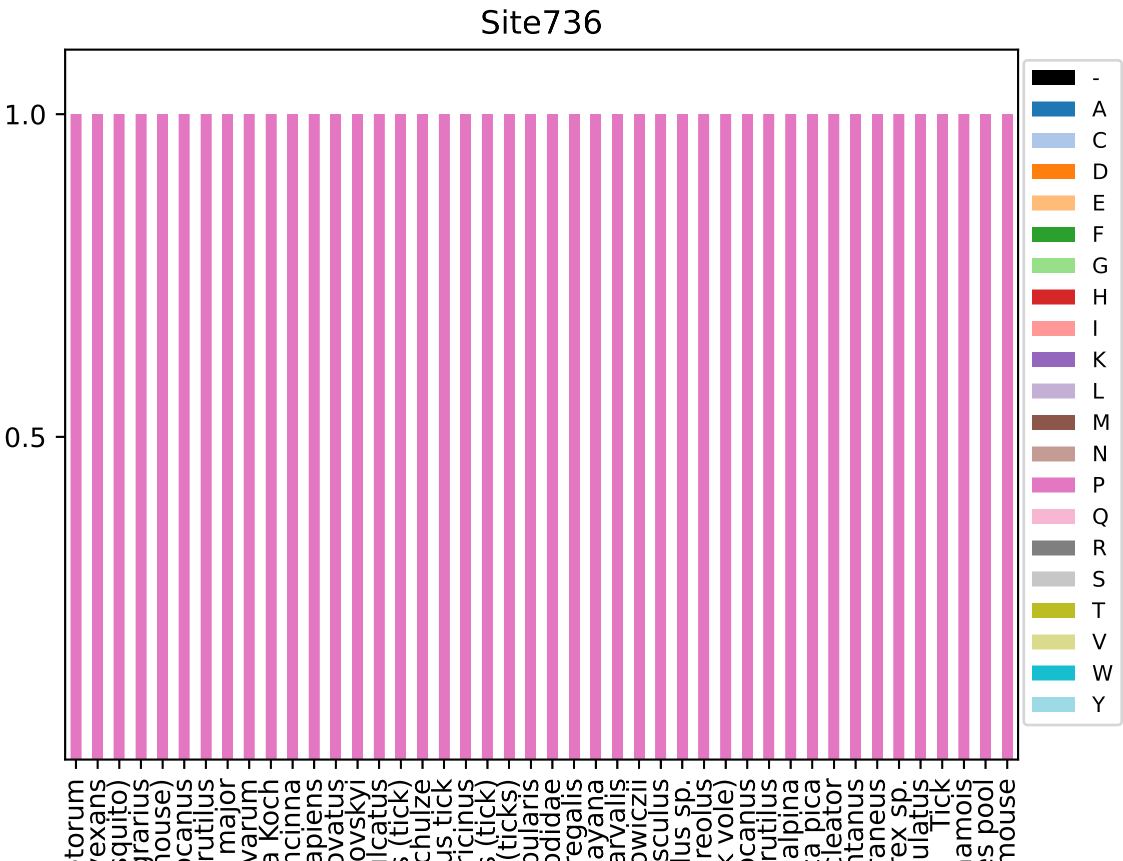

Site736
1.0
0.5
-
A
C
D
E
F
G
H
I
K
L
M
N
P
Q
R
S
T
V
W
Y
Tick

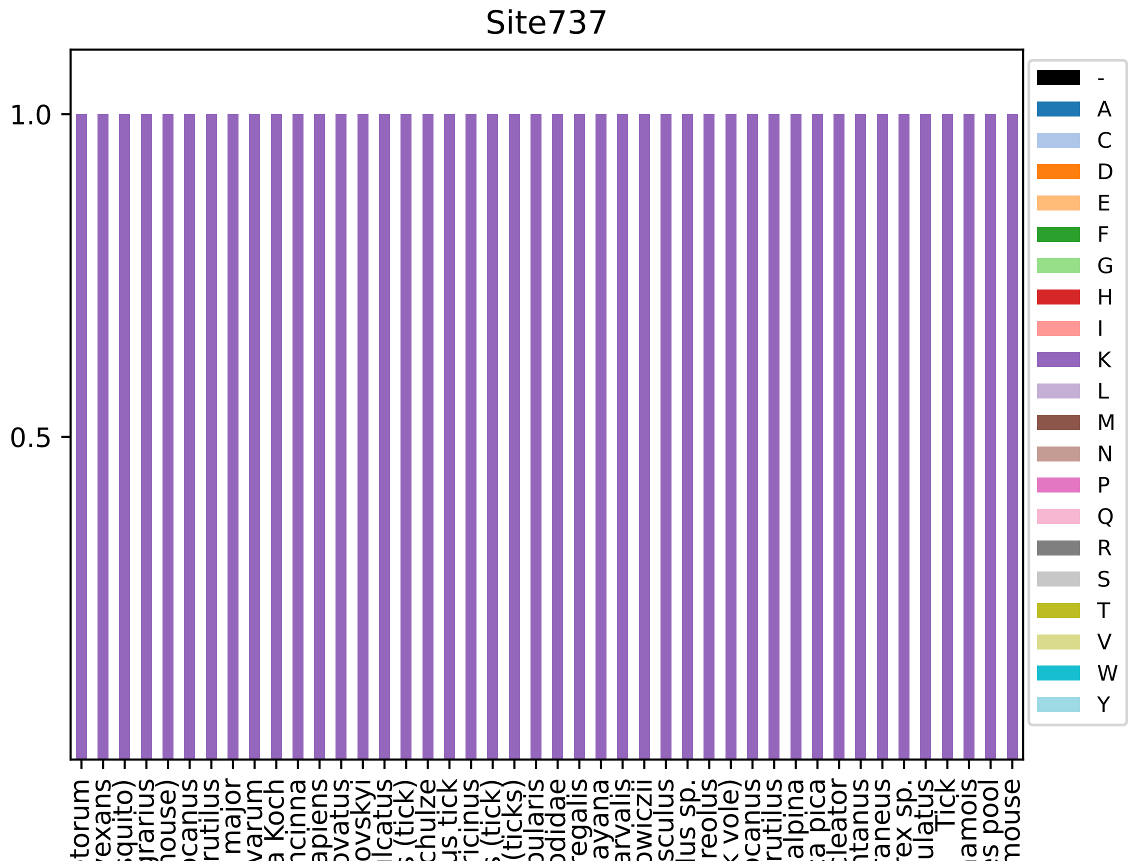

Site737
1.0
0.5
-
A
C
D
E
F
G
H
I
K
L
M
N
P
Q
R
S
T
V
W
Y

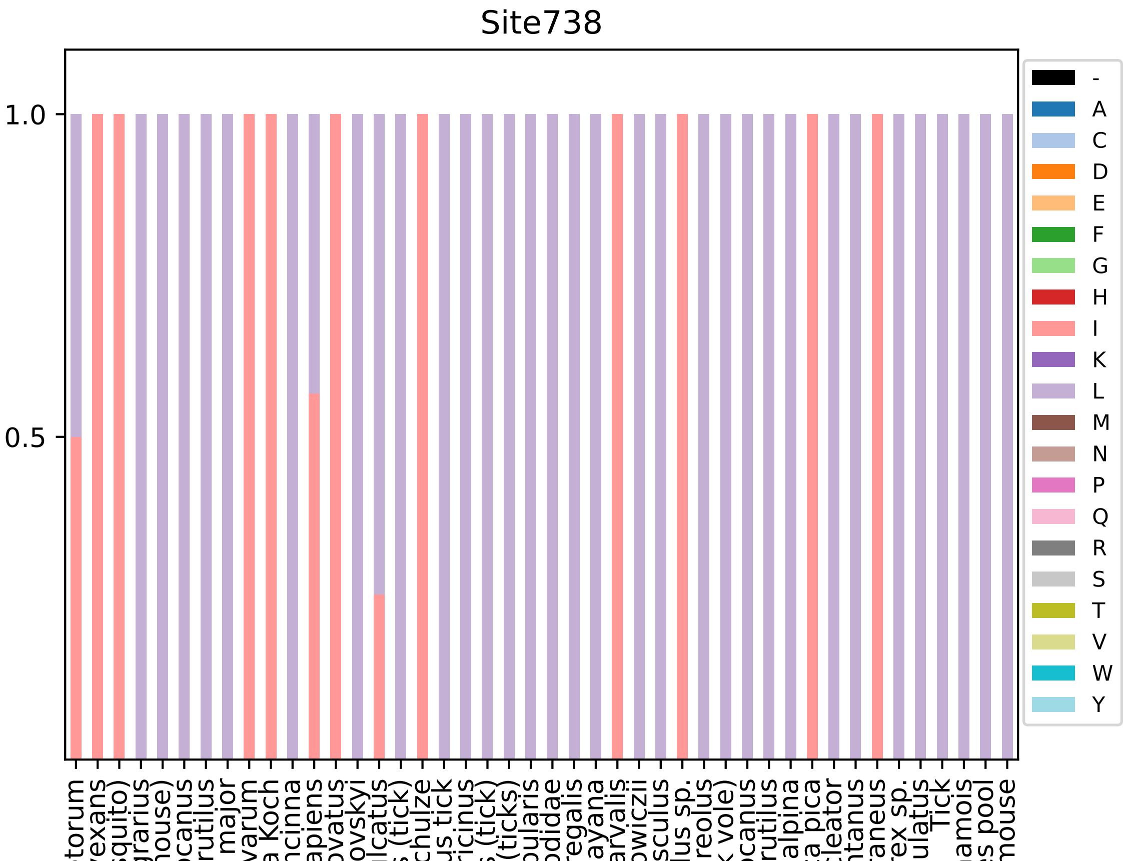
Site738
1.0
0.5
-
A
C
D
E
F
G
H
I
K
L
M
N
P
Q
R
S
T
V
W
Y

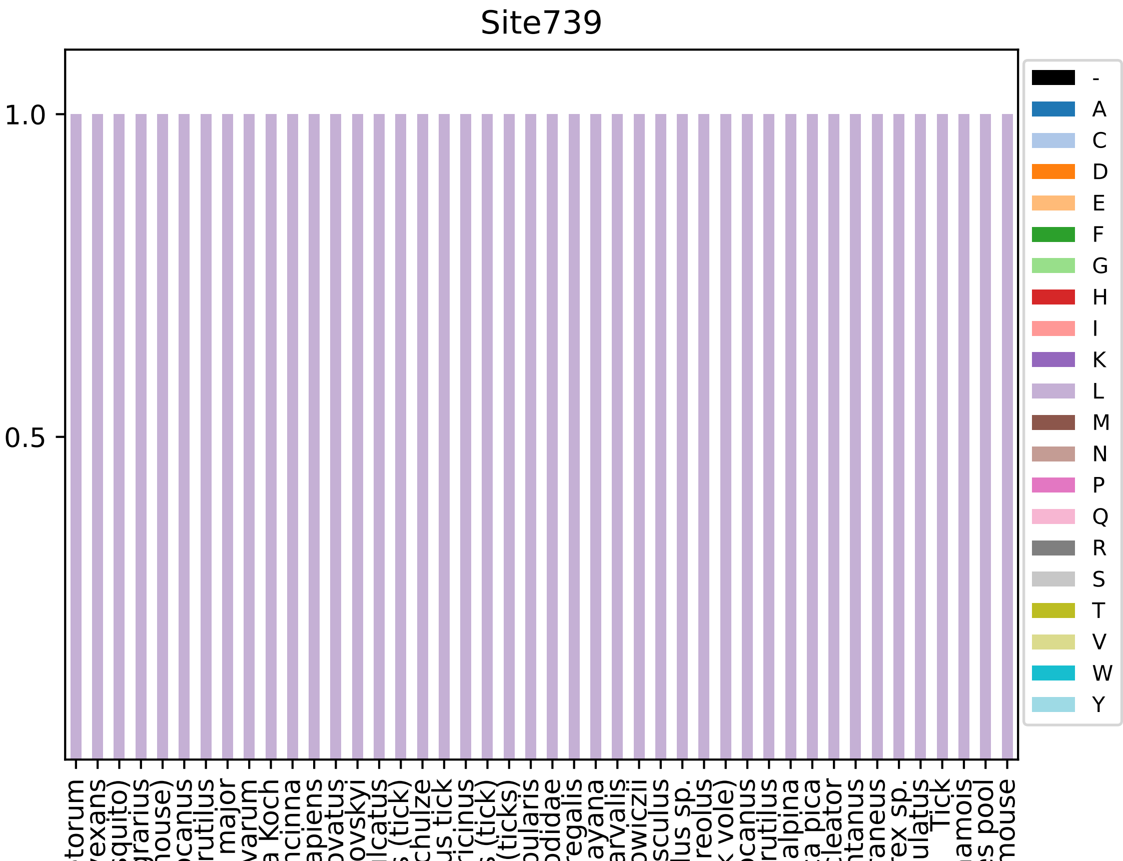
Site739
1.0
0.5
-
A
C
D
E
F
G
H
I
K
L
M
N
P
Q
R
S
T
V
W
Y

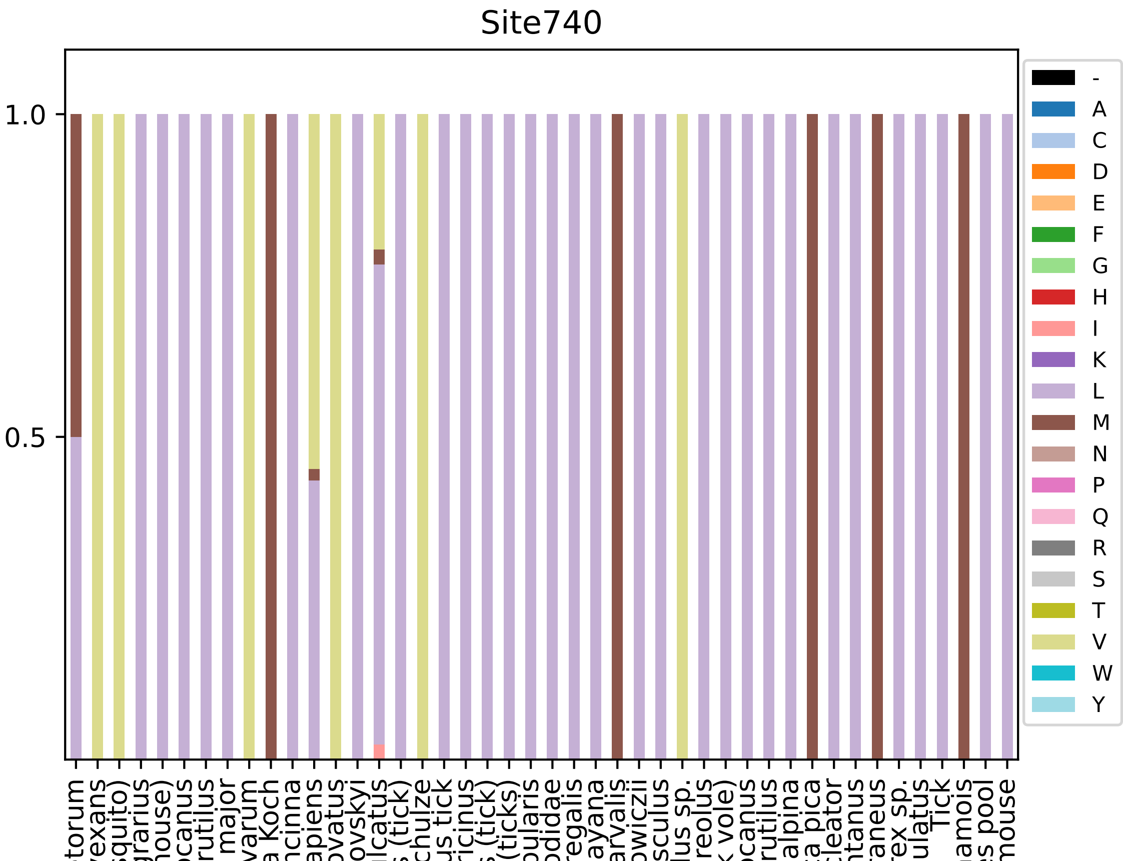
Site740
1.0
0.5
-
A
C
D
E
F
G
H
I
K
L
M
N
P
Q
R
S
T
V
W
Y

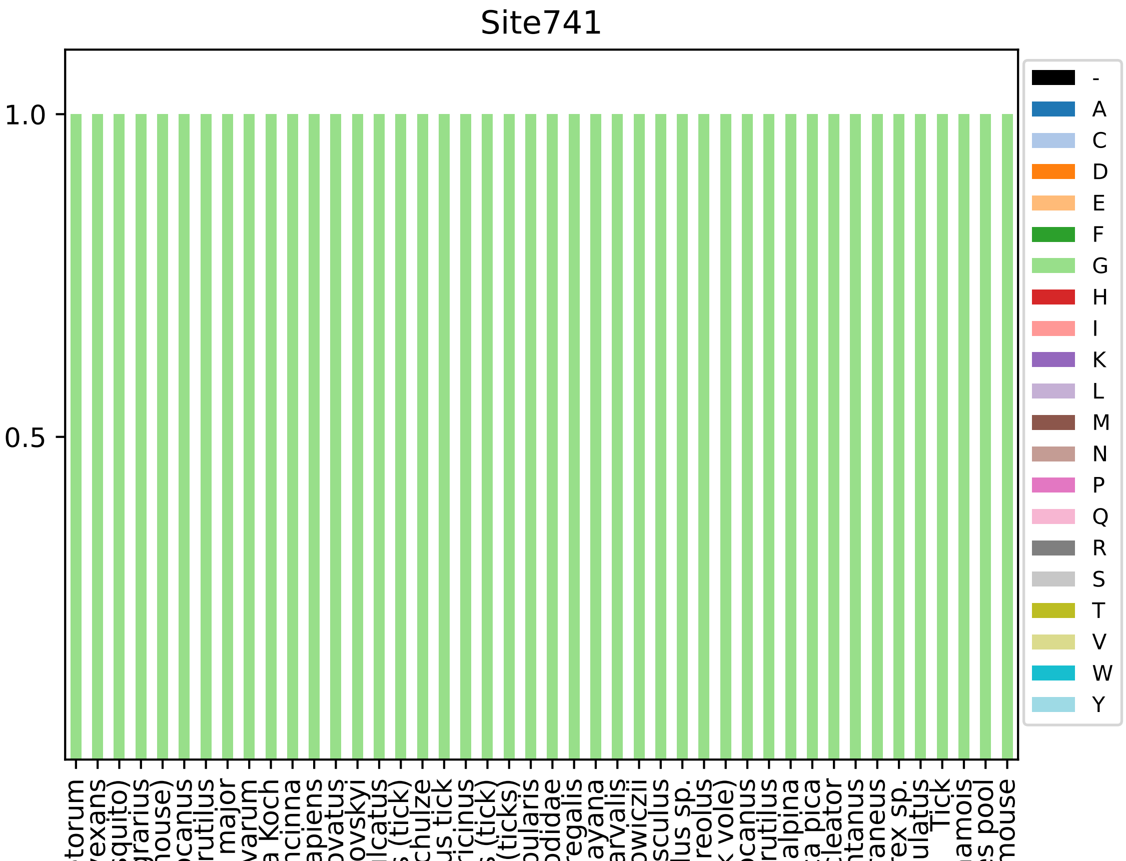
Site741
1.0
0.5
-
A
C
D
E
F
G
H
I
K
L
M
N
P
Q
R
S
T
V
W
Y
Tick

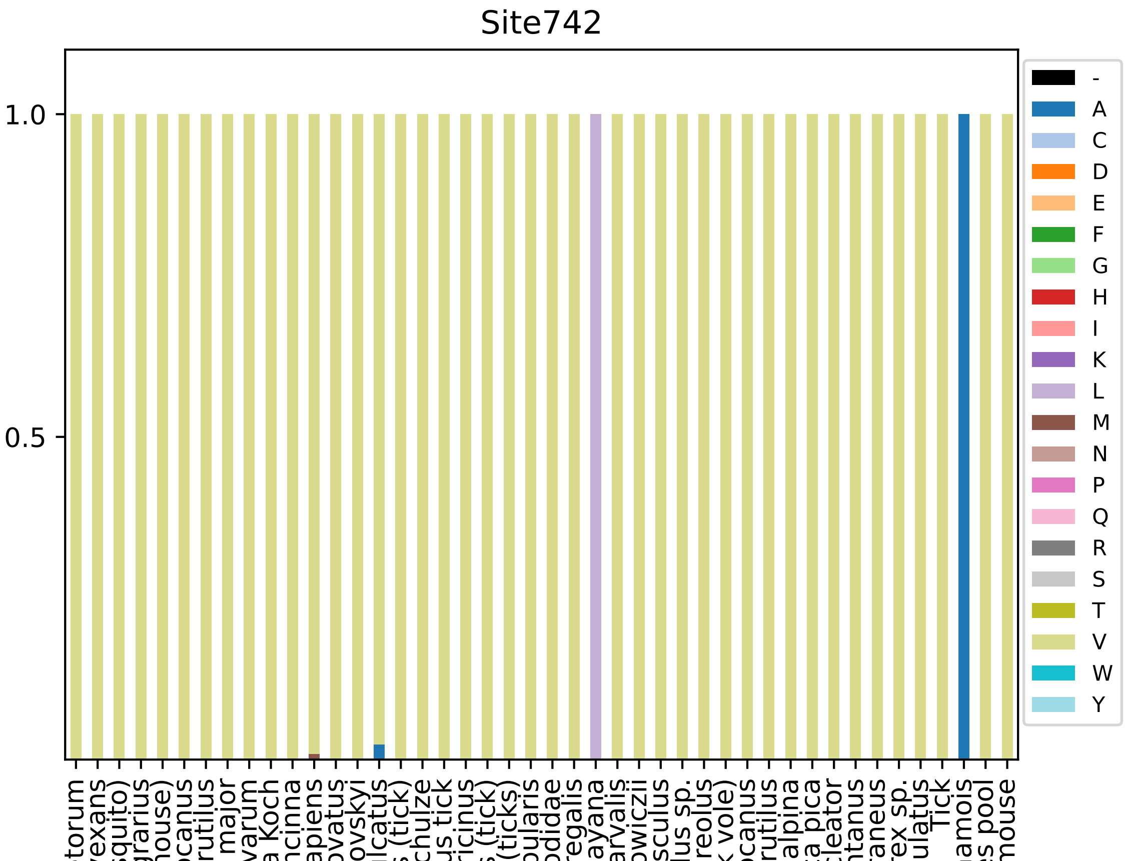
Site742
1.0
0.5
-
A
C
D
E
F
G
H
I
K
L
M
N
P
Q
R
S
T
V
W
Y

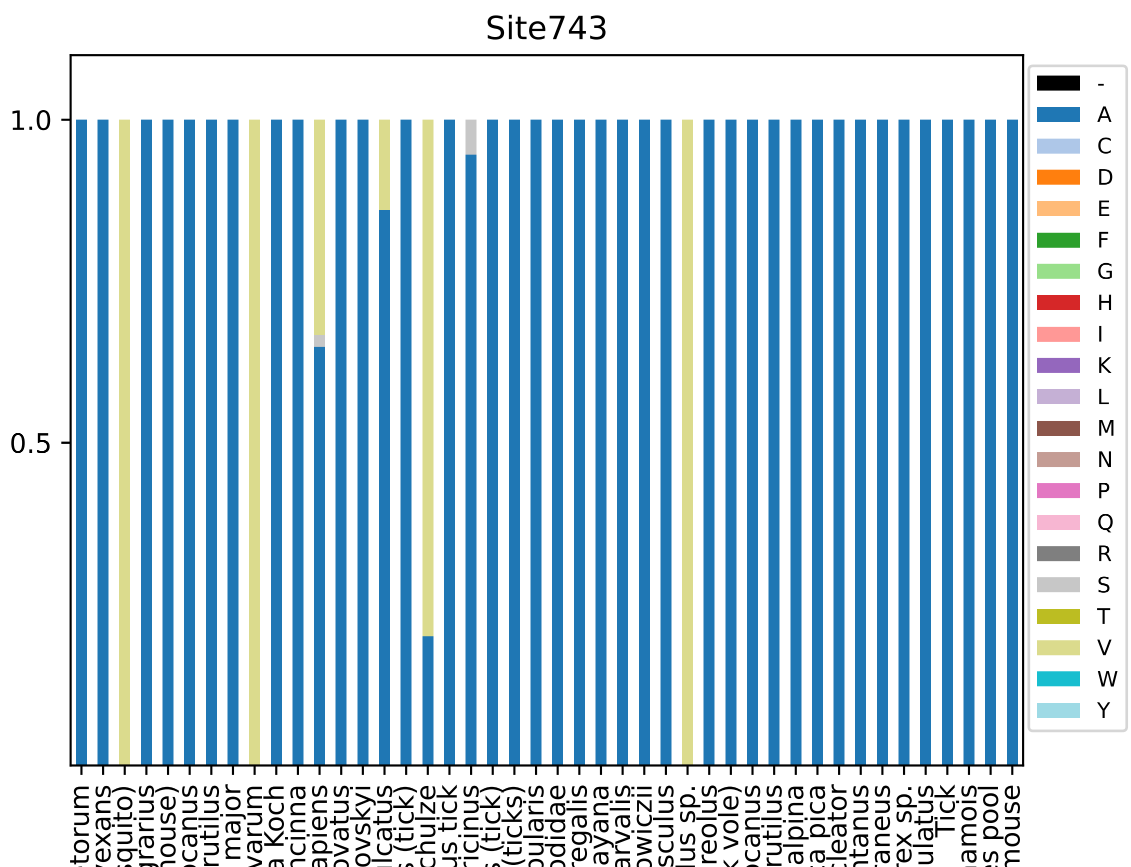

Site743
1.0
0.5
-
A
C
D
E
F
G
H
I
K
L
M
N
P
Q
R
S
T
V
W
Y

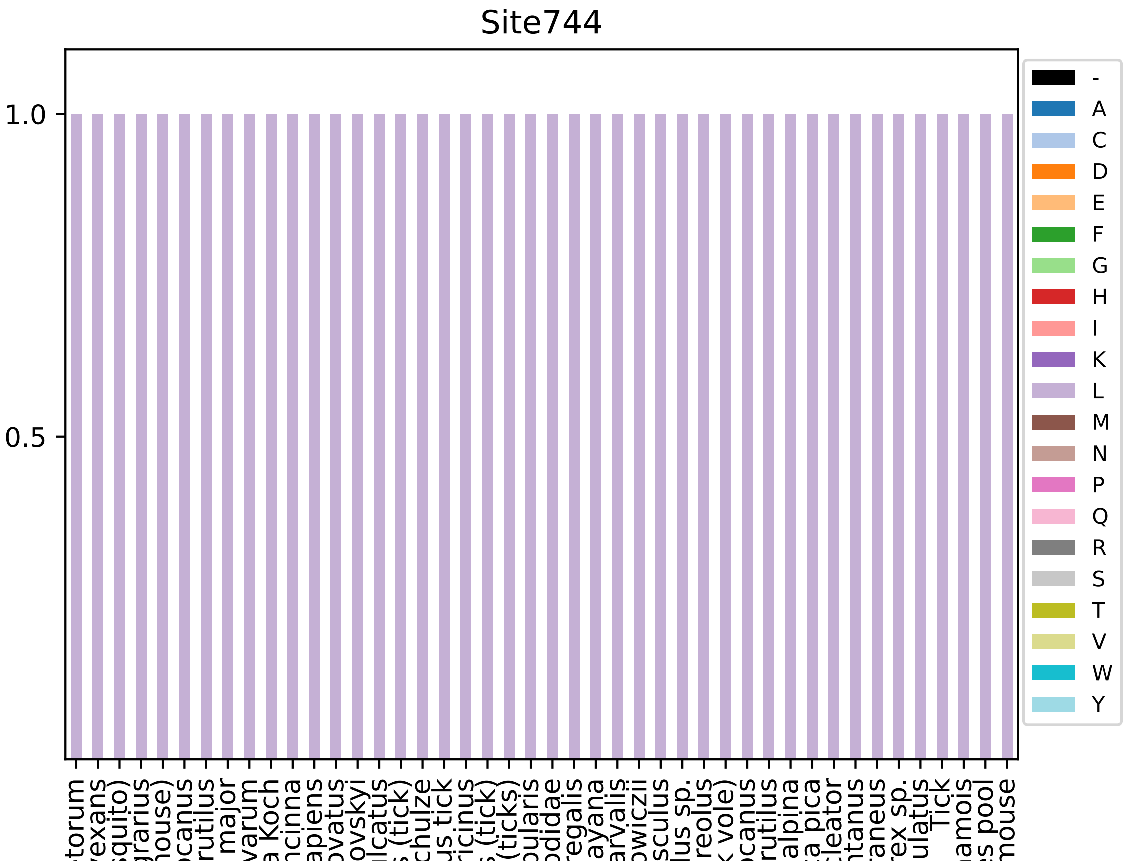
Site744
1.0
0.5
-
A
C
D
E
F
G
H
I
K
L
M
N
P
Q
R
S
T
V
W
Y

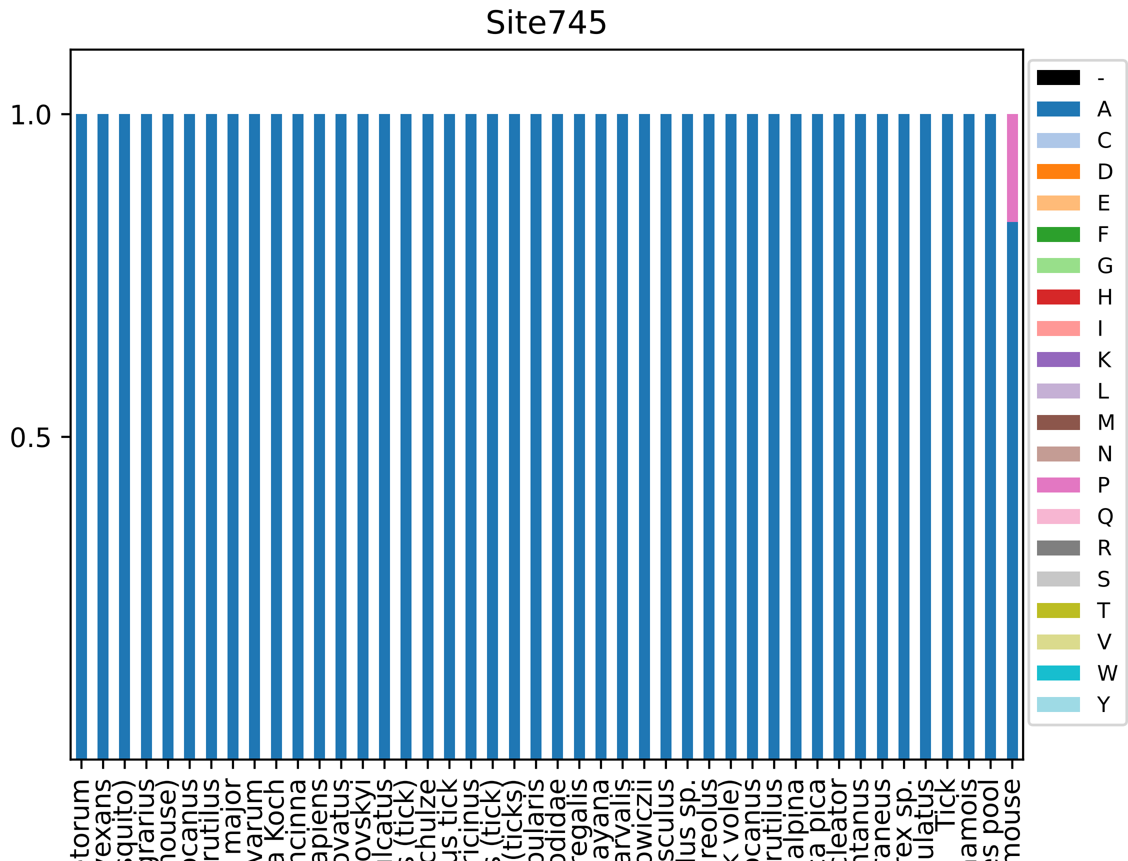
Site745
1.0
0.5
-
A
C
D
E
F
G
H
I
K
L
M
N
P
Q
R
S
T
V
W
Y

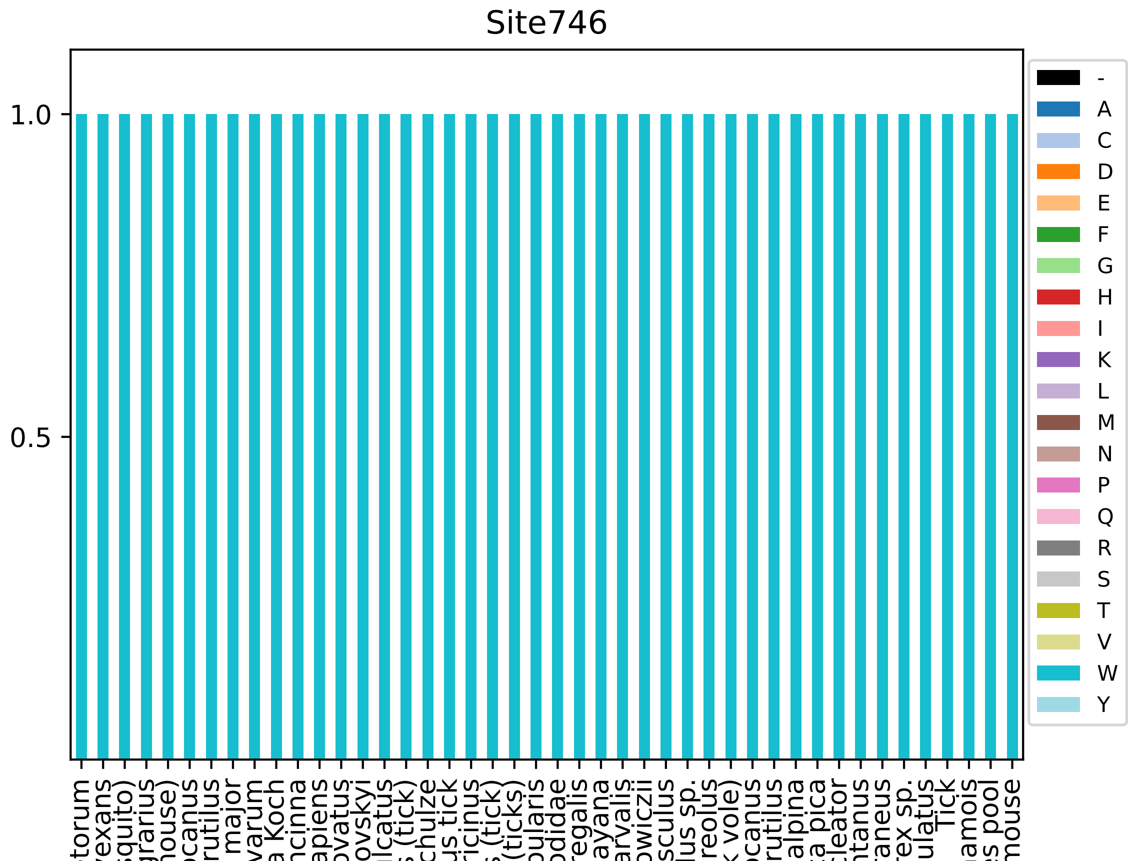

Site746
1.0
0.5
-
A
C
D
E
F
G
H
I
K
L
M
N
P
Q
R
S
T
V
W
Y

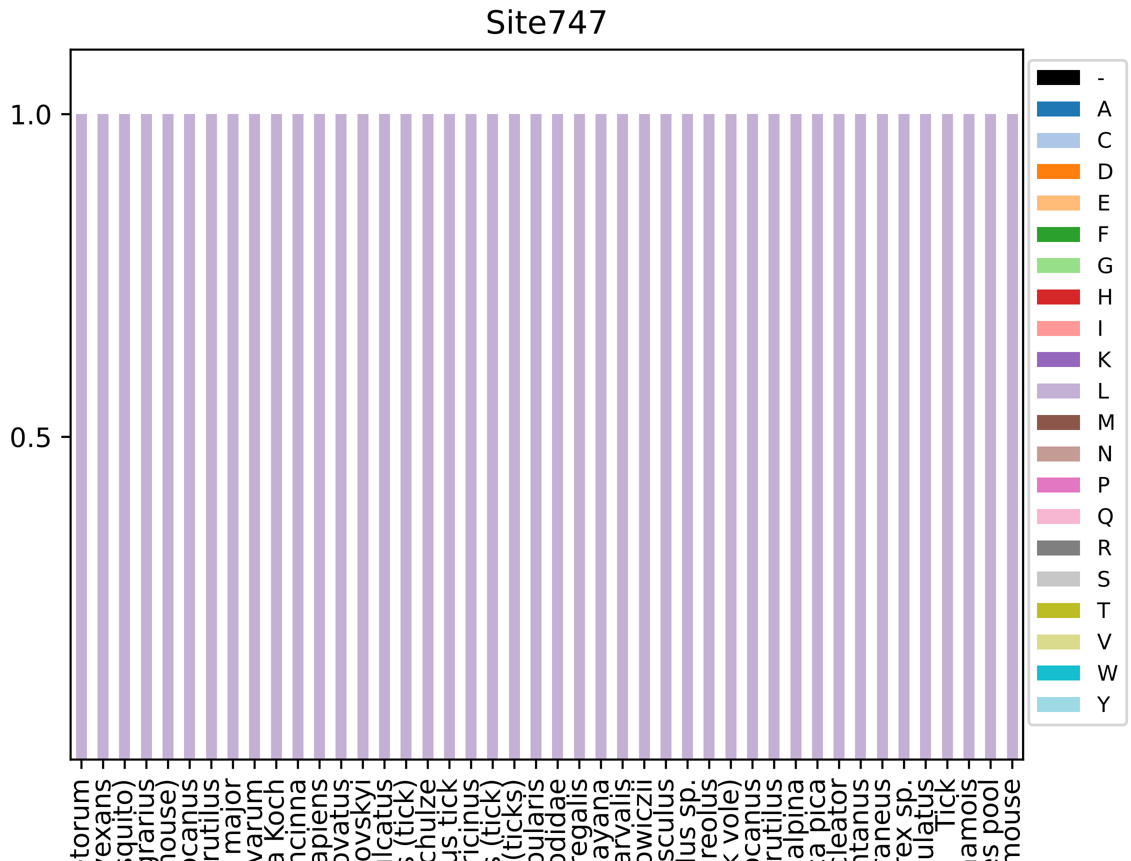

Site747
1.0
0.5
-
A
C
D
E
F
G
H
I
K
L
M
N
P
Q
R
S
T
V
W
Y

Site748

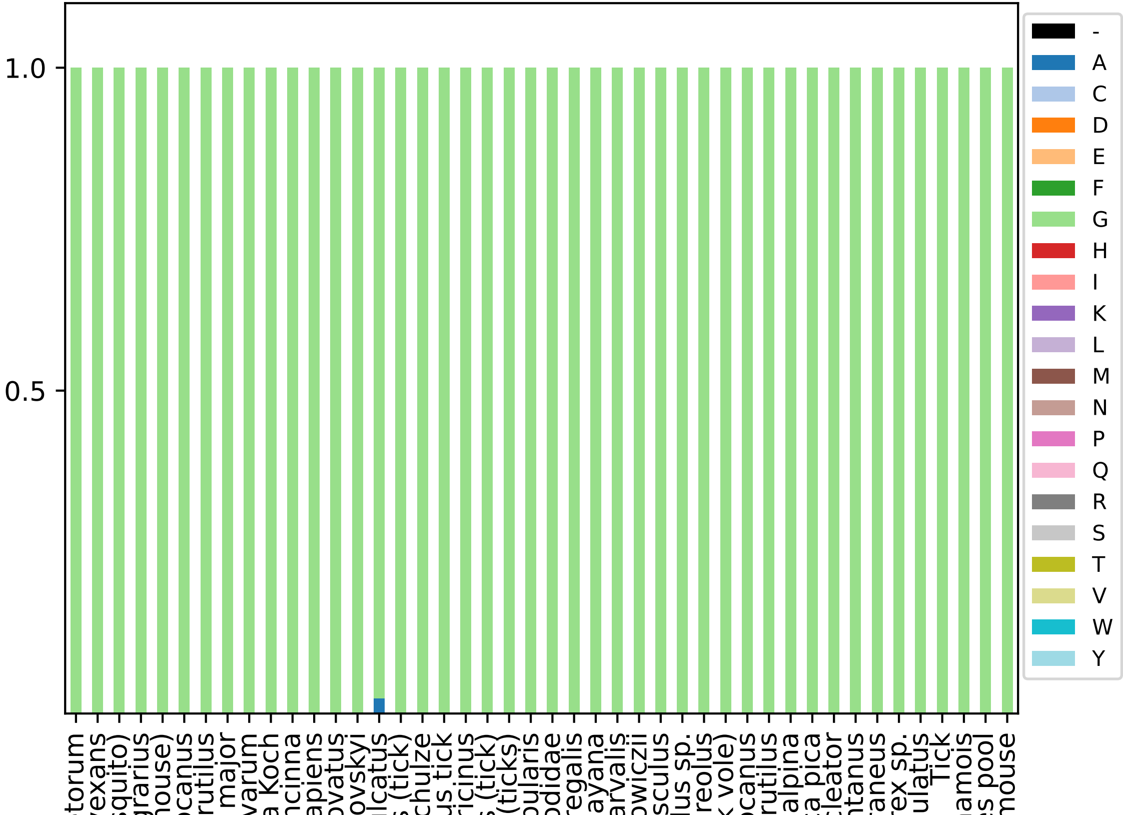

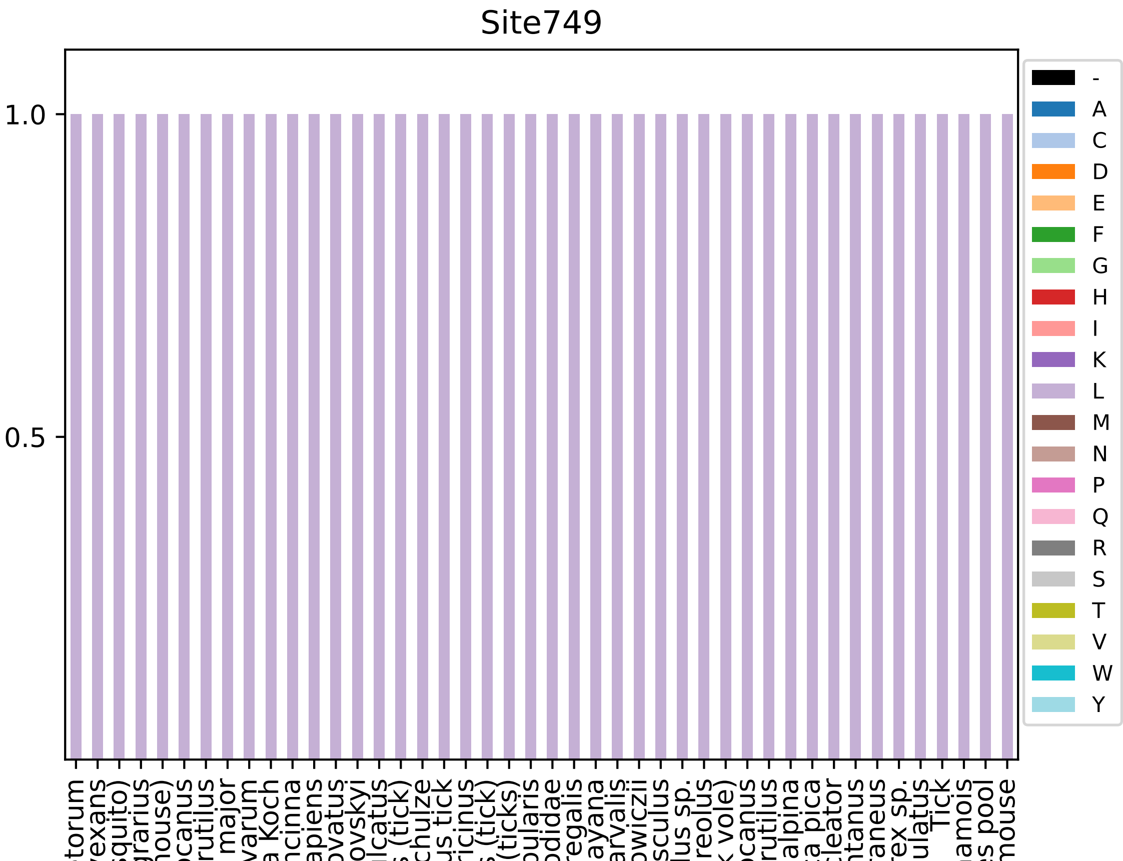
Site749
1.0
0.5
-
A
C
D
E
F
G
H
I
K
L
M
N
P
Q
R
S
T
V
W
Y

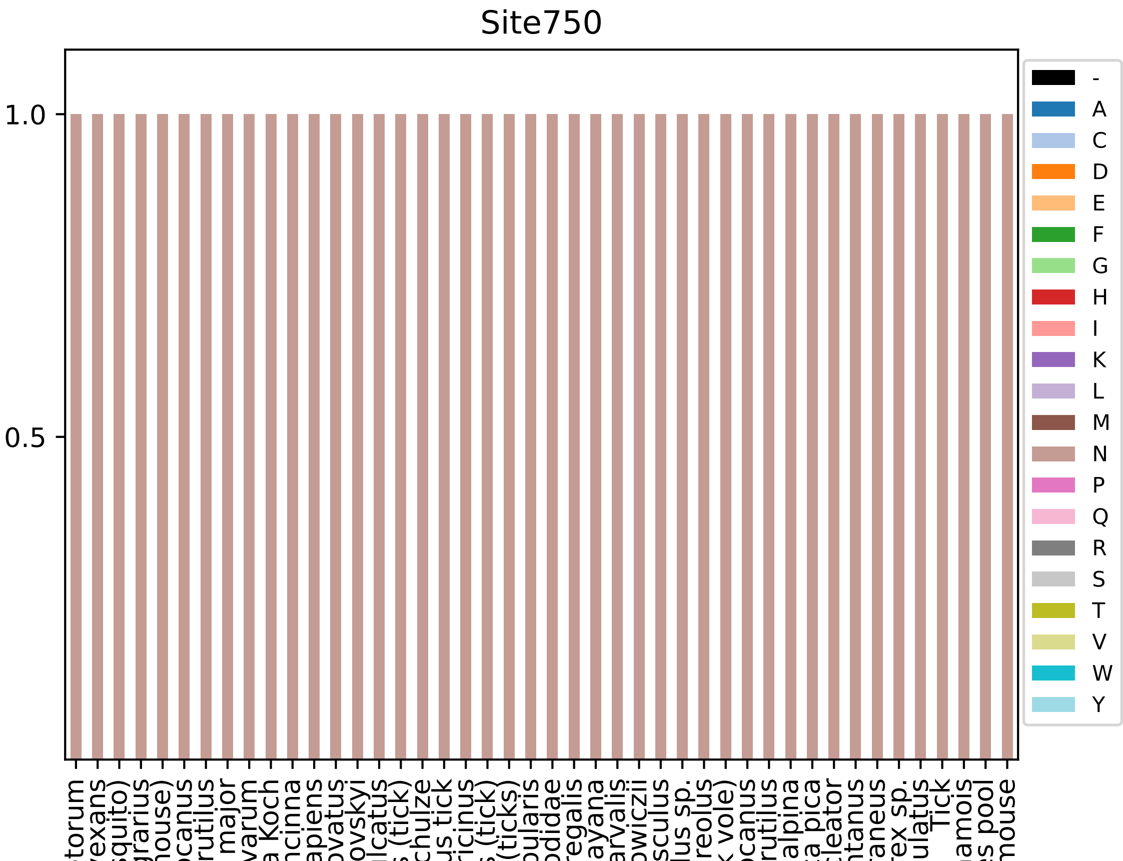
Site750
1.0
0.5
-
A
C
D
E
F
G
H
I
K
L
M
N
P
Q
R
S
T
V
W
Y
Tick

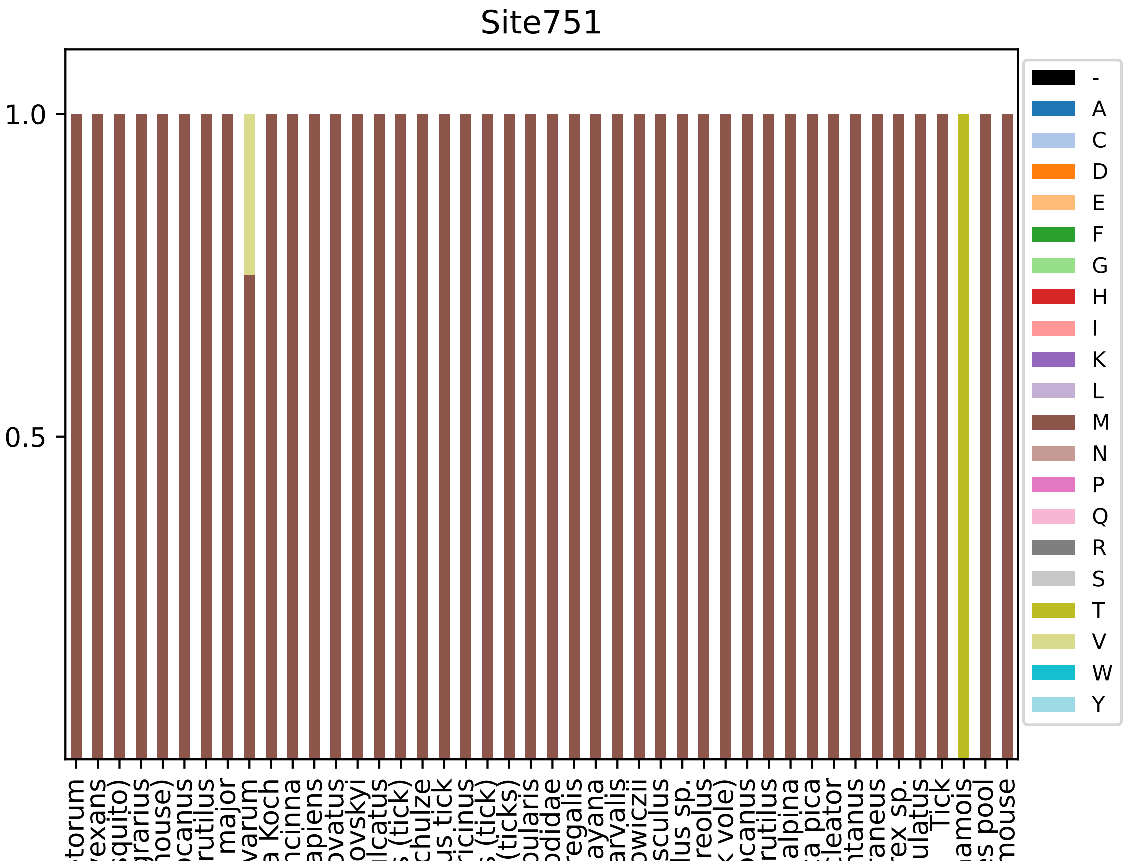
Site751
1.0
0.5
-
A
C
D
E
F
G
H
I
K
L
M
N
P
Q
R
S
T
V
W
Y

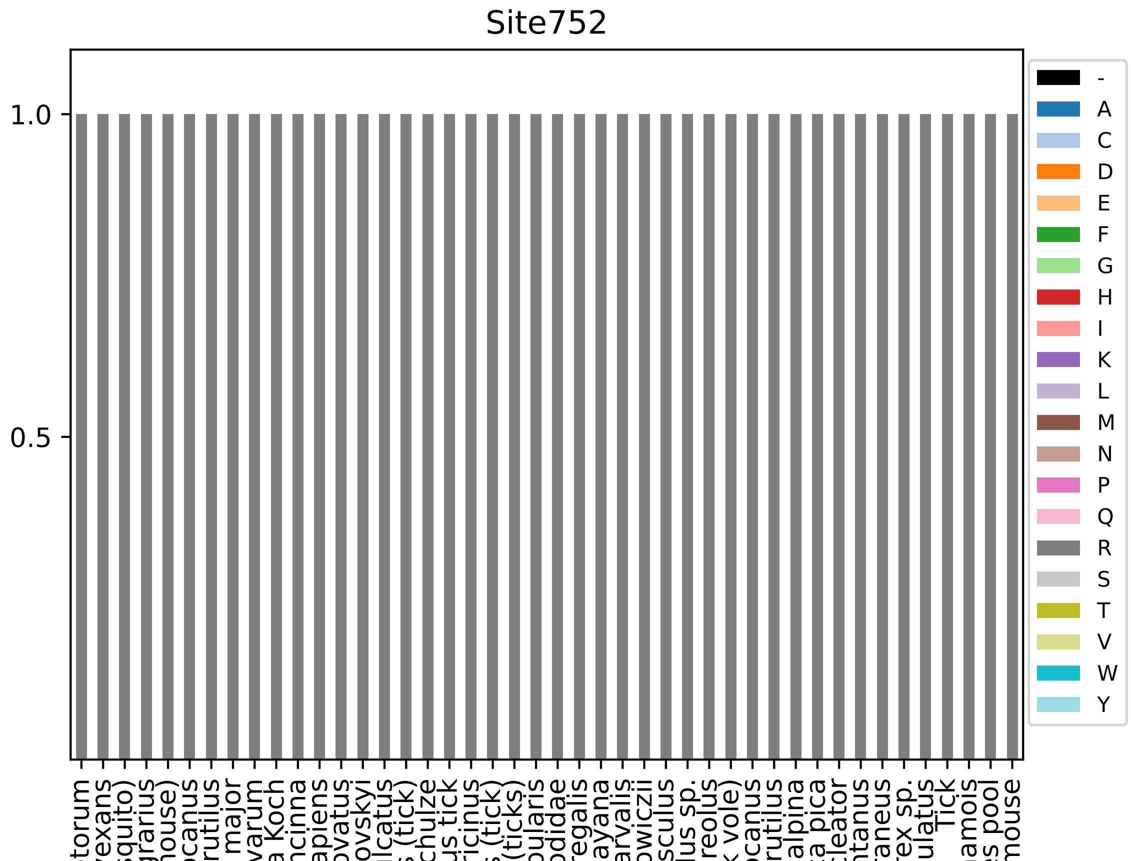
Site752
1.0
0.5
-
A
C
D
E
F
G
H
I
K
L
M
N
P
Q
R
S
T
V
W
Y

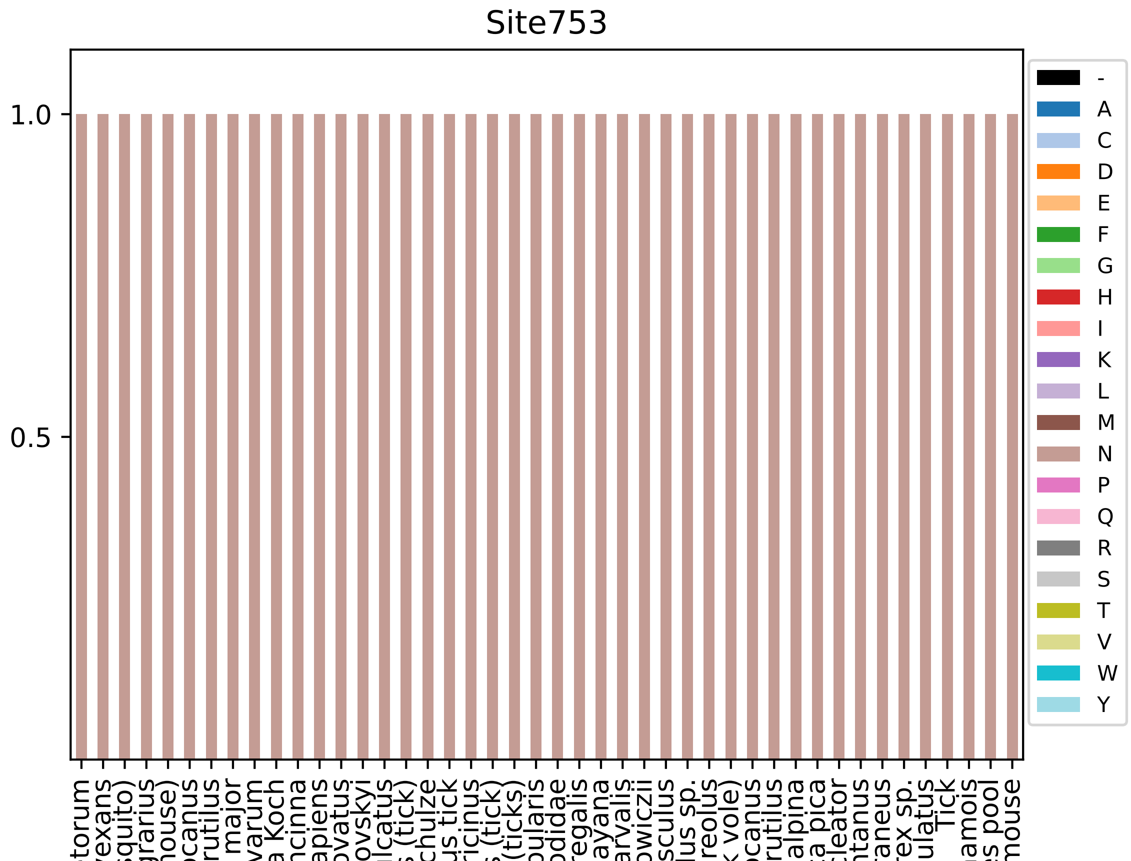

Site753
1.0
0.5
-
A
C
D
E
F
G
H
I
K
L
M
N
P
Q
R
S
T
V
W
Y

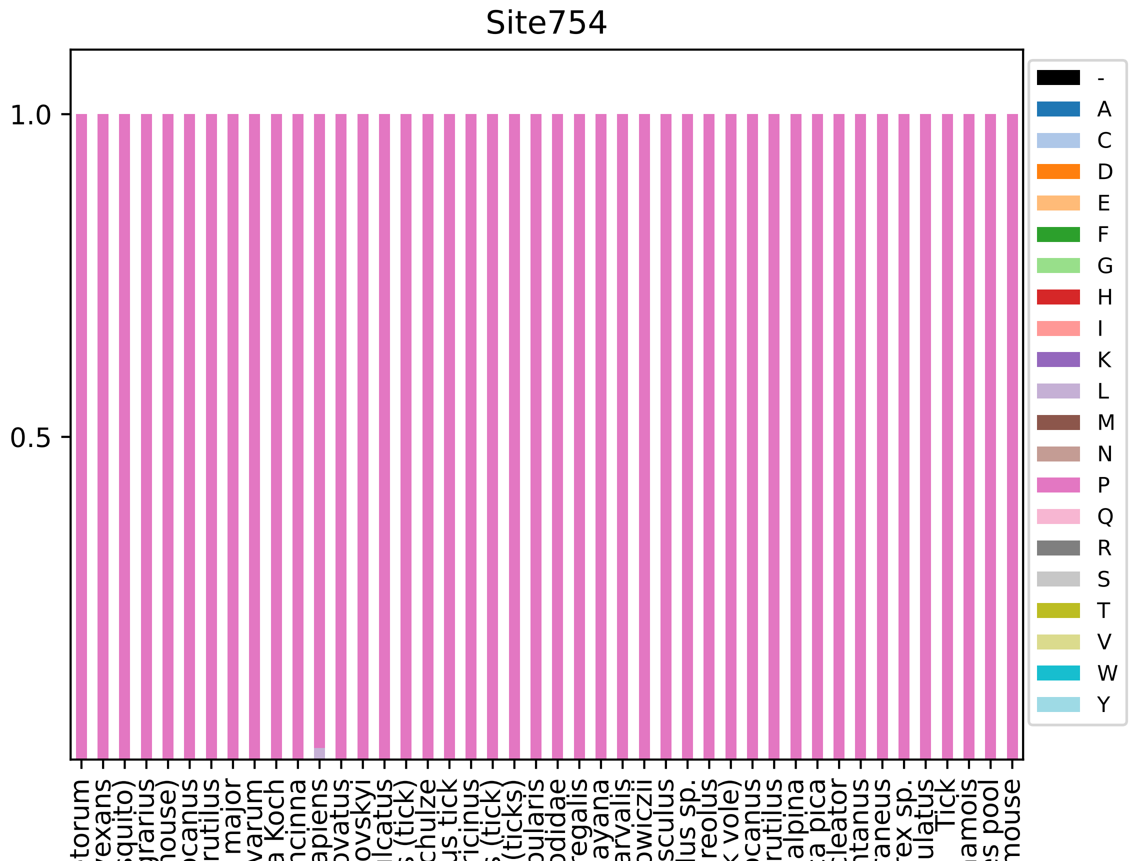
Site754
1.0
0.5
-
A
C
D
E
F
G
H
I
K
L
M
N
P
Q
R
S
T
V
W
Y

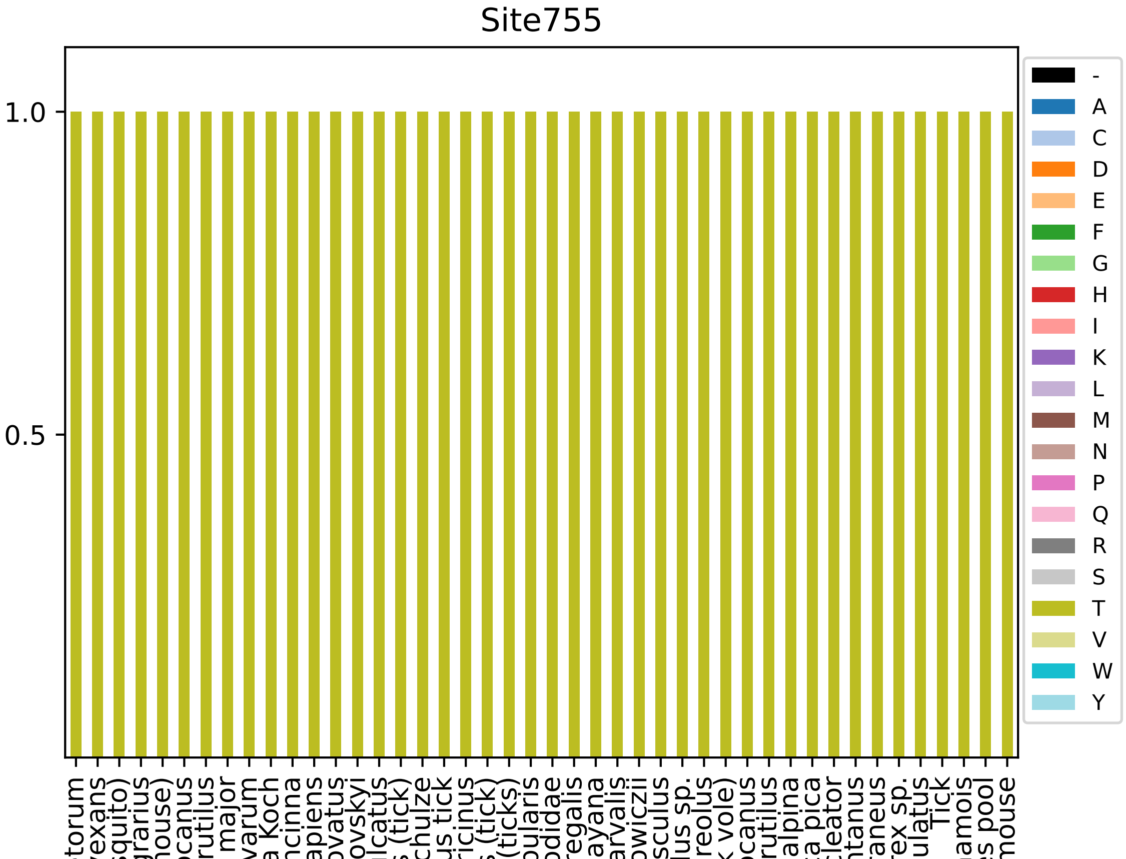

Site755
1.0
0.5
-
A
C
D
E
F
G
H
I
K
L
M
N
P
Q
R
S
T
V
W
Y

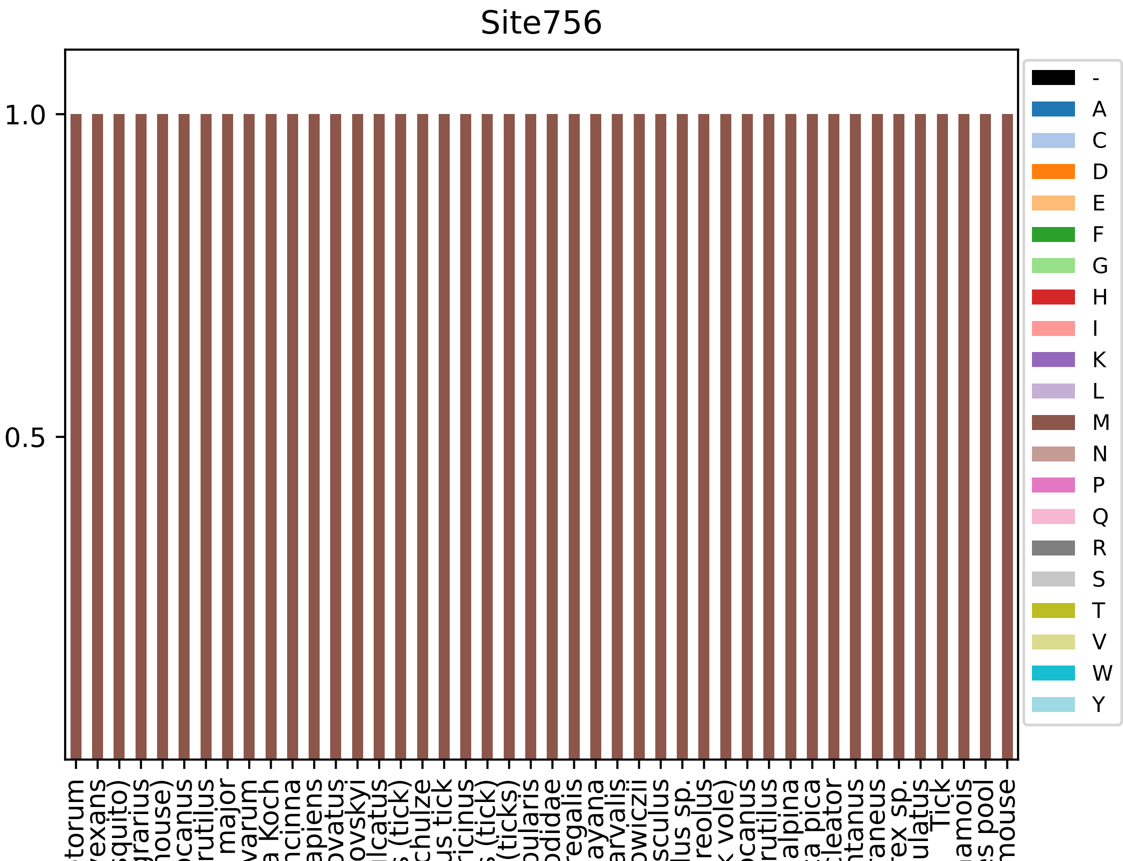

Site756
1.0
0.5
-
A
C
D
E
F
G
H
I
K
L
M
N
P
Q
R
S
T
V
W
Y

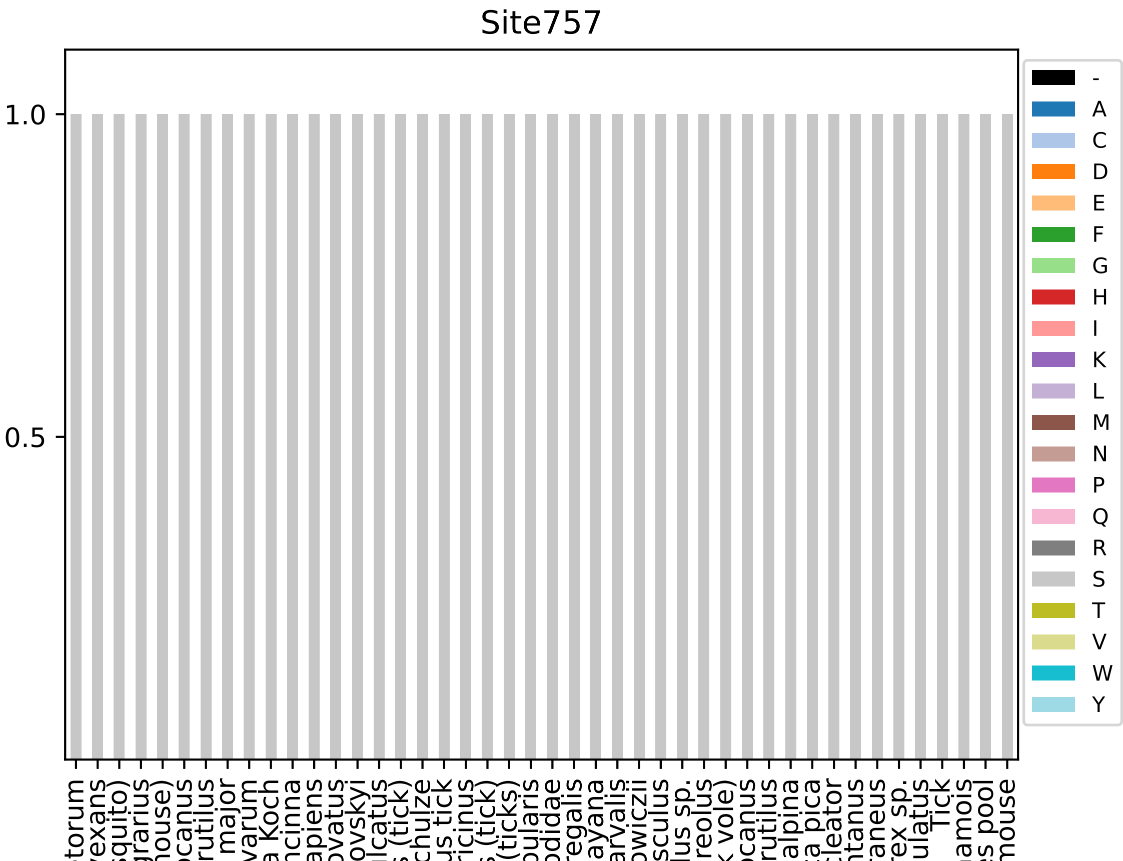

Site757
1.0
0.5
-
A
C
D
E
F
G
H
I
K
L
M
N
P
Q
R
S
T
V
W
Y

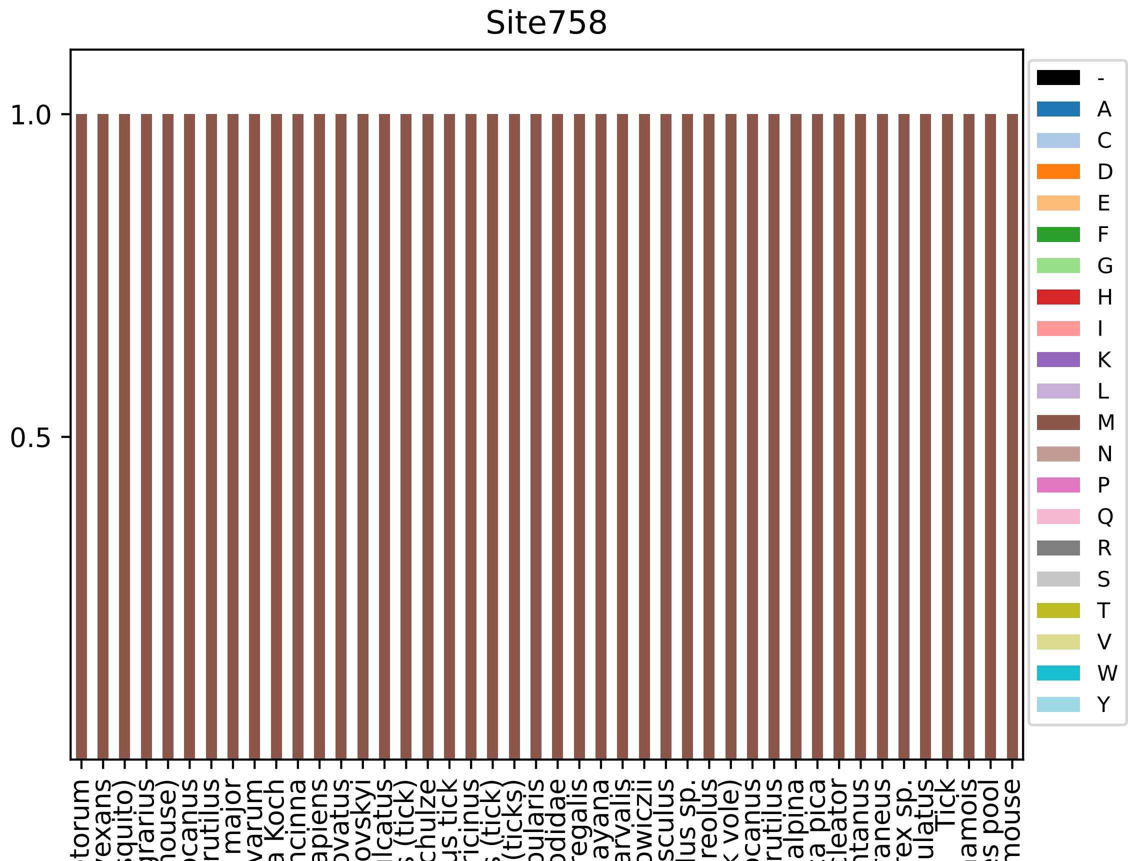
Site758
1.0
0.5
-
A
C
D
E
F
G
H
I
K
L
M
N
P
Q
R
S
T
V
W
Y

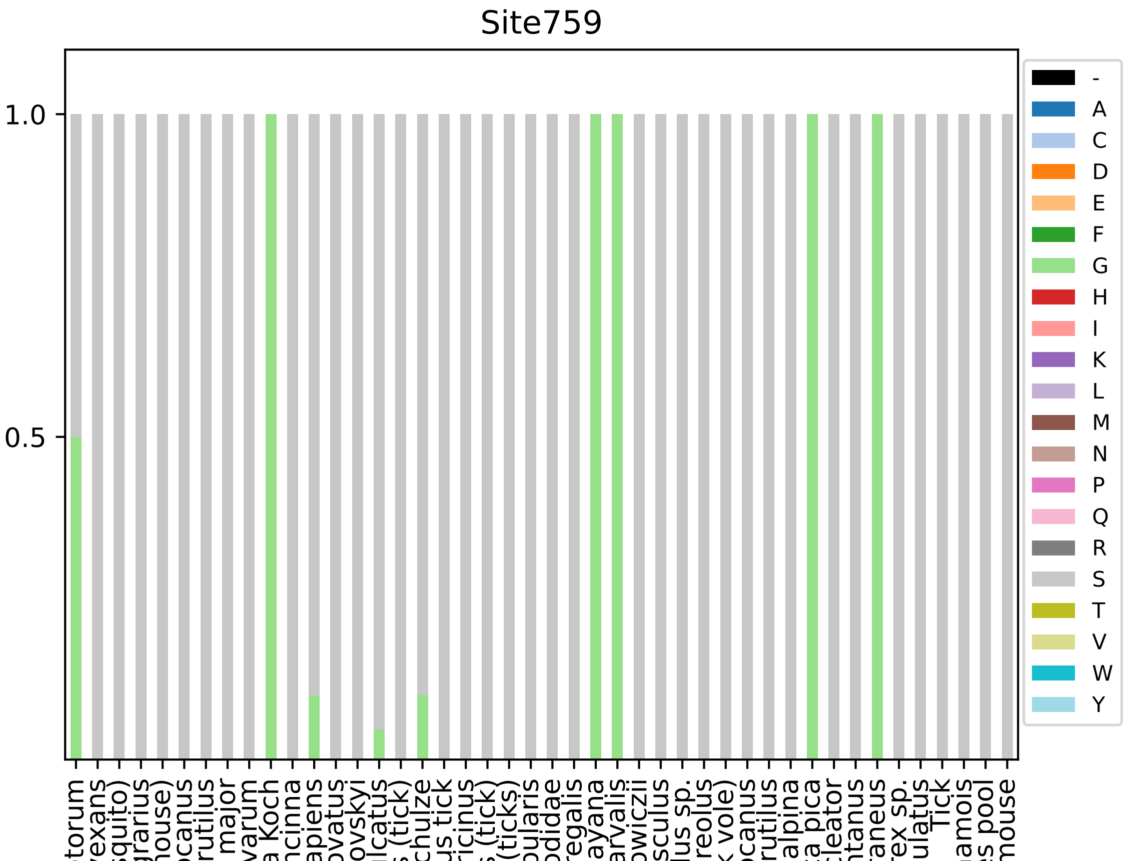
Site759
1.0
0.5
-
A
C
D
E
F
G
H
I
K
L
M
N
P
Q
R
S
T
V
W
Y
Tick

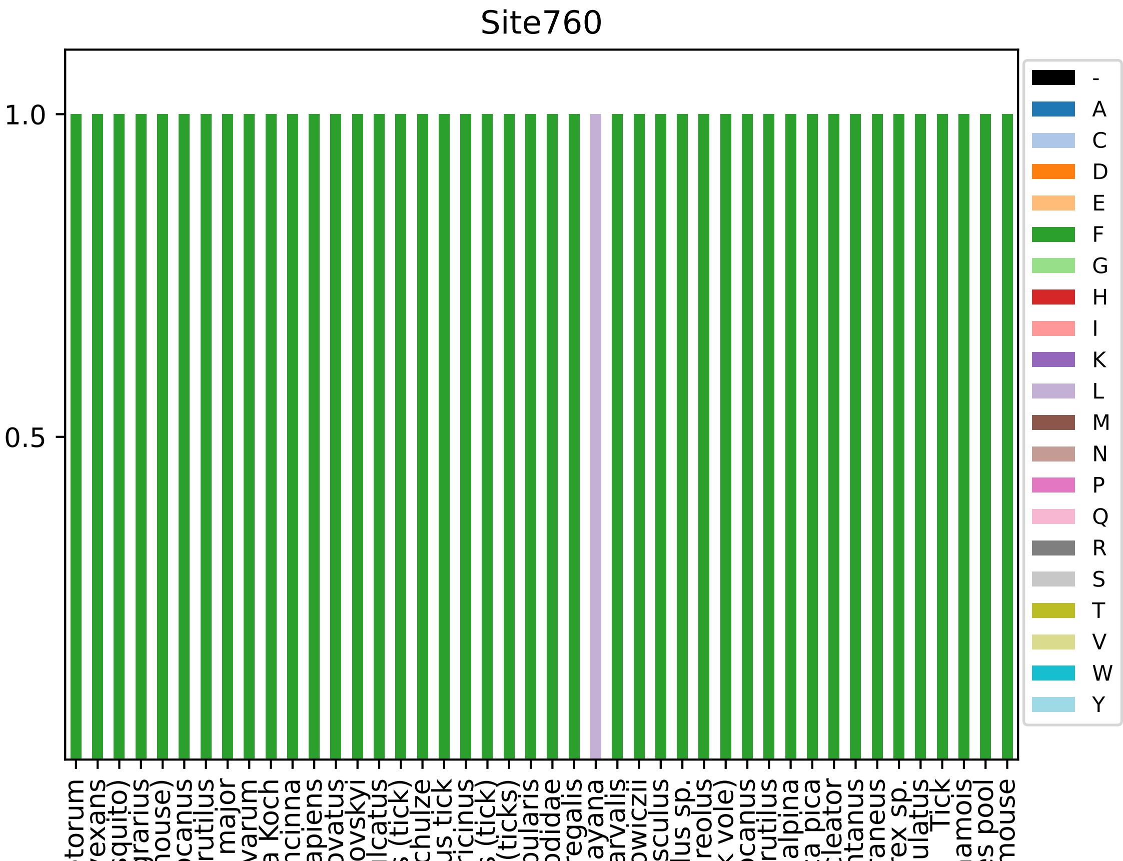
Site760
1.0
0.5
-
A
C
D
E
F
G
H
I
K
L
M
N
P
Q
R
S
T
V
W
Y

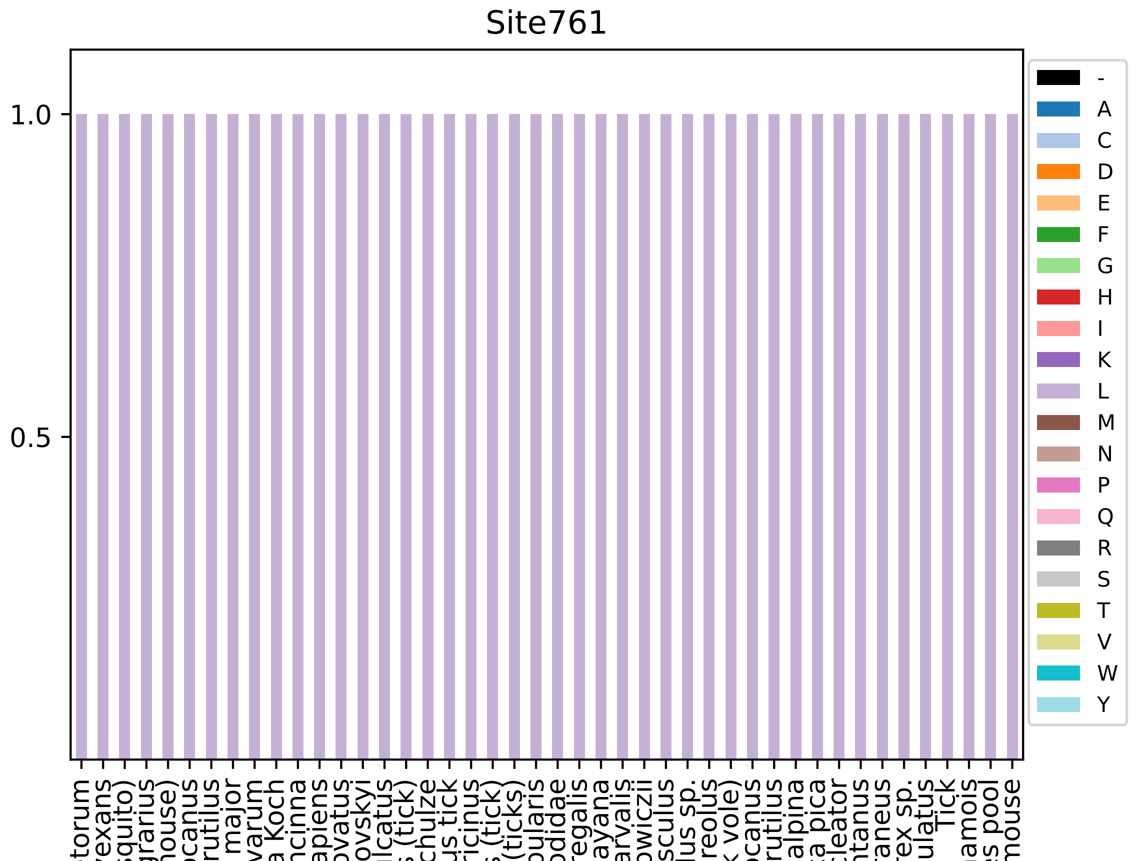
Site761
1.0
0.5
-
A
C
D
E
F
G
H
I
K
L
M
N
P
Q
R
S
T
V
W
Y

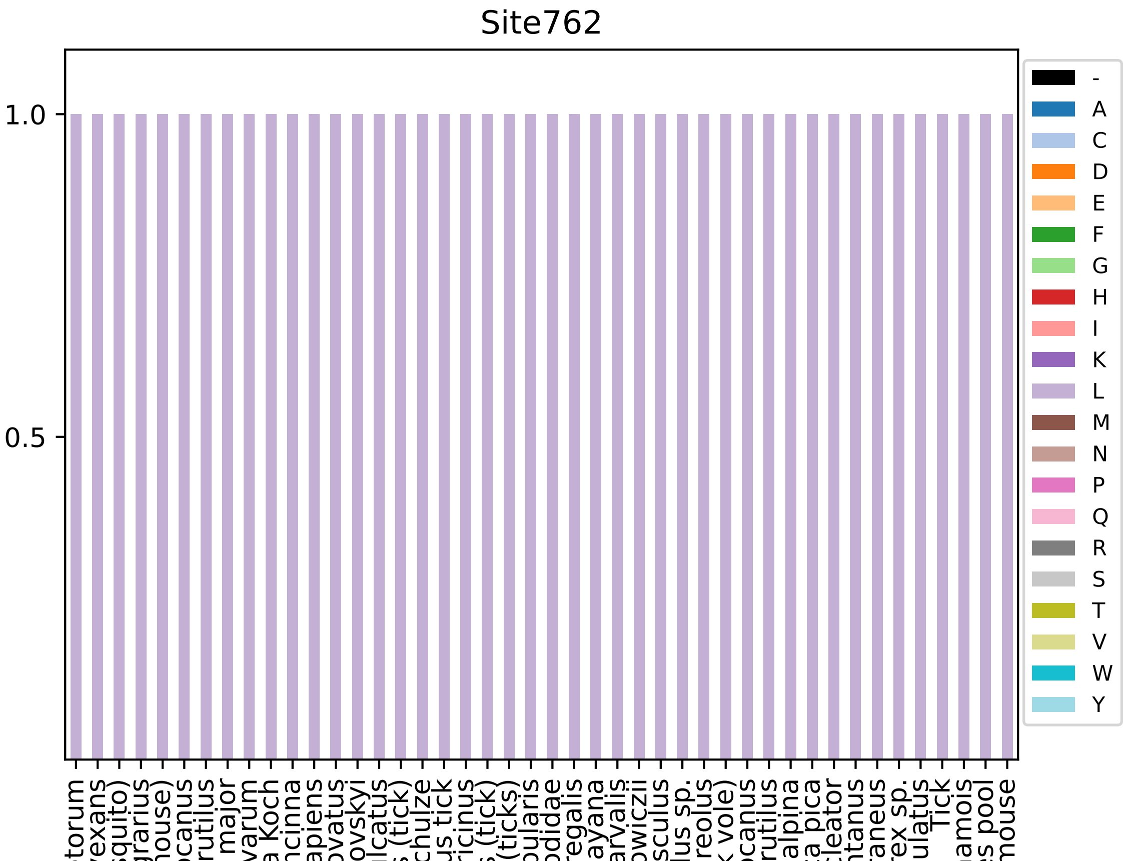

Site762
1.0
0.5
-
A
C
D
E
F
G
H
I
K
L
M
N
P
Q
R
S
T
V
W
Y

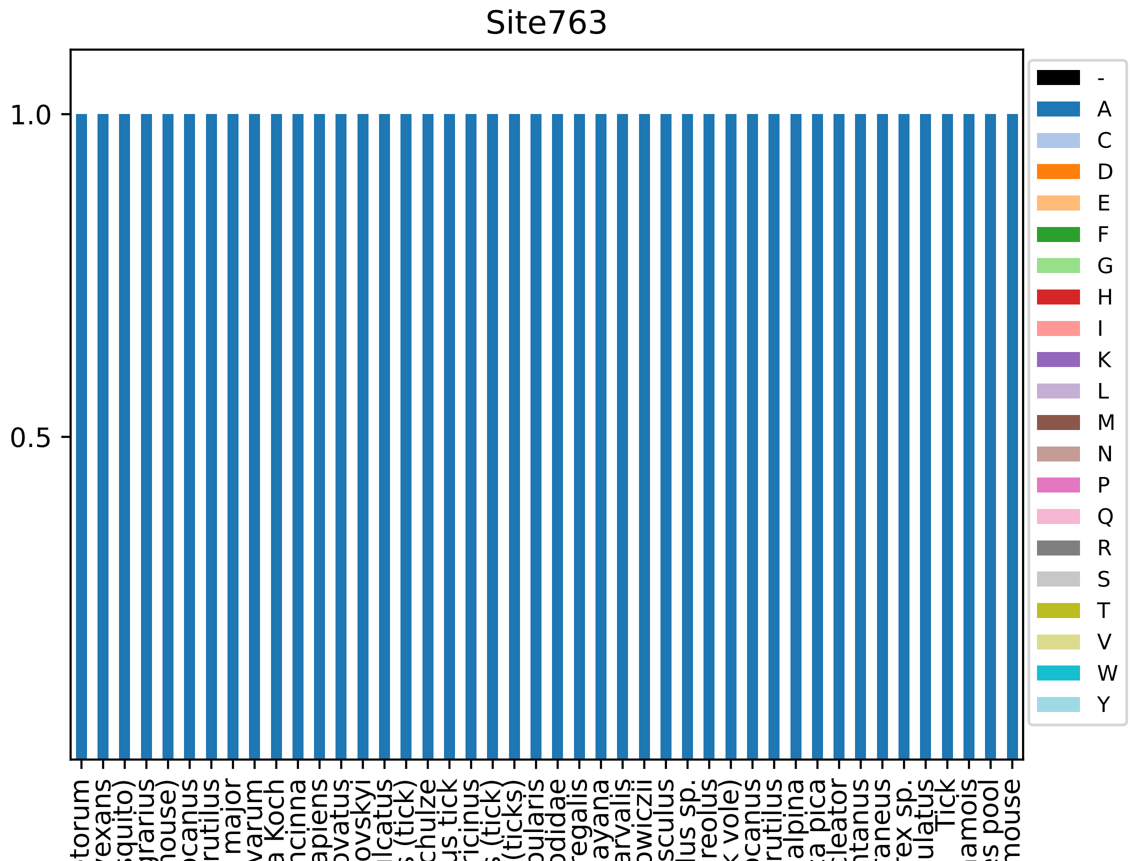
Site763
1.0
0.5
-
A
C
D
E
F
G
H
I
K
L
M
N
P
Q
R
S
T
V
W
Y

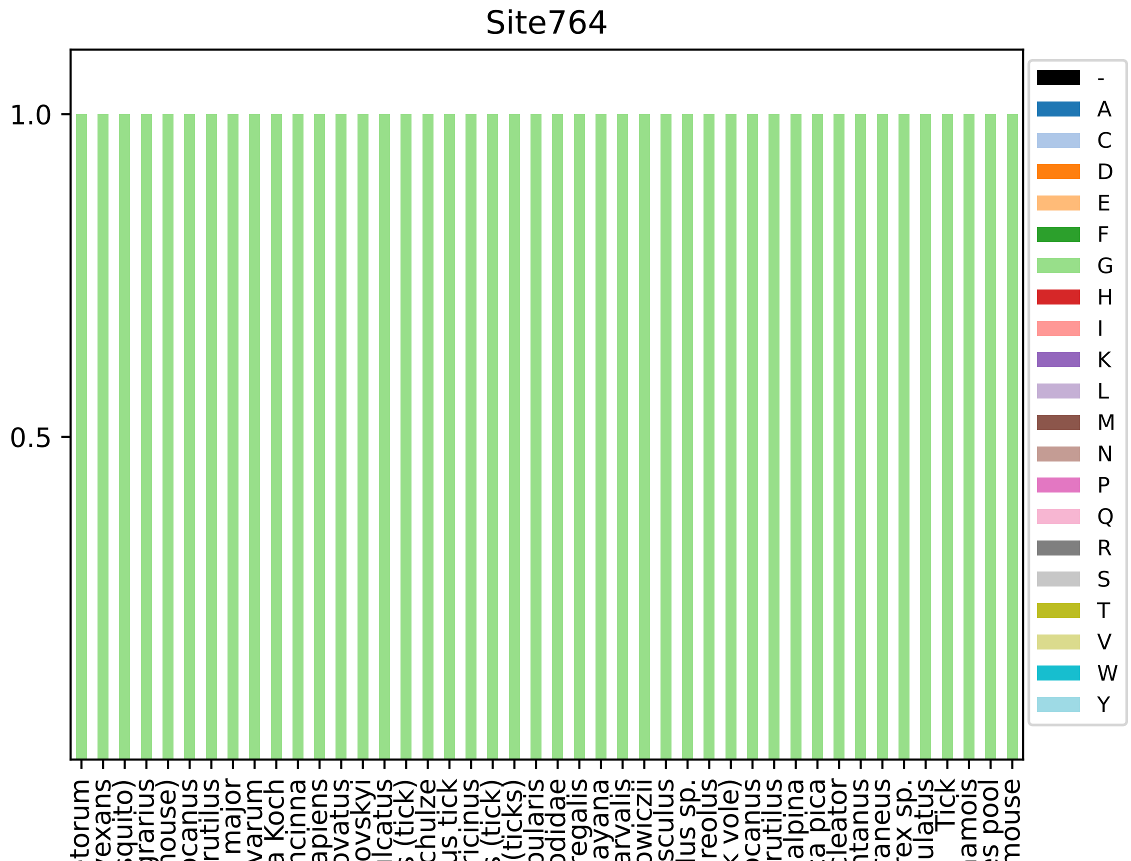
Site764
1.0
0.5
-
A
C
D
E
F
G
H
I
K
L
M
N
P
Q
R
S
T
V
W
Y

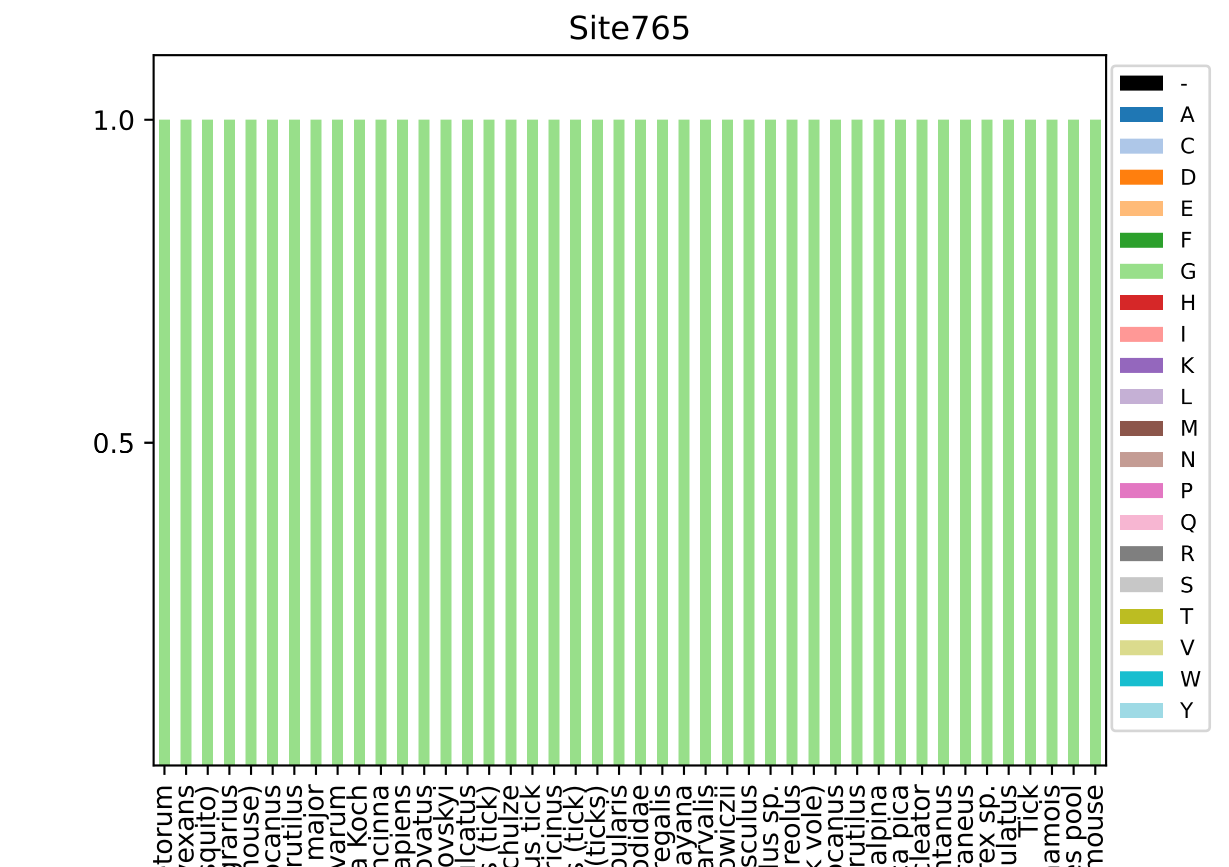

Site765
1.0
0.5
-
A
C
D
E
F
G
H
I
K
L
M
N
P
Q
R
S
T
V
W
Y

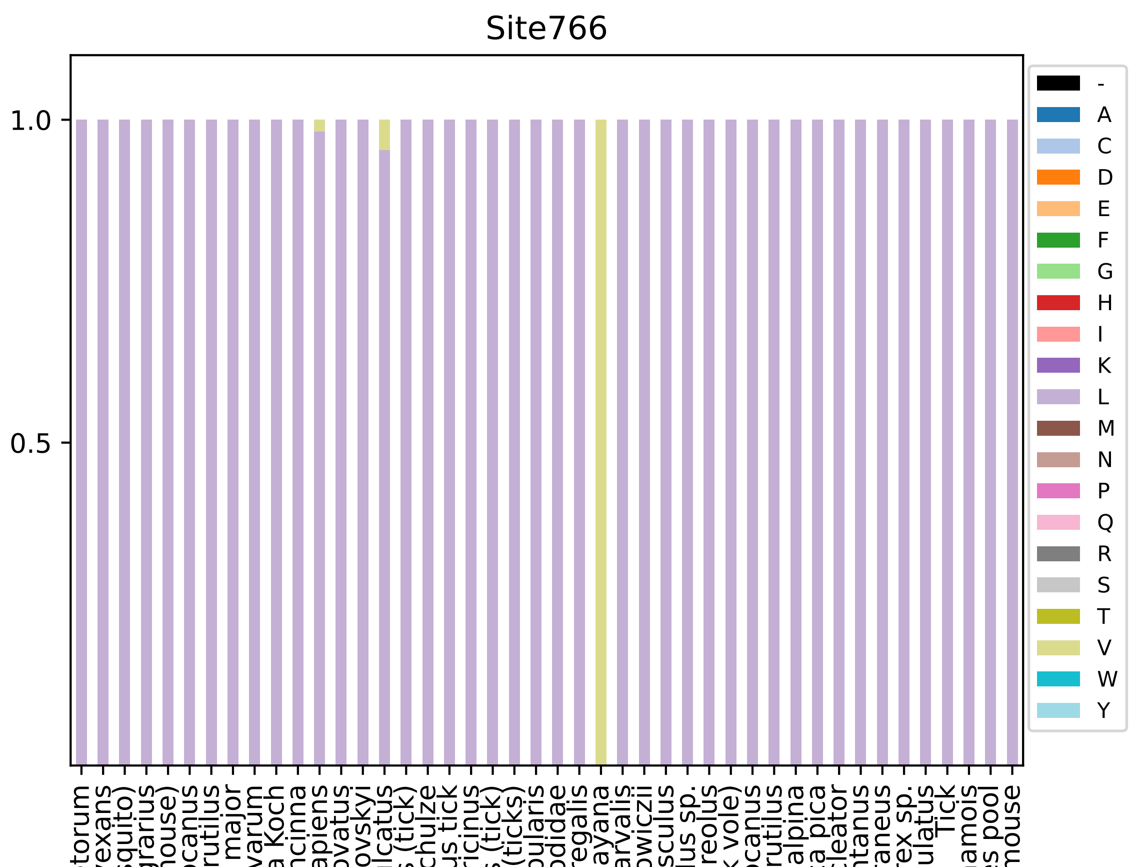

Site766
1.0
0.5
-
A
C
D
E
F
G
H
I
K
L
M
N
P
Q
R
S
T
V
W
Y

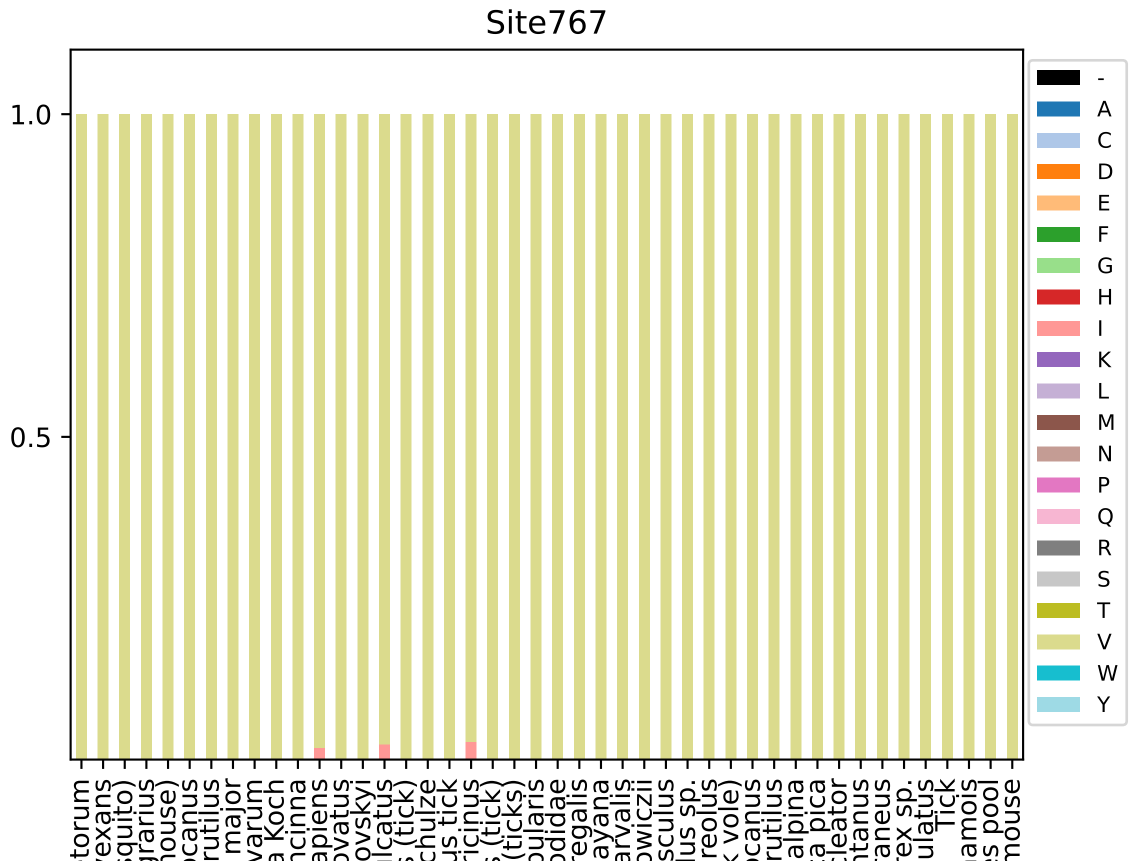
Site767
1.0
0.5
-
A
C
D
E
F
G
H
I
K
L
M
N
P
Q
R
S
T
V
W
Y

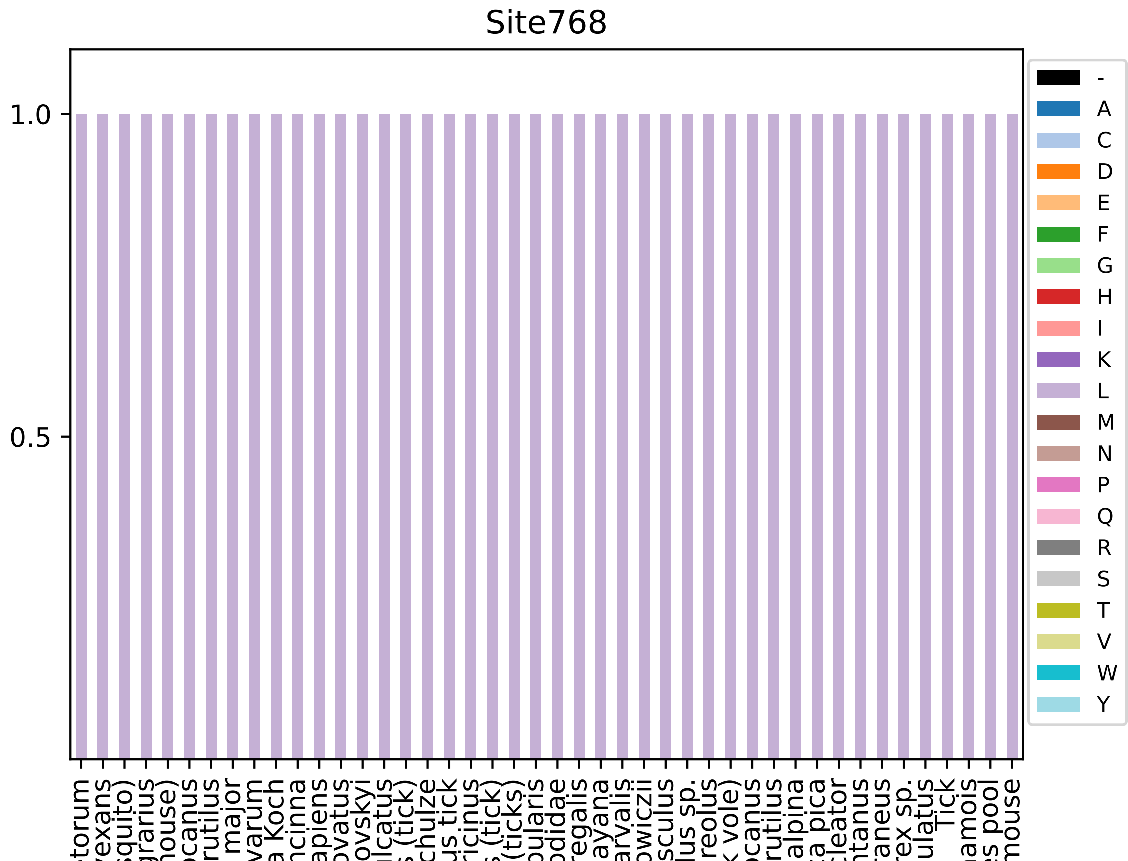
Site768
1.0
0.5
-
A
C
D
E
F
G
H
I
K
L
M
N
P
Q
R
S
T
V
W
Y

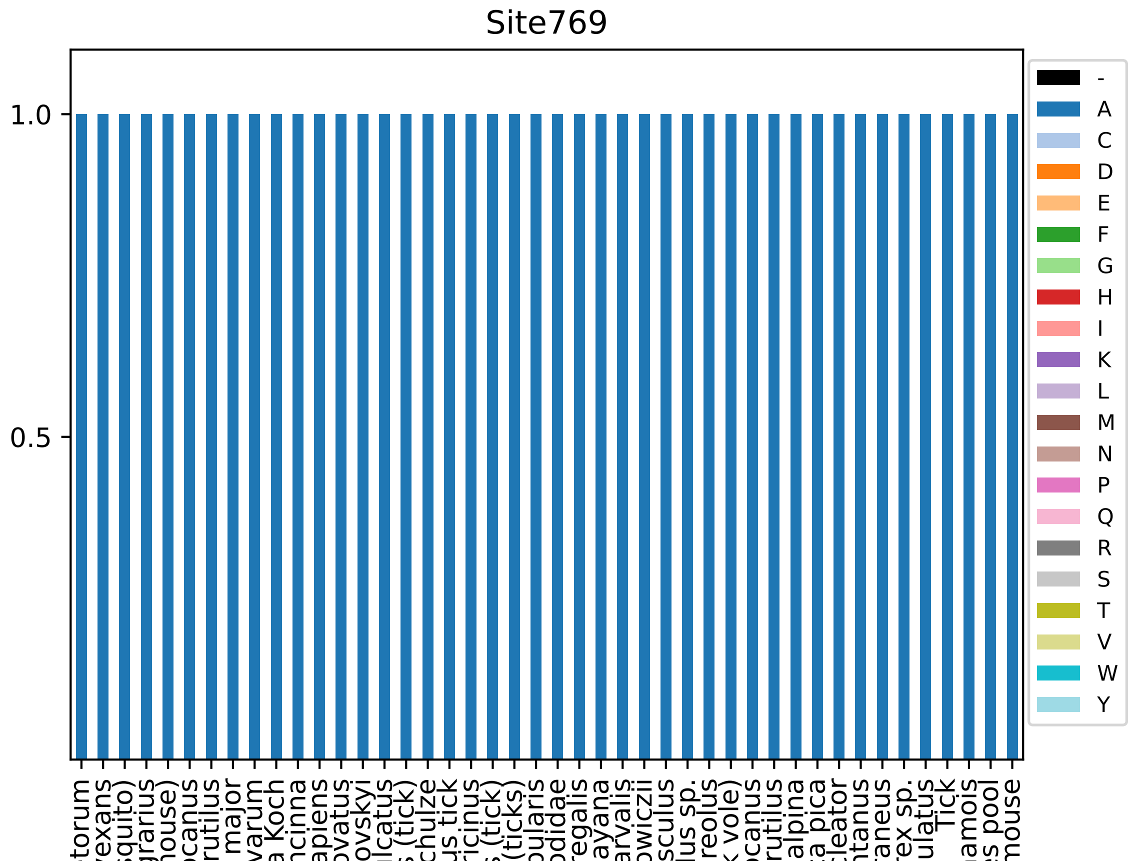
Site769
1.0
0.5
-
A
C
D
E
F
G
H
I
K
L
M
N
P
Q
R
S
T
V
W
Y

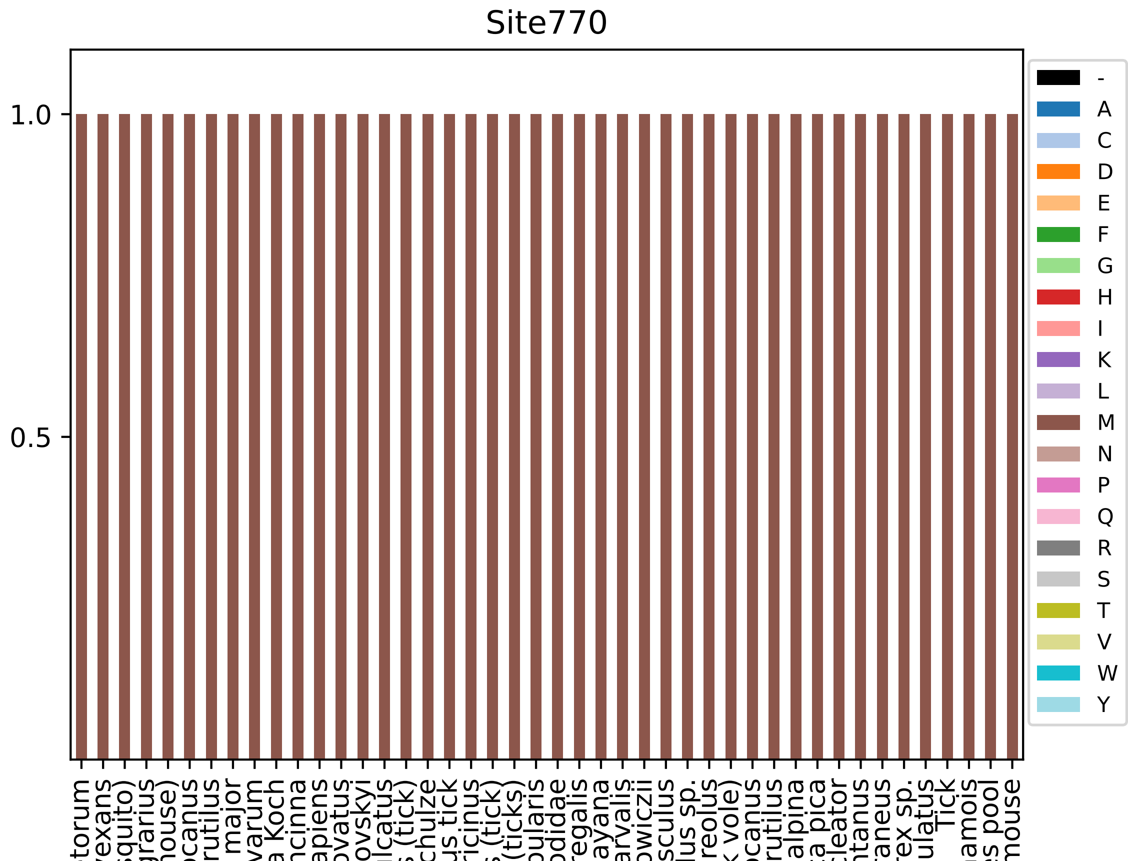
Site770
1.0
0.5
-
A
C
D
E
F
G
H
I
K
L
M
N
P
Q
R
S
T
V
W
Y

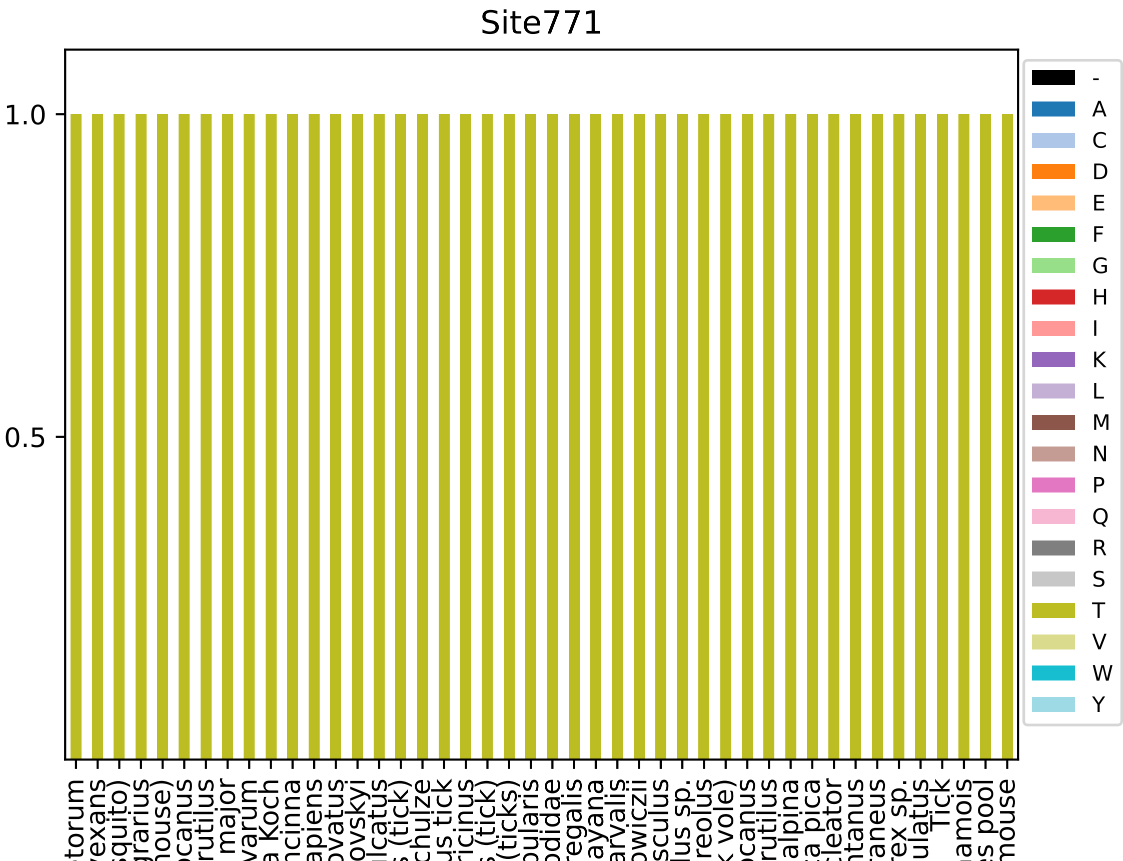
Site771
1.0
0.5
-
A
C
D
E
F
G
H
I
K
L
M
N
P
Q
R
S
T
V
W
Y

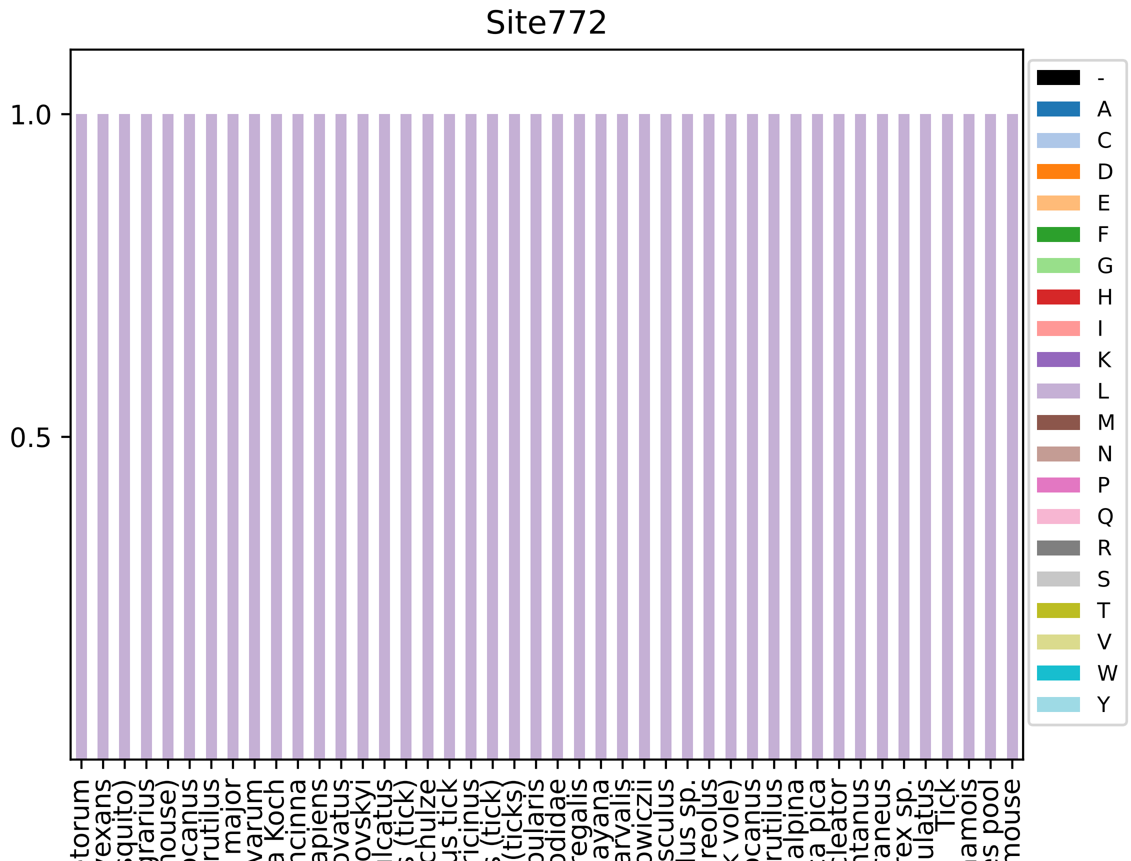
Site772
1.0
0.5
-
A
C
D
E
F
G
H
I
K
L
M
N
P
Q
R
S
T
V
W
Y

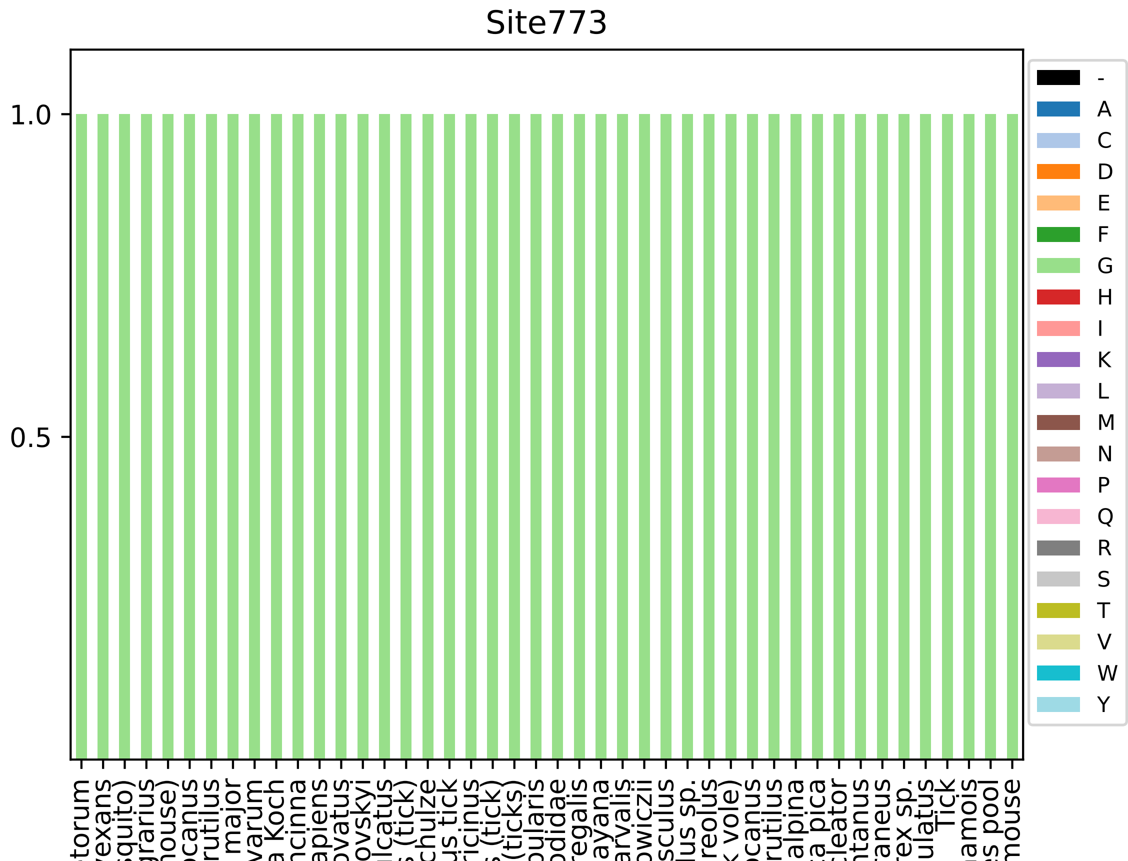
Site773
1.0
0.5
-
A
C
D
E
F
G
H
I
K
L
M
N
P
Q
R
S
T
V
W
Y

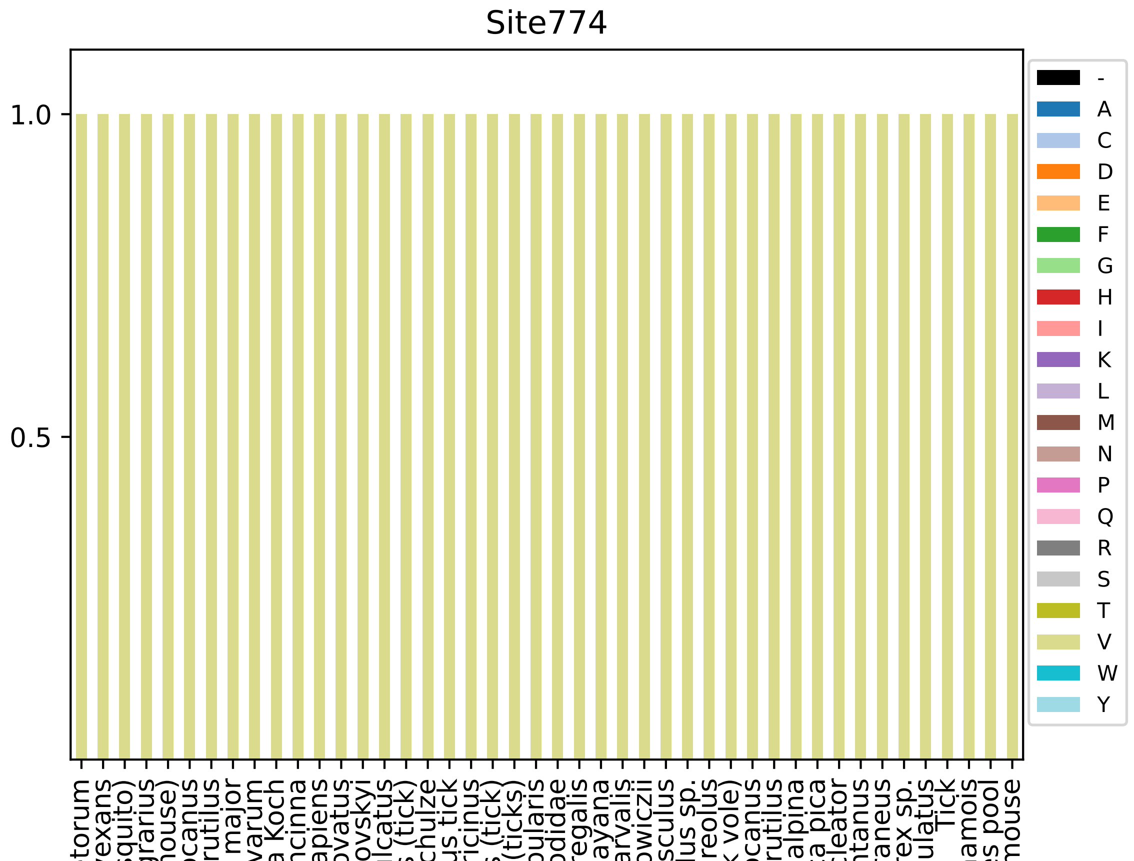

Site774
1.0
0.5
-
A
C
D
E
F
G
H
I
K
L
M
N
P
Q
R
S
T
V
W
Y

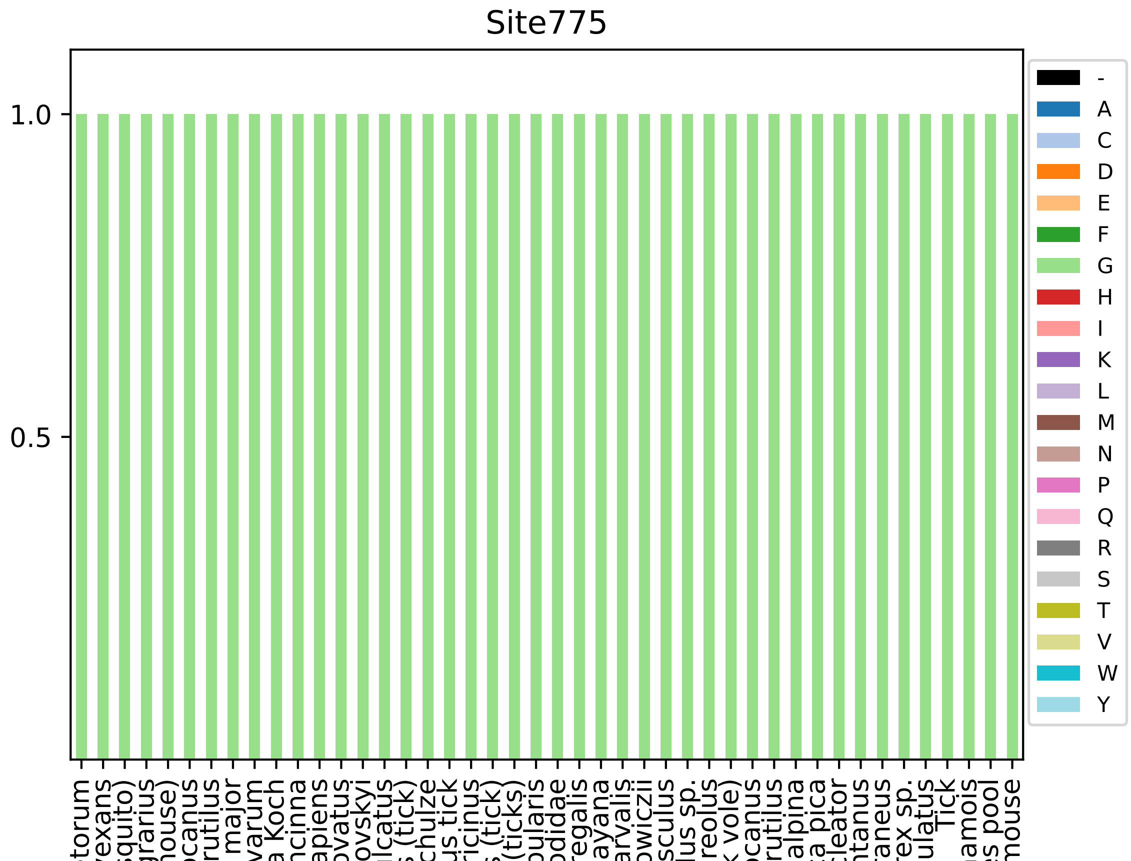
Site775
1.0
0.5
-
A
C
D
E
F
G
H
I
K
L
M
N
P
Q
R
S
T
V
W
Y

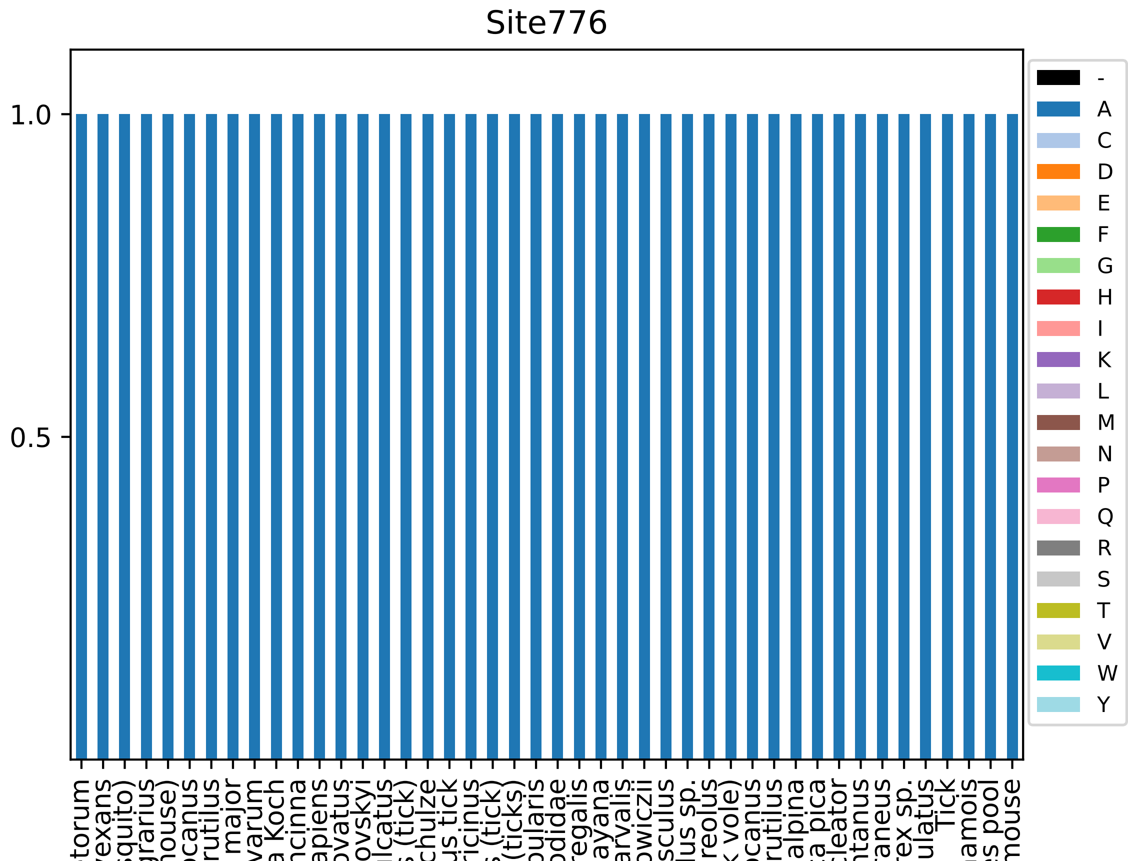

Site776
1.0
0.5
-
A
C
D
E
F
G
H
I
K
L
M
N
P
Q
R
S
T
V
W
Y

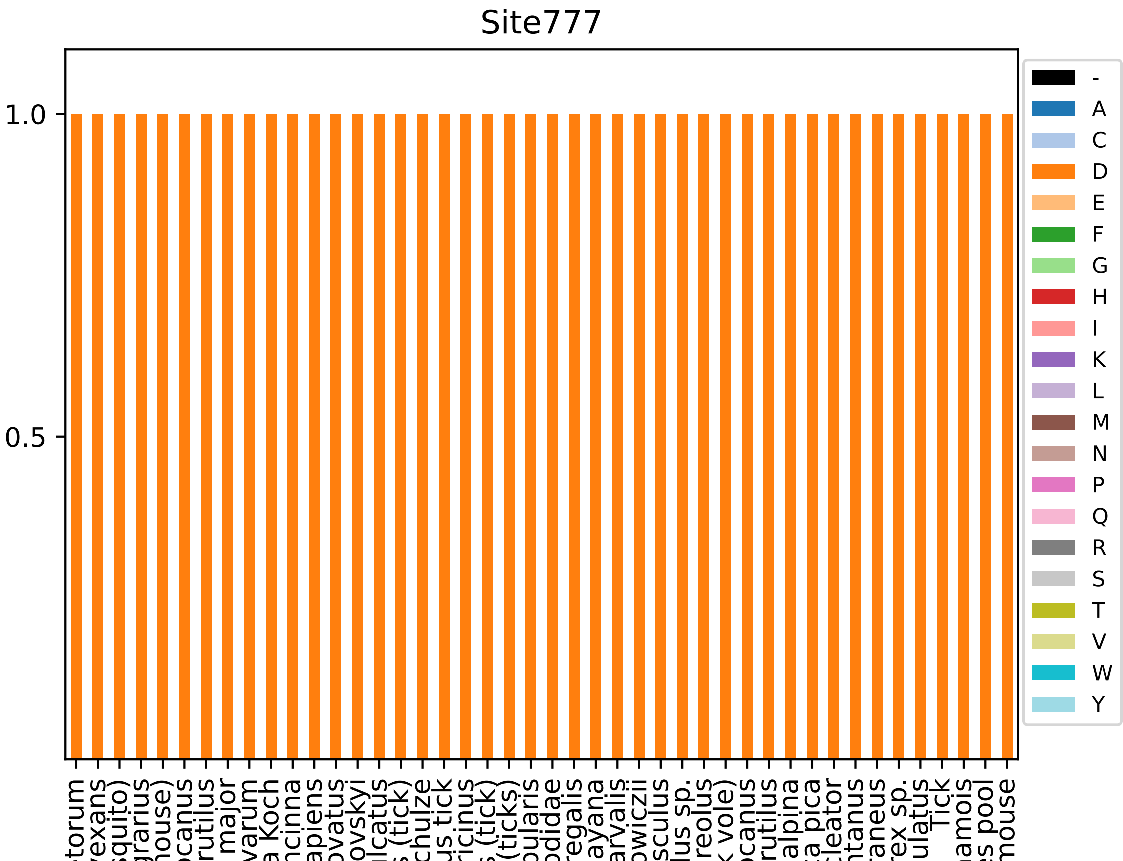
Site777
1.0
0.5
-
A
C
D
E
F
G
H
I
K
L
M
N
P
Q
R
S
T
V
W
Y

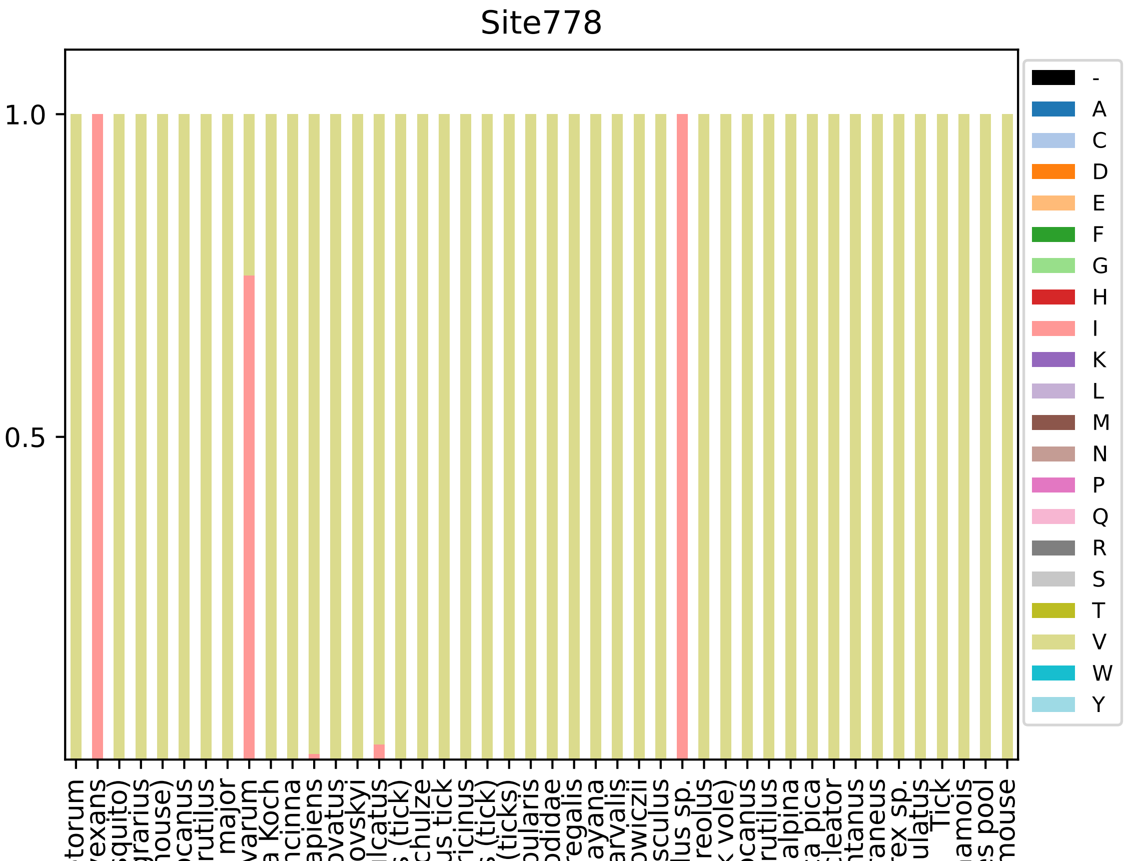
Site778
1.0
0.5
-
A
C
D
E
F
G
H
I
K
L
M
N
P
Q
R
S
T
V
W
Y
torum
vexans
squito)
garius
nouse)
ocanus
rutilus
major
varum
a Koch
ncinna
apiens
ovatus
ovskyi
ulcatus
s (tick)
chulze
us tick
ricinus
s (tick)
(ticks)
pularis
podidae
regalis
ayana
arvalis
owiczii
sculus
lus sp.
reolus
k vole)
ocanus
rutilus
alpina
a pica
cleator
ntanus
raneus
rex sp.
ulatus
Tick
amois
s pool
mouse

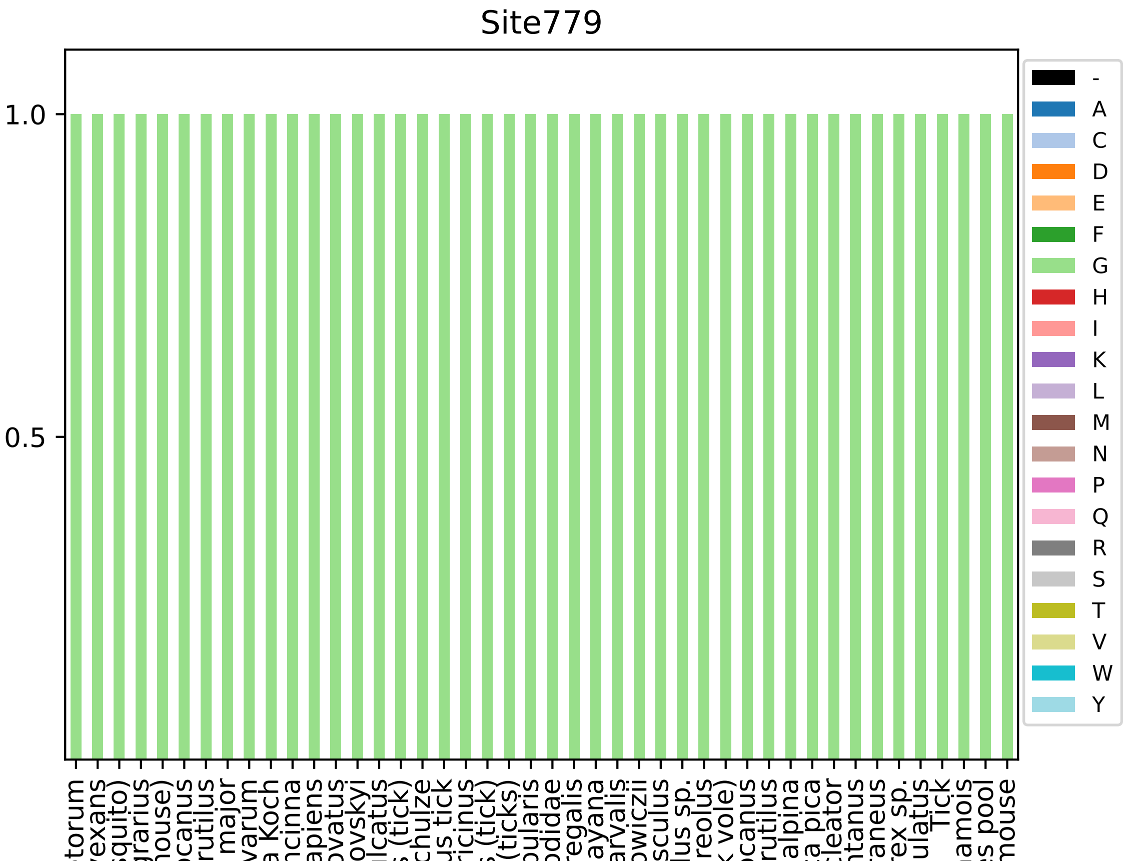
Site779
1.0
0.5
-
A
C
D
E
F
G
H
I
K
L
M
N
P
Q
R
S
T
V
W
Y

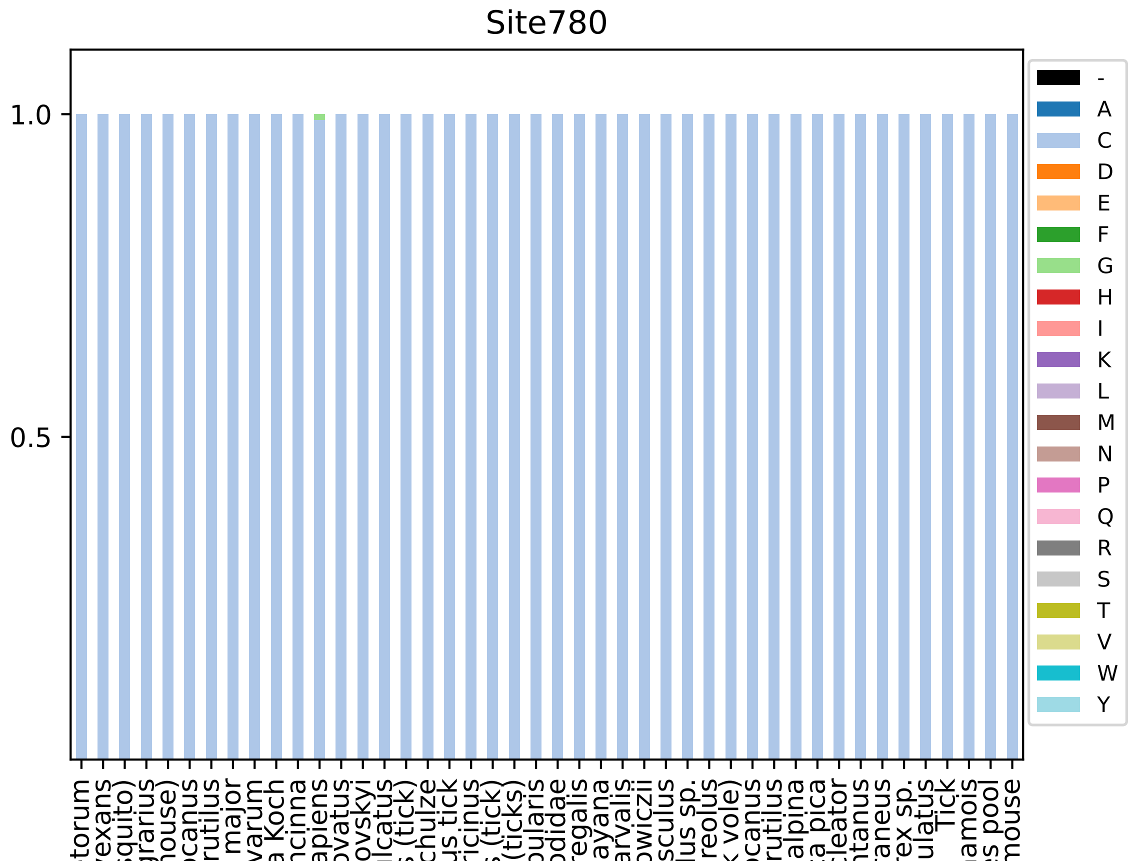
Site780
1.0
0.5
-
A
C
D
E
F
G
H
I
K
L
M
N
P
Q
R
S
T
V
W
Y

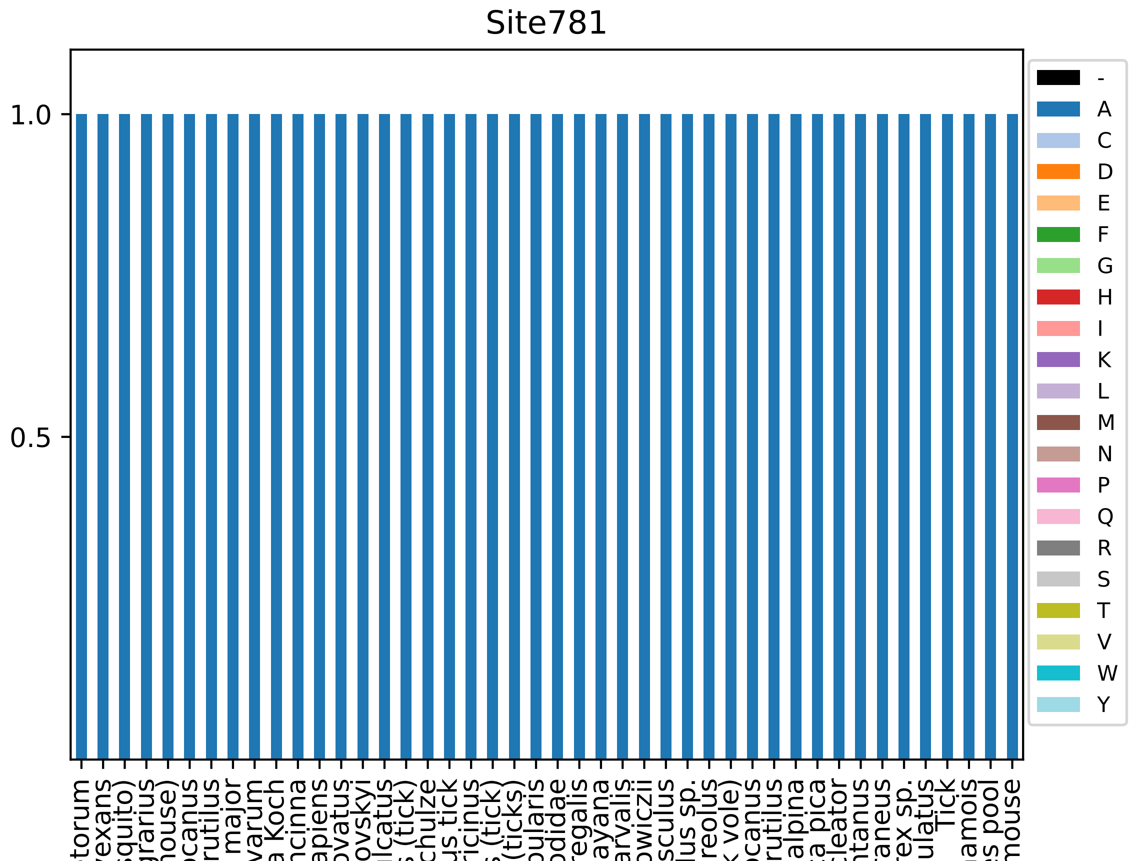
Site781
1.0
0.5
-
A
C
D
E
F
G
H
I
K
L
M
N
P
Q
R
S
T
V
W
Y

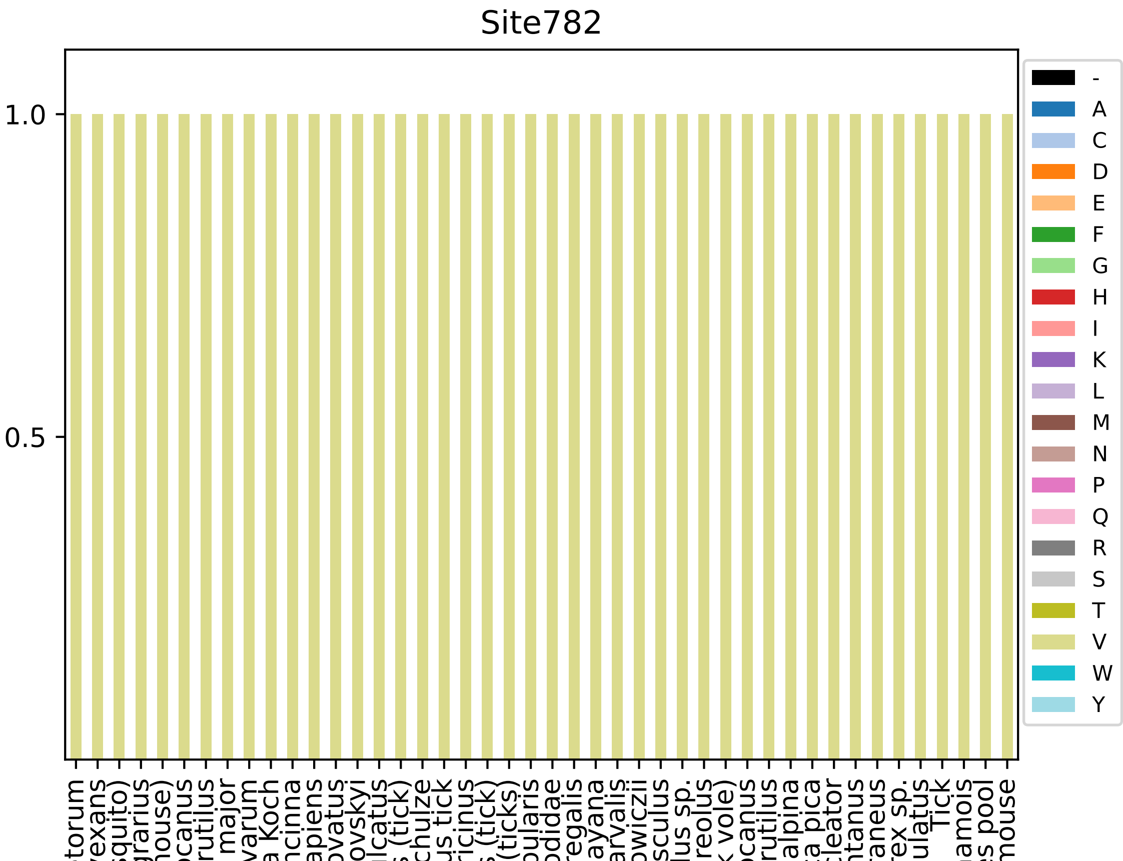
Site782
1.0
0.5
-
A
C
D
E
F
G
H
I
K
L
M
N
P
Q
R
S
T
V
W
Y

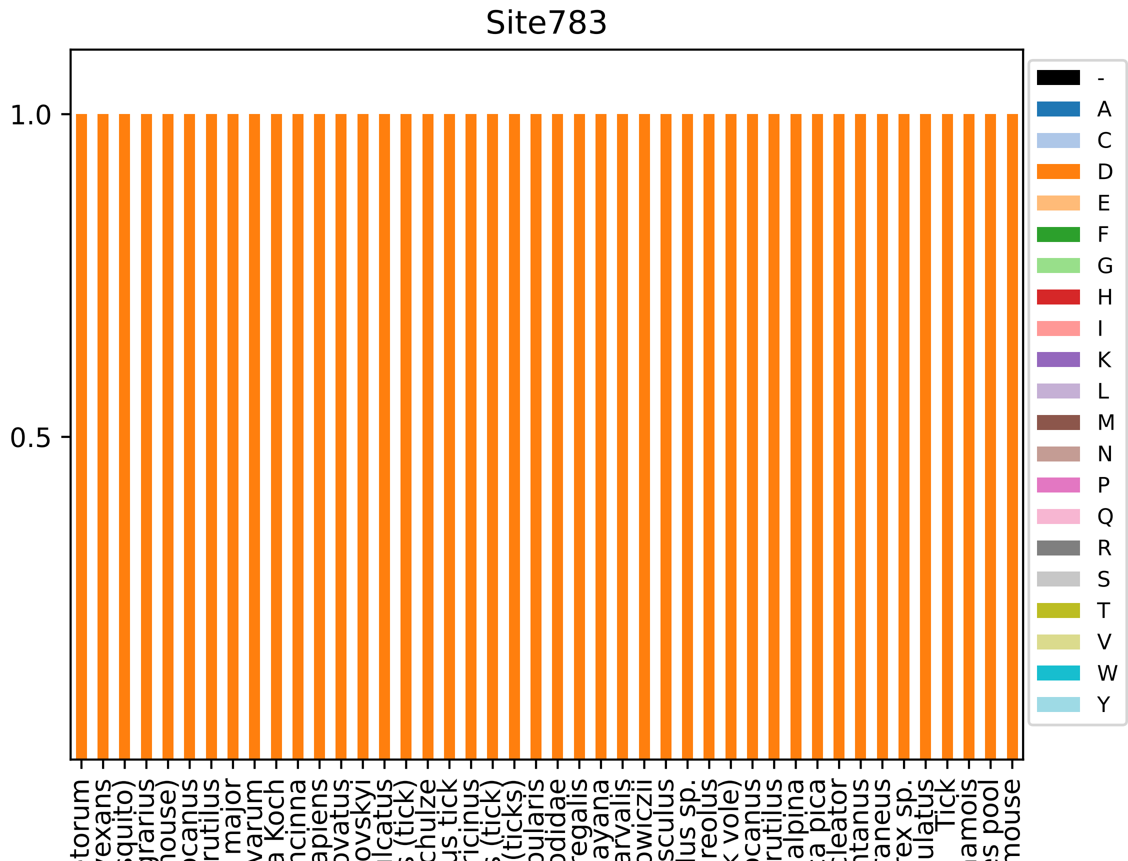

Site783
1.0
0.5
-
A
C
D
E
F
G
H
I
K
L
M
N
P
Q
R
S
T
V
W
Y

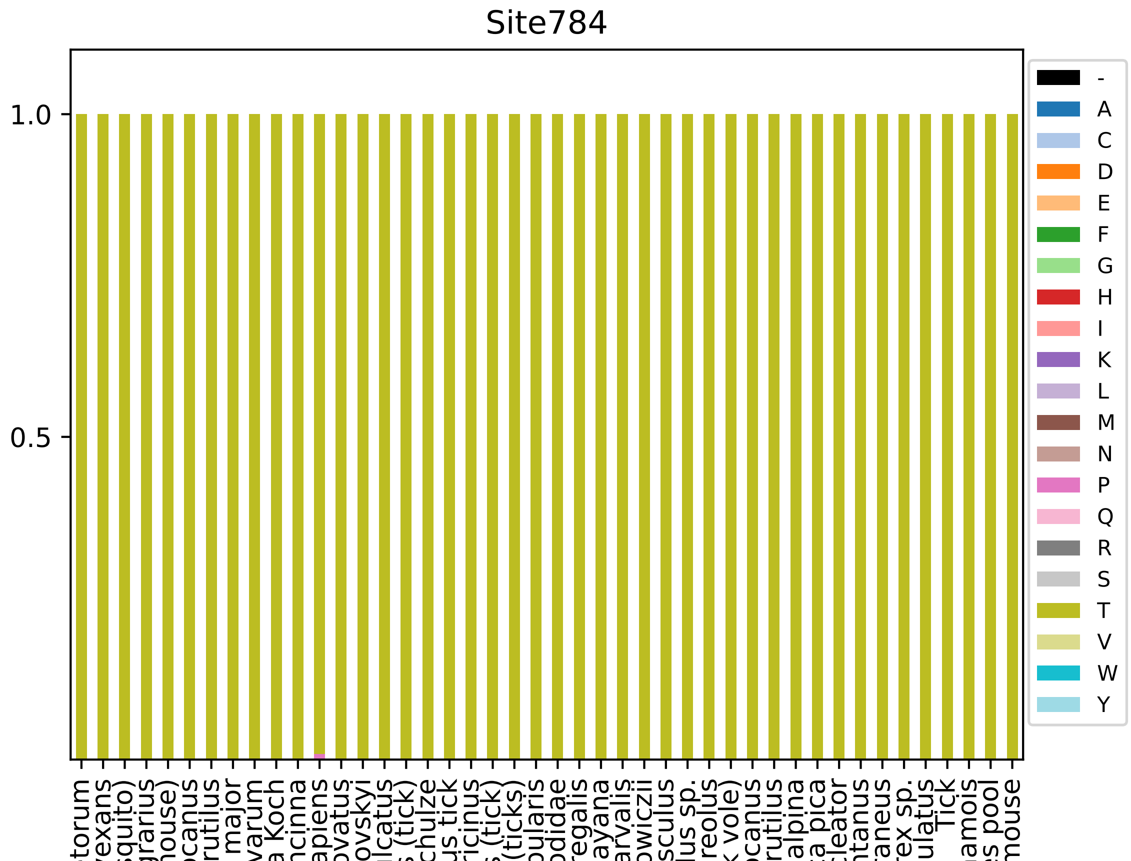
Site784
1.0
0.5
-
A
C
D
E
F
G
H
I
K
L
M
N
P
Q
R
S
T
V
W
Y

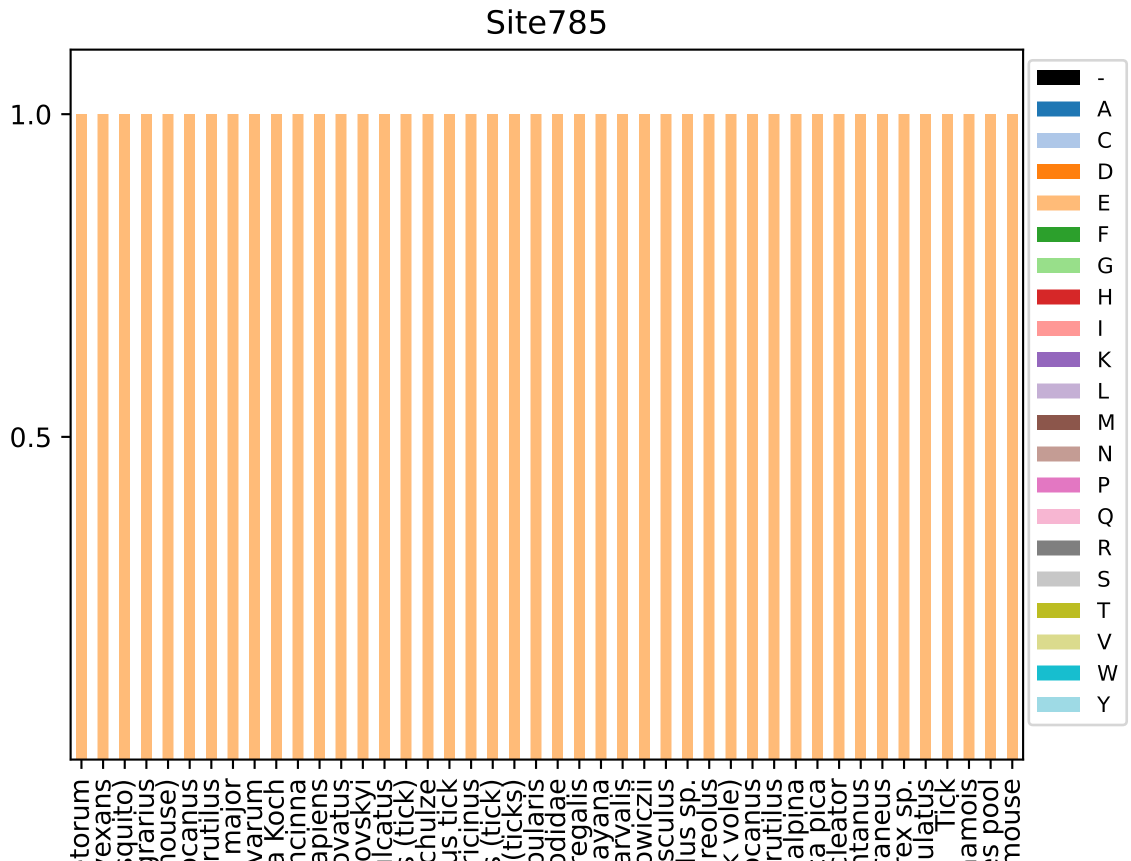
Site785
1.0
0.5
-
A
C
D
E
F
G
H
I
K
L
M
N
P
Q
R
S
T
V
W
Y
Tick

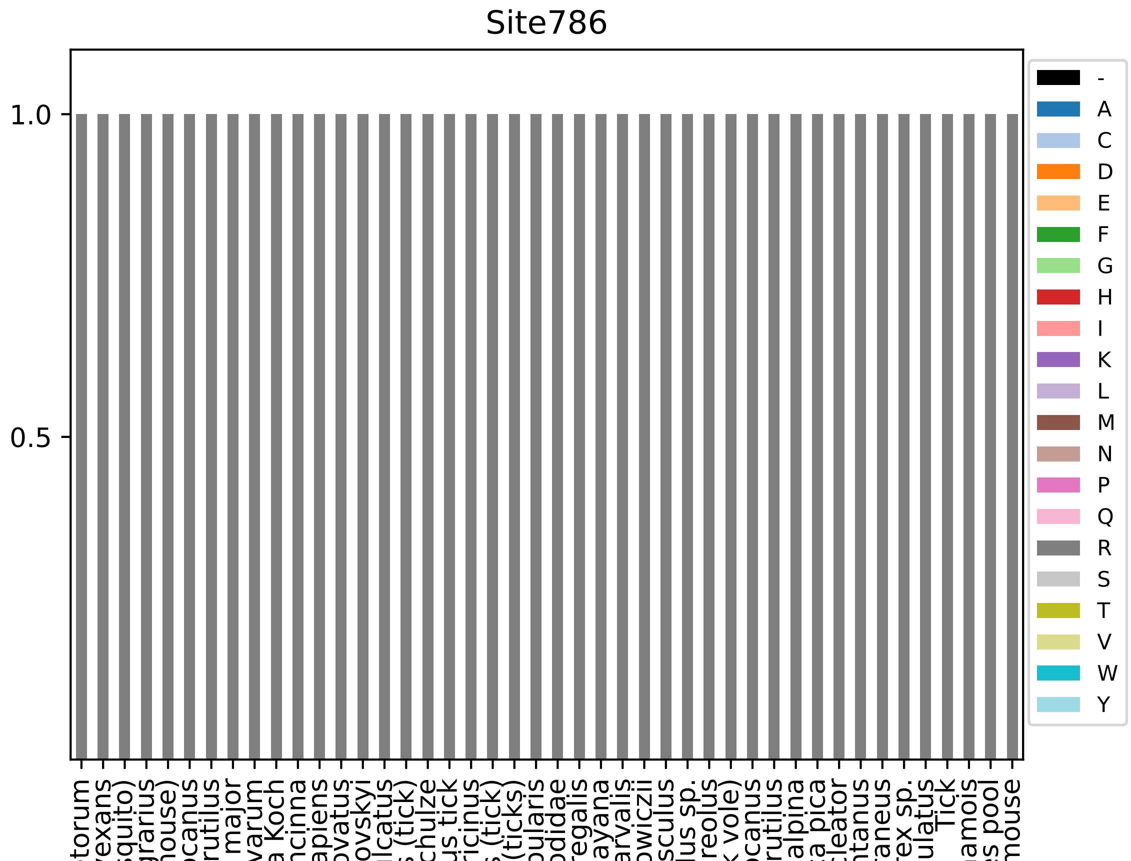
Site786
1.0
0.5
-
A
C
D
E
F
G
H
I
K
L
M
N
P
Q
R
S
T
V
W
Y

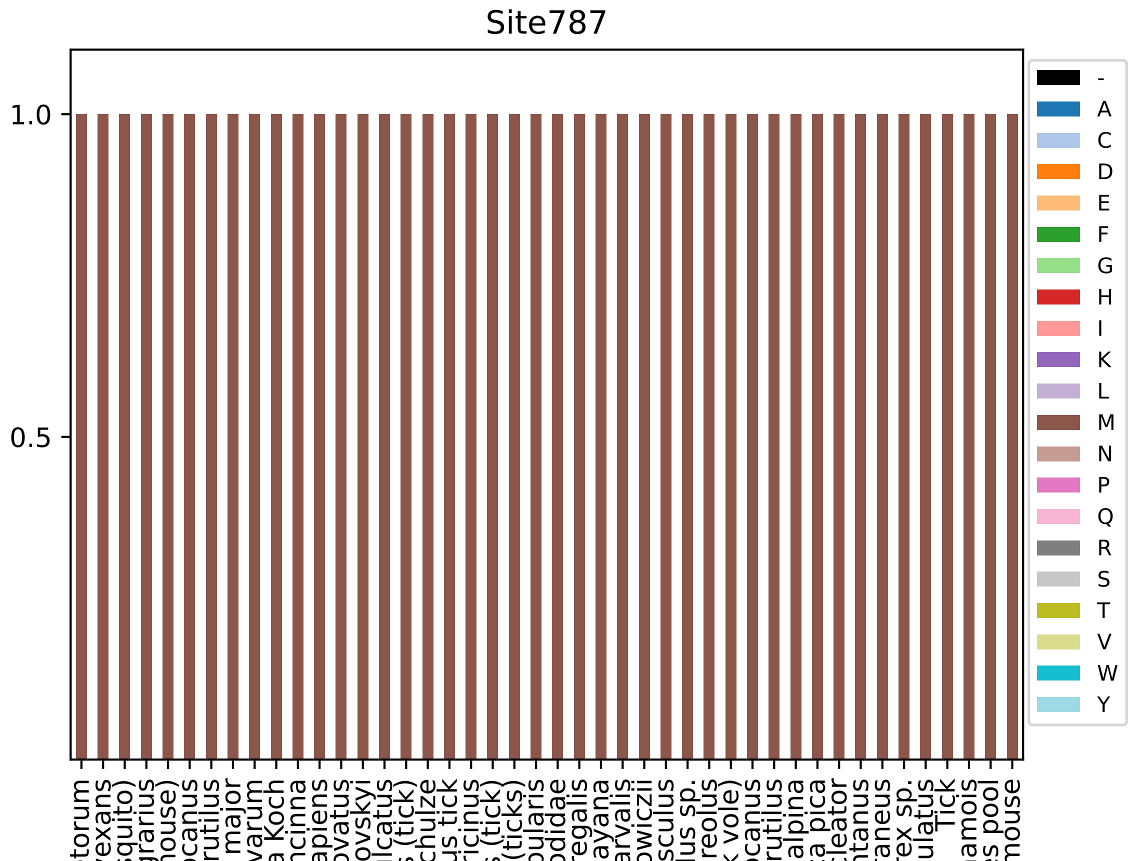
Site787
1.0
0.5
-
A
C
D
E
F
G
H
I
K
L
M
N
P
Q
R
S
T
V
W
Y

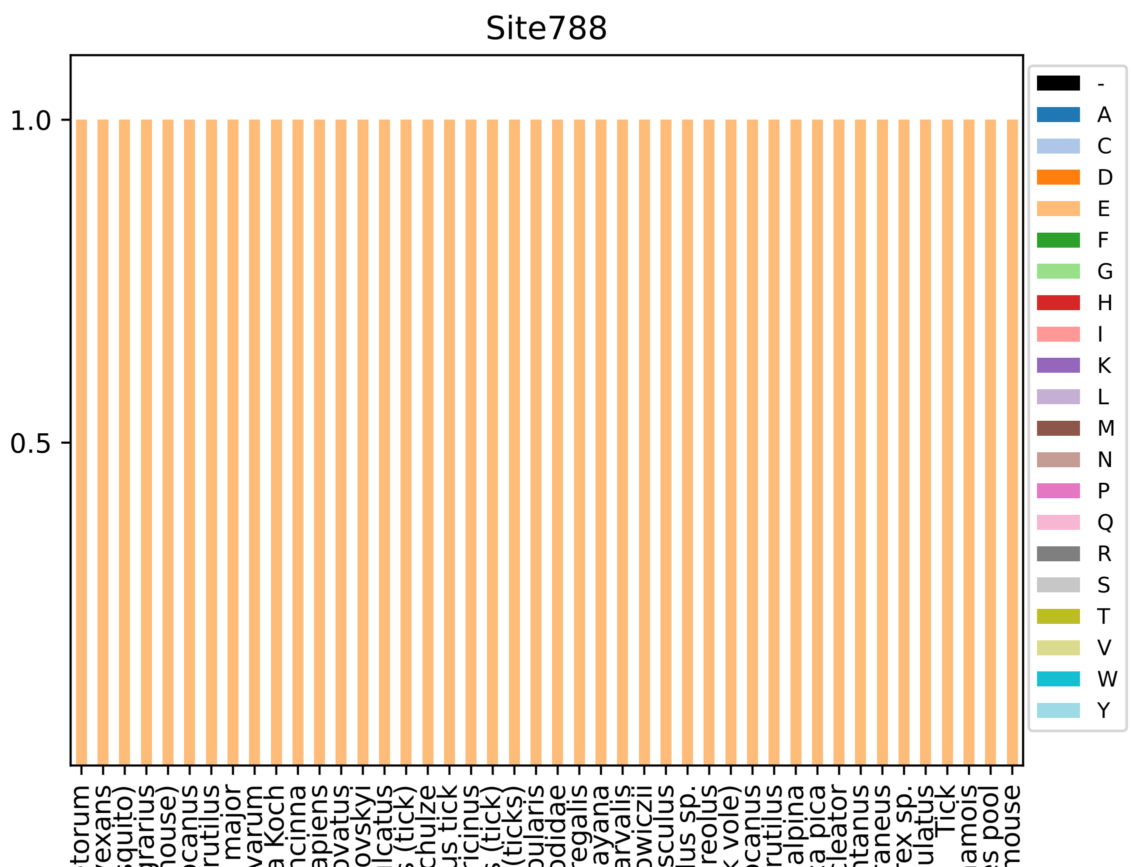
Site788
1.0
0.5
-
A
C
D
E
F
G
H
I
K
L
M
N
P
Q
R
S
T
V
W
Y

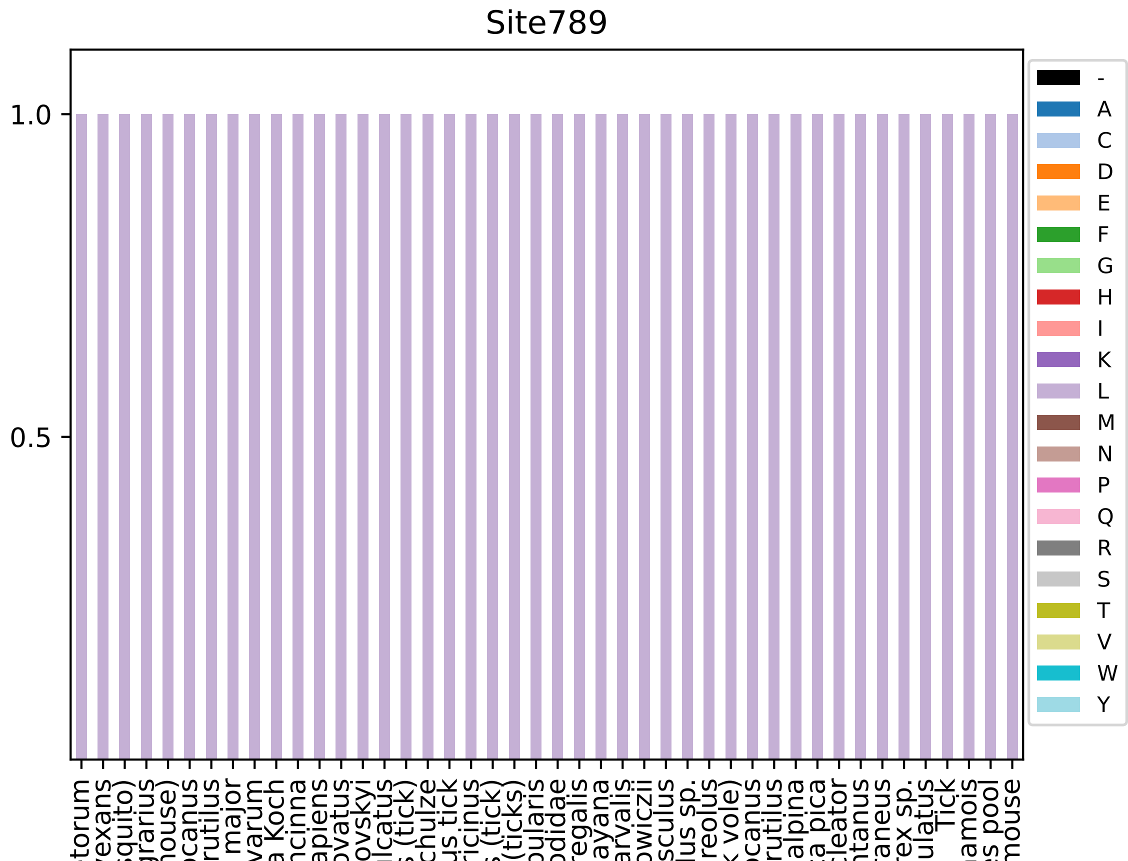
Site789
1.0
0.5
-
A
C
D
E
F
G
H
I
K
L
M
N
P
Q
R
S
T
V
W
Y

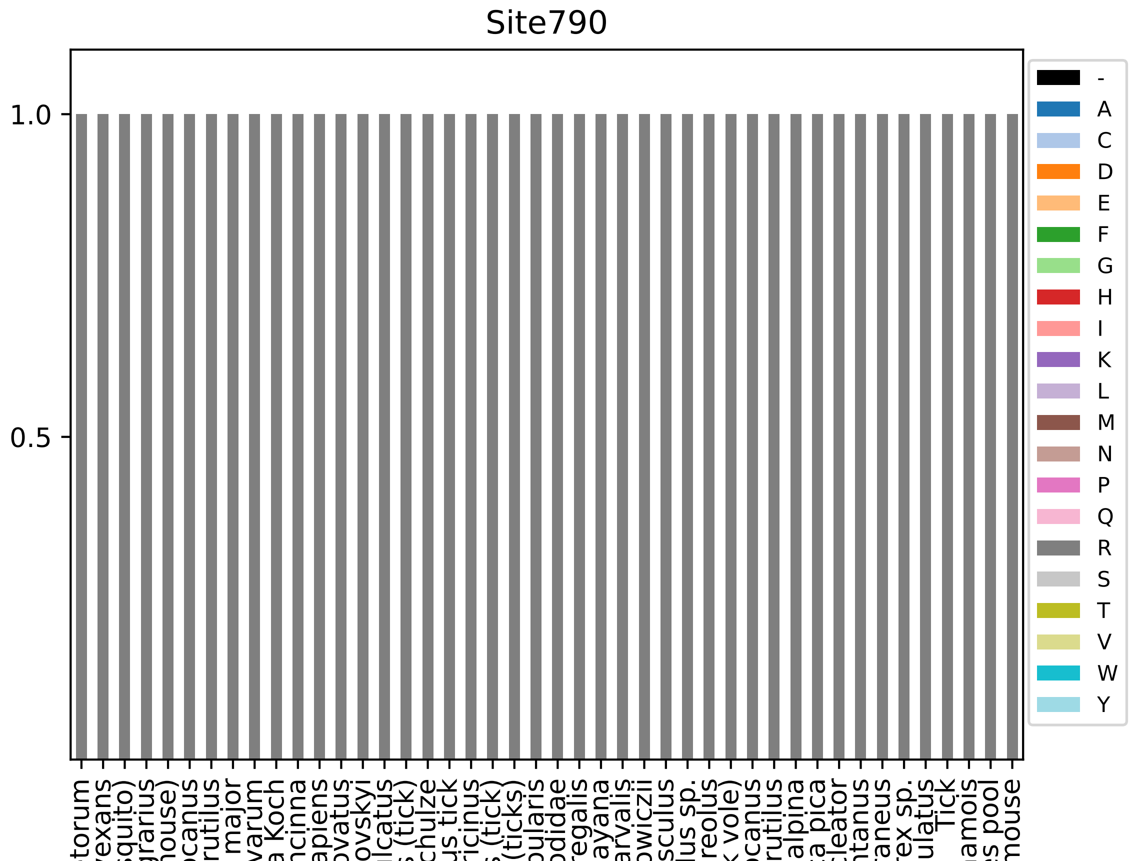
Site790
1.0
0.5
-
A
C
D
E
F
G
H
I
K
L
M
N
P
Q
R
S
T
V
W
Y
Tick

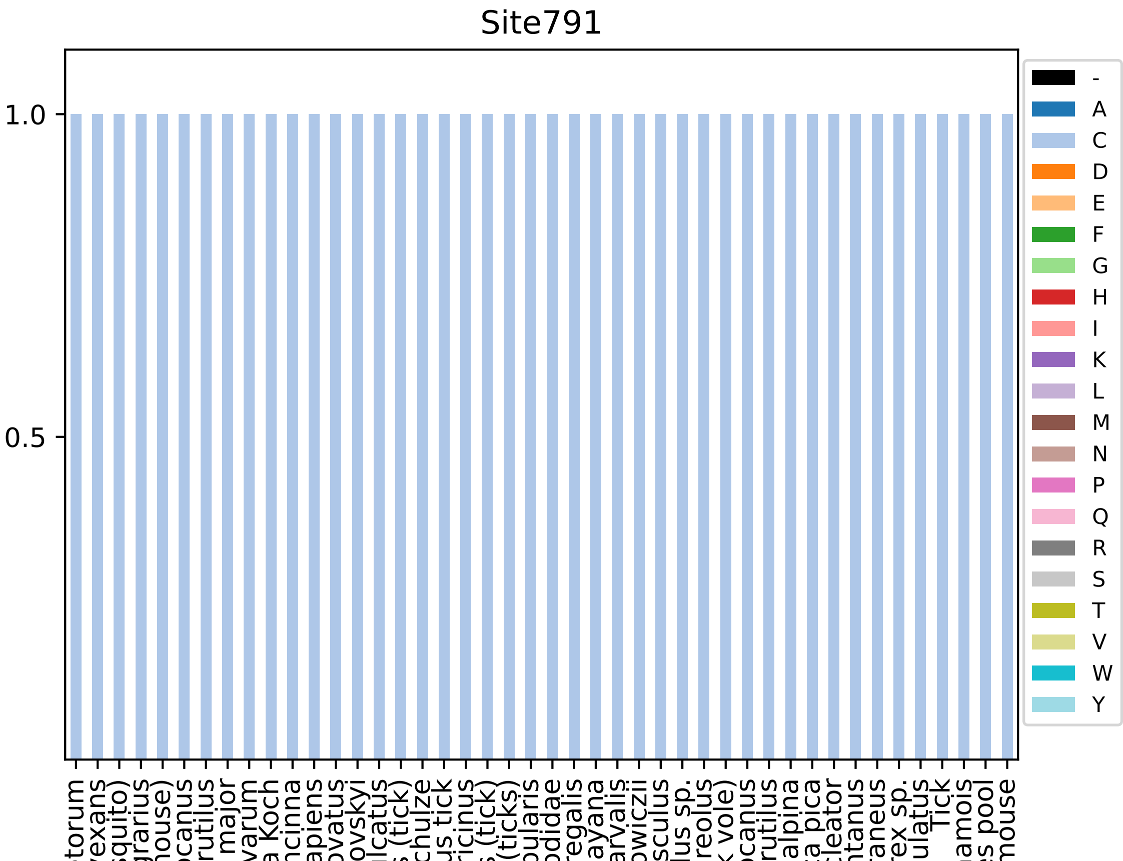
Site791
1.0
0.5
-
A
C
D
E
F
G
H
I
K
L
M
N
P
Q
R
S
T
V
W
Y

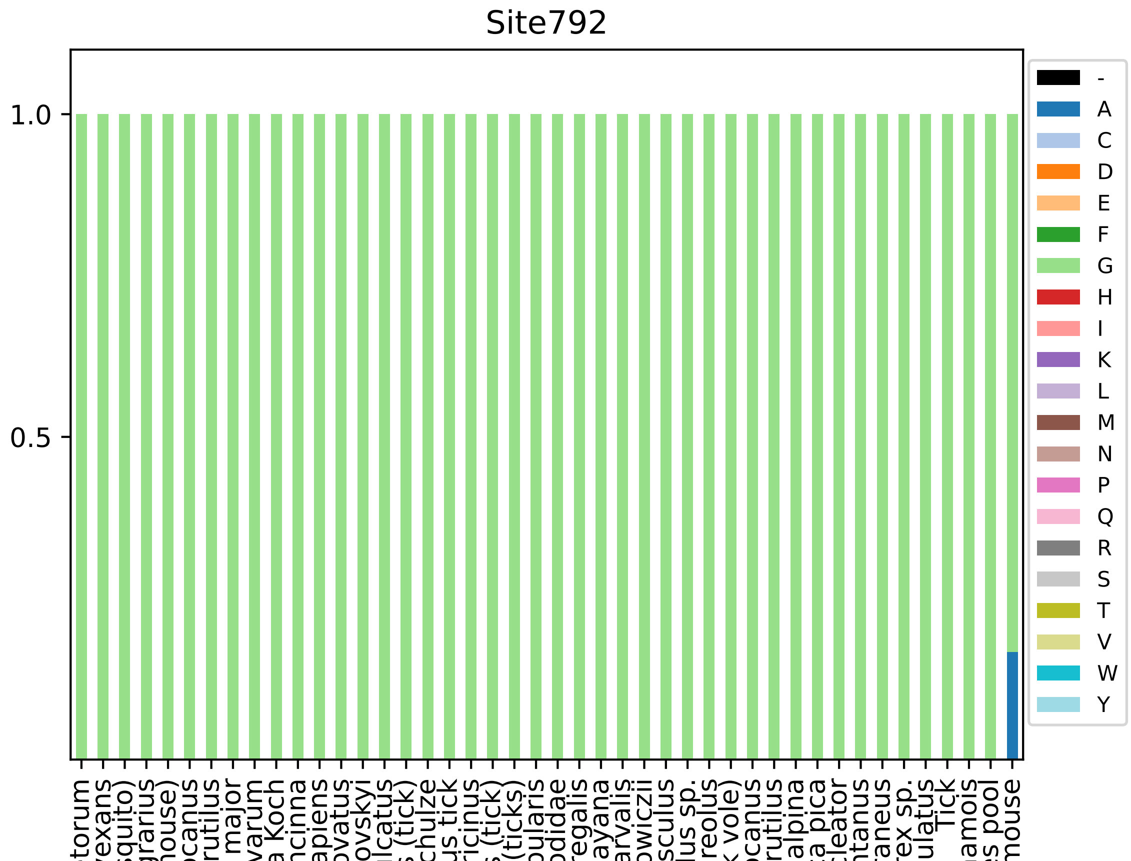
Site792
1.0
0.5
-
A
C
D
E
F
G
H
I
K
L
M
N
P
Q
R
S
T
V
W
Y

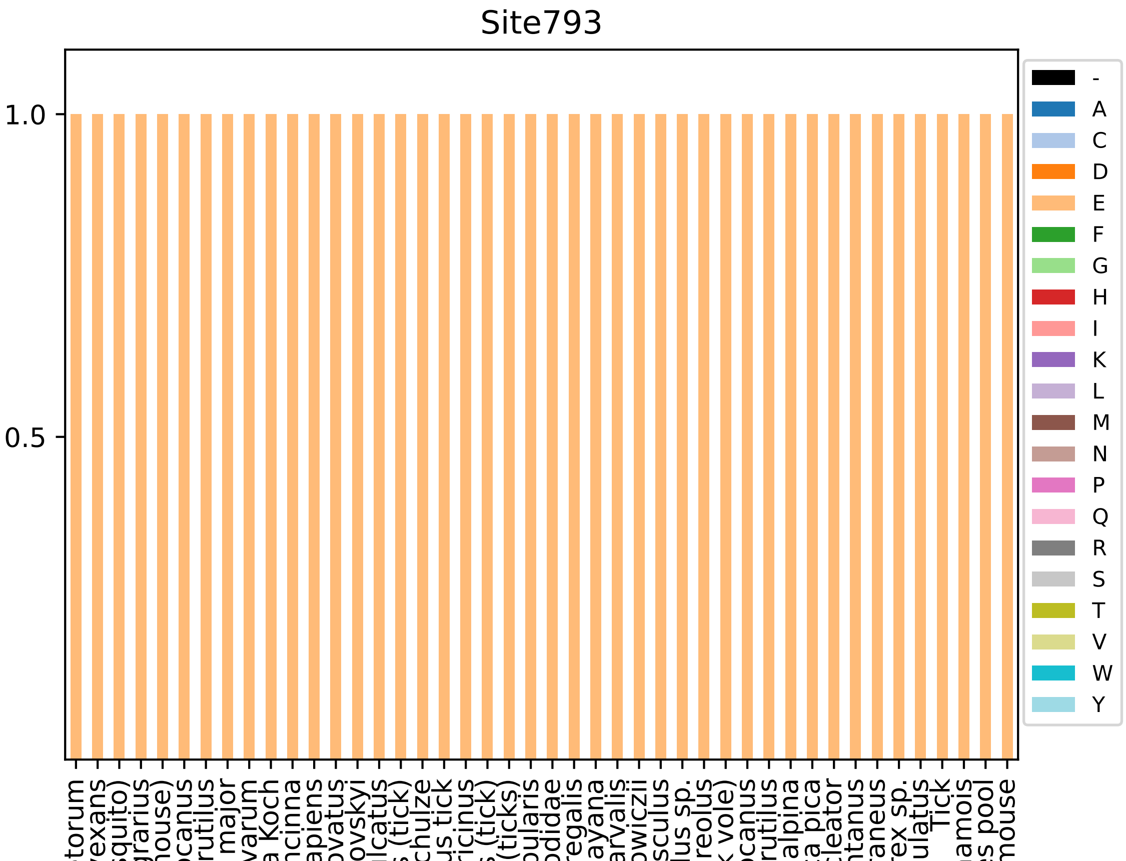
Site793
1.0
0.5
-
A
C
D
E
F
G
H
I
K
L
M
N
P
Q
R
S
T
V
W
Y

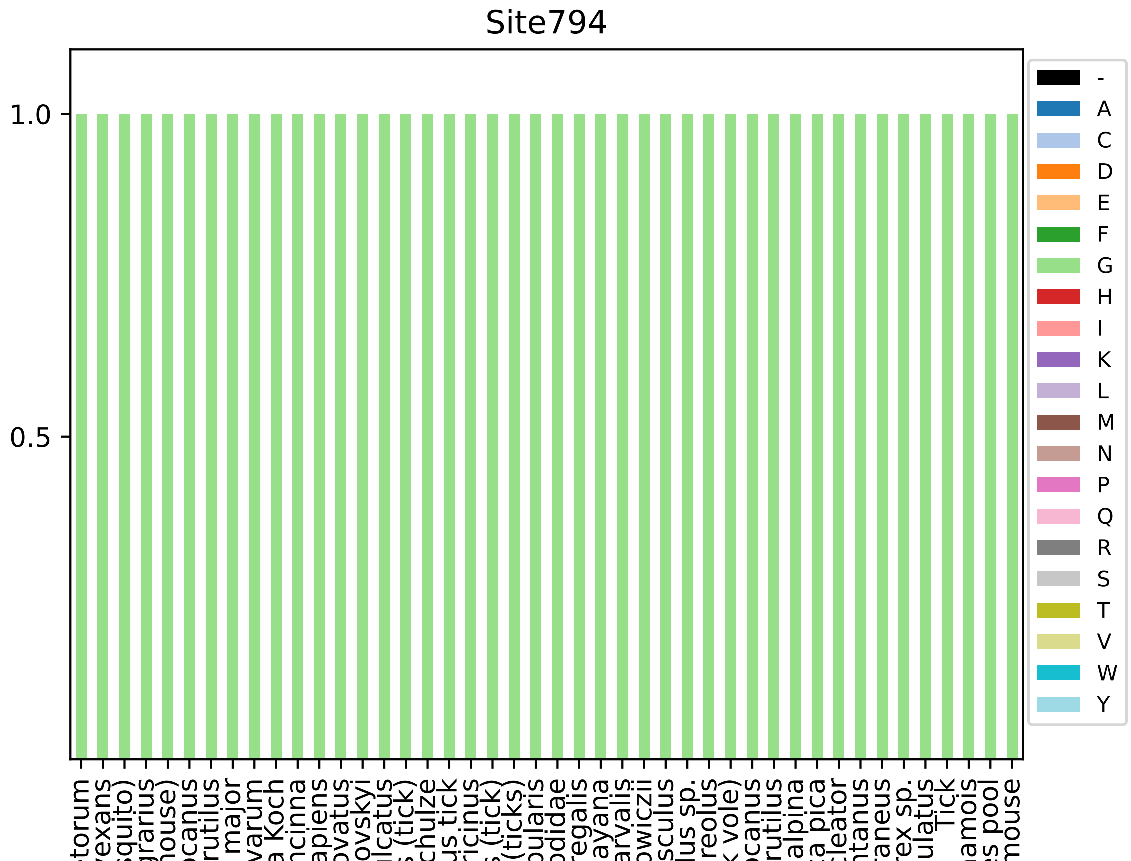
Site794
1.0
0.5
-
A
C
D
E
F
G
H
I
K
L
M
N
P
Q
R
S
T
V
W
Y

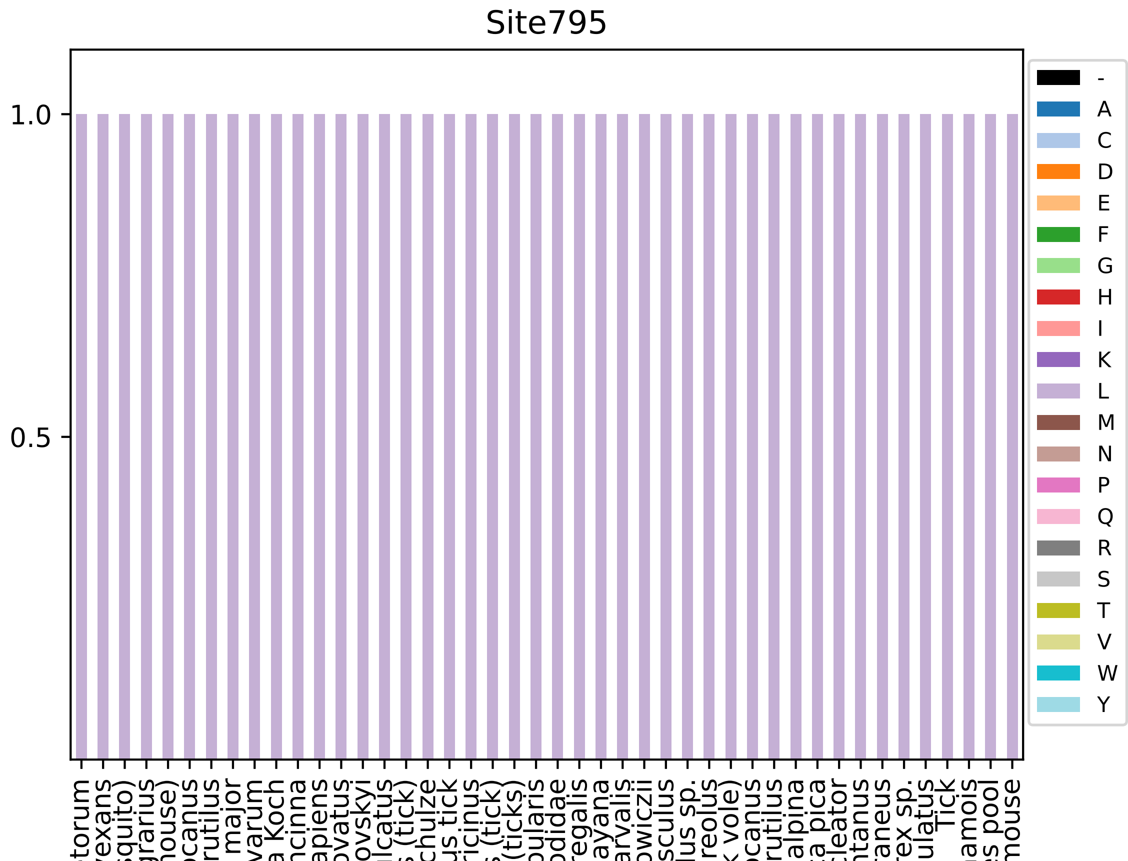
Site795
1.0
0.5
-
A
C
D
E
F
G
H
I
K
L
M
N
P
Q
R
S
T
V
W
Y

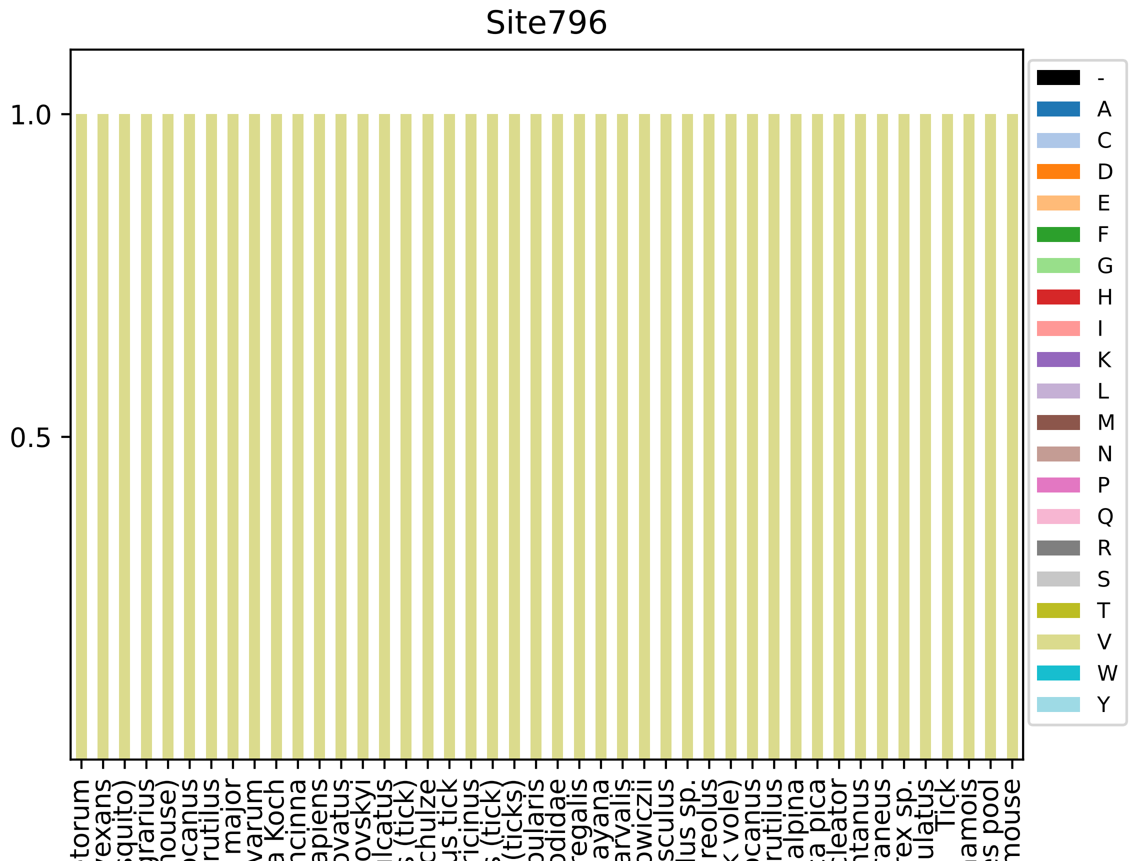

Site796
1.0
0.5
-
A
C
D
E
F
G
H
I
K
L
M
N
P
Q
R
S
T
V
W
Y

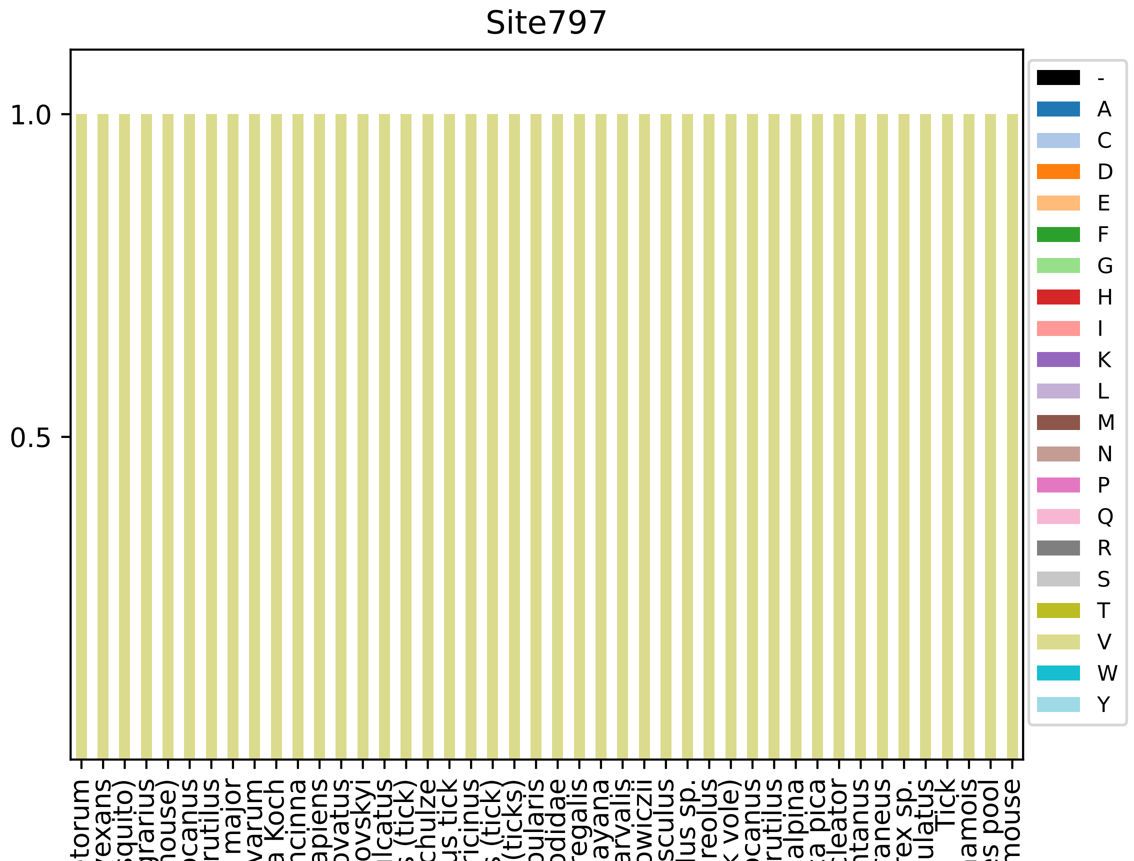

Site797
1.0
0.5
-
A
C
D
E
F
G
H
I
K
L
M
N
P
Q
R
S
T
V
W
Y

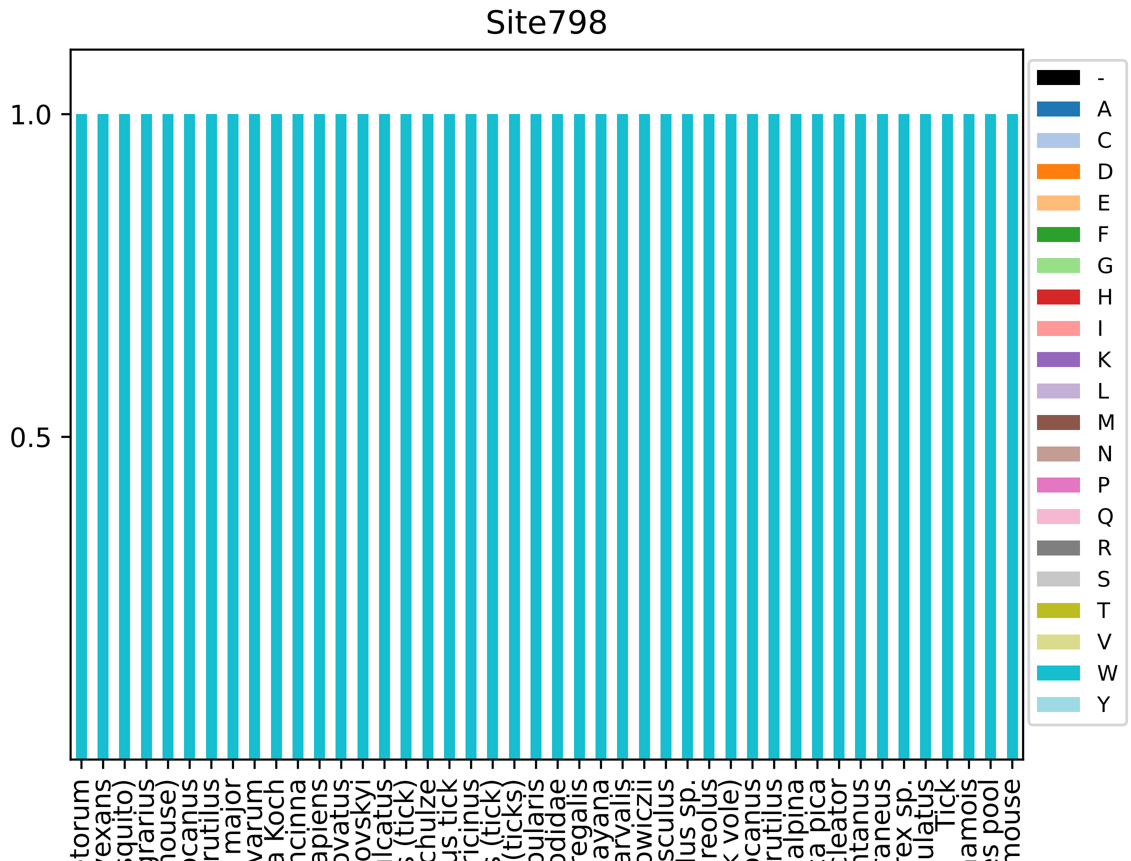
Site798
1.0
0.5
-
A
C
D
E
F
G
H
I
K
L
M
N
P
Q
R
S
T
V
W
Y

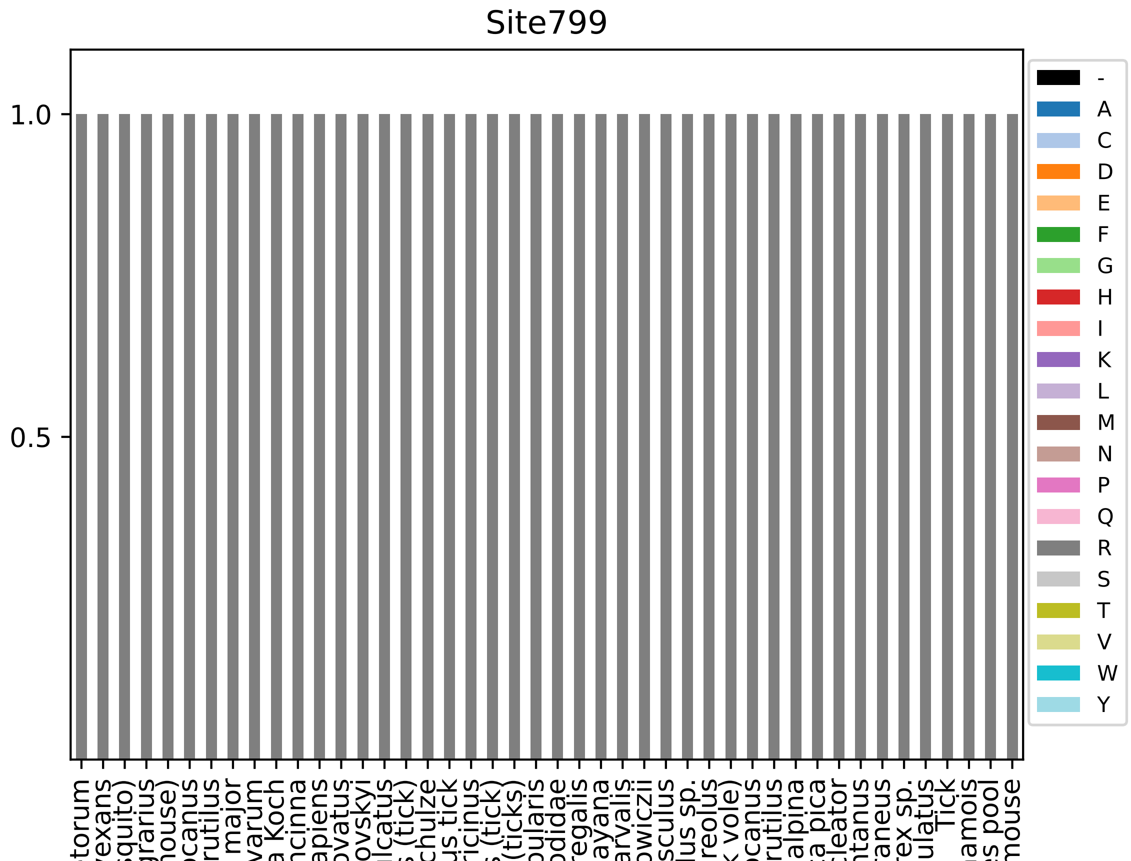
Site799
1.0
0.5
-
A
C
D
E
F
G
H
I
K
L
M
N
P
Q
R
S
T
V
W
Y

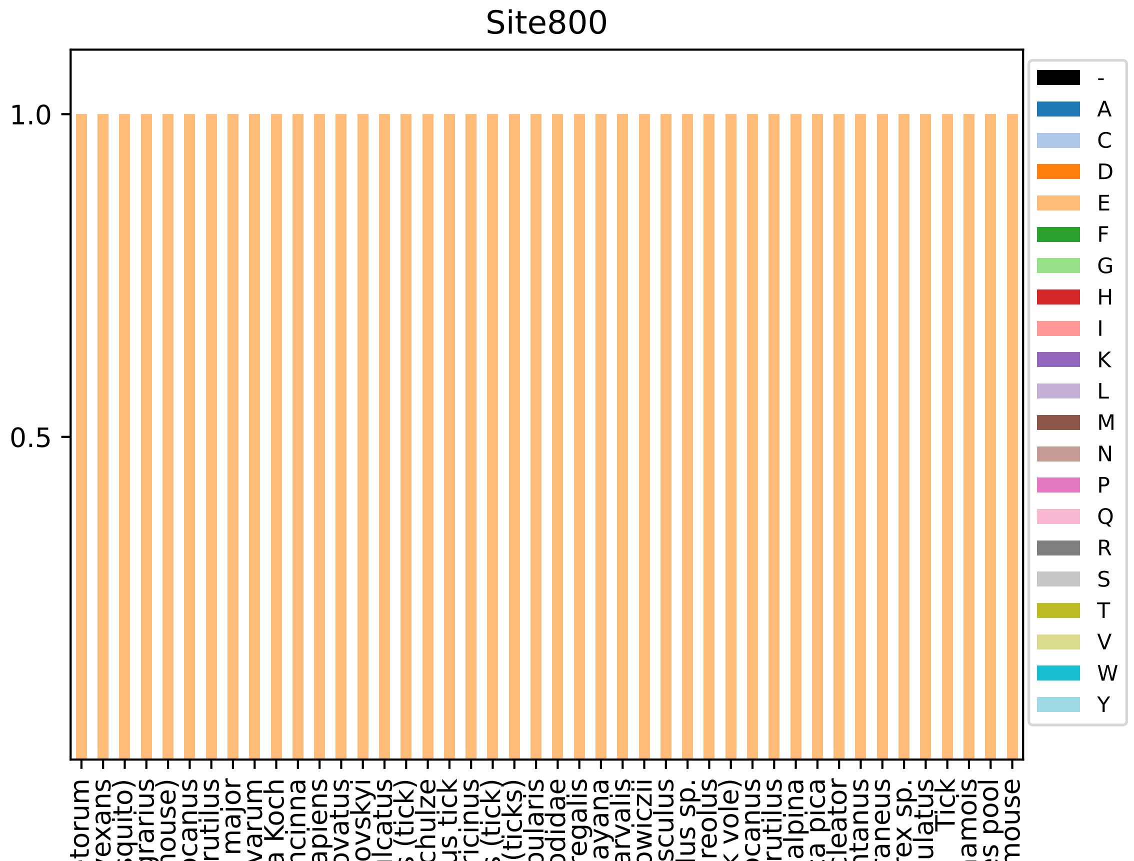

Site800
1.0
0.5
-
A
C
D
E
F
G
H
I
K
L
M
N
P
Q
R
S
T
V
W
Y

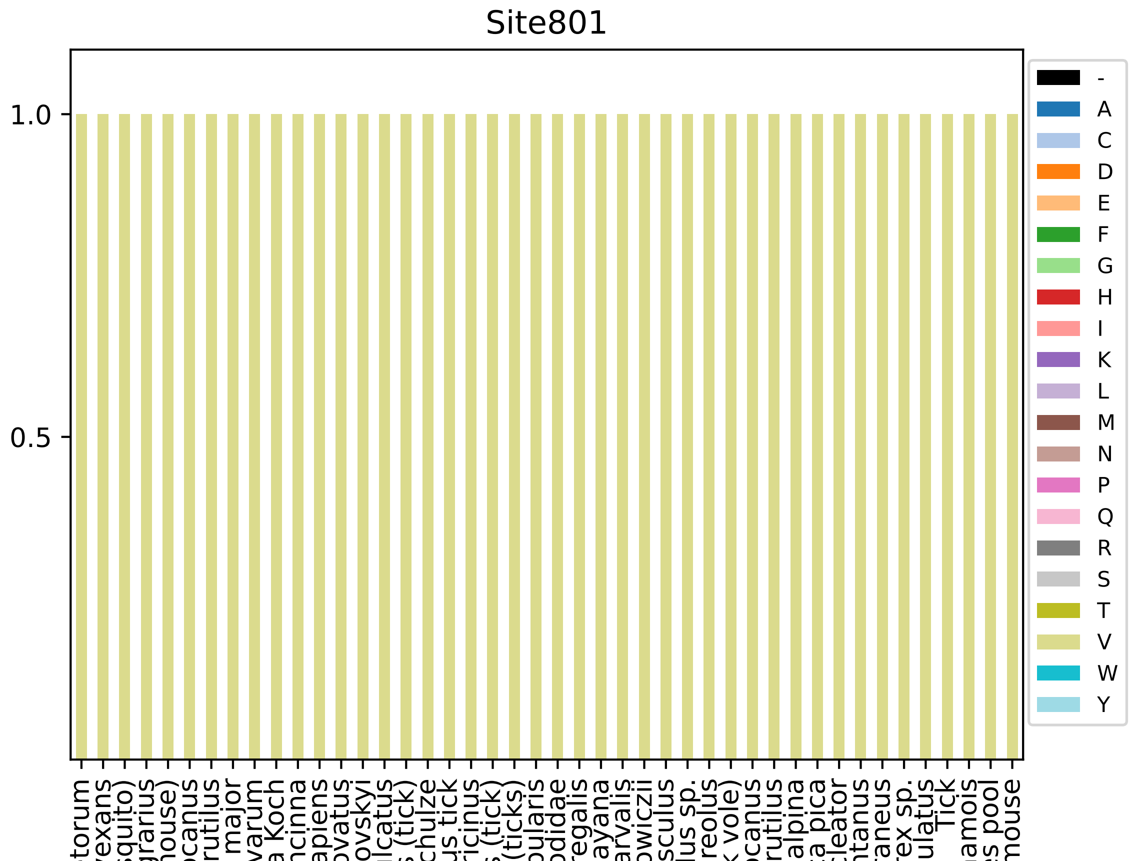
Site801
1.0
0.5
-
A
C
D
E
F
G
H
I
K
L
M
N
P
Q
R
S
T
V
W
Y

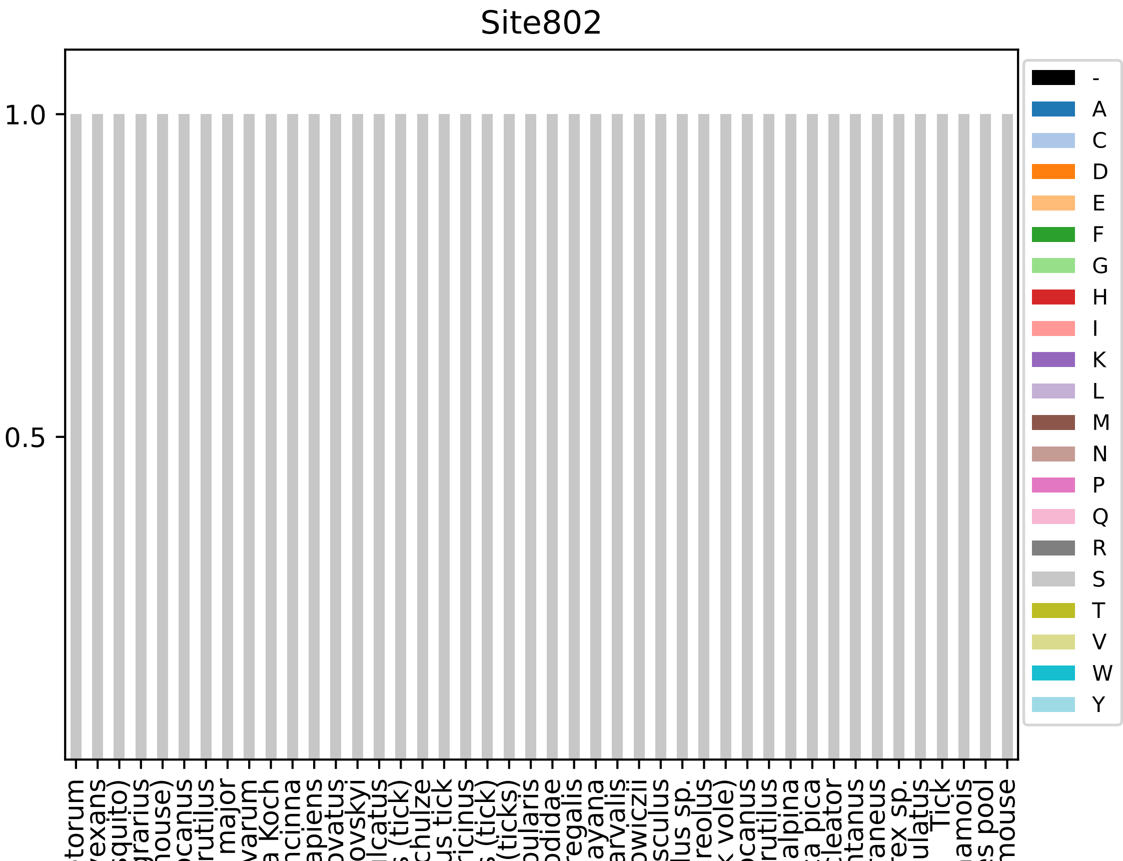
Site802
1.0
0.5
-
A
C
D
E
F
G
H
I
K
L
M
N
P
Q
R
S
T
V
W
Y

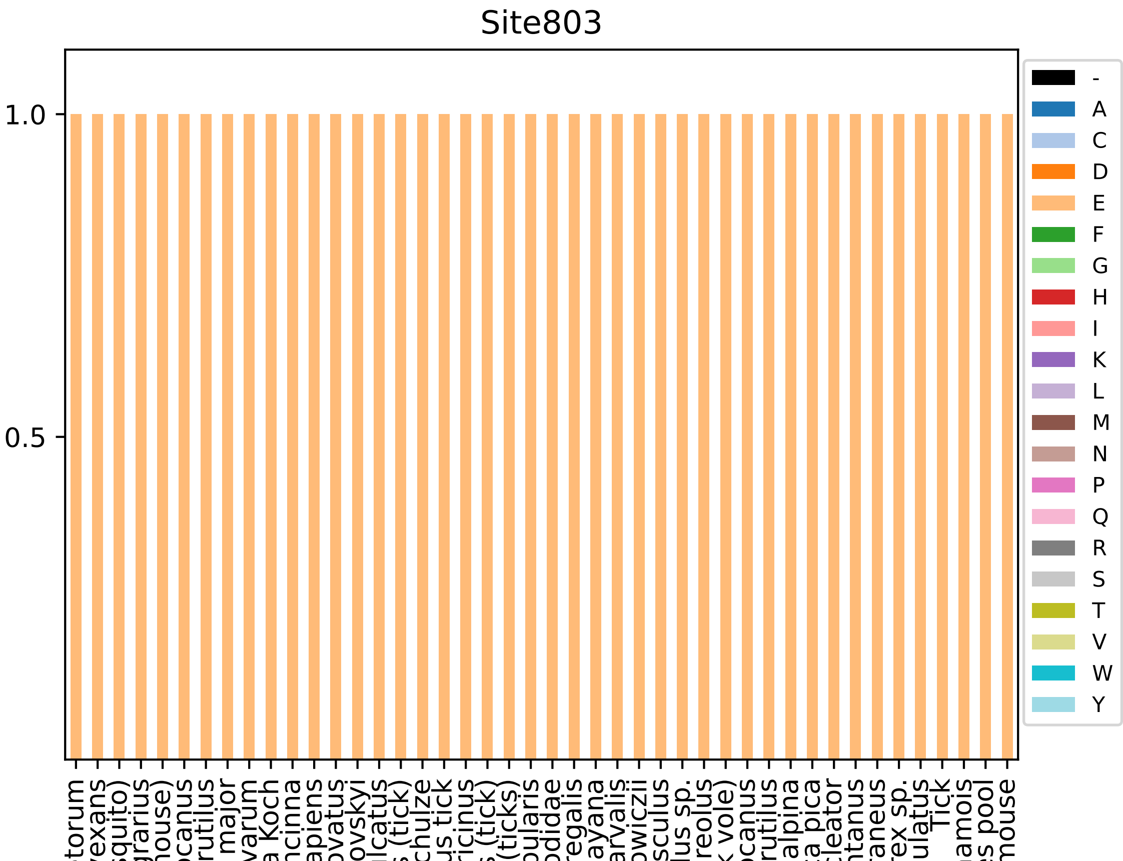
Site803
1.0
0.5
-
A
C
D
E
F
G
H
I
K
L
M
N
P
Q
R
S
T
V
W
Y

Site804

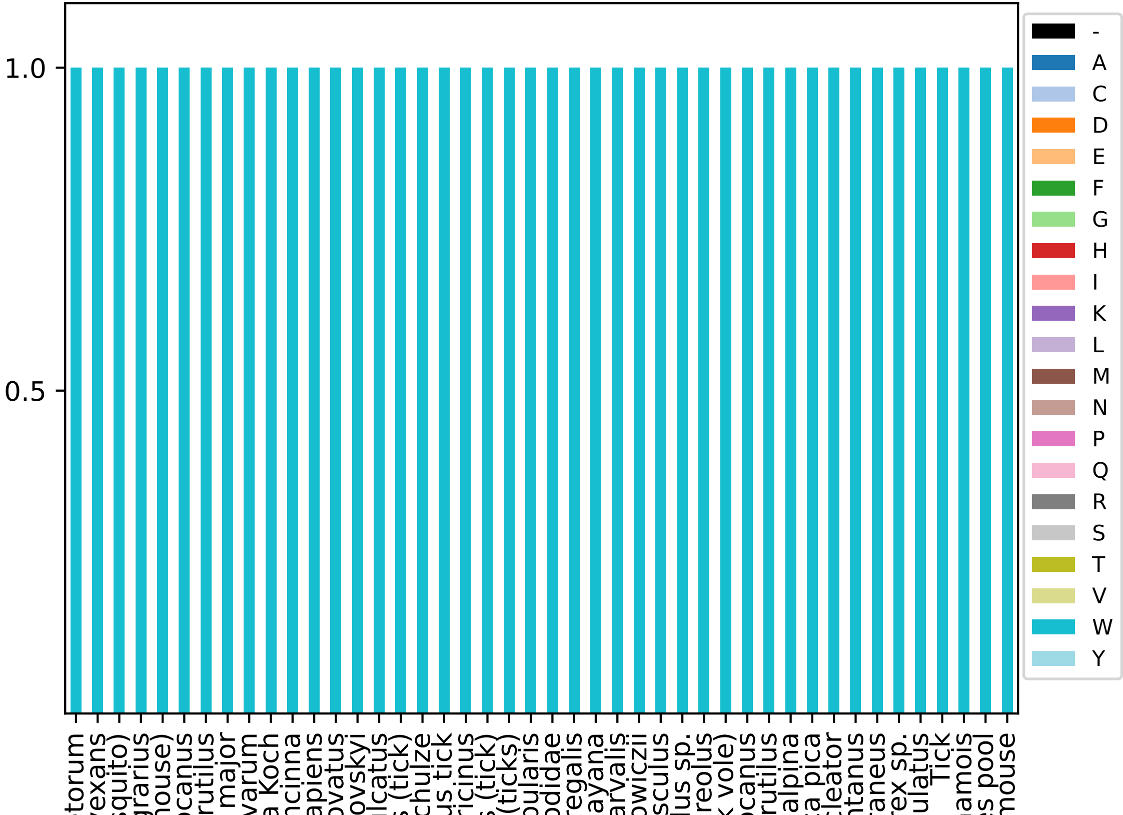

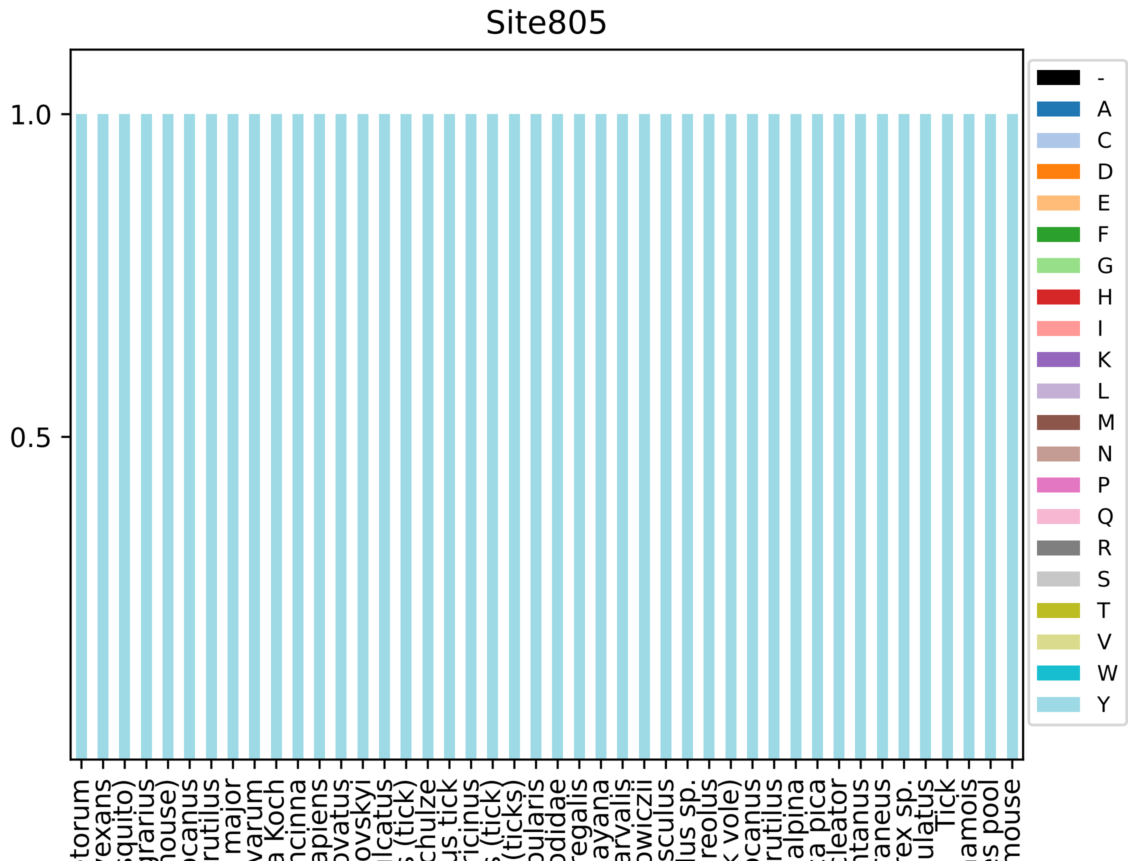
Site805
1.0
0.5
-
A
C
D
E
F
G
H
I
K
L
M
N
P
Q
R
S
T
V
W
Y

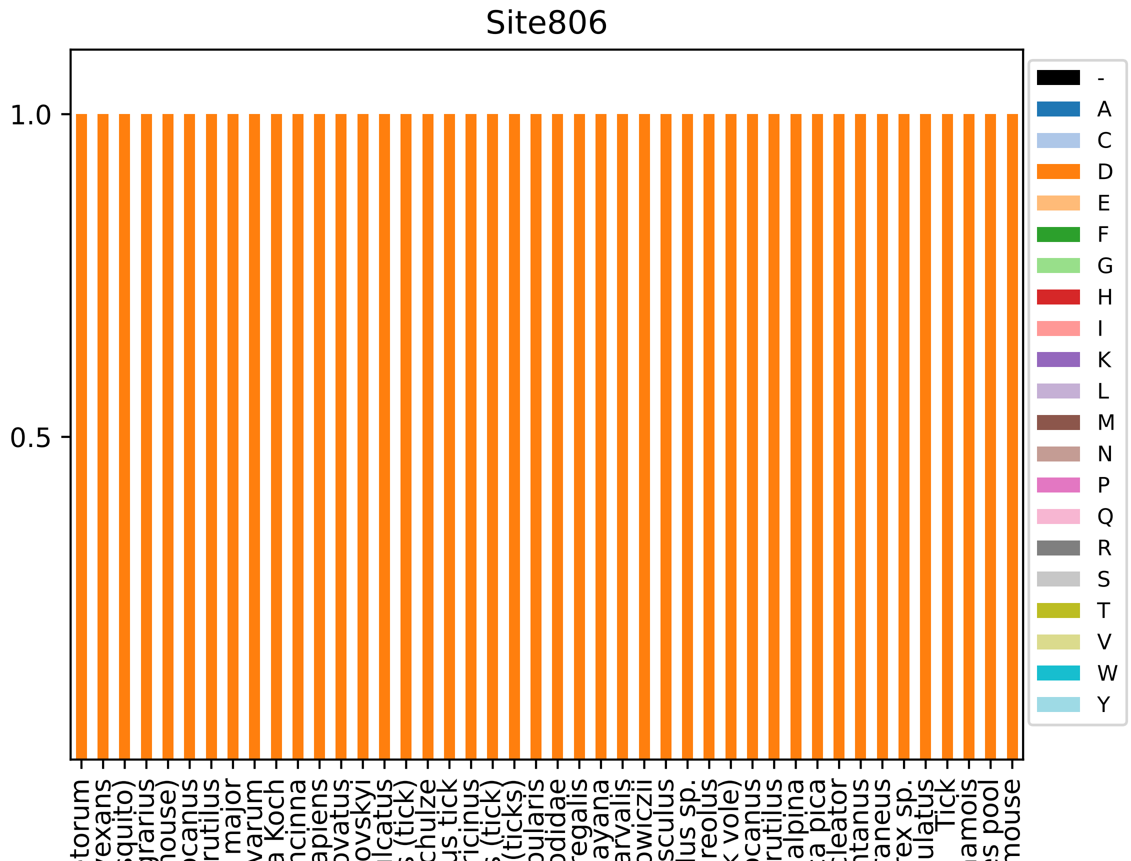
Site806
1.0
0.5
-
A
C
D
E
F
G
H
I
K
L
M
N
P
Q
R
S
T
V
W
Y

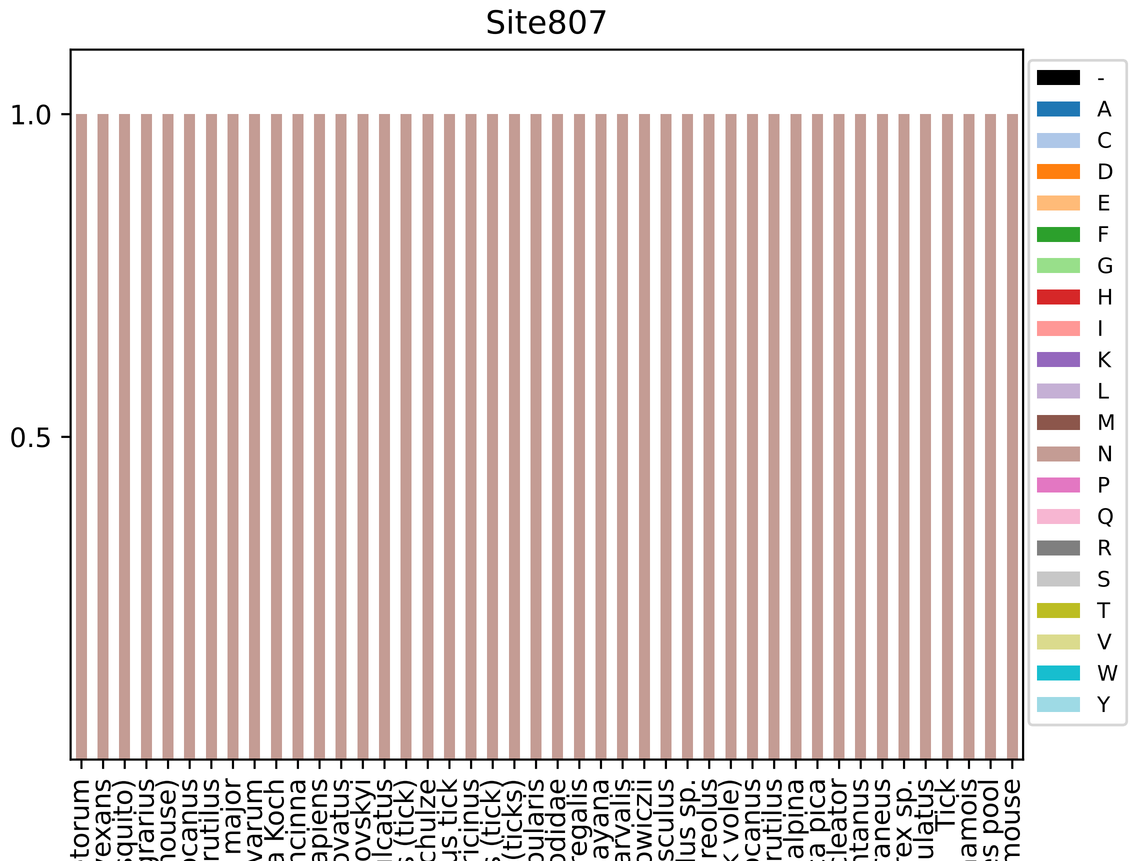

Site807
1.0
0.5
-
A
C
D
E
F
G
H
I
K
L
M
N
P
Q
R
S
T
V
W
Y
torum
vexans
squito)
garius
nouse)
ocanus
rutilus
major
varum
a Koch
ncinna
apiens
ovatus
ovskyi
ulcatus
s (tick)
chulze
us tick
ricinus
s (tick)
(ticks)
pularis
podidae
regalis
ayana
arvalis
owiczii
sculus
lus sp.
reolus
x vole)
ocanus
rutilus
alpina
a pica
cleator
ntanus
raneus
rex sp.
ulatus
Tick
amois
s pool
mouse

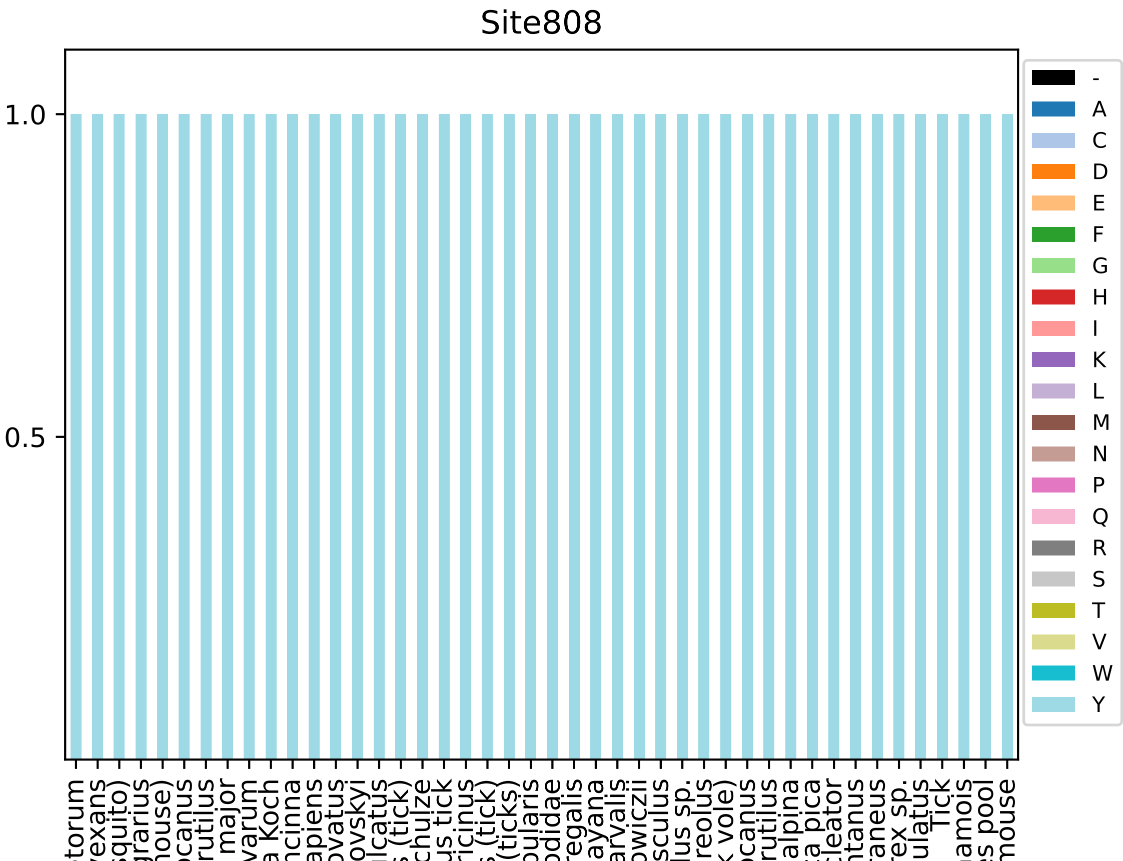
Site808
1.0
0.5
-
A
C
D
E
F
G
H
I
K
L
M
N
P
Q
R
S
T
V
W
Y

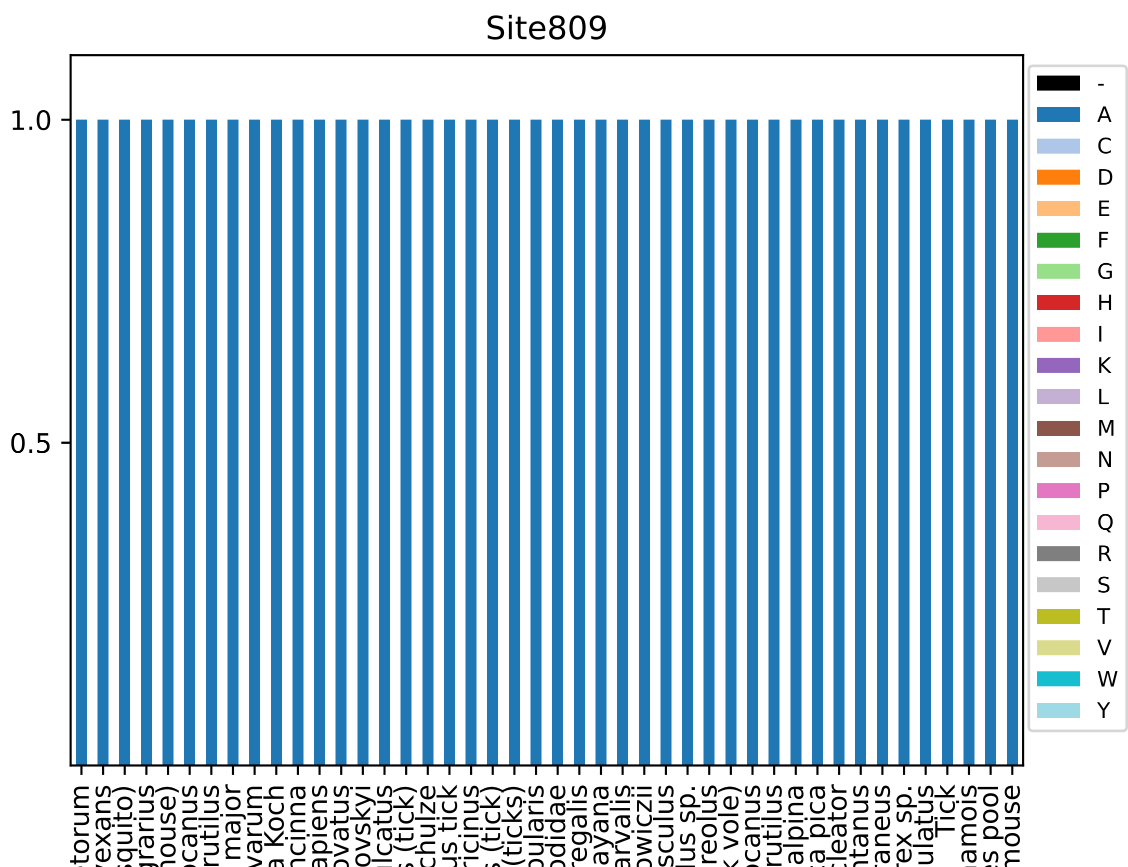

Site809
1.0
0.5
-
A
C
D
E
F
G
H
I
K
L
M
N
P
Q
R
S
T
V
W
Y

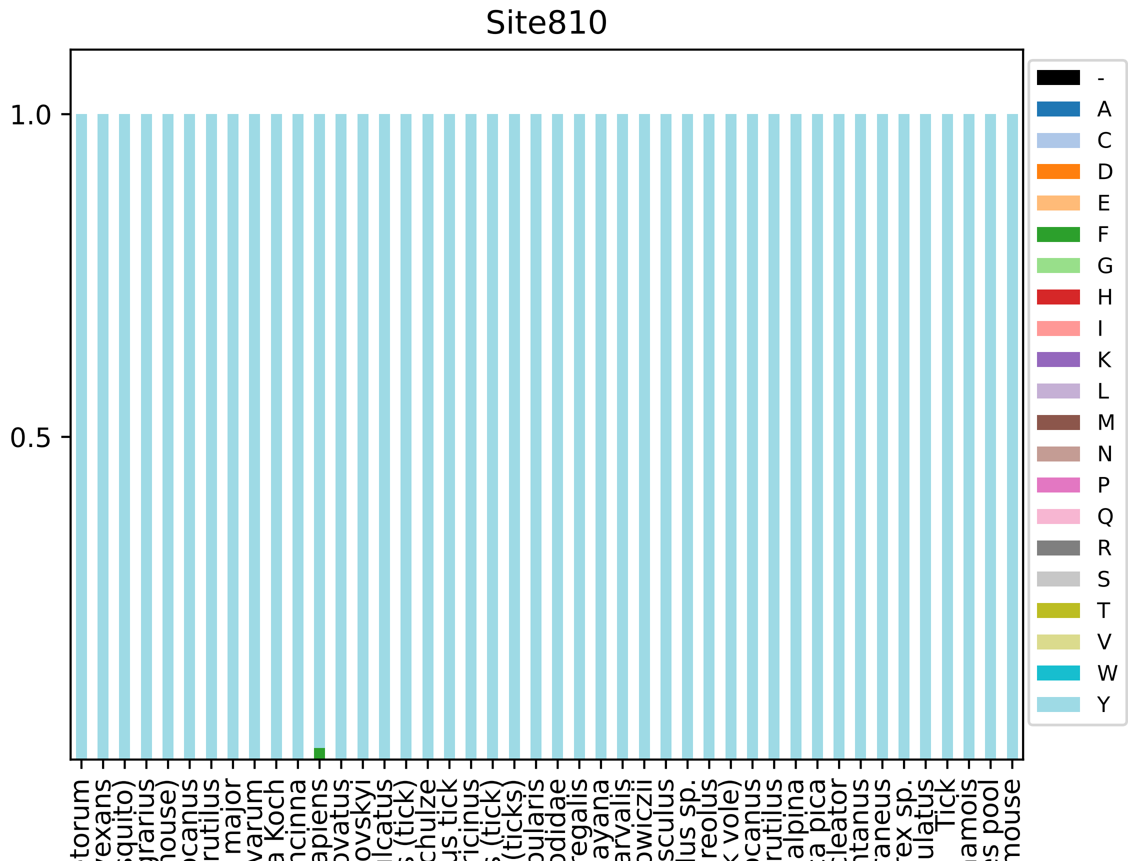
Site810
1.0
0.5
-
A
C
D
E
F
G
H
I
K
L
M
N
P
Q
R
S
T
V
W
Y

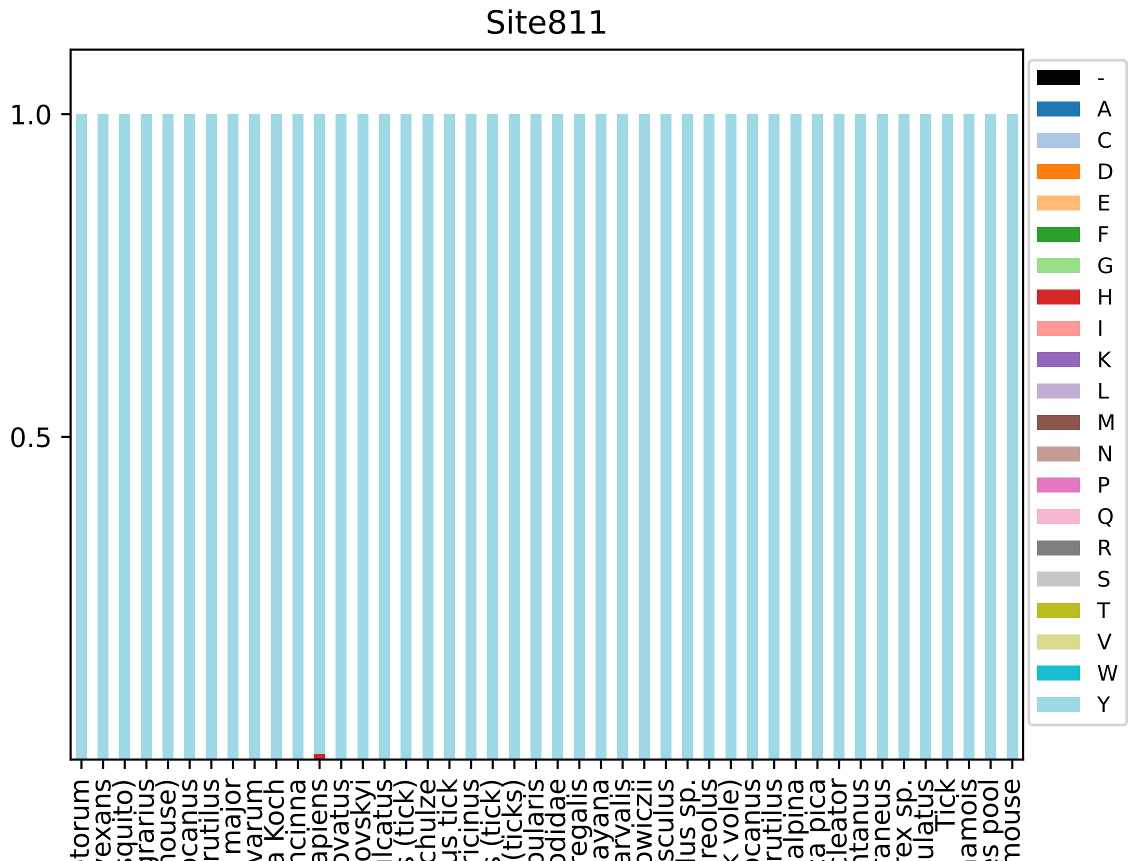
Site811
1.0
0.5
-
A
C
D
E
F
G
H
I
K
L
M
N
P
Q
R
S
T
V
W
Y

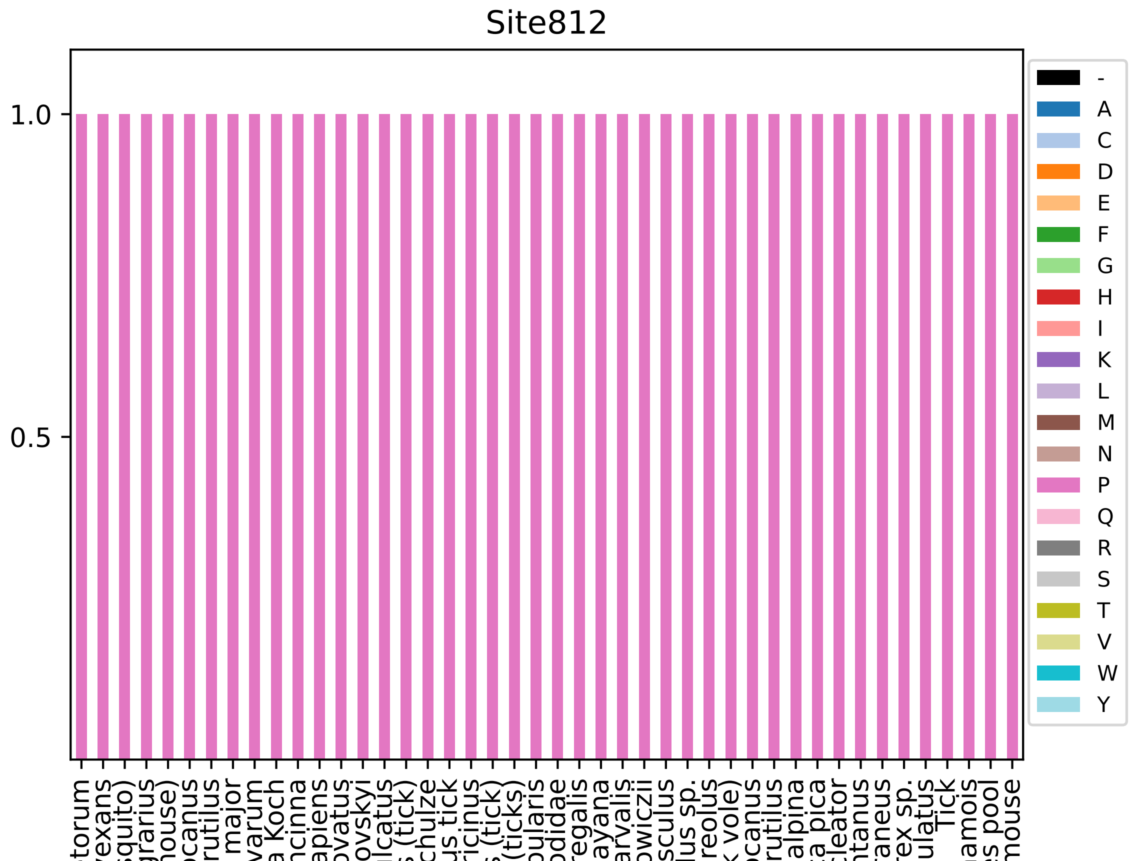
Site812
1.0
0.5
-
A
C
D
E
F
G
H
I
K
L
M
N
P
Q
R
S
T
V
W
Y

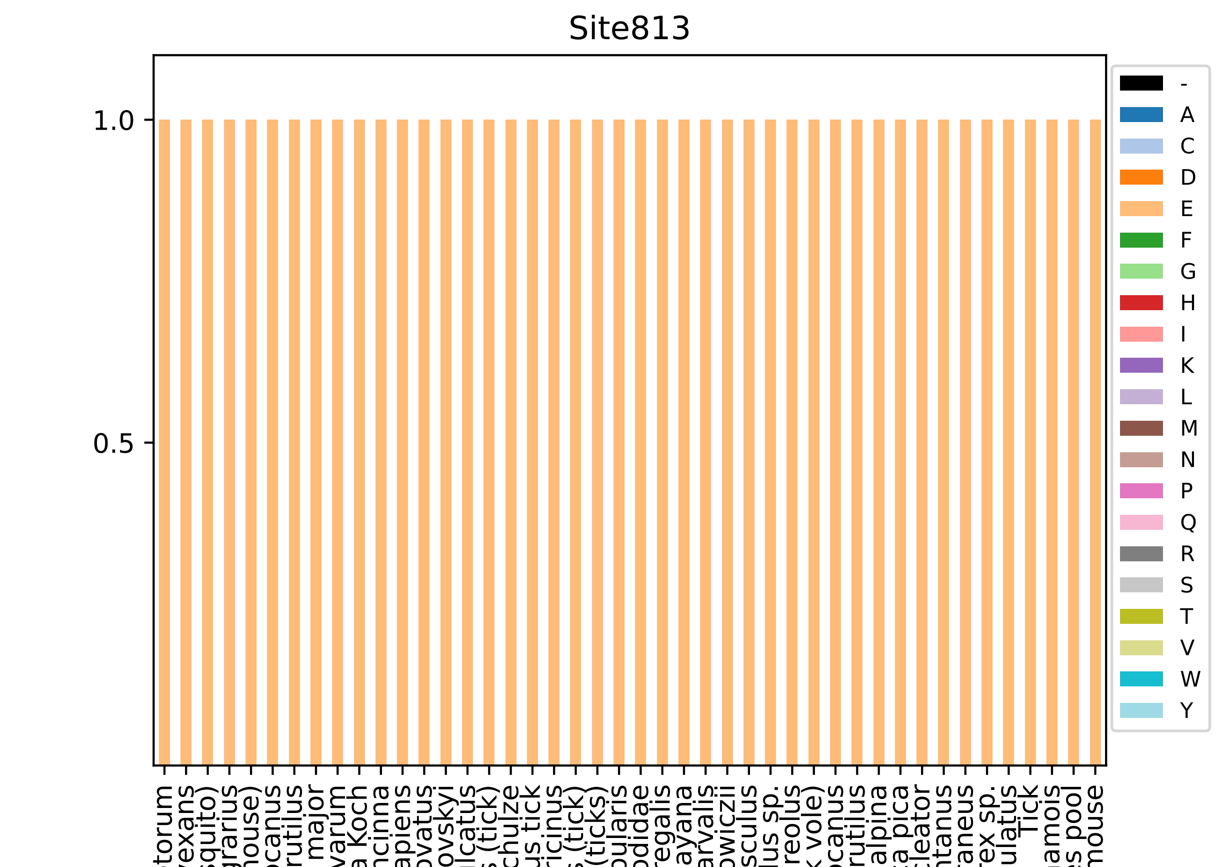
Site813
1.0
0.5
-
A
C
D
E
F
G
H
I
K
L
M
N
P
Q
R
S
T
V
W
Y

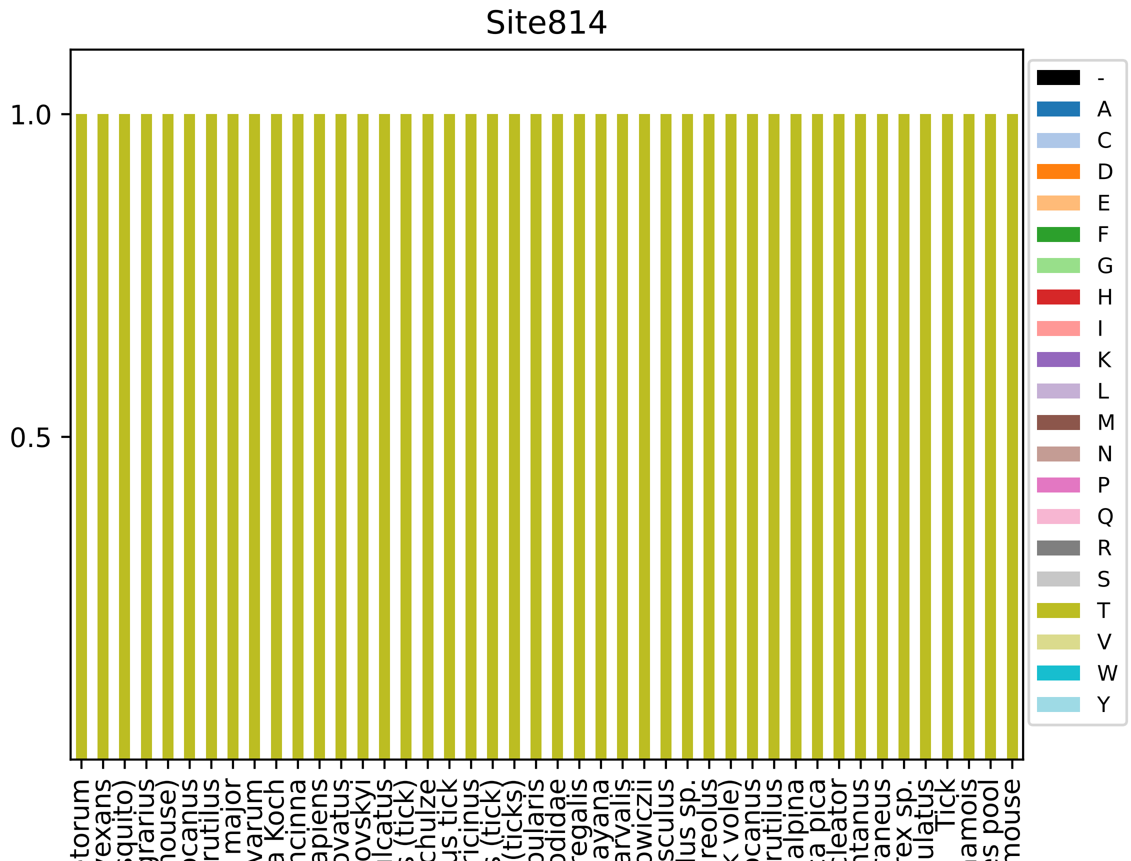
Site814
1.0
0.5
-
A
C
D
E
F
G
H
I
K
L
M
N
P
Q
R
S
T
V
W
Y

Site815

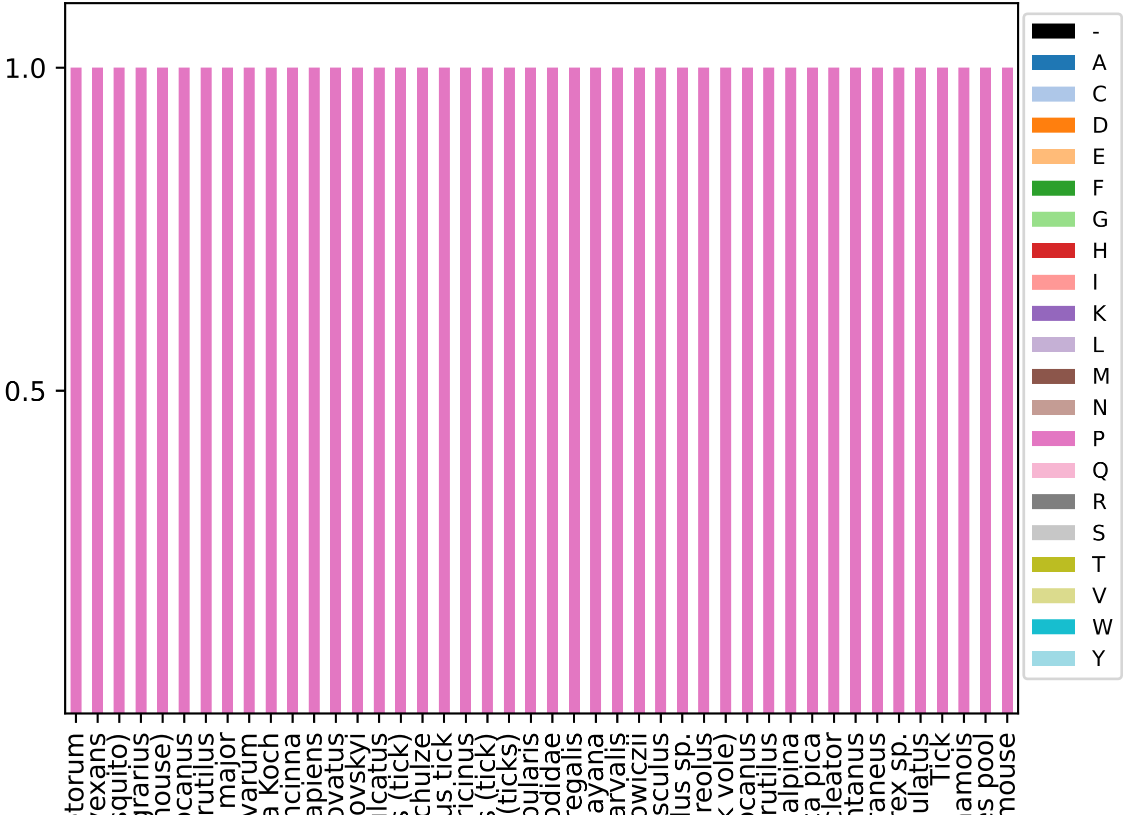

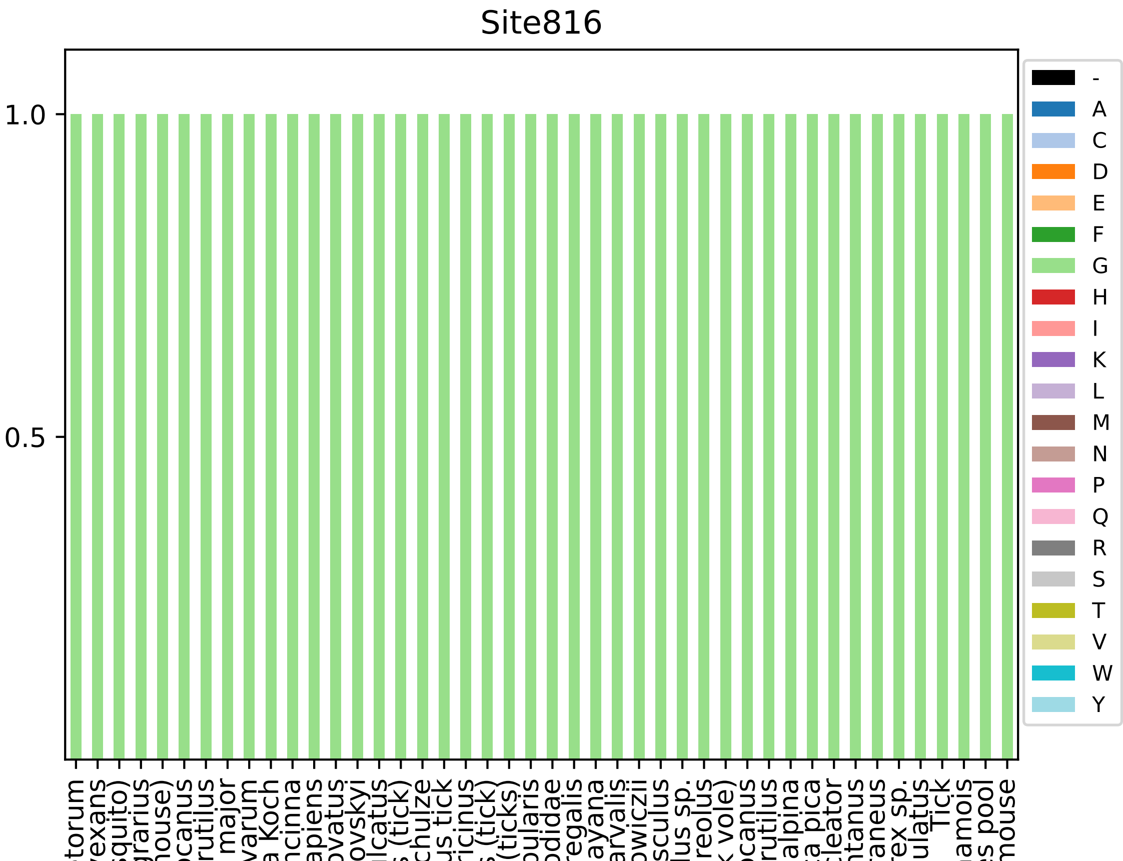
Site816
1.0
0.5
-
A
C
D
E
F
G
H
I
K
L
M
N
P
Q
R
S
T
V
W
Y

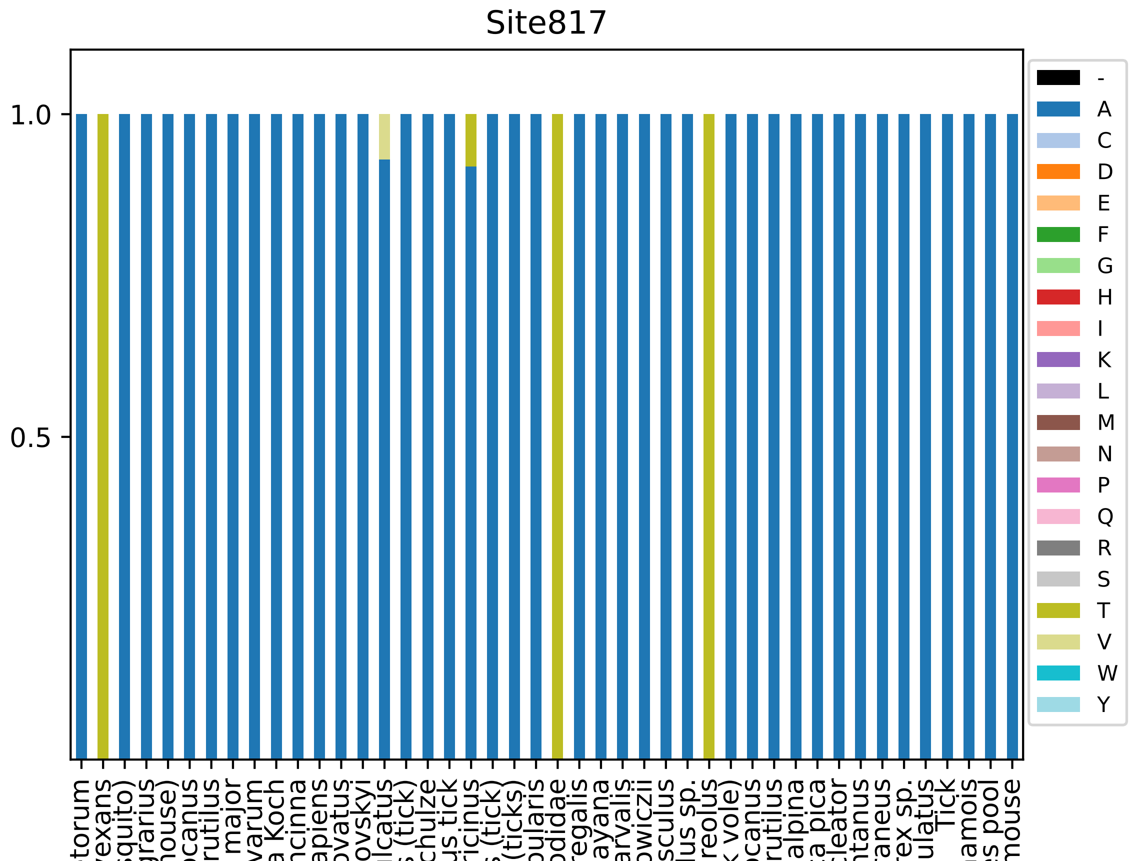
Site817
1.0
0.5
-
A
C
D
E
F
G
H
I
K
L
M
N
P
Q
R
S
T
V
W
Y

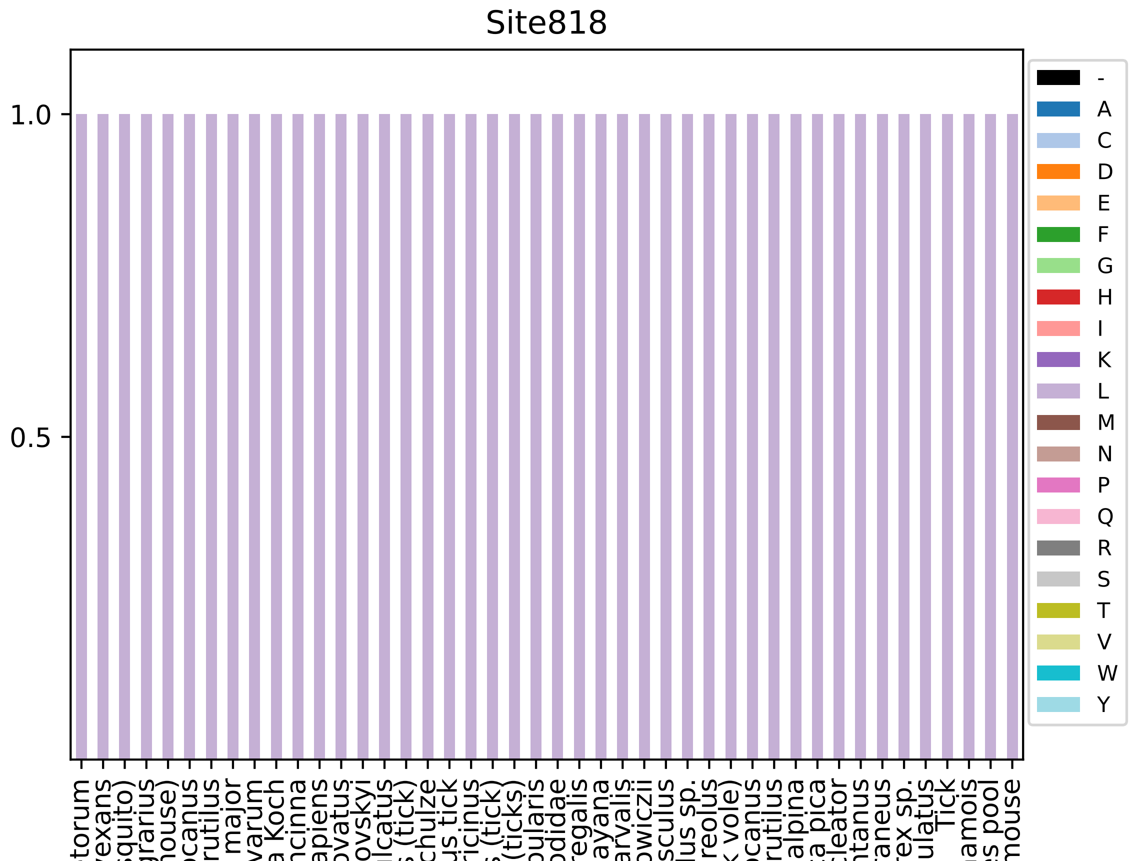
Site818
1.0
0.5
-
A
C
D
E
F
G
H
I
K
L
M
N
P
Q
R
S
T
V
W
Y

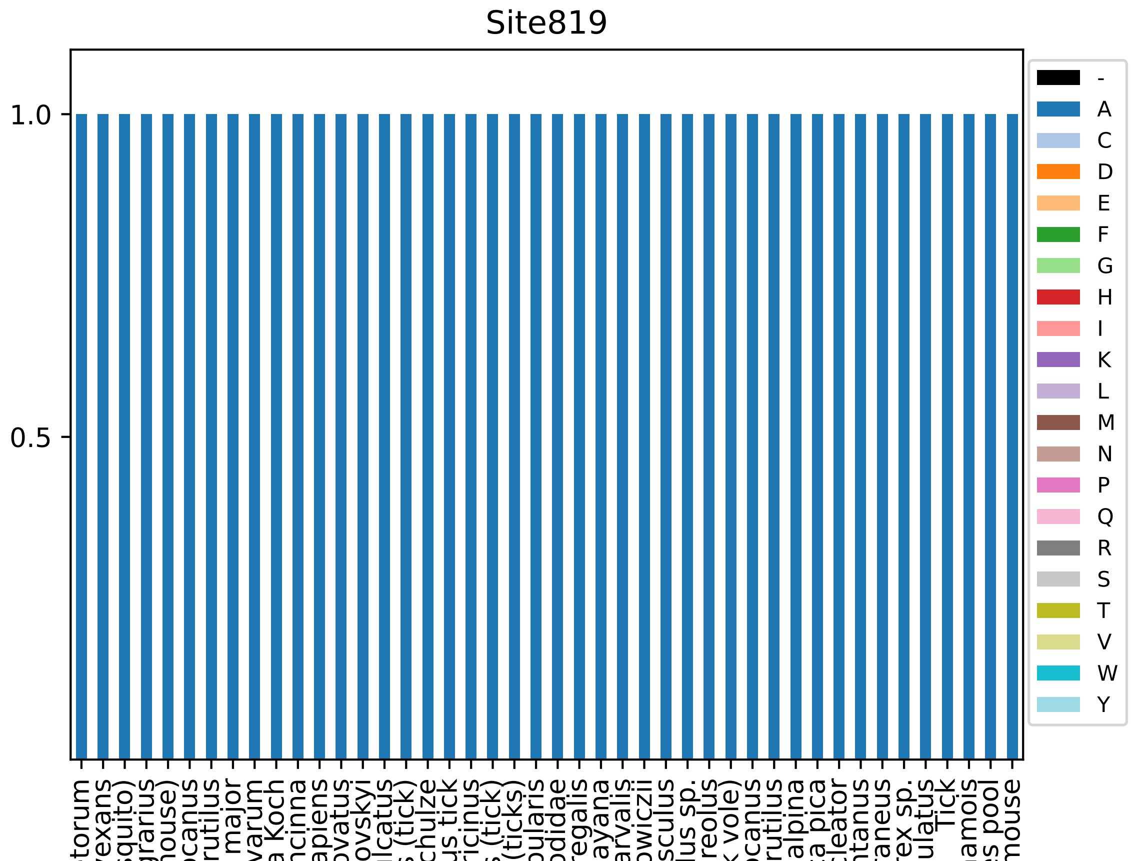

Site819
1.0
0.5
-
A
C
D
E
F
G
H
I
K
L
M
N
P
Q
R
S
T
V
W
Y

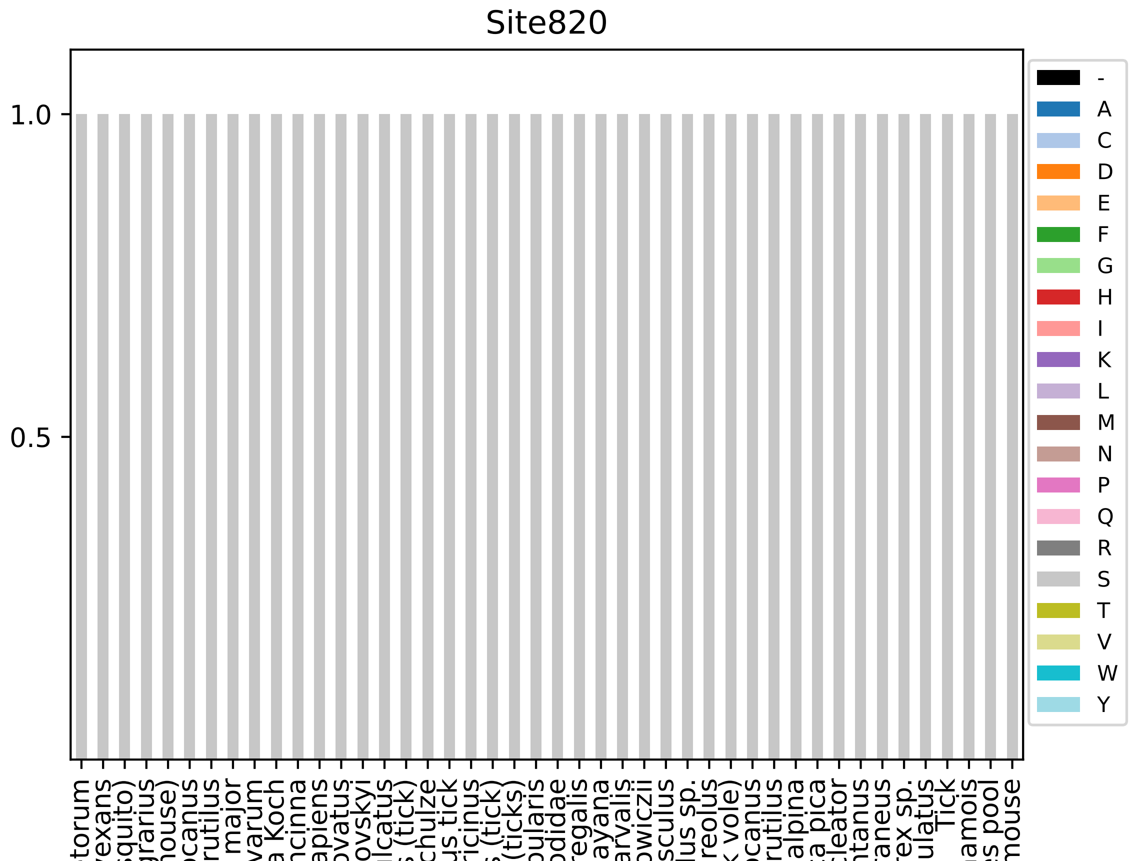
Site820
1.0
0.5
-
A
C
D
E
F
G
H
I
K
L
M
N
P
Q
R
S
T
V
W
Y

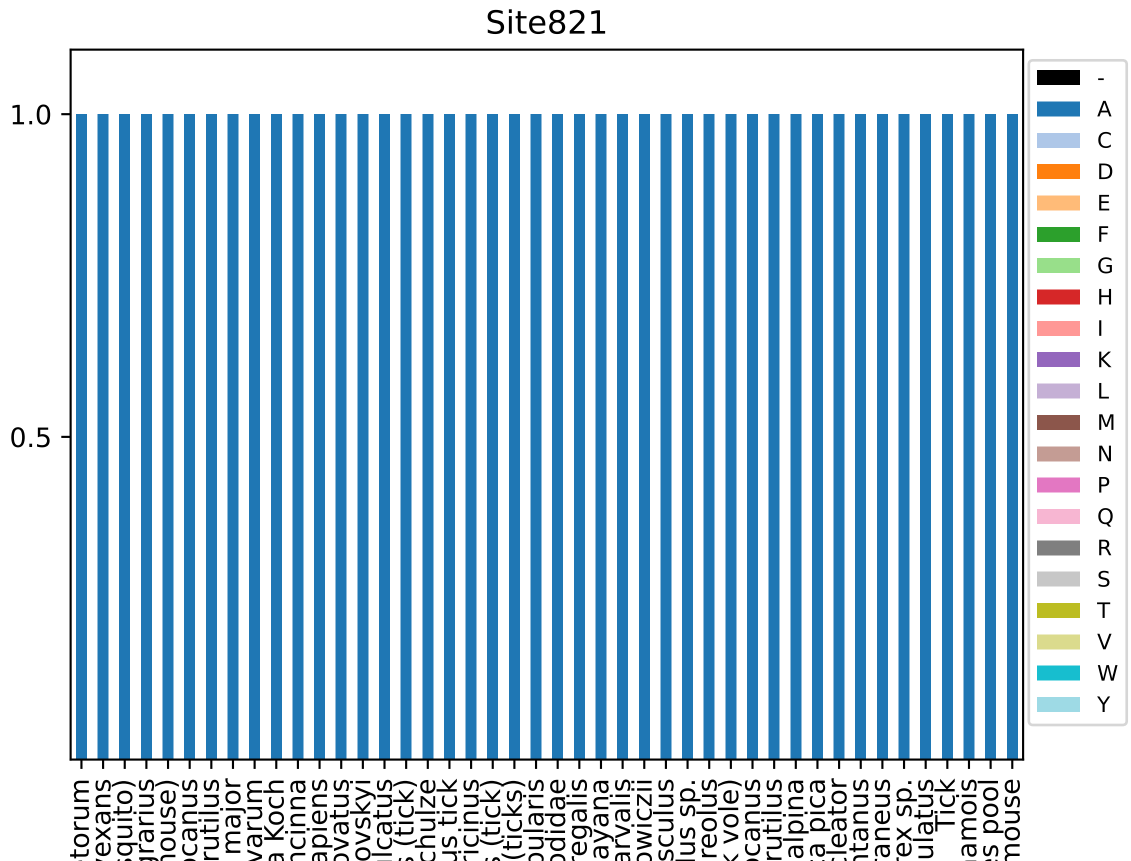
Site821
1.0
0.5
-
A
C
D
E
F
G
H
I
K
L
M
N
P
Q
R
S
T
V
W
Y

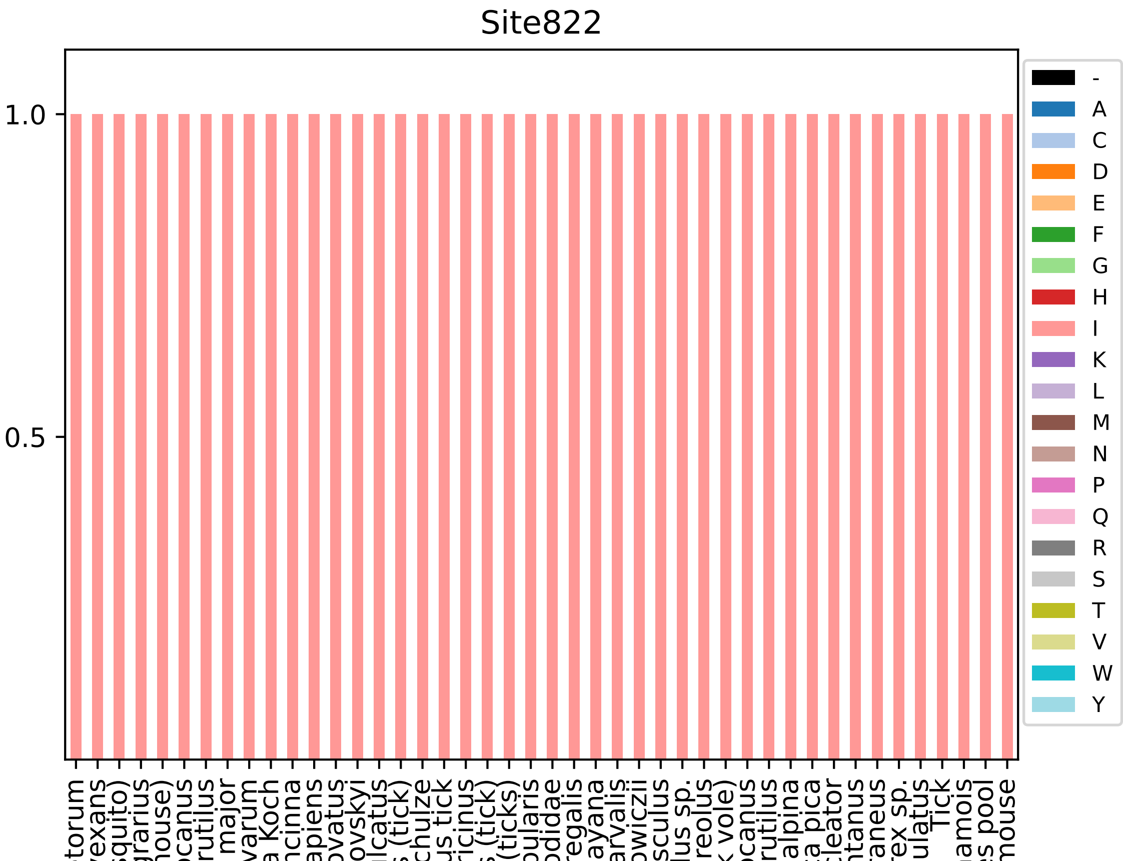
Site822
1.0
0.5
-
A
C
D
E
F
G
H
I
K
L
M
N
P
Q
R
S
T
V
W
Y

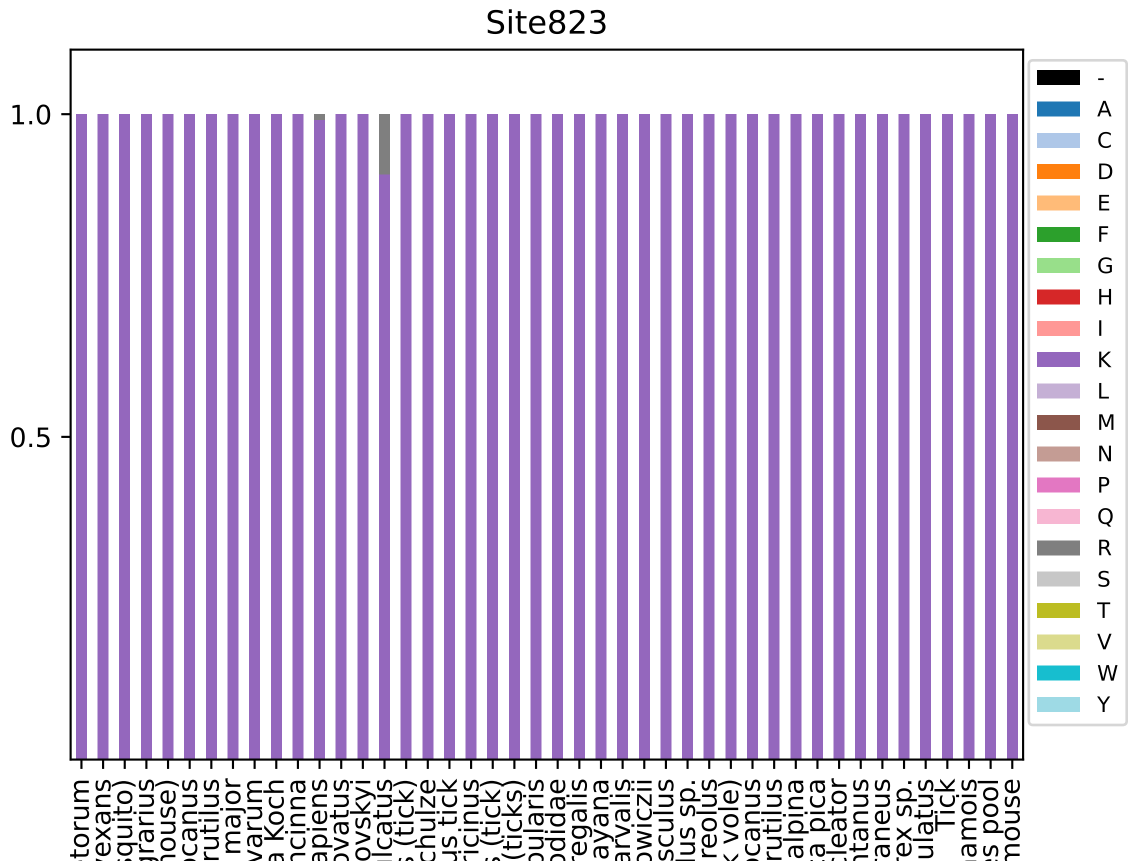
Site823
1.0
0.5
-
A
C
D
E
F
G
H
I
K
L
M
N
P
Q
R
S
T
V
W
Y

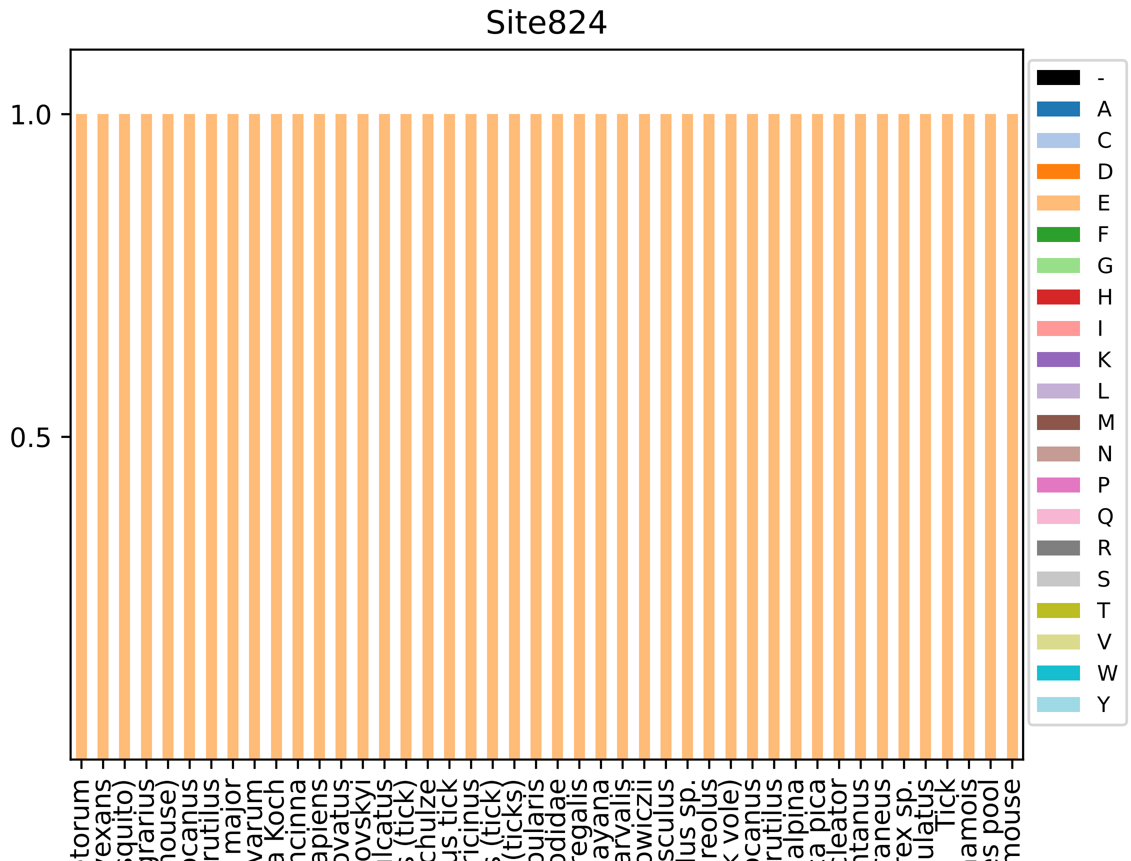
Site824
1.0
0.5
-
A
C
D
E
F
G
H
I
K
L
M
N
P
Q
R
S
T
V
W
Y

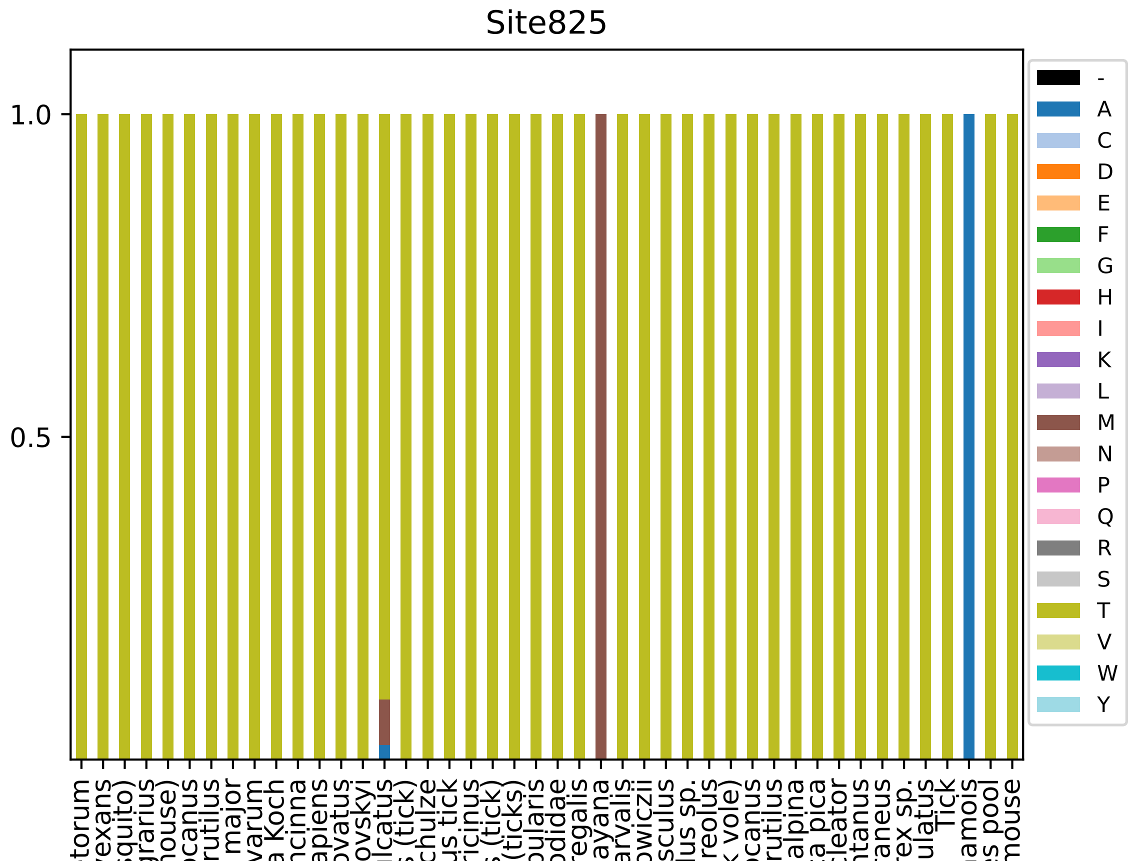
Site825
1.0
0.5
-
A
C
D
E
F
G
H
I
K
L
M
N
P
Q
R
S
T
V
W
Y

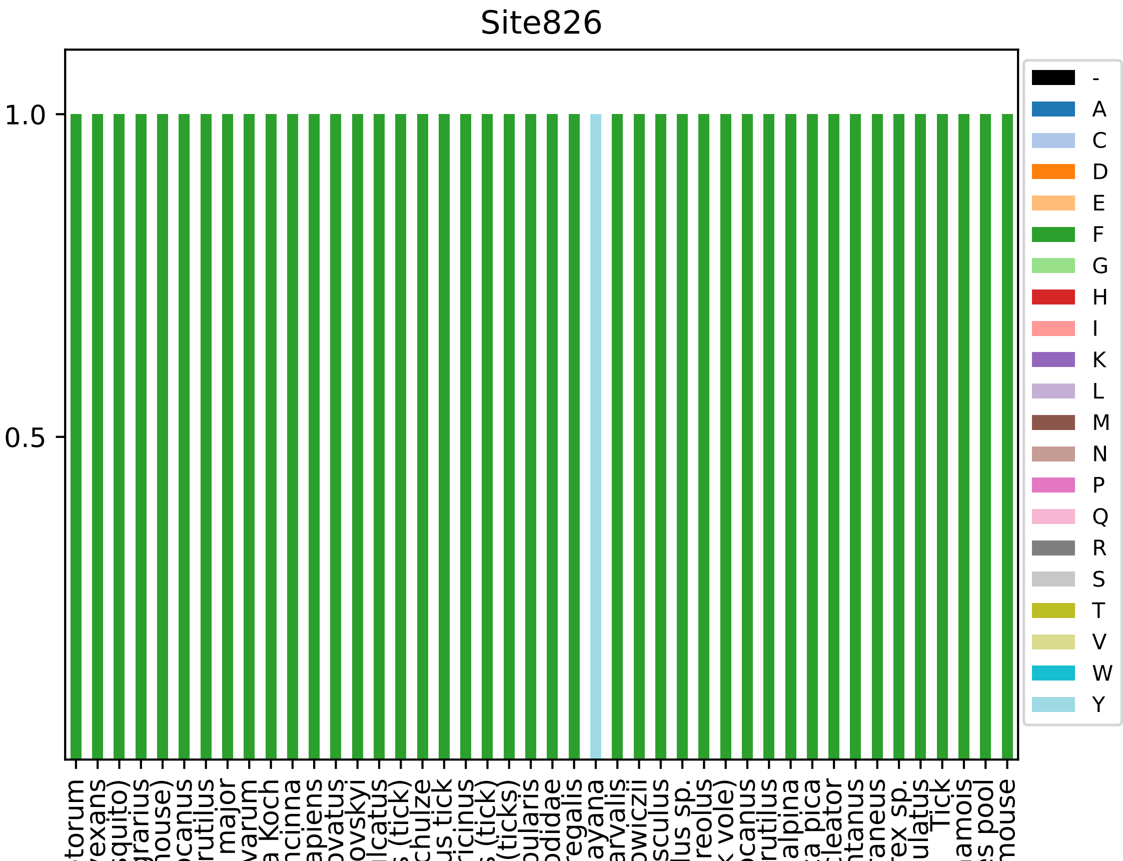
Site826
1.0
0.5
-
A
C
D
E
F
G
H
I
K
L
M
N
P
Q
R
S
T
V
W
Y

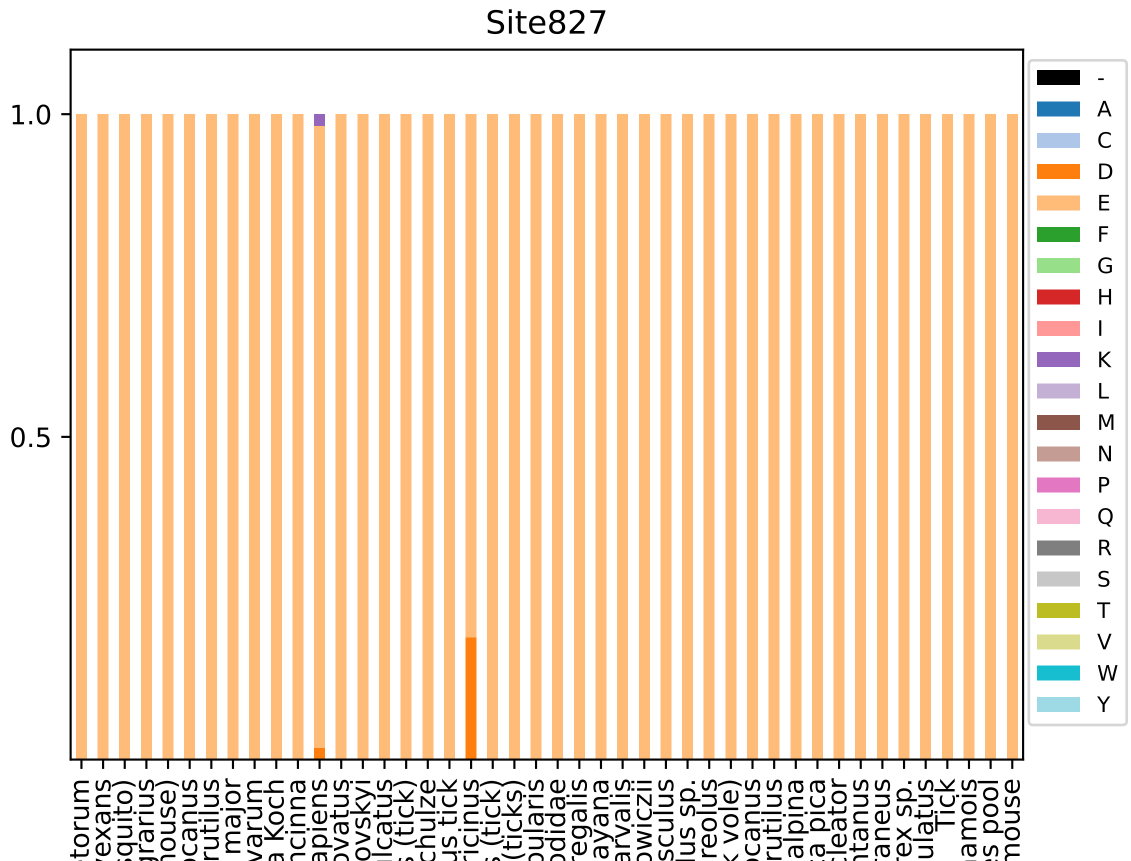
Site827
1.0
0.5
-
A
C
D
E
F
G
H
I
K
L
M
N
P
Q
R
S
T
V
W
Y

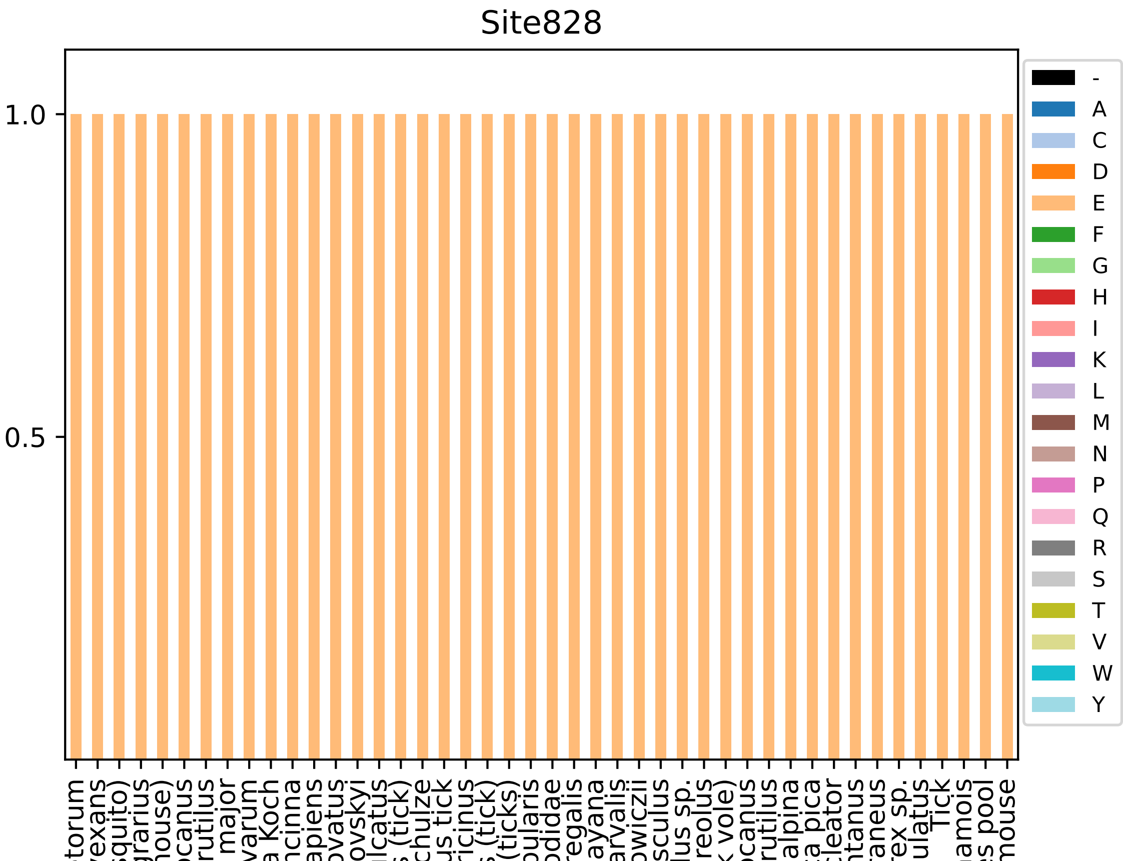

Site828
1.0
0.5
-
A
C
D
E
F
G
H
I
K
L
M
N
P
Q
R
S
T
V
W
Y

Site829

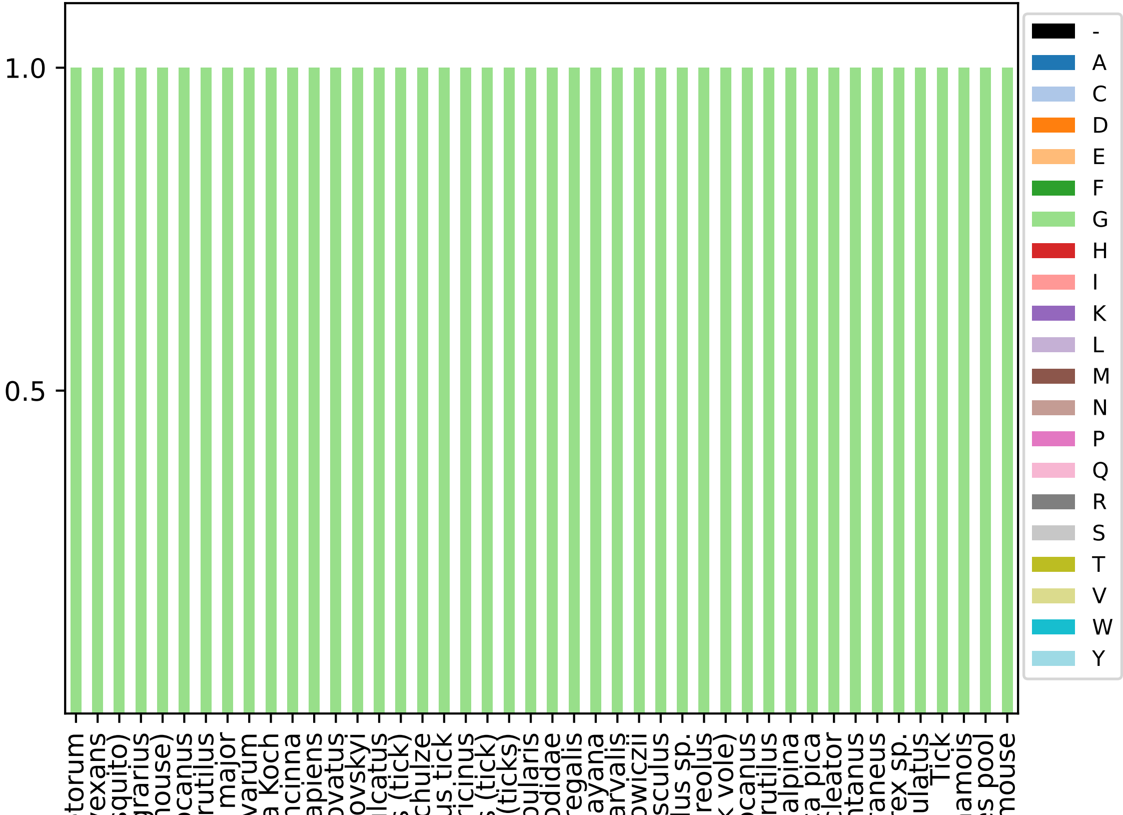

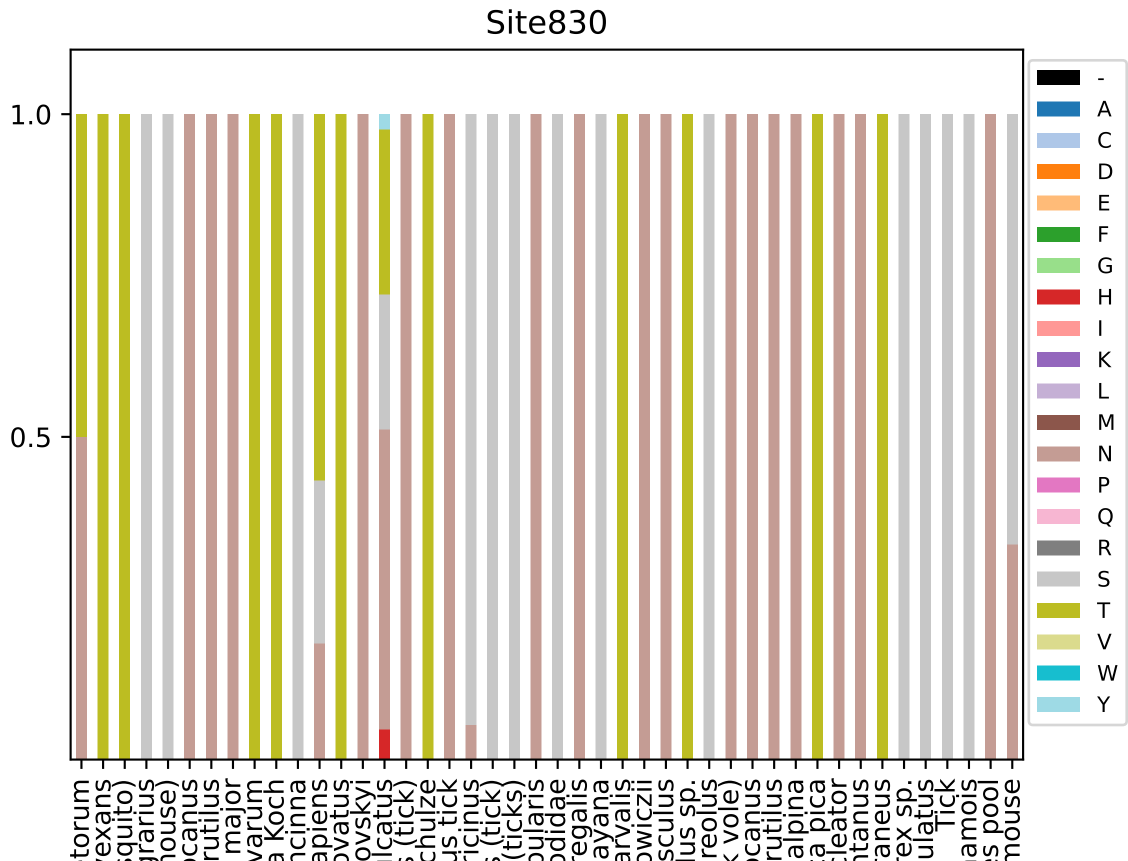

Site830
1.0
0.5
-
A
C
D
E
F
G
H
I
K
L
M
N
P
Q
R
S
T
V
W
Y

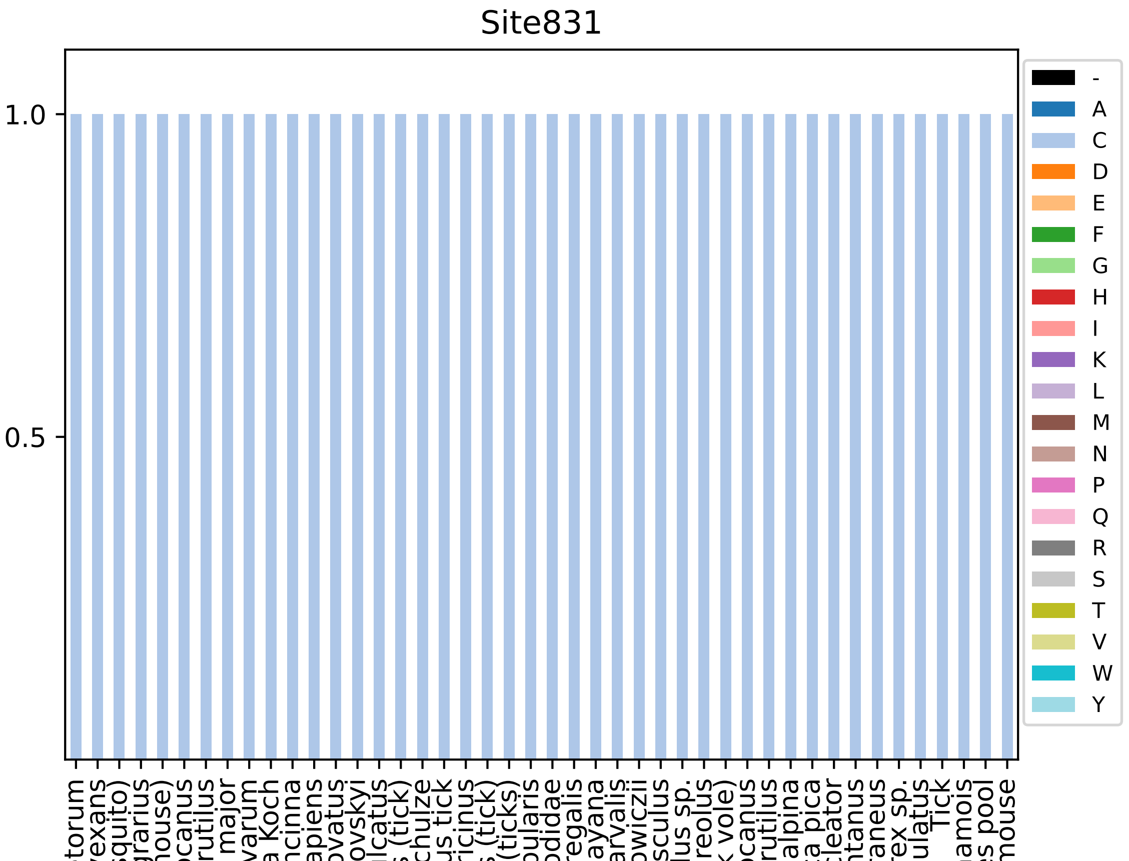
Site831
1.0
0.5
-
A
C
D
E
F
G
H
I
K
L
M
N
P
Q
R
S
T
V
W
Y

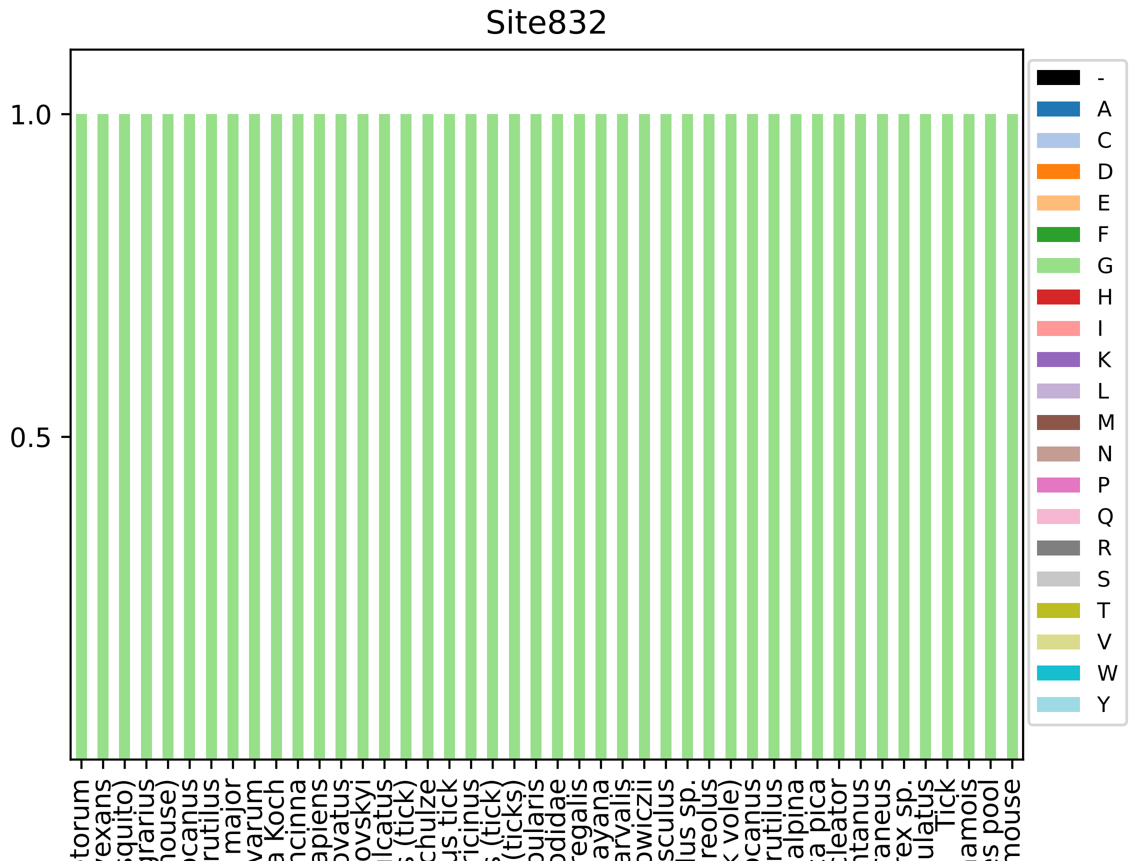
Site832
1.0
0.5
-
A
C
D
E
F
G
H
I
K
L
M
N
P
Q
R
S
T
V
W
Y

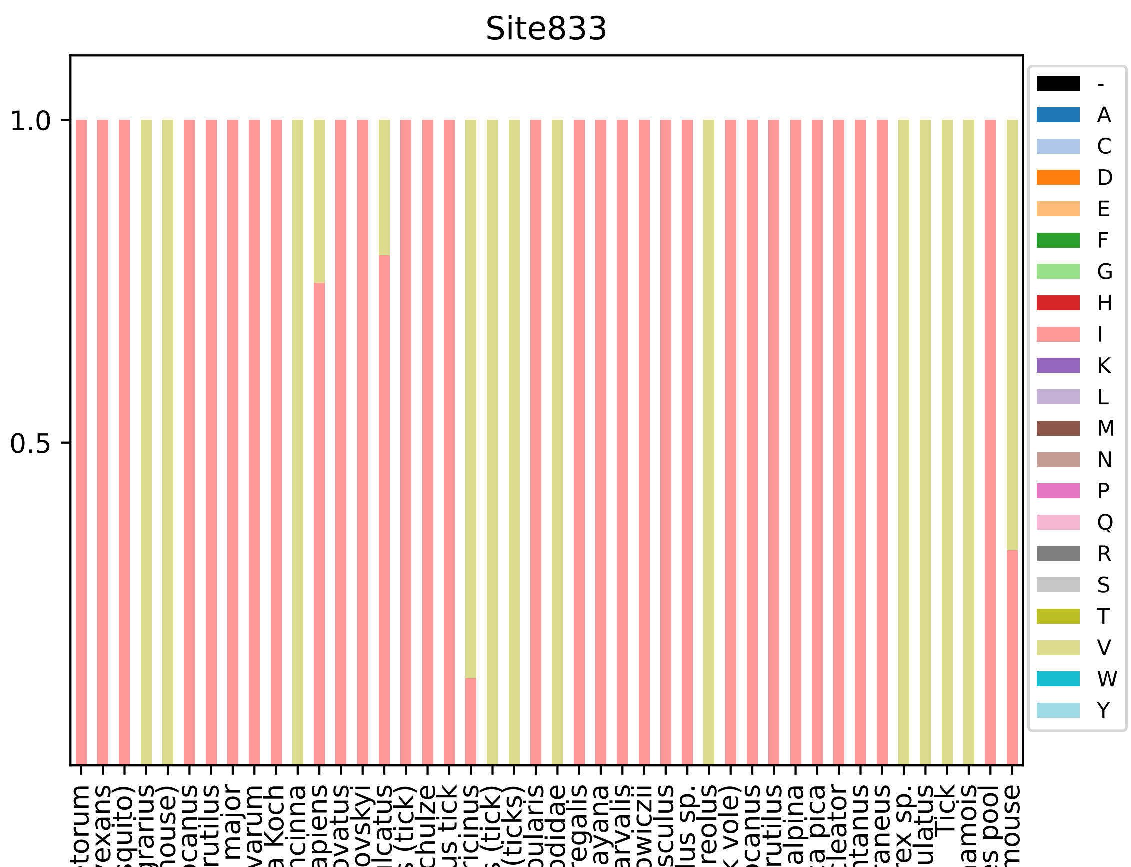
Site833
1.0
0.5
-
A
C
D
E
F
G
H
I
K
L
M
N
P
Q
R
S
T
V
W
Y

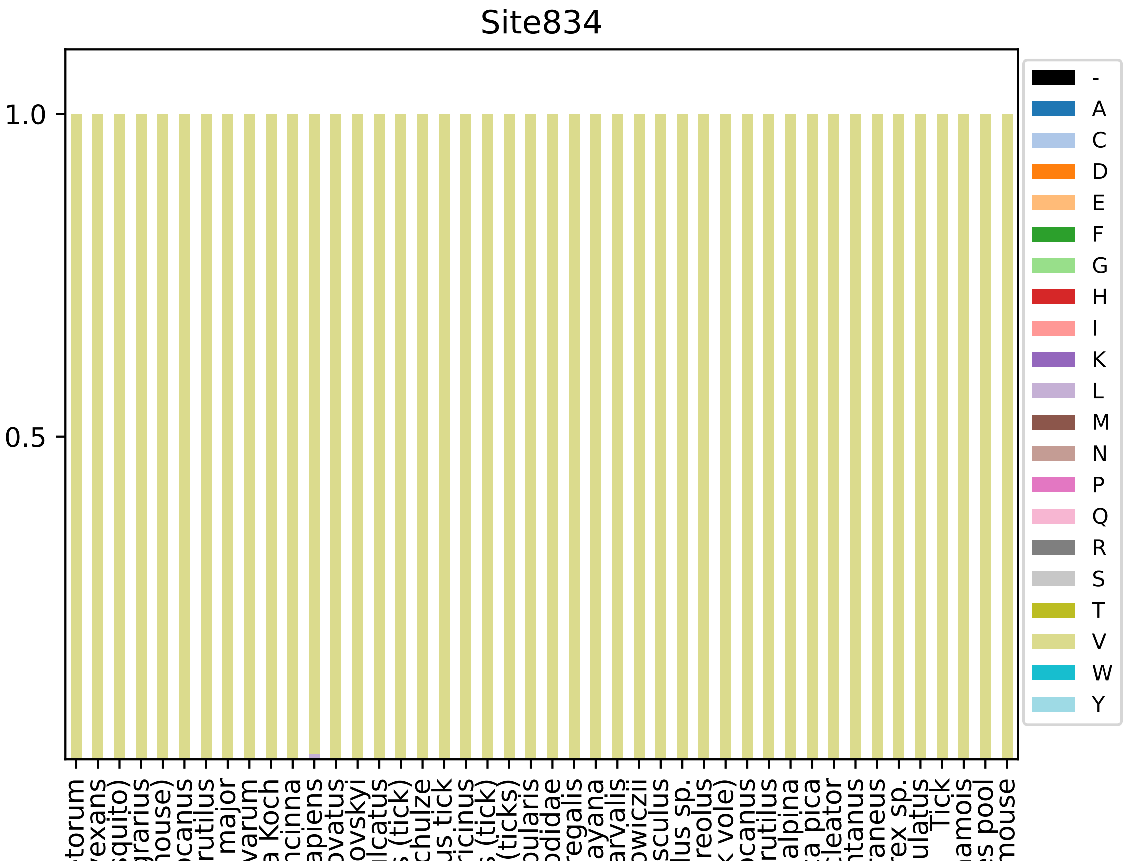

Site834
1.0
0.5
-
A
C
D
E
F
G
H
I
K
L
M
N
P
Q
R
S
T
V
W
Y

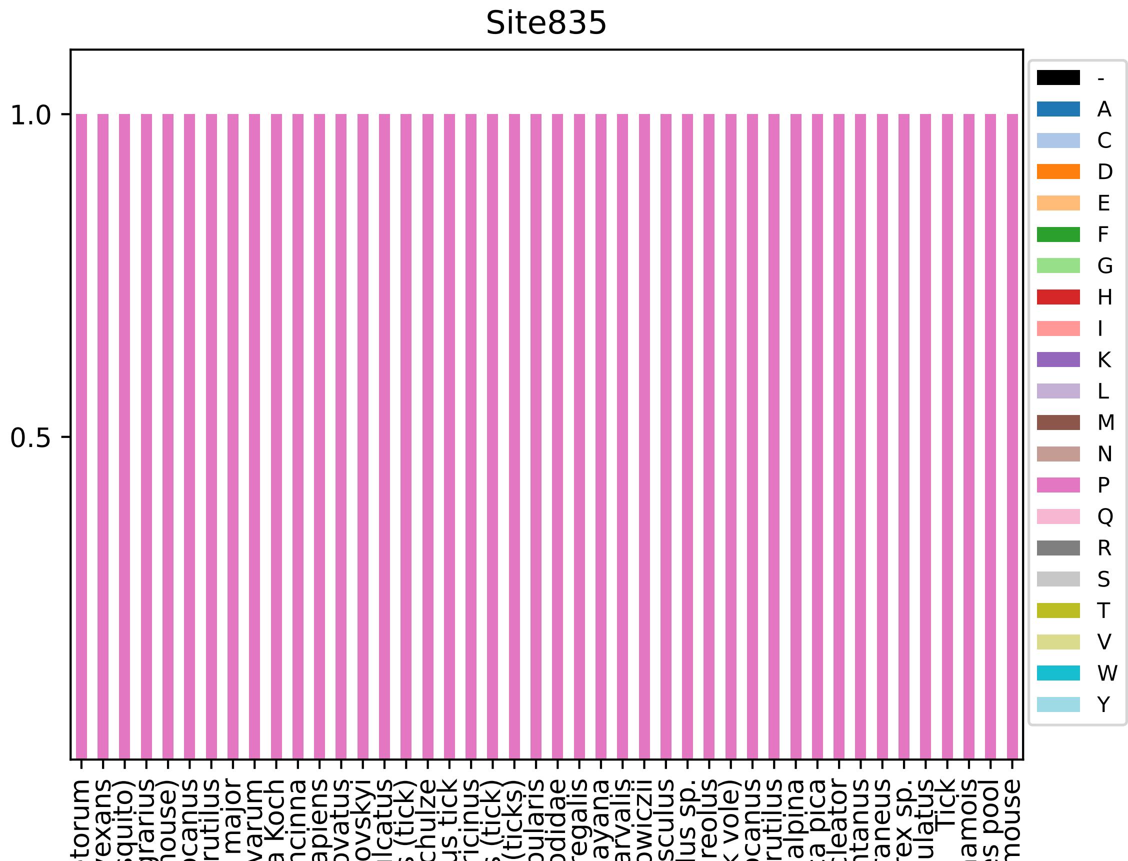

Site835
1.0
0.5
-
A
C
D
E
F
G
H
I
K
L
M
N
P
Q
R
S
T
V
W
Y

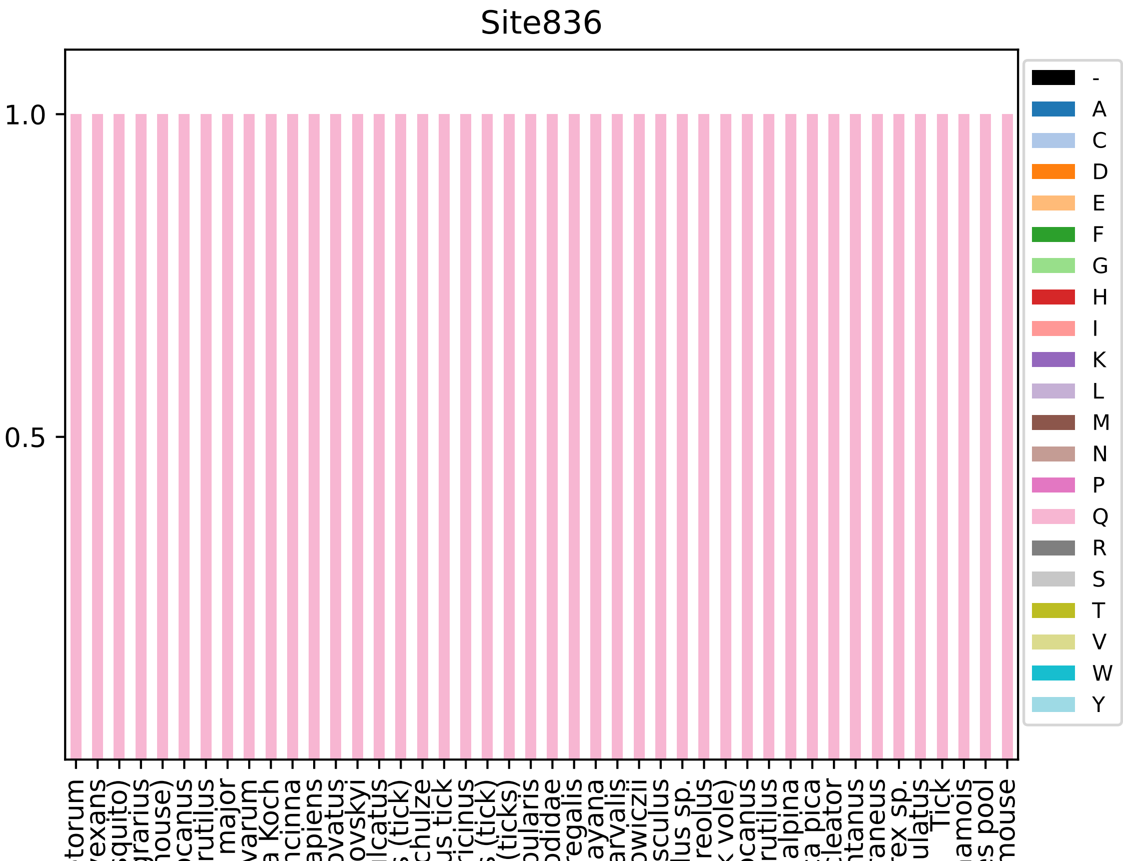
Site836
1.0
0.5
-
A
C
D
E
F
G
H
I
K
L
M
N
P
Q
R
S
T
V
W
Y

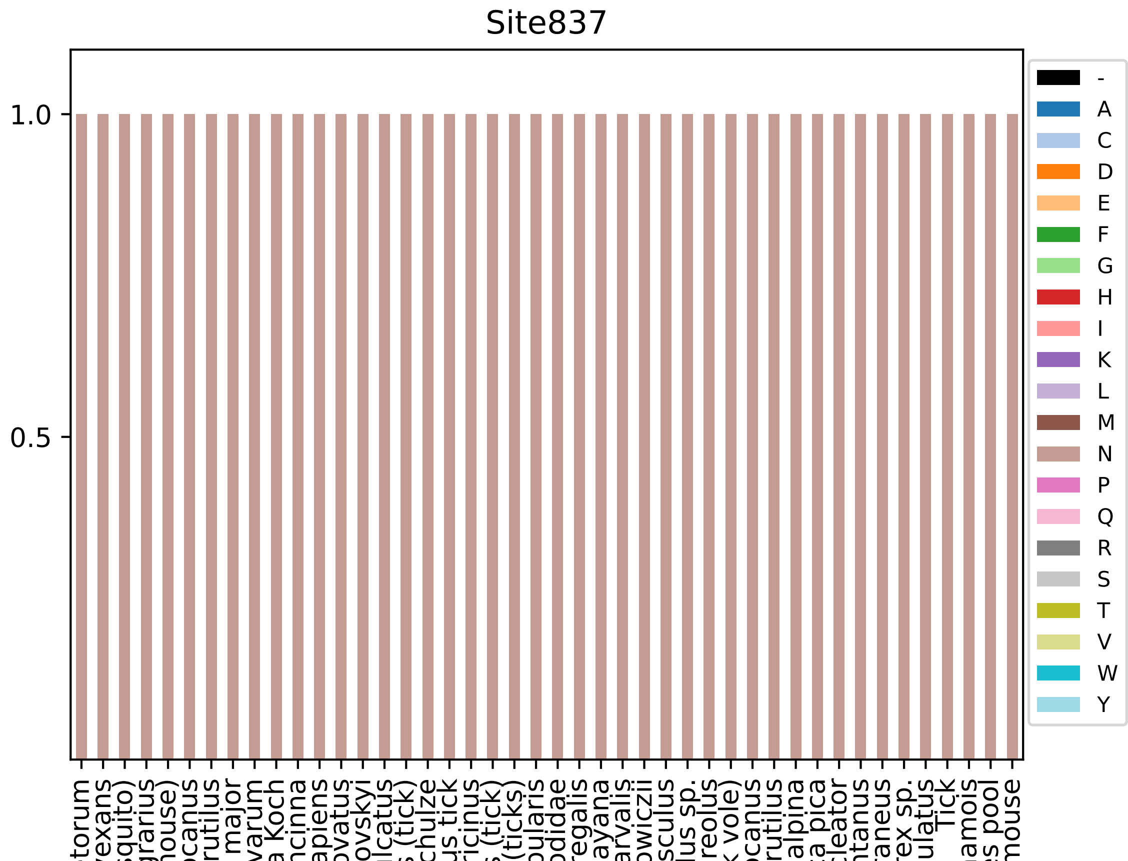
Site837
1.0
0.5
-
A
C
D
E
F
G
H
I
K
L
M
N
P
Q
R
S
T
V
W
Y

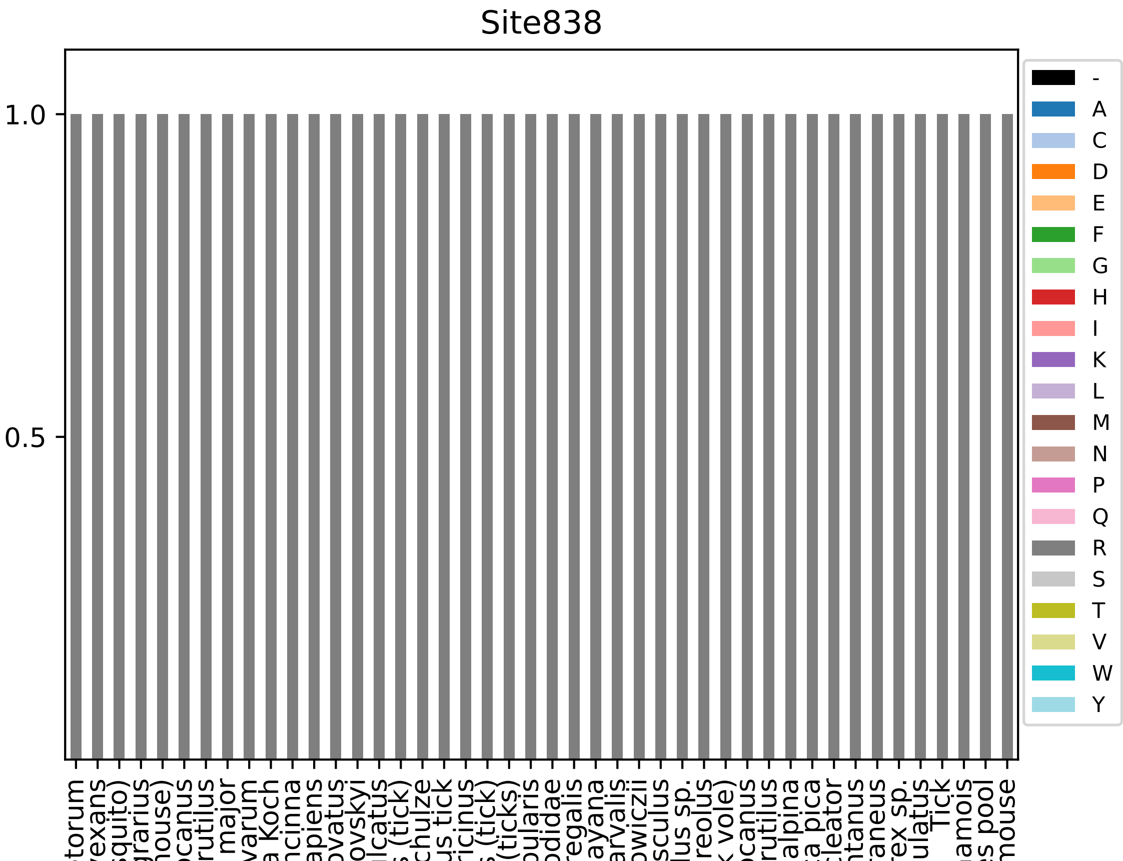
Site838
1.0
0.5
-
A
C
D
E
F
G
H
I
K
L
M
N
P
Q
R
S
T
V
W
Y
Tick

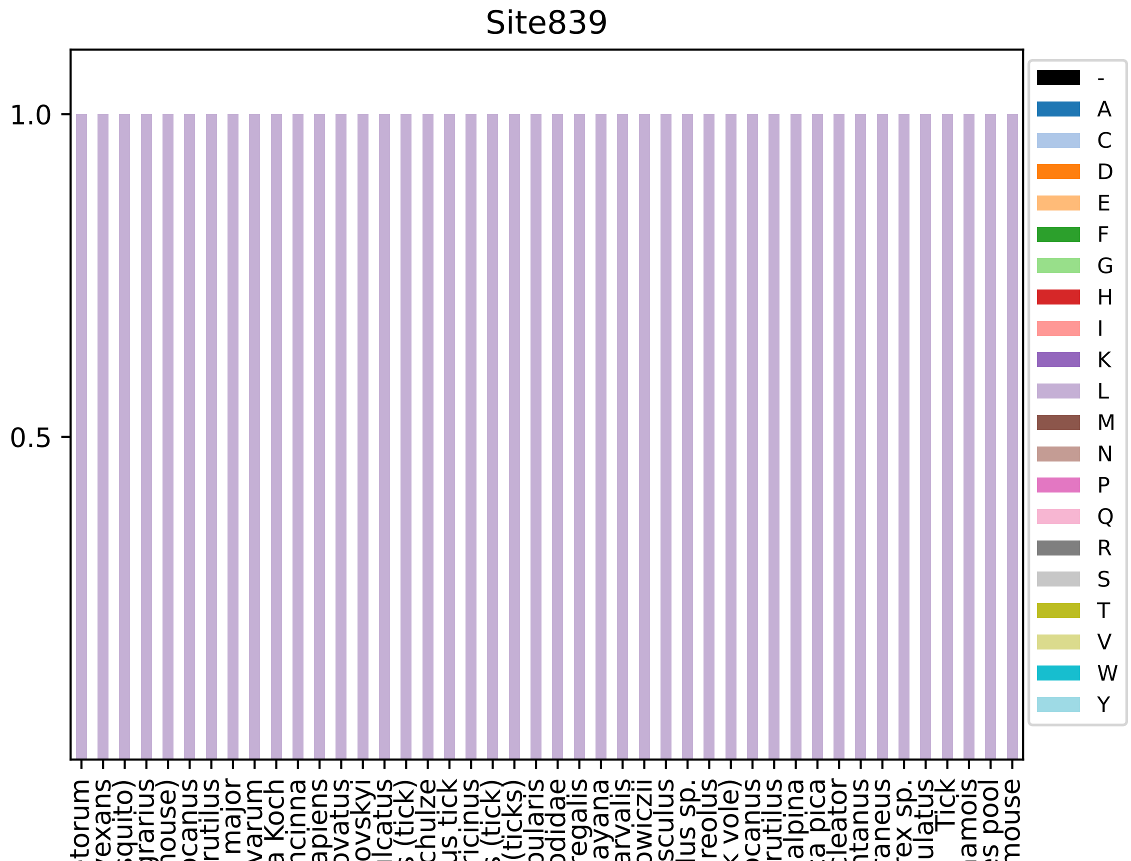

Site839
1.0
0.5
-
A
C
D
E
F
G
H
I
K
L
M
N
P
Q
R
S
T
V
W
Y

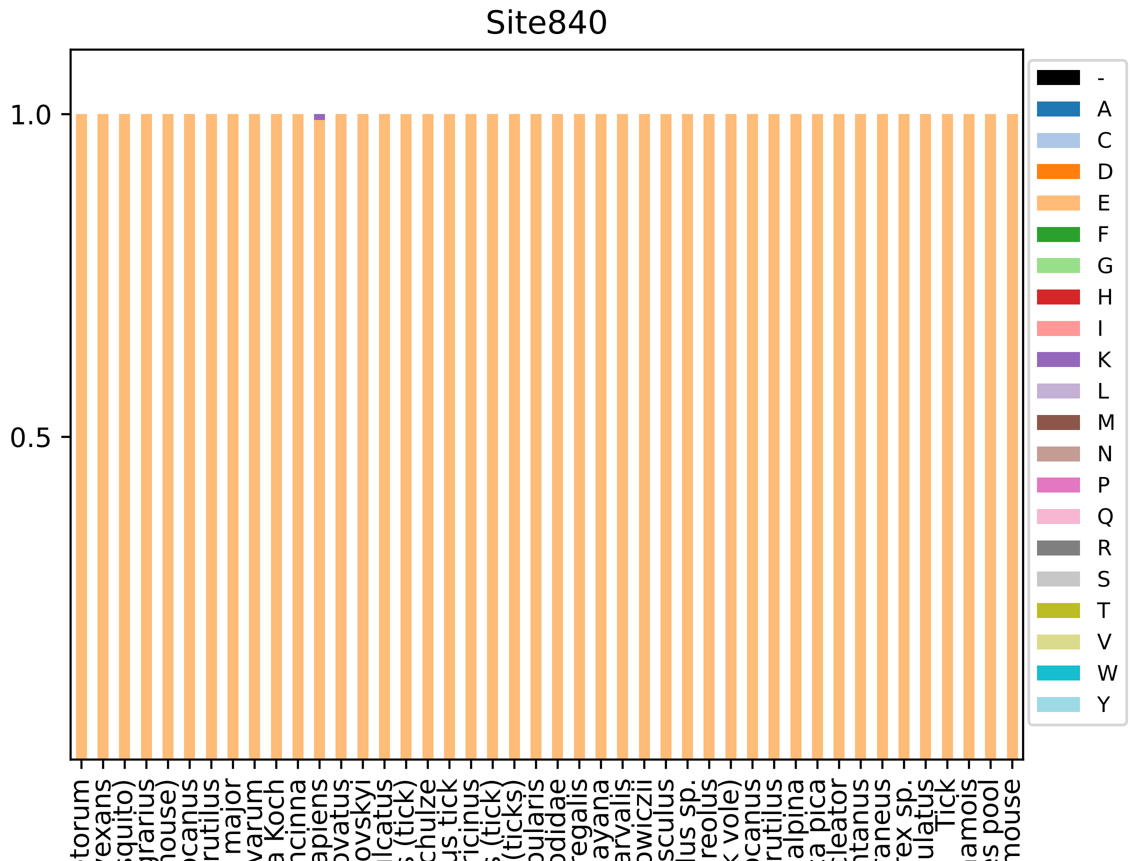

Site840
1.0
0.5
-
A
C
D
E
F
G
H
I
K
L
M
N
P
Q
R
S
T
V
W
Y

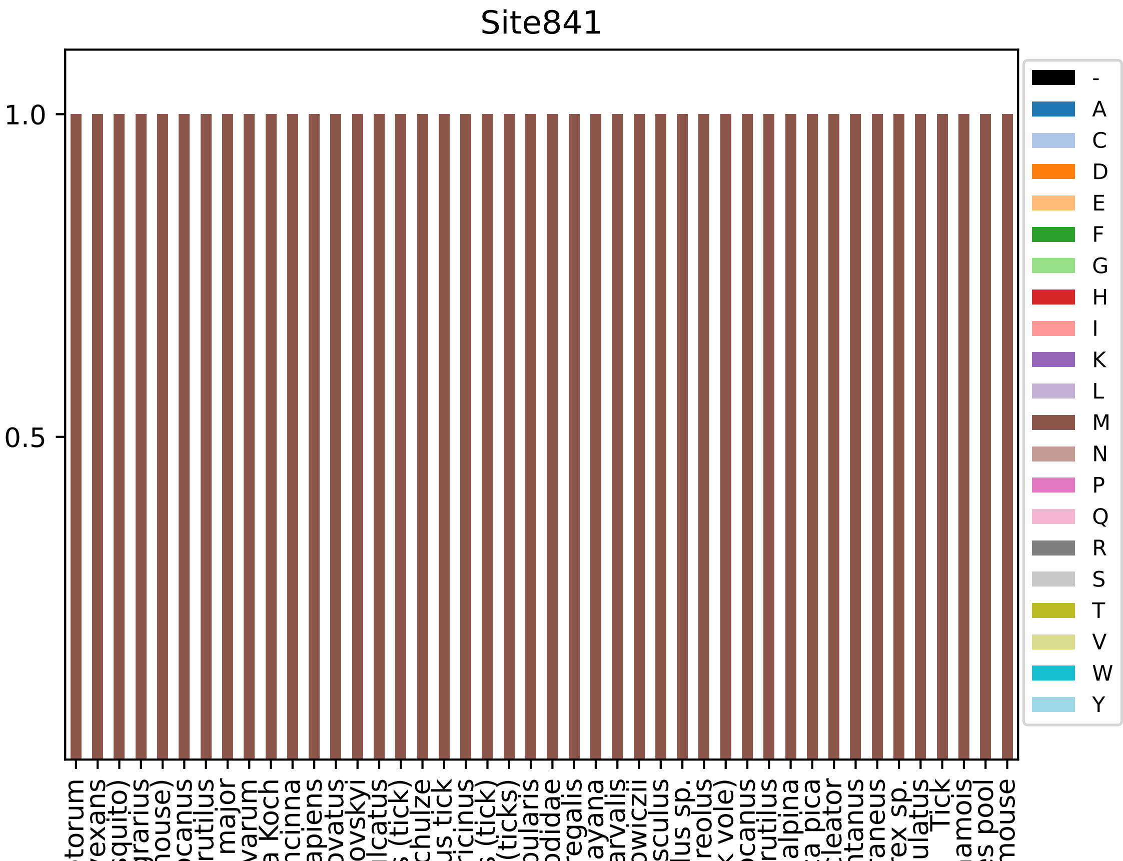
Site841
1.0
0.5
-
A
C
D
E
F
G
H
I
K
L
M
N
P
Q
R
S
T
V
W
Y

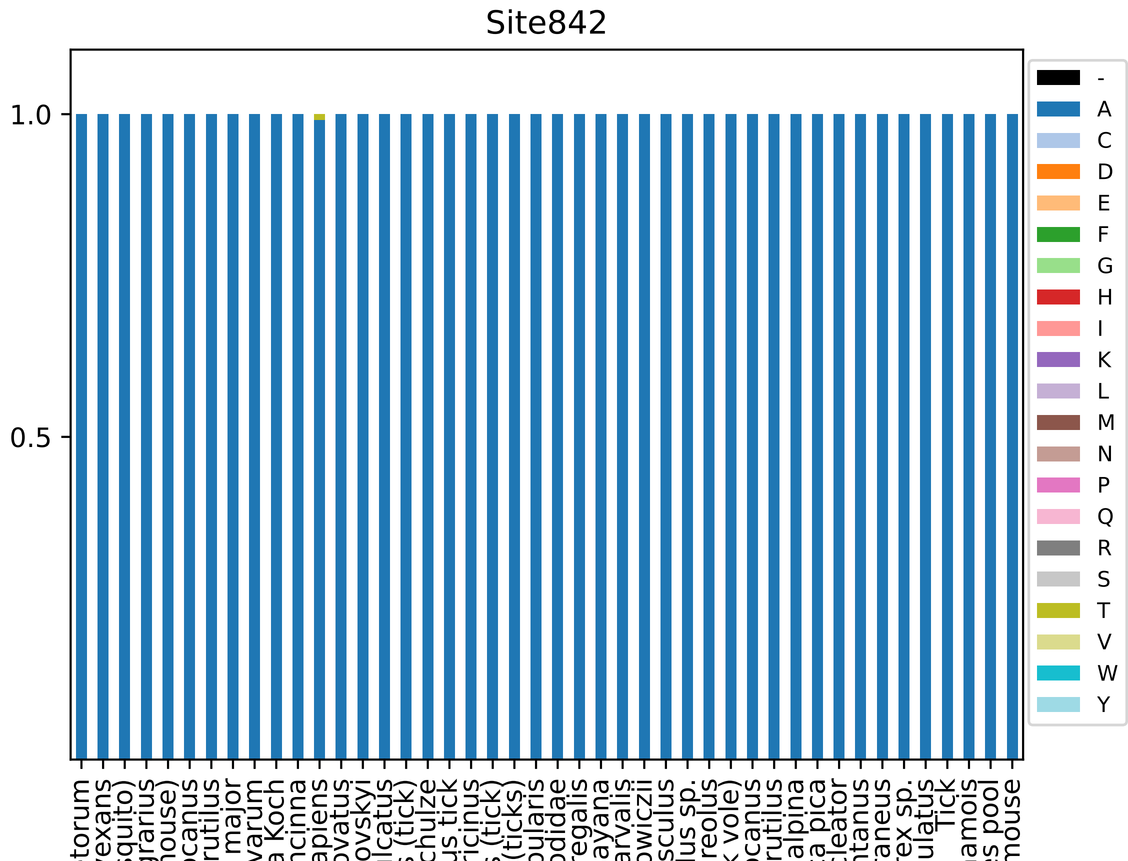

Site842
1.0
0.5
-
A
C
D
E
F
G
H
I
K
L
M
N
P
Q
R
S
T
V
W
Y

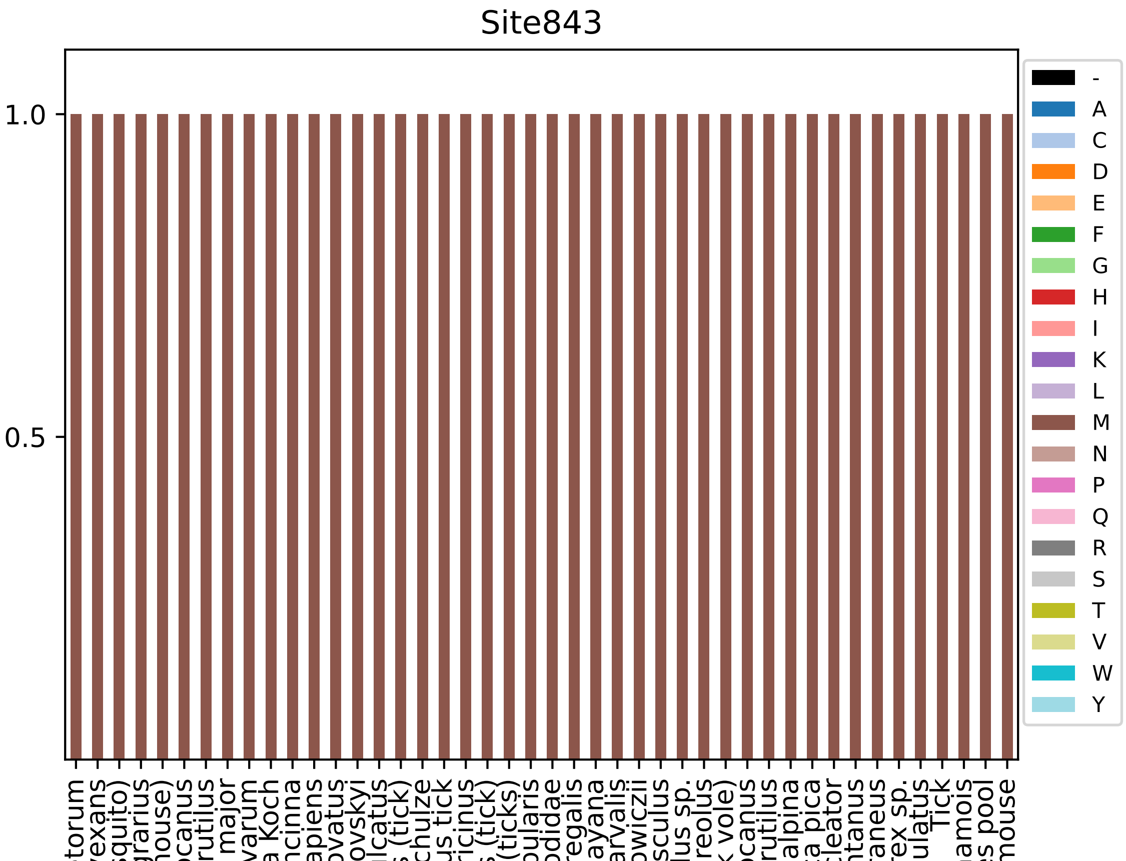
Site843
1.0
0.5
-
A
C
D
E
F
G
H
I
K
L
M
N
P
Q
R
S
T
V
W
Y

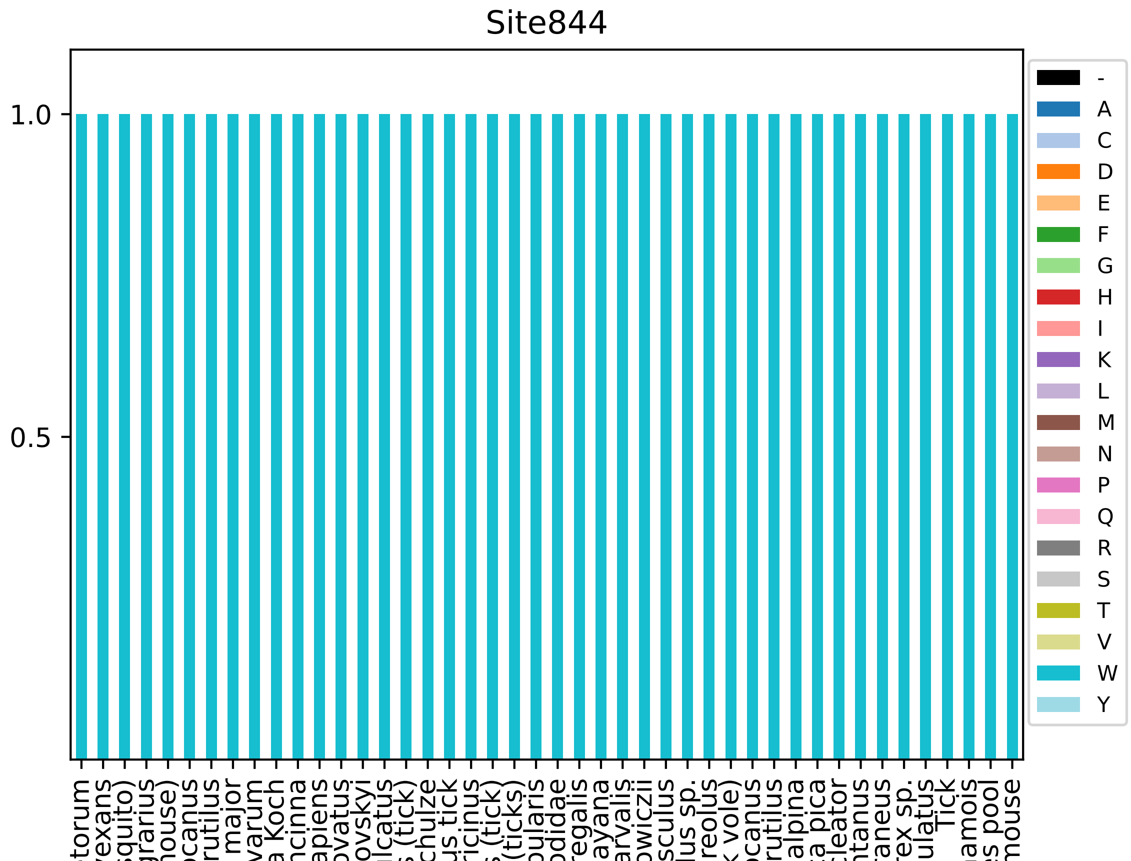
Site844
1.0
0.5
-
A
C
D
E
F
G
H
I
K
L
M
N
P
Q
R
S
T
V
W
Y
Tick

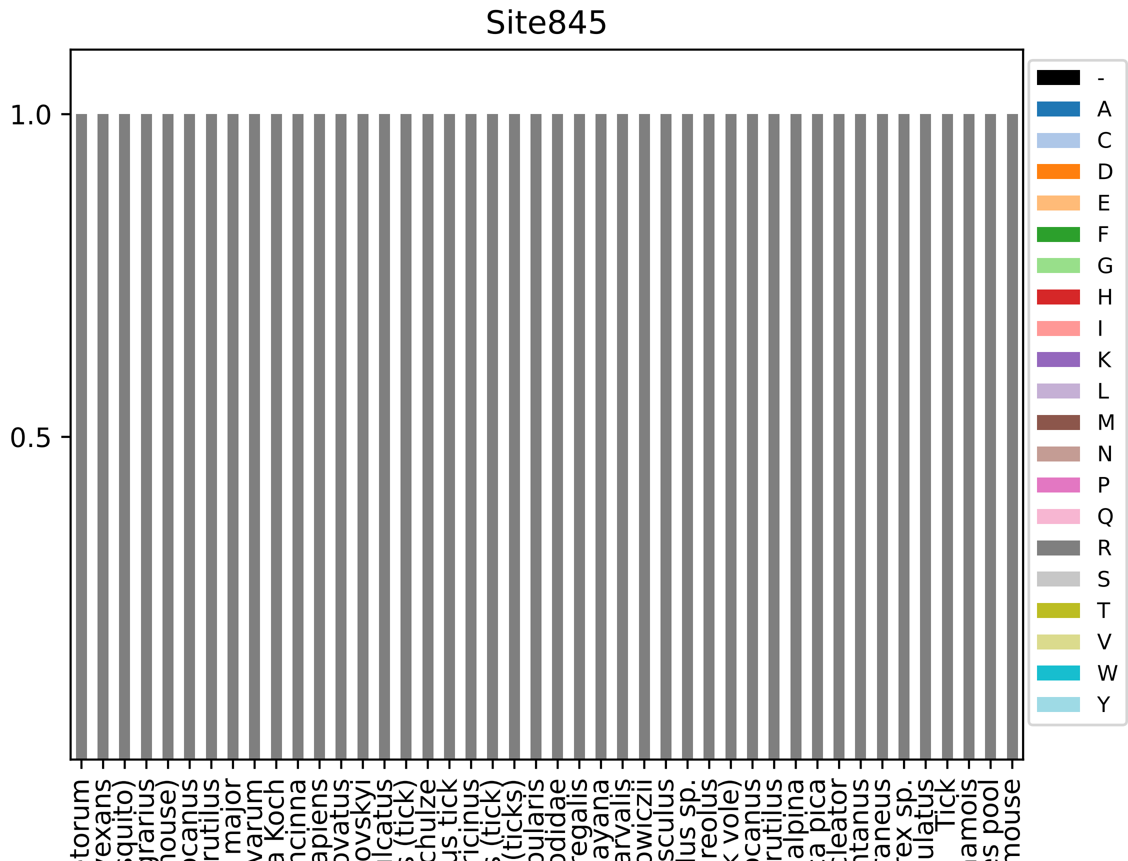

Site845
1.0
0.5
-
A
C
D
E
F
G
H
I
K
L
M
N
P
Q
R
S
T
V
W
Y

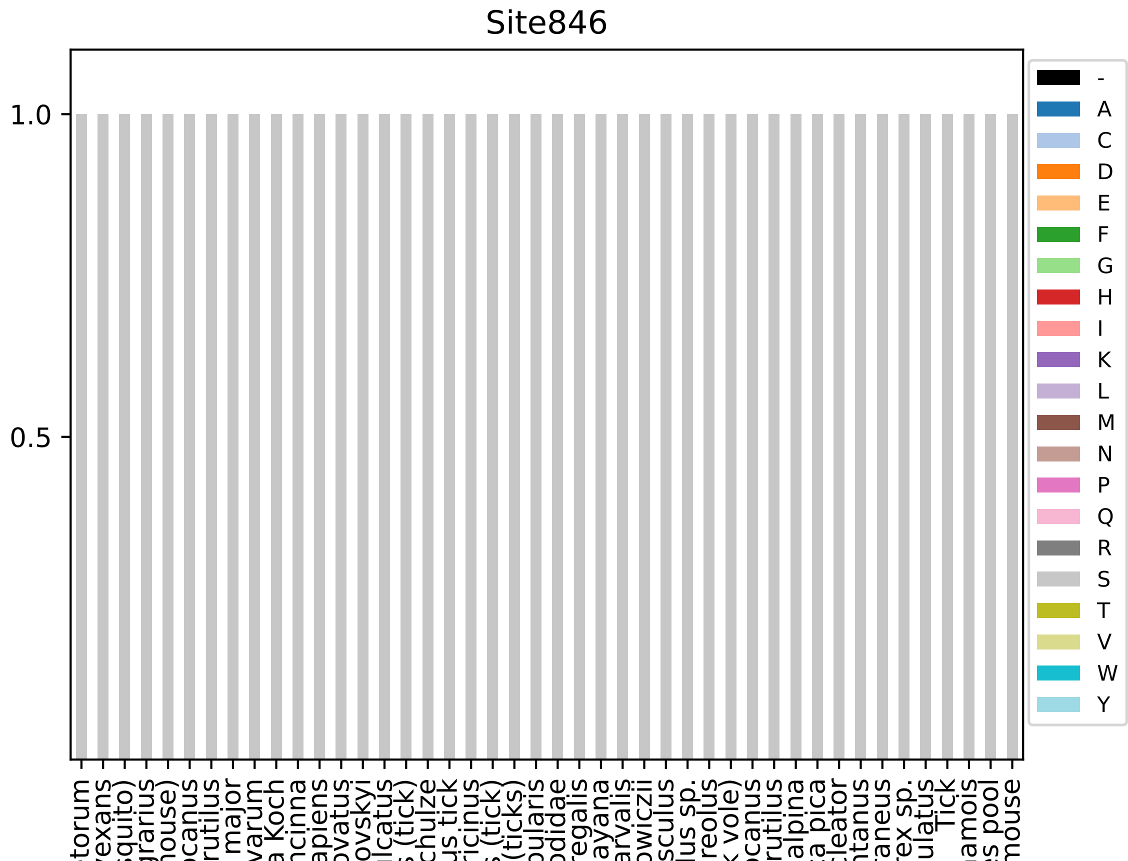
Site846
1.0
0.5
-
A
C
D
E
F
G
H
I
K
L
M
N
P
Q
R
S
T
V
W
Y

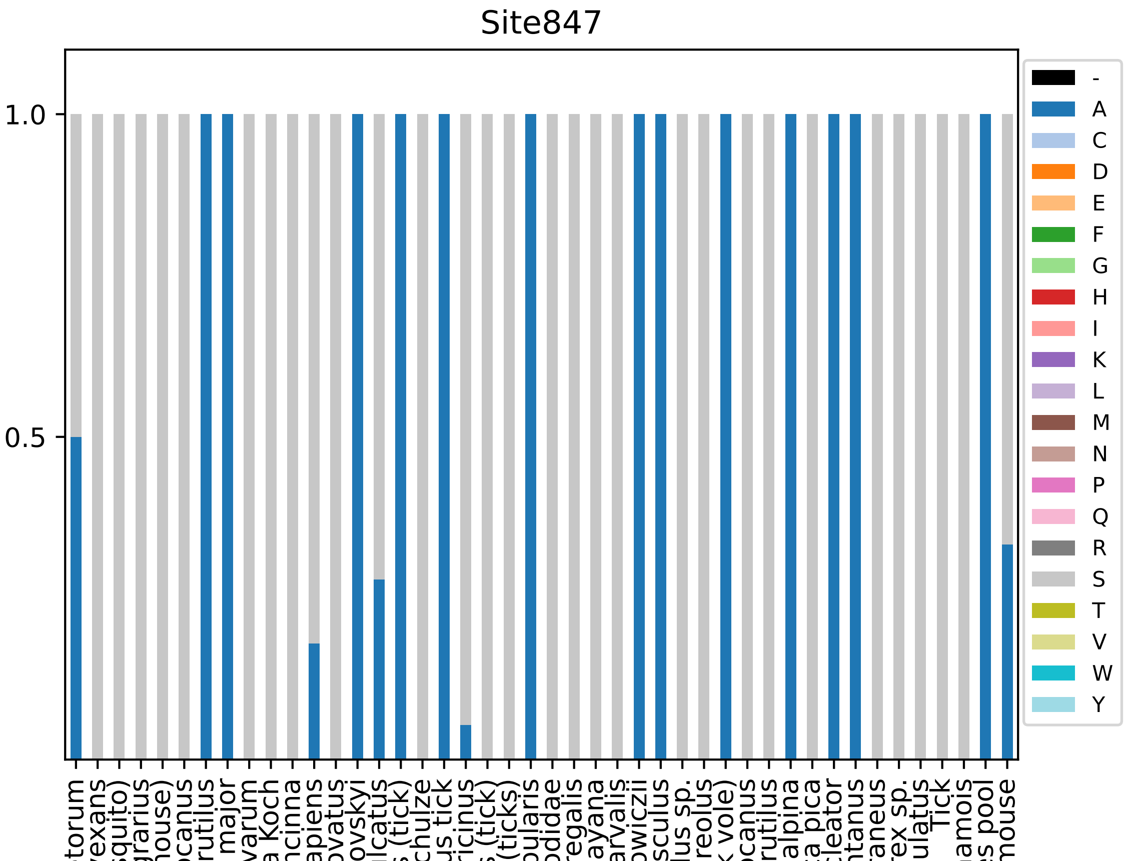

Site847
1.0
0.5
-
A
C
D
E
F
G
H
I
K
L
M
N
P
Q
R
S
T
V
W
Y

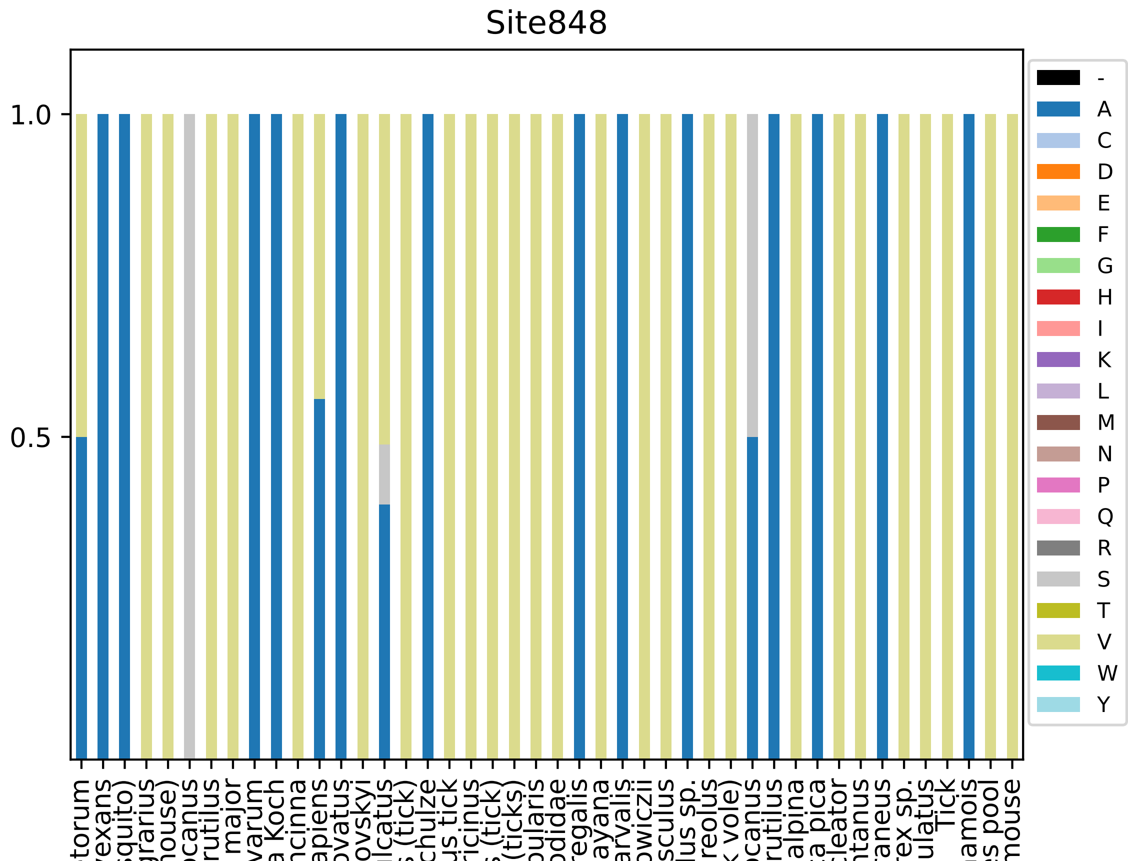

Site848
1.0
0.5
-
A
C
D
E
F
G
H
I
K
L
M
N
P
Q
R
S
T
V
W
Y

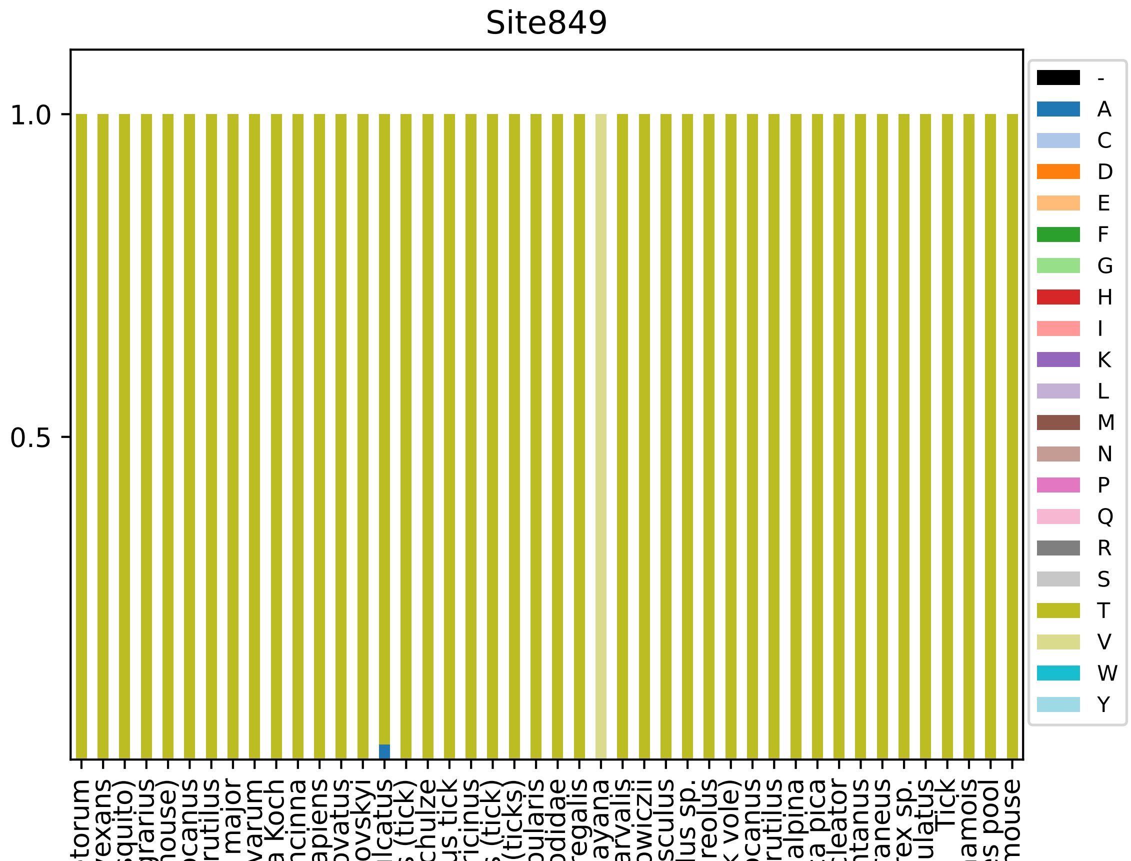

Site849
1.0
0.5
-
A
C
D
E
F
G
H
I
K
L
M
N
P
Q
R
S
T
V
W
Y

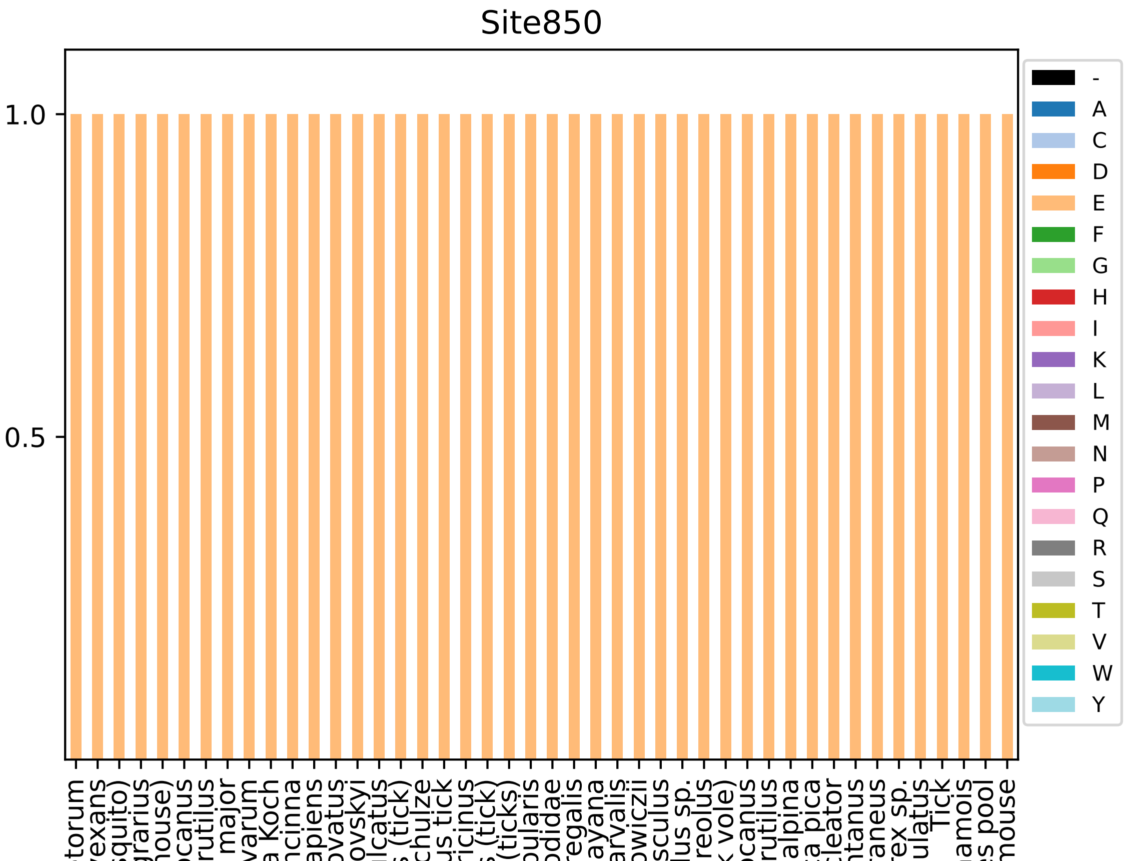
Site850
1.0
0.5
-
A
C
D
E
F
G
H
I
K
L
M
N
P
Q
R
S
T
V
W
Y

Site851

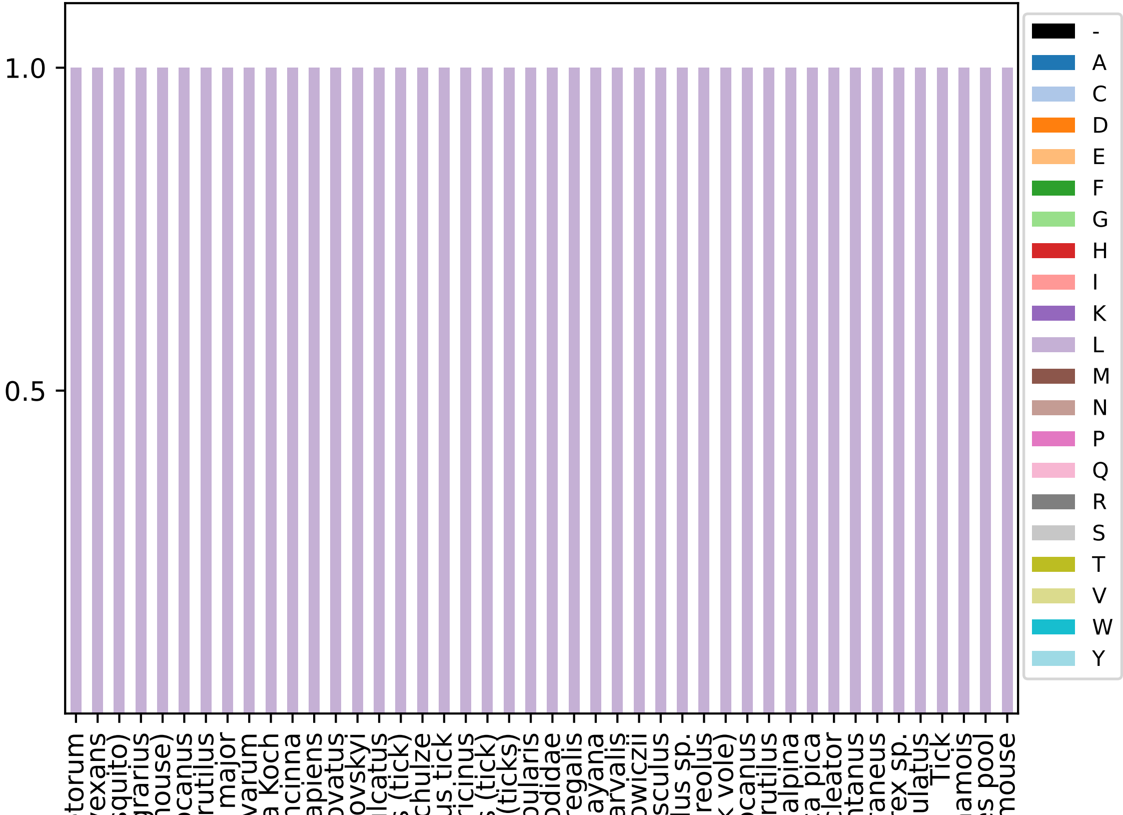

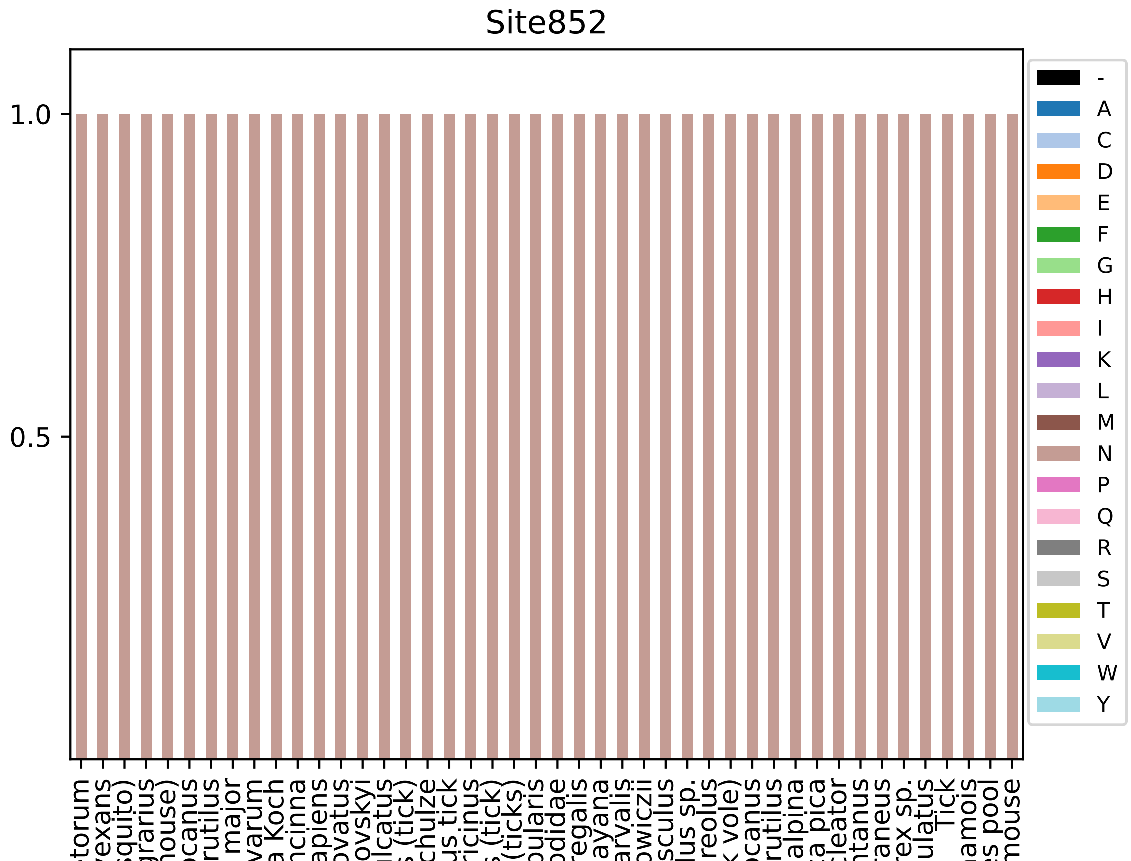
Site852
1.0
0.5
-
A
C
D
E
F
G
H
I
K
L
M
N
P
Q
R
S
T
V
W
Y

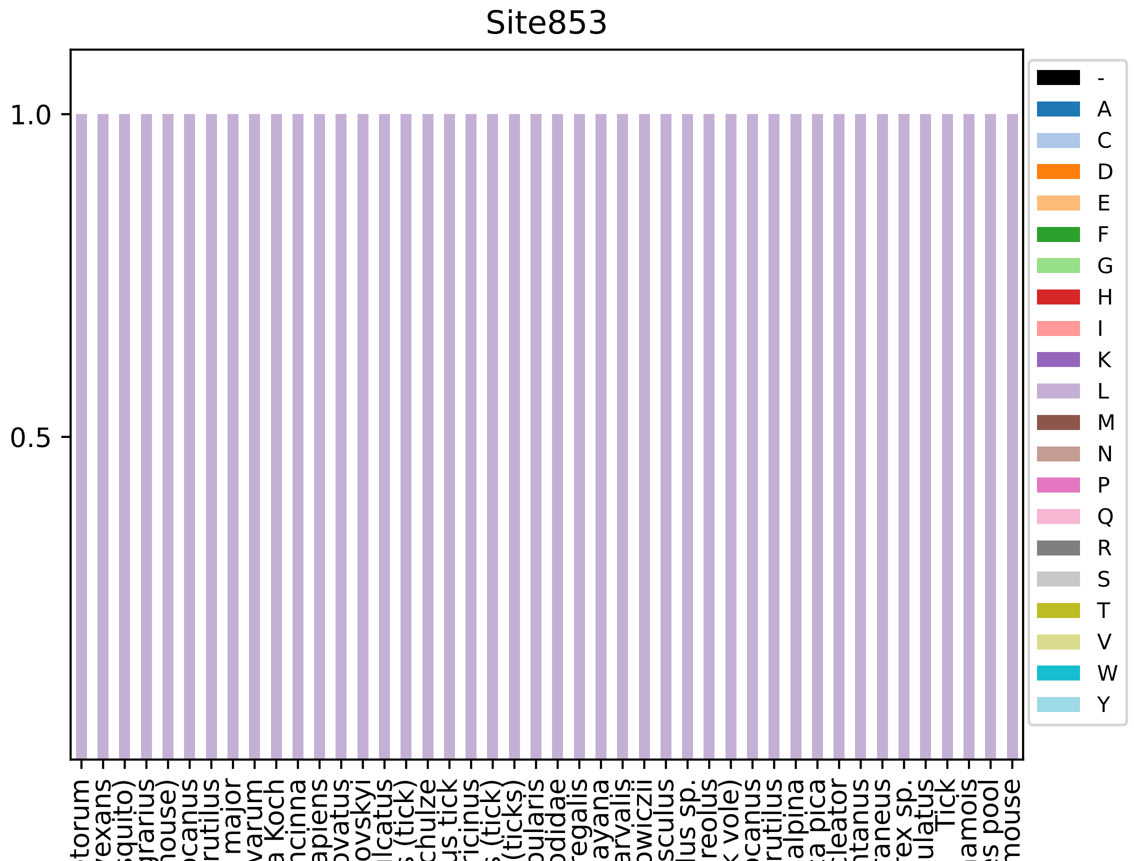
Site853
1.0
0.5
-
A
C
D
E
F
G
H
I
K
L
M
N
P
Q
R
S
T
V
W
Y

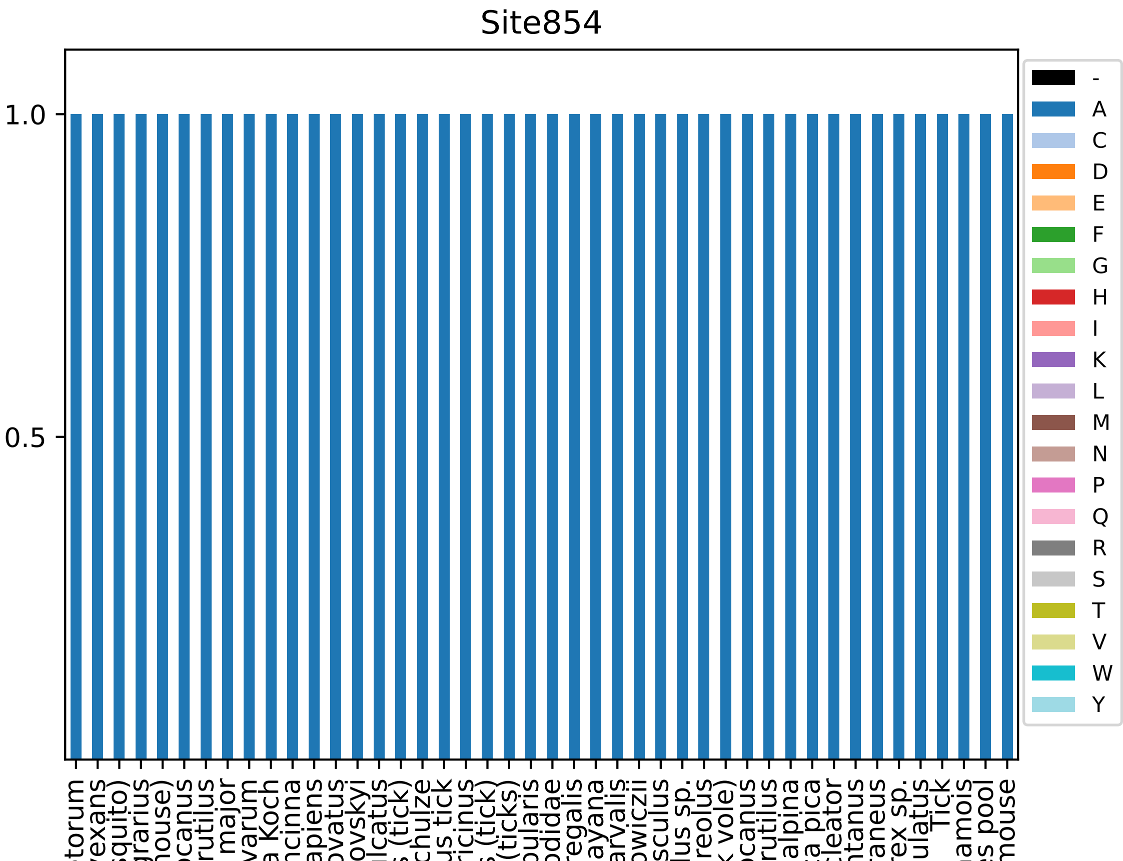

Site854
1.0
0.5
-
A
C
D
E
F
G
H
I
K
L
M
N
P
Q
R
S
T
V
W
Y

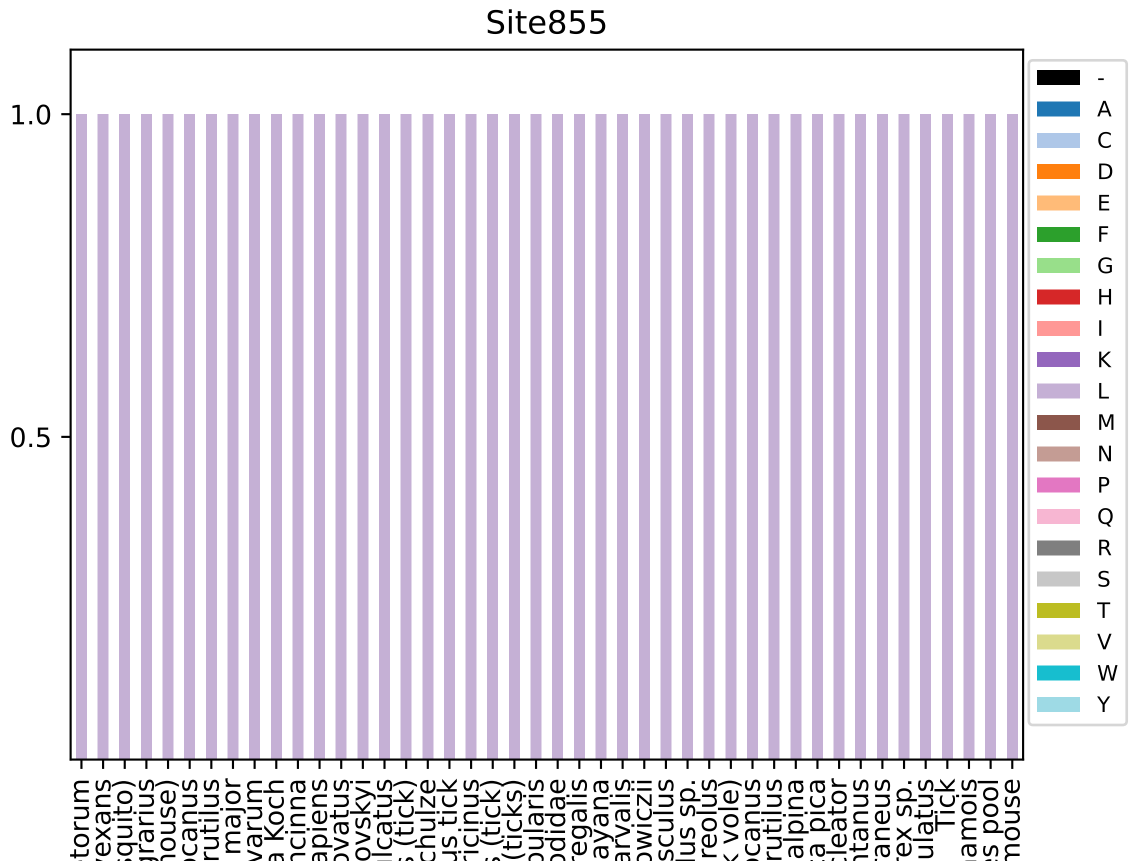

Site855
1.0
0.5
-
A
C
D
E
F
G
H
I
K
L
M
N
P
Q
R
S
T
V
W
Y

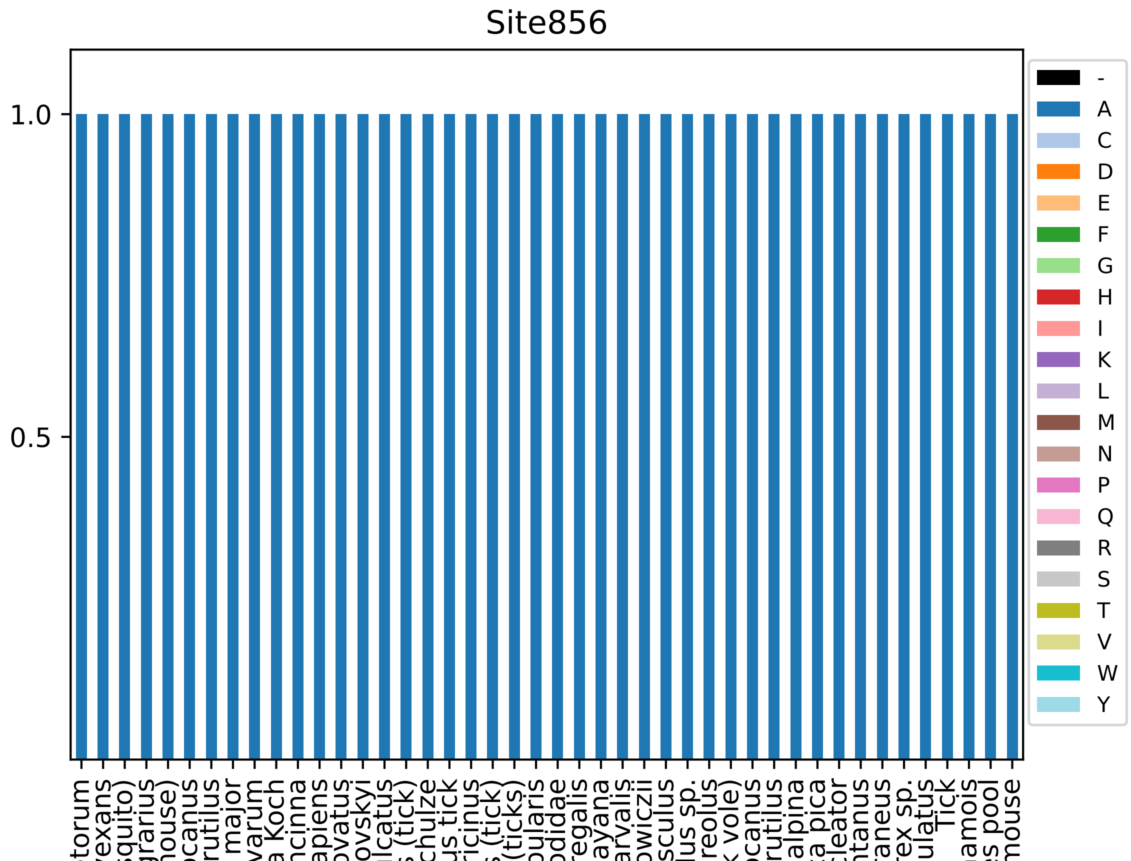

Site856
1.0
0.5
-
A
C
D
E
F
G
H
I
K
L
M
N
P
Q
R
S
T
V
W
Y
Tick

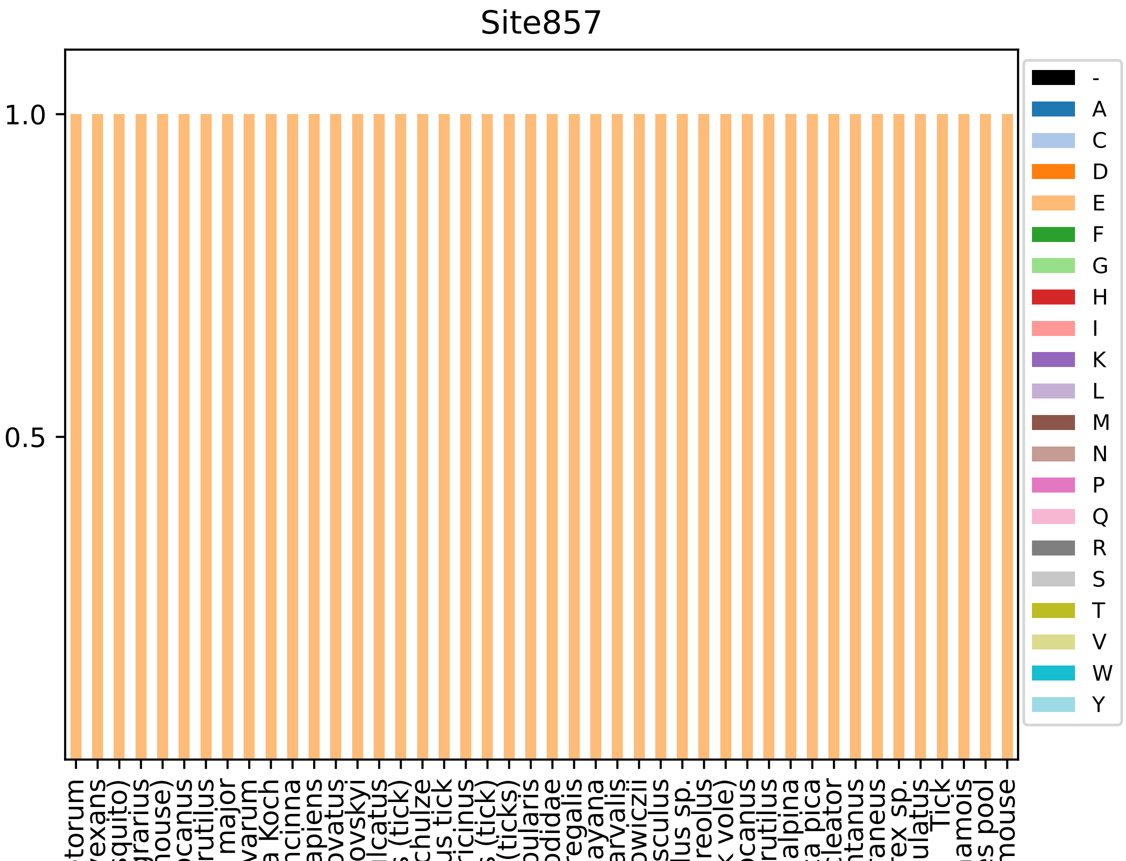
Site857
1.0
0.5
-
A
C
D
E
F
G
H
I
K
L
M
N
P
Q
R
S
T
V
W
Y

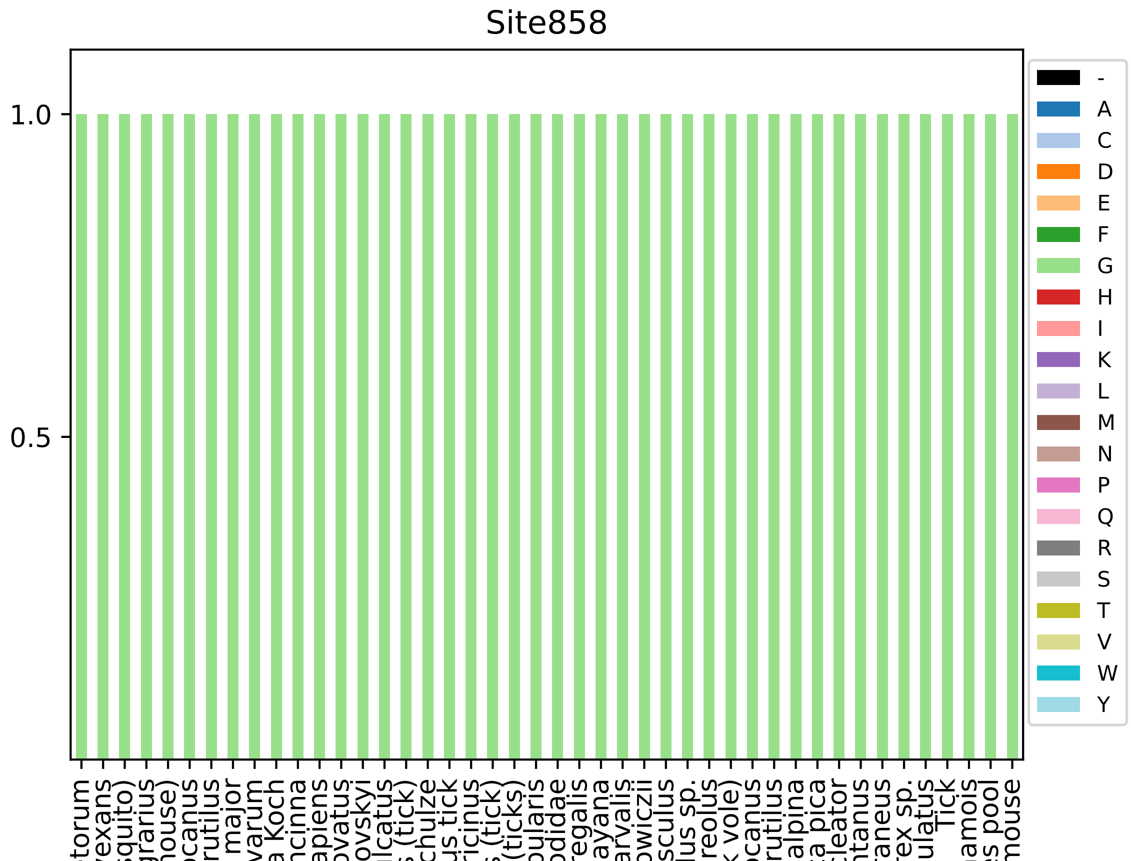

Site858
1.0
0.5
-
A
C
D
E
F
G
H
I
K
L
M
N
P
Q
R
S
T
V
W
Y

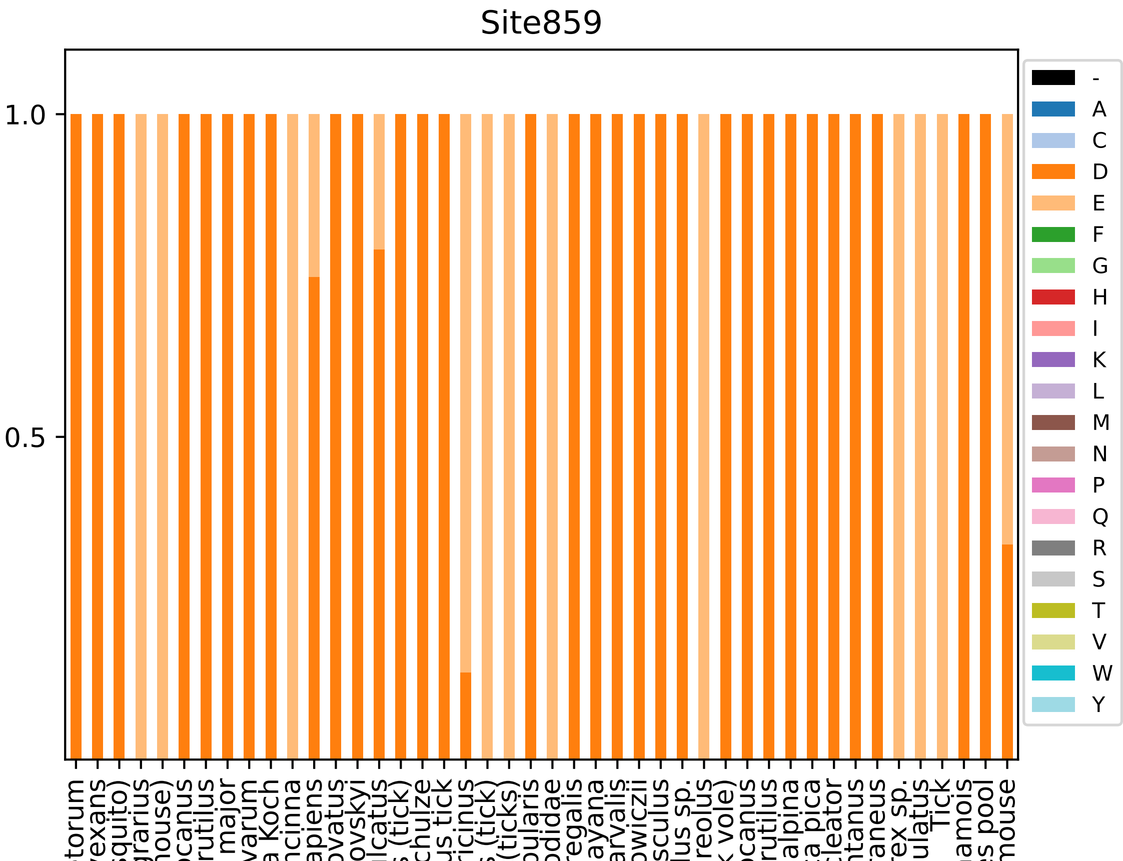
Site859
1.0
0.5
-
A
C
D
E
F
G
H
I
K
L
M
N
P
Q
R
S
T
V
W
Y

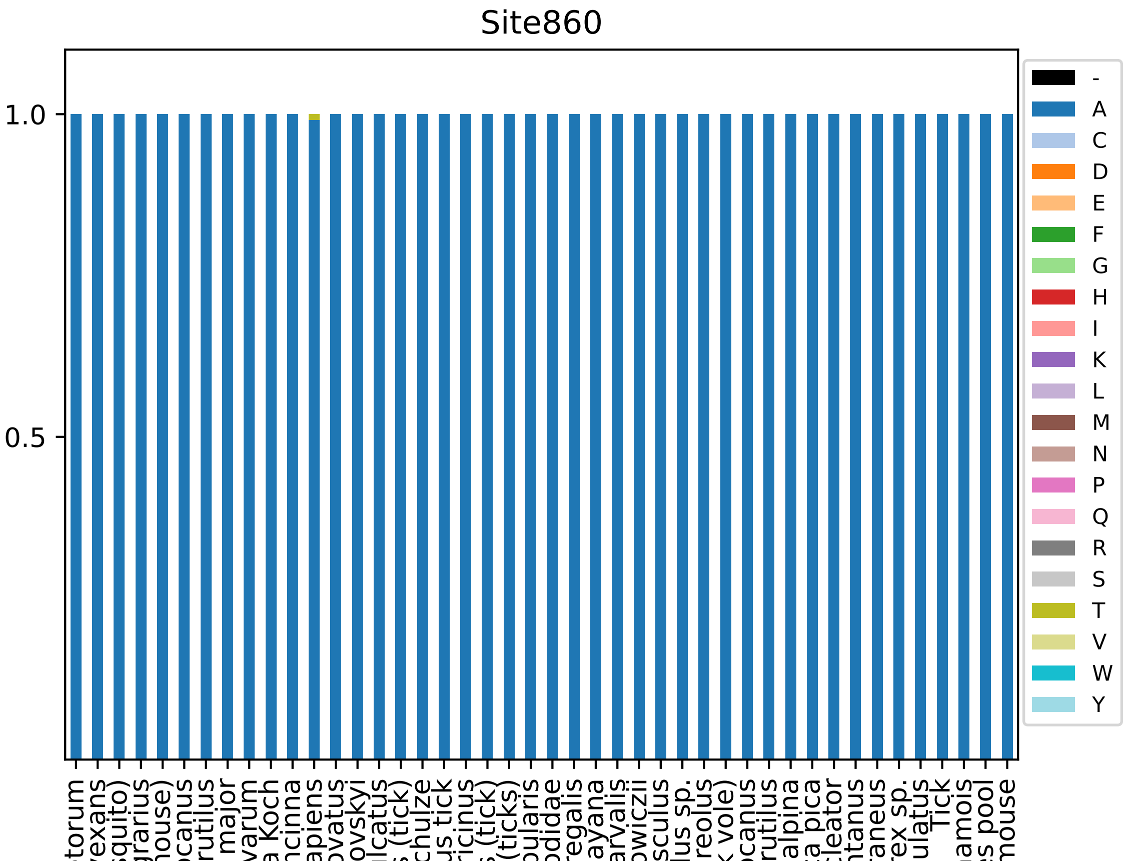
Site860
1.0
0.5
-
A
C
D
E
F
G
H
I
K
L
M
N
P
Q
R
S
T
V
W
Y

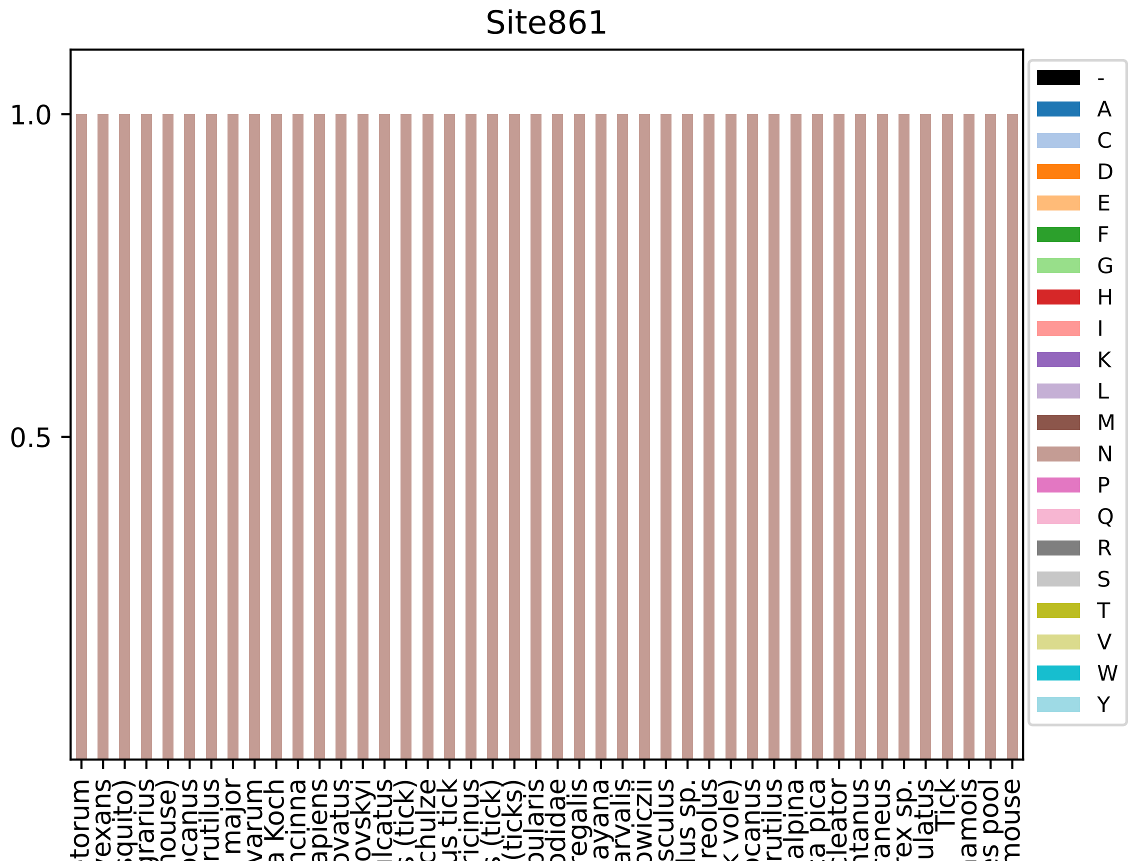
Site861
1.0
0.5
-
A
C
D
E
F
G
H
I
K
L
M
N
P
Q
R
S
T
V
W
Y
Tick

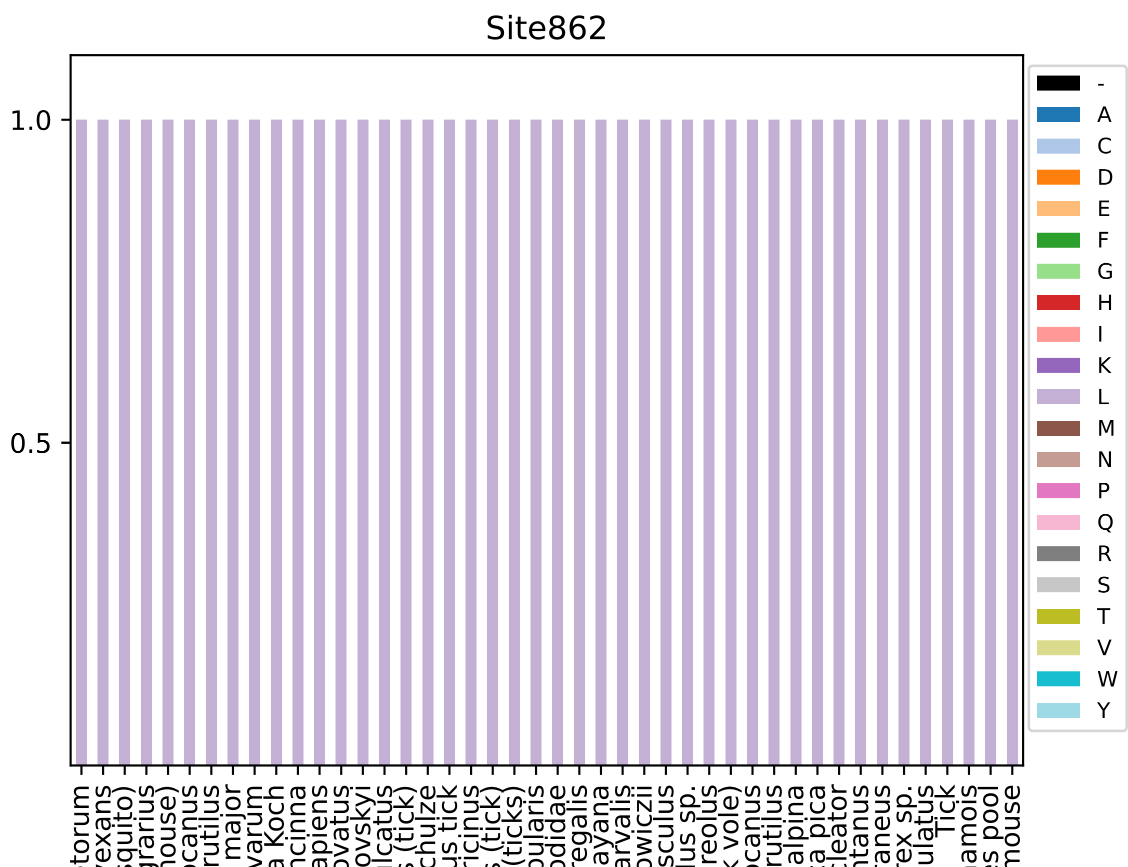
Site862
1.0
0.5
-
A
C
D
E
F
G
H
I
K
L
M
N
P
Q
R
S
T
V
W
Y

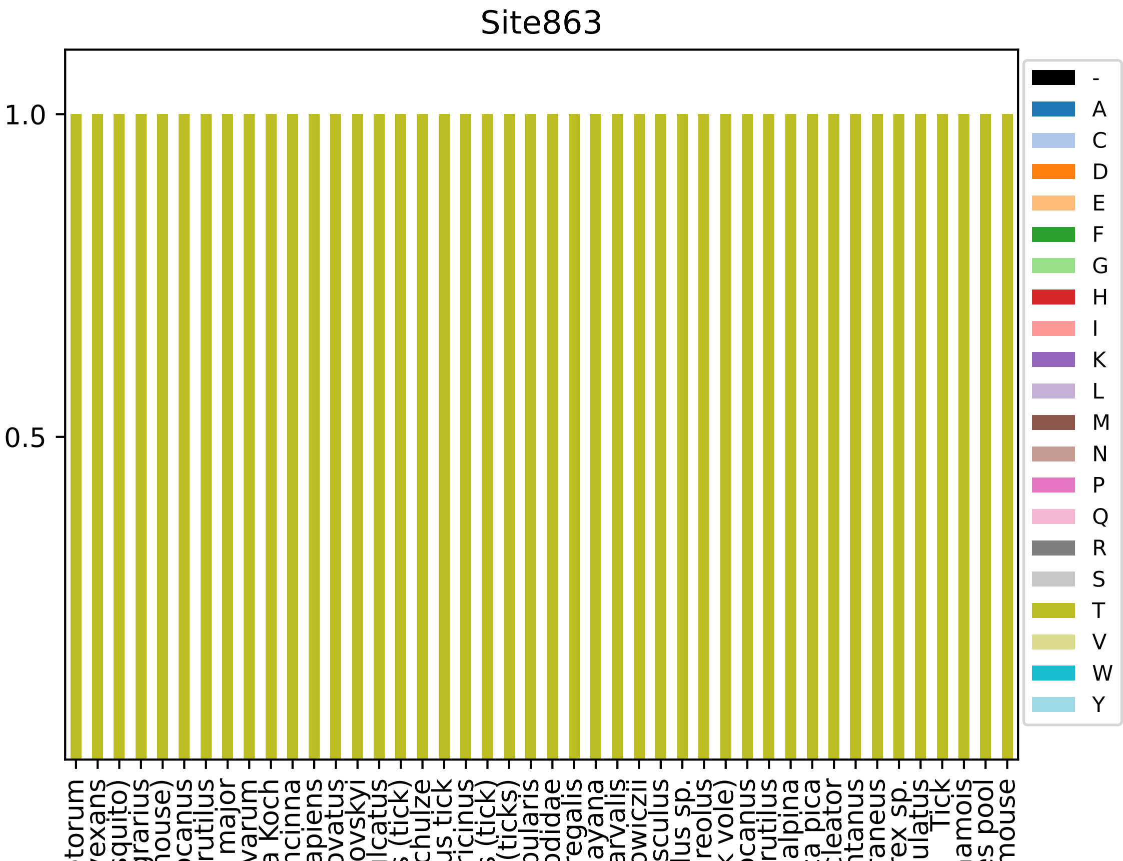

Site863
1.0
0.5
-
A
C
D
E
F
G
H
I
K
L
M
N
P
Q
R
S
T
V
W
Y

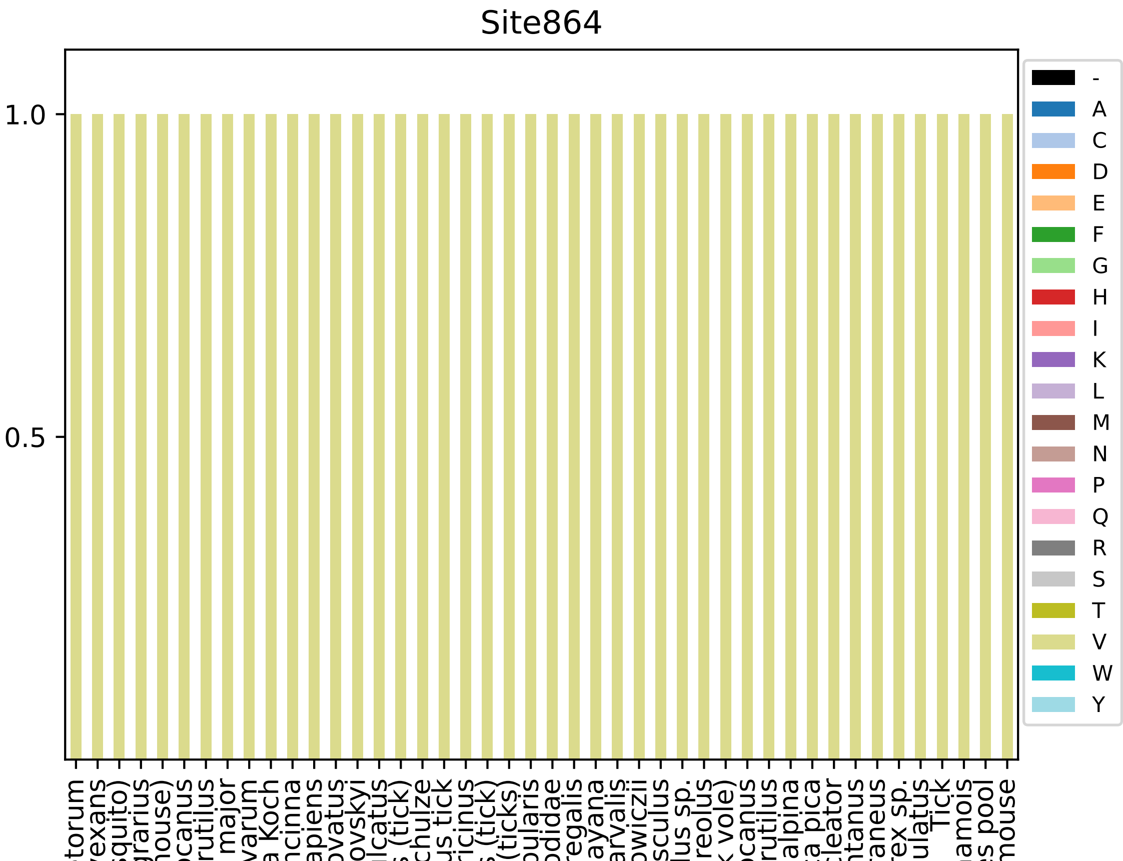

Site864
1.0
0.5
-
A
C
D
E
F
G
H
I
K
L
M
N
P
Q
R
S
T
V
W
Y

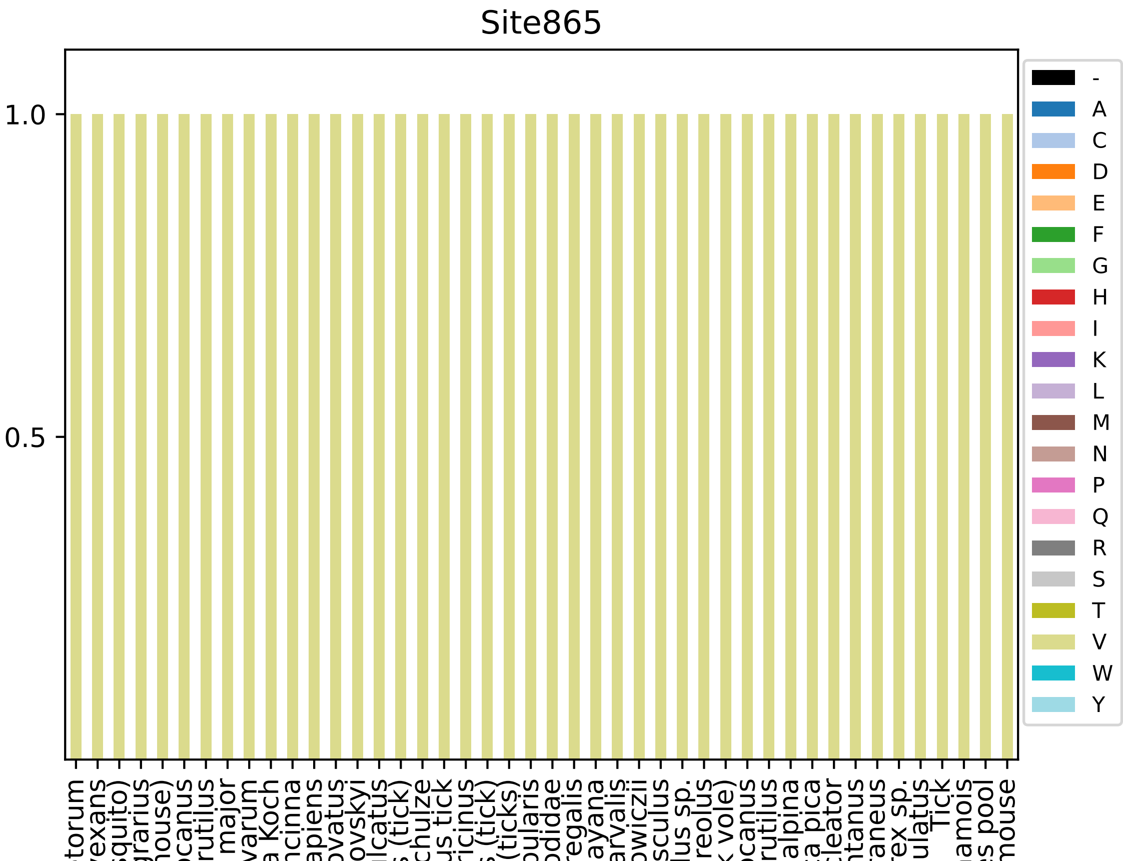
Site865
1.0
0.5
-
A
C
D
E
F
G
H
I
K
L
M
N
P
Q
R
S
T
V
W
Y

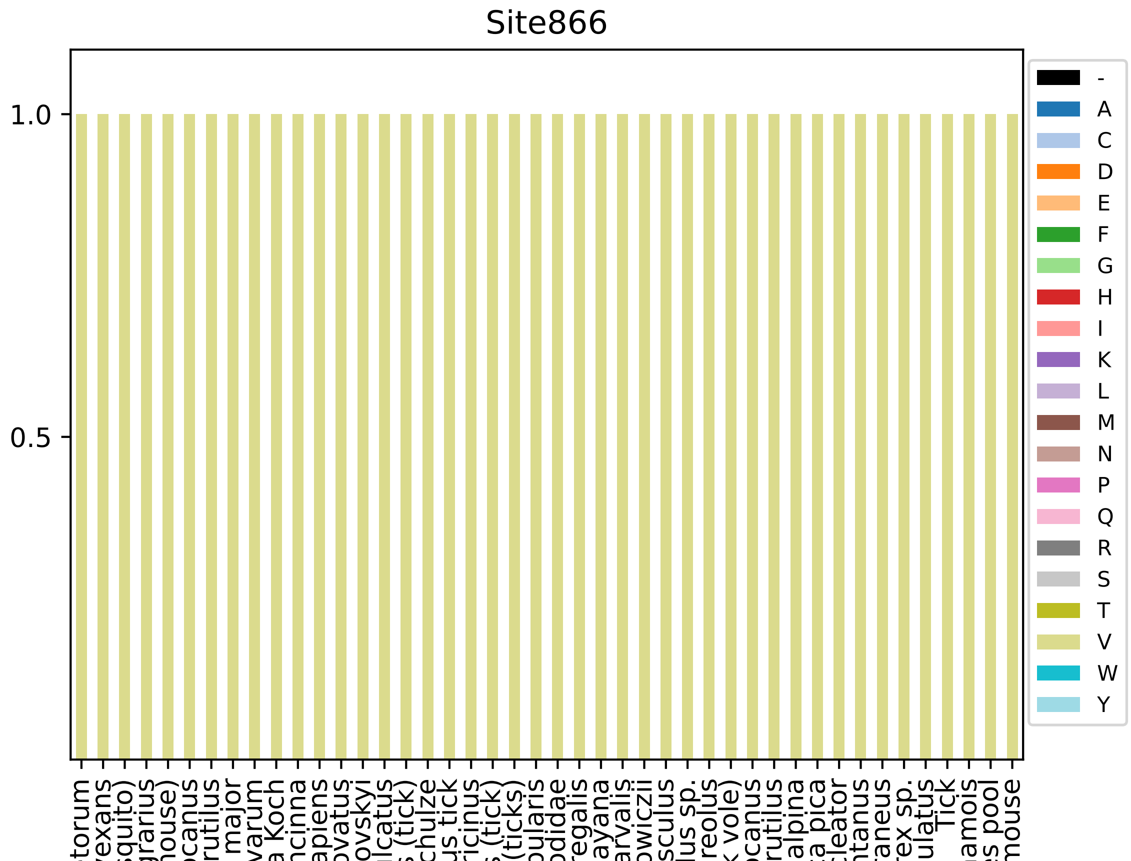

Site866
1.0
0.5
-
A
C
D
E
F
G
H
I
K
L
M
N
P
Q
R
S
T
V
W
Y

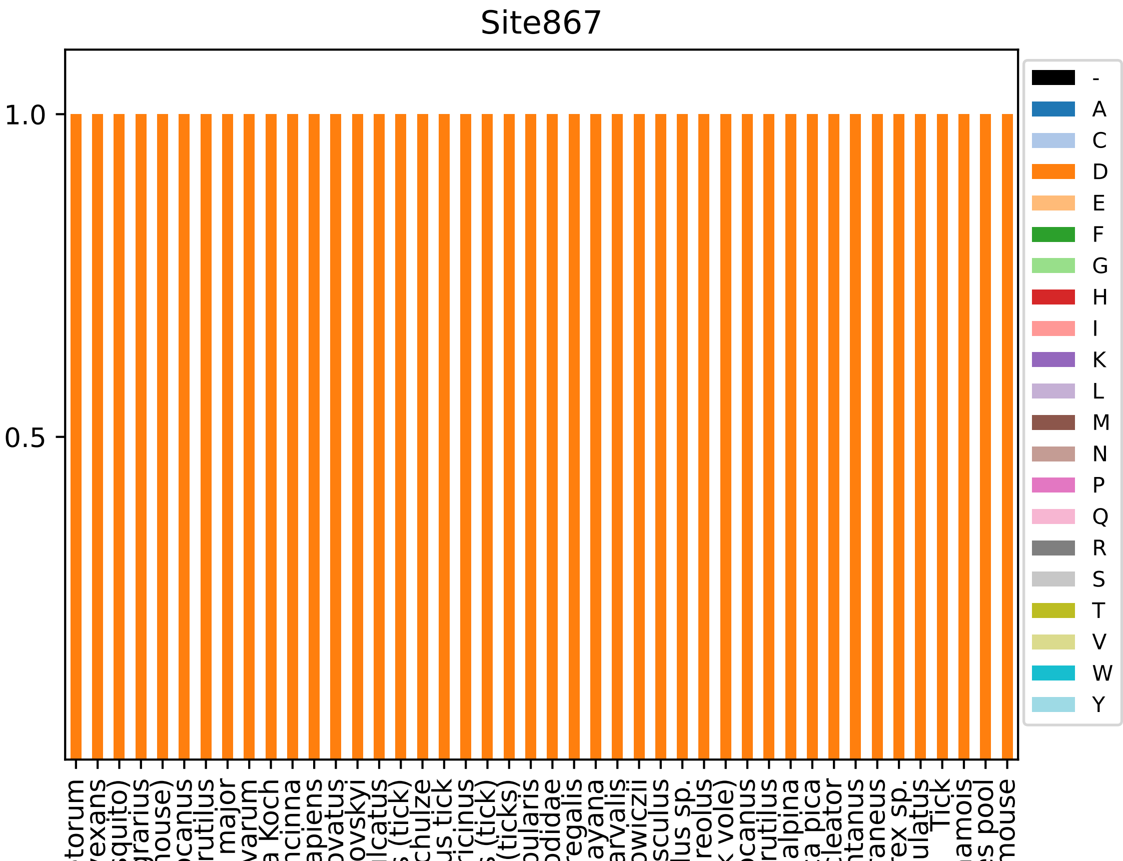
Site867
1.0
0.5
-
A
C
D
E
F
G
H
I
K
L
M
N
P
Q
R
S
T
V
W
Y

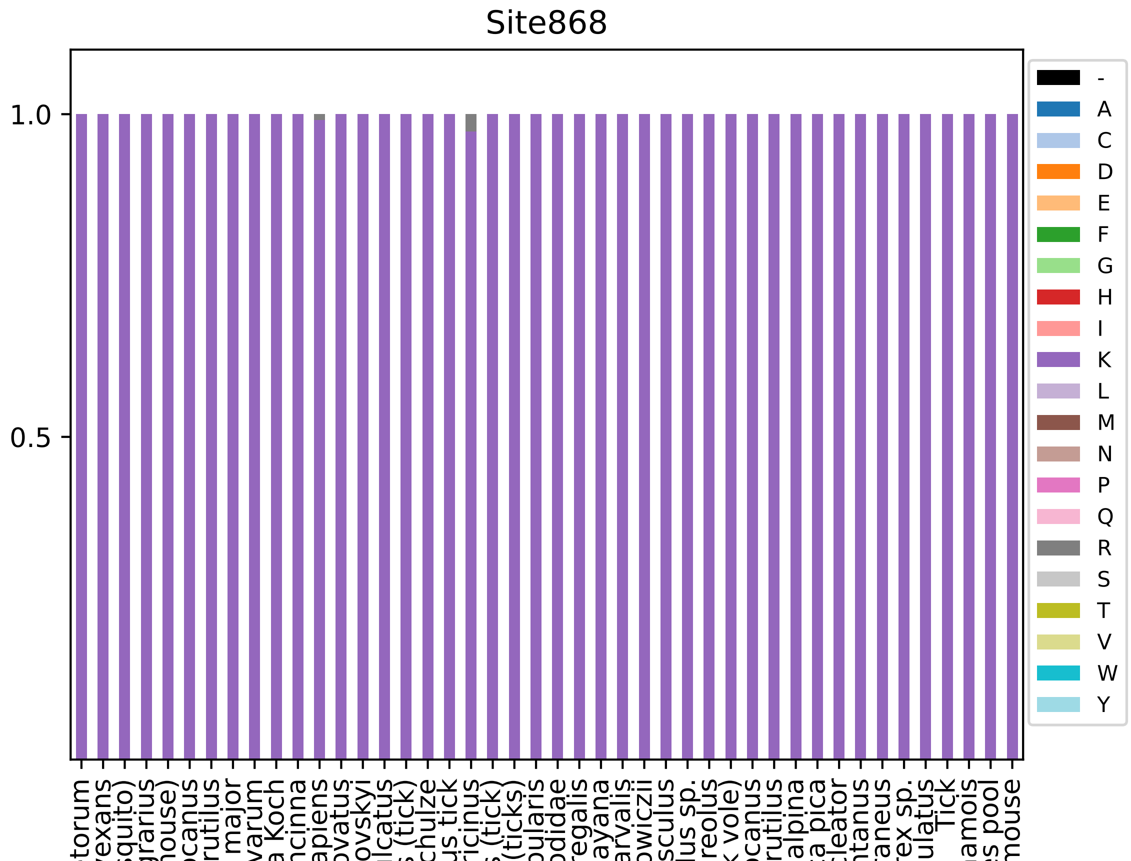
Site868
1.0
0.5
-
A
C
D
E
F
G
H
I
K
L
M
N
P
Q
R
S
T
V
W
Y

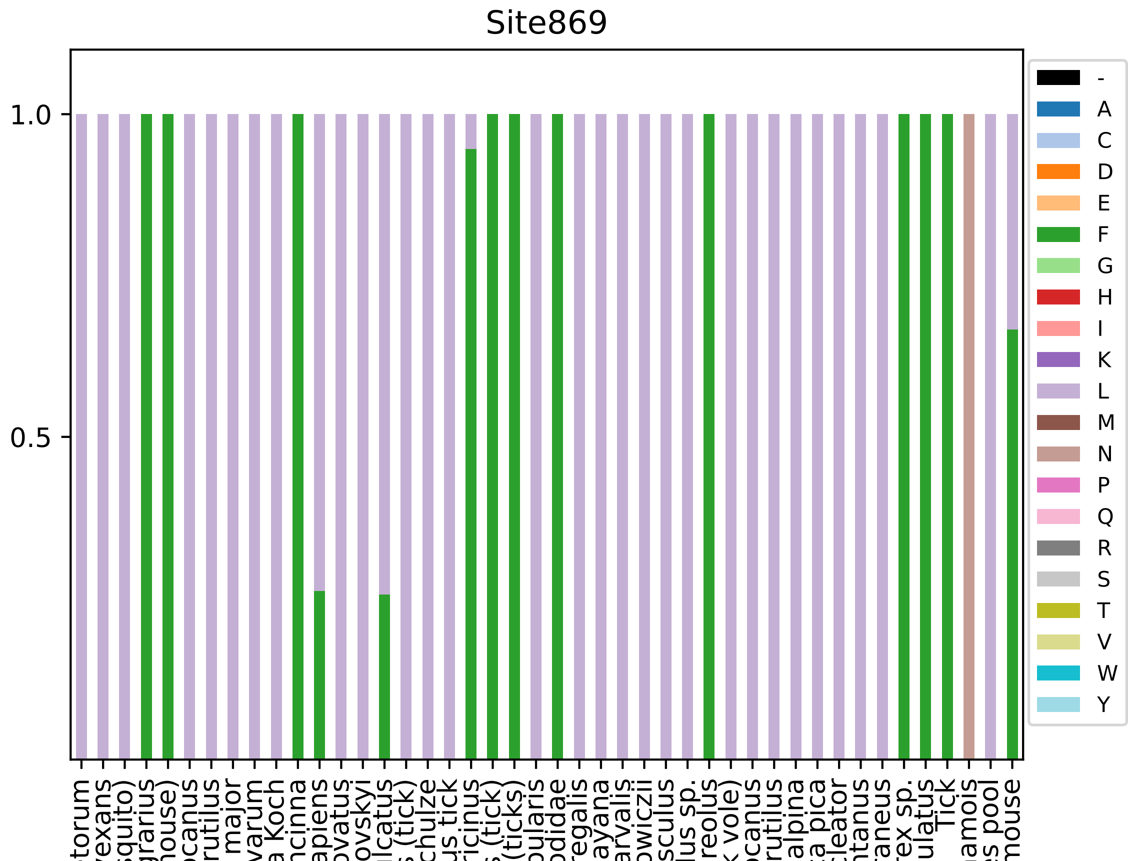
Site869
1.0
0.5
-
A
C
D
E
F
G
H
I
K
L
M
N
P
Q
R
S
T
V
W
Y

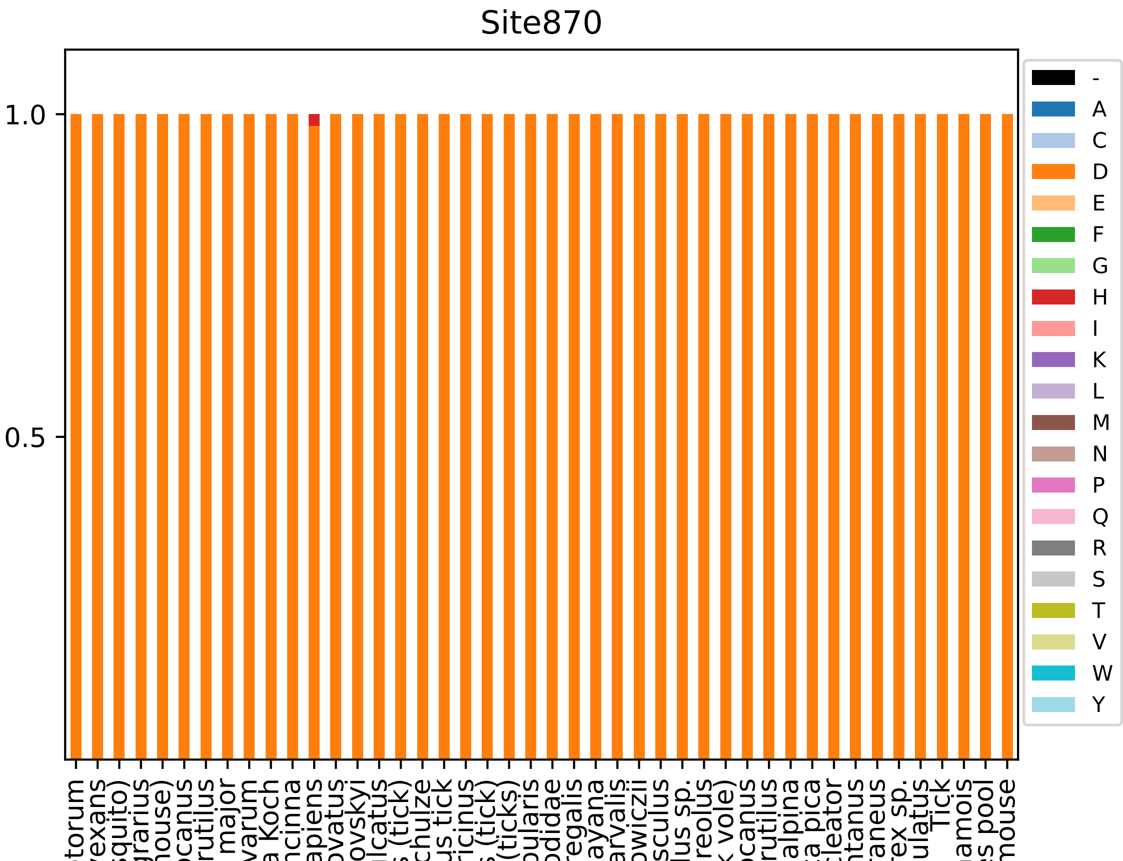
Site870
1.0
0.5
-
A
C
D
E
F
G
H
I
K
L
M
N
P
Q
R
S
T
V
W
Y

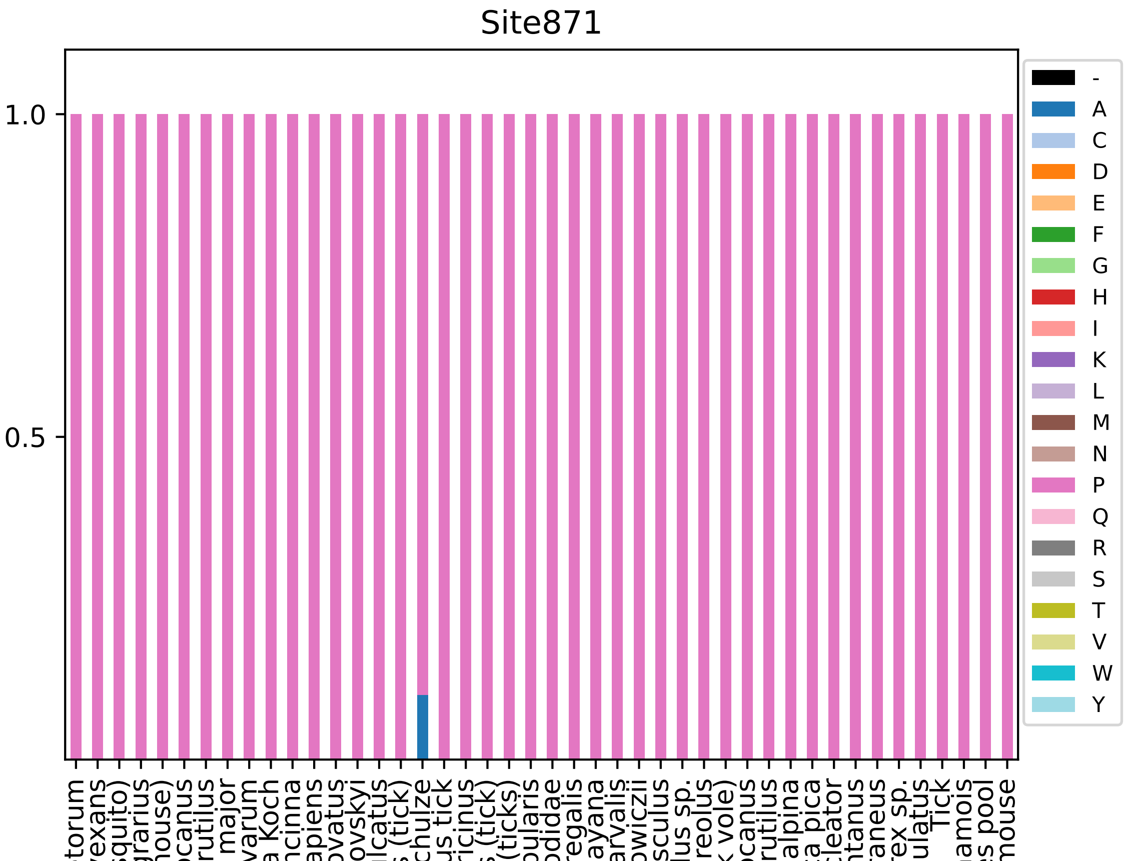
Site871
1.0
0.5
-
A
C
D
E
F
G
H
I
K
L
M
N
P
Q
R
S
T
V
W
Y

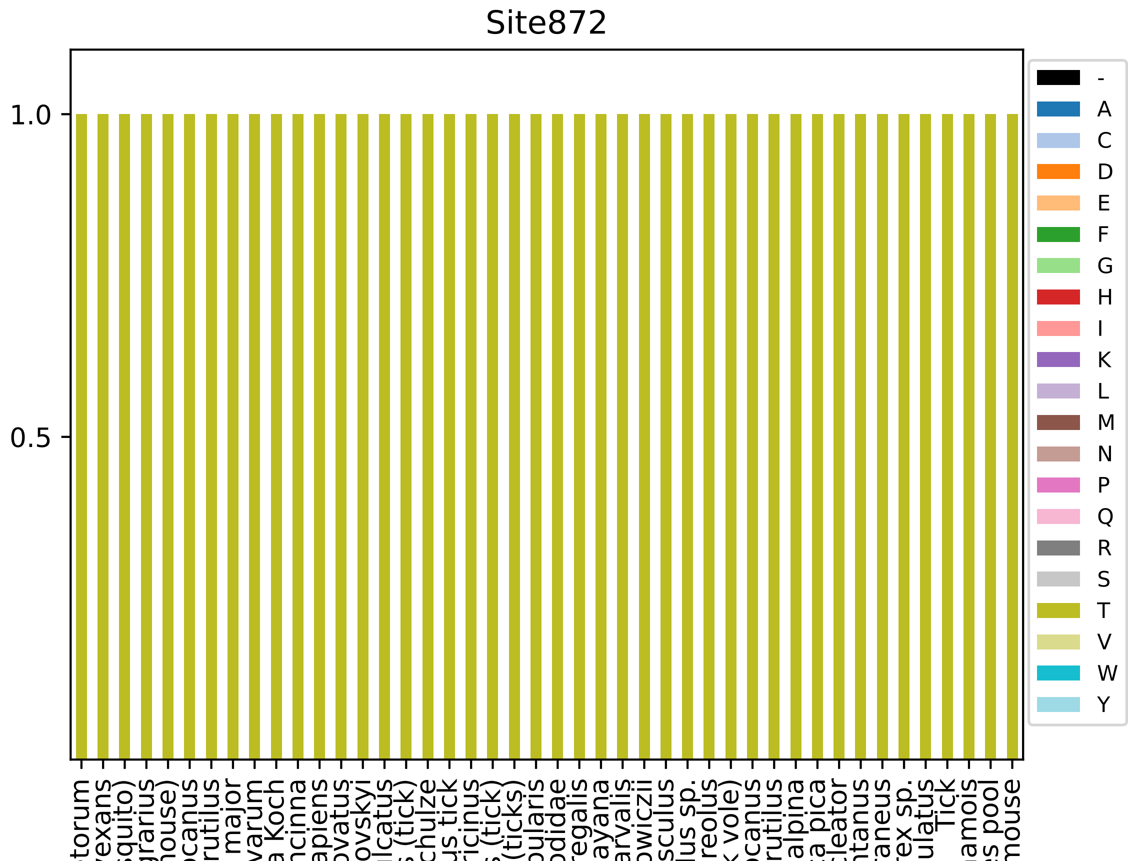

Site872
1.0
0.5
-
A
C
D
E
F
G
H
I
K
L
M
N
P
Q
R
S
T
V
W
Y

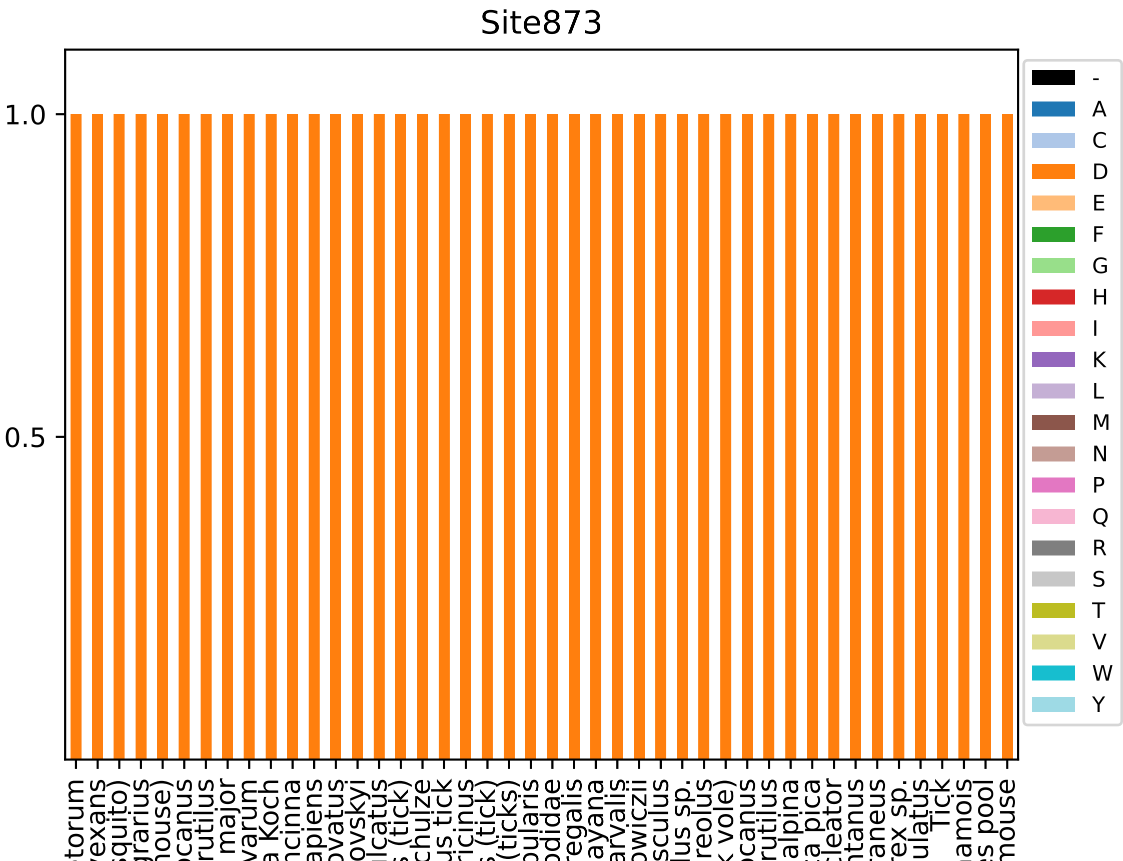
Site873
1.0
0.5
-
A
C
D
E
F
G
H
I
K
L
M
N
P
Q
R
S
T
V
W
Y

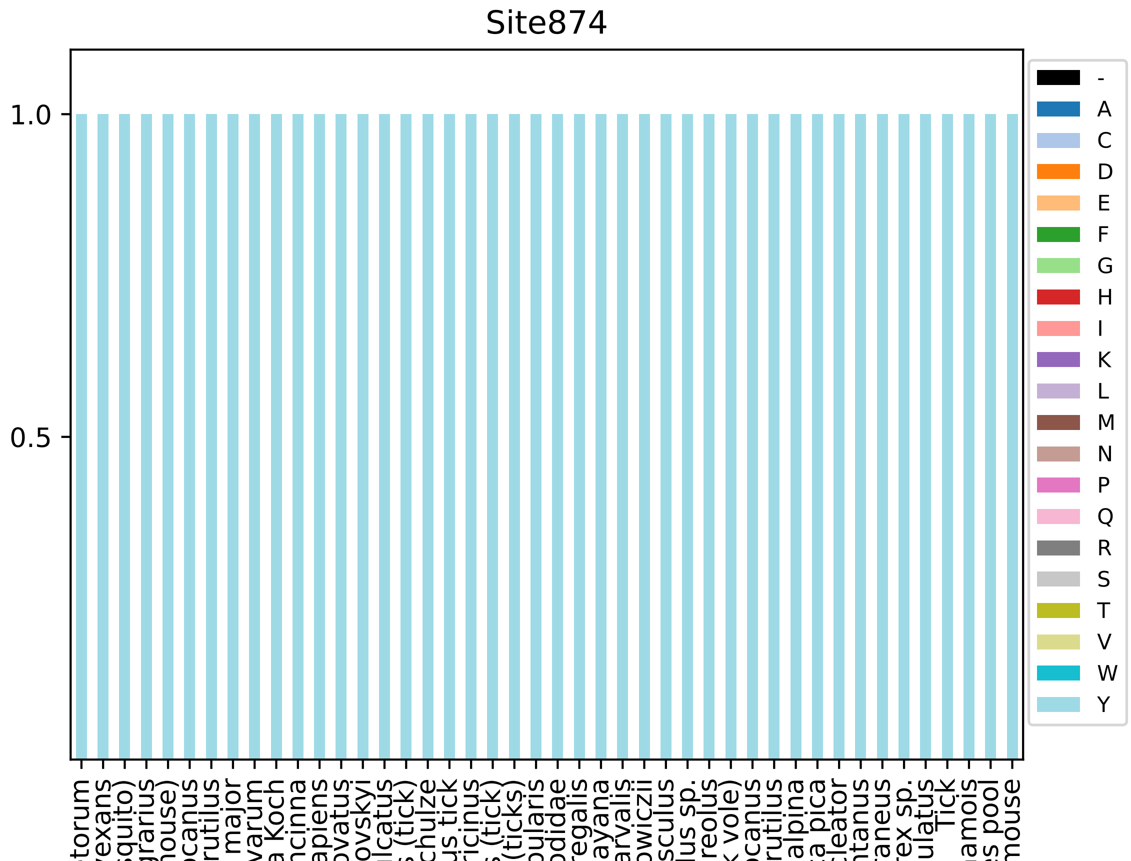
Site874
1.0
0.5
-
A
C
D
E
F
G
H
I
K
L
M
N
P
Q
R
S
T
V
W
Y

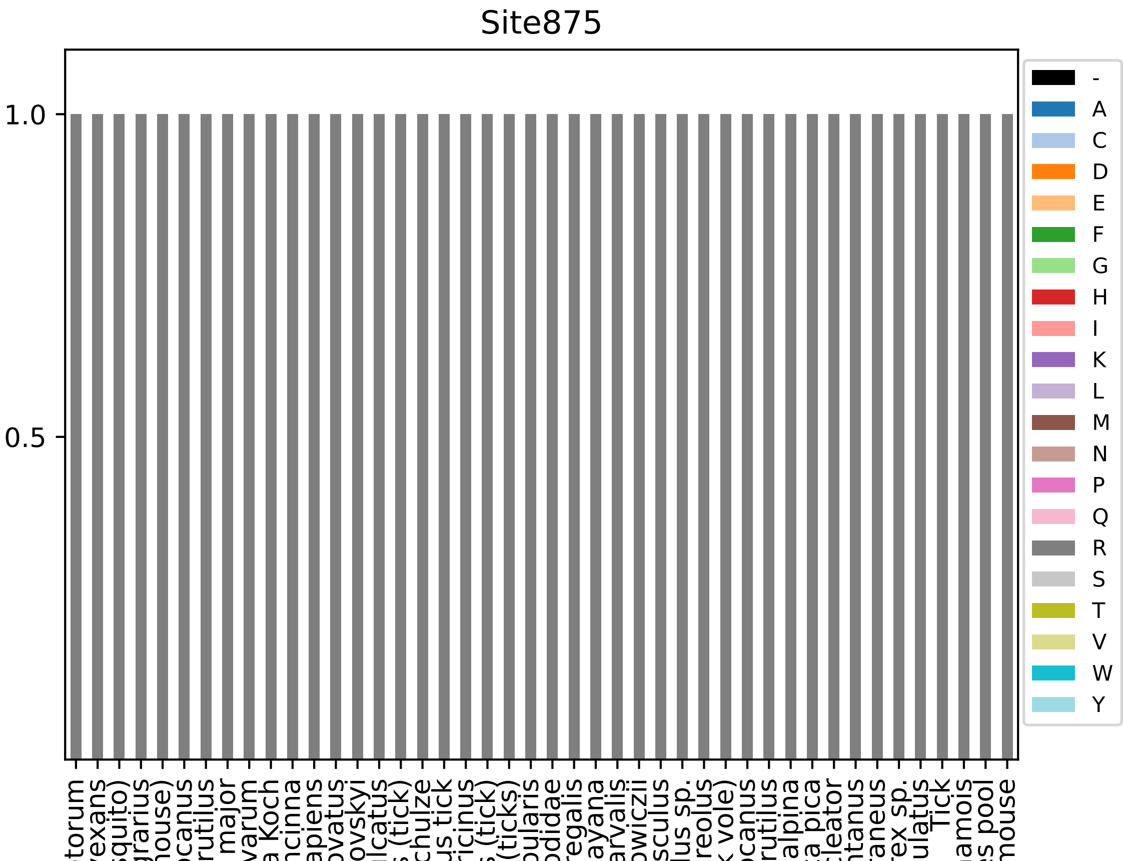
Site875
1.0
0.5
-
A
C
D
E
F
G
H
I
K
L
M
N
P
Q
R
S
T
V
W
Y
Tick

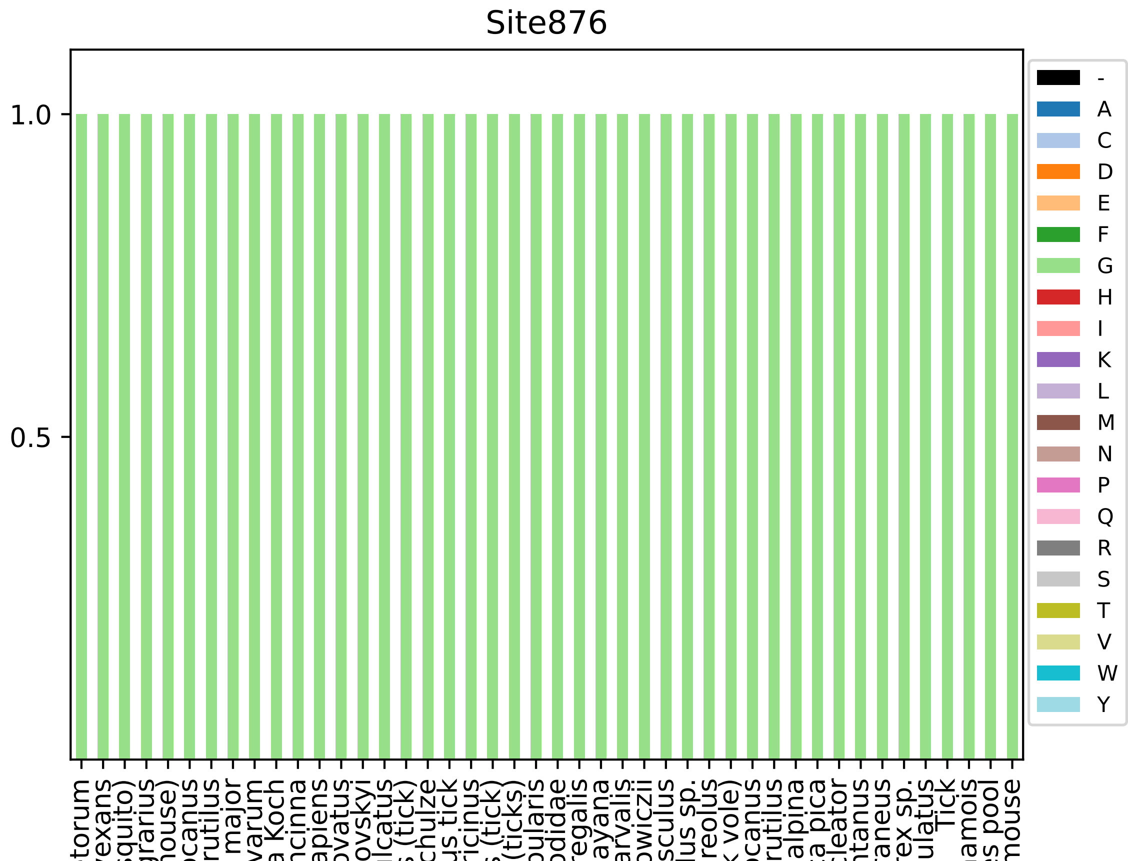
Site876
1.0
0.5
-
A
C
D
E
F
G
H
I
K
L
M
N
P
Q
R
S
T
V
W
Y

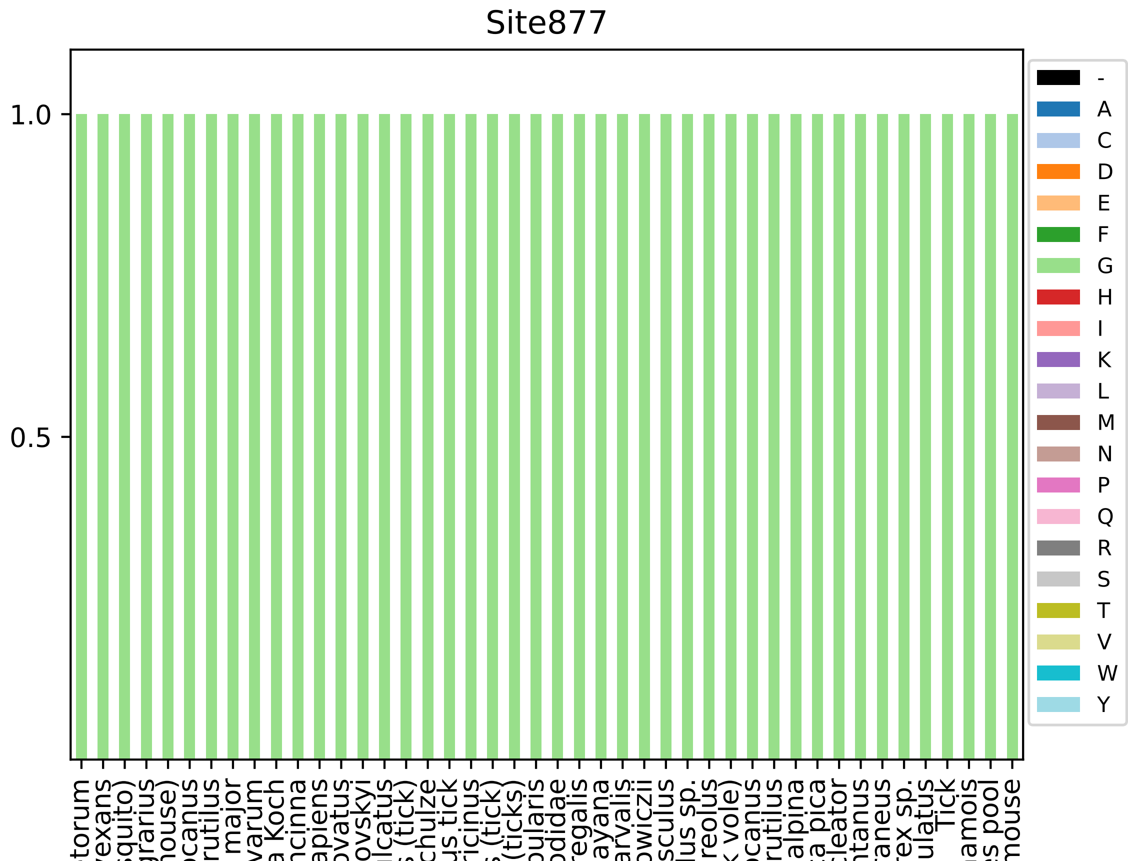
Site877
1.0
0.5
-
A
C
D
E
F
G
H
I
K
L
M
N
P
Q
R
S
T
V
W
Y

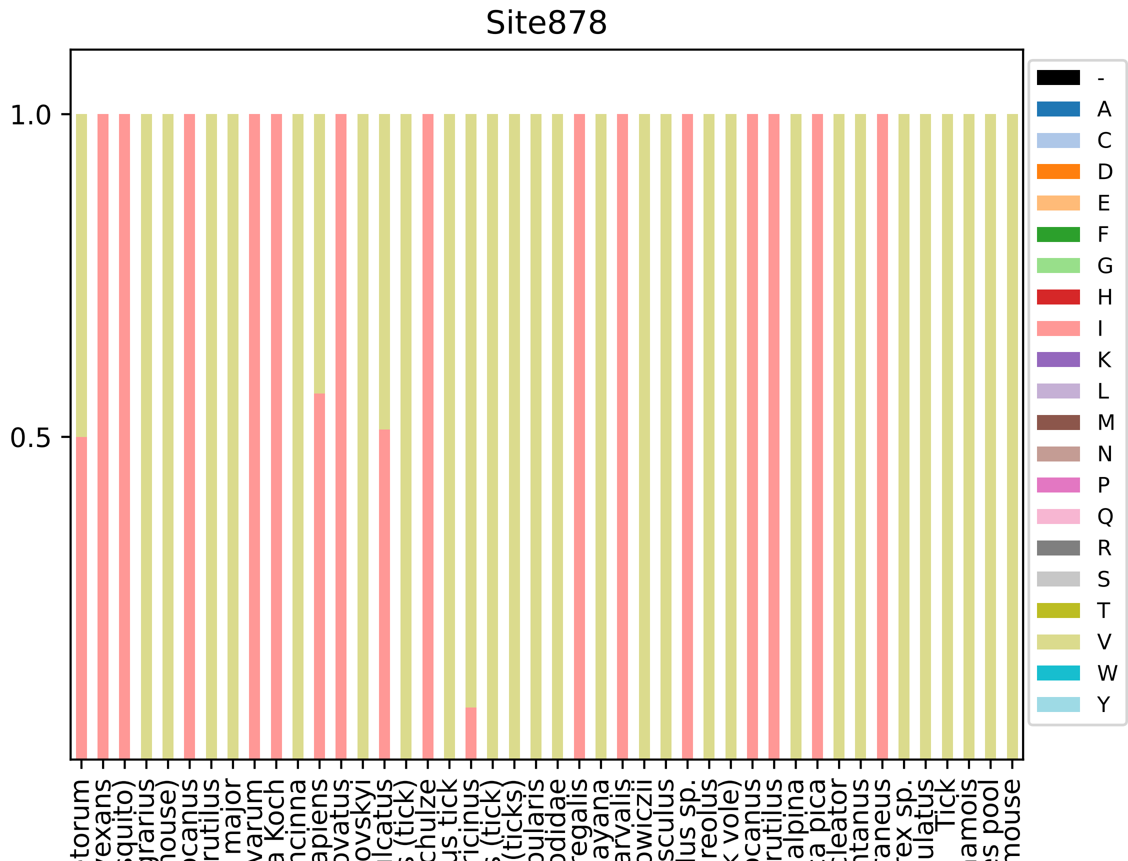

Site878
1.0
0.5
-
A
C
D
E
F
G
H
I
K
L
M
N
P
Q
R
S
T
V
W
Y

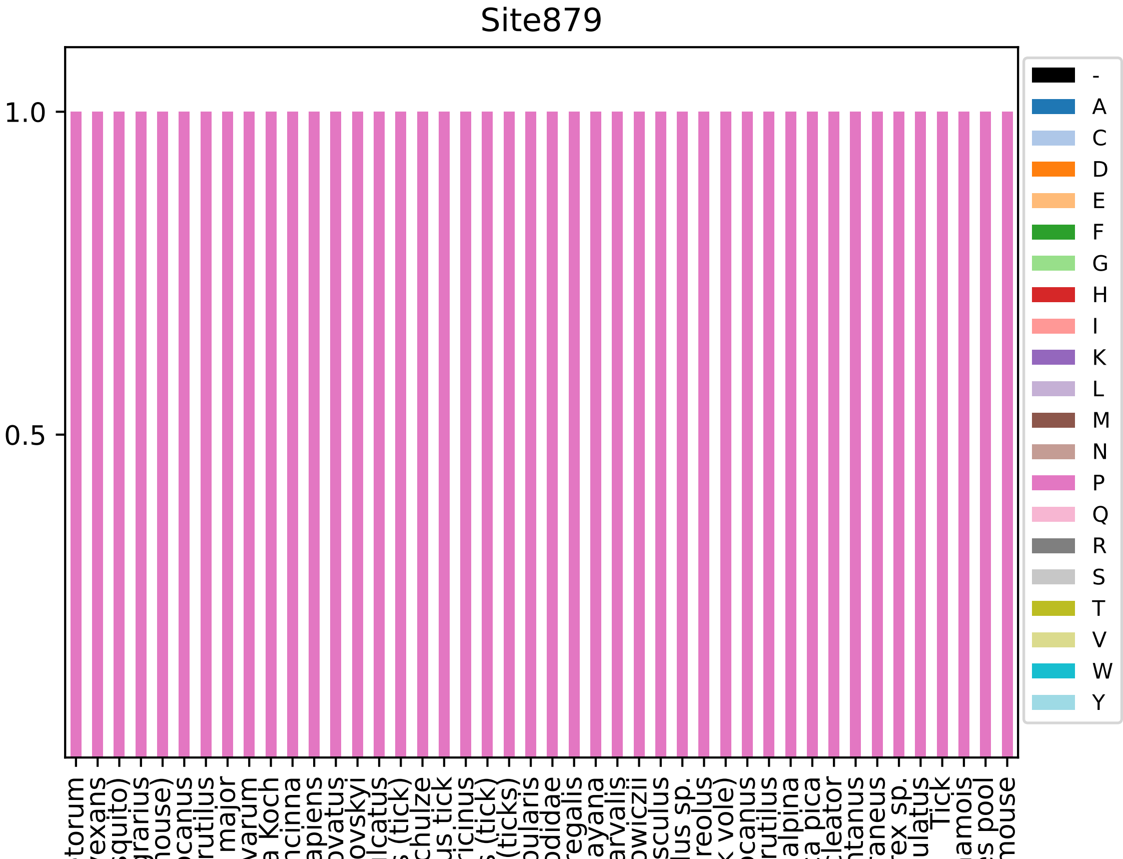

Site879
1.0
0.5
-
A
C
D
E
F
G
H
I
K
L
M
N
P
Q
R
S
T
V
W
Y

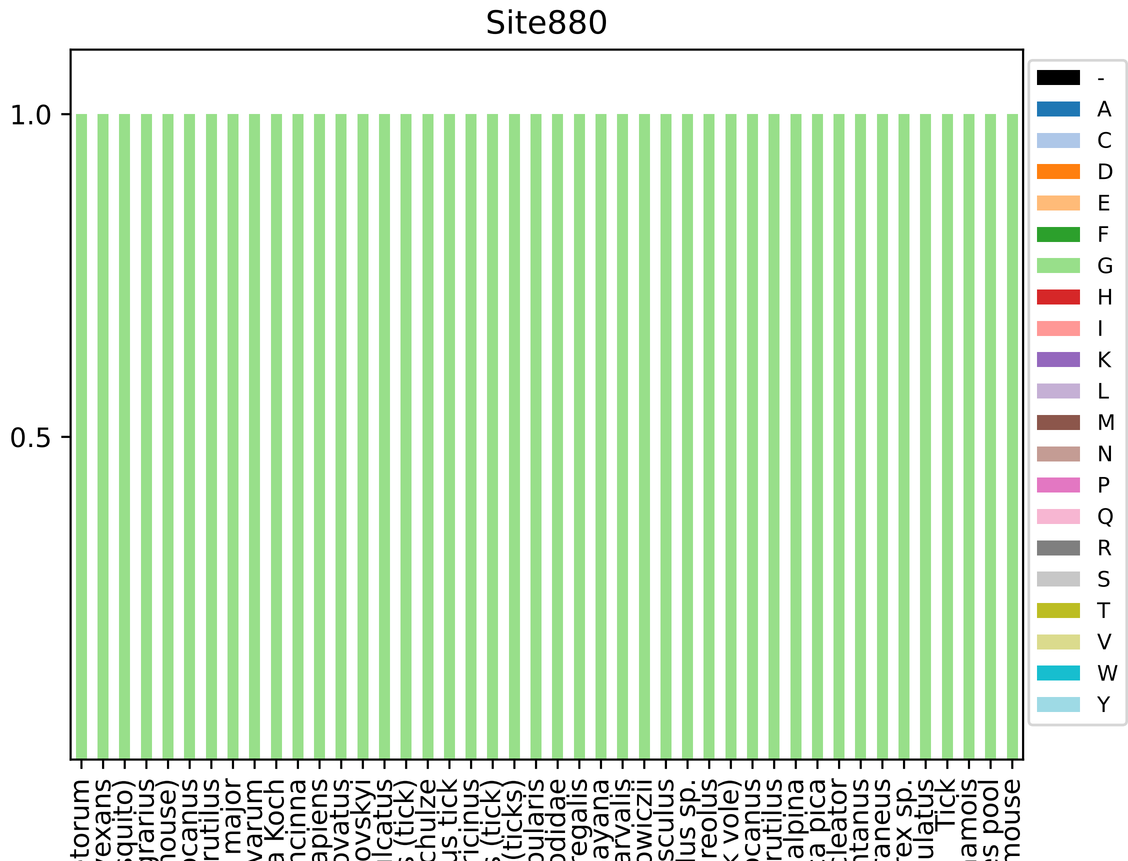
Site880
1.0
0.5
-
A
C
D
E
F
G
H
I
K
L
M
N
P
Q
R
S
T
V
W
Y
Tick

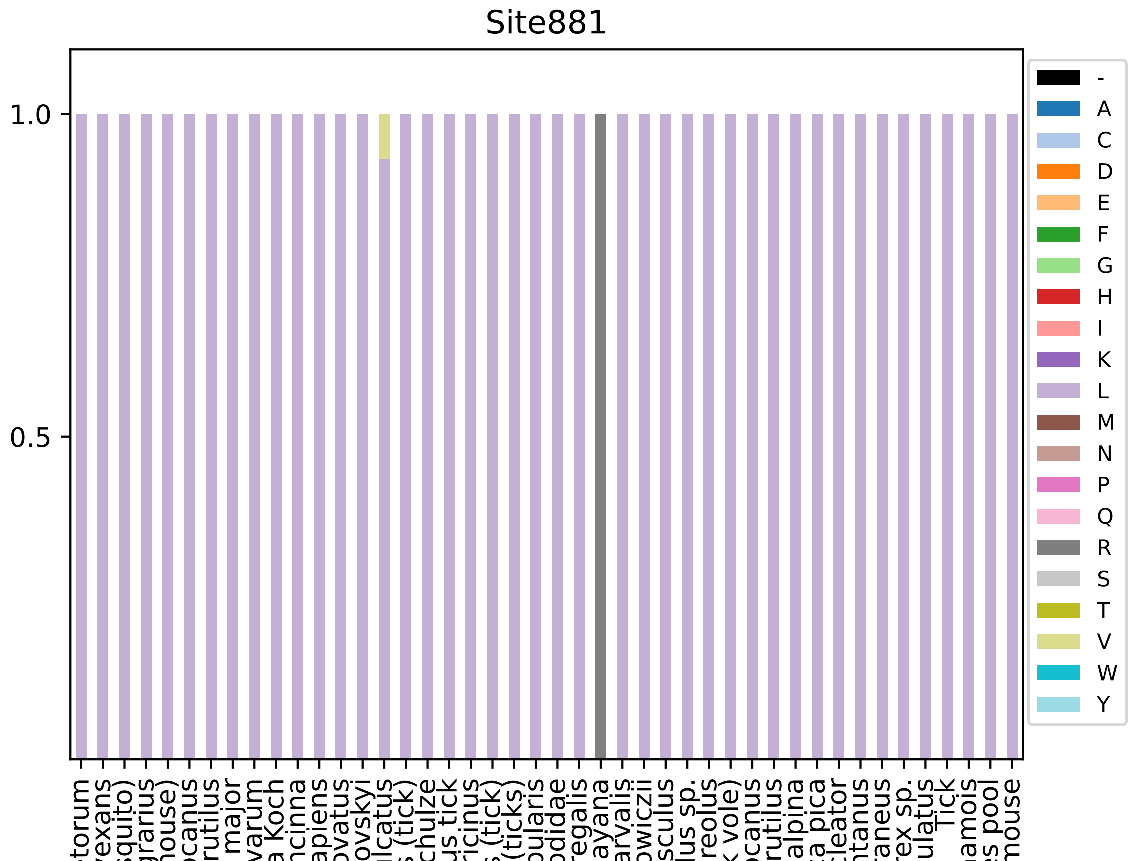

Site881
1.0
0.5
-
A
C
D
E
F
G
H
I
K
L
M
N
P
Q
R
S
T
V
W
Y

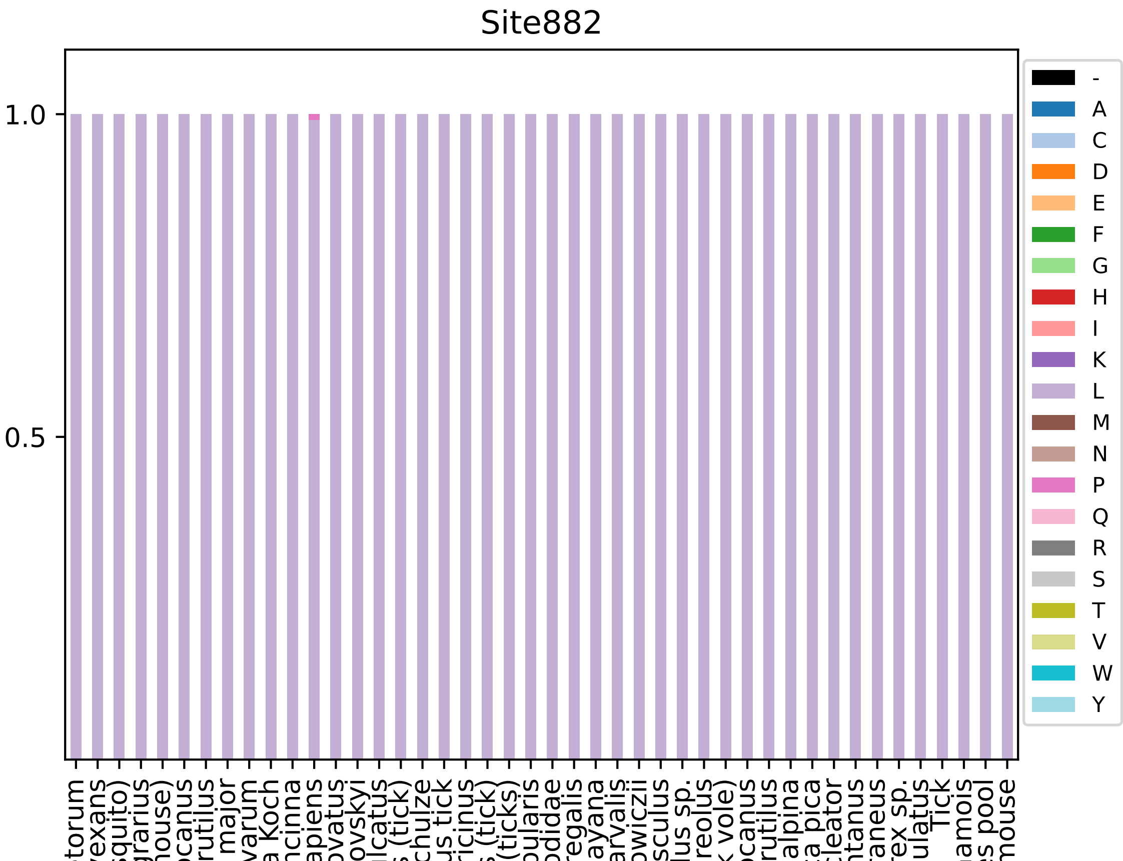
Site882
1.0
0.5
-
A
C
D
E
F
G
H
I
K
L
M
N
P
Q
R
S
T
V
W
Y

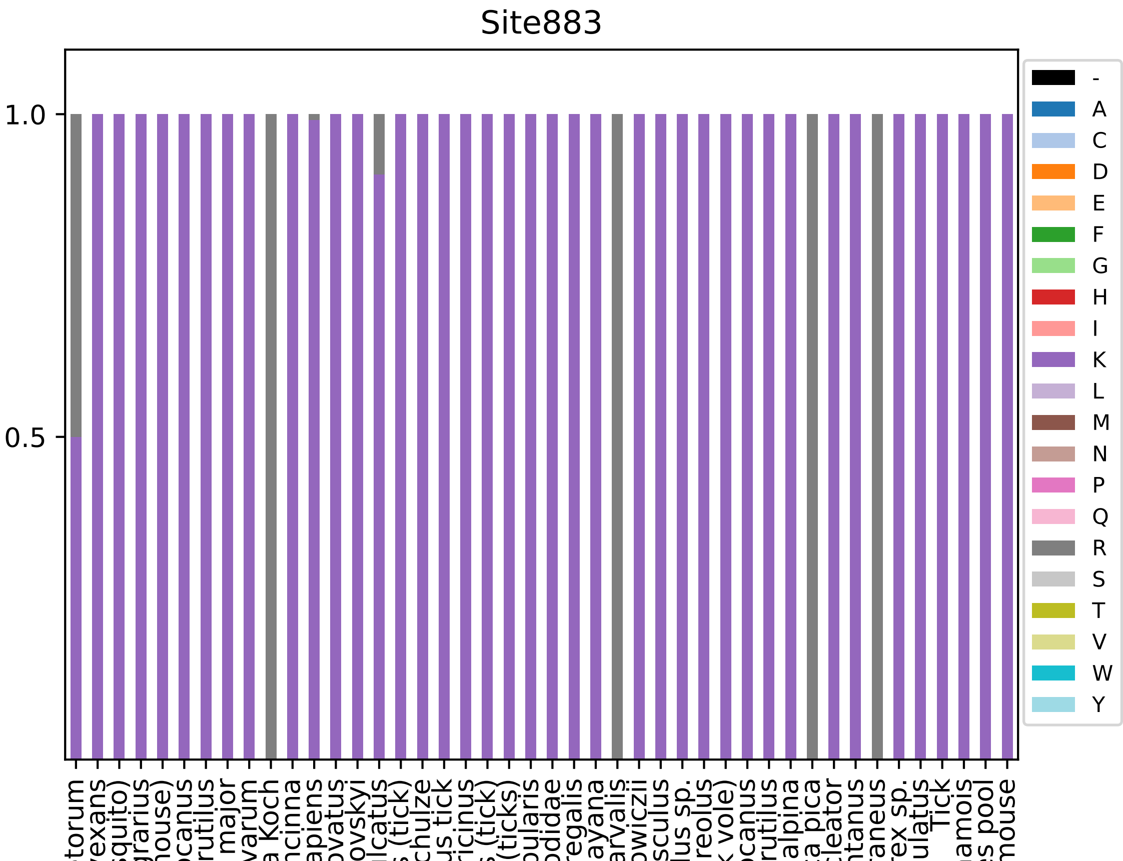

Site883
1.0
0.5
-
A
C
D
E
F
G
H
I
K
L
M
N
P
Q
R
S
T
V
W
Y

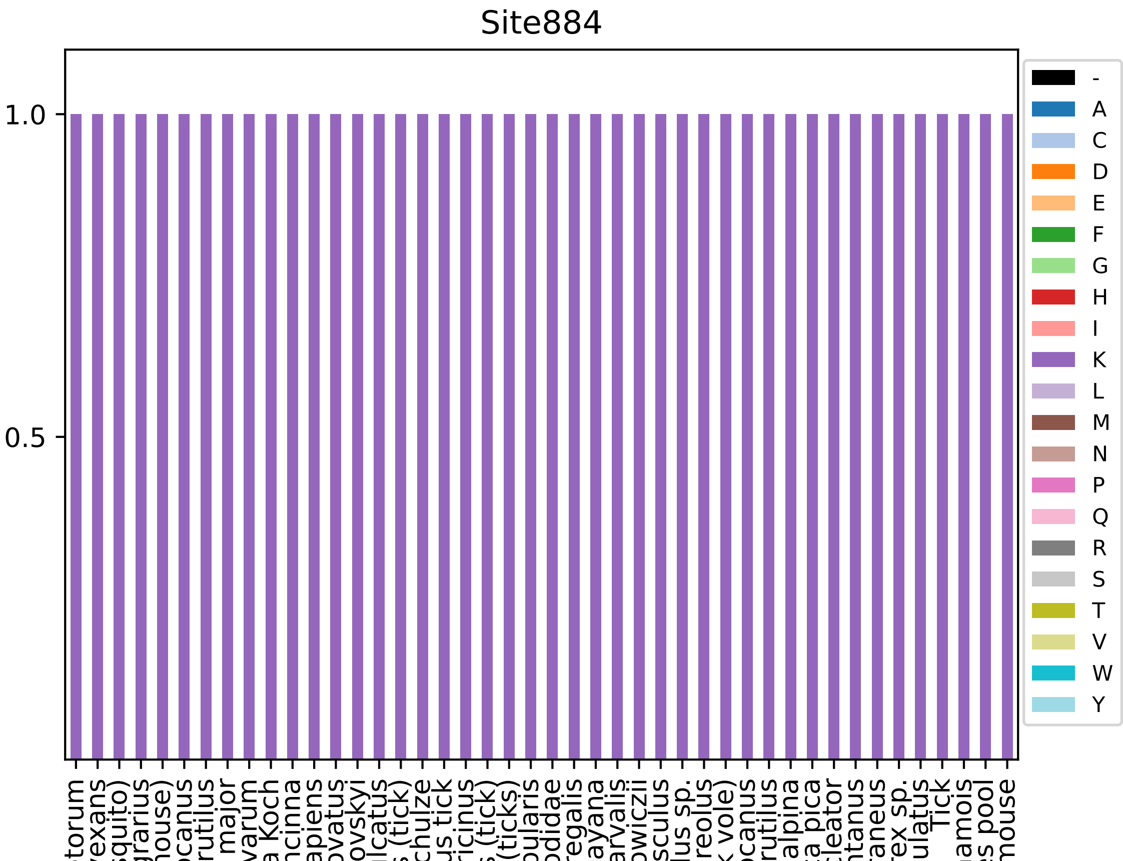

Site884
1.0
0.5
-
A
C
D
E
F
G
H
I
K
L
M
N
P
Q
R
S
T
V
W
Y

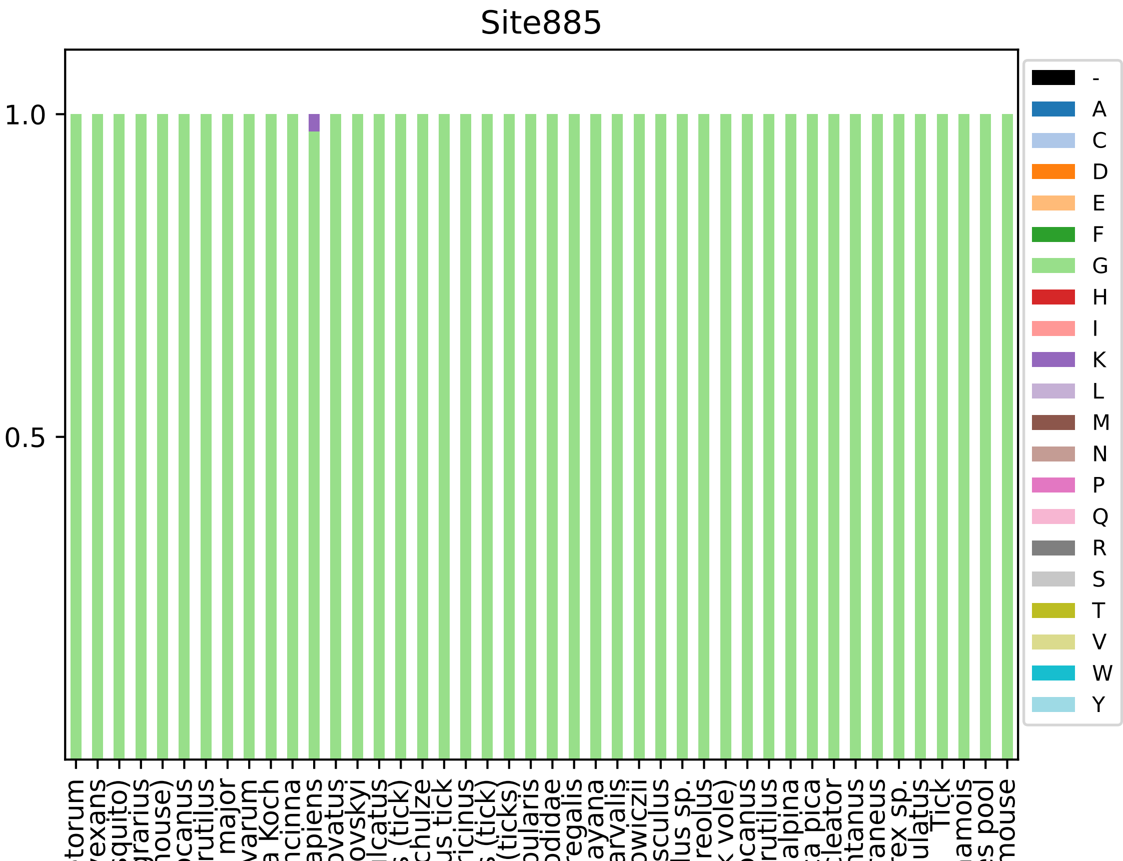
Site885
1.0
0.5
-
A
C
D
E
F
G
H
I
K
L
M
N
P
Q
R
S
T
V
W
Y

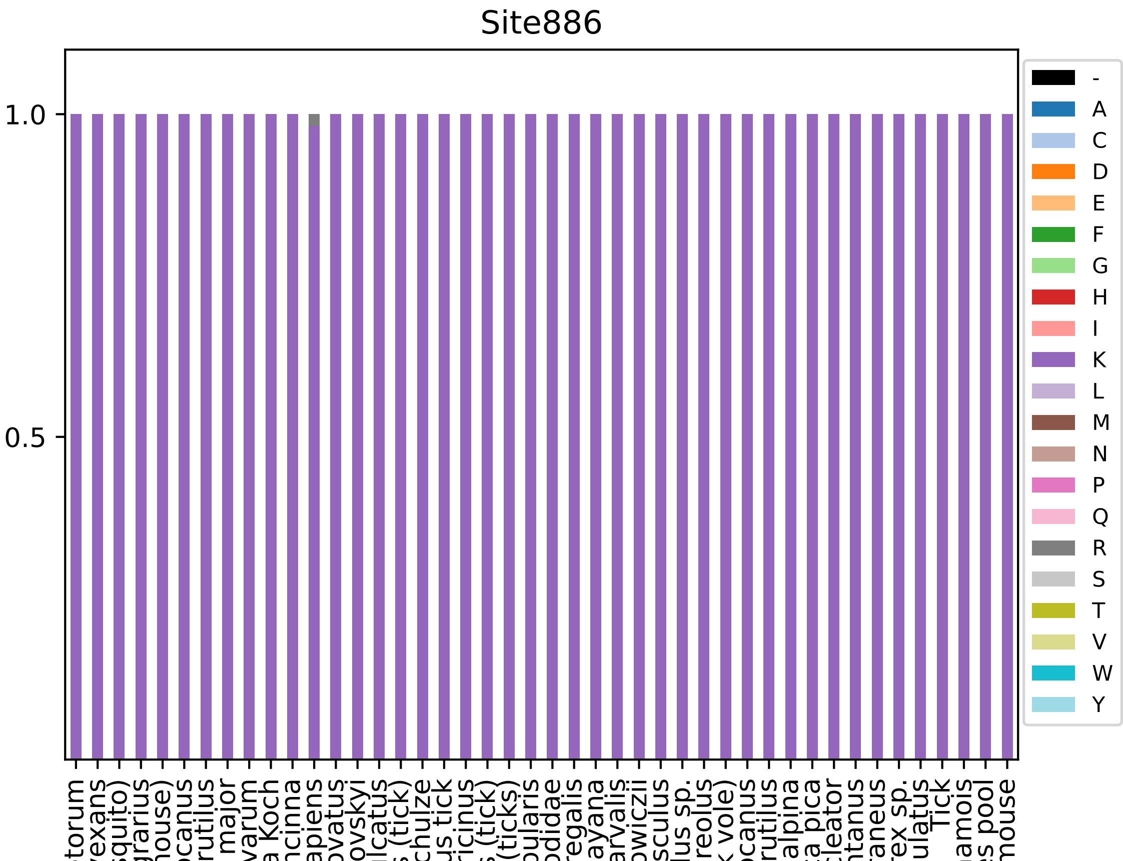
Site886
1.0
0.5
-
A
C
D
E
F
G
H
I
K
L
M
N
P
Q
R
S
T
V
W
Y

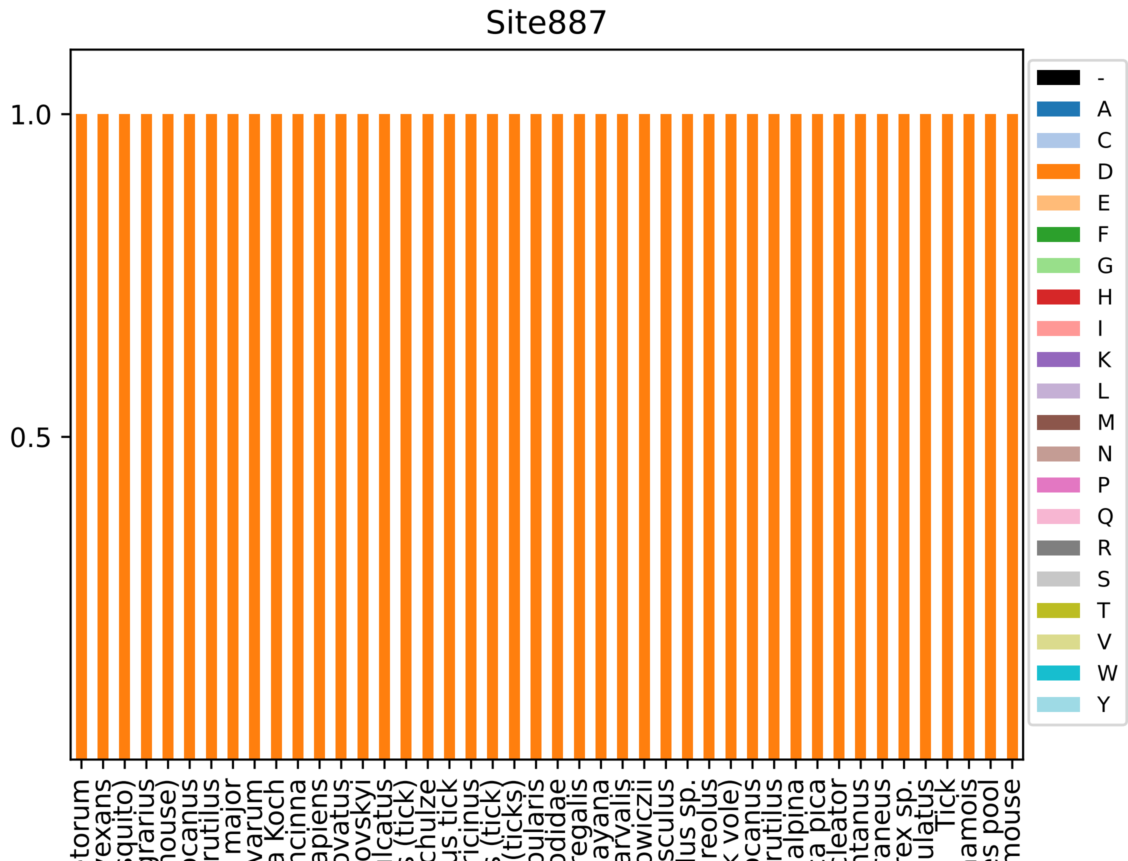
Site887
1.0
0.5
-
A
C
D
E
F
G
H
I
K
L
M
N
P
Q
R
S
T
V
W
Y

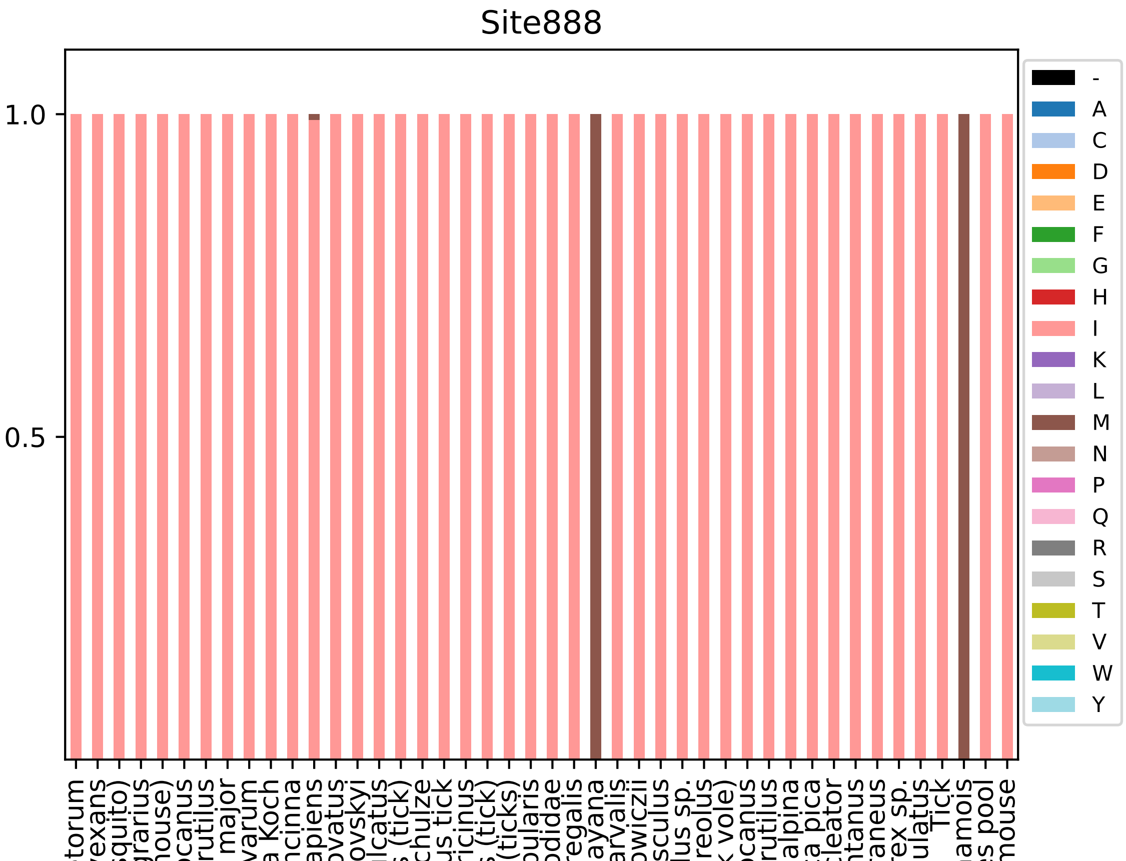
Site888
1.0
0.5
-
A
C
D
E
F
G
H
I
K
L
M
N
P
Q
R
S
T
V
W
Y

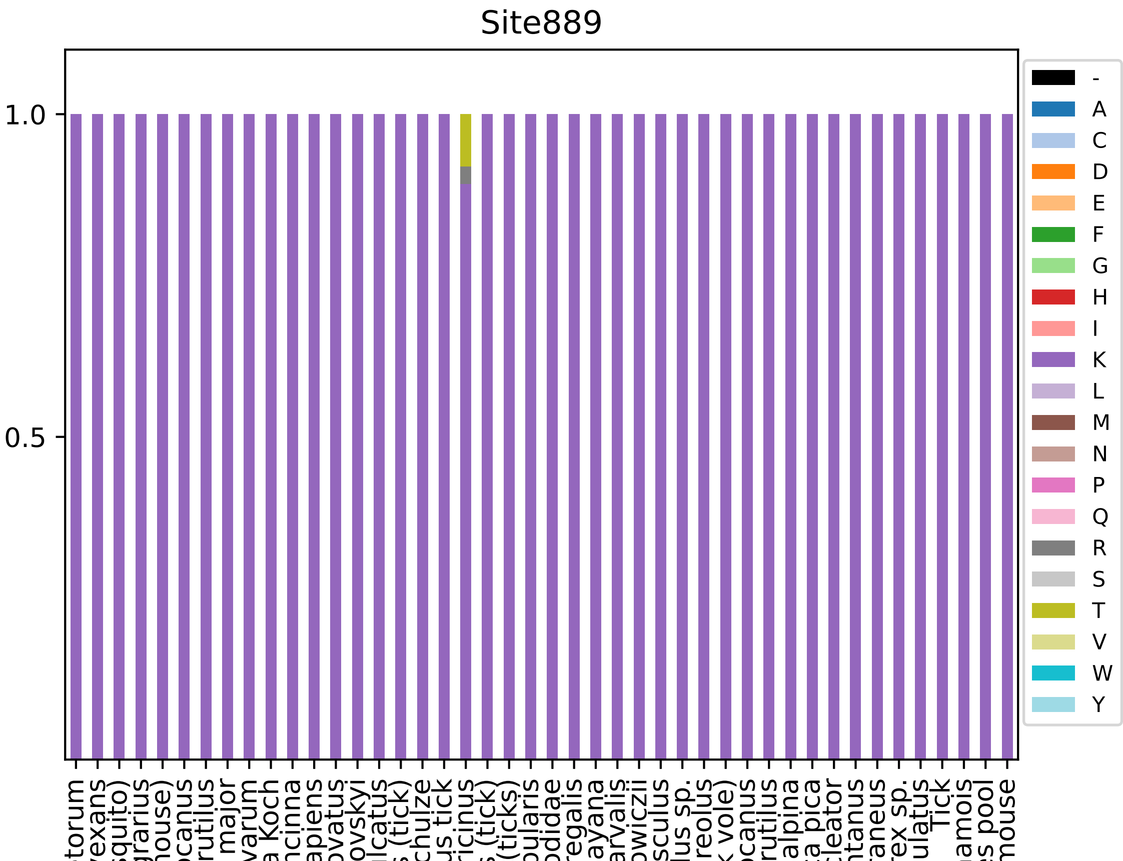
Site889
1.0
0.5
-
A
C
D
E
F
G
H
I
K
L
M
N
P
Q
R
S
T
V
W
Y

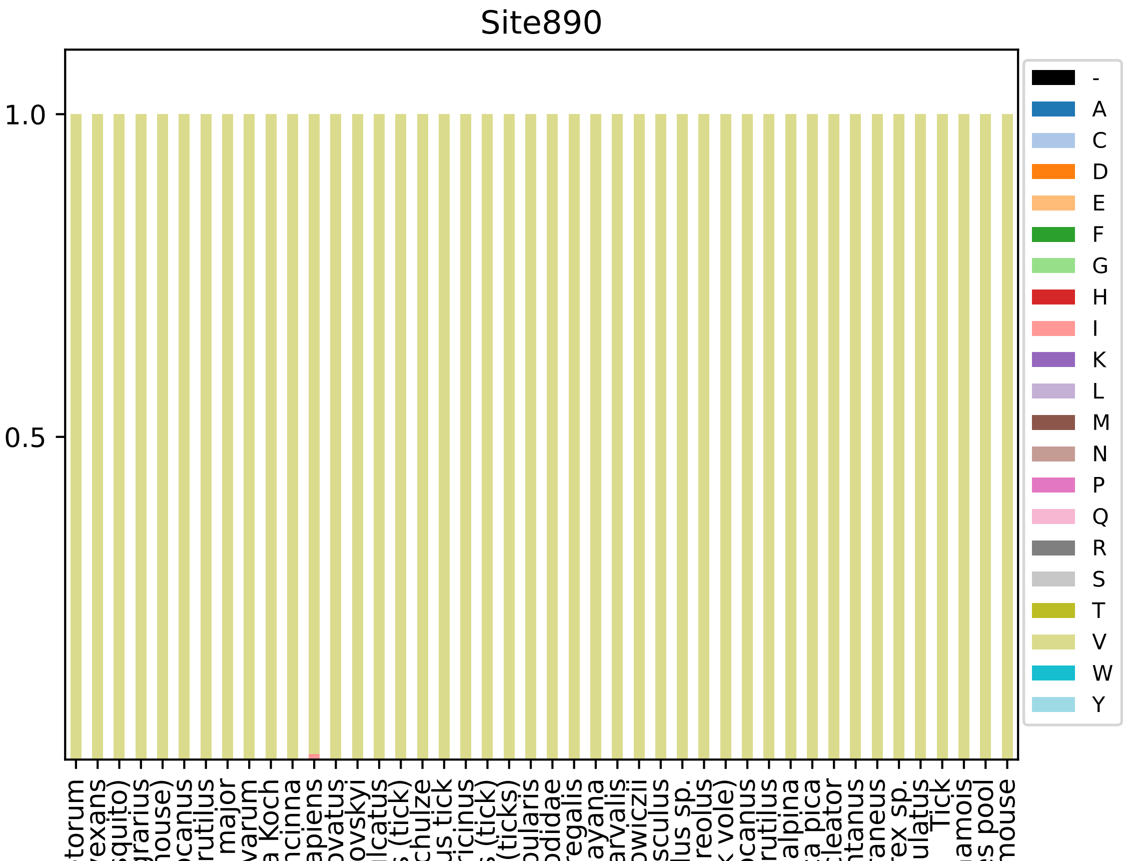
Site890
1.0
0.5
-
A
C
D
E
F
G
H
I
K
L
M
N
P
Q
R
S
T
V
W
Y

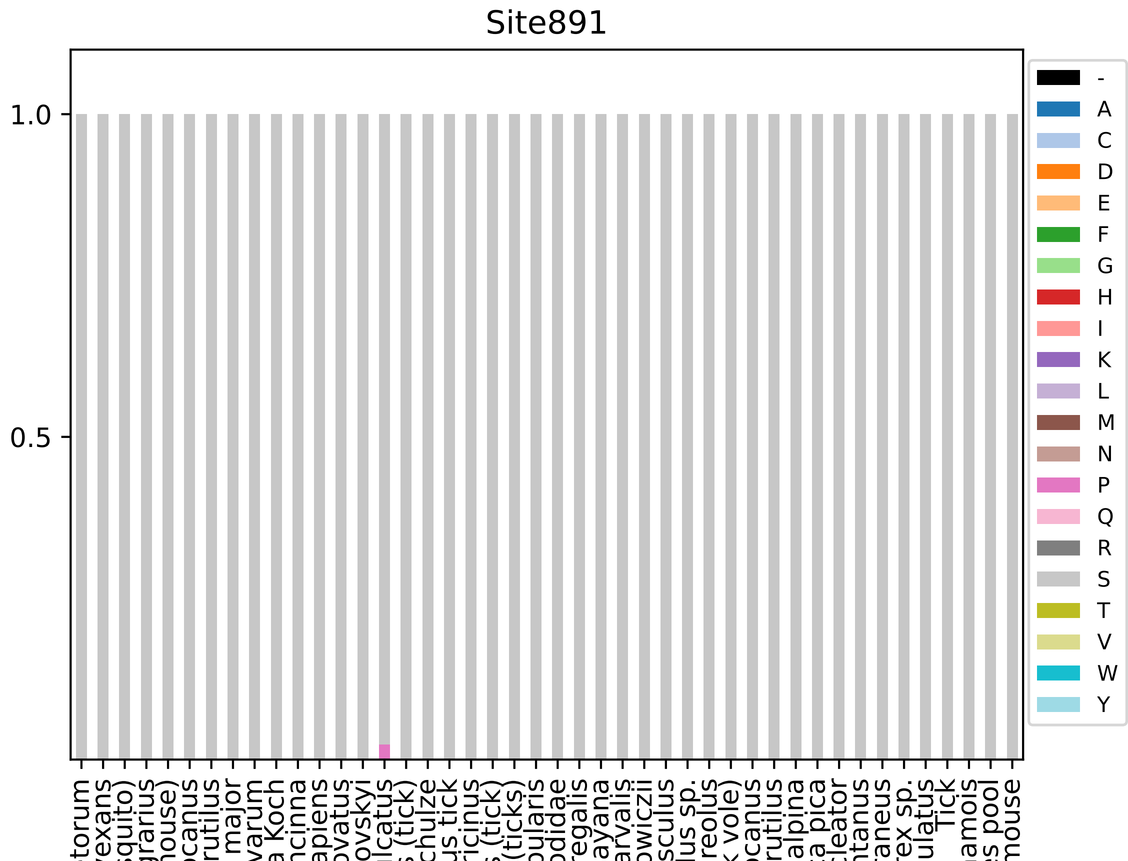

Site891
1.0
0.5
-
A
C
D
E
F
G
H
I
K
L
M
N
P
Q
R
S
T
V
W
Y

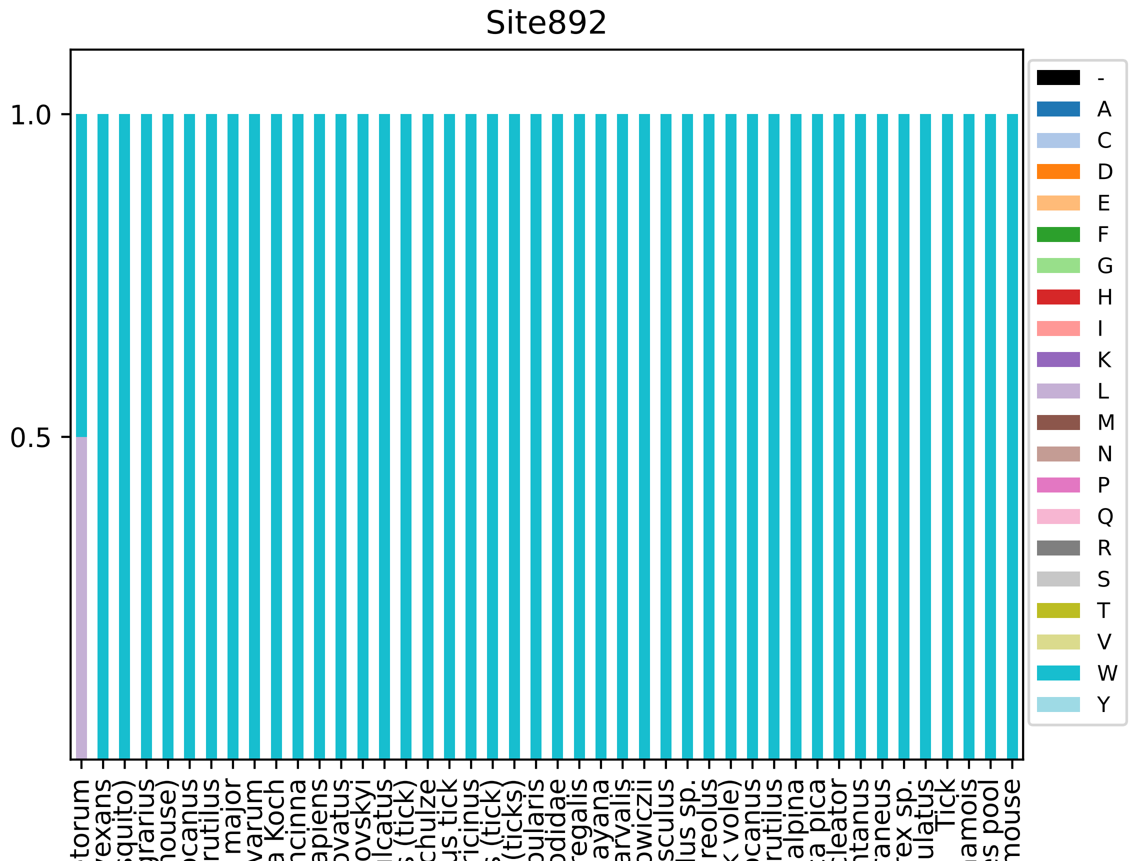
Site892
1.0
0.5
torum
vexans
squito)
grarius
nouse)
ocanus
rutilus
major
varum
a Koch
ncinna
apiens
ovatus
ovskyi
ulcatus
s (tick)
chulze
us tick
ricinus
s (tick)
(ticks)
pularis
podidae
regalis
ayana
arvalis
owiczii
sculus
lus sp.
reolus
k vole)
ocanus
rutilus
alpina
ca pica
cleator
ntanus
raneus
rex sp.
ulatus
Tick
amois
es pool
mouse
-
A
C
D
E
F
G
H
I
K
L
M
N
P
Q
R
S
T
V
W
Y

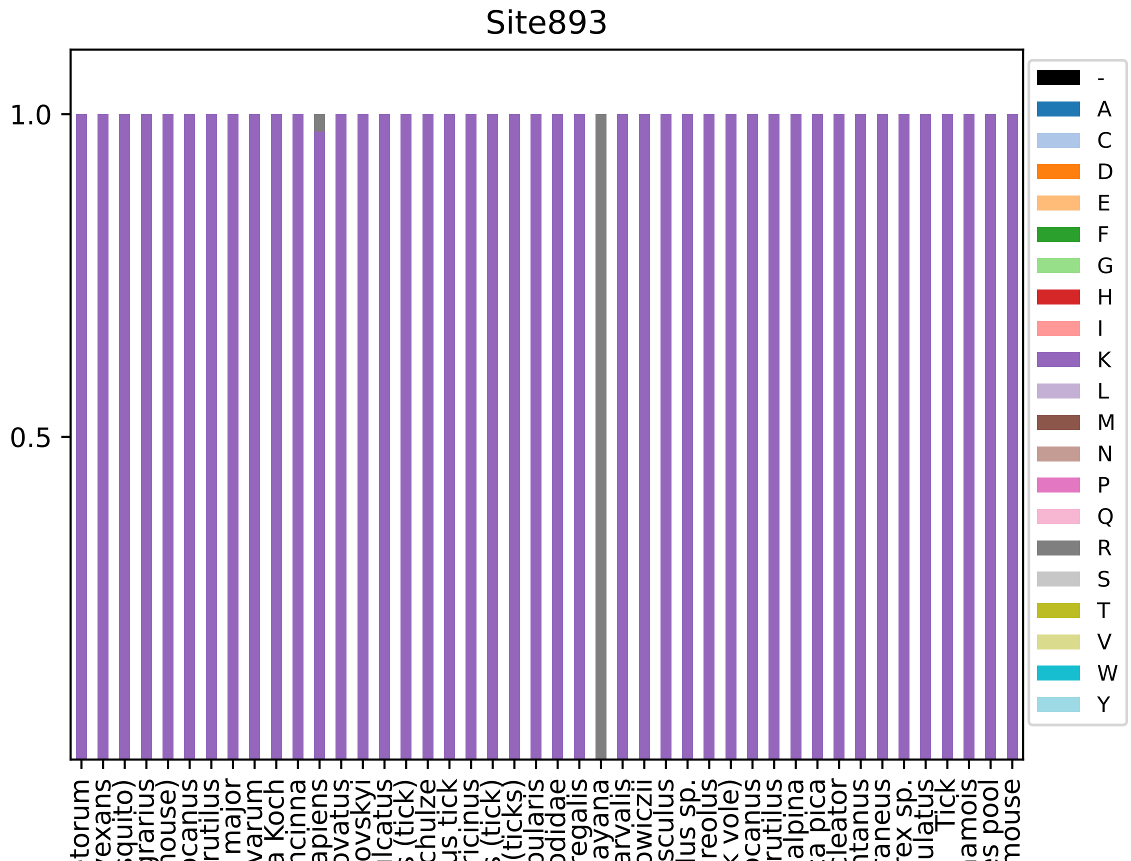
Site893
1.0
0.5
-
A
C
D
E
F
G
H
I
K
L
M
N
P
Q
R
S
T
V
W
Y

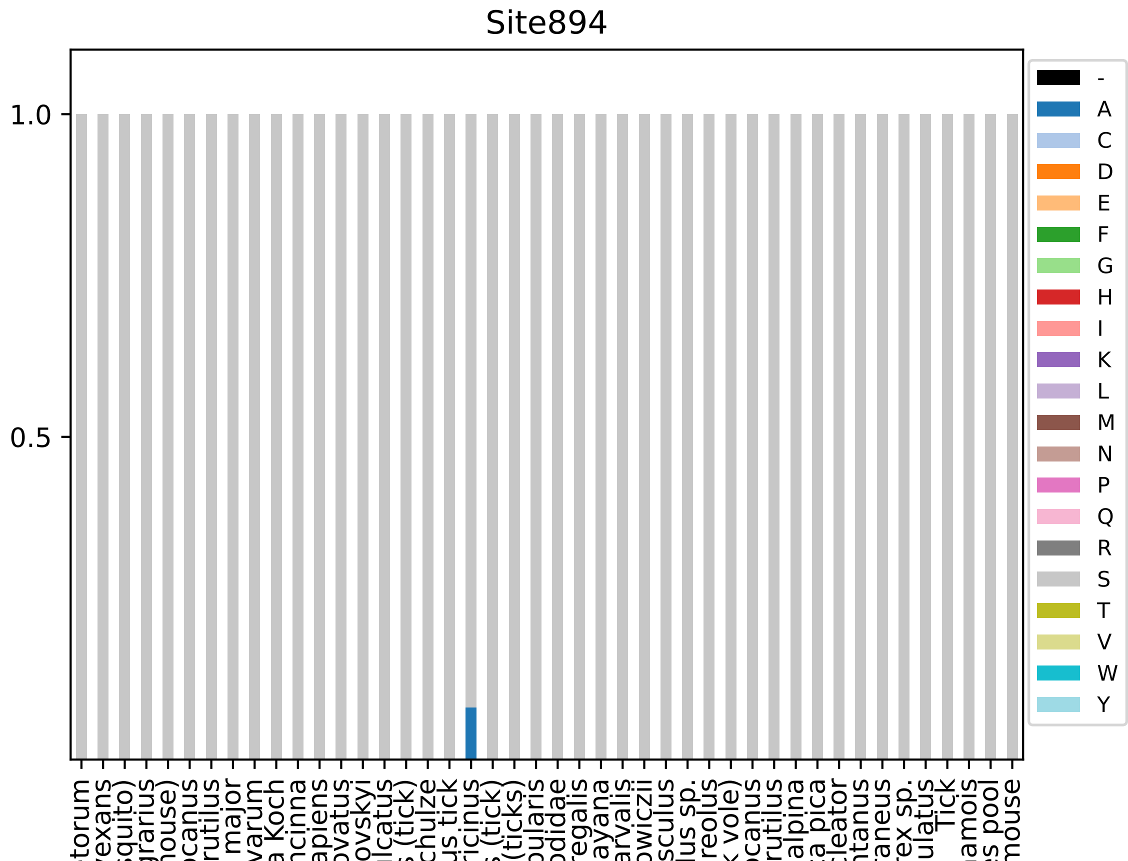
Site894
1.0
0.5
-
A
C
D
E
F
G
H
I
K
L
M
N
P
Q
R
S
T
V
W
Y

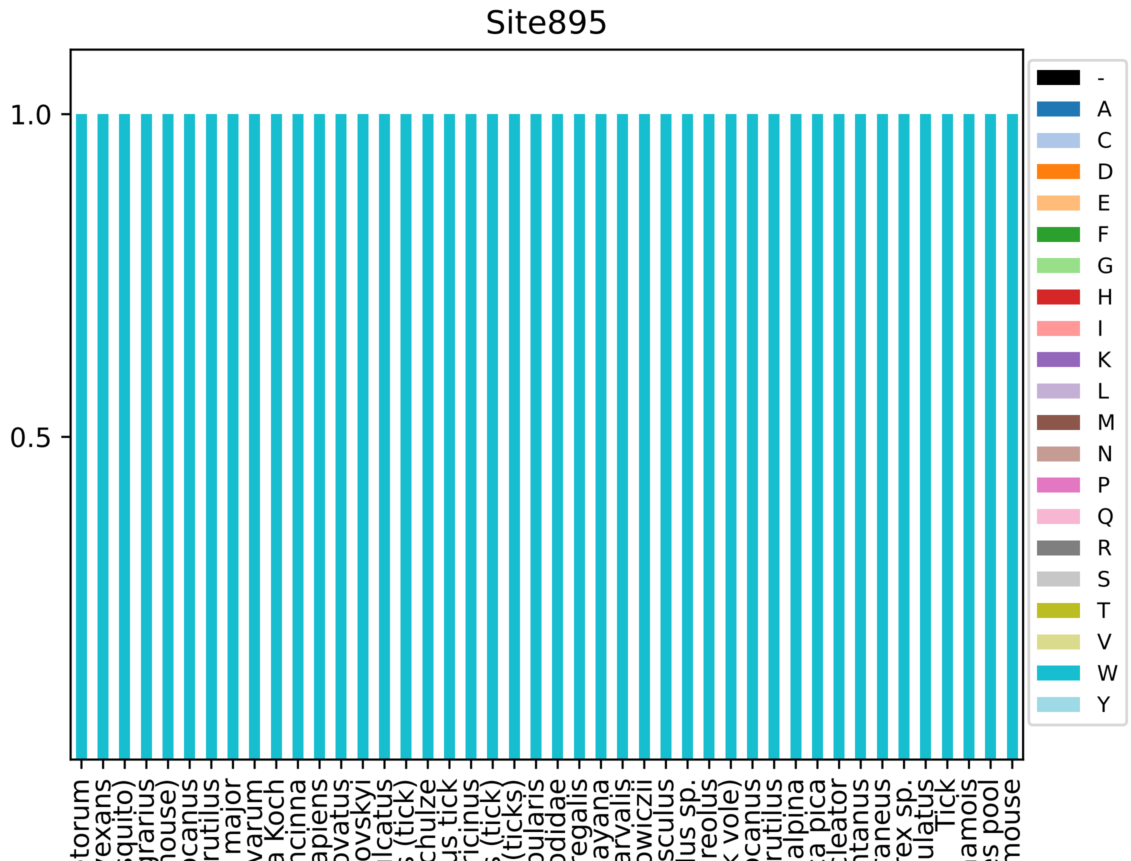

Site895
1.0
0.5
-
A
C
D
E
F
G
H
I
K
L
M
N
P
Q
R
S
T
V
W
Y

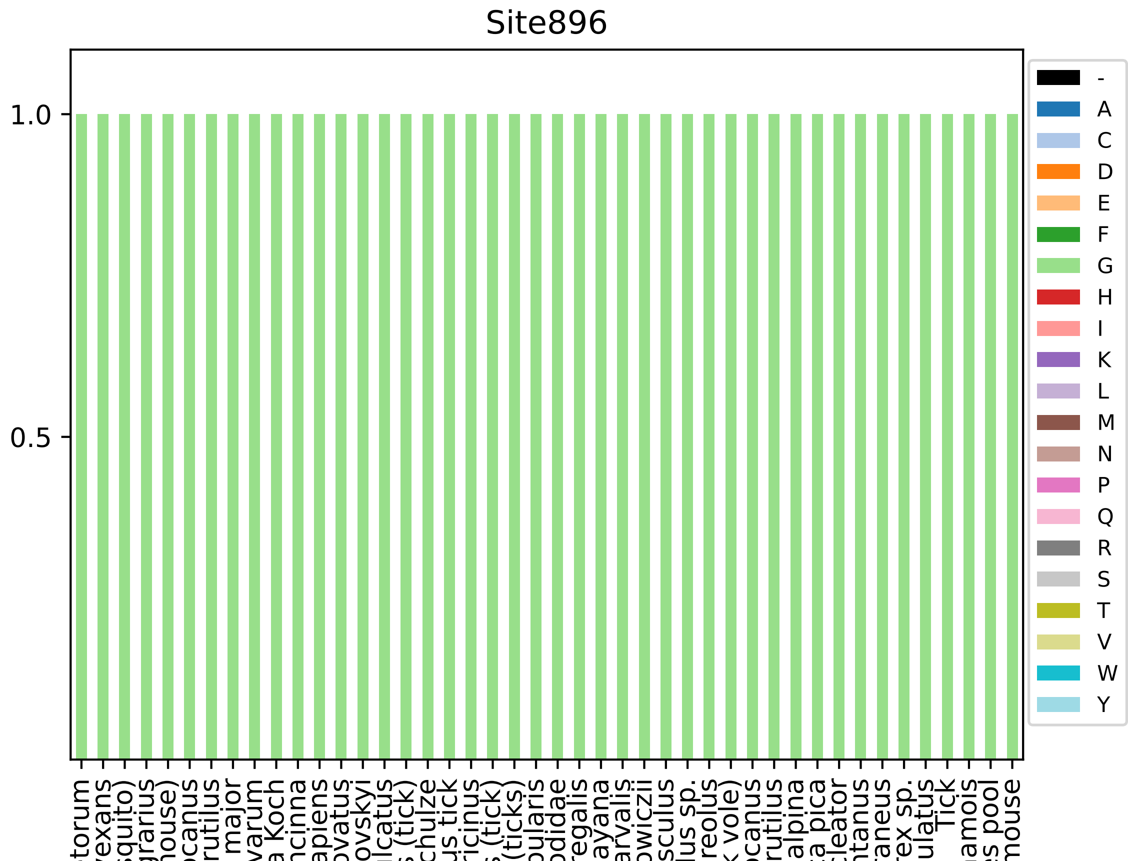
Site896
1.0
0.5
-
A
C
D
E
F
G
H
I
K
L
M
N
P
Q
R
S
T
V
W
Y

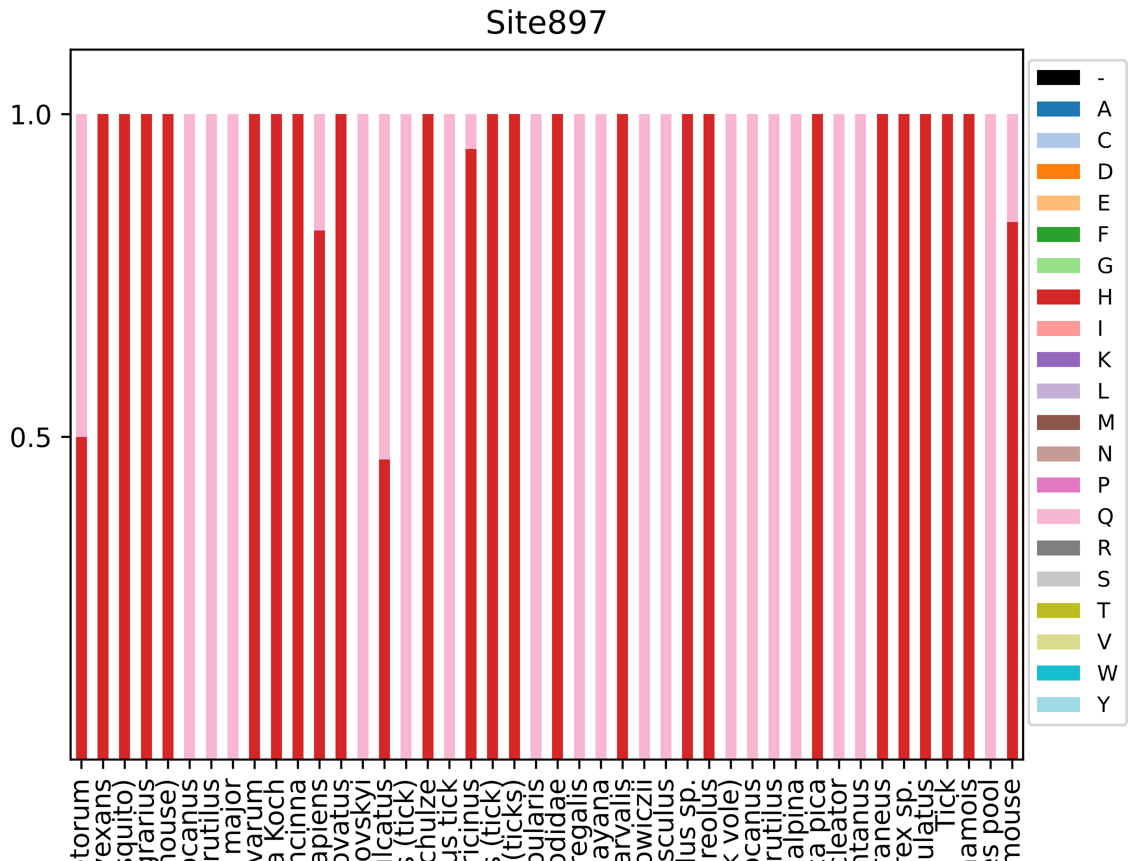

Site897
1.0
0.5
-
A
C
D
E
F
G
H
I
K
L
M
N
P
Q
R
S
T
V
W
Y

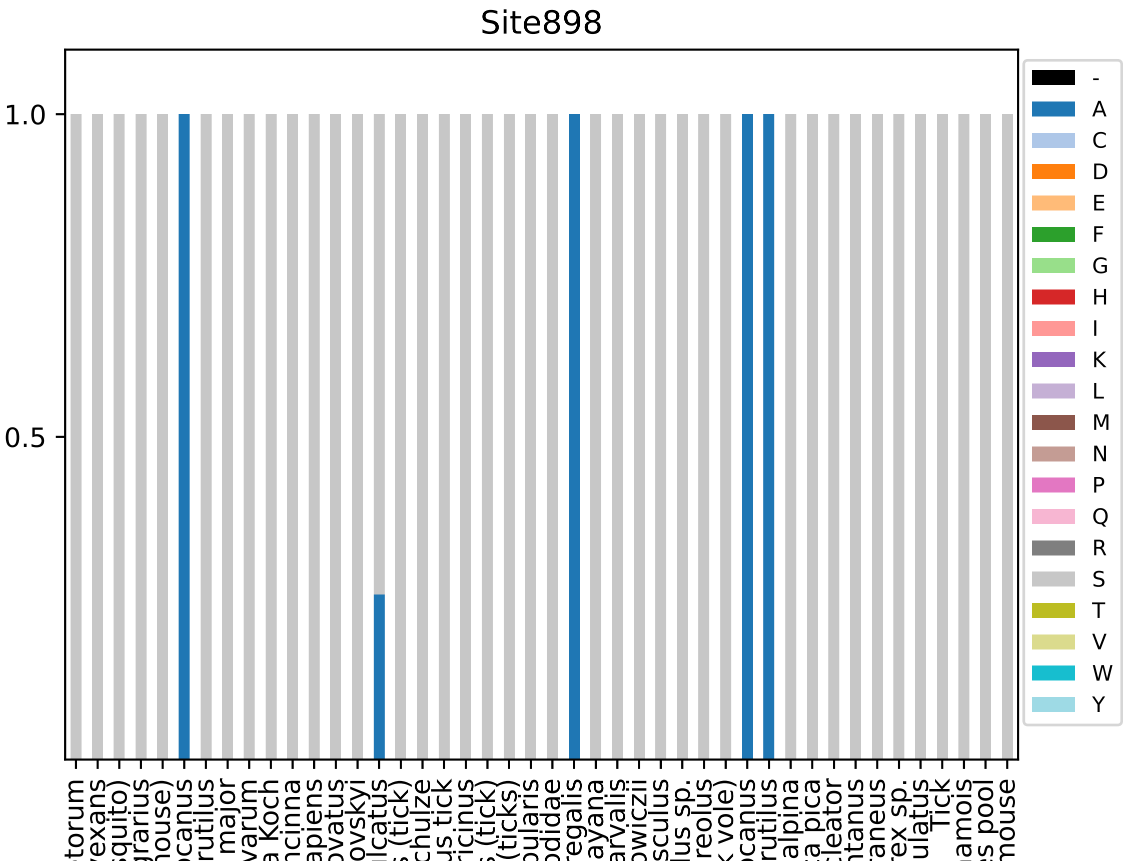

Site898
1.0
0.5
-
A
C
D
E
F
G
H
I
K
L
M
N
P
Q
R
S
T
V
W
Y

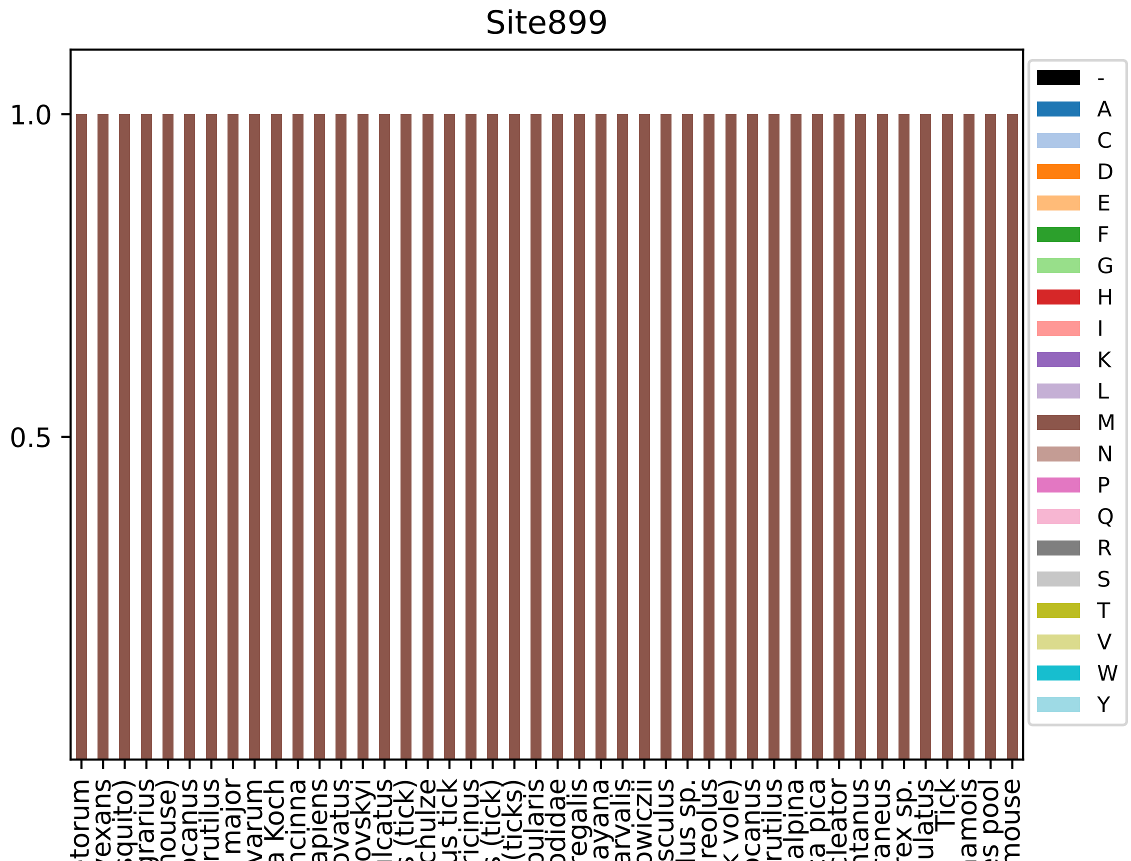
Site899
1.0
0.5
-
A
C
D
E
F
G
H
I
K
L
M
N
P
Q
R
S
T
V
W
Y

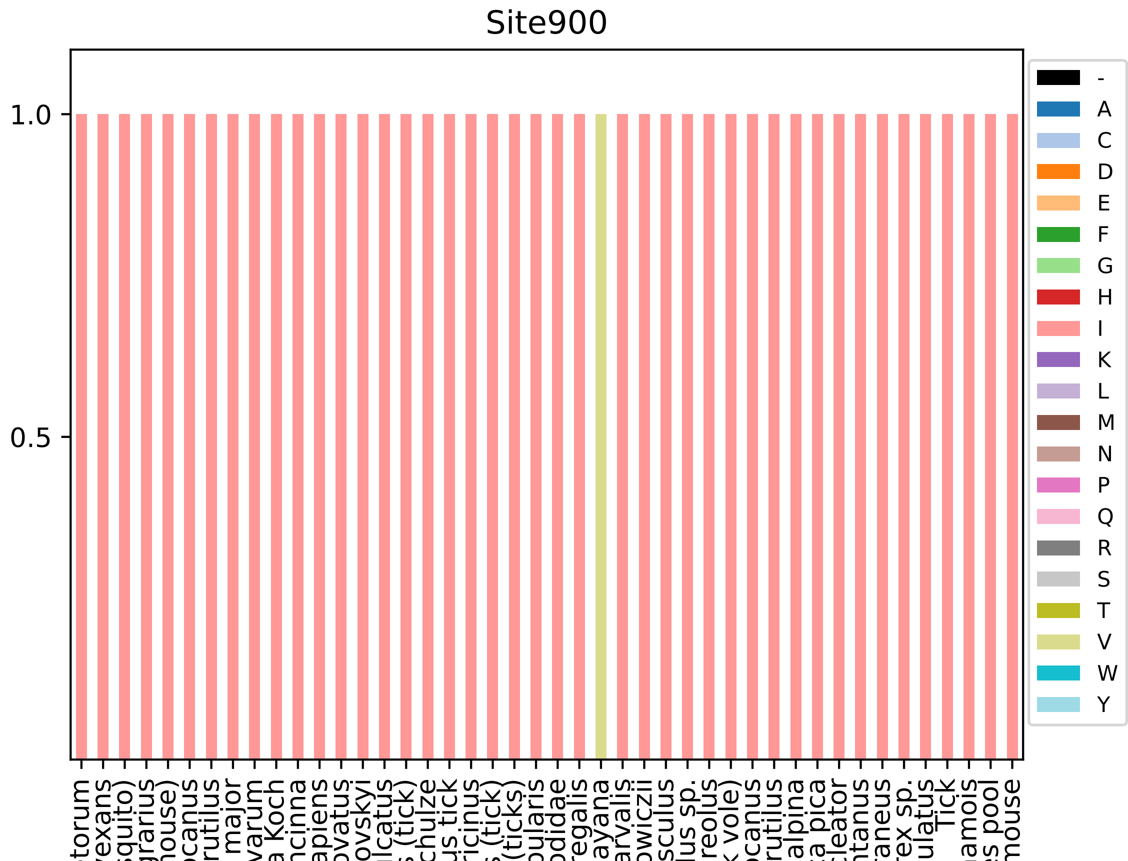
Site900
1.0
0.5
-
A
C
D
E
F
G
H
I
K
L
M
N
P
Q
R
S
T
V
W
Y

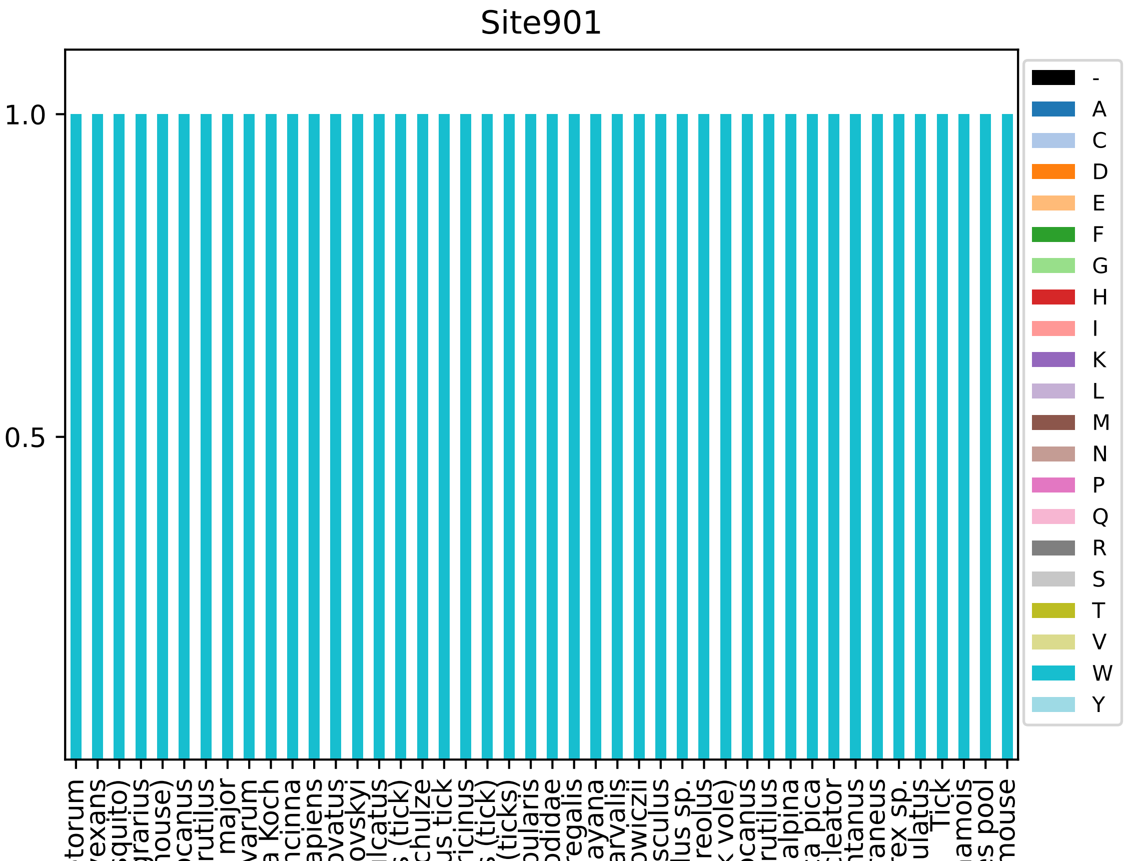
Site901
1.0
0.5
-
A
C
D
E
F
G
H
I
K
L
M
N
P
Q
R
S
T
V
W
Y

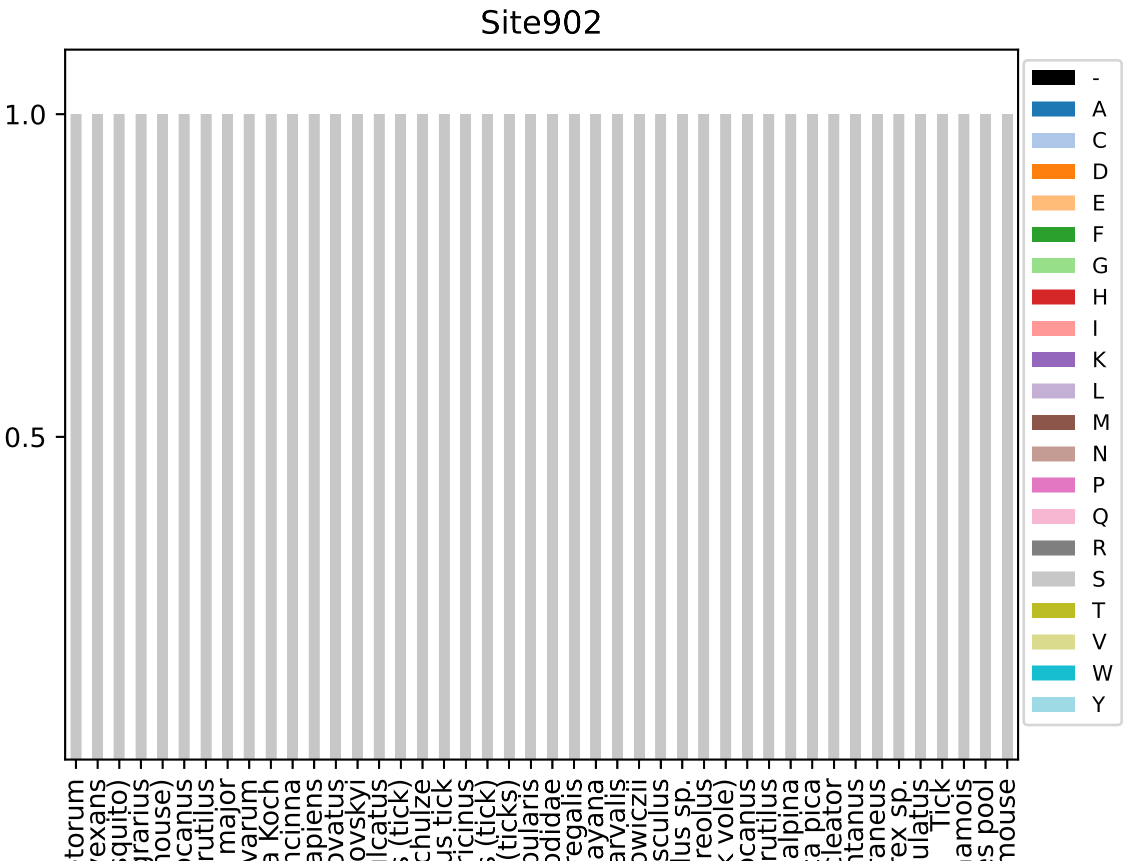
Site902
1.0
0.5
-
A
C
D
E
F
G
H
I
K
L
M
N
P
Q
R
S
T
V
W
Y

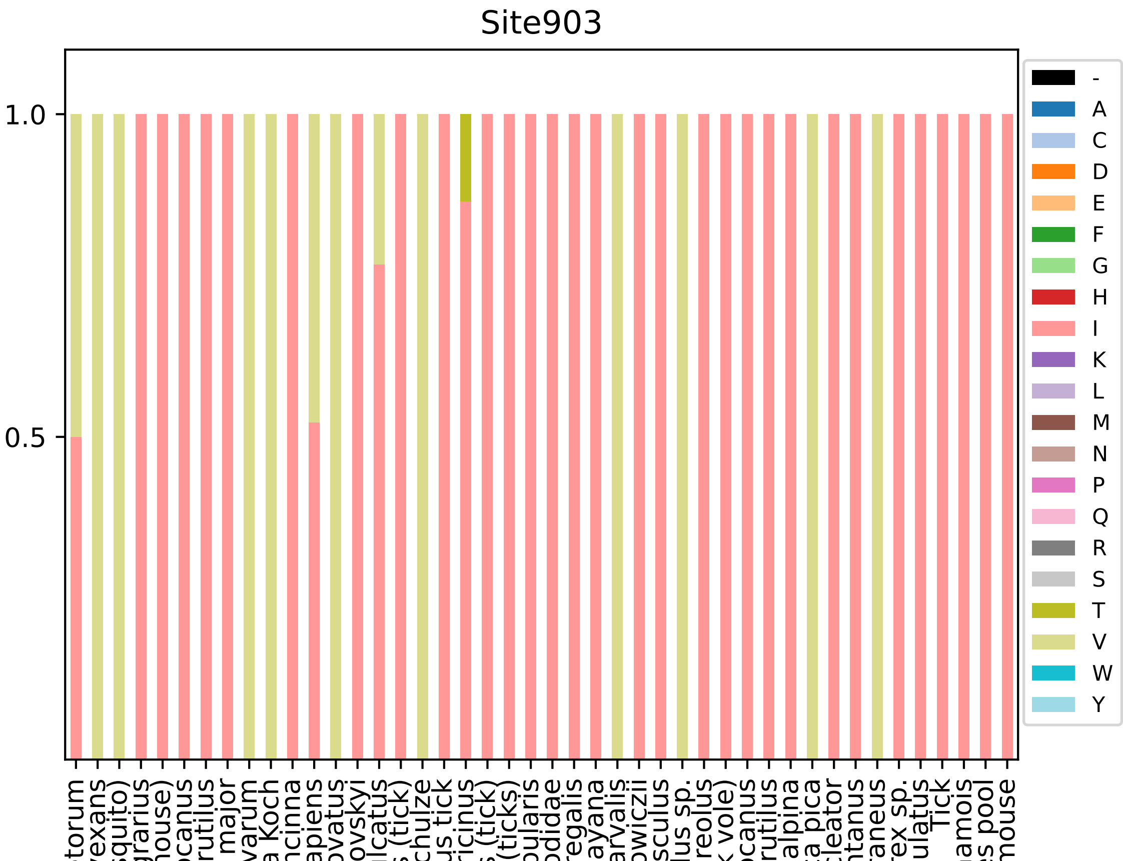
Site903
1.0
0.5
-
A
C
D
E
F
G
H
I
K
L
M
N
P
Q
R
S
T
V
W
Y

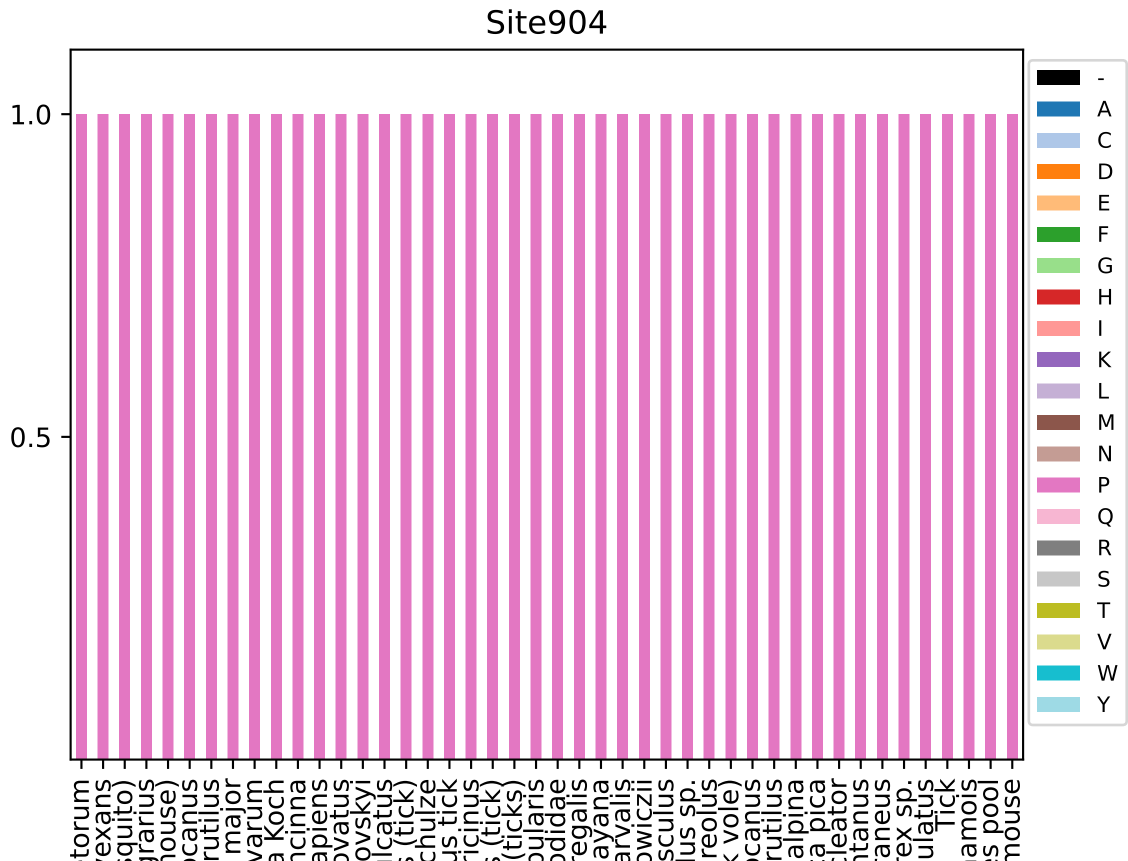
Site904
1.0
0.5
-
A
C
D
E
F
G
H
I
K
L
M
N
P
Q
R
S
T
V
W
Y

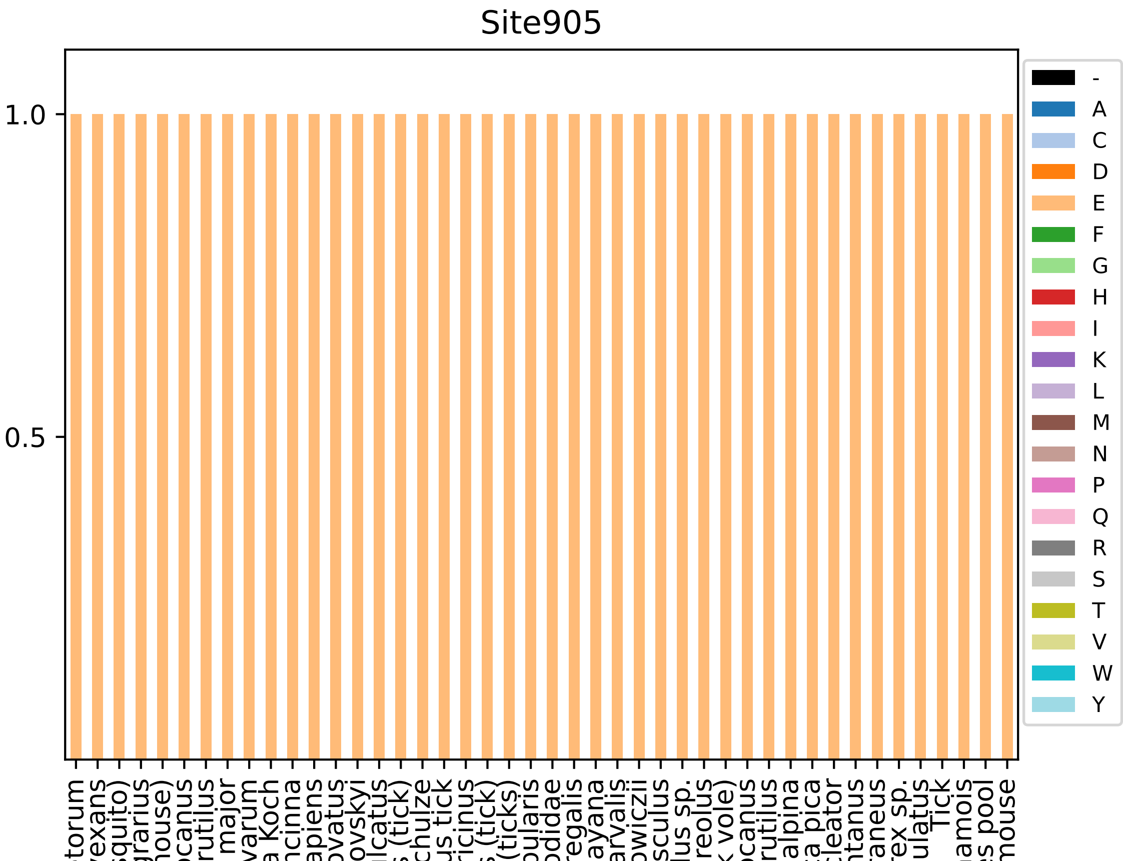
Site905
1.0
0.5
-
A
C
D
E
F
G
H
I
K
L
M
N
P
Q
R
S
T
V
W
Y

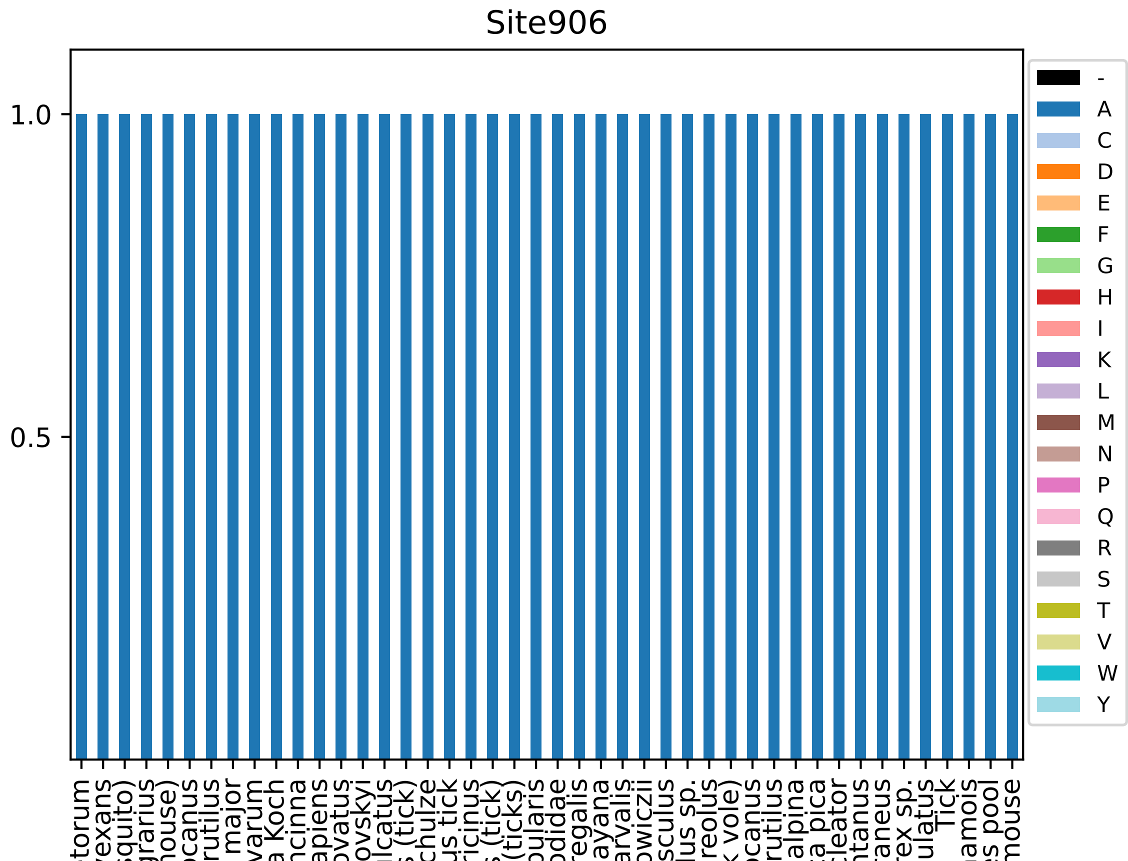
Site906
1.0
0.5
-
A
C
D
E
F
G
H
I
K
L
M
N
P
Q
R
S
T
V
W
Y

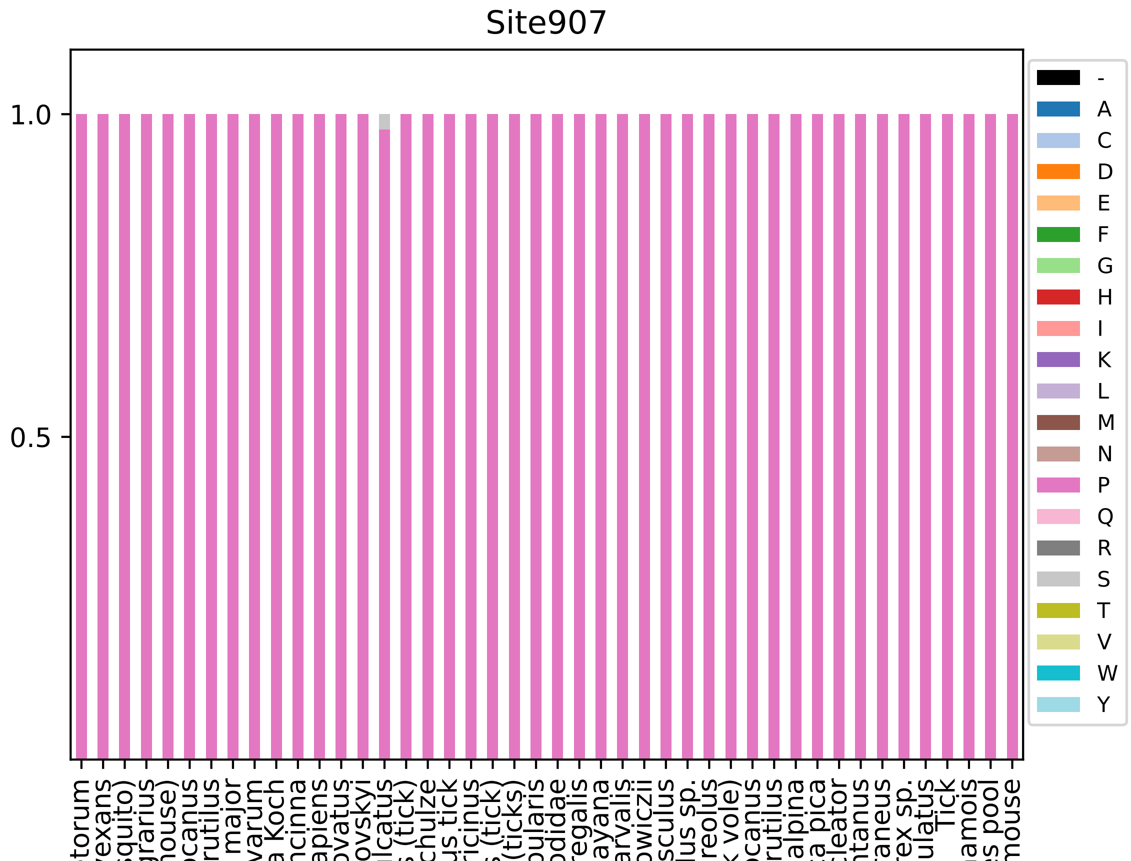
Site907
1.0
0.5
-
A
C
D
E
F
G
H
I
K
L
M
N
P
Q
R
S
T
V
W
Y

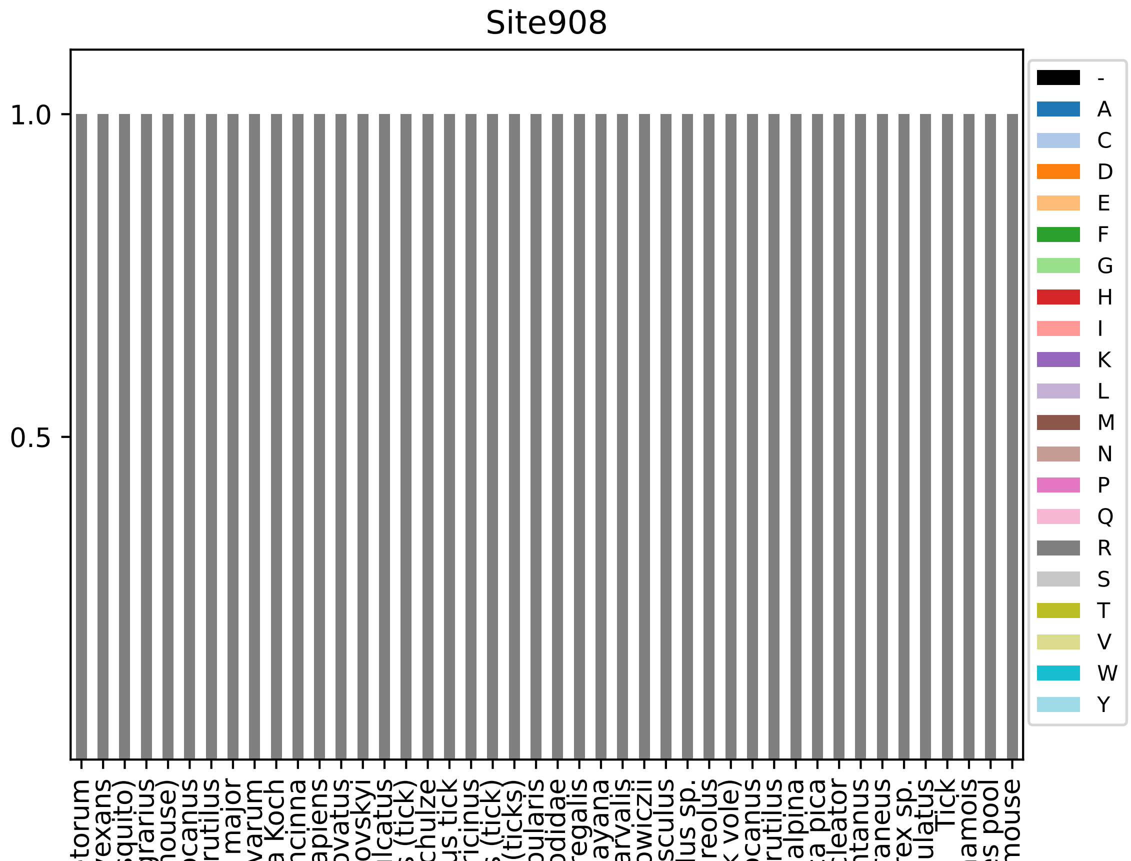
Site908
1.0
0.5
-
A
C
D
E
F
G
H
I
K
L
M
N
P
Q
R
S
T
V
W
Y

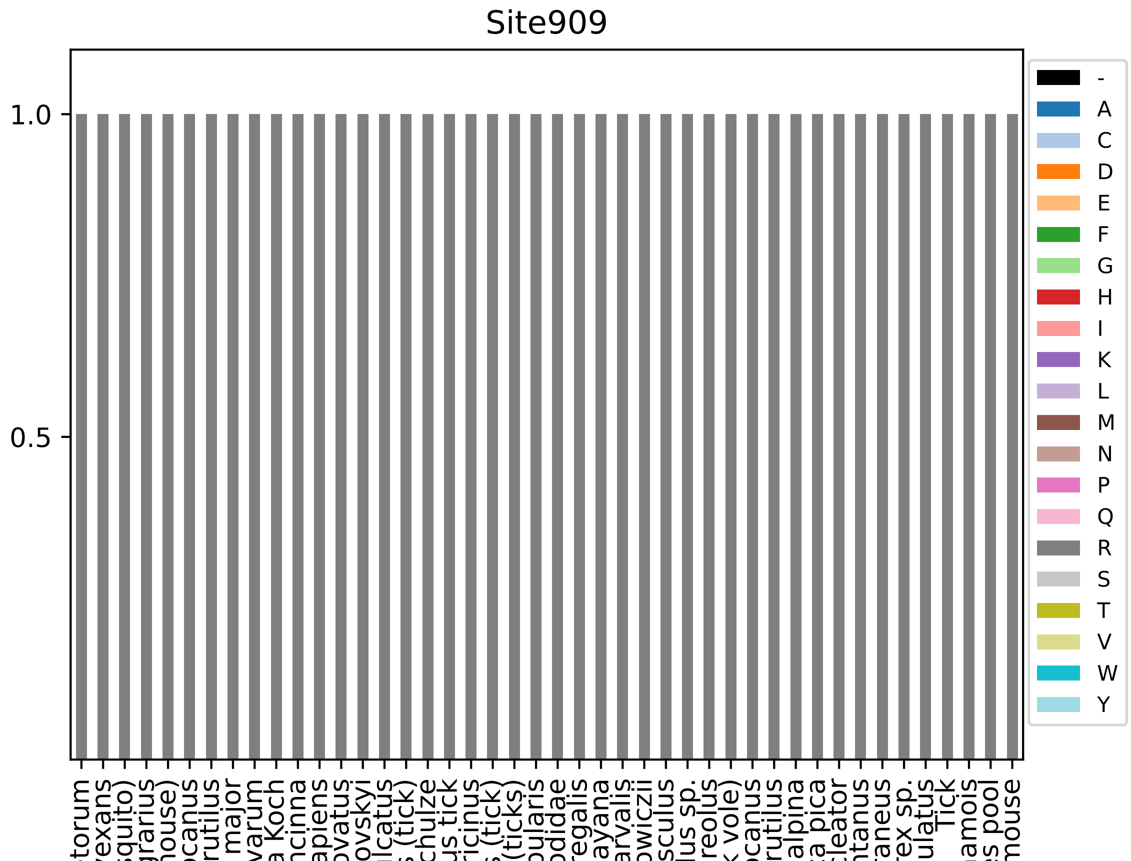
Site909
1.0
0.5
-
A
C
D
E
F
G
H
I
K
L
M
N
P
Q
R
S
T
V
W
Y
Tick

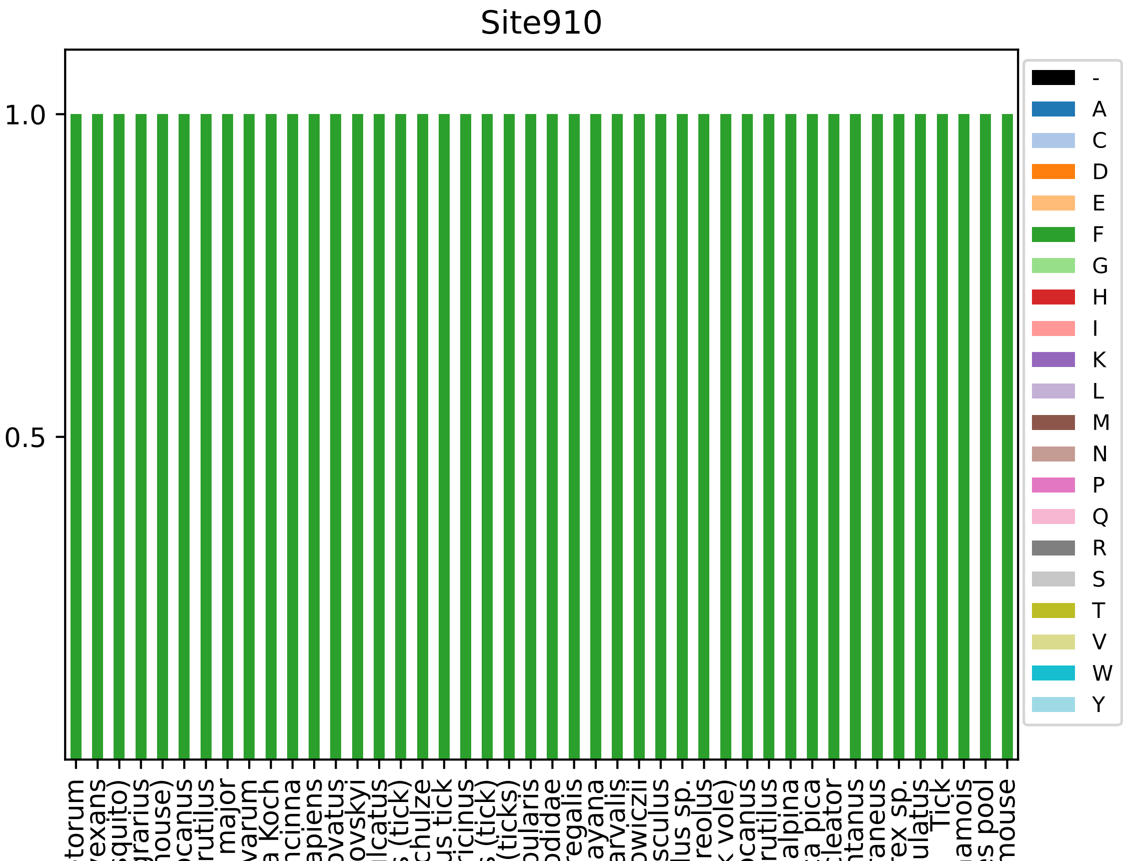
Site910
1.0
0.5
-
A
C
D
E
F
G
H
I
K
L
M
N
P
Q
R
S
T
V
W
Y
Tick

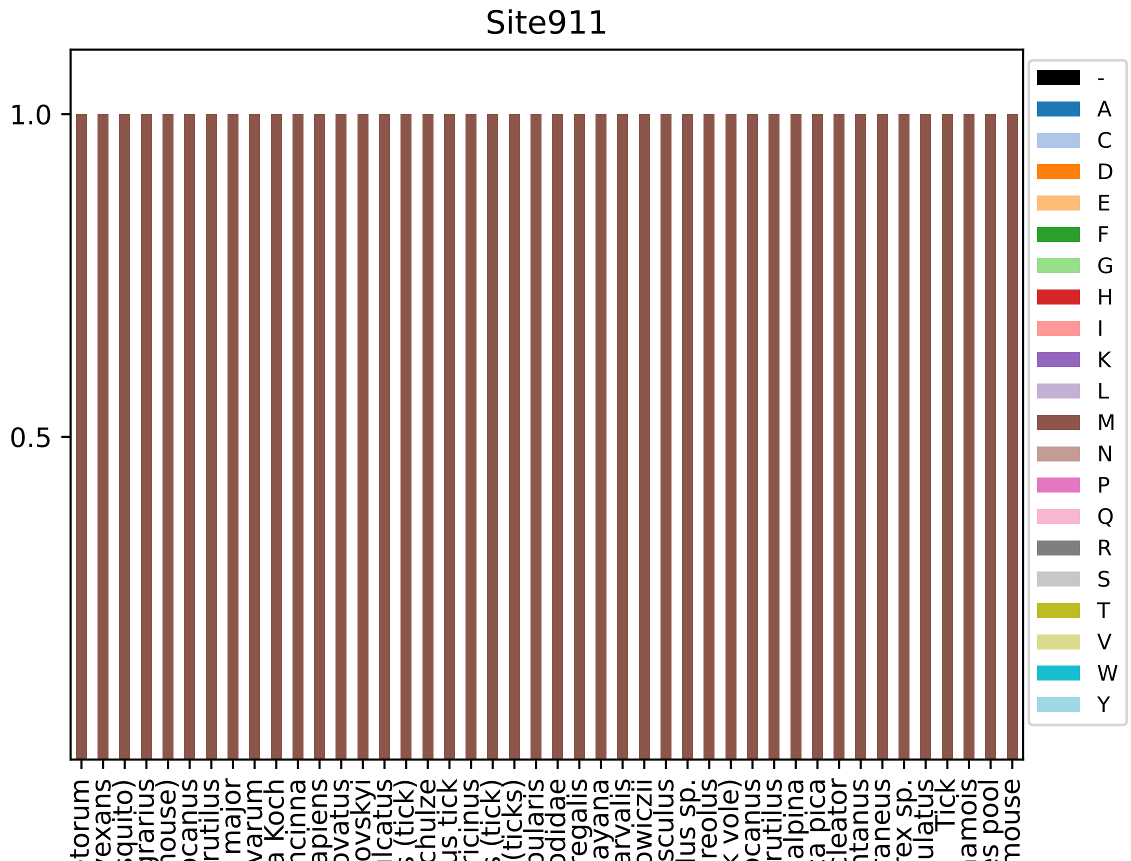
Site911
1.0
0.5
-
A
C
D
E
F
G
H
I
K
L
M
N
P
Q
R
S
T
V
W
Y

Site912

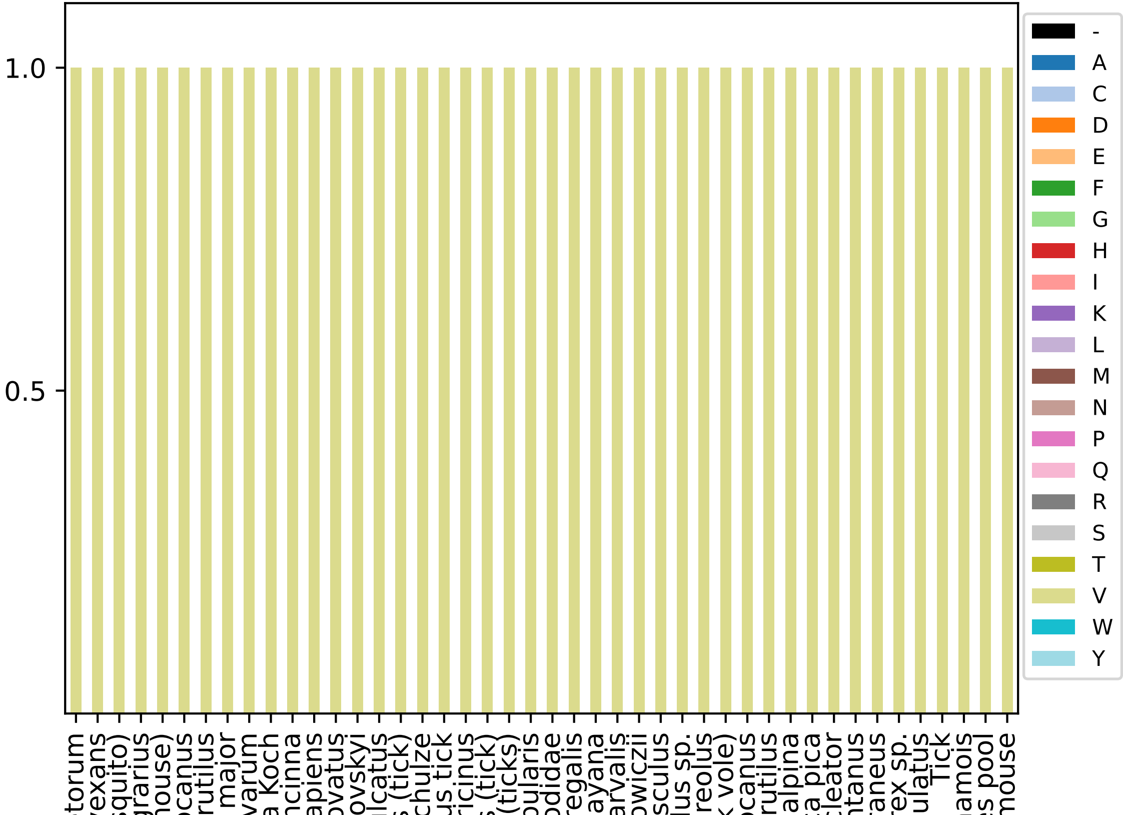

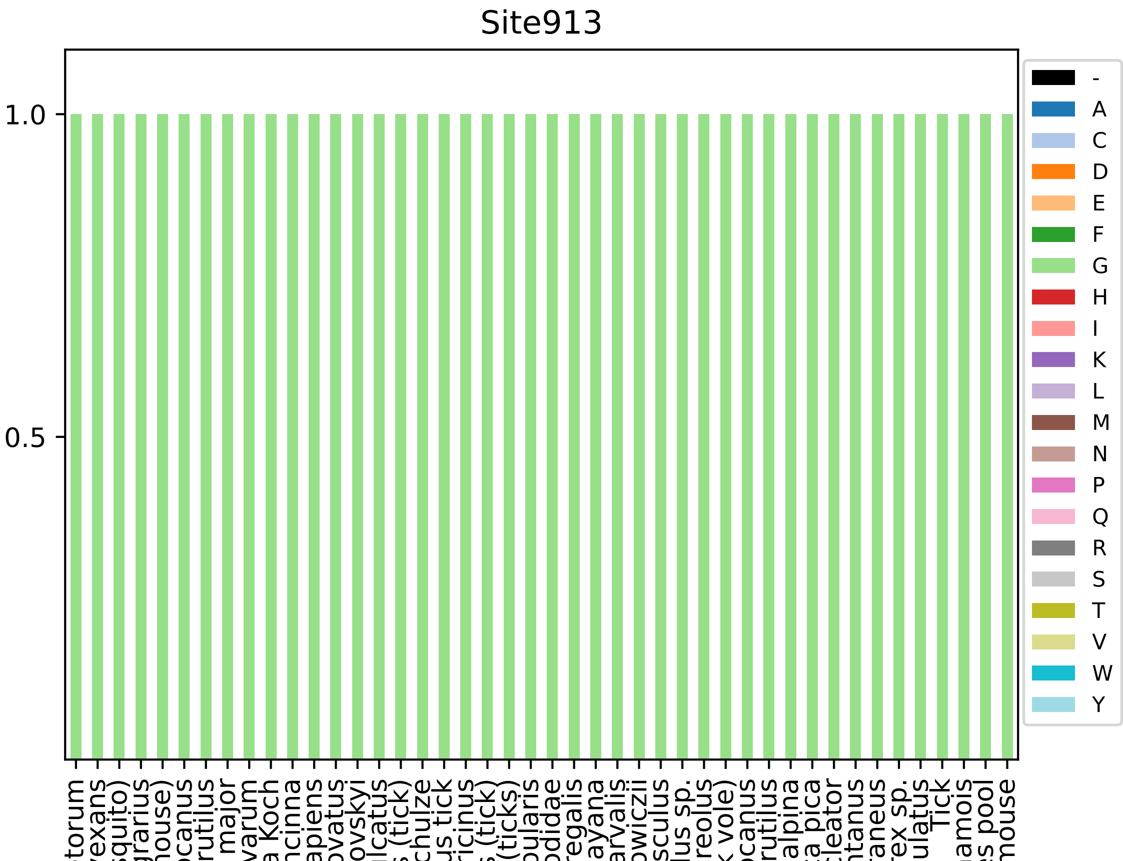
Site913
1.0
0.5
-
A
C
D
E
F
G
H
I
K
L
M
N
P
Q
R
S
T
V
W
Y

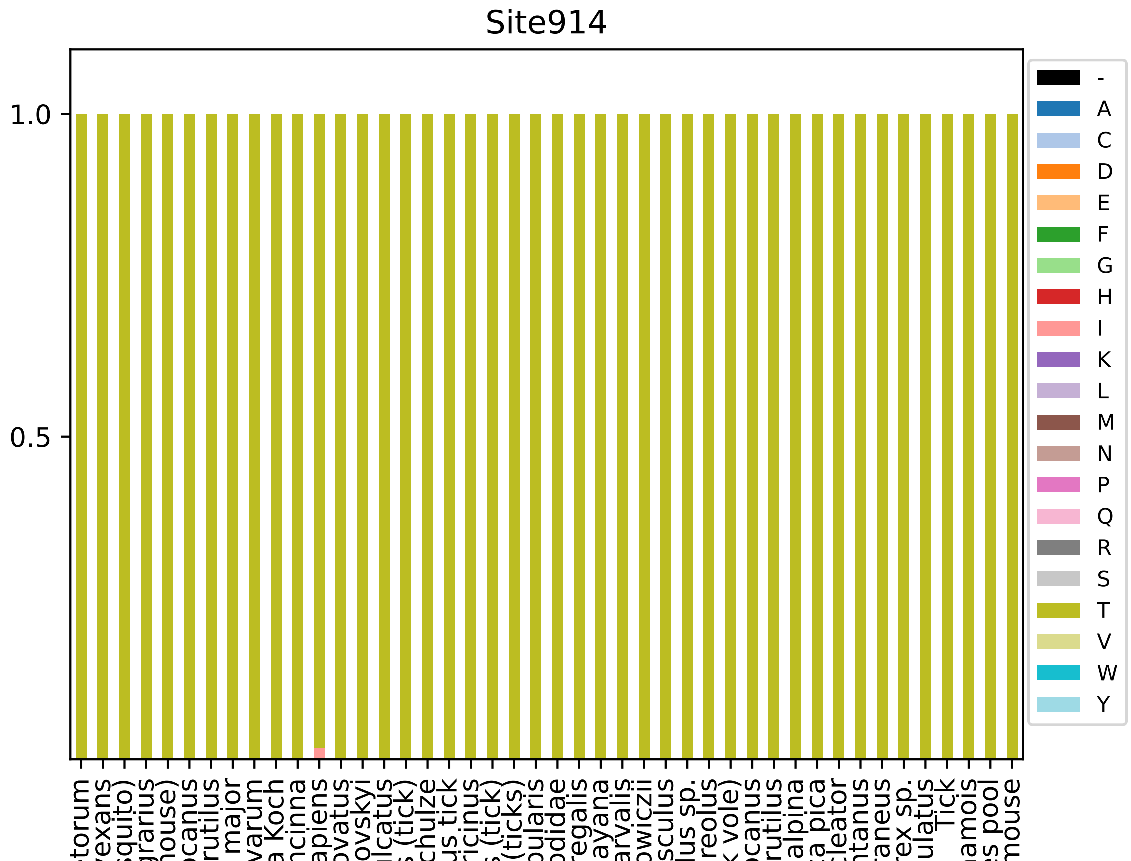

Site914
1.0
0.5
-
A
C
D
E
F
G
H
I
K
L
M
N
P
Q
R
S
T
V
W
Y

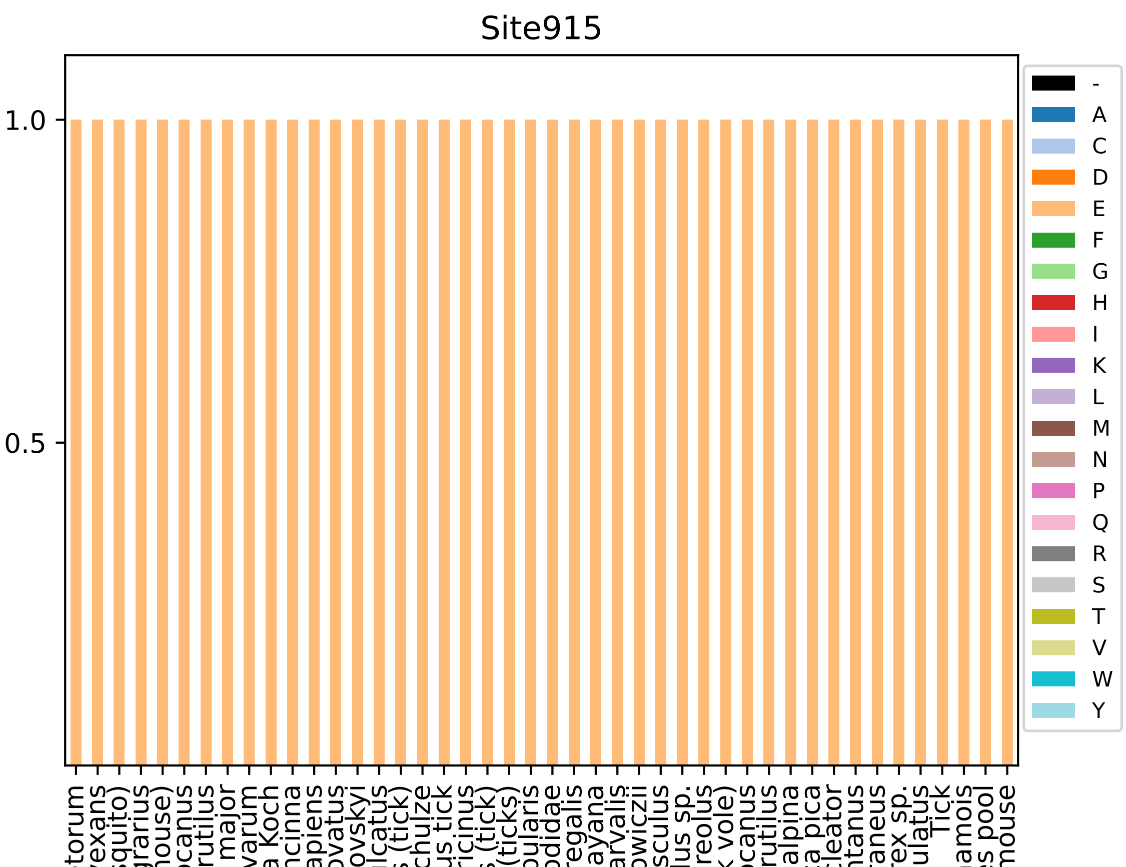
Site915
1.0
0.5
-
A
C
D
E
F
G
H
I
K
L
M
N
P
Q
R
S
T
V
W
Y

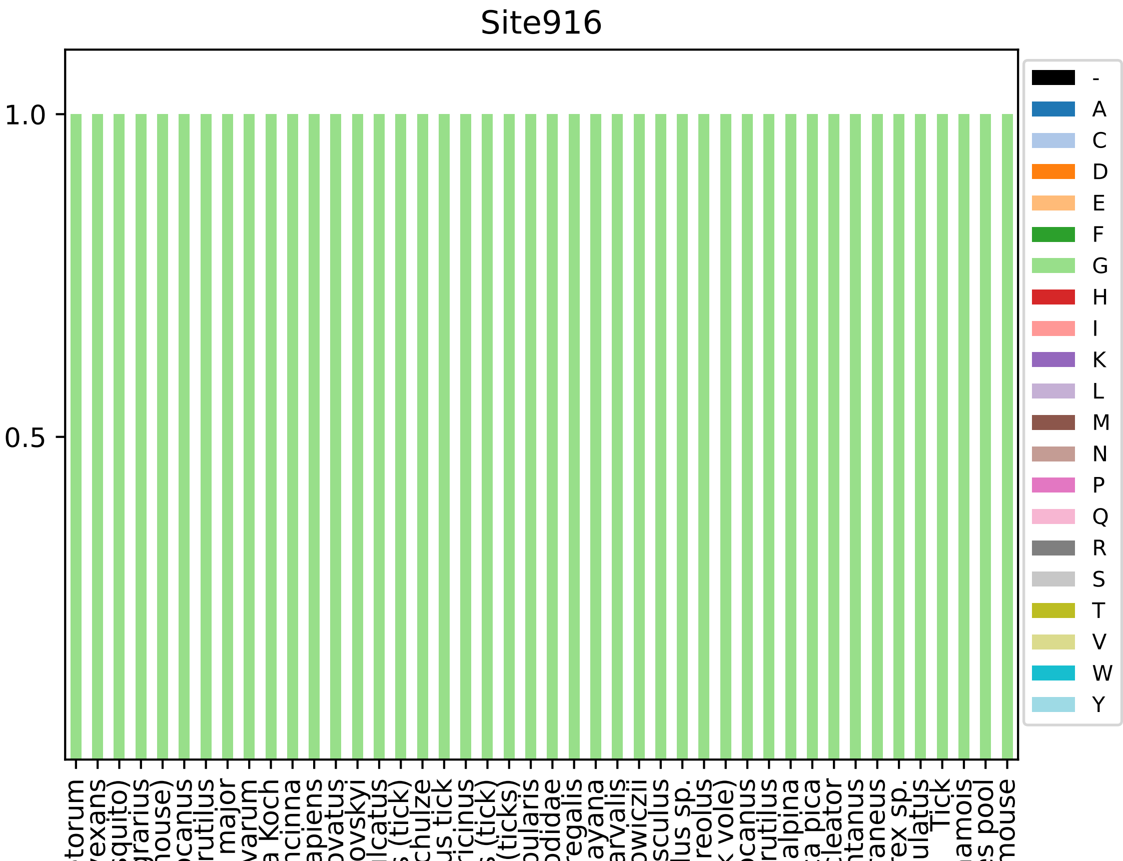

Site916
1.0
0.5
-
A
C
D
E
F
G
H
I
K
L
M
N
P
Q
R
S
T
V
W
Y

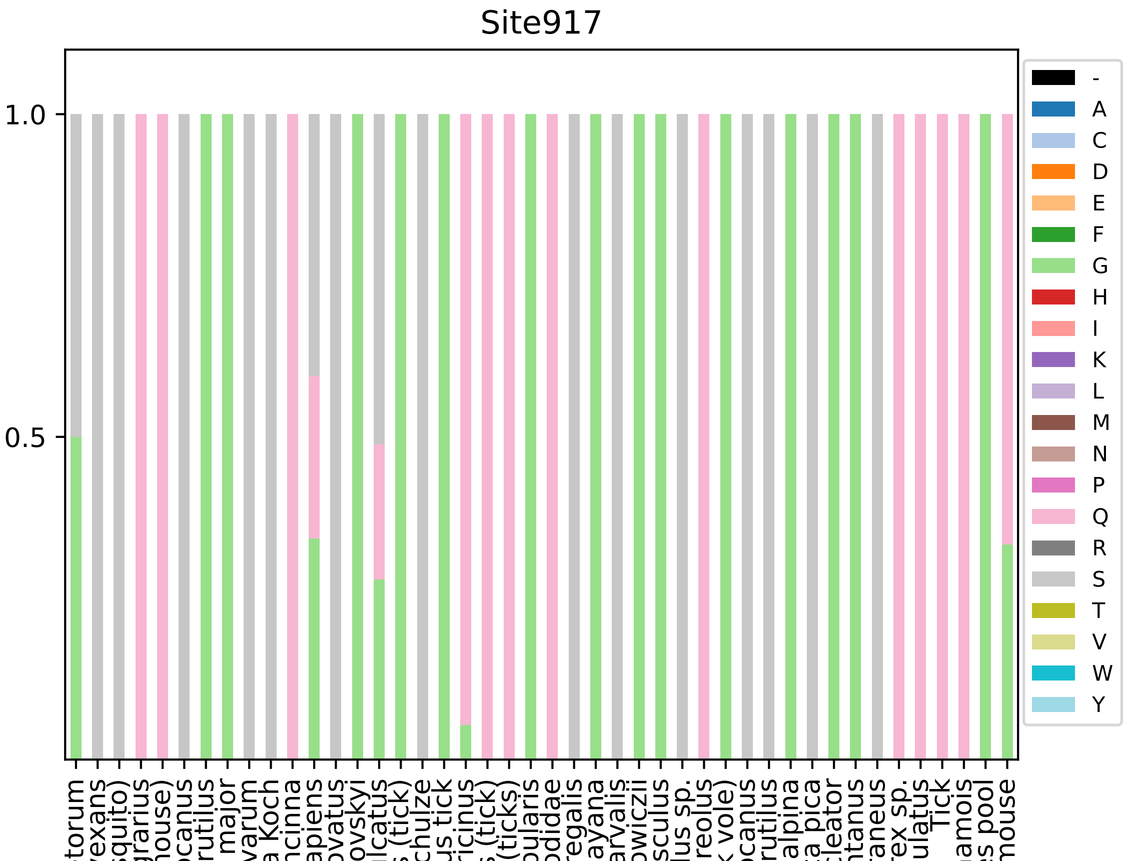
Site917
1.0
0.5
-
A
C
D
E
F
G
H
I
K
L
M
N
P
Q
R
S
T
V
W
Y

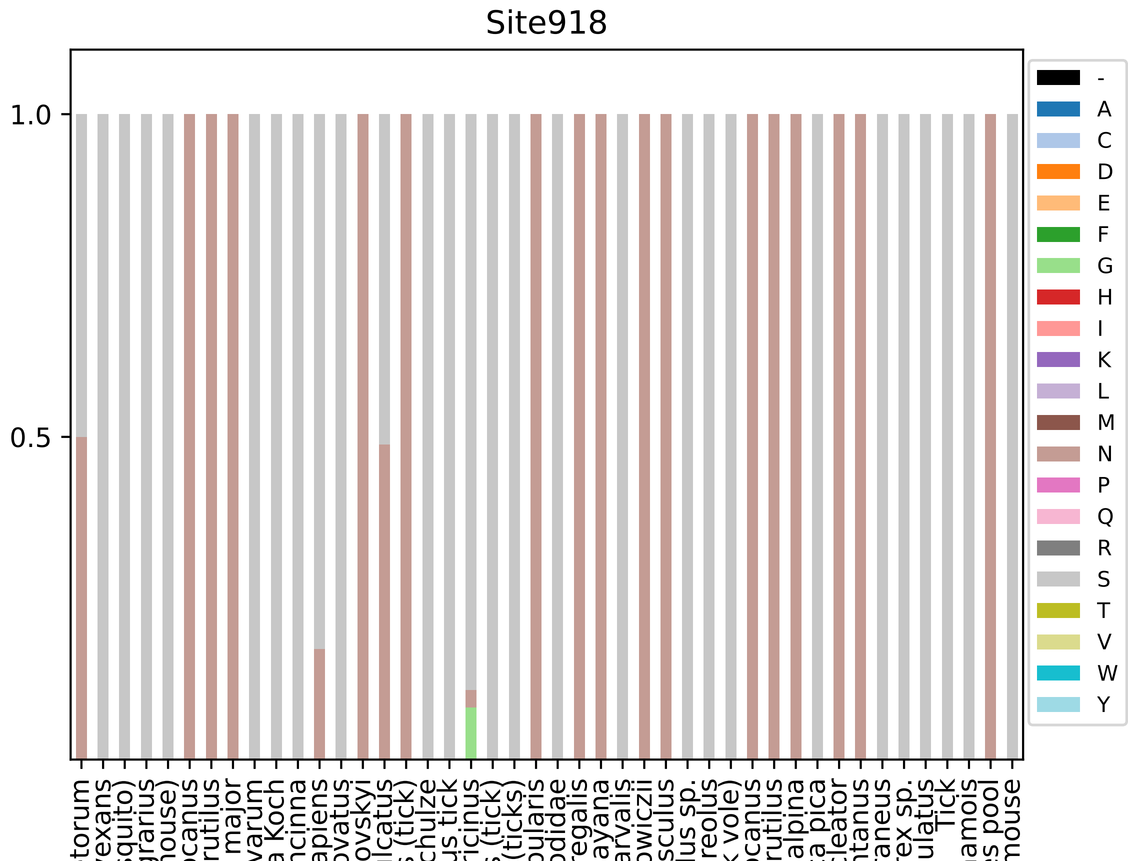
Site918
1.0
0.5
-
A
C
D
E
F
G
H
I
K
L
M
N
P
Q
R
S
T
V
W
Y

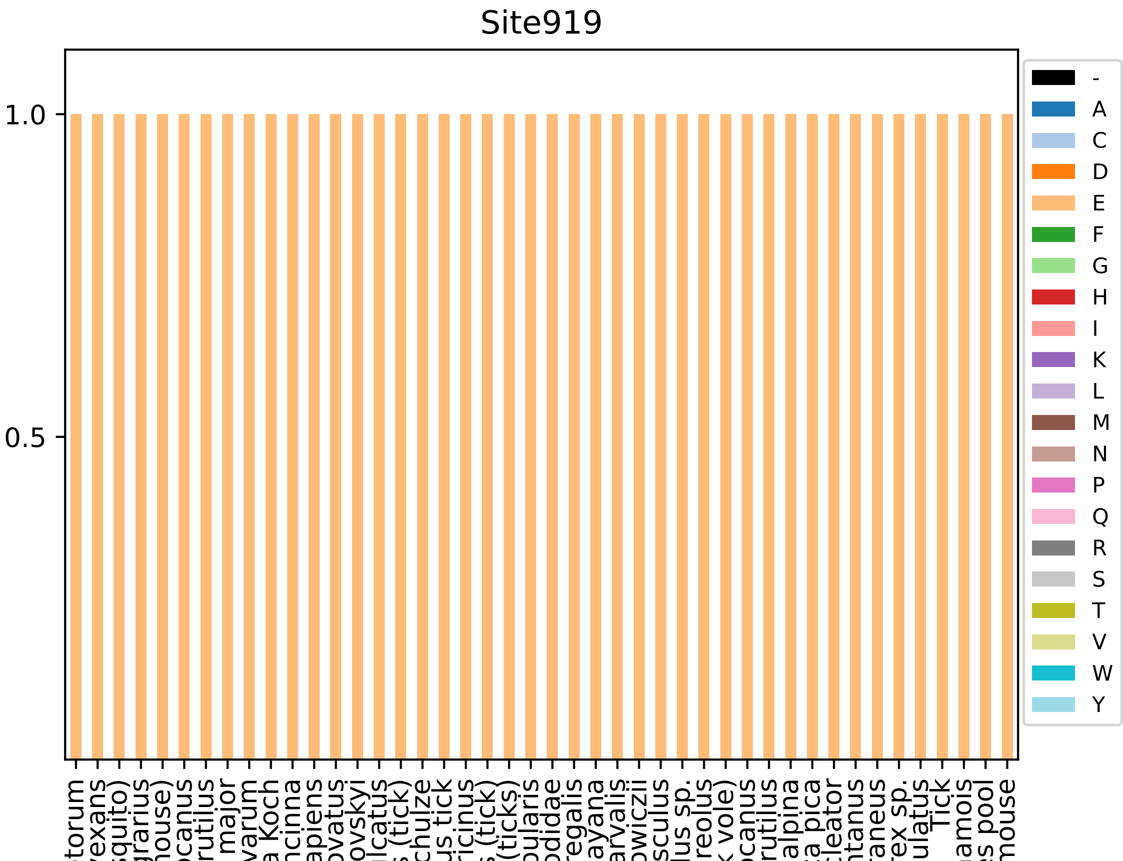
Site919
1.0
0.5
-
A
C
D
E
F
G
H
I
K
L
M
N
P
Q
R
S
T
V
W
Y

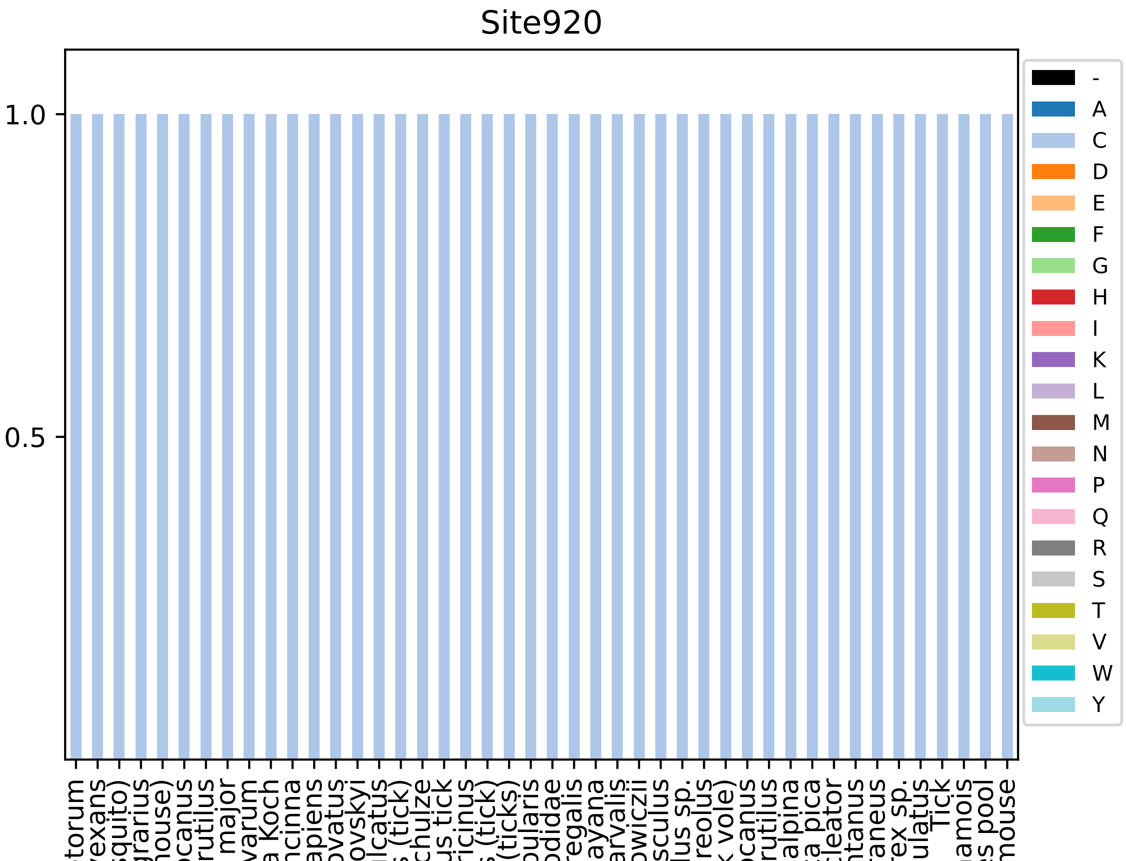
Site920
1.0
0.5
-
A
C
D
E
F
G
H
I
K
L
M
N
P
Q
R
S
T
V
W
Y

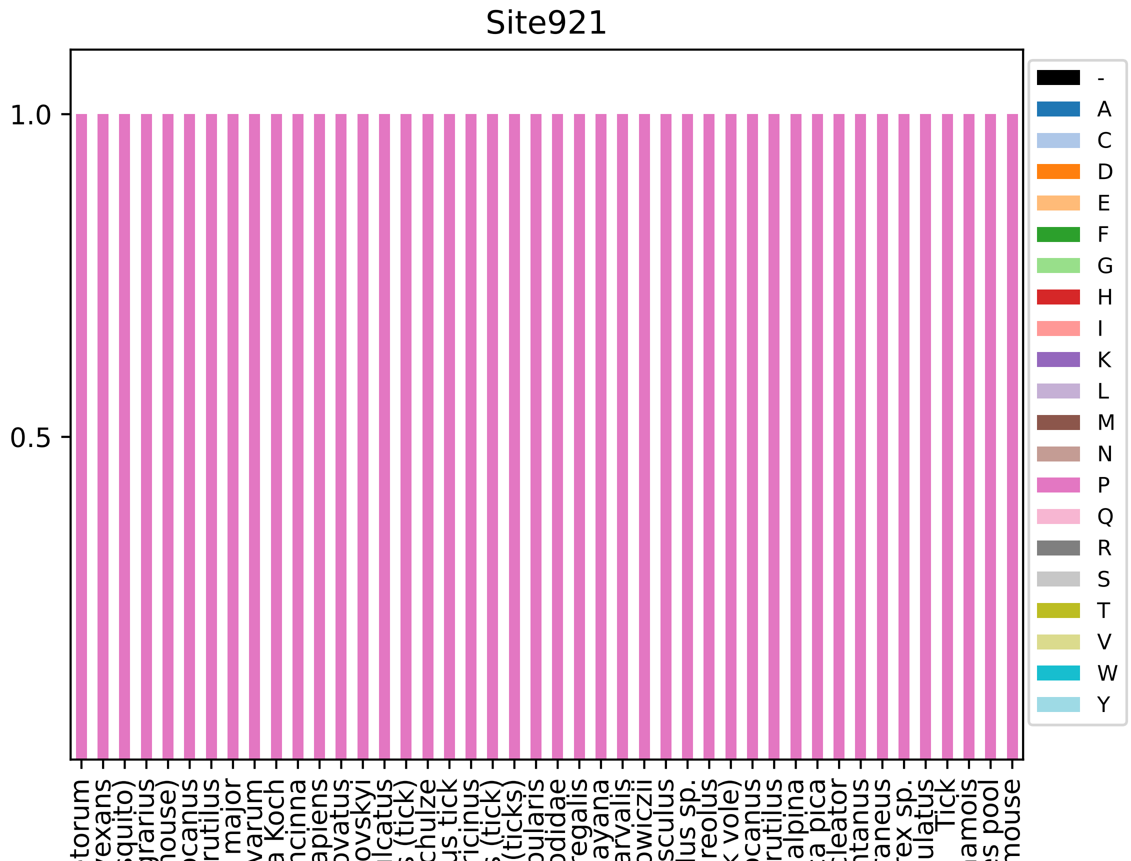
Site921
1.0
0.5
-
A
C
D
E
F
G
H
I
K
L
M
N
P
Q
R
S
T
V
W
Y

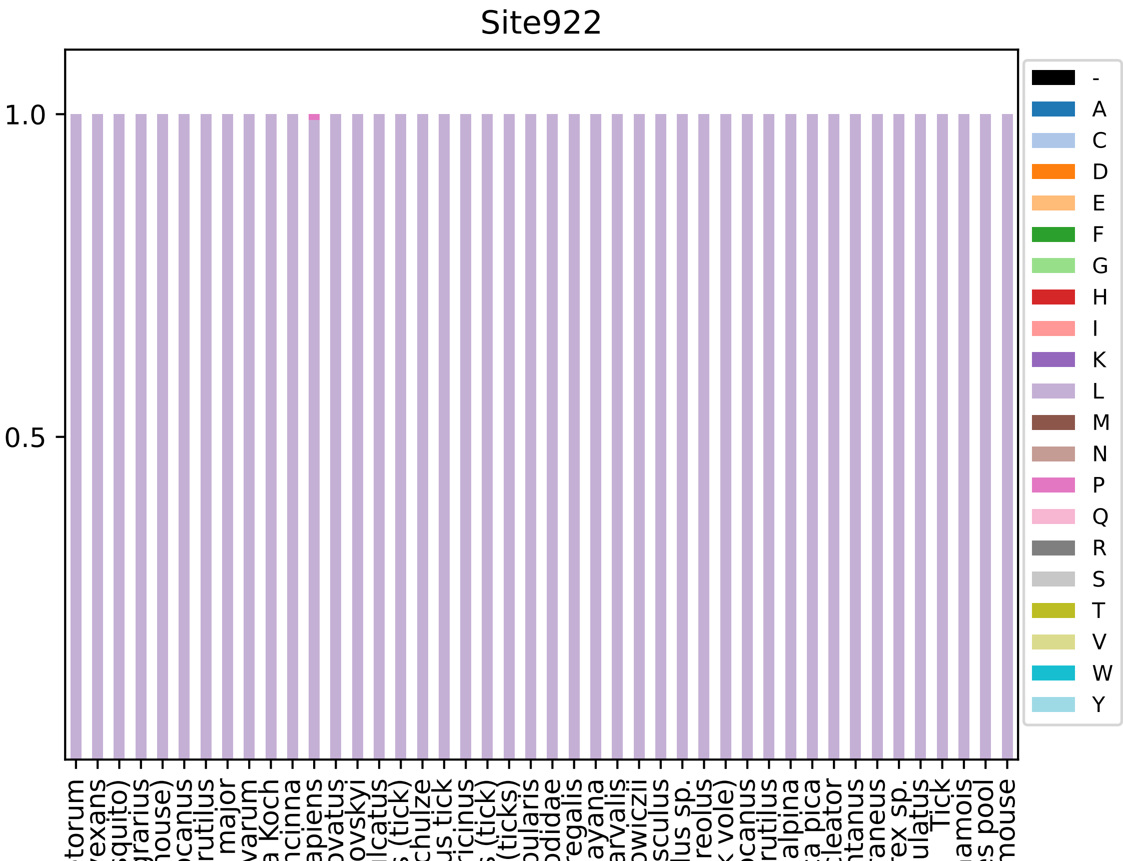
Site922
1.0
0.5
-
A
C
D
E
F
G
H
I
K
L
M
N
P
Q
R
S
T
V
W
Y

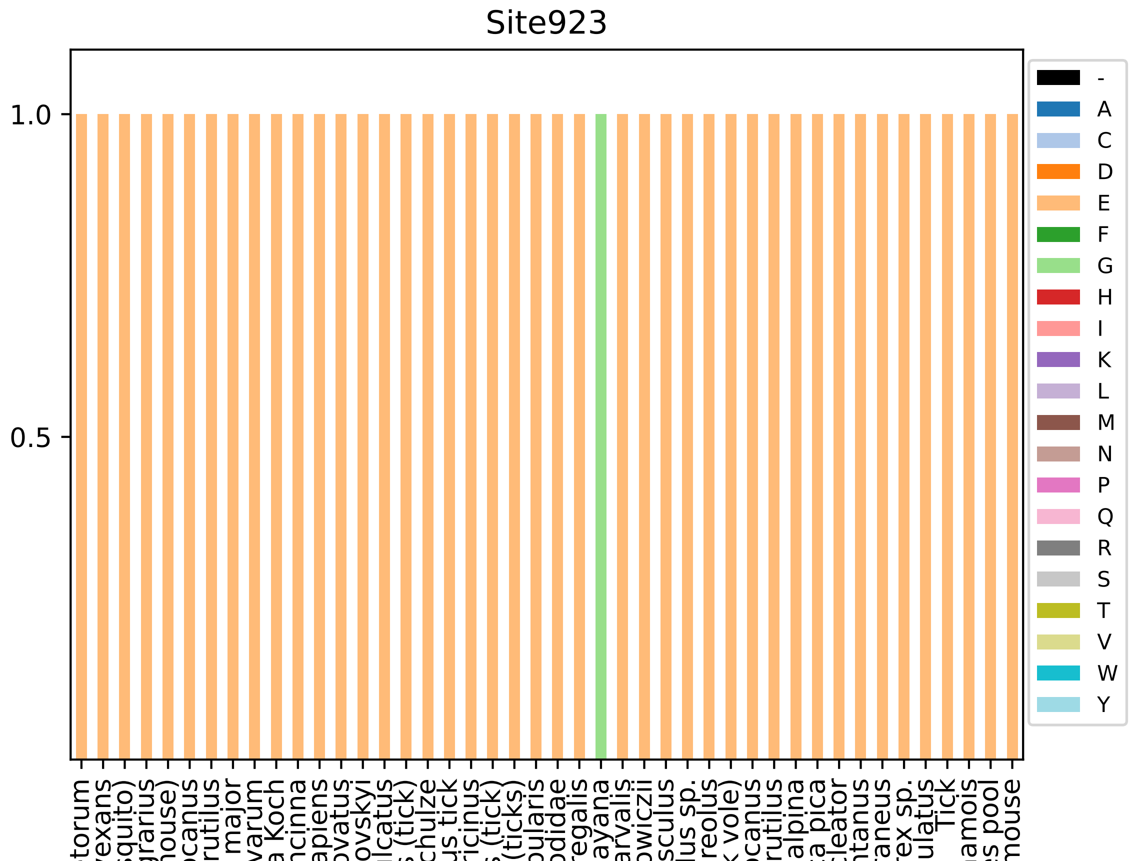

Site923
1.0
0.5
-
A
C
D
E
F
G
H
I
K
L
M
N
P
Q
R
S
T
V
W
Y

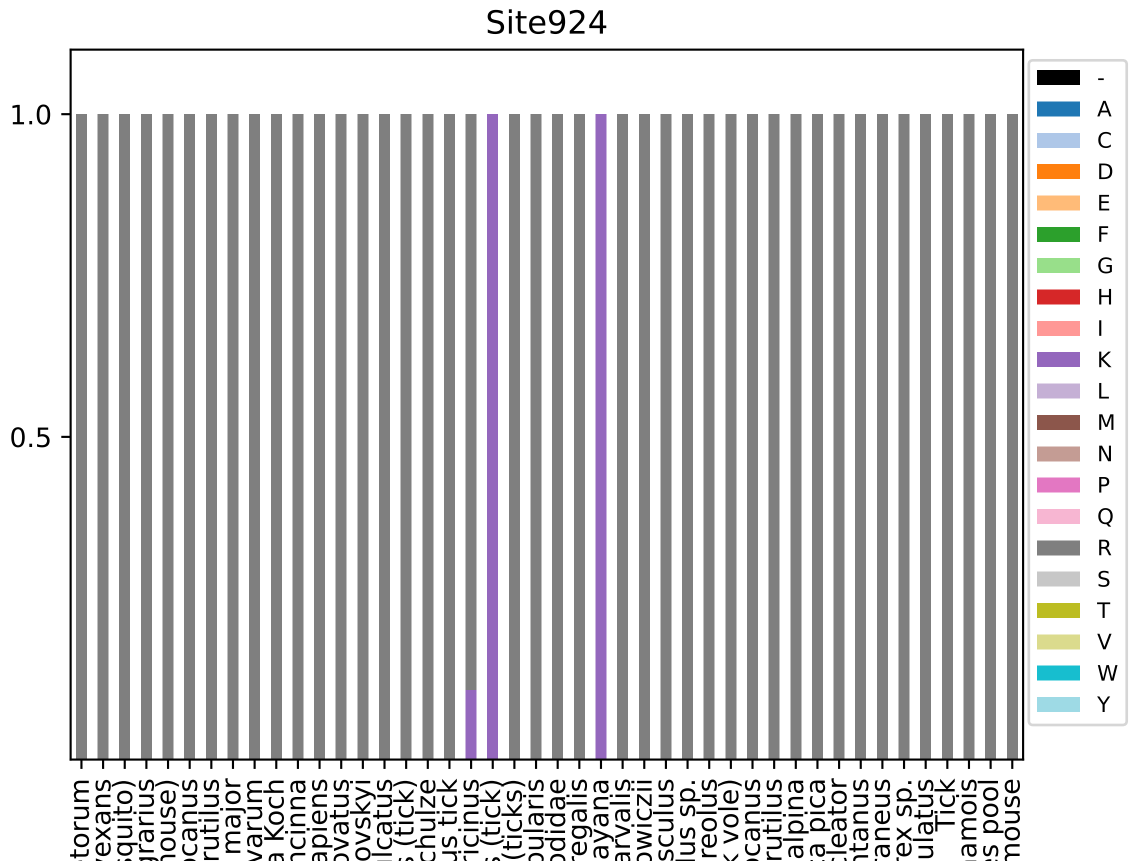

Site924
1.0
0.5
-
A
C
D
E
F
G
H
I
K
L
M
N
P
Q
R
S
T
V
W
Y

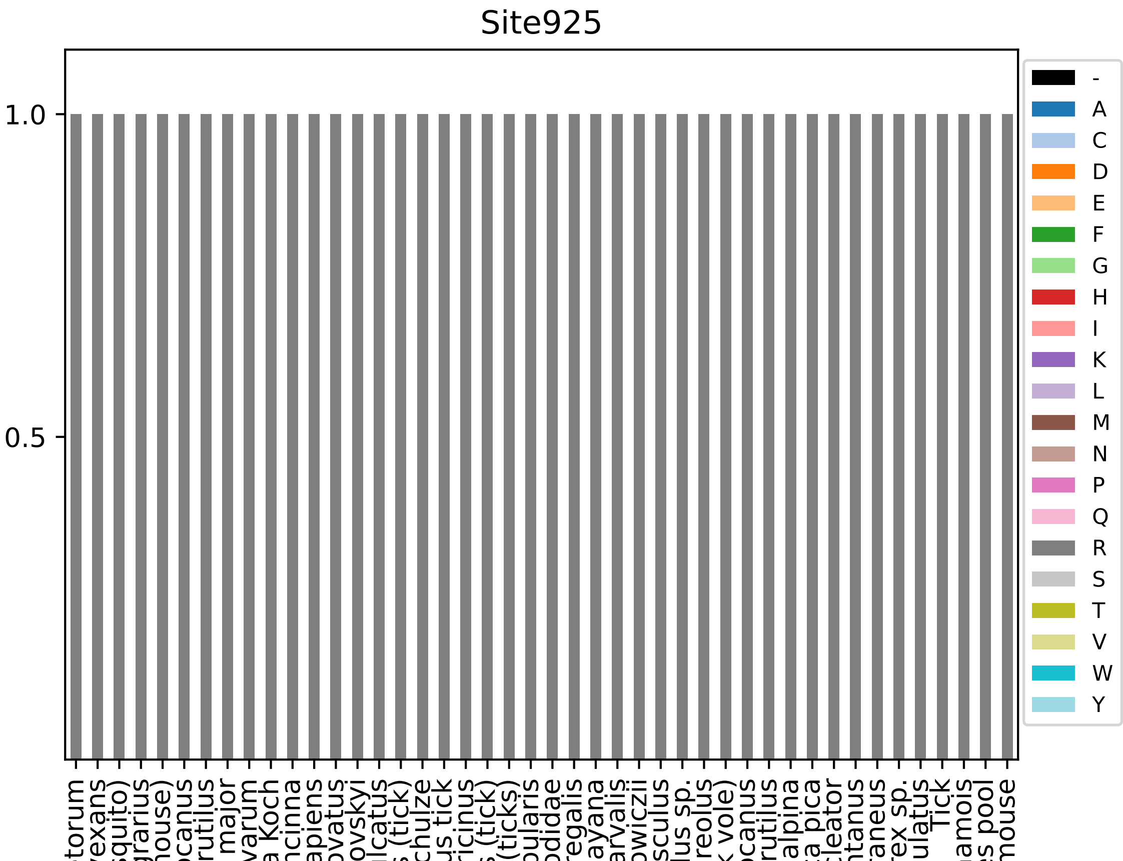

Site925
1.0
0.5
-
A
C
D
E
F
G
H
I
K
L
M
N
P
Q
R
S
T
V
W
Y

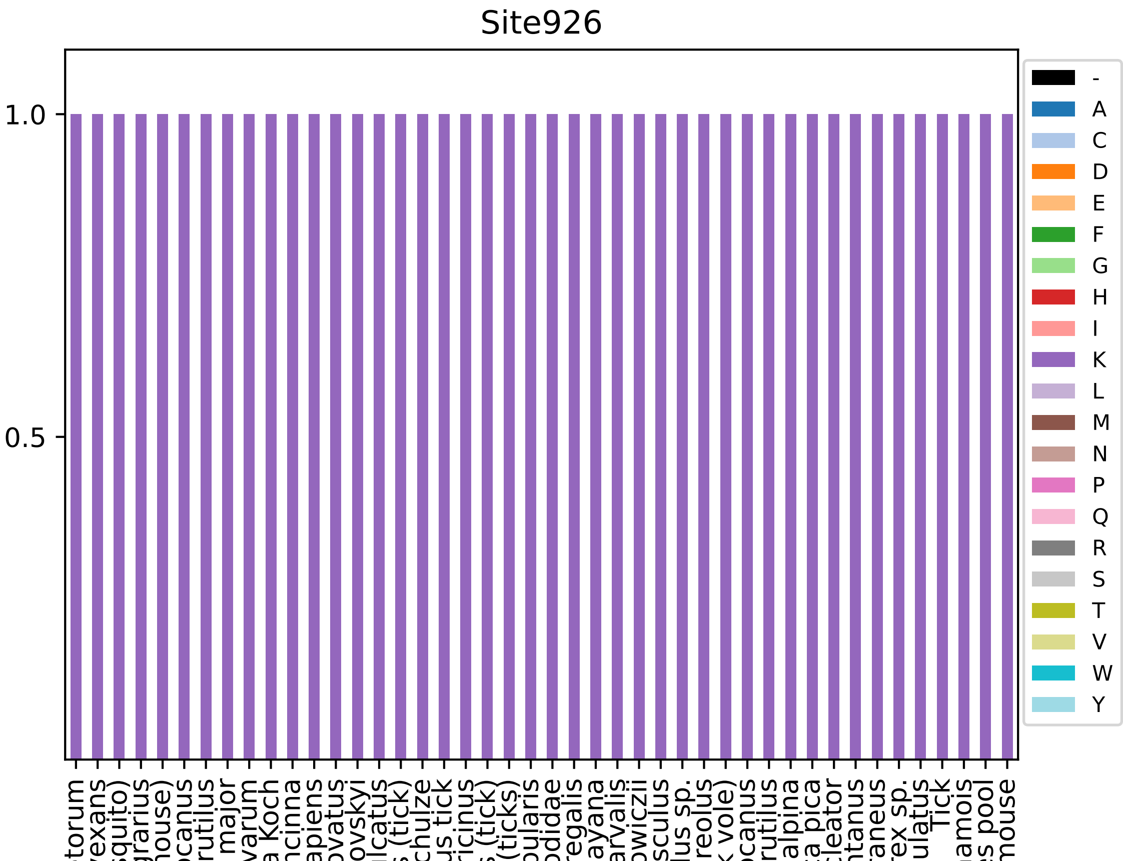
Site926
1.0
0.5
-
A
C
D
E
F
G
H
I
K
L
M
N
P
Q
R
S
T
V
W
Y
Tick

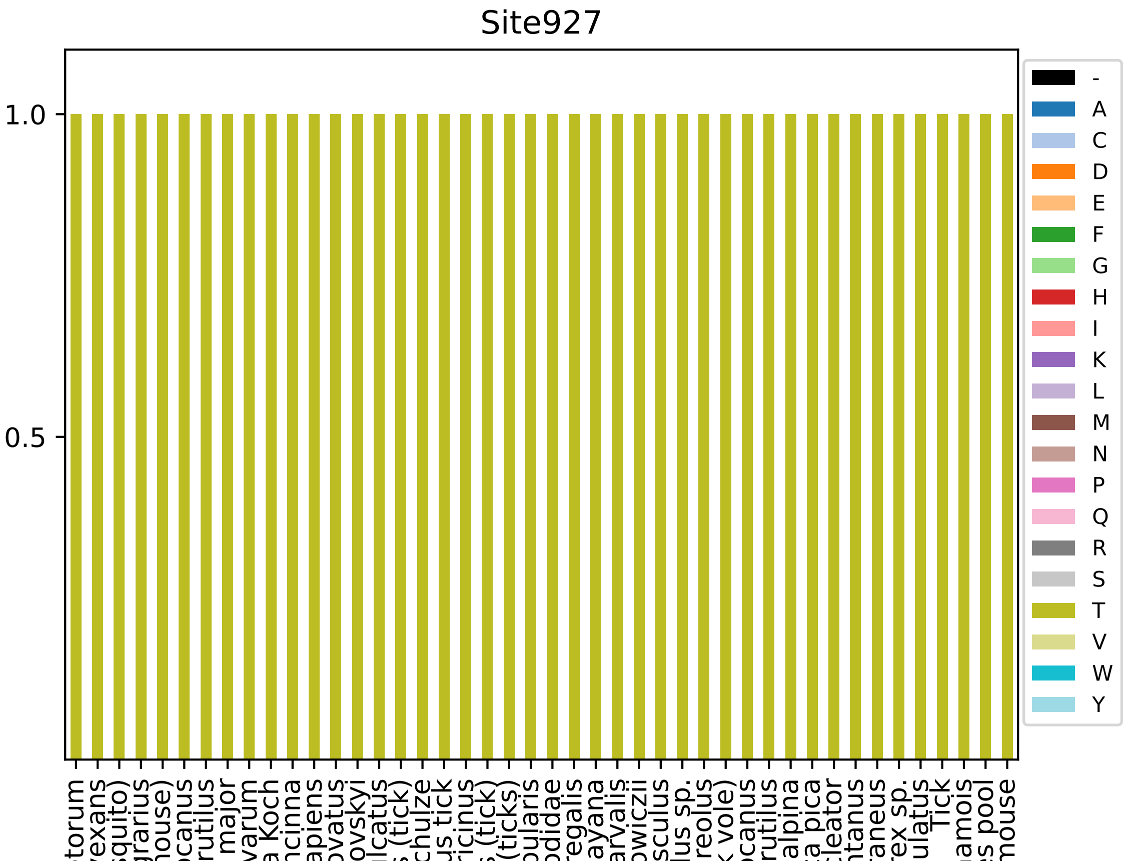
Site927
1.0
0.5
-
A
C
D
E
F
G
H
I
K
L
M
N
P
Q
R
S
T
V
W
Y

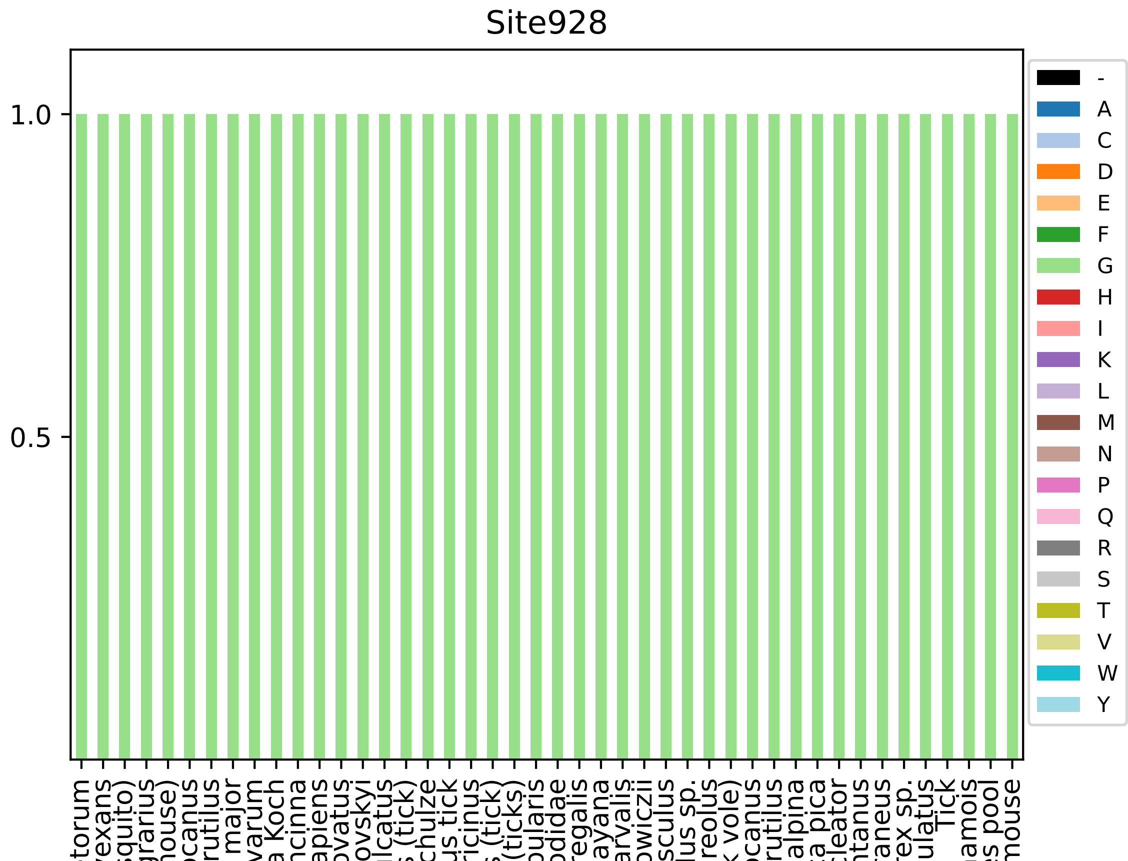
Site928
1.0
0.5
-
A
C
D
E
F
G
H
I
K
L
M
N
P
Q
R
S
T
V
W
Y

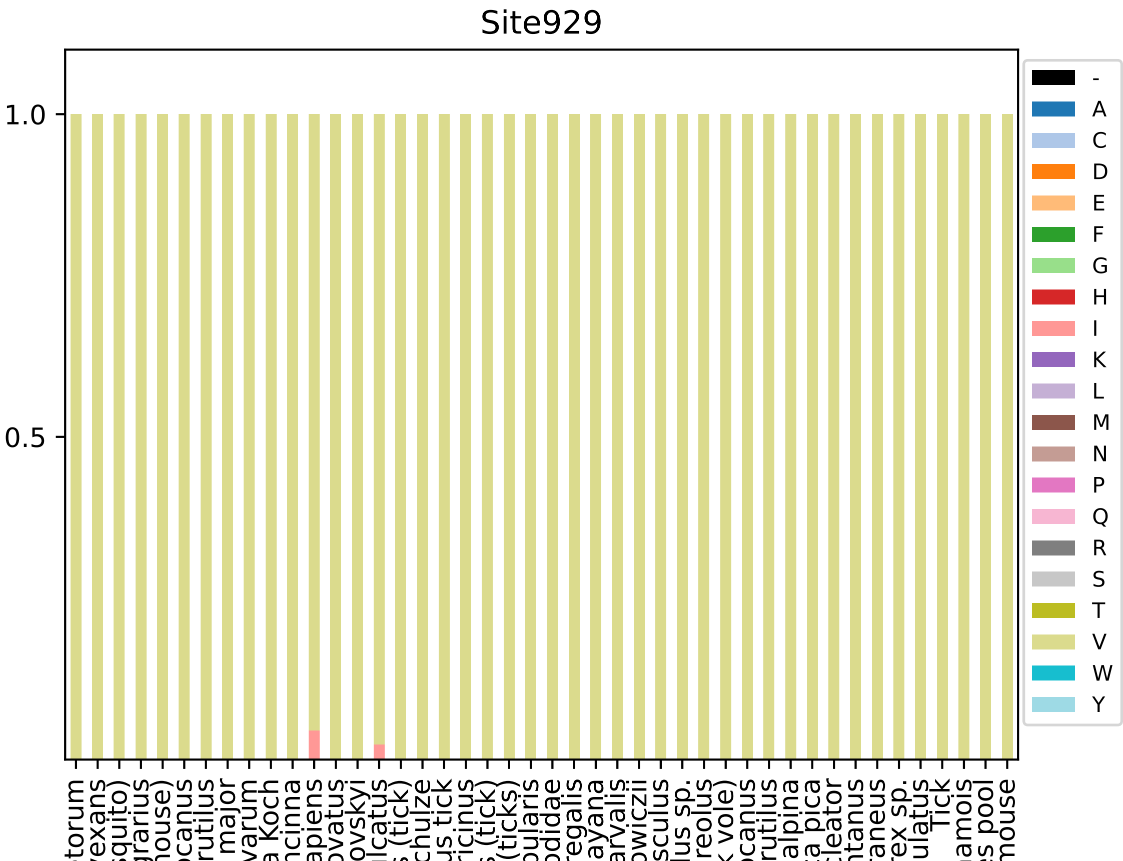

Site929
1.0
0.5
-
A
C
D
E
F
G
H
I
K
L
M
N
P
Q
R
S
T
V
W
Y

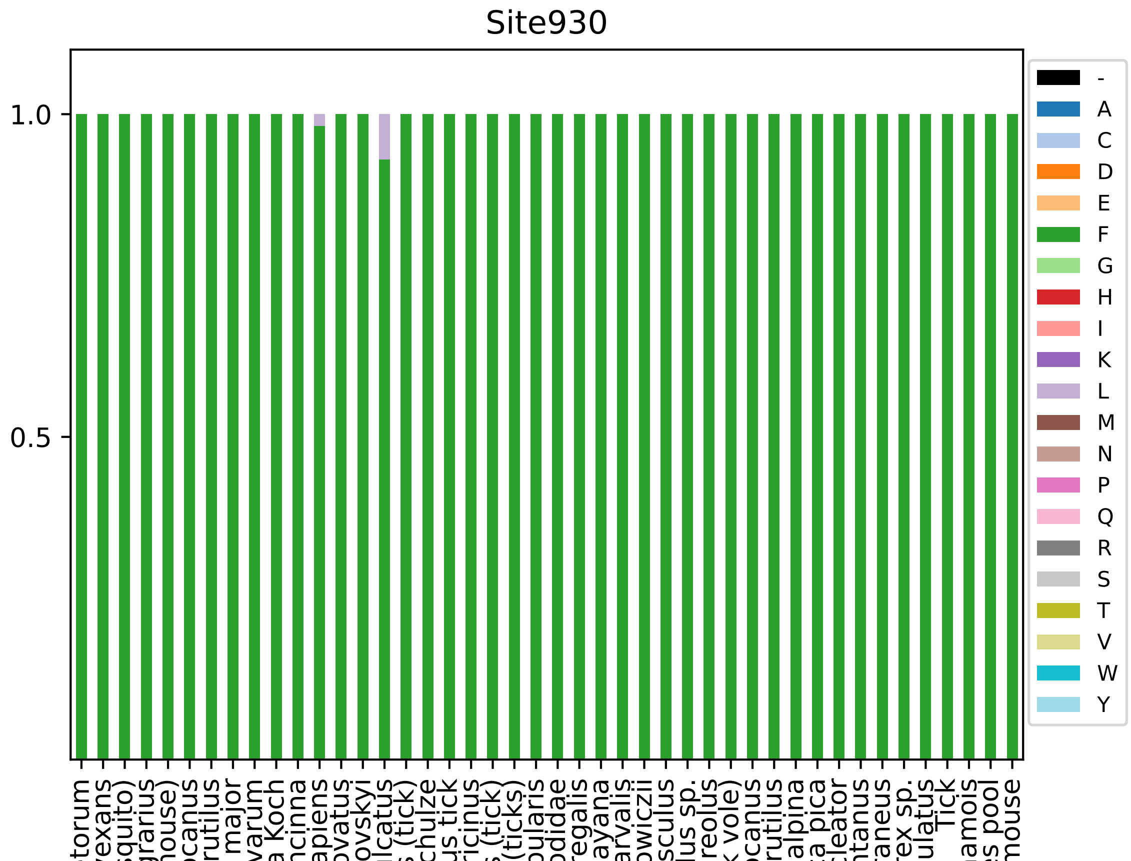
Site930
1.0
0.5
-
A
C
D
E
F
G
H
I
K
L
M
N
P
Q
R
S
T
V
W
Y

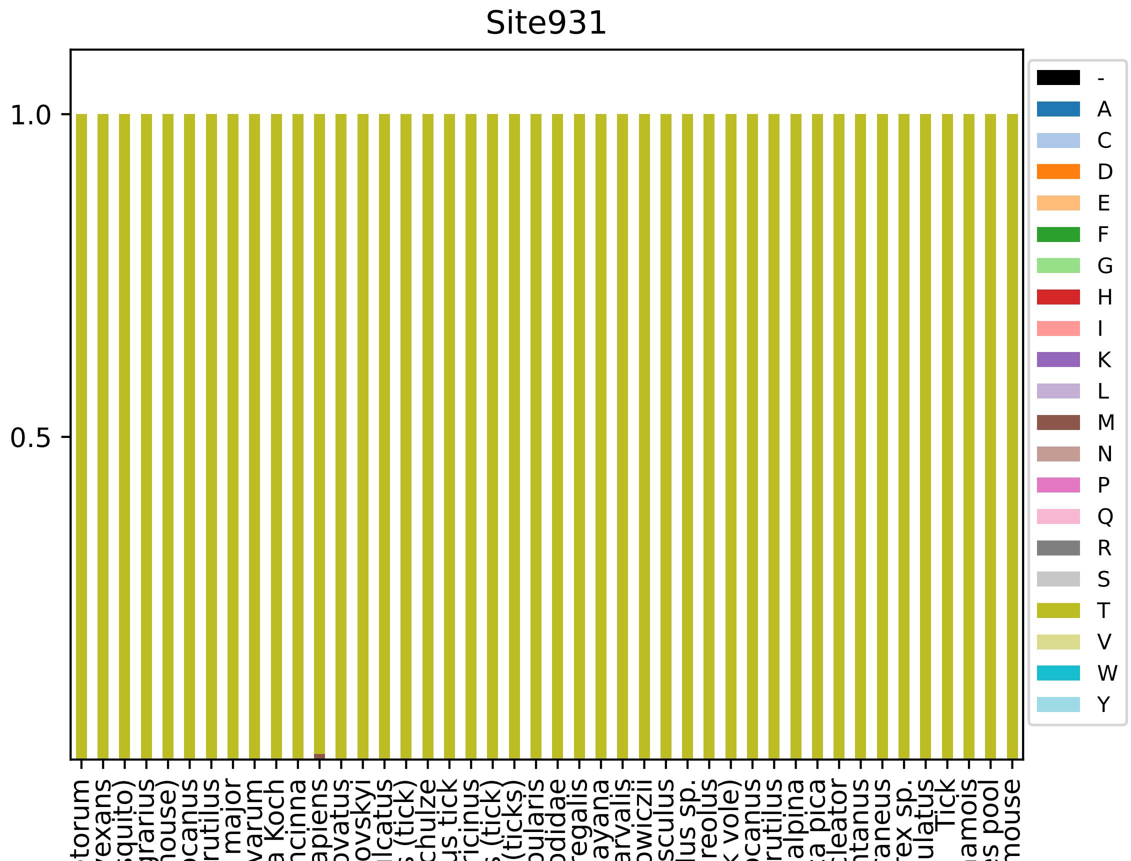

Site931
1.0
0.5
-
A
C
D
E
F
G
H
I
K
L
M
N
P
Q
R
S
T
V
W
Y

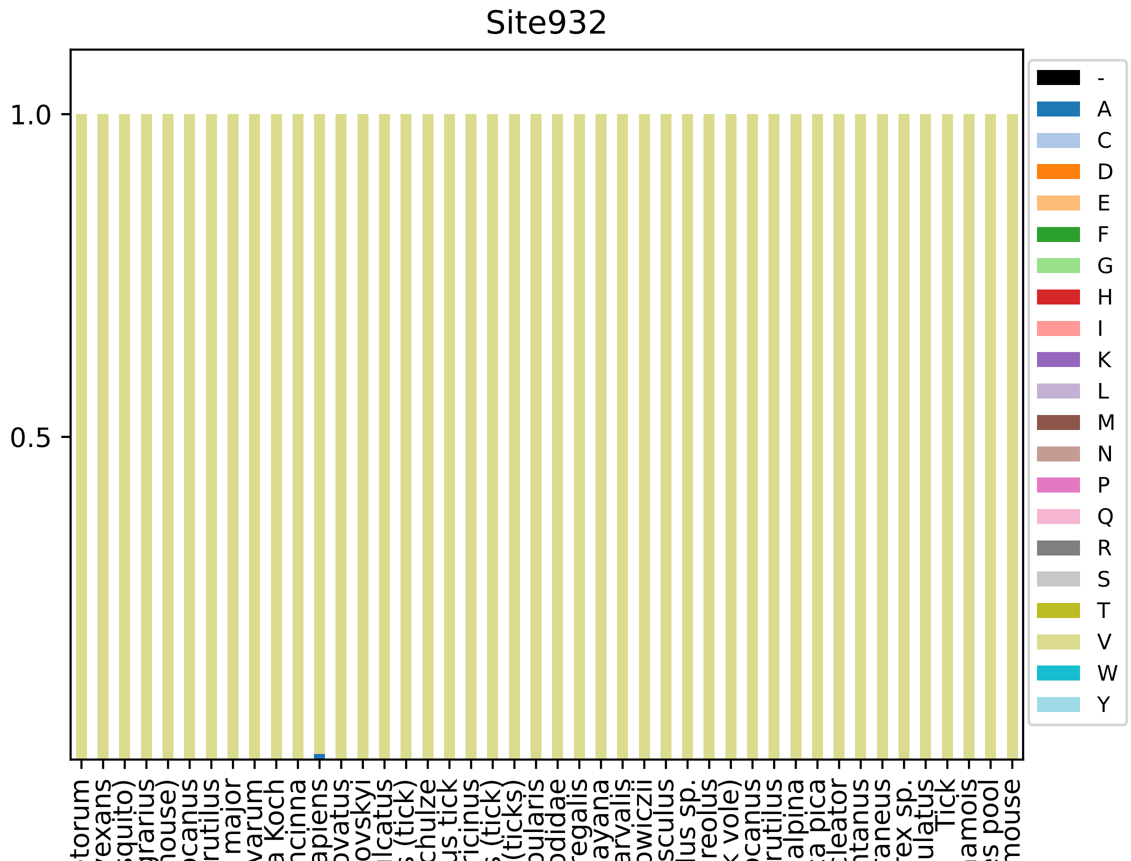
Site932
1.0
0.5
-
A
C
D
E
F
G
H
I
K
L
M
N
P
Q
R
S
T
V
W
Y

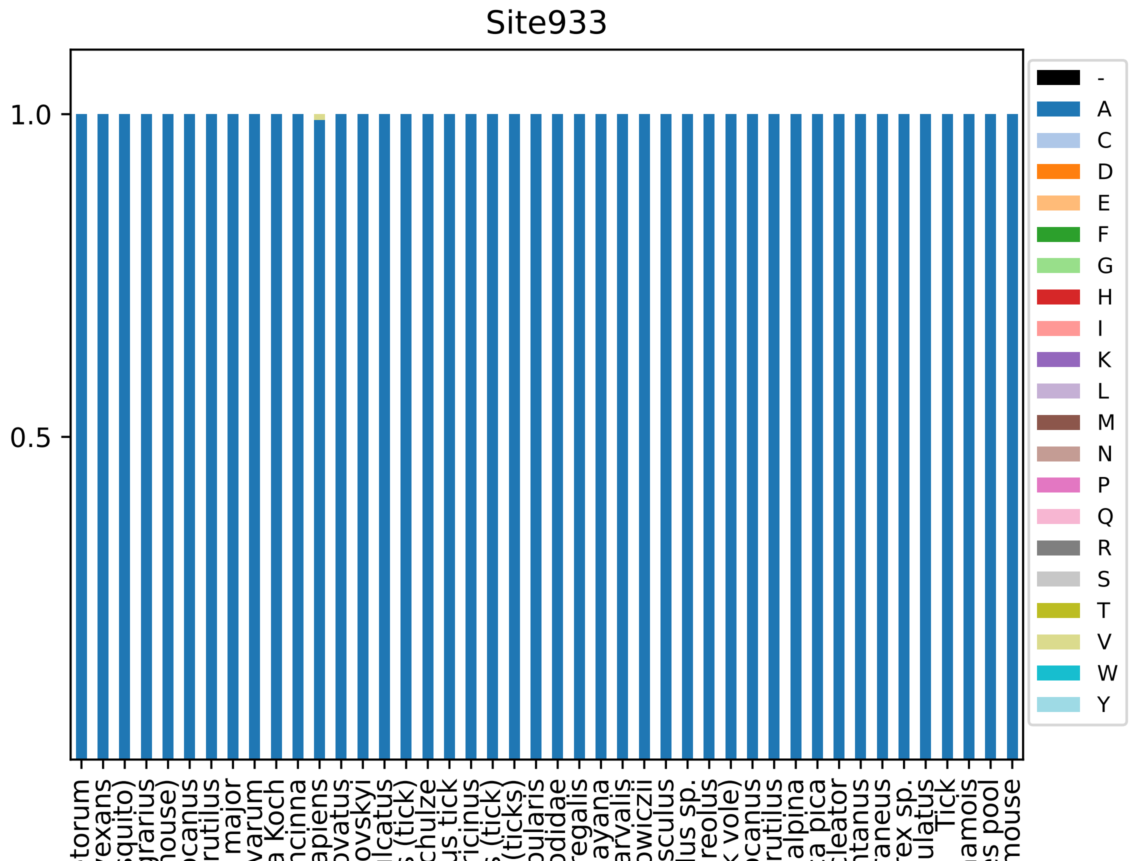

Site933
1.0
0.5
-
A
C
D
E
F
G
H
I
K
L
M
N
P
Q
R
S
T
V
W
Y

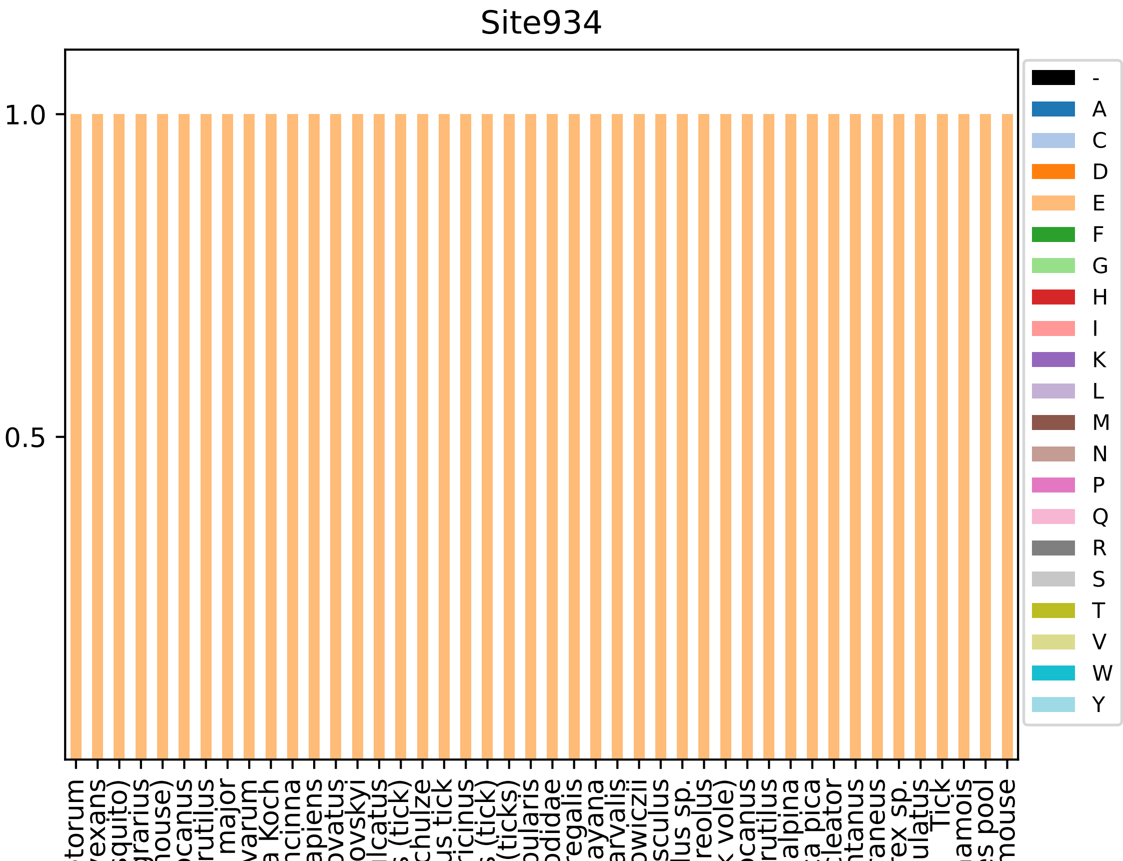

Site934
1.0
0.5
-
A
C
D
E
F
G
H
I
K
L
M
N
P
Q
R
S
T
V
W
Y

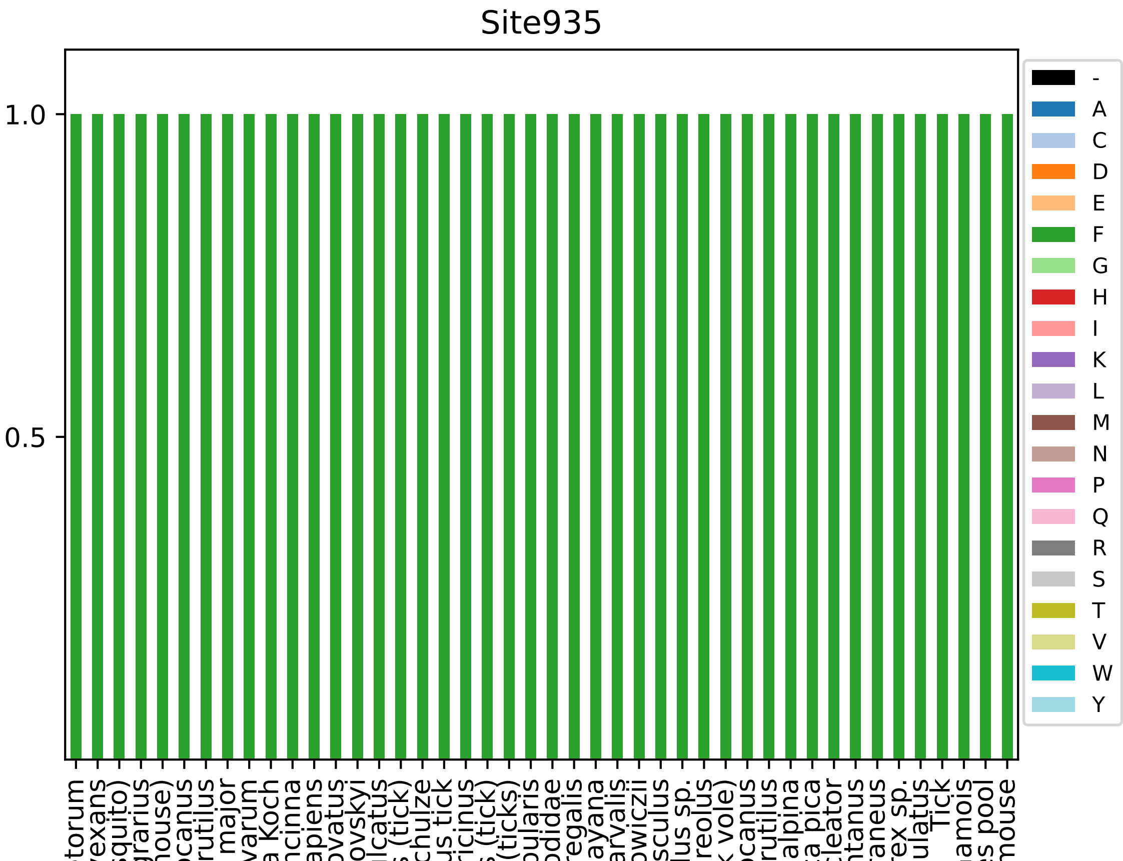

Site935
1.0
0.5
-
A
C
D
E
F
G
H
I
K
L
M
N
P
Q
R
S
T
V
W
Y

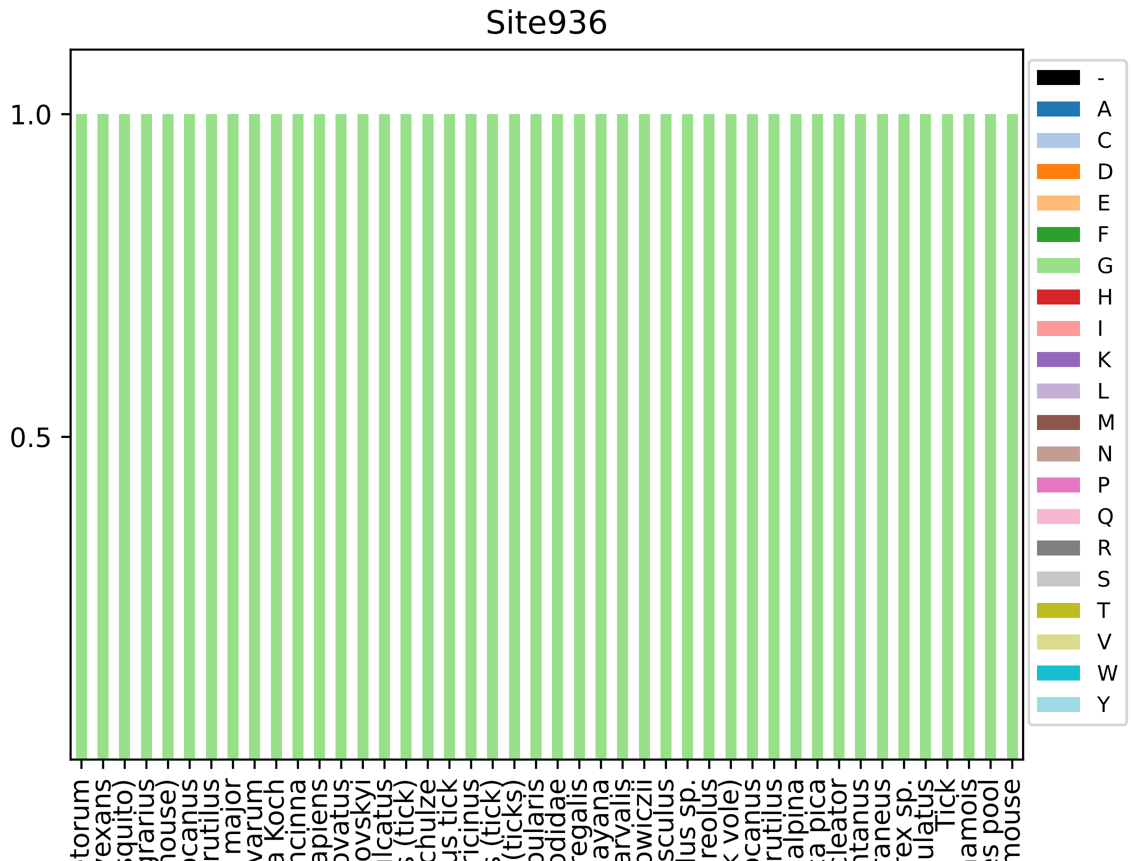
Site936
1.0
0.5
-
A
C
D
E
F
G
H
I
K
L
M
N
P
Q
R
S
T
V
W
Y

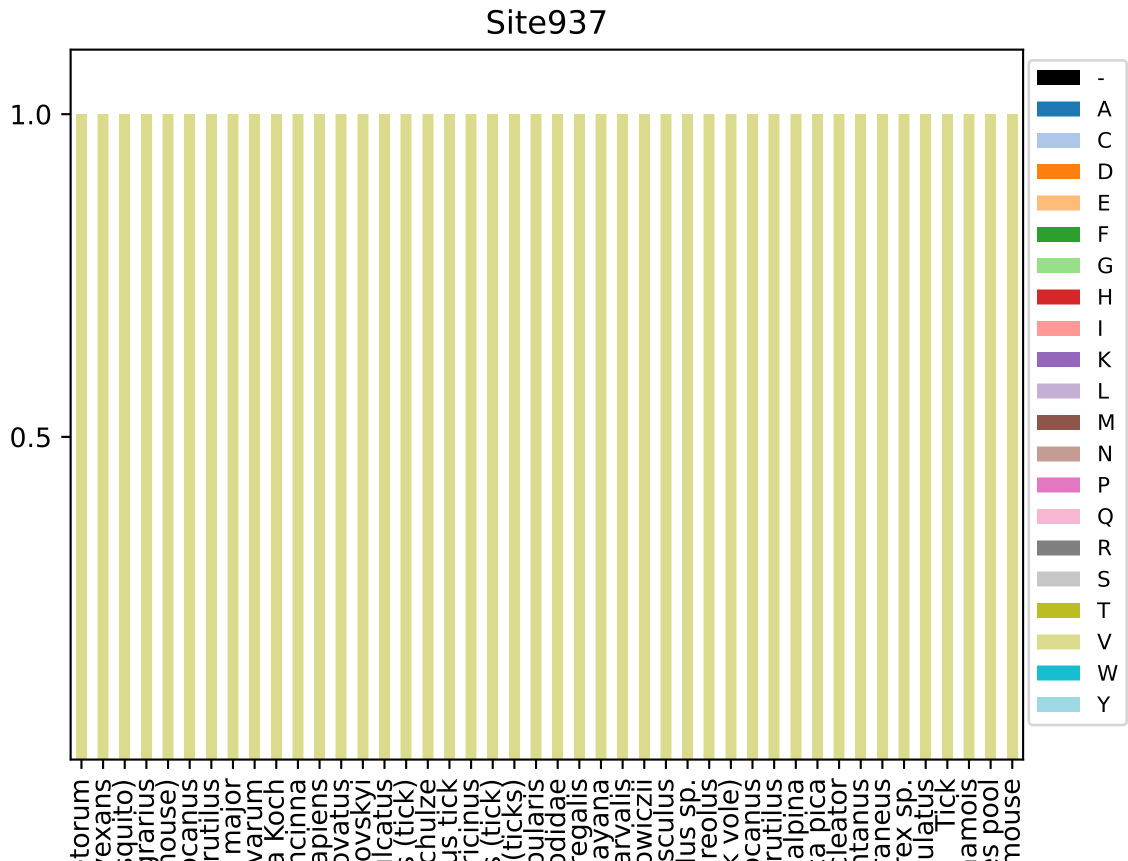

Site937
1.0
0.5
-
A
C
D
E
F
G
H
I
K
L
M
N
P
Q
R
S
T
V
W
Y

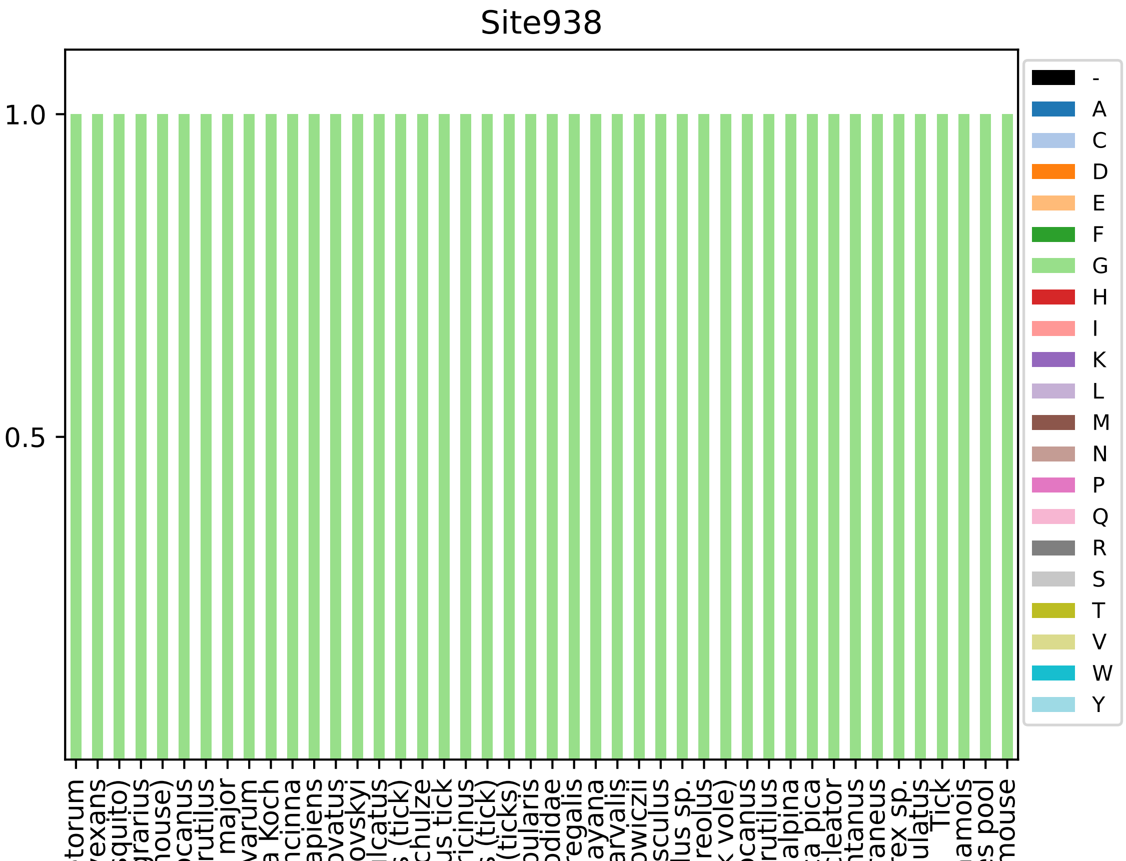

Site938
1.0
0.5
-
A
C
D
E
F
G
H
I
K
L
M
N
P
Q
R
S
T
V
W
Y

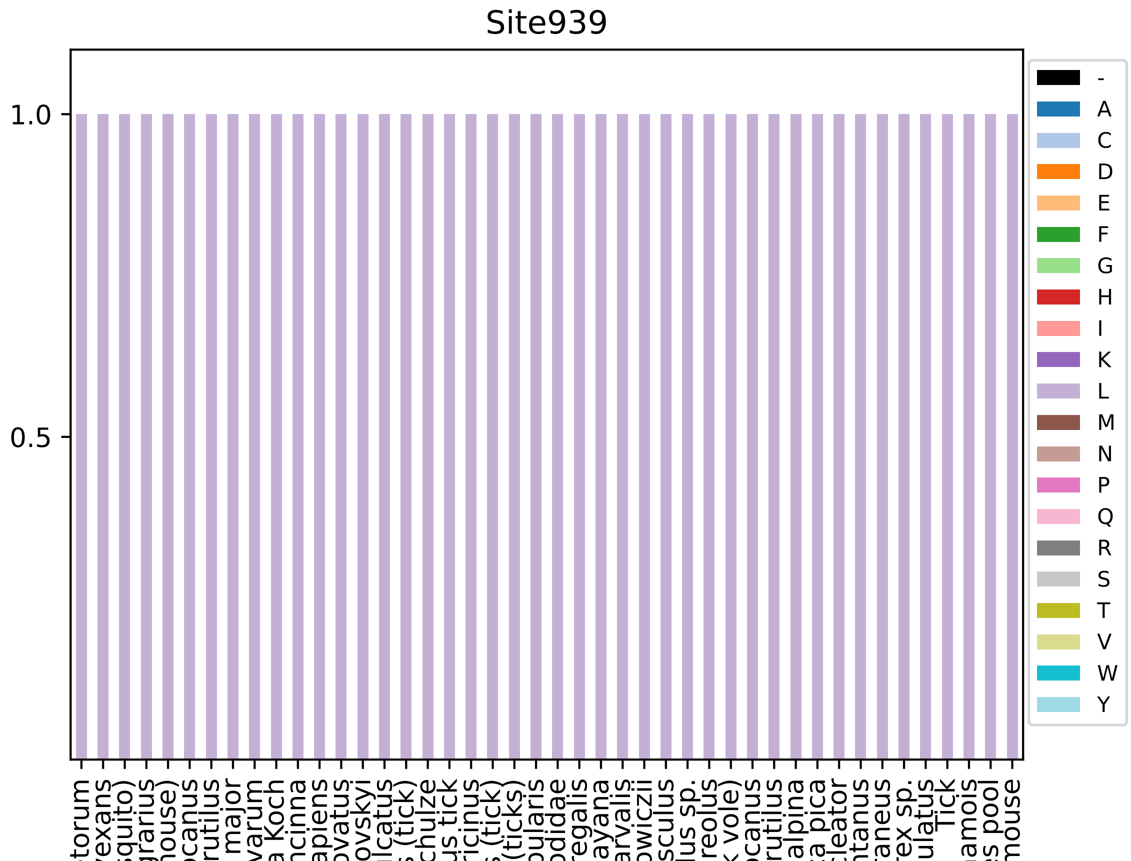
Site939
1.0
0.5
-
A
C
D
E
F
G
H
I
K
L
M
N
P
Q
R
S
T
V
W
Y

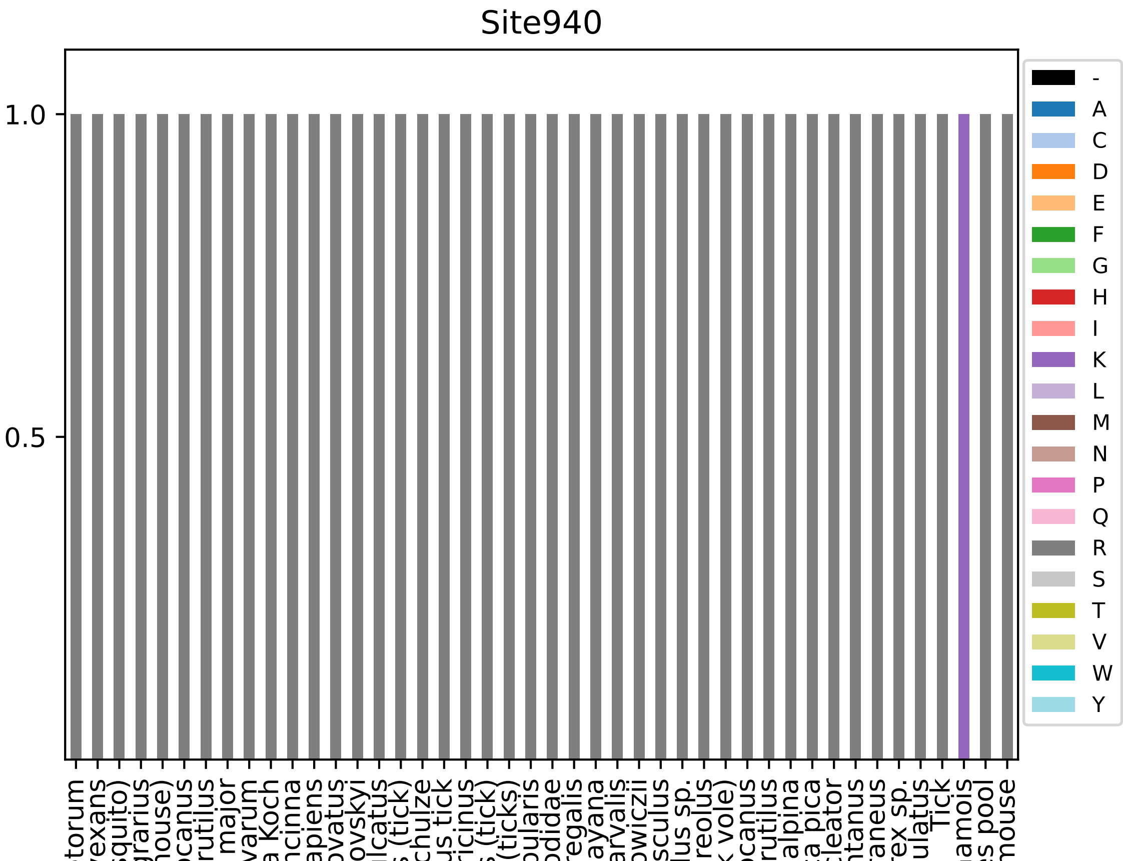

Site940
1.0
0.5
-
A
C
D
E
F
G
H
I
K
L
M
N
P
Q
R
S
T
V
W
Y
Tick

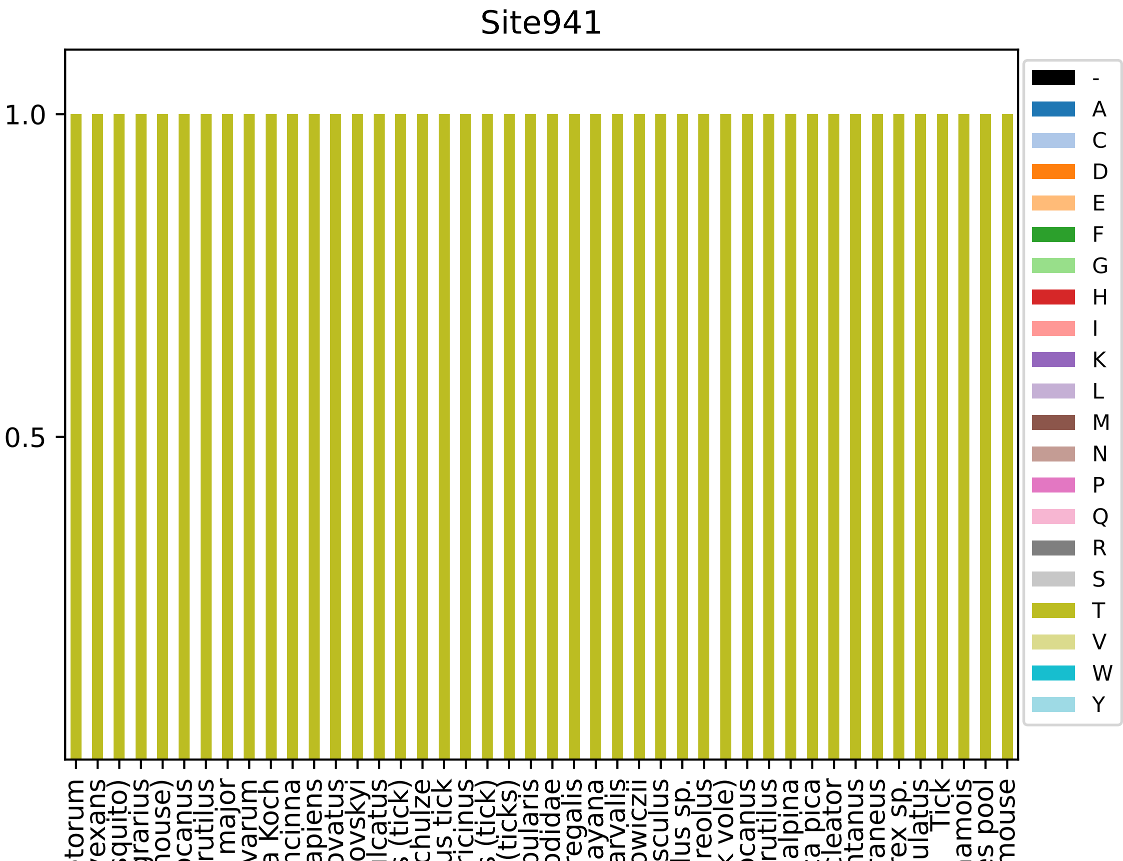

Site941
1.0
0.5
-
A
C
D
E
F
G
H
I
K
L
M
N
P
Q
R
S
T
V
W
Y

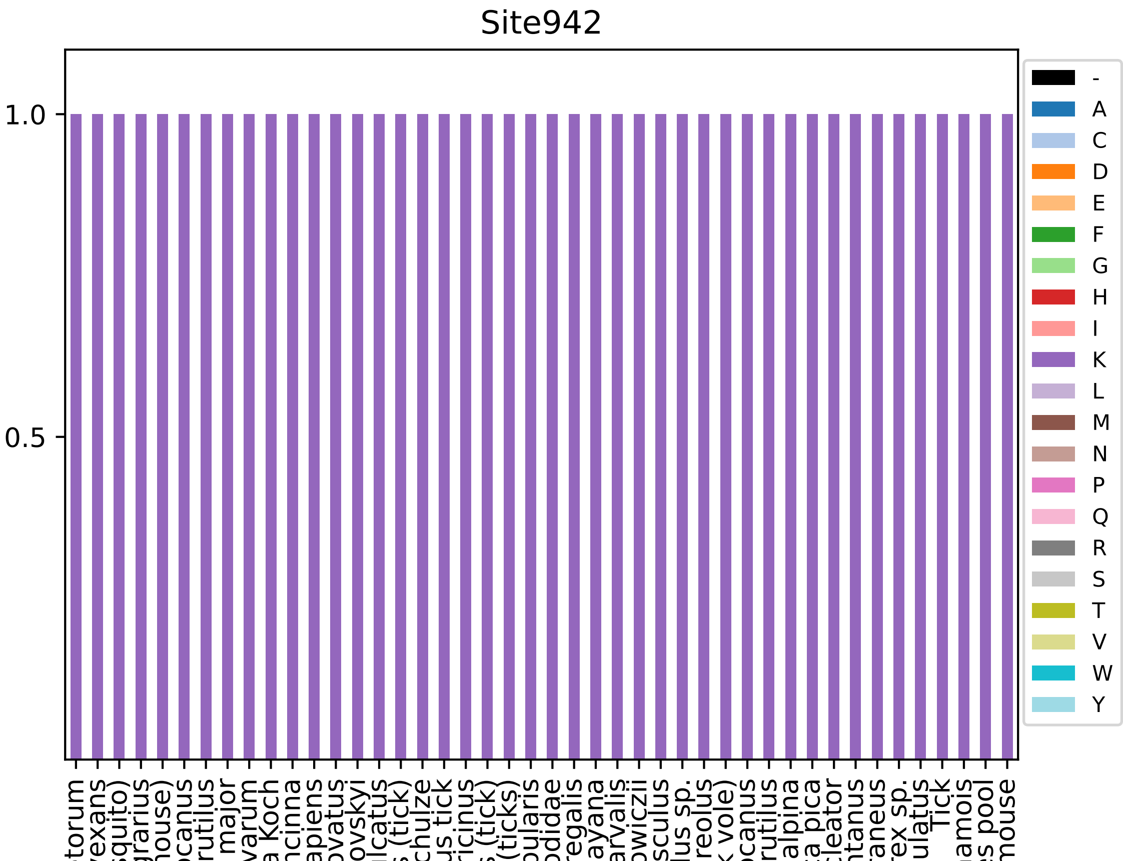
Site942
1.0
0.5
-
A
C
D
E
F
G
H
I
K
L
M
N
P
Q
R
S
T
V
W
Y

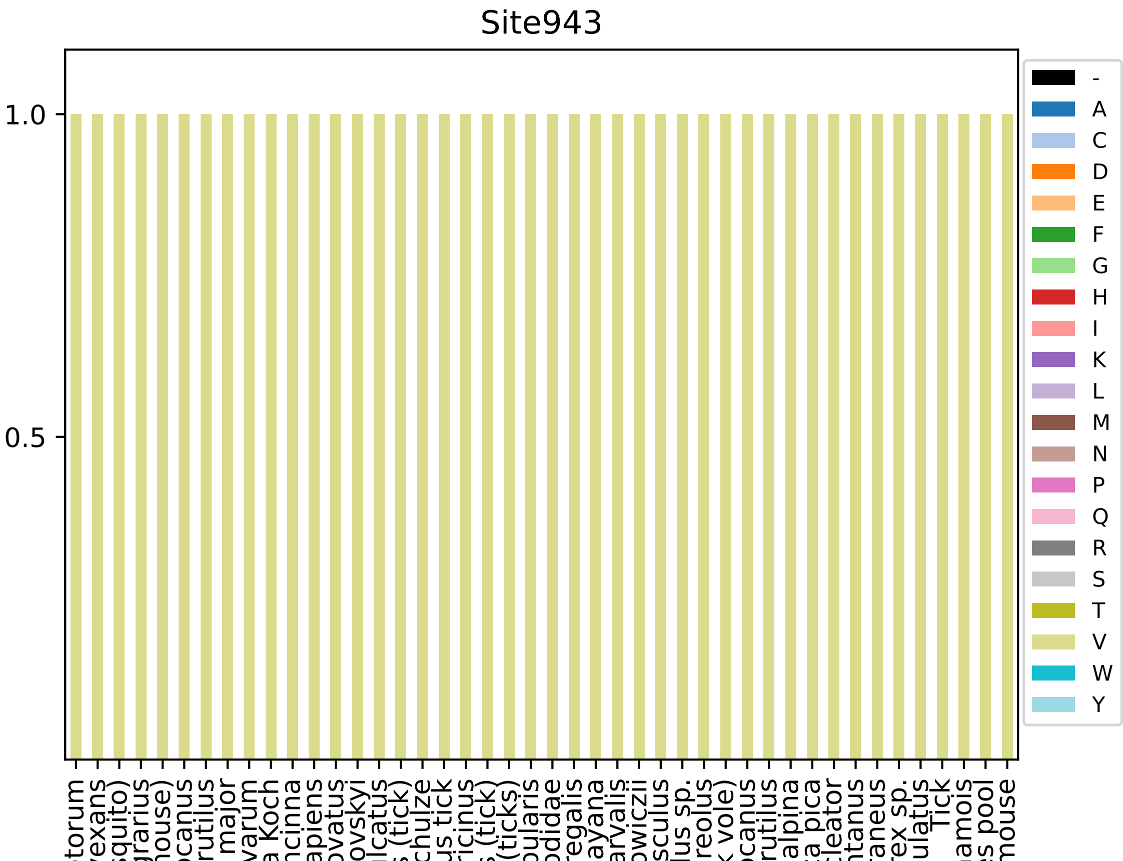
Site943
1.0
0.5
-
A
C
D
E
F
G
H
I
K
L
M
N
P
Q
R
S
T
V
W
Y

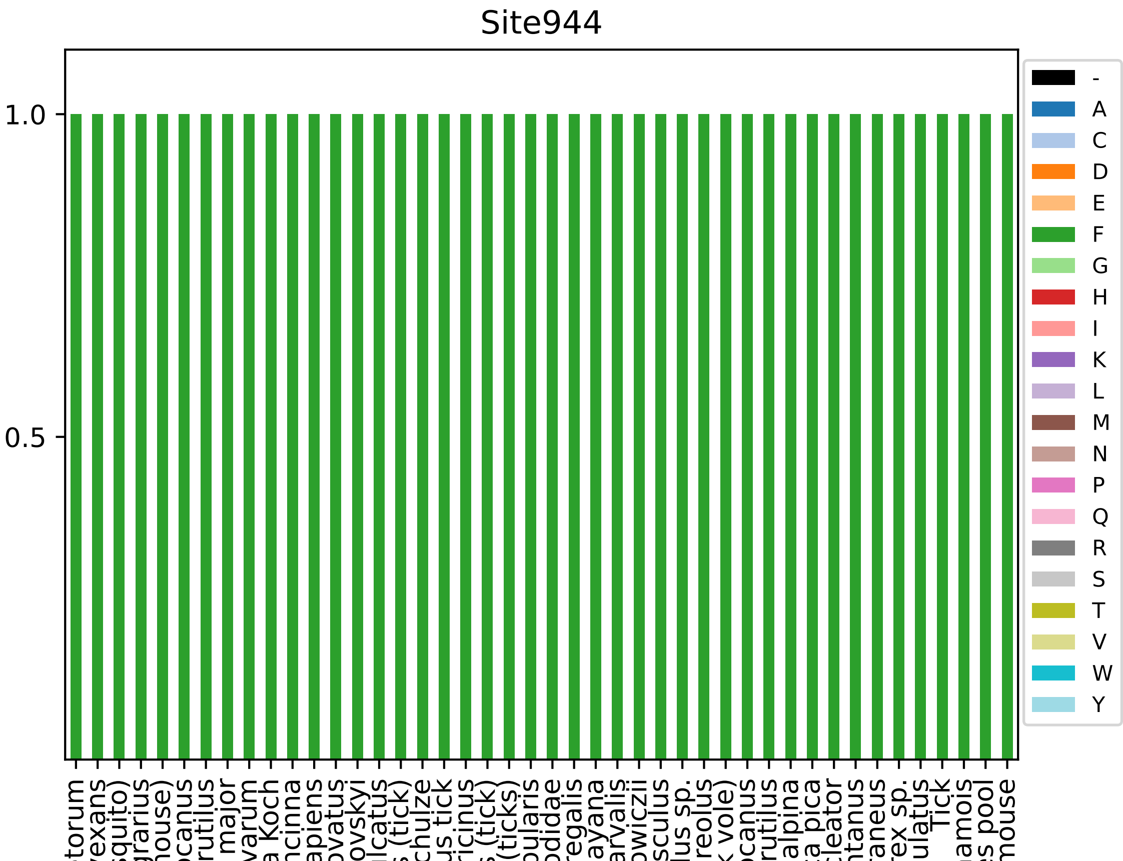

Site944
1.0
0.5
-
A
C
D
E
F
G
H
I
K
L
M
N
P
Q
R
S
T
V
W
Y

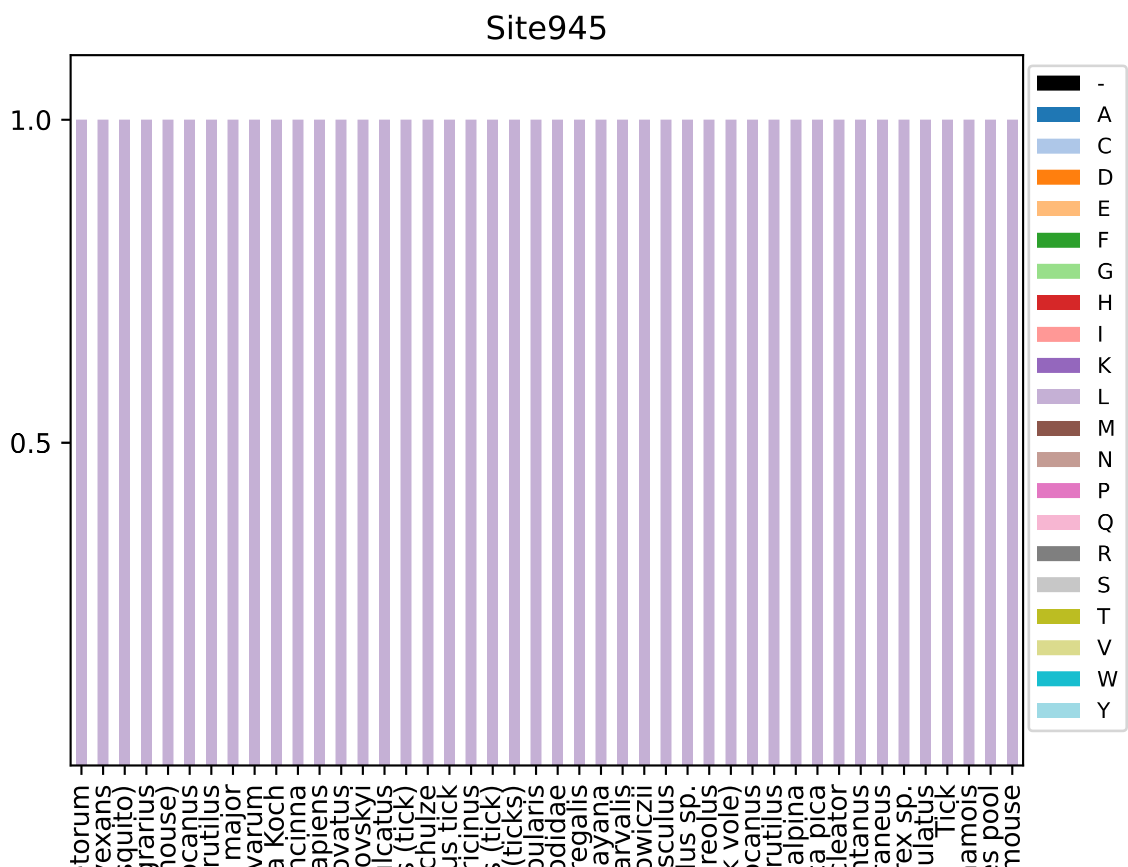
Site945
1.0
0.5
-
A
C
D
E
F
G
H
I
K
L
M
N
P
Q
R
S
T
V
W
Y

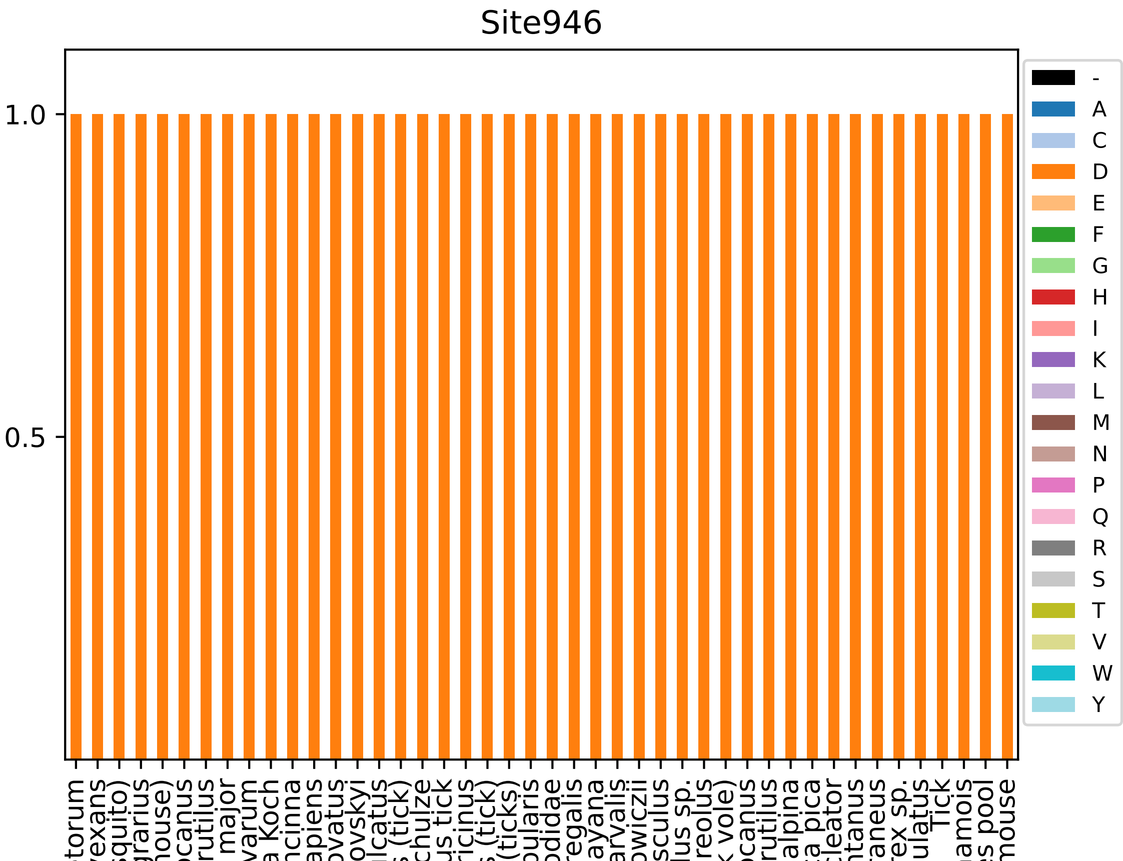

Site946
1.0
0.5
-
A
C
D
E
F
G
H
I
K
L
M
N
P
Q
R
S
T
V
W
Y

Site947

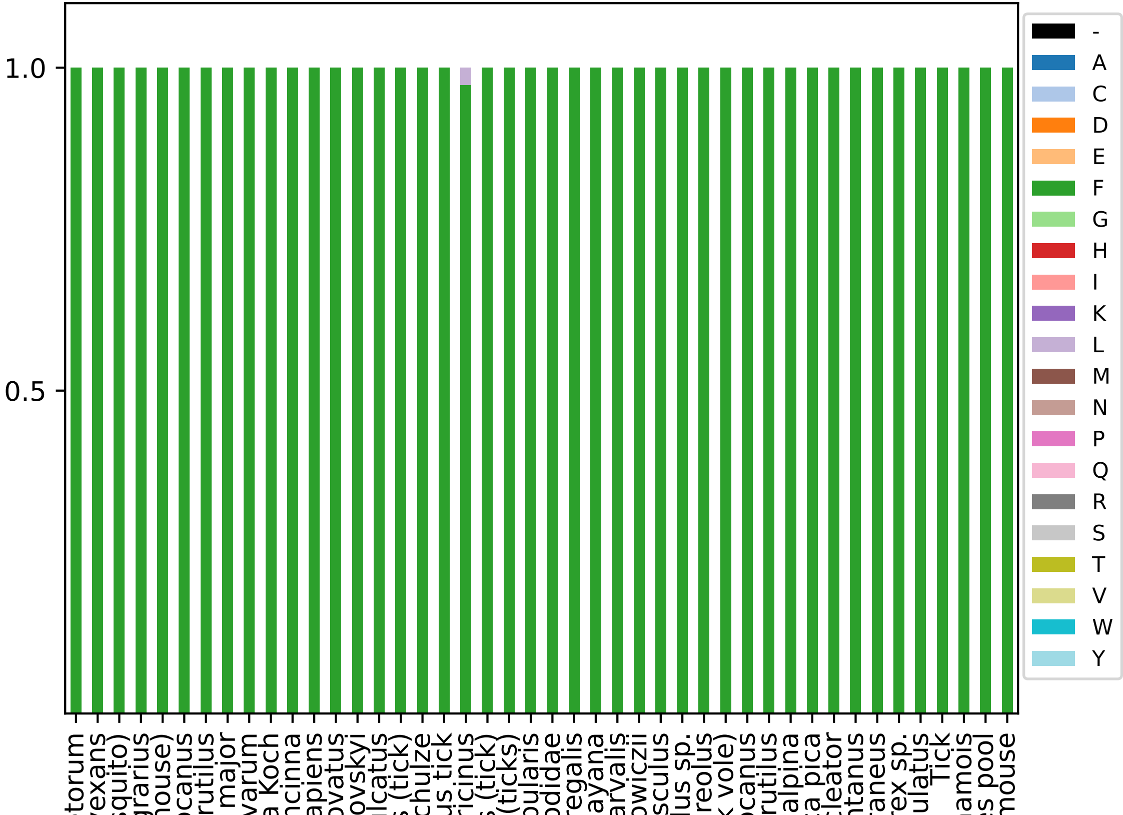

Site948

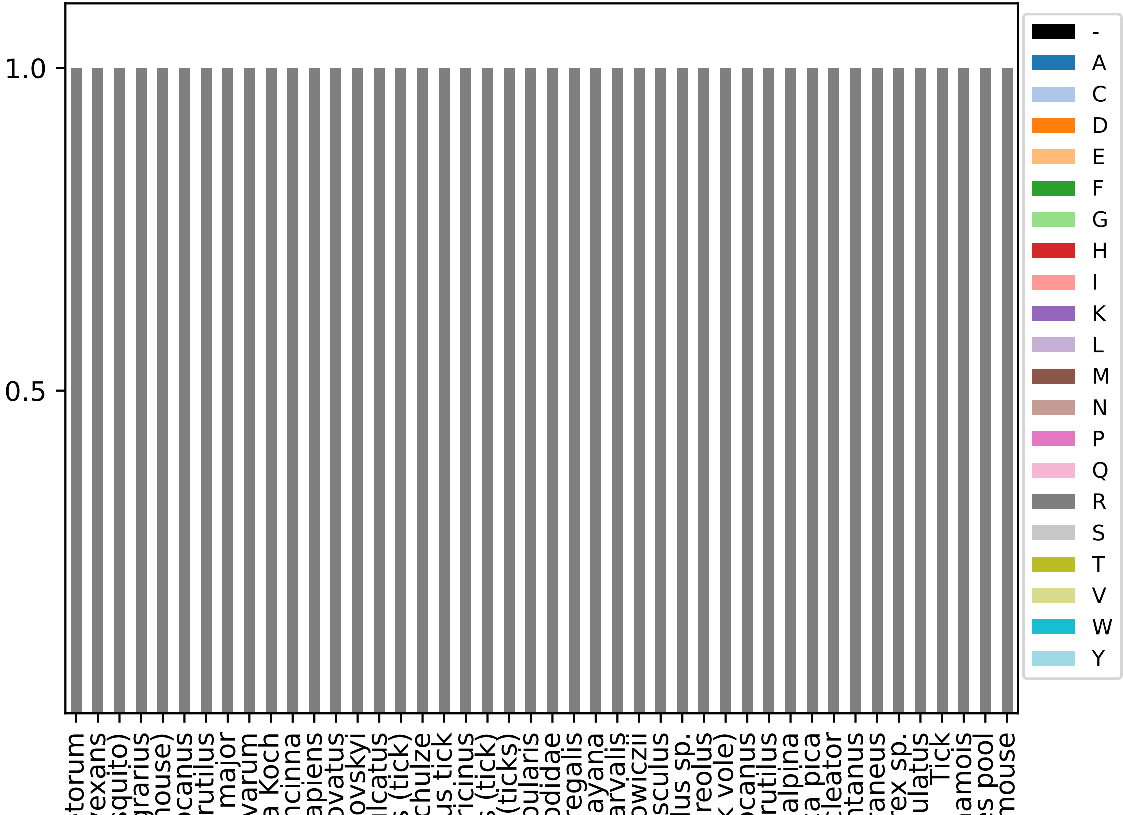

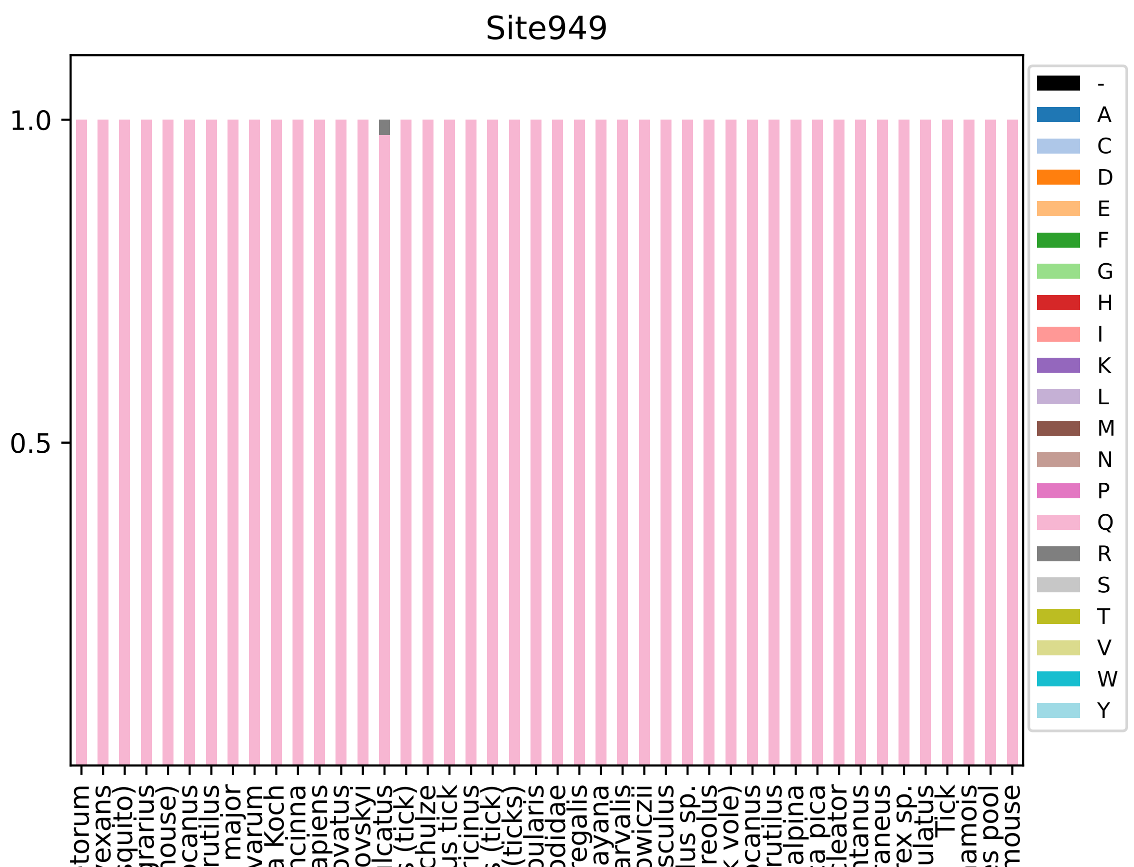
Site949
1.0
0.5
-
A
C
D
E
F
G
H
I
K
L
M
N
P
Q
R
S
T
V
W
Y

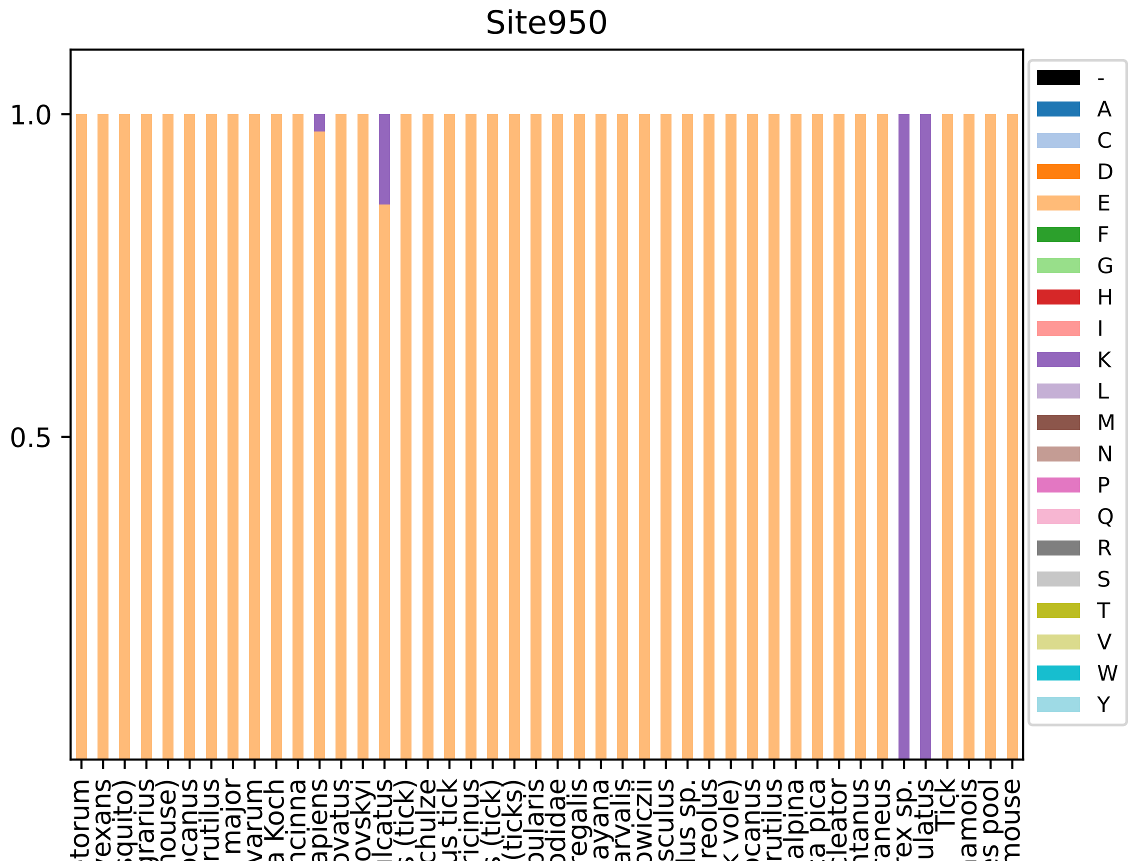
Site950
1.0
0.5
-
A
C
D
E
F
G
H
I
K
L
M
N
P
Q
R
S
T
V
W
Y

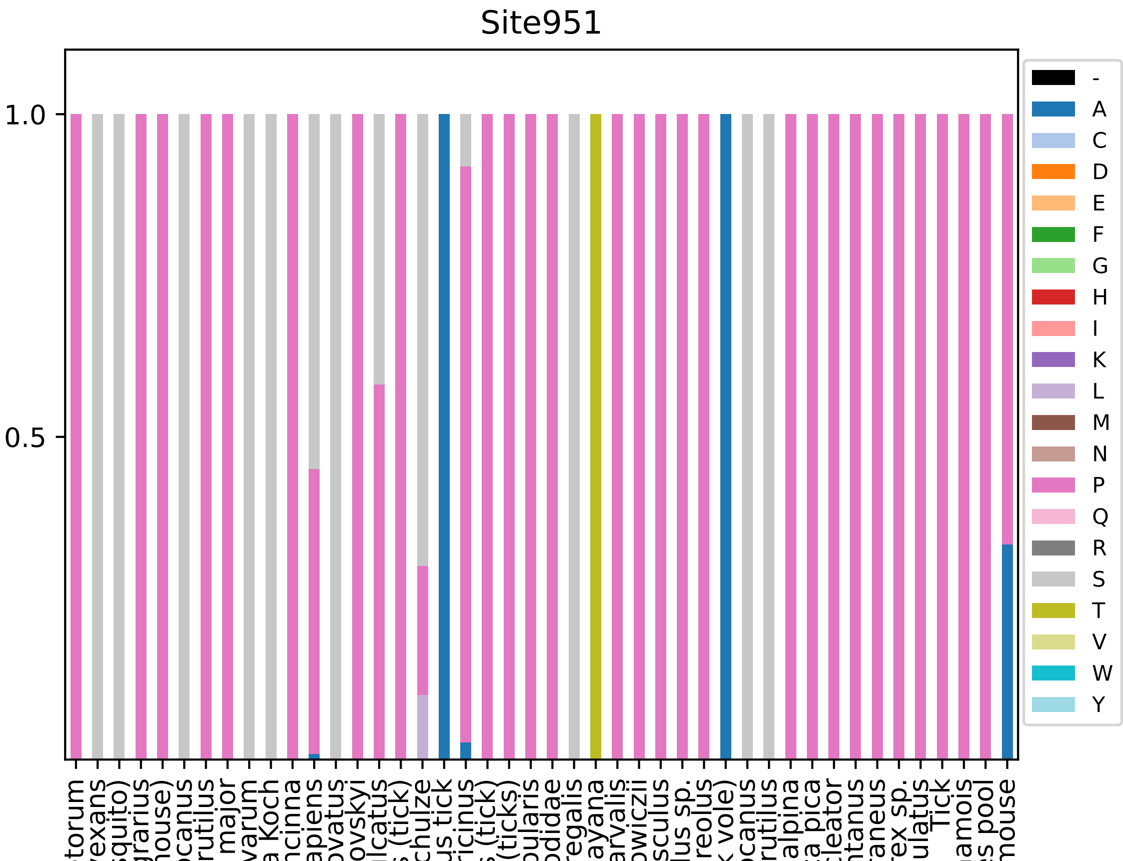

Site951
1.0
0.5
-
A
C
D
E
F
G
H
I
K
L
M
N
P
Q
R
S
T
V
W
Y

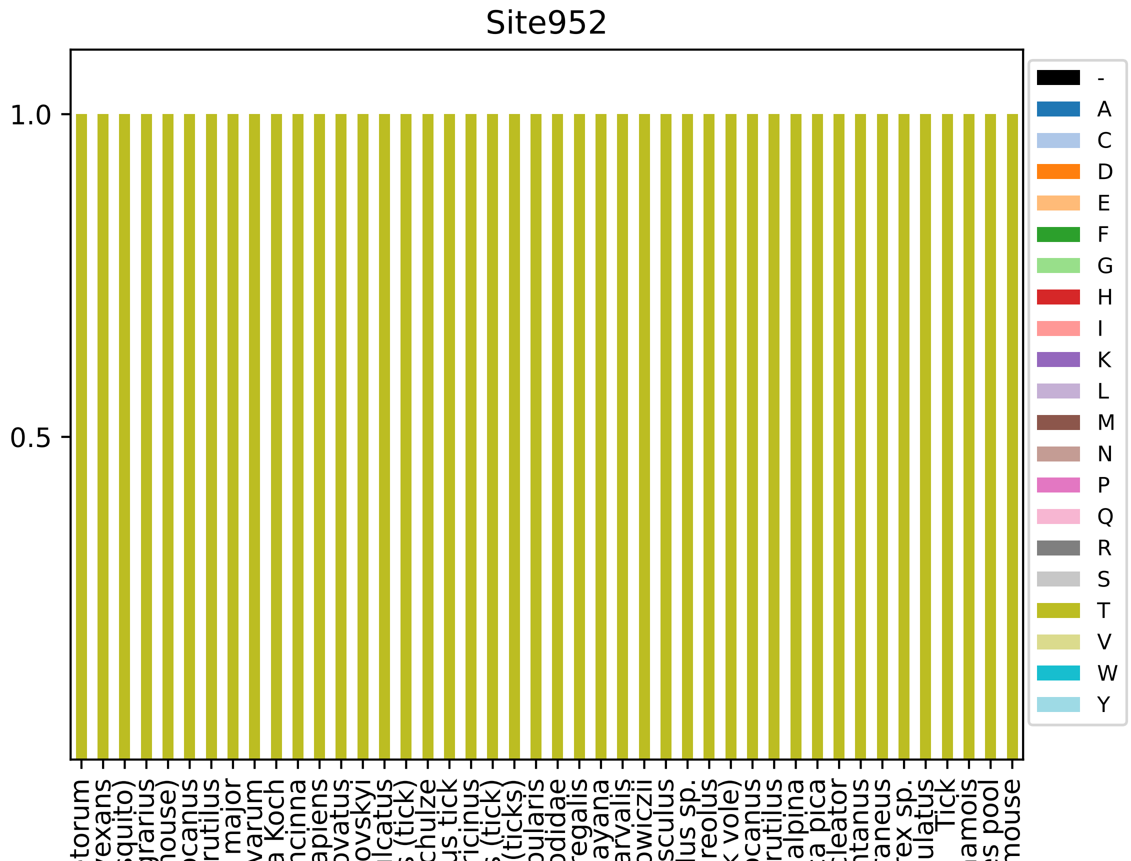
Site952
1.0
0.5
-
A
C
D
E
F
G
H
I
K
L
M
N
P
Q
R
S
T
V
W
Y

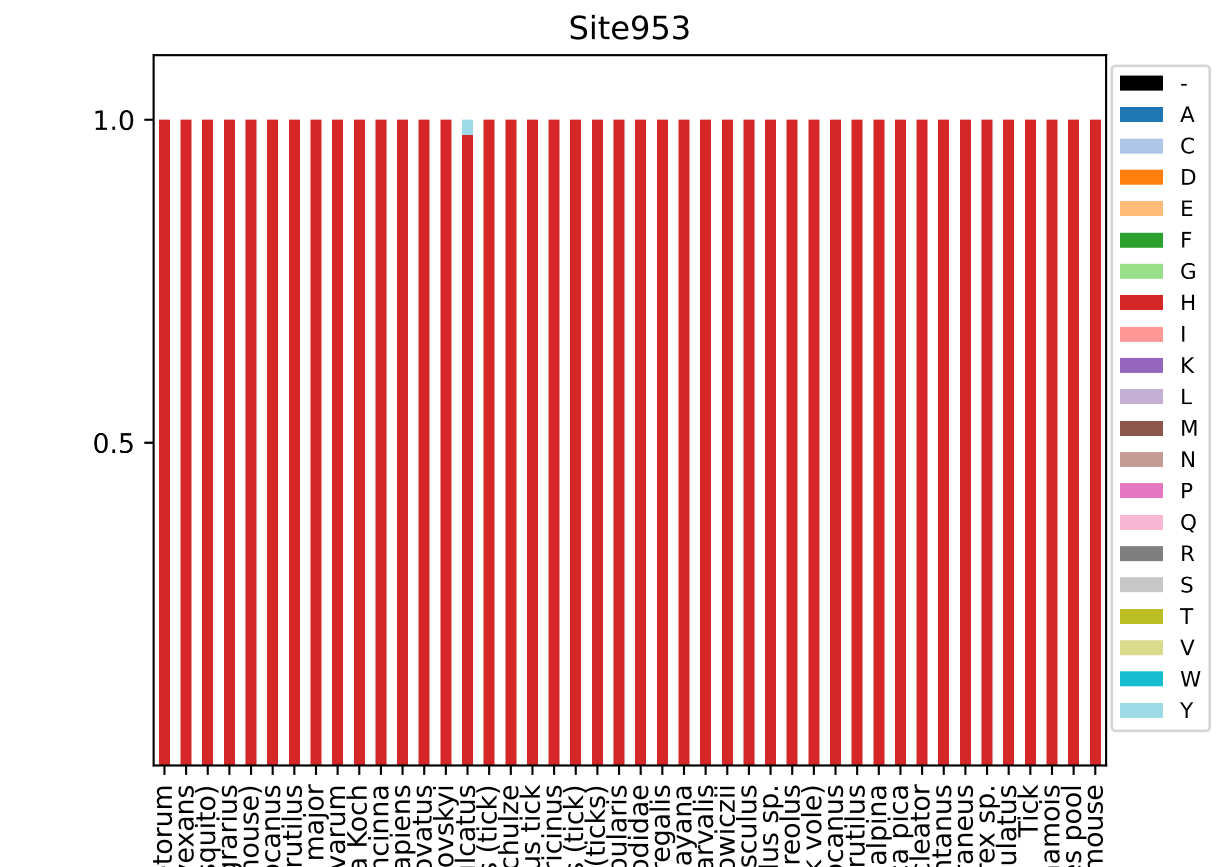

Site953
1.0
0.5
-
A
C
D
E
F
G
H
I
K
L
M
N
P
Q
R
S
T
V
W
Y

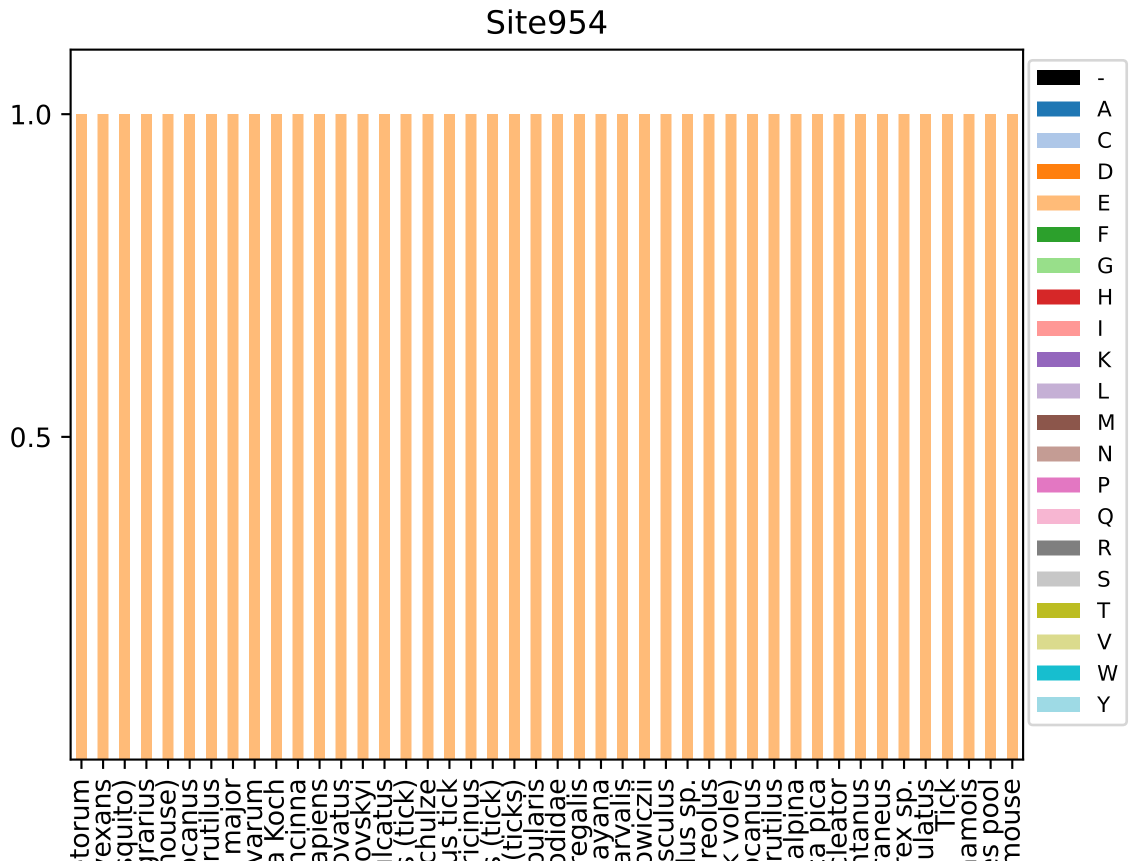
Site954
1.0
0.5
-
A
C
D
E
F
G
H
I
K
L
M
N
P
Q
R
S
T
V
W
Y
Tick

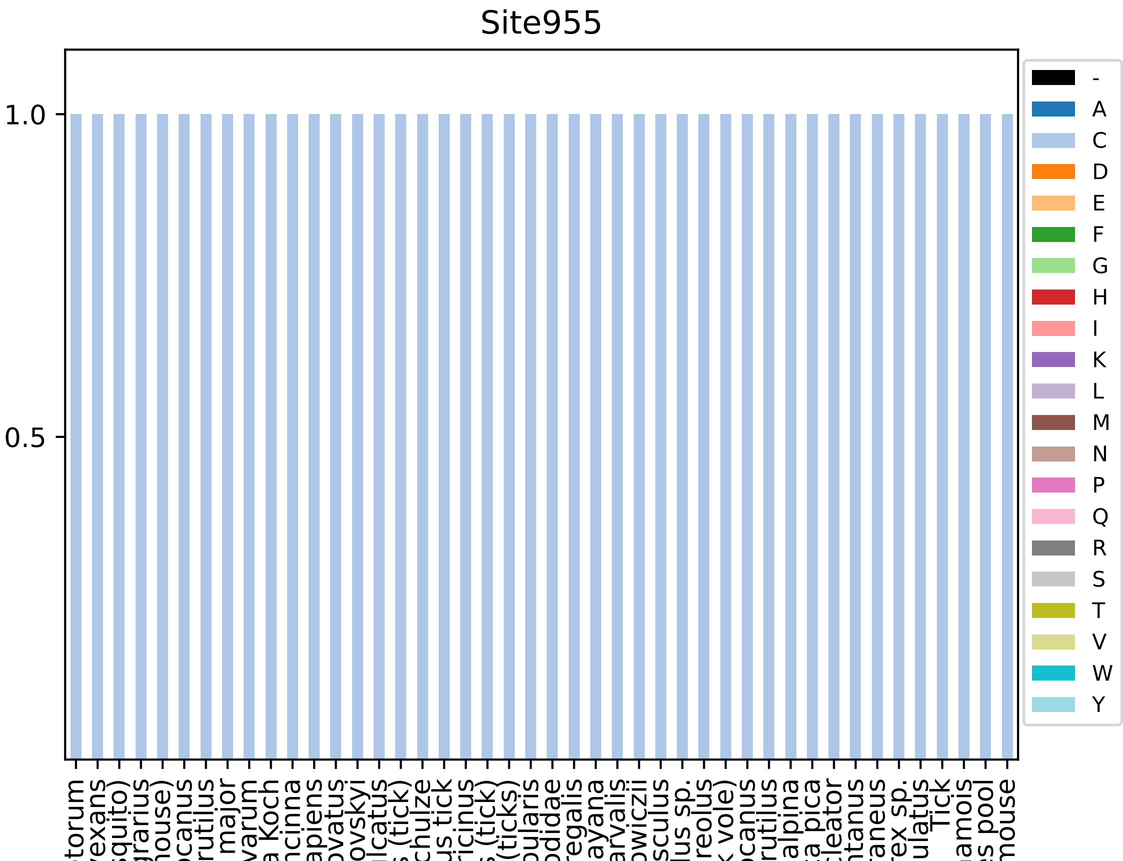
Site955
1.0
0.5
-
A
C
D
E
F
G
H
I
K
L
M
N
P
Q
R
S
T
V
W
Y
torum
vexans
squito)
grarius
nouse)
ocanus
rutilus
major
varum
a Koch
ncinna
apiens
ovatus
ovskyi
ulcatus
s (tick)
chulze
us tick
ricinus
s (tick)
(ticks)
pularis
podidae
regalis
ayana
arvalis
owiczii
isculus
lus sp.
reolus
k vole)
ocanus
rutilus
alpina
a pica
cleator
ntanus
raneus
rex sp.
ulatus
Tick
amois
es pool
mouse

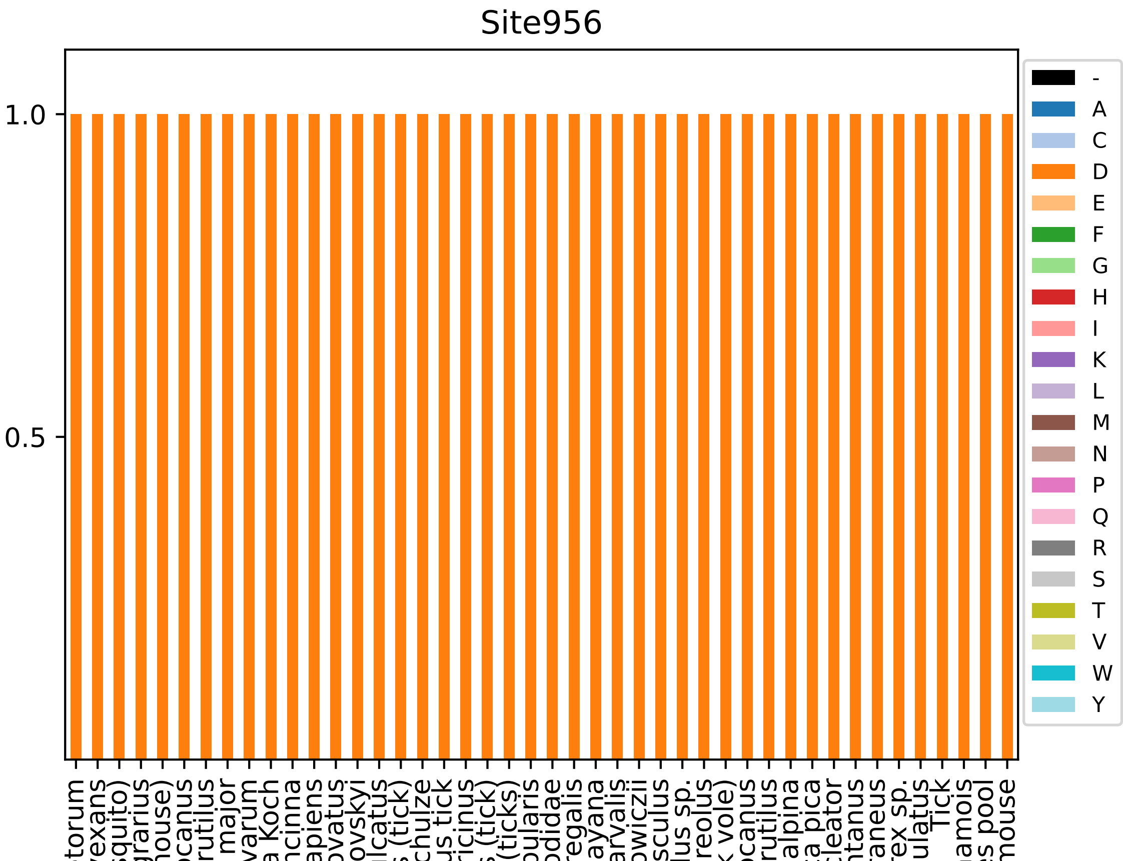
Site956
1.0
0.5
-
A
C
D
E
F
G
H
I
K
L
M
N
P
Q
R
S
T
V
W
Y

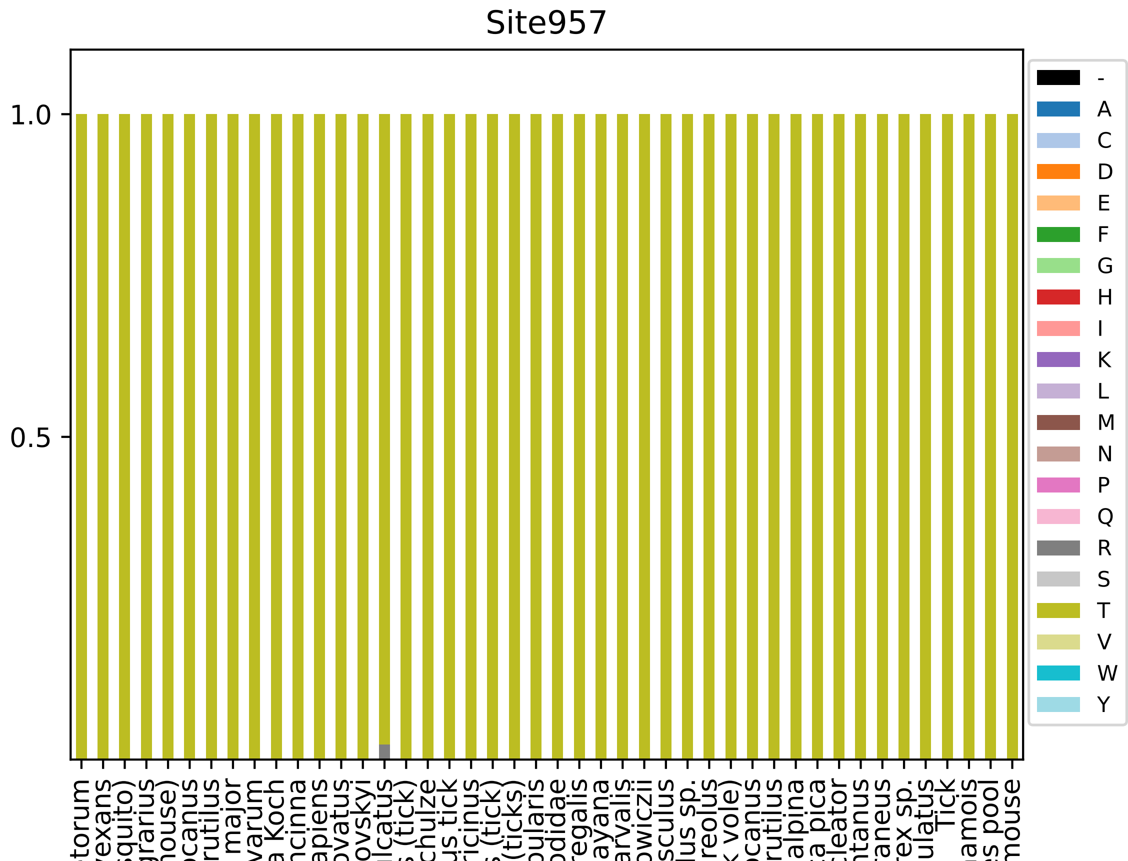
Site957
1.0
0.5
-
A
C
D
E
F
G
H
I
K
L
M
N
P
Q
R
S
T
V
W
Y

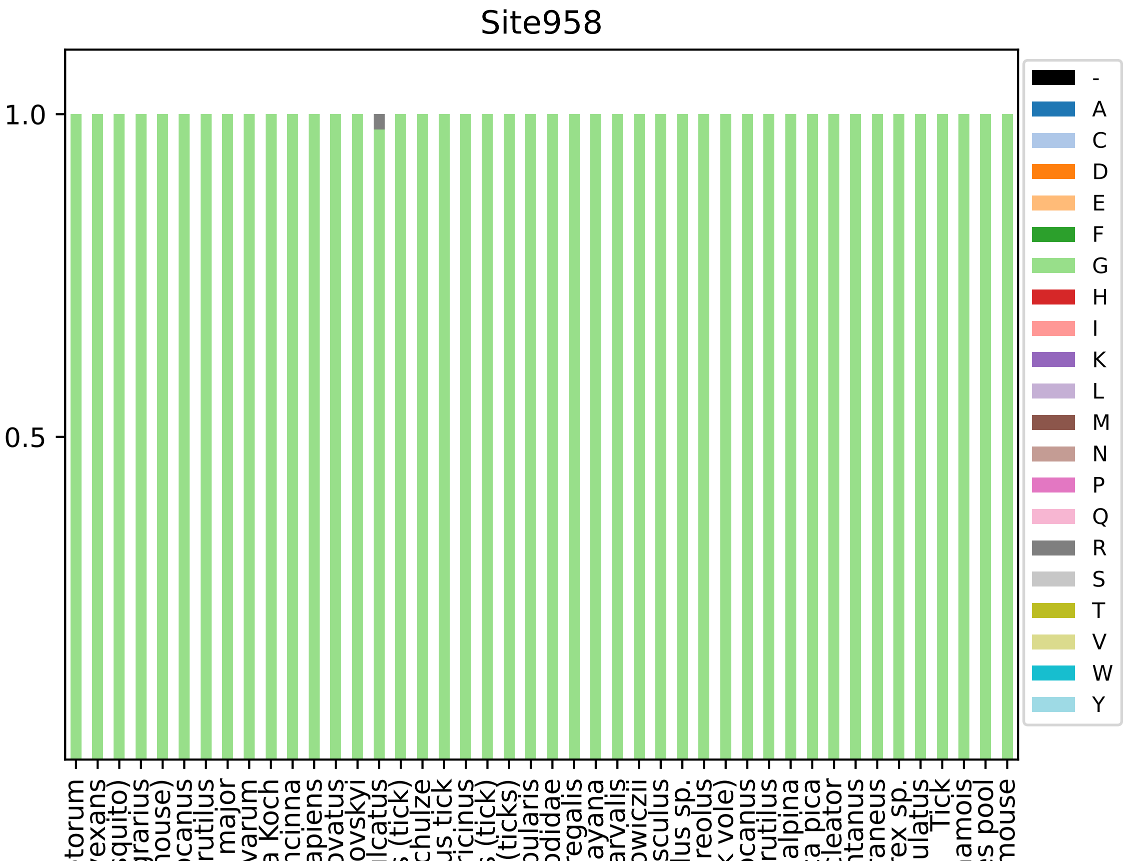

Site958
1.0
0.5
-
A
C
D
E
F
G
H
I
K
L
M
N
P
Q
R
S
T
V
W
Y

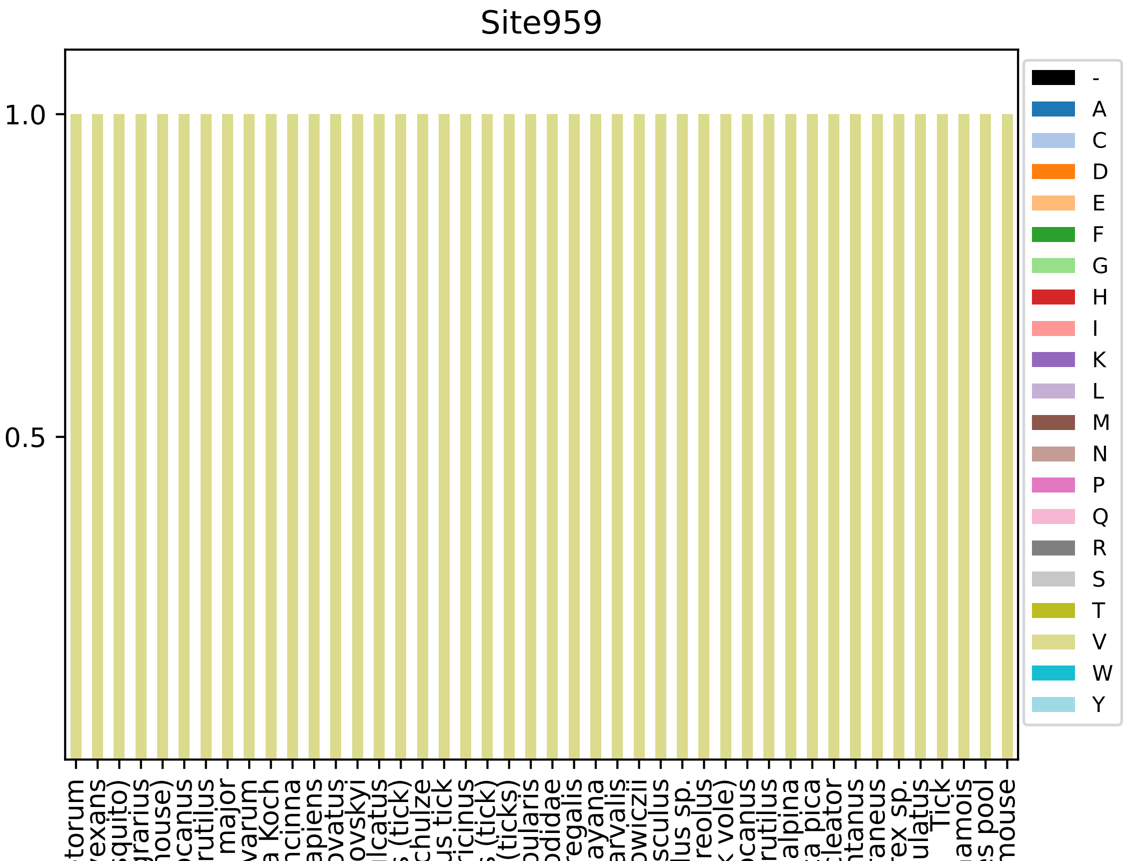
Site959
1.0
0.5
-
A
C
D
E
F
G
H
I
K
L
M
N
P
Q
R
S
T
V
W
Y

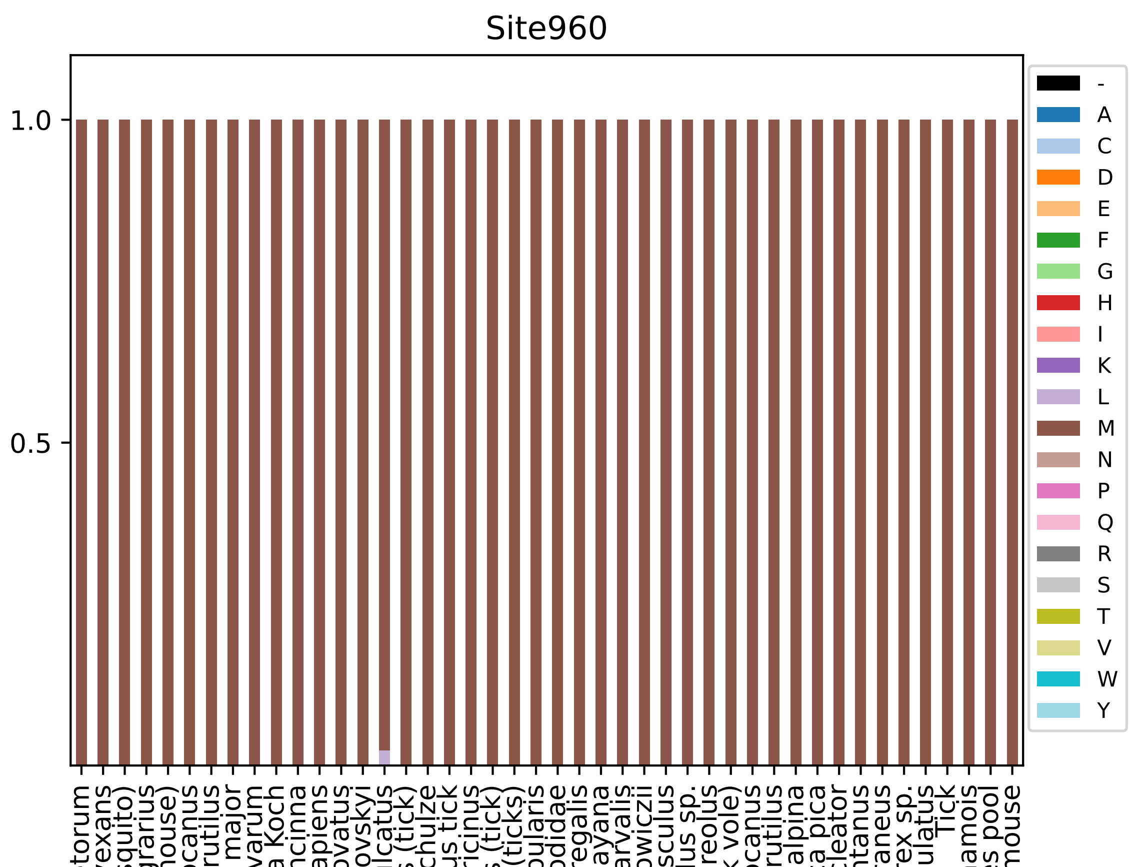
Site960
1.0
0.5
-
A
C
D
E
F
G
H
I
K
L
M
N
P
Q
R
S
T
V
W
Y

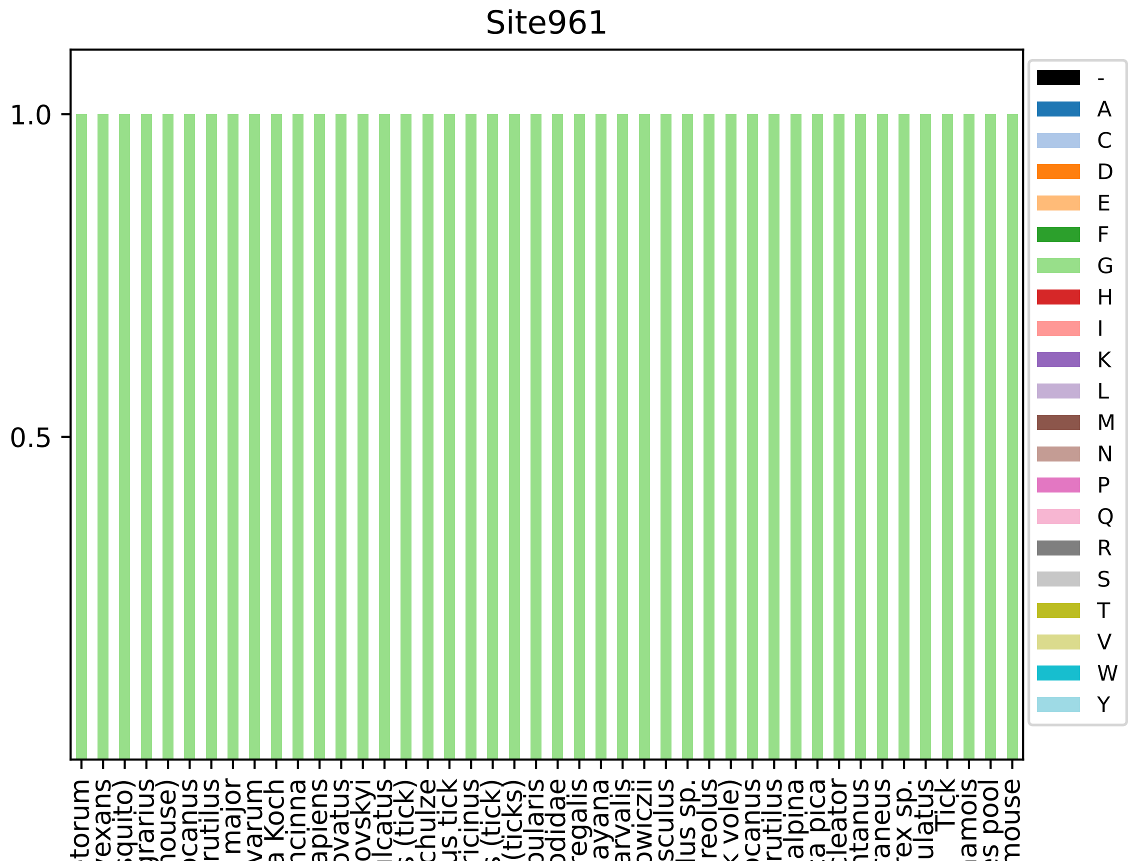
Site961
1.0
0.5
-
A
C
D
E
F
G
H
I
K
L
M
N
P
Q
R
S
T
V
W
Y

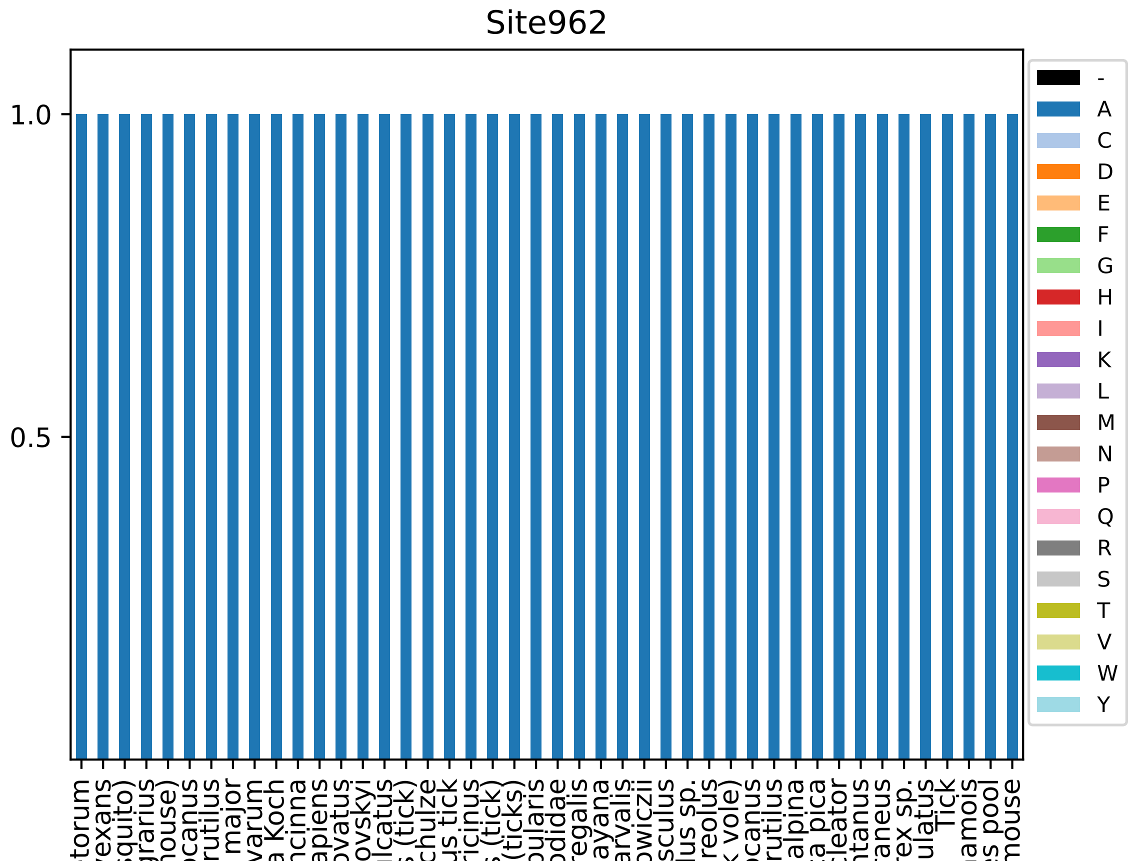
Site962
1.0
0.5
-
A
C
D
E
F
G
H
I
K
L
M
N
P
Q
R
S
T
V
W
Y

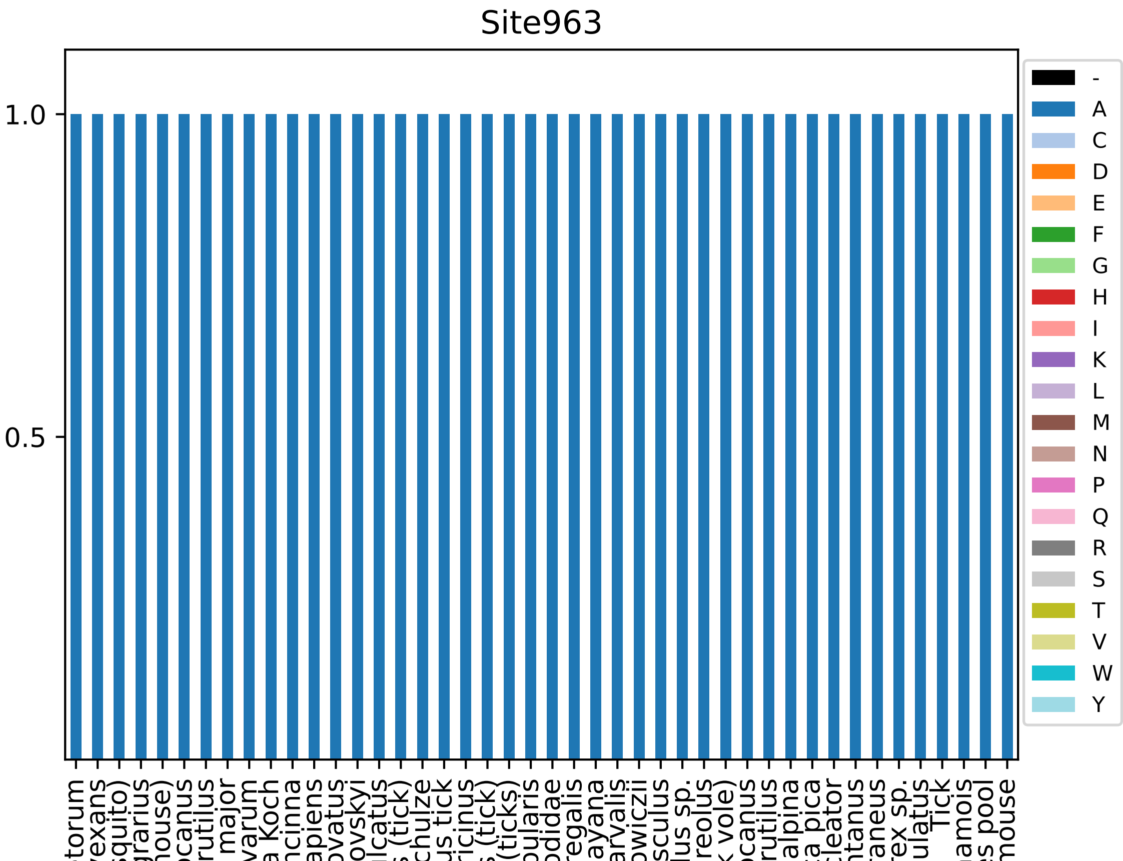
Site963
1.0
0.5
-
A
C
D
E
F
G
H
I
K
L
M
N
P
Q
R
S
T
V
W
Y

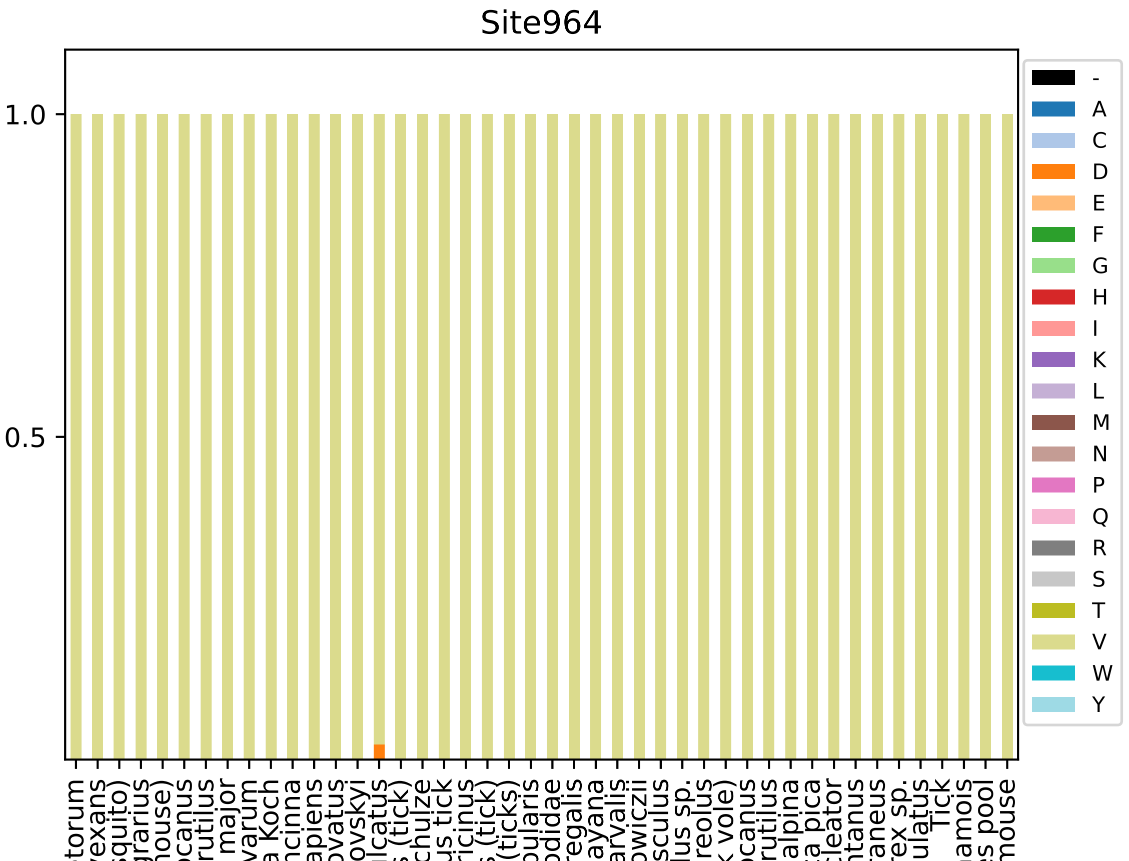
Site964
1.0
0.5
-
A
C
D
E
F
G
H
I
K
L
M
N
P
Q
R
S
T
V
W
Y

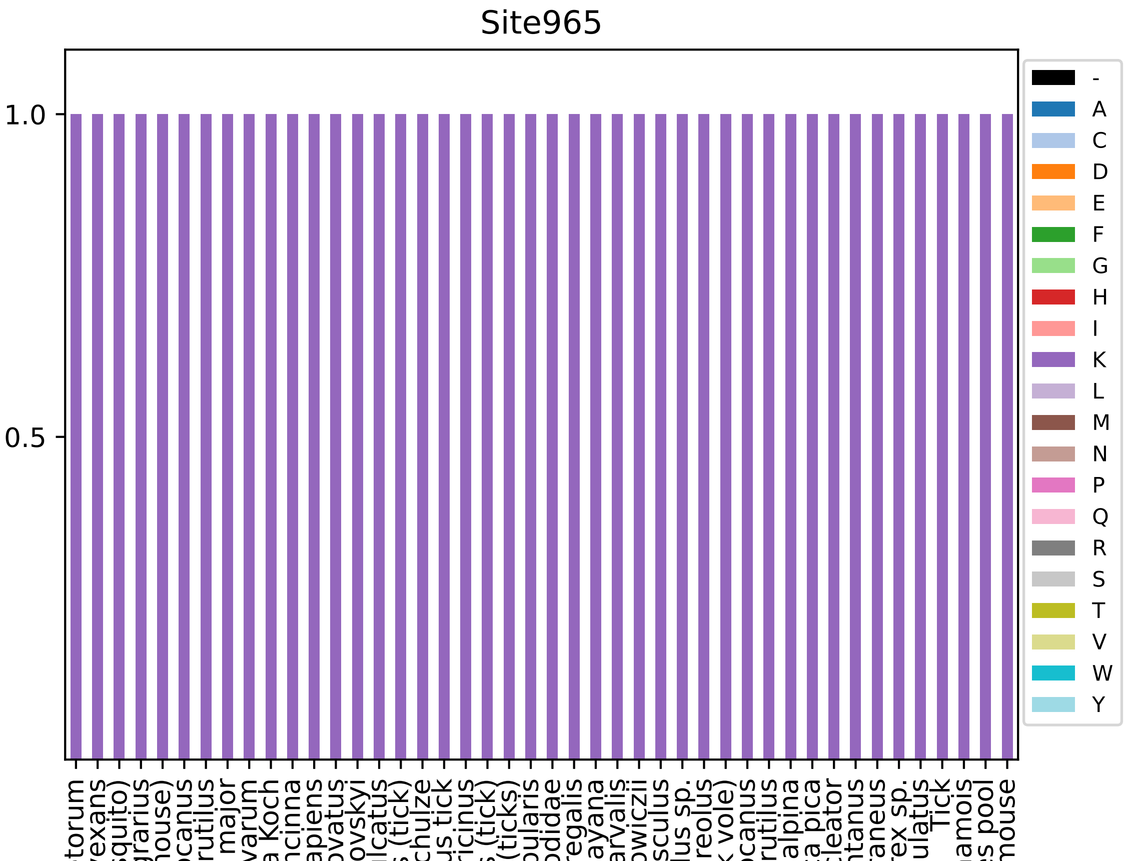
Site965
1.0
0.5
-
A
C
D
E
F
G
H
I
K
L
M
N
P
Q
R
S
T
V
W
Y
Tick

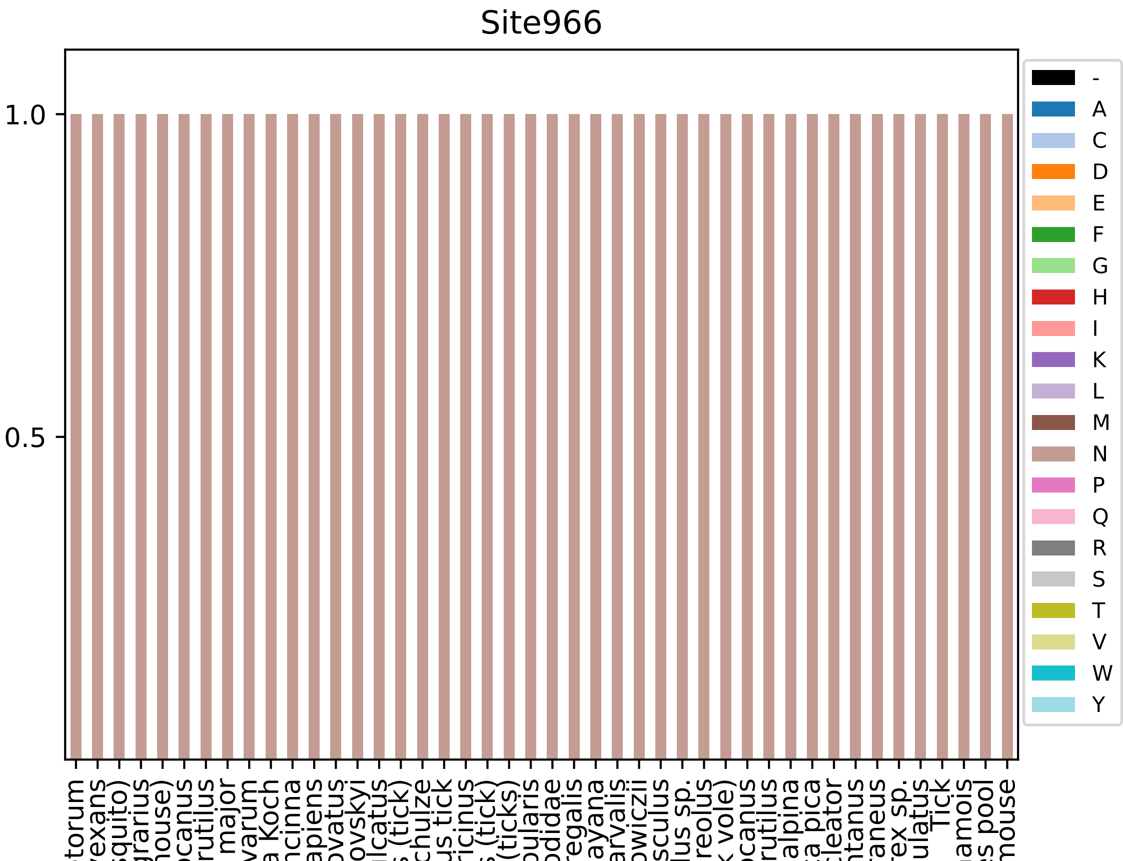
Site966
1.0
0.5
-
A
C
D
E
F
G
H
I
K
L
M
N
P
Q
R
S
T
V
W
Y

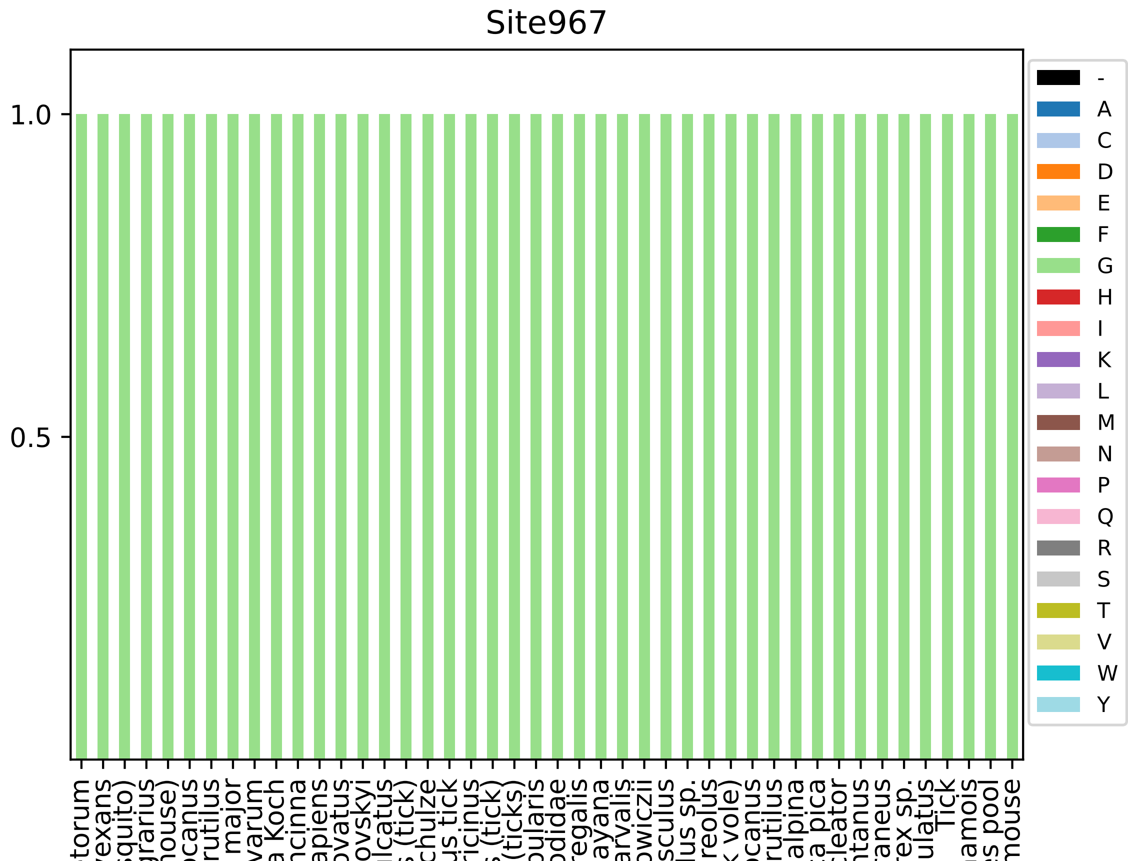
Site967
1.0
0.5
-
A
C
D
E
F
G
H
I
K
L
M
N
P
Q
R
S
T
V
W
Y

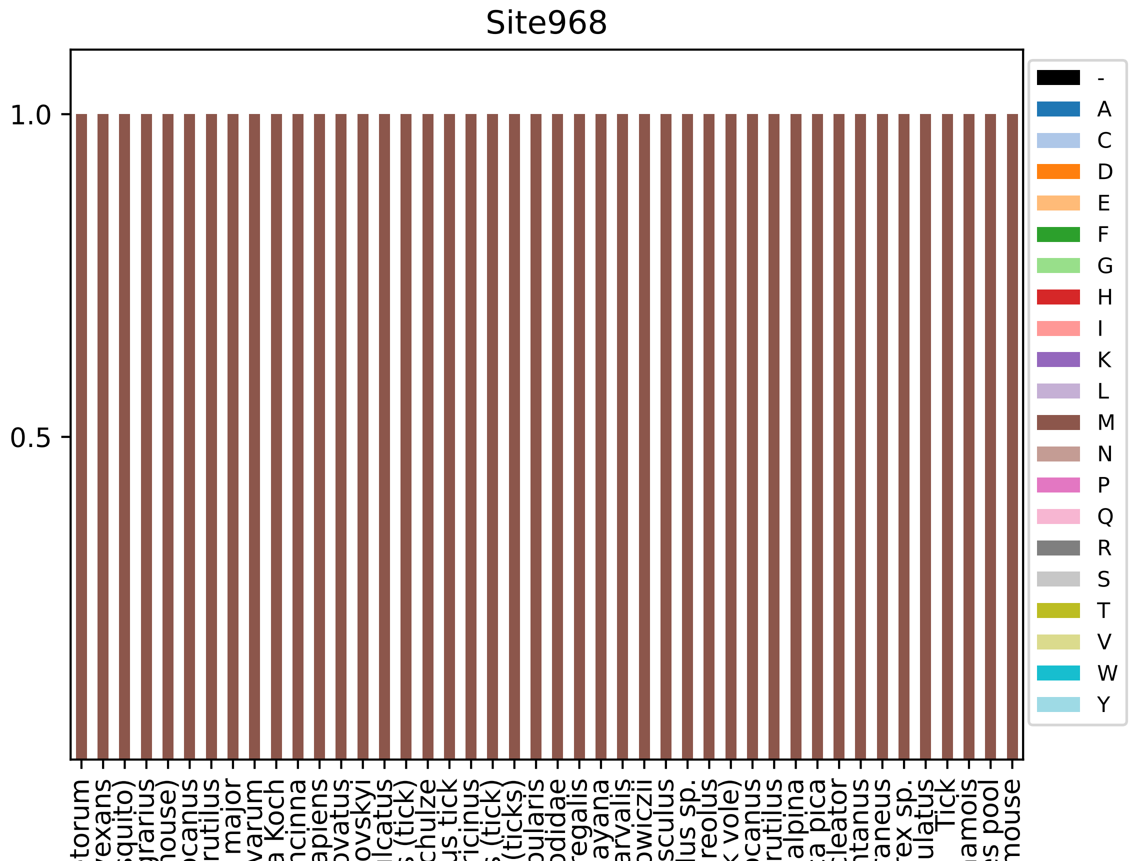
Site968
1.0
0.5
-
A
C
D
E
F
G
H
I
K
L
M
N
P
Q
R
S
T
V
W
Y

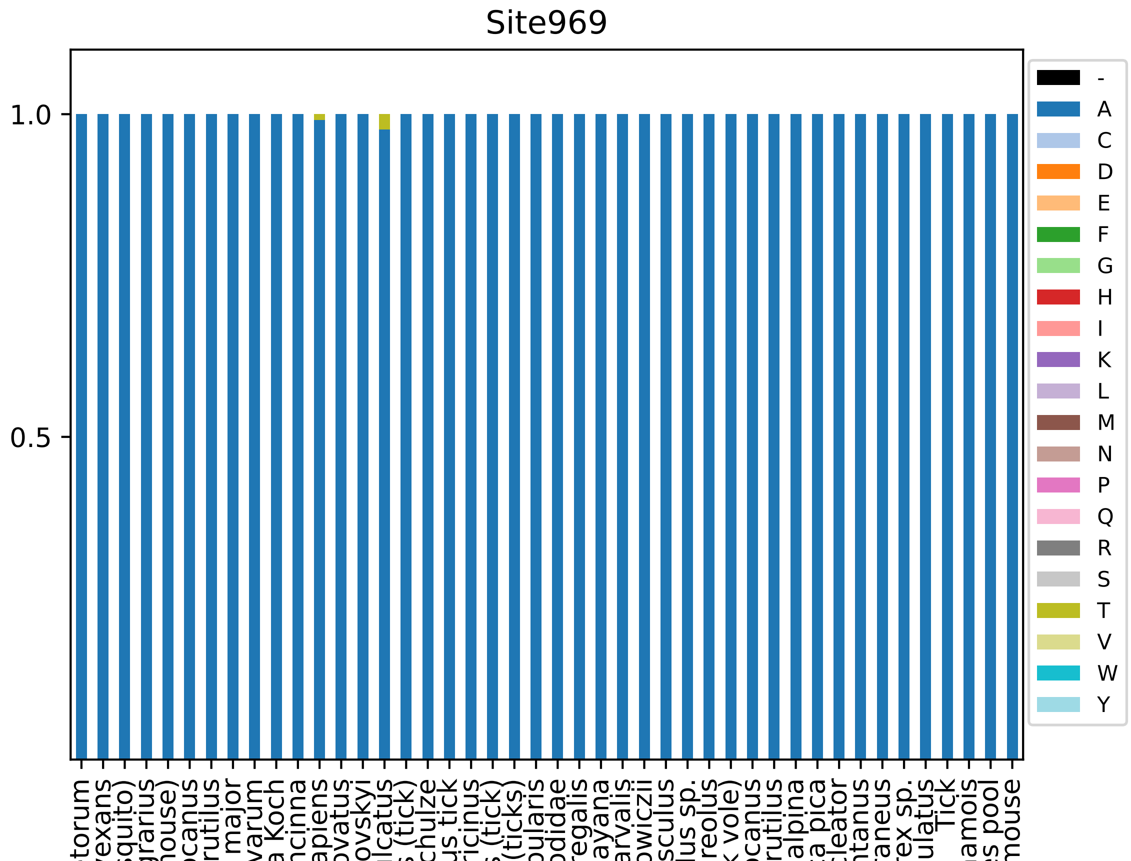
Site969
1.0
0.5
-
A
C
D
E
F
G
H
I
K
L
M
N
P
Q
R
S
T
V
W
Y

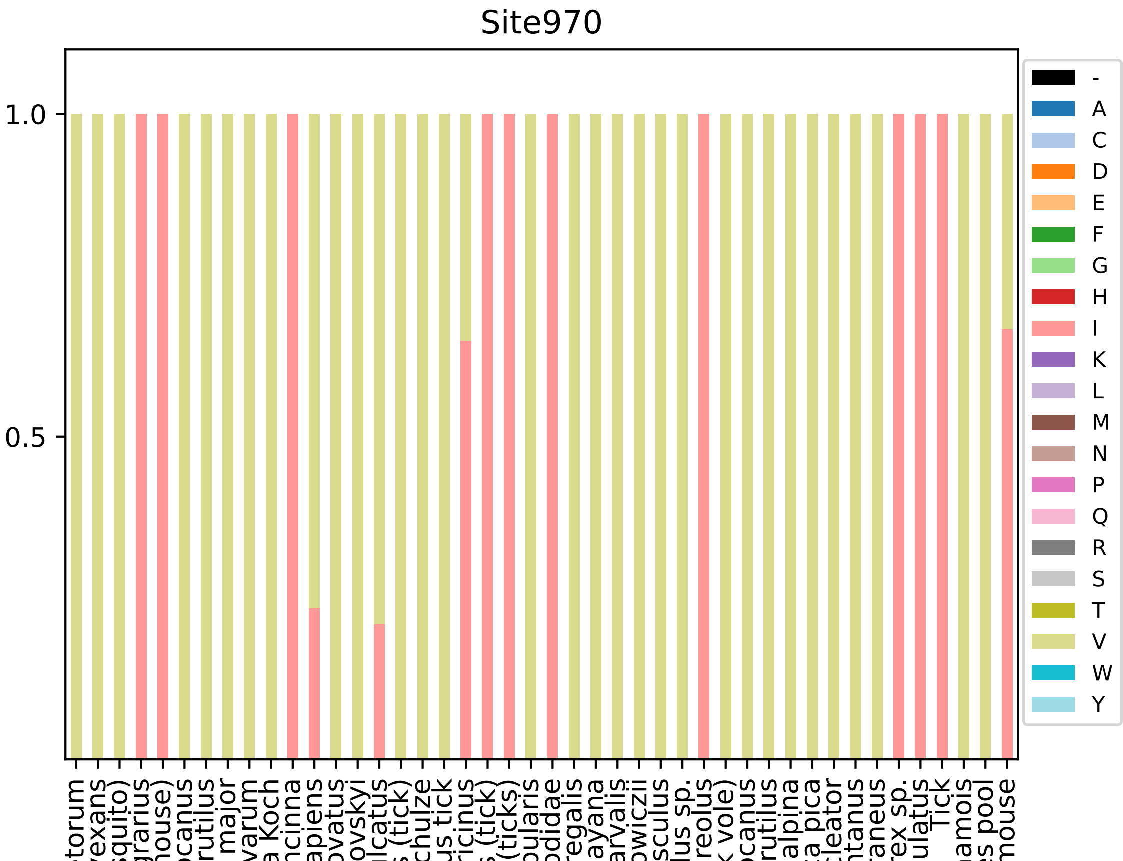
Site970
1.0
0.5
-
A
C
D
E
F
G
H
I
K
L
M
N
P
Q
R
S
T
V
W
Y
Tick

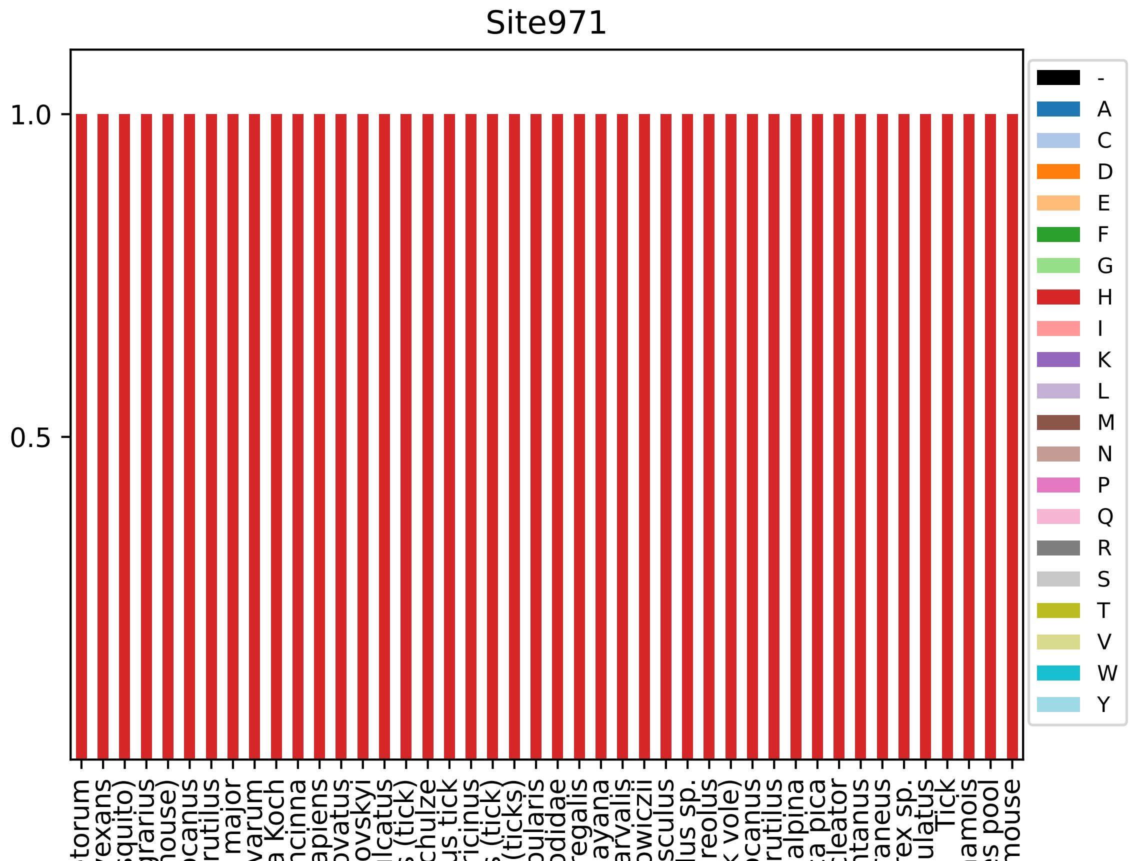
Site971
1.0
0.5
-
A
C
D
E
F
G
H
I
K
L
M
N
P
Q
R
S
T
V
W
Y

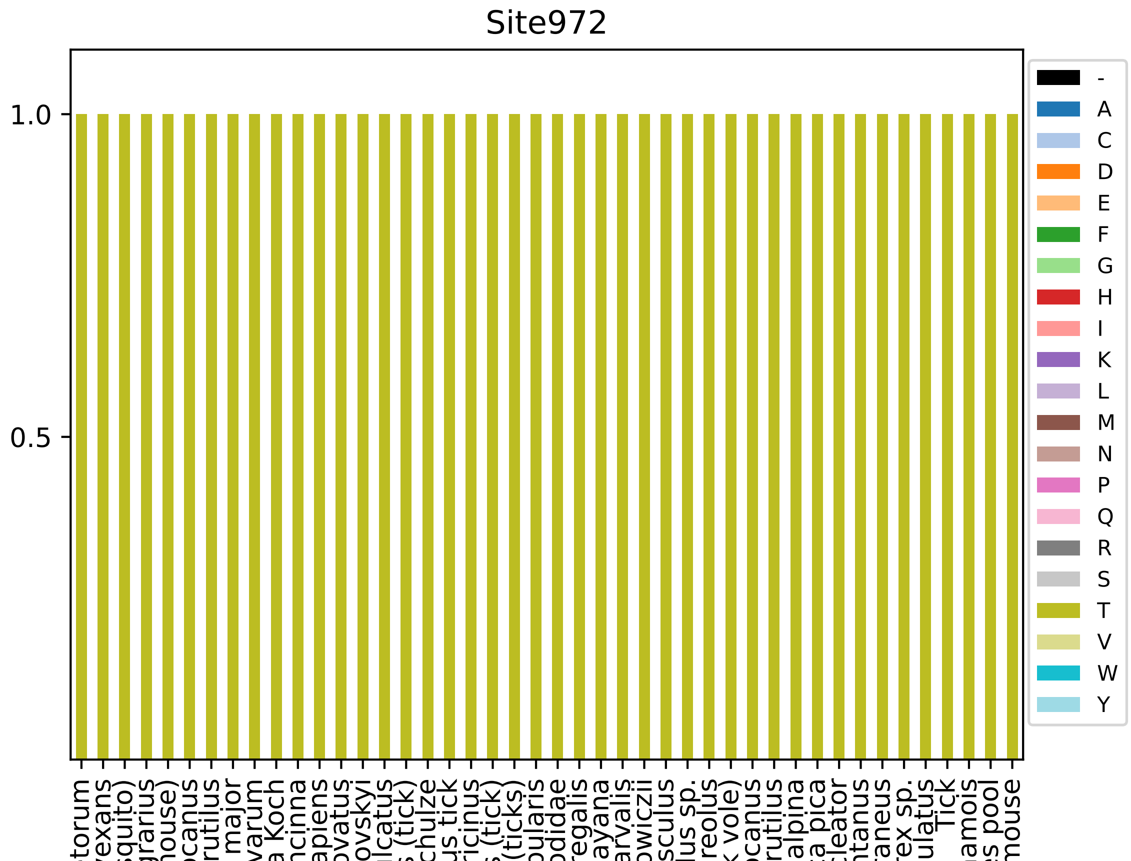
Site972
1.0
0.5
-
A
C
D
E
F
G
H
I
K
L
M
N
P
Q
R
S
T
V
W
Y

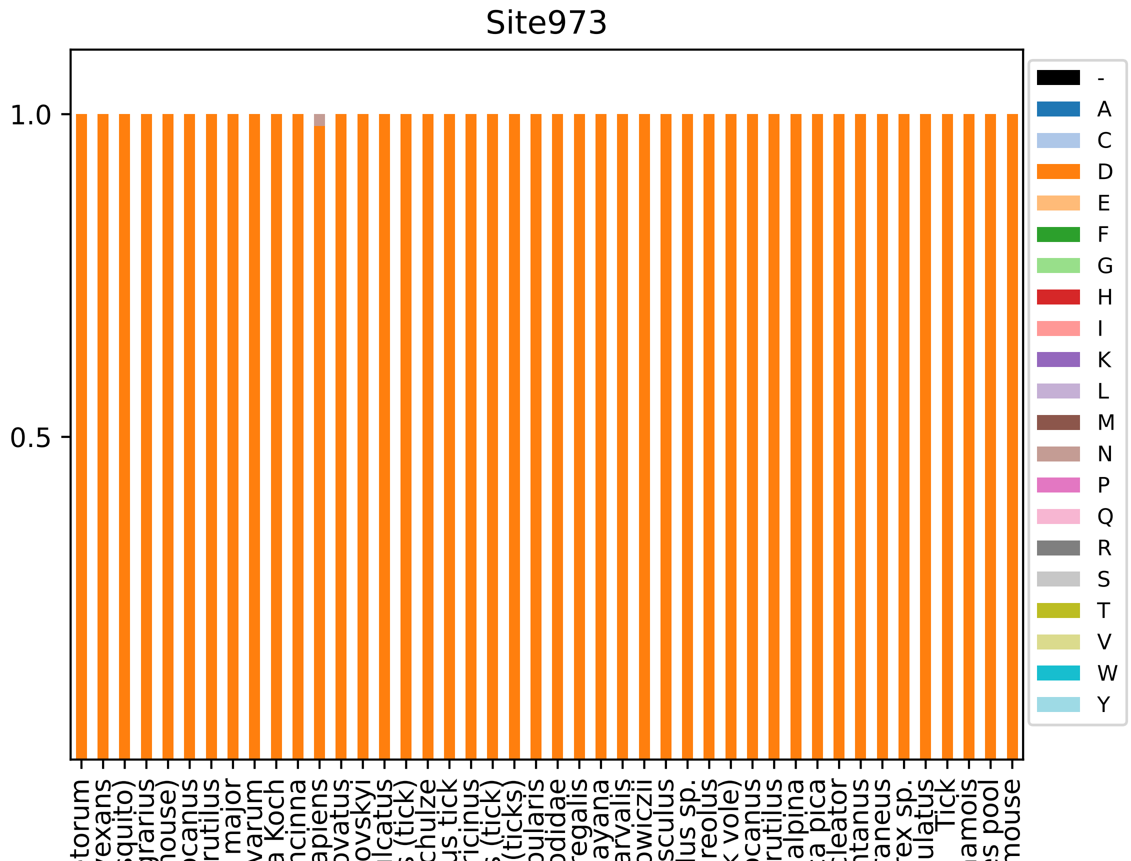
Site973
1.0
0.5
-
A
C
D
E
F
G
H
I
K
L
M
N
P
Q
R
S
T
V
W
Y

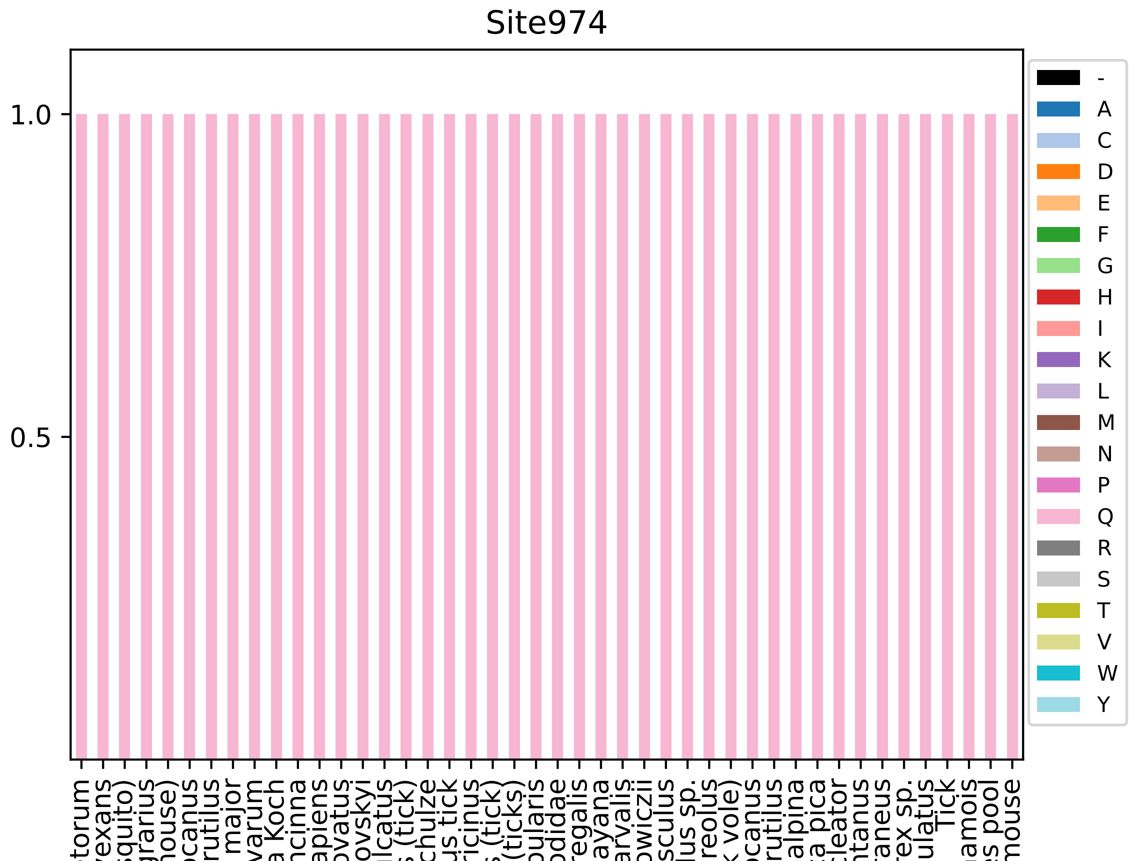

Site974
1.0
0.5
-
A
C
D
E
F
G
H
I
K
L
M
N
P
Q
R
S
T
V
W
Y

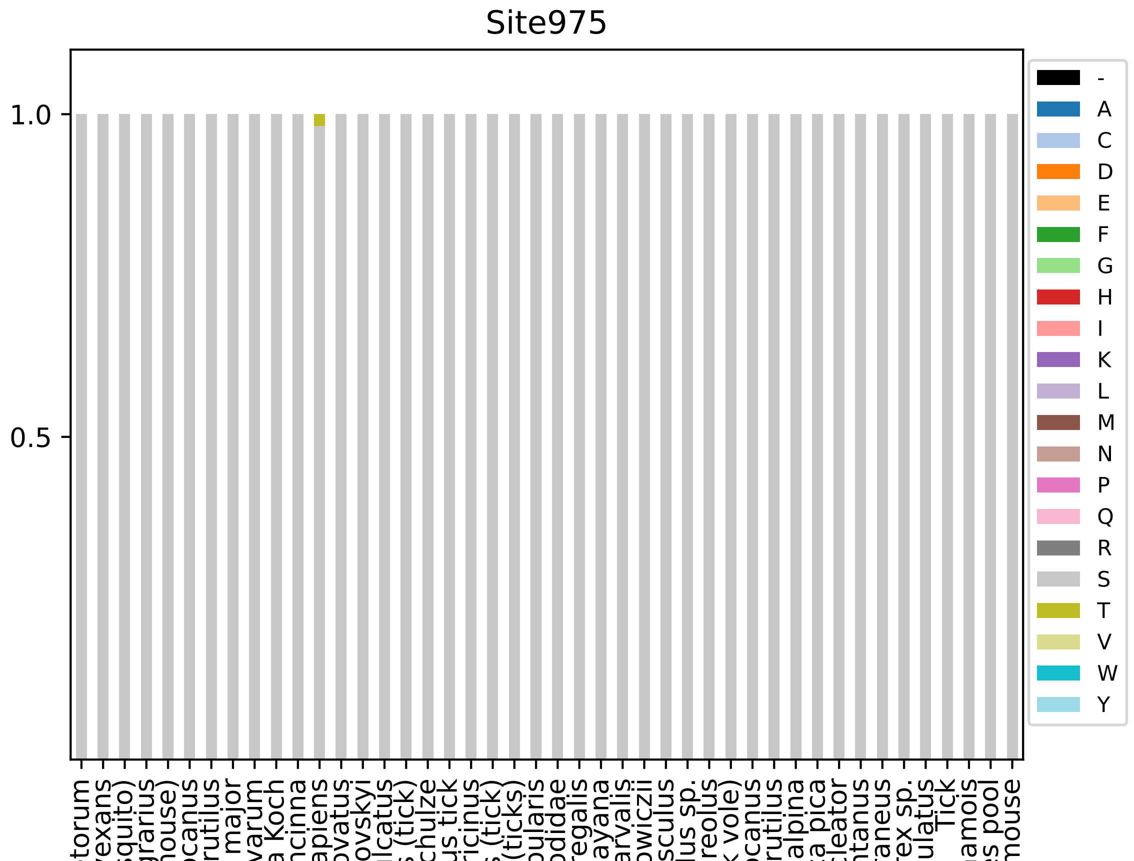

Site975
1.0
0.5
-
A
C
D
E
F
G
H
I
K
L
M
N
P
Q
R
S
T
V
W
Y

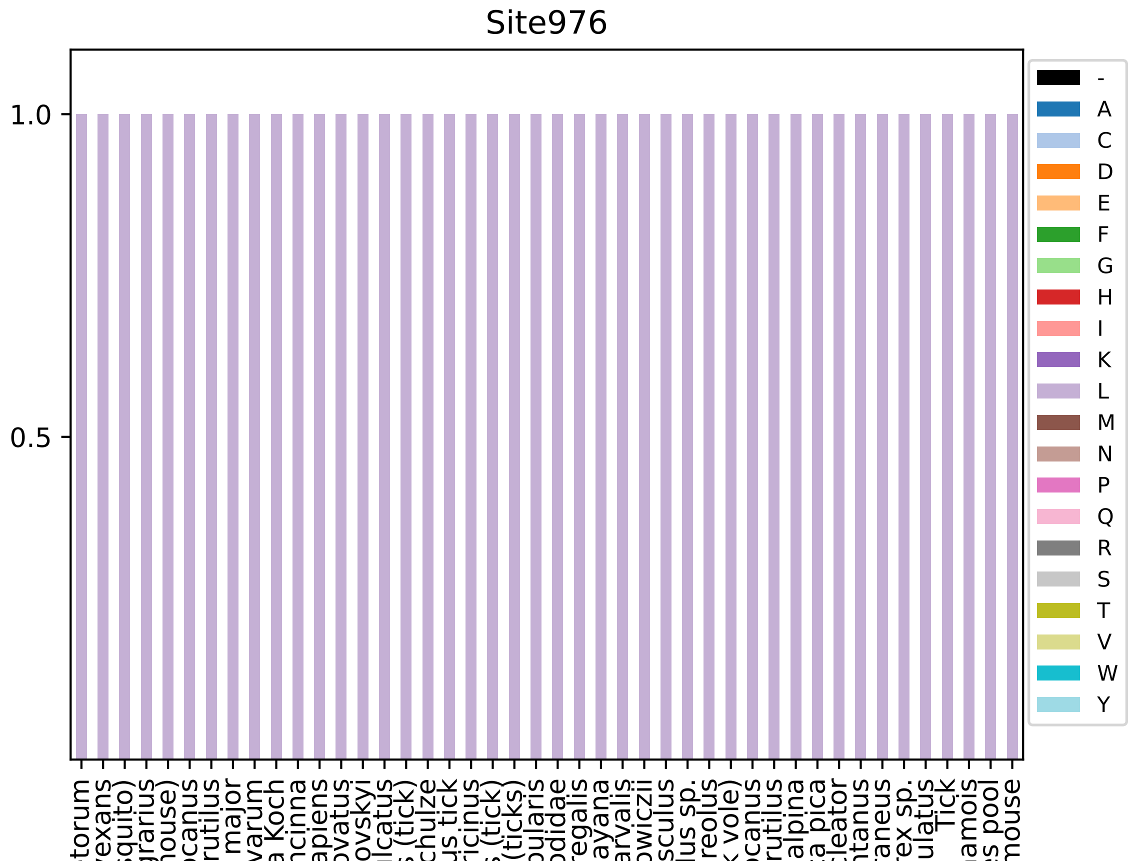

Site976
1.0
0.5
-
A
C
D
E
F
G
H
I
K
L
M
N
P
Q
R
S
T
V
W
Y

Site977

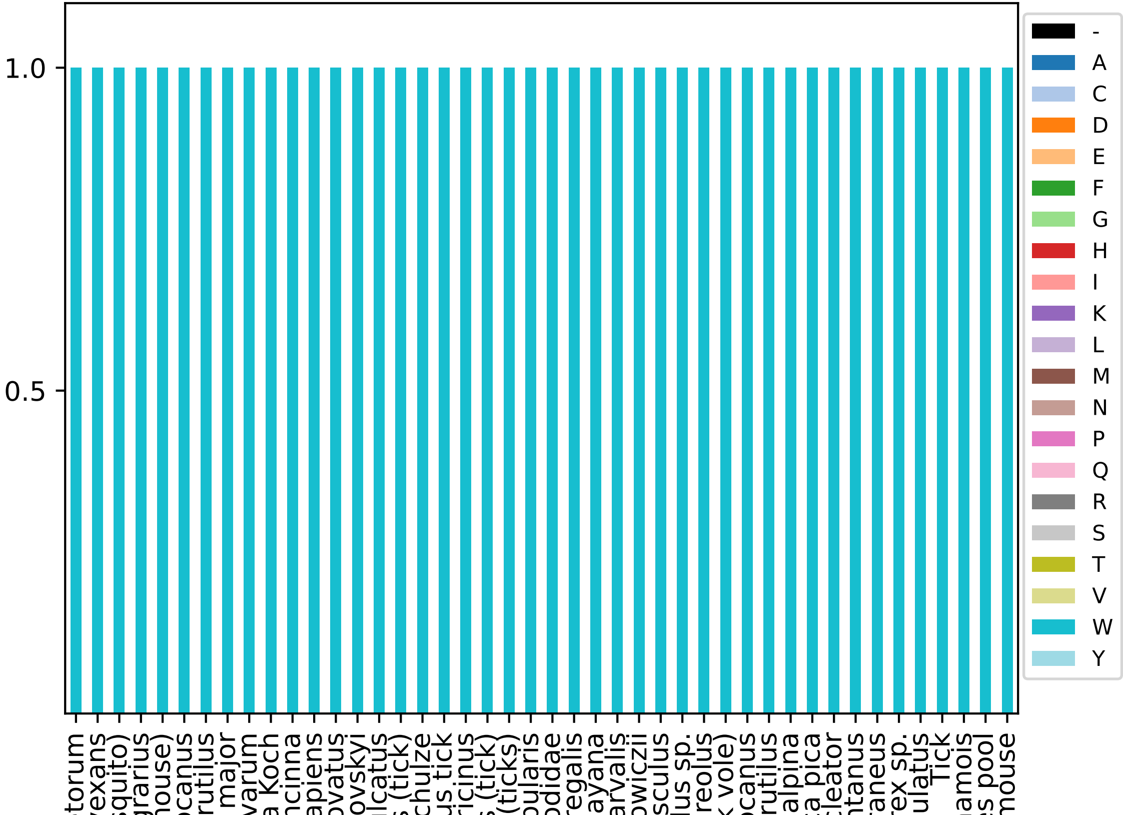

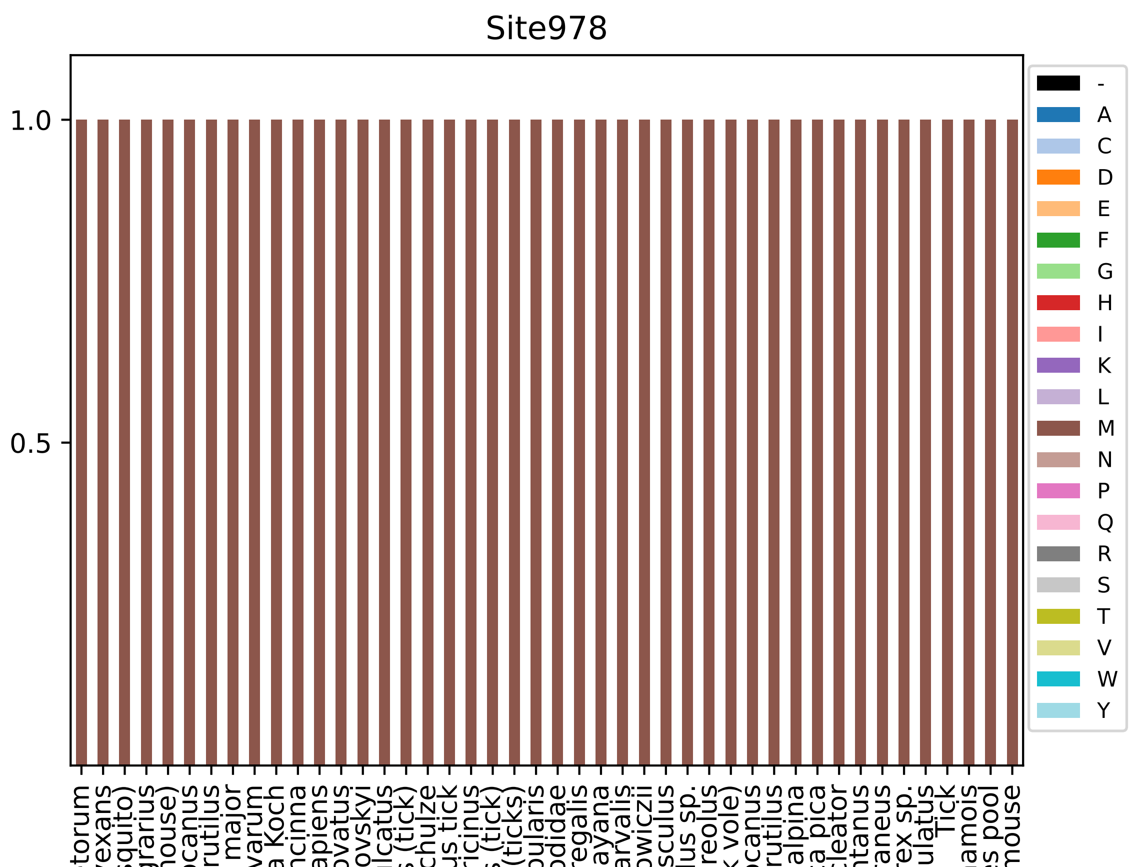
Site978
1.0
0.5
-
A
C
D
E
F
G
H
I
K
L
M
N
P
Q
R
S
T
V
W
Y

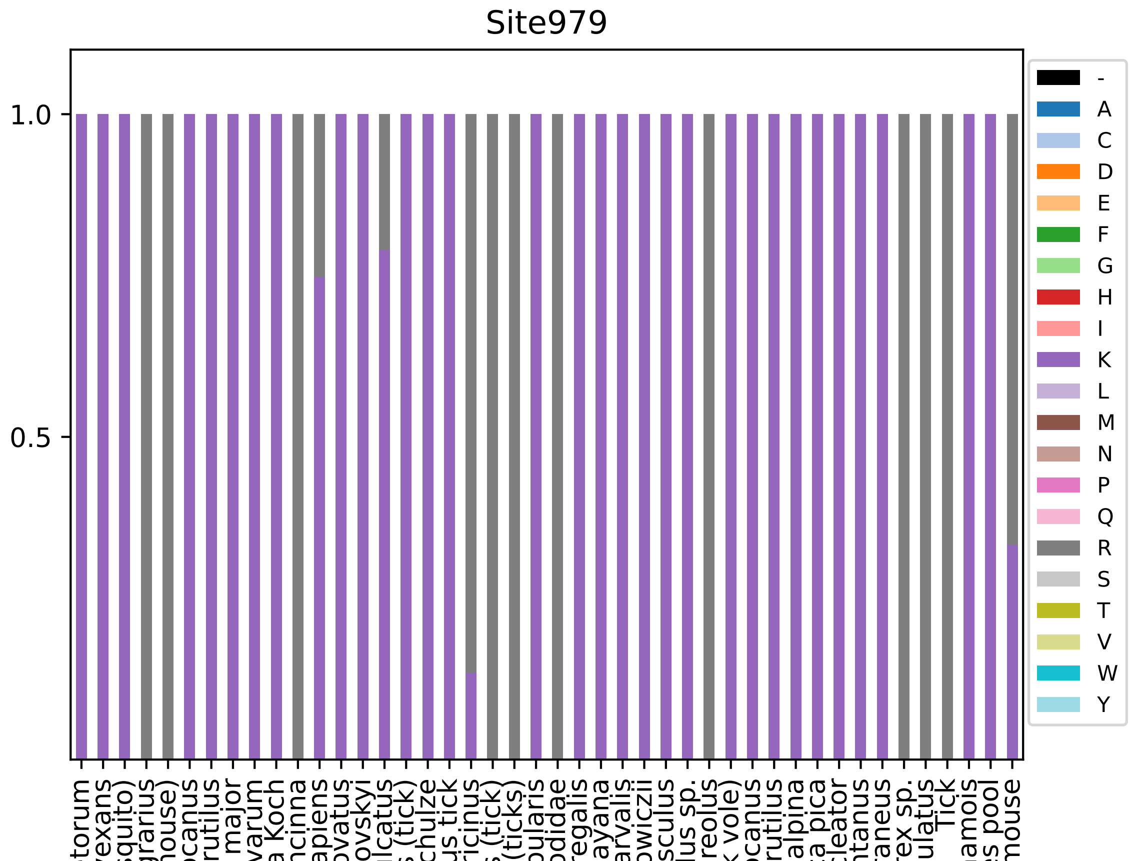
Site979
1.0
0.5
-
A
C
D
E
F
G
H
I
K
L
M
N
P
Q
R
S
T
V
W
Y
Tick

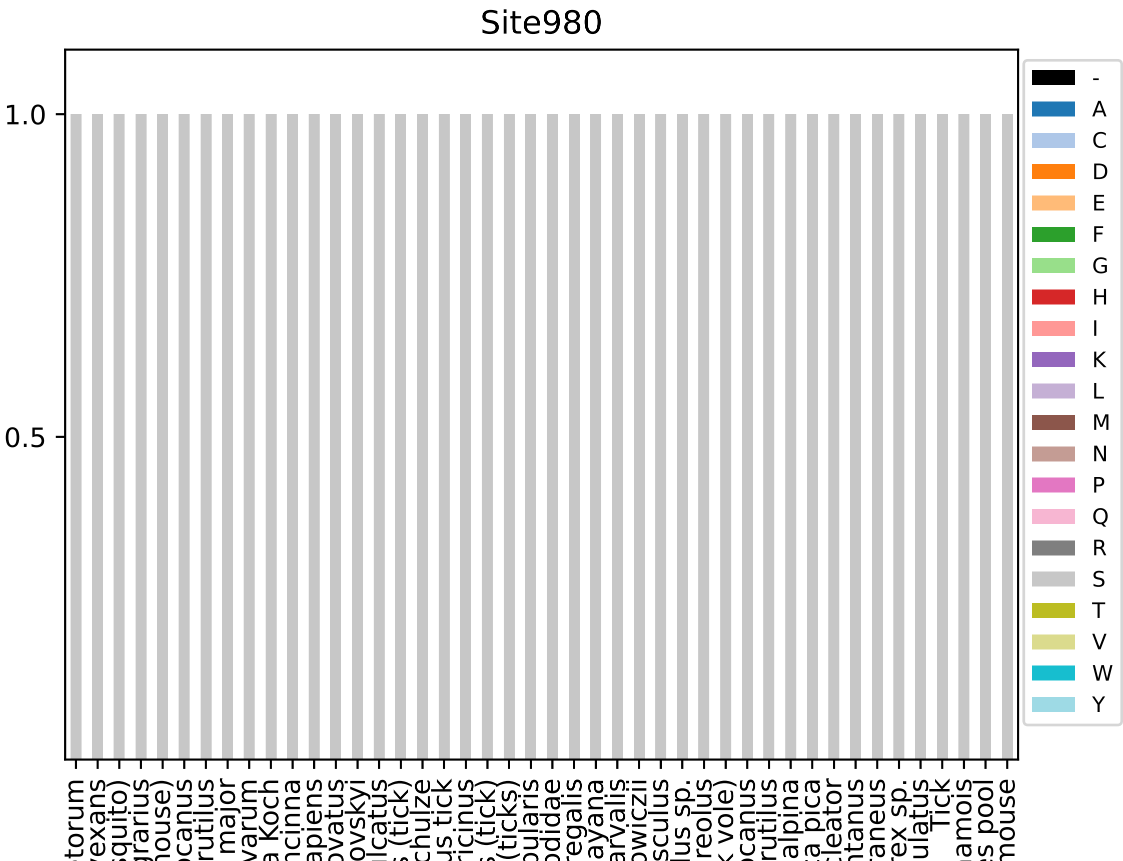

Site980
1.0
0.5
-
A
C
D
E
F
G
H
I
K
L
M
N
P
Q
R
S
T
V
W
Y

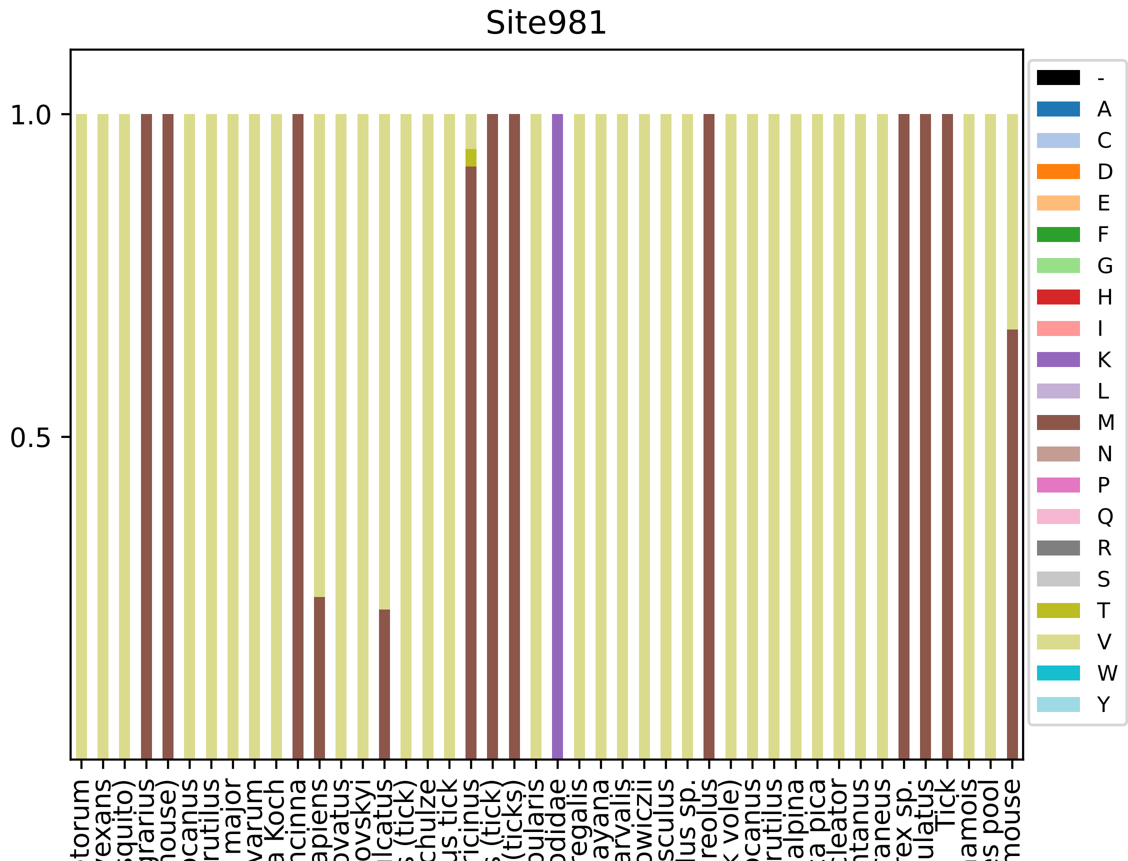

Site981
1.0
0.5
-
A
C
D
E
F
G
H
I
K
L
M
N
P
Q
R
S
T
V
W
Y

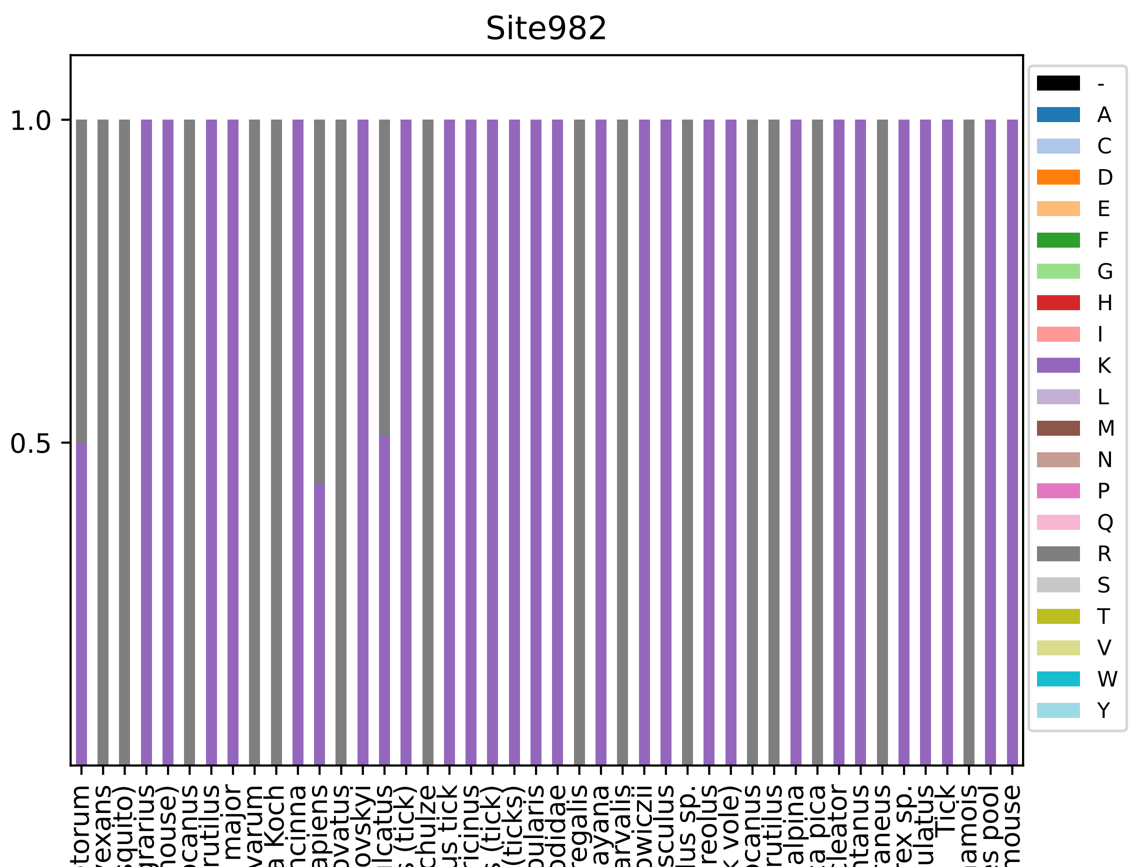

Site982
1.0
0.5
-
A
C
D
E
F
G
H
I
K
L
M
N
P
Q
R
S
T
V
W
Y

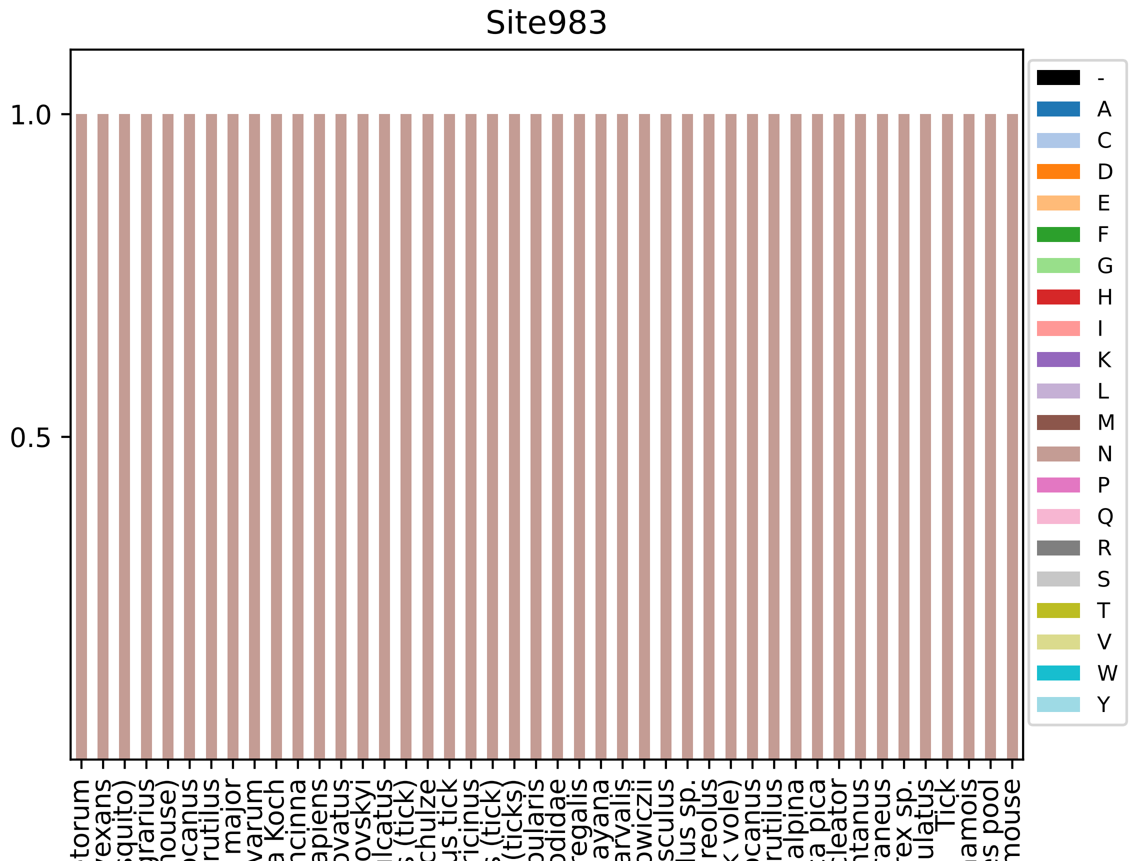
Site983
1.0
0.5
-
A
C
D
E
F
G
H
I
K
L
M
N
P
Q
R
S
T
V
W
Y
Tick

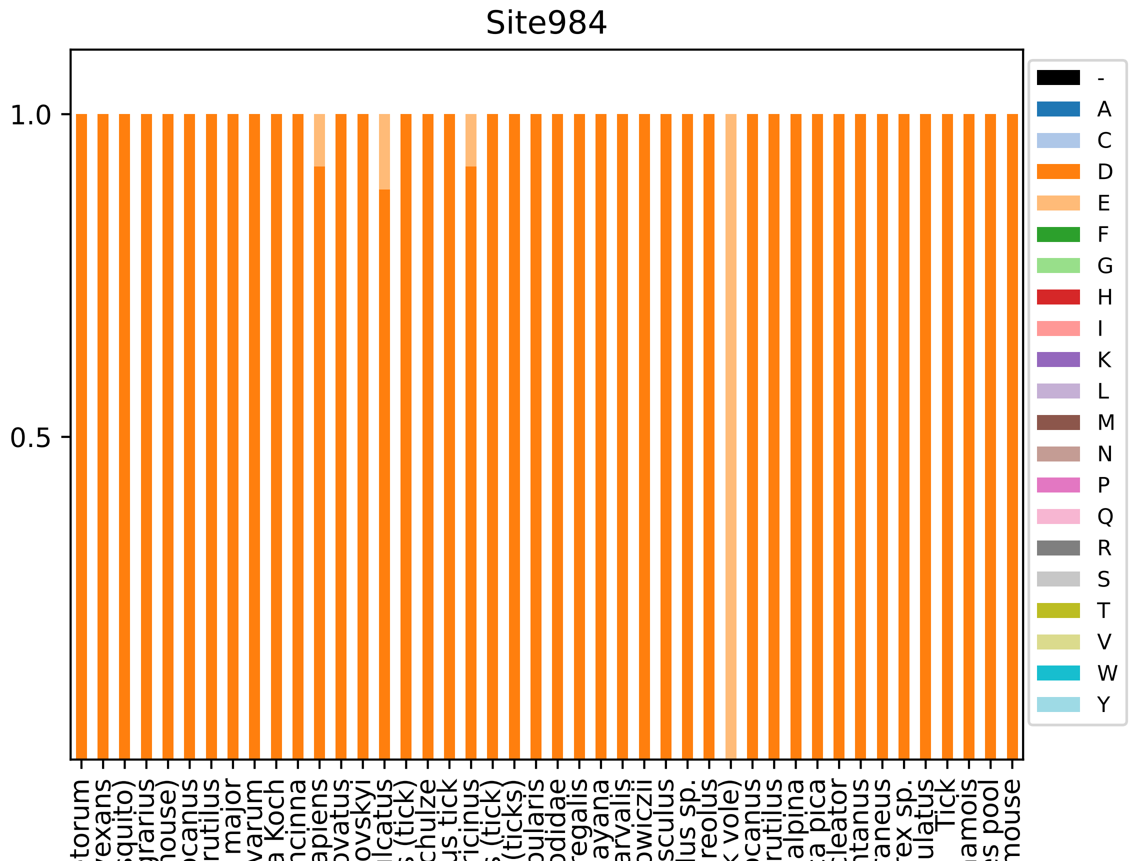

Site984
1.0
0.5
-
A
C
D
E
F
G
H
I
K
L
M
N
P
Q
R
S
T
V
W
Y

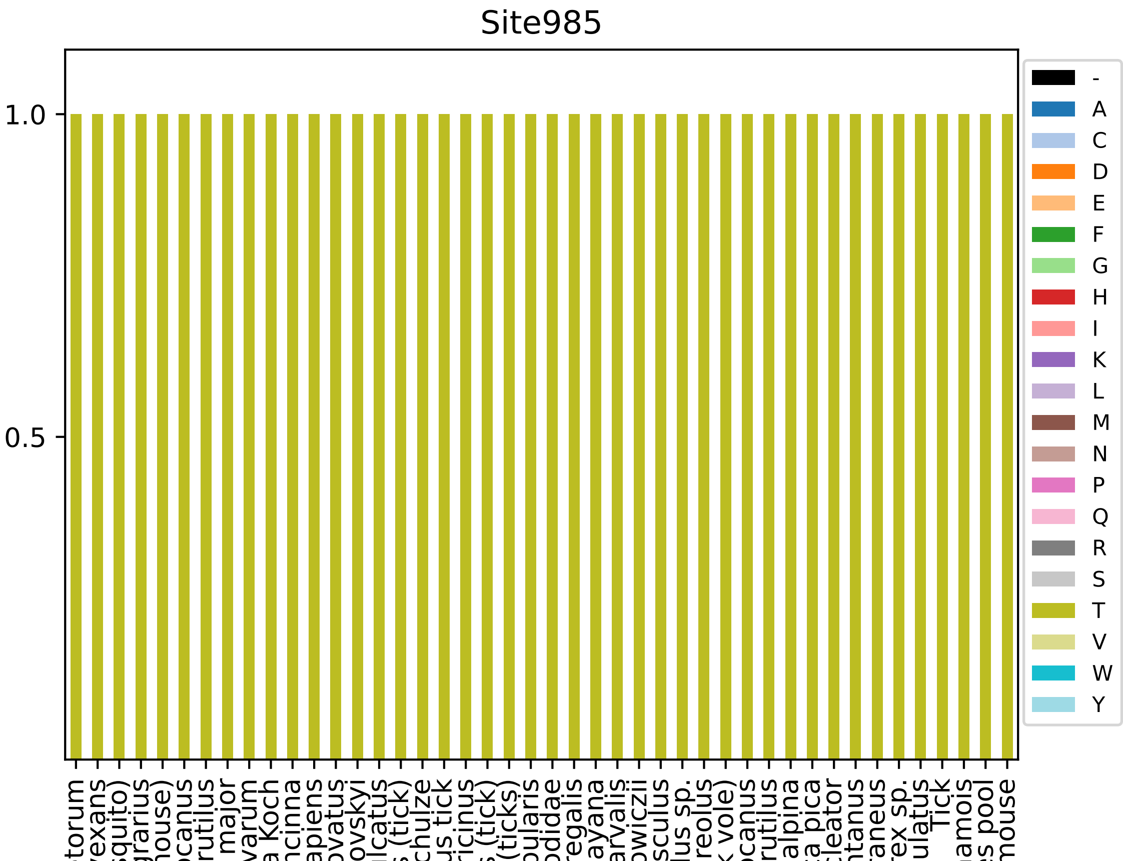
Site985
1.0
0.5
-
A
C
D
E
F
G
H
I
K
L
M
N
P
Q
R
S
T
V
W
Y
Tick

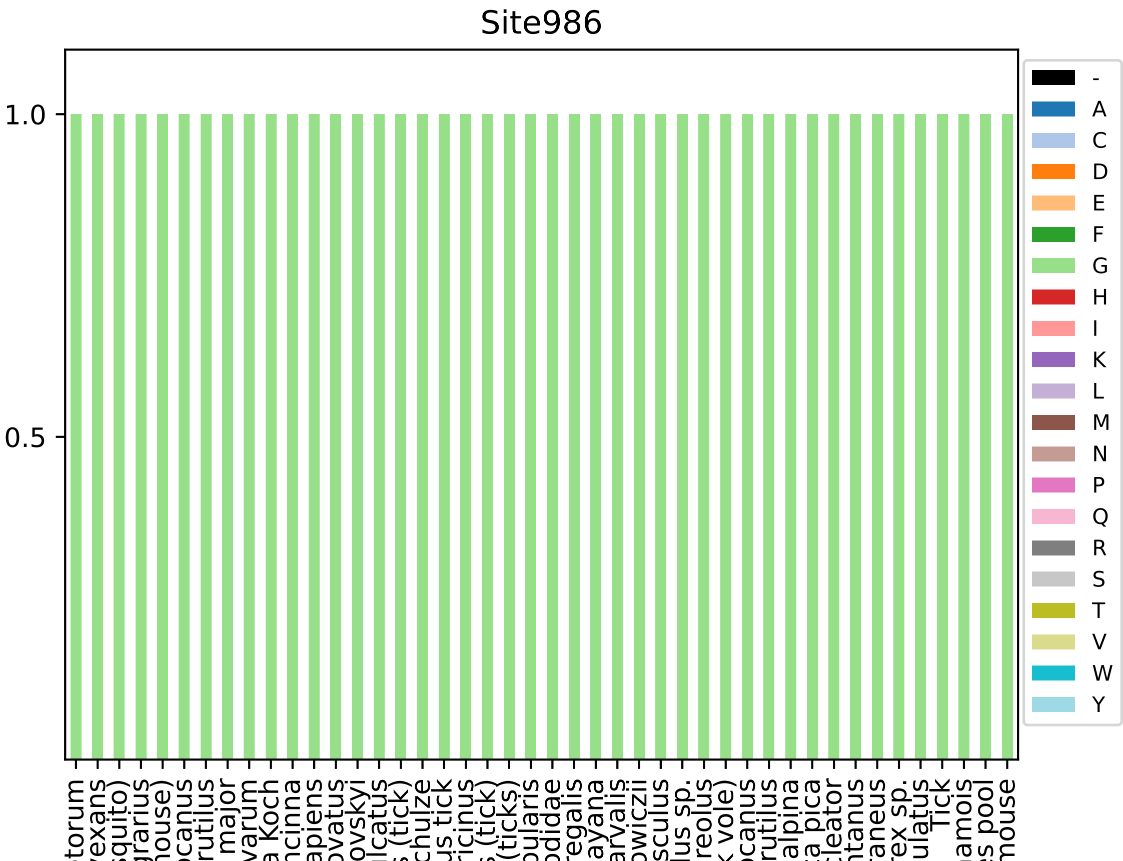
Site986
1.0
0.5
-
A
C
D
E
F
G
H
I
K
L
M
N
P
Q
R
S
T
V
W
Y

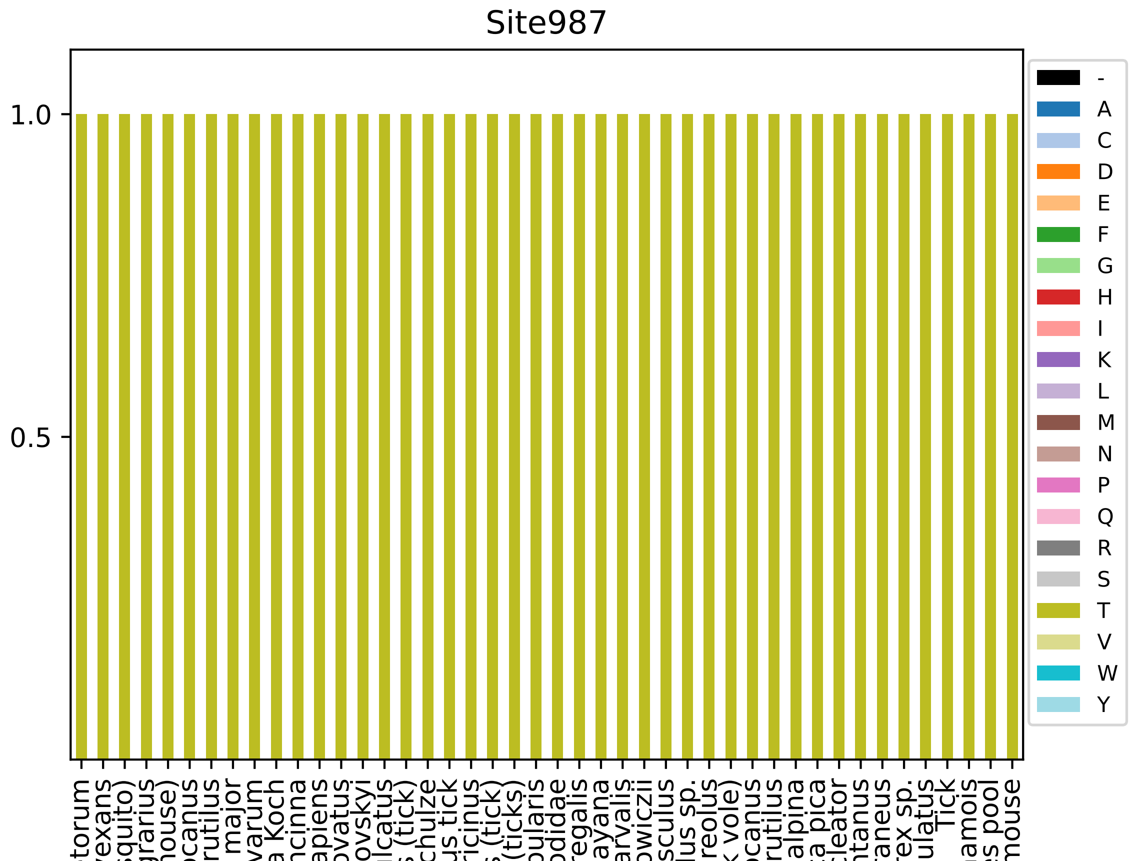
Site987
1.0
0.5
-
A
C
D
E
F
G
H
I
K
L
M
N
P
Q
R
S
T
V
W
Y

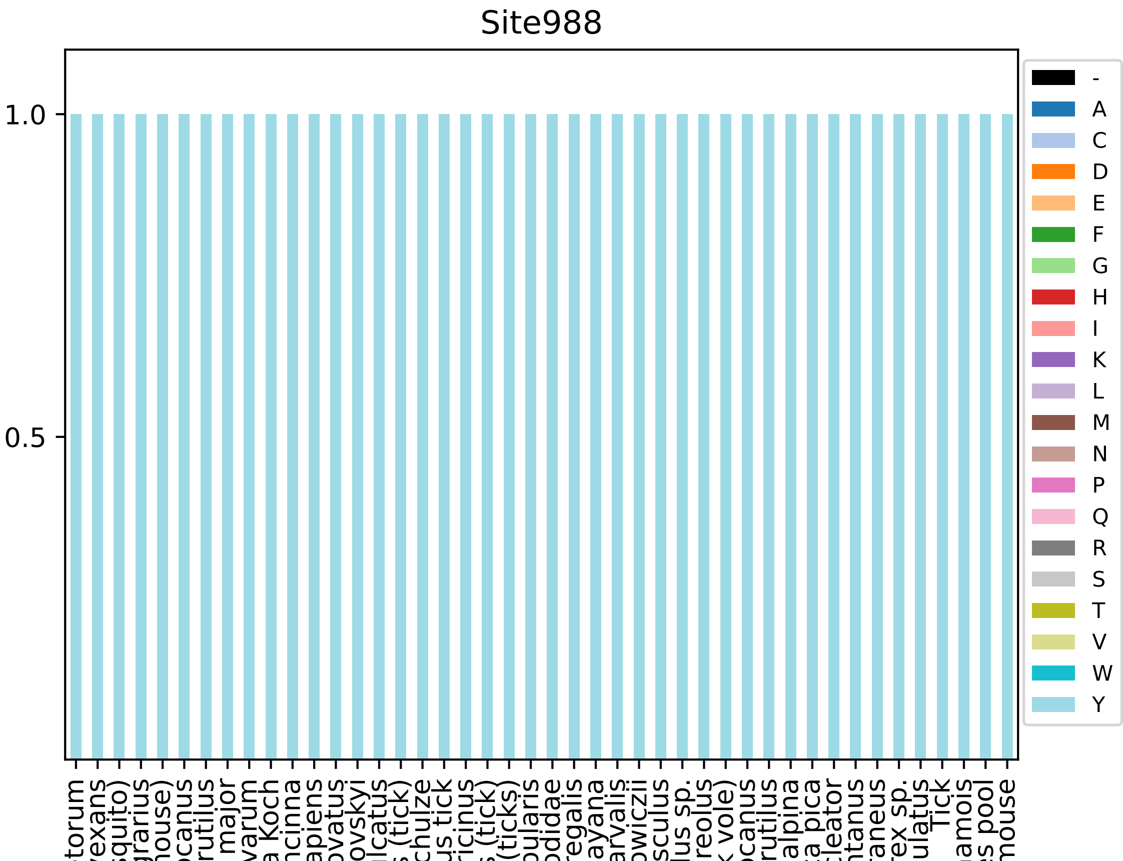

Site988
1.0
0.5
-
A
C
D
E
F
G
H
I
K
L
M
N
P
Q
R
S
T
V
W
Y

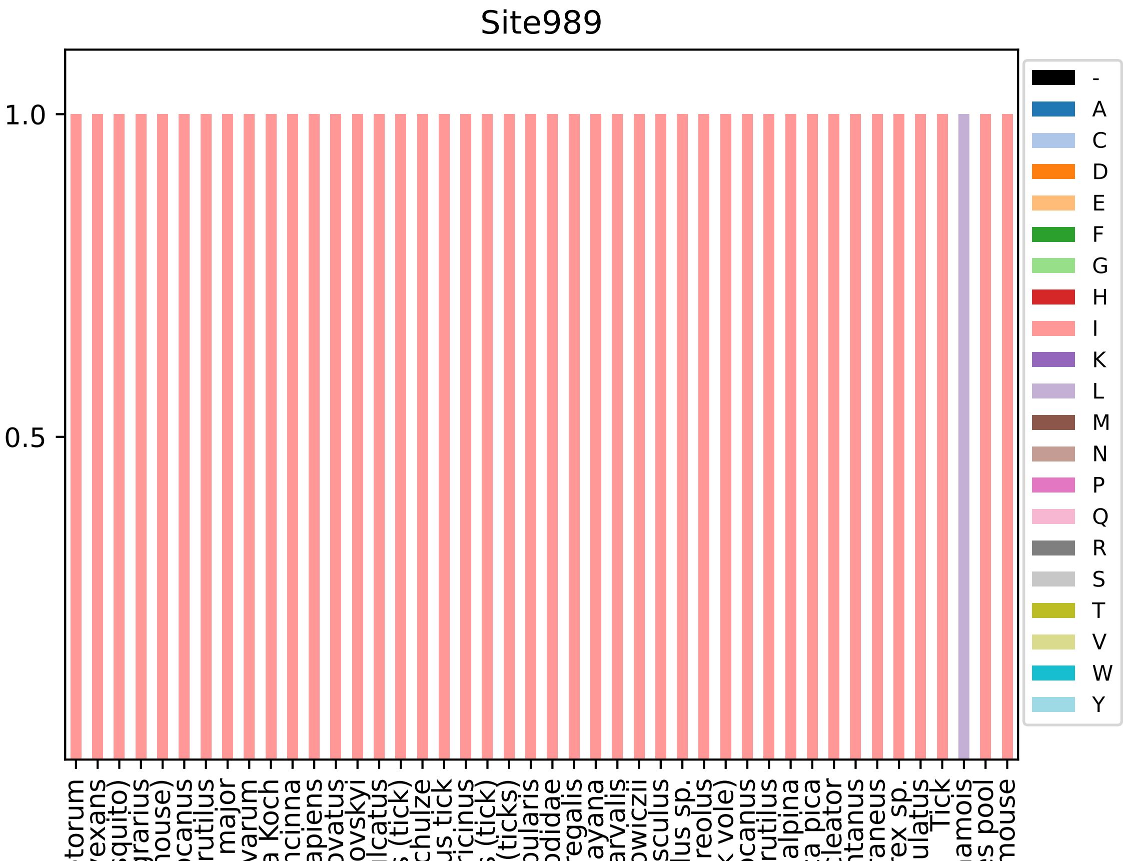
Site989
1.0
0.5
-
A
C
D
E
F
G
H
I
K
L
M
N
P
Q
R
S
T
V
W
Y

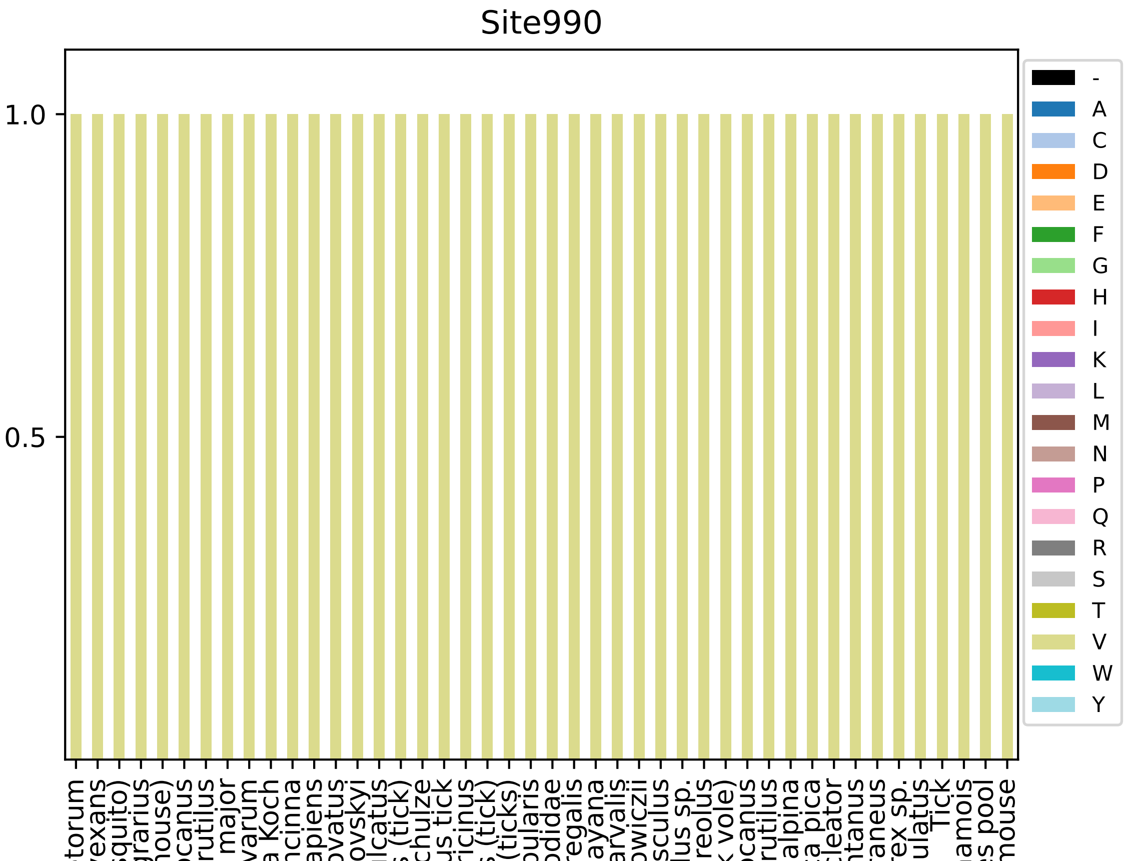

Site990
1.0
0.5
-
A
C
D
E
F
G
H
I
K
L
M
N
P
Q
R
S
T
V
W
Y

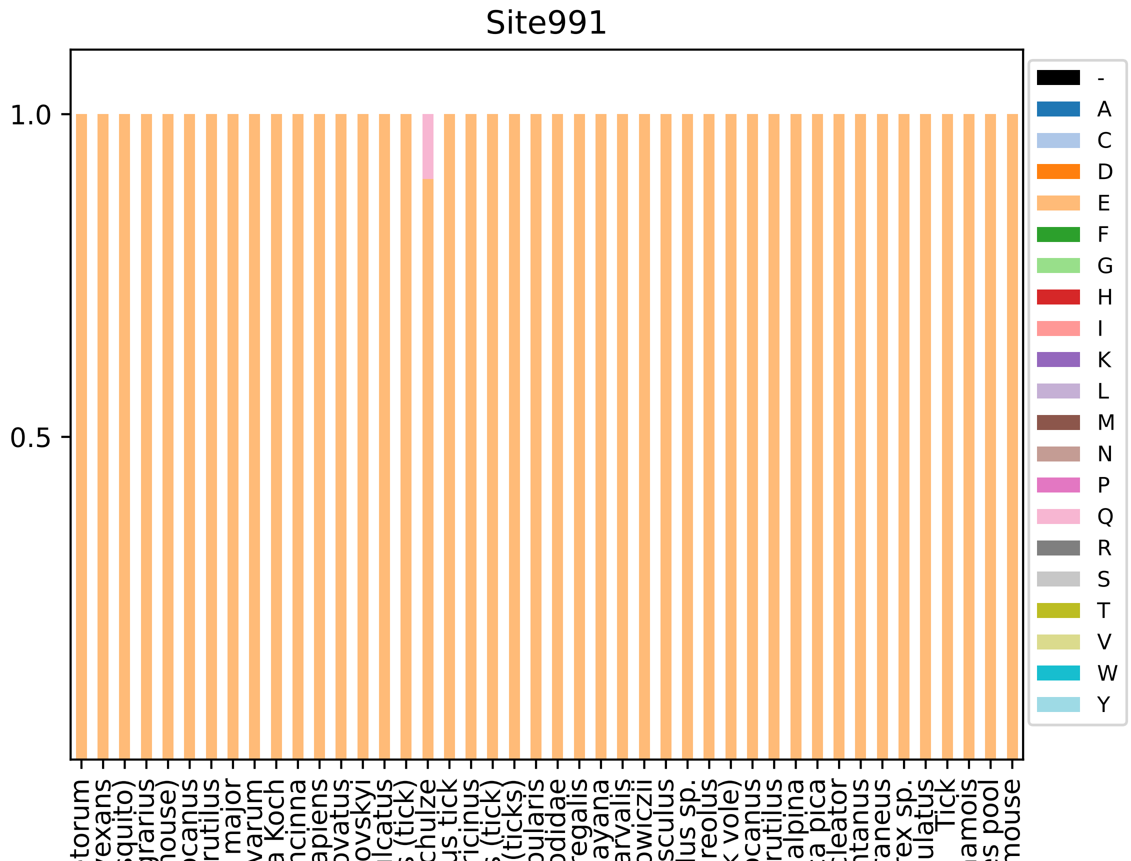

Site991
1.0
0.5
-
A
C
D
E
F
G
H
I
K
L
M
N
P
Q
R
S
T
V
W
Y

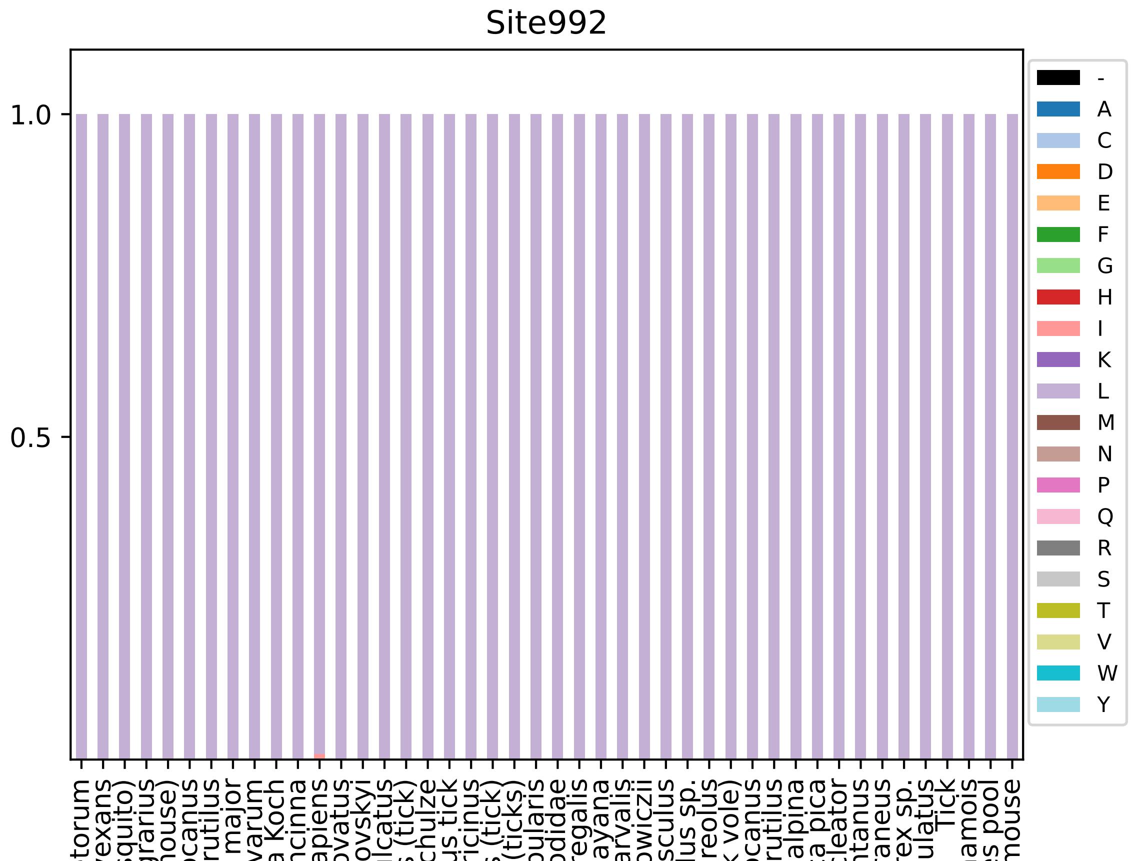
Site992
1.0
0.5
-
A
C
D
E
F
G
H
I
K
L
M
N
P
Q
R
S
T
V
W
Y

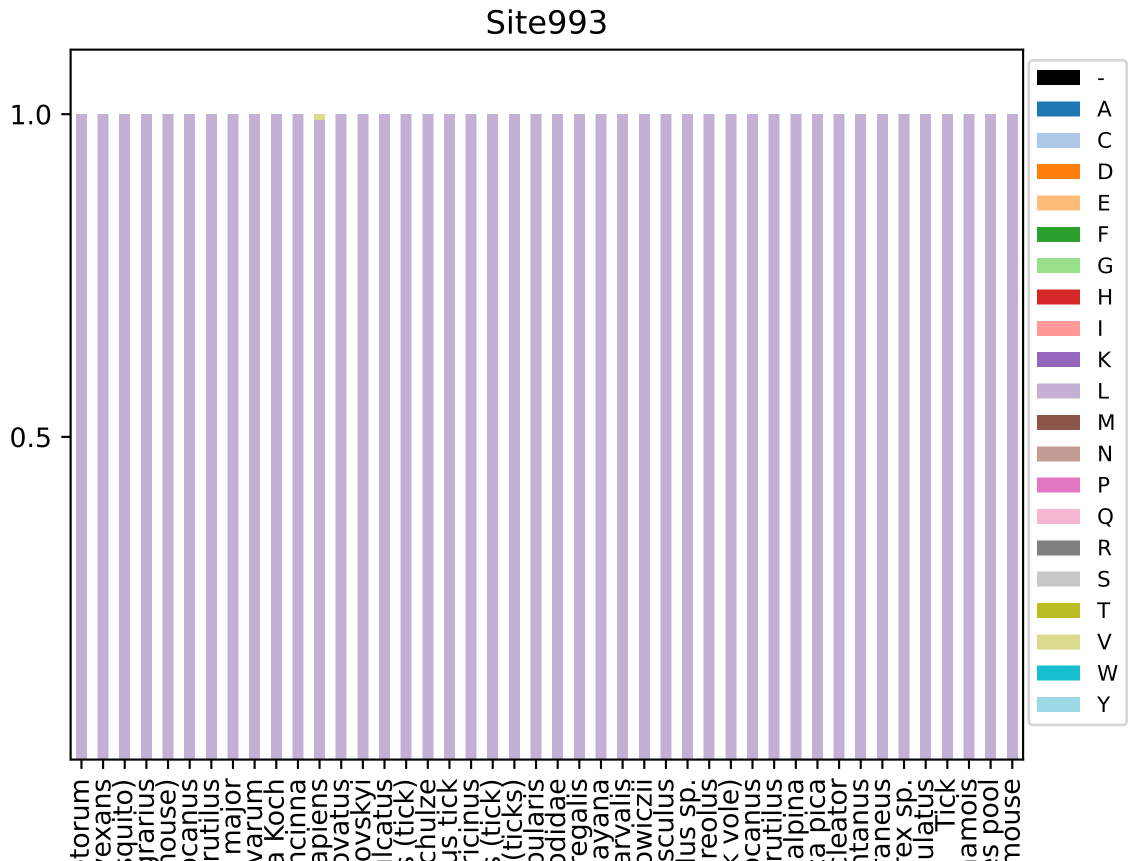

Site993
1.0
0.5
-
A
C
D
E
F
G
H
I
K
L
M
N
P
Q
R
S
T
V
W
Y

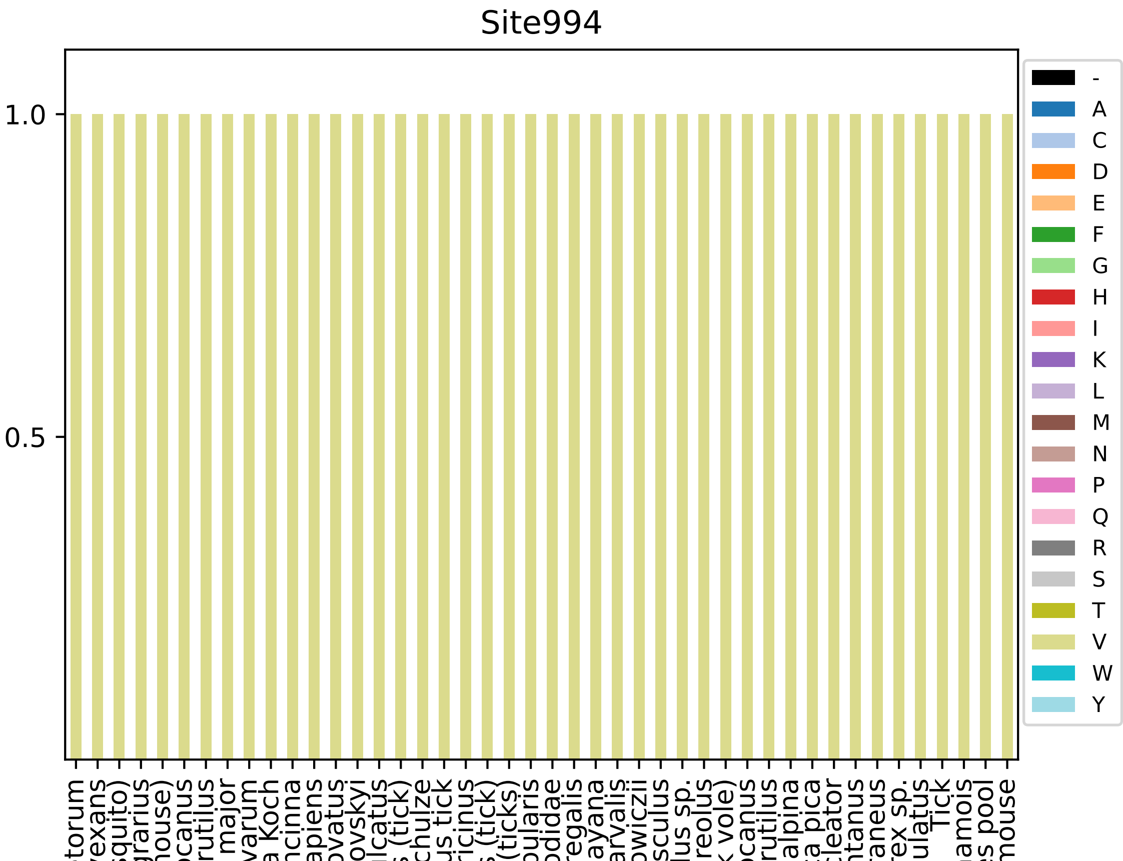

Site994
1.0
0.5
-
A
C
D
E
F
G
H
I
K
L
M
N
P
Q
R
S
T
V
W
Y

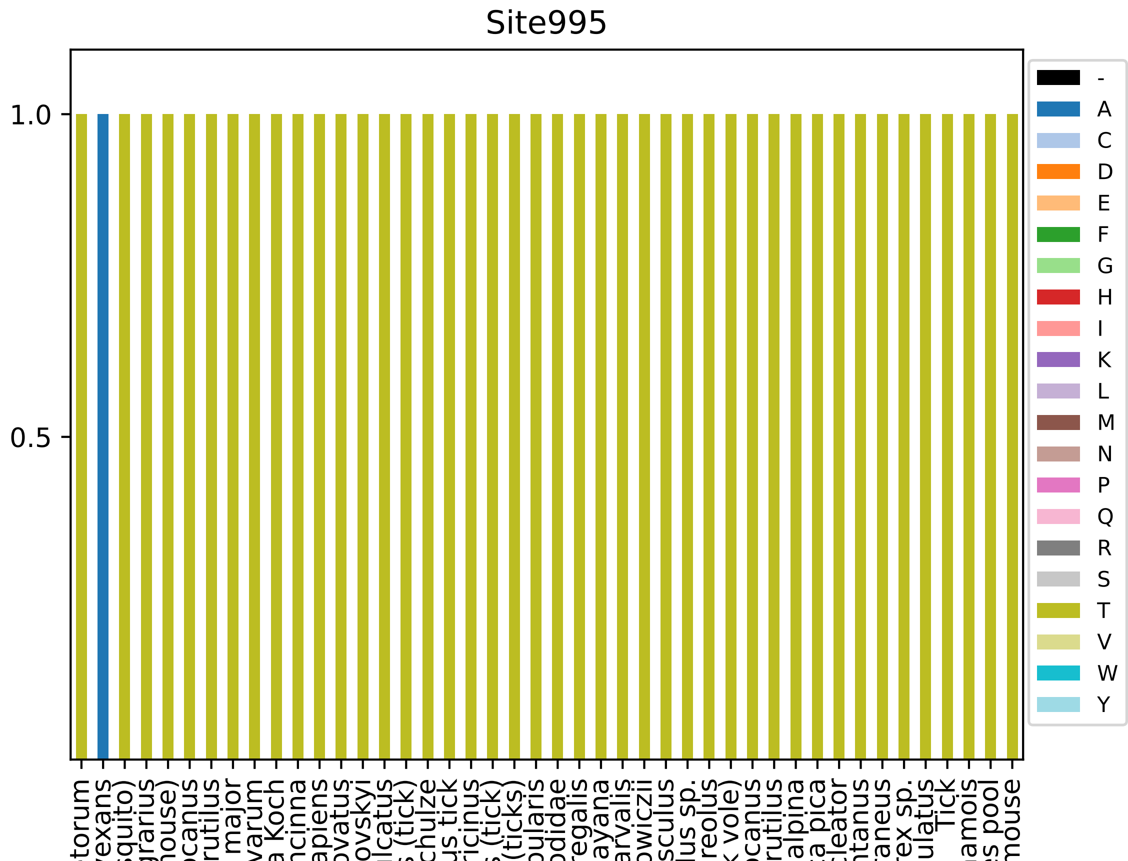

Site995
1.0
0.5
-
A
C
D
E
F
G
H
I
K
L
M
N
P
Q
R
S
T
V
W
Y

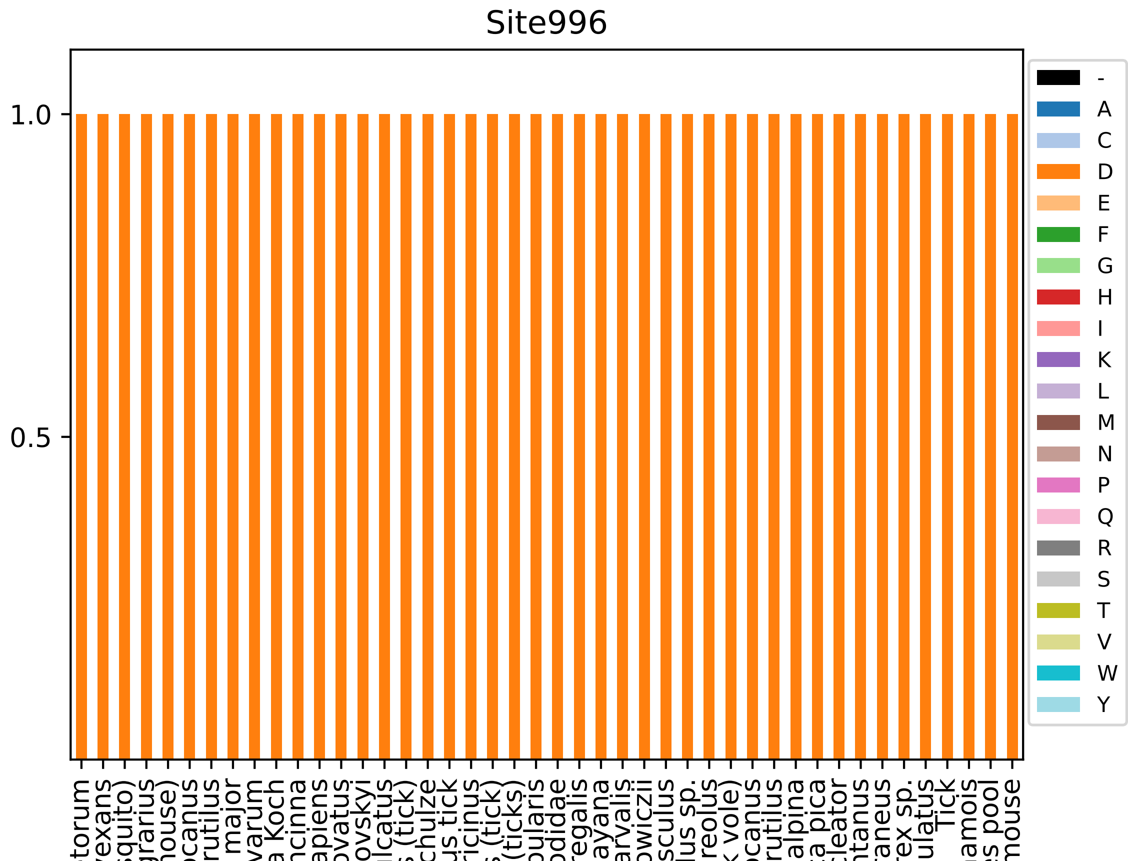
Site996
1.0
0.5
-
A
C
D
E
F
G
H
I
K
L
M
N
P
Q
R
S
T
V
W
Y

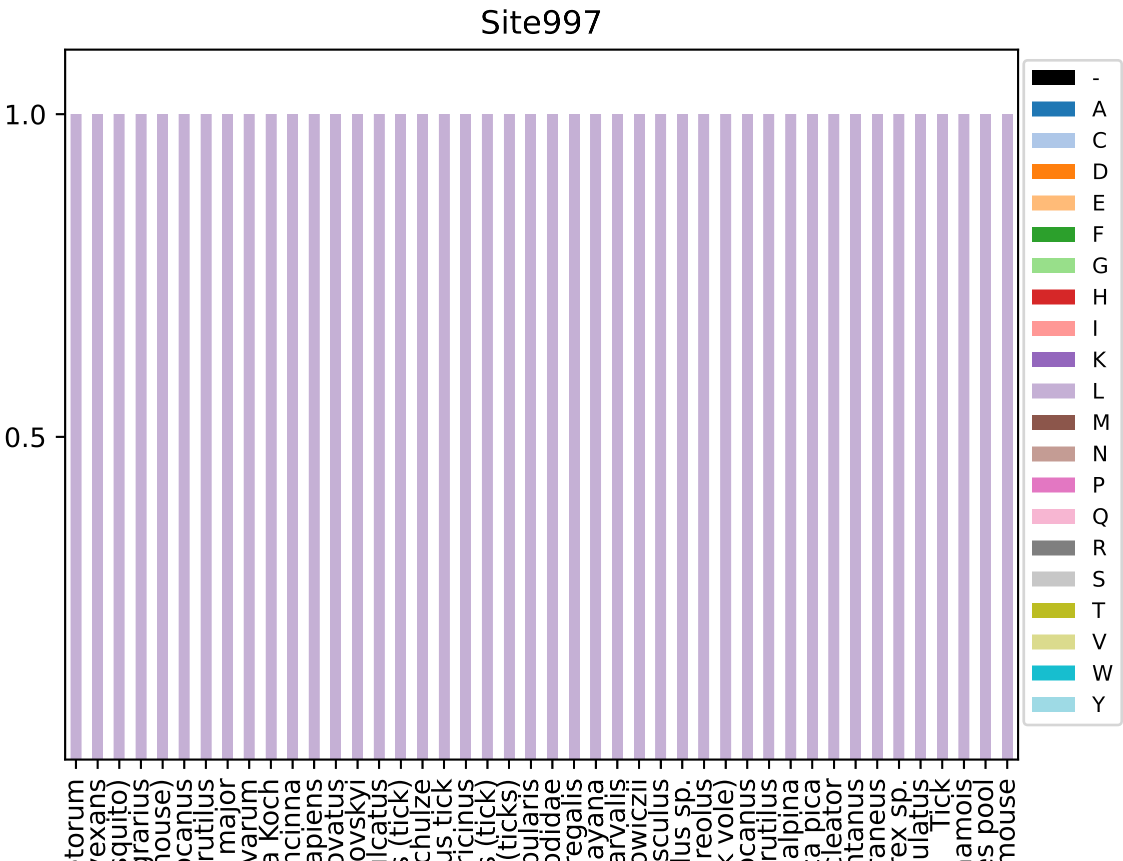

Site997
1.0
0.5
-
A
C
D
E
F
G
H
I
K
L
M
N
P
Q
R
S
T
V
W
Y

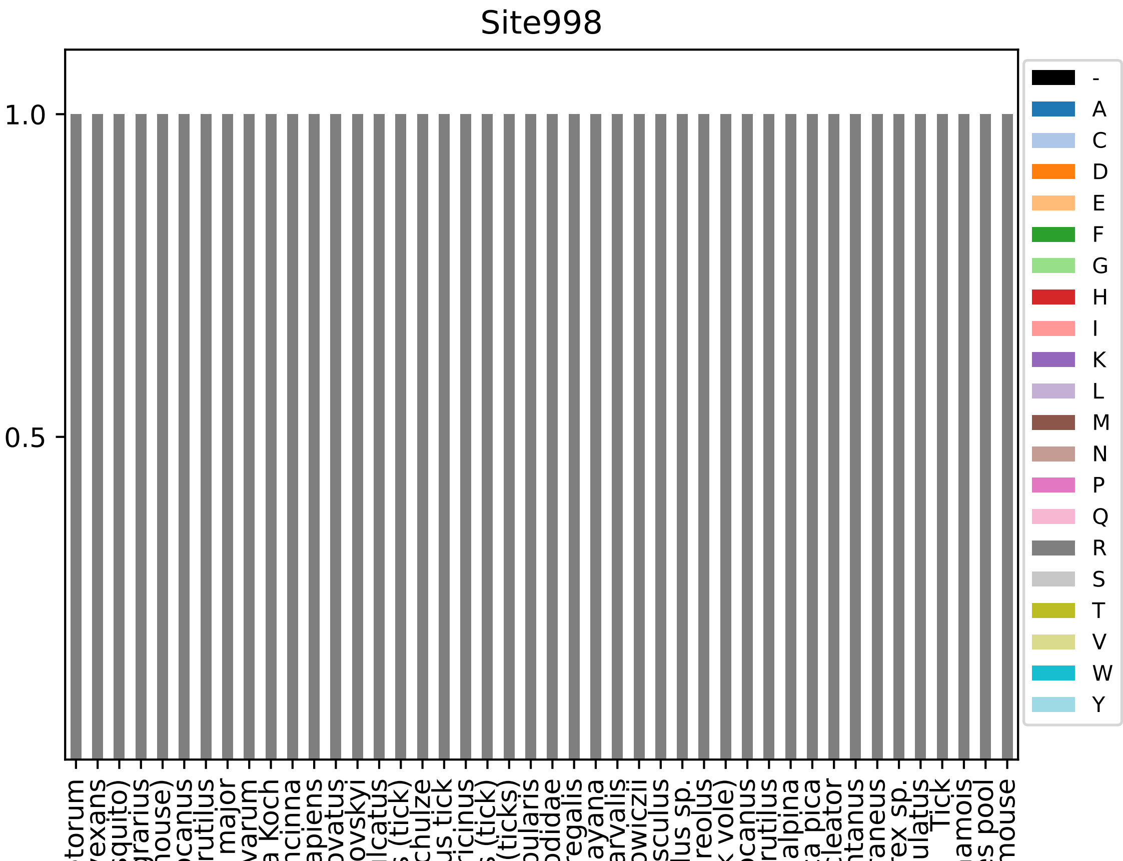
Site998
1.0
0.5
-
A
C
D
E
F
G
H
I
K
L
M
N
P
Q
R
S
T
V
W
Y

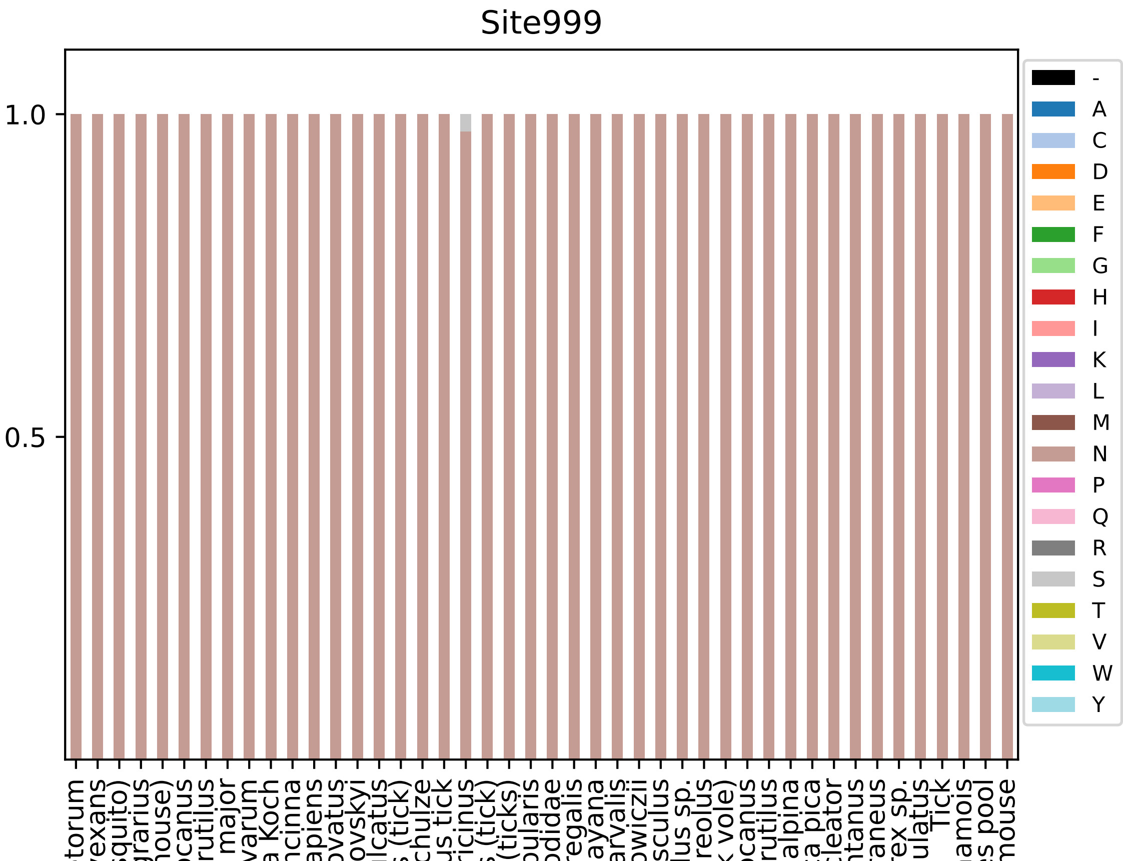

Site999
1.0
0.5
-
A
C
D
E
F
G
H
I
K
L
M
N
P
Q
R
S
T
V
W
Y

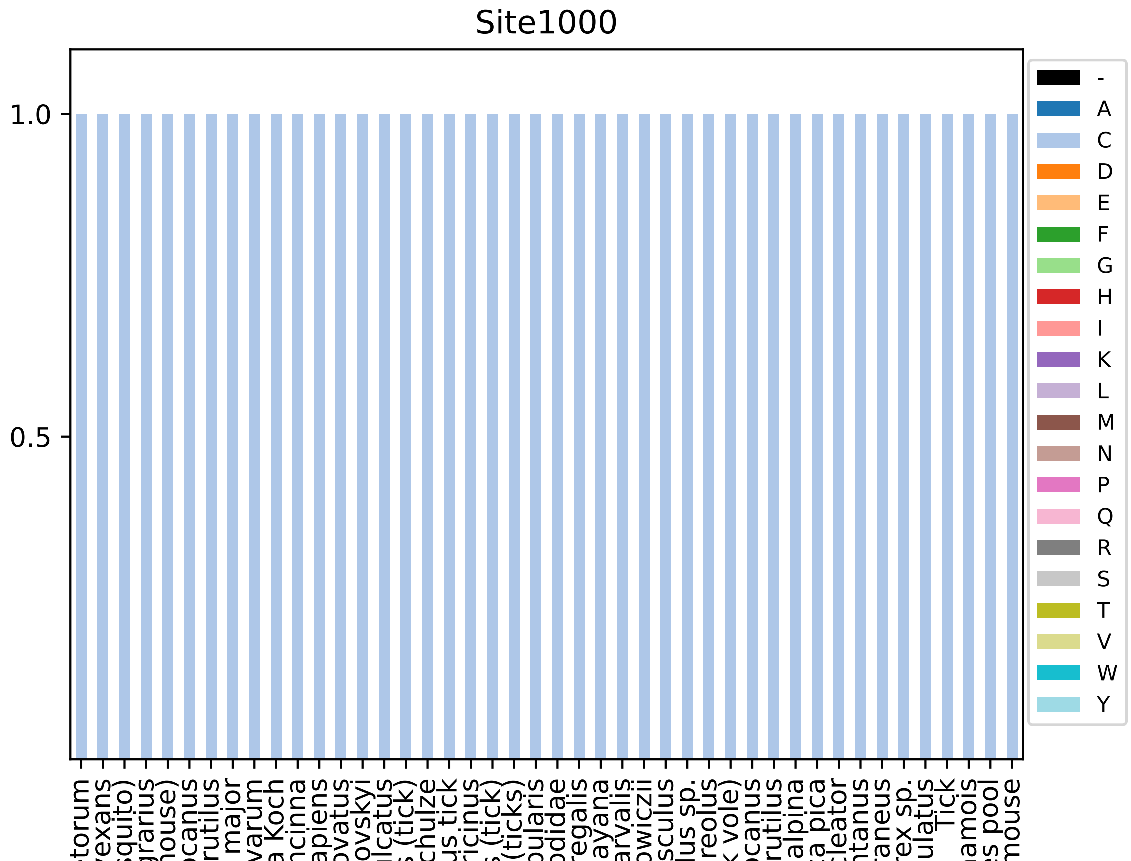
Site1000
1.0
0.5
-
A
C
D
E
F
G
H
I
K
L
M
N
P
Q
R
S
T
V
W
Y

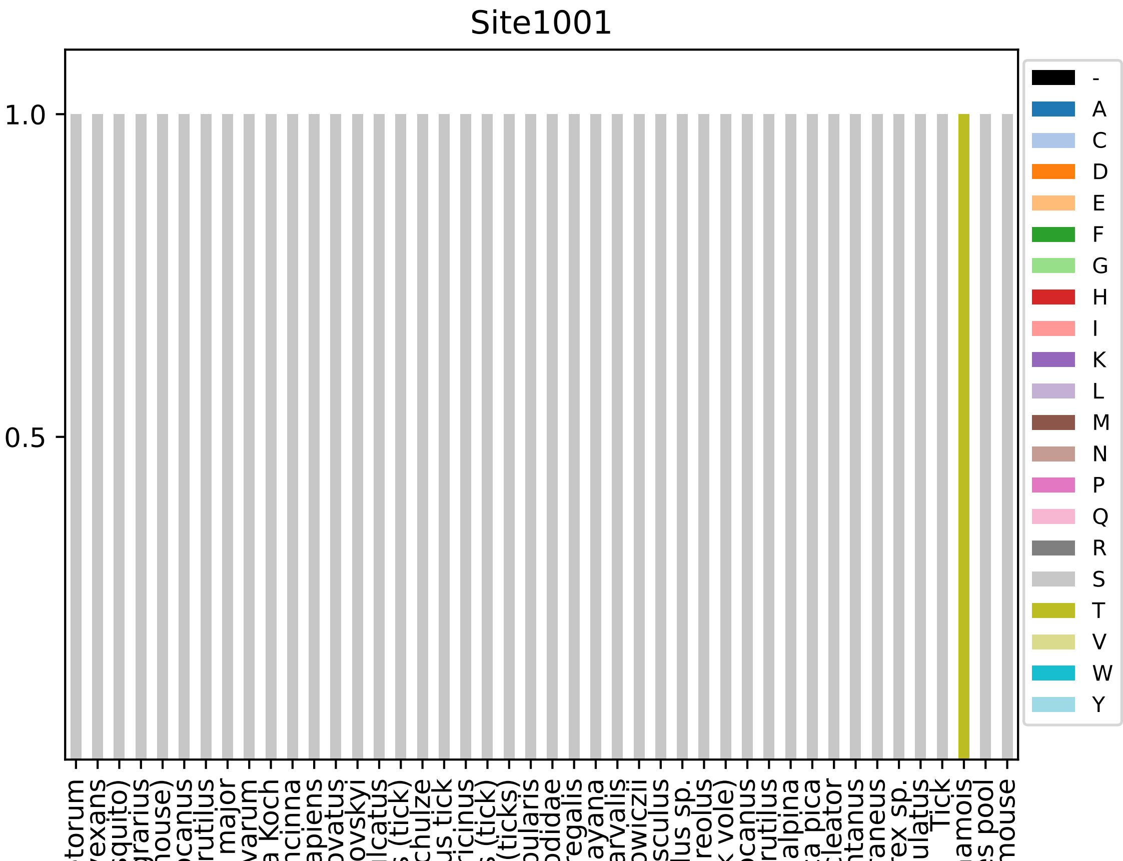

Site1001
1.0
0.5
-
A
C
D
E
F
G
H
I
K
L
M
N
P
Q
R
S
T
V
W
Y

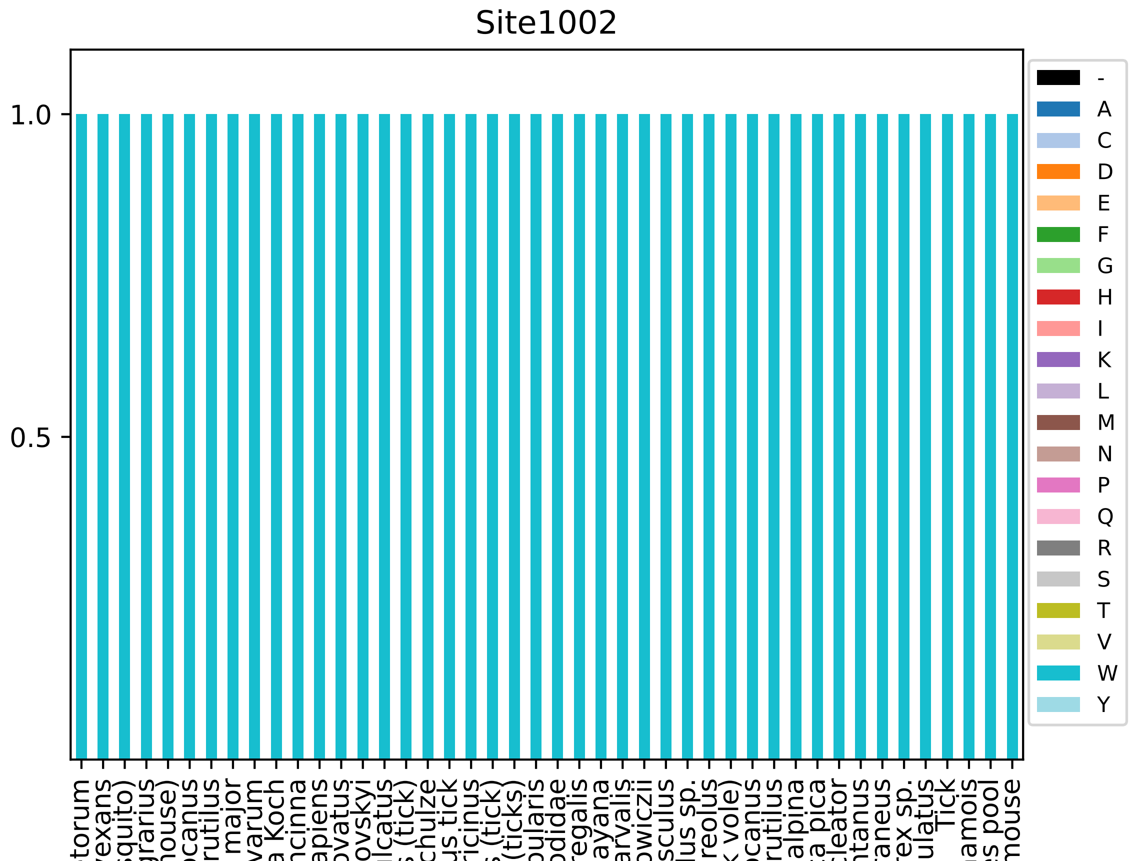
Site1002
1.0
0.5
-
A
C
D
E
F
G
H
I
K
L
M
N
P
Q
R
S
T
V
W
Y

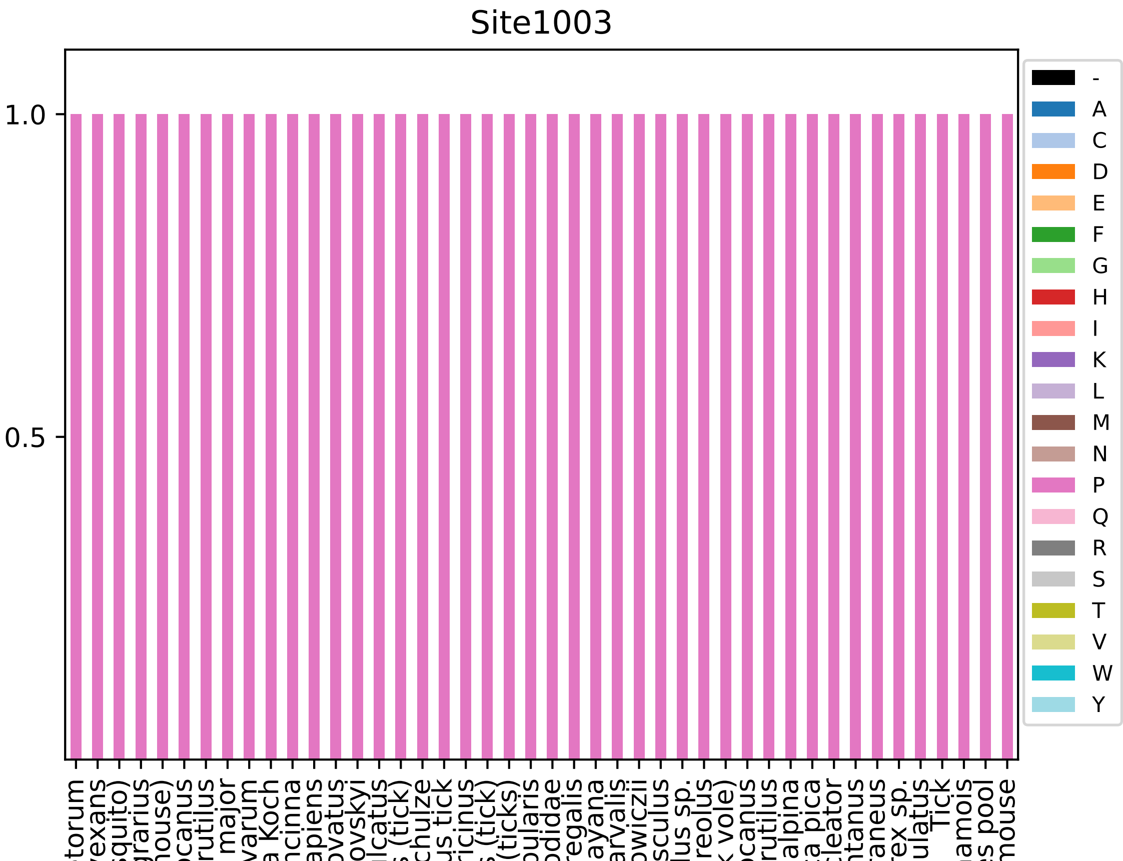
Site1003
1.0
0.5
-
A
C
D
E
F
G
H
I
K
L
M
N
P
Q
R
S
T
V
W
Y

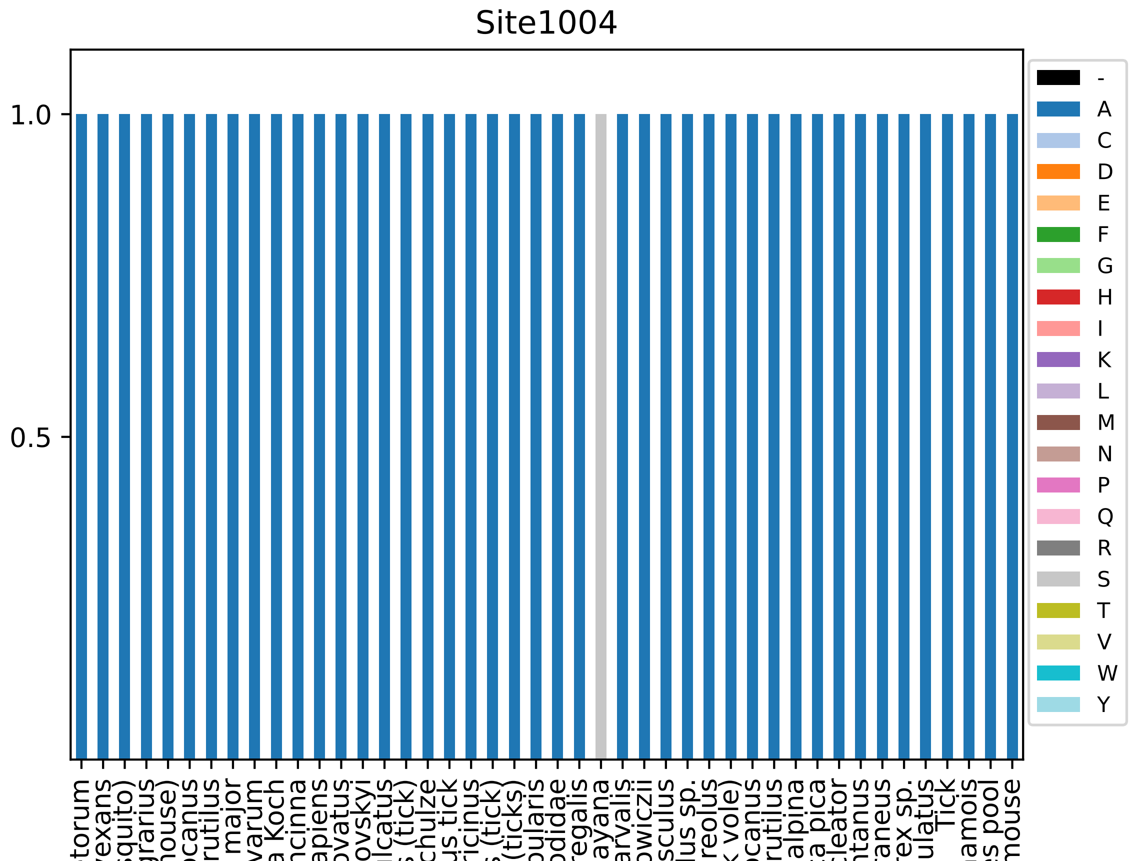
Site1004
1.0
0.5
-
A
C
D
E
F
G
H
I
K
L
M
N
P
Q
R
S
T
V
W
Y

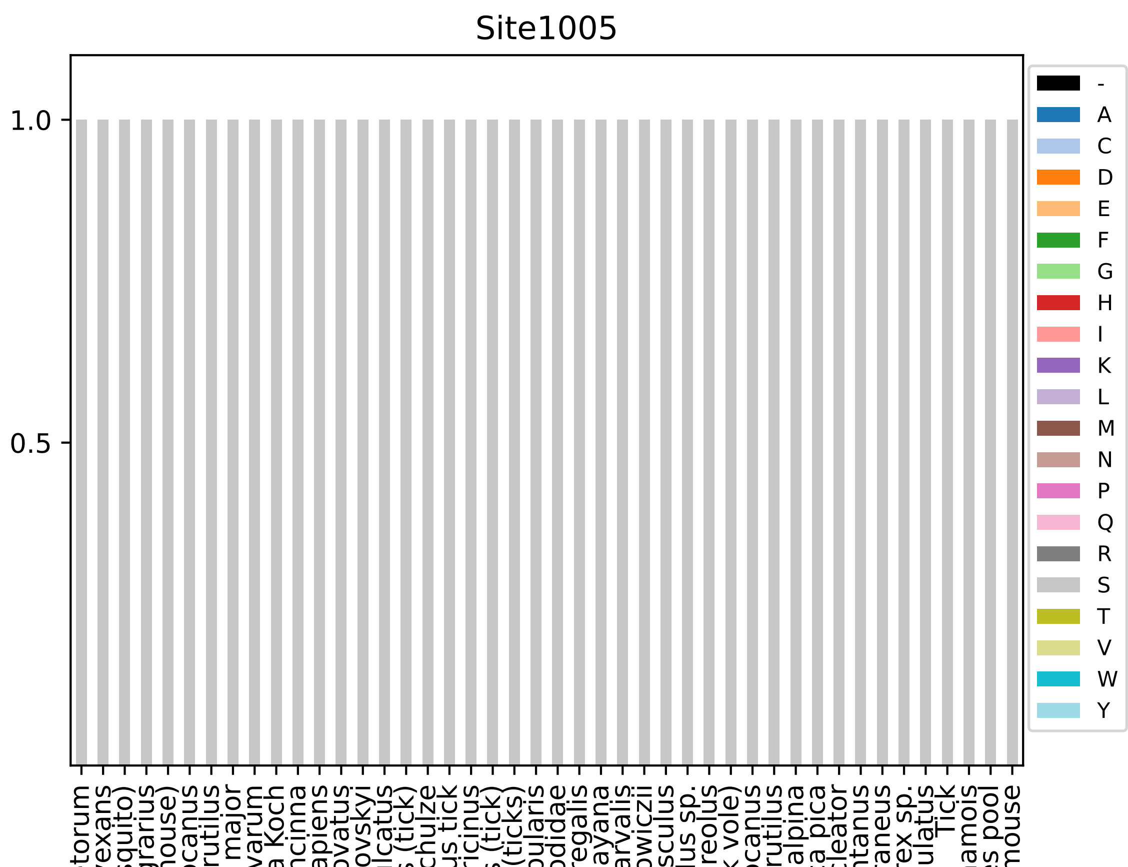
Site1005
1.0
0.5
-
A
C
D
E
F
G
H
I
K
L
M
N
P
Q
R
S
T
V
W
Y

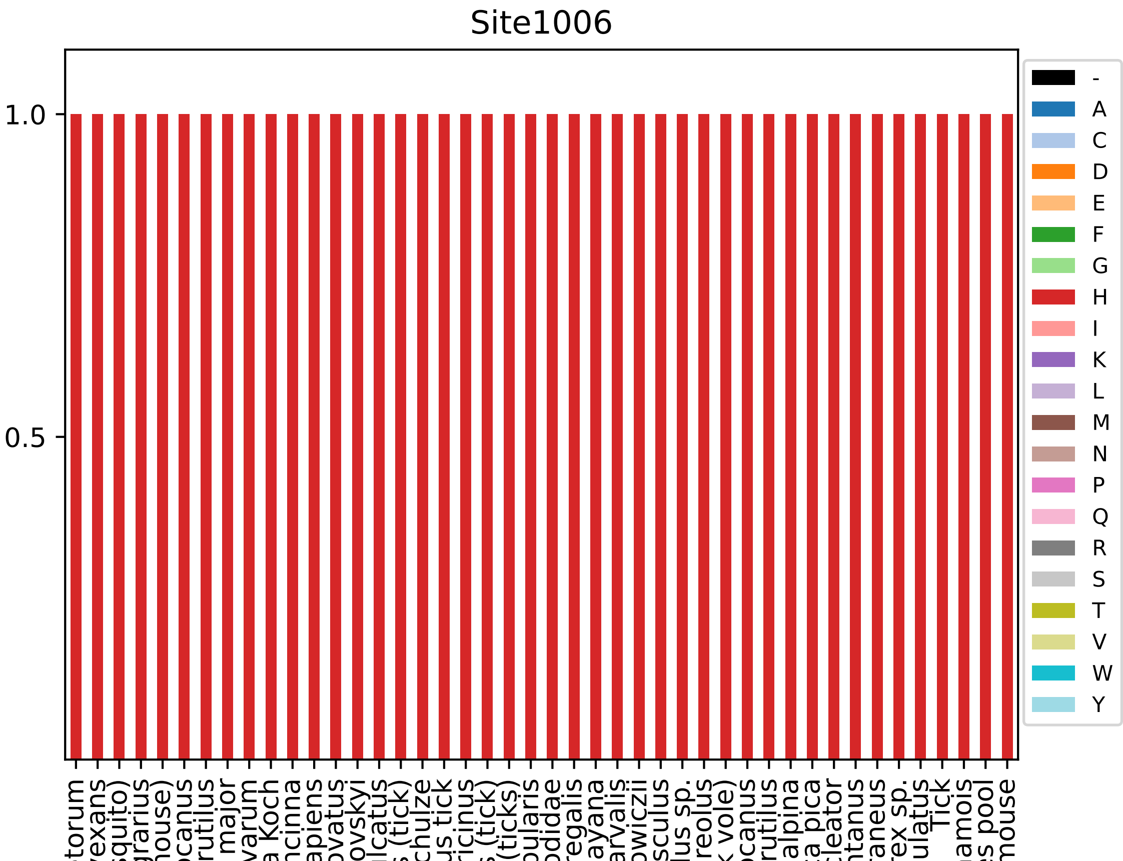
Site1006
1.0
0.5
-
A
C
D
E
F
G
H
I
K
L
M
N
P
Q
R
S
T
V
W
Y

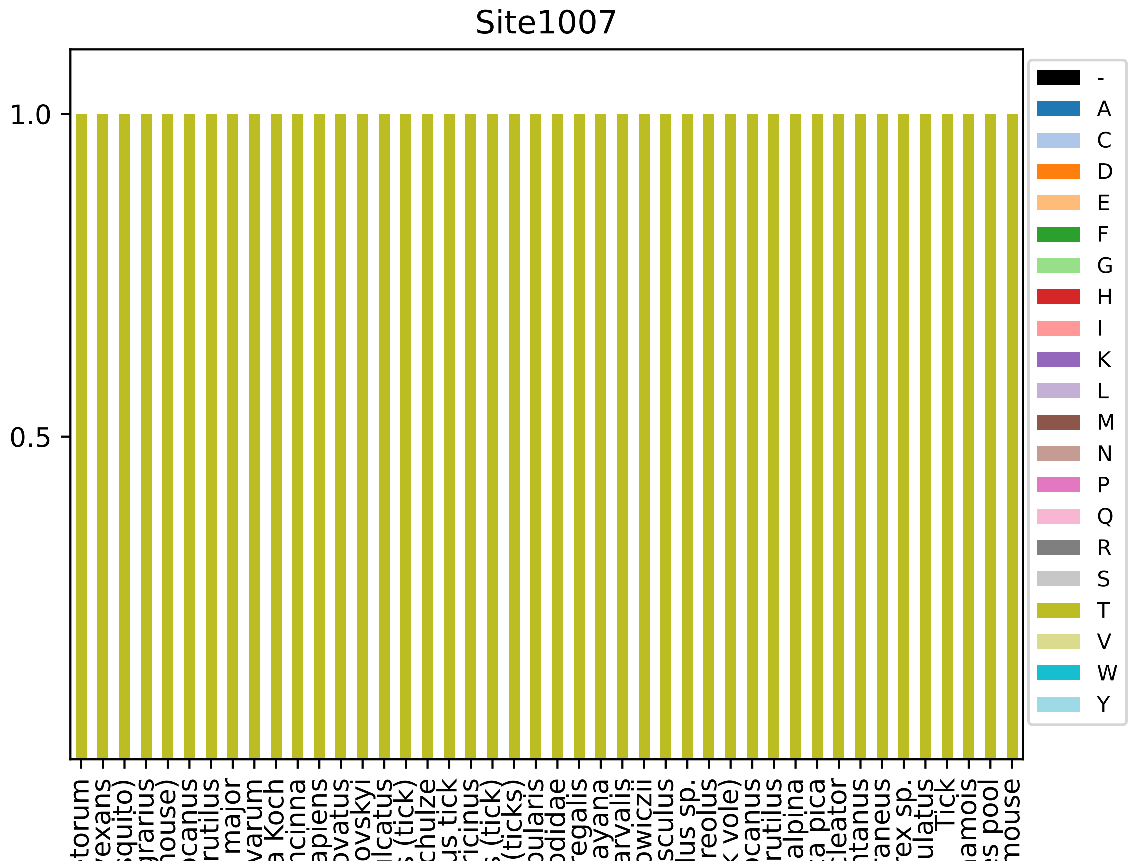
Site1007
1.0
0.5
-
A
C
D
E
F
G
H
I
K
L
M
N
P
Q
R
S
T
V
W
Y

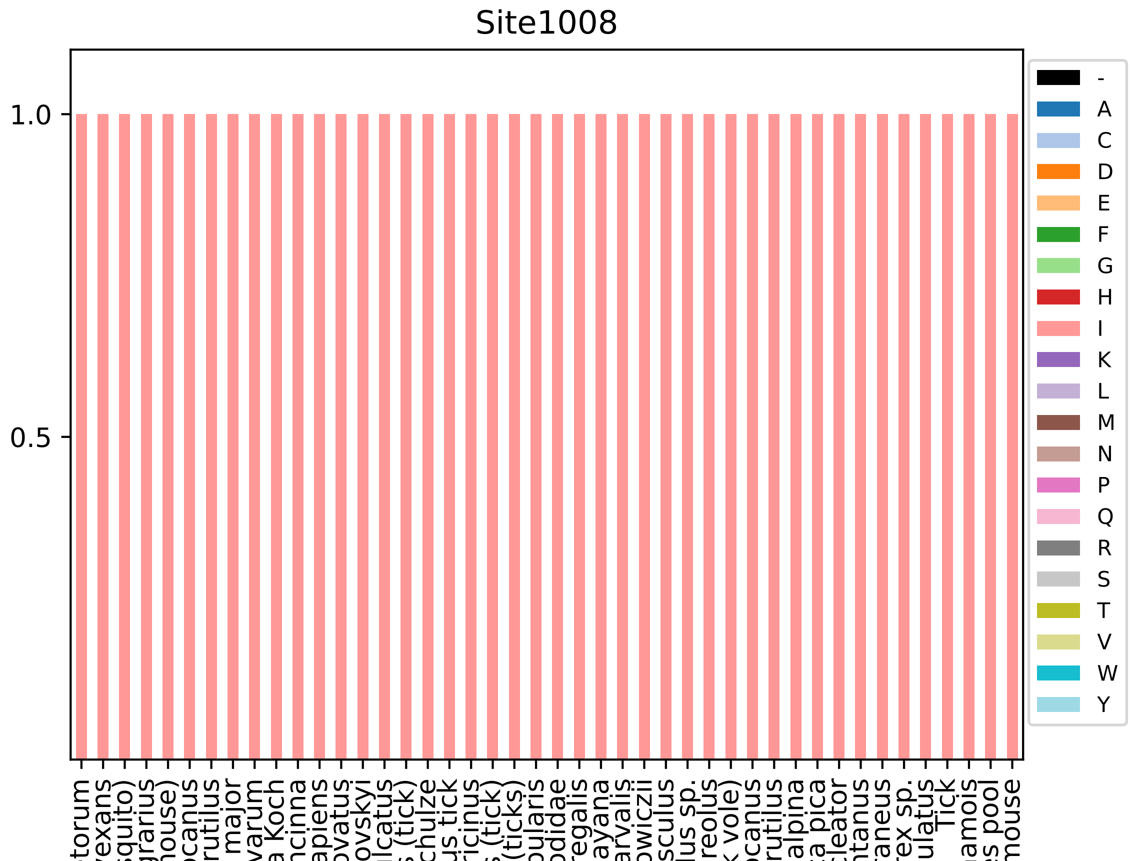
Site1008
1.0
0.5
-
A
C
D
E
F
G
H
I
K
L
M
N
P
Q
R
S
T
V
W
Y

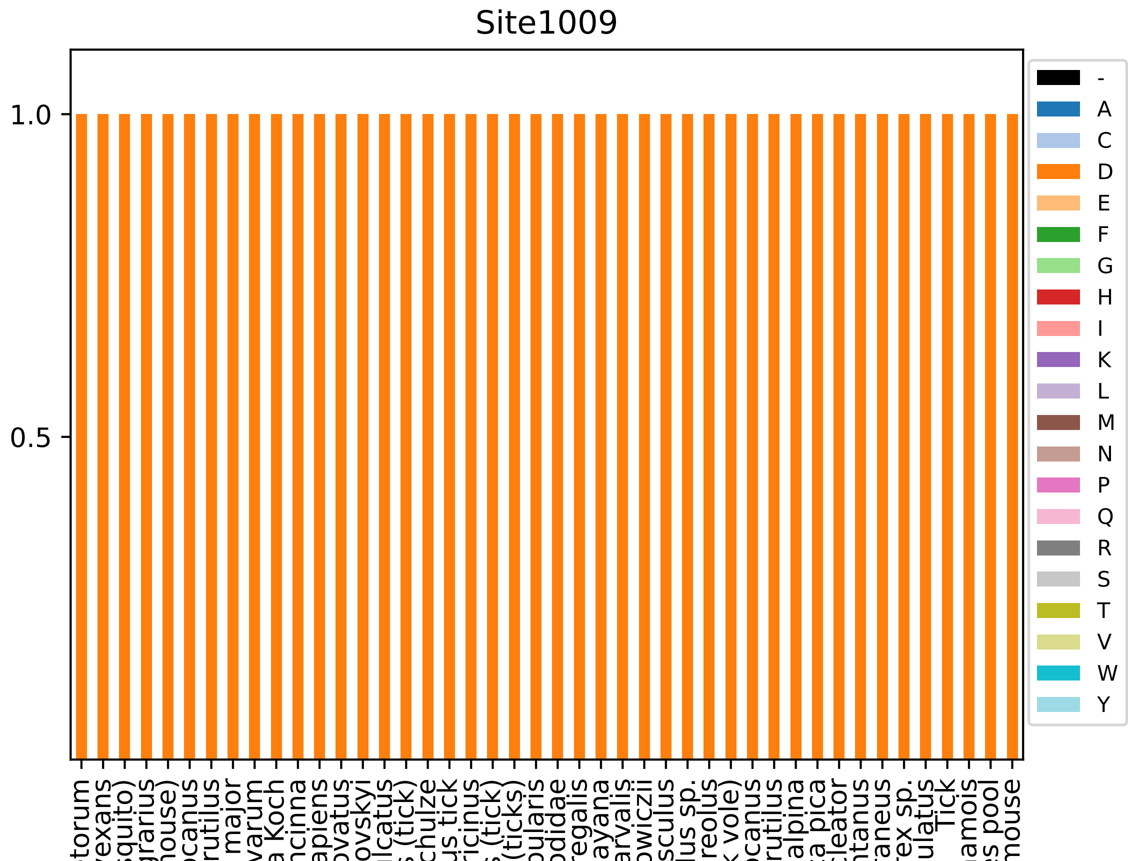
Site1009
1.0
0.5
-
A
C
D
E
F
G
H
I
K
L
M
N
P
Q
R
S
T
V
W
Y

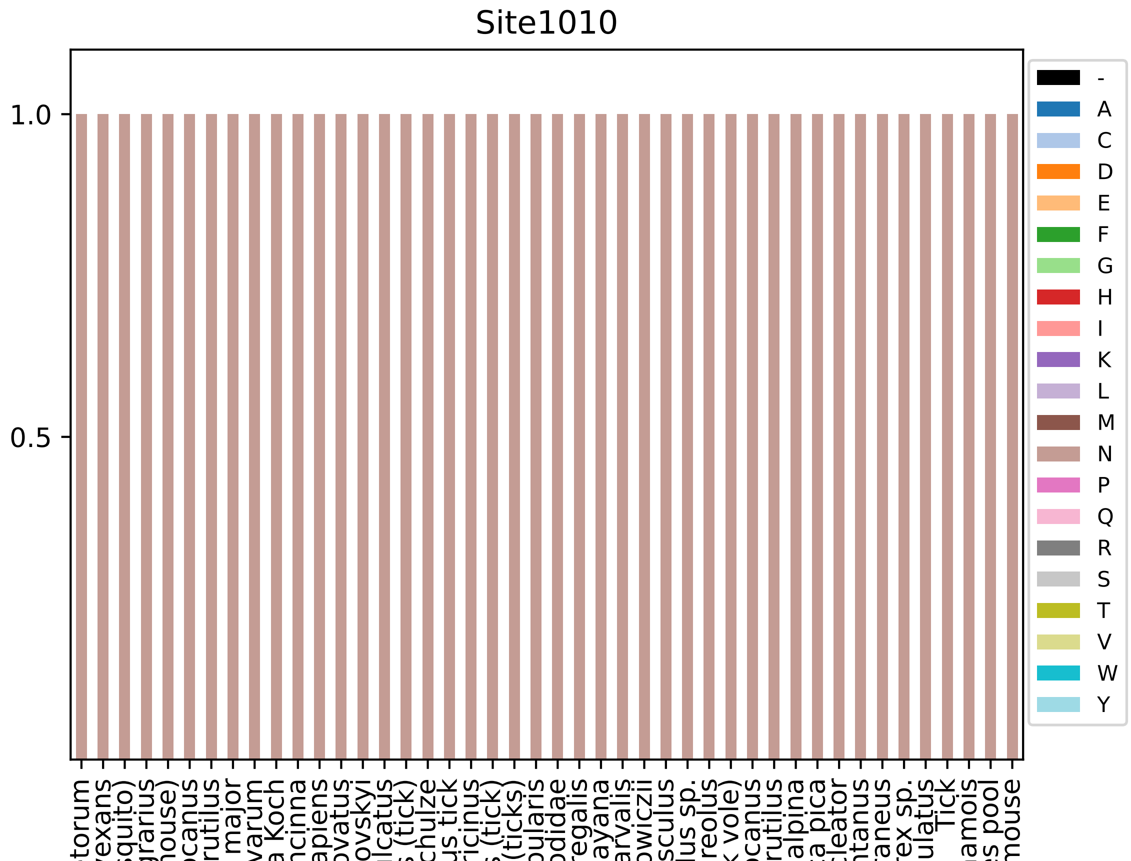
Site1010
1.0
0.5
-
A
C
D
E
F
G
H
I
K
L
M
N
P
Q
R
S
T
V
W
Y

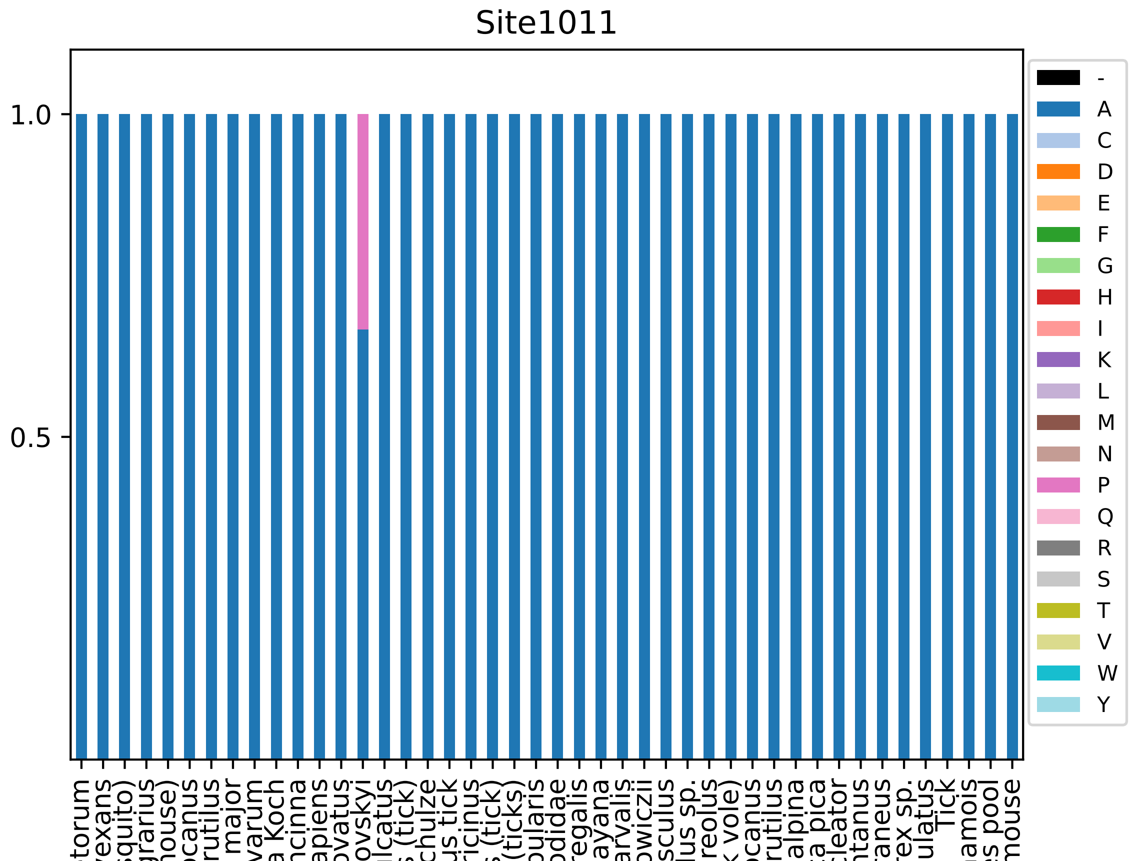
Site1011
1.0
0.5
-
A
C
D
E
F
G
H
I
K
L
M
N
P
Q
R
S
T
V
W
Y

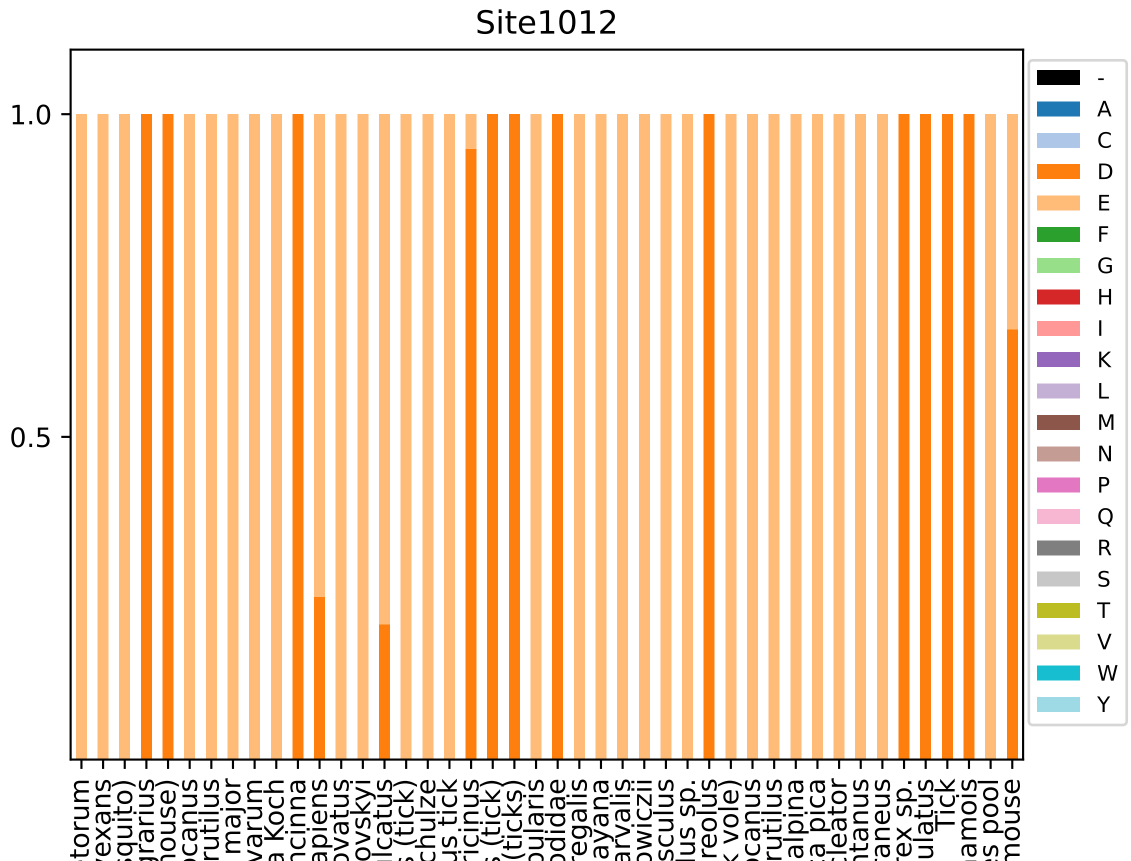
Site1012
1.0
0.5
-
A
C
D
E
F
G
H
I
K
L
M
N
P
Q
R
S
T
V
W
Y

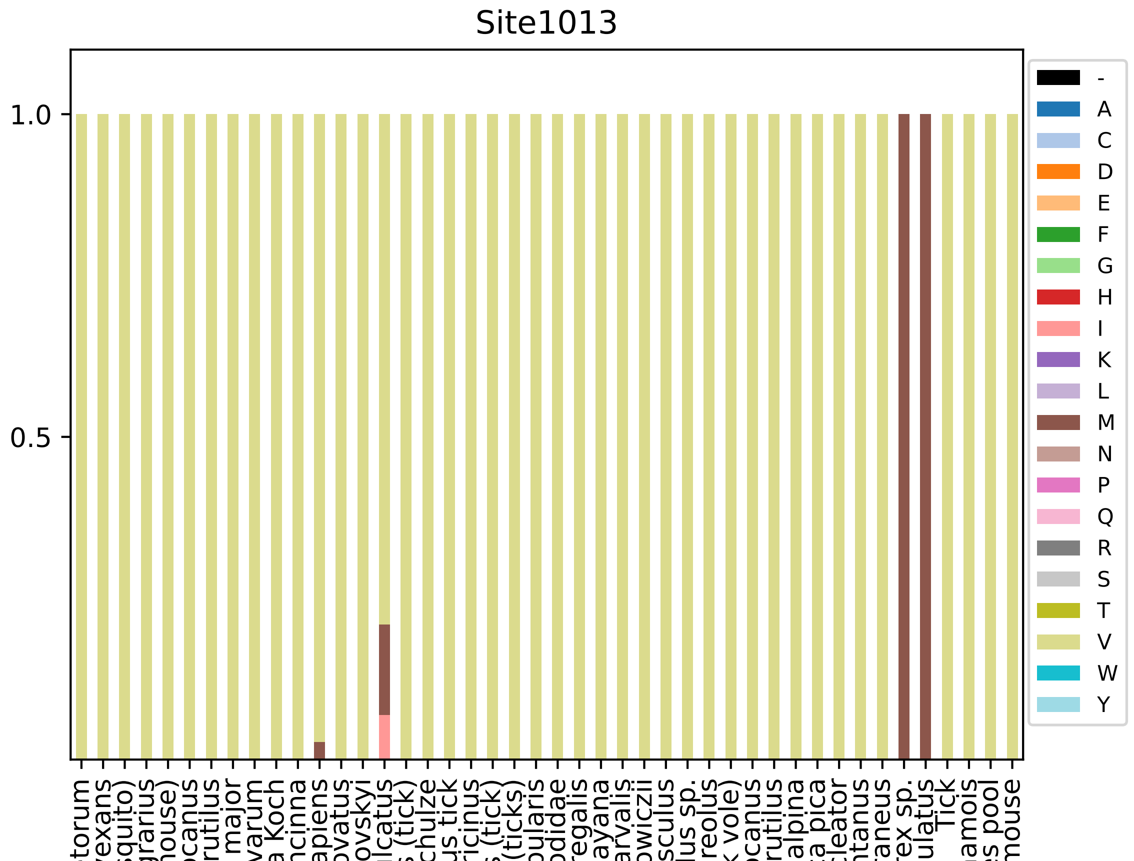

Site1013
1.0
0.5
-
A
C
D
E
F
G
H
I
K
L
M
N
P
Q
R
S
T
V
W
Y

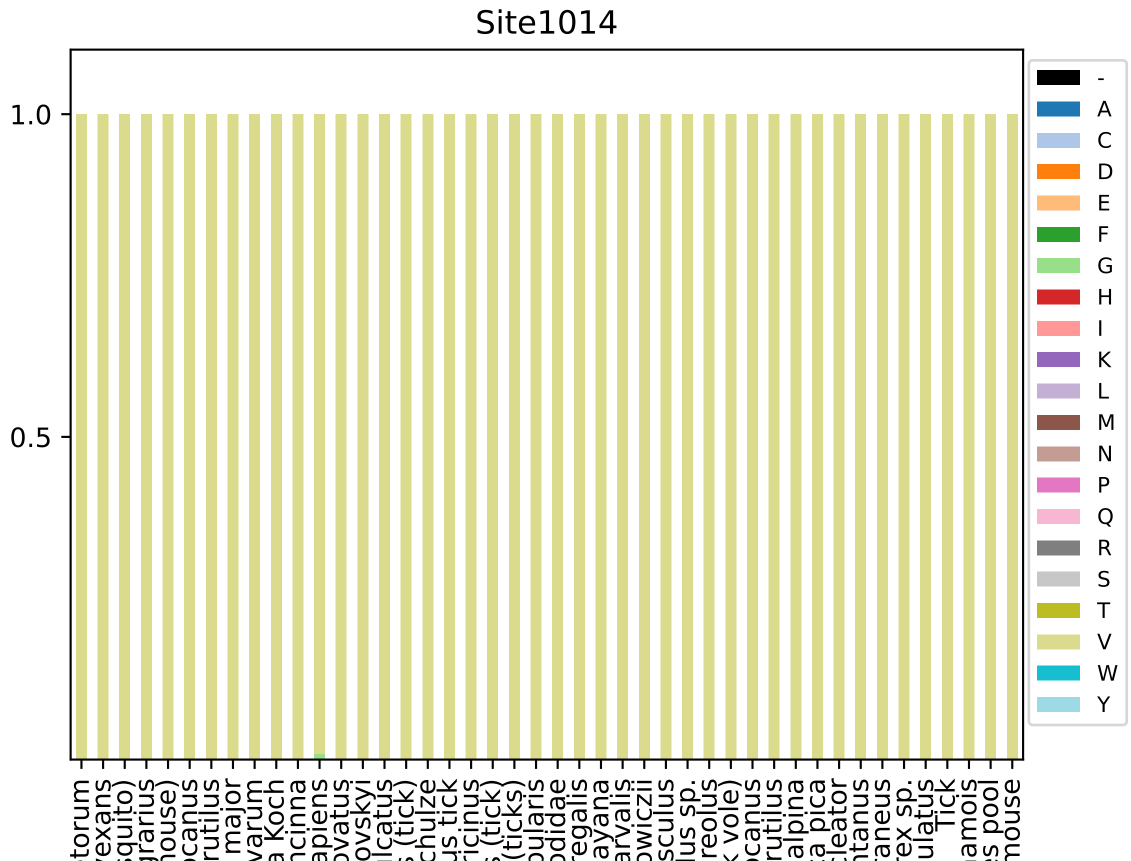

Site1014
1.0
0.5
-
A
C
D
E
F
G
H
I
K
L
M
N
P
Q
R
S
T
V
W
Y

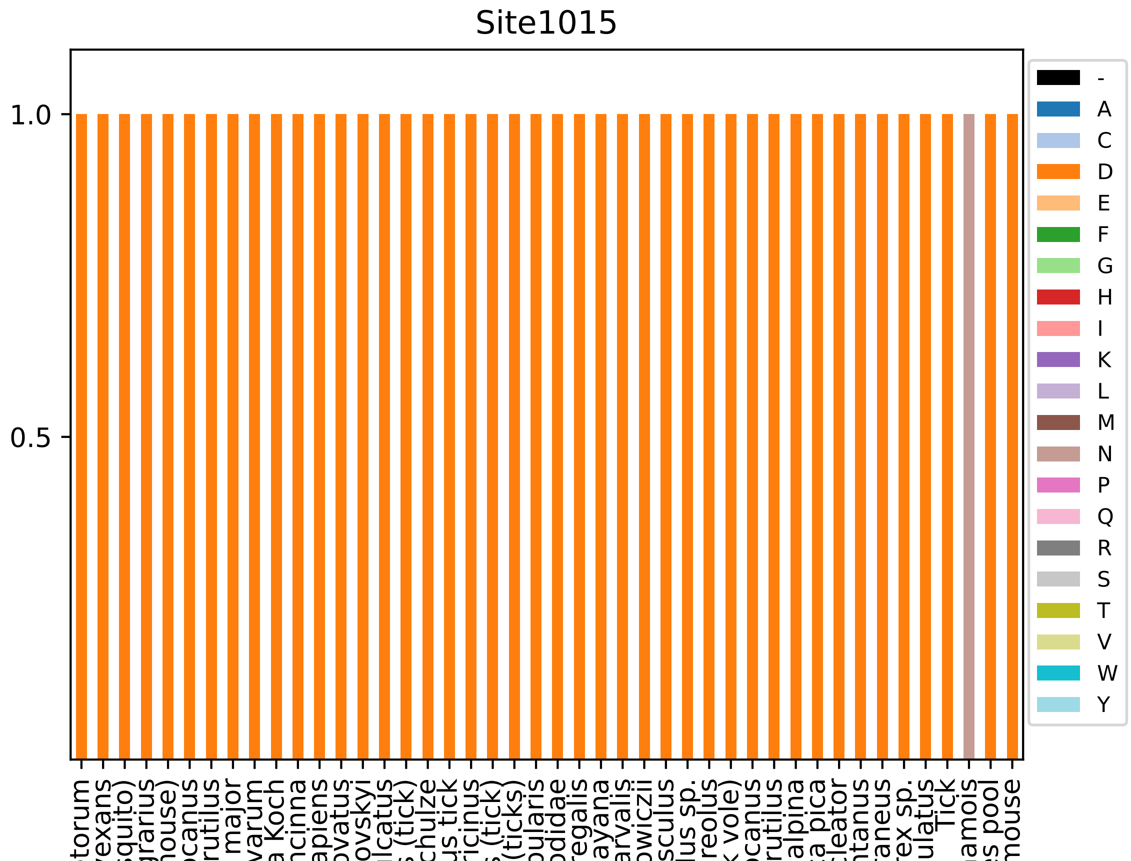
Site1015
1.0
0.5
-
A
C
D
E
F
G
H
I
K
L
M
N
P
Q
R
S
T
V
W
Y

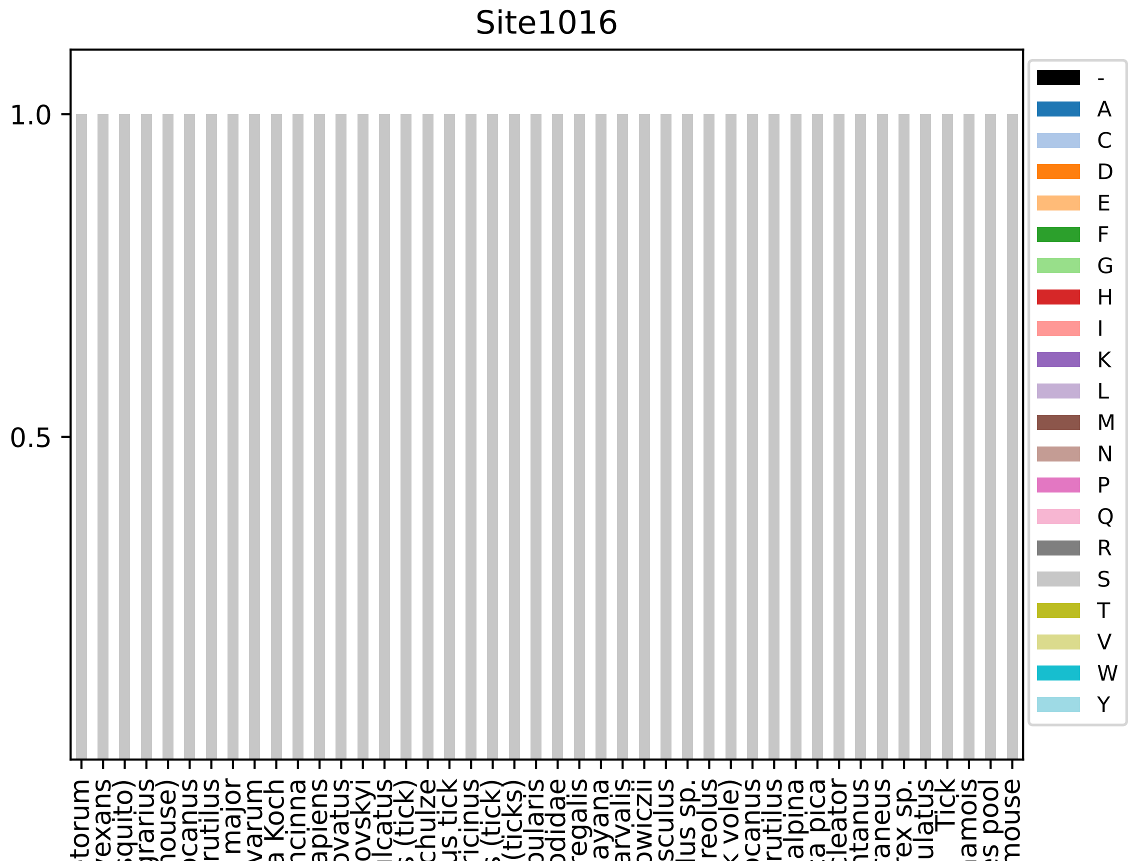

Site1016
1.0
0.5
-
A
C
D
E
F
G
H
I
K
L
M
N
P
Q
R
S
T
V
W
Y

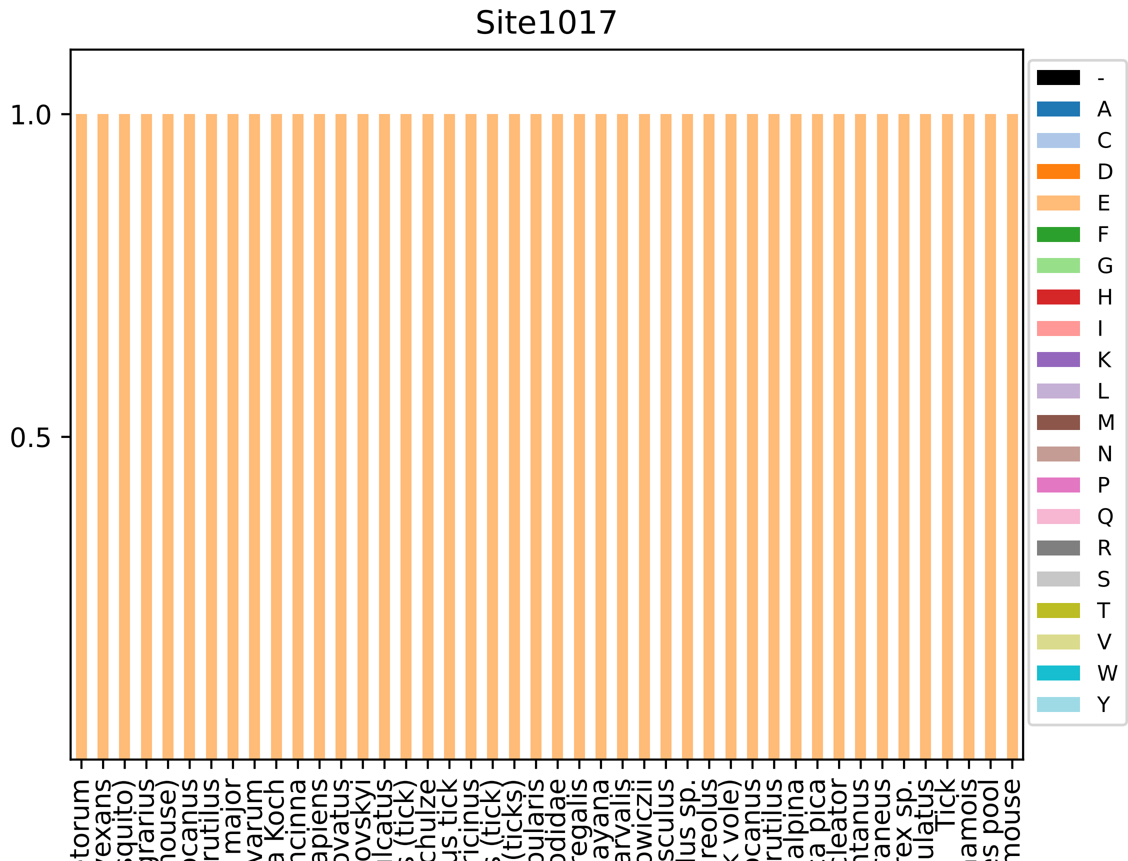
Site1017
1.0
0.5
-
A
C
D
E
F
G
H
I
K
L
M
N
P
Q
R
S
T
V
W
Y
Tick

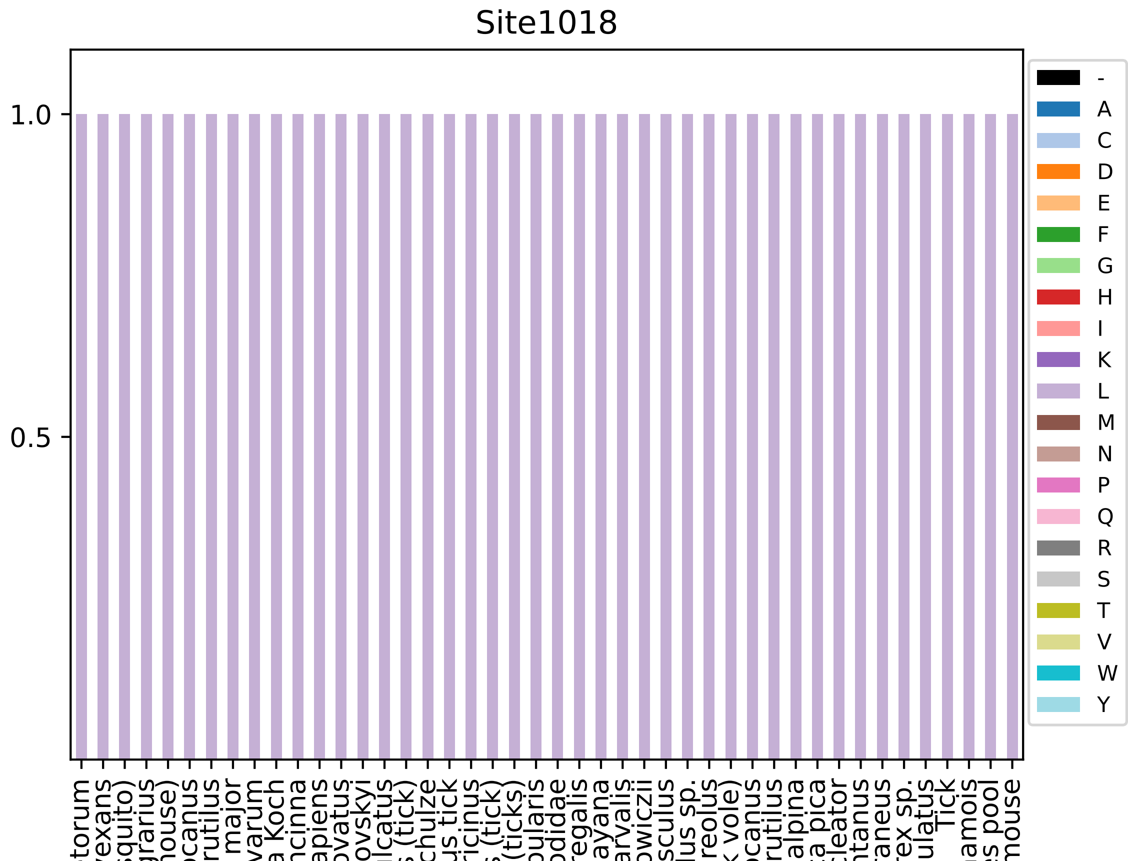
Site1018
1.0
0.5
-
A
C
D
E
F
G
H
I
K
L
M
N
P
Q
R
S
T
V
W
Y

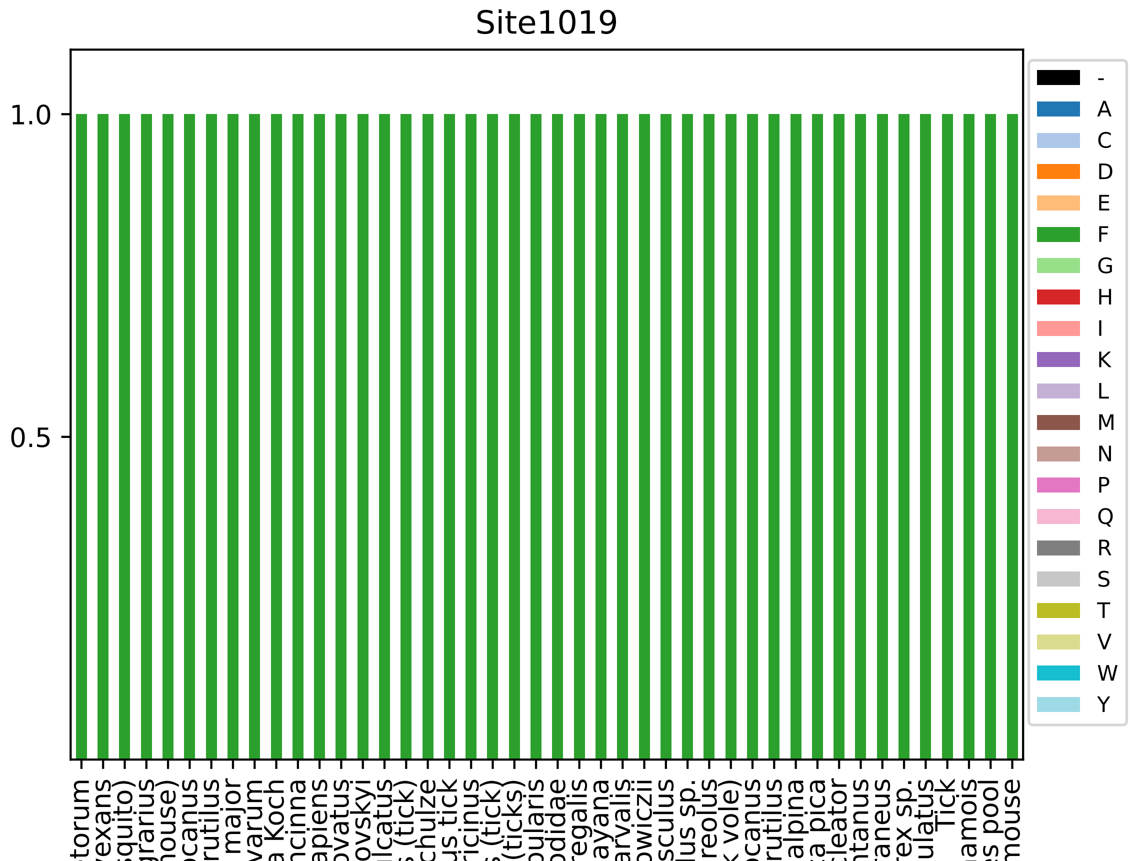
Site1019
1.0
0.5
-
A
C
D
E
F
G
H
I
K
L
M
N
P
Q
R
S
T
V
W
Y

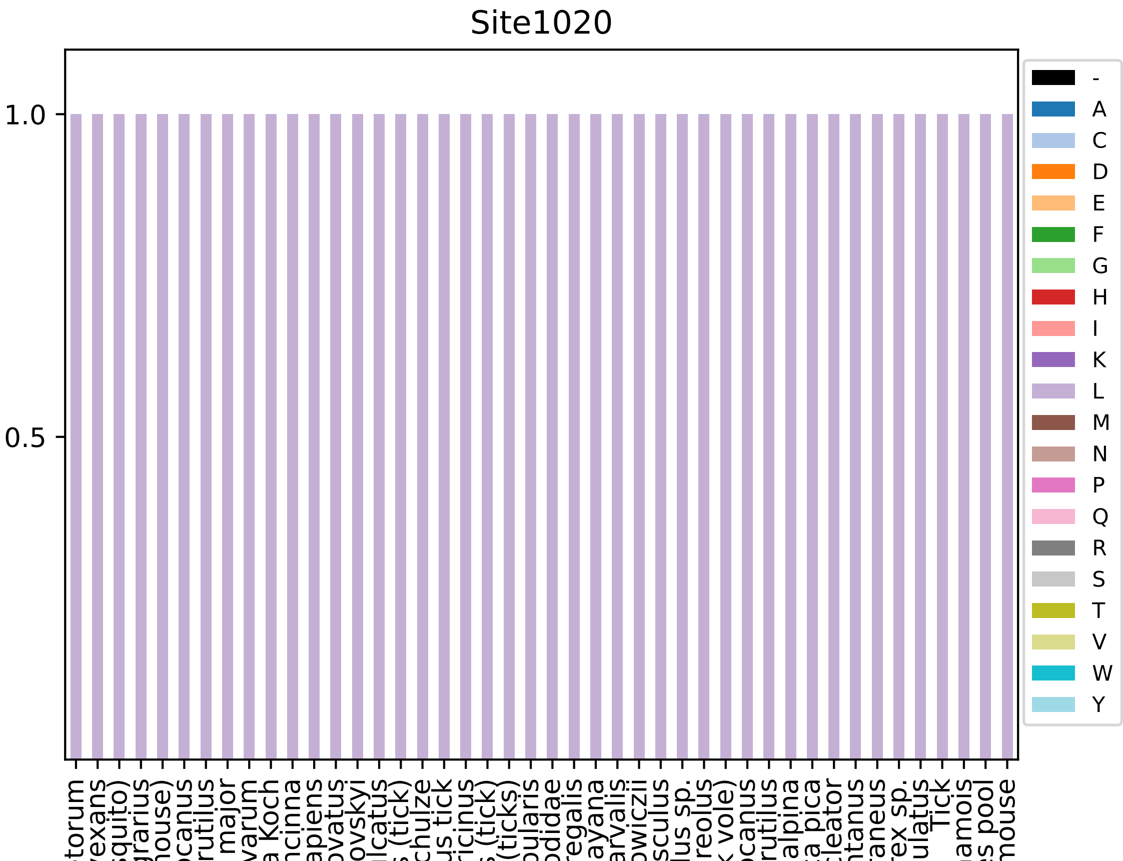

Site1020
1.0
0.5
-
A
C
D
E
F
G
H
I
K
L
M
N
P
Q
R
S
T
V
W
Y
Tick

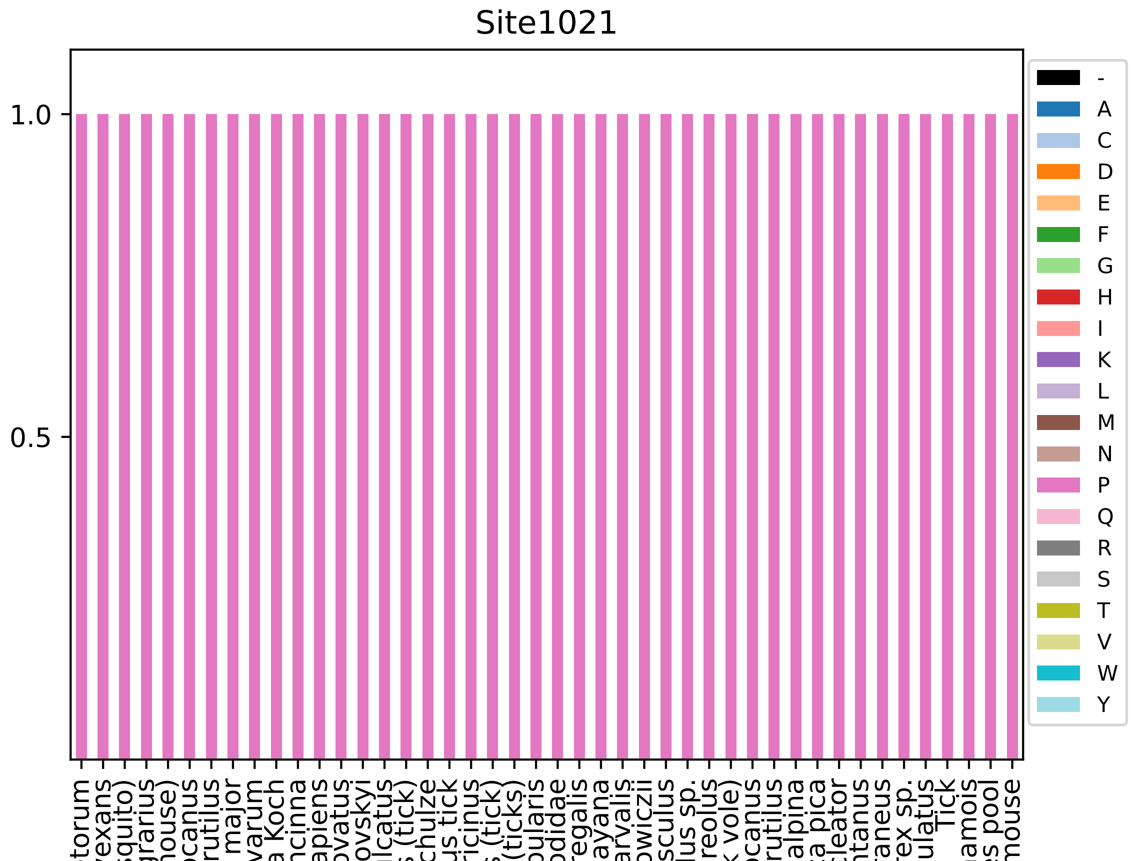

Site1021
1.0
0.5
-
A
C
D
E
F
G
H
I
K
L
M
N
P
Q
R
S
T
V
W
Y

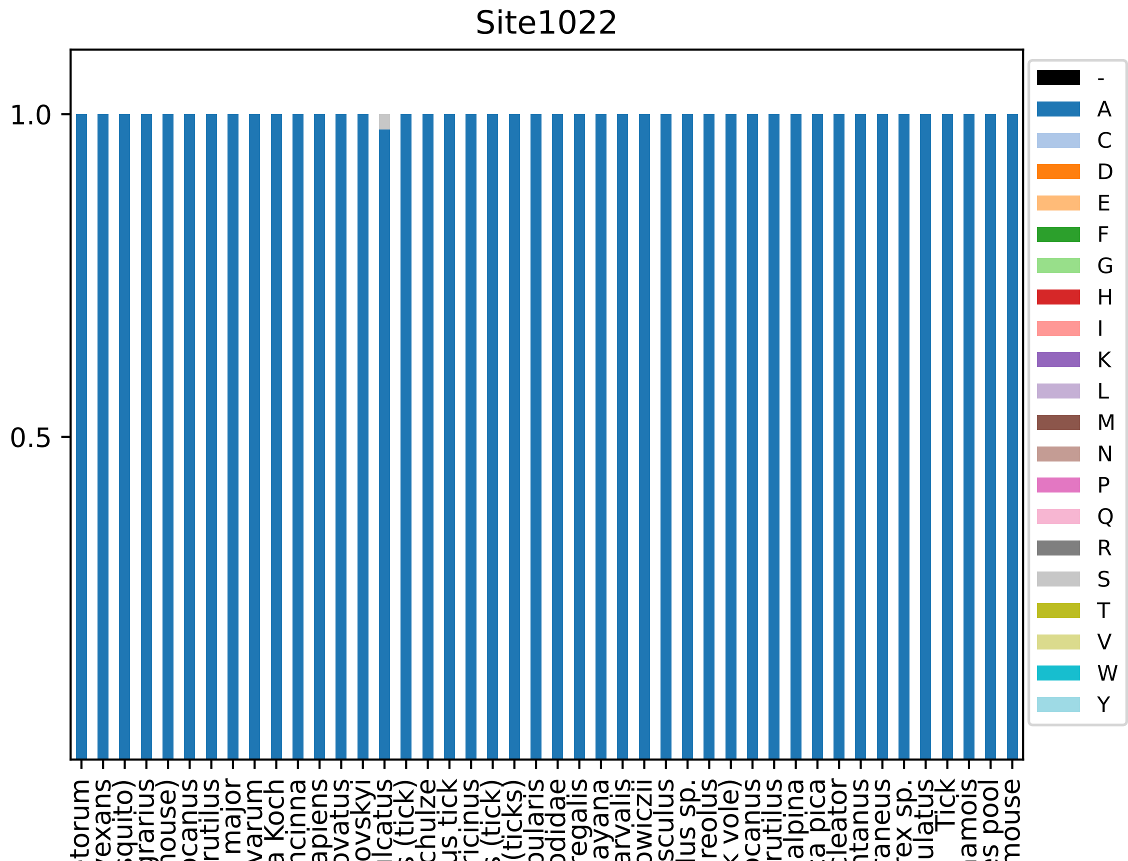

Site1022
1.0
0.5
-
A
C
D
E
F
G
H
I
K
L
M
N
P
Q
R
S
T
V
W
Y

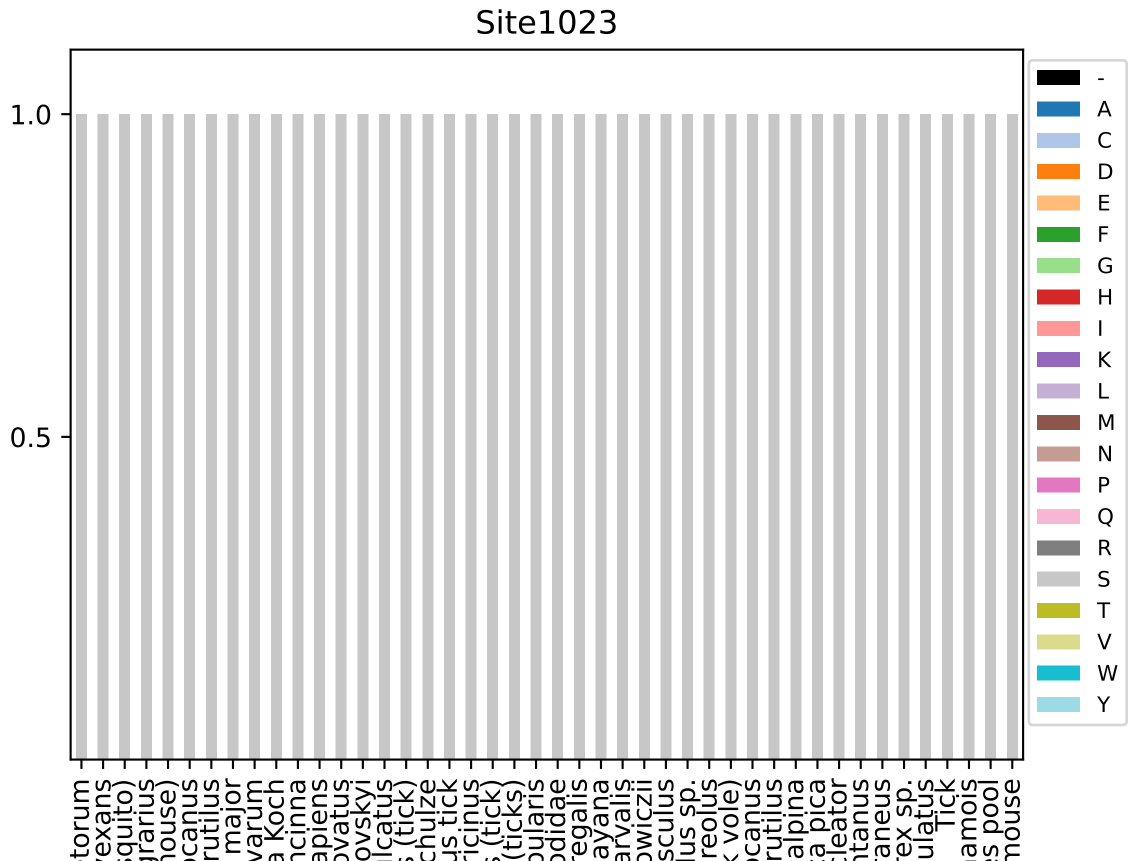
Site1023
1.0
0.5
-
A
C
D
E
F
G
H
I
K
L
M
N
P
Q
R
S
T
V
W
Y

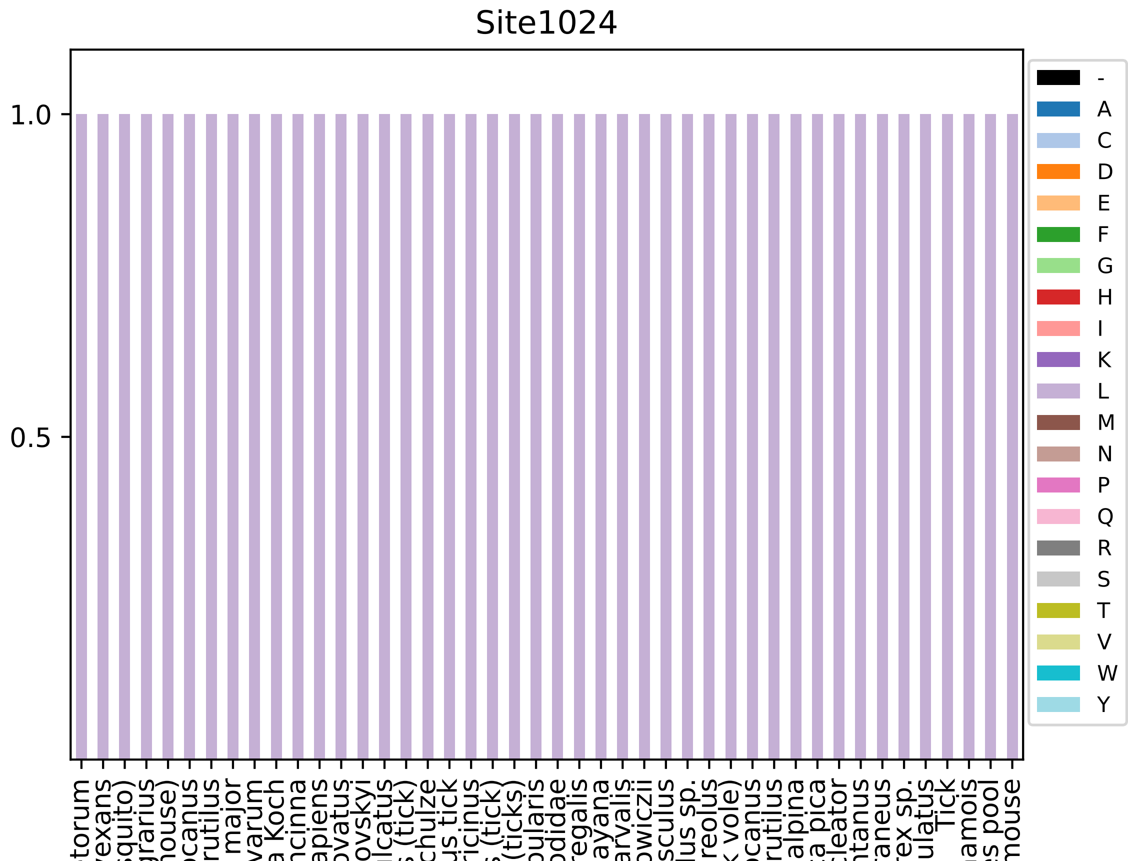

Site1024
1.0
0.5
-
A
C
D
E
F
G
H
I
K
L
M
N
P
Q
R
S
T
V
W
Y

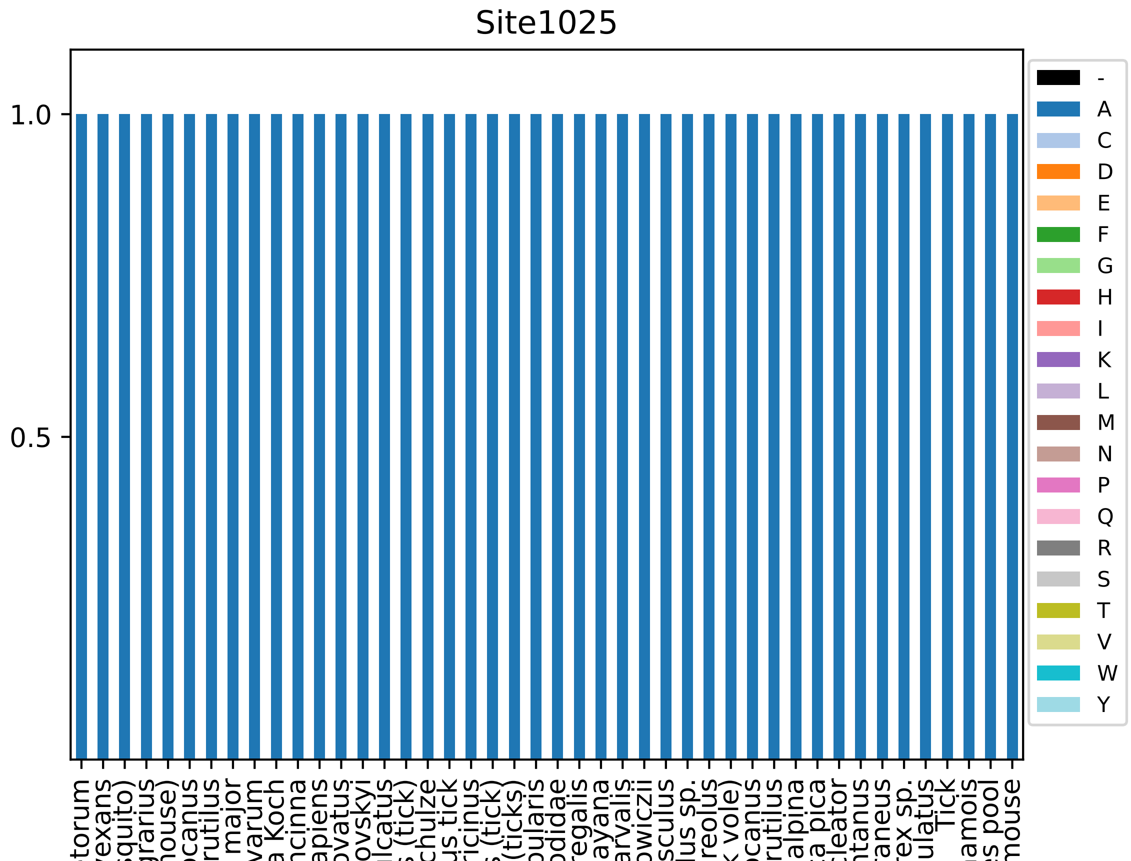
Site1025
1.0
0.5
-
A
C
D
E
F
G
H
I
K
L
M
N
P
Q
R
S
T
V
W
Y

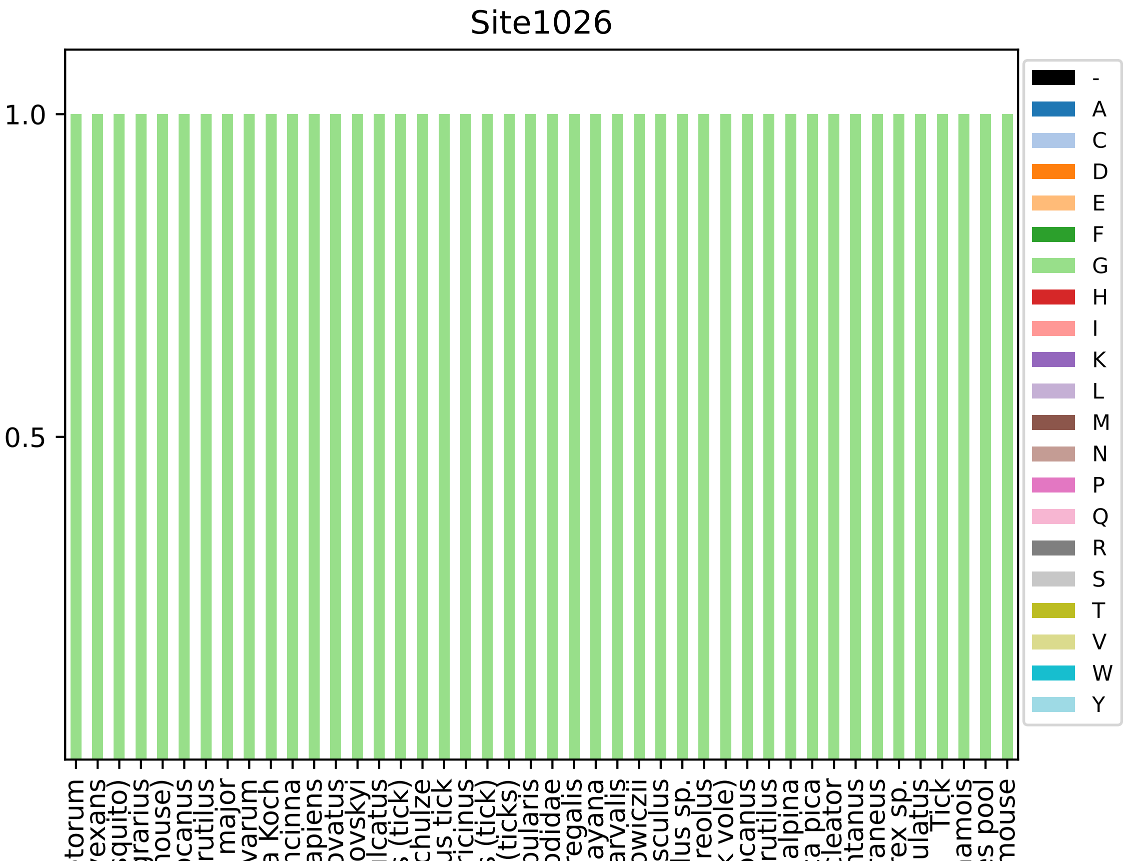

Site1026
1.0
0.5
-
A
C
D
E
F
G
H
I
K
L
M
N
P
Q
R
S
T
V
W
Y

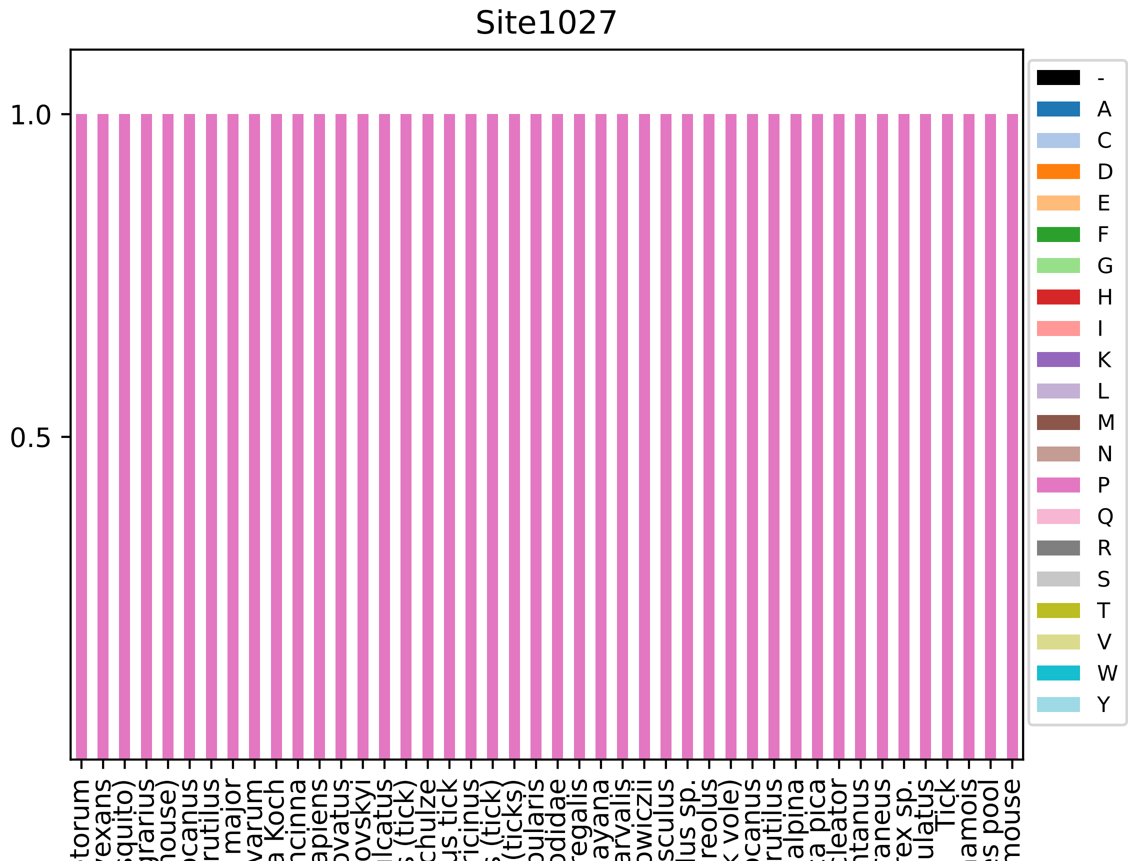

Site1027
1.0
0.5
-
A
C
D
E
F
G
H
I
K
L
M
N
P
Q
R
S
T
V
W
Y

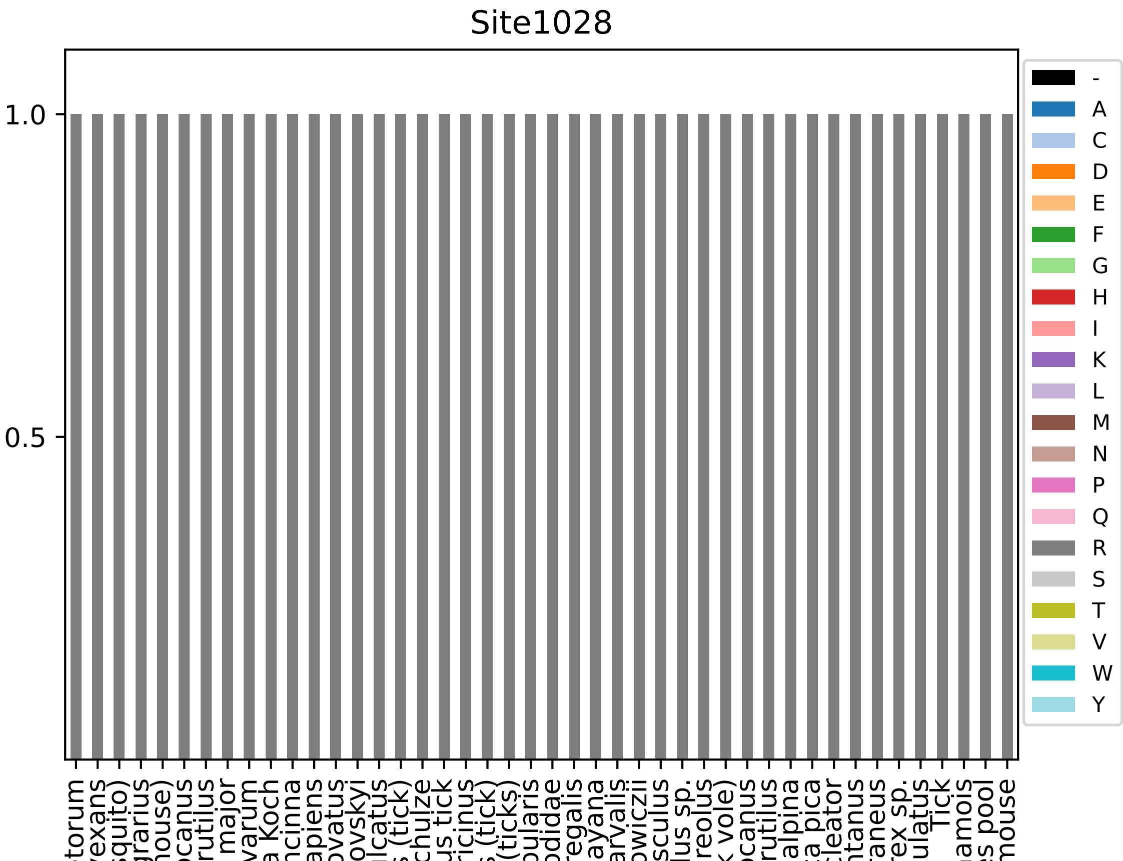
Site1028
1.0
0.5
-
A
C
D
E
F
G
H
I
K
L
M
N
P
Q
R
S
T
V
W
Y

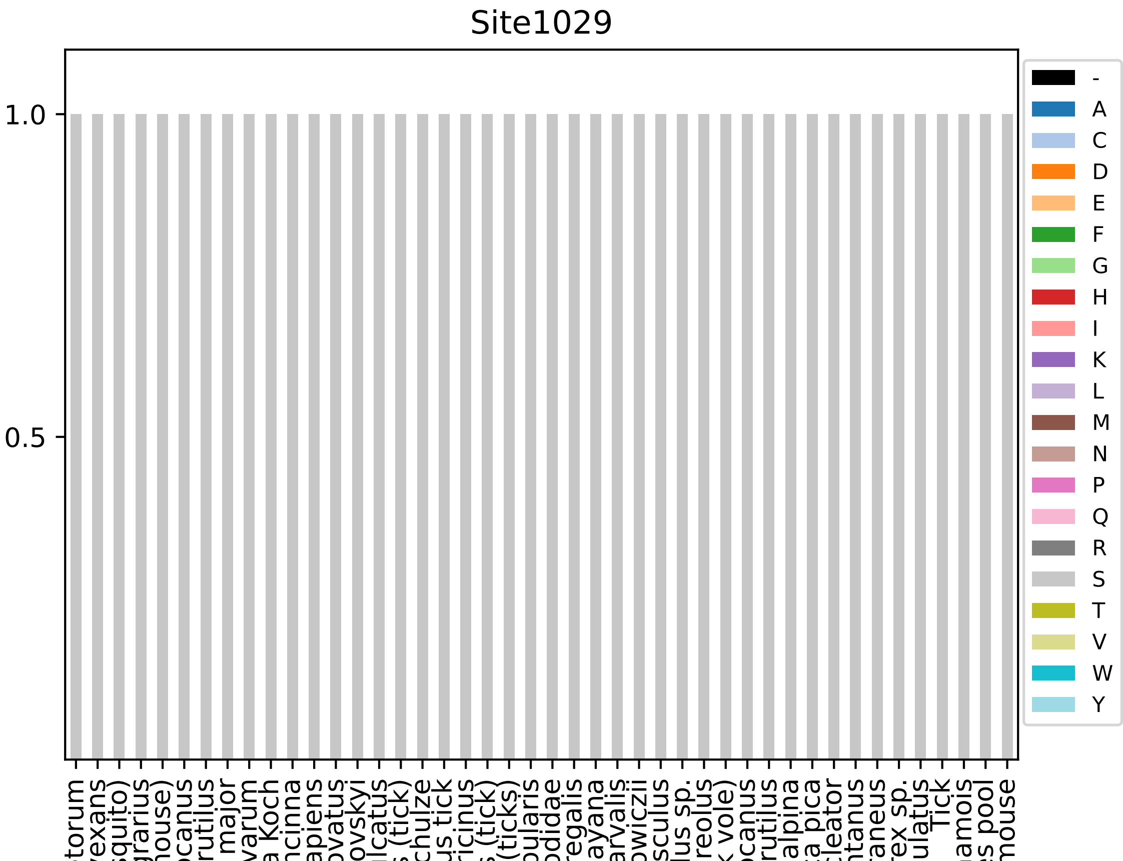
Site1029
1.0
0.5
-
A
C
D
E
F
G
H
I
K
L
M
N
P
Q
R
S
T
V
W
Y

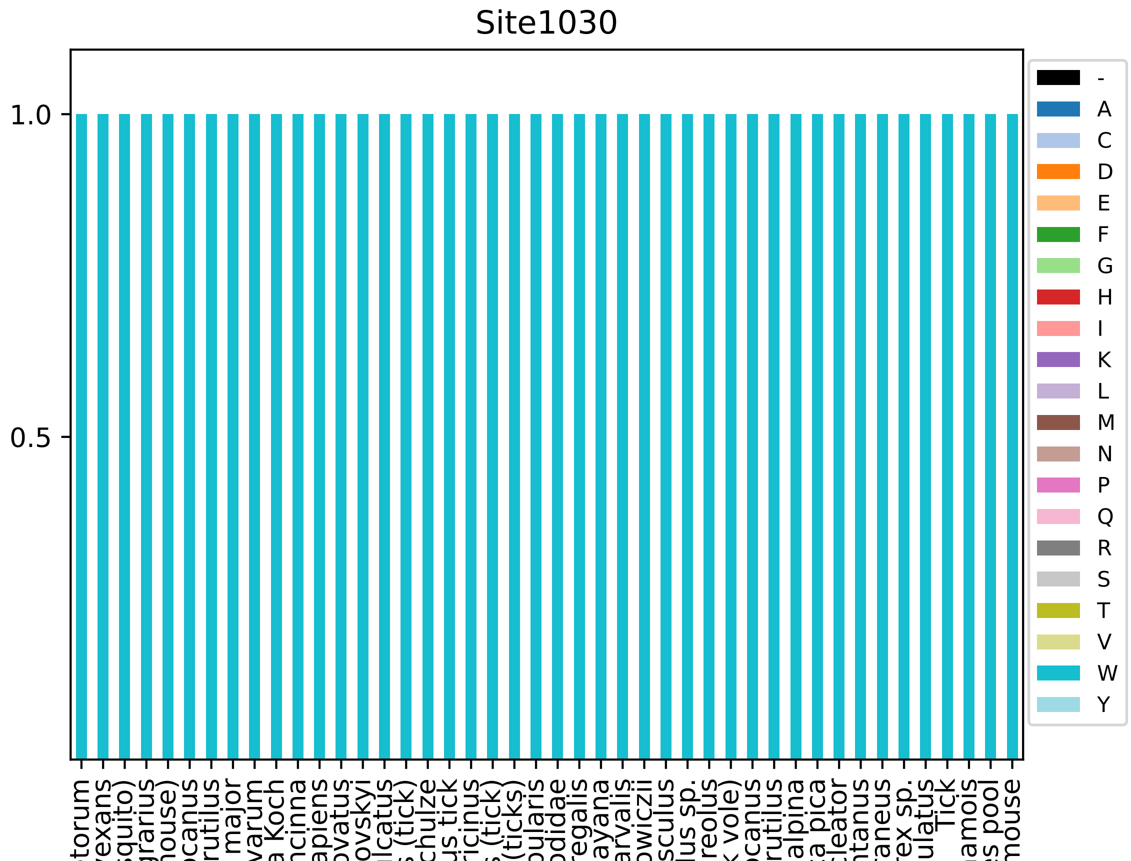
Site1030
1.0
0.5
-
A
C
D
E
F
G
H
I
K
L
M
N
P
Q
R
S
T
V
W
Y

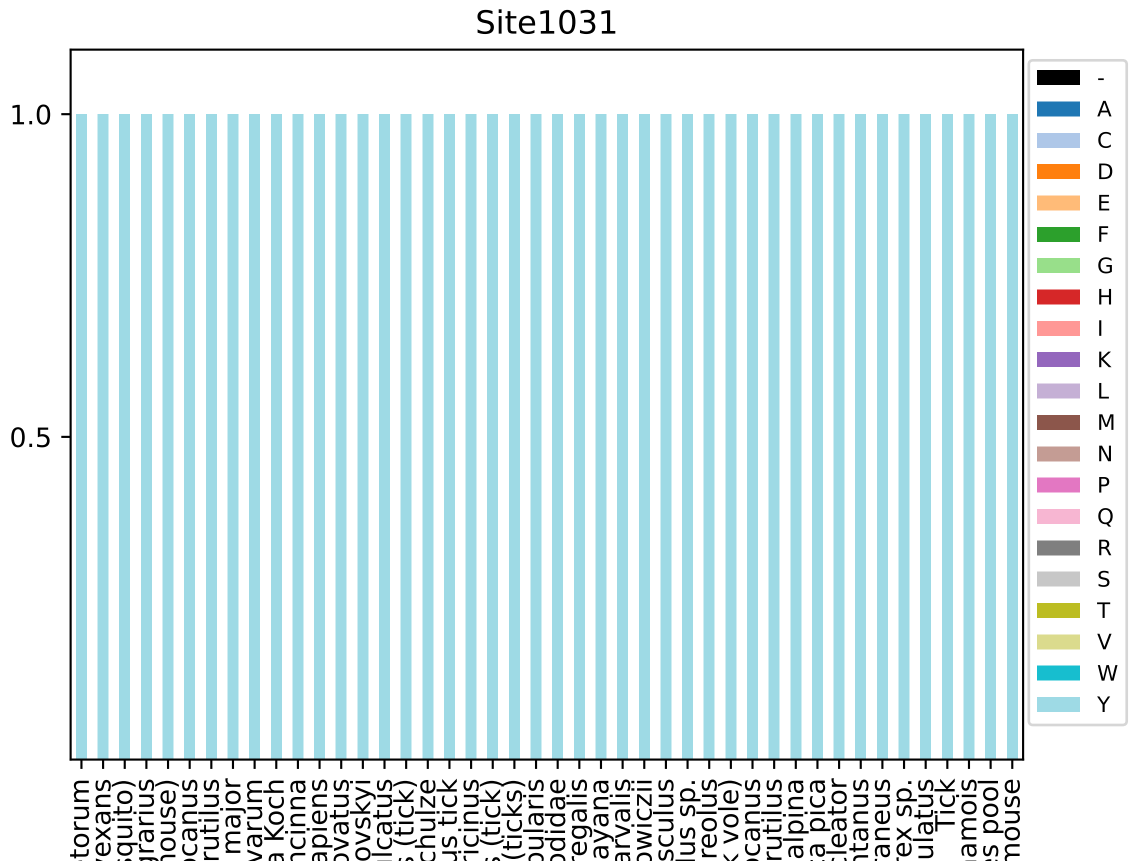

Site1031
1.0
0.5
-
A
C
D
E
F
G
H
I
K
L
M
N
P
Q
R
S
T
V
W
Y

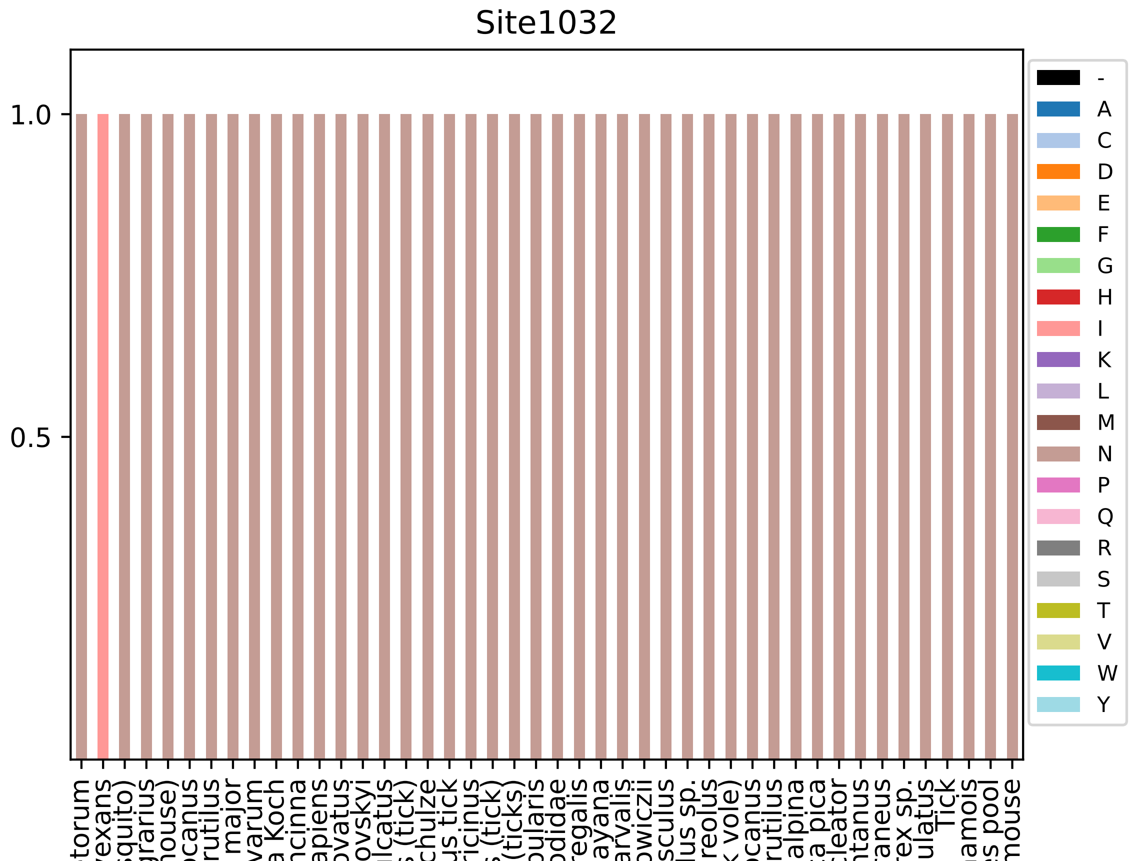

Site1032
1.0
0.5
-
A
C
D
E
F
G
H
I
K
L
M
N
P
Q
R
S
T
V
W
Y

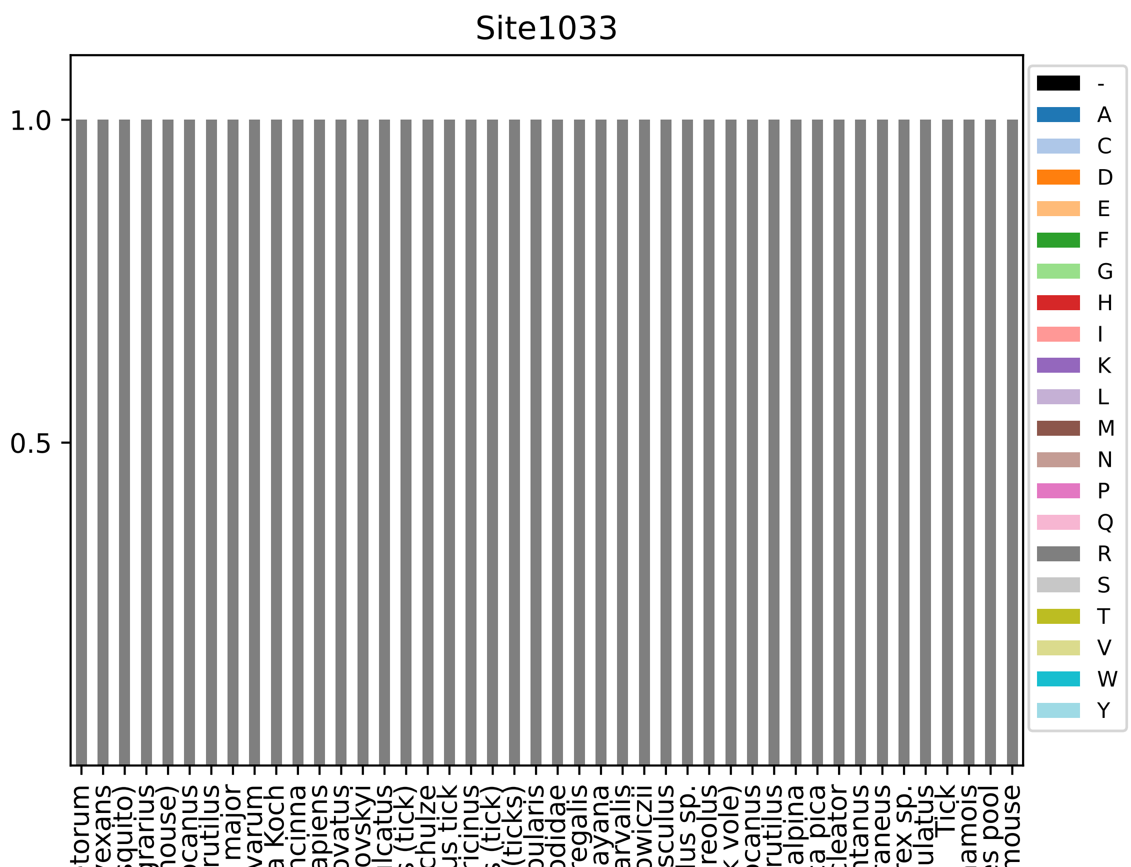
Site1033
1.0
0.5
-
A
C
D
E
F
G
H
I
K
L
M
N
P
Q
R
S
T
V
W
Y

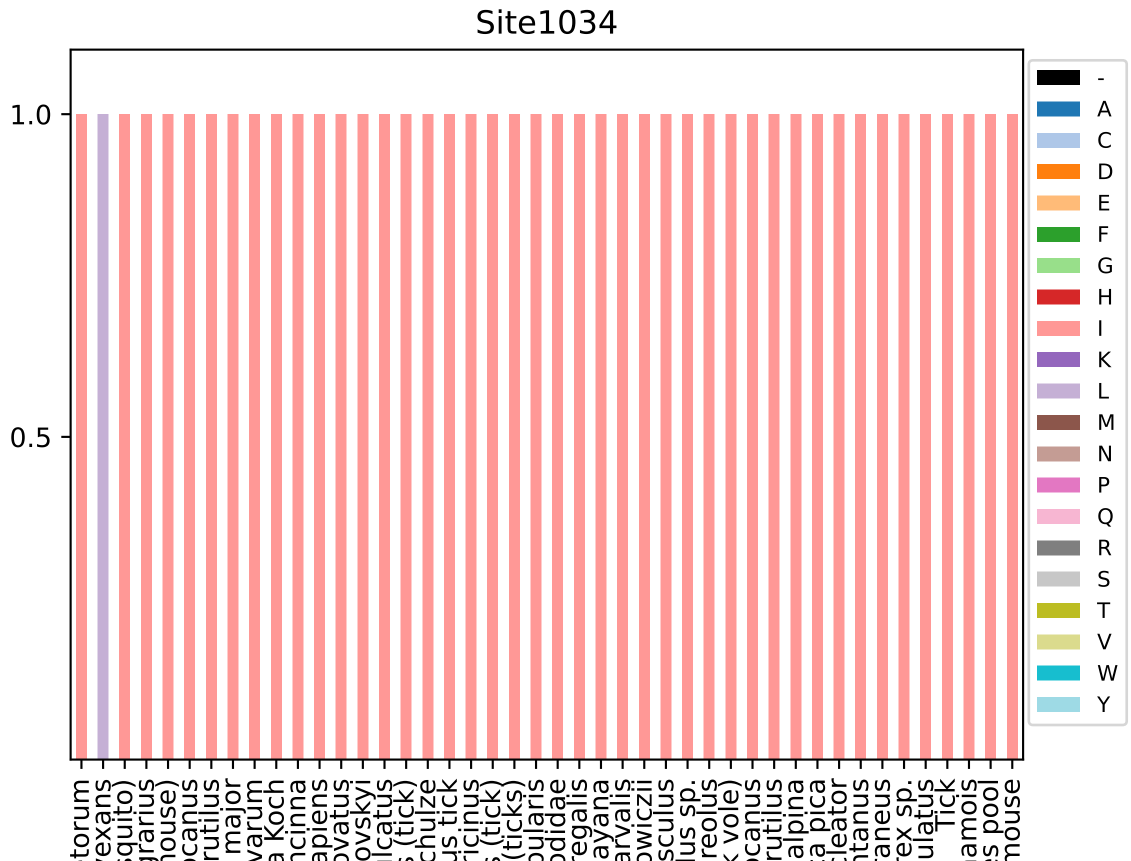

Site1034
1.0
0.5
-
A
C
D
E
F
G
H
I
K
L
M
N
P
Q
R
S
T
V
W
Y

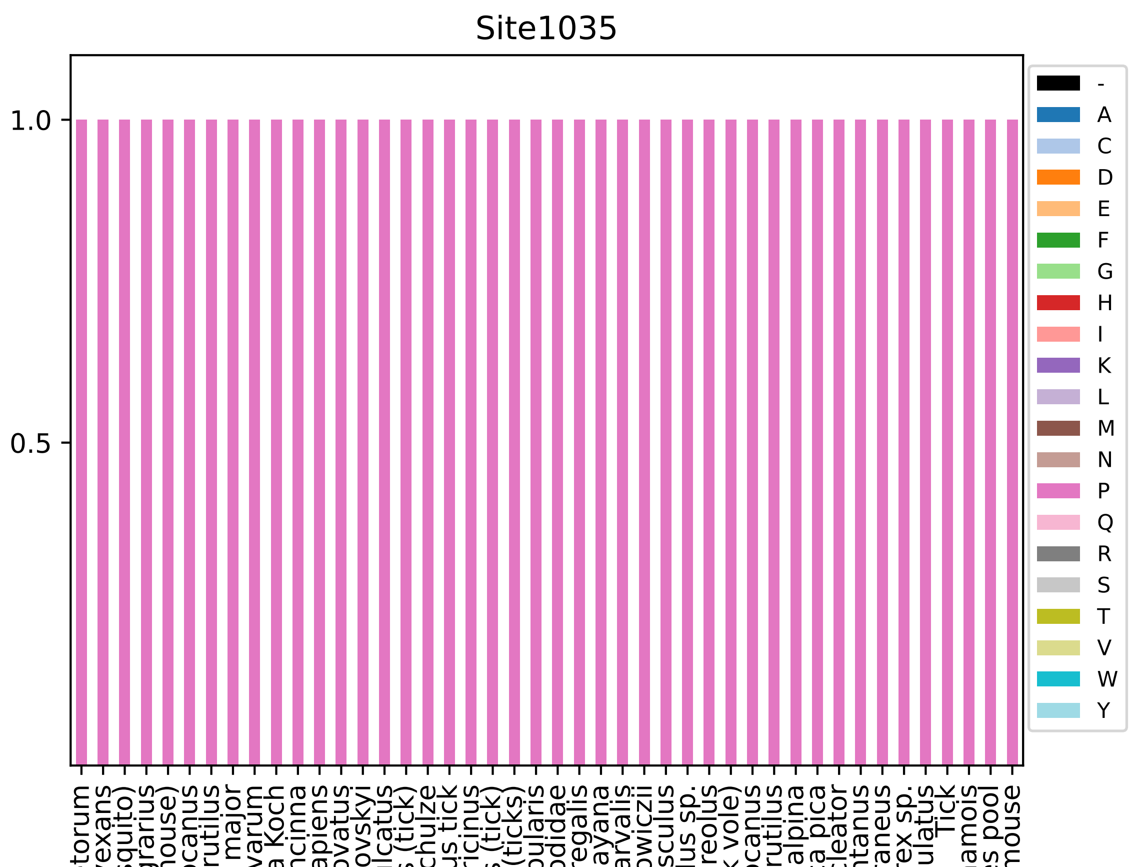

Site1035
1.0
0.5
-
A
C
D
E
F
G
H
I
K
L
M
N
P
Q
R
S
T
V
W
Y

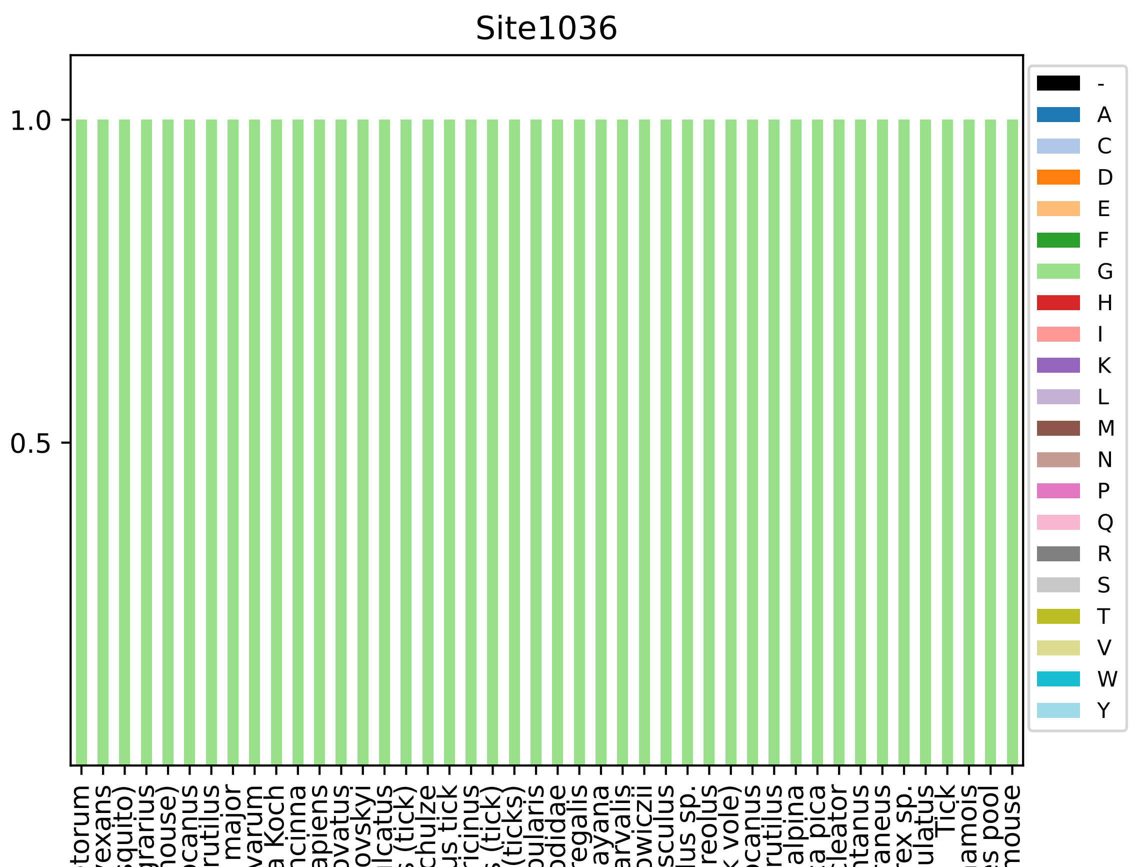
Site1036
1.0
0.5
-
A
C
D
E
F
G
H
I
K
L
M
N
P
Q
R
S
T
V
W
Y

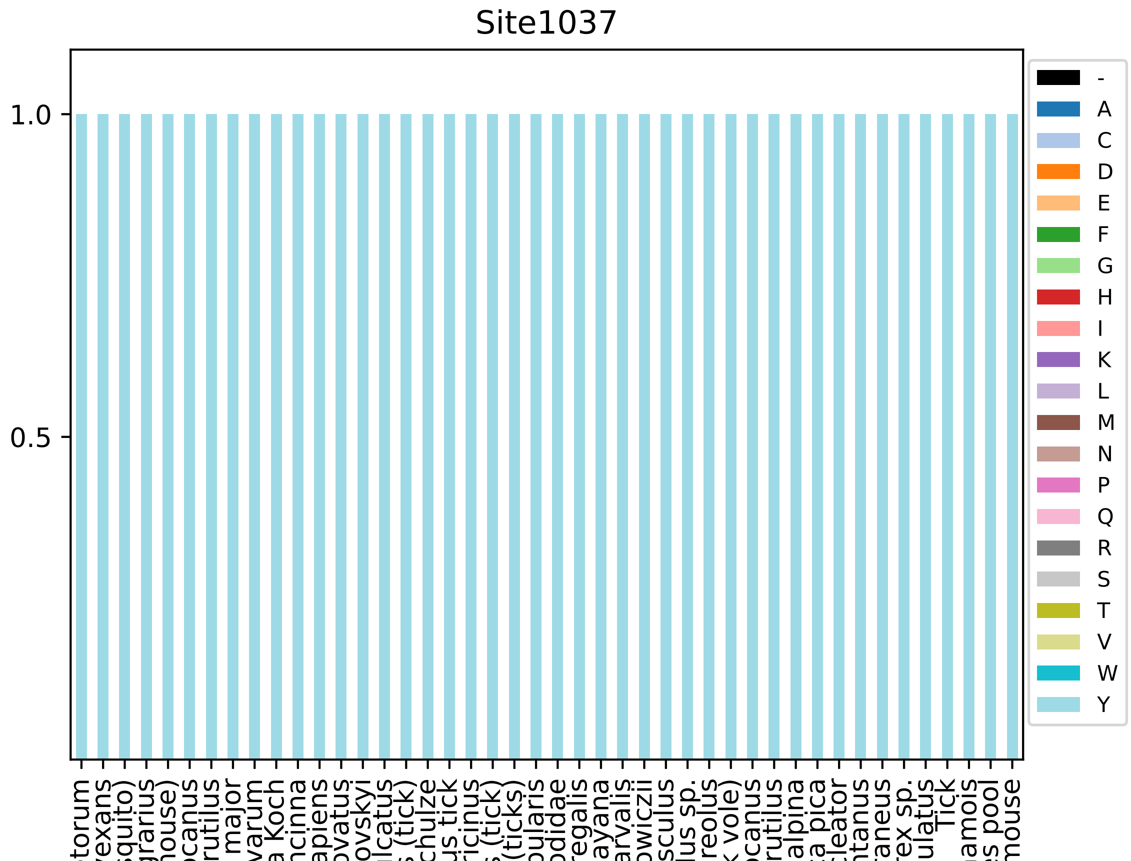
Site1037
1.0
0.5
-
A
C
D
E
F
G
H
I
K
L
M
N
P
Q
R
S
T
V
W
Y

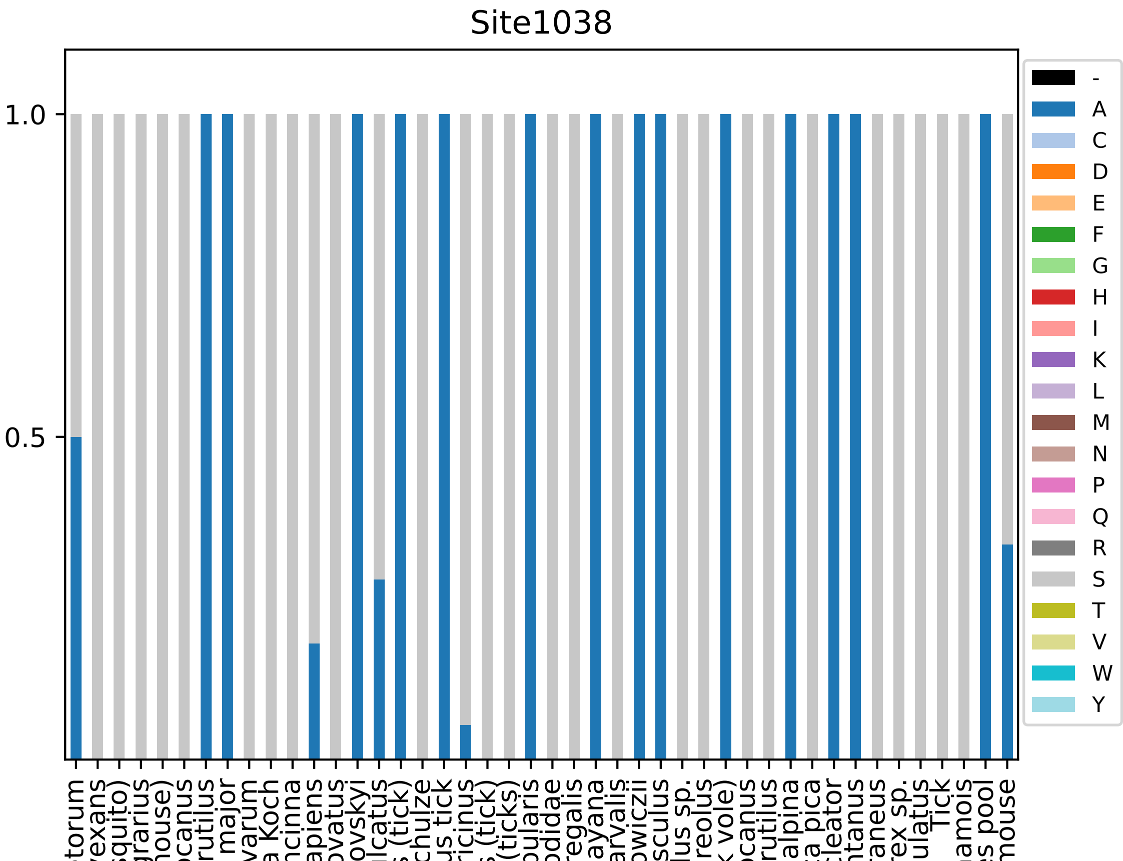
Site1038
1.0
0.5
-
A
C
D
E
F
G
H
I
K
L
M
N
P
Q
R
S
T
V
W
Y

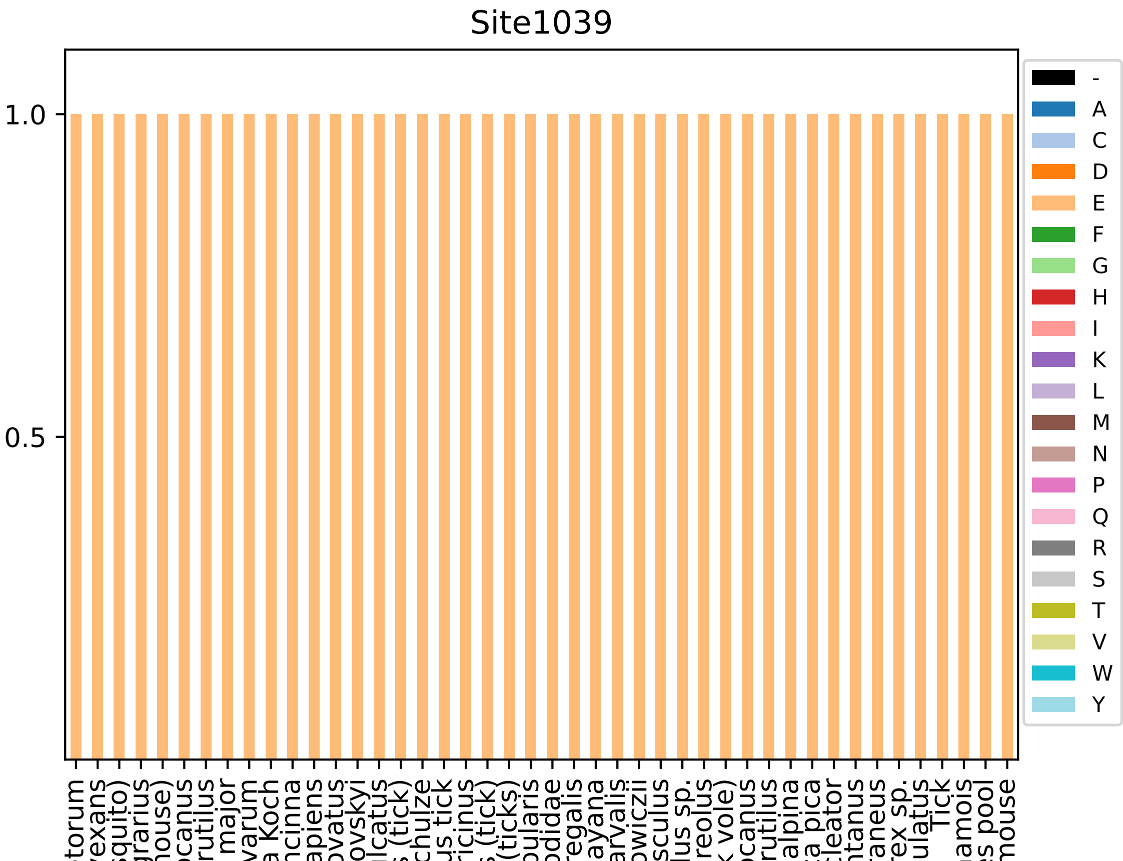

Site1039
1.0
0.5
-
A
C
D
E
F
G
H
I
K
L
M
N
P
Q
R
S
T
V
W
Y

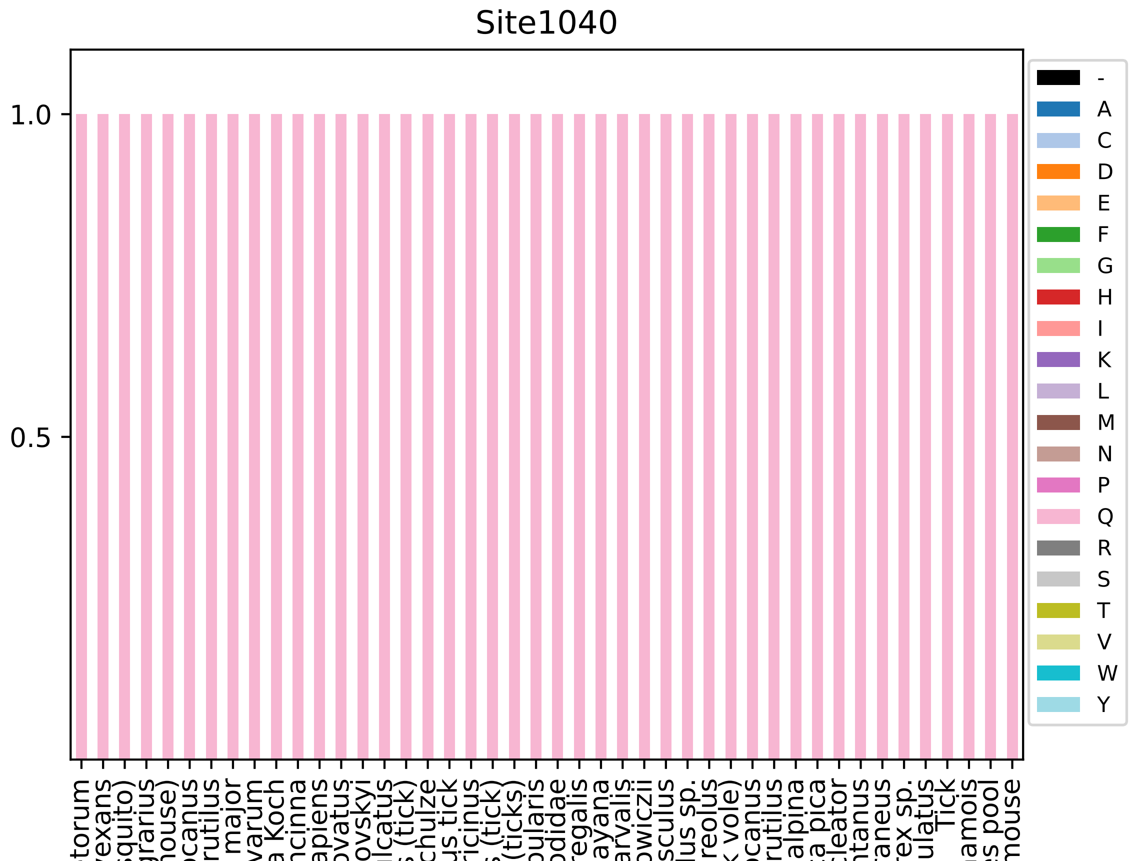
Site1040
1.0
0.5
-
A
C
D
E
F
G
H
I
K
L
M
N
P
Q
R
S
T
V
W
Y

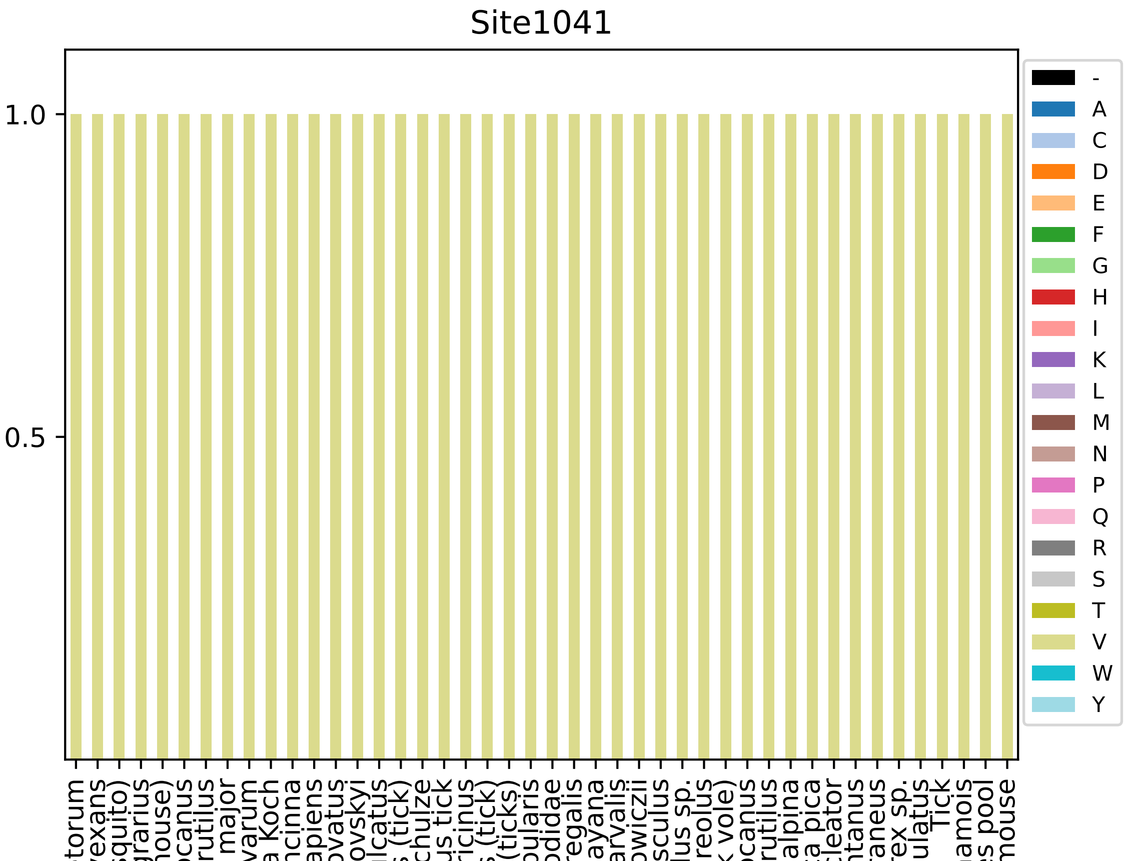

Site1041
1.0
0.5
-
A
C
D
E
F
G
H
I
K
L
M
N
P
Q
R
S
T
V
W
Y

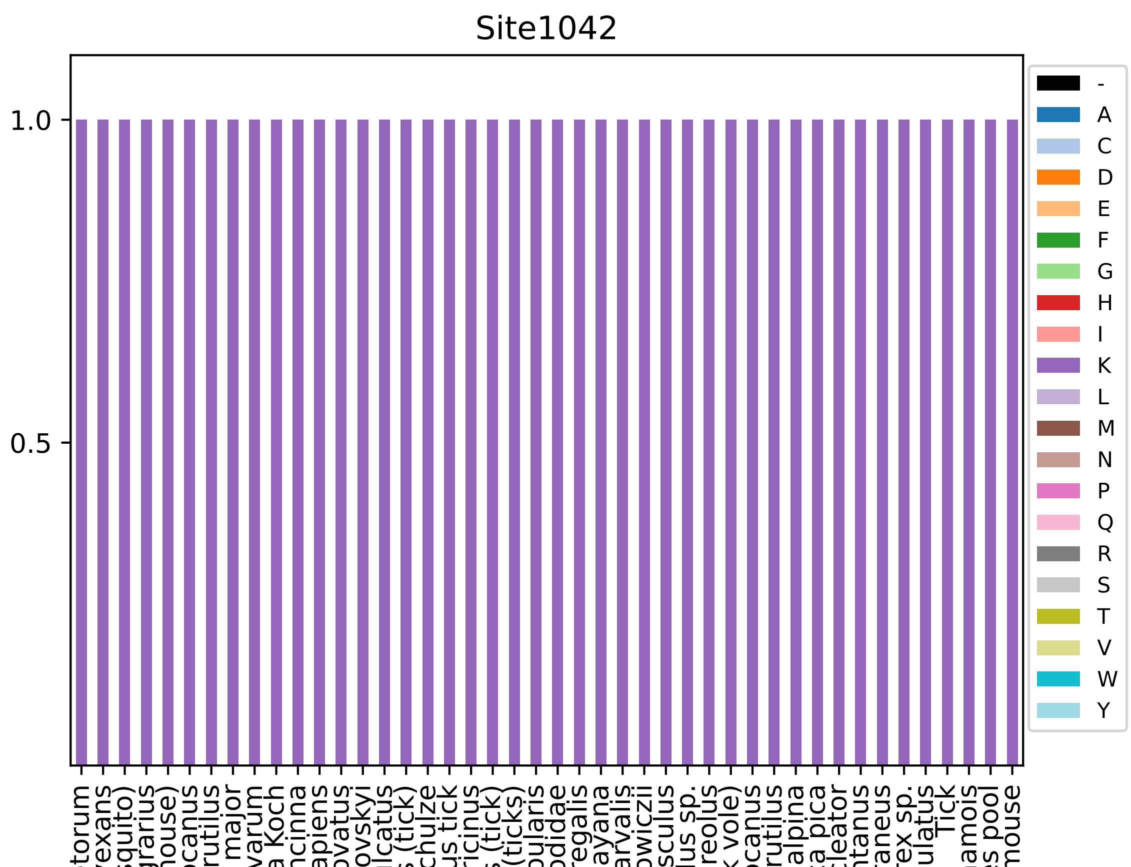
Site1042
1.0
0.5
-
A
C
D
E
F
G
H
I
K
L
M
N
P
Q
R
S
T
V
W
Y

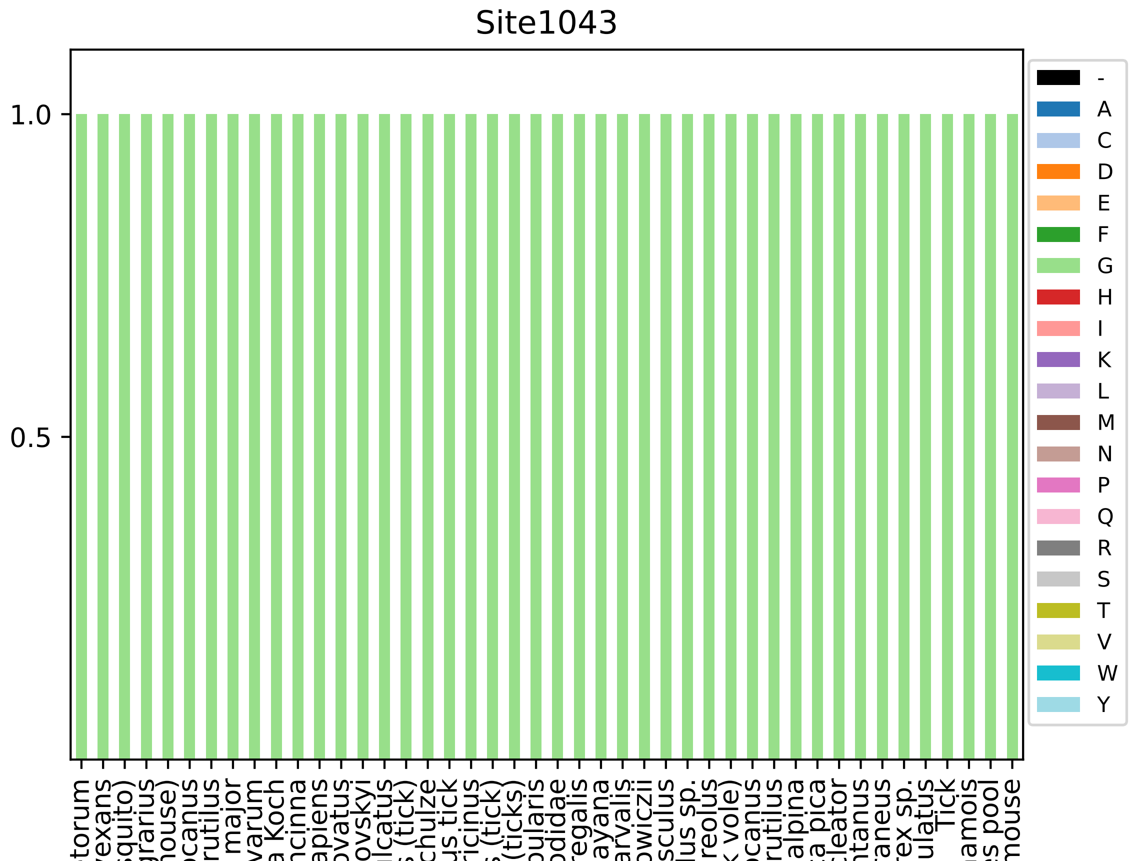
Site1043
1.0
0.5
-
A
C
D
E
F
G
H
I
K
L
M
N
P
Q
R
S
T
V
W
Y

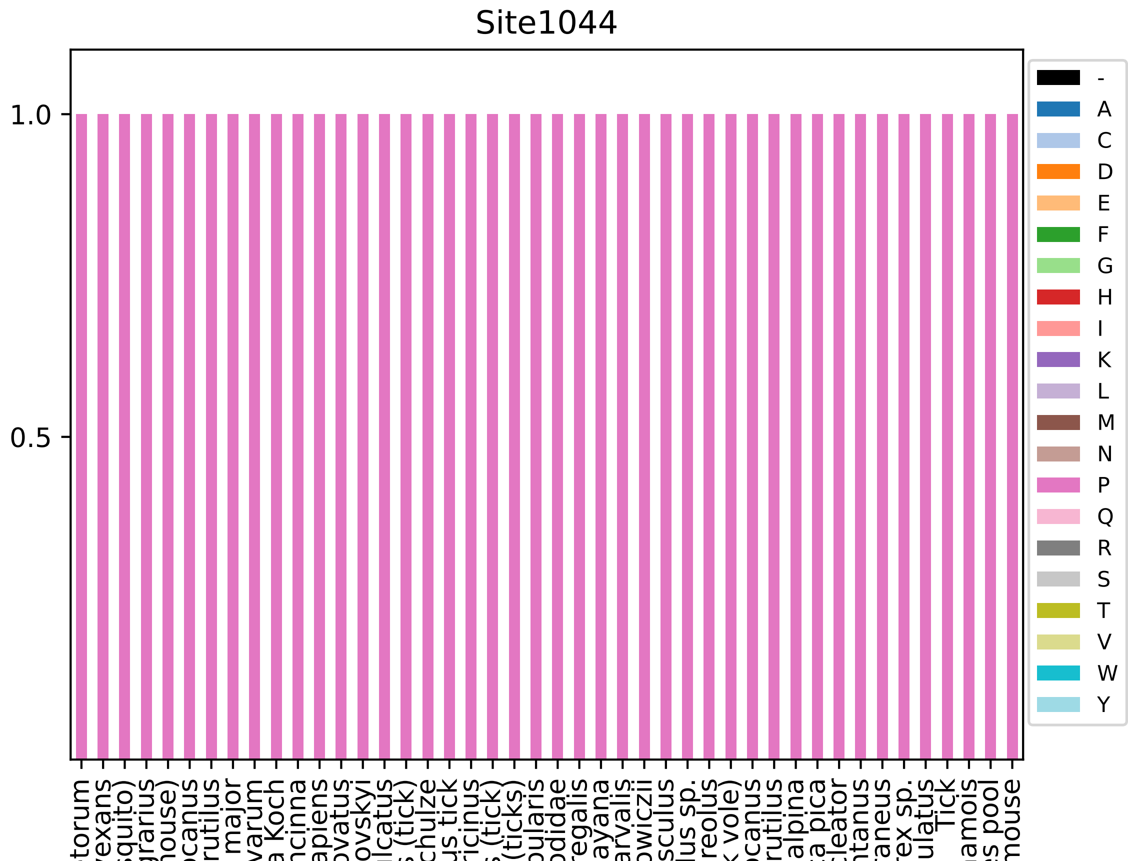
Site1044
1.0
0.5
-
A
C
D
E
F
G
H
I
K
L
M
N
P
Q
R
S
T
V
W
Y

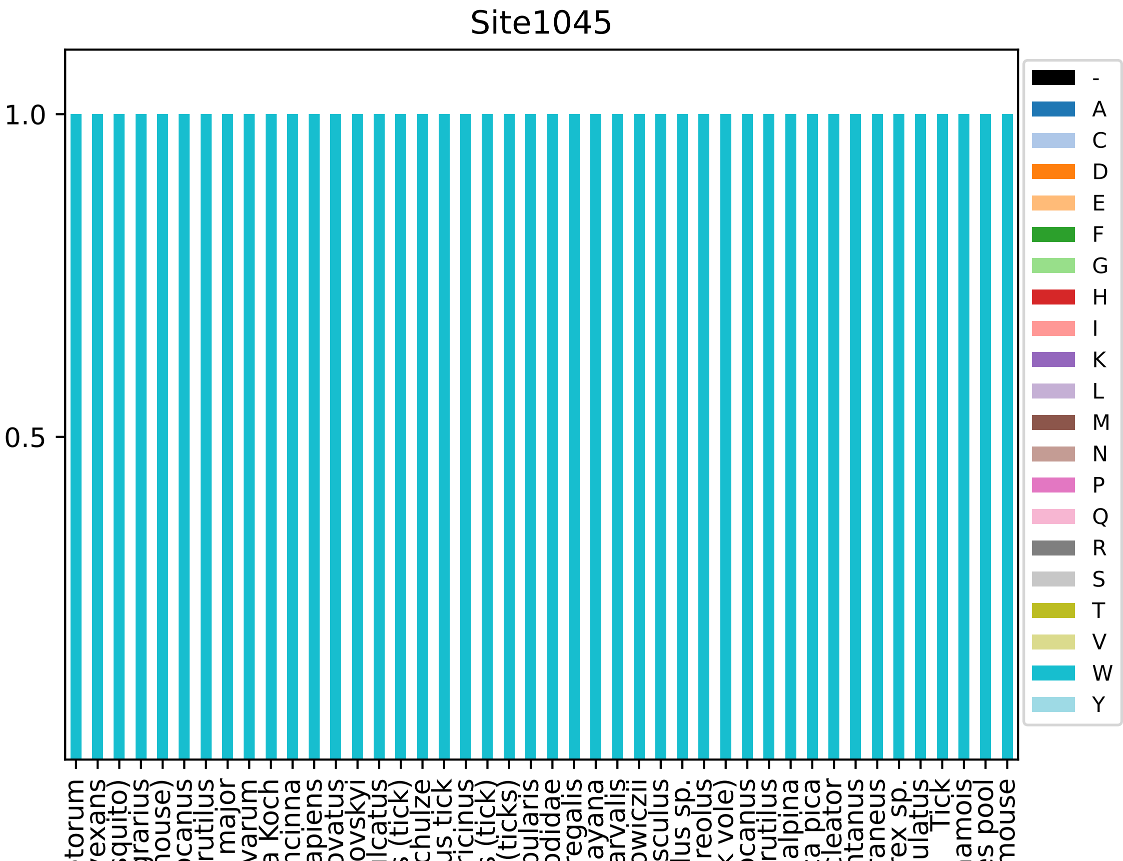
Site1045
1.0
0.5
-
A
C
D
E
F
G
H
I
K
L
M
N
P
Q
R
S
T
V
W
Y

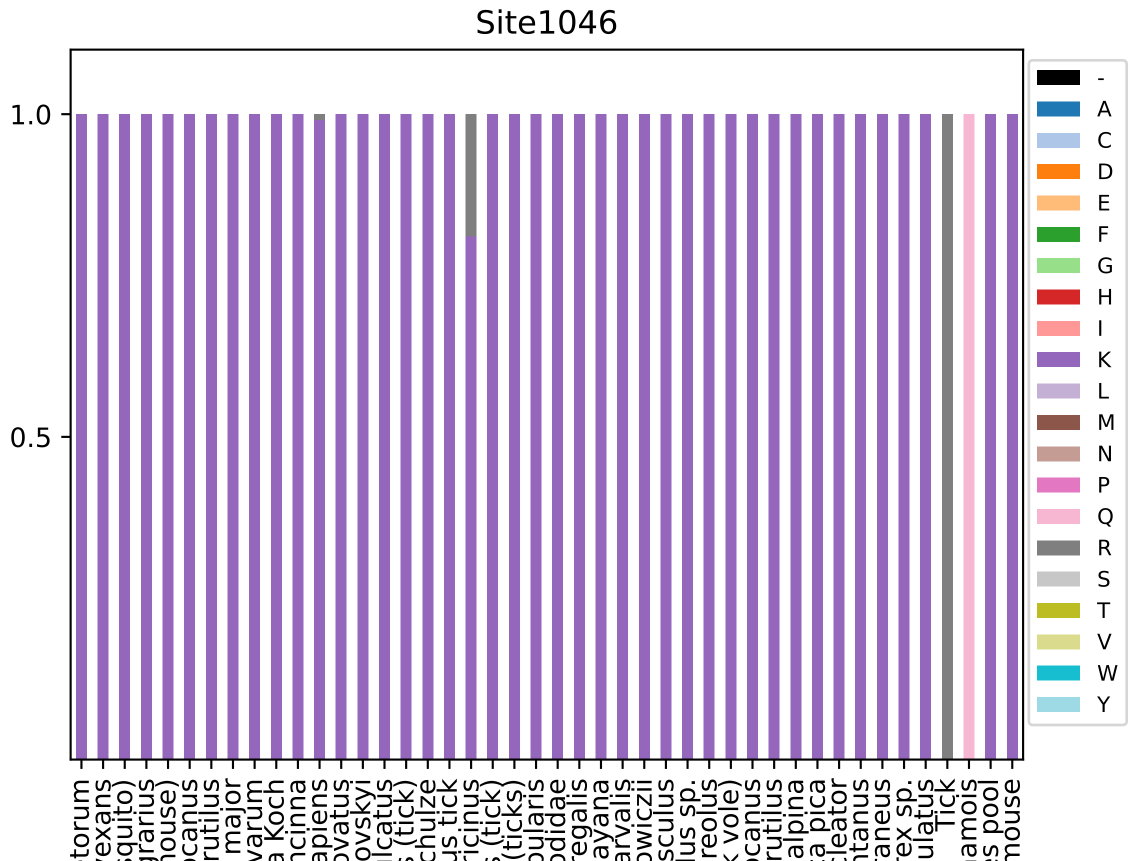
Site1046
1.0
0.5
-
A
C
D
E
F
G
H
I
K
L
M
N
P
Q
R
S
T
V
W
Y
Tick

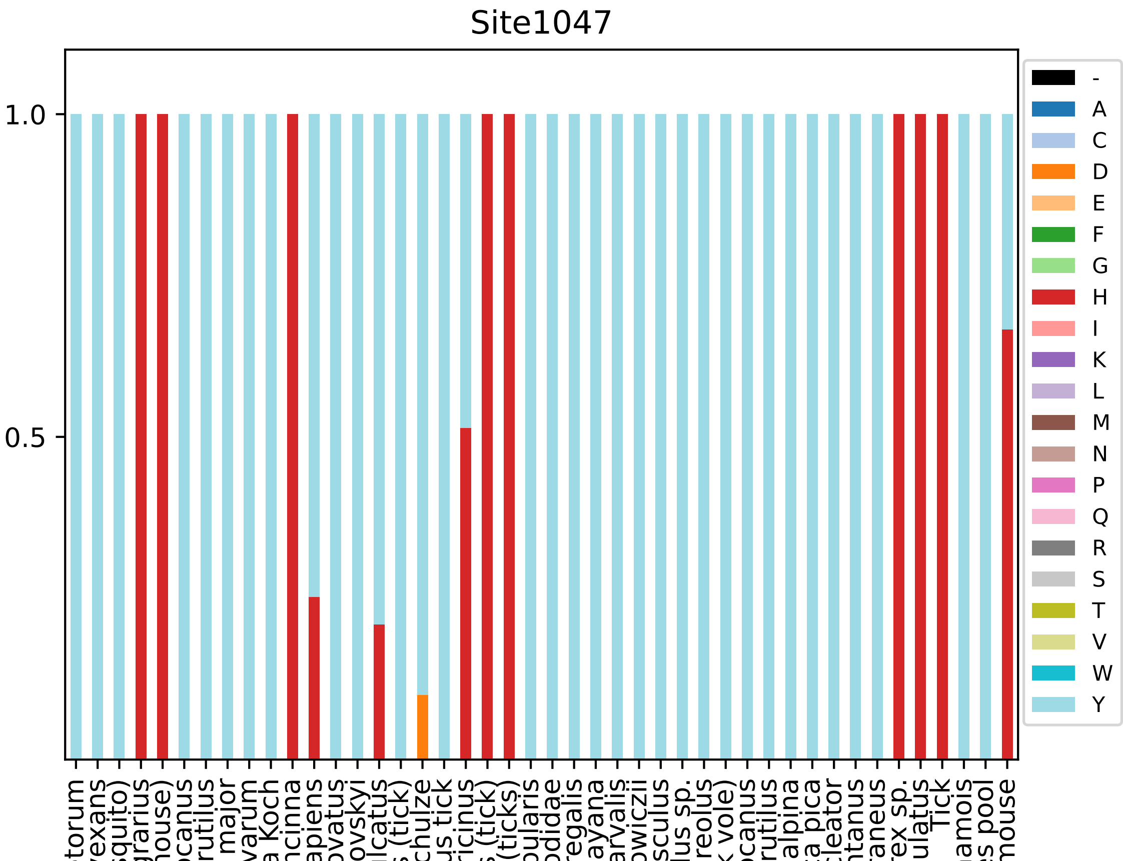

Site1047
1.0
0.5
-
A
C
D
E
F
G
H
I
K
L
M
N
P
Q
R
S
T
V
W
Y

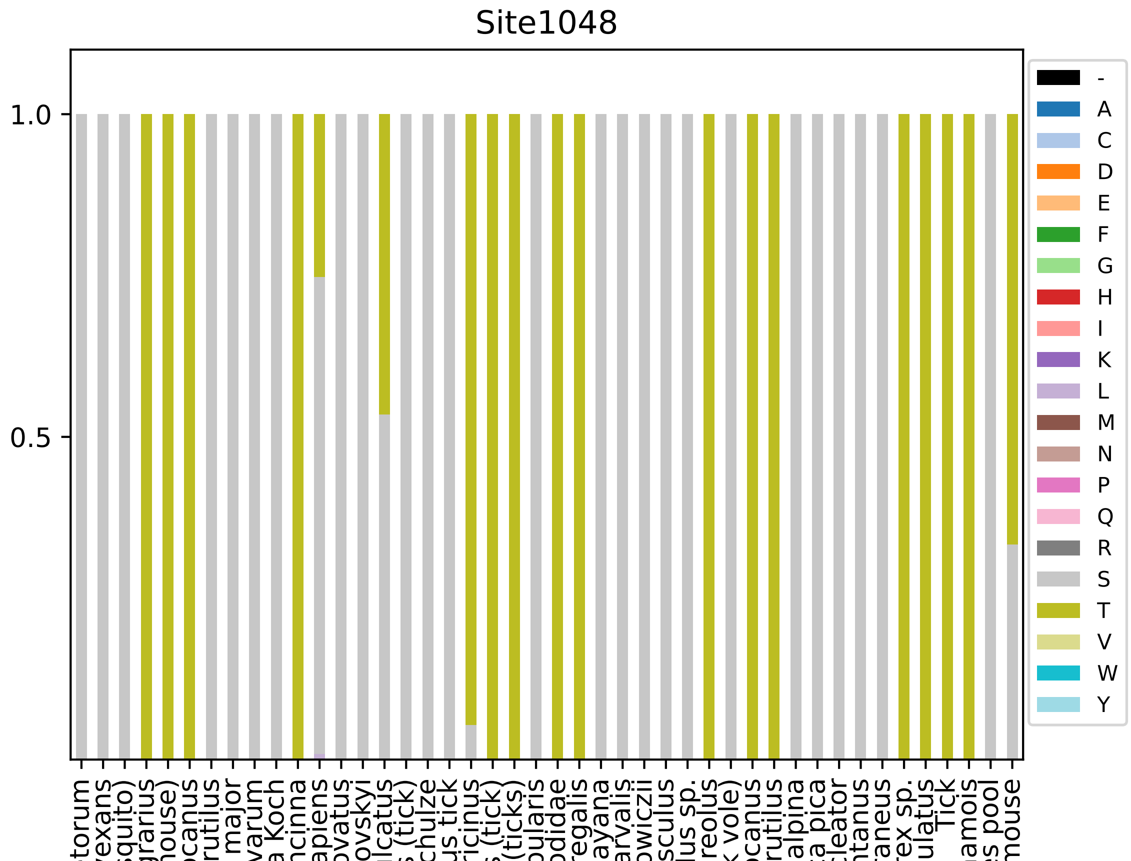

Site1048
1.0
0.5
-
A
C
D
E
F
G
H
I
K
L
M
N
P
Q
R
S
T
V
W
Y
Tick

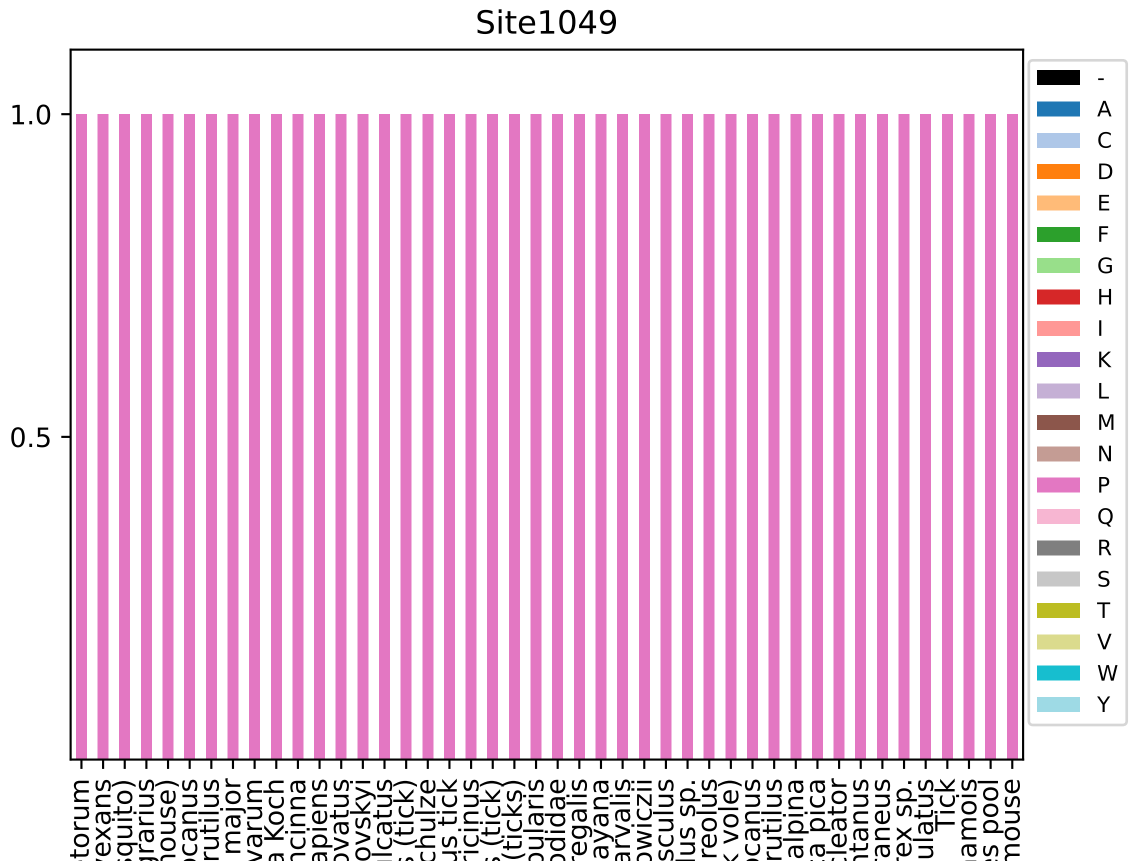
Site1049
1.0
0.5
-
A
C
D
E
F
G
H
I
K
L
M
N
P
Q
R
S
T
V
W
Y
Tick

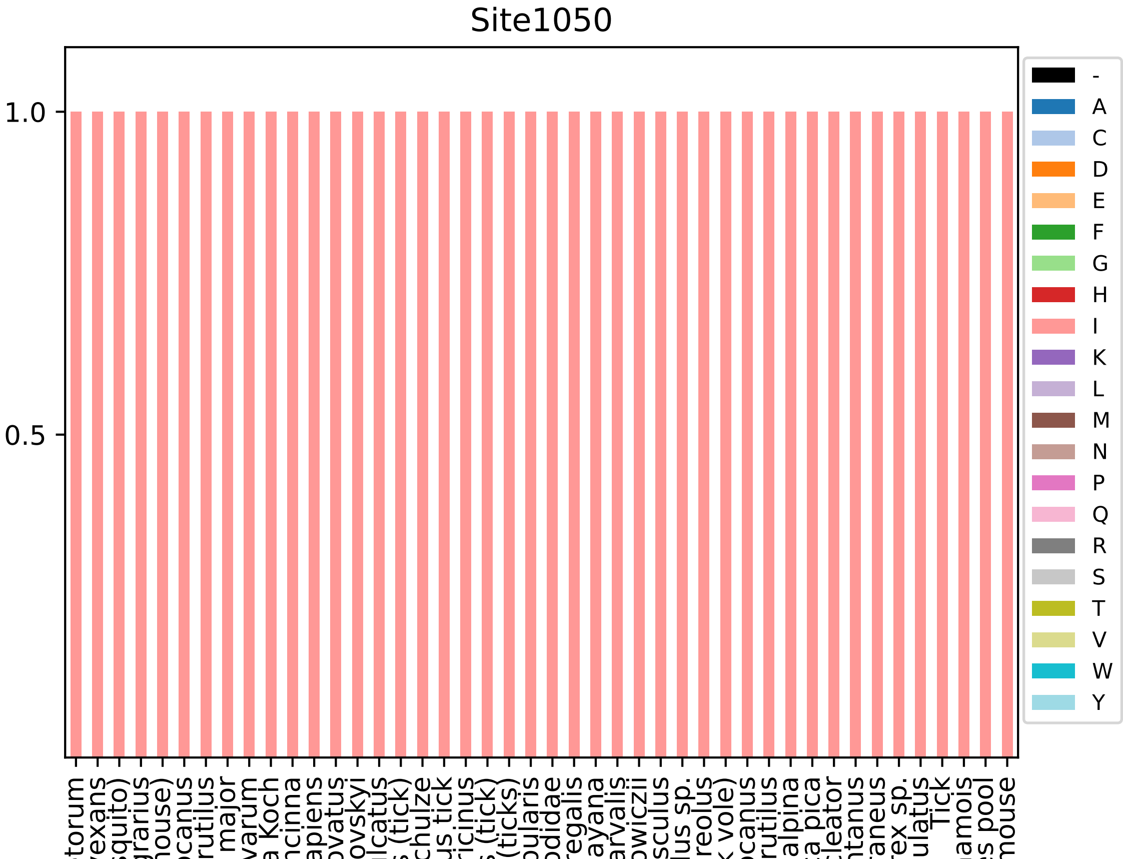
Site1050
1.0
0.5
-
A
C
D
E
F
G
H
I
K
L
M
N
P
Q
R
S
T
V
W
Y

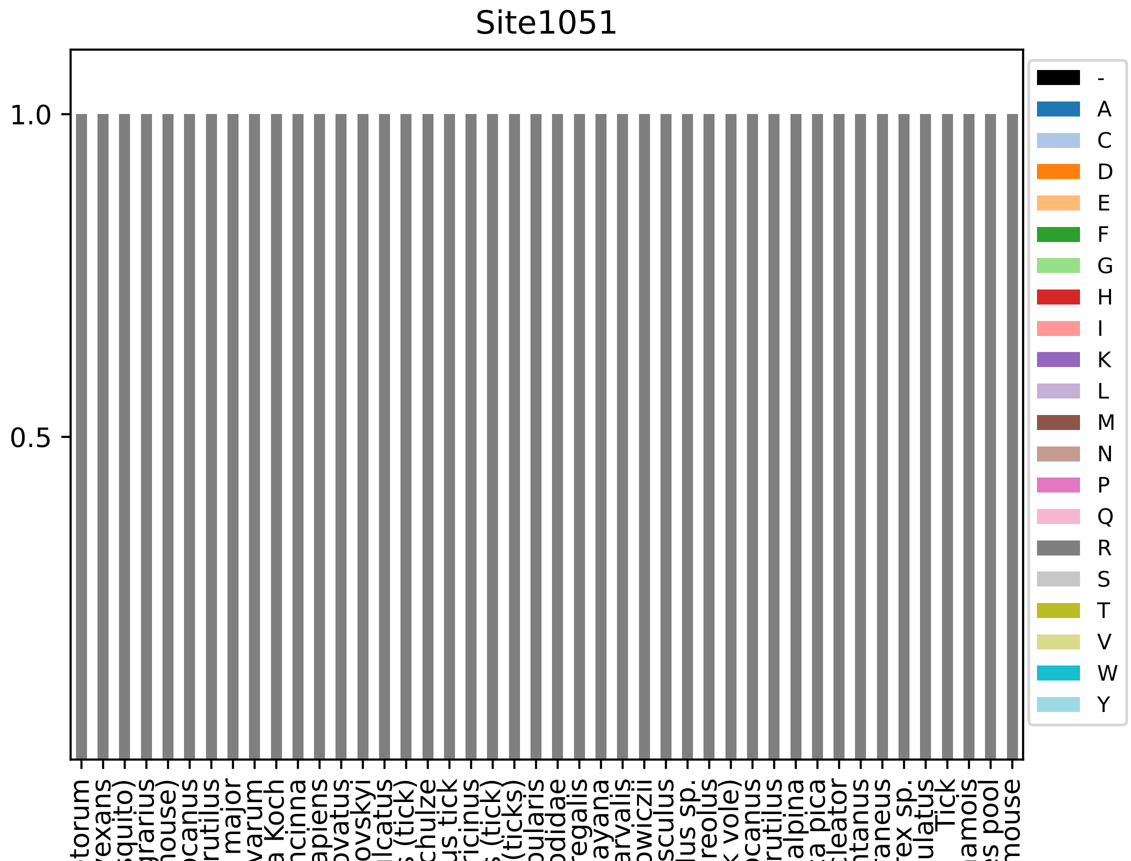

Site1051
1.0
0.5
-
A
C
D
E
F
G
H
I
K
L
M
N
P
Q
R
S
T
V
W
Y
Tick

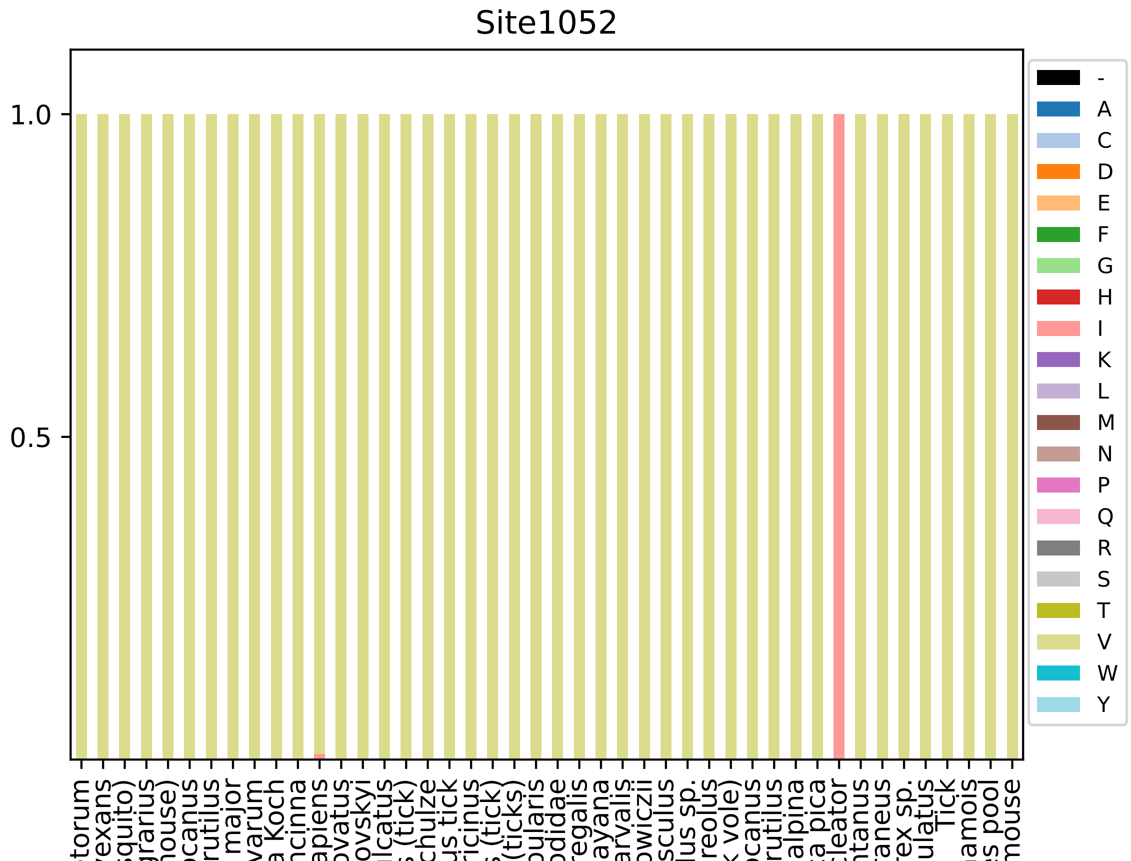

Site1052
1.0
0.5
-
A
C
D
E
F
G
H
I
K
L
M
N
P
Q
R
S
T
V
W
Y

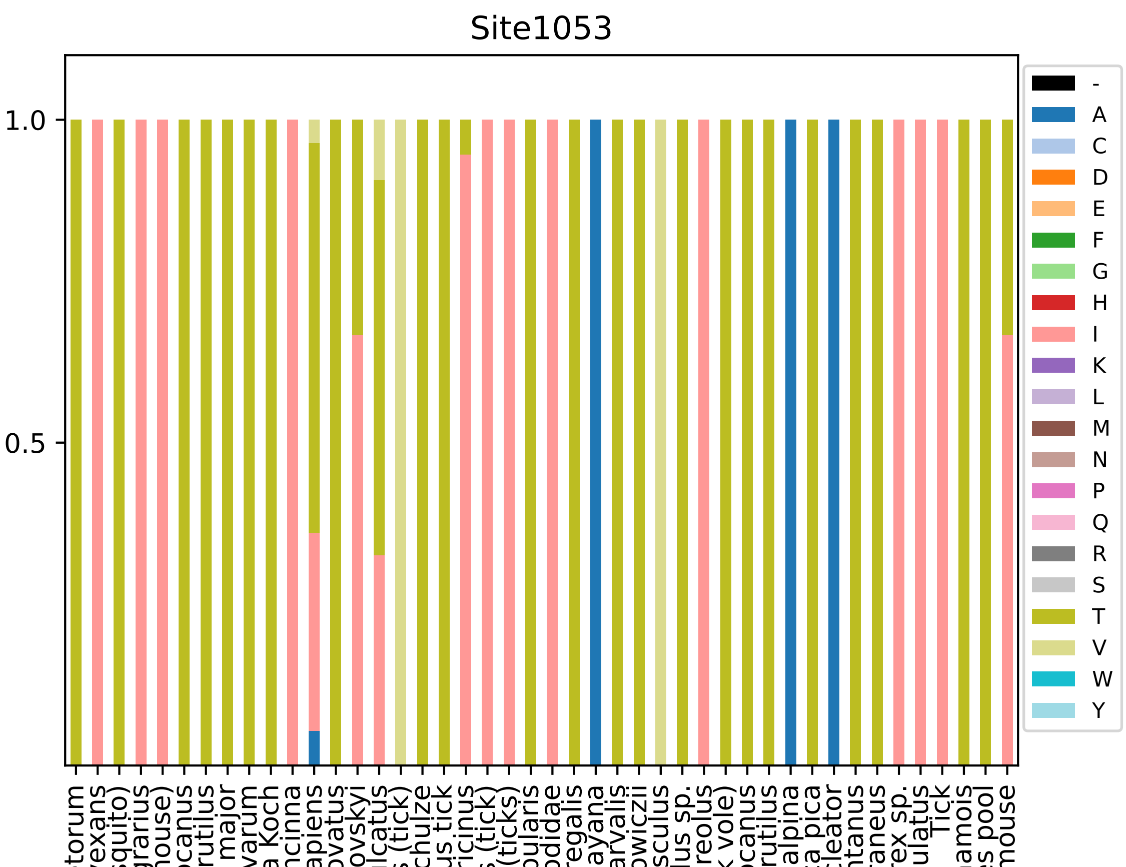
Site1053
1.0
0.5
-
A
C
D
E
F
G
H
I
K
L
M
N
P
Q
R
S
T
V
W
Y
Tick

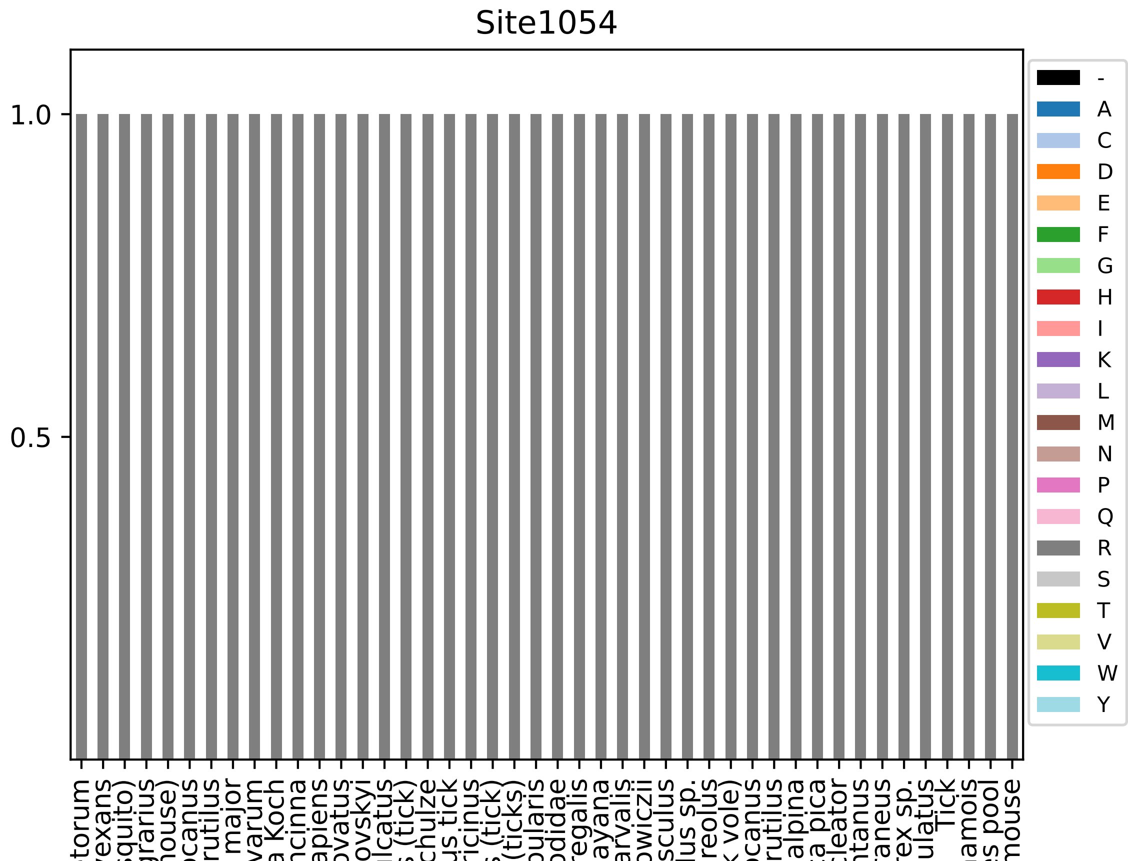
Site1054
1.0
0.5
-
A
C
D
E
F
G
H
I
K
L
M
N
P
Q
R
S
T
V
W
Y

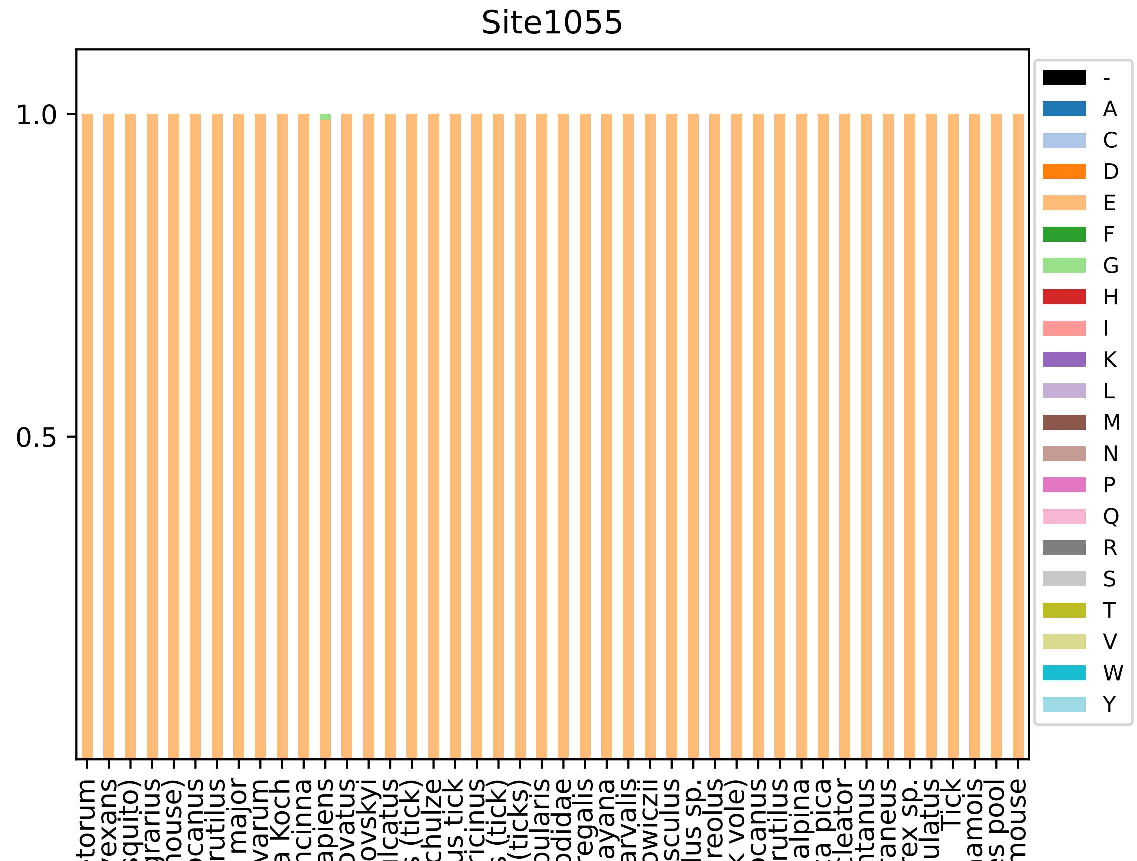
Site1055
1.0
0.5
-
A
C
D
E
F
G
H
I
K
L
M
N
P
Q
R
S
T
V
W
Y

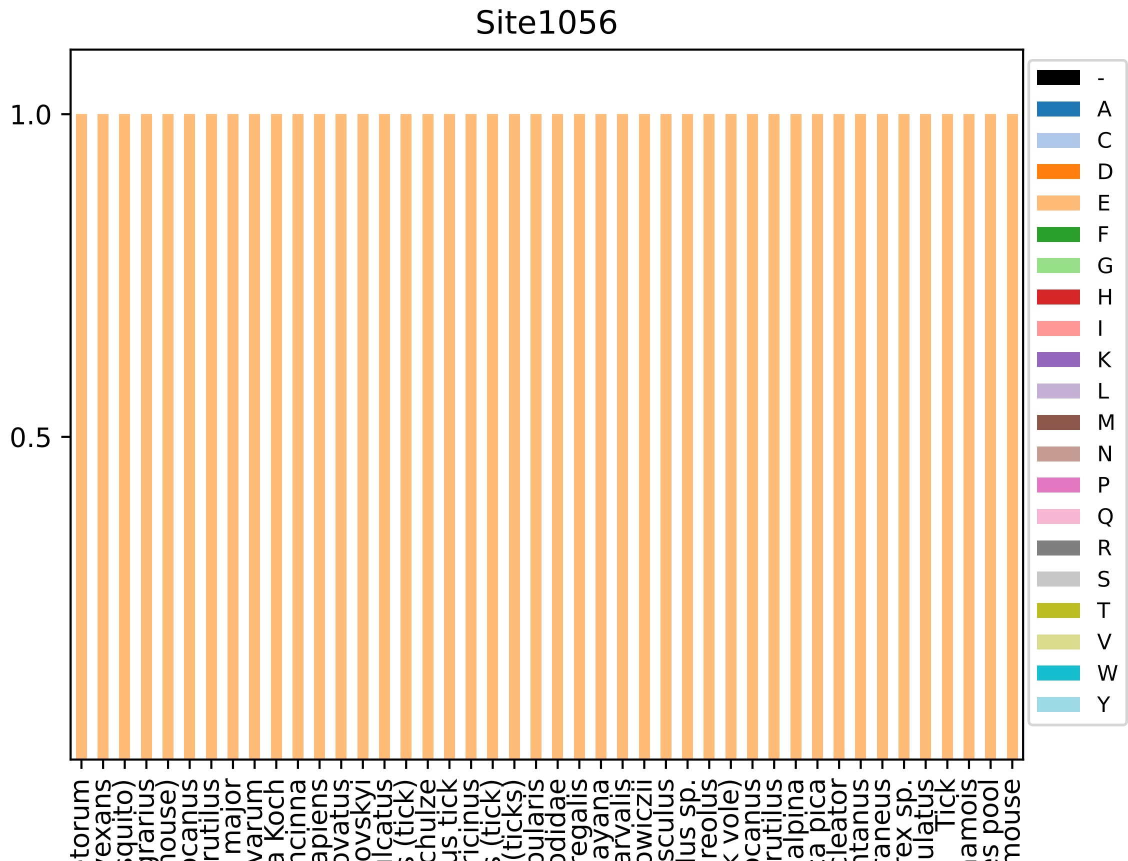

Site1056
1.0
0.5
-
A
C
D
E
F
G
H
I
K
L
M
N
P
Q
R
S
T
V
W
Y

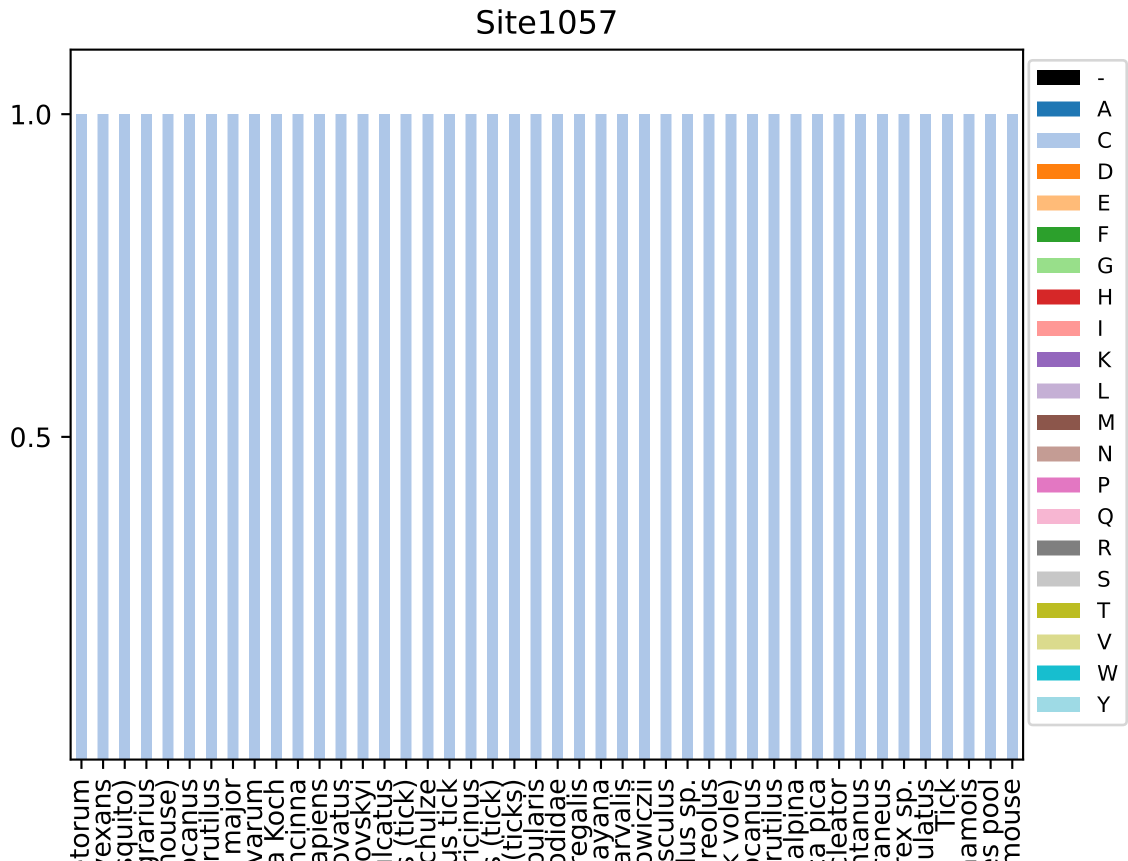
Site1057
1.0
0.5
-
A
C
D
E
F
G
H
I
K
L
M
N
P
Q
R
S
T
V
W
Y

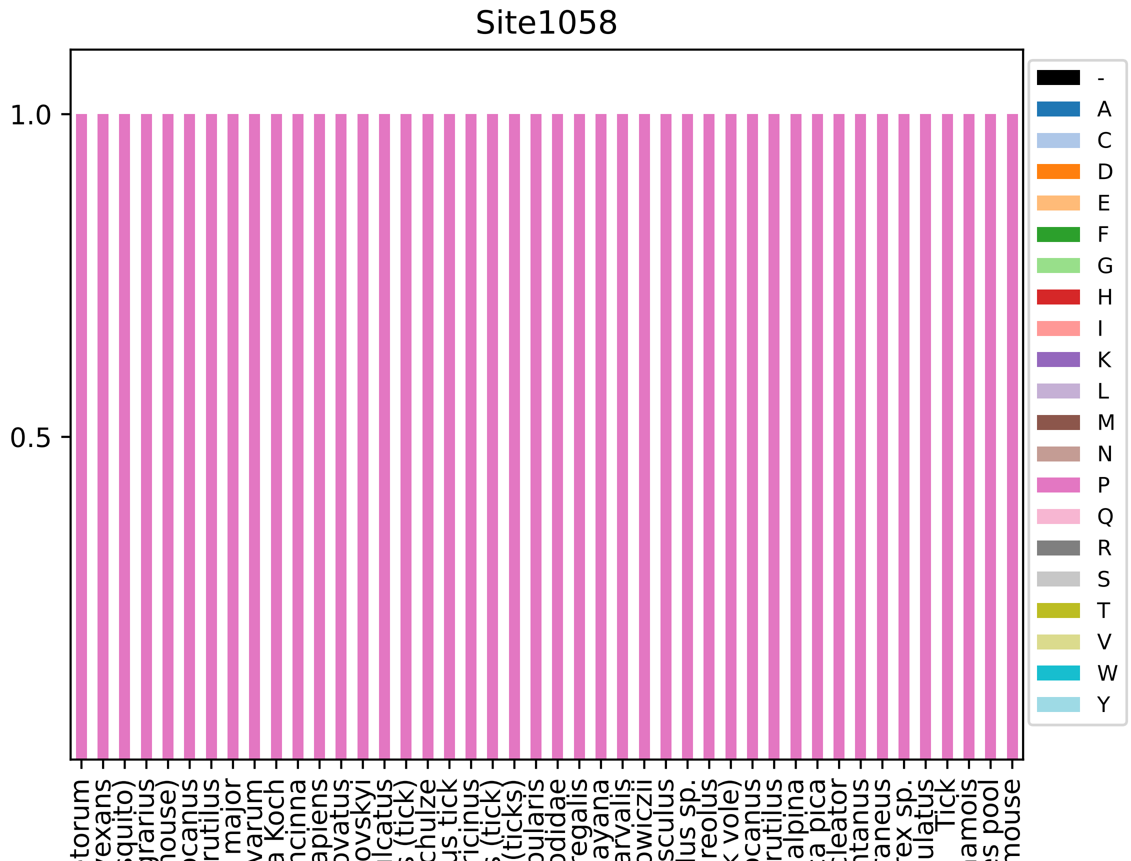

Site1058
1.0
0.5
-
A
C
D
E
F
G
H
I
K
L
M
N
P
Q
R
S
T
V
W
Y

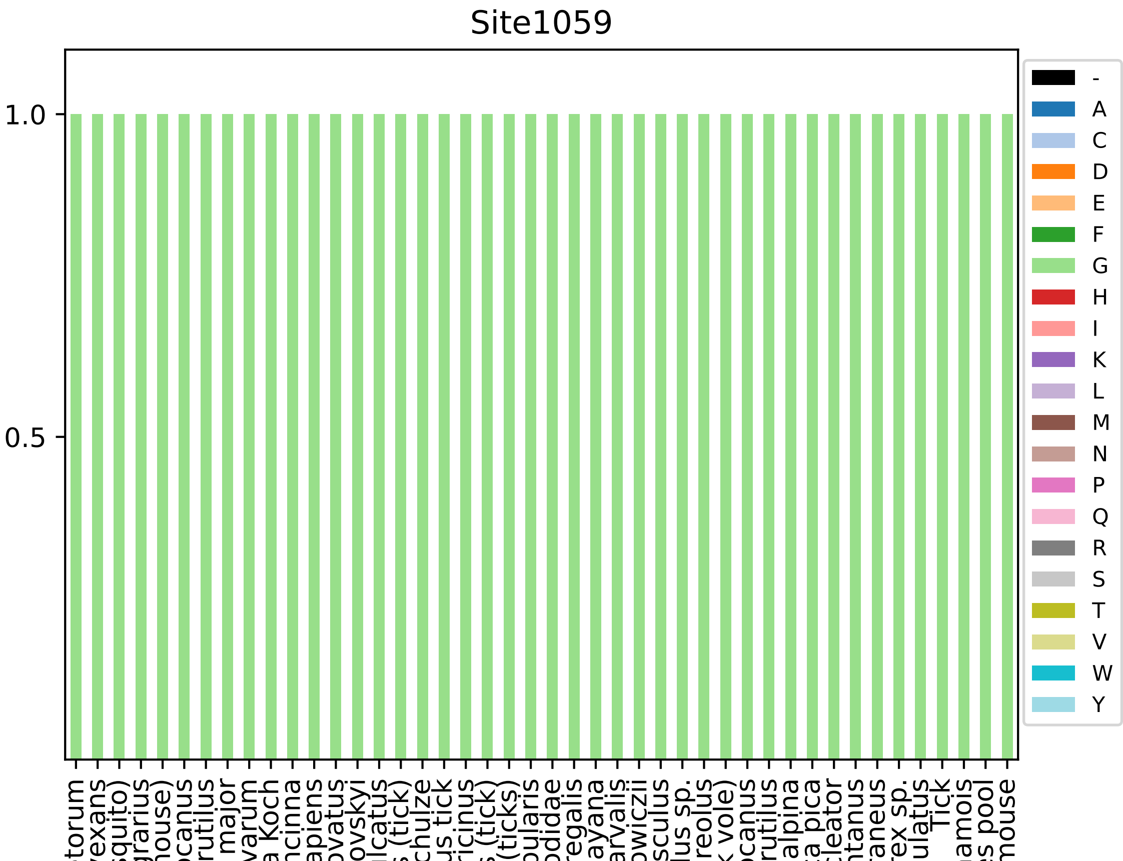
Site1059
1.0
0.5
-
A
C
D
E
F
G
H
I
K
L
M
N
P
Q
R
S
T
V
W
Y
Tick

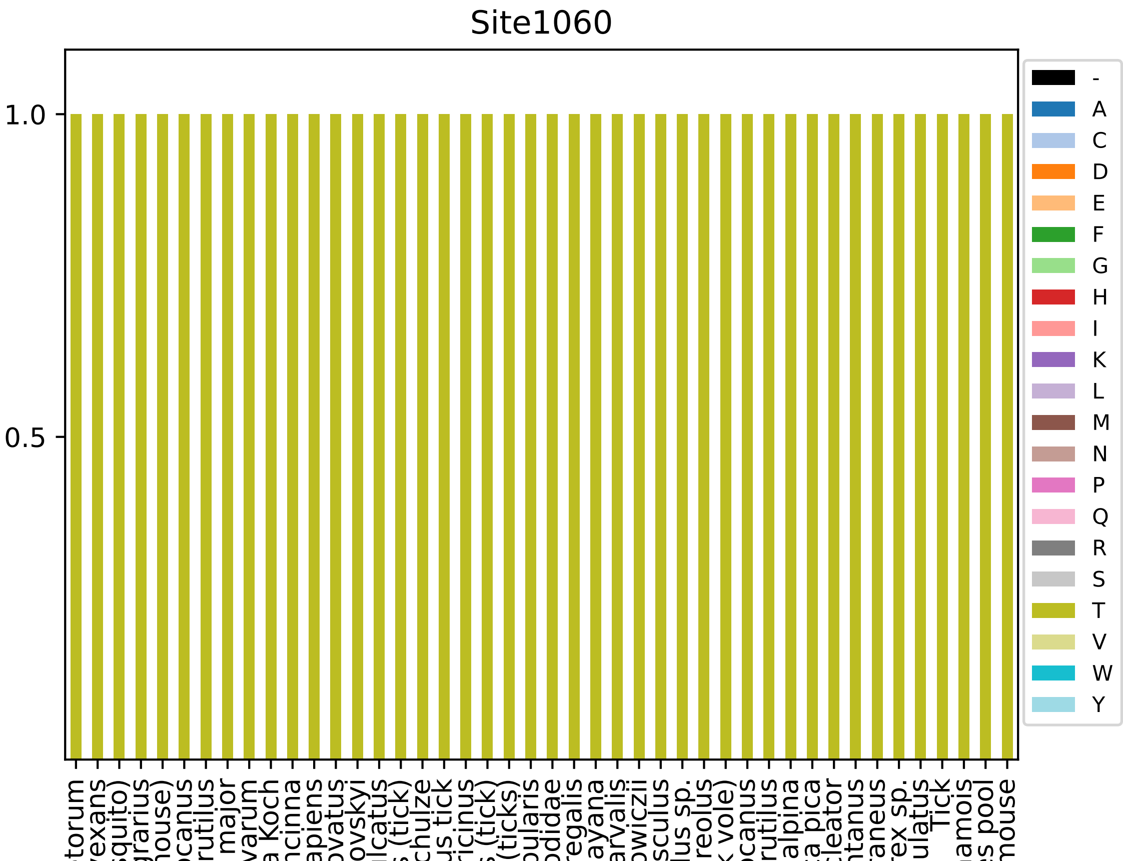
Site1060
1.0
0.5
-
A
C
D
E
F
G
H
I
K
L
M
N
P
Q
R
S
T
V
W
Y

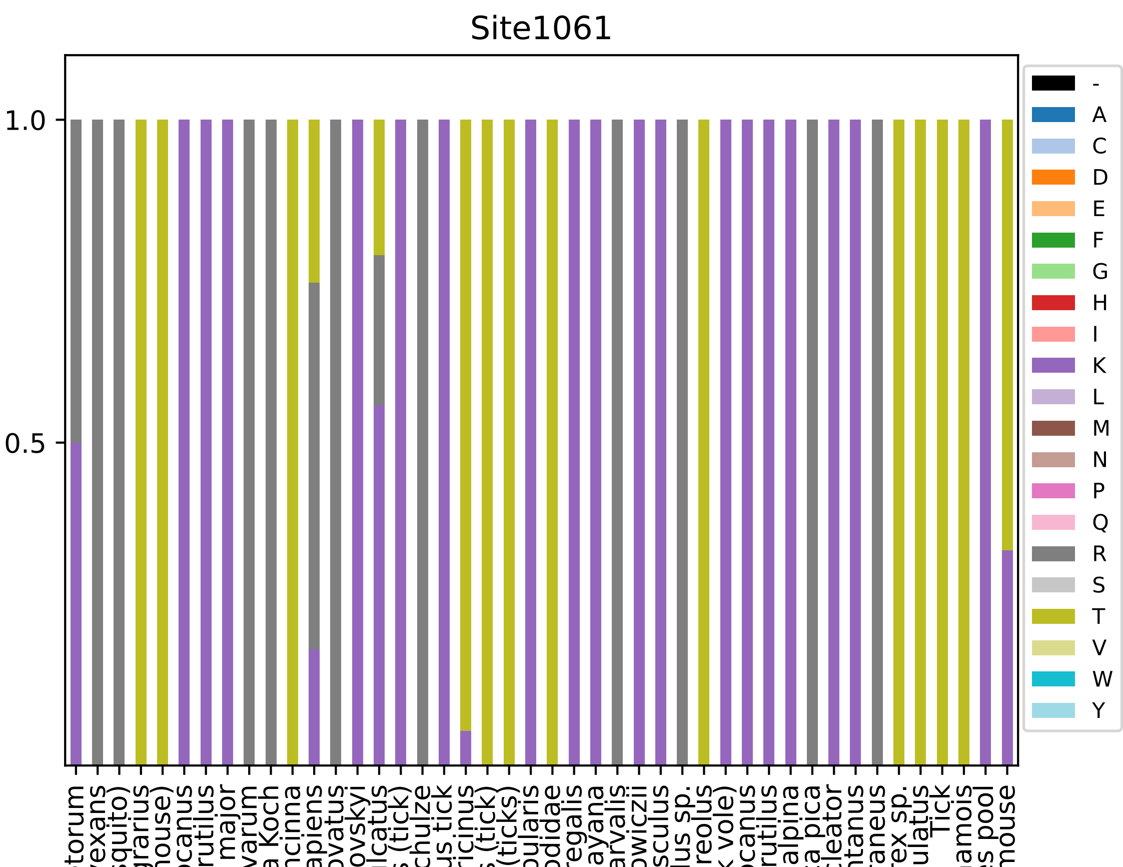

Site1061
1.0
0.5
-
A
C
D
E
F
G
H
I
K
L
M
N
P
Q
R
S
T
V
W
Y
torum
vexans
squito)
garius
nouse)
ocanus
rutilus
major
varum
a Koch
ncinna
apiens
ovatus
ovskyi
ulcatus
s (tick)
chulze
us tick
ricinus
s (tick)
(ticks)
pularis
podidae
regalis
ayana
arvalis
owiczii
sculus
lus sp.
reolus
k vole)
ocanus
rutilus
alpina
a pica
cleator
ntanus
raneus
rex sp.
ulatus
Tick
amois
es pool
mouse

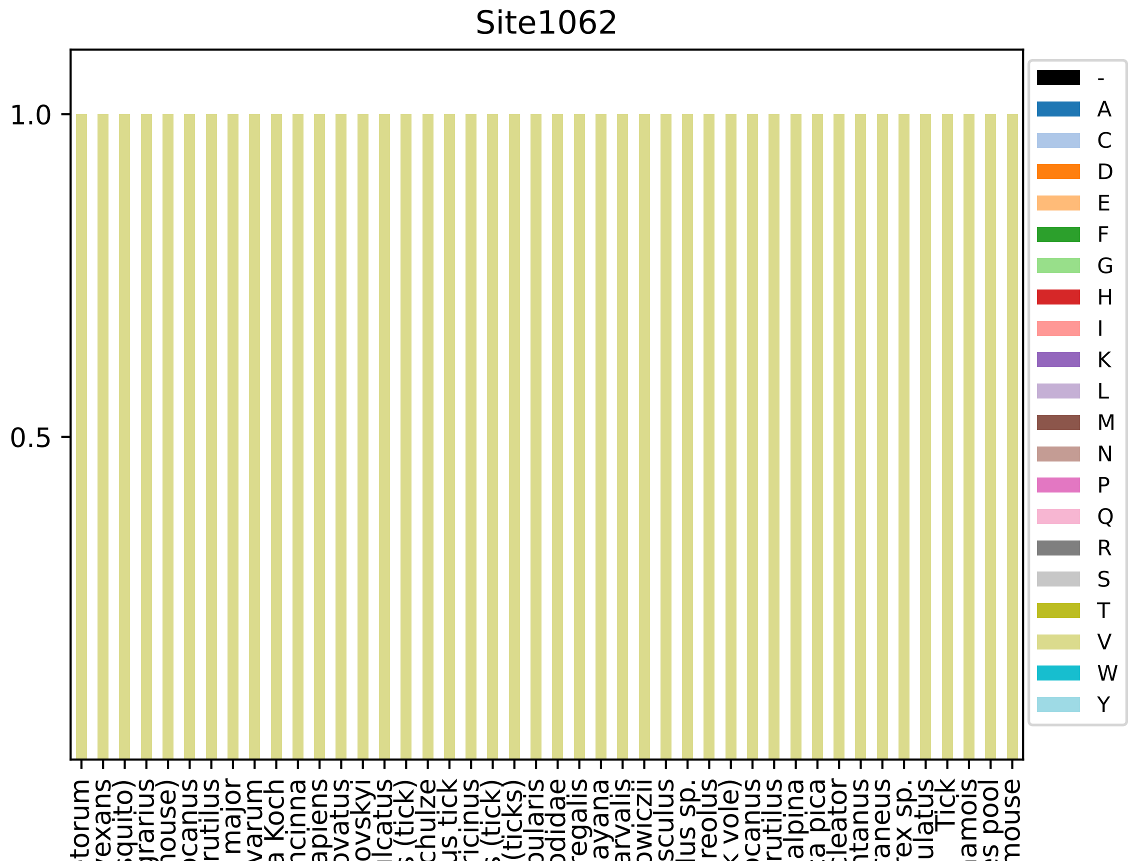

Site1062
1.0
0.5
-
A
C
D
E
F
G
H
I
K
L
M
N
P
Q
R
S
T
V
W
Y

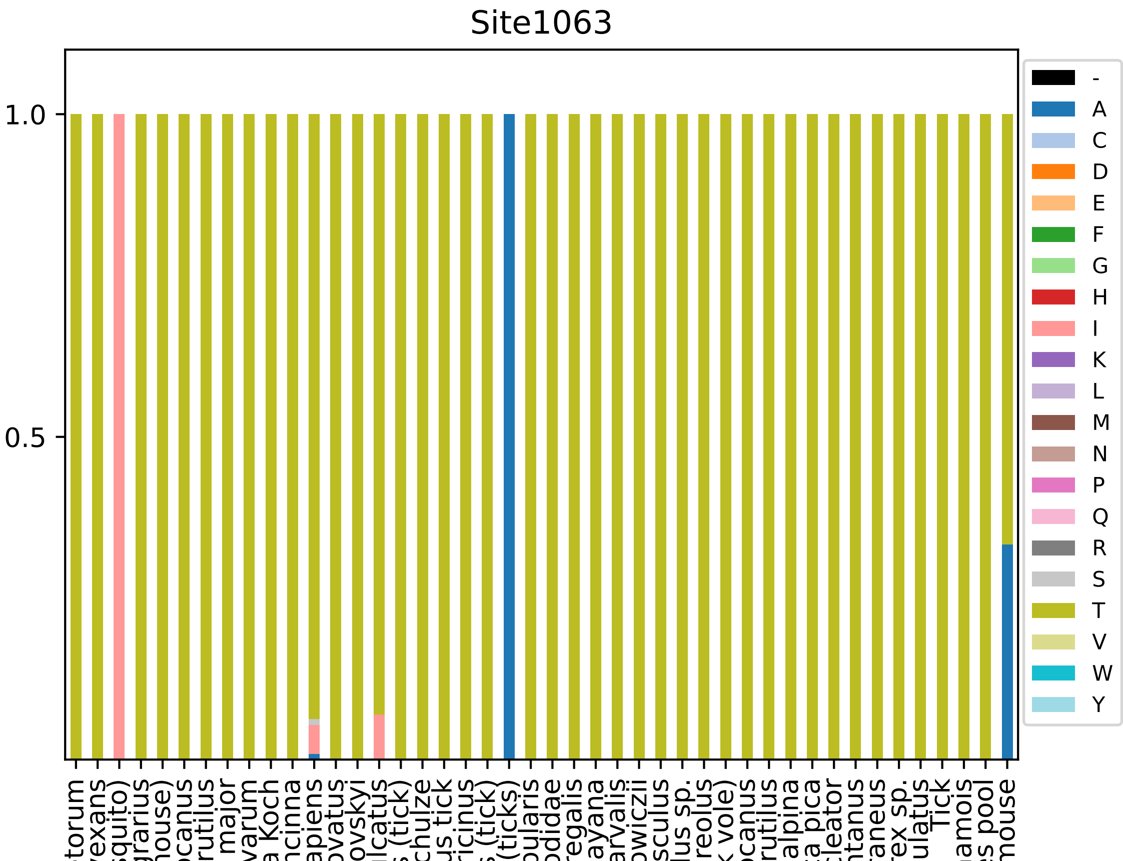
Site1063
1.0
0.5
-
A
C
D
E
F
G
H
I
K
L
M
N
P
Q
R
S
T
V
W
Y
torum
vexans
squito)
garius
house)
ocanus
rutilus
major
varum
a Koch
ncinna
apiens
ovatus
ovskyi
lcatus
(tick)
chulze
us tick
ricinus
(tick)
(ticks)
pularis
podidae
regalis
ayana
arvalis
owiczii
sculus
lus sp.
reolus
k vole)
ocanus
rutilus
alpina
a pica
leator
ntanus
raneus
ex sp.
ulatus
Tick
amois
es pool
mouse

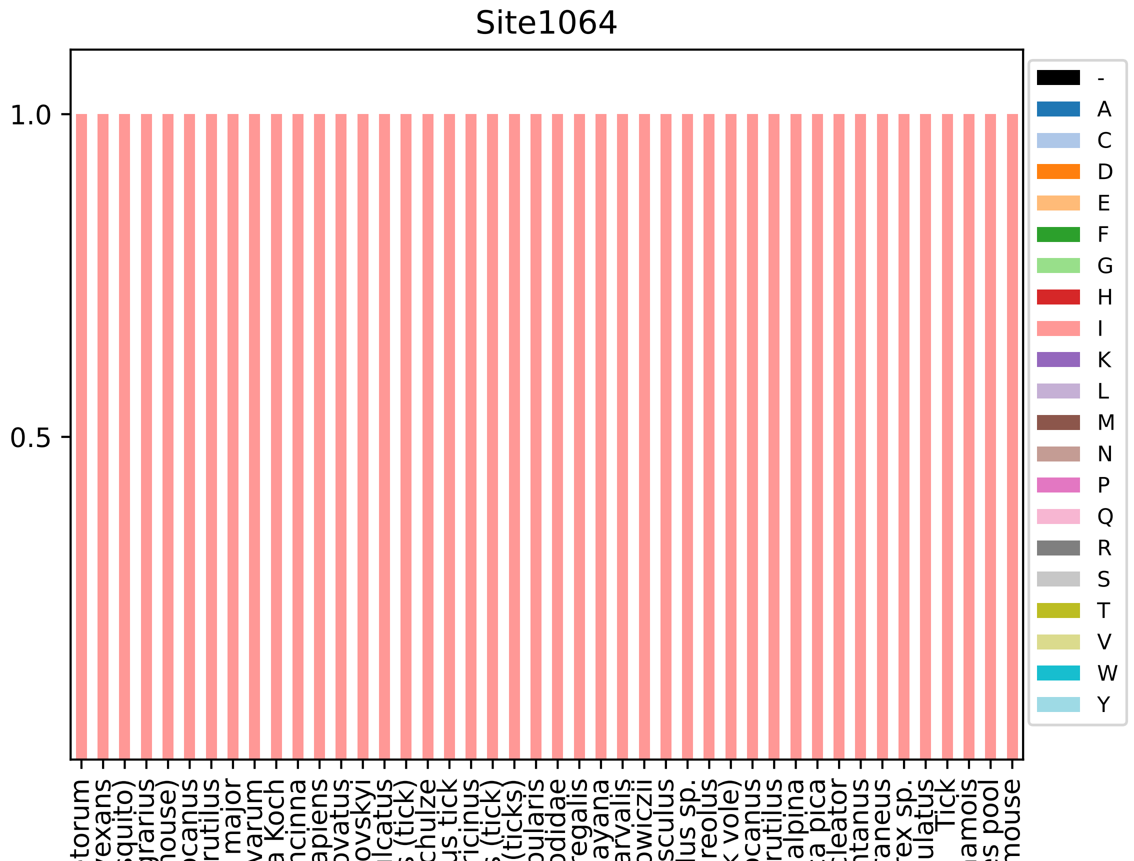

Site1064
1.0
0.5
-
A
C
D
E
F
G
H
I
K
L
M
N
P
Q
R
S
T
V
W
Y

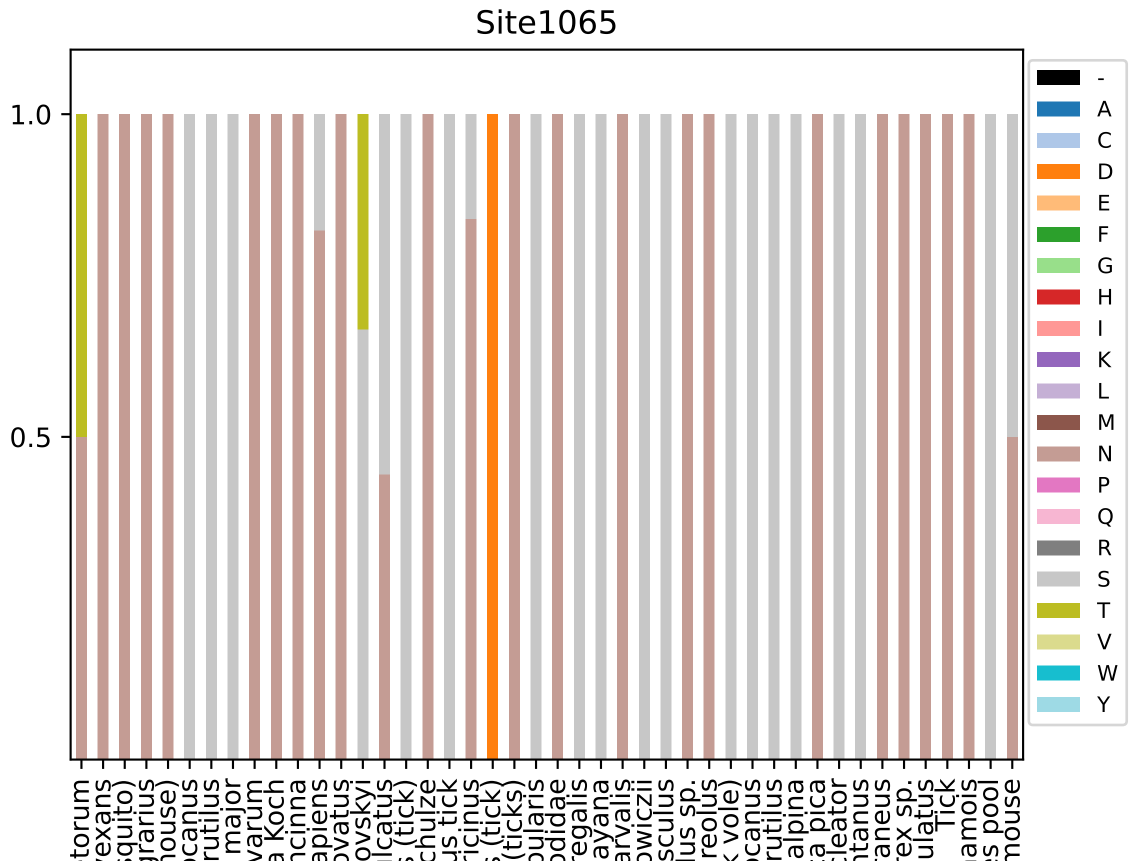
Site1065
1.0
0.5
-
A
C
D
E
F
G
H
I
K
L
M
N
P
Q
R
S
T
V
W
Y

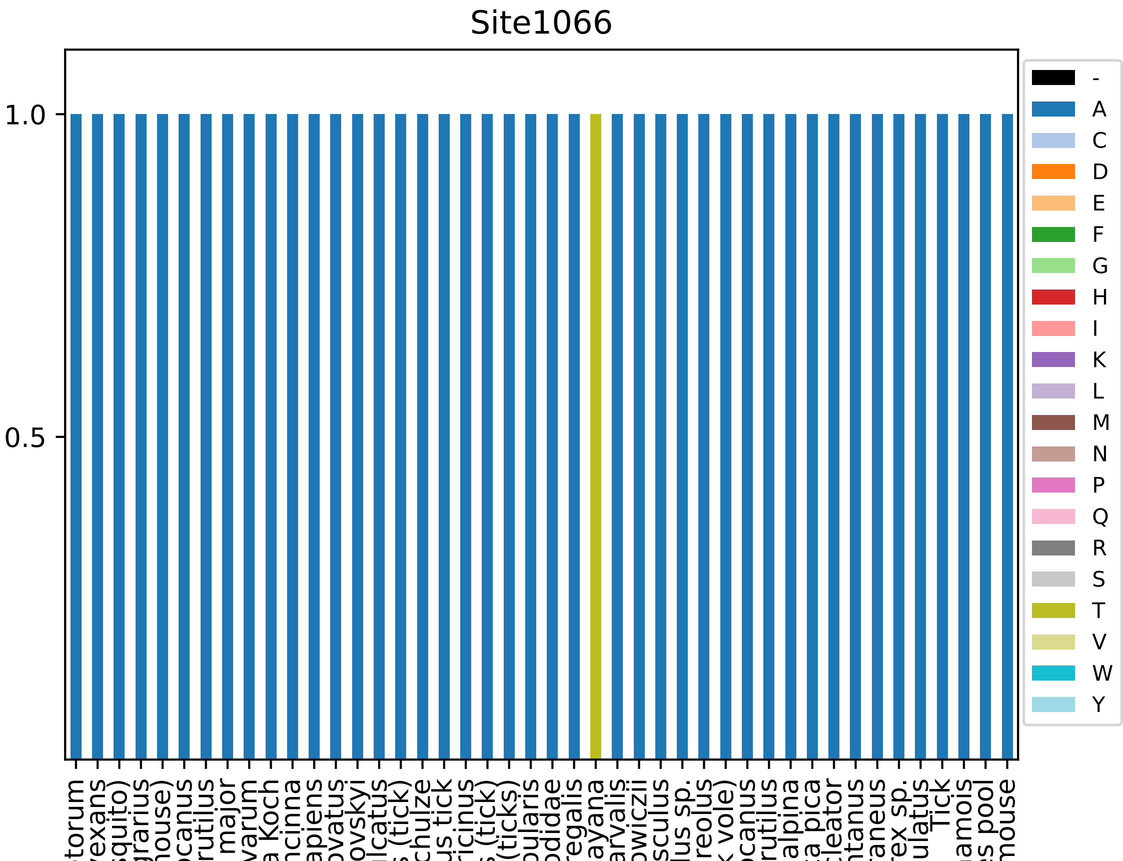
Site1066
1.0
0.5
-
A
C
D
E
F
G
H
I
K
L
M
N
P
Q
R
S
T
V
W
Y

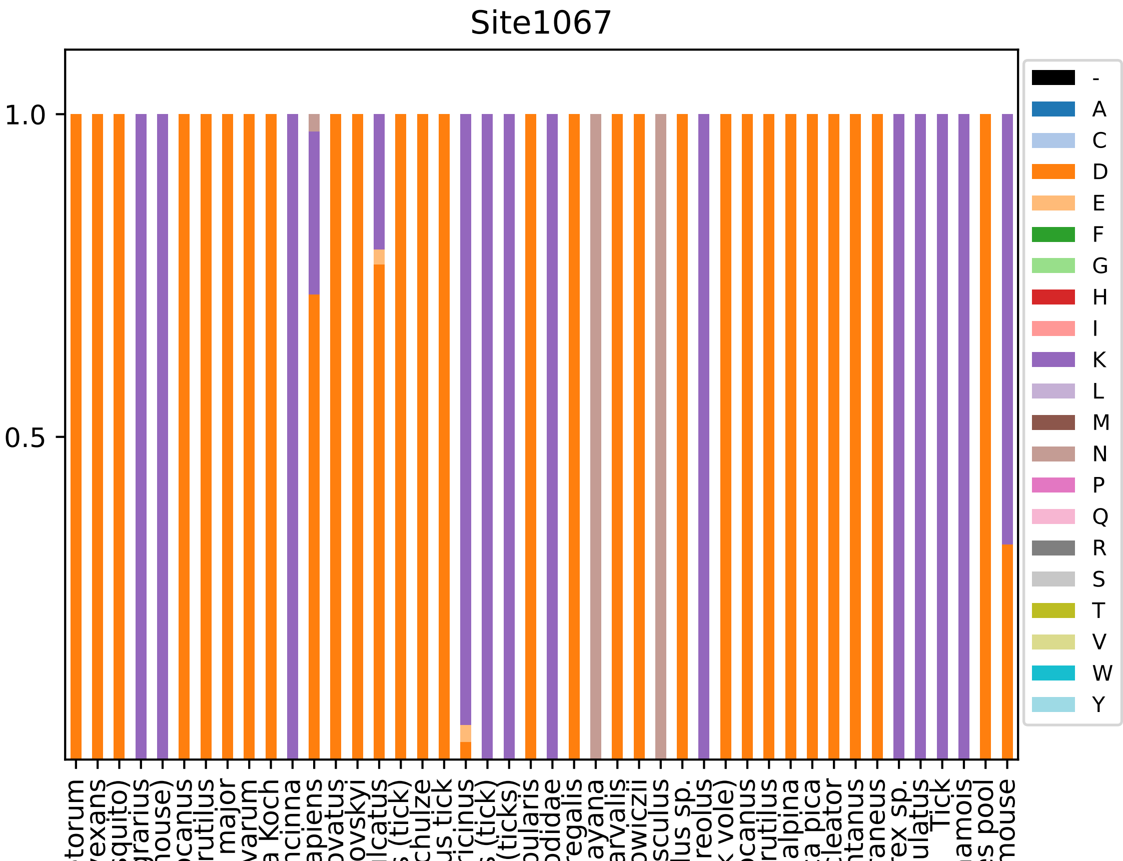

Site1067
1.0
0.5
-
A
C
D
E
F
G
H
I
K
L
M
N
P
Q
R
S
T
V
W
Y

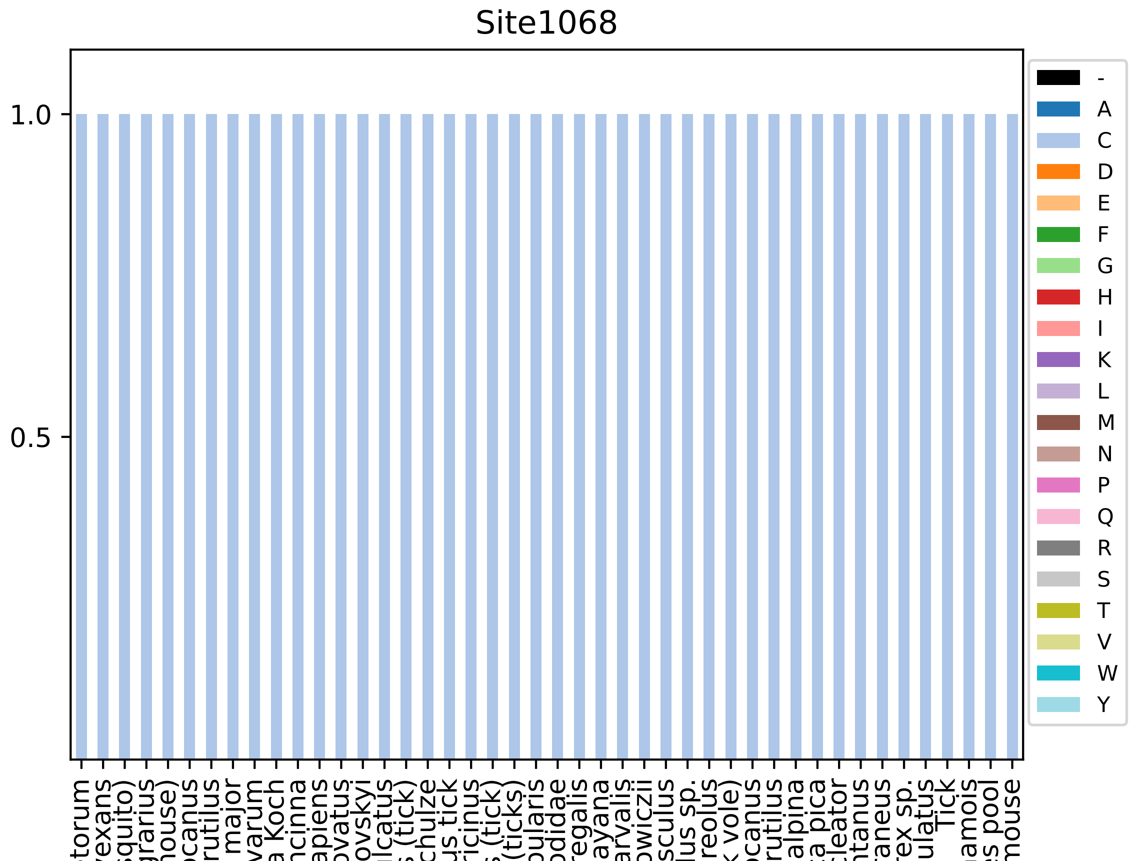
Site1068
1.0
0.5
-
A
C
D
E
F
G
H
I
K
L
M
N
P
Q
R
S
T
V
W
Y

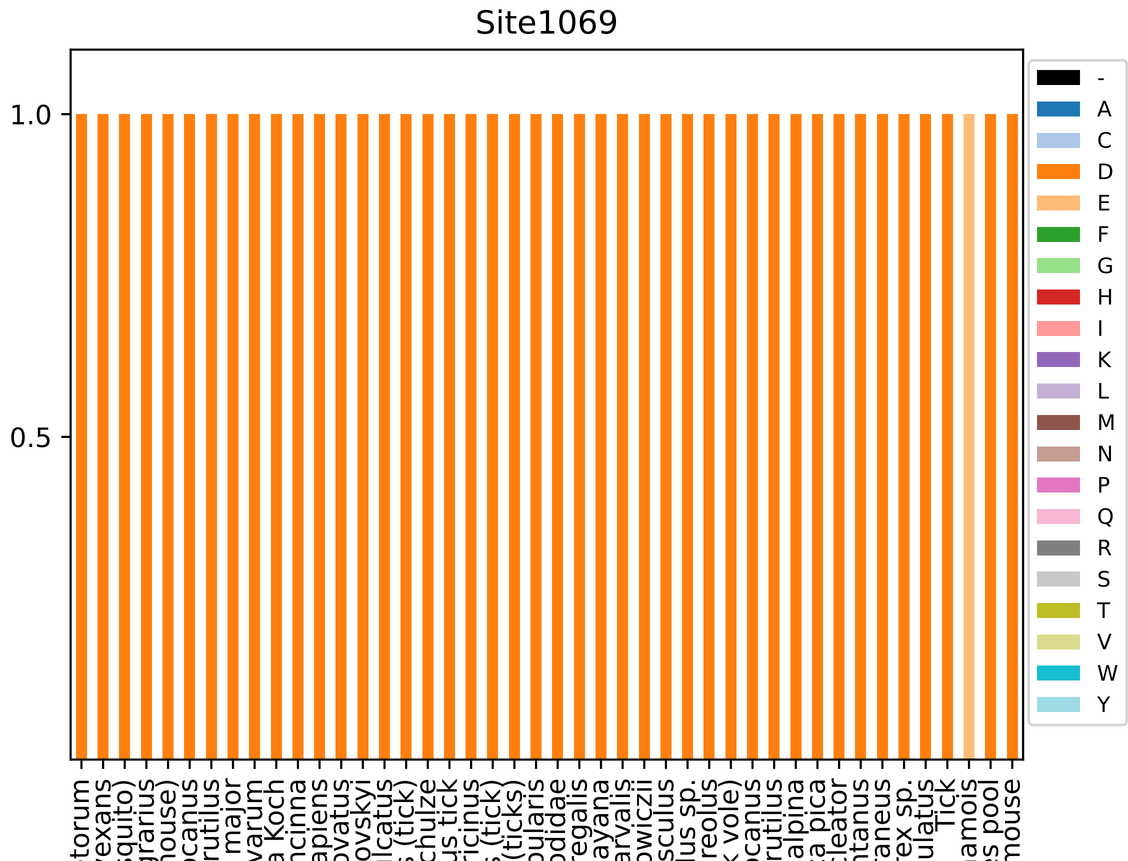

Site1069
1.0
0.5
-
A
C
D
E
F
G
H
I
K
L
M
N
P
Q
R
S
T
V
W
Y

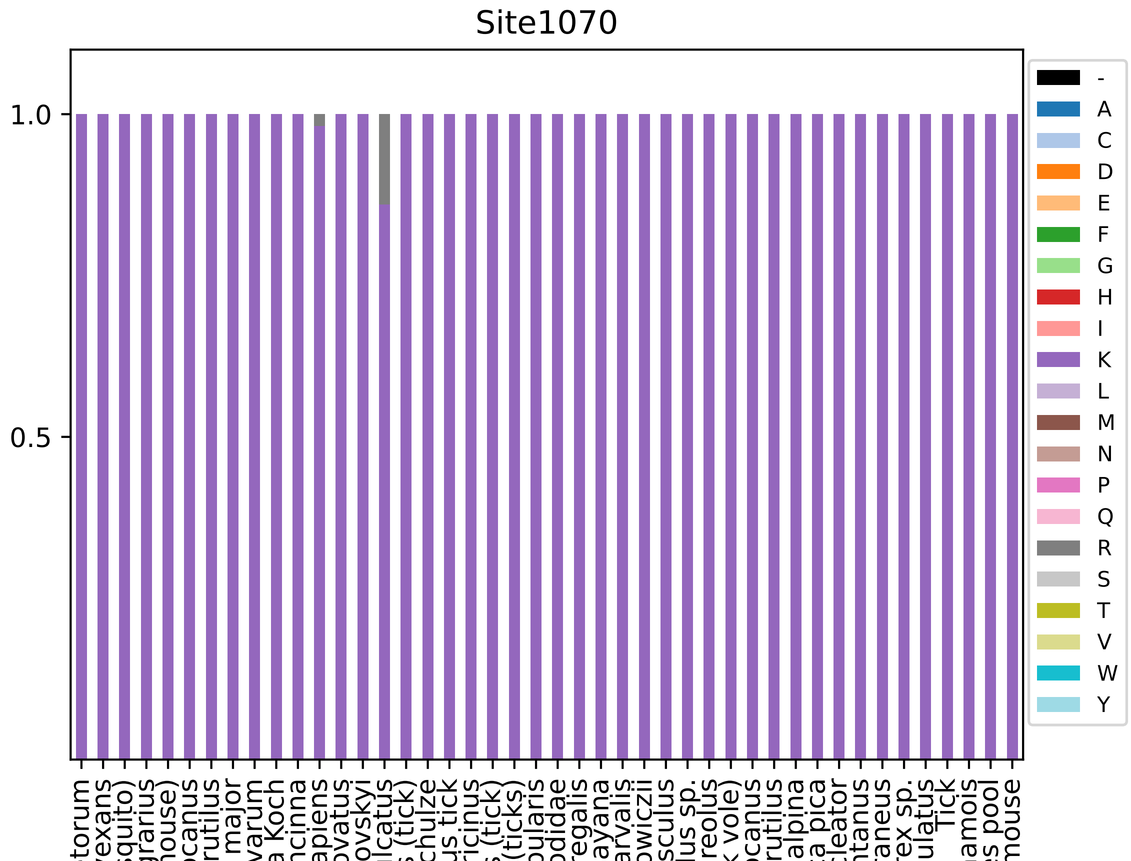

Site1070
1.0
0.5
-
A
C
D
E
F
G
H
I
K
L
M
N
P
Q
R
S
T
V
W
Y

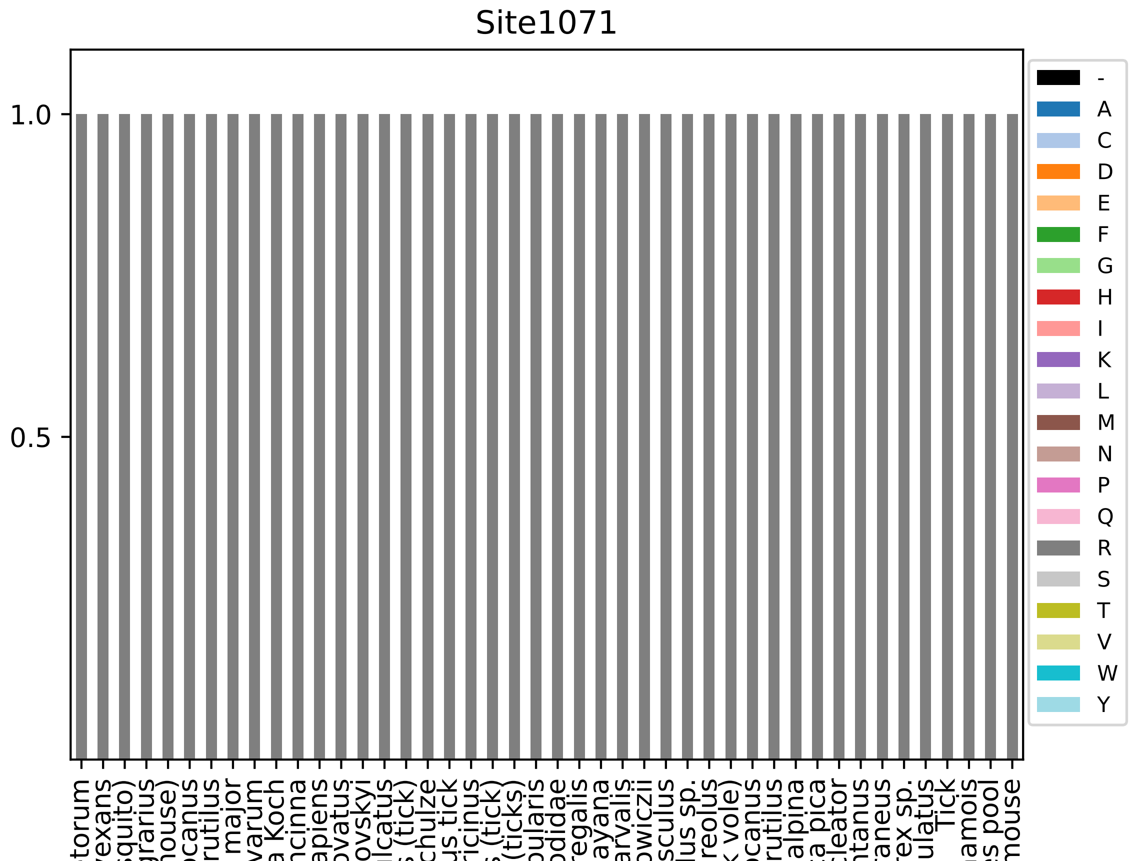

Site1071
1.0
0.5
-
A
C
D
E
F
G
H
I
K
L
M
N
P
Q
R
S
T
V
W
Y
Tick

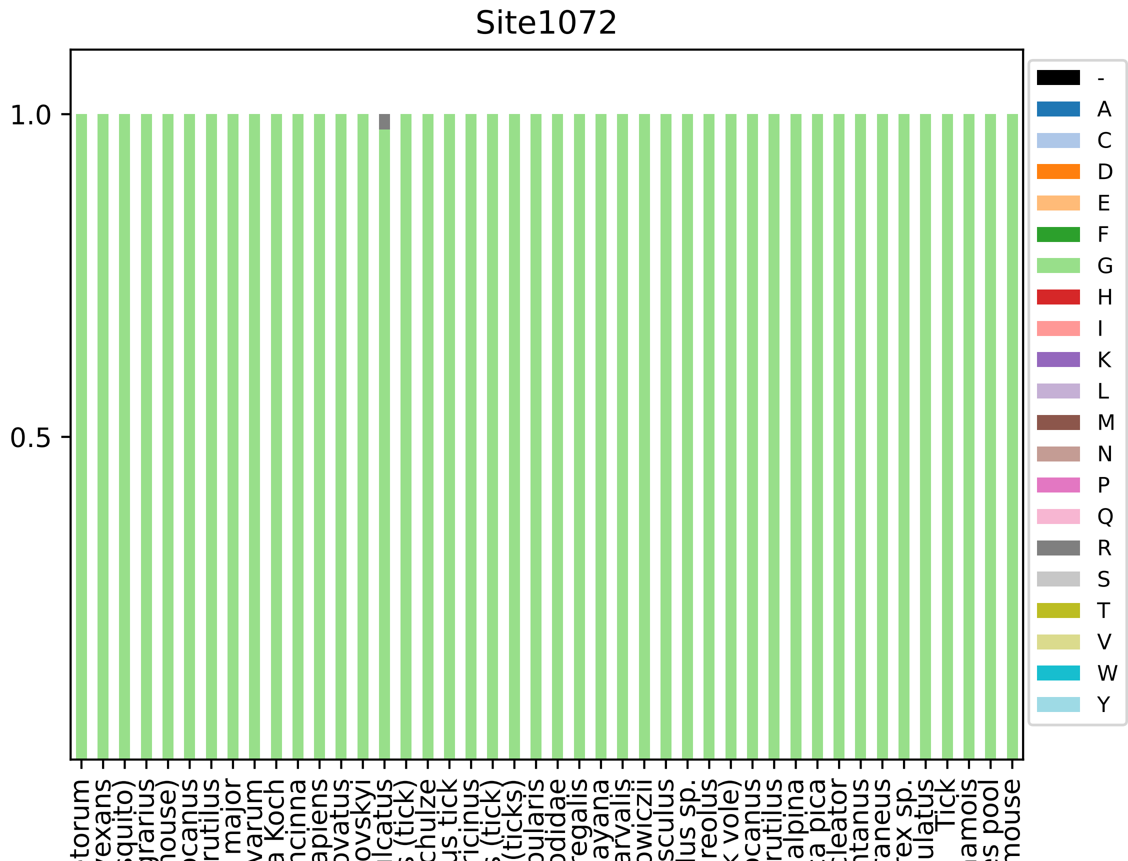
Site1072
1.0
0.5
-
A
C
D
E
F
G
H
I
K
L
M
N
P
Q
R
S
T
V
W
Y

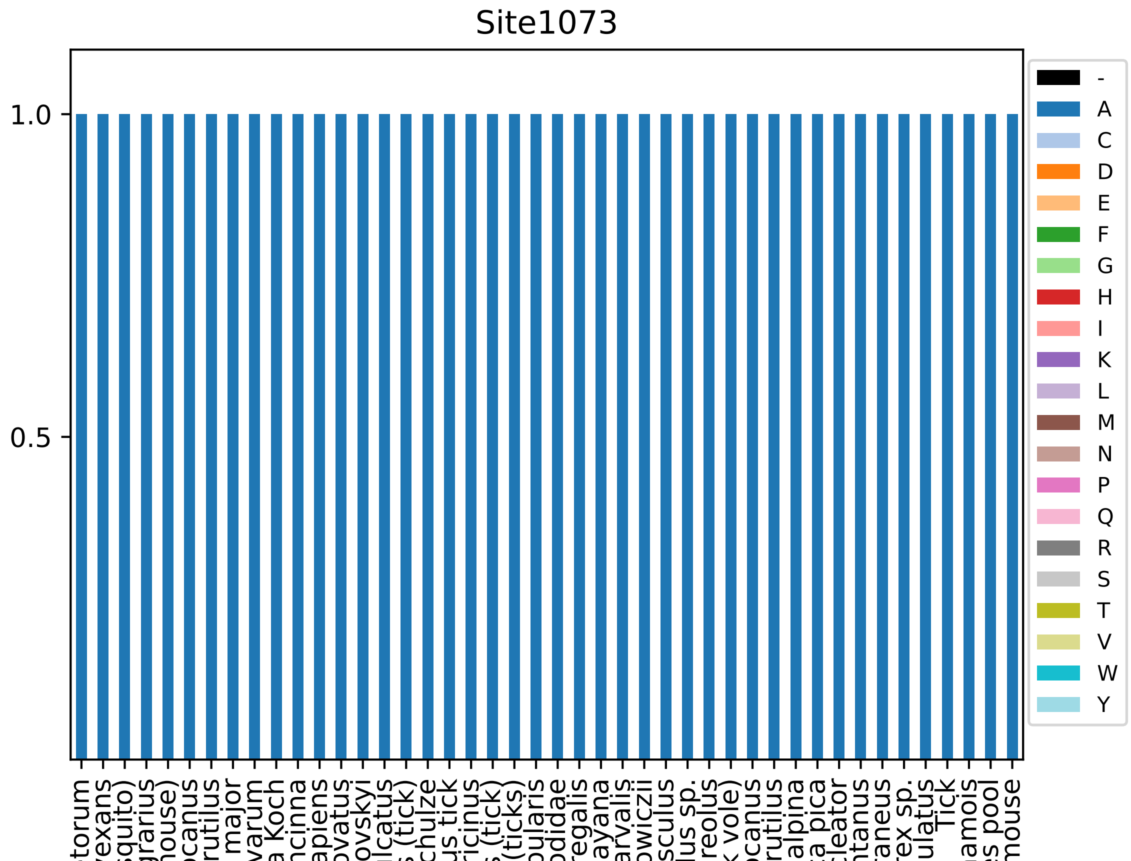
Site1073
1.0
0.5
-
A
C
D
E
F
G
H
I
K
L
M
N
P
Q
R
S
T
V
W
Y

Site1074

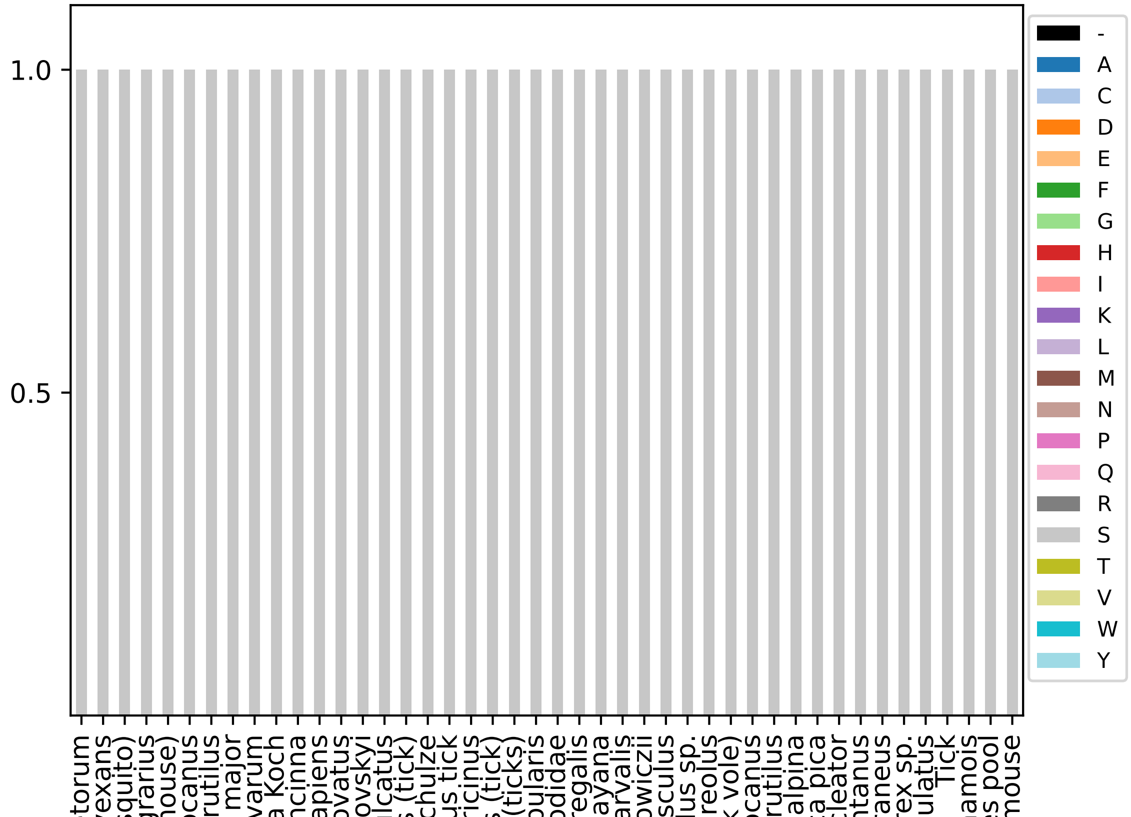

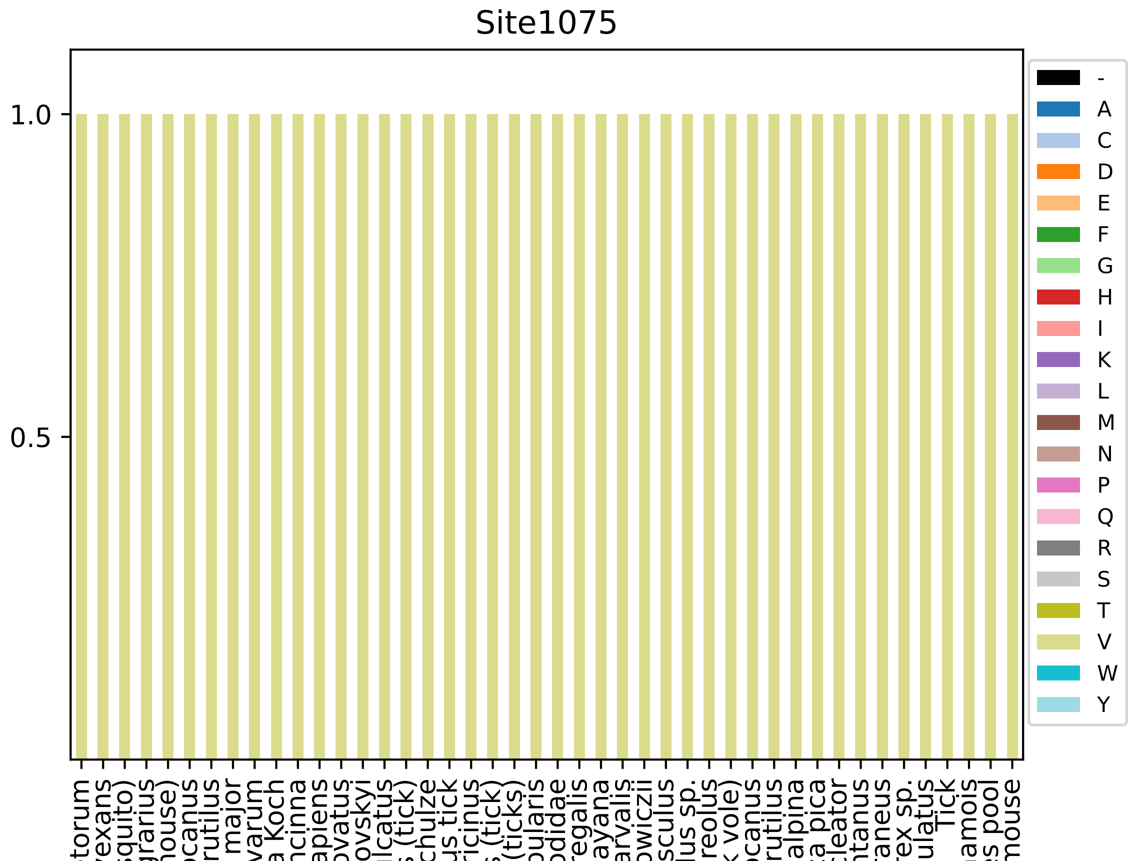
Site1075
1.0
0.5
-
A
C
D
E
F
G
H
I
K
L
M
N
P
Q
R
S
T
V
W
Y

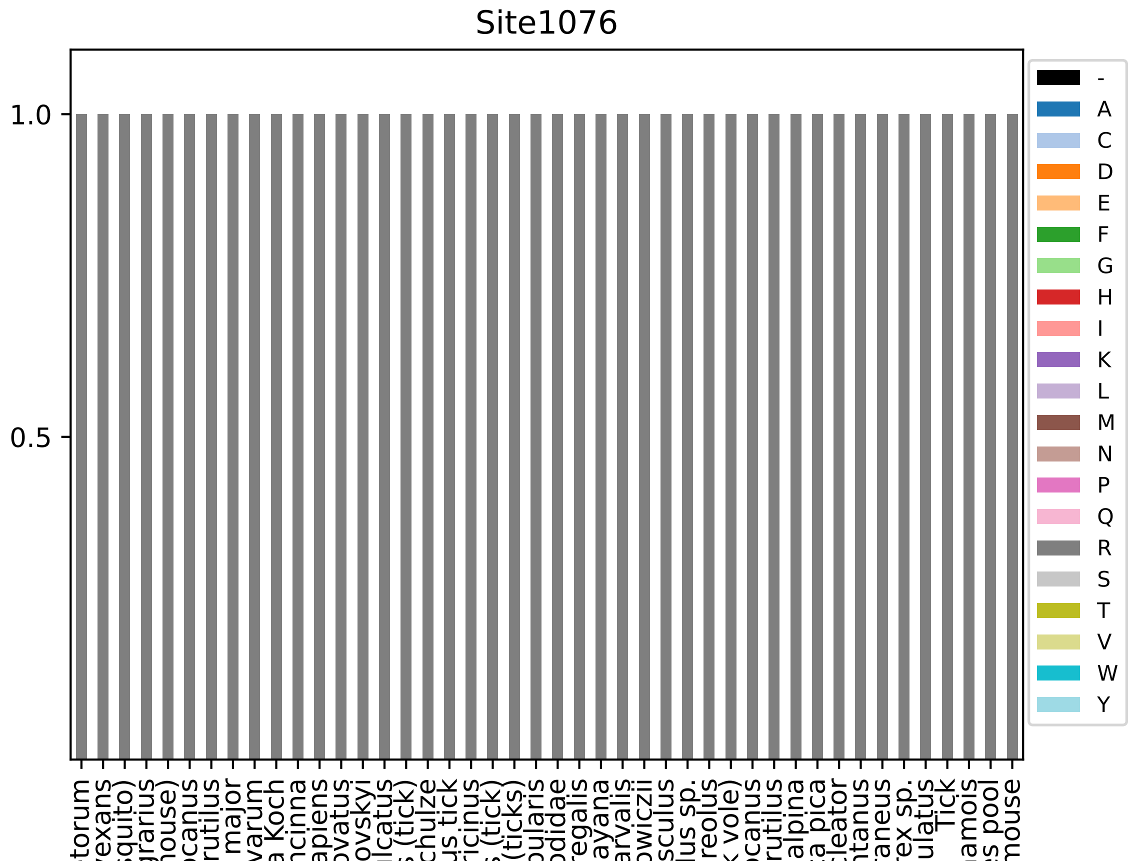
Site1076
1.0
0.5
-
A
C
D
E
F
G
H
I
K
L
M
N
P
Q
R
S
T
V
W
Y

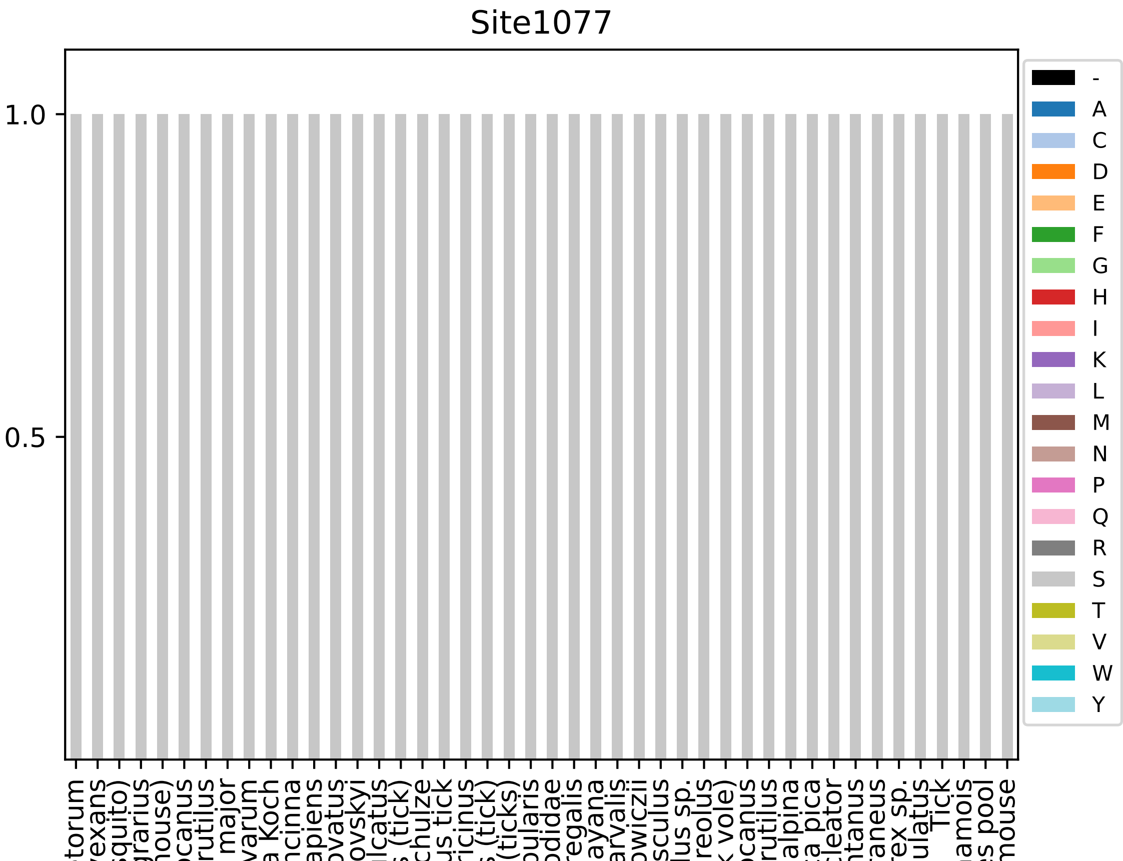
Site1077
1.0
0.5
-
A
C
D
E
F
G
H
I
K
L
M
N
P
Q
R
S
T
V
W
Y

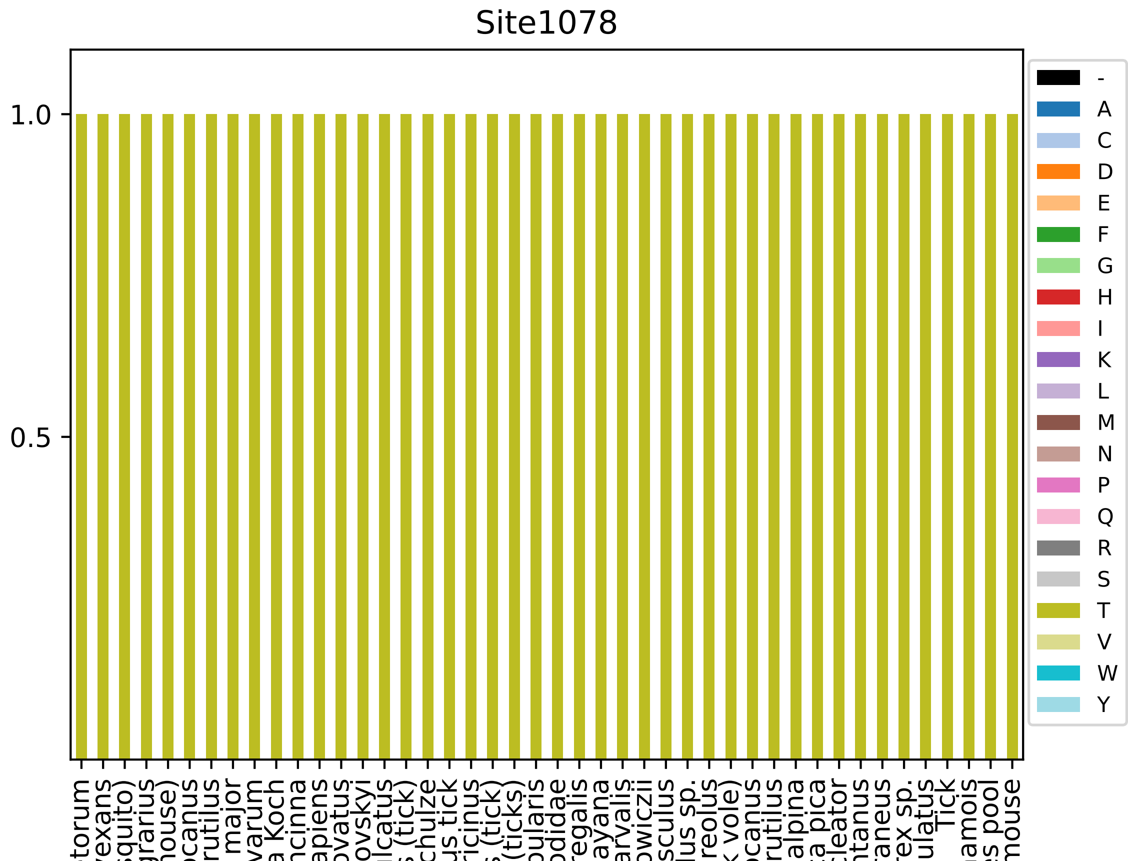

Site1078
1.0
0.5
-
A
C
D
E
F
G
H
I
K
L
M
N
P
Q
R
S
T
V
W
Y

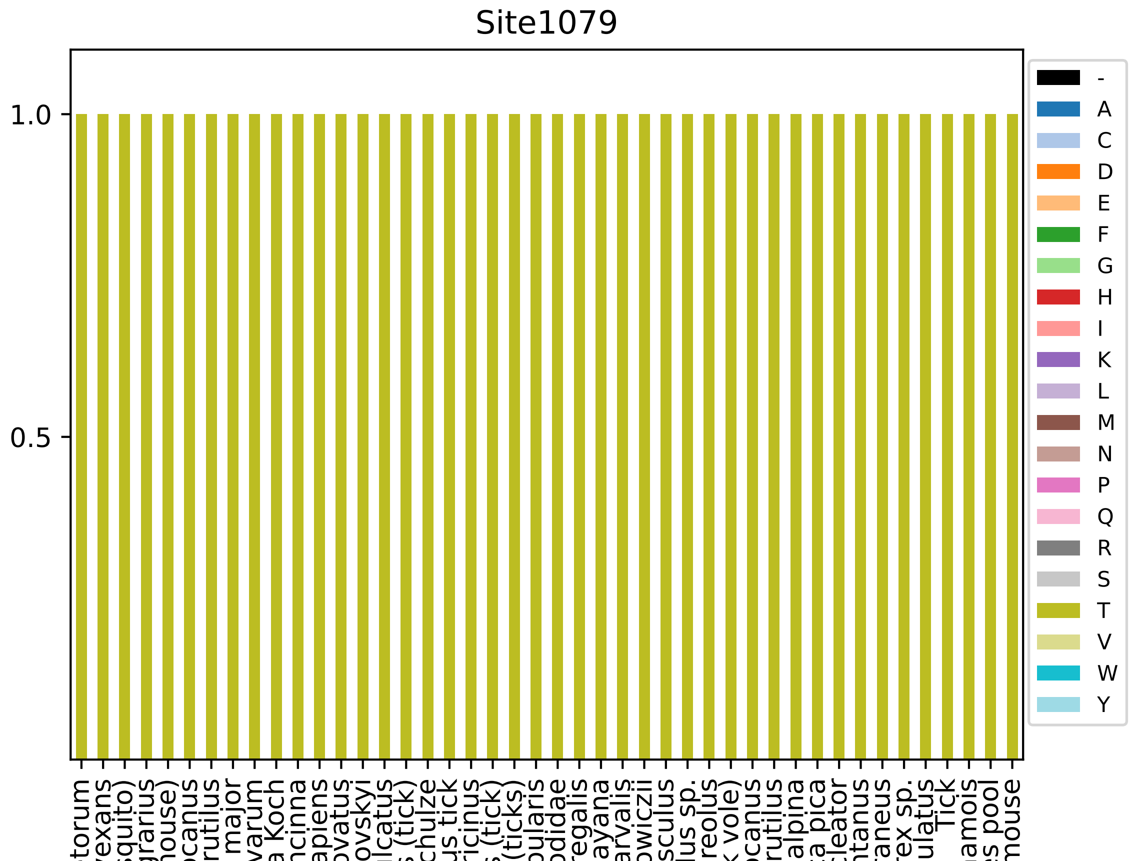
Site1079
1.0
0.5
-
A
C
D
E
F
G
H
I
K
L
M
N
P
Q
R
S
T
V
W
Y

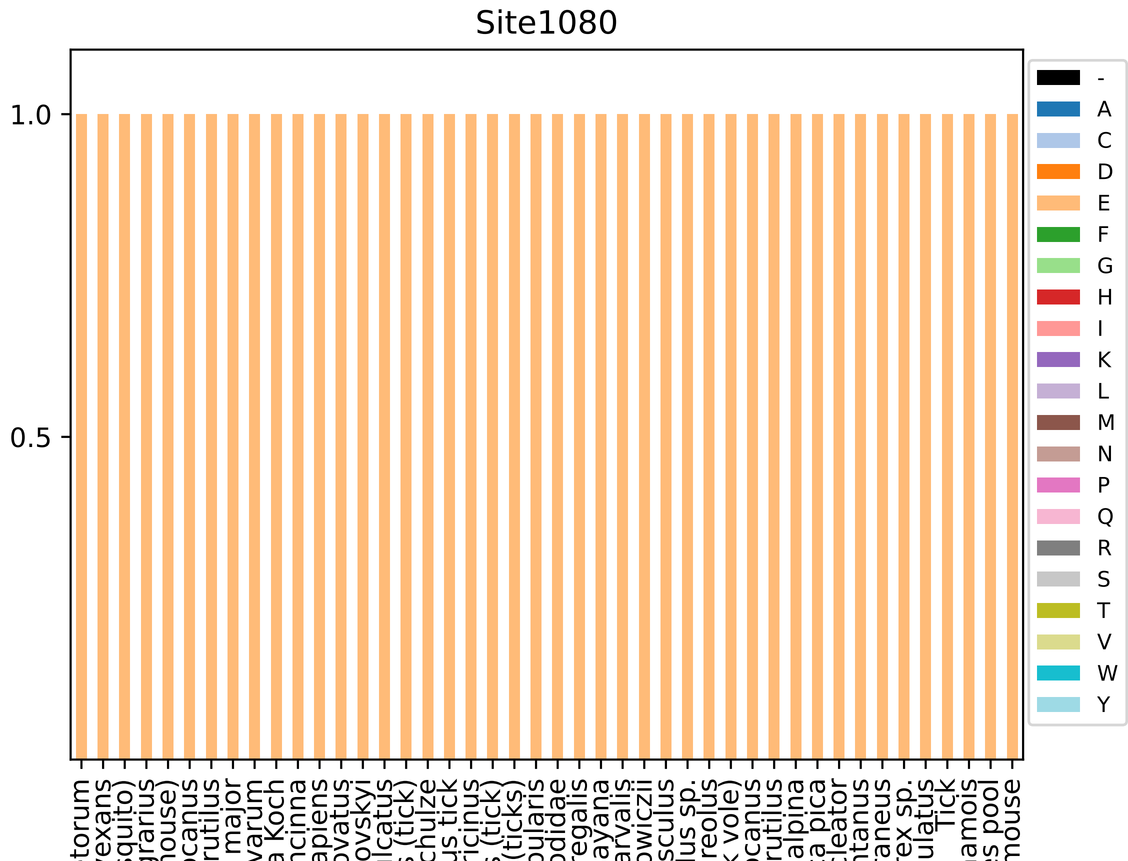
Site1080
1.0
0.5
-
A
C
D
E
F
G
H
I
K
L
M
N
P
Q
R
S
T
V
W
Y

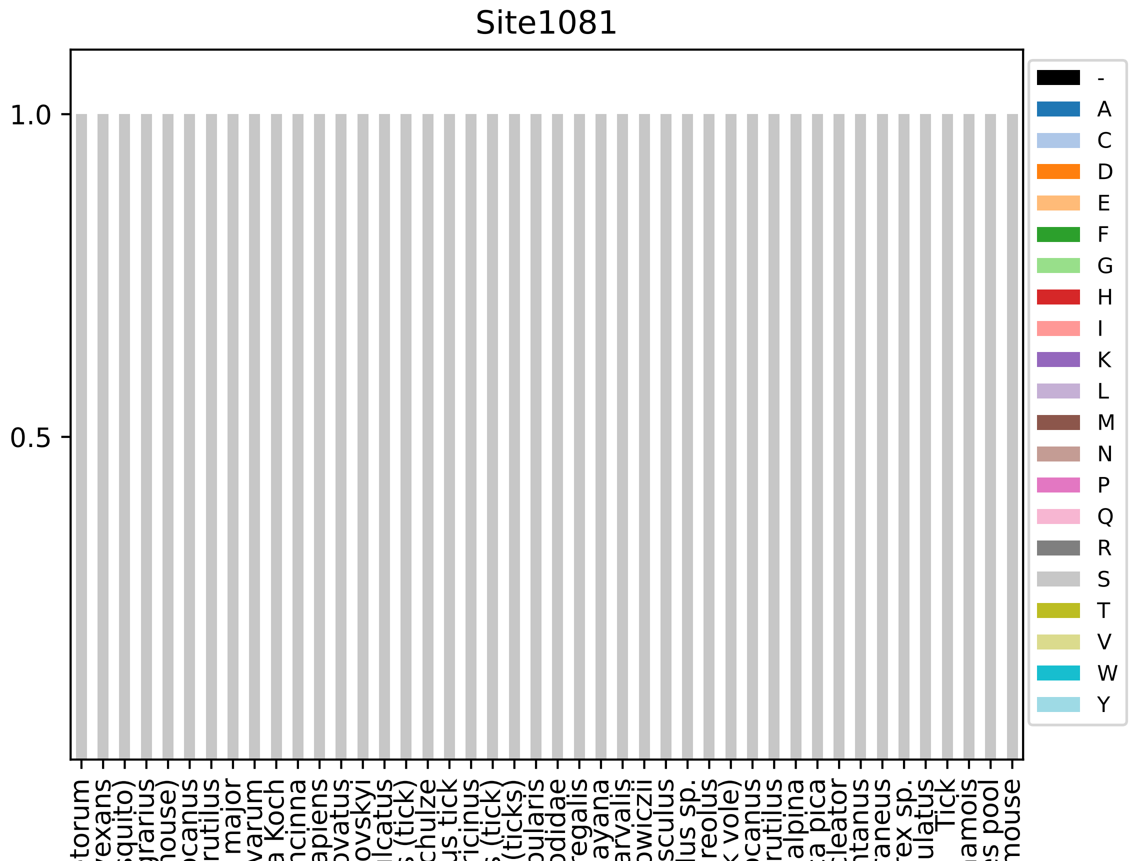

Site1081
1.0
0.5
-
A
C
D
E
F
G
H
I
K
L
M
N
P
Q
R
S
T
V
W
Y

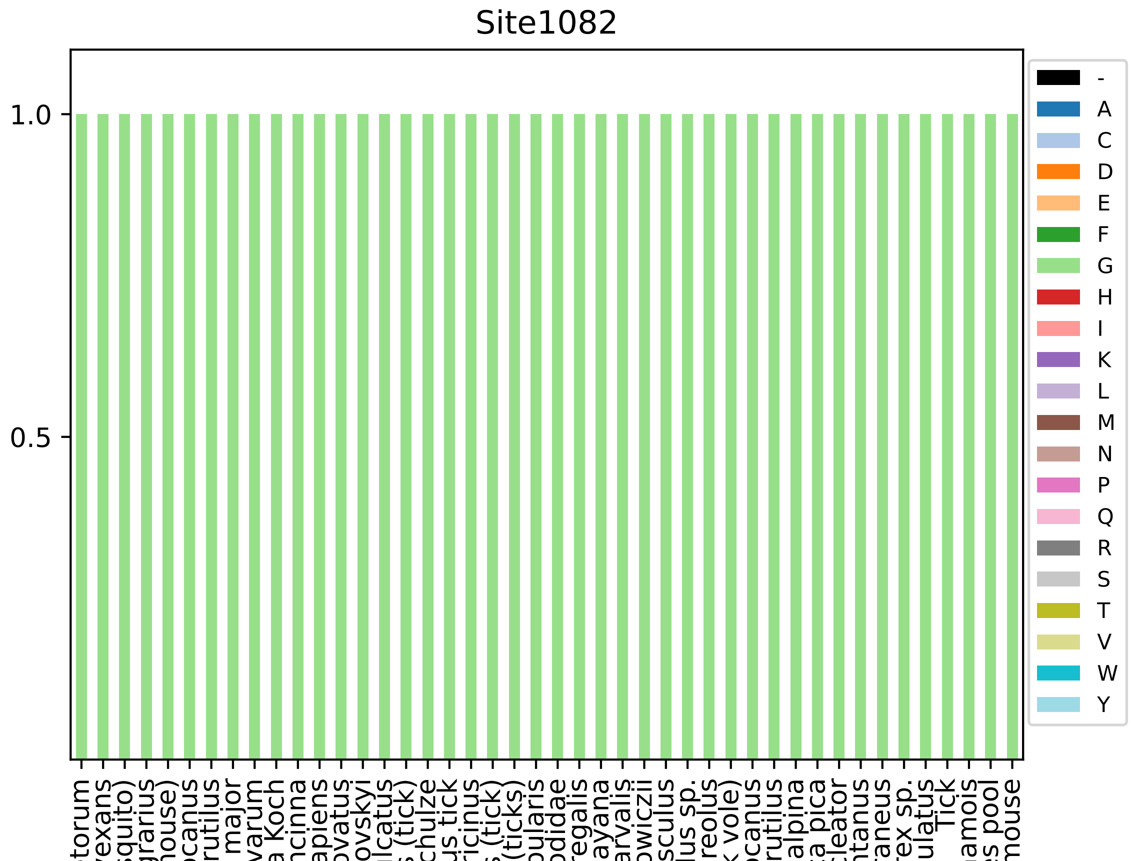
Site1082
1.0
0.5
-
A
C
D
E
F
G
H
I
K
L
M
N
P
Q
R
S
T
V
W
Y

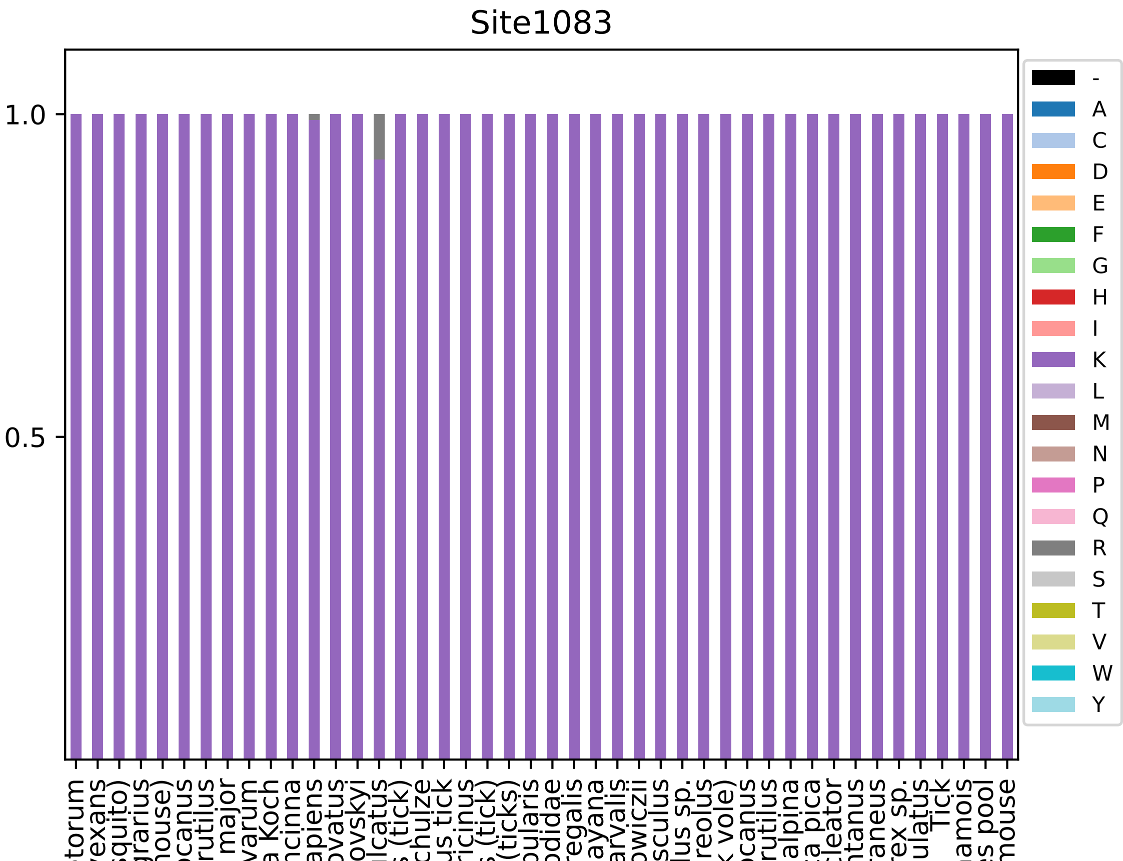
Site1083
1.0
0.5
-
A
C
D
E
F
G
H
I
K
L
M
N
P
Q
R
S
T
V
W
Y

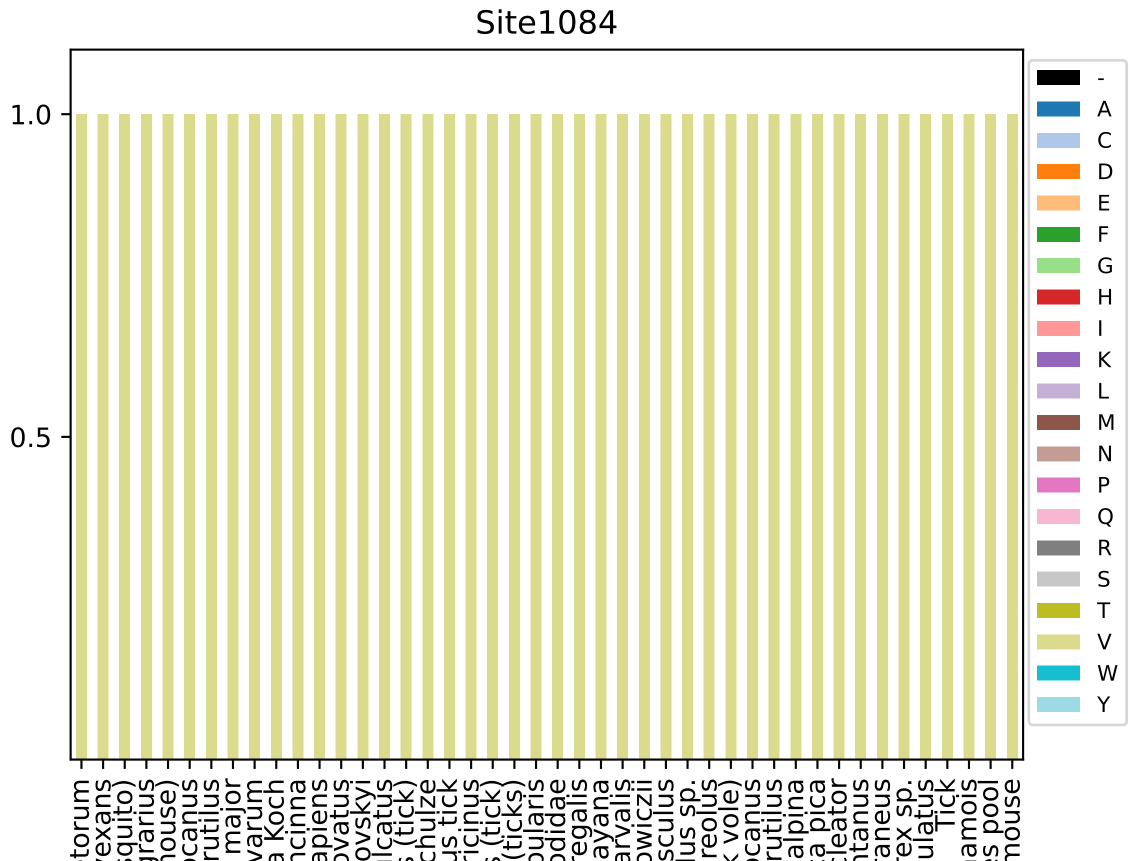

Site1084
1.0
0.5
-
A
C
D
E
F
G
H
I
K
L
M
N
P
Q
R
S
T
V
W
Y

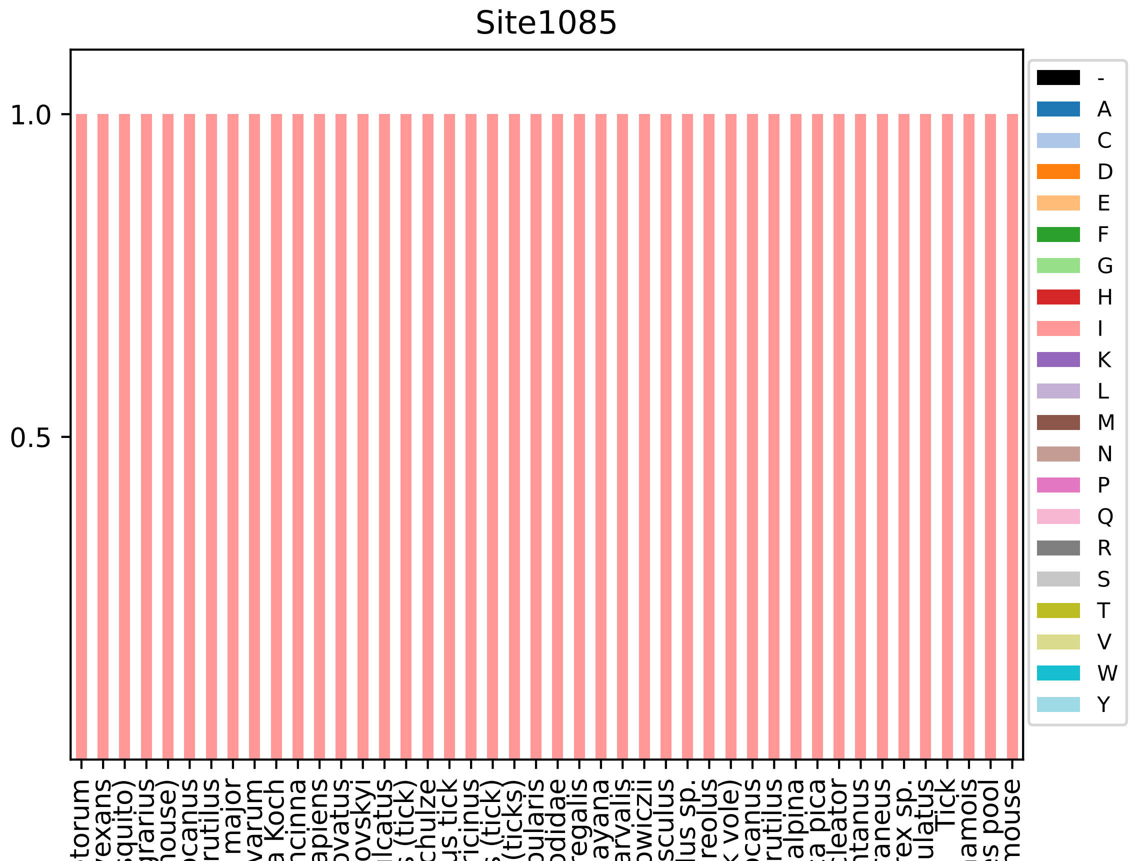
Site1085
1.0
0.5
-
A
C
D
E
F
G
H
I
K
L
M
N
P
Q
R
S
T
V
W
Y

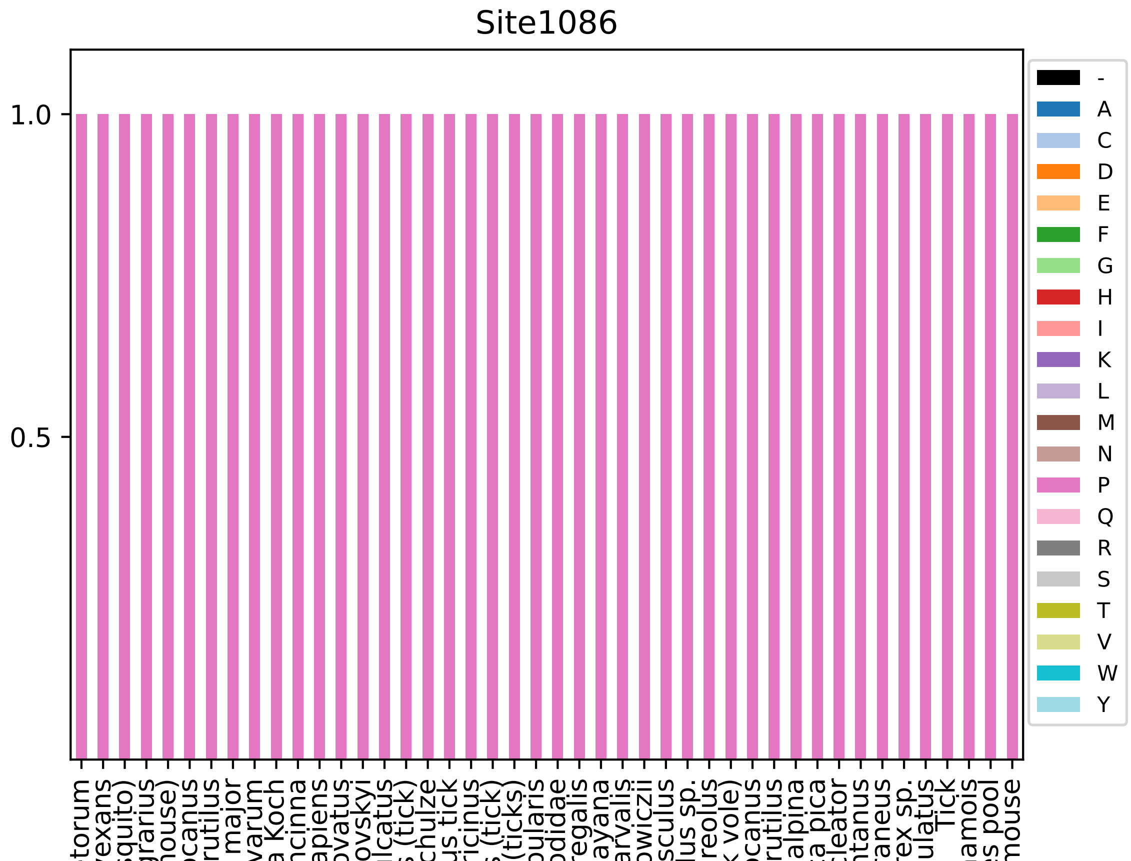
Site1086
1.0
0.5
-
A
C
D
E
F
G
H
I
K
L
M
N
P
Q
R
S
T
V
W
Y

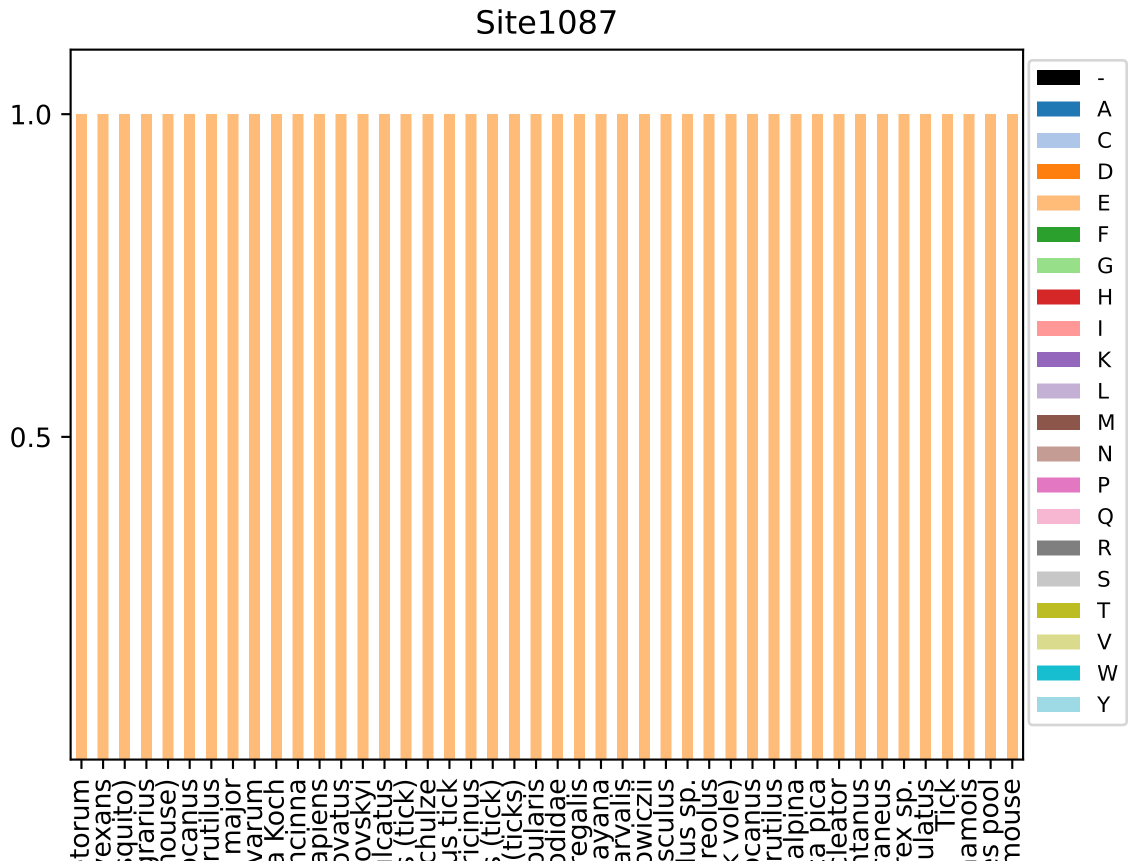

Site1087
1.0
0.5
-
A
C
D
E
F
G
H
I
K
L
M
N
P
Q
R
S
T
V
W
Y

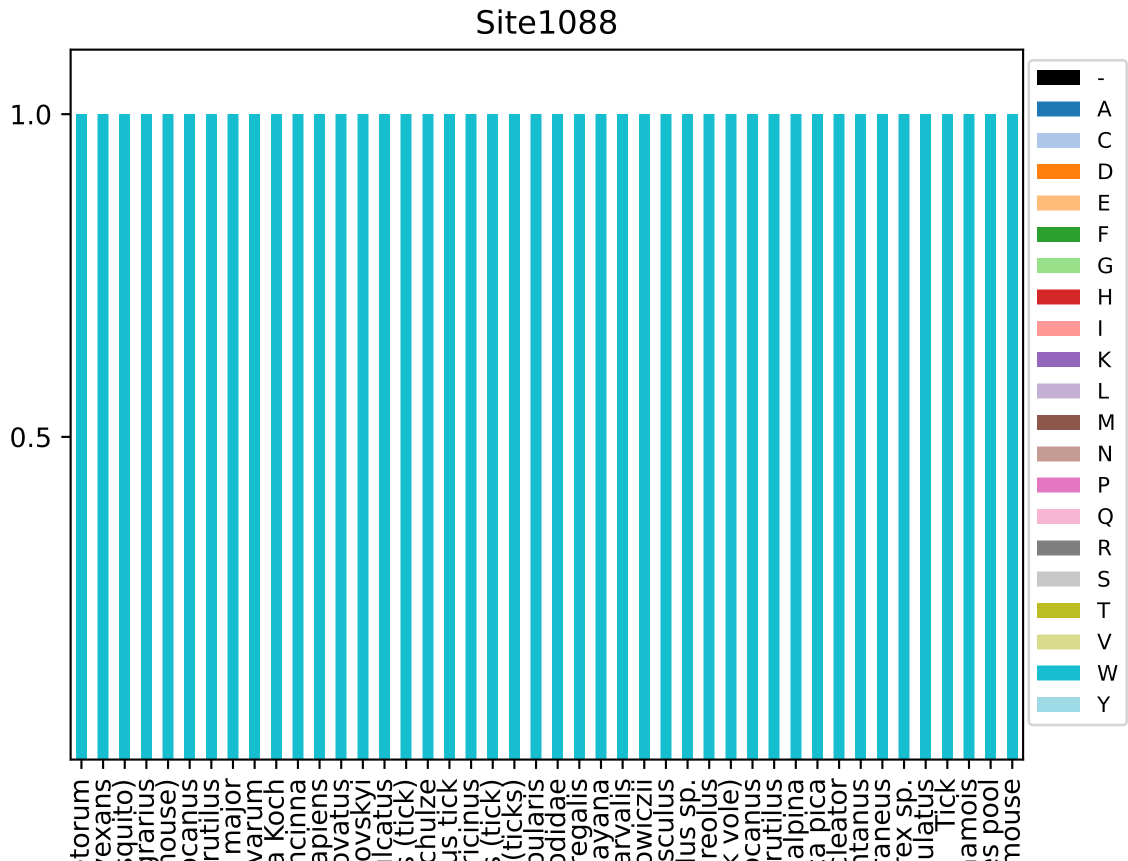
Site1088
1.0
0.5
-
A
C
D
E
F
G
H
I
K
L
M
N
P
Q
R
S
T
V
W
Y

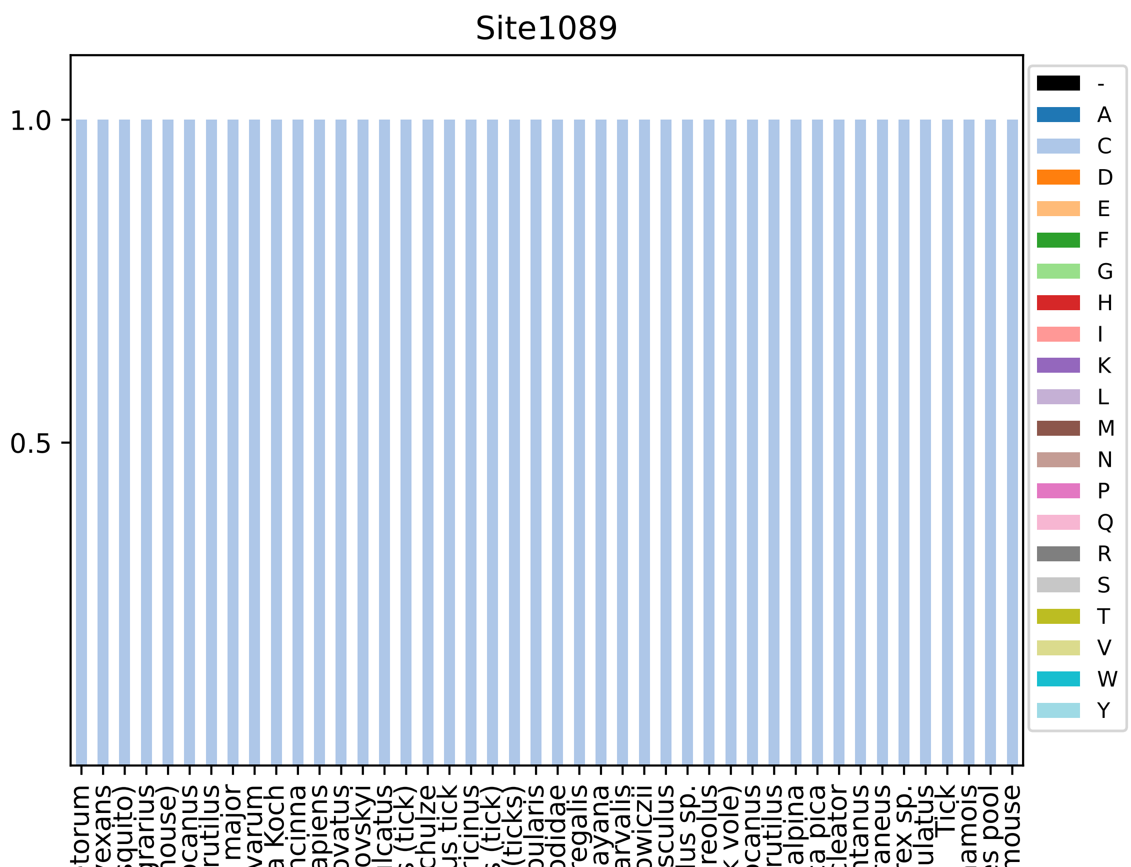
Site1089
1.0
0.5
-
A
C
D
E
F
G
H
I
K
L
M
N
P
Q
R
S
T
V
W
Y

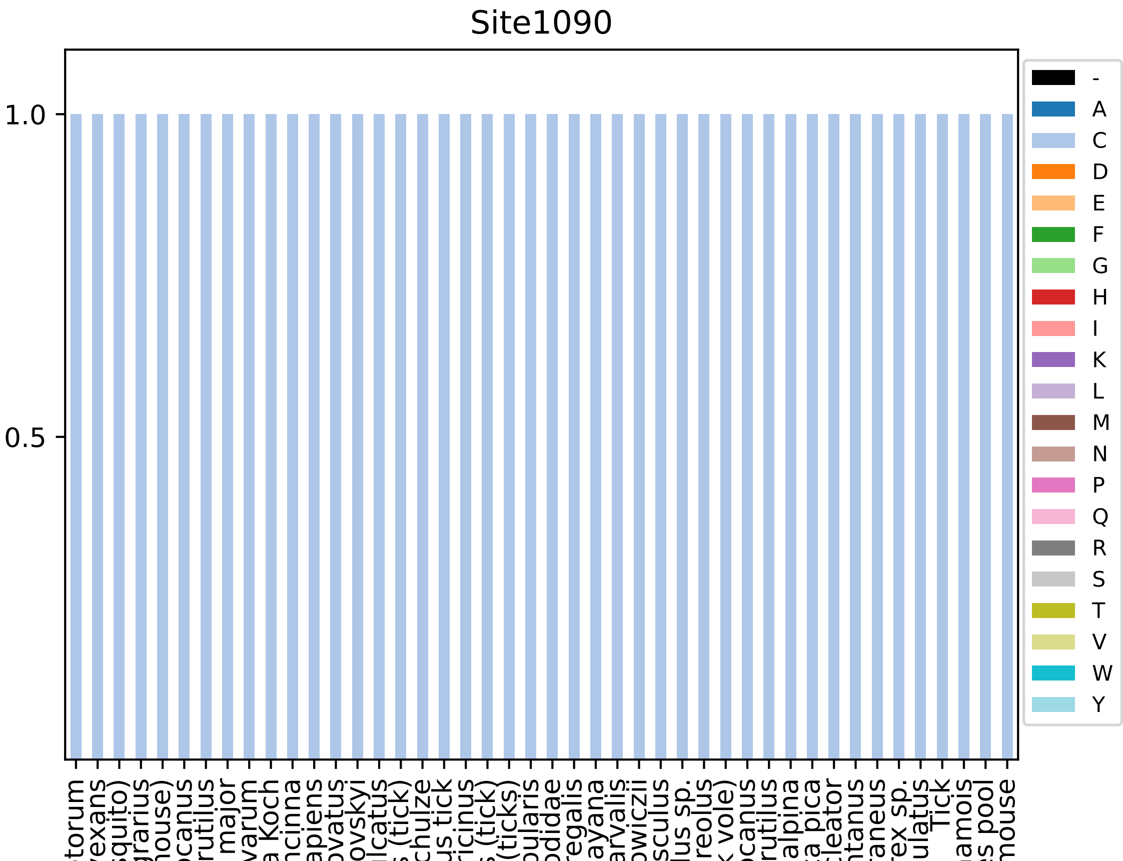
Site1090
1.0
0.5
-
A
C
D
E
F
G
H
I
K
L
M
N
P
Q
R
S
T
V
W
Y
torum
vexans
squito)
garius
nouse)
ocanus
rutilus
major
varum
a Koch
ncinna
apiens
ovatus
ovskyi
ulcatus
s (tick)
chulze
us tick
ricinus
s (tick)
(ticks)
pularis
odidae
regalis
ayana
arvalis
owiczii
sculus
lus sp.
reolus
x vole)
ocanus
rutilus
alpina
a pica
cleator
ntanus
raneus
rex sp.
ulatus
Tick
amois
es pool
mouse

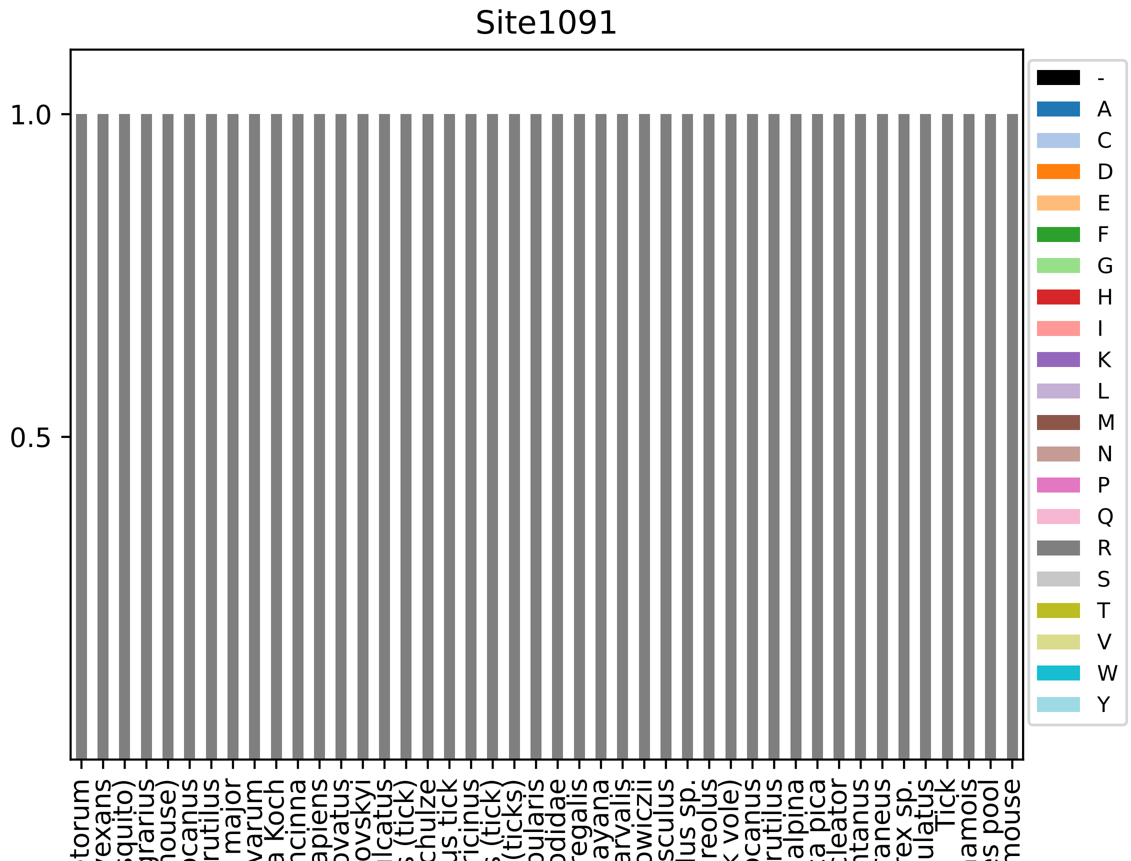
Site1091
1.0
0.5
-
A
C
D
E
F
G
H
I
K
L
M
N
P
Q
R
S
T
V
W
Y
Tick

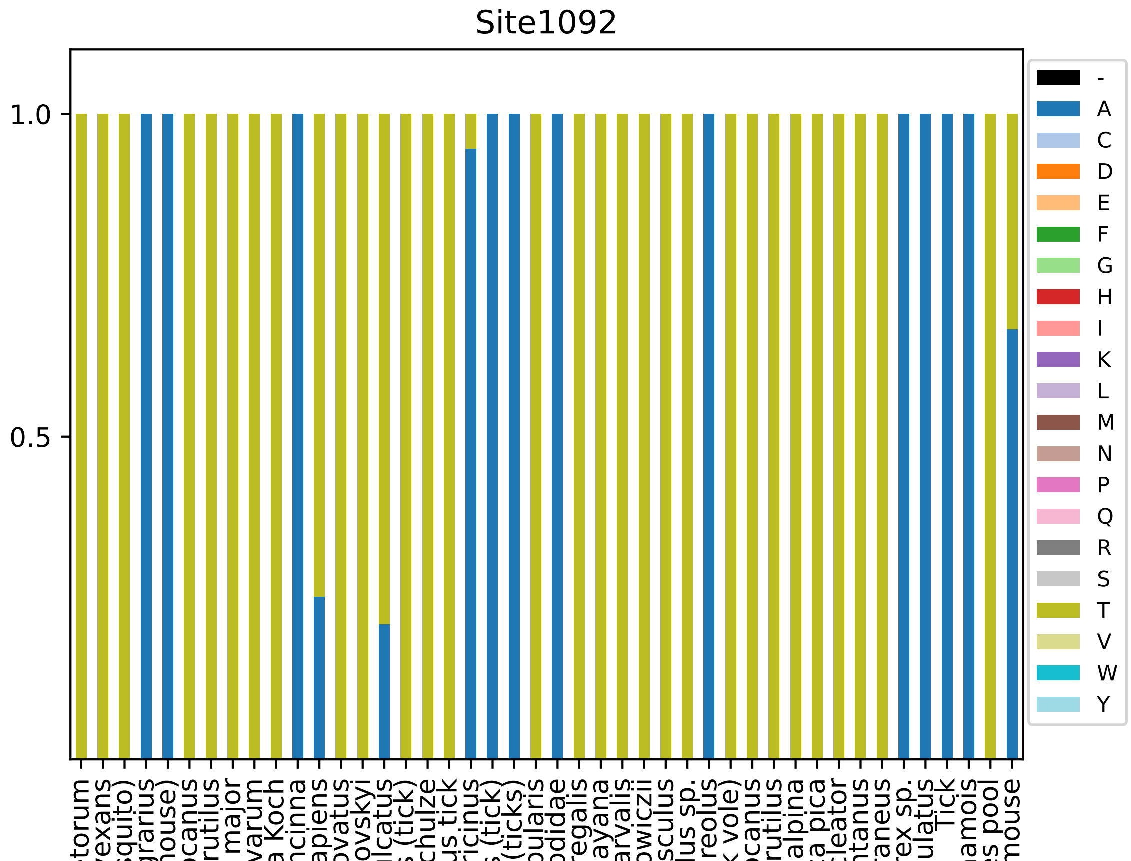
Site1092
1.0
0.5
-
A
C
D
E
F
G
H
I
K
L
M
N
P
Q
R
S
T
V
W
Y
Tick

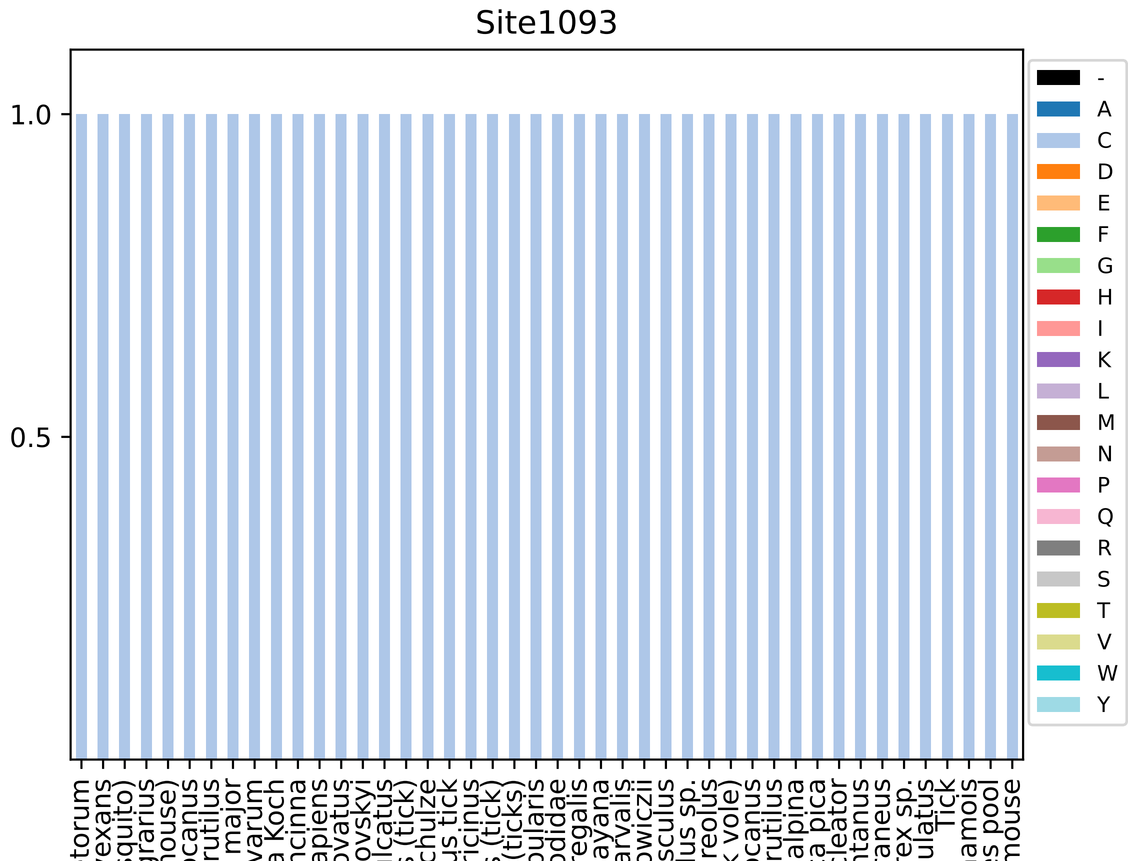

Site1093
1.0
0.5
-
A
C
D
E
F
G
H
I
K
L
M
N
P
Q
R
S
T
V
W
Y

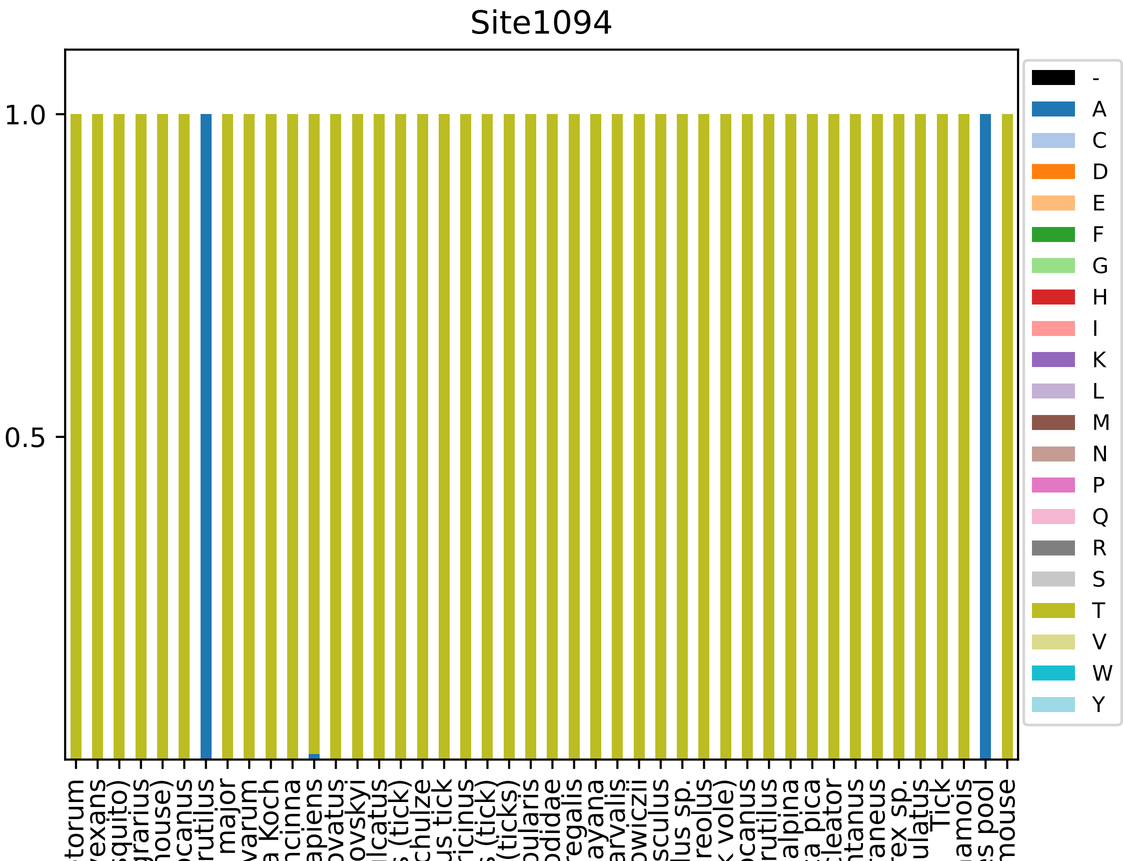

Site1094
1.0
0.5
-
A
C
D
E
F
G
H
I
K
L
M
N
P
Q
R
S
T
V
W
Y

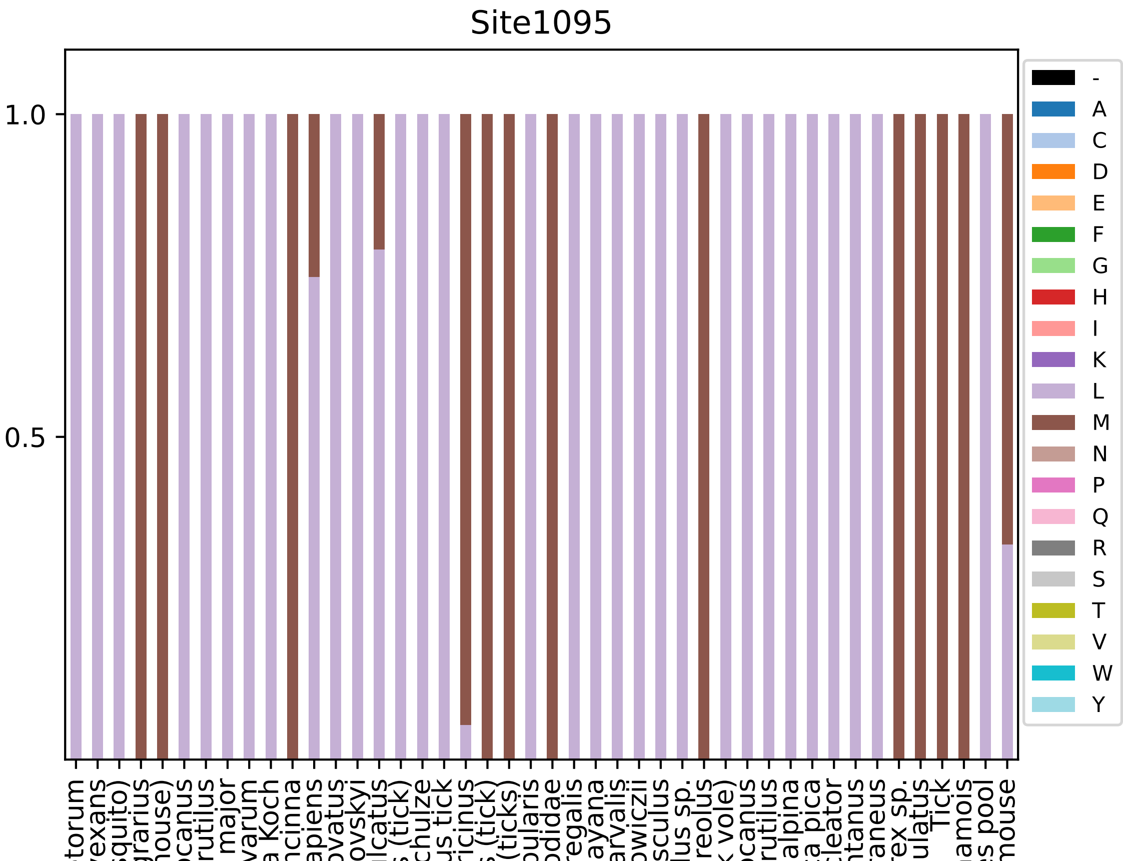

Site1095
1.0
0.5
-
A
C
D
E
F
G
H
I
K
L
M
N
P
Q
R
S
T
V
W
Y

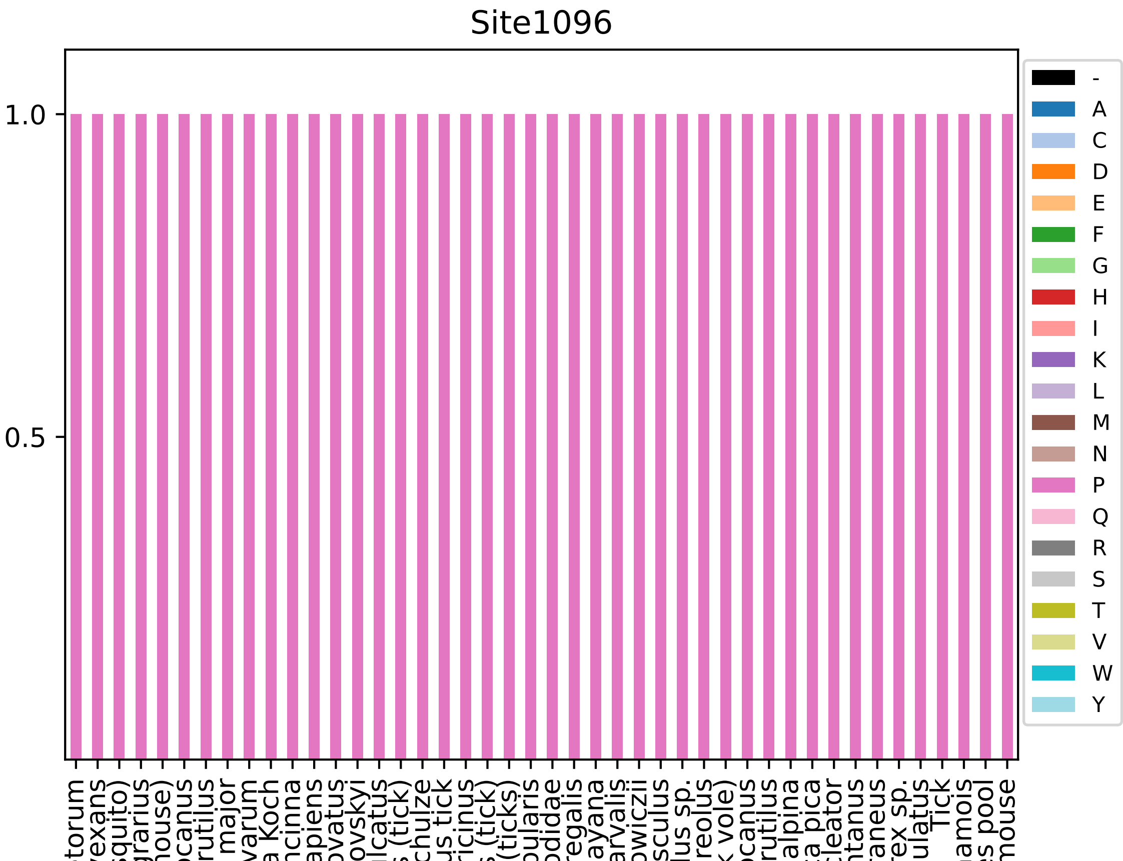

Site1096
1.0
0.5
-
A
C
D
E
F
G
H
I
K
L
M
N
P
Q
R
S
T
V
W
Y

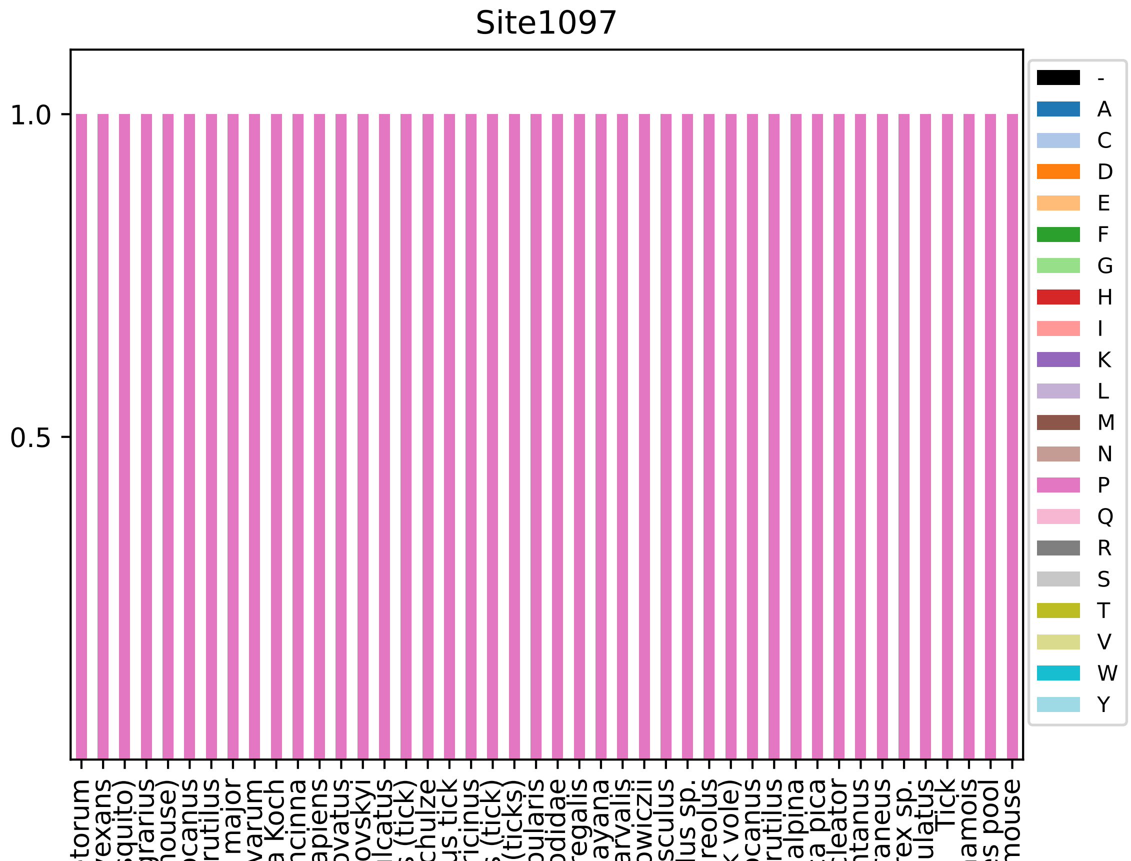
Site1097
1.0
0.5
-
A
C
D
E
F
G
H
I
K
L
M
N
P
Q
R
S
T
V
W
Y

Site1098

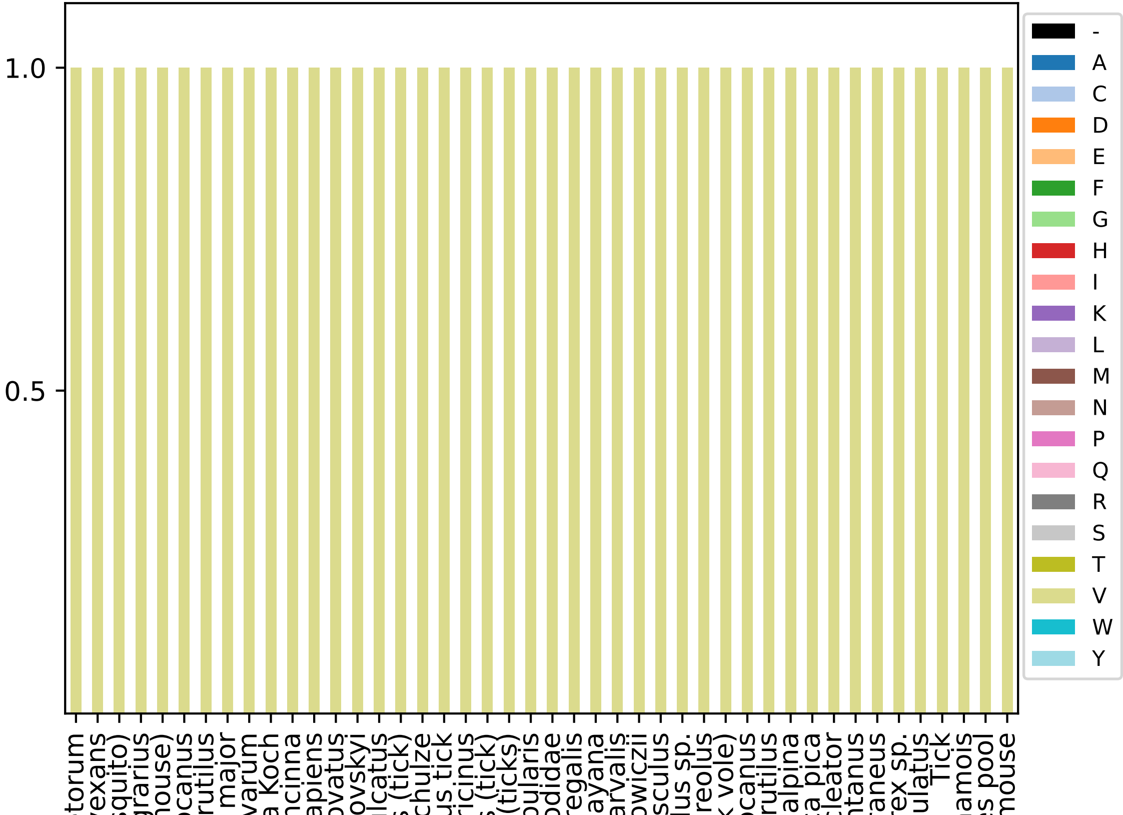

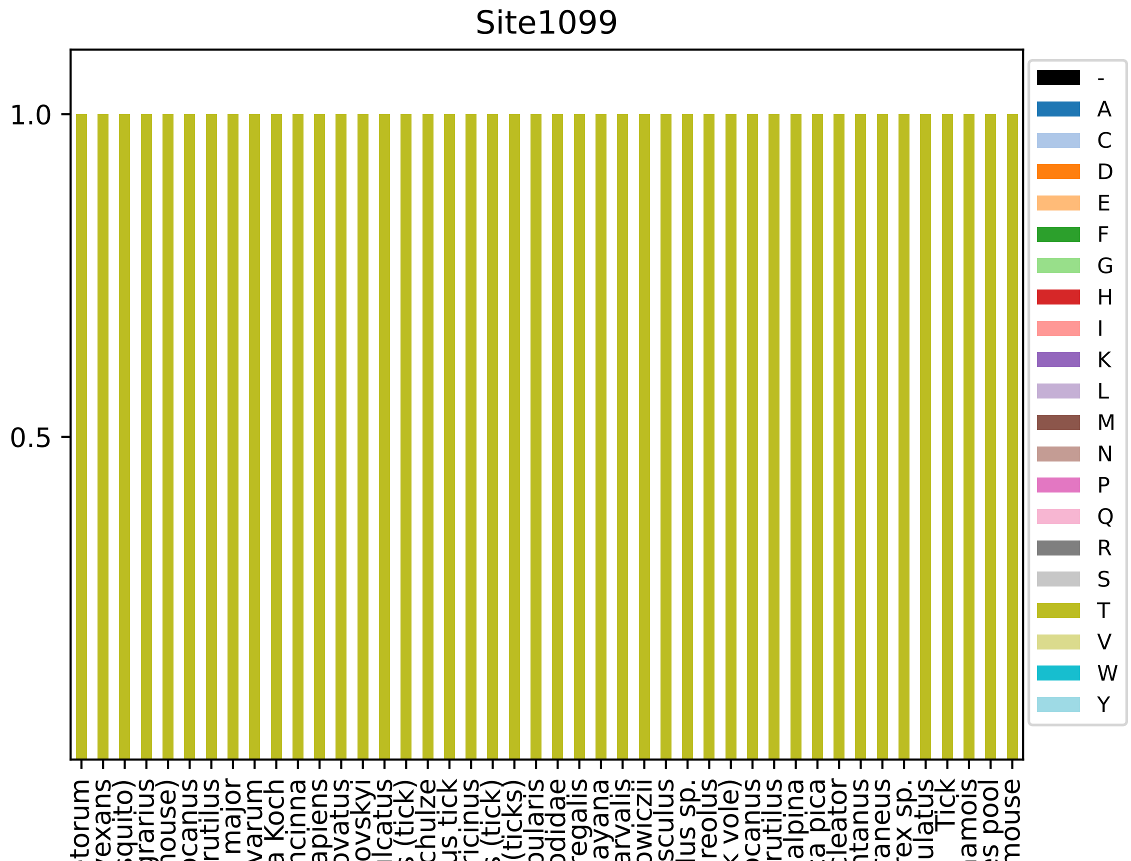

Site1099
1.0
0.5
-
A
C
D
E
F
G
H
I
K
L
M
N
P
Q
R
S
T
V
W
Y

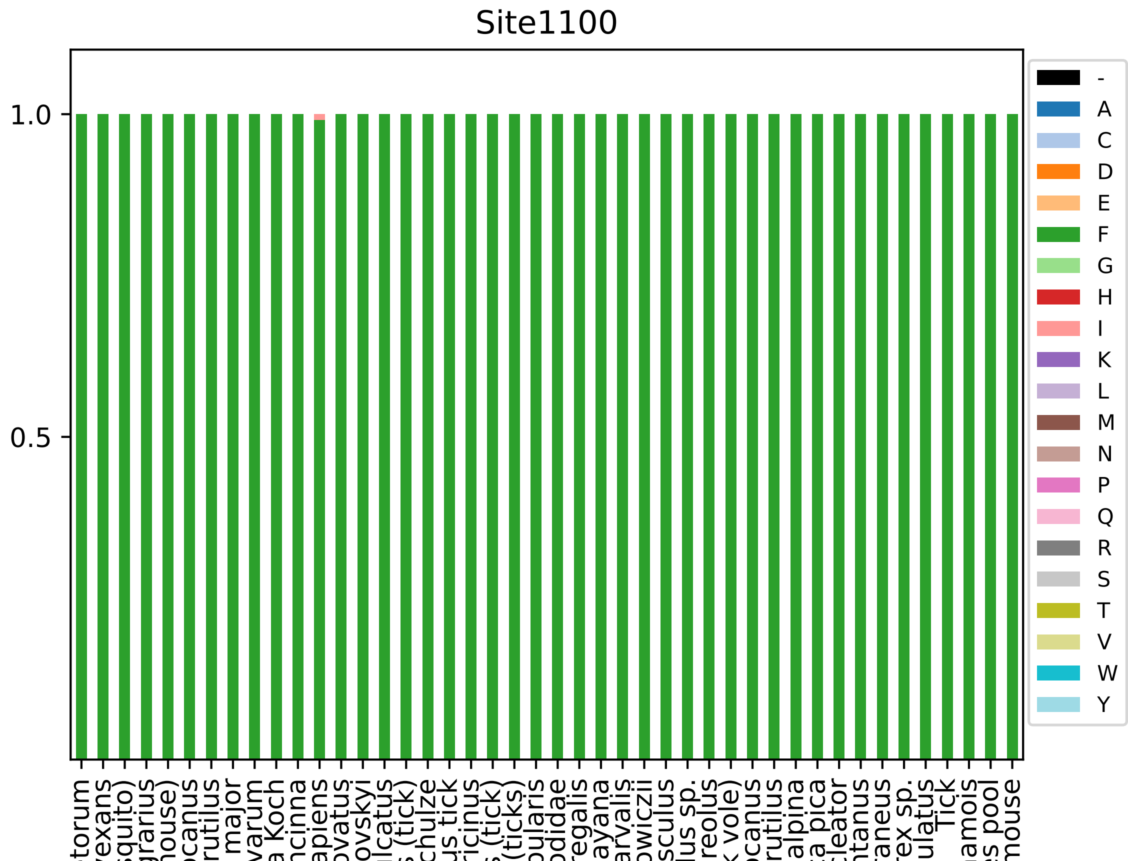
Site1100
1.0
0.5
-
A
C
D
E
F
G
H
I
K
L
M
N
P
Q
R
S
T
V
W
Y

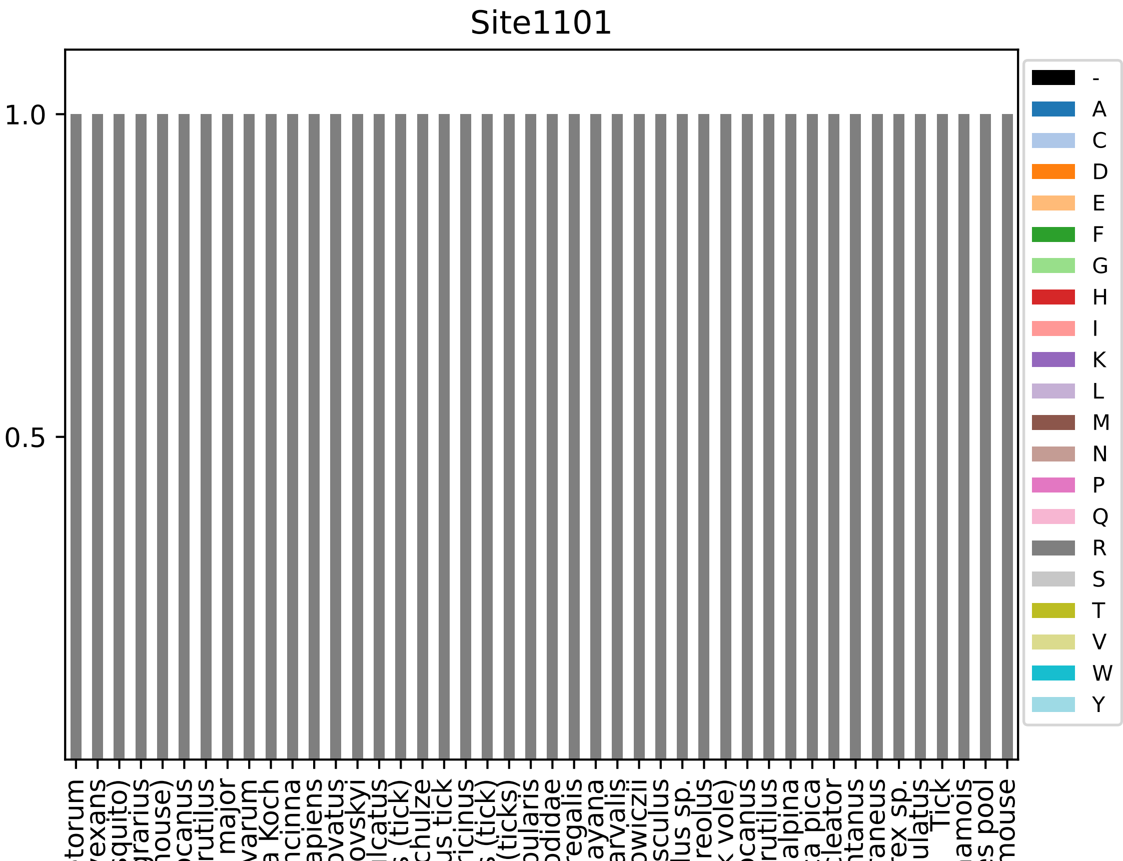

Site1101
1.0
0.5
-
A
C
D
E
F
G
H
I
K
L
M
N
P
Q
R
S
T
V
W
Y

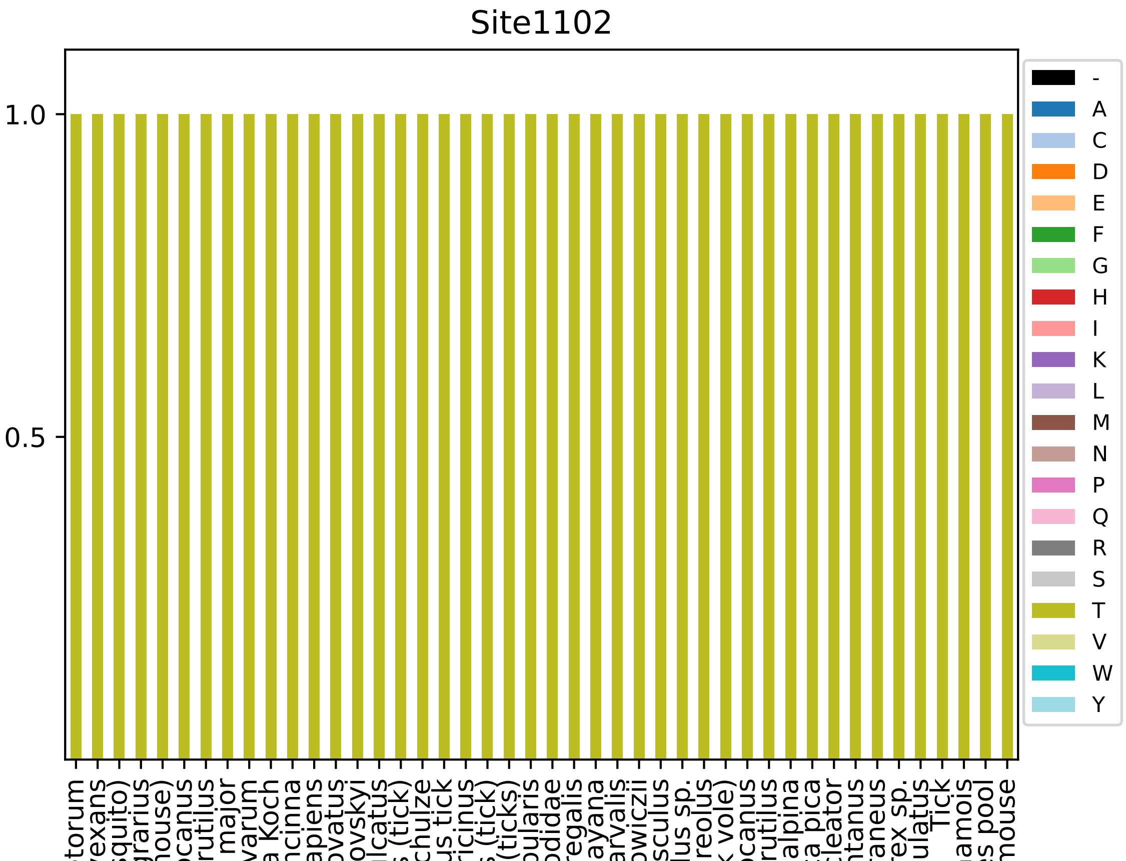
Site1102
1.0
0.5
-
A
C
D
E
F
G
H
I
K
L
M
N
P
Q
R
S
T
V
W
Y
torum
vexans
squito)
grarius
nouse)
ocanus
rutilus
major
varum
a Koch
ncinna
apiens
ovatus
ovskyi
ulcatus
s (tick)
chulze
us tick
ricinus
s (tick)
(ticks)
pularis
odidae
regalis
ayana
arvalis
owiczii
sculus
lus sp.
reolus
x vole)
ocanus
rutilus
alpina
a pica
cleator
ntanus
raneus
rex sp.
ulatus
Tick
amois
es pool
mouse

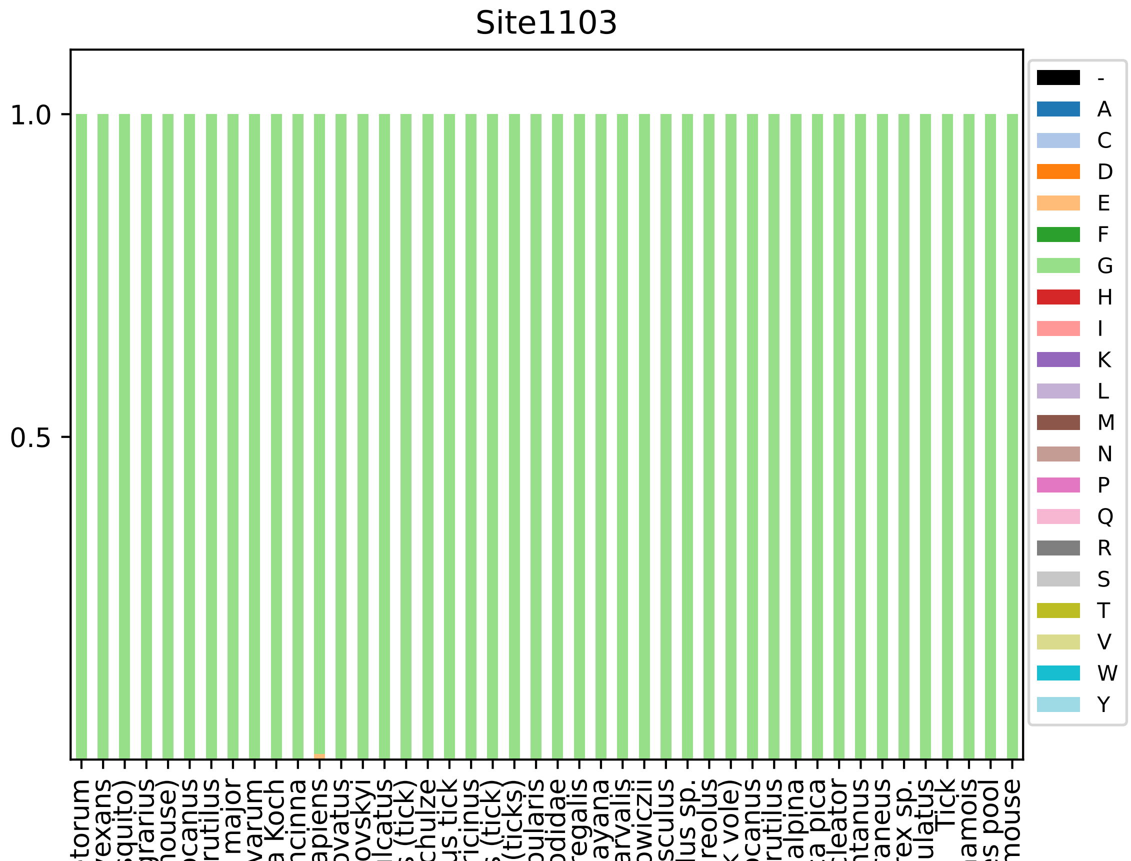
Site1103
1.0
0.5
-
A
C
D
E
F
G
H
I
K
L
M
N
P
Q
R
S
T
V
W
Y

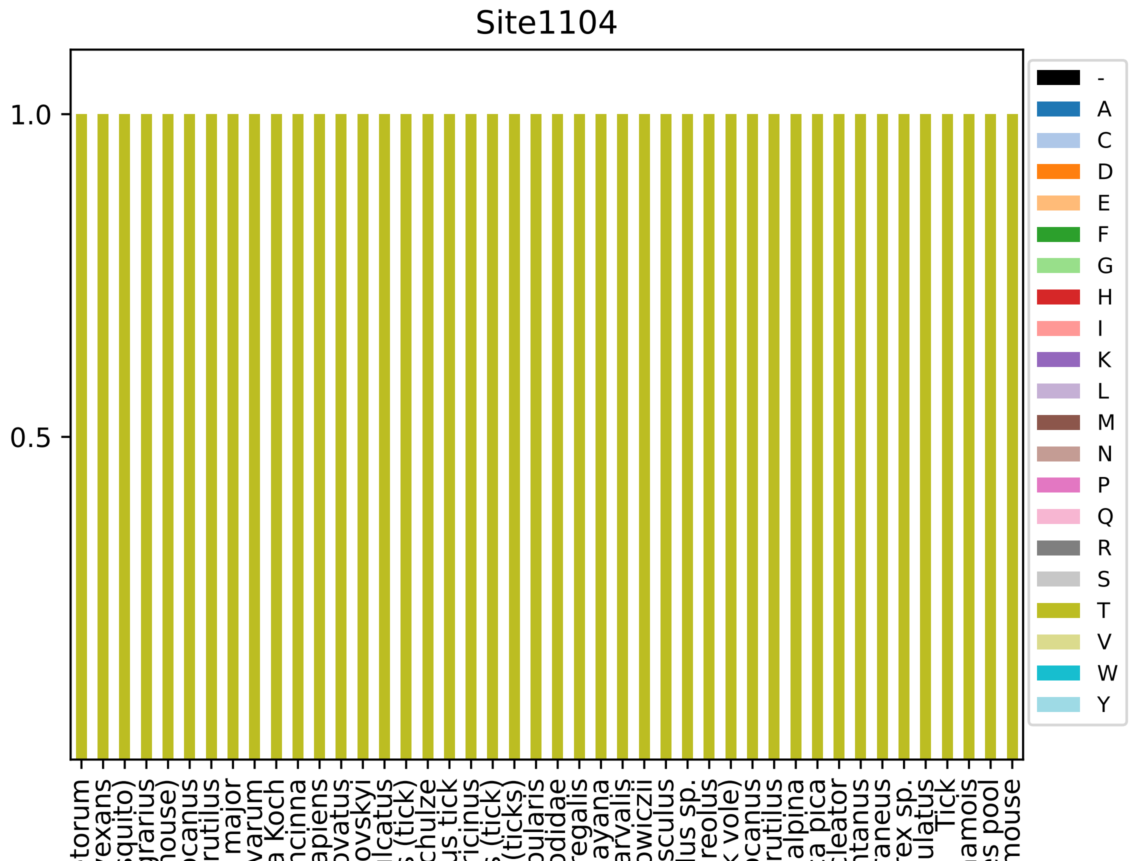

Site1104
1.0
0.5
-
A
C
D
E
F
G
H
I
K
L
M
N
P
Q
R
S
T
V
W
Y

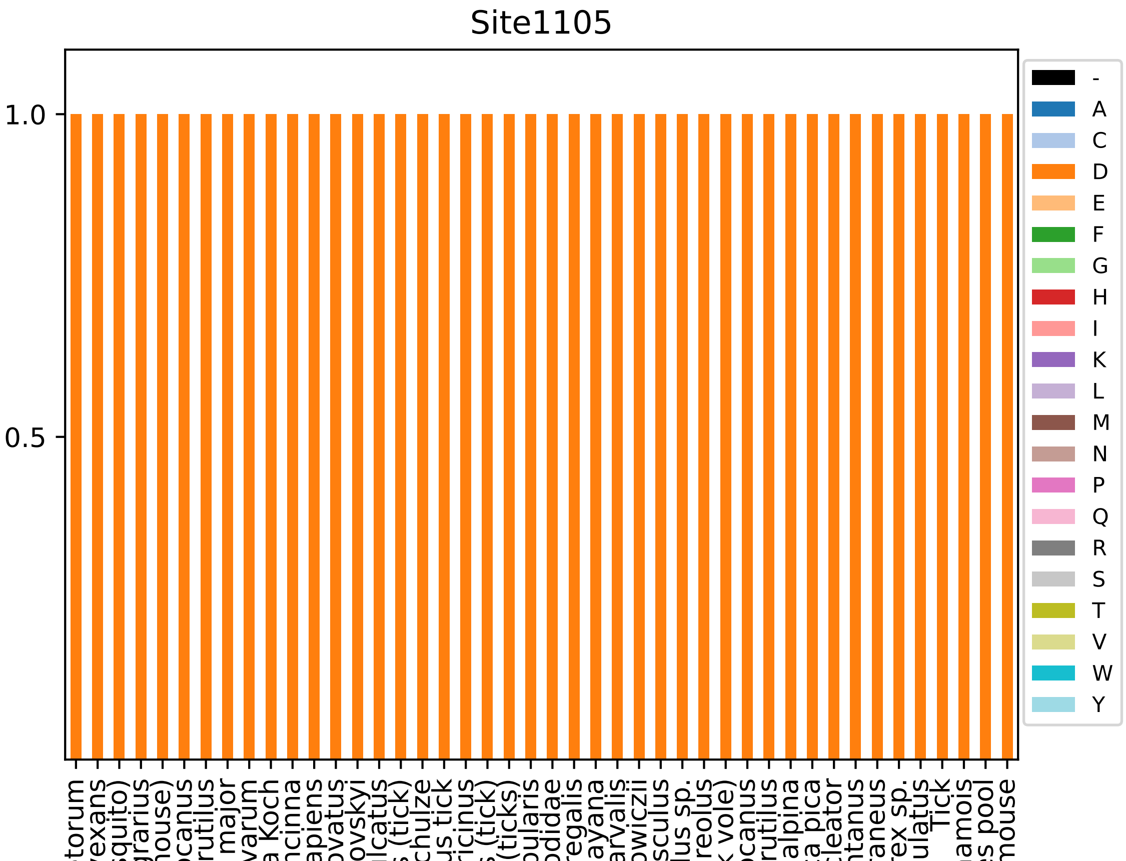
Site1105
1.0
0.5
-
A
C
D
E
F
G
H
I
K
L
M
N
P
Q
R
S
T
V
W
Y

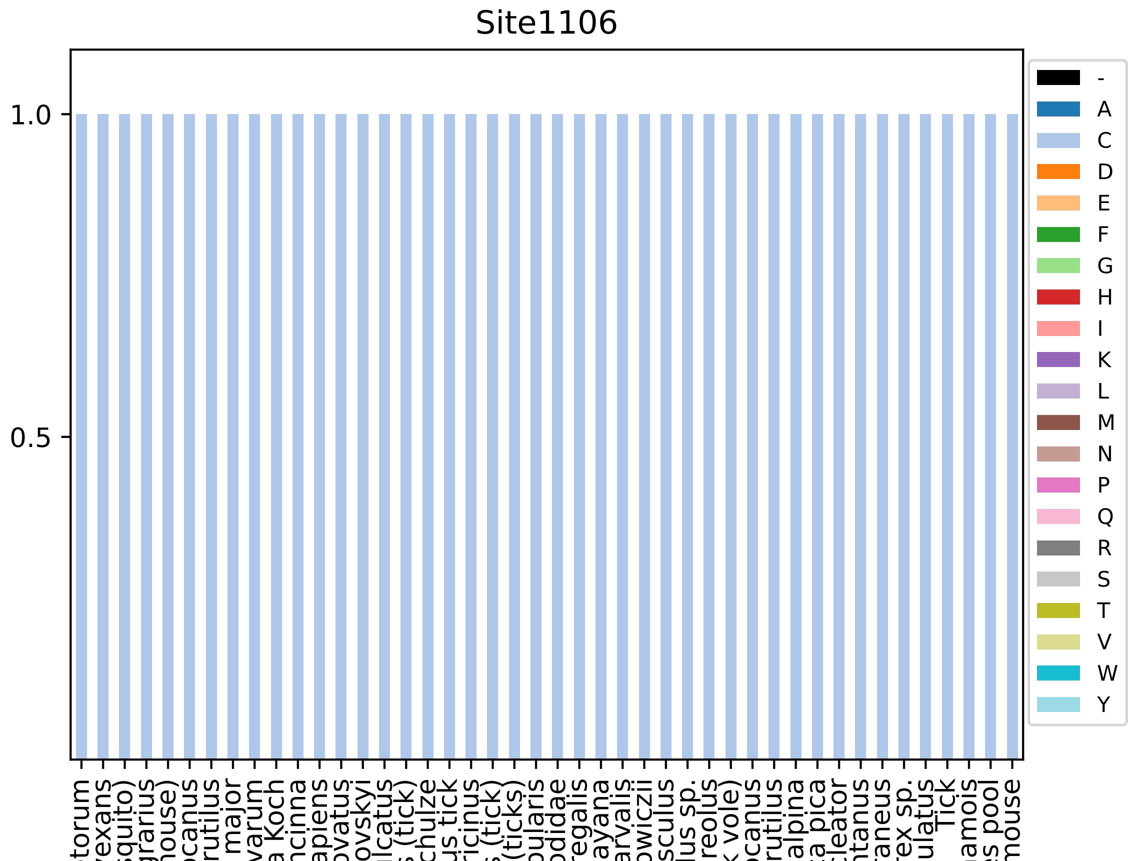

Site1106
1.0
0.5
-
A
C
D
E
F
G
H
I
K
L
M
N
P
Q
R
S
T
V
W
Y

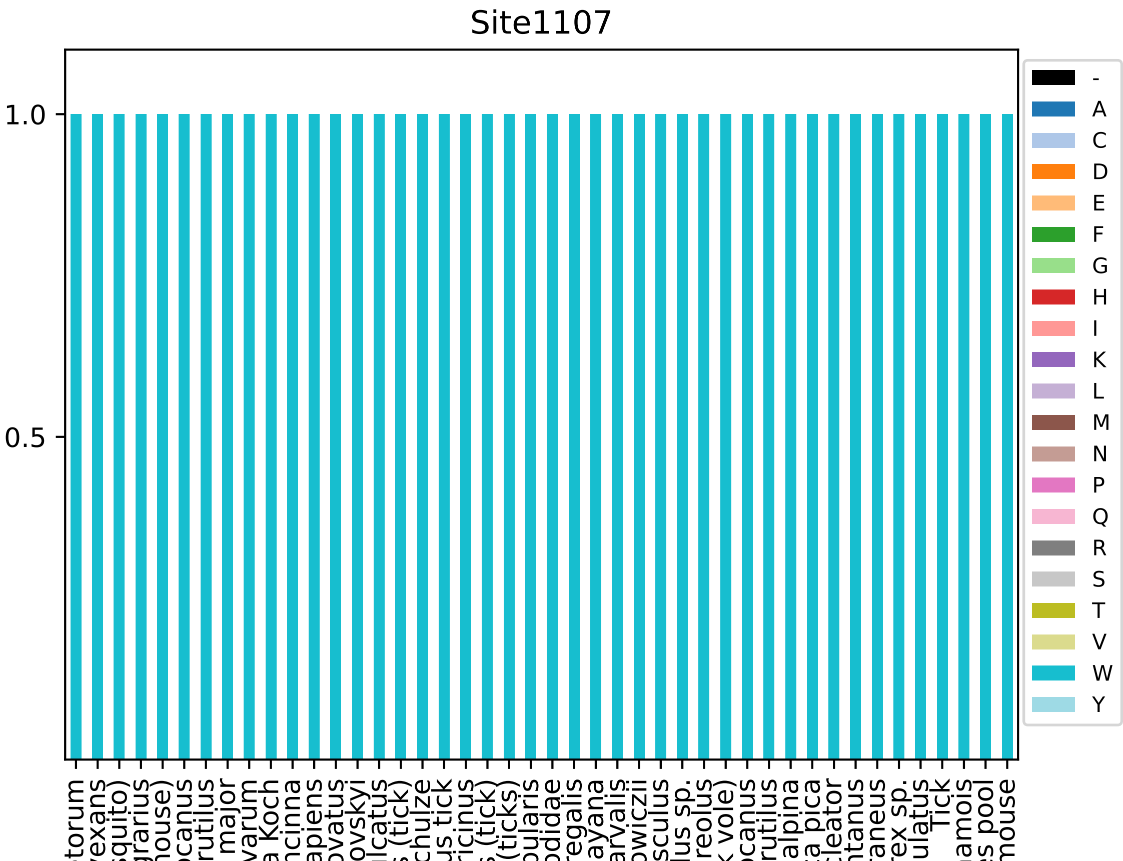

Site1107
1.0
0.5
-
A
C
D
E
F
G
H
I
K
L
M
N
P
Q
R
S
T
V
W
Y

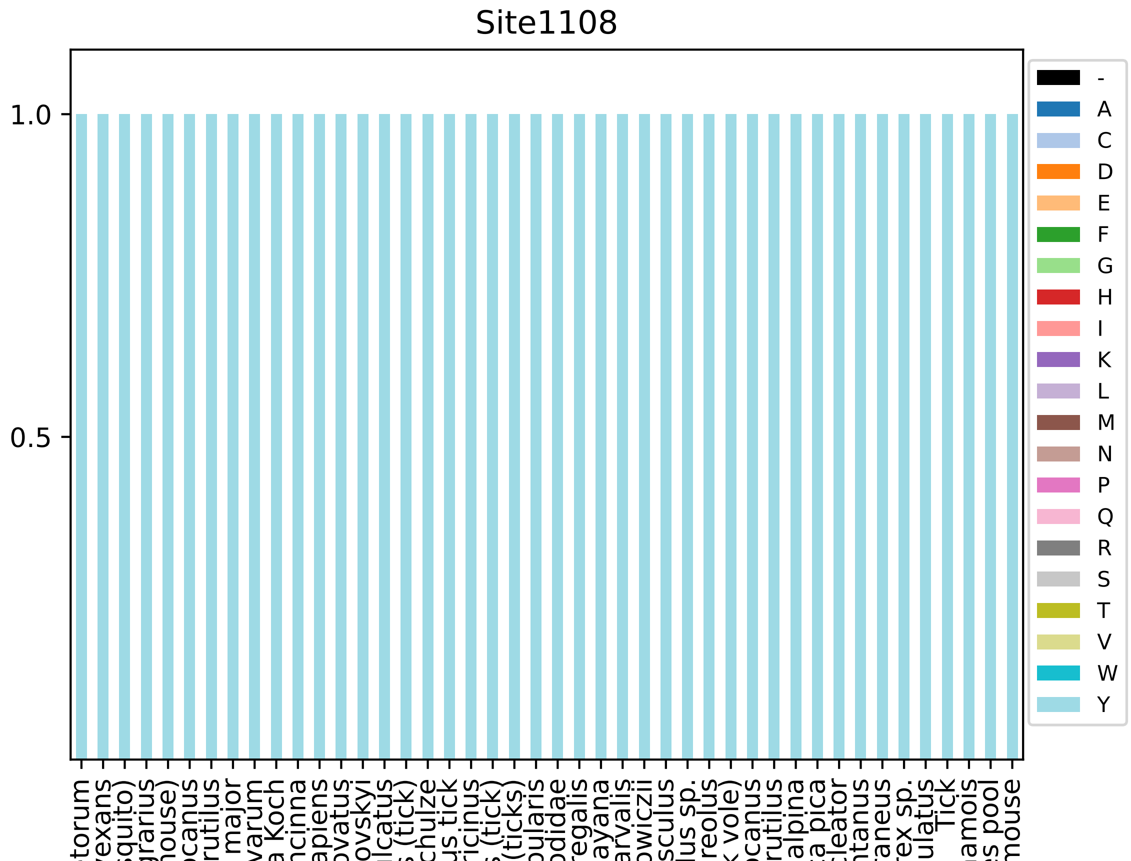
Site1108
1.0
0.5
-
A
C
D
E
F
G
H
I
K
L
M
N
P
Q
R
S
T
V
W
Y

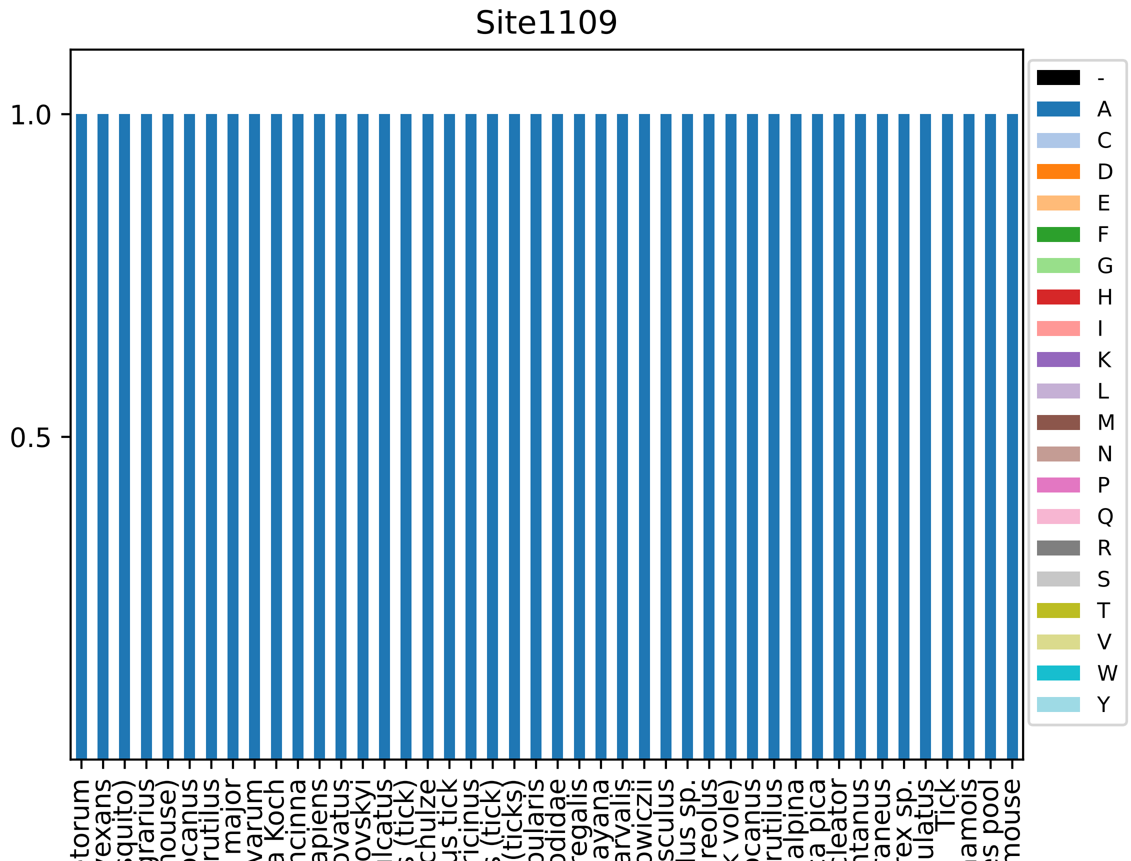
Site1109
1.0
0.5
-
A
C
D
E
F
G
H
I
K
L
M
N
P
Q
R
S
T
V
W
Y
Tick

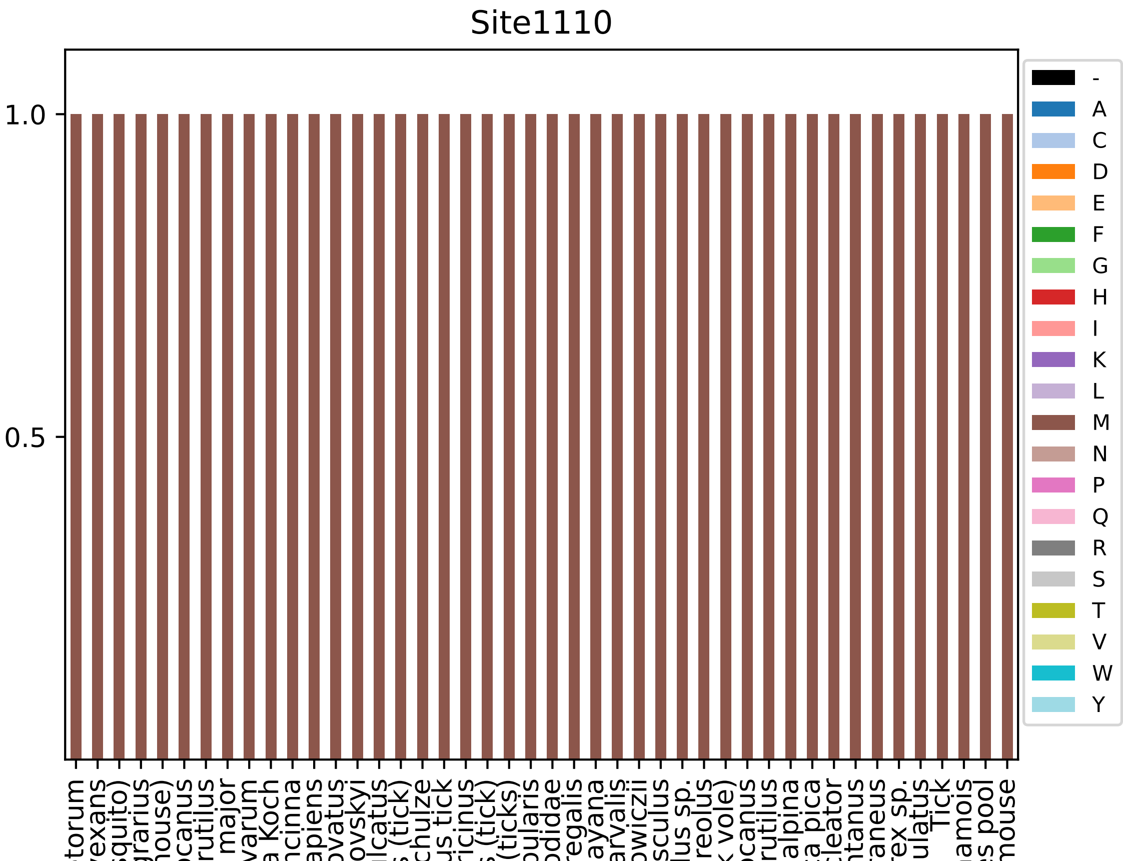
Site1110
1.0
0.5
-
A
C
D
E
F
G
H
I
K
L
M
N
P
Q
R
S
T
V
W
Y

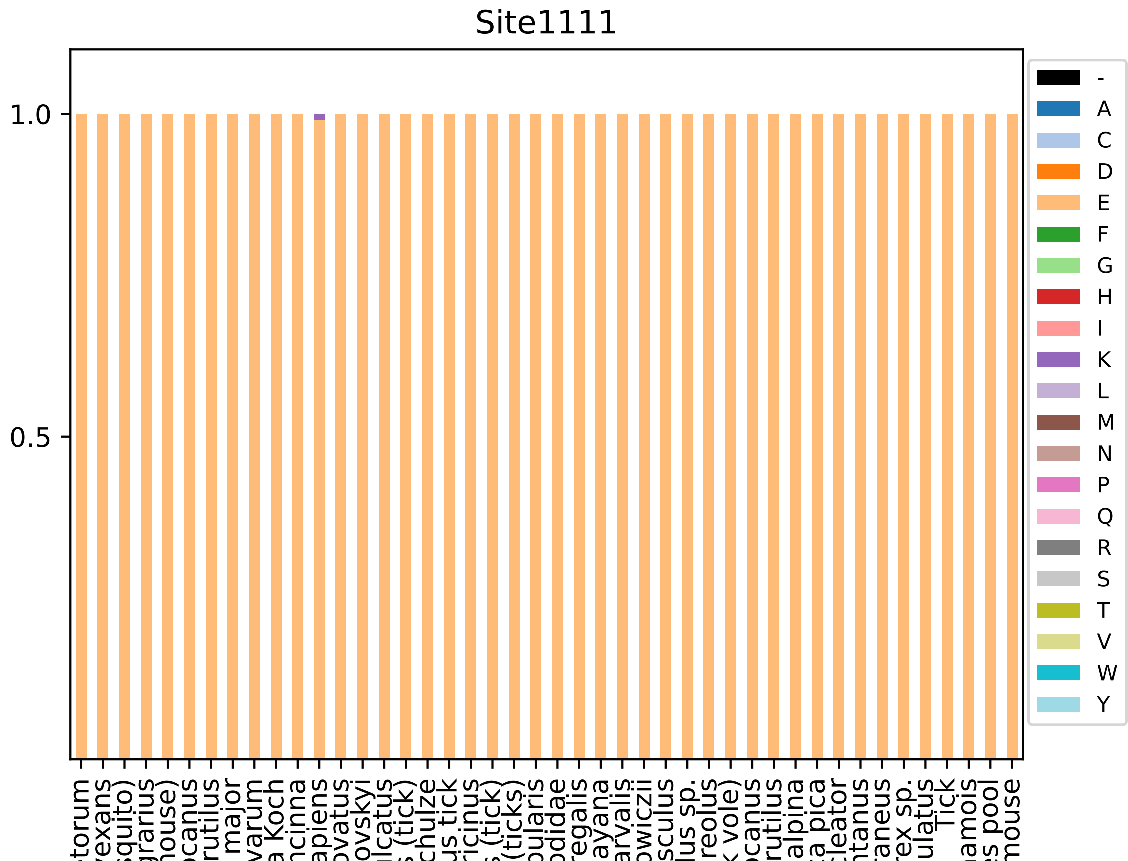
Site1111
1.0
0.5
-
A
C
D
E
F
G
H
I
K
L
M
N
P
Q
R
S
T
V
W
Y

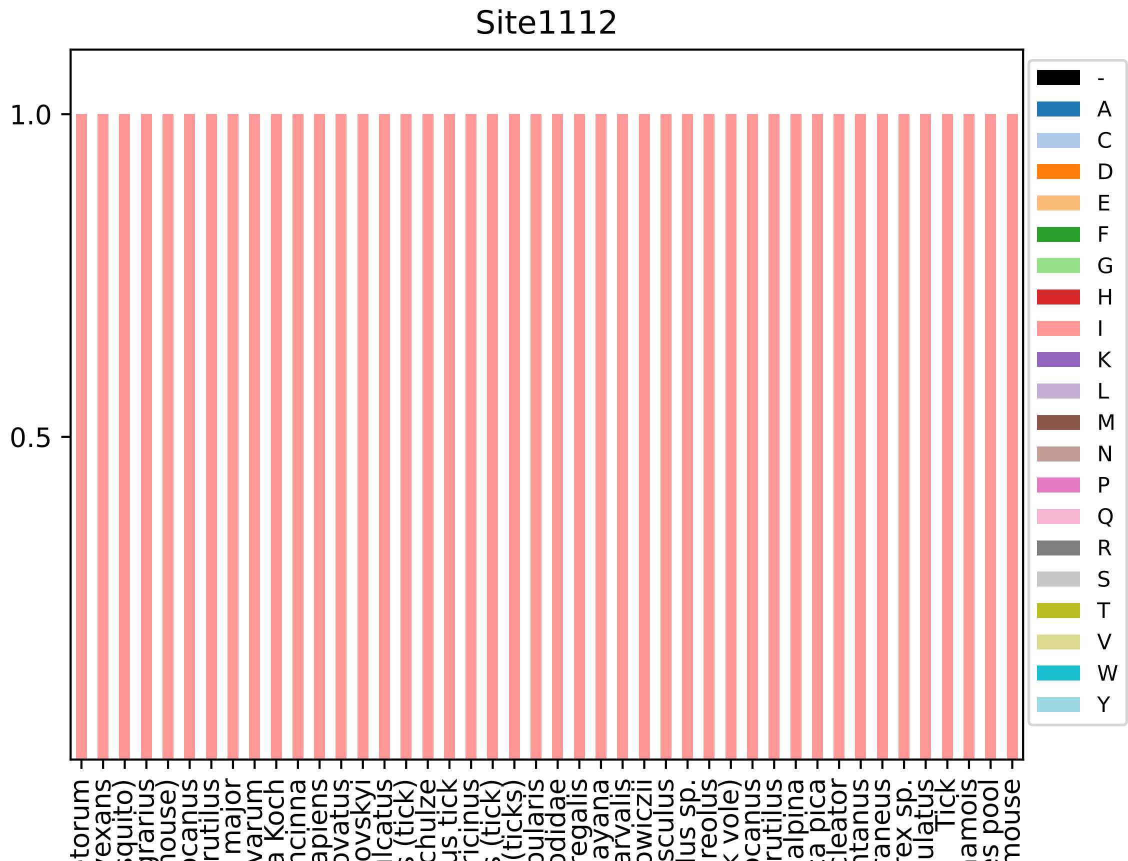
Site1112
1.0
0.5
-
A
C
D
E
F
G
H
I
K
L
M
N
P
Q
R
S
T
V
W
Y
torum
vexans
squito)
garius
nouse)
pcanus
rutilus
major
varum
a Koch
ncinna
apiens
ovatus
ovskyi
ulcatus
s (tick)
chulze
us tick
ricinus
s (tick)
(ticks)
pularis
podidae
regalis
ayana
arvalis
owiczii
sculus
lus sp.
reolus
x vole)
pcanus
rutilus
alpina
a pica
cleator
ntanus
raneus
rex sp.
ulatus
Tick
amois
es pool
mouse

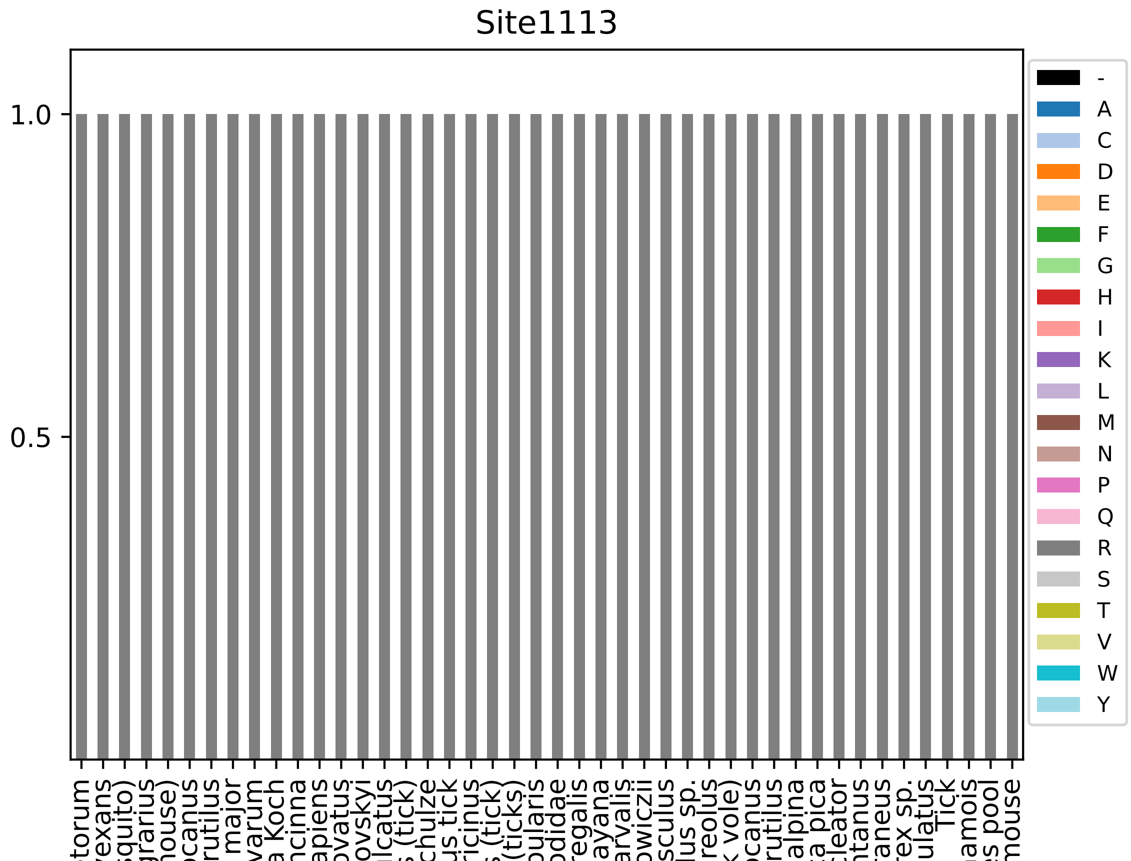
Site1113
1.0
0.5
-
A
C
D
E
F
G
H
I
K
L
M
N
P
Q
R
S
T
V
W
Y

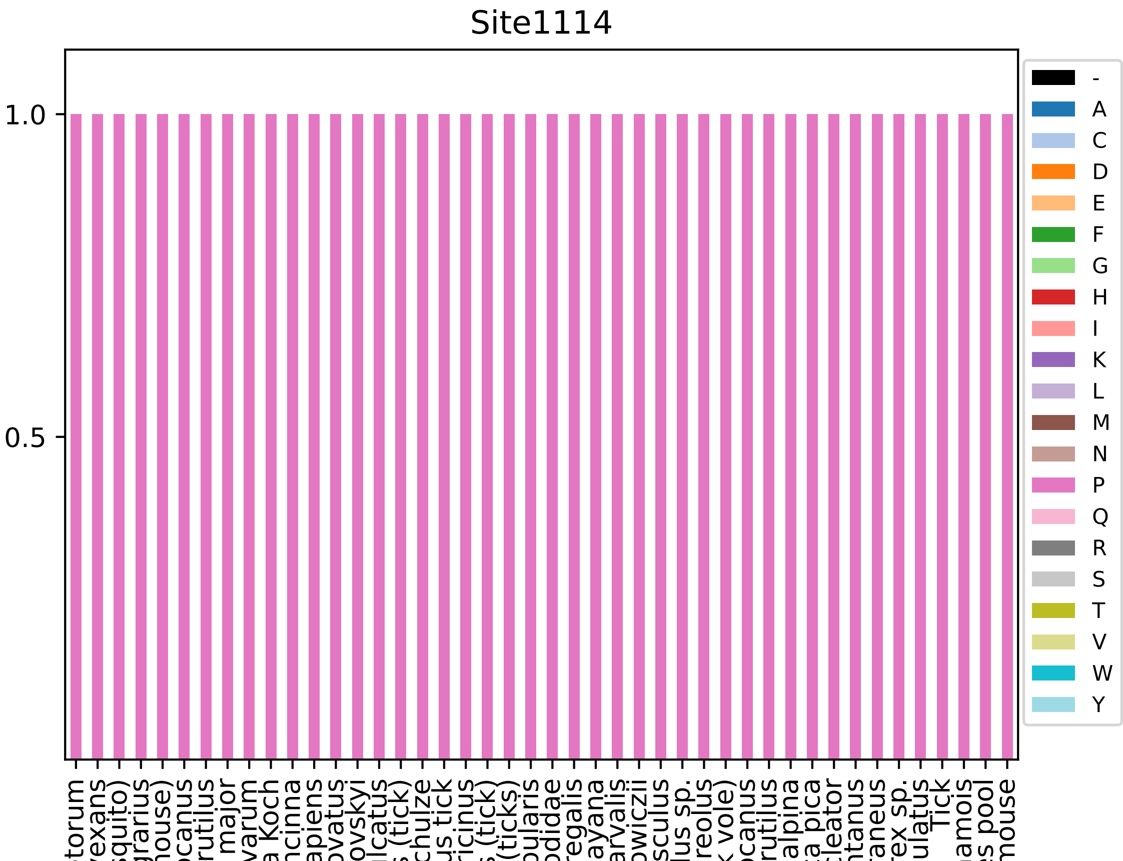
Site1114
1.0
0.5
-
A
C
D
E
F
G
H
I
K
L
M
N
P
Q
R
S
T
V
W
Y

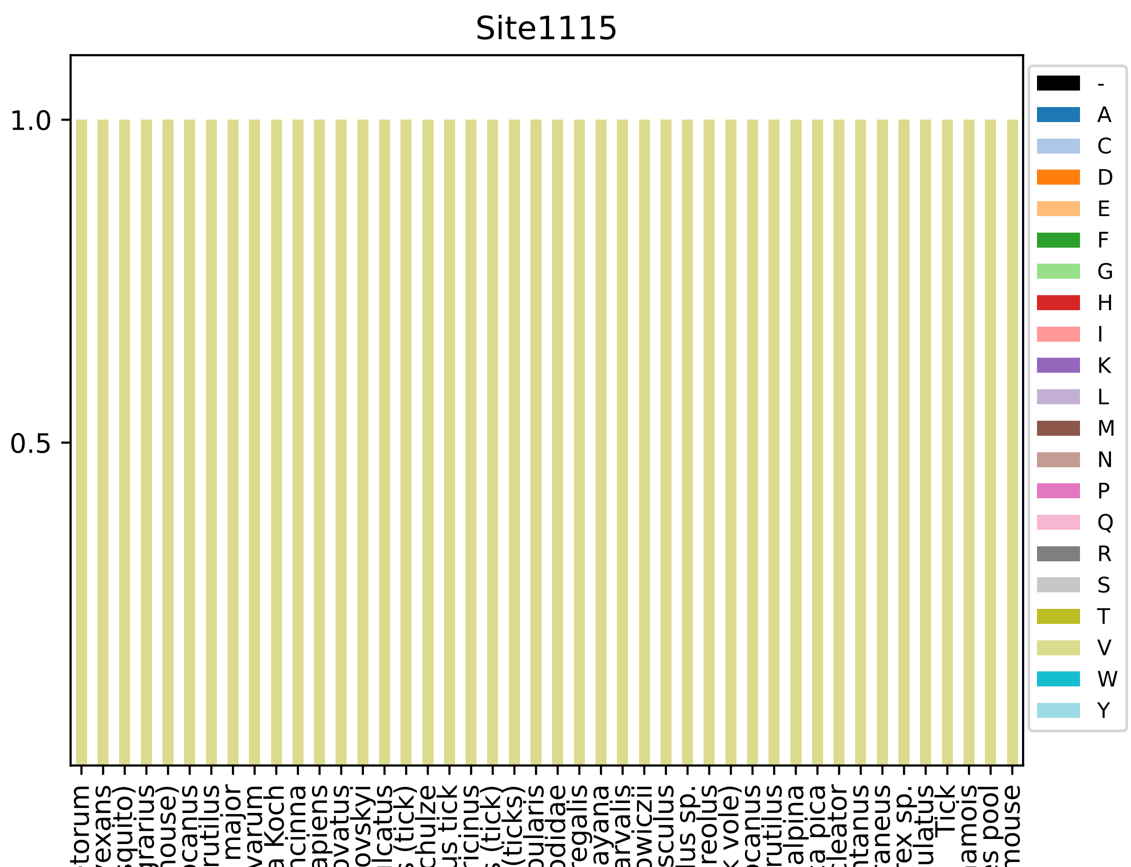

Site1115
1.0
0.5
-
A
C
D
E
F
G
H
I
K
L
M
N
P
Q
R
S
T
V
W
Y

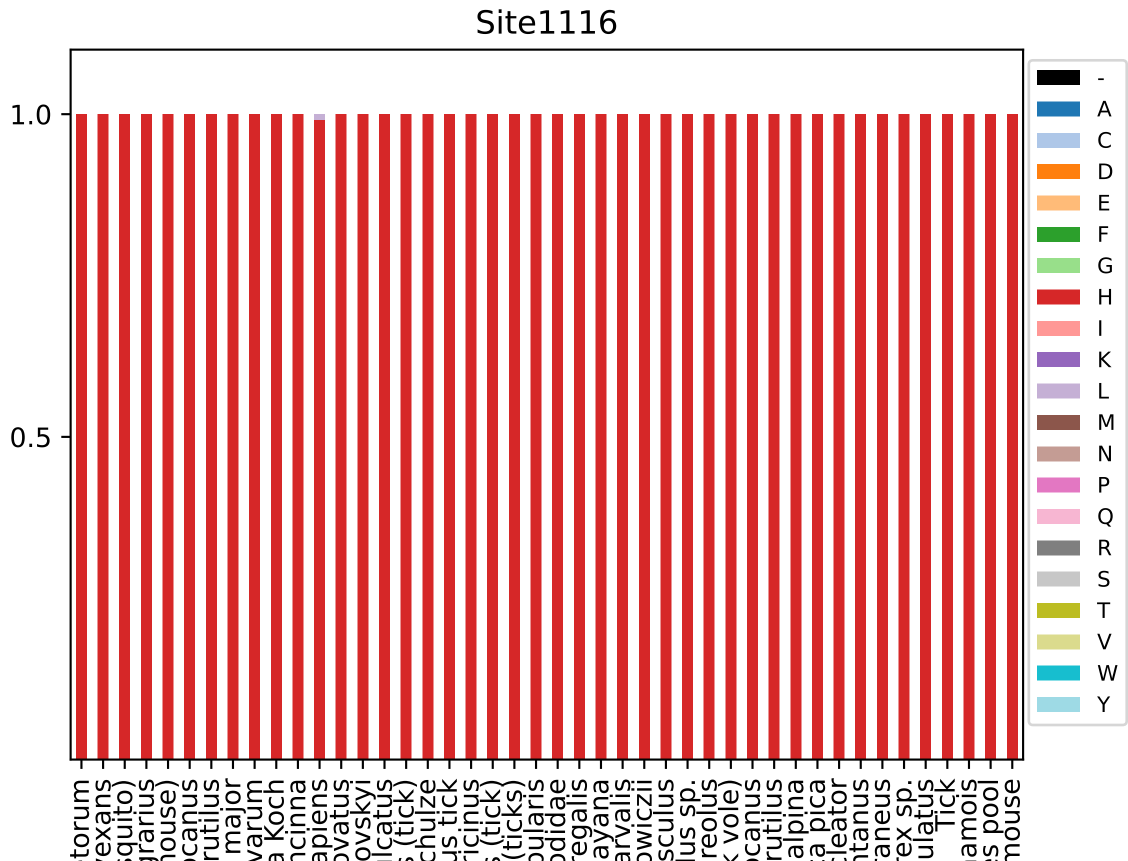
Site1116
1.0
0.5
-
A
C
D
E
F
G
H
I
K
L
M
N
P
Q
R
S
T
V
W
Y

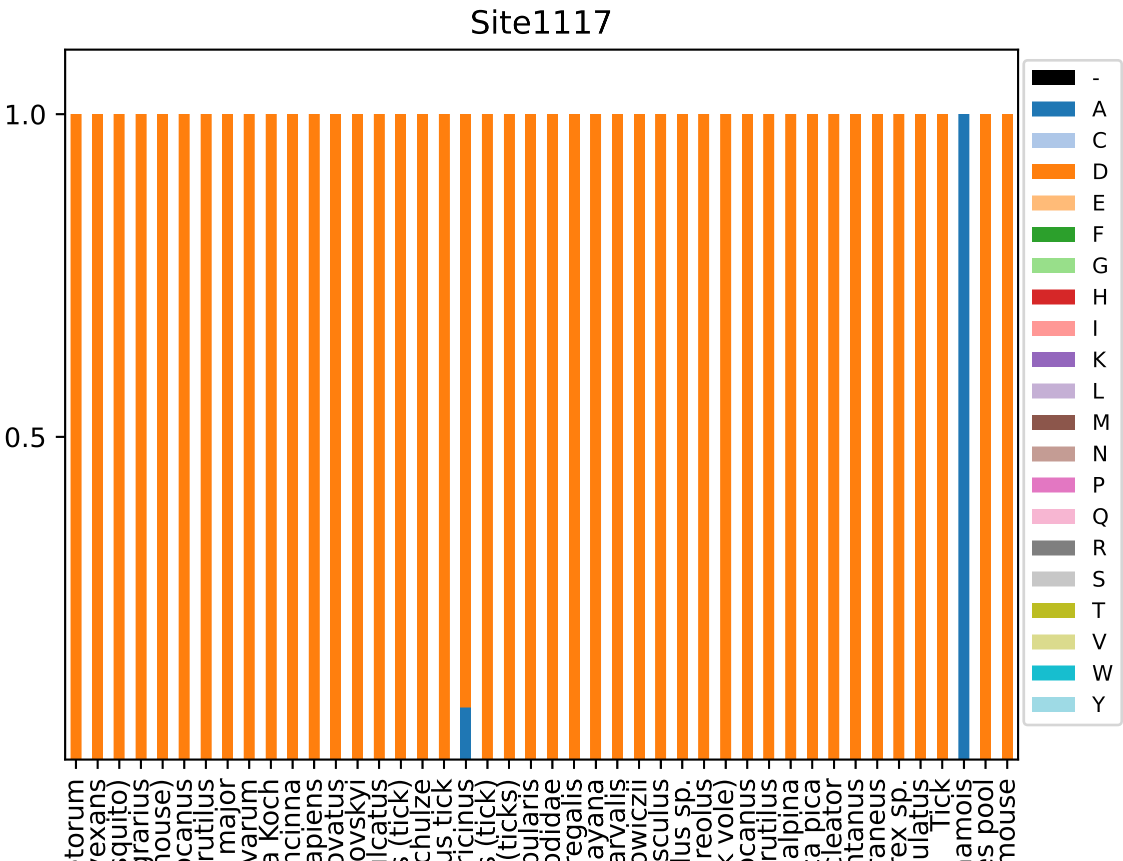
Site1117
1.0
0.5
-
A
C
D
E
F
G
H
I
K
L
M
N
P
Q
R
S
T
V
W
Y

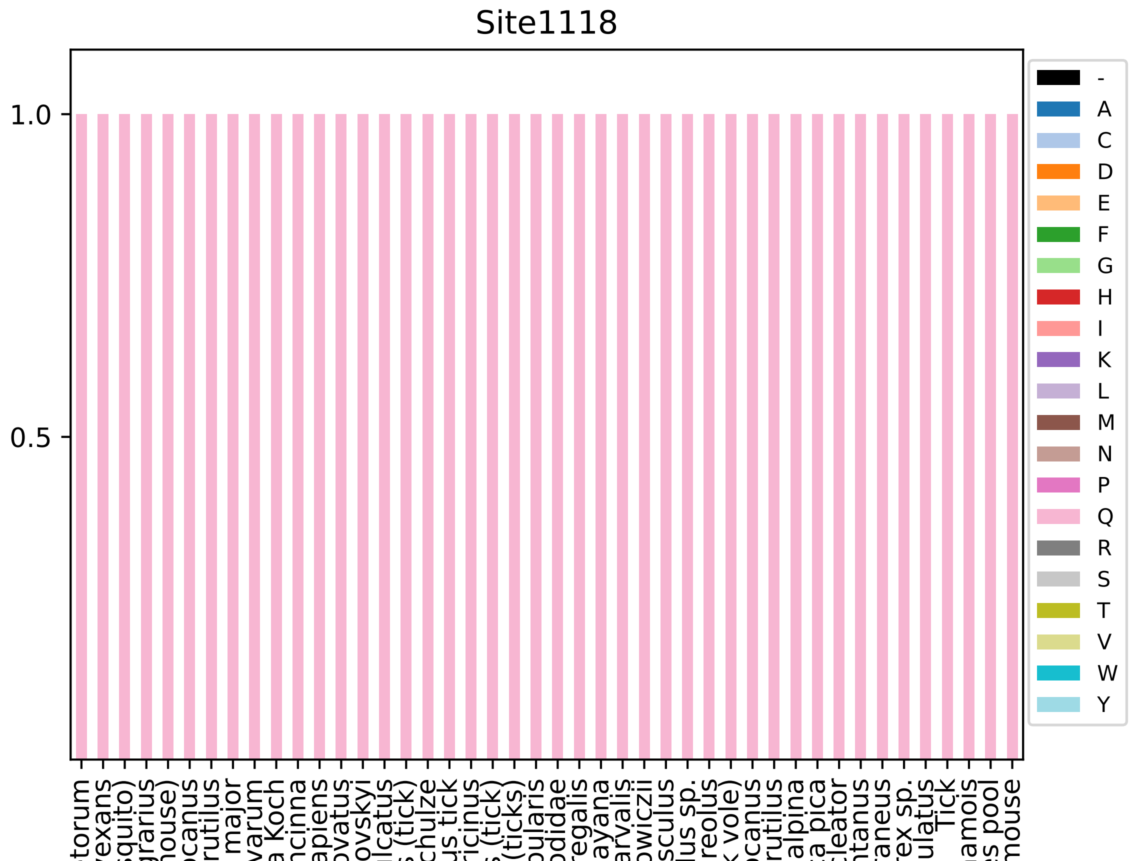

Site1118
1.0
0.5
-
A
C
D
E
F
G
H
I
K
L
M
N
P
Q
R
S
T
V
W
Y

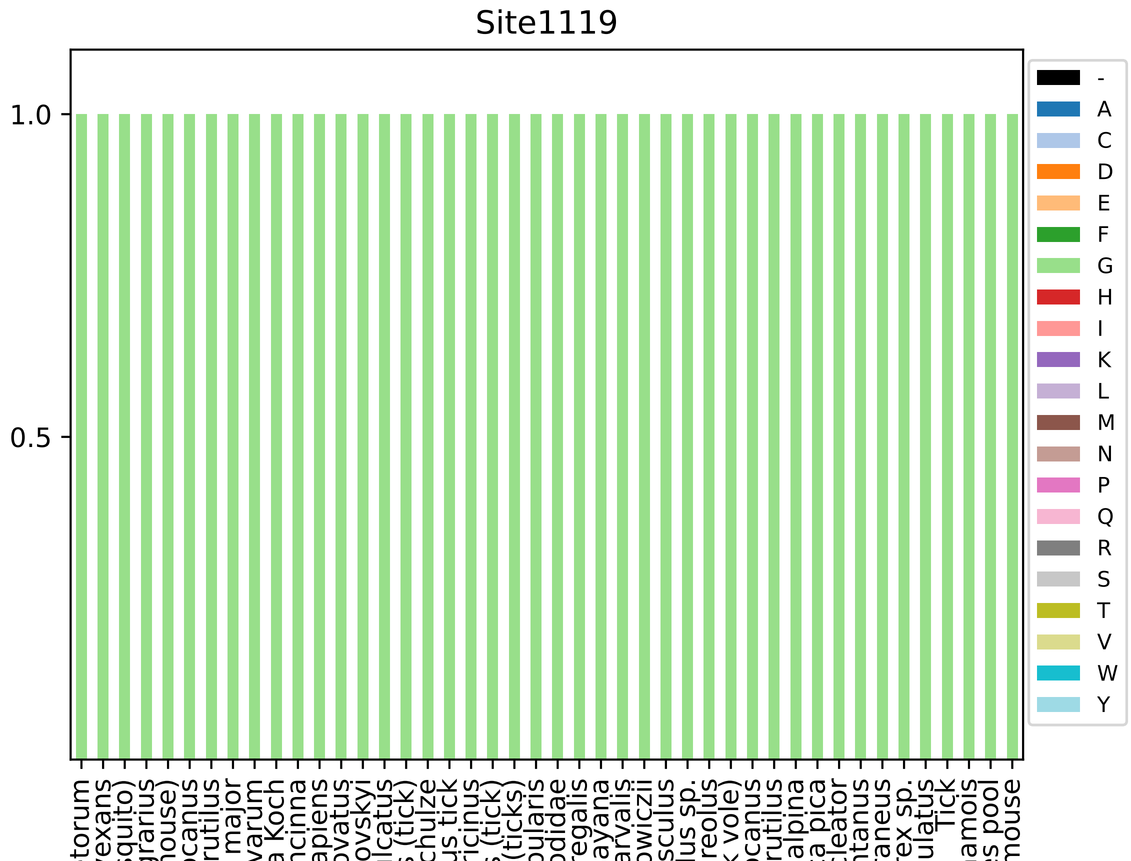
Site1119
1.0
0.5
-
A
C
D
E
F
G
H
I
K
L
M
N
P
Q
R
S
T
V
W
Y

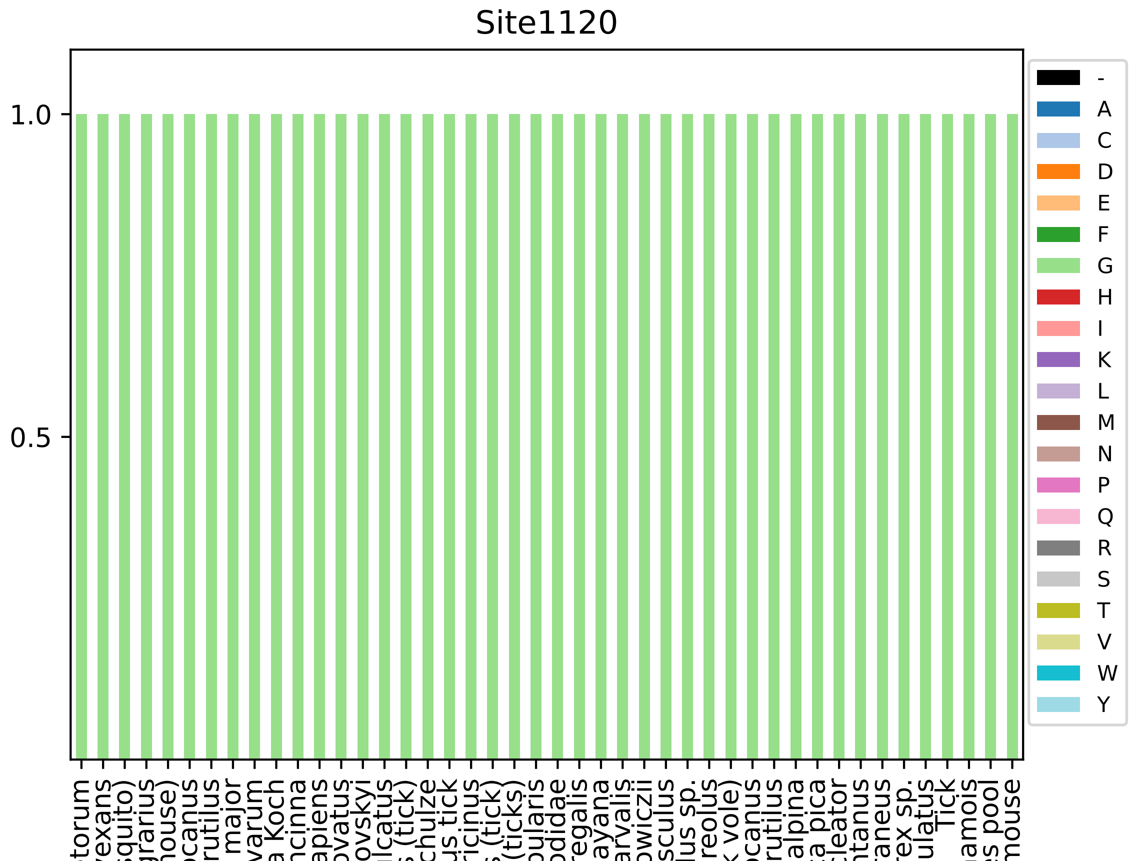
Site1120
1.0
0.5
-
A
C
D
E
F
G
H
I
K
L
M
N
P
Q
R
S
T
V
W
Y

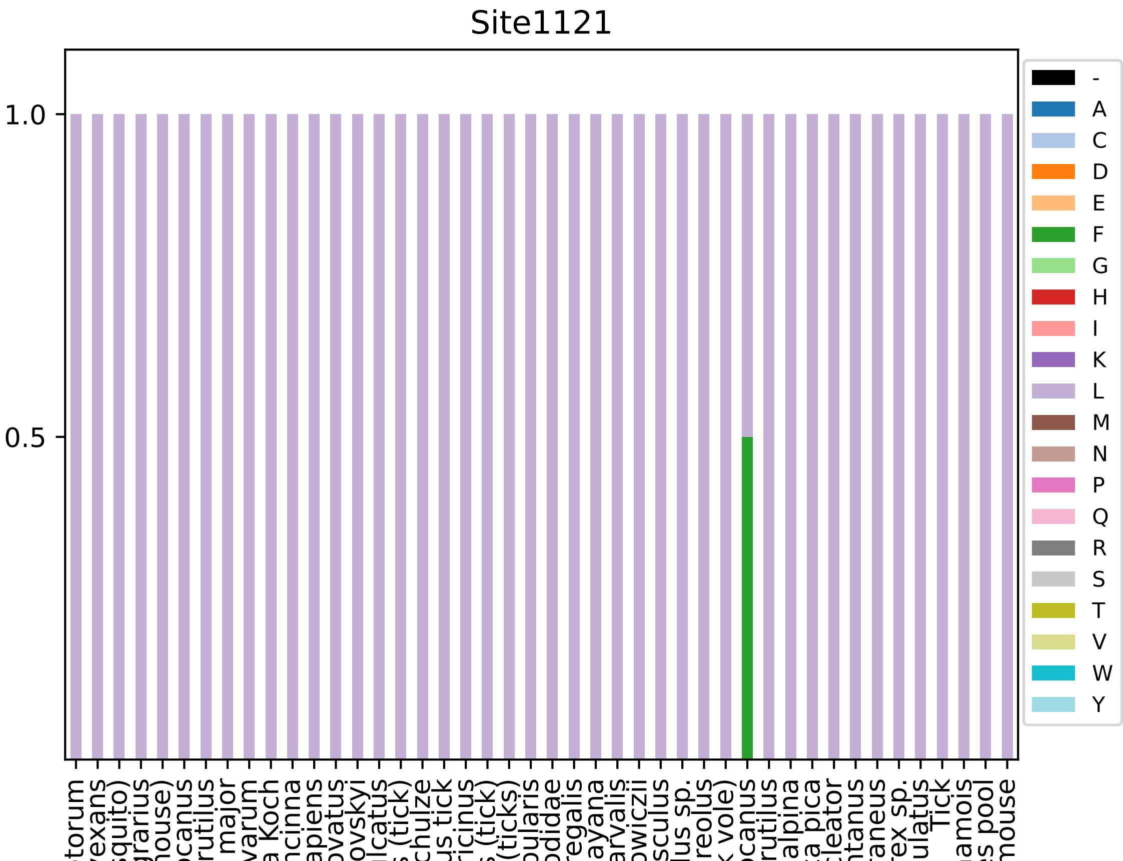
Site1121
1.0
0.5
-
A
C
D
E
F
G
H
I
K
L
M
N
P
Q
R
S
T
V
W
Y

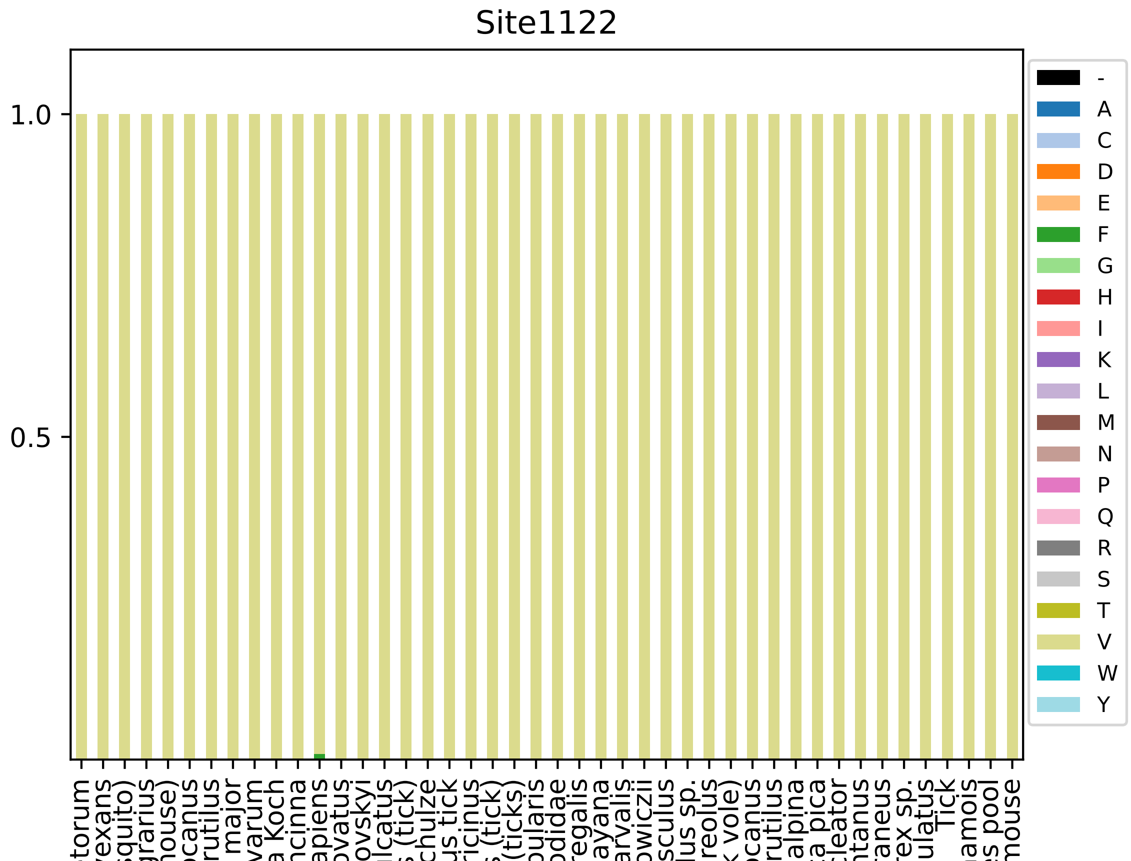

Site1122
1.0
0.5
-
A
C
D
E
F
G
H
I
K
L
M
N
P
Q
R
S
T
V
W
Y

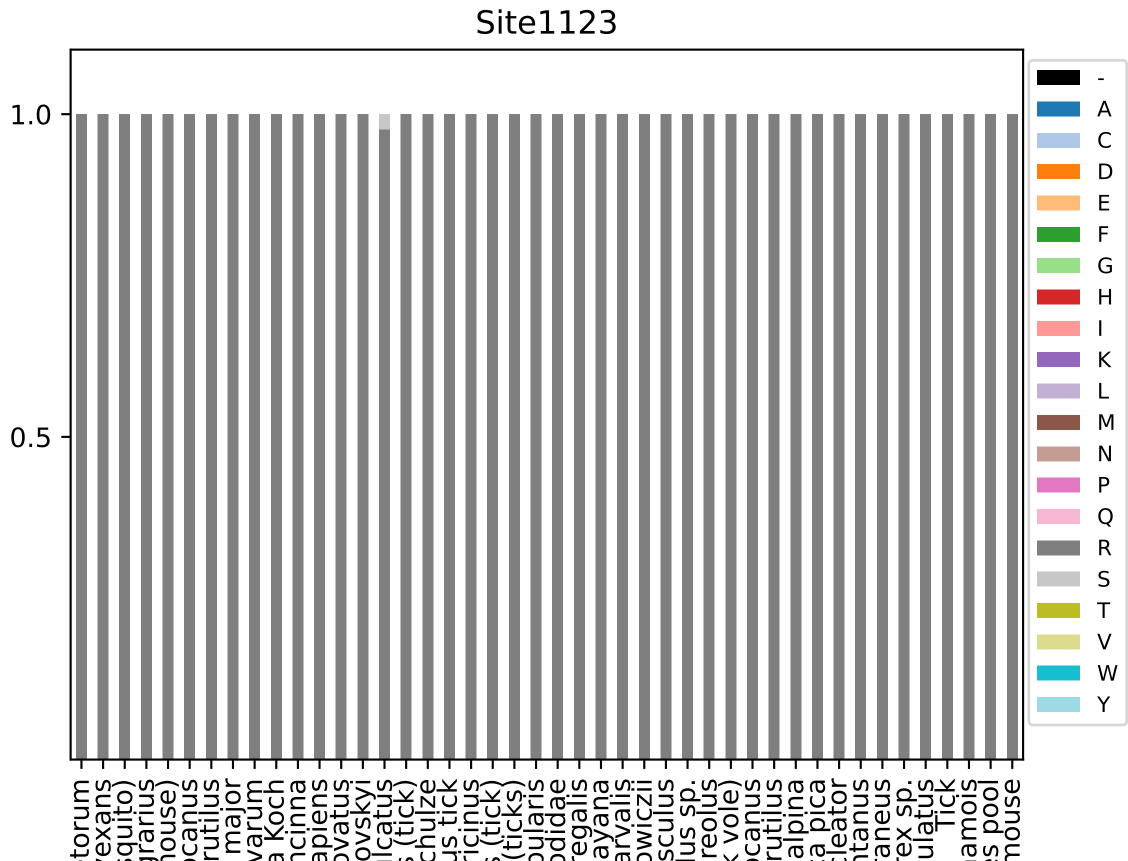
Site1123
1.0
0.5
-
A
C
D
E
F
G
H
I
K
L
M
N
P
Q
R
S
T
V
W
Y

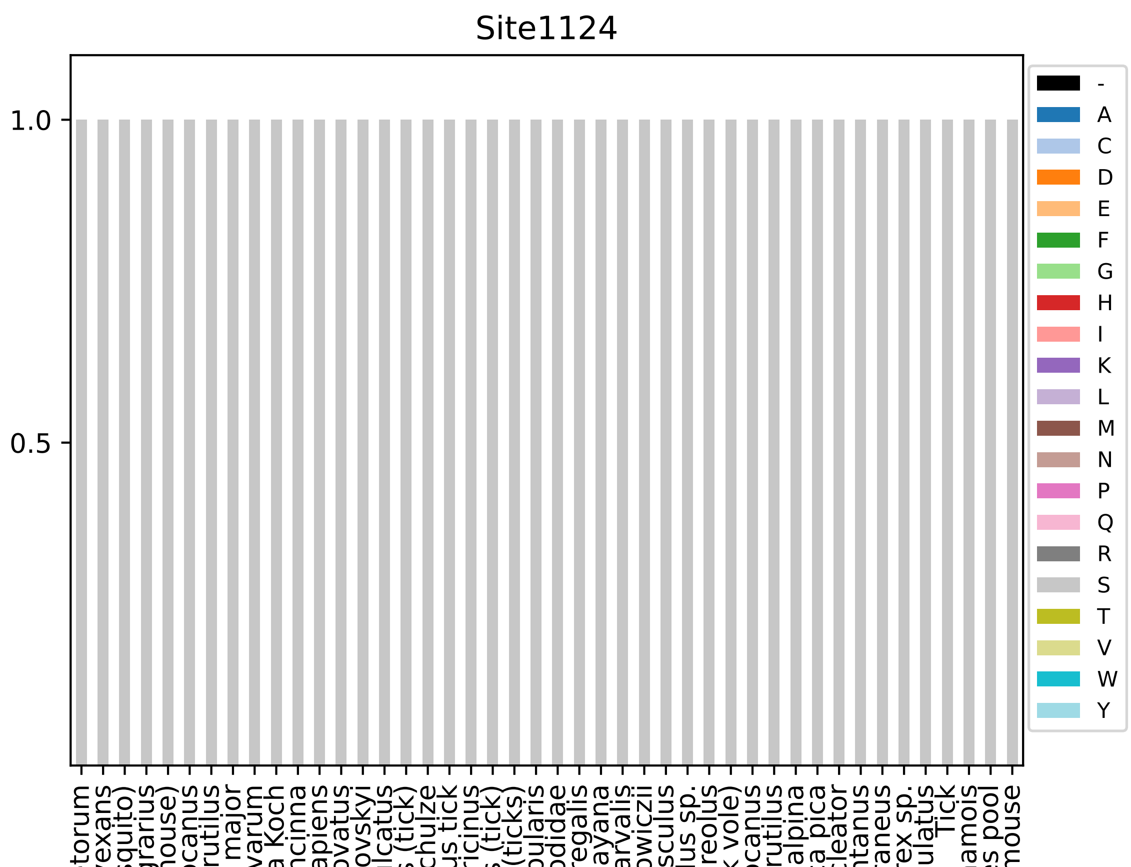
Site1124
1.0
0.5
-
A
C
D
E
F
G
H
I
K
L
M
N
P
Q
R
S
T
V
W
Y

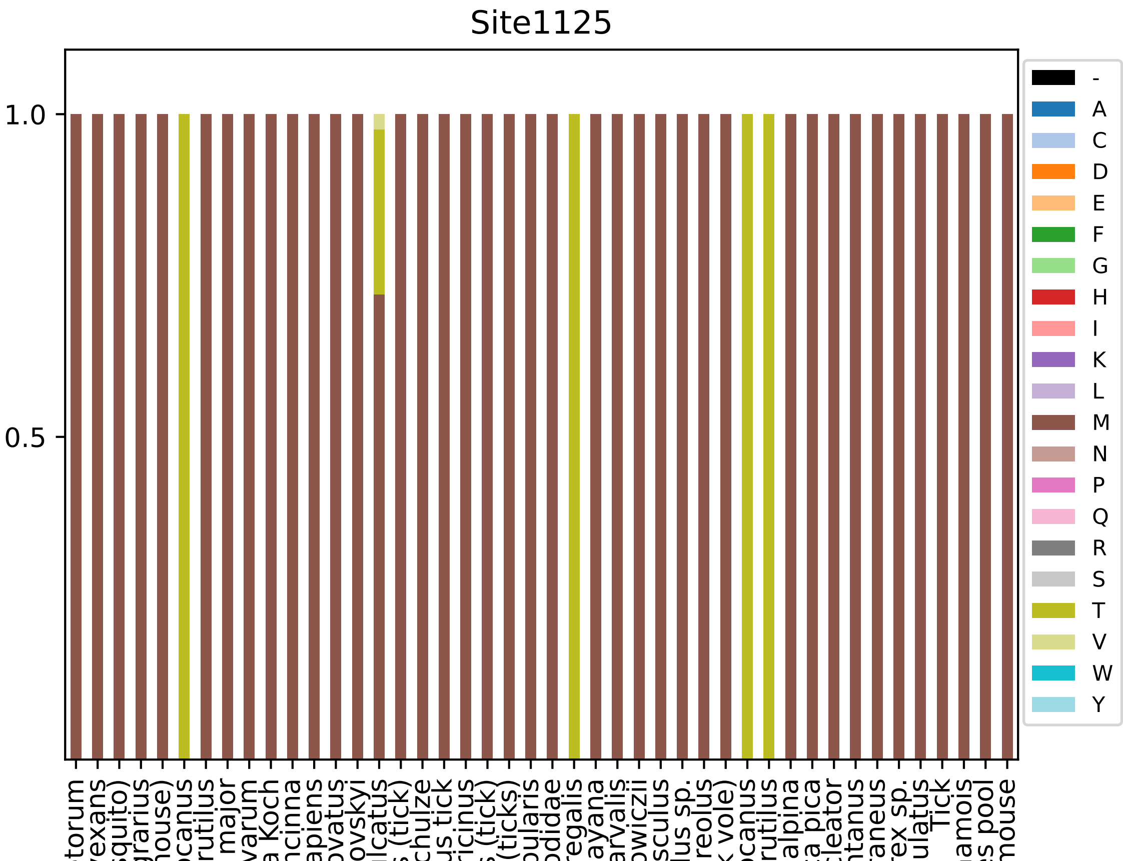

Site1125
1.0
0.5
-
A
C
D
E
F
G
H
I
K
L
M
N
P
Q
R
S
T
V
W
Y

Site1126

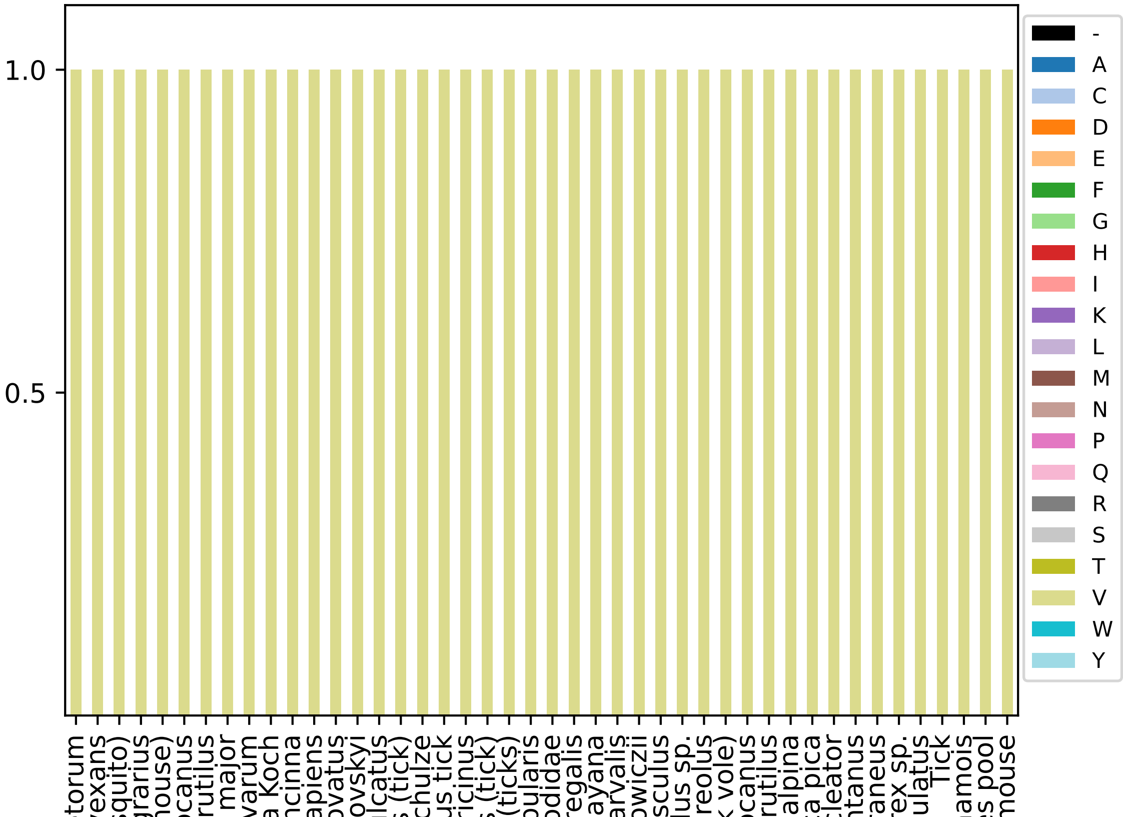

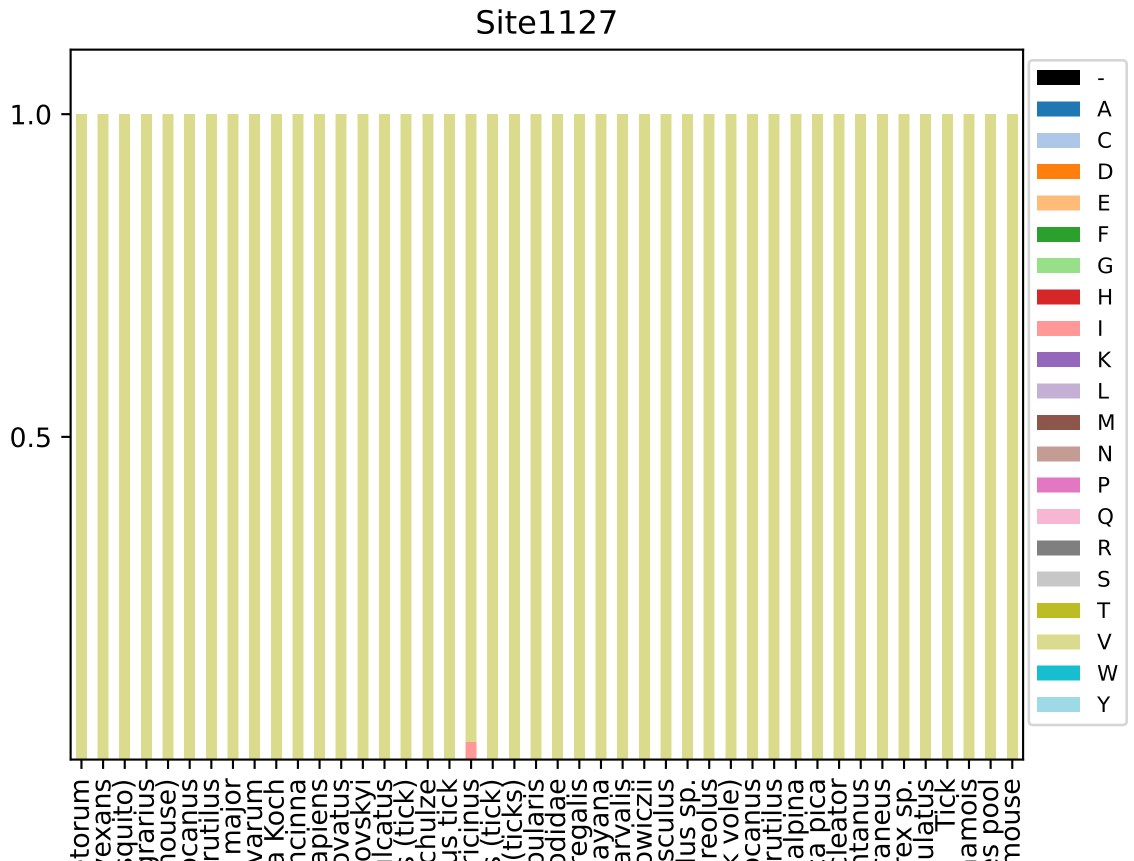
Site1127
1.0
0.5
-
A
C
D
E
F
G
H
I
K
L
M
N
P
Q
R
S
T
V
W
Y
Tick

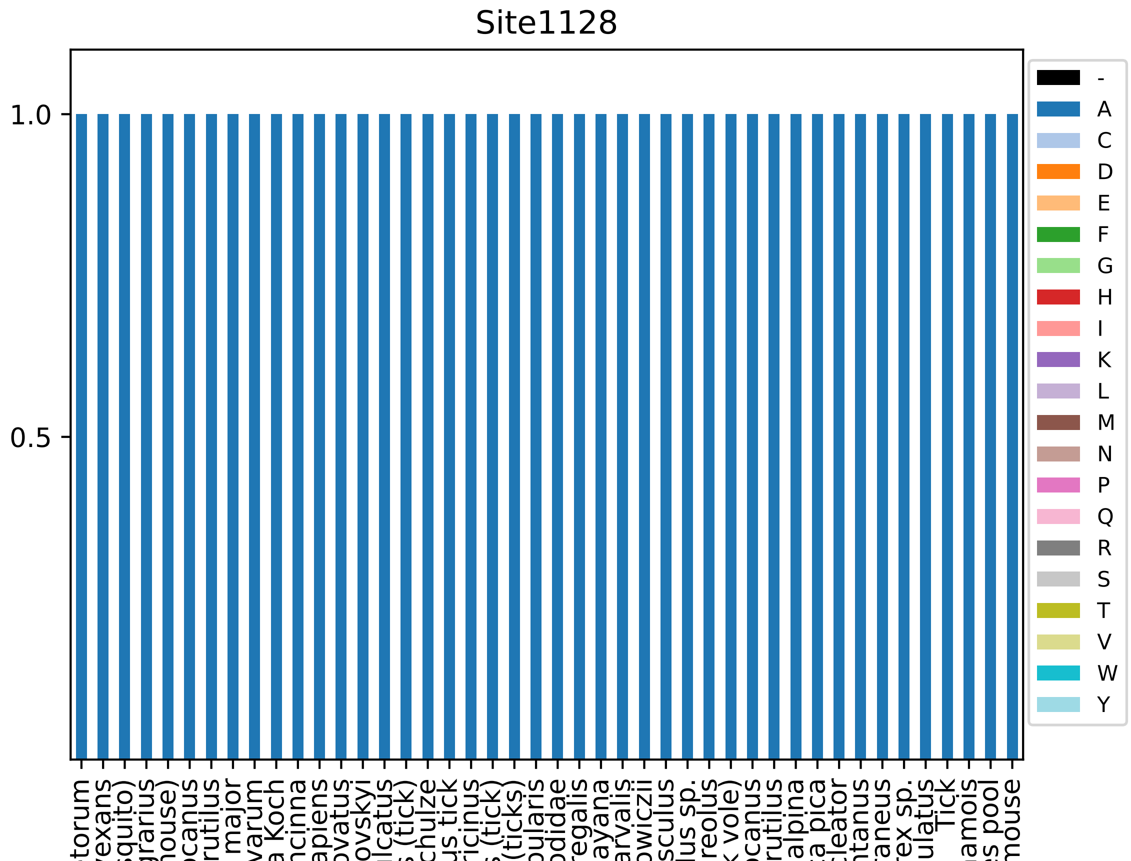
Site1128
1.0
0.5
-
A
C
D
E
F
G
H
I
K
L
M
N
P
Q
R
S
T
V
W
Y
Tick

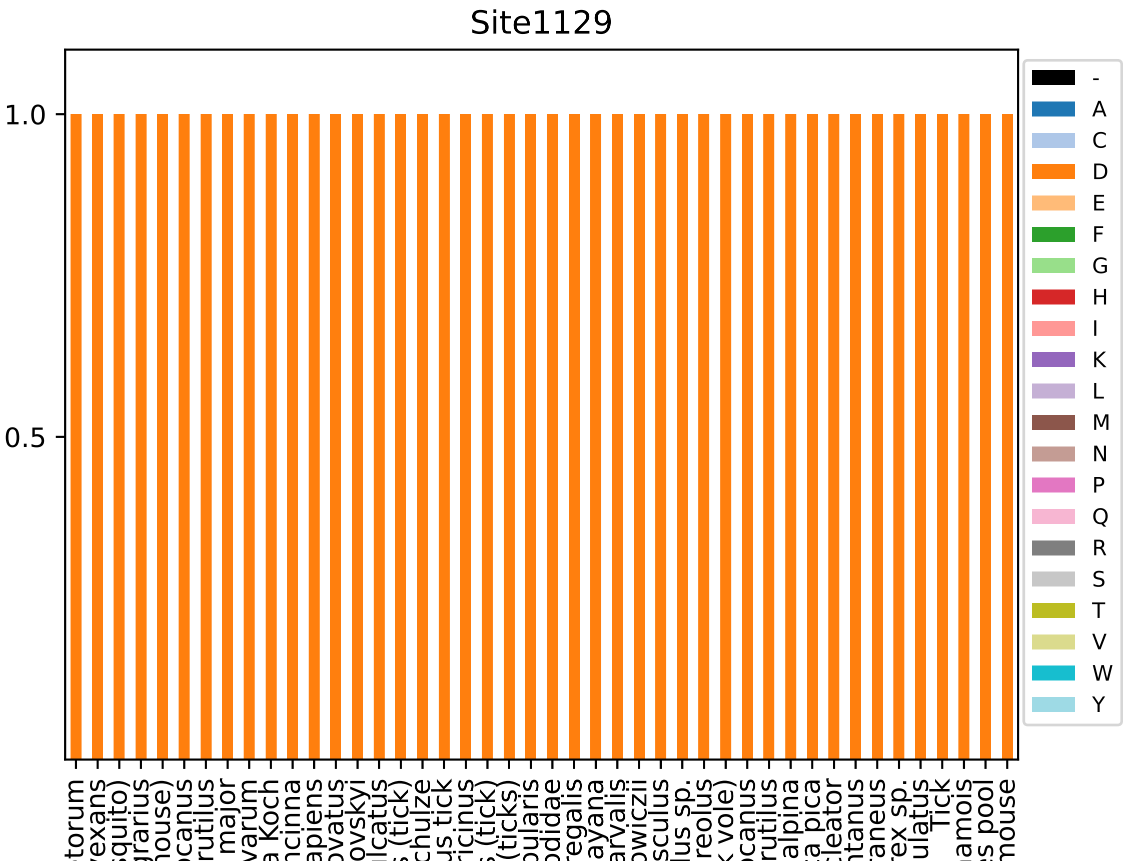
Site1129
1.0
0.5
-
A
C
D
E
F
G
H
I
K
L
M
N
P
Q
R
S
T
V
W
Y

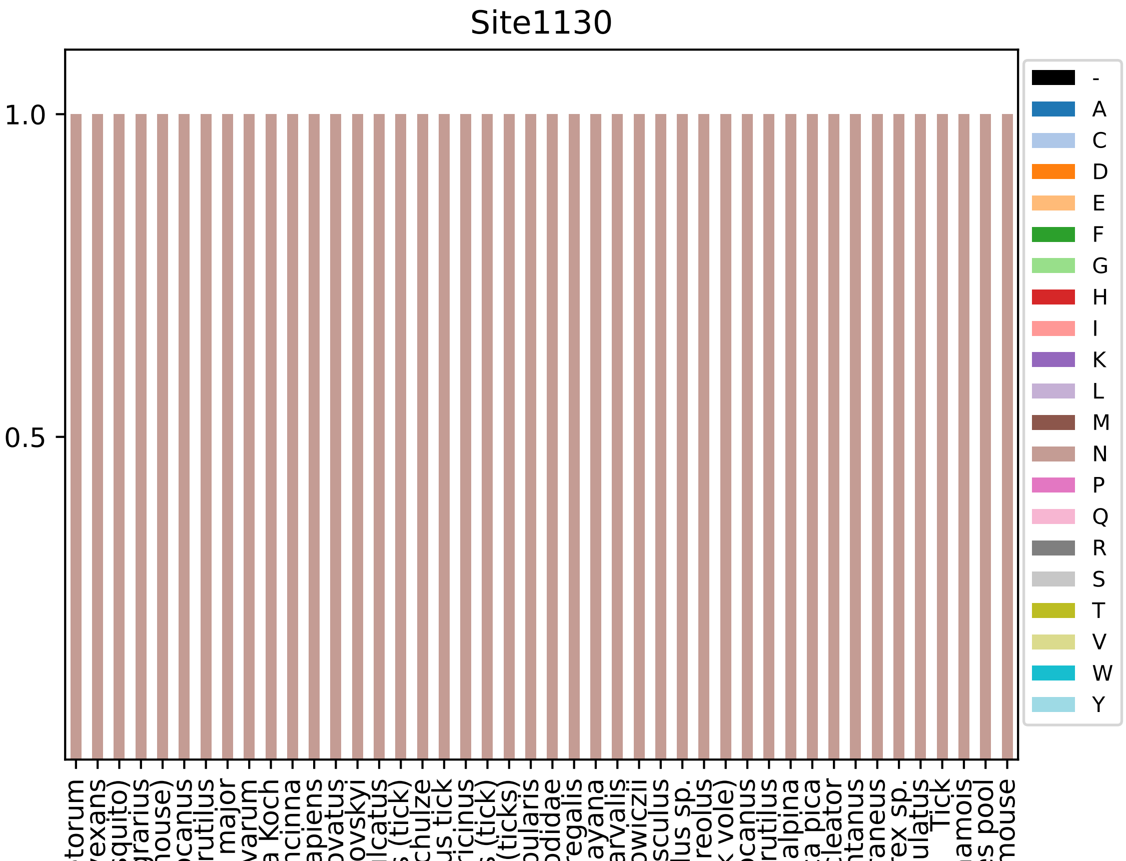
Site1130
1.0
0.5
-
A
C
D
E
F
G
H
I
K
L
M
N
P
Q
R
S
T
V
W
Y

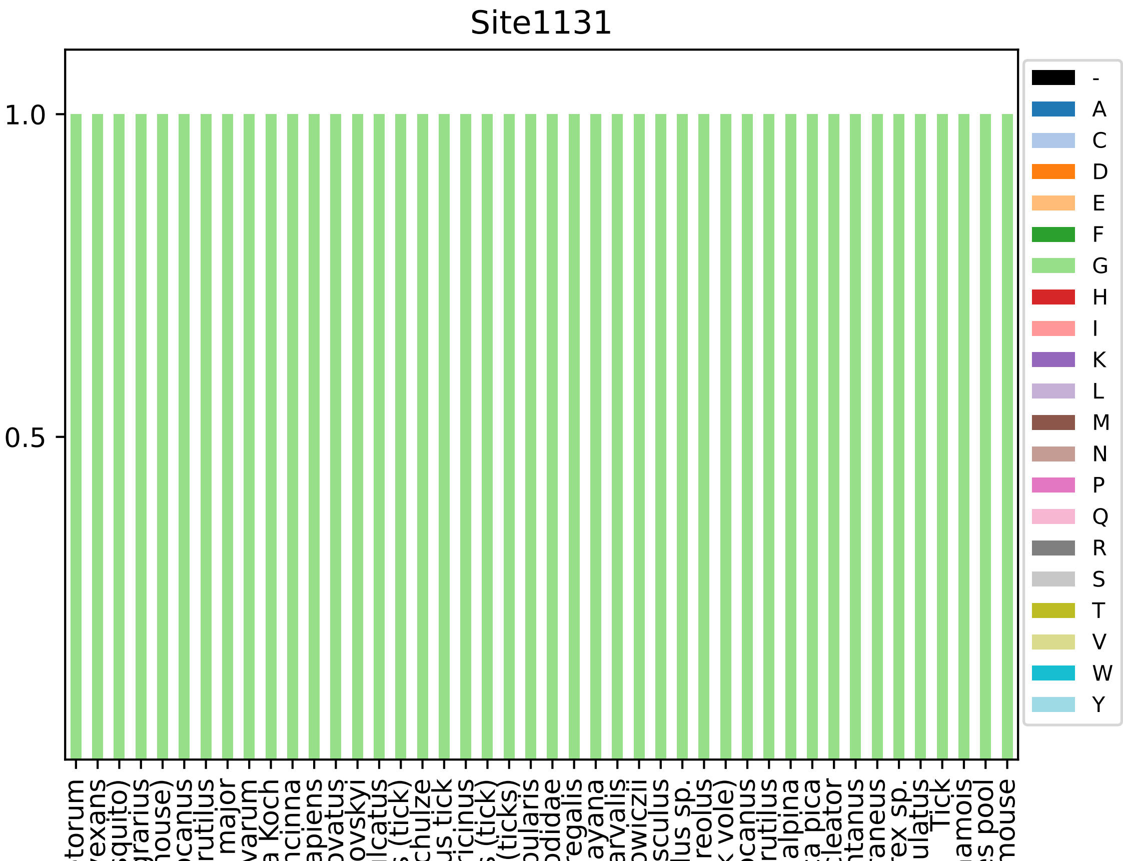
Site1131
1.0
0.5
-
A
C
D
E
F
G
H
I
K
L
M
N
P
Q
R
S
T
V
W
Y

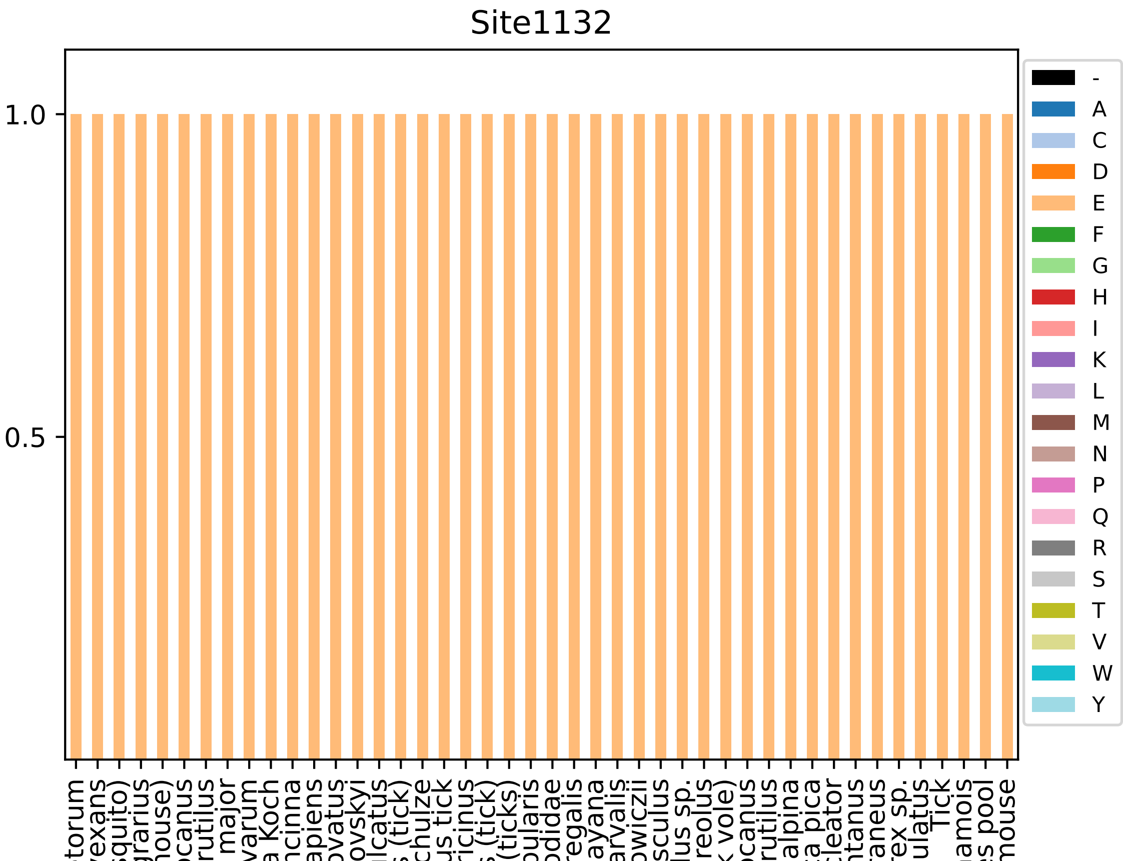
Site1132
1.0
0.5
-
A
C
D
E
F
G
H
I
K
L
M
N
P
Q
R
S
T
V
W
Y

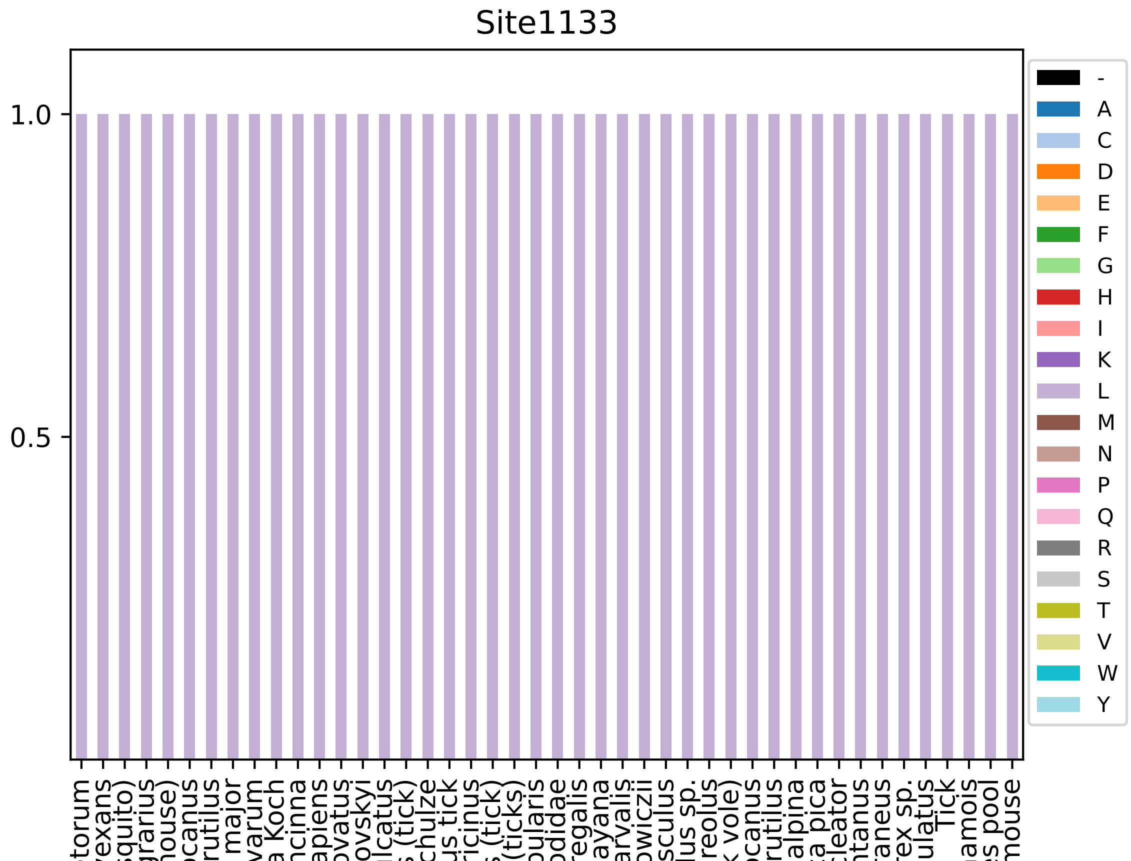

Site1133
1.0
0.5
-
A
C
D
E
F
G
H
I
K
L
M
N
P
Q
R
S
T
V
W
Y

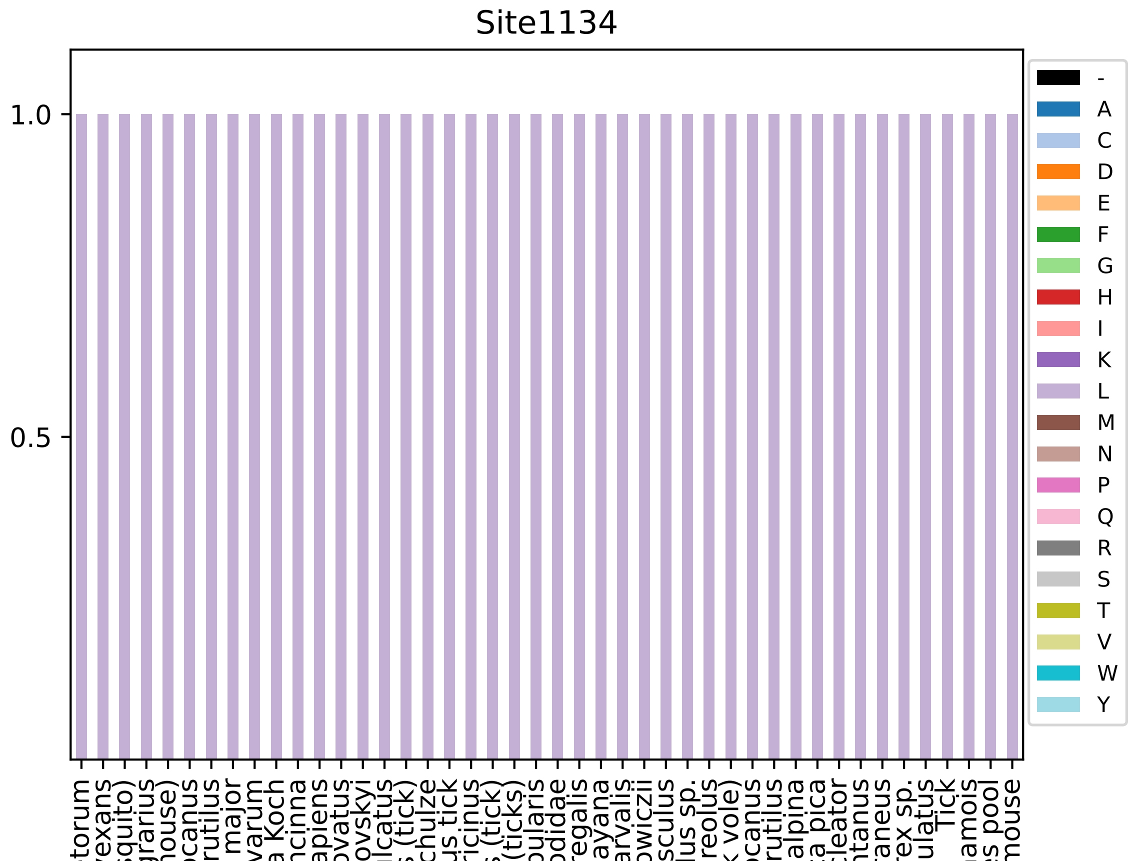

Site1134
1.0
0.5
-
A
C
D
E
F
G
H
I
K
L
M
N
P
Q
R
S
T
V
W
Y

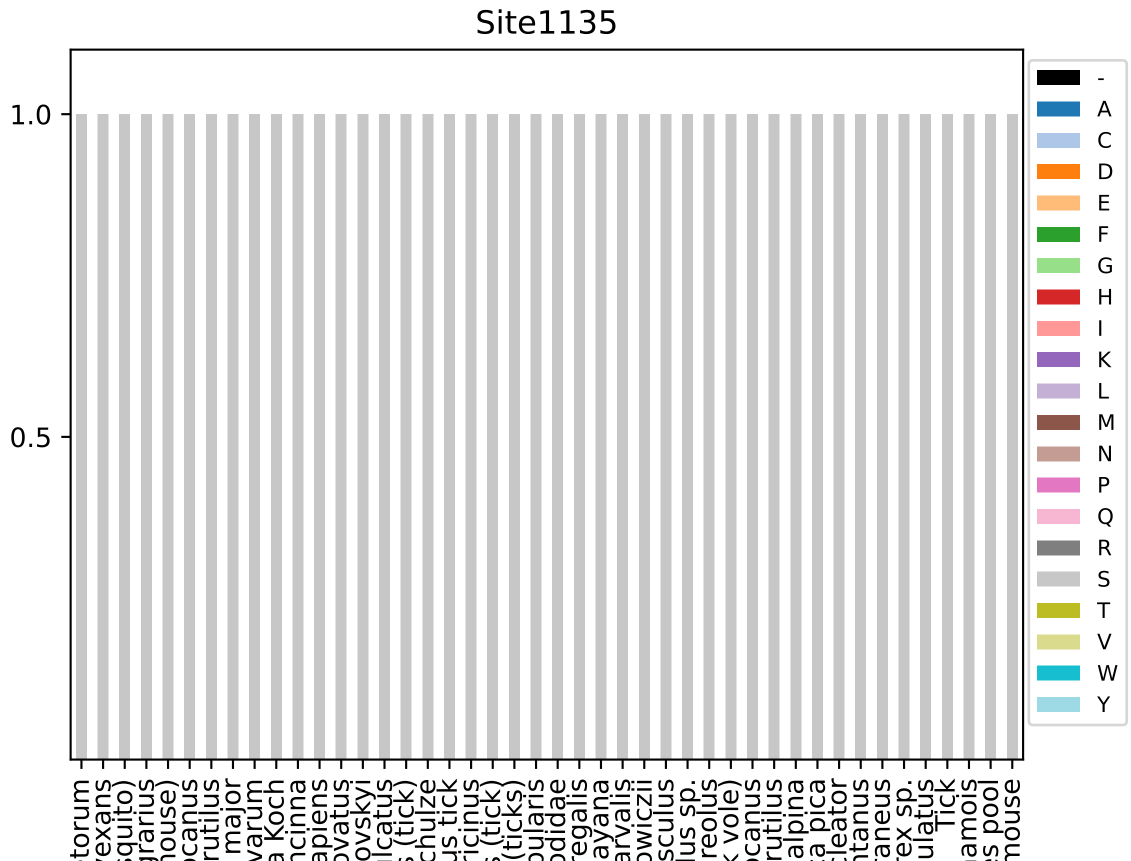
Site1135
1.0
0.5
-
A
C
D
E
F
G
H
I
K
L
M
N
P
Q
R
S
T
V
W
Y

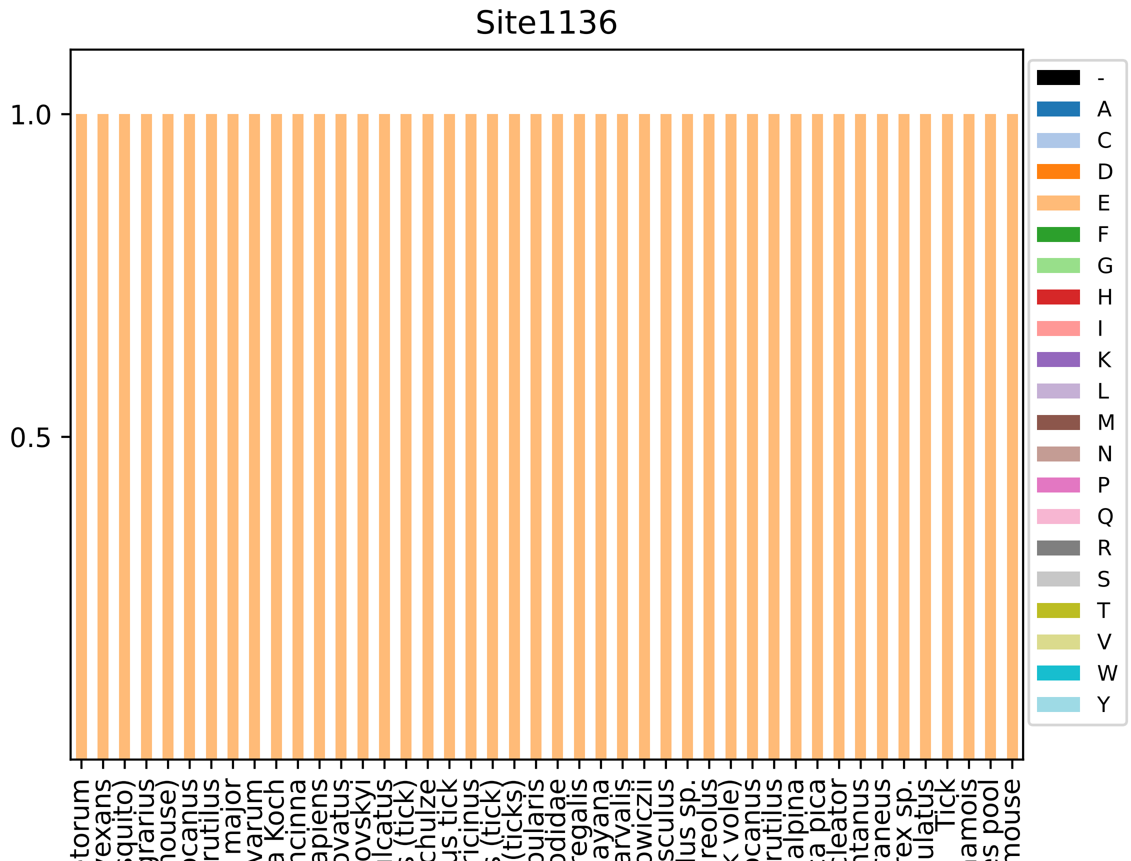
Site1136
1.0
0.5
-
A
C
D
E
F
G
H
I
K
L
M
N
P
Q
R
S
T
V
W
Y

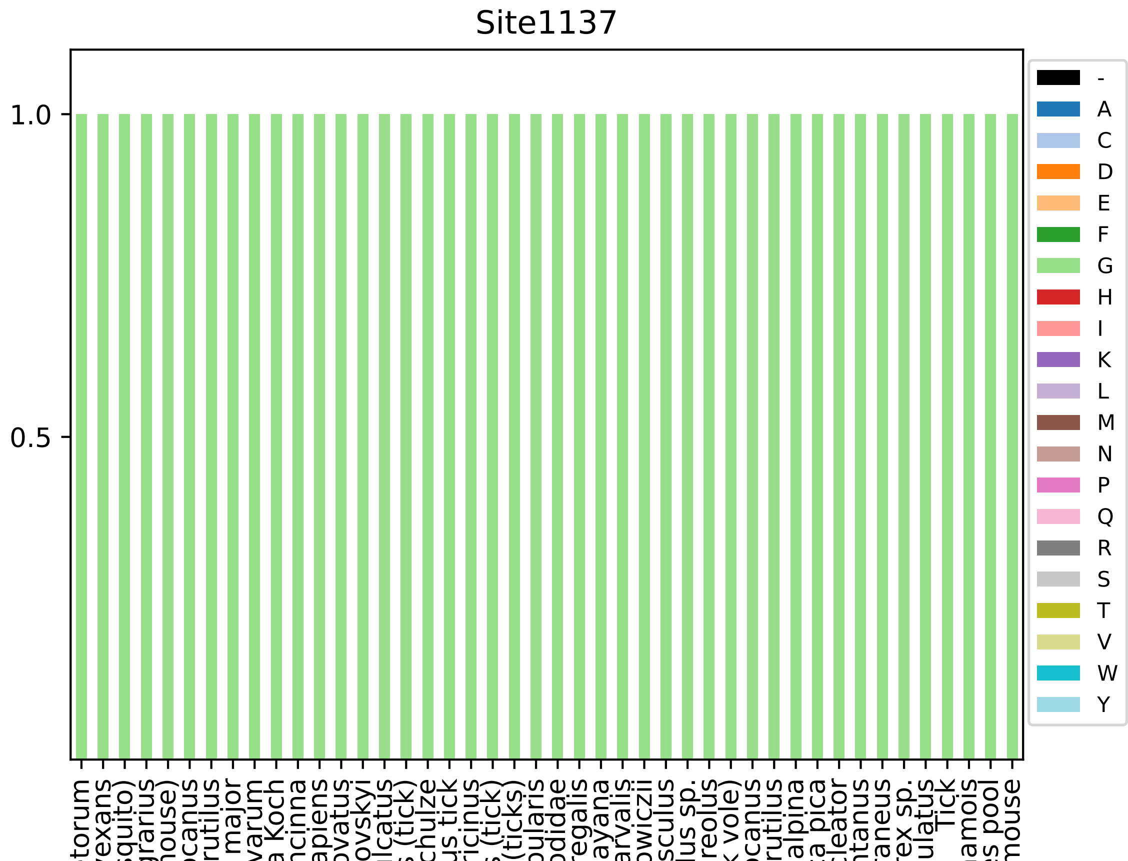
Site1137
1.0
0.5
-
A
C
D
E
F
G
H
I
K
L
M
N
P
Q
R
S
T
V
W
Y

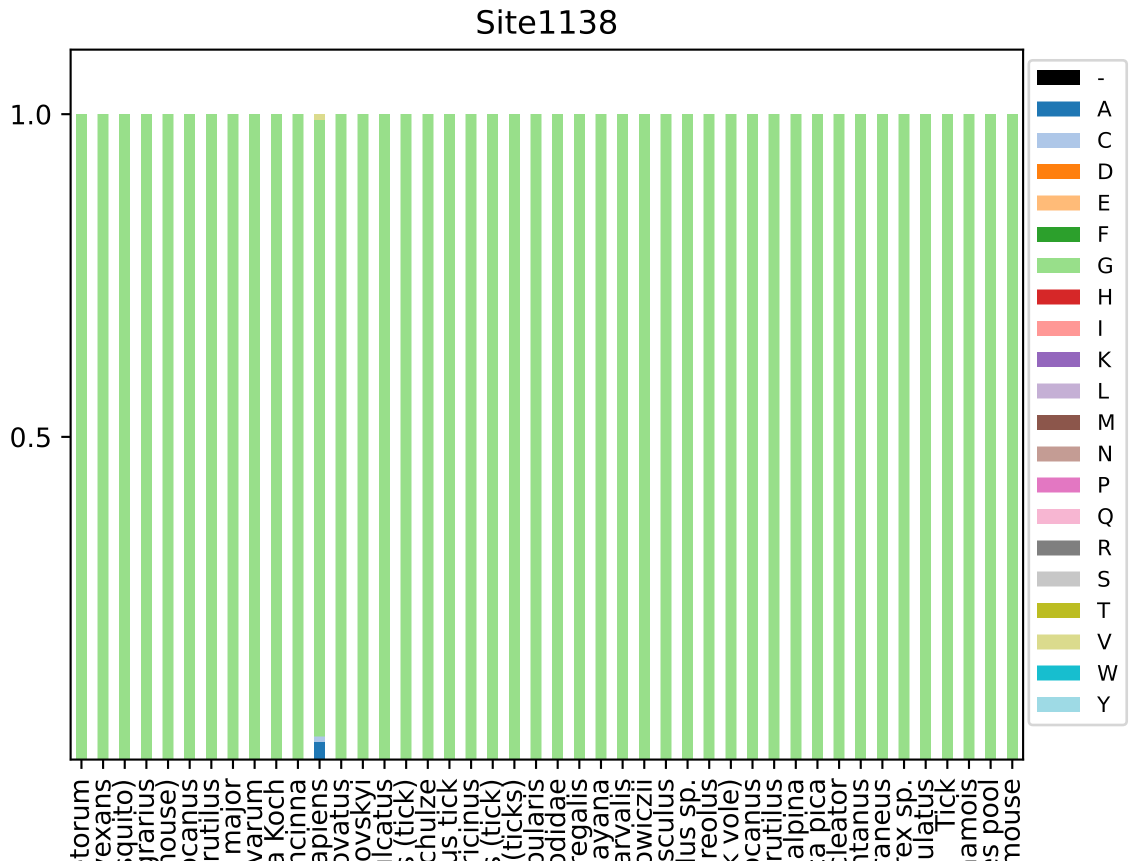
Site1138
1.0
0.5
-
A
C
D
E
F
G
H
I
K
L
M
N
P
Q
R
S
T
V
W
Y

Site1139

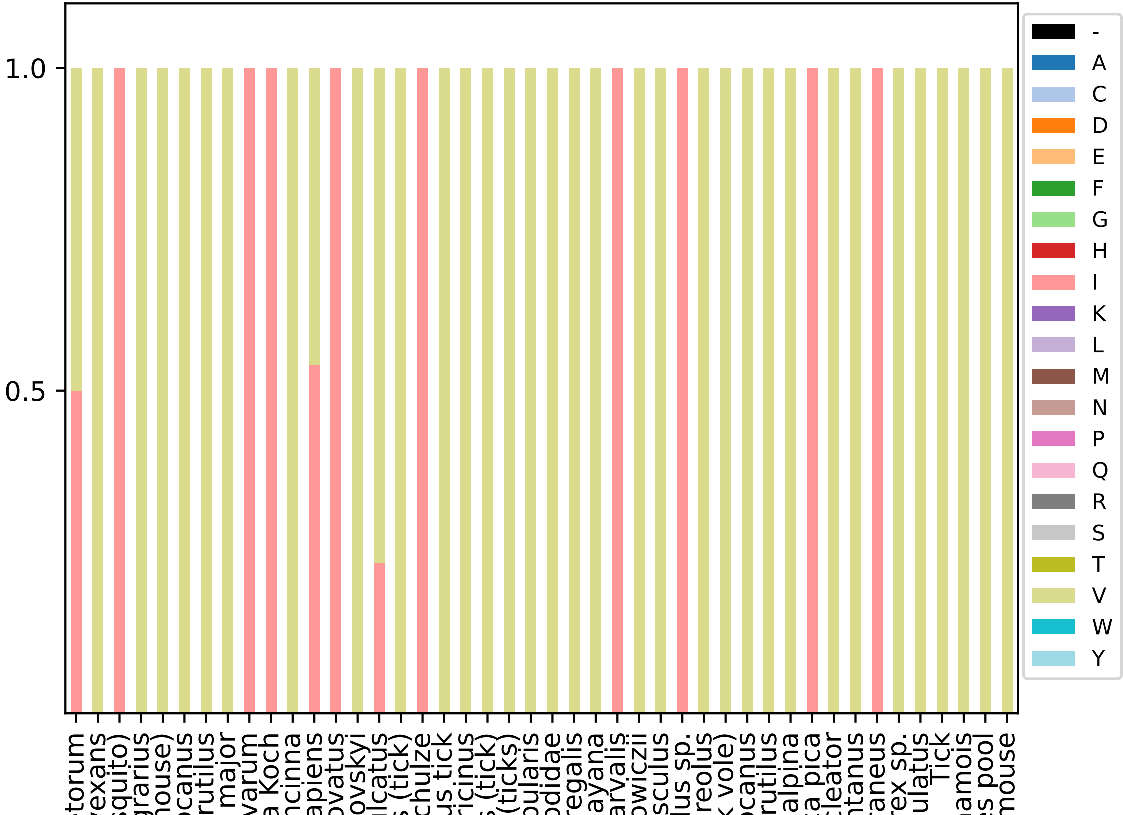

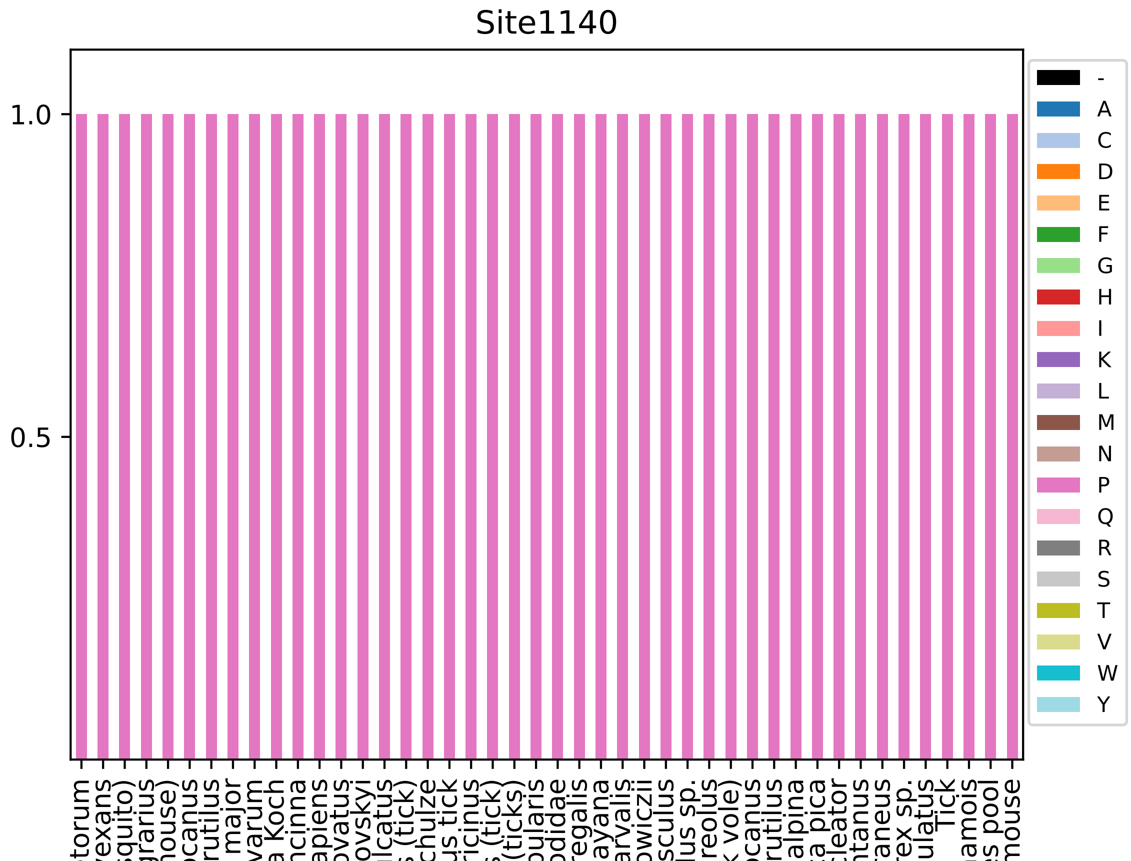
Site1140
1.0
0.5
-
A
C
D
E
F
G
H
I
K
L
M
N
P
Q
R
S
T
V
W
Y

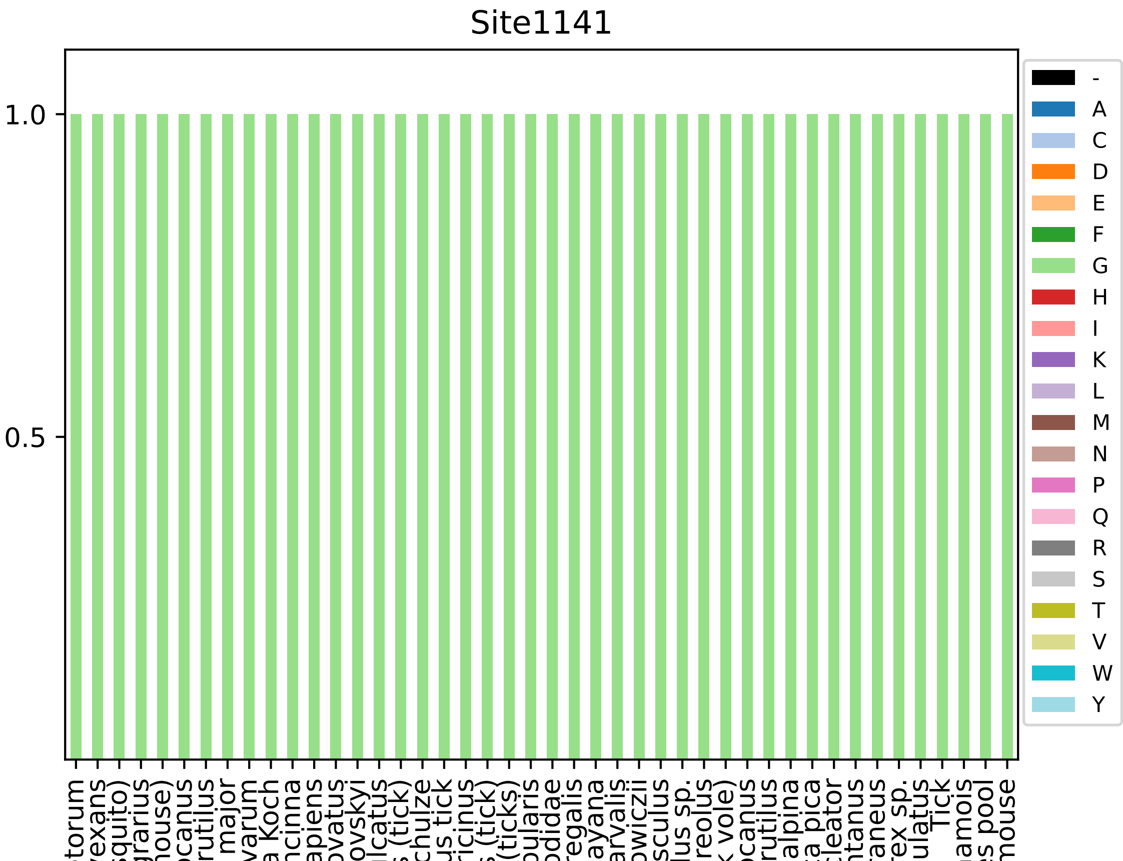

Site1141
1.0
0.5
-
A
C
D
E
F
G
H
I
K
L
M
N
P
Q
R
S
T
V
W
Y

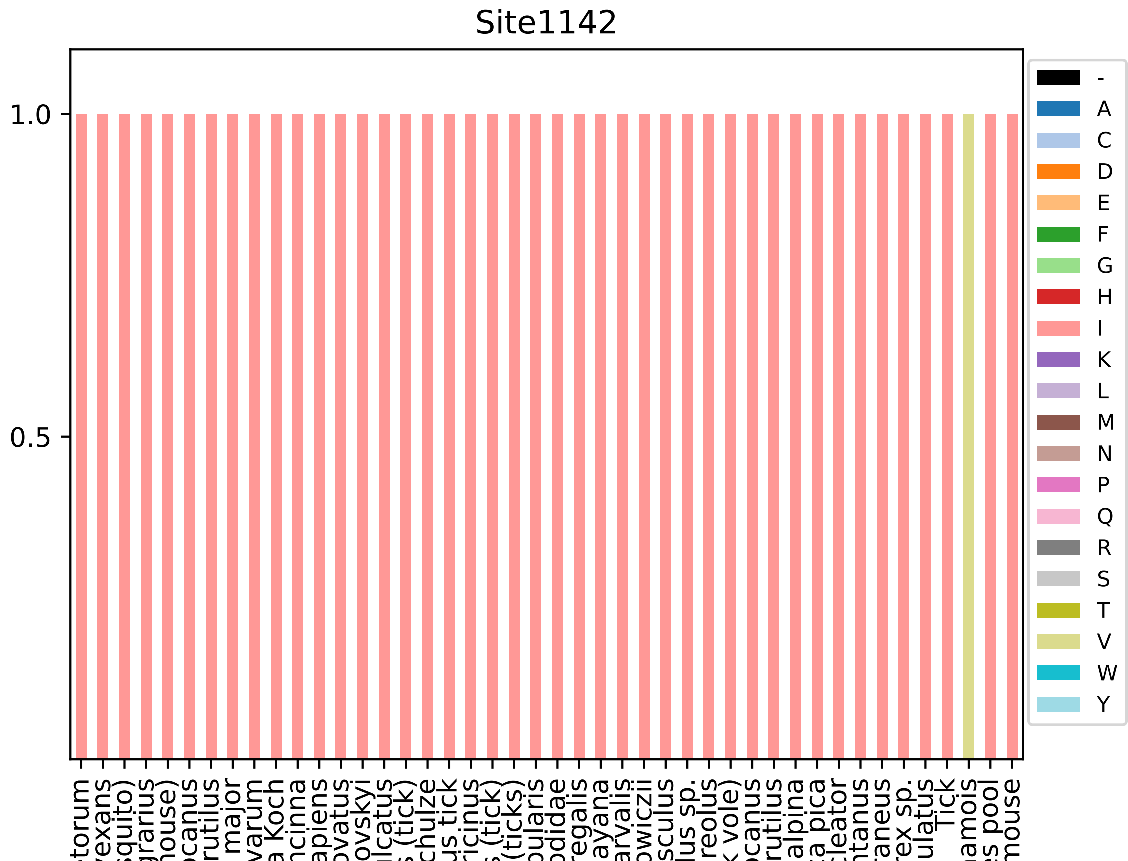

Site1142
1.0
0.5
-
A
C
D
E
F
G
H
I
K
L
M
N
P
Q
R
S
T
V
W
Y
Tick

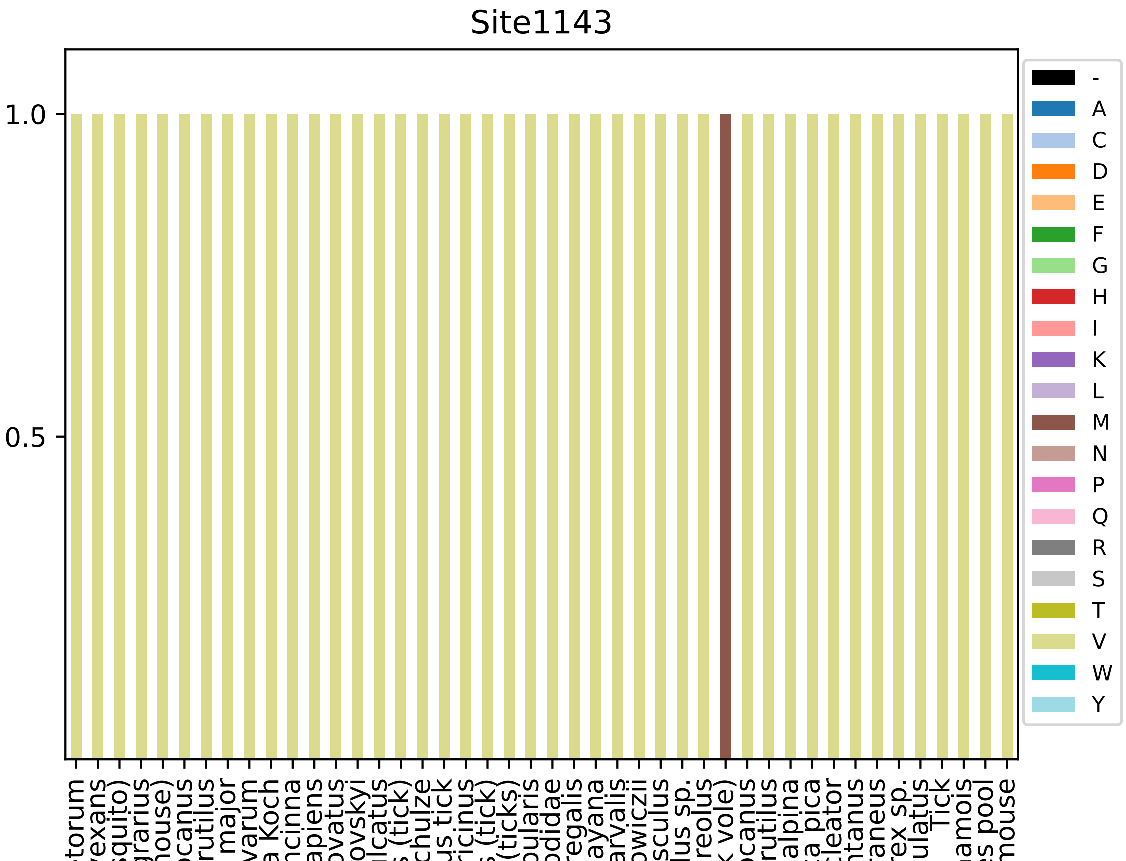
Site1143
1.0
0.5
-
A
C
D
E
F
G
H
I
K
L
M
N
P
Q
R
S
T
V
W
Y
Tick

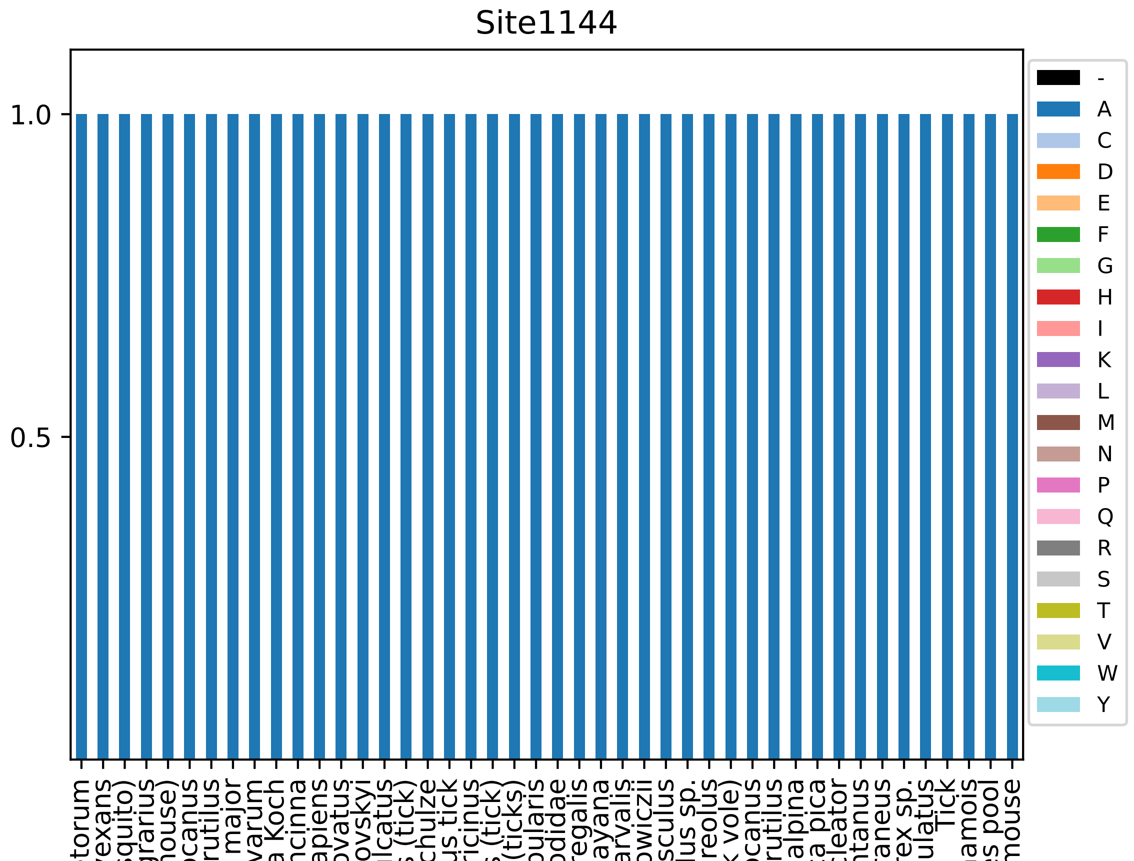

Site1144
1.0
0.5
-
A
C
D
E
F
G
H
I
K
L
M
N
P
Q
R
S
T
V
W
Y

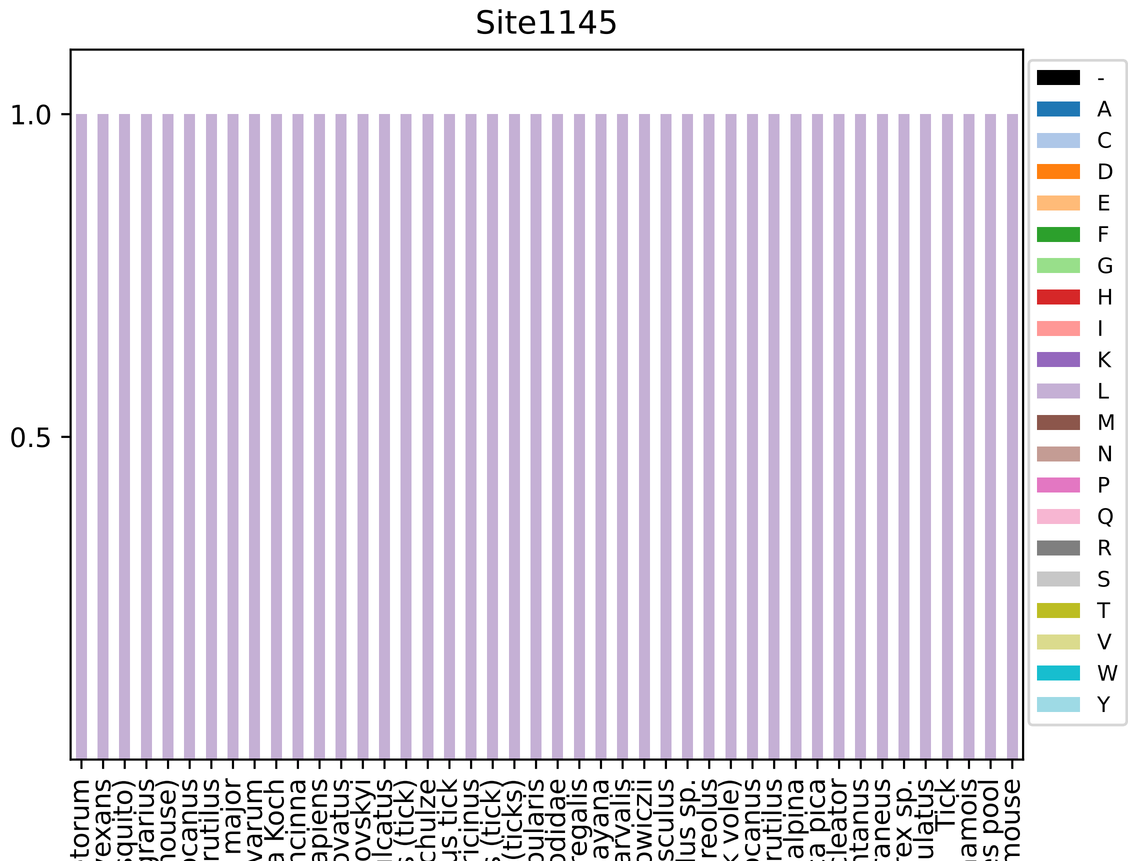
Site1145
1.0
0.5
-
A
C
D
E
F
G
H
I
K
L
M
N
P
Q
R
S
T
V
W
Y

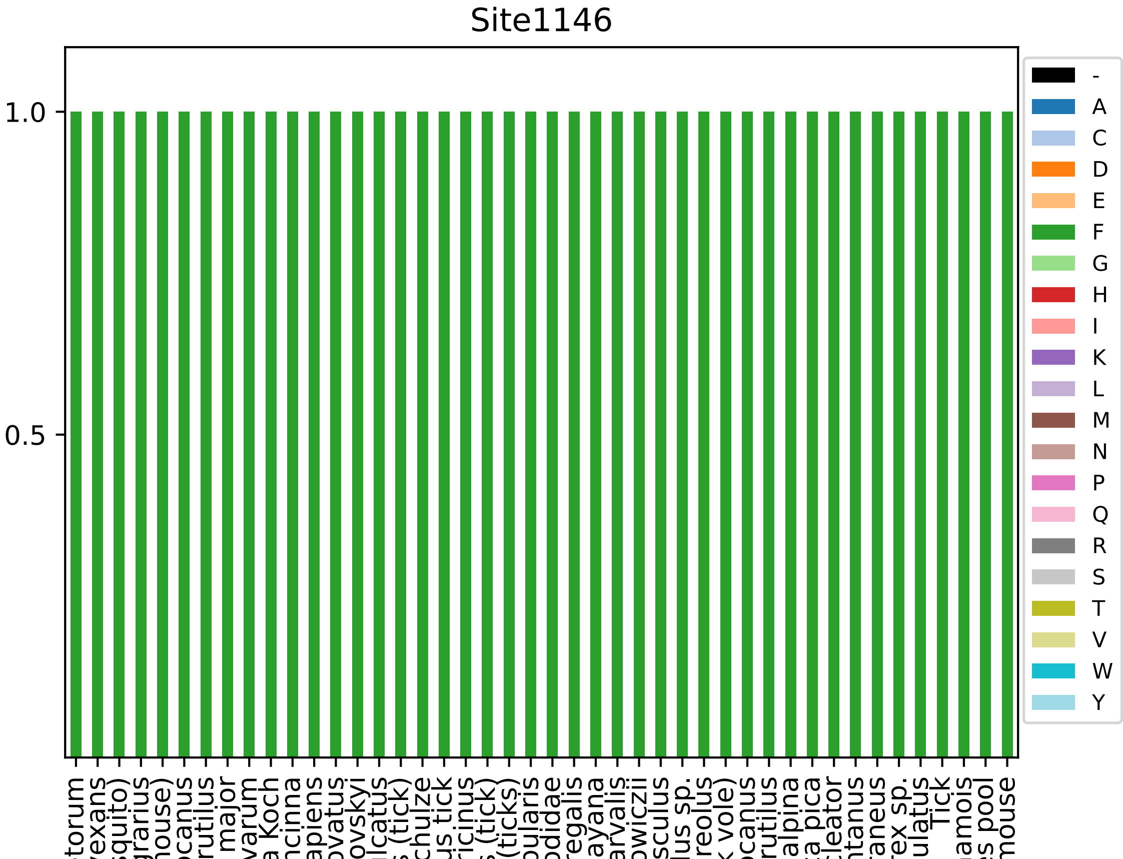
Site1146
1.0
0.5
-
A
C
D
E
F
G
H
I
K
L
M
N
P
Q
R
S
T
V
W
Y
Tick

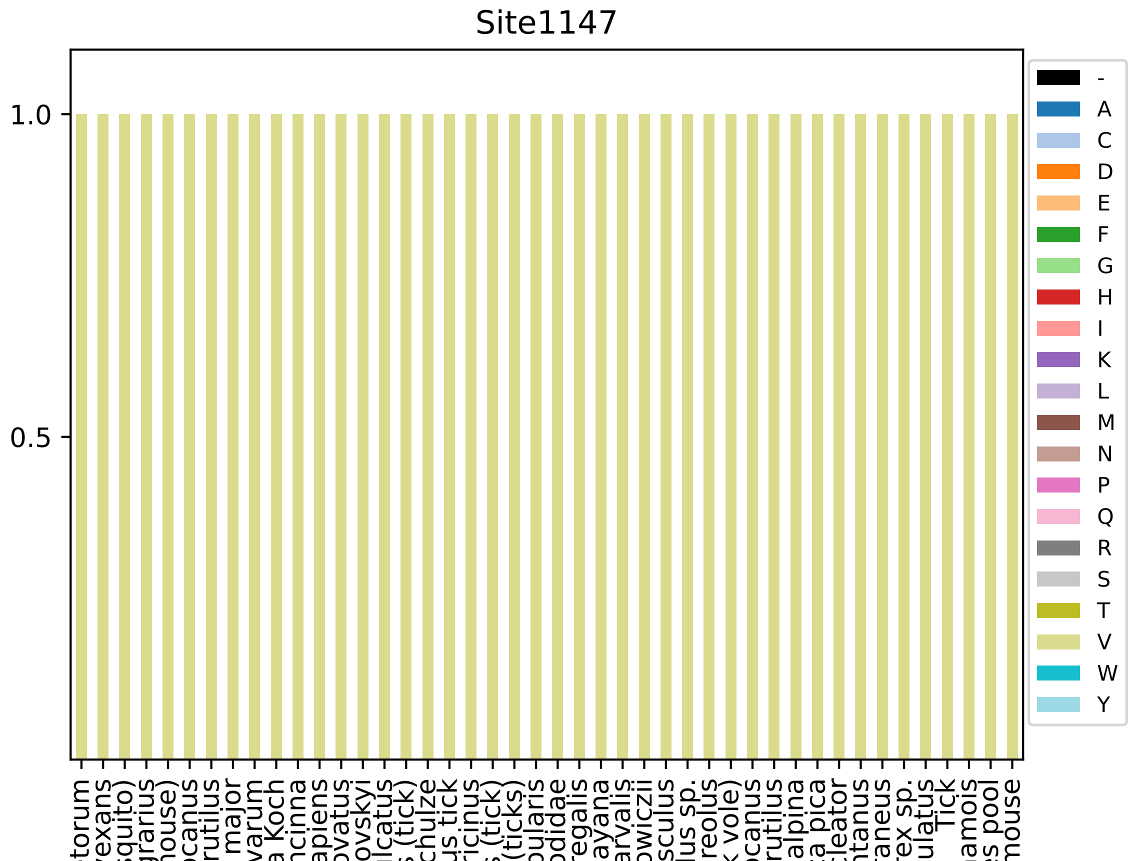
Site1147
1.0
0.5
-
A
C
D
E
F
G
H
I
K
L
M
N
P
Q
R
S
T
V
W
Y

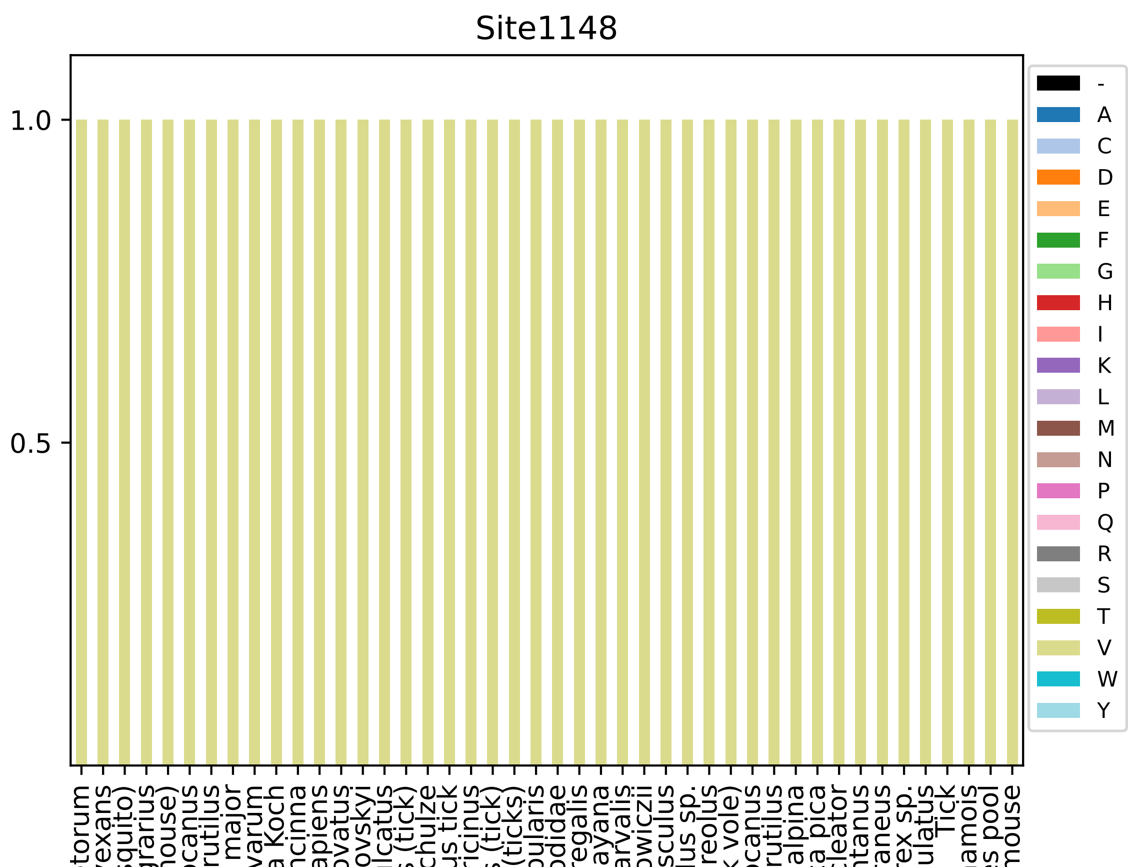
Site1148
1.0
0.5
-
A
C
D
E
F
G
H
I
K
L
M
N
P
Q
R
S
T
V
W
Y

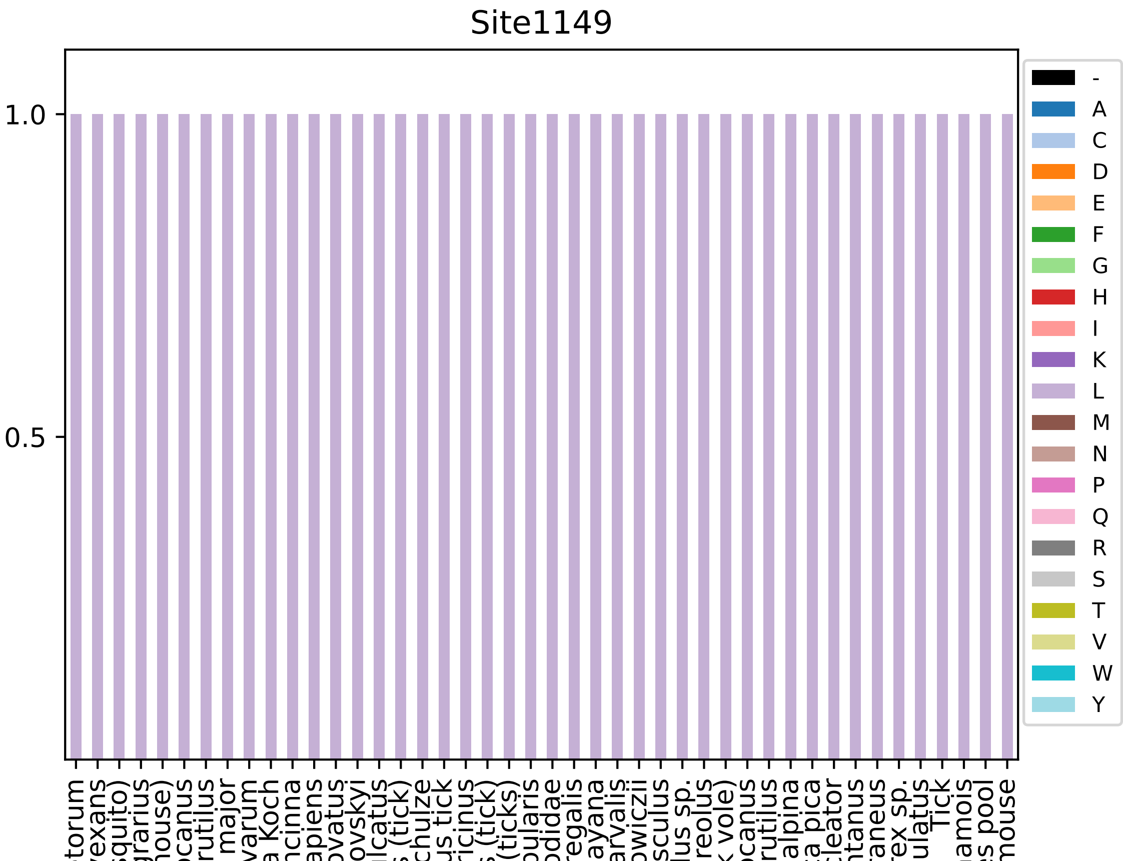
Site1149
1.0
0.5
-
A
C
D
E
F
G
H
I
K
L
M
N
P
Q
R
S
T
V
W
Y

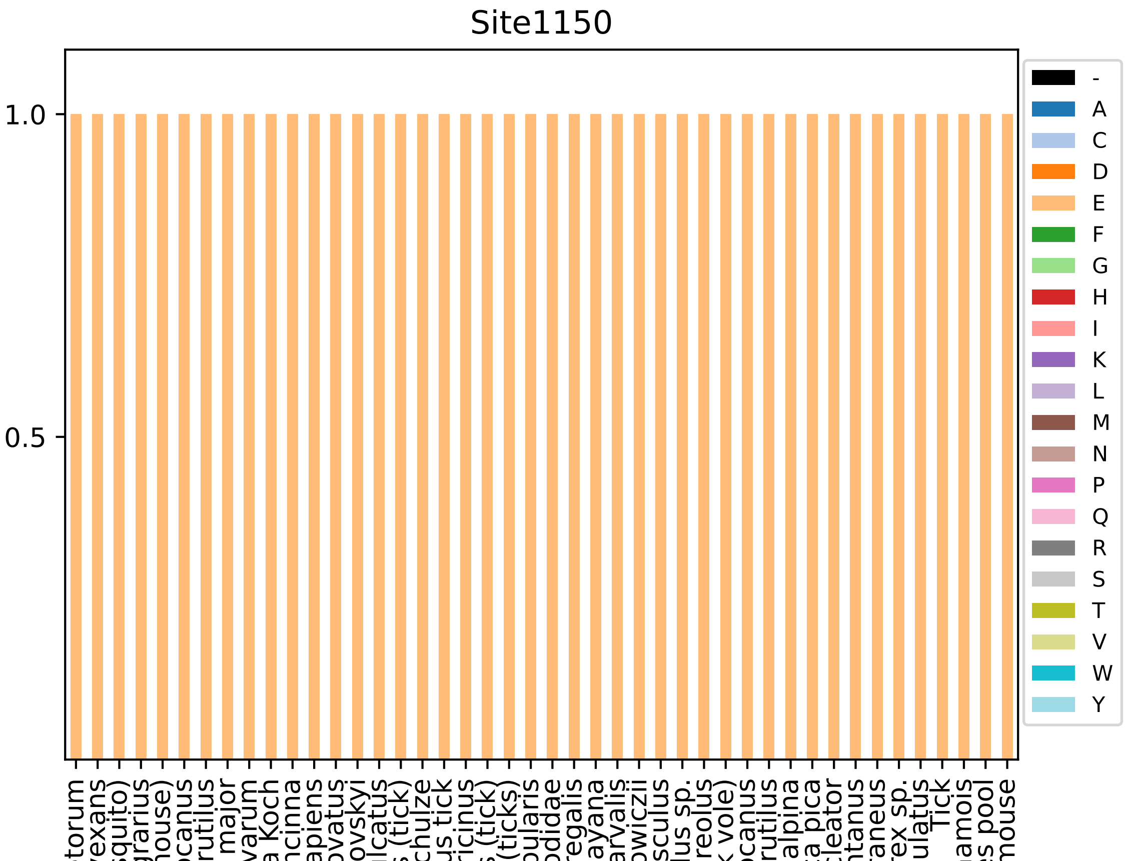
Site1150
1.0
0.5
-
A
C
D
E
F
G
H
I
K
L
M
N
P
Q
R
S
T
V
W
Y

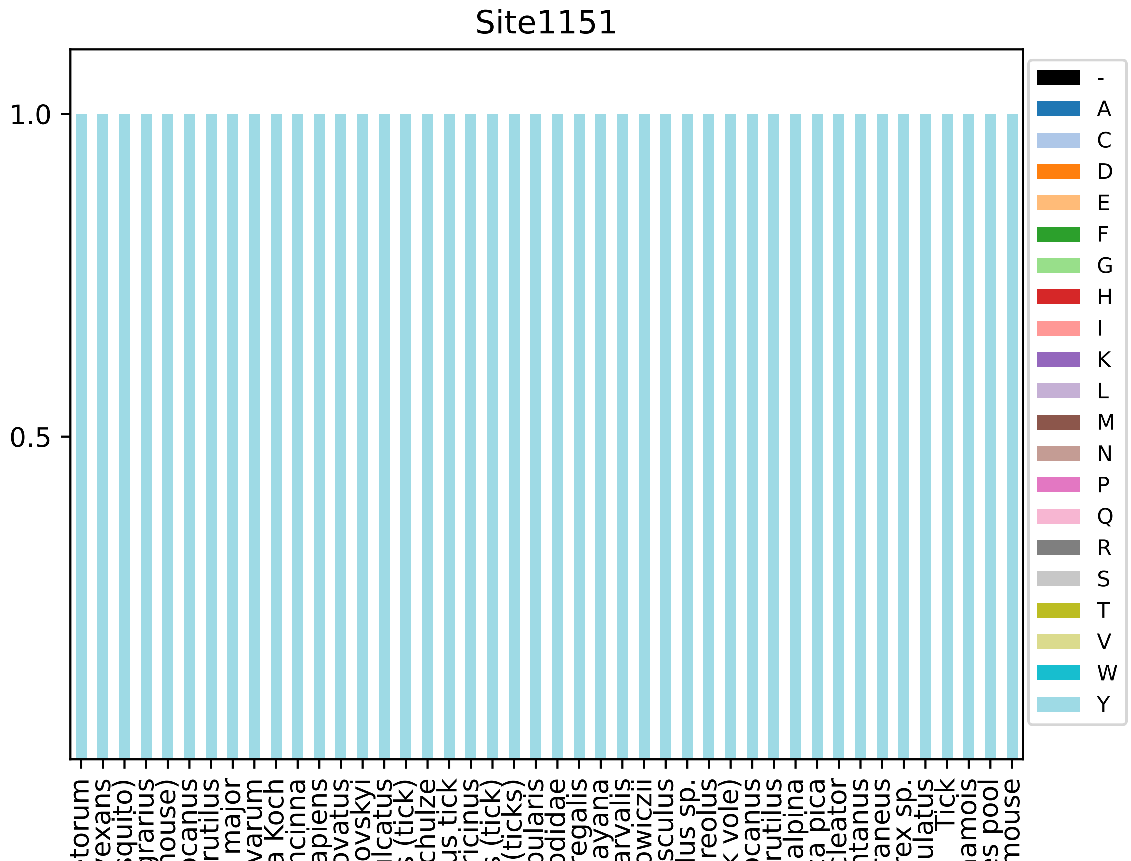
Site1151
1.0
0.5
-
A
C
D
E
F
G
H
I
K
L
M
N
P
Q
R
S
T
V
W
Y

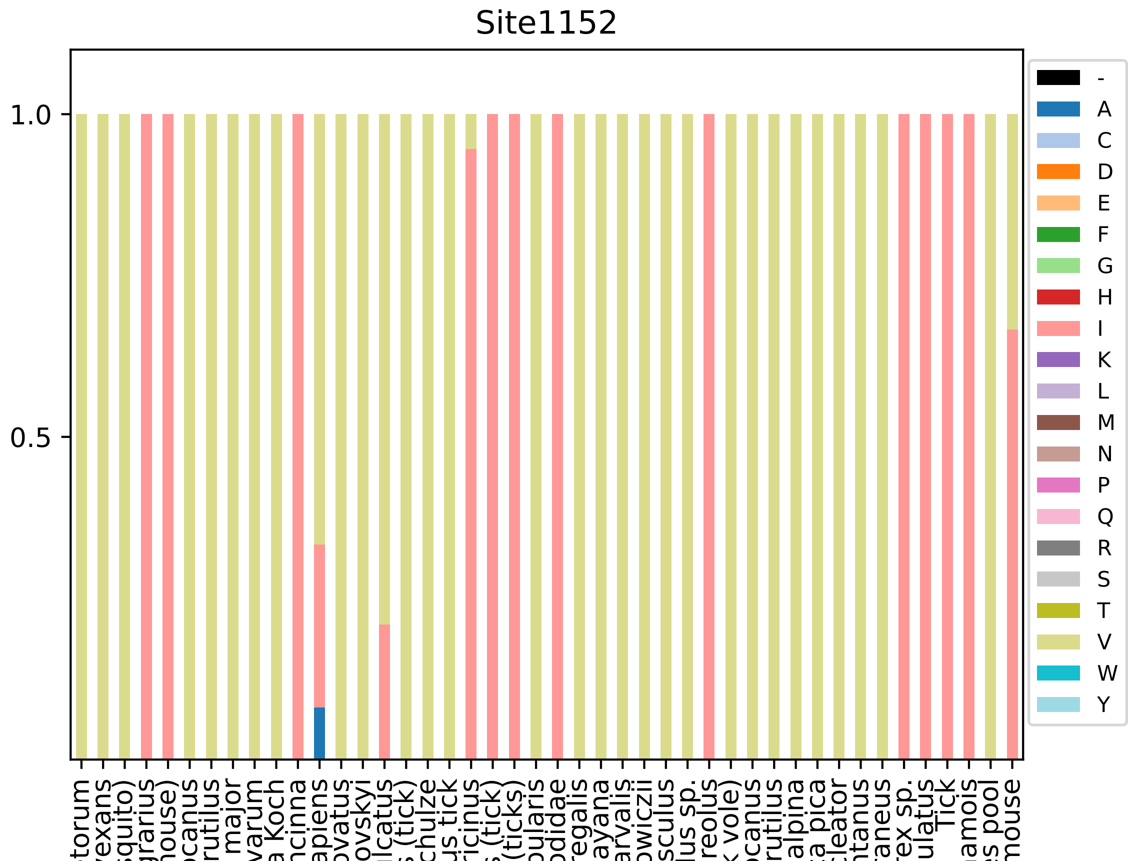
Site1152
1.0
0.5
-
A
C
D
E
F
G
H
I
K
L
M
N
P
Q
R
S
T
V
W
Y

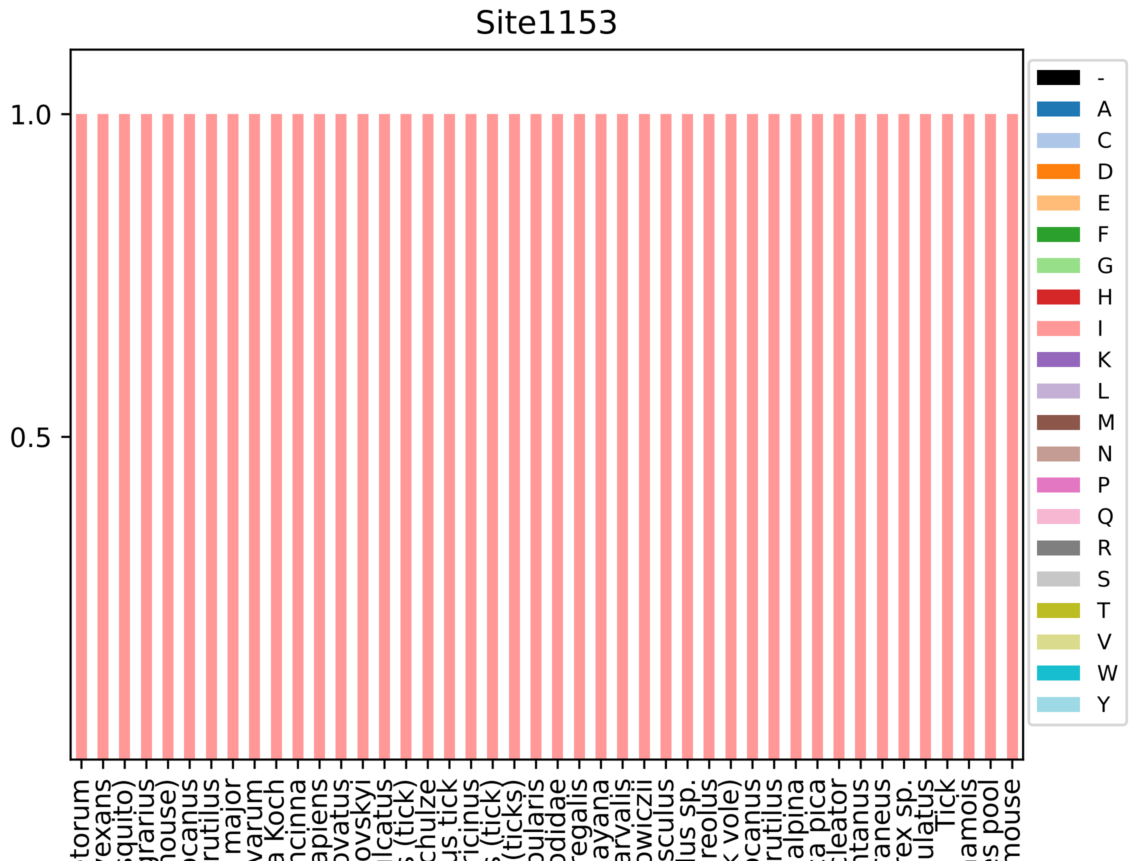
Site1153
1.0
0.5
-
A
C
D
E
F
G
H
I
K
L
M
N
P
Q
R
S
T
V
W
Y

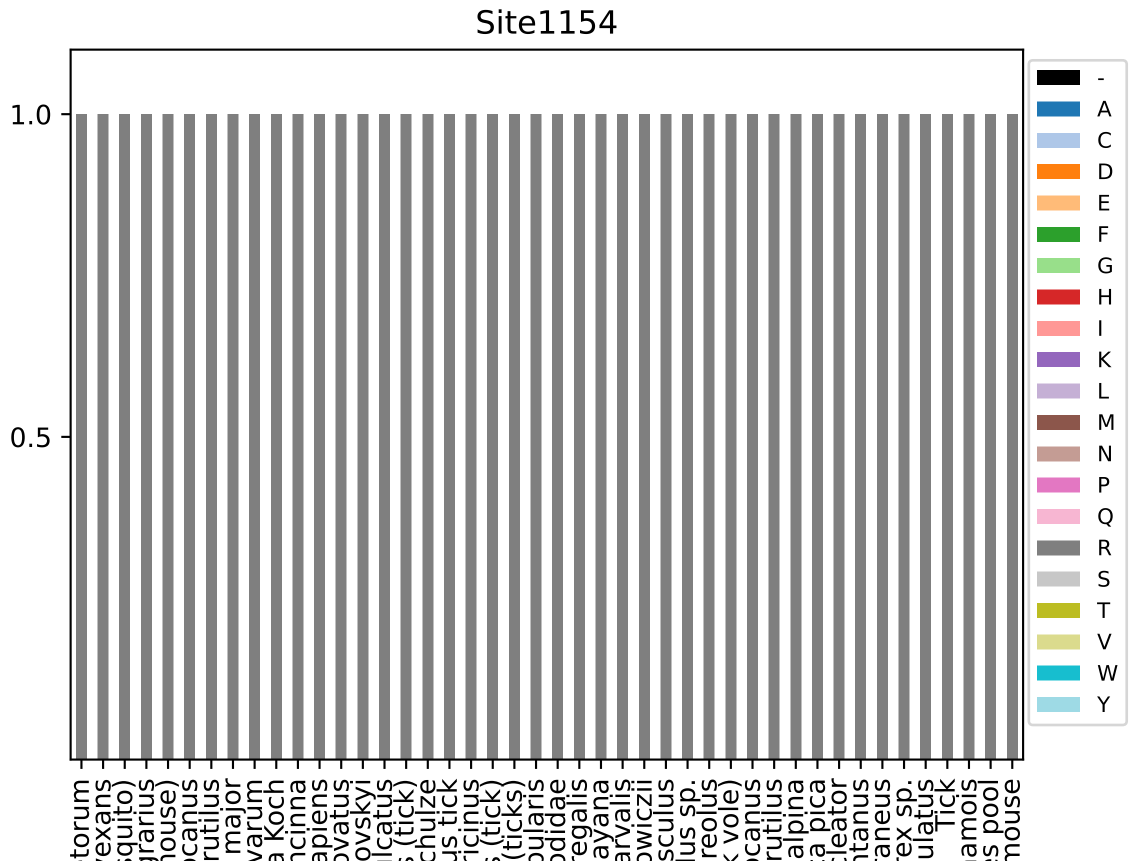
Site1154
1.0
0.5
-
A
C
D
E
F
G
H
I
K
L
M
N
P
Q
R
S
T
V
W
Y
Tick

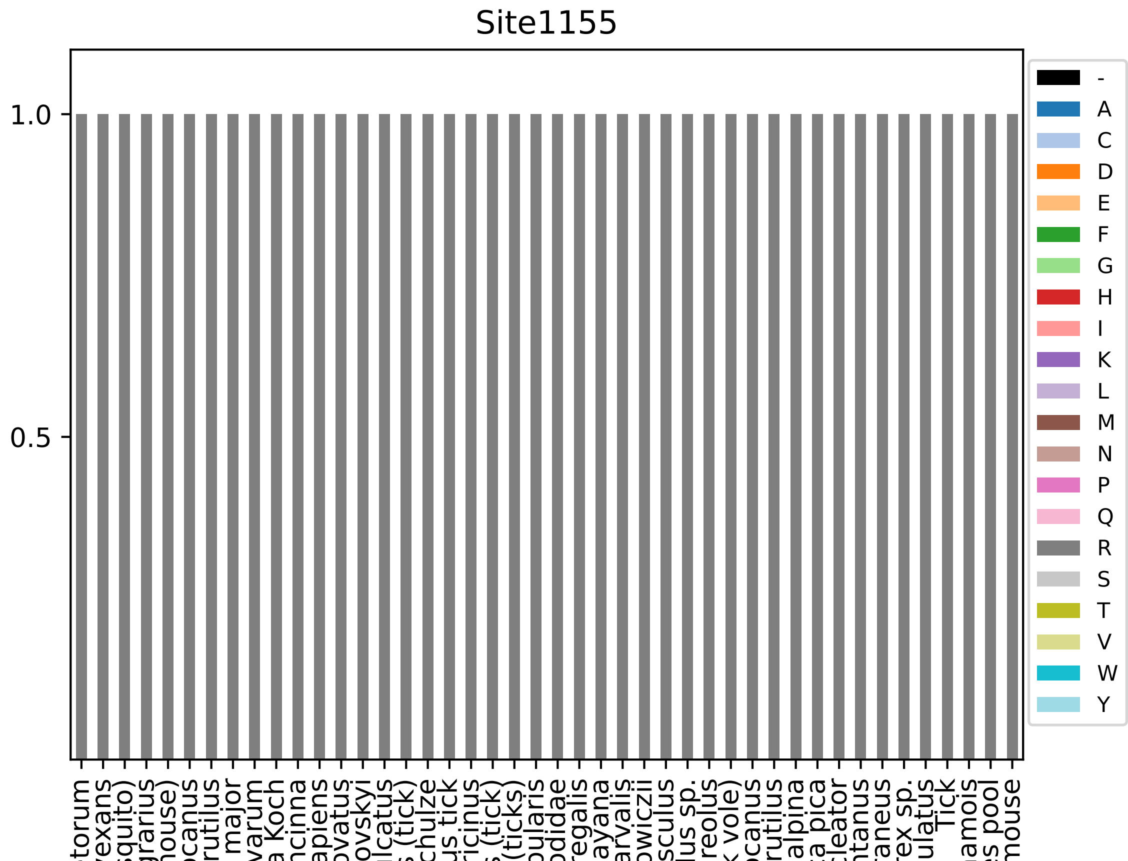
Site1155
1.0
0.5
-
A
C
D
E
F
G
H
I
K
L
M
N
P
Q
R
S
T
V
W
Y
Tick

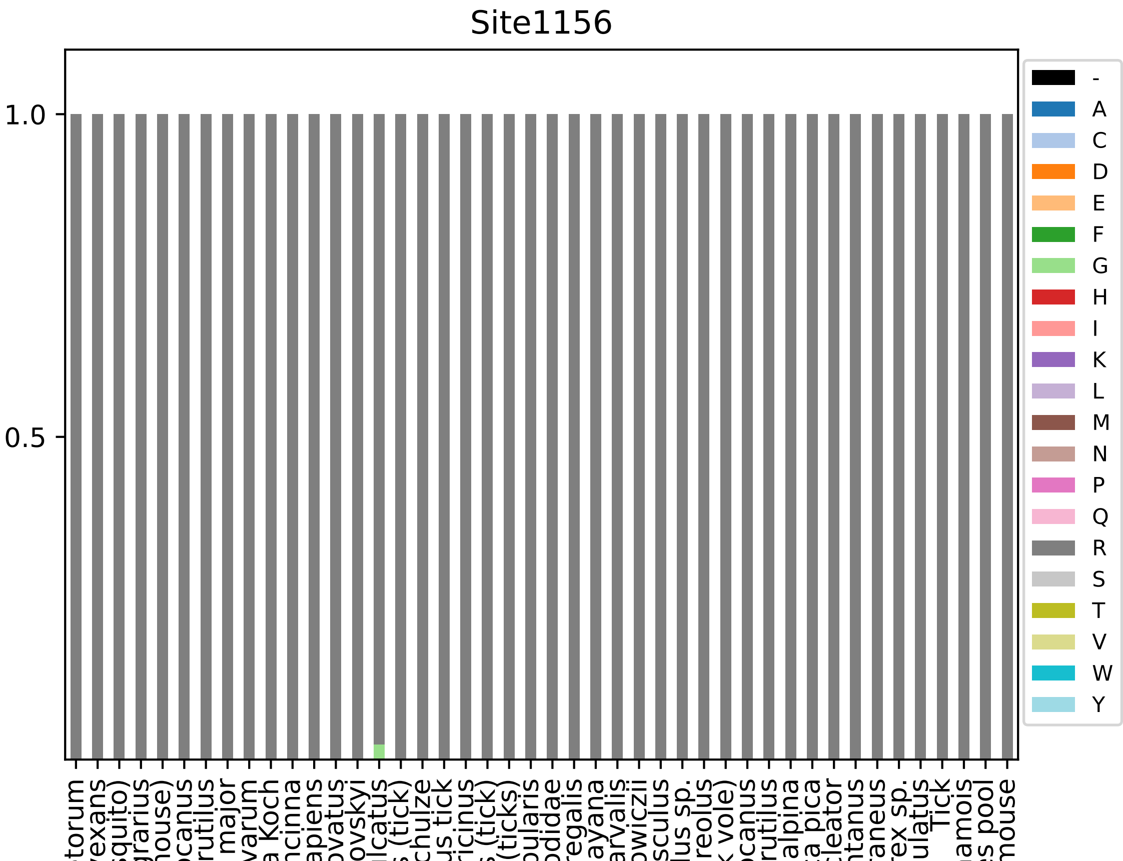
Site1156
1.0
0.5
-
A
C
D
E
F
G
H
I
K
L
M
N
P
Q
R
S
T
V
W
Y

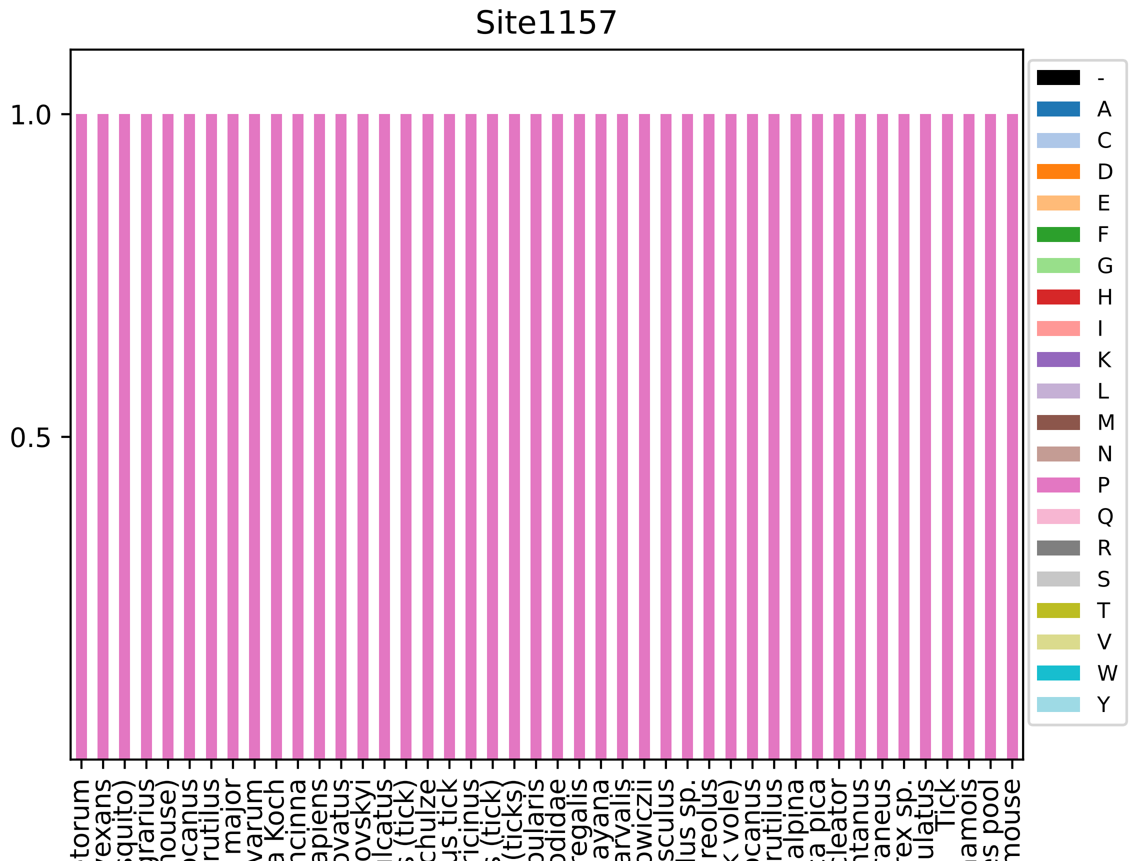
Site1157
1.0
0.5
-
A
C
D
E
F
G
H
I
K
L
M
N
P
Q
R
S
T
V
W
Y

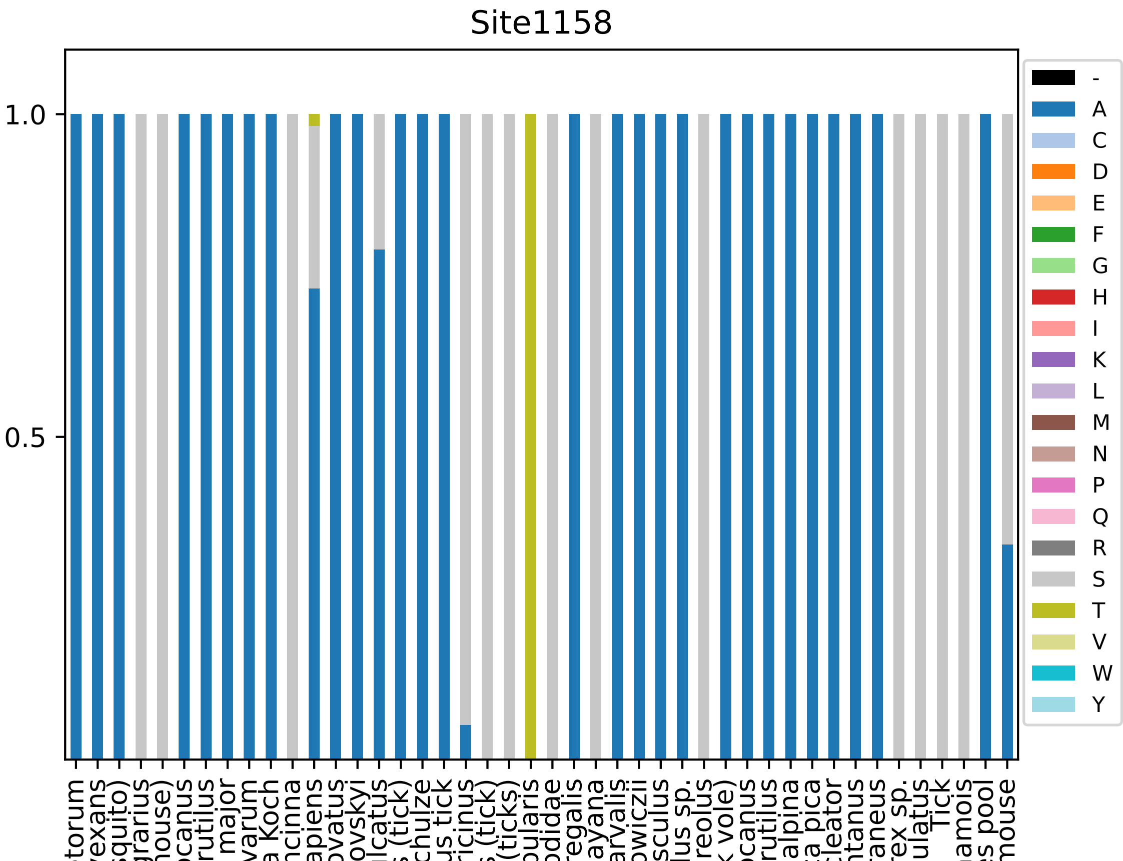
Site1158
1.0
0.5
-
A
C
D
E
F
G
H
I
K
L
M
N
P
Q
R
S
T
V
W
Y

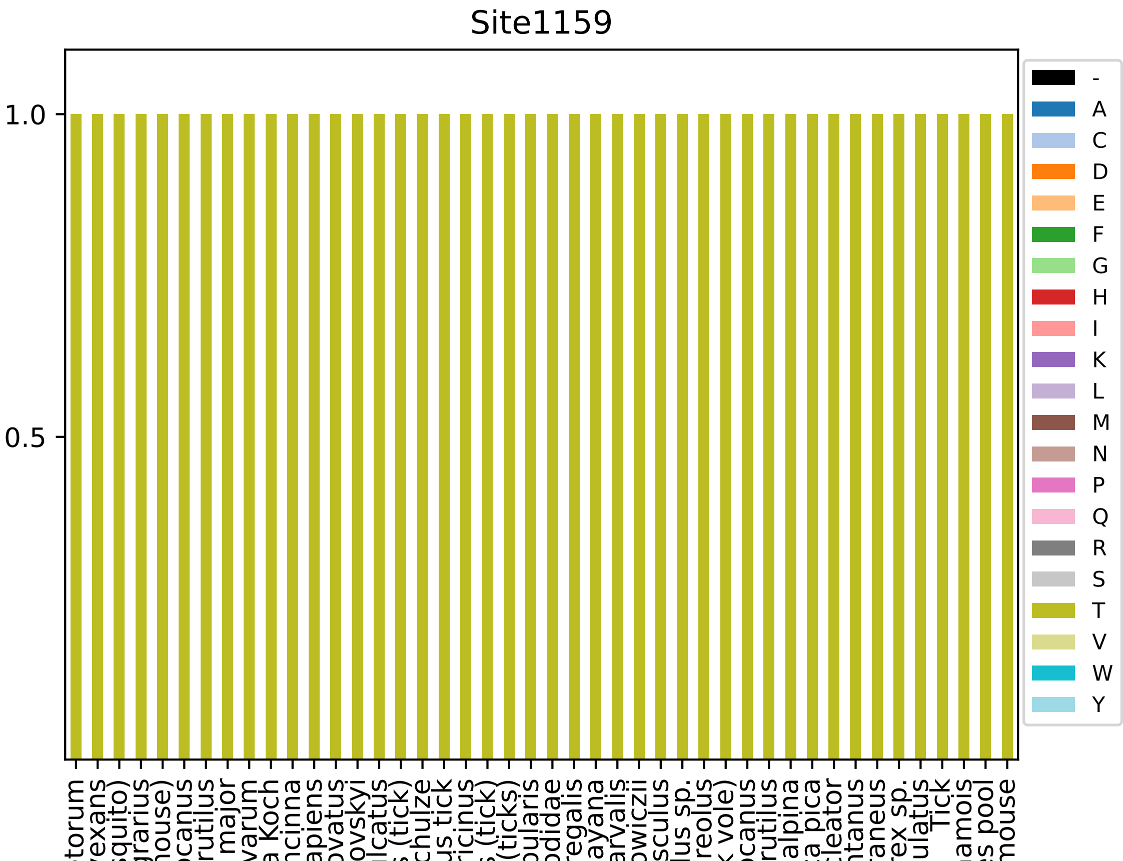
Site1159
1.0
0.5
-
A
C
D
E
F
G
H
I
K
L
M
N
P
Q
R
S
T
V
W
Y

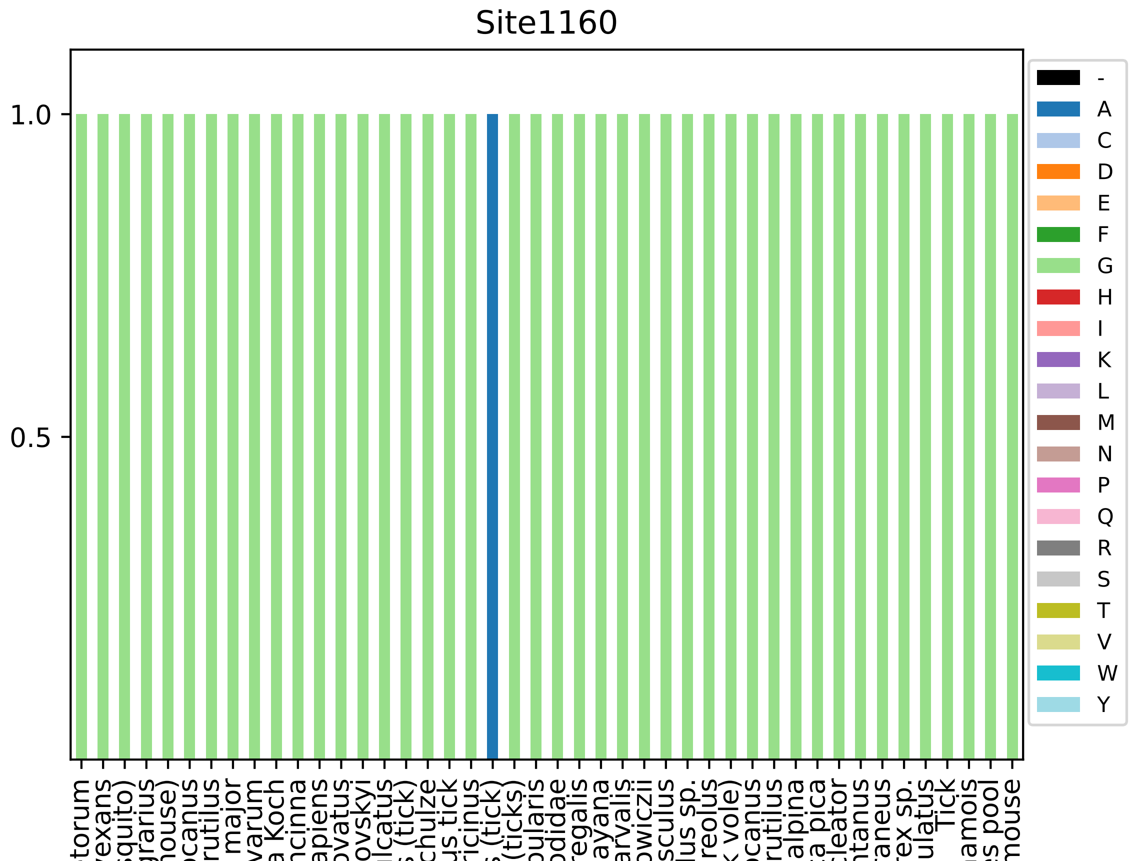
Site1160
1.0
0.5
-
A
C
D
E
F
G
H
I
K
L
M
N
P
Q
R
S
T
V
W
Y

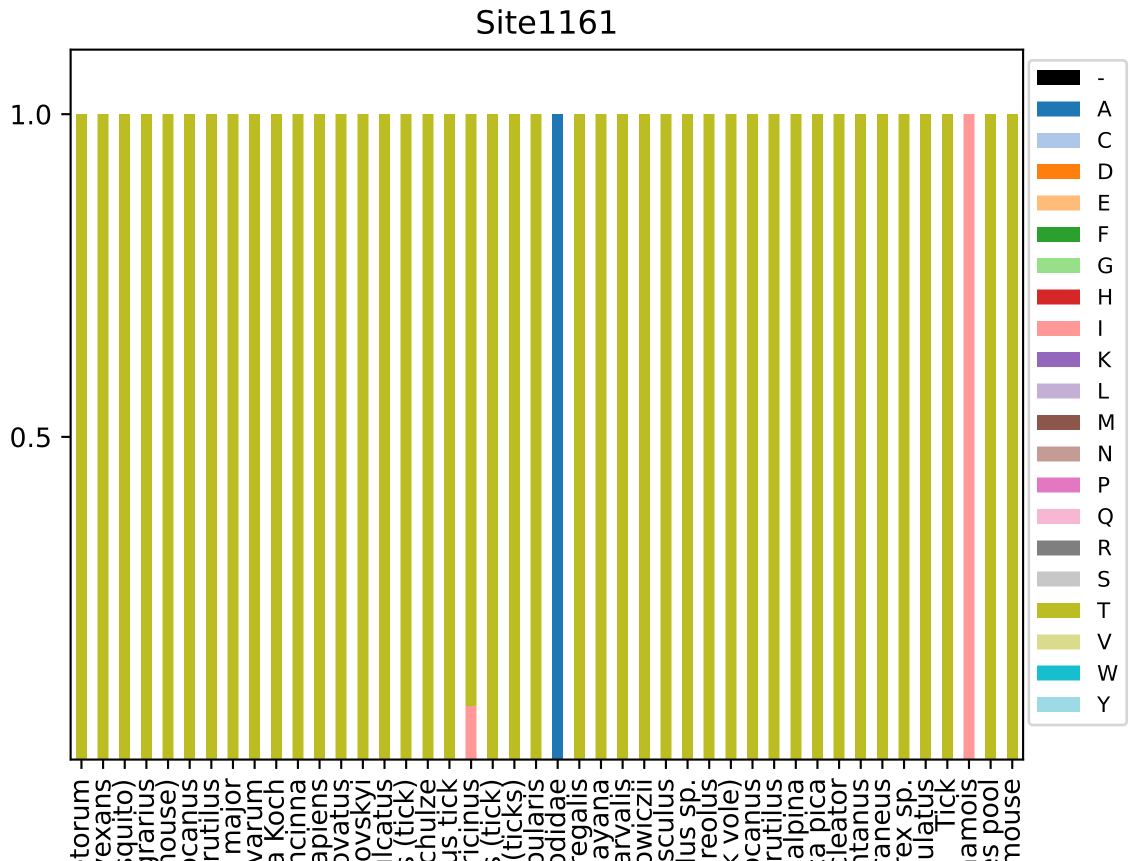
Site1161
1.0
0.5
-
A
C
D
E
F
G
H
I
K
L
M
N
P
Q
R
S
T
V
W
Y
Tick

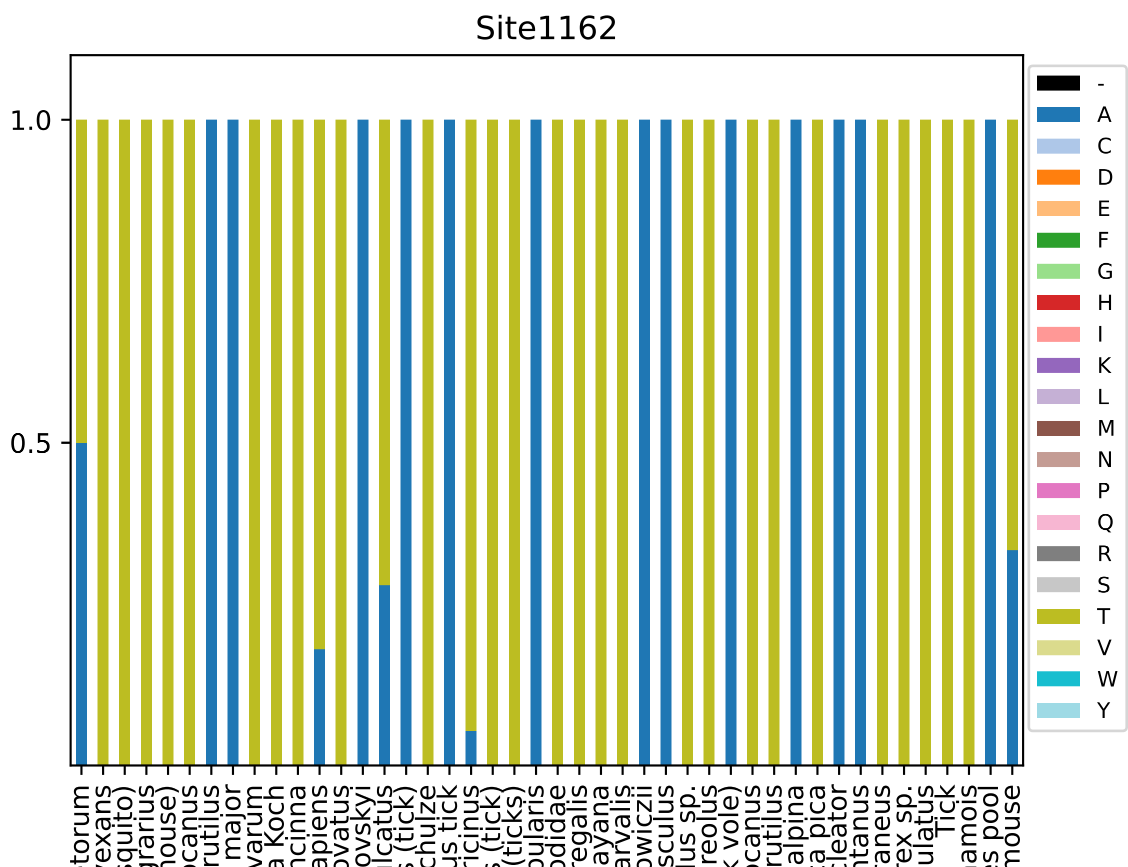

Site1162
1.0
0.5
-
A
C
D
E
F
G
H
I
K
L
M
N
P
Q
R
S
T
V
W
Y

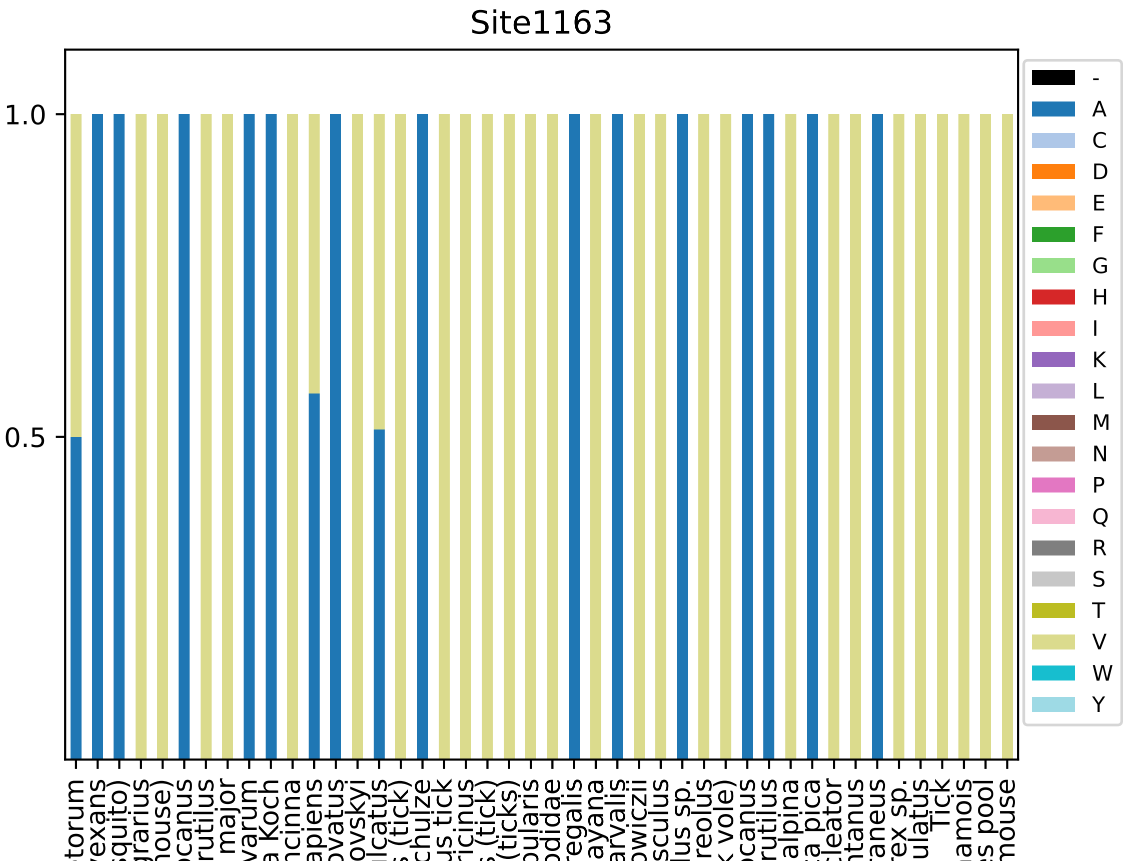
Site1163
1.0
0.5
-
A
C
D
E
F
G
H
I
K
L
M
N
P
Q
R
S
T
V
W
Y

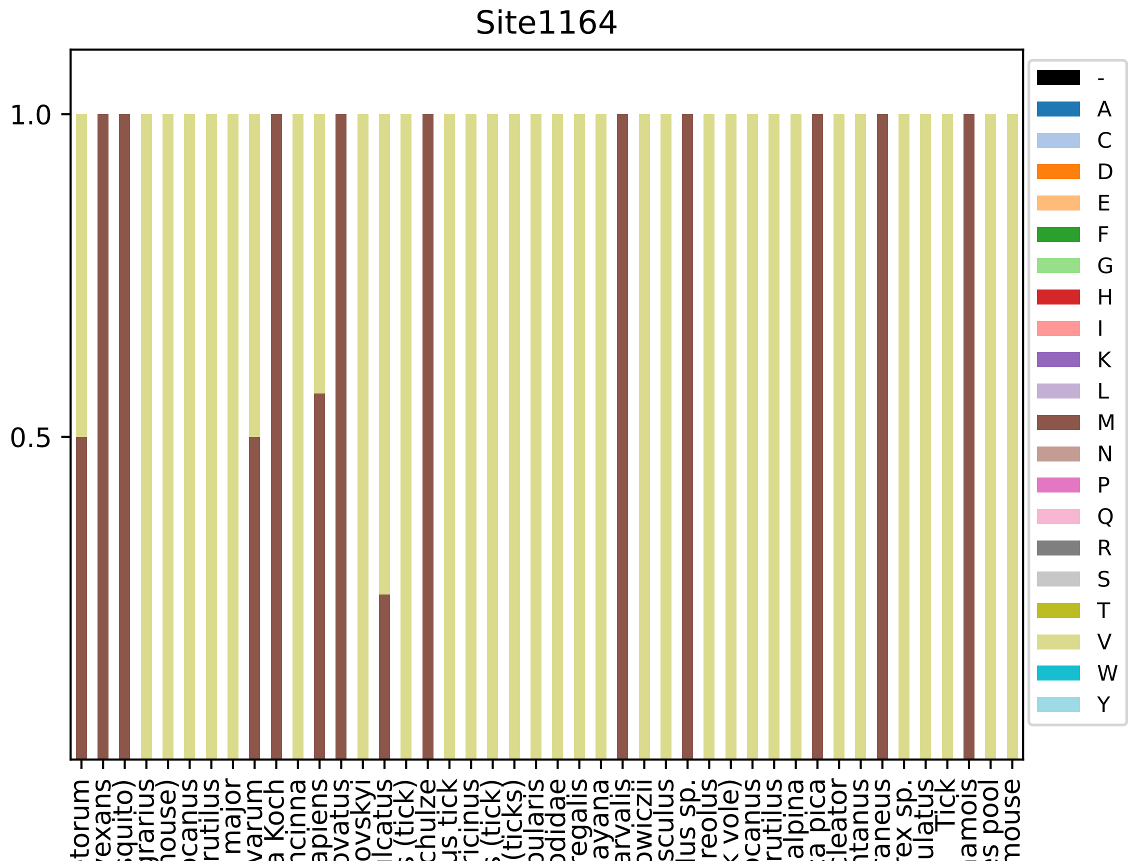
Site1164
1.0
0.5
-
A
C
D
E
F
G
H
I
K
L
M
N
P
Q
R
S
T
V
W
Y

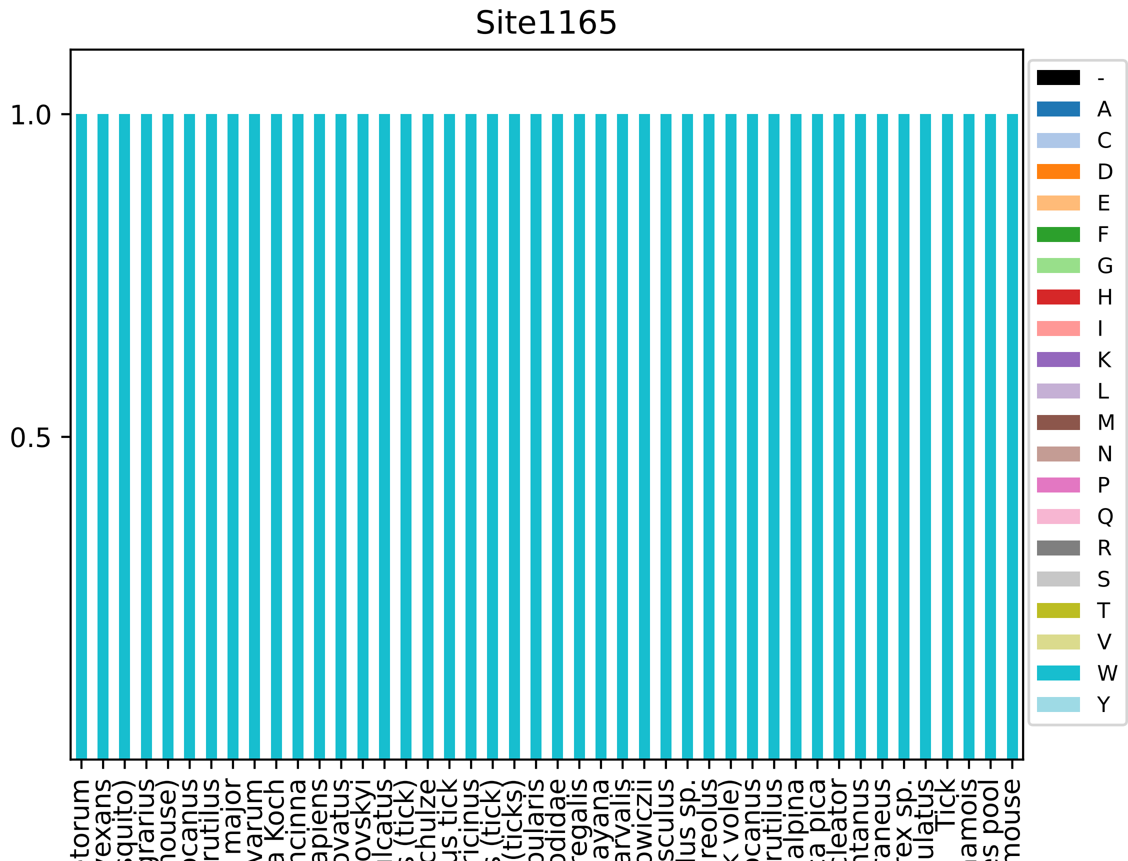
Site1165
1.0
0.5
-
A
C
D
E
F
G
H
I
K
L
M
N
P
Q
R
S
T
V
W
Y

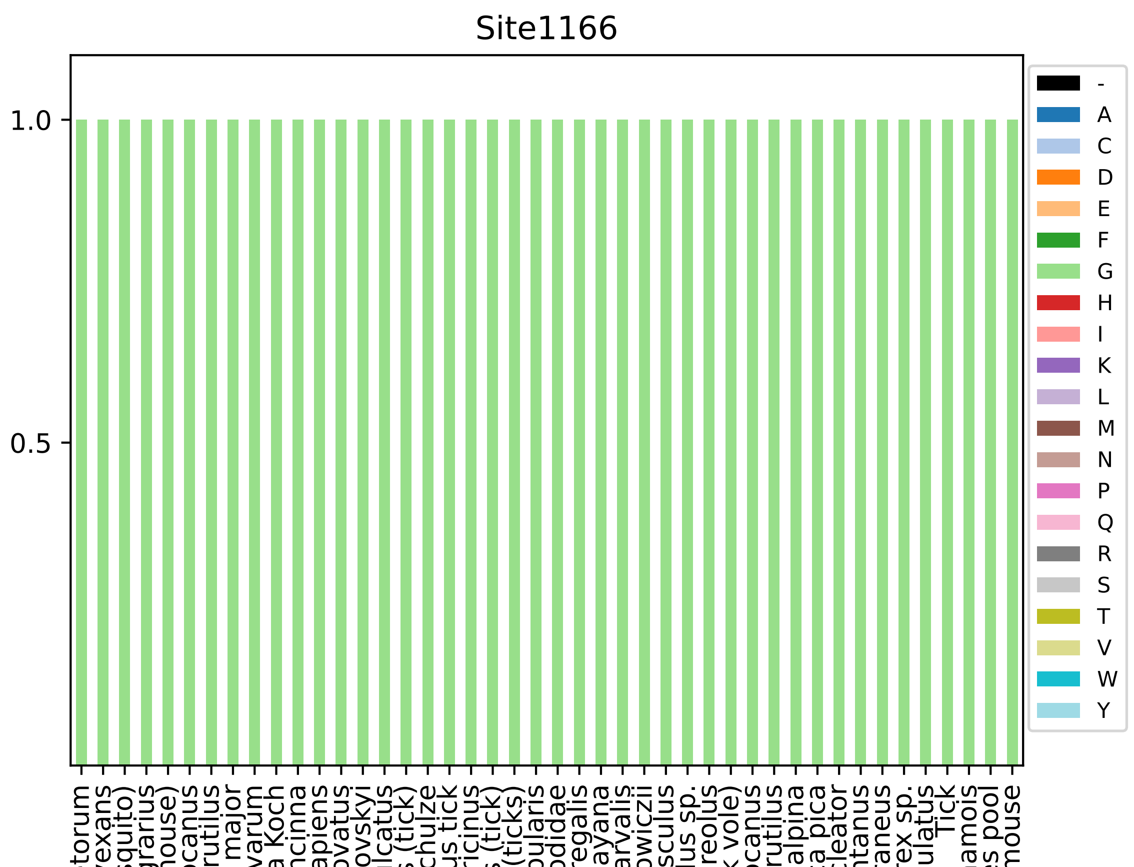
Site1166
1.0
0.5
-
A
C
D
E
F
G
H
I
K
L
M
N
P
Q
R
S
T
V
W
Y

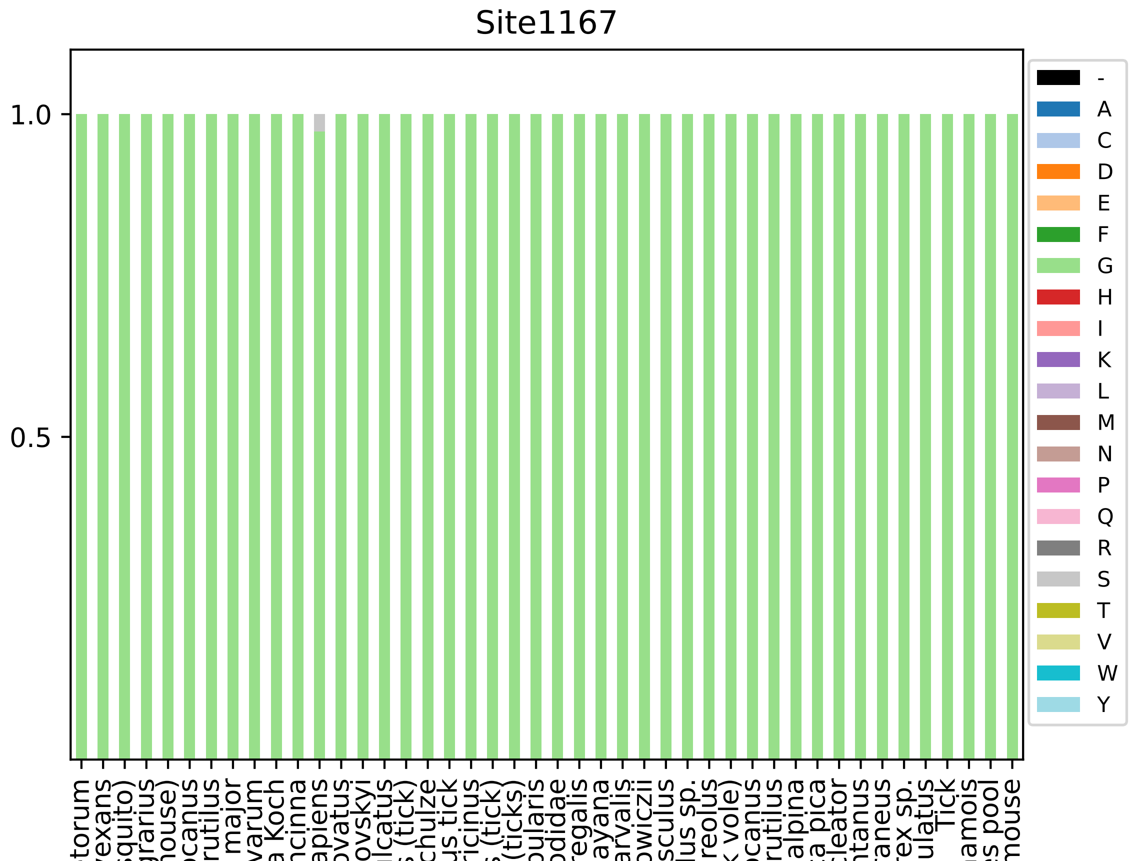

Site1167
1.0
0.5
-
A
C
D
E
F
G
H
I
K
L
M
N
P
Q
R
S
T
V
W
Y

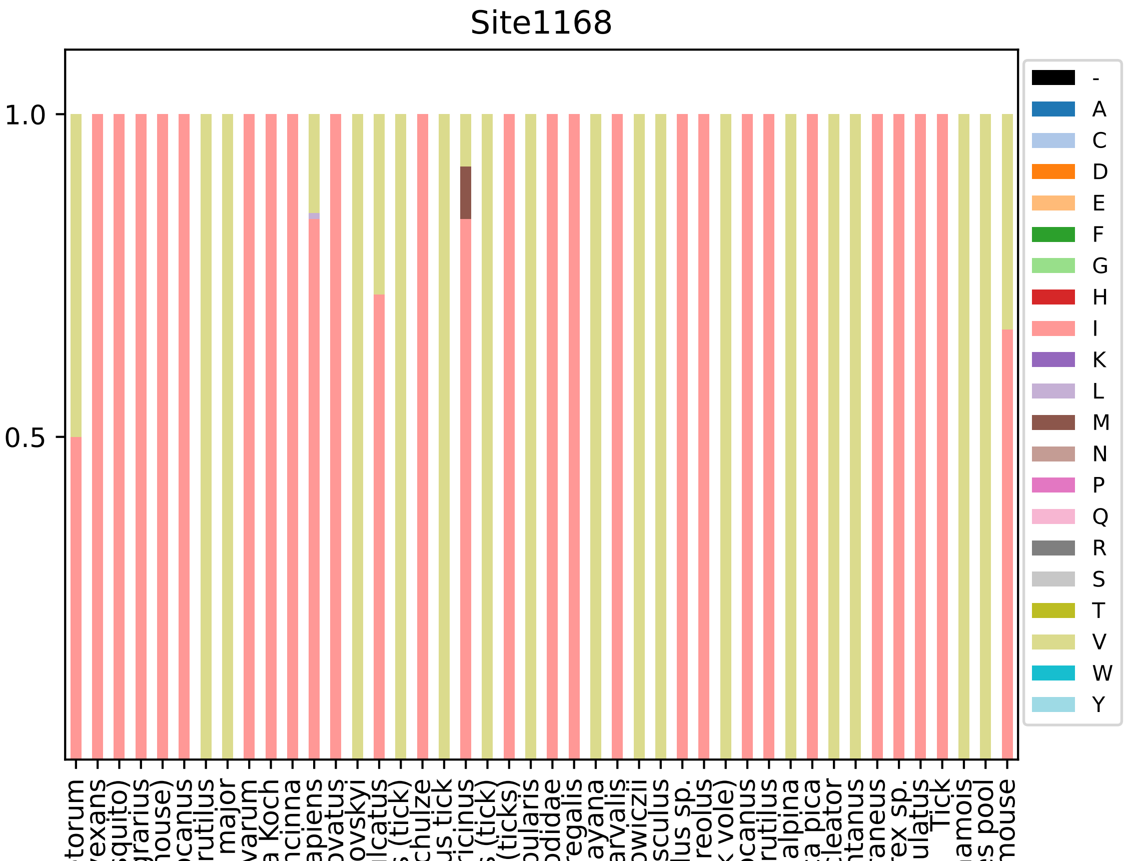

Site1168
1.0
0.5
-
A
C
D
E
F
G
H
I
K
L
M
N
P
Q
R
S
T
V
W
Y
torum
vexans
squito)
garius
nouse)
ocanus
rutilus
major
varum
a Koch
ncinna
apiens
ovatus
ovskyi
ulcatus
s (tick)
chulze
us tick
ricinus
s (tick)
s (ticks)
pularis
podidae
regalis
ayana
arvalis
owiczii
sculus
lus sp.
reolus
k vole)
ocanus
rutilus
alpina
a pica
leator
ntanus
aneus
rex sp.
ulatus
Tick
amois
es pool
mouse

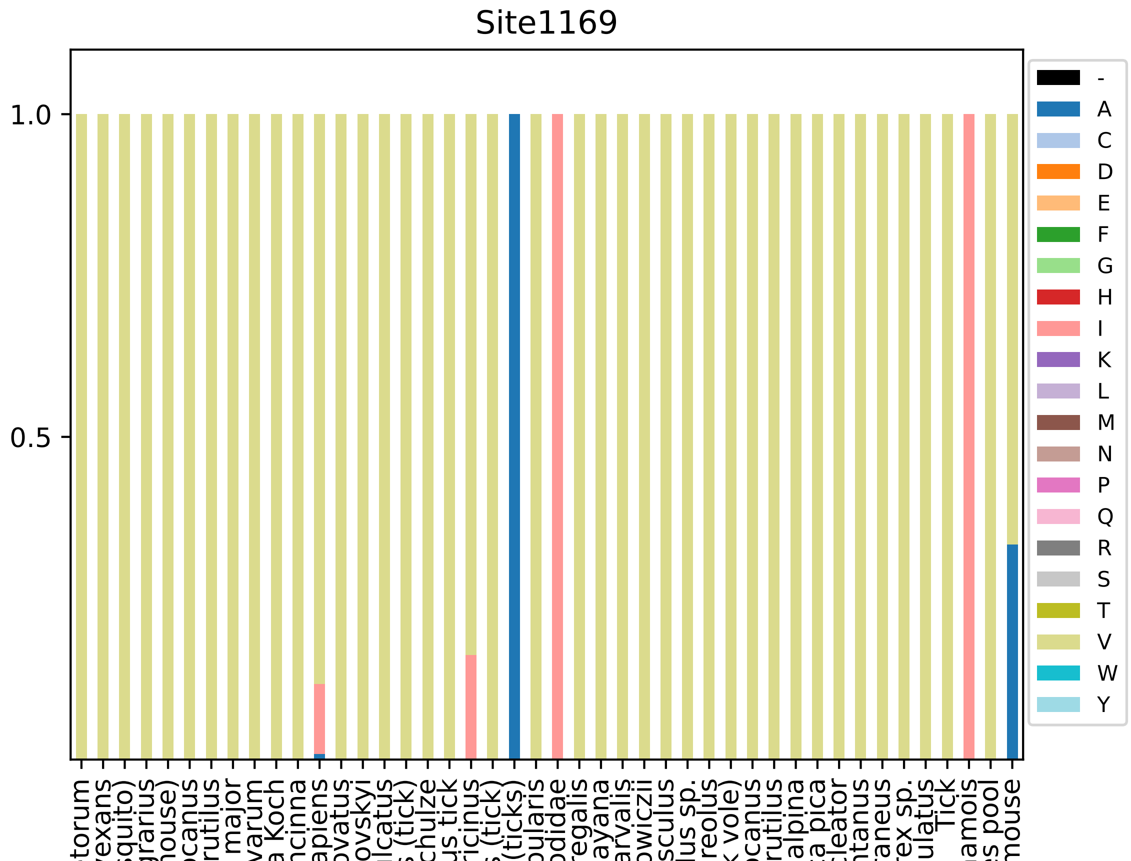
Site1169
1.0
0.5
-
A
C
D
E
F
G
H
I
K
L
M
N
P
Q
R
S
T
V
W
Y

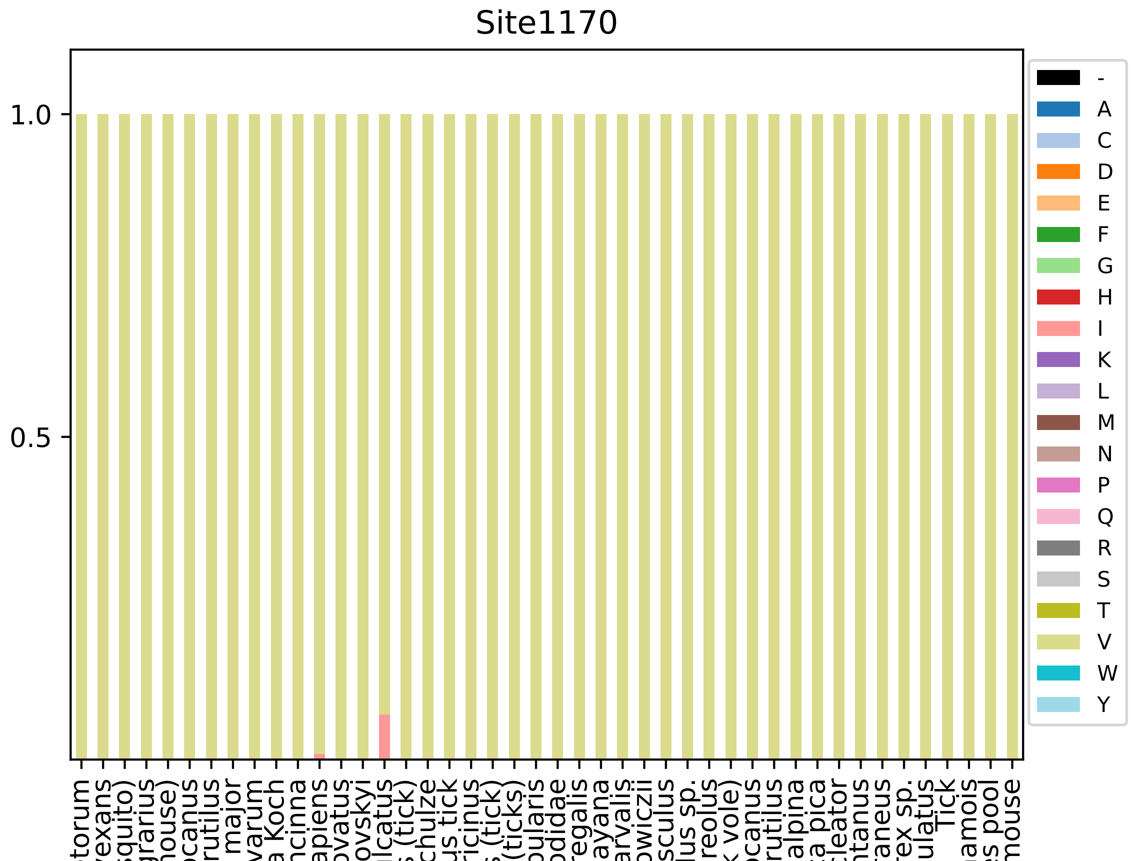
Site1170
1.0
0.5
-
A
C
D
E
F
G
H
I
K
L
M
N
P
Q
R
S
T
V
W
Y

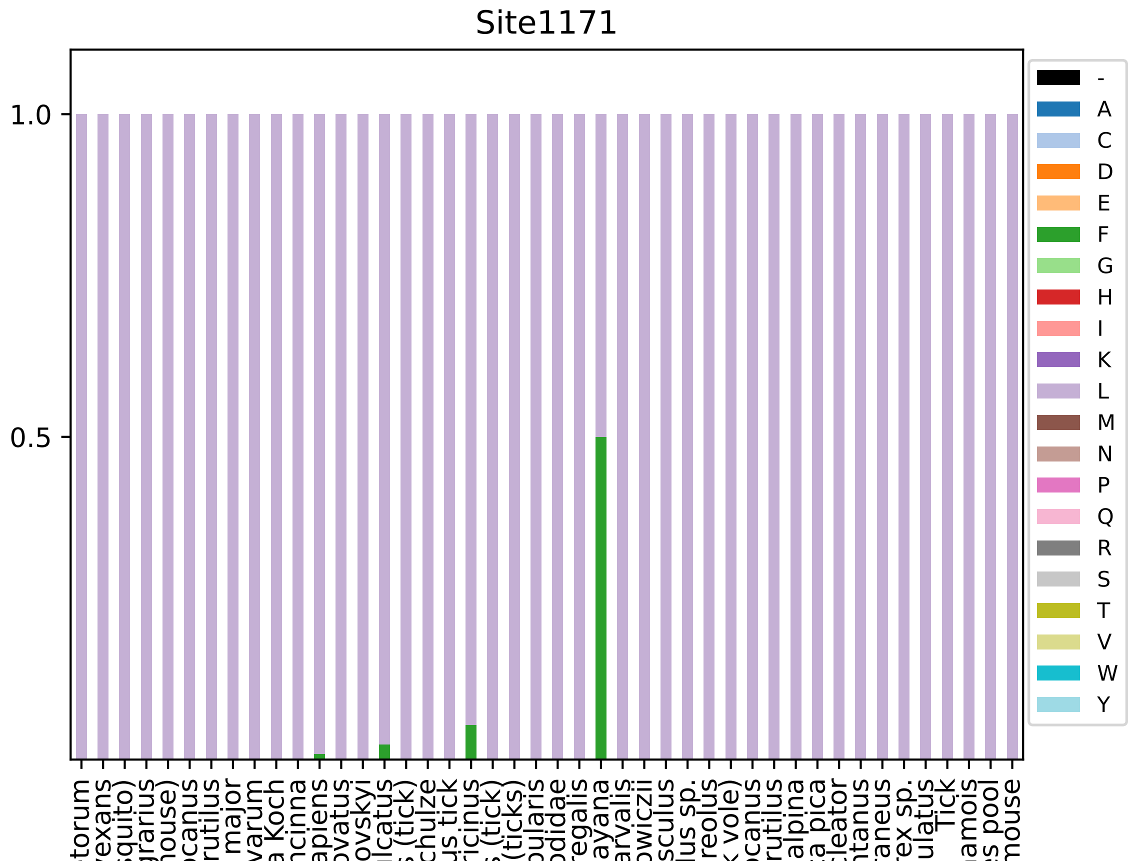
Site1171
1.0
0.5
-
A
C
D
E
F
G
H
I
K
L
M
N
P
Q
R
S
T
V
W
Y

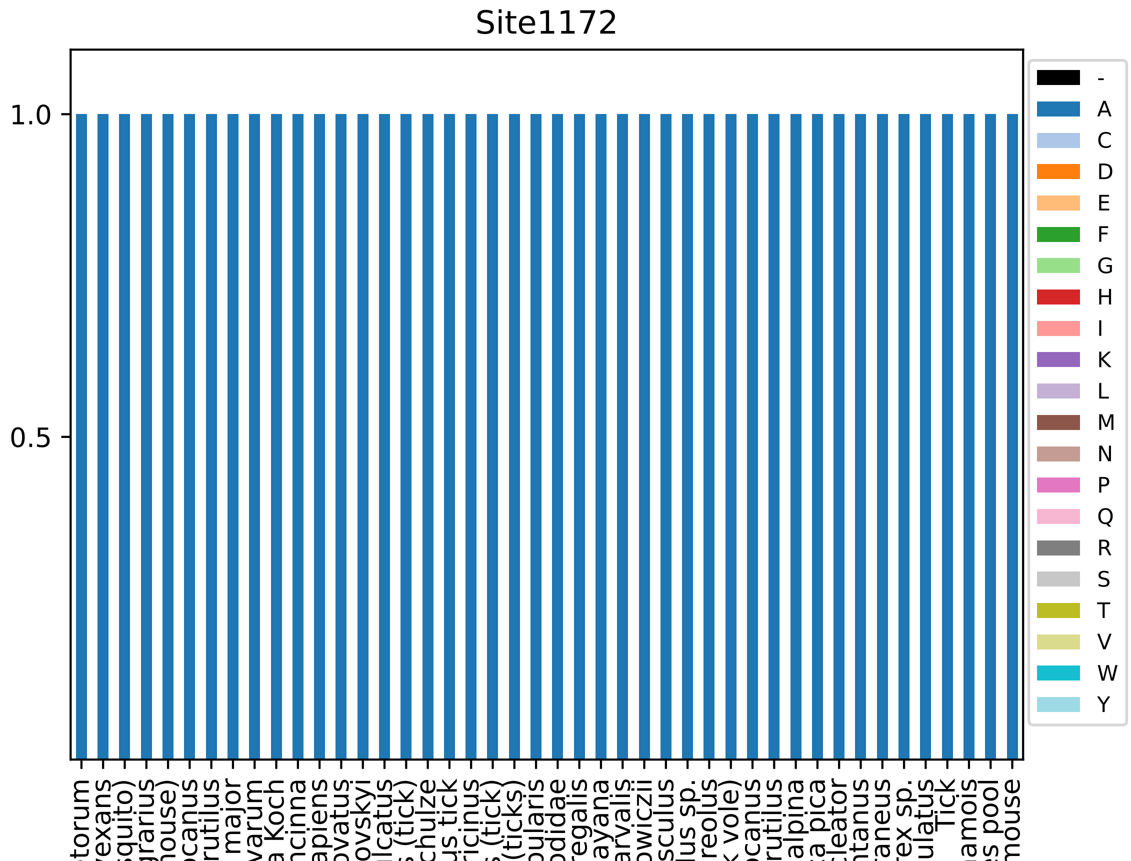
Site1172
1.0
0.5
-
A
C
D
E
F
G
H
I
K
L
M
N
P
Q
R
S
T
V
W
Y

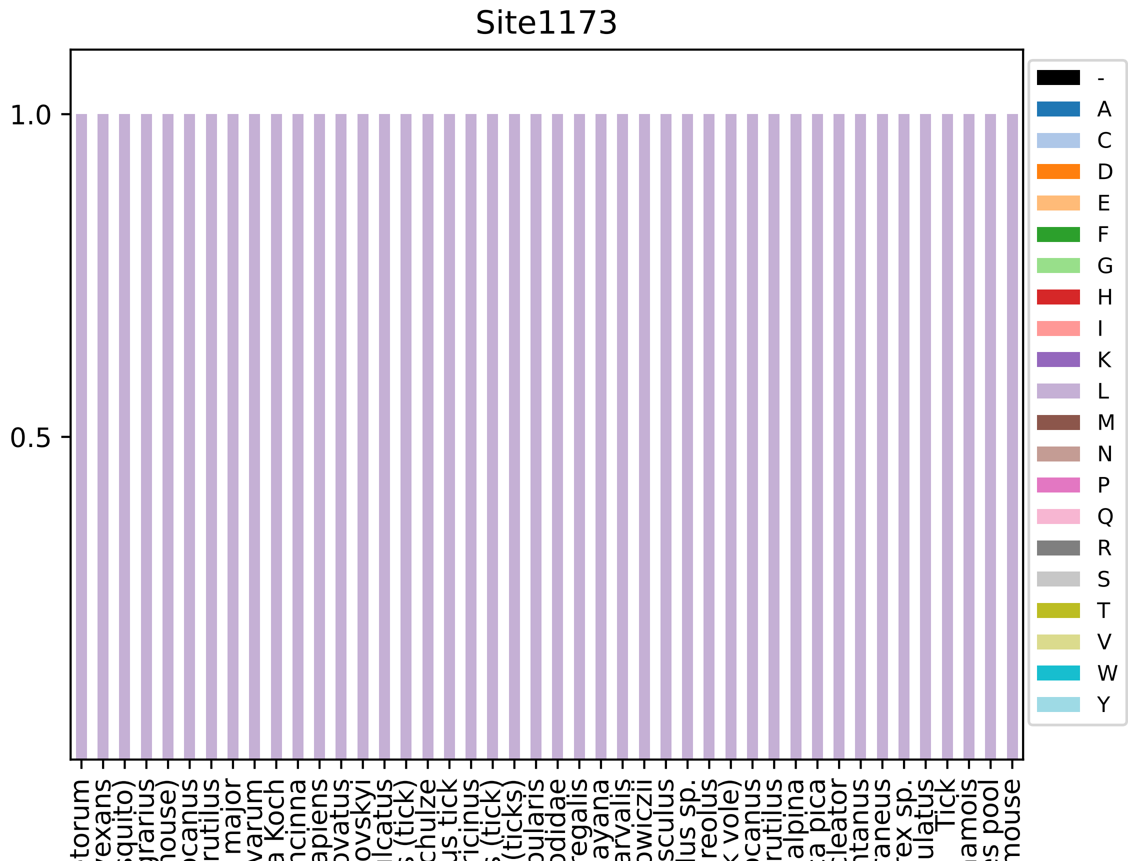
Site1173
1.0
0.5
-
A
C
D
E
F
G
H
I
K
L
M
N
P
Q
R
S
T
V
W
Y

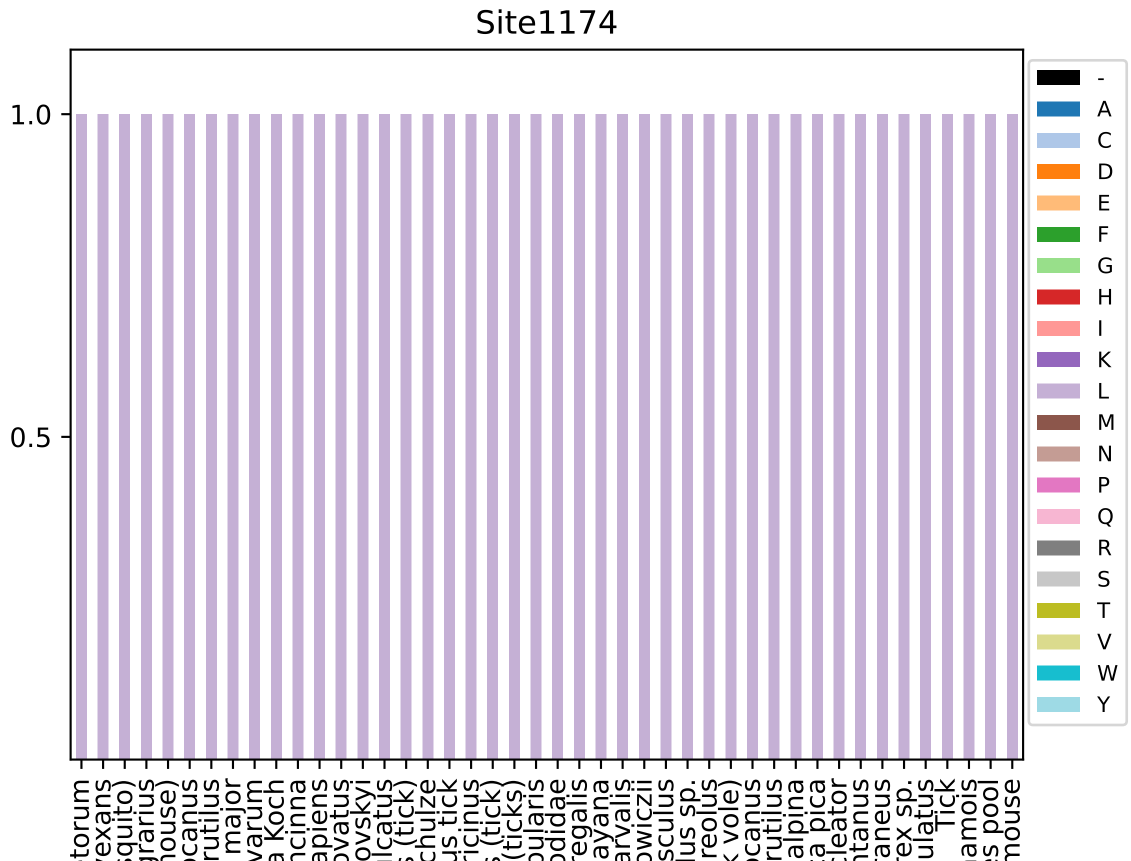
Site1174
1.0
0.5
-
A
C
D
E
F
G
H
I
K
L
M
N
P
Q
R
S
T
V
W
Y

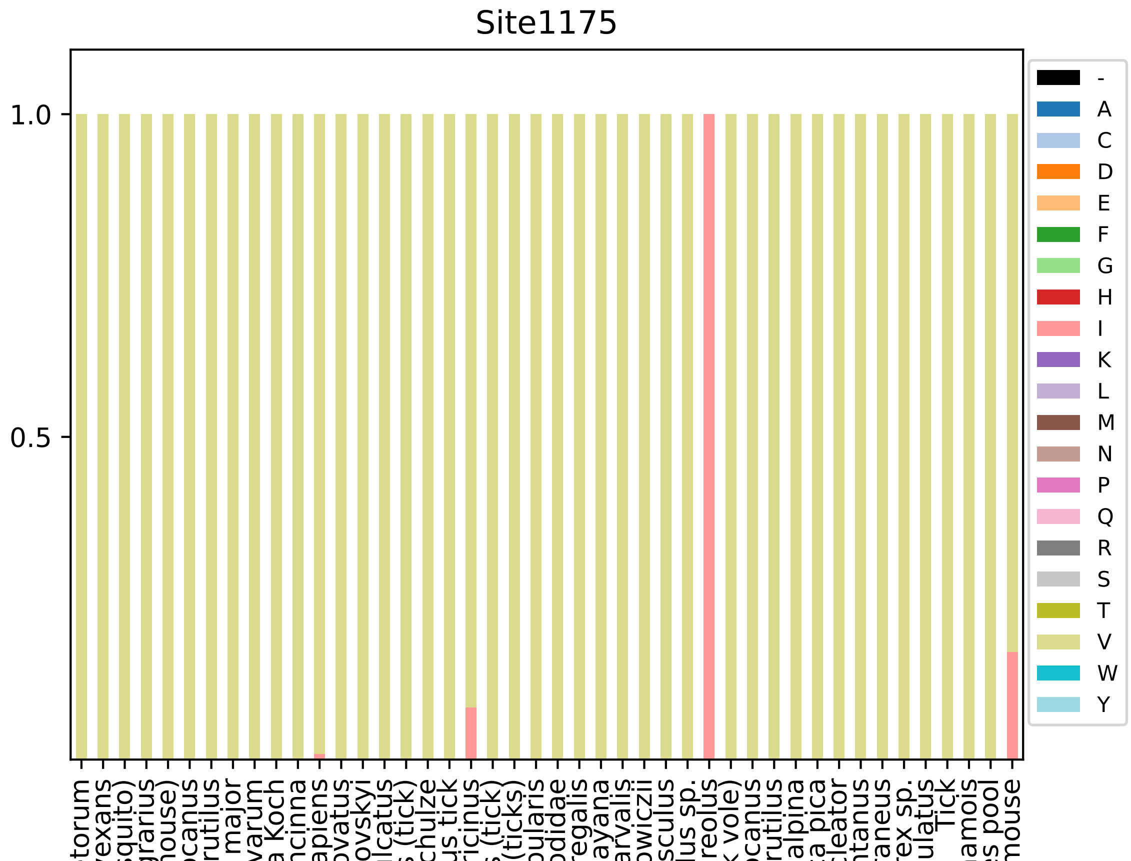

Site1175
1.0
0.5
-
A
C
D
E
F
G
H
I
K
L
M
N
P
Q
R
S
T
V
W
Y

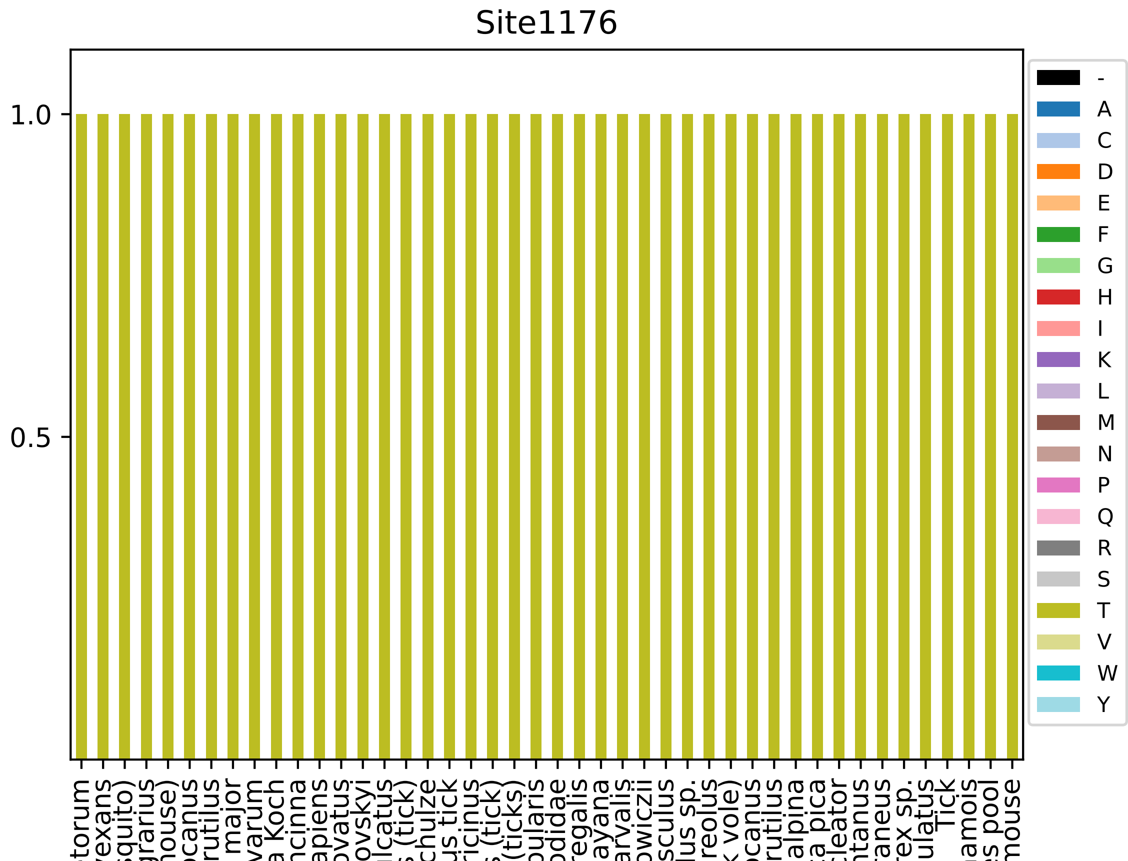
Site1176
1.0
0.5
-
A
C
D
E
F
G
H
I
K
L
M
N
P
Q
R
S
T
V
W
Y

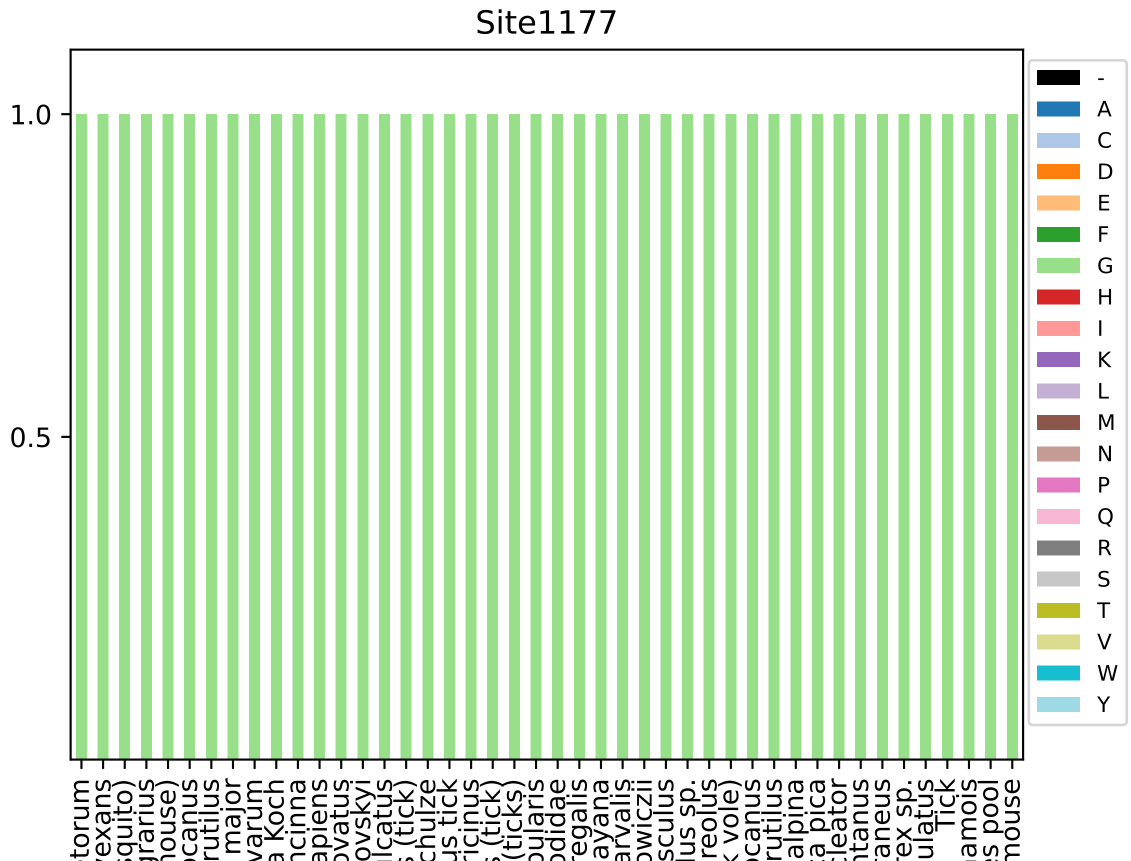
Site1177
1.0
0.5
-
A
C
D
E
F
G
H
I
K
L
M
N
P
Q
R
S
T
V
W
Y

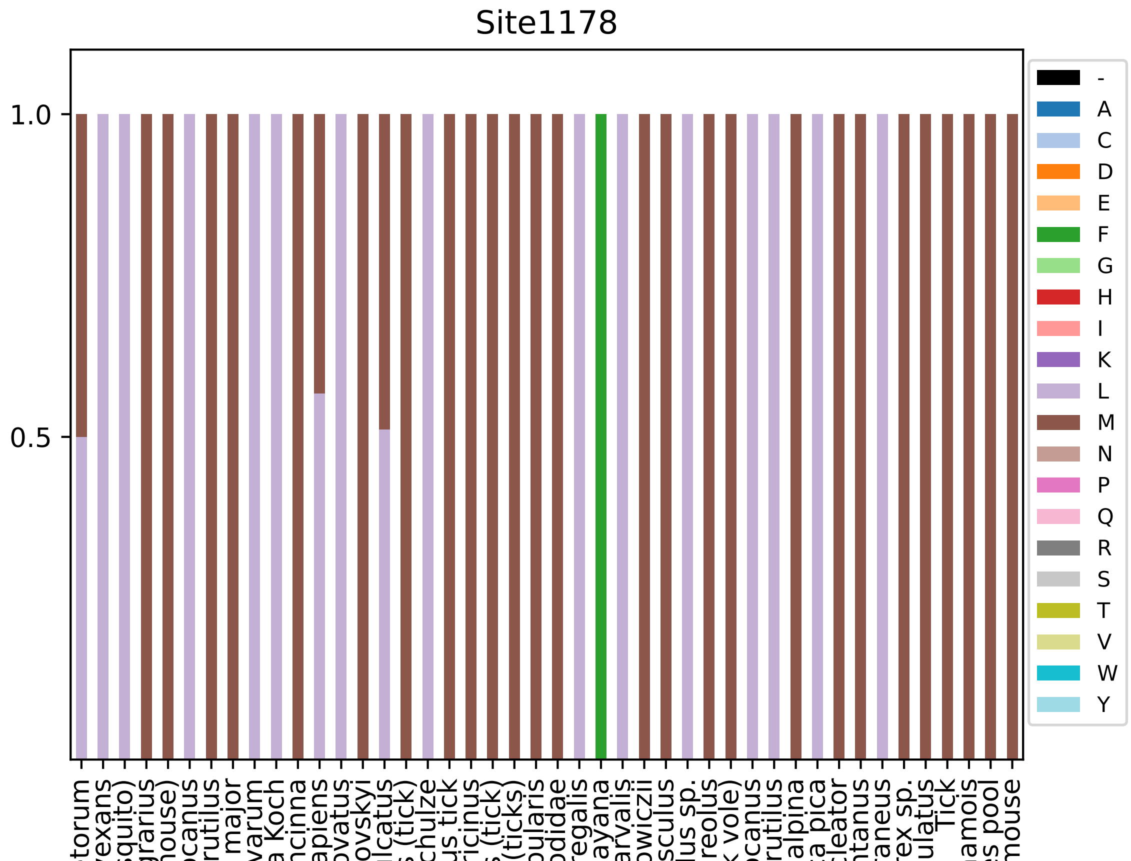
Site1178
1.0
0.5
-
A
C
D
E
F
G
H
I
K
L
M
N
P
Q
R
S
T
V
W
Y

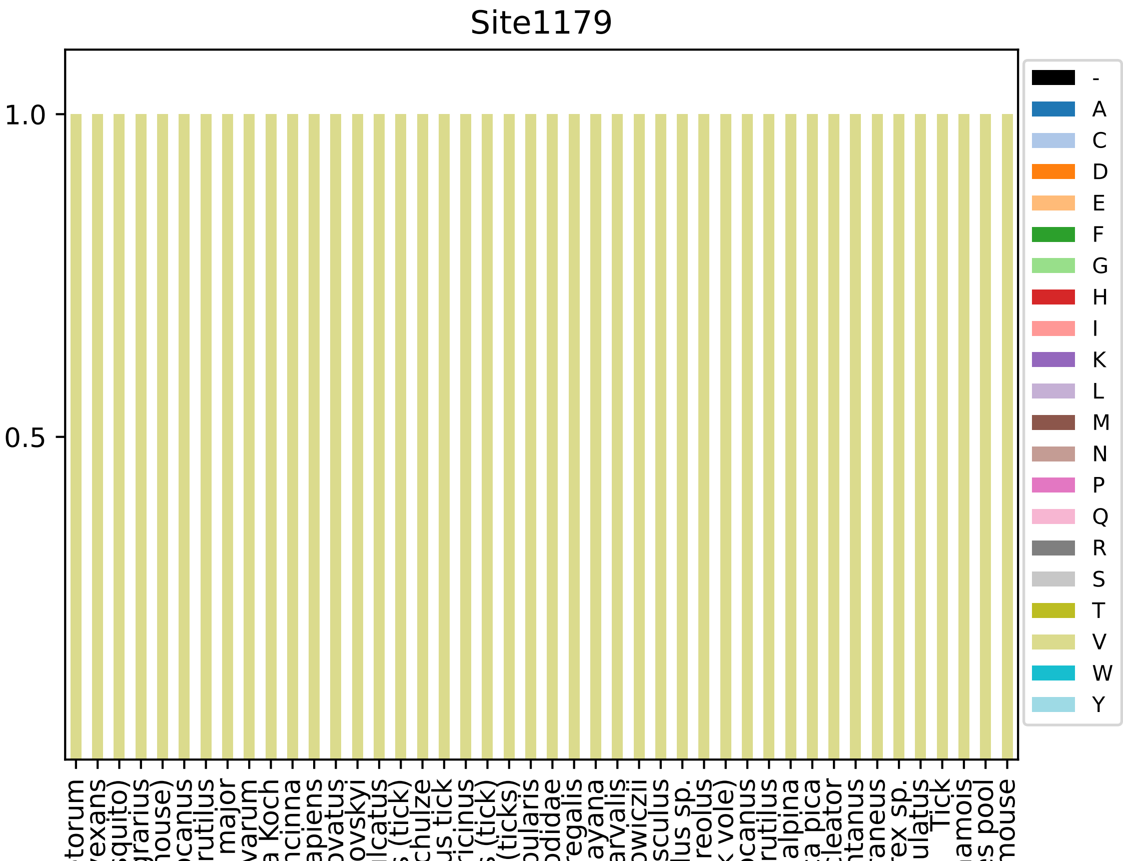
Site1179
1.0
0.5
-
A
C
D
E
F
G
H
I
K
L
M
N
P
Q
R
S
T
V
W
Y

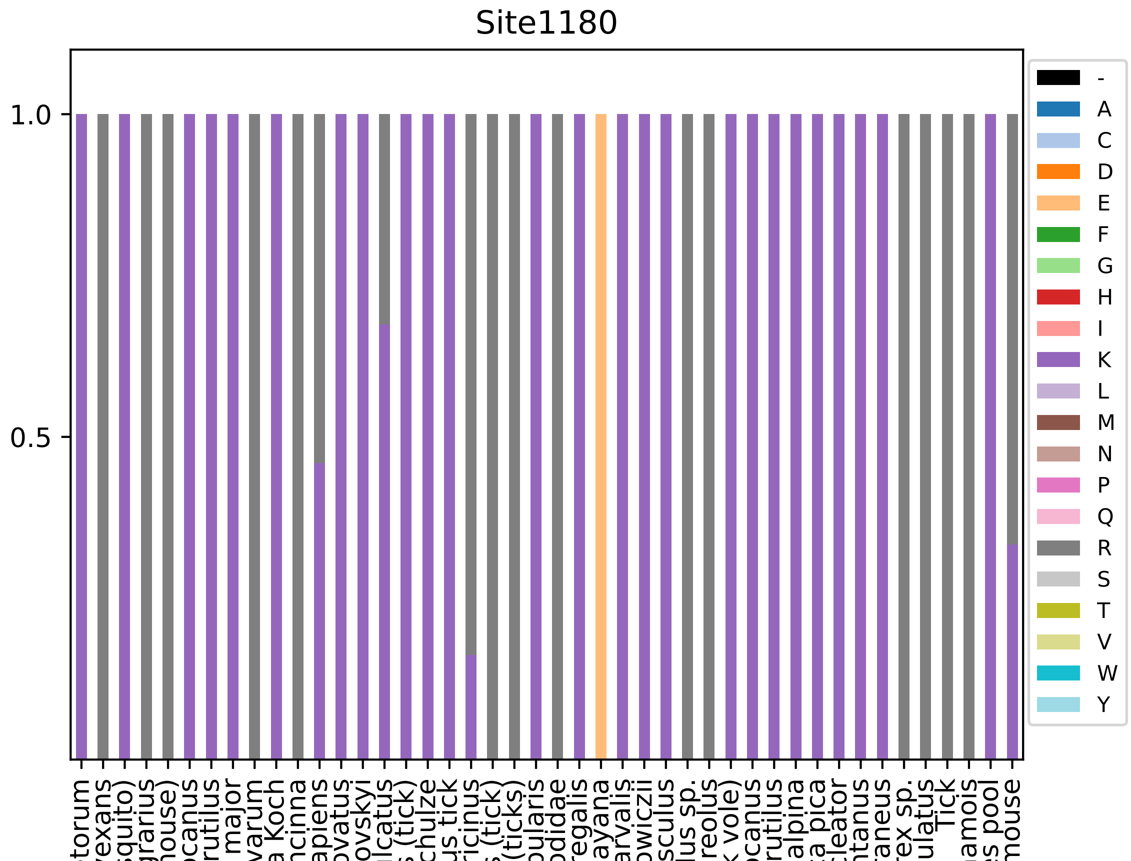
Site1180
1.0
0.5
-
A
C
D
E
F
G
H
I
K
L
M
N
P
Q
R
S
T
V
W
Y

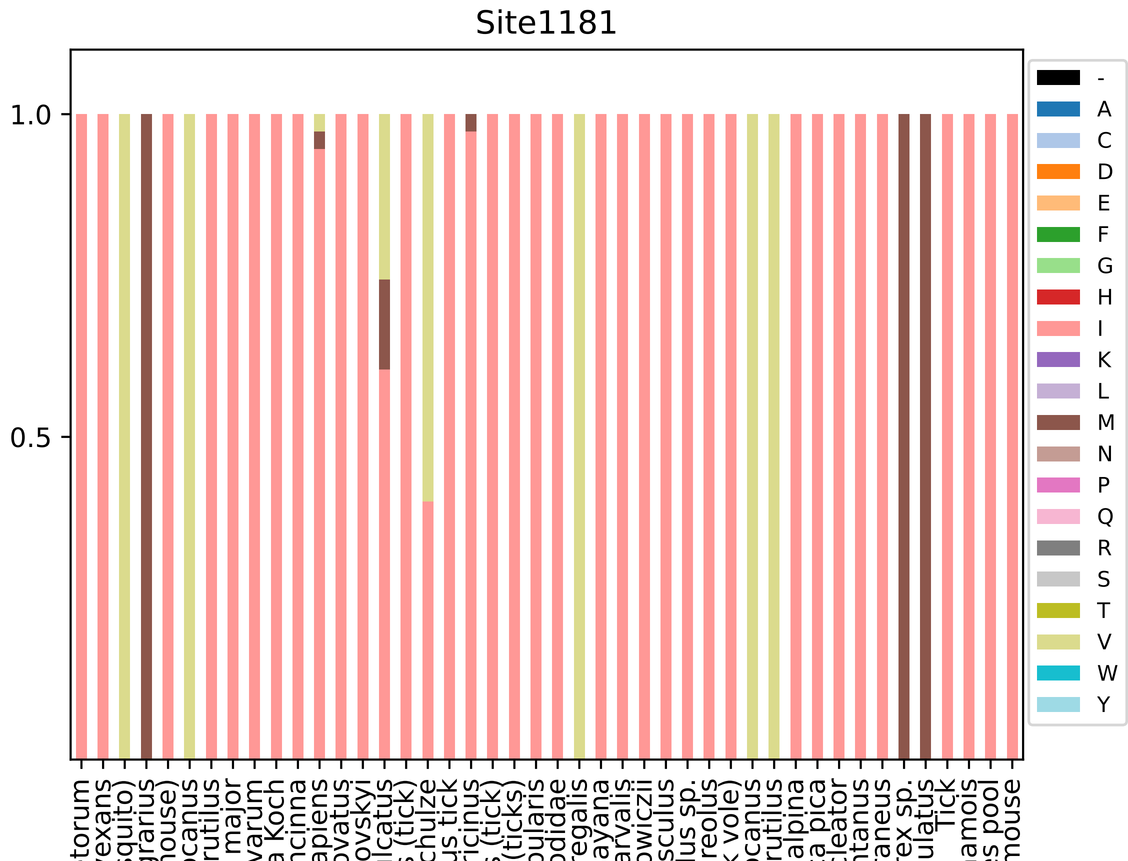

Site1181
1.0
0.5
-
A
C
D
E
F
G
H
I
K
L
M
N
P
Q
R
S
T
V
W
Y

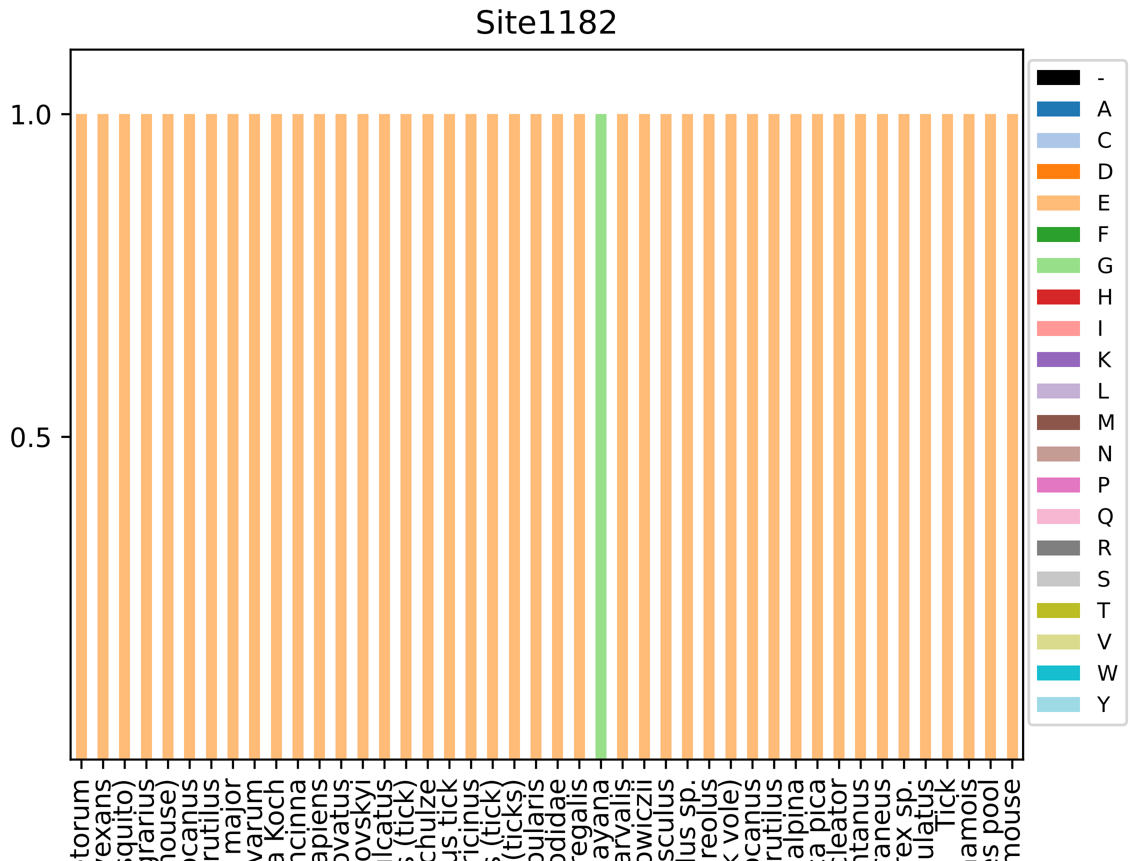

Site1182
1.0
0.5
-
A
C
D
E
F
G
H
I
K
L
M
N
P
Q
R
S
T
V
W
Y

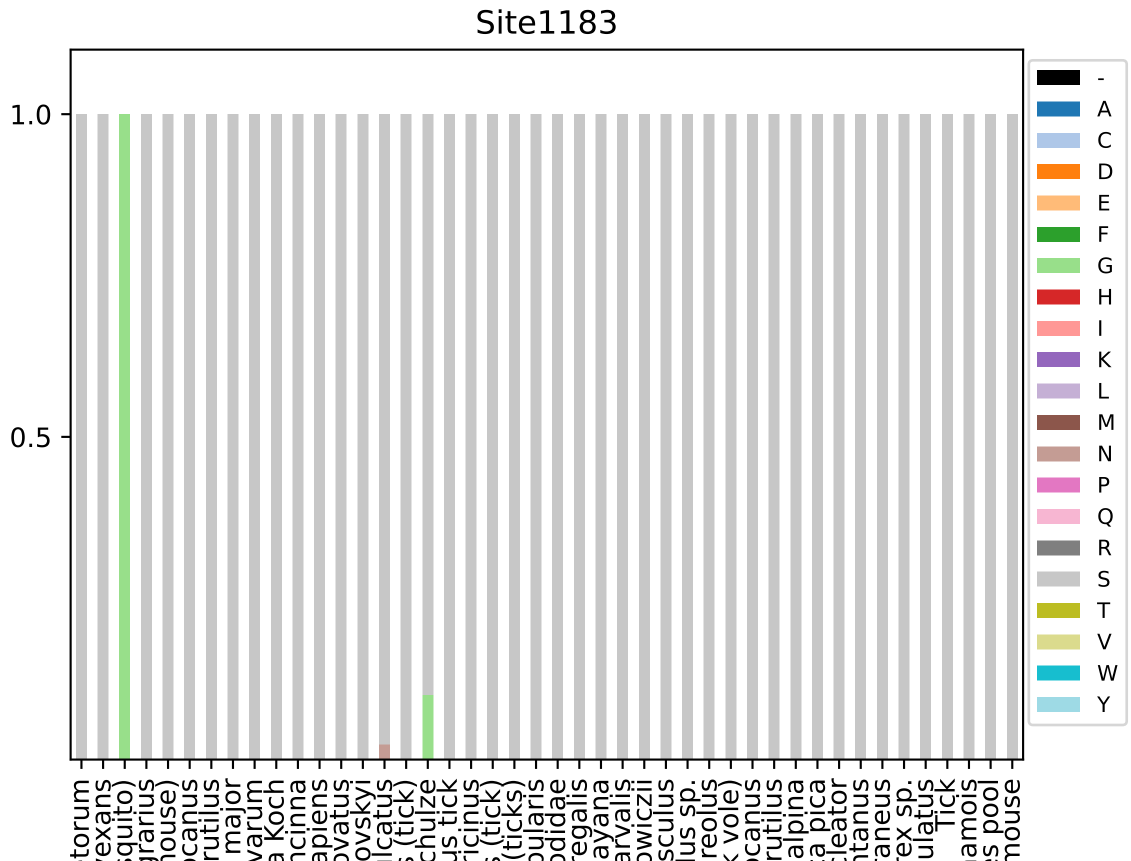
Site1183
1.0
0.5
-
A
C
D
E
F
G
H
I
K
L
M
N
P
Q
R
S
T
V
W
Y

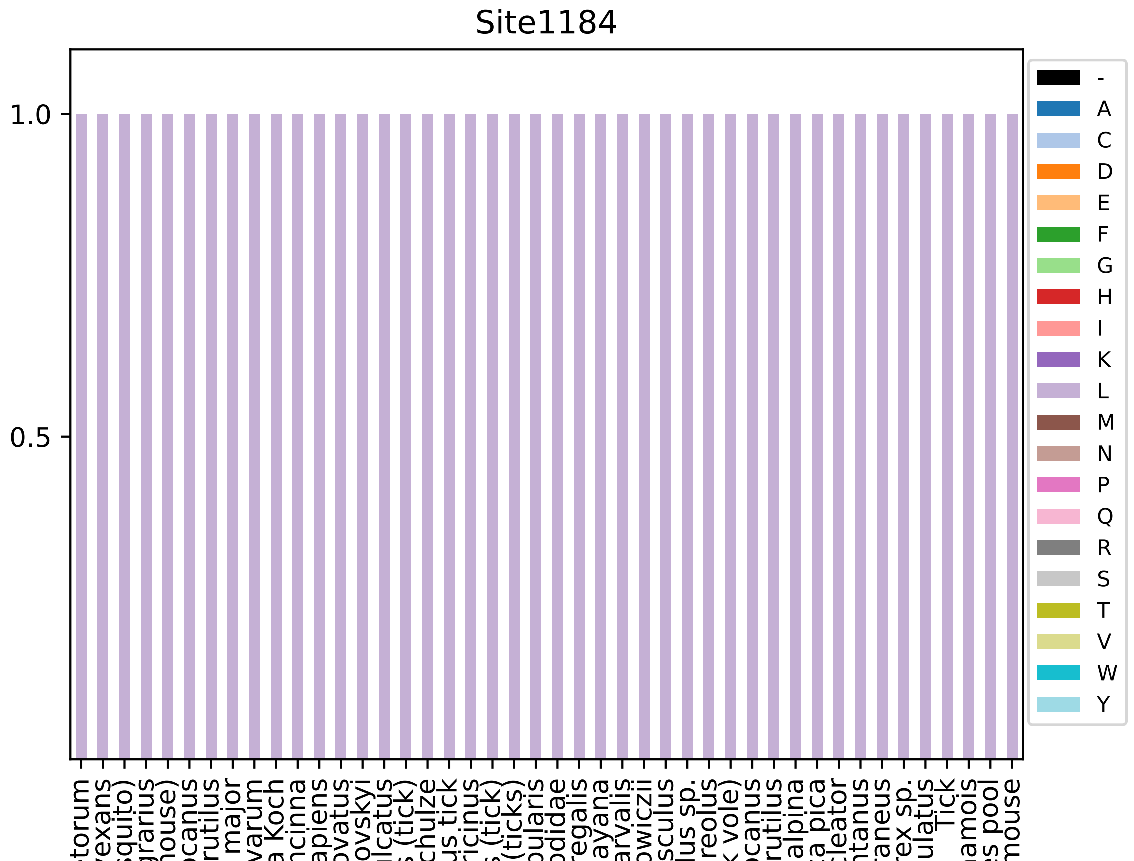
Site1184
1.0
0.5
-
A
C
D
E
F
G
H
I
K
L
M
N
P
Q
R
S
T
V
W
Y

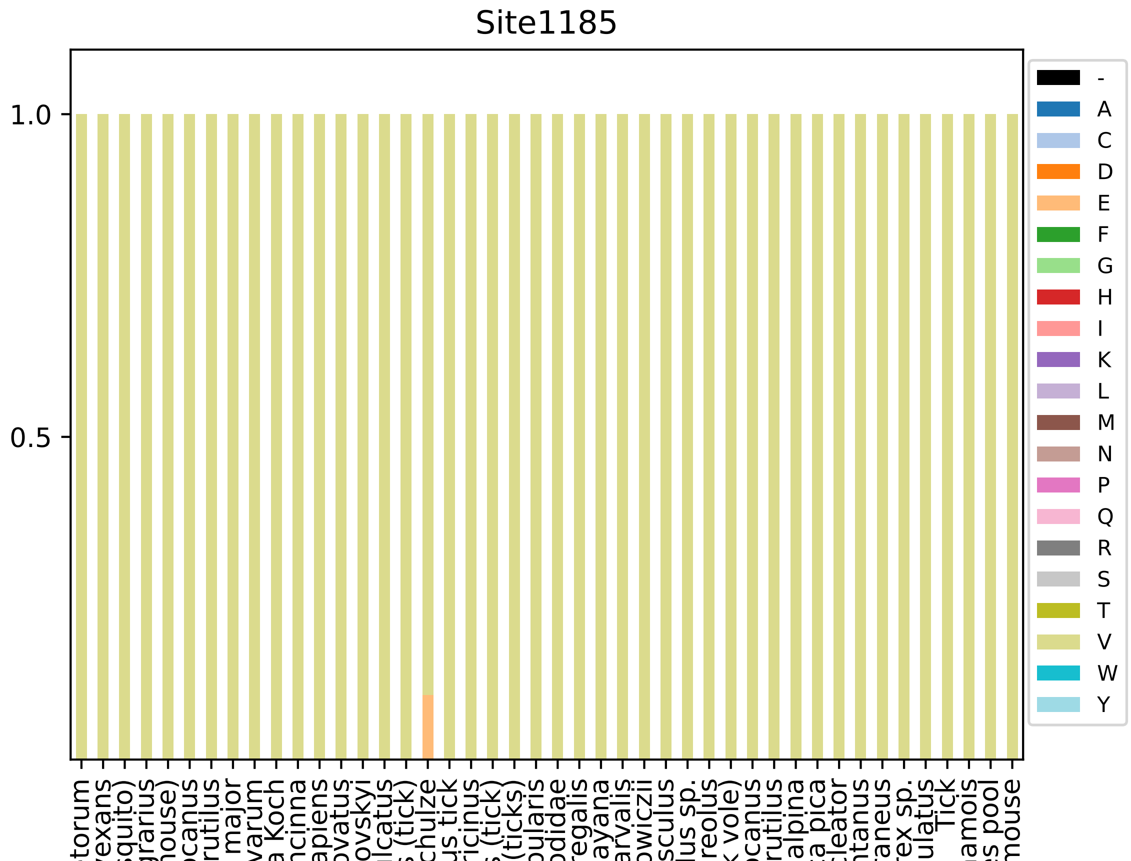
Site1185
1.0
0.5
-
A
C
D
E
F
G
H
I
K
L
M
N
P
Q
R
S
T
V
W
Y

Site1186

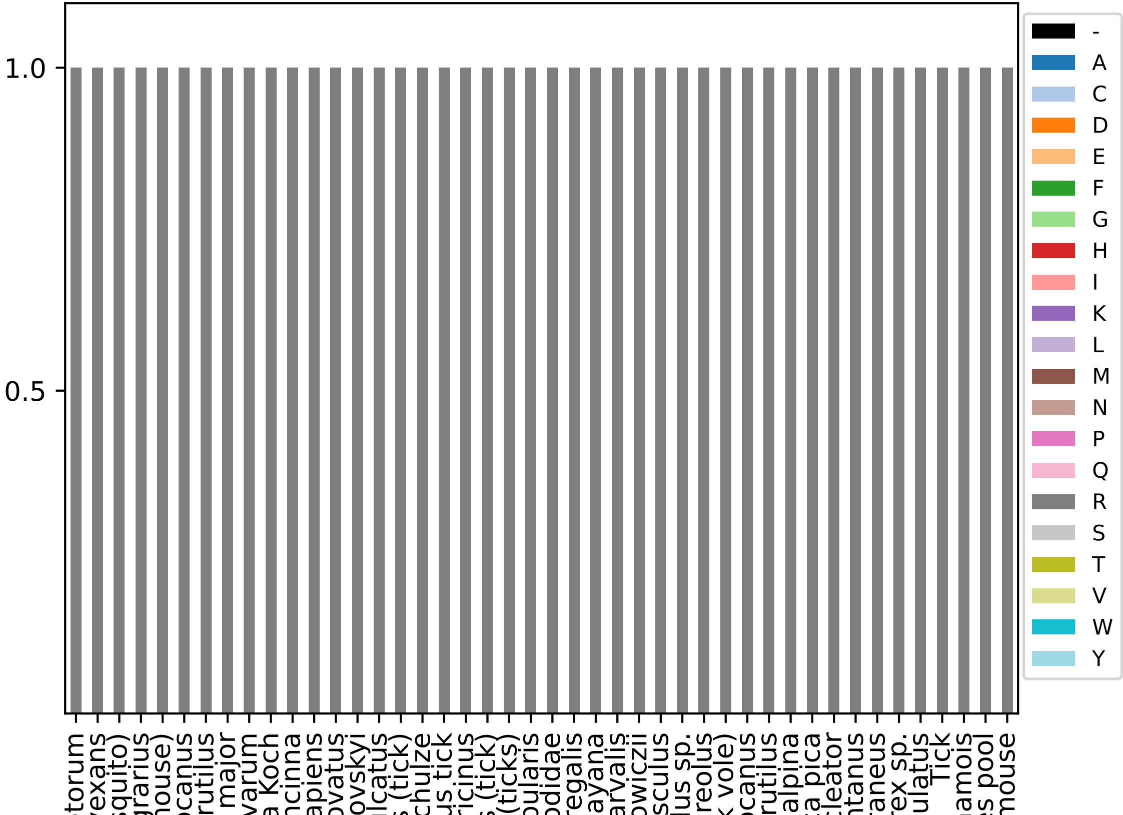

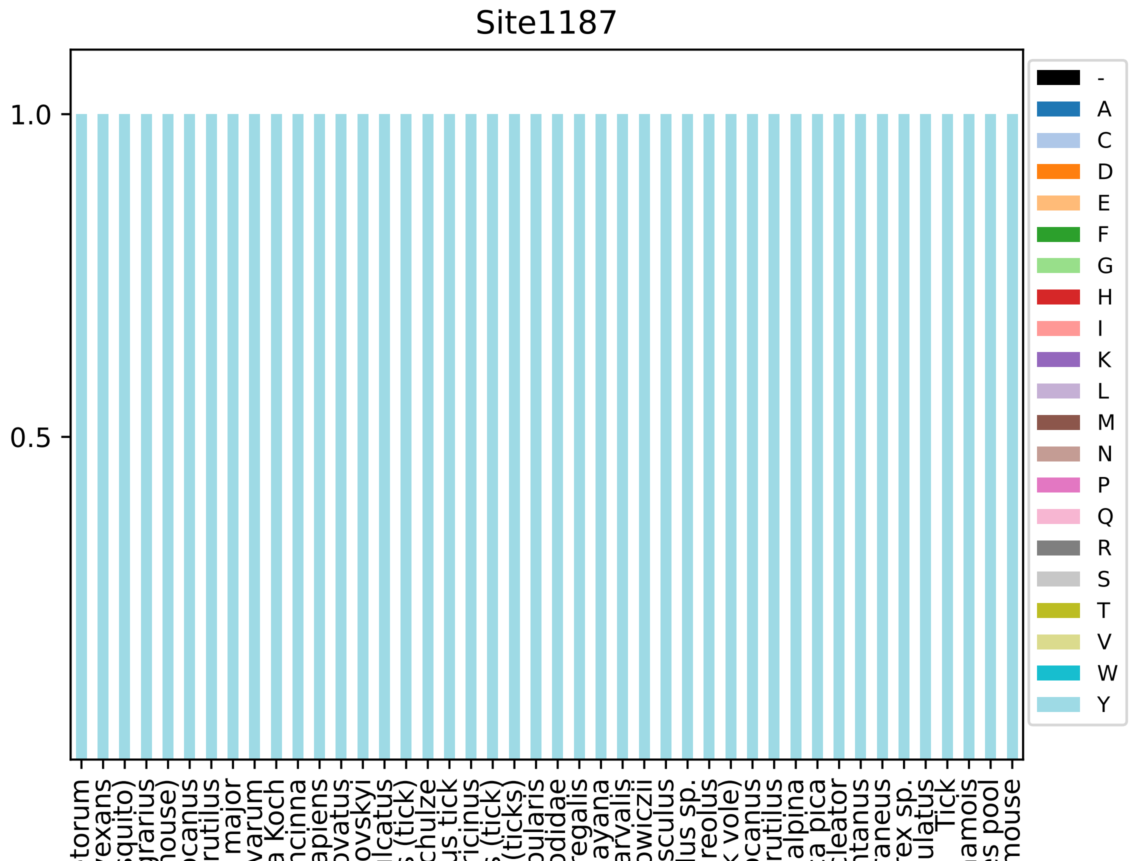
Site1187
1.0
0.5
-
A
C
D
E
F
G
H
I
K
L
M
N
P
Q
R
S
T
V
W
Y

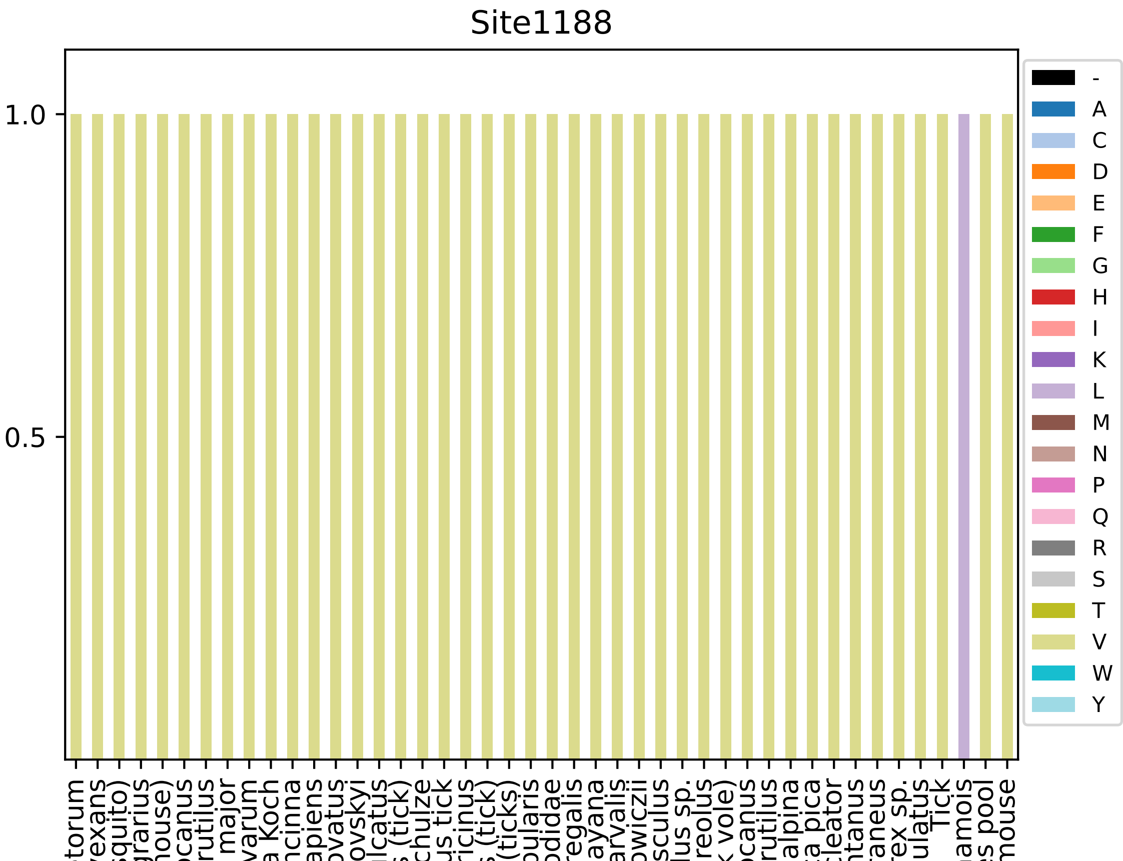
Site1188
1.0
0.5
-
A
C
D
E
F
G
H
I
K
L
M
N
P
Q
R
S
T
V
W
Y

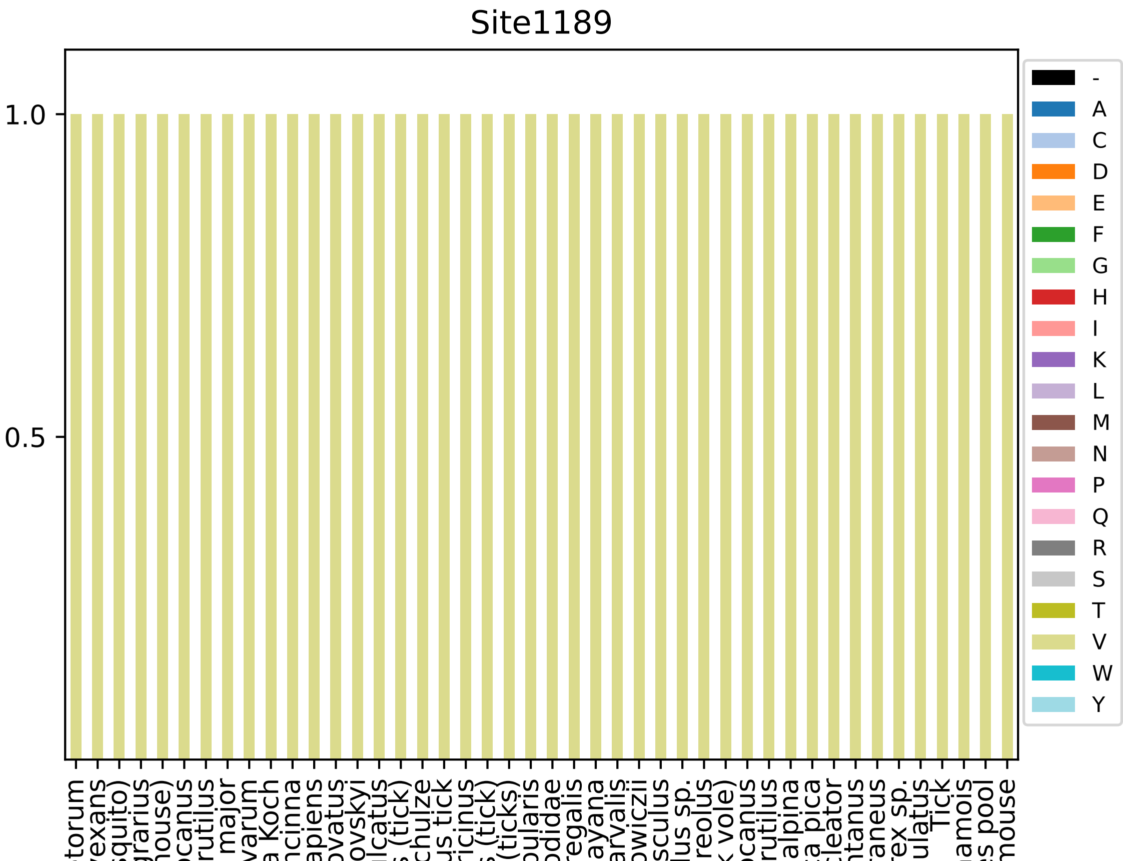
Site1189
1.0
0.5
-
A
C
D
E
F
G
H
I
K
L
M
N
P
Q
R
S
T
V
W
Y

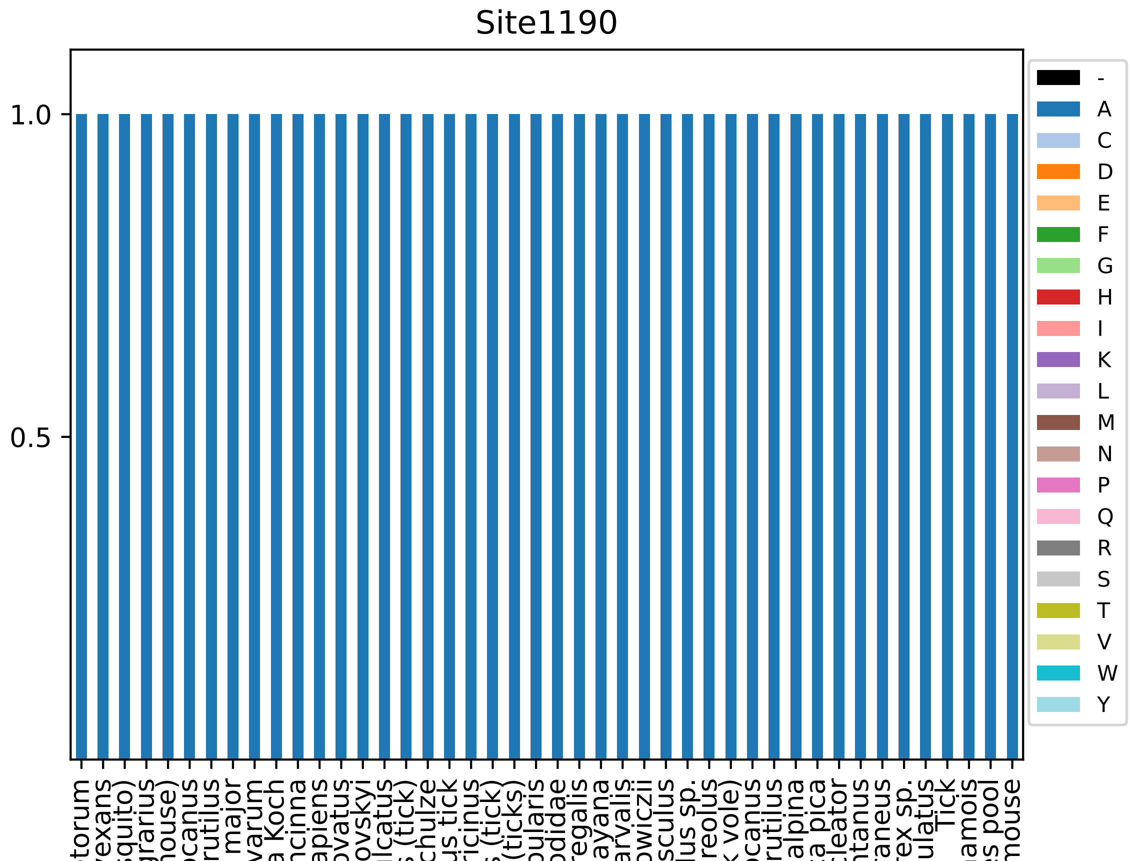
Site1190
1.0
0.5
-
A
C
D
E
F
G
H
I
K
L
M
N
P
Q
R
S
T
V
W
Y

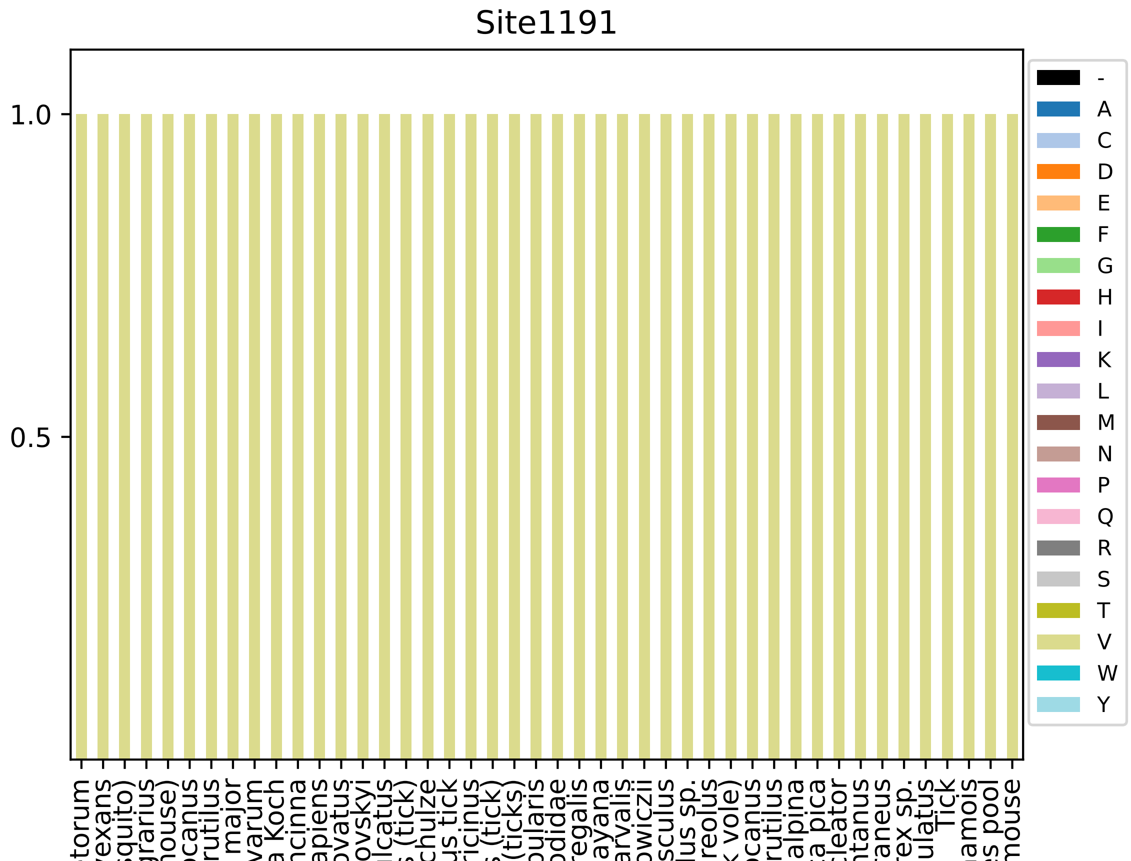
Site1191
1.0
0.5
-
A
C
D
E
F
G
H
I
K
L
M
N
P
Q
R
S
T
V
W
Y

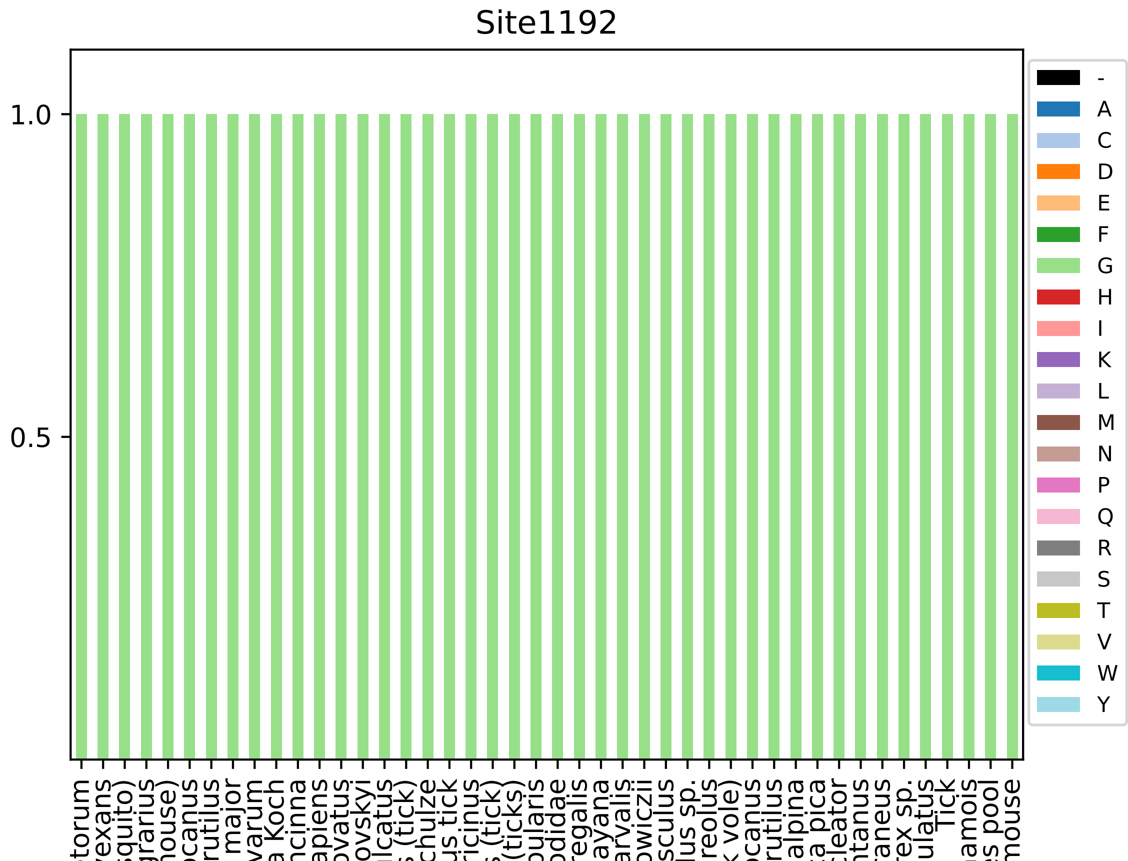
Site1192
1.0
0.5
-
A
C
D
E
F
G
H
I
K
L
M
N
P
Q
R
S
T
V
W
Y

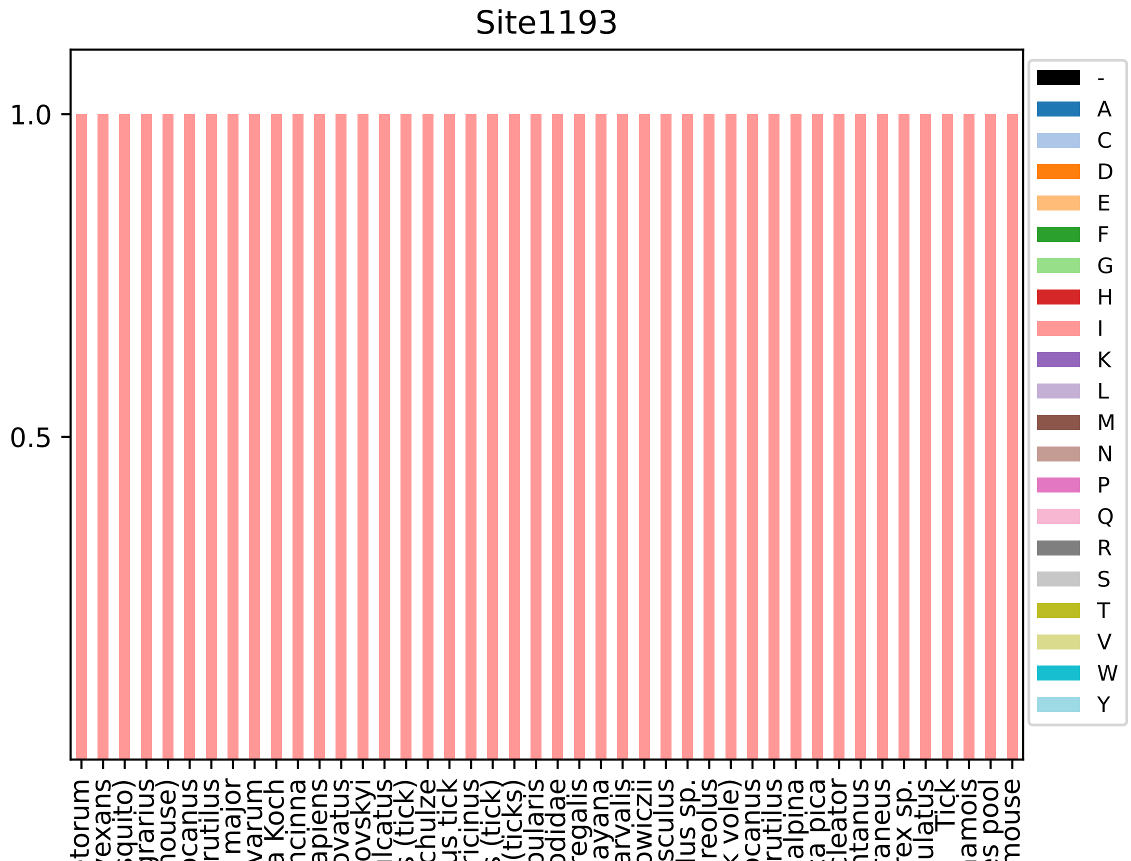
Site1193
1.0
0.5
-
A
C
D
E
F
G
H
I
K
L
M
N
P
Q
R
S
T
V
W
Y

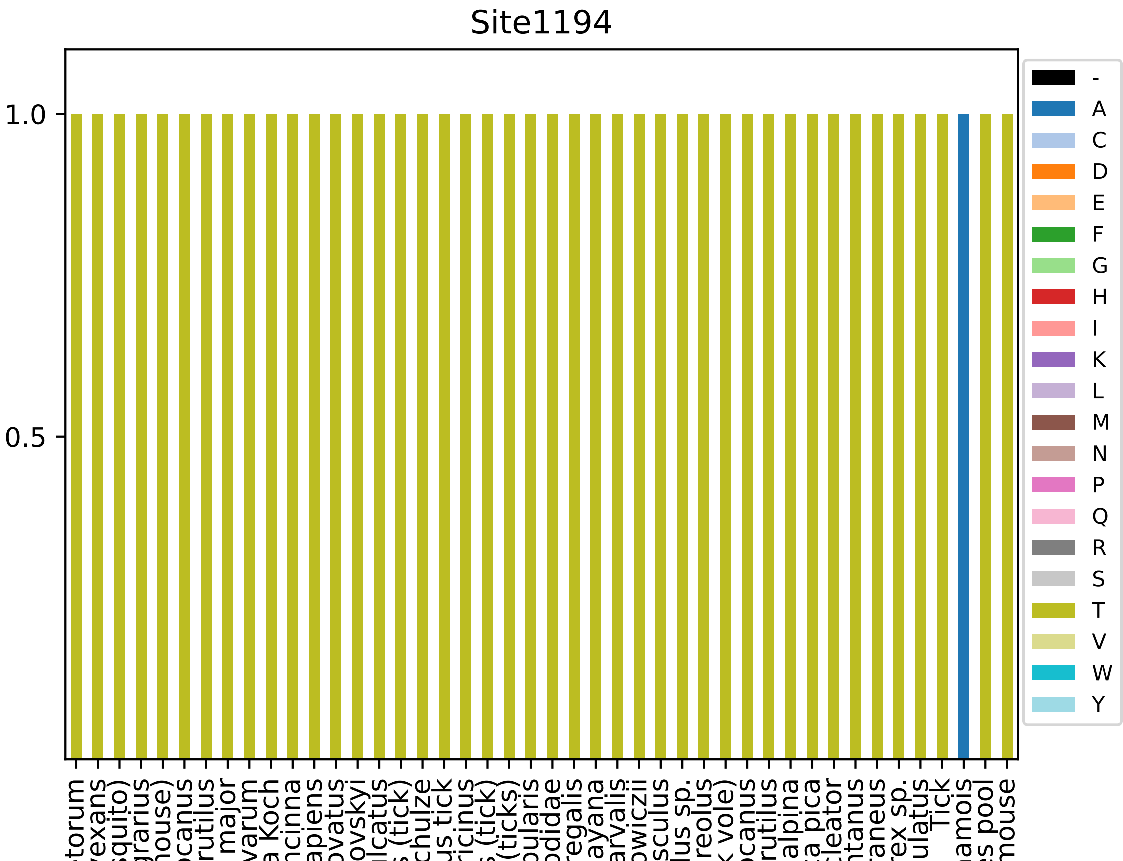
Site1194
1.0
0.5
-
A
C
D
E
F
G
H
I
K
L
M
N
P
Q
R
S
T
V
W
Y

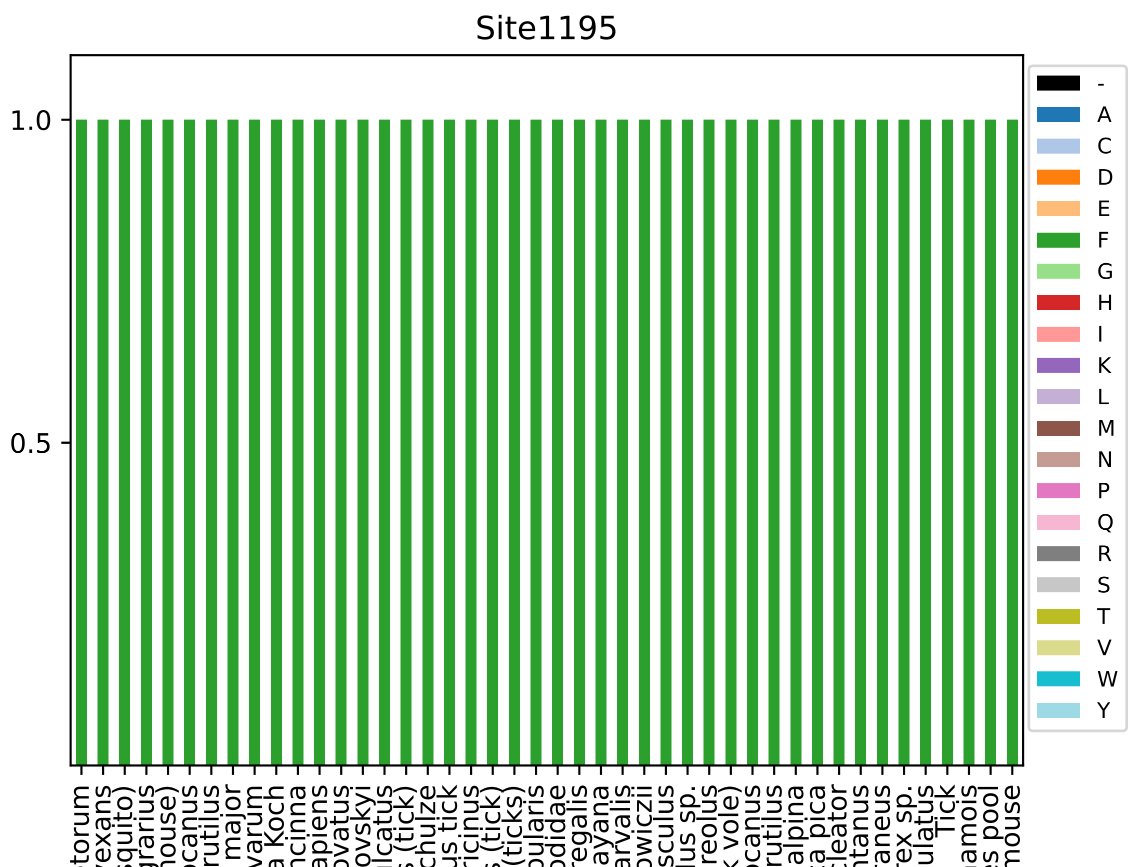
Site1195
1.0
0.5
-
A
C
D
E
F
G
H
I
K
L
M
N
P
Q
R
S
T
V
W
Y

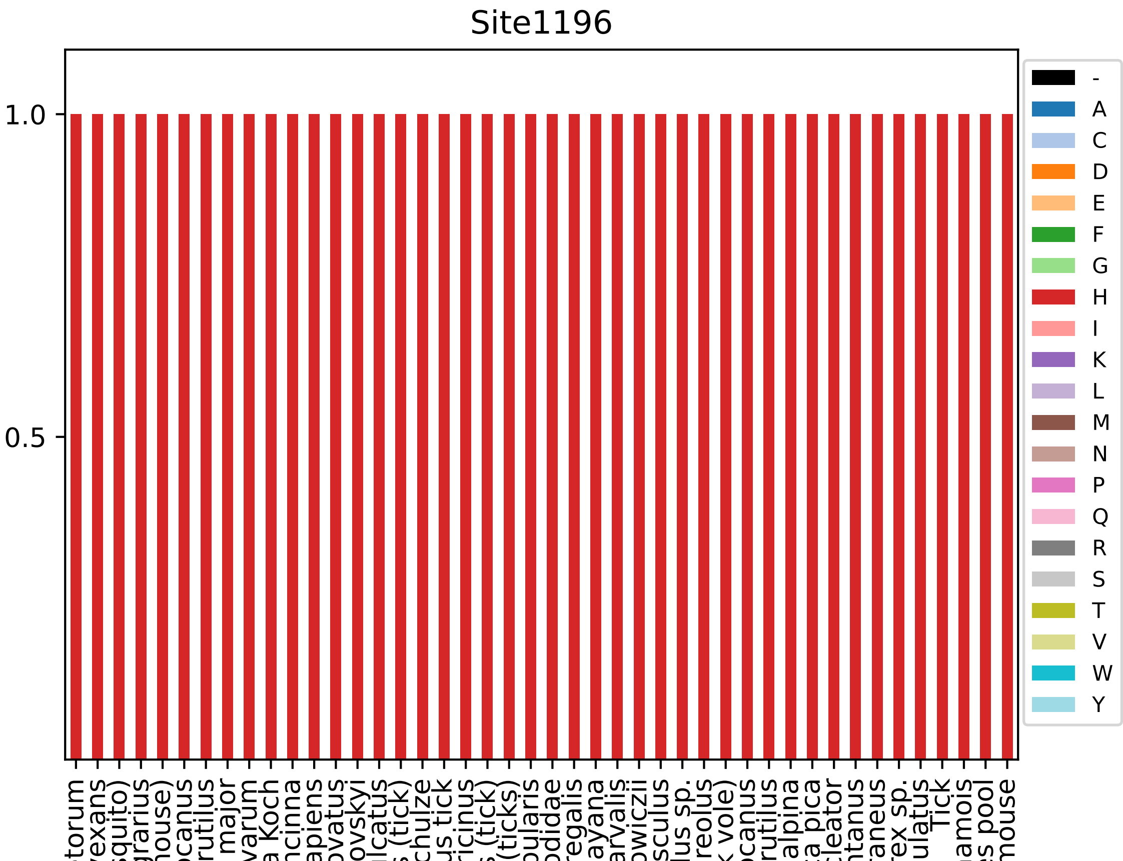

Site1196
1.0
0.5
-
A
C
D
E
F
G
H
I
K
L
M
N
P
Q
R
S
T
V
W
Y

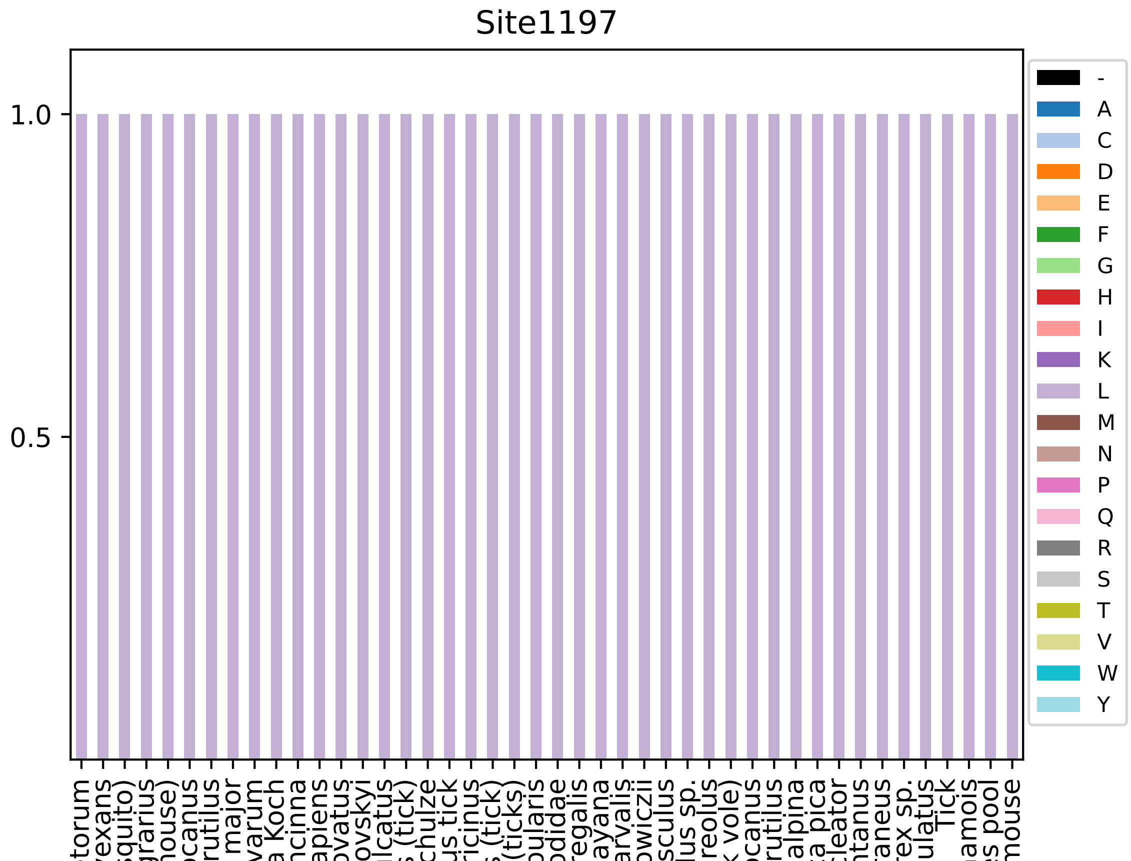
Site1197
1.0
0.5
-
A
C
D
E
F
G
H
I
K
L
M
N
P
Q
R
S
T
V
W
Y

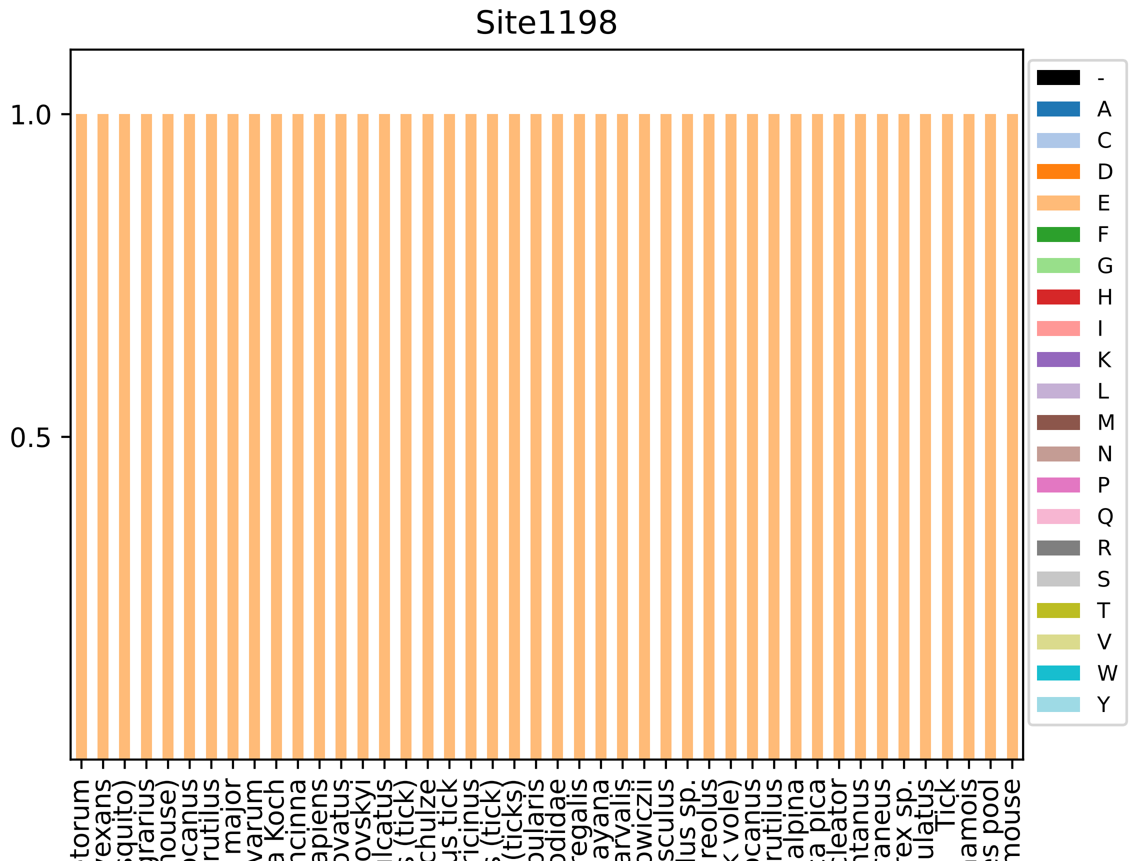
Site1198
1.0
0.5
-
A
C
D
E
F
G
H
I
K
L
M
N
P
Q
R
S
T
V
W
Y

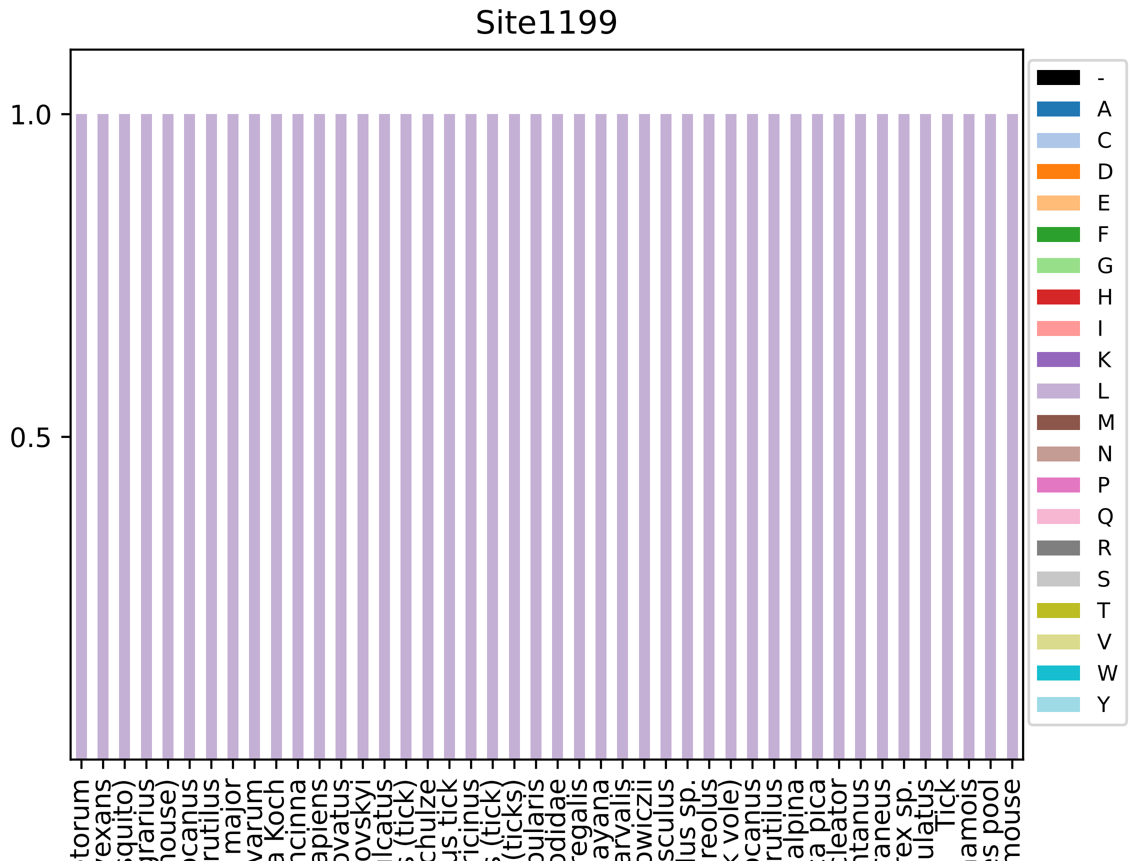
Site1199
1.0
0.5
-
A
C
D
E
F
G
H
I
K
L
M
N
P
Q
R
S
T
V
W
Y
Tick

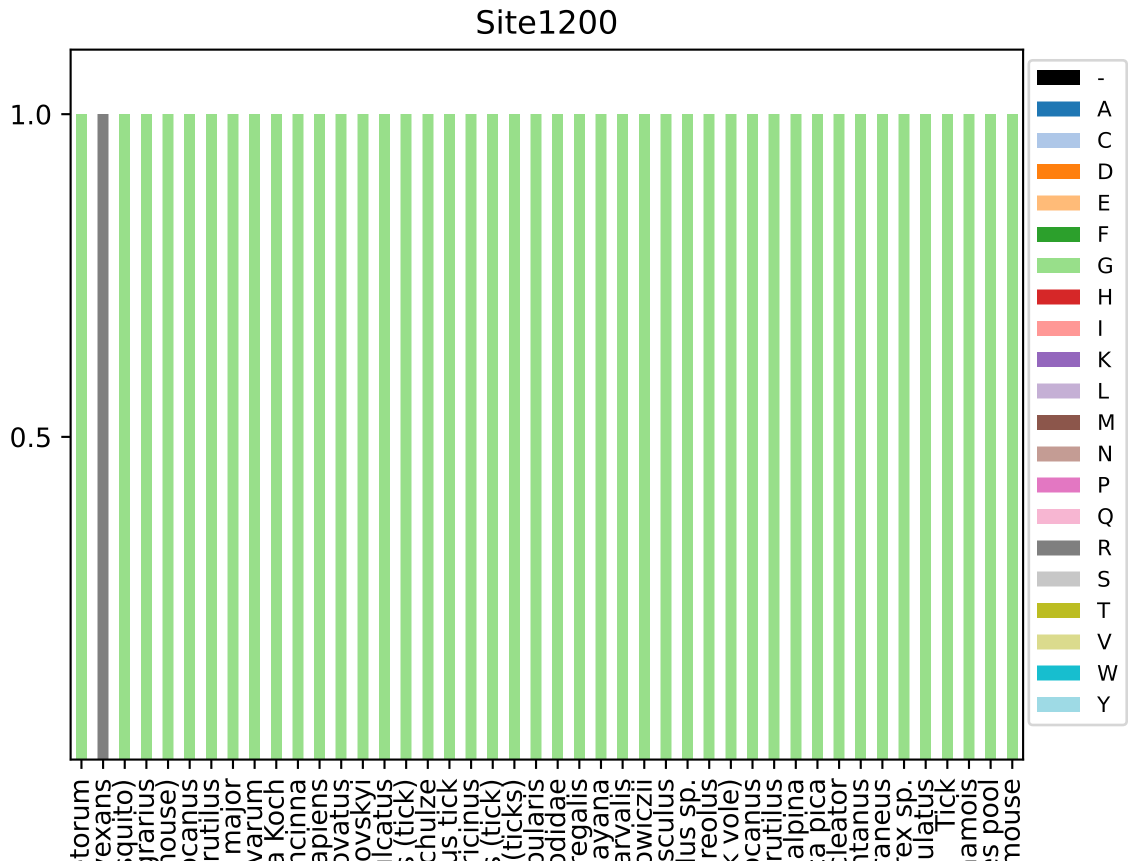
Site1200
1.0
0.5
-
A
C
D
E
F
G
H
I
K
L
M
N
P
Q
R
S
T
V
W
Y

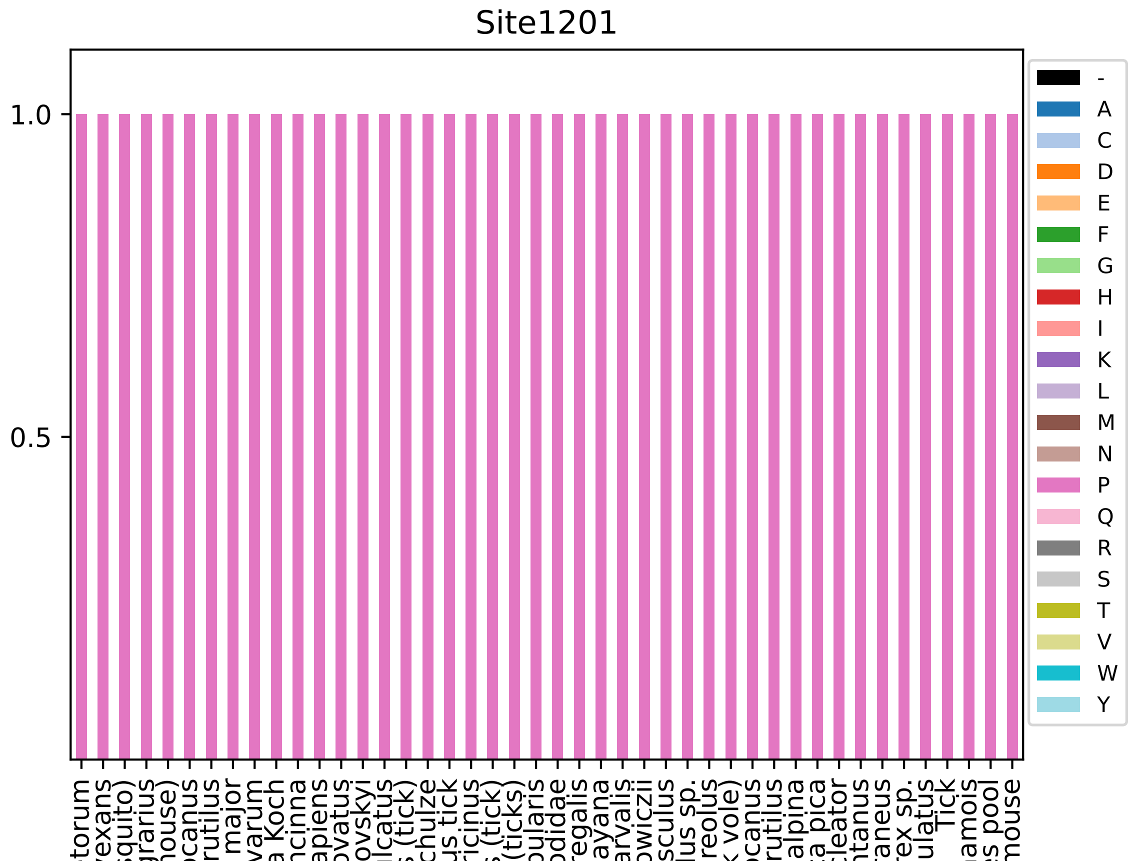
Site1201
1.0
0.5
-
A
C
D
E
F
G
H
I
K
L
M
N
P
Q
R
S
T
V
W
Y

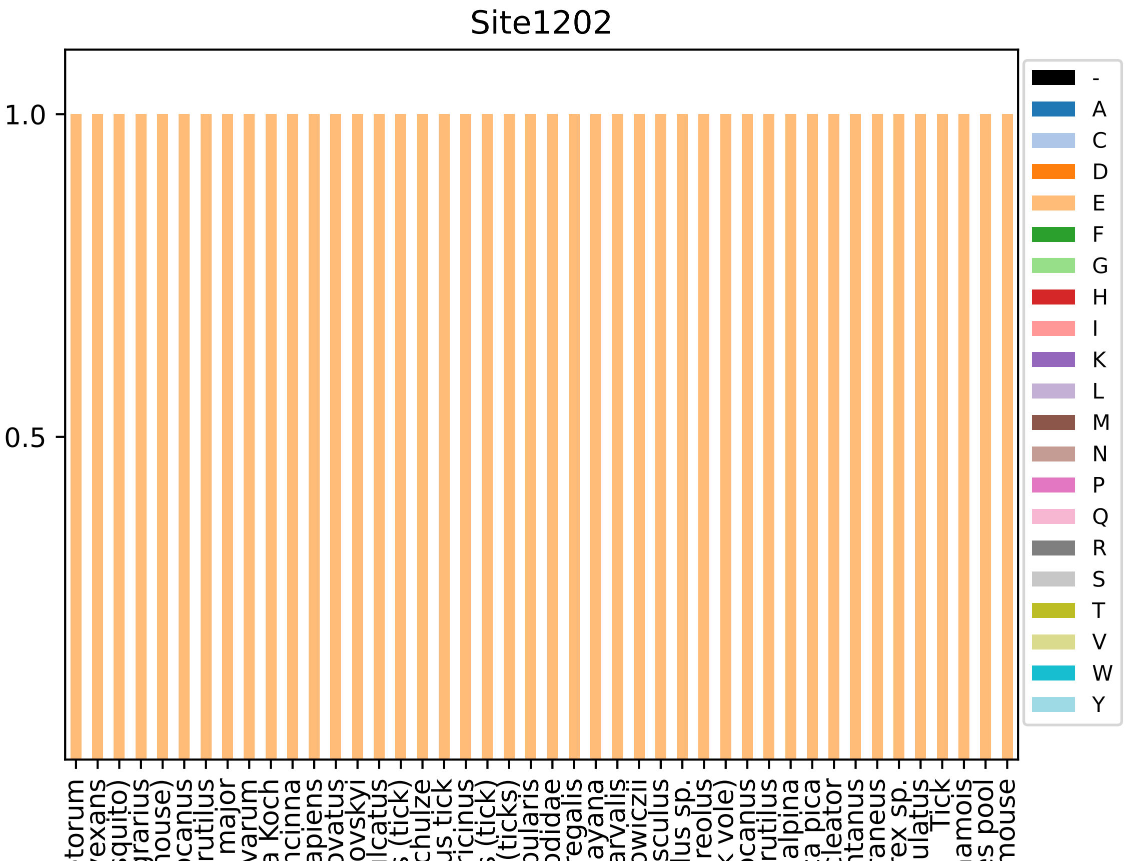
Site1202
1.0
0.5
-
A
C
D
E
F
G
H
I
K
L
M
N
P
Q
R
S
T
V
W
Y

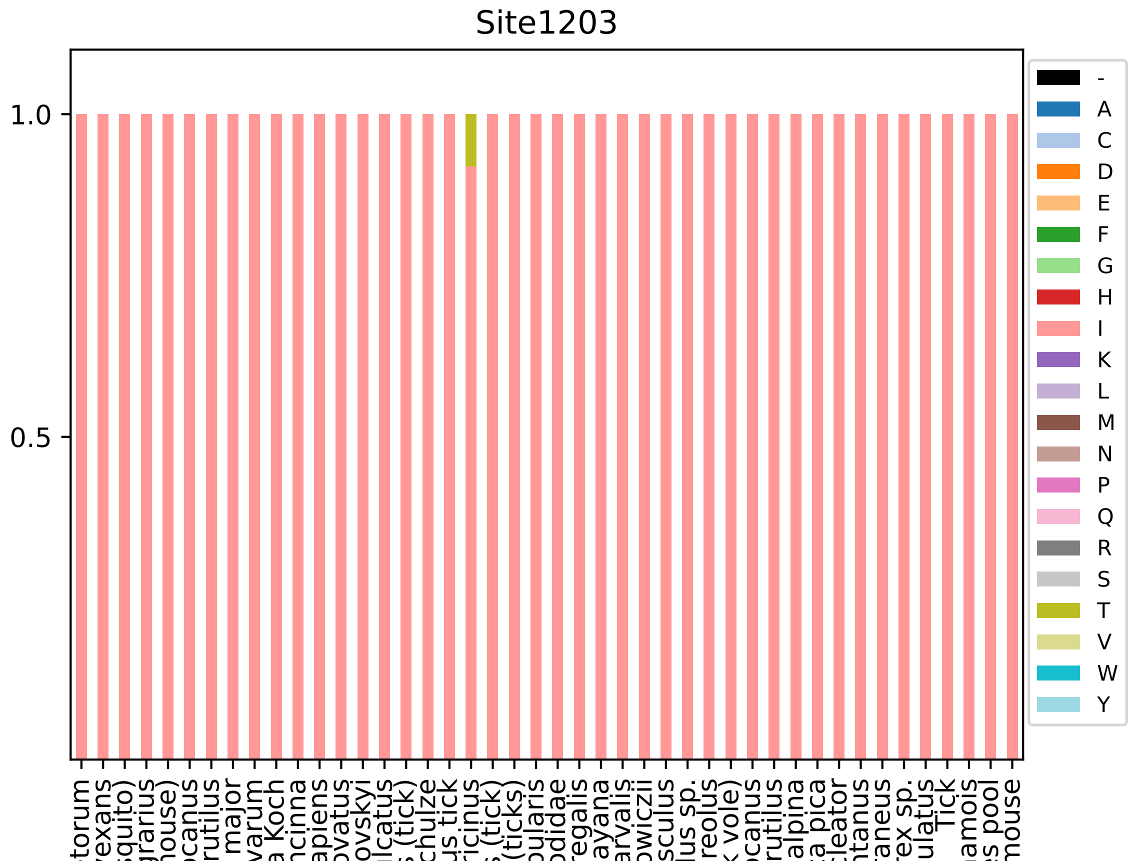
Site1203
1.0
0.5
-
A
C
D
E
F
G
H
I
K
L
M
N
P
Q
R
S
T
V
W
Y

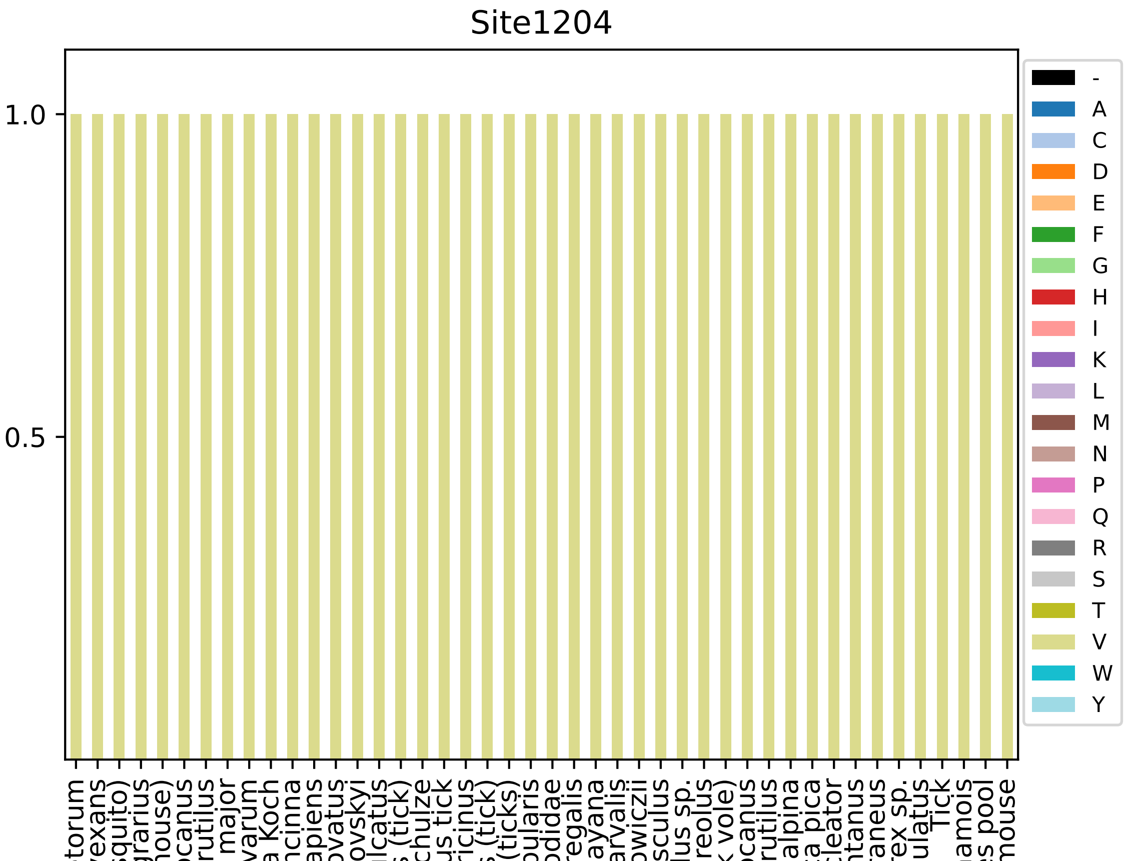

Site1204
1.0
0.5
-
A
C
D
E
F
G
H
I
K
L
M
N
P
Q
R
S
T
V
W
Y

Site1205

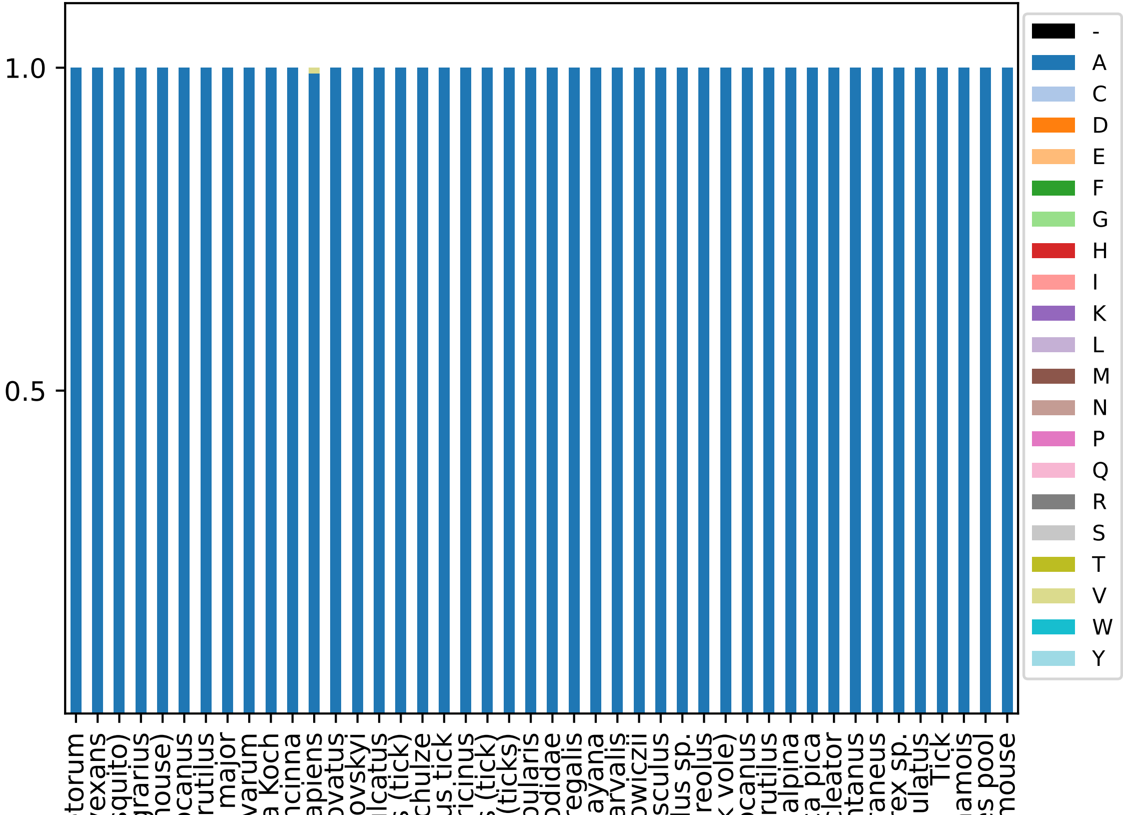

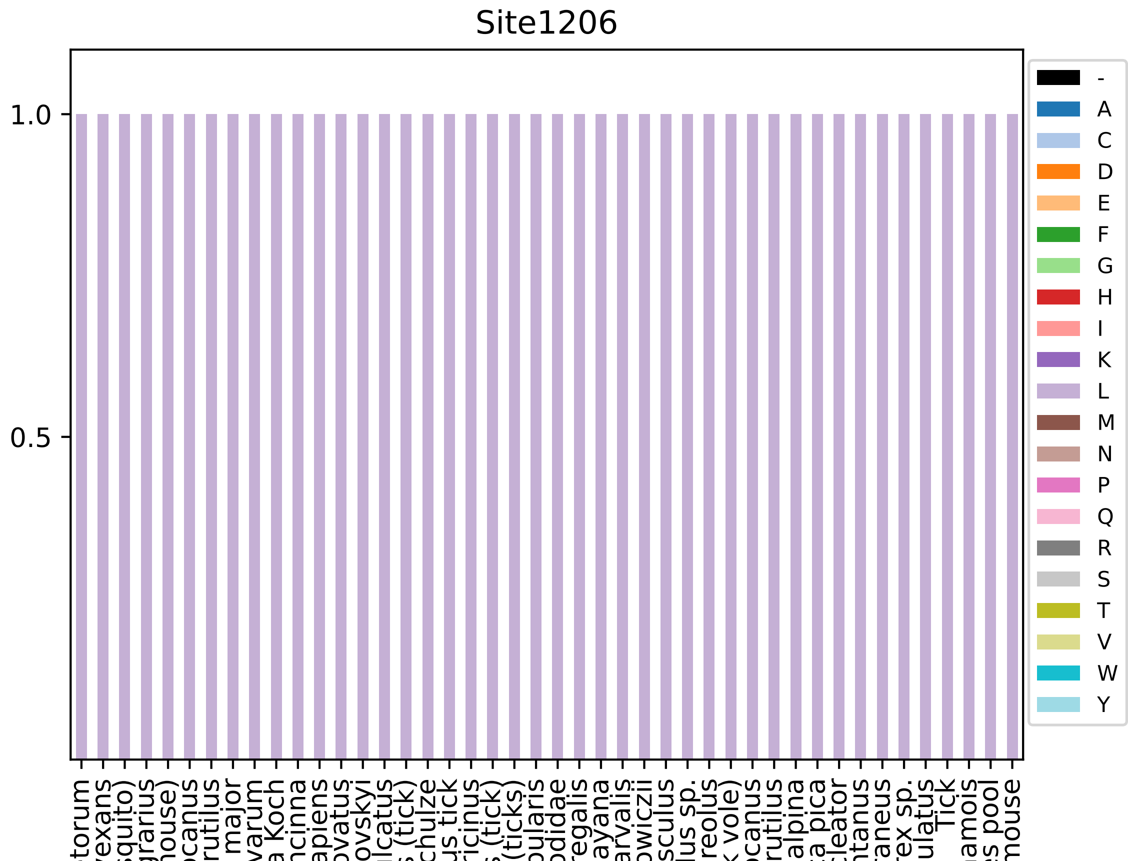

Site1206
1.0
0.5
-
A
C
D
E
F
G
H
I
K
L
M
N
P
Q
R
S
T
V
W
Y

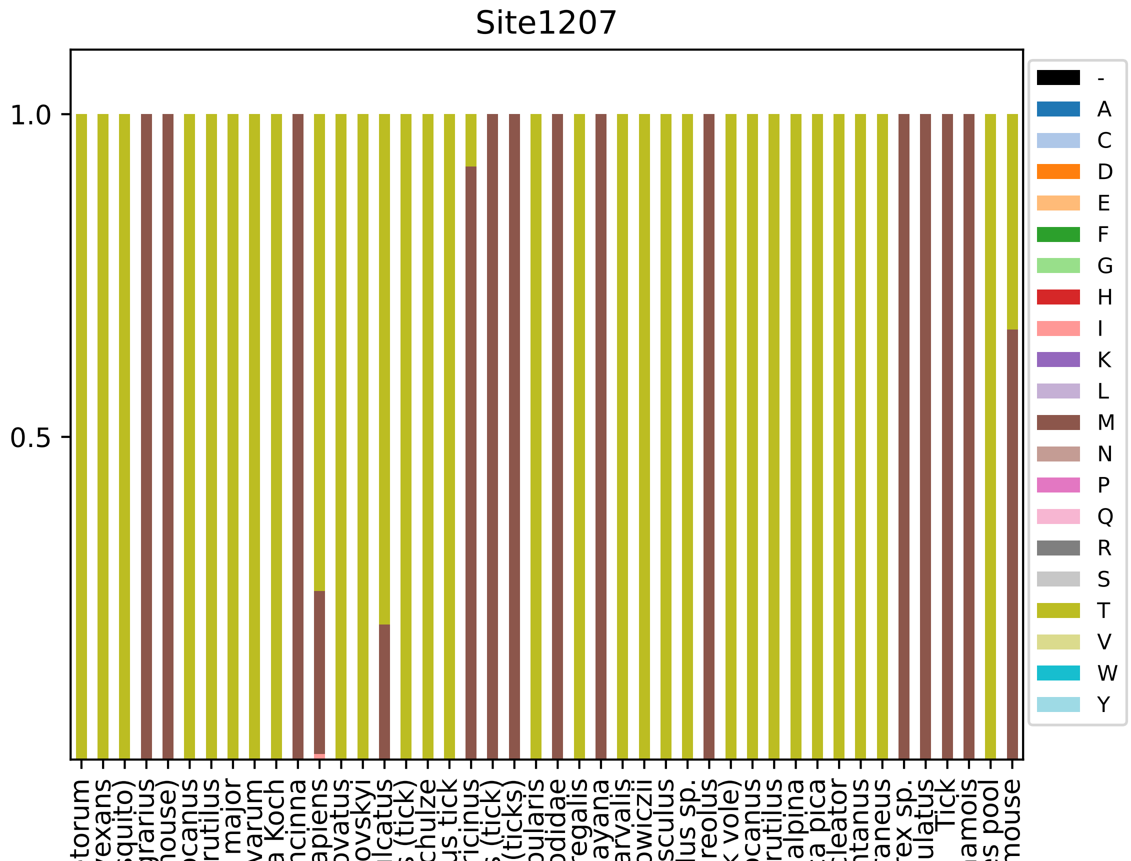
Site1207
1.0
0.5
-
A
C
D
E
F
G
H
I
K
L
M
N
P
Q
R
S
T
V
W
Y

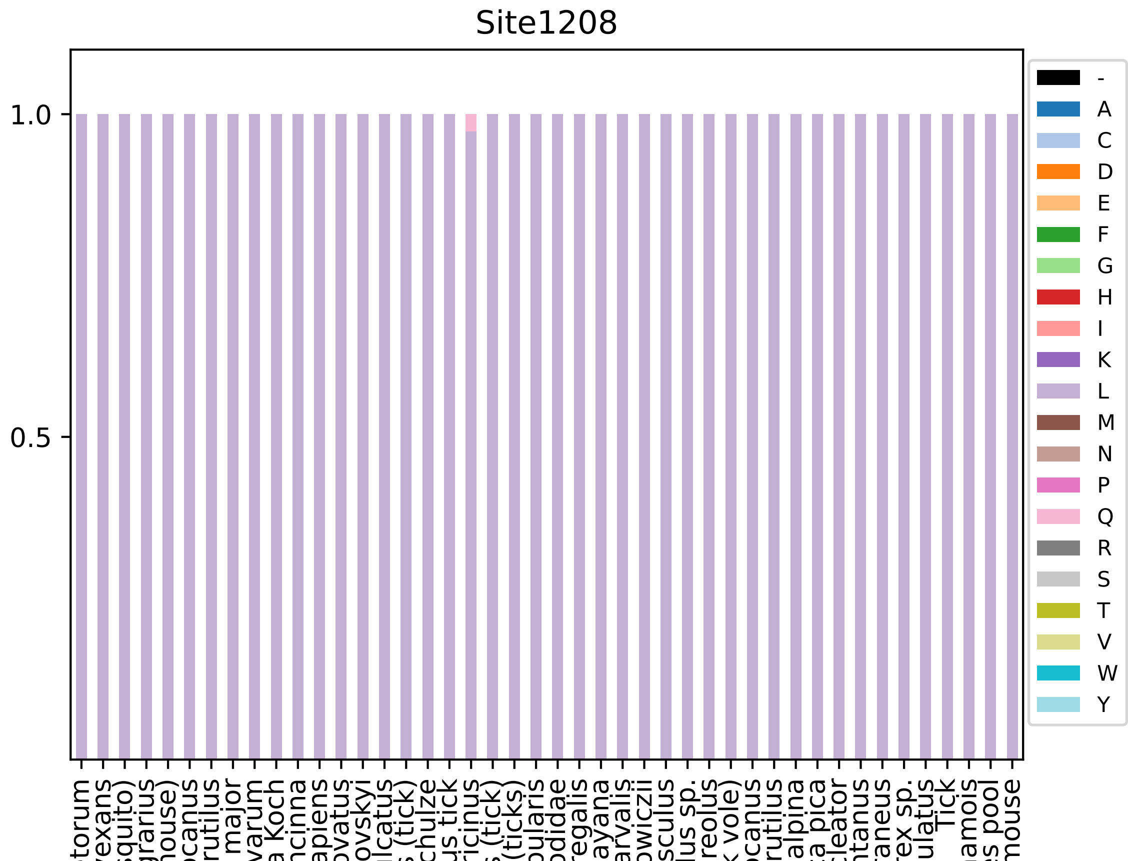
Site1208
1.0
0.5
-
A
C
D
E
F
G
H
I
K
L
M
N
P
Q
R
S
T
V
W
Y

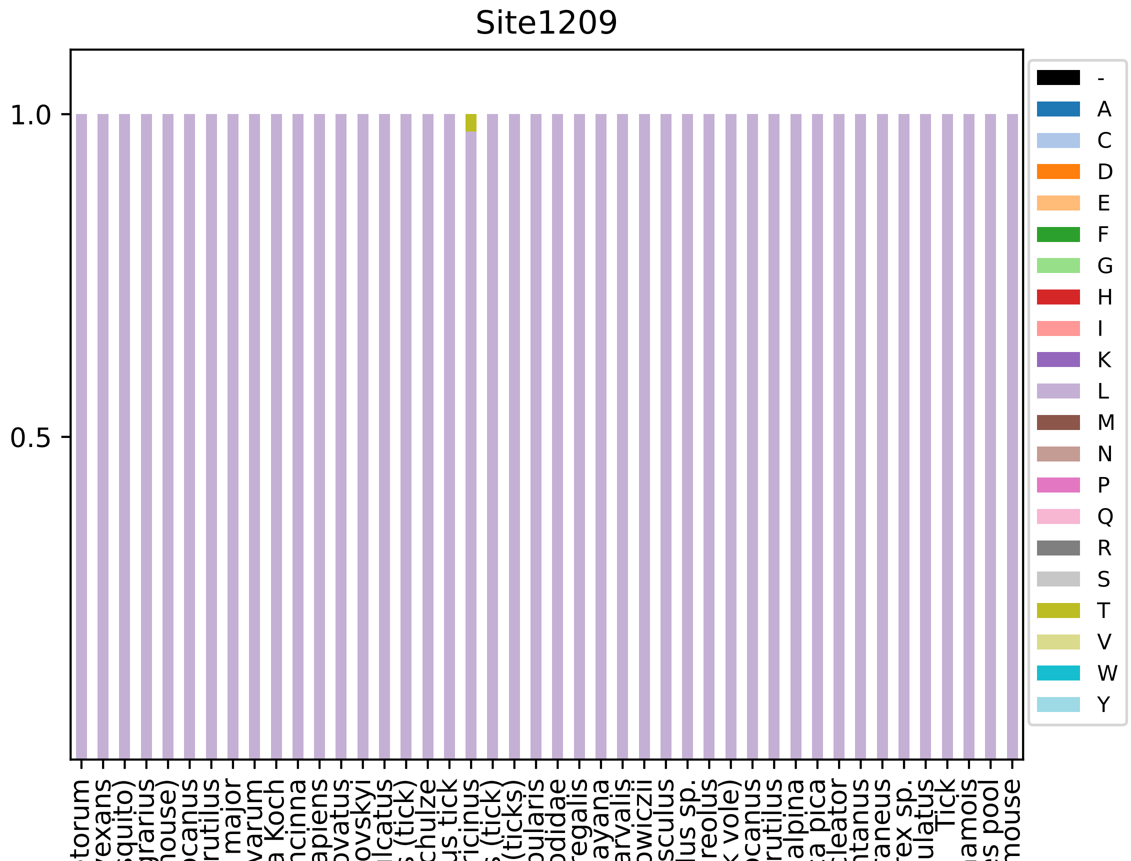
Site1209
1.0
0.5
-
A
C
D
E
F
G
H
I
K
L
M
N
P
Q
R
S
T
V
W
Y

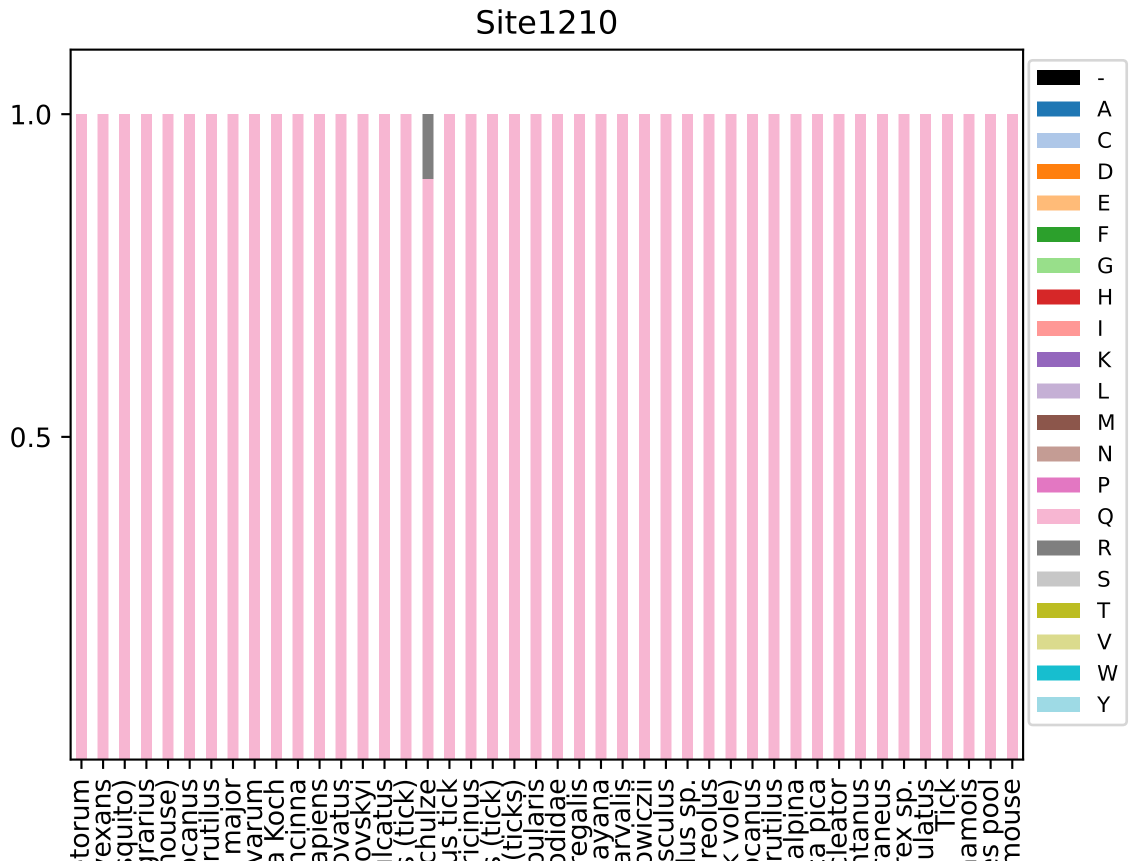
Site1210
1.0
0.5
-
A
C
D
E
F
G
H
I
K
L
M
N
P
Q
R
S
T
V
W
Y

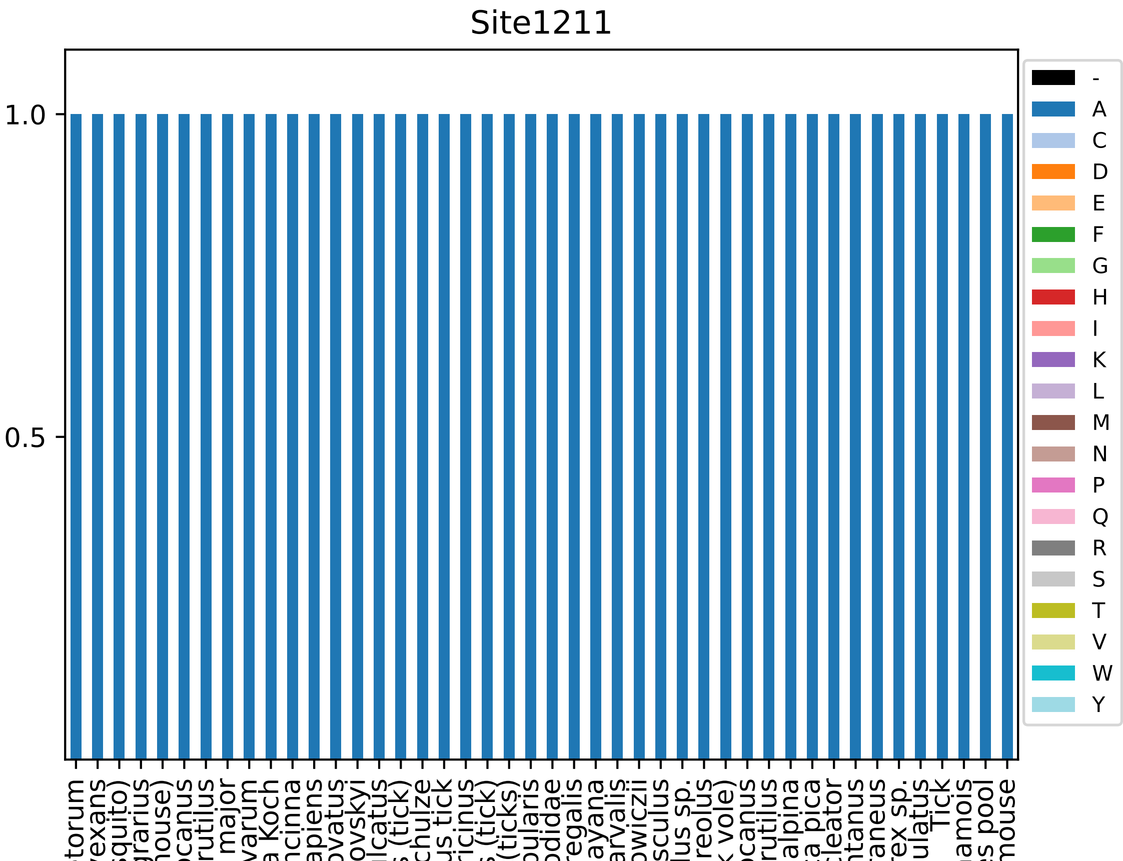
Site1211
1.0
0.5
-
A
C
D
E
F
G
H
I
K
L
M
N
P
Q
R
S
T
V
W
Y
Tick

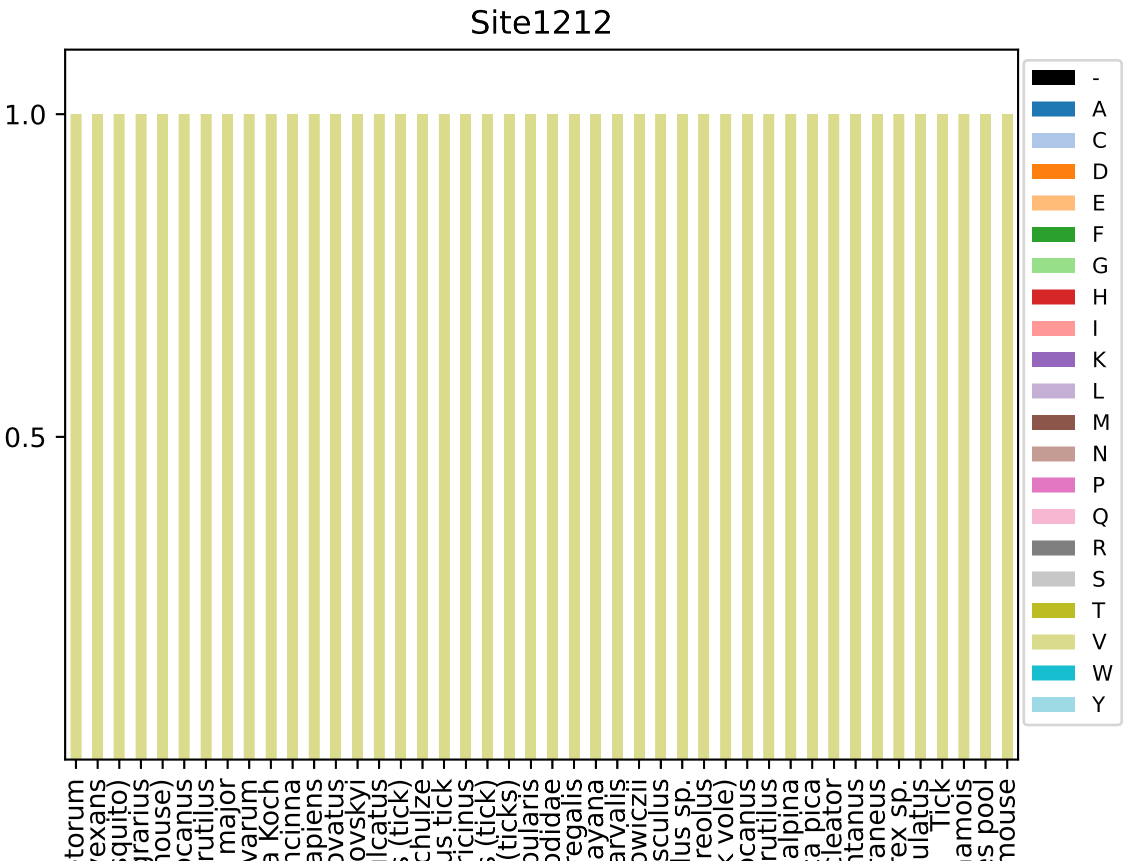
Site1212
1.0
0.5
-
A
C
D
E
F
G
H
I
K
L
M
N
P
Q
R
S
T
V
W
Y

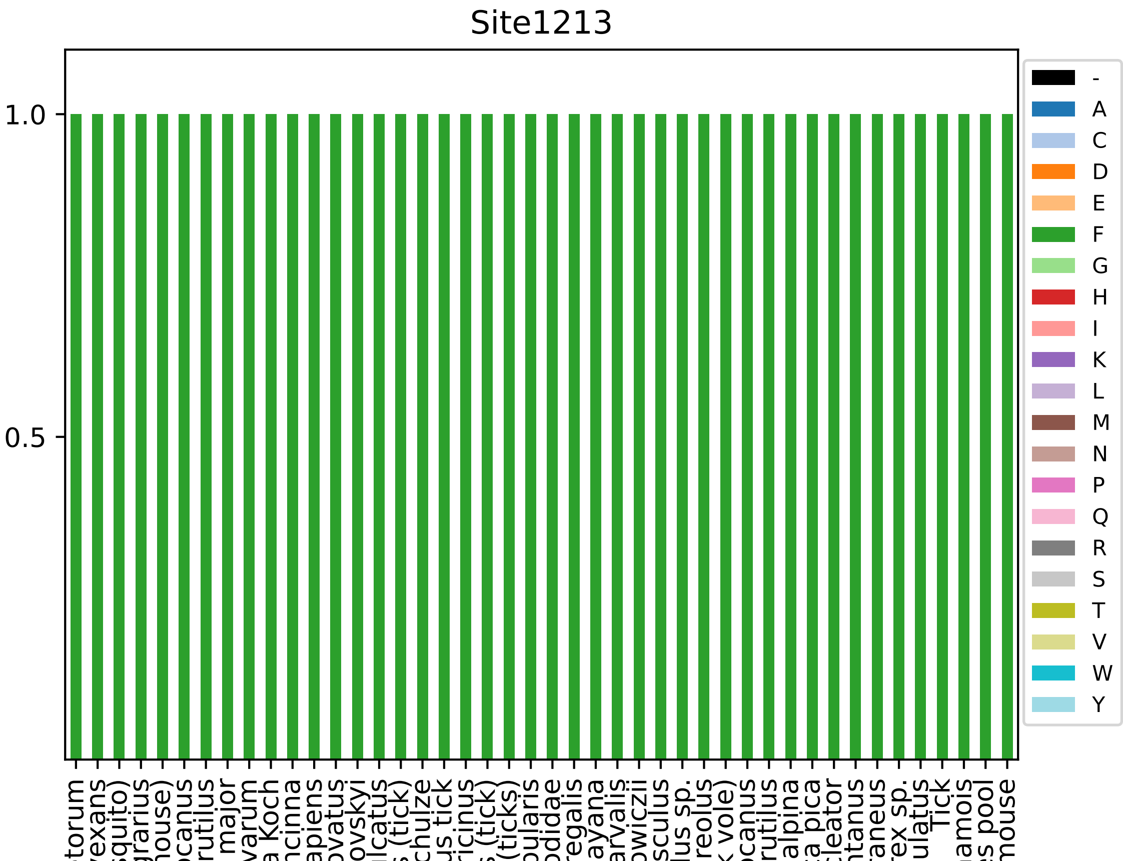
Site1213
1.0
0.5
-
A
C
D
E
F
G
H
I
K
L
M
N
P
Q
R
S
T
V
W
Y

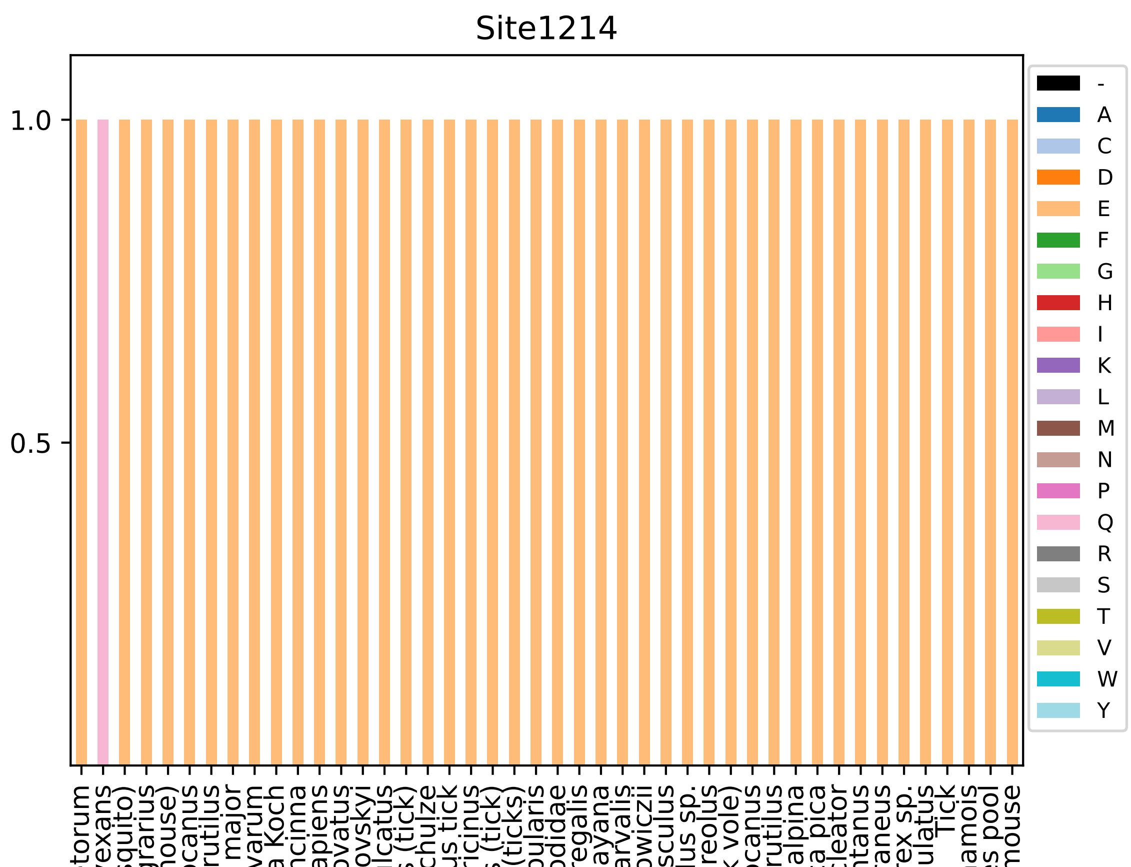
Site1214
1.0
0.5
-
A
C
D
E
F
G
H
I
K
L
M
N
P
Q
R
S
T
V
W
Y

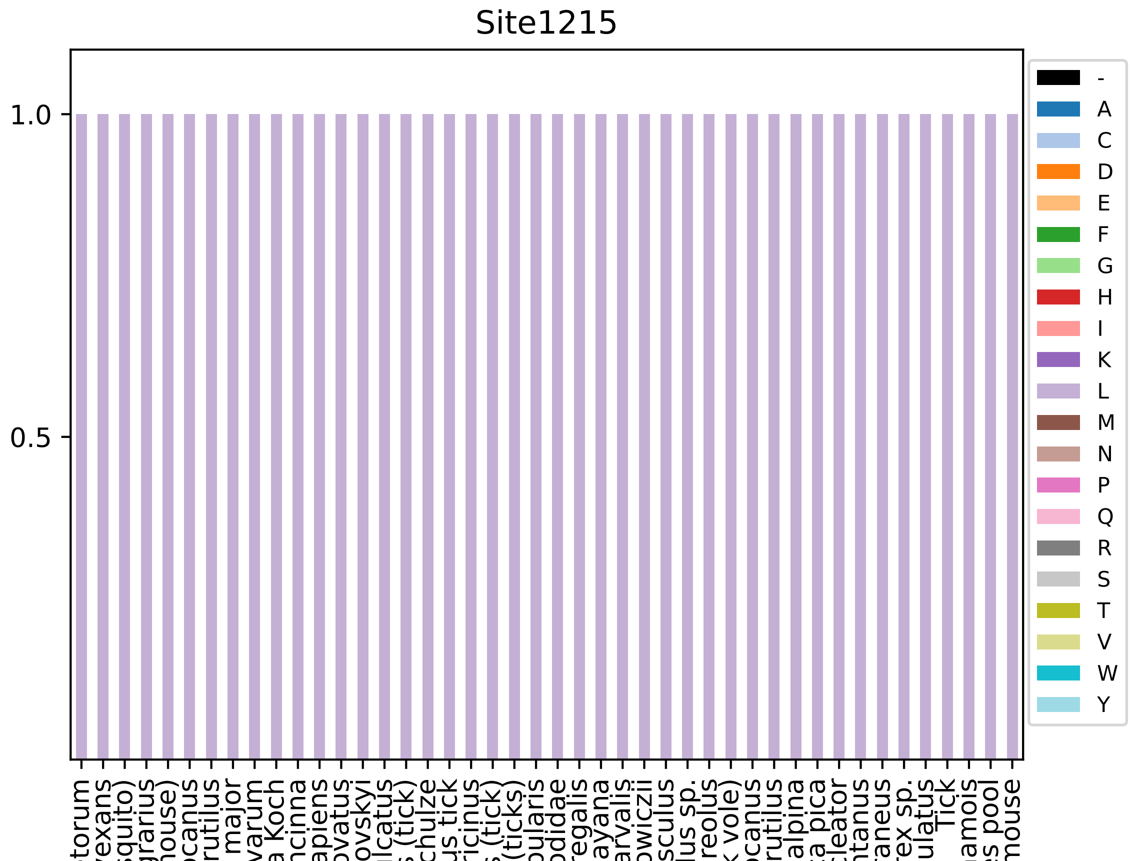
Site1215
1.0
0.5
-
A
C
D
E
F
G
H
I
K
L
M
N
P
Q
R
S
T
V
W
Y

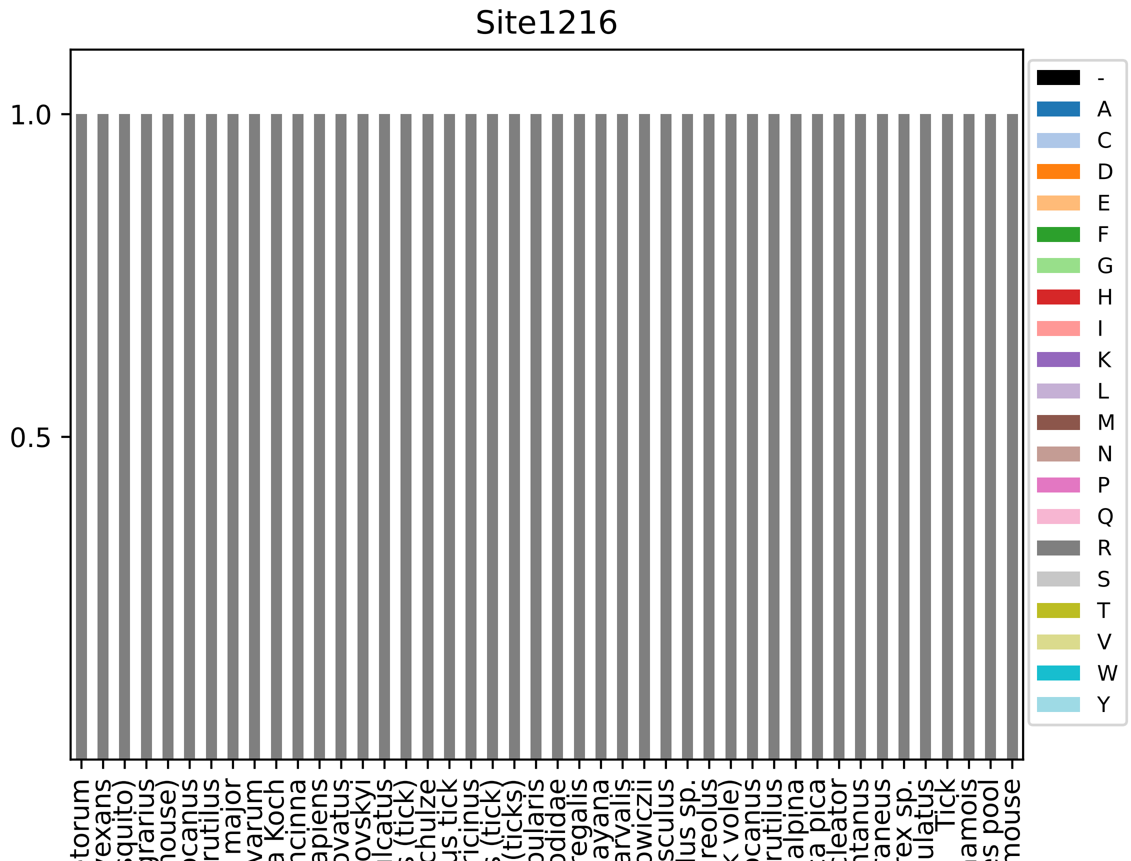
Site1216
1.0
0.5
-
A
C
D
E
F
G
H
I
K
L
M
N
P
Q
R
S
T
V
W
Y

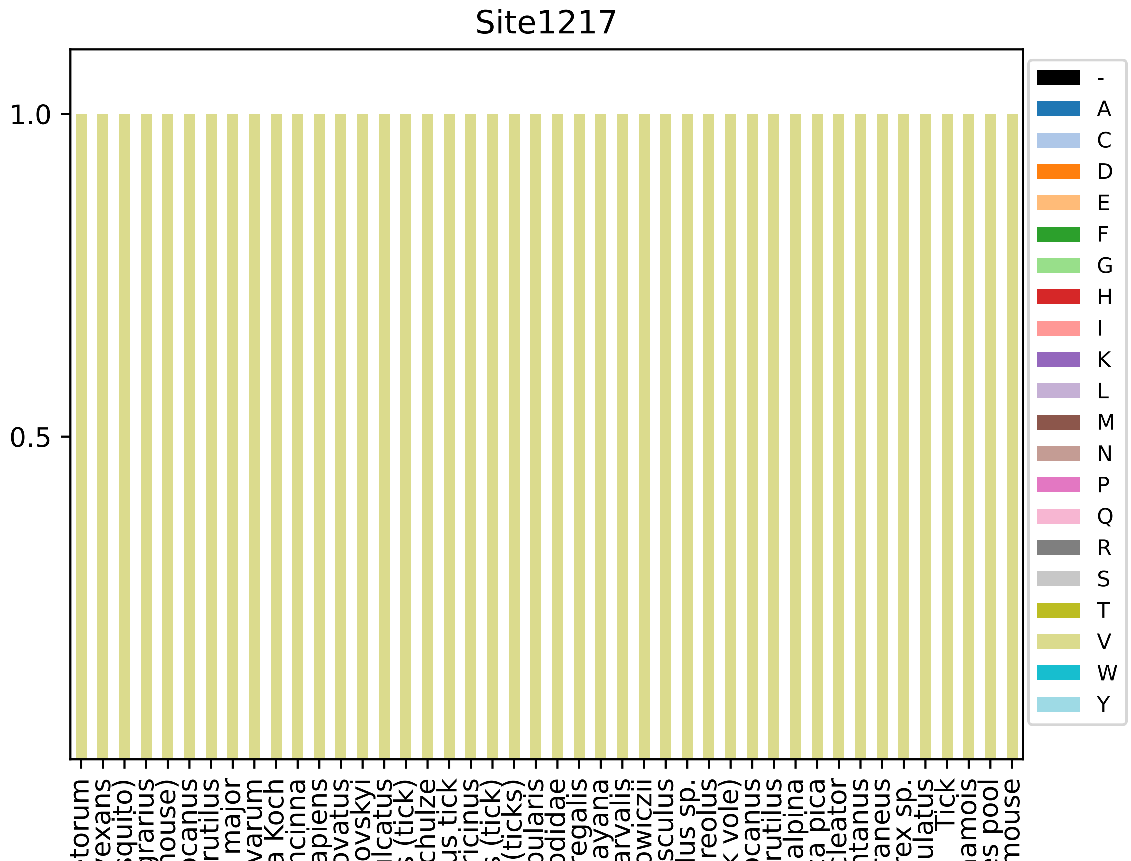
Site1217
1.0
0.5
-
A
C
D
E
F
G
H
I
K
L
M
N
P
Q
R
S
T
V
W
Y

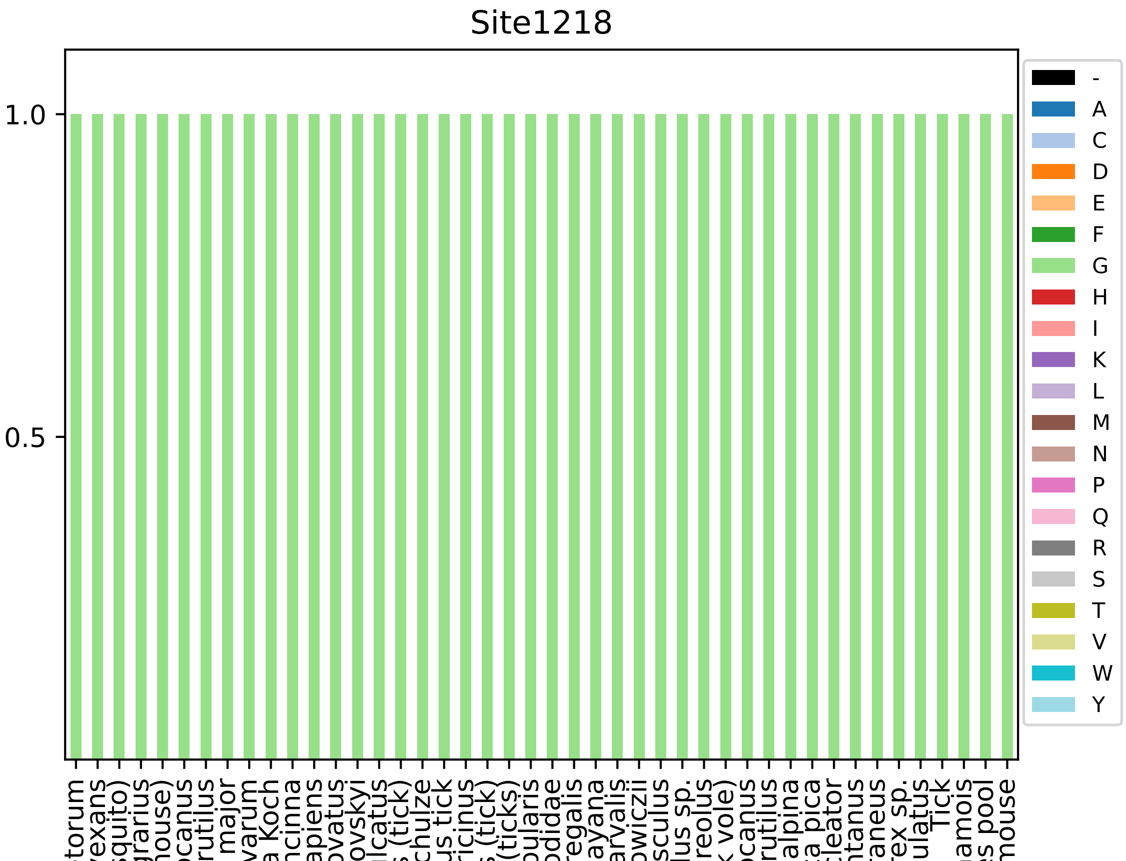
Site1218
1.0
0.5
-
A
C
D
E
F
G
H
I
K
L
M
N
P
Q
R
S
T
V
W
Y

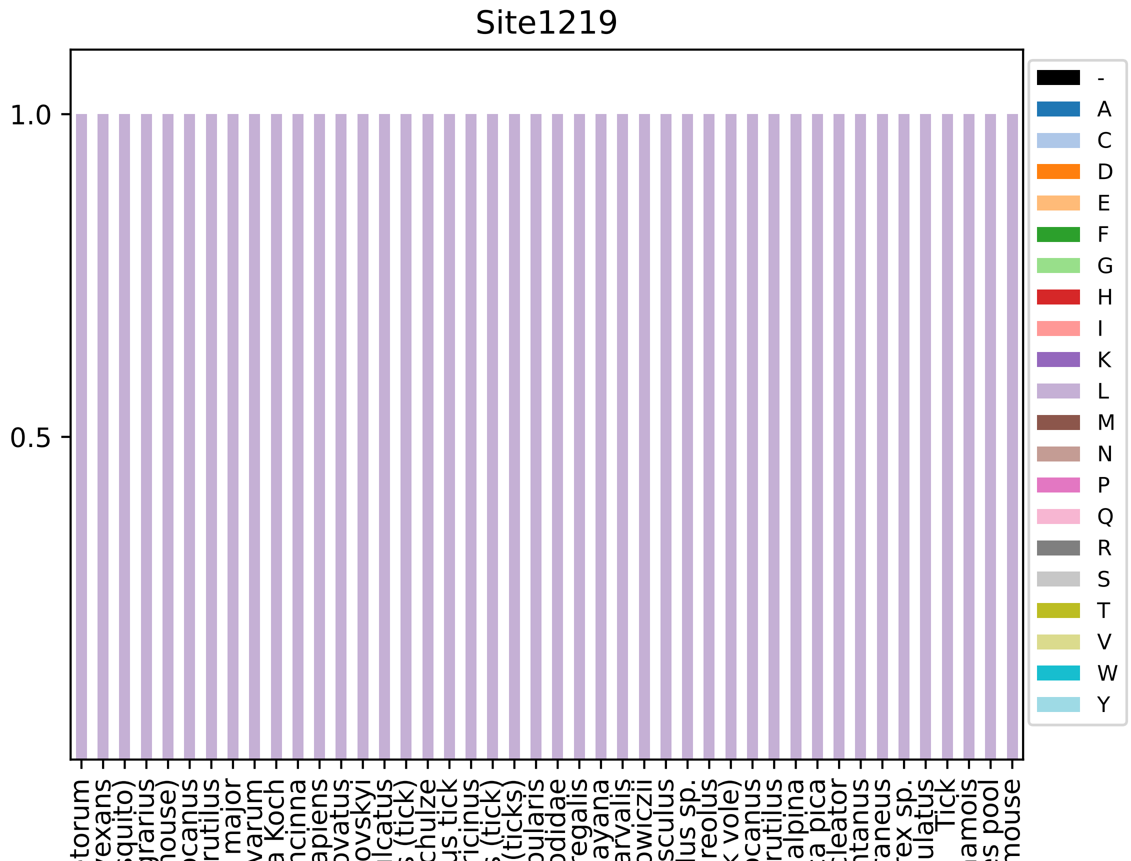
Site1219
1.0
0.5
-
A
C
D
E
F
G
H
I
K
L
M
N
P
Q
R
S
T
V
W
Y

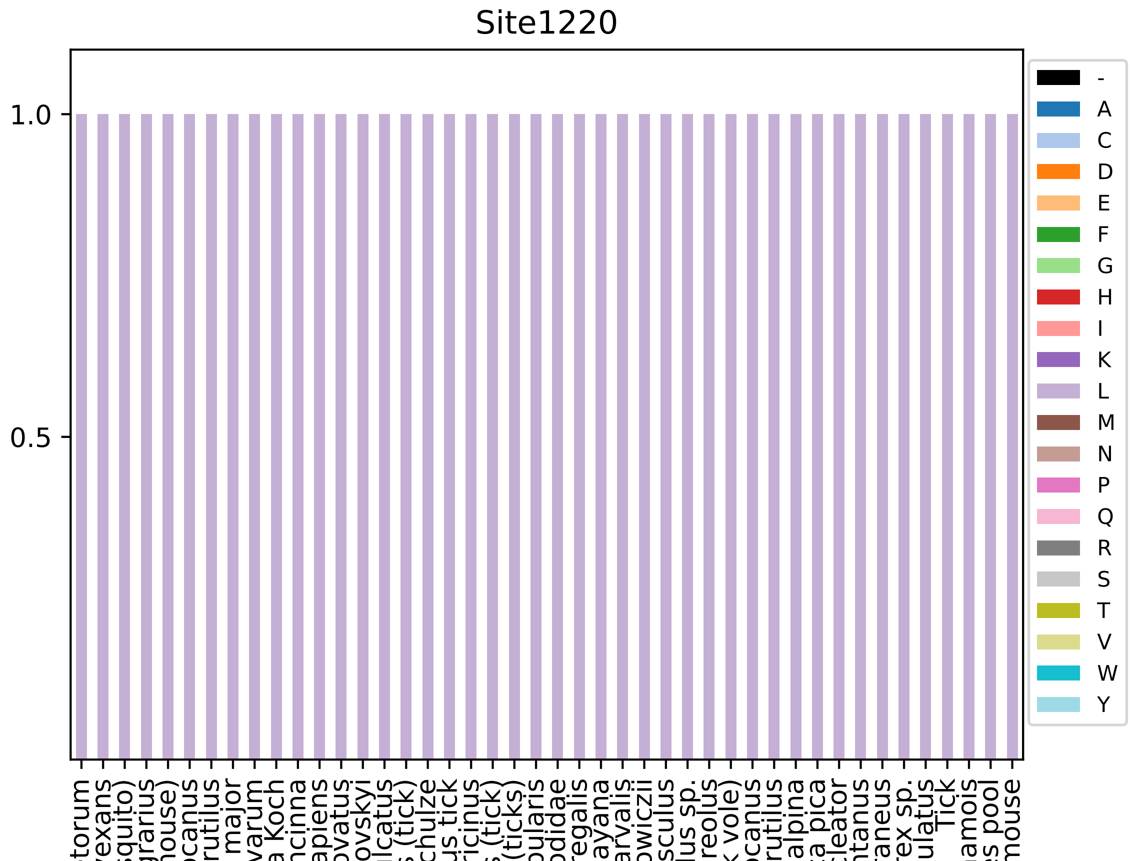

Site1220
1.0
0.5
-
A
C
D
E
F
G
H
I
K
L
M
N
P
Q
R
S
T
V
W
Y

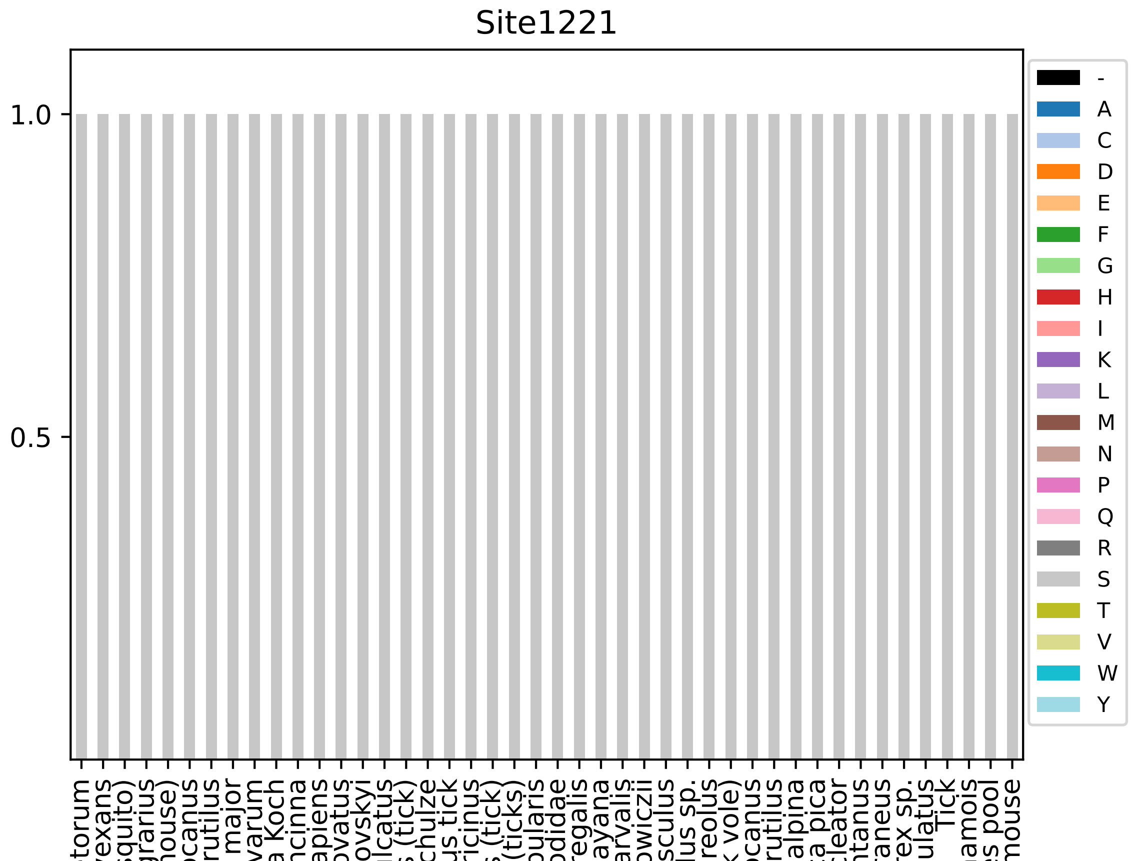

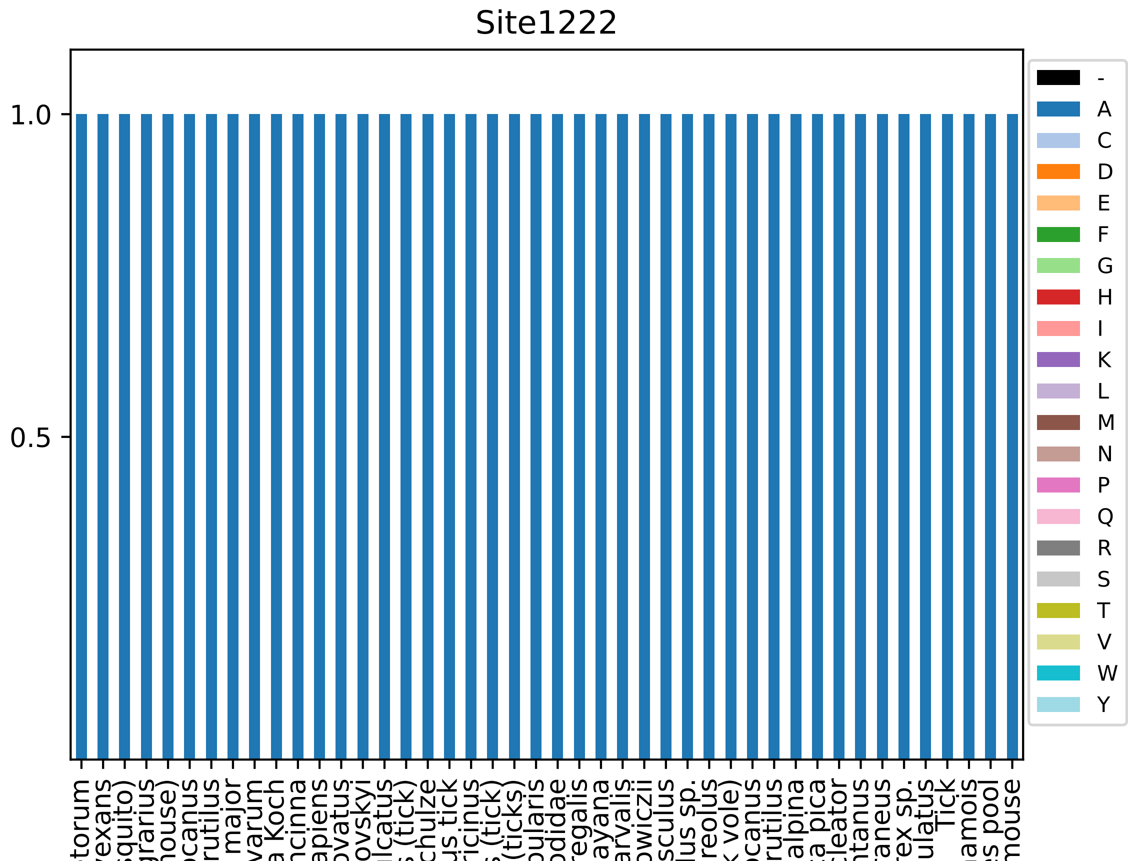

Site1222
1.0
0.5
-
A
C
D
E
F
G
H
I
K
L
M
N
P
Q
R
S
T
V
W
Y

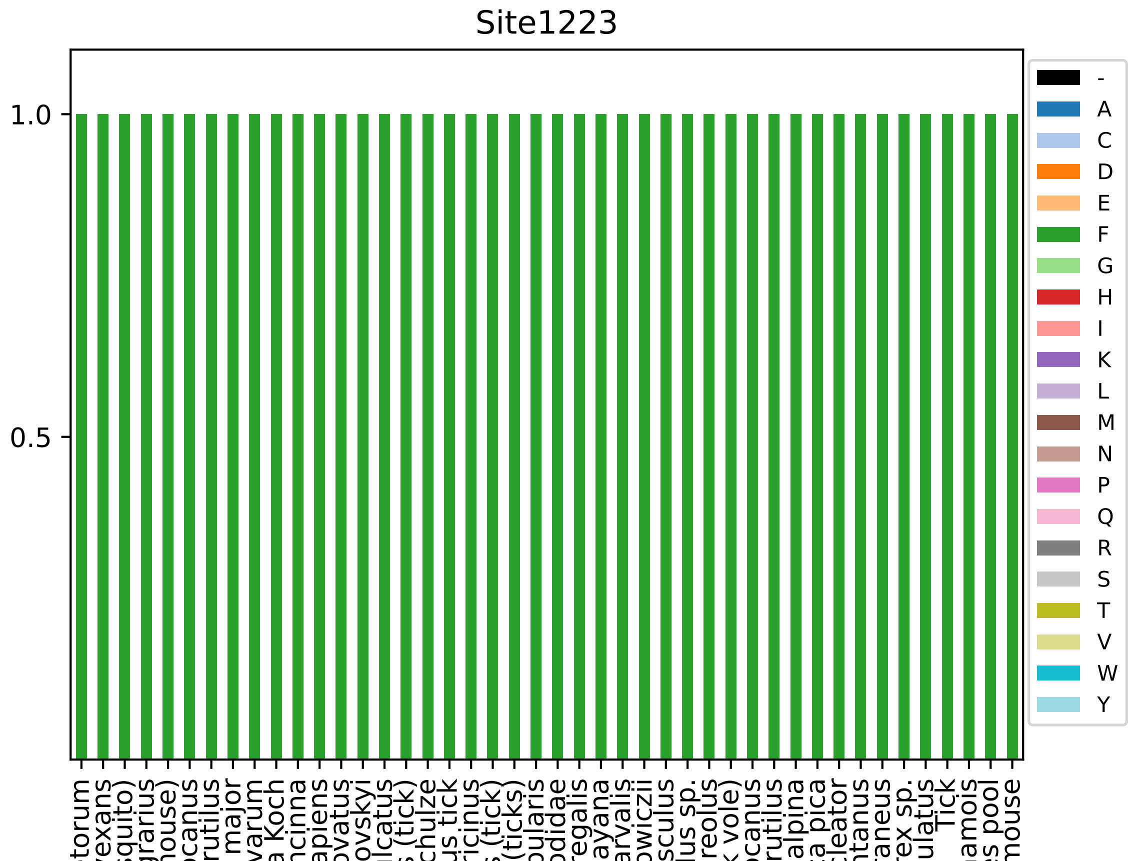
Site1223
1.0
0.5
-
A
C
D
E
F
G
H
I
K
L
M
N
P
Q
R
S
T
V
W
Y

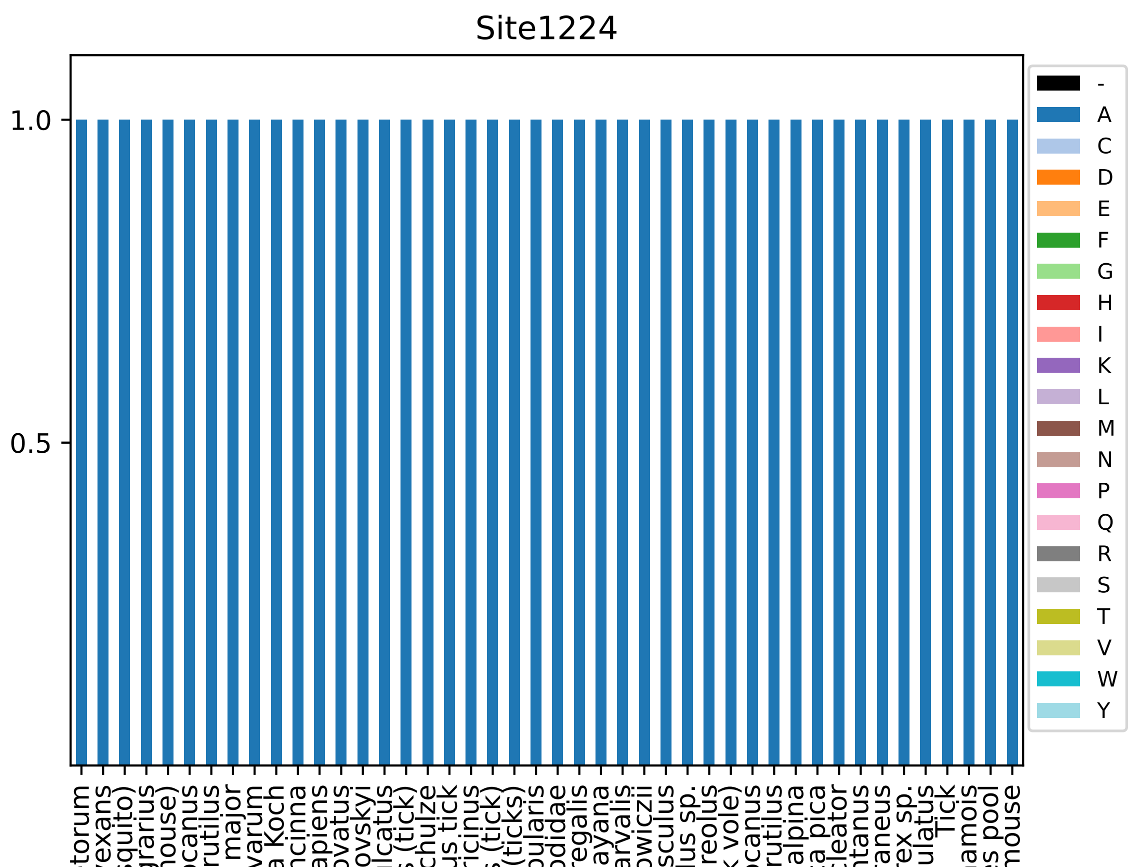
Site1224
1.0
0.5
-
A
C
D
E
F
G
H
I
K
L
M
N
P
Q
R
S
T
V
W
Y

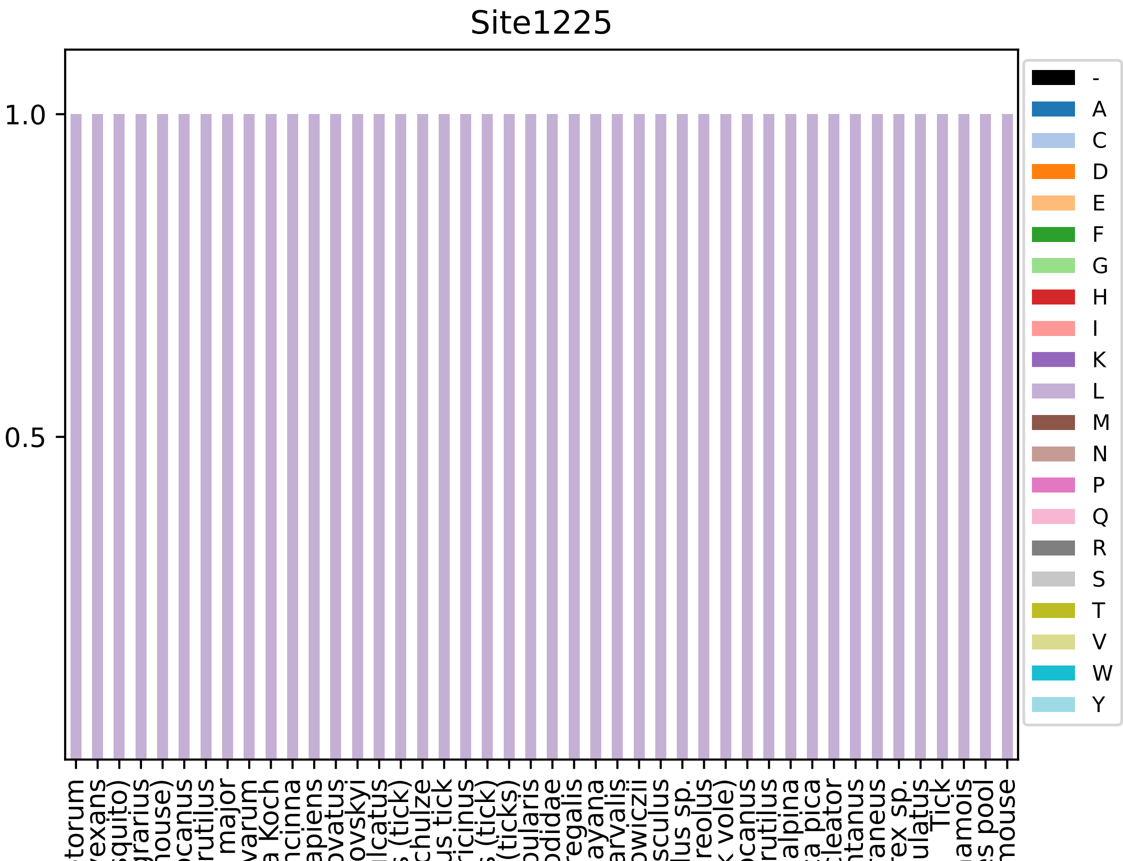

Site1225
1.0
0.5
-
A
C
D
E
F
G
H
I
K
L
M
N
P
Q
R
S
T
V
W
Y

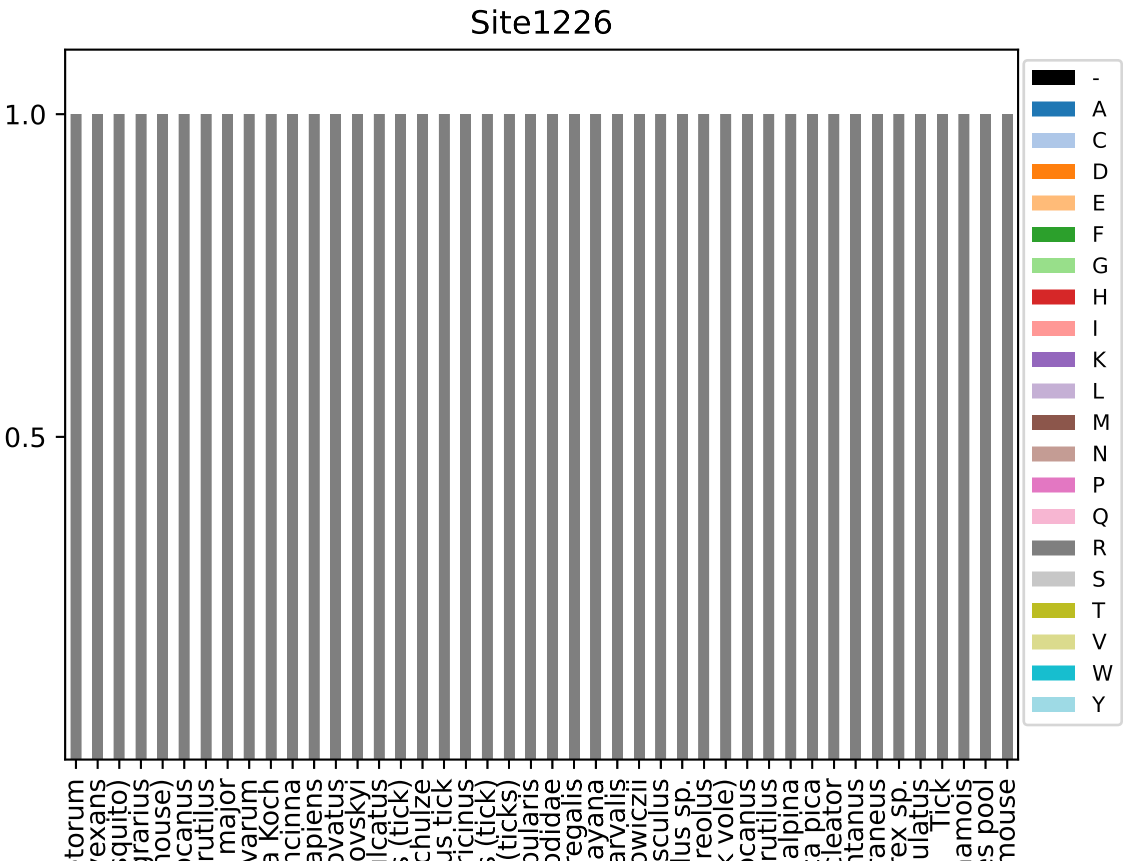

Site1226
1.0
0.5
-
A
C
D
E
F
G
H
I
K
L
M
N
P
Q
R
S
T
V
W
Y

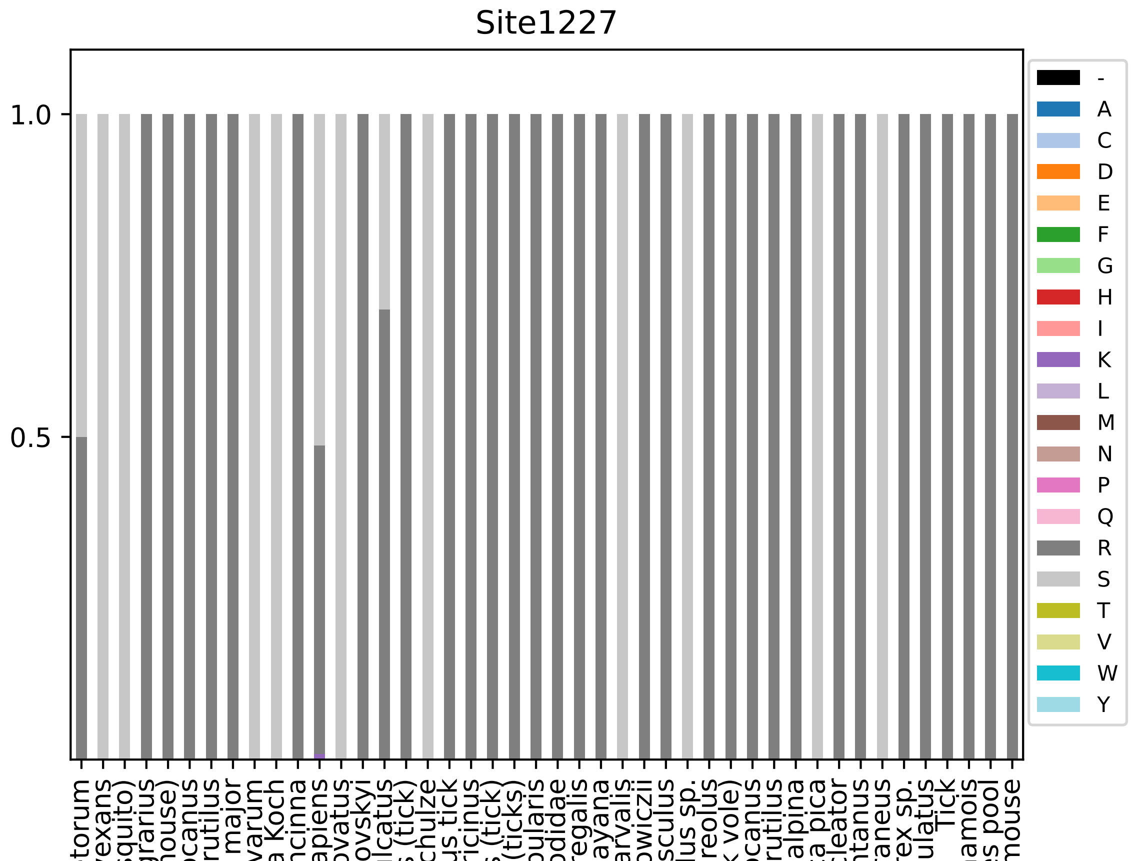

Site1227
1.0
0.5
-
A
C
D
E
F
G
H
I
K
L
M
N
P
Q
R
S
T
V
W
Y

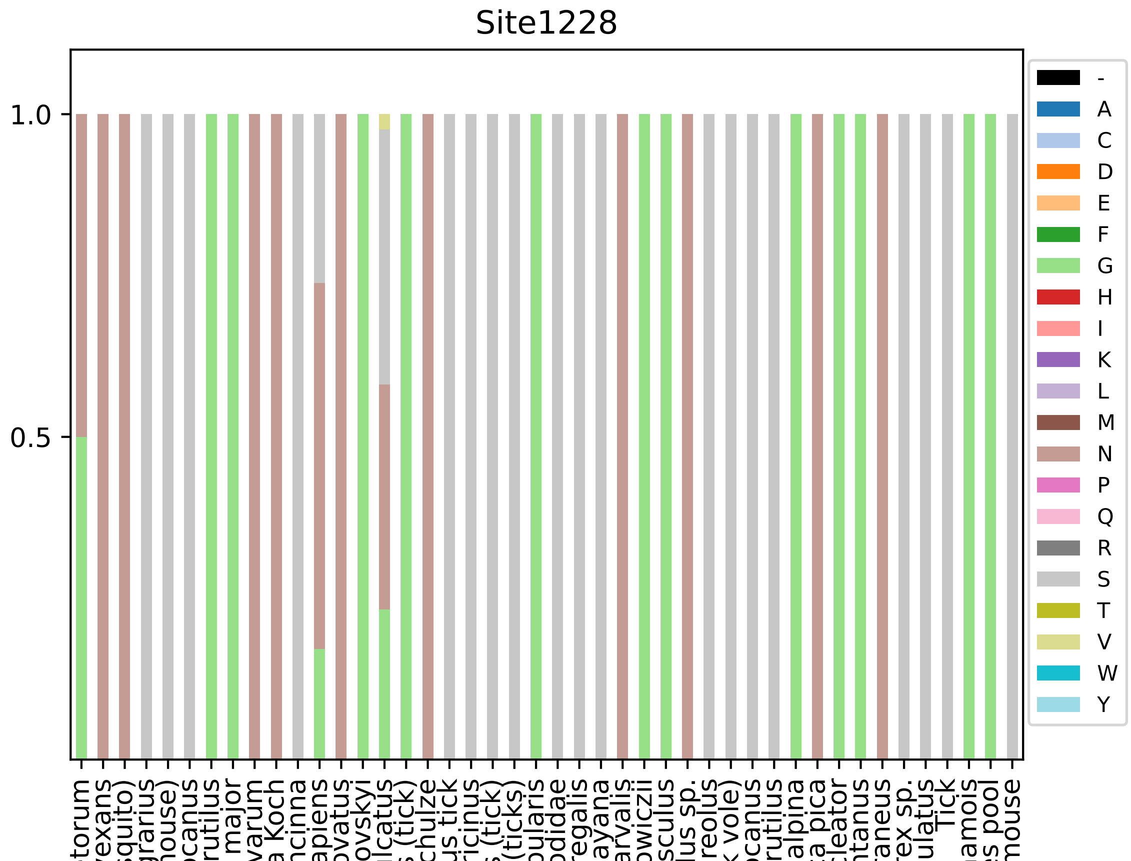

Site1228
1.0
0.5
-
A
C
D
E
F
G
H
I
K
L
M
N
P
Q
R
S
T
V
W
Y
torum
vexans
squito)
garius
nouse)
pcanus
rutilus
major
varum
a Koch
ncinna
apiens
ovatus
ovskyi
ulcatus
s (tick)
chulze
us tick
ricinus
s (ticks)
pularis
podidae
regalis
ayana
arvalis
owiczii
sculus
lus sp.
reolus
x vole)
pcanus
rutilus
alpina
a pica
cleator
ntanus
raneus
ex sp.
ulatus
Tick
amois
s pool
mouse

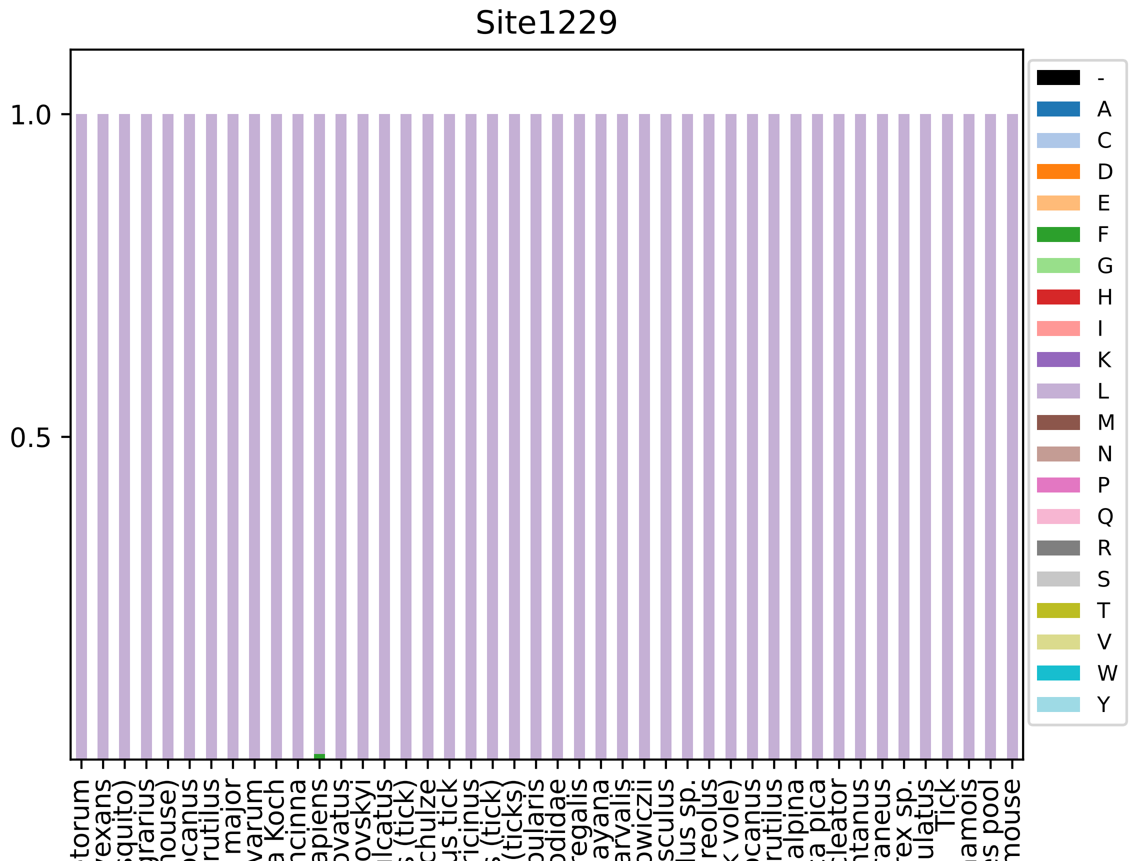
Site1229
1.0
0.5
-
A
C
D
E
F
G
H
I
K
L
M
N
P
Q
R
S
T
V
W
Y

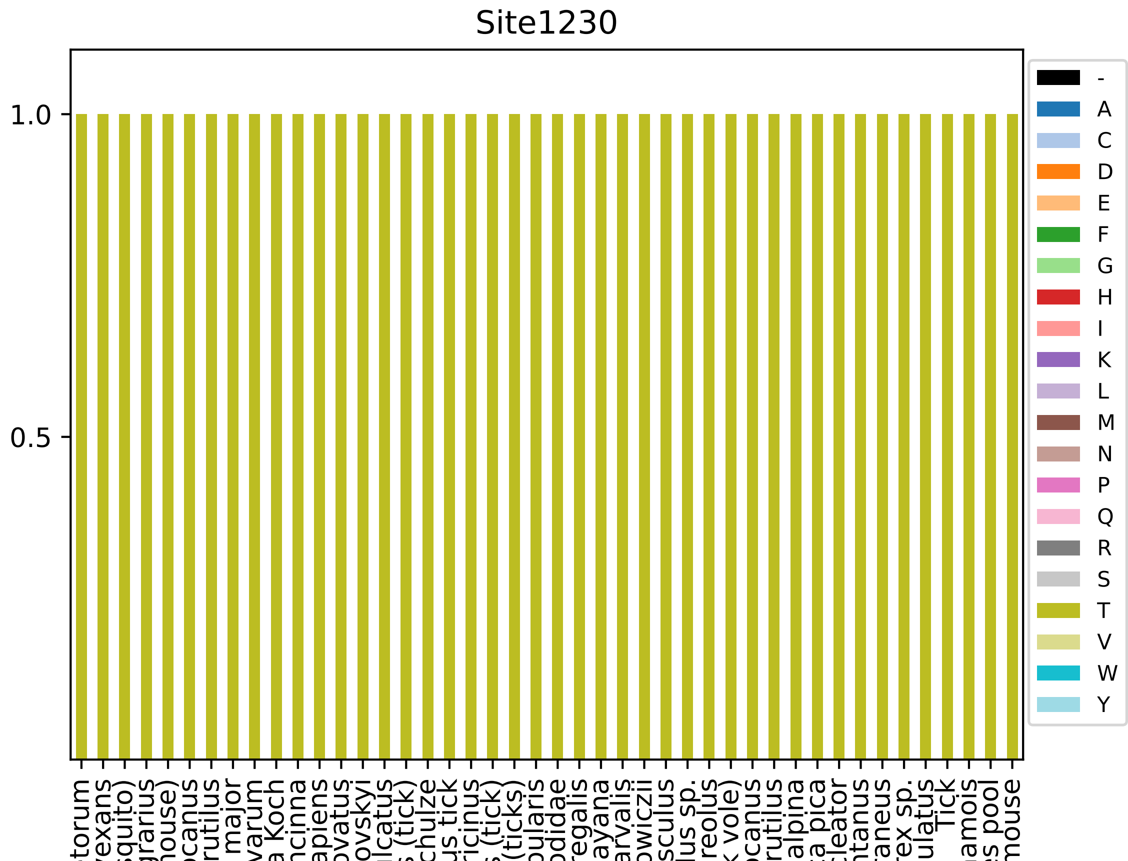
Site1230
1.0
0.5
-
A
C
D
E
F
G
H
I
K
L
M
N
P
Q
R
S
T
V
W
Y
torum
exans
squito)
grarius
house)
ocanus
rutilus
major
varum
a Koch
ncinna
apiens
ovatus
ovskyi
ulcatus
s (tick)
chulze
us tick
ricinus
s (tick)
(ticks)
oularis
odidae
regalis
ayana
arvalis
owiczii
sculus
lus sp.
reolus
k vole)
ocanus
rutilus
alpina
a pica
cleator
ntanus
raneus
rex sp.
ulatus
Tick
amois
es pool
mouse

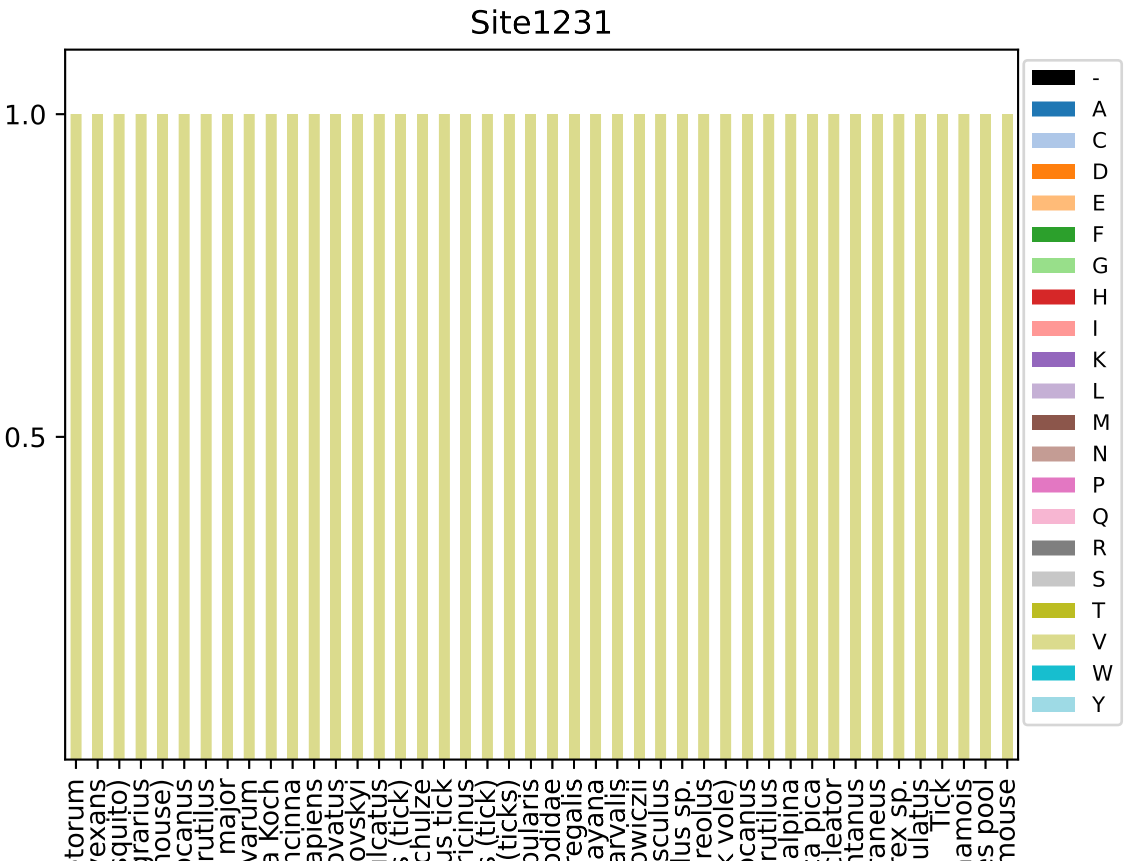
Site1231
1.0
0.5
-
A
C
D
E
F
G
H
I
K
L
M
N
P
Q
R
S
T
V
W
Y
torum
vexans
squito)
grarius
nouse)
ocanus
rutilus
major
varum
a Koch
ncinna
apiens
ovatus
ovskyi
ulcatus
s (tick)
chulze
us tick
ricinus
s (tick)
(ticks)
pularis
podidae
regalis
ayana
arvalis
owiczii
sculus
lus sp.
reolus
k vole)
ocanus
rutilus
alpina
a pica
leator
ntanus
raneus
rex sp.
ulatus
Tick
amois
es pool
mouse

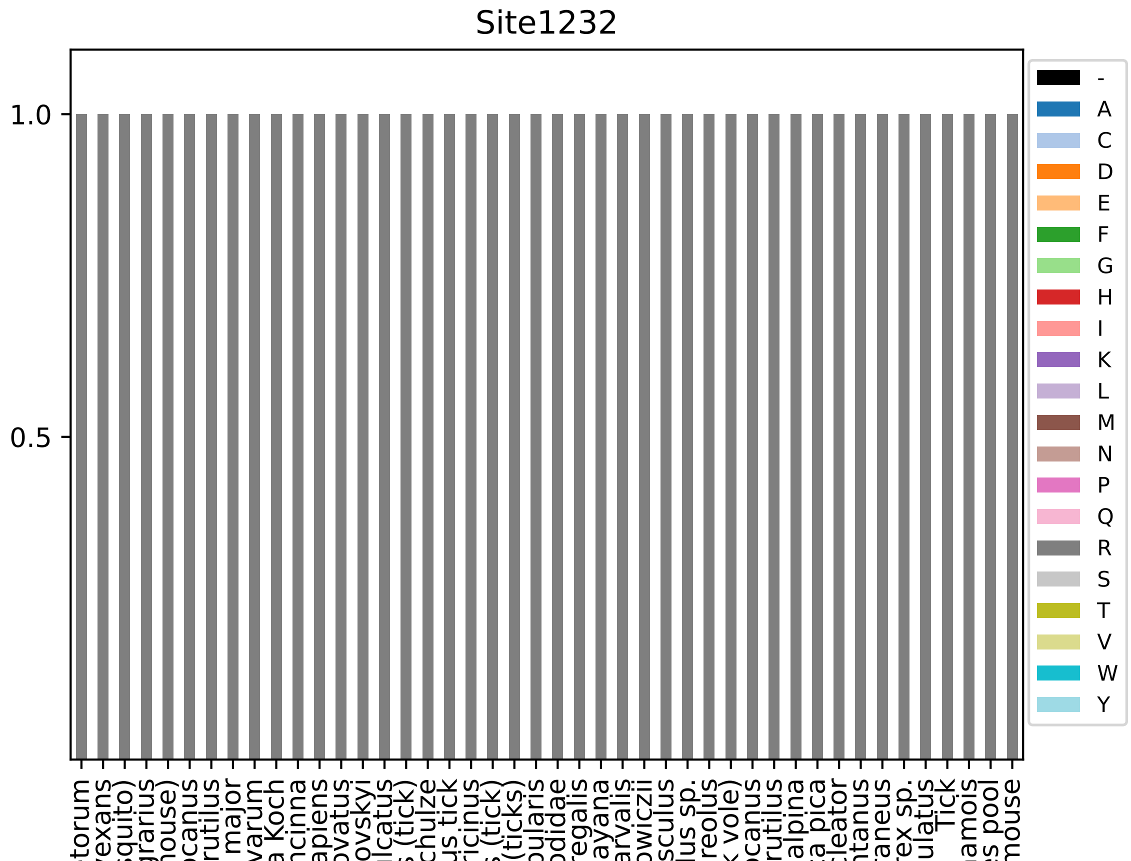
Site1232
1.0
0.5
-
A
C
D
E
F
G
H
I
K
L
M
N
P
Q
R
S
T
V
W
Y

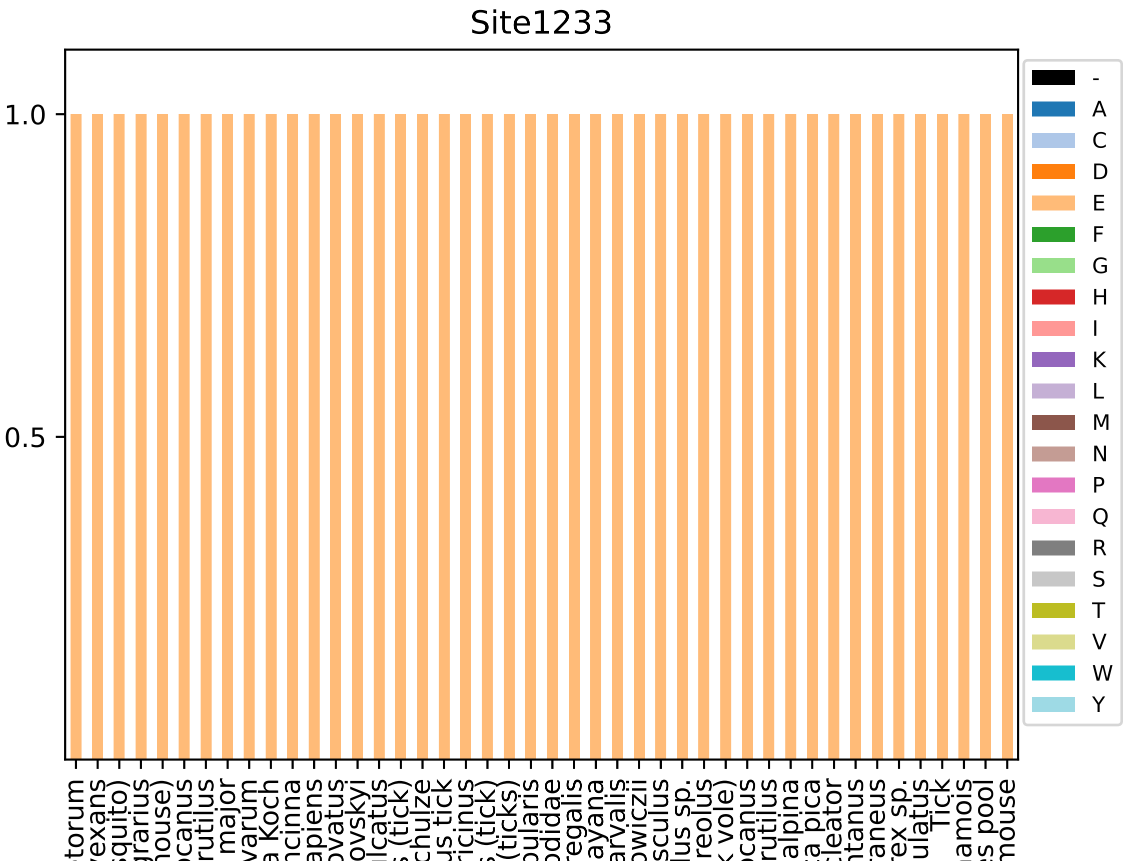
Site1233
1.0
0.5
-
A
C
D
E
F
G
H
I
K
L
M
N
P
Q
R
S
T
V
W
Y

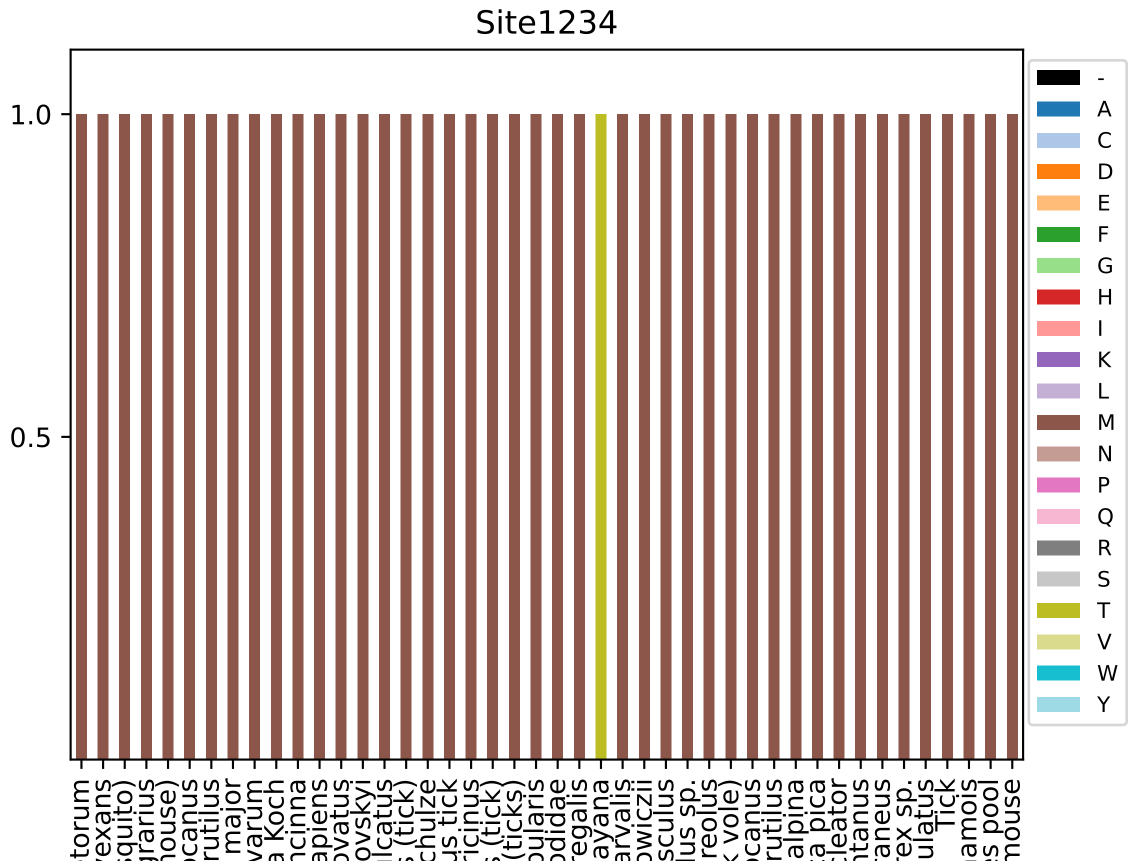
Site1234
1.0
0.5
-
A
C
D
E
F
G
H
I
K
L
M
N
P
Q
R
S
T
V
W
Y

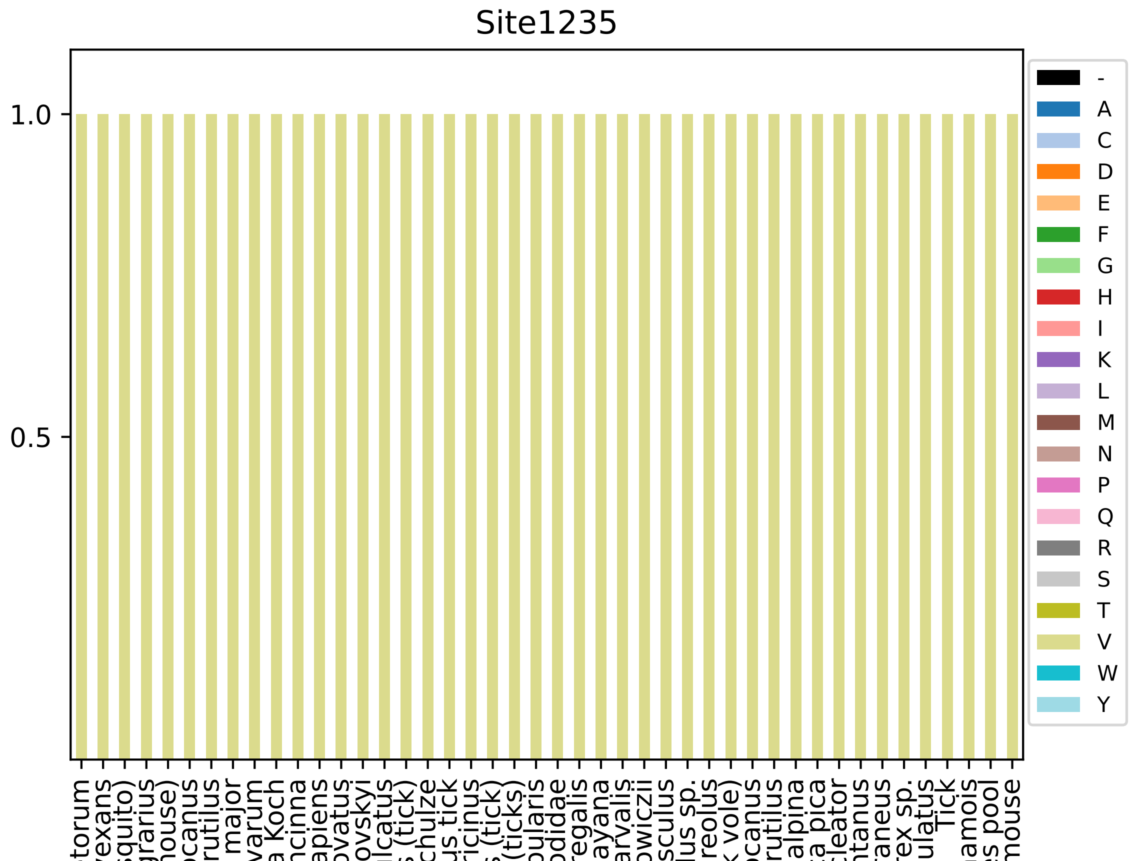
Site1235
1.0
0.5
-
A
C
D
E
F
G
H
I
K
L
M
N
P
Q
R
S
T
V
W
Y

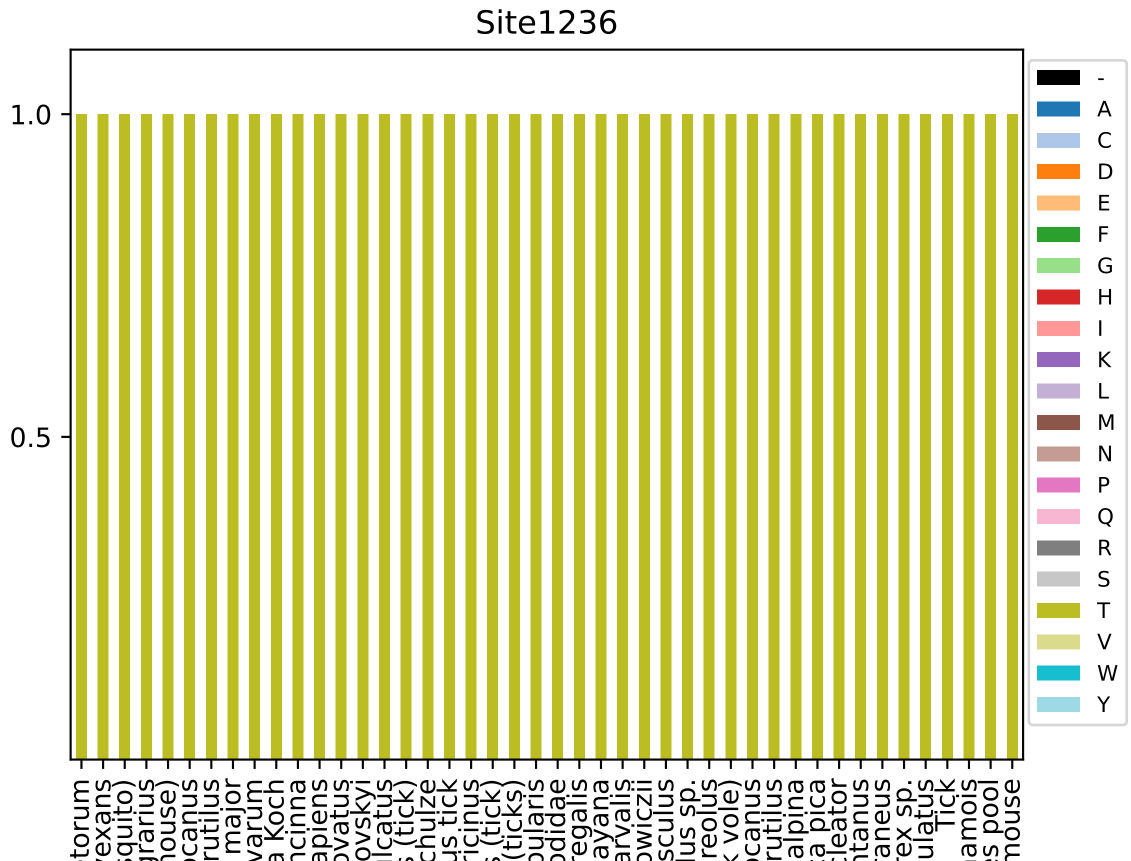
Site1236
1.0
0.5
-
A
C
D
E
F
G
H
I
K
L
M
N
P
Q
R
S
T
V
W
Y

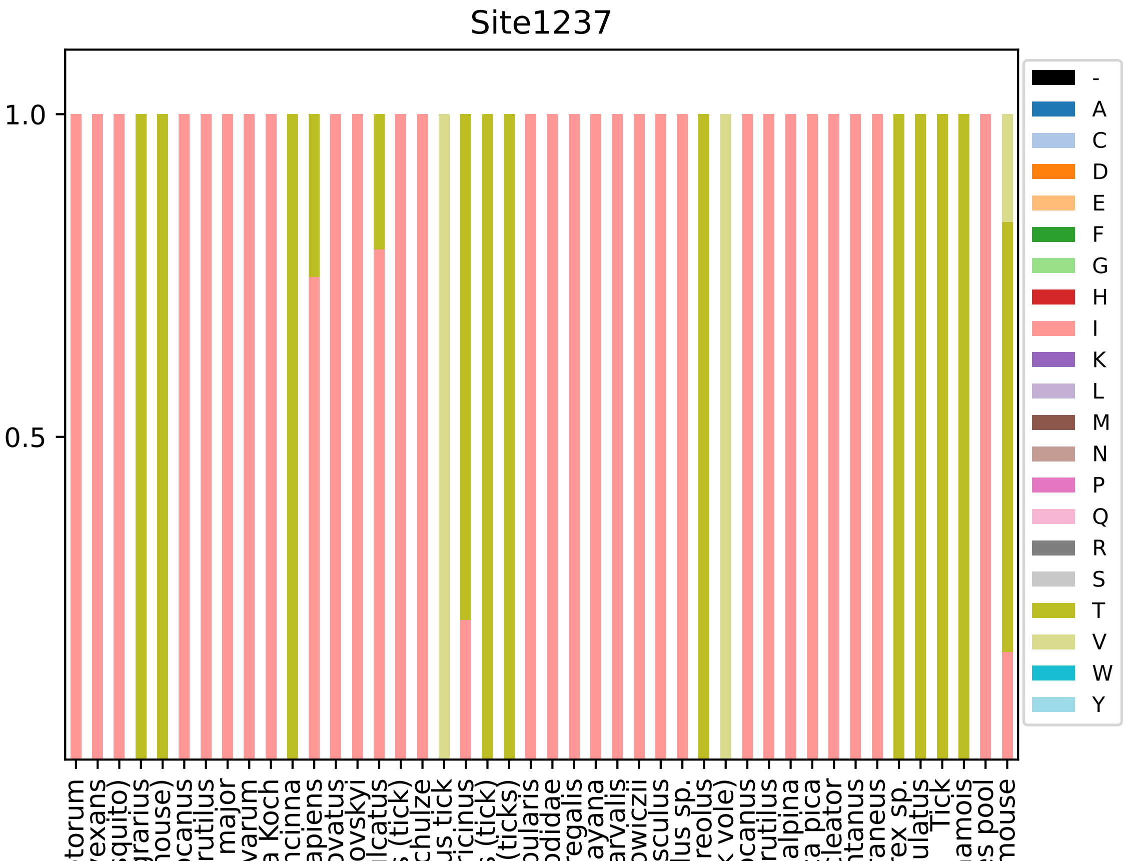
Site1237
1.0
0.5
-
A
C
D
E
F
G
H
I
K
L
M
N
P
Q
R
S
T
V
W
Y

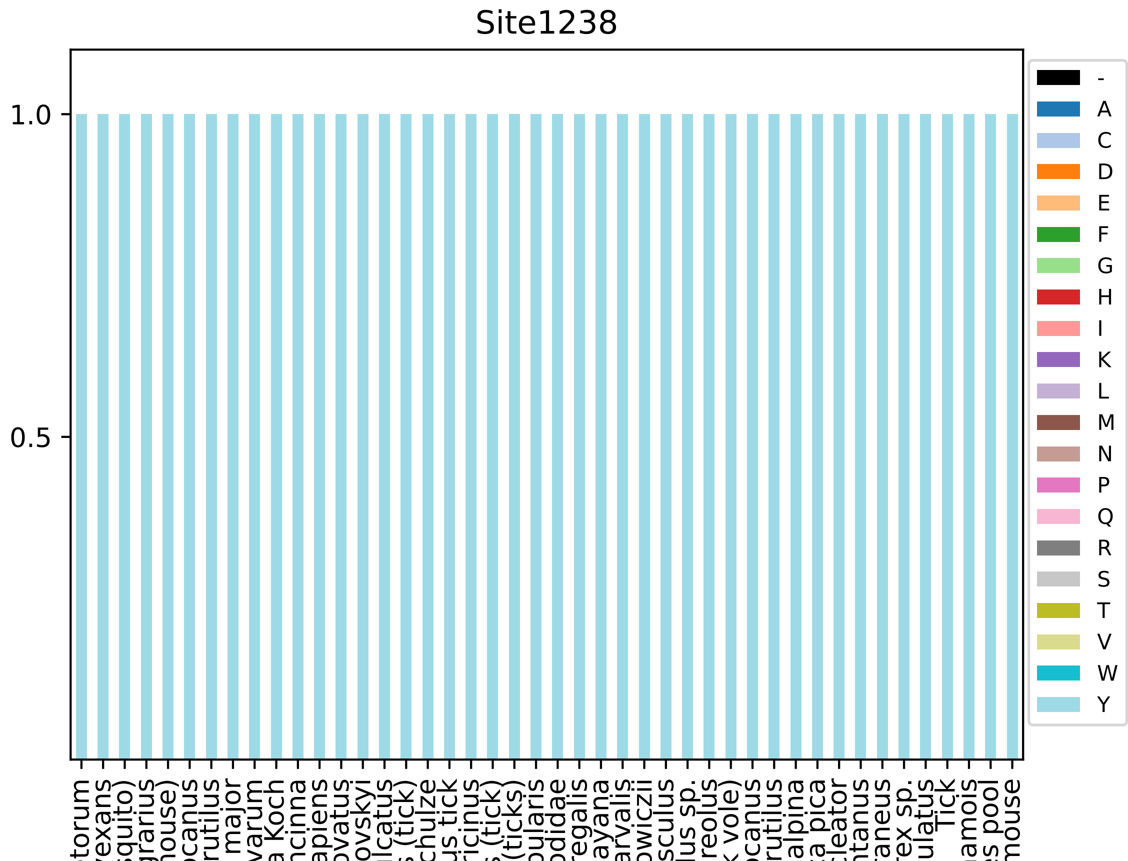
Site1238
1.0
0.5
-
A
C
D
E
F
G
H
I
K
L
M
N
P
Q
R
S
T
V
W
Y

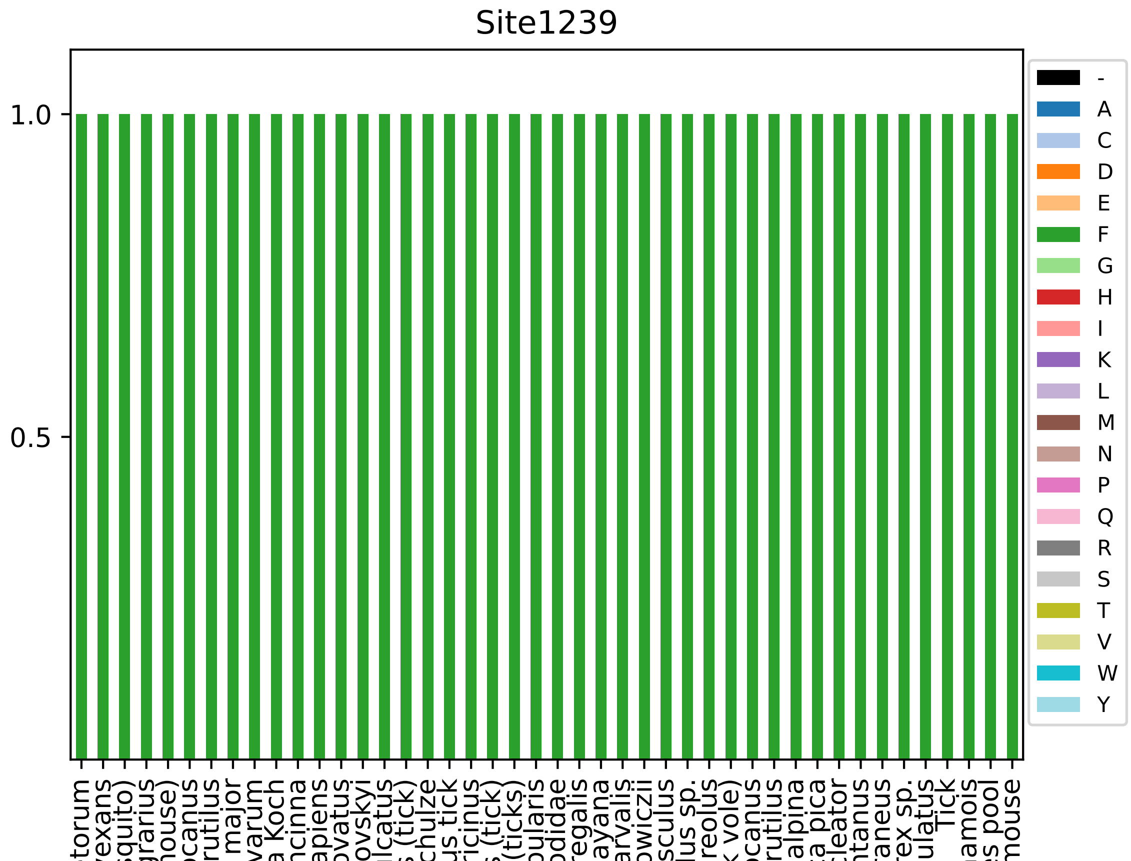
Site1239
1.0
0.5
-
A
C
D
E
F
G
H
I
K
L
M
N
P
Q
R
S
T
V
W
Y

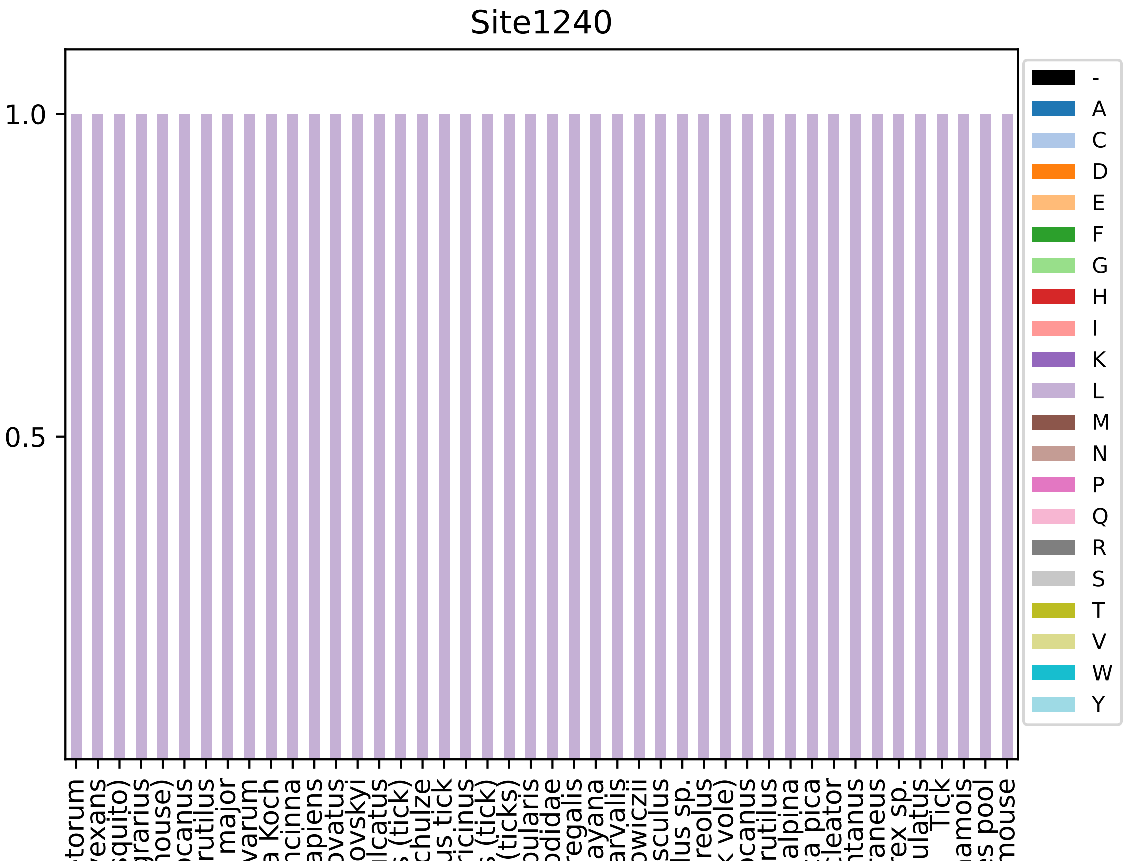
Site1240
1.0
0.5
-
A
C
D
E
F
G
H
I
K
L
M
N
P
Q
R
S
T
V
W
Y

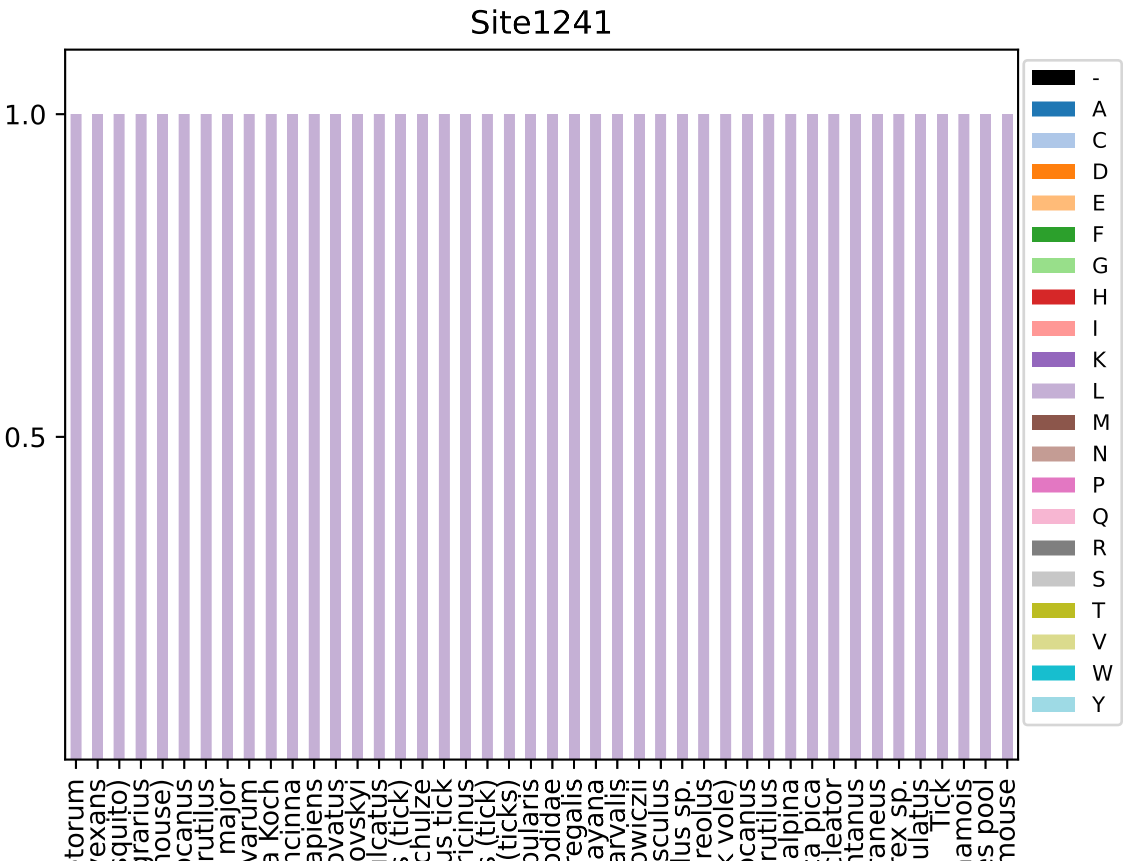
Site1241
1.0
0.5
-
A
C
D
E
F
G
H
I
K
L
M
N
P
Q
R
S
T
V
W
Y

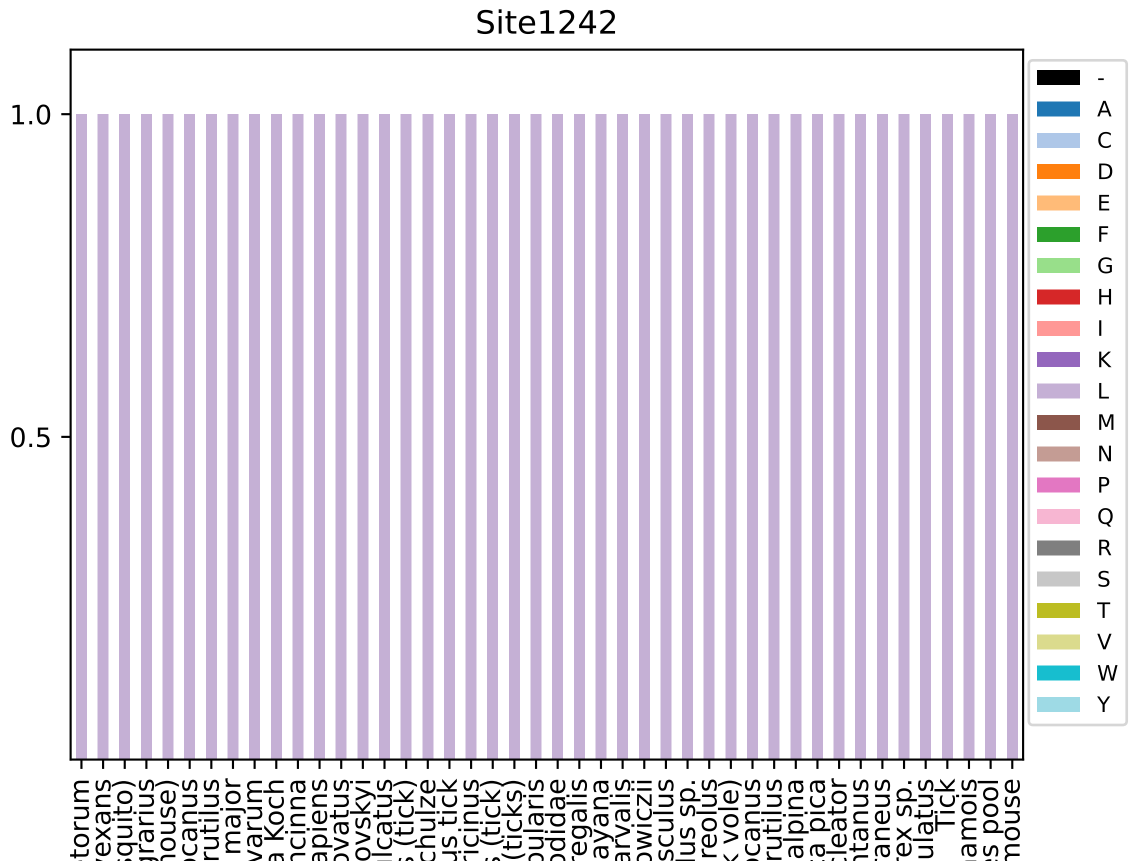
Site1242
1.0
0.5
-
A
C
D
E
F
G
H
I
K
L
M
N
P
Q
R
S
T
V
W
Y

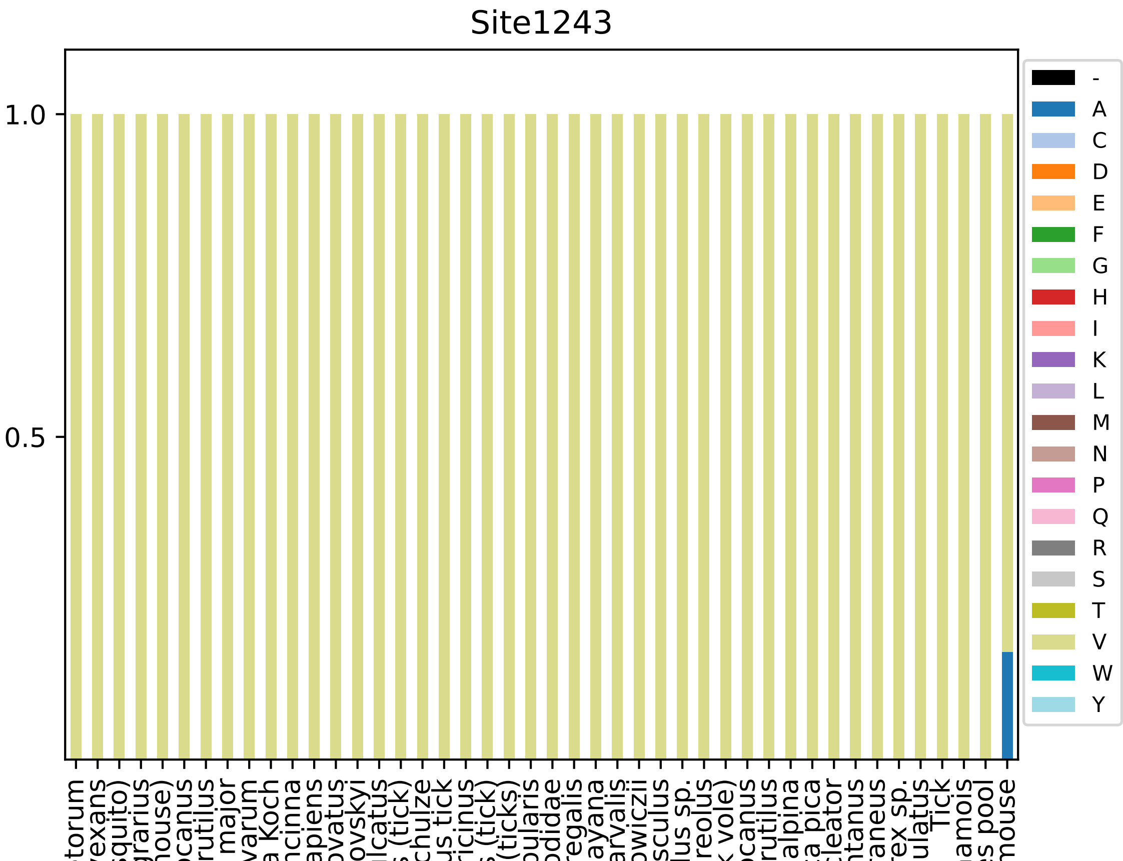
Site1243
1.0
0.5
-
A
C
D
E
F
G
H
I
K
L
M
N
P
Q
R
S
T
V
W
Y

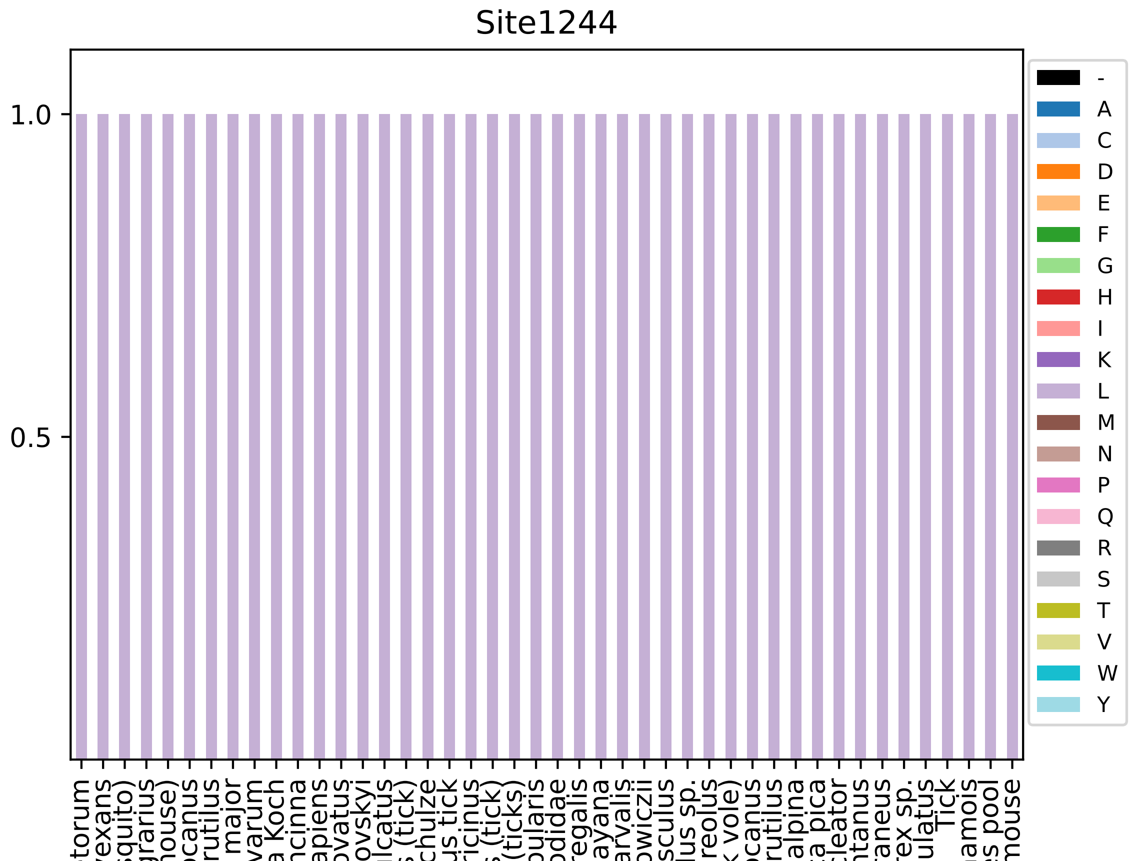

Site1244
1.0
0.5
-
A
C
D
E
F
G
H
I
K
L
M
N
P
Q
R
S
T
V
W
Y

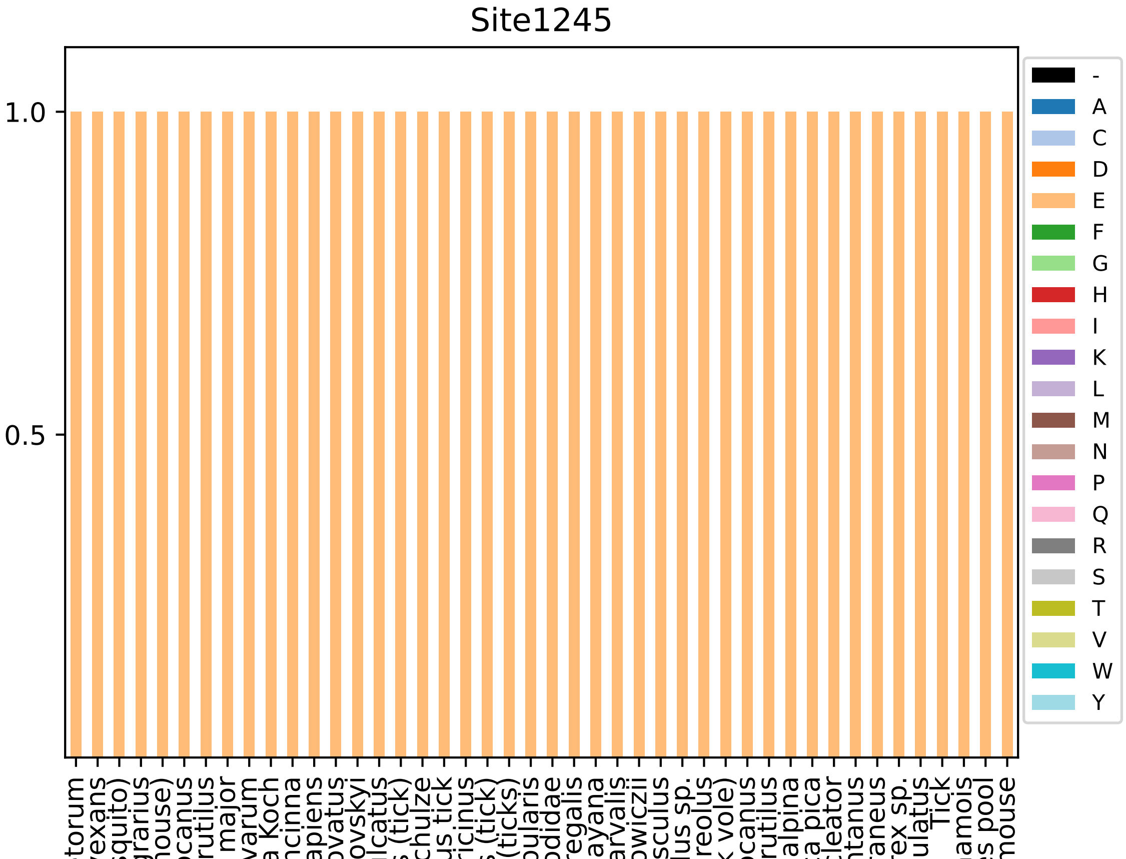
Site1245
1.0
0.5
-
A
C
D
E
F
G
H
I
K
L
M
N
P
Q
R
S
T
V
W
Y

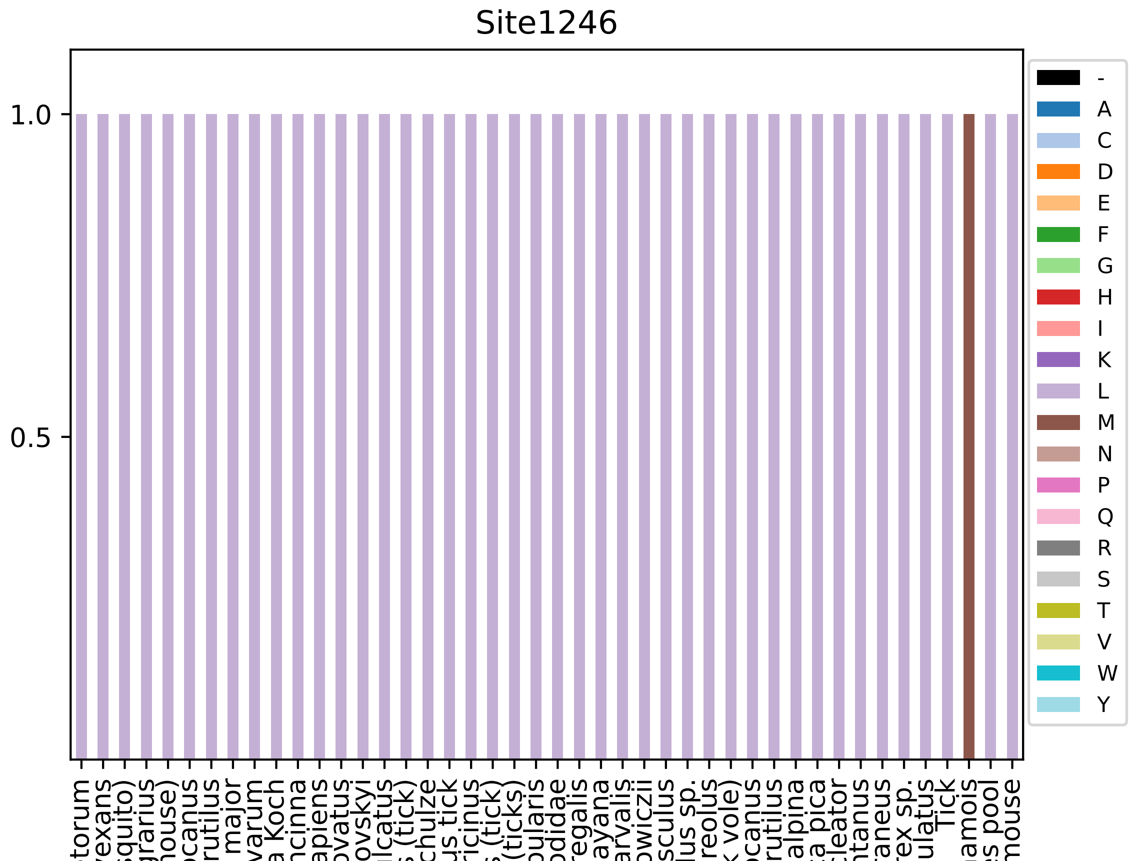

Site1246
1.0
0.5
-
A
C
D
E
F
G
H
I
K
L
M
N
P
Q
R
S
T
V
W
Y

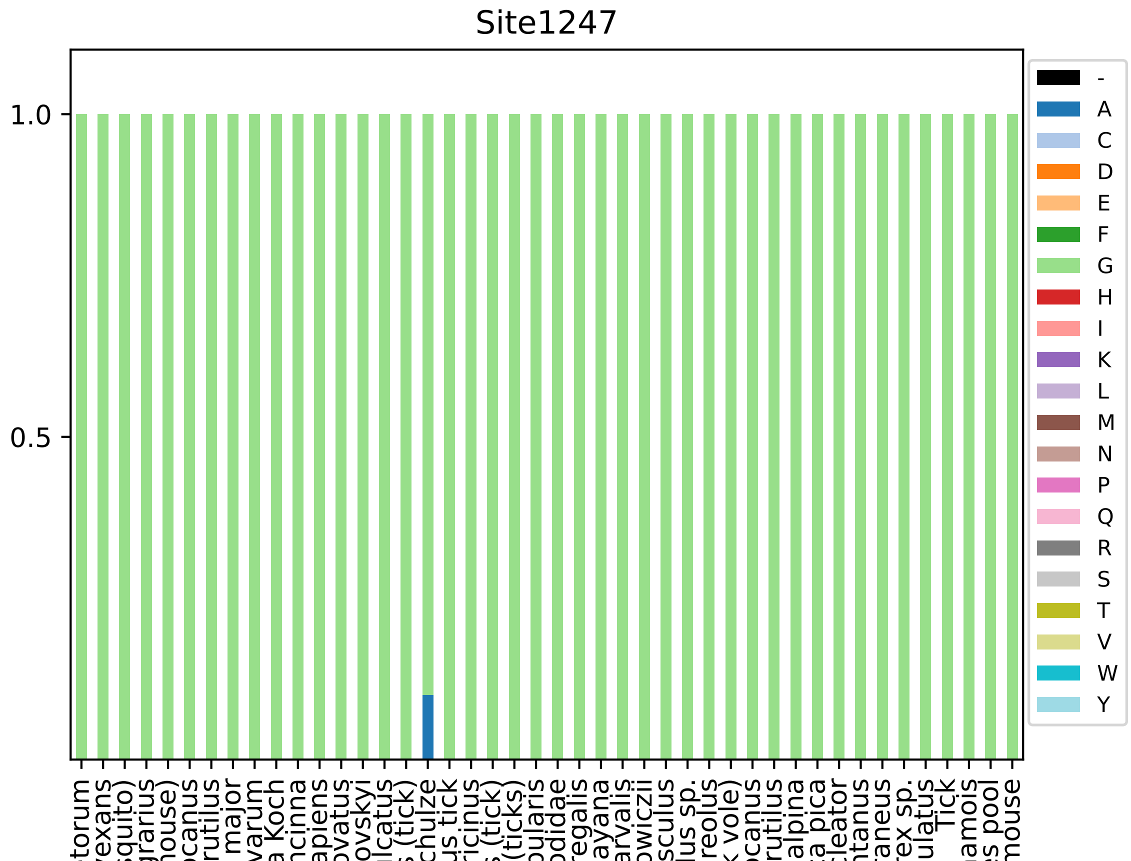

Site1247
1.0
0.5
-
A
C
D
E
F
G
H
I
K
L
M
N
P
Q
R
S
T
V
W
Y

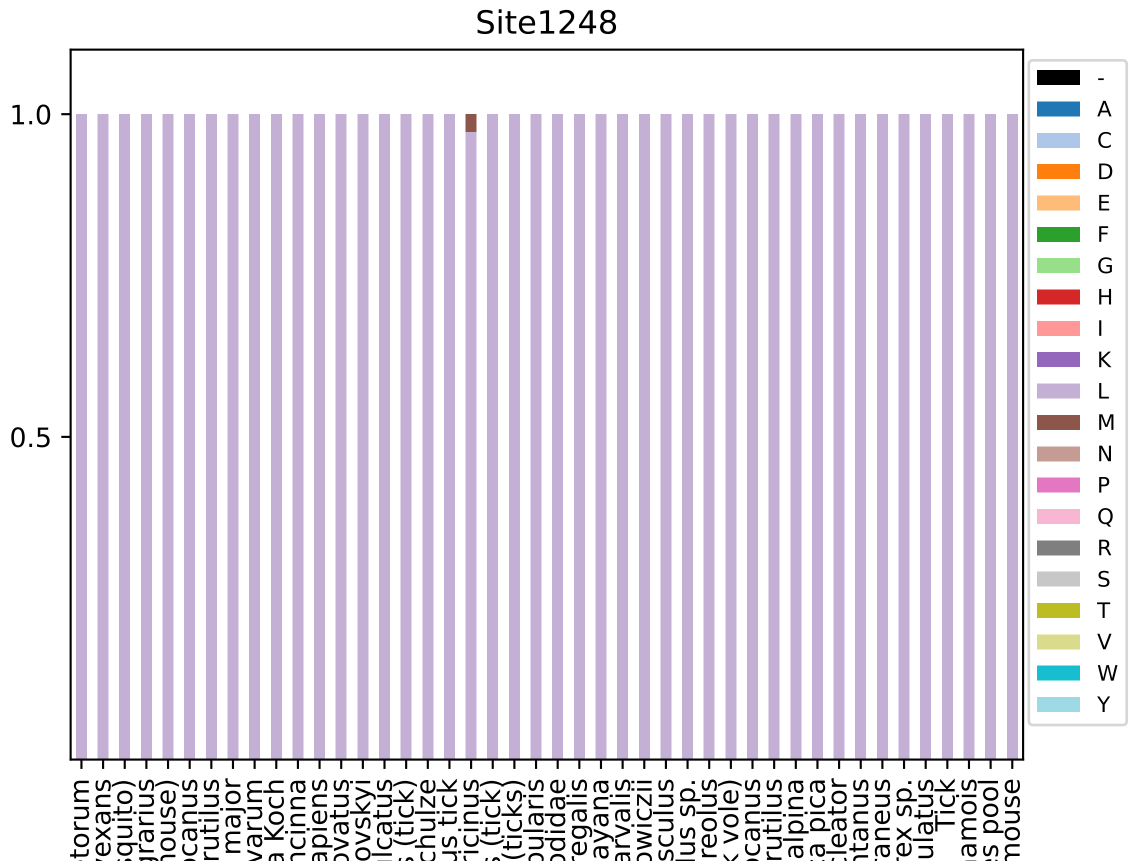

Site1248
1.0
0.5
-
A
C
D
E
F
G
H
I
K
L
M
N
P
Q
R
S
T
V
W
Y

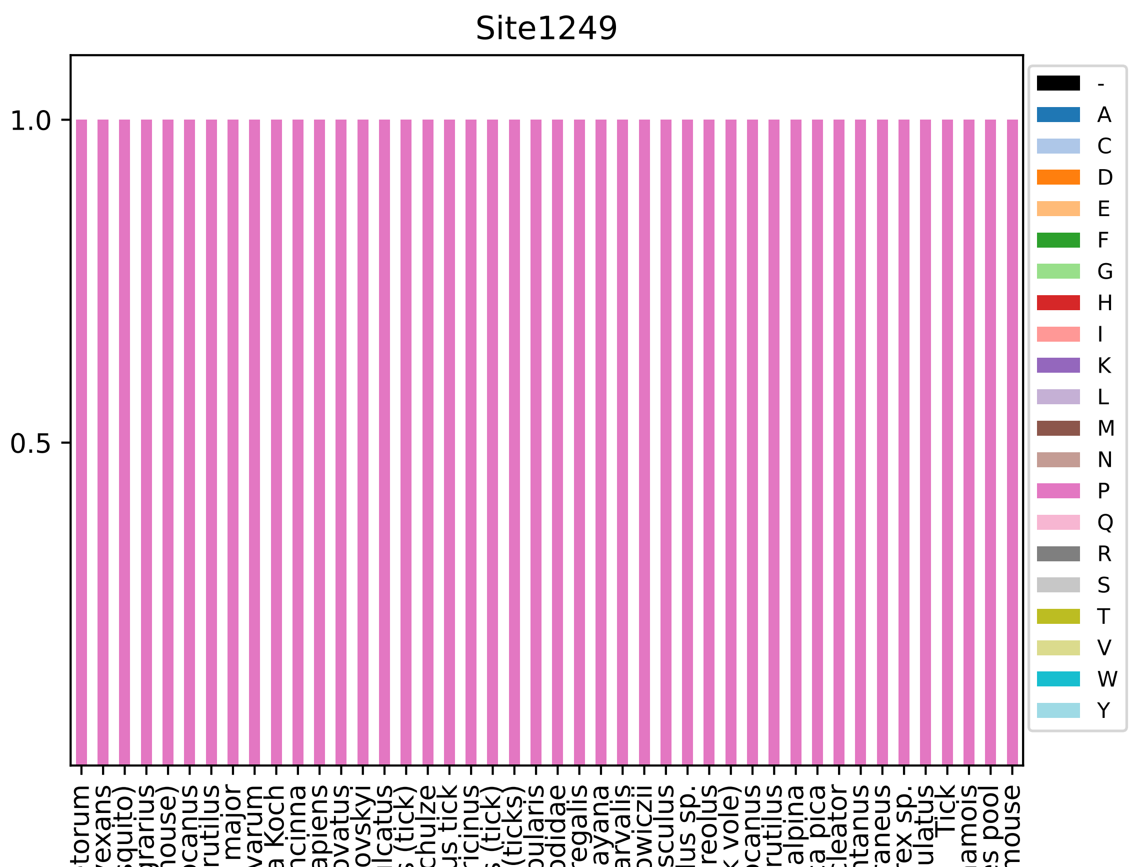
Site1249
1.0
0.5
-
A
C
D
E
F
G
H
I
K
L
M
N
P
Q
R
S
T
V
W
Y

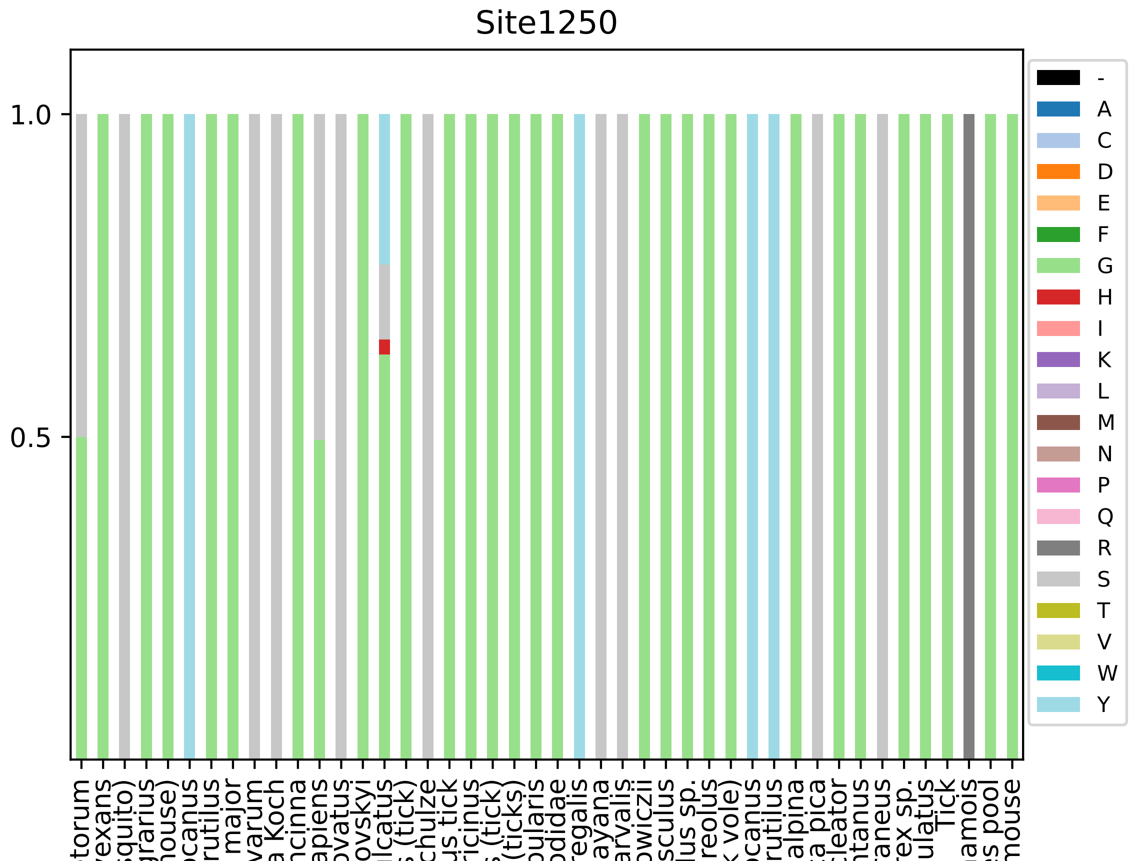

Site1250
1.0
0.5
-
A
C
D
E
F
G
H
I
K
L
M
N
P
Q
R
S
T
V
W
Y

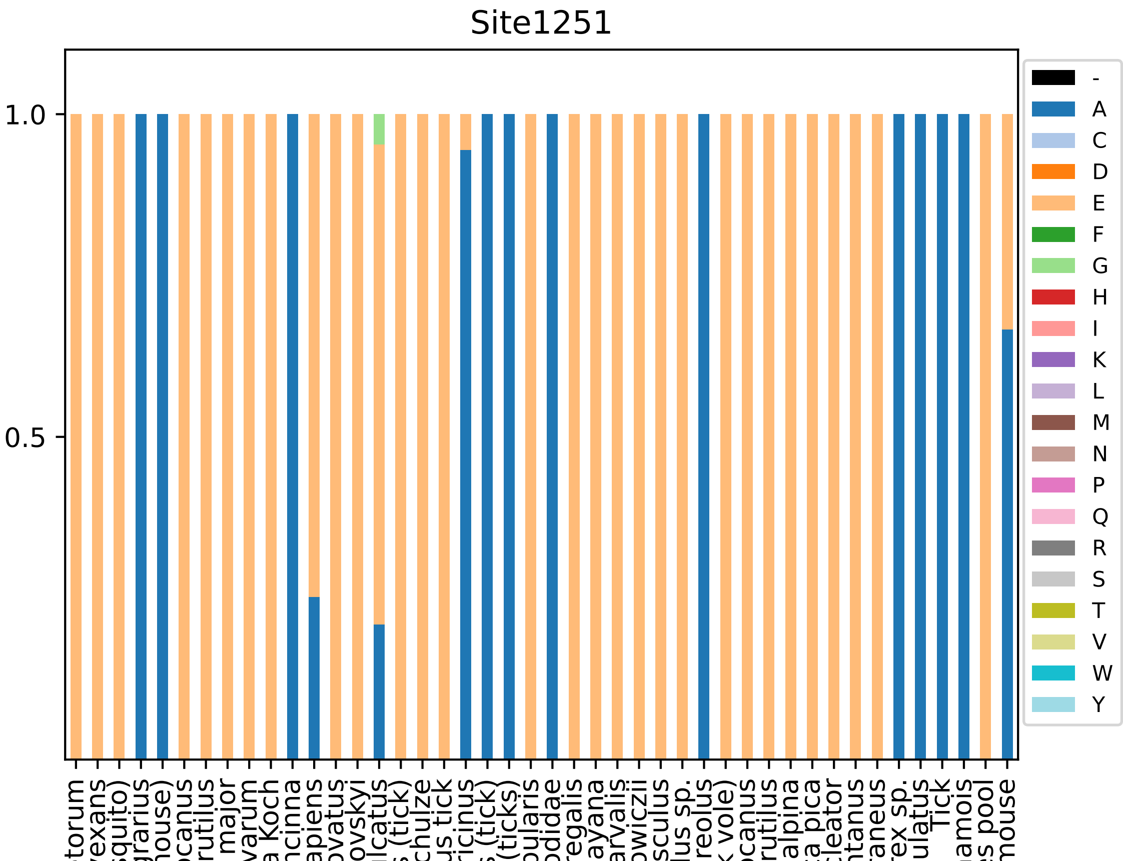

Site1251
1.0
0.5
-
A
C
D
E
F
G
H
I
K
L
M
N
P
Q
R
S
T
V
W
Y
torum
vexans
squito)
garius
nouse)
ocanus
rutilus
major
varum
a Koch
ncinna
apiens
ovatus
ovskyi
ulcatus
s (tick)
chulze
us tick
ricinus
s (tick)
(ticks)
pularis
podidae
regalis
ayana
arvalis
owiczii
sculus
lus sp.
reolus
k vole)
ocanus
rutilus
alpina
a pica
cleator
ntanus
raneus
rex sp.
ulatus
Tick
amois
es pool
mouse

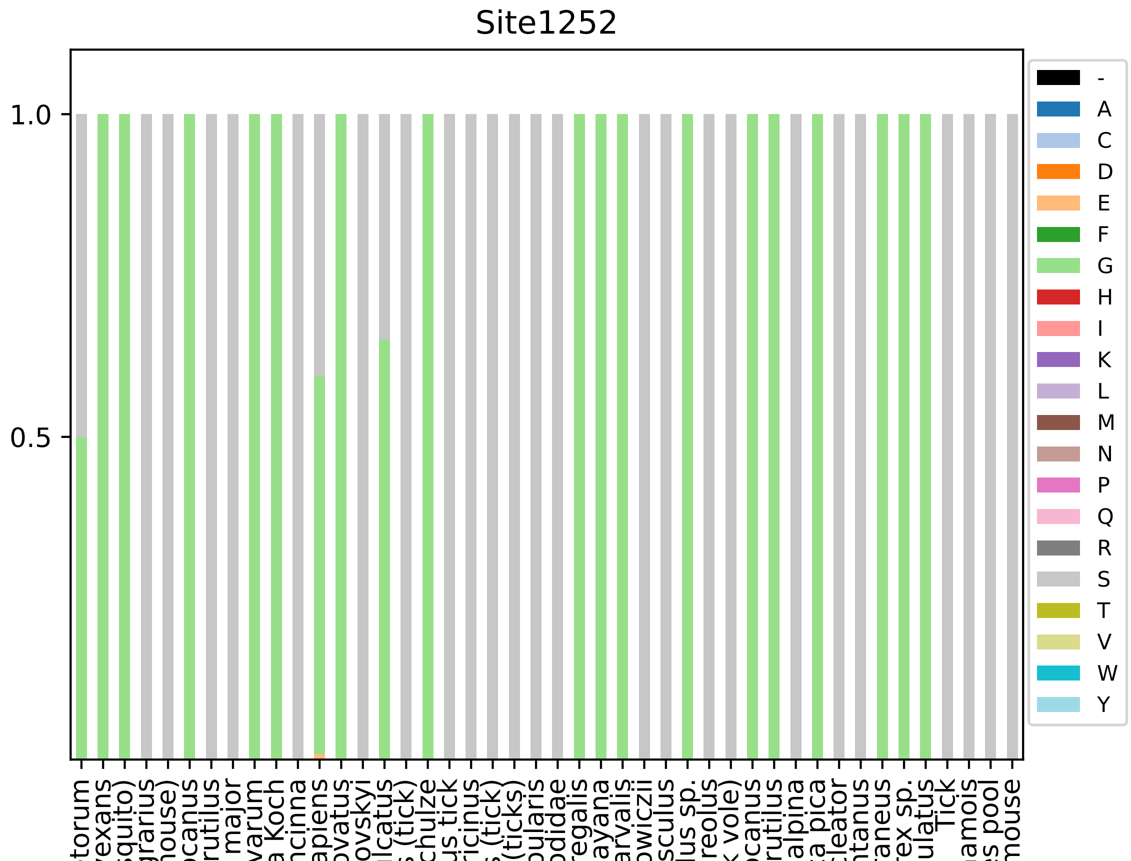
Site1252
1.0
0.5
-
A
C
D
E
F
G
H
I
K
L
M
N
P
Q
R
S
T
V
W
Y

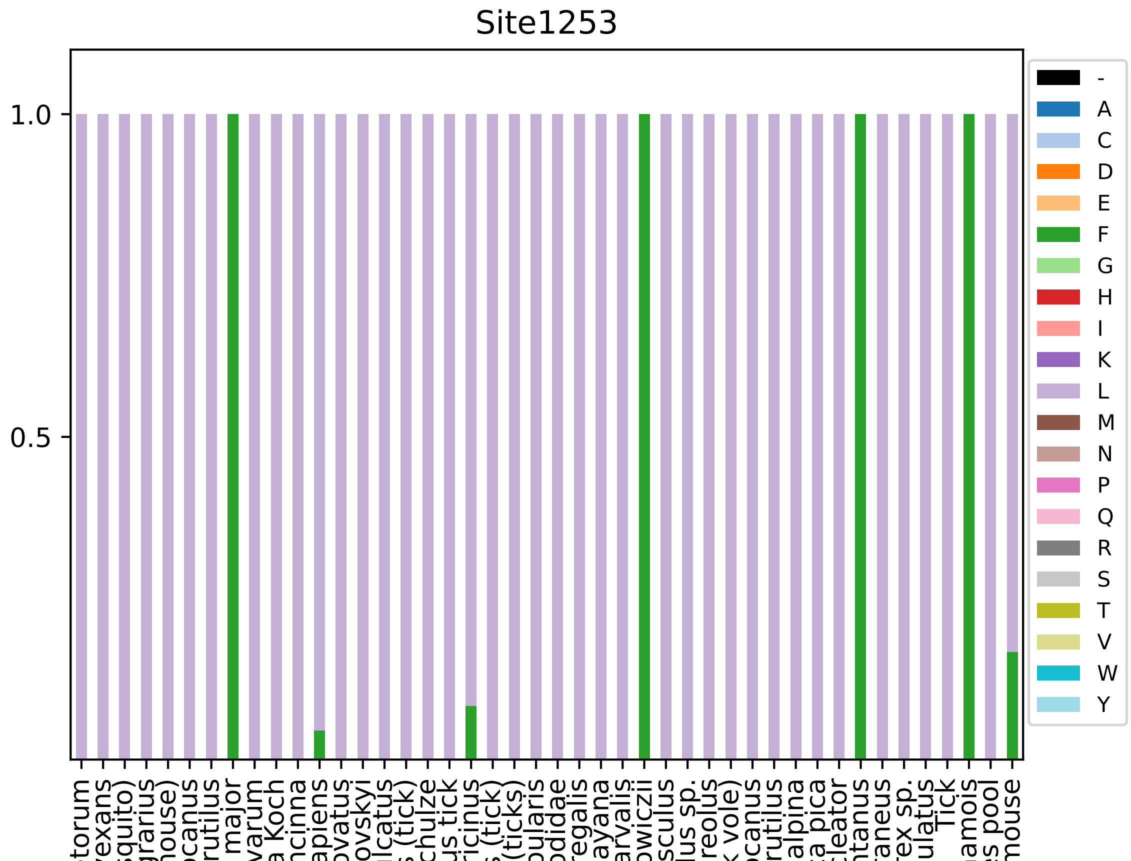

Site1253
1.0
0.5
-
A
C
D
E
F
G
H
I
K
L
M
N
P
Q
R
S
T
V
W
Y

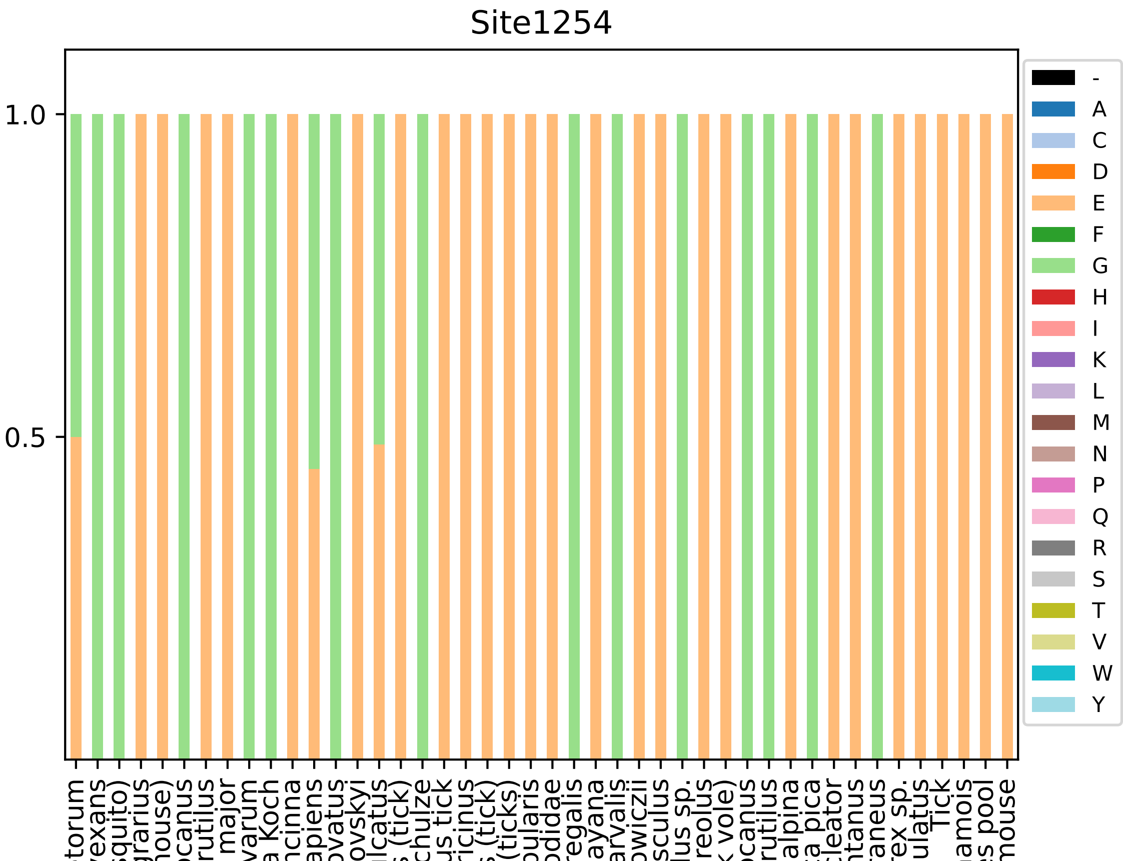

Site1254
1.0
0.5
-
A
C
D
E
F
G
H
I
K
L
M
N
P
Q
R
S
T
V
W
Y

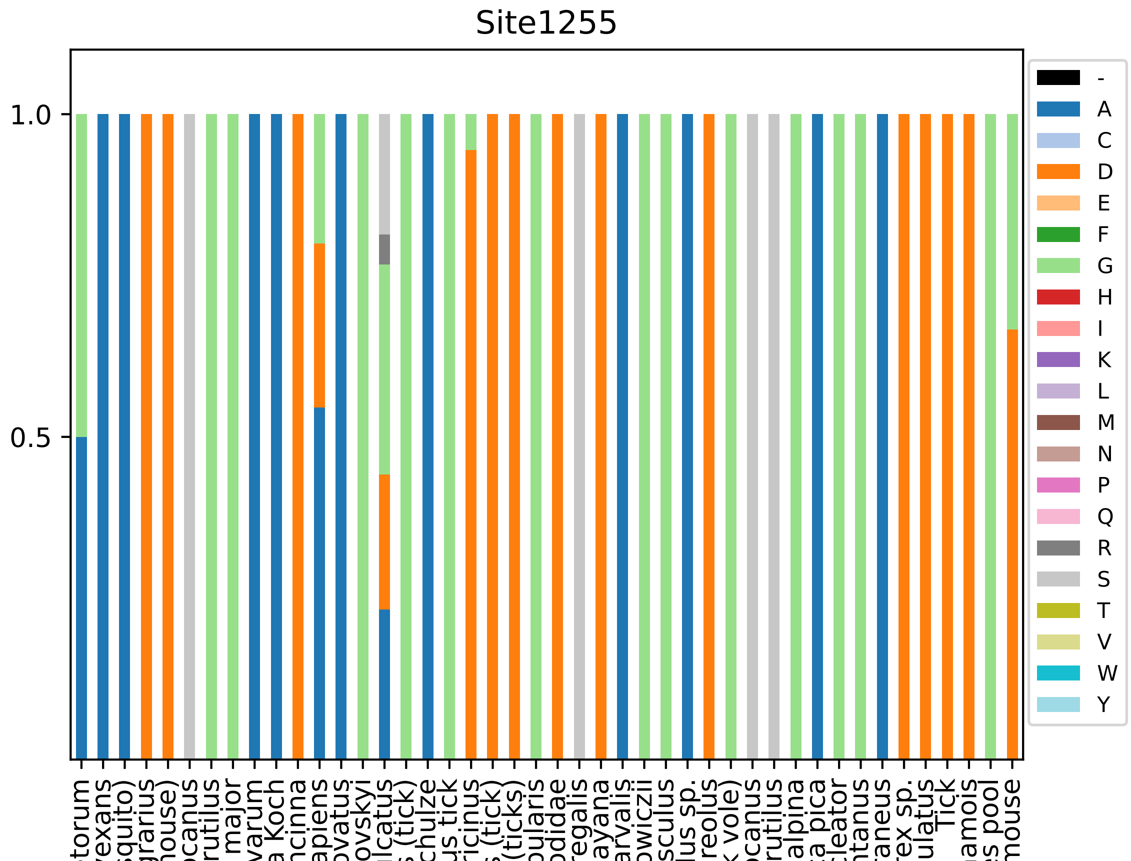
Site1255
1.0
0.5
-
A
C
D
E
F
G
H
I
K
L
M
N
P
Q
R
S
T
V
W
Y

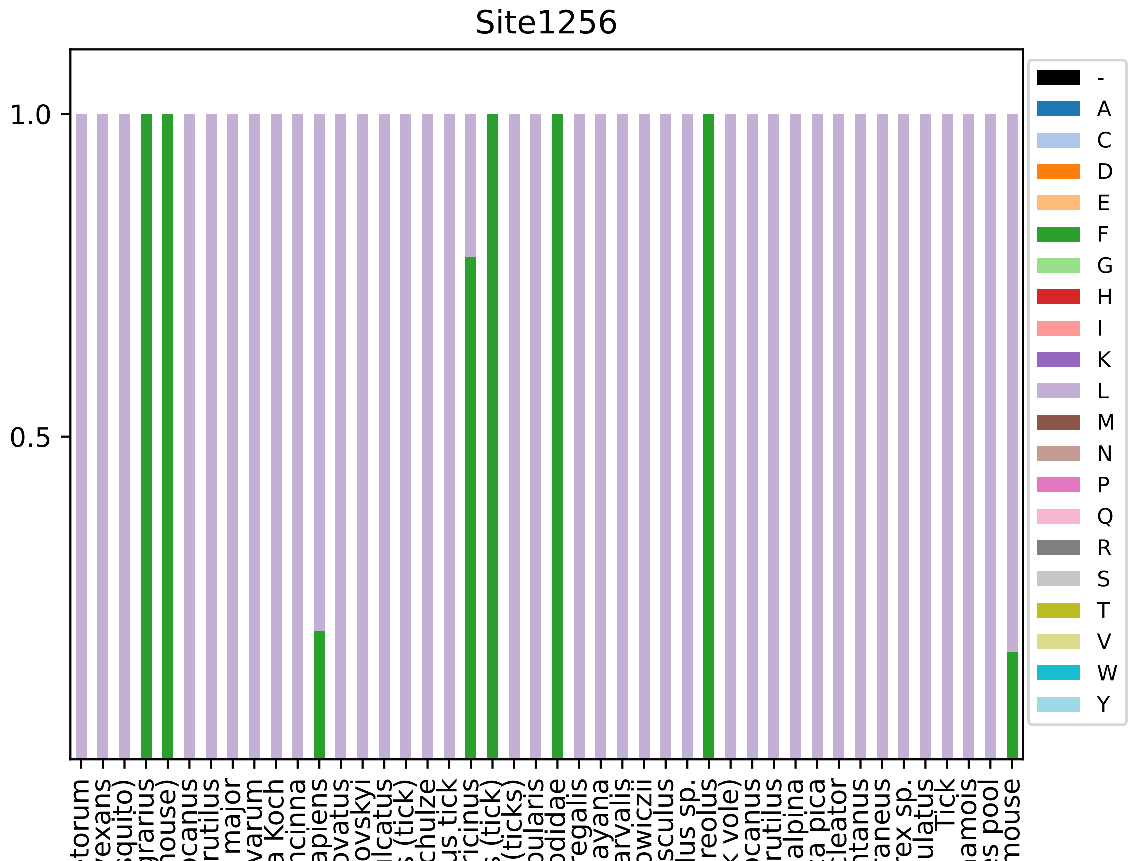
Site1256
1.0
0.5
-
A
C
D
E
F
G
H
I
K
L
M
N
P
Q
R
S
T
V
W
Y
Tick

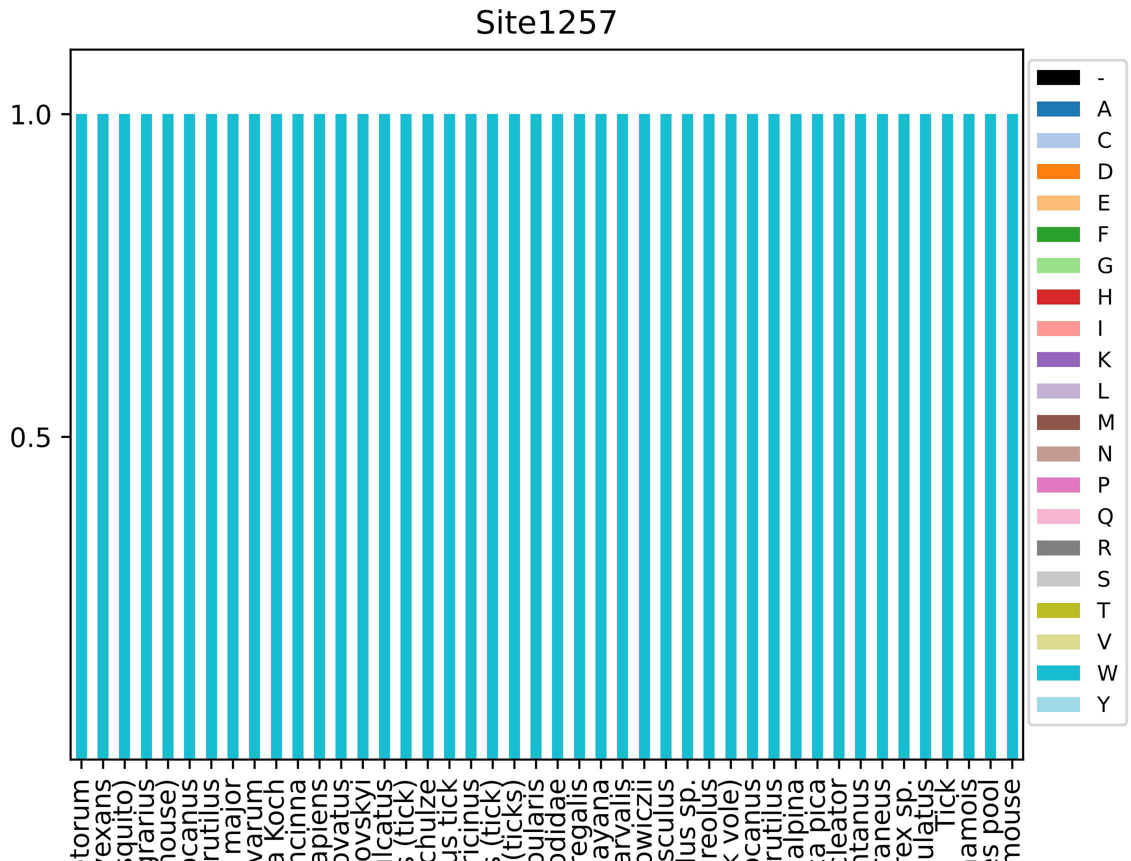

Site1257
1.0
0.5
-
A
C
D
E
F
G
H
I
K
L
M
N
P
Q
R
S
T
V
W
Y
torum
xexans
squito)
grarius
nouse)
ocanus
rutilus
major
varum
a Koch
ncinna
apiens
ovatus
ovskyi
ulcatus
s (tick)
chulze
us tick
ricinus
s (tick)
s (ticks)
pularis
podidae
regalis
ayana
arvalis
owiczii
sculus
lus sp.
reolus
k vole)
ocanus
rutilus
alpina
a pica
leator
ntanus
raneus
rex sp.
ulatus
Tick
amois
es pool
mouse

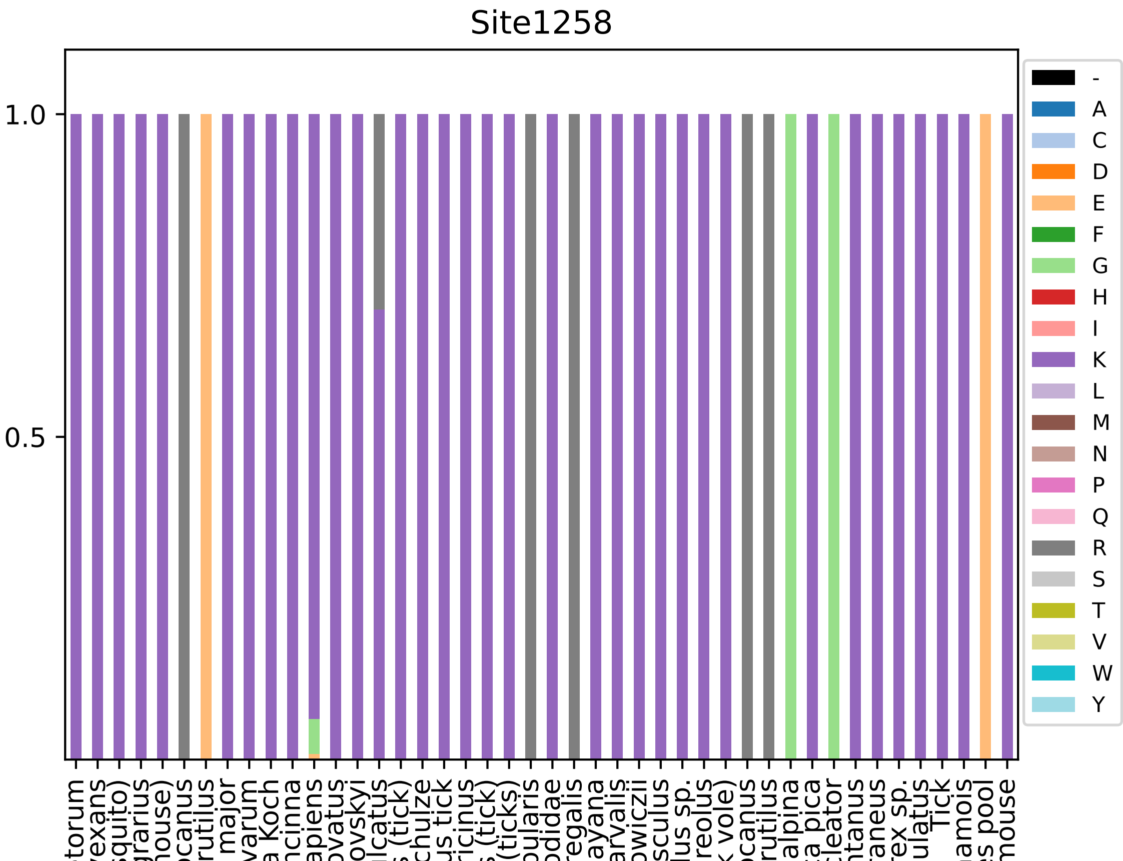

Site1258
1.0
0.5
-
A
C
D
E
F
G
H
I
K
L
M
N
P
Q
R
S
T
V
W
Y

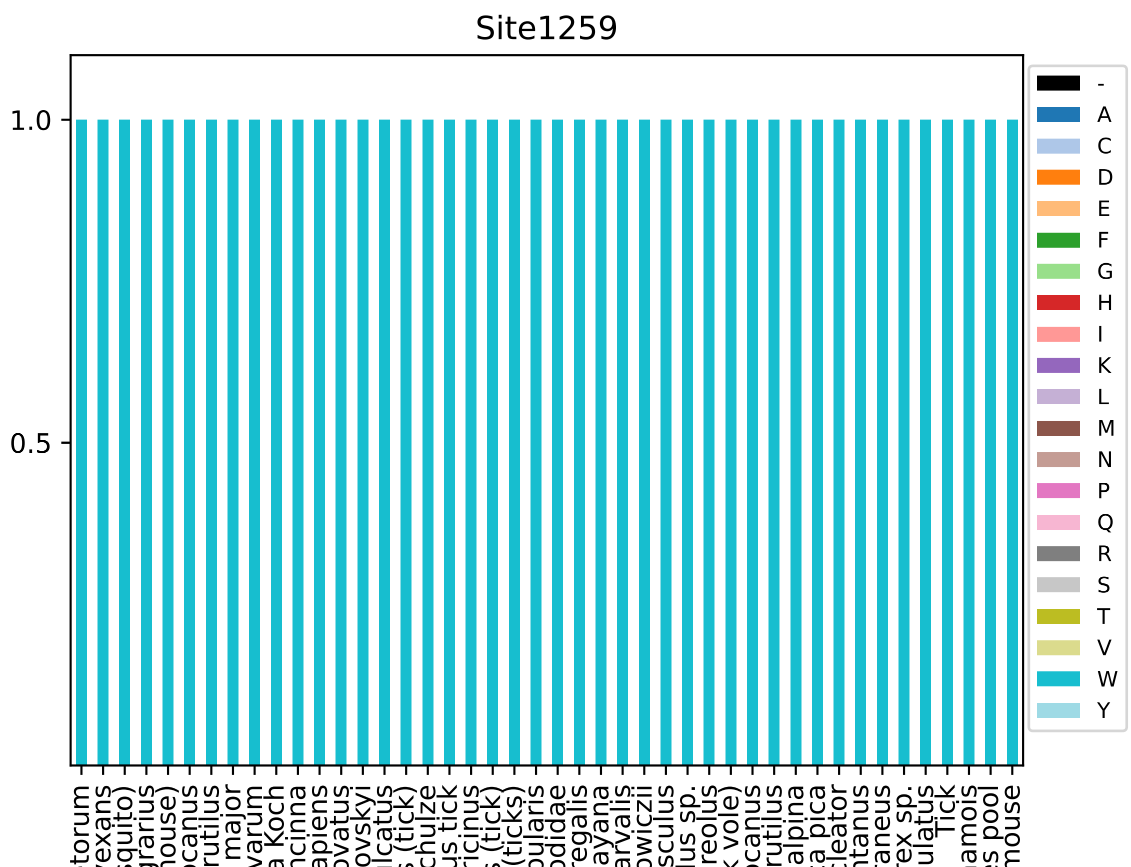

Site1259
1.0
0.5
-
A
C
D
E
F
G
H
I
K
L
M
N
P
Q
R
S
T
V
W
Y

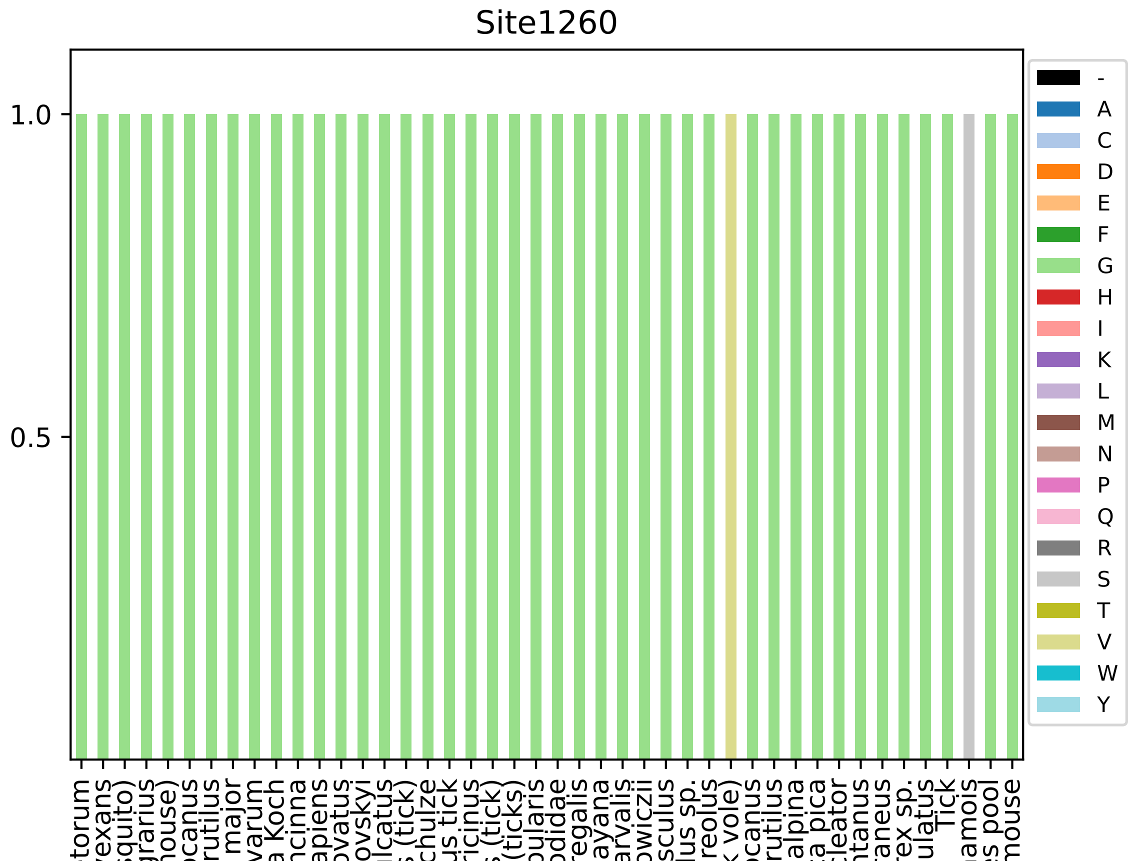
Site1260
1.0
0.5
-
A
C
D
E
F
G
H
I
K
L
M
N
P
Q
R
S
T
V
W
Y

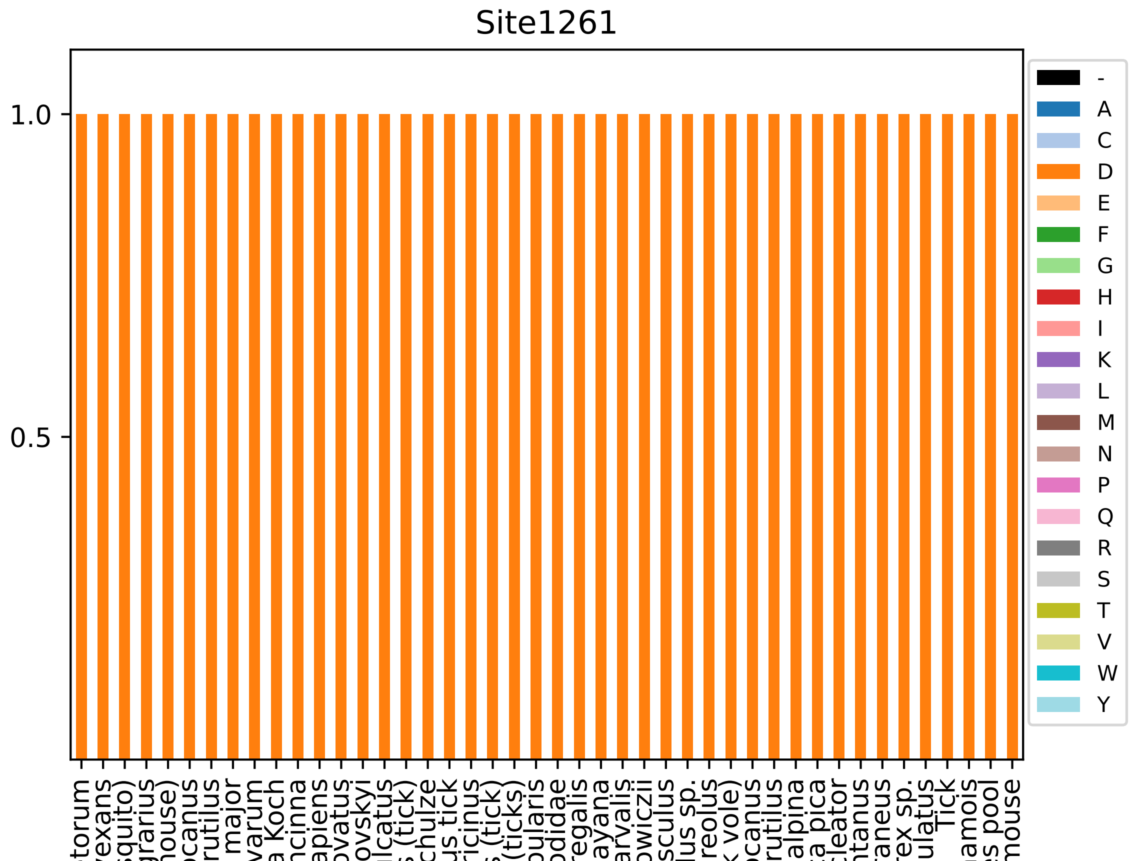
Site1261
1.0
0.5
-
A
C
D
E
F
G
H
I
K
L
M
N
P
Q
R
S
T
V
W
Y

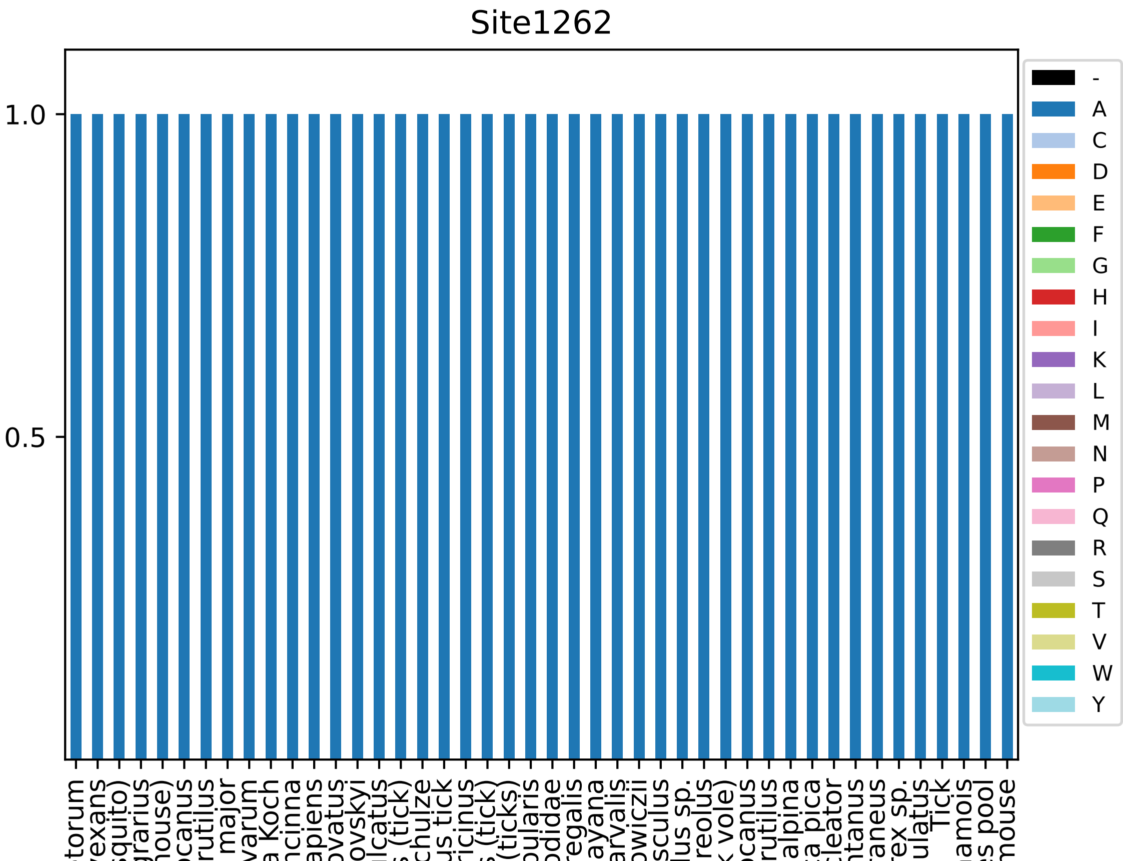
Site1262
1.0
0.5
-
A
C
D
E
F
G
H
I
K
L
M
N
P
Q
R
S
T
V
W
Y

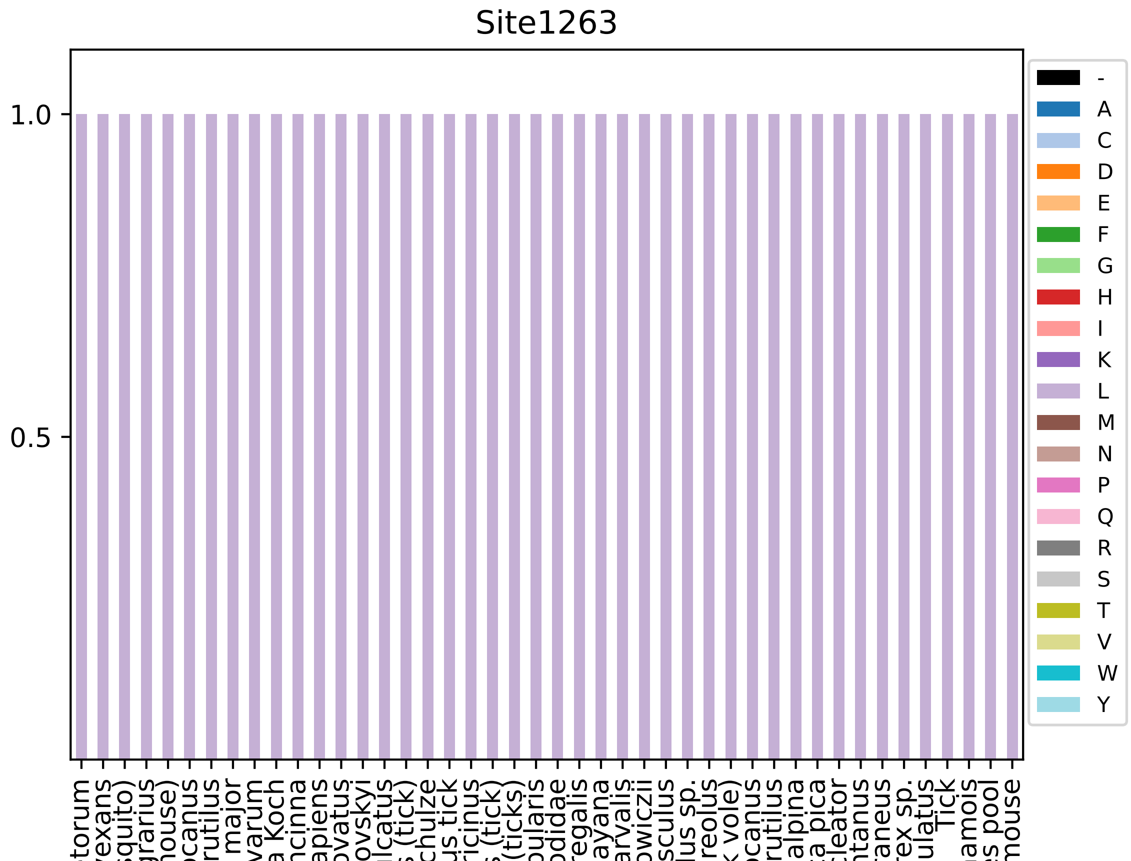
Site1263
1.0
0.5
-
A
C
D
E
F
G
H
I
K
L
M
N
P
Q
R
S
T
V
W
Y
torum
vexans
squito)
grarius
nouse)
ocanus
rutilus
major
varum
a Koch
ncinna
apiens
ovatus
ovskyi
ulcatus
s (tick)
chulze
us tick
ricinus
s (tick)
(ticks)
pularis
podidae
regalis
ayana
arvalis
owiczii
sculus
lus sp.
reolus
x vole)
ocanus
rutilus
alpina
a pica
cleator
ntanus
raneus
rex sp.
ulatus
Tick
amois
es pool
mouse

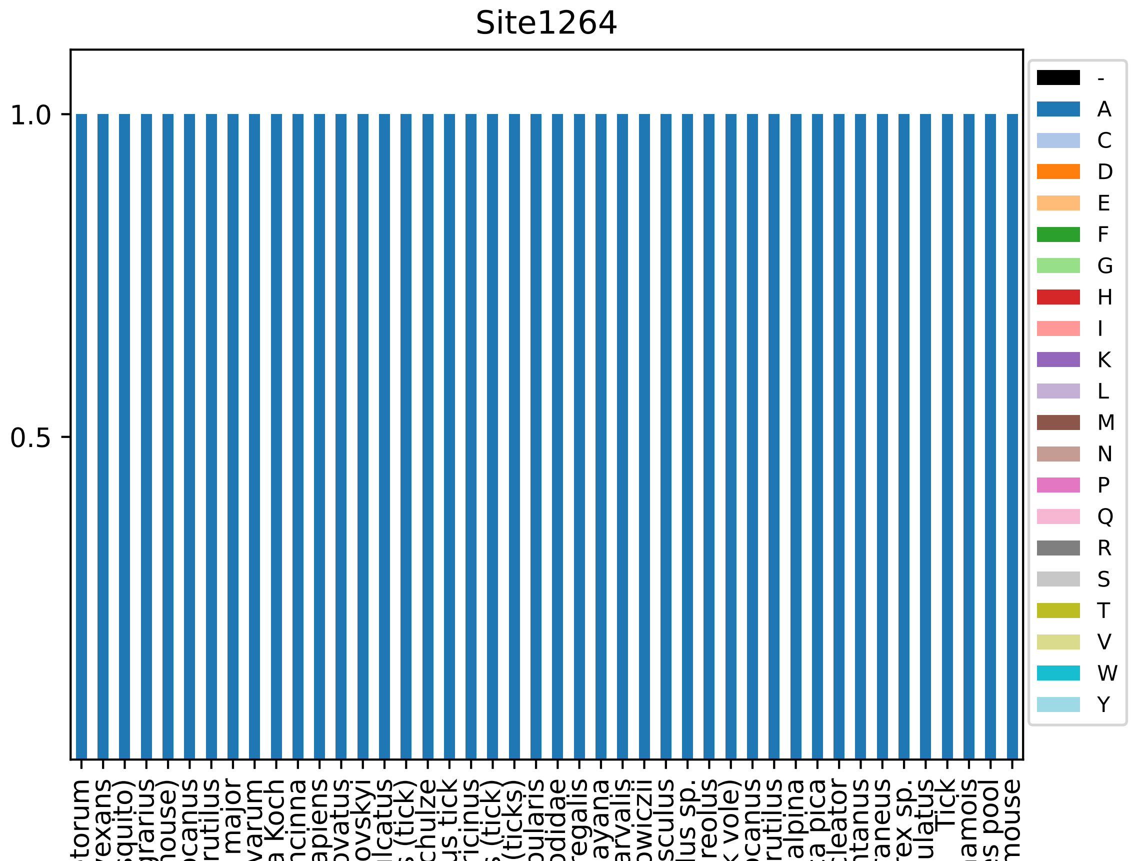
Site1264
1.0
0.5
-
A
C
D
E
F
G
H
I
K
L
M
N
P
Q
R
S
T
V
W
Y

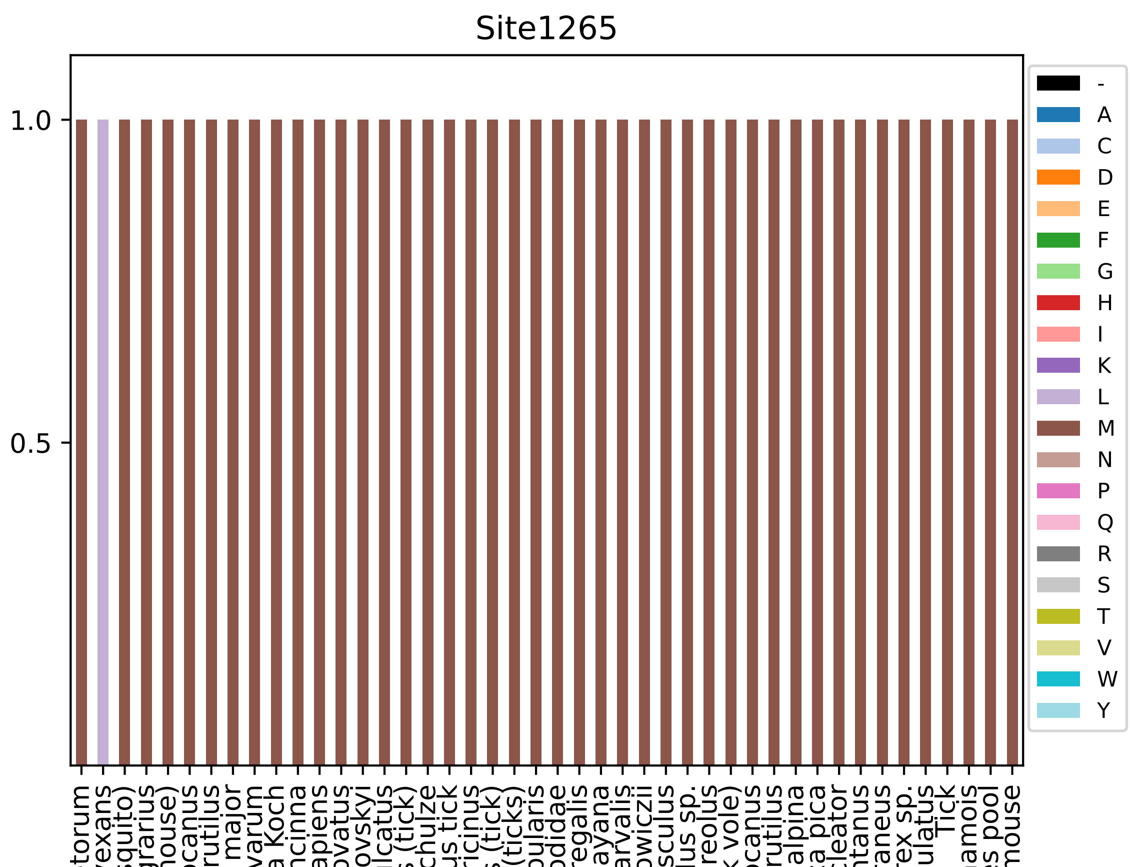
Site1265
1.0
0.5
-
A
C
D
E
F
G
H
I
K
L
M
N
P
Q
R
S
T
V
W
Y

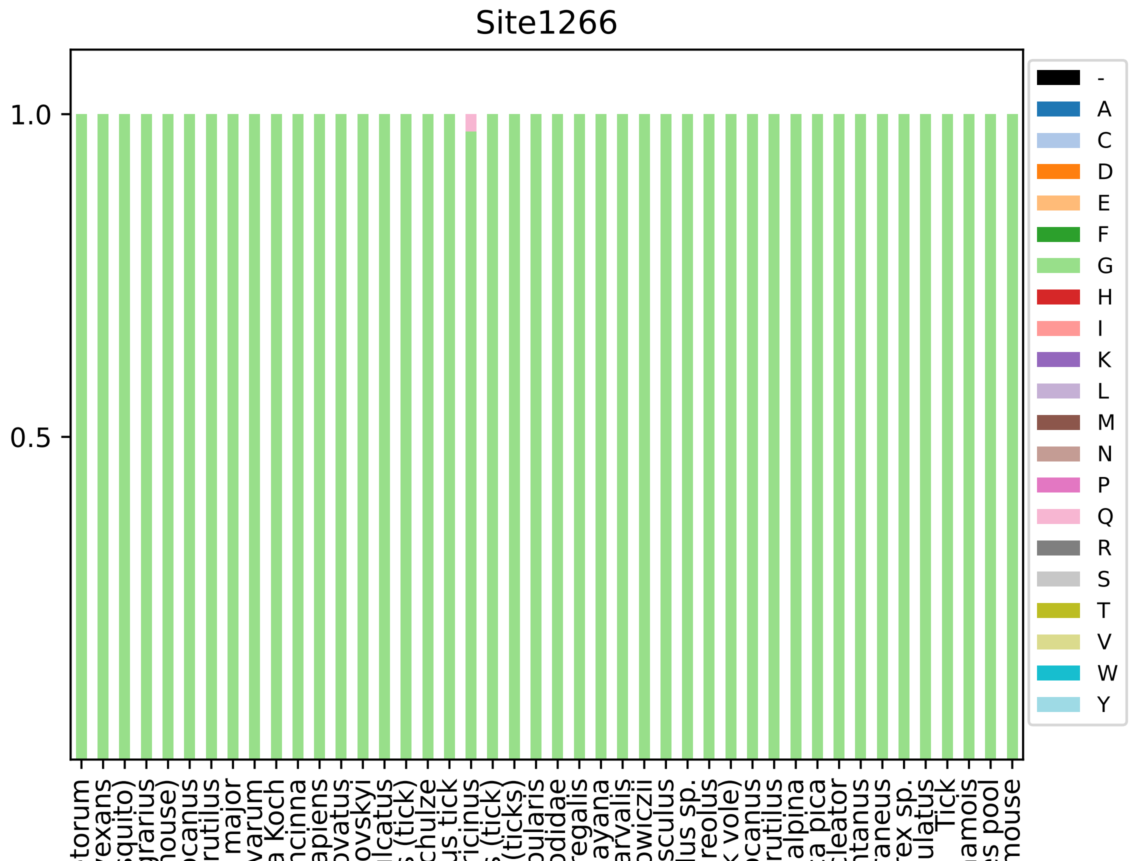

Site1266
1.0
0.5
-
A
C
D
E
F
G
H
I
K
L
M
N
P
Q
R
S
T
V
W
Y
Tick

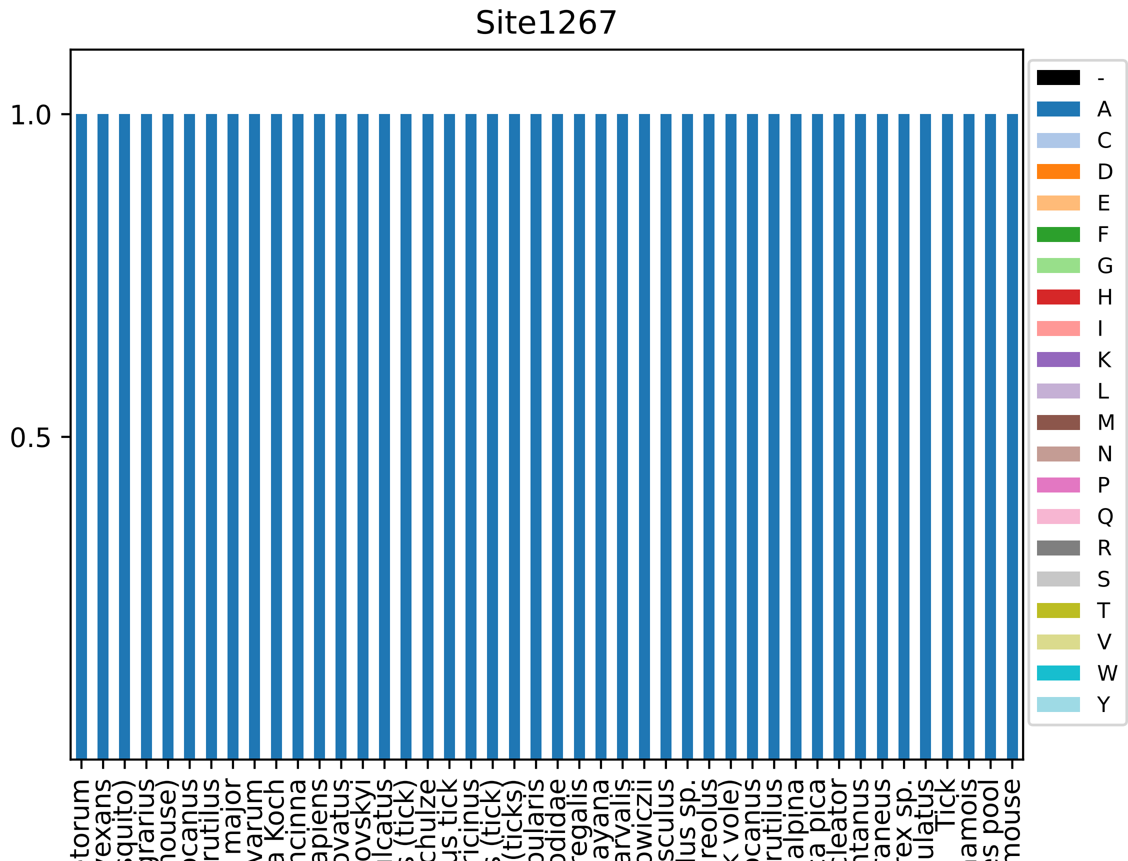
Site1267
1.0
0.5
-
A
C
D
E
F
G
H
I
K
L
M
N
P
Q
R
S
T
V
W
Y

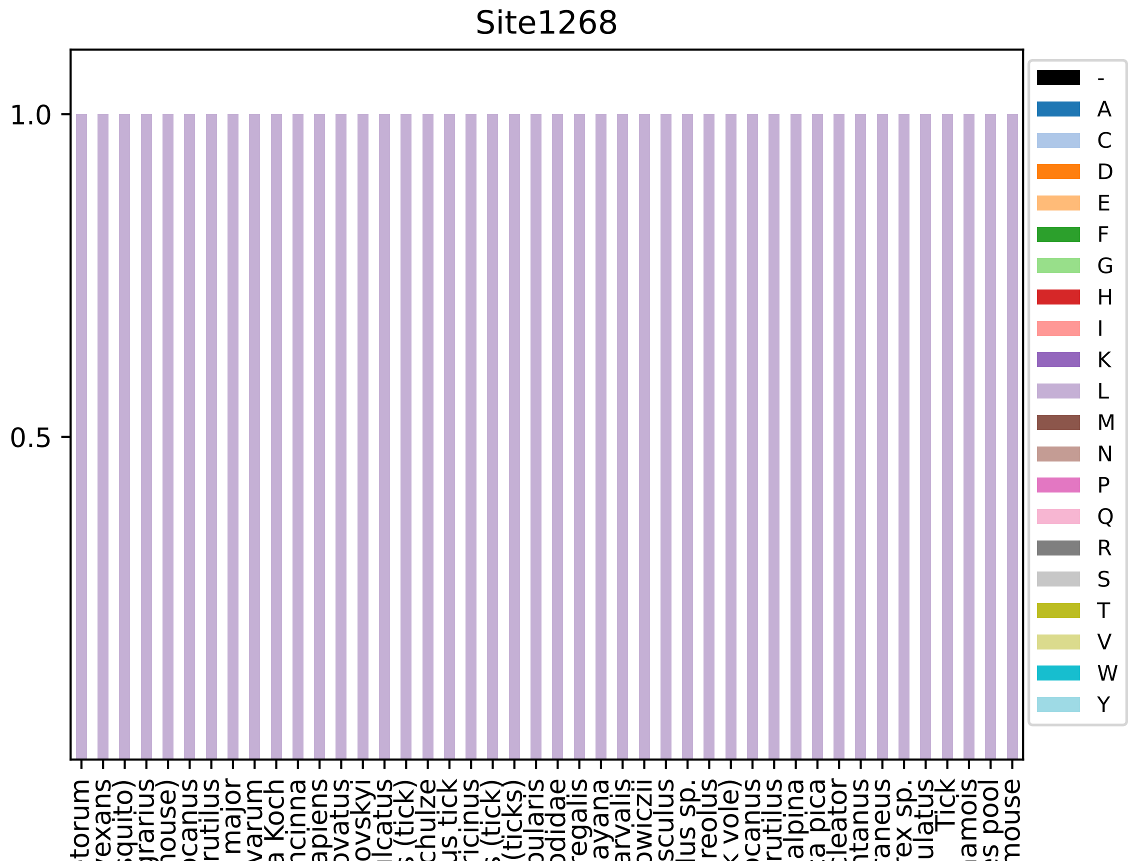
Site1268
1.0
0.5
-
A
C
D
E
F
G
H
I
K
L
M
N
P
Q
R
S
T
V
W
Y

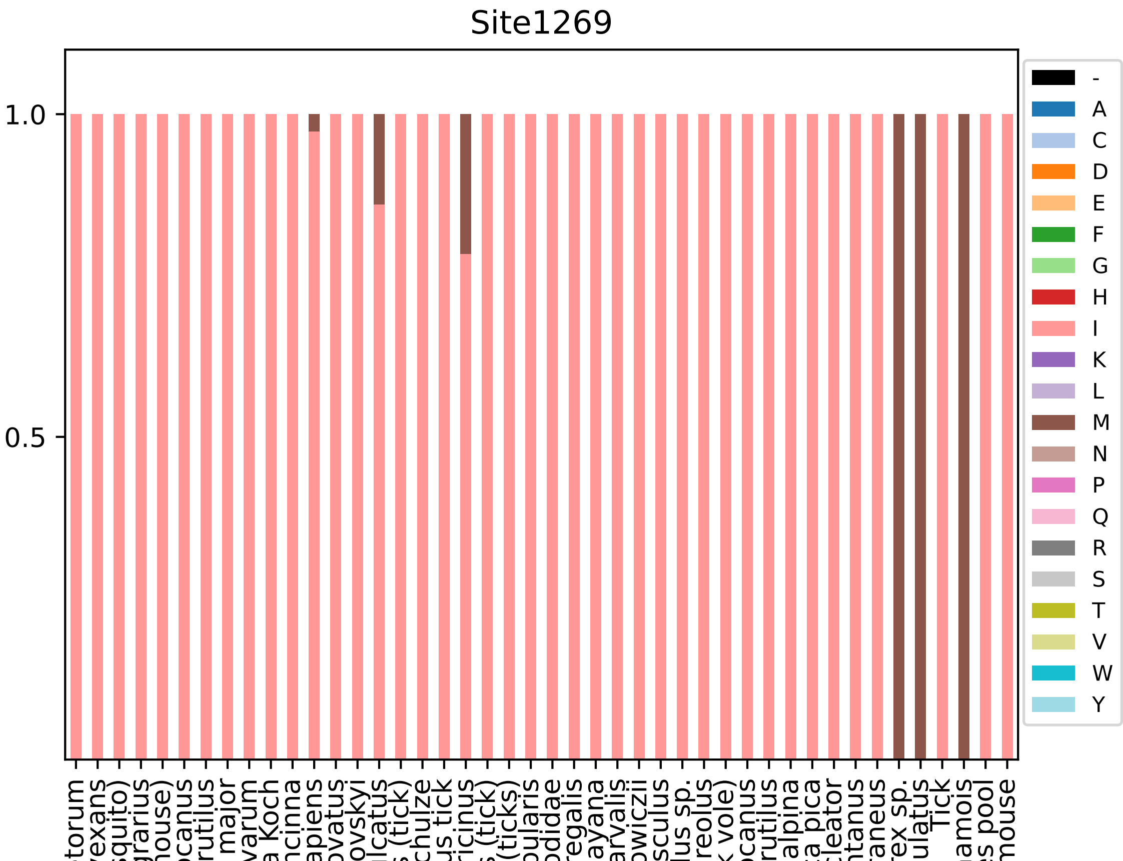

Site1269
1.0
0.5
-
A
C
D
E
F
G
H
I
K
L
M
N
P
Q
R
S
T
V
W
Y

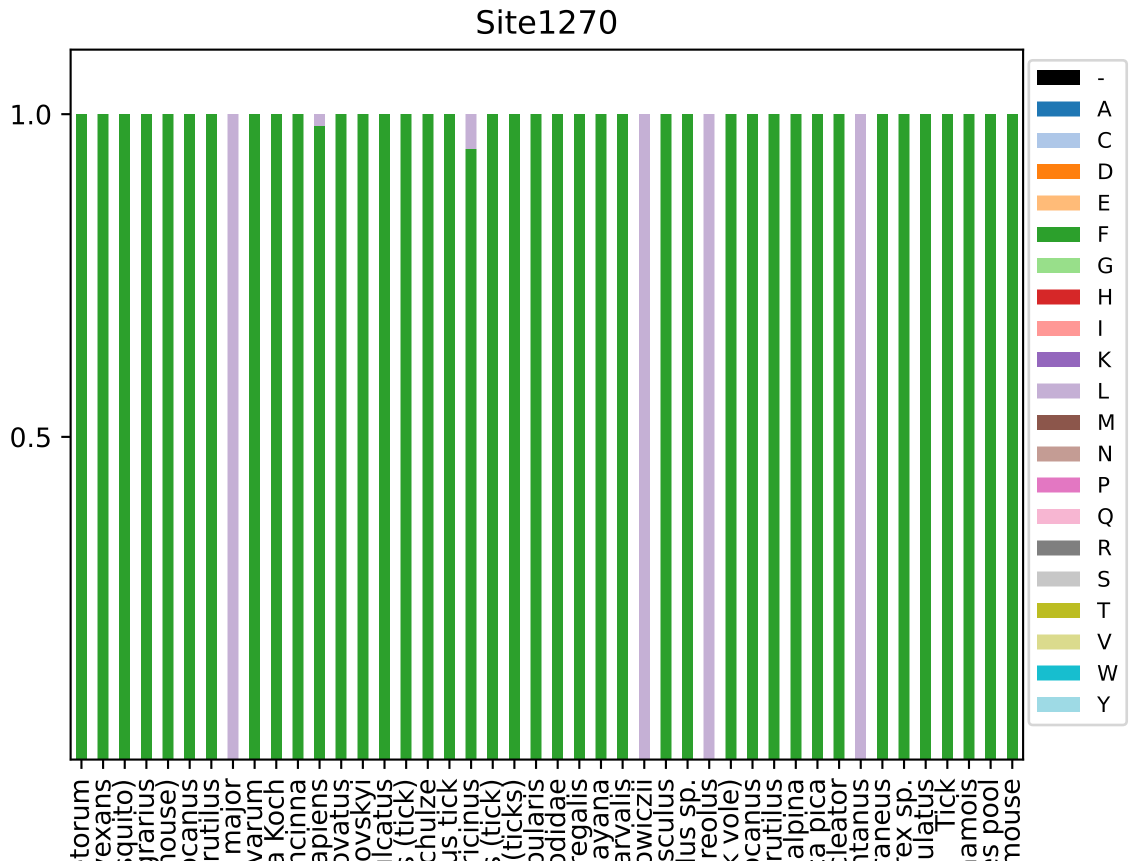
Site1270
1.0
0.5
-
A
C
D
E
F
G
H
I
K
L
M
N
P
Q
R
S
T
V
W
Y
torum
vexans
squito)
garius
nouse)
ocanus
rutilus
major
varum
a Koch
ncinna
apiens
ovatus
ovskyi
ulcatus
s (tick)
chulze
us tick
ricinus
s (tick)
(ticks)
pularis
podidae
regalis
ayana
arvalis
owiczii
sculus
lus sp.
reolus
x vole)
ocanus
rutilus
alpina
a pica
cleator
ntanus
raneus
rex sp.
ulatus
Tick
amois
es pool
mouse

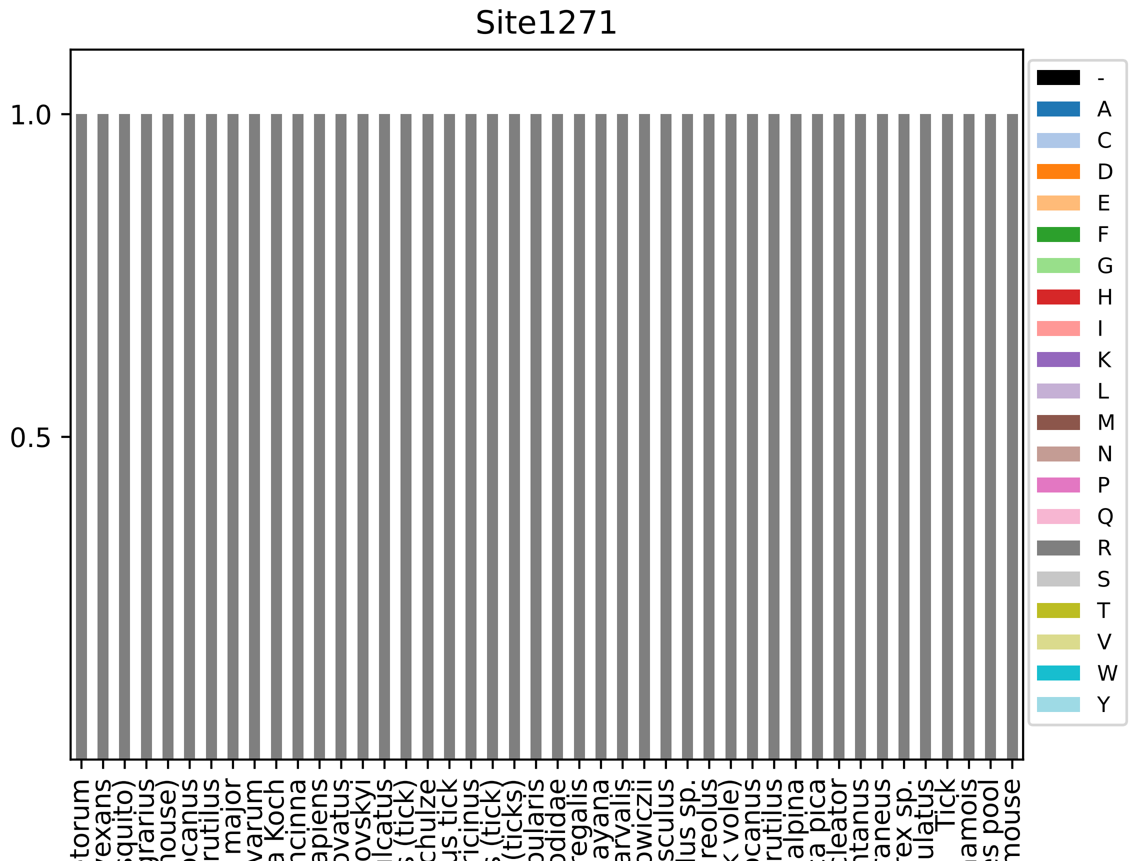
Site1271
1.0
0.5
-
A
C
D
E
F
G
H
I
K
L
M
N
P
Q
R
S
T
V
W
Y

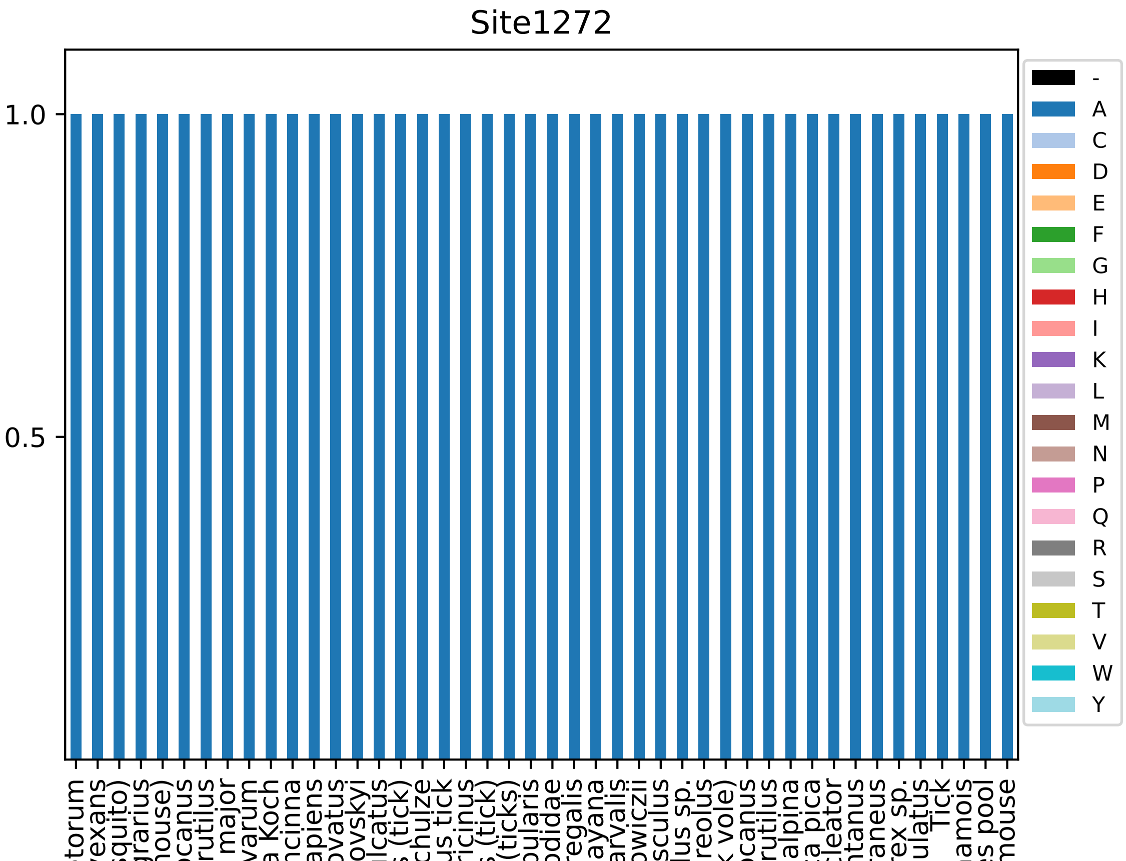

Site1272
1.0
0.5
-
A
C
D
E
F
G
H
I
K
L
M
N
P
Q
R
S
T
V
W
Y
torum
vexans
squito)
grarius
nouse)
ocanus
rutilus
major
varum
a Koch
ncinna
apiens
ovatus
ovskyi
lcatus
(tick)
chulze
us tick
ricinus
(tick)
(ticks)
pularis
podidae
regalis
ayana
arvalis
owiczii
sculus
lus sp.
reolus
x vole)
ocanus
rutilus
alpina
a pica
cleator
ntanus
raneus
rex sp.
ulatus
Tick
amois
es pool
mouse

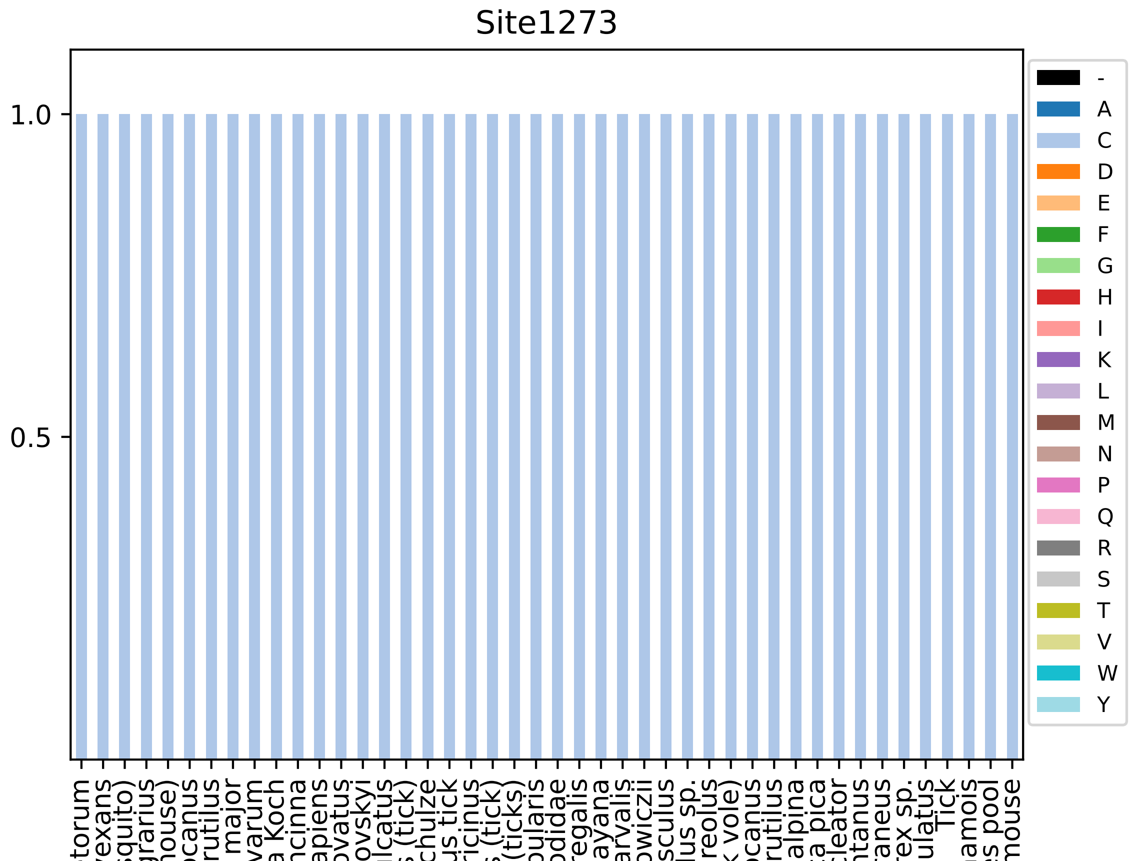
Site1273
1.0
0.5
-
A
C
D
E
F
G
H
I
K
L
M
N
P
Q
R
S
T
V
W
Y

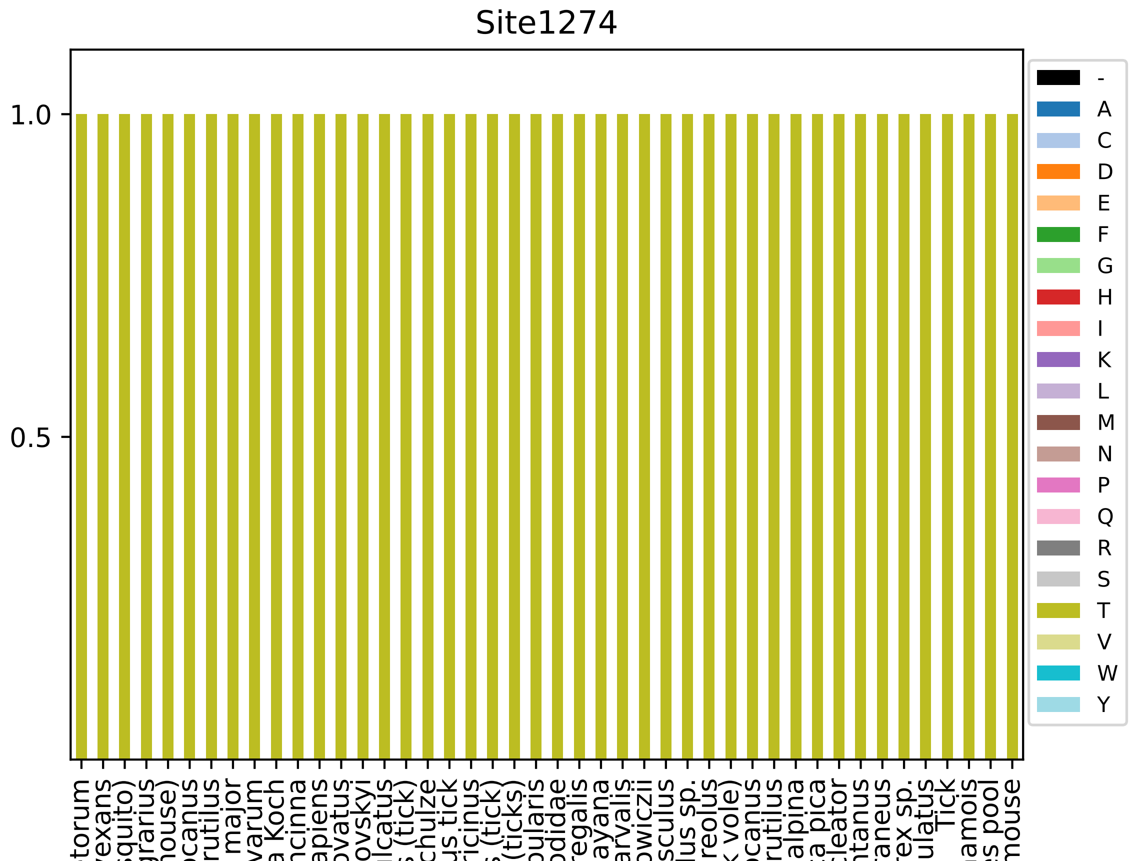
Site1274
1.0
0.5
-
A
C
D
E
F
G
H
I
K
L
M
N
P
Q
R
S
T
V
W
Y

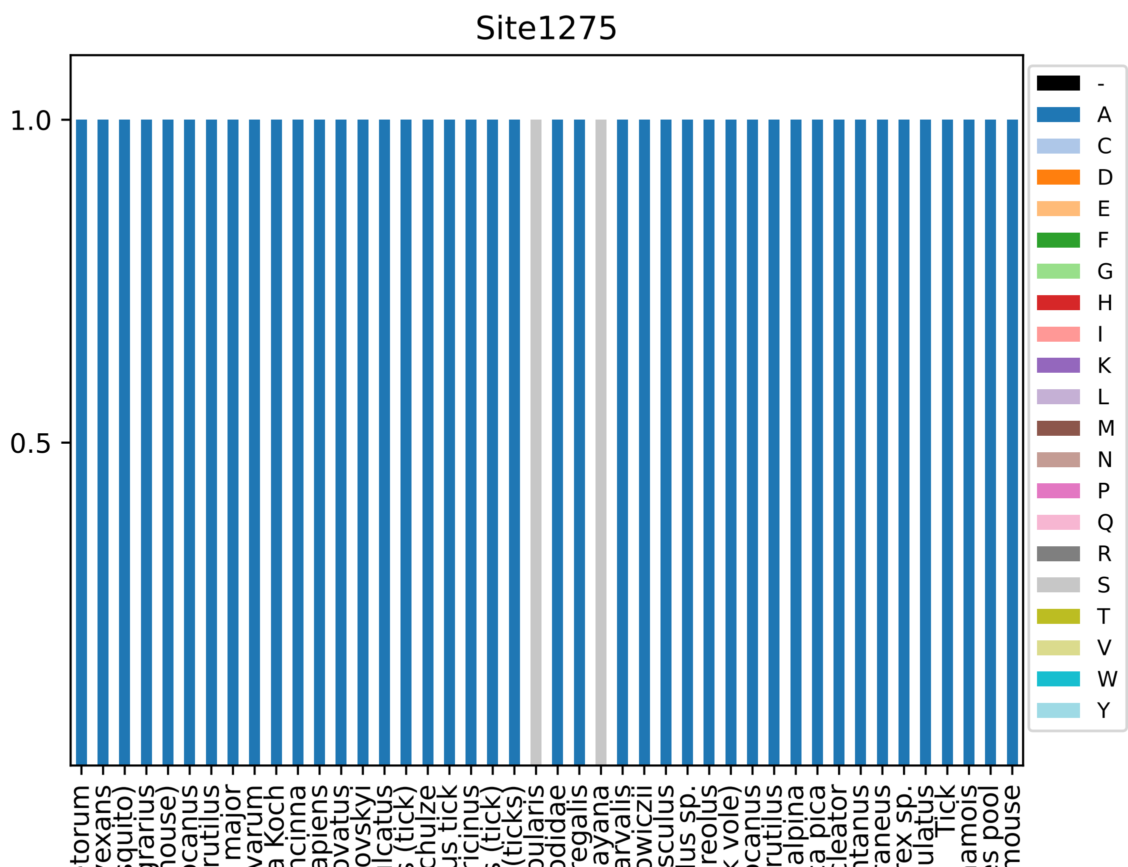
Site1275
1.0
0.5
-
A
C
D
E
F
G
H
I
K
L
M
N
P
Q
R
S
T
V
W
Y

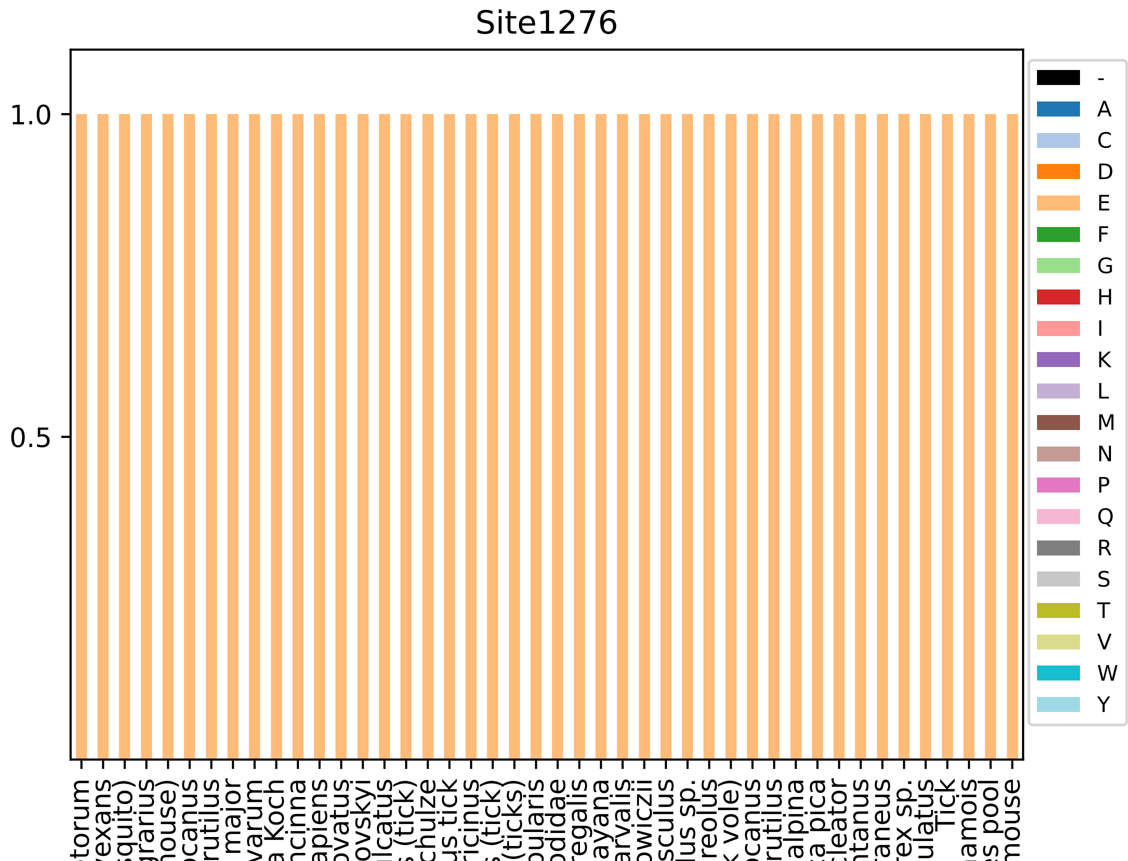
Site1276
1.0
0.5
-
A
C
D
E
F
G
H
I
K
L
M
N
P
Q
R
S
T
V
W
Y
Tick

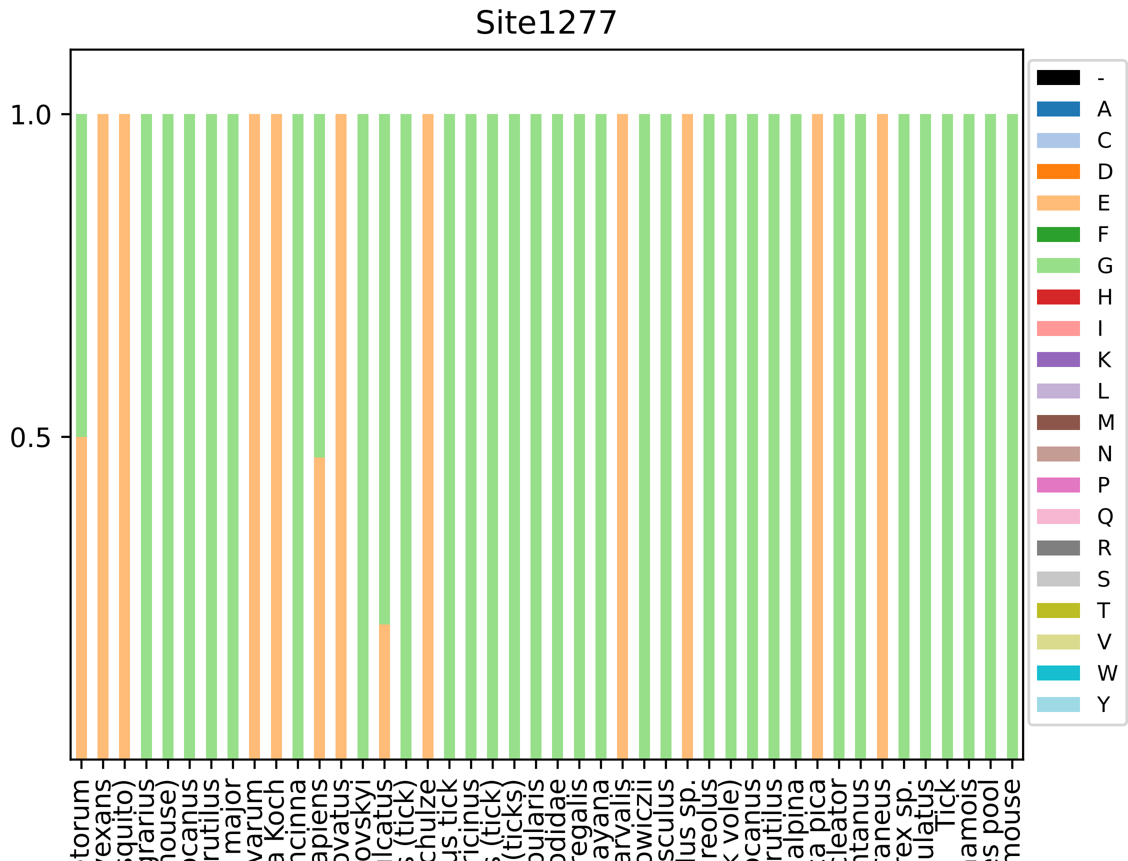

Site1277
1.0
0.5
-
A
C
D
E
F
G
H
I
K
L
M
N
P
Q
R
S
T
V
W
Y
torum
vexans
squito)
garius
nouse)
ocanus
rutilus
major
varum
a Koch
ncinna
apiens
ovatus
ovskyi
ulcatus
s (tick)
chulze
us tick
ricinus
s (ticks)
pularis
podidae
regalis
ayana
arvalis
owiczii
sculus
lus sp.
reolus
k vole)
ocanus
rutilus
alpina
a pica
cleator
ntanus
raneus
rex sp.
ulatus
Tick
amois
es pool
mouse

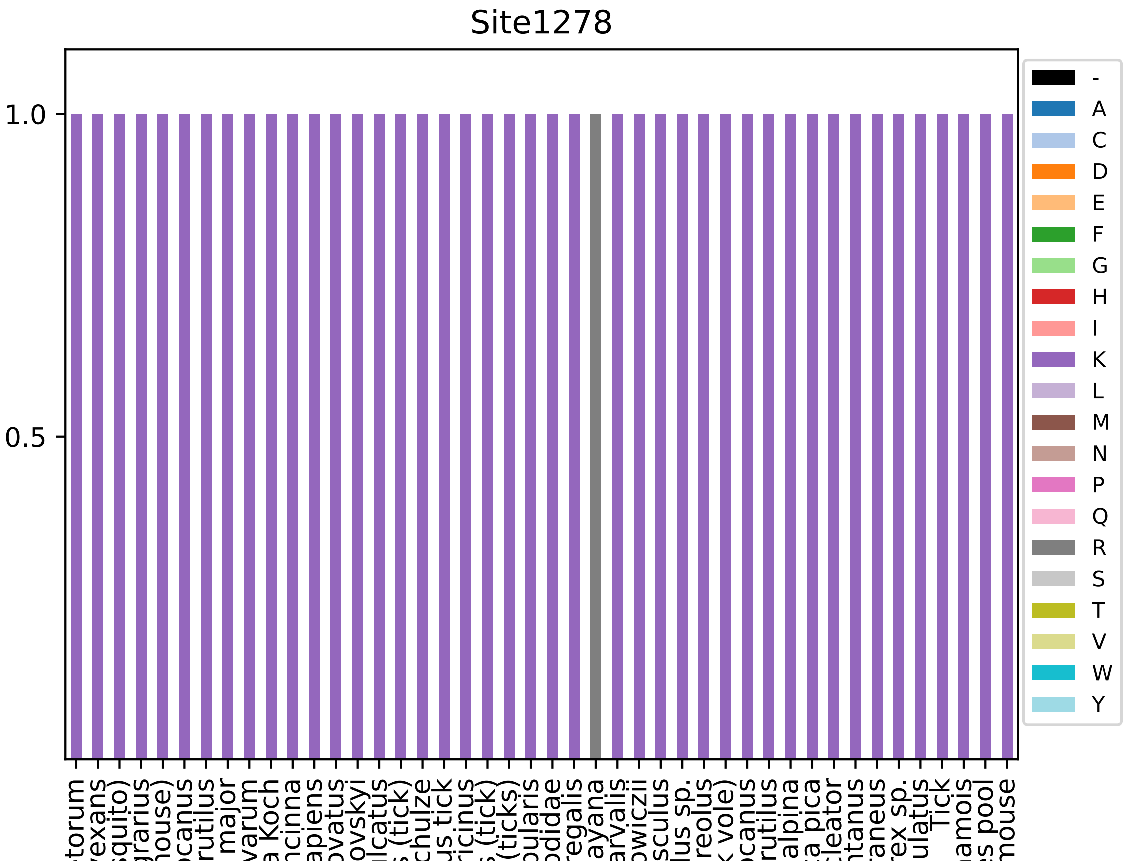

Site1278
1.0
0.5
-
A
C
D
E
F
G
H
I
K
L
M
N
P
Q
R
S
T
V
W
Y

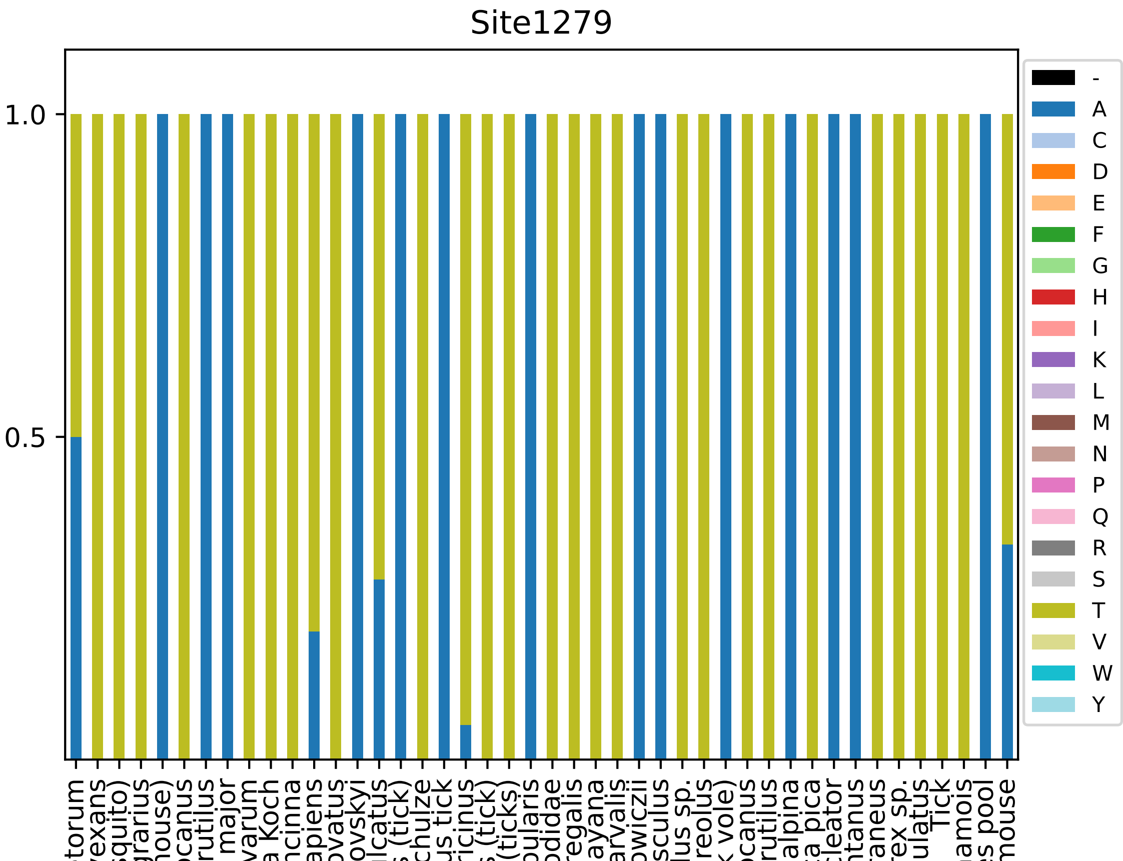
Site1279
1.0
0.5
-
A
C
D
E
F
G
H
I
K
L
M
N
P
Q
R
S
T
V
W
Y

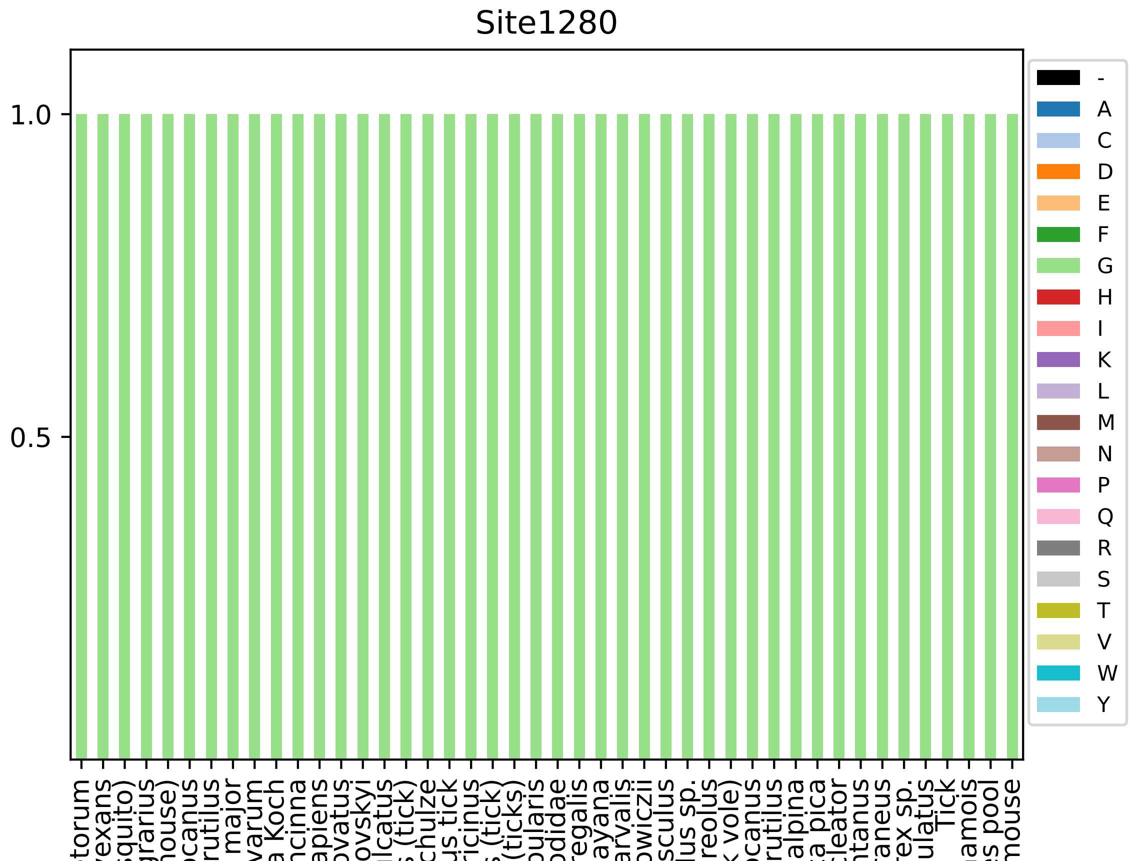
Site1280
1.0
0.5
-
A
C
D
E
F
G
H
I
K
L
M
N
P
Q
R
S
T
V
W
Y

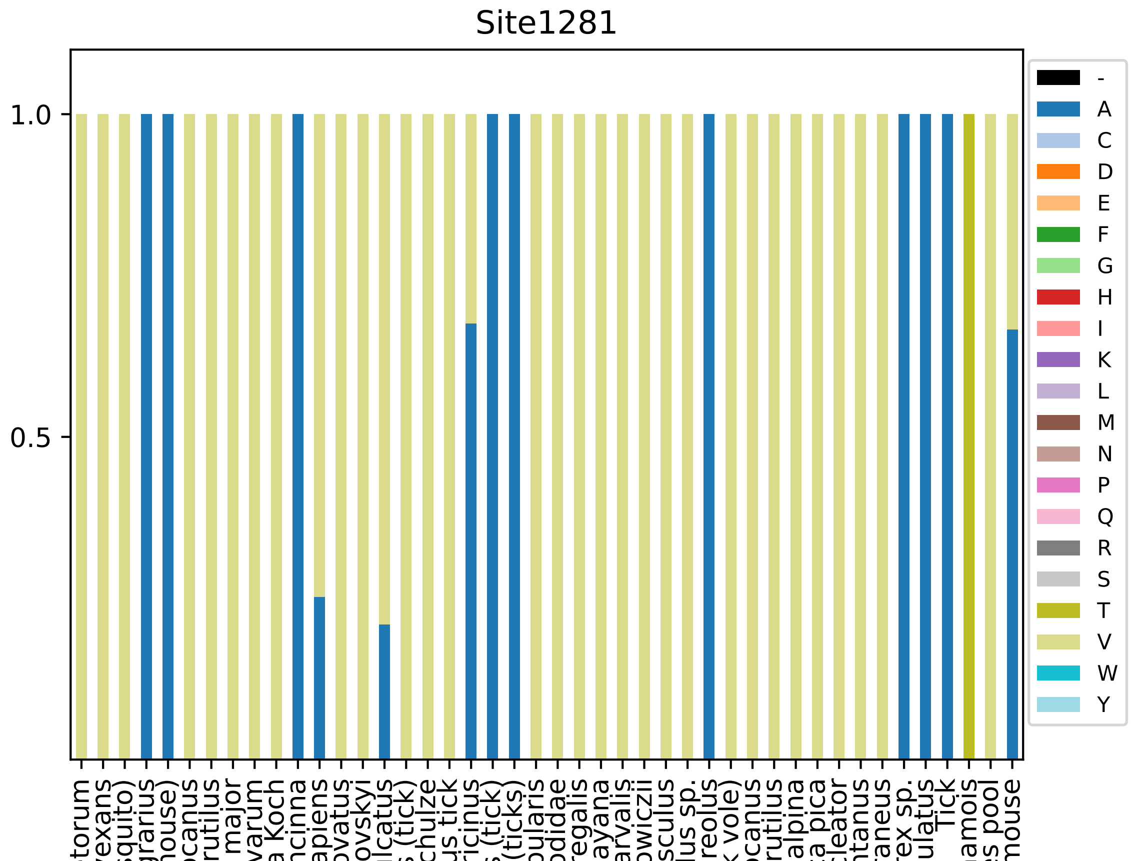
Site1281
1.0
0.5
-
A
C
D
E
F
G
H
I
K
L
M
N
P
Q
R
S
T
V
W
Y

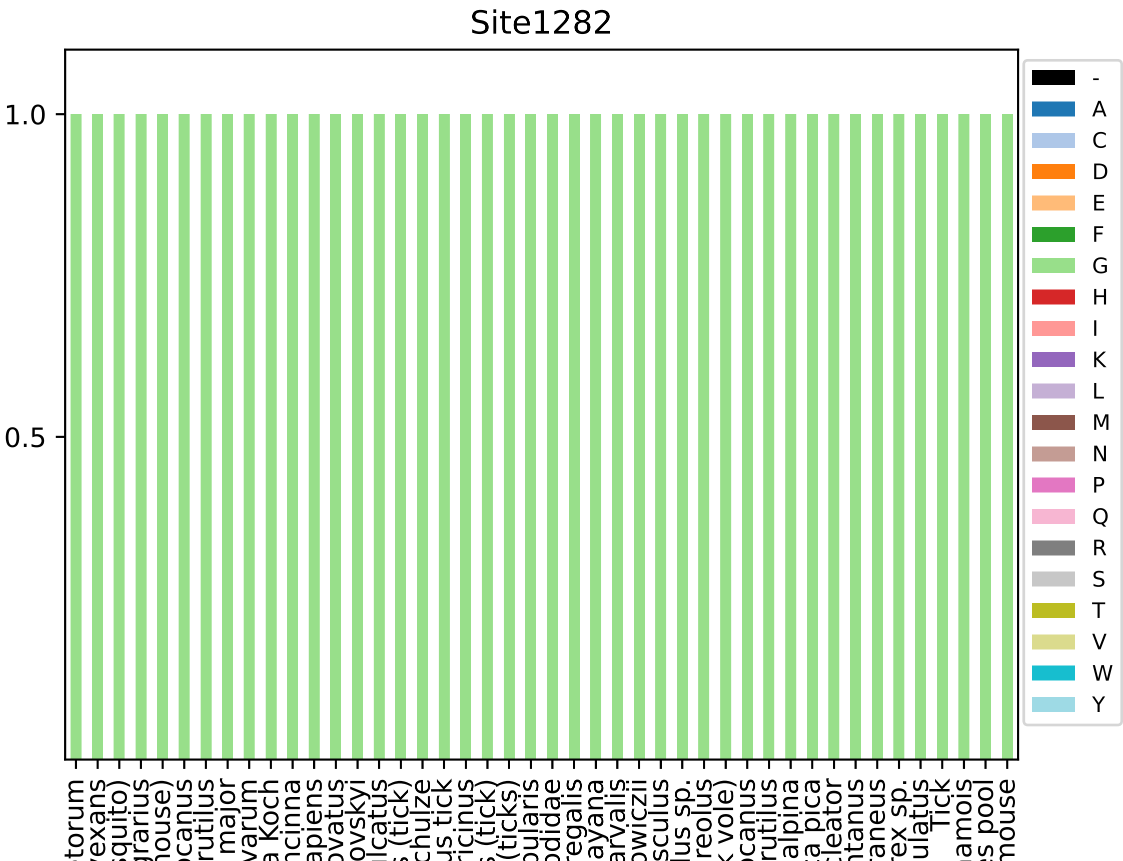
Site1282
1.0
0.5
-
A
C
D
E
F
G
H
I
K
L
M
N
P
Q
R
S
T
V
W
Y

Site1283

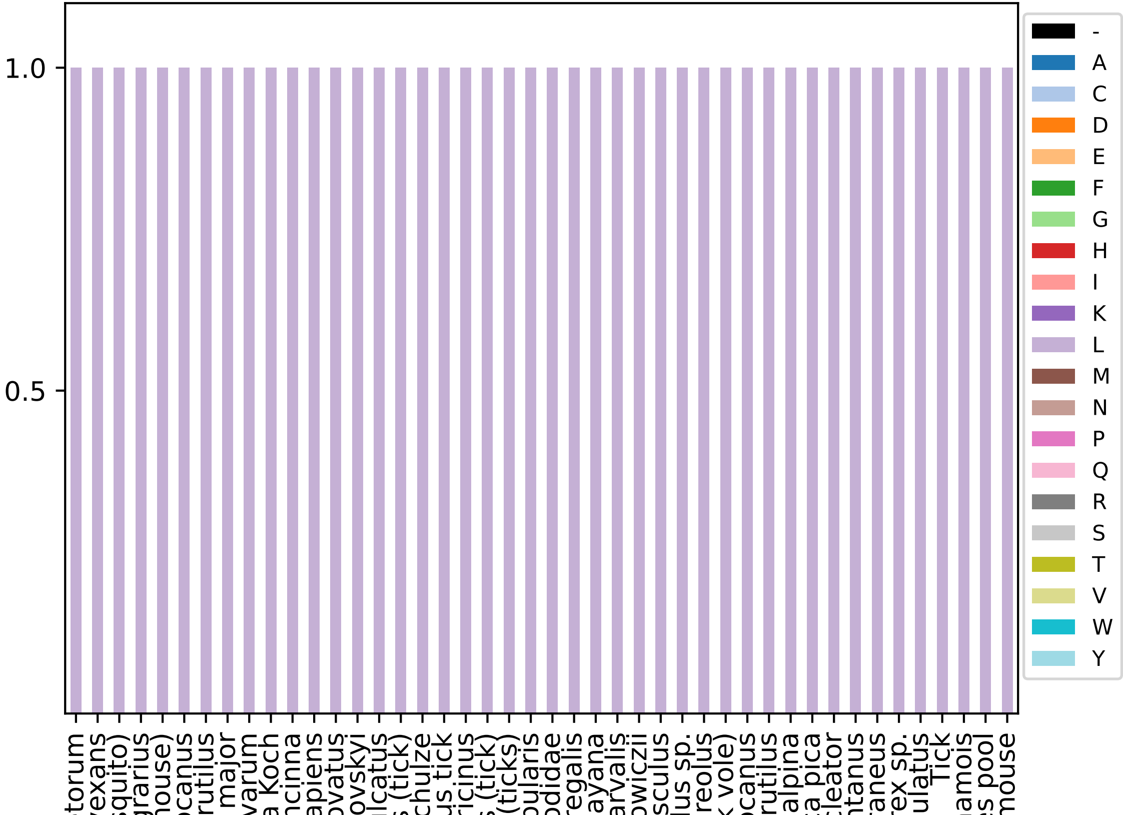

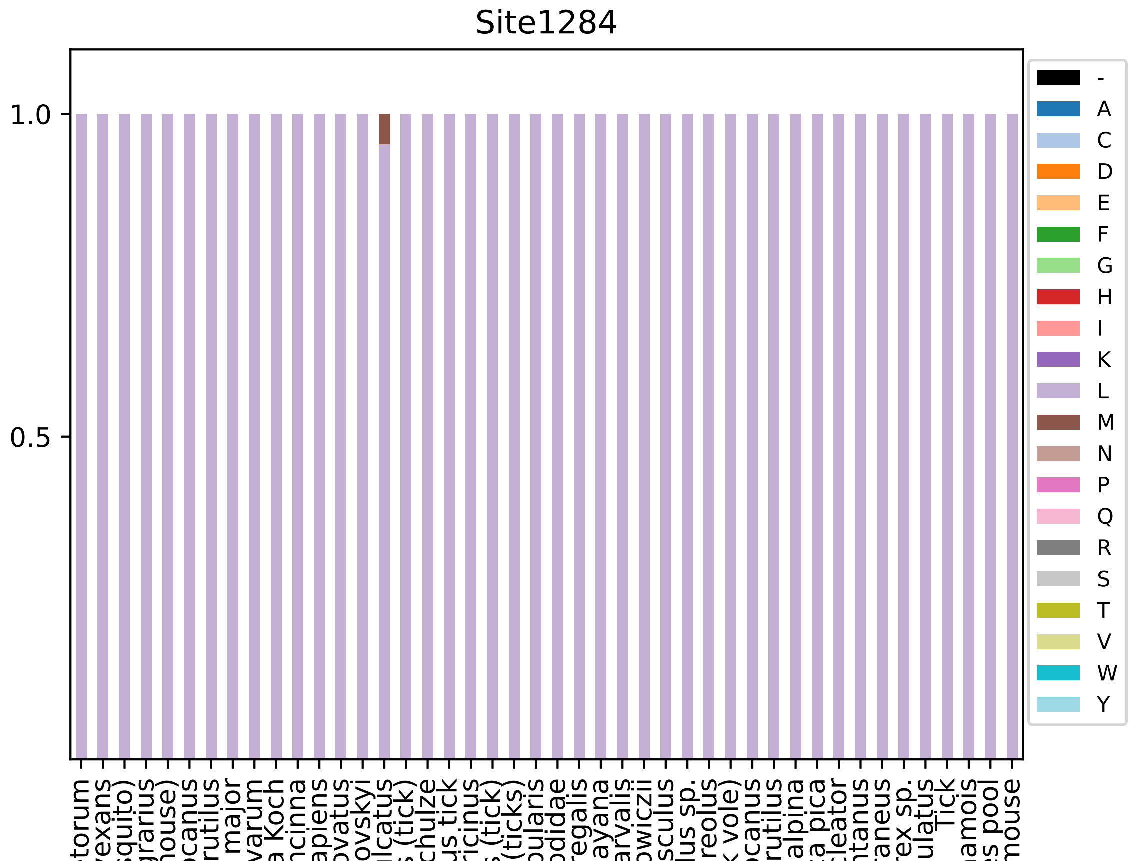

Site1284
1.0
0.5
-
A
C
D
E
F
G
H
I
K
L
M
N
P
Q
R
S
T
V
W
Y

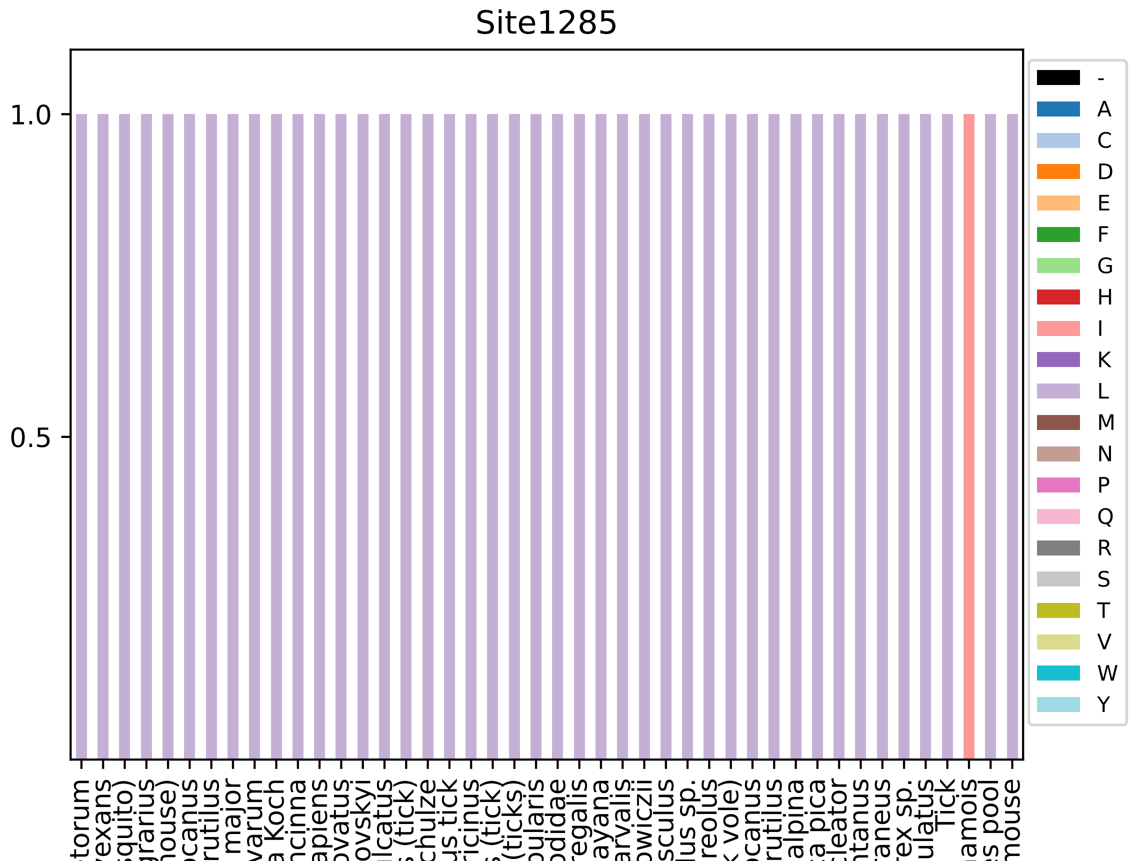

Site1285
1.0
0.5
-
A
C
D
E
F
G
H
I
K
L
M
N
P
Q
R
S
T
V
W
Y

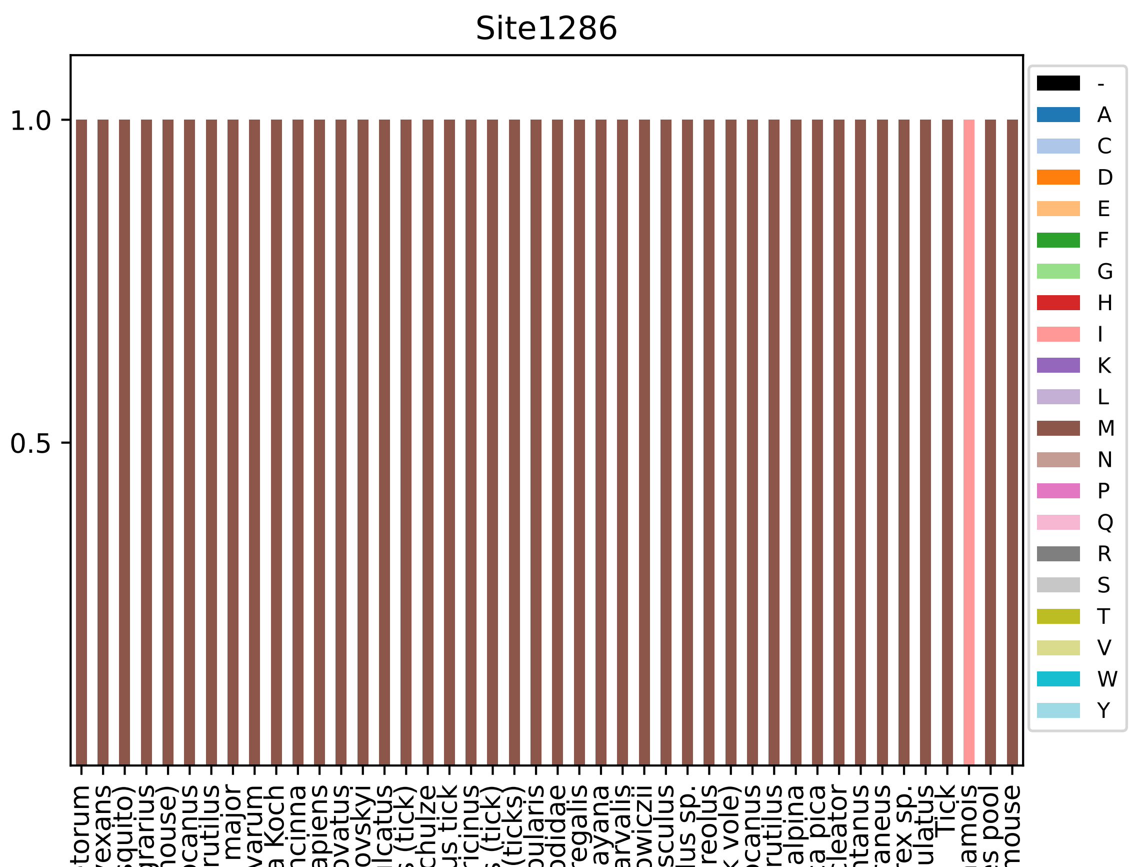
Site1286
1.0
0.5
-
A
C
D
E
F
G
H
I
K
L
M
N
P
Q
R
S
T
V
W
Y

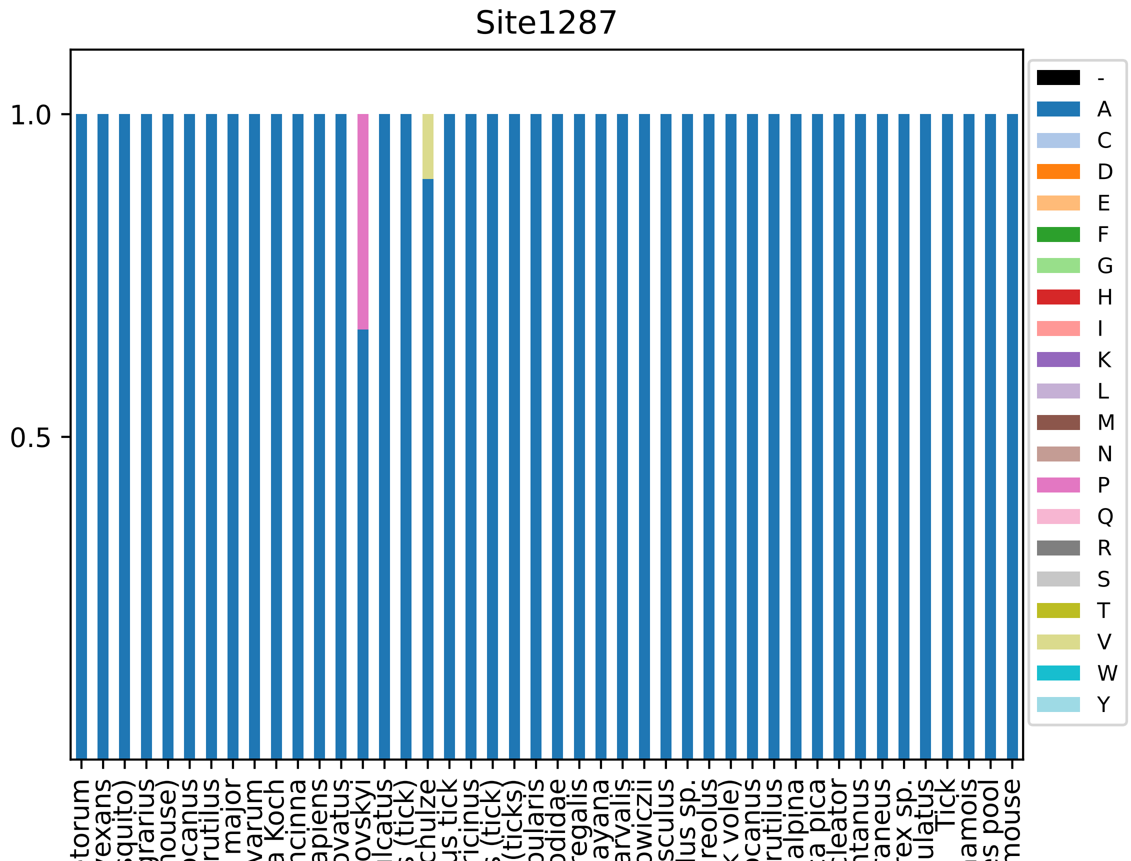
Site1287
1.0
0.5
-
A
C
D
E
F
G
H
I
K
L
M
N
P
Q
R
S
T
V
W
Y

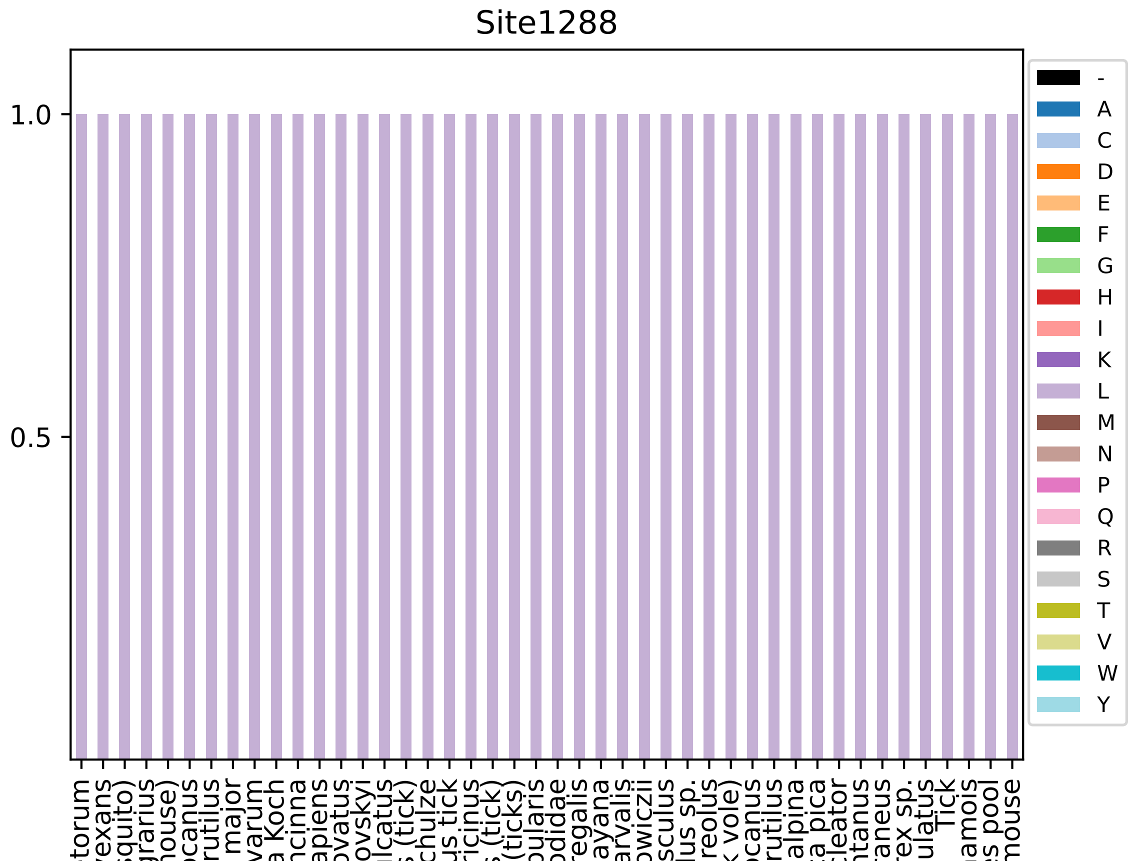
Site1288
1.0
0.5
-
A
C
D
E
F
G
H
I
K
L
M
N
P
Q
R
S
T
V
W
Y

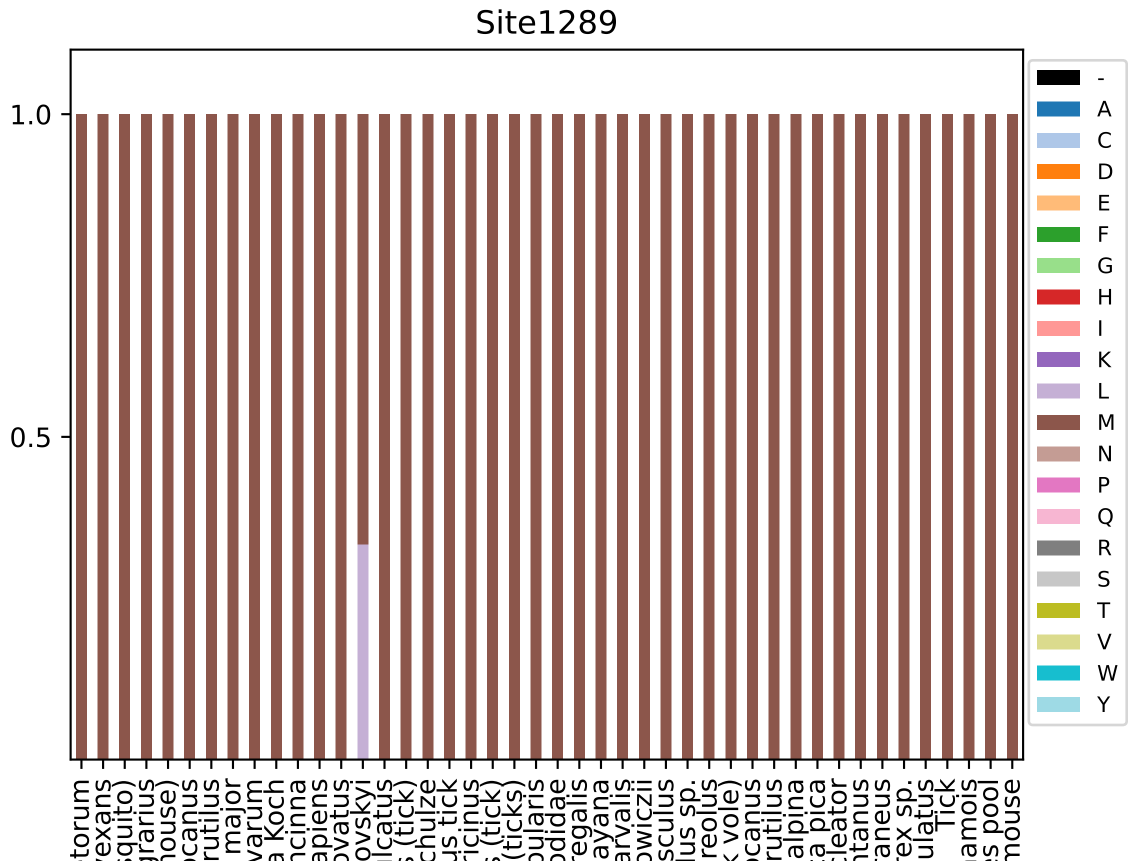
Site1289
1.0
0.5
-
A
C
D
E
F
G
H
I
K
L
M
N
P
Q
R
S
T
V
W
Y

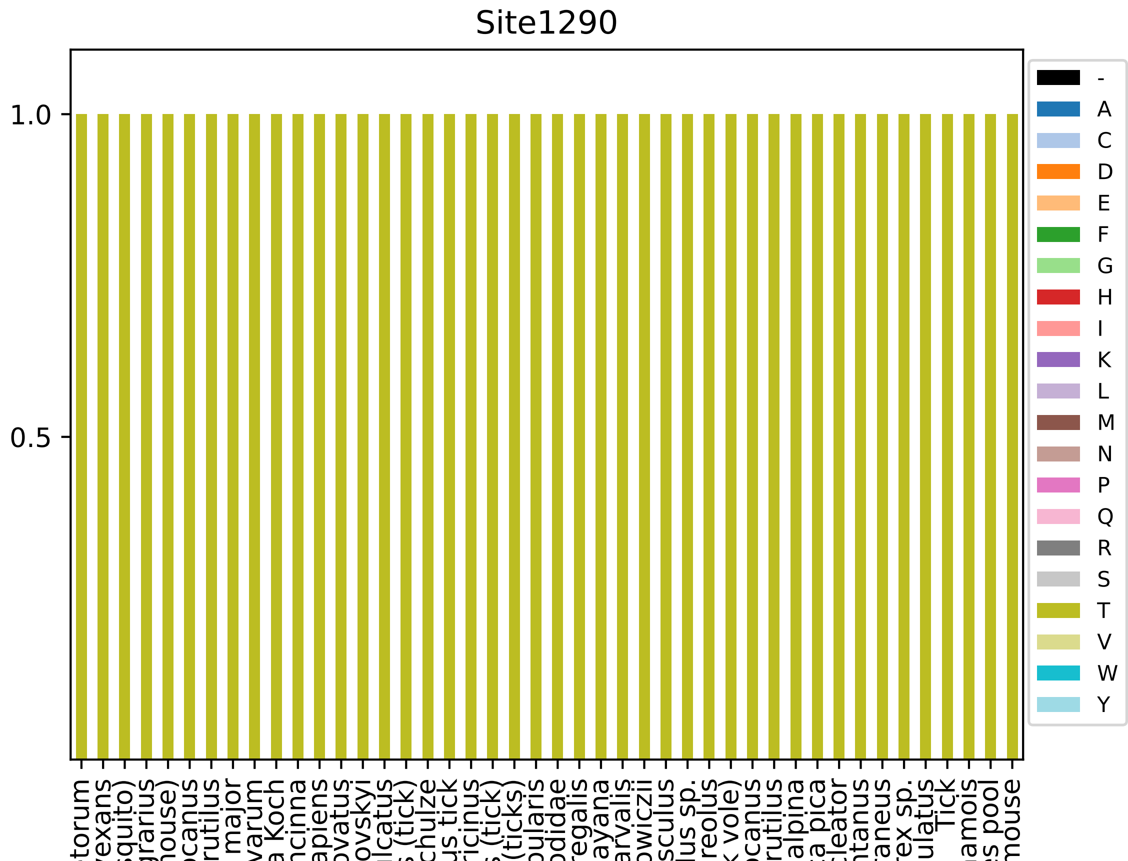

Site1290
1.0
0.5
-
A
C
D
E
F
G
H
I
K
L
M
N
P
Q
R
S
T
V
W
Y

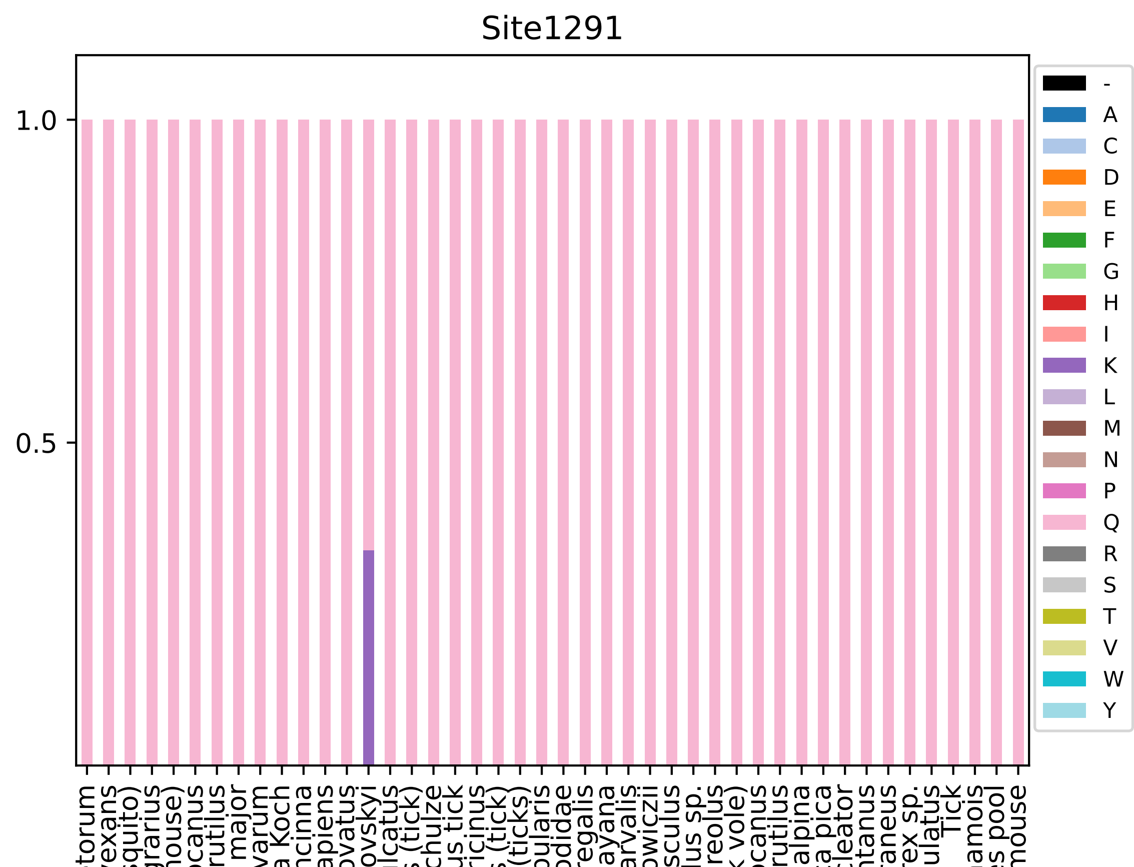

Site1291
1.0
0.5
-
A
C
D
E
F
G
H
I
K
L
M
N
P
Q
R
S
T
V
W
Y

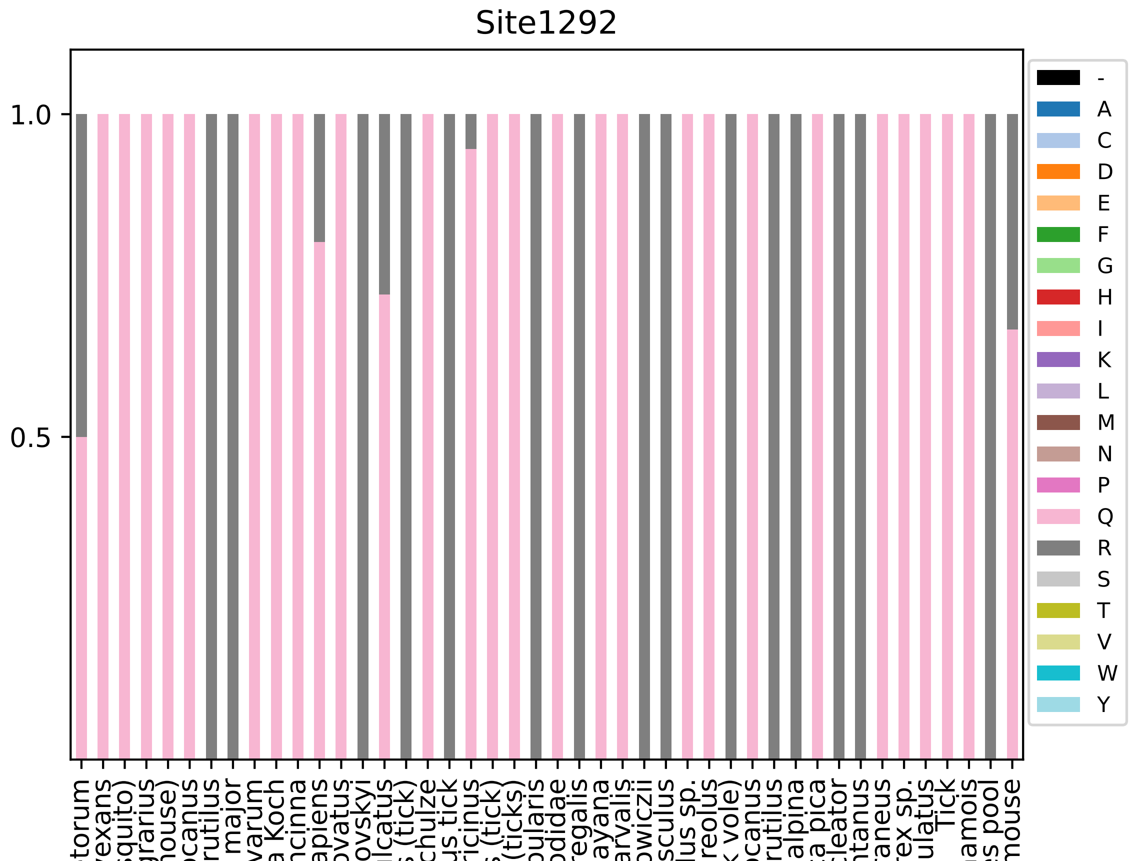

Site1292
1.0
0.5
-
A
C
D
E
F
G
H
I
K
L
M
N
P
Q
R
S
T
V
W
Y

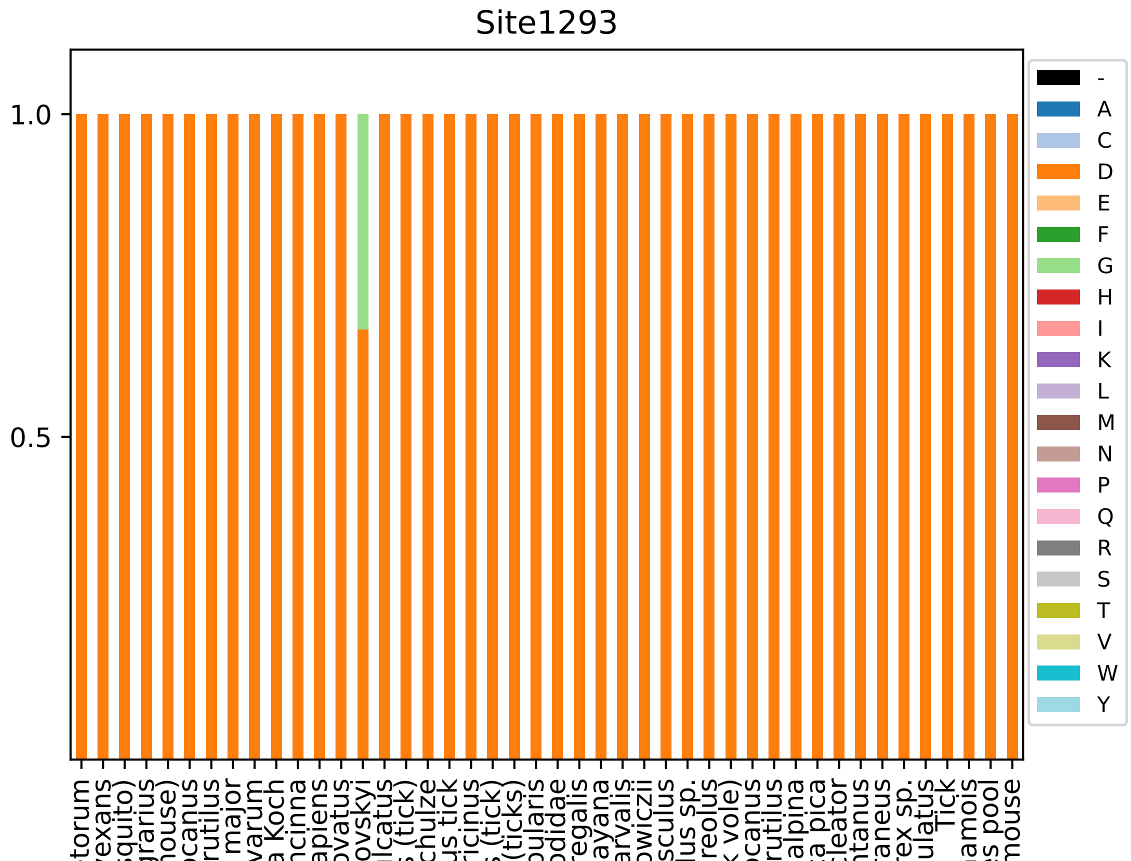
Site1293
1.0
0.5
-
A
C
D
E
F
G
H
I
K
L
M
N
P
Q
R
S
T
V
W
Y

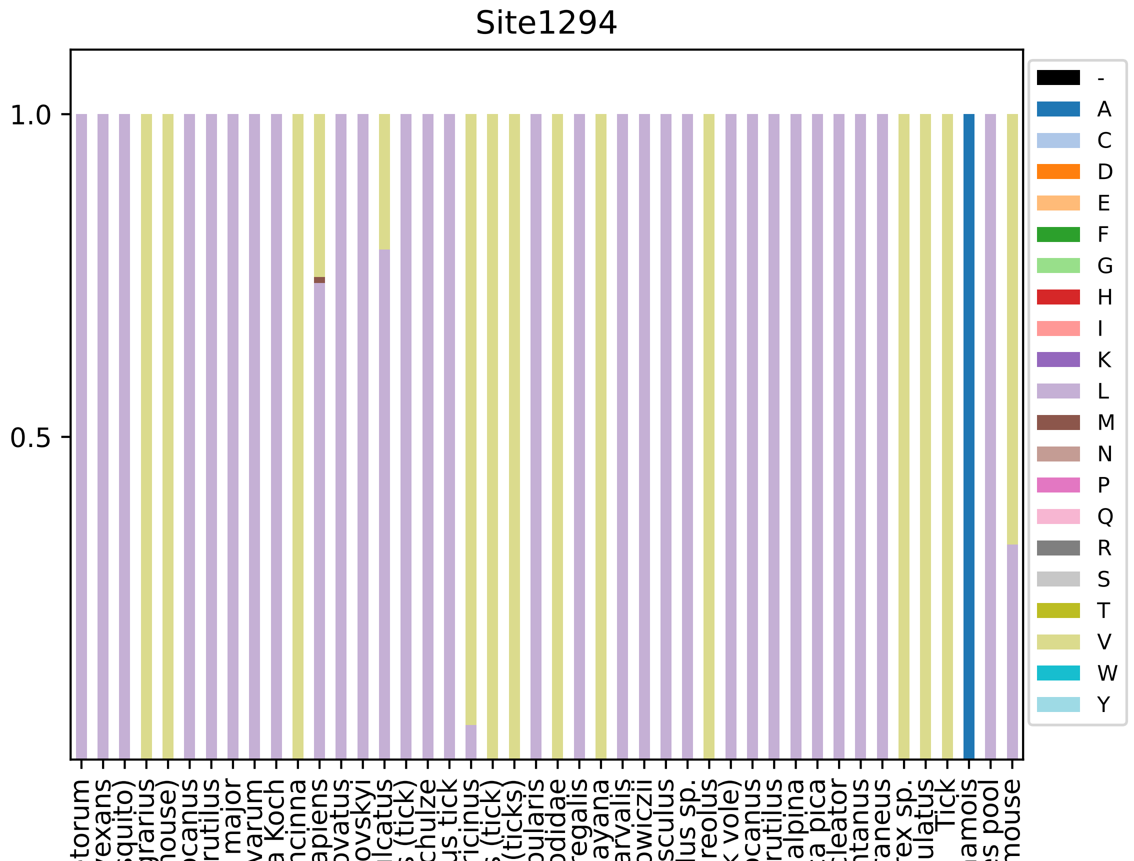
Site1294
1.0
0.5
-
A
C
D
E
F
G
H
I
K
L
M
N
P
Q
R
S
T
V
W
Y

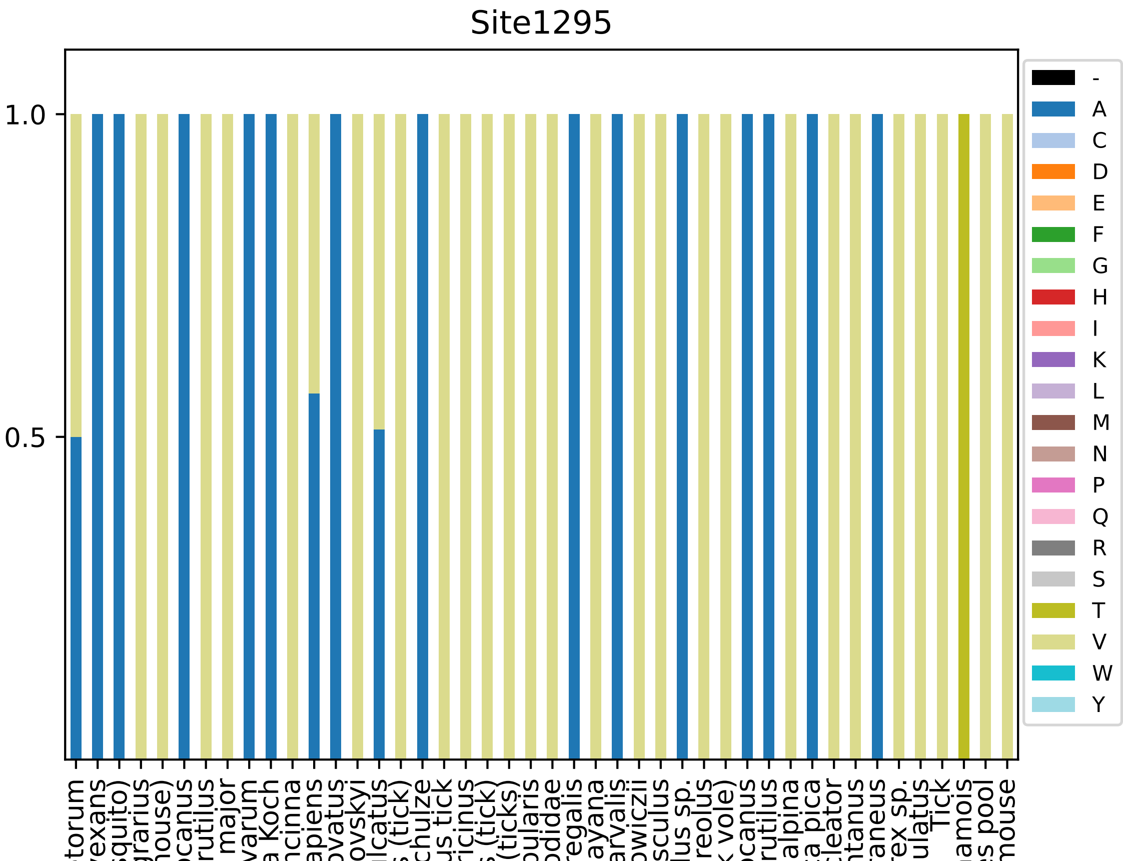

Site1295
1.0
0.5
-
A
C
D
E
F
G
H
I
K
L
M
N
P
Q
R
S
T
V
W
Y

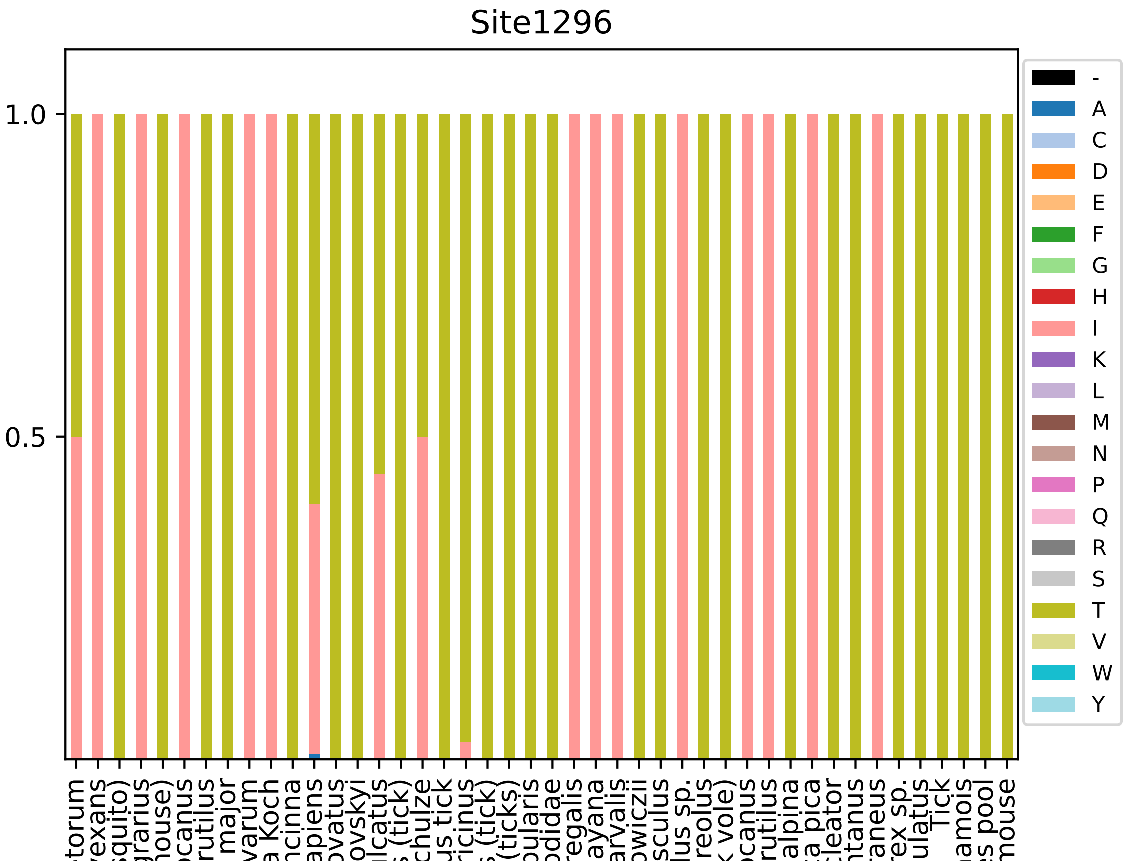
Site1296
1.0
0.5
-
A
C
D
E
F
G
H
I
K
L
M
N
P
Q
R
S
T
V
W
Y

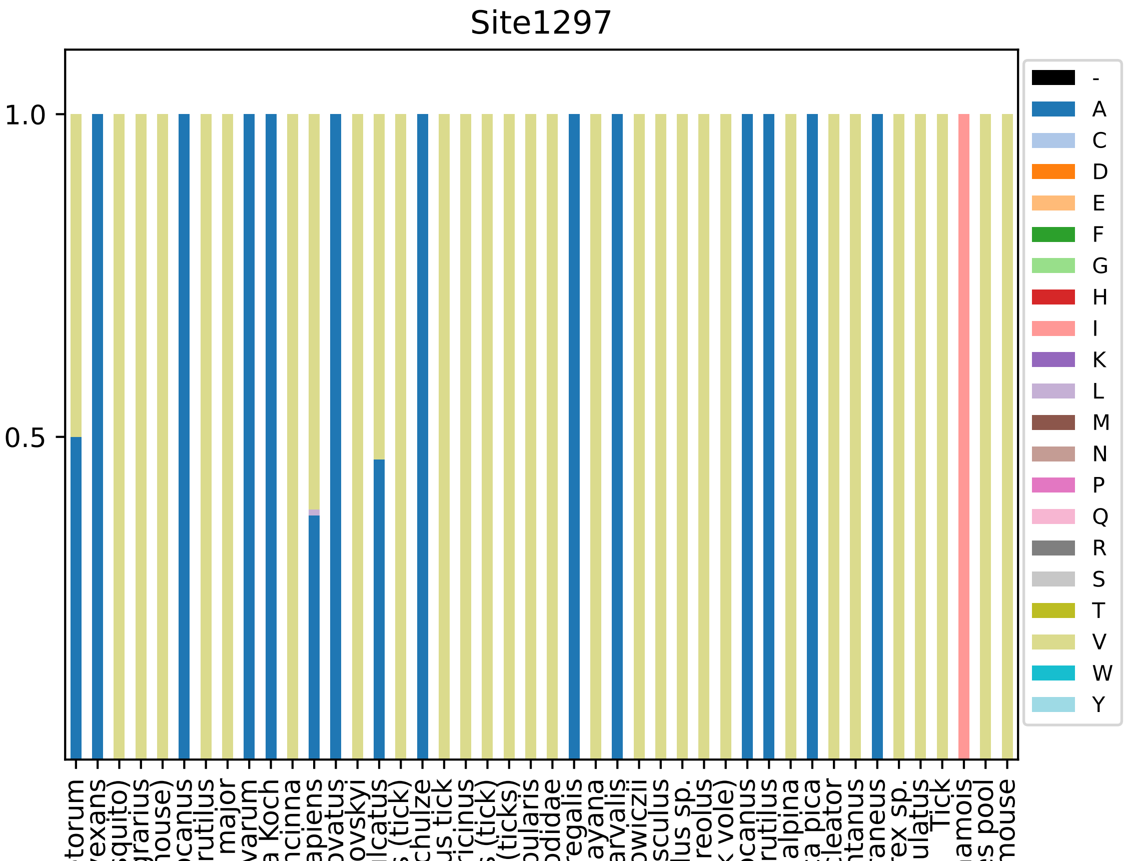
Site1297
1.0
0.5
-
A
C
D
E
F
G
H
I
K
L
M
N
P
Q
R
S
T
V
W
Y

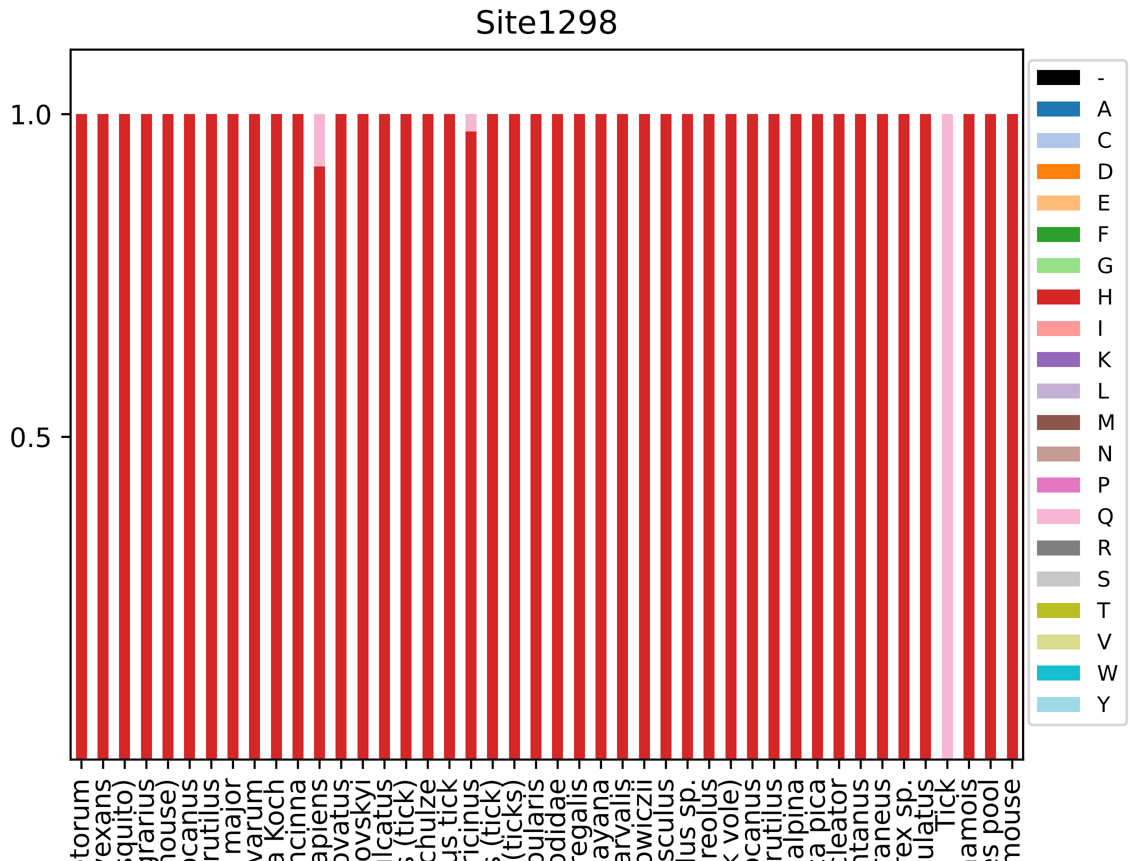

Site1298
1.0
0.5
-
A
C
D
E
F
G
H
I
K
L
M
N
P
Q
R
S
T
V
W
Y

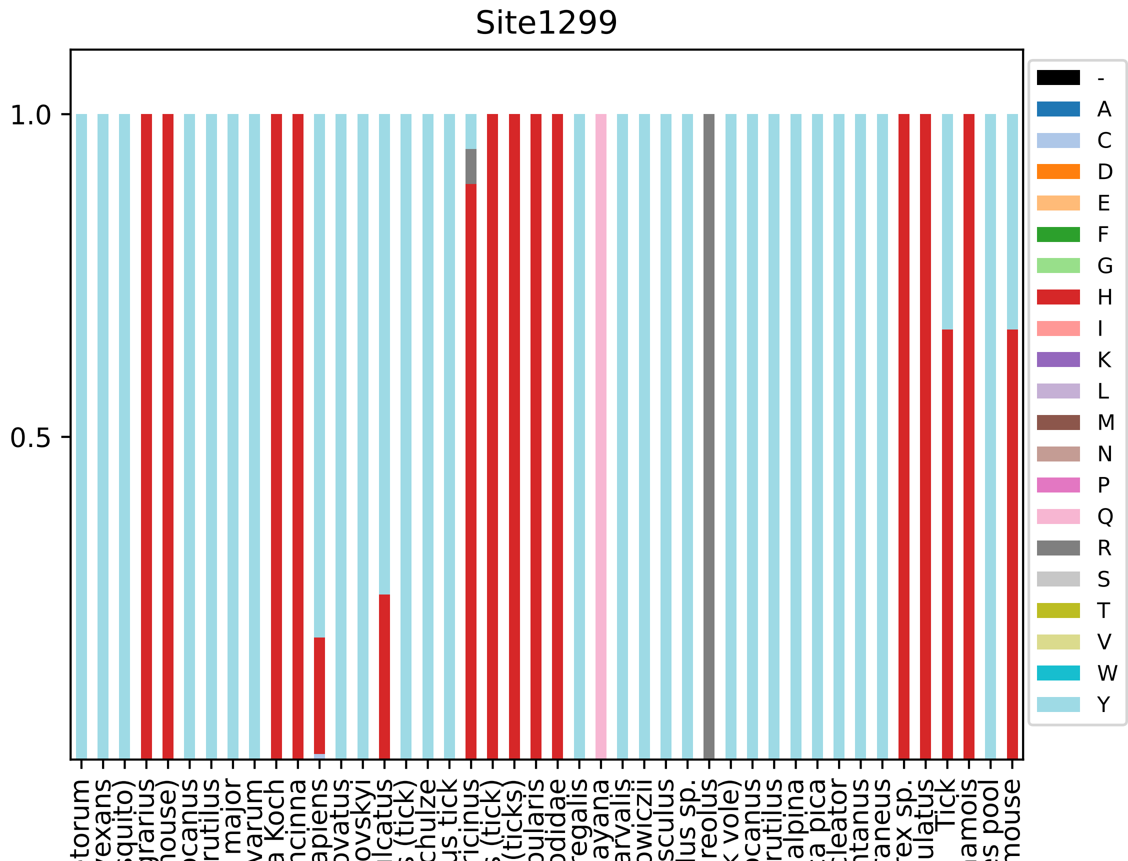
Site1299
1.0
0.5
-
A
C
D
E
F
G
H
I
K
L
M
N
P
Q
R
S
T
V
W
Y
Tick

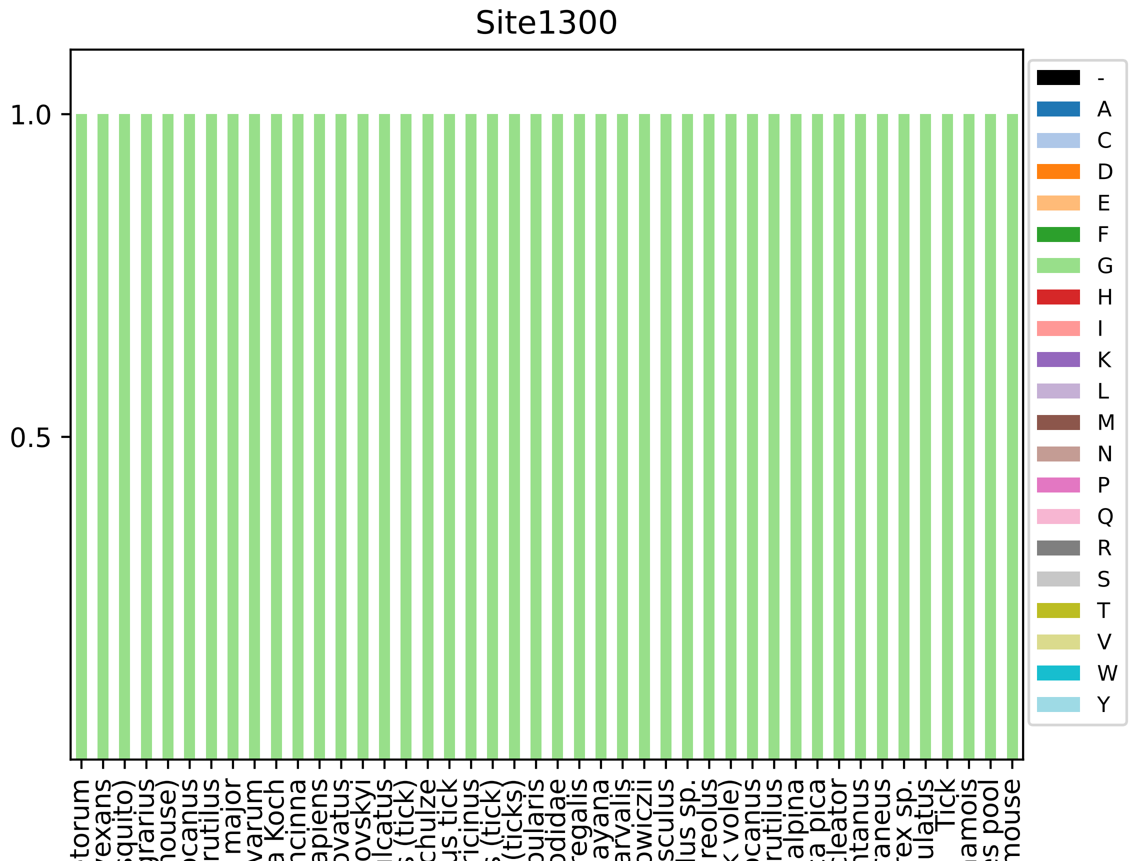

Site1300
1.0
0.5
-
A
C
D
E
F
G
H
I
K
L
M
N
P
Q
R
S
T
V
W
Y

Site1301

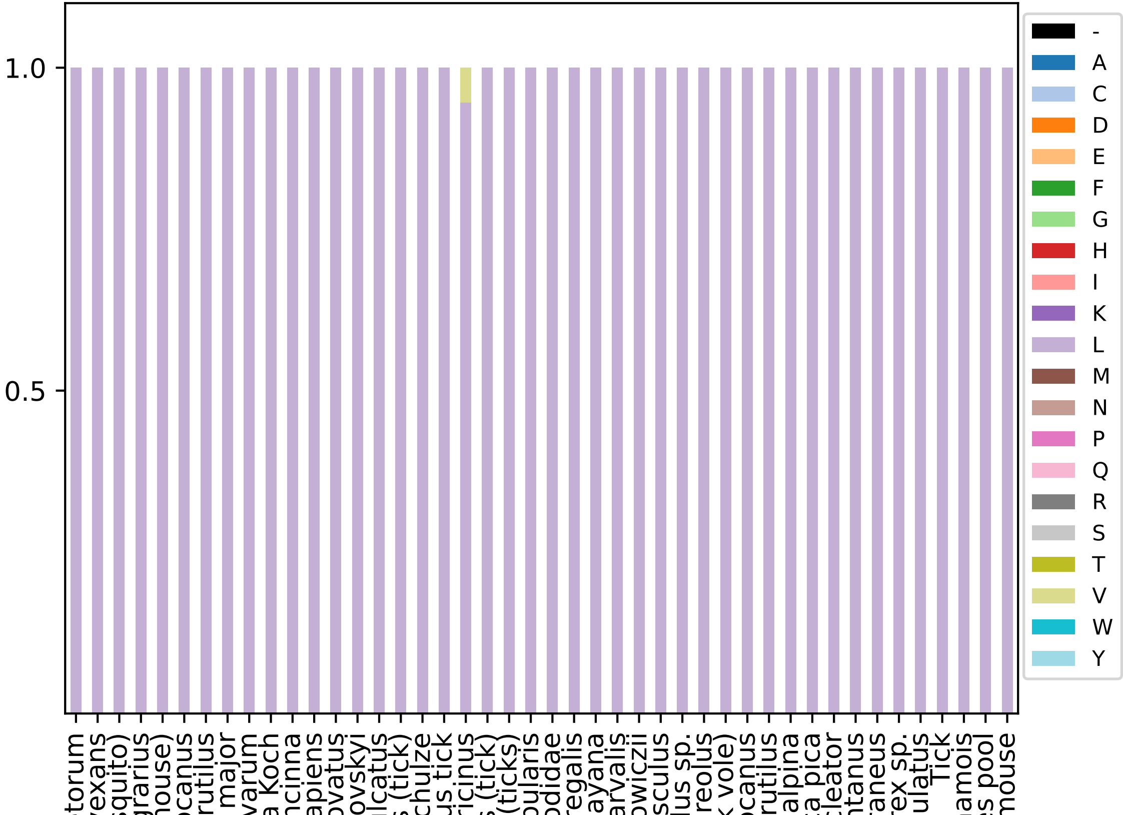

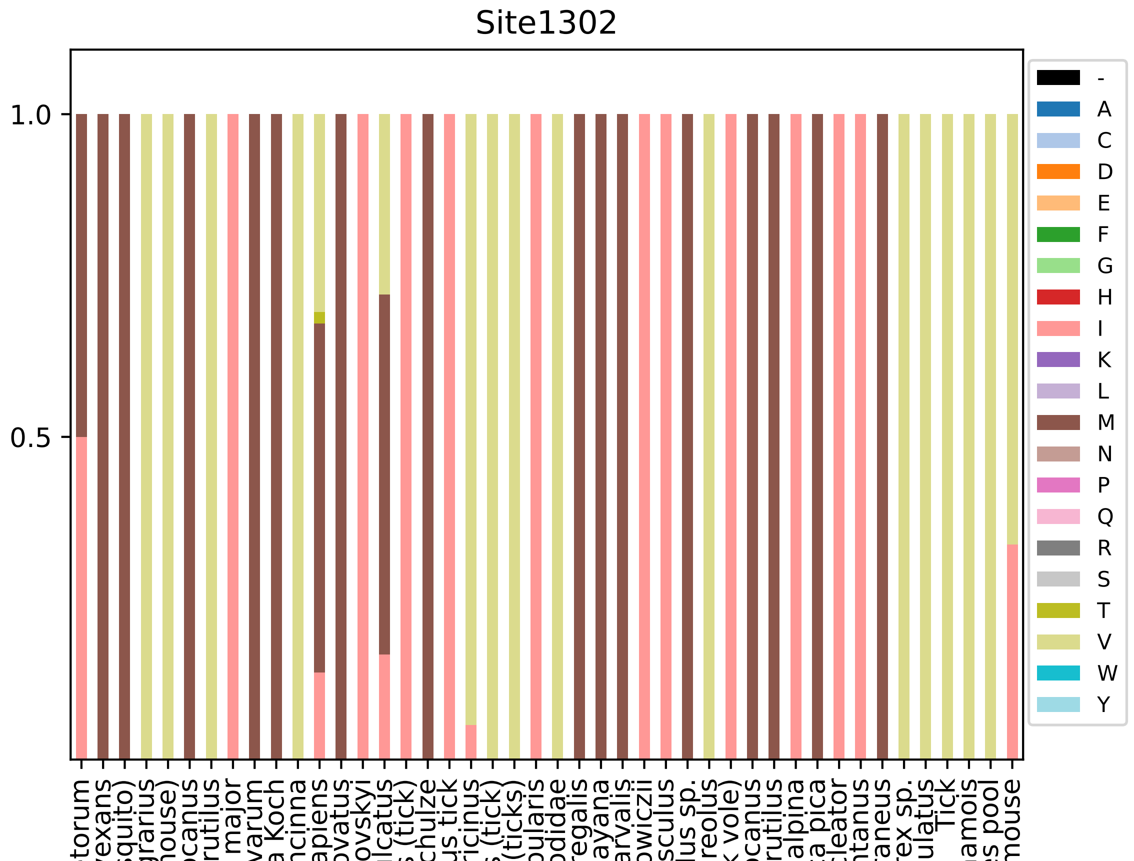
Site1302
1.0
0.5
-
A
C
D
E
F
G
H
I
K
L
M
N
P
Q
R
S
T
V
W
Y

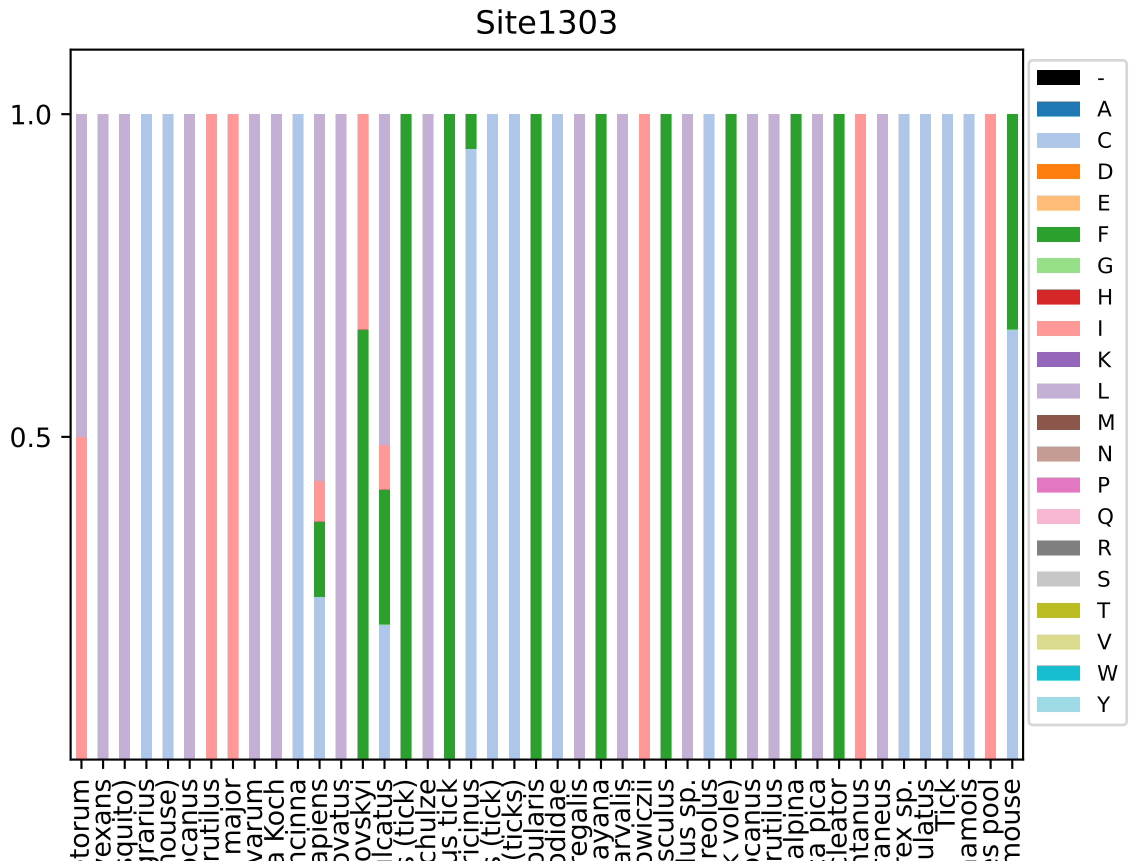
Site1303
1.0
0.5
-
A
C
D
E
F
G
H
I
K
L
M
N
P
Q
R
S
T
V
W
Y
Tick

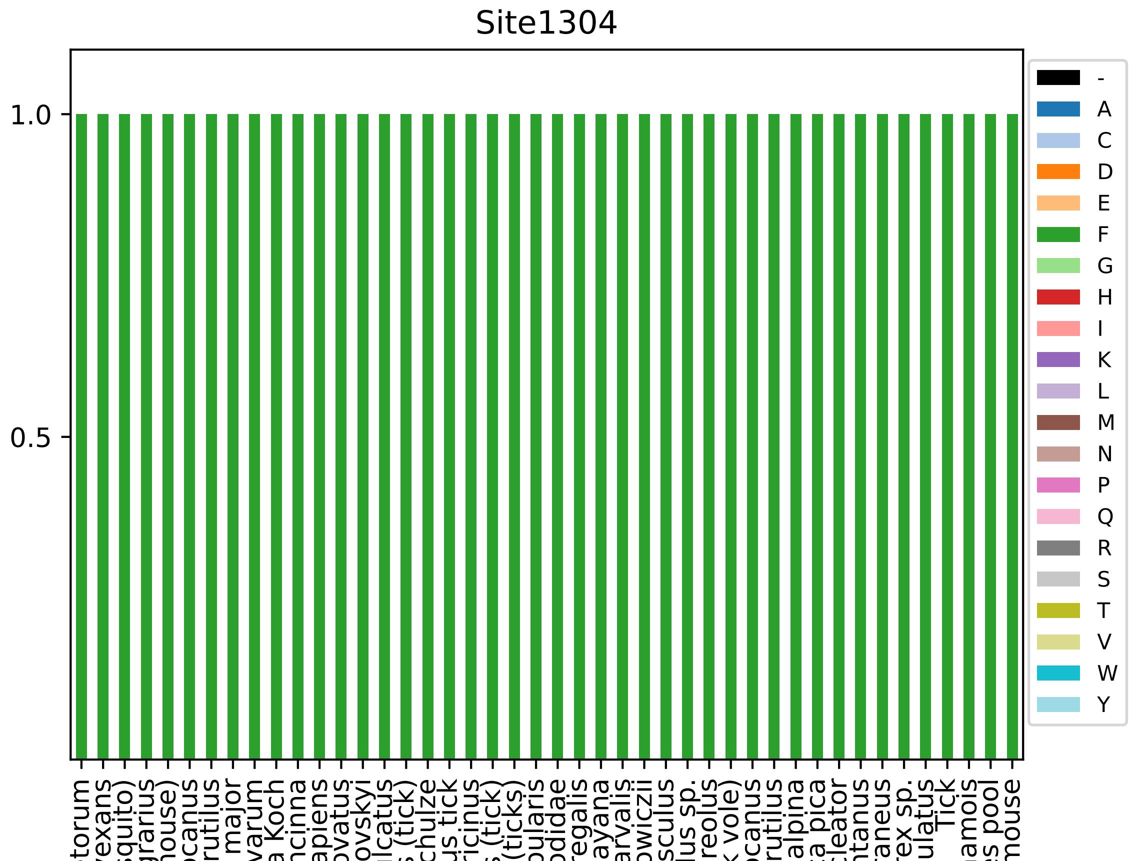
Site1304
1.0
0.5
-
A
C
D
E
F
G
H
I
K
L
M
N
P
Q
R
S
T
V
W
Y

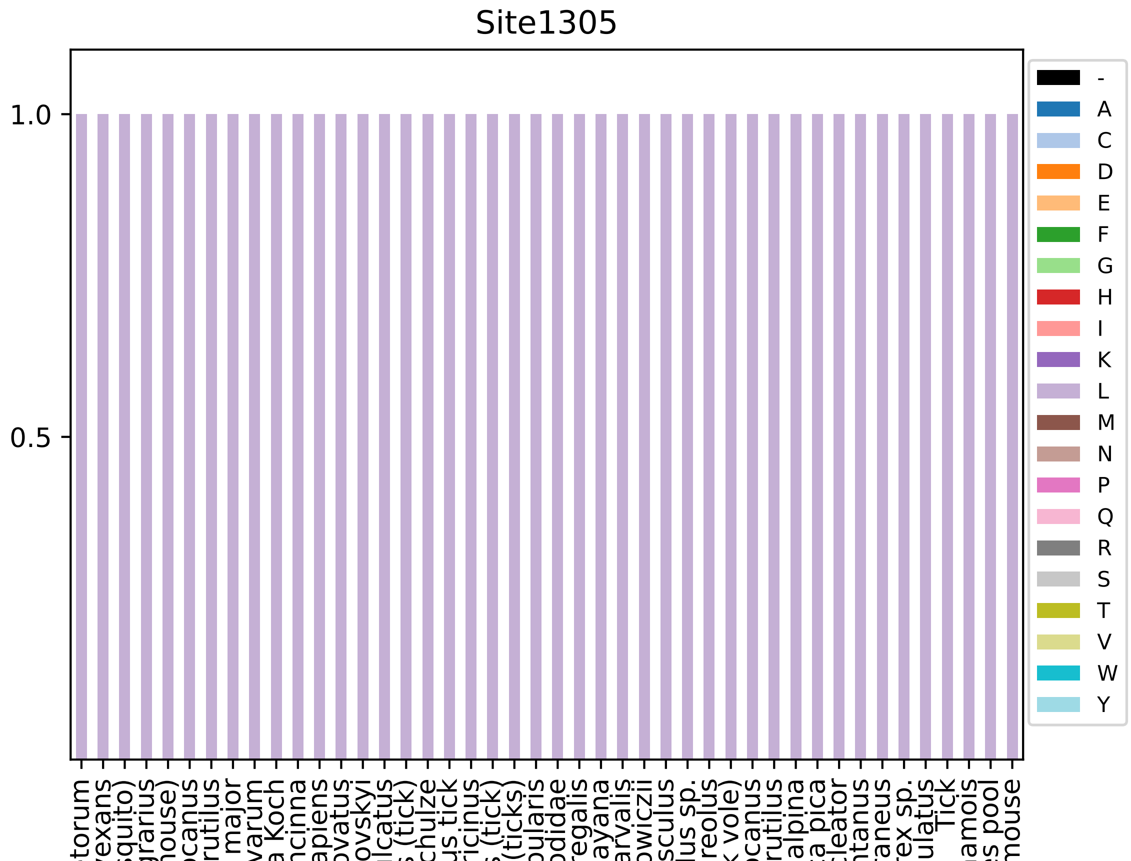

Site1305
1.0
0.5
-
A
C
D
E
F
G
H
I
K
L
M
N
P
Q
R
S
T
V
W
Y

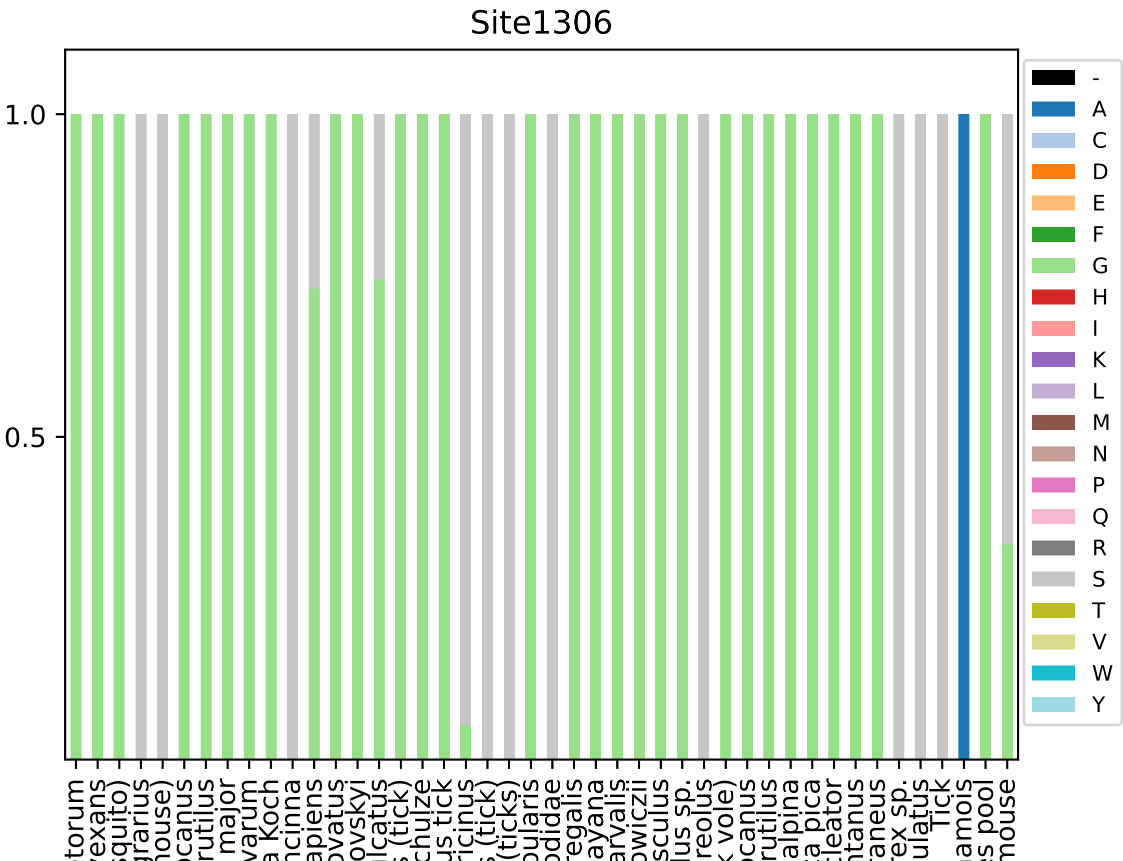
Site1306
1.0
0.5
-
A
C
D
E
F
G
H
I
K
L
M
N
P
Q
R
S
T
V
W
Y

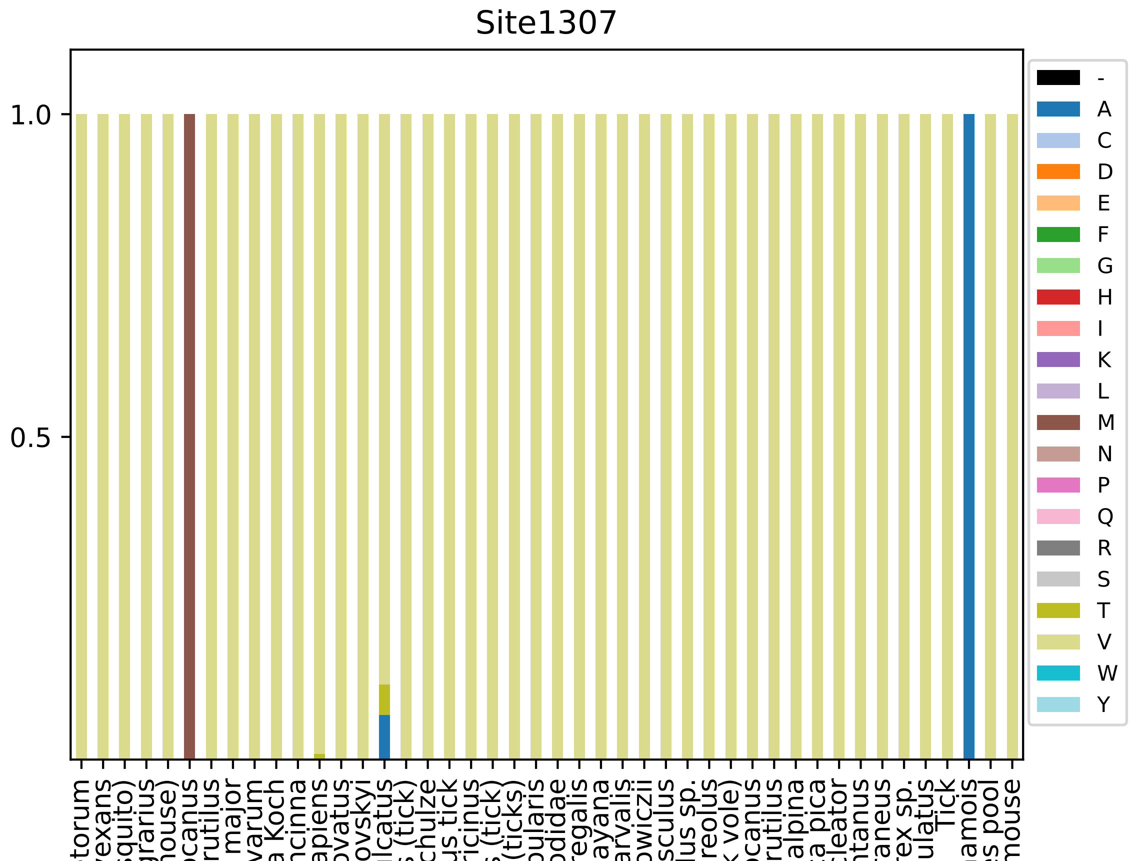
Site1307
1.0
0.5
-
A
C
D
E
F
G
H
I
K
L
M
N
P
Q
R
S
T
V
W
Y
torum
vexans
squito)
grarius
nouse)
pcanus
rutilus
major
varum
a Koch
ncinna
apiens
ovatus
ovskyi
ulcatus
s (tick)
chulze
us tick
ricinus
s (tick)
(ticks)
pularis
podidae
regalis
ayana
arvalis
owiczii
sculus
lus sp.
reolus
k vole)
ocanus
rutilus
alpina
a pica
cleator
ntanus
raneus
rex sp.
ulatus
Tick
amois
s pool
mouse

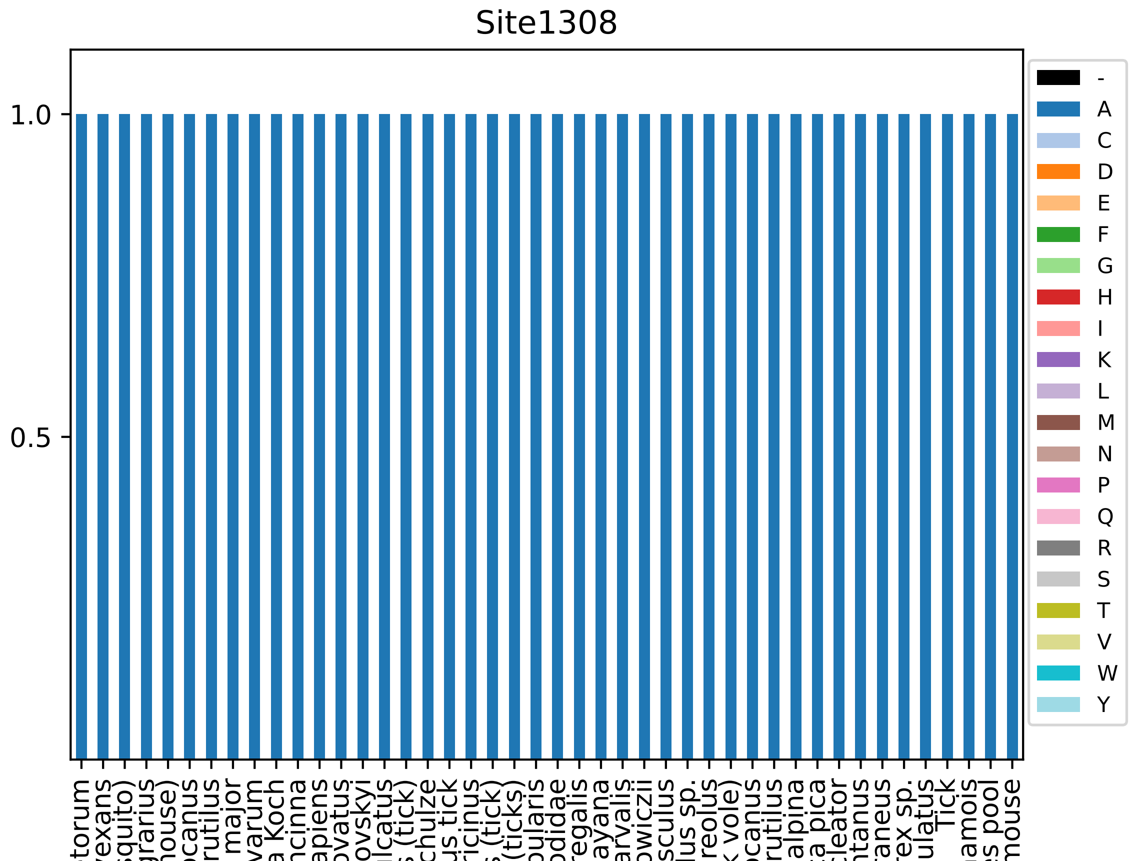
Site1308
1.0
0.5
-
A
C
D
E
F
G
H
I
K
L
M
N
P
Q
R
S
T
V
W
Y

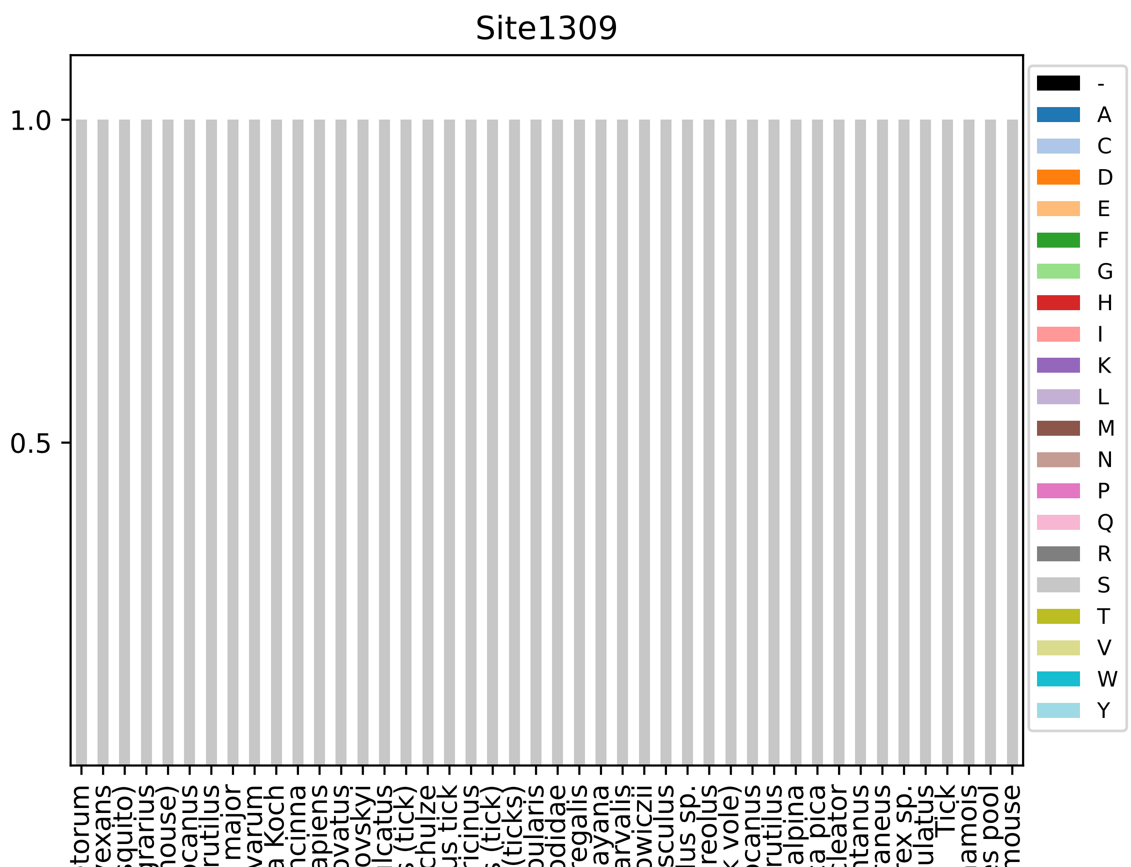
Site1309
1.0
0.5
-
A
C
D
E
F
G
H
I
K
L
M
N
P
Q
R
S
T
V
W
Y

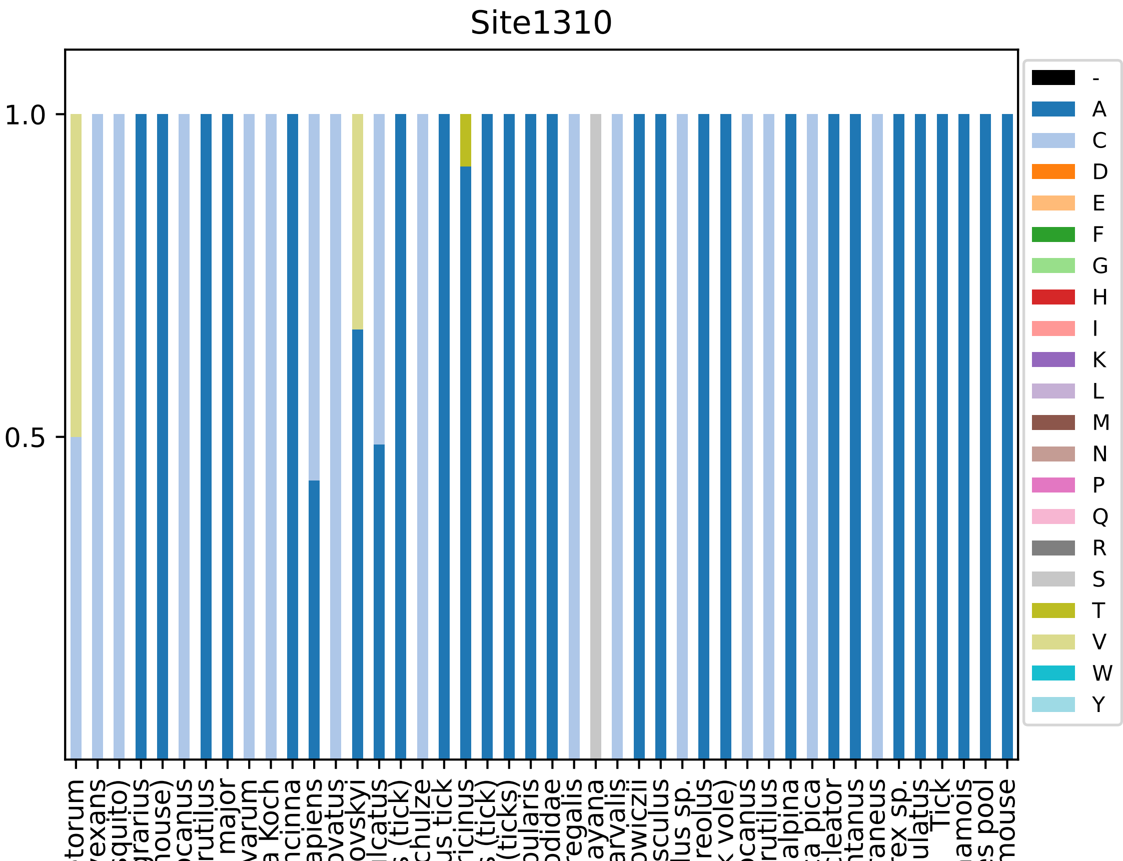
Site1310
1.0
0.5
-
A
C
D
E
F
G
H
I
K
L
M
N
P
Q
R
S
T
V
W
Y
Tick

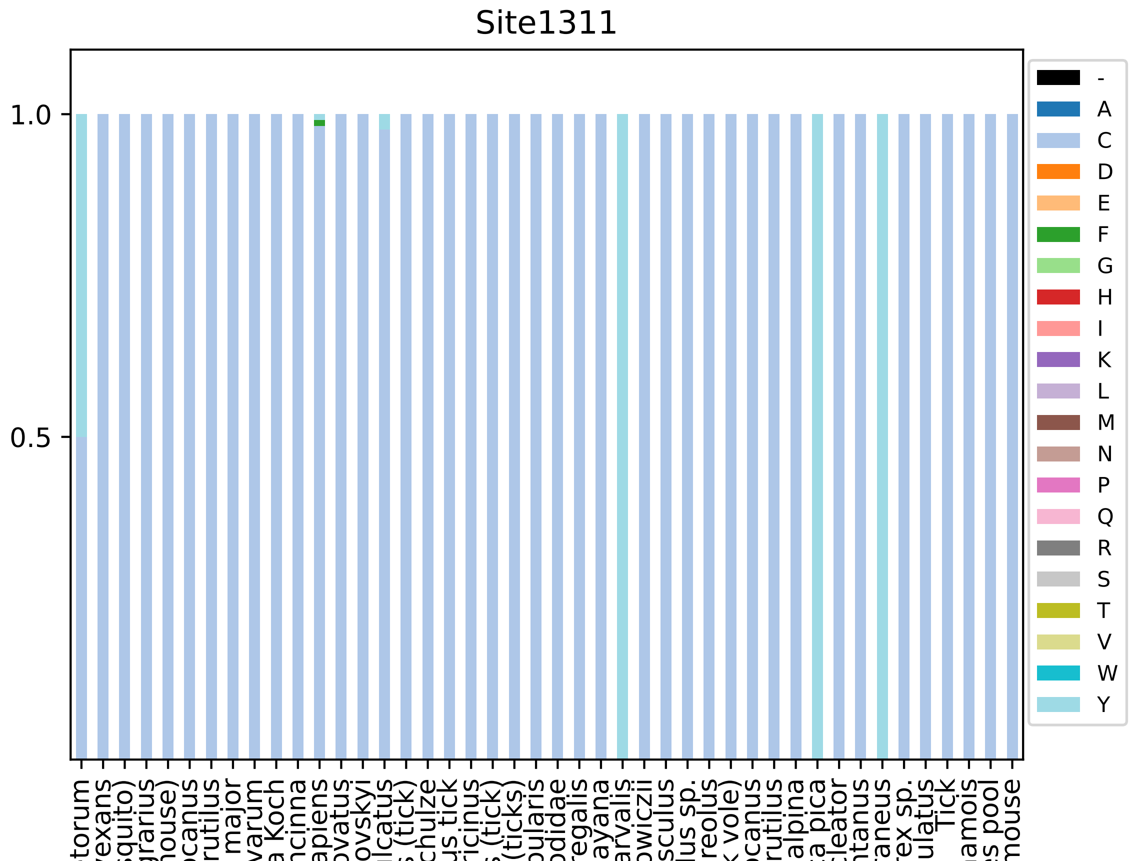

Site1311
1.0
0.5
-
A
C
D
E
F
G
H
I
K
L
M
N
P
Q
R
S
T
V
W
Y

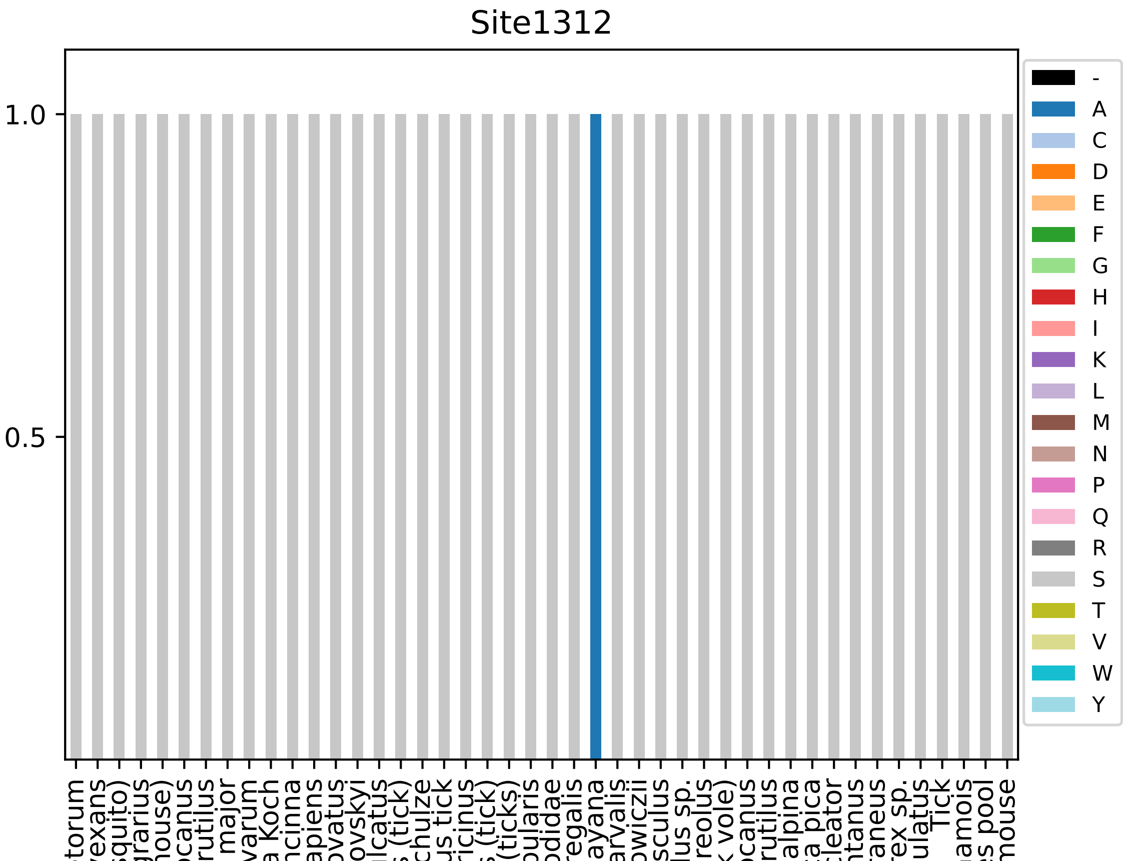

Site1312
1.0
0.5
-
A
C
D
E
F
G
H
I
K
L
M
N
P
Q
R
S
T
V
W
Y

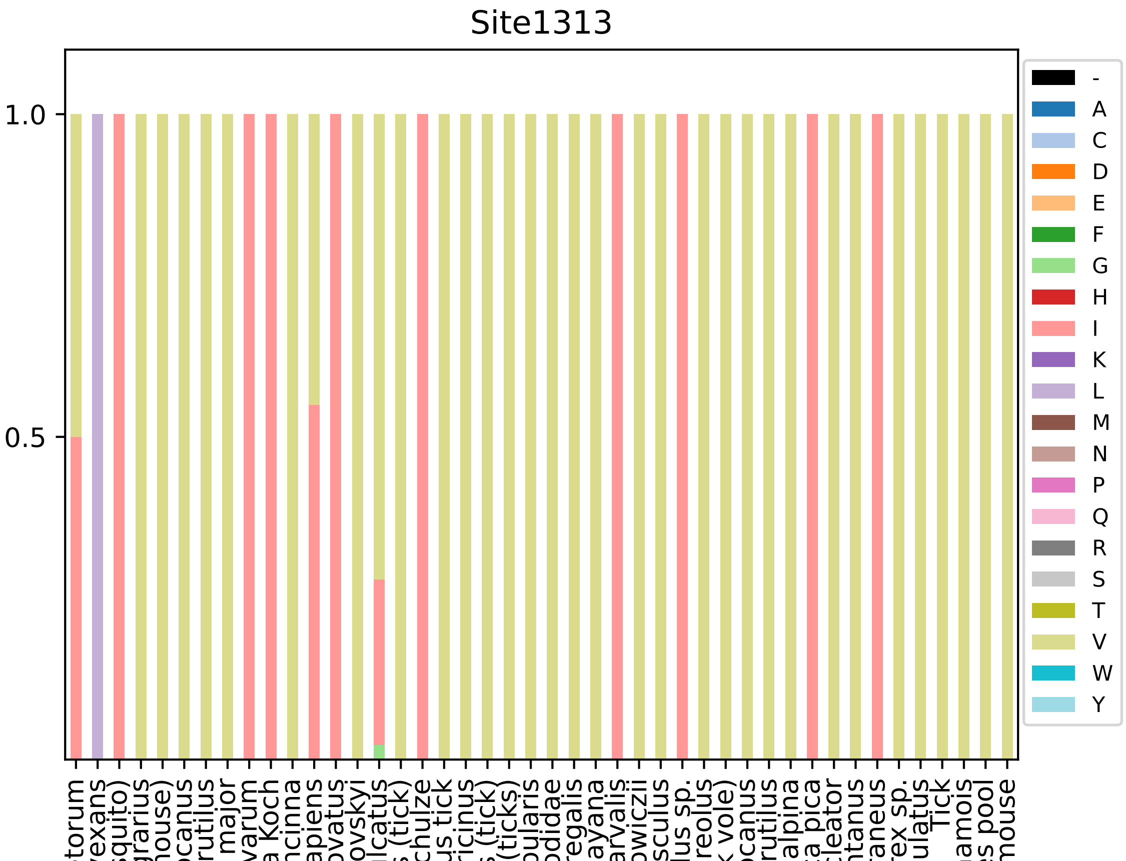

Site1313
1.0
0.5
-
A
C
D
E
F
G
H
I
K
L
M
N
P
Q
R
S
T
V
W
Y

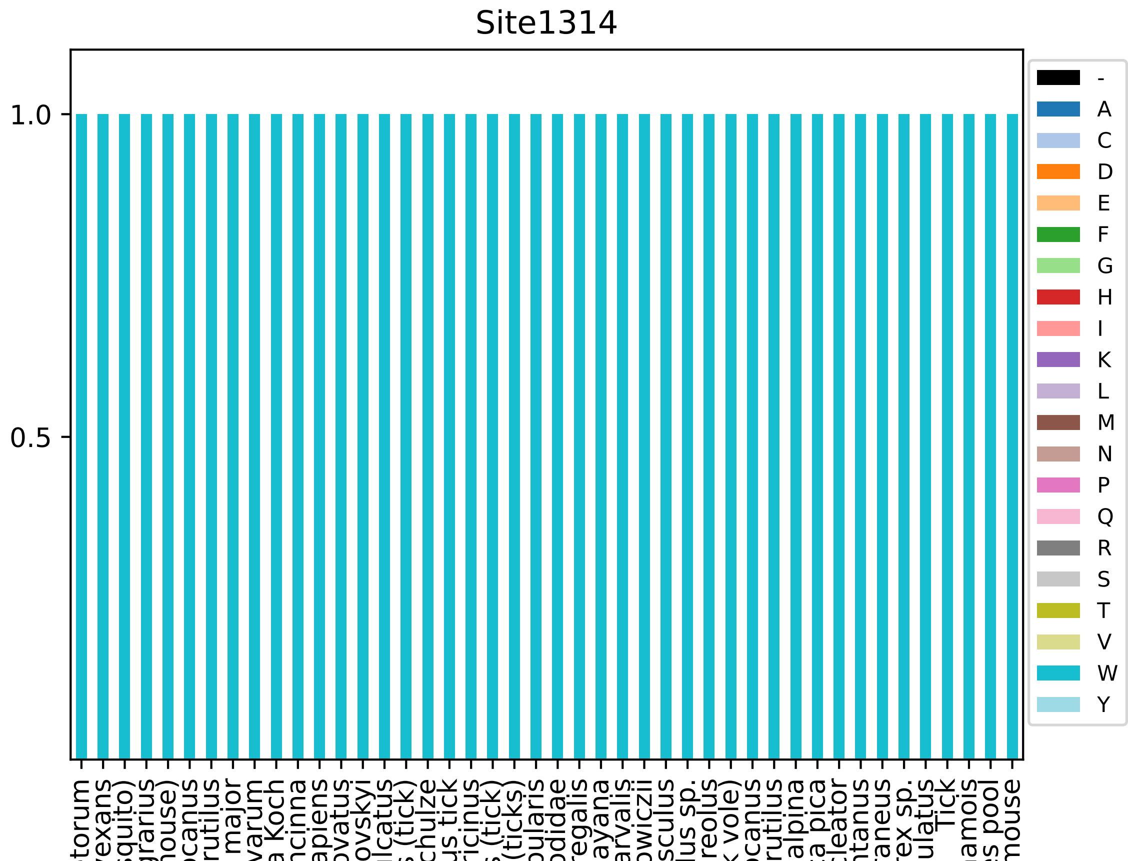
Site1314
1.0
0.5
-
A
C
D
E
F
G
H
I
K
L
M
N
P
Q
R
S
T
V
W
Y

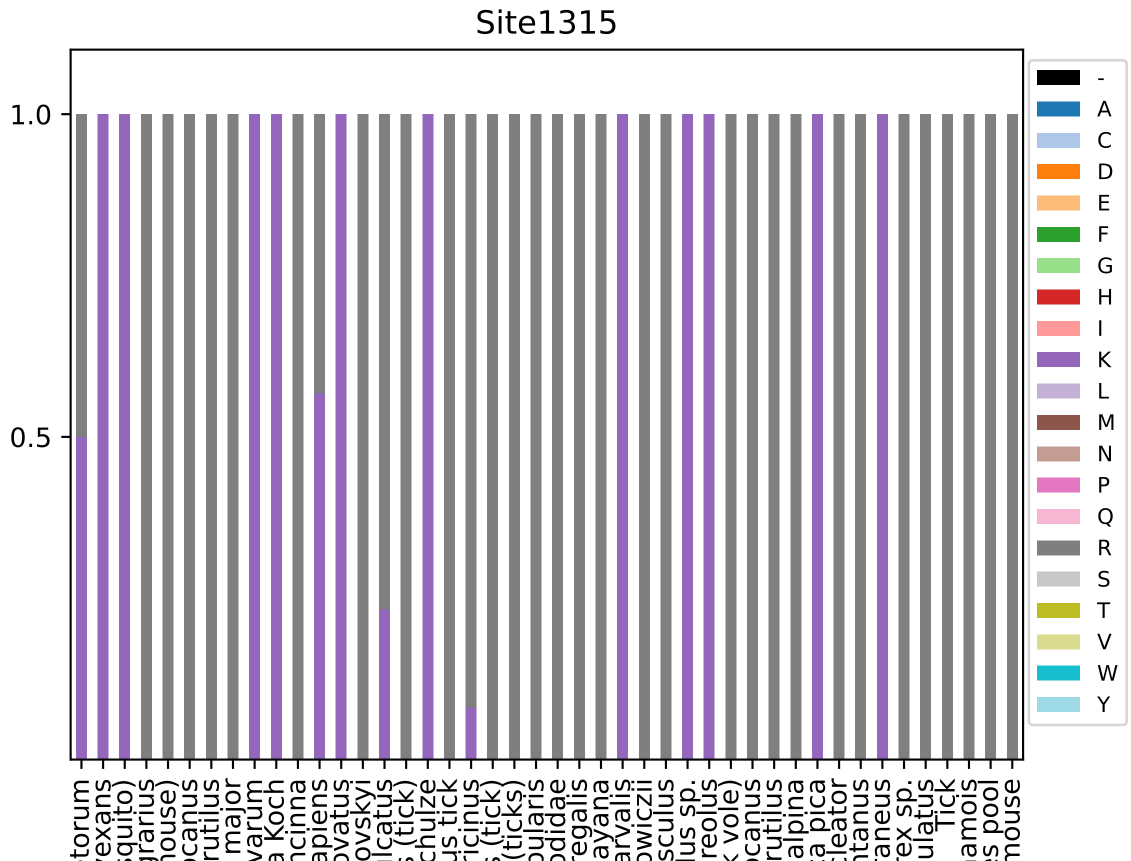
Site1315
1.0
0.5
-
A
C
D
E
F
G
H
I
K
L
M
N
P
Q
R
S
T
V
W
Y

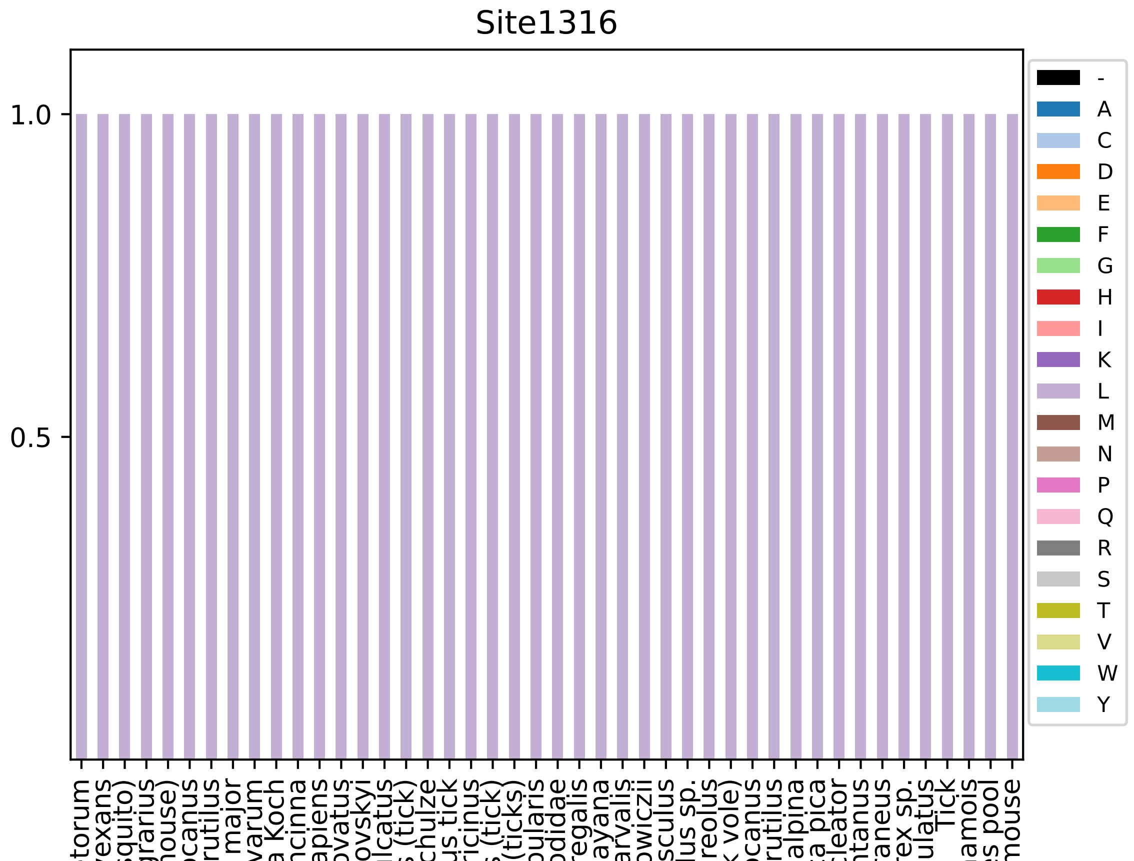

Site1316
1.0
0.5
-
A
C
D
E
F
G
H
I
K
L
M
N
P
Q
R
S
T
V
W
Y

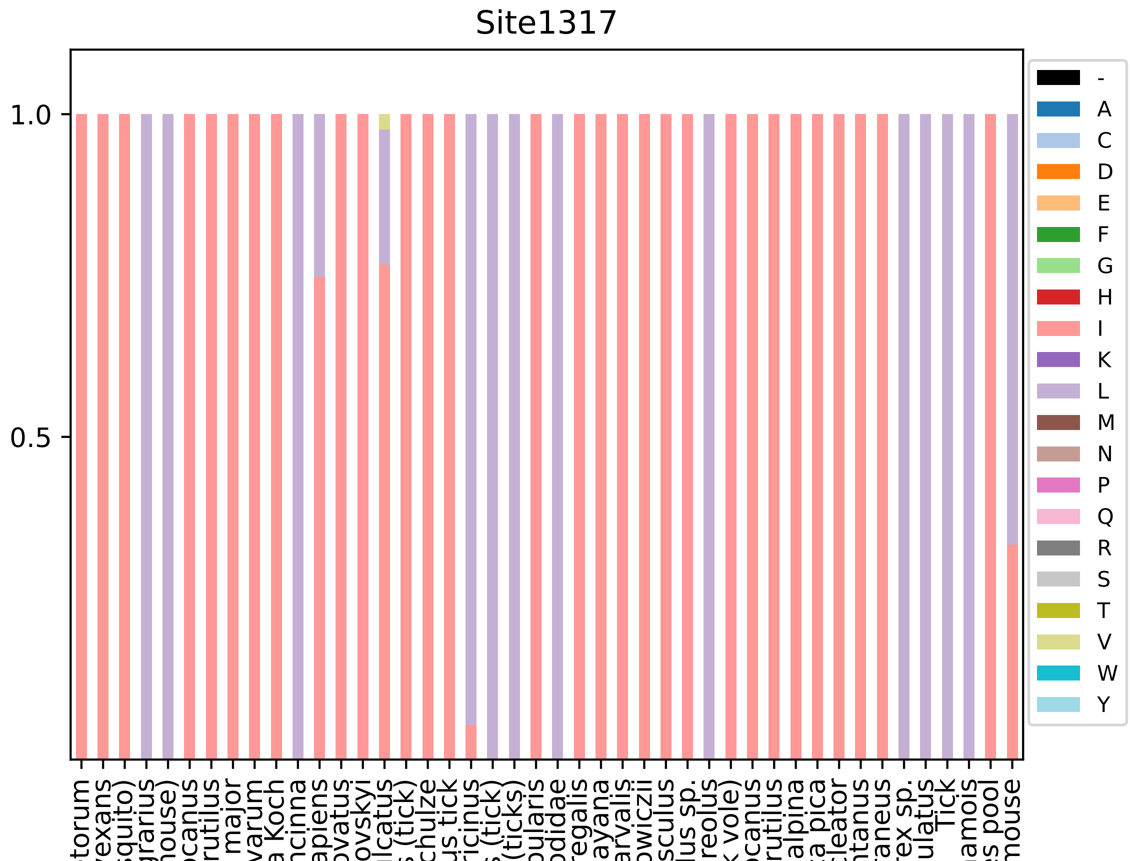

Site1317
1.0
0.5
-
A
C
D
E
F
G
H
I
K
L
M
N
P
Q
R
S
T
V
W
Y
Tick

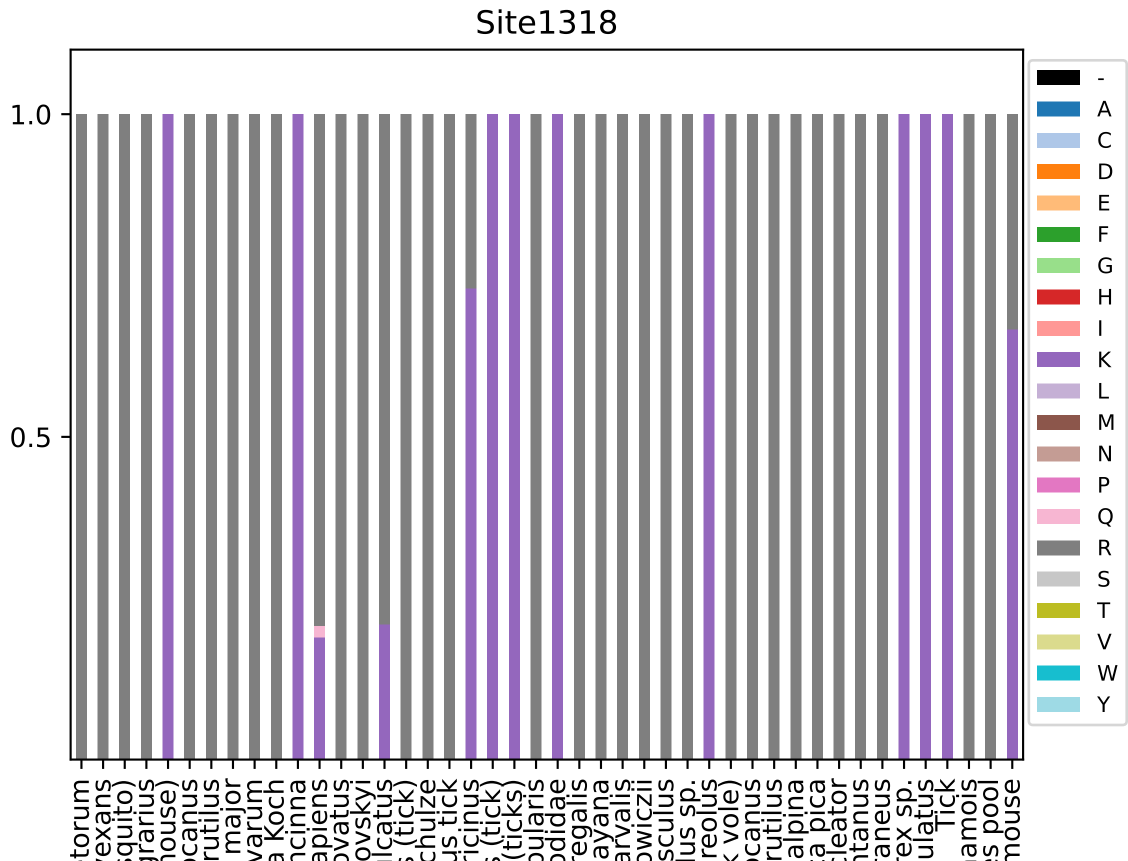
Site1318
1.0
0.5
-
A
C
D
E
F
G
H
I
K
L
M
N
P
Q
R
S
T
V
W
Y
torum
exans
squito)
garius
house)
canus
rutilus
major
varum
a Koch
ncinna
apiens
ovatus
ovskyi
lcatus
s (tick)
chulze
us tick
ricinus
s (ticks)
pularis
odidae
regalis
ayana
arvalis
owiczii
sculus
lus sp.
reolus
k vole)
canus
rutilus
alpina
a pica
leator
ntanus
raneus
rex sp.
ulatus
Tick
amois
es pool
mouse

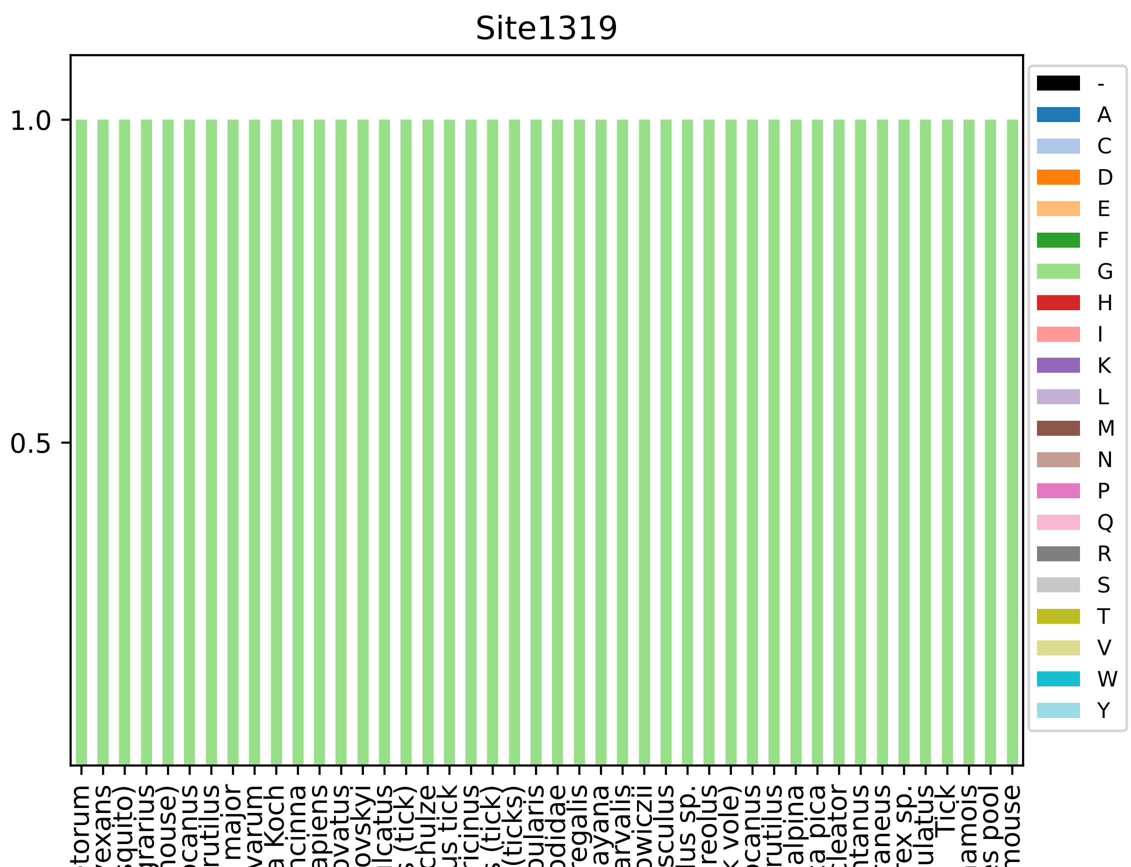
Site1319
1.0
0.5
-
A
C
D
E
F
G
H
I
K
L
M
N
P
Q
R
S
T
V
W
Y

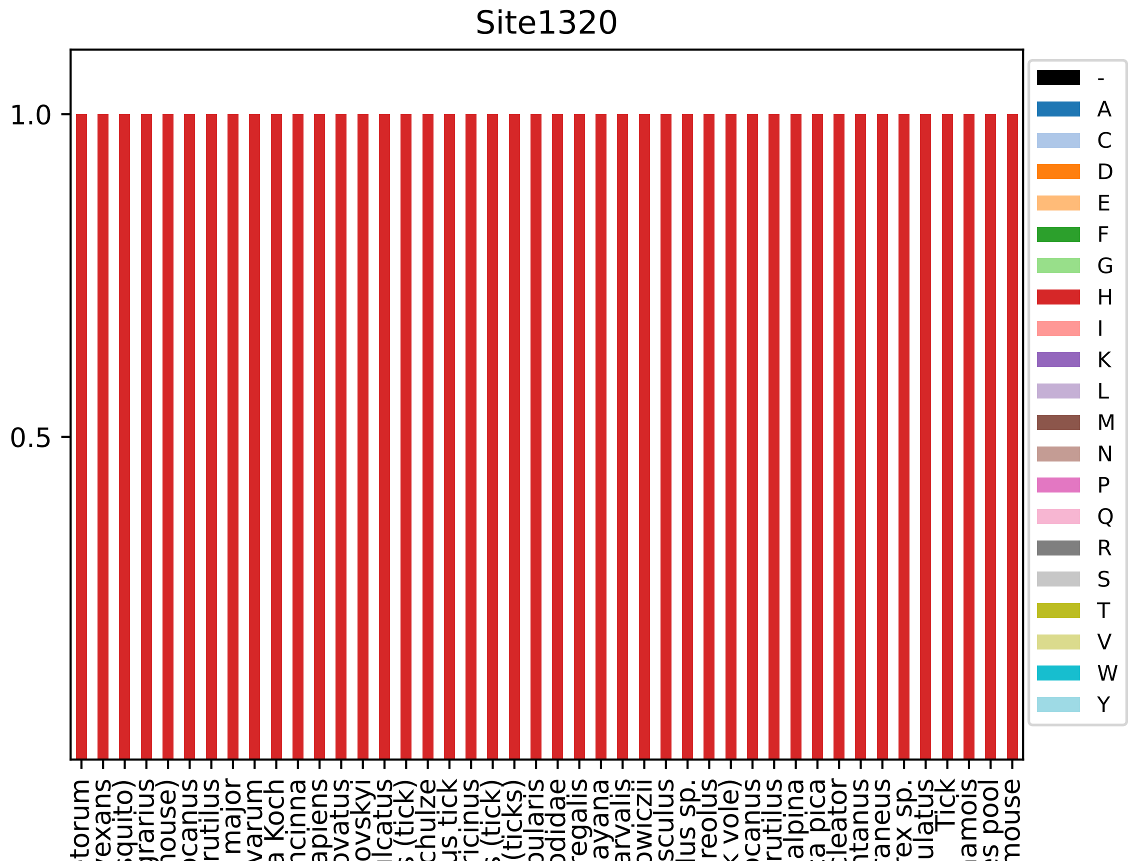
Site1320
1.0
0.5
-
A
C
D
E
F
G
H
I
K
L
M
N
P
Q
R
S
T
V
W
Y

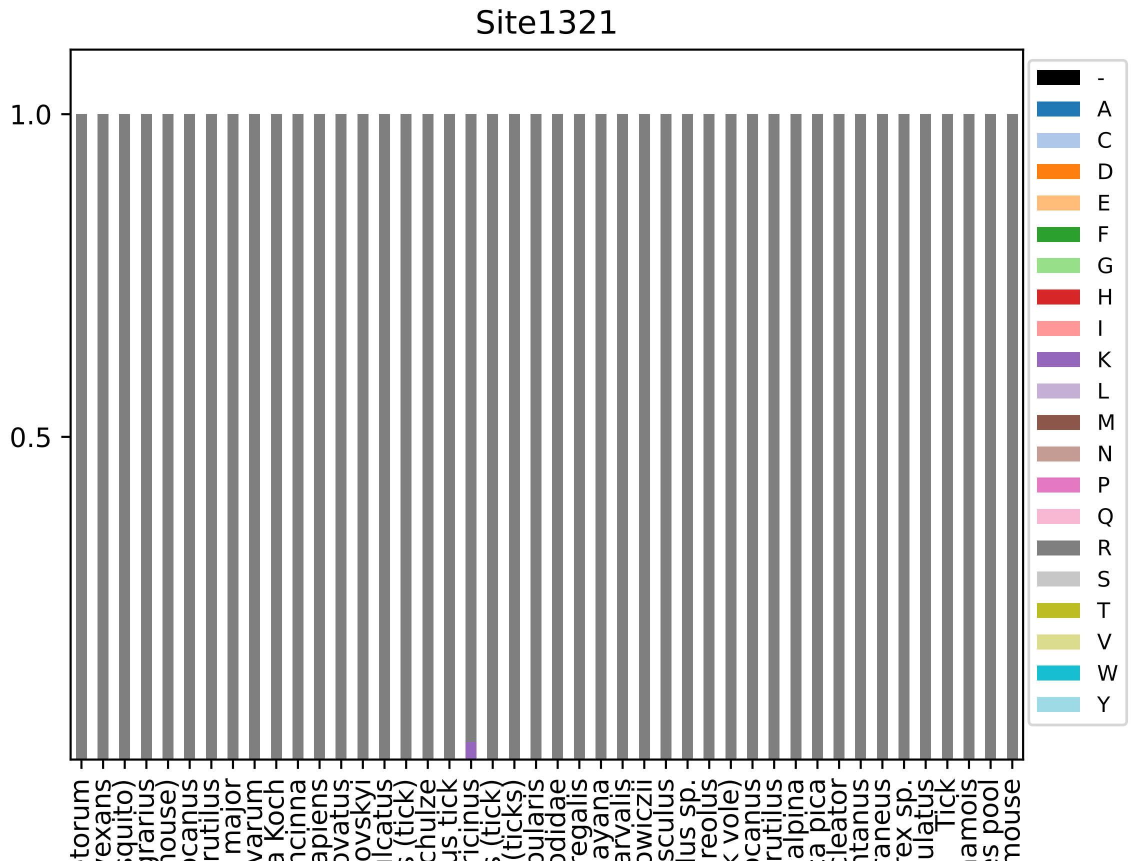
Site1321
1.0
0.5
-
A
C
D
E
F
G
H
I
K
L
M
N
P
Q
R
S
T
V
W
Y

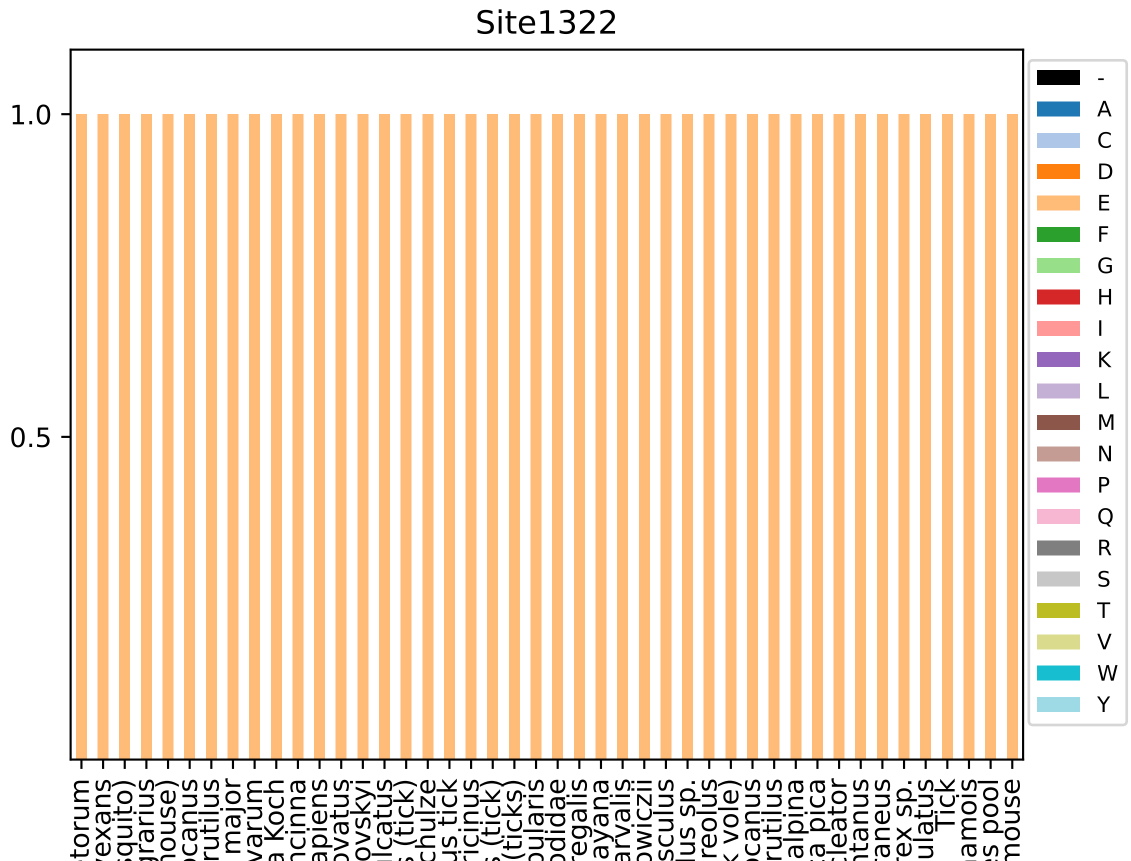
Site1322
1.0
0.5
-
A
C
D
E
F
G
H
I
K
L
M
N
P
Q
R
S
T
V
W
Y

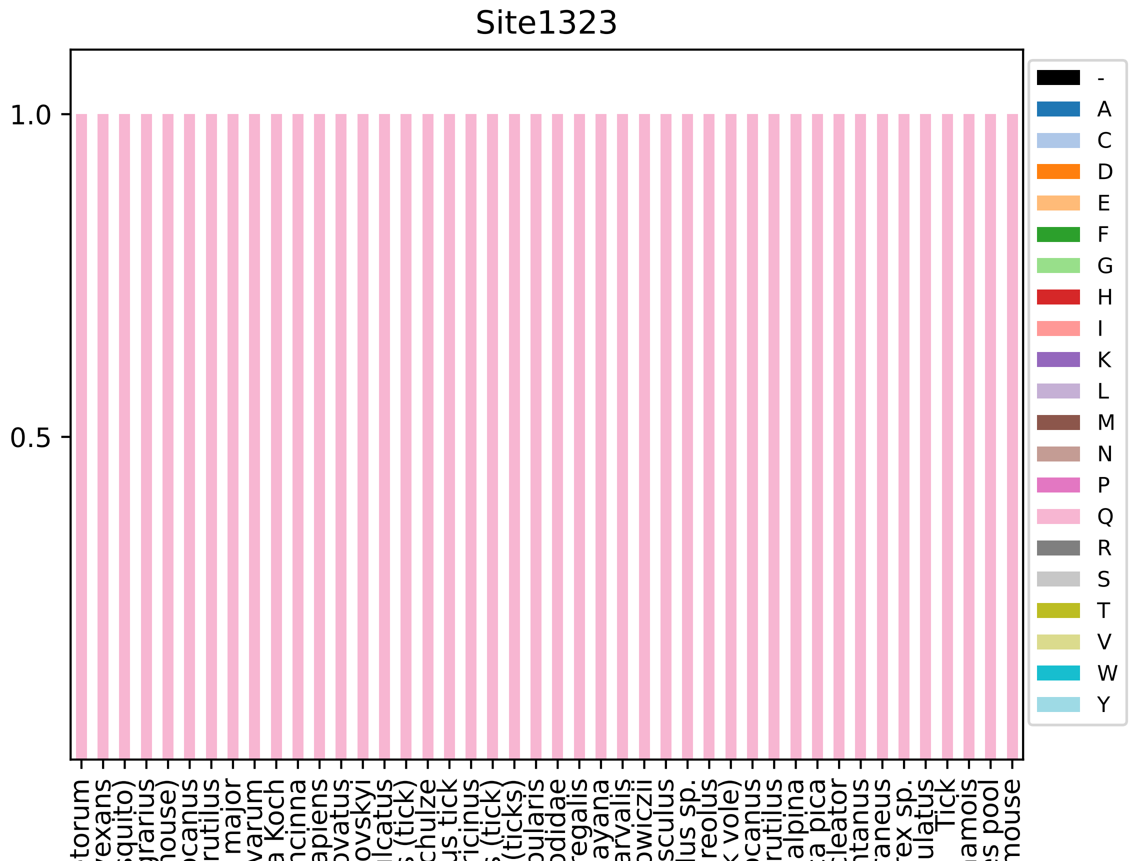
Site1323
1.0
0.5
-
A
C
D
E
F
G
H
I
K
L
M
N
P
Q
R
S
T
V
W
Y

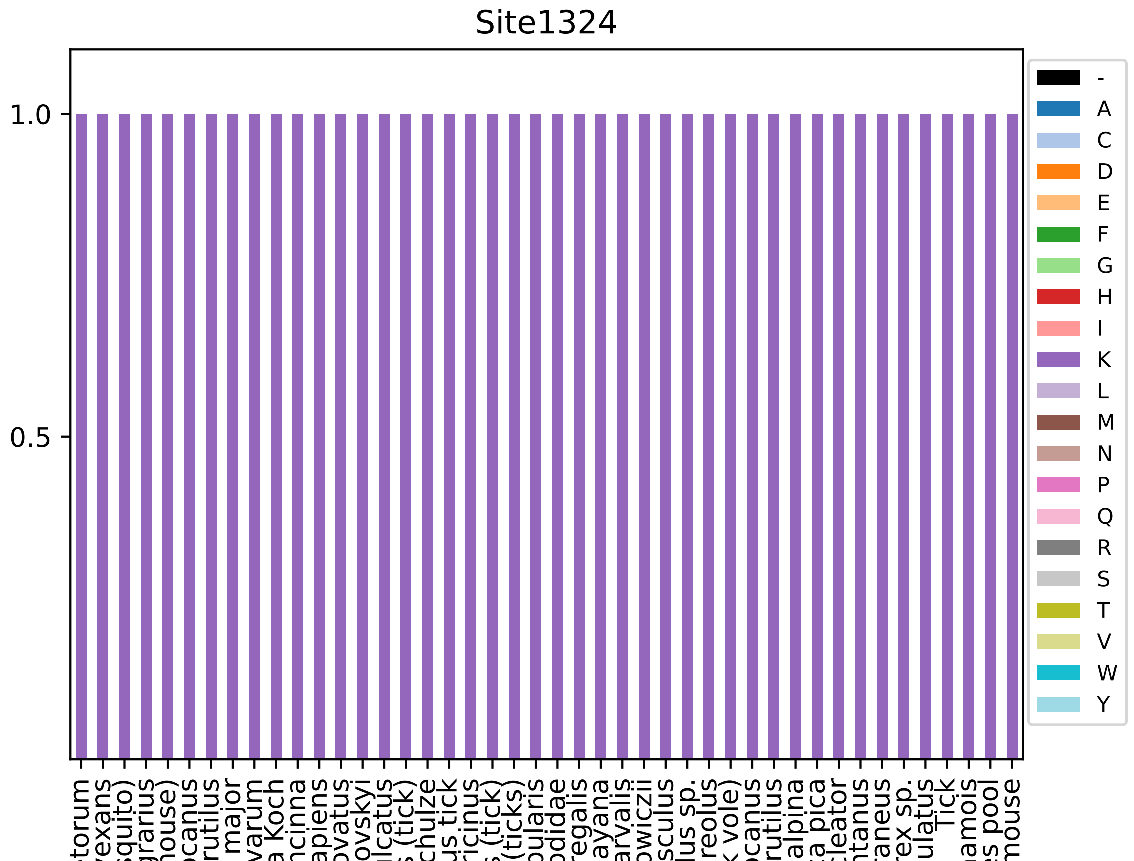
Site1324
1.0
0.5
-
A
C
D
E
F
G
H
I
K
L
M
N
P
Q
R
S
T
V
W
Y

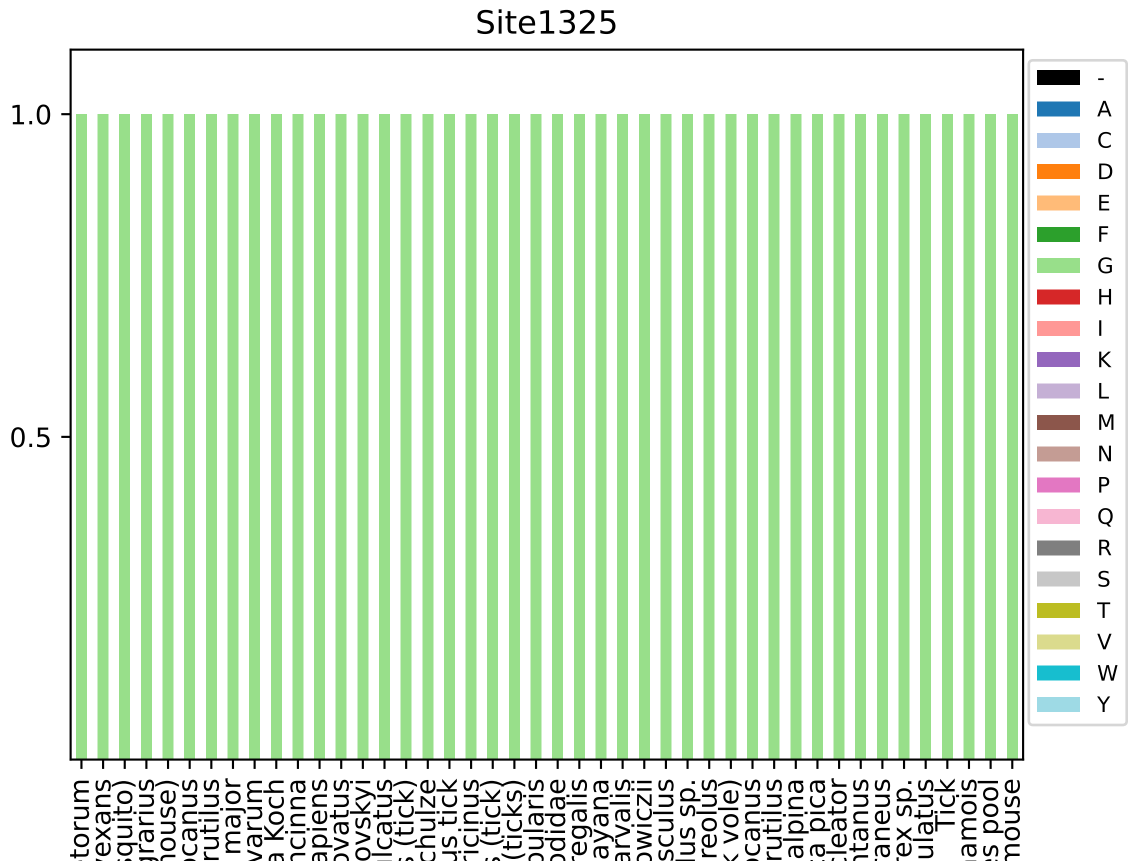
Site1325
1.0
0.5
-
A
C
D
E
F
G
H
I
K
L
M
N
P
Q
R
S
T
V
W
Y

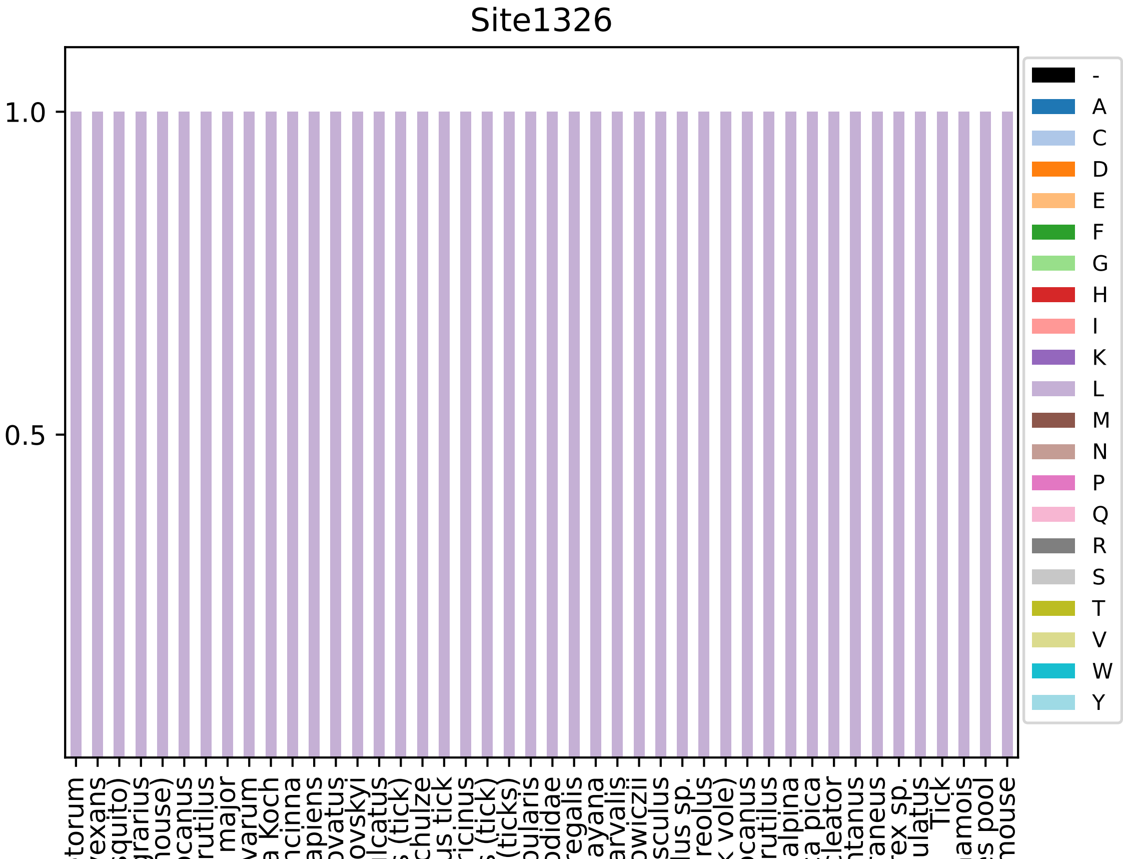
Site1326
1.0
0.5
-
A
C
D
E
F
G
H
I
K
L
M
N
P
Q
R
S
T
V
W
Y
Tick

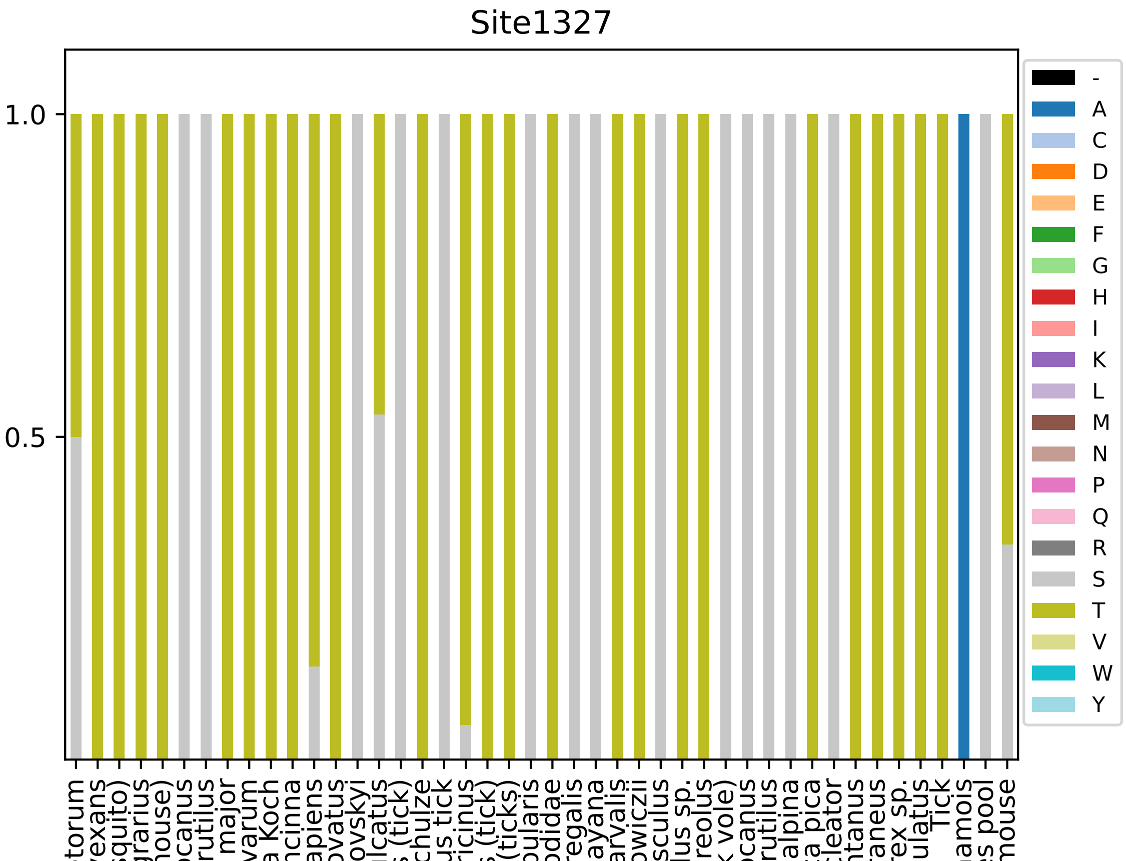
Site1327
1.0
0.5
-
A
C
D
E
F
G
H
I
K
L
M
N
P
Q
R
S
T
V
W
Y
Tick

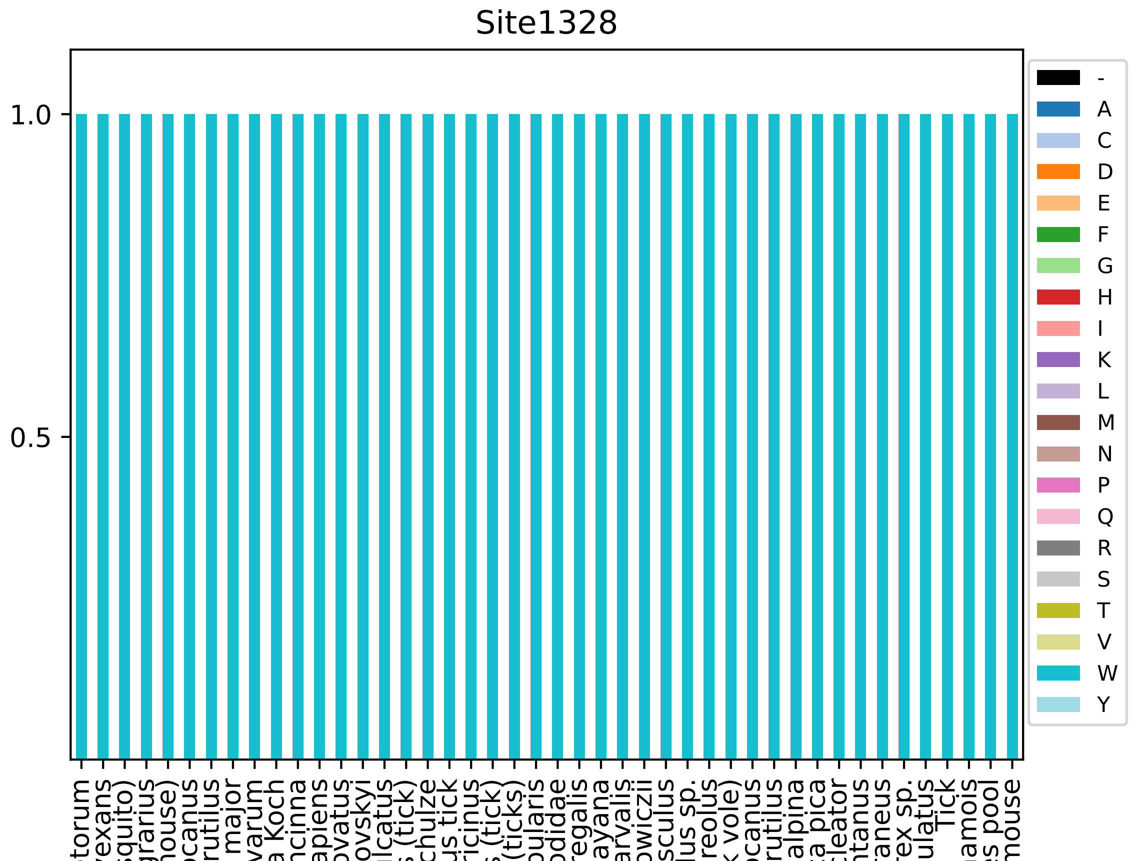
Site1328
1.0
0.5
-
A
C
D
E
F
G
H
I
K
L
M
N
P
Q
R
S
T
V
W
Y

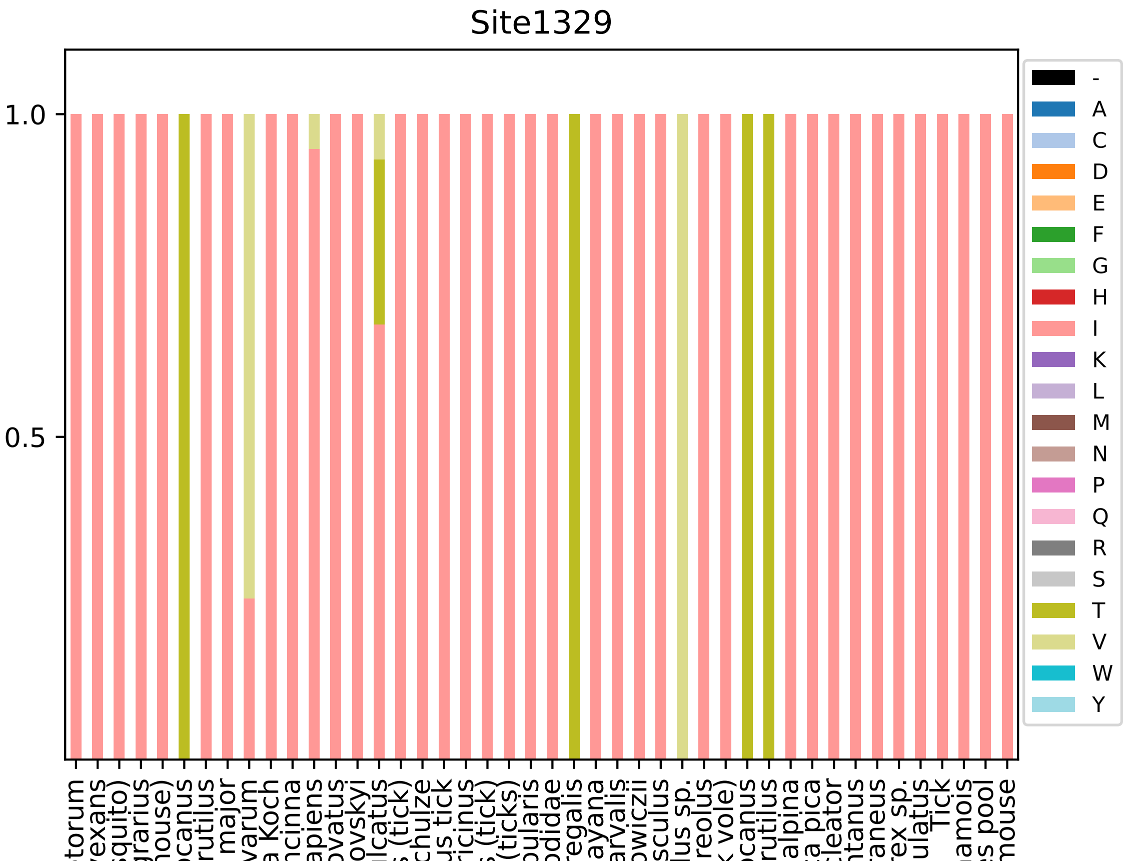

Site1329
1.0
0.5
-
A
C
D
E
F
G
H
I
K
L
M
N
P
Q
R
S
T
V
W
Y

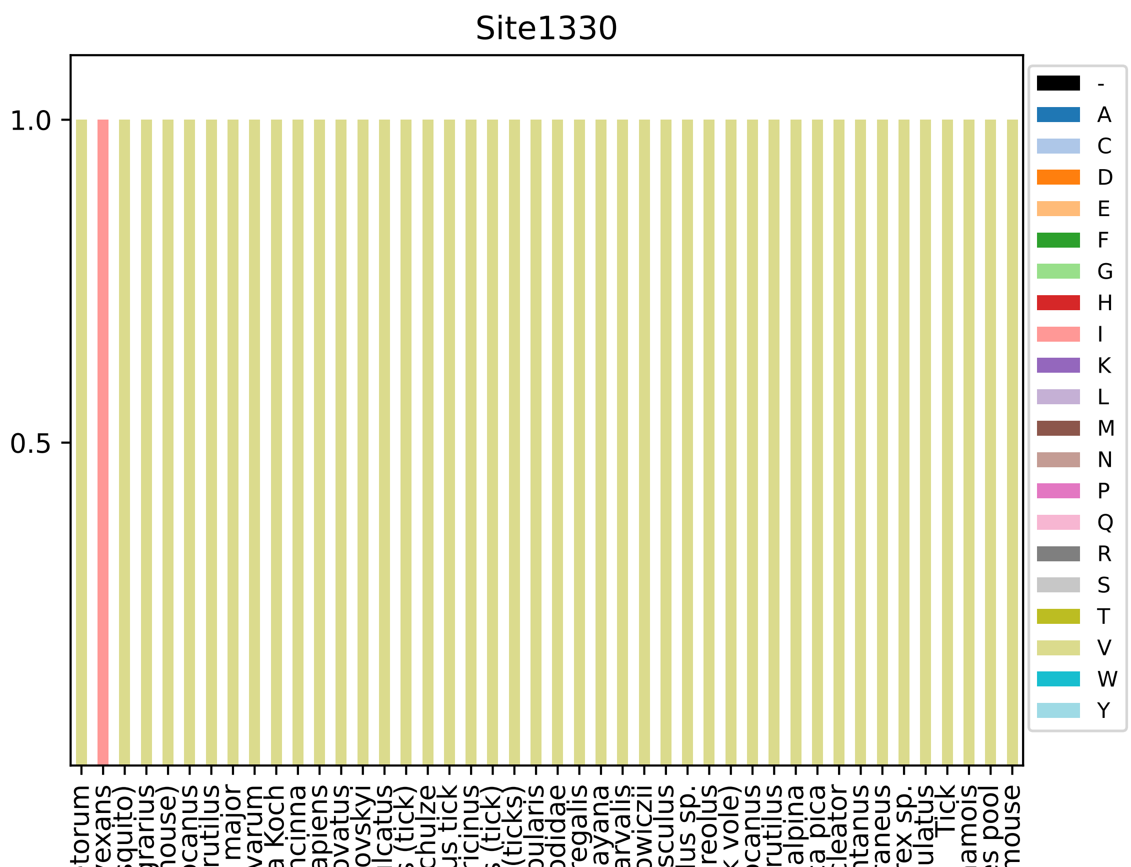

Site1330
1.0
0.5
-
A
C
D
E
F
G
H
I
K
L
M
N
P
Q
R
S
T
V
W
Y

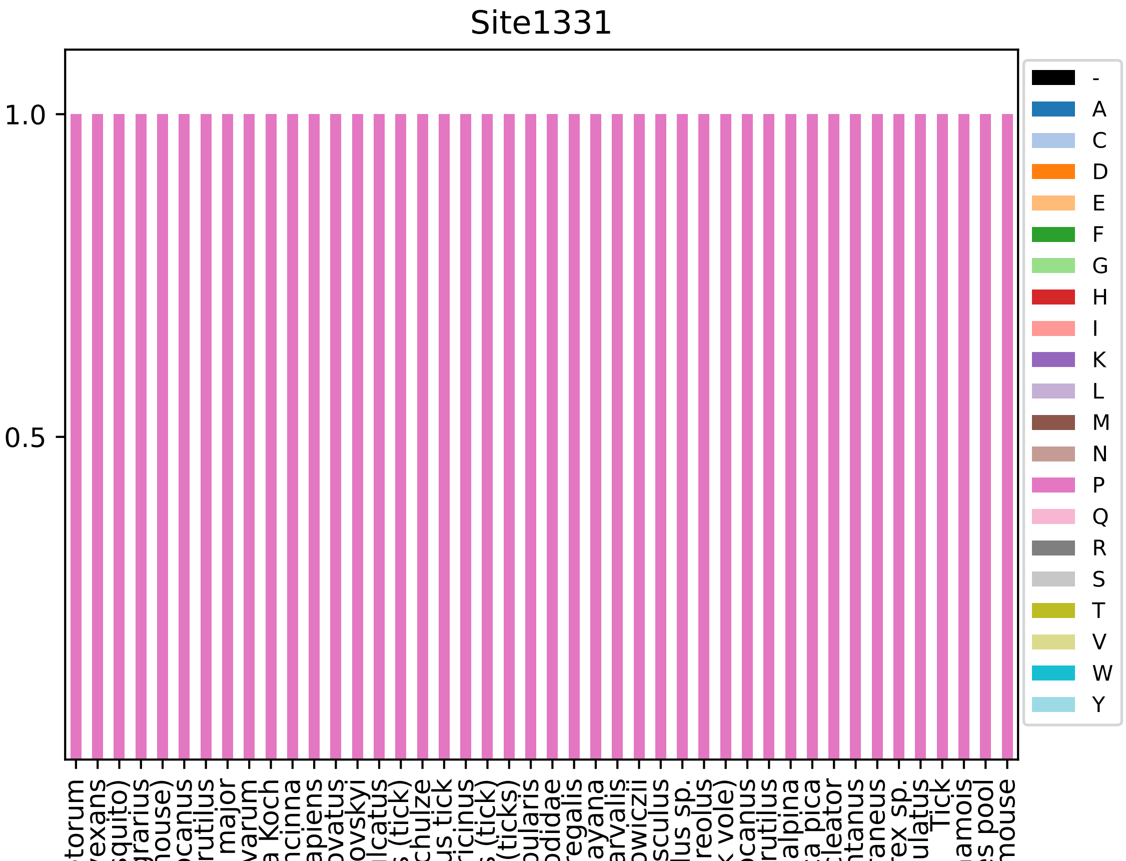
Site1331
1.0
0.5
-
A
C
D
E
F
G
H
I
K
L
M
N
P
Q
R
S
T
V
W
Y

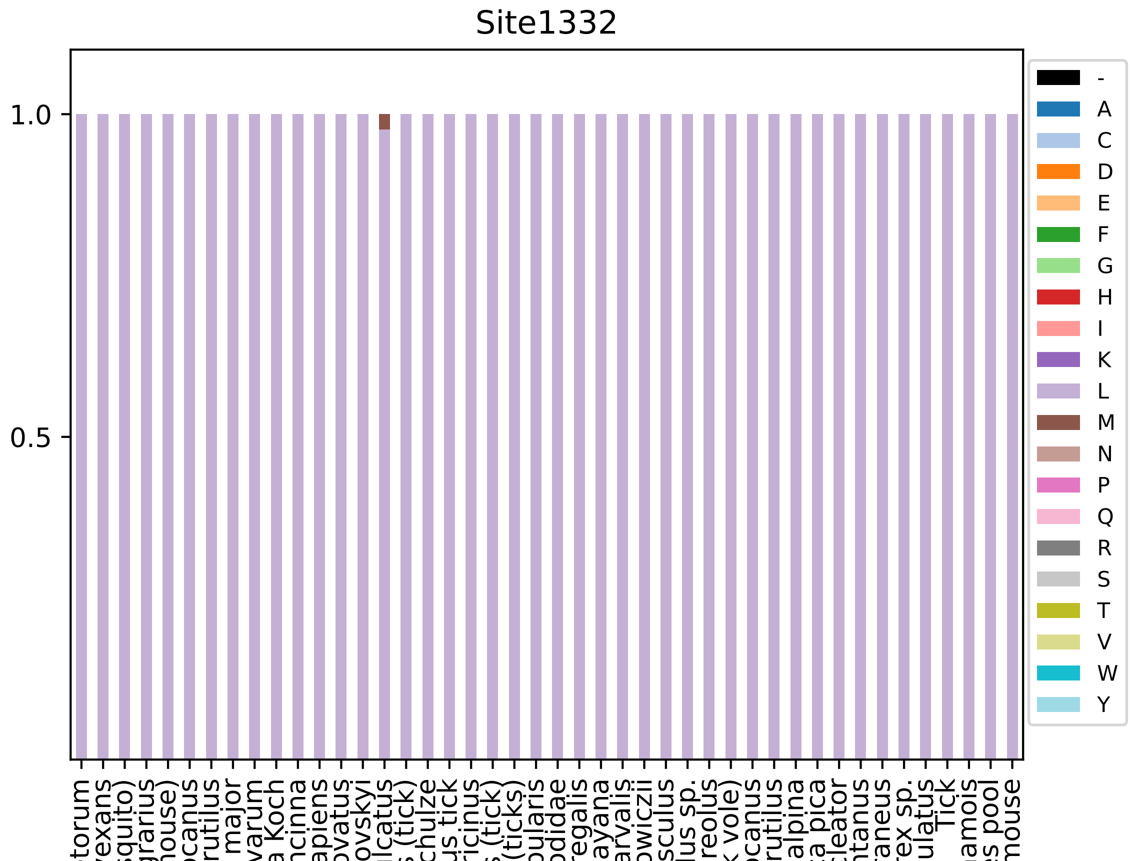
Site1332
1.0
0.5
-
A
C
D
E
F
G
H
I
K
L
M
N
P
Q
R
S
T
V
W
Y

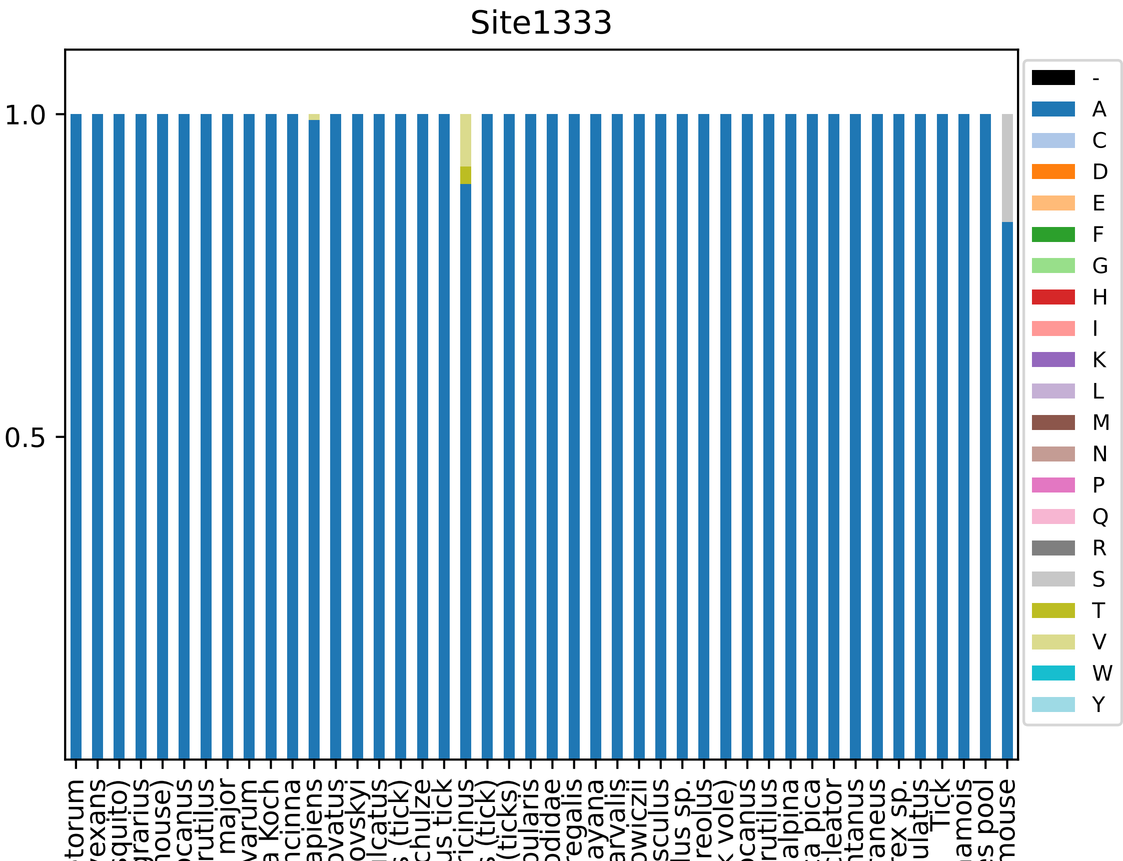

Site1333
1.0
0.5
-
A
C
D
E
F
G
H
I
K
L
M
N
P
Q
R
S
T
V
W
Y

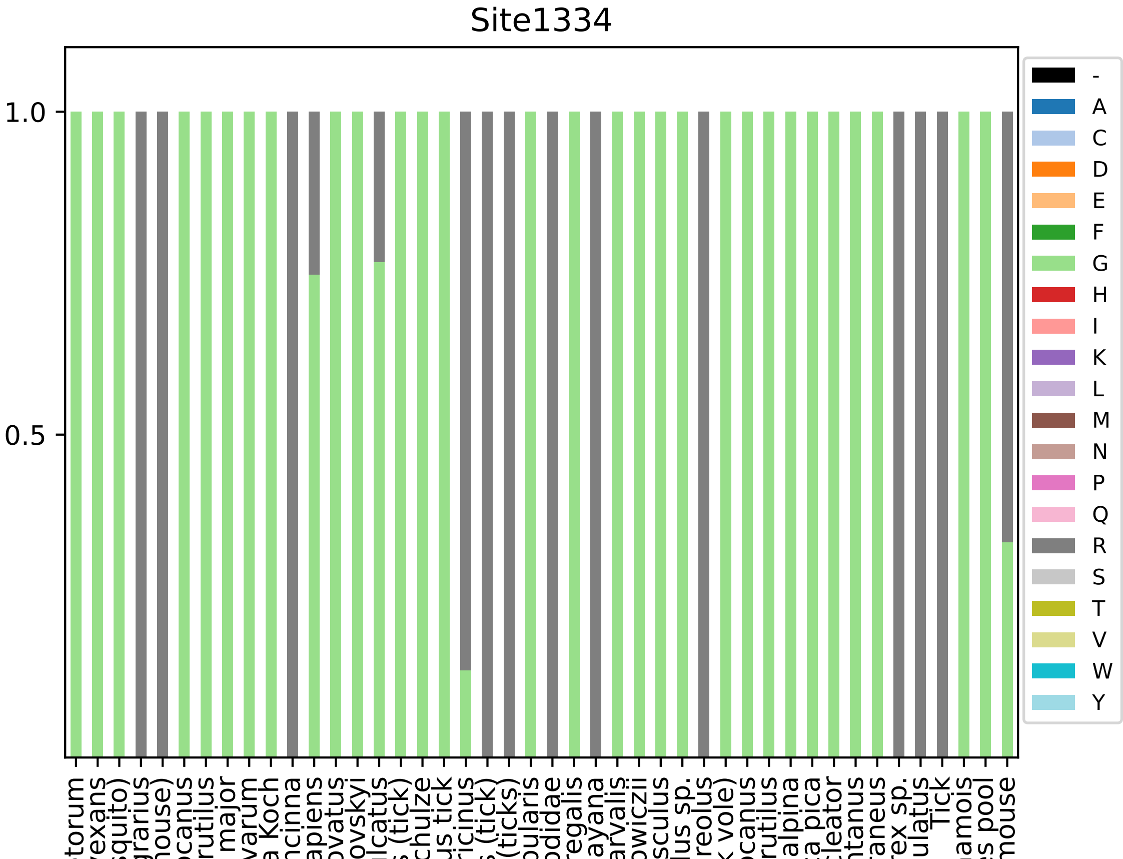
Site1334
1.0
0.5
-
A
C
D
E
F
G
H
I
K
L
M
N
P
Q
R
S
T
V
W
Y

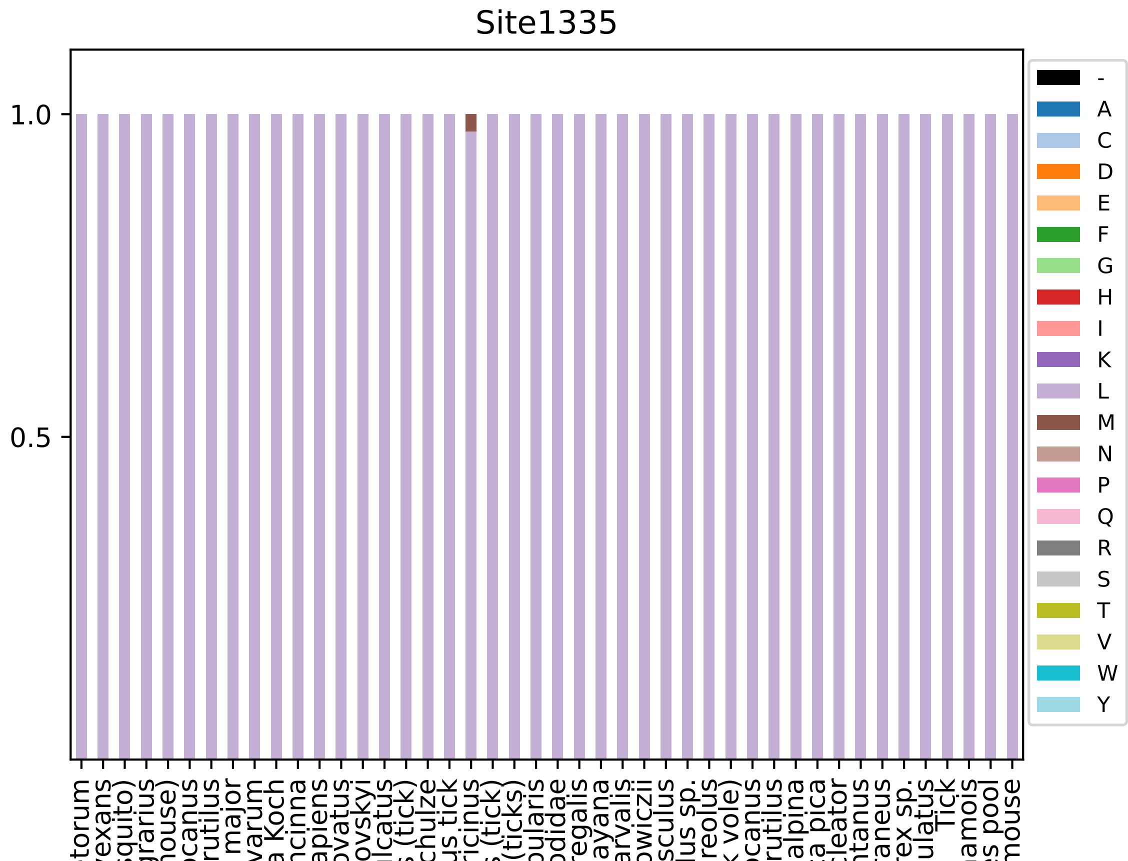
Site1335
1.0
0.5
-
A
C
D
E
F
G
H
I
K
L
M
N
P
Q
R
S
T
V
W
Y
Tick

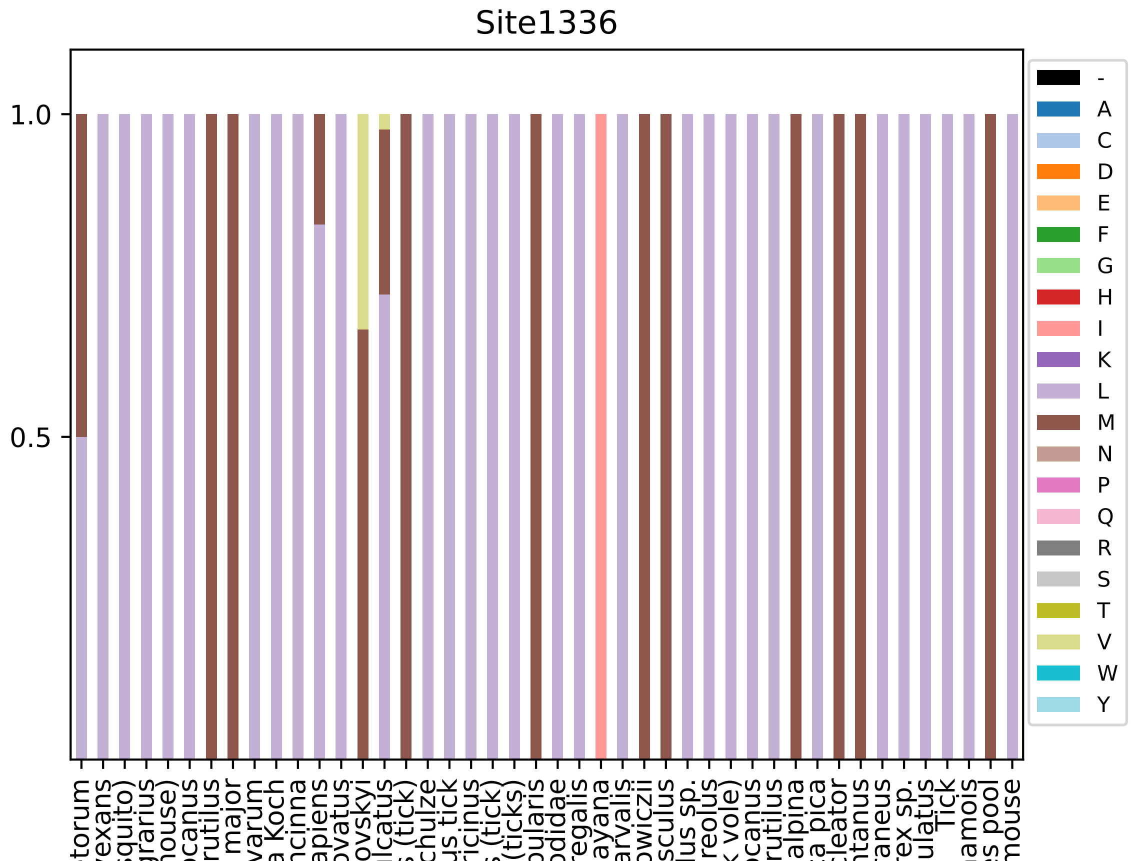
Site1336
1.0
0.5
-
A
C
D
E
F
G
H
I
K
L
M
N
P
Q
R
S
T
V
W
Y

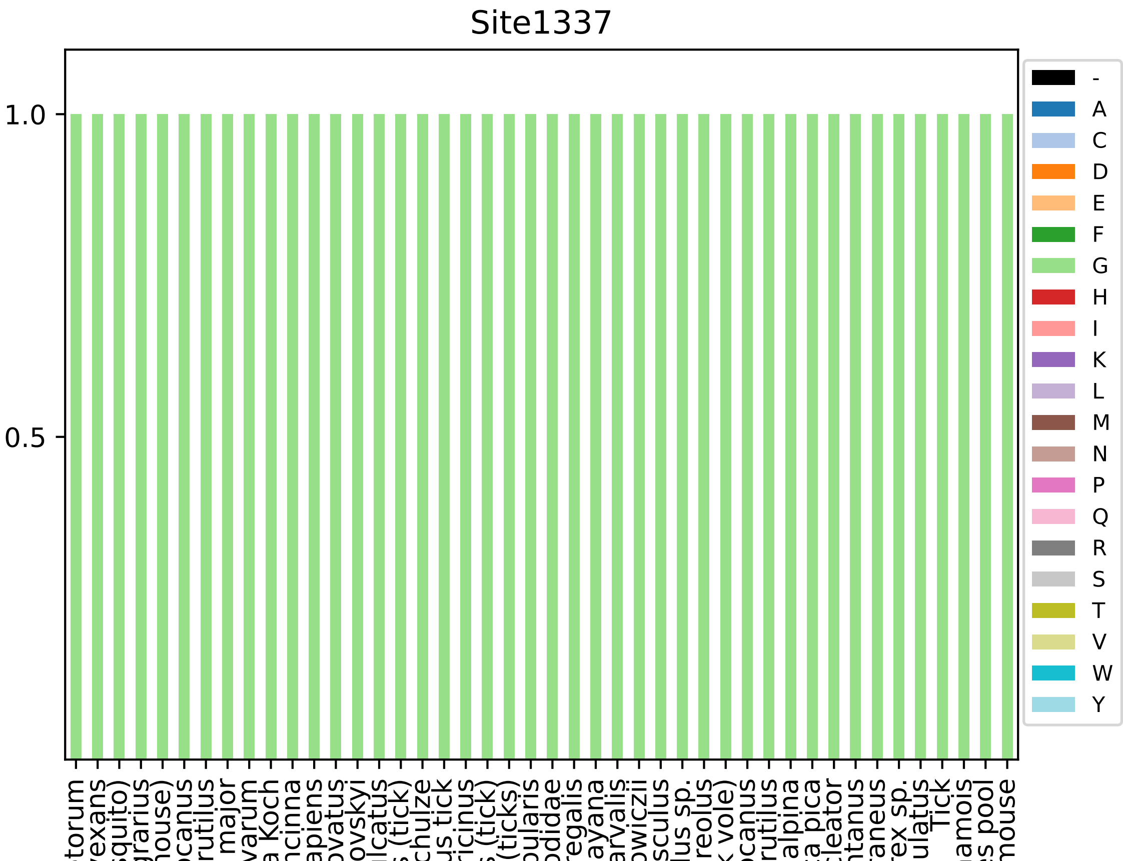
Site1337
1.0
0.5
-
A
C
D
E
F
G
H
I
K
L
M
N
P
Q
R
S
T
V
W
Y

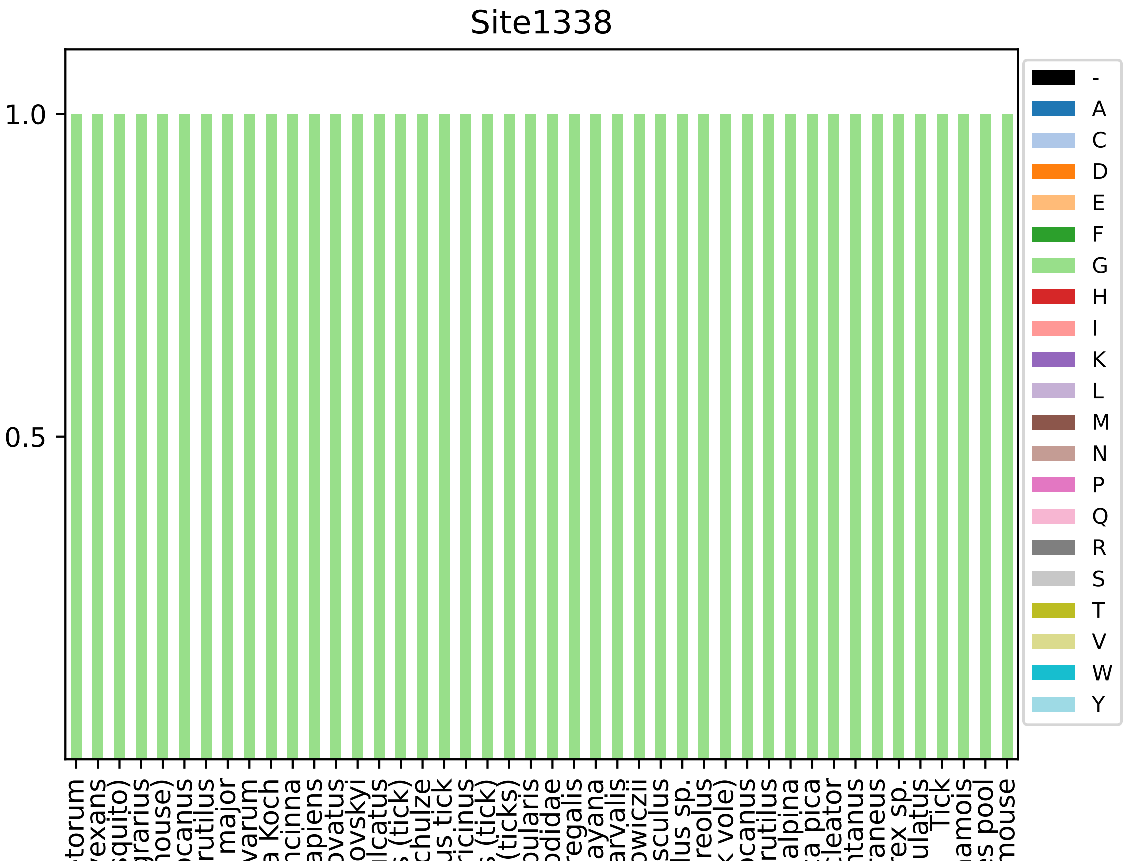
Site1338
1.0
0.5
-
A
C
D
E
F
G
H
I
K
L
M
N
P
Q
R
S
T
V
W
Y

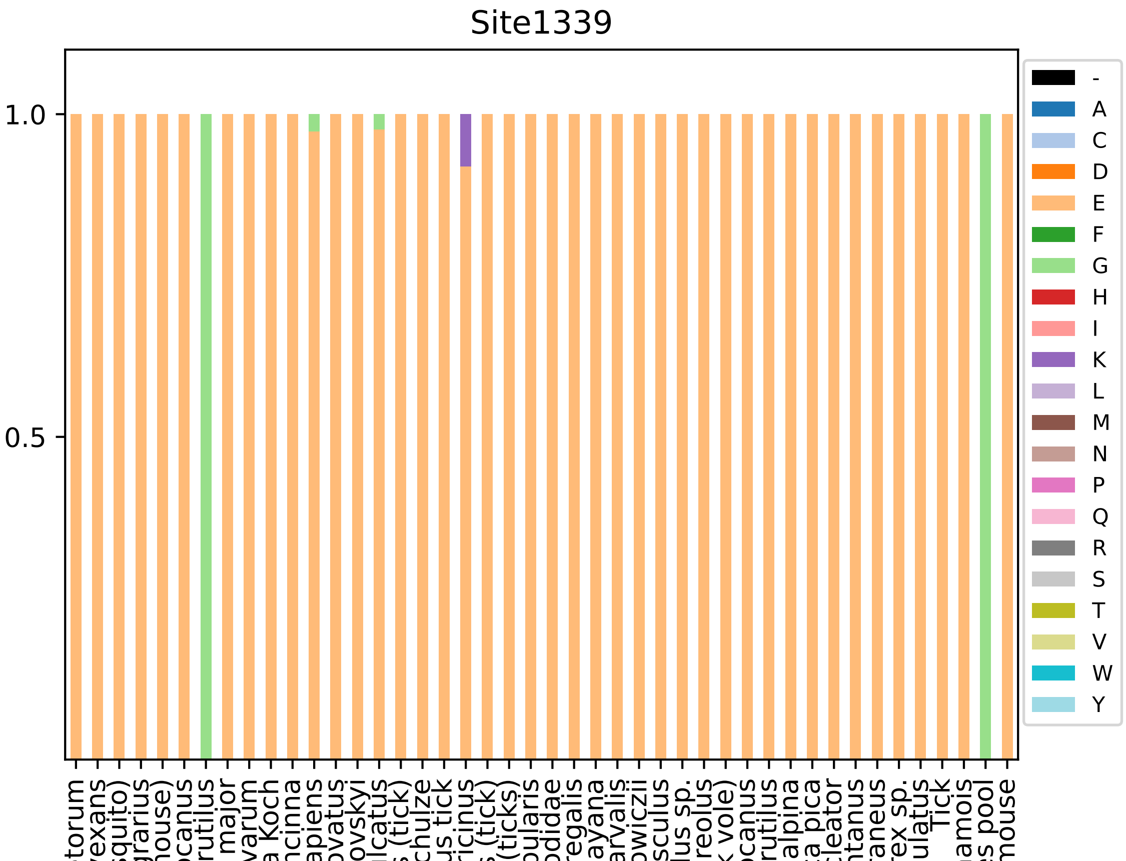
Site1339
1.0
0.5
-
A
C
D
E
F
G
H
I
K
L
M
N
P
Q
R
S
T
V
W
Y

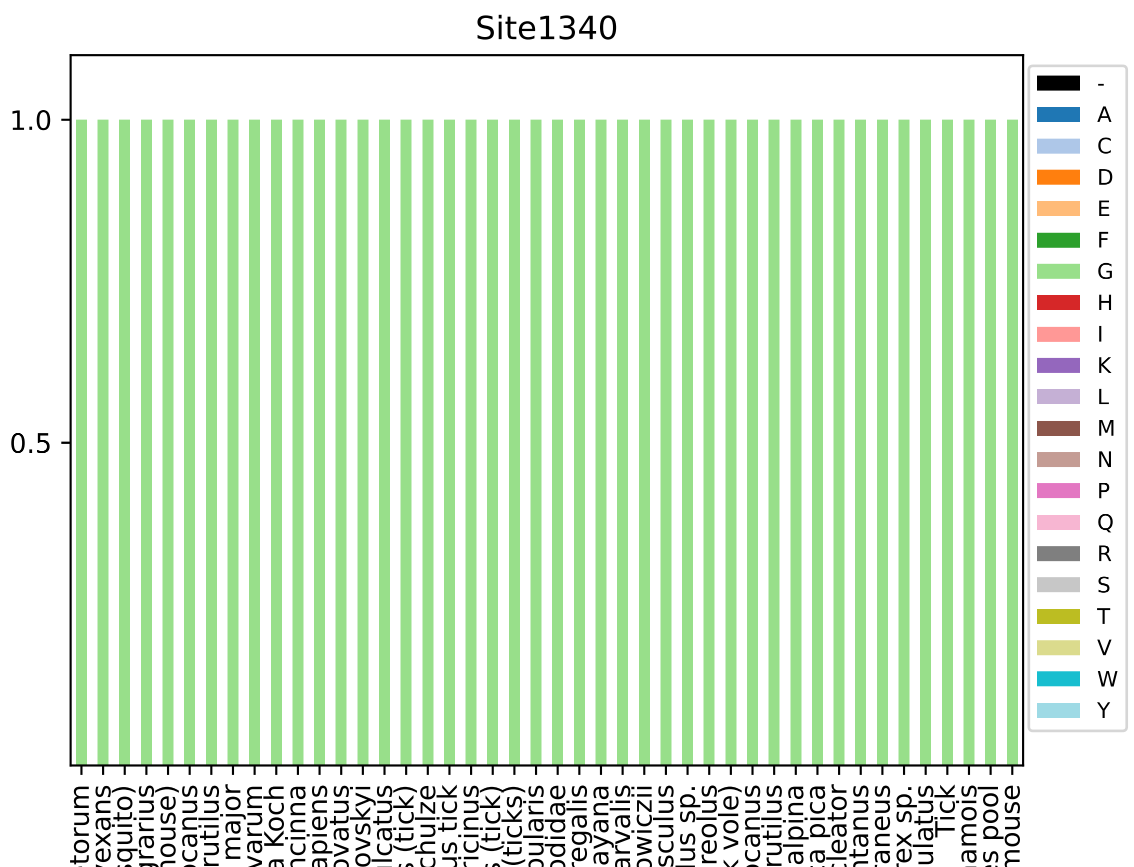
Site1340
1.0
0.5
-
A
C
D
E
F
G
H
I
K
L
M
N
P
Q
R
S
T
V
W
Y

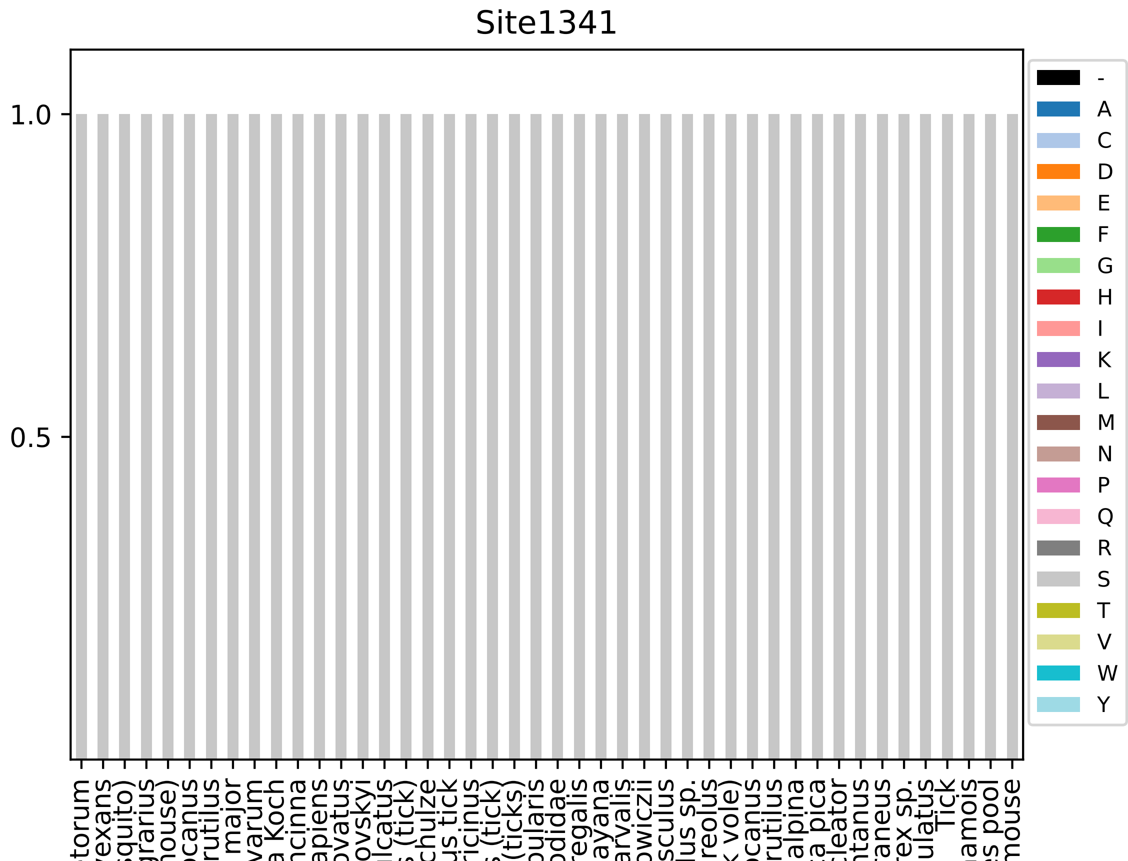
Site1341
1.0
0.5
-
A
C
D
E
F
G
H
I
K
L
M
N
P
Q
R
S
T
V
W
Y

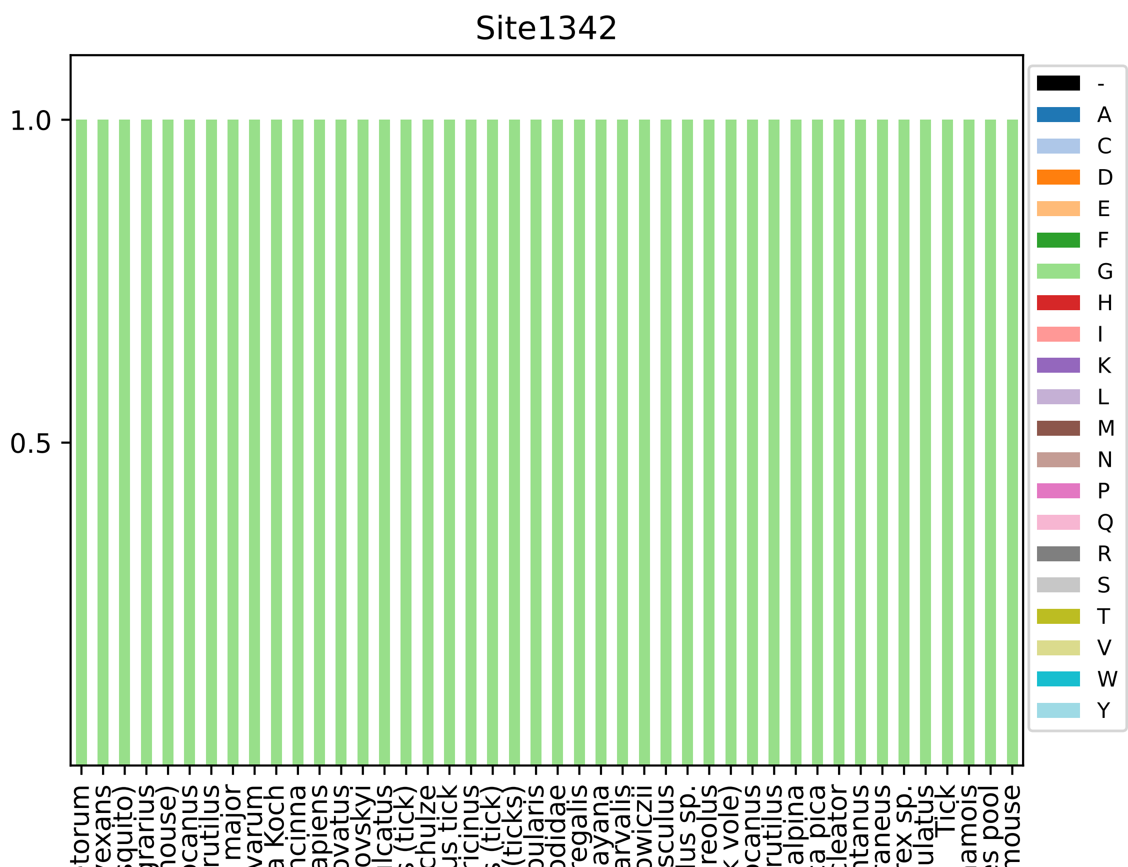
Site1342
1.0
0.5
-
A
C
D
E
F
G
H
I
K
L
M
N
P
Q
R
S
T
V
W
Y
Tick

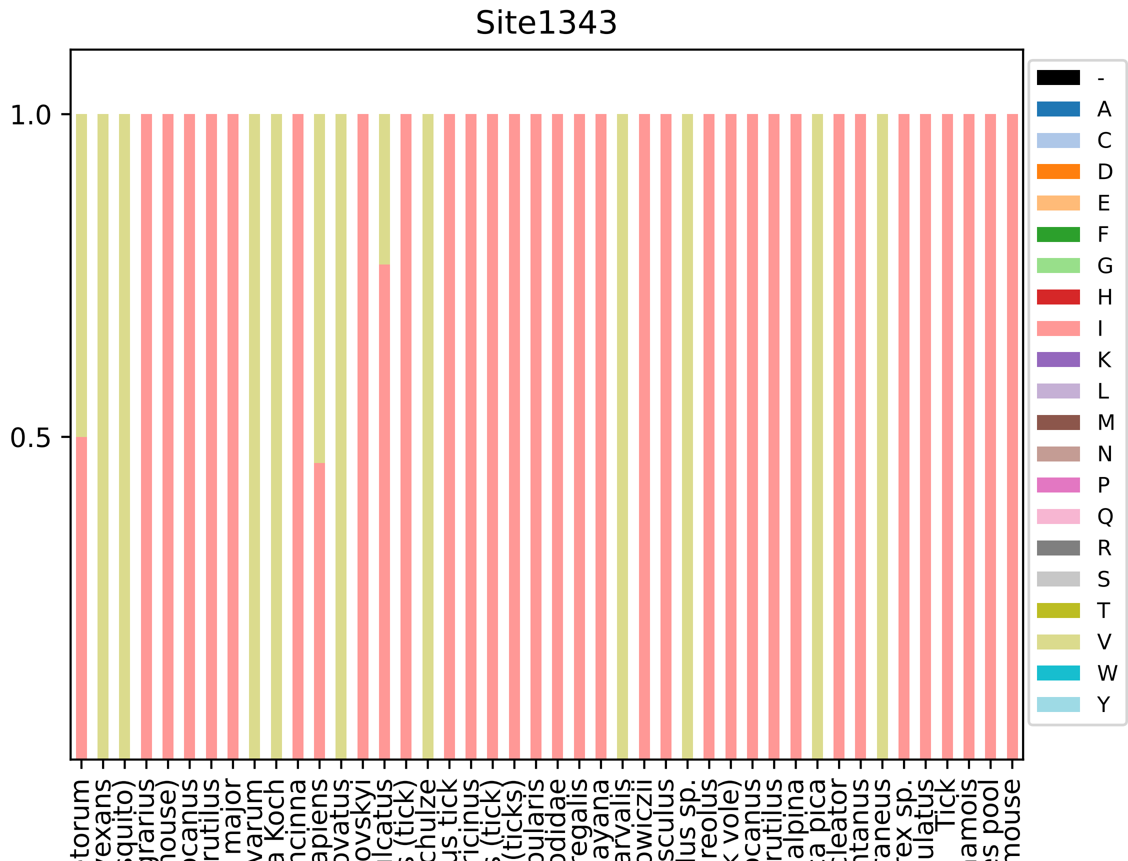

Site1343
1.0
0.5
-
A
C
D
E
F
G
H
I
K
L
M
N
P
Q
R
S
T
V
W
Y

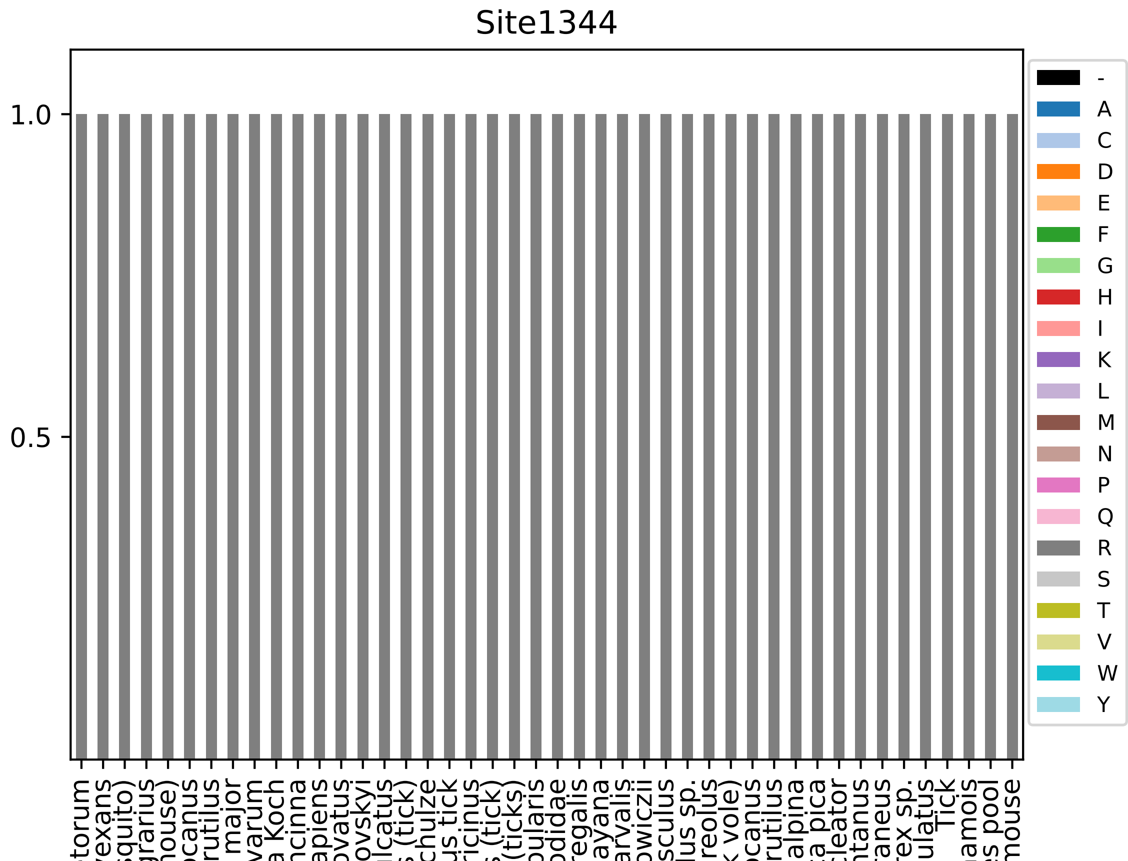
Site1344
1.0
0.5
-
A
C
D
E
F
G
H
I
K
L
M
N
P
Q
R
S
T
V
W
Y
Tick

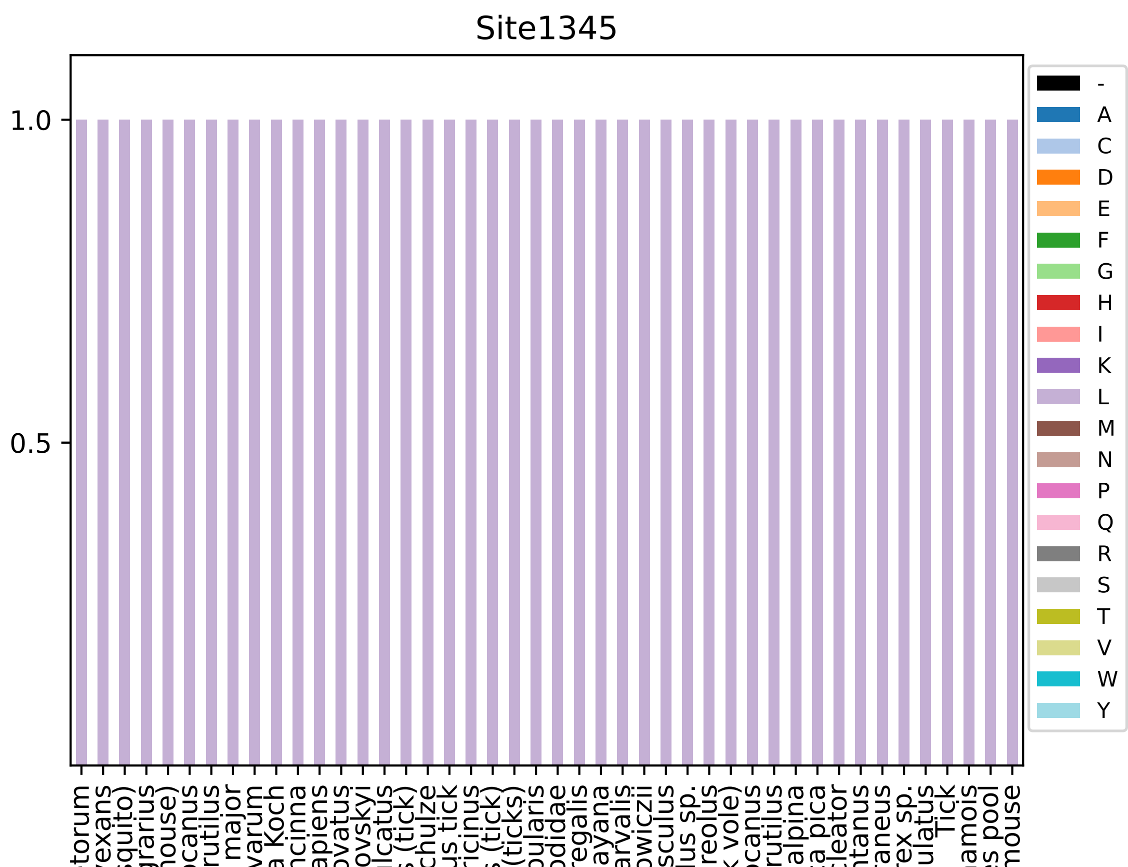

Site1345
1.0
0.5
-
A
C
D
E
F
G
H
I
K
L
M
N
P
Q
R
S
T
V
W
Y

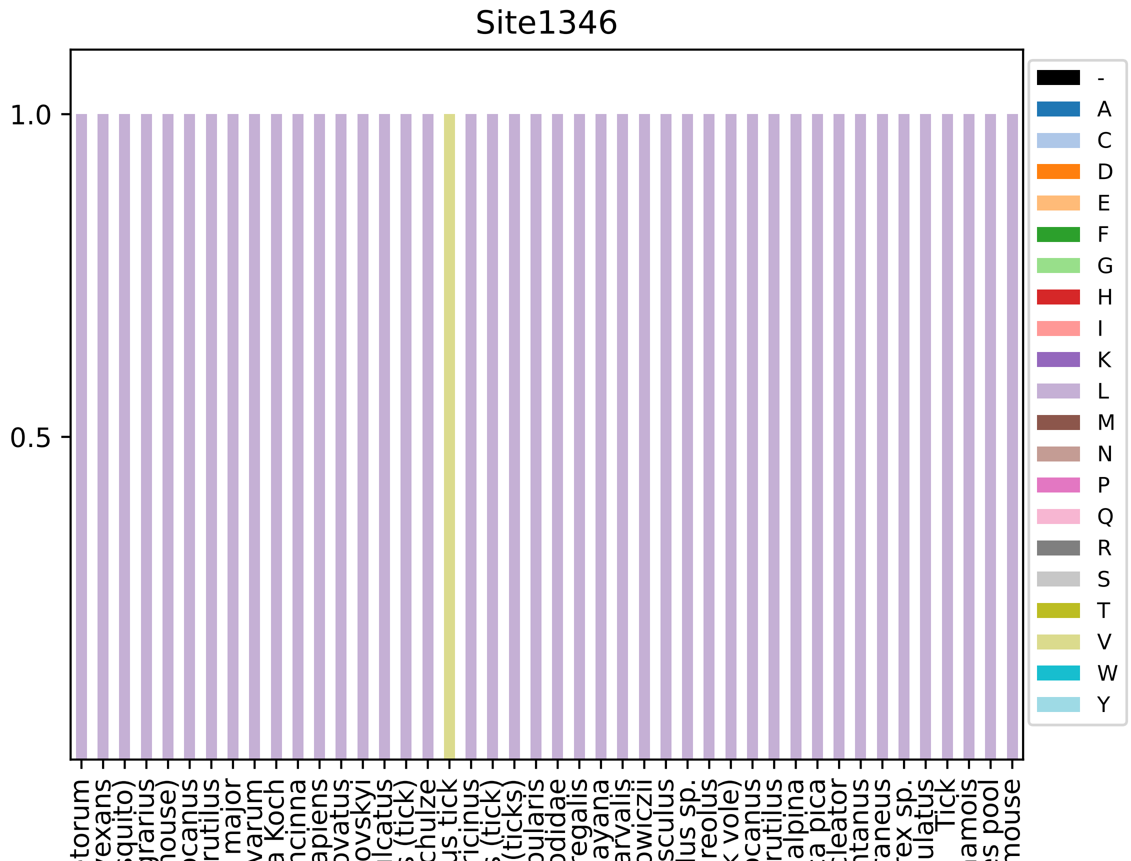
Site1346
1.0
0.5
-
A
C
D
E
F
G
H
I
K
L
M
N
P
Q
R
S
T
V
W
Y

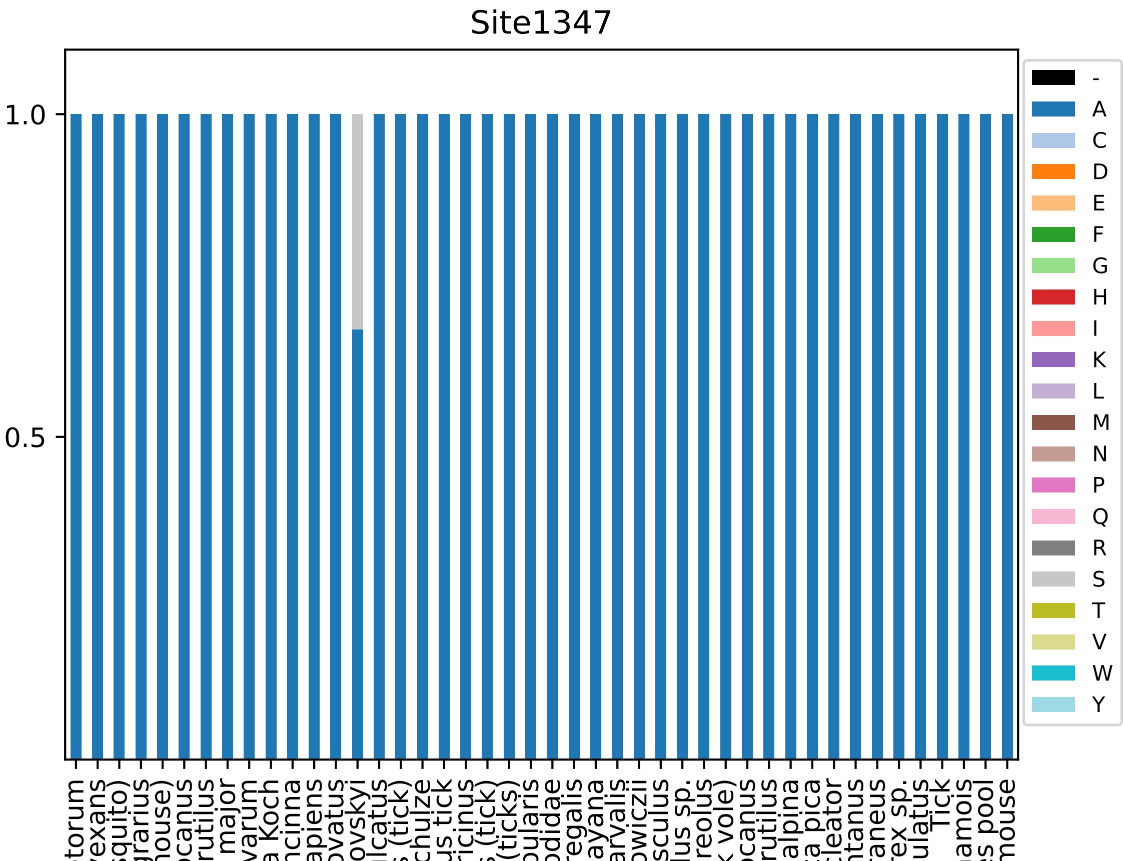
Site1347
1.0
0.5
-
A
C
D
E
F
G
H
I
K
L
M
N
P
Q
R
S
T
V
W
Y

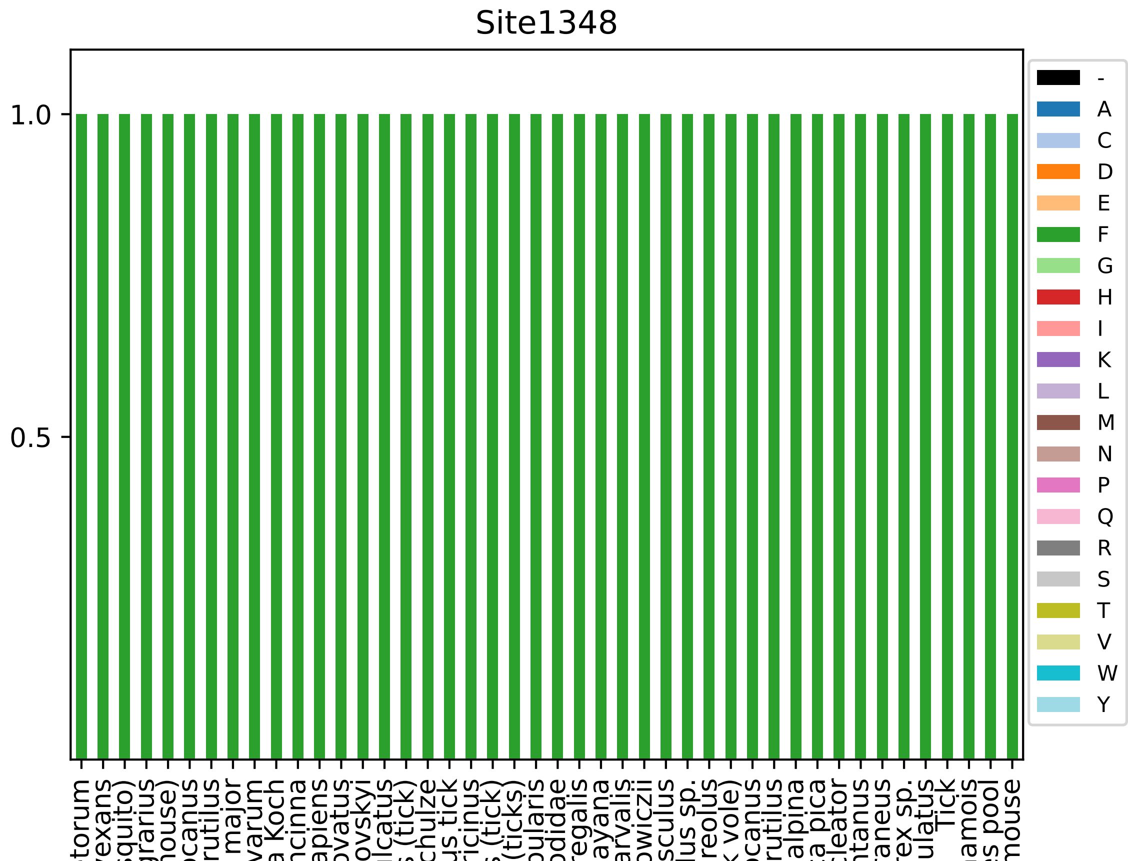
Site1348
1.0
0.5
-
A
C
D
E
F
G
H
I
K
L
M
N
P
Q
R
S
T
V
W
Y

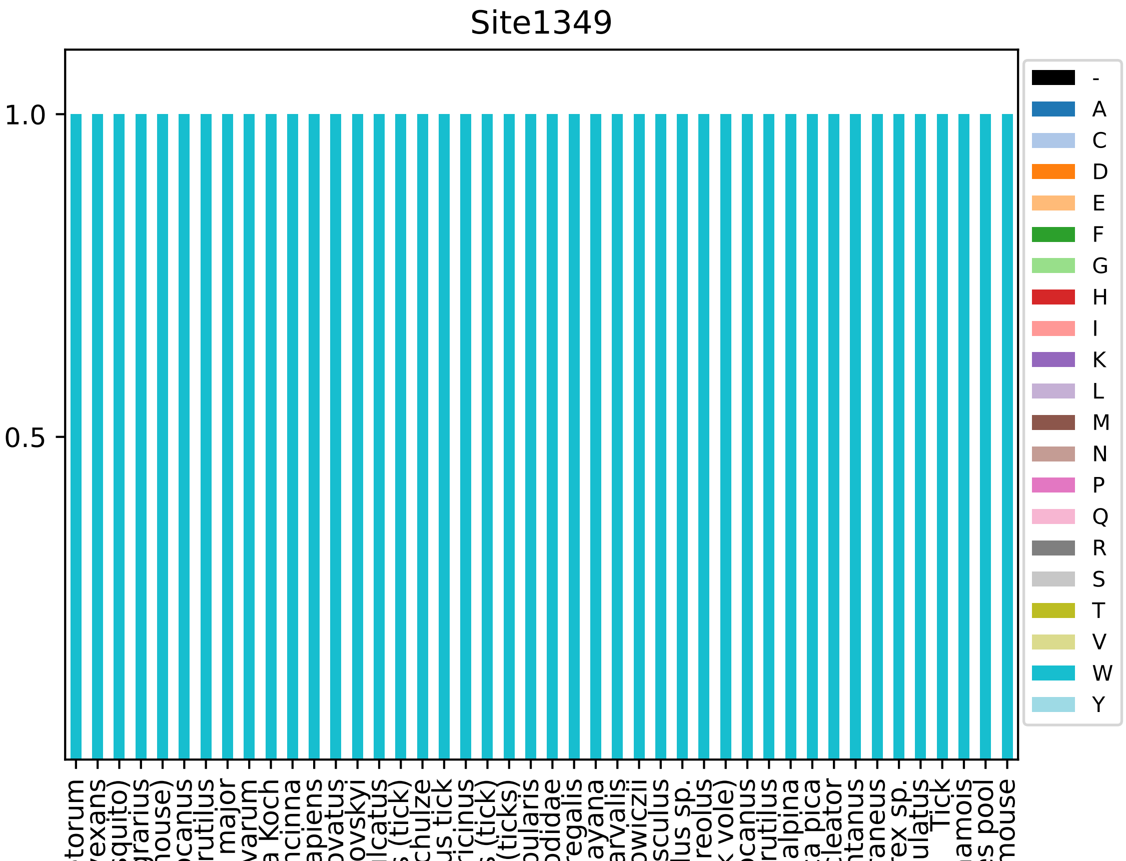

Site1349
1.0
0.5
-
A
C
D
E
F
G
H
I
K
L
M
N
P
Q
R
S
T
V
W
Y

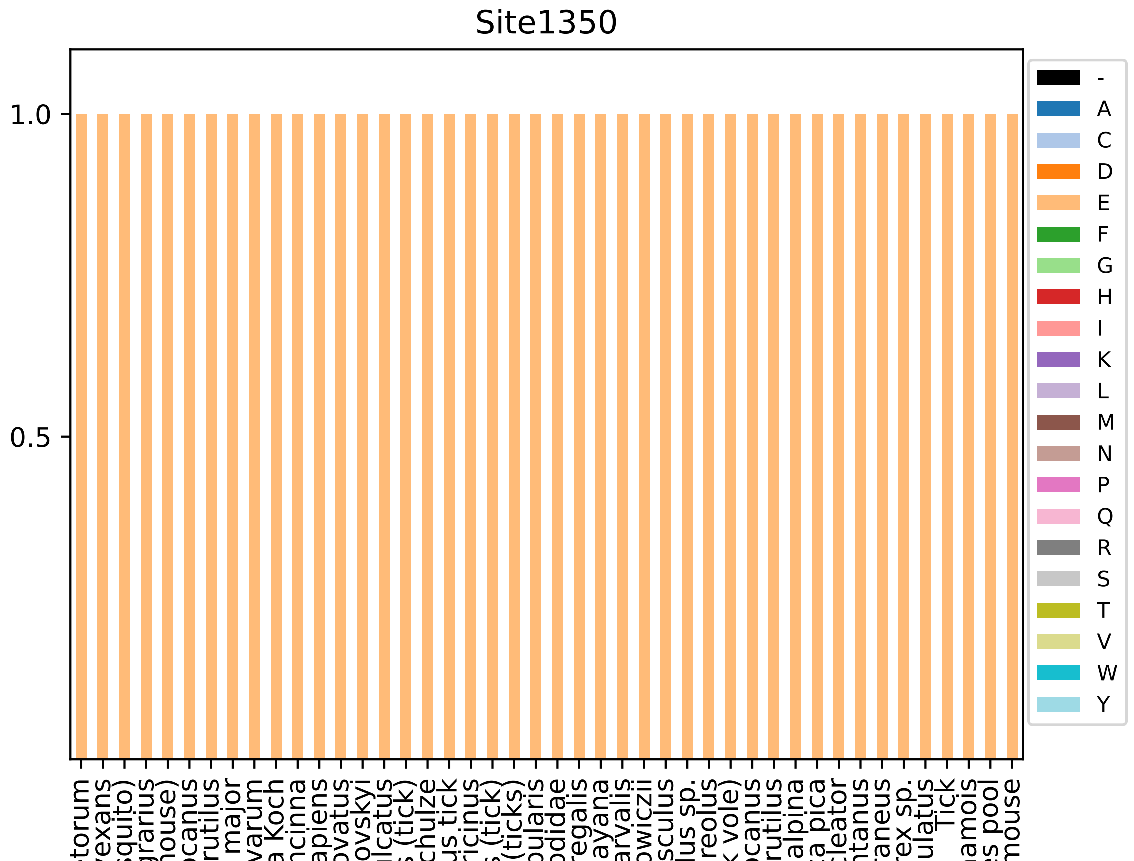

Site1350
1.0
0.5
-
A
C
D
E
F
G
H
I
K
L
M
N
P
Q
R
S
T
V
W
Y

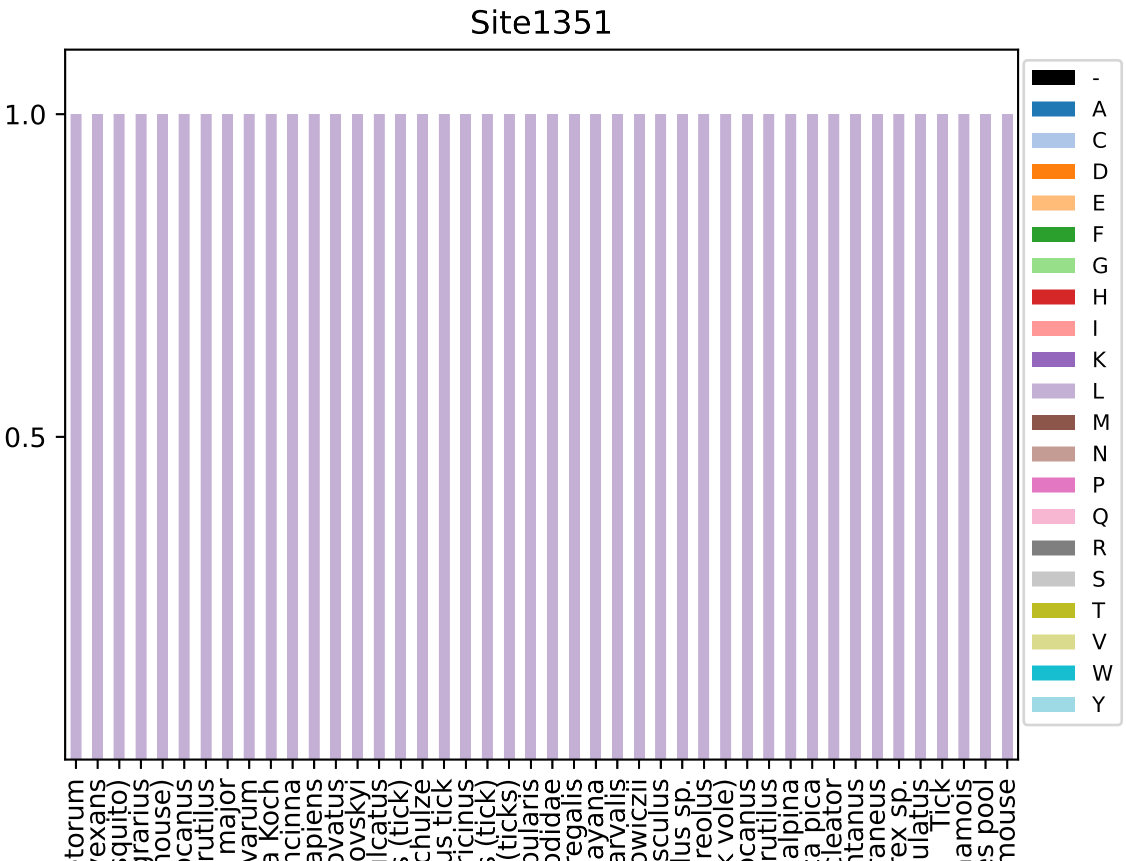
Site1351
1.0
0.5
-
A
C
D
E
F
G
H
I
K
L
M
N
P
Q
R
S
T
V
W
Y

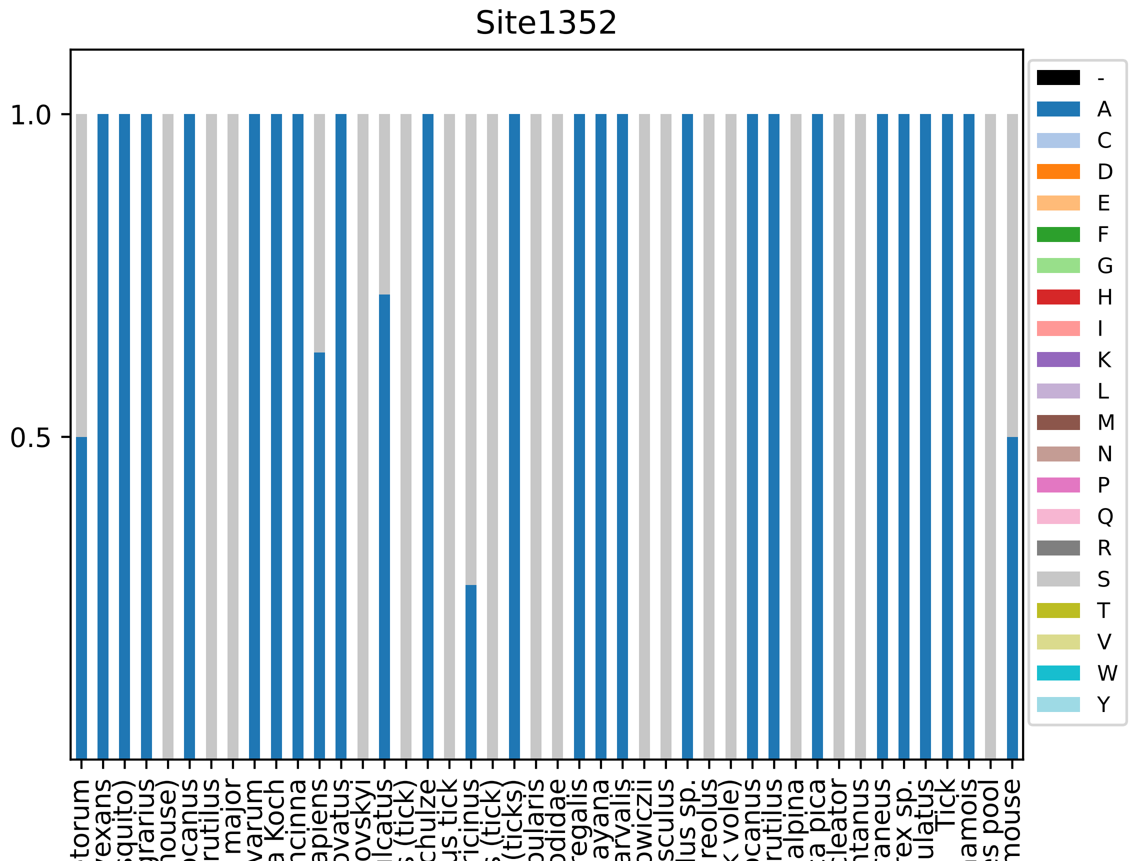
Site1352
1.0
0.5
-
A
C
D
E
F
G
H
I
K
L
M
N
P
Q
R
S
T
V
W
Y

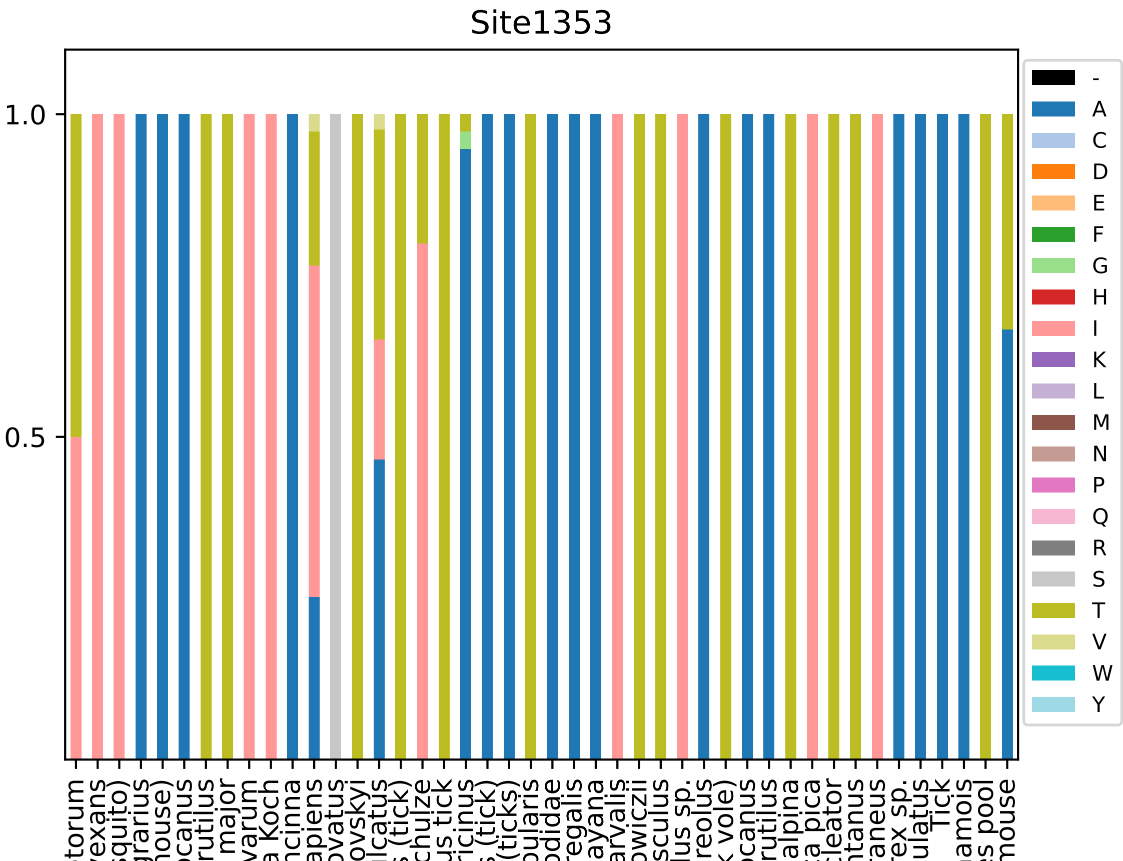
Site1353
1.0
0.5
-
A
C
D
E
F
G
H
I
K
L
M
N
P
Q
R
S
T
V
W
Y

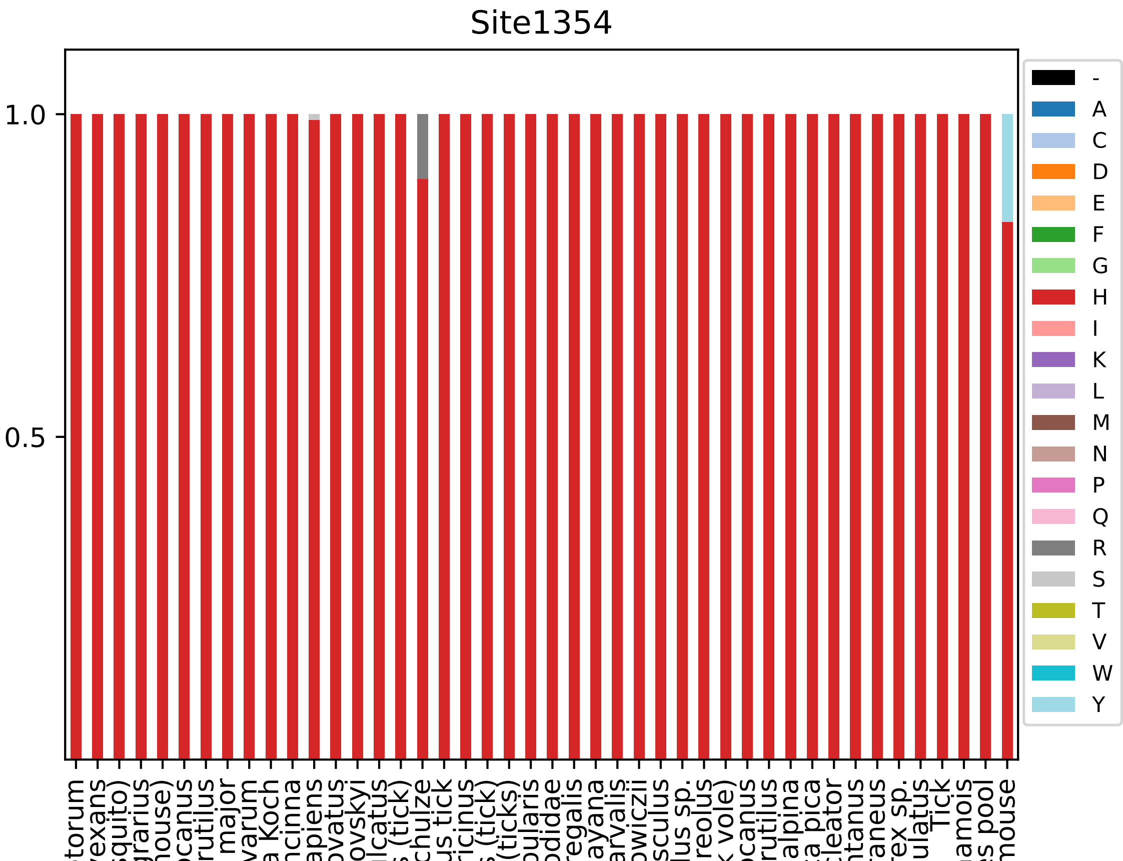
Site1354
1.0
0.5
-
A
C
D
E
F
G
H
I
K
L
M
N
P
Q
R
S
T
V
W
Y

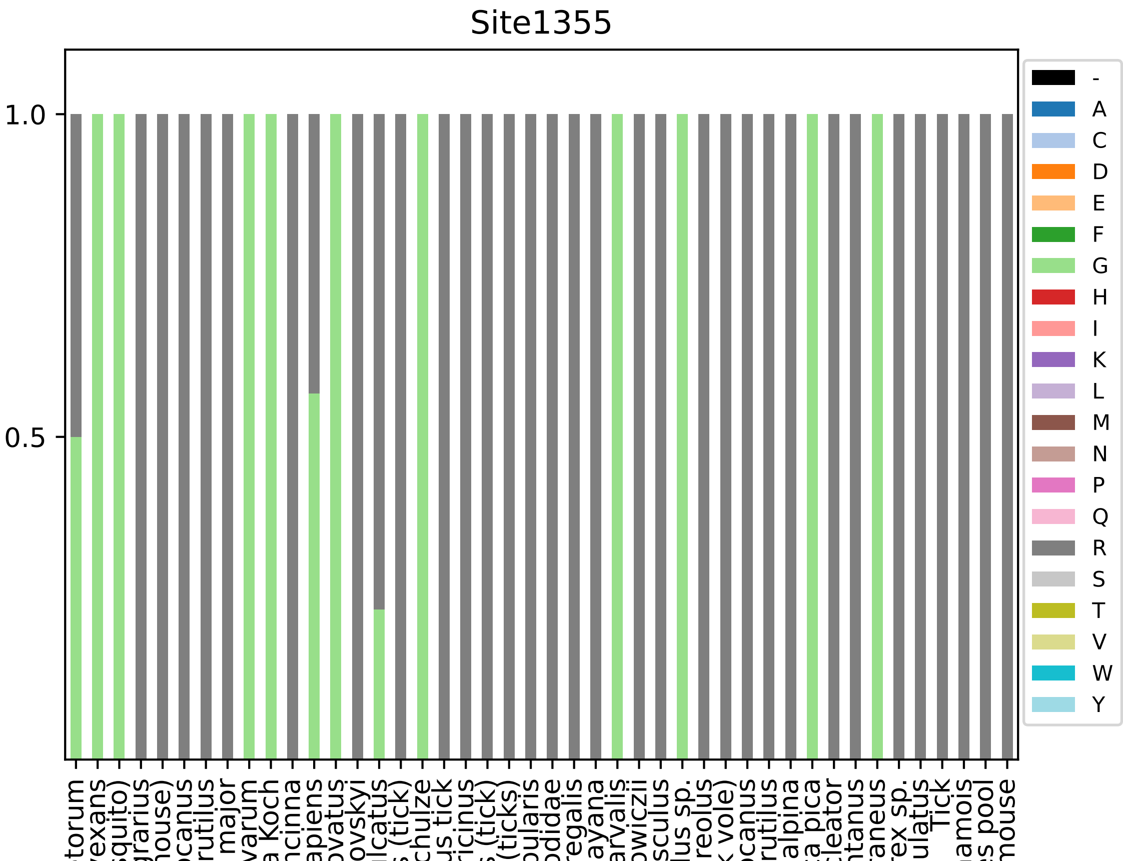

Site1355
1.0
0.5
-
A
C
D
E
F
G
H
I
K
L
M
N
P
Q
R
S
T
V
W
Y

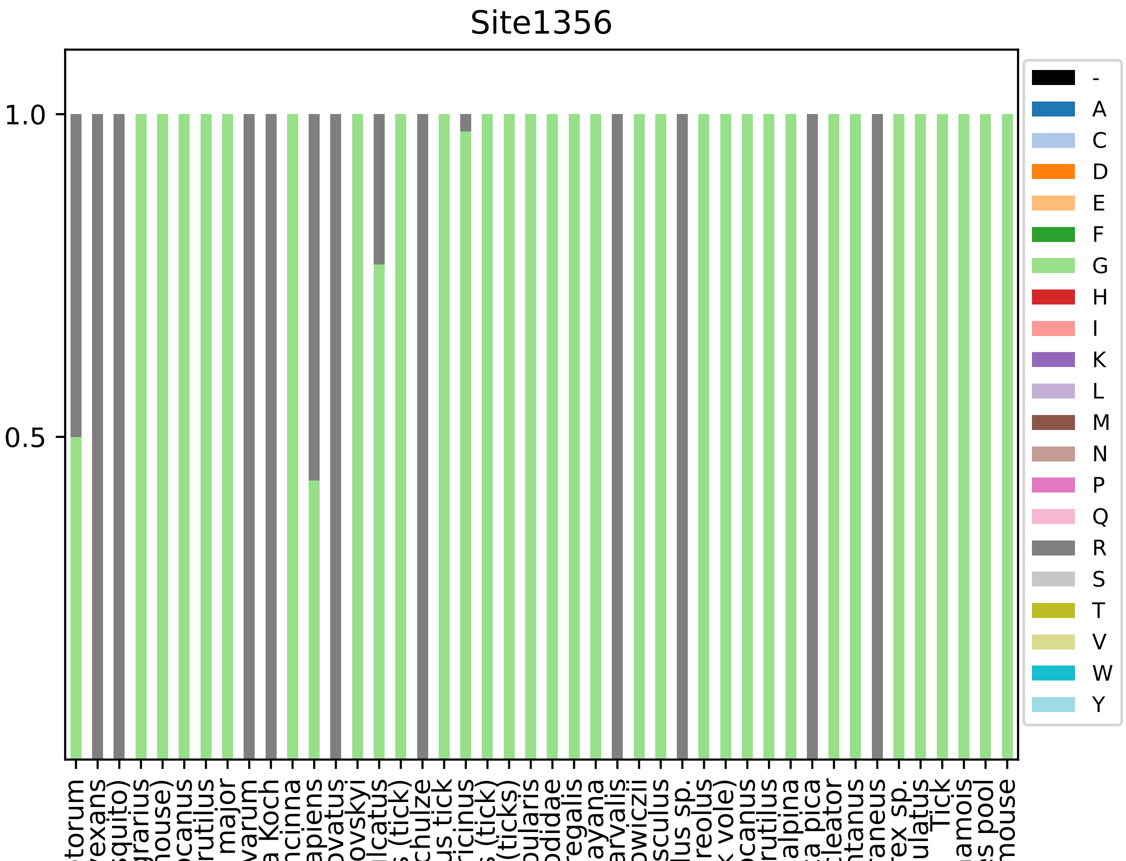

Site1356
1.0
0.5
-
A
C
D
E
F
G
H
I
K
L
M
N
P
Q
R
S
T
V
W
Y

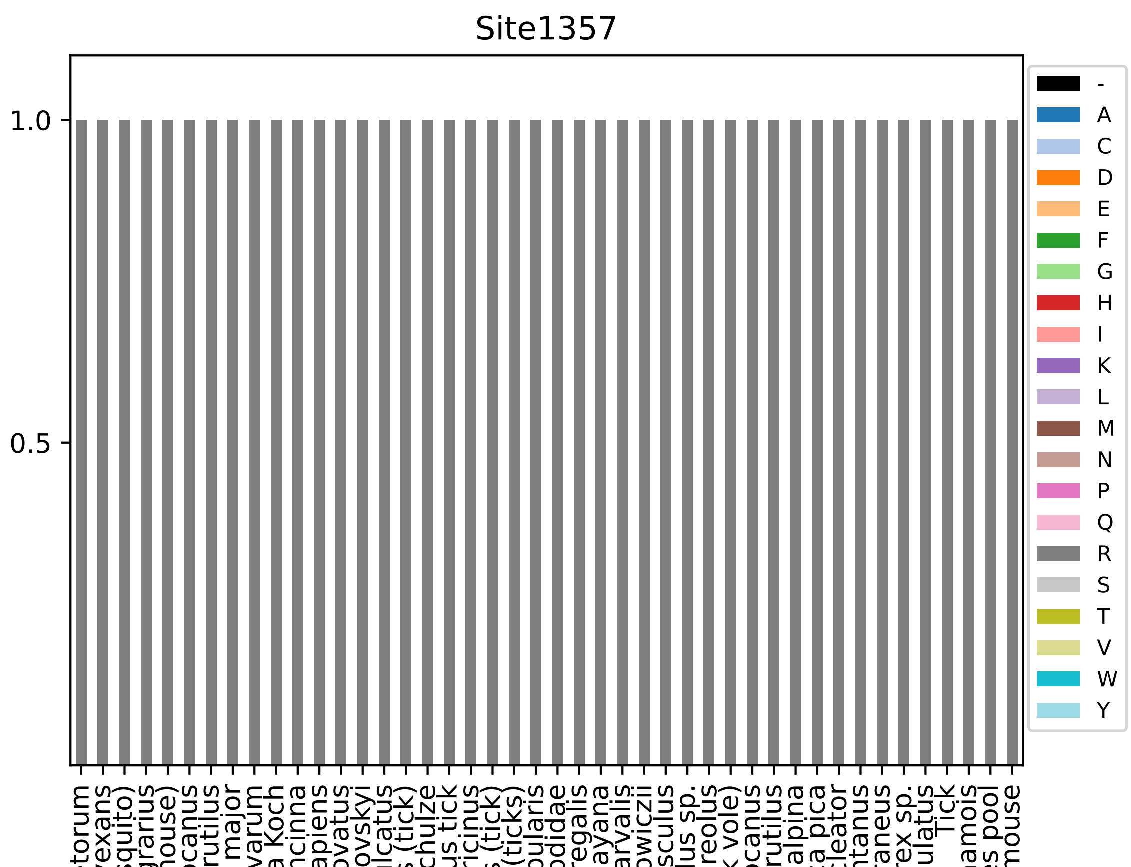
Site1357
1.0
0.5
-
A
C
D
E
F
G
H
I
K
L
M
N
P
Q
R
S
T
V
W
Y

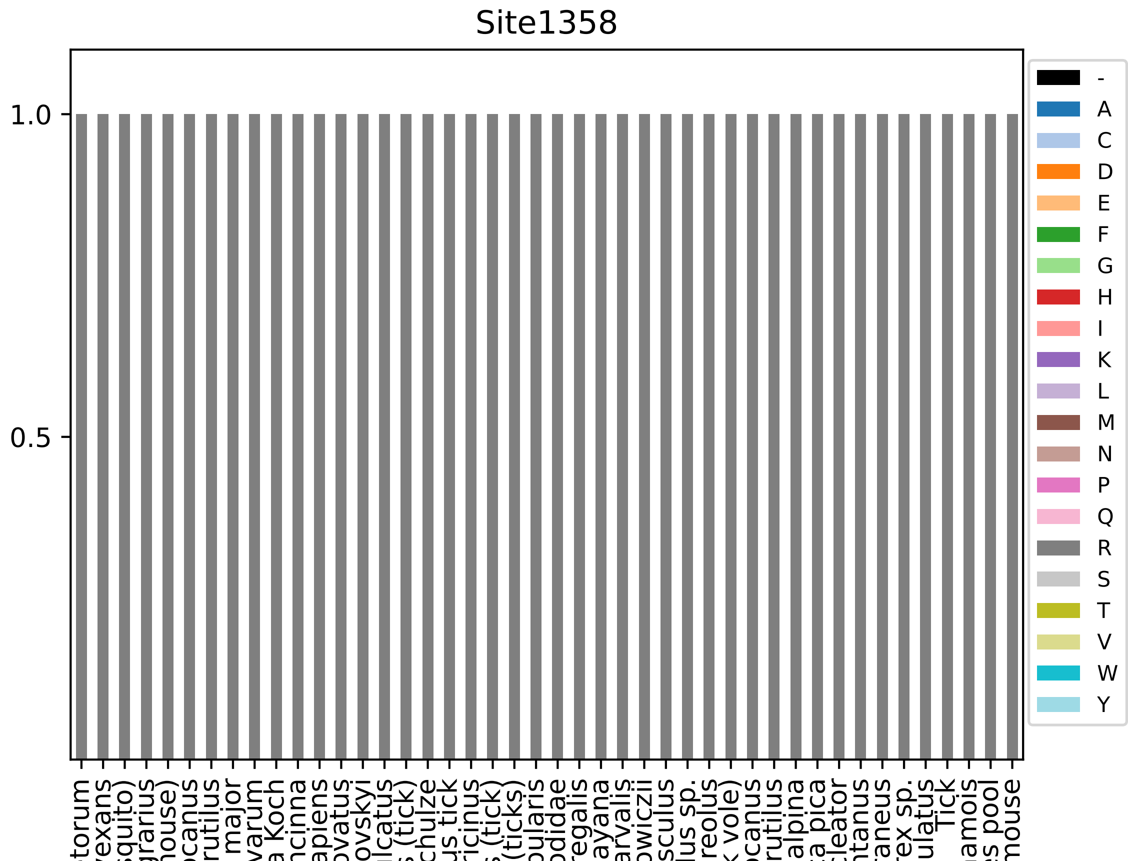
Site1358
1.0
0.5
-
A
C
D
E
F
G
H
I
K
L
M
N
P
Q
R
S
T
V
W
Y

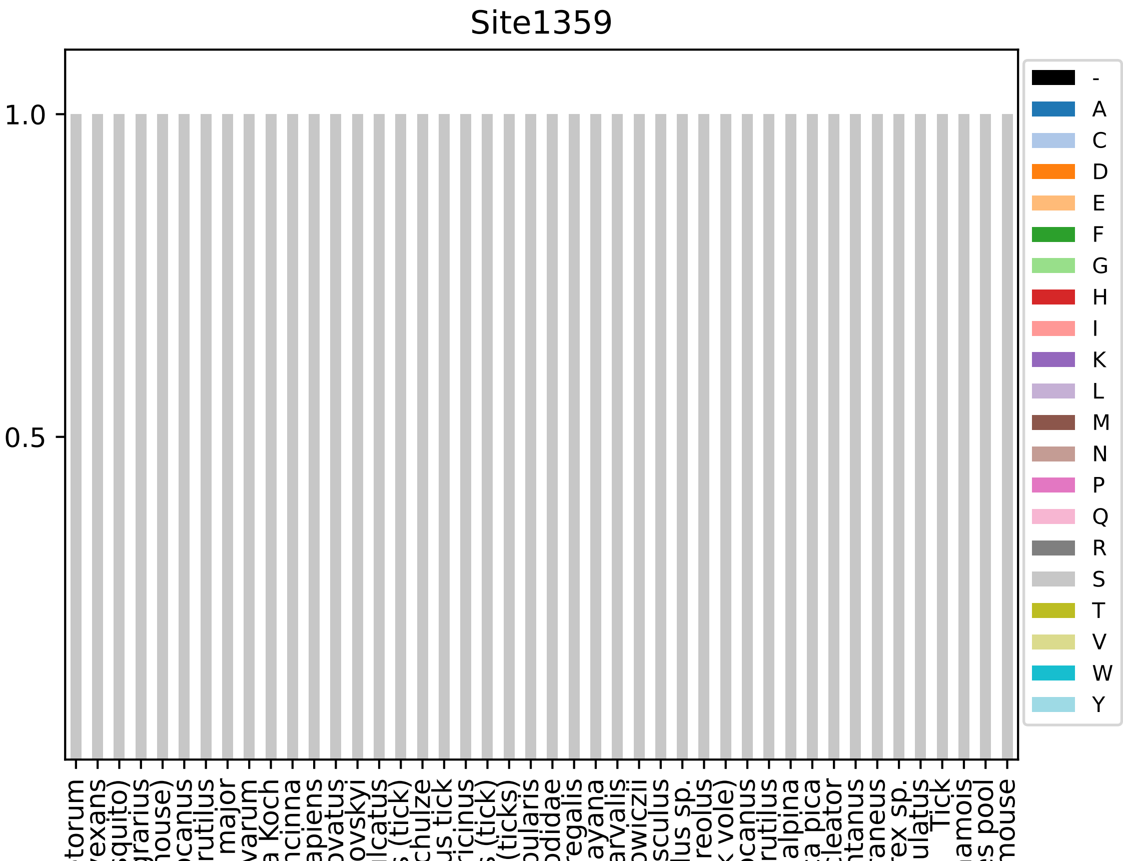

Site1359
1.0
0.5
-
A
C
D
E
F
G
H
I
K
L
M
N
P
Q
R
S
T
V
W
Y

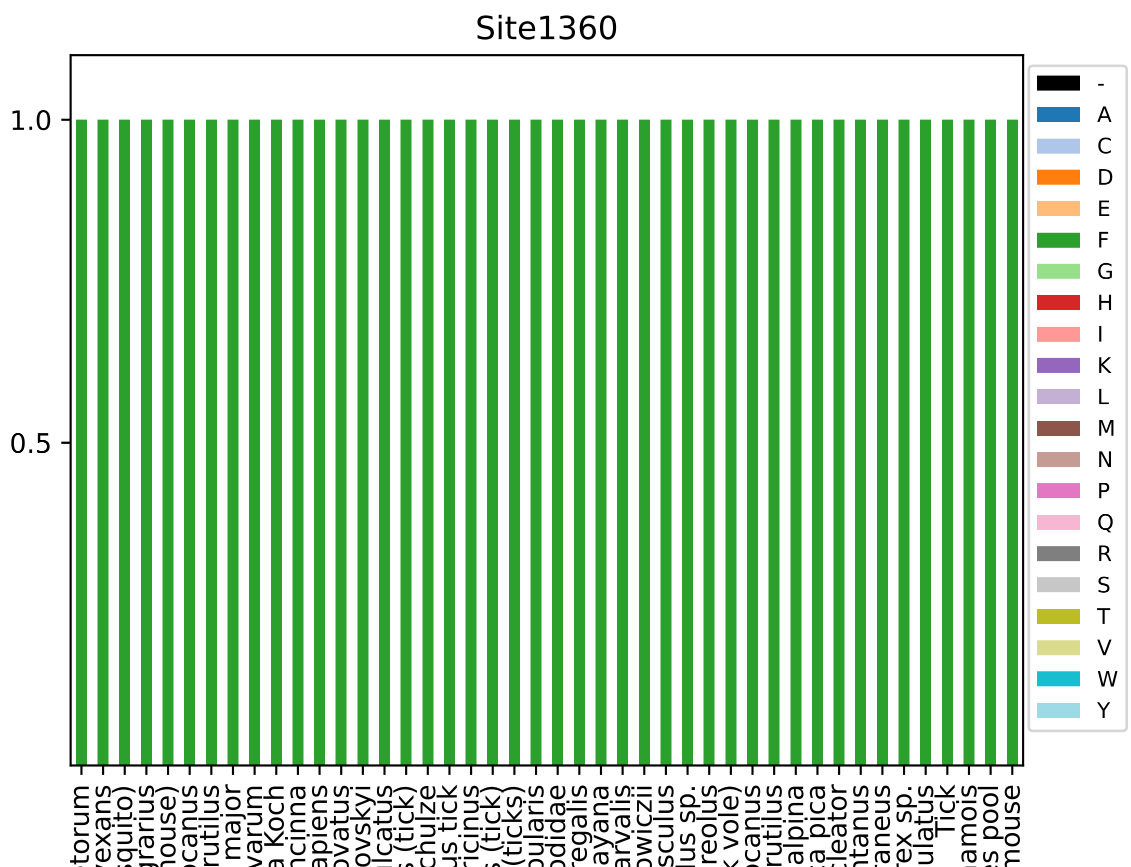
Site1360
1.0
0.5
-
A
C
D
E
F
G
H
I
K
L
M
N
P
Q
R
S
T
V
W
Y

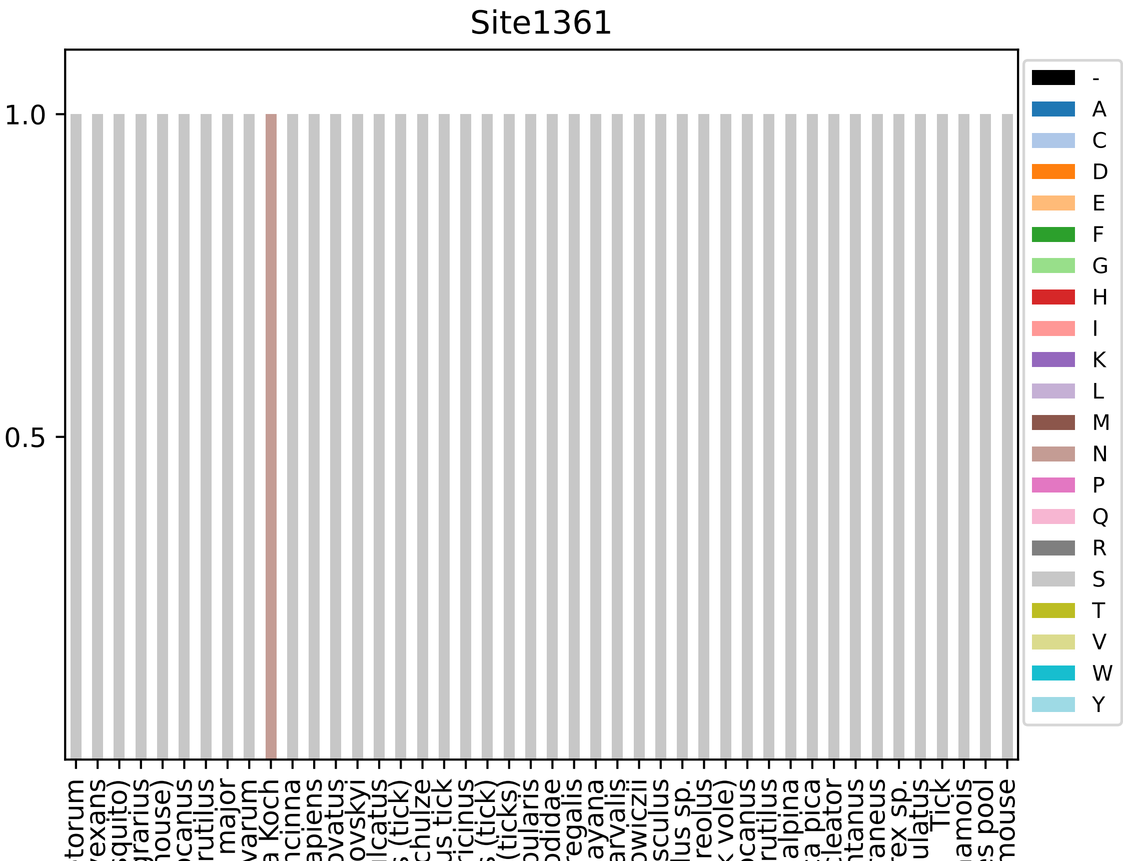
Site1361
1.0
0.5
-
A
C
D
E
F
G
H
I
K
L
M
N
P
Q
R
S
T
V
W
Y

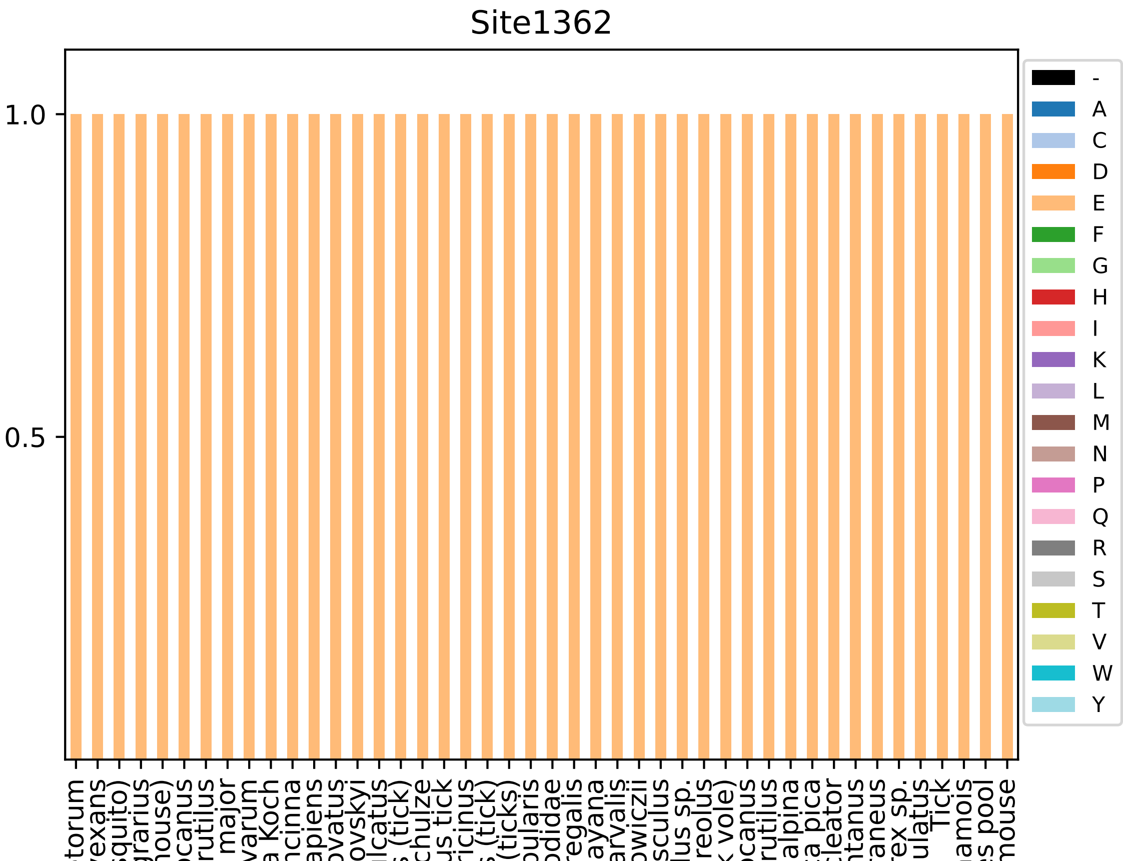
Site1362
1.0
0.5
-
A
C
D
E
F
G
H
I
K
L
M
N
P
Q
R
S
T
V
W
Y

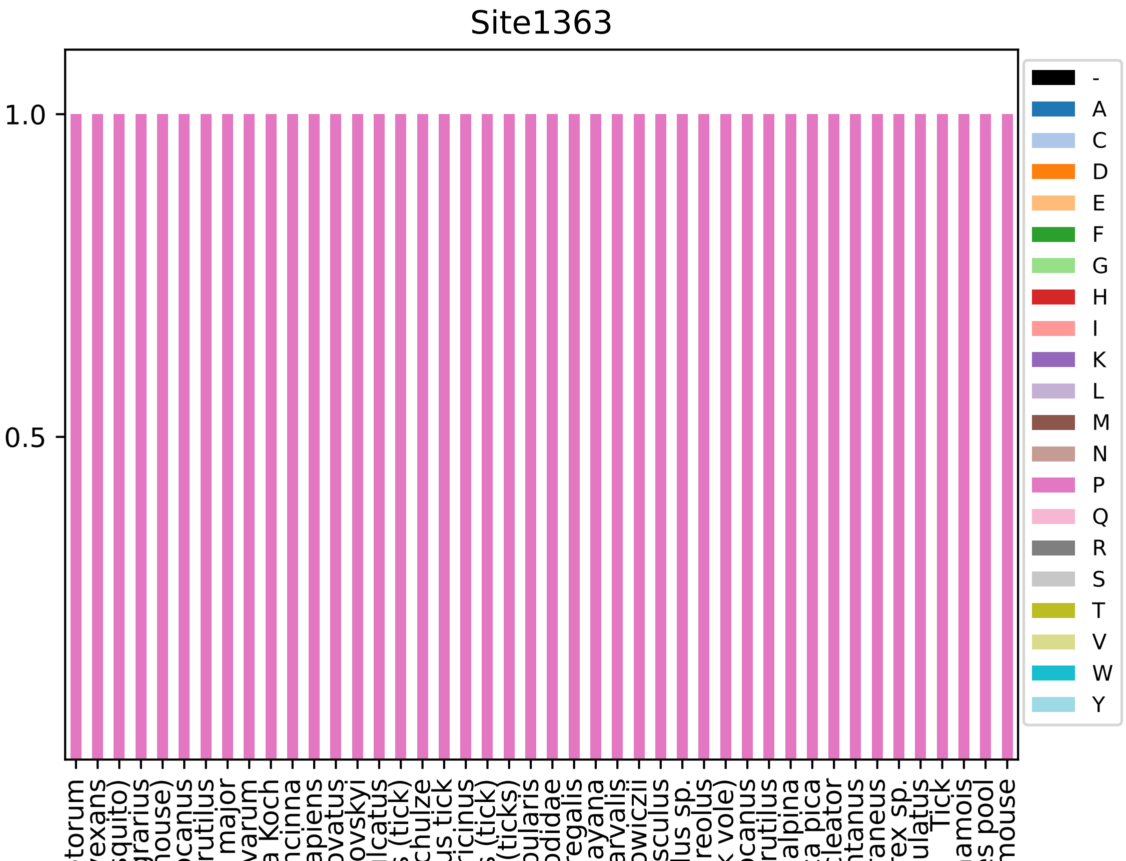
Site1363
1.0
0.5
-
A
C
D
E
F
G
H
I
K
L
M
N
P
Q
R
S
T
V
W
Y

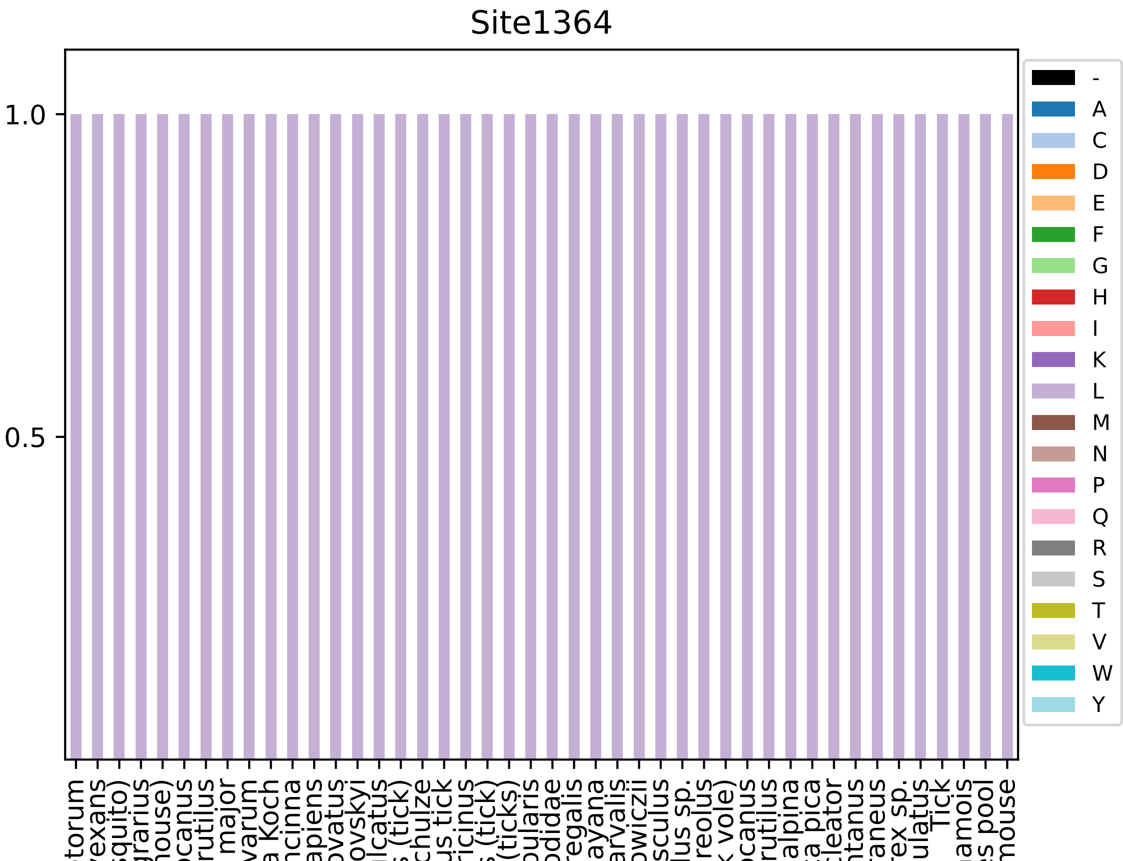
Site1364
1.0
0.5
-
A
C
D
E
F
G
H
I
K
L
M
N
P
Q
R
S
T
V
W
Y

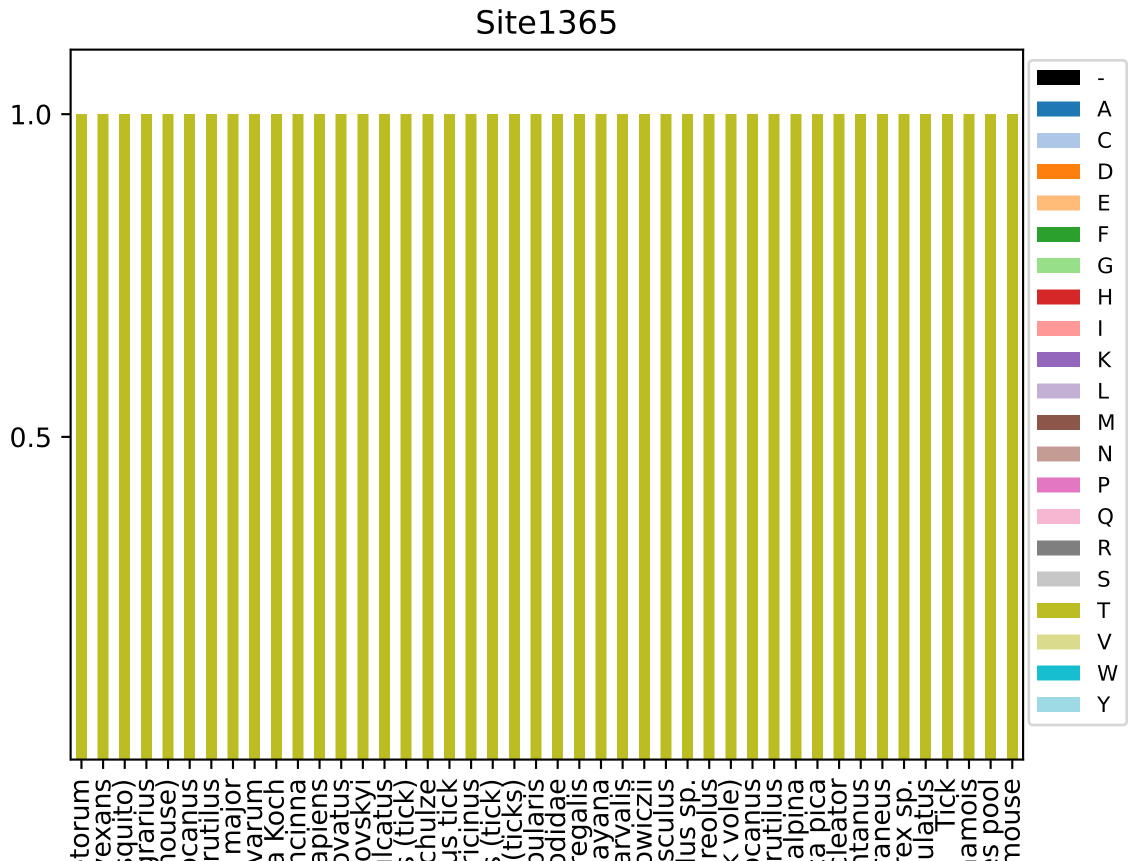

Site1365
1.0
0.5
-
A
C
D
E
F
G
H
I
K
L
M
N
P
Q
R
S
T
V
W
Y

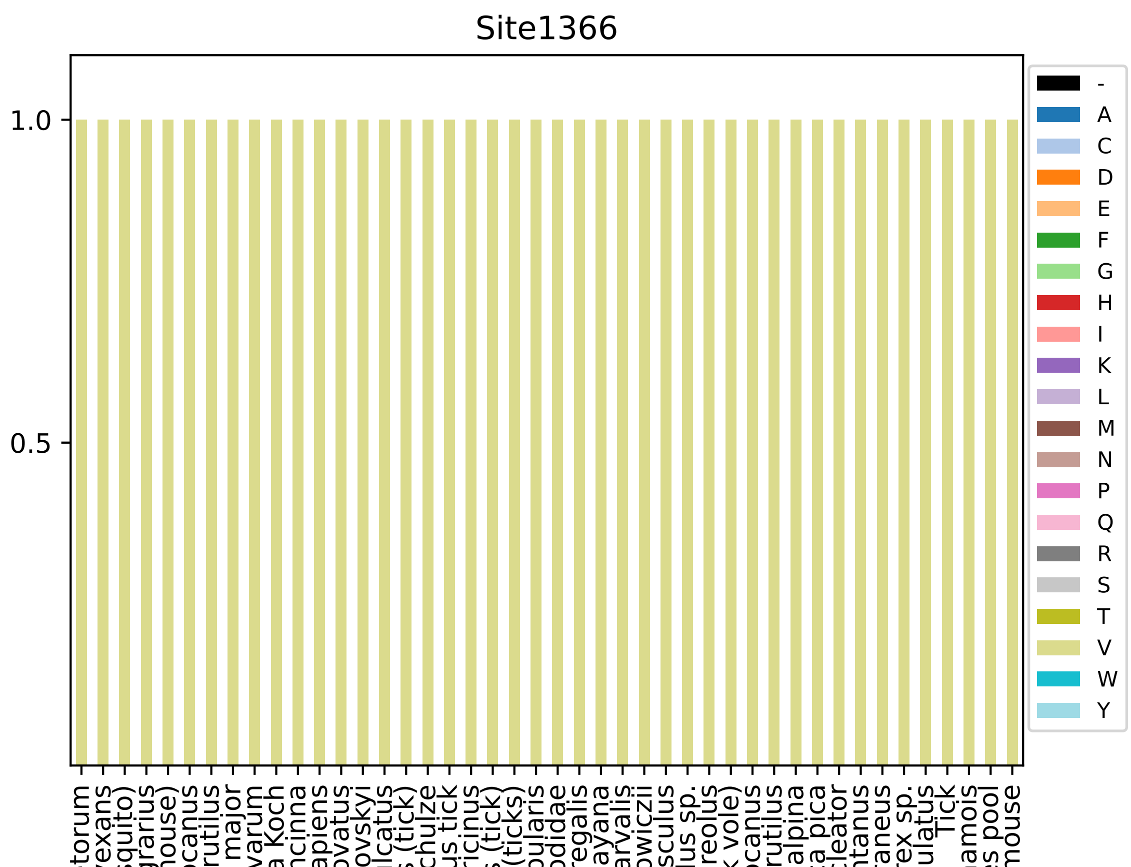

Site1366
1.0
0.5
-
A
C
D
E
F
G
H
I
K
L
M
N
P
Q
R
S
T
V
W
Y

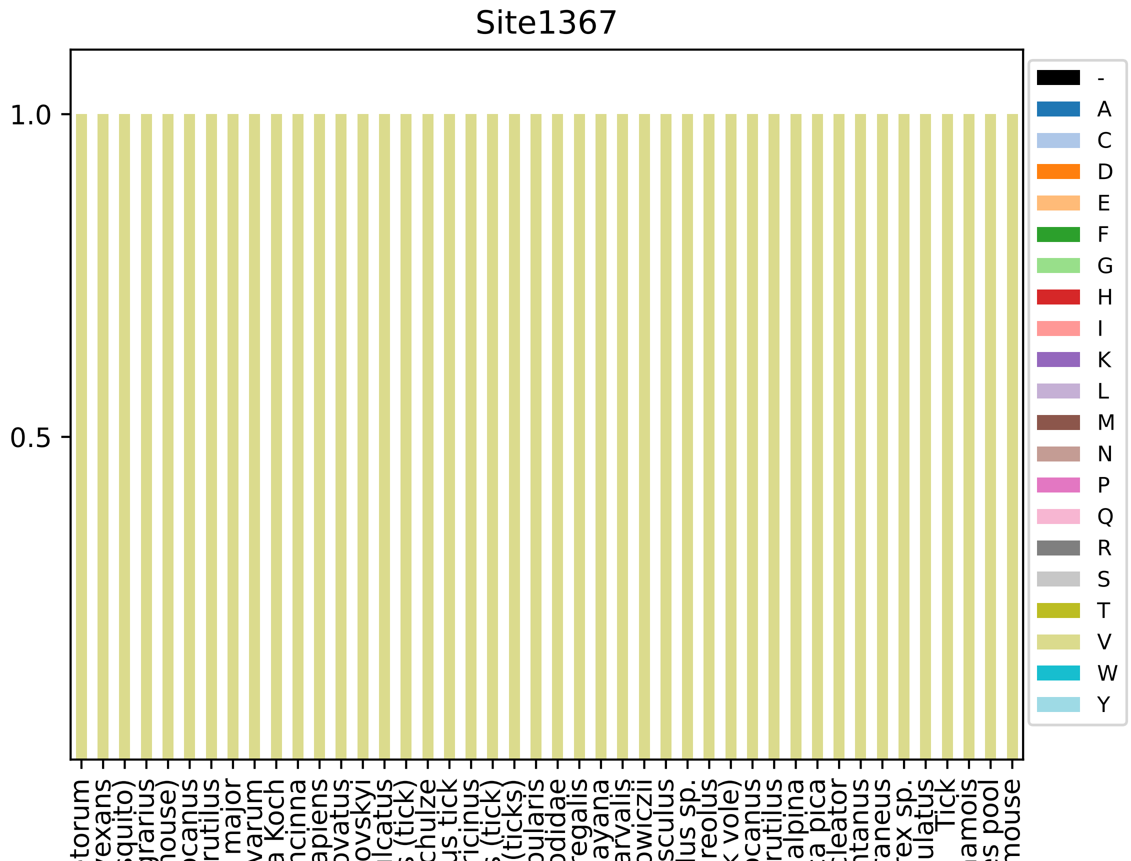
Site1367
1.0
0.5
-
A
C
D
E
F
G
H
I
K
L
M
N
P
Q
R
S
T
V
W
Y

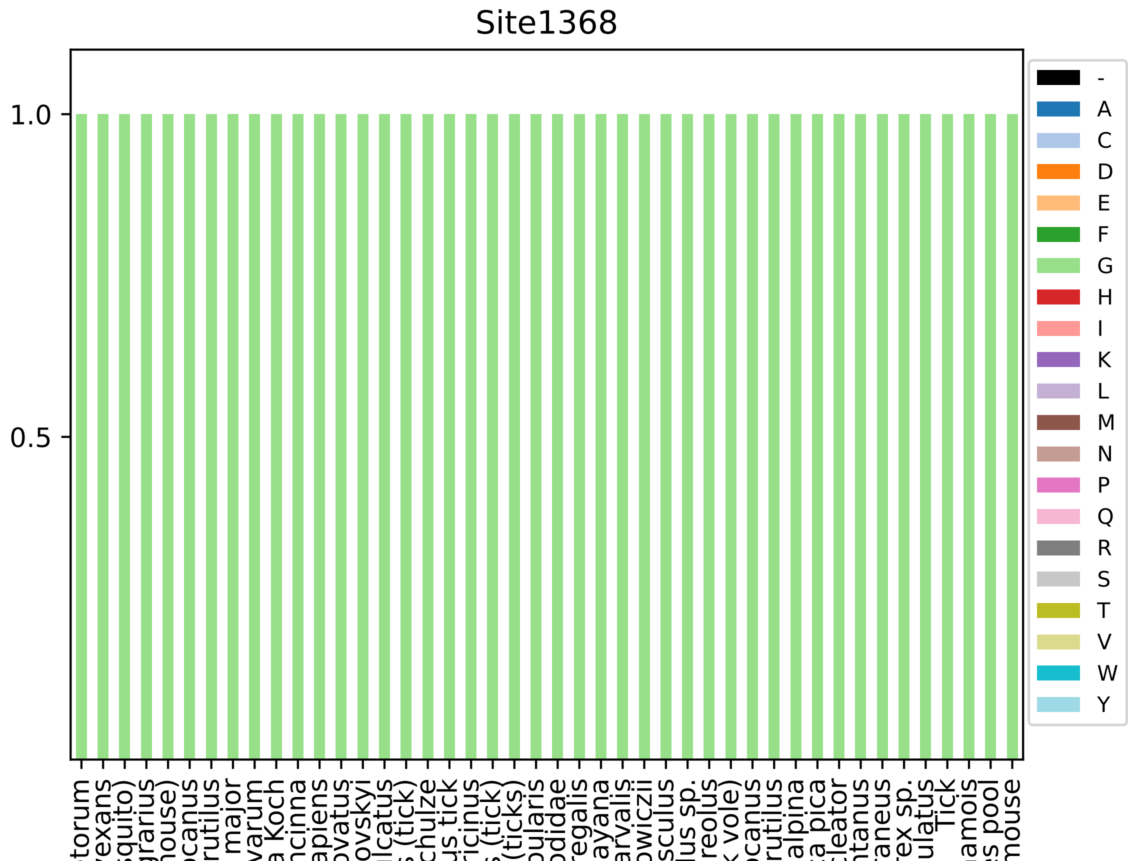
Site1368
1.0
0.5
-
A
C
D
E
F
G
H
I
K
L
M
N
P
Q
R
S
T
V
W
Y

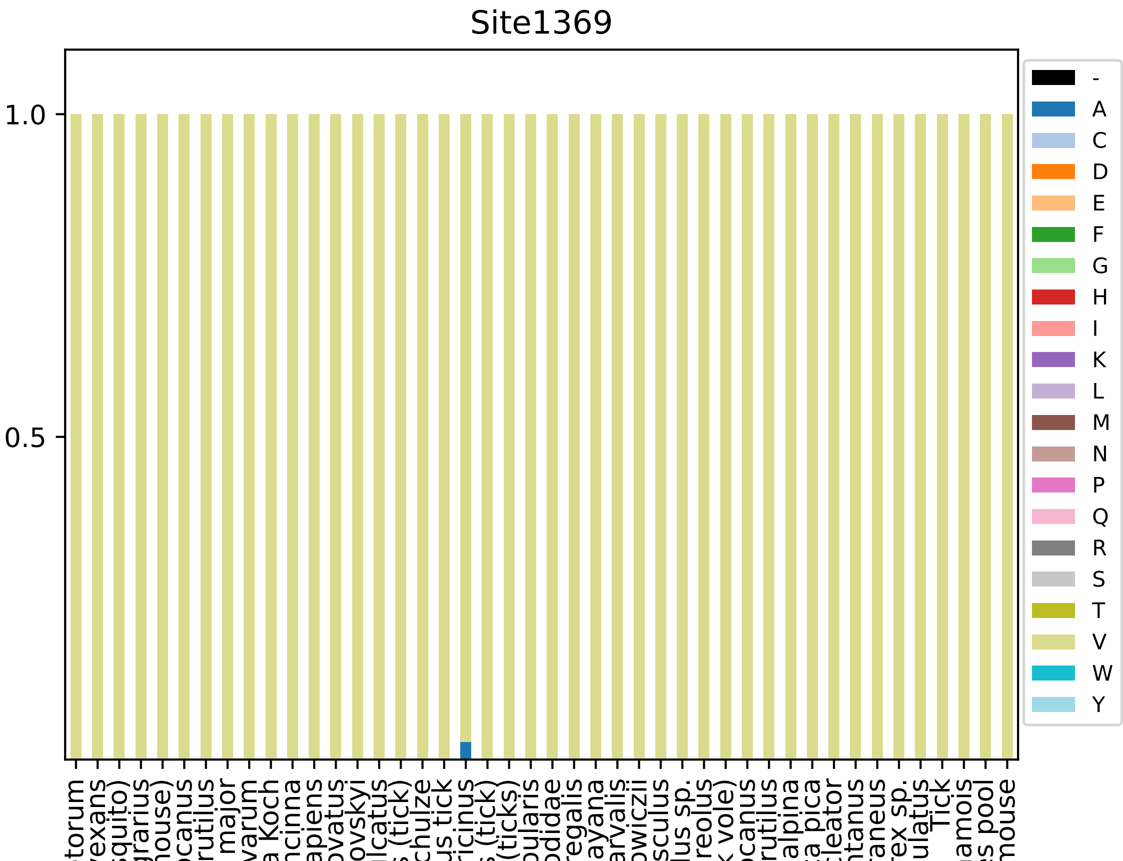
Site1369
1.0
0.5
-
A
C
D
E
F
G
H
I
K
L
M
N
P
Q
R
S
T
V
W
Y
Tick

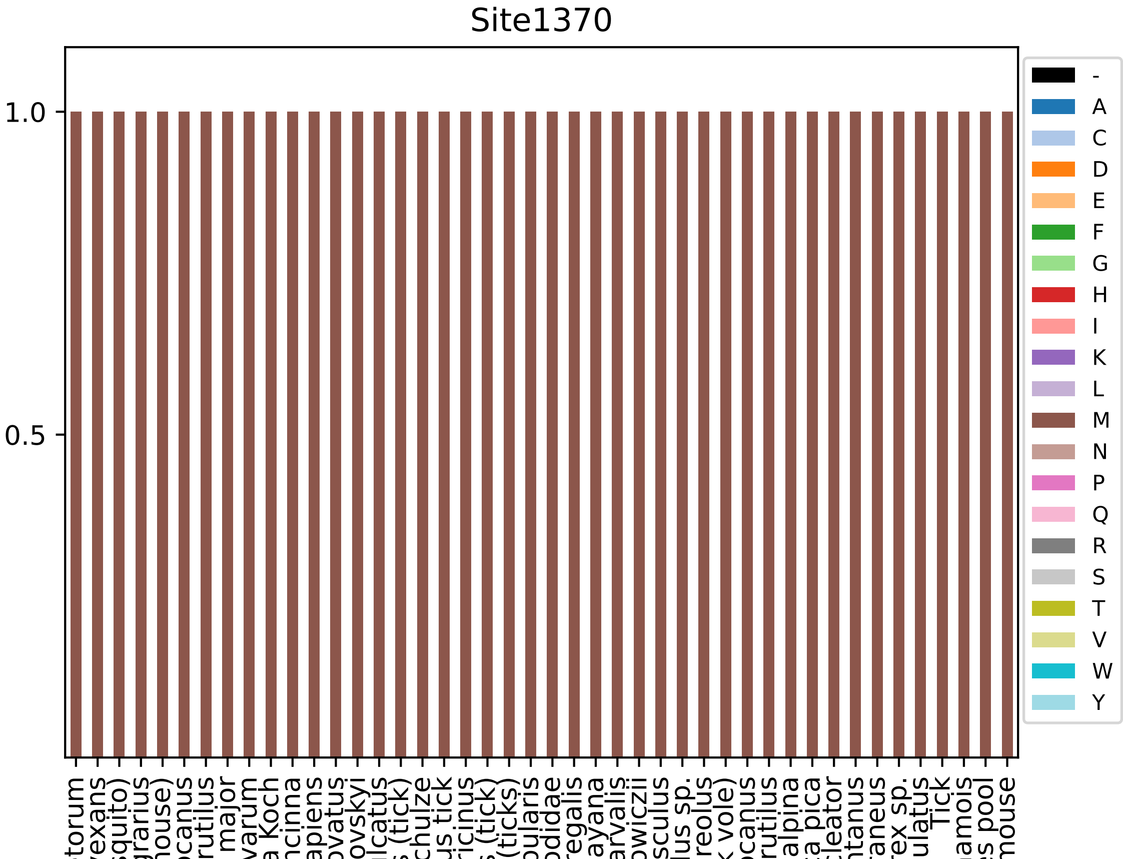
Site1370
1.0
0.5
-
A
C
D
E
F
G
H
I
K
L
M
N
P
Q
R
S
T
V
W
Y

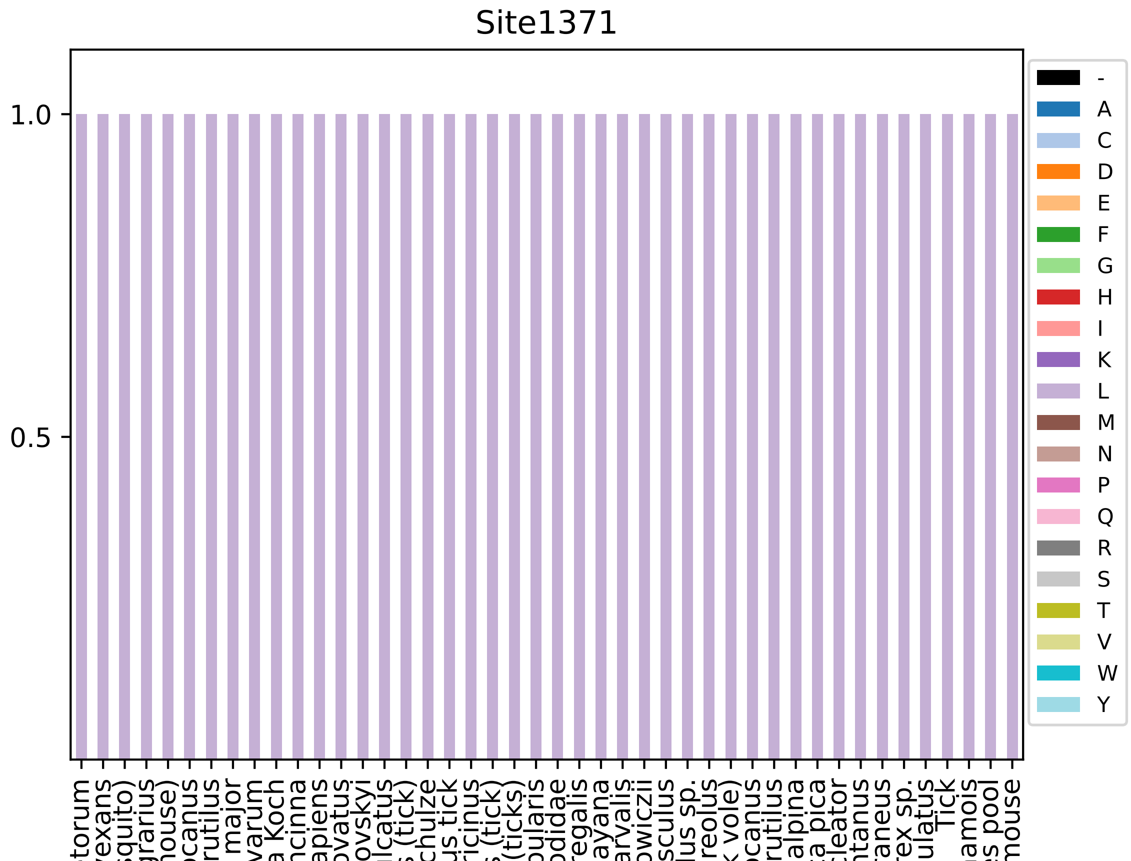
Site1371
1.0
0.5
-
A
C
D
E
F
G
H
I
K
L
M
N
P
Q
R
S
T
V
W
Y

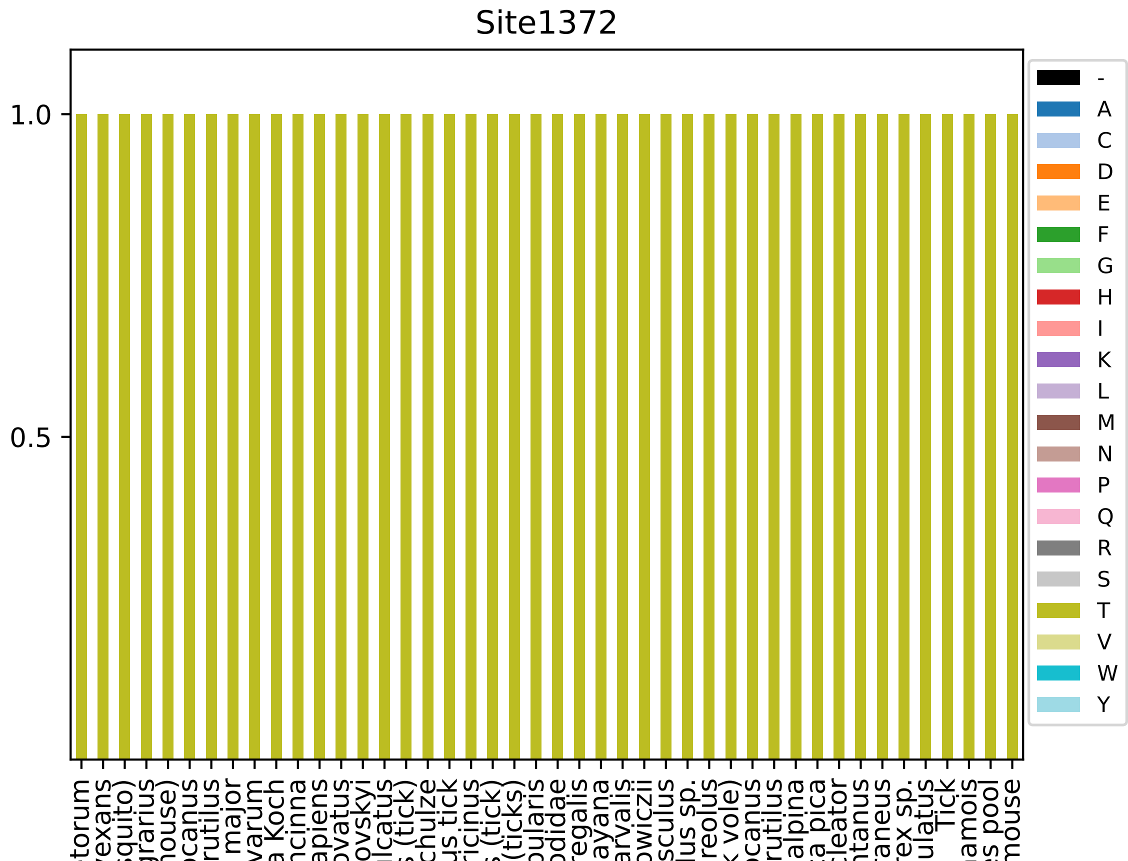
Site1372
1.0
0.5
-
A
C
D
E
F
G
H
I
K
L
M
N
P
Q
R
S
T
V
W
Y

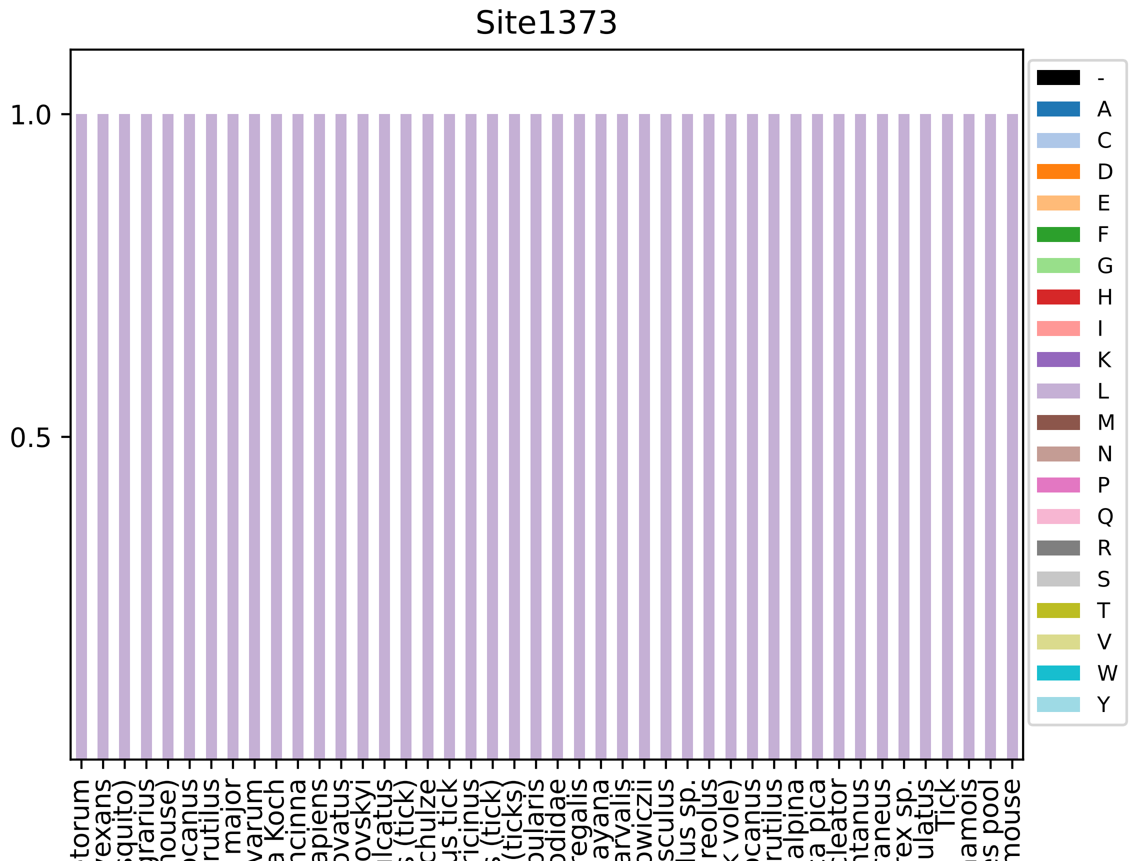
Site1373
1.0
0.5
-
A
C
D
E
F
G
H
I
K
L
M
N
P
Q
R
S
T
V
W
Y

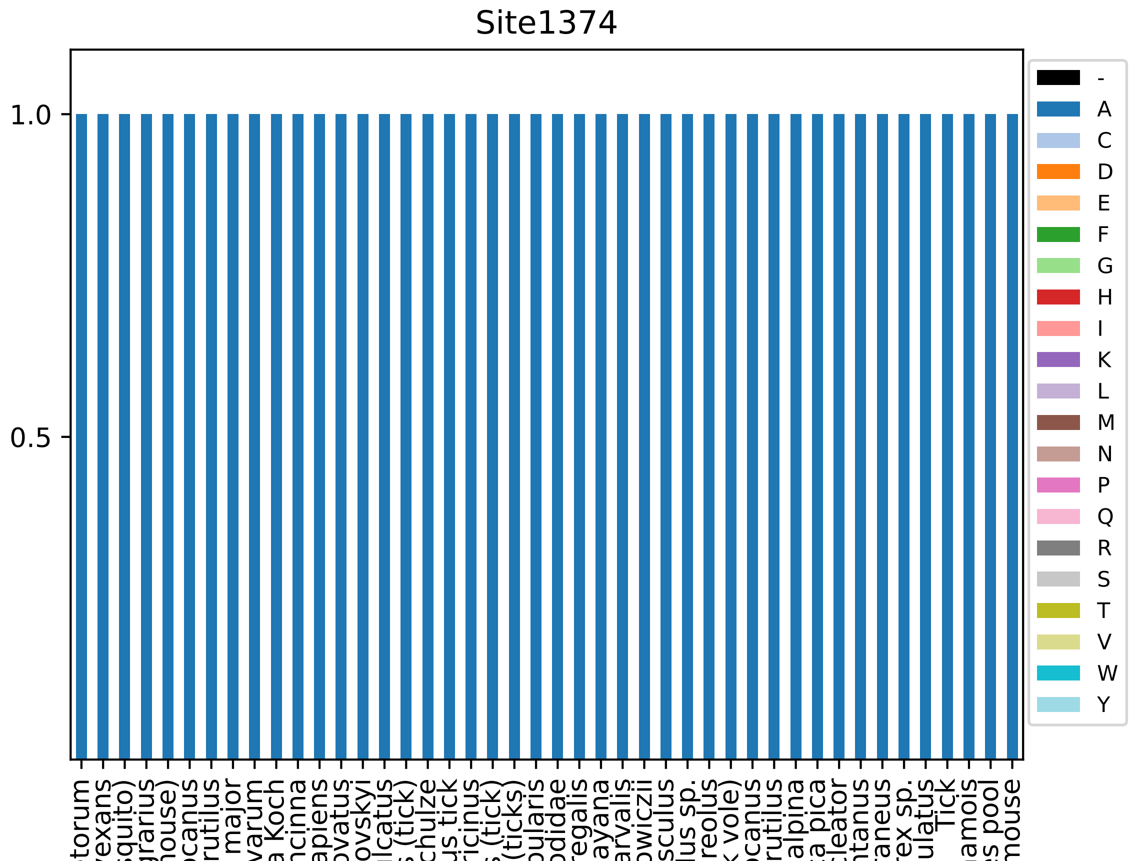
Site1374
1.0
0.5
-
A
C
D
E
F
G
H
I
K
L
M
N
P
Q
R
S
T
V
W
Y

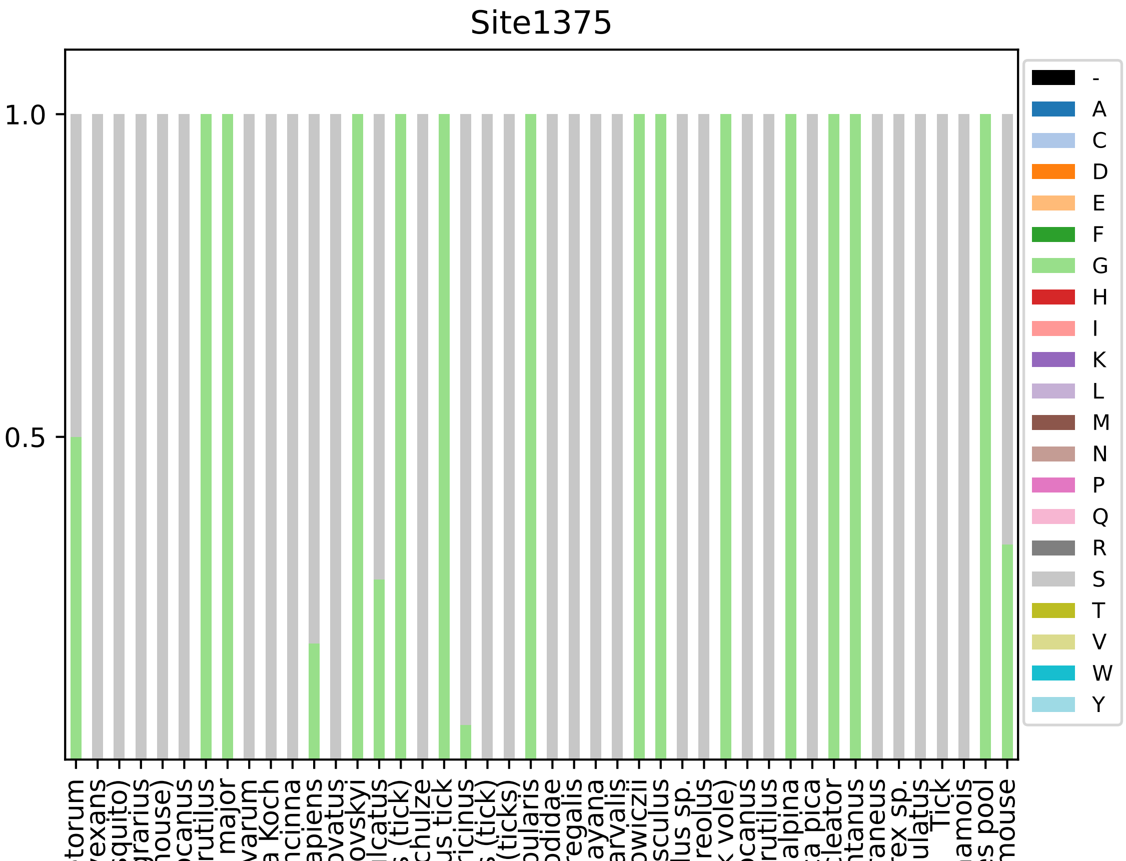
Site1375
1.0
0.5
-
A
C
D
E
F
G
H
I
K
L
M
N
P
Q
R
S
T
V
W
Y

Site1376

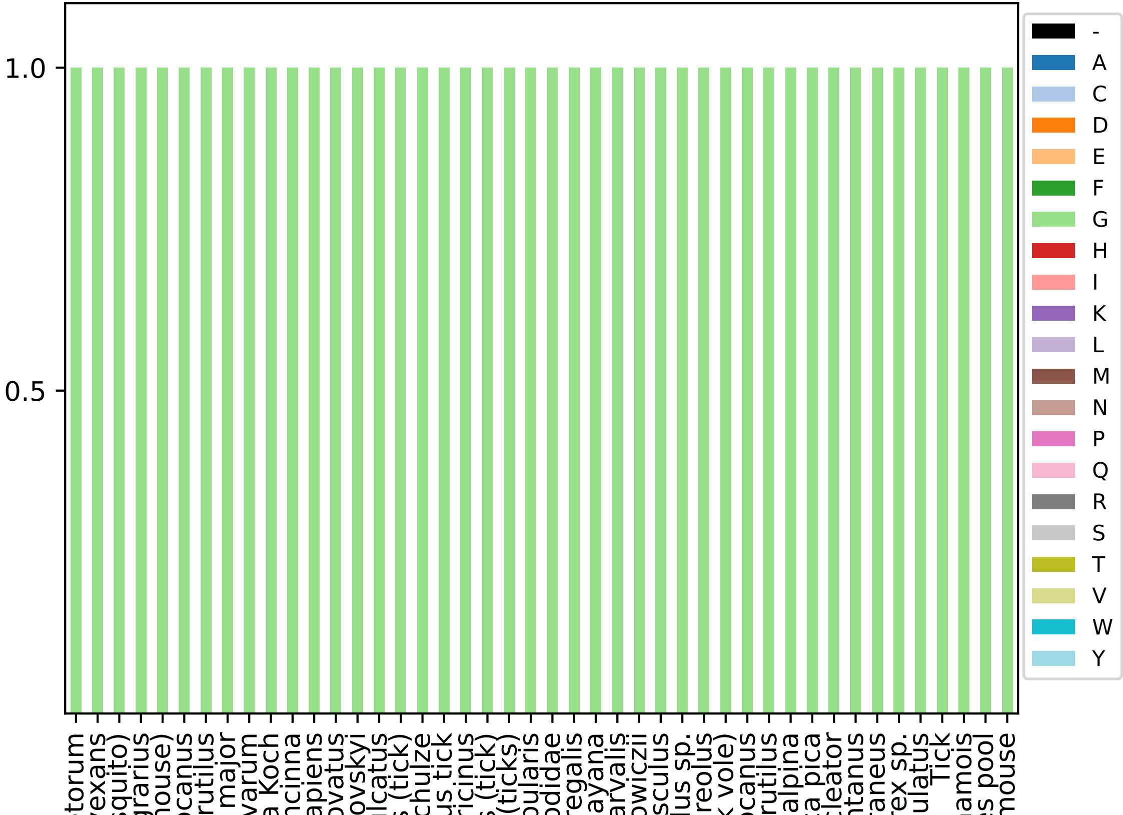

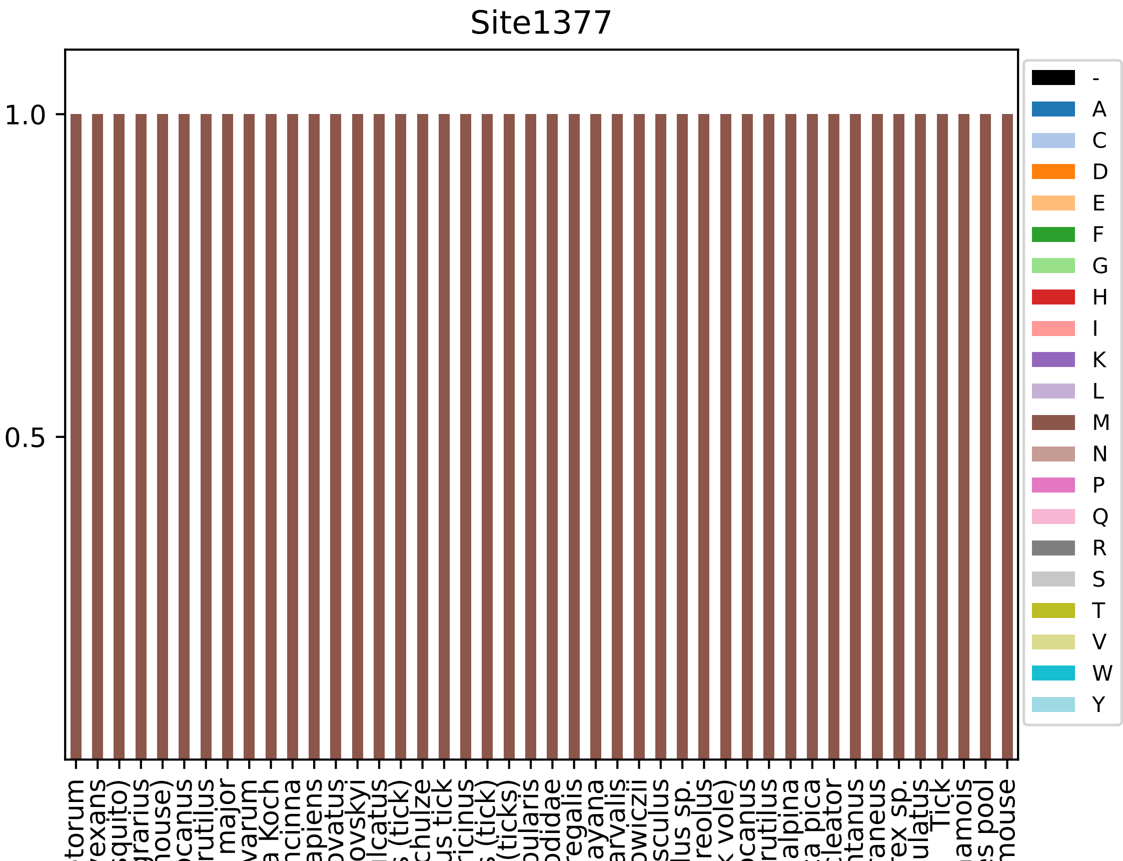
Site1377
1.0
0.5
-
A
C
D
E
F
G
H
I
K
L
M
N
P
Q
R
S
T
V
W
Y

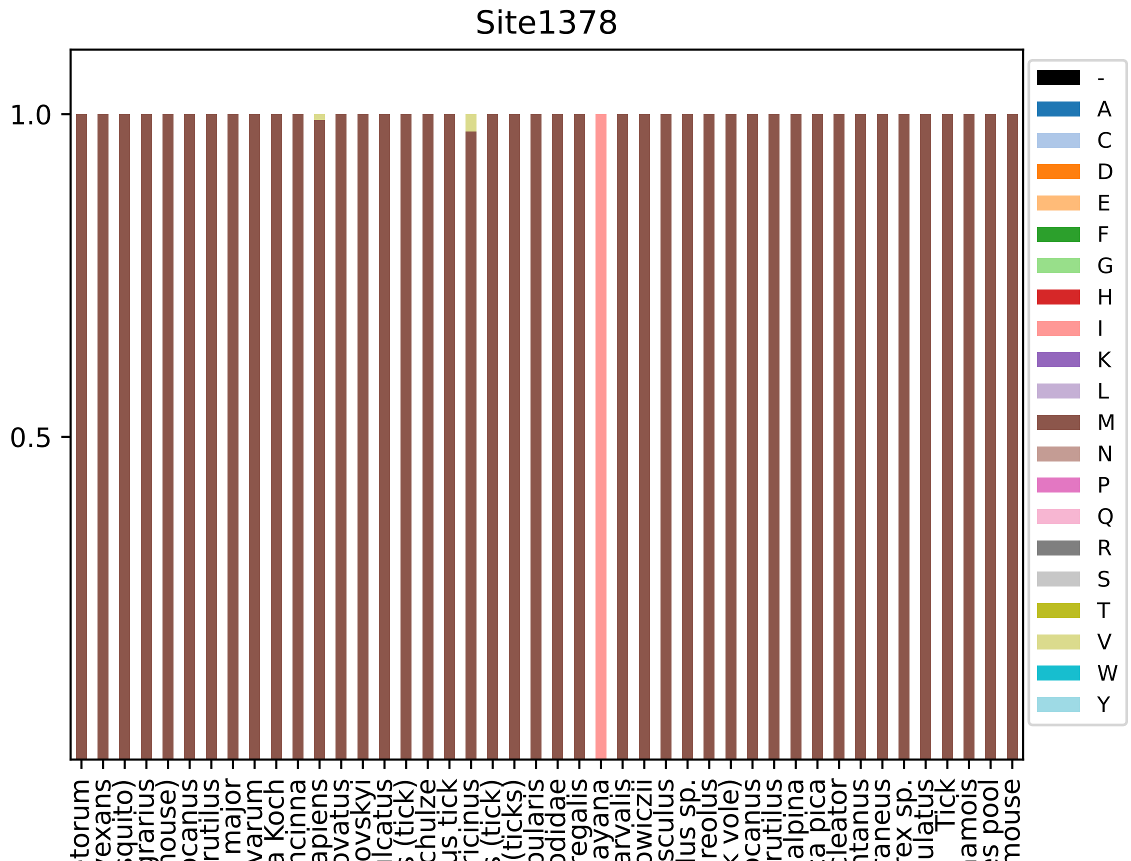
Site1378
1.0
0.5
-
A
C
D
E
F
G
H
I
K
L
M
N
P
Q
R
S
T
V
W
Y

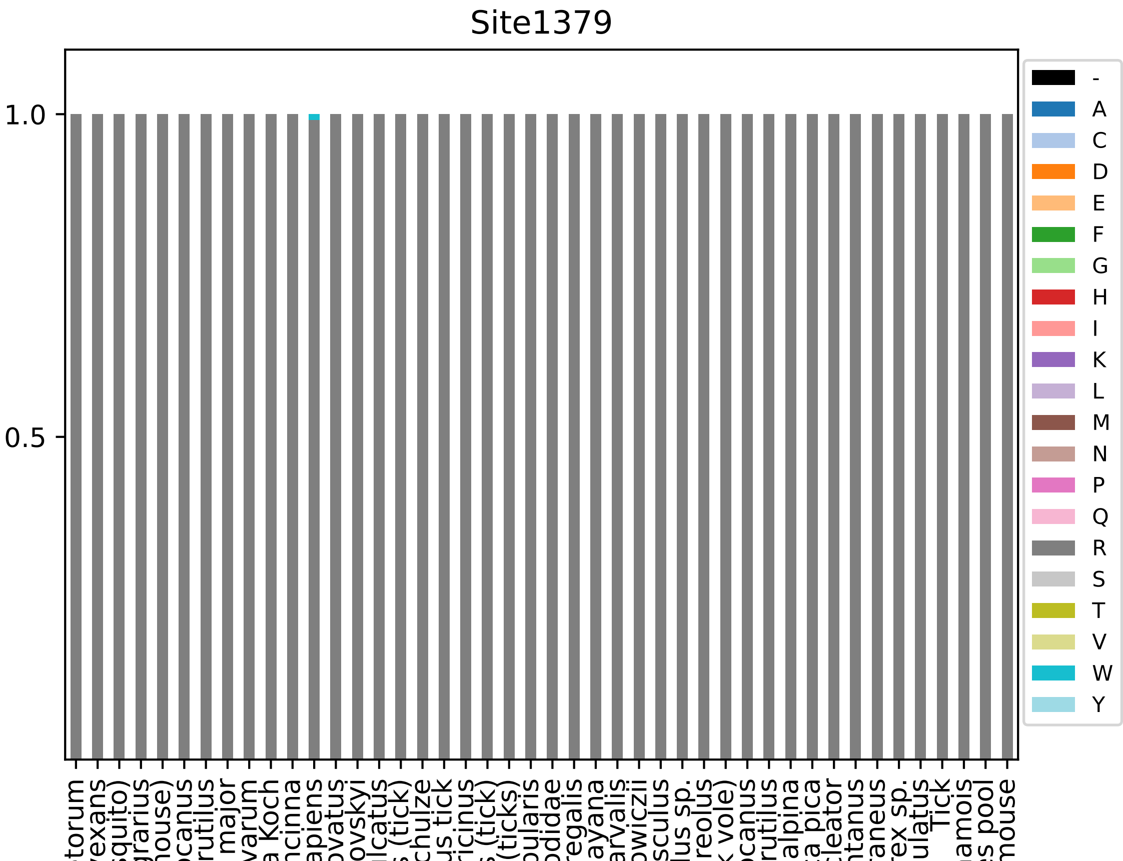
Site1379
1.0
0.5
-
A
C
D
E
F
G
H
I
K
L
M
N
P
Q
R
S
T
V
W
Y

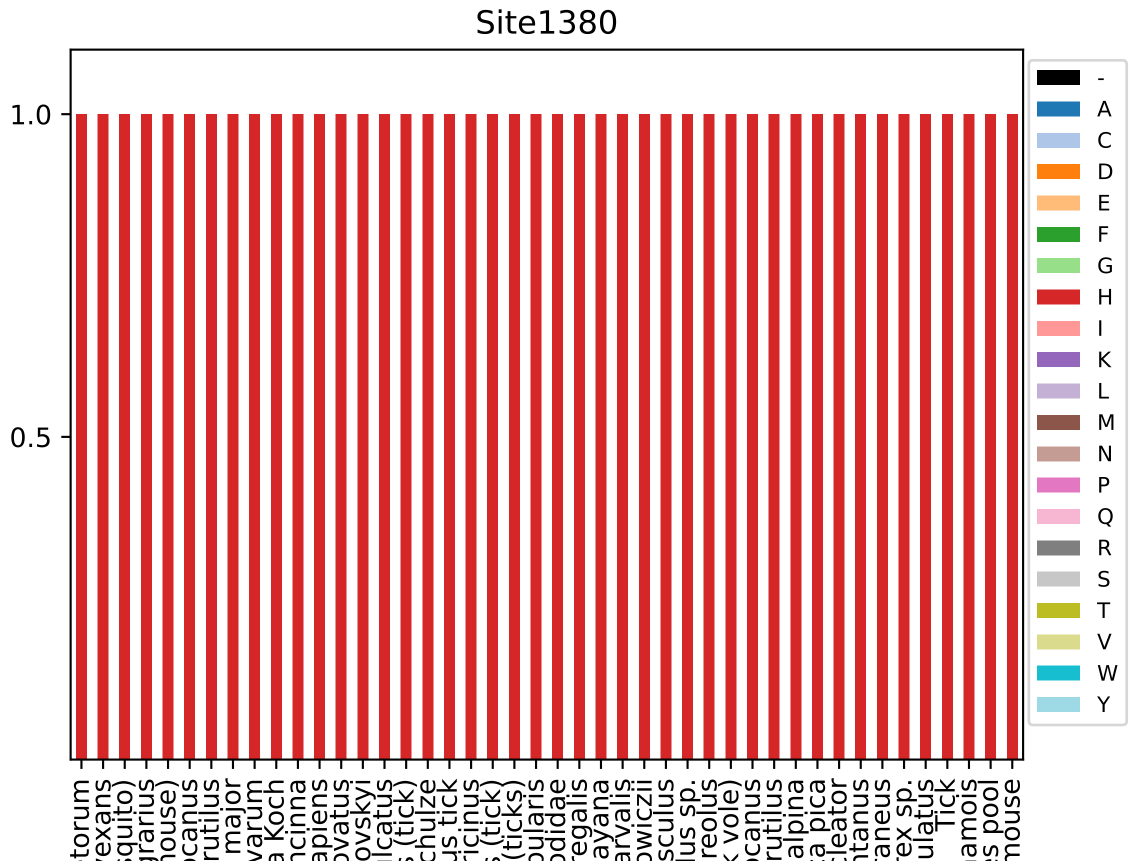

Site1380
1.0
0.5
-
A
C
D
E
F
G
H
I
K
L
M
N
P
Q
R
S
T
V
W
Y

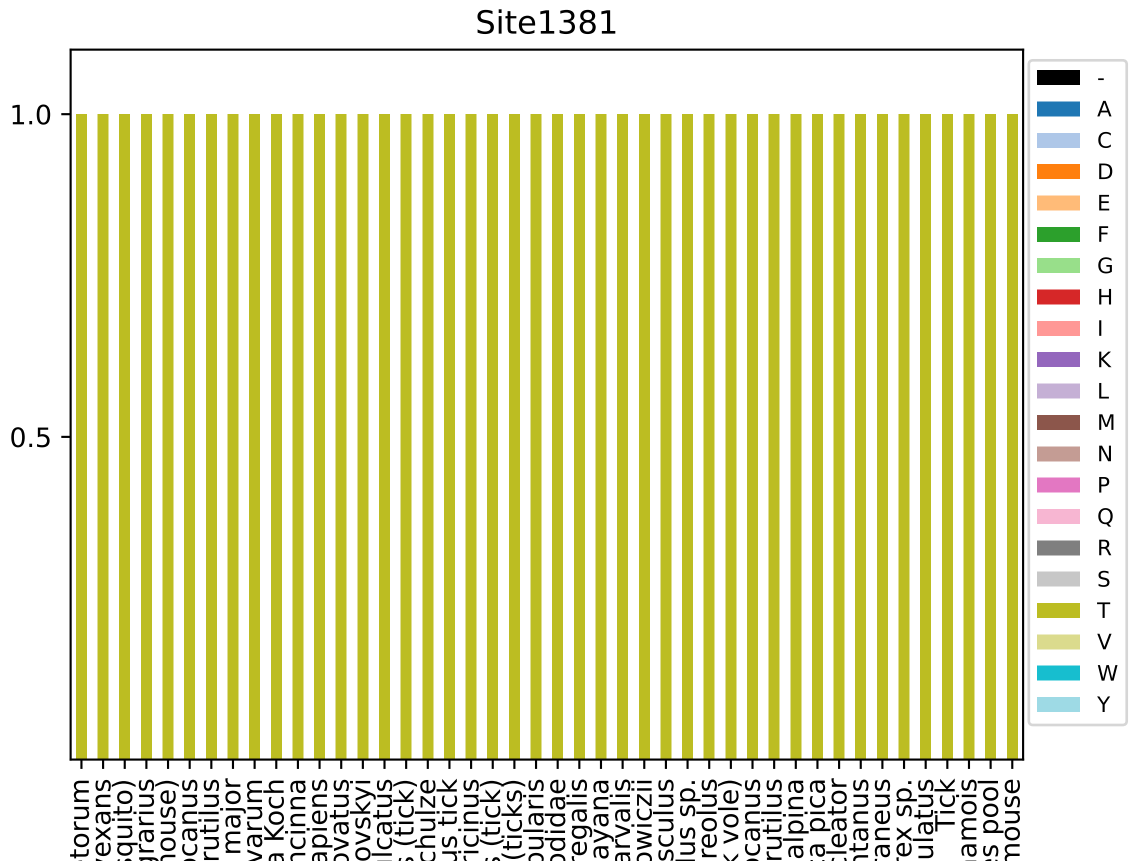
Site1381
1.0
0.5
-
A
C
D
E
F
G
H
I
K
L
M
N
P
Q
R
S
T
V
W
Y

Site1382

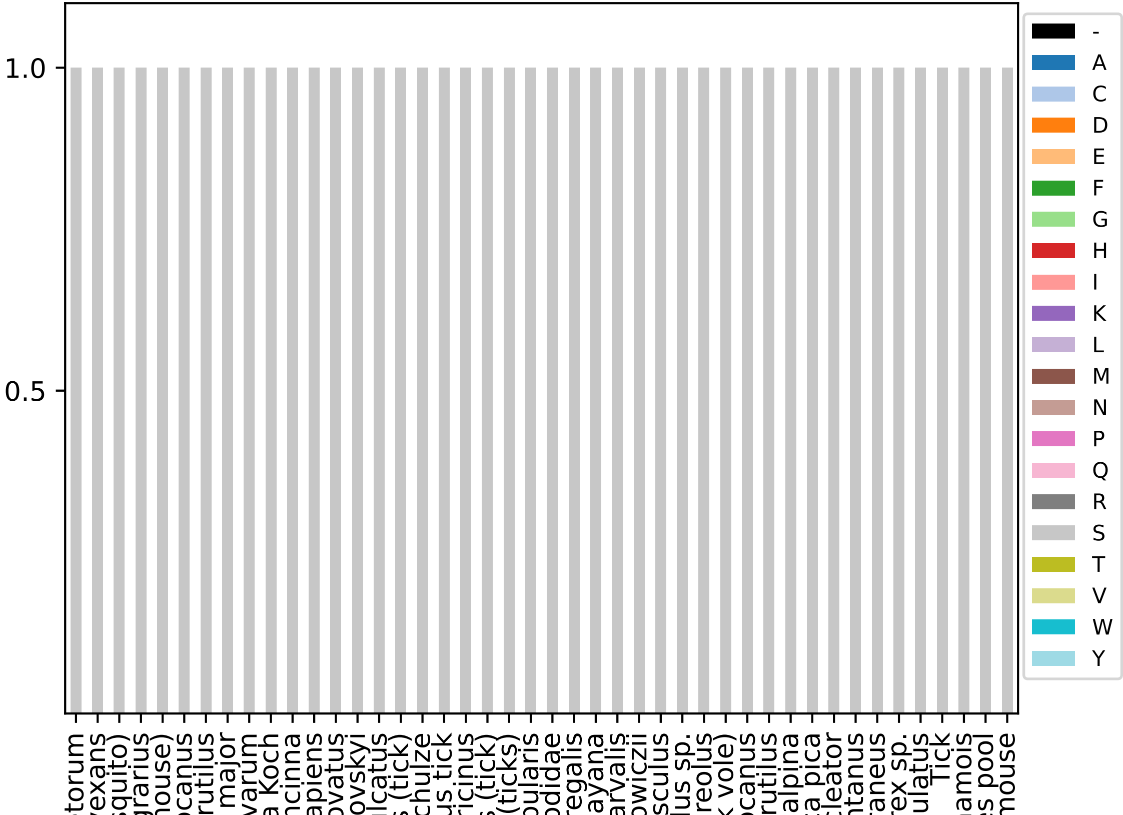

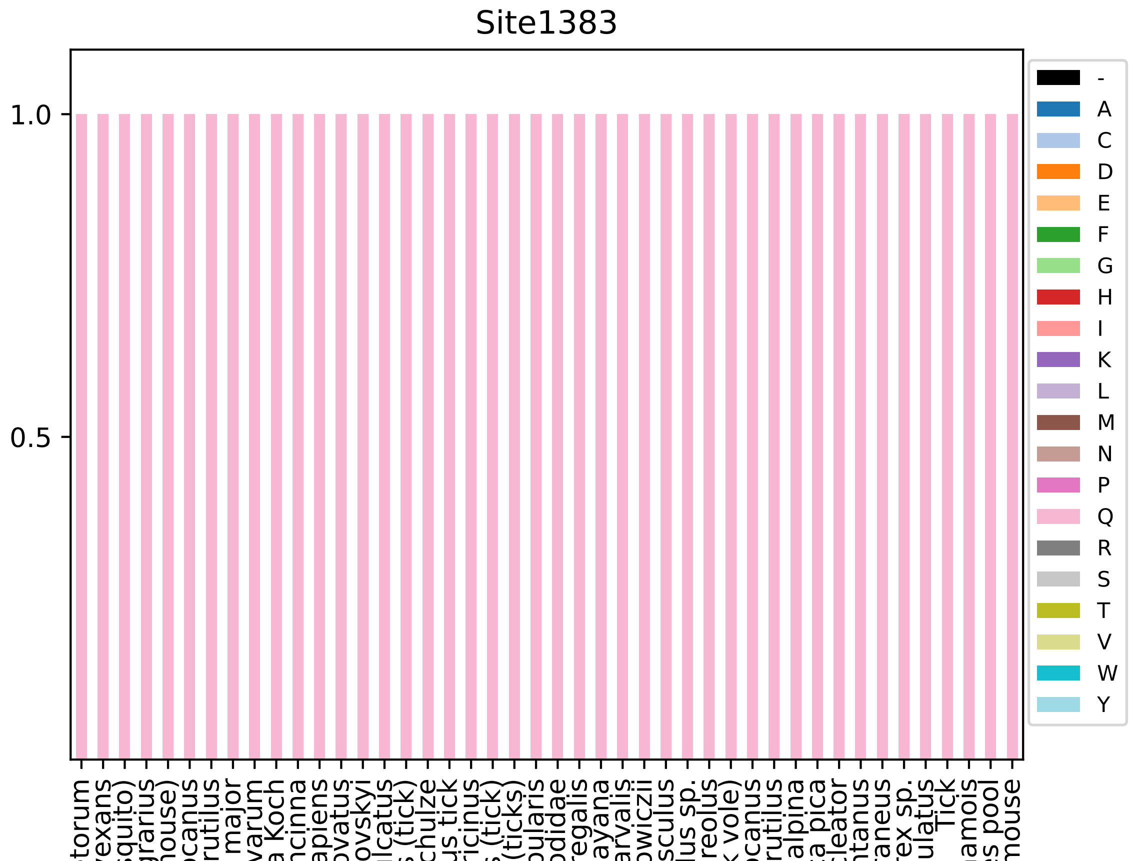
Site1383
1.0
0.5
-
A
C
D
E
F
G
H
I
K
L
M
N
P
Q
R
S
T
V
W
Y

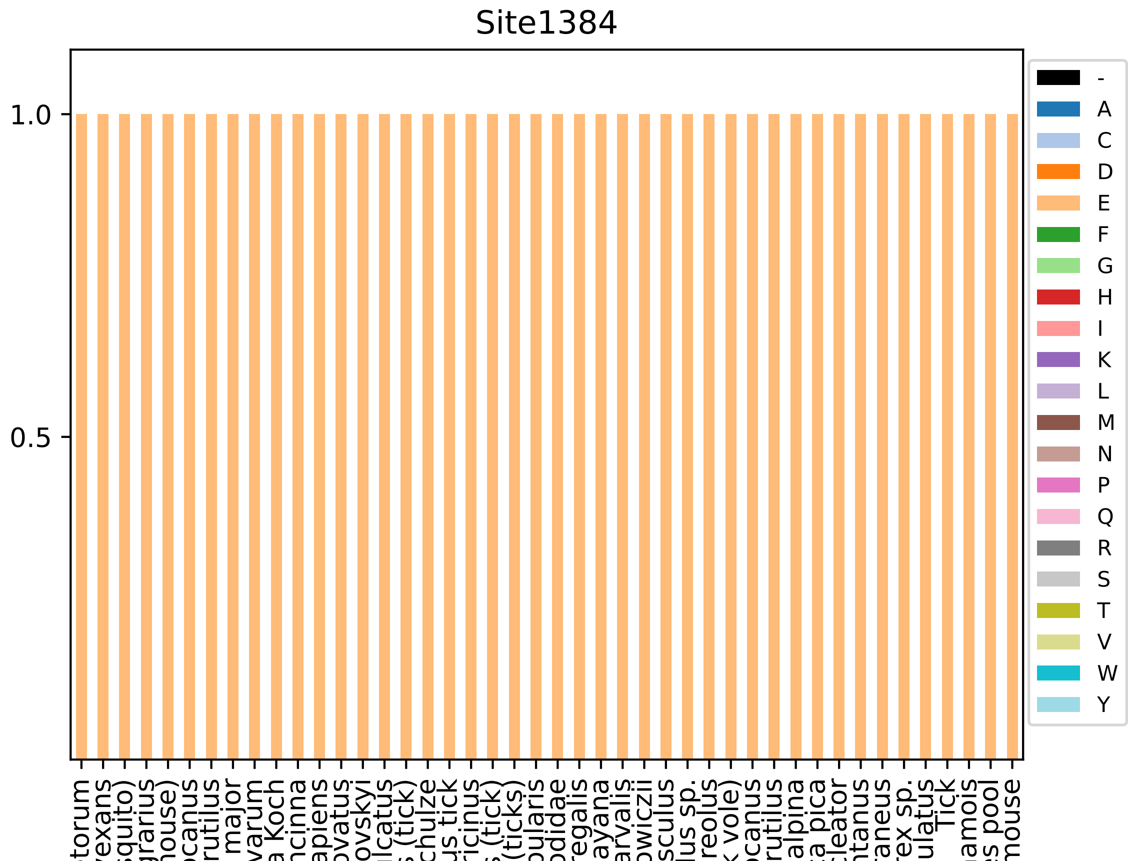
Site1384
1.0
0.5
-
A
C
D
E
F
G
H
I
K
L
M
N
P
Q
R
S
T
V
W
Y

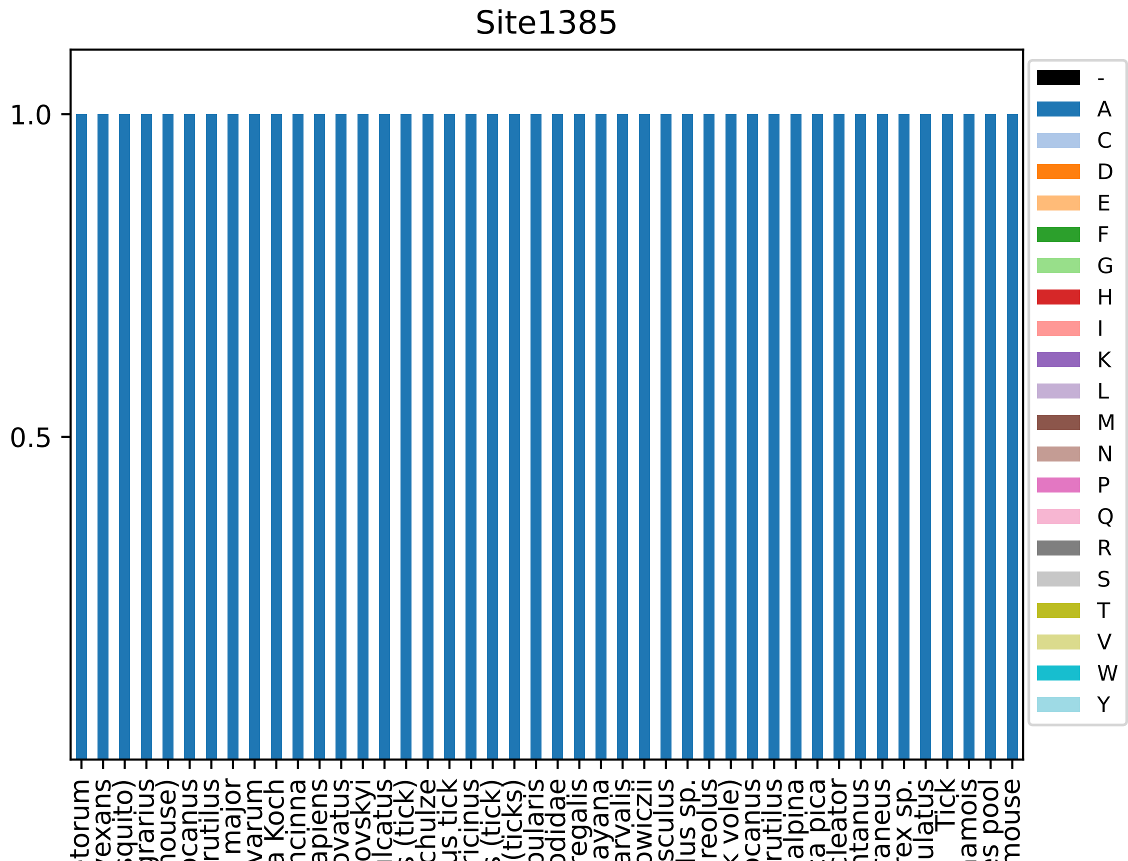

Site1385
1.0
0.5
-
A
C
D
E
F
G
H
I
K
L
M
N
P
Q
R
S
T
V
W
Y
Tick

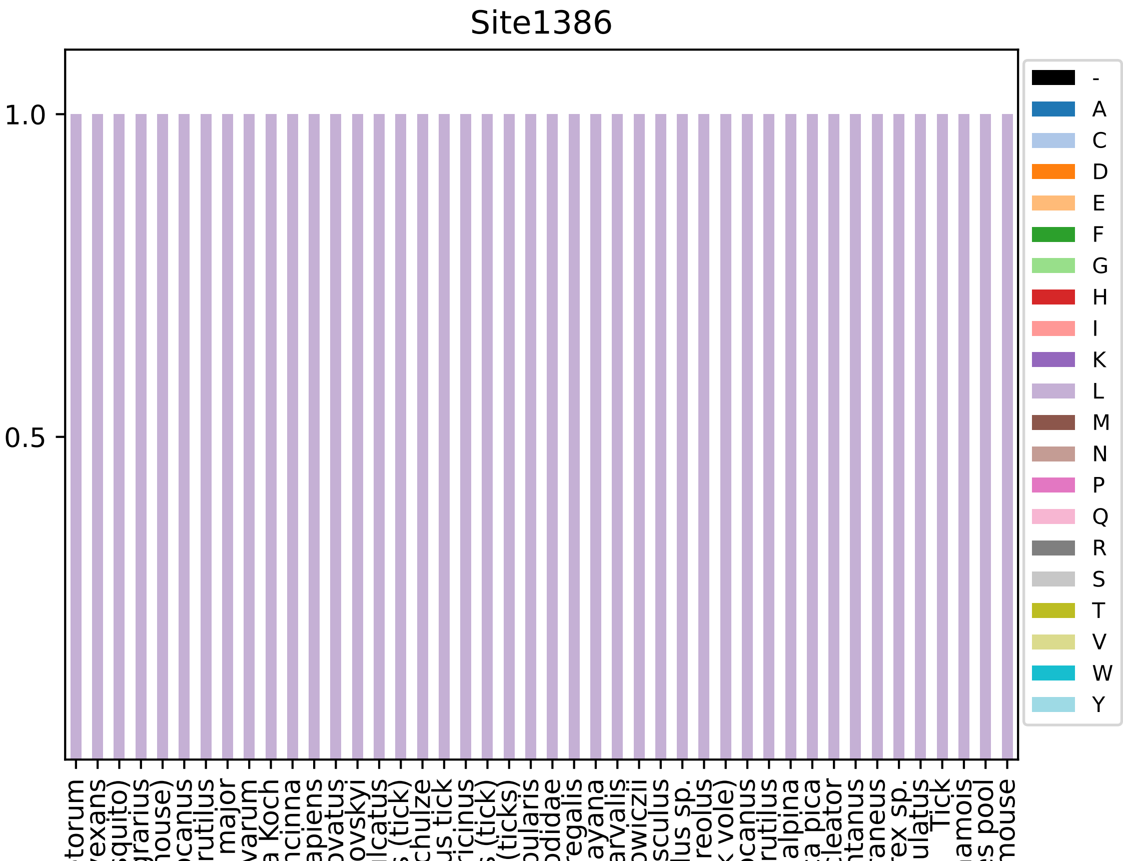

Site1386
1.0
0.5
-
A
C
D
E
F
G
H
I
K
L
M
N
P
Q
R
S
T
V
W
Y

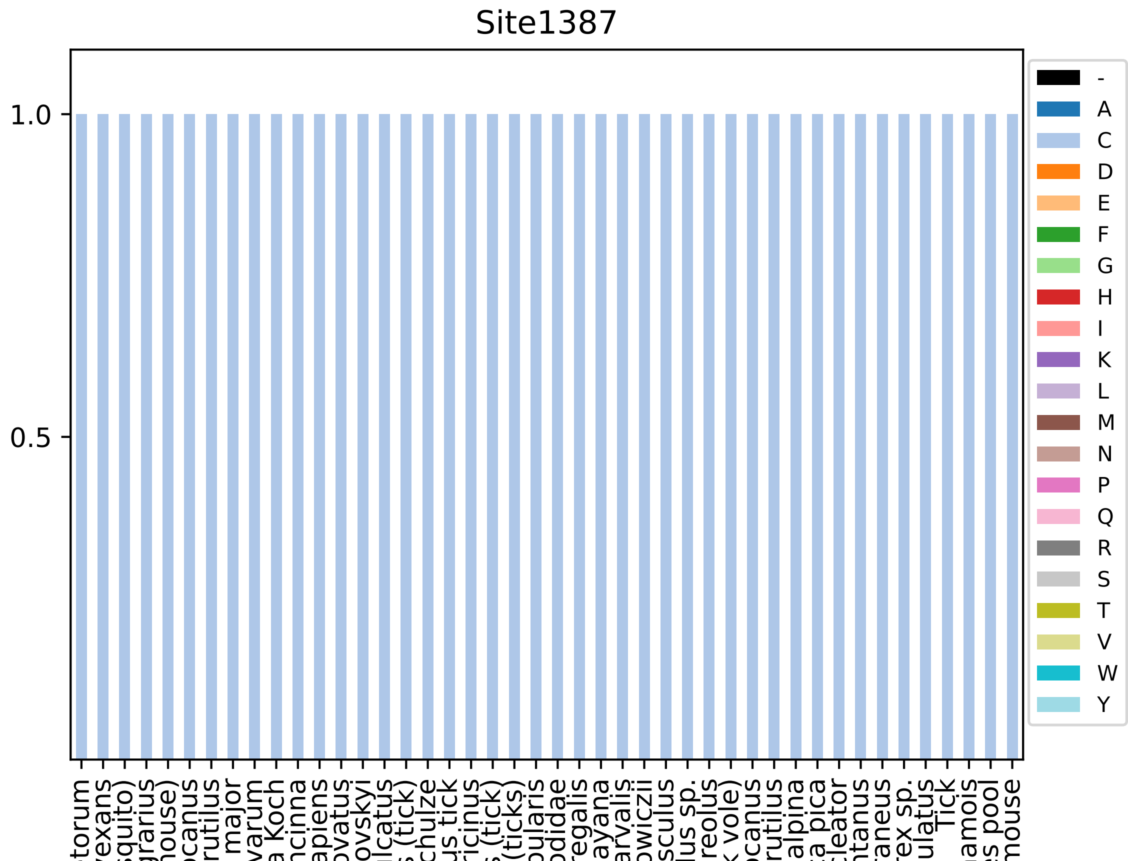
Site1387
1.0
0.5
-
A
C
D
E
F
G
H
I
K
L
M
N
P
Q
R
S
T
V
W
Y
Tick

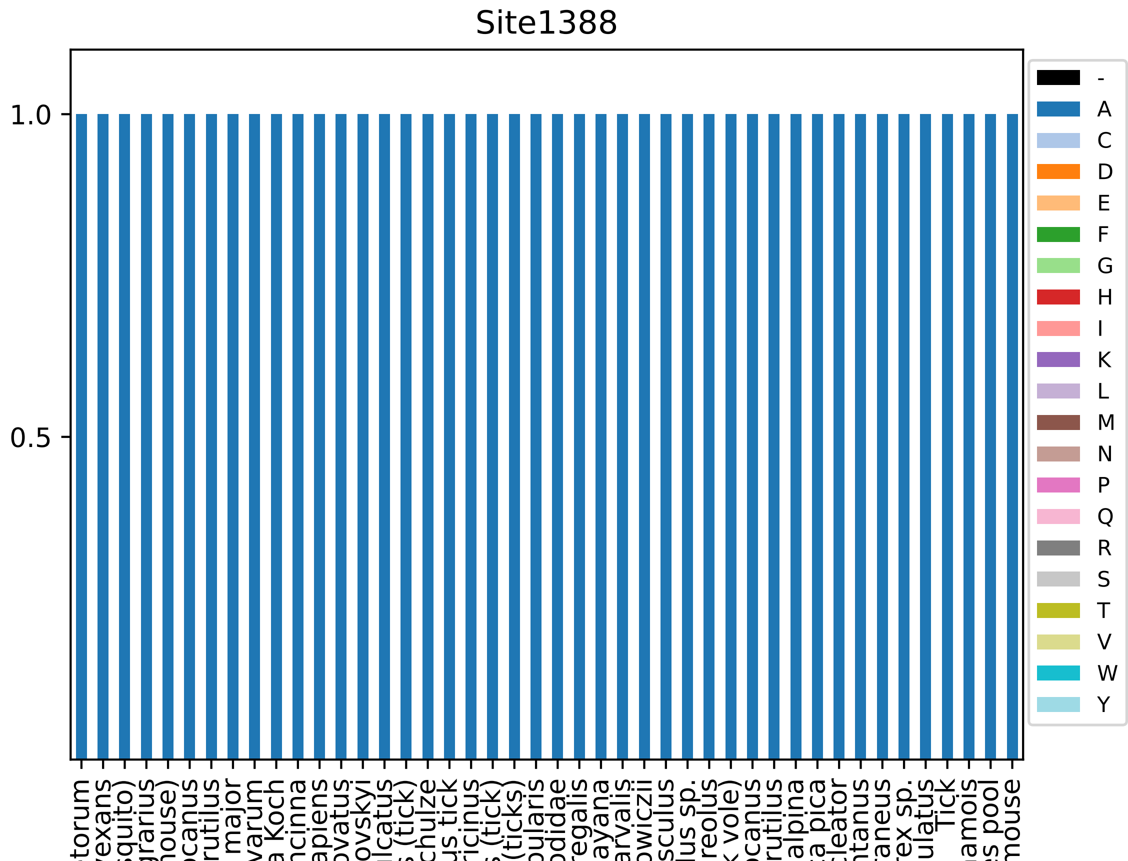
Site1388
1.0
0.5
-
A
C
D
E
F
G
H
I
K
L
M
N
P
Q
R
S
T
V
W
Y

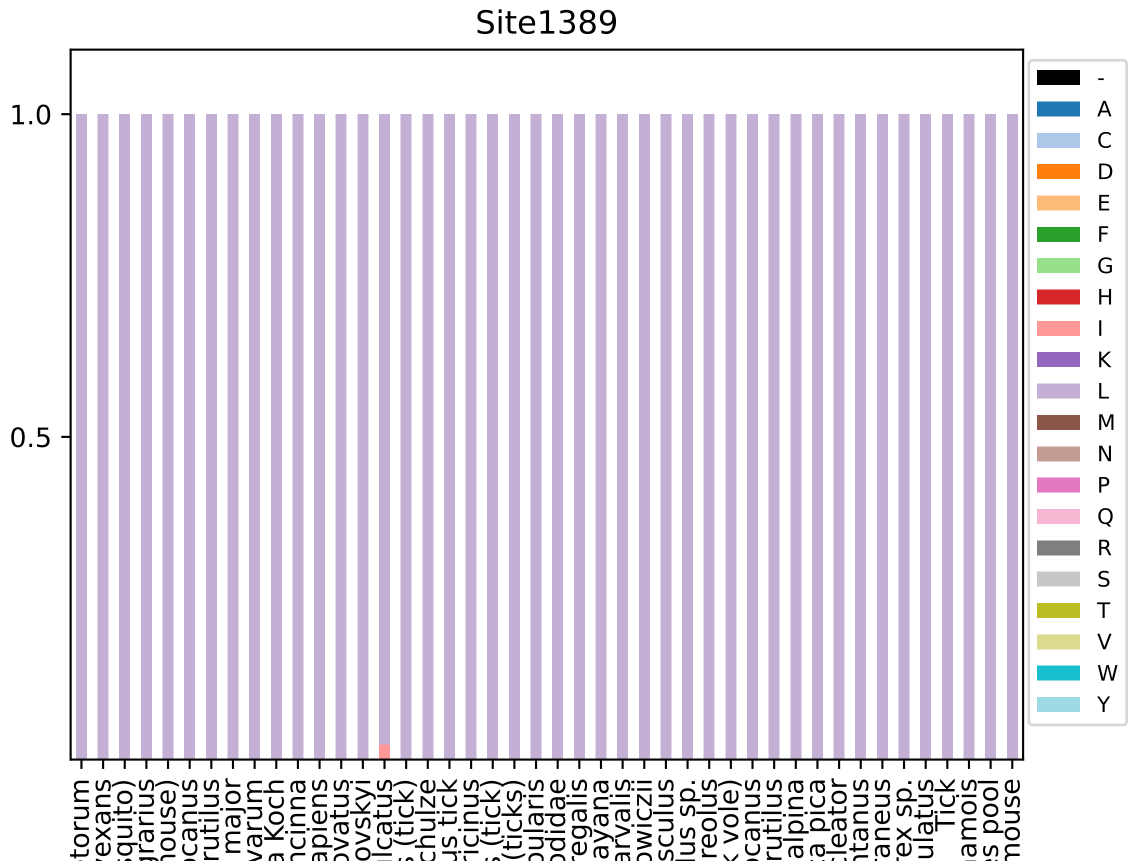

Site1389
1.0
0.5
-
A
C
D
E
F
G
H
I
K
L
M
N
P
Q
R
S
T
V
W
Y

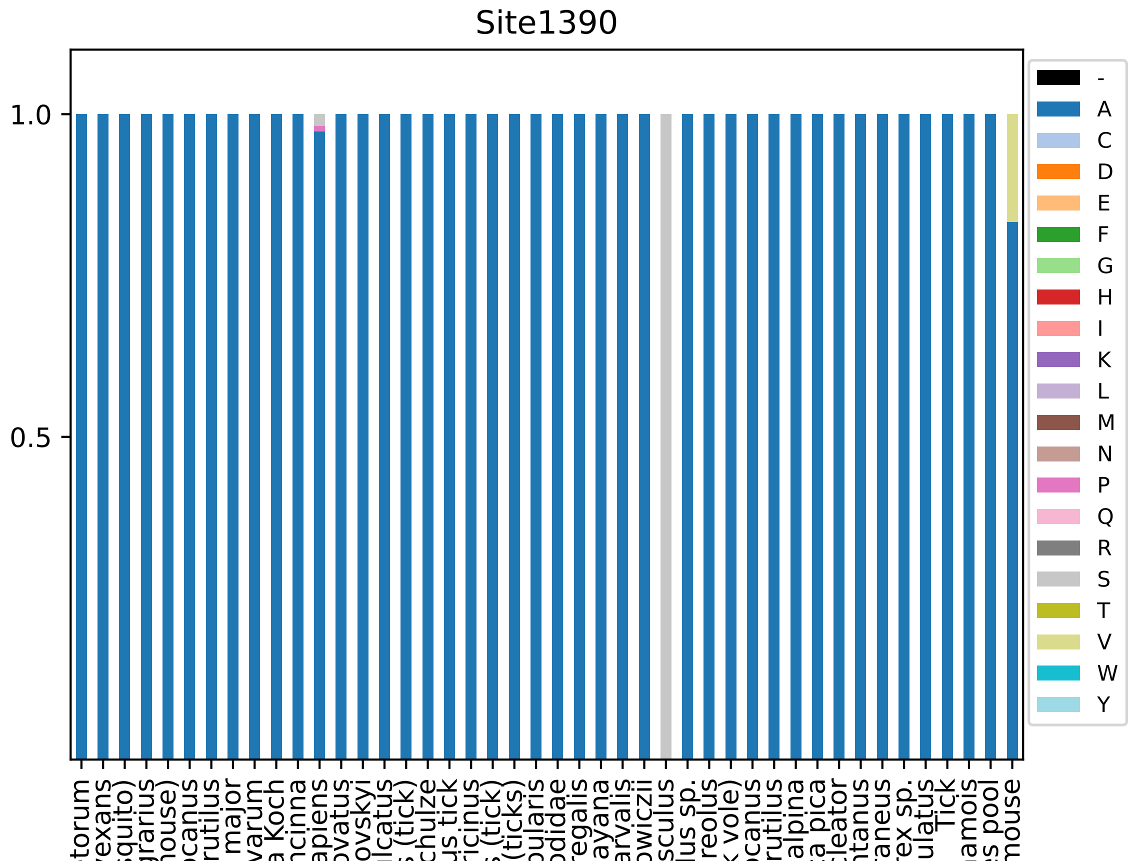
Site1390
1.0
0.5
-
A
C
D
E
F
G
H
I
K
L
M
N
P
Q
R
S
T
V
W
Y

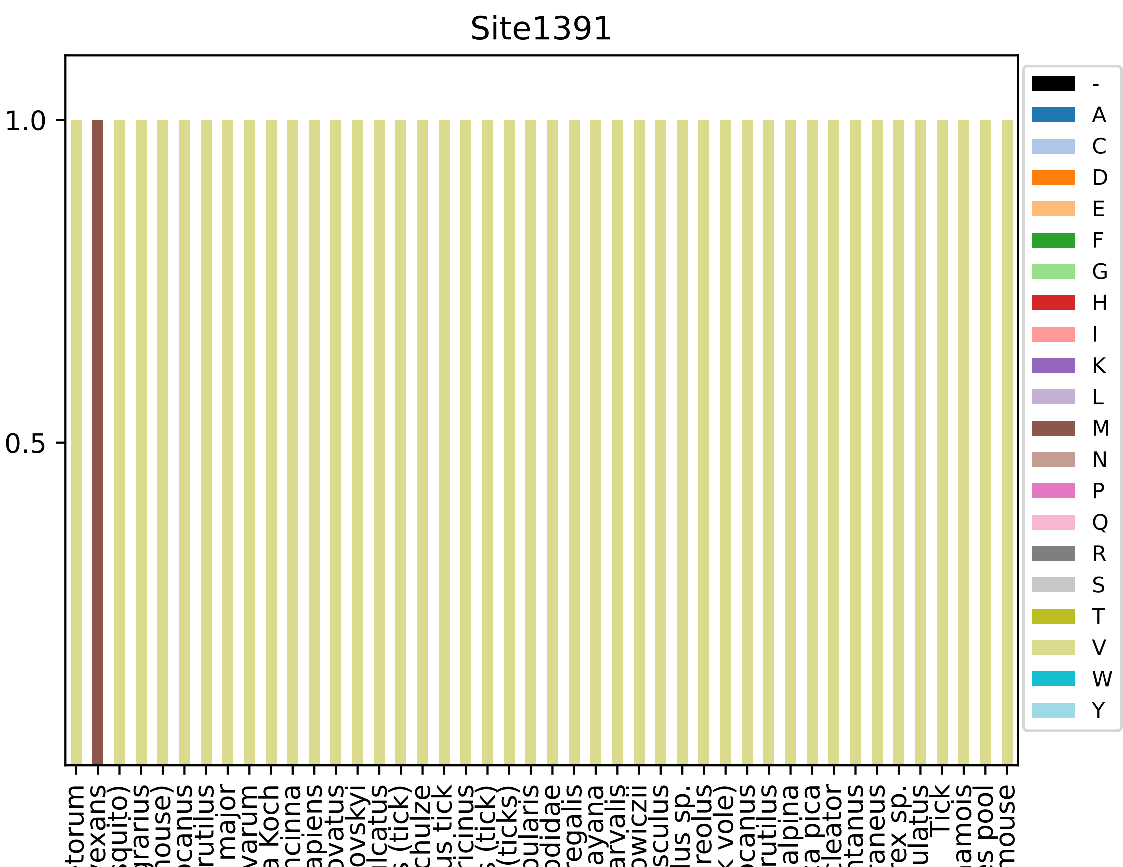
Site1391
1.0
0.5
-
A
C
D
E
F
G
H
I
K
L
M
N
P
Q
R
S
T
V
W
Y
torum
vexans
squito)
grarius
nouse)
canus
rutilus
major
varum
a Koch
ncinna
apiens
ovatus
ovskyi
ulcatus
s (tick)
chulze
us tick
icinus
s (tick)
(ticks)
pularis
odidae
regalis
ayana
arvalis
owiczii
sculus
lus sp.
reolus
k vole)
canus
rutilus
alpina
a pica
leator
ntanus
raneus
rex sp.
ulatus
Tick
amois
es pool
mouse

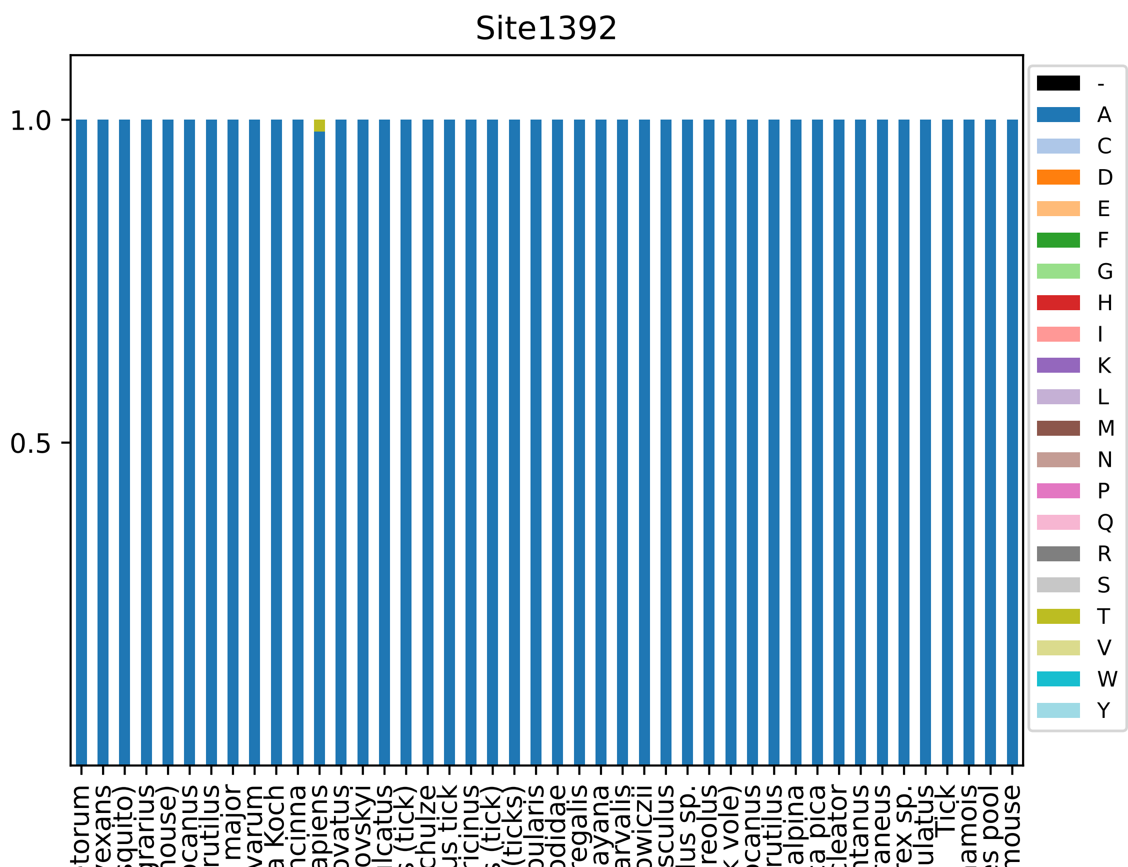

Site1392
1.0
0.5
-
A
C
D
E
F
G
H
I
K
L
M
N
P
Q
R
S
T
V
W
Y

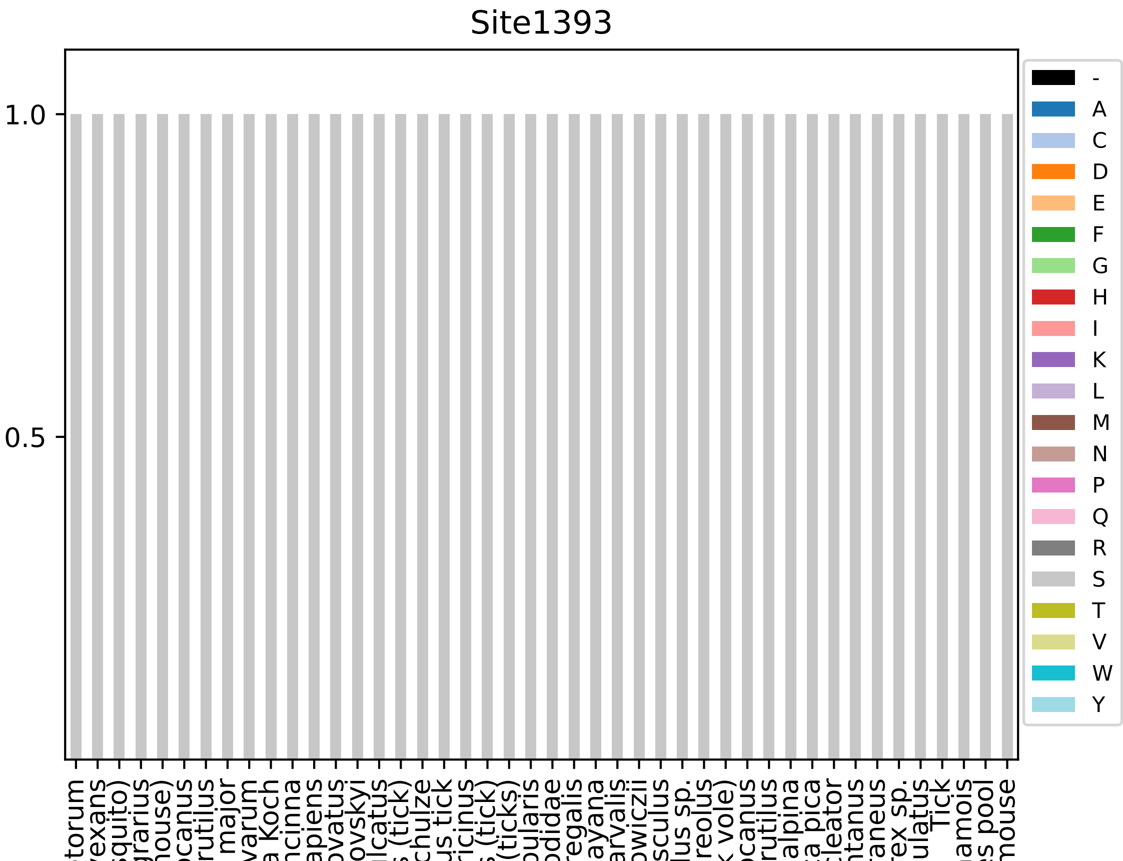
Site1393
1.0
0.5
-
A
C
D
E
F
G
H
I
K
L
M
N
P
Q
R
S
T
V
W
Y

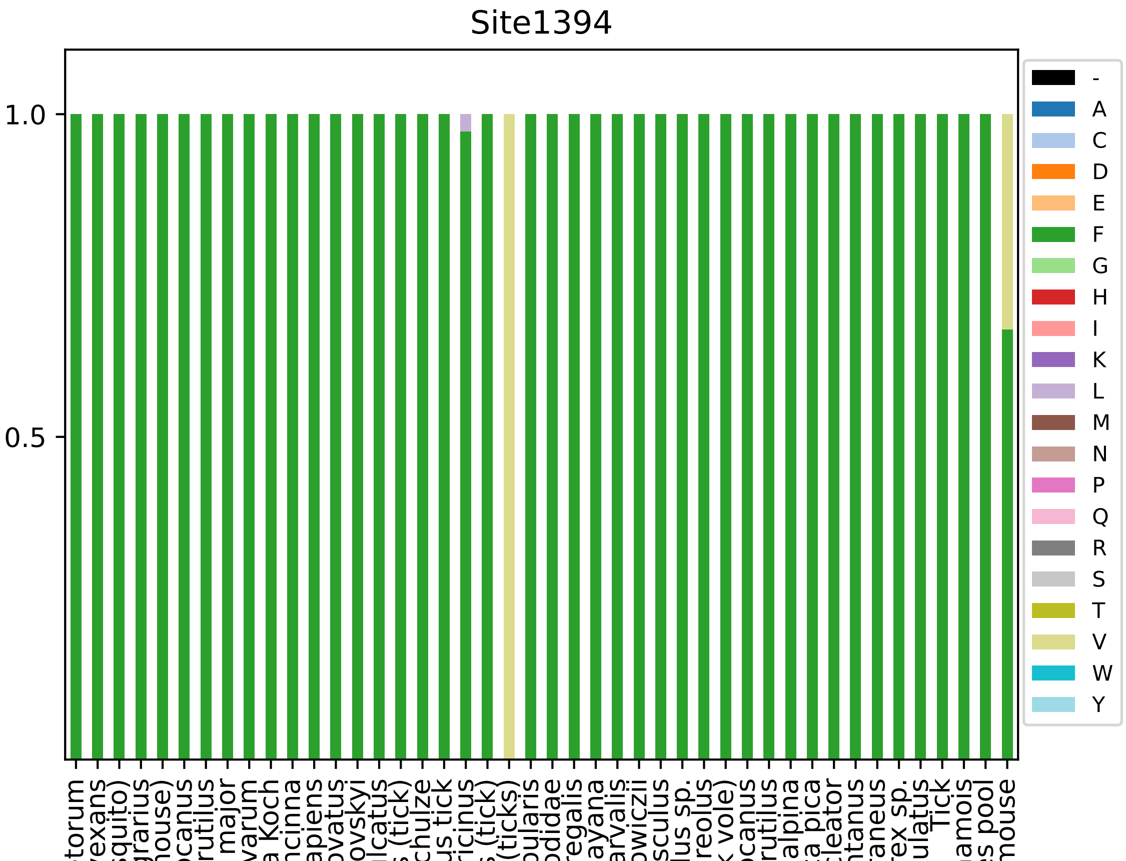
Site1394
1.0
0.5
-
A
C
D
E
F
G
H
I
K
L
M
N
P
Q
R
S
T
V
W
Y

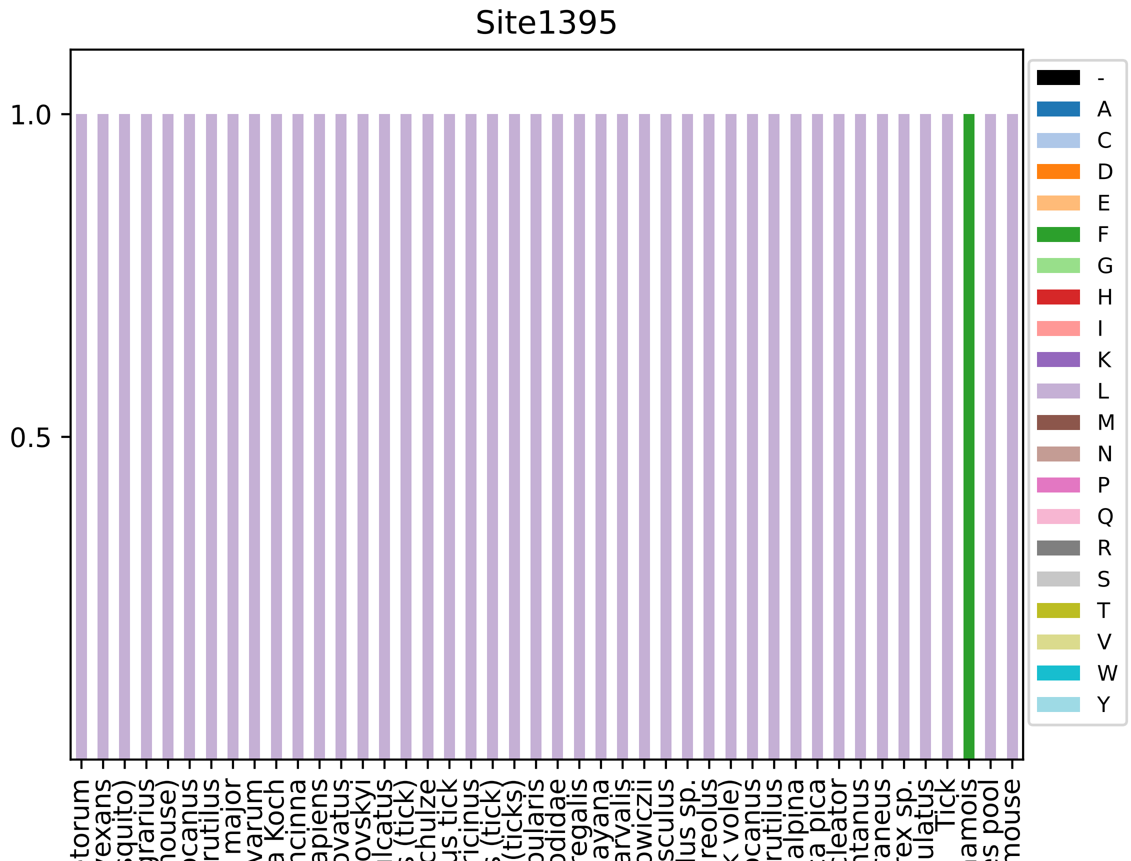
Site1395
1.0
0.5
-
A
C
D
E
F
G
H
I
K
L
M
N
P
Q
R
S
T
V
W
Y

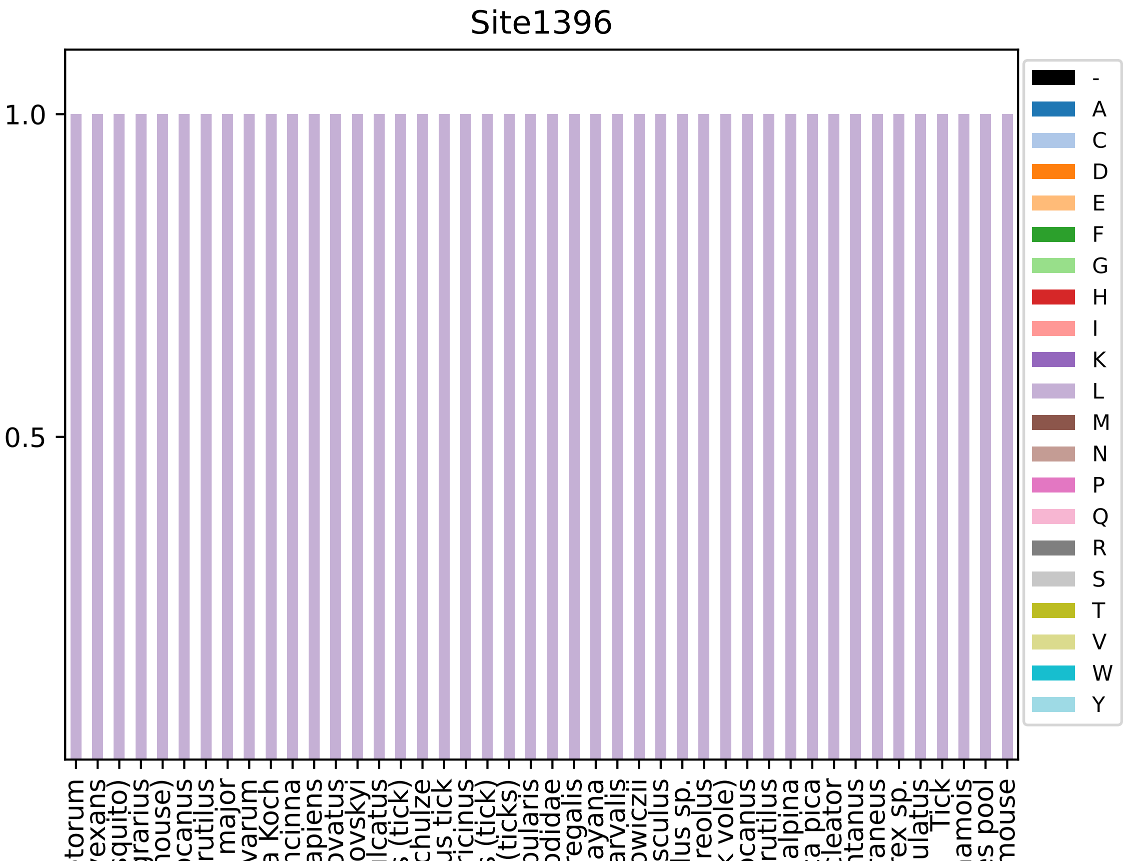
Site1396
1.0
0.5
-
A
C
D
E
F
G
H
I
K
L
M
N
P
Q
R
S
T
V
W
Y

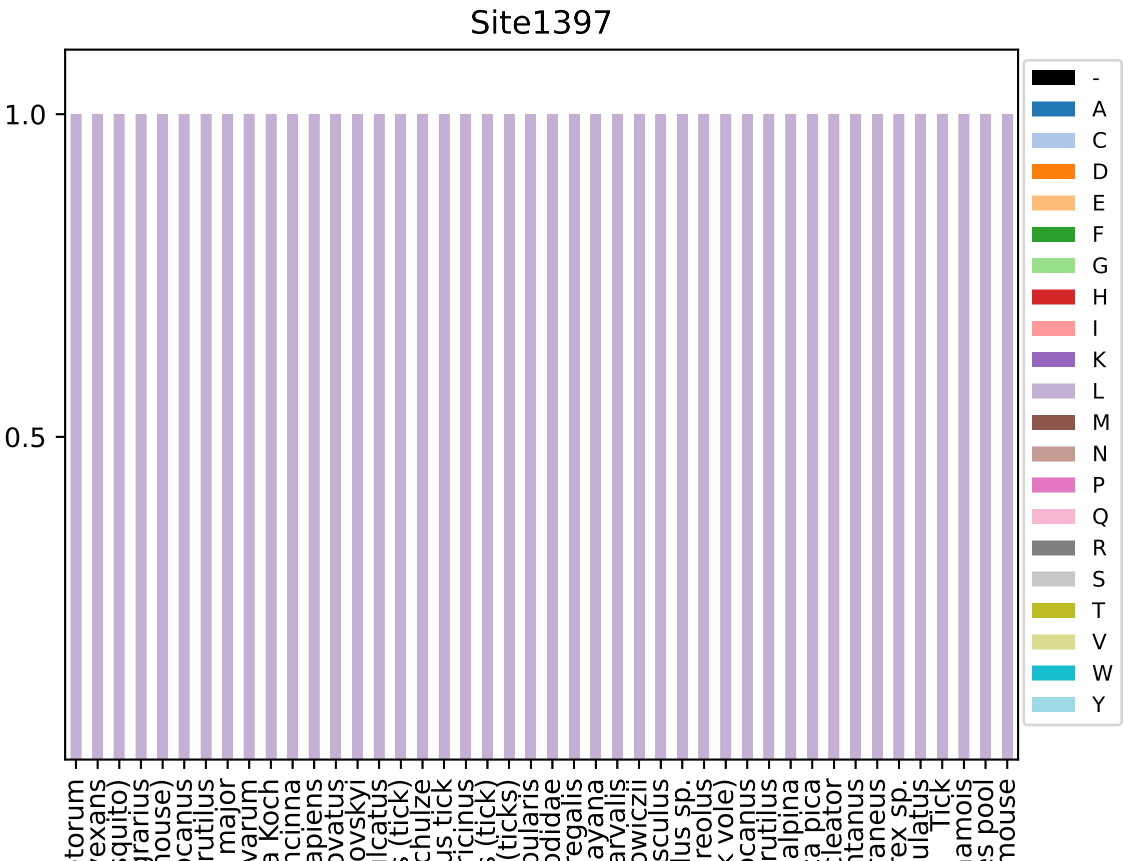
Site1397
1.0
0.5
-
A
C
D
E
F
G
H
I
K
L
M
N
P
Q
R
S
T
V
W
Y
Tick

Site1398

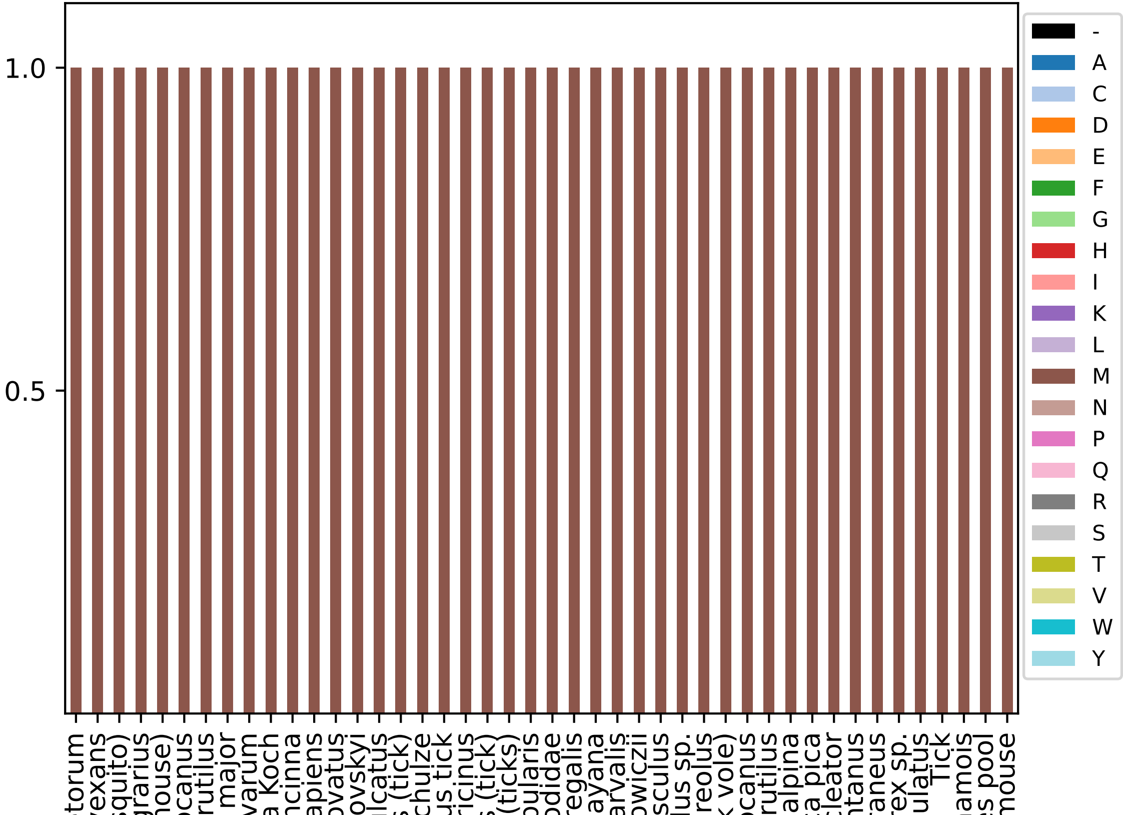

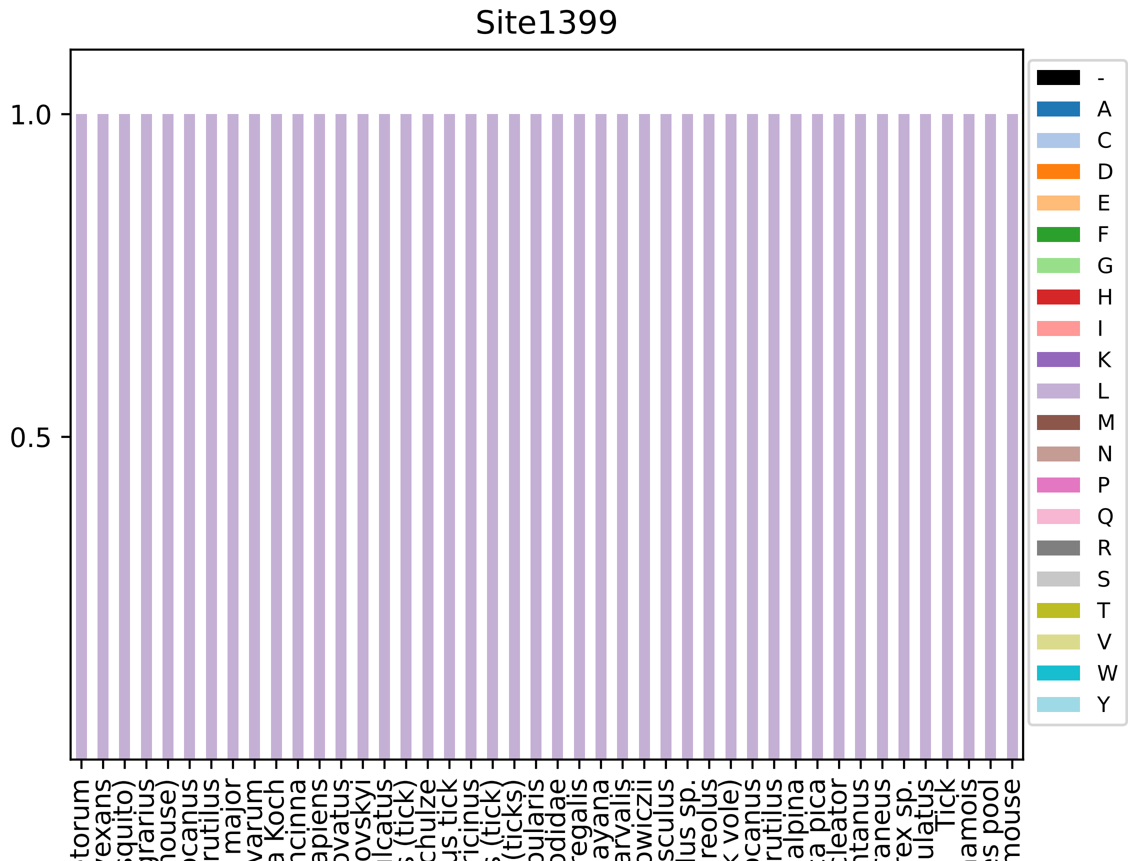
Site1399
1.0
0.5
-
A
C
D
E
F
G
H
I
K
L
M
N
P
Q
R
S
T
V
W
Y

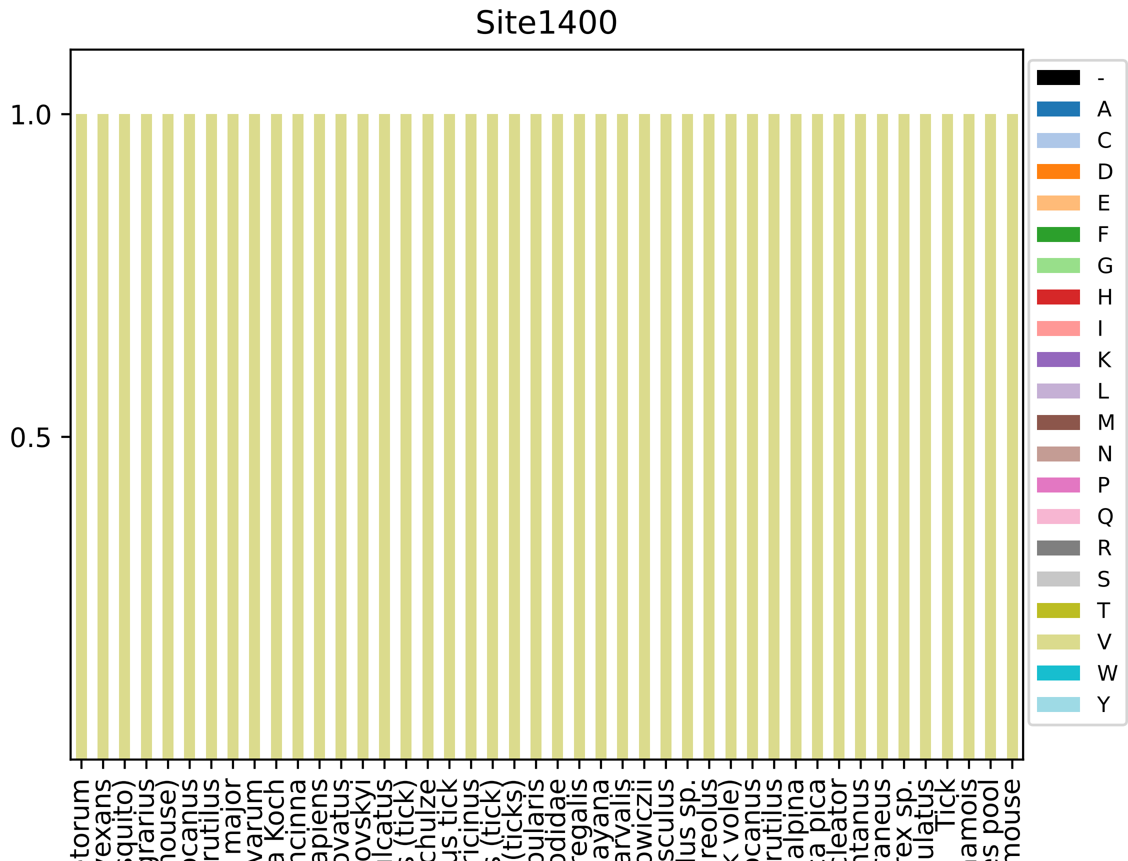
Site1400
1.0
0.5
-
A
C
D
E
F
G
H
I
K
L
M
N
P
Q
R
S
T
V
W
Y
Tick

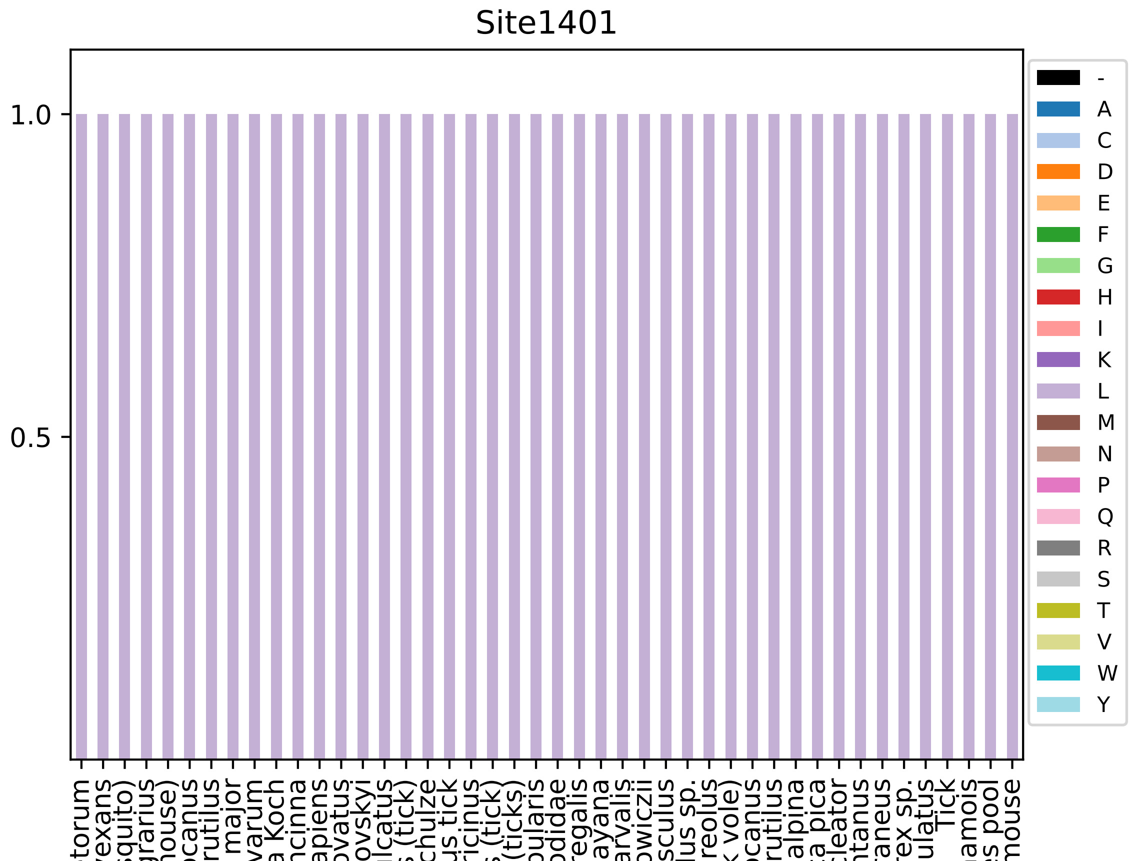

Site1401
1.0
0.5
-
A
C
D
E
F
G
H
I
K
L
M
N
P
Q
R
S
T
V
W
Y

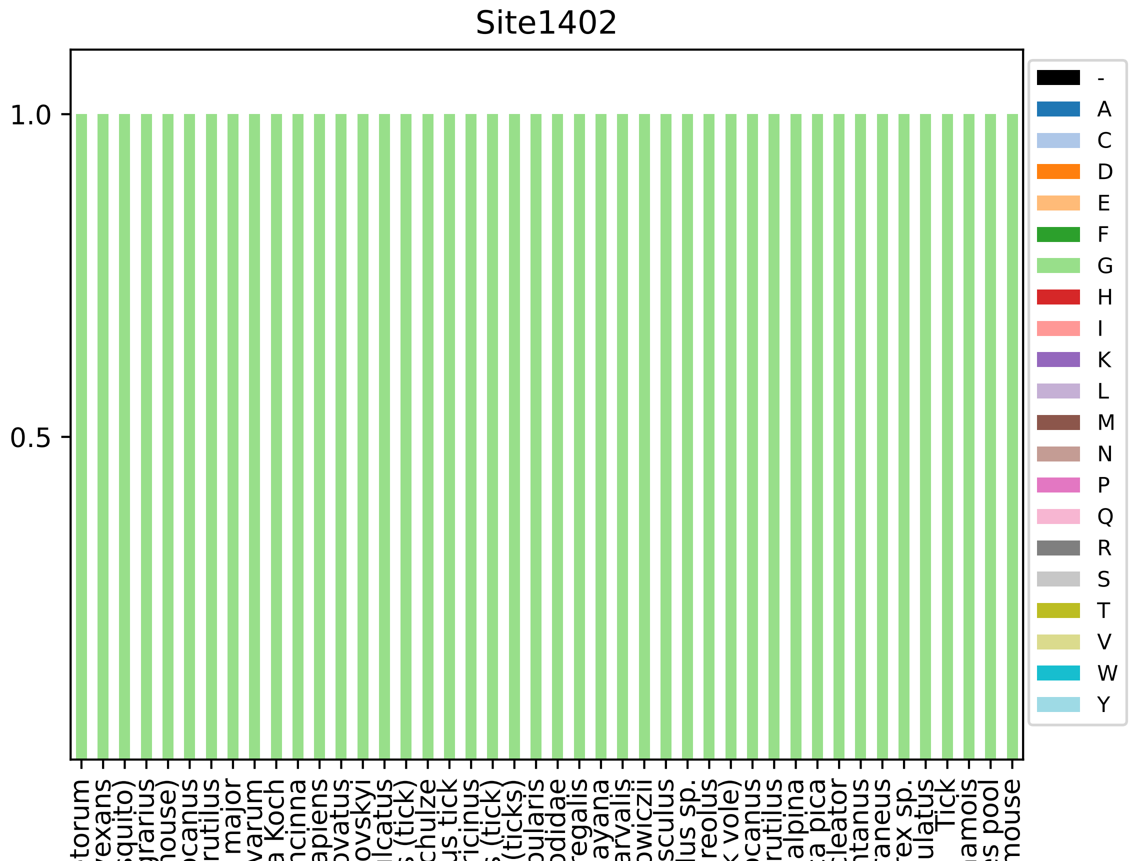
Site1402
1.0
0.5
-
A
C
D
E
F
G
H
I
K
L
M
N
P
Q
R
S
T
V
W
Y

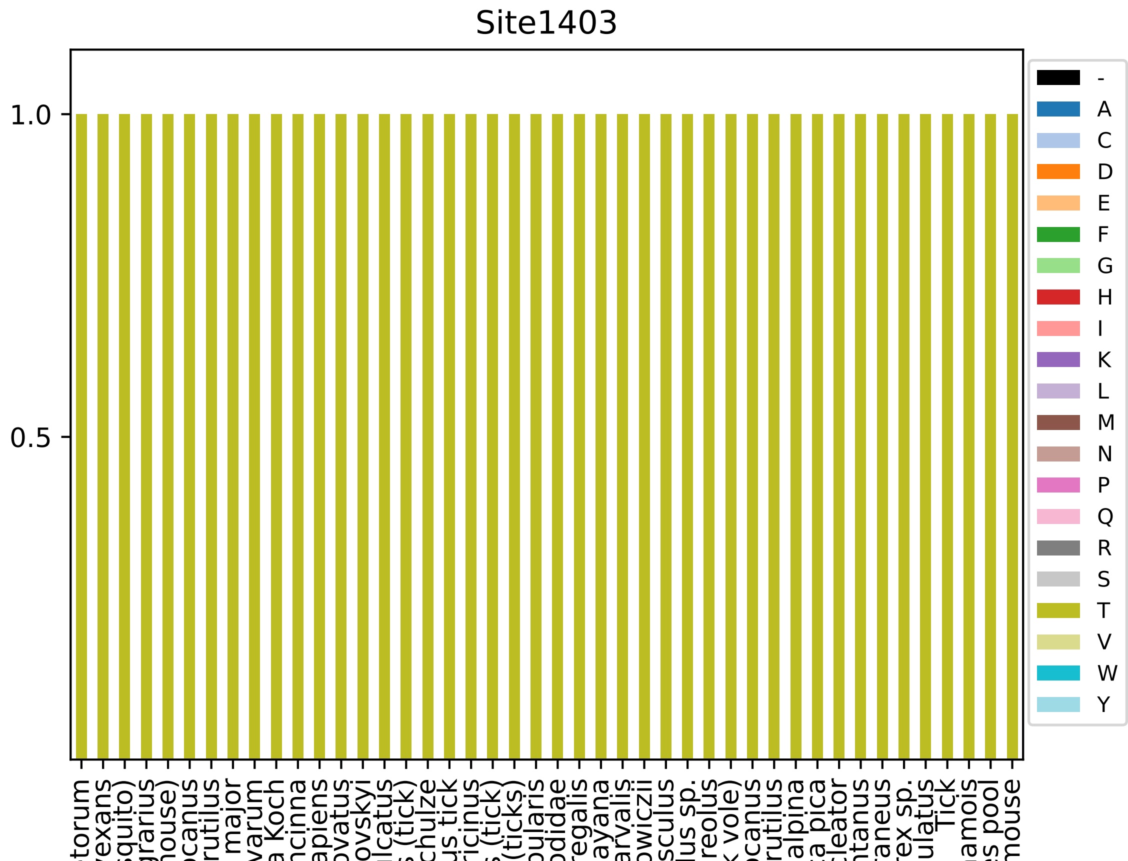
Site1403
1.0
0.5
-
A
C
D
E
F
G
H
I
K
L
M
N
P
Q
R
S
T
V
W
Y

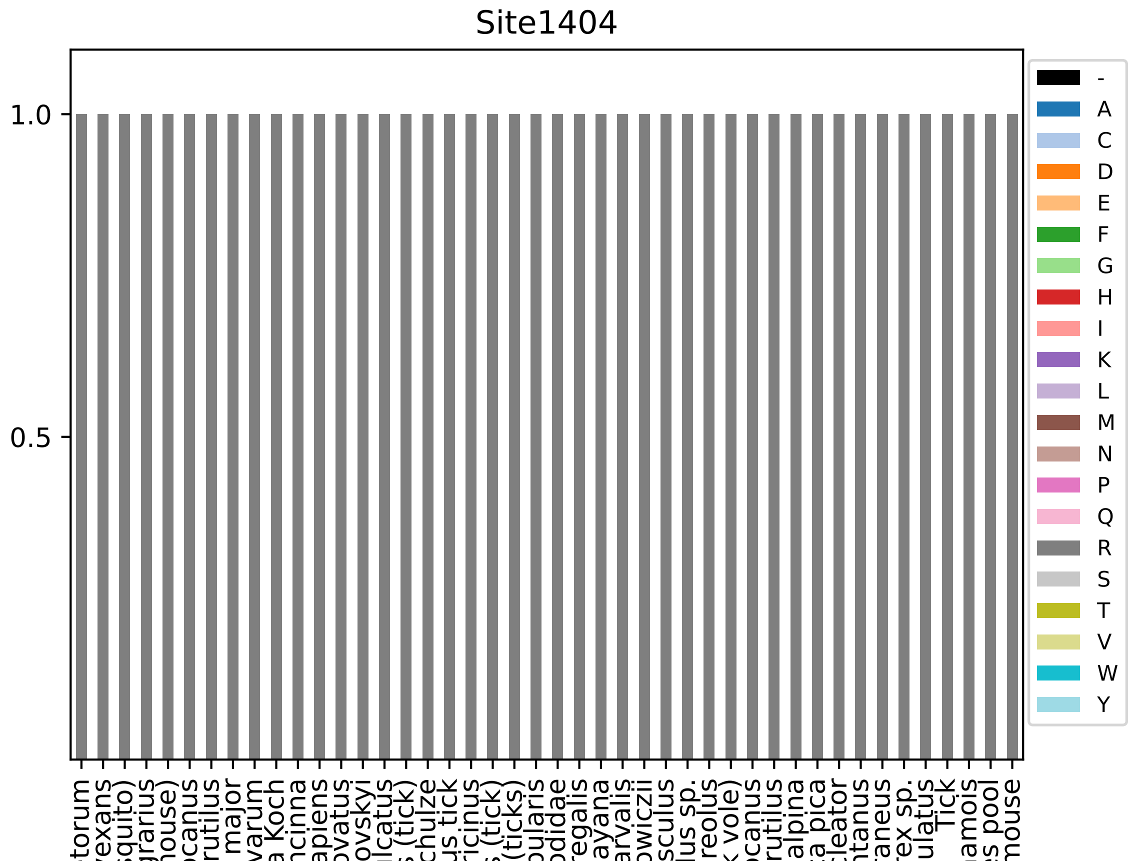
Site1404
1.0
0.5
-
A
C
D
E
F
G
H
I
K
L
M
N
P
Q
R
S
T
V
W
Y

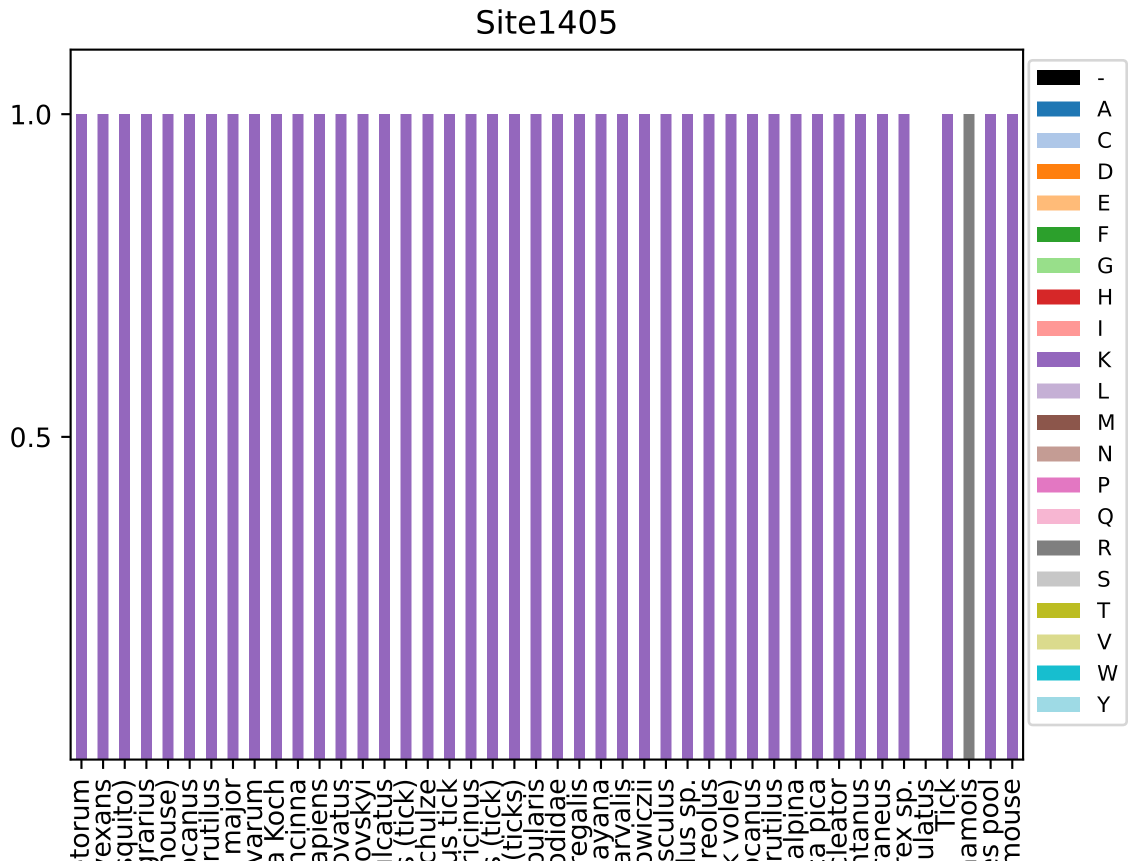
Site1405
1.0
0.5
-
A
C
D
E
F
G
H
I
K
L
M
N
P
Q
R
S
T
V
W
Y
Tick

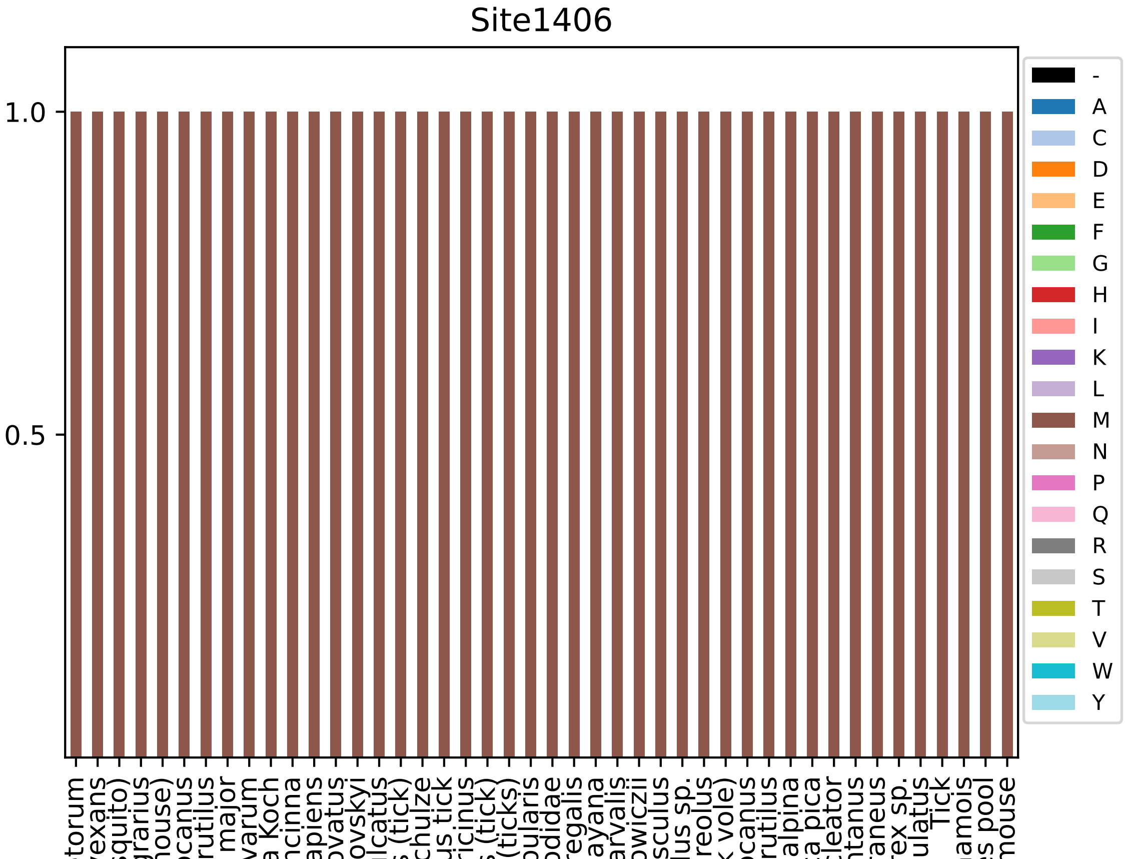
Site1406
1.0
0.5
-
A
C
D
E
F
G
H
I
K
L
M
N
P
Q
R
S
T
V
W
Y

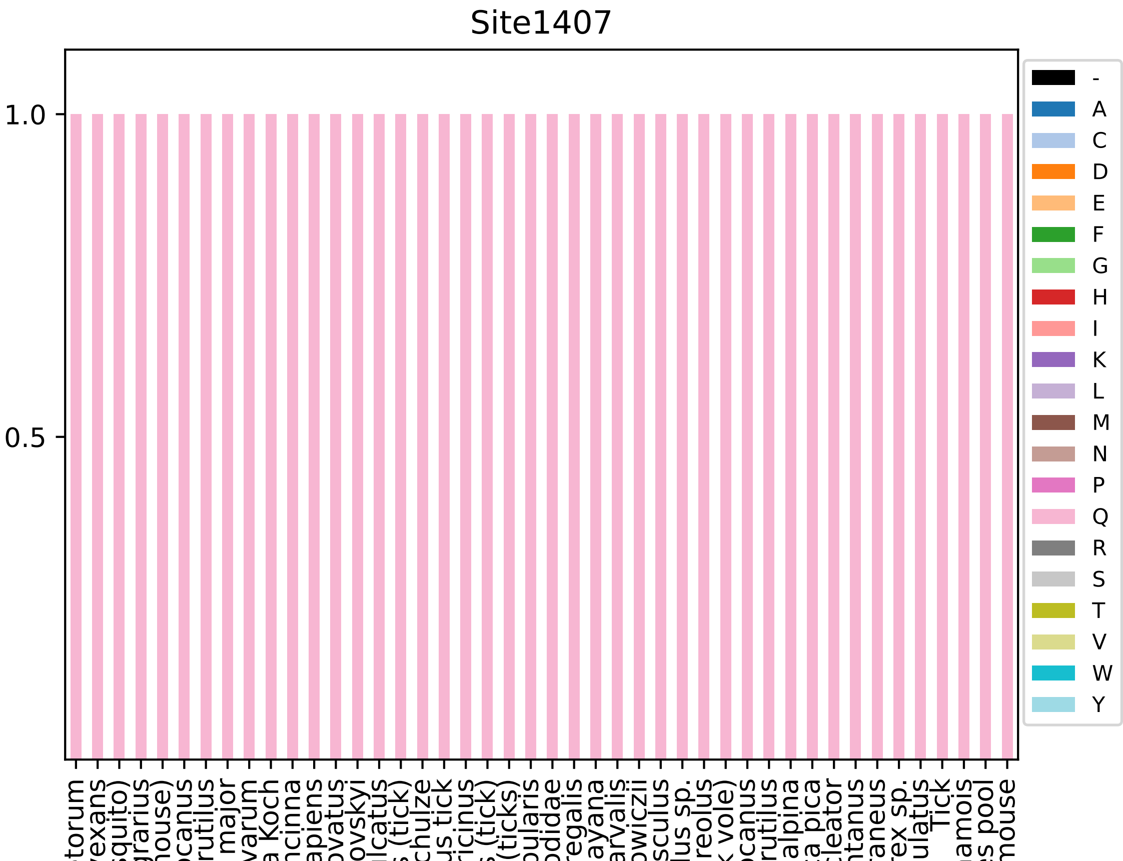
Site1407
1.0
0.5
-
A
C
D
E
F
G
H
I
K
L
M
N
P
Q
R
S
T
V
W
Y

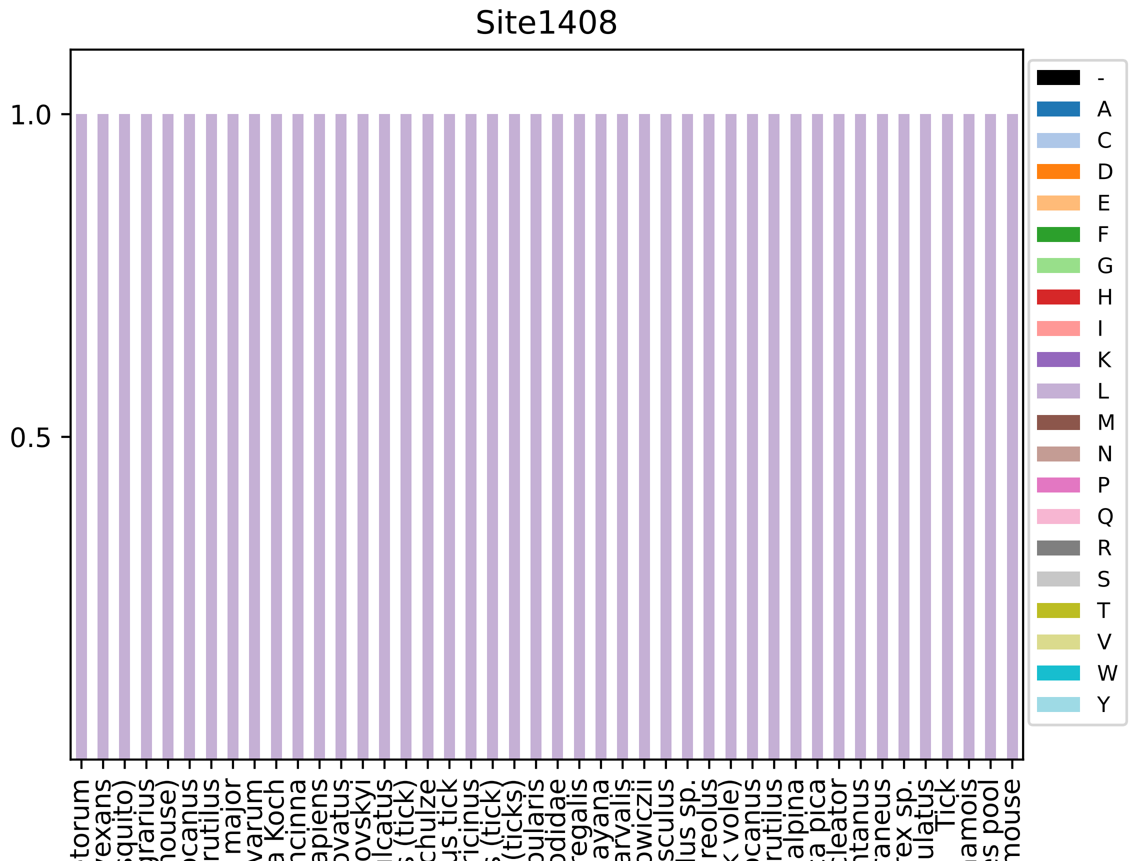

Site1408
1.0
0.5
-
A
C
D
E
F
G
H
I
K
L
M
N
P
Q
R
S
T
V
W
Y

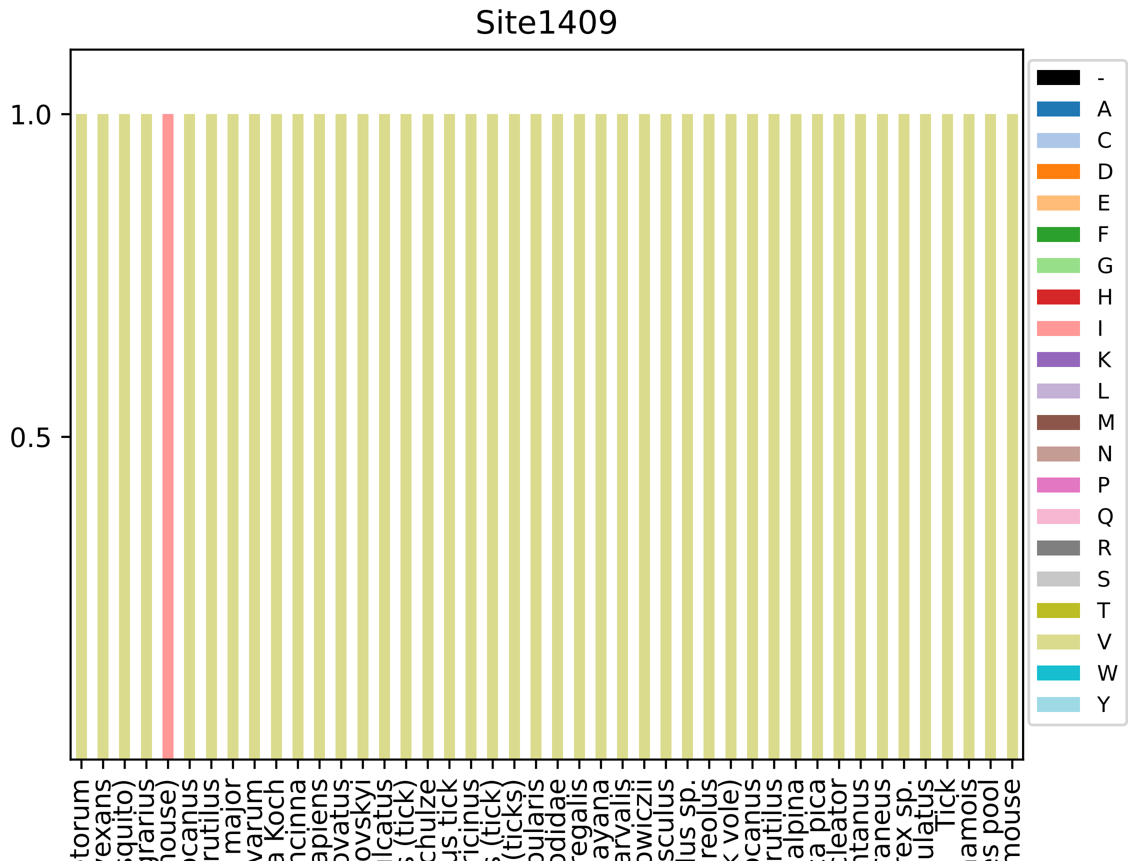
Site1409
1.0
0.5
-
A
C
D
E
F
G
H
I
K
L
M
N
P
Q
R
S
T
V
W
Y
Tick
tick

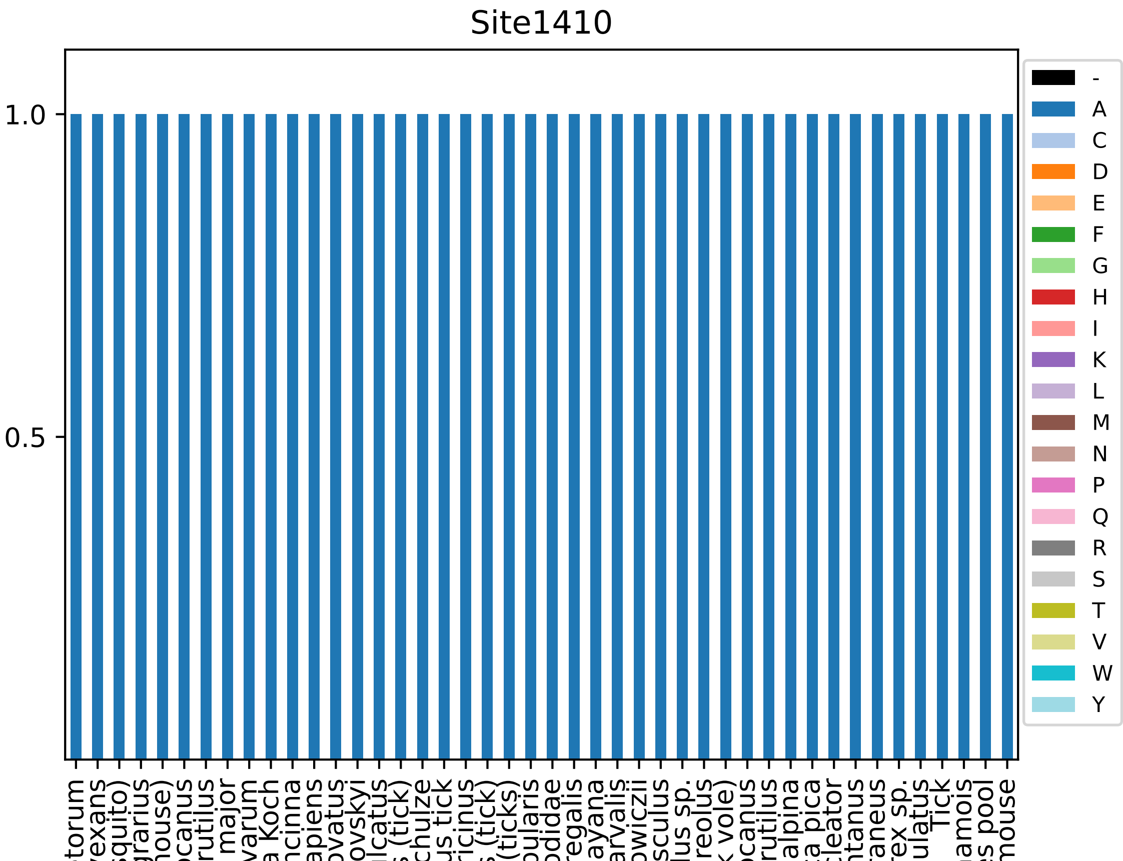
Site1410
1.0
0.5
-
A
C
D
E
F
G
H
I
K
L
M
N
P
Q
R
S
T
V
W
Y

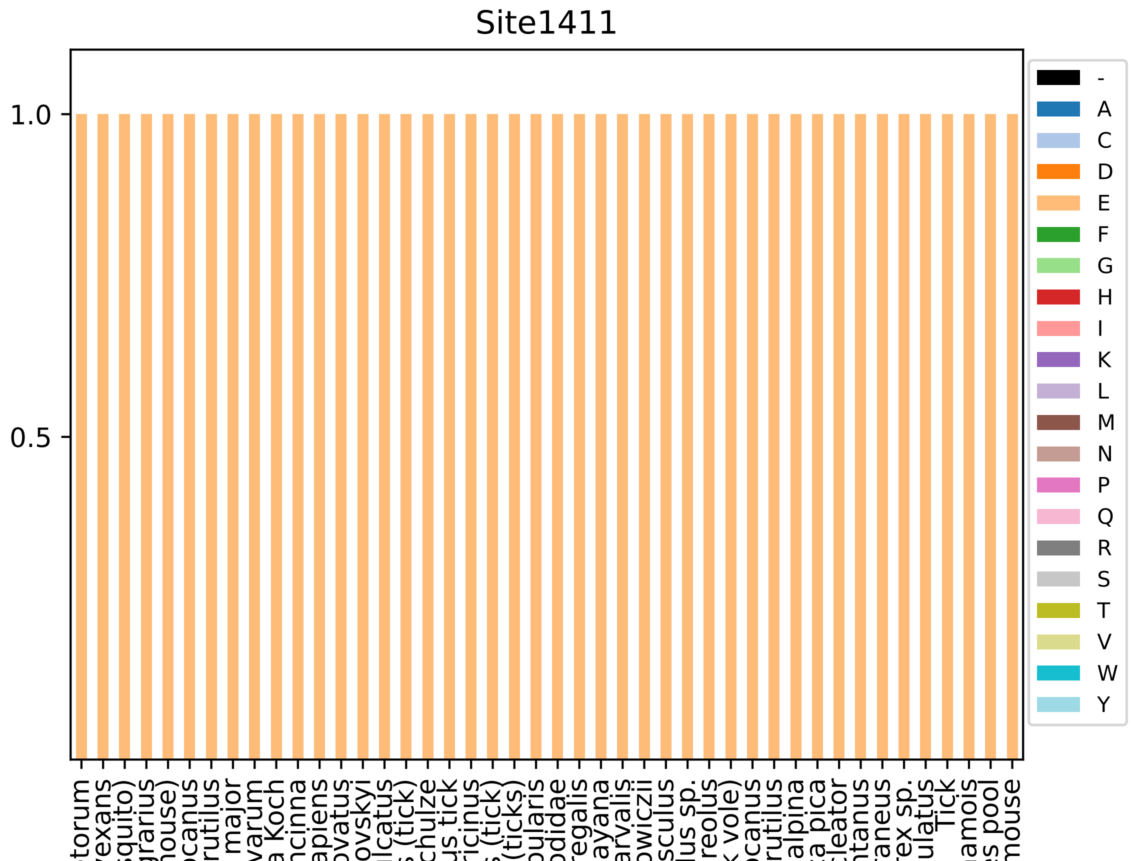

Site1411
1.0
0.5
-
A
C
D
E
F
G
H
I
K
L
M
N
P
Q
R
S
T
V
W
Y

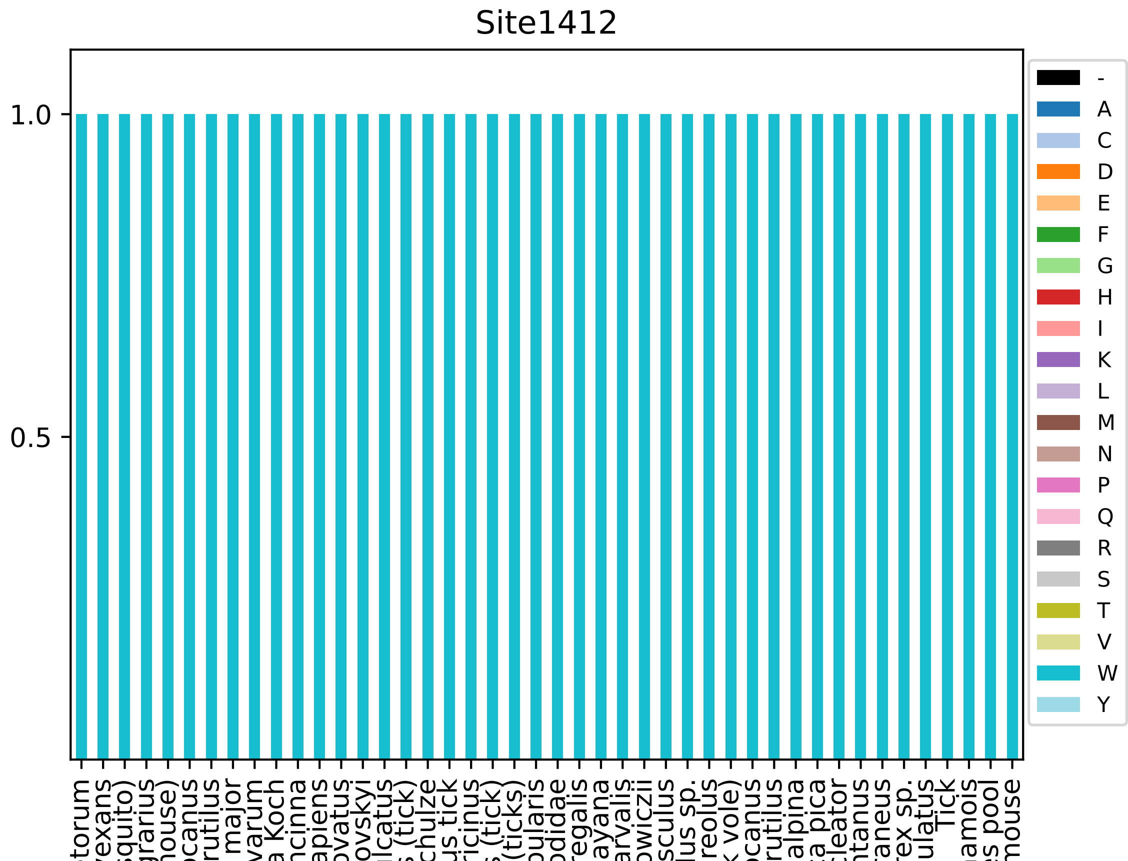
Site1412
1.0
0.5
-
A
C
D
E
F
G
H
I
K
L
M
N
P
Q
R
S
T
V
W
Y

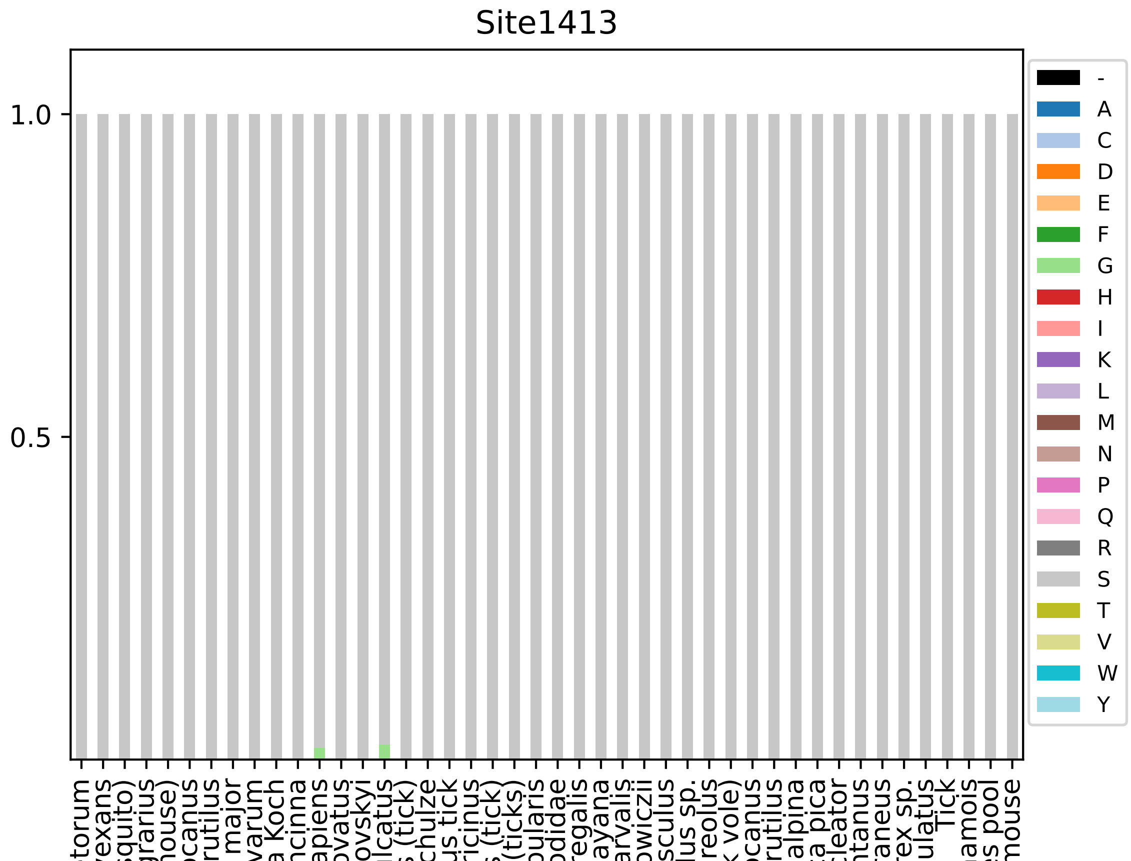

Site1413
1.0
0.5
-
A
C
D
E
F
G
H
I
K
L
M
N
P
Q
R
S
T
V
W
Y

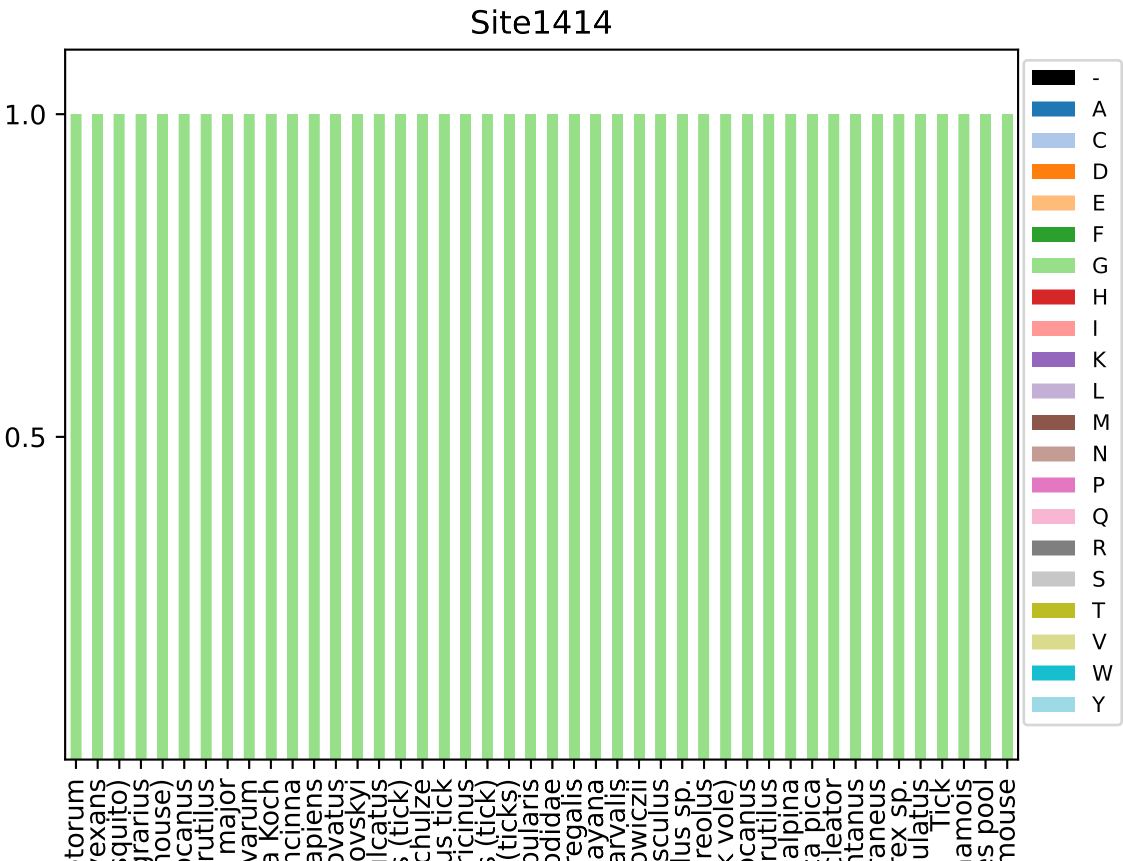
Site1414
1.0
0.5
-
A
C
D
E
F
G
H
I
K
L
M
N
P
Q
R
S
T
V
W
Y

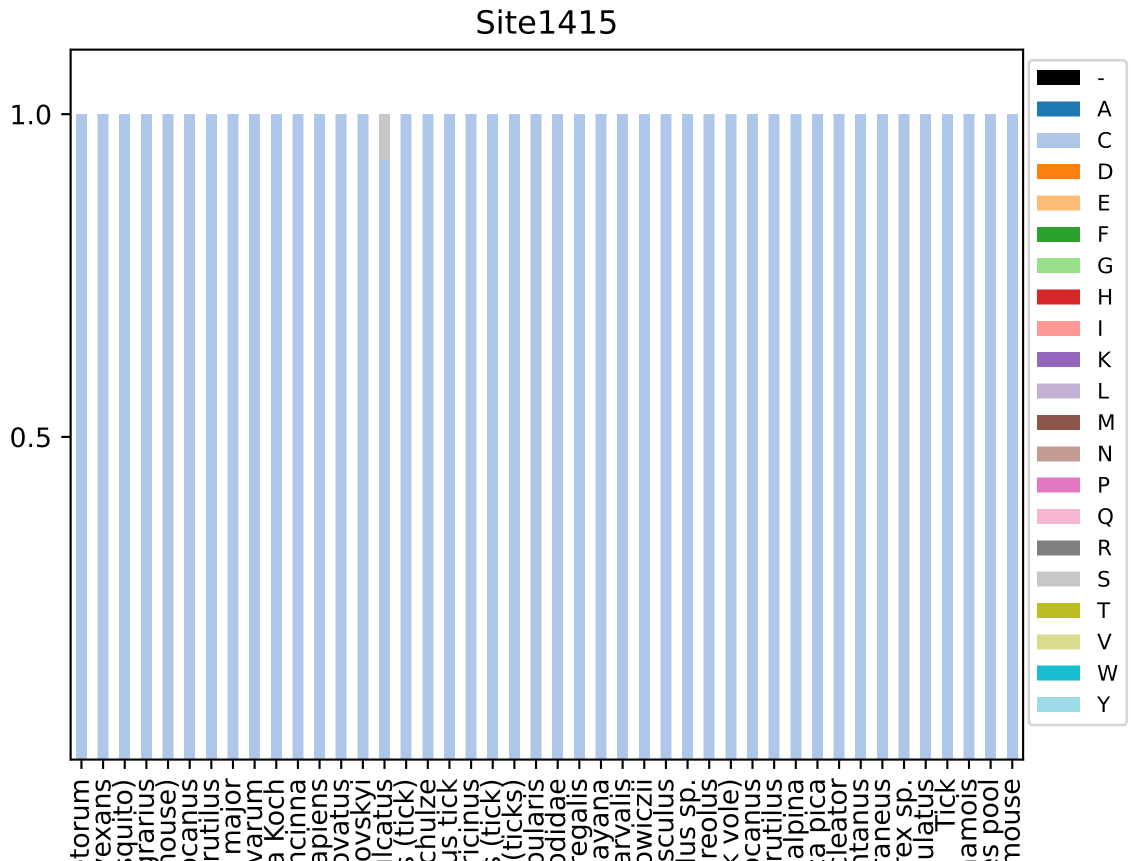
Site1415
1.0
0.5
-
A
C
D
E
F
G
H
I
K
L
M
N
P
Q
R
S
T
V
W
Y

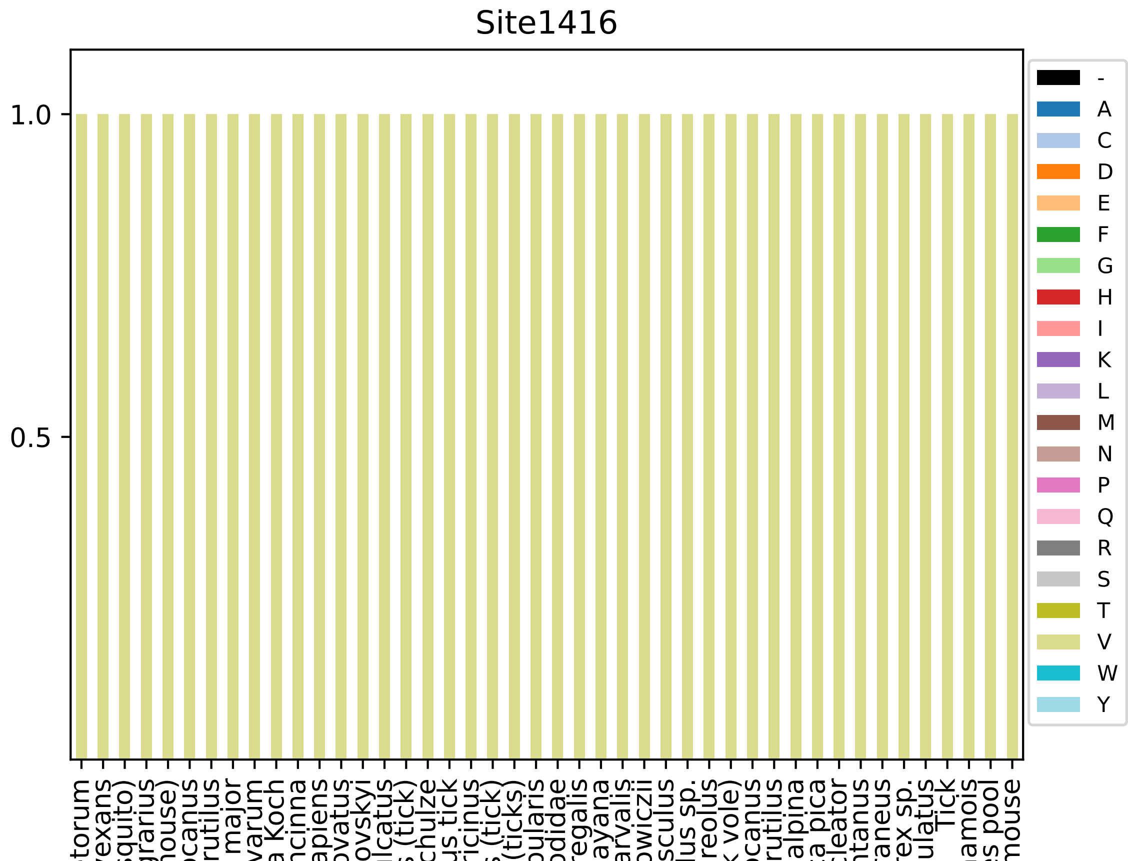
Site1416
1.0
0.5
-
A
C
D
E
F
G
H
I
K
L
M
N
P
Q
R
S
T
V
W
Y

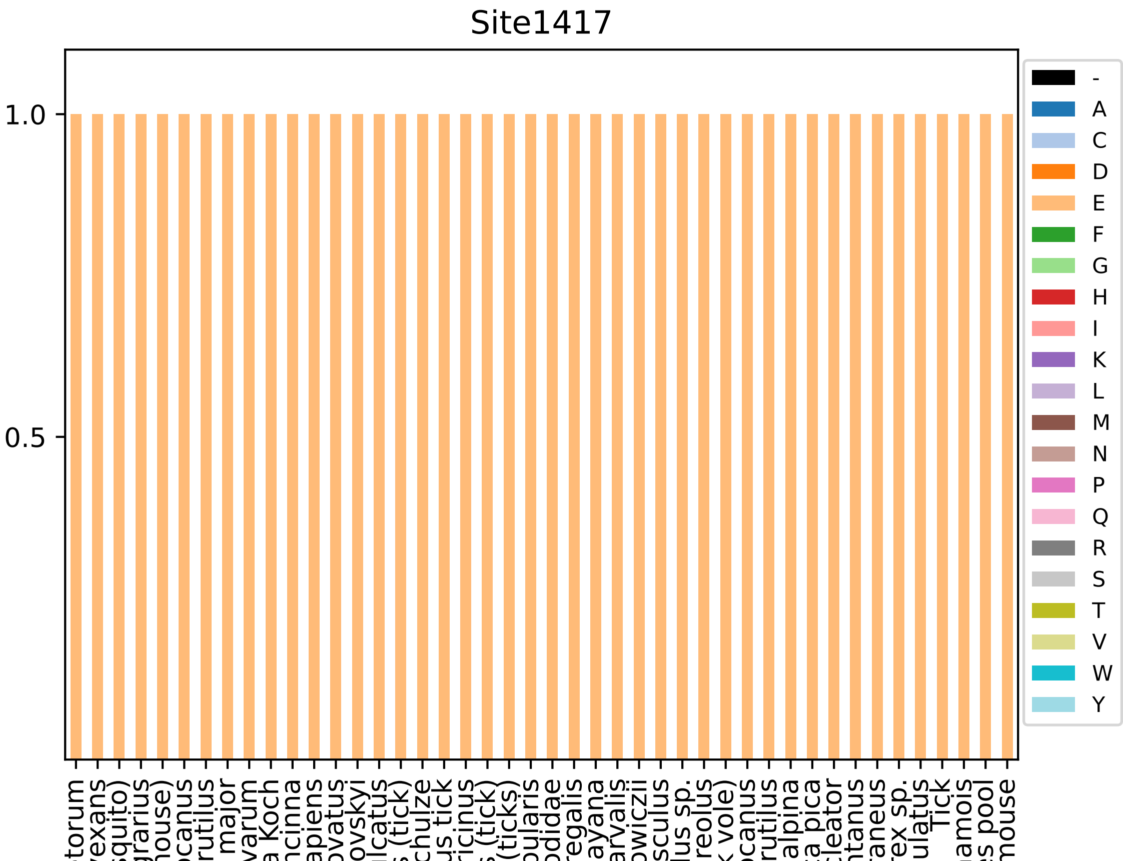
Site1417
1.0
0.5
-
A
C
D
E
F
G
H
I
K
L
M
N
P
Q
R
S
T
V
W
Y

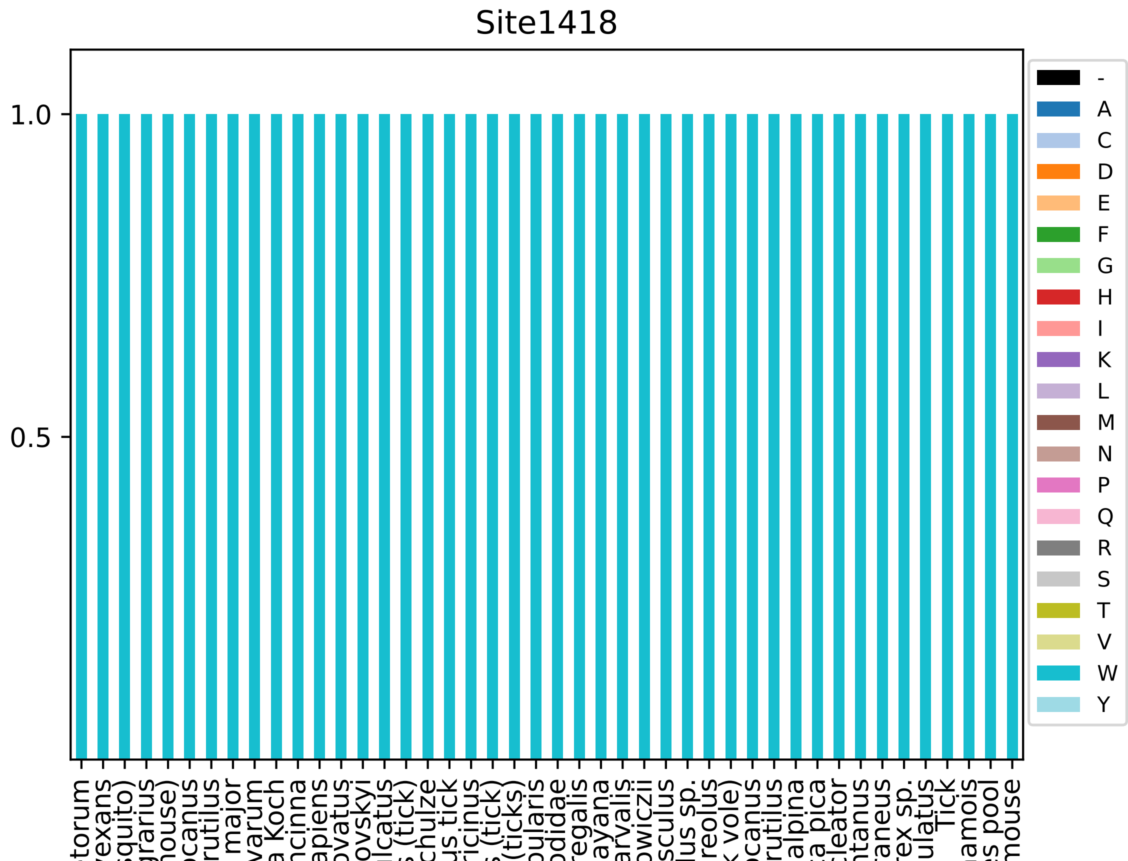
Site1418
1.0
0.5
-
A
C
D
E
F
G
H
I
K
L
M
N
P
Q
R
S
T
V
W
Y

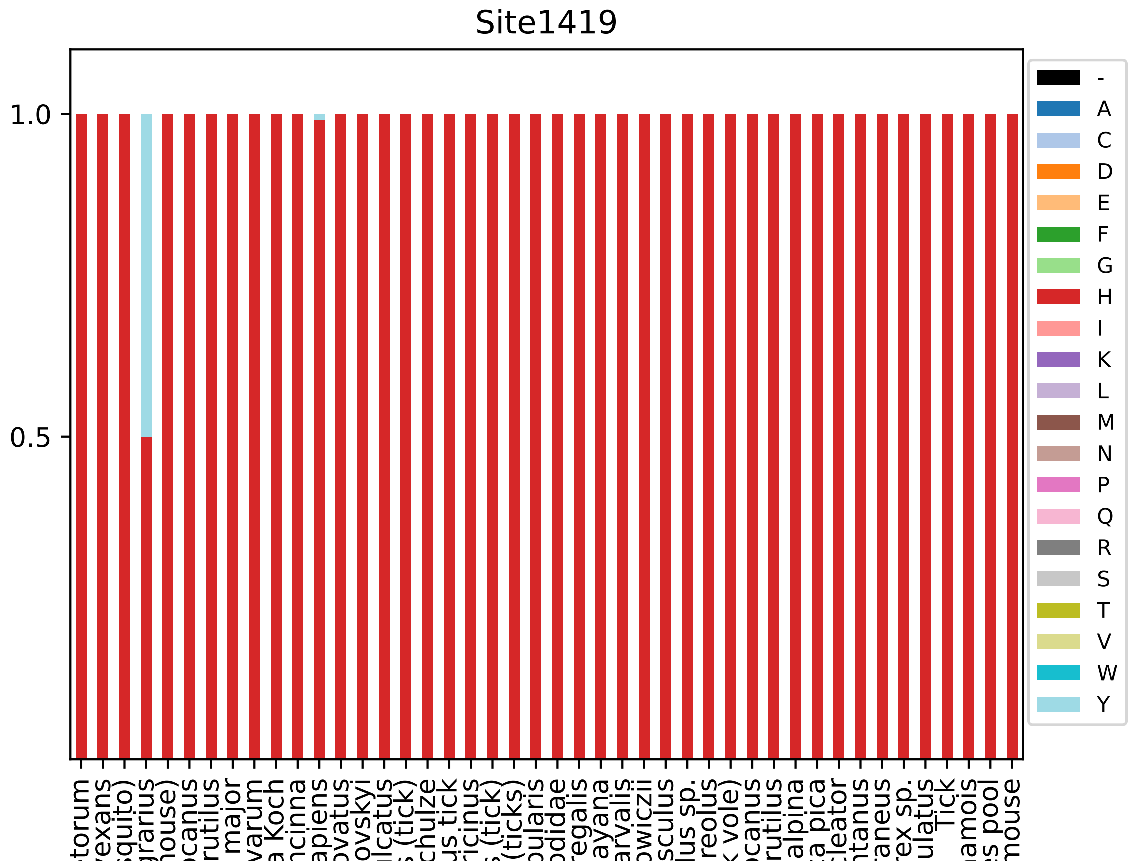

Site1419
1.0
0.5
-
A
C
D
E
F
G
H
I
K
L
M
N
P
Q
R
S
T
V
W
Y

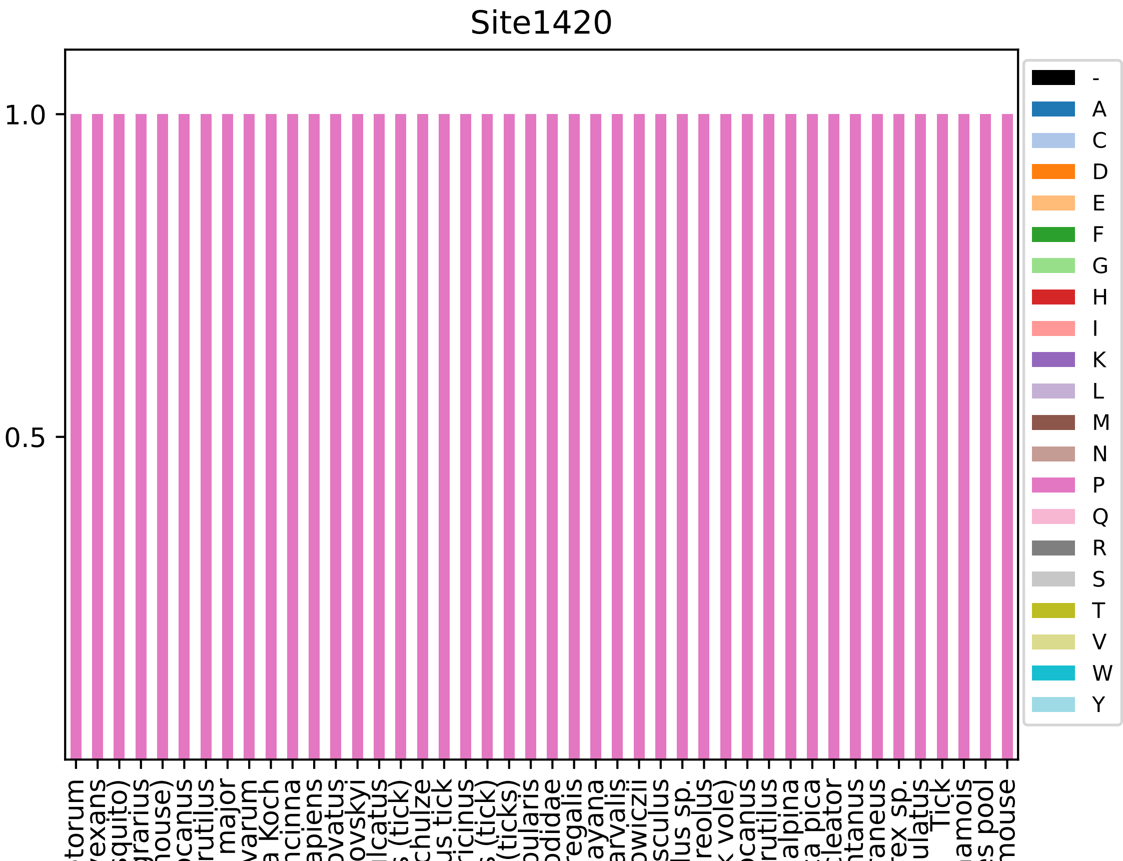
Site1420
1.0
0.5
-
A
C
D
E
F
G
H
I
K
L
M
N
P
Q
R
S
T
V
W
Y

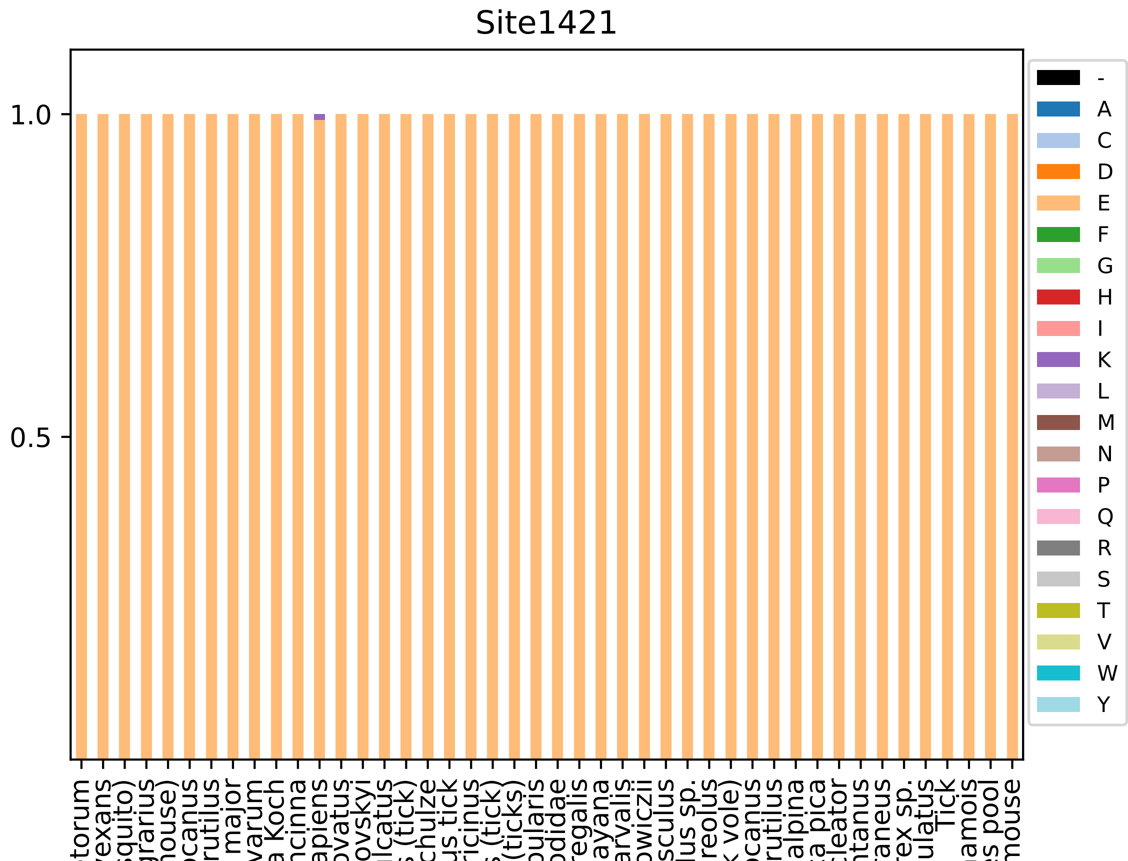
Site1421
1.0
0.5
-
A
C
D
E
F
G
H
I
K
L
M
N
P
Q
R
S
T
V
W
Y

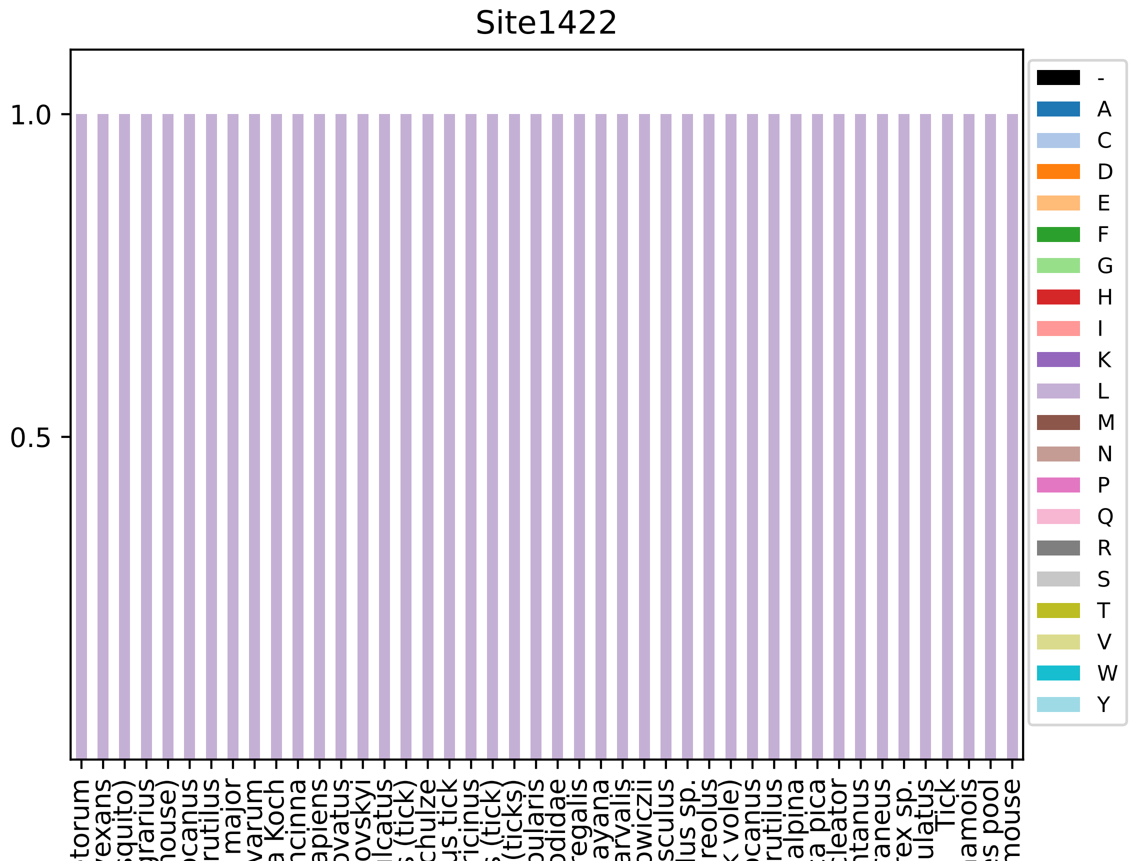
Site1422
1.0
0.5
-
A
C
D
E
F
G
H
I
K
L
M
N
P
Q
R
S
T
V
W
Y
Tick

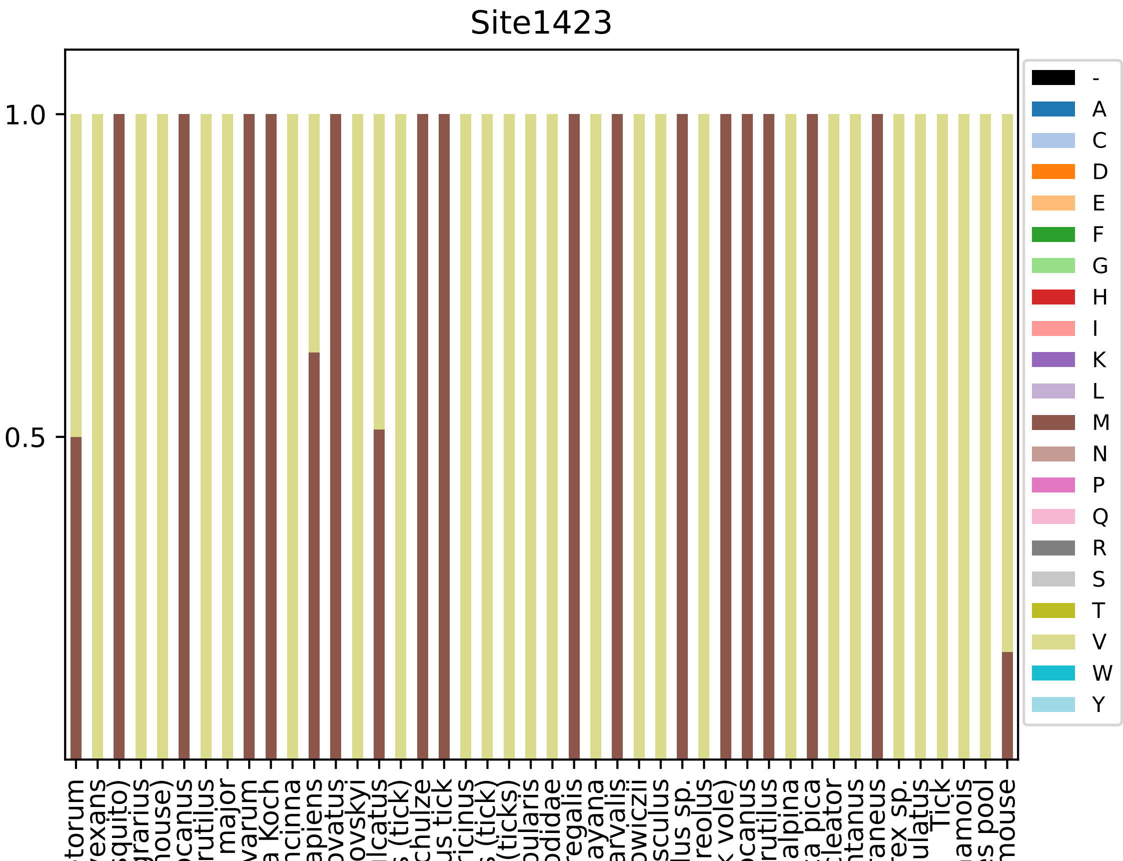
Site1423
1.0
0.5
-
A
C
D
E
F
G
H
I
K
L
M
N
P
Q
R
S
T
V
W
Y
Tick

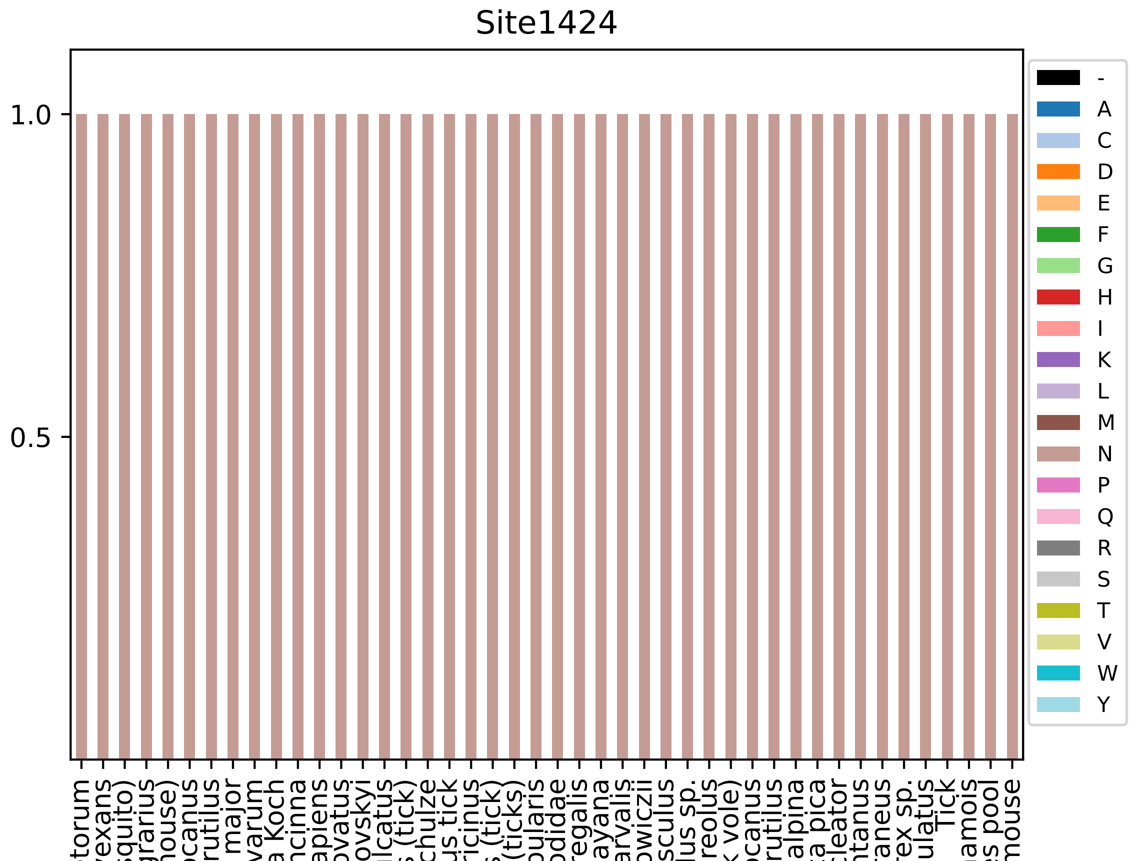
Site1424
1.0
0.5
-
A
C
D
E
F
G
H
I
K
L
M
N
P
Q
R
S
T
V
W
Y

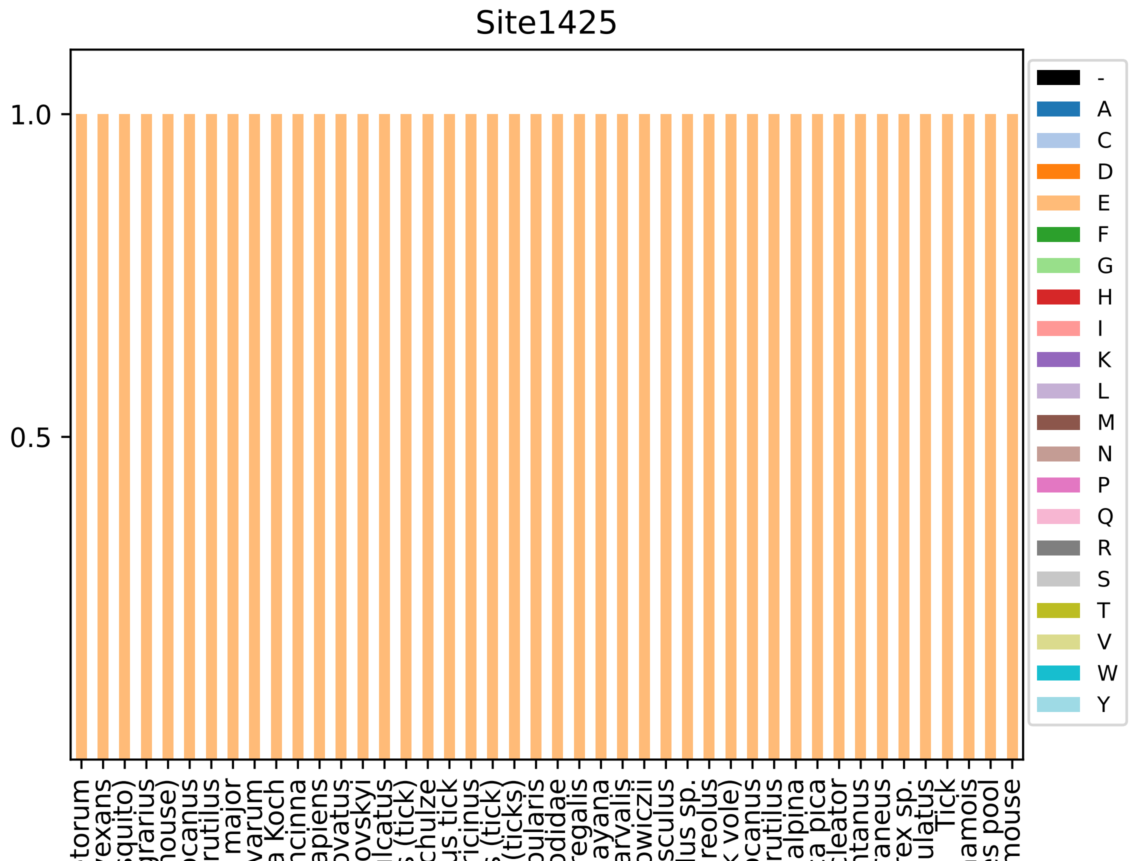
Site1425
1.0
0.5
-
A
C
D
E
F
G
H
I
K
L
M
N
P
Q
R
S
T
V
W
Y

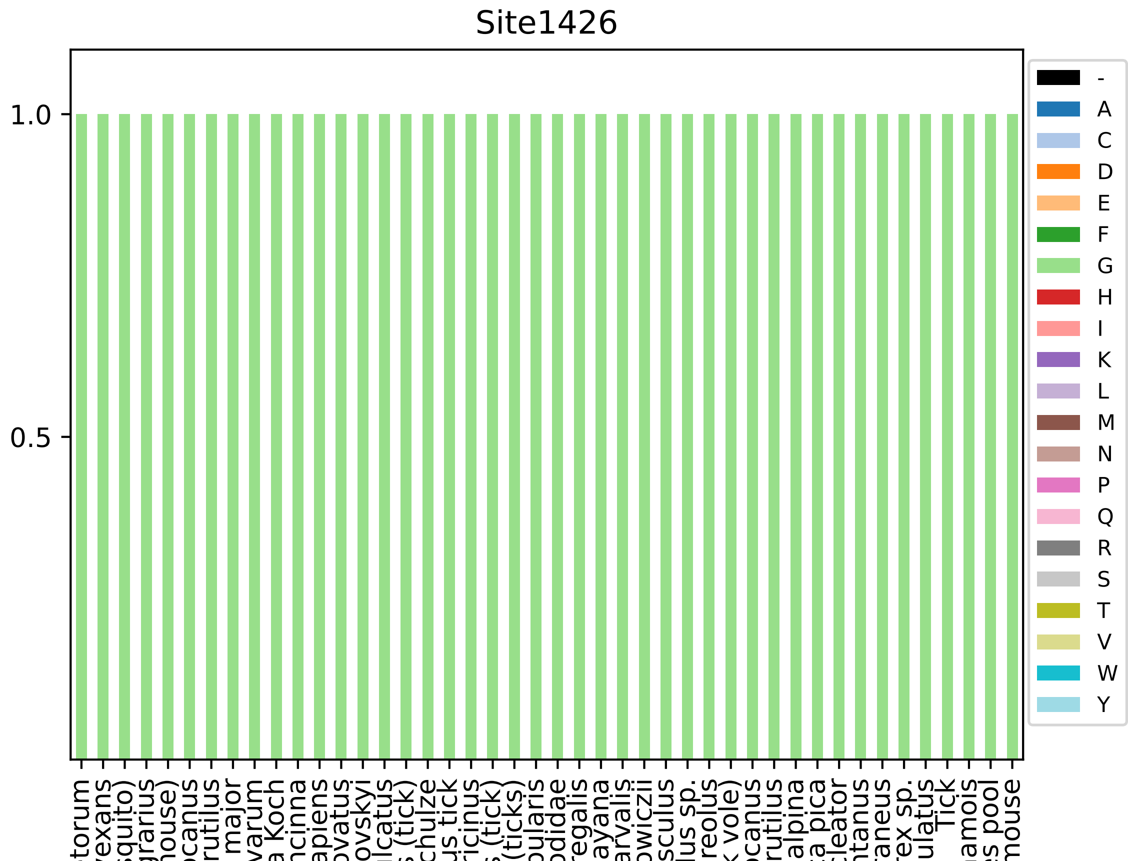
Site1426
1.0
0.5
-
A
C
D
E
F
G
H
I
K
L
M
N
P
Q
R
S
T
V
W
Y

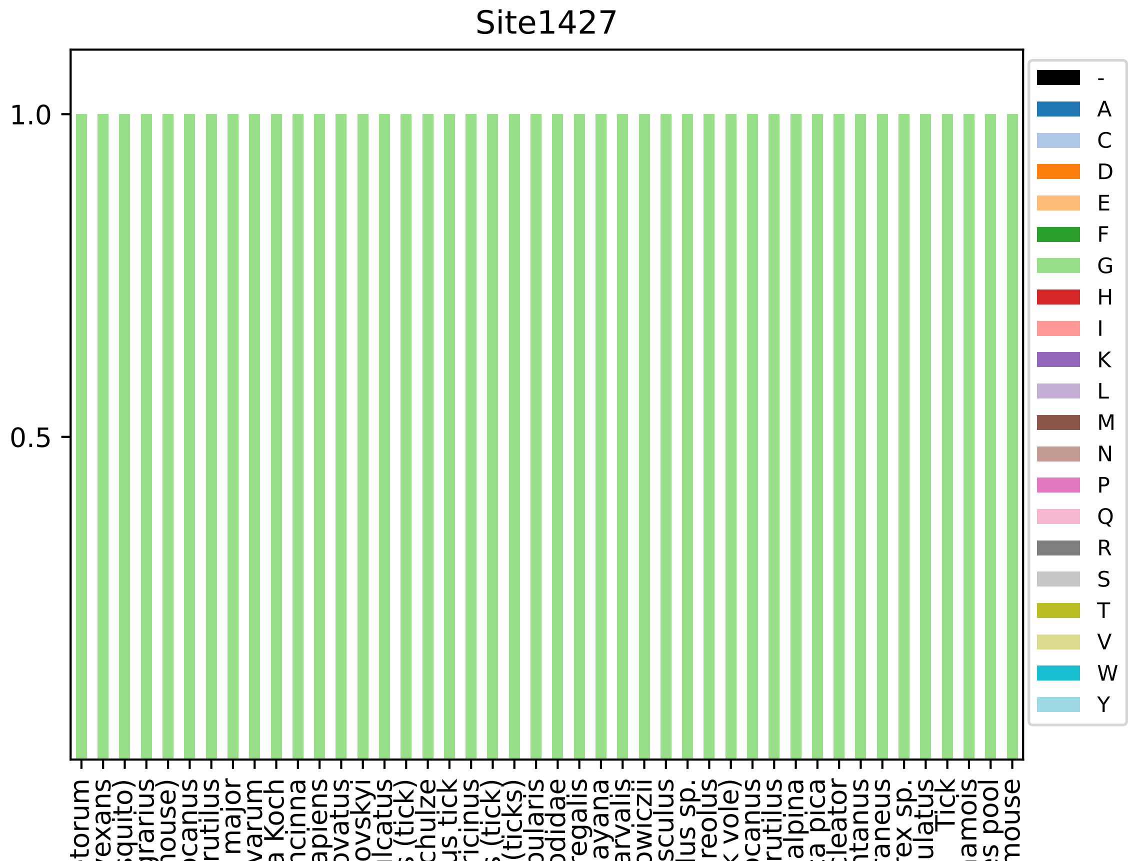
Site1427
1.0
0.5
-
A
C
D
E
F
G
H
I
K
L
M
N
P
Q
R
S
T
V
W
Y

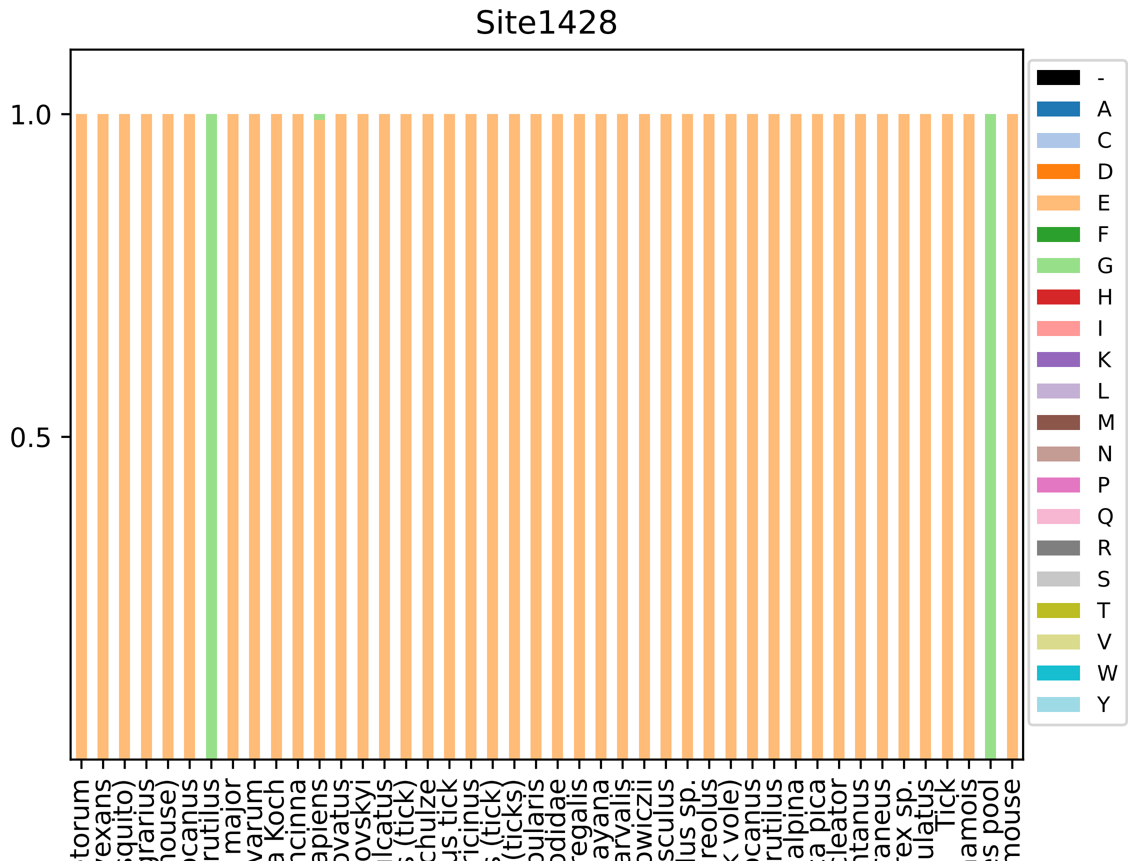
Site1428
1.0
0.5
-
A
C
D
E
F
G
H
I
K
L
M
N
P
Q
R
S
T
V
W
Y

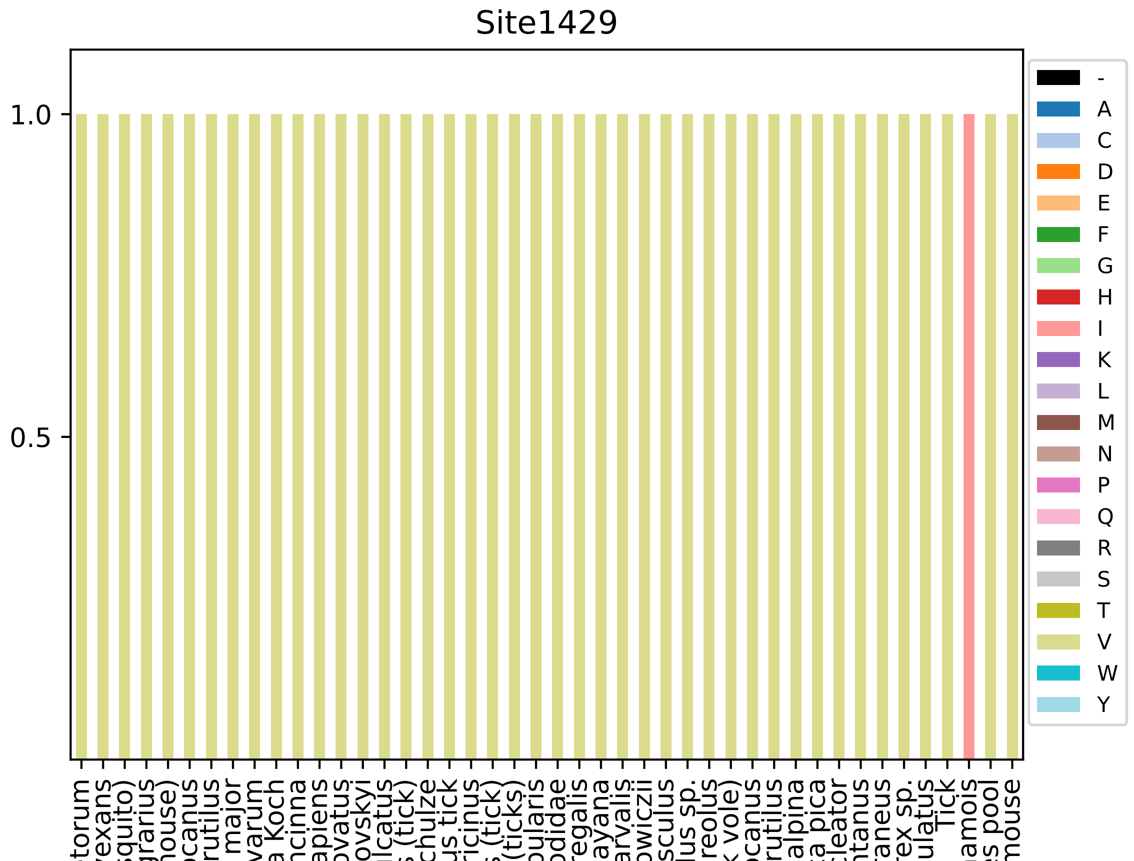

Site1429
1.0
0.5
-
A
C
D
E
F
G
H
I
K
L
M
N
P
Q
R
S
T
V
W
Y
Tick

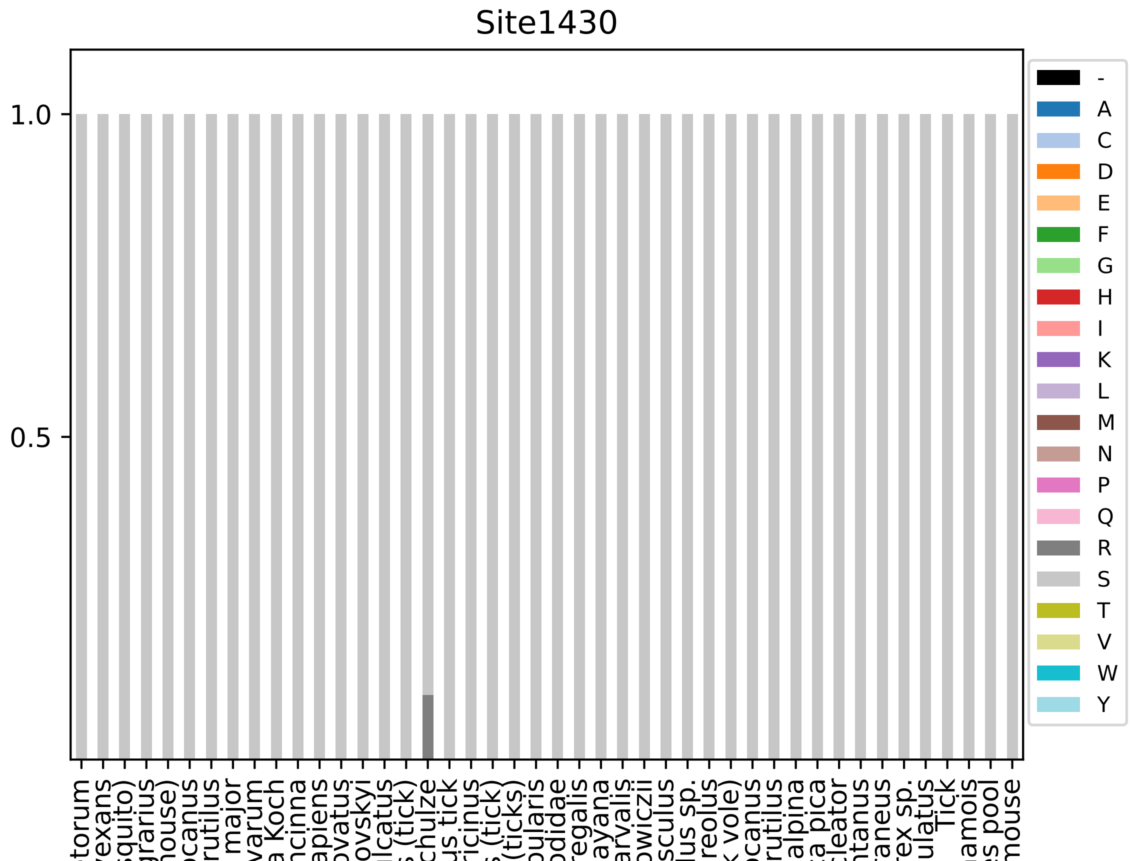

Site1430
1.0
0.5
-
A
C
D
E
F
G
H
I
K
L
M
N
P
Q
R
S
T
V
W
Y

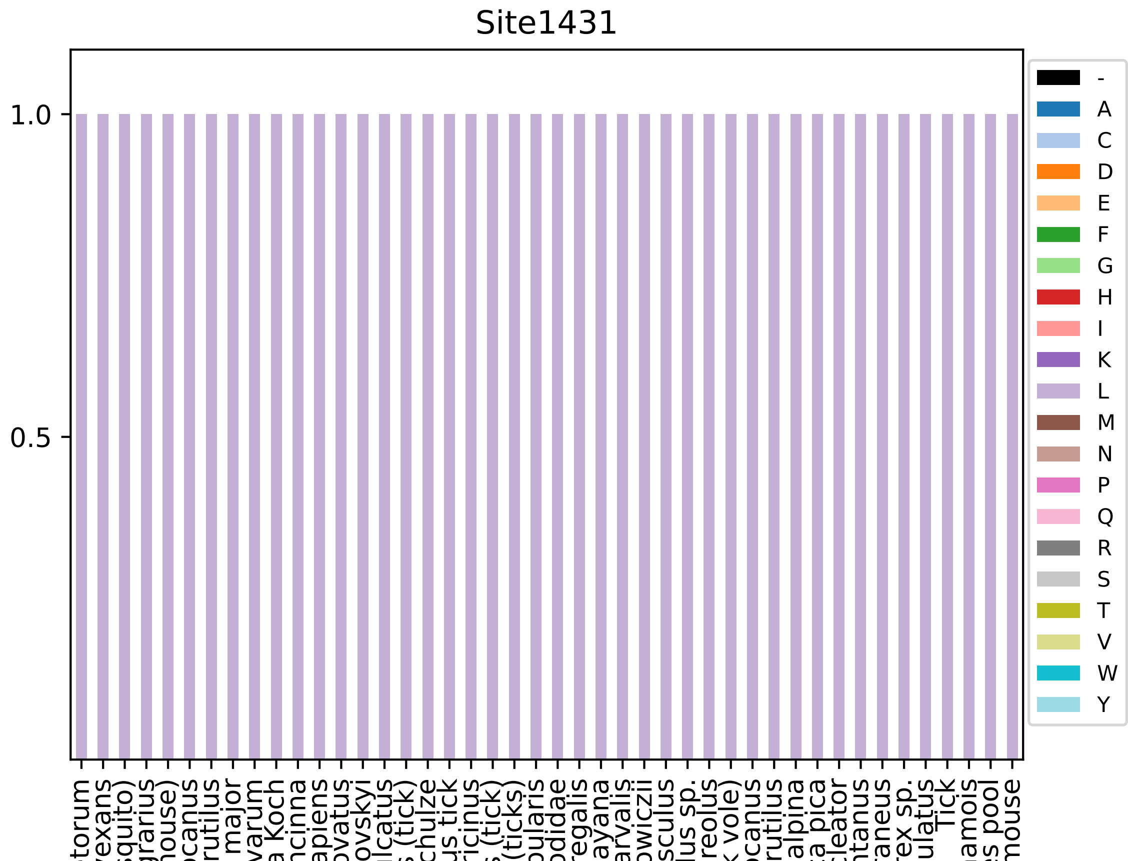
Site1431
1.0
0.5
-
A
C
D
E
F
G
H
I
K
L
M
N
P
Q
R
S
T
V
W
Y

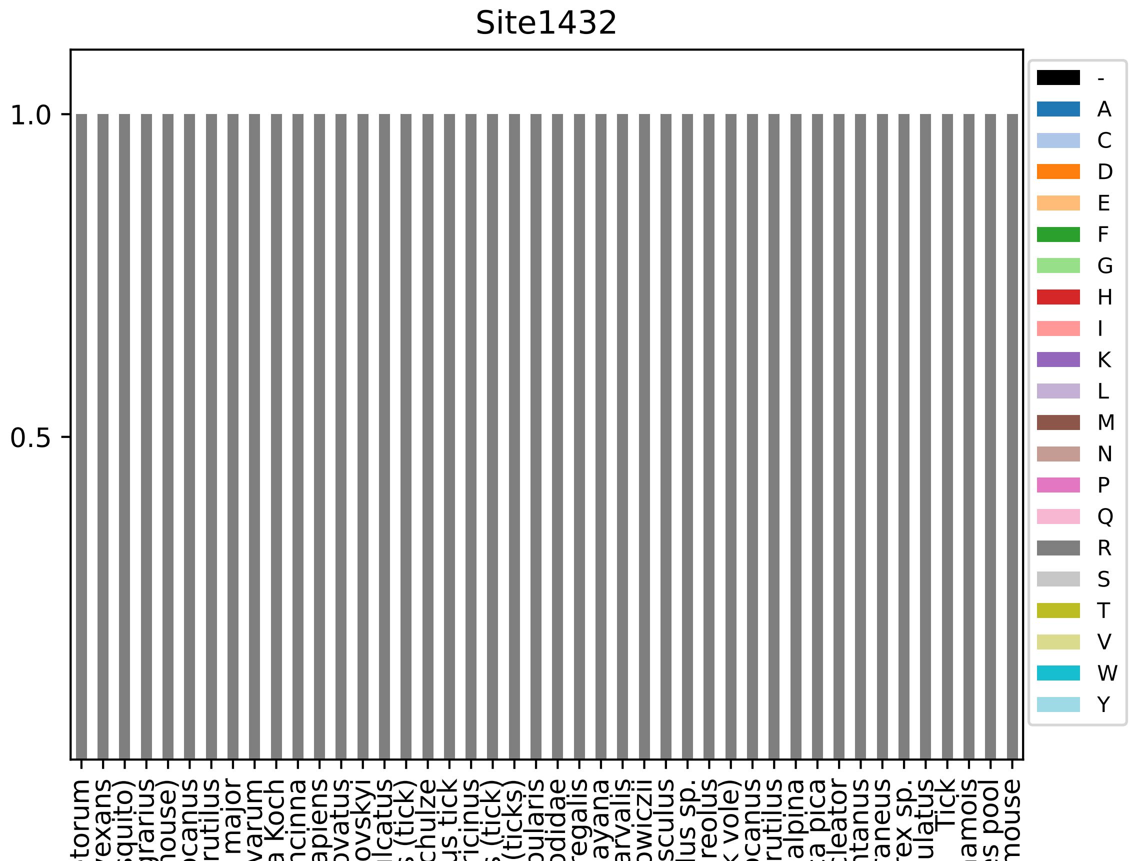
Site1432
1.0
0.5
-
A
C
D
E
F
G
H
I
K
L
M
N
P
Q
R
S
T
V
W
Y

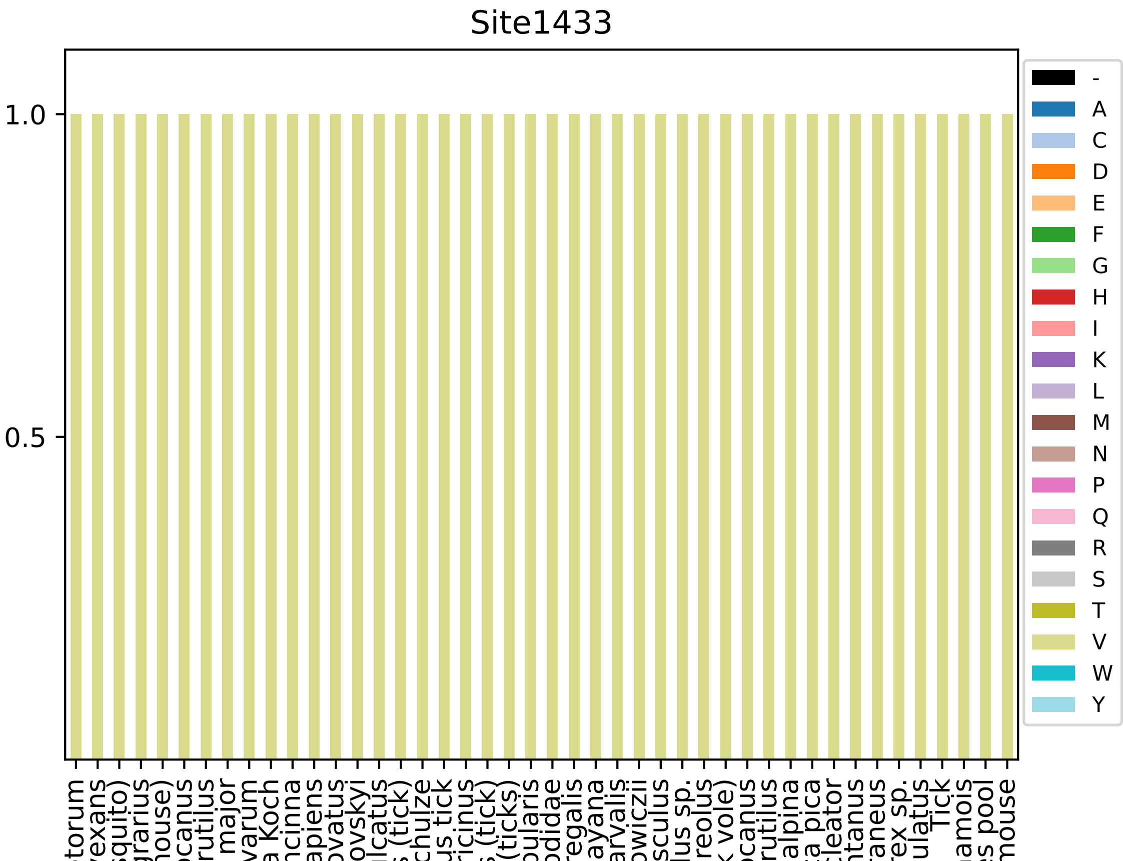
Site1433
1.0
0.5
-
A
C
D
E
F
G
H
I
K
L
M
N
P
Q
R
S
T
V
W
Y

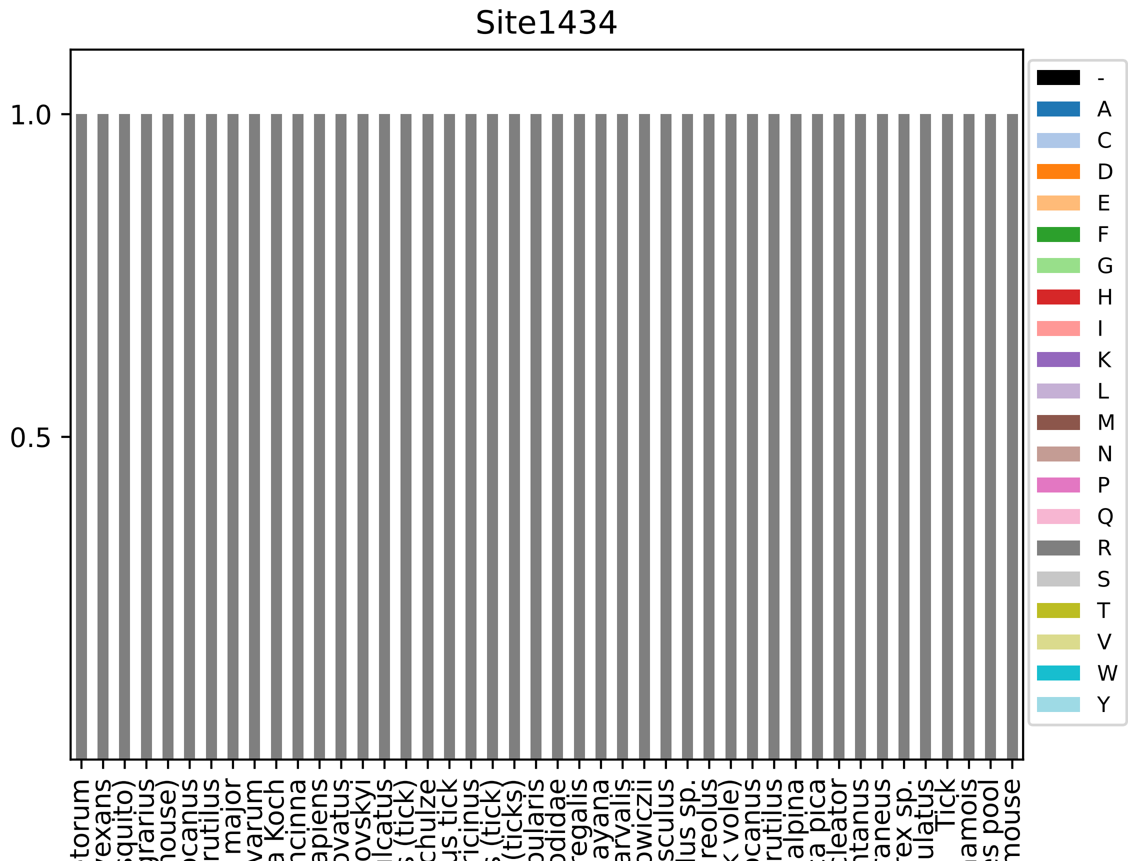
Site1434
1.0
0.5
-
A
C
D
E
F
G
H
I
K
L
M
N
P
Q
R
S
T
V
W
Y

Site1435

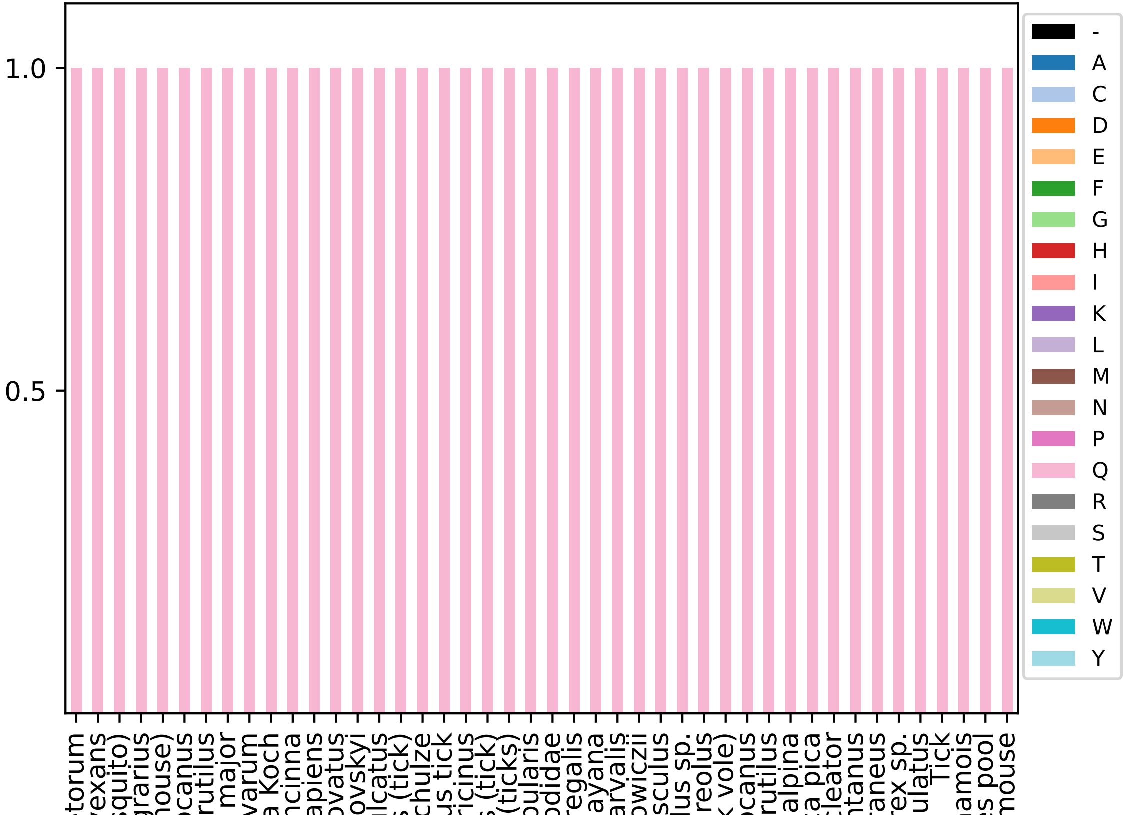

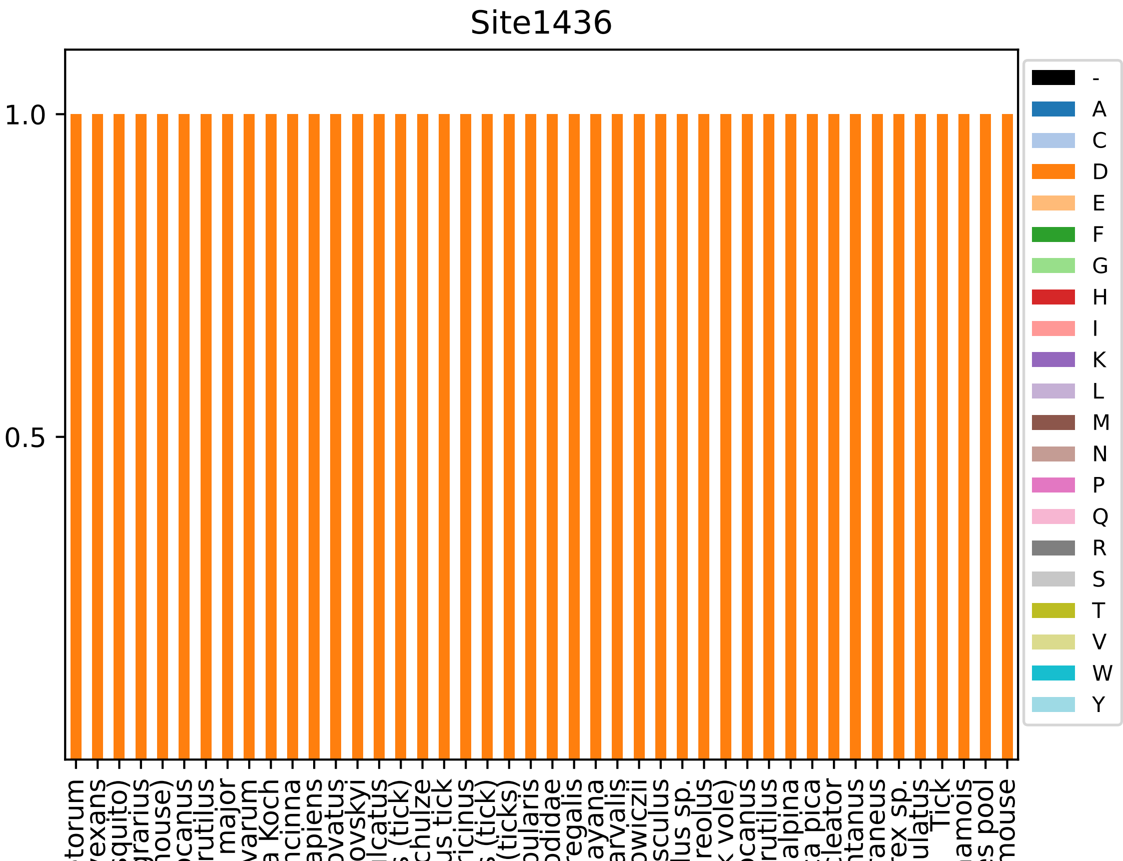

Site1436
1.0
0.5
-
A
C
D
E
F
G
H
I
K
L
M
N
P
Q
R
S
T
V
W
Y

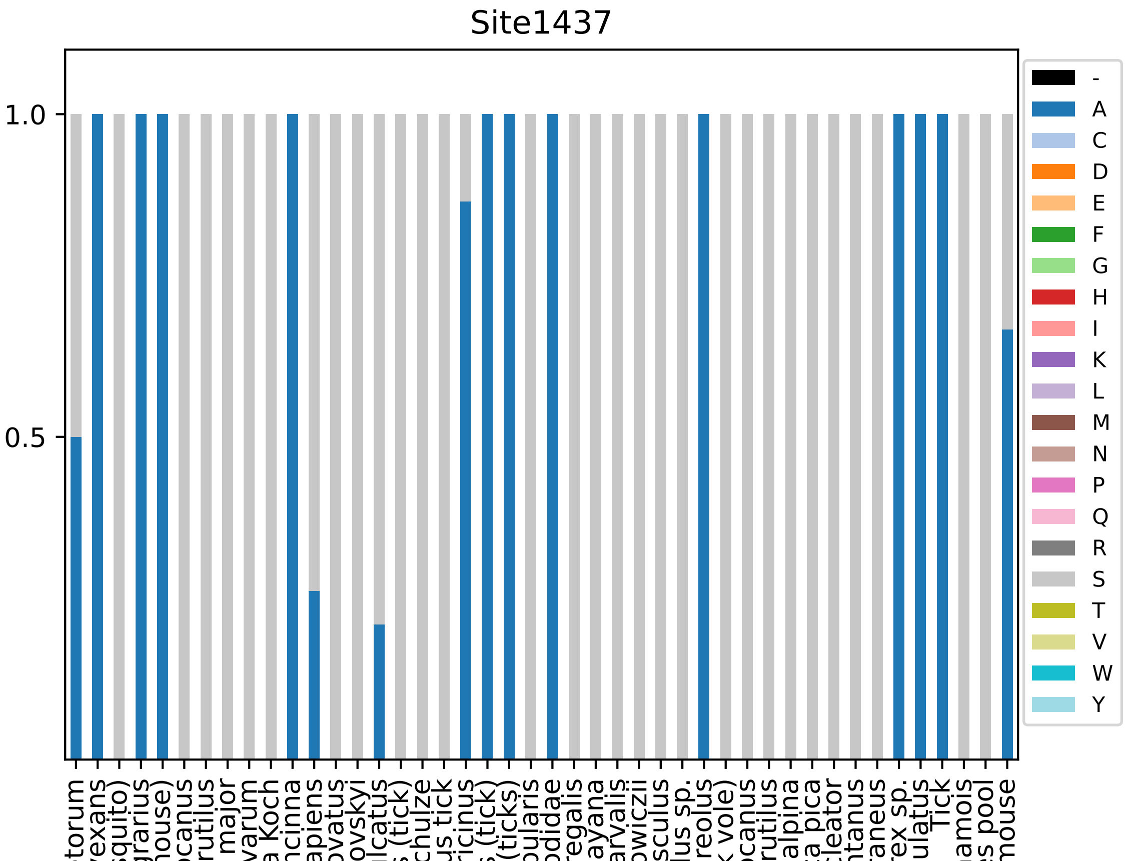
Site1437
1.0
0.5
-
A
C
D
E
F
G
H
I
K
L
M
N
P
Q
R
S
T
V
W
Y

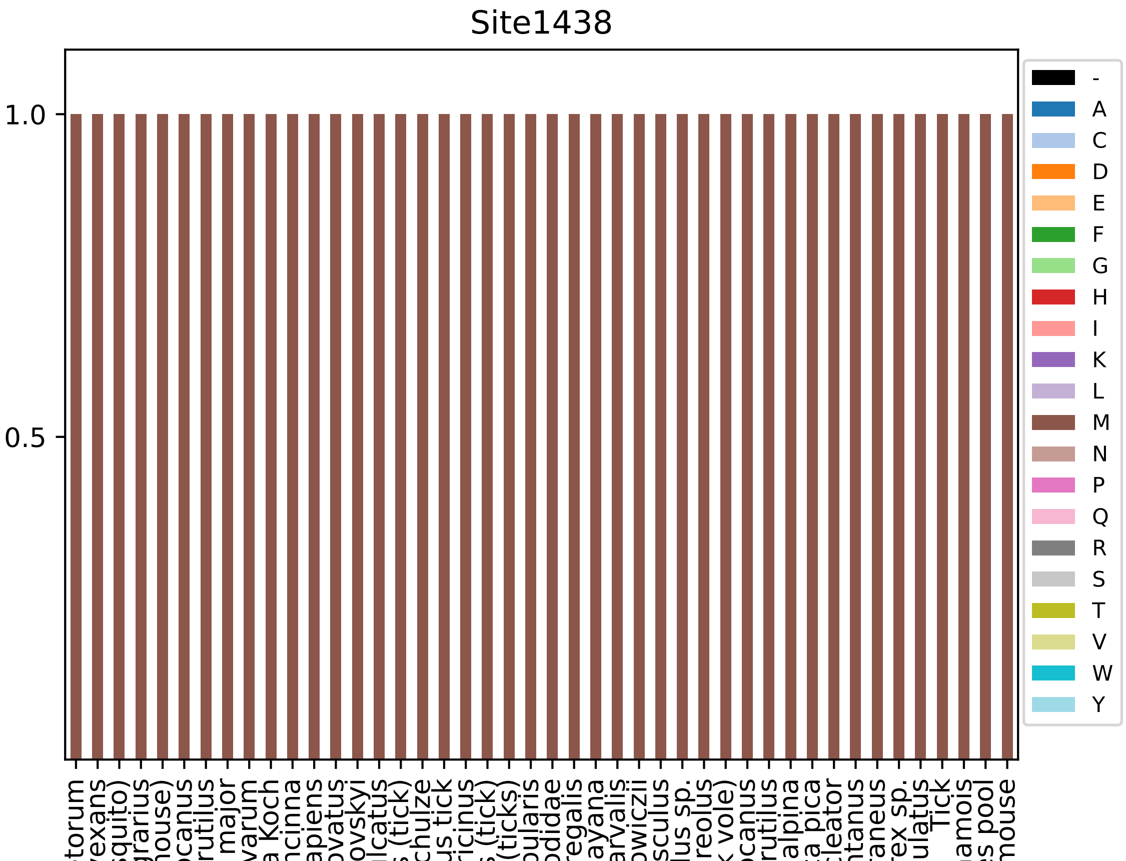

Site1438
1.0
0.5
-
A
C
D
E
F
G
H
I
K
L
M
N
P
Q
R
S
T
V
W
Y

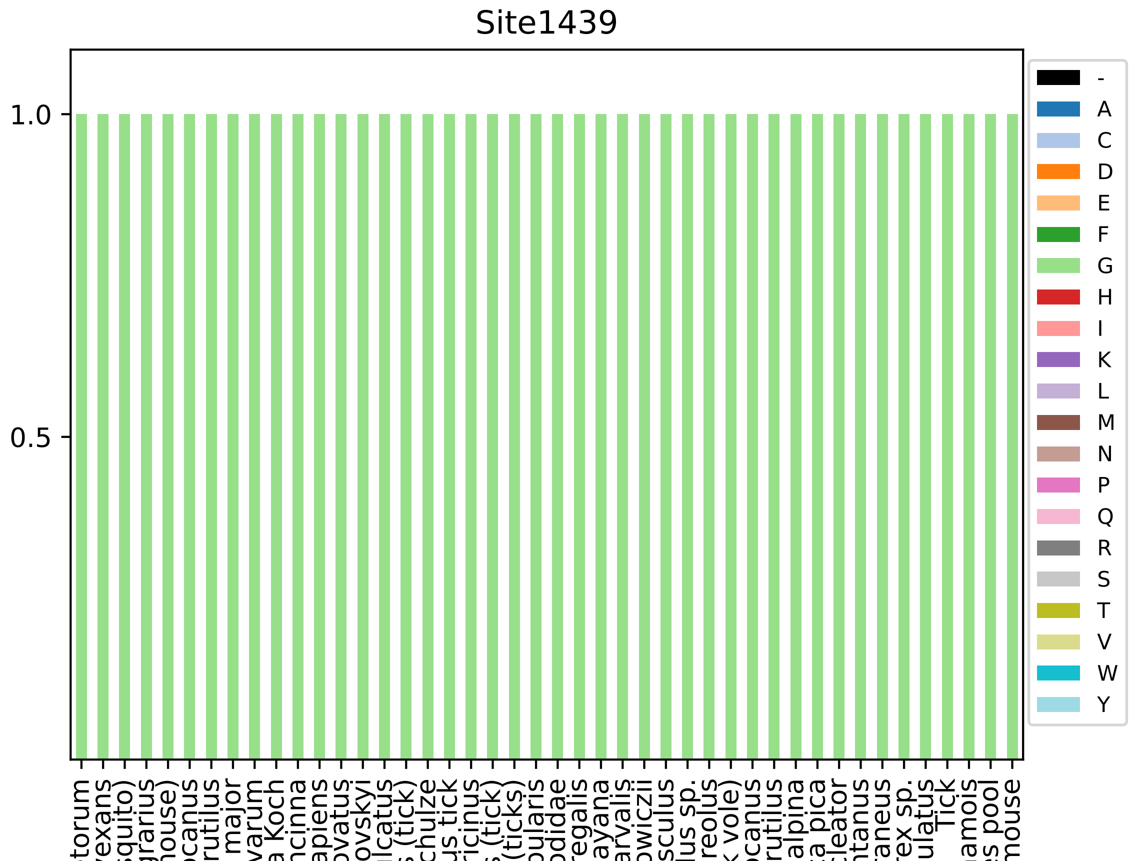
Site1439
1.0
0.5
-
A
C
D
E
F
G
H
I
K
L
M
N
P
Q
R
S
T
V
W
Y

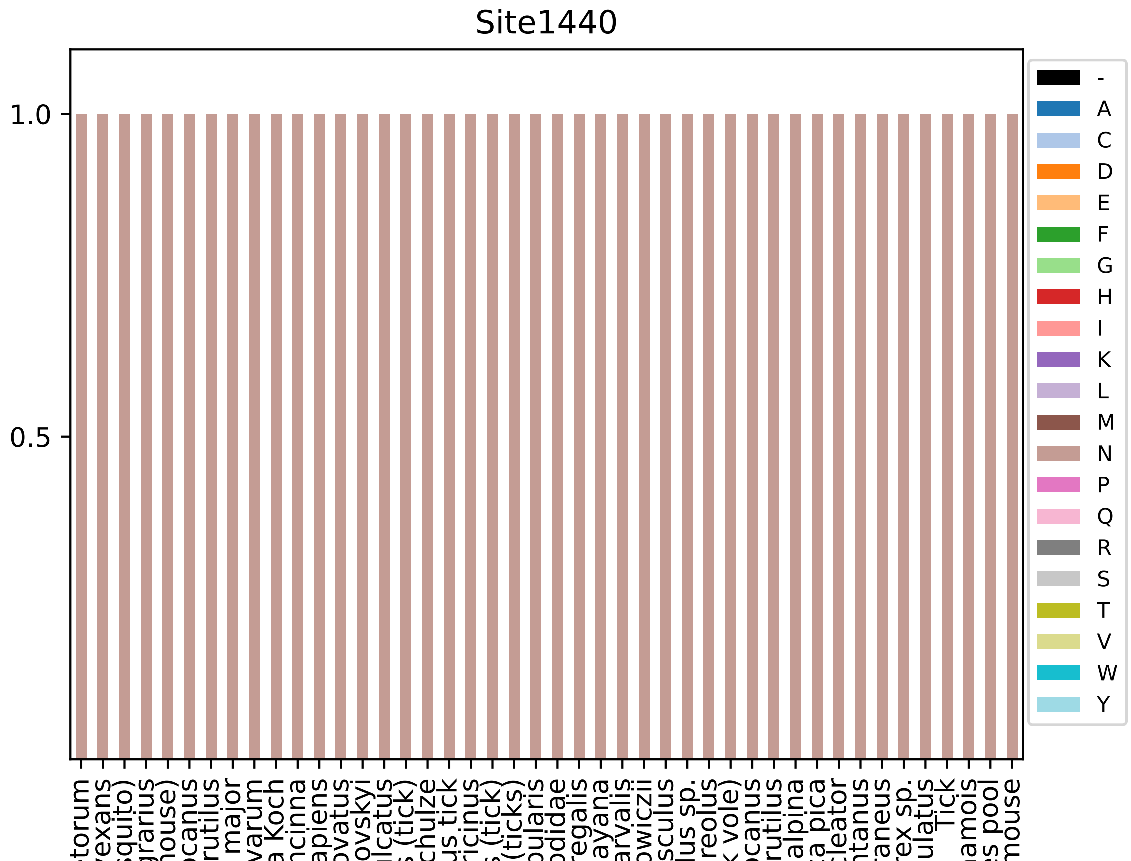
Site1440
1.0
0.5
-
A
C
D
E
F
G
H
I
K
L
M
N
P
Q
R
S
T
V
W
Y

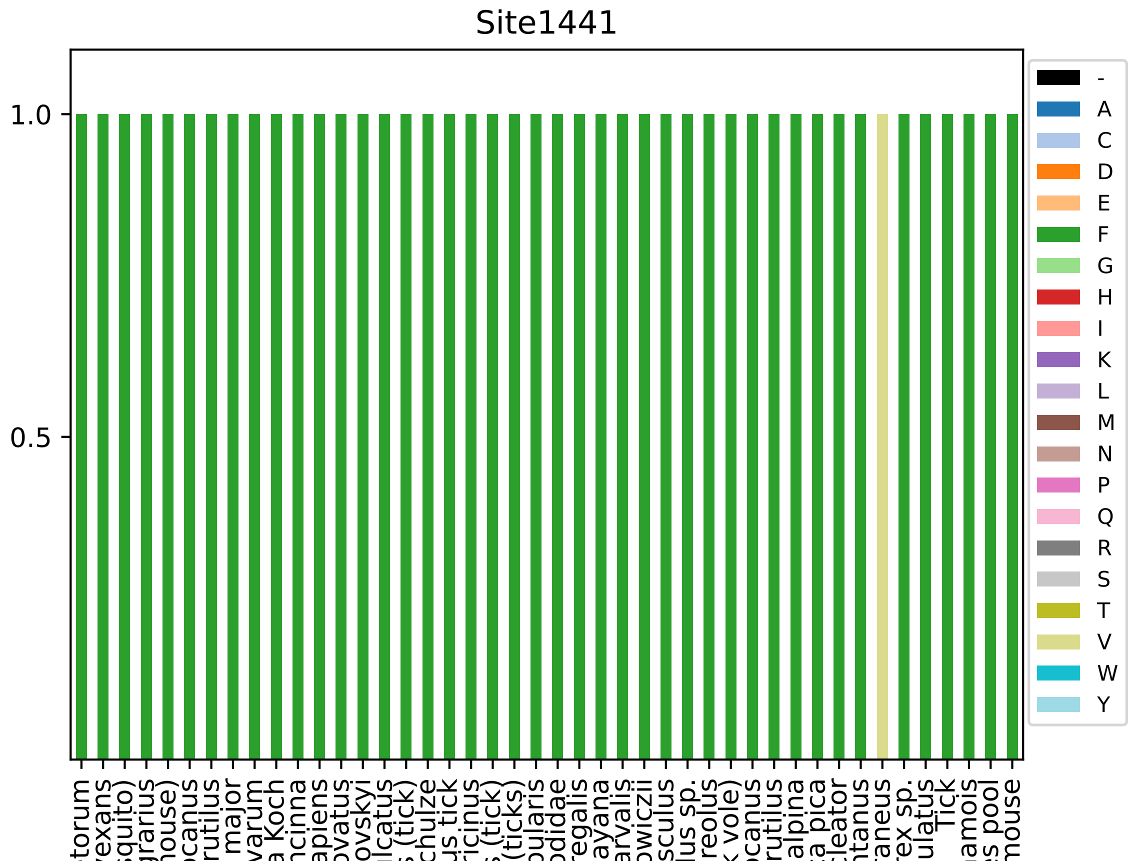

Site1441
1.0
0.5
-
A
C
D
E
F
G
H
I
K
L
M
N
P
Q
R
S
T
V
W
Y

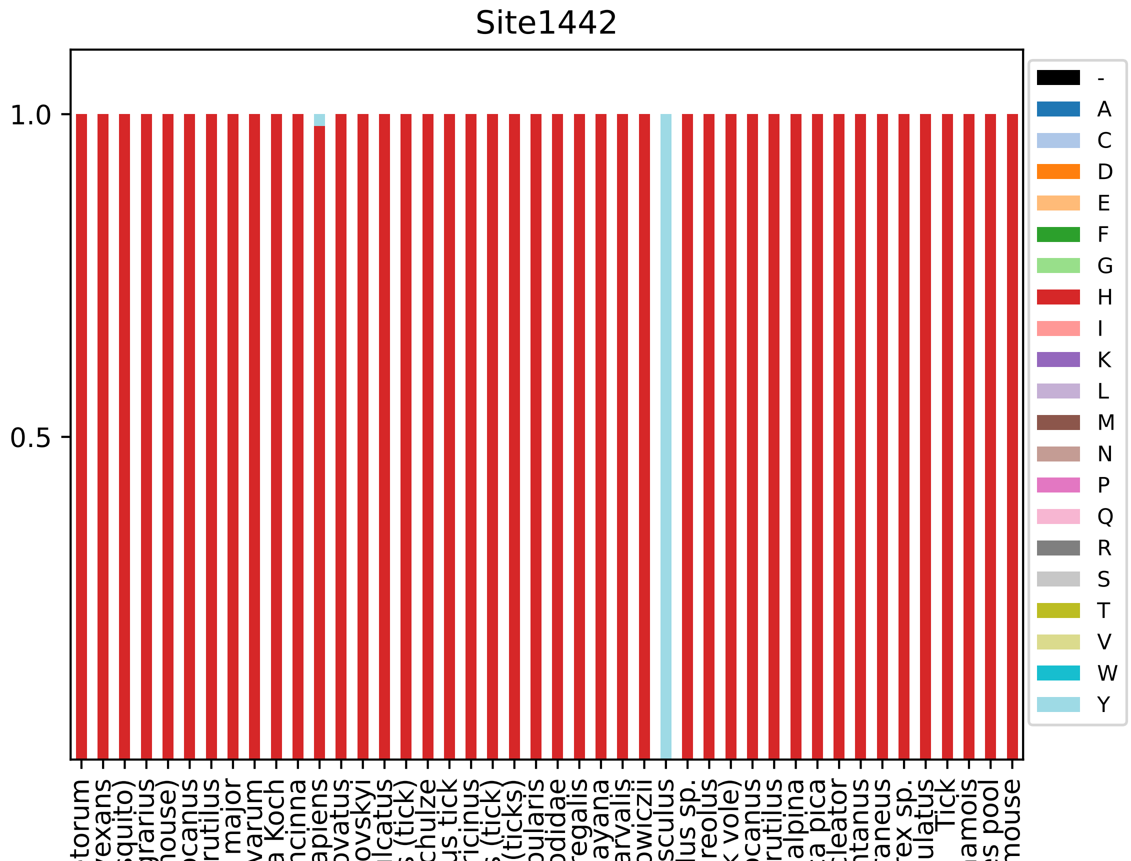
Site1442
1.0
0.5
-
A
C
D
E
F
G
H
I
K
L
M
N
P
Q
R
S
T
V
W
Y

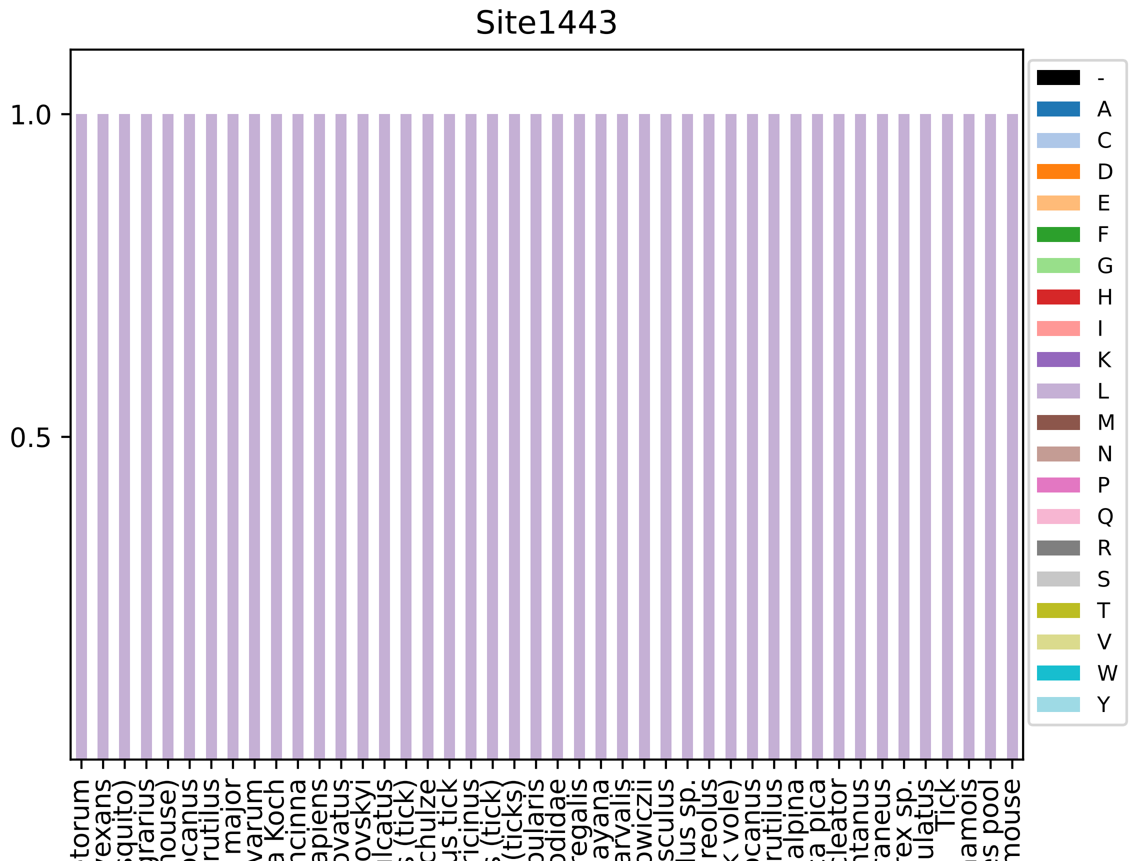
Site1443
1.0
0.5
-
A
C
D
E
F
G
H
I
K
L
M
N
P
Q
R
S
T
V
W
Y

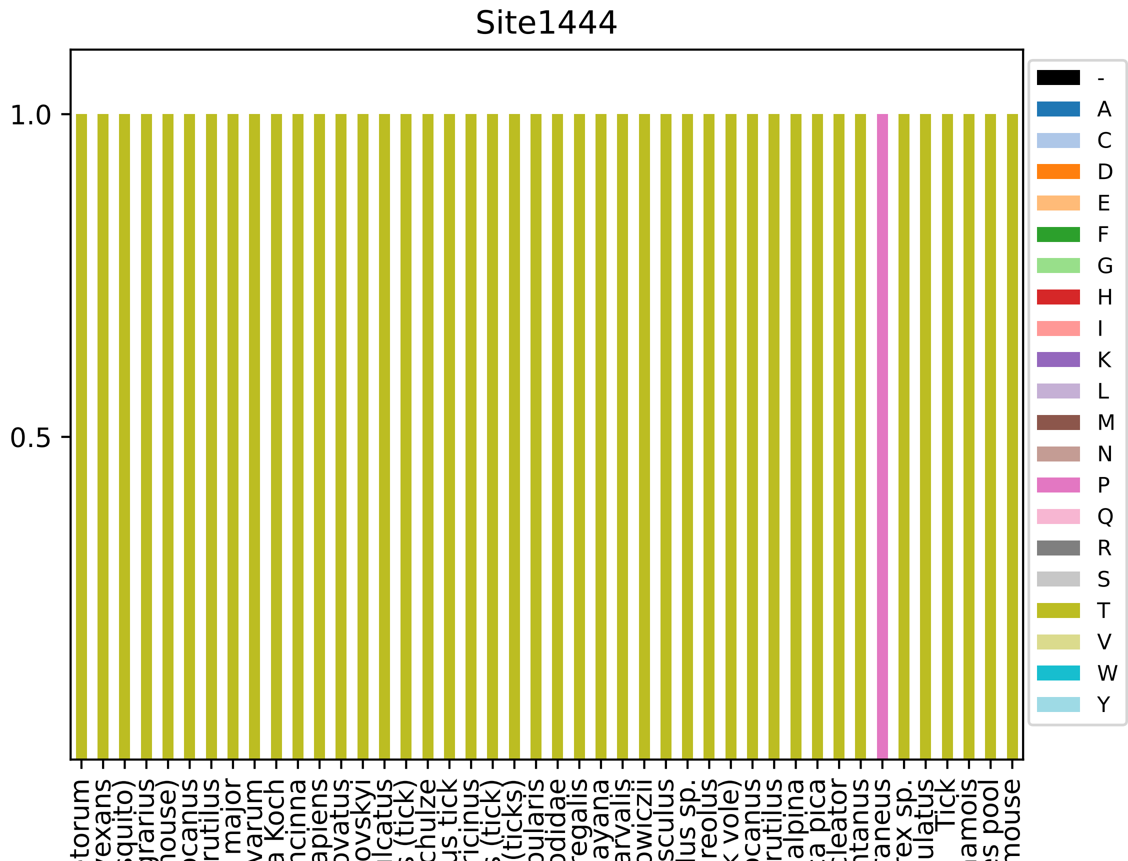
Site1444
1.0
0.5
-
A
C
D
E
F
G
H
I
K
L
M
N
P
Q
R
S
T
V
W
Y

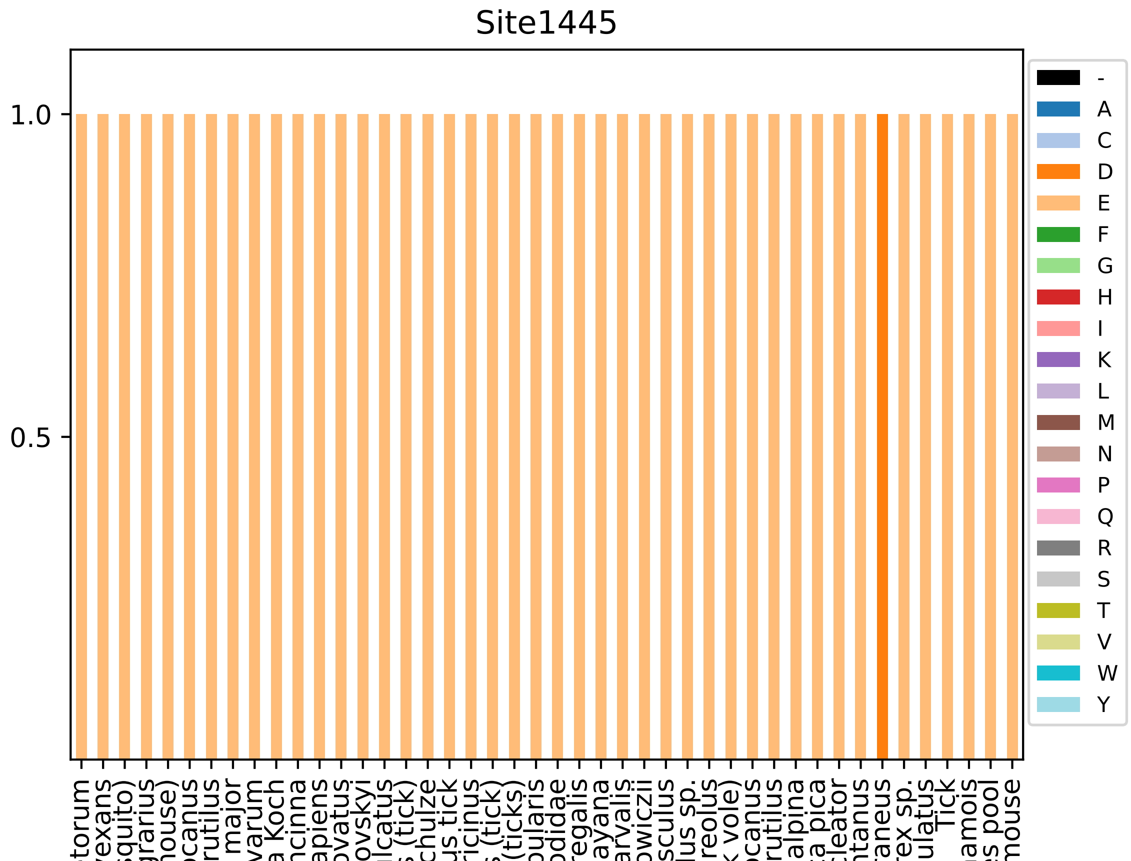
Site1445
1.0
0.5
-
A
C
D
E
F
G
H
I
K
L
M
N
P
Q
R
S
T
V
W
Y

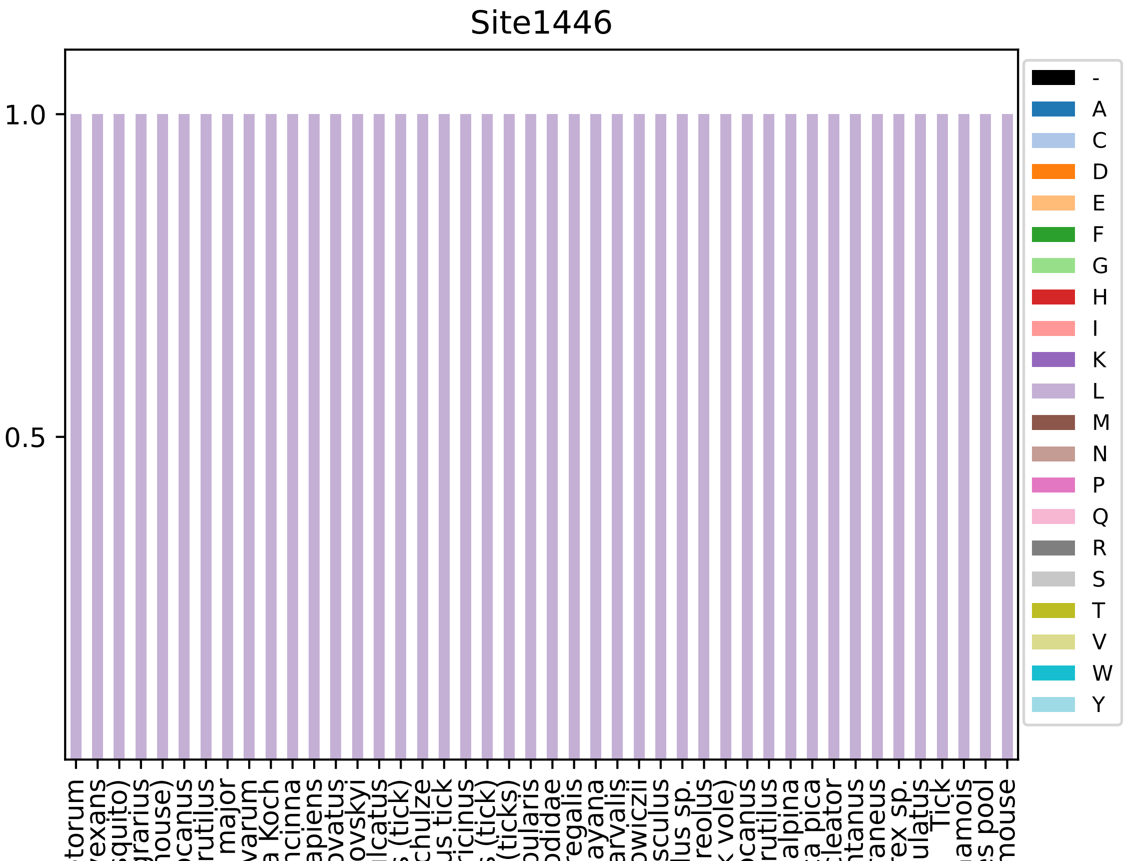
Site1446
1.0
0.5
-
A
C
D
E
F
G
H
I
K
L
M
N
P
Q
R
S
T
V
W
Y

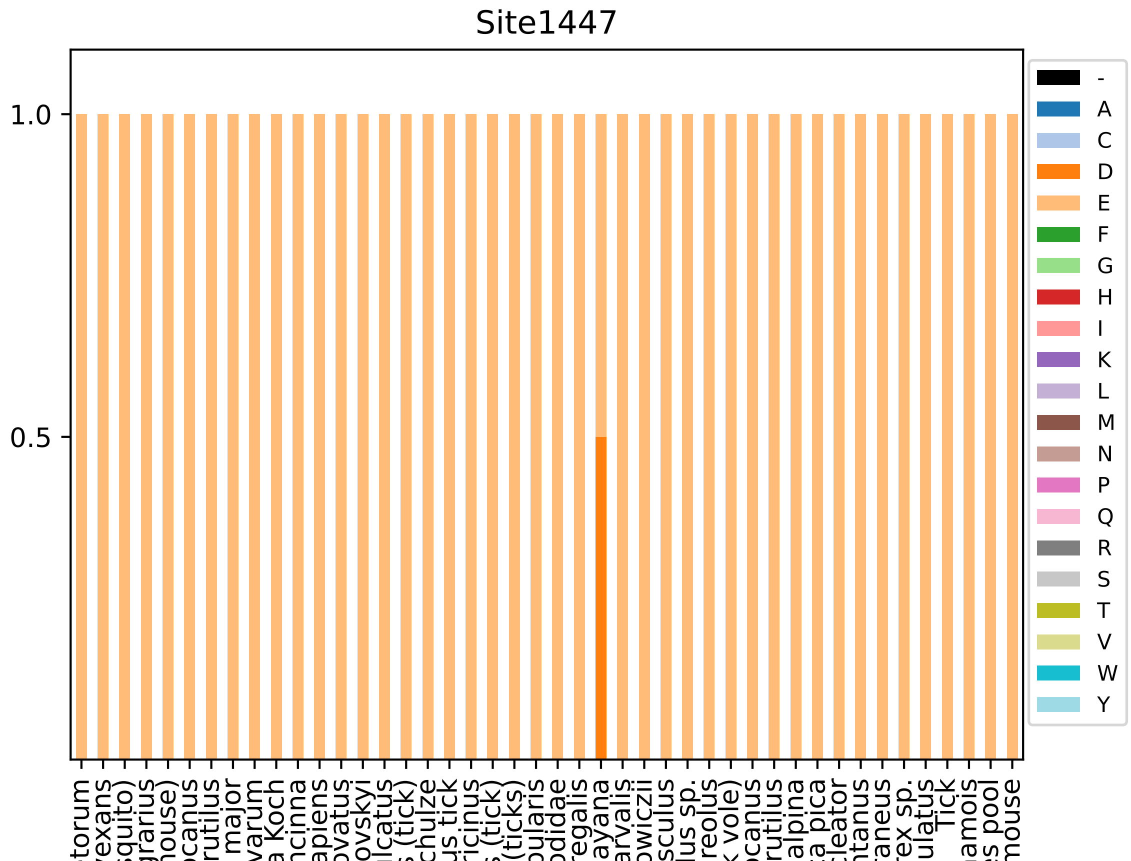

Site1447
1.0
0.5
-
A
C
D
E
F
G
H
I
K
L
M
N
P
Q
R
S
T
V
W
Y

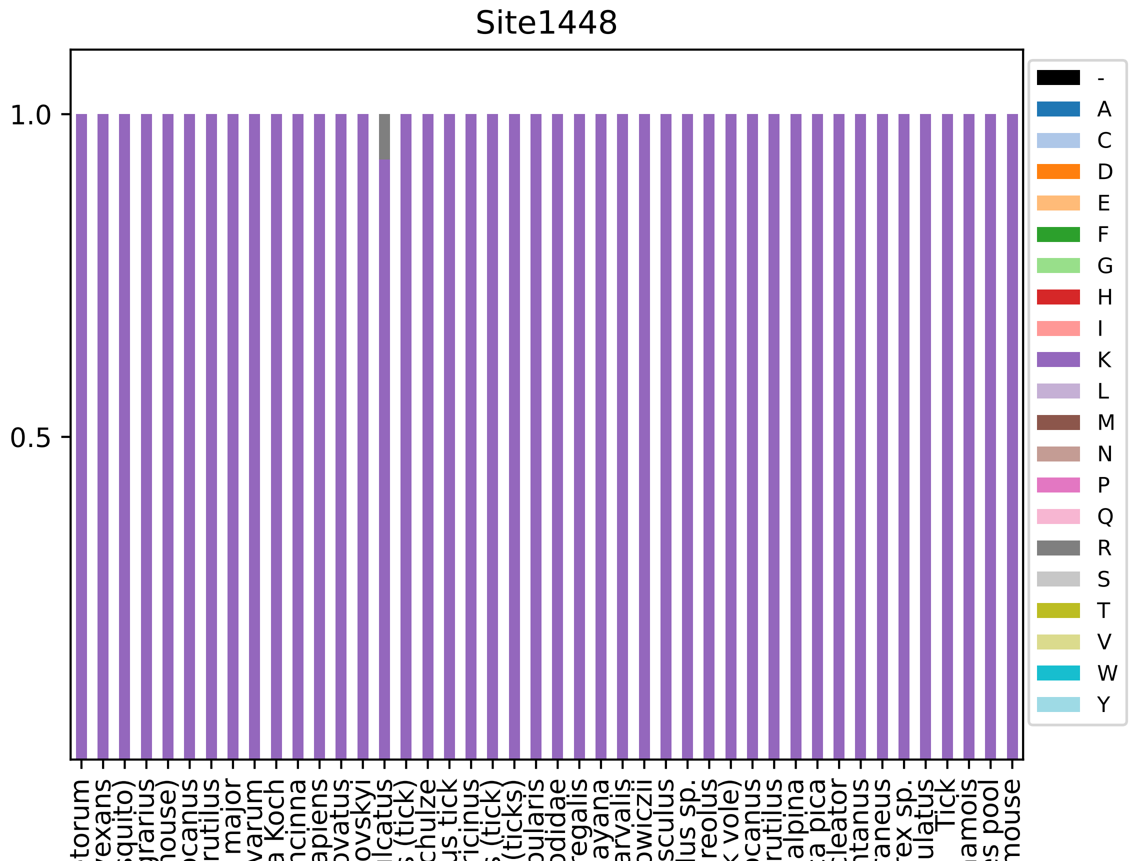
Site1448
1.0
0.5
-
A
C
D
E
F
G
H
I
K
L
M
N
P
Q
R
S
T
V
W
Y

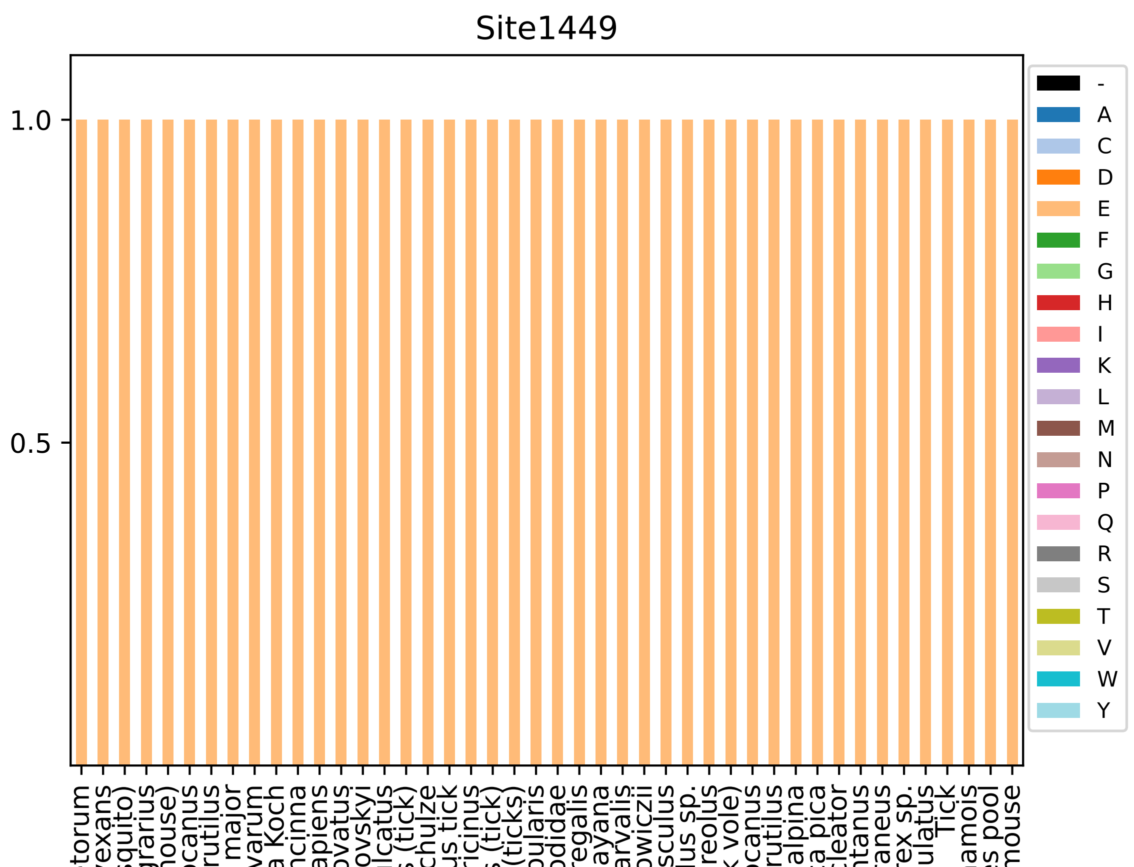

Site1449
1.0
0.5
-
A
C
D
E
F
G
H
I
K
L
M
N
P
Q
R
S
T
V
W
Y

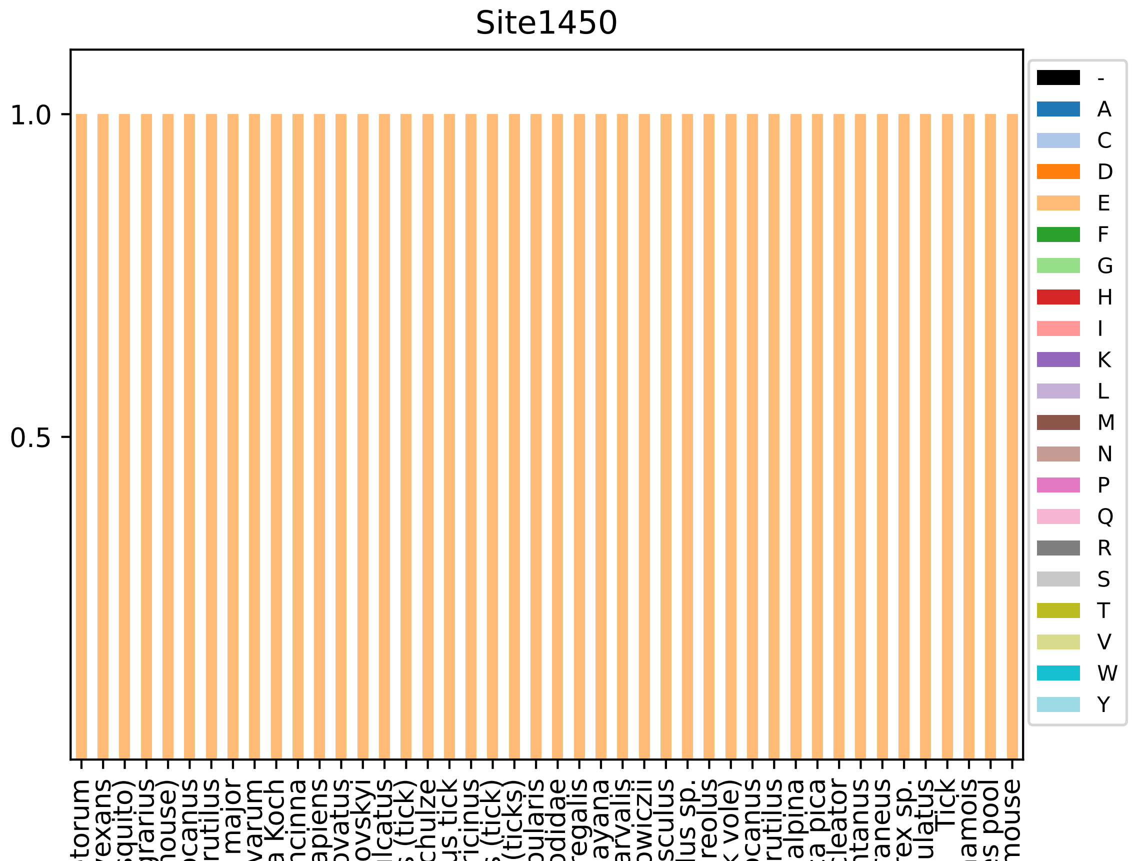
Site1450
1.0
0.5
-
A
C
D
E
F
G
H
I
K
L
M
N
P
Q
R
S
T
V
W
Y

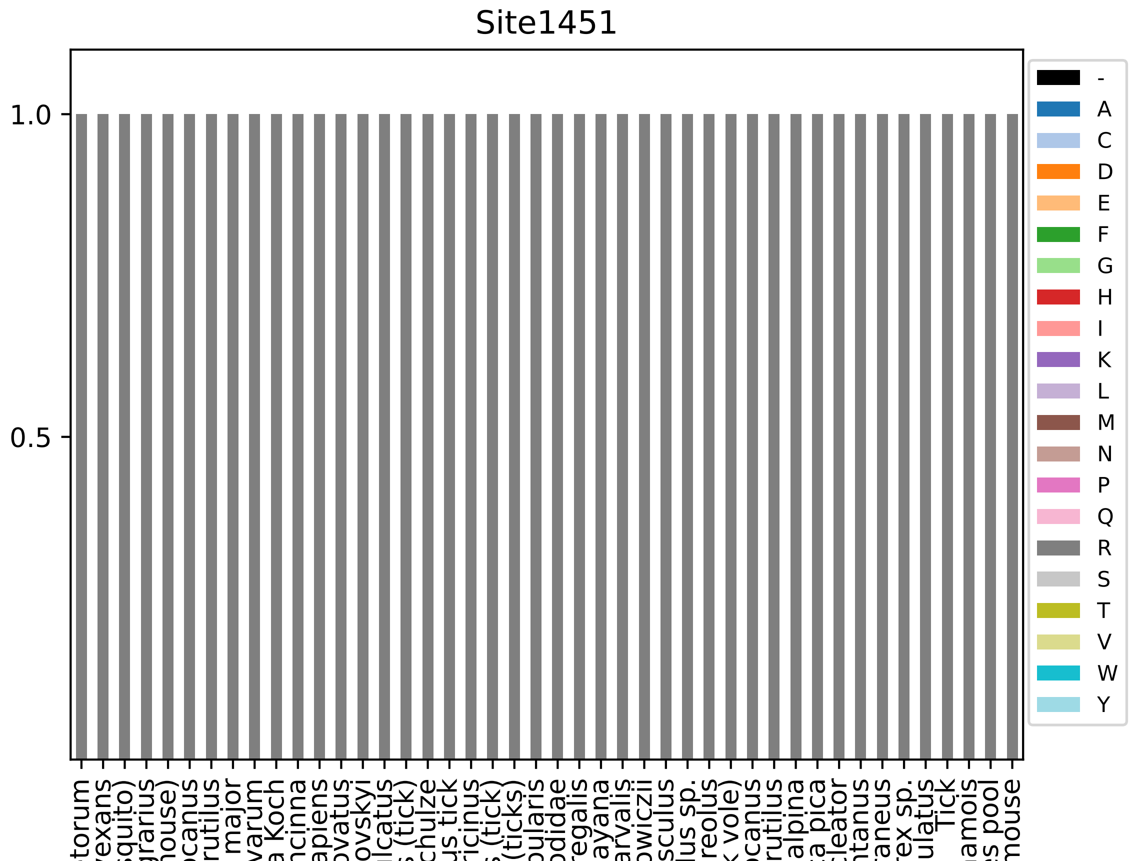
Site1451
1.0
0.5
-
A
C
D
E
F
G
H
I
K
L
M
N
P
Q
R
S
T
V
W
Y

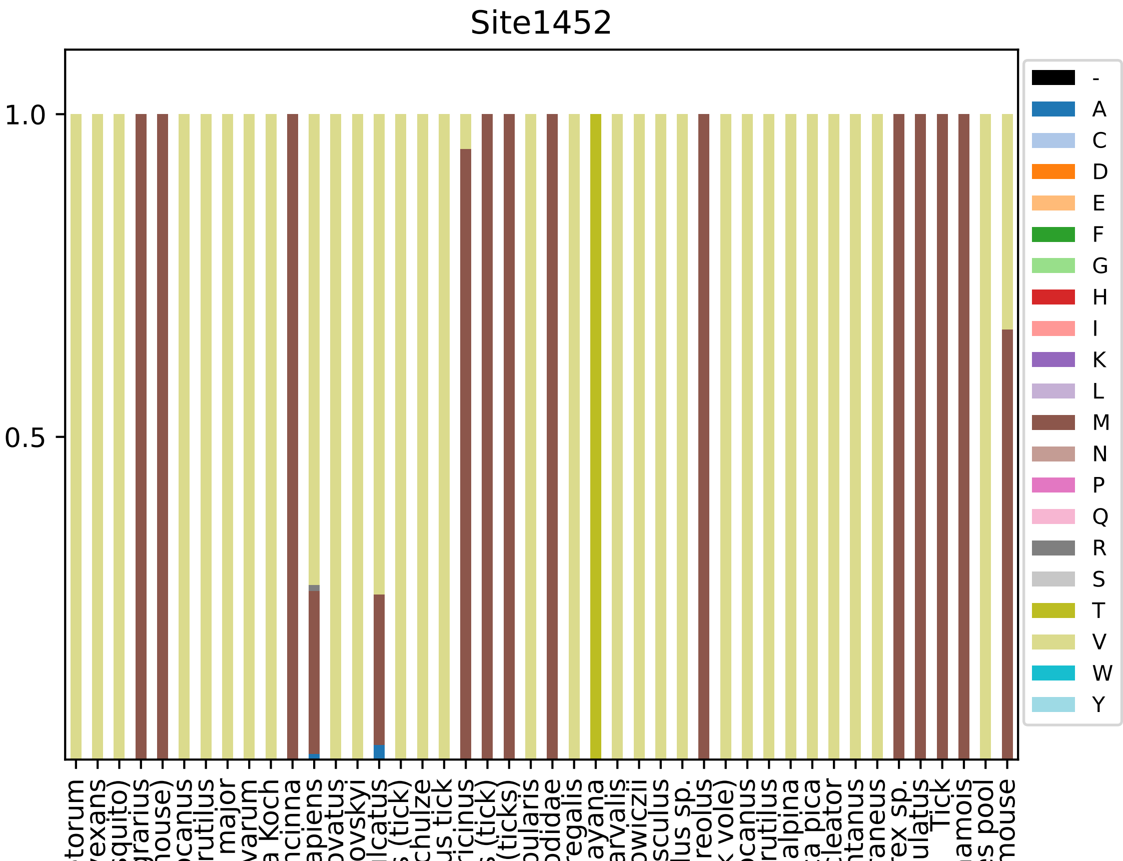

Site1452
1.0
0.5
-
A
C
D
E
F
G
H
I
K
L
M
N
P
Q
R
S
T
V
W
Y

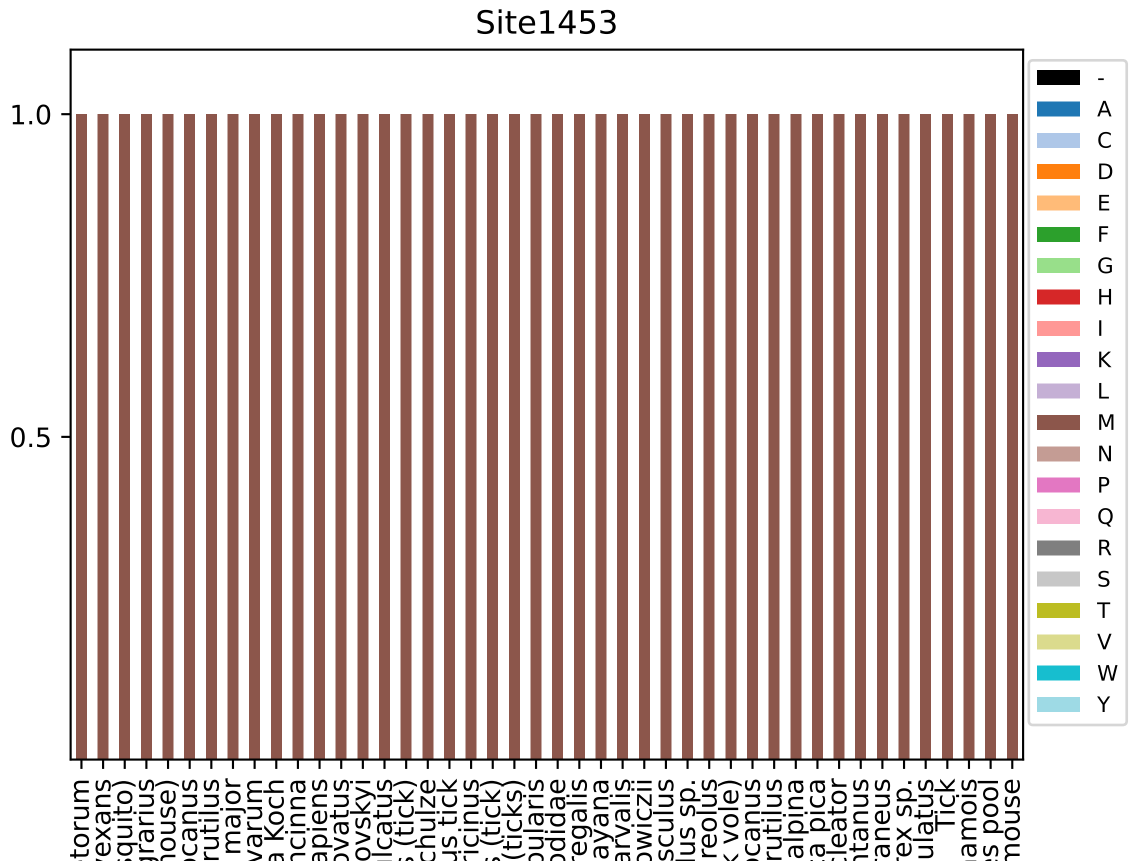
Site1453
1.0
0.5
-
A
C
D
E
F
G
H
I
K
L
M
N
P
Q
R
S
T
V
W
Y

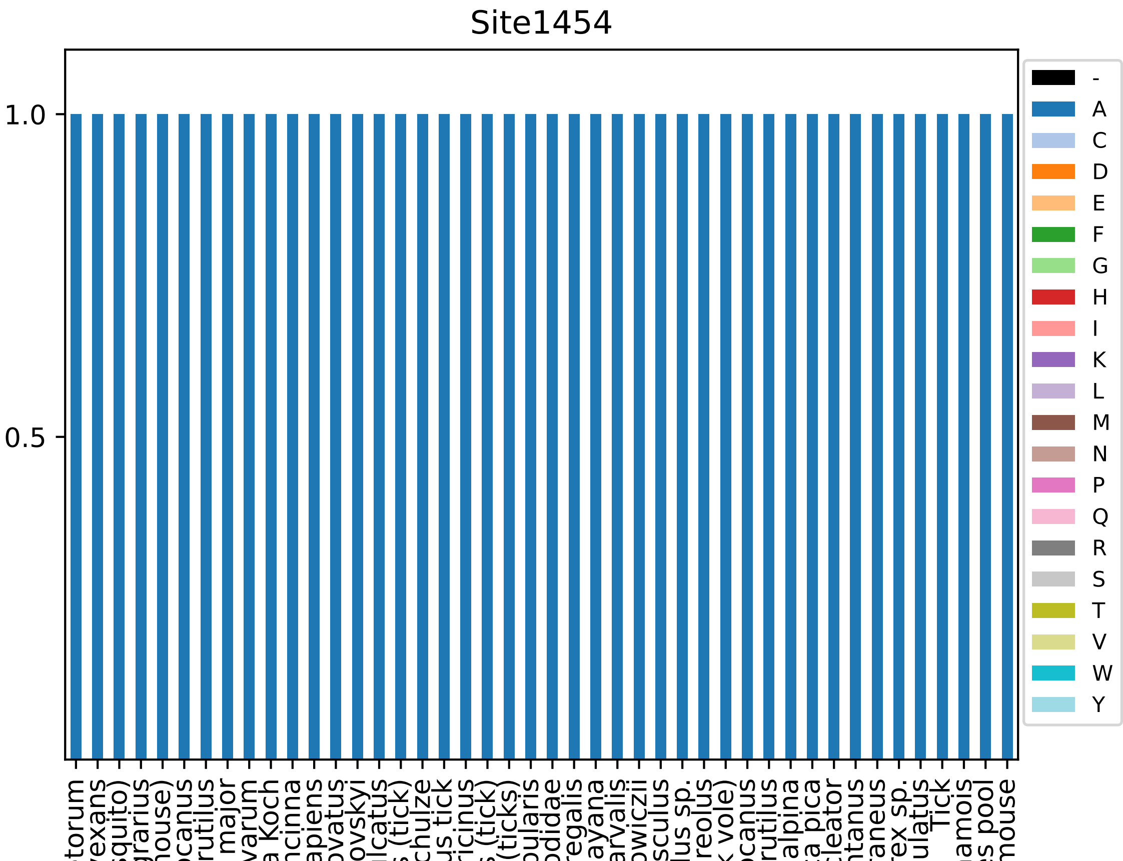

Site1454
1.0
0.5
-
A
C
D
E
F
G
H
I
K
L
M
N
P
Q
R
S
T
V
W
Y

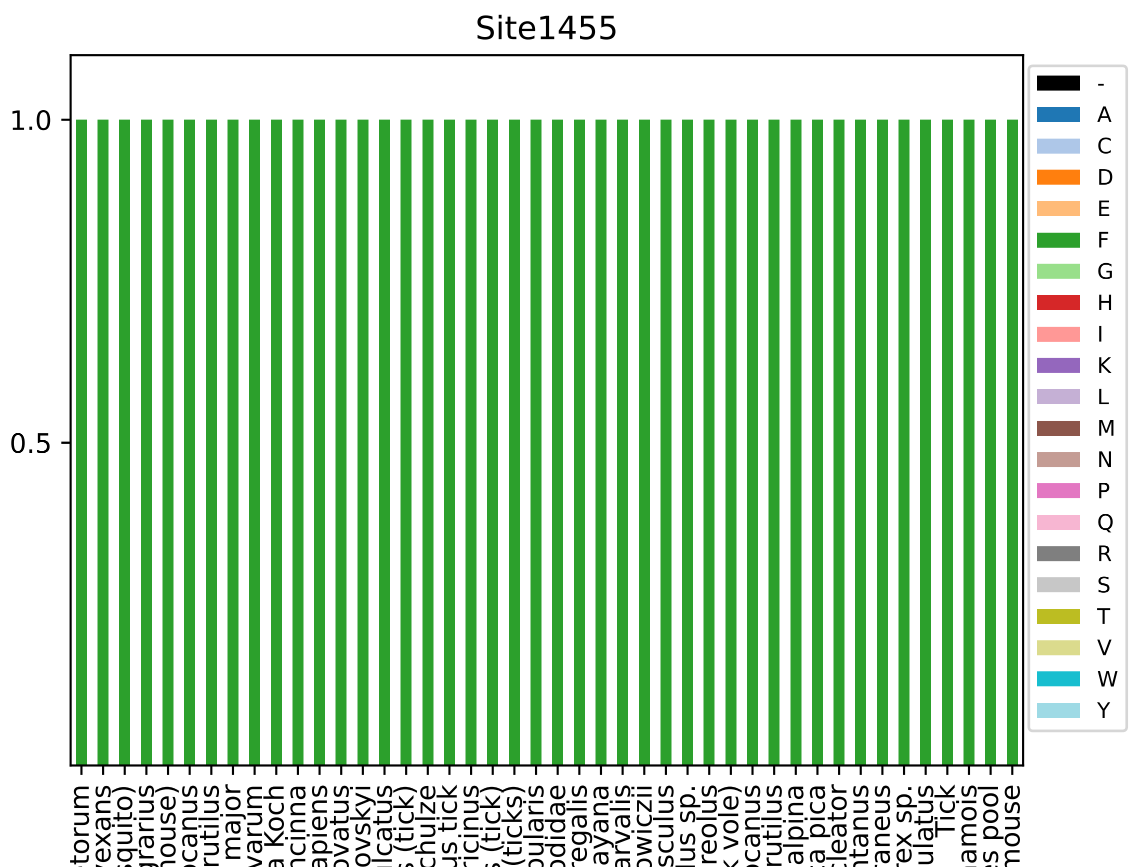
Site1455
1.0
0.5
-
A
C
D
E
F
G
H
I
K
L
M
N
P
Q
R
S
T
V
W
Y

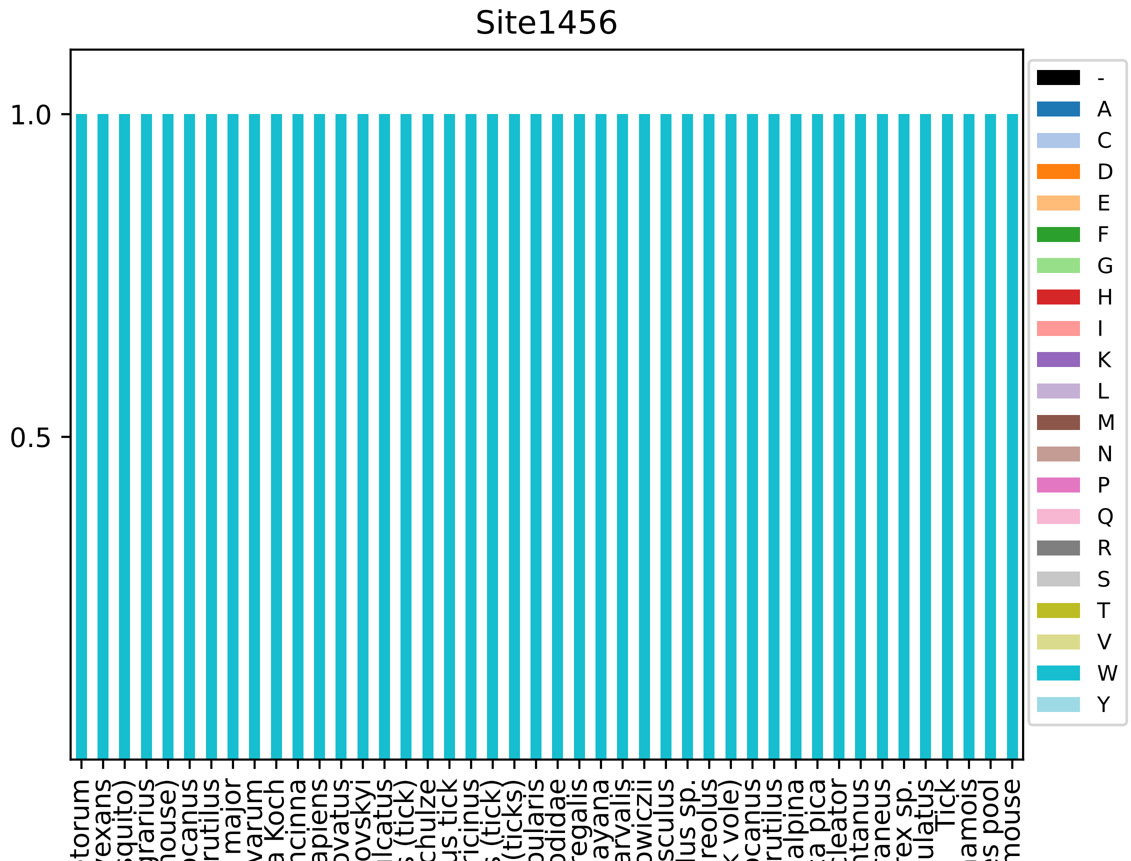
Site1456
1.0
0.5
-
A
C
D
E
F
G
H
I
K
L
M
N
P
Q
R
S
T
V
W
Y

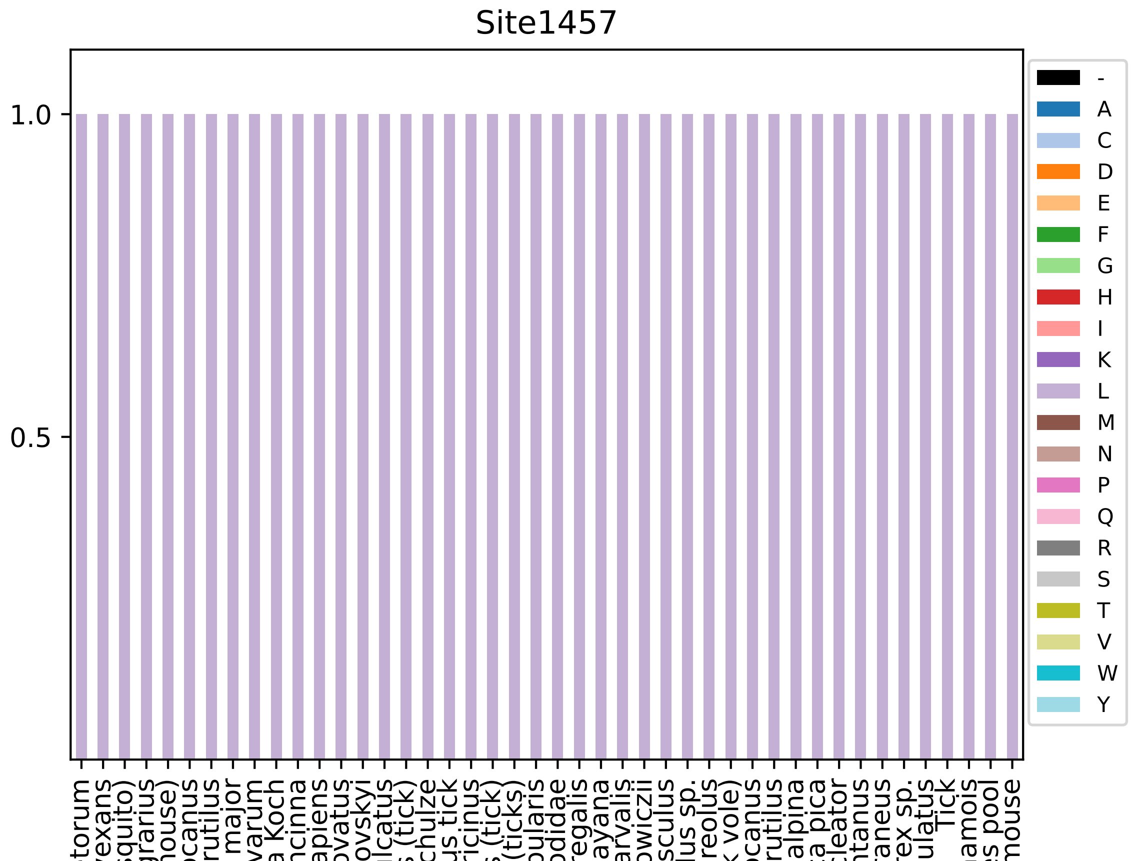

Site1457
1.0
0.5
-
A
C
D
E
F
G
H
I
K
L
M
N
P
Q
R
S
T
V
W
Y

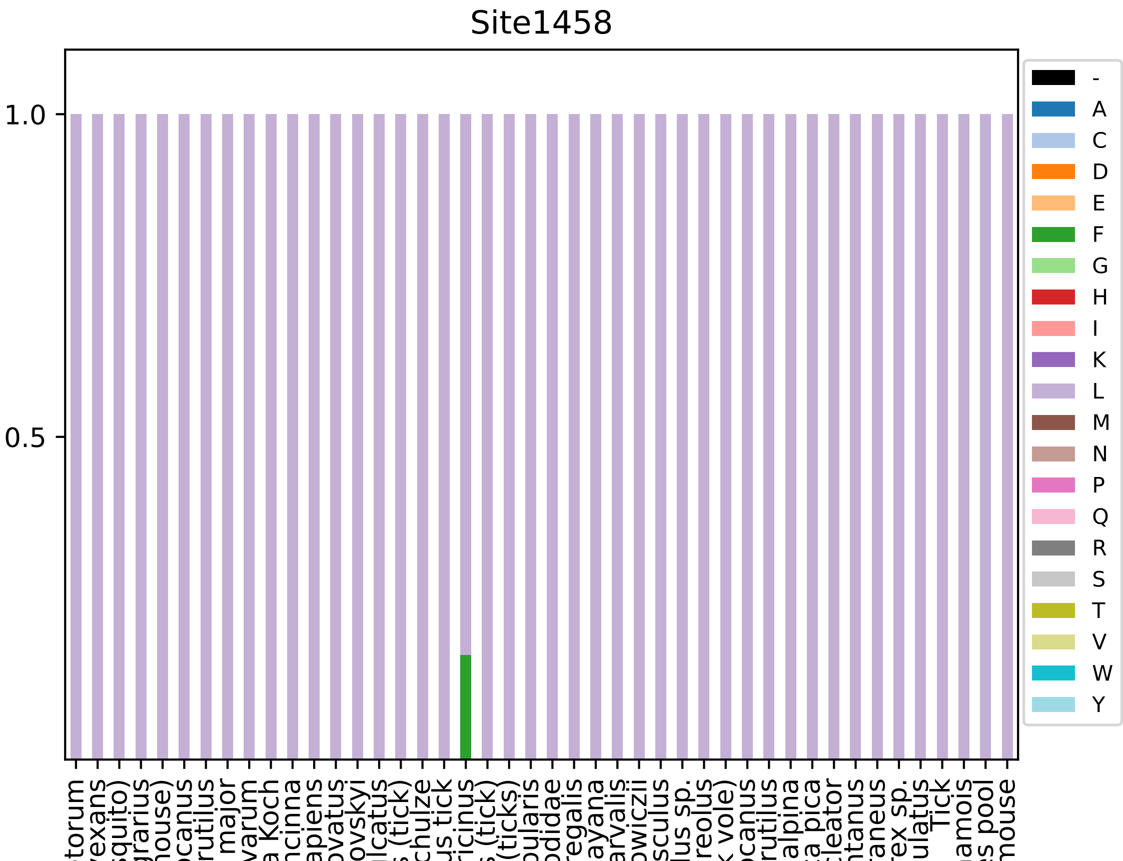

Site1458
1.0
0.5
-
A
C
D
E
F
G
H
I
K
L
M
N
P
Q
R
S
T
V
W
Y

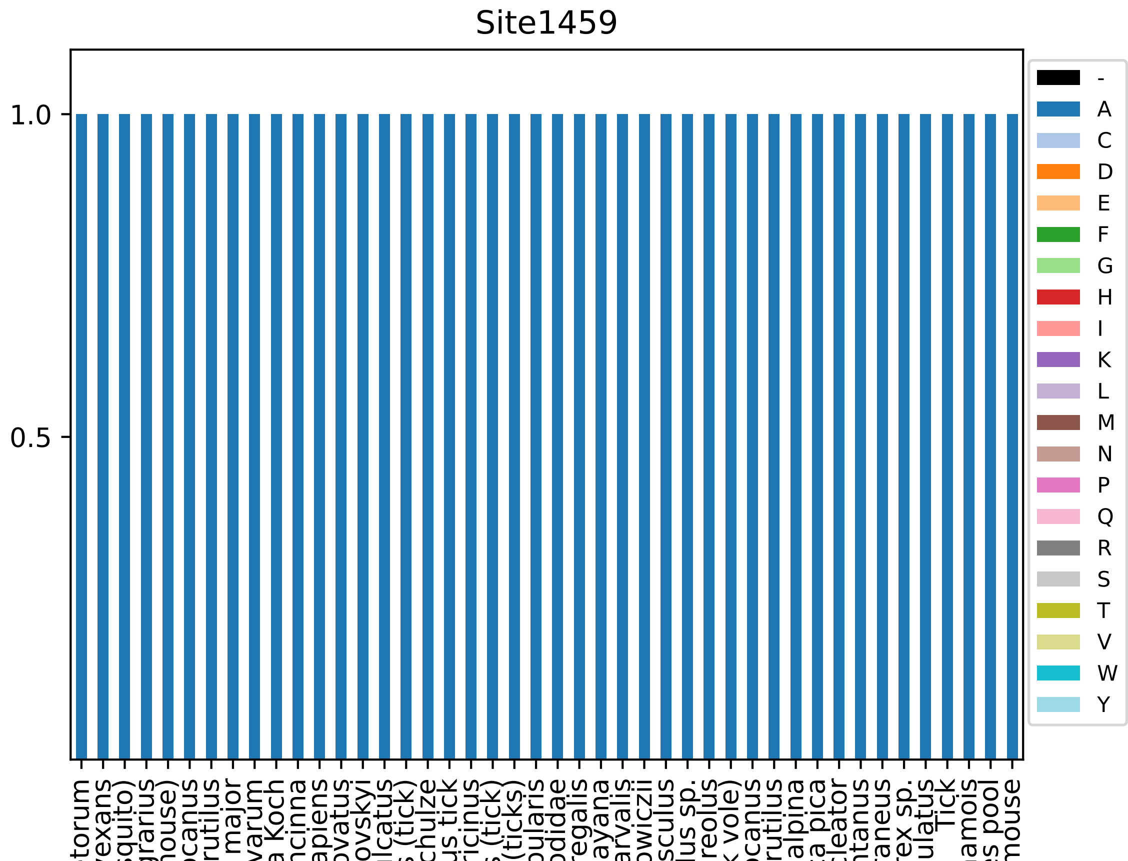
Site1459
1.0
0.5
-
A
C
D
E
F
G
H
I
K
L
M
N
P
Q
R
S
T
V
W
Y

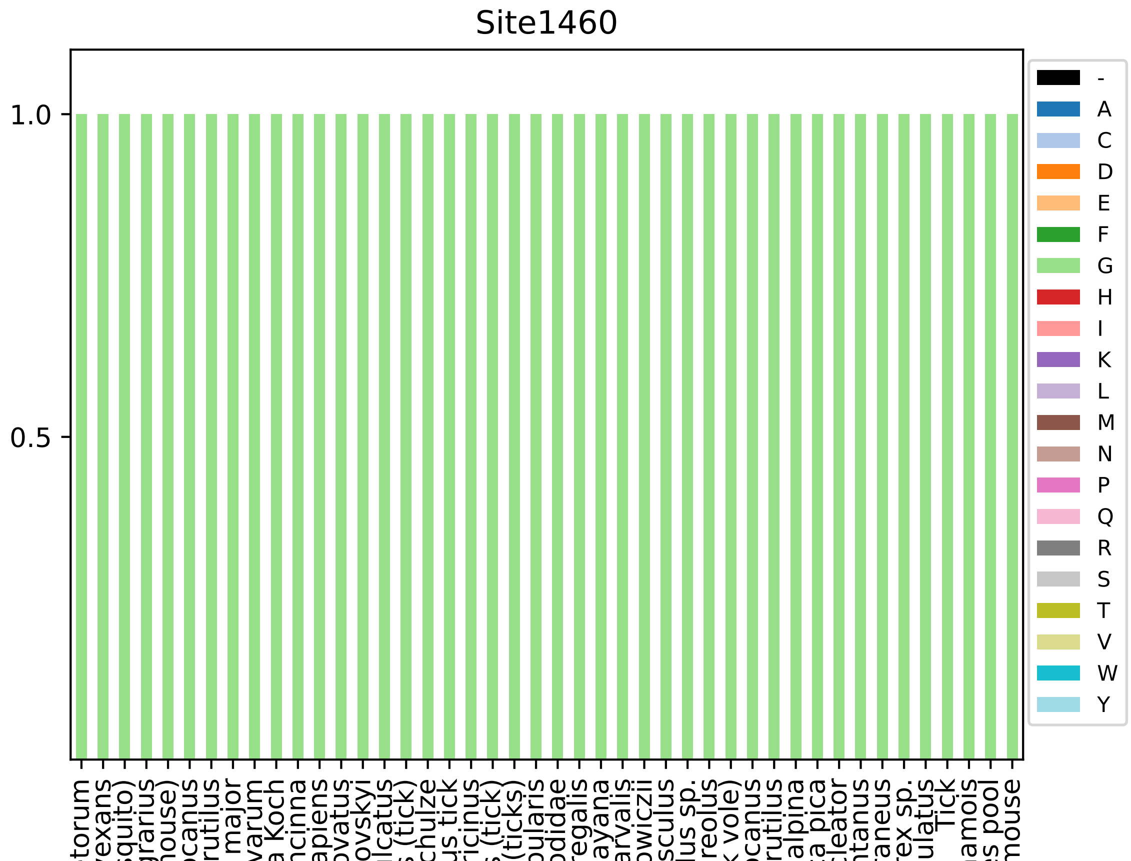
Site1460
1.0
0.5
-
A
C
D
E
F
G
H
I
K
L
M
N
P
Q
R
S
T
V
W
Y

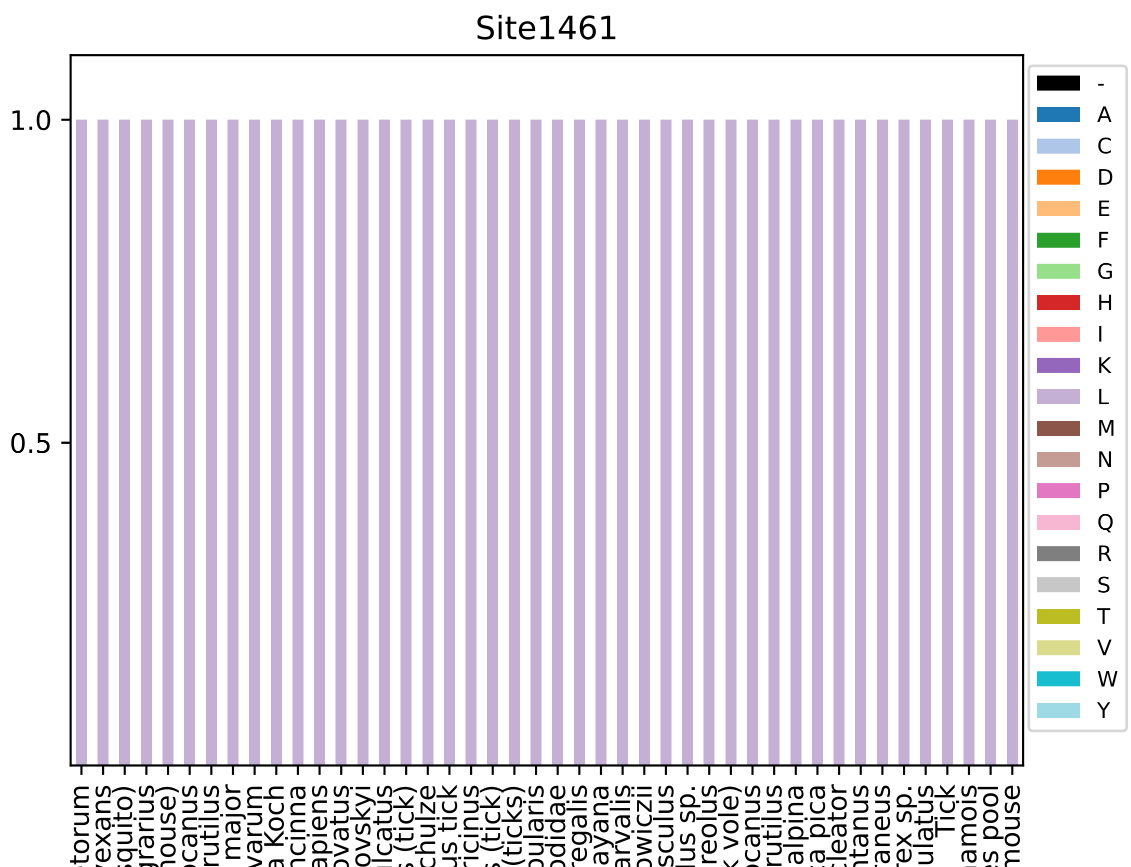
Site1461
1.0
0.5
-
A
C
D
E
F
G
H
I
K
L
M
N
P
Q
R
S
T
V
W
Y

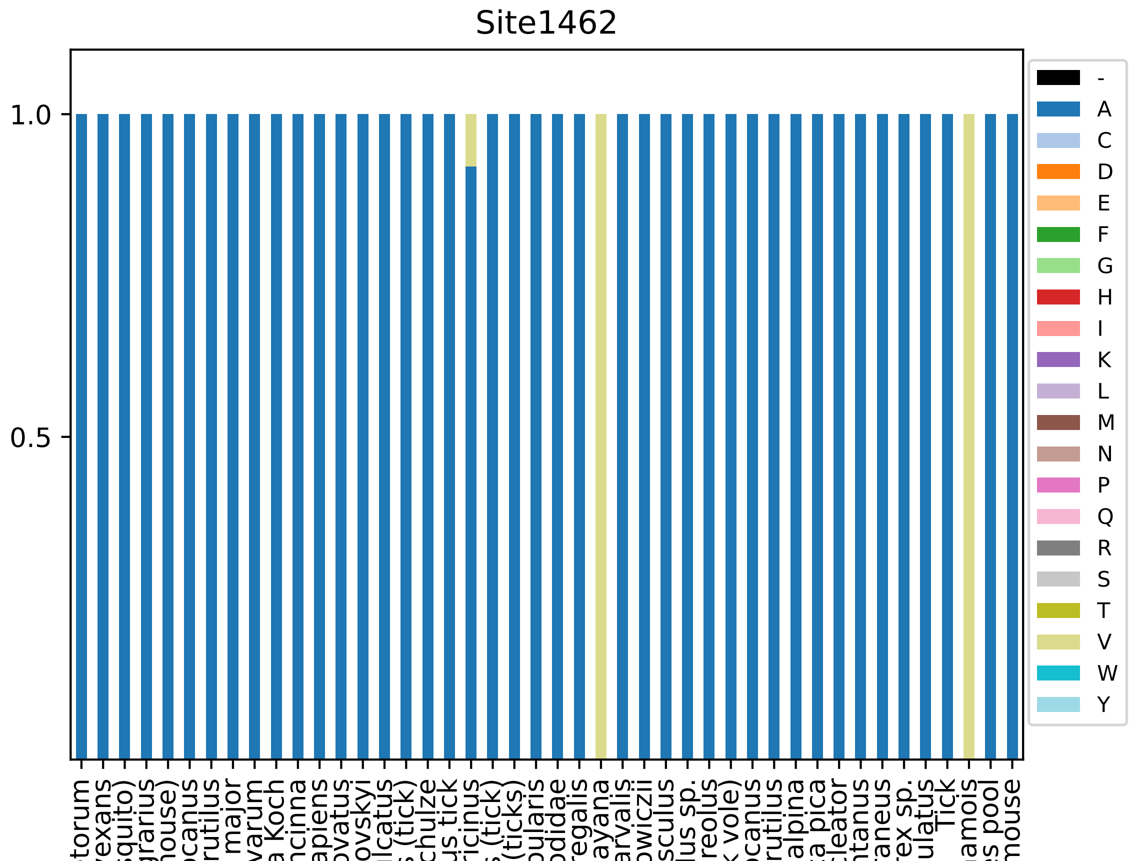
Site1462
1.0
0.5
-
A
C
D
E
F
G
H
I
K
L
M
N
P
Q
R
S
T
V
W
Y

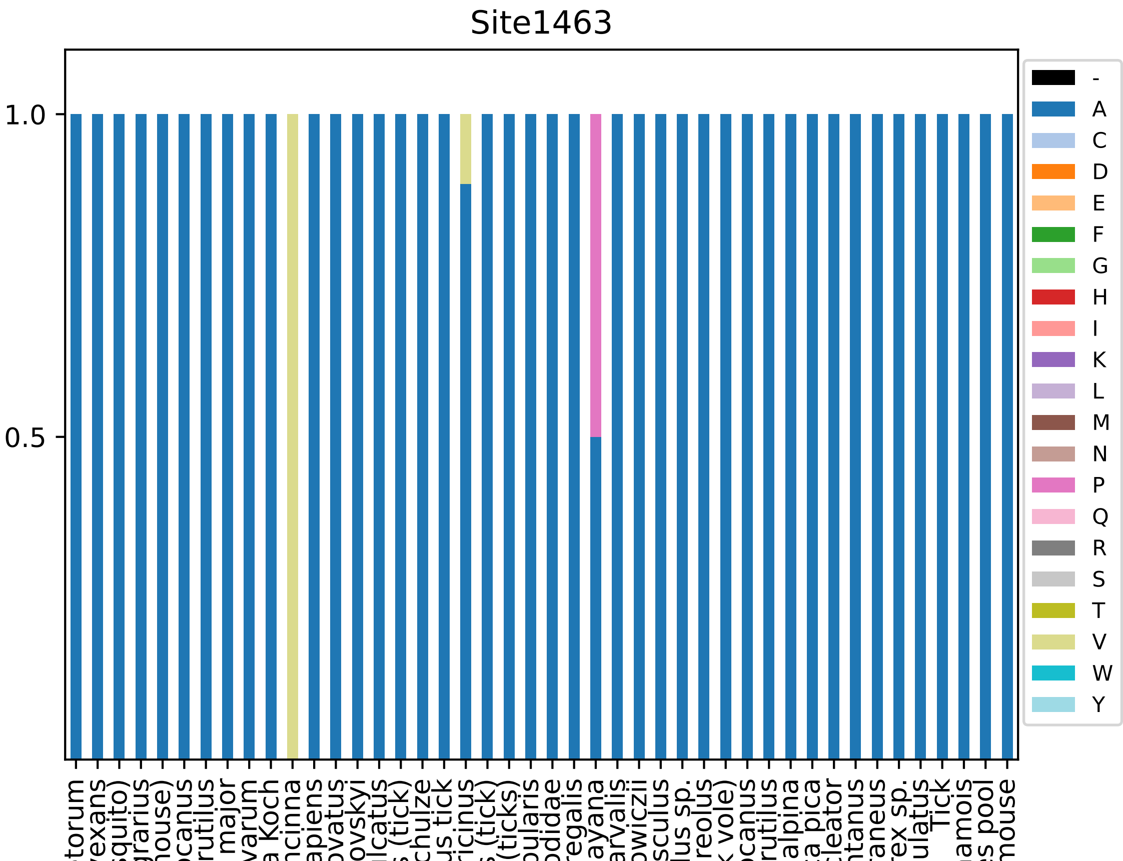
Site1463
1.0
0.5
-
A
C
D
E
F
G
H
I
K
L
M
N
P
Q
R
S
T
V
W
Y

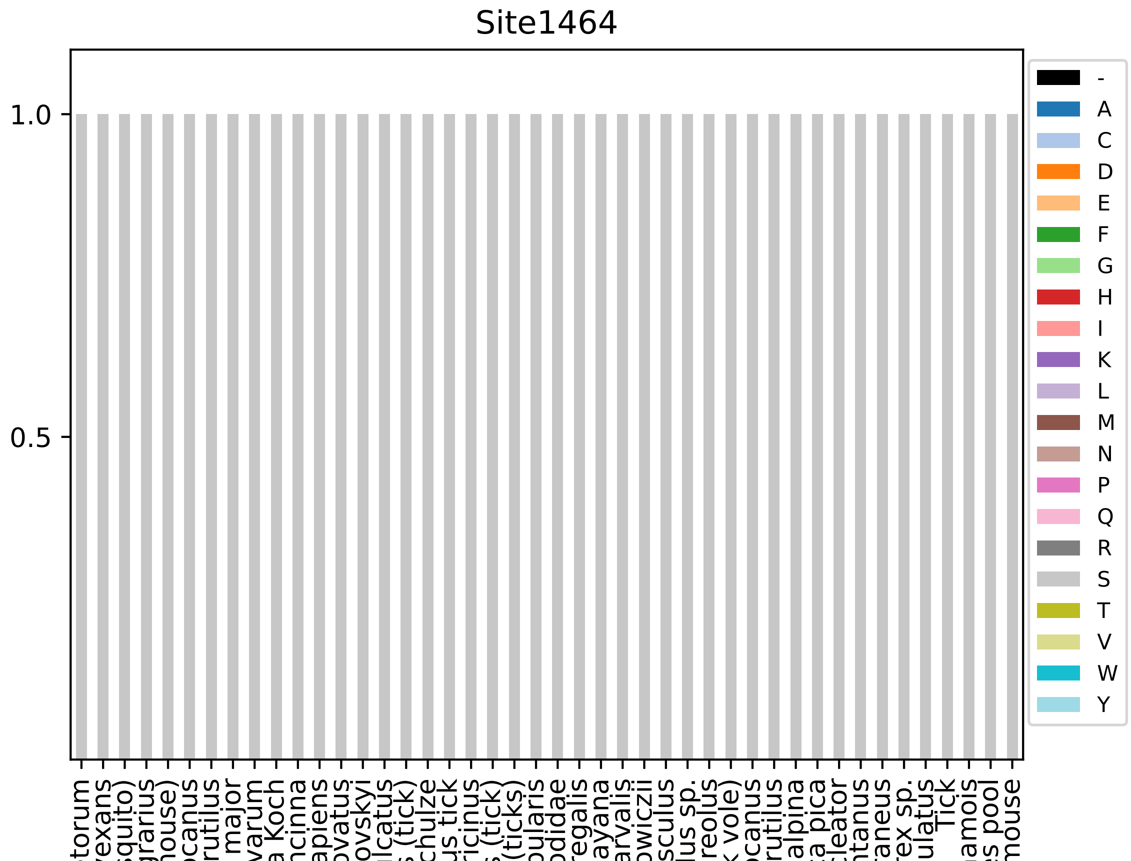

Site1464
1.0
0.5
-
A
C
D
E
F
G
H
I
K
L
M
N
P
Q
R
S
T
V
W
Y

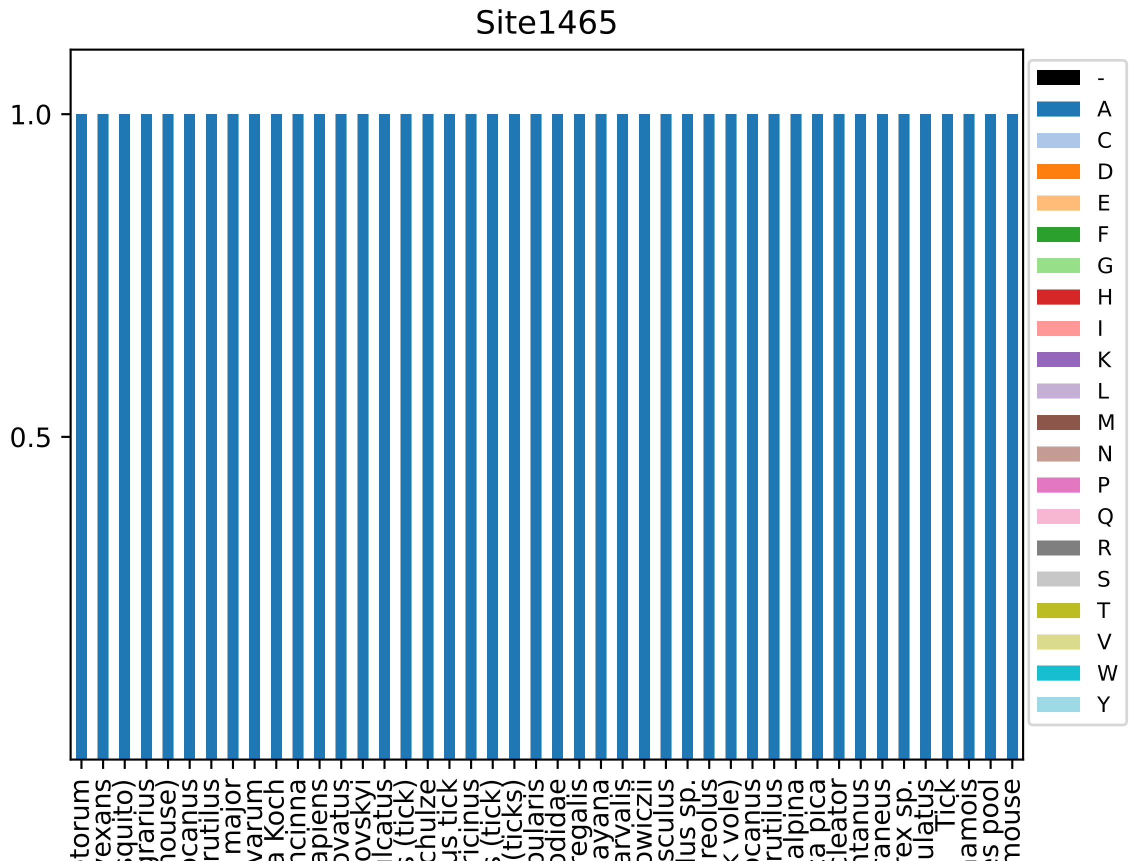
Site1465
1.0
0.5
-
A
C
D
E
F
G
H
I
K
L
M
N
P
Q
R
S
T
V
W
Y

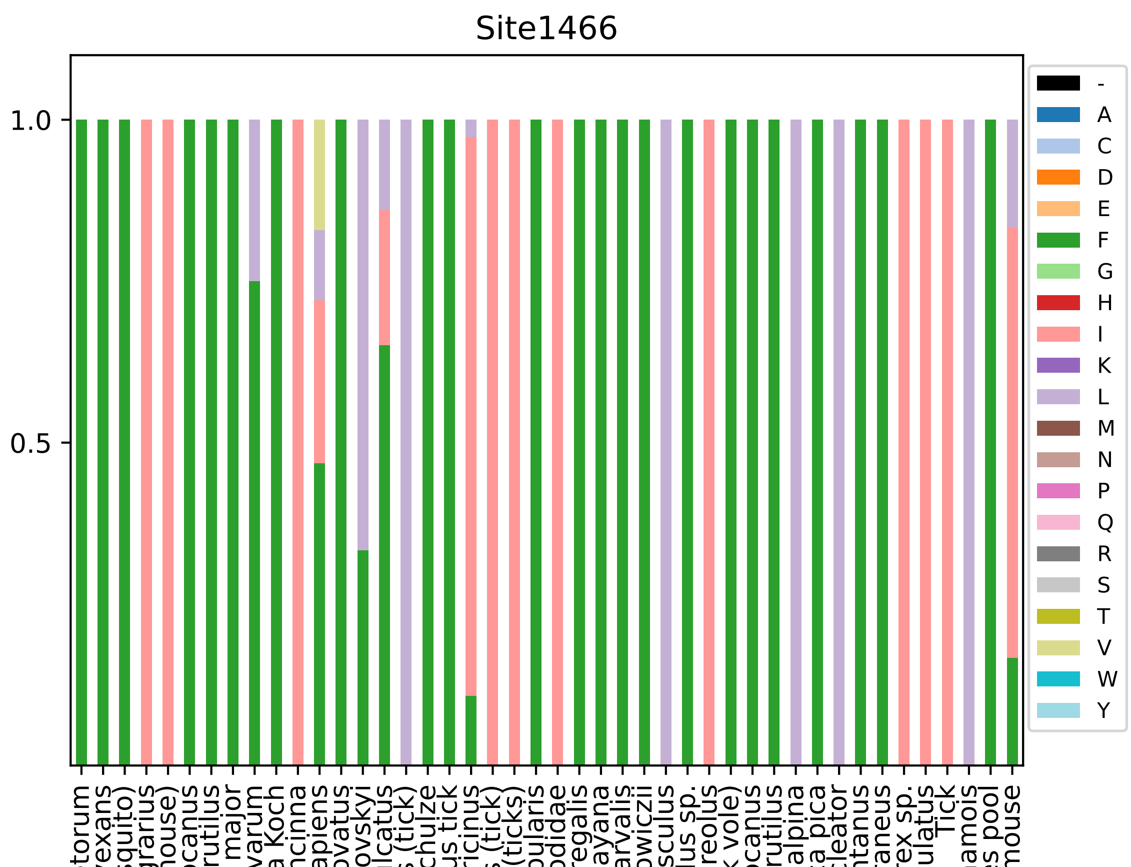
Site1466
1.0
0.5
-
A
C
D
E
F
G
H
I
K
L
M
N
P
Q
R
S
T
V
W
Y
torum
vexans
squito)
grarius
nouse)
ocanus
rutilus
major
varum
a Koch
ncinna
apiens
ovatus
ovskyi
ulcatus
s (tick)
chulze
us tick
ricinus
s (tick)
(ticks)
pularis
odidae
regalis
ayana
arvalis
owiczii
sculus
lus sp.
reolus
x vole)
ocanus
rutilus
alpina
a pica
leator
ntanus
raneus
rex sp.
ulatus
Tick
amois
es pool
mouse

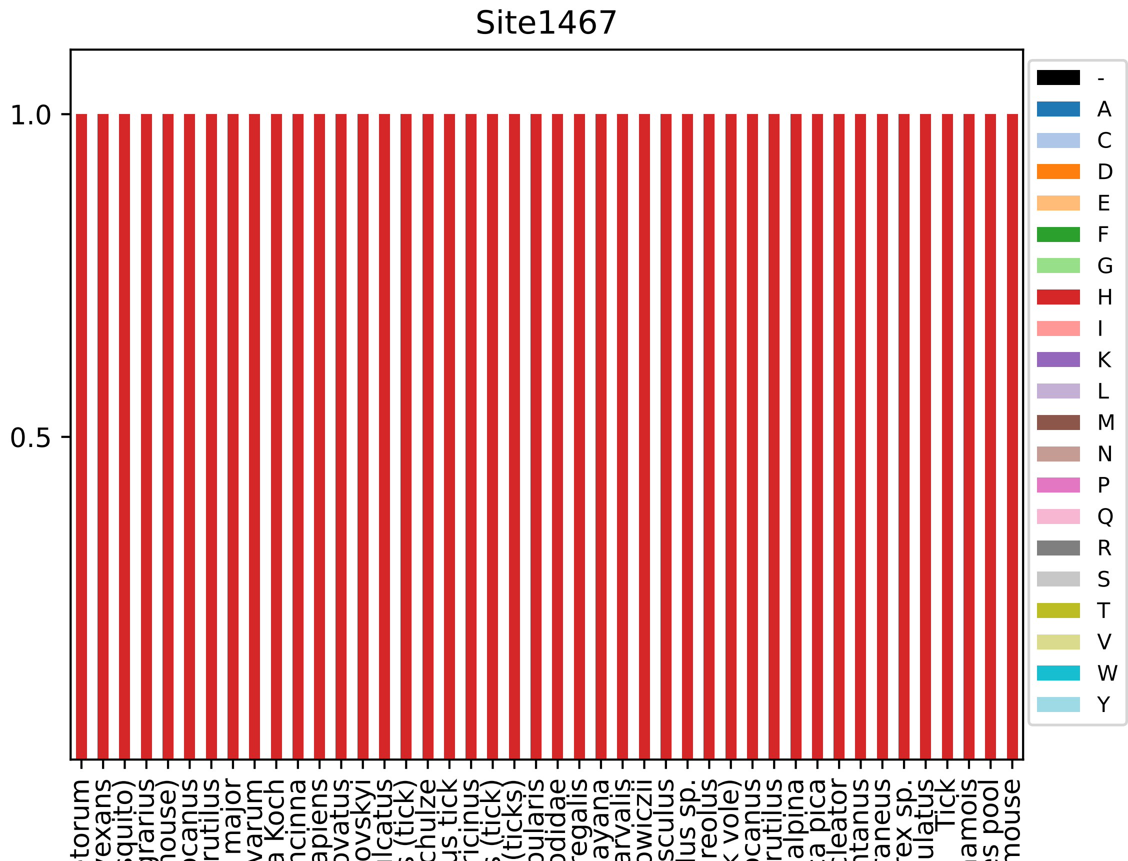
Site1467
1.0
0.5
-
A
C
D
E
F
G
H
I
K
L
M
N
P
Q
R
S
T
V
W
Y

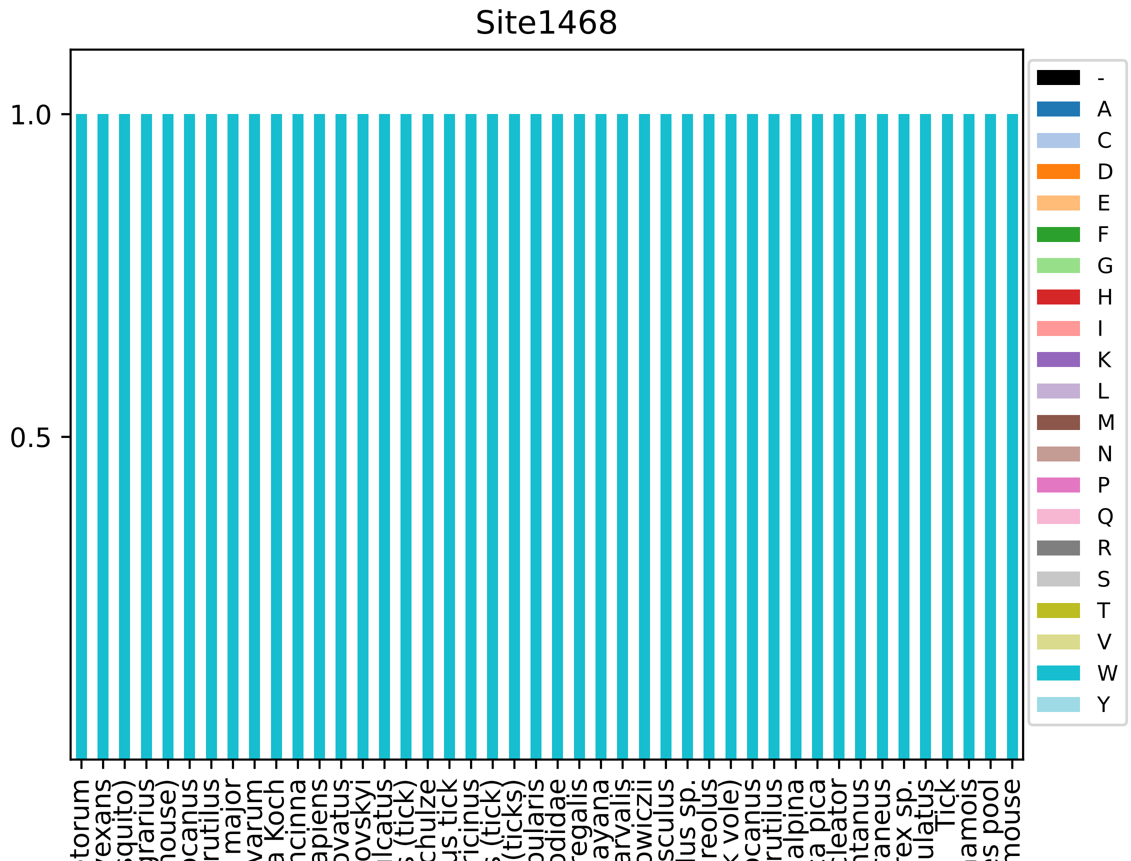

Site1468
1.0
0.5
-
A
C
D
E
F
G
H
I
K
L
M
N
P
Q
R
S
T
V
W
Y

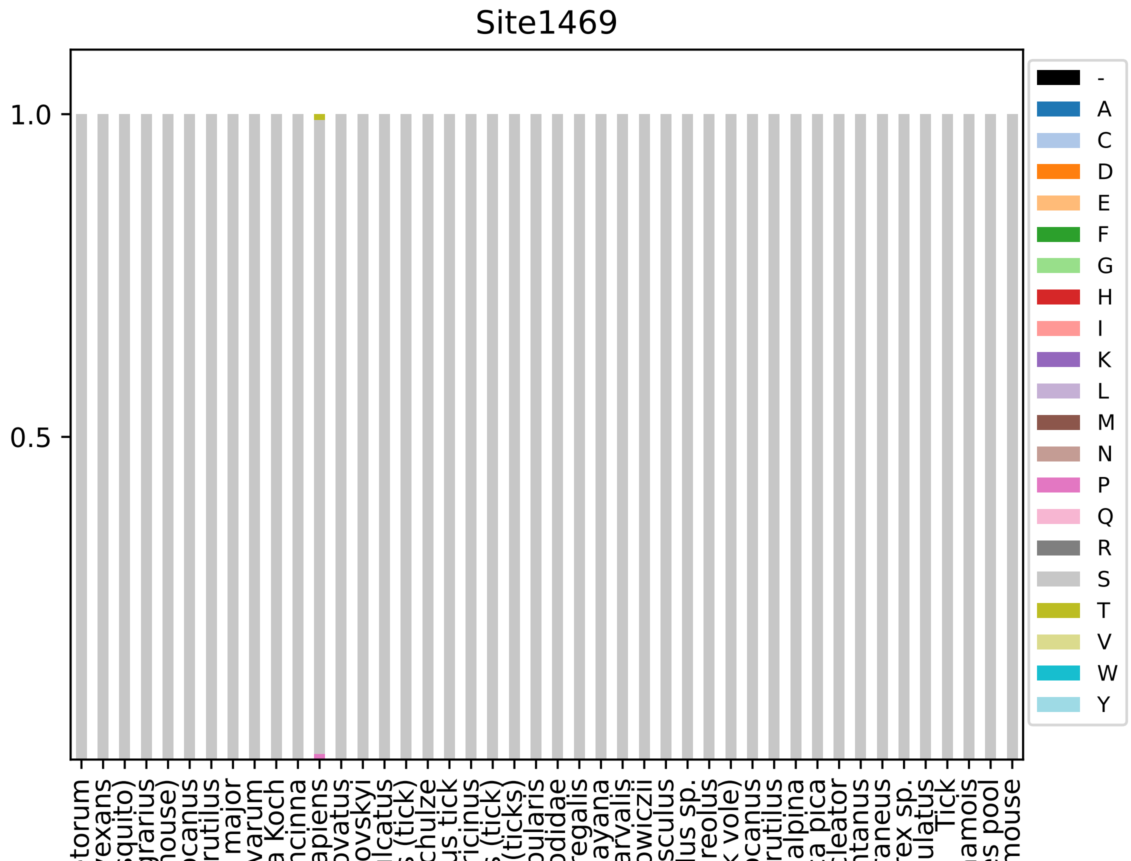

Site1469
1.0
0.5
-
A
C
D
E
F
G
H
I
K
L
M
N
P
Q
R
S
T
V
W
Y

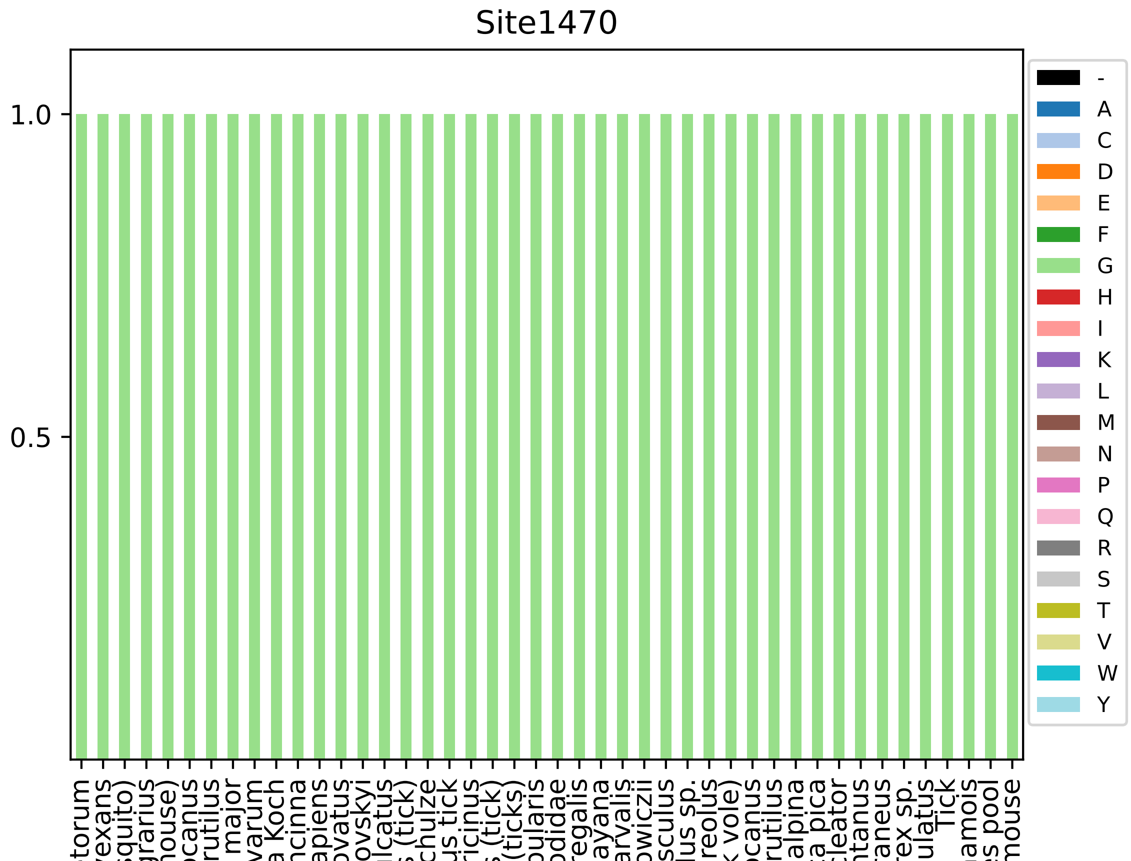

Site1470
1.0
0.5
-
A
C
D
E
F
G
H
I
K
L
M
N
P
Q
R
S
T
V
W
Y

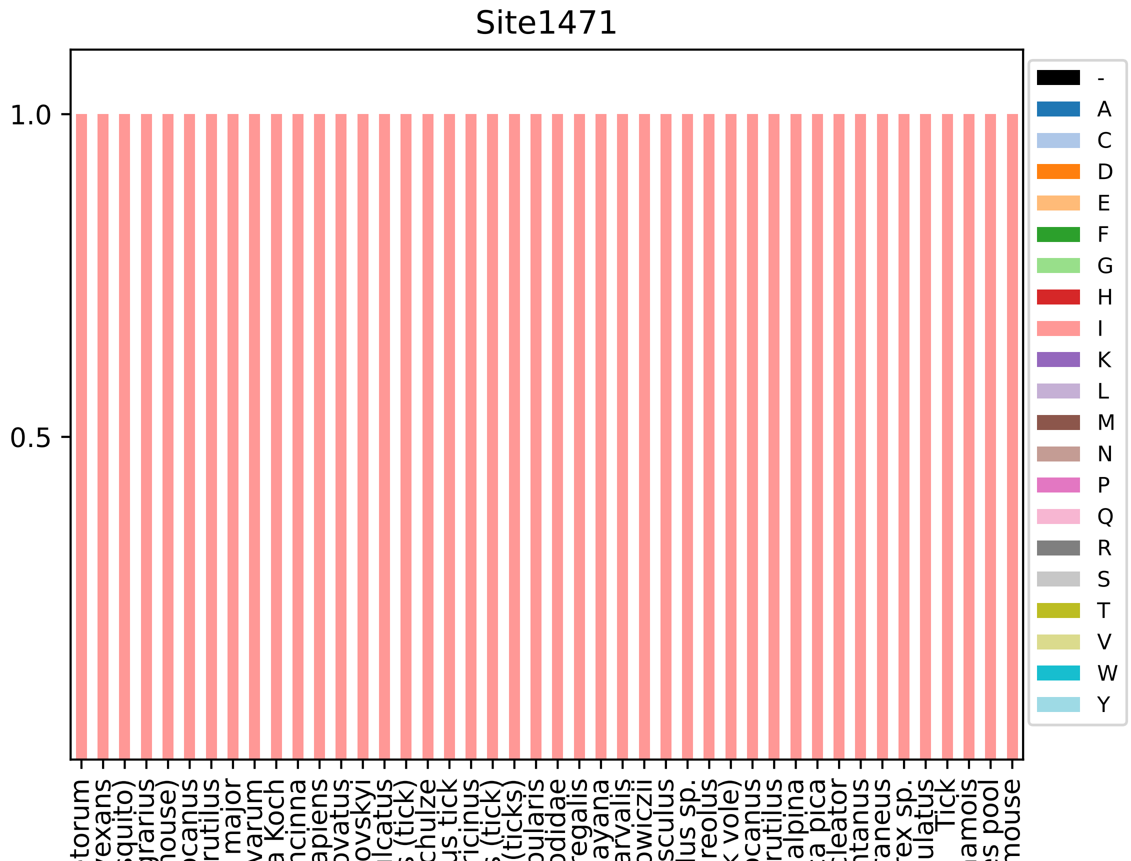

Site1471
1.0
0.5
-
A
C
D
E
F
G
H
I
K
L
M
N
P
Q
R
S
T
V
W
Y

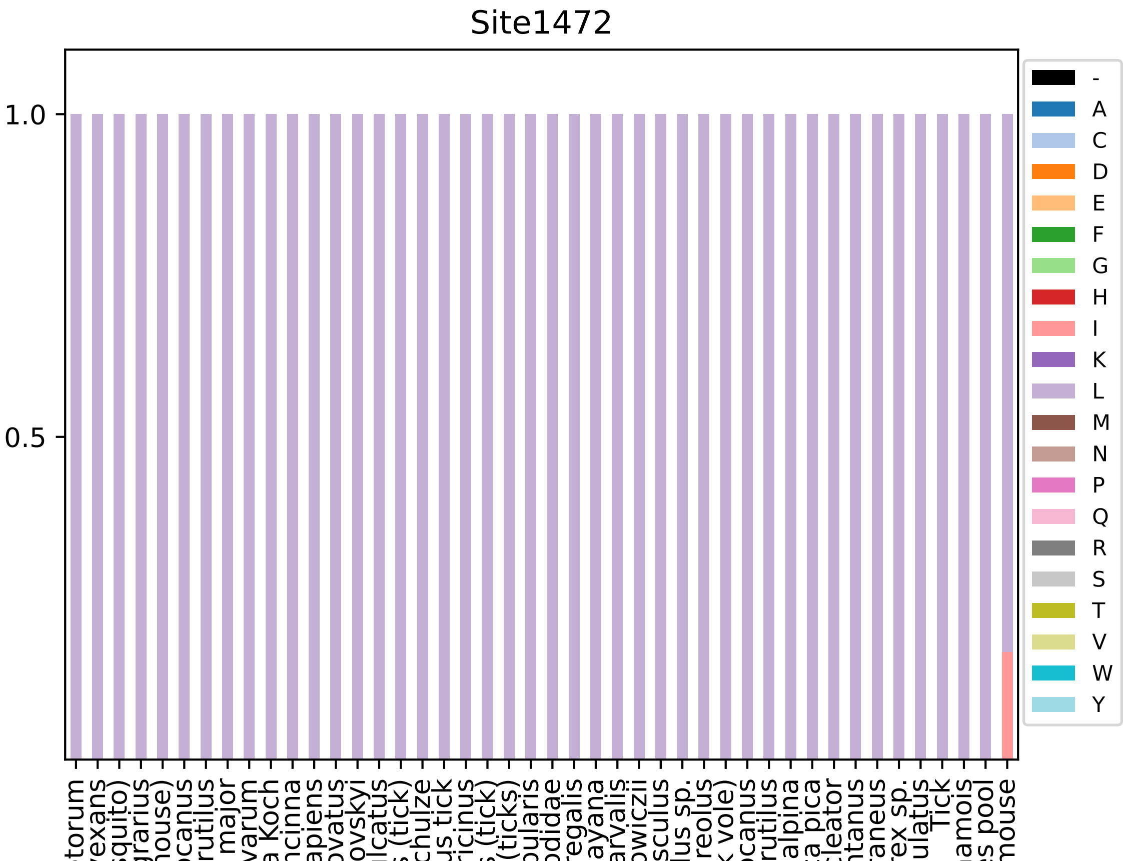
Site1472
1.0
0.5
-
A
C
D
E
F
G
H
I
K
L
M
N
P
Q
R
S
T
V
W
Y

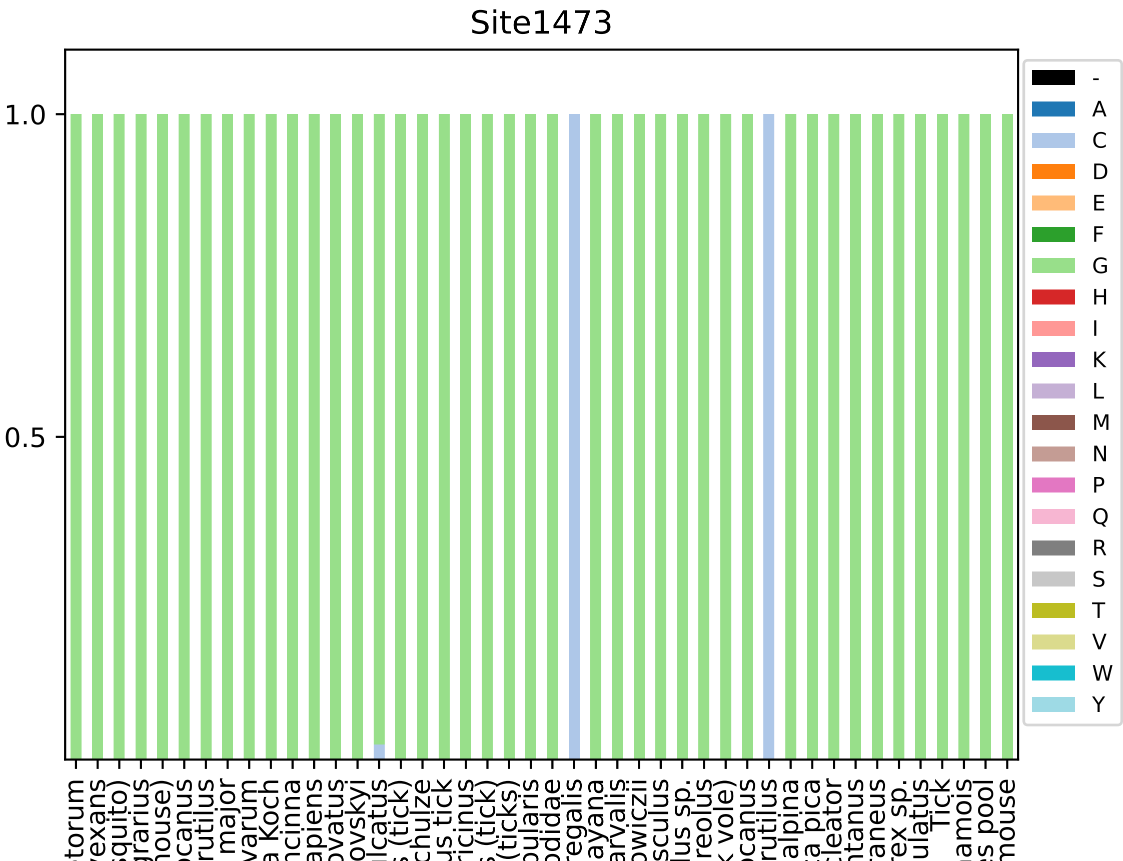
Site1473
1.0
0.5
-
A
C
D
E
F
G
H
I
K
L
M
N
P
Q
R
S
T
V
W
Y

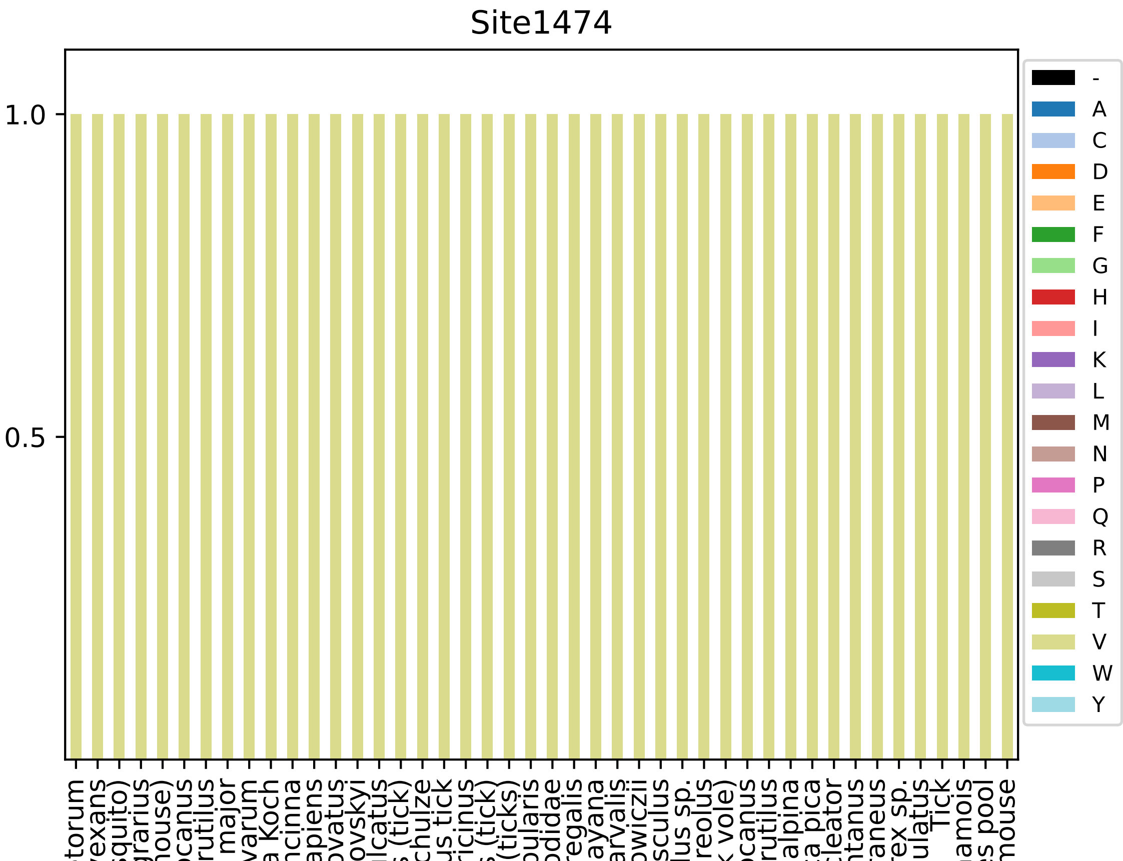
Site1474
1.0
0.5
-
A
C
D
E
F
G
H
I
K
L
M
N
P
Q
R
S
T
V
W
Y

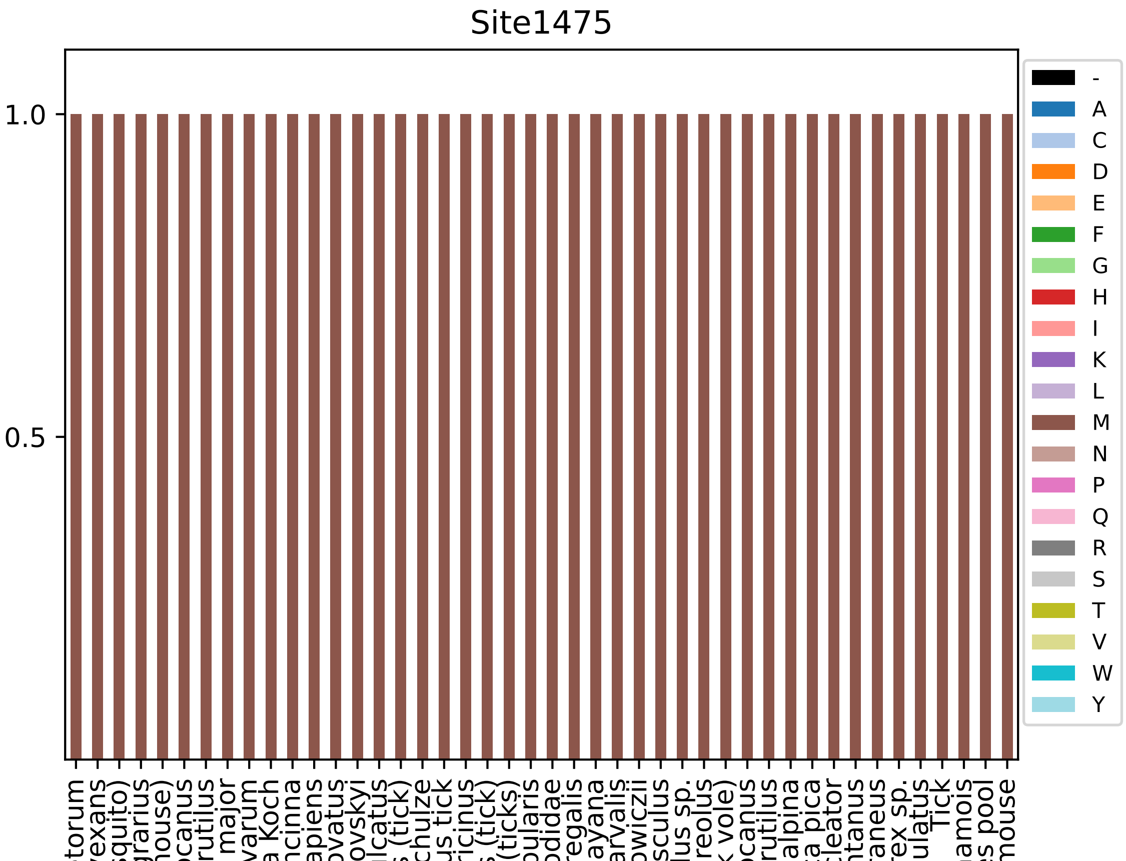
Site1475
1.0
0.5
-
A
C
D
E
F
G
H
I
K
L
M
N
P
Q
R
S
T
V
W
Y

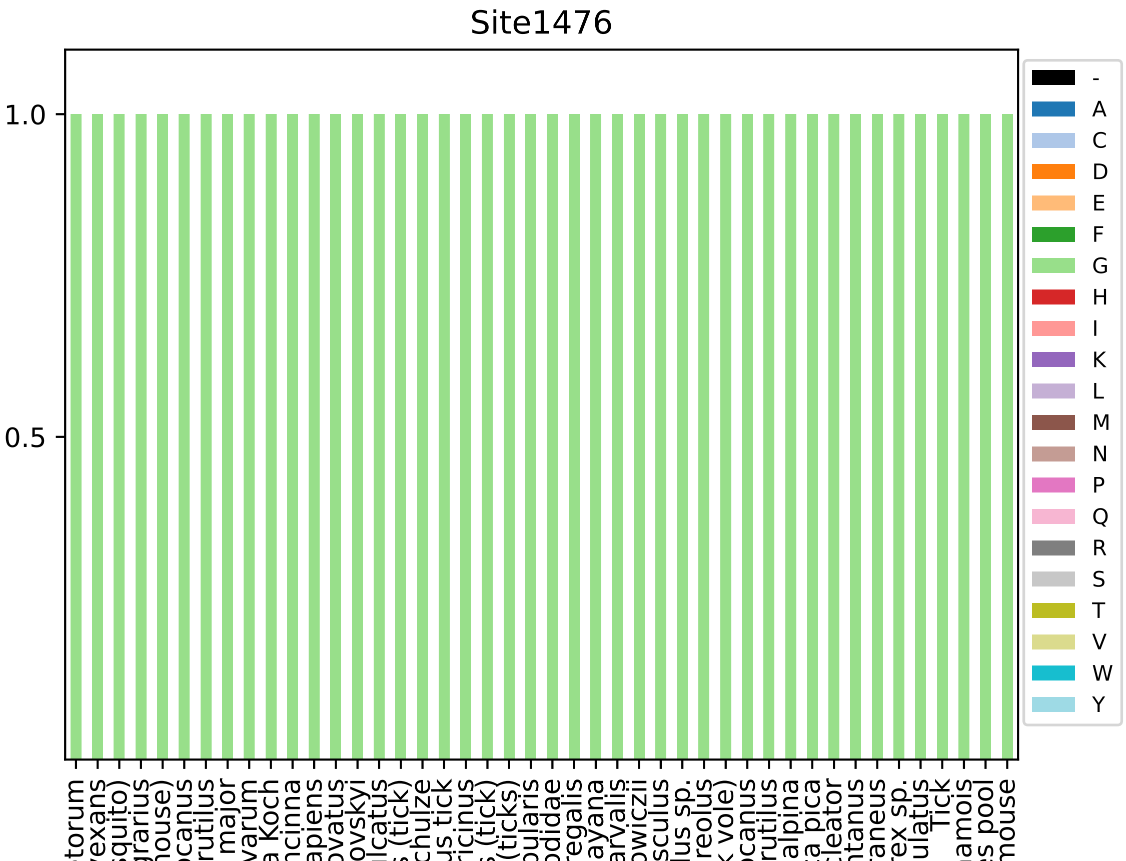
Site1476
1.0
0.5
-
A
C
D
E
F
G
H
I
K
L
M
N
P
Q
R
S
T
V
W
Y

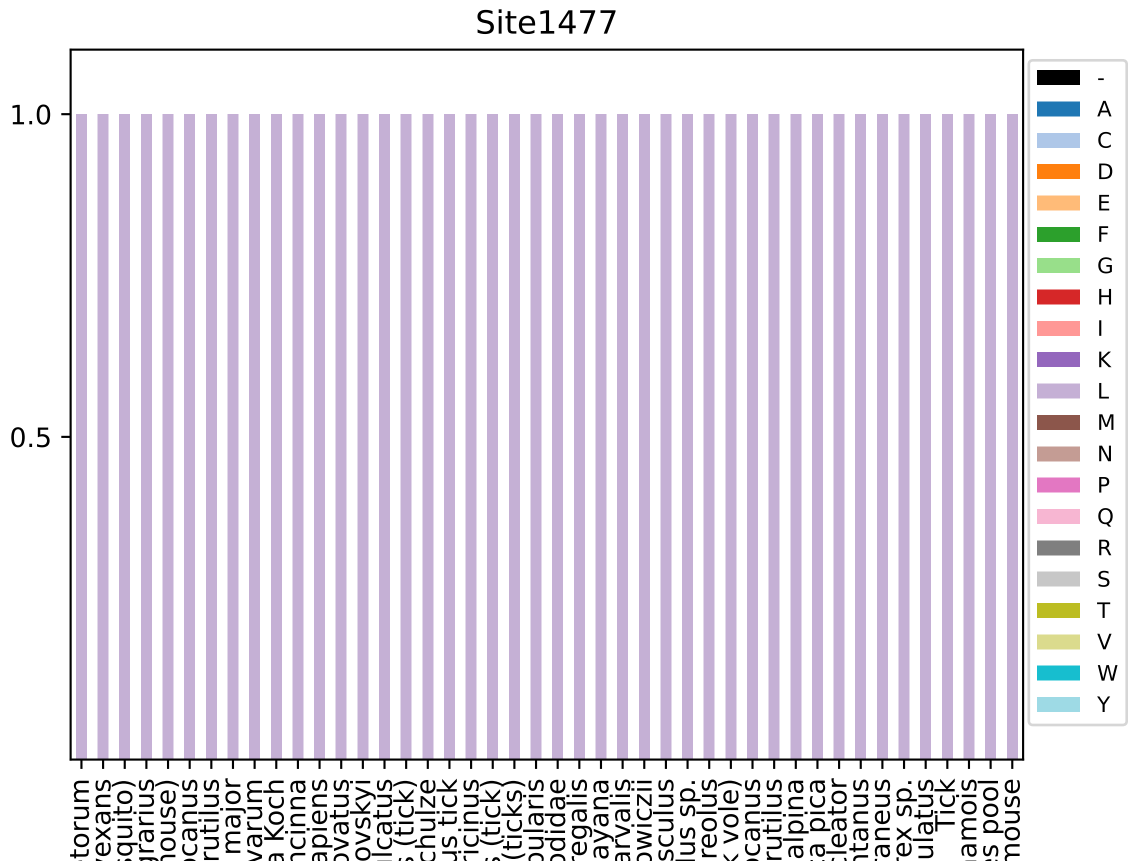

Site1477
1.0
0.5
-
A
C
D
E
F
G
H
I
K
L
M
N
P
Q
R
S
T
V
W
Y
torum
vexans
squito)
garius
nouse)
ocanus
rutilus
major
varum
a Koch
ncinna
apiens
ovatus
ovskyi
lcatus
(tick)
chulze
us tick
ricinus
(tick)
(ticks)
pularis
podidae
regalis
ayana
arvalis
owiczii
sculus
lus sp.
reolus
x vole)
ocanus
rutilus
alpina
a pica
leator
ntanus
raneus
ex sp.
ulatus
Tick
amois
s pool
mouse

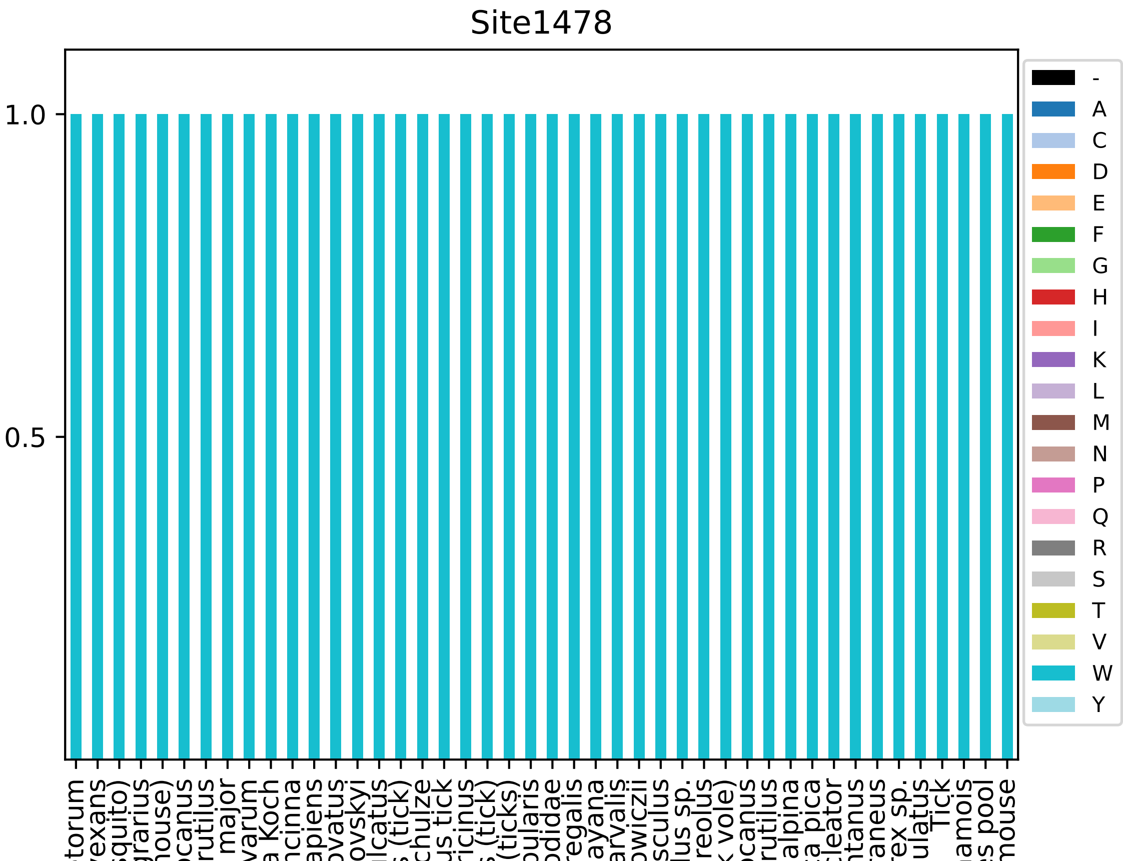
Site1478
1.0
0.5
-
A
C
D
E
F
G
H
I
K
L
M
N
P
Q
R
S
T
V
W
Y

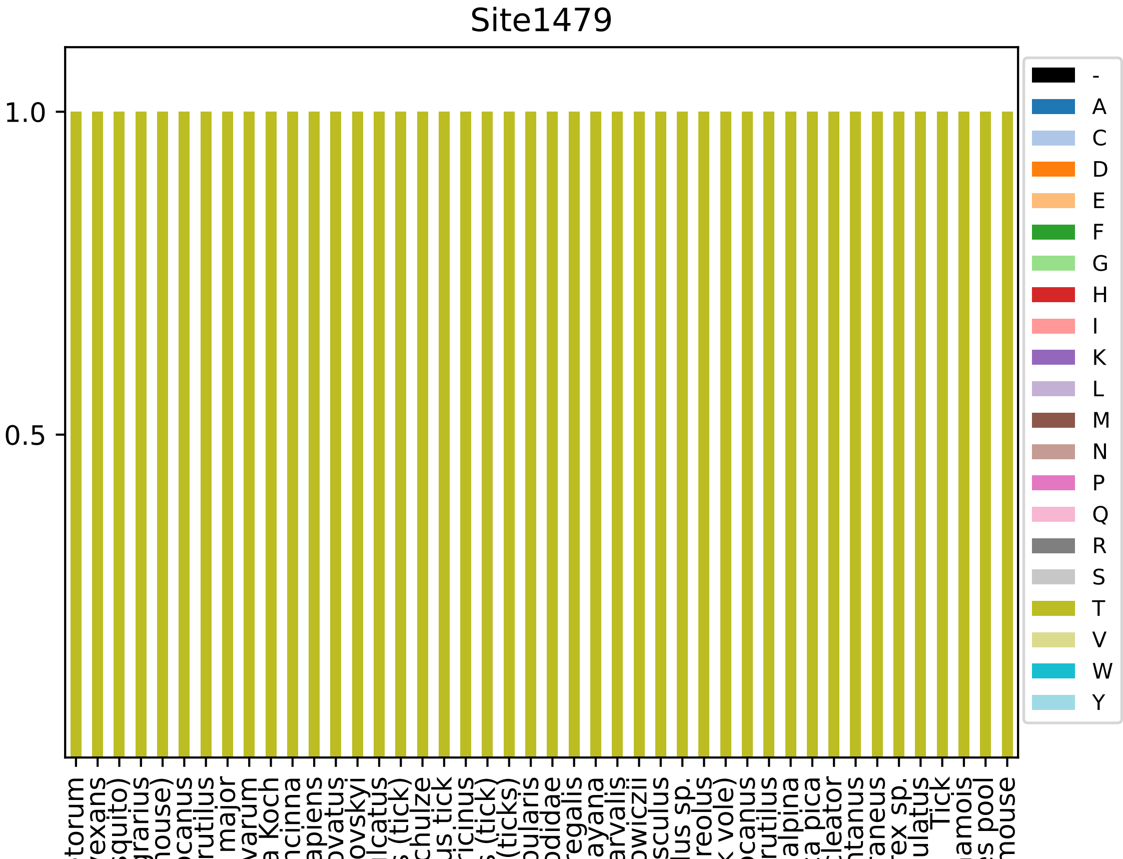

Site1479
1.0
0.5
-
A
C
D
E
F
G
H
I
K
L
M
N
P
Q
R
S
T
V
W
Y

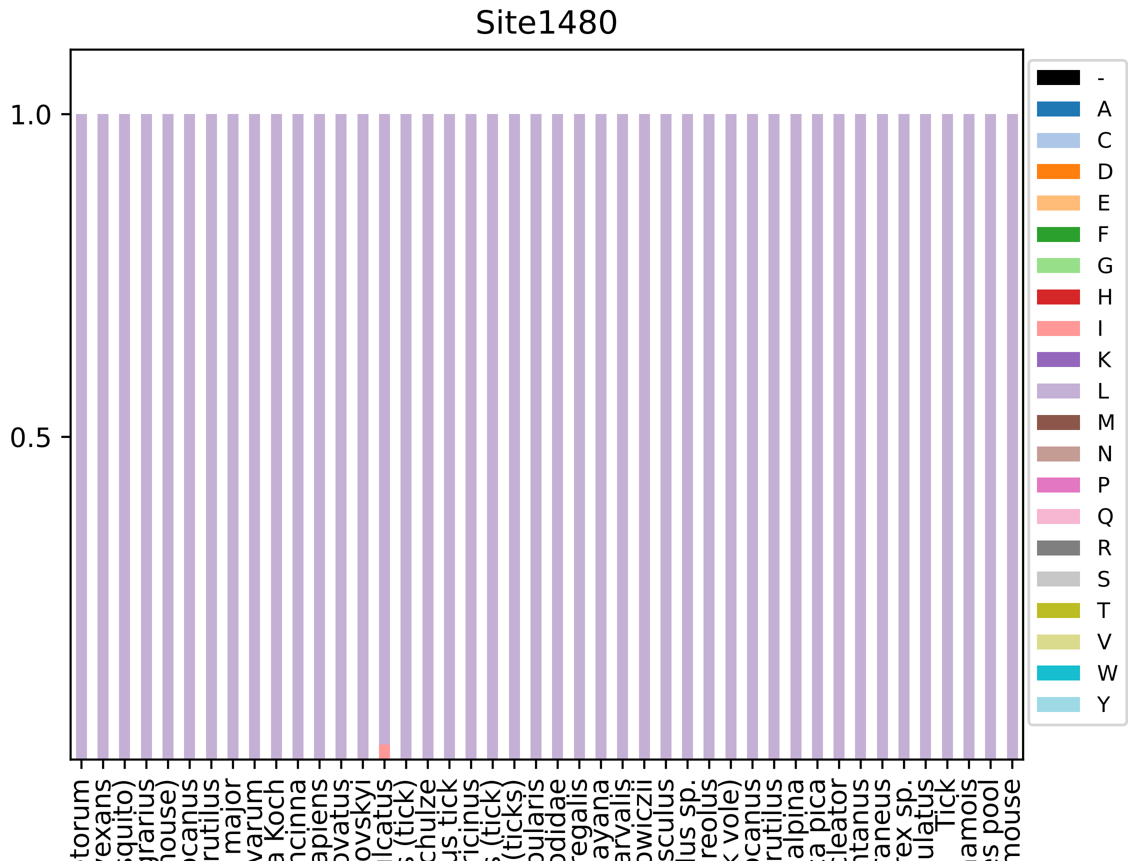
Site1480
1.0
0.5
-
A
C
D
E
F
G
H
I
K
L
M
N
P
Q
R
S
T
V
W
Y

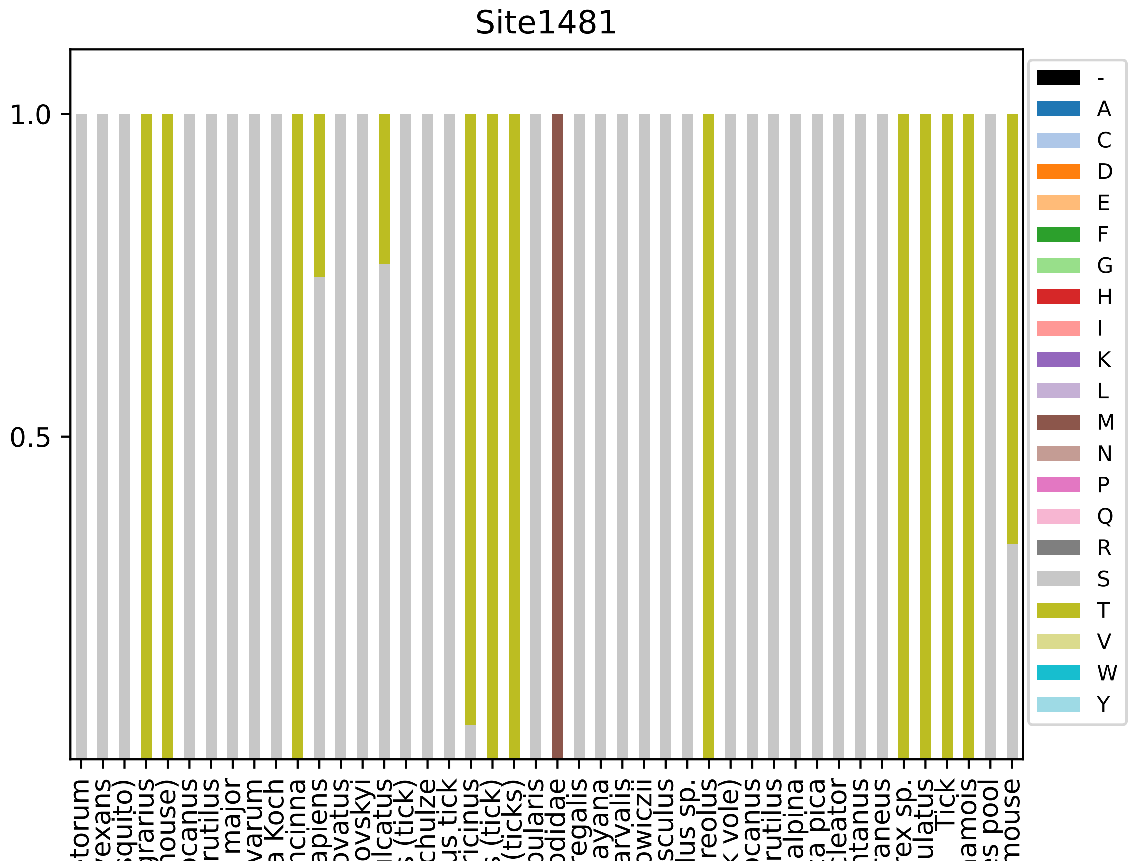

Site1481
1.0
0.5
-
A
C
D
E
F
G
H
I
K
L
M
N
P
Q
R
S
T
V
W
Y
torum
exans
squito)
garius
house)
ocanus
rutilus
major
varum
a Koch
ncinna
apiens
ovatus
ovskyi
lcatus
s (tick)
chulze
us tick
ricinus
s (tick)
(ticks)
pularis
podidae
regalis
ayana
arvalis
owiczii
sculus
lus sp.
reolus
k vole)
ocanus
rutilus
alpina
a pica
cleator
ntanus
aneus
rex sp.
ulatus
Tick
amois
es pool
mouse

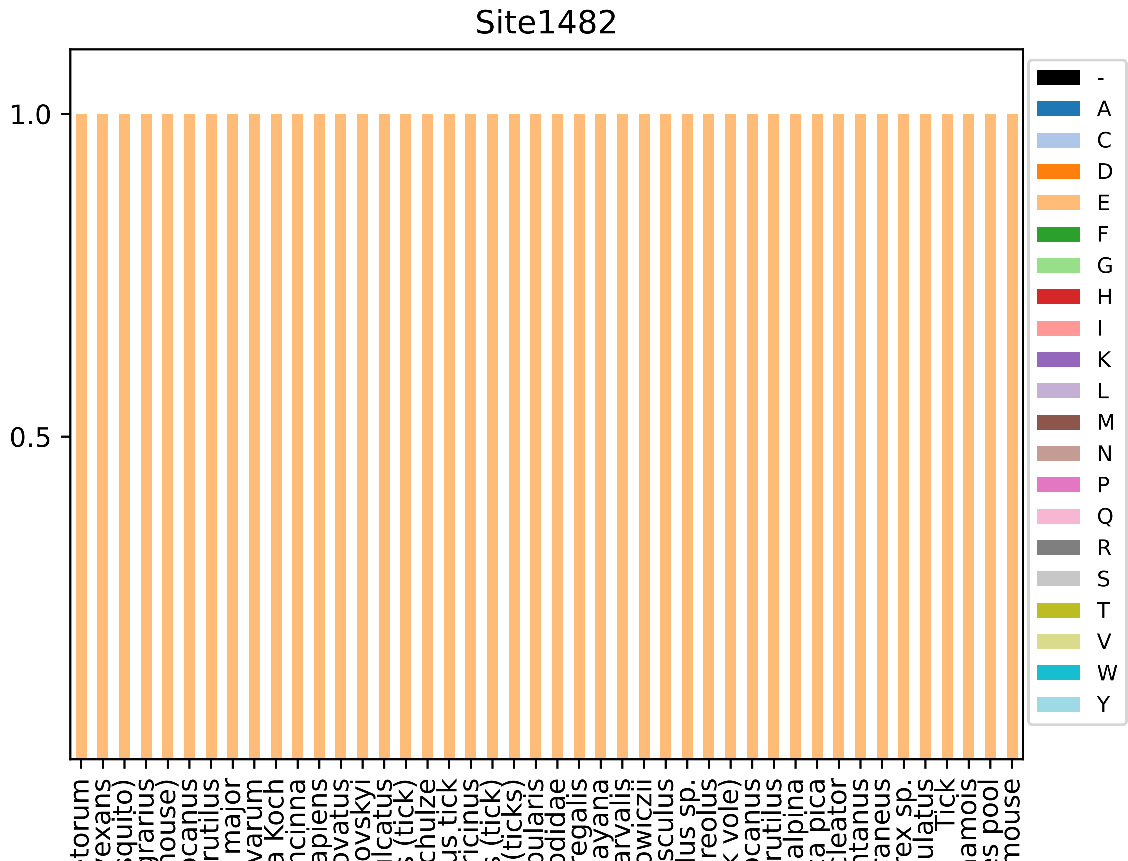
Site1482
1.0
0.5
-
A
C
D
E
F
G
H
I
K
L
M
N
P
Q
R
S
T
V
W
Y

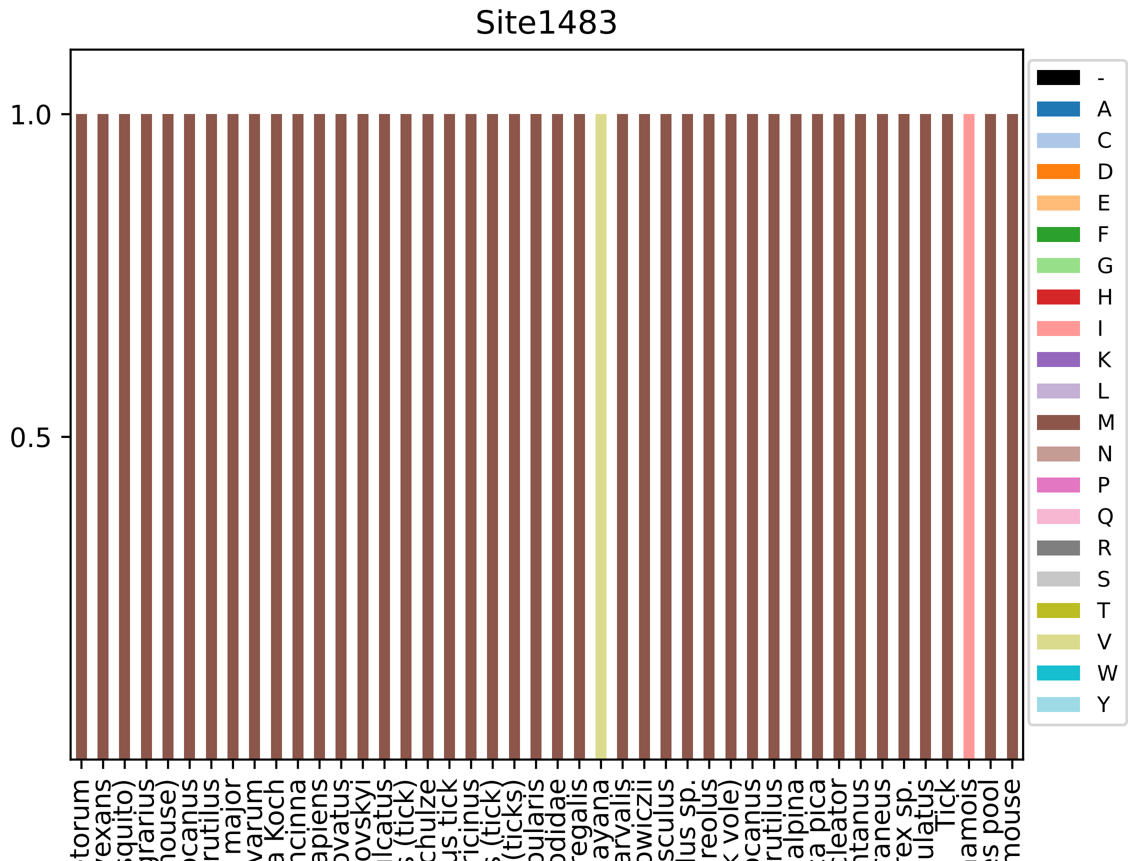
Site1483
1.0
0.5
-
A
C
D
E
F
G
H
I
K
L
M
N
P
Q
R
S
T
V
W
Y

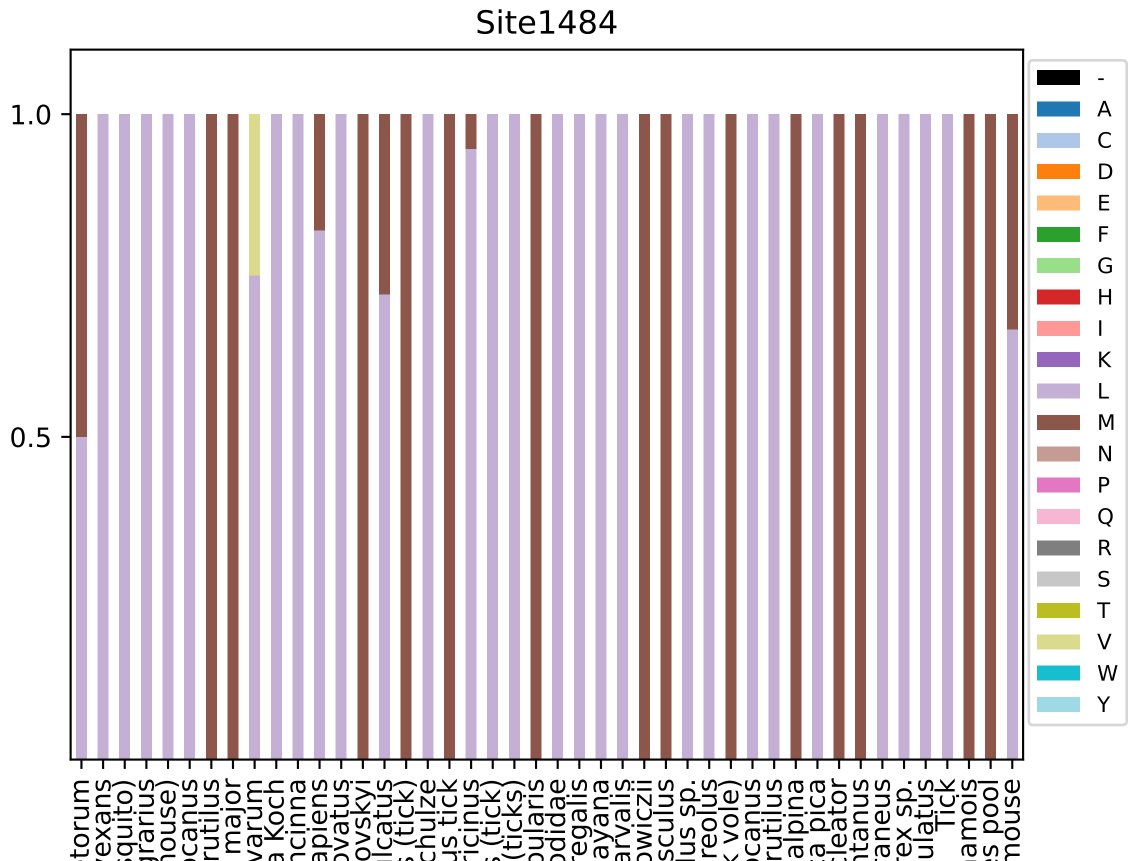

Site1484
1.0
0.5
-
A
C
D
E
F
G
H
I
K
L
M
N
P
Q
R
S
T
V
W
Y

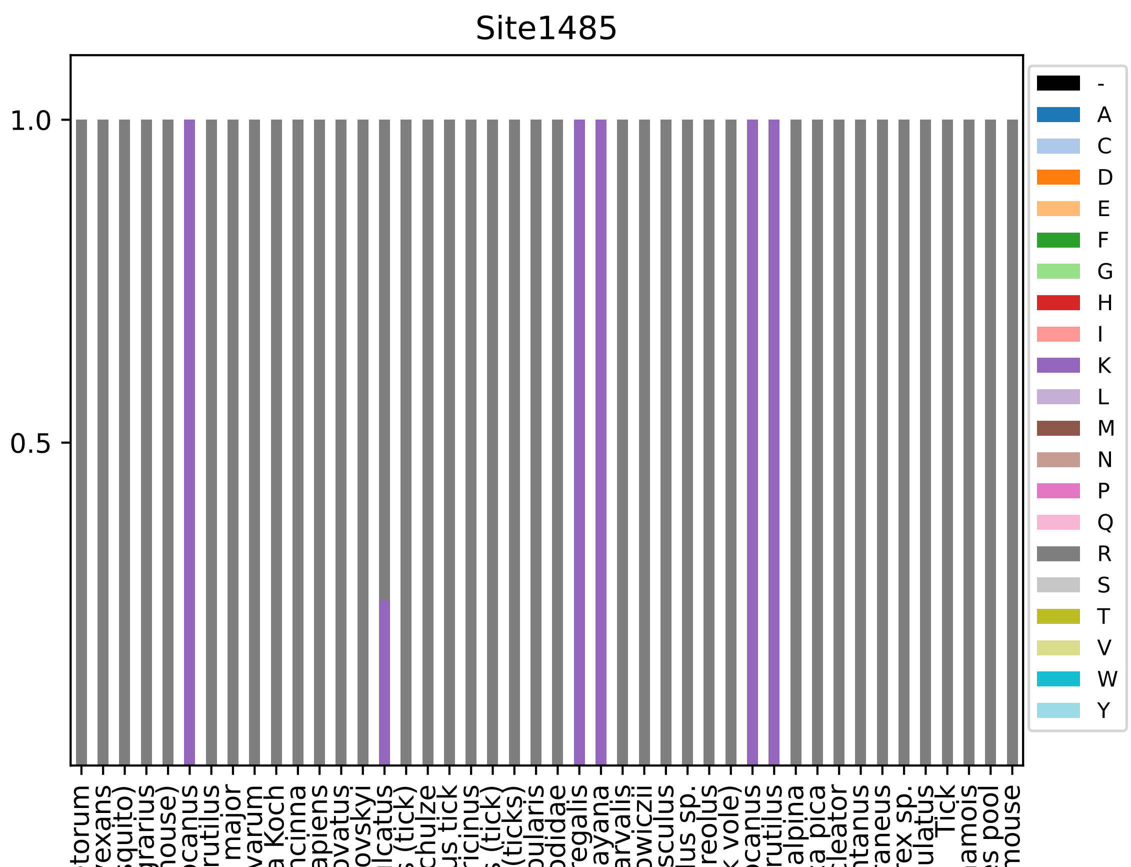
Site1485
1.0
0.5
-
A
C
D
E
F
G
H
I
K
L
M
N
P
Q
R
S
T
V
W
Y

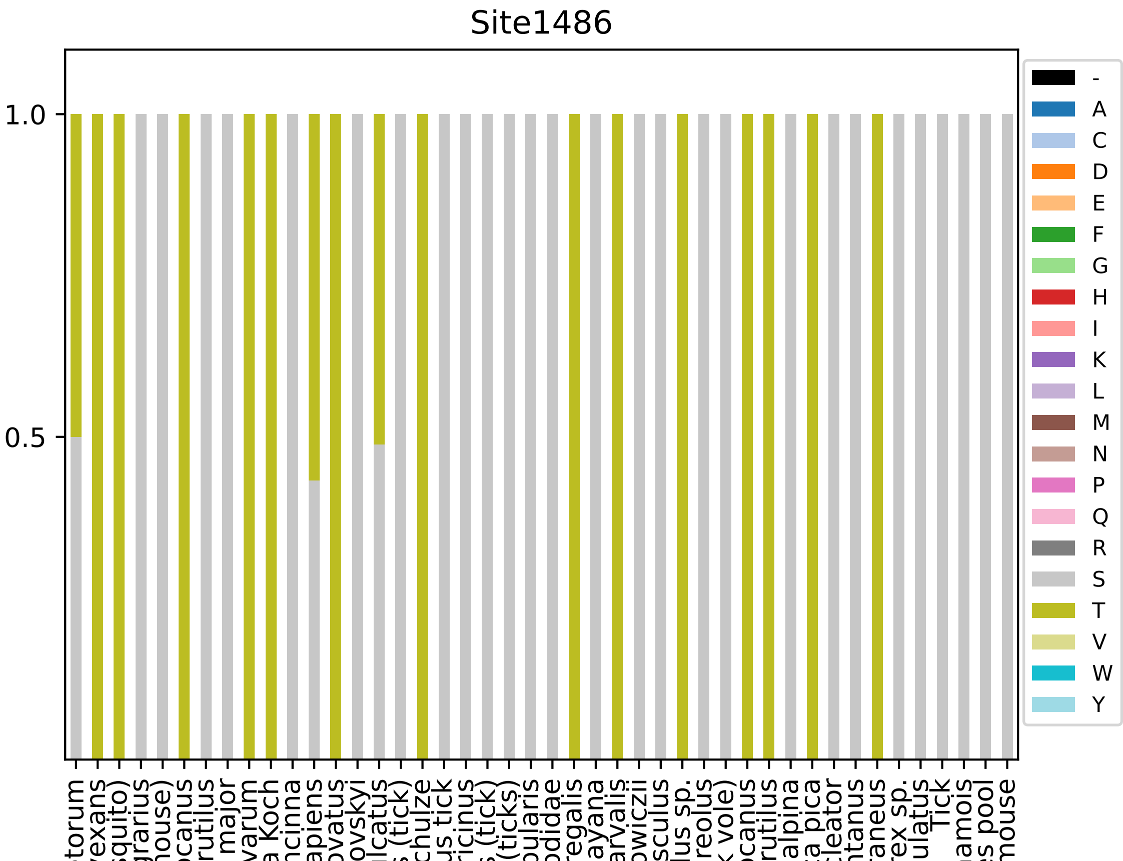

Site1486
1.0
0.5
-
A
C
D
E
F
G
H
I
K
L
M
N
P
Q
R
S
T
V
W
Y
Tick

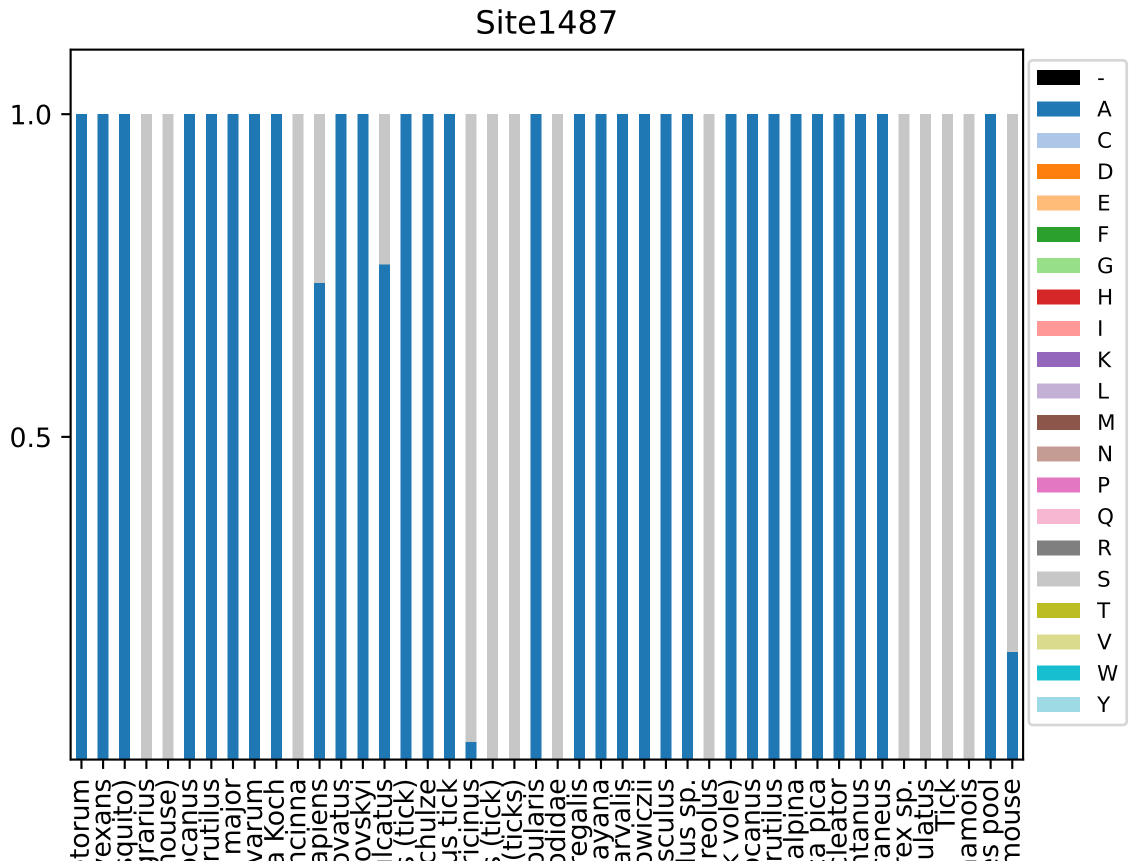
Site1487
1.0
0.5
-
A
C
D
E
F
G
H
I
K
L
M
N
P
Q
R
S
T
V
W
Y

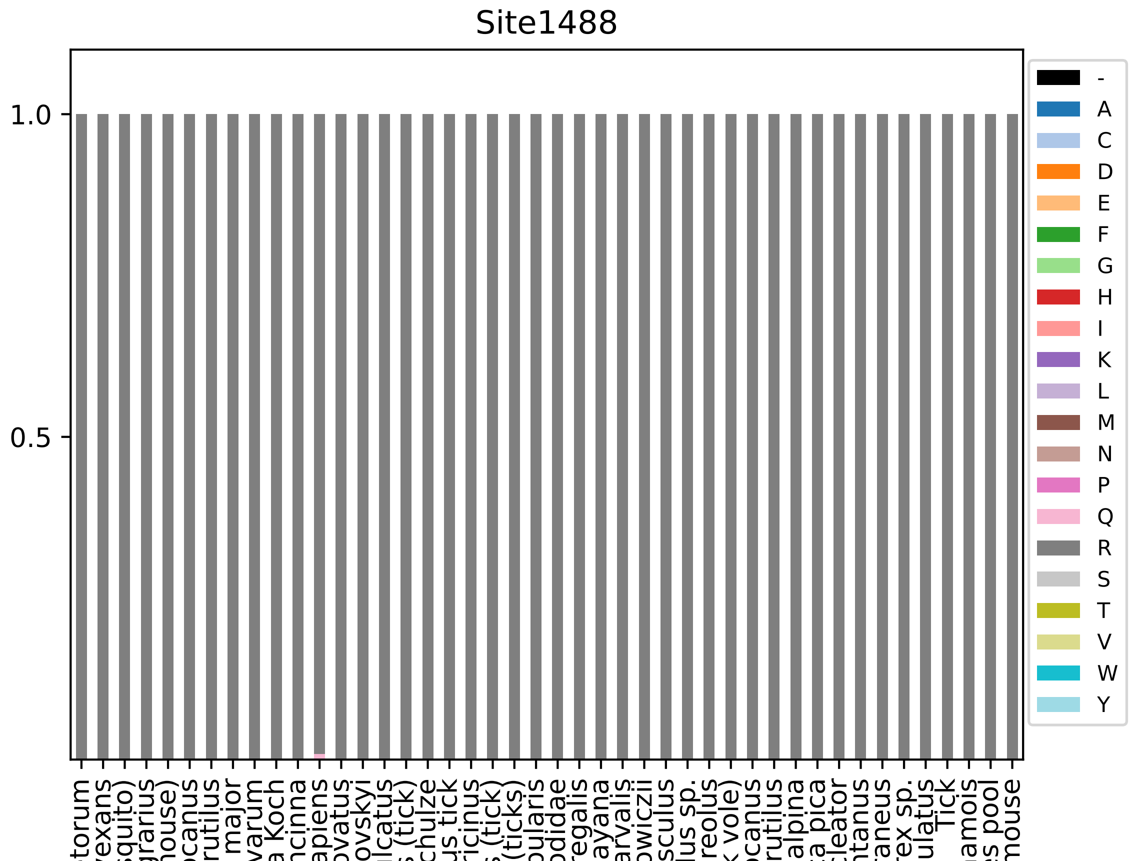

Site1488
1.0
0.5
-
A
C
D
E
F
G
H
I
K
L
M
N
P
Q
R
S
T
V
W
Y

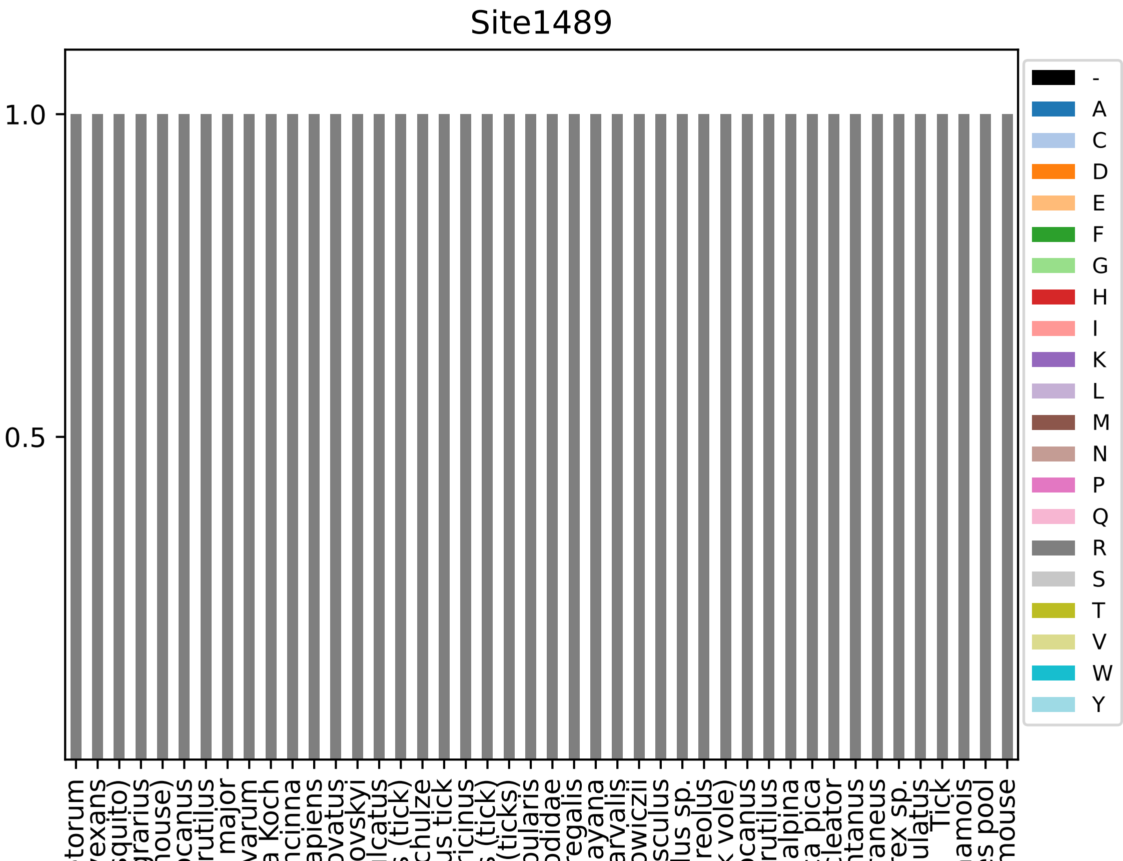
Site1489
1.0
0.5
-
A
C
D
E
F
G
H
I
K
L
M
N
P
Q
R
S
T
V
W
Y

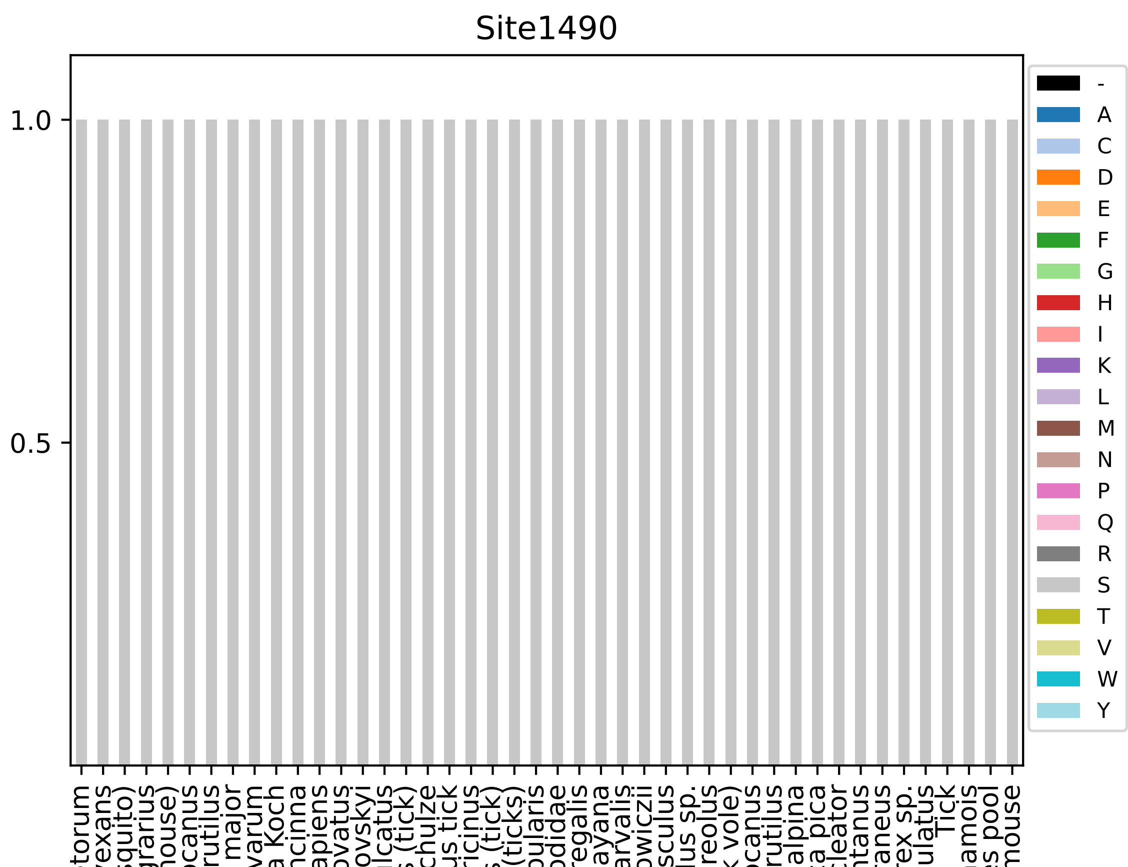

Site1490
1.0
0.5
-
A
C
D
E
F
G
H
I
K
L
M
N
P
Q
R
S
T
V
W
Y

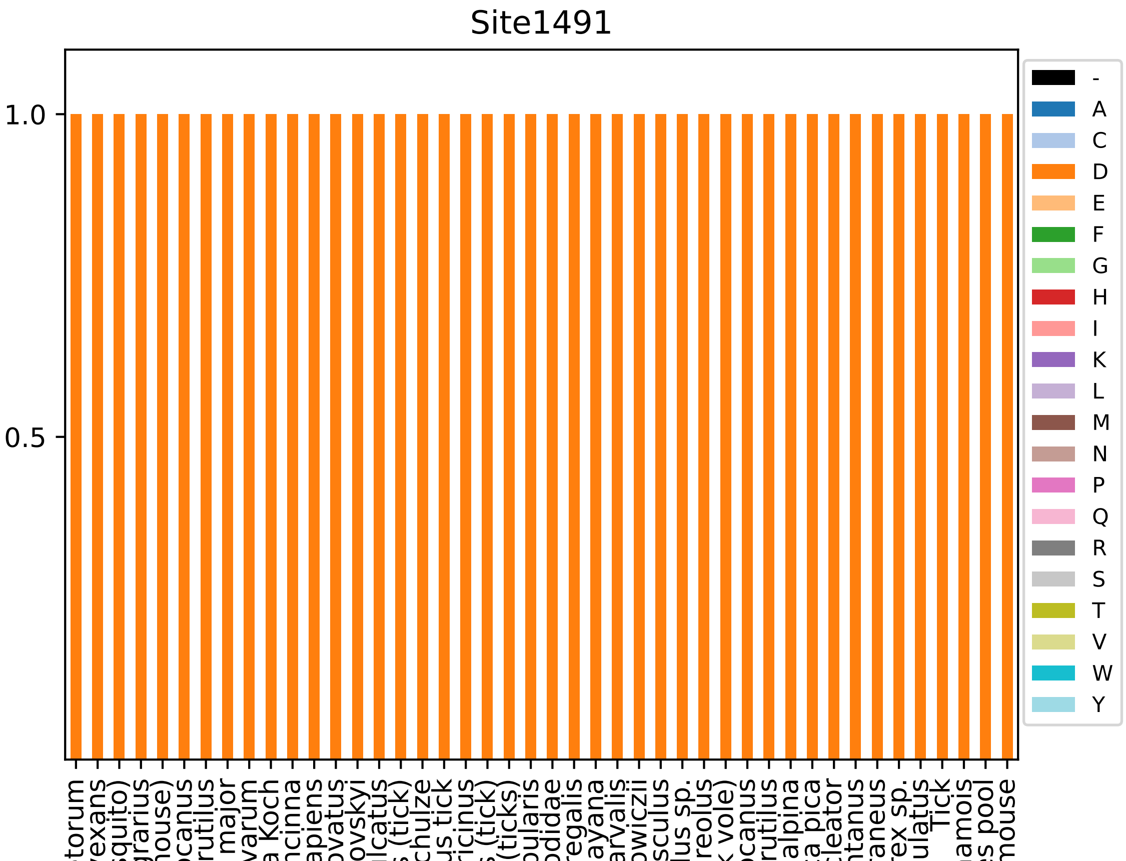
Site1491
1.0
0.5
-
A
C
D
E
F
G
H
I
K
L
M
N
P
Q
R
S
T
V
W
Y

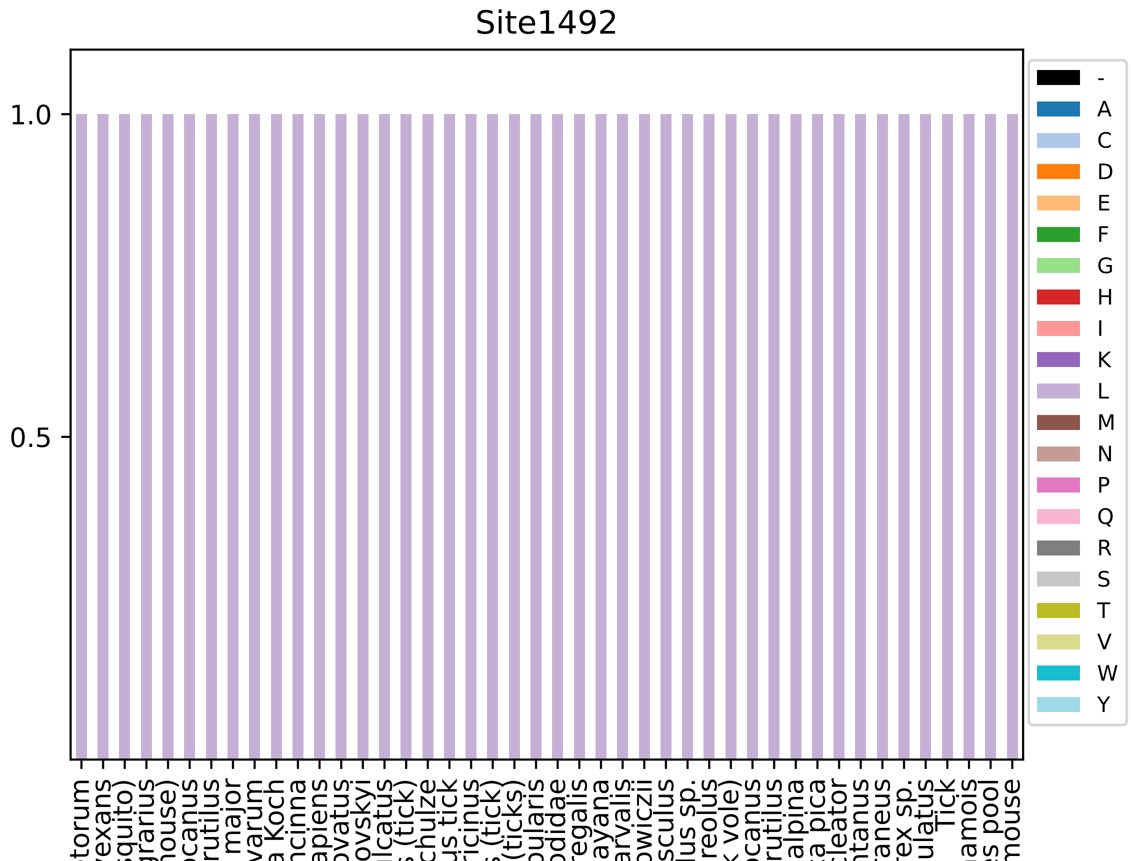
Site1492
1.0
0.5
-
A
C
D
E
F
G
H
I
K
L
M
N
P
Q
R
S
T
V
W
Y

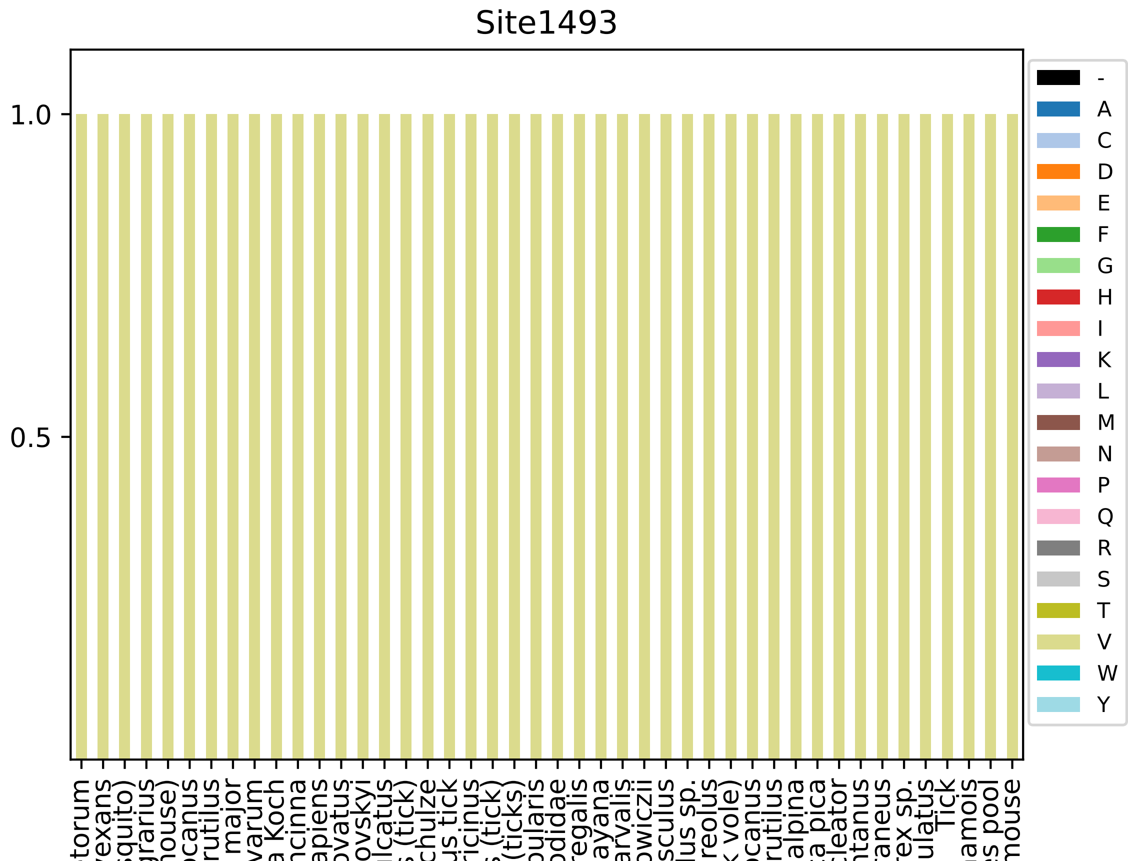
Site1493
1.0
0.5
-
A
C
D
E
F
G
H
I
K
L
M
N
P
Q
R
S
T
V
W
Y

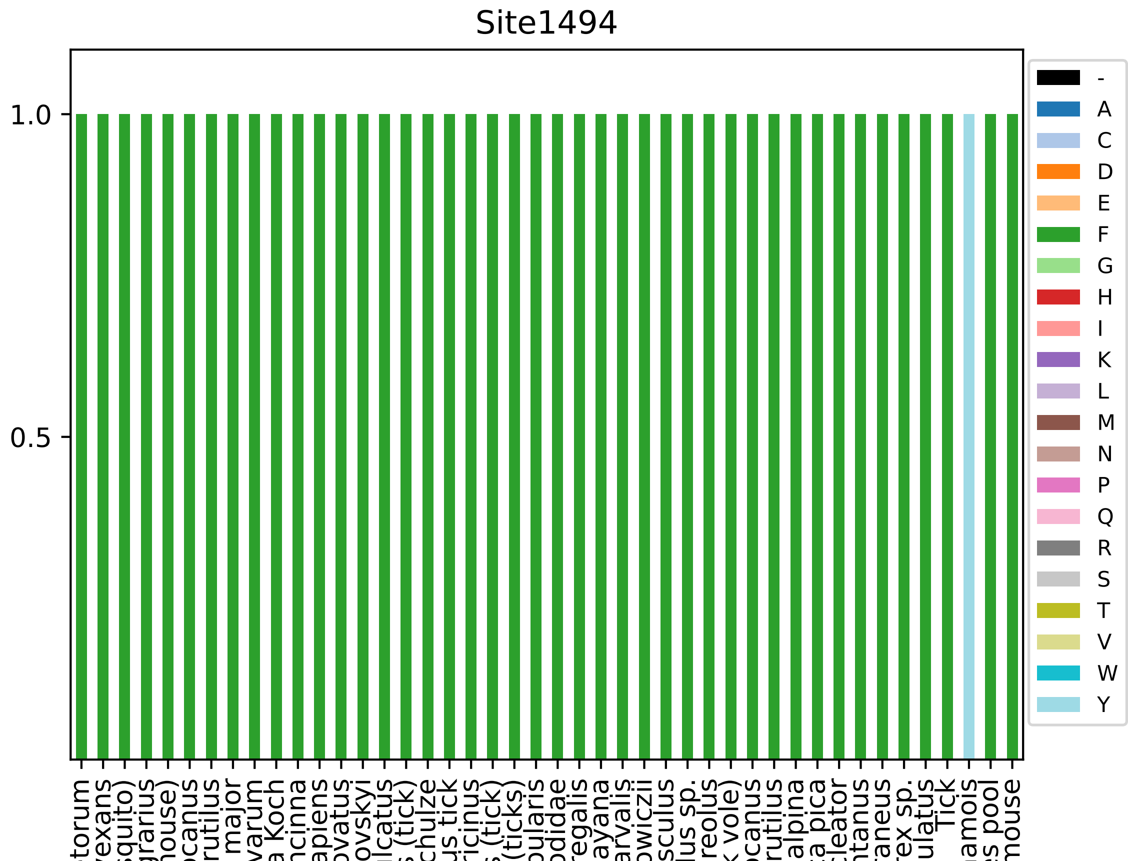
Site1494
1.0
0.5
-
A
C
D
E
F
G
H
I
K
L
M
N
P
Q
R
S
T
V
W
Y

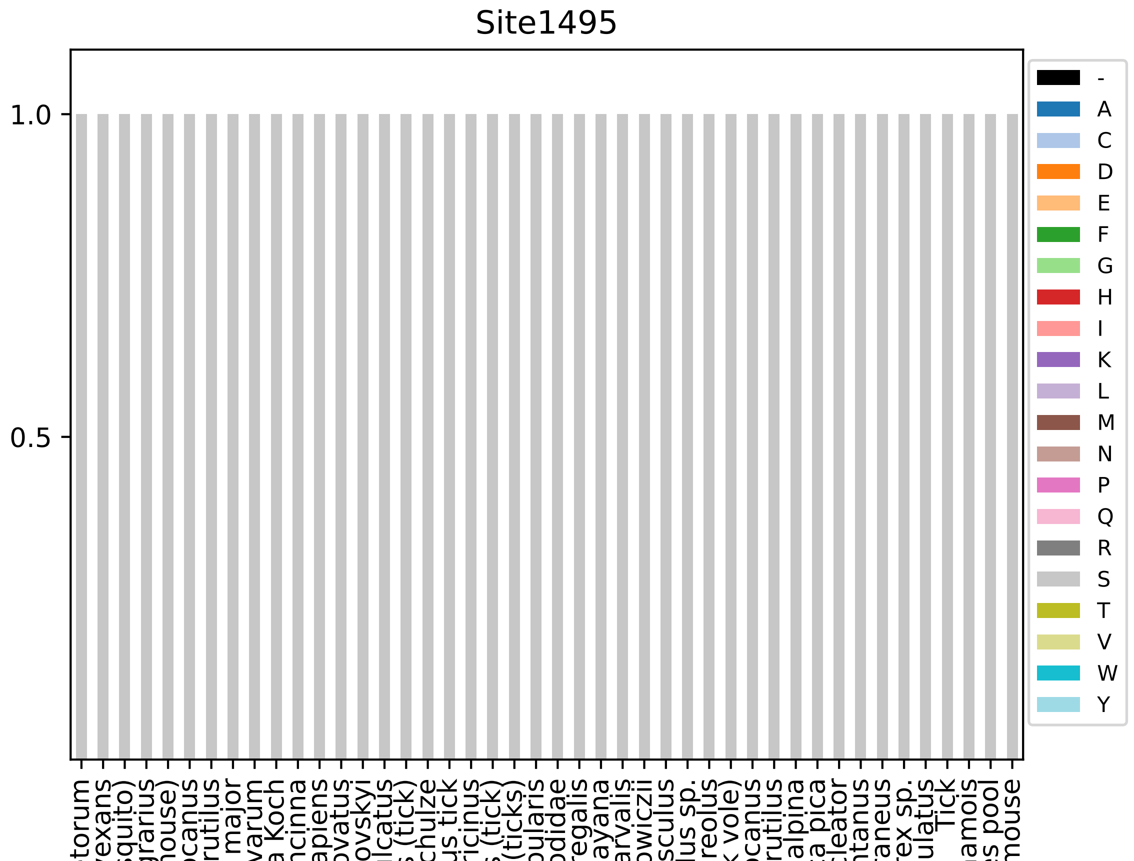

Site1495
1.0
0.5
-
A
C
D
E
F
G
H
I
K
L
M
N
P
Q
R
S
T
V
W
Y

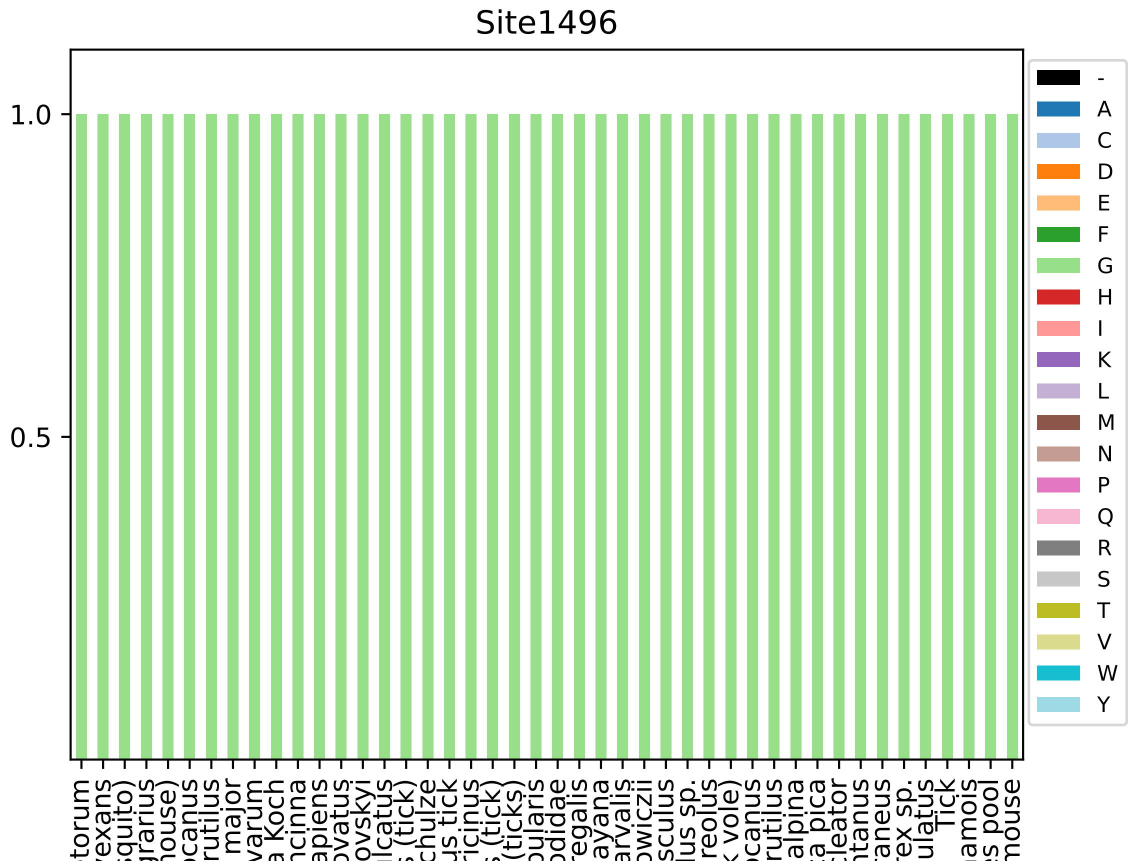
Site1496
1.0
0.5
-
A
C
D
E
F
G
H
I
K
L
M
N
P
Q
R
S
T
V
W
Y

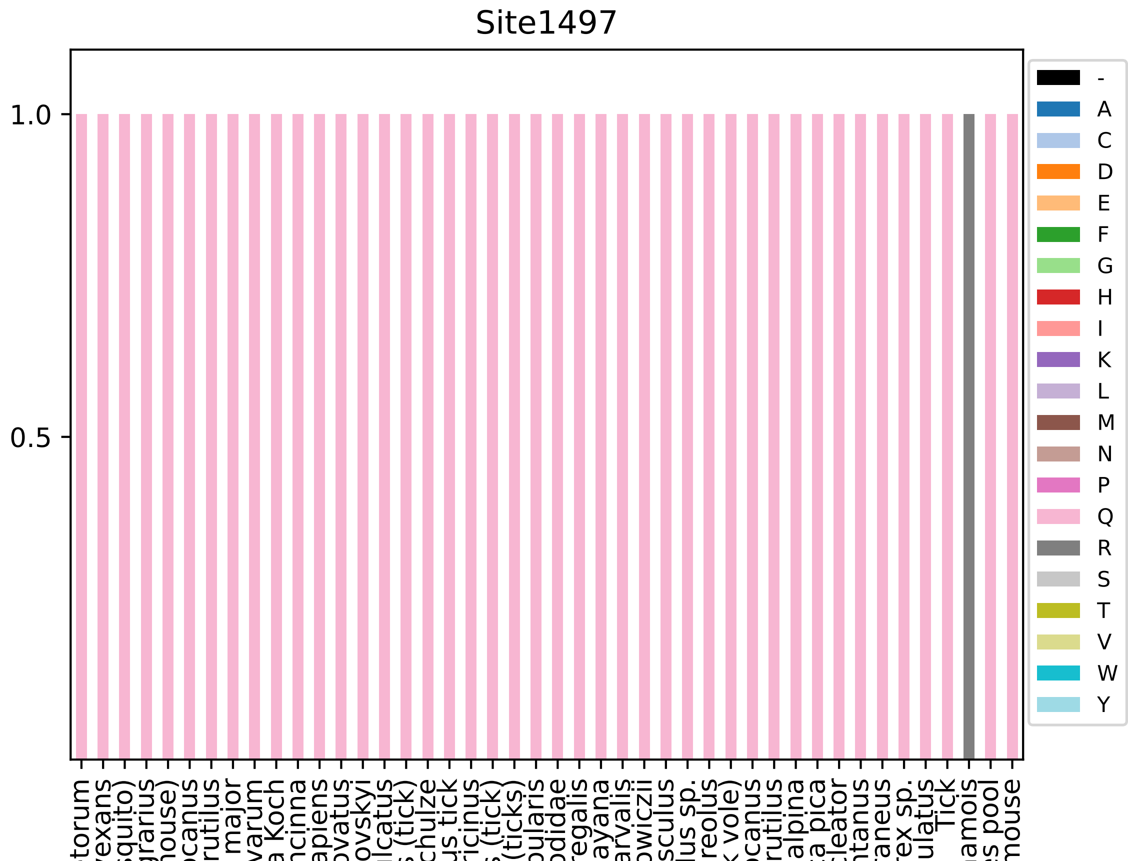

Site1497
1.0
0.5
-
A
C
D
E
F
G
H
I
K
L
M
N
P
Q
R
S
T
V
W
Y

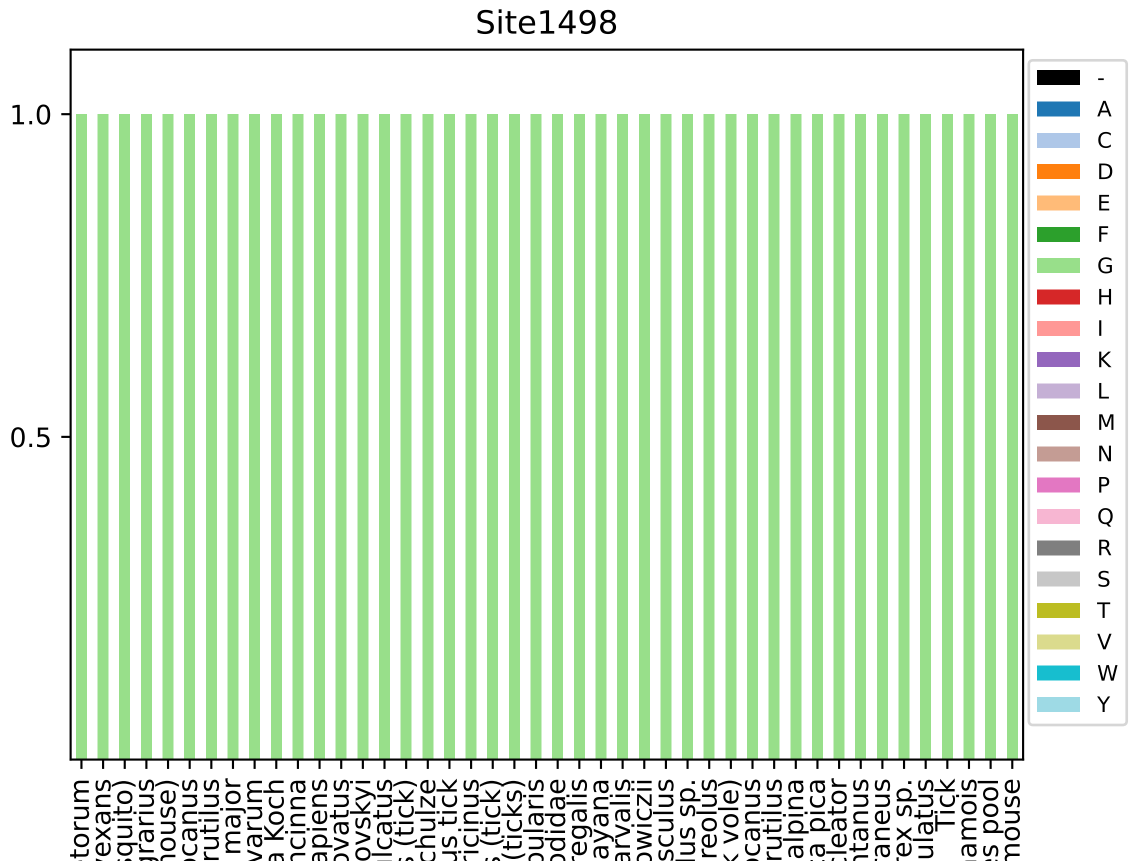

Site1498
1.0
0.5
-
A
C
D
E
F
G
H
I
K
L
M
N
P
Q
R
S
T
V
W
Y

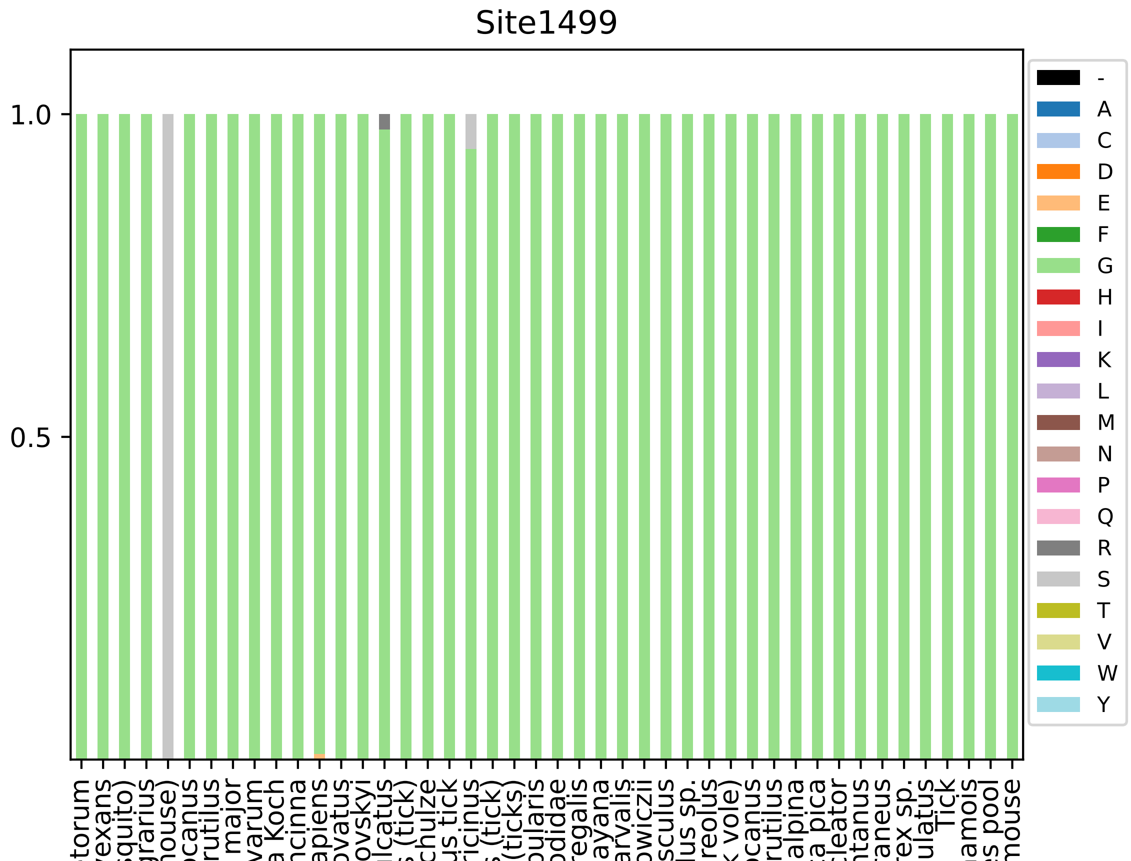

Site1499
1.0
0.5
-
A
C
D
E
F
G
H
I
K
L
M
N
P
Q
R
S
T
V
W
Y

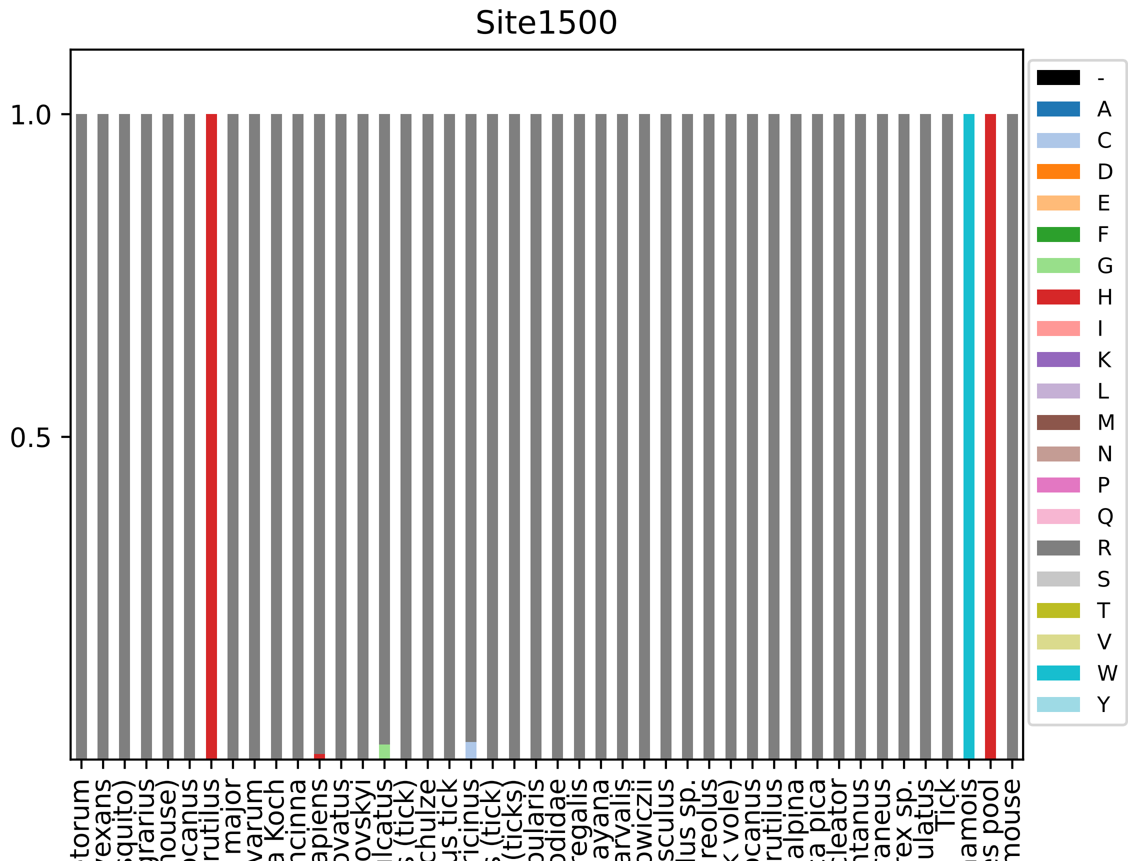

Site1500
1.0
0.5
-
A
C
D
E
F
G
H
I
K
L
M
N
P
Q
R
S
T
V
W
Y
torum
vexans
squito)
grarius
nouse)
ocanus
rutilus
major
varum
a Koch
ncinna
apiens
ovatus
ovskyi
lcatus
s (tick)
chulze
us tick
ricinus
s (tick)
(ticks)
pularis
podidae
regalis
ayana
arvalis
owiczii
sculus
lus sp.
reolus
k vole)
ocanus
rutilus
alpina
a pica
cleator
ntanus
raneus
rex sp.
ulatus
Tick
amois
es pool
mouse

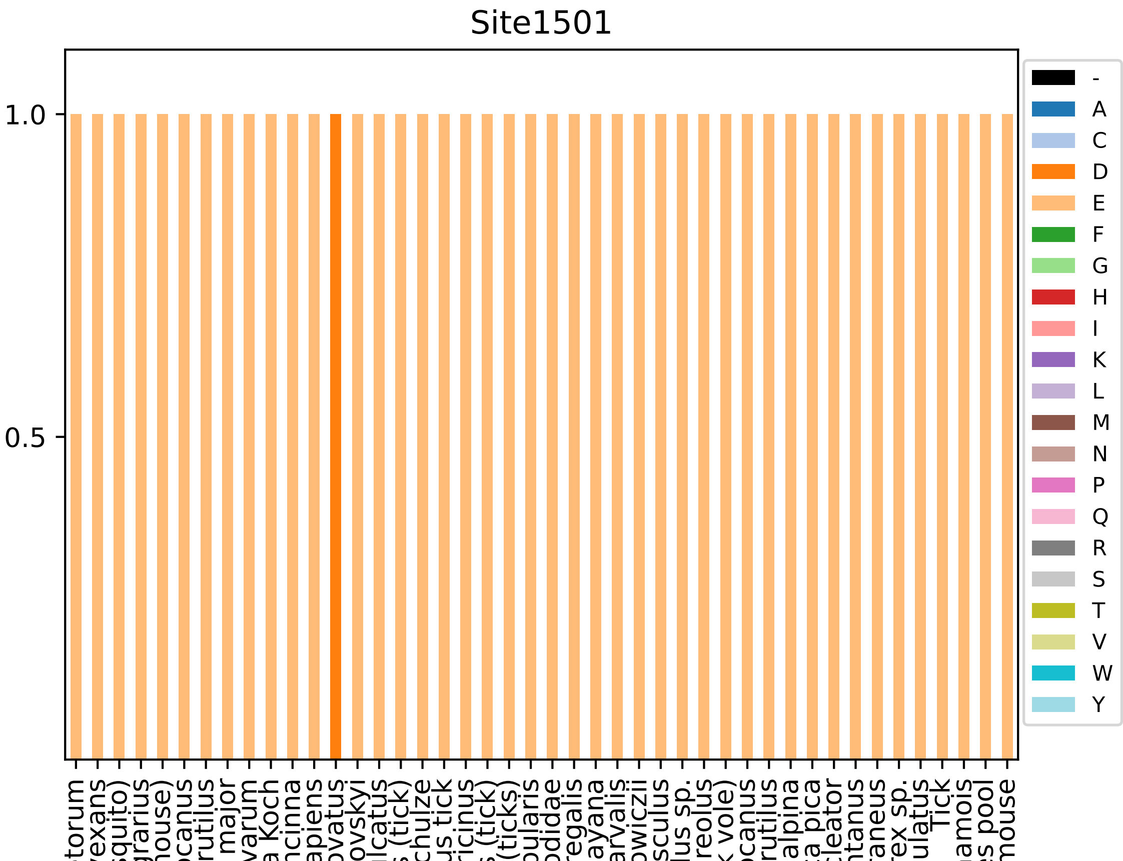
Site1501
1.0
0.5
-
A
C
D
E
F
G
H
I
K
L
M
N
P
Q
R
S
T
V
W
Y

Site1502

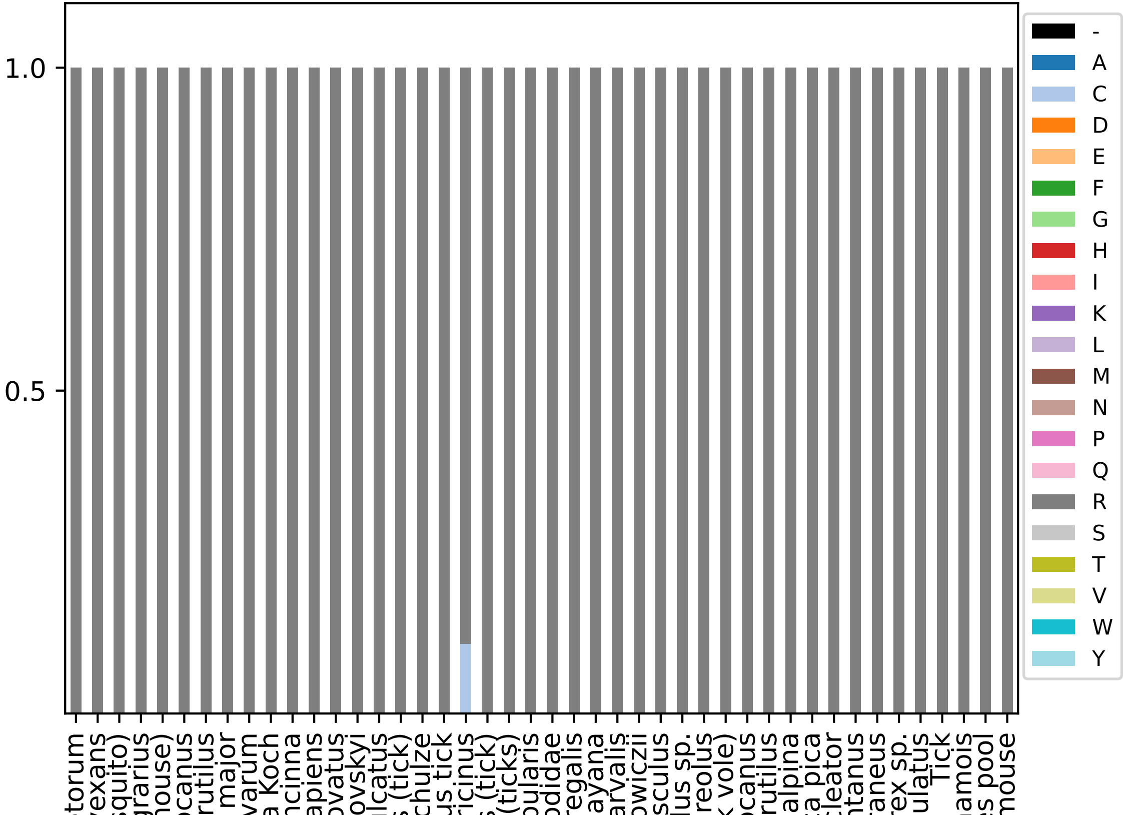

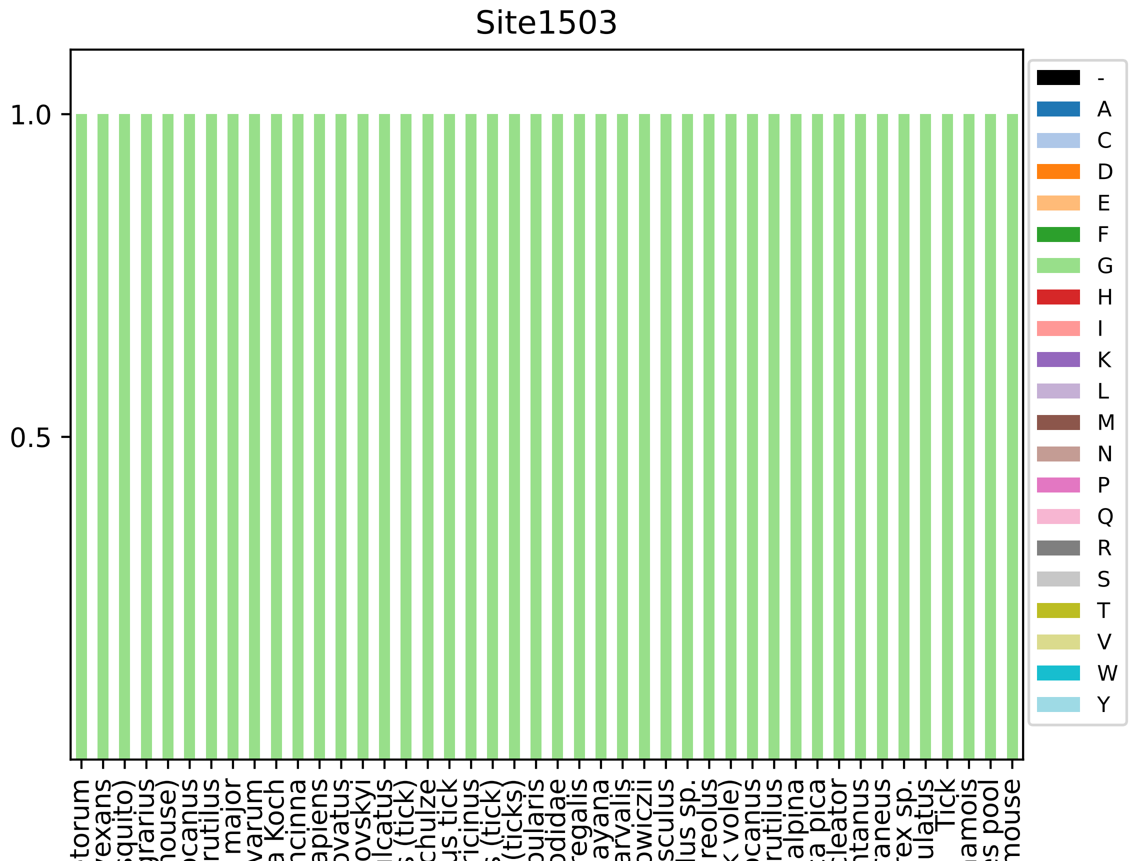
Site1503
1.0
0.5
-
A
C
D
E
F
G
H
I
K
L
M
N
P
Q
R
S
T
V
W
Y

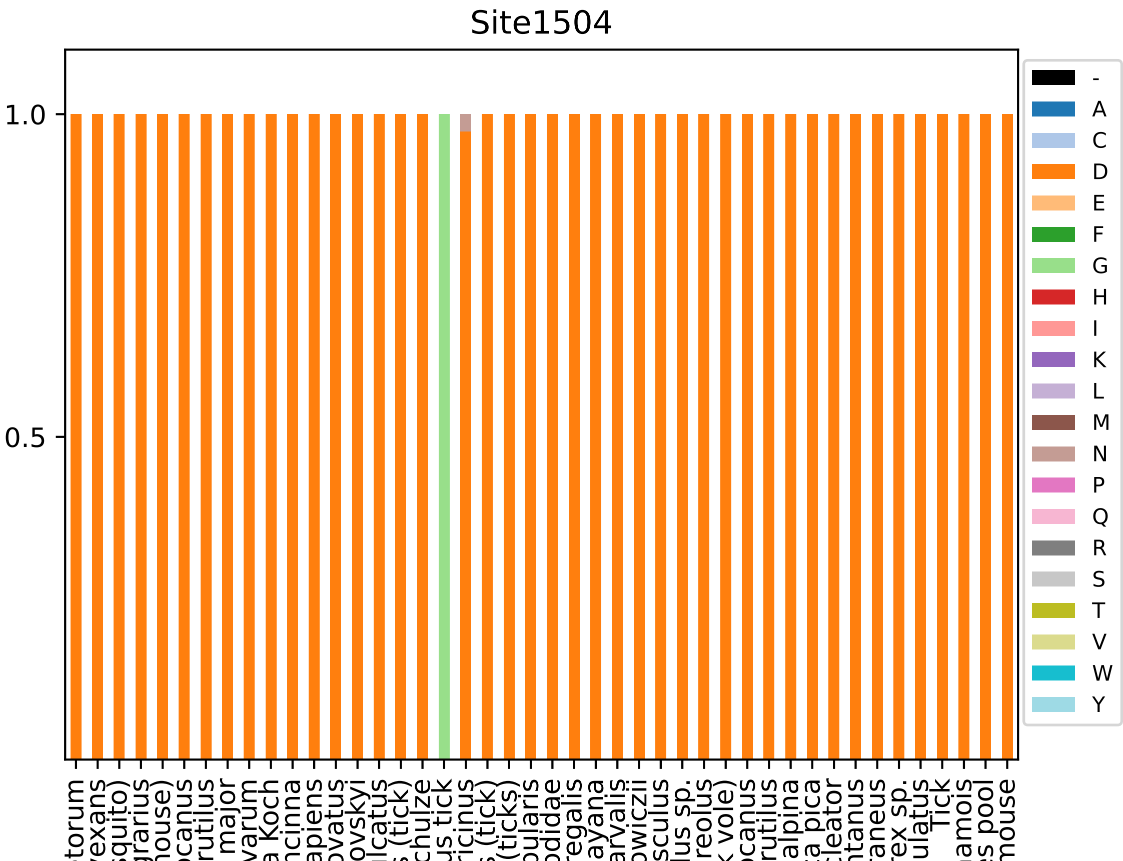

Site1504
1.0
0.5
-
A
C
D
E
F
G
H
I
K
L
M
N
P
Q
R
S
T
V
W
Y
Tick

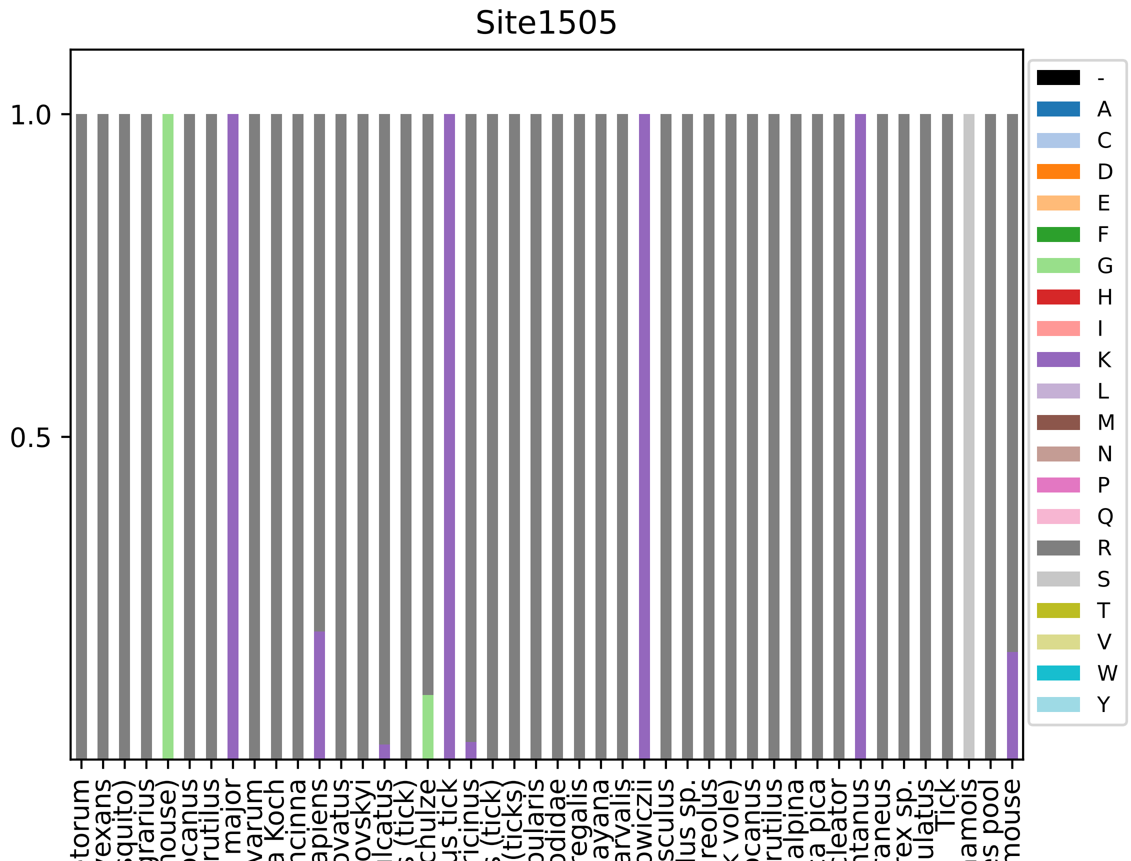

Site1505
1.0
0.5
-
A
C
D
E
F
G
H
I
K
L
M
N
P
Q
R
S
T
V
W
Y

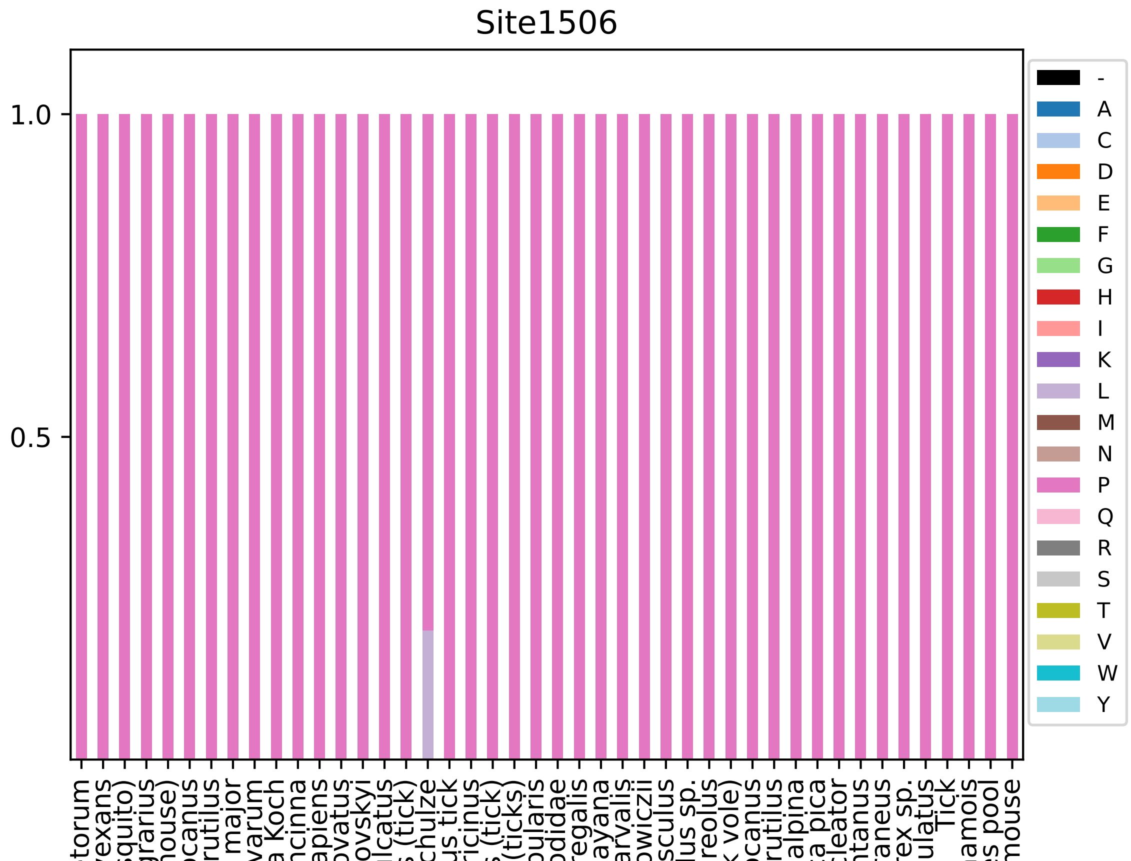
Site1506
1.0
0.5
-
A
C
D
E
F
G
H
I
K
L
M
N
P
Q
R
S
T
V
W
Y

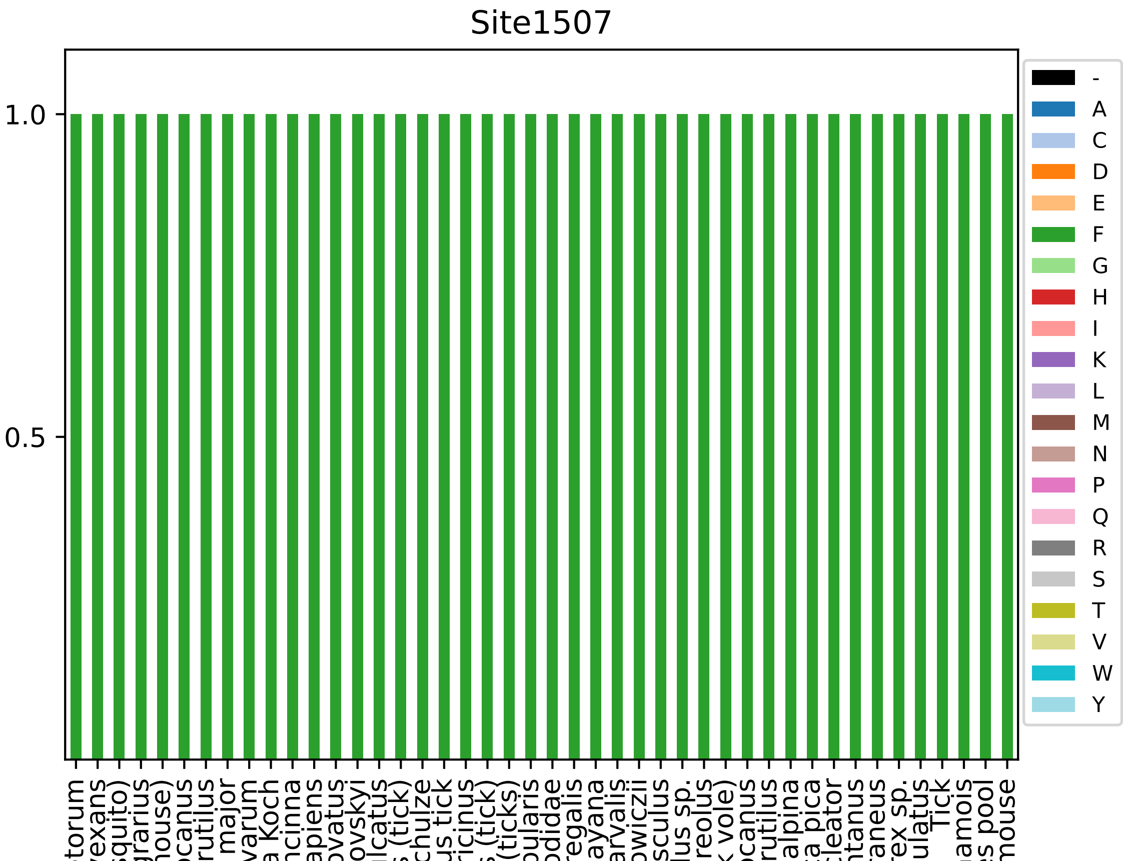
Site1507
1.0
0.5
-
A
C
D
E
F
G
H
I
K
L
M
N
P
Q
R
S
T
V
W
Y

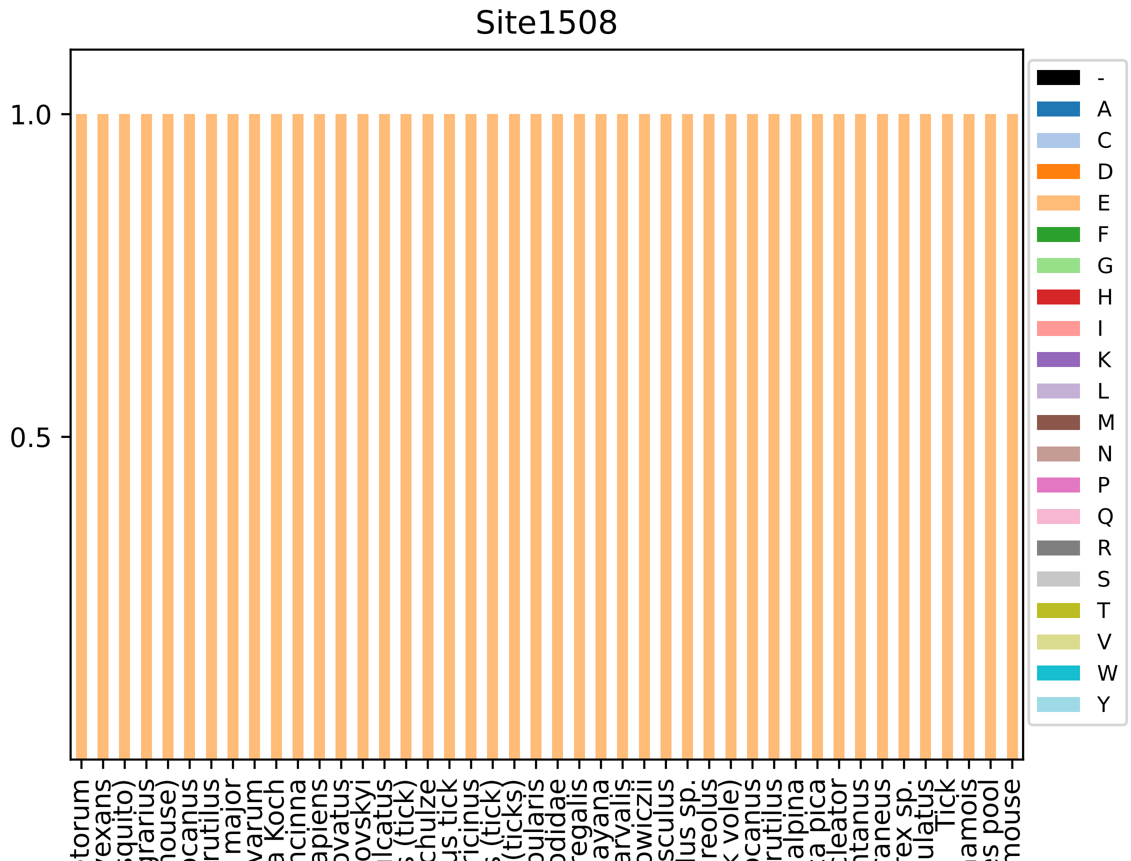
Site1508
1.0
0.5
-
A
C
D
E
F
G
H
I
K
L
M
N
P
Q
R
S
T
V
W
Y

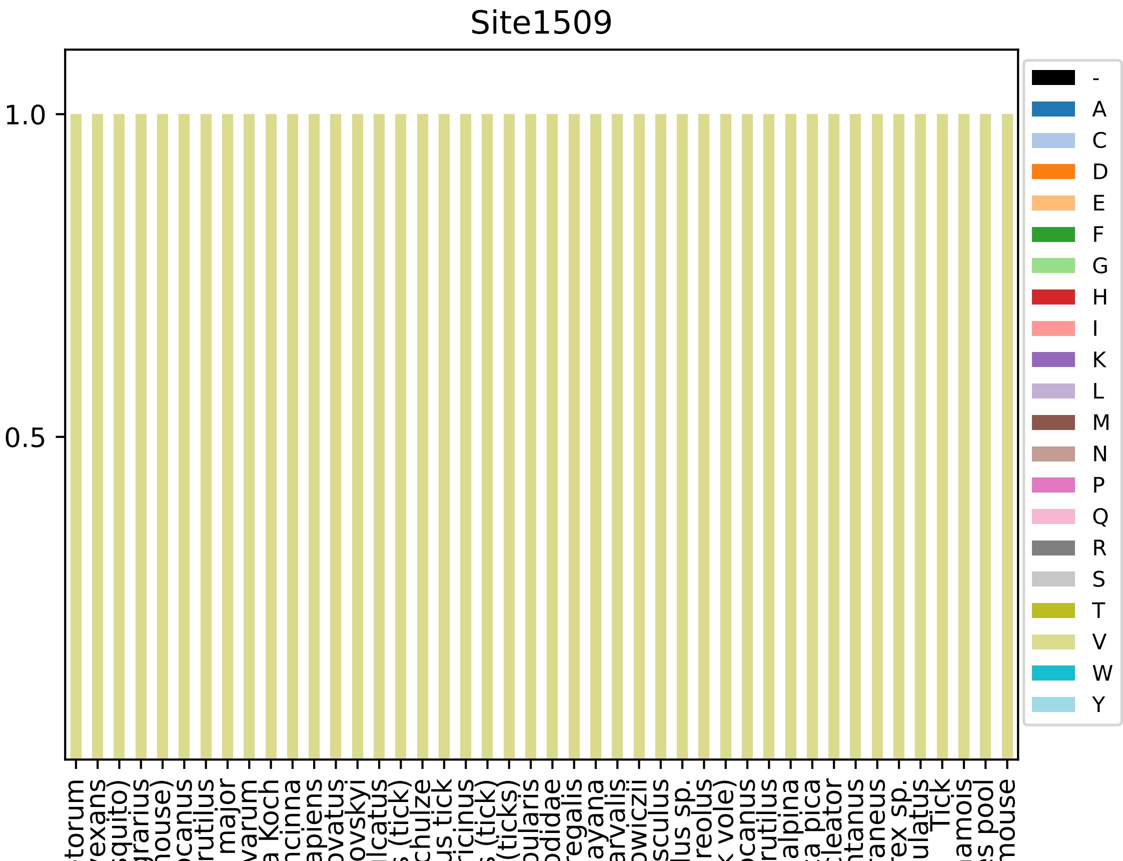
Site1509
1.0
0.5
-
A
C
D
E
F
G
H
I
K
L
M
N
P
Q
R
S
T
V
W
Y

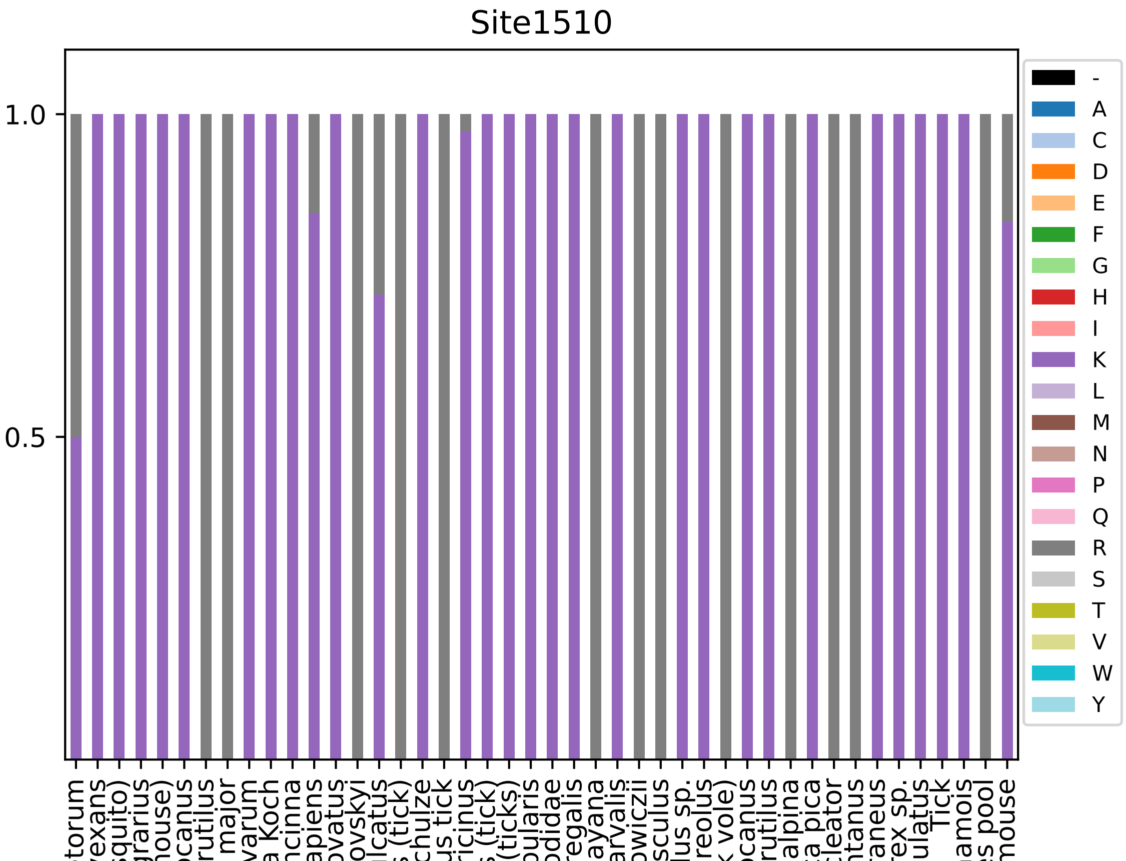
Site1510
1.0
0.5
-
A
C
D
E
F
G
H
I
K
L
M
N
P
Q
R
S
T
V
W
Y

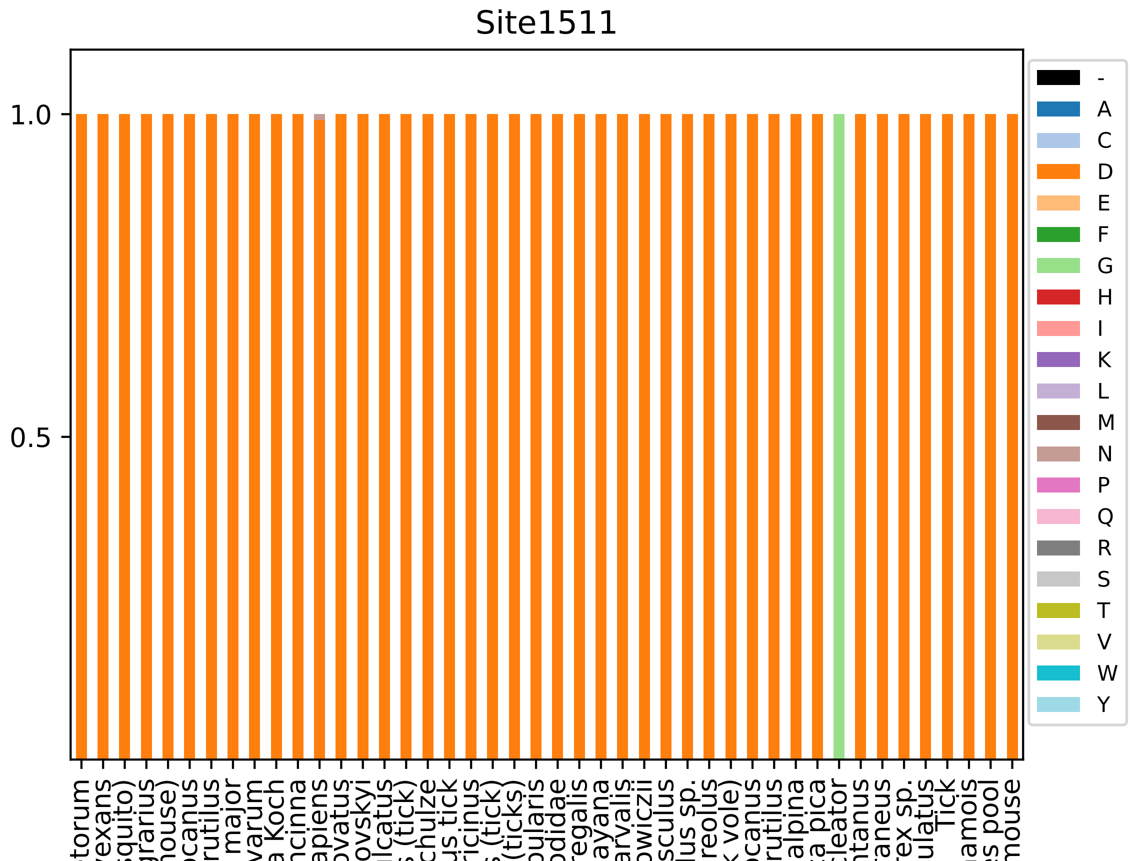
Site1511
1.0
0.5
-
A
C
D
E
F
G
H
I
K
L
M
N
P
Q
R
S
T
V
W
Y

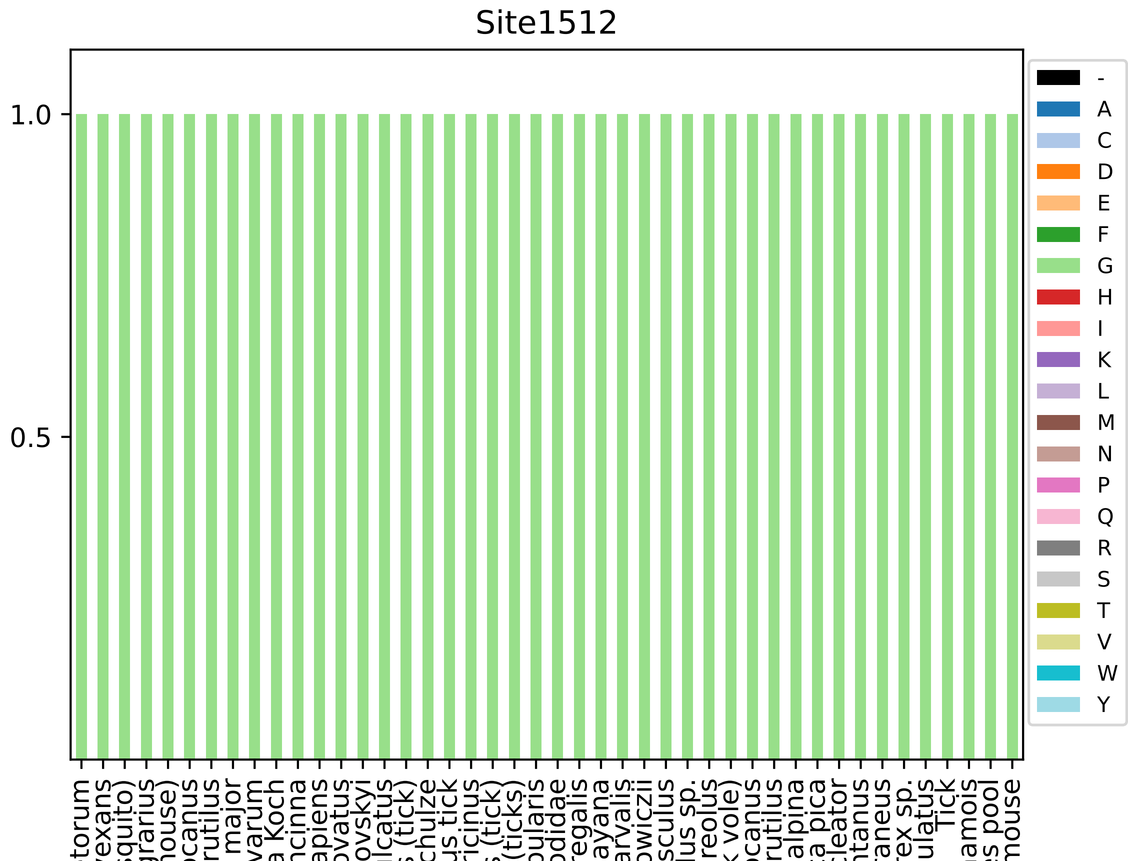
Site1512
1.0
0.5
-
A
C
D
E
F
G
H
I
K
L
M
N
P
Q
R
S
T
V
W
Y

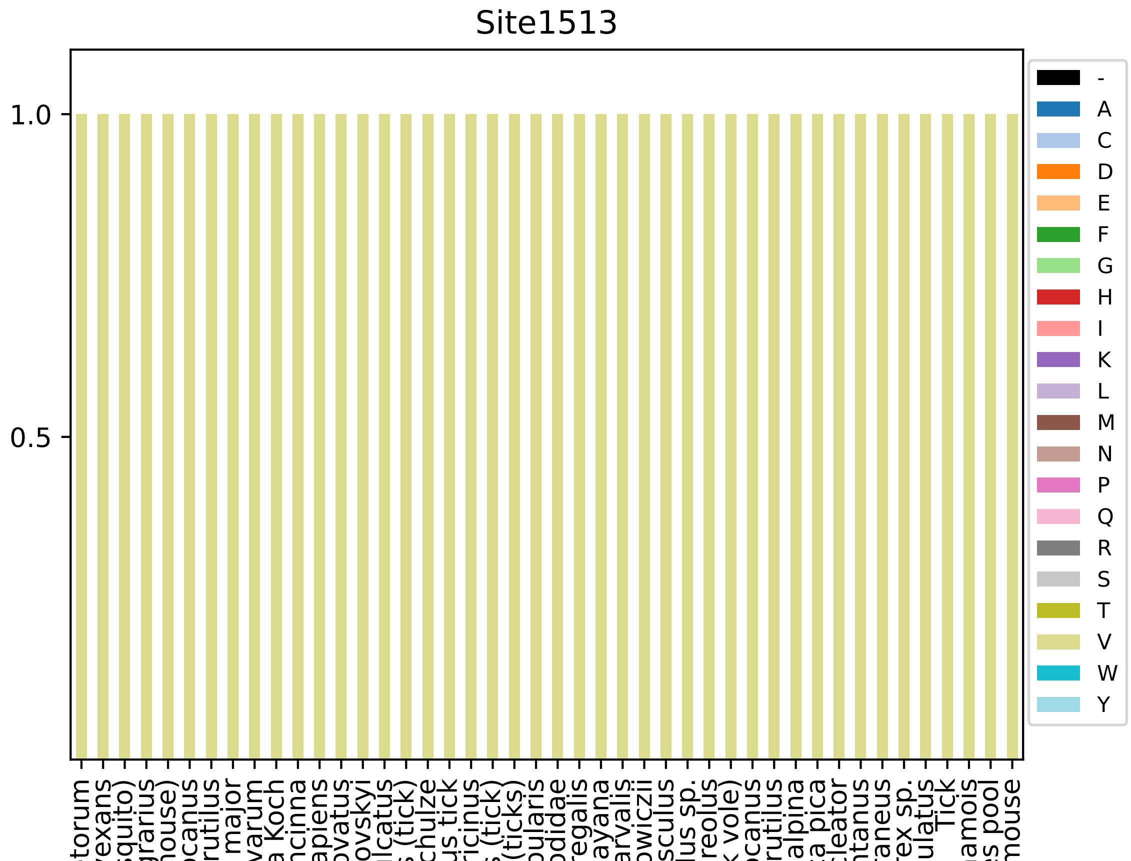
Site1513
1.0
0.5
-
A
C
D
E
F
G
H
I
K
L
M
N
P
Q
R
S
T
V
W
Y

Site1514

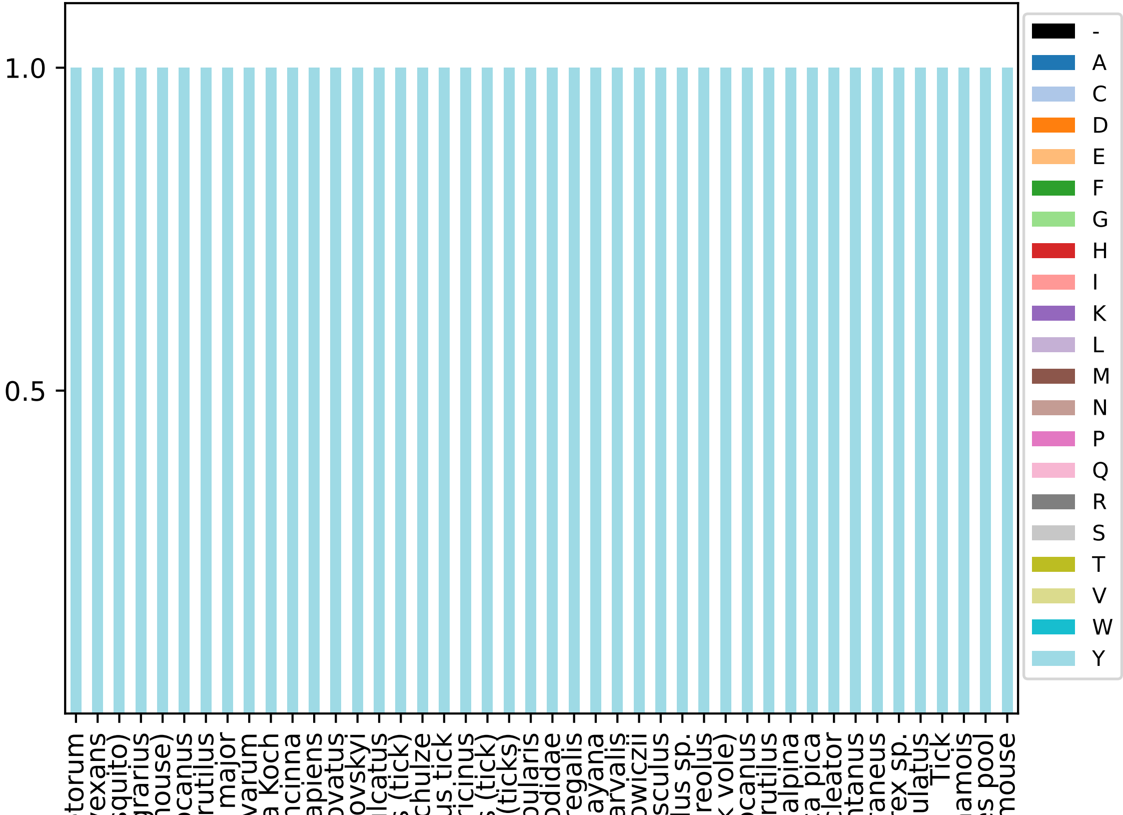

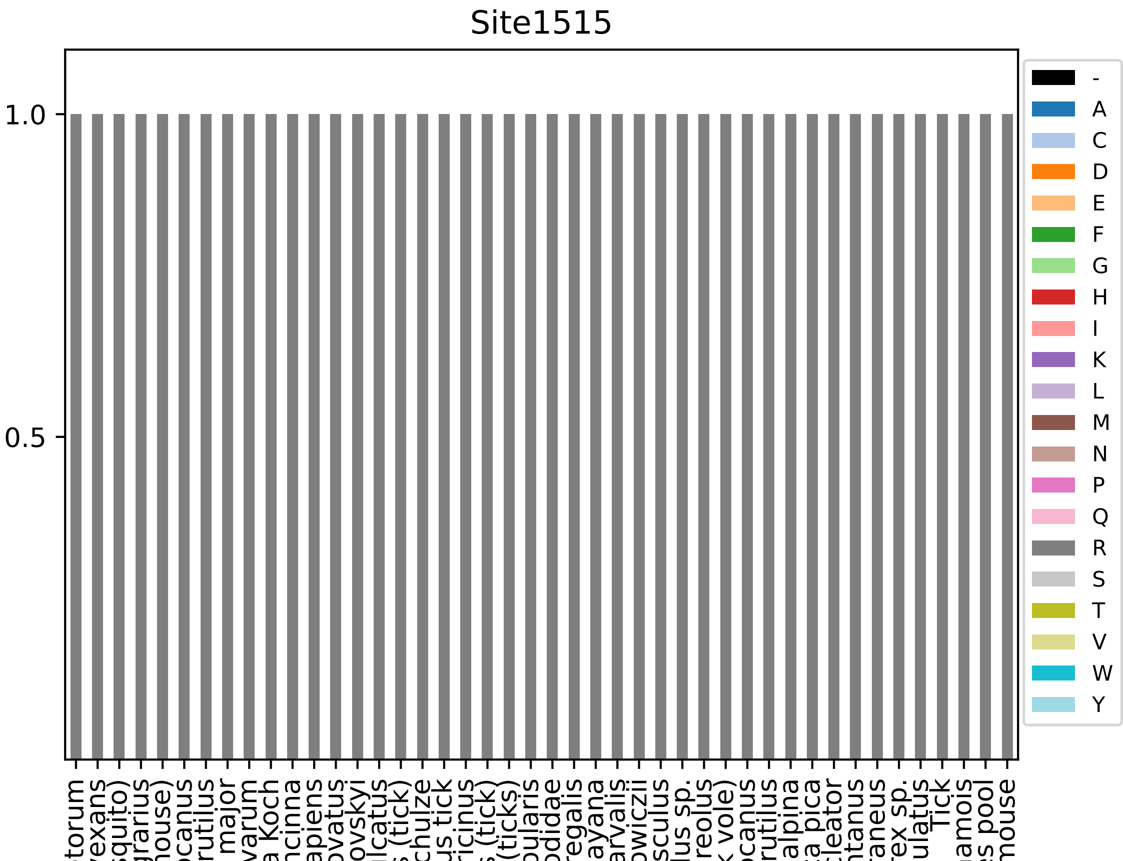
Site1515
1.0
0.5
-
A
C
D
E
F
G
H
I
K
L
M
N
P
Q
R
S
T
V
W
Y

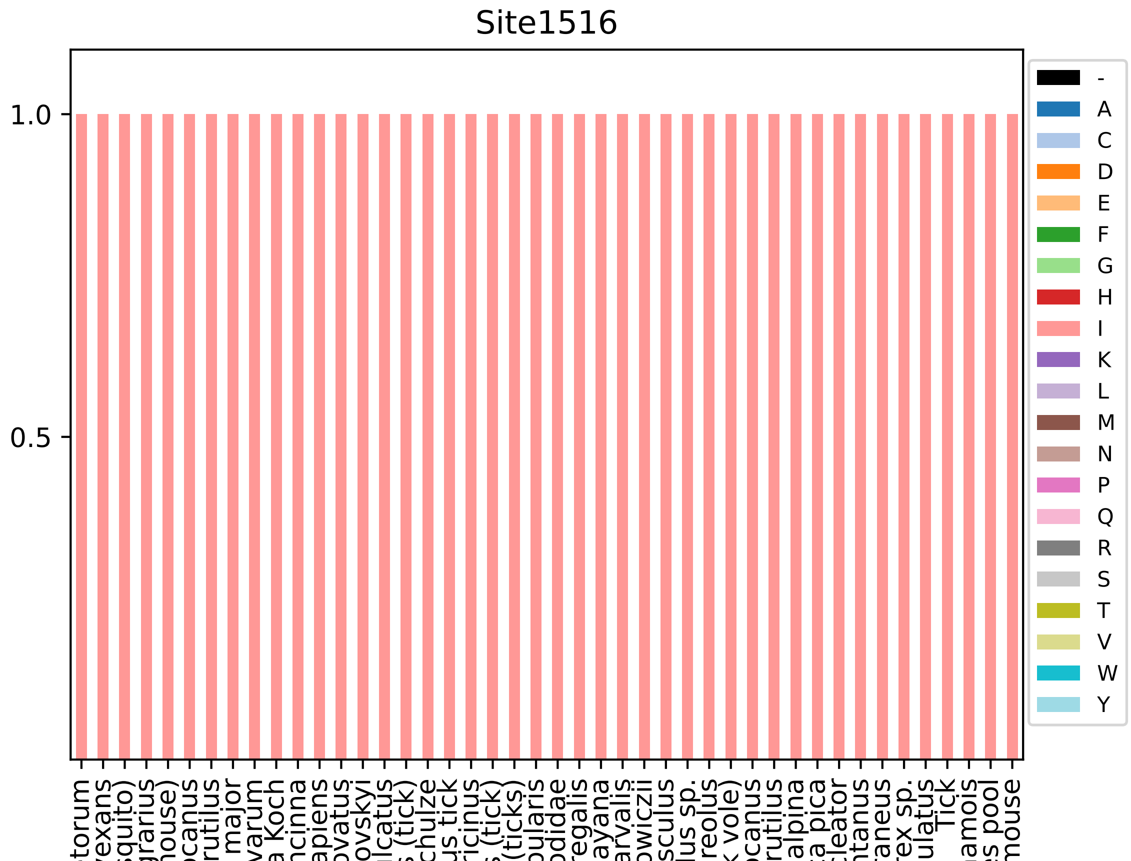
Site1516
1.0
0.5
-
A
C
D
E
F
G
H
I
K
L
M
N
P
Q
R
S
T
V
W
Y

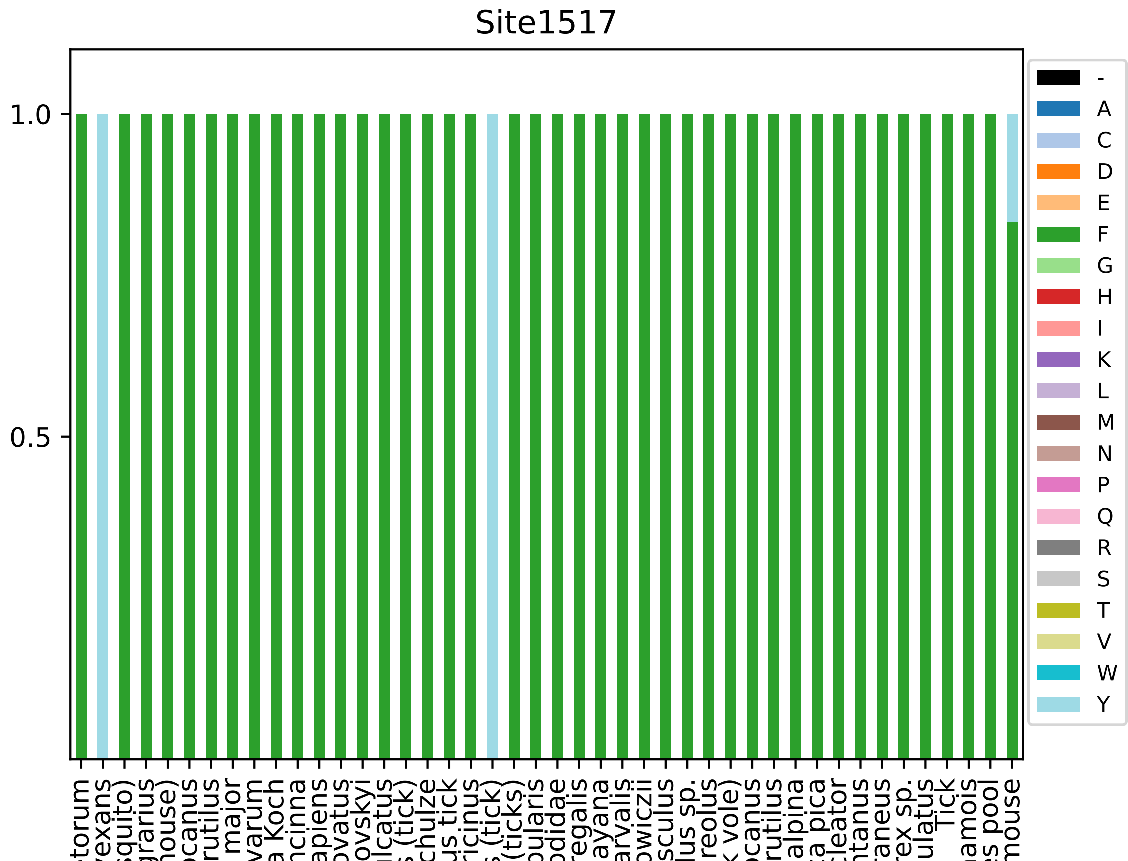
Site1517
1.0
0.5
-
A
C
D
E
F
G
H
I
K
L
M
N
P
Q
R
S
T
V
W
Y

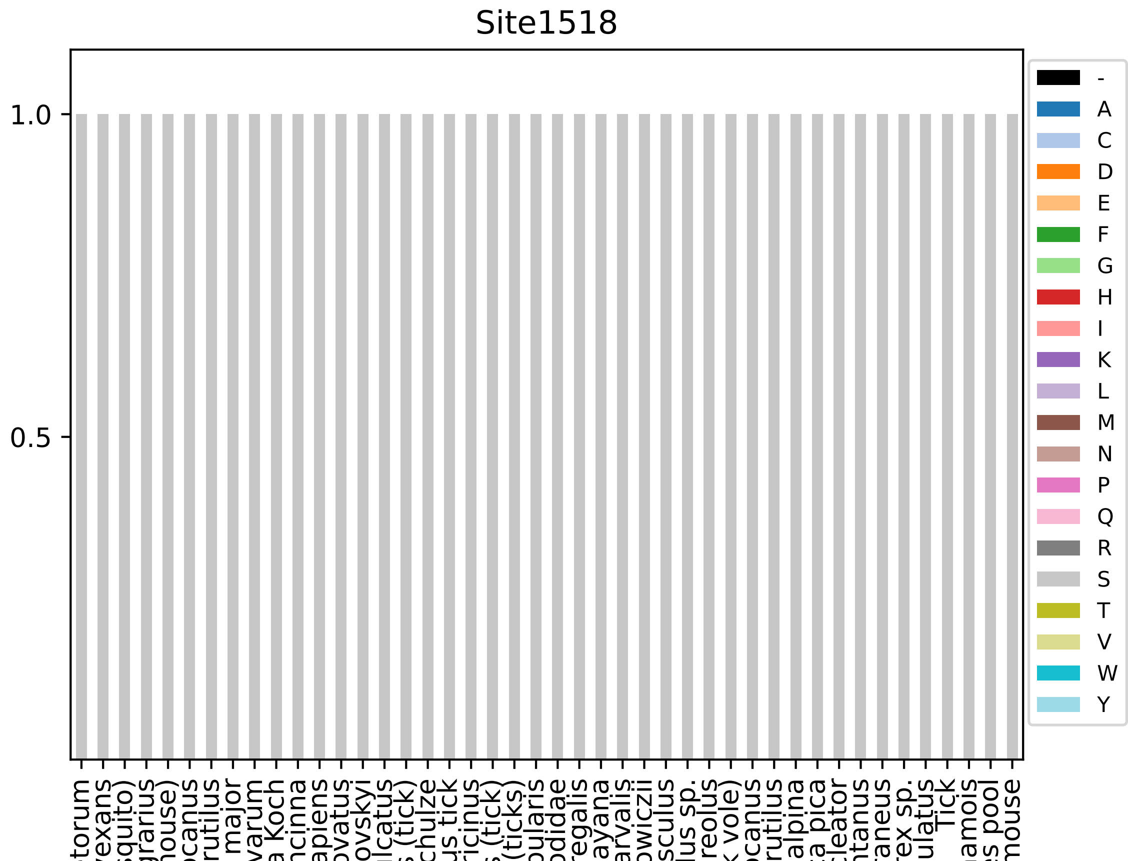
Site1518
1.0
0.5
-
A
C
D
E
F
G
H
I
K
L
M
N
P
Q
R
S
T
V
W
Y

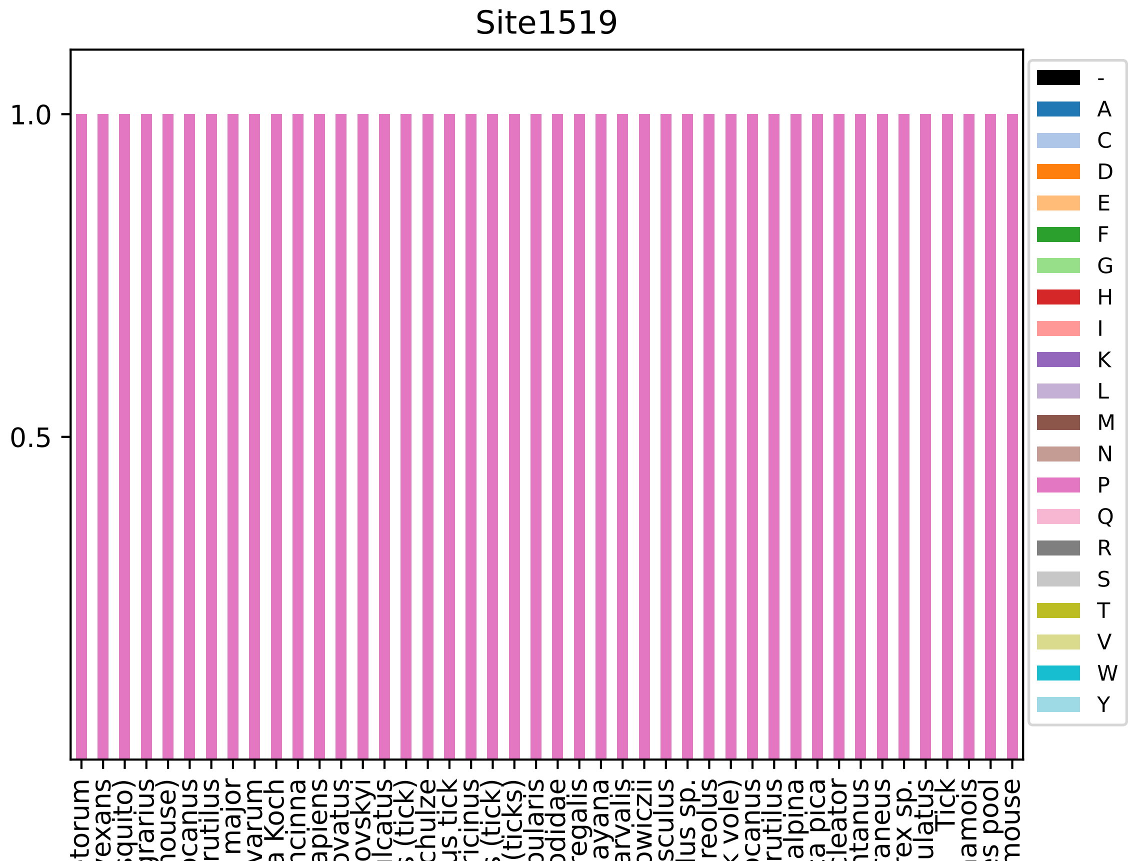
Site1519
1.0
0.5
-
A
C
D
E
F
G
H
I
K
L
M
N
P
Q
R
S
T
V
W
Y

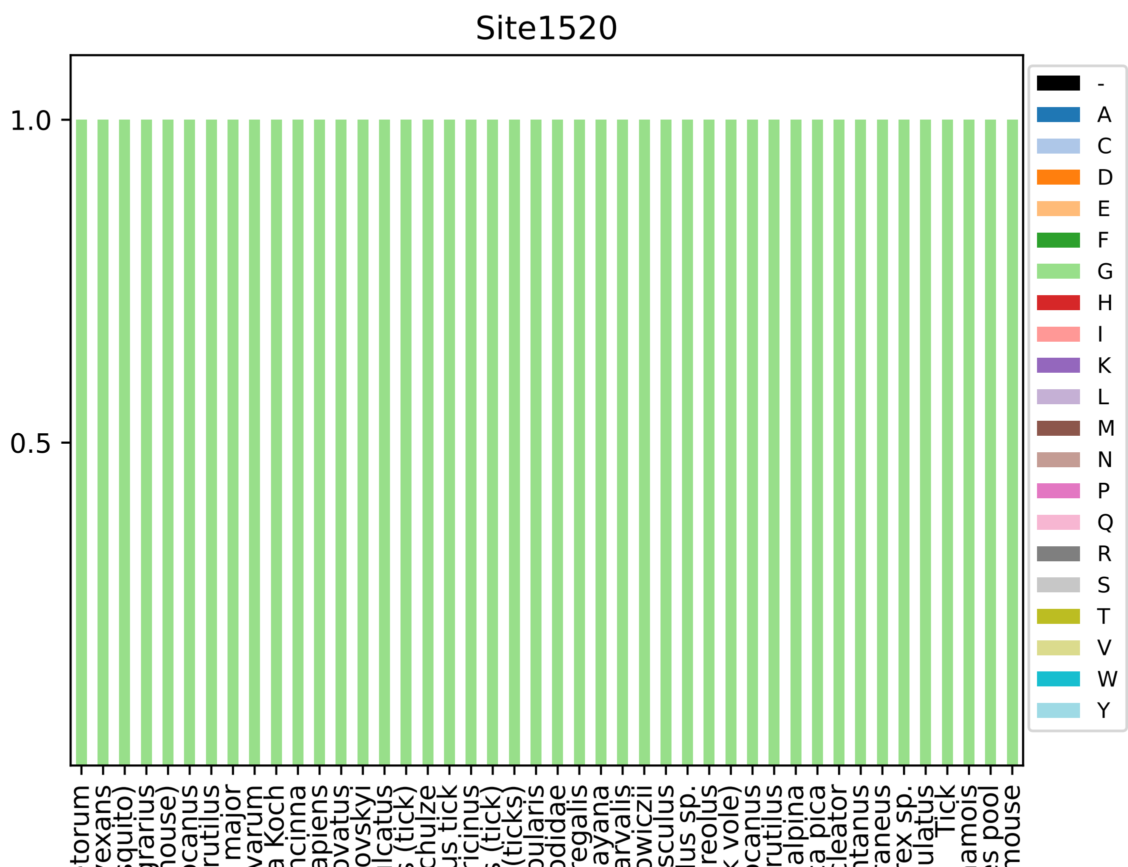

Site1520
1.0
0.5
-
A
C
D
E
F
G
H
I
K
L
M
N
P
Q
R
S
T
V
W
Y

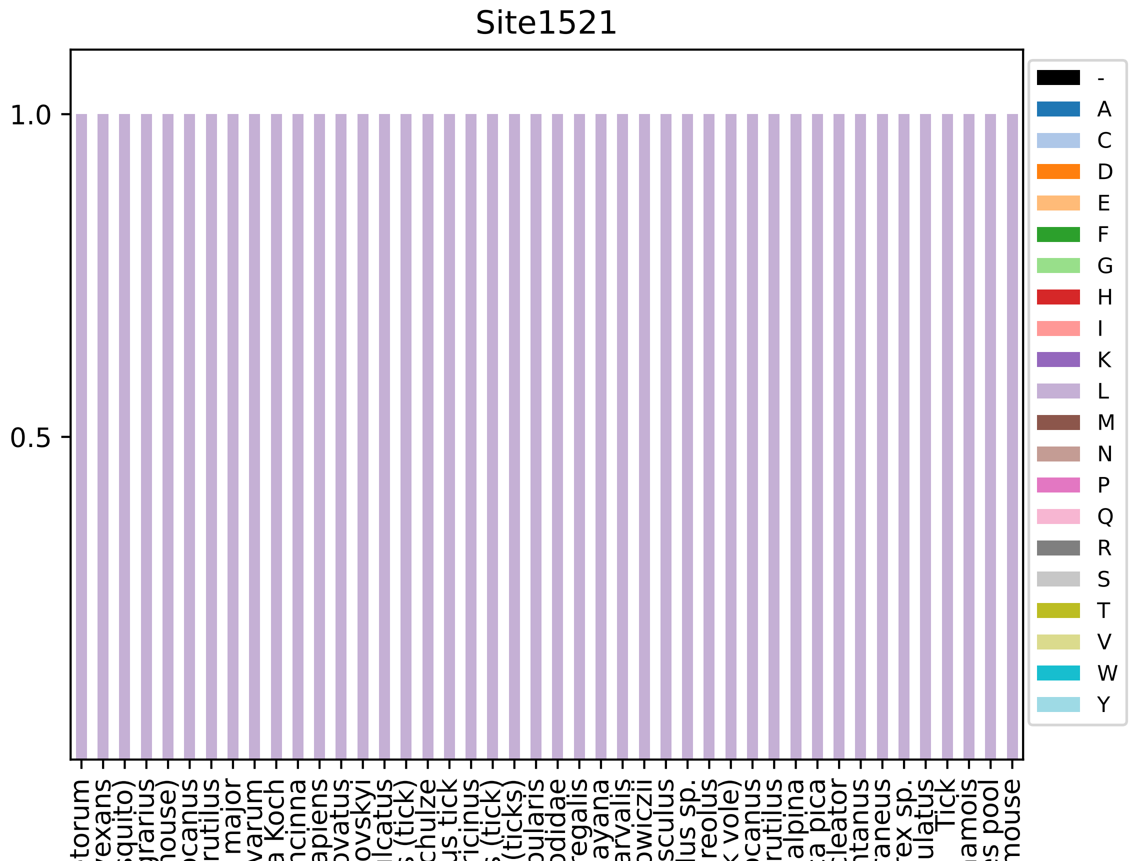
Site1521
1.0
0.5
-
A
C
D
E
F
G
H
I
K
L
M
N
P
Q
R
S
T
V
W
Y
Tick

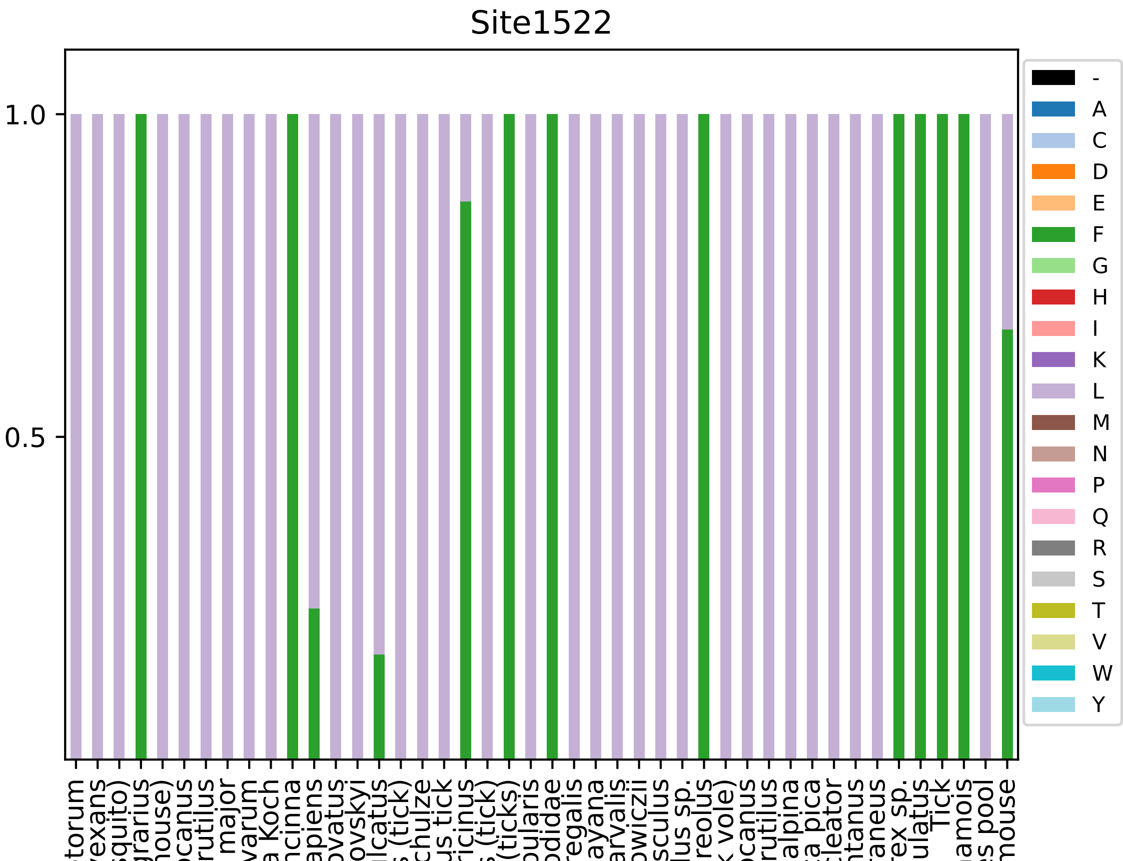
Site1522
1.0
0.5
-
A
C
D
E
F
G
H
I
K
L
M
N
P
Q
R
S
T
V
W
Y

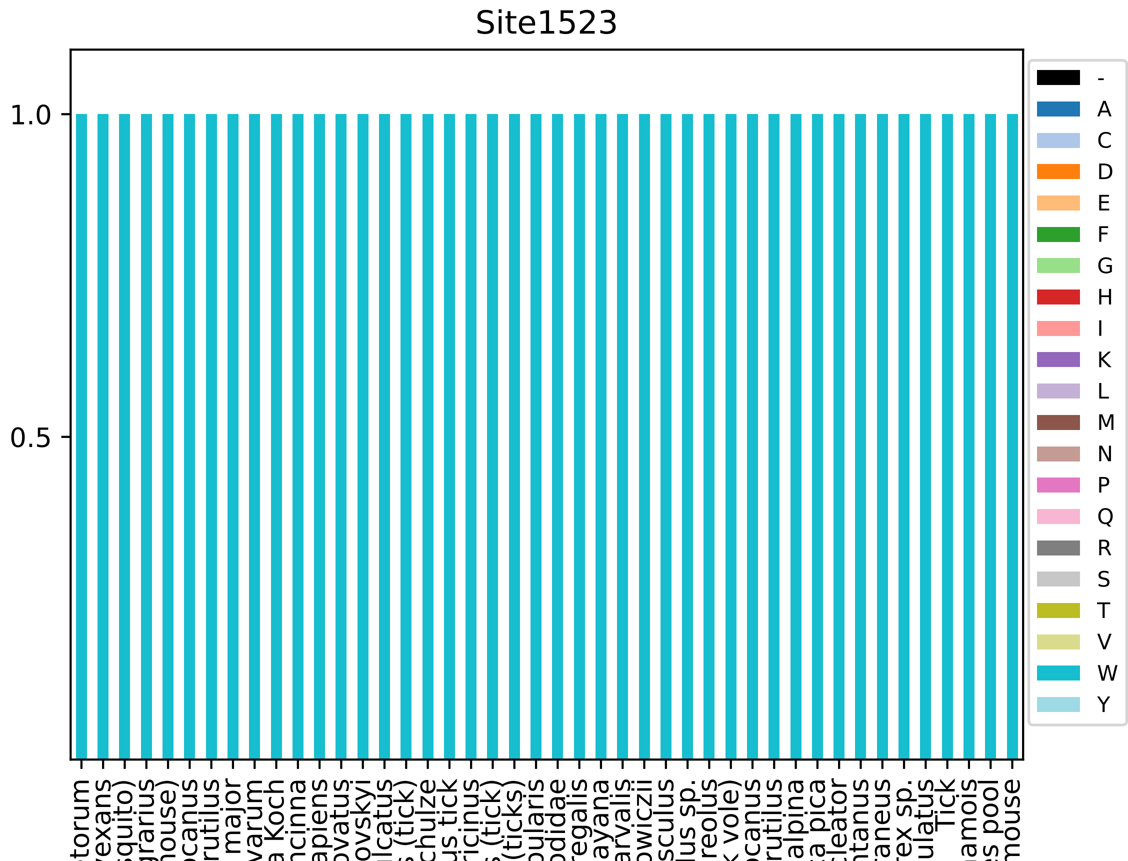
Site1523
1.0
0.5
-
A
C
D
E
F
G
H
I
K
L
M
N
P
Q
R
S
T
V
W
Y

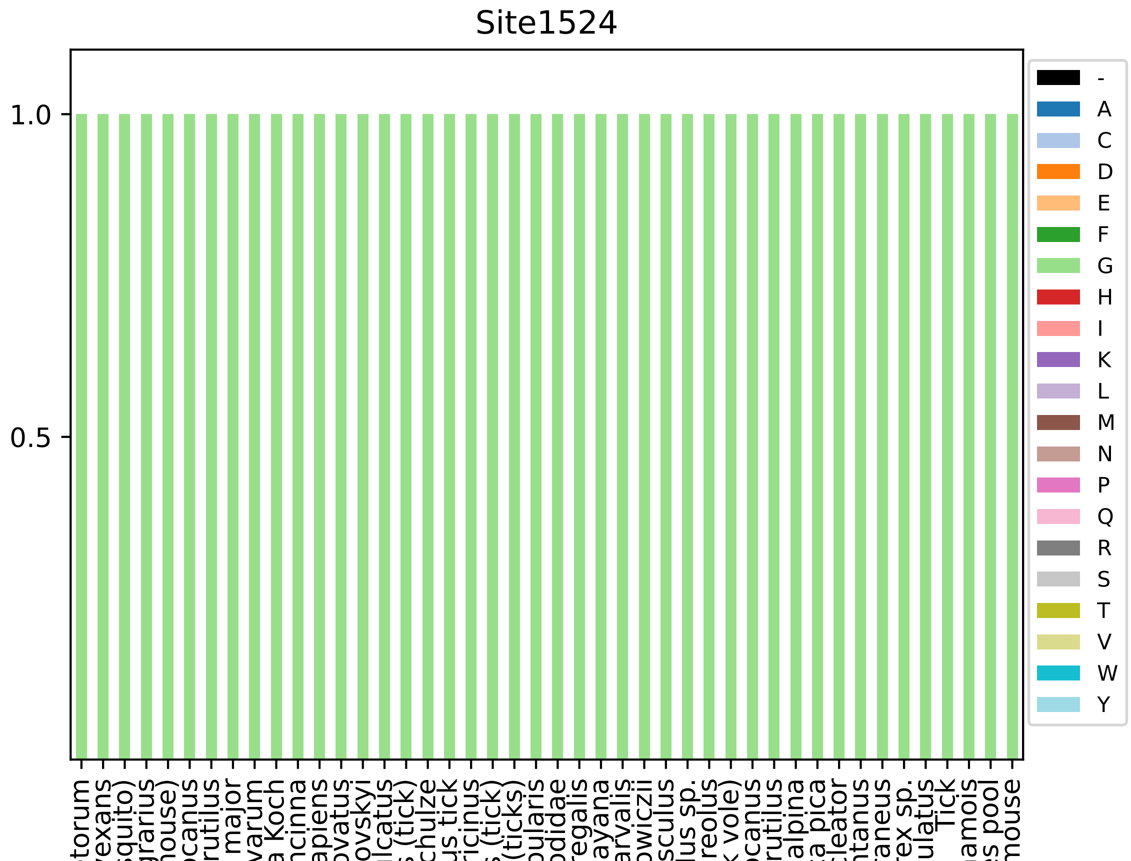
Site1524
1.0
0.5
-
A
C
D
E
F
G
H
I
K
L
M
N
P
Q
R
S
T
V
W
Y

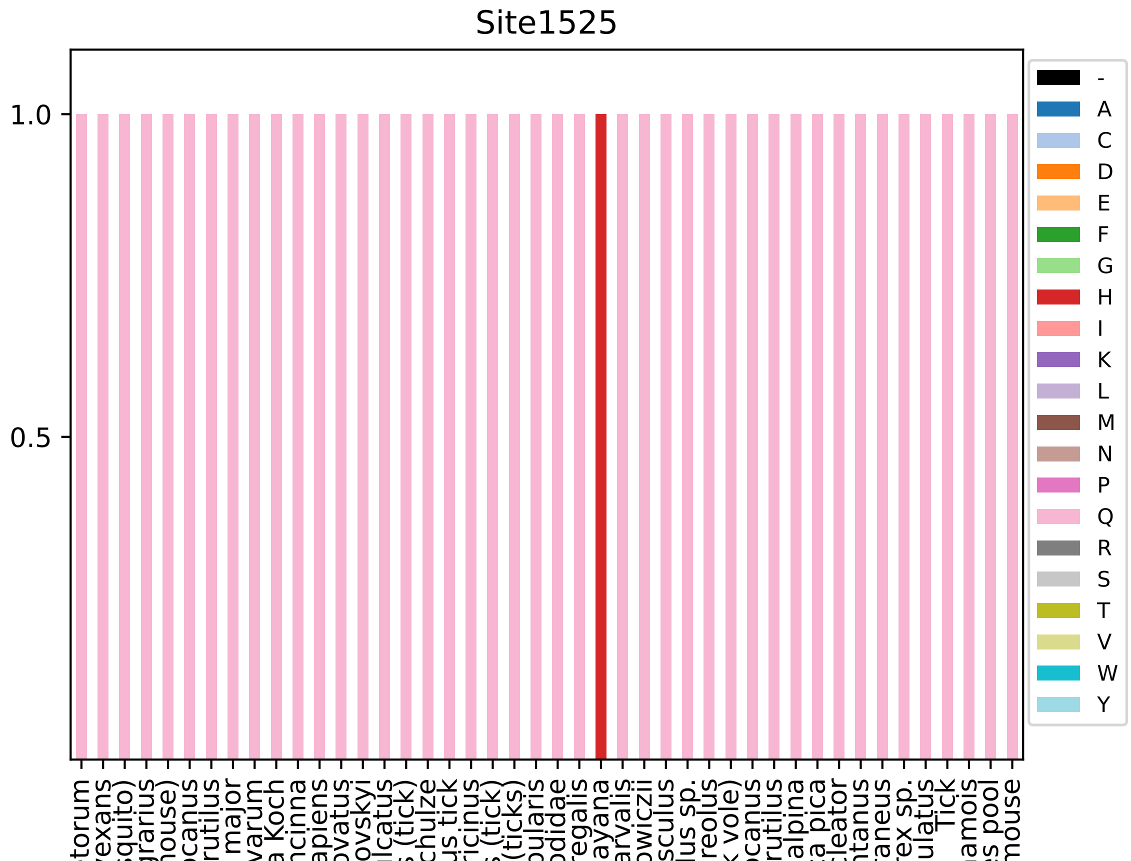
Site1525
1.0
0.5
-
A
C
D
E
F
G
H
I
K
L
M
N
P
Q
R
S
T
V
W
Y

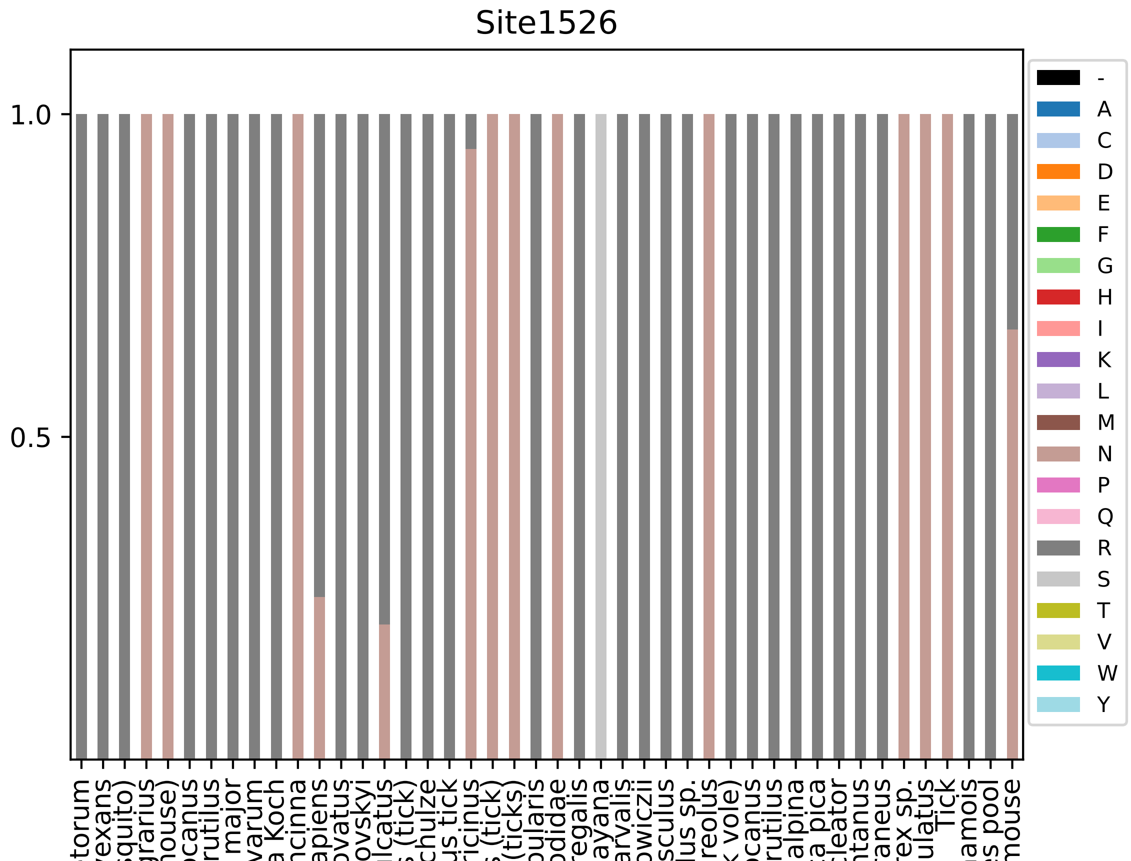
Site1526
1.0
0.5
-
A
C
D
E
F
G
H
I
K
L
M
N
P
Q
R
S
T
V
W
Y

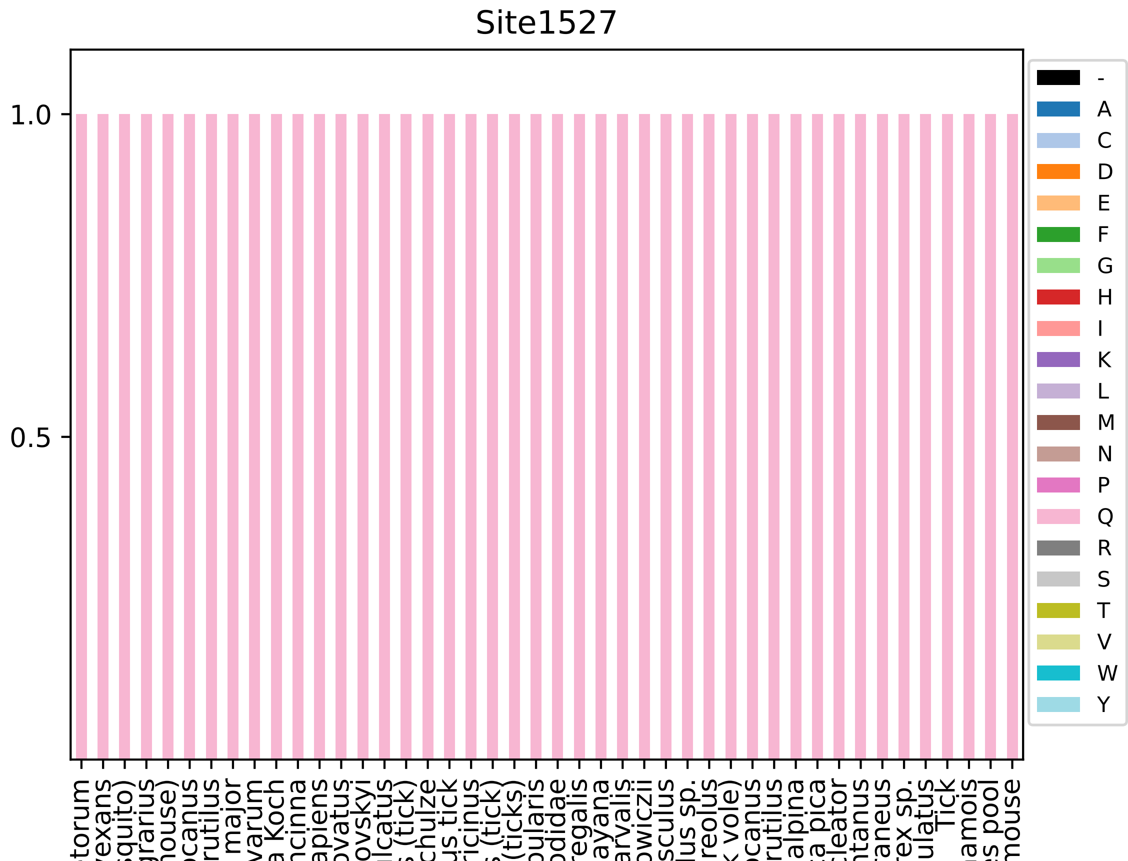

Site1527
1.0
0.5
-
A
C
D
E
F
G
H
I
K
L
M
N
P
Q
R
S
T
V
W
Y
Tick

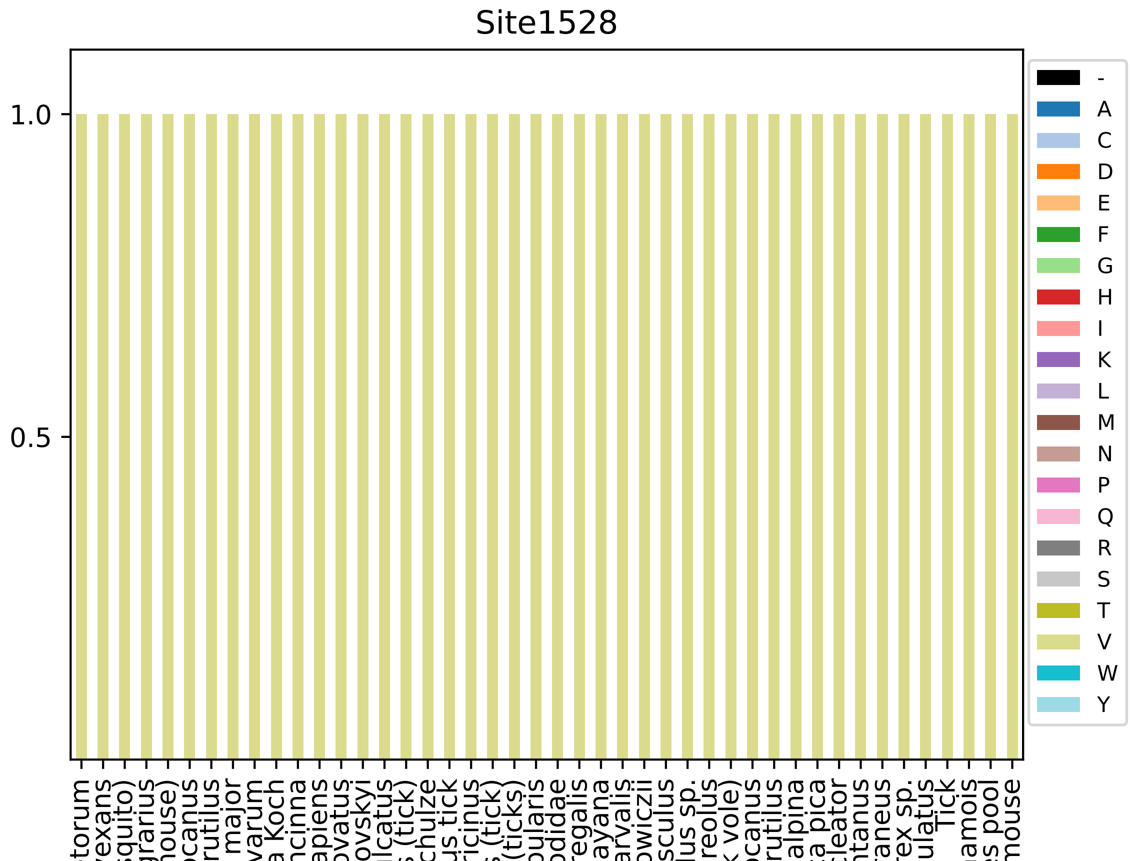
Site1528
1.0
0.5
-
A
C
D
E
F
G
H
I
K
L
M
N
P
Q
R
S
T
V
W
Y

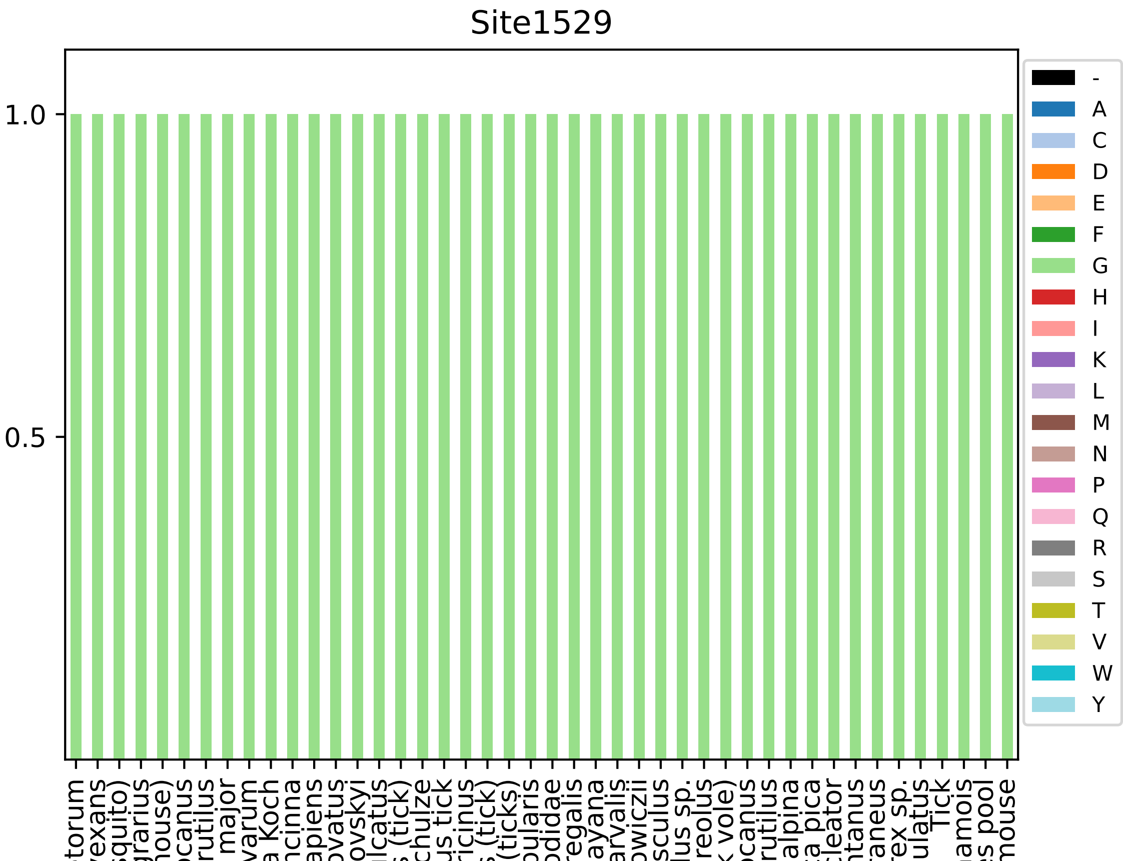
Site1529
1.0
0.5
-
A
C
D
E
F
G
H
I
K
L
M
N
P
Q
R
S
T
V
W
Y

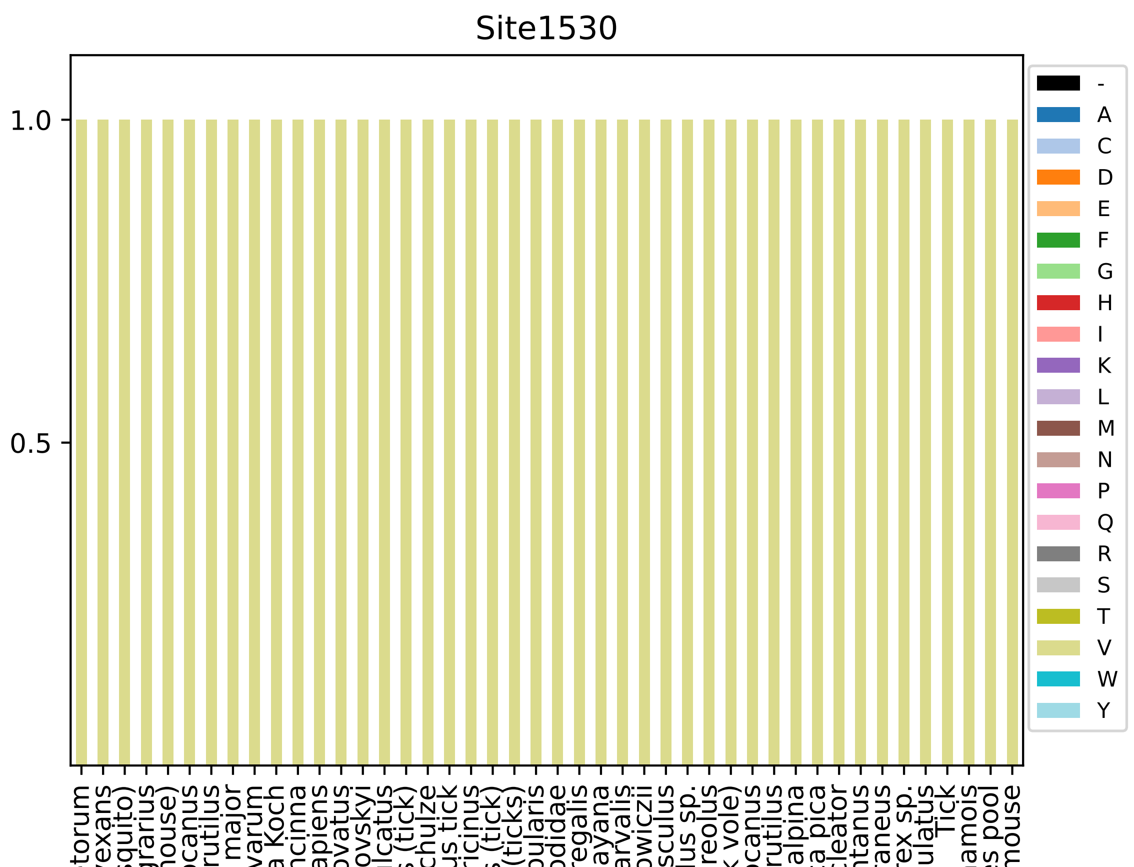

Site1530
1.0
0.5
-
A
C
D
E
F
G
H
I
K
L
M
N
P
Q
R
S
T
V
W
Y

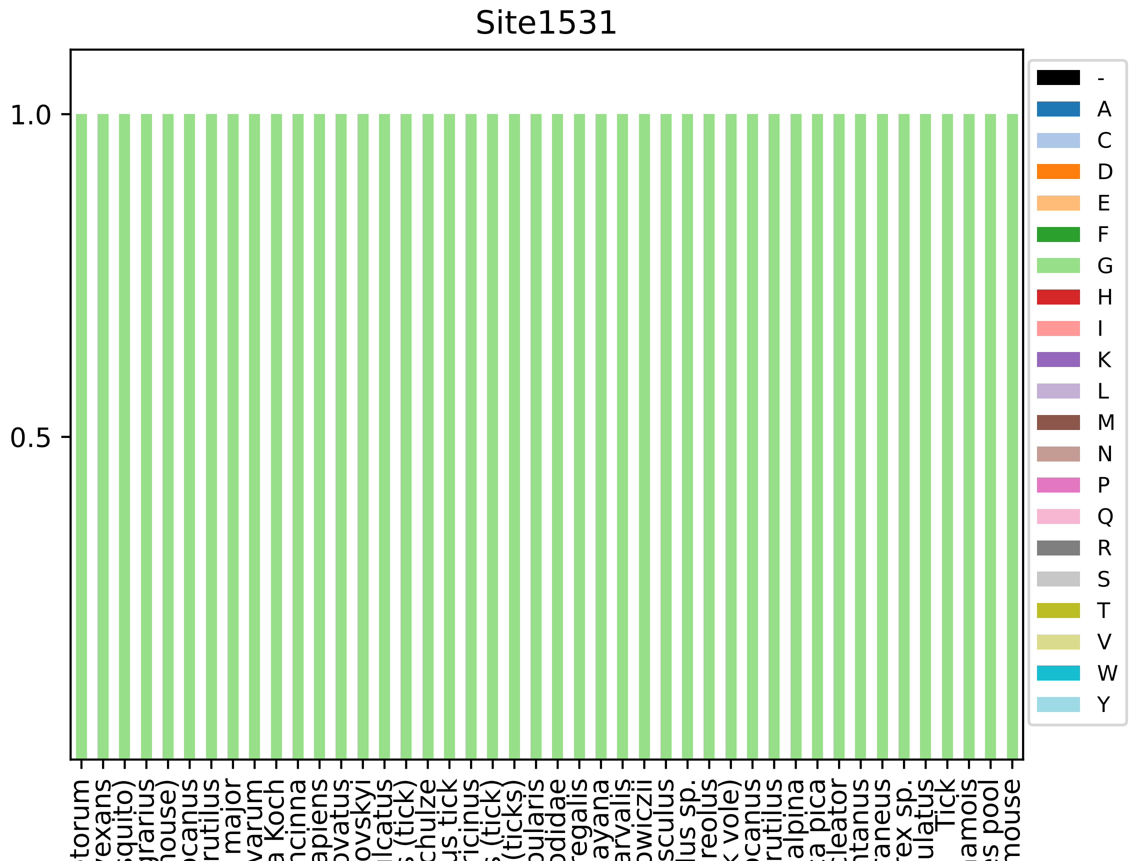

Site1531
1.0
0.5
-
A
C
D
E
F
G
H
I
K
L
M
N
P
Q
R
S
T
V
W
Y

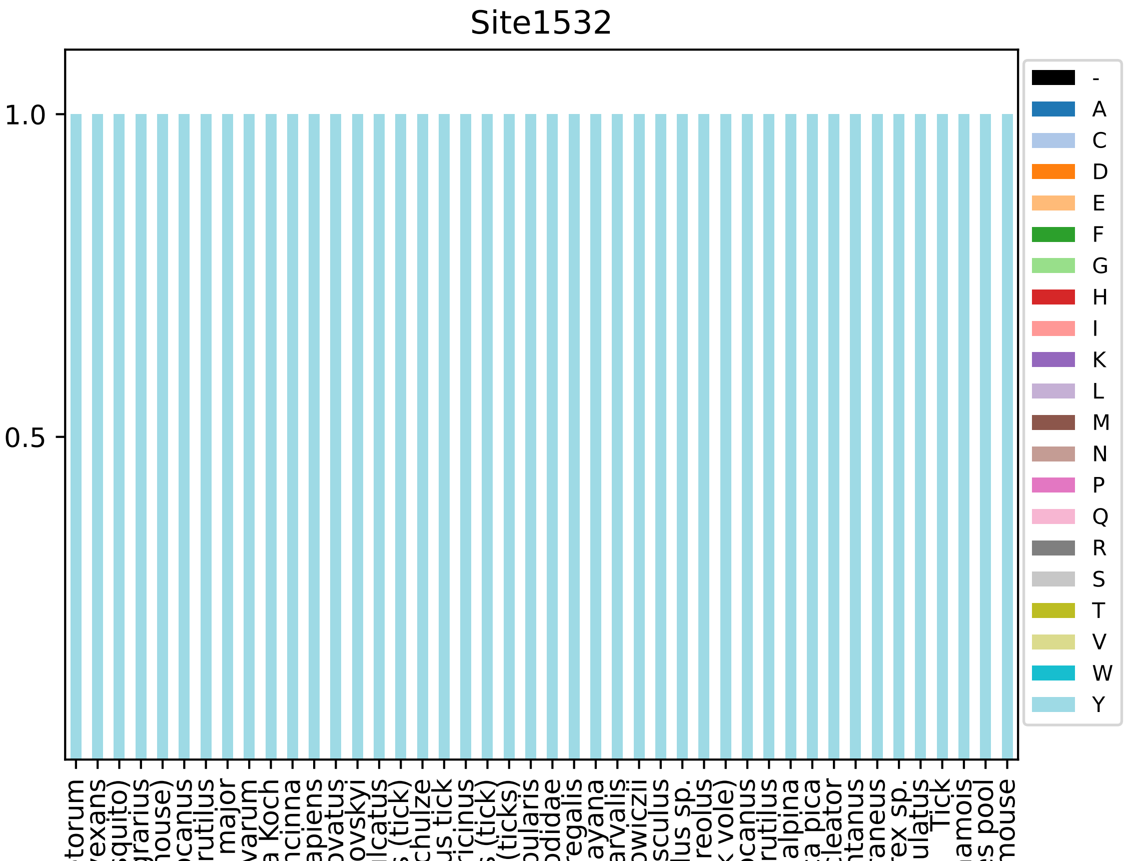
Site1532
1.0
0.5
-
A
C
D
E
F
G
H
I
K
L
M
N
P
Q
R
S
T
V
W
Y

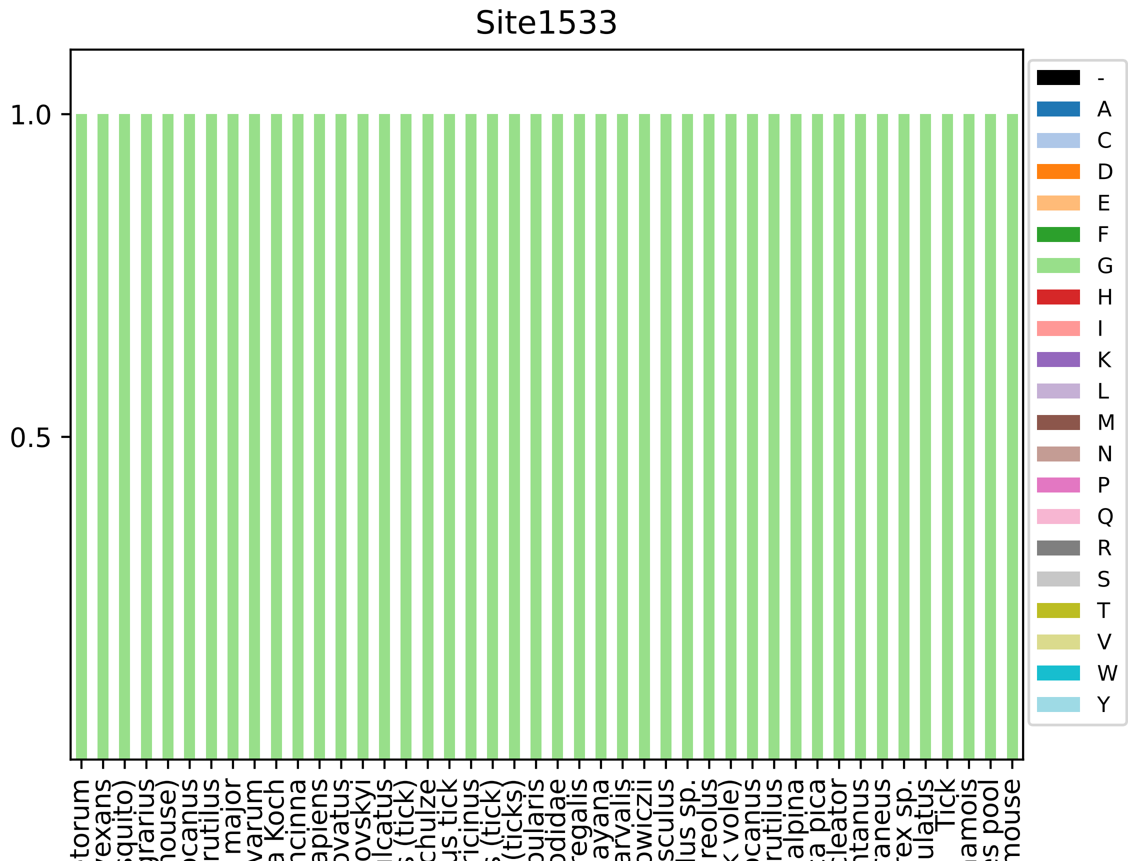

Site1533
1.0
0.5
-
A
C
D
E
F
G
H
I
K
L
M
N
P
Q
R
S
T
V
W
Y

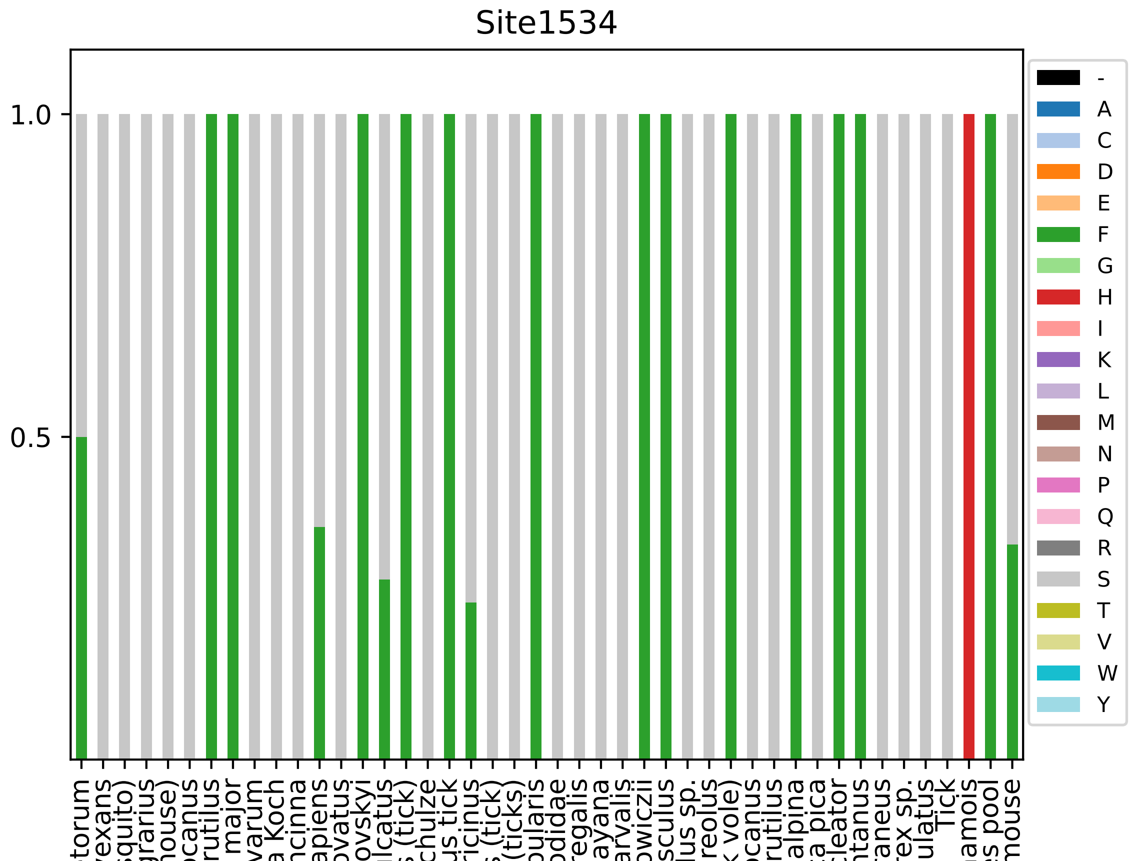

Site1534
1.0
0.5
-
A
C
D
E
F
G
H
I
K
L
M
N
P
Q
R
S
T
V
W
Y

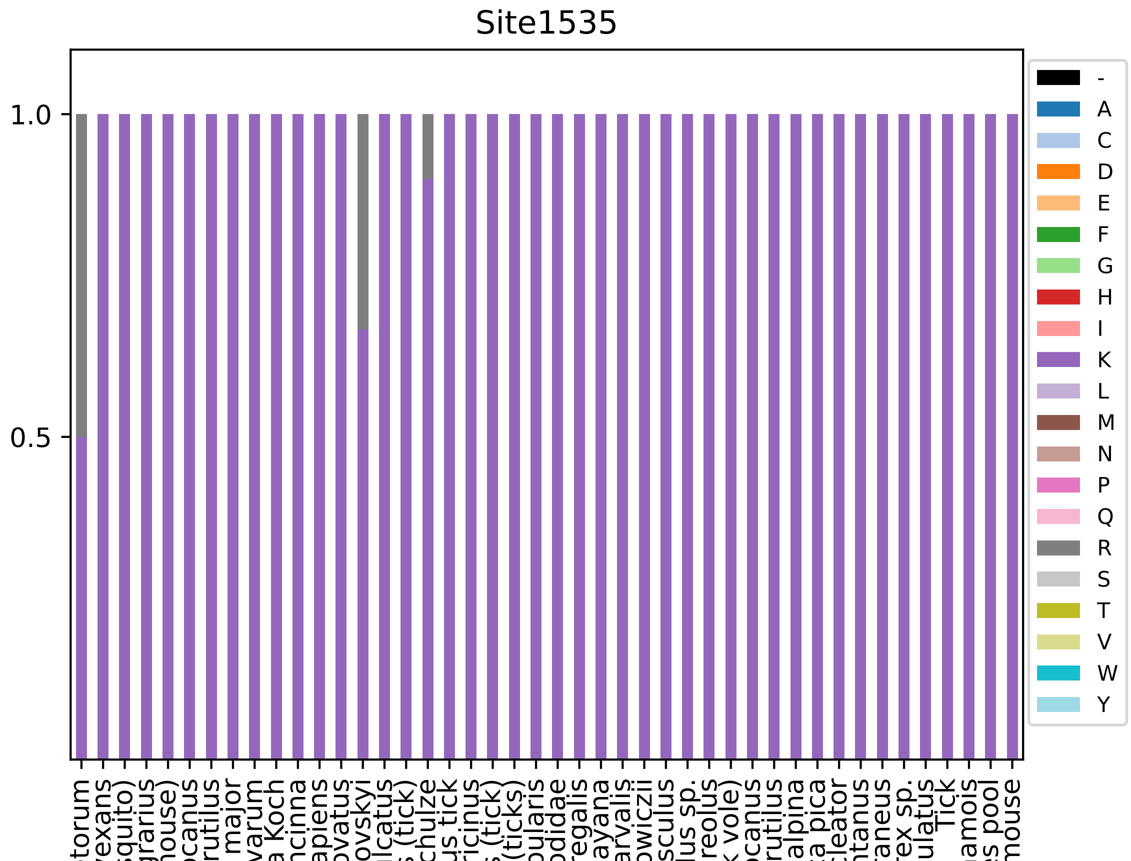

Site1535
1.0
0.5
-
A
C
D
E
F
G
H
I
K
L
M
N
P
Q
R
S
T
V
W
Y

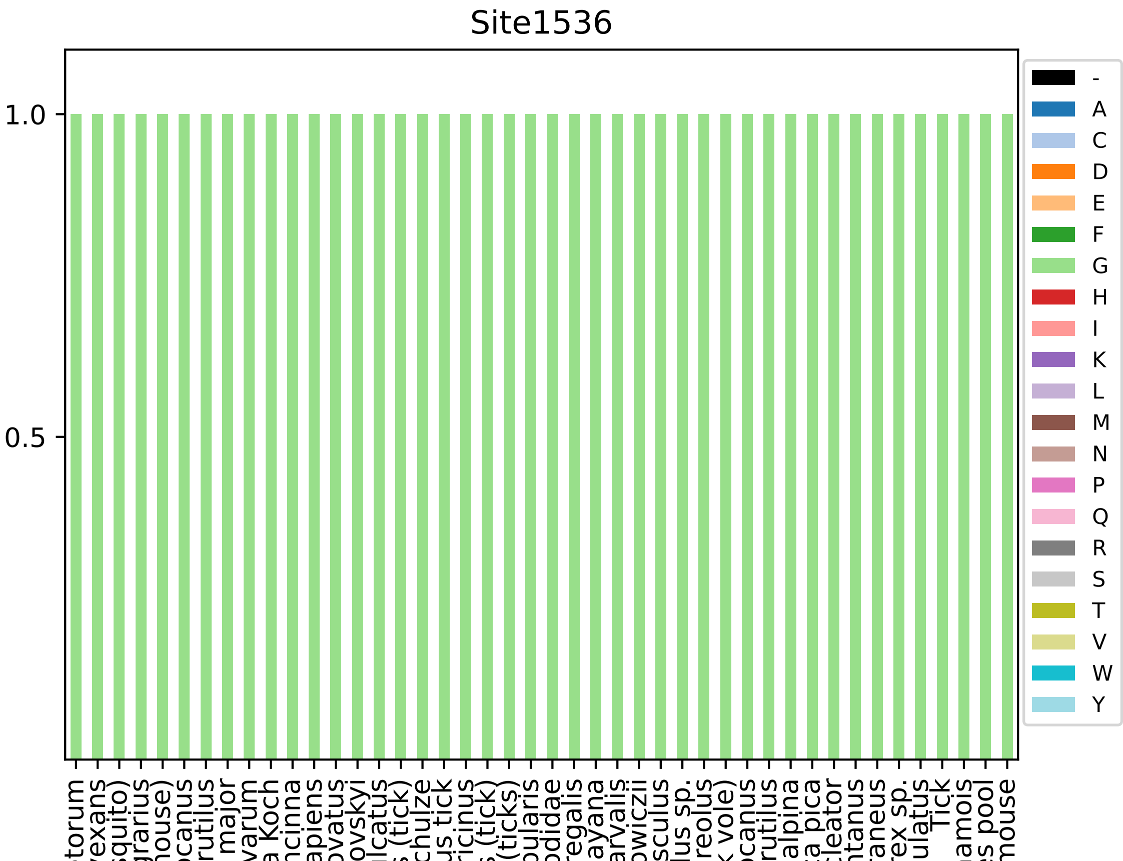
Site1536
1.0
0.5
-
A
C
D
E
F
G
H
I
K
L
M
N
P
Q
R
S
T
V
W
Y

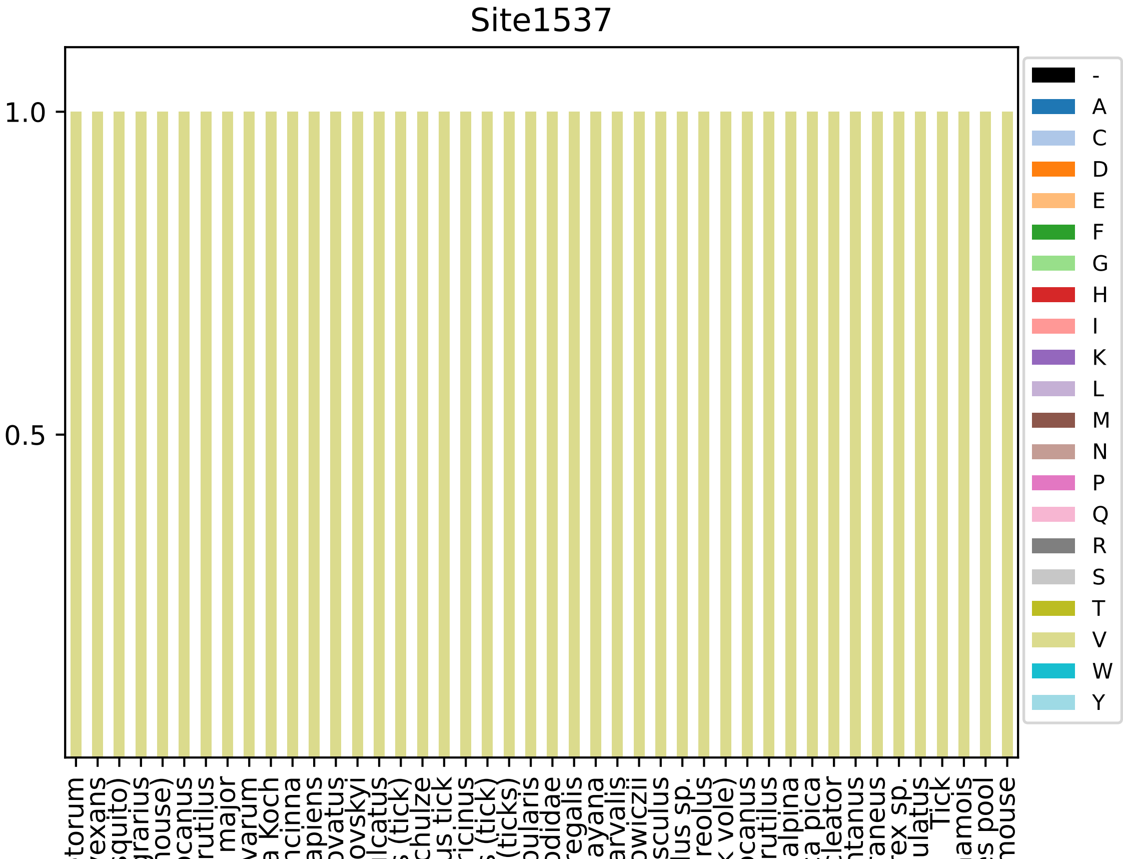
Site1537
1.0
0.5
-
A
C
D
E
F
G
H
I
K
L
M
N
P
Q
R
S
T
V
W
Y

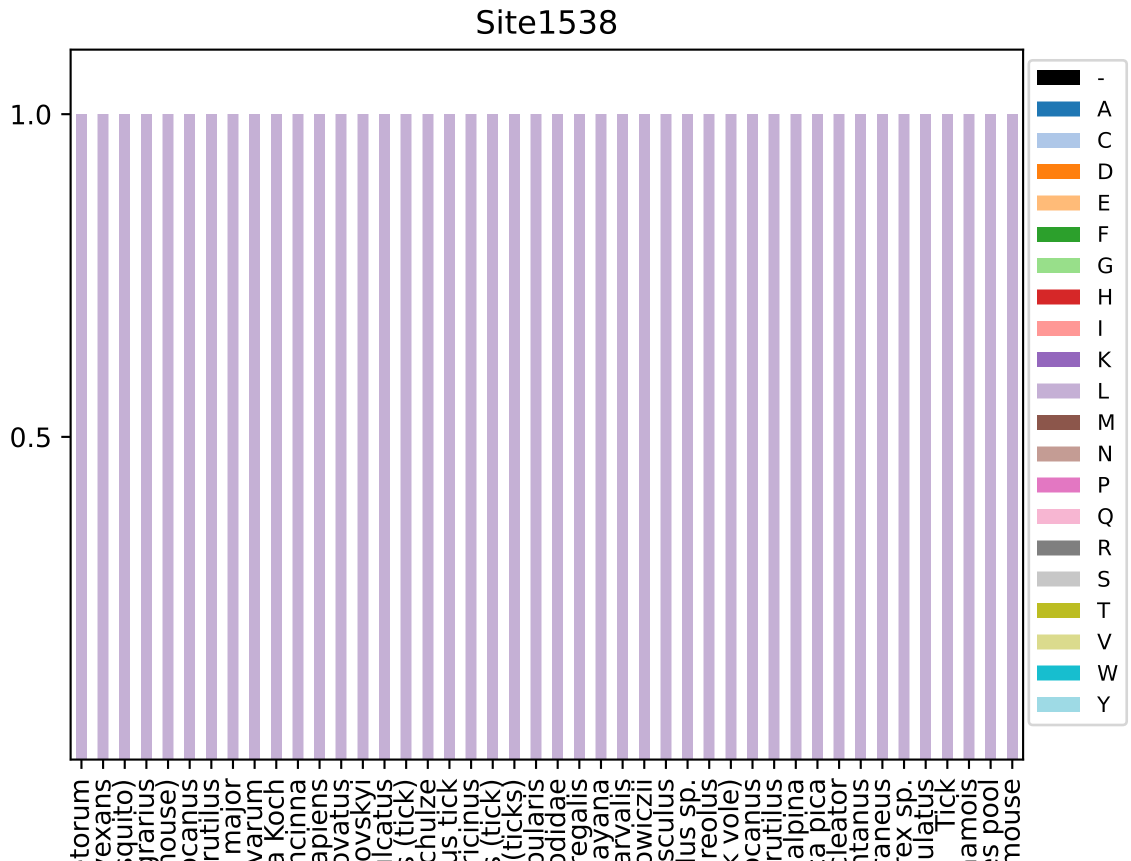
Site1538
1.0
0.5
-
A
C
D
E
F
G
H
I
K
L
M
N
P
Q
R
S
T
V
W
Y

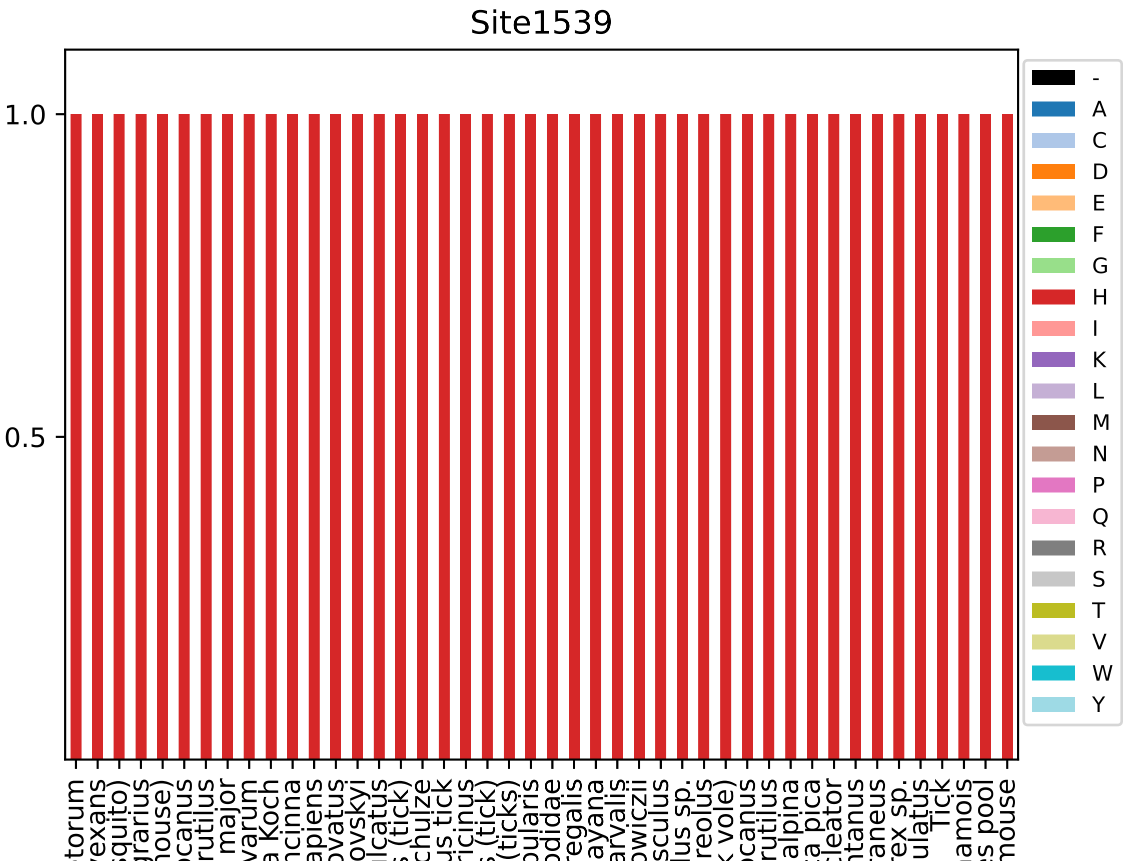

Site1539
1.0
0.5
-
A
C
D
E
F
G
H
I
K
L
M
N
P
Q
R
S
T
V
W
Y

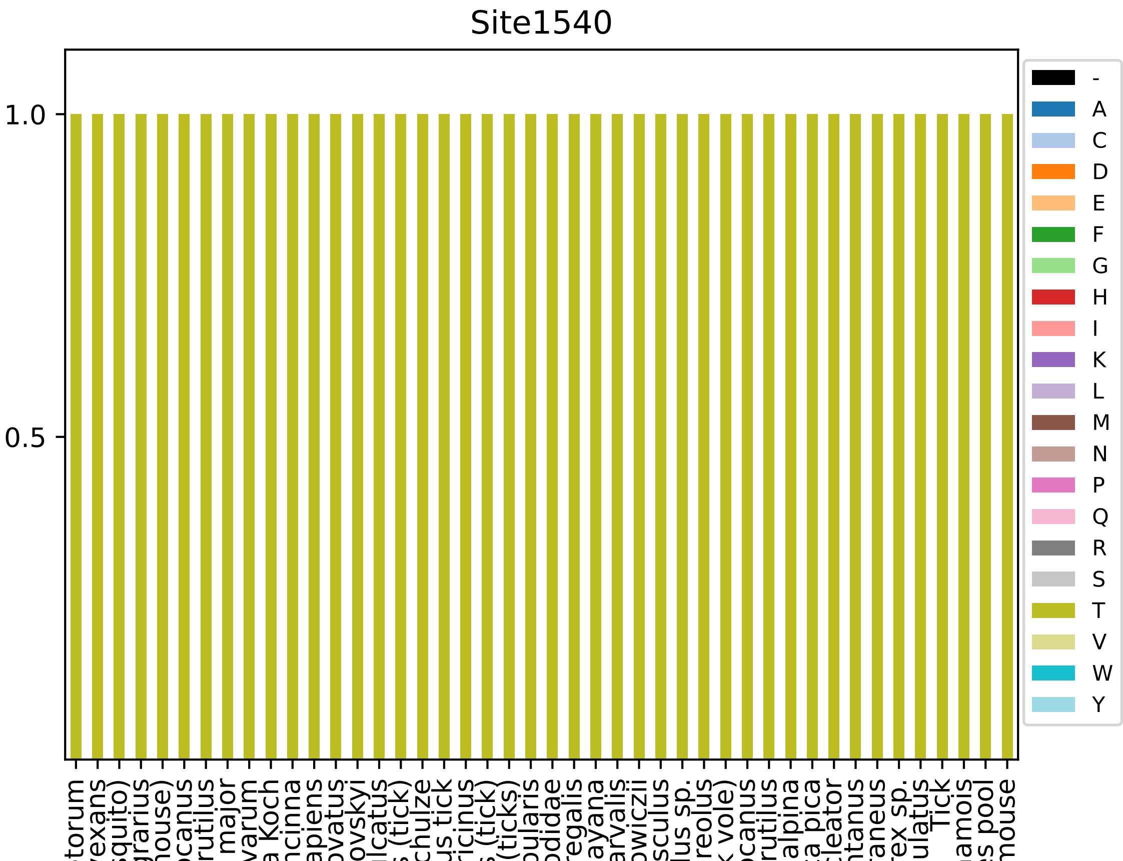

Site1540
1.0
0.5
-
A
C
D
E
F
G
H
I
K
L
M
N
P
Q
R
S
T
V
W
Y

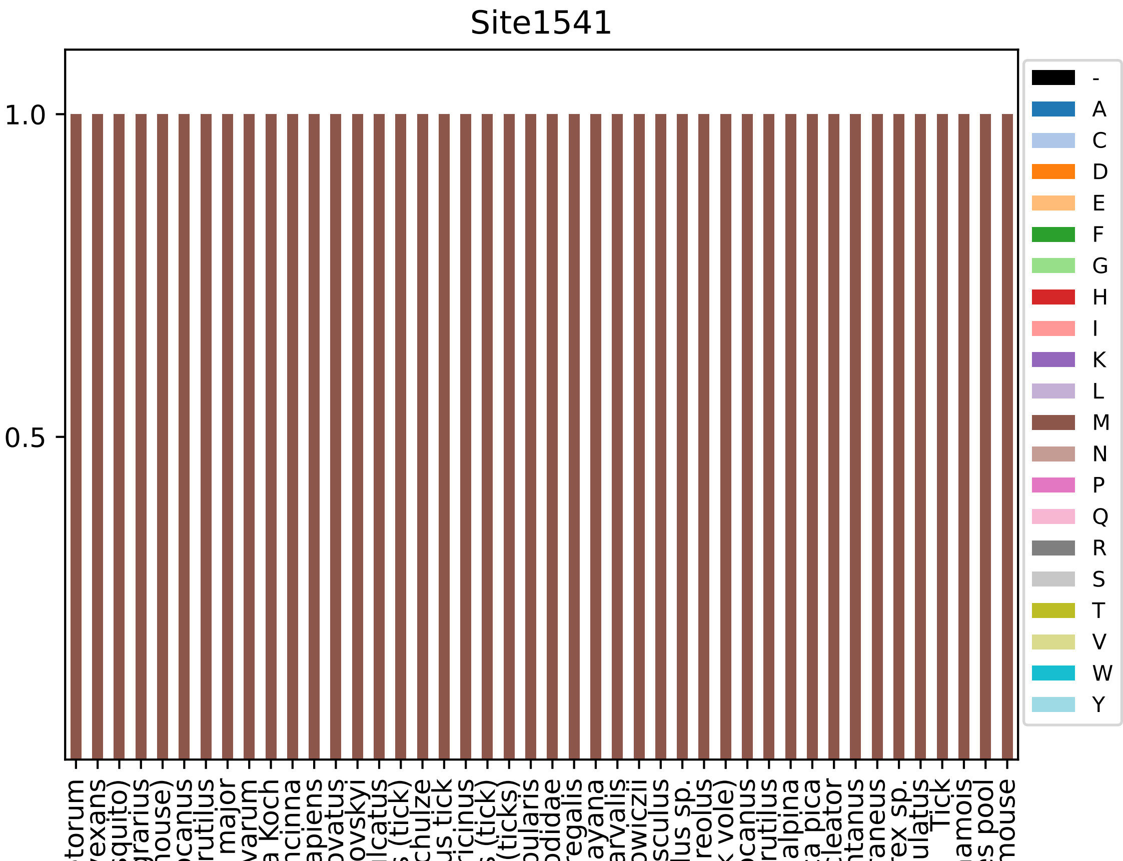
Site1541
1.0
0.5
-
A
C
D
E
F
G
H
I
K
L
M
N
P
Q
R
S
T
V
W
Y

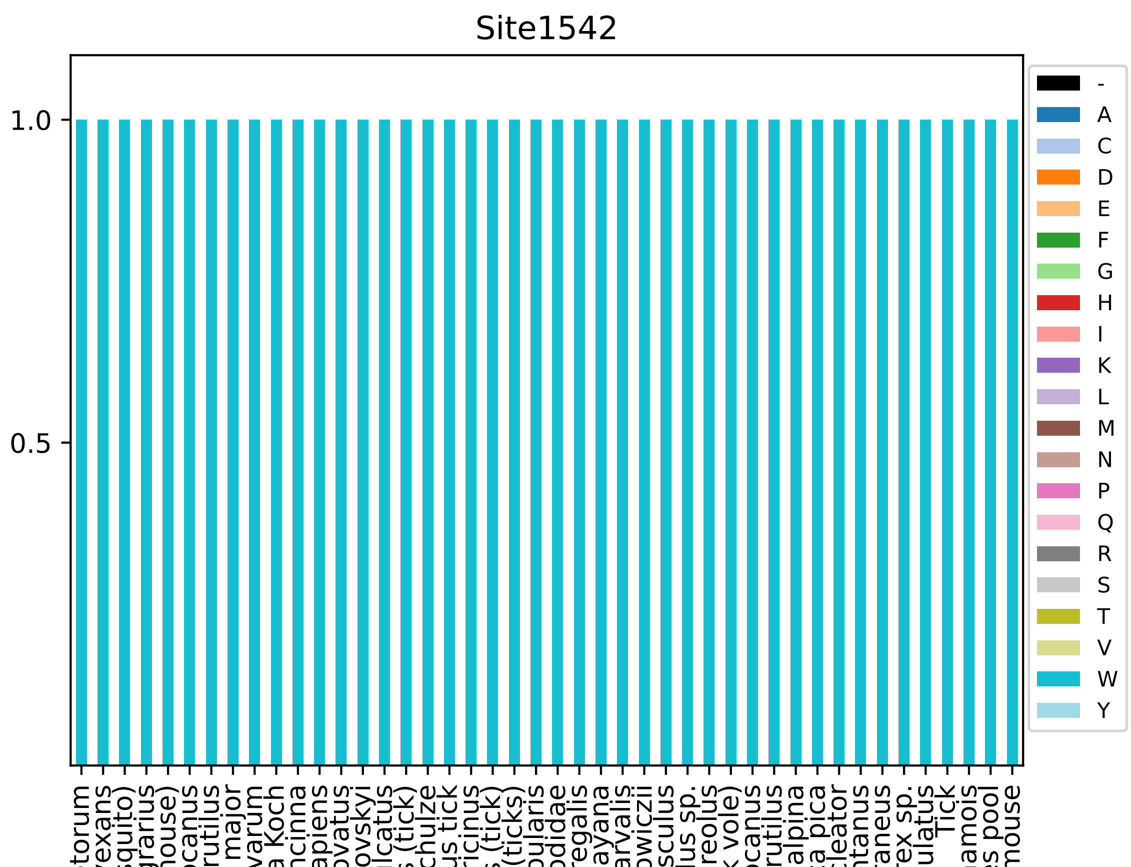
Site1542
1.0
0.5
-
A
C
D
E
F
G
H
I
K
L
M
N
P
Q
R
S
T
V
W
Y

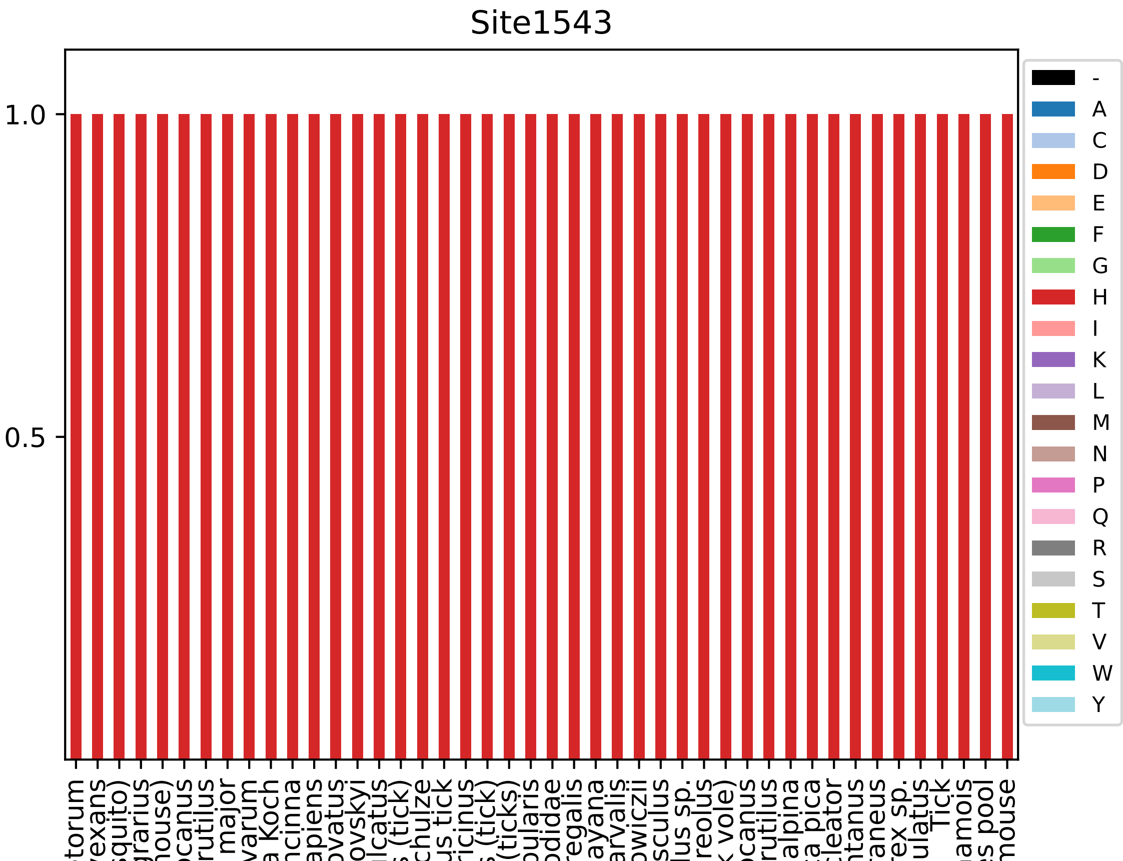
Site1543
1.0
0.5
-
A
C
D
E
F
G
H
I
K
L
M
N
P
Q
R
S
T
V
W
Y

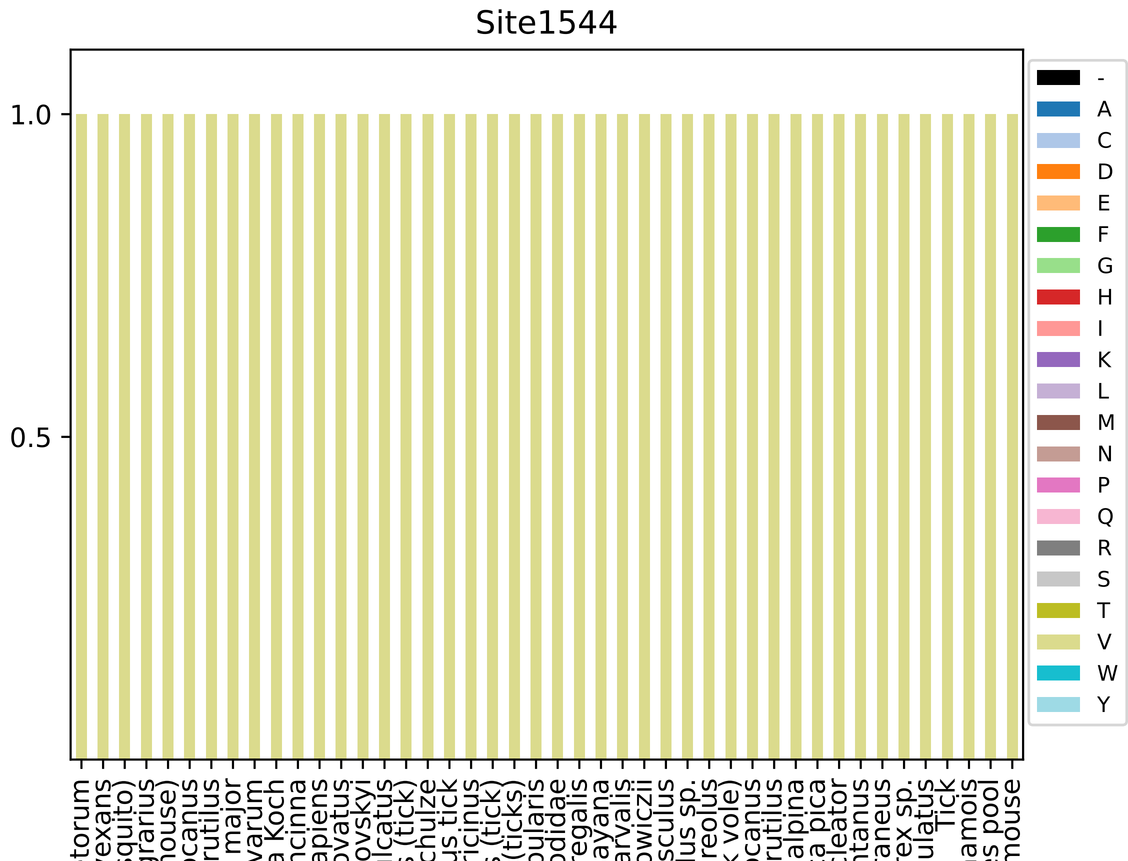
Site1544
1.0
0.5
-
A
C
D
E
F
G
H
I
K
L
M
N
P
Q
R
S
T
V
W
Y

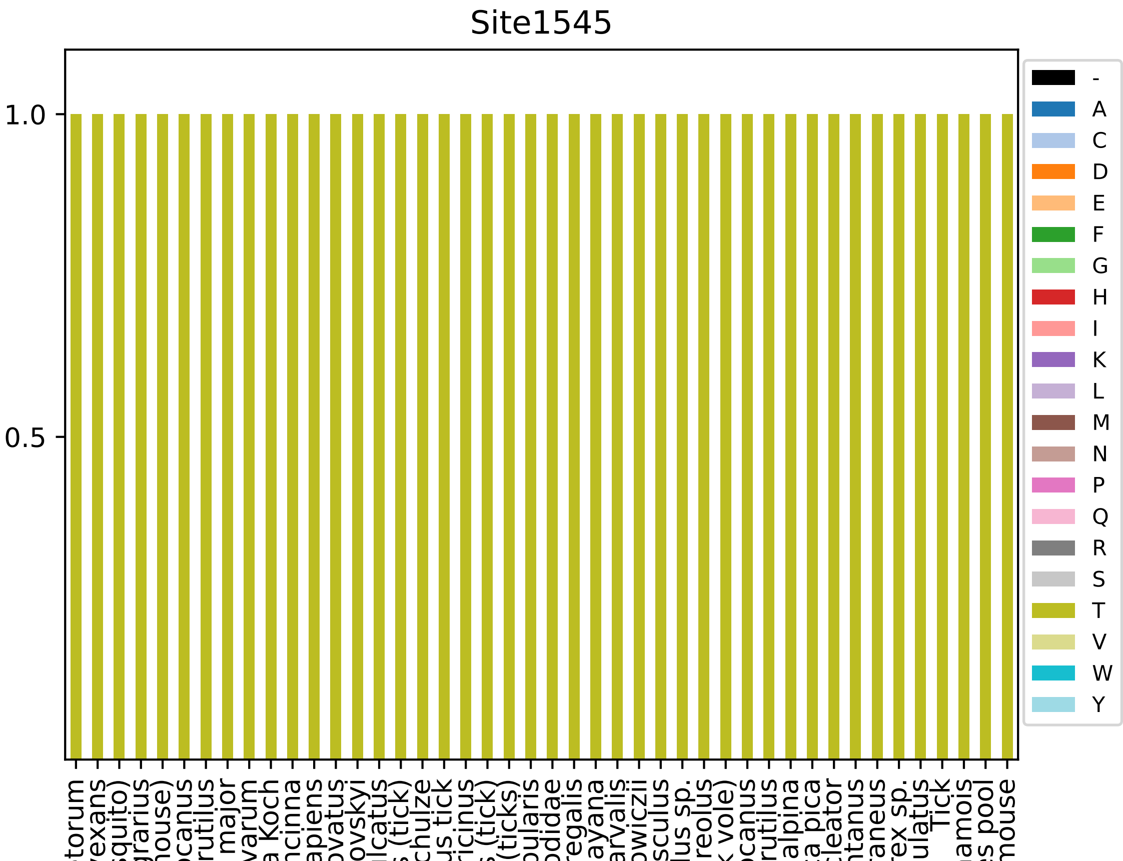
Site1545
1.0
0.5
-
A
C
D
E
F
G
H
I
K
L
M
N
P
Q
R
S
T
V
W
Y

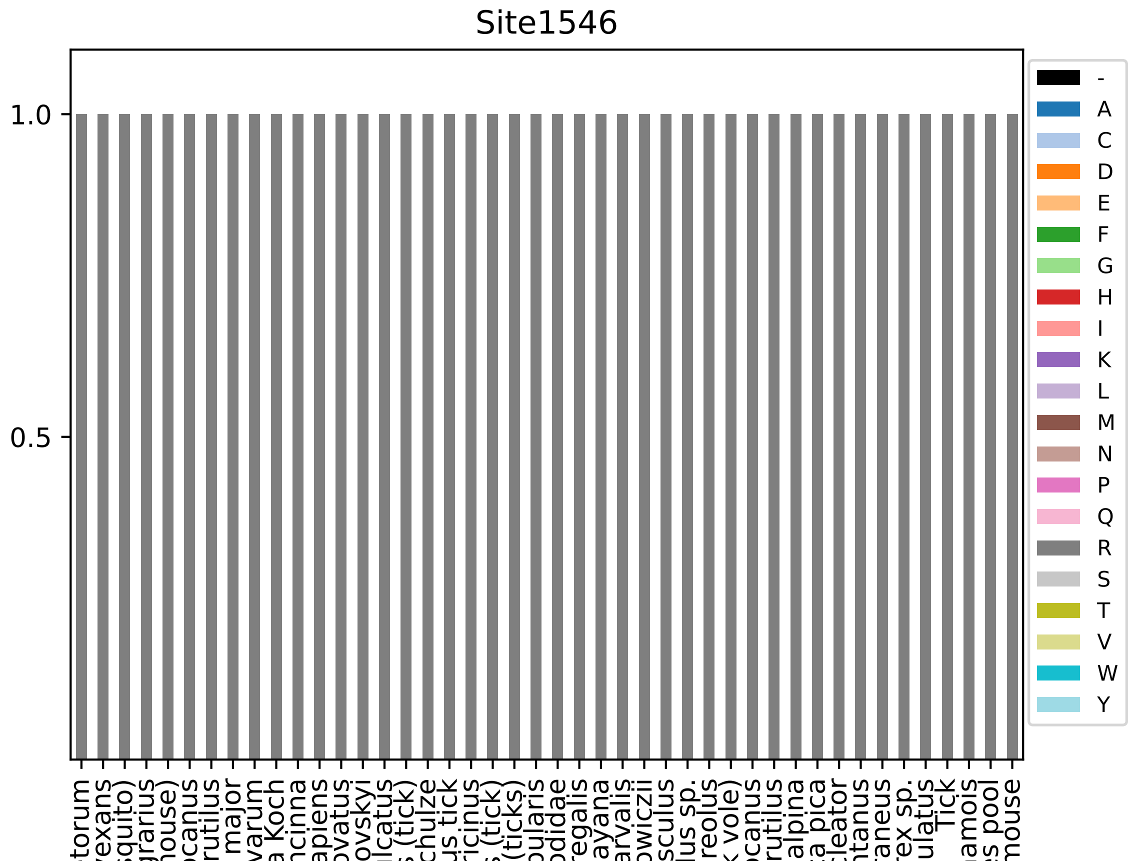
Site1546
1.0
0.5
-
A
C
D
E
F
G
H
I
K
L
M
N
P
Q
R
S
T
V
W
Y

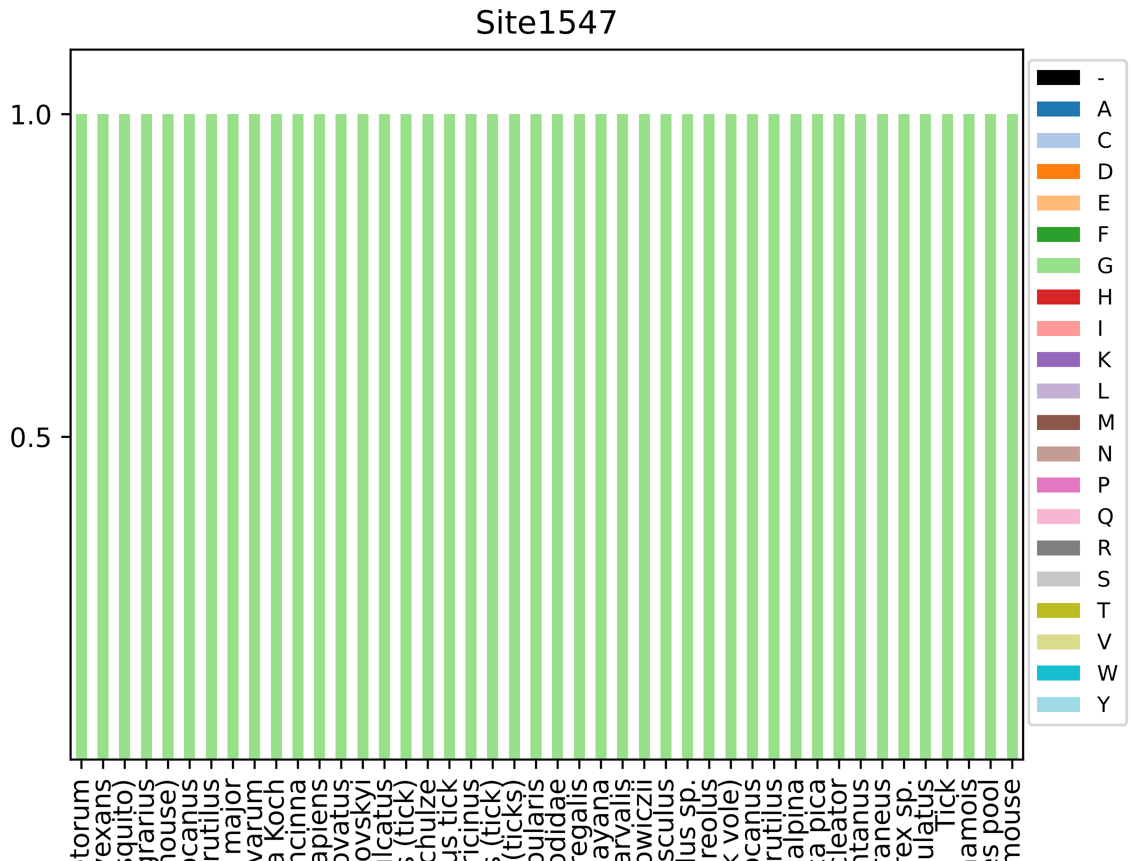
Site1547
1.0
0.5
-
A
C
D
E
F
G
H
I
K
L
M
N
P
Q
R
S
T
V
W
Y

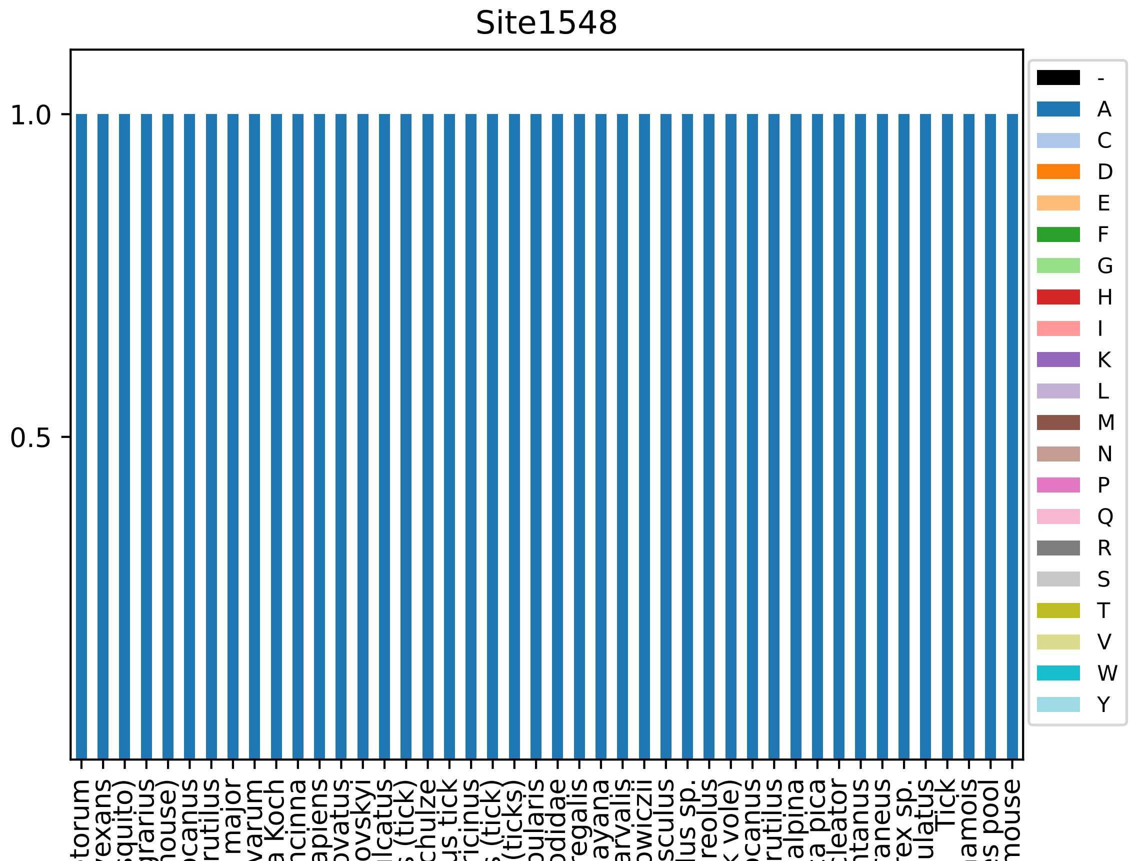
Site1548
1.0
0.5
-
A
C
D
E
F
G
H
I
K
L
M
N
P
Q
R
S
T
V
W
Y

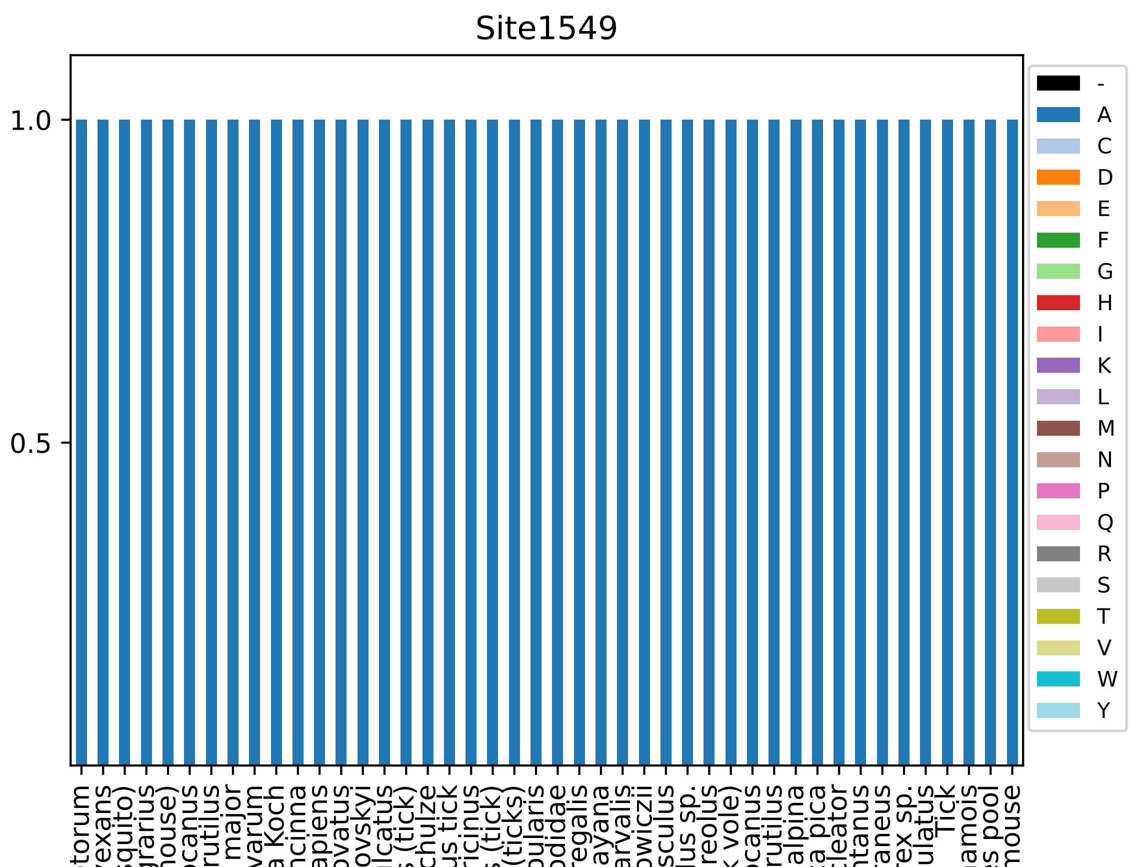
Site1549
1.0
0.5
-
A
C
D
E
F
G
H
I
K
L
M
N
P
Q
R
S
T
V
W
Y

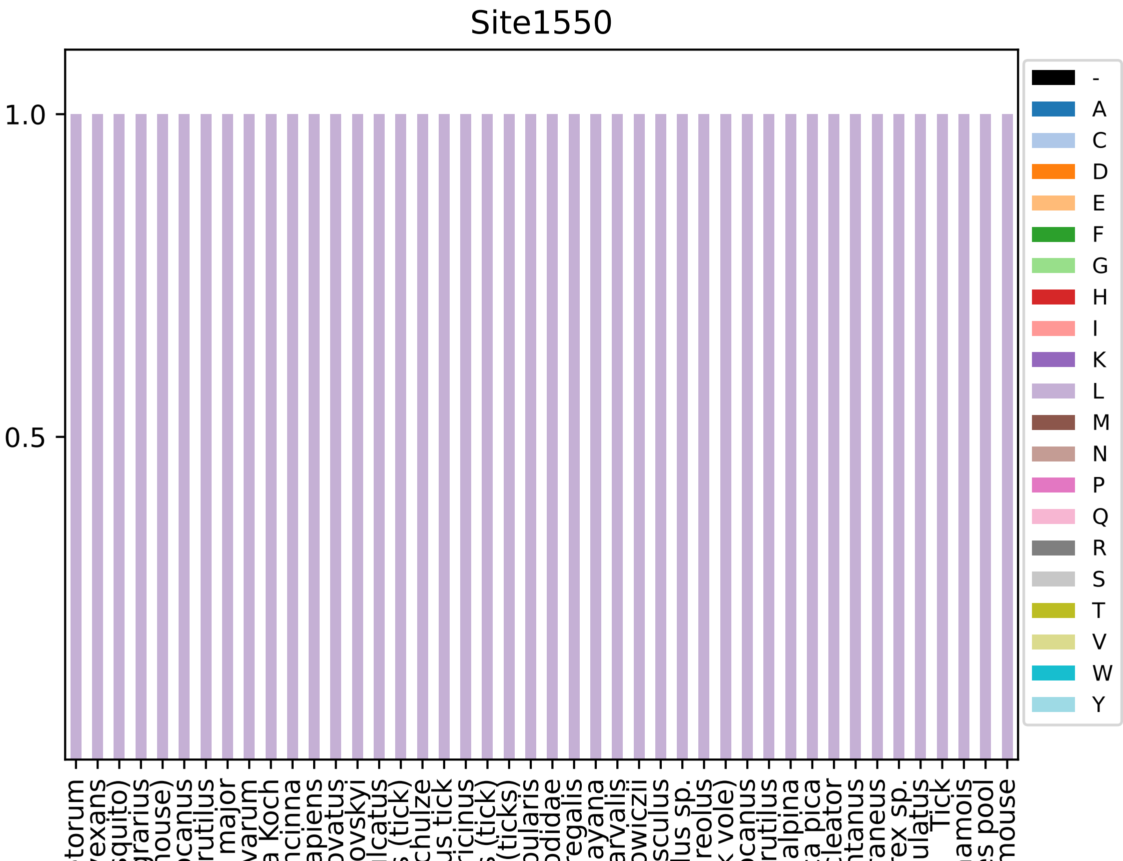
Site1550
1.0
0.5
-
A
C
D
E
F
G
H
I
K
L
M
N
P
Q
R
S
T
V
W
Y

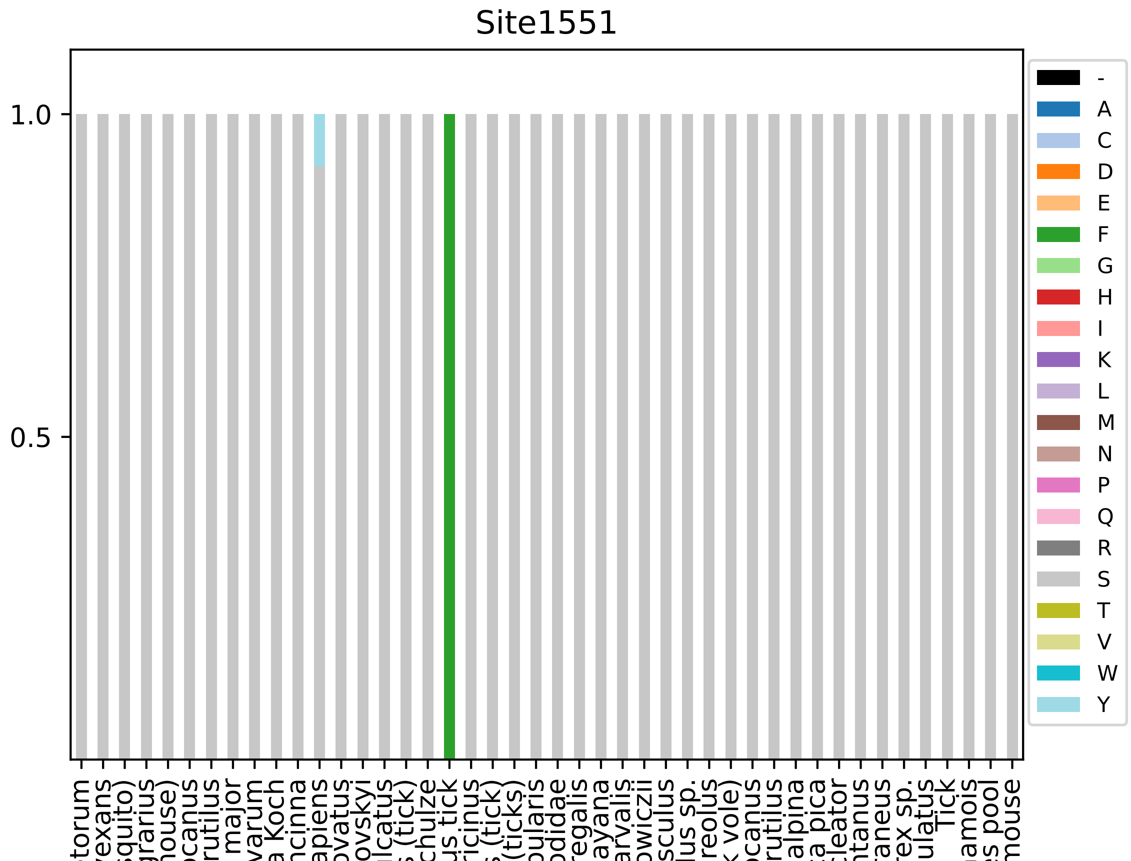

Site1551
1.0
0.5
-
A
C
D
E
F
G
H
I
K
L
M
N
P
Q
R
S
T
V
W
Y

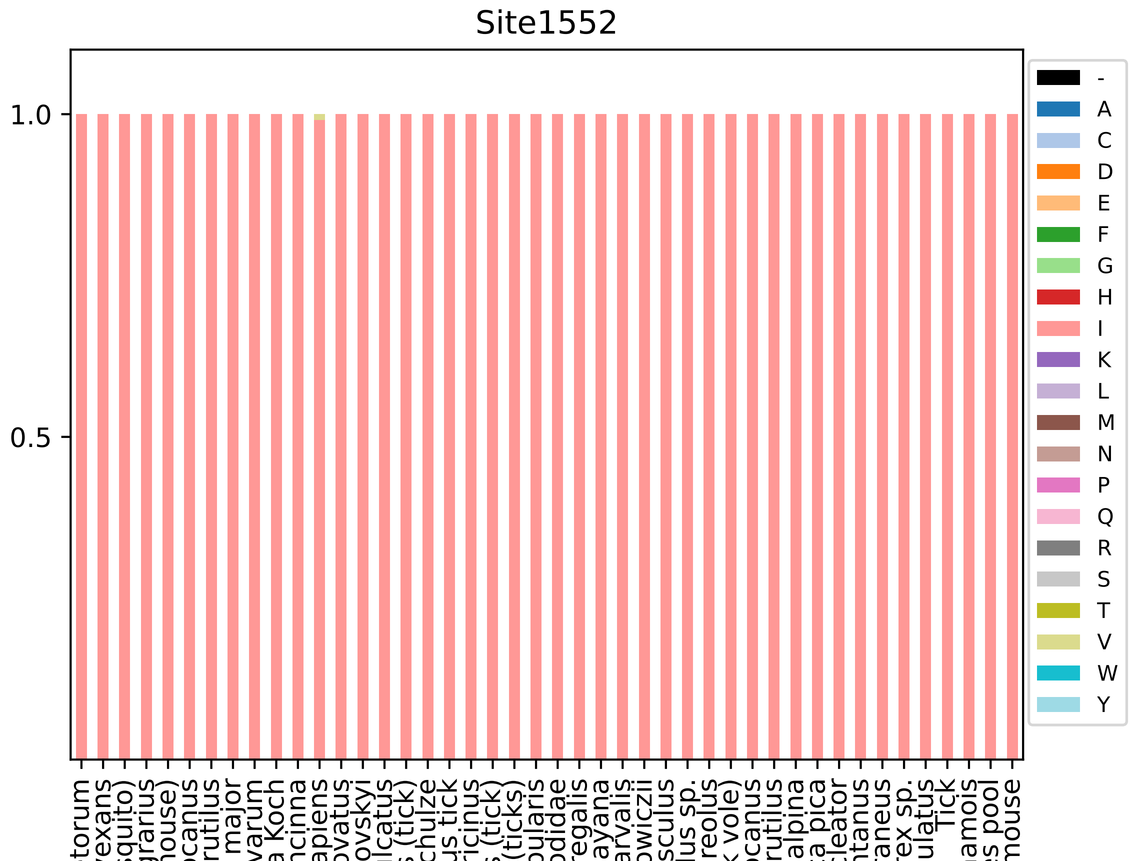
Site1552
1.0
0.5
-
A
C
D
E
F
G
H
I
K
L
M
N
P
Q
R
S
T
V
W
Y

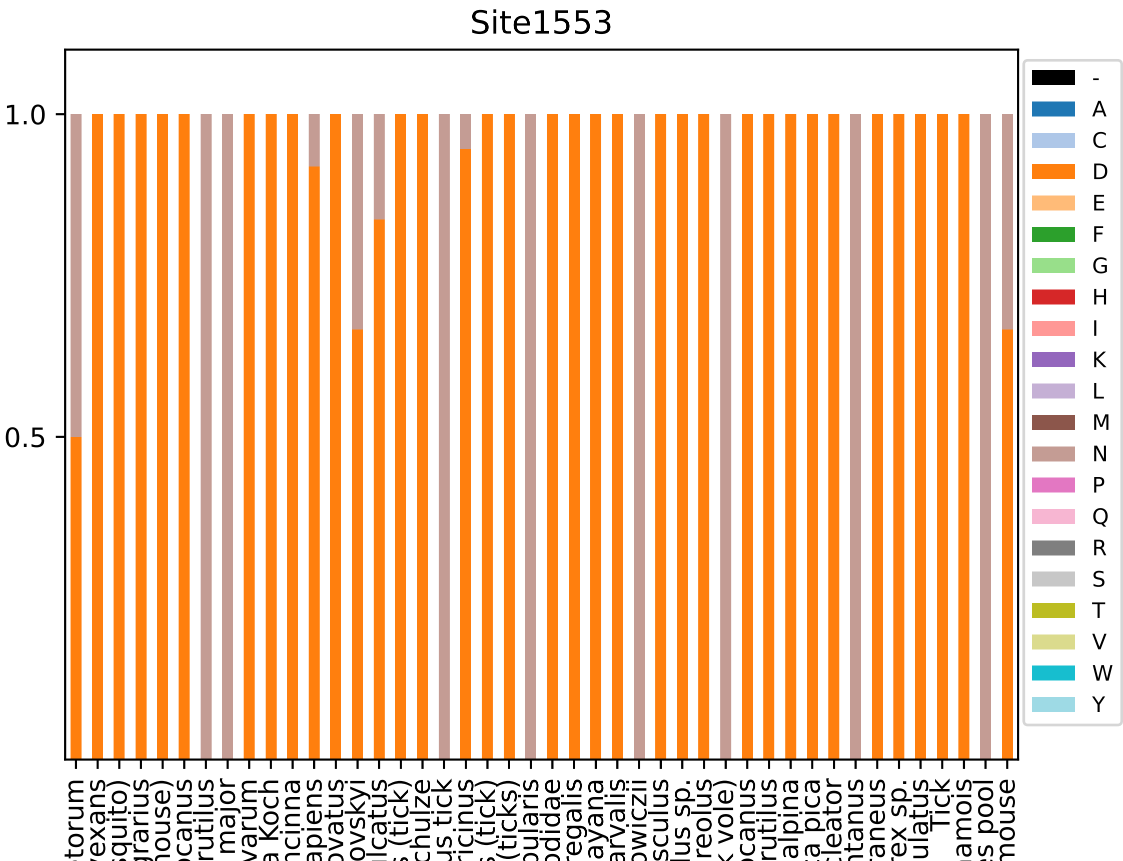
Site1553
1.0
0.5
-
A
C
D
E
F
G
H
I
K
L
M
N
P
Q
R
S
T
V
W
Y

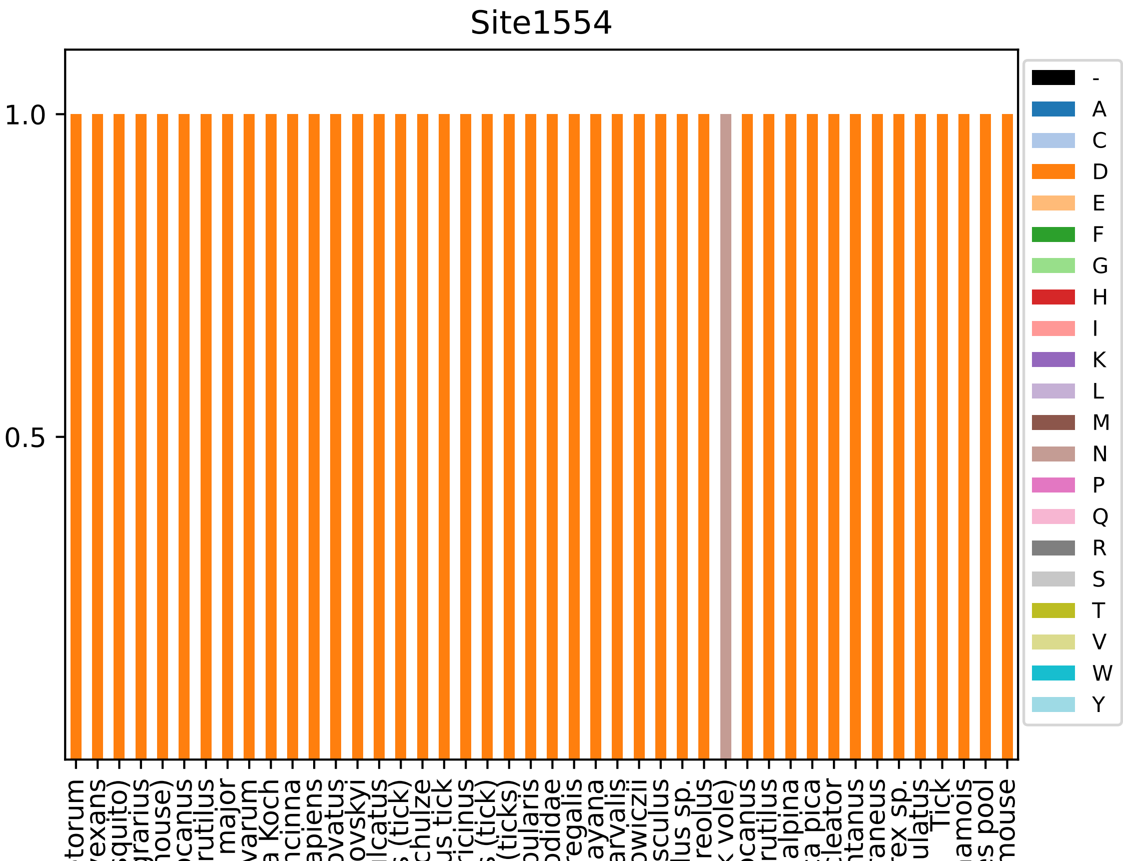
Site1554
1.0
0.5
-
A
C
D
E
F
G
H
I
K
L
M
N
P
Q
R
S
T
V
W
Y

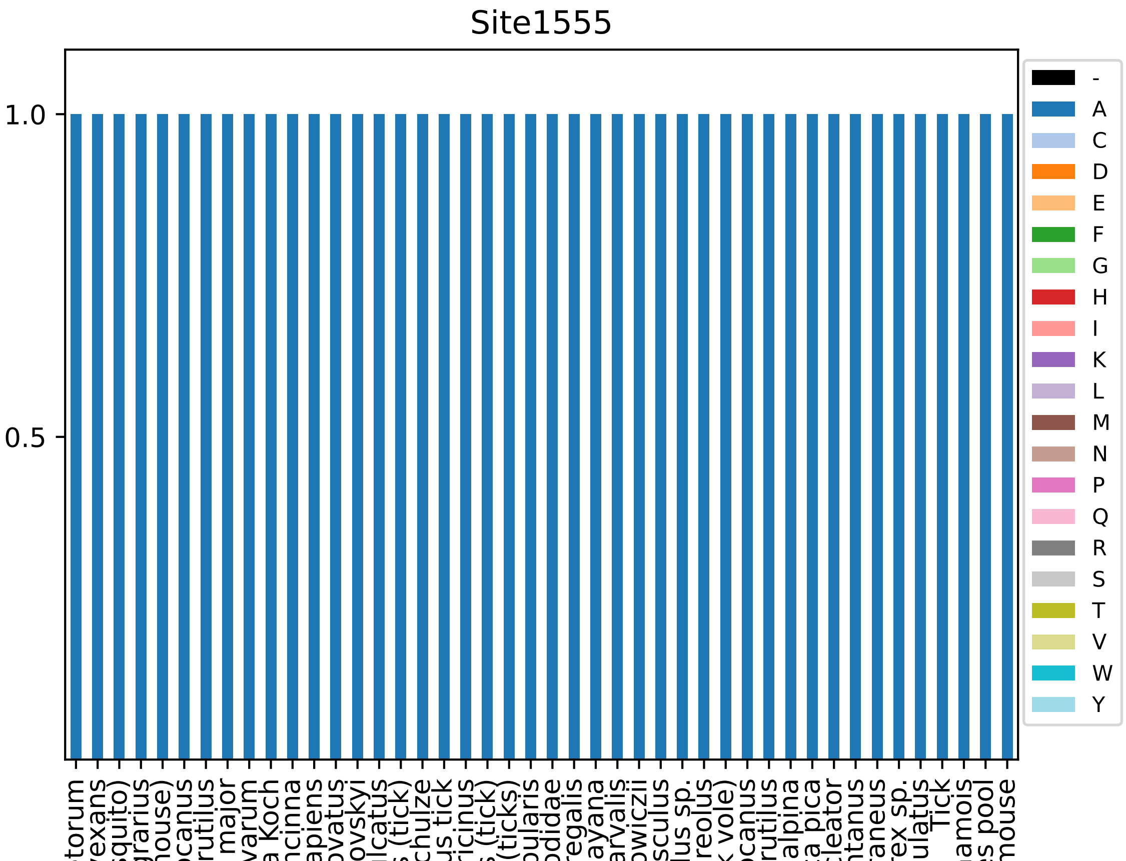

Site1555
1.0
0.5
-
A
C
D
E
F
G
H
I
K
L
M
N
P
Q
R
S
T
V
W
Y

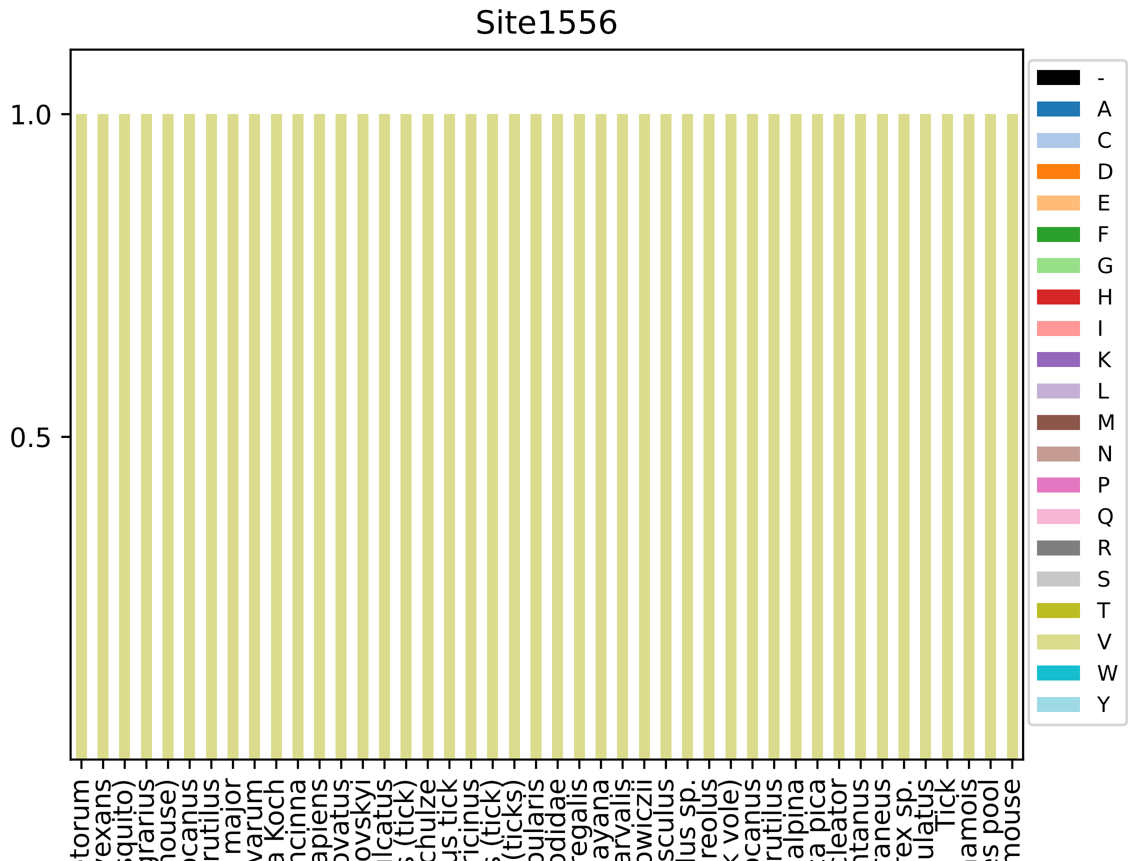

Site1556
1.0
0.5
-
A
C
D
E
F
G
H
I
K
L
M
N
P
Q
R
S
T
V
W
Y

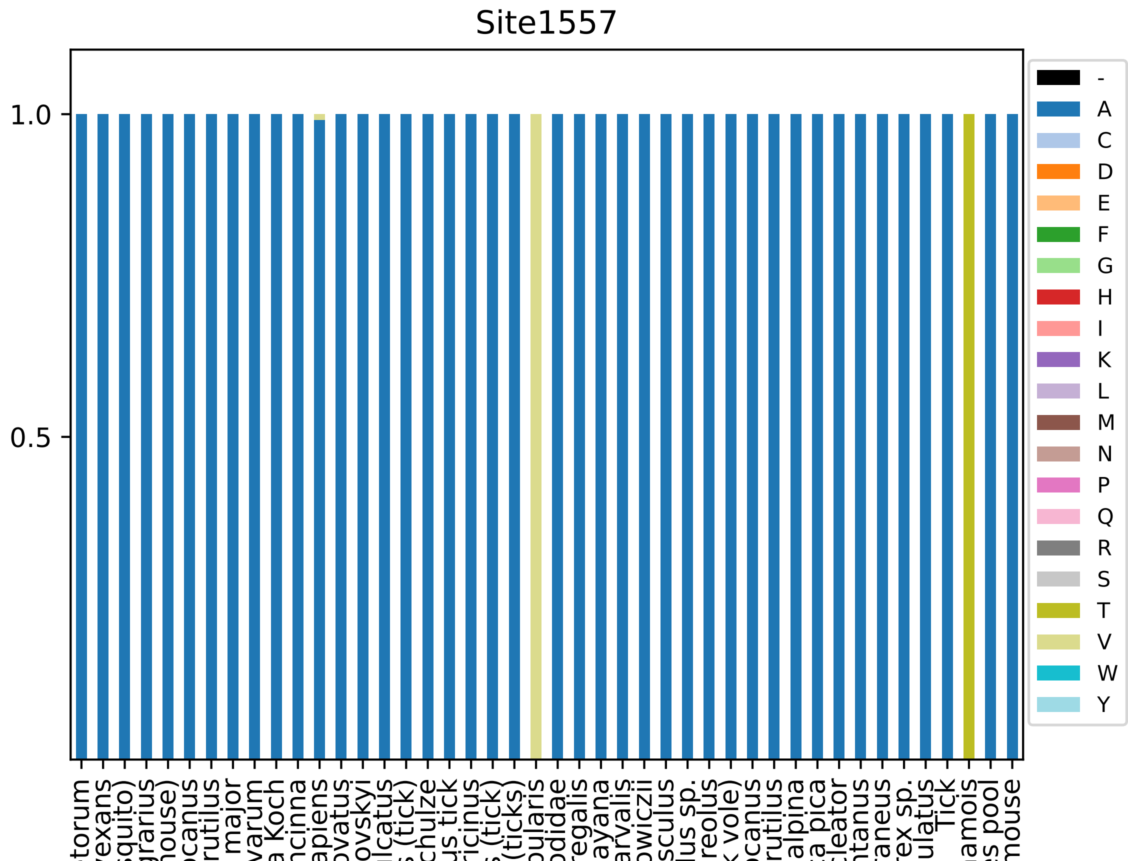
Site1557
1.0
0.5
-
A
C
D
E
F
G
H
I
K
L
M
N
P
Q
R
S
T
V
W
Y

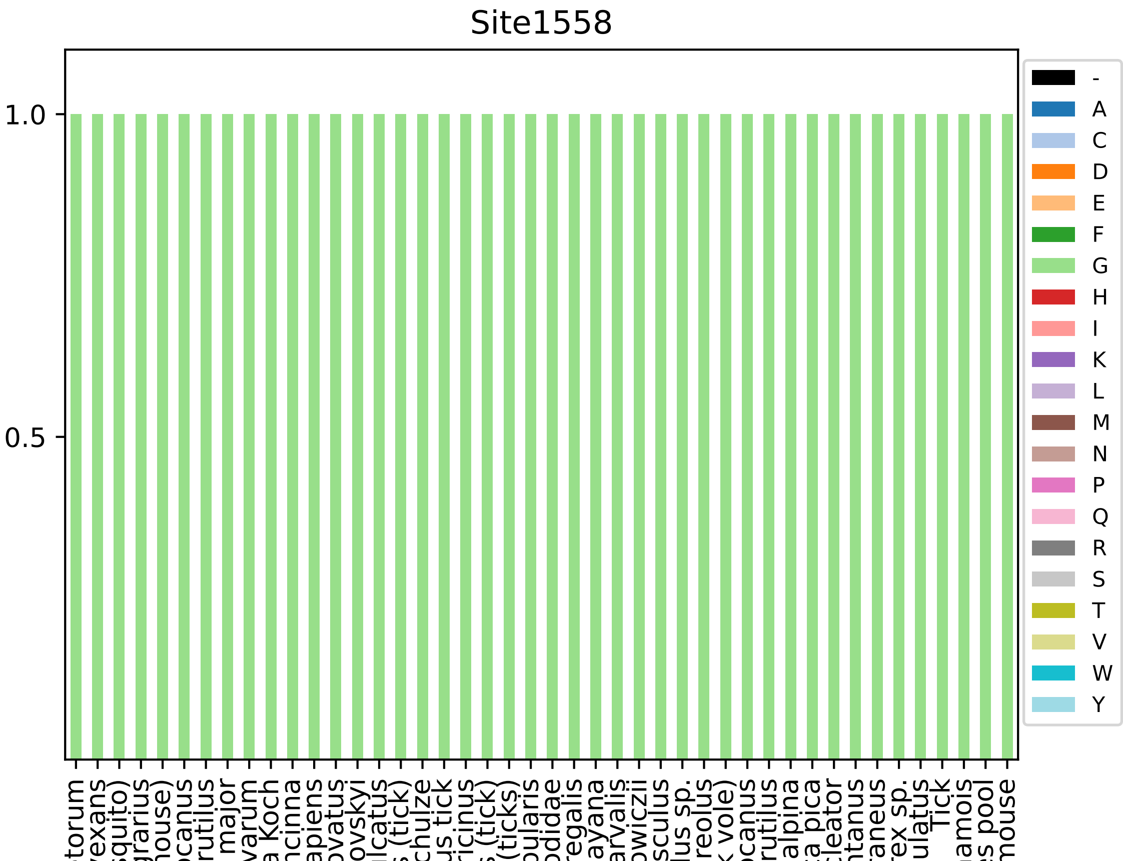

Site1558
1.0
0.5
-
A
C
D
E
F
G
H
I
K
L
M
N
P
Q
R
S
T
V
W
Y

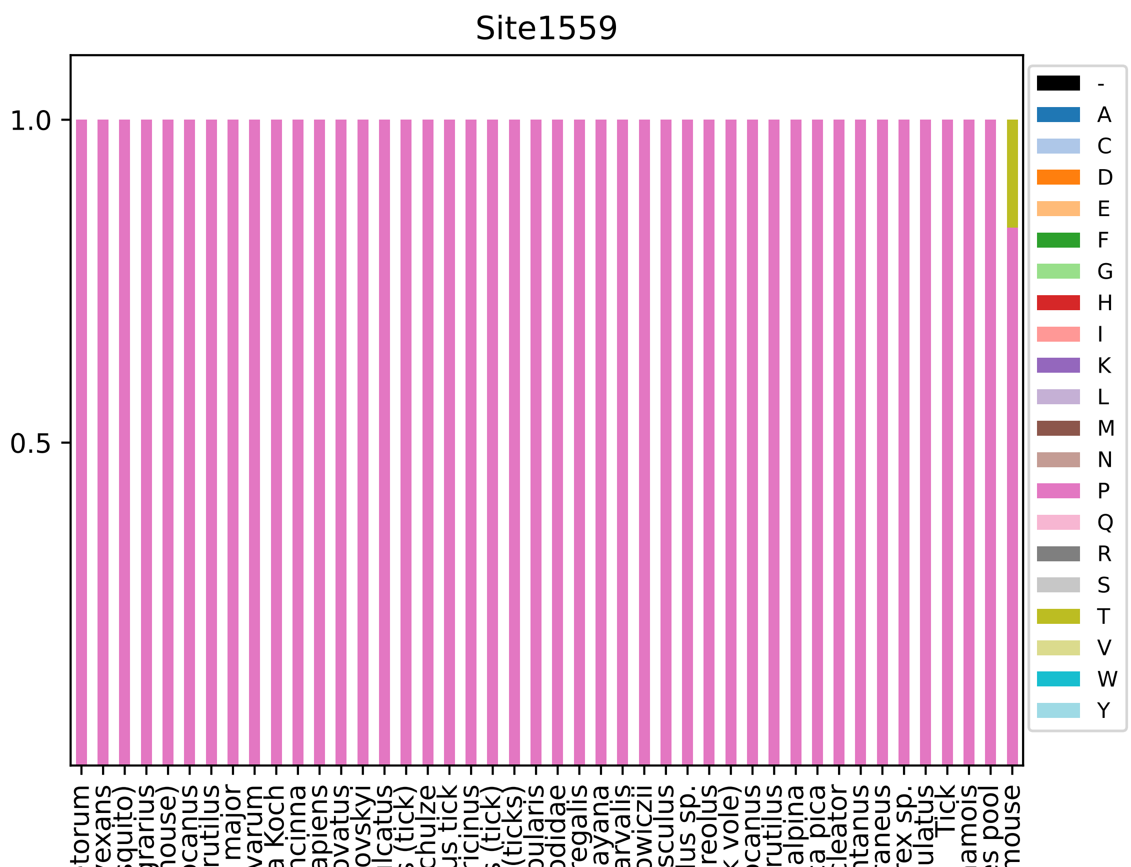
Site1559
1.0
0.5
-
A
C
D
E
F
G
H
I
K
L
M
N
P
Q
R
S
T
V
W
Y

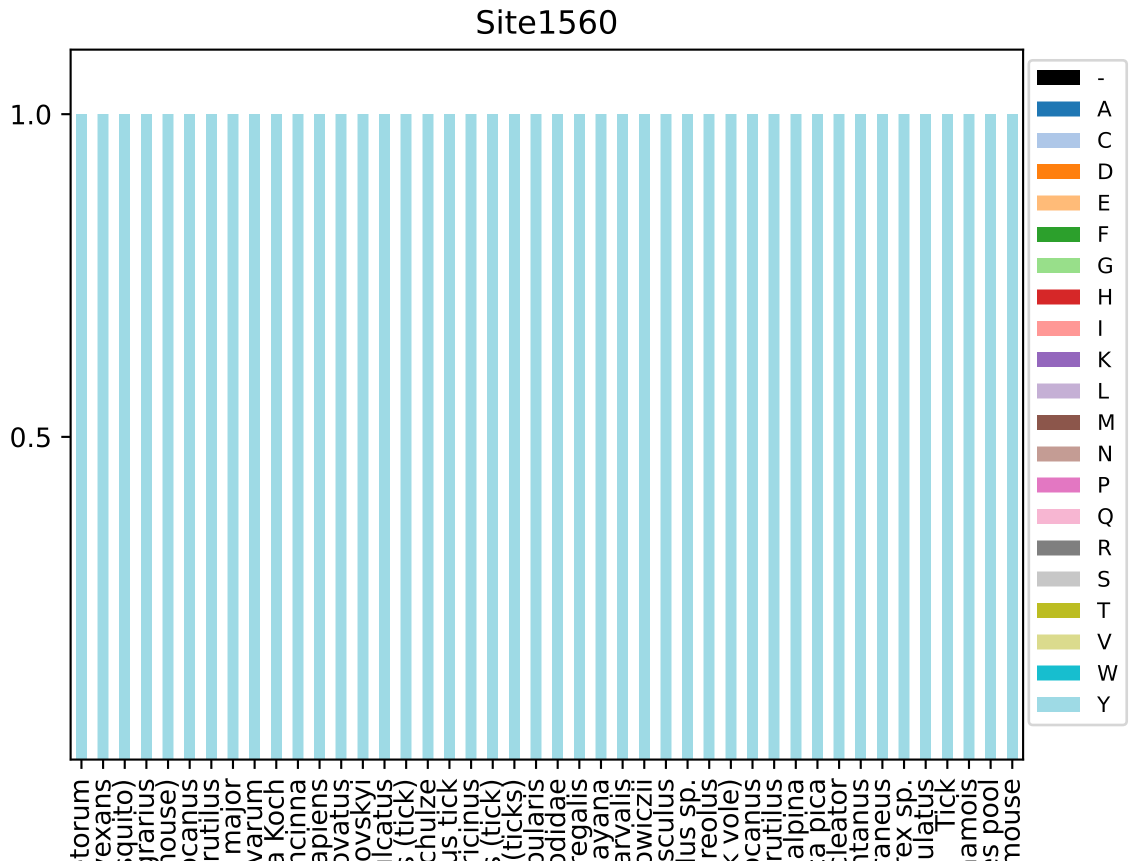
Site1560
1.0
0.5
-
A
C
D
E
F
G
H
I
K
L
M
N
P
Q
R
S
T
V
W
Y

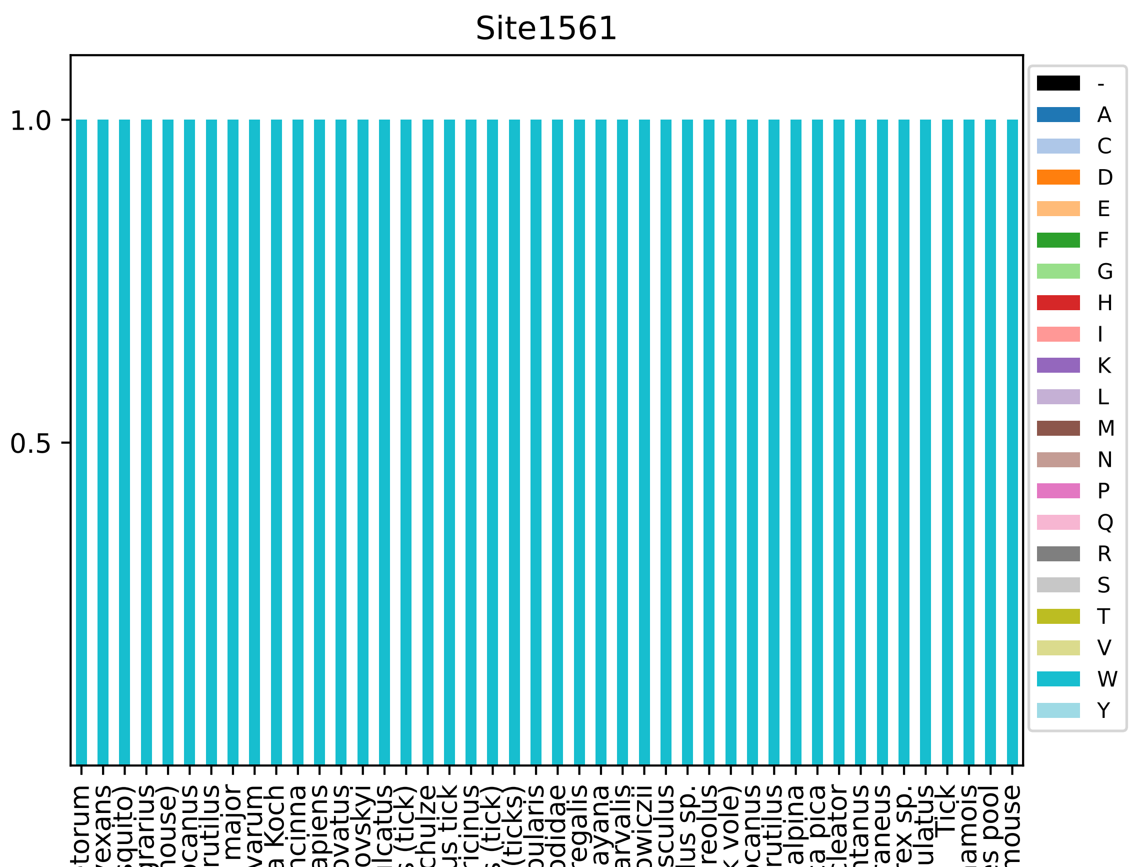

Site1561
1.0
0.5
-
A
C
D
E
F
G
H
I
K
L
M
N
P
Q
R
S
T
V
W
Y

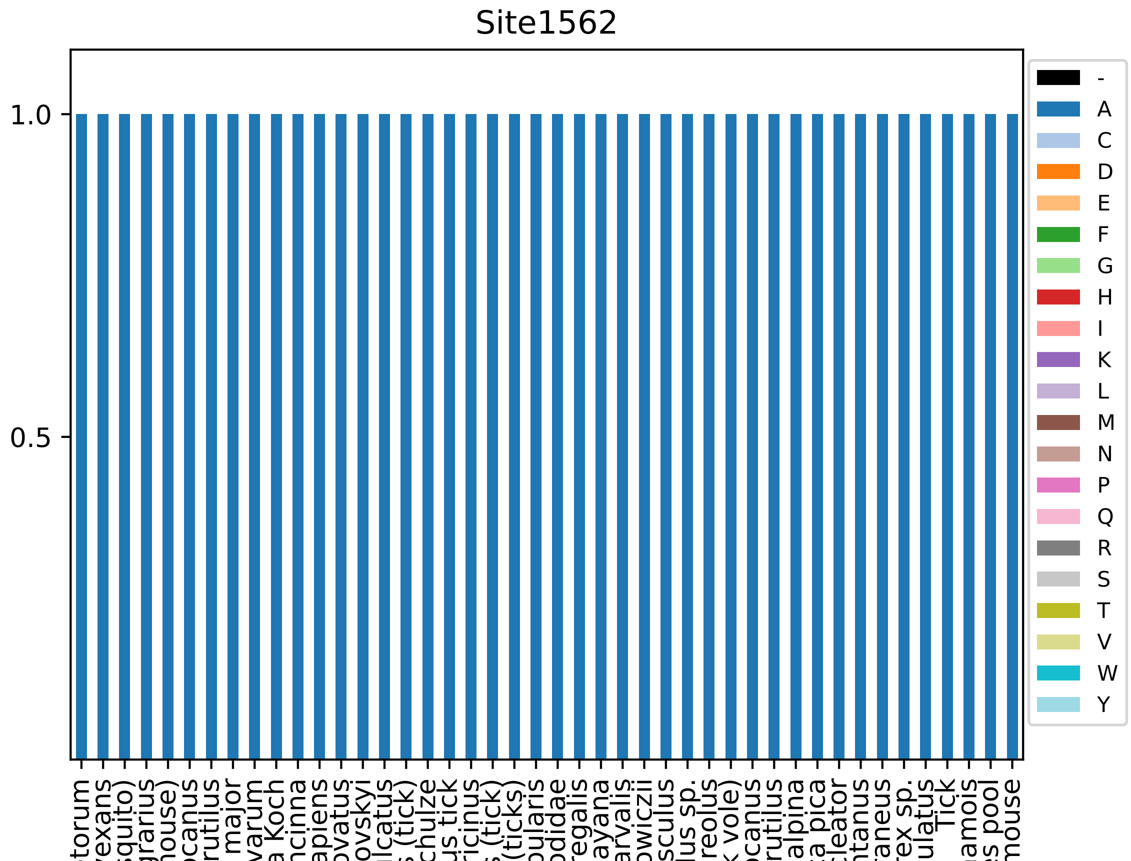
Site1562
1.0
0.5
-
A
C
D
E
F
G
H
I
K
L
M
N
P
Q
R
S
T
V
W
Y

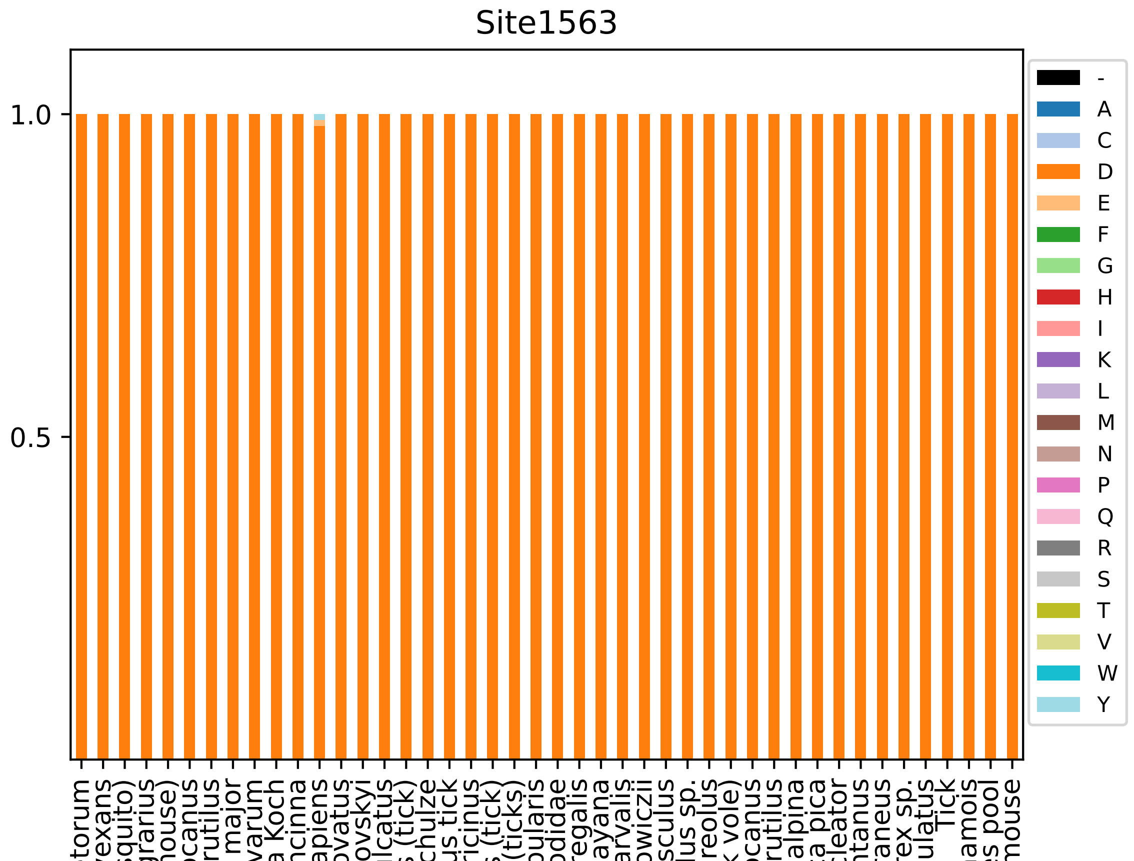

Site1563
1.0
0.5
-
A
C
D
E
F
G
H
I
K
L
M
N
P
Q
R
S
T
V
W
Y

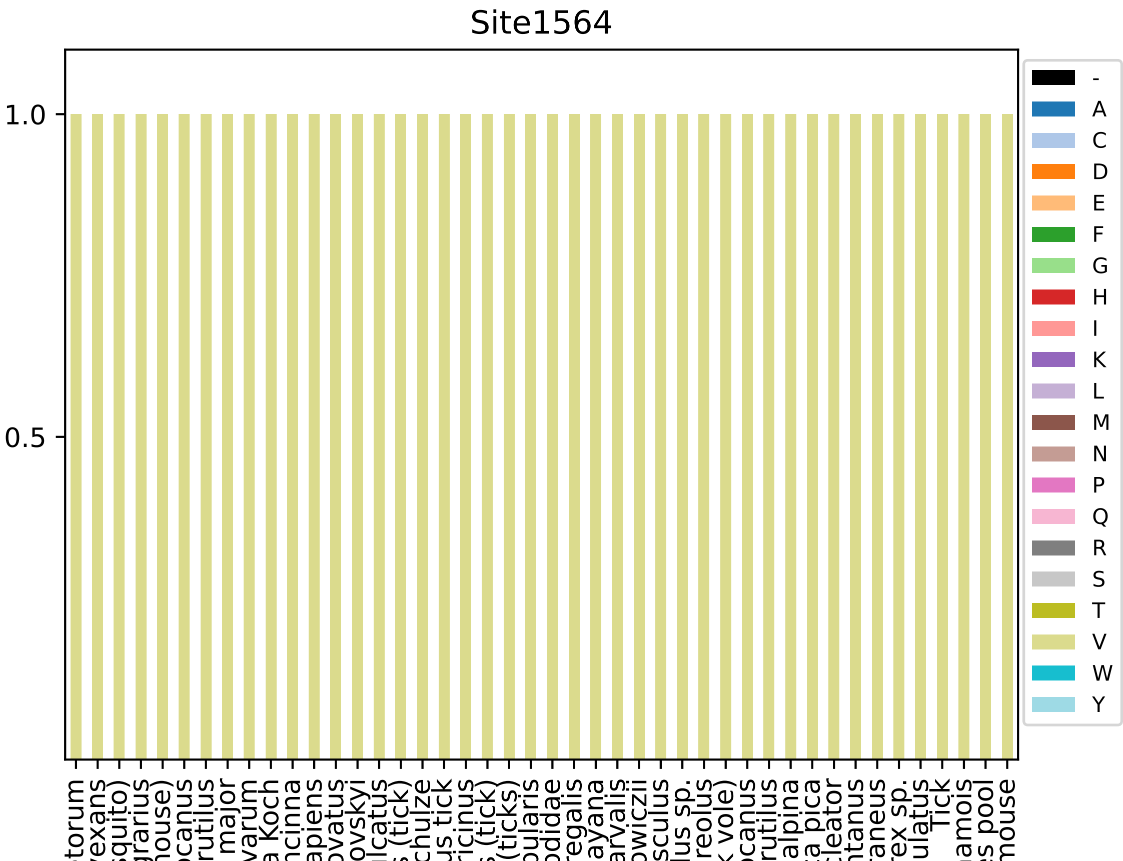

Site1564
1.0
0.5
-
A
C
D
E
F
G
H
I
K
L
M
N
P
Q
R
S
T
V
W
Y

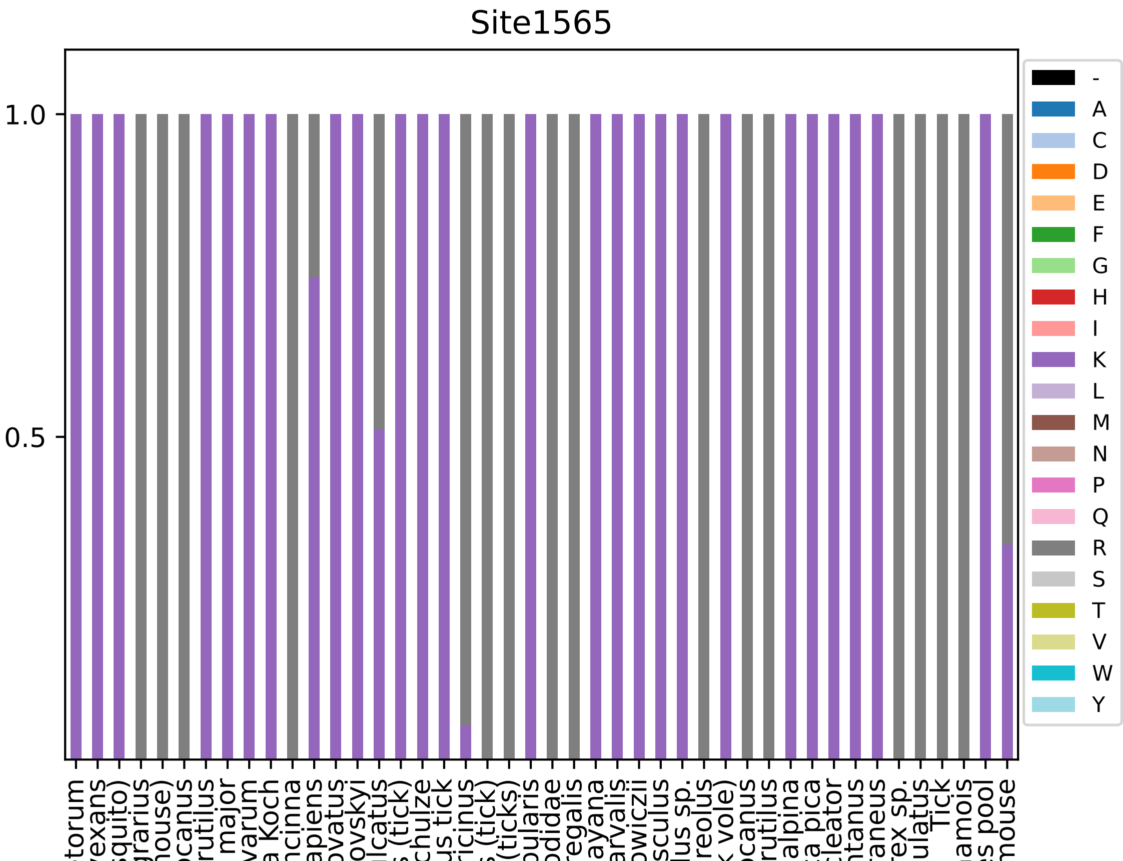

Site1565
1.0
0.5
-
A
C
D
E
F
G
H
I
K
L
M
N
P
Q
R
S
T
V
W
Y
Tick

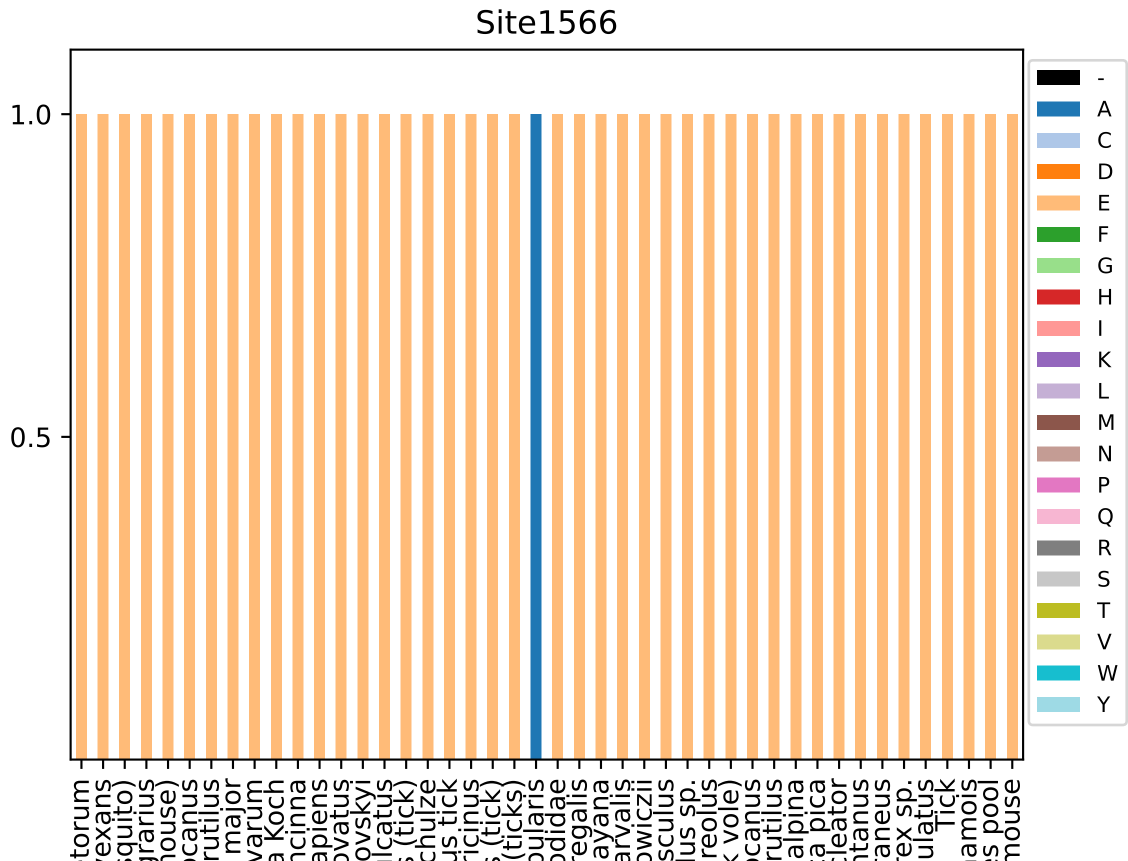
Site1566
1.0
0.5
-
A
C
D
E
F
G
H
I
K
L
M
N
P
Q
R
S
T
V
W
Y
Tick

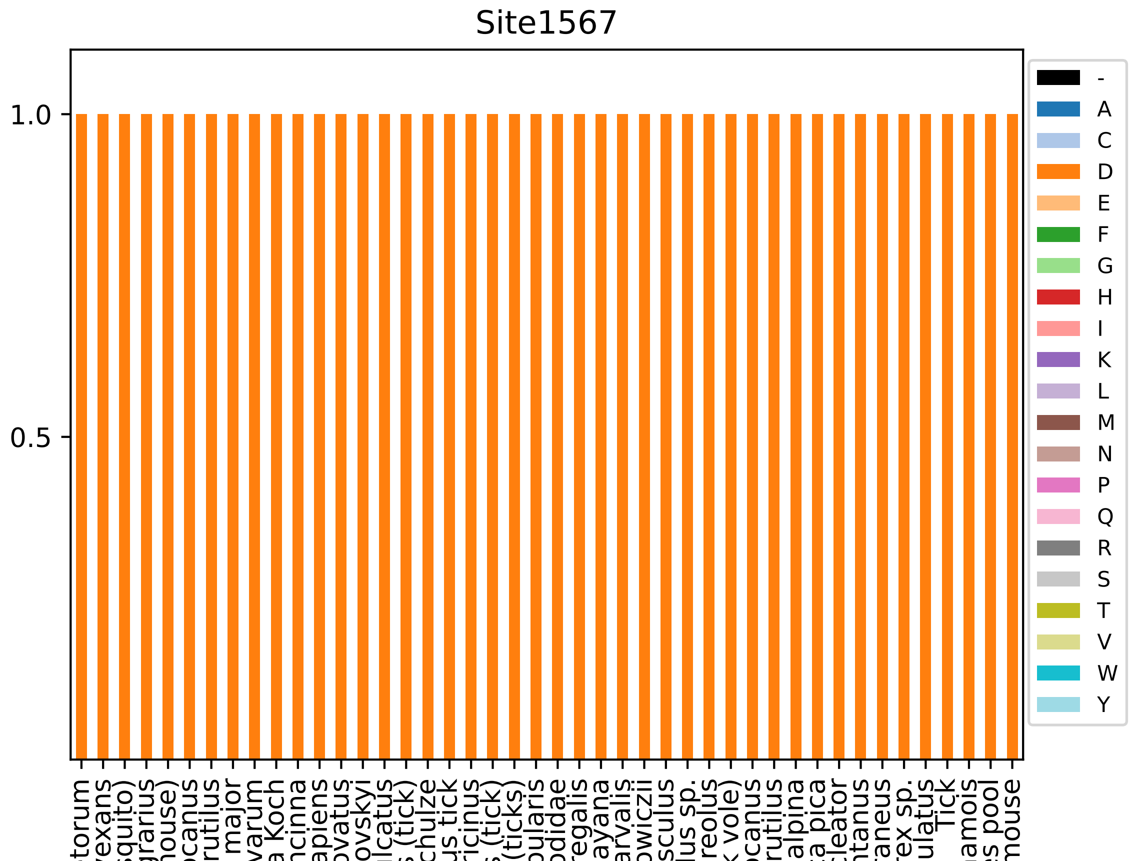
Site1567
1.0
0.5
-
A
C
D
E
F
G
H
I
K
L
M
N
P
Q
R
S
T
V
W
Y

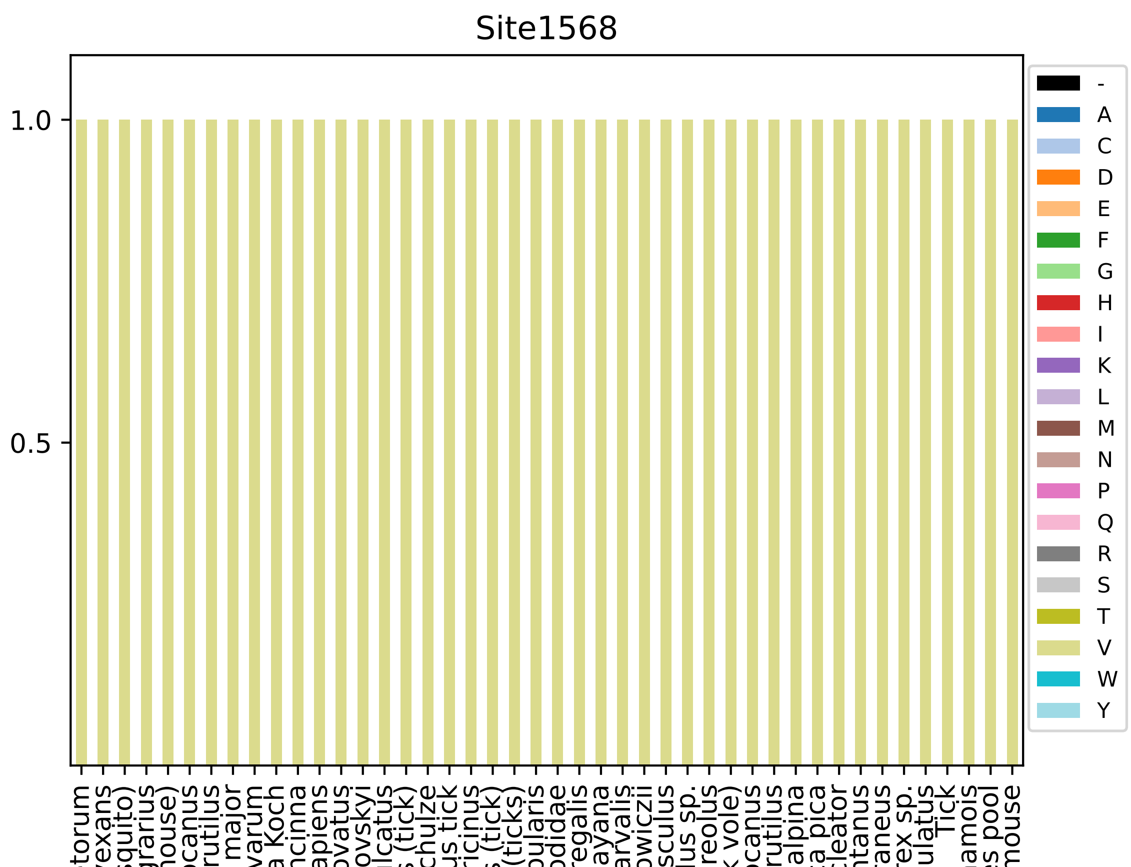
Site1568
1.0
0.5
-
A
C
D
E
F
G
H
I
K
L
M
N
P
Q
R
S
T
V
W
Y

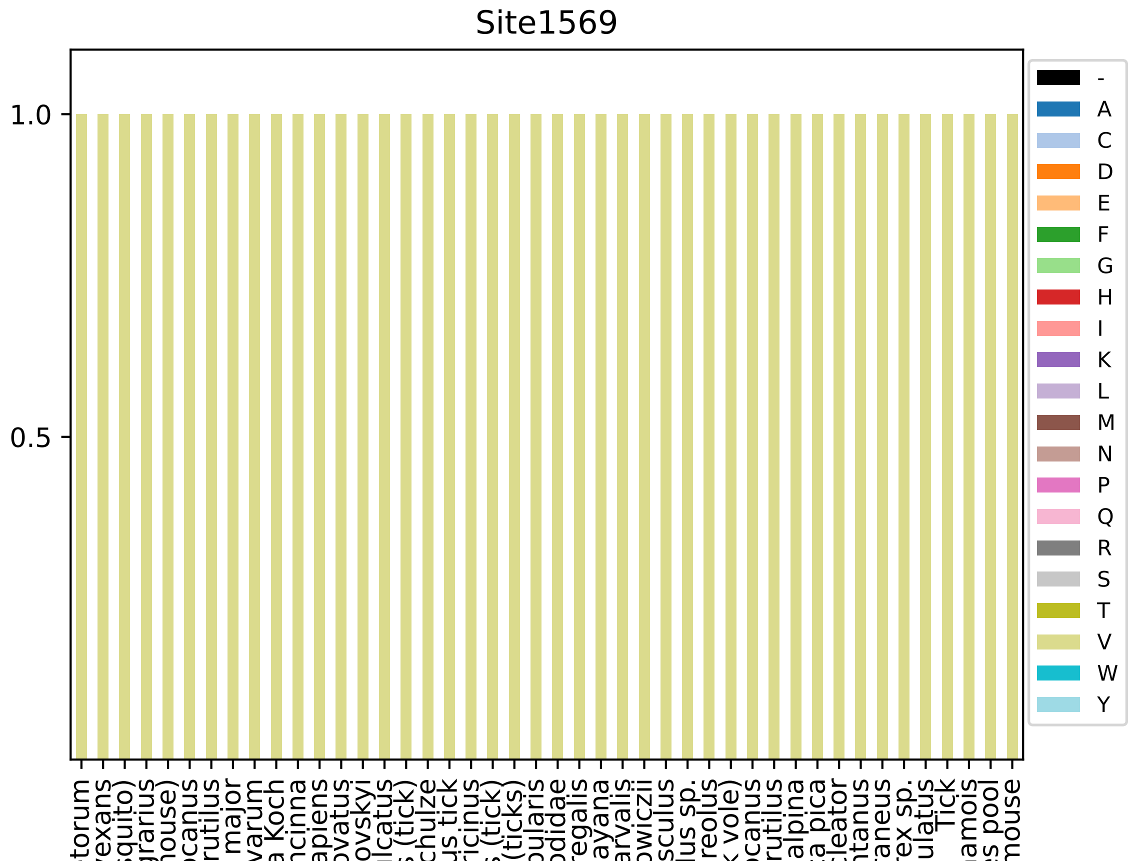
Site1569
1.0
0.5
-
A
C
D
E
F
G
H
I
K
L
M
N
P
Q
R
S
T
V
W
Y

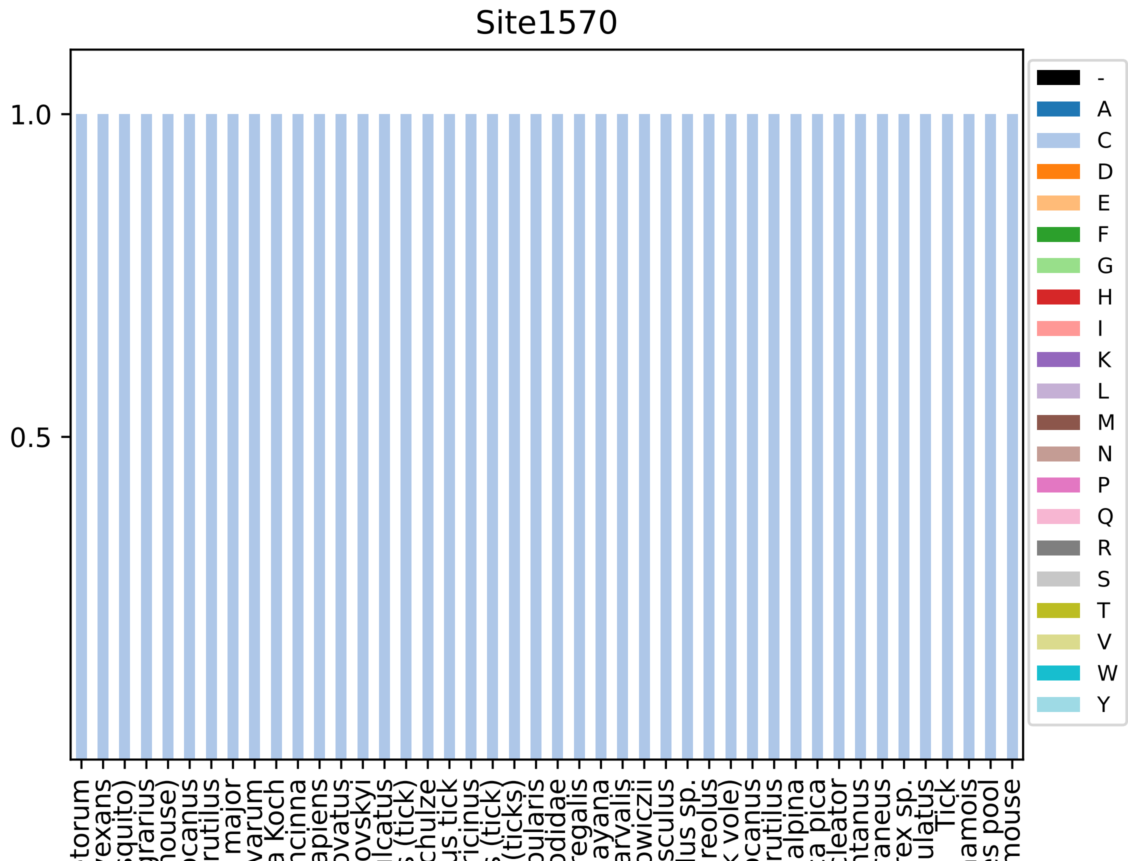

Site1570
1.0
0.5
-
A
C
D
E
F
G
H
I
K
L
M
N
P
Q
R
S
T
V
W
Y

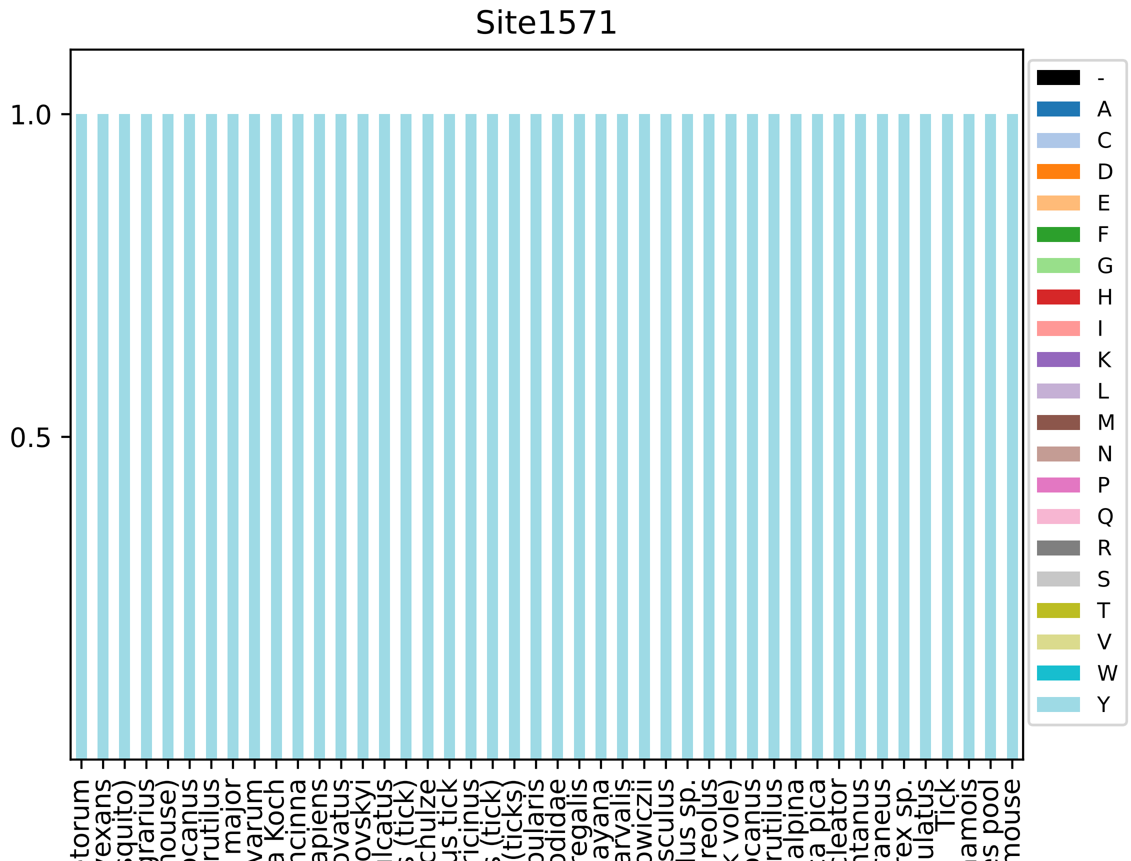

Site1571
1.0
0.5
-
A
C
D
E
F
G
H
I
K
L
M
N
P
Q
R
S
T
V
W
Y

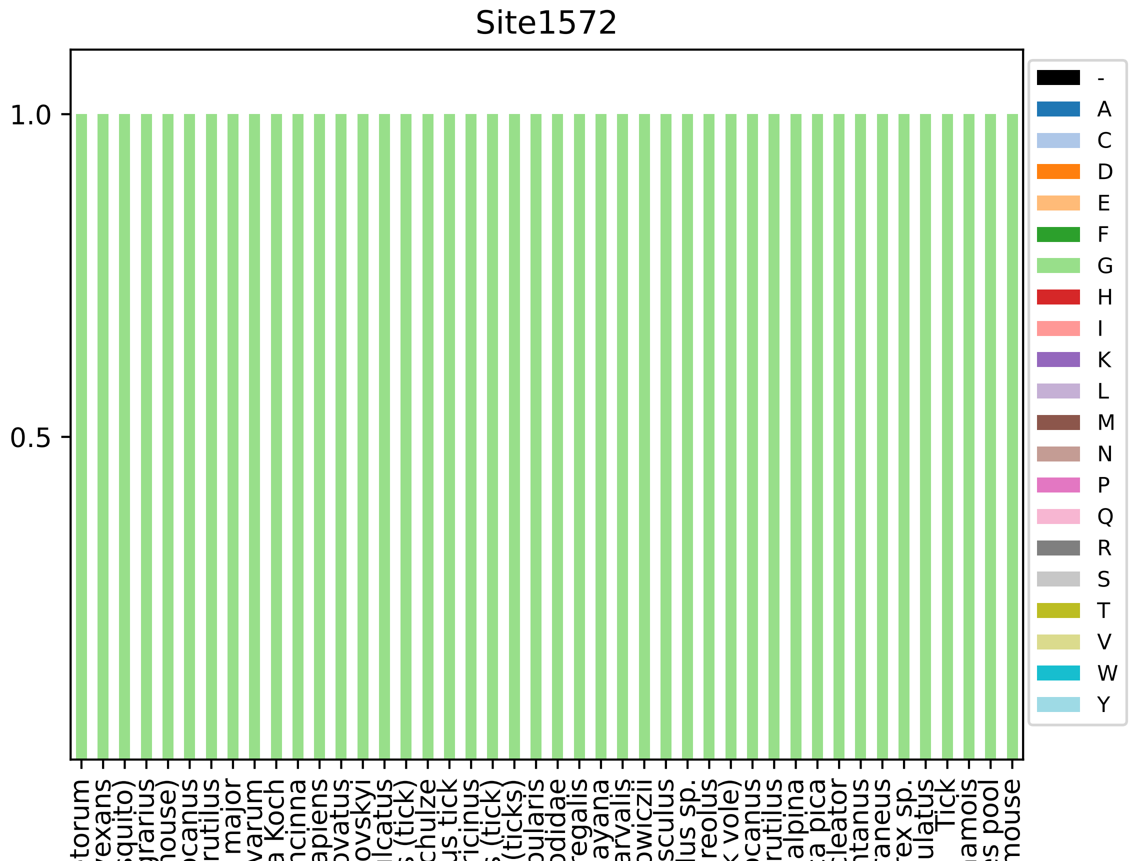
Site1572
1.0
0.5
-
A
C
D
E
F
G
H
I
K
L
M
N
P
Q
R
S
T
V
W
Y

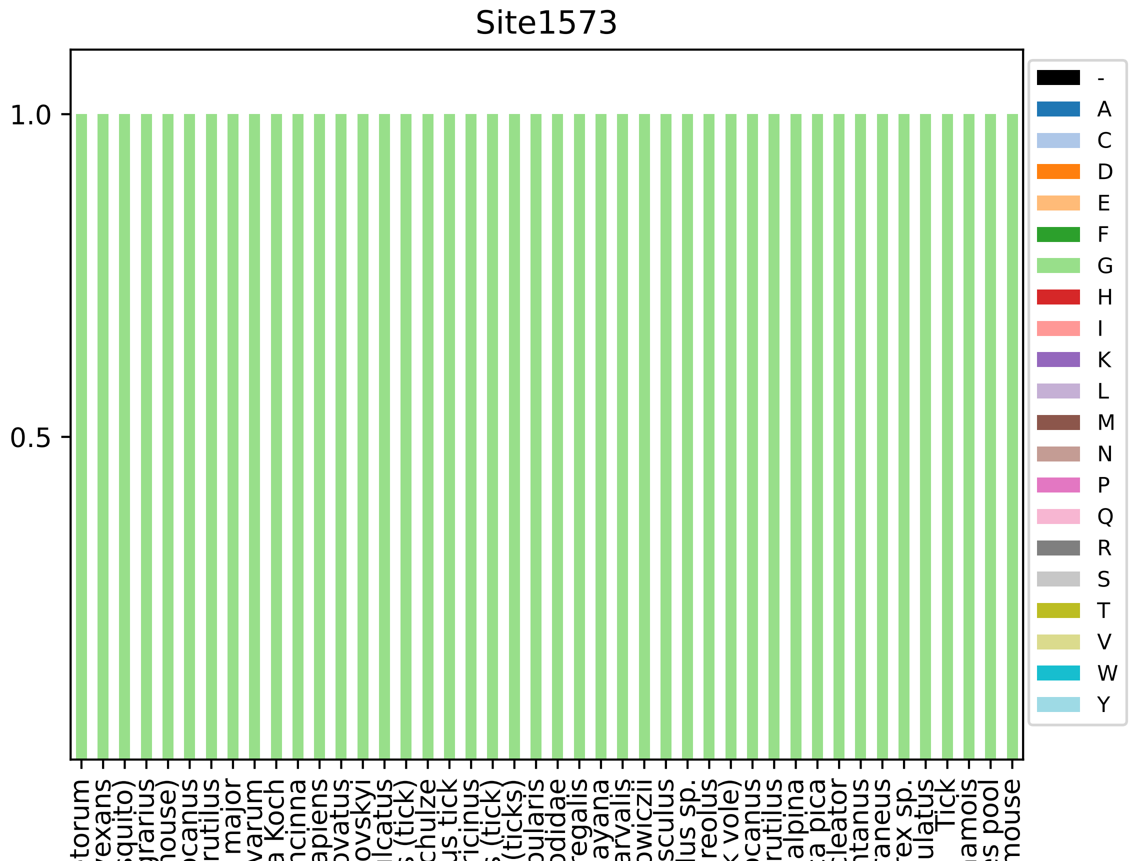
Site1573
1.0
0.5
-
A
C
D
E
F
G
H
I
K
L
M
N
P
Q
R
S
T
V
W
Y

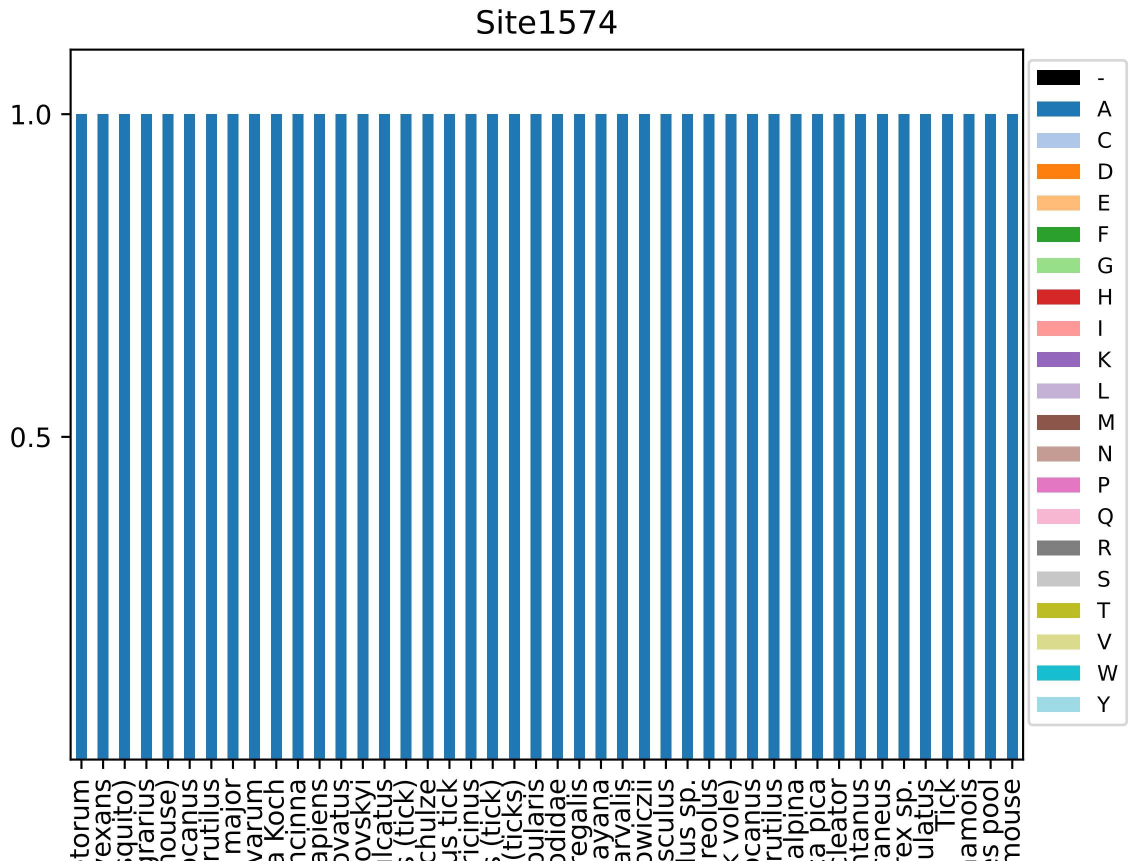
Site1574
1.0
0.5
-
A
C
D
E
F
G
H
I
K
L
M
N
P
Q
R
S
T
V
W
Y

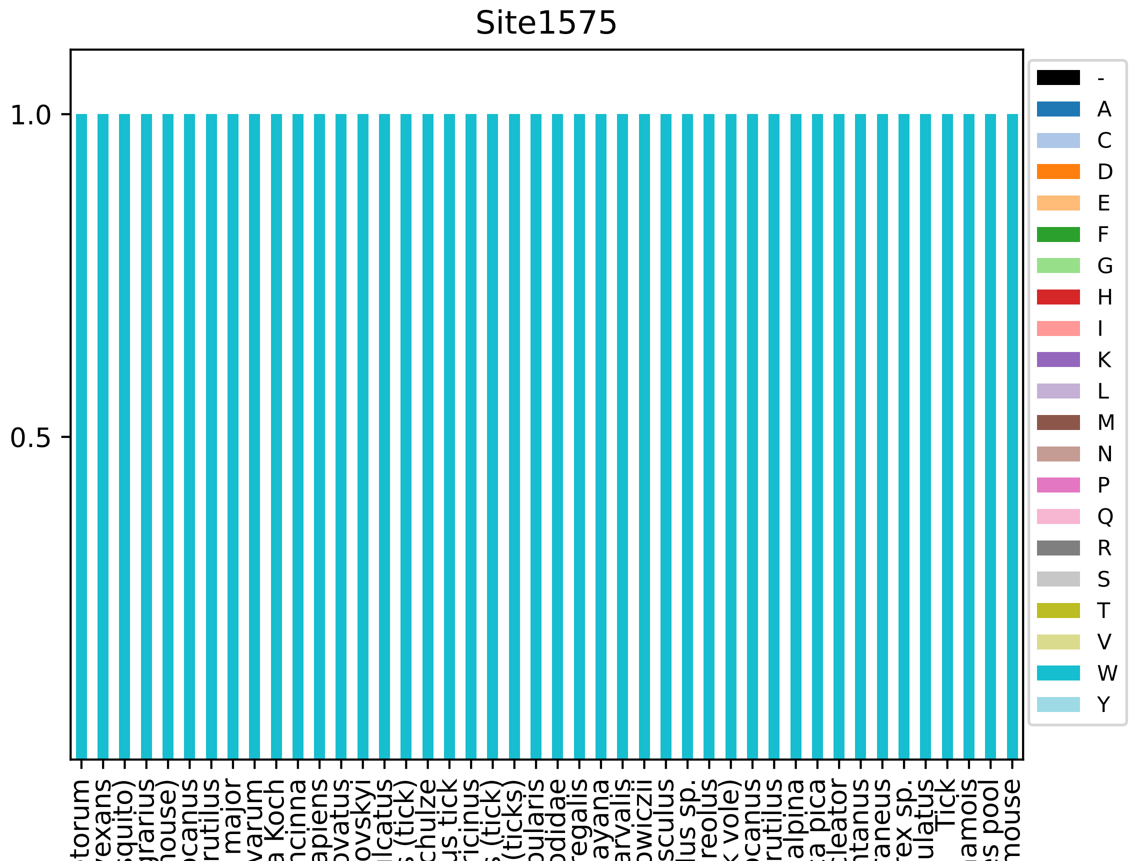
Site1575
1.0
0.5
-
A
C
D
E
F
G
H
I
K
L
M
N
P
Q
R
S
T
V
W
Y

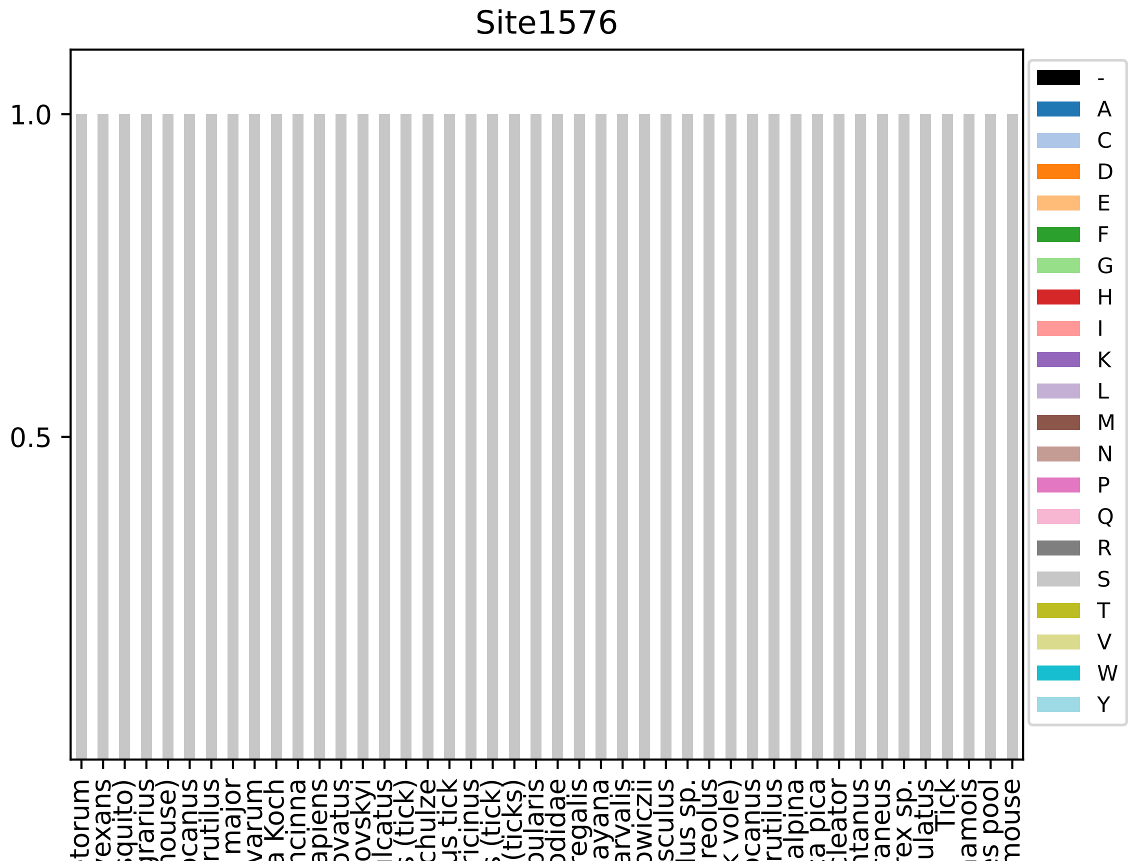
Site1576
1.0
0.5
-
A
C
D
E
F
G
H
I
K
L
M
N
P
Q
R
S
T
V
W
Y

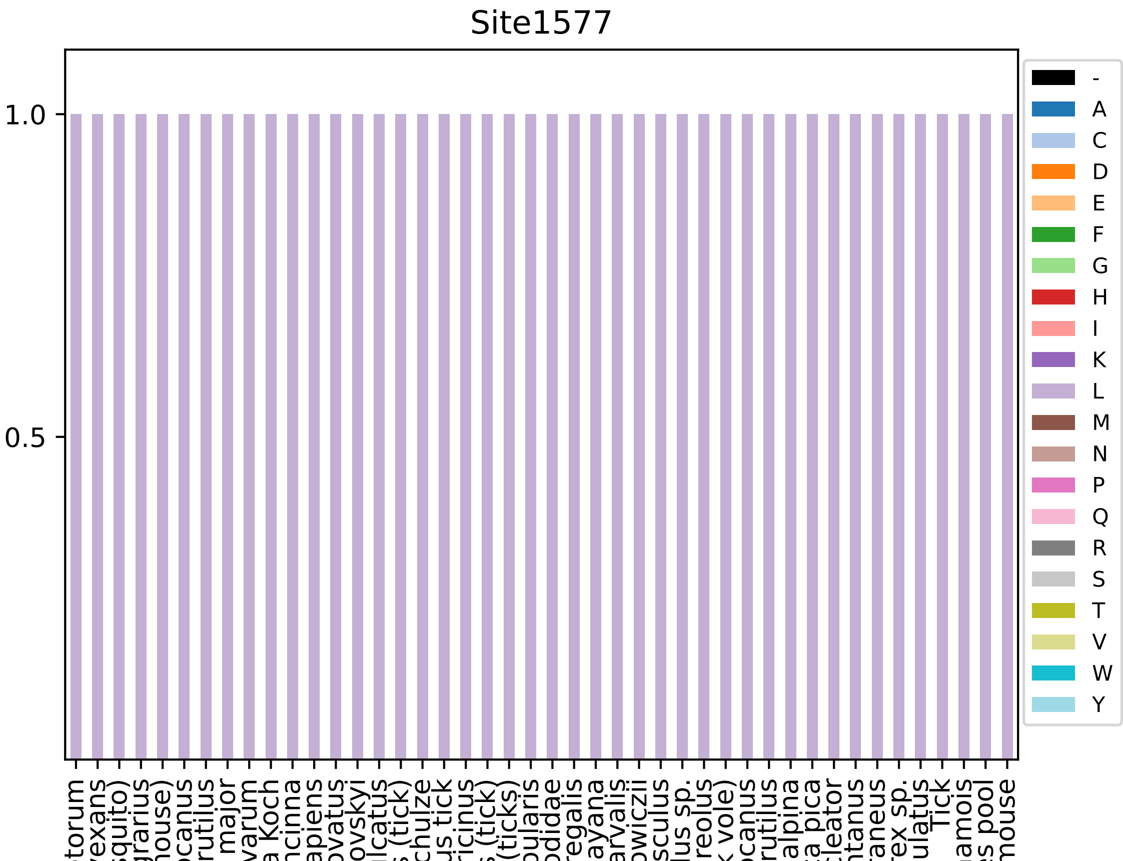

Site1577
1.0
0.5
-
A
C
D
E
F
G
H
I
K
L
M
N
P
Q
R
S
T
V
W
Y

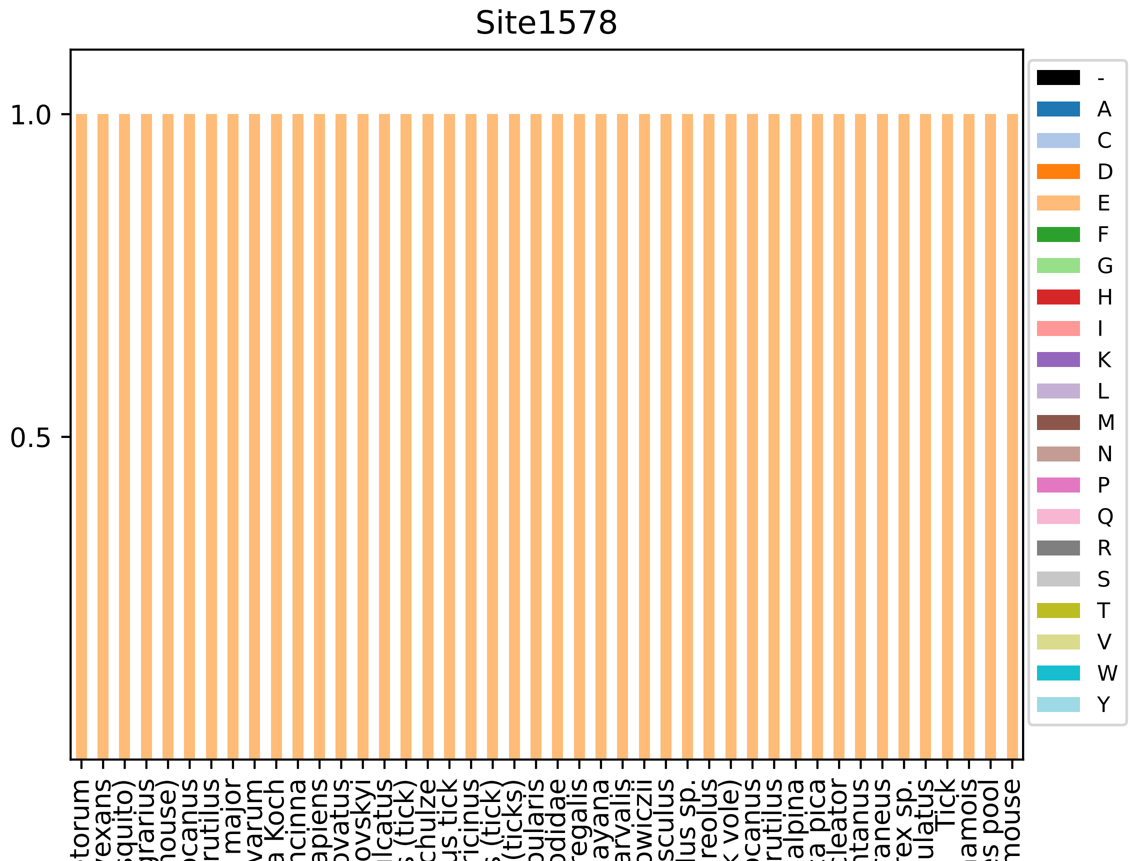
Site1578
1.0
0.5
-
A
C
D
E
F
G
H
I
K
L
M
N
P
Q
R
S
T
V
W
Y

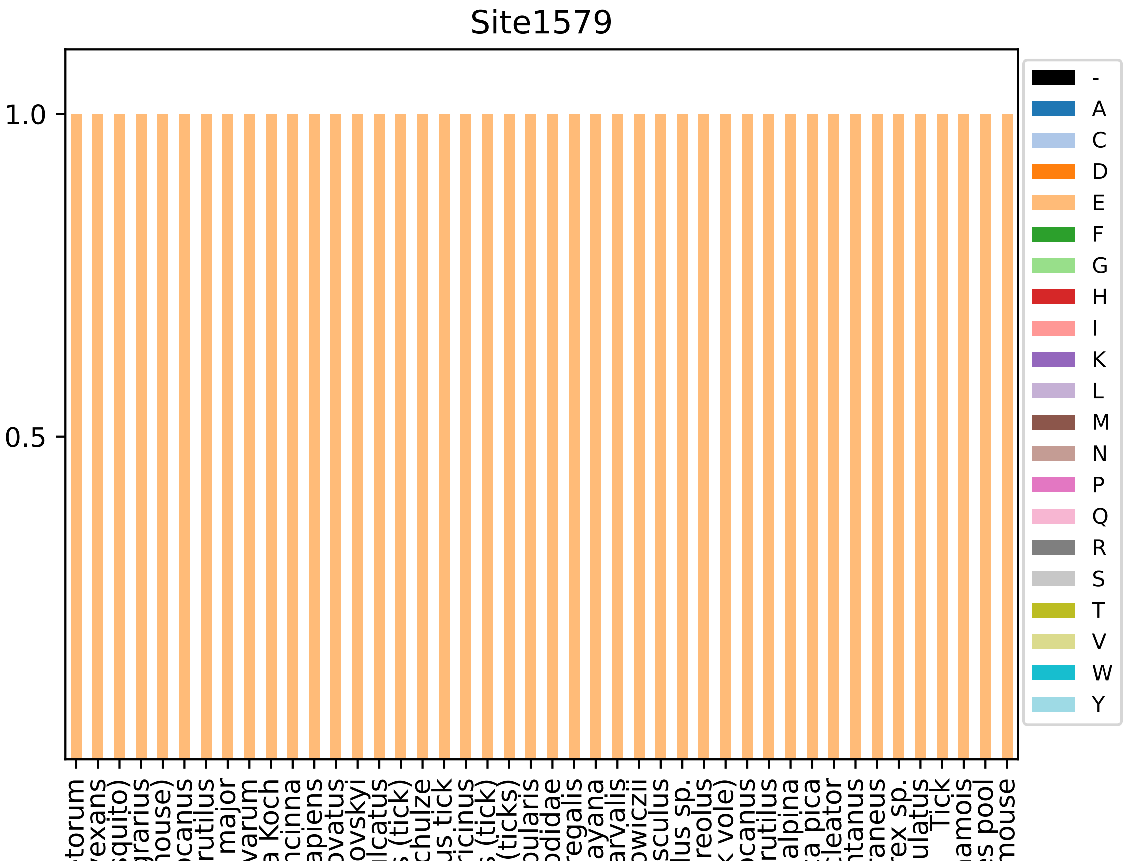
Site1579
1.0
0.5
-
A
C
D
E
F
G
H
I
K
L
M
N
P
Q
R
S
T
V
W
Y

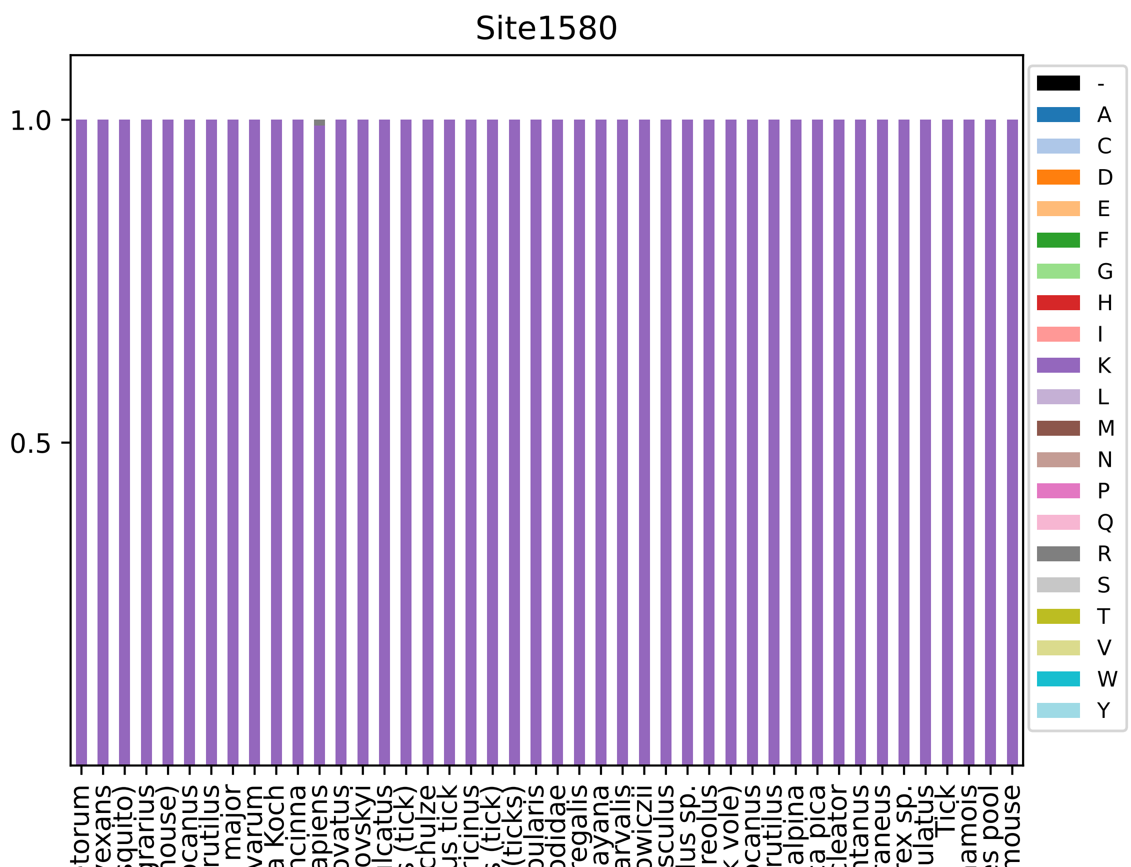

Site1580
1.0
0.5
-
A
C
D
E
F
G
H
I
K
L
M
N
P
Q
R
S
T
V
W
Y

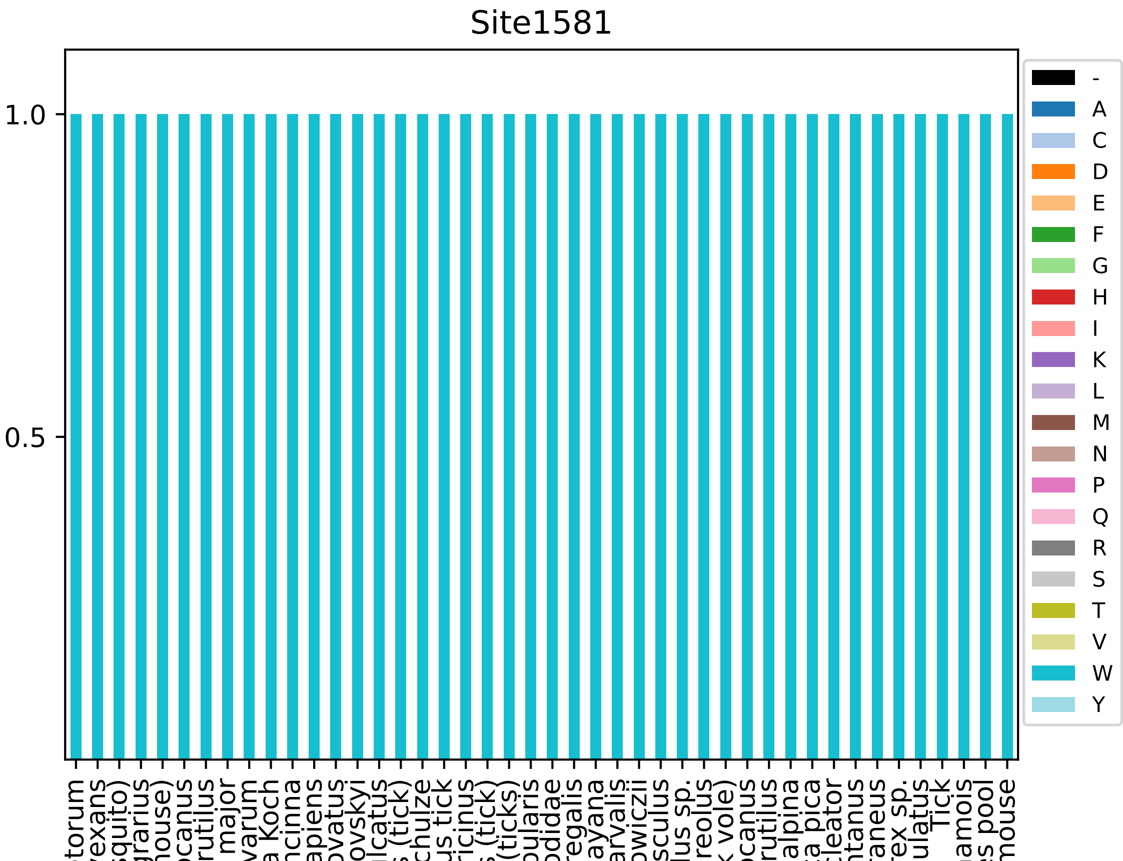
Site1581
1.0
0.5
-
A
C
D
E
F
G
H
I
K
L
M
N
P
Q
R
S
T
V
W
Y

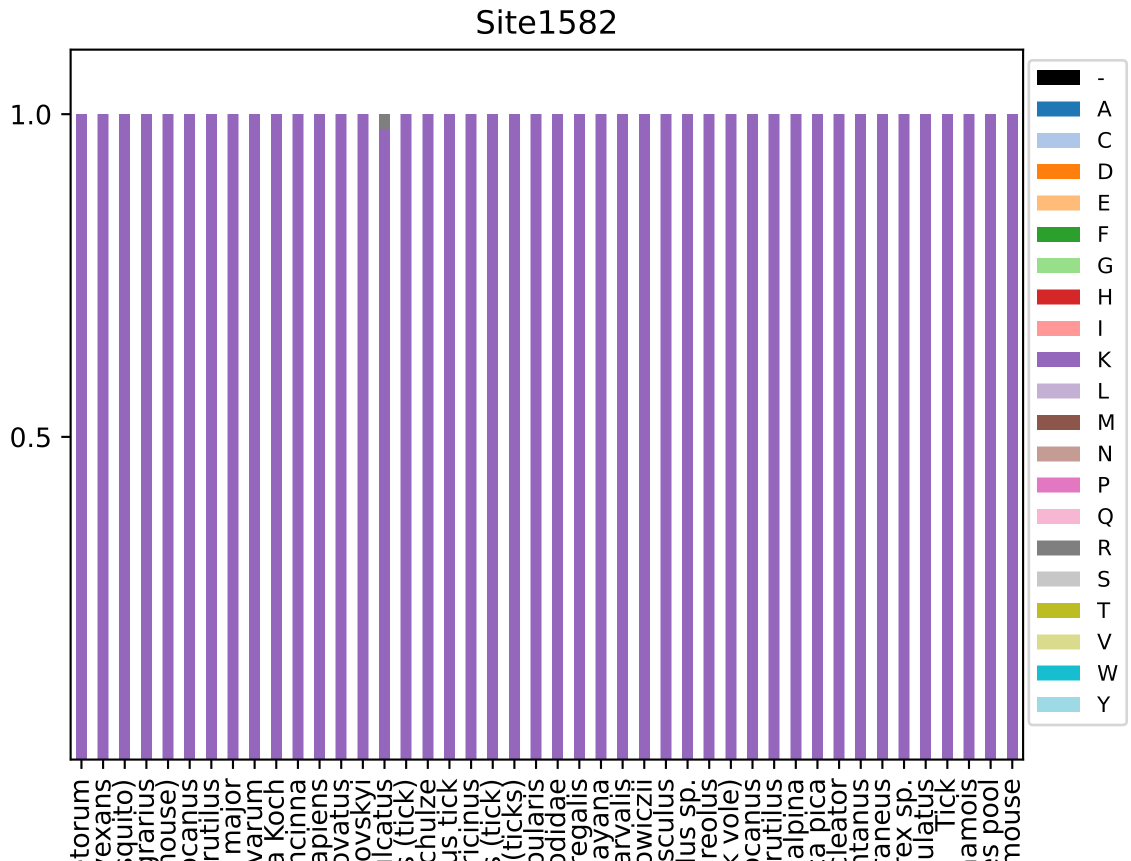
Site1582
1.0
0.5
-
A
C
D
E
F
G
H
I
K
L
M
N
P
Q
R
S
T
V
W
Y

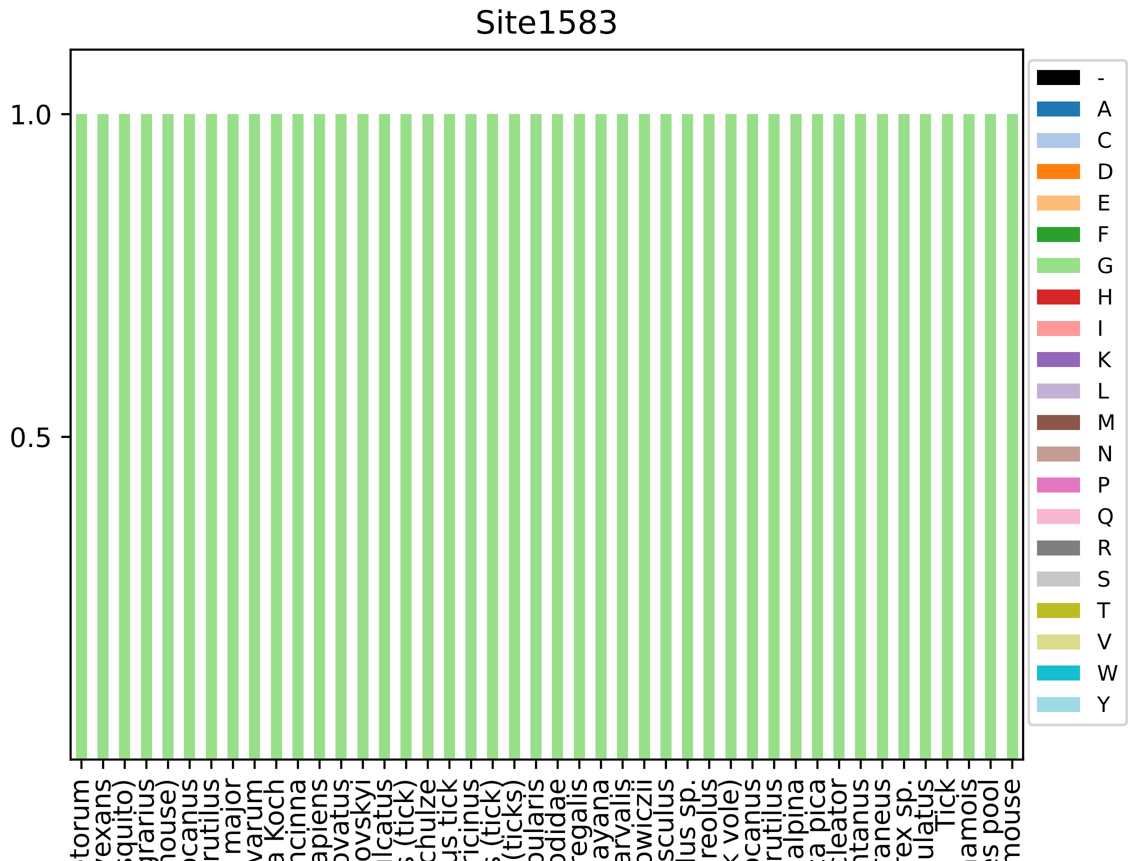
Site1583
1.0
0.5
-
A
C
D
E
F
G
H
I
K
L
M
N
P
Q
R
S
T
V
W
Y
Tick

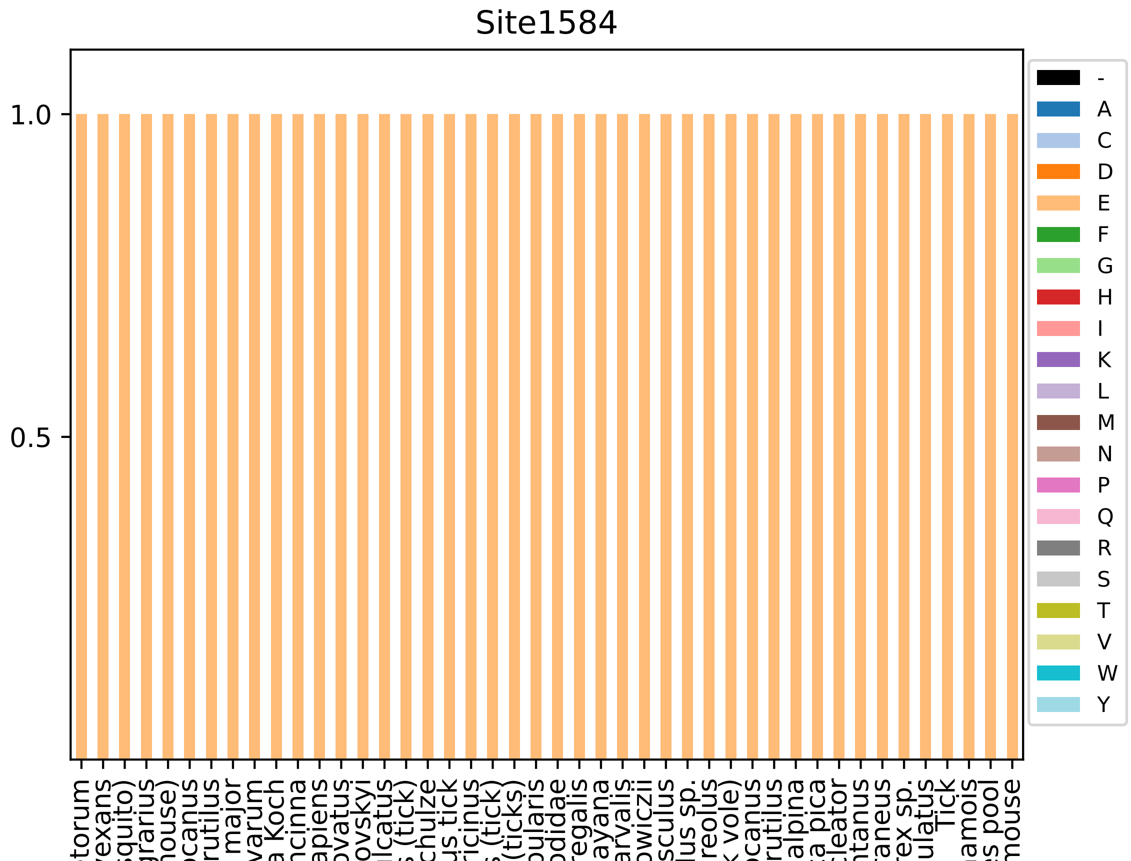
Site1584
1.0
0.5
-
A
C
D
E
F
G
H
I
K
L
M
N
P
Q
R
S
T
V
W
Y

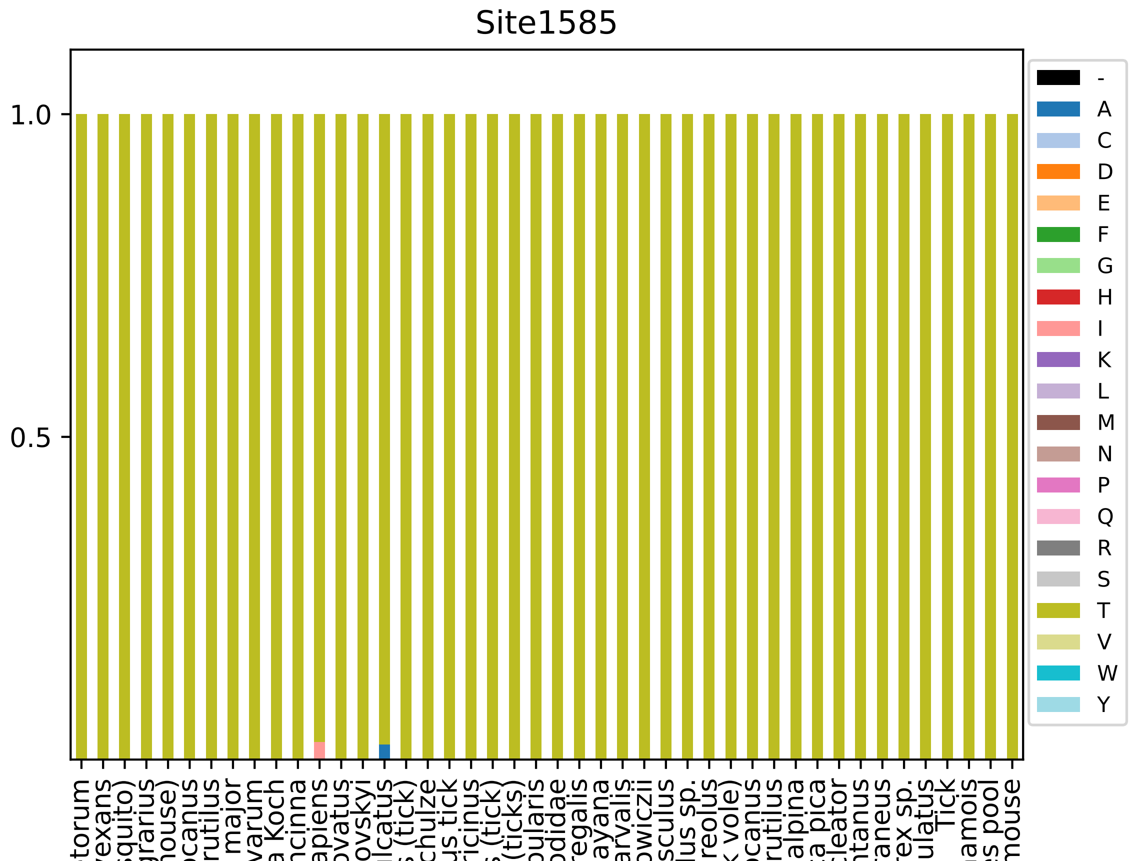
Site1585
1.0
0.5
-
A
C
D
E
F
G
H
I
K
L
M
N
P
Q
R
S
T
V
W
Y

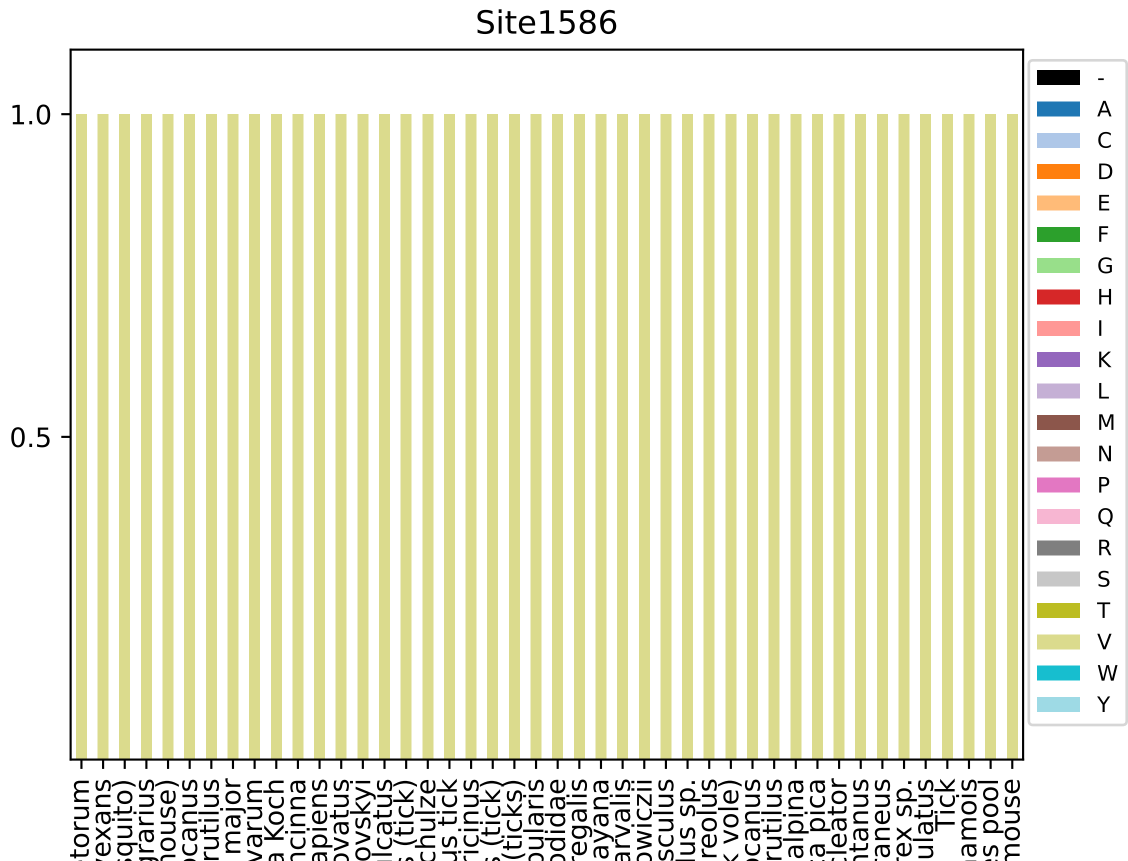

Site1586
1.0
0.5
-
A
C
D
E
F
G
H
I
K
L
M
N
P
Q
R
S
T
V
W
Y

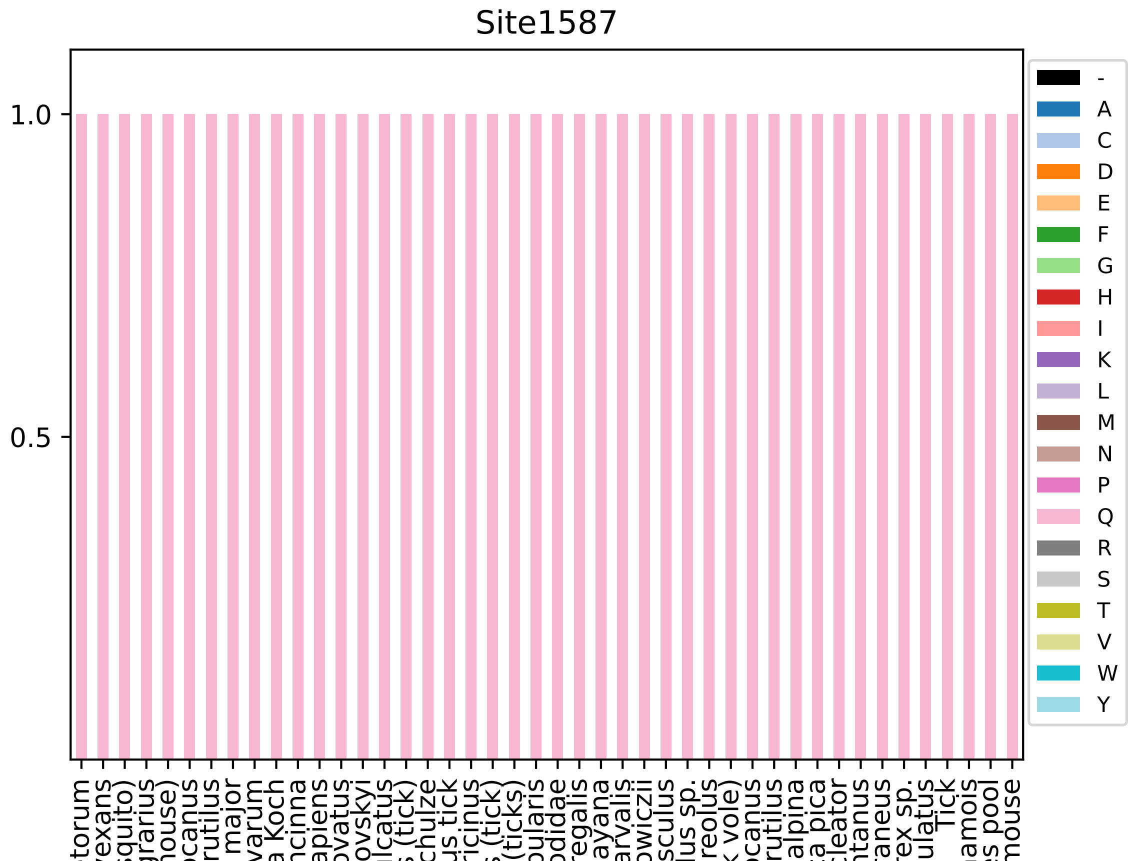

Site1587
1.0
0.5
-
A
C
D
E
F
G
H
I
K
L
M
N
P
Q
R
S
T
V
W
Y
Tick

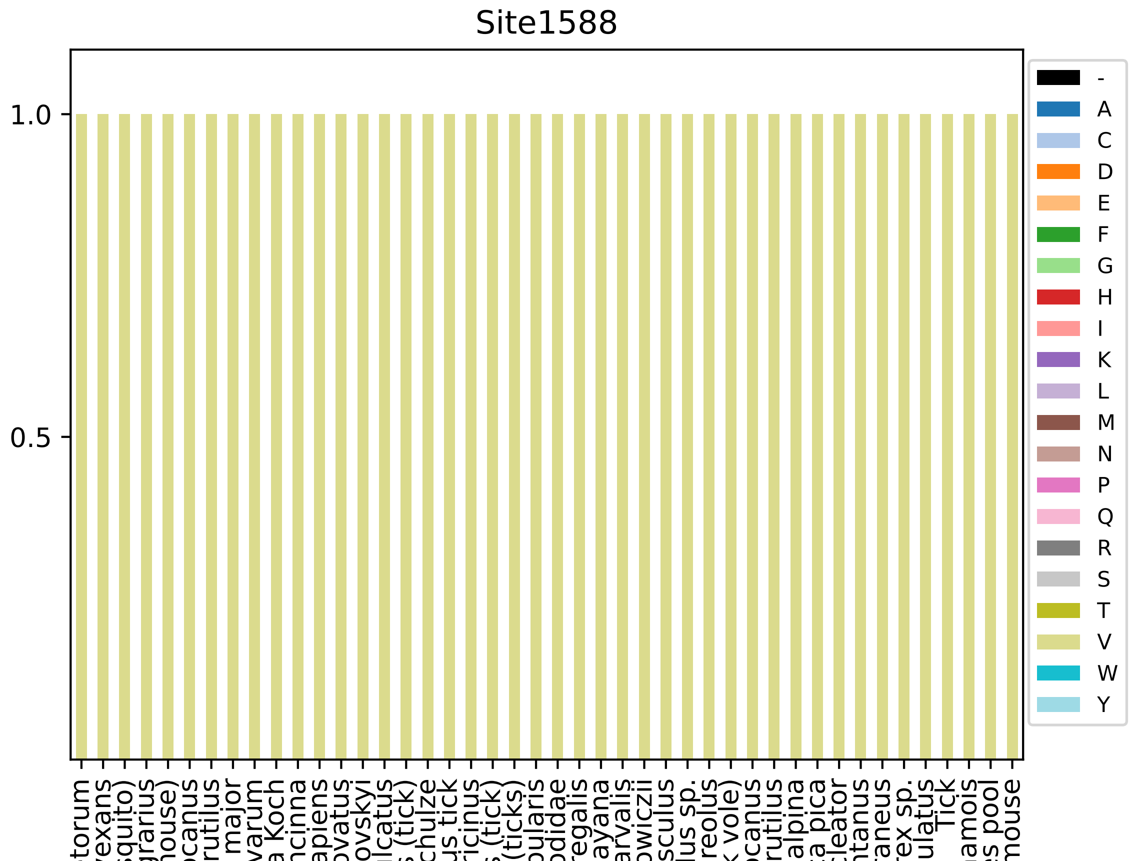
Site1588
1.0
0.5
-
A
C
D
E
F
G
H
I
K
L
M
N
P
Q
R
S
T
V
W
Y

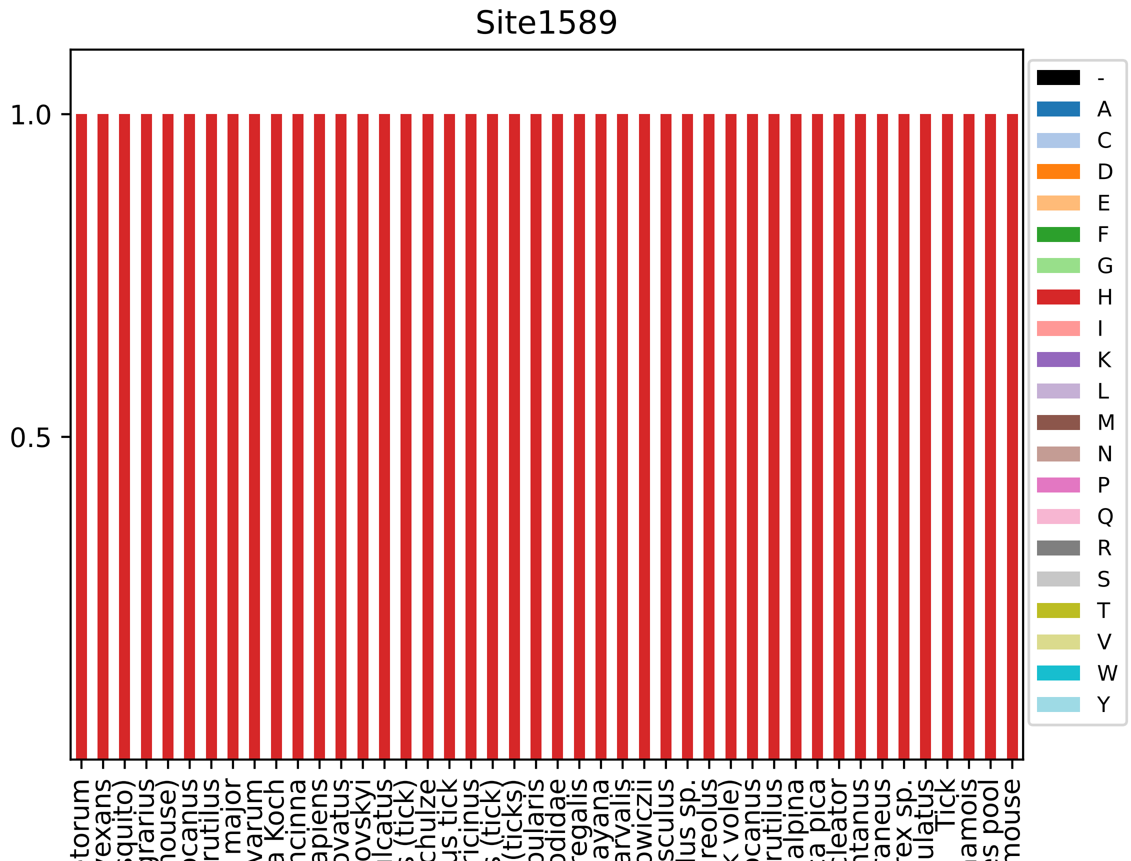
Site1589
1.0
0.5
-
A
C
D
E
F
G
H
I
K
L
M
N
P
Q
R
S
T
V
W
Y

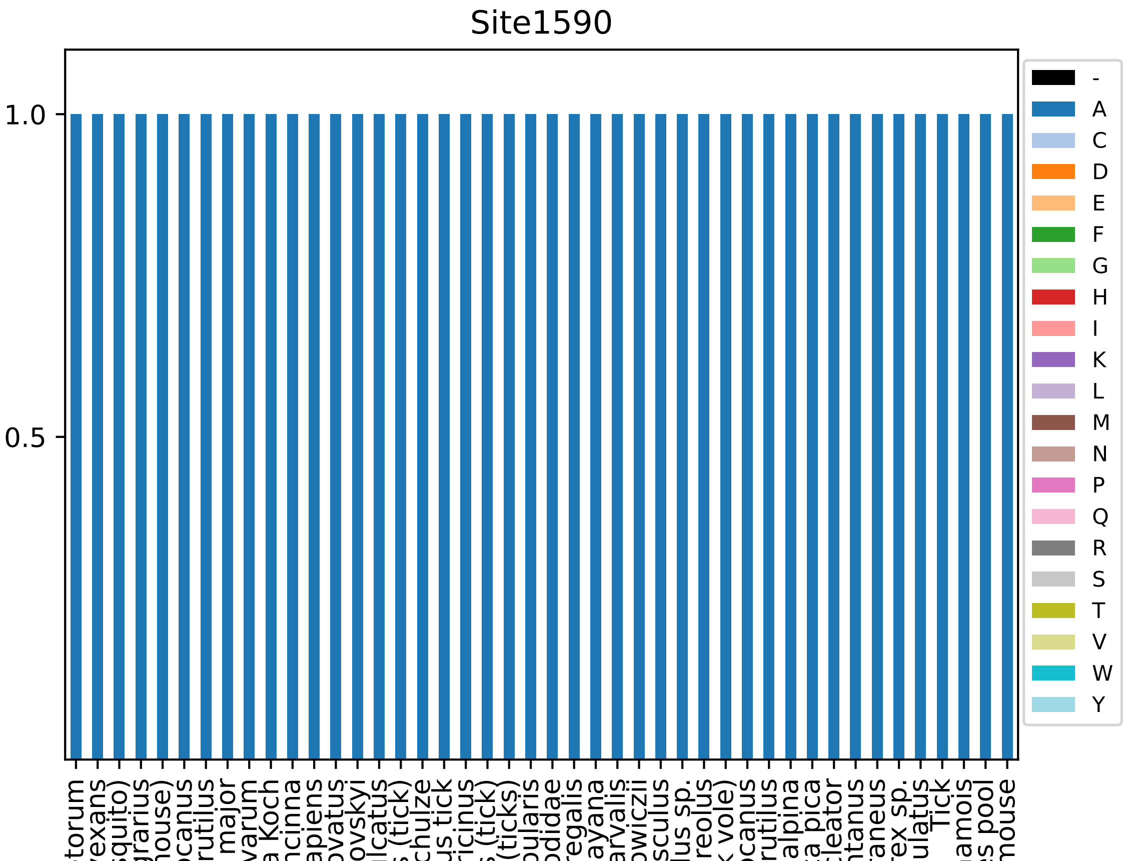

Site1590
1.0
0.5
-
A
C
D
E
F
G
H
I
K
L
M
N
P
Q
R
S
T
V
W
Y

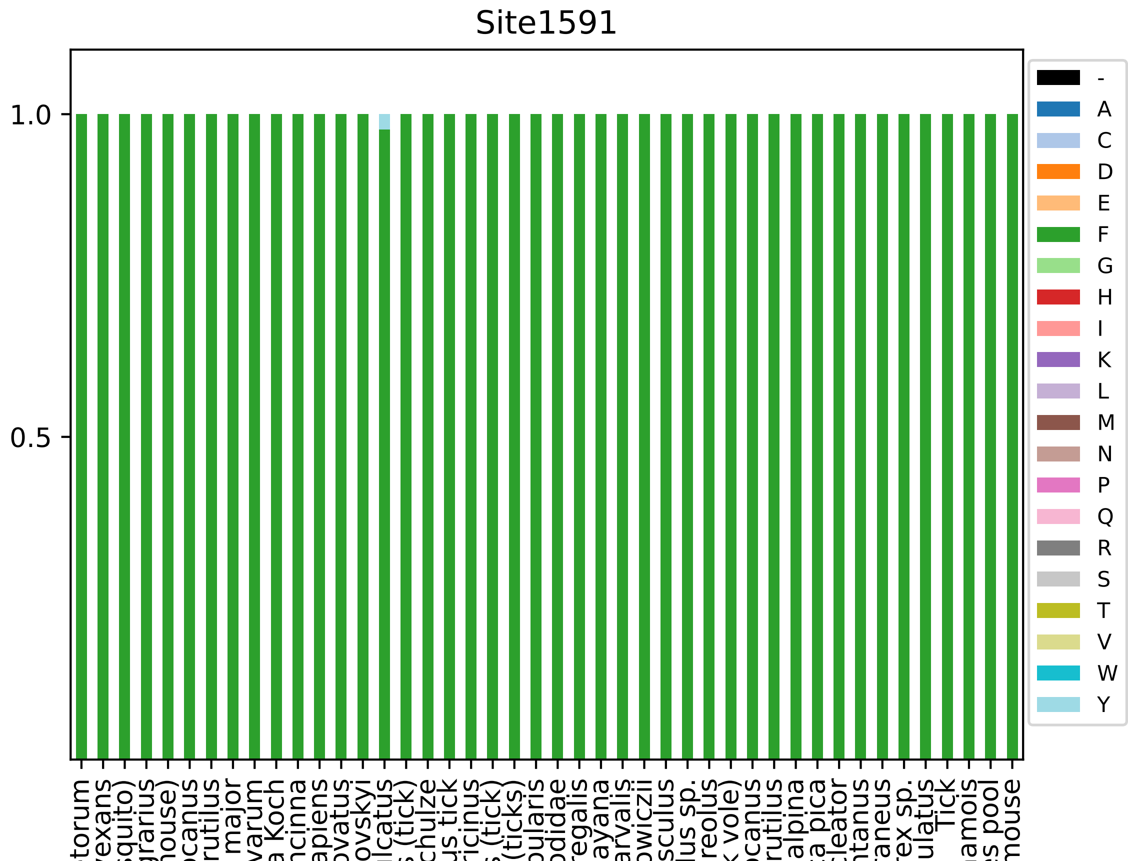
Site1591
1.0
0.5
-
A
C
D
E
F
G
H
I
K
L
M
N
P
Q
R
S
T
V
W
Y

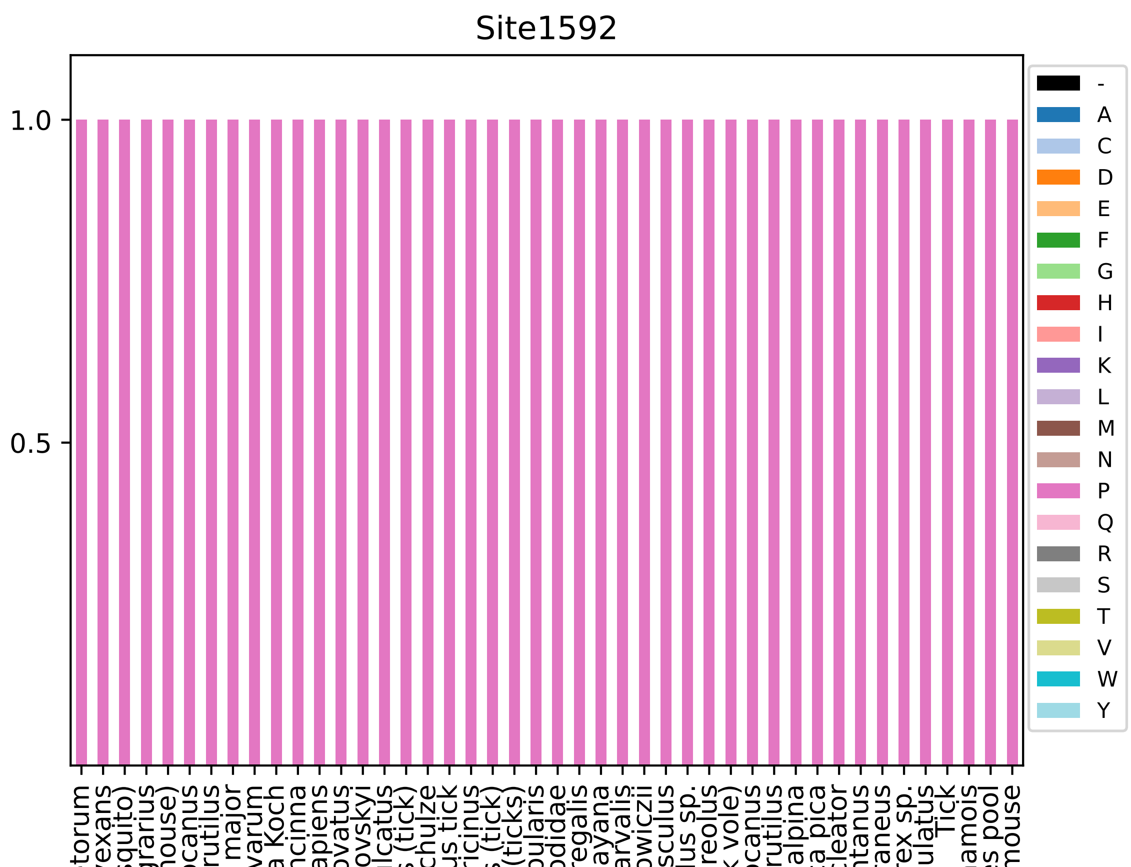
Site1592
1.0
0.5
-
A
C
D
E
F
G
H
I
K
L
M
N
P
Q
R
S
T
V
W
Y
Tick

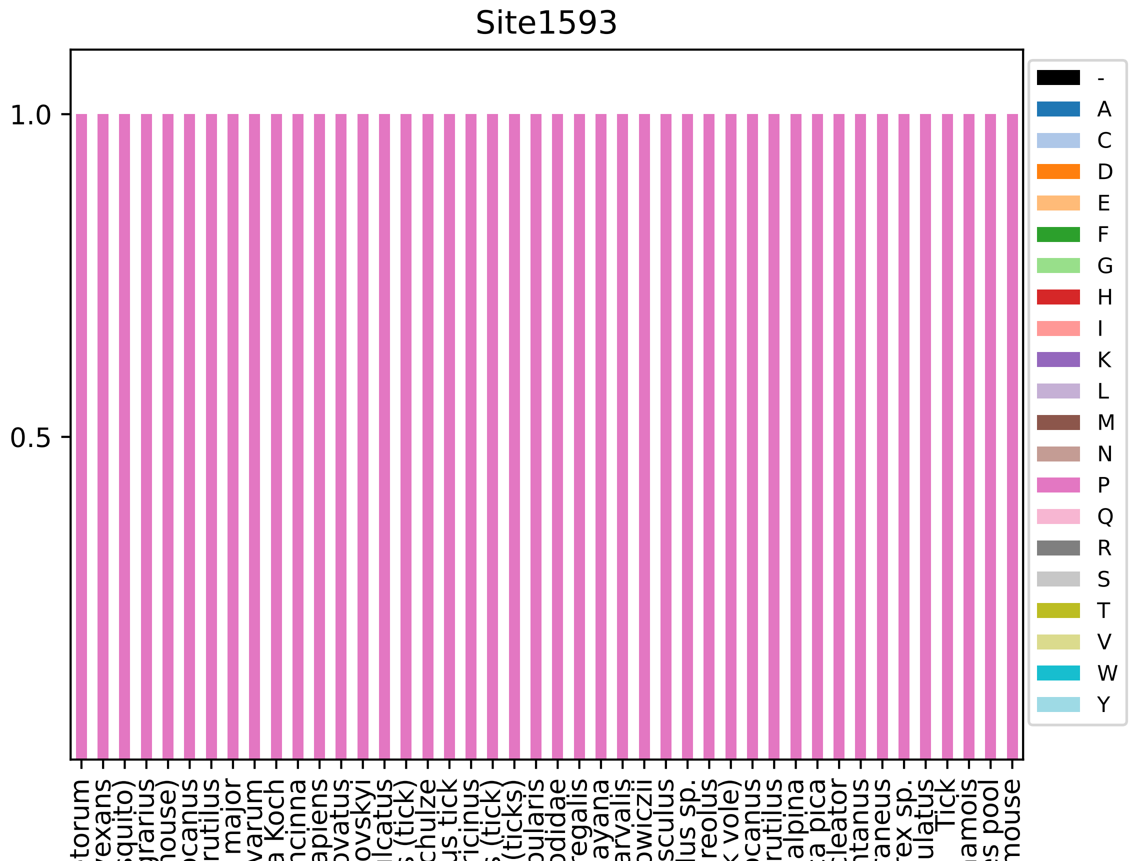

Site1593
1.0
0.5
-
A
C
D
E
F
G
H
I
K
L
M
N
P
Q
R
S
T
V
W
Y

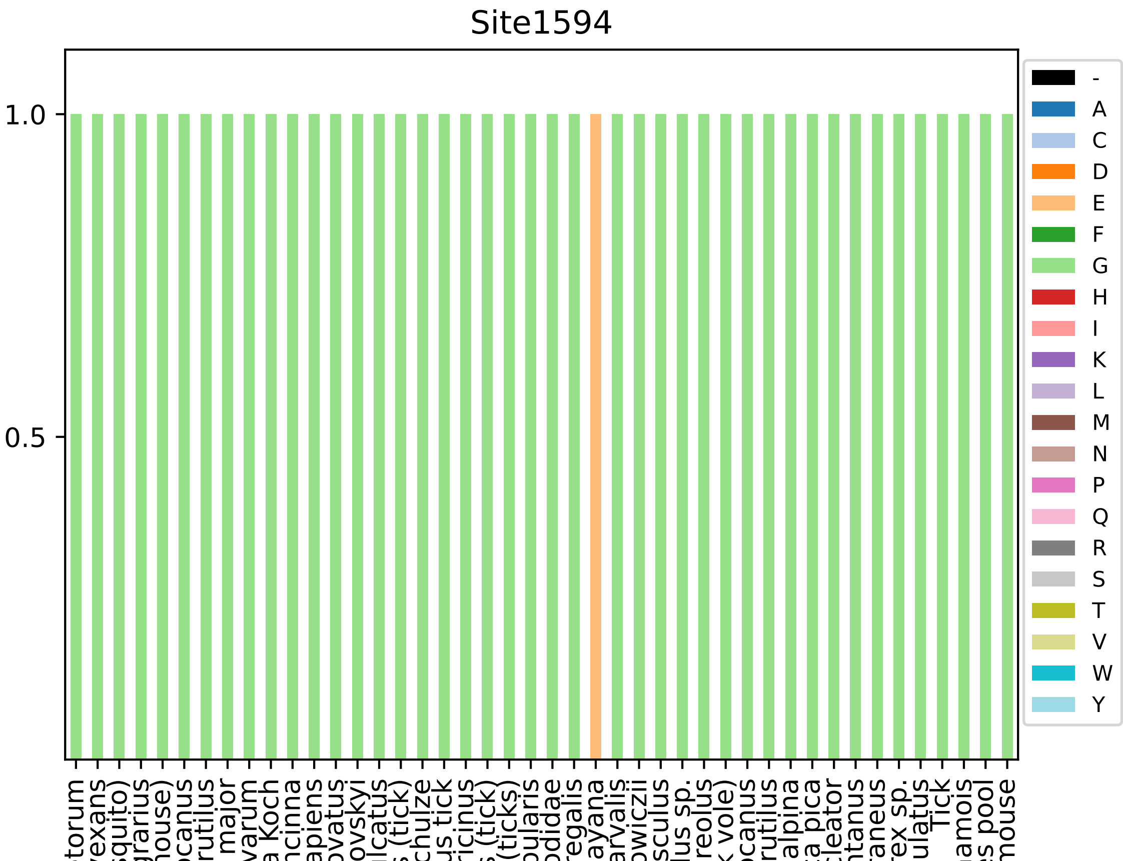
Site1594
1.0
0.5
-
A
C
D
E
F
G
H
I
K
L
M
N
P
Q
R
S
T
V
W
Y

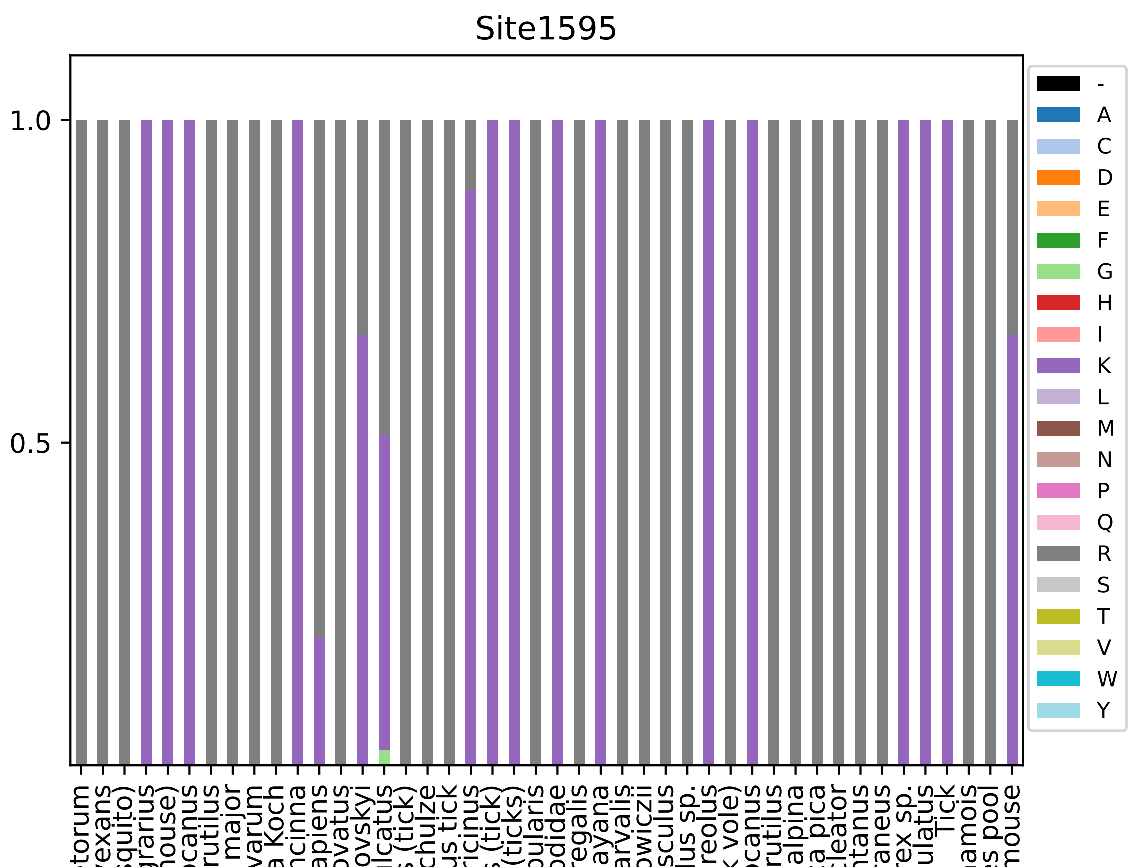

Site1595
1.0
0.5
-
A
C
D
E
F
G
H
I
K
L
M
N
P
Q
R
S
T
V
W
Y

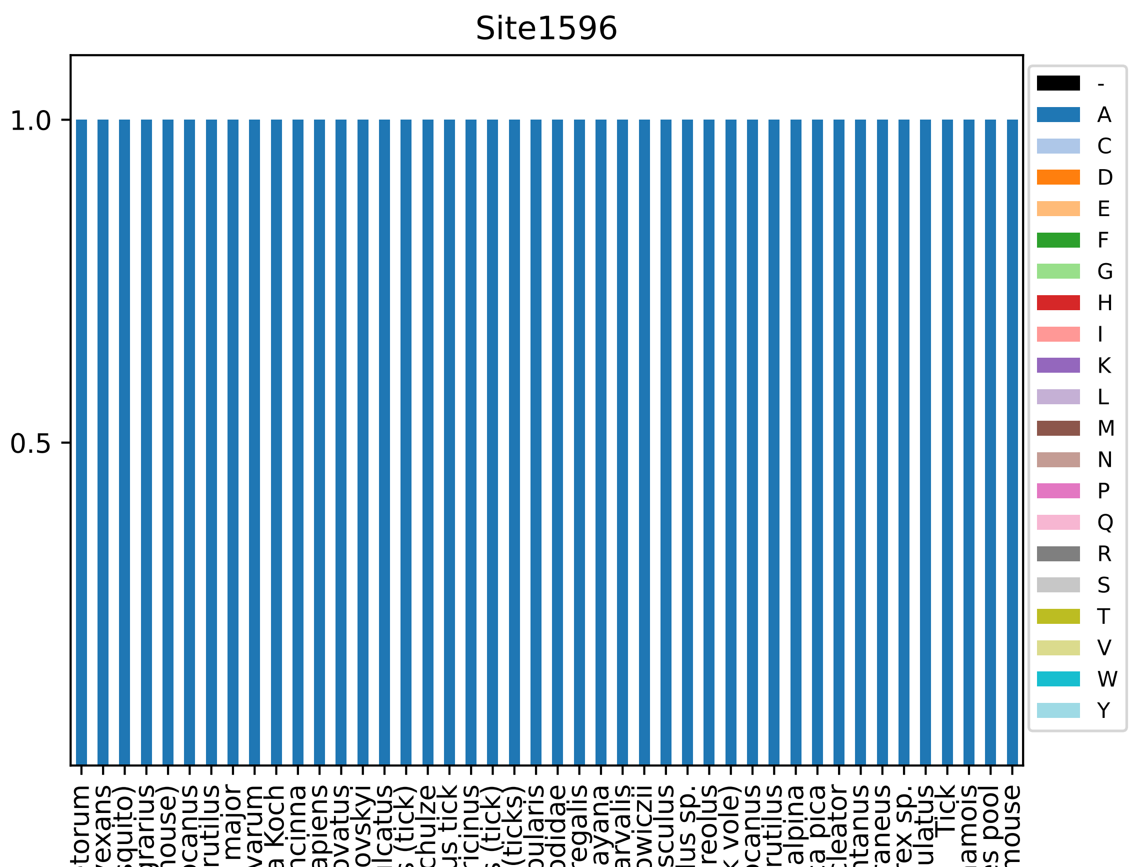
Site1596
1.0
0.5
-
A
C
D
E
F
G
H
I
K
L
M
N
P
Q
R
S
T
V
W
Y

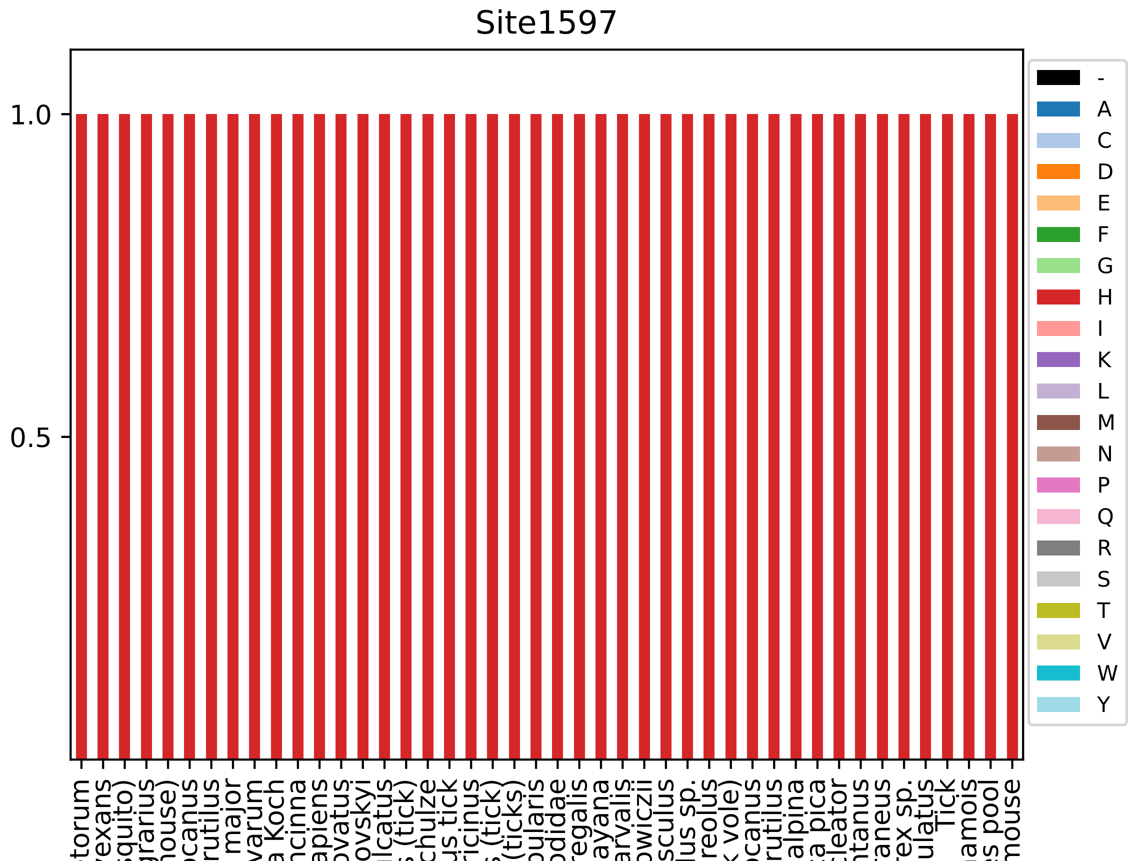
Site1597
1.0
0.5
-
A
C
D
E
F
G
H
I
K
L
M
N
P
Q
R
S
T
V
W
Y
Tick

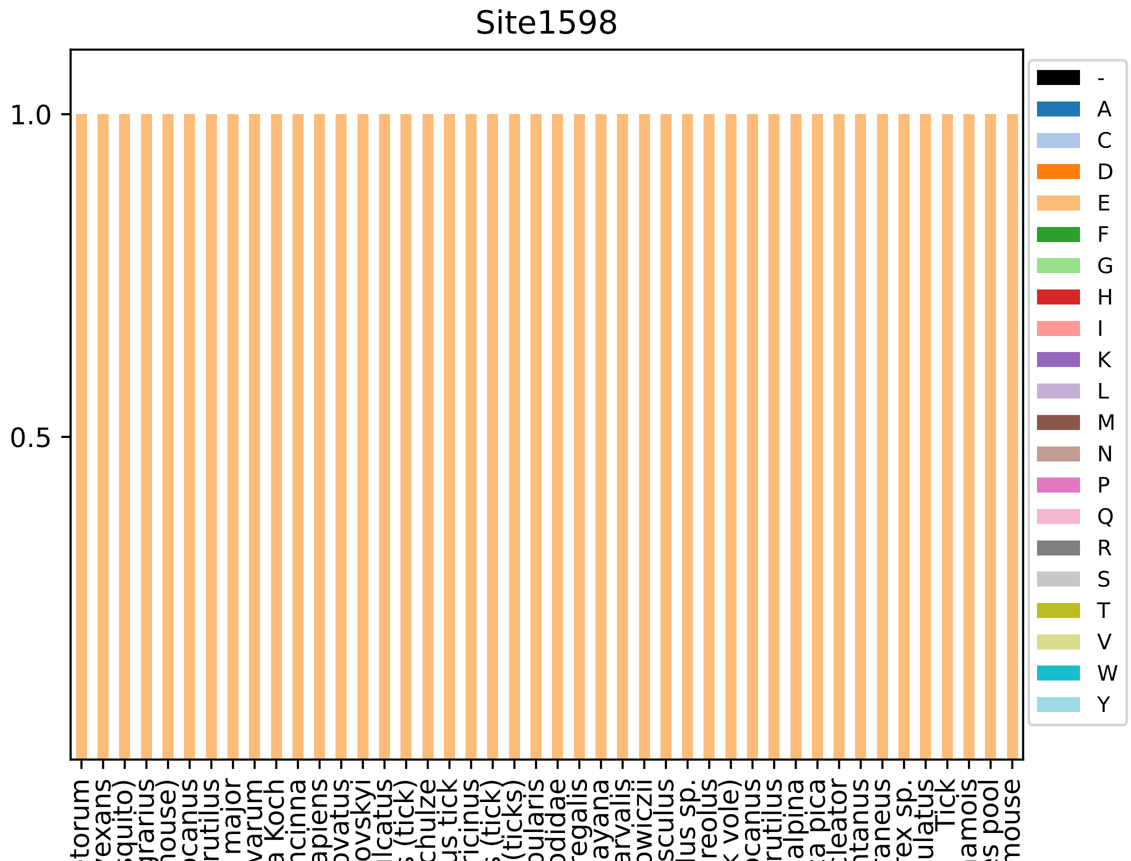
Site1598
1.0
0.5
-
A
C
D
E
F
G
H
I
K
L
M
N
P
Q
R
S
T
V
W
Y

Site1599

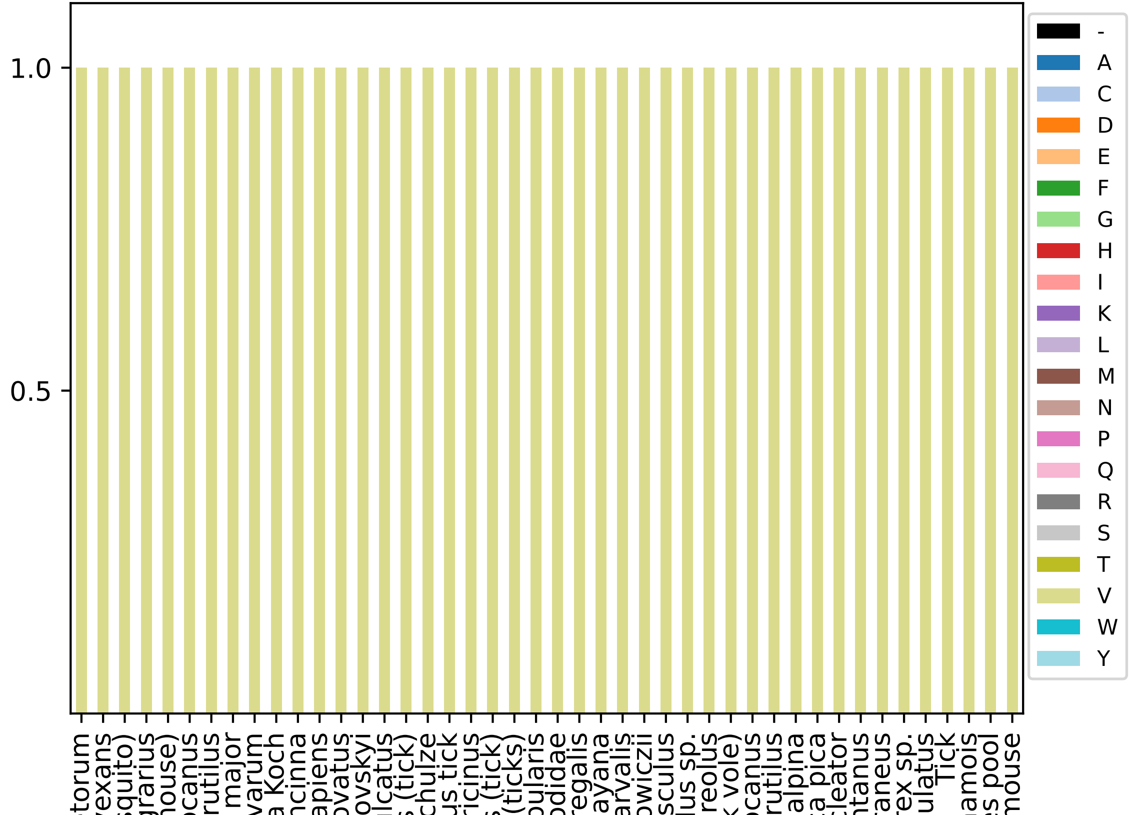

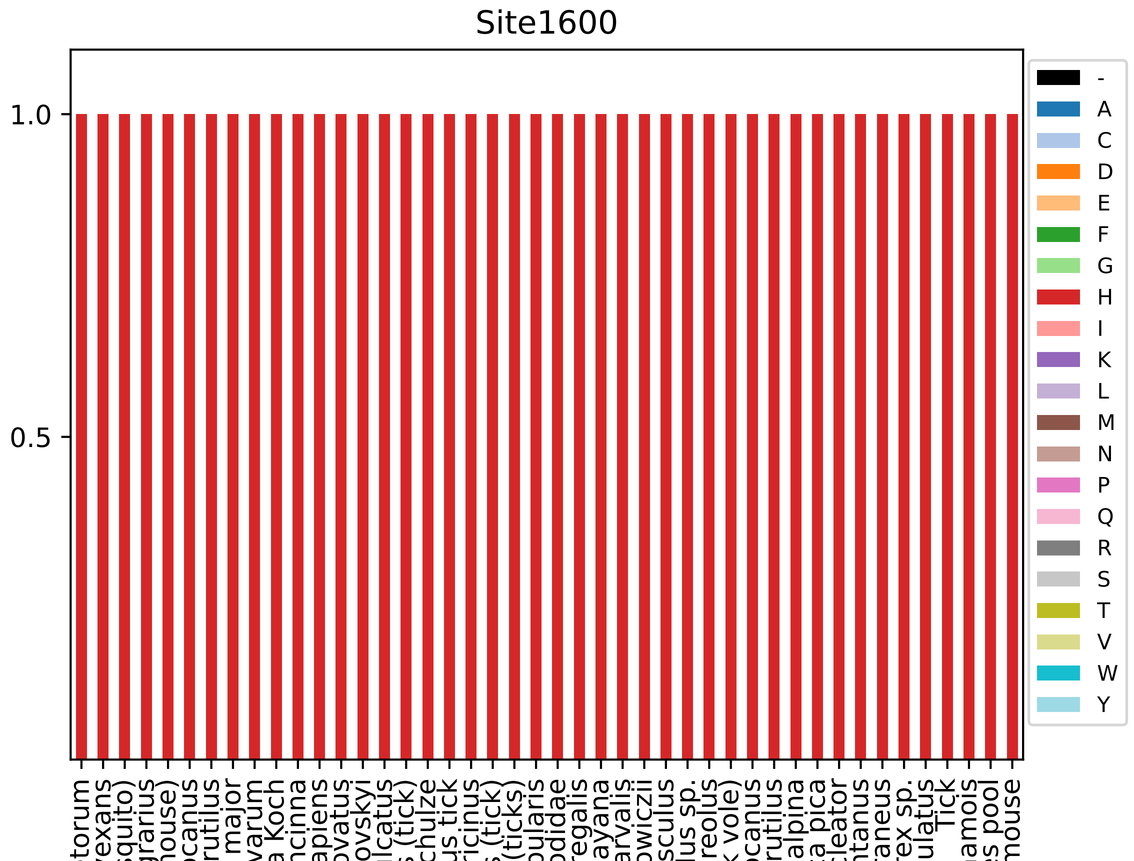

Site1600
1.0
0.5
-
A
C
D
E
F
G
H
I
K
L
M
N
P
Q
R
S
T
V
W
Y

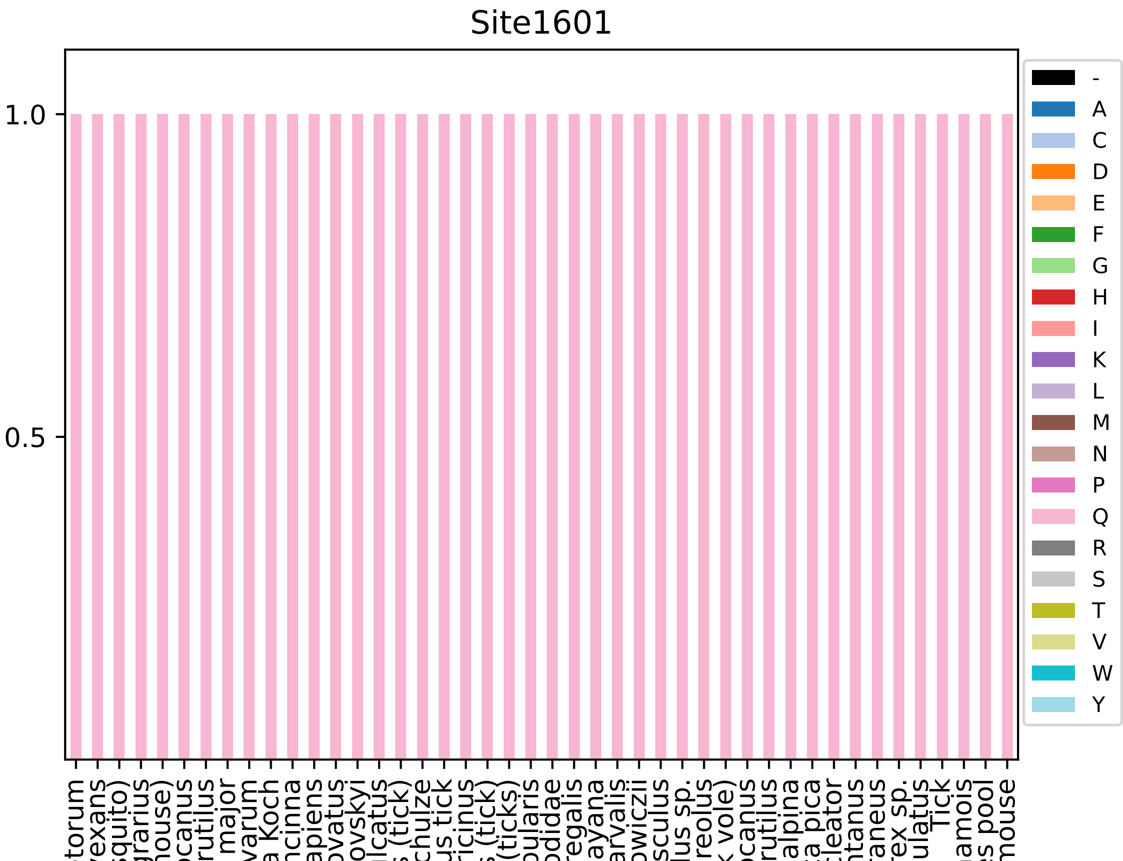
Site1601
1.0
0.5
-
A
C
D
E
F
G
H
I
K
L
M
N
P
Q
R
S
T
V
W
Y

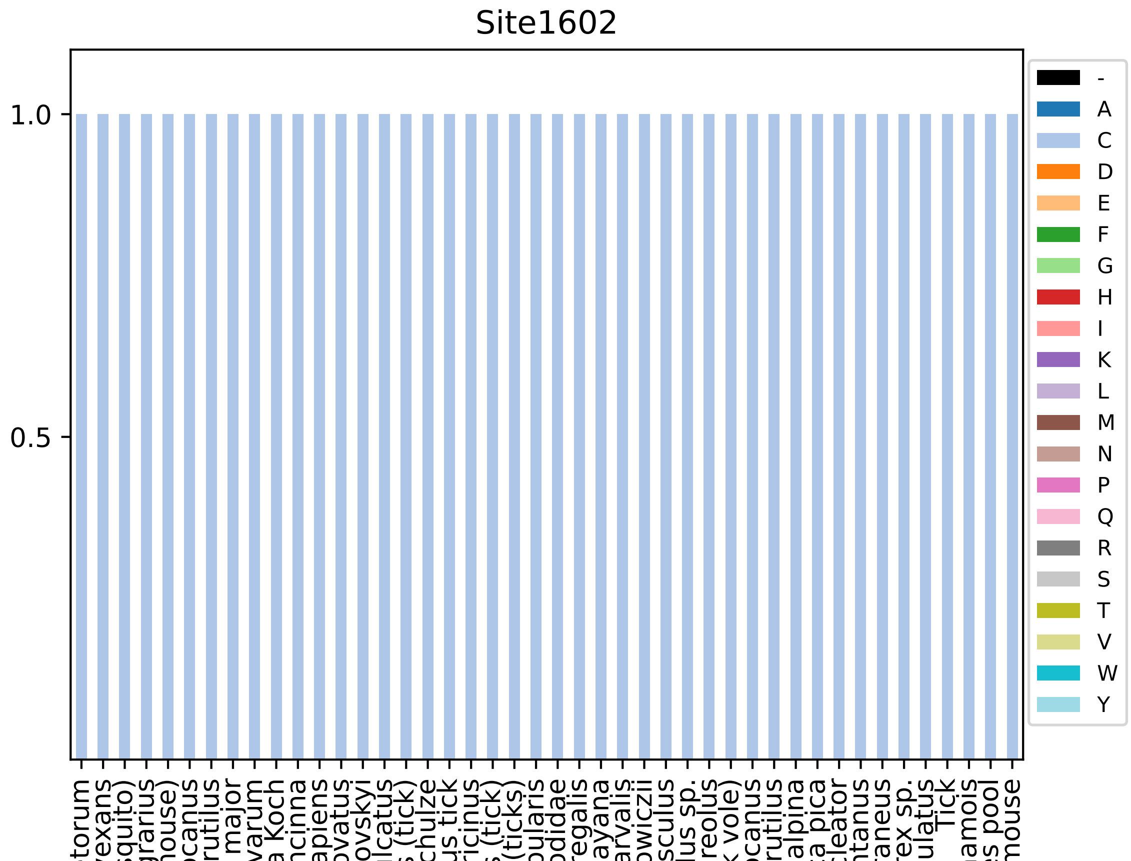

Site1602
1.0
0.5
-
A
C
D
E
F
G
H
I
K
L
M
N
P
Q
R
S
T
V
W
Y

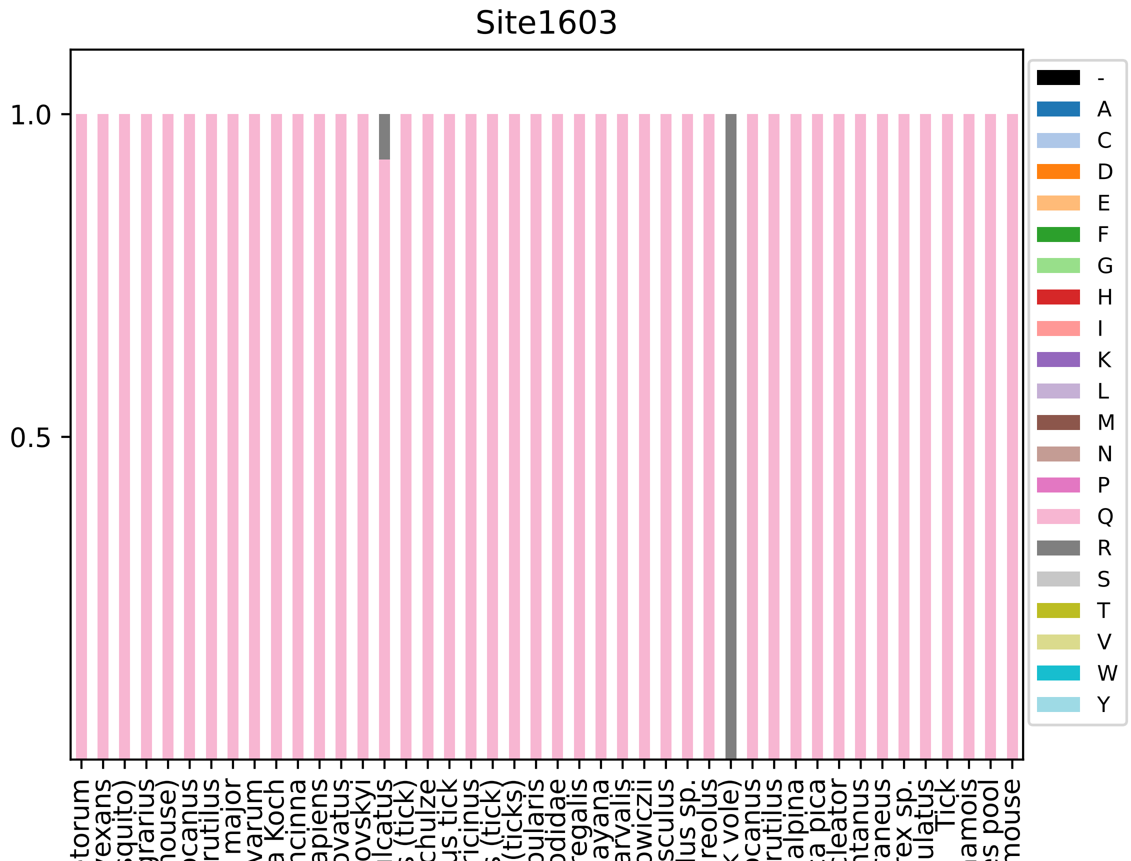

Site1603
1.0
0.5
-
A
C
D
E
F
G
H
I
K
L
M
N
P
Q
R
S
T
V
W
Y

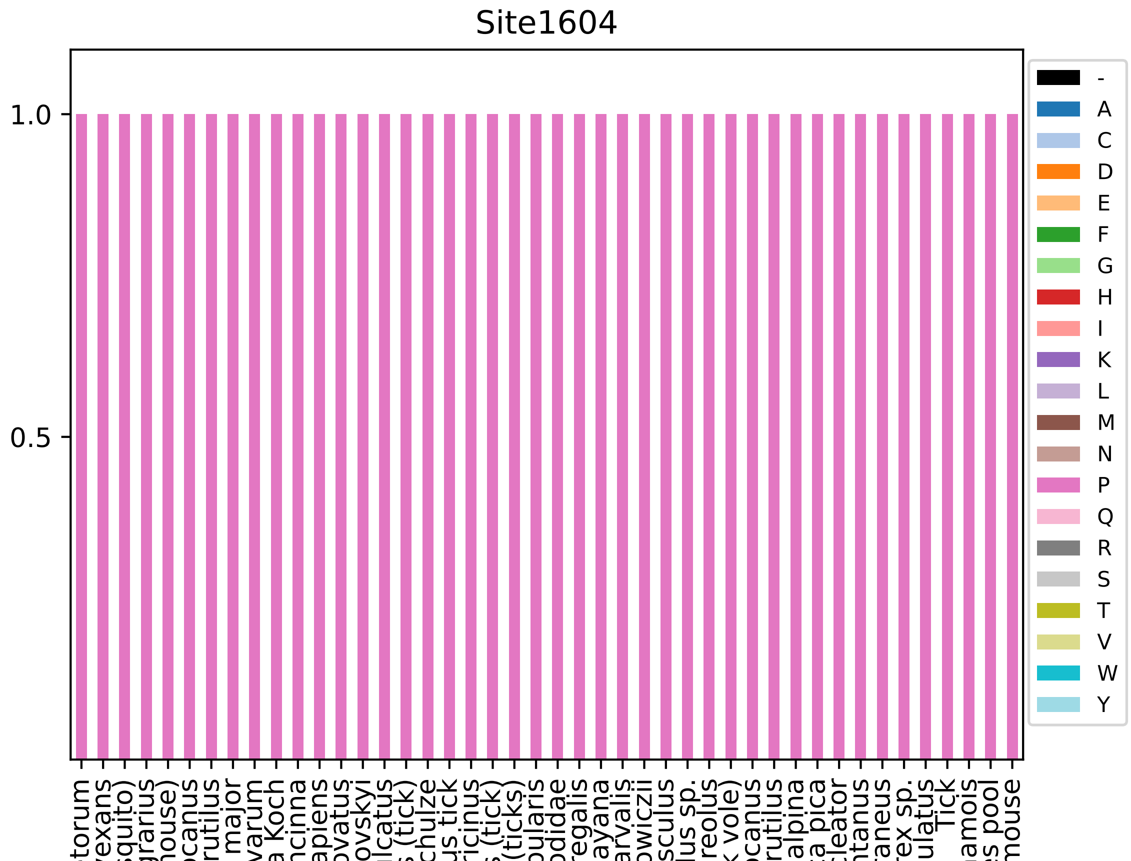
Site1604
1.0
0.5
-
A
C
D
E
F
G
H
I
K
L
M
N
P
Q
R
S
T
V
W
Y

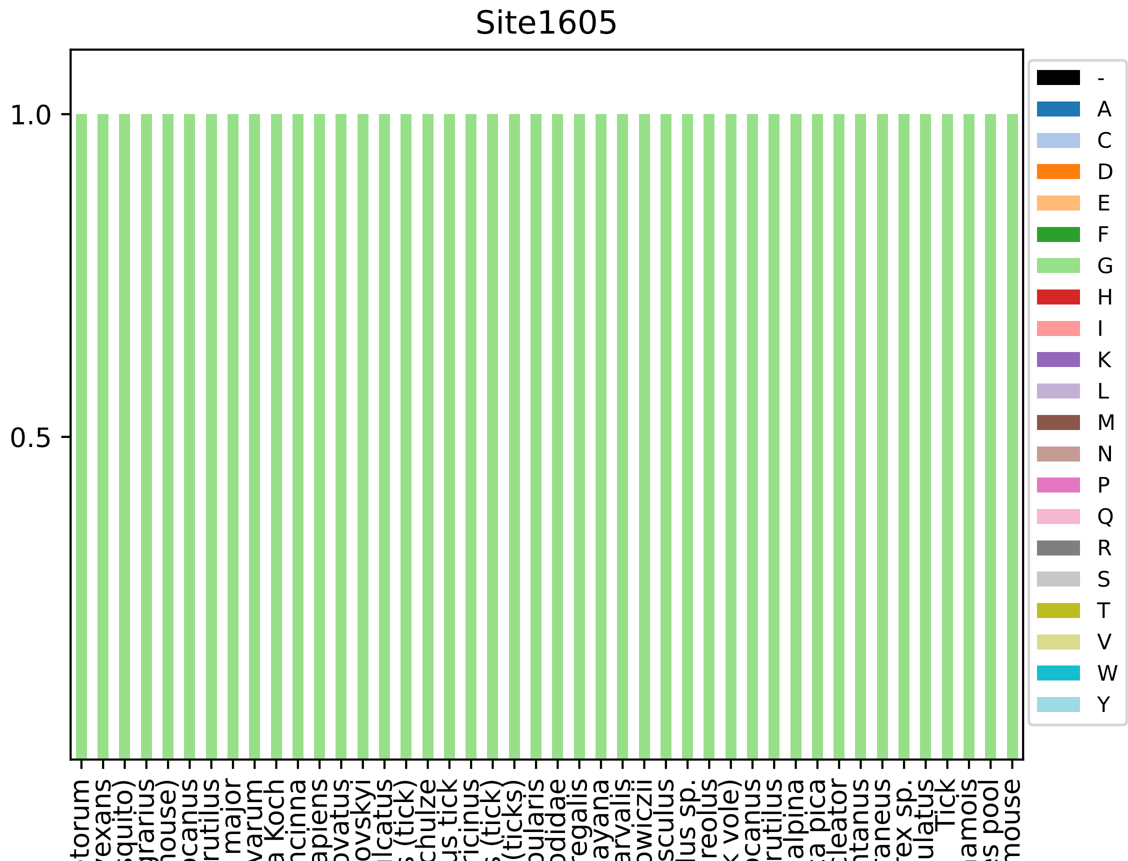
Site1605
1.0
0.5
-
A
C
D
E
F
G
H
I
K
L
M
N
P
Q
R
S
T
V
W
Y

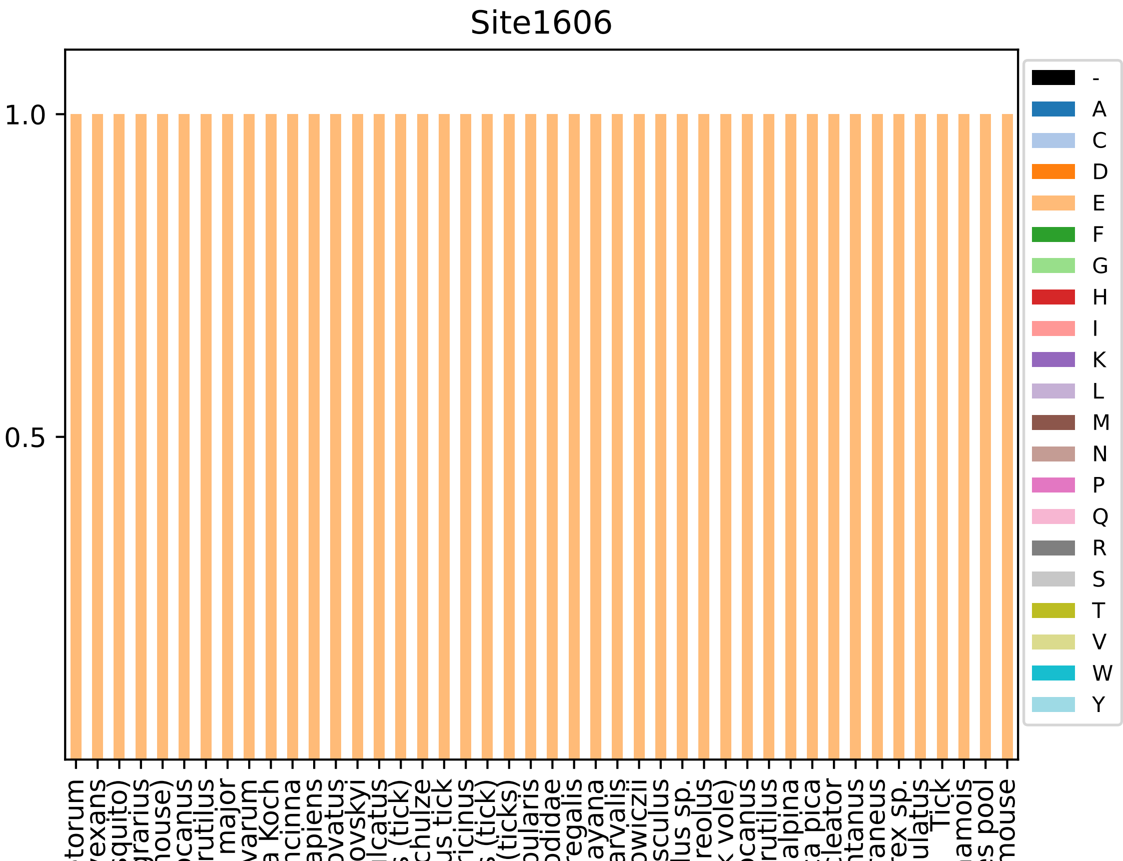
Site1606
1.0
0.5
-
A
C
D
E
F
G
H
I
K
L
M
N
P
Q
R
S
T
V
W
Y

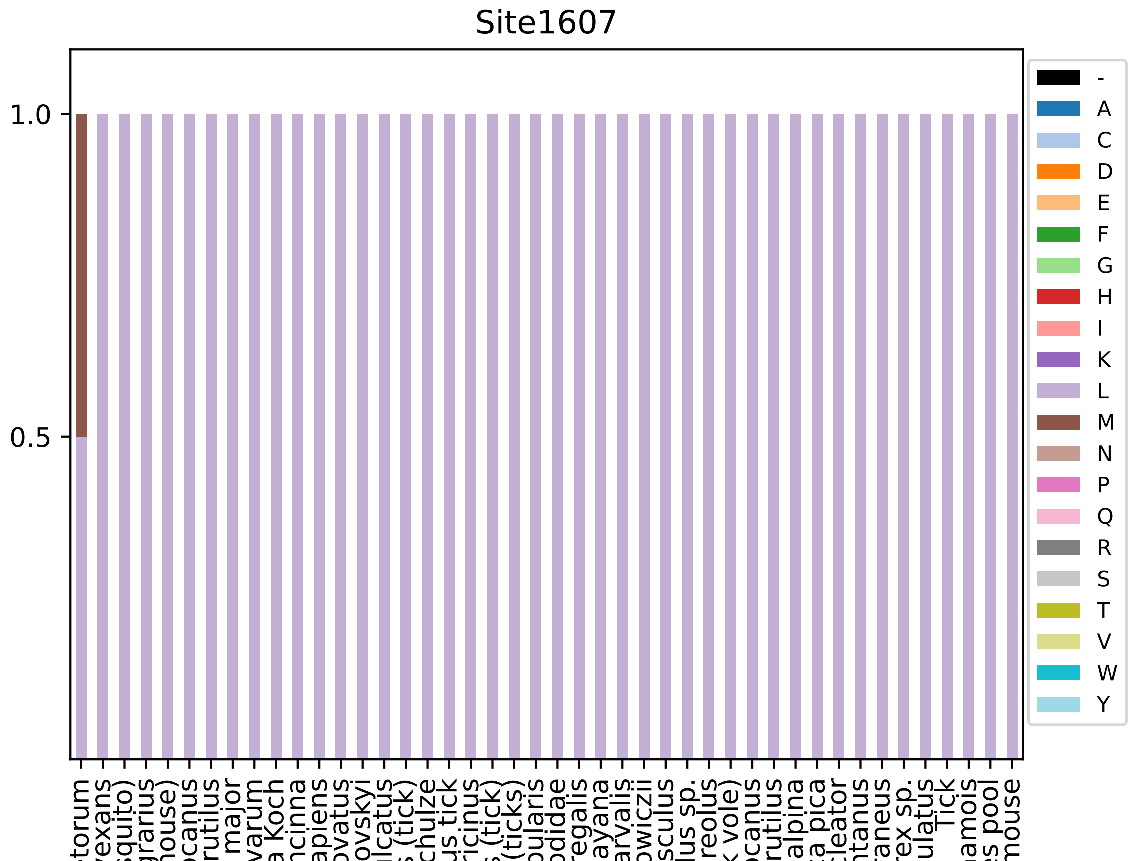

Site1607
1.0
0.5
-
A
C
D
E
F
G
H
I
K
L
M
N
P
Q
R
S
T
V
W
Y

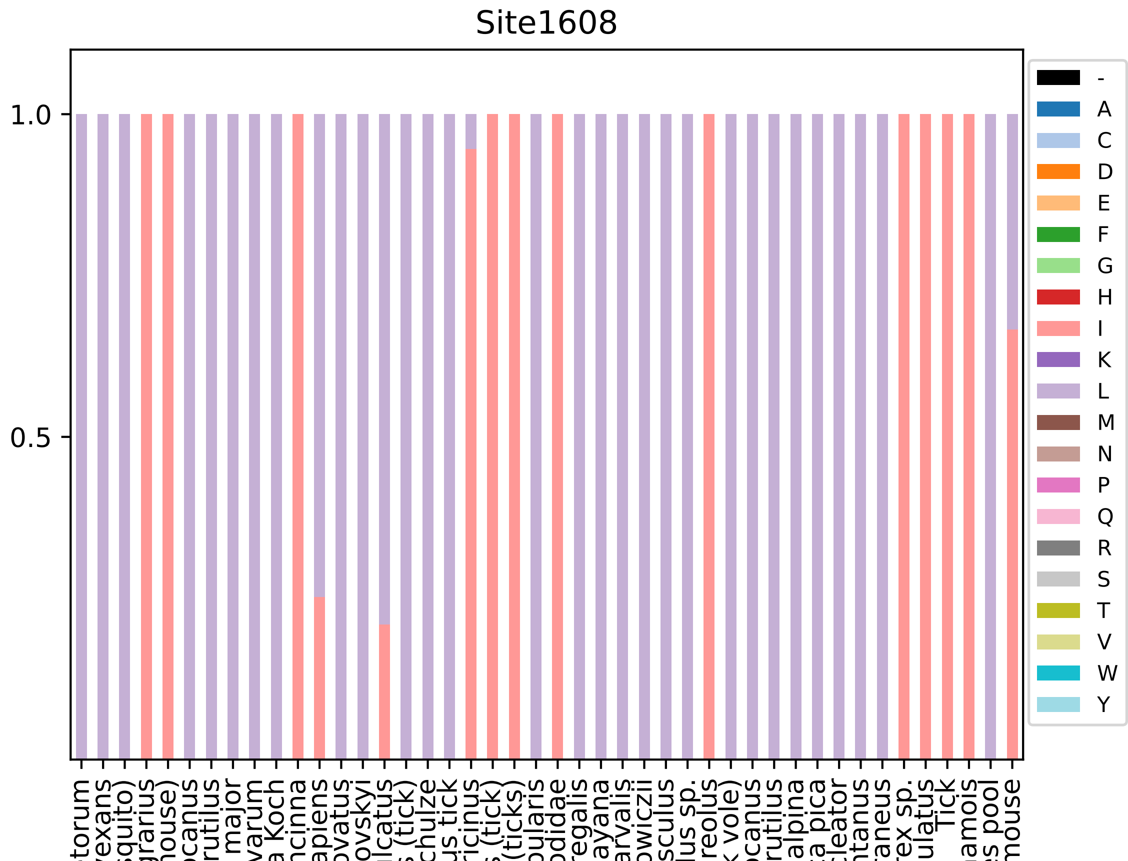

Site1608
1.0
0.5
-
A
C
D
E
F
G
H
I
K
L
M
N
P
Q
R
S
T
V
W
Y

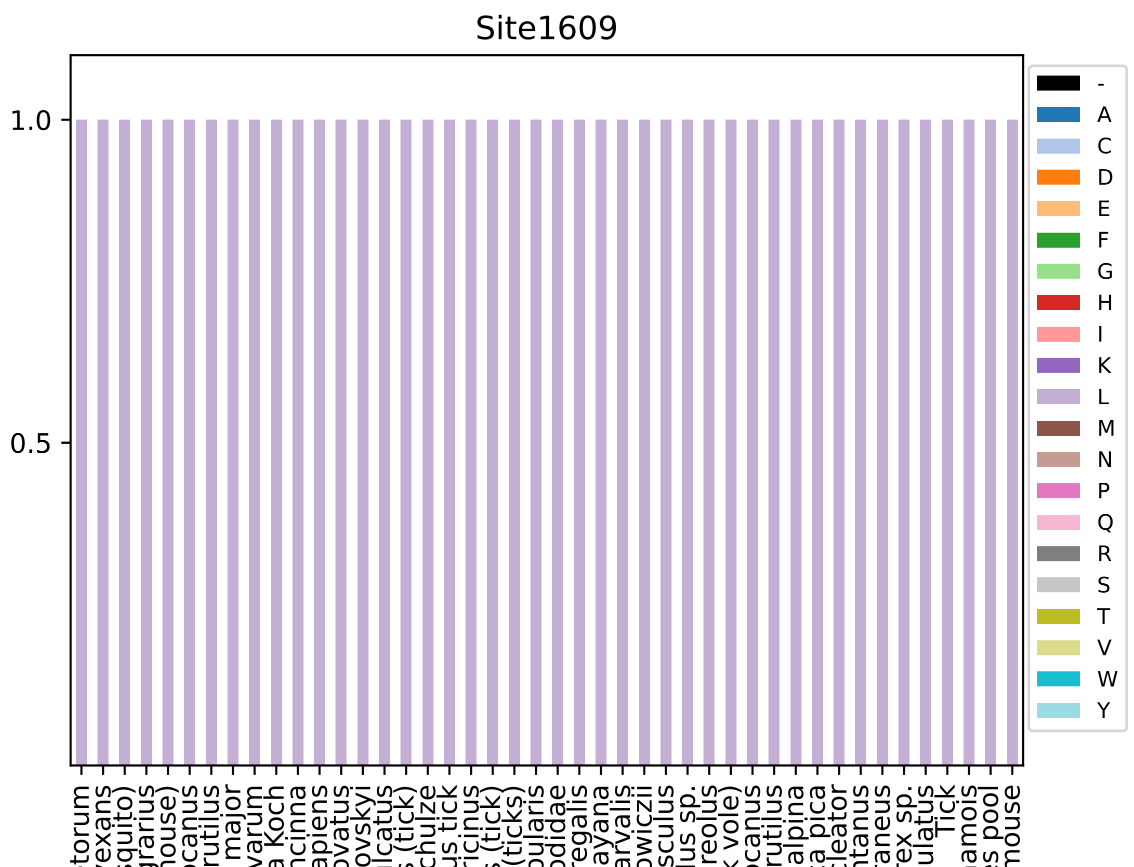

Site1609
1.0
0.5
-
A
C
D
E
F
G
H
I
K
L
M
N
P
Q
R
S
T
V
W
Y

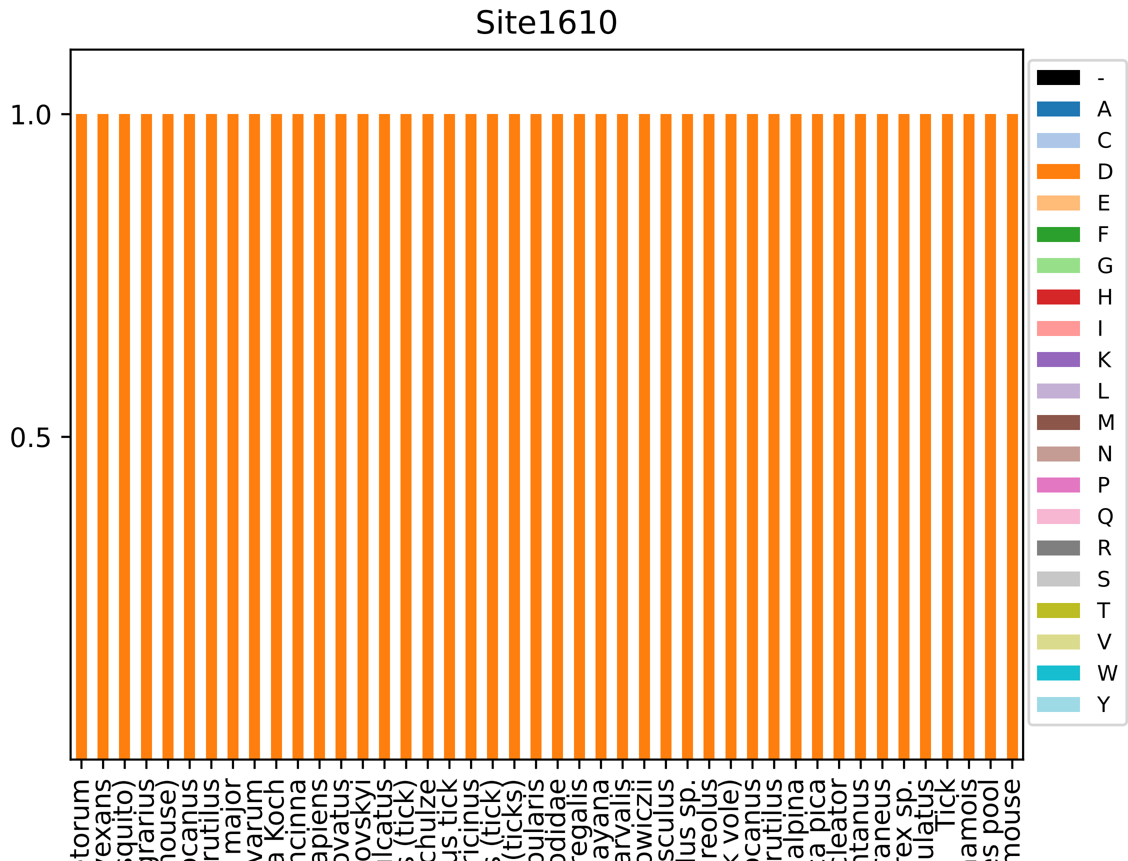

Site1610
1.0
0.5
-
A
C
D
E
F
G
H
I
K
L
M
N
P
Q
R
S
T
V
W
Y

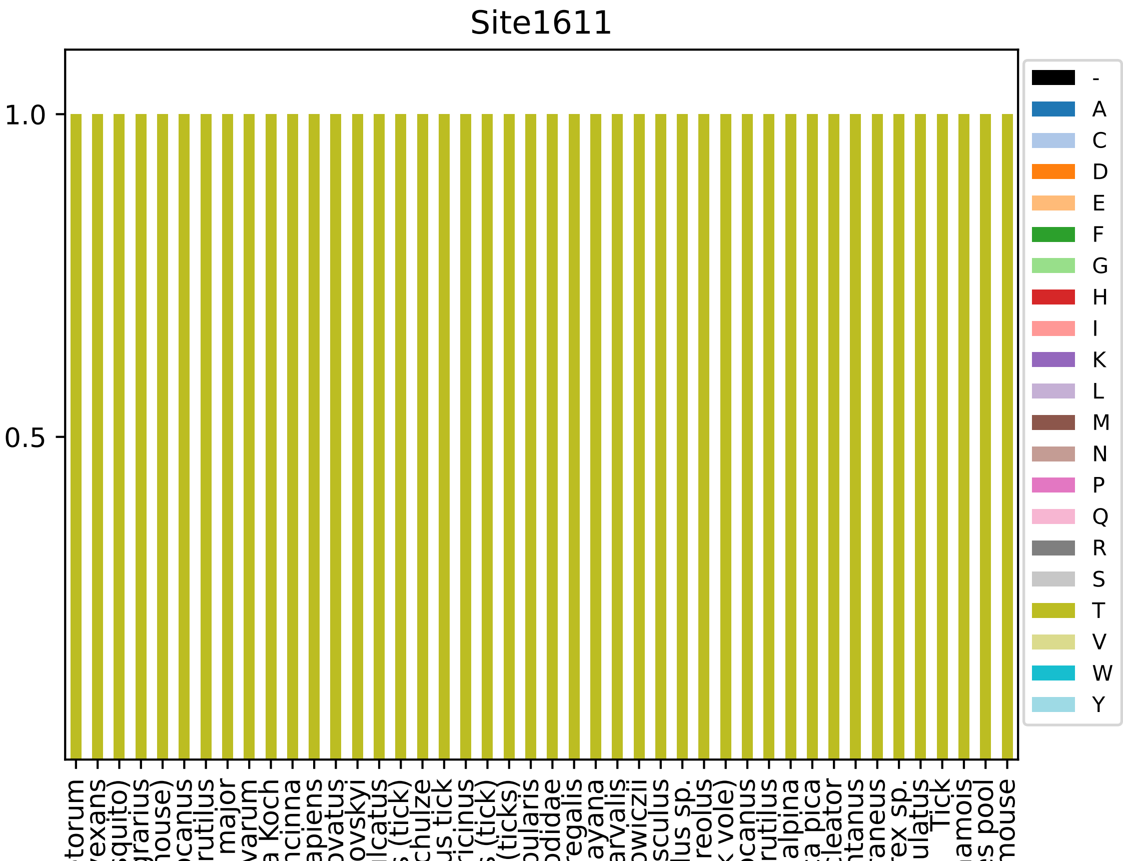

Site1611
1.0
0.5
-
A
C
D
E
F
G
H
I
K
L
M
N
P
Q
R
S
T
V
W
Y

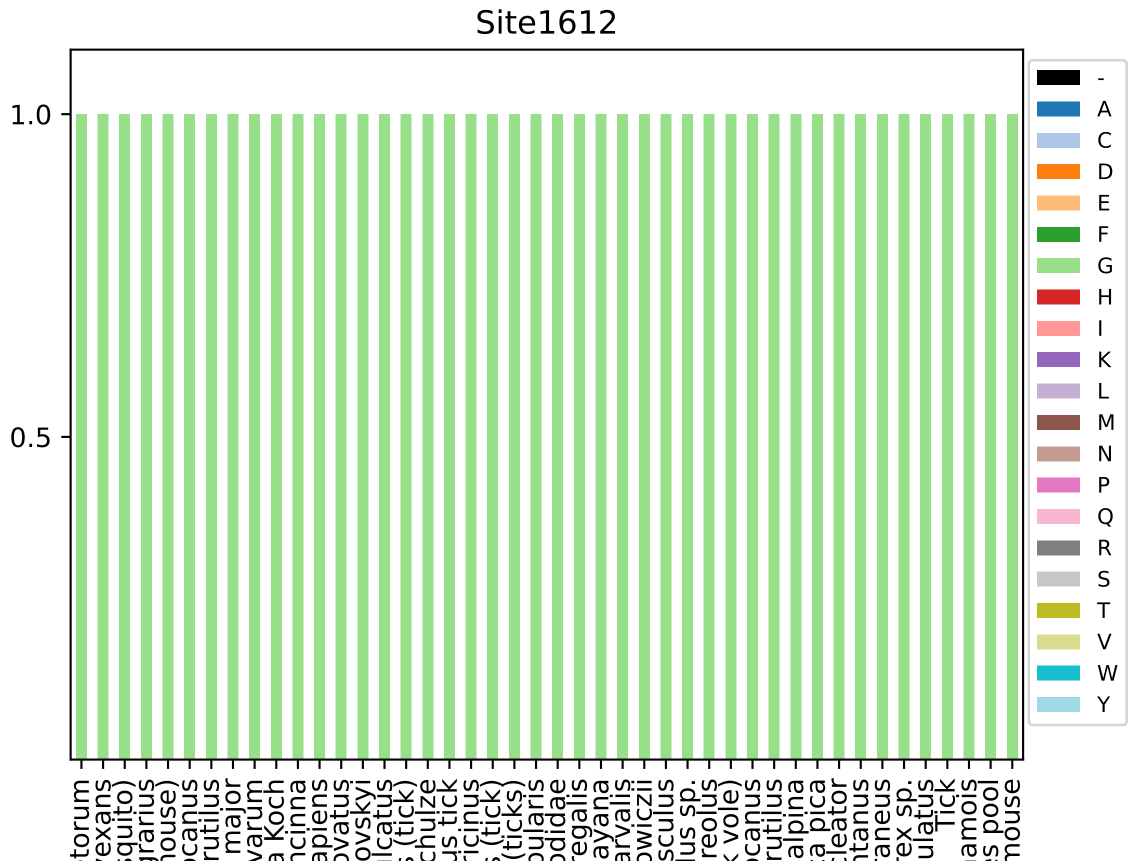
Site1612
1.0
0.5
-
A
C
D
E
F
G
H
I
K
L
M
N
P
Q
R
S
T
V
W
Y

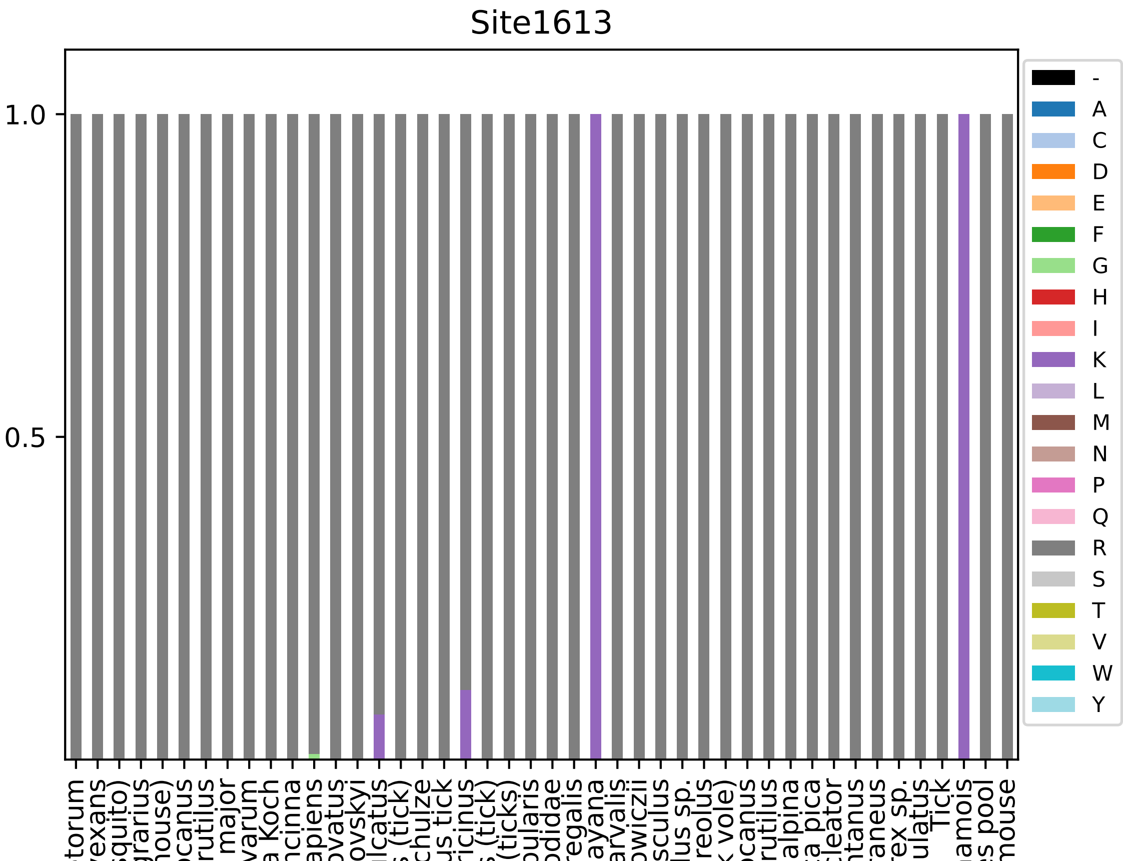
Site1613
1.0
0.5
-
A
C
D
E
F
G
H
I
K
L
M
N
P
Q
R
S
T
V
W
Y

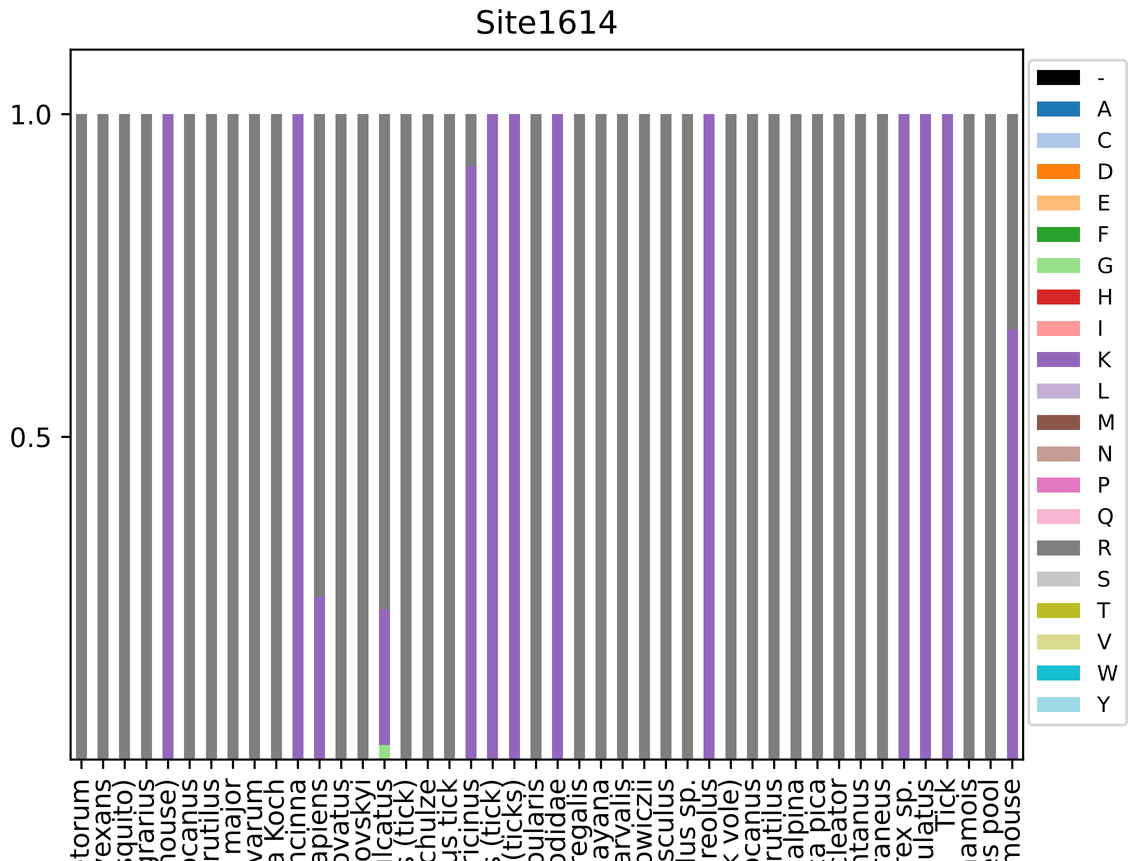

Site1614
1.0
0.5
-
A
C
D
E
F
G
H
I
K
L
M
N
P
Q
R
S
T
V
W
Y

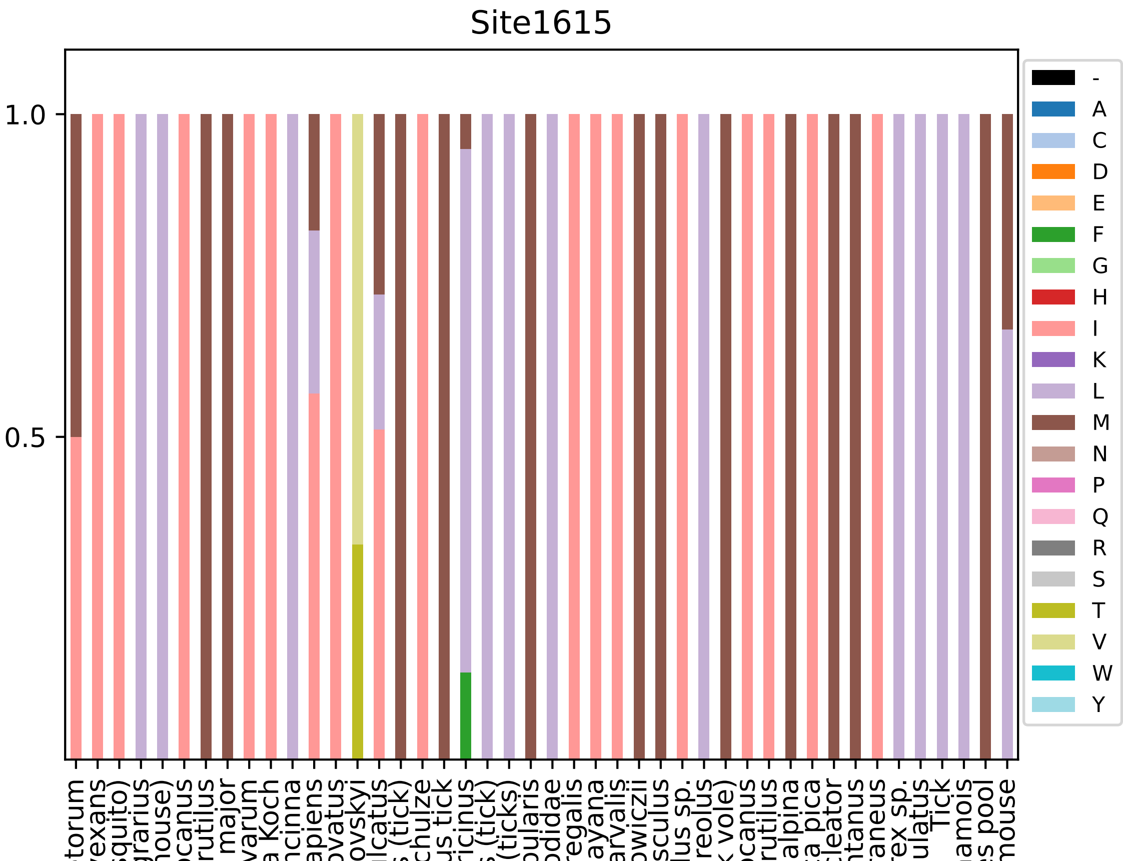
Site1615
1.0
0.5
-
A
C
D
E
F
G
H
I
K
L
M
N
P
Q
R
S
T
V
W
Y

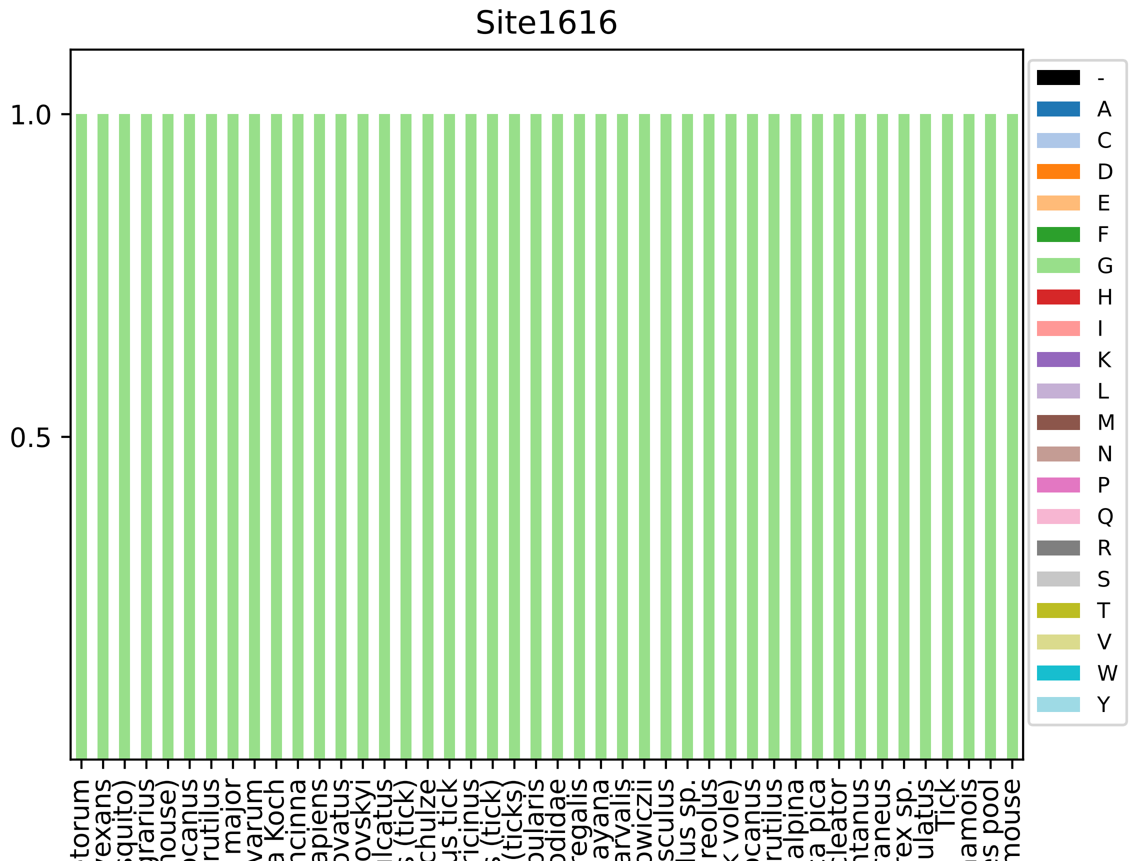
Site1616
1.0
0.5
-
A
C
D
E
F
G
H
I
K
L
M
N
P
Q
R
S
T
V
W
Y

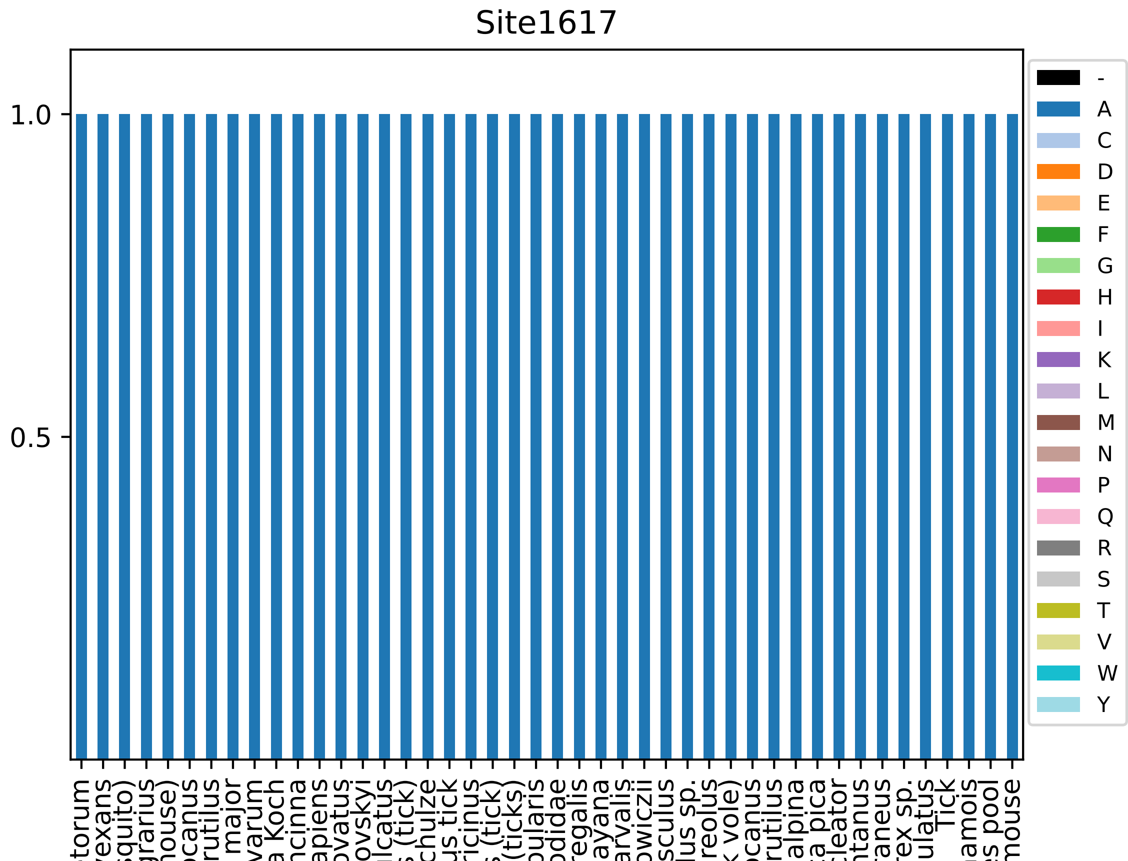
Site1617
1.0
0.5
-
A
C
D
E
F
G
H
I
K
L
M
N
P
Q
R
S
T
V
W
Y

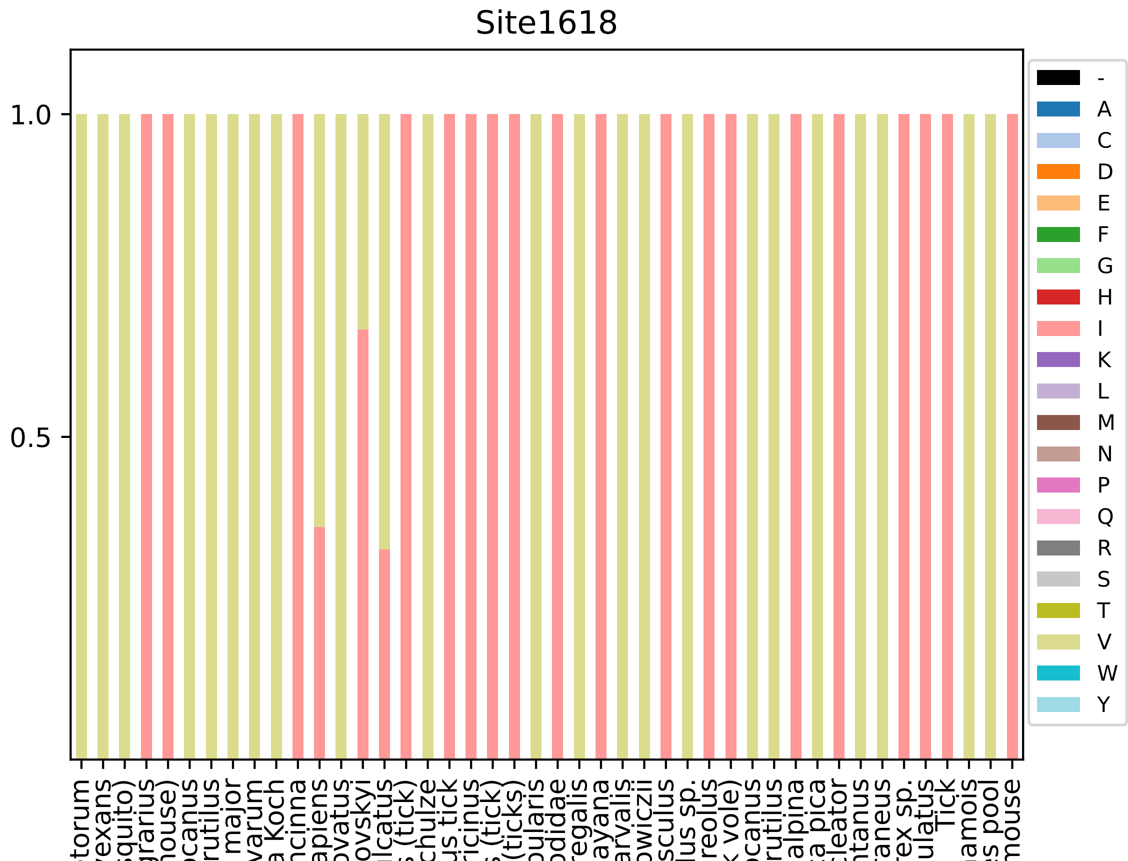

Site1618
1.0
0.5
-
A
C
D
E
F
G
H
I
K
L
M
N
P
Q
R
S
T
V
W
Y

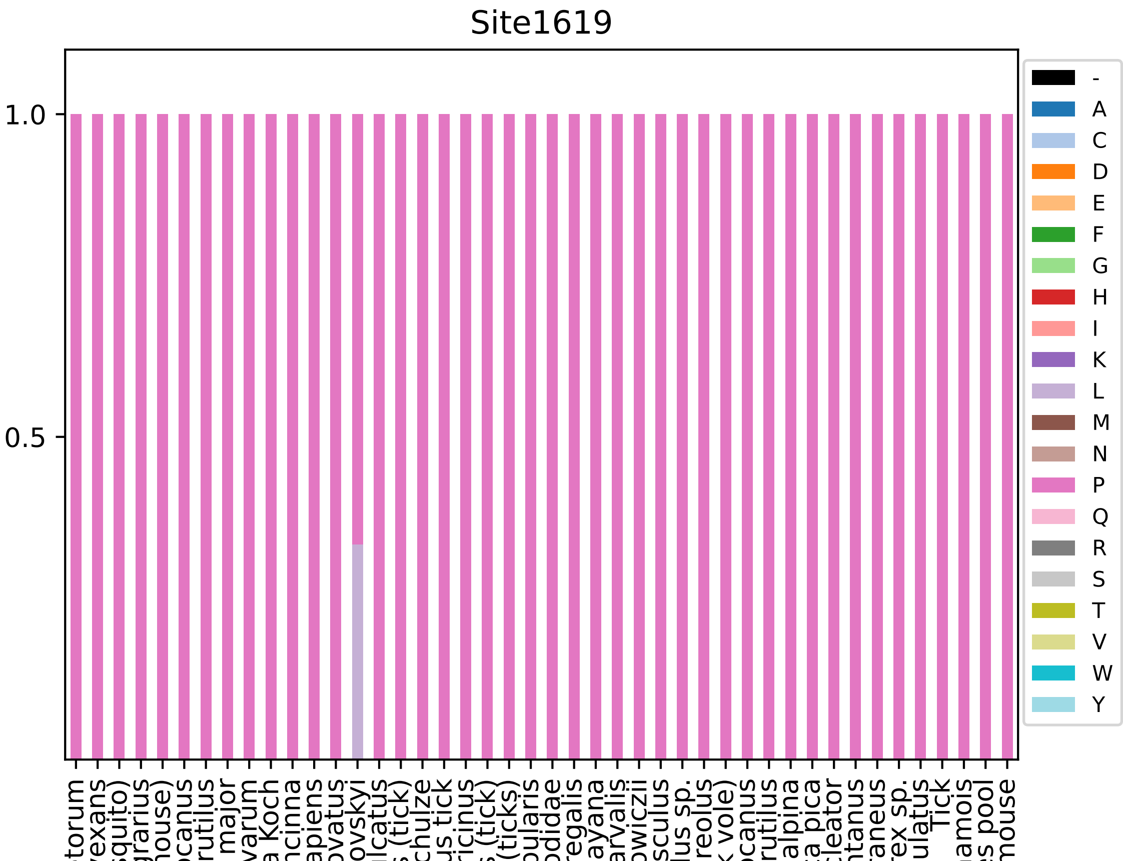
Site1619
1.0
0.5
-
A
C
D
E
F
G
H
I
K
L
M
N
P
Q
R
S
T
V
W
Y

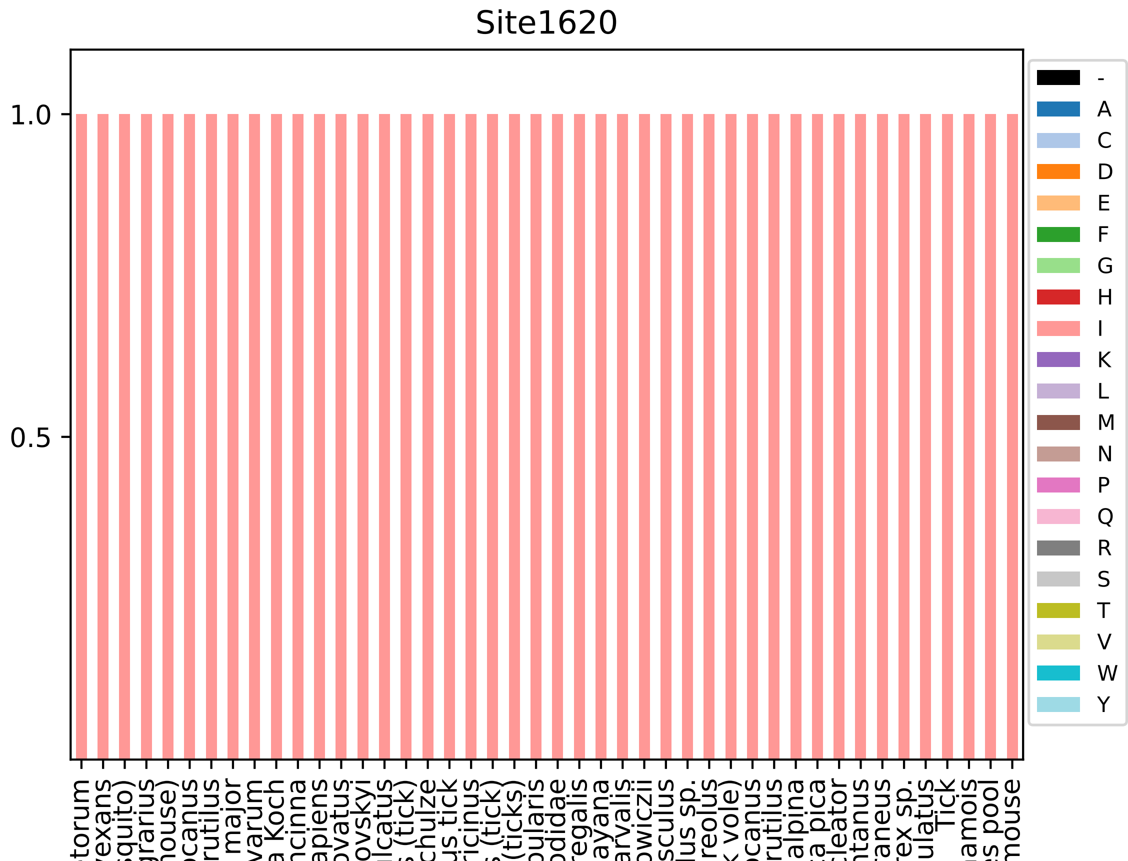

Site1620
1.0
0.5
-
A
C
D
E
F
G
H
I
K
L
M
N
P
Q
R
S
T
V
W
Y

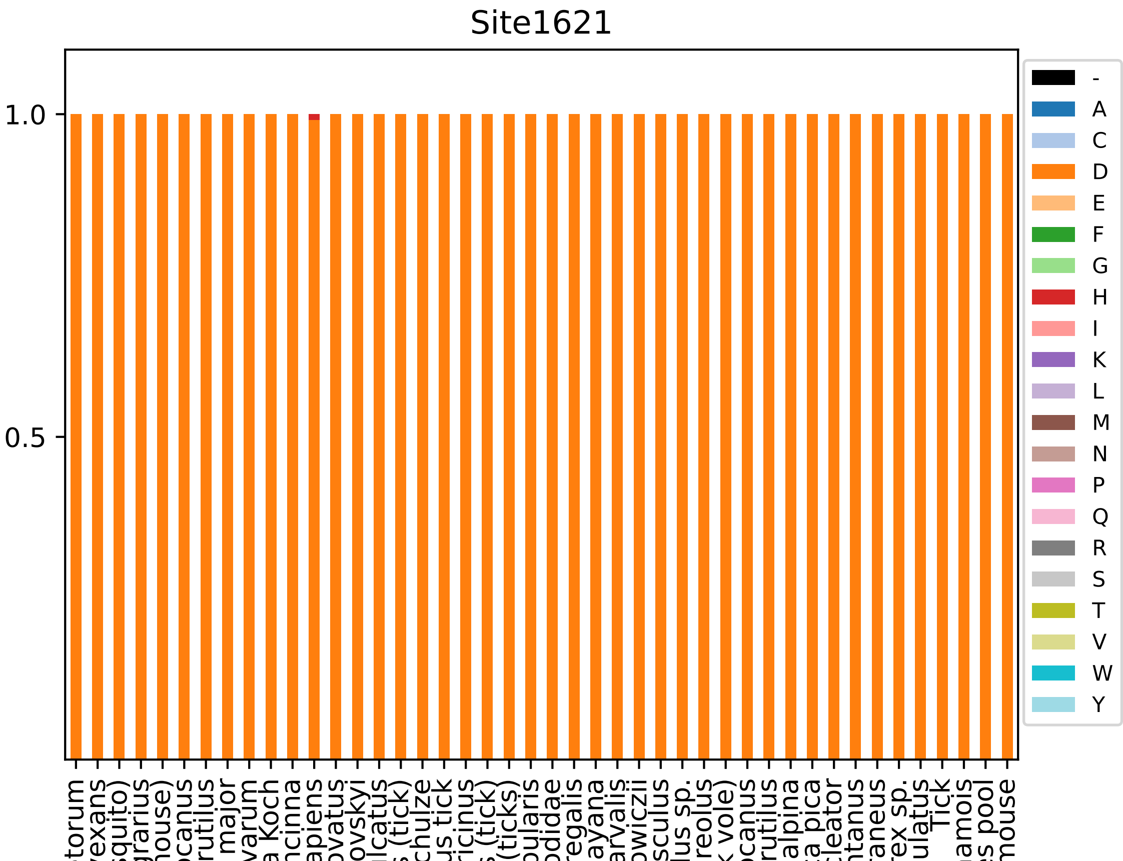
Site1621
1.0
0.5
-
A
C
D
E
F
G
H
I
K
L
M
N
P
Q
R
S
T
V
W
Y

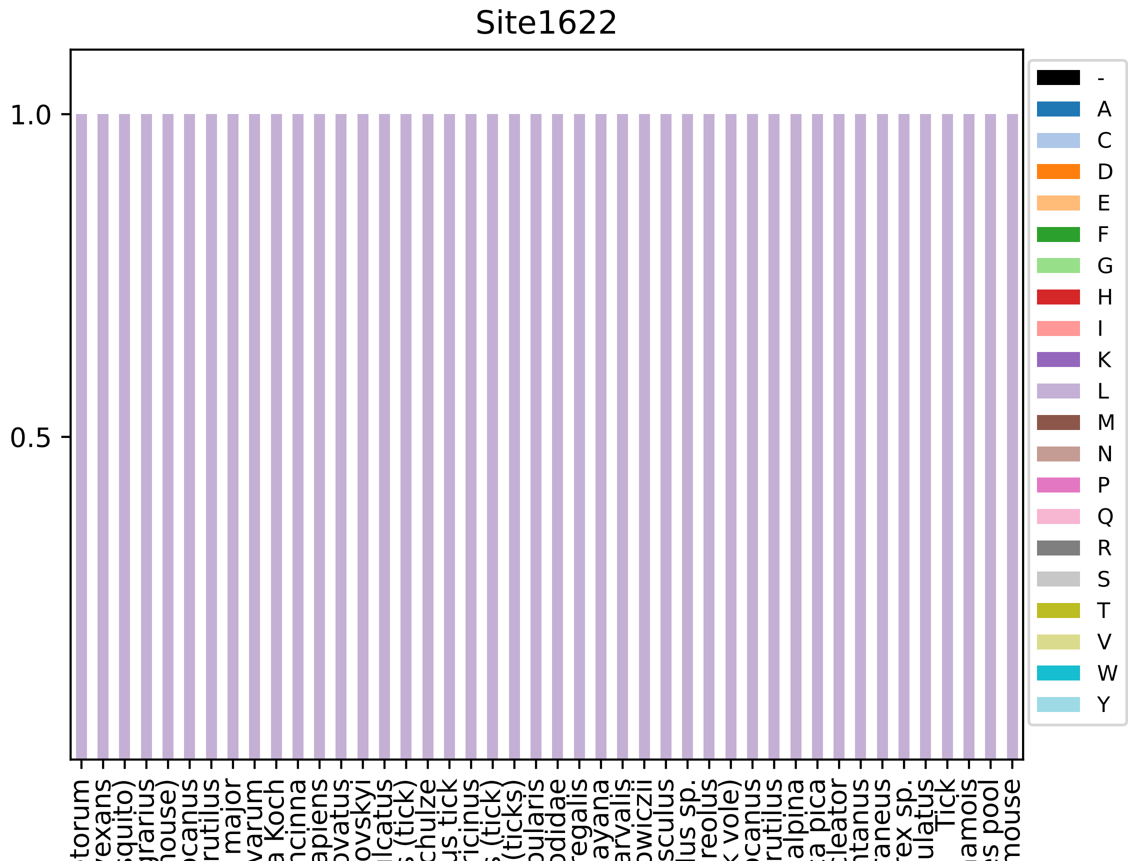
Site1622
1.0
0.5
-
A
C
D
E
F
G
H
I
K
L
M
N
P
Q
R
S
T
V
W
Y

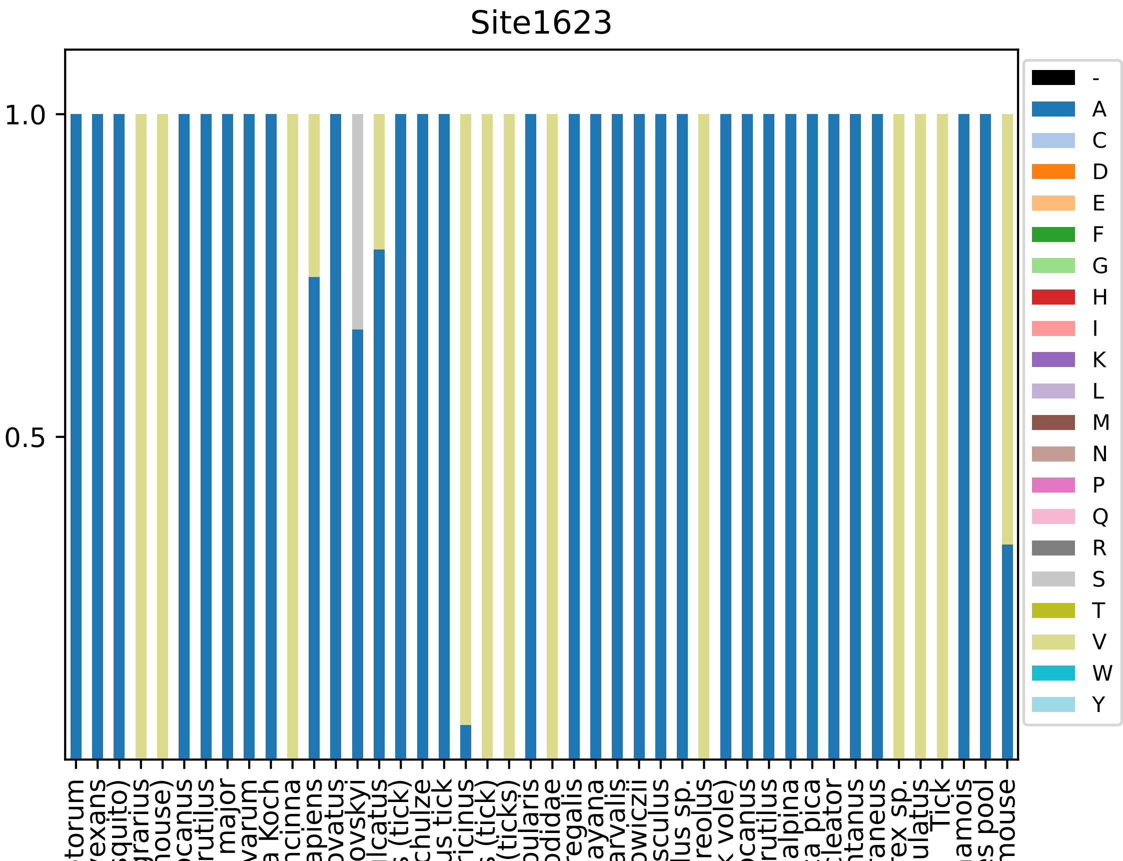
Site1623
1.0
0.5
-
A
C
D
E
F
G
H
I
K
L
M
N
P
Q
R
S
T
V
W
Y

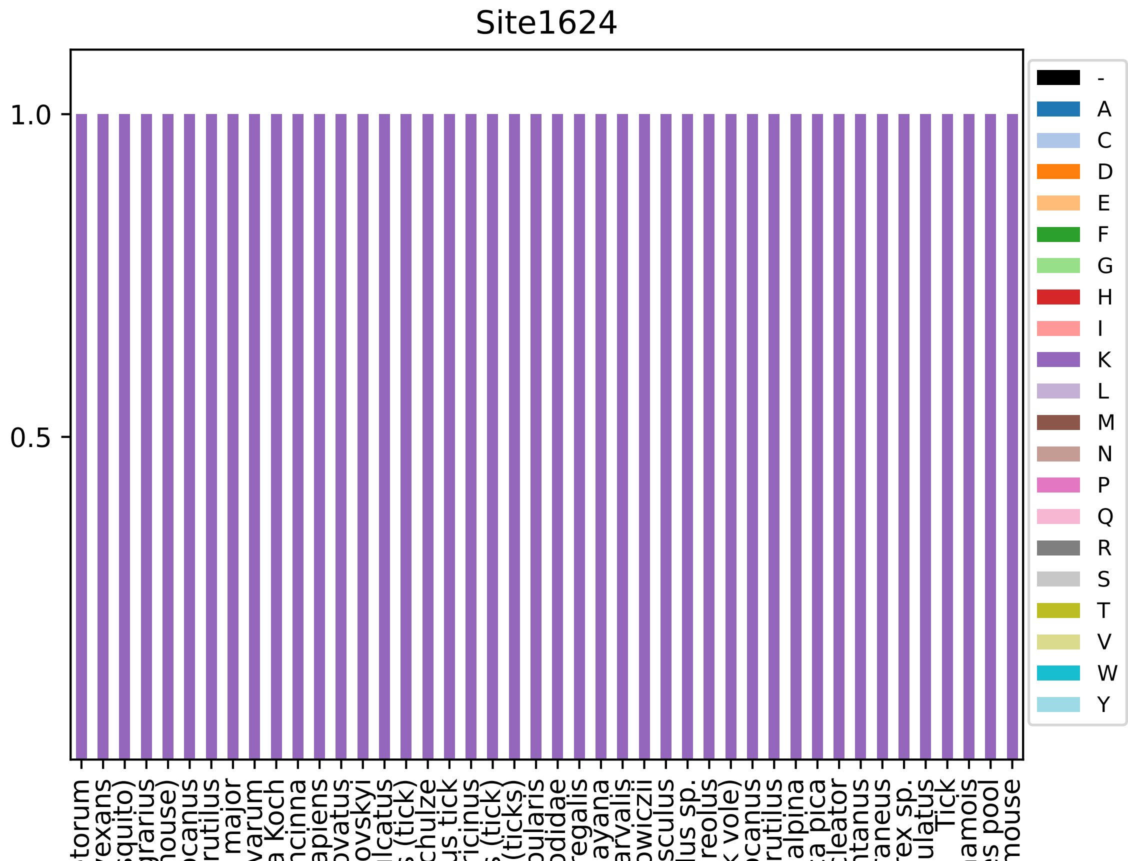
Site1624
1.0
0.5
-
A
C
D
E
F
G
H
I
K
L
M
N
P
Q
R
S
T
V
W
Y

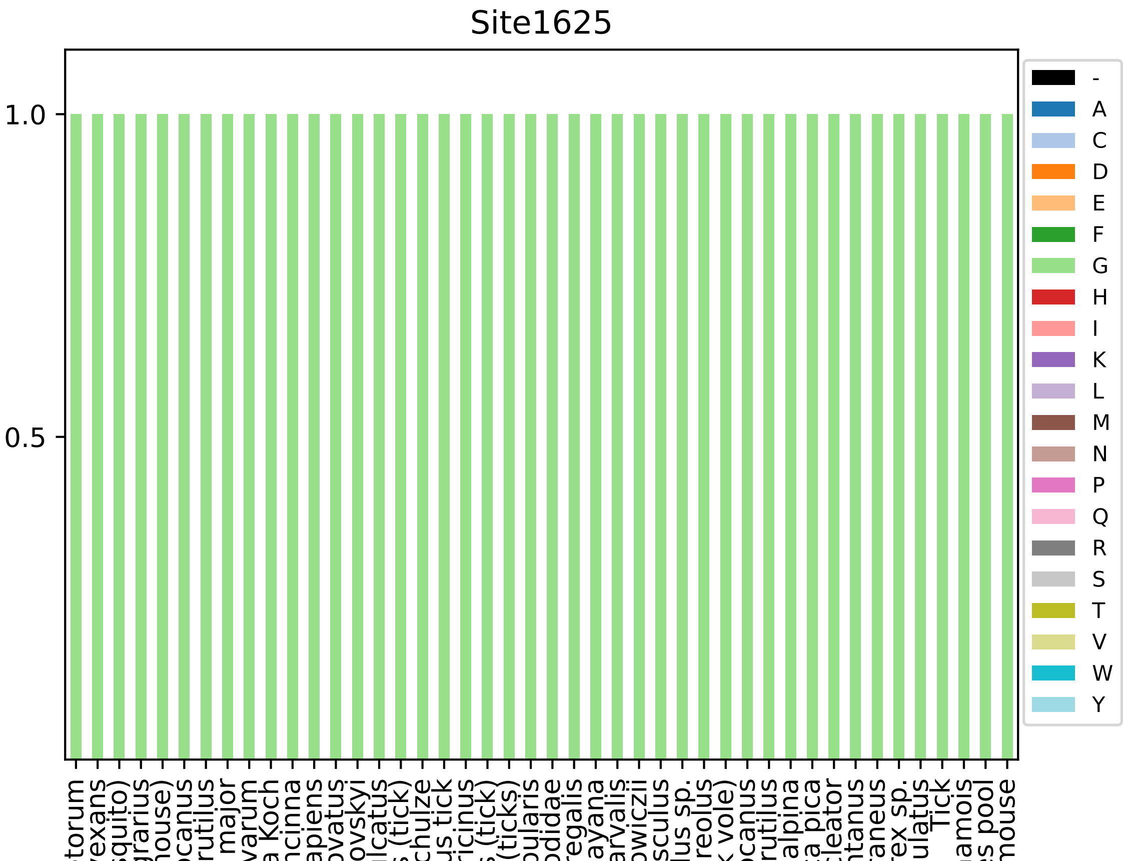

Site1625
1.0
0.5
-
A
C
D
E
F
G
H
I
K
L
M
N
P
Q
R
S
T
V
W
Y

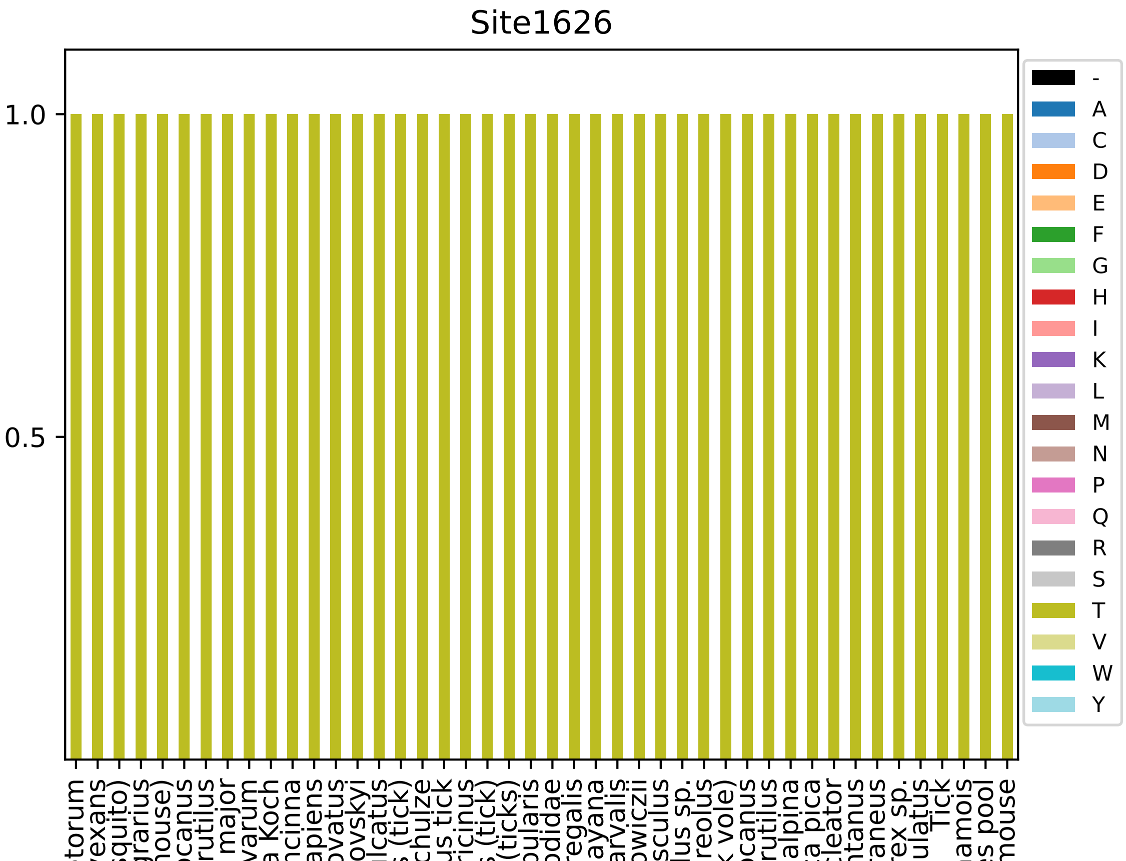

Site1626
1.0
0.5
-
A
C
D
E
F
G
H
I
K
L
M
N
P
Q
R
S
T
V
W
Y

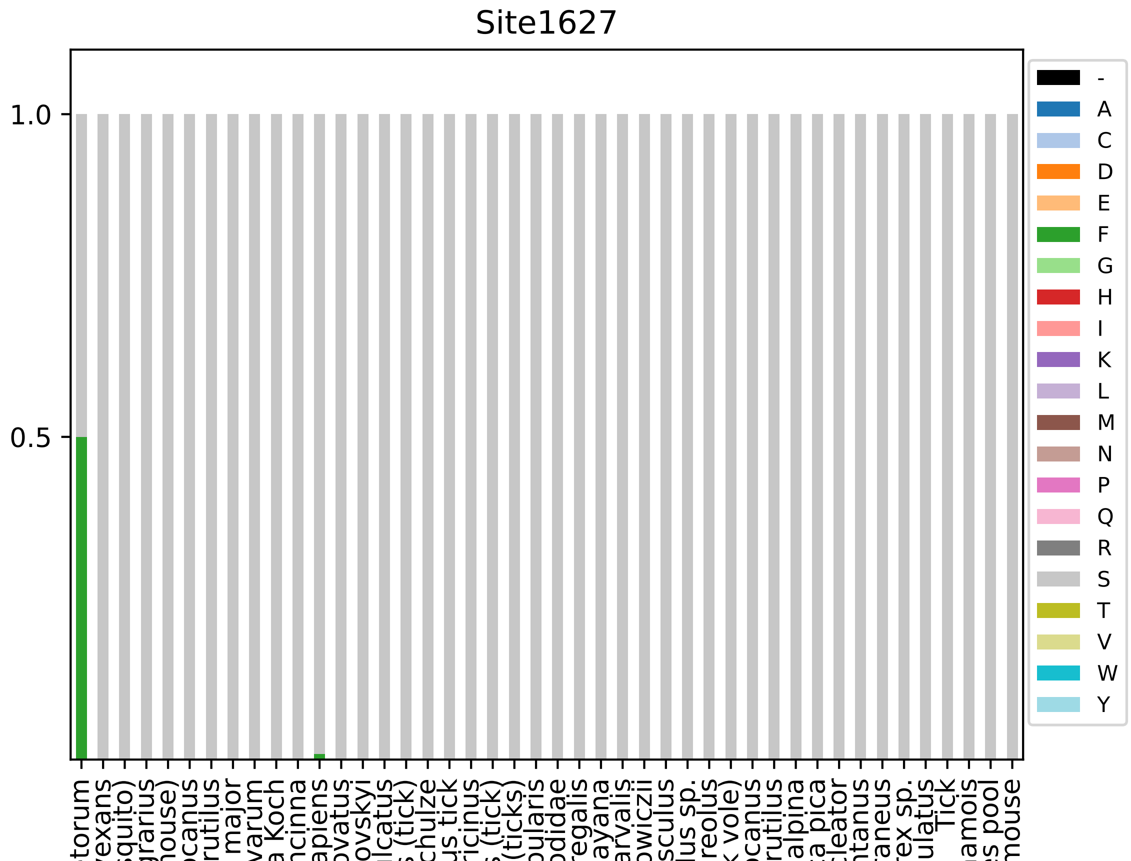
Site1627
1.0
0.5
-
A
C
D
E
F
G
H
I
K
L
M
N
P
Q
R
S
T
V
W
Y

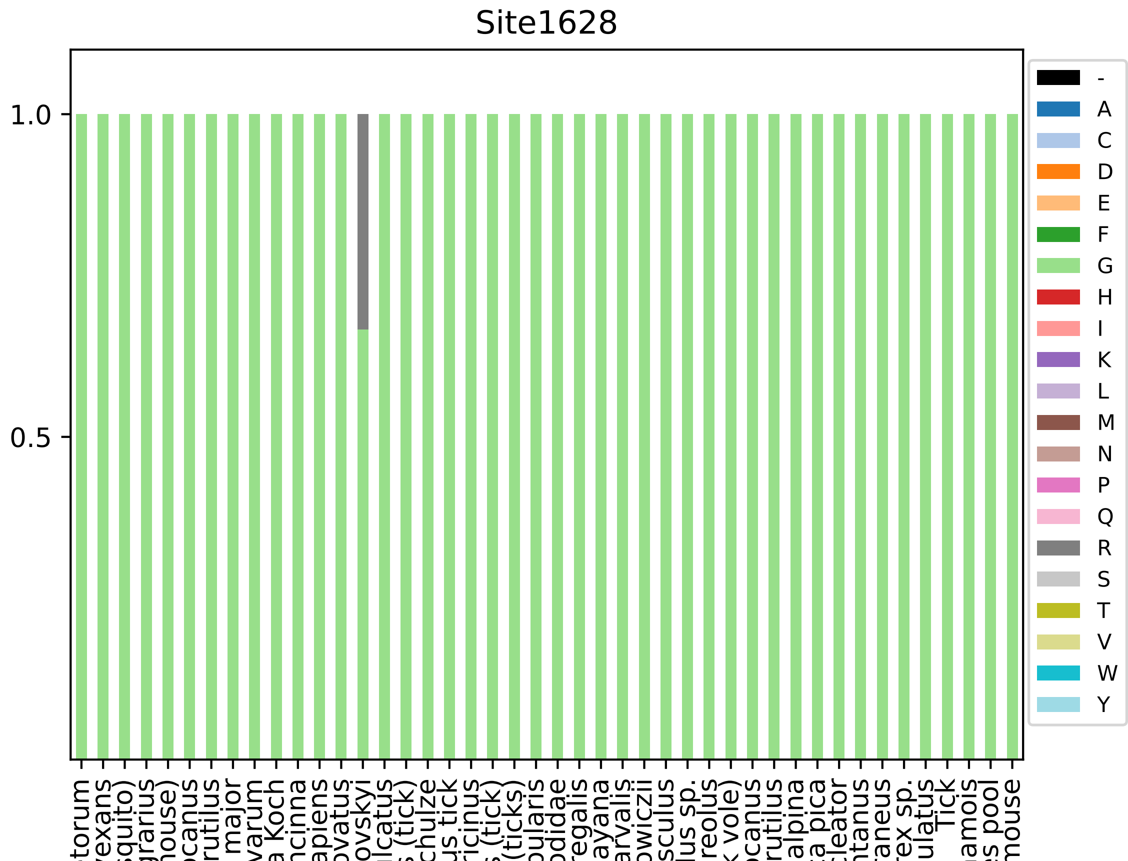
Site1628
1.0
0.5
-
A
C
D
E
F
G
H
I
K
L
M
N
P
Q
R
S
T
V
W
Y

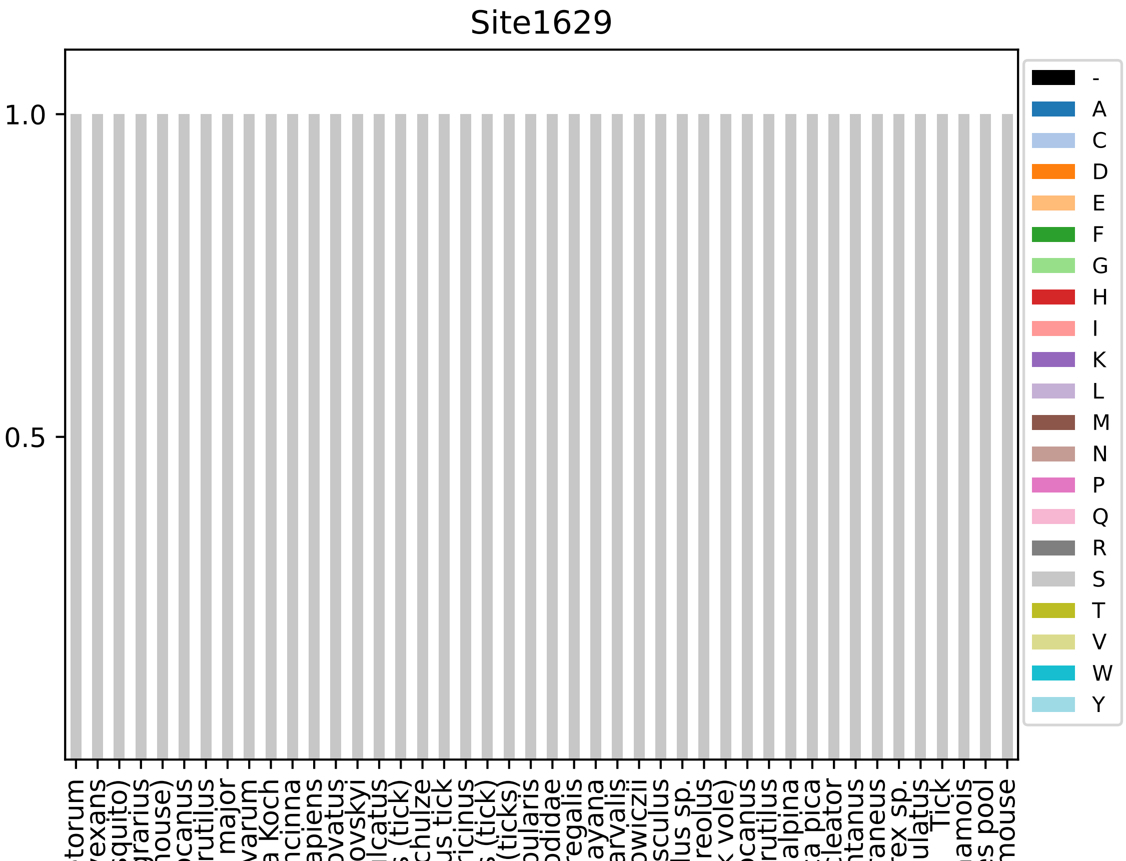
Site1629
1.0
0.5
-
A
C
D
E
F
G
H
I
K
L
M
N
P
Q
R
S
T
V
W
Y

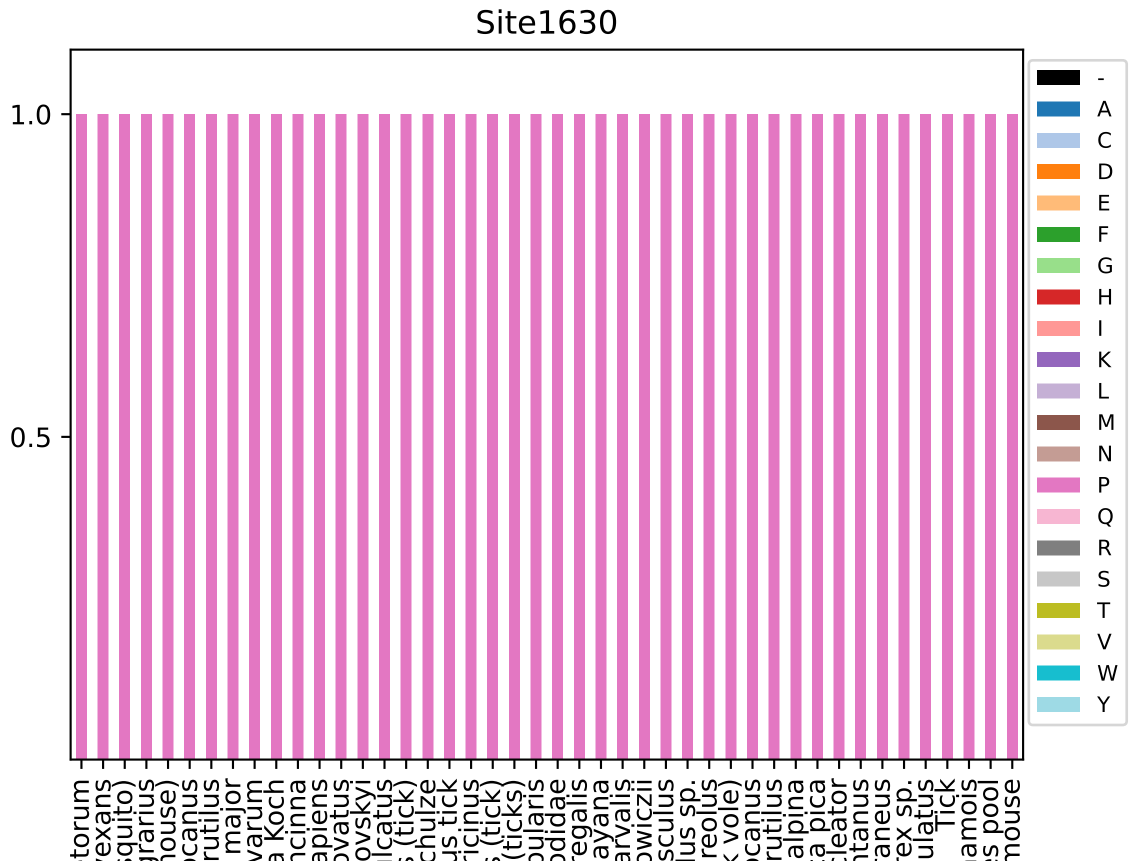
Site1630
1.0
0.5
-
A
C
D
E
F
G
H
I
K
L
M
N
P
Q
R
S
T
V
W
Y

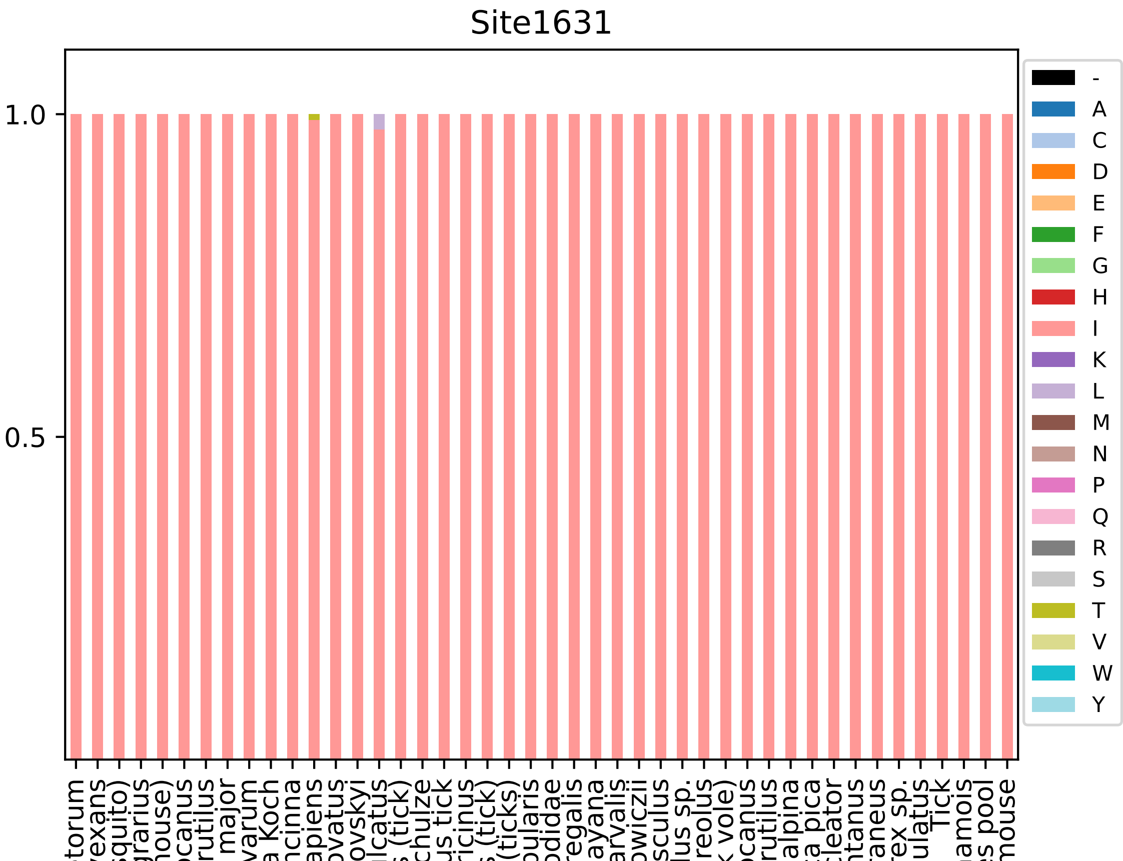

Site1631
1.0
0.5
-
A
C
D
E
F
G
H
I
K
L
M
N
P
Q
R
S
T
V
W
Y

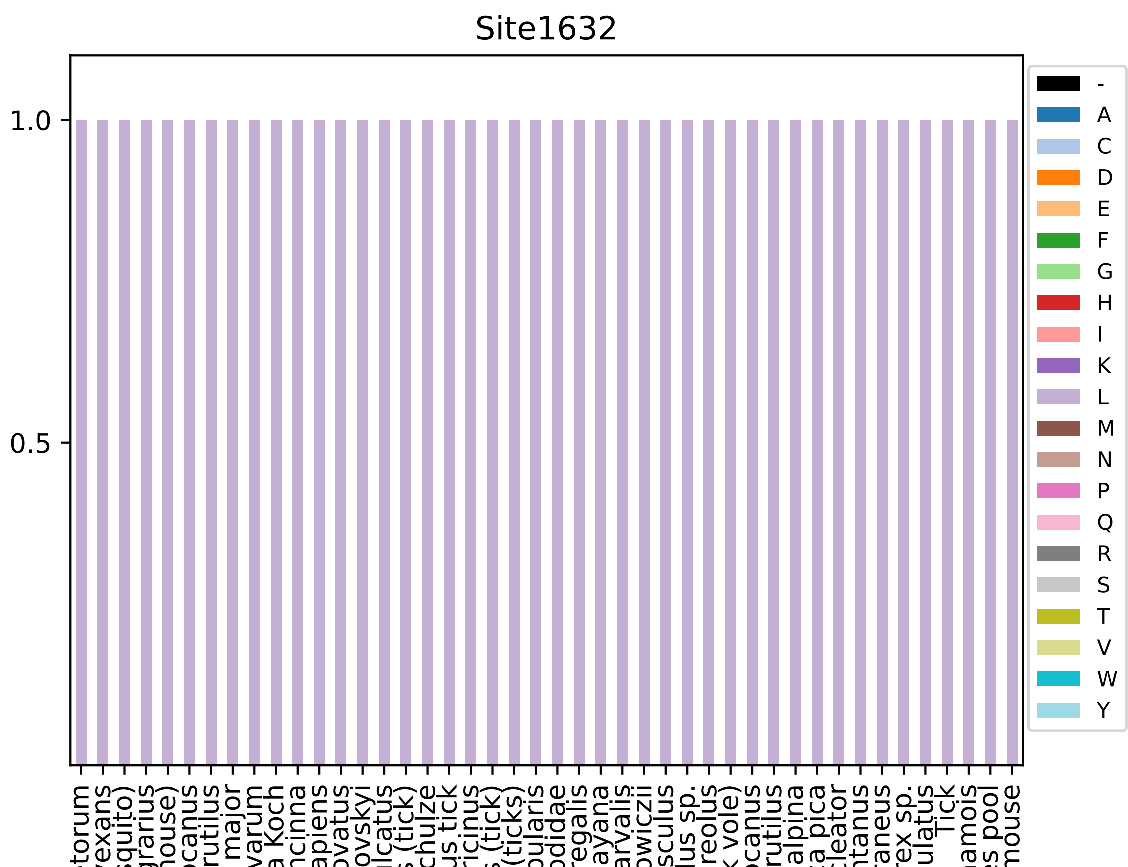
Site1632
1.0
0.5
-
A
C
D
E
F
G
H
I
K
L
M
N
P
Q
R
S
T
V
W
Y

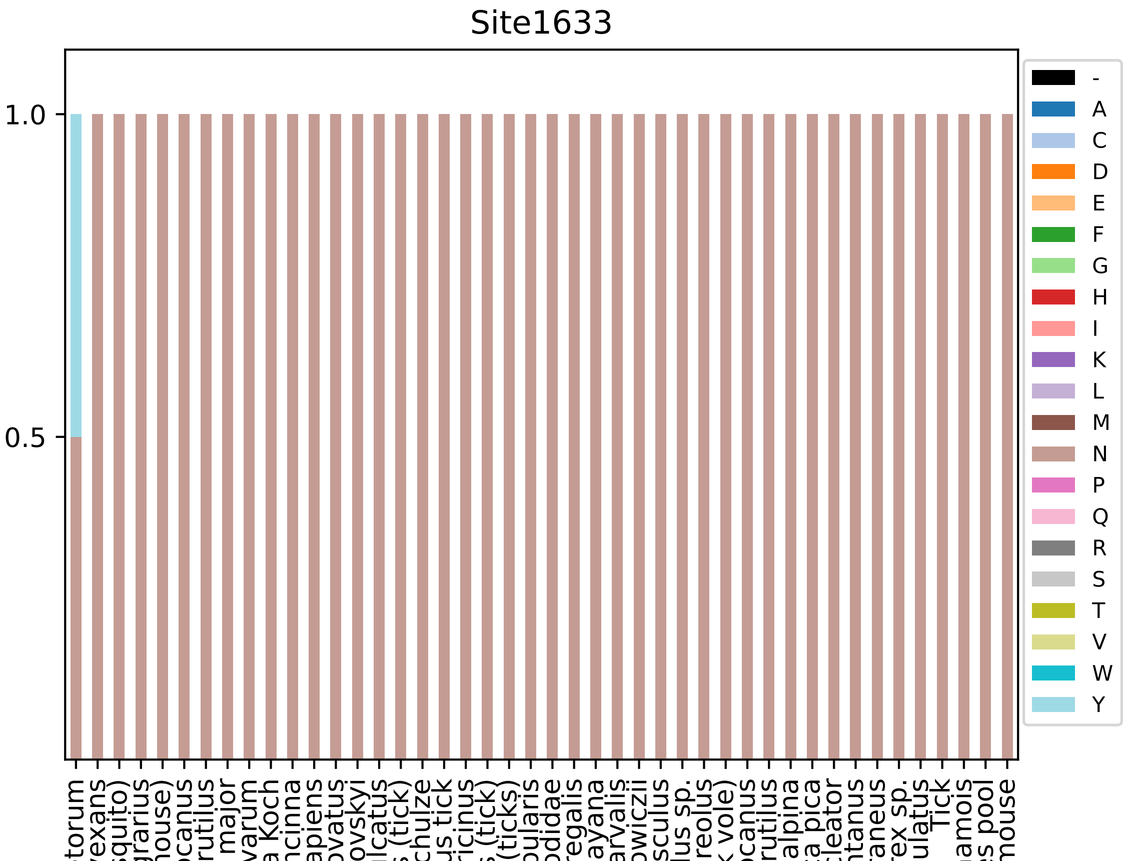
Site1633
1.0
0.5
-
A
C
D
E
F
G
H
I
K
L
M
N
P
Q
R
S
T
V
W
Y

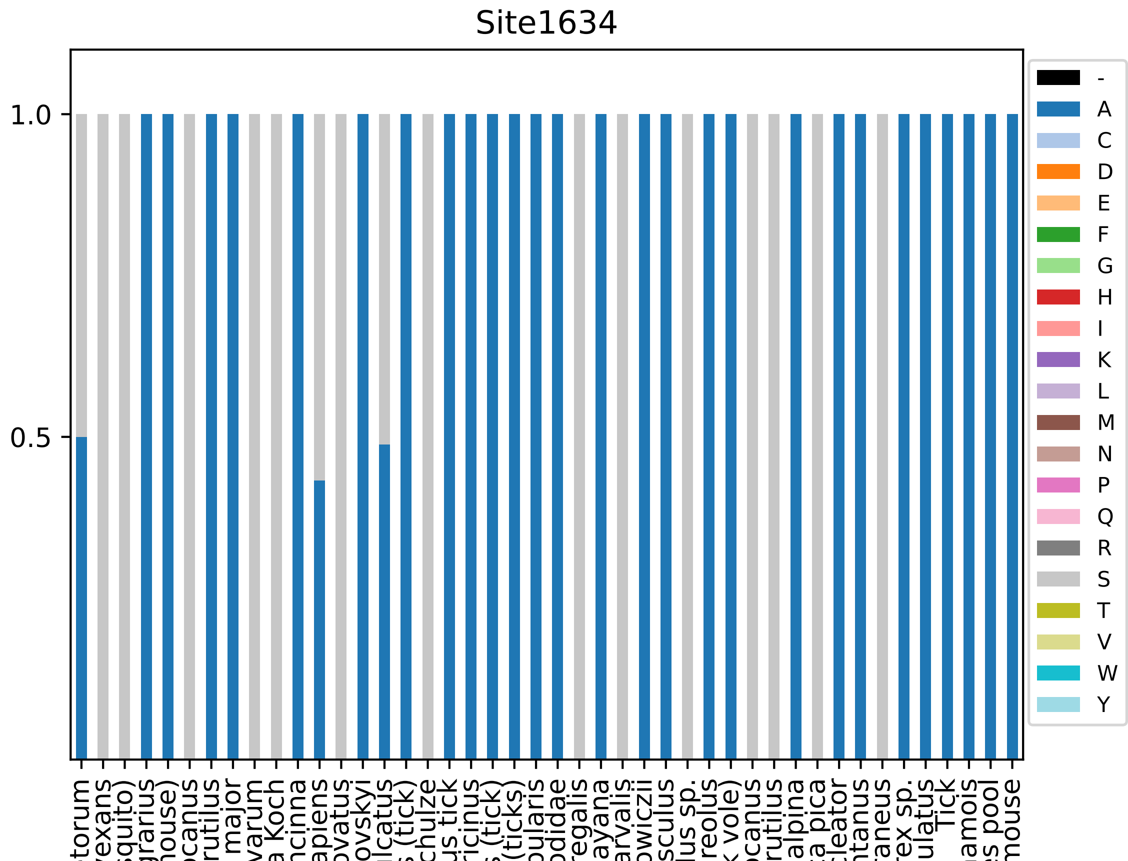
Site1634
1.0
0.5
-
A
C
D
E
F
G
H
I
K
L
M
N
P
Q
R
S
T
V
W
Y

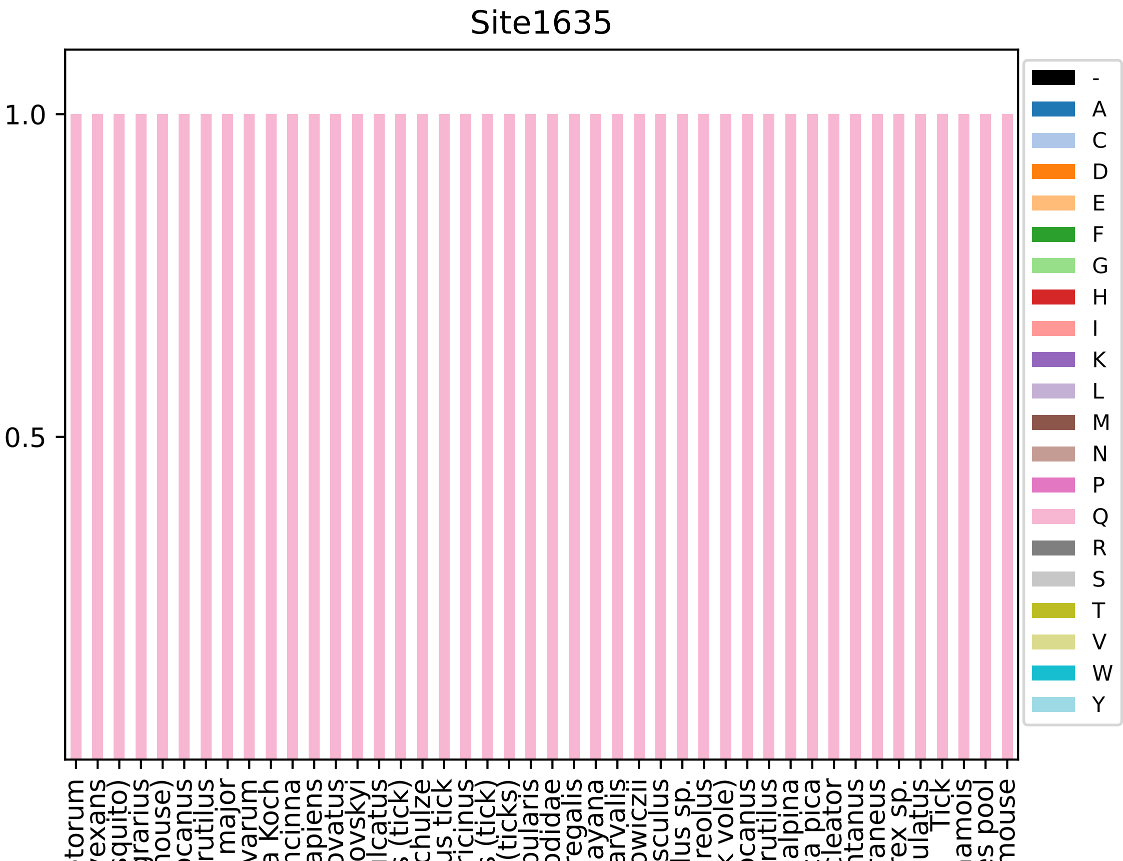

Site1635
1.0
0.5
-
A
C
D
E
F
G
H
I
K
L
M
N
P
Q
R
S
T
V
W
Y

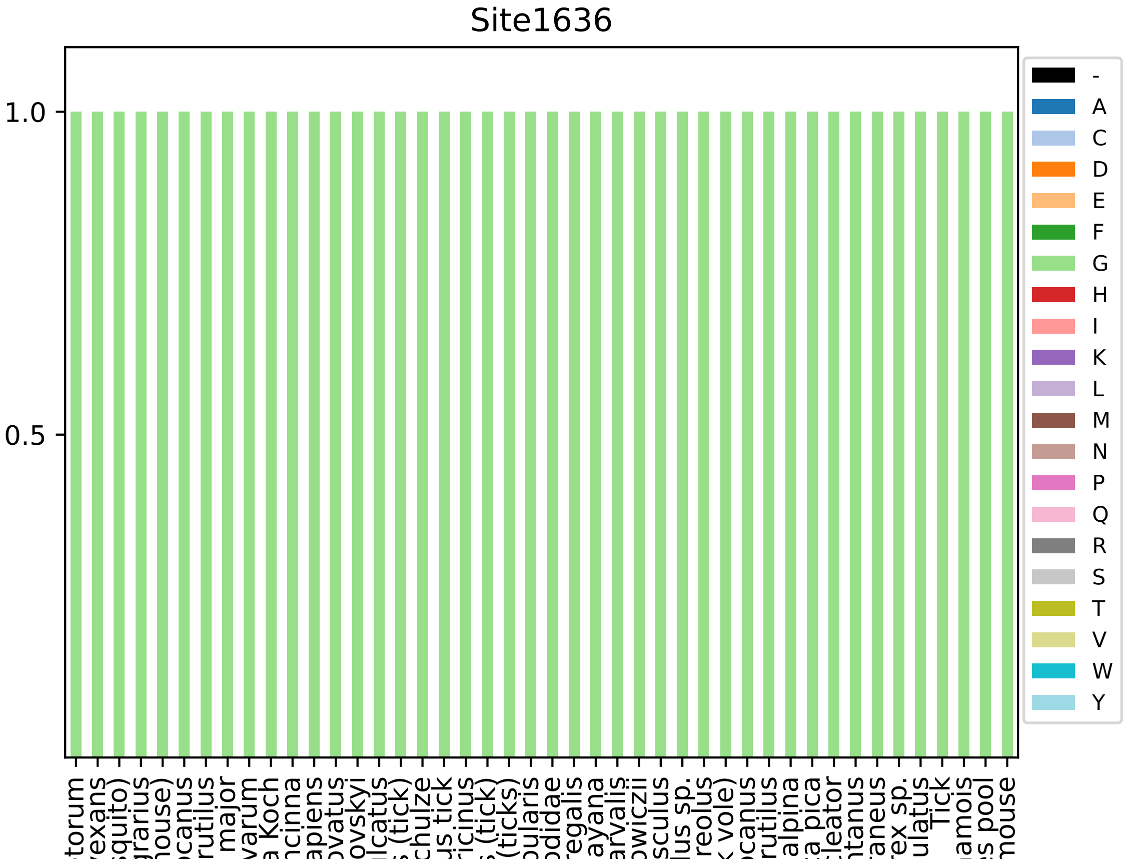

Site1636
1.0
0.5
-
A
C
D
E
F
G
H
I
K
L
M
N
P
Q
R
S
T
V
W
Y

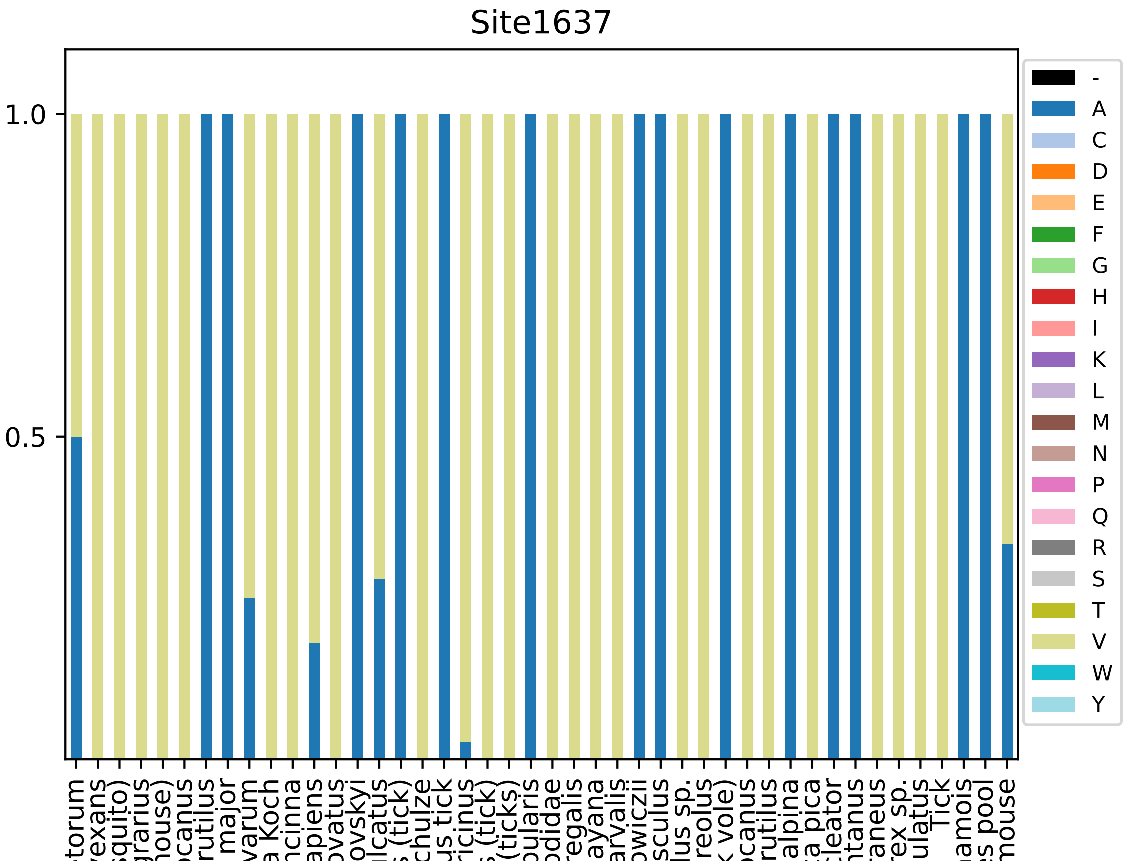
Site1637
1.0
0.5
-
A
C
D
E
F
G
H
I
K
L
M
N
P
Q
R
S
T
V
W
Y

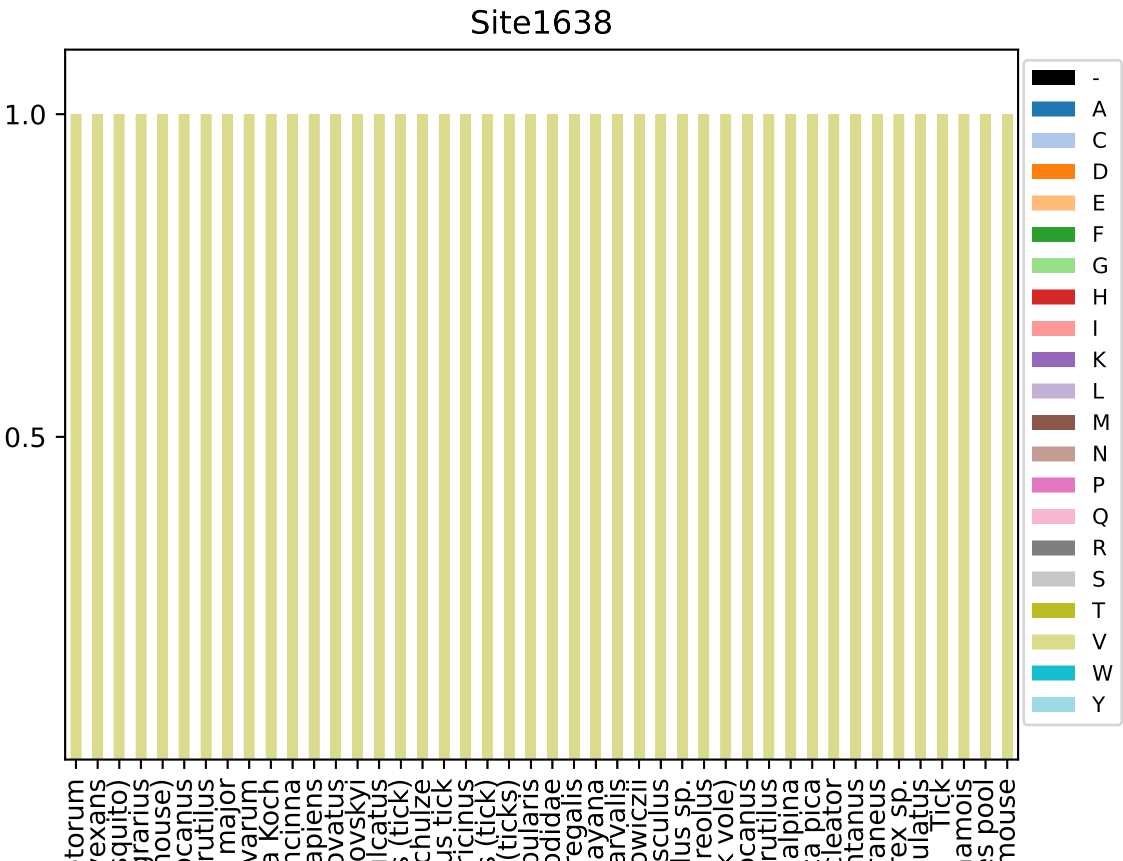

Site1638
1.0
0.5
-
A
C
D
E
F
G
H
I
K
L
M
N
P
Q
R
S
T
V
W
Y

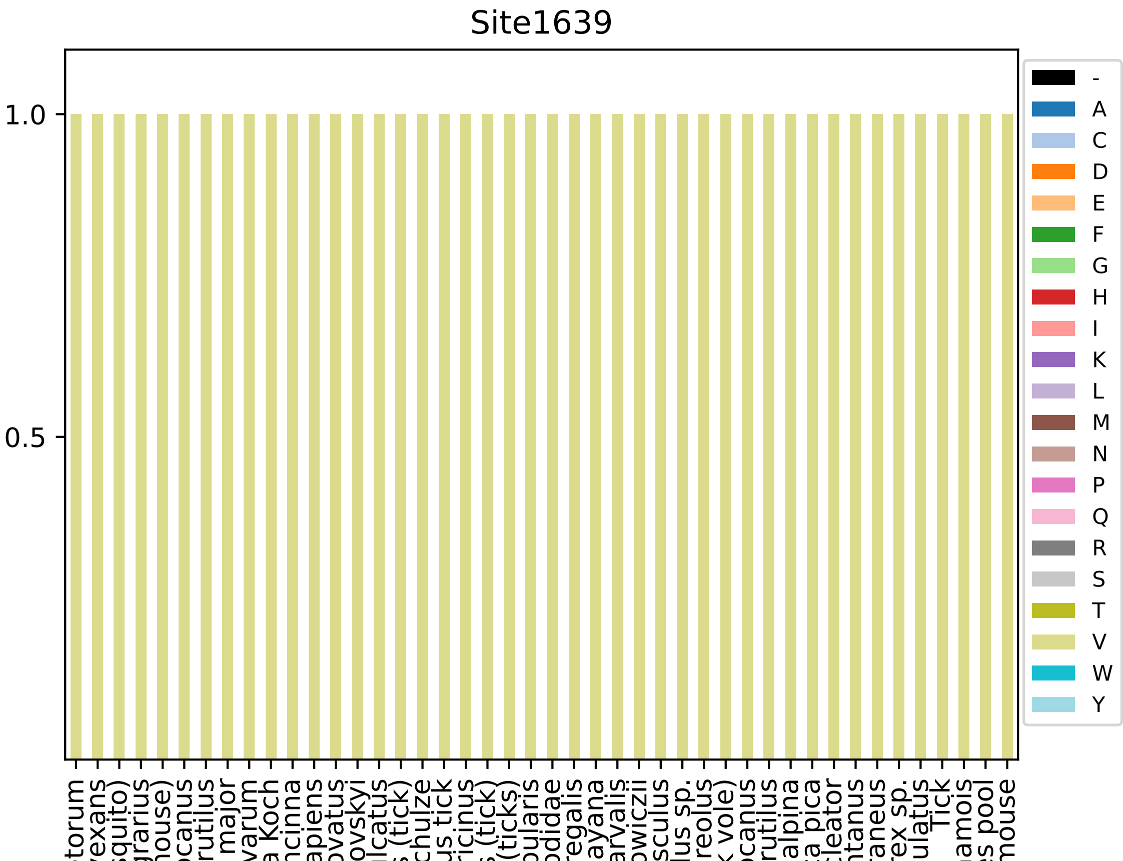
Site1639
1.0
0.5
-
A
C
D
E
F
G
H
I
K
L
M
N
P
Q
R
S
T
V
W
Y

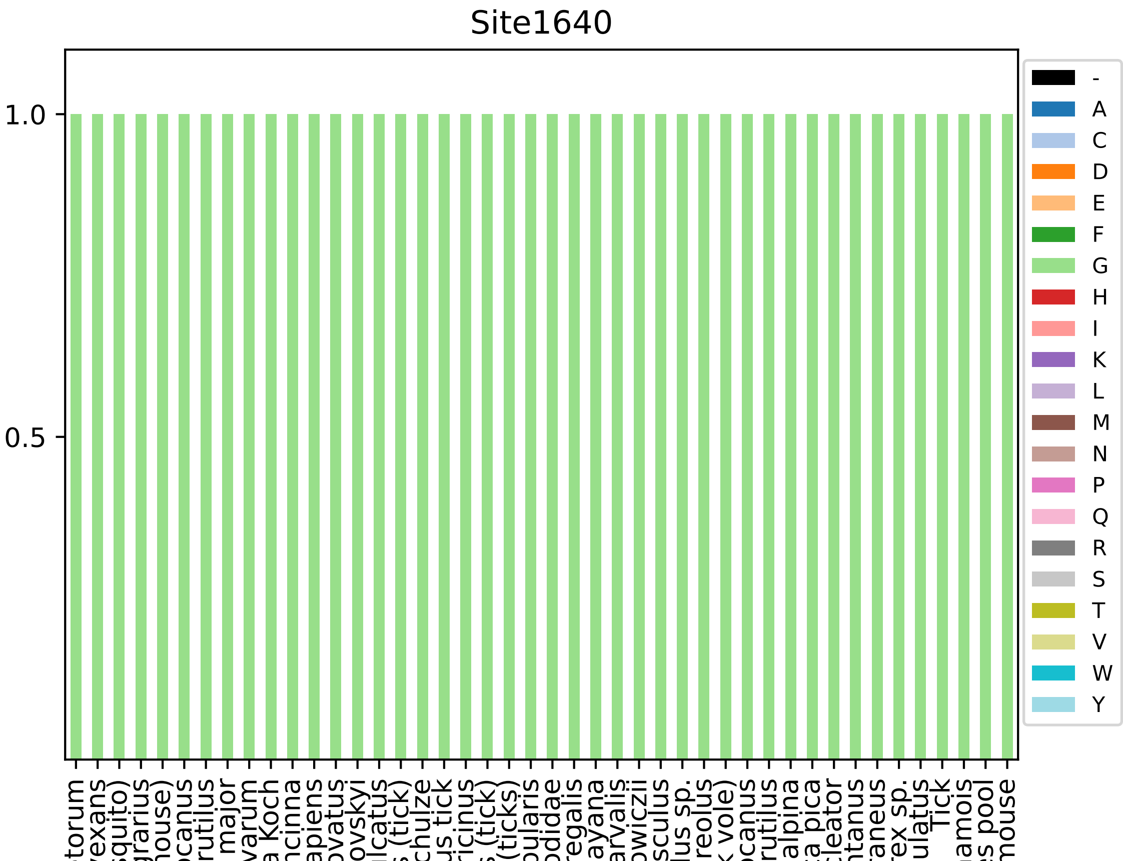
Site1640
1.0
0.5
-
A
C
D
E
F
G
H
I
K
L
M
N
P
Q
R
S
T
V
W
Y

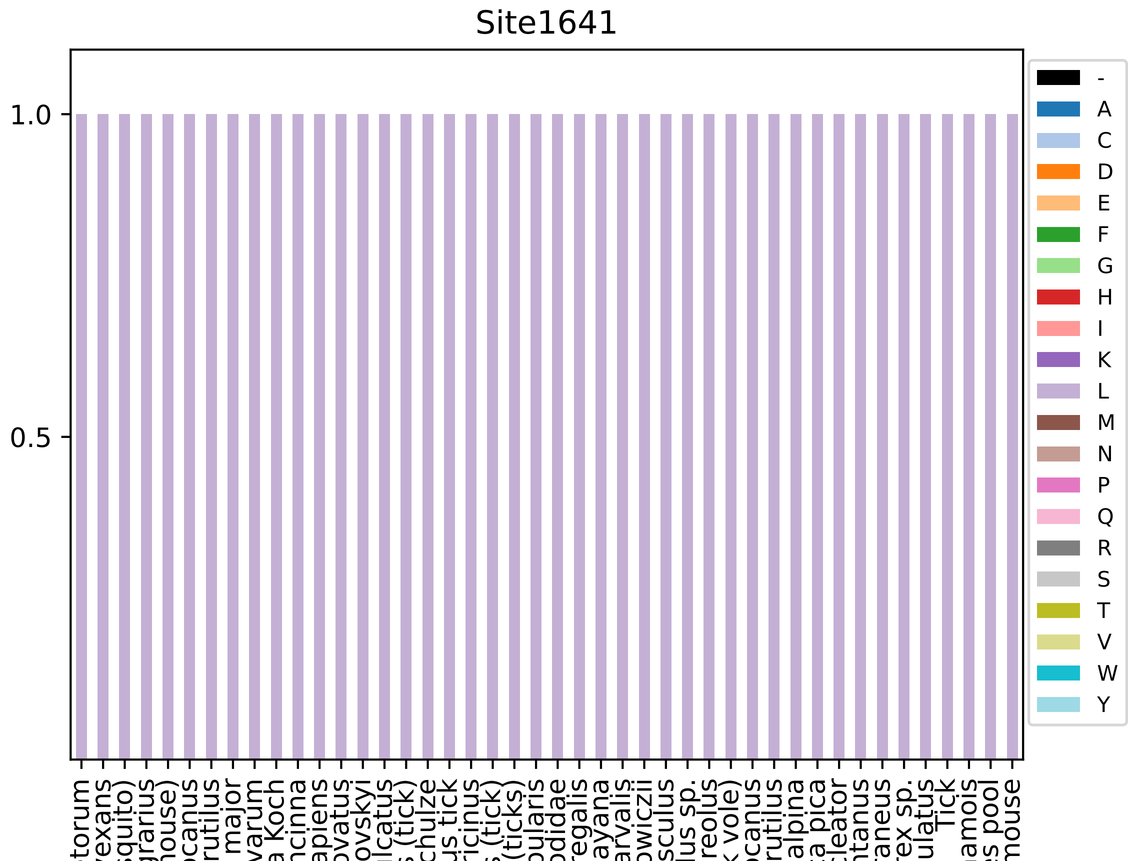
Site1641
1.0
0.5
-
A
C
D
E
F
G
H
I
K
L
M
N
P
Q
R
S
T
V
W
Y

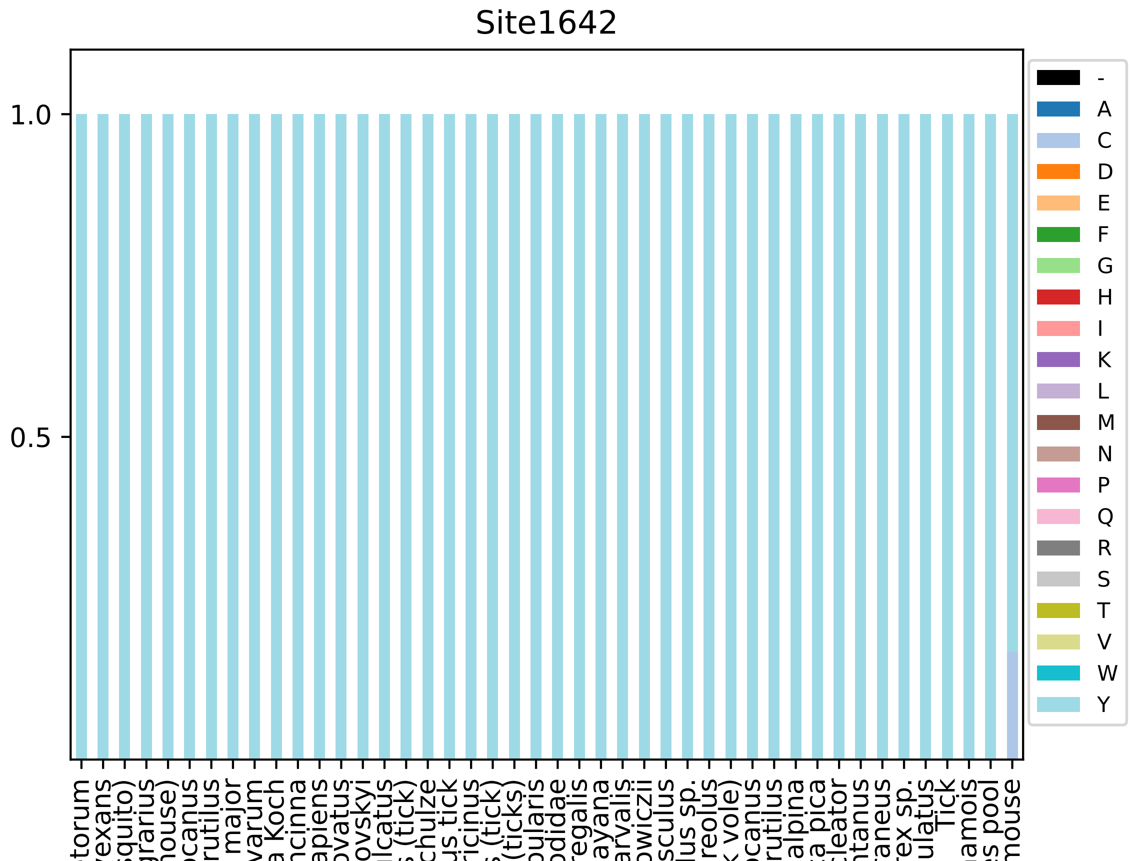

Site1642
1.0
0.5
-
A
C
D
E
F
G
H
I
K
L
M
N
P
Q
R
S
T
V
W
Y

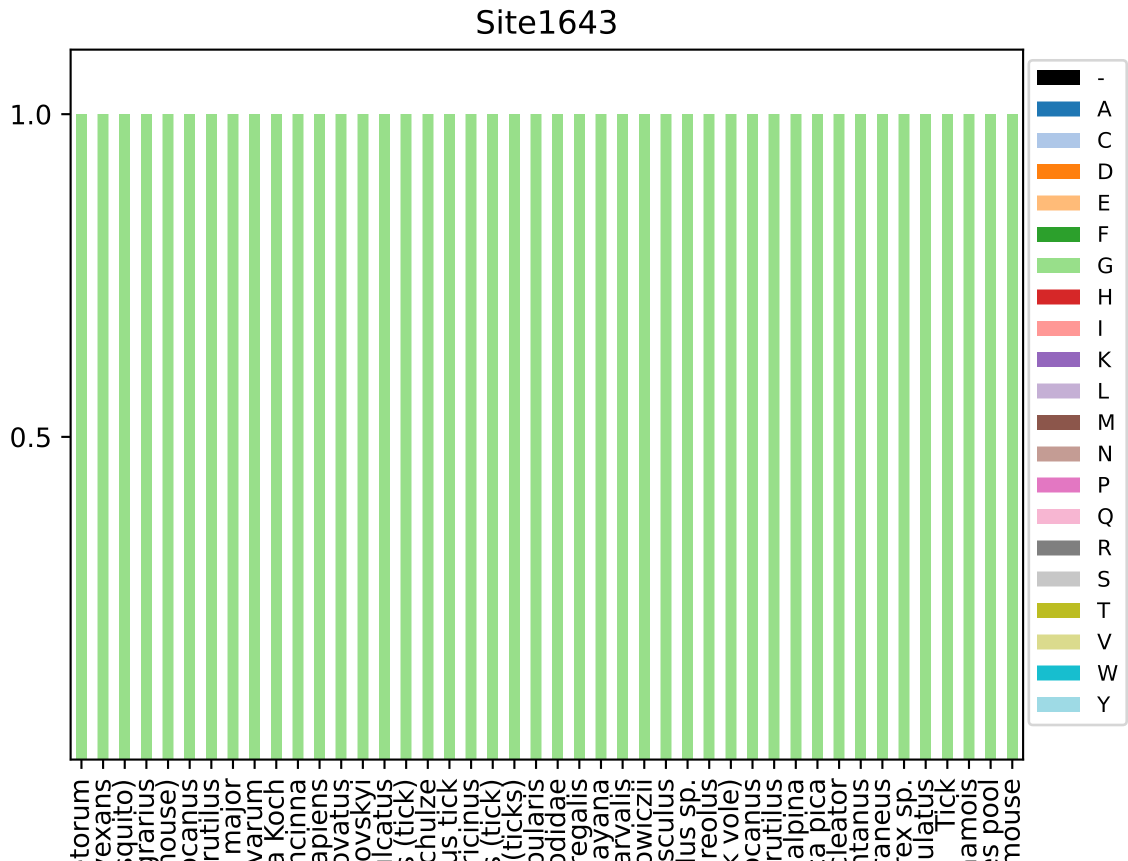
Site1643
1.0
0.5
-
A
C
D
E
F
G
H
I
K
L
M
N
P
Q
R
S
T
V
W
Y

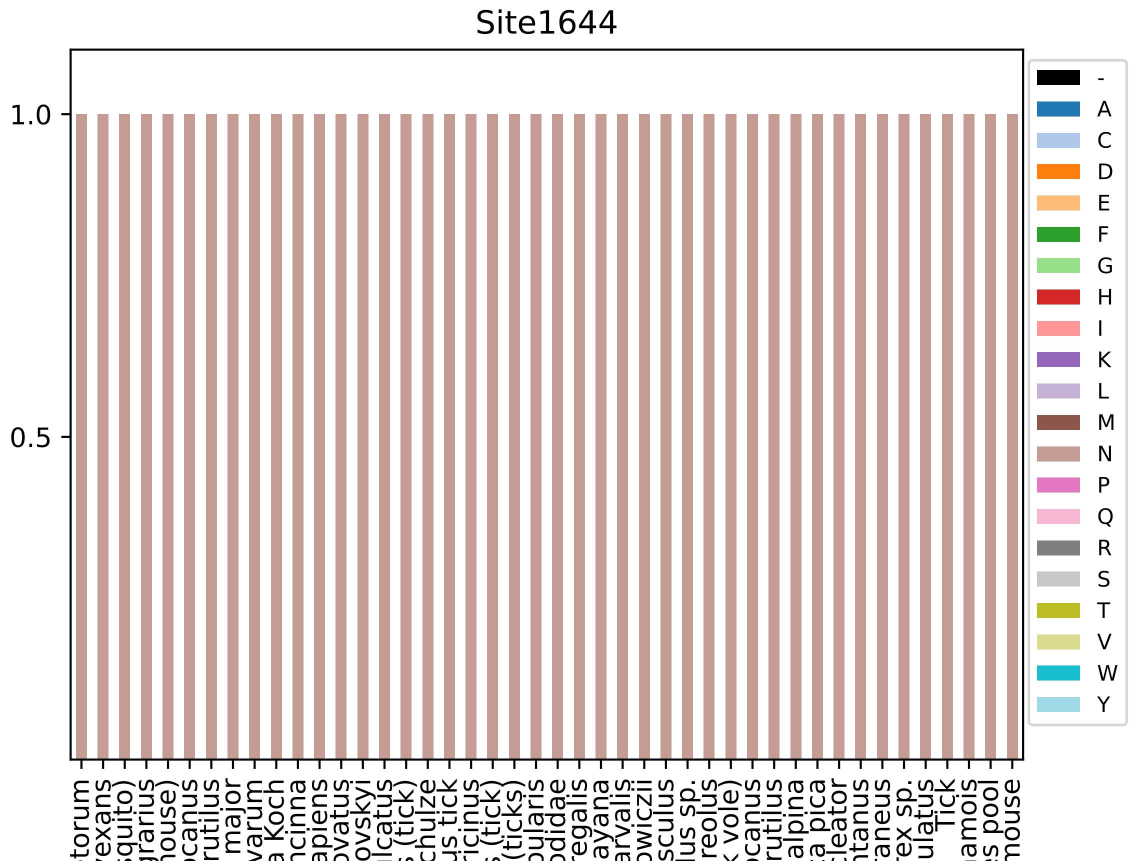

Site1644
1.0
0.5
-
A
C
D
E
F
G
H
I
K
L
M
N
P
Q
R
S
T
V
W
Y

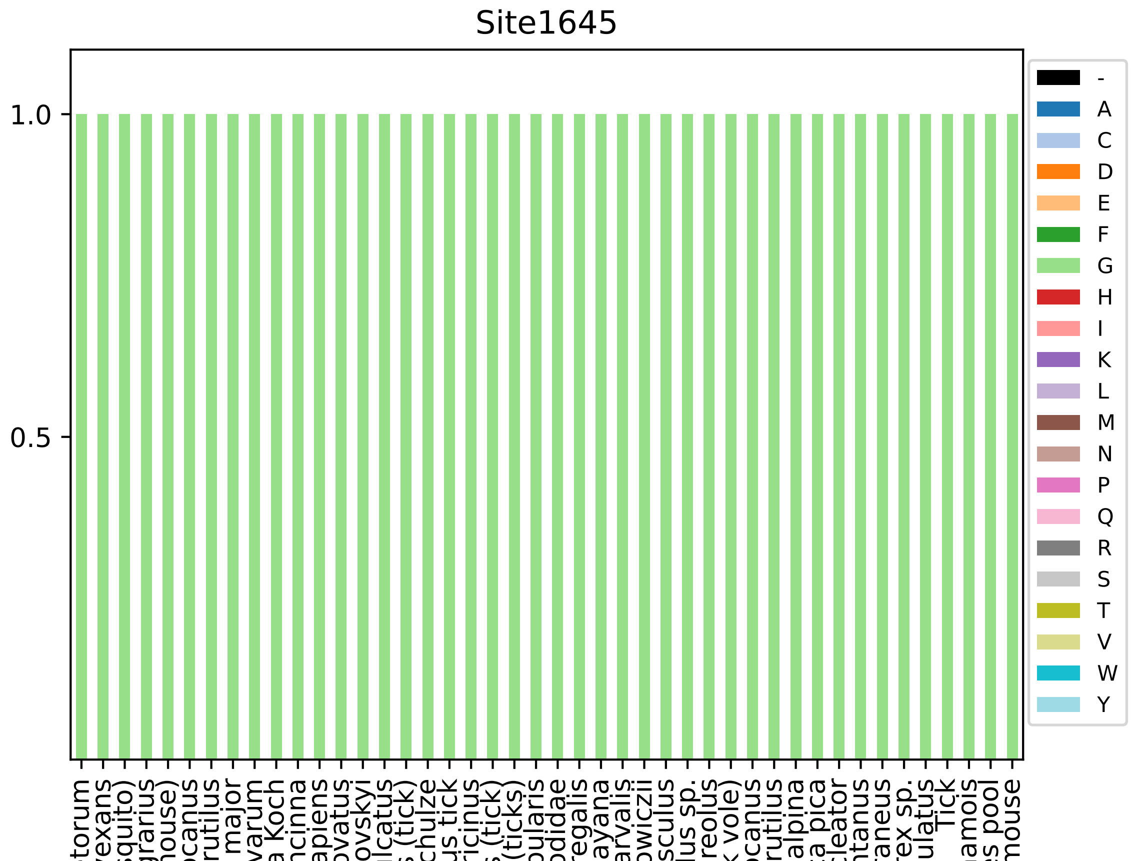
Site1645
1.0
0.5
-
A
C
D
E
F
G
H
I
K
L
M
N
P
Q
R
S
T
V
W
Y

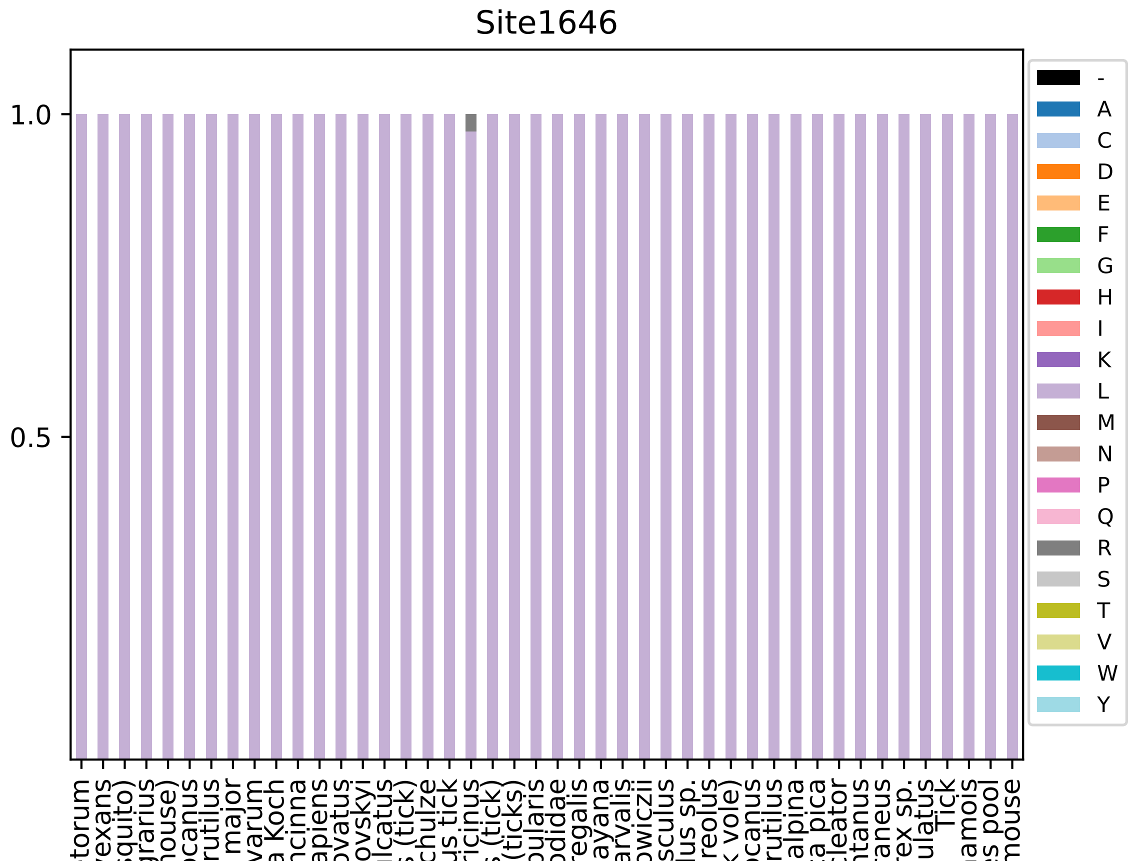
Site1646
1.0
0.5
-
A
C
D
E
F
G
H
I
K
L
M
N
P
Q
R
S
T
V
W
Y

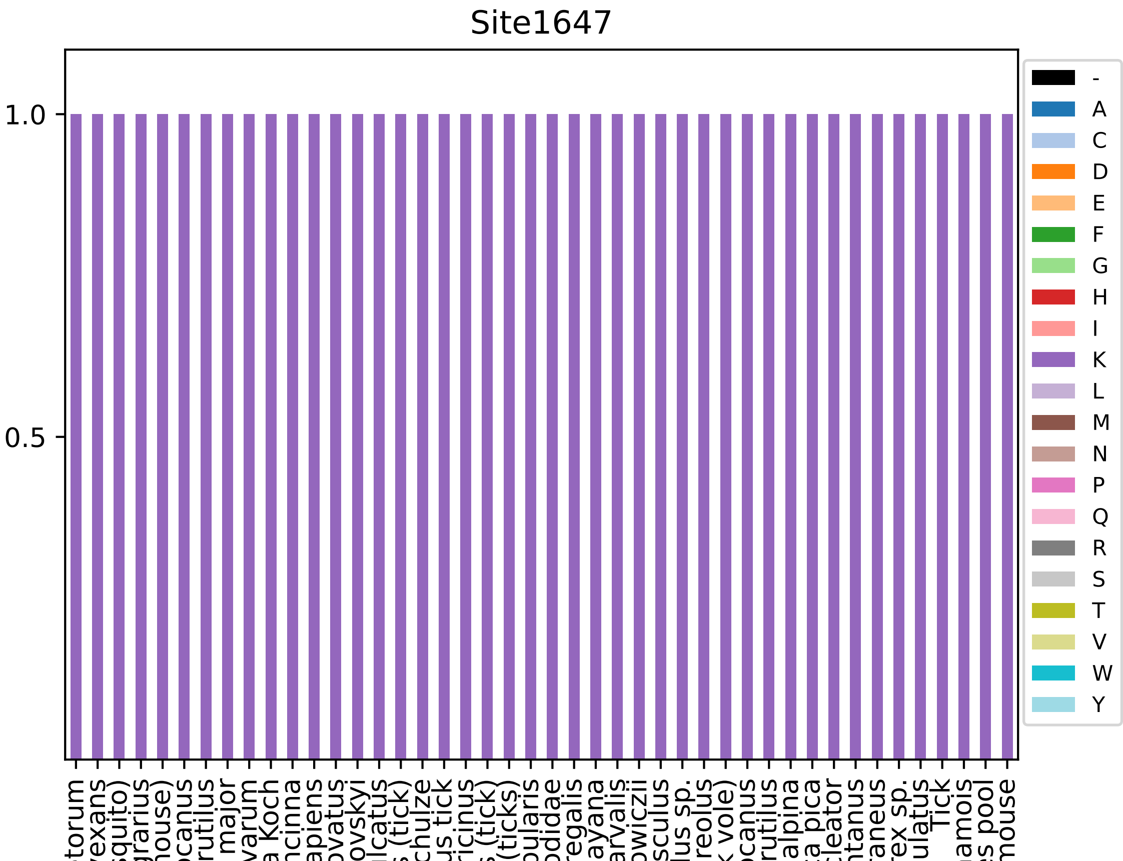
Site1647
1.0
0.5
-
A
C
D
E
F
G
H
I
K
L
M
N
P
Q
R
S
T
V
W
Y

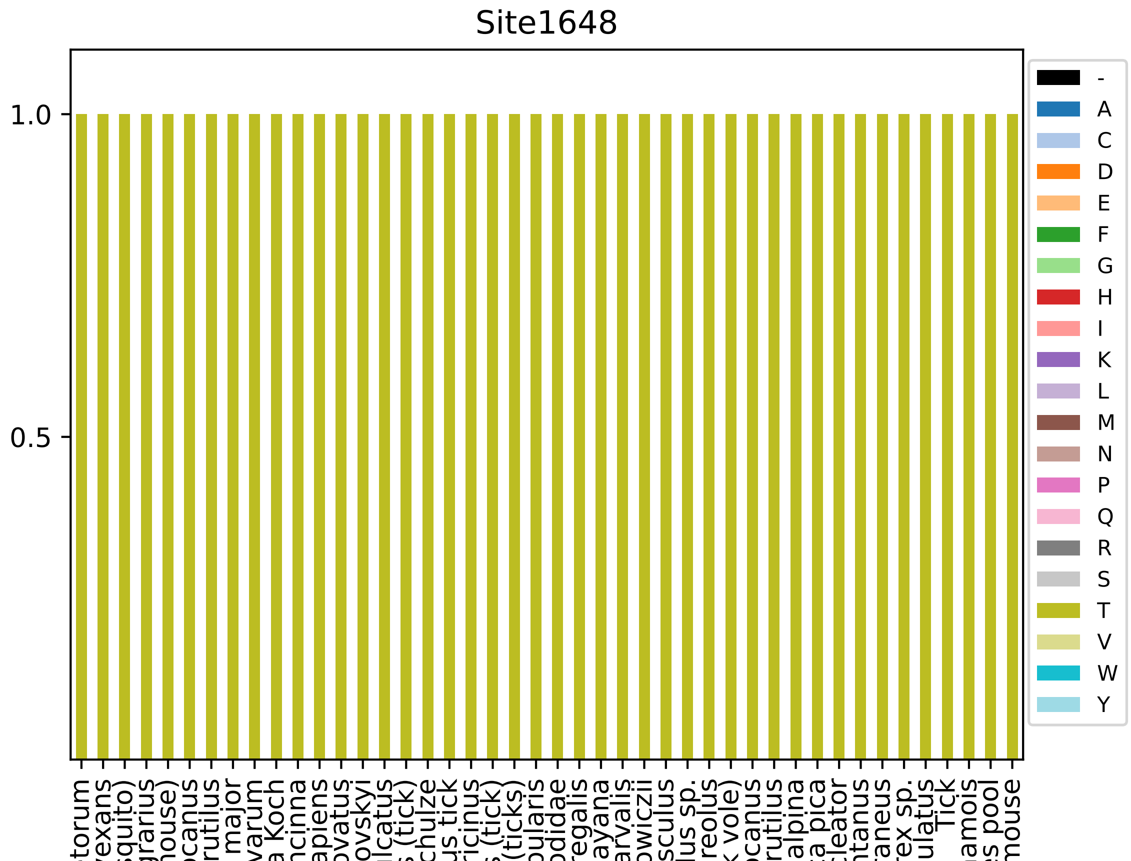

Site1648
1.0
0.5
-
A
C
D
E
F
G
H
I
K
L
M
N
P
Q
R
S
T
V
W
Y

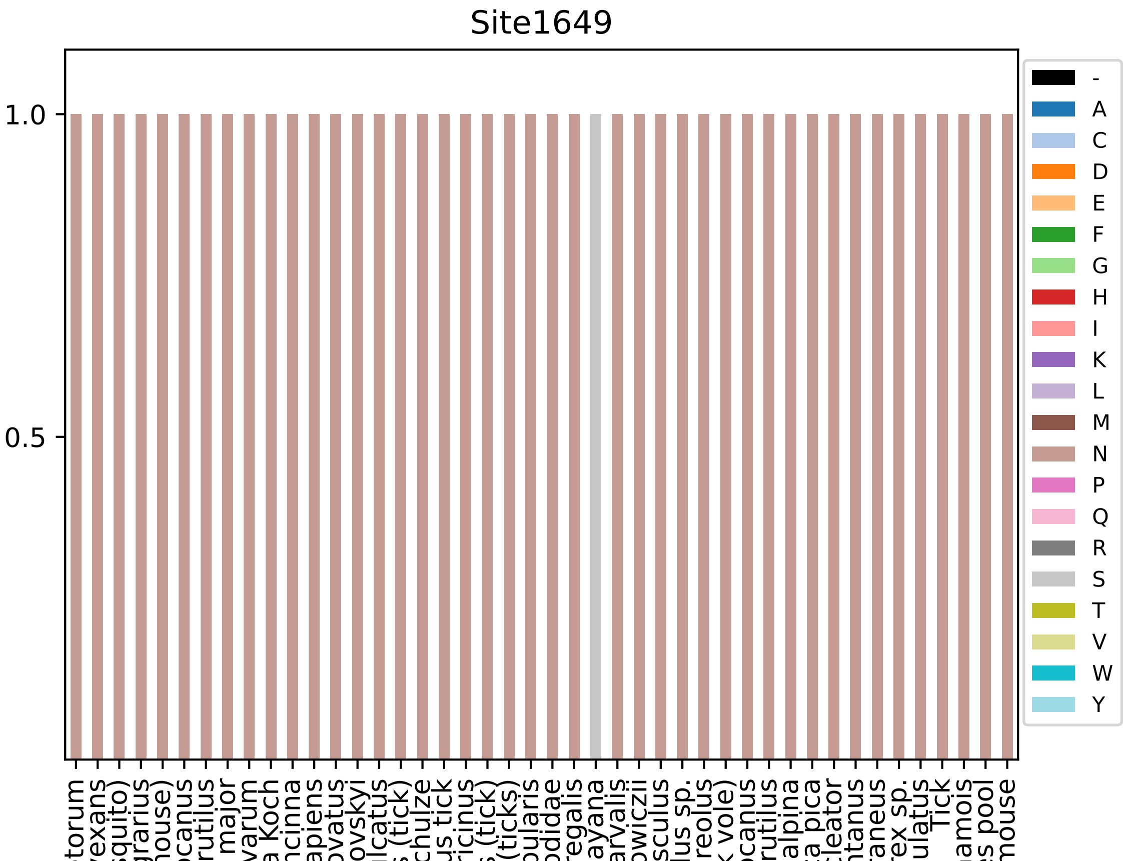
Site1649
1.0
0.5
-
A
C
D
E
F
G
H
I
K
L
M
N
P
Q
R
S
T
V
W
Y

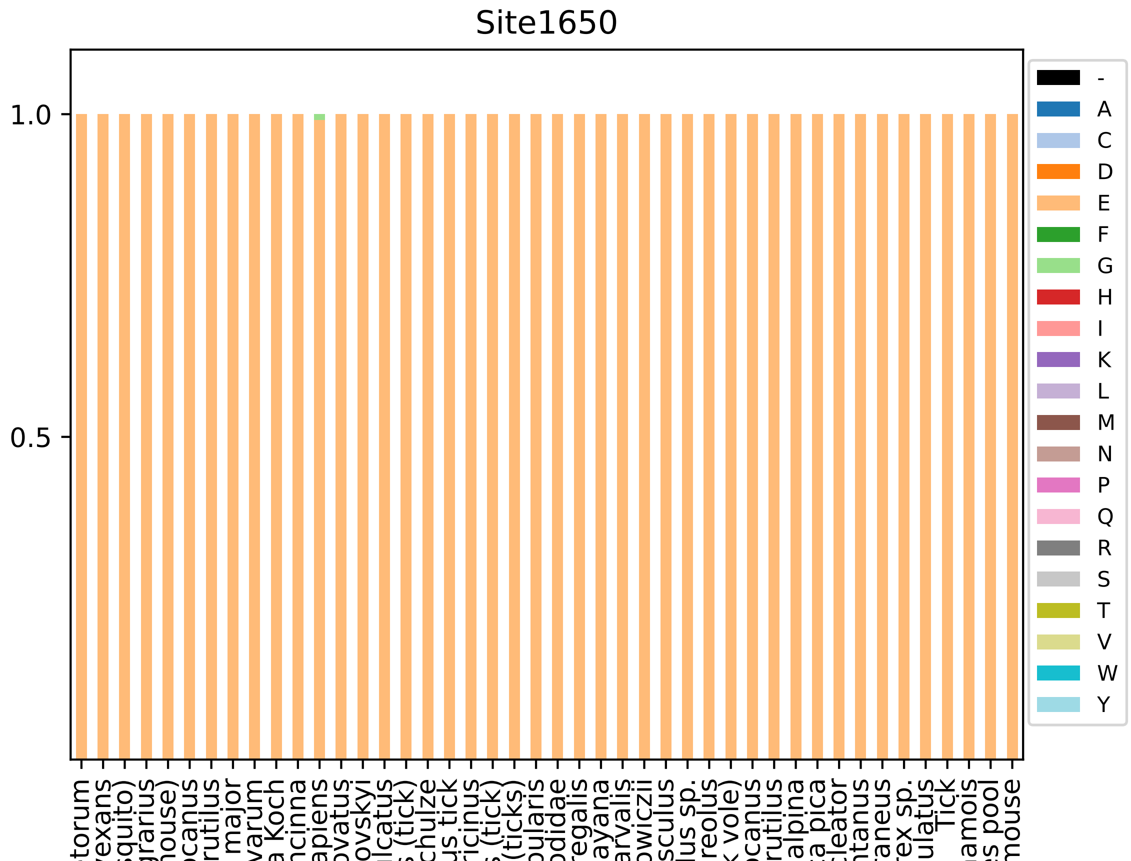
Site1650
1.0
0.5
-
A
C
D
E
F
G
H
I
K
L
M
N
P
Q
R
S
T
V
W
Y

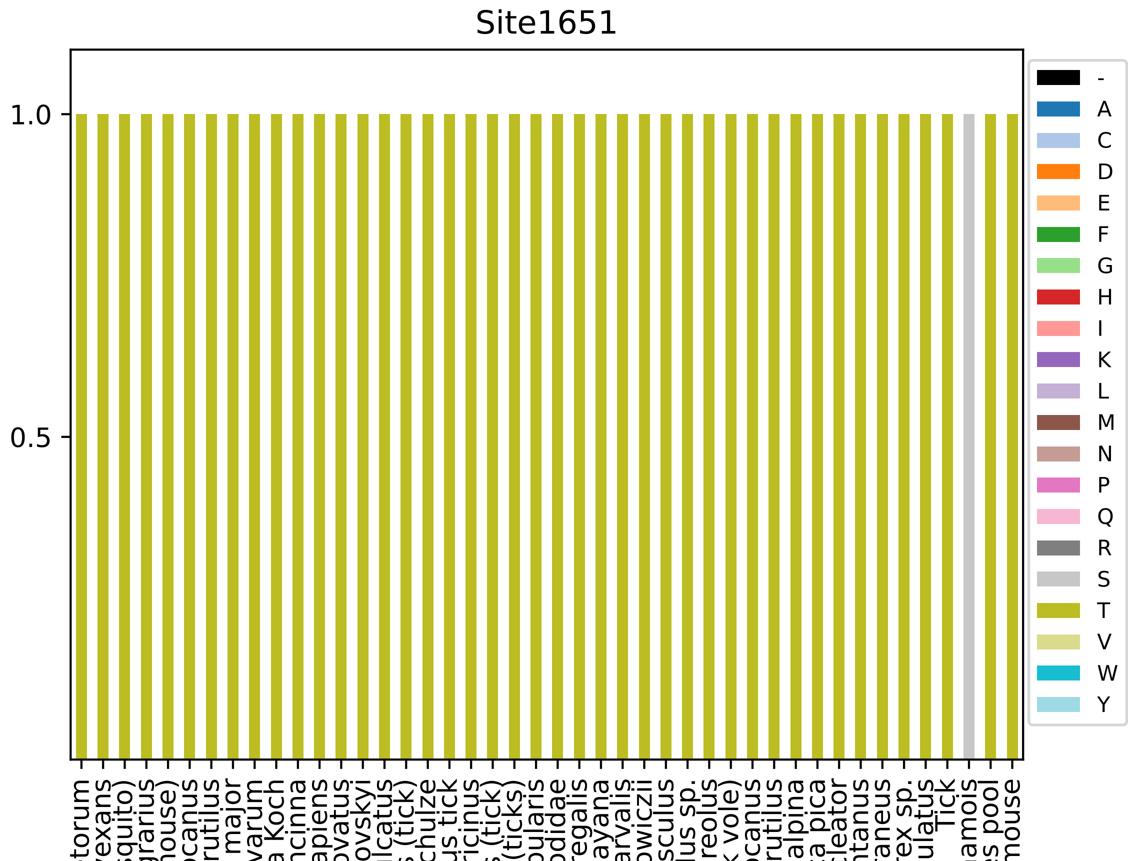

Site1651
1.0
0.5
-
A
C
D
E
F
G
H
I
K
L
M
N
P
Q
R
S
T
V
W
Y
torum
vexans
squito)
grarius
nouse)
ocanus
rutilus
major
varum
a Koch
ncinna
apiens
ovatus
ovskyi
ulcatus
s (tick)
chulze
us tick
ricinus
s (tick)
(ticks)
pularis
podidae
regalis
ayana
arvalis
owiczii
sculus
lus sp.
reolus
k vole)
ocanus
rutilus
alpina
a pica
cleator
ntanus
raneus
rex sp.
ulatus
Tick
amois
es pool
mouse

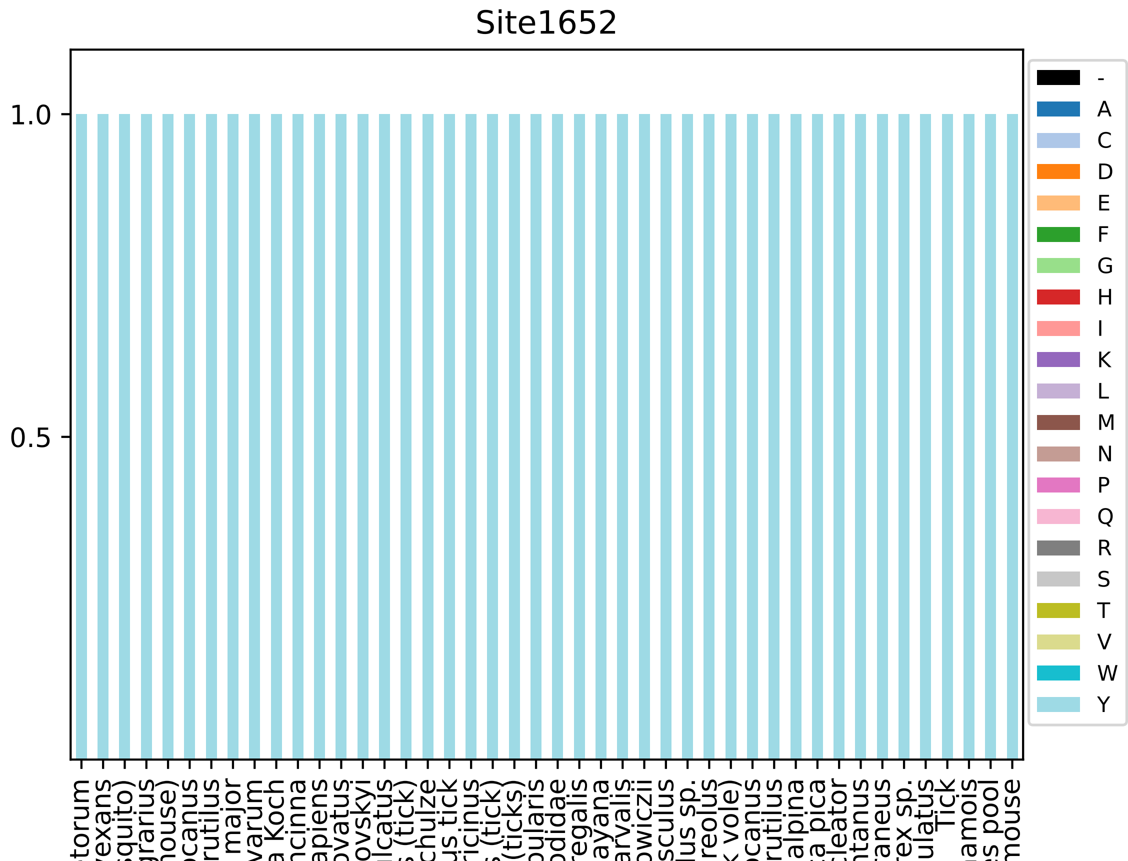
Site1652
1.0
0.5
-
A
C
D
E
F
G
H
I
K
L
M
N
P
Q
R
S
T
V
W
Y

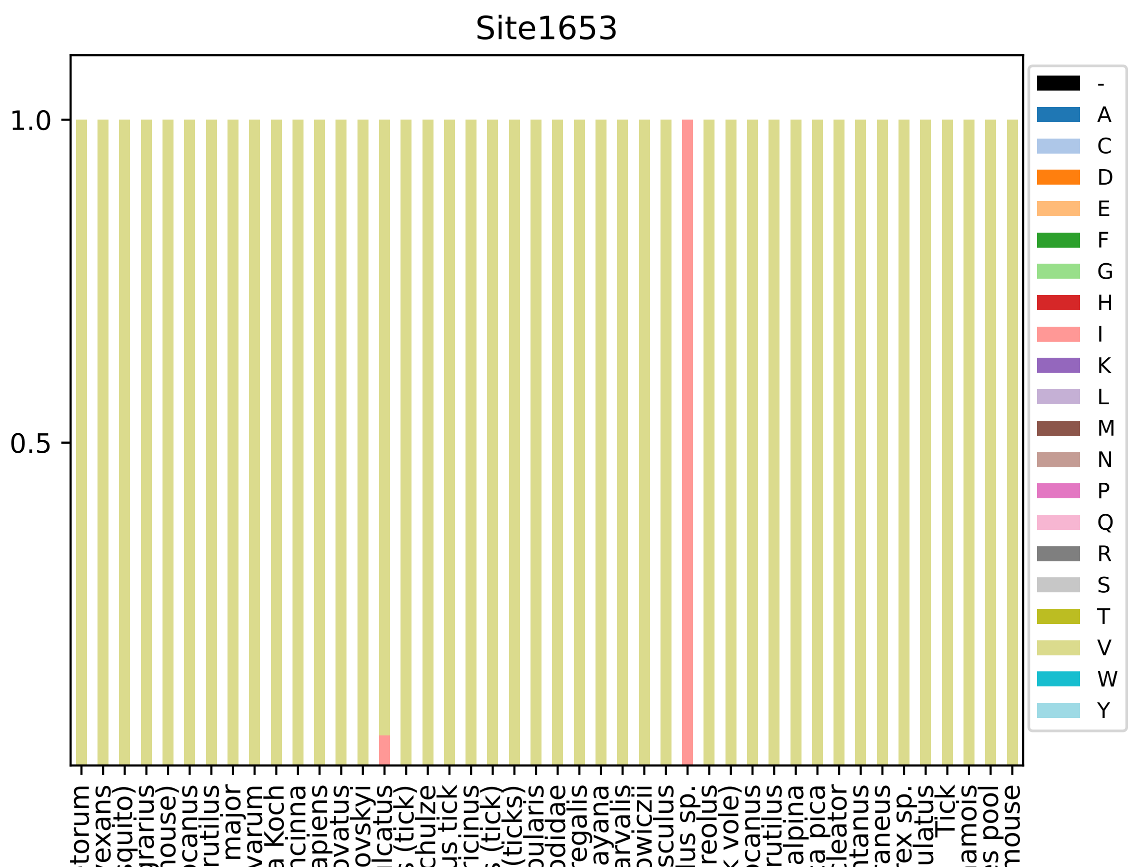
Site1653
1.0
0.5
-
A
C
D
E
F
G
H
I
K
L
M
N
P
Q
R
S
T
V
W
Y

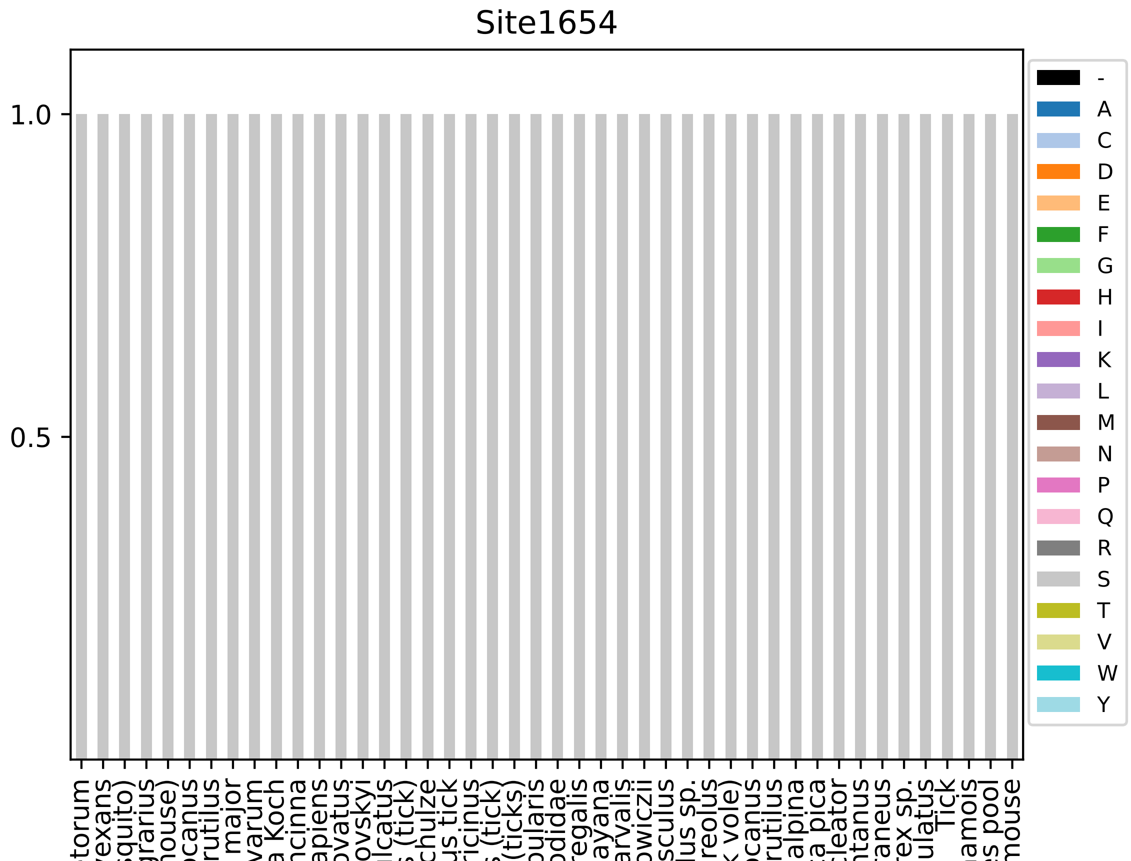
Site1654
1.0
0.5
-
A
C
D
E
F
G
H
I
K
L
M
N
P
Q
R
S
T
V
W
Y

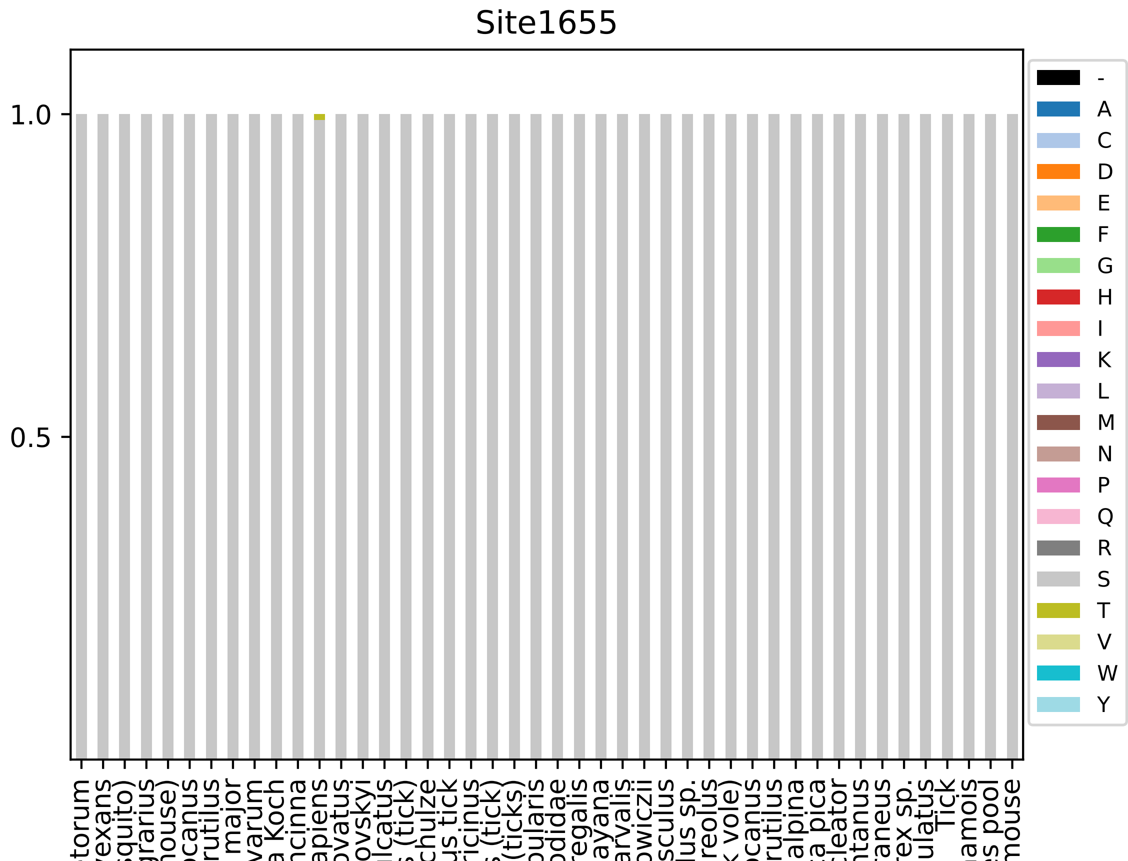
Site1655
1.0
0.5
-
A
C
D
E
F
G
H
I
K
L
M
N
P
Q
R
S
T
V
W
Y
torum
vexans
squito)
garius
nouse)
ocanus
rutilus
major
varum
a Koch
ncinna
apiens
ovatus
ovskyi
lcatus
s (tick)
chulze
us tick
ricinus
s (tick)
(ticks)
pularis
odidae
regalis
ayana
arvalis
owiczii
sculus
lus sp.
reolus
k vole)
ocanus
rutilus
alpina
a pica
cleator
ntanus
raneus
rex sp.
ulatus
Tick
amois
es pool
mouse

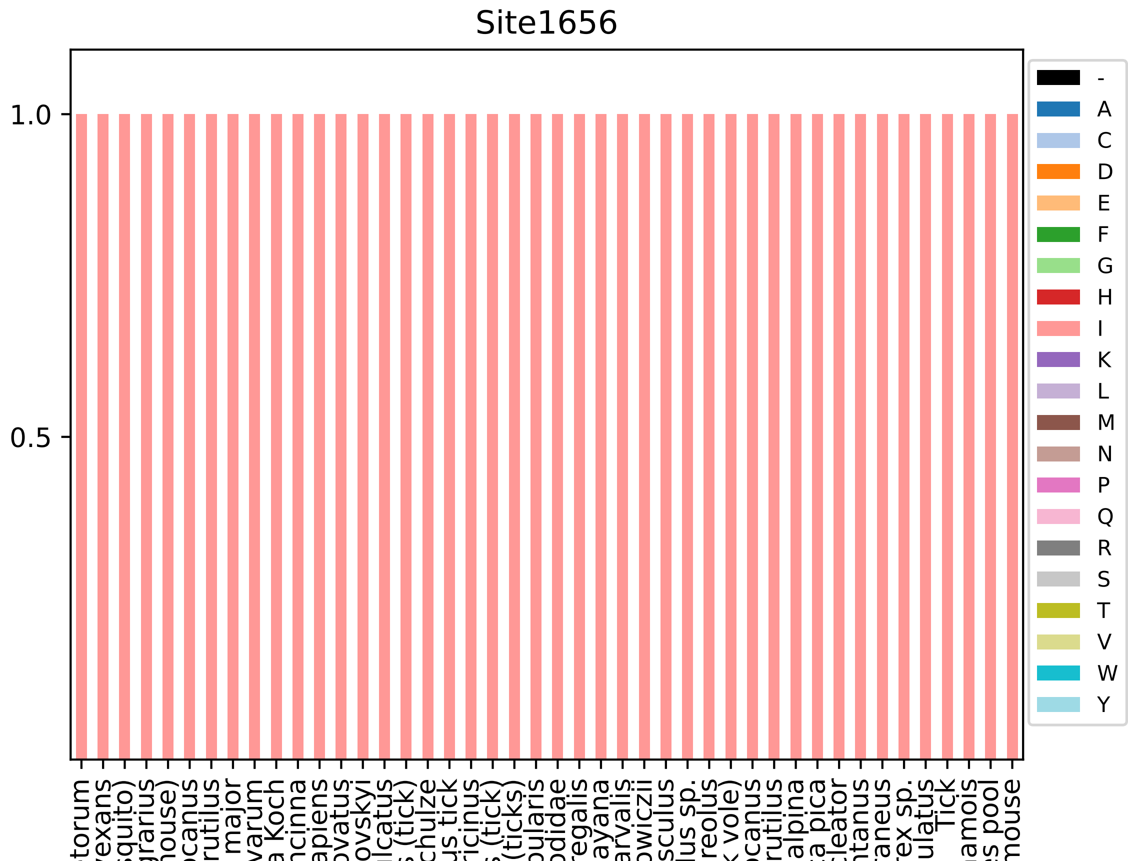
Site1656
1.0
0.5
-
A
C
D
E
F
G
H
I
K
L
M
N
P
Q
R
S
T
V
W
Y

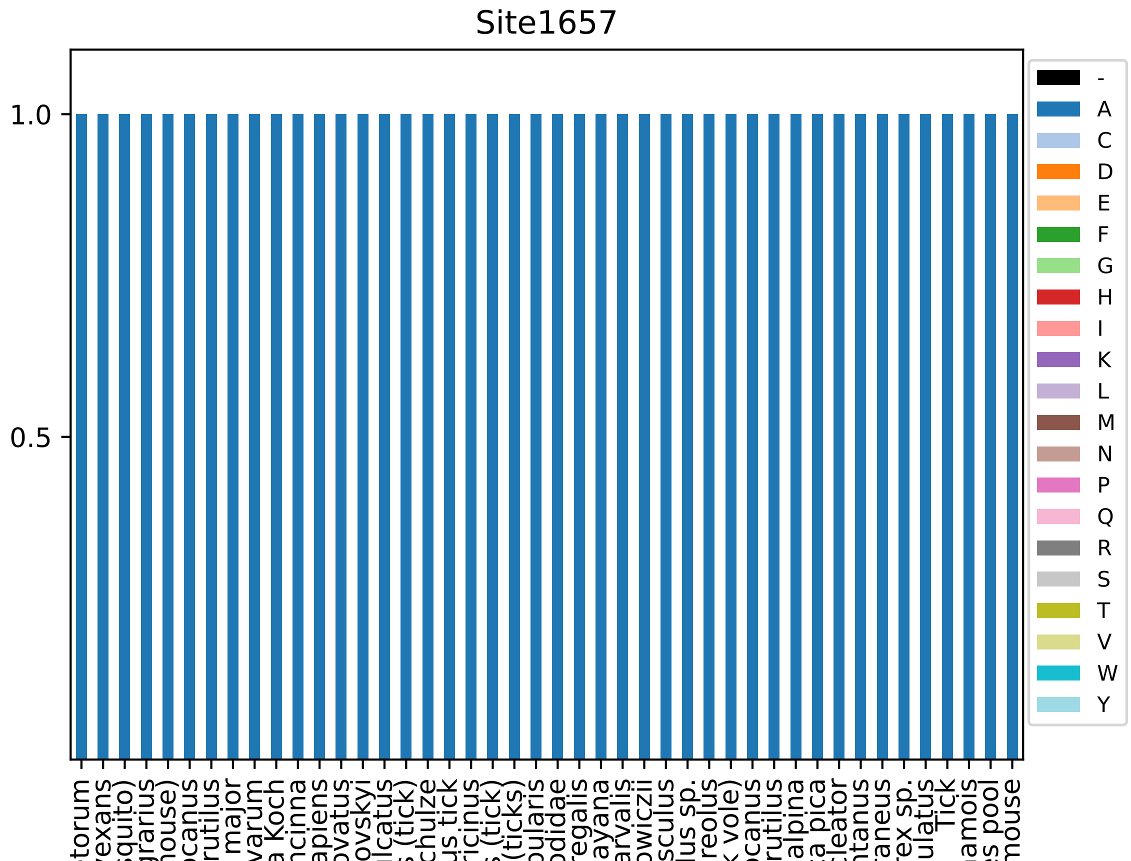
Site1657
1.0
0.5
-
A
C
D
E
F
G
H
I
K
L
M
N
P
Q
R
S
T
V
W
Y

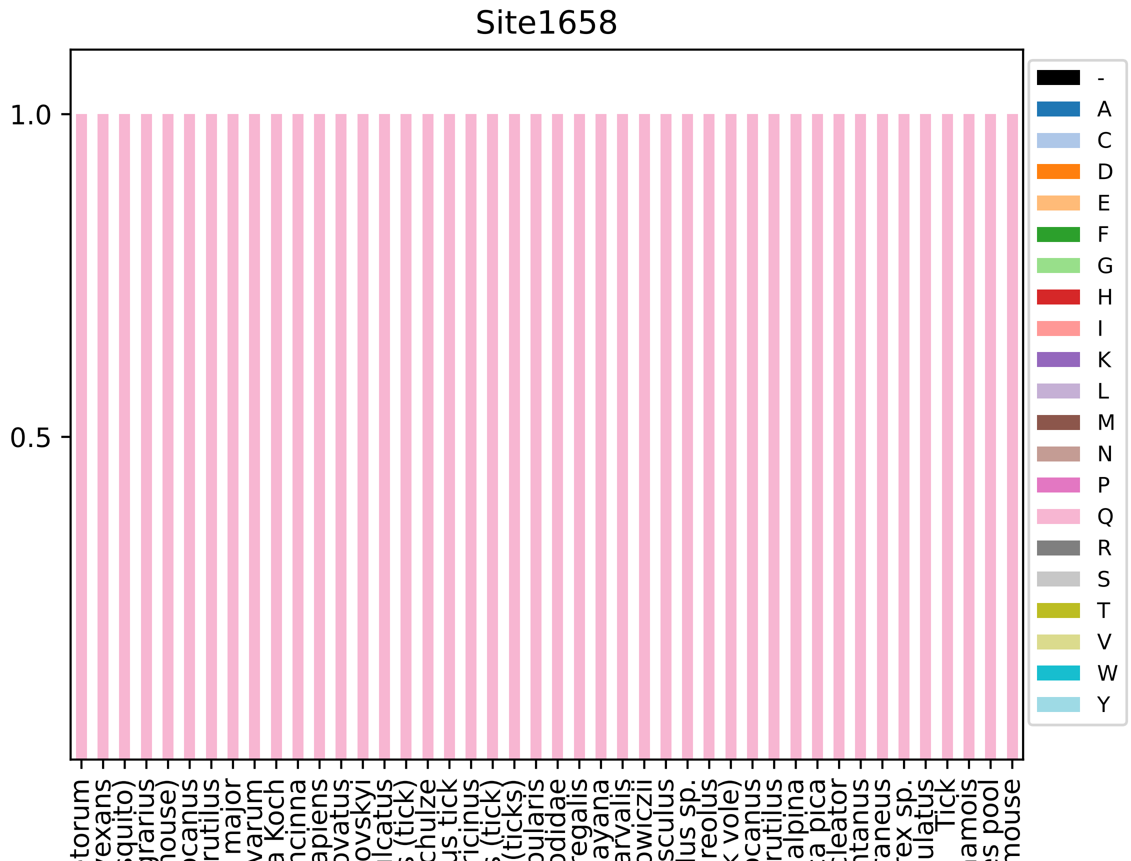

Site1658
1.0
0.5
-
A
C
D
E
F
G
H
I
K
L
M
N
P
Q
R
S
T
V
W
Y

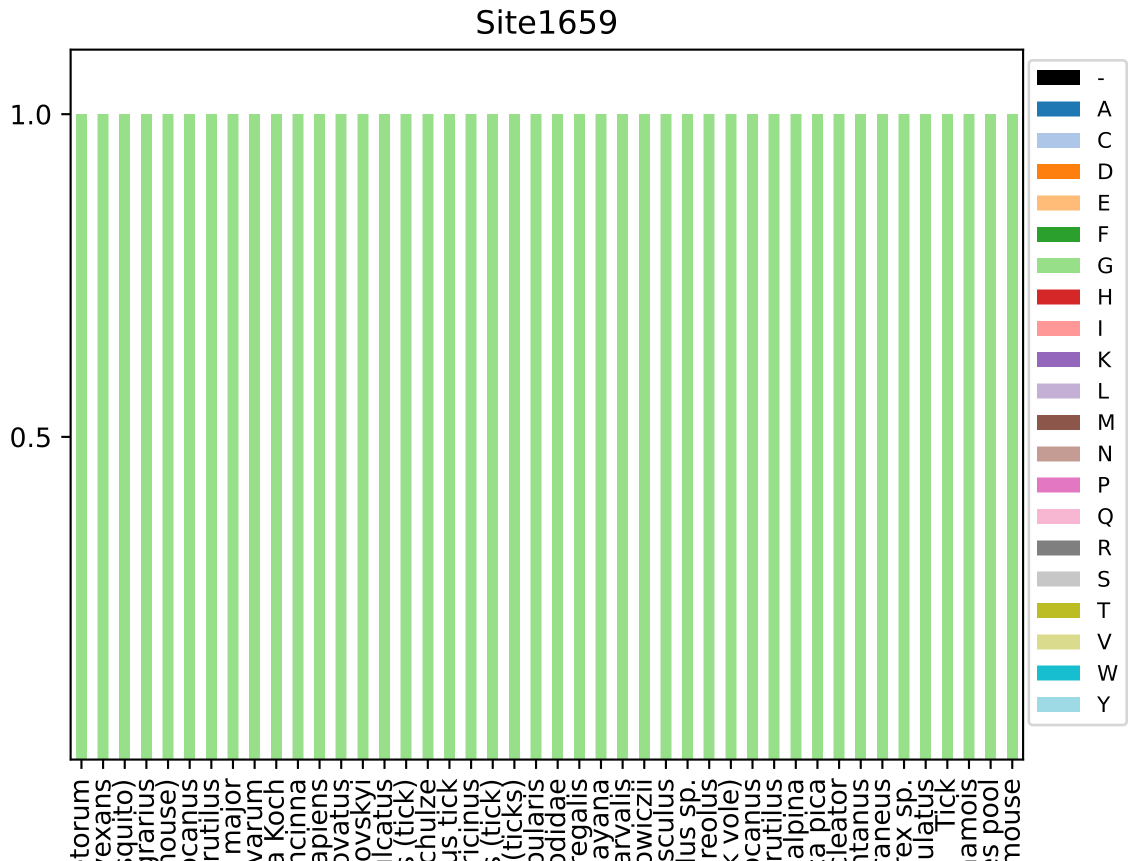
Site1659
1.0
0.5
-
A
C
D
E
F
G
H
I
K
L
M
N
P
Q
R
S
T
V
W
Y

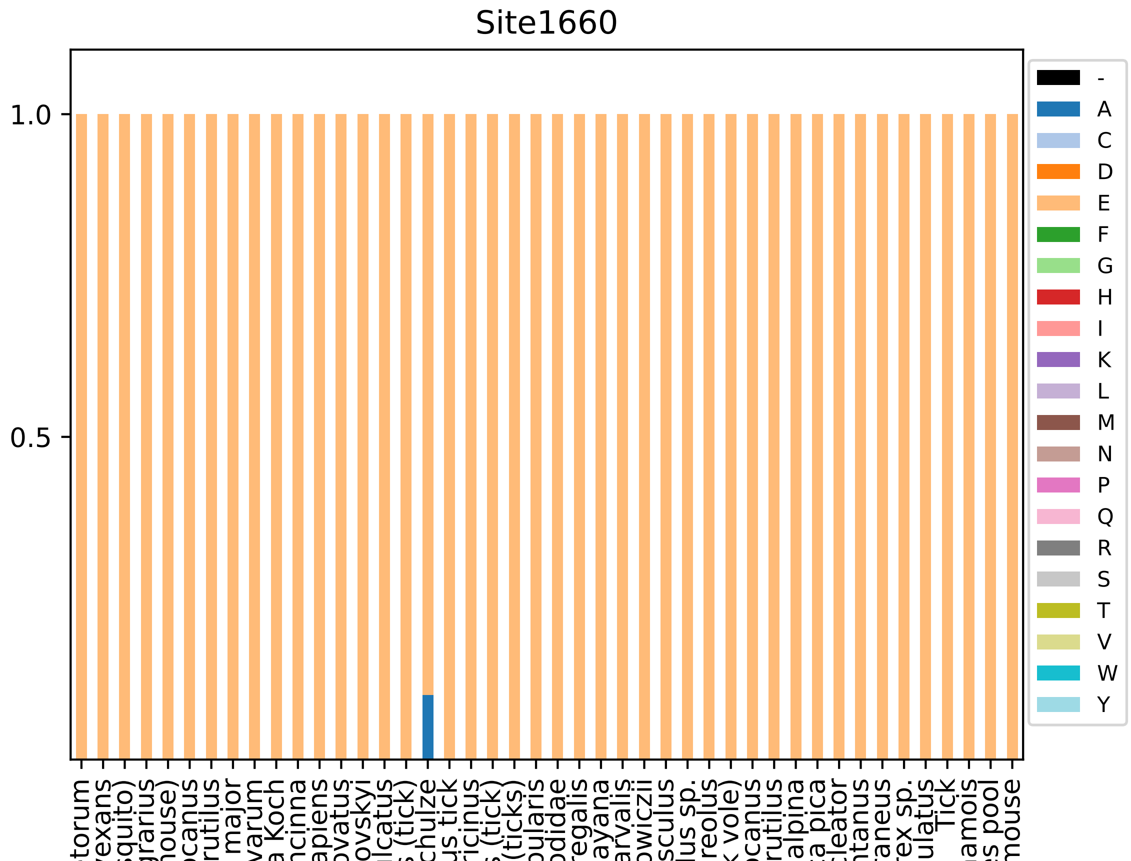
Site1660
1.0
0.5
-
A
C
D
E
F
G
H
I
K
L
M
N
P
Q
R
S
T
V
W
Y

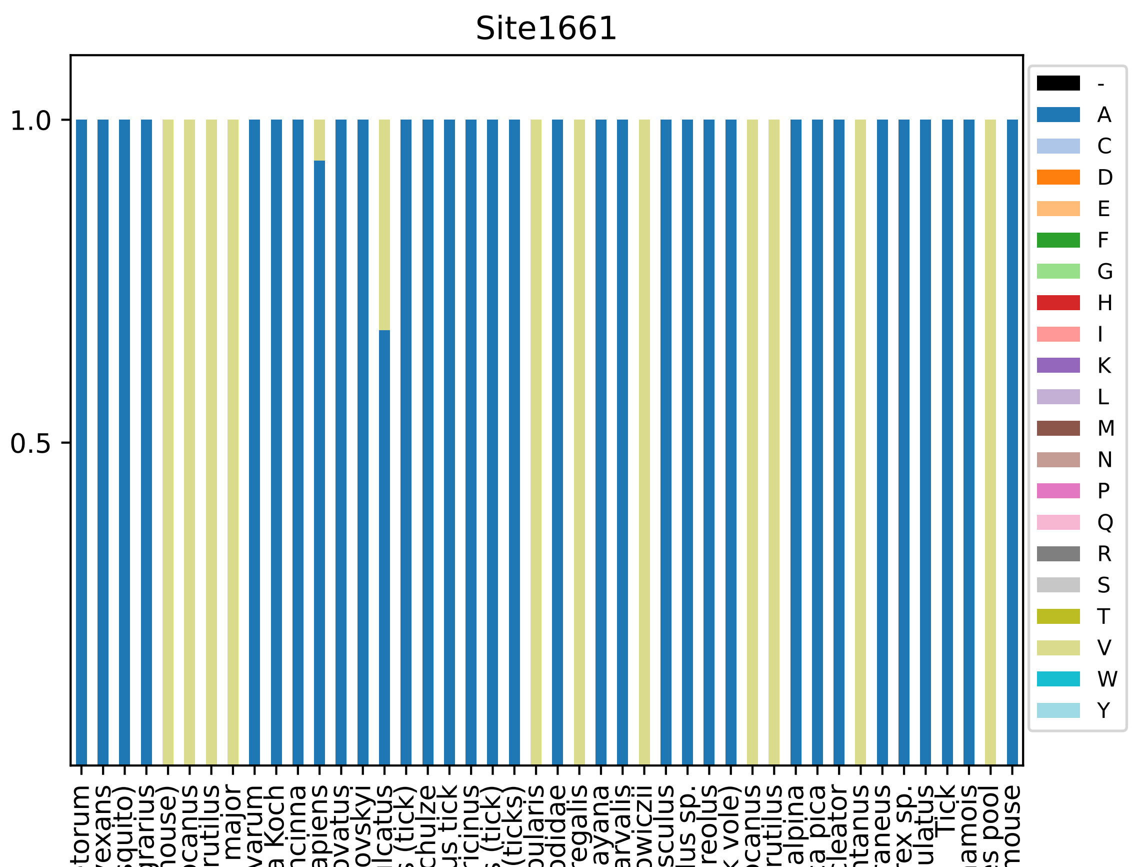

Site1661
1.0
0.5
-
A
C
D
E
F
G
H
I
K
L
M
N
P
Q
R
S
T
V
W
Y

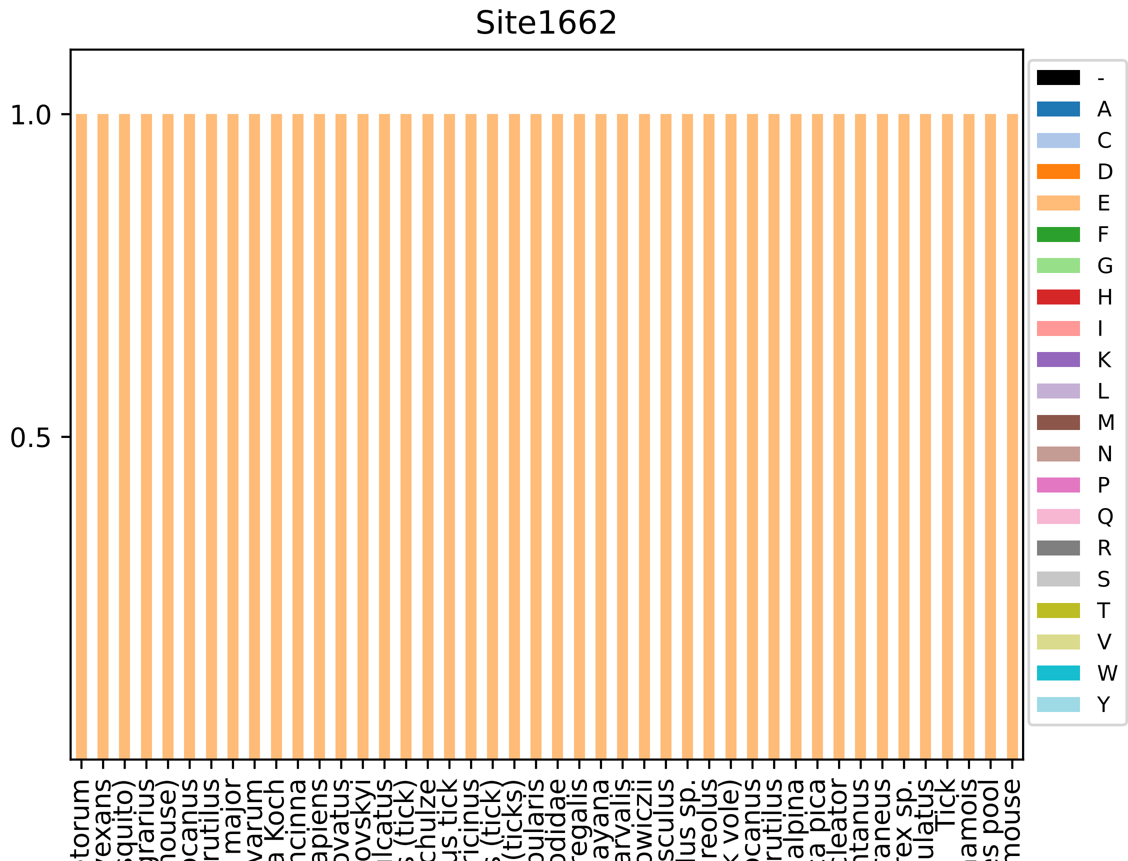
Site1662
1.0
0.5
-
A
C
D
E
F
G
H
I
K
L
M
N
P
Q
R
S
T
V
W
Y

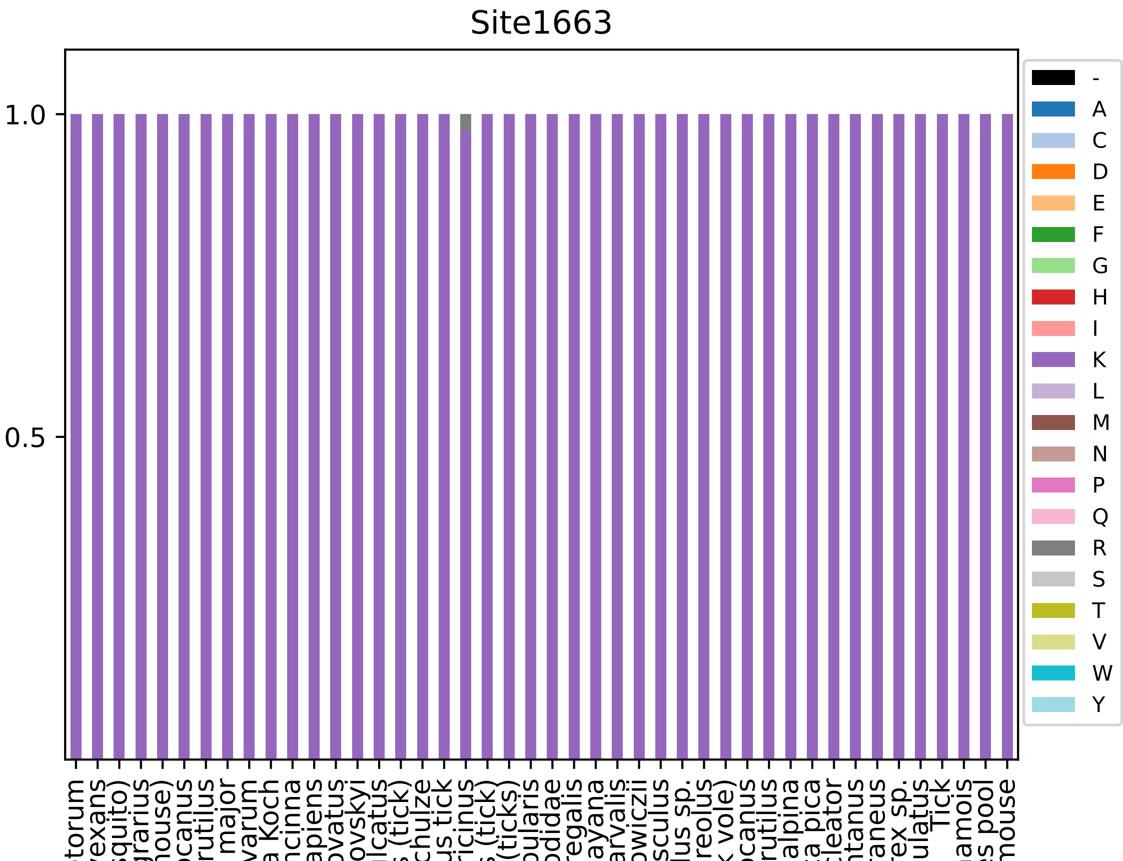
Site1663
1.0
0.5
-
A
C
D
E
F
G
H
I
K
L
M
N
P
Q
R
S
T
V
W
Y

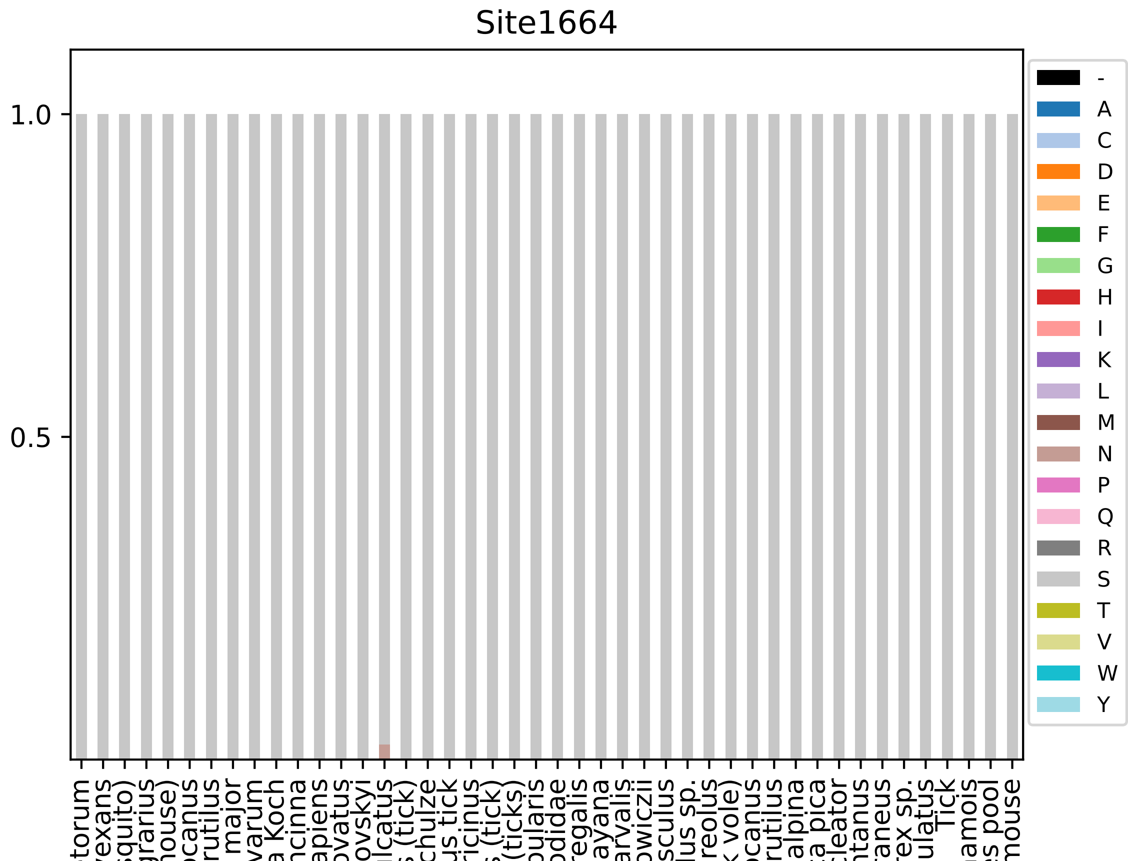
Site1664
1.0
0.5
-
A
C
D
E
F
G
H
I
K
L
M
N
P
Q
R
S
T
V
W
Y

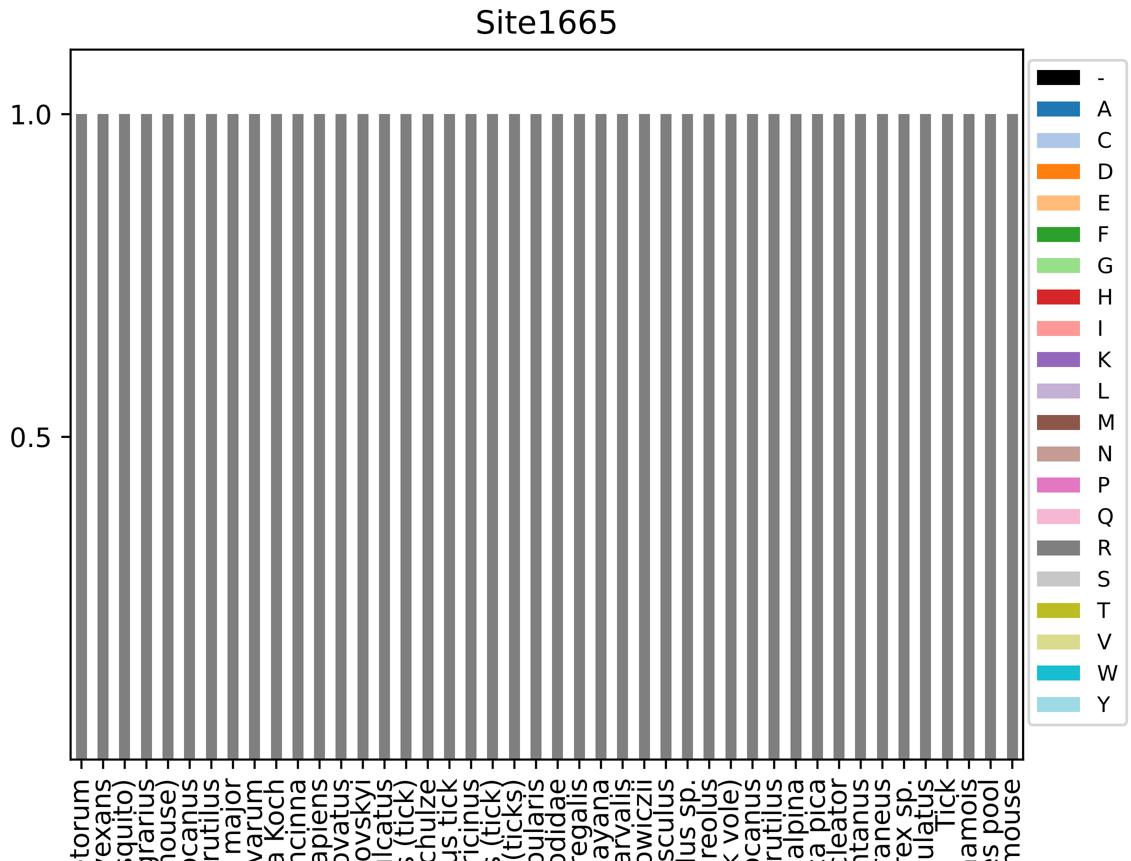
Site1665
1.0
0.5
-
A
C
D
E
F
G
H
I
K
L
M
N
P
Q
R
S
T
V
W
Y

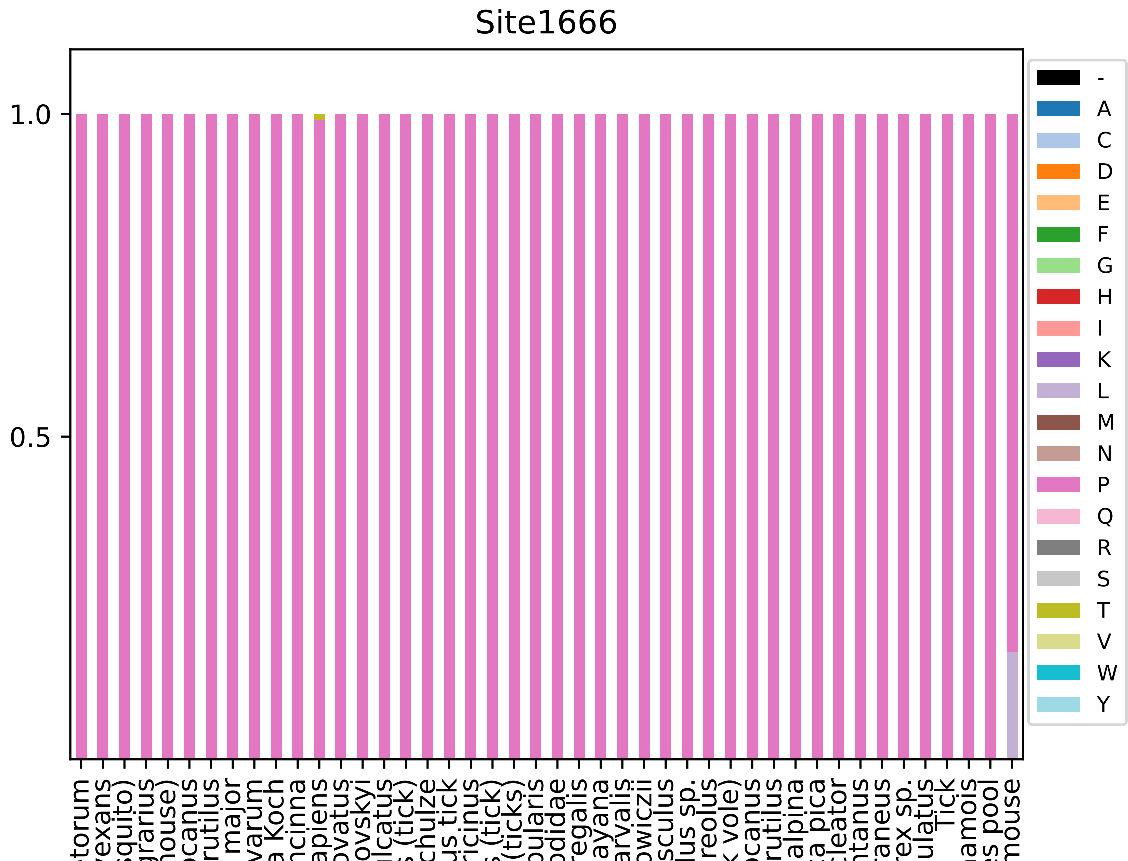
Site1666
1.0
0.5
-
A
C
D
E
F
G
H
I
K
L
M
N
P
Q
R
S
T
V
W
Y

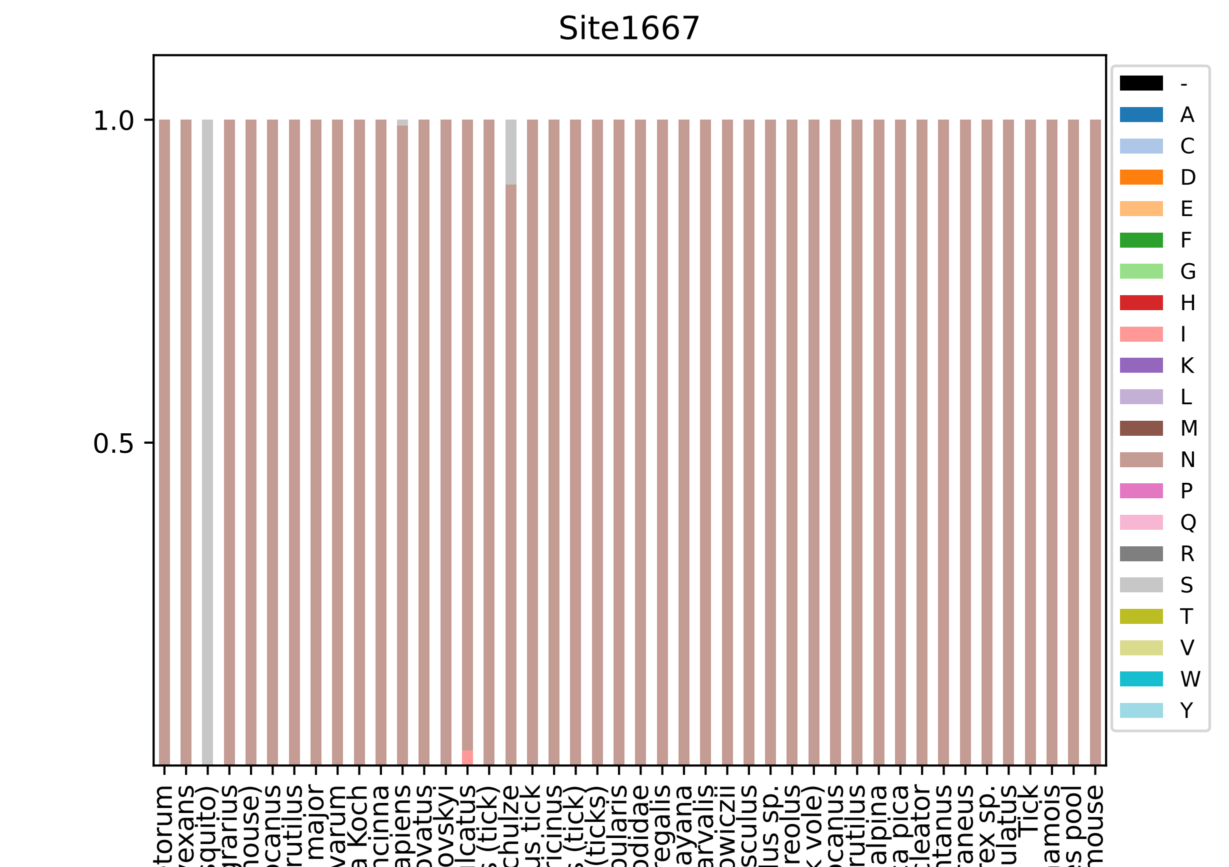
Site1667
1.0
0.5
-
A
C
D
E
F
G
H
I
K
L
M
N
P
Q
R
S
T
V
W
Y

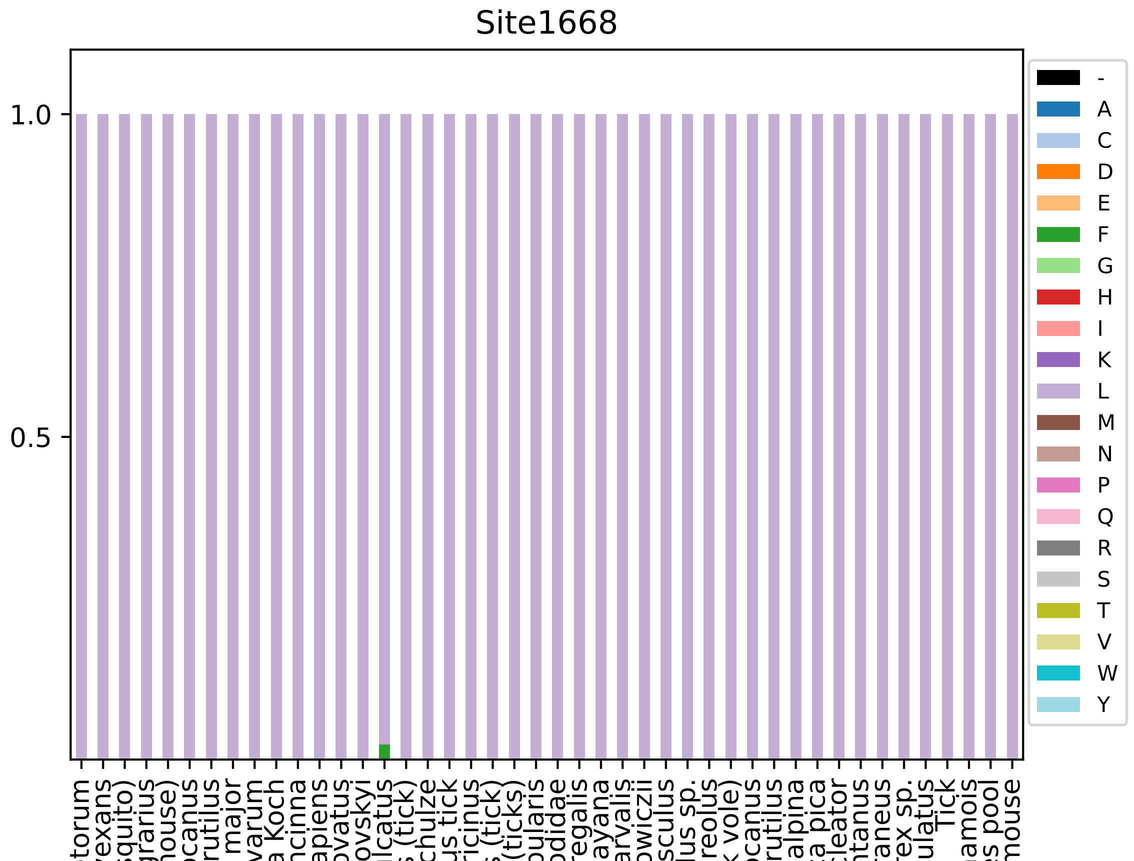

Site1668
1.0
0.5
-
A
C
D
E
F
G
H
I
K
L
M
N
P
Q
R
S
T
V
W
Y

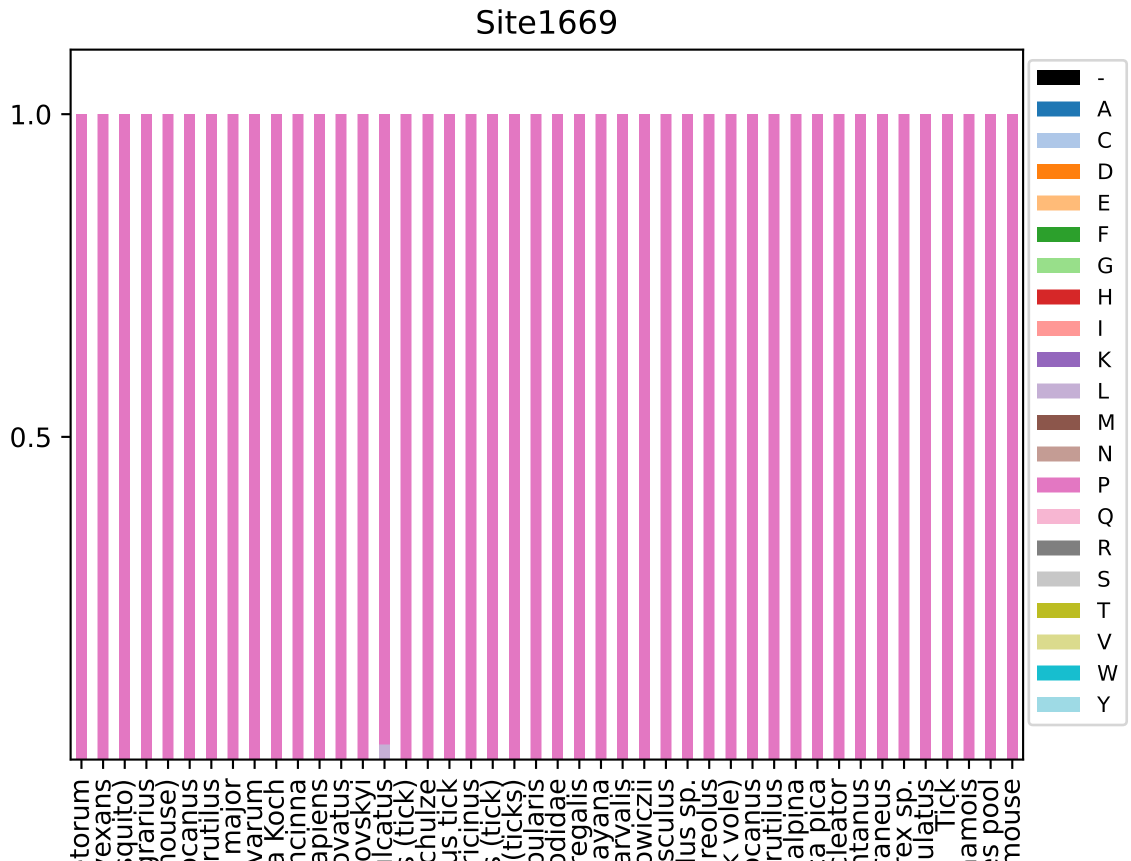
Site1669
1.0
0.5
-
A
C
D
E
F
G
H
I
K
L
M
N
P
Q
R
S
T
V
W
Y

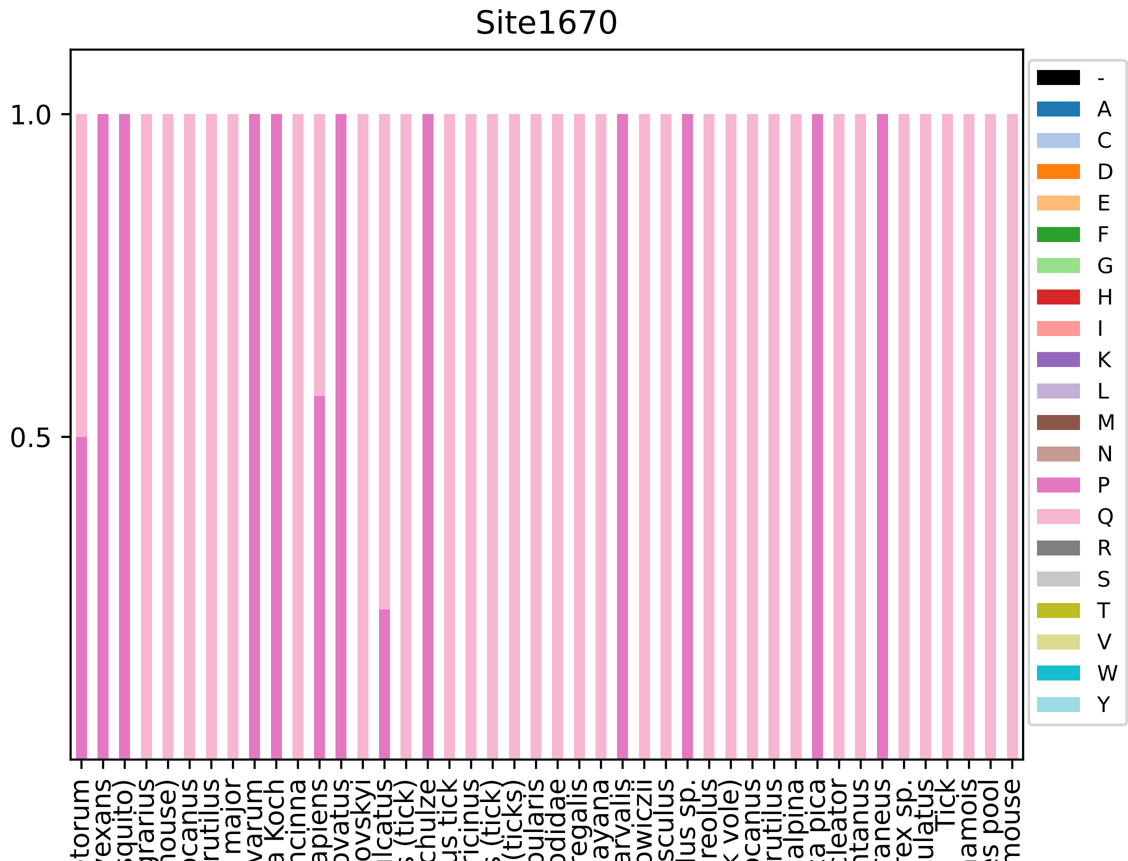

Site1670
1.0
0.5
-
A
C
D
E
F
G
H
I
K
L
M
N
P
Q
R
S
T
V
W
Y

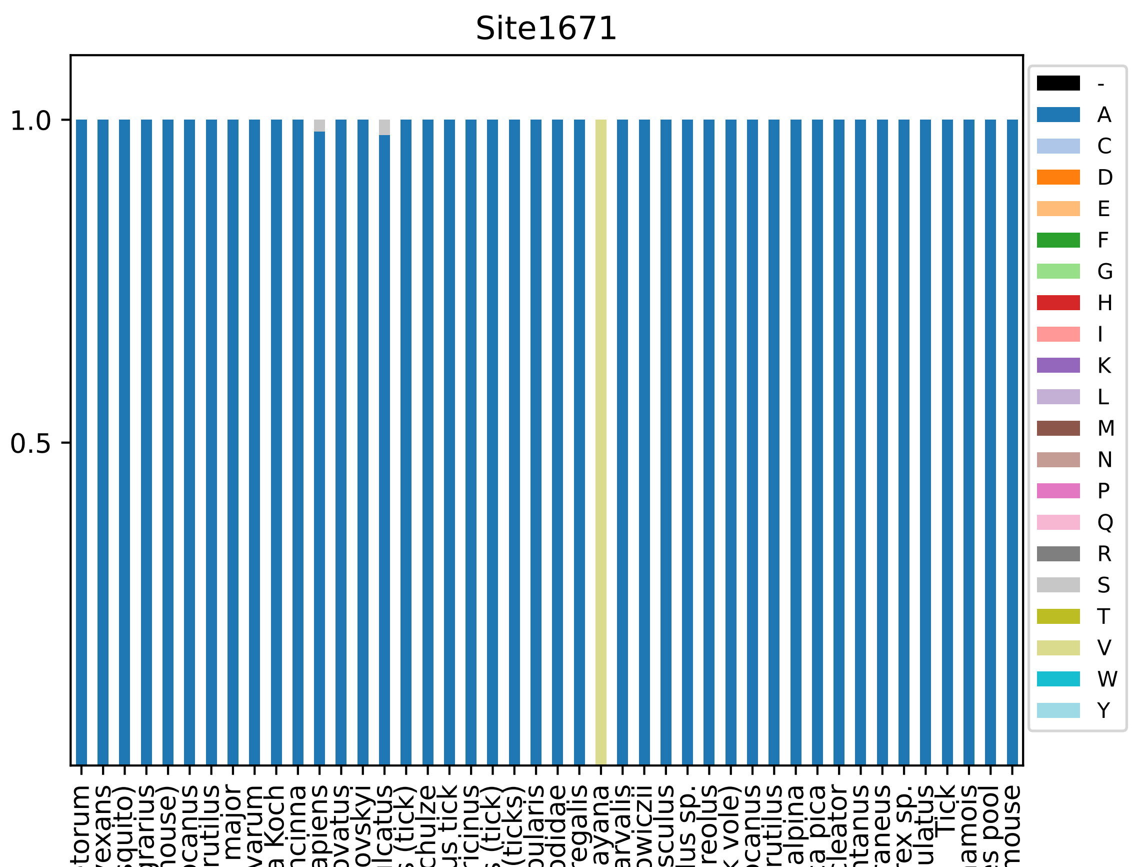

Site1671
1.0
0.5
-
A
C
D
E
F
G
H
I
K
L
M
N
P
Q
R
S
T
V
W
Y

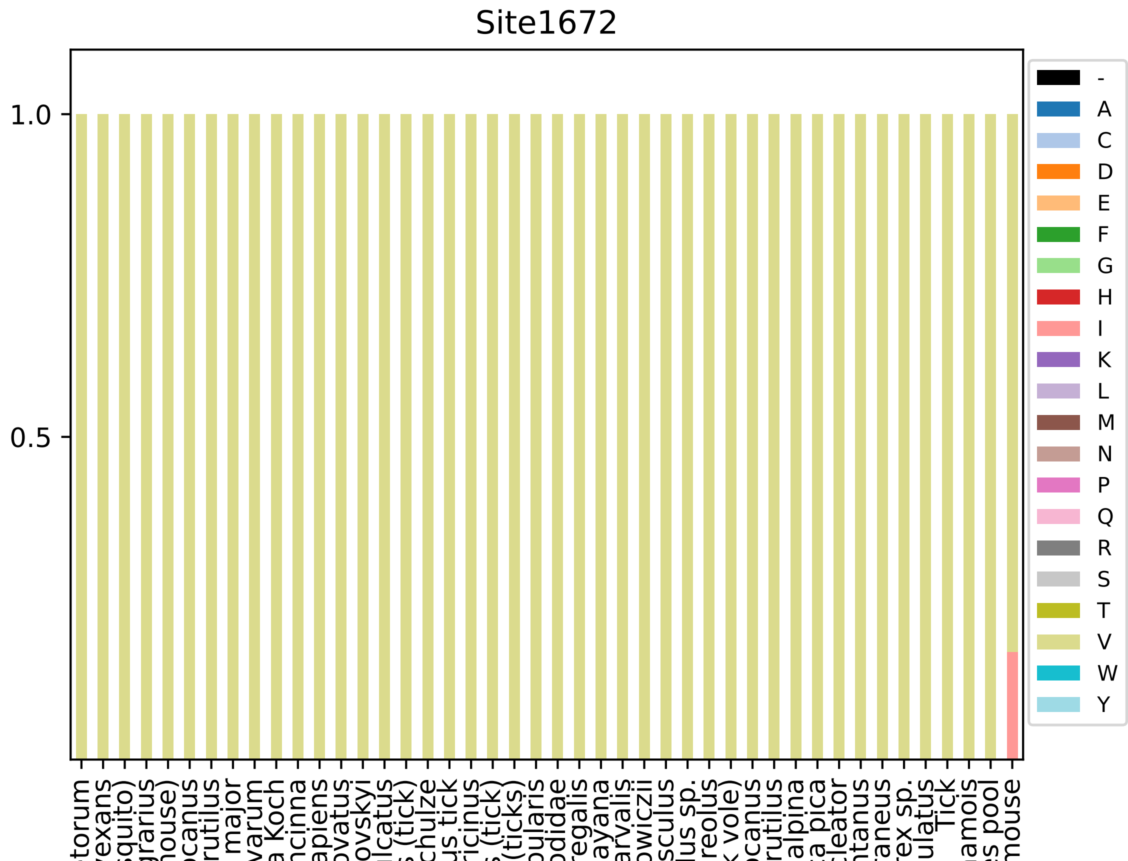
Site1672
1.0
0.5
-
A
C
D
E
F
G
H
I
K
L
M
N
P
Q
R
S
T
V
W
Y

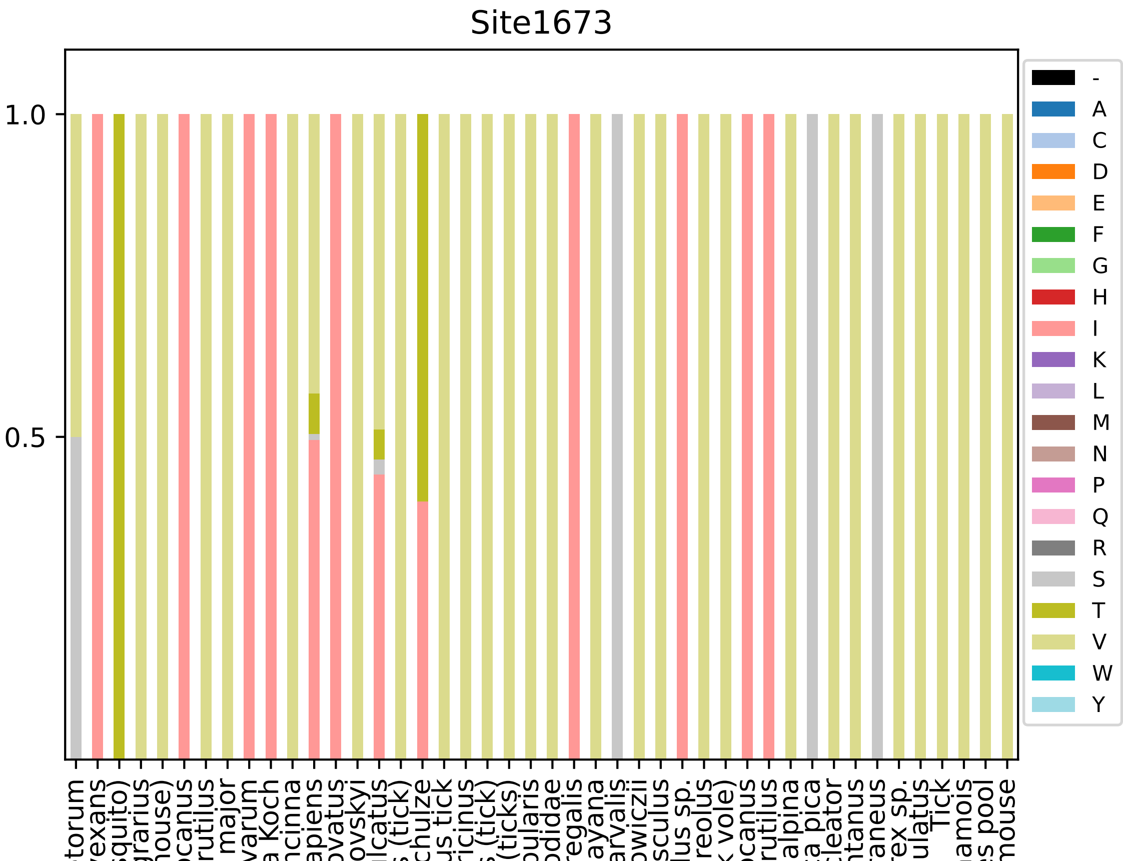
Site1673
1.0
0.5
-
A
C
D
E
F
G
H
I
K
L
M
N
P
Q
R
S
T
V
W
Y

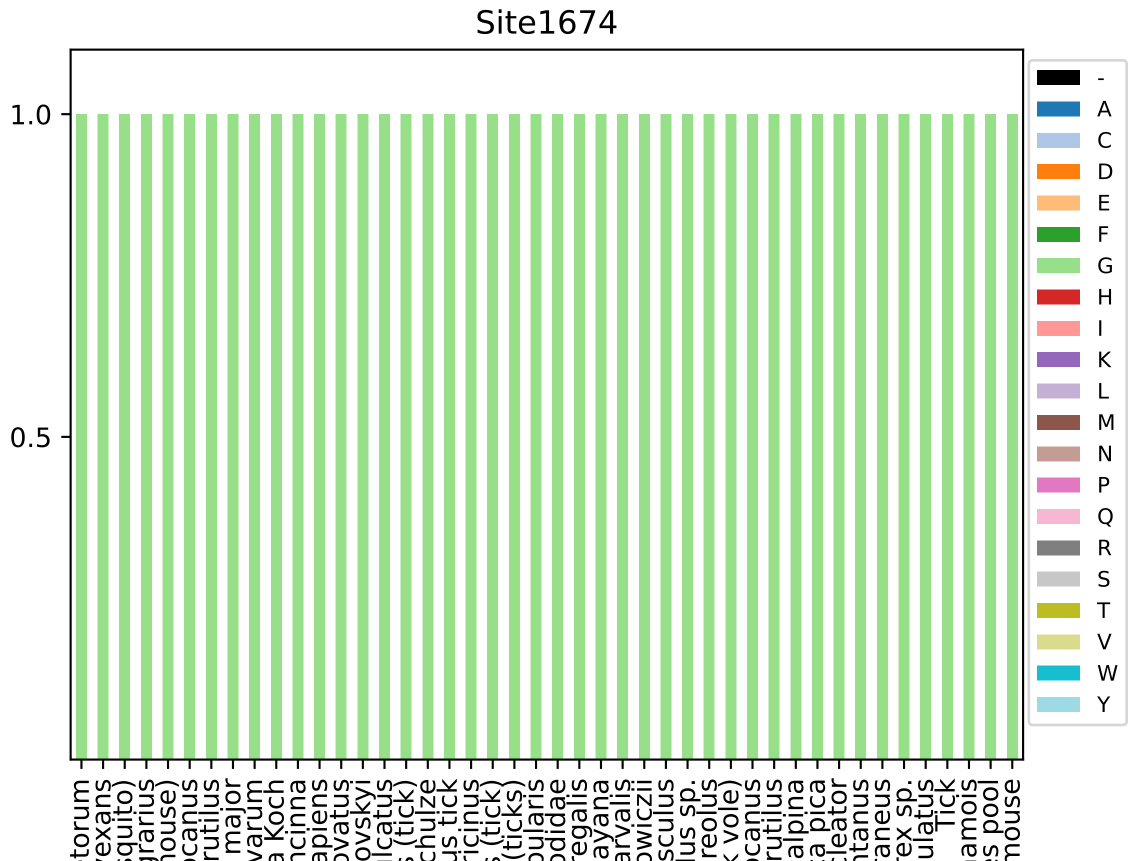

Site1674
1.0
0.5
-
A
C
D
E
F
G
H
I
K
L
M
N
P
Q
R
S
T
V
W
Y

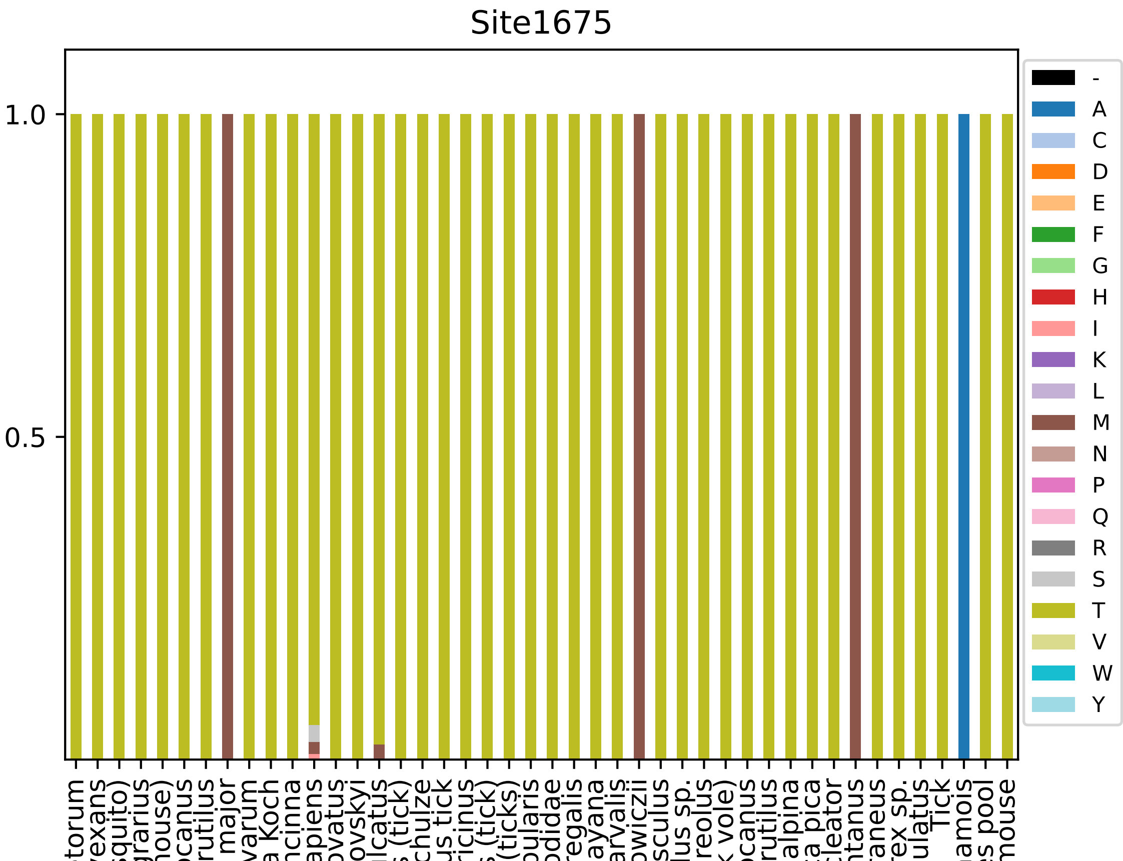
Site1675
1.0
0.5
-
A
C
D
E
F
G
H
I
K
L
M
N
P
Q
R
S
T
V
W
Y

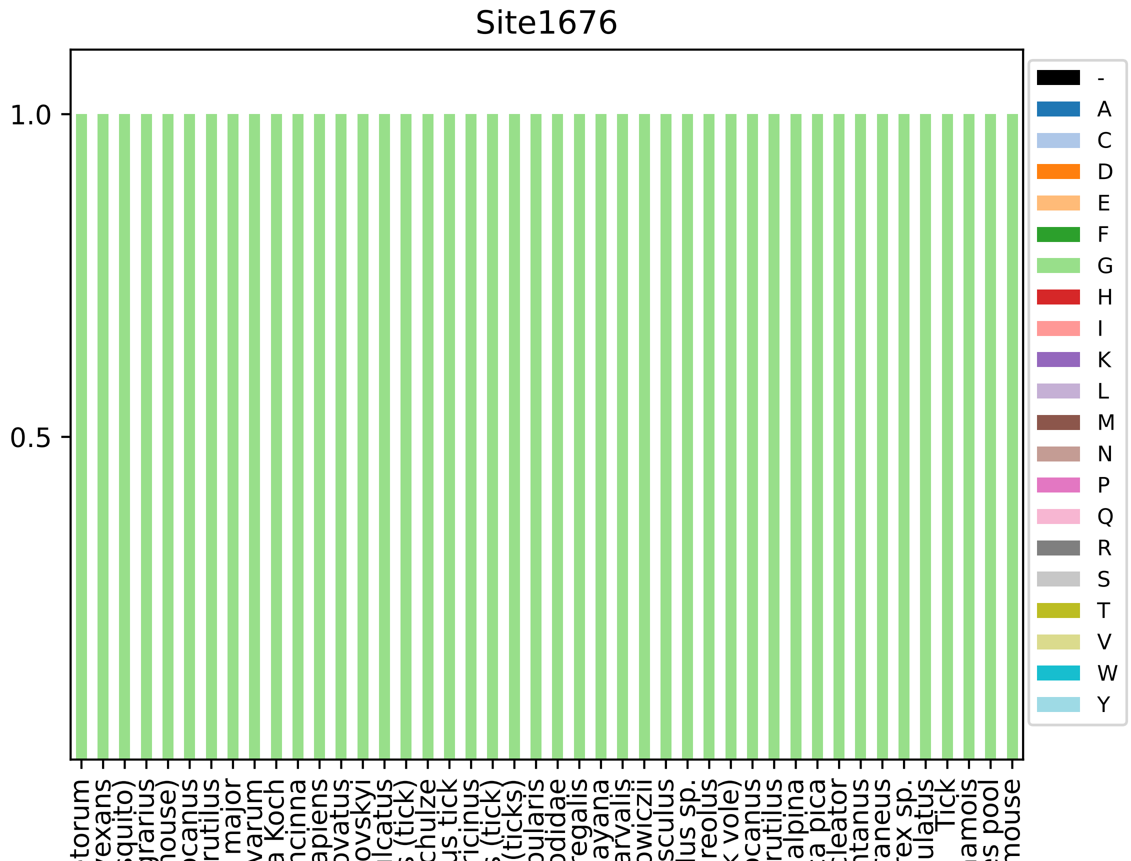
Site1676
1.0
0.5
-
A
C
D
E
F
G
H
I
K
L
M
N
P
Q
R
S
T
V
W
Y

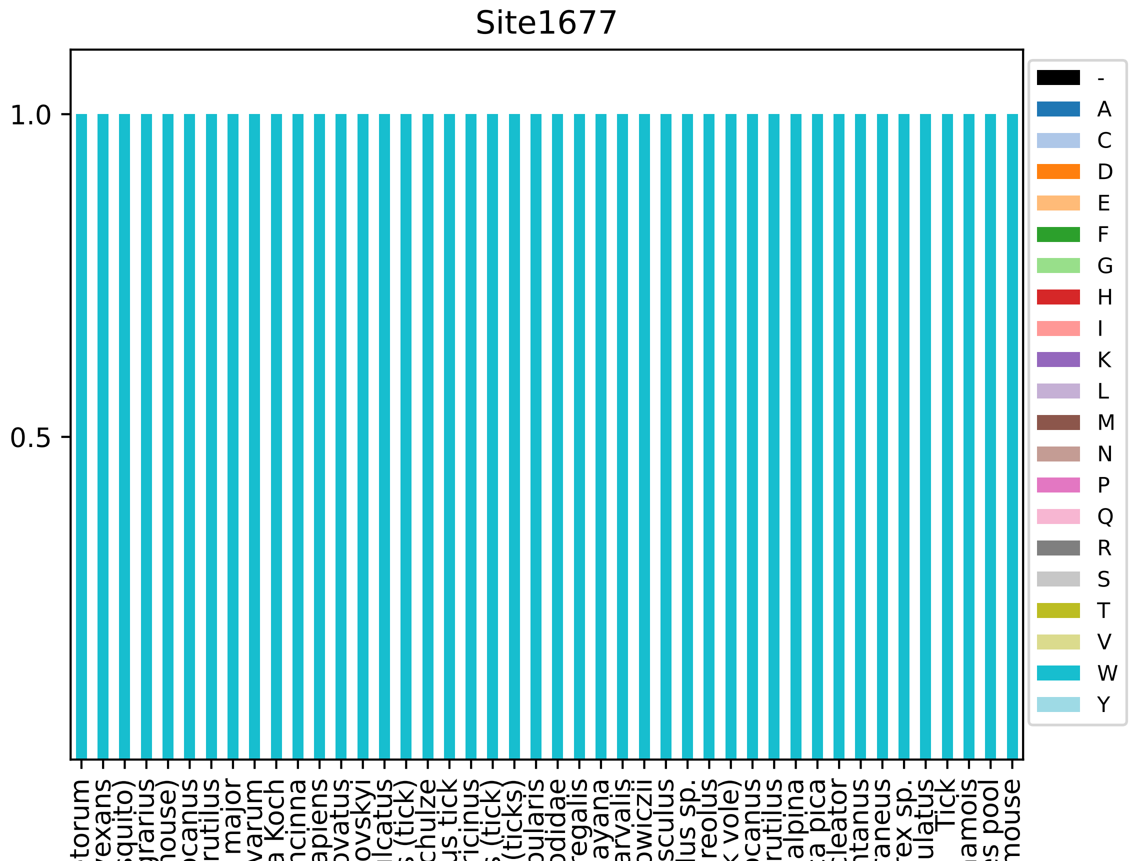
Site1677
1.0
0.5
-
A
C
D
E
F
G
H
I
K
L
M
N
P
Q
R
S
T
V
W
Y
Tick

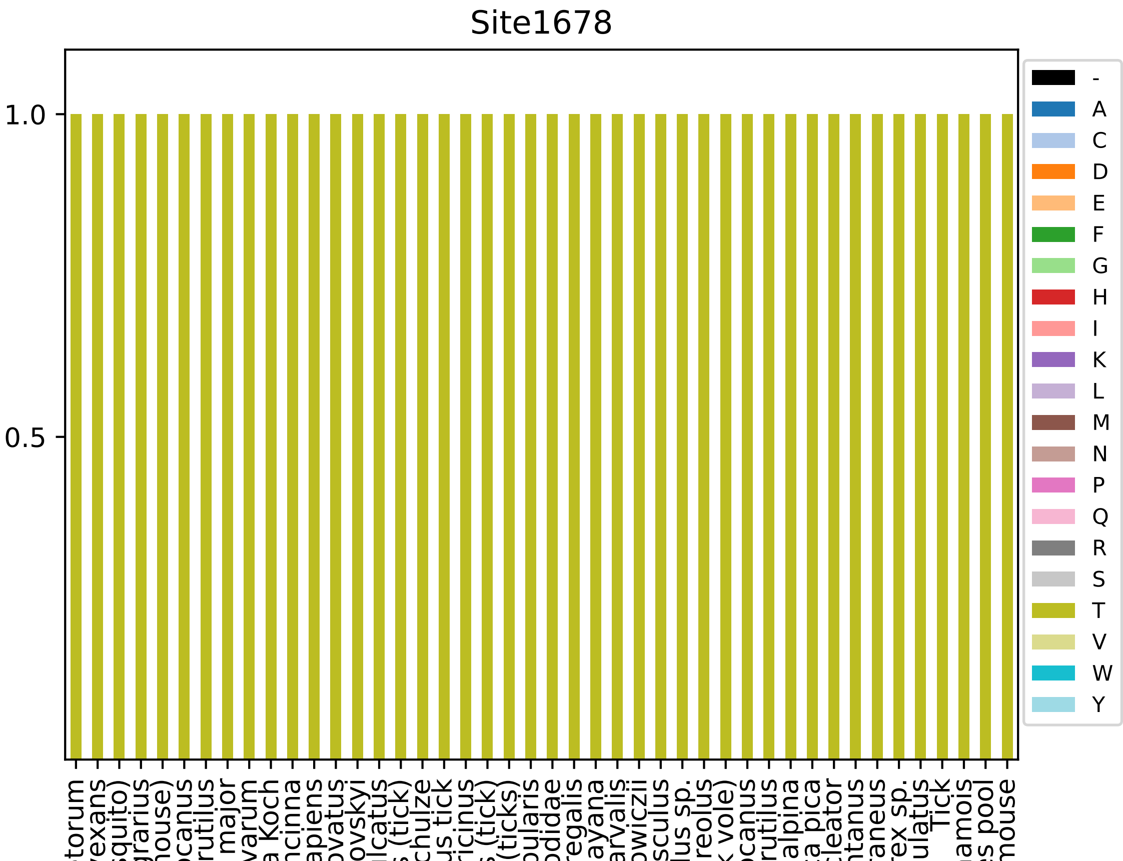
Site1678
1.0
0.5
-
A
C
D
E
F
G
H
I
K
L
M
N
P
Q
R
S
T
V
W
Y

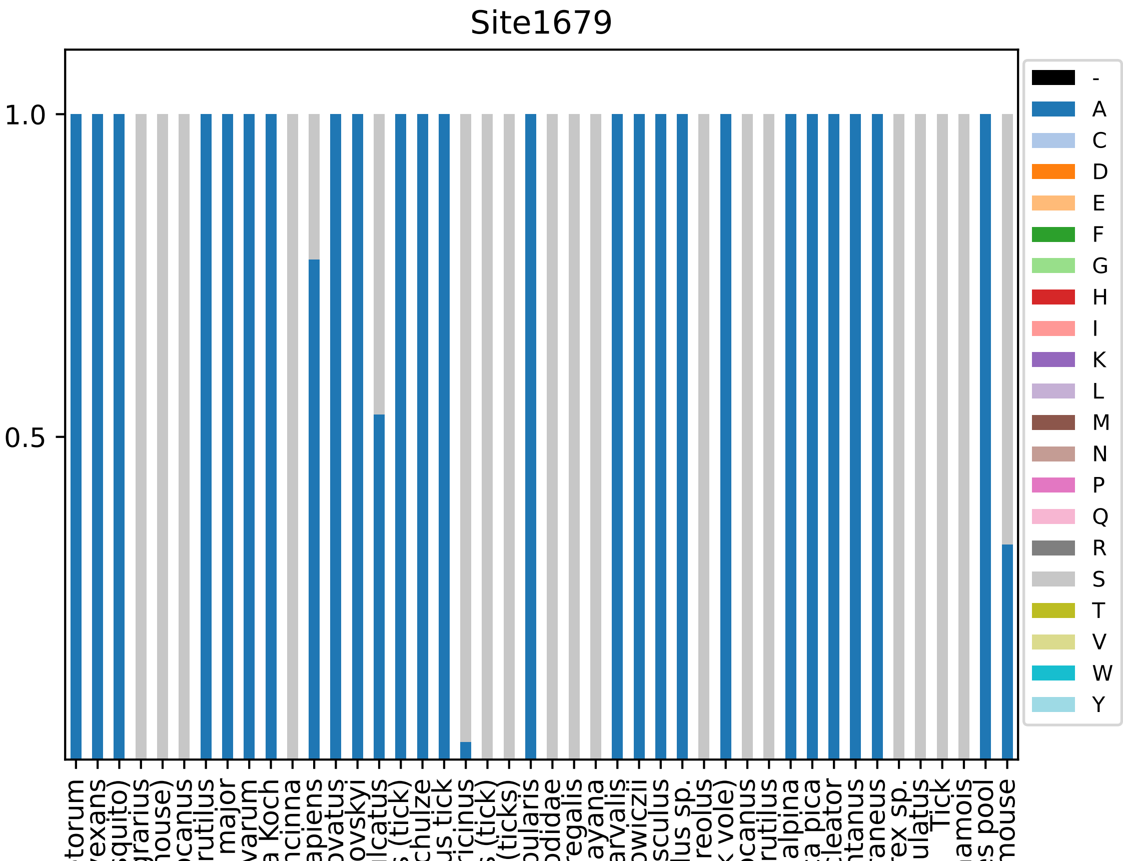
Site1679
1.0
0.5
-
A
C
D
E
F
G
H
I
K
L
M
N
P
Q
R
S
T
V
W
Y

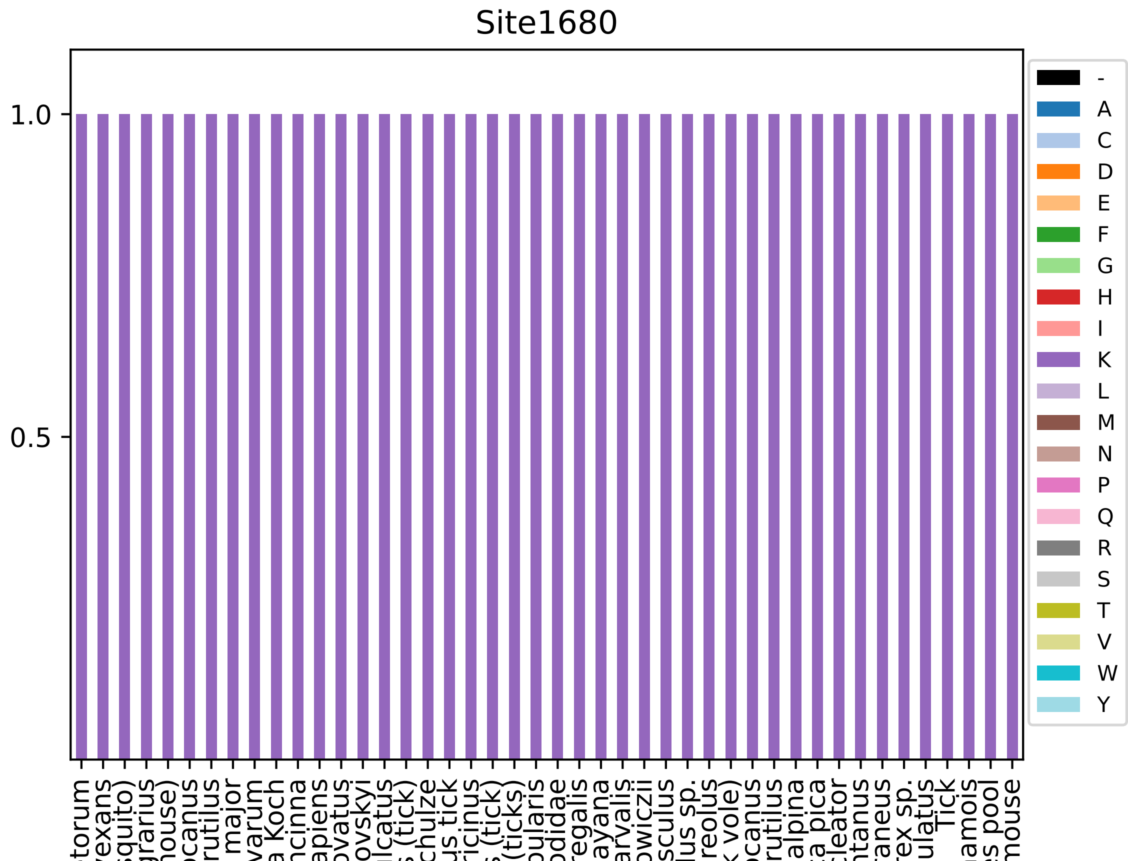
Site1680
1.0
0.5
-
A
C
D
E
F
G
H
I
K
L
M
N
P
Q
R
S
T
V
W
Y

Site1681

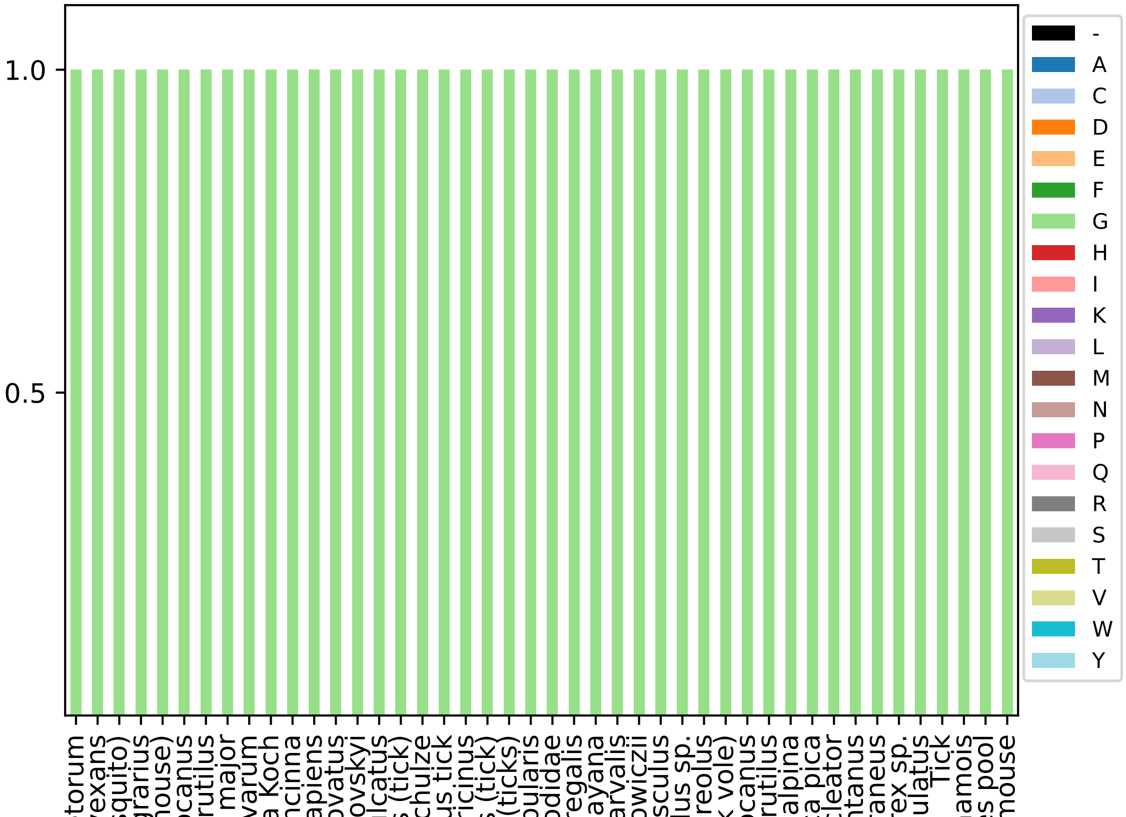

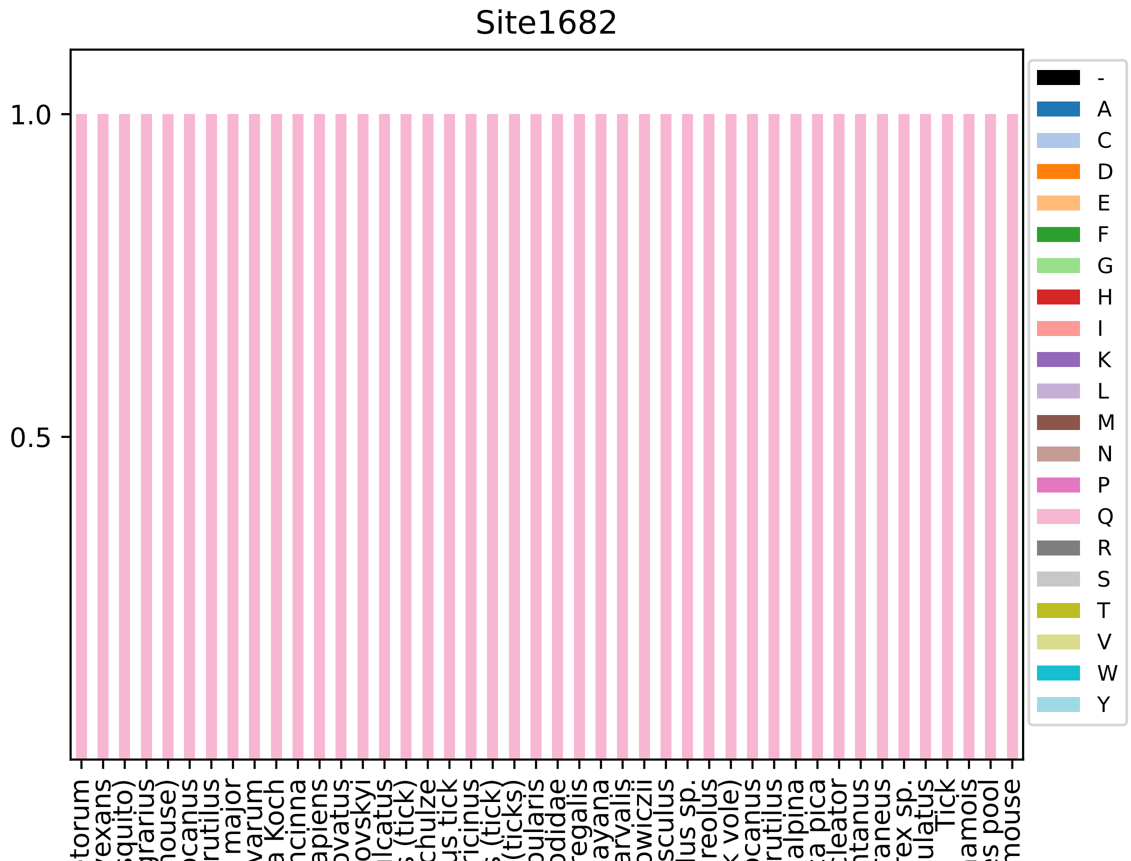
Site1682
1.0
0.5
-
A
C
D
E
F
G
H
I
K
L
M
N
P
Q
R
S
T
V
W
Y

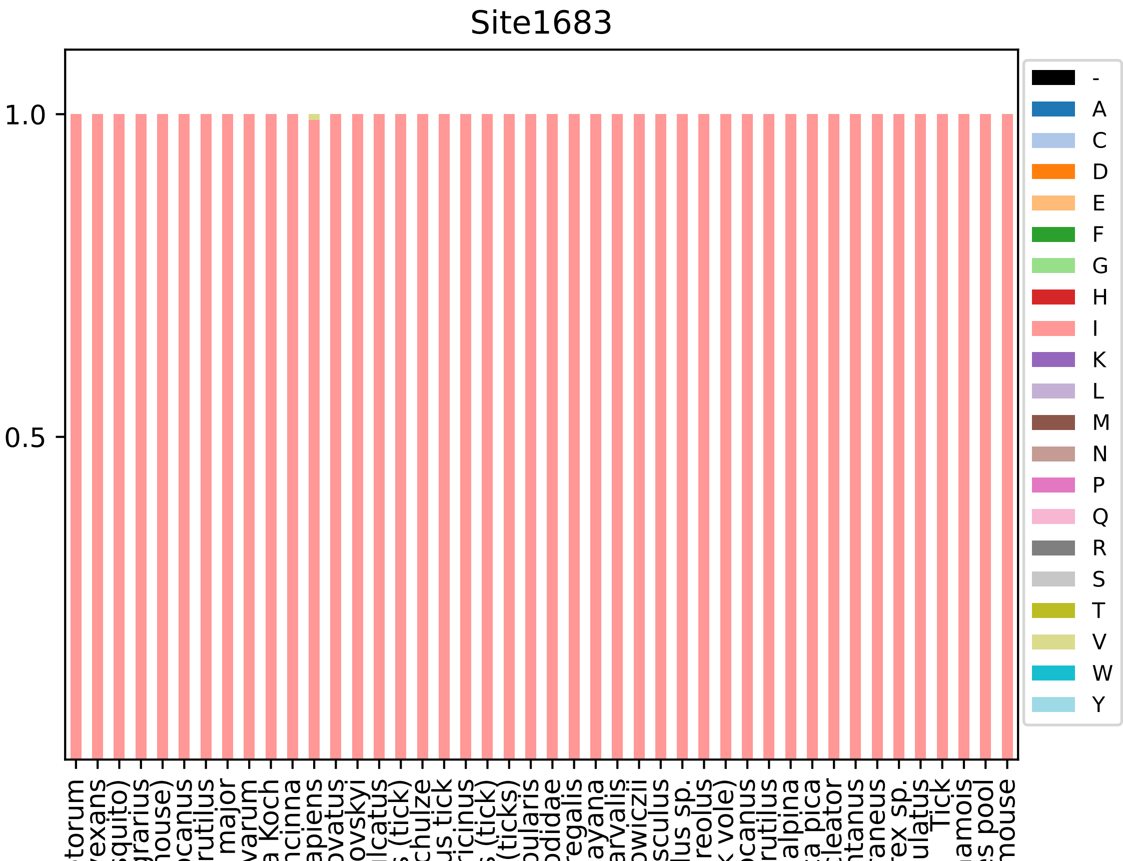

Site1683
1.0
0.5
-
A
C
D
E
F
G
H
I
K
L
M
N
P
Q
R
S
T
V
W
Y

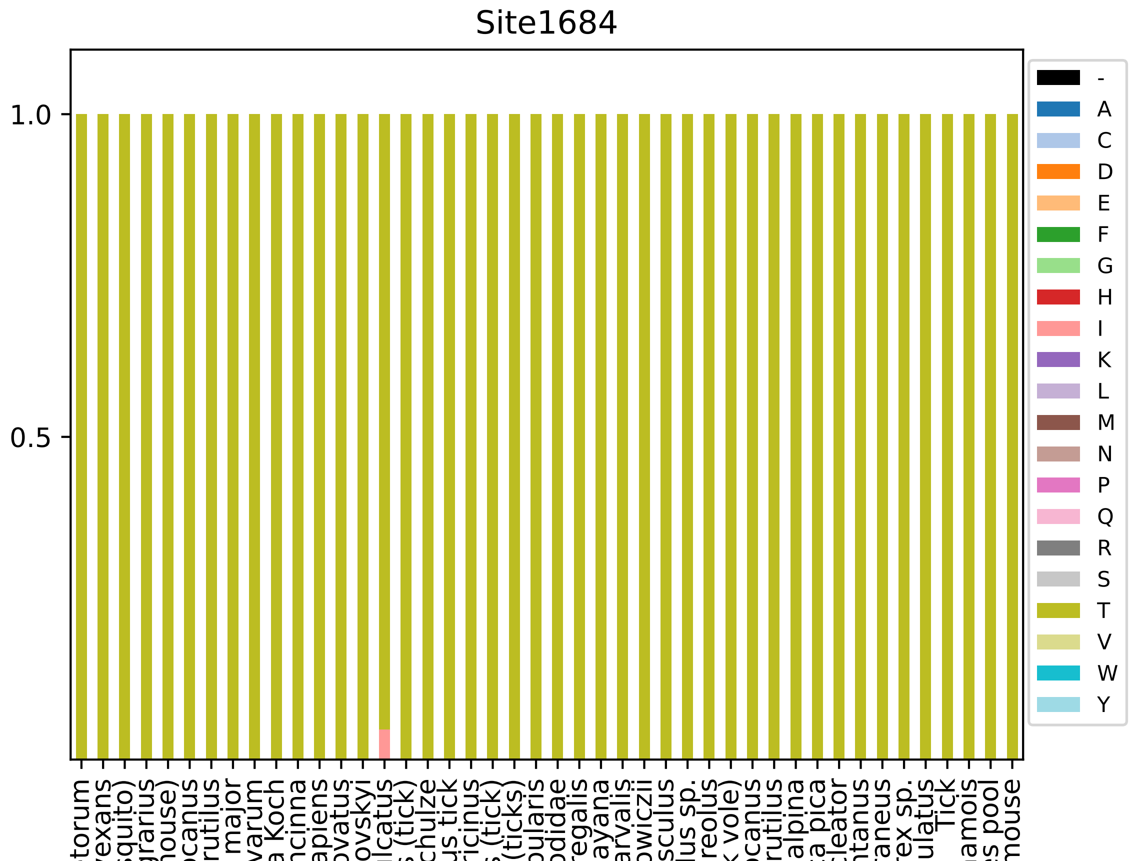

Site1684
1.0
0.5
-
A
C
D
E
F
G
H
I
K
L
M
N
P
Q
R
S
T
V
W
Y
torum
vexans
squito)
garius
nouse)
ocanus
rutilus
major
varum
a Koch
ncinna
apiens
ovatus
ovskyi
lcatus
(tick)
chulze
us tick
ricinus
(tick)
(ticks)
pularis
podidae
regalis
ayana
arvalis
owiczii
sculus
lus sp.
reolus
k vole)
ocanus
rutilus
alpina
a pica
leator
ntanus
raneus
rex sp.
ulatus
Tick
amois
es pool
mouse

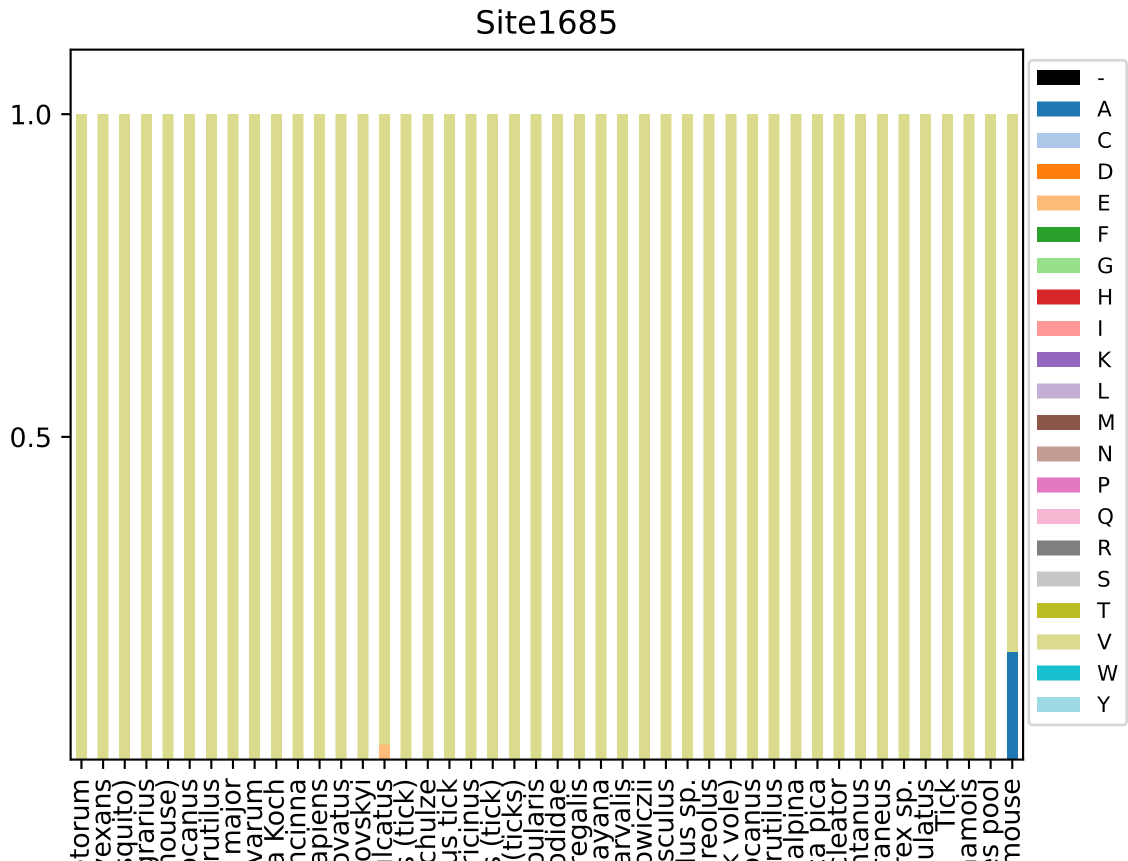

Site1685
1.0
0.5
-
A
C
D
E
F
G
H
I
K
L
M
N
P
Q
R
S
T
V
W
Y
torum
vexans
squito)
grarius
nouse)
ocanus
rutilus
major
varum
a Koch
ncinna
apiens
ovatus
ovskyi
ulcatus
s (tick)
chulze
us tick
ricinus
s (tick)
(ticks)
pularis
podidae
regalis
ayana
arvalis
owiczii
sculus
lus sp.
reolus
k vole)
ocanus
rutilus
alpina
a pica
cleator
ntanus
raneus
rex sp.
ulatus
Tick
amois
es pool
mouse

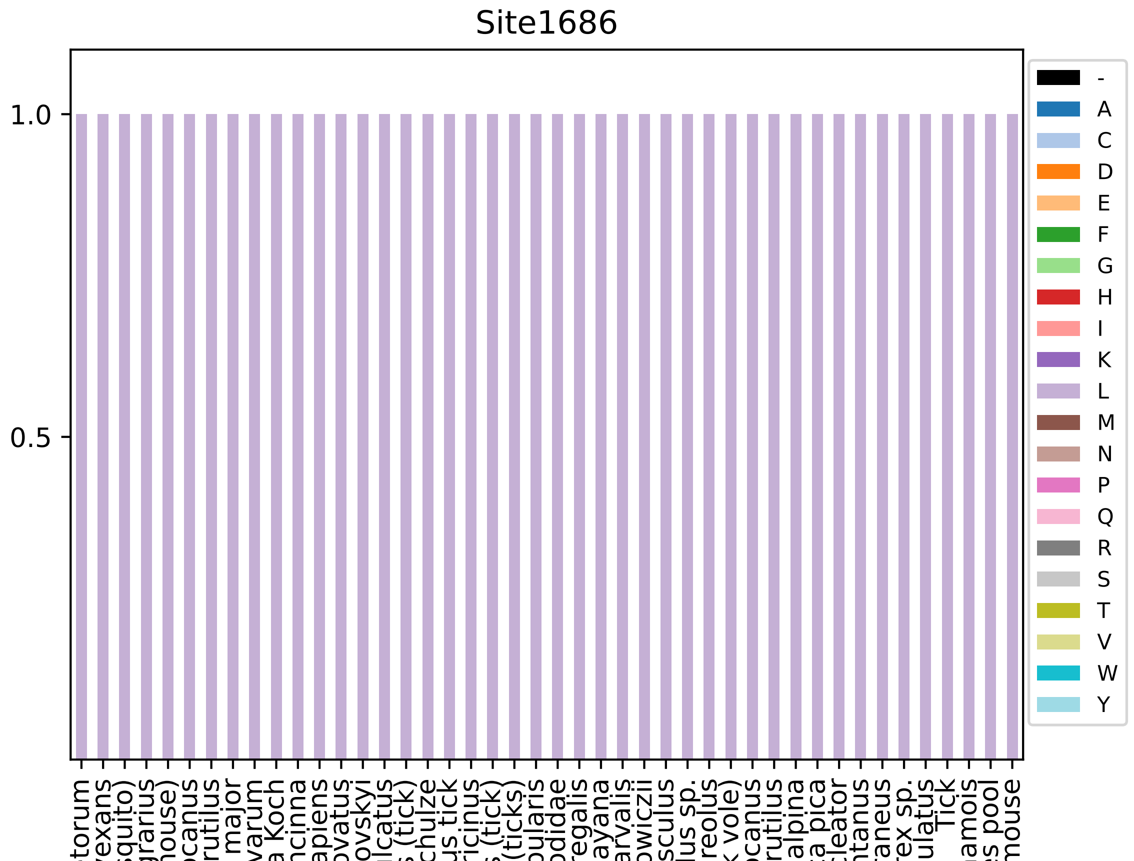
Site1686
1.0
0.5
-
A
C
D
E
F
G
H
I
K
L
M
N
P
Q
R
S
T
V
W
Y

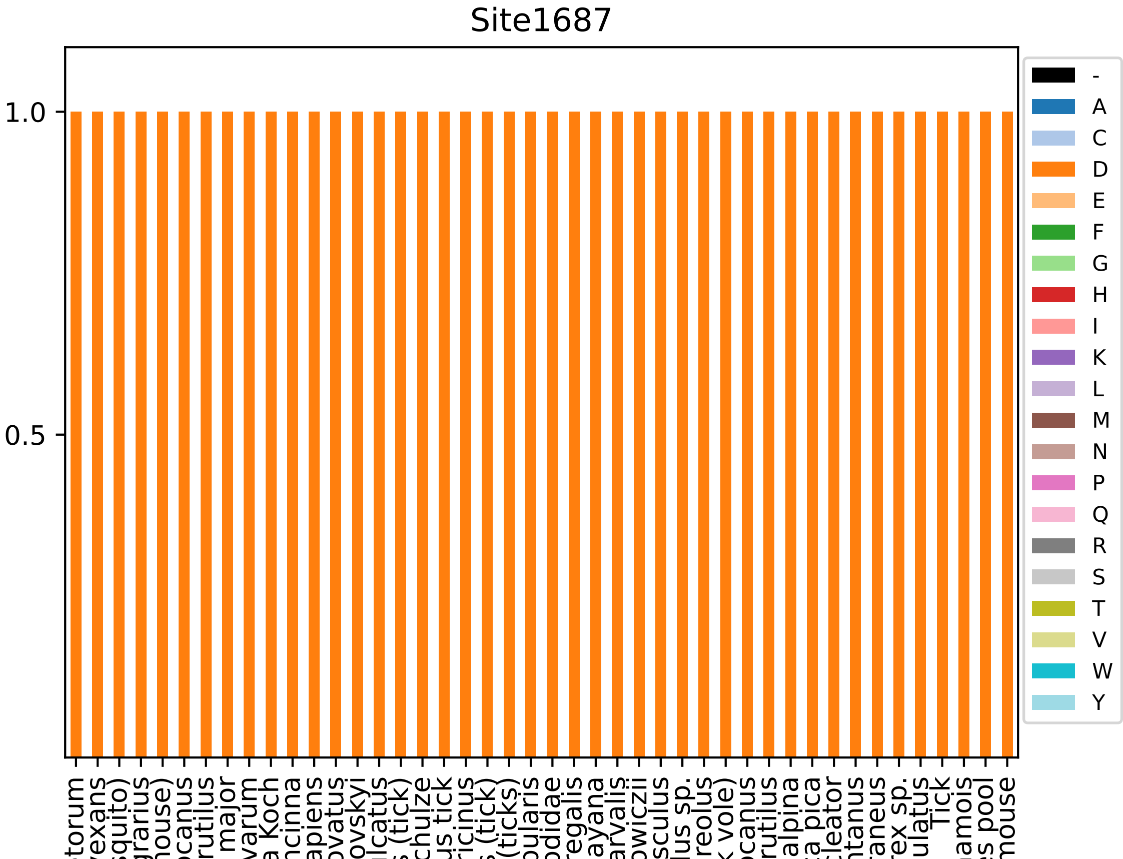
Site1687
1.0
0.5
-
A
C
D
E
F
G
H
I
K
L
M
N
P
Q
R
S
T
V
W
Y

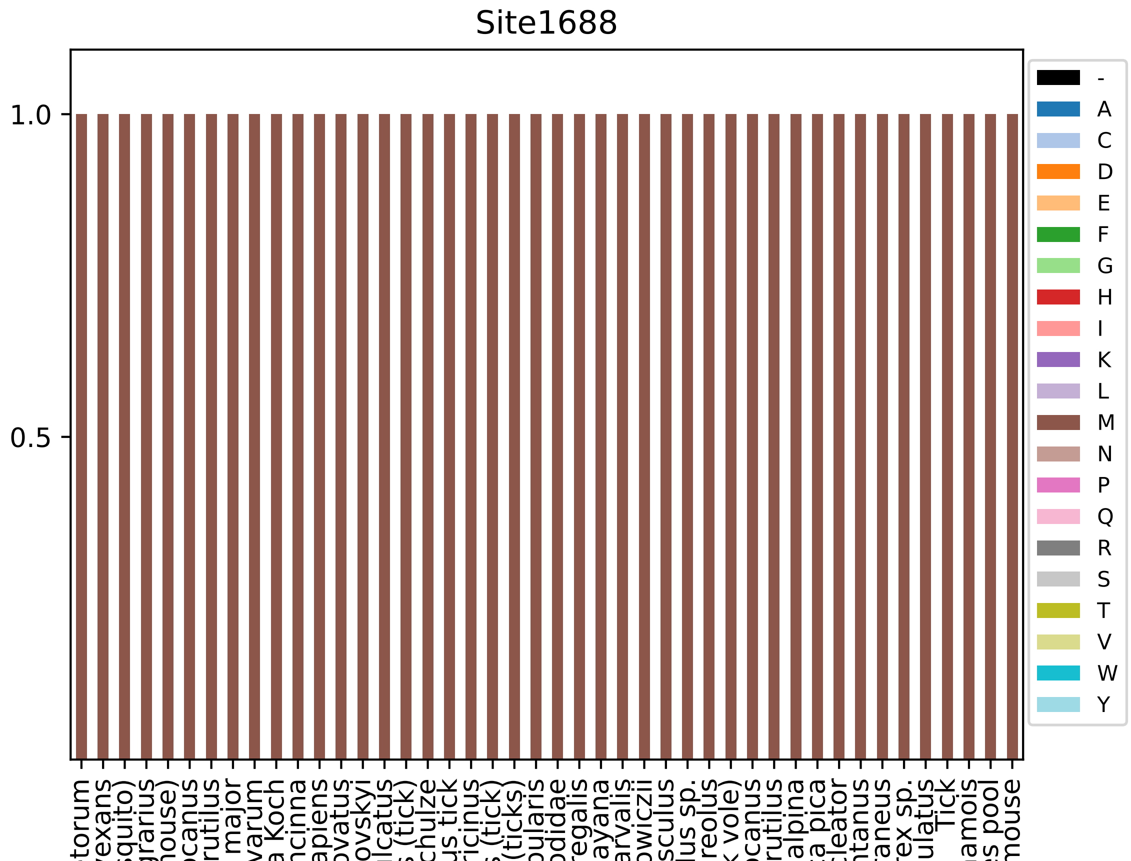
Site1688
1.0
0.5
-
A
C
D
E
F
G
H
I
K
L
M
N
P
Q
R
S
T
V
W
Y

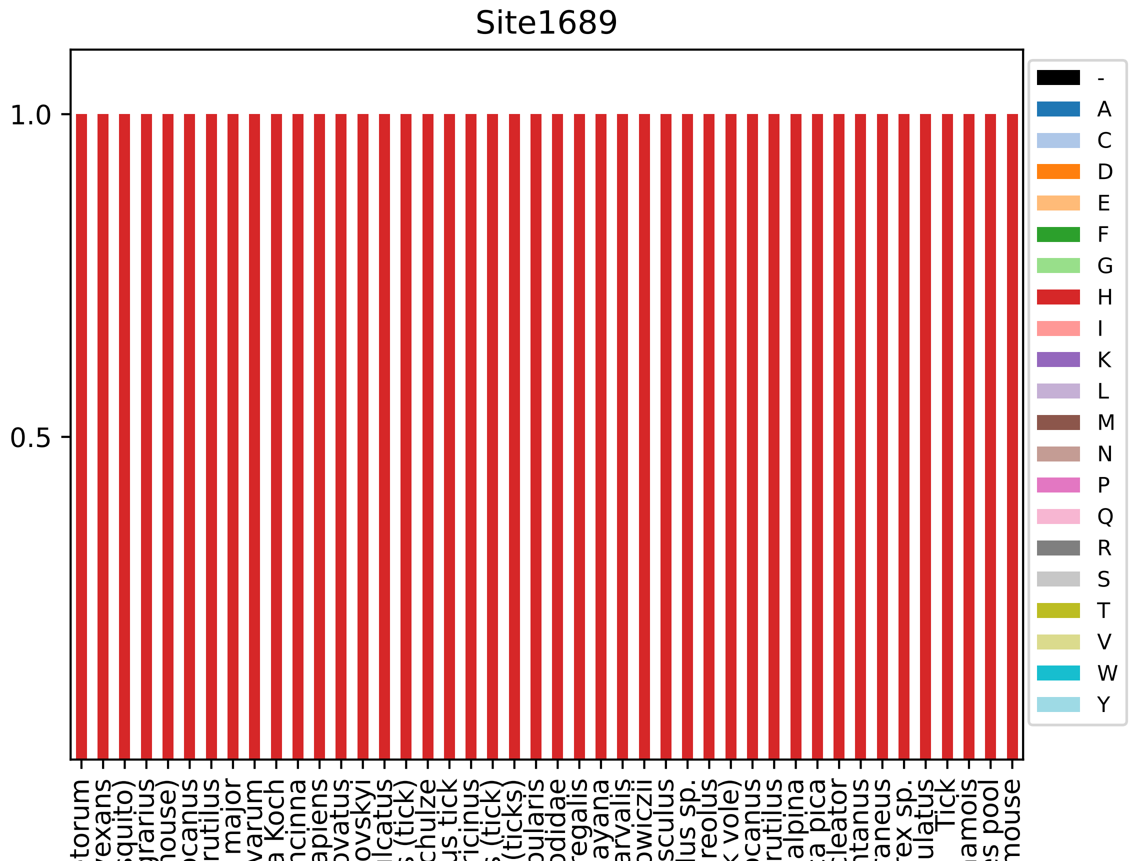
Site1689
1.0
0.5
-
A
C
D
E
F
G
H
I
K
L
M
N
P
Q
R
S
T
V
W
Y

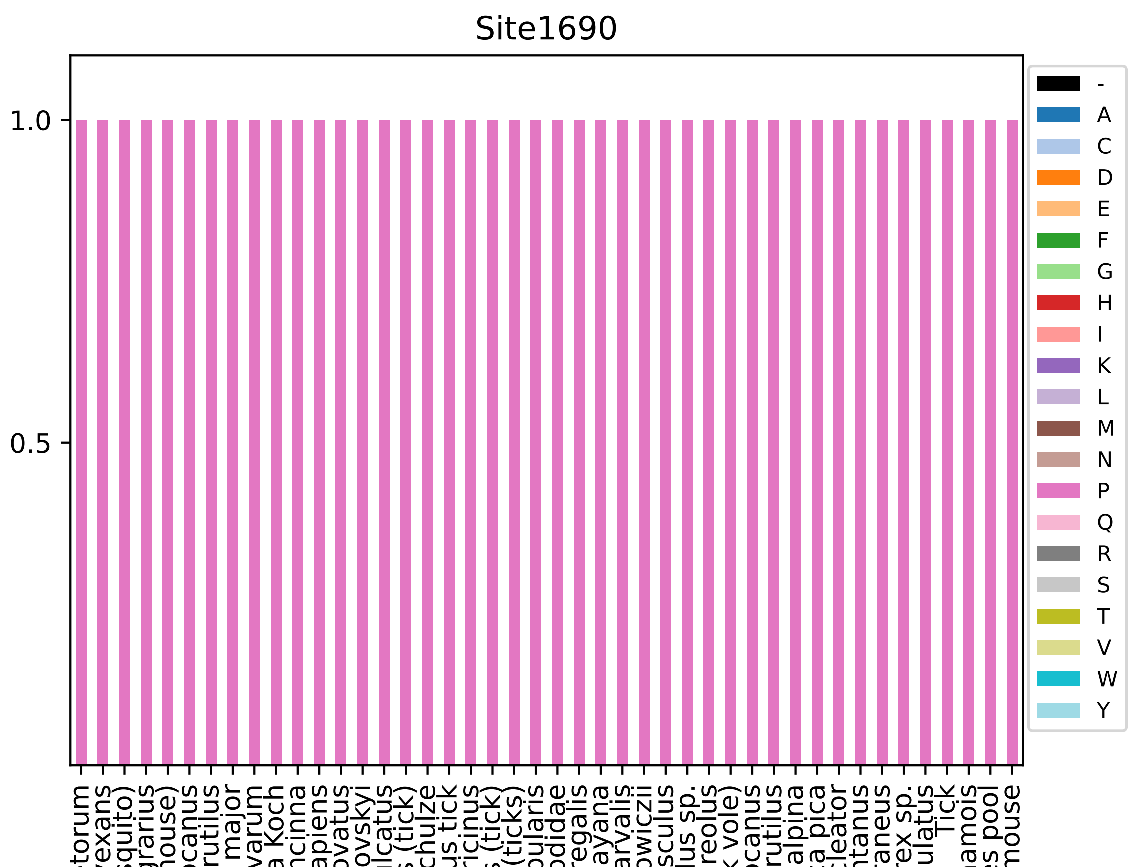
Site1690
1.0
0.5
-
A
C
D
E
F
G
H
I
K
L
M
N
P
Q
R
S
T
V
W
Y

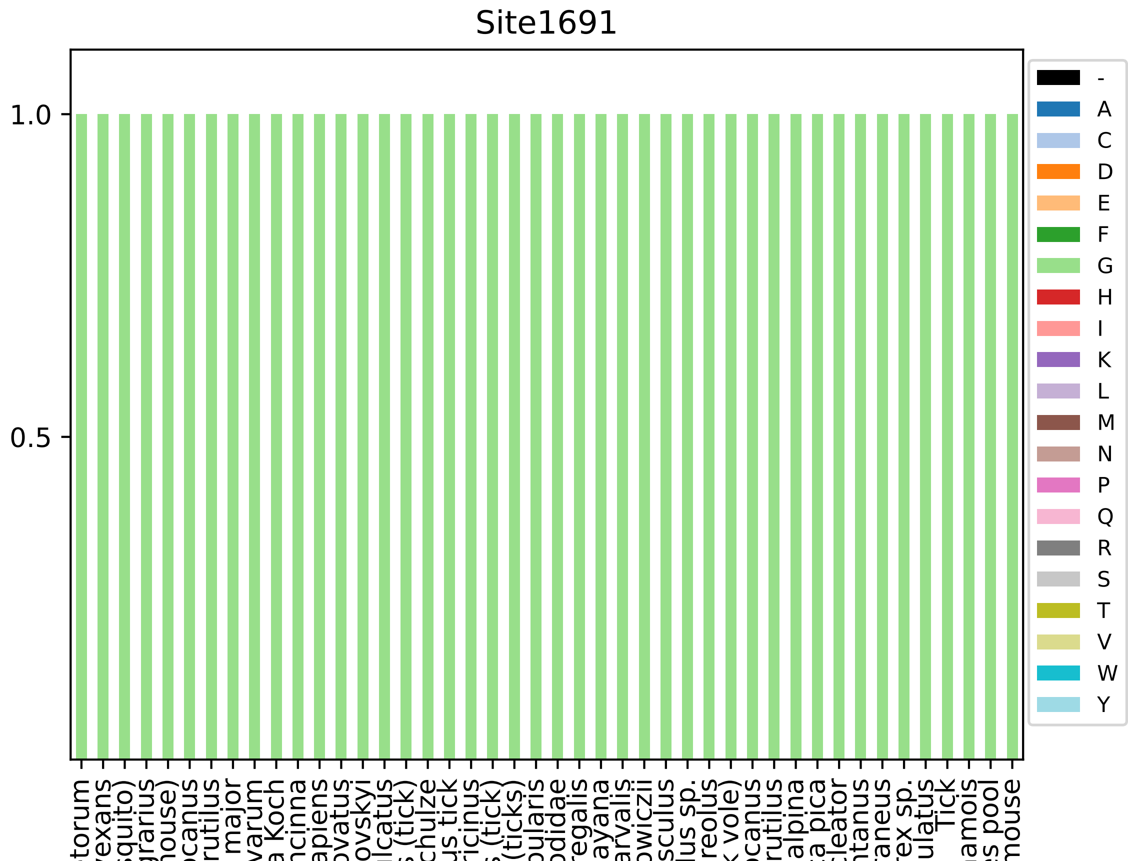
Site1691
1.0
0.5
-
A
C
D
E
F
G
H
I
K
L
M
N
P
Q
R
S
T
V
W
Y

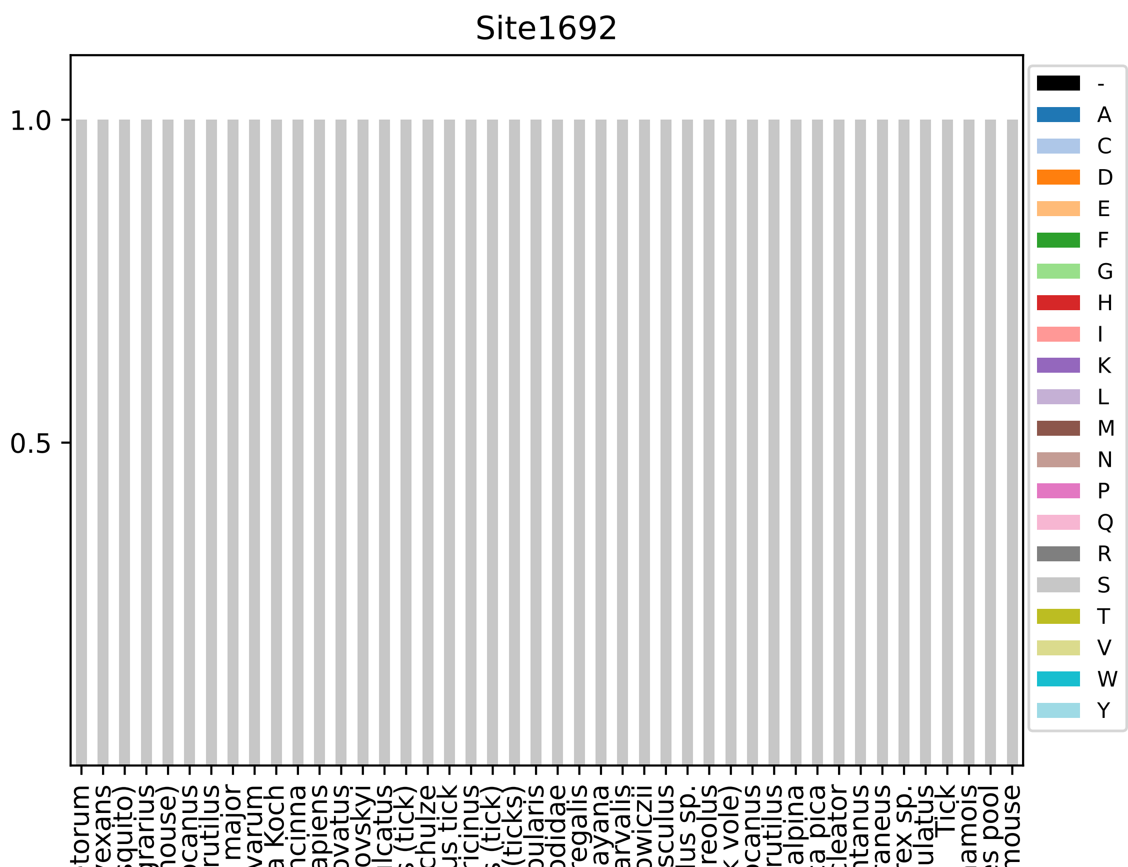

Site1692
1.0
0.5
-
A
C
D
E
F
G
H
I
K
L
M
N
P
Q
R
S
T
V
W
Y

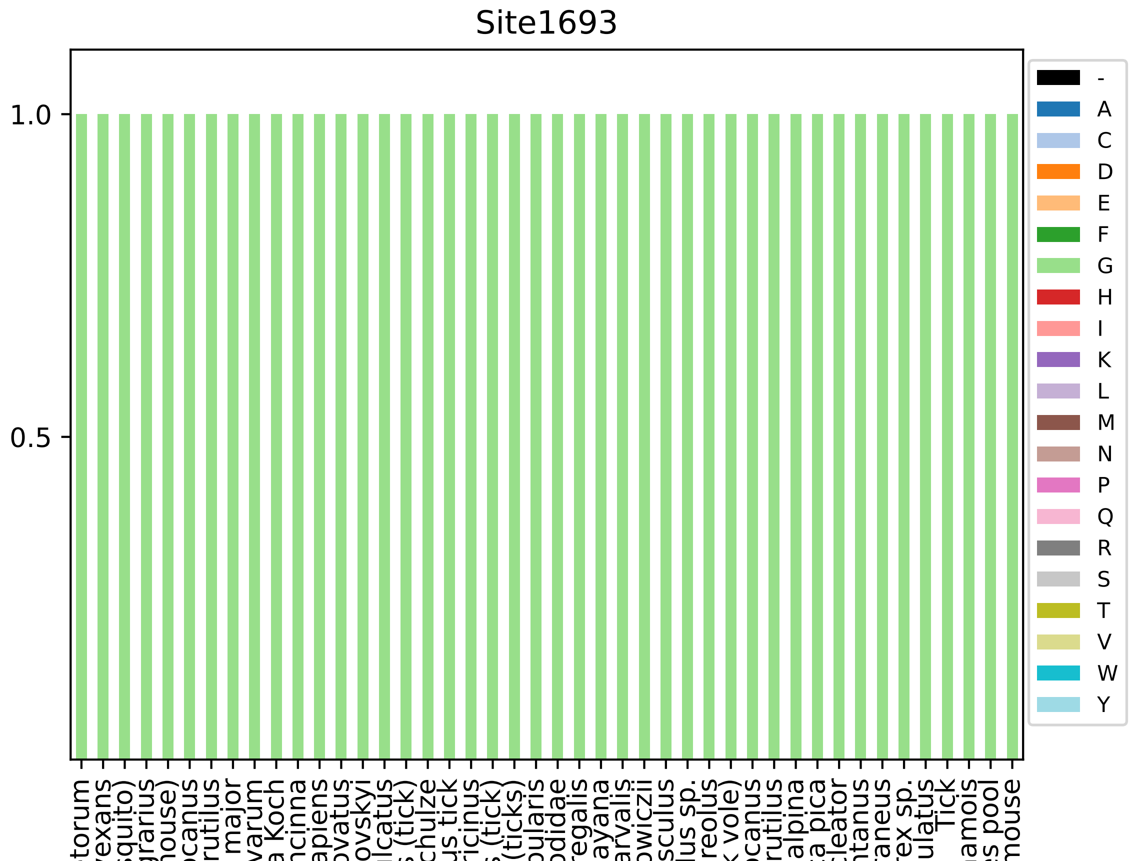

Site1693
1.0
0.5
-
A
C
D
E
F
G
H
I
K
L
M
N
P
Q
R
S
T
V
W
Y

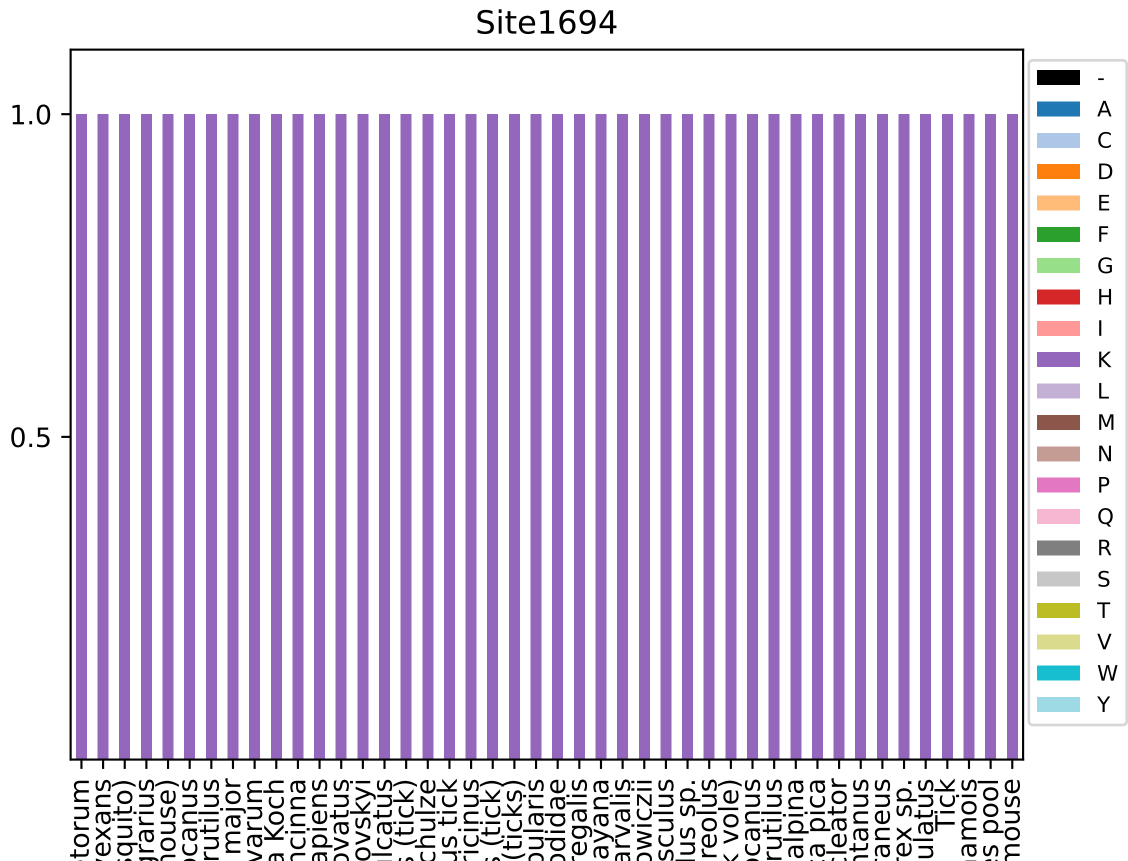

Site1694
1.0
0.5
-
A
C
D
E
F
G
H
I
K
L
M
N
P
Q
R
S
T
V
W
Y

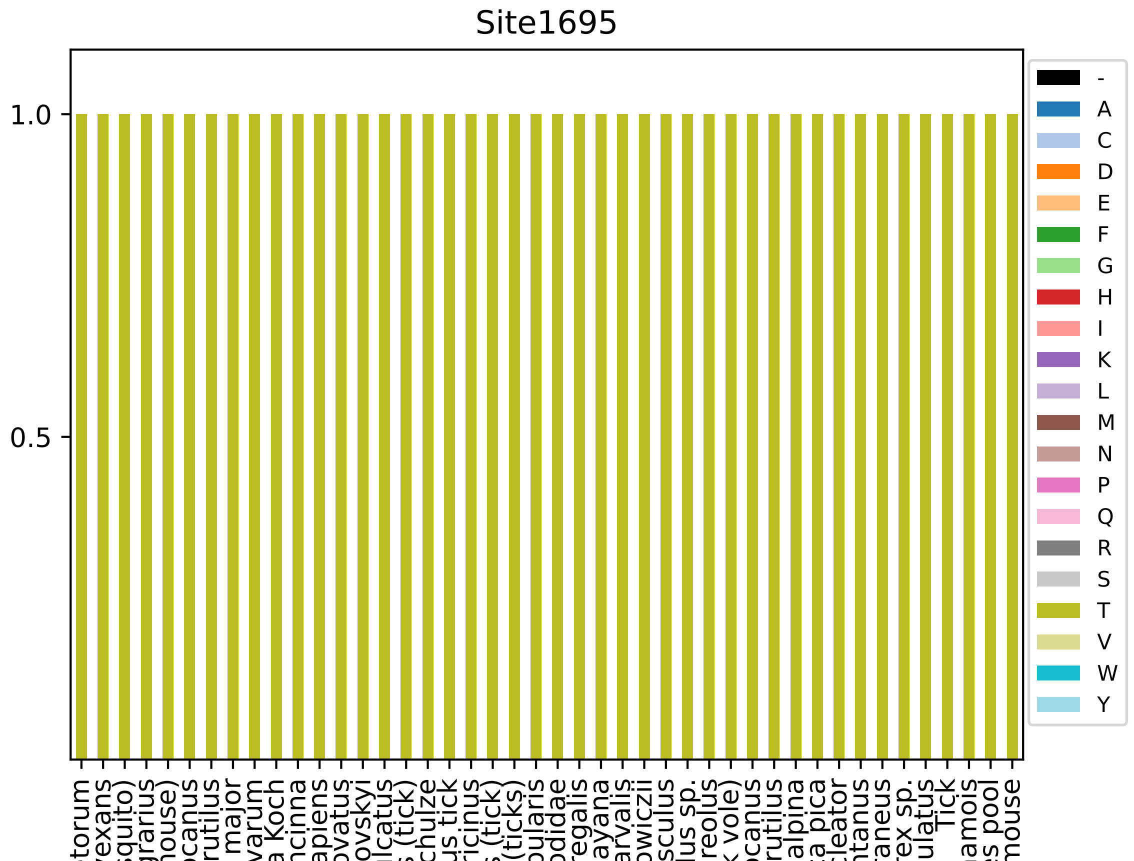

Site1695
1.0
0.5
-
A
C
D
E
F
G
H
I
K
L
M
N
P
Q
R
S
T
V
W
Y

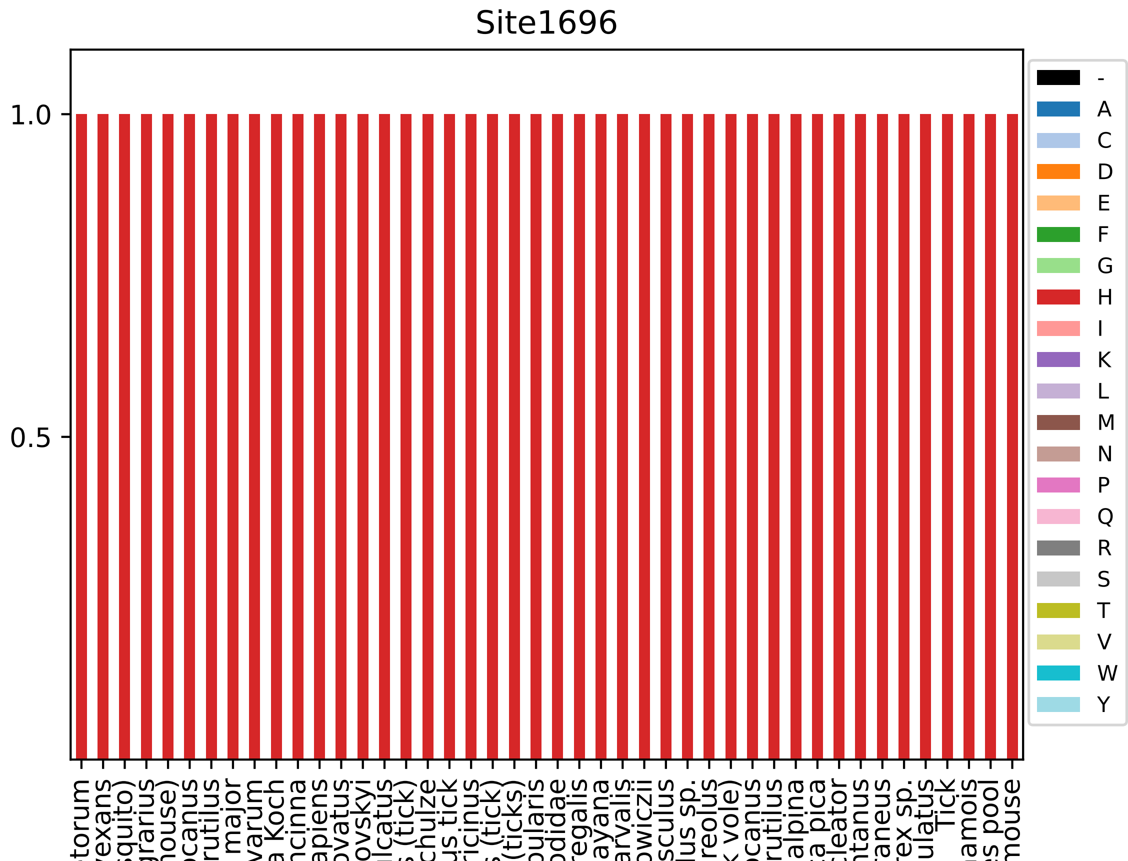
Site1696
1.0
0.5
-
A
C
D
E
F
G
H
I
K
L
M
N
P
Q
R
S
T
V
W
Y

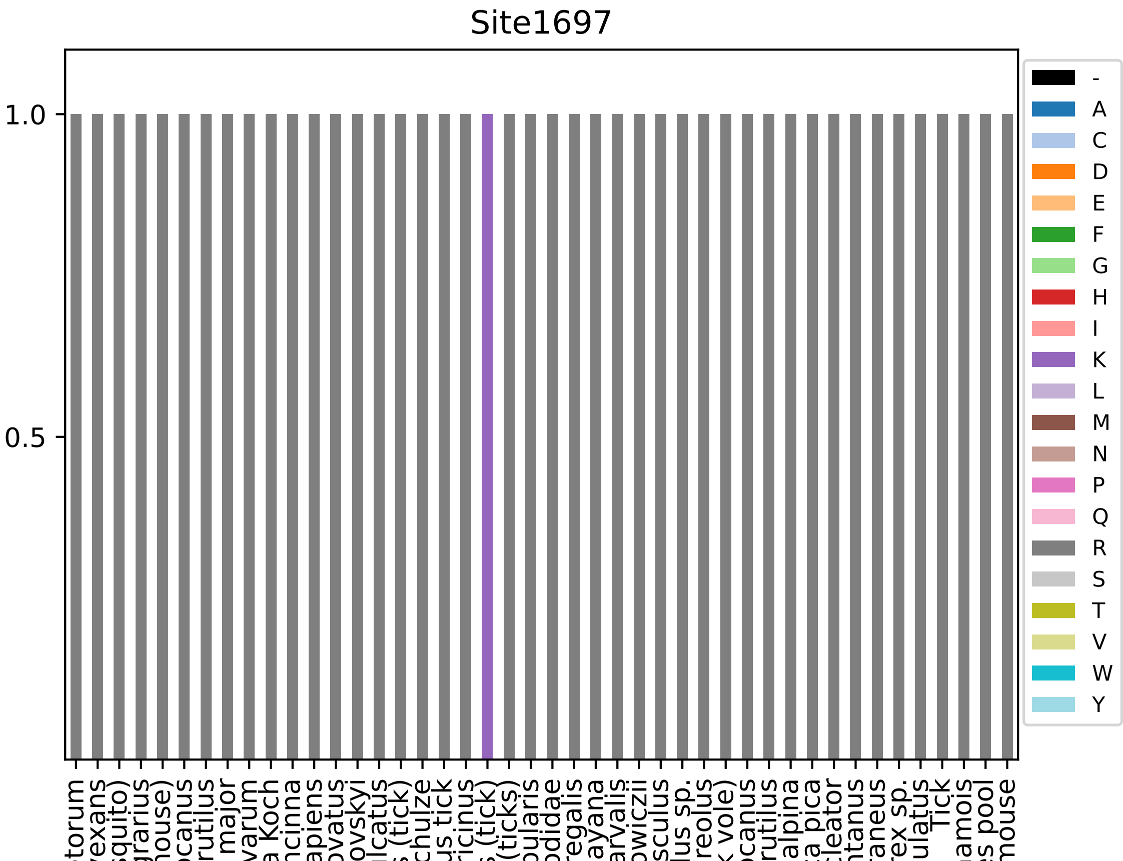
Site1697
1.0
0.5
-
A
C
D
E
F
G
H
I
K
L
M
N
P
Q
R
S
T
V
W
Y
torum
vexans
squito)
grarius
nouse)
ocanus
rutilus
major
varum
a Koch
ncinna
apiens
ovatus
ovskyi
ulcatus
s (tick)
chulze
us tick
ricinus
s (tick)
(ticks)
pularis
odidae
regalis
ayana
arvalis
owiczii
isculus
lus sp.
reolus
k vole)
ocanus
rutilus
alpina
a pica
cleator
ntanus
raneus
rex sp.
ulatus
Tick
amois
es pool
mouse

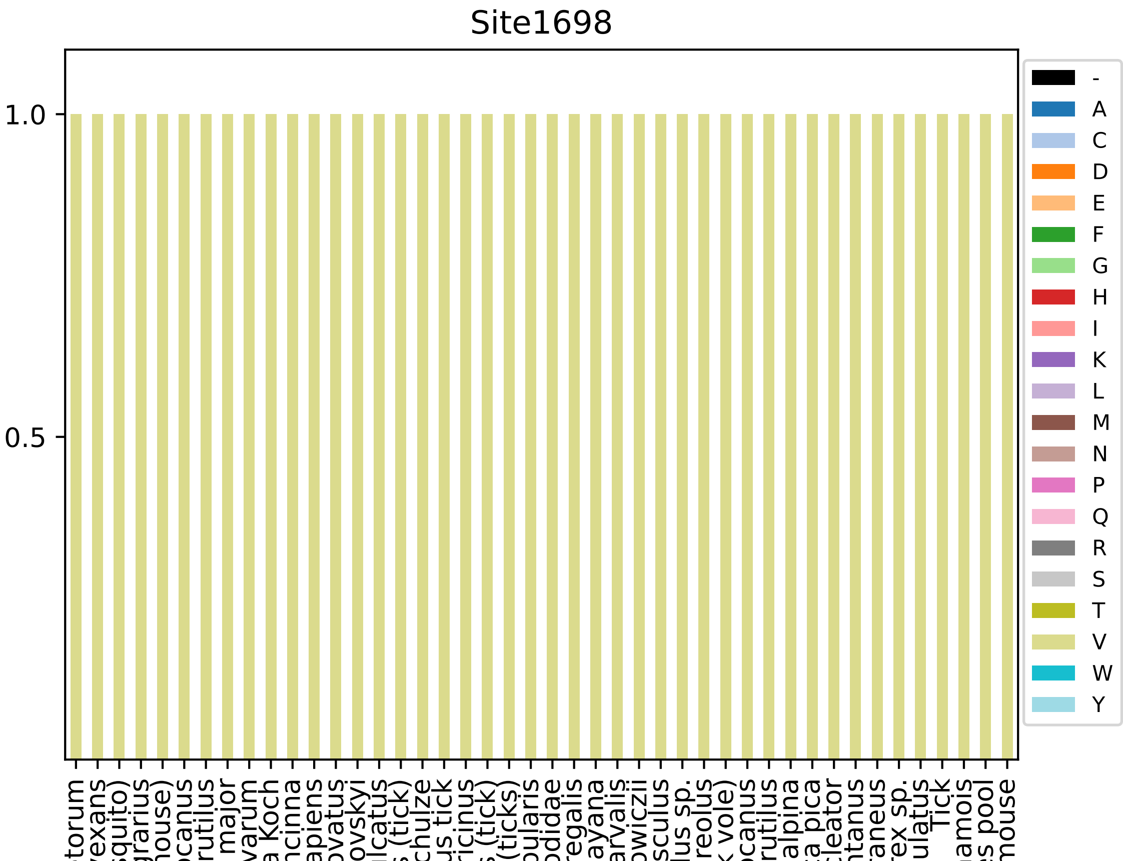

Site1698
1.0
0.5
-
A
C
D
E
F
G
H
I
K
L
M
N
P
Q
R
S
T
V
W
Y
Tick

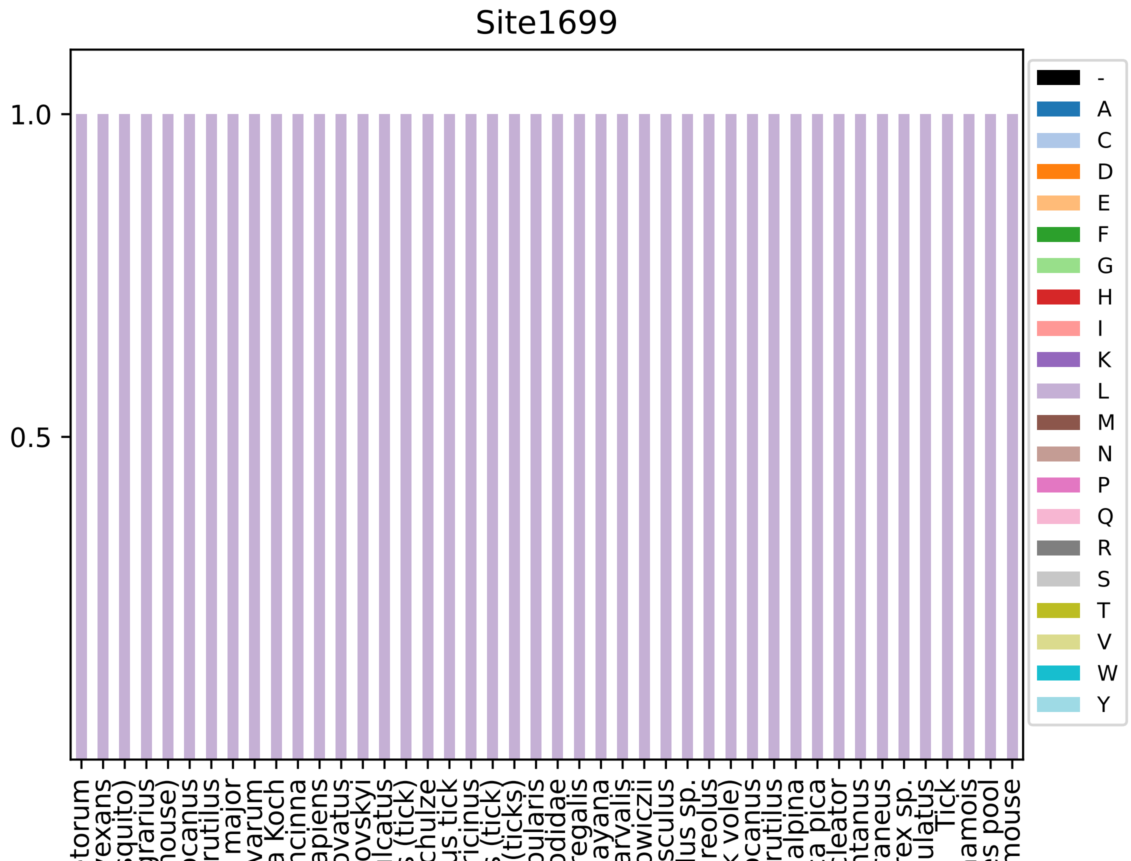
Site1699
1.0
0.5
-
A
C
D
E
F
G
H
I
K
L
M
N
P
Q
R
S
T
V
W
Y

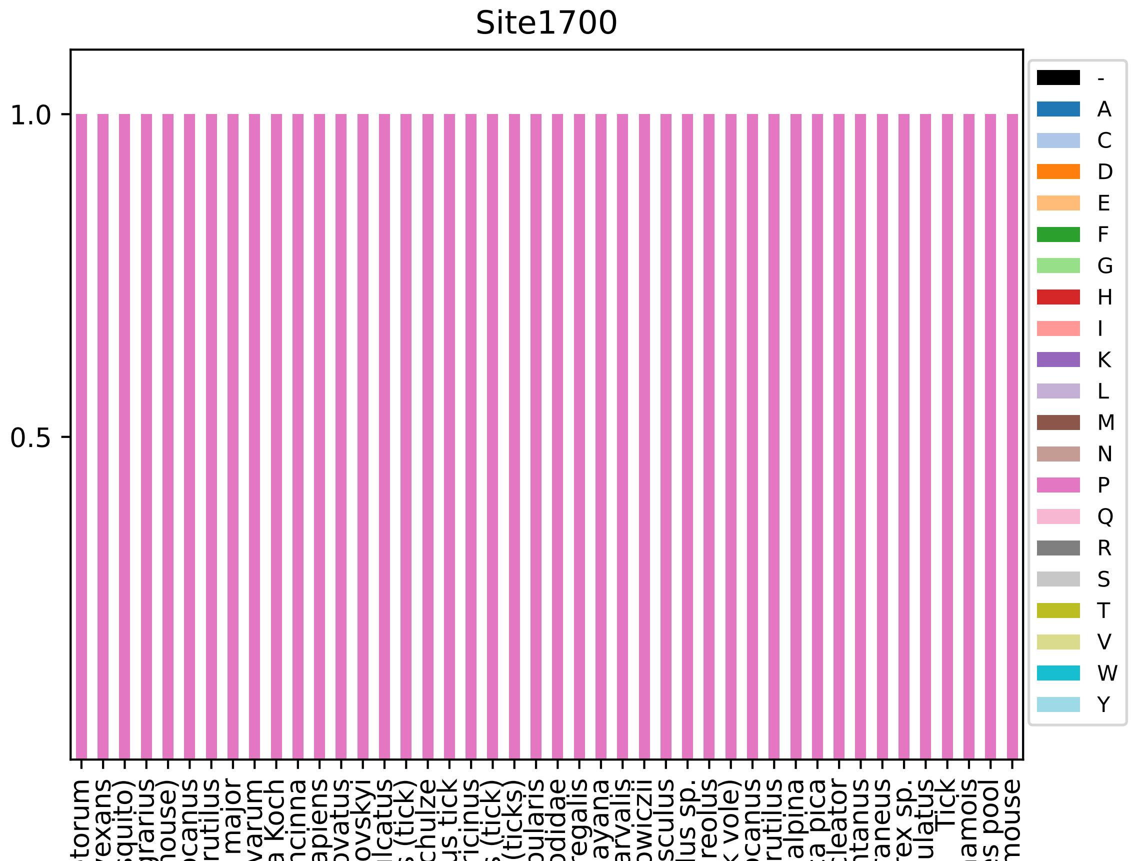
Site1700
1.0
0.5
-
A
C
D
E
F
G
H
I
K
L
M
N
P
Q
R
S
T
V
W
Y

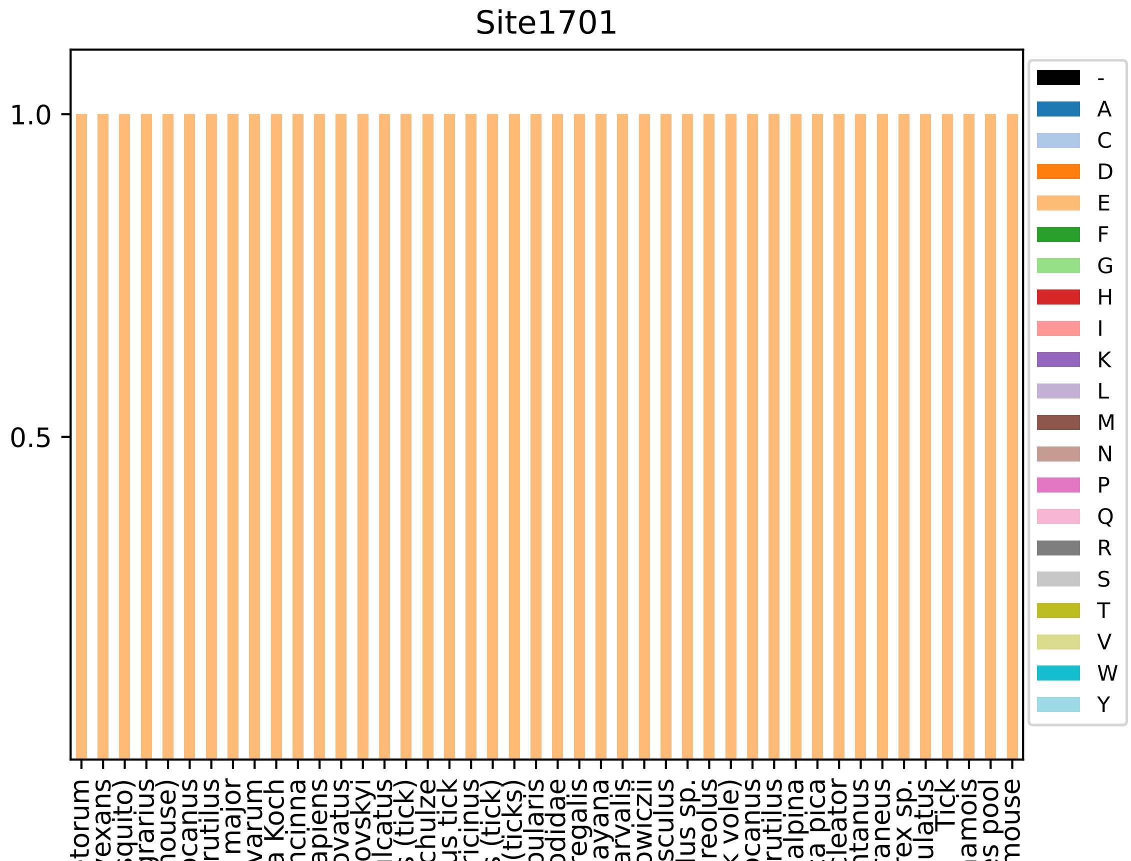

Site1701
1.0
0.5
-
A
C
D
E
F
G
H
I
K
L
M
N
P
Q
R
S
T
V
W
Y

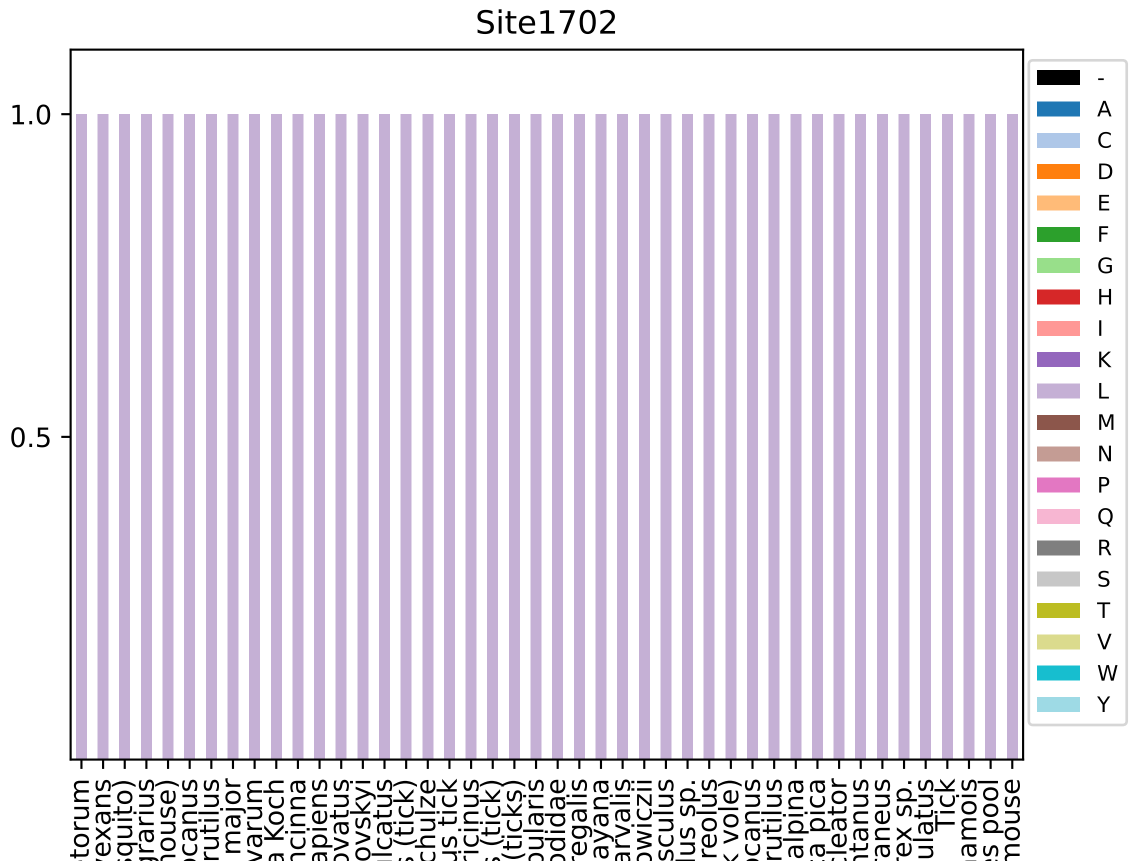
Site1702
1.0
0.5
-
A
C
D
E
F
G
H
I
K
L
M
N
P
Q
R
S
T
V
W
Y

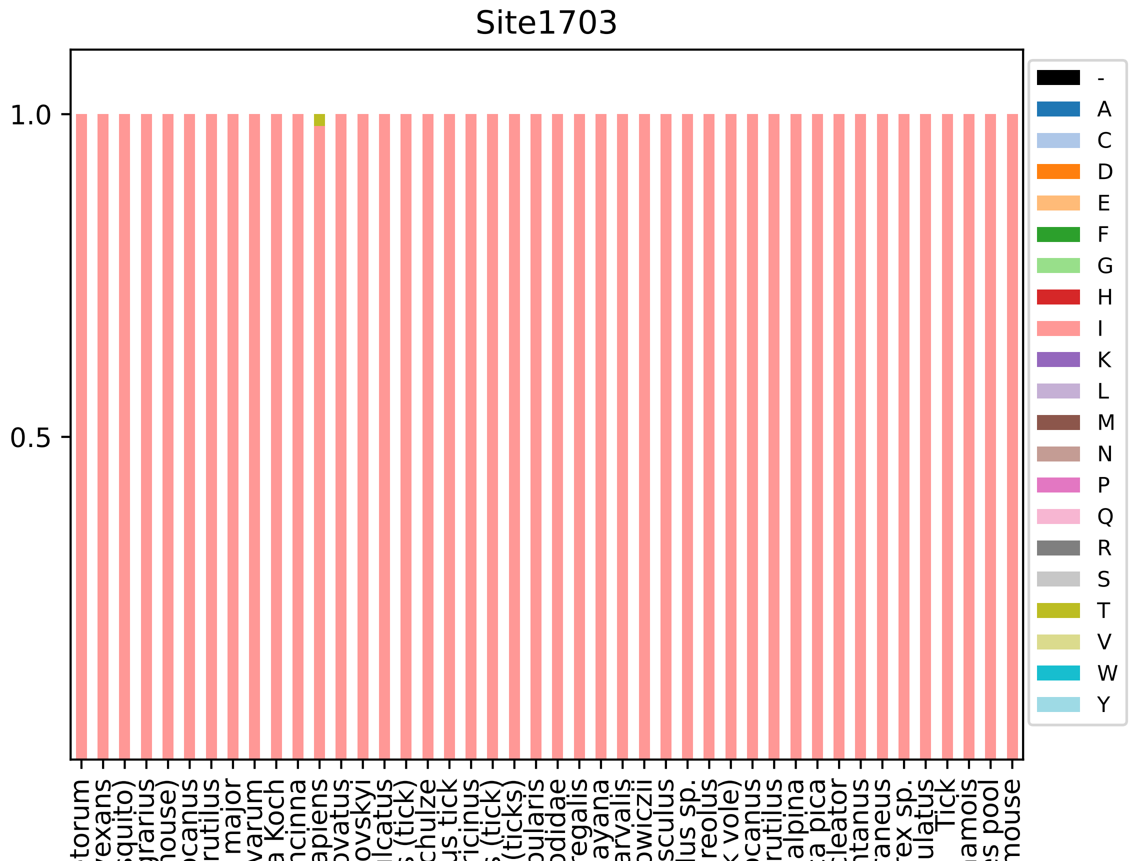
Site1703
1.0
0.5
-
A
C
D
E
F
G
H
I
K
L
M
N
P
Q
R
S
T
V
W
Y

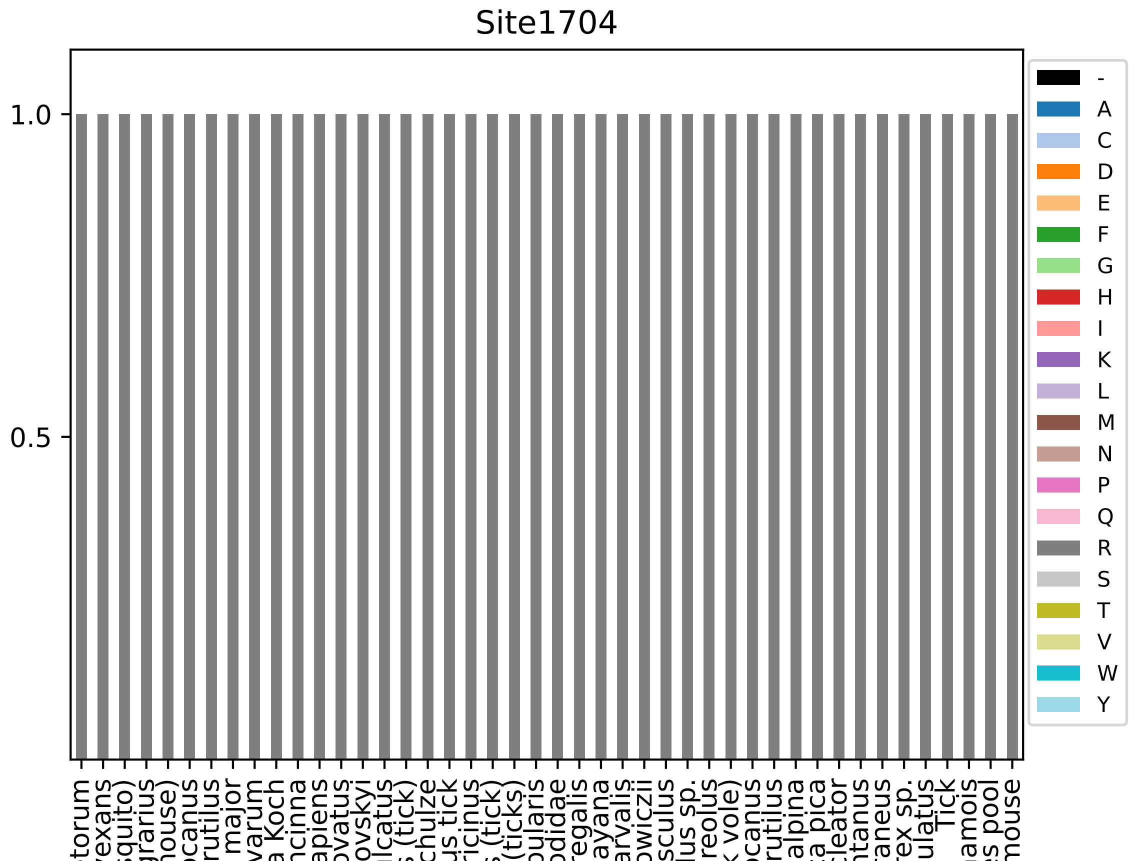
Site1704
1.0
0.5
-
A
C
D
E
F
G
H
I
K
L
M
N
P
Q
R
S
T
V
W
Y

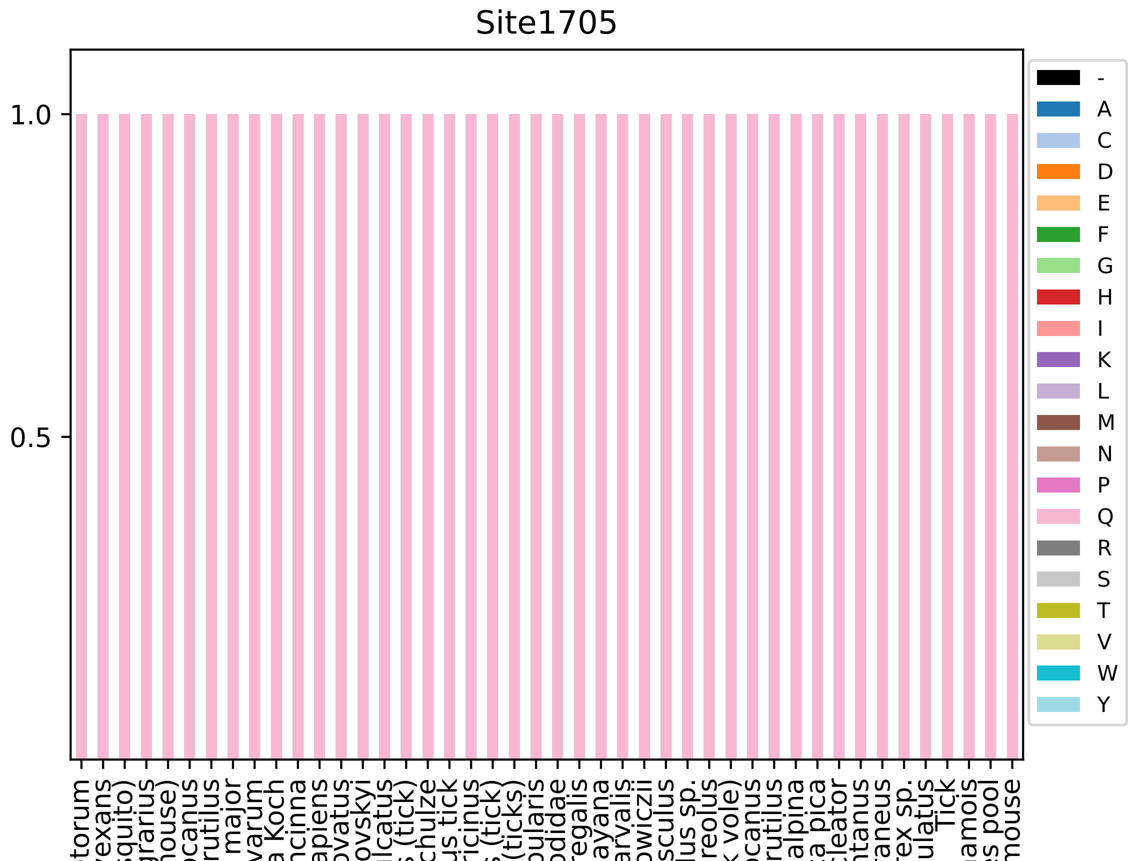
Site1705
1.0
0.5
-
A
C
D
E
F
G
H
I
K
L
M
N
P
Q
R
S
T
V
W
Y

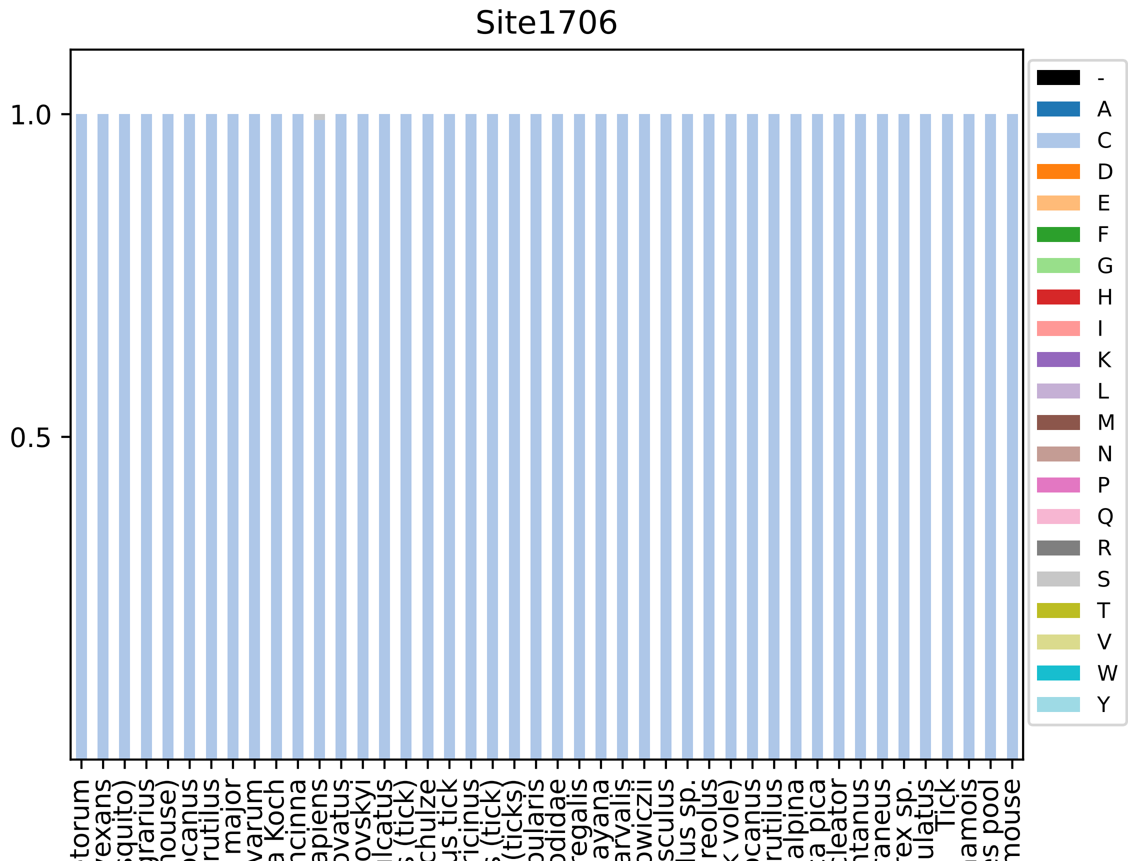
Site1706
1.0
0.5
-
A
C
D
E
F
G
H
I
K
L
M
N
P
Q
R
S
T
V
W
Y
torum
vexans
squito)
grarius
nouse)
ocanus
rutilus
major
varum
a Koch
ncinna
apiens
ovatus
ovskyi
ulcatus
s (tick)
chulze
us tick
ricinus
s (tick)
(ticks)
pularis
podidae
regalis
ayana
arvalis
owiczii
sculus
lus sp.
reolus
x vole)
ocanus
rutilus
alpina
a pica
cleator
ntanus
raneus
rex sp.
ulatus
Tick
amois
es pool
mouse

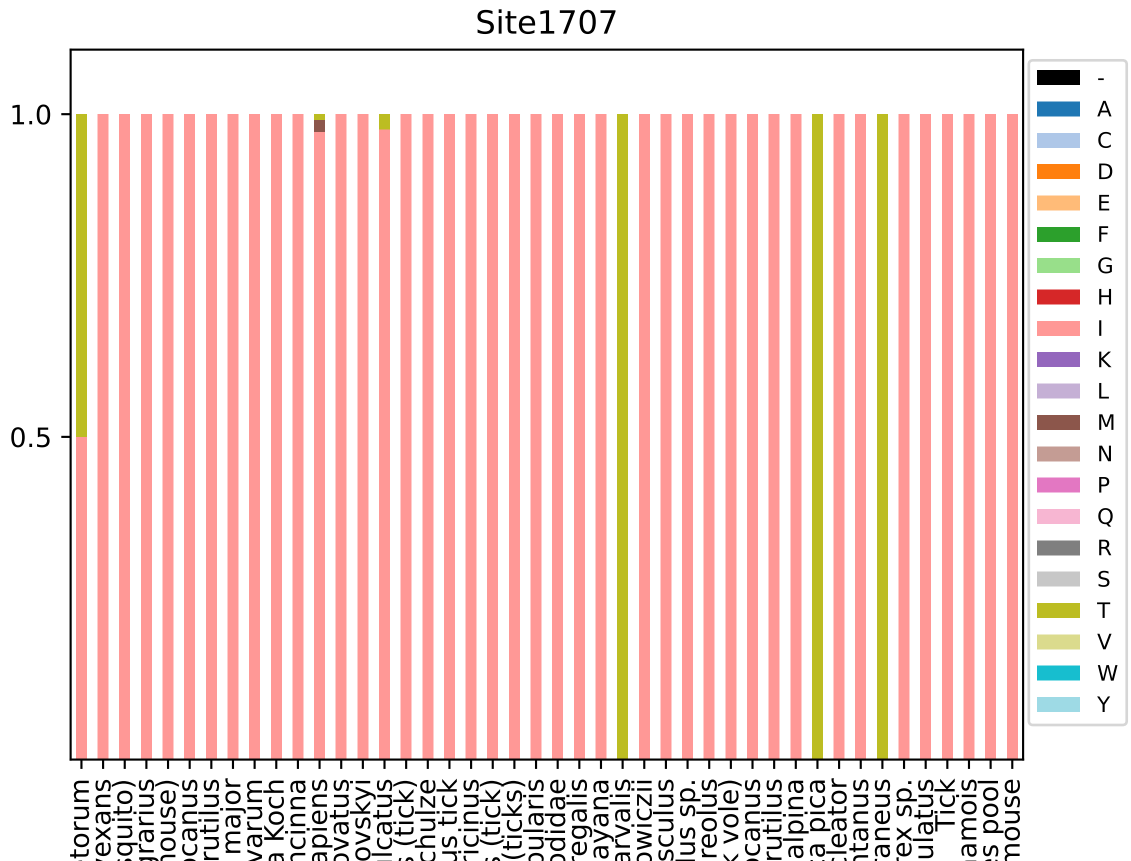

Site1707
1.0
0.5
-
A
C
D
E
F
G
H
I
K
L
M
N
P
Q
R
S
T
V
W
Y

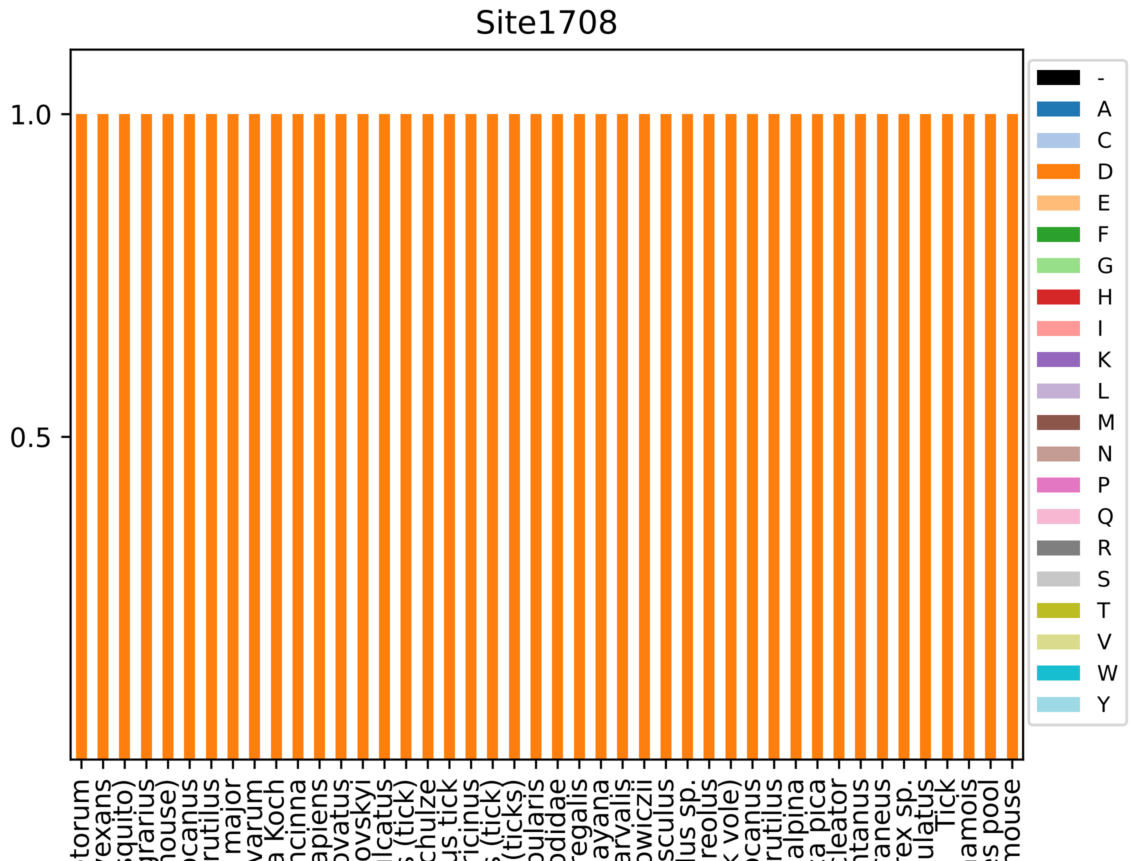

Site1708
1.0
0.5
-
A
C
D
E
F
G
H
I
K
L
M
N
P
Q
R
S
T
V
W
Y

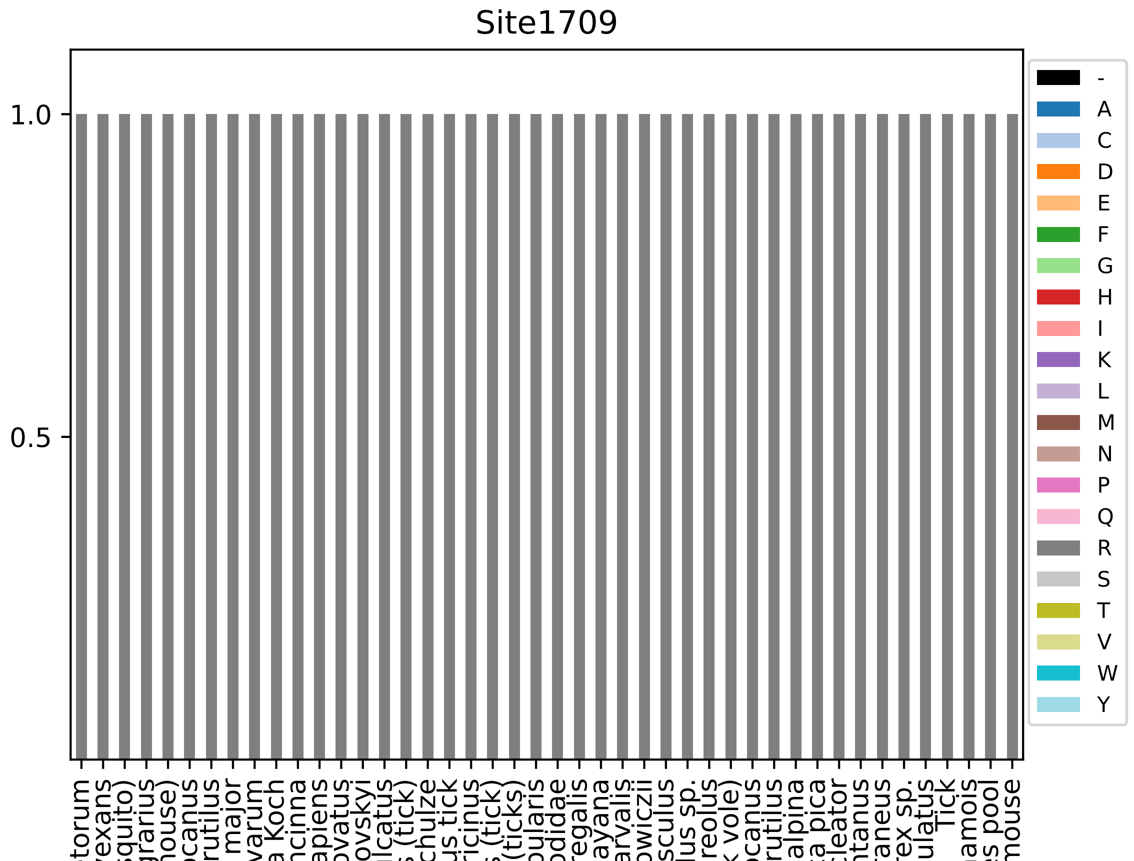

Site1709
1.0
0.5
-
A
C
D
E
F
G
H
I
K
L
M
N
P
Q
R
S
T
V
W
Y

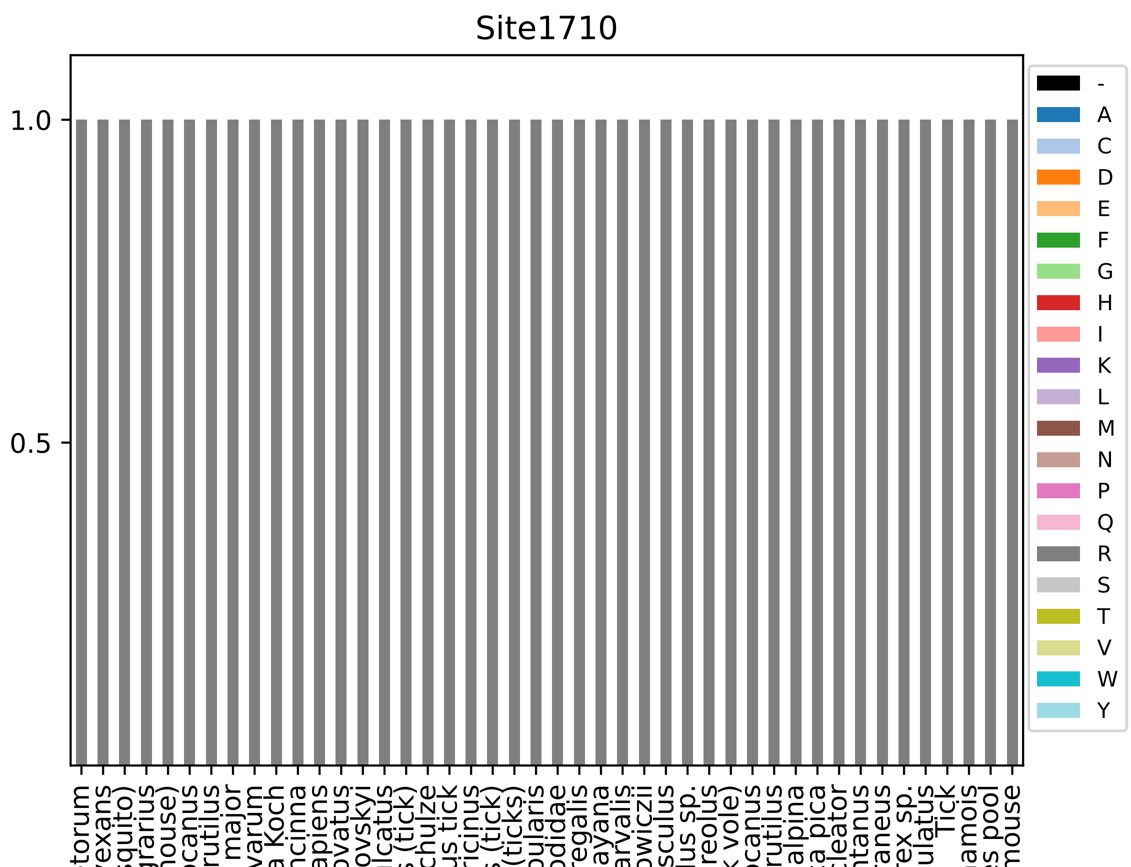
Site1710
1.0
0.5
-
A
C
D
E
F
G
H
I
K
L
M
N
P
Q
R
S
T
V
W
Y

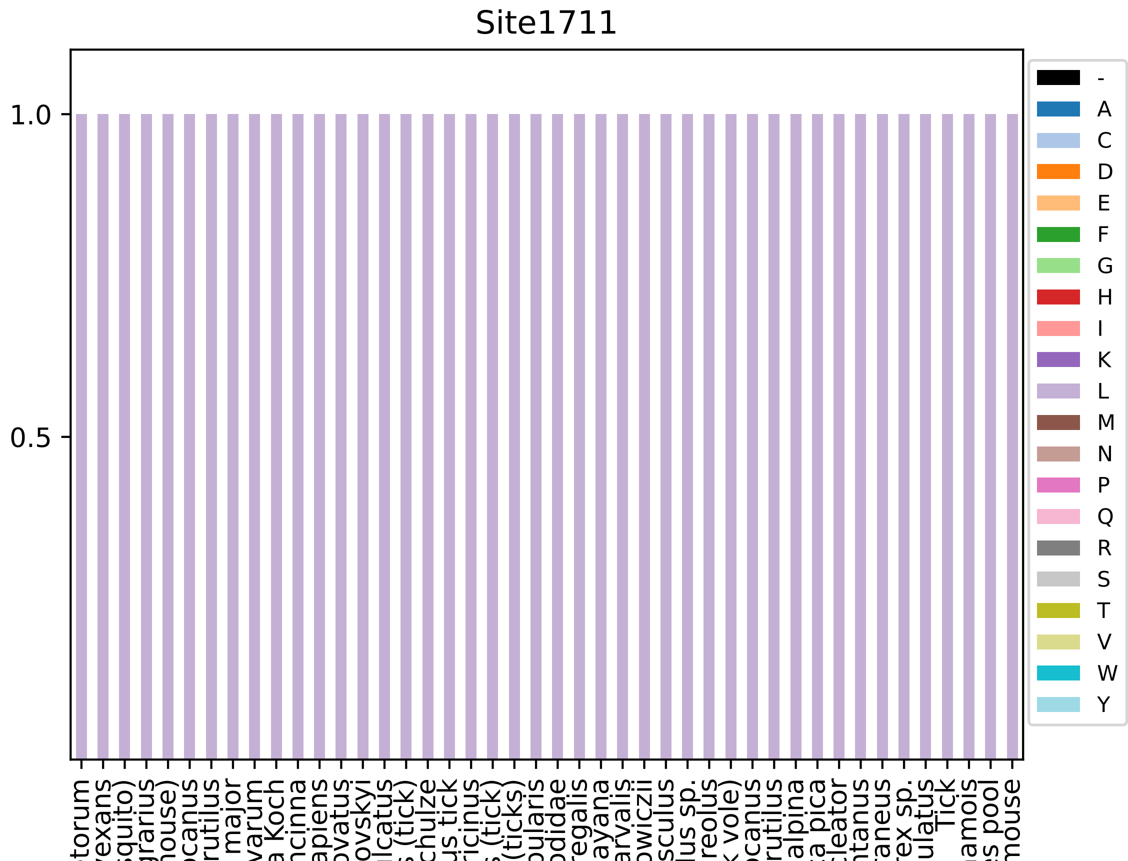
Site1711
1.0
0.5
-
A
C
D
E
F
G
H
I
K
L
M
N
P
Q
R
S
T
V
W
Y

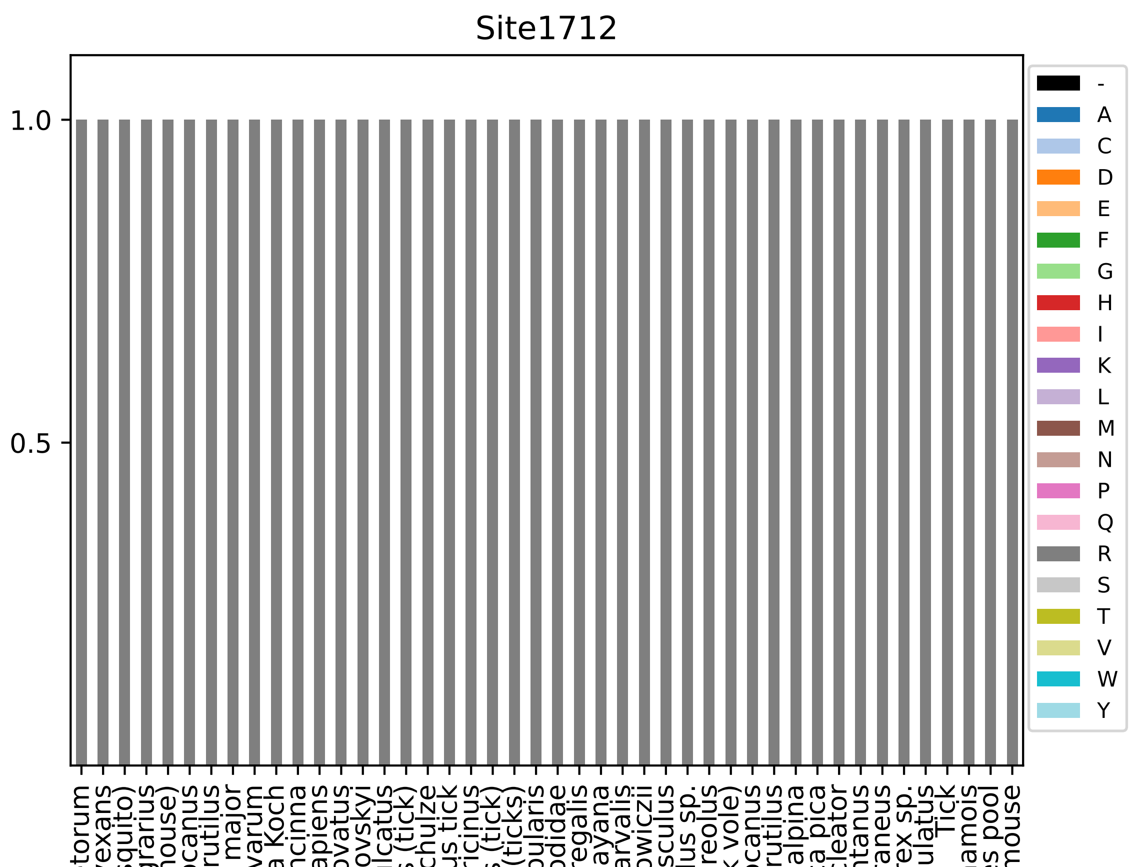
Site1712
1.0
0.5
-
A
C
D
E
F
G
H
I
K
L
M
N
P
Q
R
S
T
V
W
Y

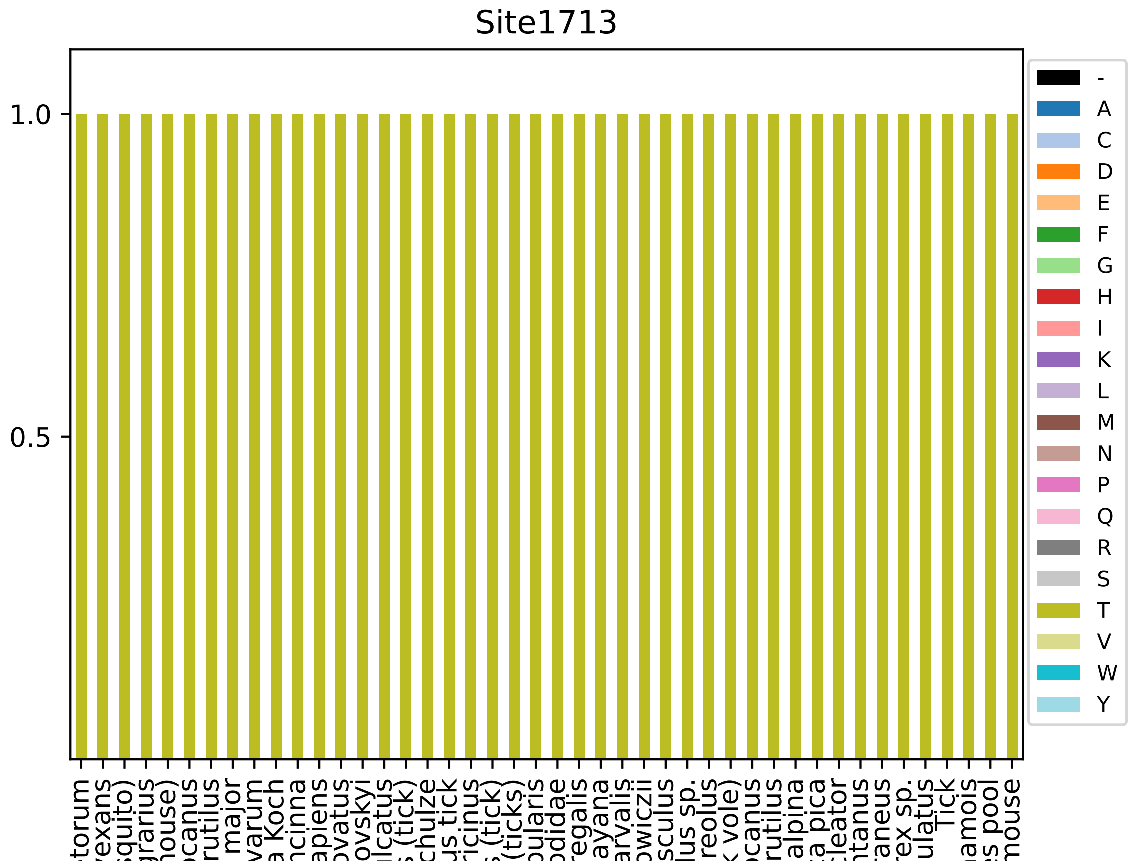

Site1713
1.0
0.5
-
A
C
D
E
F
G
H
I
K
L
M
N
P
Q
R
S
T
V
W
Y

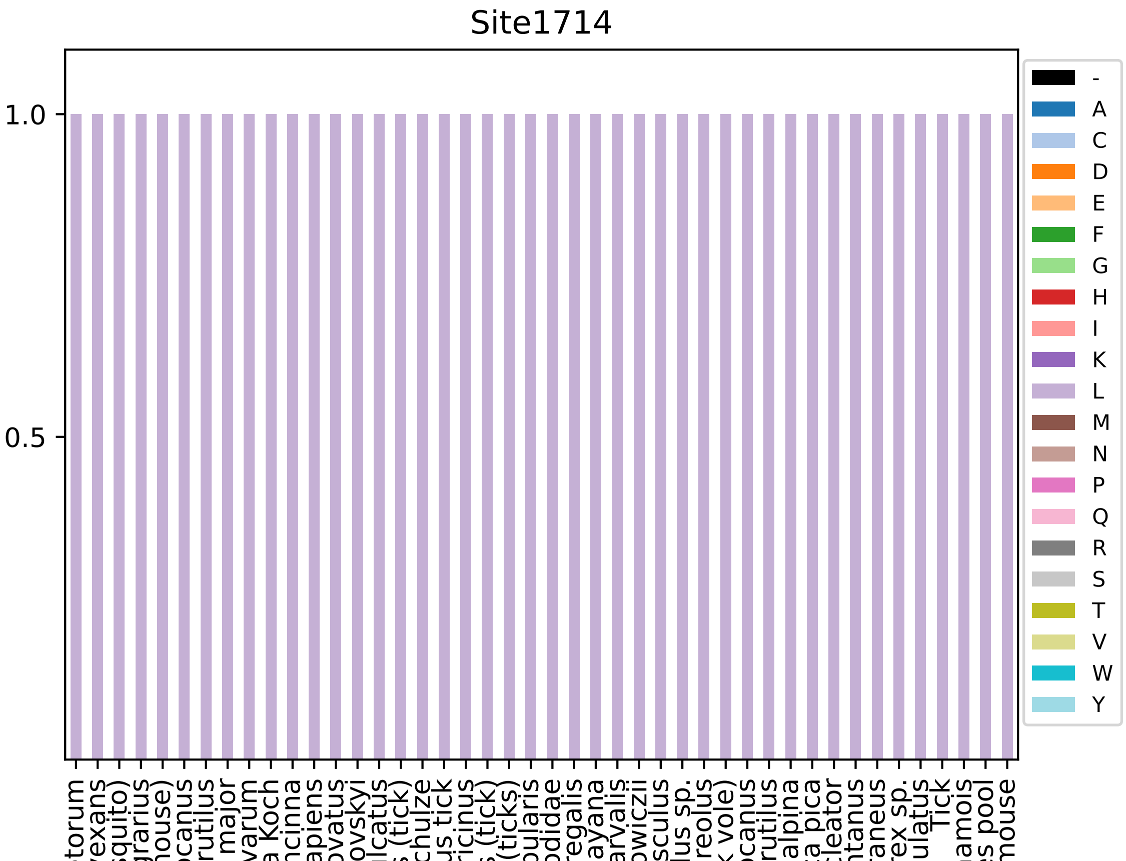
Site1714
1.0
0.5
-
A
C
D
E
F
G
H
I
K
L
M
N
P
Q
R
S
T
V
W
Y

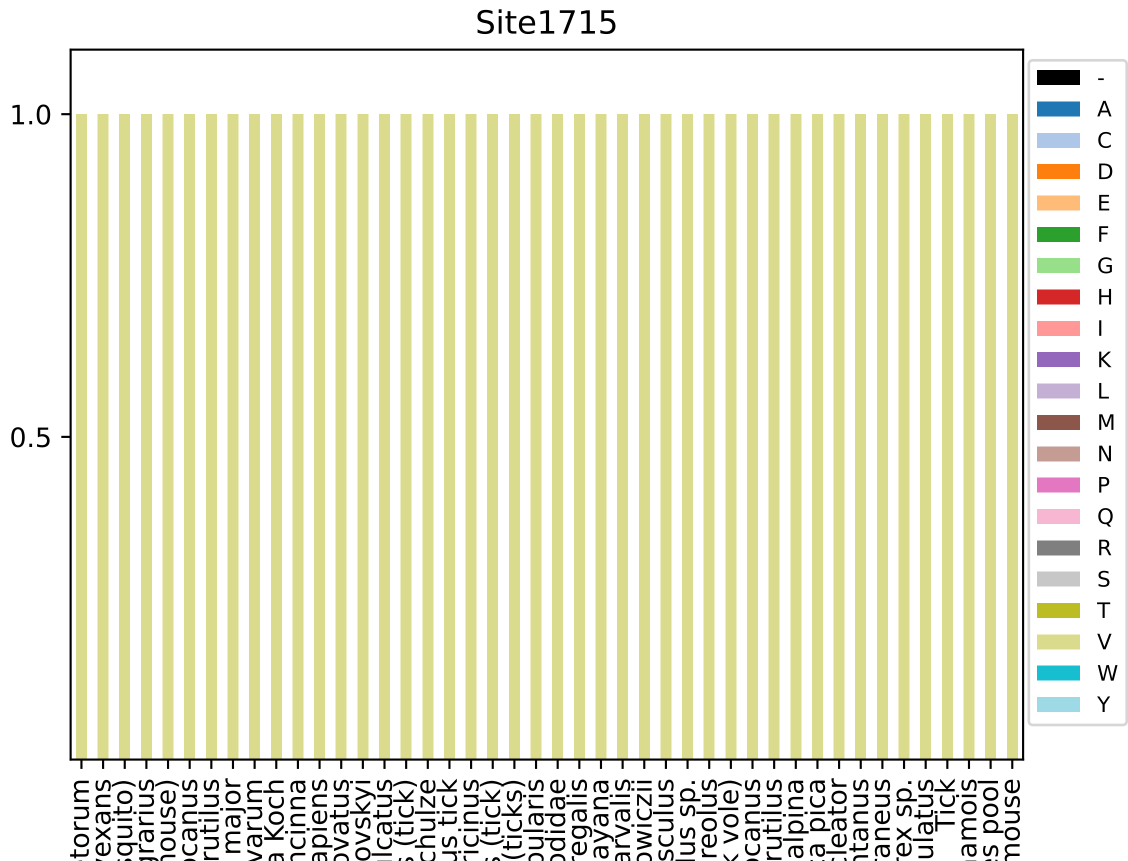
Site1715
1.0
0.5
-
A
C
D
E
F
G
H
I
K
L
M
N
P
Q
R
S
T
V
W
Y

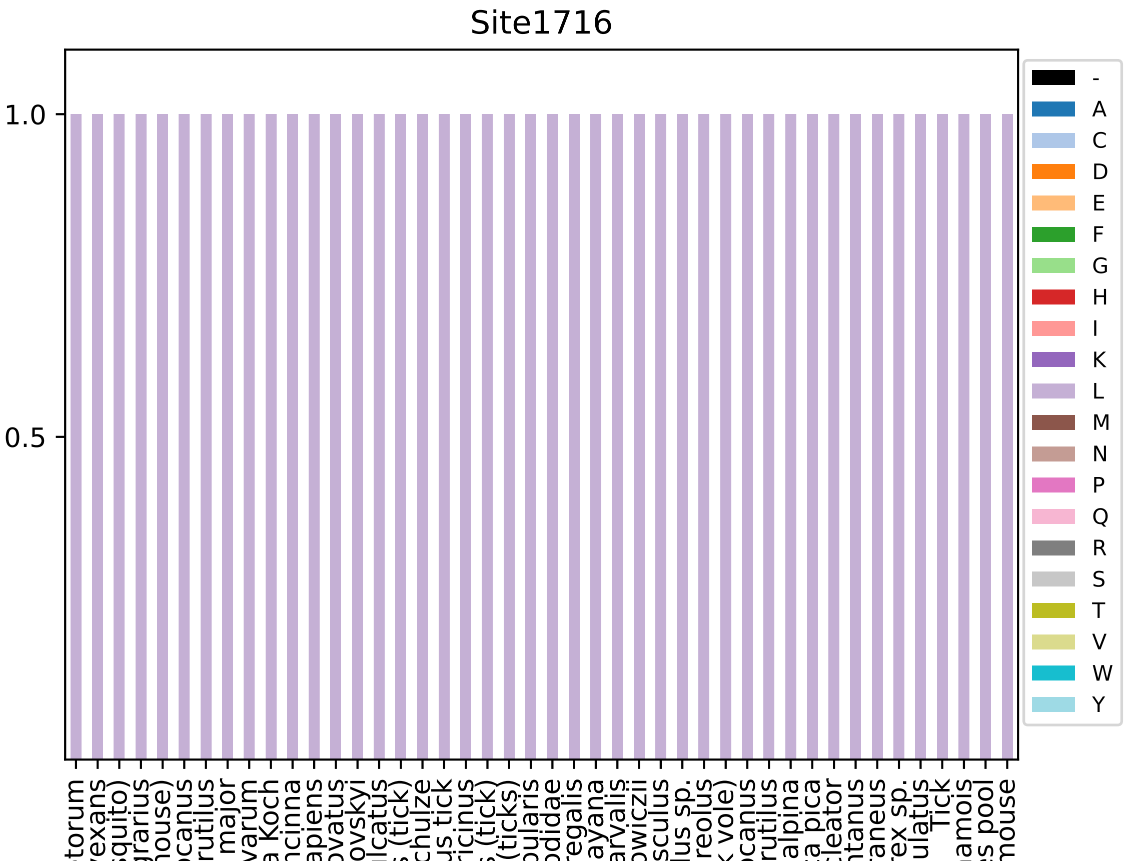
Site1716
1.0
0.5
-
A
C
D
E
F
G
H
I
K
L
M
N
P
Q
R
S
T
V
W
Y

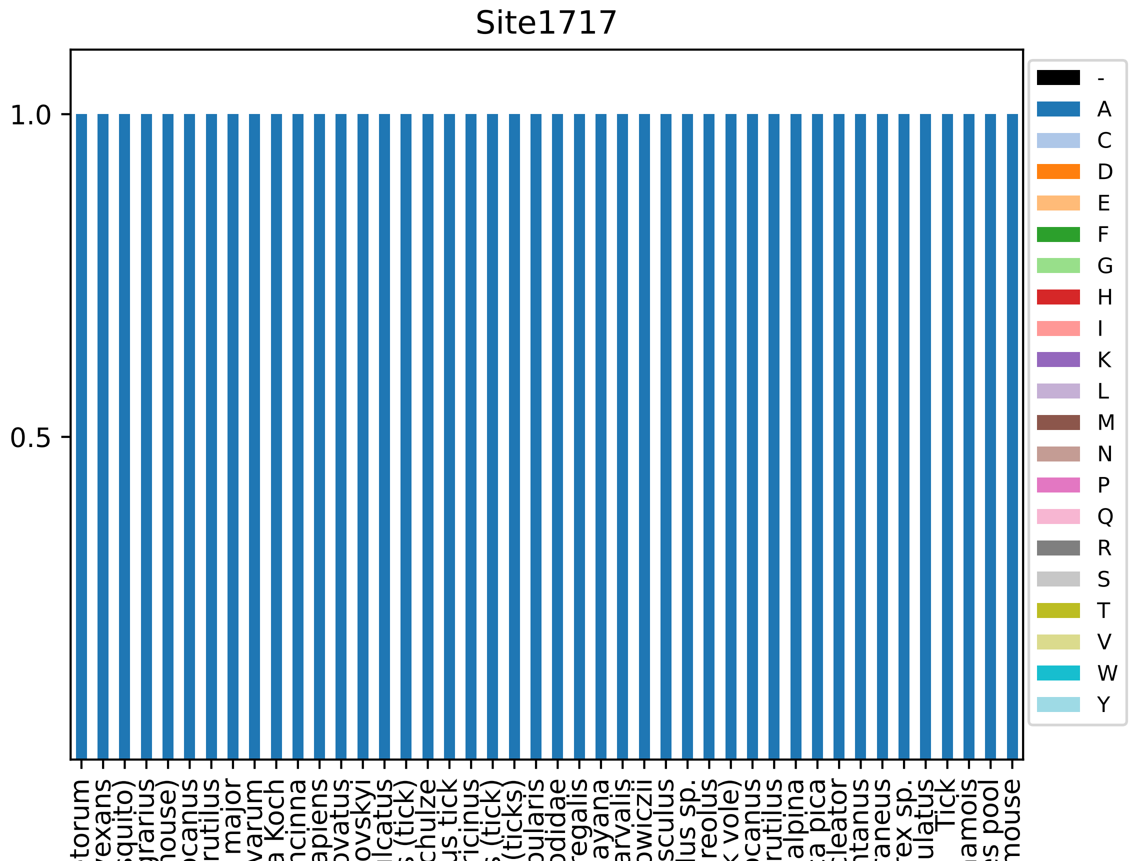
Site1717
1.0
0.5
-
A
C
D
E
F
G
H
I
K
L
M
N
P
Q
R
S
T
V
W
Y

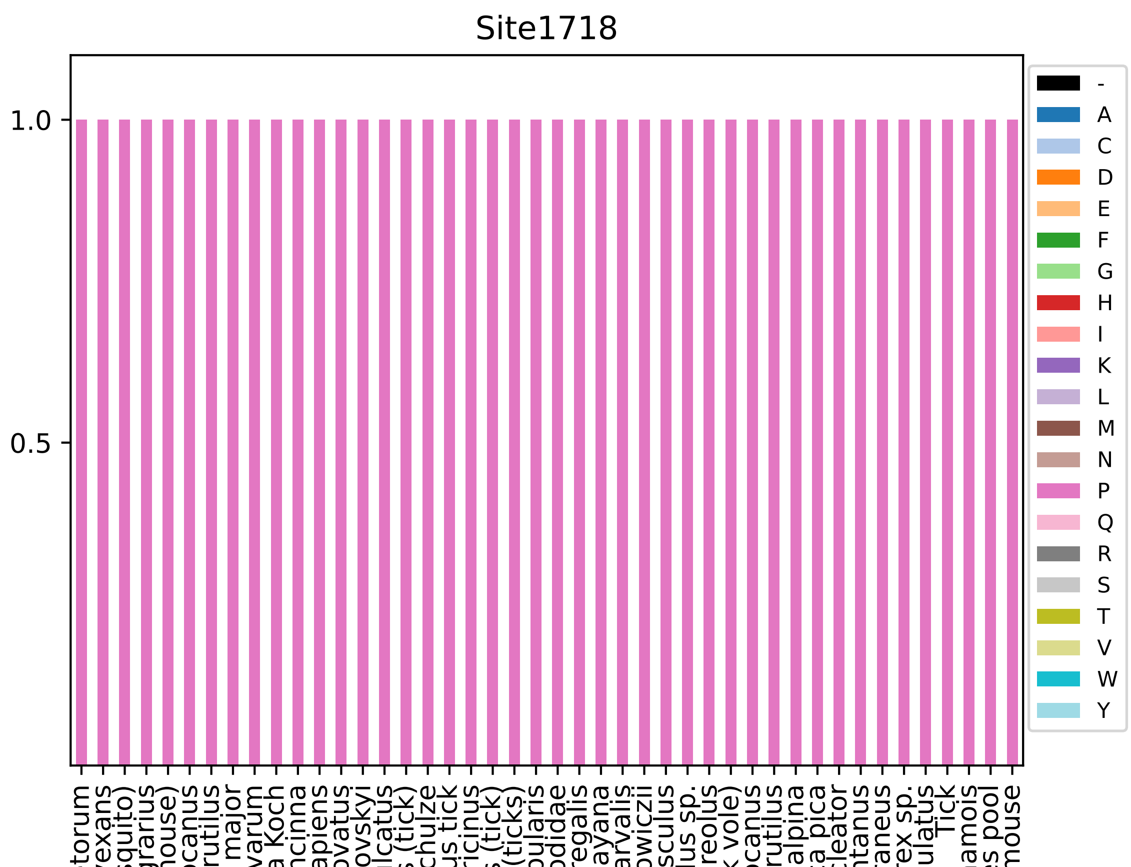

Site1718
1.0
0.5
-
A
C
D
E
F
G
H
I
K
L
M
N
P
Q
R
S
T
V
W
Y

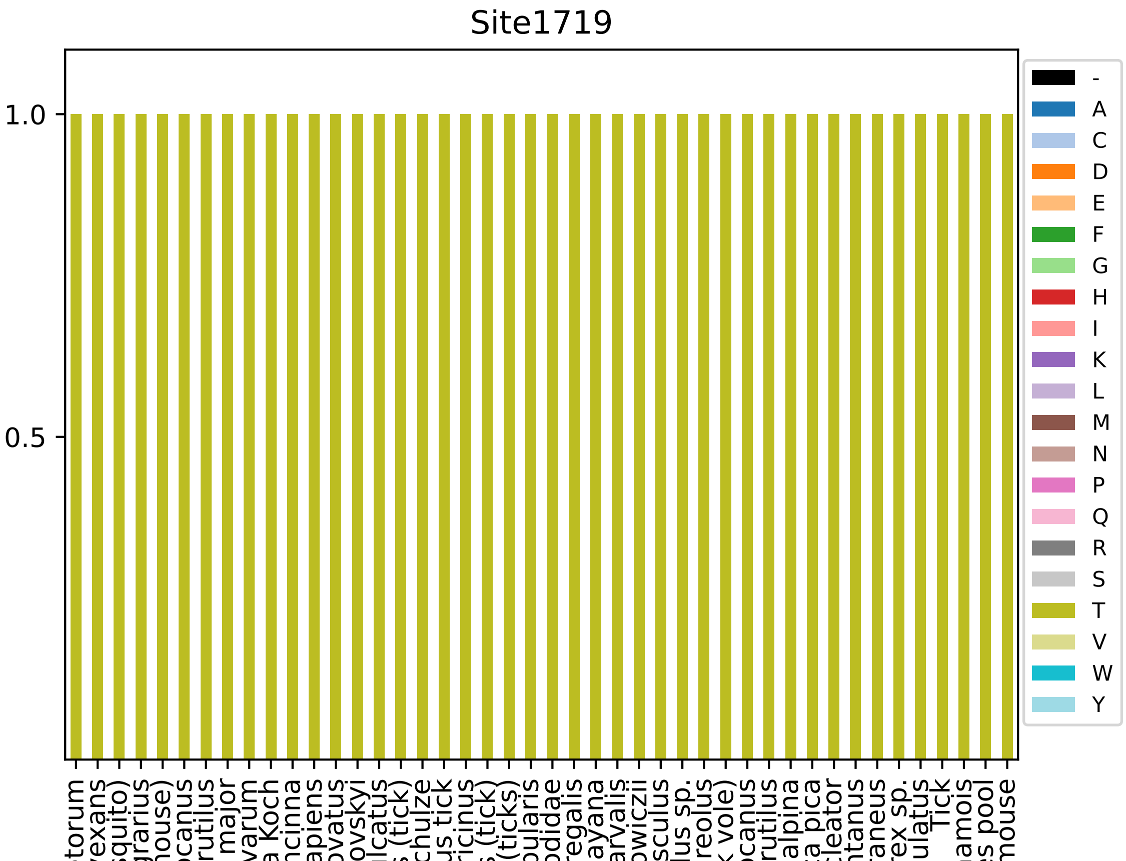
Site1719
1.0
0.5
-
A
C
D
E
F
G
H
I
K
L
M
N
P
Q
R
S
T
V
W
Y

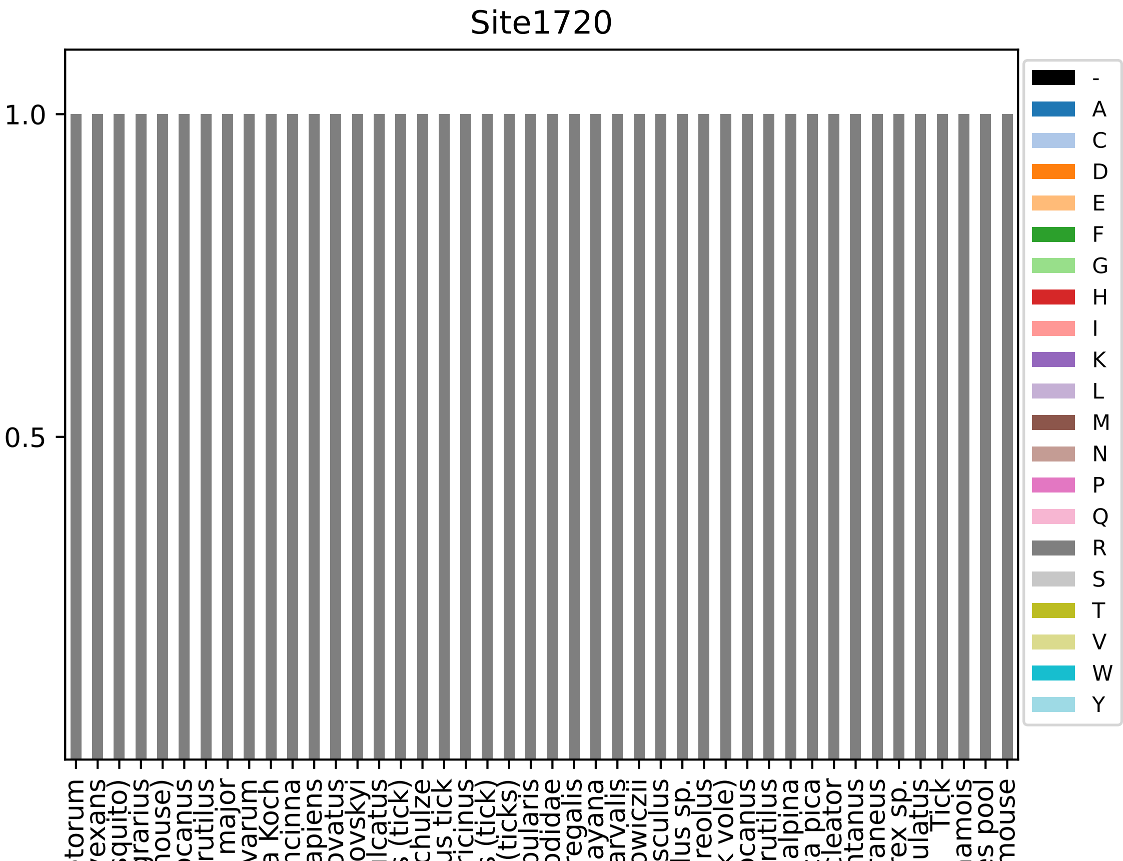
Site1720
1.0
0.5
-
A
C
D
E
F
G
H
I
K
L
M
N
P
Q
R
S
T
V
W
Y

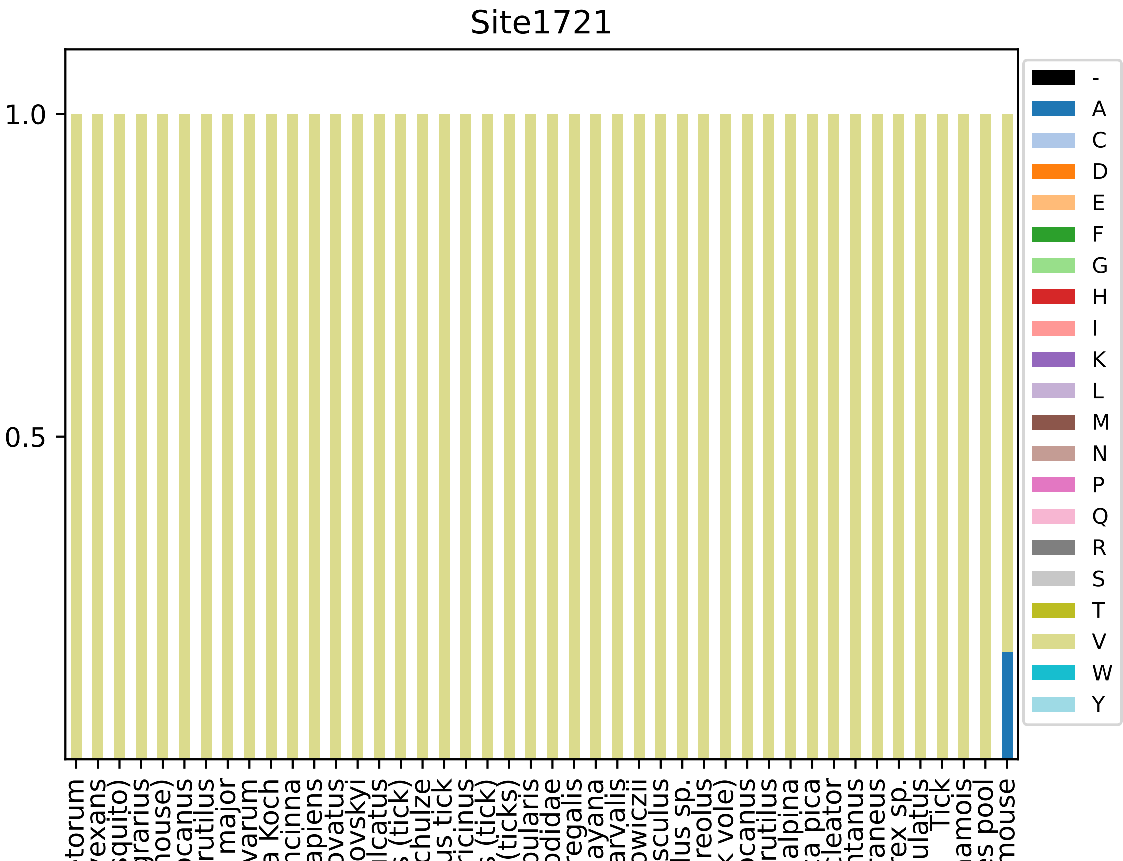
Site1721
1.0
0.5
-
A
C
D
E
F
G
H
I
K
L
M
N
P
Q
R
S
T
V
W
Y

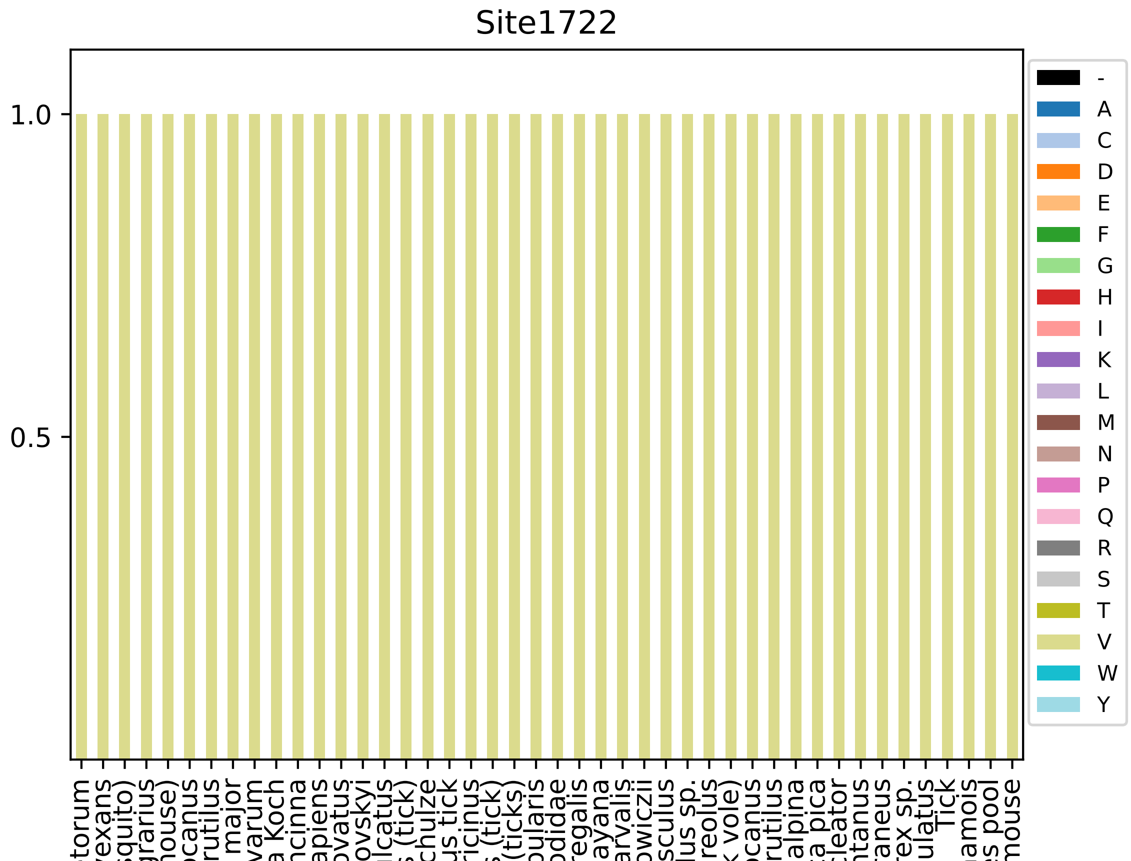
Site1722
1.0
0.5
-
A
C
D
E
F
G
H
I
K
L
M
N
P
Q
R
S
T
V
W
Y

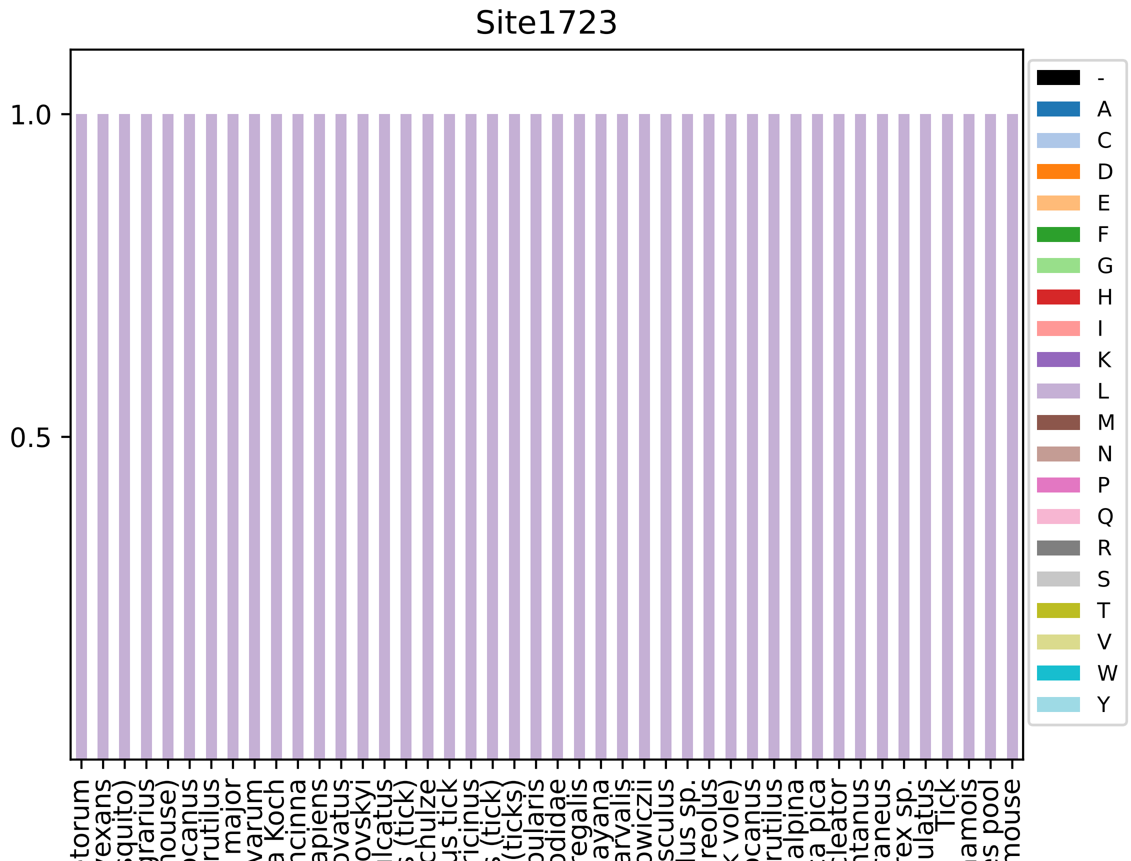
Site1723
1.0
0.5
-
A
C
D
E
F
G
H
I
K
L
M
N
P
Q
R
S
T
V
W
Y

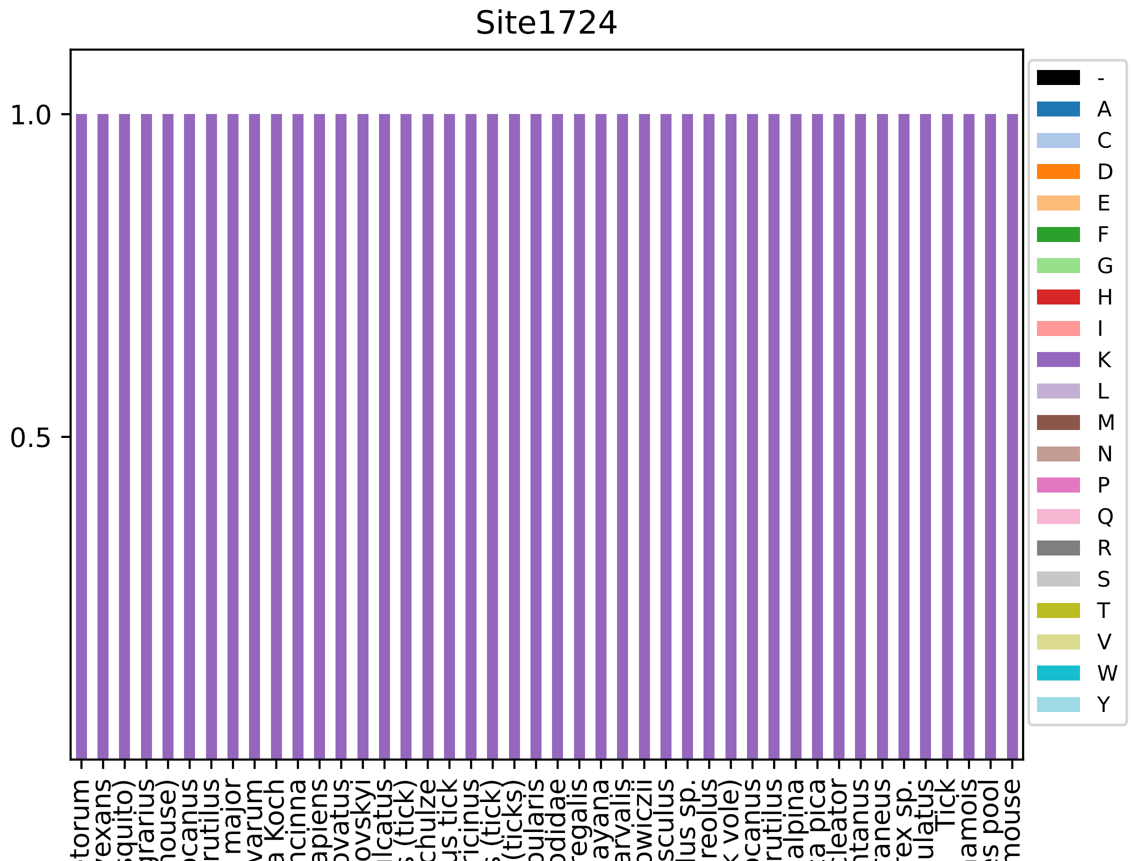
Site1724
1.0
0.5
-
A
C
D
E
F
G
H
I
K
L
M
N
P
Q
R
S
T
V
W
Y

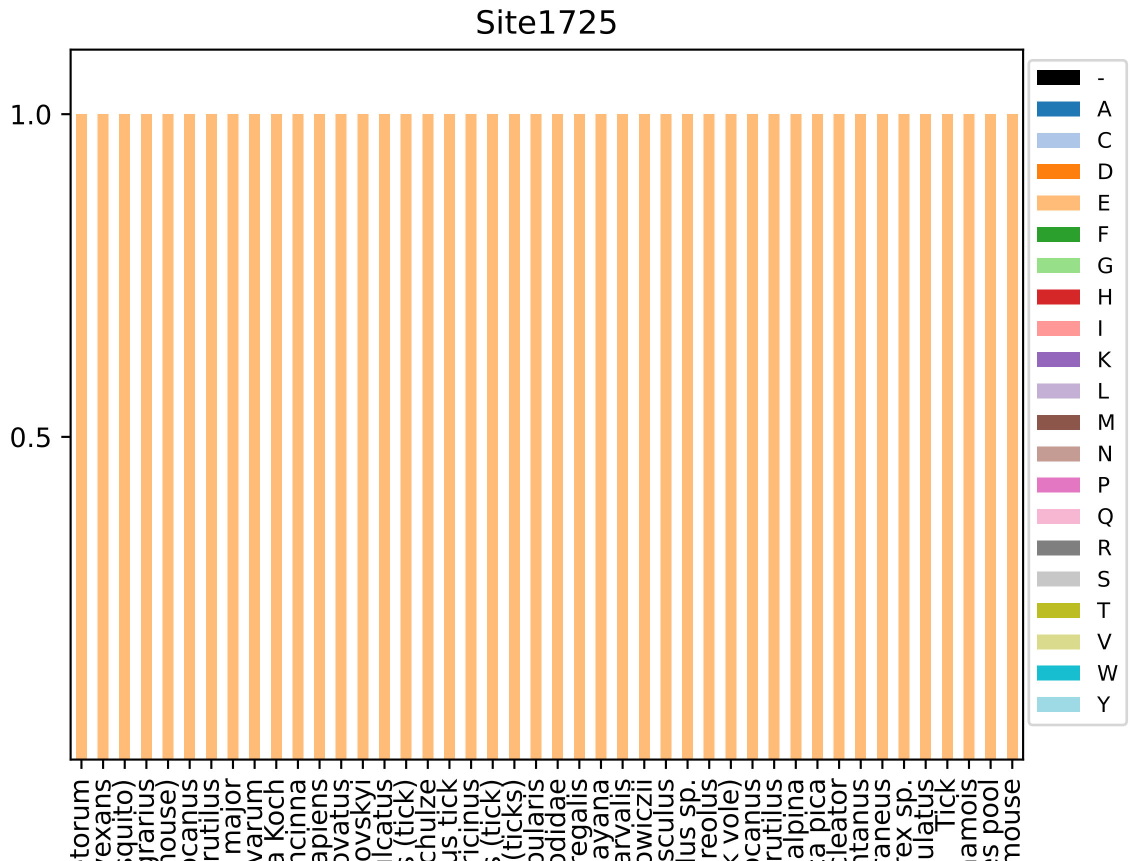
Site1725
1.0
0.5
-
A
C
D
E
F
G
H
I
K
L
M
N
P
Q
R
S
T
V
W
Y

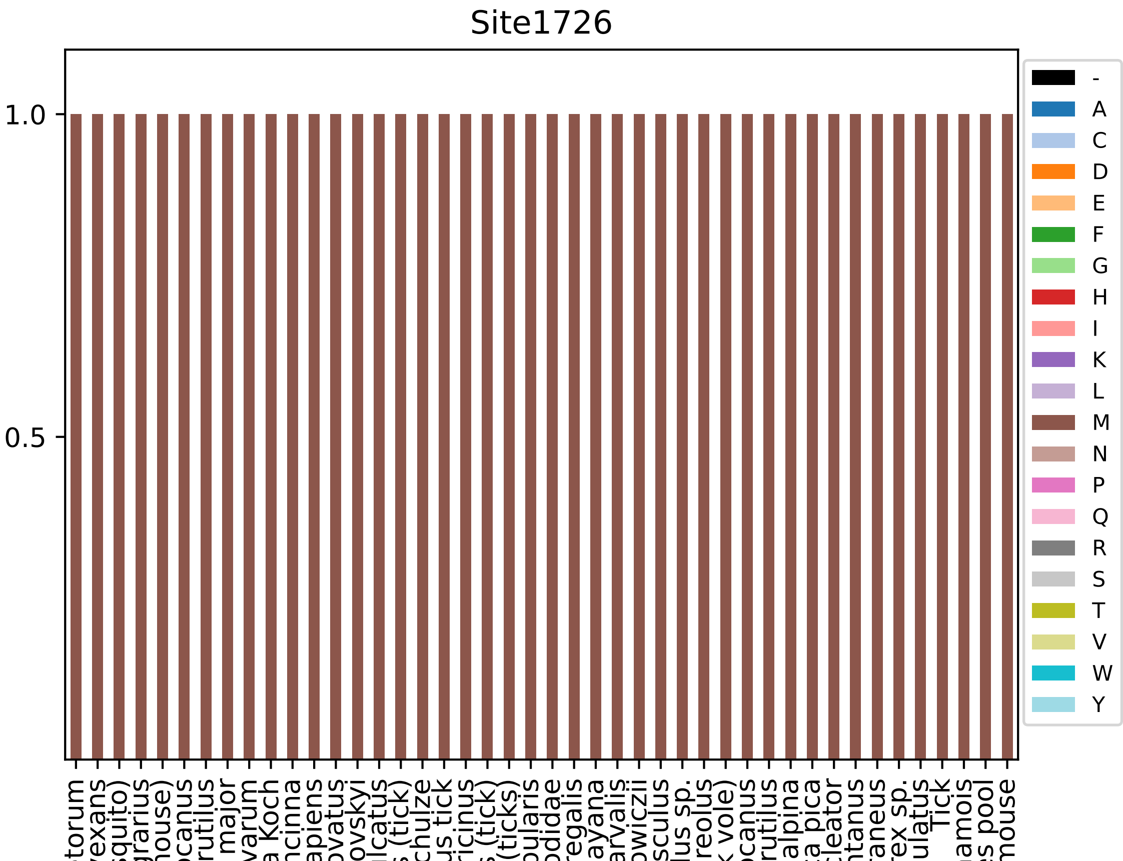
Site1726
1.0
0.5
-
A
C
D
E
F
G
H
I
K
L
M
N
P
Q
R
S
T
V
W
Y

Site1727

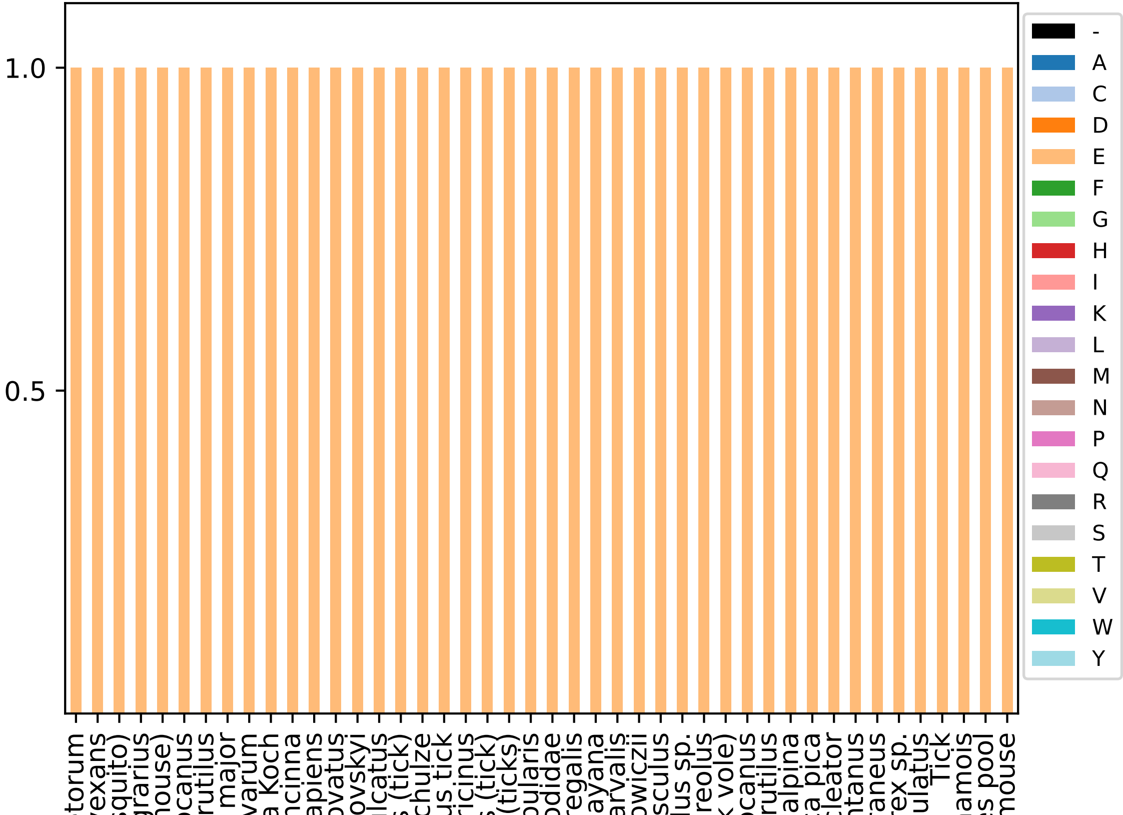

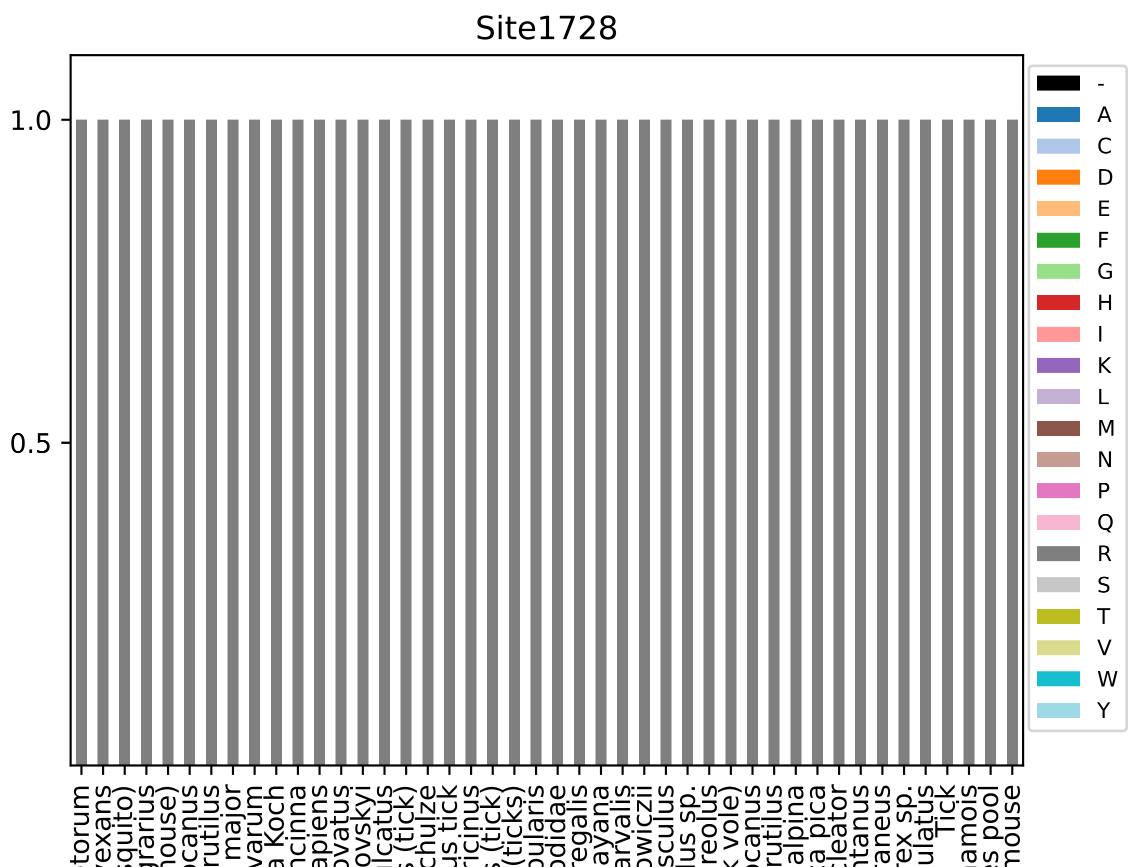

Site1728
1.0
0.5
-
A
C
D
E
F
G
H
I
K
L
M
N
P
Q
R
S
T
V
W
Y

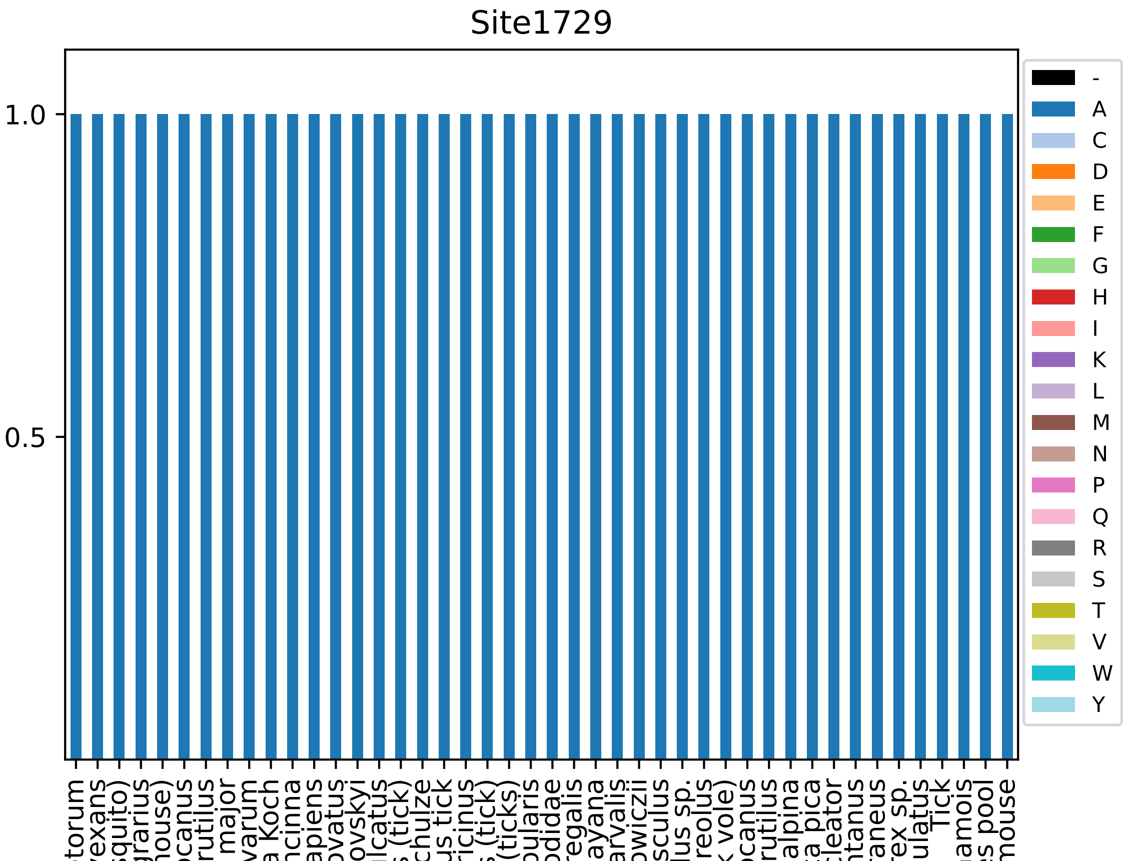
Site1729
1.0
0.5
-
A
C
D
E
F
G
H
I
K
L
M
N
P
Q
R
S
T
V
W
Y

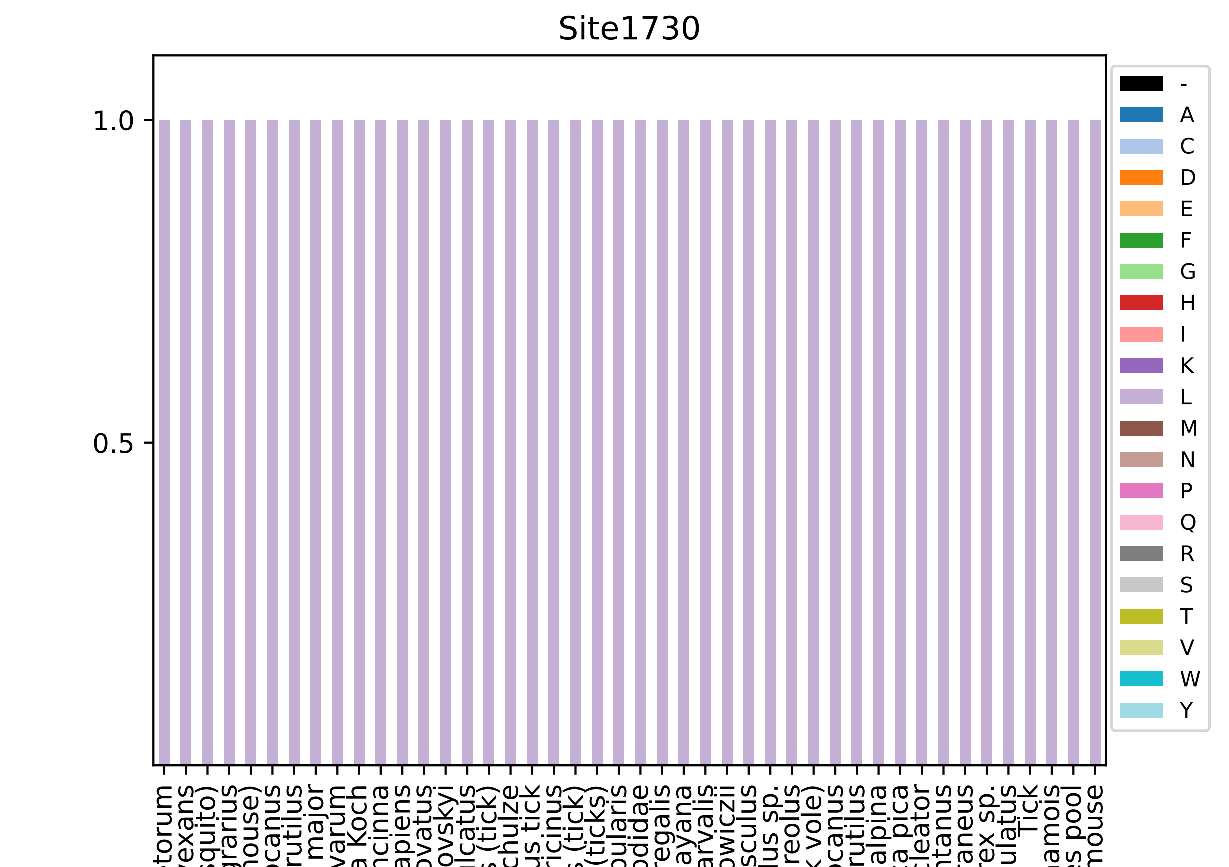

Site1730
1.0
0.5
-
A
C
D
E
F
G
H
I
K
L
M
N
P
Q
R
S
T
V
W
Y

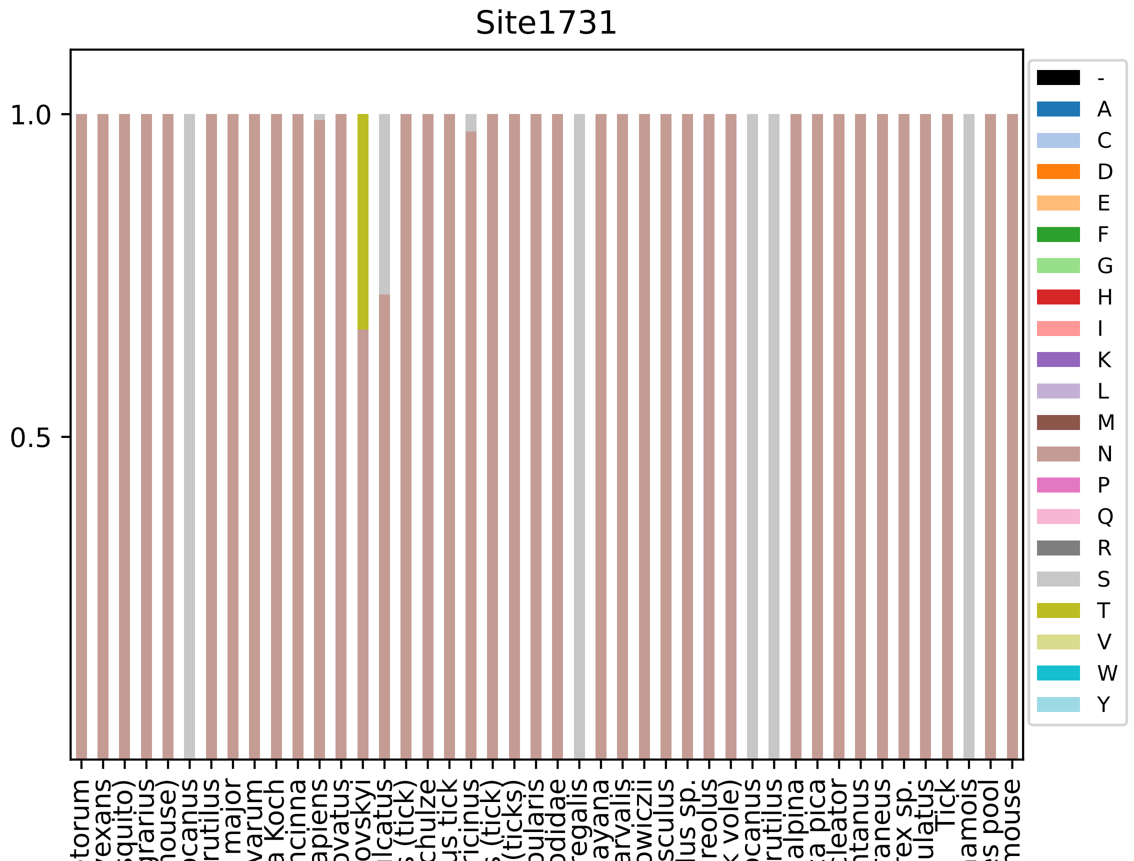

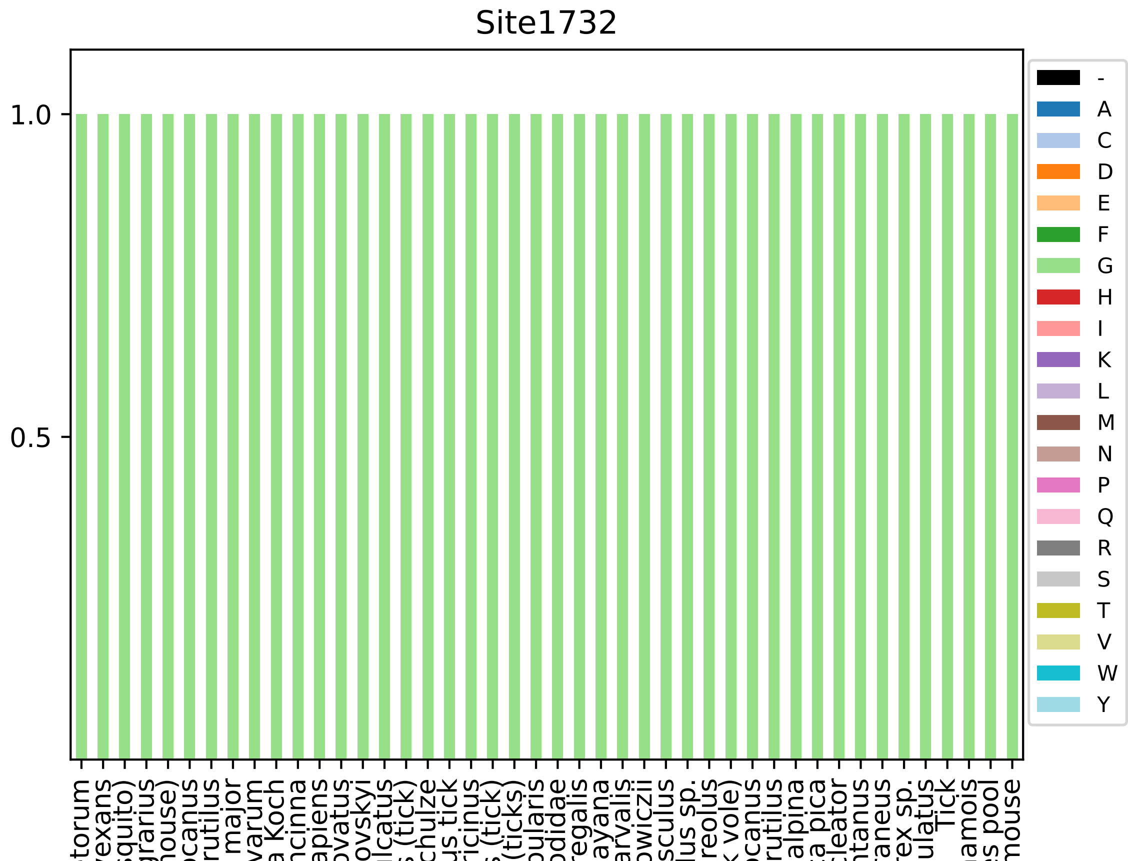
Site1732
1.0
0.5
-
A
C
D
E
F
G
H
I
K
L
M
N
P
Q
R
S
T
V
W
Y

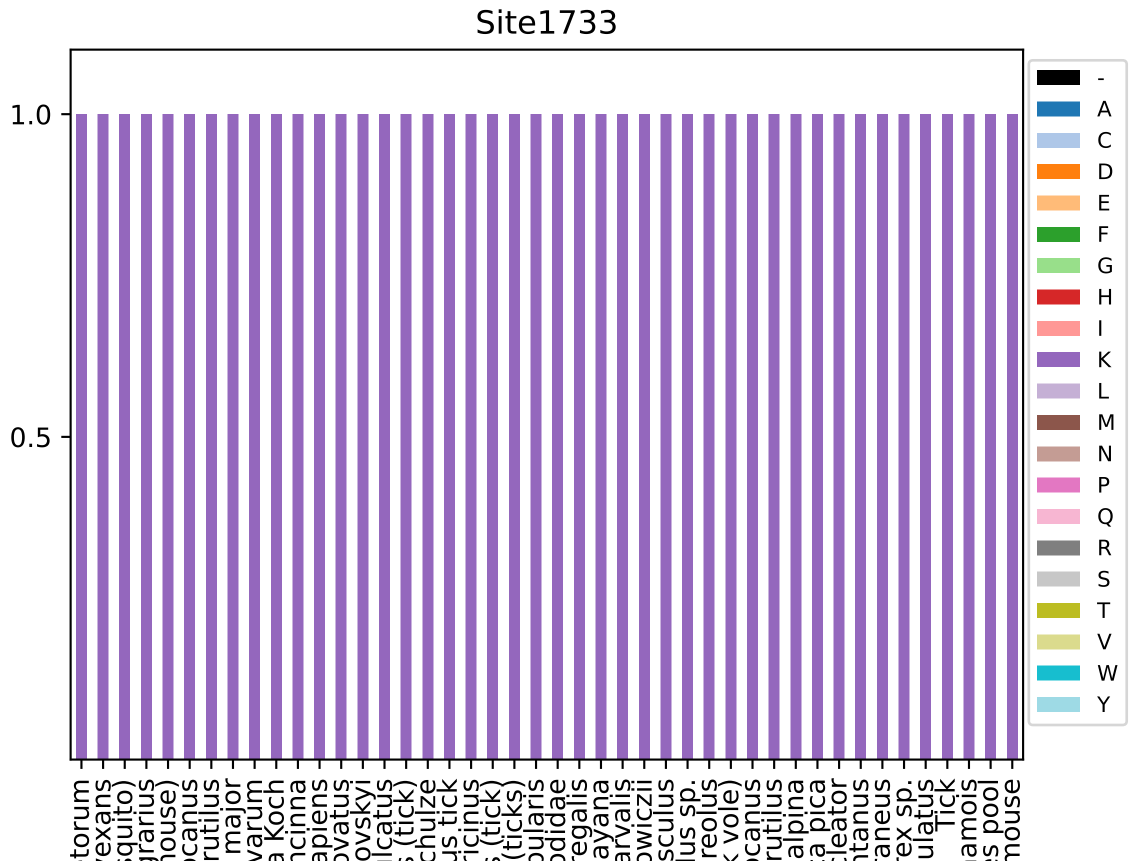
Site1733
1.0
0.5
-
A
C
D
E
F
G
H
I
K
L
M
N
P
Q
R
S
T
V
W
Y

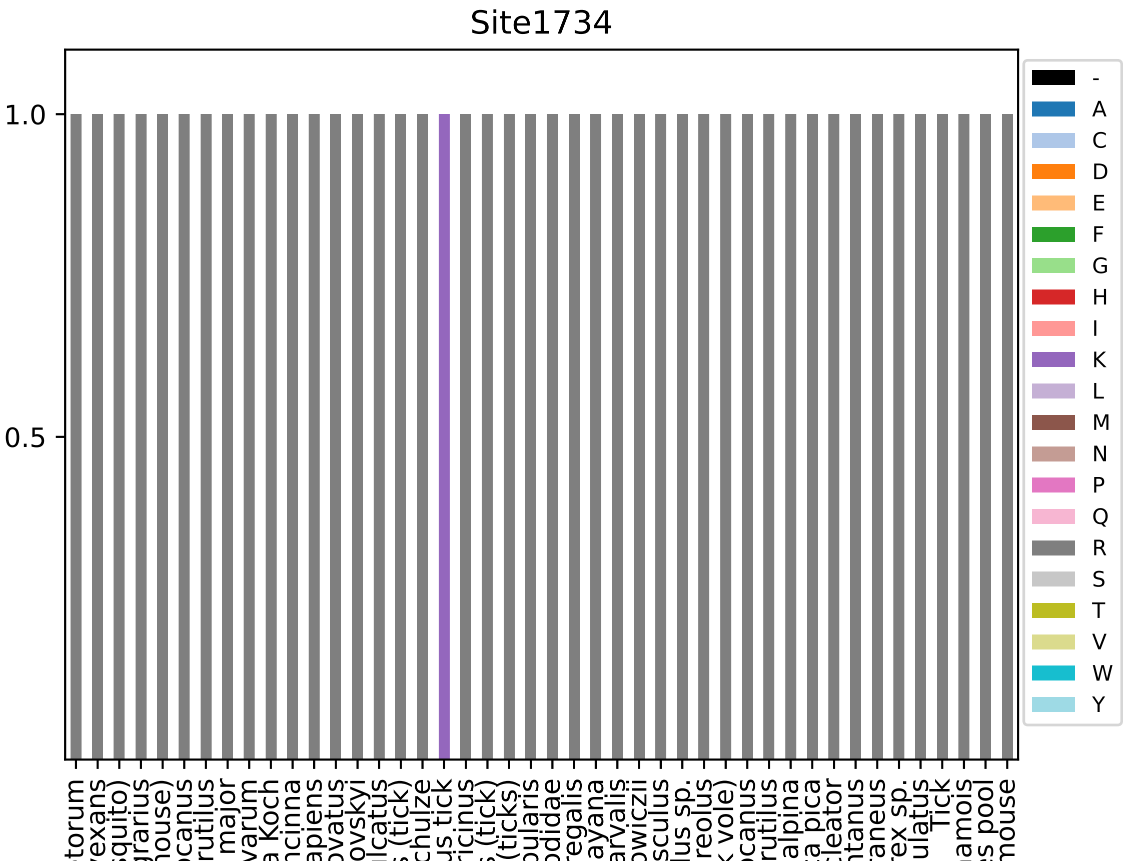
Site1734
1.0
0.5
-
A
C
D
E
F
G
H
I
K
L
M
N
P
Q
R
S
T
V
W
Y

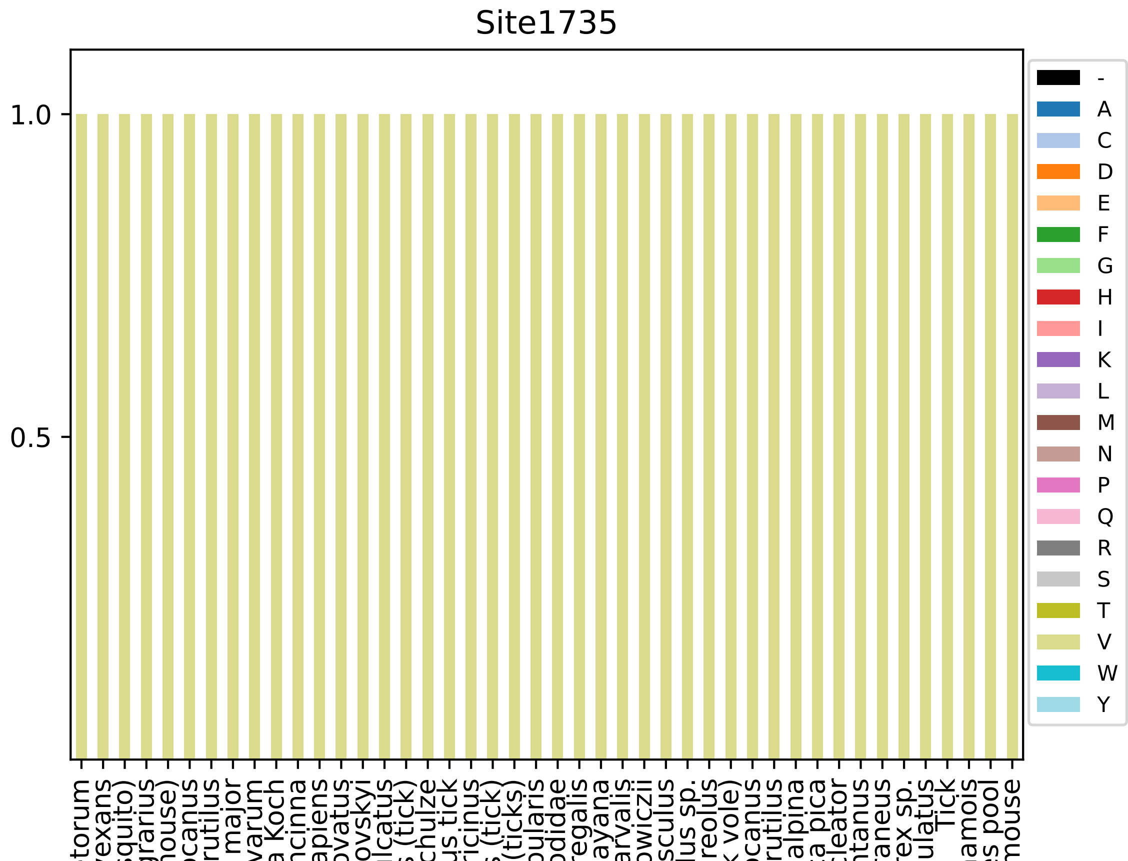
Site1735
1.0
0.5
-
A
C
D
E
F
G
H
I
K
L
M
N
P
Q
R
S
T
V
W
Y

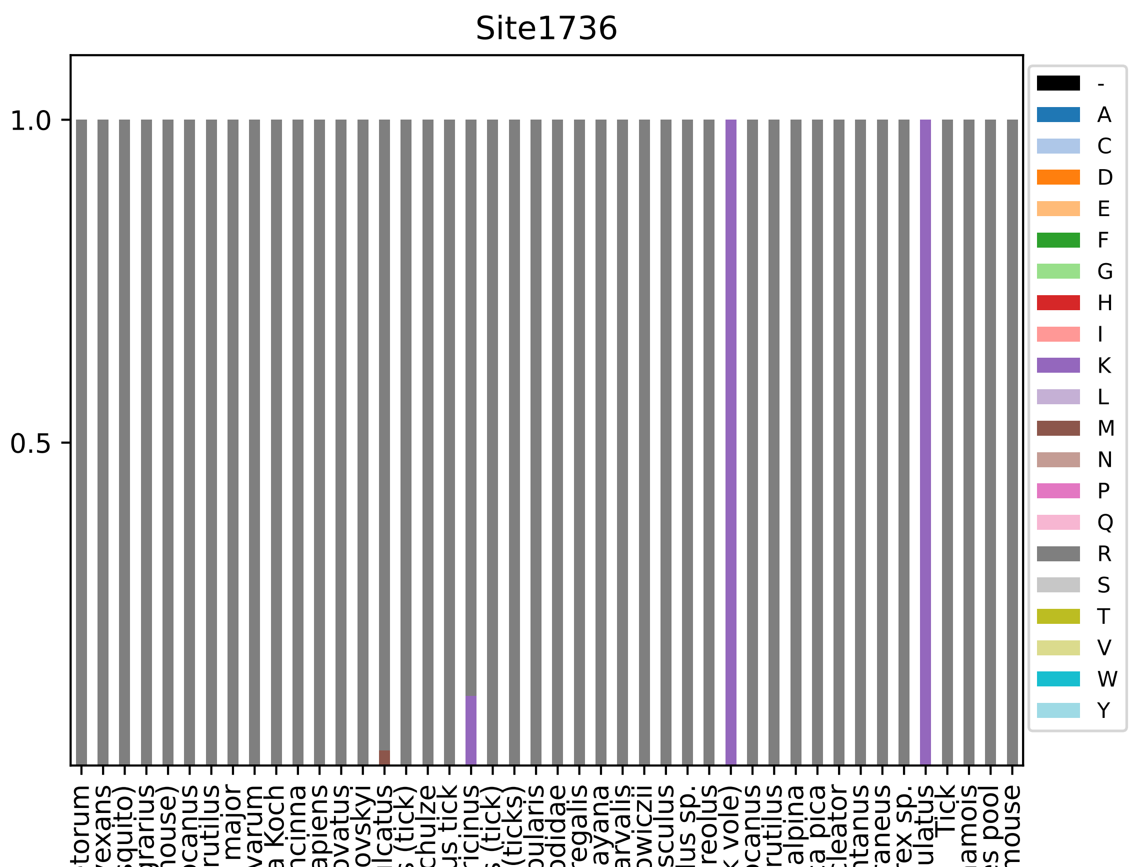

Site1736
1.0
0.5
-
A
C
D
E
F
G
H
I
K
L
M
N
P
Q
R
S
T
V
W
Y

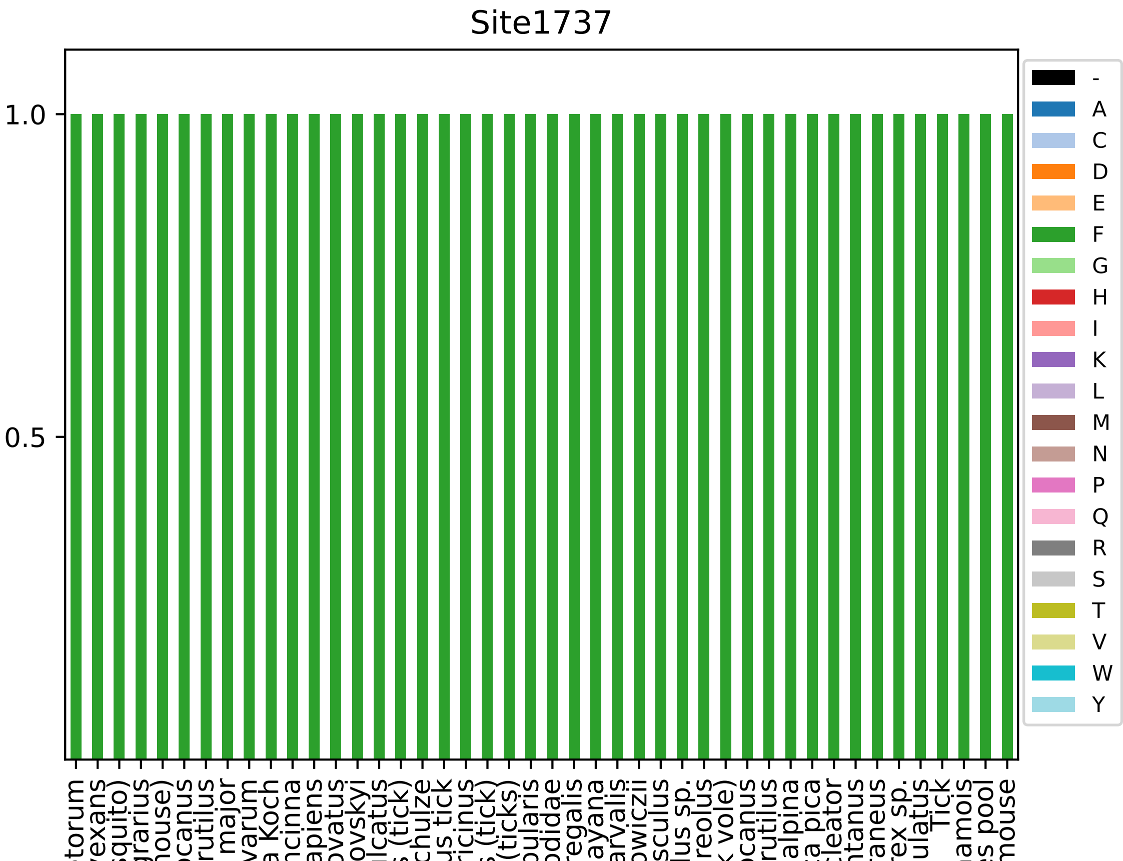
Site1737
1.0
0.5
-
A
C
D
E
F
G
H
I
K
L
M
N
P
Q
R
S
T
V
W
Y

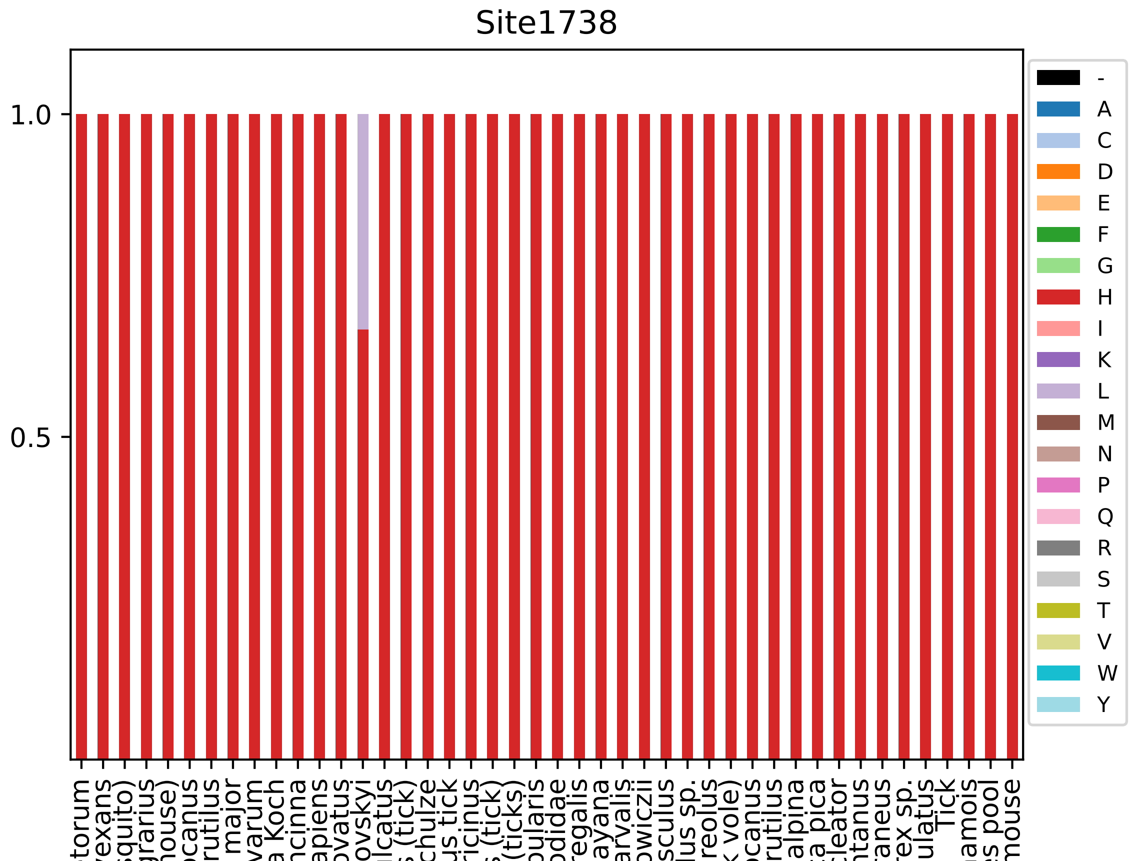

Site1738
1.0
0.5
-
A
C
D
E
F
G
H
I
K
L
M
N
P
Q
R
S
T
V
W
Y

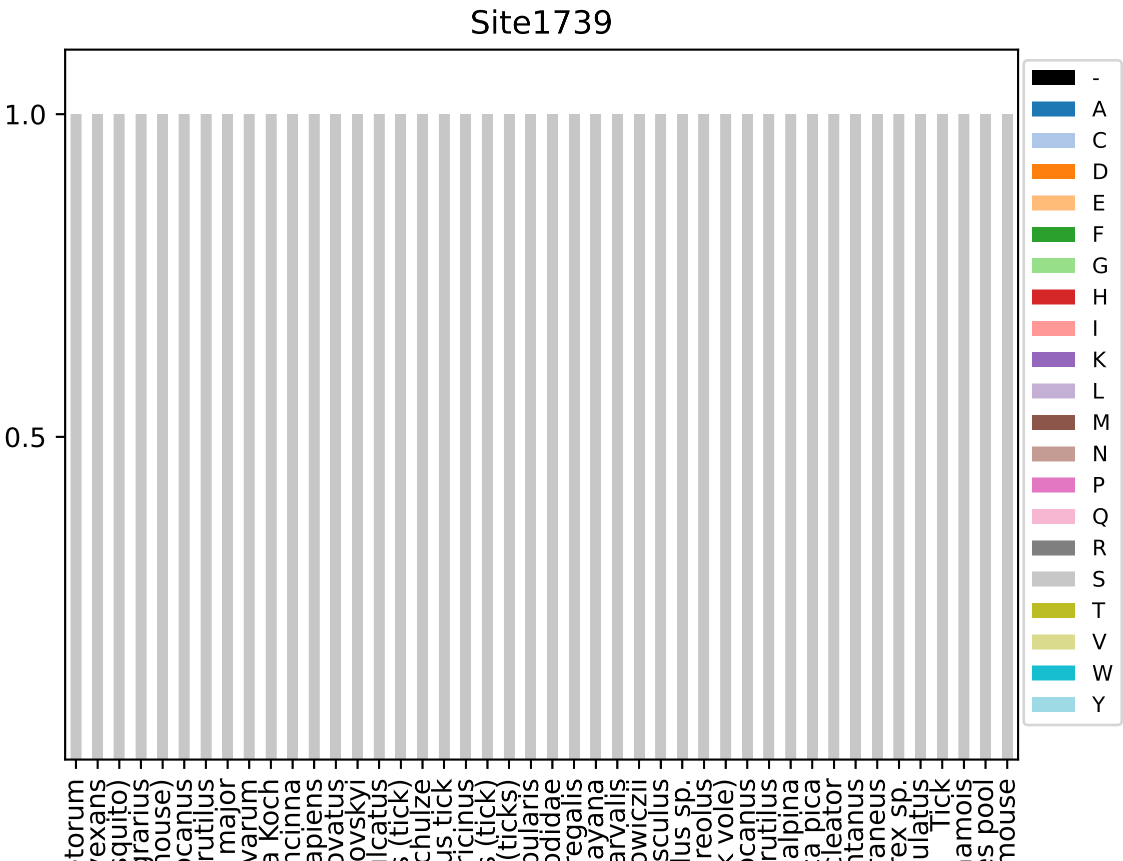
Site1739
1.0
0.5
-
A
C
D
E
F
G
H
I
K
L
M
N
P
Q
R
S
T
V
W
Y

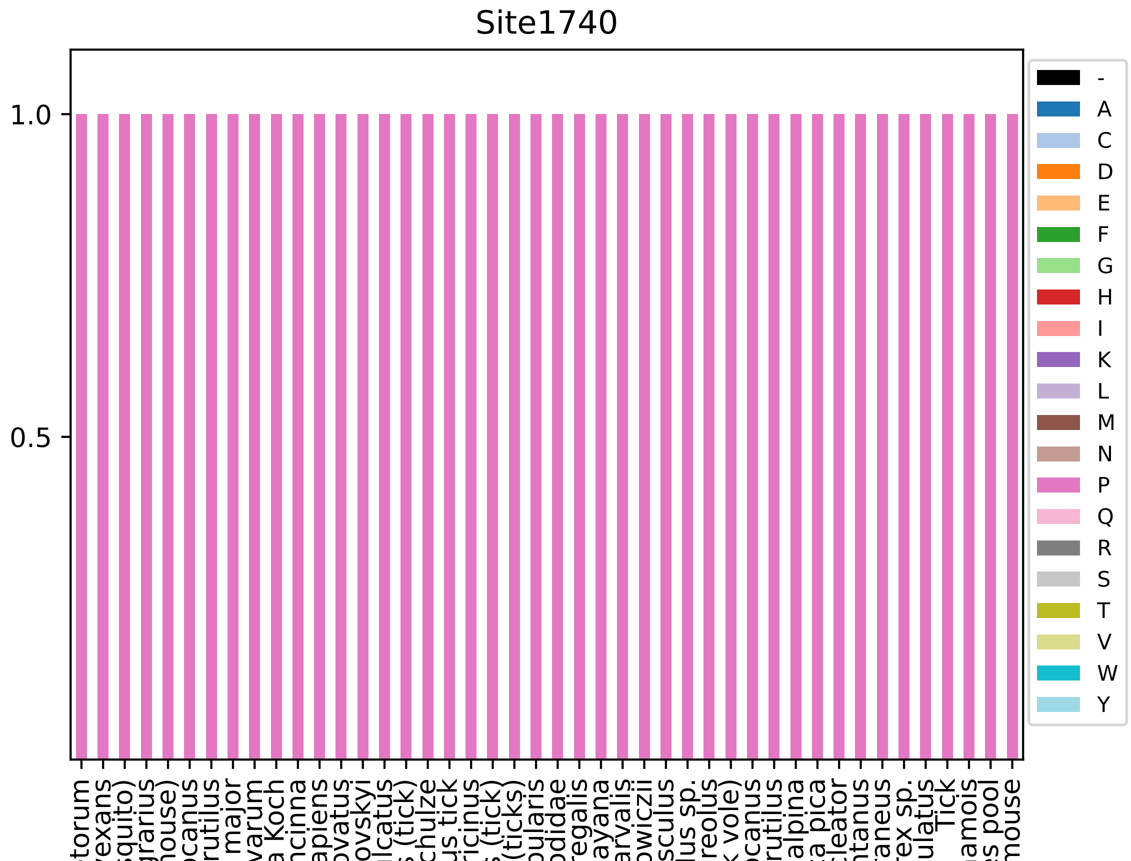
Site1740
1.0
0.5
-
A
C
D
E
F
G
H
I
K
L
M
N
P
Q
R
S
T
V
W
Y

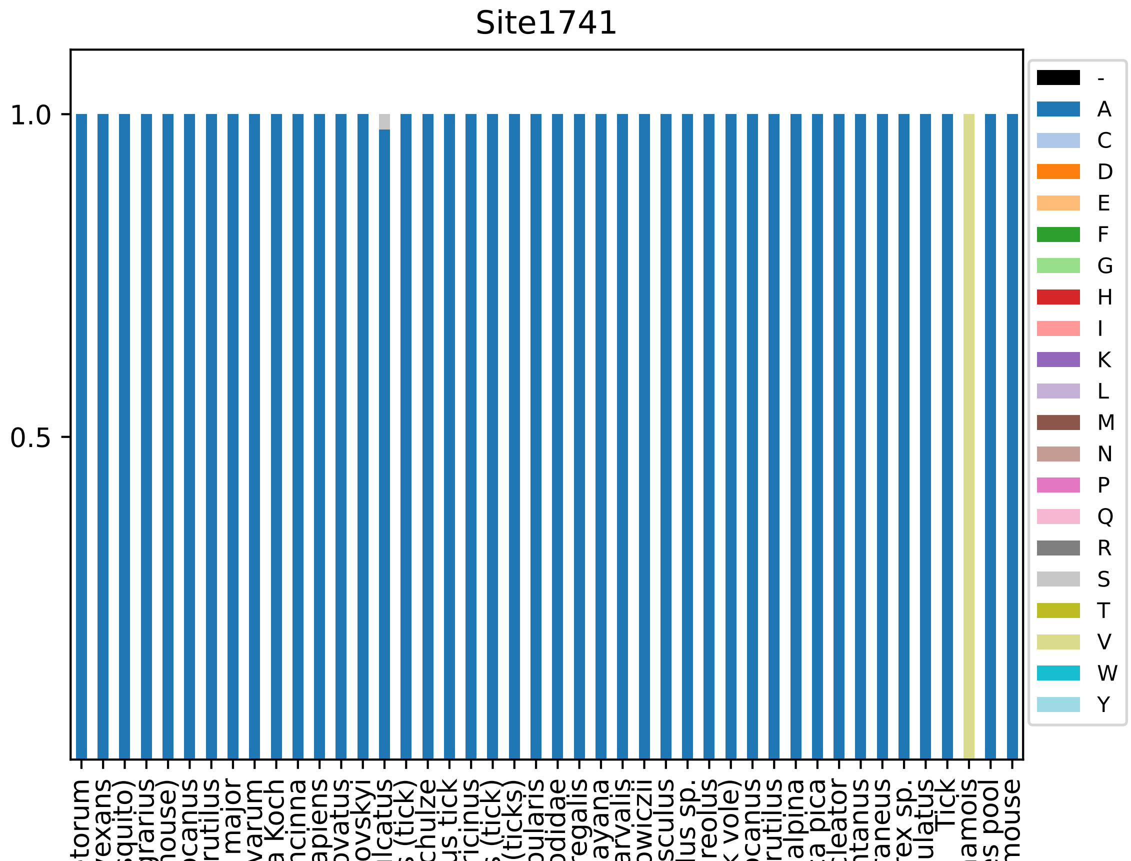
Site1741
1.0
0.5
-
A
C
D
E
F
G
H
I
K
L
M
N
P
Q
R
S
T
V
W
Y

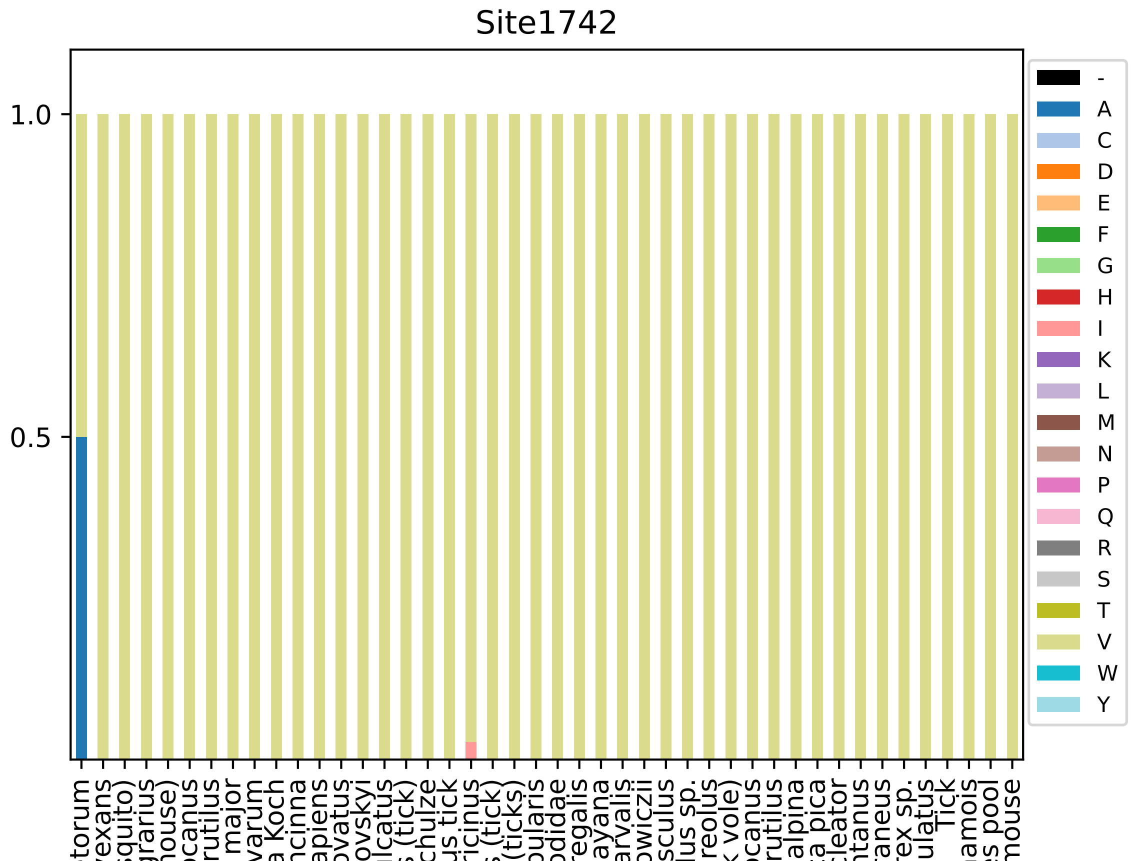
Site1742
1.0
0.5
-
A
C
D
E
F
G
H
I
K
L
M
N
P
Q
R
S
T
V
W
Y

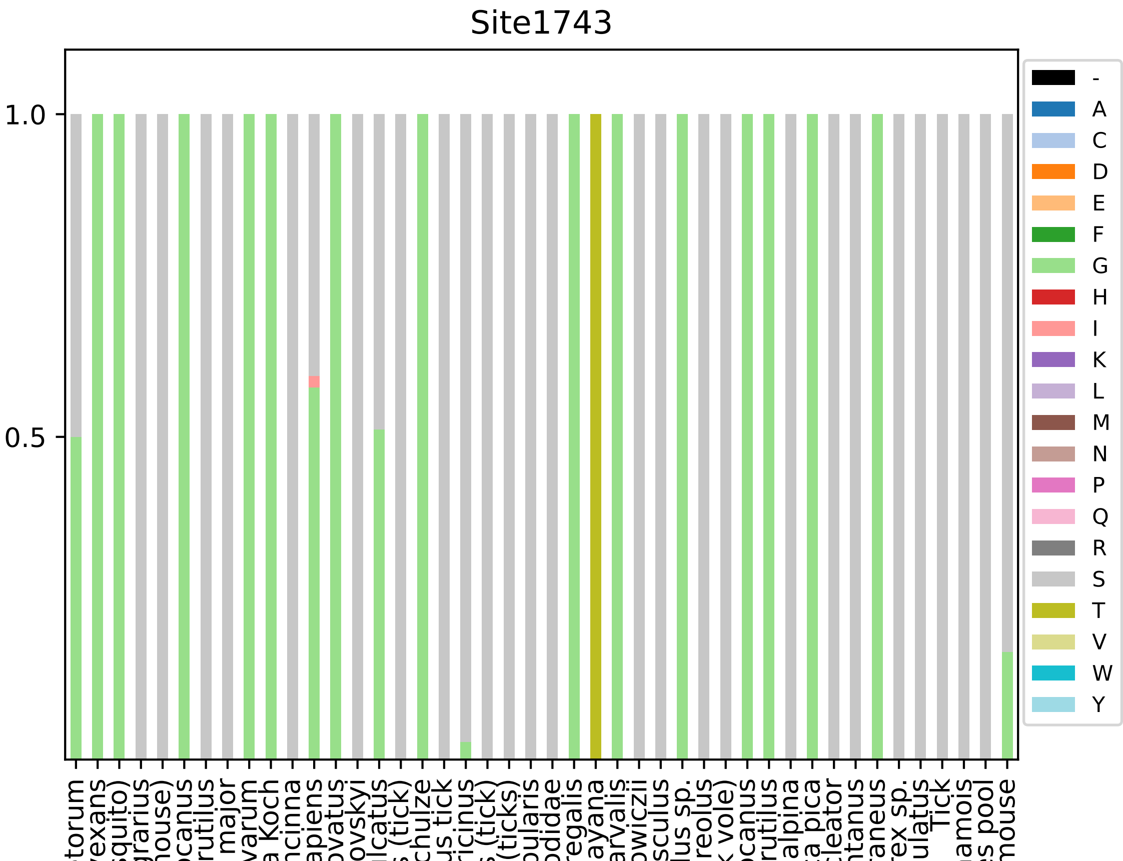

Site1743
1.0
0.5
-
A
C
D
E
F
G
H
I
K
L
M
N
P
Q
R
S
T
V
W
Y

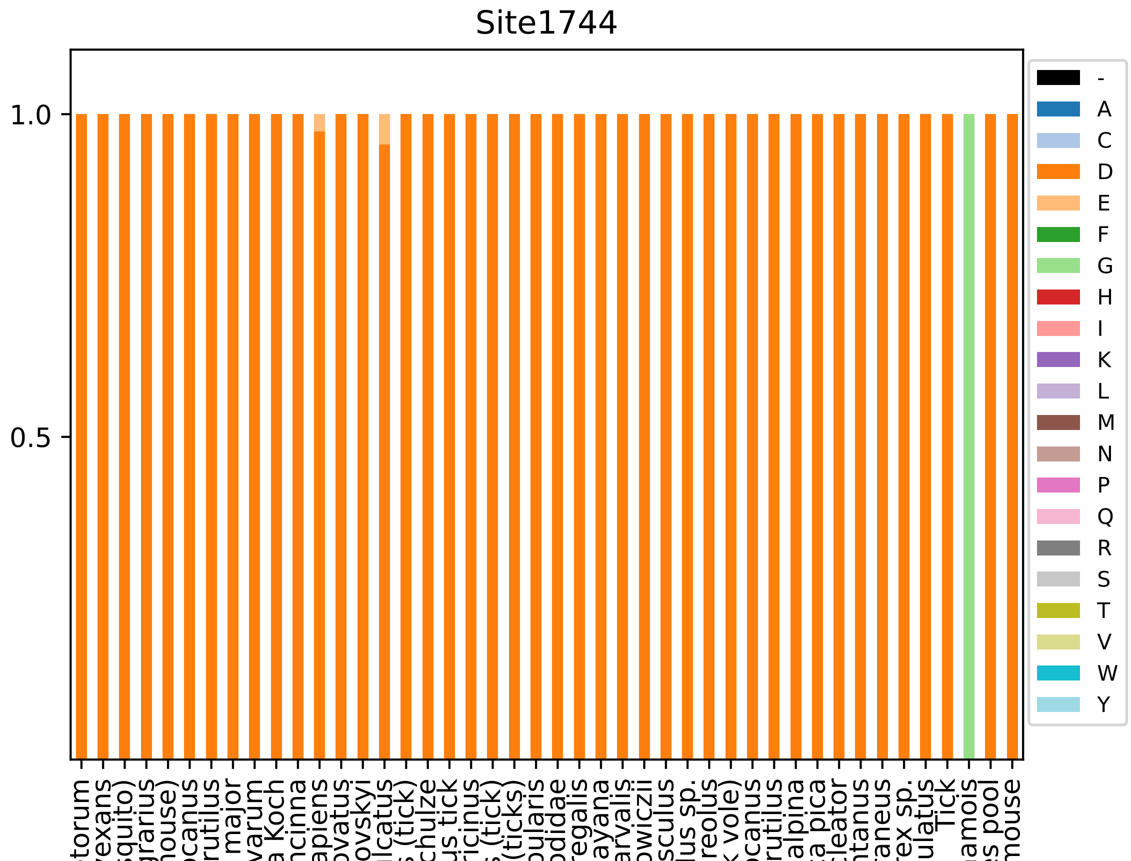
Site1744
1.0
0.5
-
A
C
D
E
F
G
H
I
K
L
M
N
P
Q
R
S
T
V
W
Y

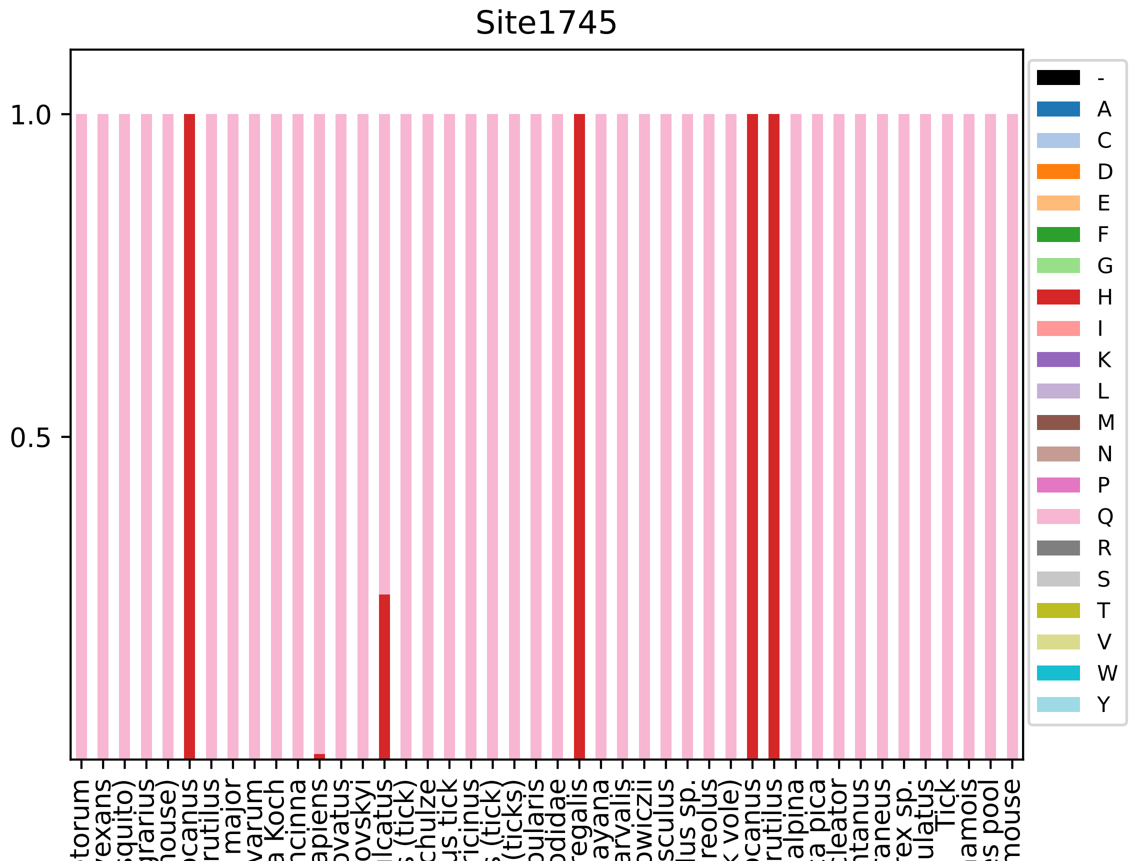

Site1745
1.0
0.5
-
A
C
D
E
F
G
H
I
K
L
M
N
P
Q
R
S
T
V
W
Y
Tick

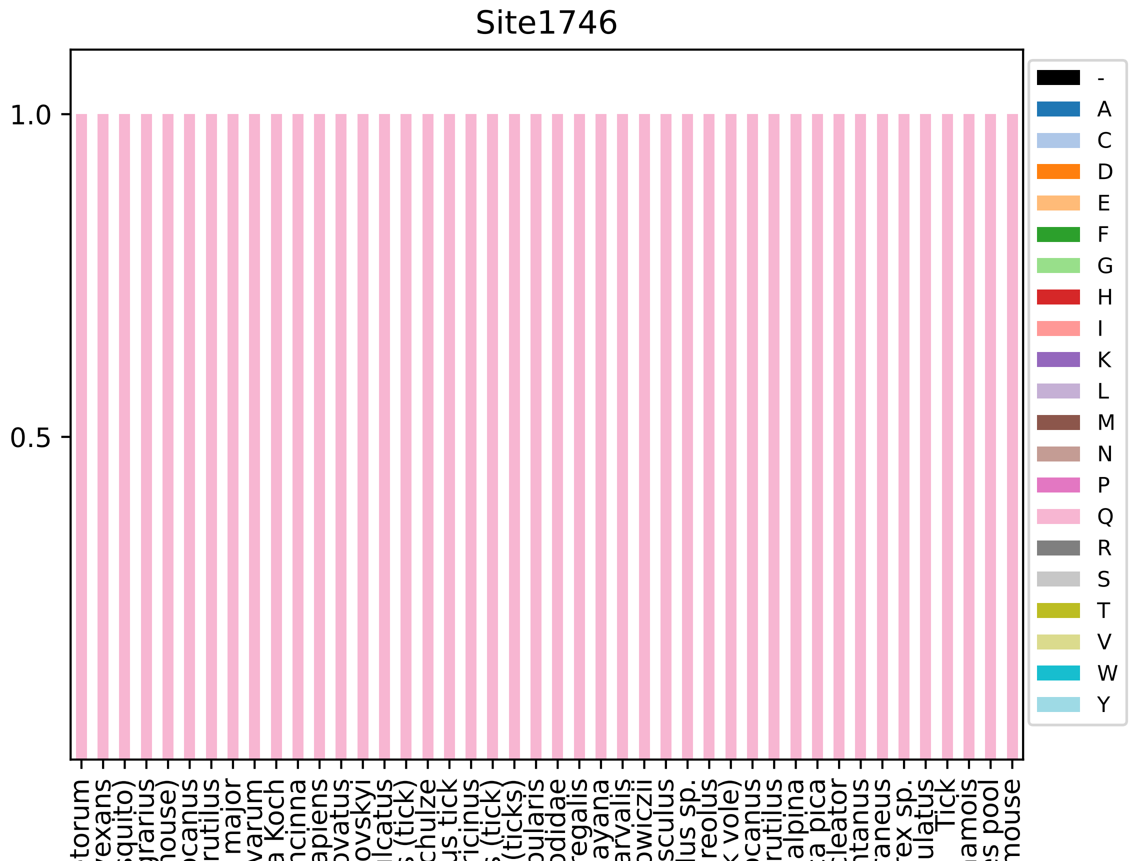
Site1746
1.0
0.5
-
A
C
D
E
F
G
H
I
K
L
M
N
P
Q
R
S
T
V
W
Y

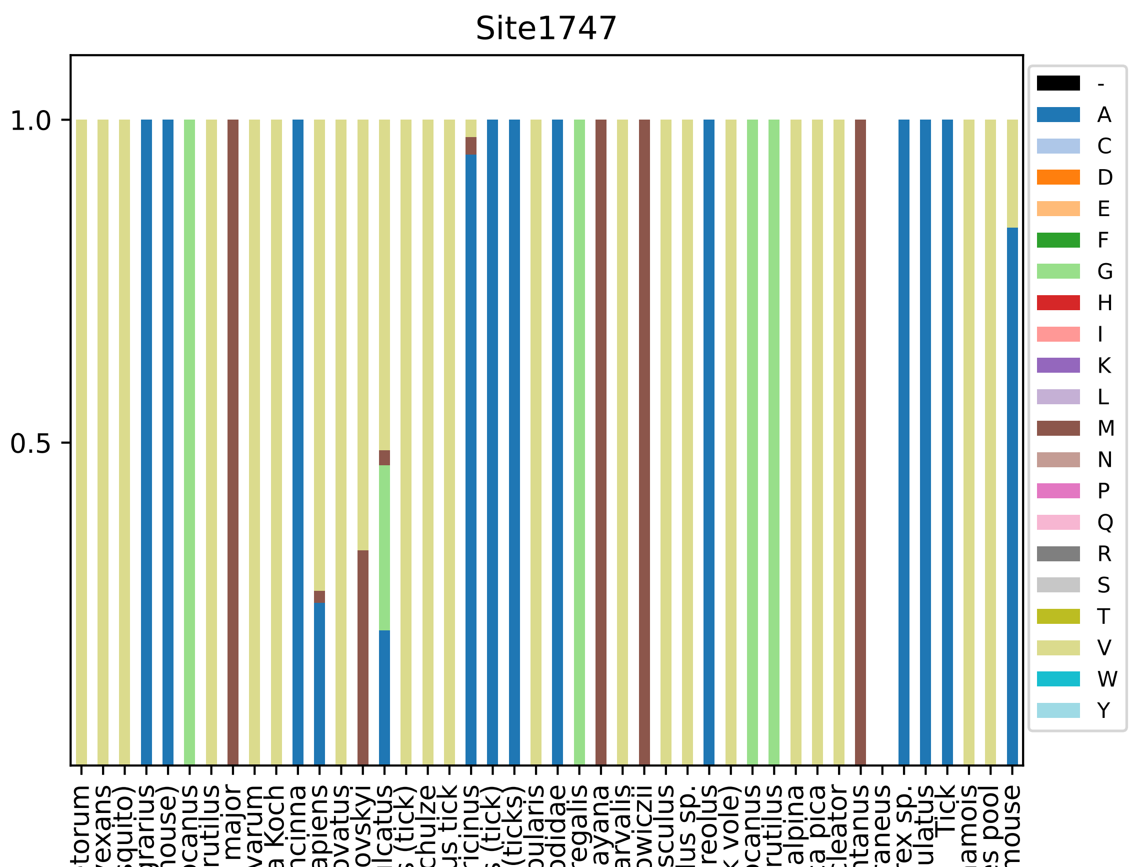

Site1747
1.0
0.5
-
A
C
D
E
F
G
H
I
K
L
M
N
P
Q
R
S
T
V
W
Y

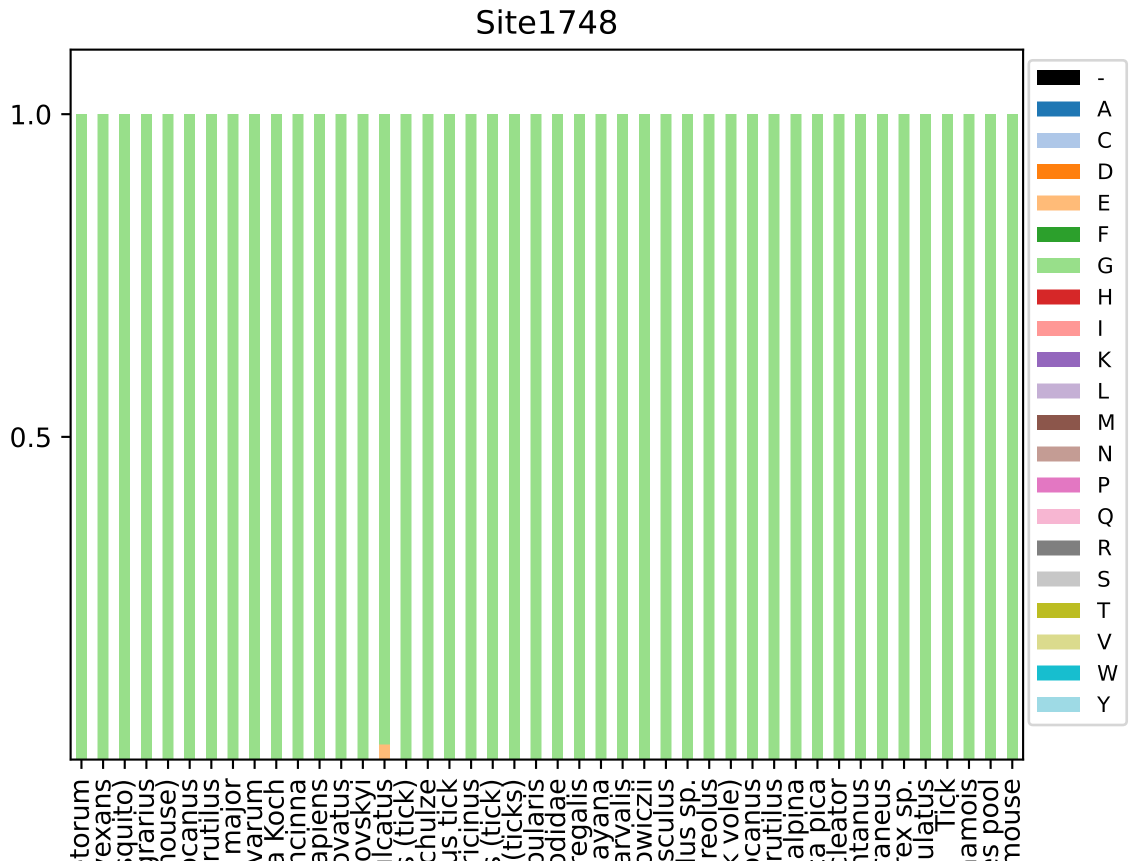
Site1748
1.0
0.5
-
A
C
D
E
F
G
H
I
K
L
M
N
P
Q
R
S
T
V
W
Y

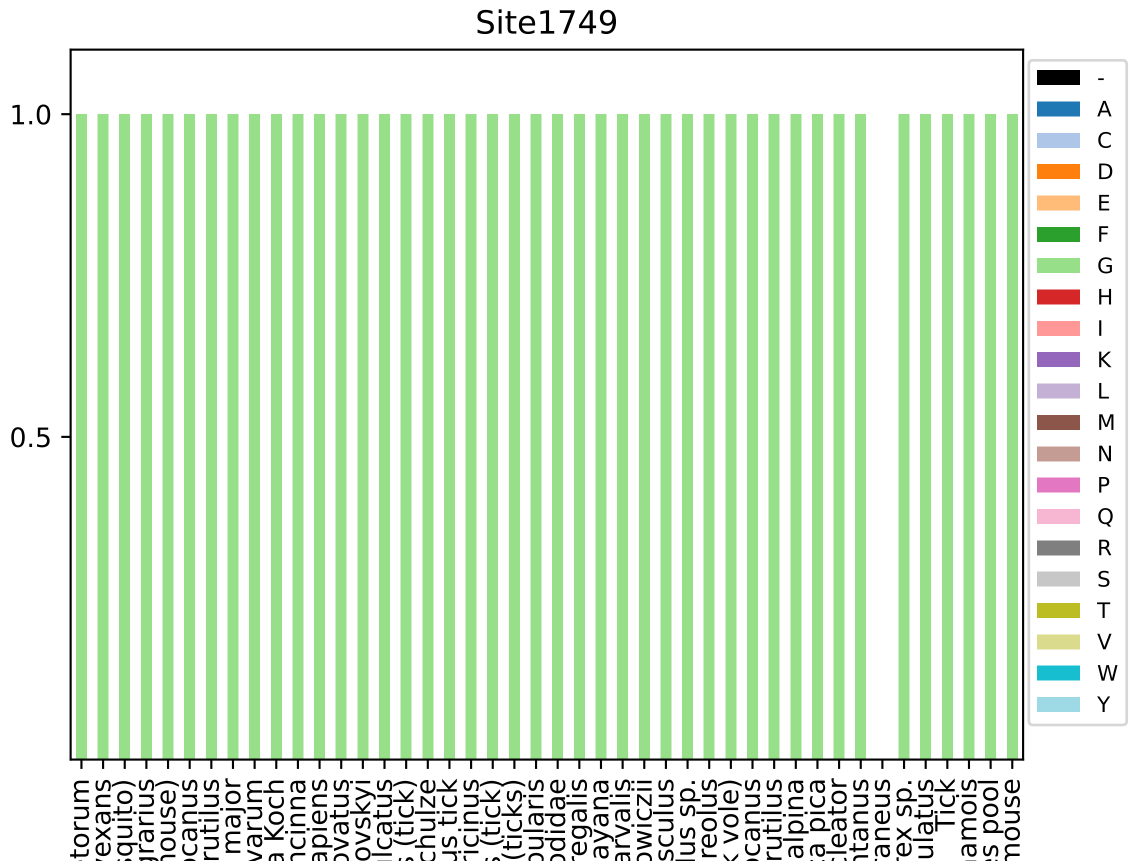

Site1749
1.0
0.5
-
A
C
D
E
F
G
H
I
K
L
M
N
P
Q
R
S
T
V
W
Y

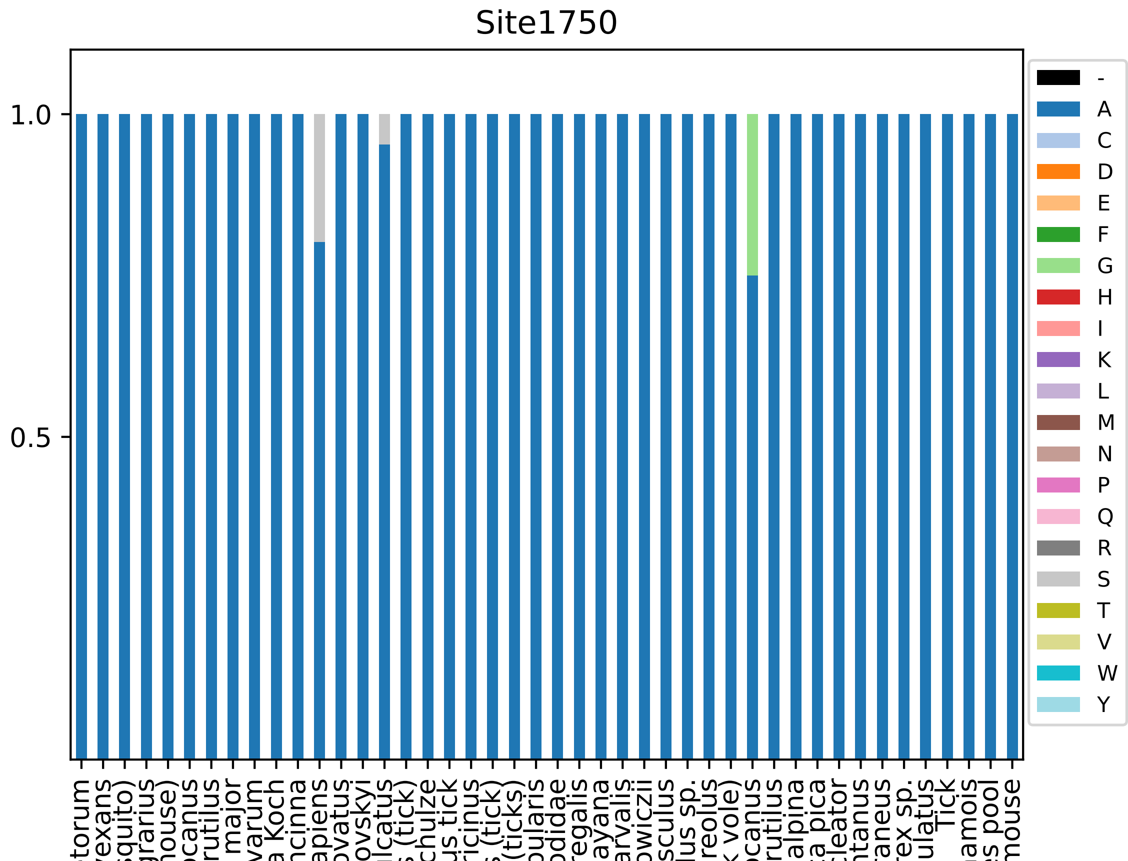
Site1750
1.0
0.5
-
A
C
D
E
F
G
H
I
K
L
M
N
P
Q
R
S
T
V
W
Y

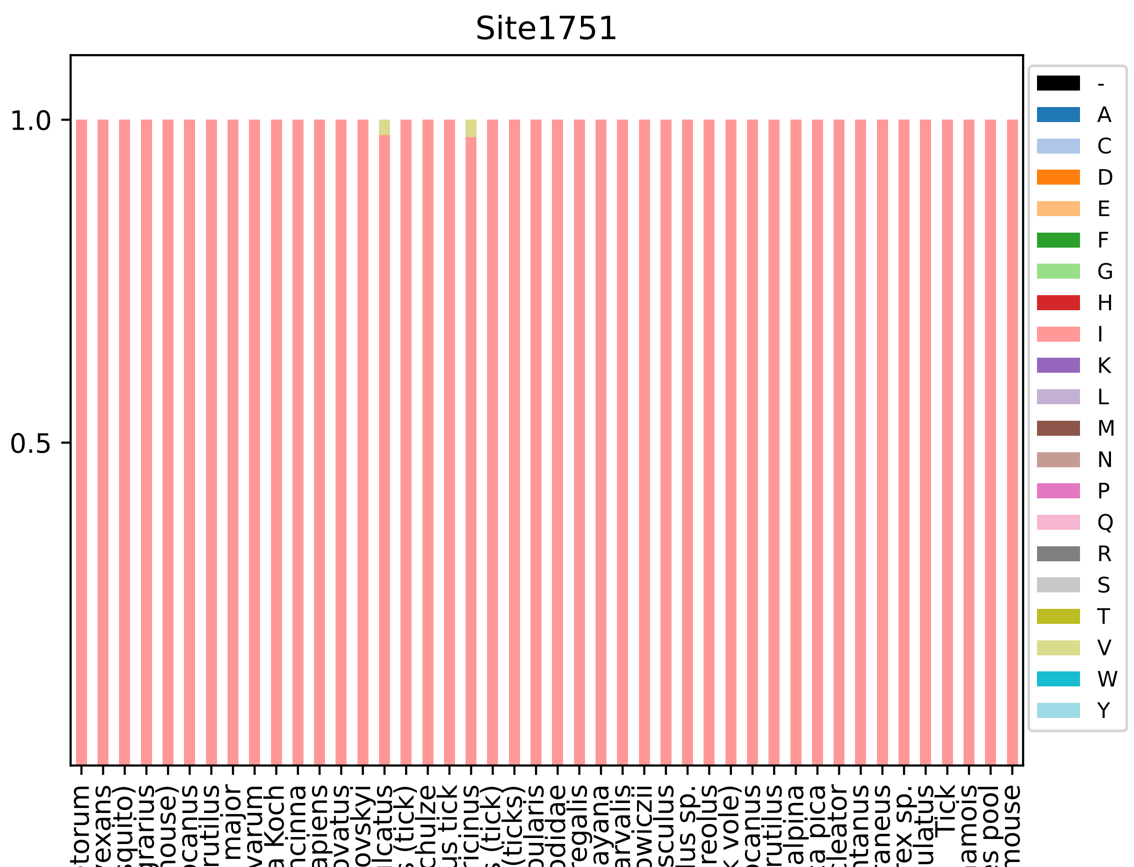

Site1751
1.0
0.5
-
A
C
D
E
F
G
H
I
K
L
M
N
P
Q
R
S
T
V
W
Y

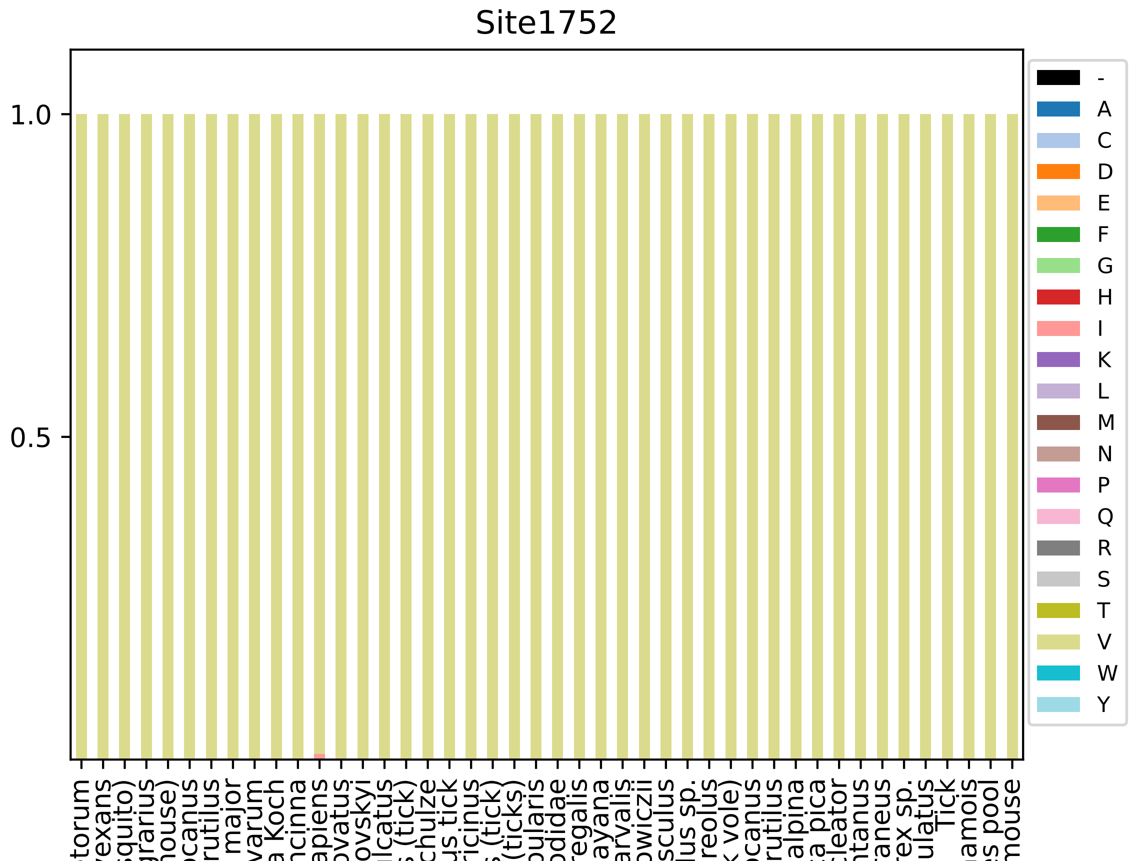
Site1752
1.0
0.5
-
A
C
D
E
F
G
H
I
K
L
M
N
P
Q
R
S
T
V
W
Y
torum
vexans
squito)
grarius
nouse)
ocanus
rutilus
major
varum
a Koch
ncinna
apiens
ovatus
ovskyi
ulcatus
s (tick)
chulze
us tick
ricinus
s (tick)
(ticks)
pularis
odidae
regalis
ayana
arvalis
owiczii
sculus
lus sp.
reolus
k vole)
ocanus
rutilus
alpina
a pica
cleator
ntanus
raneus
rex sp.
ulatus
Tick
amois
es pool
mouse

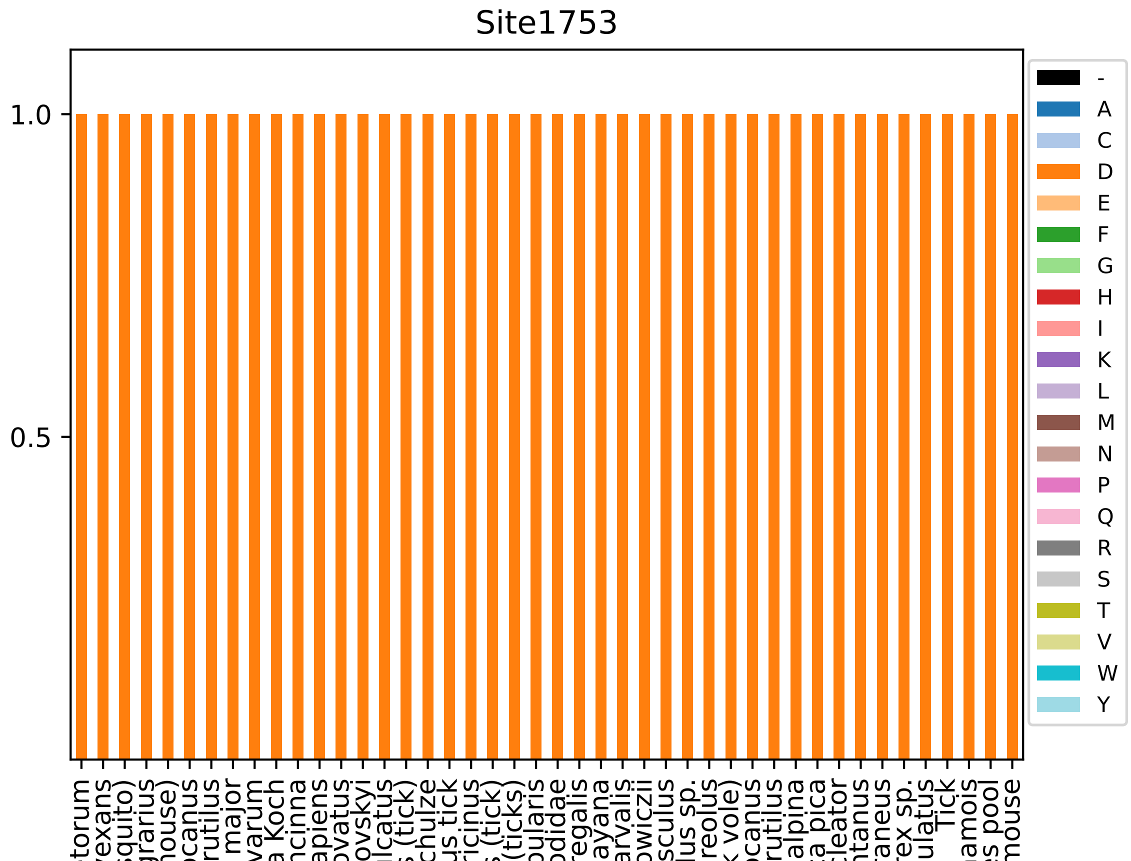
Site1753
1.0
0.5
-
A
C
D
E
F
G
H
I
K
L
M
N
P
Q
R
S
T
V
W
Y

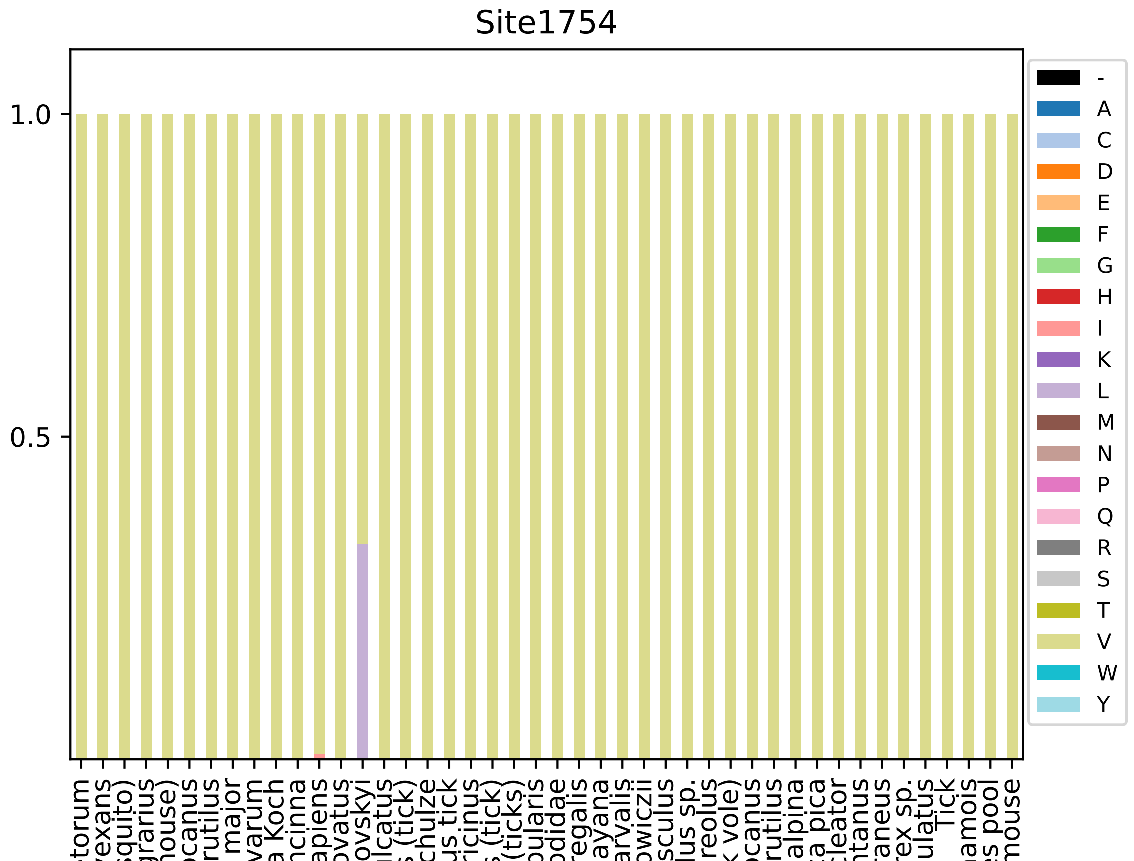
Site1754
1.0
0.5
-
A
C
D
E
F
G
H
I
K
L
M
N
P
Q
R
S
T
V
W
Y

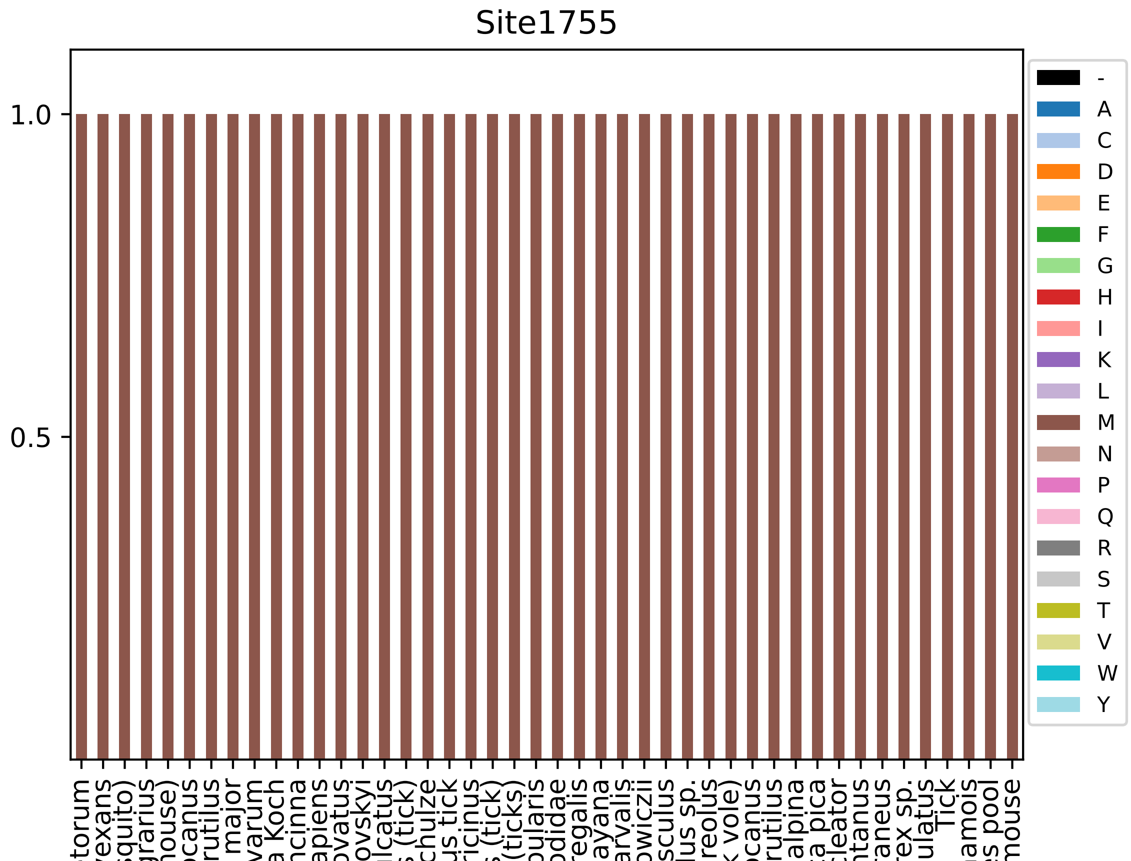
Site1755
1.0
0.5
-
A
C
D
E
F
G
H
I
K
L
M
N
P
Q
R
S
T
V
W
Y
torum
vexans
squito)
grarius
nouse)
ocanus
rutilus
major
varum
a Koch
ncinna
apiens
ovatus
ovskyi
lcatus
(tick)
chulze
us tick
ricinus
(tick)
(ticks)
pularis
podidae
regalis
ayana
arvalis
owiczii
sculus
lus sp.
reolus
k vole)
ocanus
rutilus
alpina
a pica
leator
ntanus
raneus
rex sp.
ulatus
Tick
amois
es pool
mouse

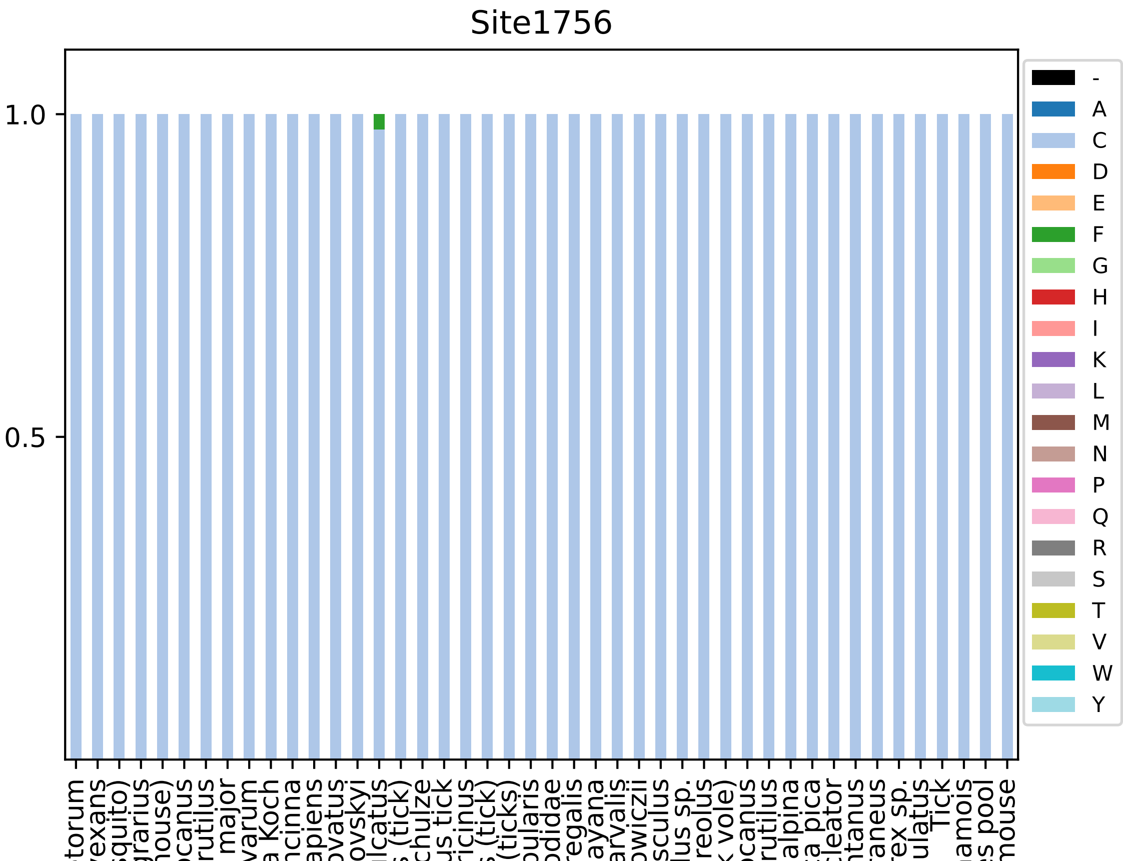
Site1756
1.0
0.5
-
A
C
D
E
F
G
H
I
K
L
M
N
P
Q
R
S
T
V
W
Y

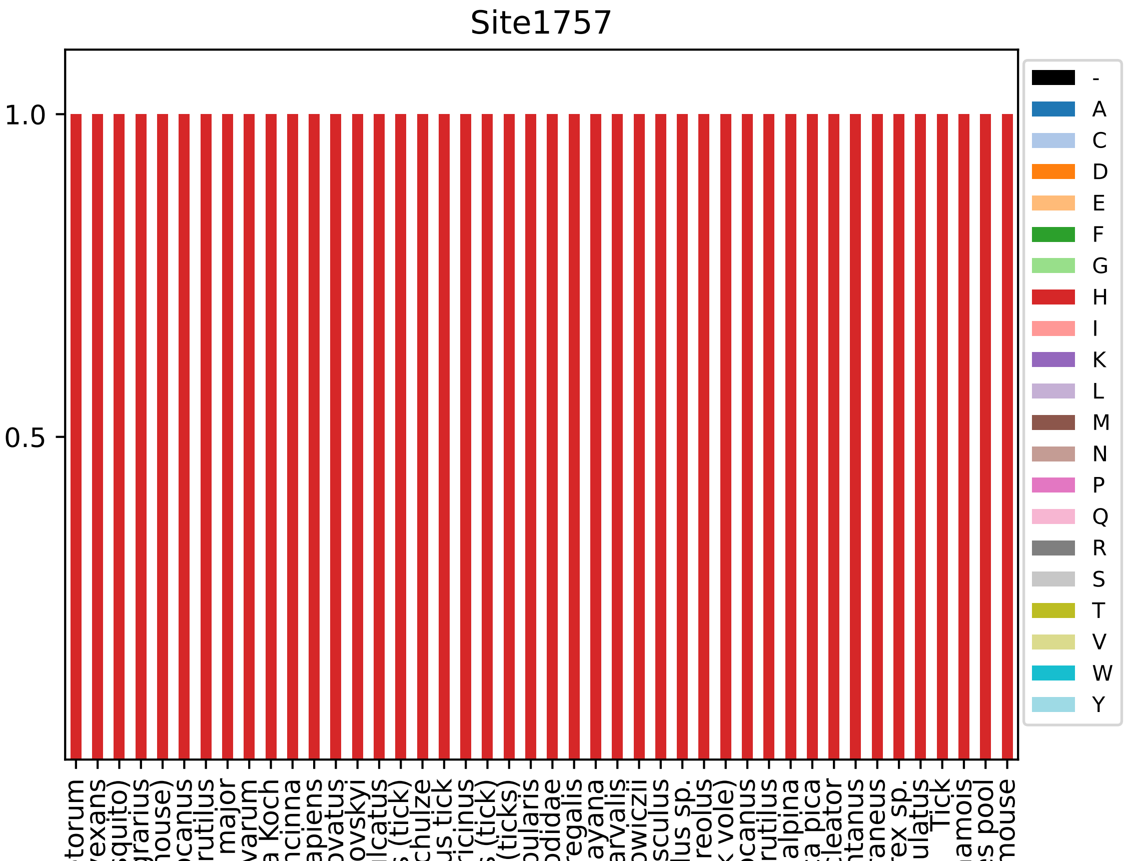

Site1757
1.0
0.5
-
A
C
D
E
F
G
H
I
K
L
M
N
P
Q
R
S
T
V
W
Y
Tick

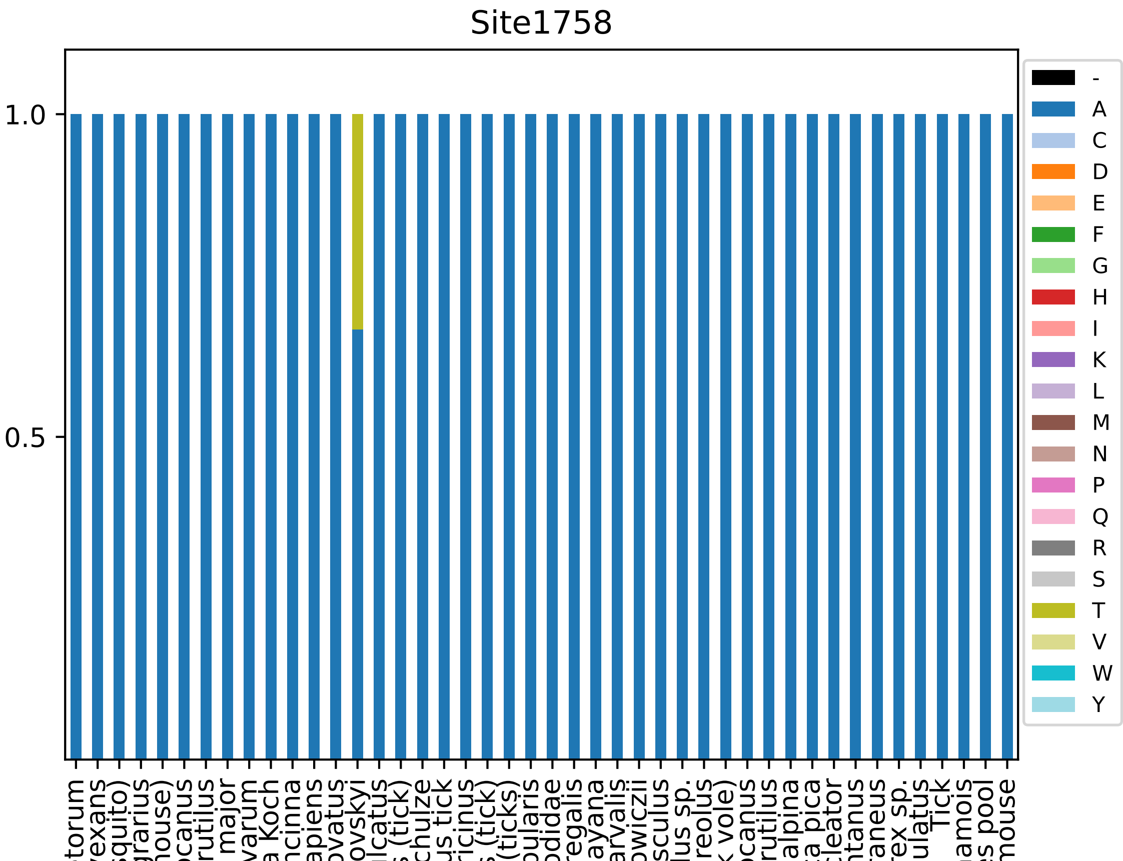

Site1758
1.0
0.5
-
A
C
D
E
F
G
H
I
K
L
M
N
P
Q
R
S
T
V
W
Y

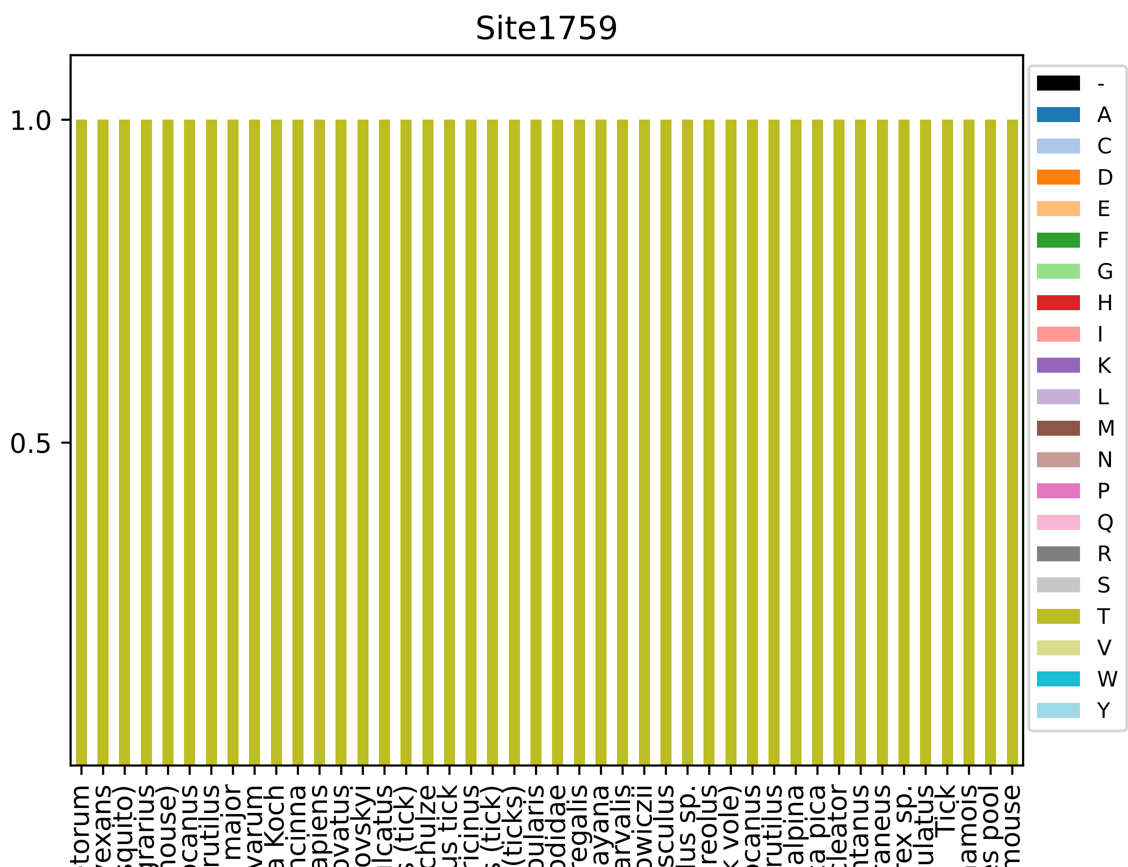
Site1759
1.0
0.5
-
A
C
D
E
F
G
H
I
K
L
M
N
P
Q
R
S
T
V
W
Y

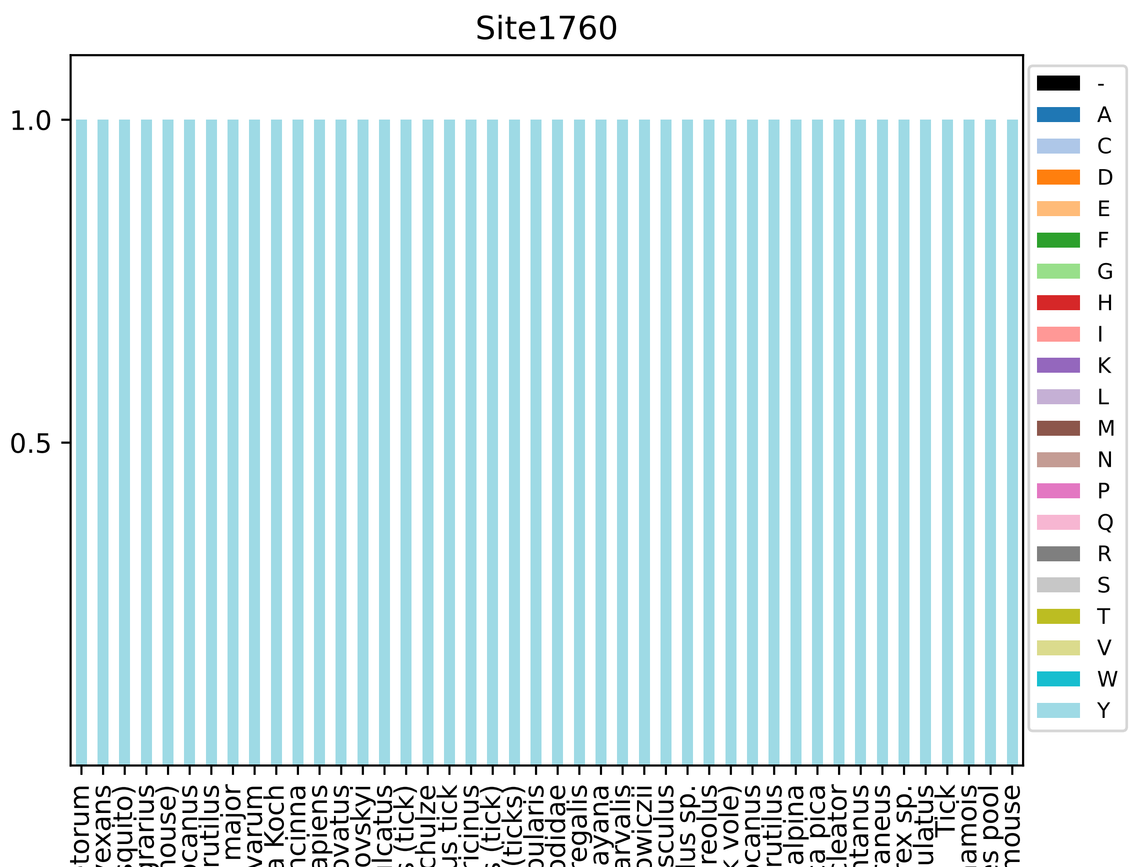
Site1760
1.0
0.5
-
A
C
D
E
F
G
H
I
K
L
M
N
P
Q
R
S
T
V
W
Y

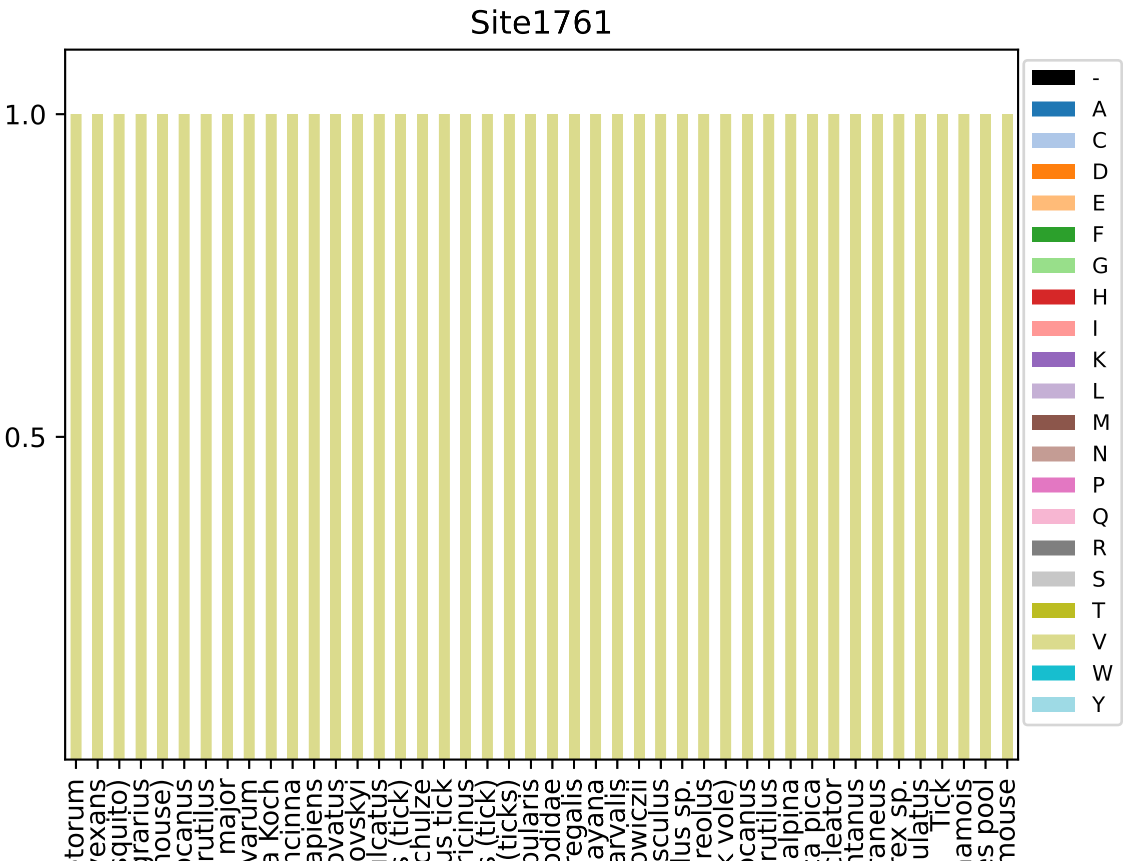
Site1761
1.0
0.5
-
A
C
D
E
F
G
H
I
K
L
M
N
P
Q
R
S
T
V
W
Y

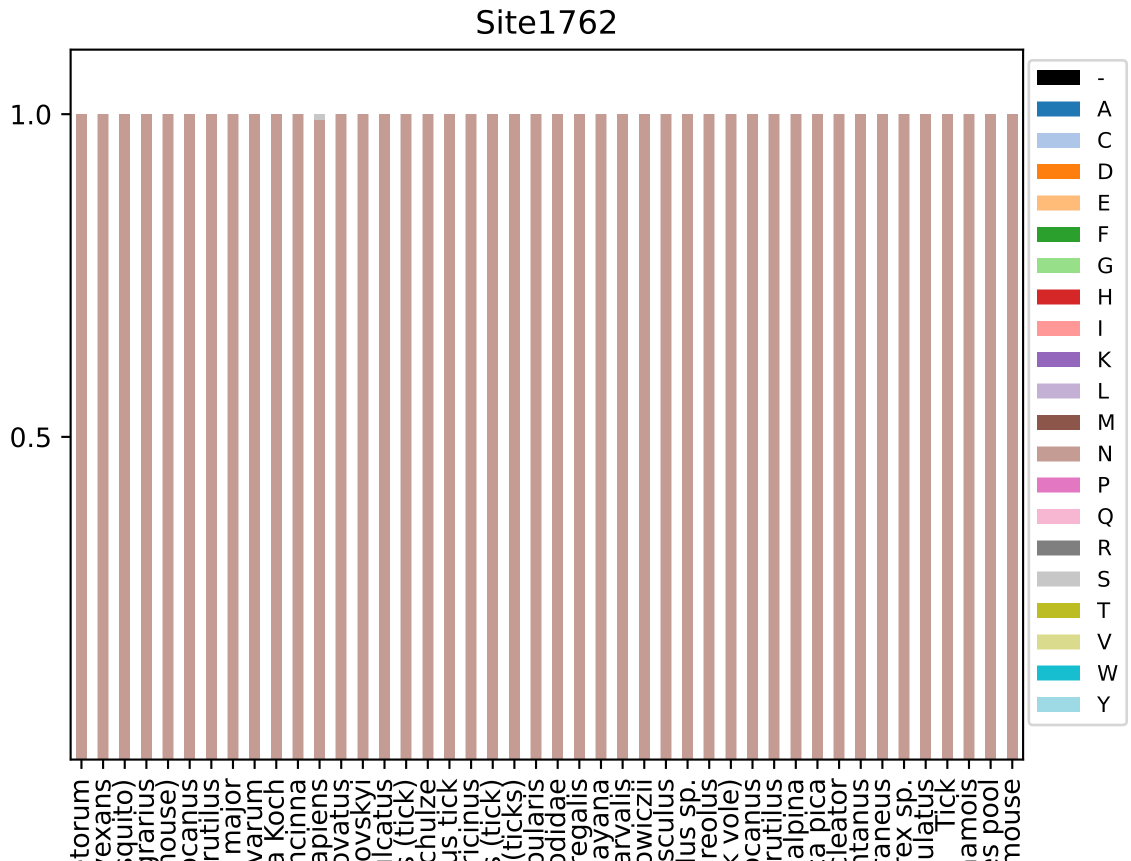
Site1762
1.0
0.5
-
A
C
D
E
F
G
H
I
K
L
M
N
P
Q
R
S
T
V
W
Y

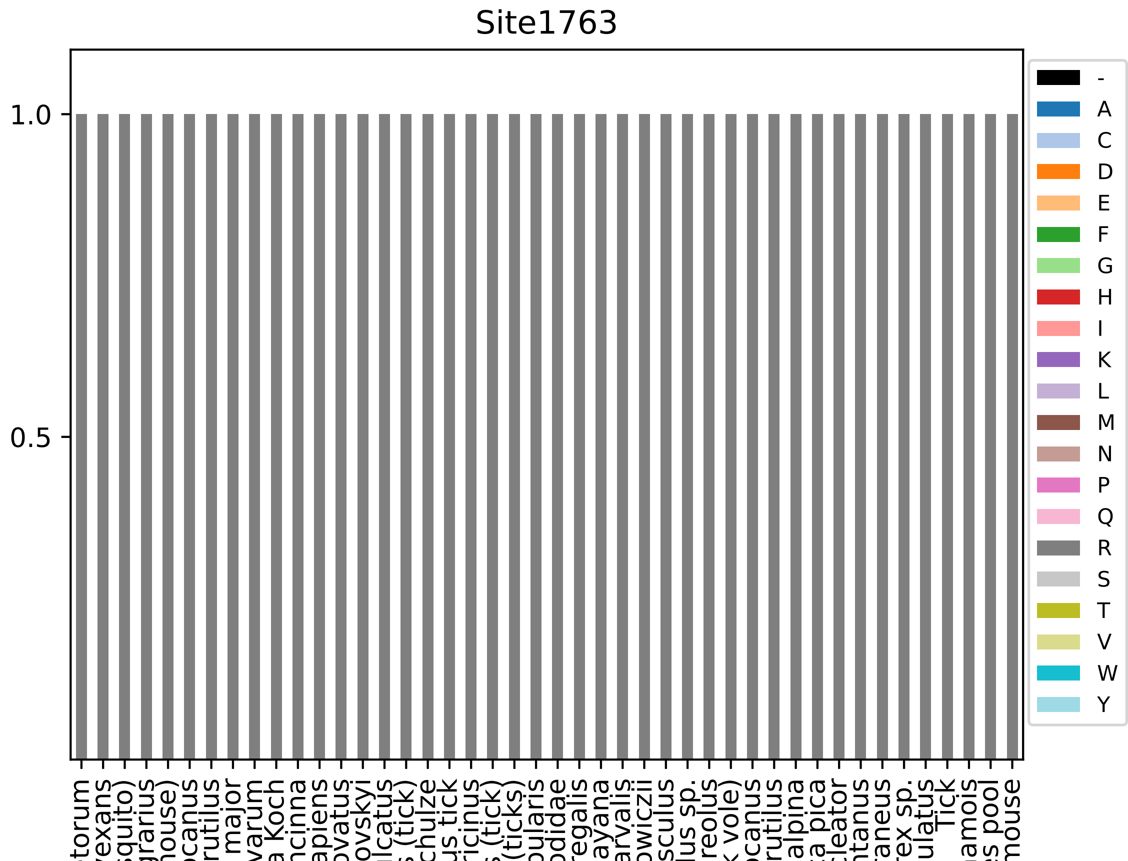
Site1763
1.0
0.5
-
A
C
D
E
F
G
H
I
K
L
M
N
P
Q
R
S
T
V
W
Y

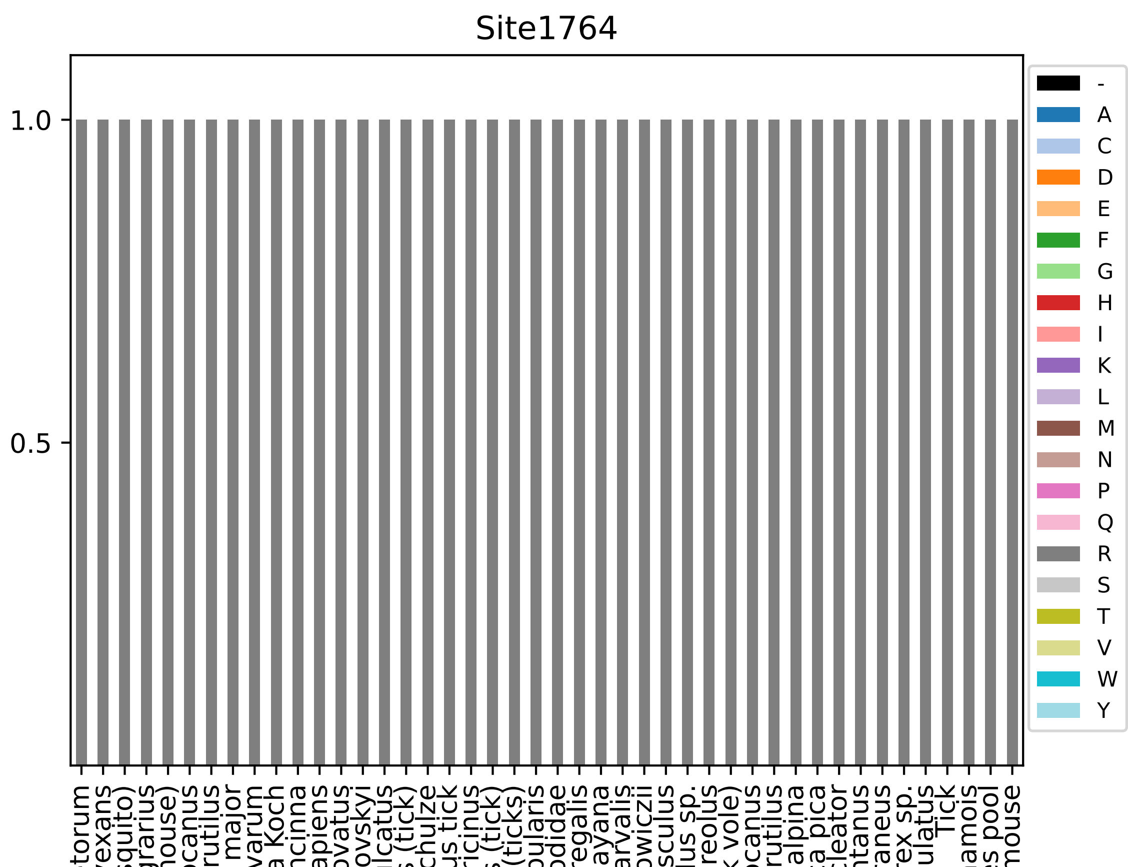
Site1764
1.0
0.5
-
A
C
D
E
F
G
H
I
K
L
M
N
P
Q
R
S
T
V
W
Y

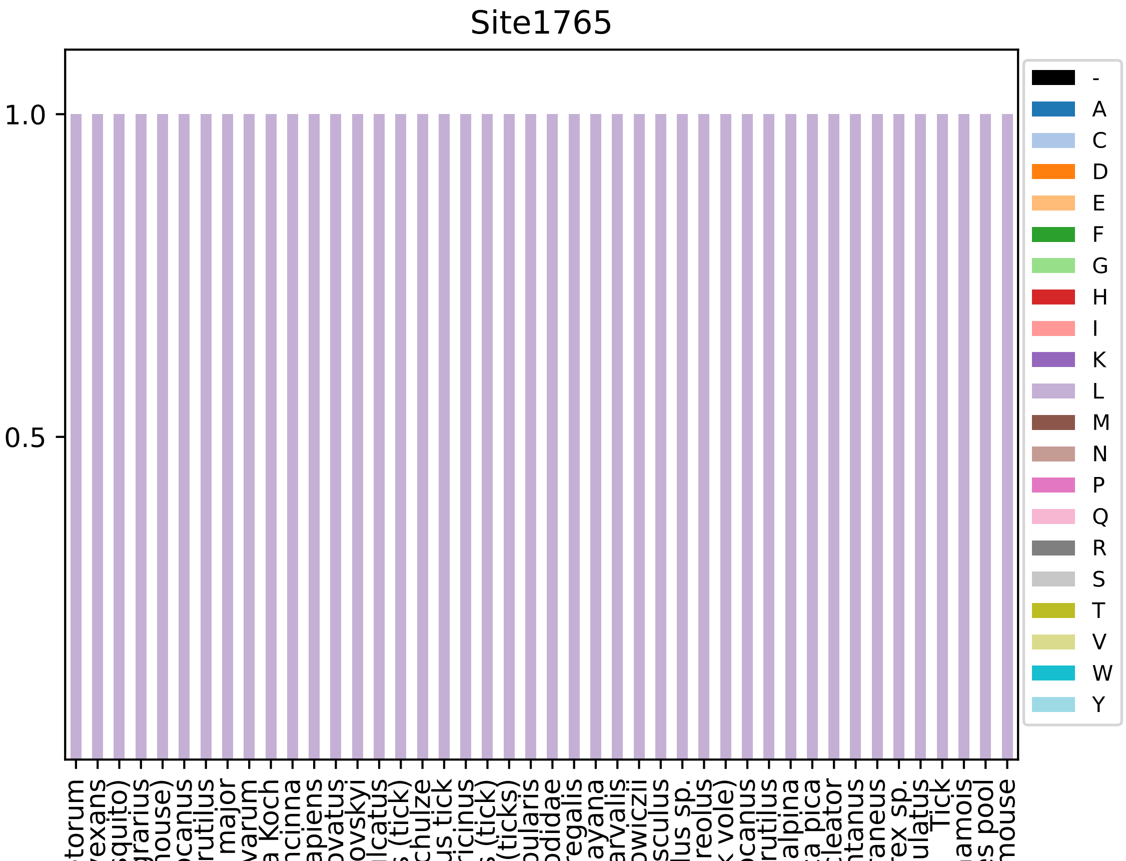
Site1765
1.0
0.5
-
A
C
D
E
F
G
H
I
K
L
M
N
P
Q
R
S
T
V
W
Y

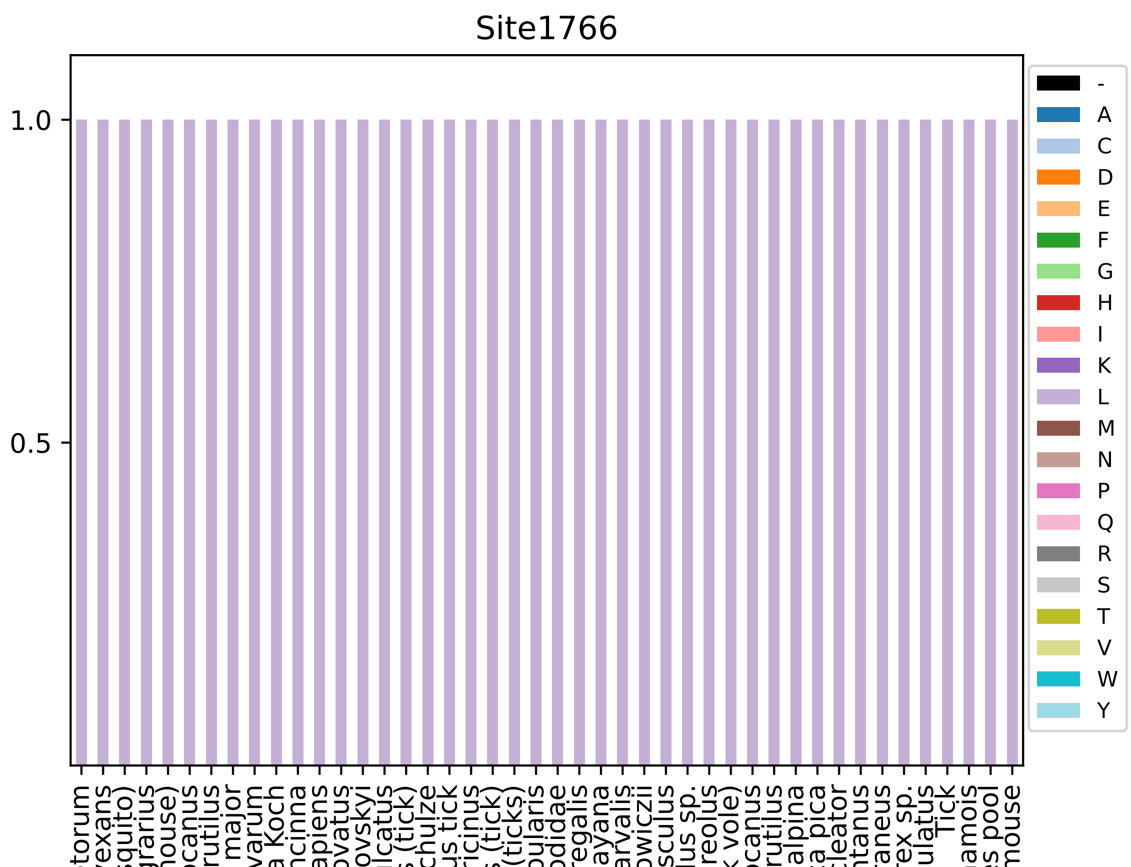
Site1766
1.0
0.5
-
A
C
D
E
F
G
H
I
K
L
M
N
P
Q
R
S
T
V
W
Y

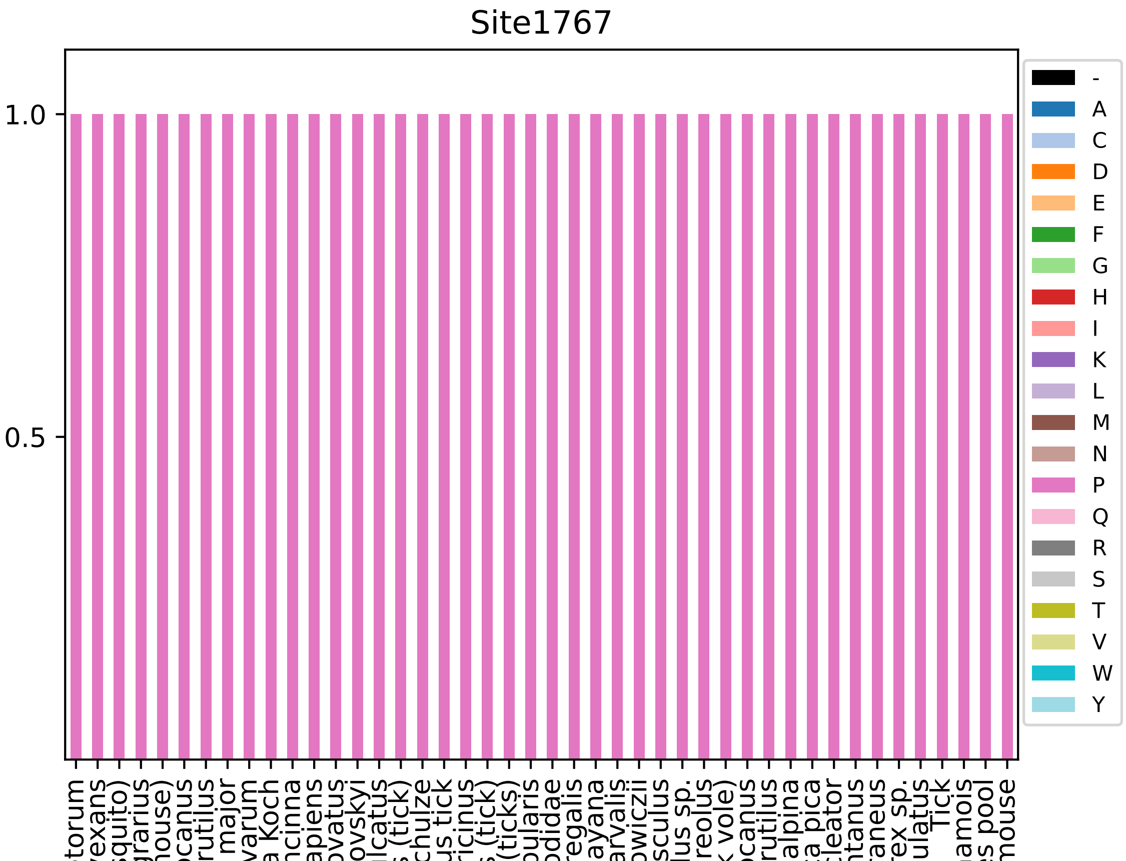

Site1767
1.0
0.5
-
A
C
D
E
F
G
H
I
K
L
M
N
P
Q
R
S
T
V
W
Y

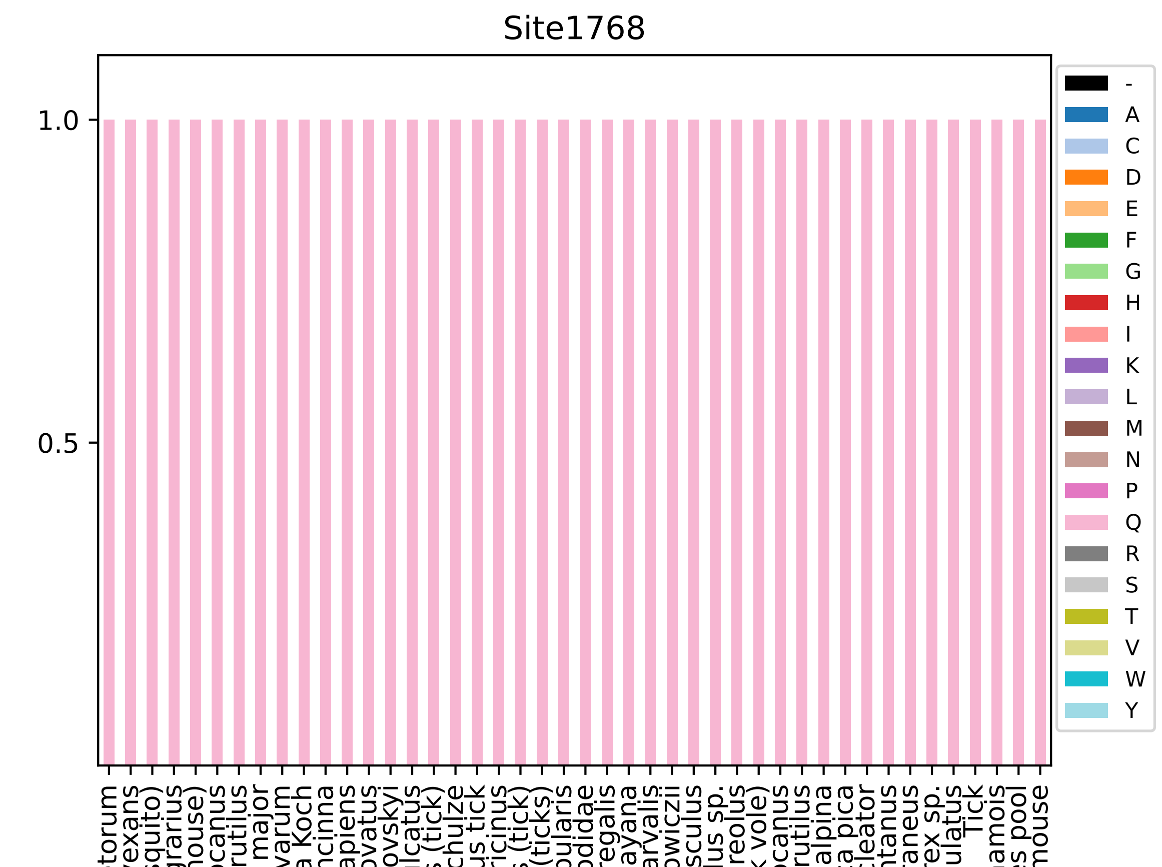
Site1768
1.0
0.5
-
A
C
D
E
F
G
H
I
K
L
M
N
P
Q
R
S
T
V
W
Y

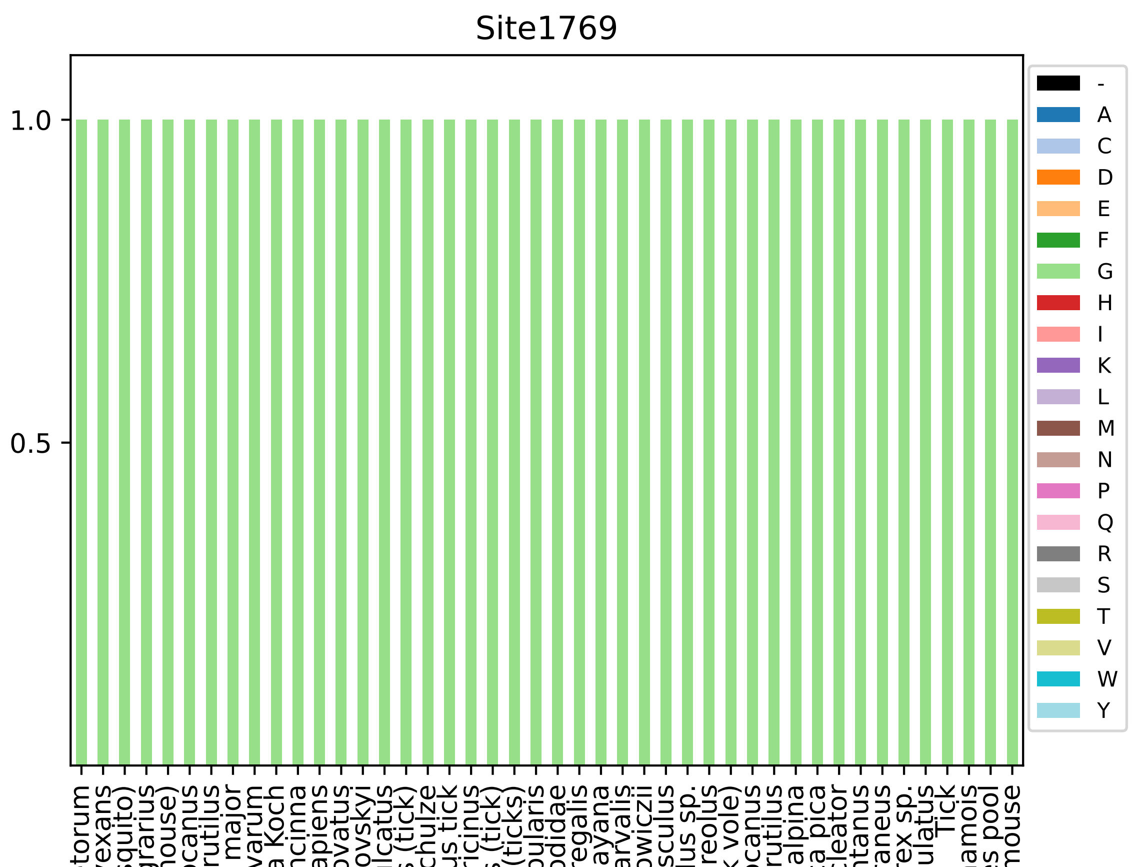
Site1769
1.0
0.5
-
A
C
D
E
F
G
H
I
K
L
M
N
P
Q
R
S
T
V
W
Y

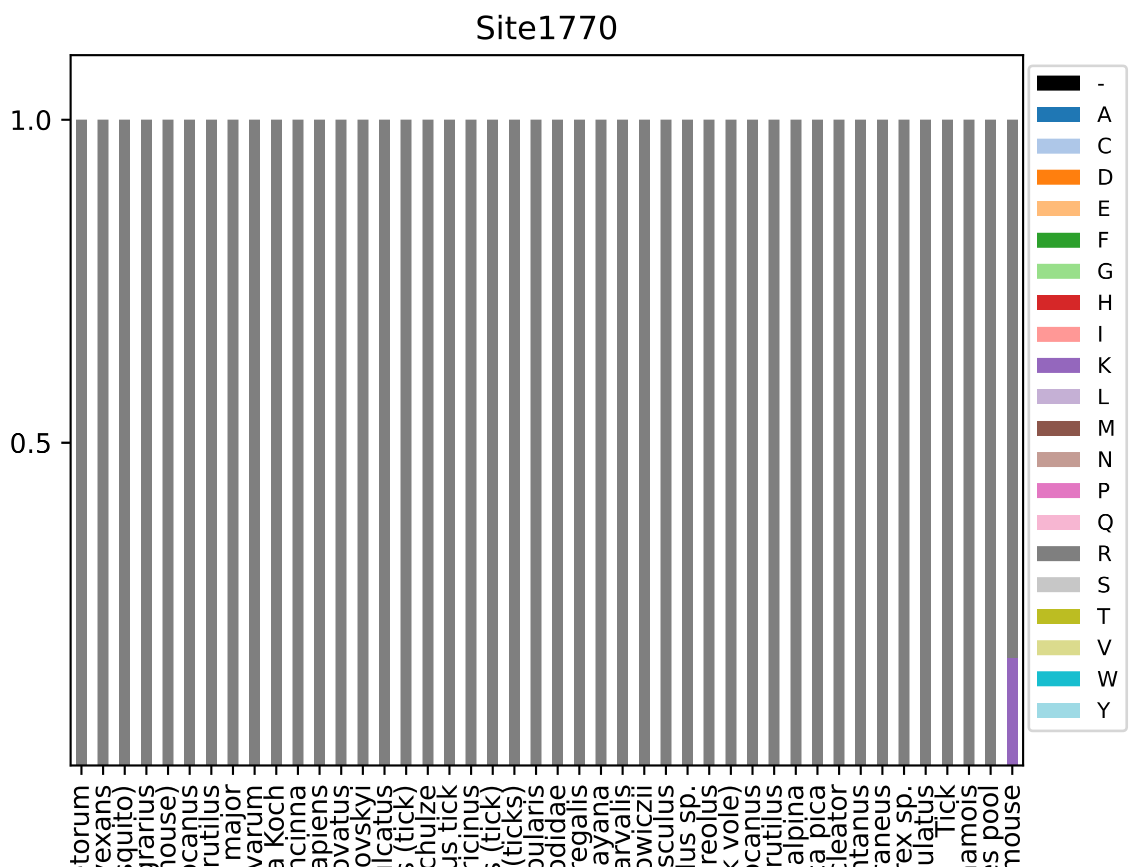

Site1770
1.0
0.5
-
A
C
D
E
F
G
H
I
K
L
M
N
P
Q
R
S
T
V
W
Y

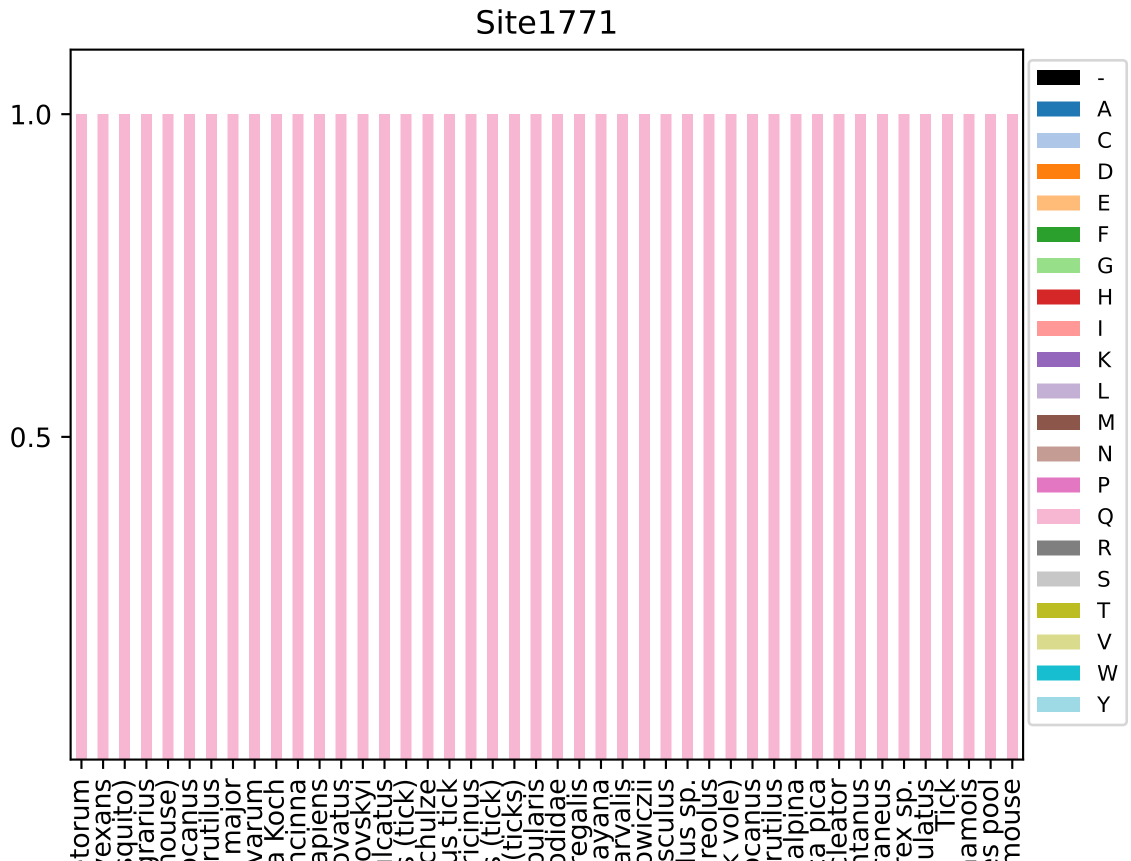

Site1771
1.0
0.5
-
A
C
D
E
F
G
H
I
K
L
M
N
P
Q
R
S
T
V
W
Y

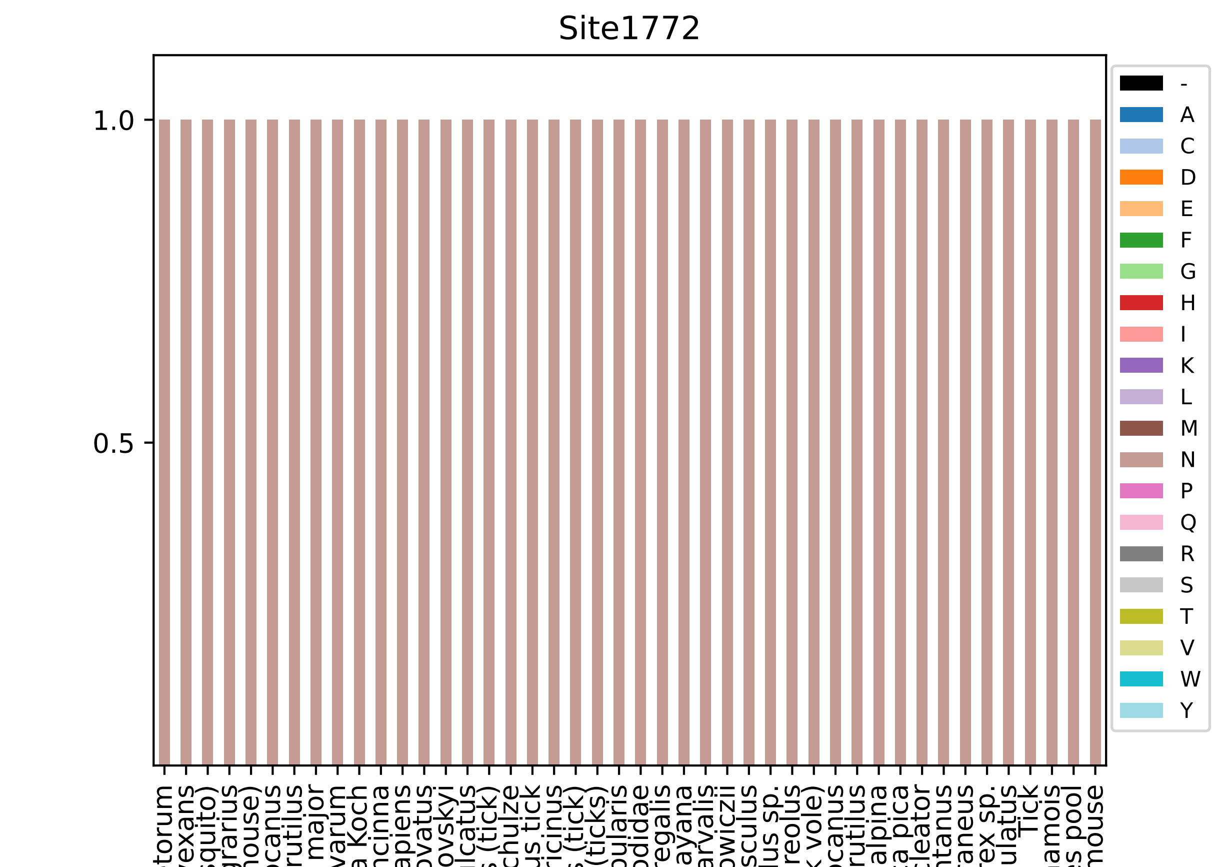

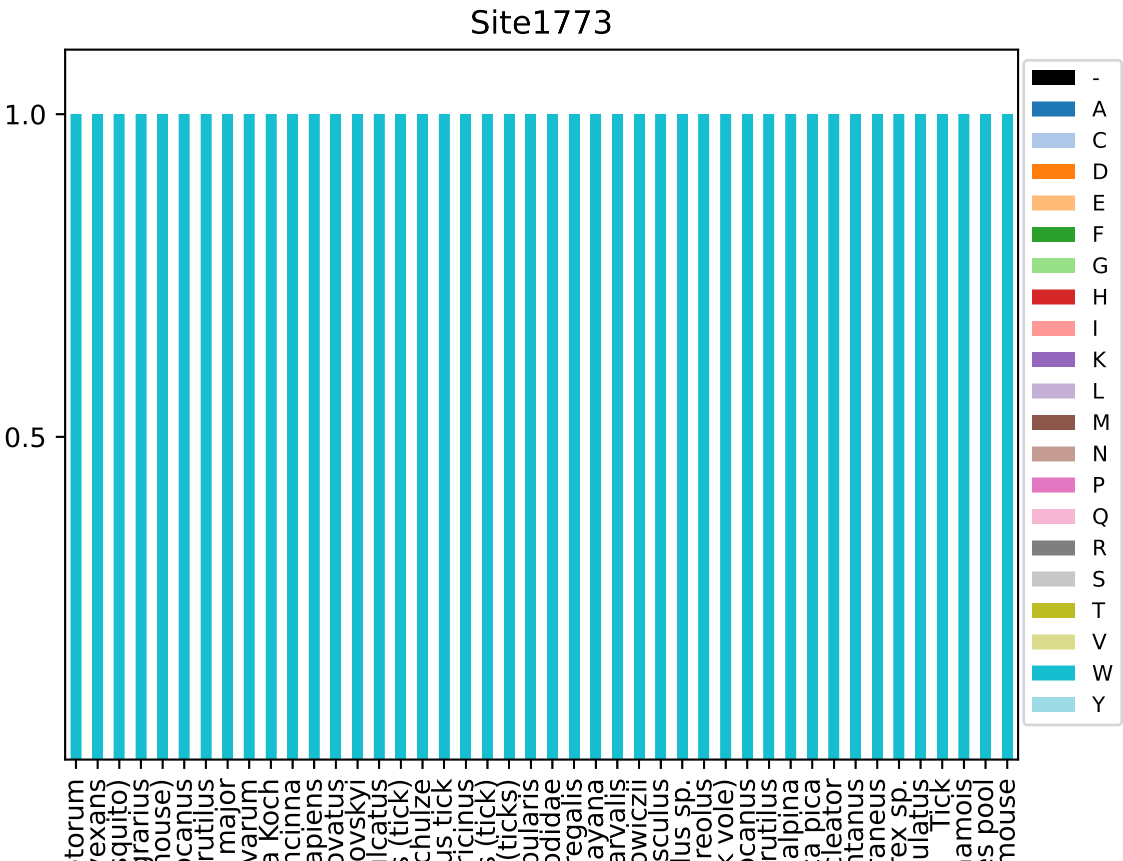
Site1773
1.0
0.5
-
A
C
D
E
F
G
H
I
K
L
M
N
P
Q
R
S
T
V
W
Y

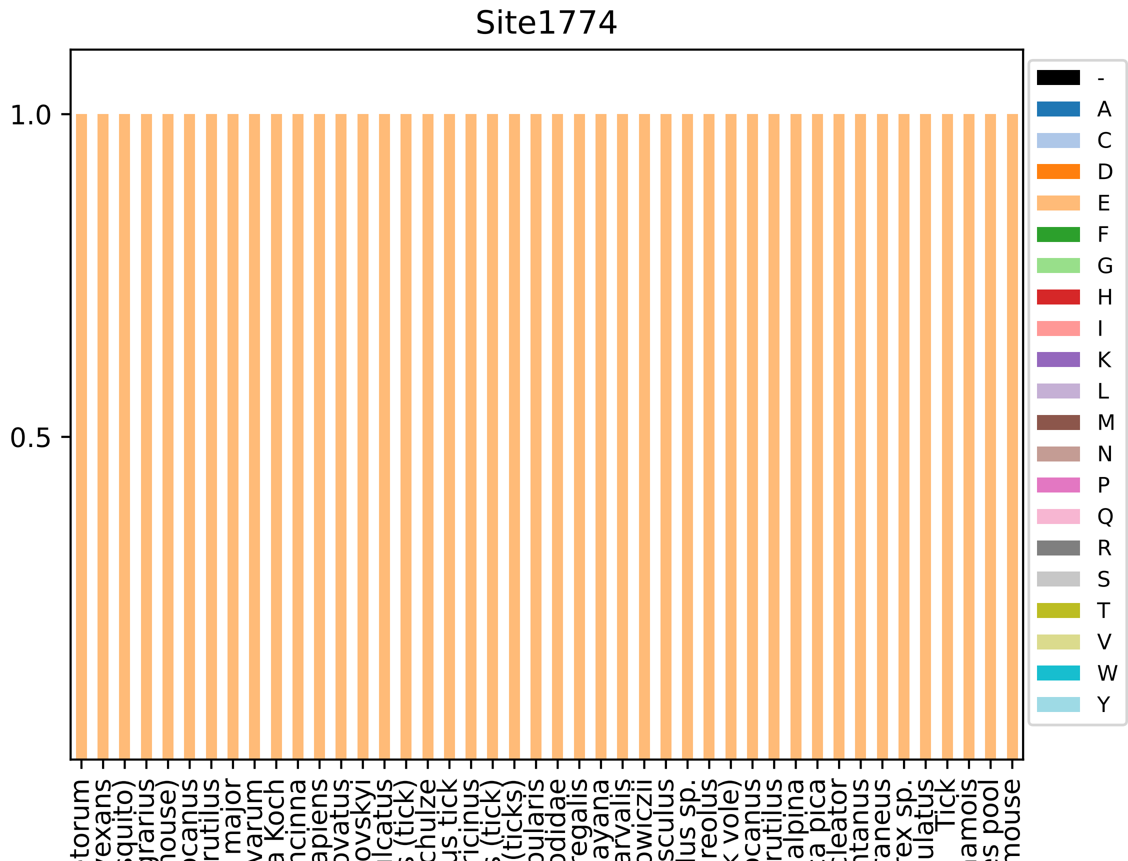
Site1774
1.0
0.5
-
A
C
D
E
F
G
H
I
K
L
M
N
P
Q
R
S
T
V
W
Y

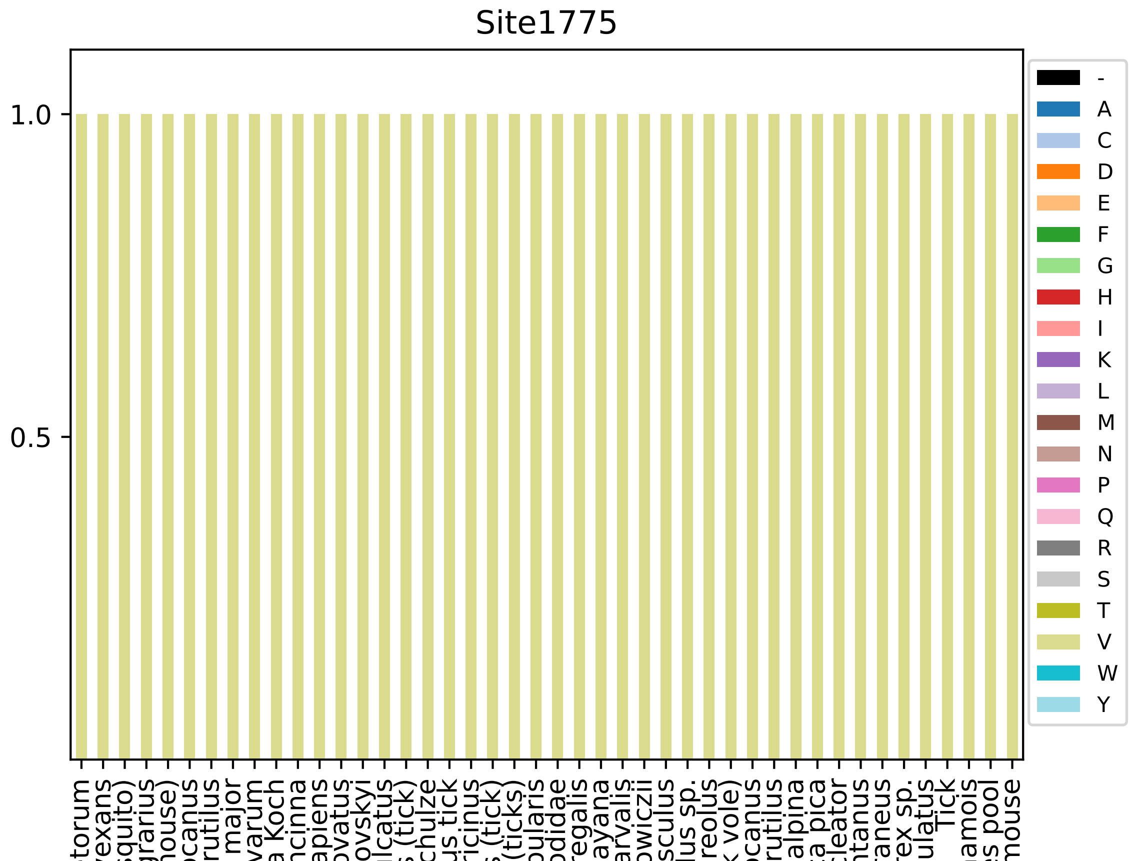
Site1775
1.0
0.5
-
A
C
D
E
F
G
H
I
K
L
M
N
P
Q
R
S
T
V
W
Y

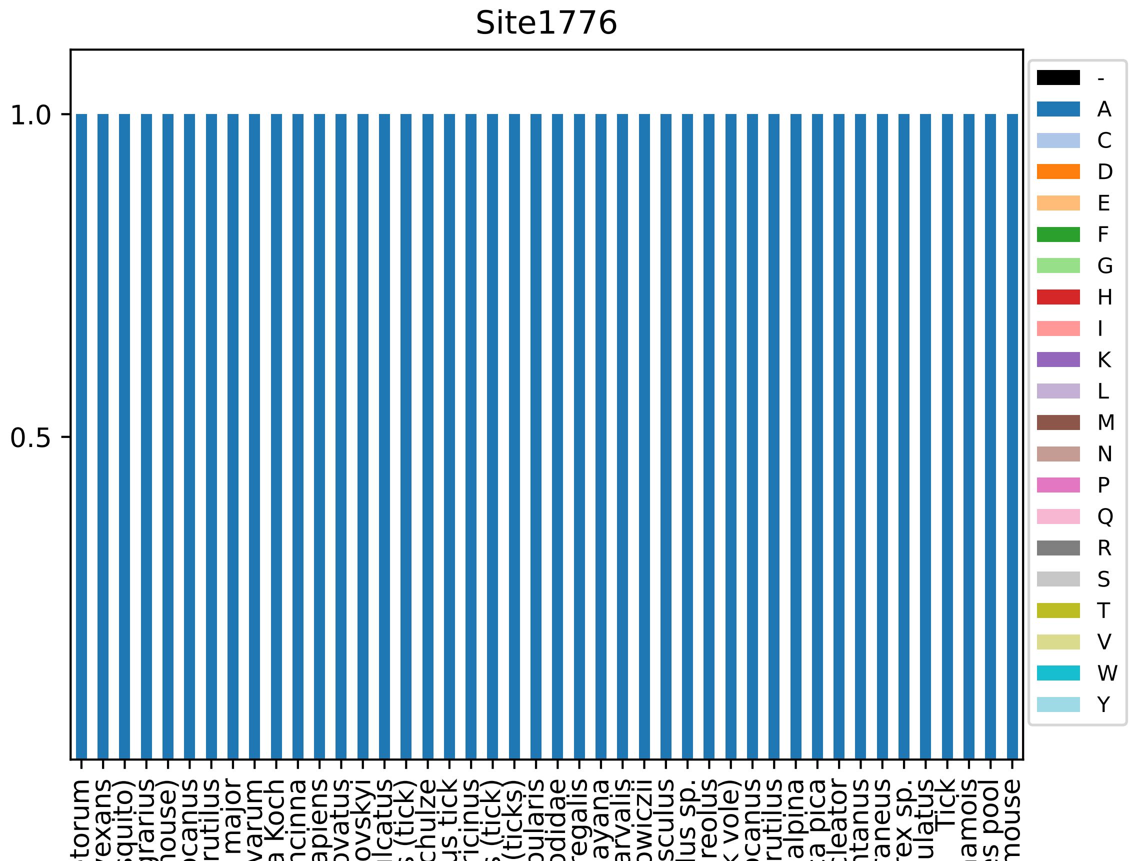

Site1776
1.0
0.5
-
A
C
D
E
F
G
H
I
K
L
M
N
P
Q
R
S
T
V
W
Y

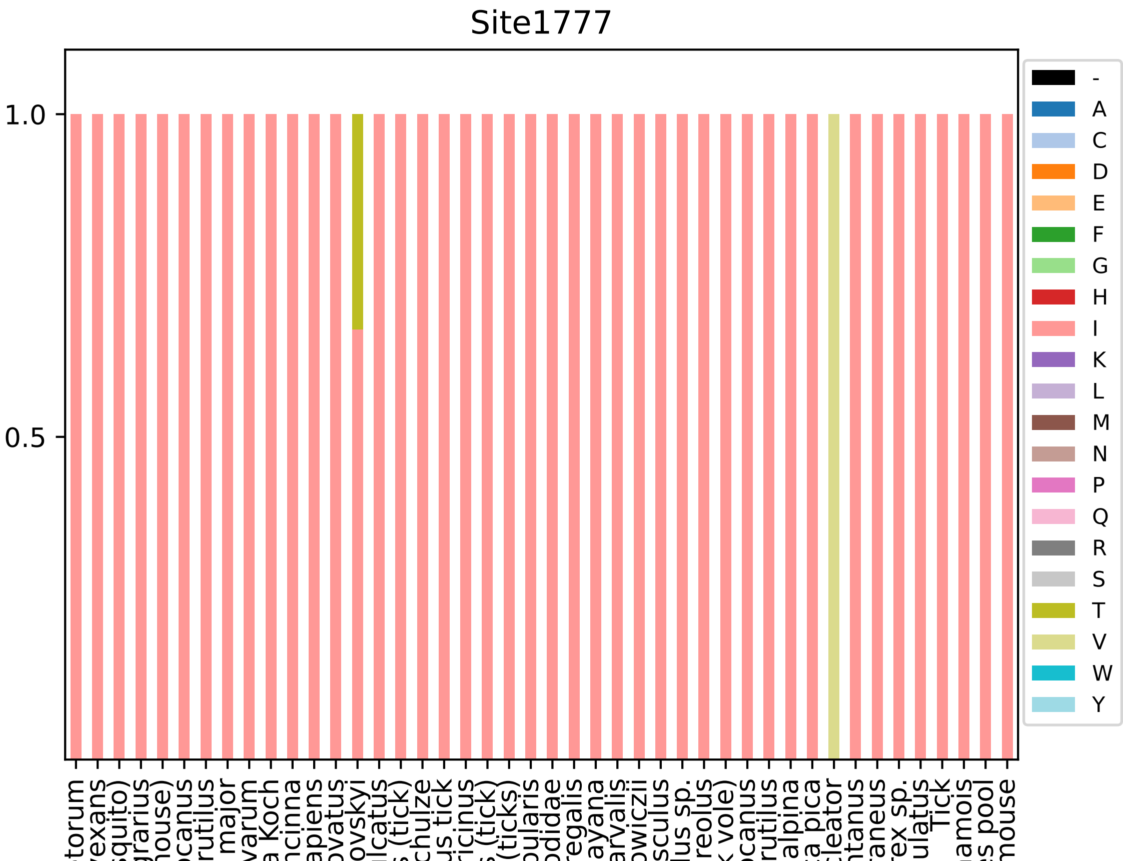
Site1777
1.0
0.5
-
A
C
D
E
F
G
H
I
K
L
M
N
P
Q
R
S
T
V
W
Y

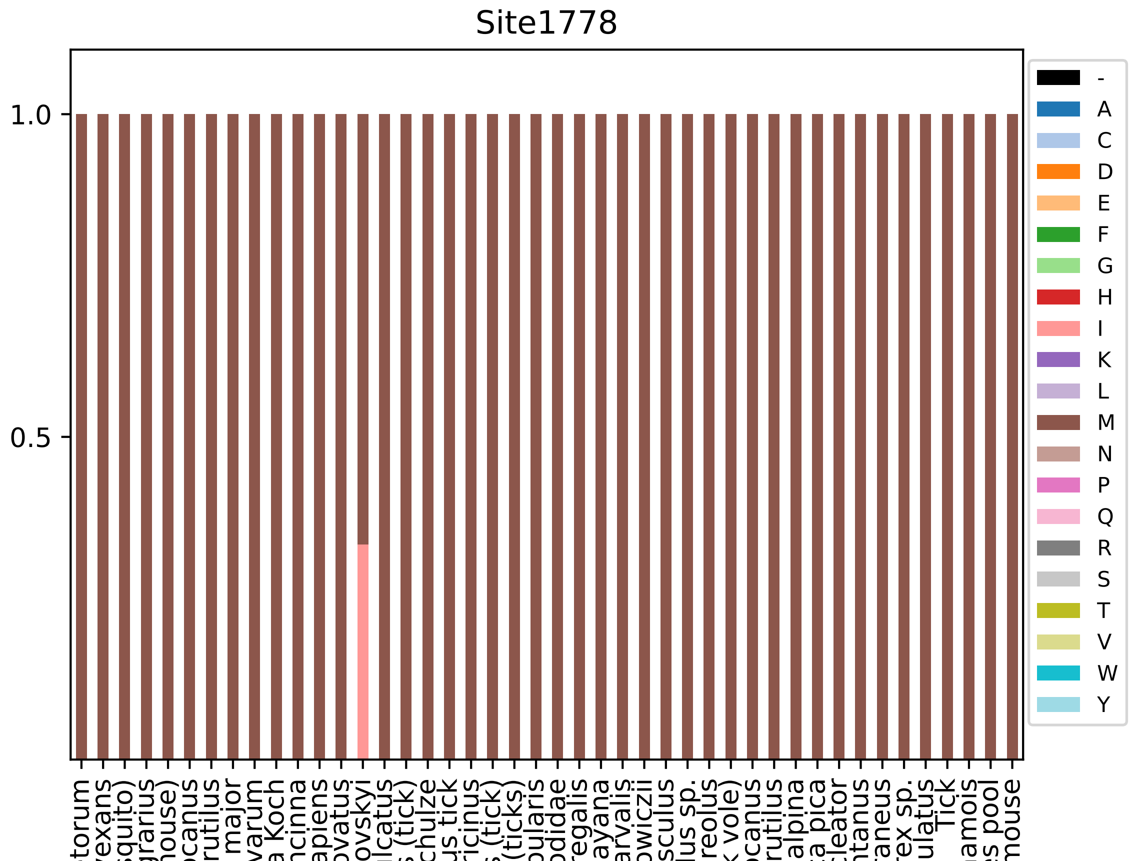
Site1778
1.0
0.5
-
A
C
D
E
F
G
H
I
K
L
M
N
P
Q
R
S
T
V
W
Y

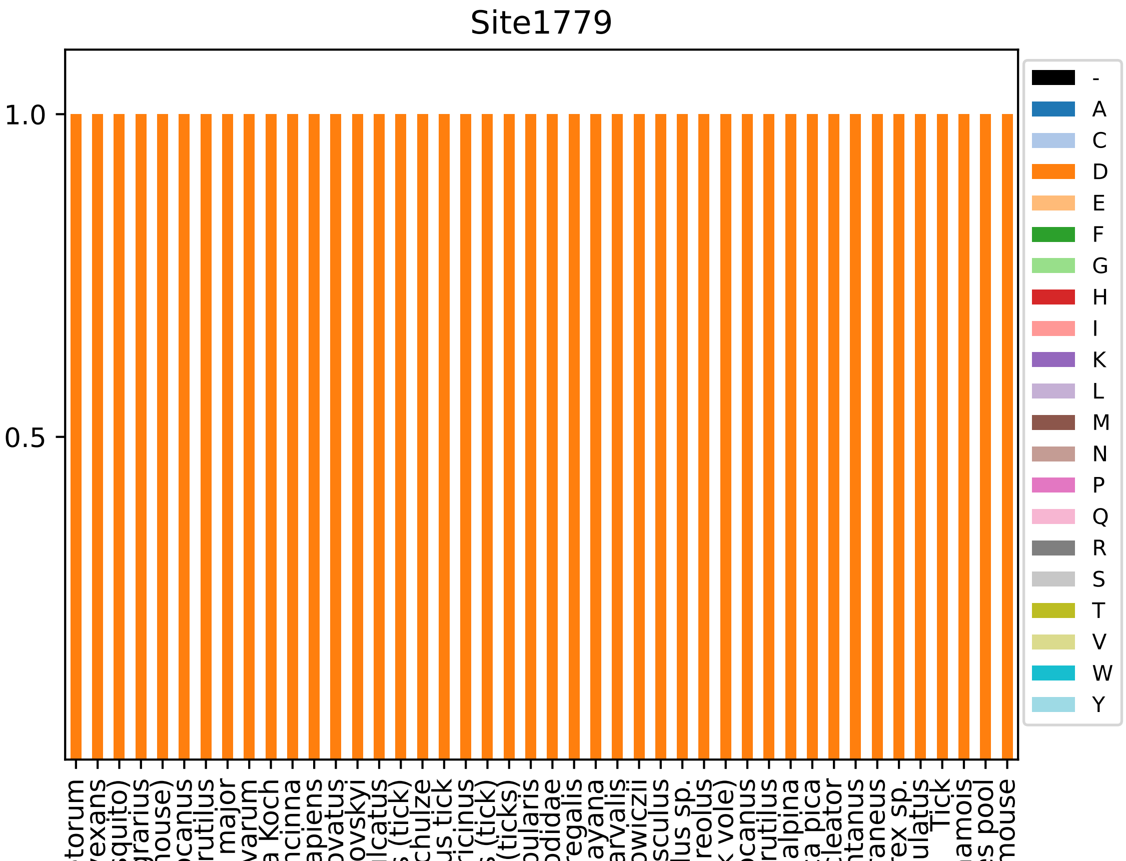

Site1779
1.0
0.5
-
A
C
D
E
F
G
H
I
K
L
M
N
P
Q
R
S
T
V
W
Y

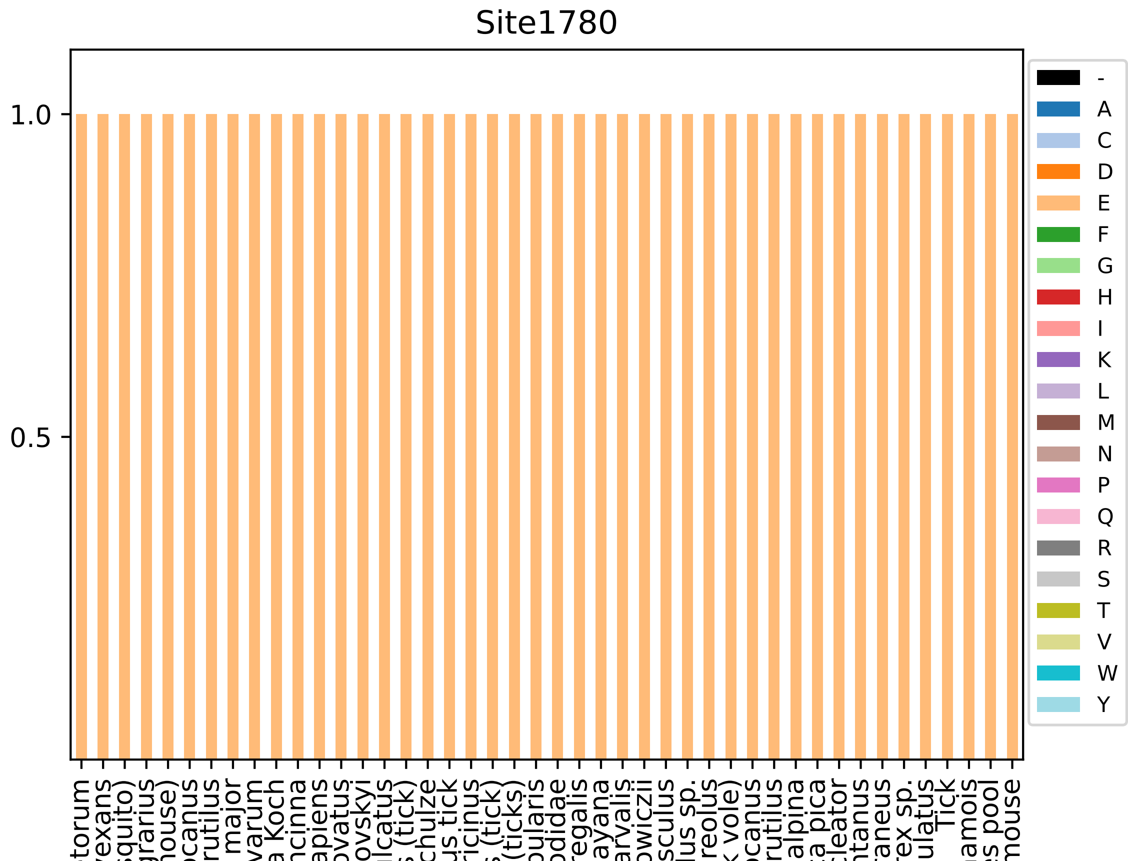

Site1780
1.0
0.5
-
A
C
D
E
F
G
H
I
K
L
M
N
P
Q
R
S
T
V
W
Y
Tick

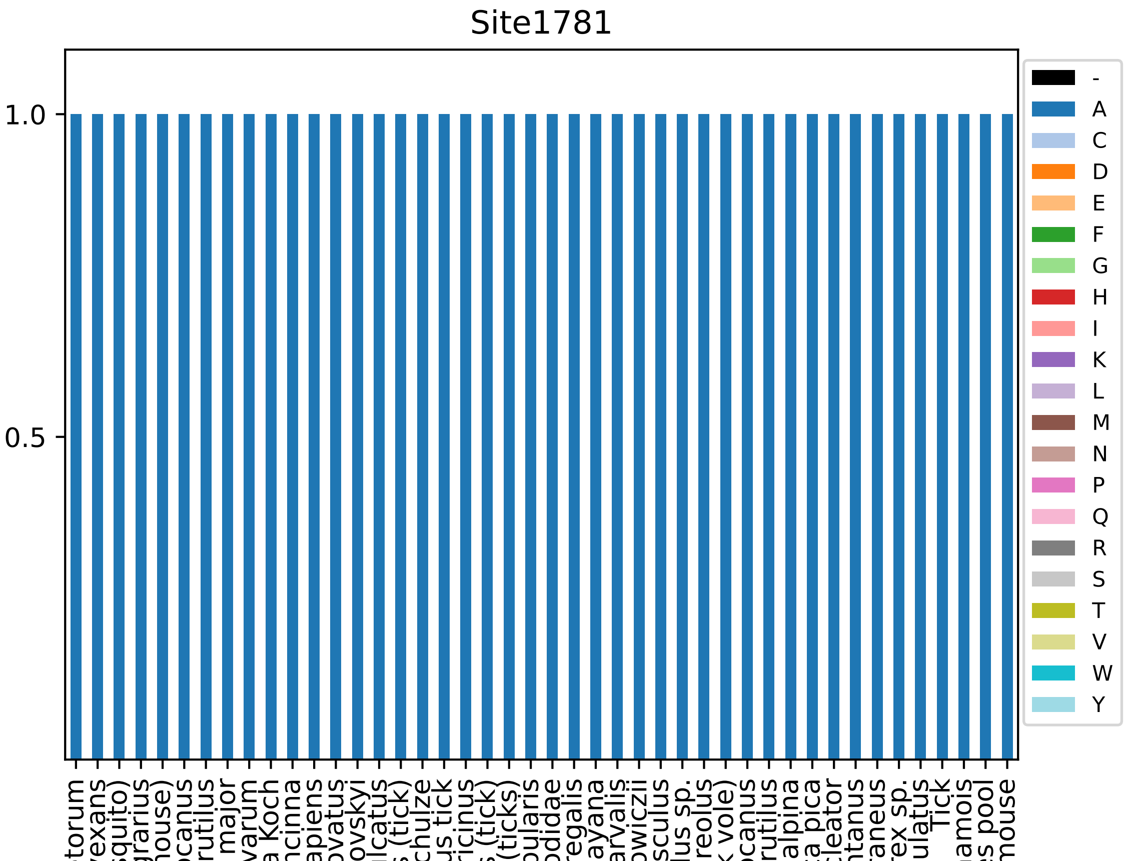
Site1781
1.0
0.5
-
A
C
D
E
F
G
H
I
K
L
M
N
P
Q
R
S
T
V
W
Y
Tick

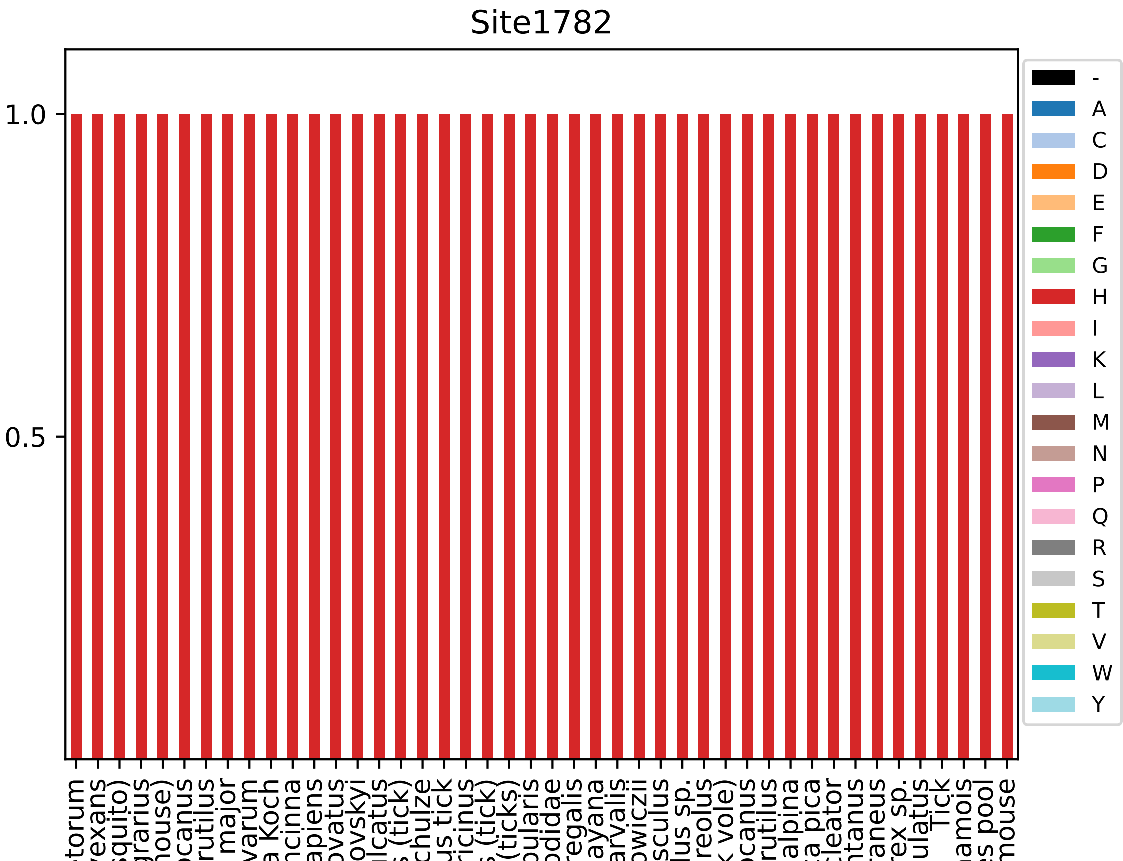
Site1782
1.0
0.5
-
A
C
D
E
F
G
H
I
K
L
M
N
P
Q
R
S
T
V
W
Y

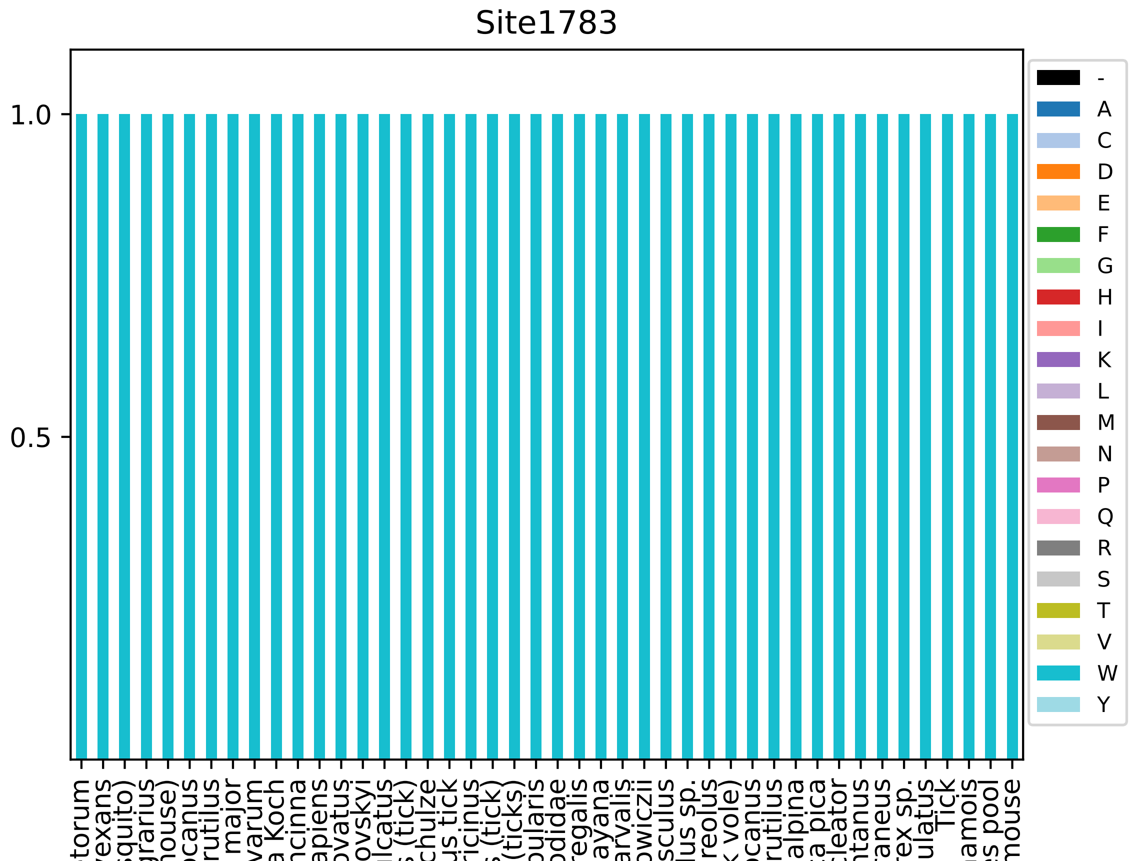
Site1783
1.0
0.5
-
A
C
D
E
F
G
H
I
K
L
M
N
P
Q
R
S
T
V
W
Y

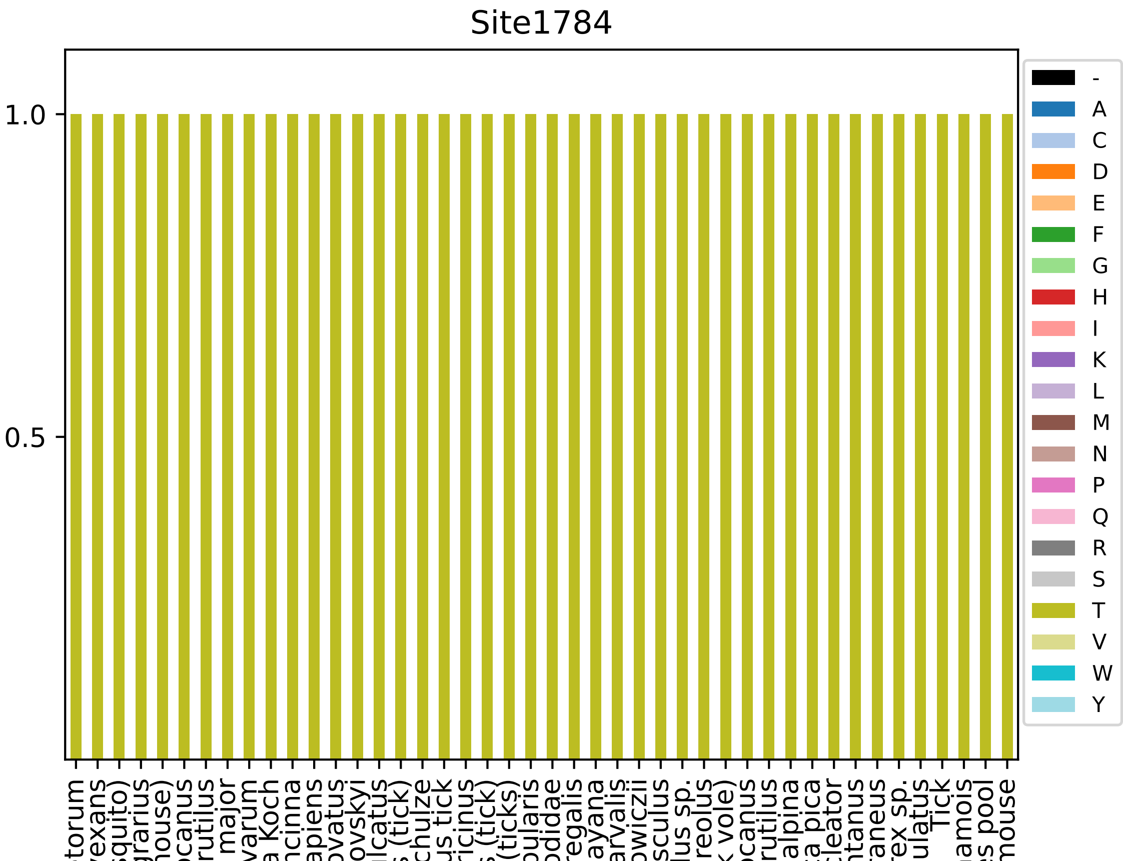
Site1784
1.0
0.5
-
A
C
D
E
F
G
H
I
K
L
M
N
P
Q
R
S
T
V
W
Y
Tick

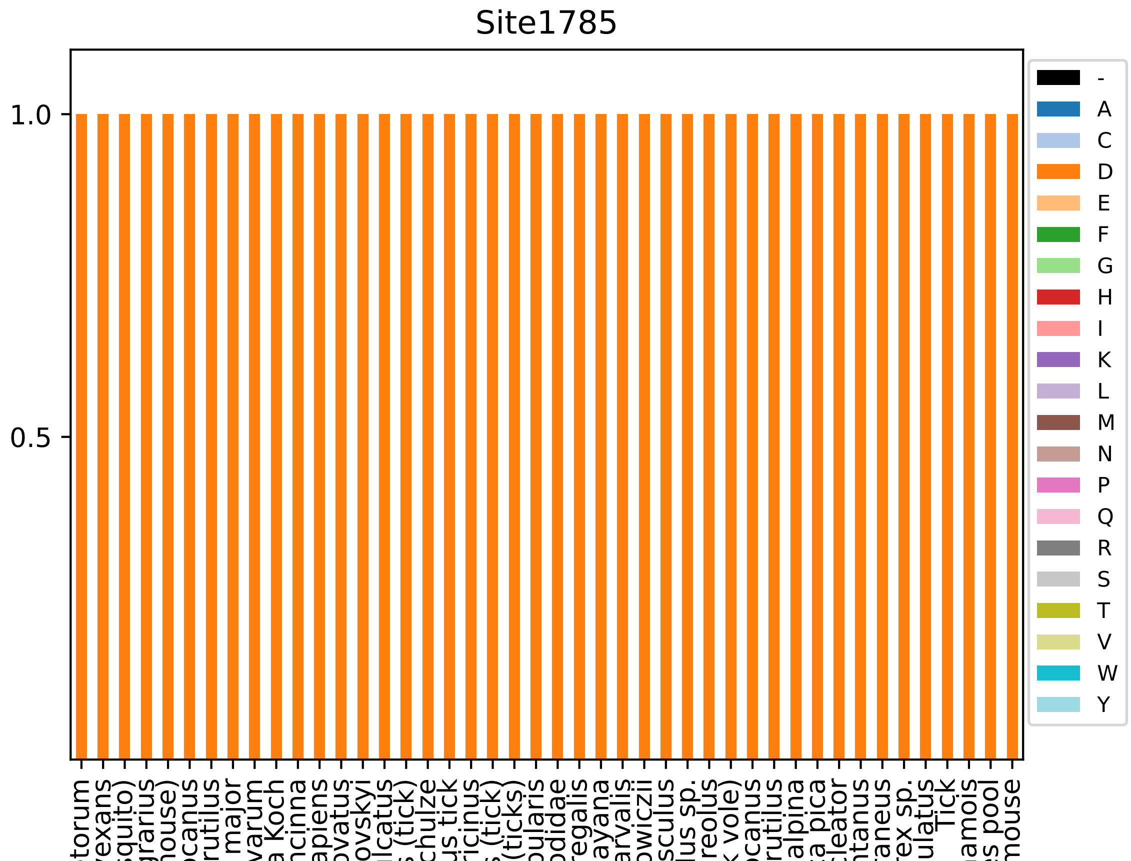
Site1785
1.0
0.5
-
A
C
D
E
F
G
H
I
K
L
M
N
P
Q
R
S
T
V
W
Y
Tick

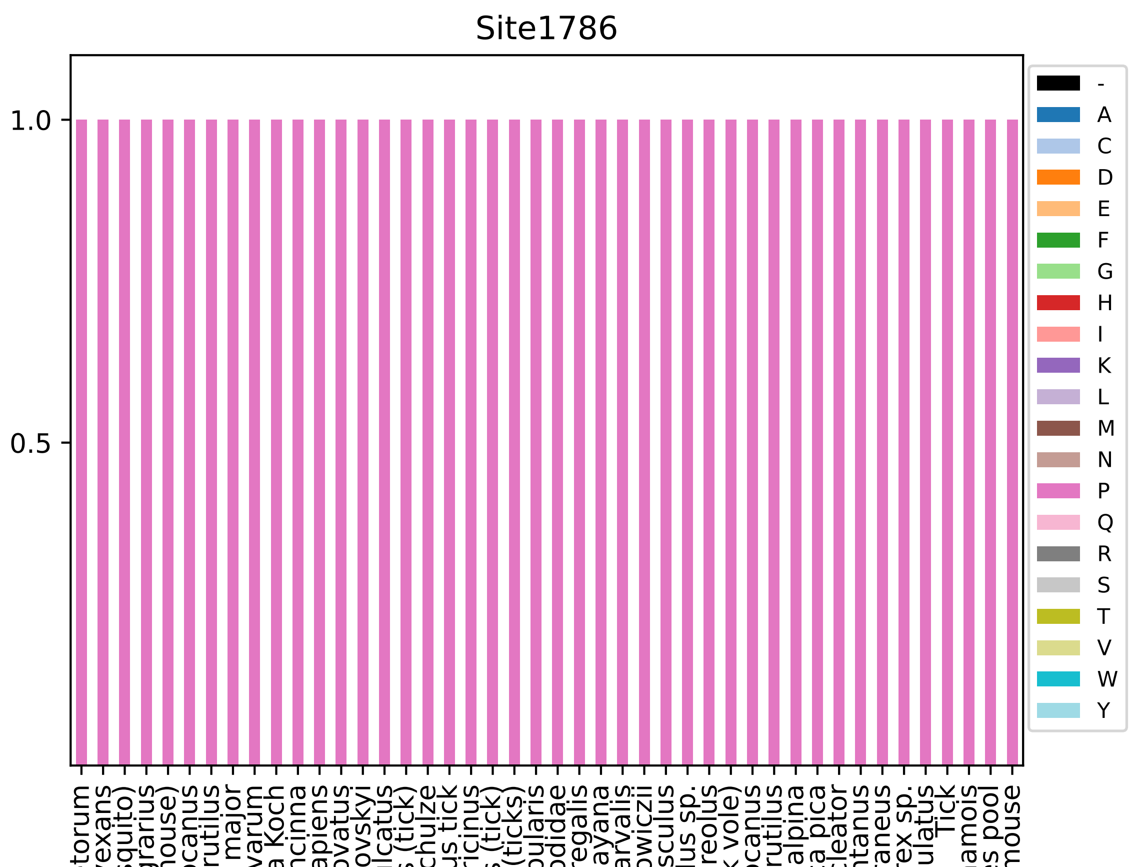
Site1786
1.0
0.5
-
A
C
D
E
F
G
H
I
K
L
M
N
P
Q
R
S
T
V
W
Y

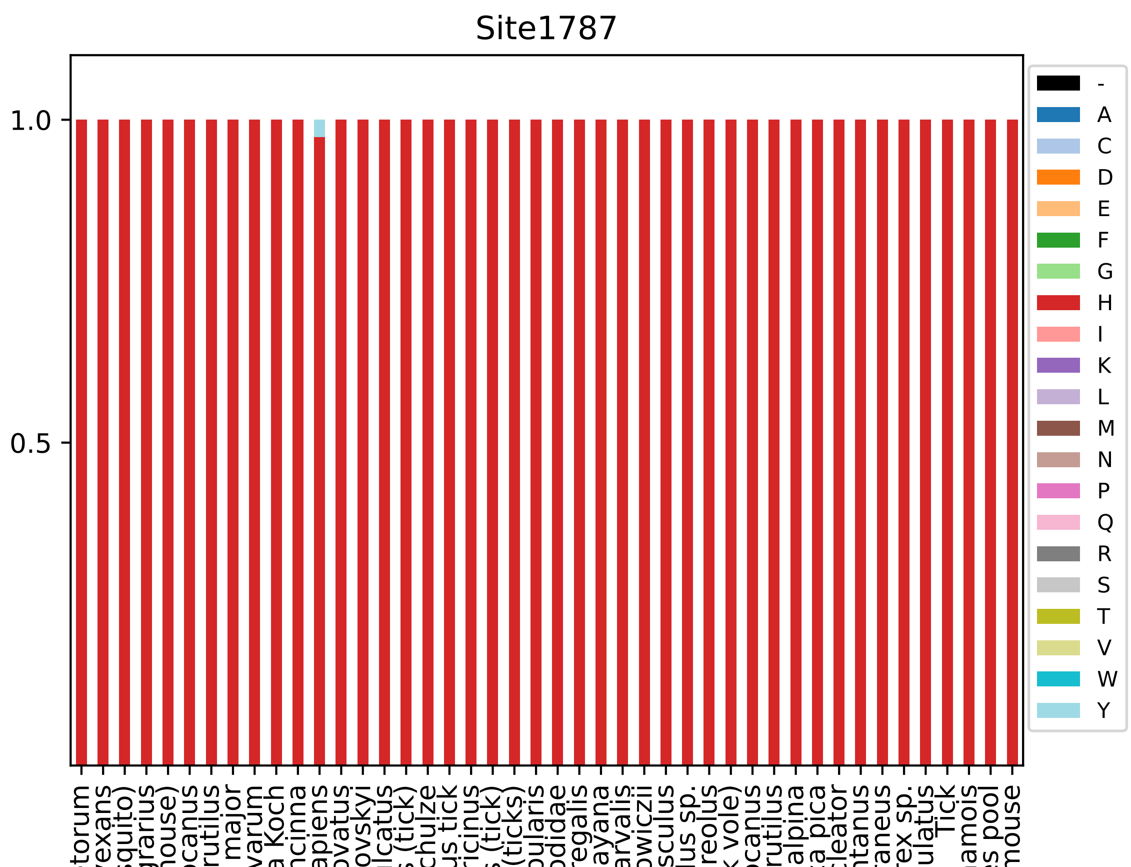
Site1787
1.0
0.5
-
A
C
D
E
F
G
H
I
K
L
M
N
P
Q
R
S
T
V
W
Y

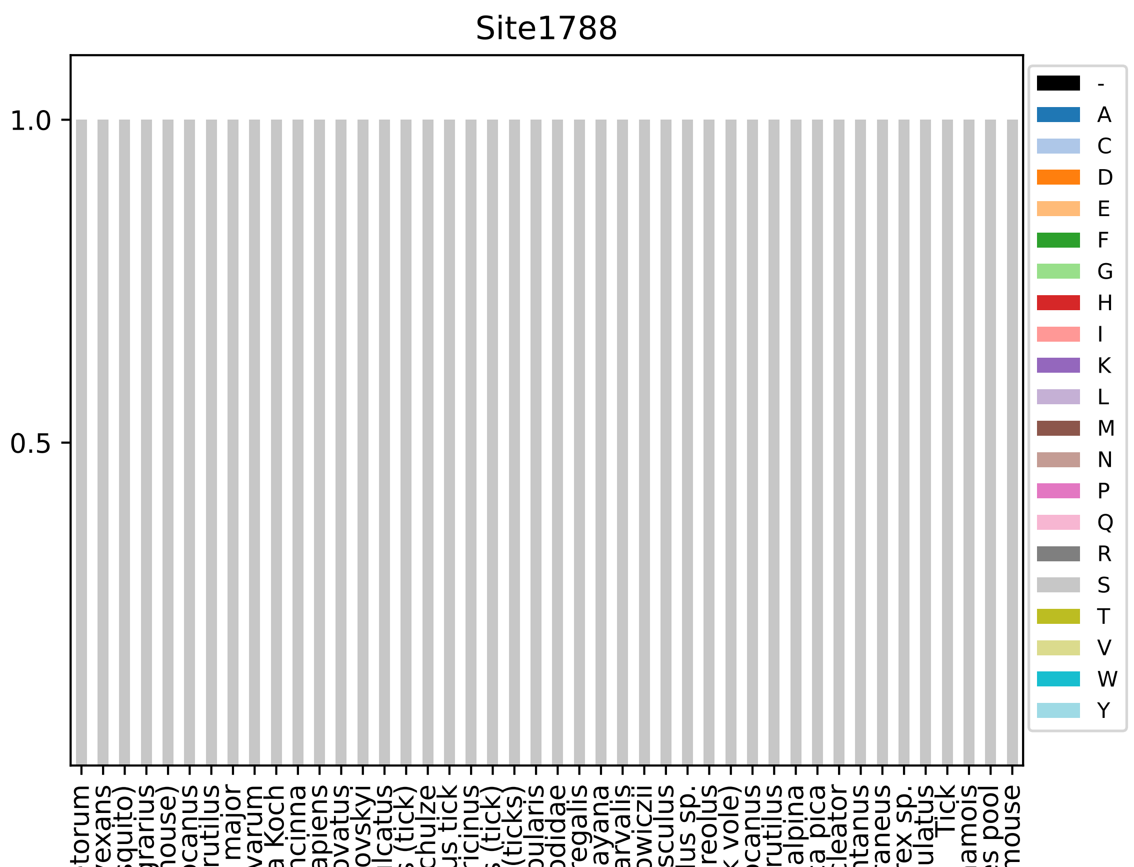

Site1788
1.0
0.5
-
A
C
D
E
F
G
H
I
K
L
M
N
P
Q
R
S
T
V
W
Y

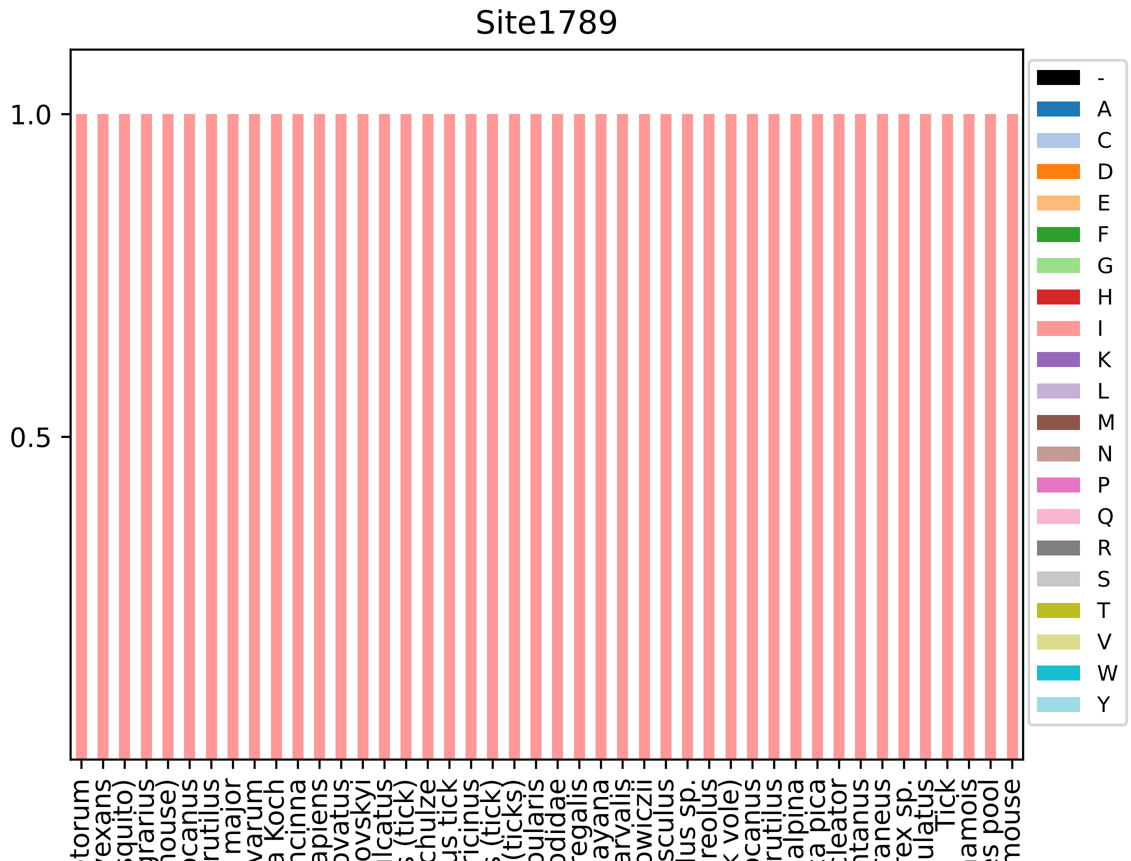

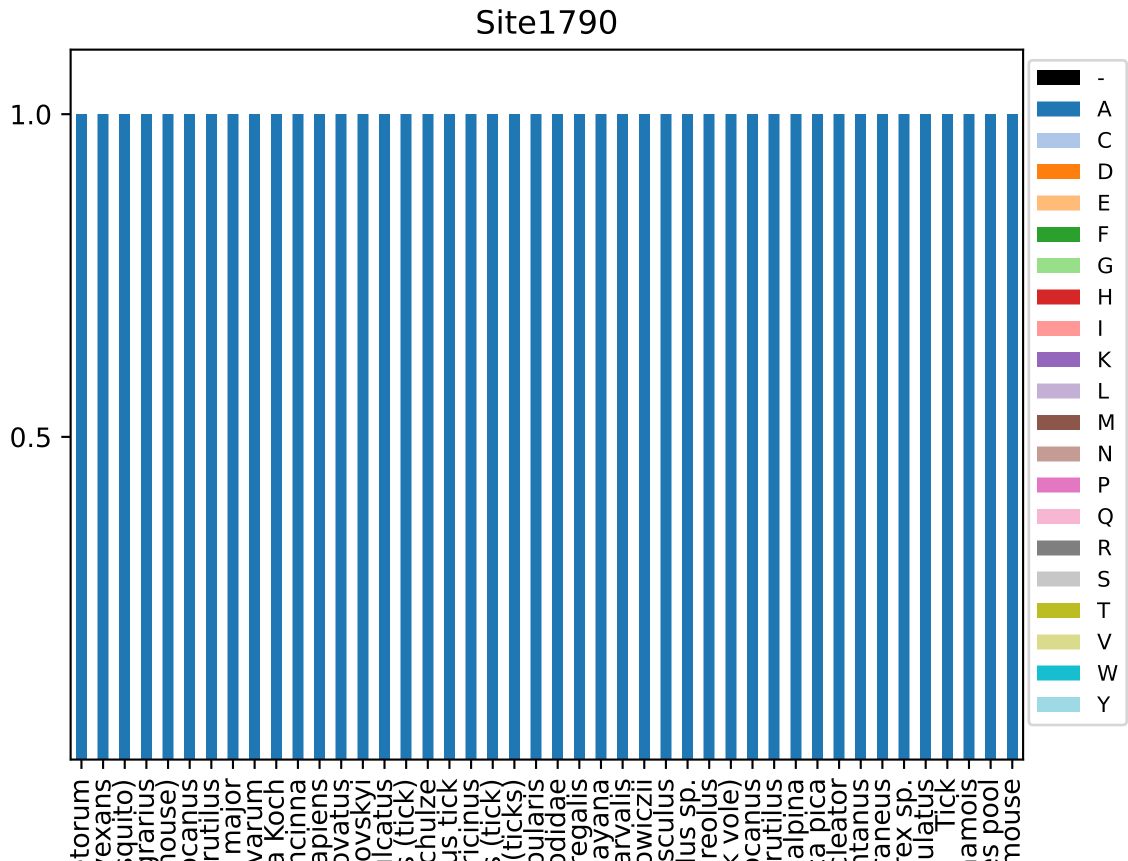
Site1790
1.0
0.5
-
A
C
D
E
F
G
H
I
K
L
M
N
P
Q
R
S
T
V
W
Y

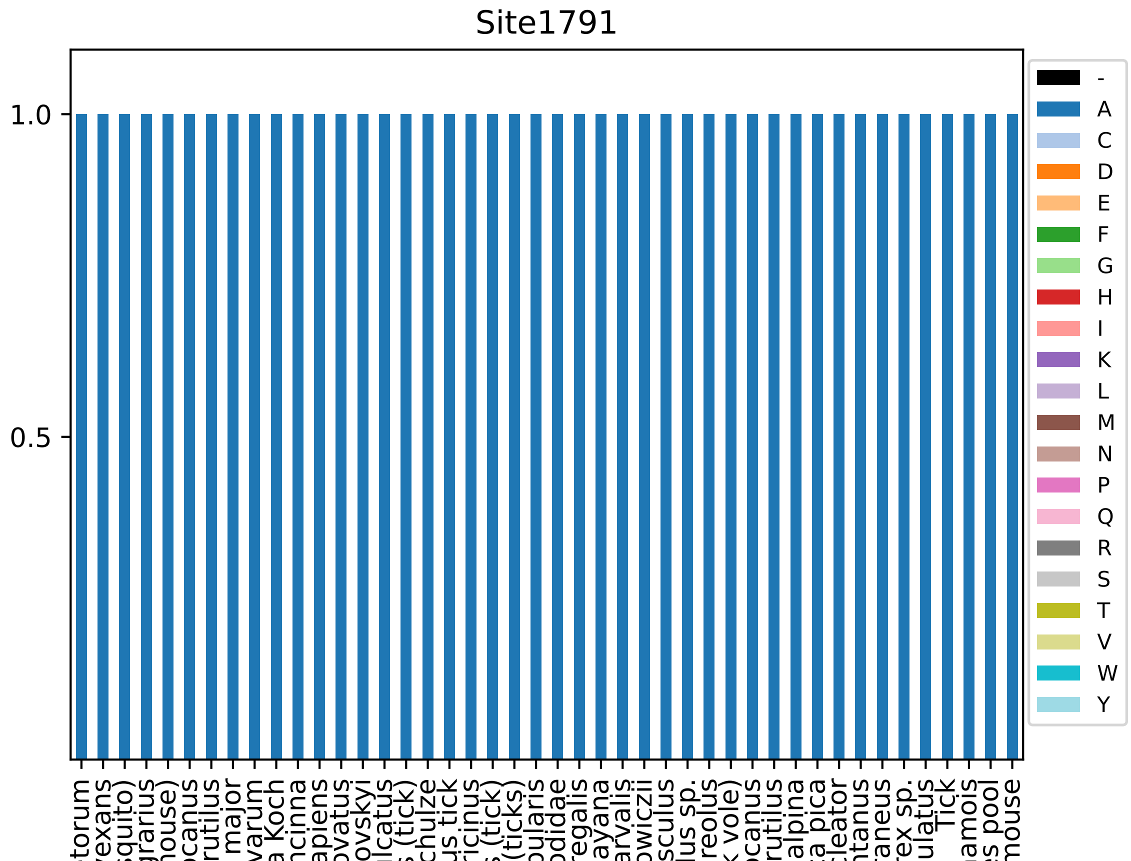
Site1791
1.0
0.5
-
A
C
D
E
F
G
H
I
K
L
M
N
P
Q
R
S
T
V
W
Y

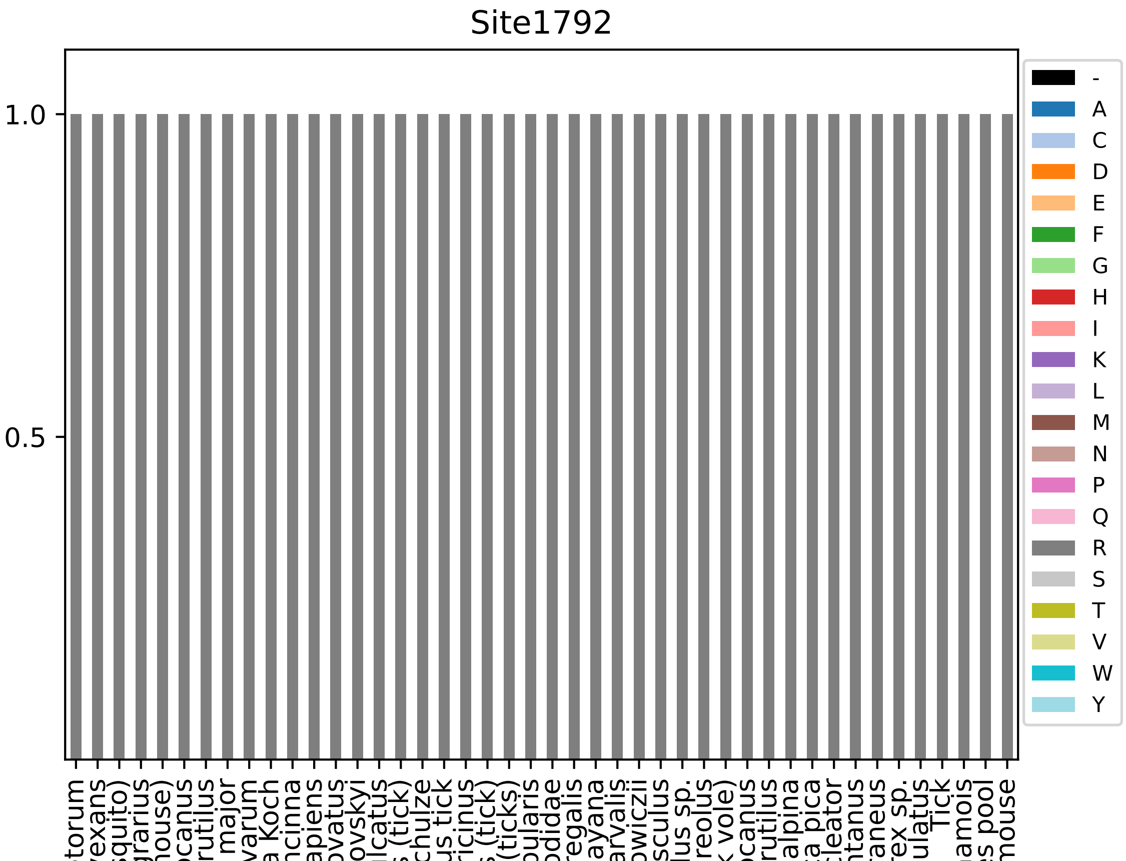
Site1792
1.0
0.5
-
A
C
D
E
F
G
H
I
K
L
M
N
P
Q
R
S
T
V
W
Y

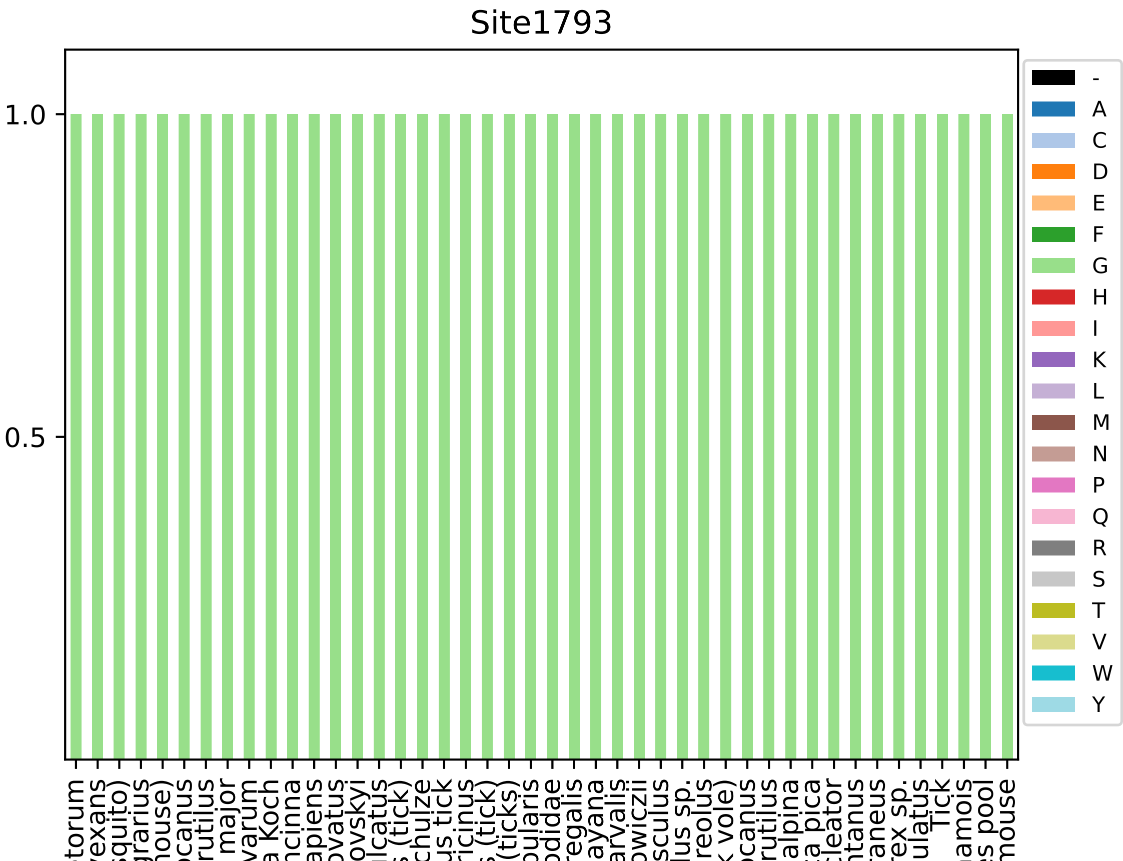
Site1793
1.0
0.5
-
A
C
D
E
F
G
H
I
K
L
M
N
P
Q
R
S
T
V
W
Y

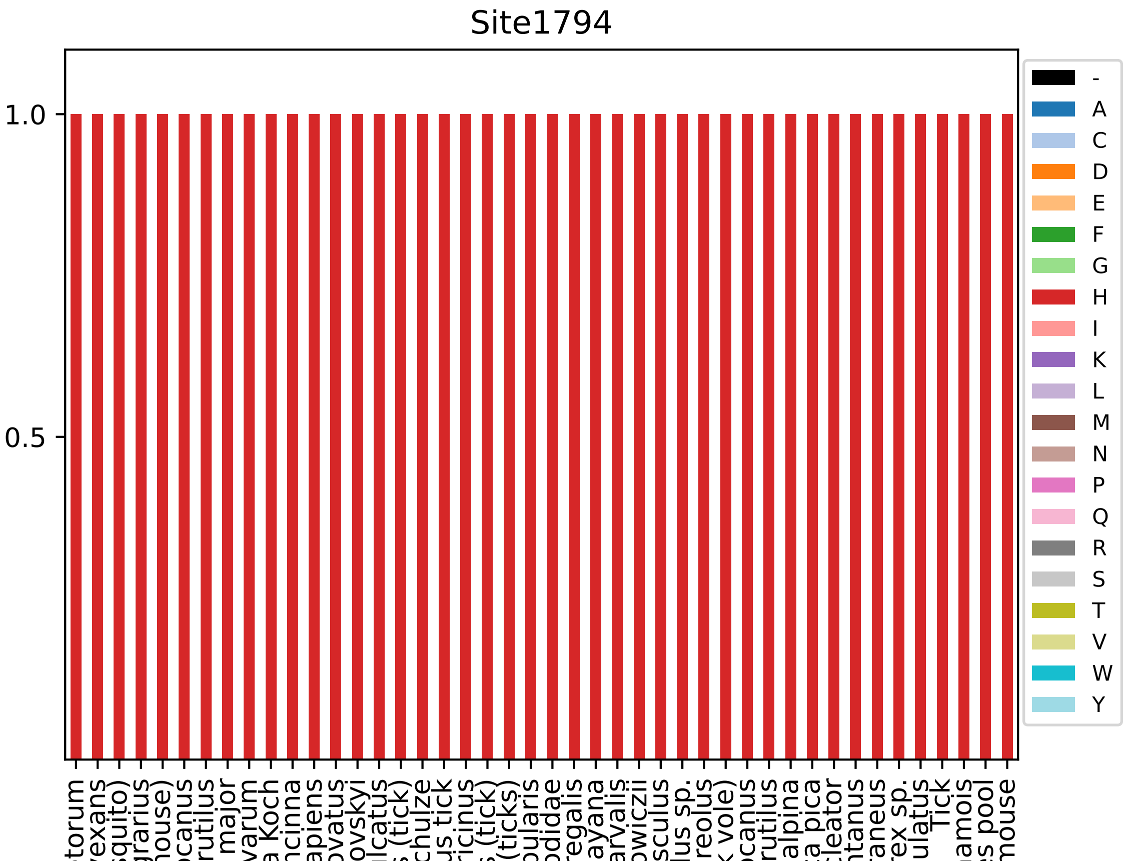

Site1794
1.0
0.5
-
A
C
D
E
F
G
H
I
K
L
M
N
P
Q
R
S
T
V
W
Y

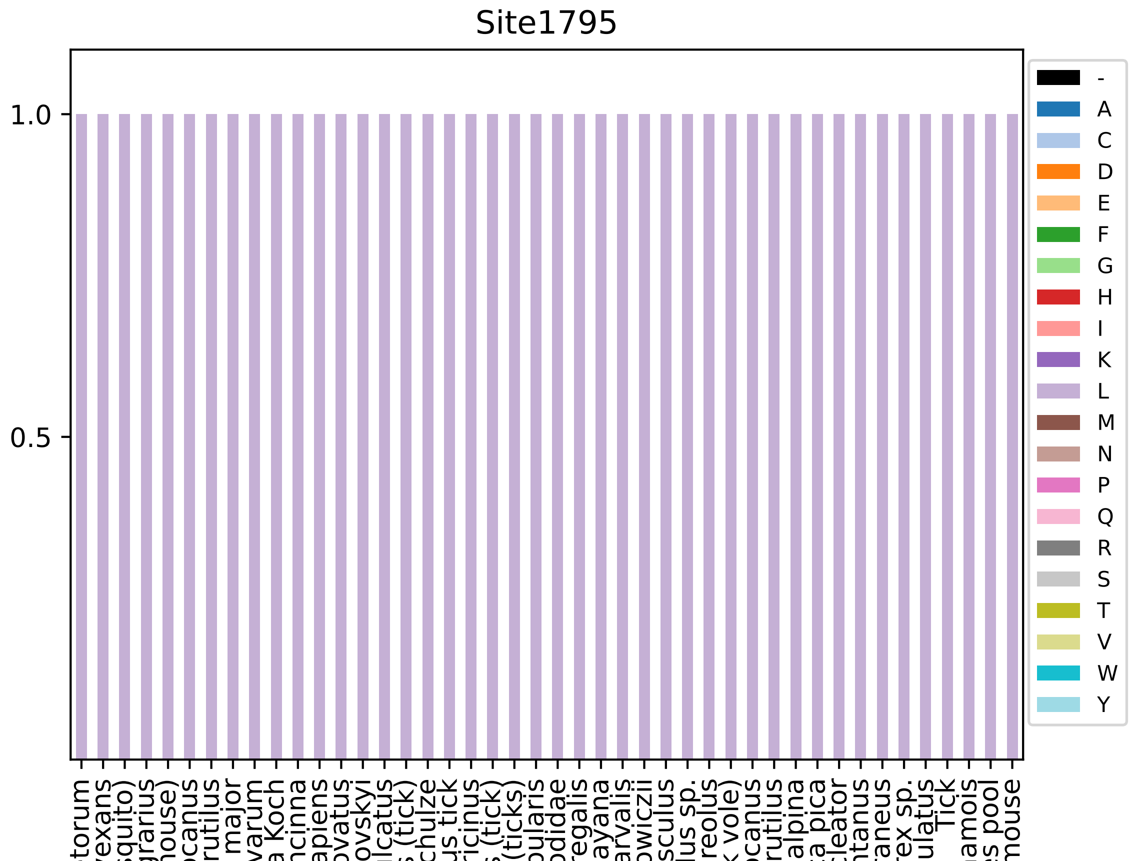
Site1795
1.0
0.5
-
A
C
D
E
F
G
H
I
K
L
M
N
P
Q
R
S
T
V
W
Y

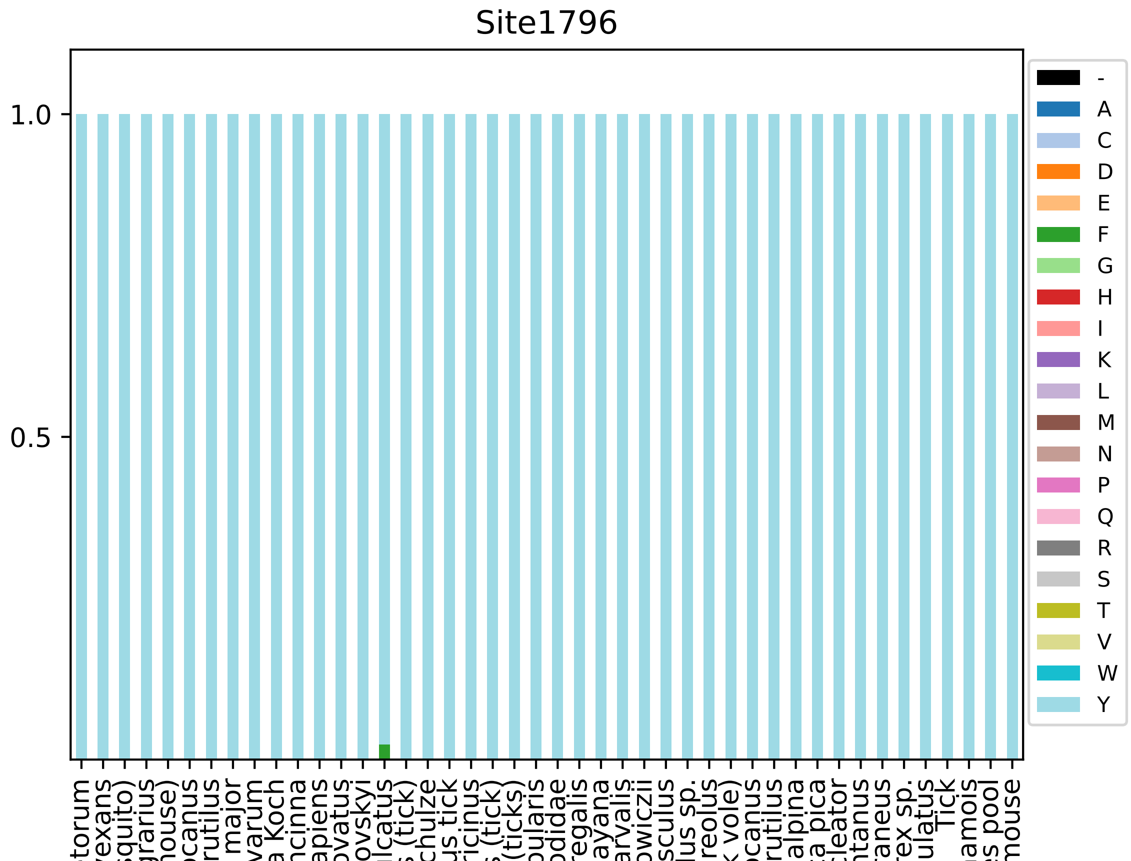
Site1796
1.0
0.5
-
A
C
D
E
F
G
H
I
K
L
M
N
P
Q
R
S
T
V
W
Y
Tick

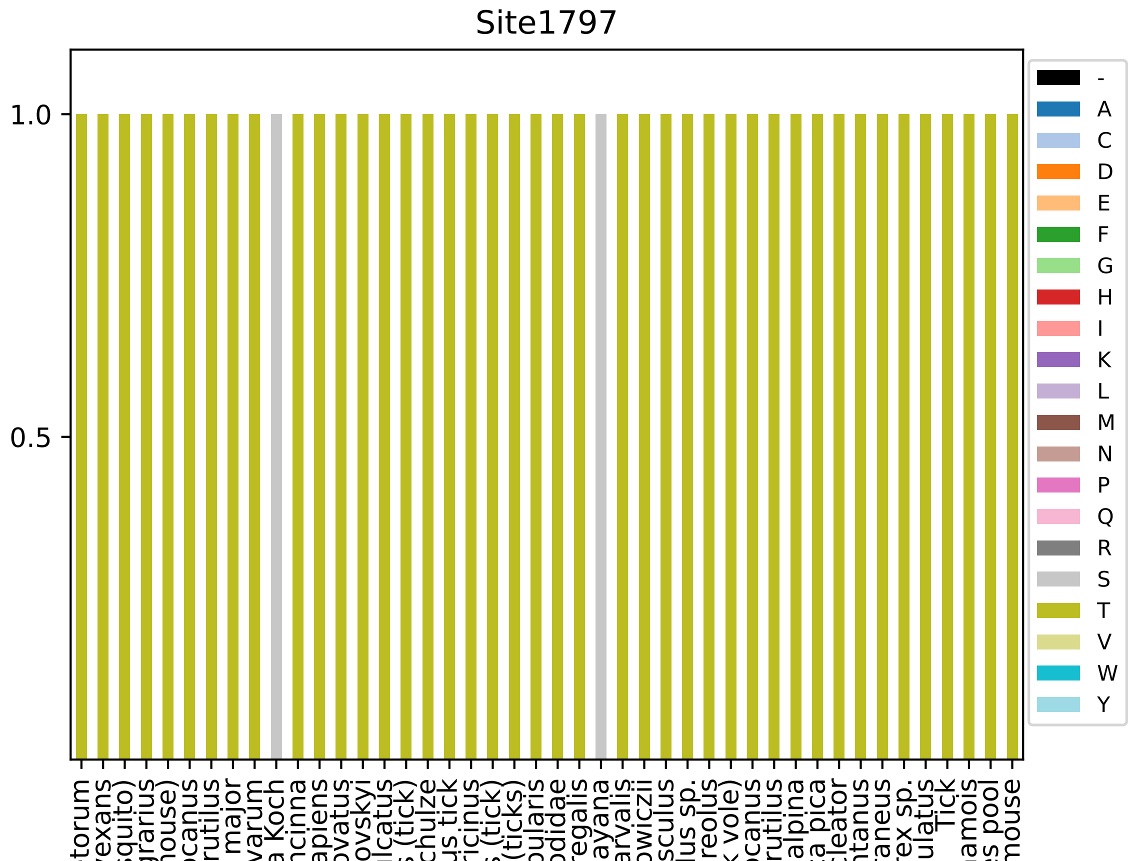

Site1797
1.0
0.5
-
A
C
D
E
F
G
H
I
K
L
M
N
P
Q
R
S
T
V
W
Y

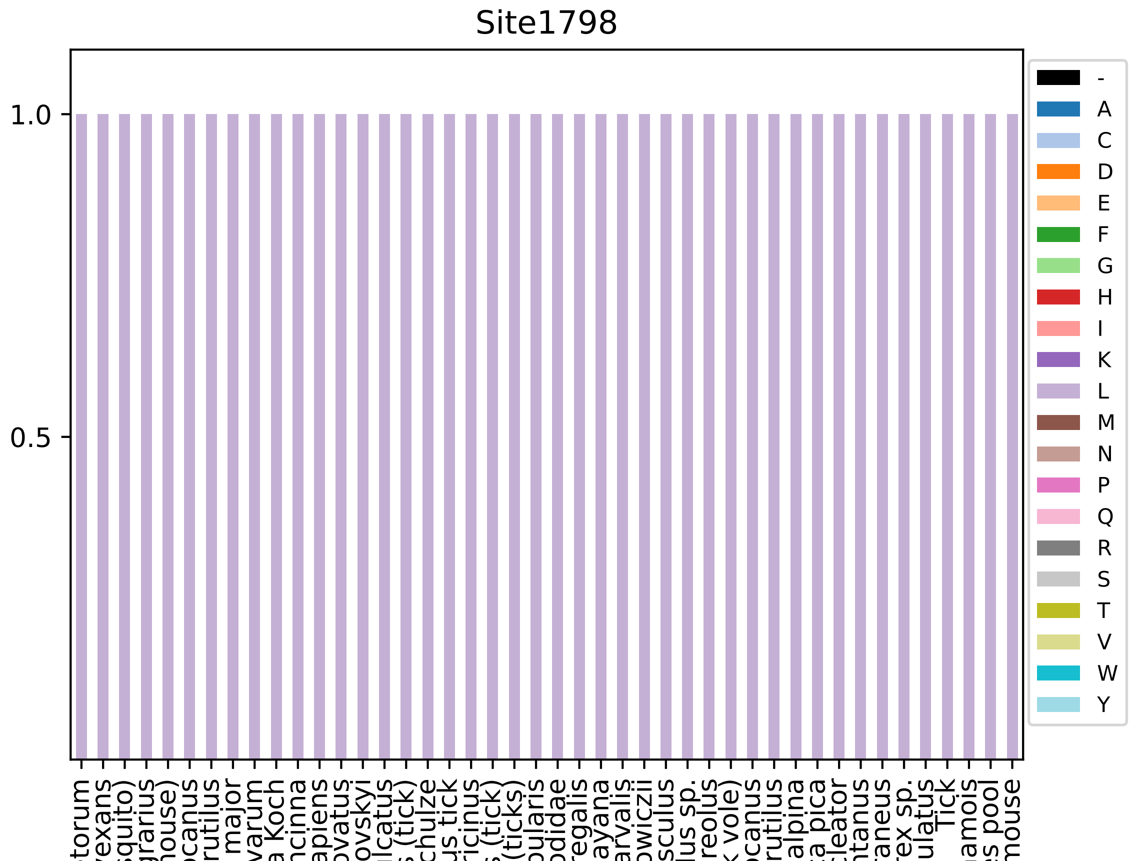
Site1798
1.0
0.5
-
A
C
D
E
F
G
H
I
K
L
M
N
P
Q
R
S
T
V
W
Y

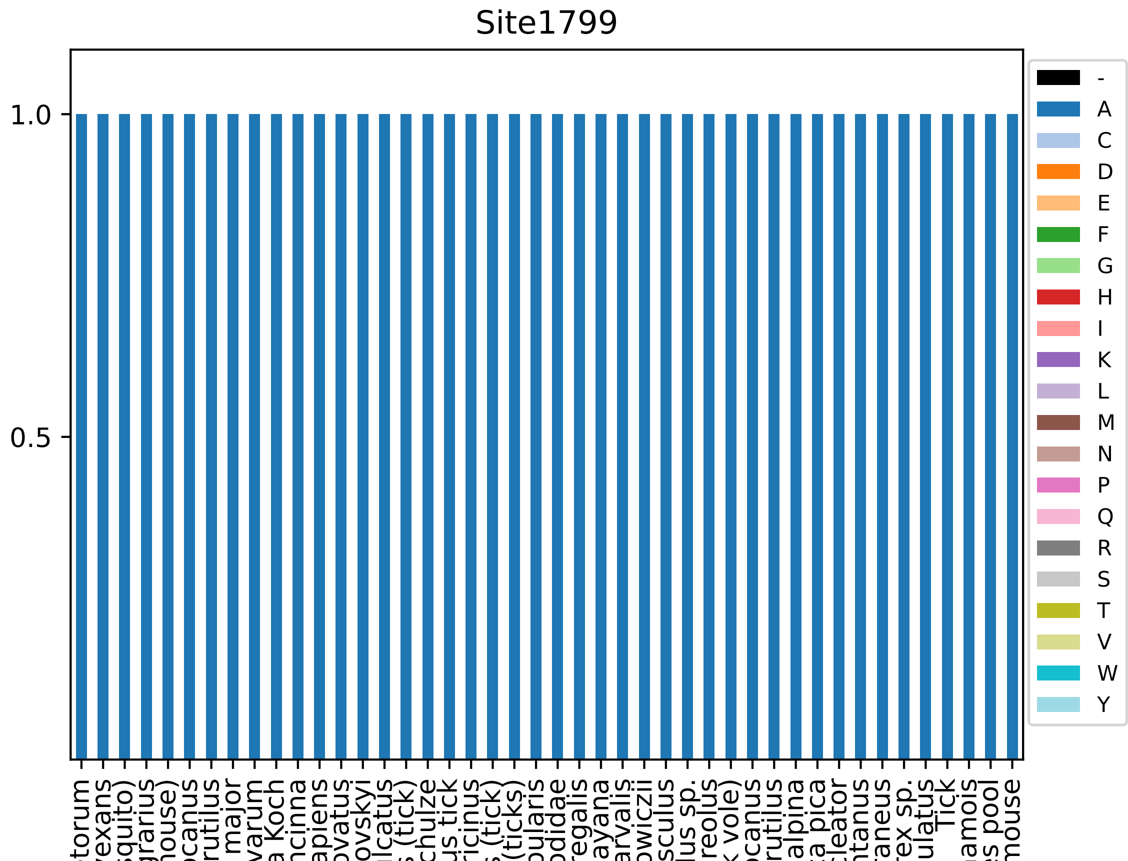
Site1799
1.0
0.5
-
A
C
D
E
F
G
H
I
K
L
M
N
P
Q
R
S
T
V
W
Y

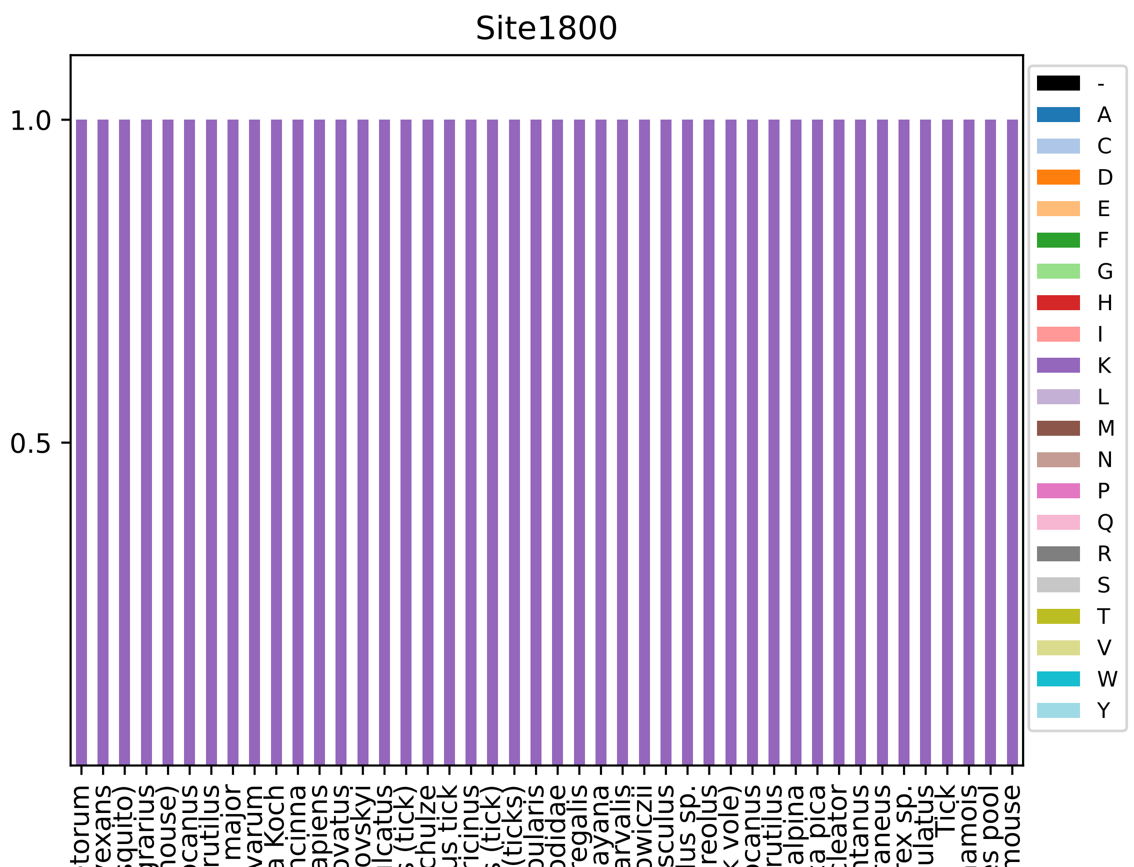
Site1800
1.0
0.5
-
A
C
D
E
F
G
H
I
K
L
M
N
P
Q
R
S
T
V
W
Y
Tick

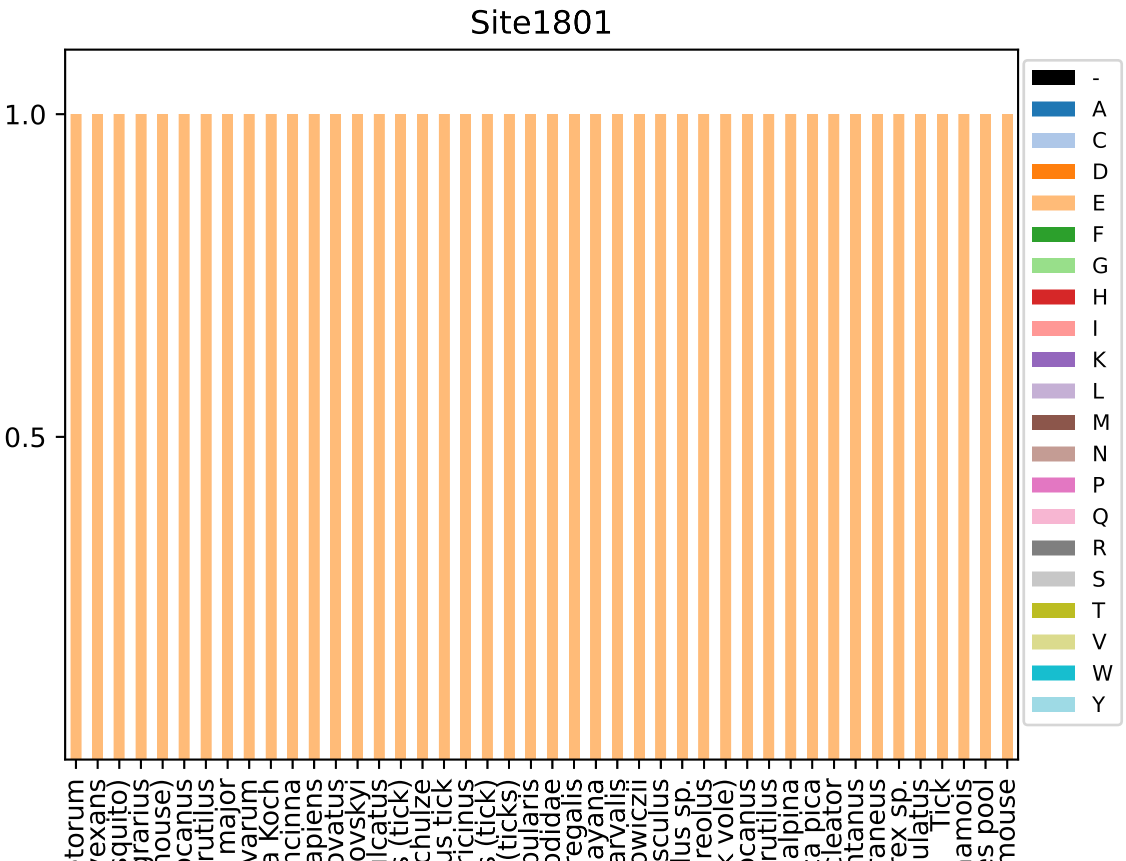

Site1801
1.0
0.5
-
A
C
D
E
F
G
H
I
K
L
M
N
P
Q
R
S
T
V
W
Y

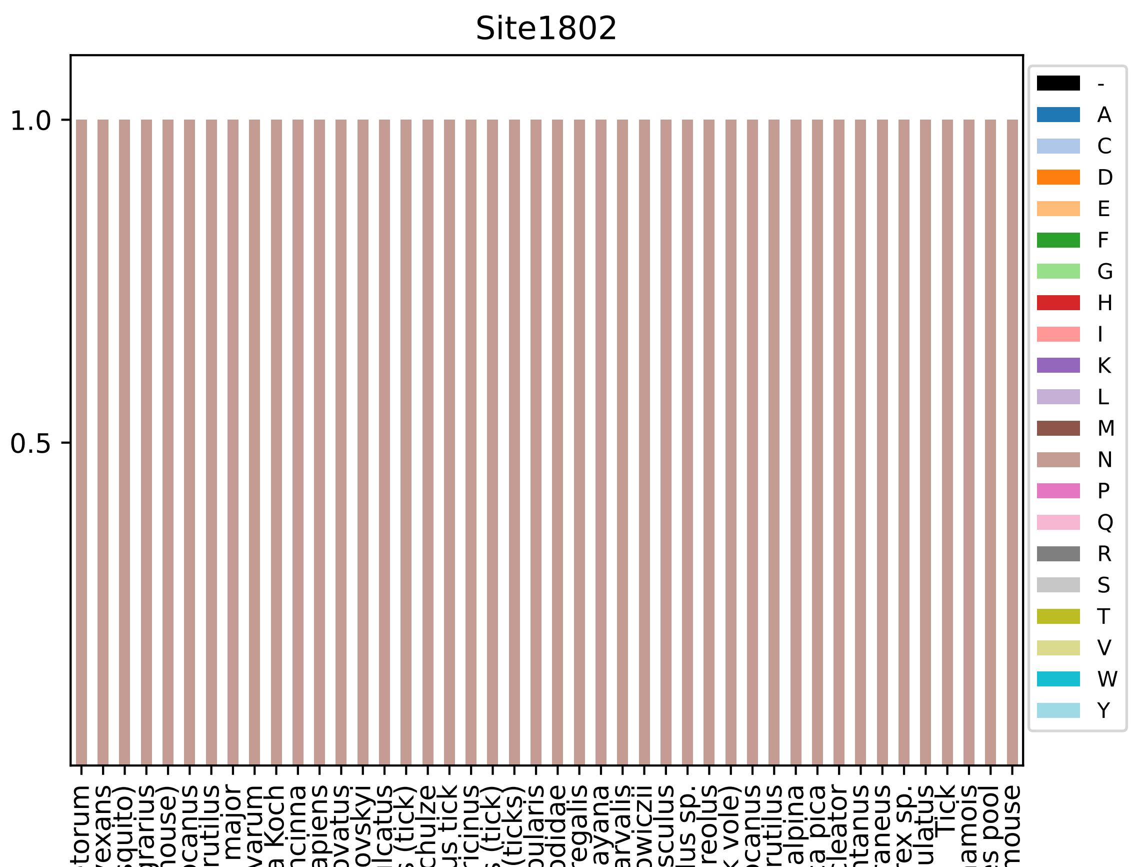
Site1802
1.0
0.5
-
A
C
D
E
F
G
H
I
K
L
M
N
P
Q
R
S
T
V
W
Y
Tick

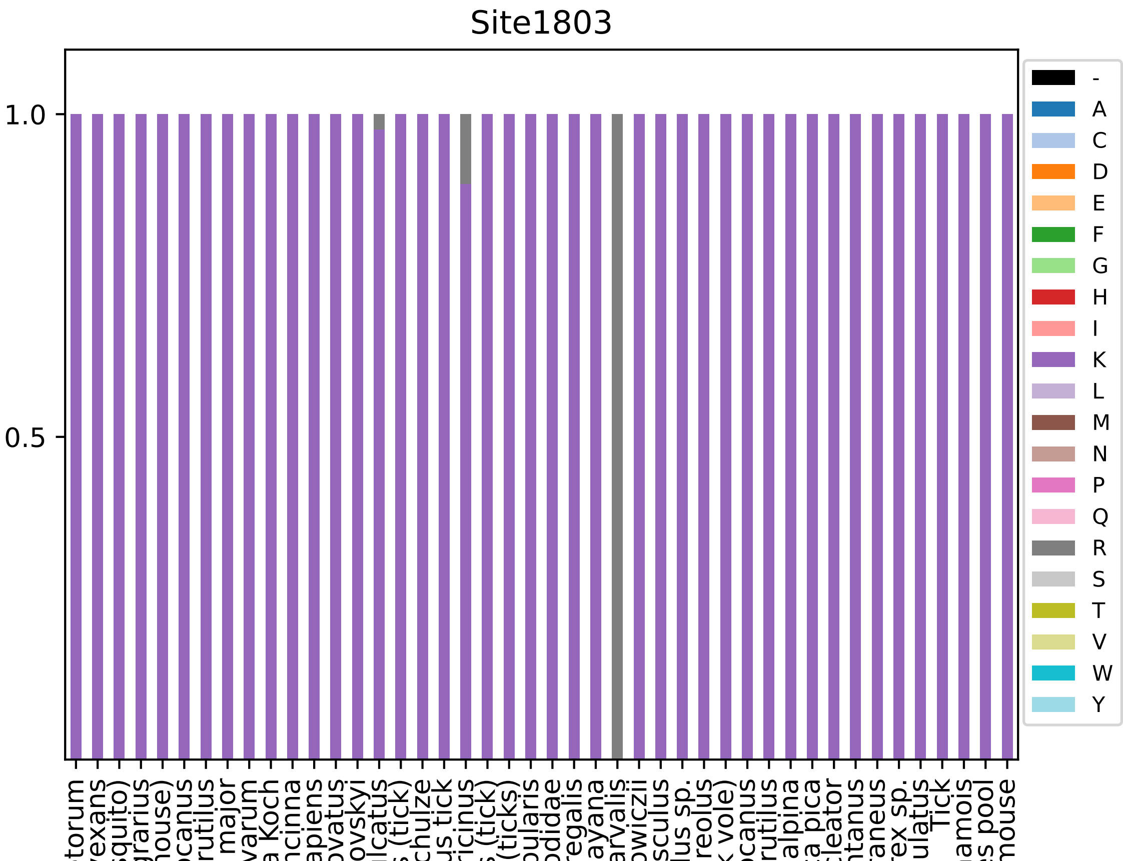

Site1803
1.0
0.5
-
A
C
D
E
F
G
H
I
K
L
M
N
P
Q
R
S
T
V
W
Y
torum
vexans
squito)
grarius
nouse)
ocanus
rutilus
major
varum
a Koch
ncinna
apiens
ovatus
ovskyi
ulcatus
s (tick)
chulze
us tick
ricinus
s (tick)
(ticks)
pularis
podidae
regalis
ayana
arvalis
owiczii
isculus
lus sp.
reolus
k vole)
ocanus
rutilus
alpina
ca pica
cleator
ntanus
raneus
rex sp.
ulatus
Tick
amois
es pool
mouse

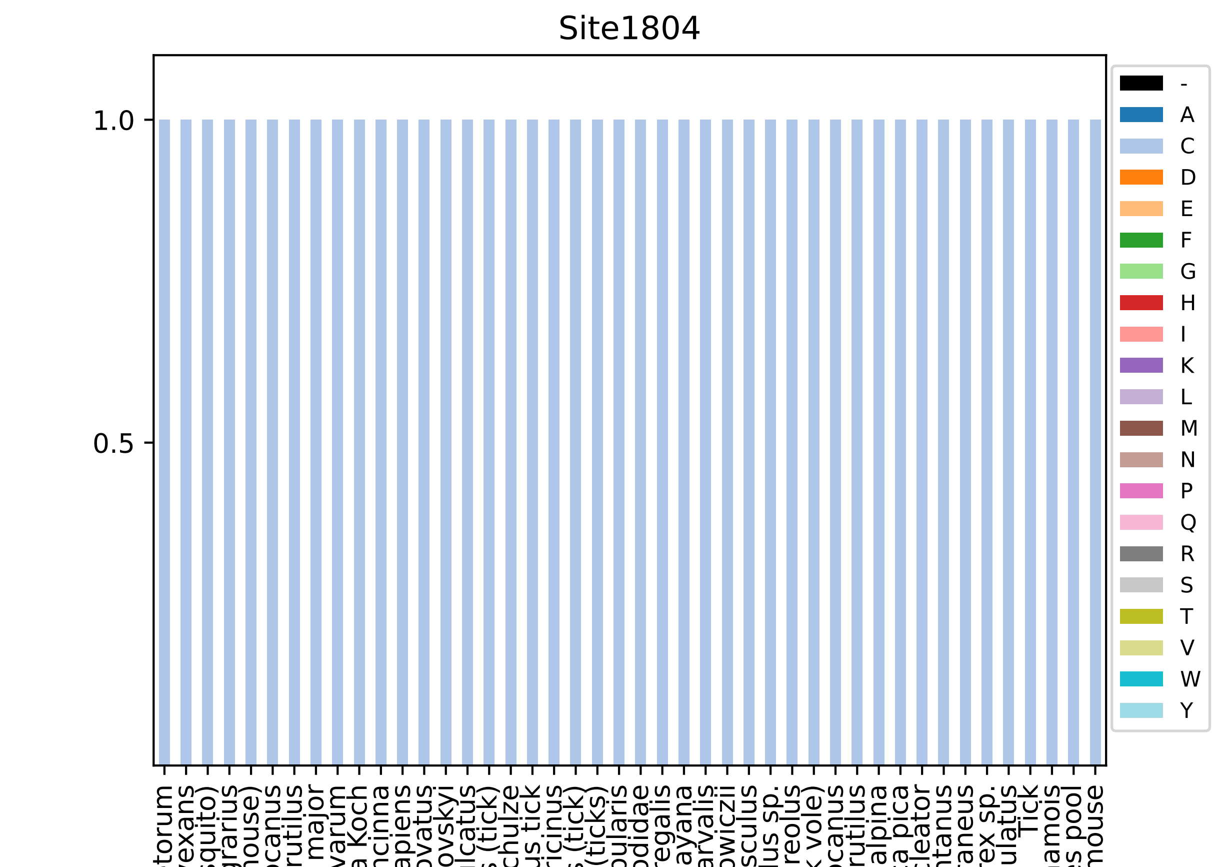
Site1804
1.0
0.5
-
A
C
D
E
F
G
H
I
K
L
M
N
P
Q
R
S
T
V
W
Y

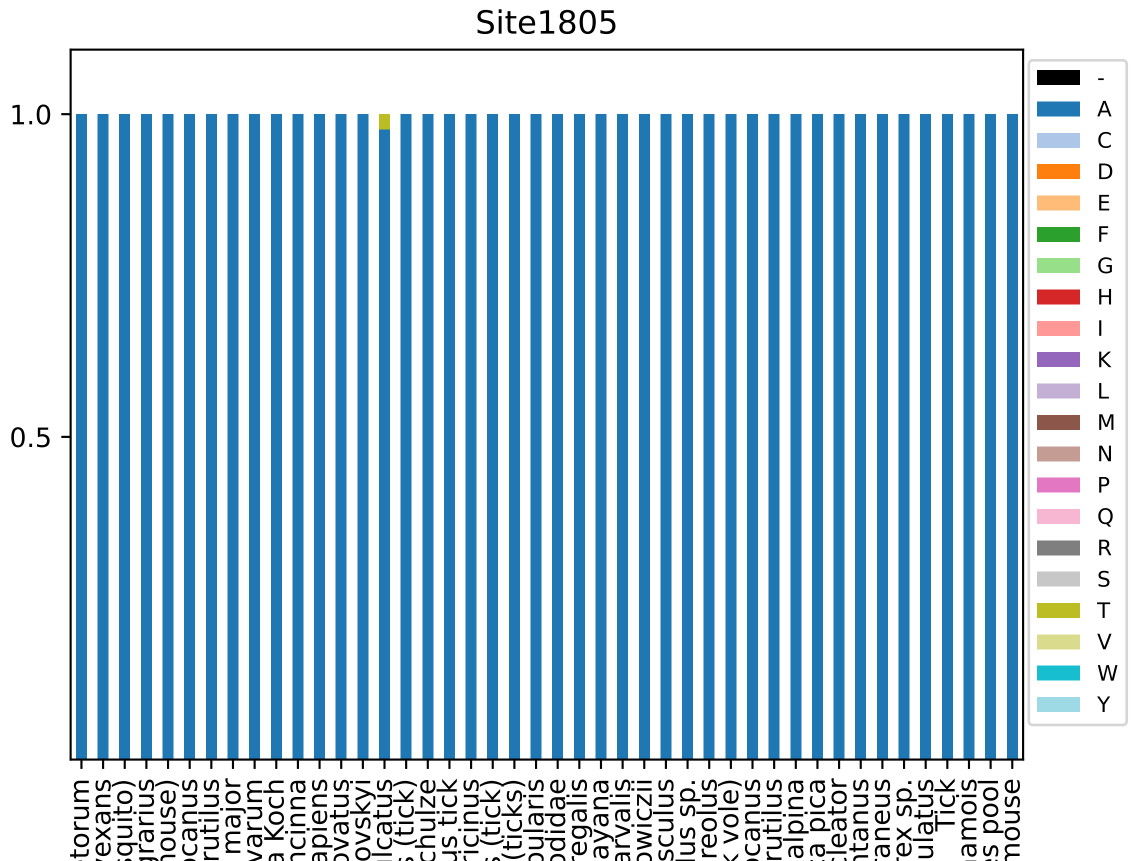
Site1805
1.0
0.5
-
A
C
D
E
F
G
H
I
K
L
M
N
P
Q
R
S
T
V
W
Y

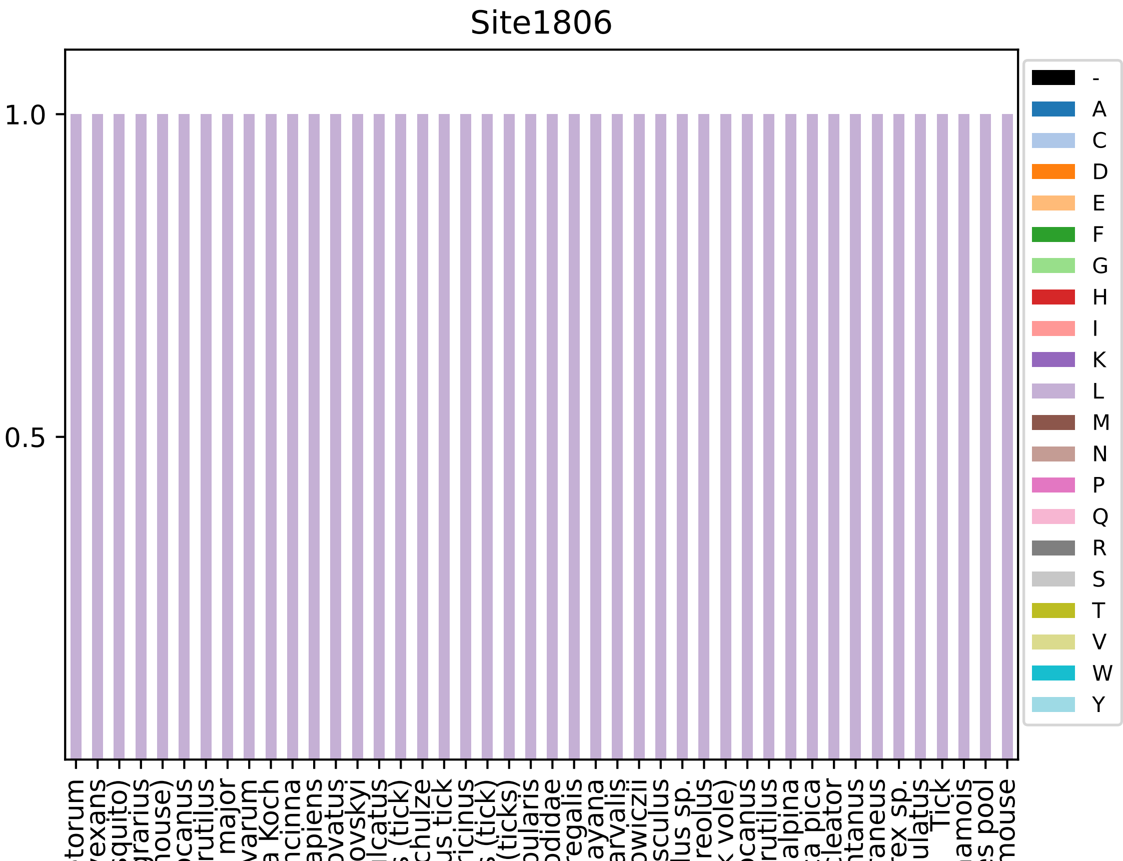

Site1806
1.0
0.5
-
A
C
D
E
F
G
H
I
K
L
M
N
P
Q
R
S
T
V
W
Y

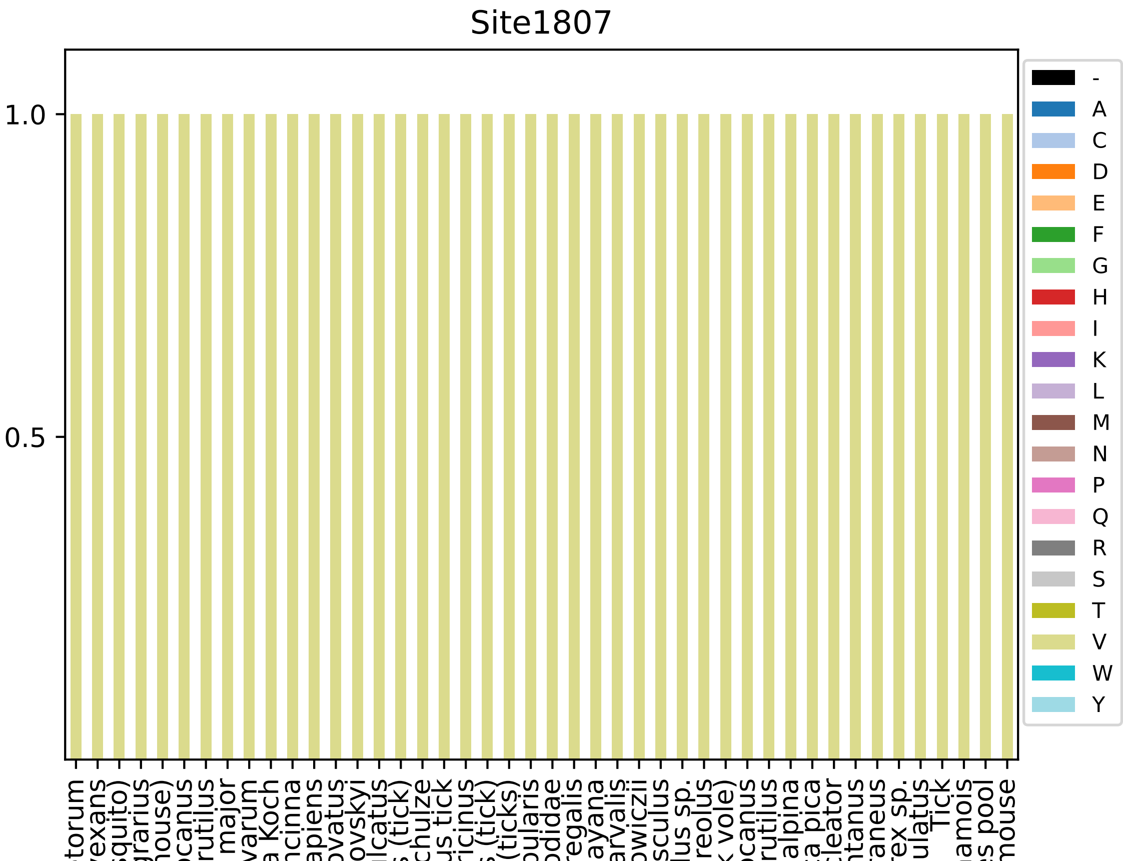
Site1807
1.0
0.5
-
A
C
D
E
F
G
H
I
K
L
M
N
P
Q
R
S
T
V
W
Y

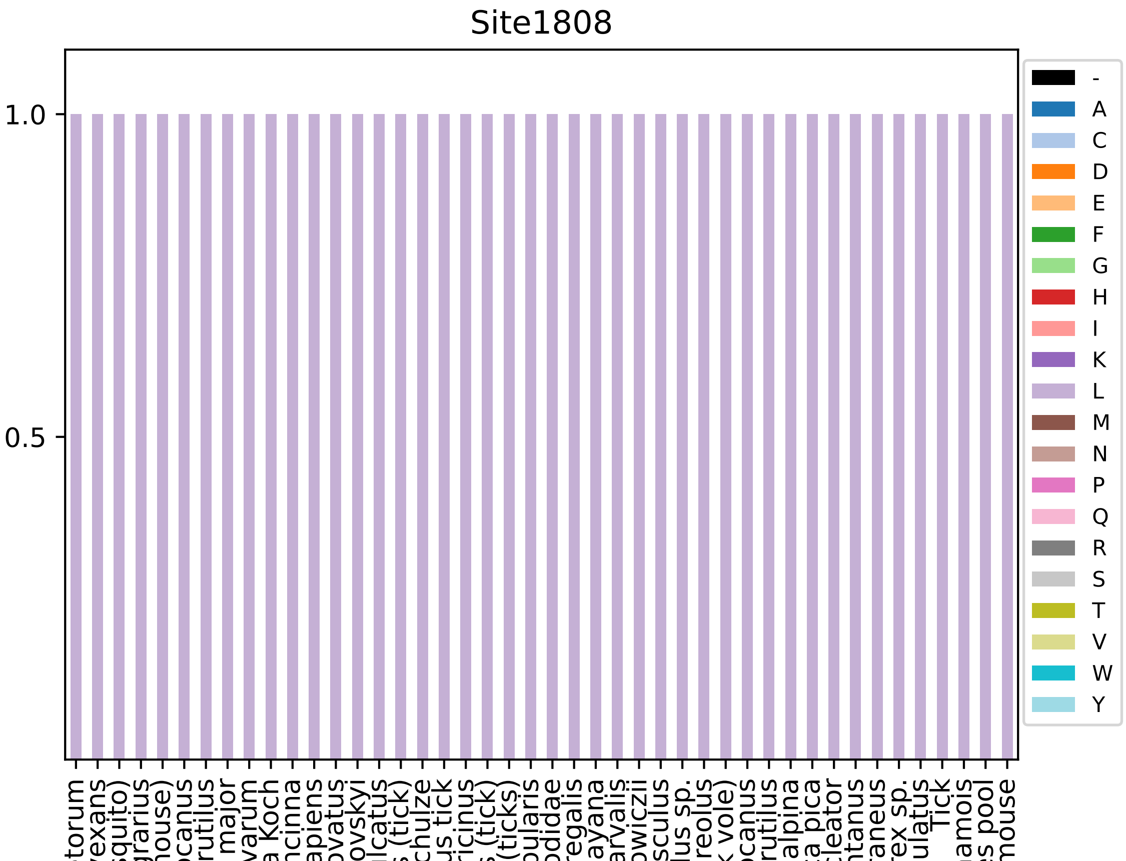

Site1808
1.0
0.5
-
A
C
D
E
F
G
H
I
K
L
M
N
P
Q
R
S
T
V
W
Y

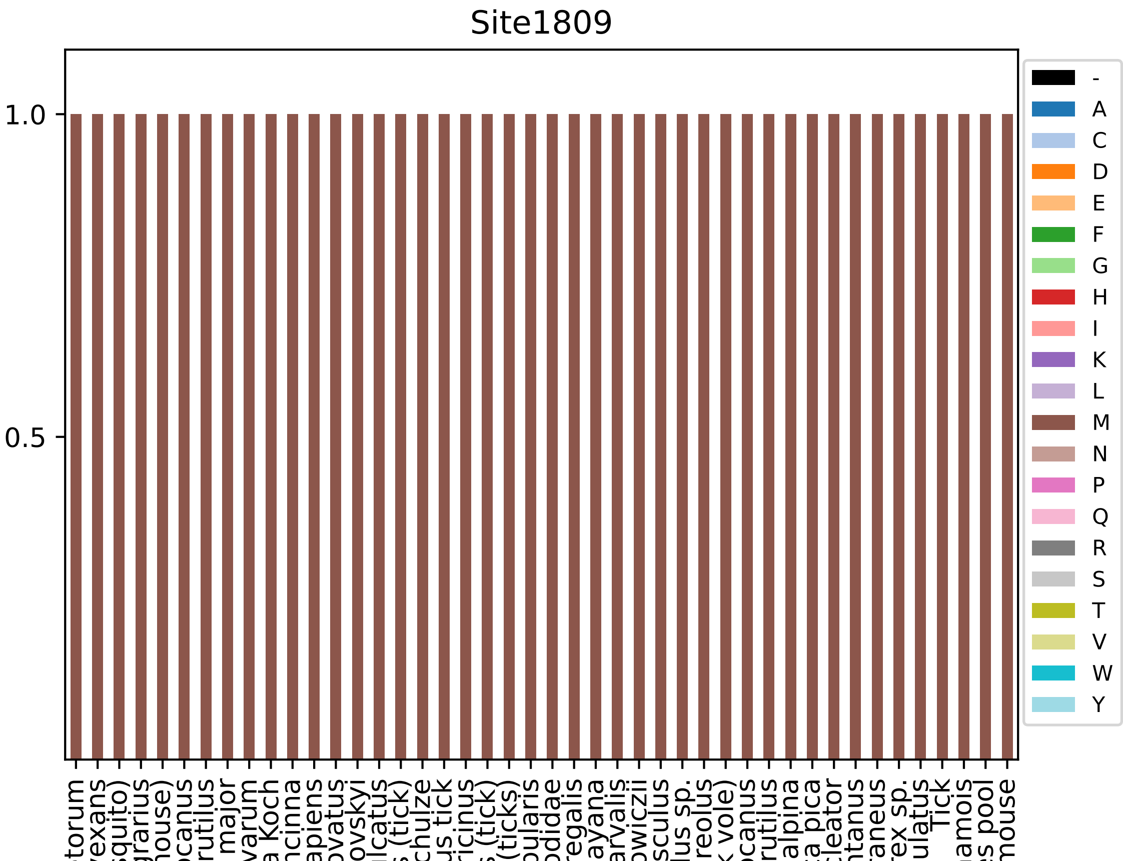
Site1809
1.0
0.5
-
A
C
D
E
F
G
H
I
K
L
M
N
P
Q
R
S
T
V
W
Y

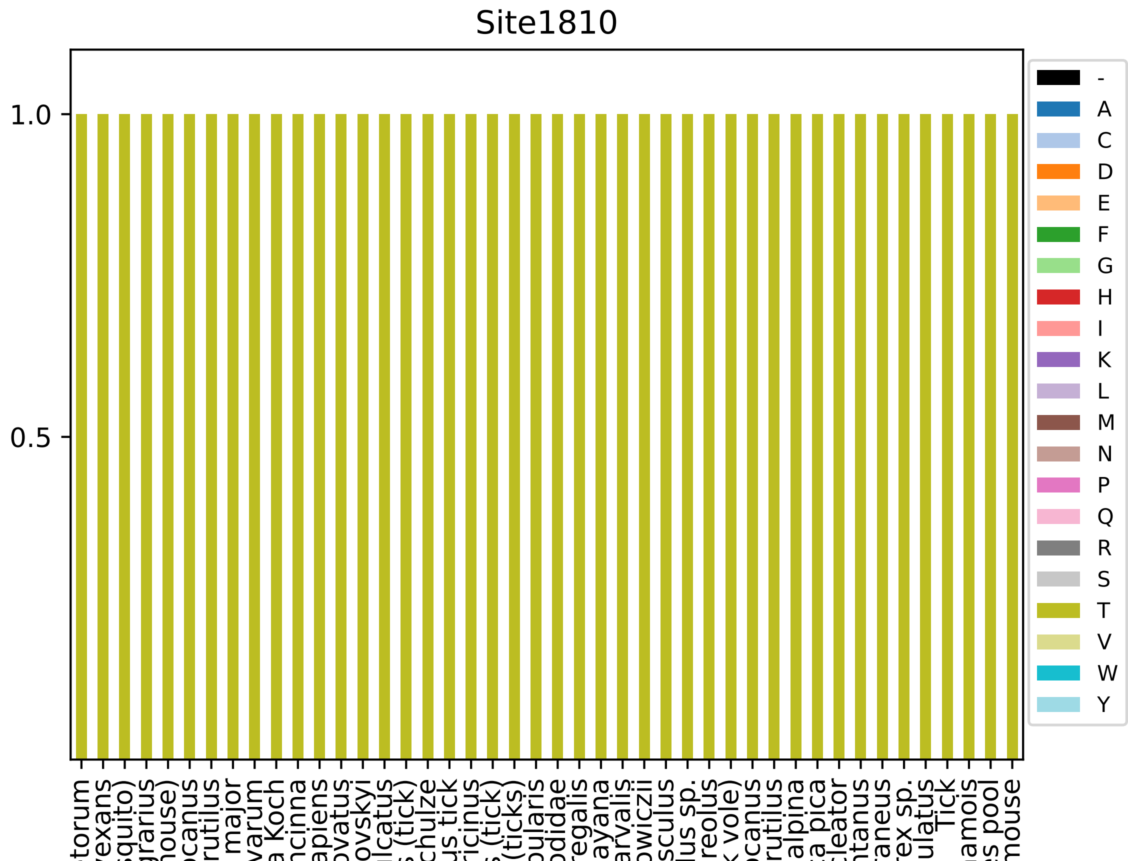
Site1810
1.0
0.5
-
A
C
D
E
F
G
H
I
K
L
M
N
P
Q
R
S
T
V
W
Y

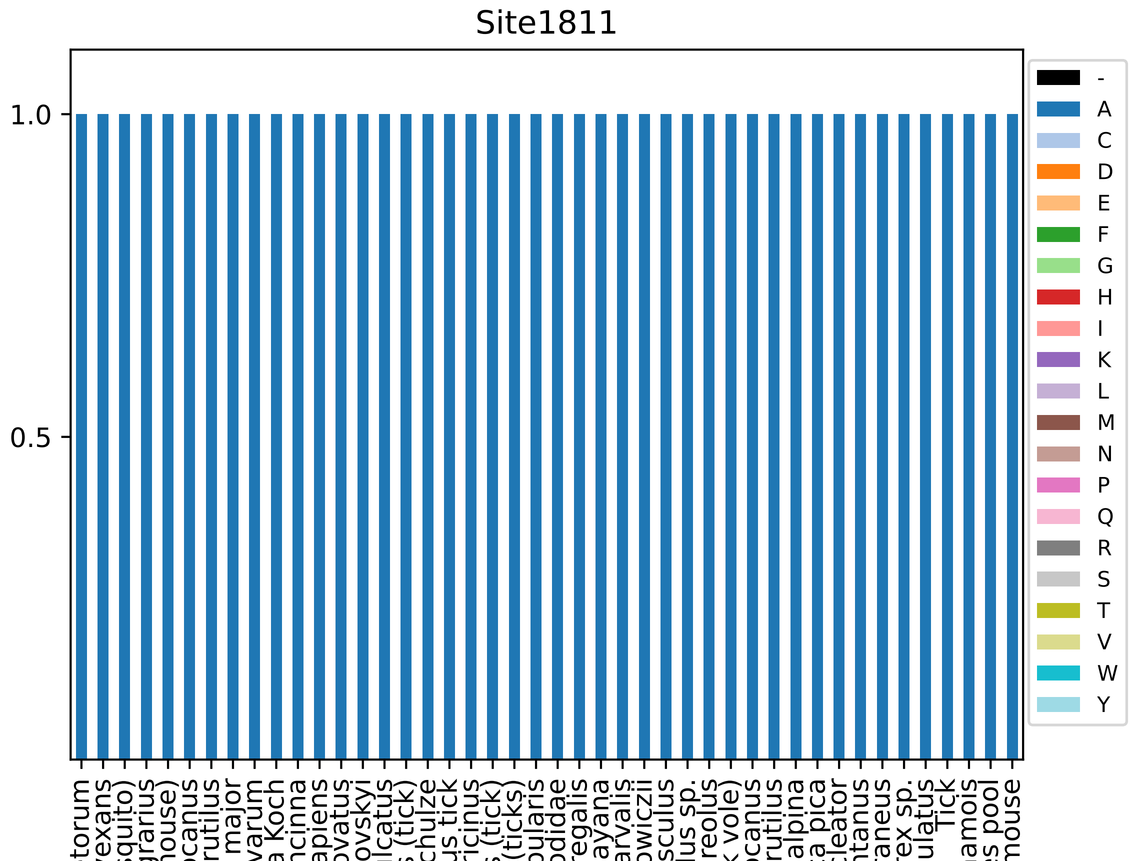
Site1811
1.0
0.5
-
A
C
D
E
F
G
H
I
K
L
M
N
P
Q
R
S
T
V
W
Y

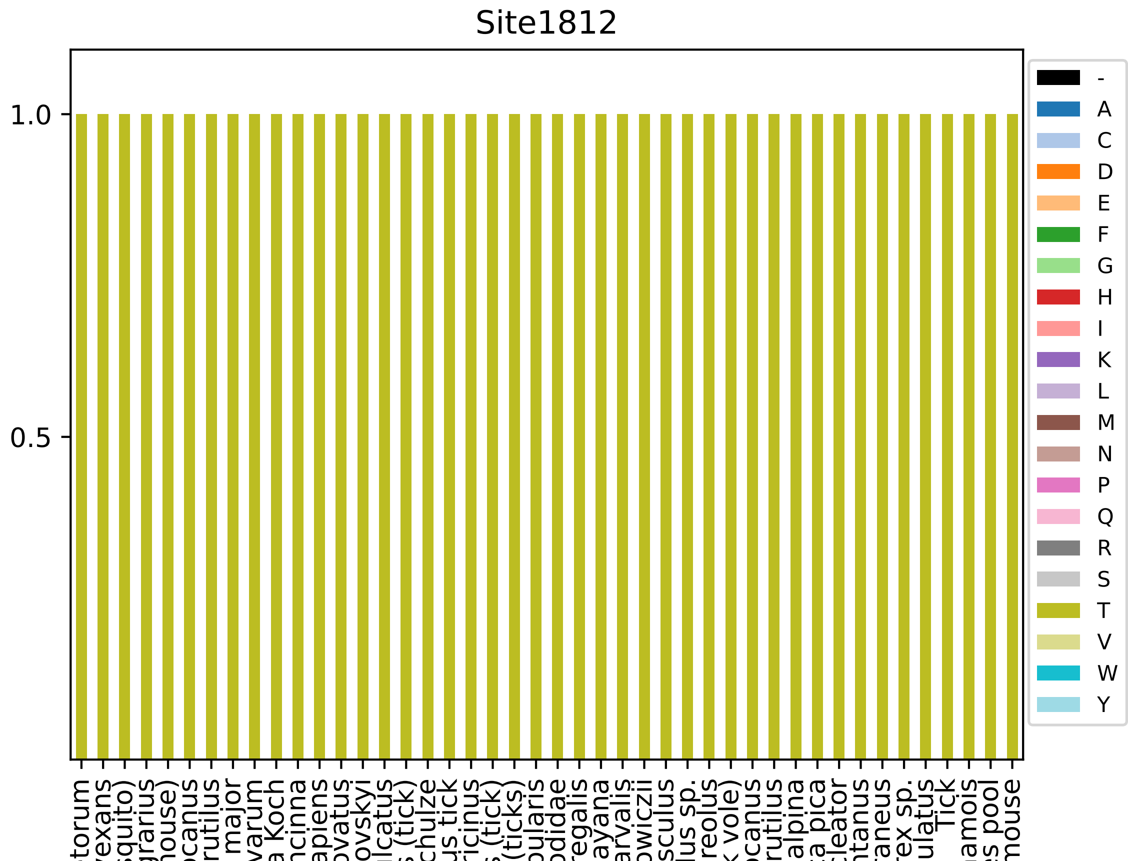
Site1812
1.0
0.5
-
A
C
D
E
F
G
H
I
K
L
M
N
P
Q
R
S
T
V
W
Y

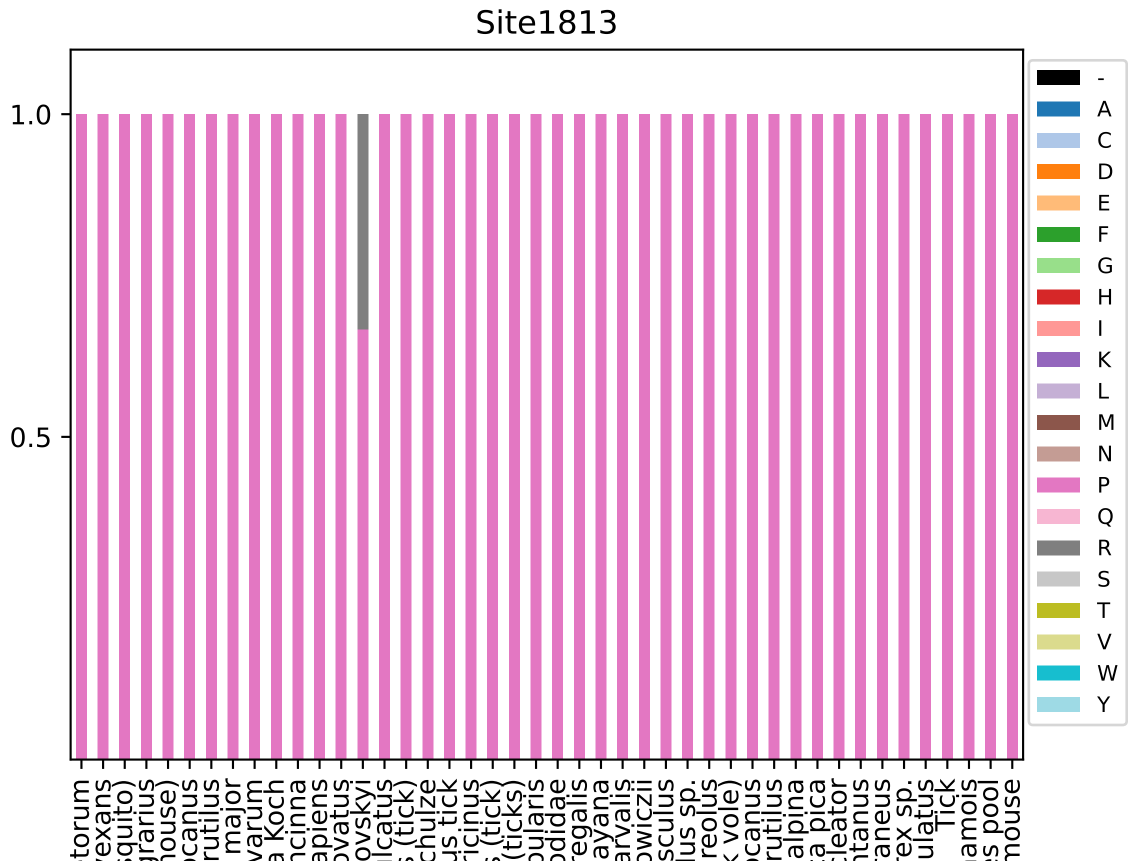
Site1813
1.0
0.5
-
A
C
D
E
F
G
H
I
K
L
M
N
P
Q
R
S
T
V
W
Y
Tick

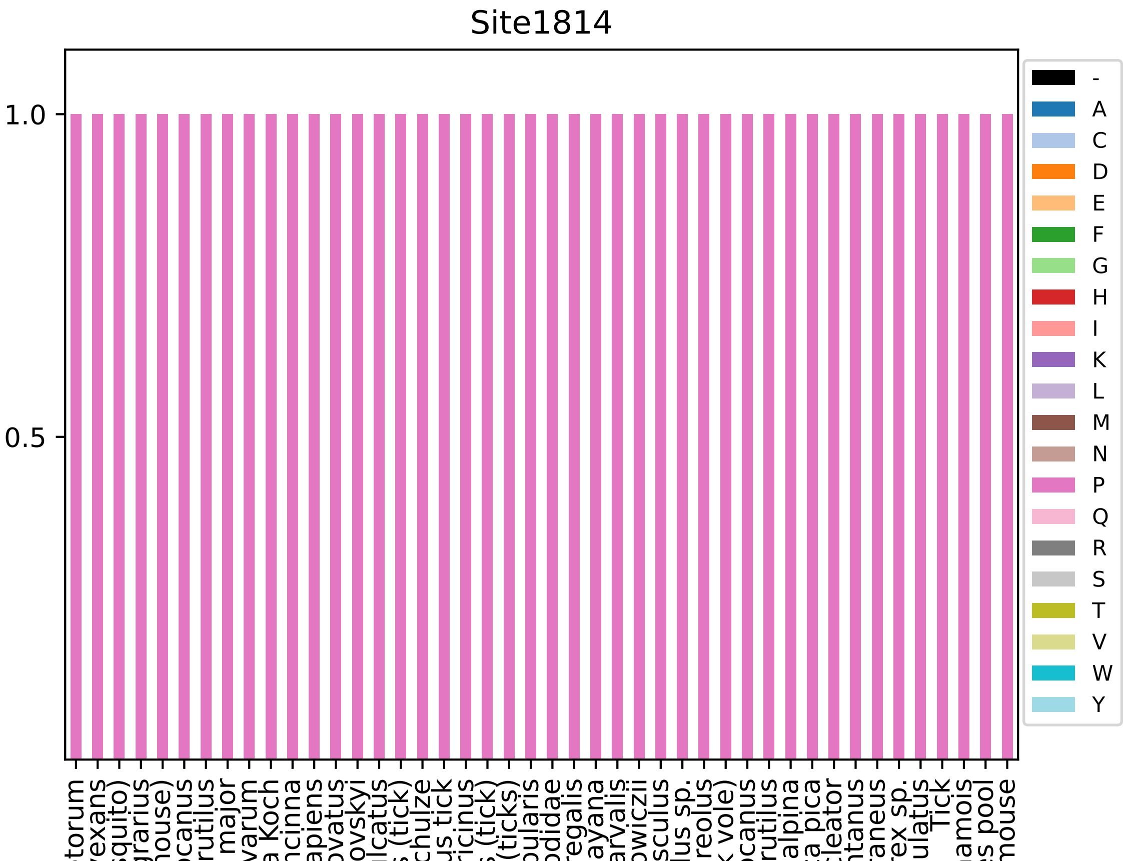
Site1814
1.0
0.5
-
A
C
D
E
F
G
H
I
K
L
M
N
P
Q
R
S
T
V
W
Y

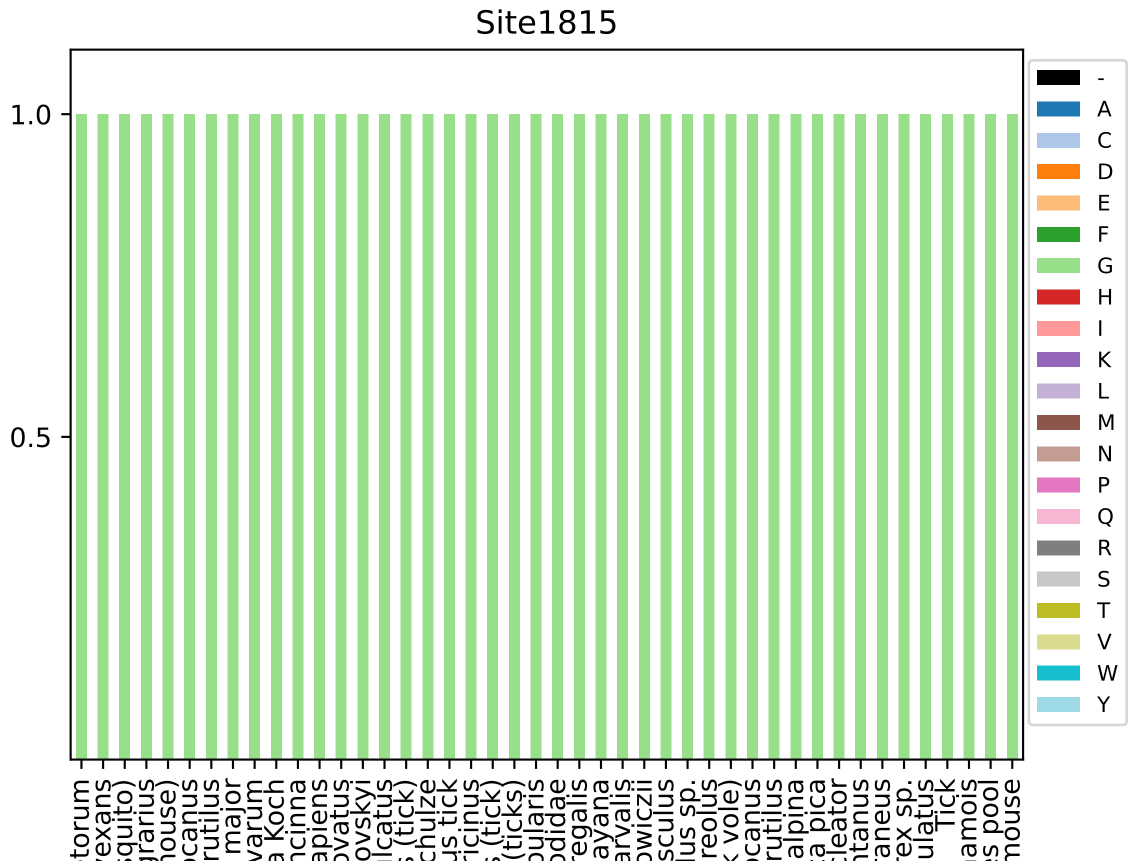

Site1815
1.0
0.5
-
A
C
D
E
F
G
H
I
K
L
M
N
P
Q
R
S
T
V
W
Y
Tick

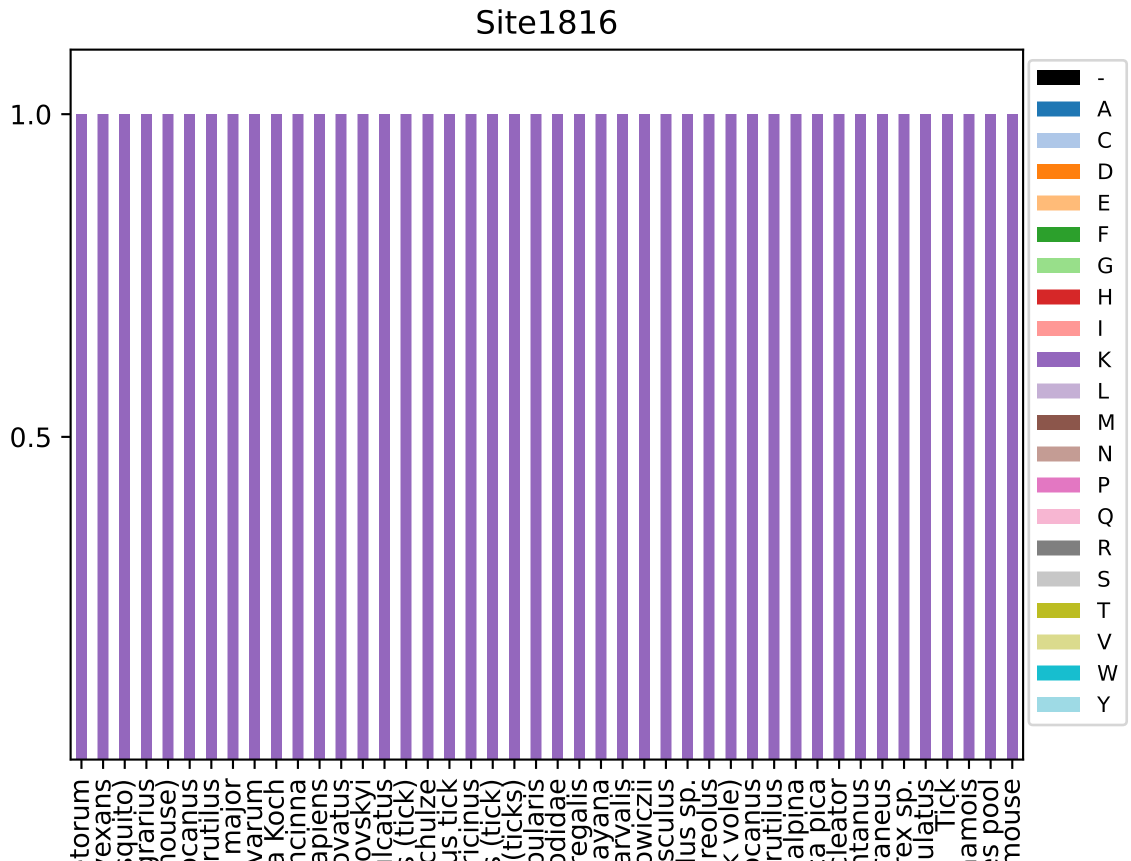

Site1816
1.0
0.5
-
A
C
D
E
F
G
H
I
K
L
M
N
P
Q
R
S
T
V
W
Y

Site1817

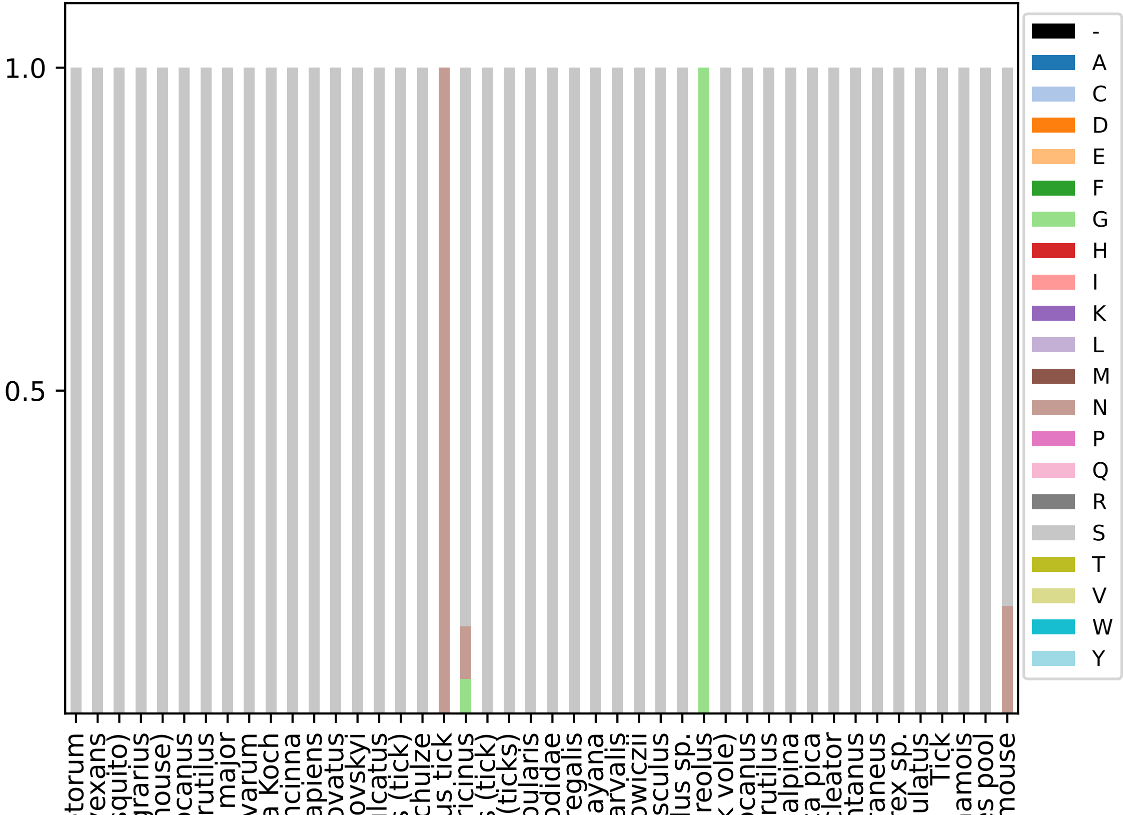

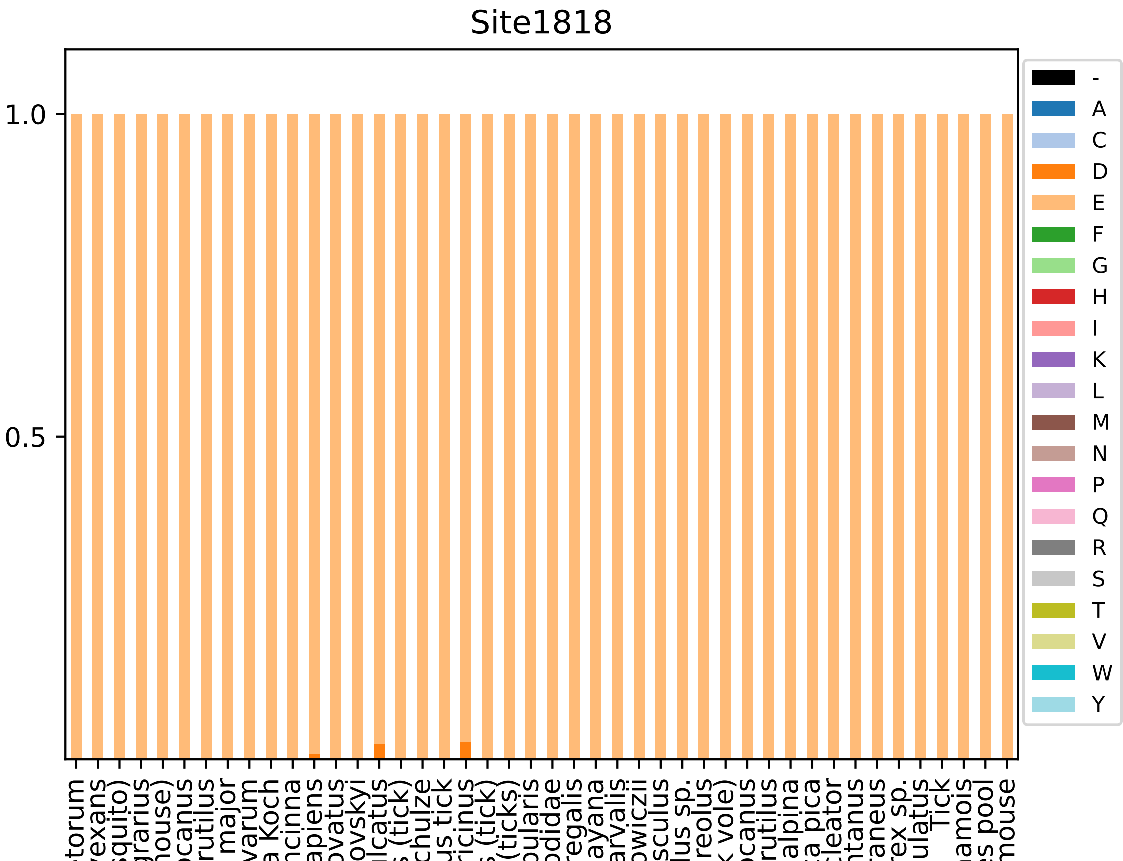
Site1818
1.0
0.5
-
A
C
D
E
F
G
H
I
K
L
M
N
P
Q
R
S
T
V
W
Y

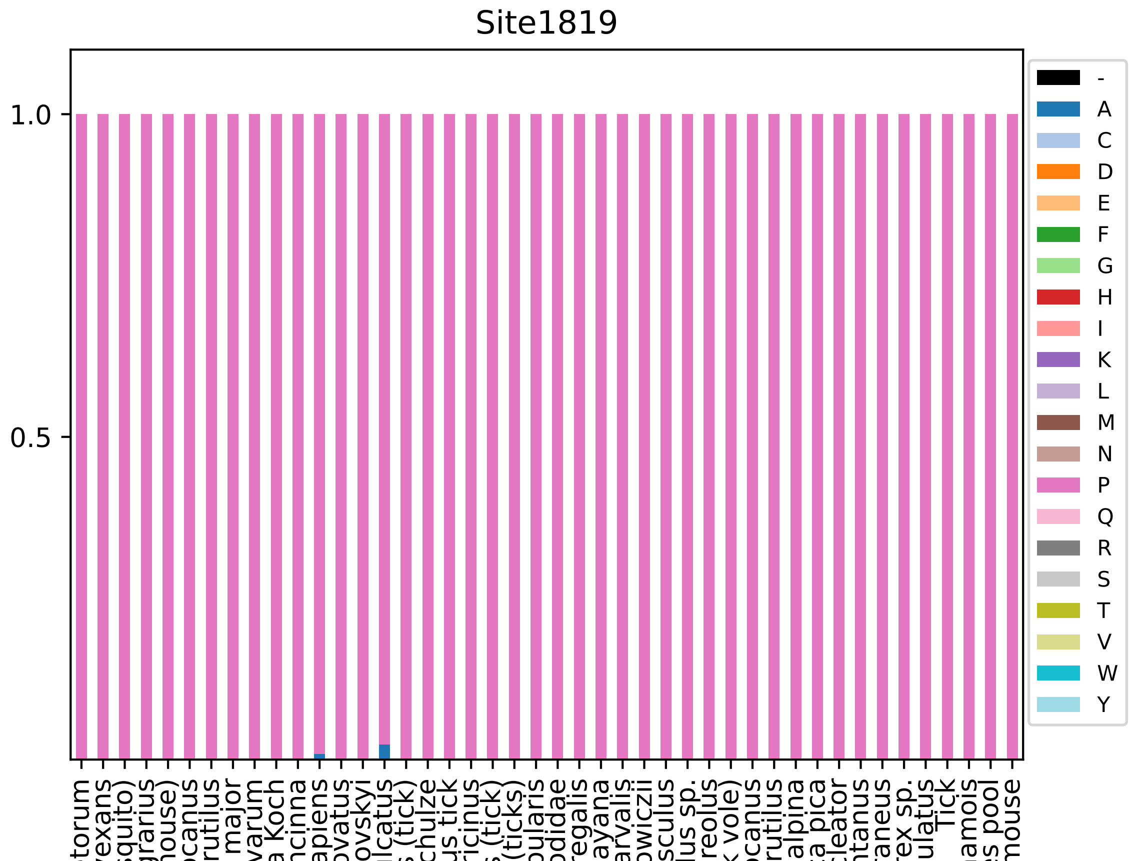
Site1819
1.0
0.5
-
A
C
D
E
F
G
H
I
K
L
M
N
P
Q
R
S
T
V
W
Y

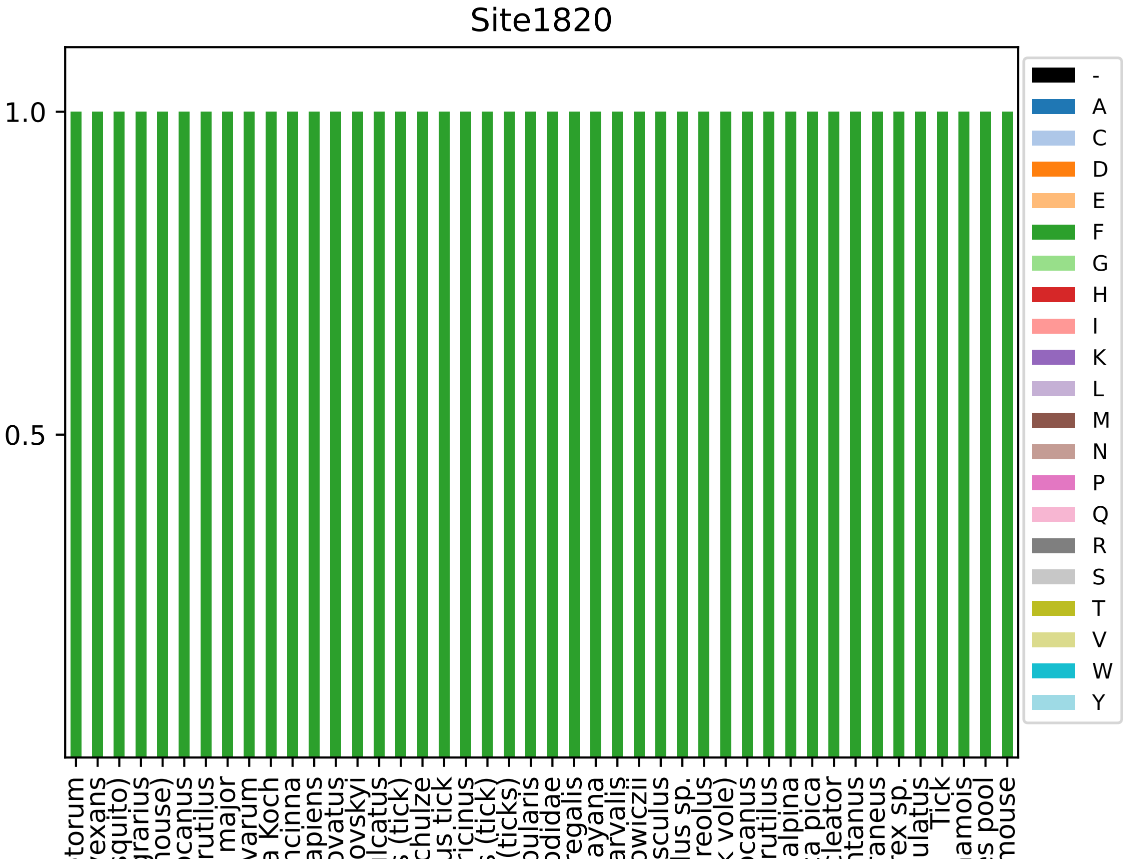
Site1820
1.0
0.5
-
A
C
D
E
F
G
H
I
K
L
M
N
P
Q
R
S
T
V
W
Y

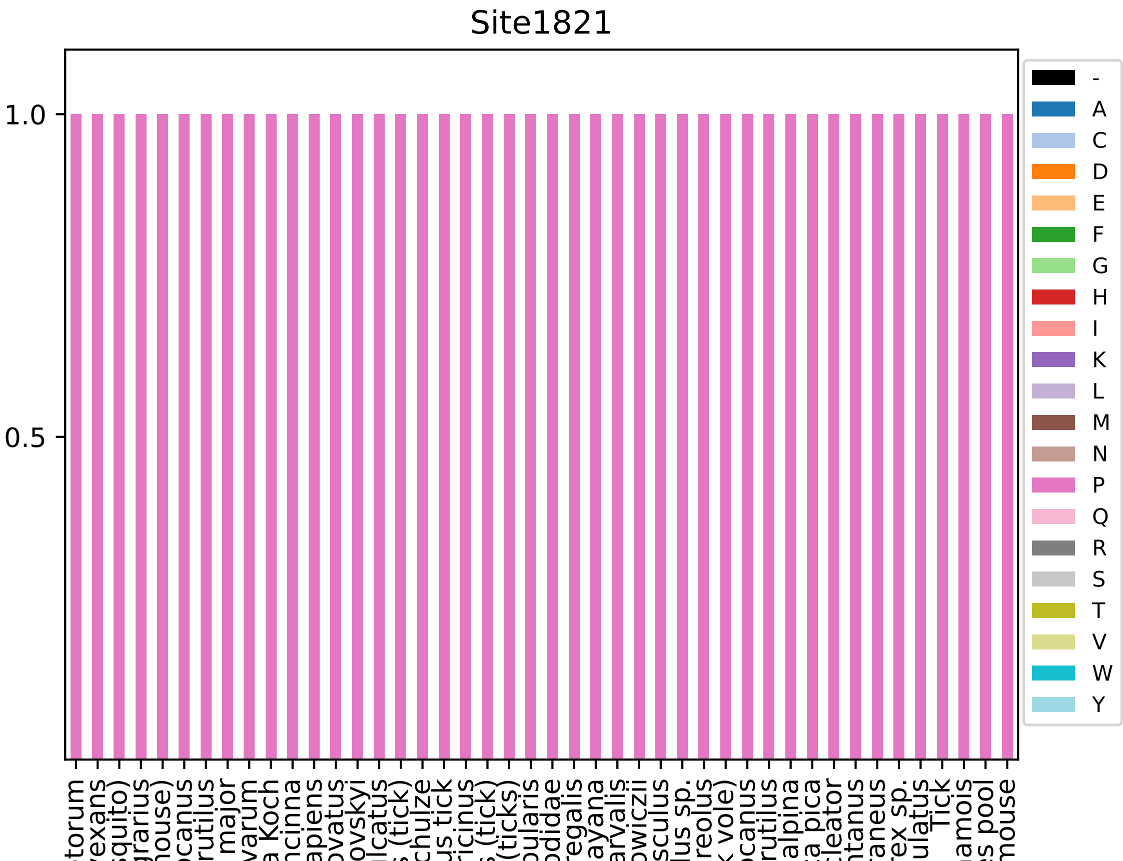
Site1821
1.0
0.5
-
A
C
D
E
F
G
H
I
K
L
M
N
P
Q
R
S
T
V
W
Y

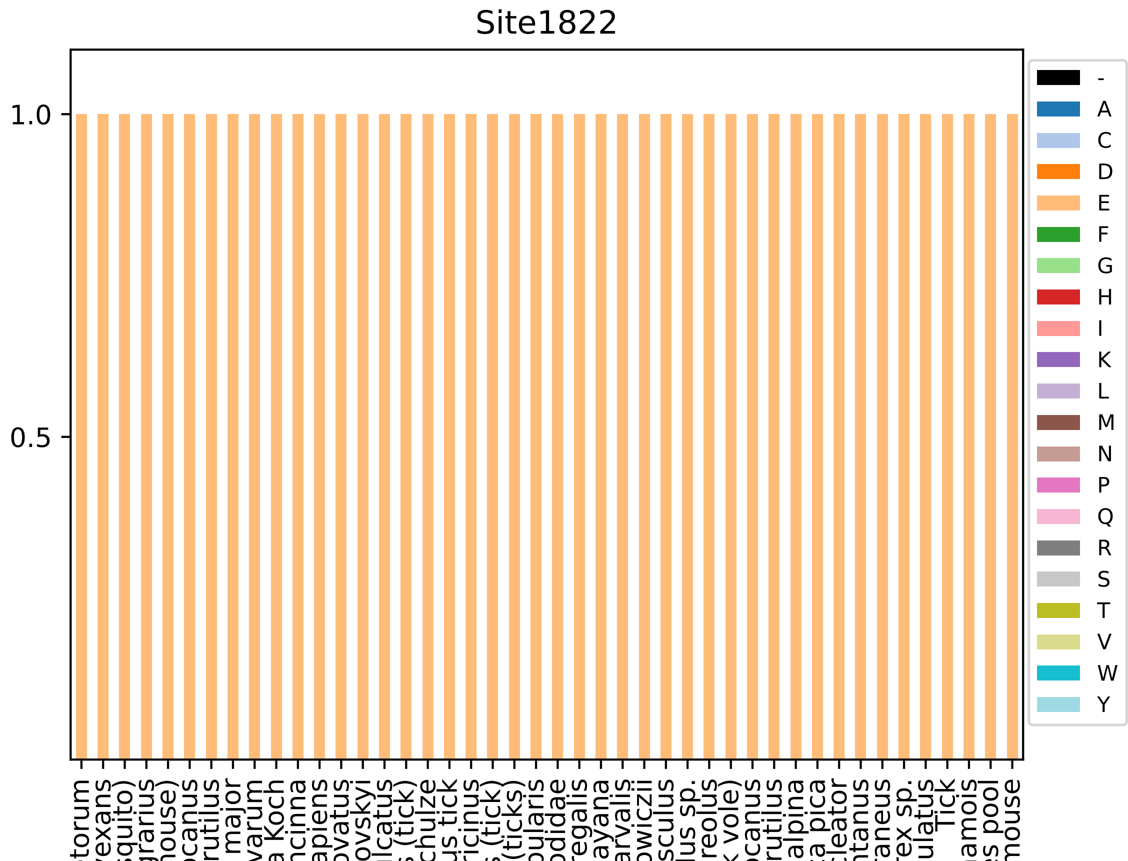
Site1822
1.0
0.5
-
A
C
D
E
F
G
H
I
K
L
M
N
P
Q
R
S
T
V
W
Y

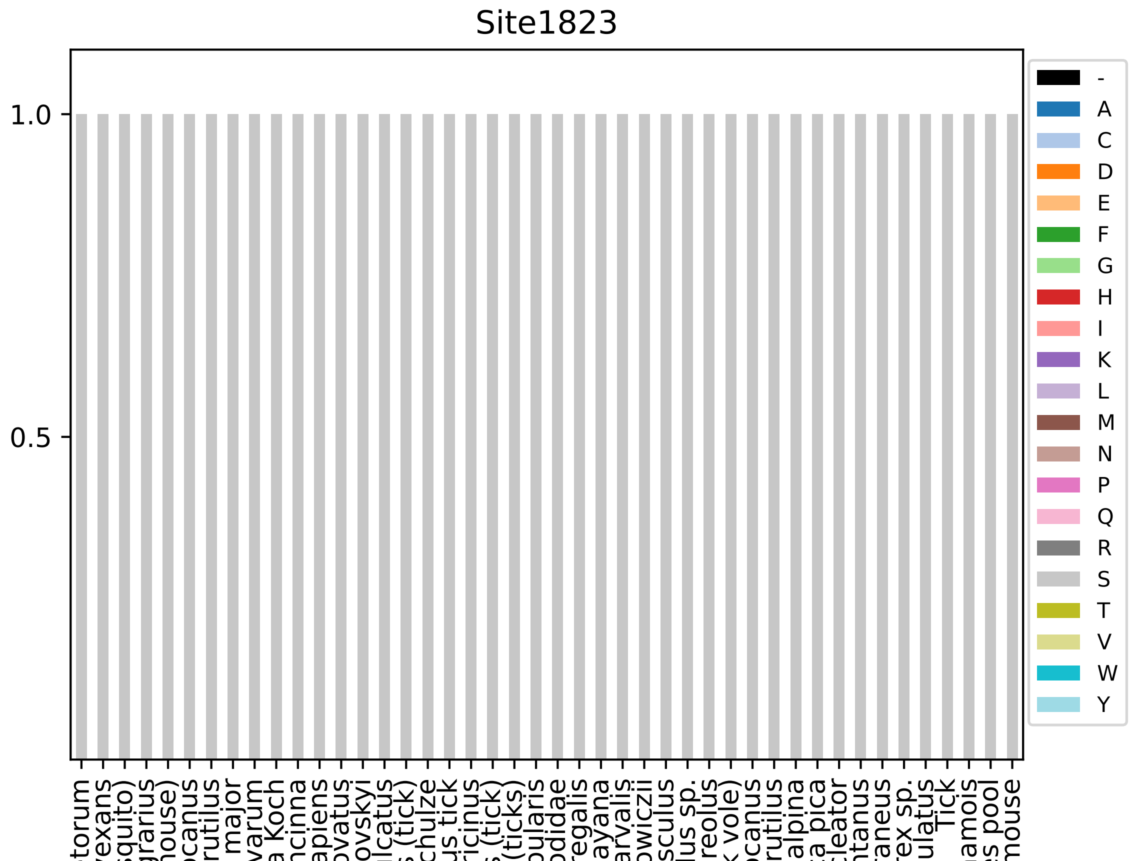
Site1823
1.0
0.5
-
A
C
D
E
F
G
H
I
K
L
M
N
P
Q
R
S
T
V
W
Y

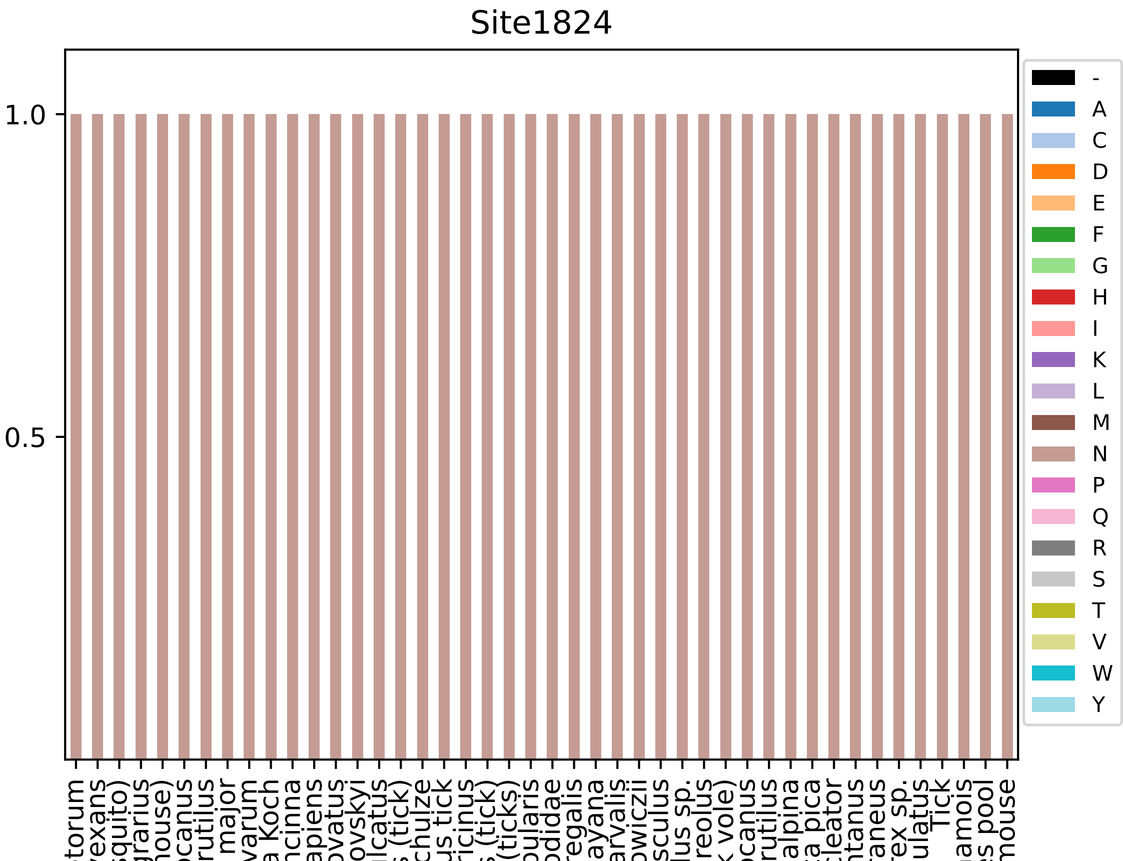
Site1824
1.0
0.5
-
A
C
D
E
F
G
H
I
K
L
M
N
P
Q
R
S
T
V
W
Y
Tick

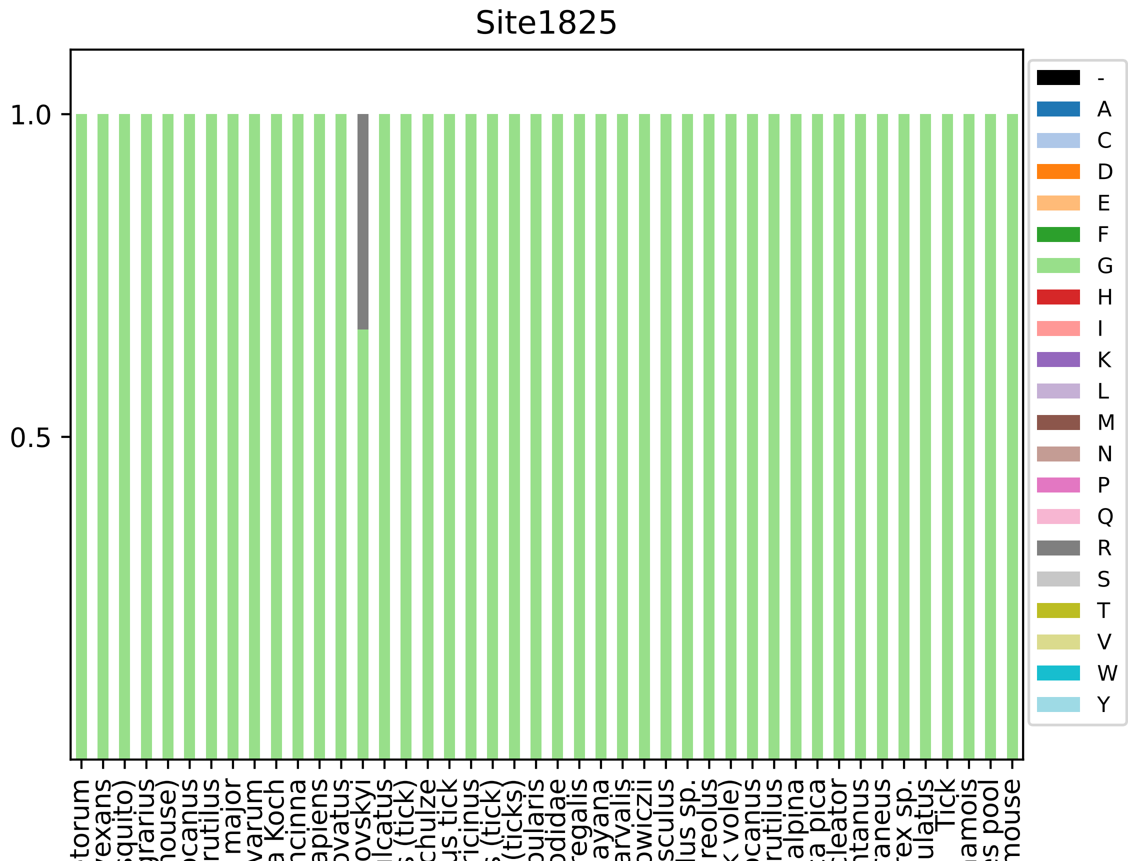
Site1825
1.0
0.5
-
A
C
D
E
F
G
H
I
K
L
M
N
P
Q
R
S
T
V
W
Y

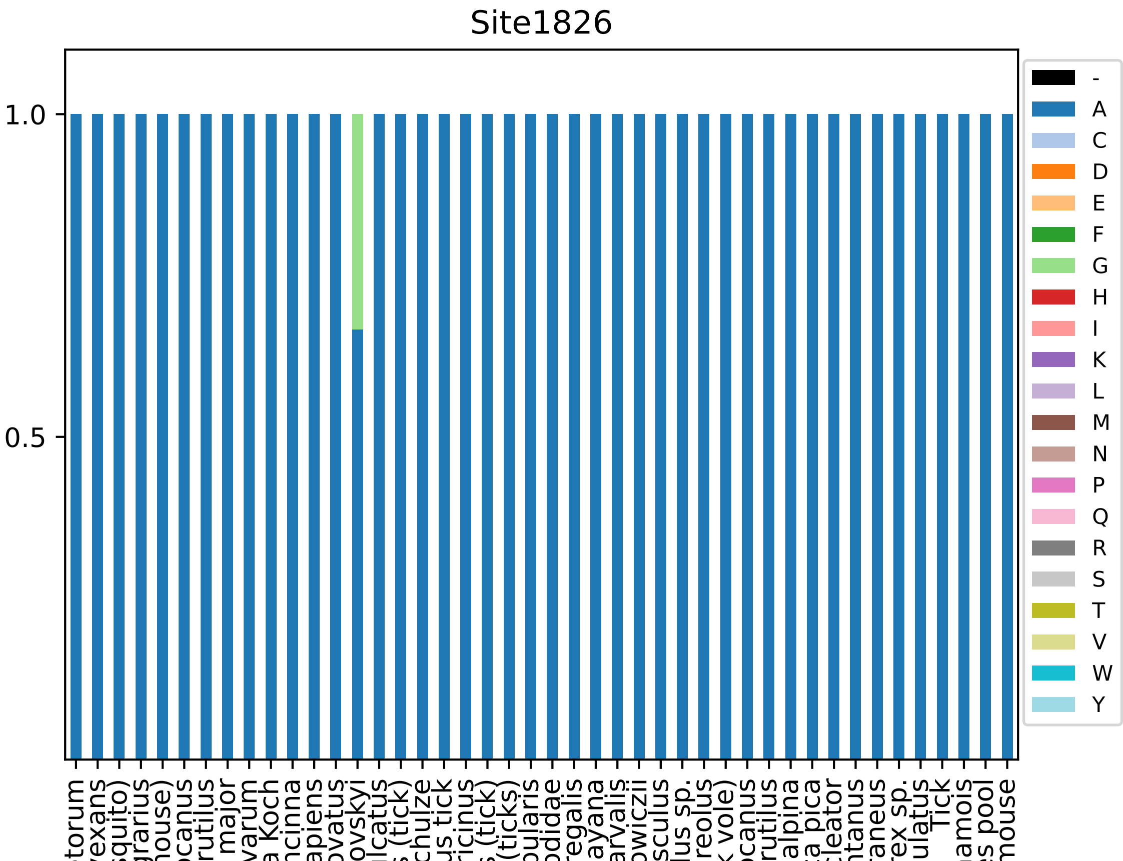
Site1826
1.0
0.5
-
A
C
D
E
F
G
H
I
K
L
M
N
P
Q
R
S
T
V
W
Y

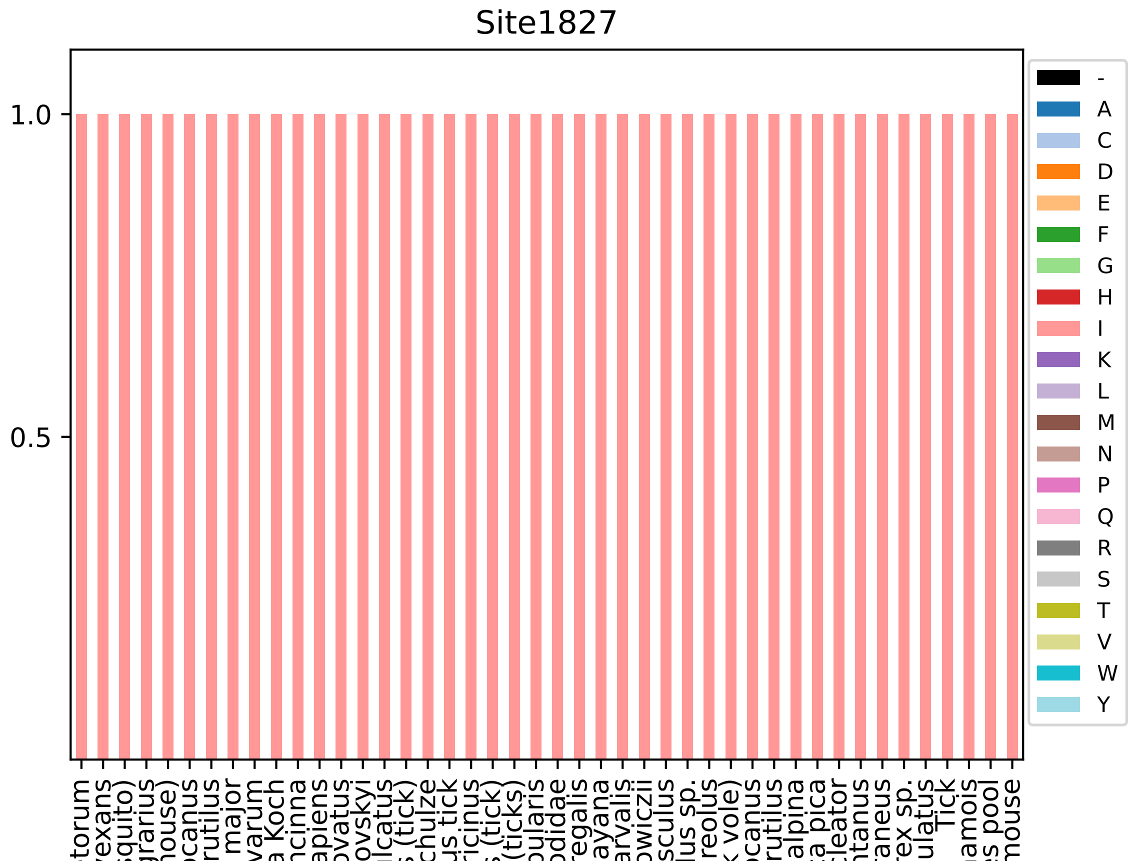

Site1827
1.0
0.5
-
A
C
D
E
F
G
H
I
K
L
M
N
P
Q
R
S
T
V
W
Y

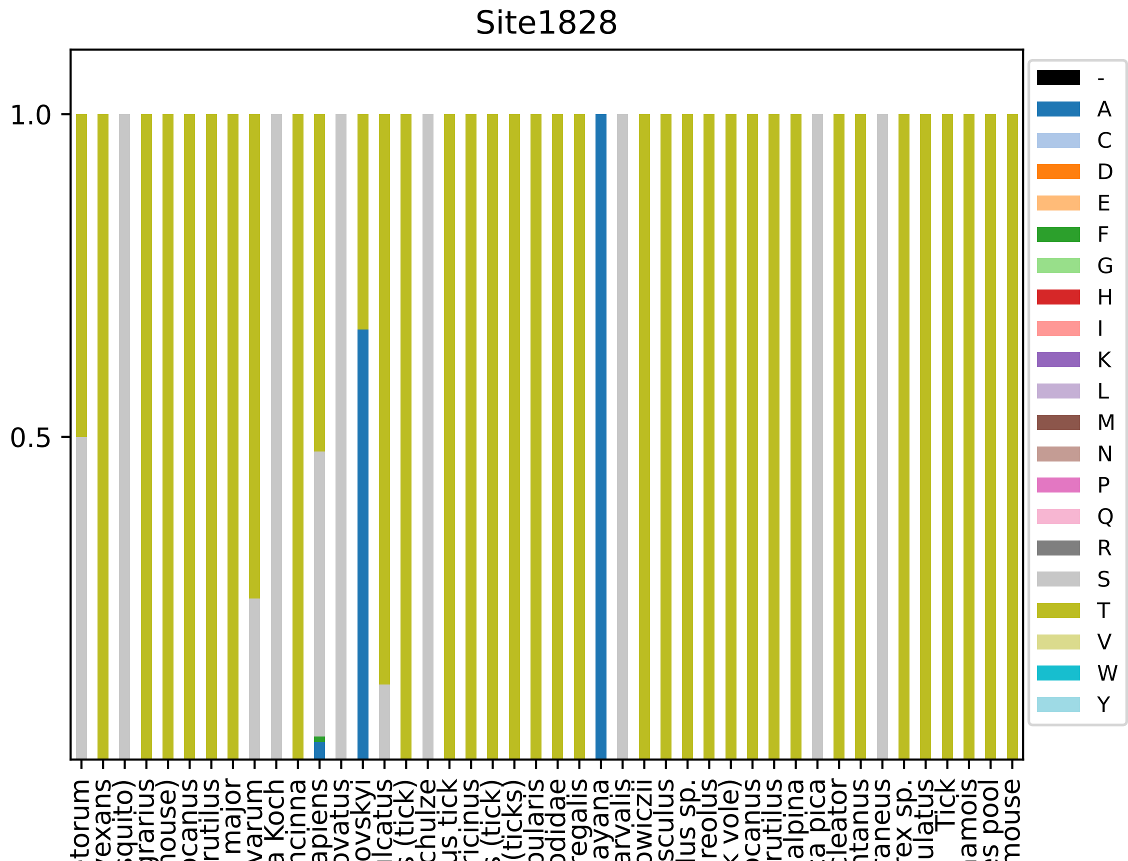
Site1828
1.0
0.5
-
A
C
D
E
F
G
H
I
K
L
M
N
P
Q
R
S
T
V
W
Y

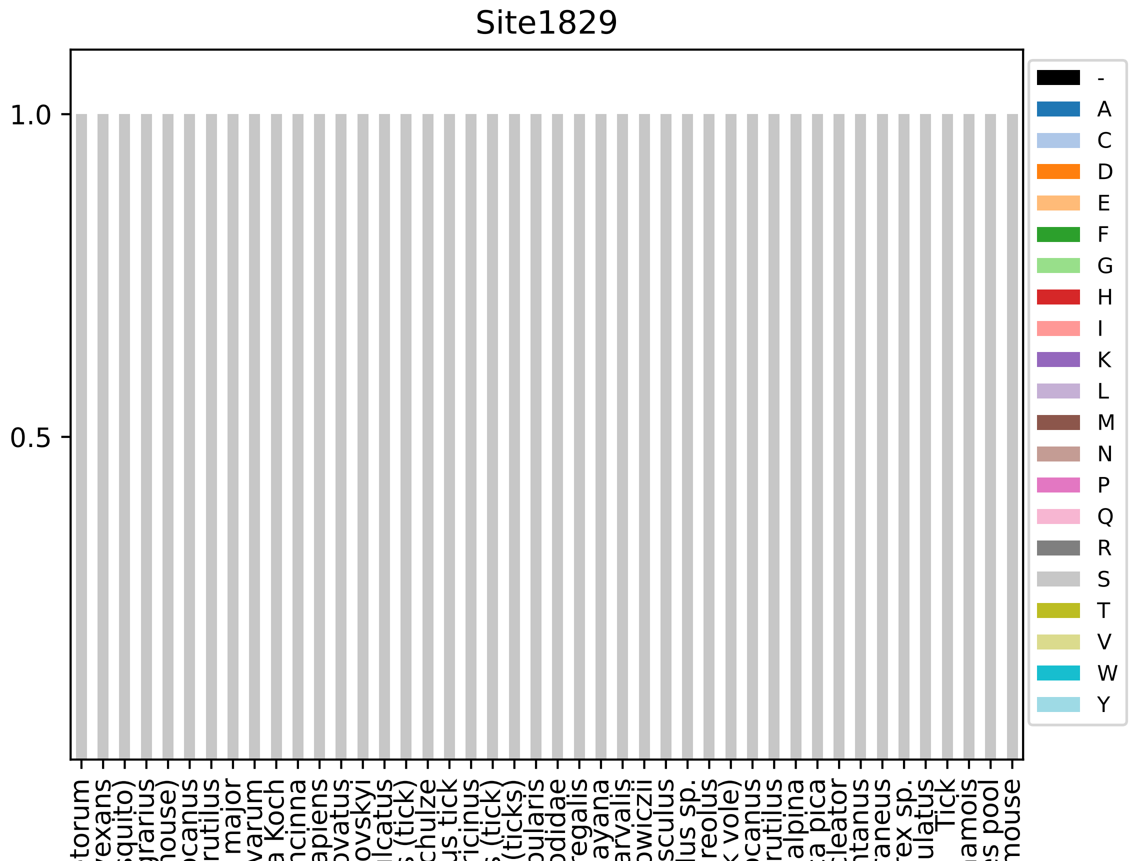
Site1829
1.0
0.5
-
A
C
D
E
F
G
H
I
K
L
M
N
P
Q
R
S
T
V
W
Y

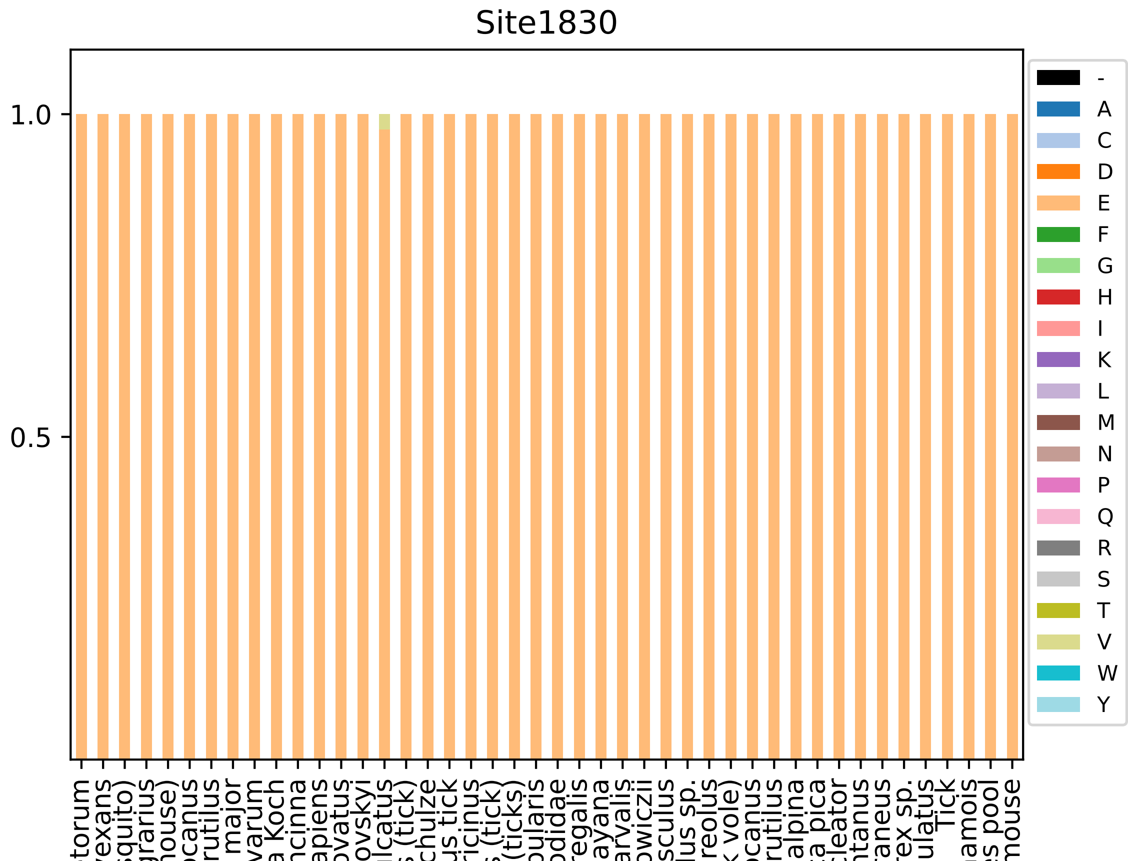
Site1830
1.0
0.5
-
A
C
D
E
F
G
H
I
K
L
M
N
P
Q
R
S
T
V
W
Y

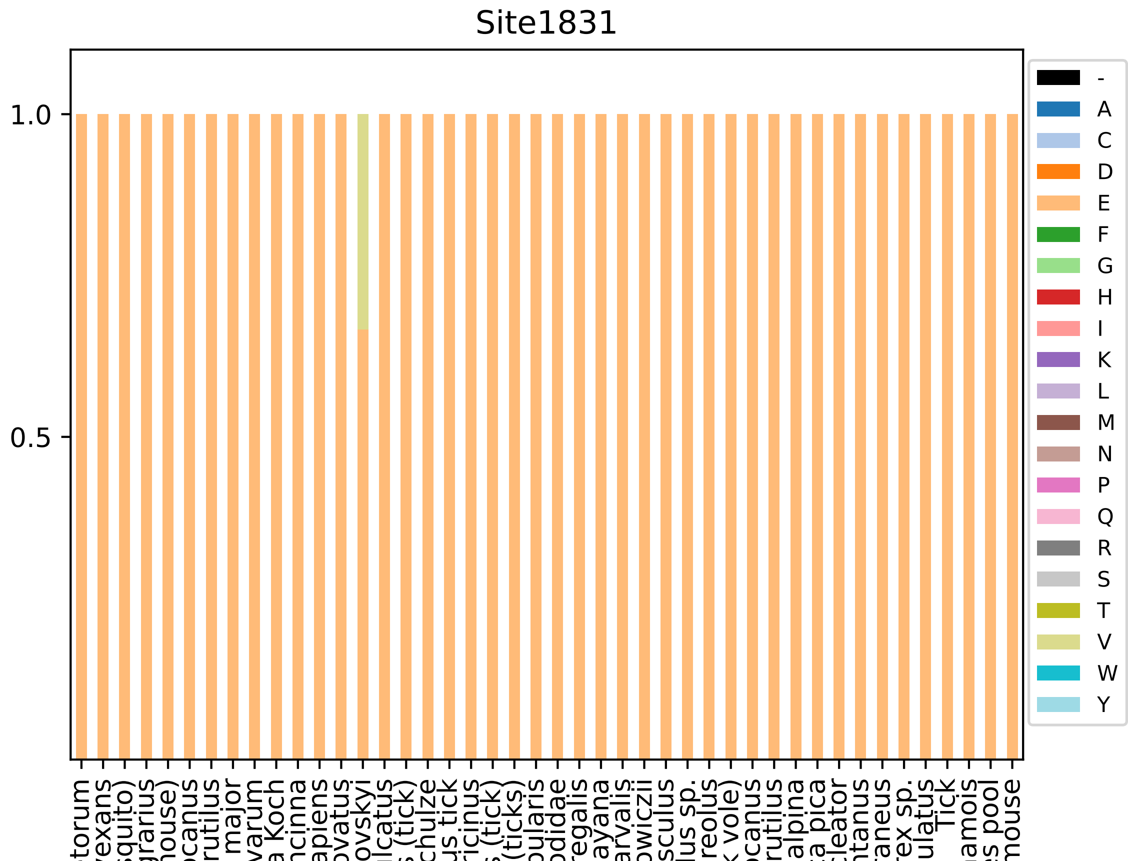
Site1831
1.0
0.5
-
A
C
D
E
F
G
H
I
K
L
M
N
P
Q
R
S
T
V
W
Y

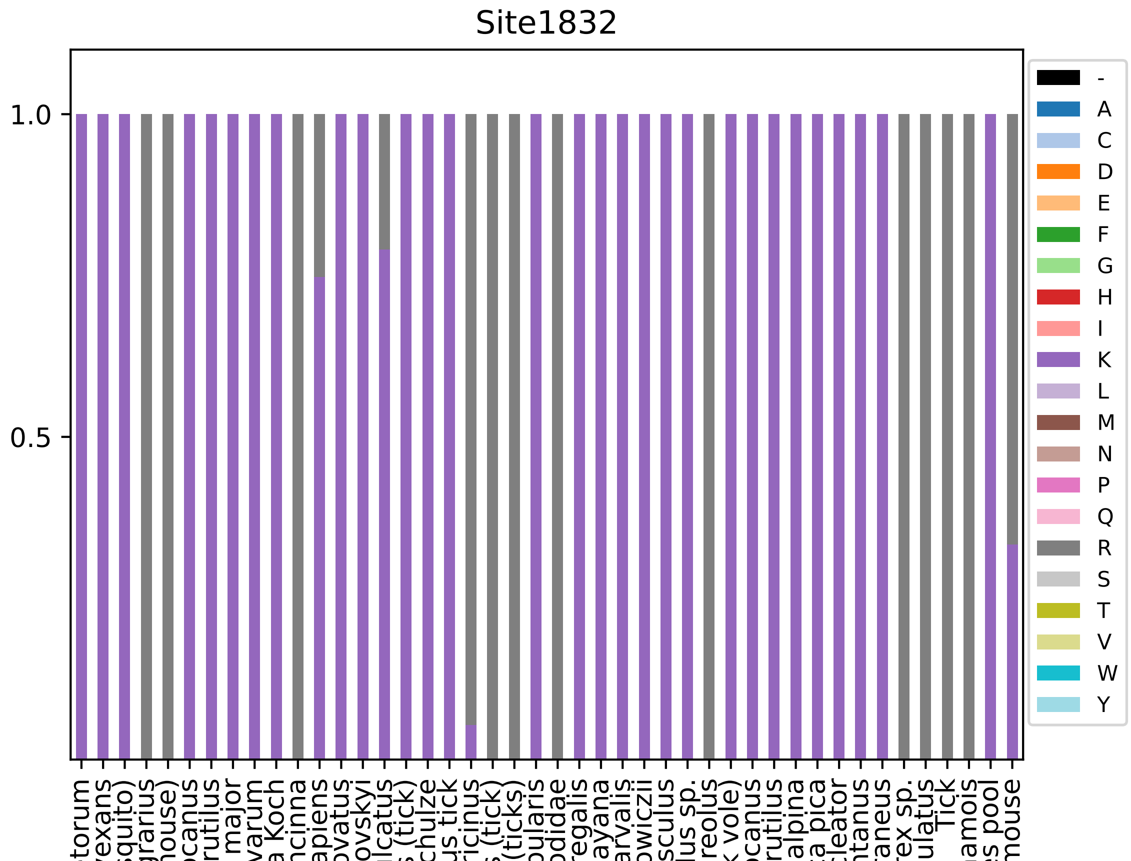
Site1832
1.0
0.5
-
A
C
D
E
F
G
H
I
K
L
M
N
P
Q
R
S
T
V
W
Y

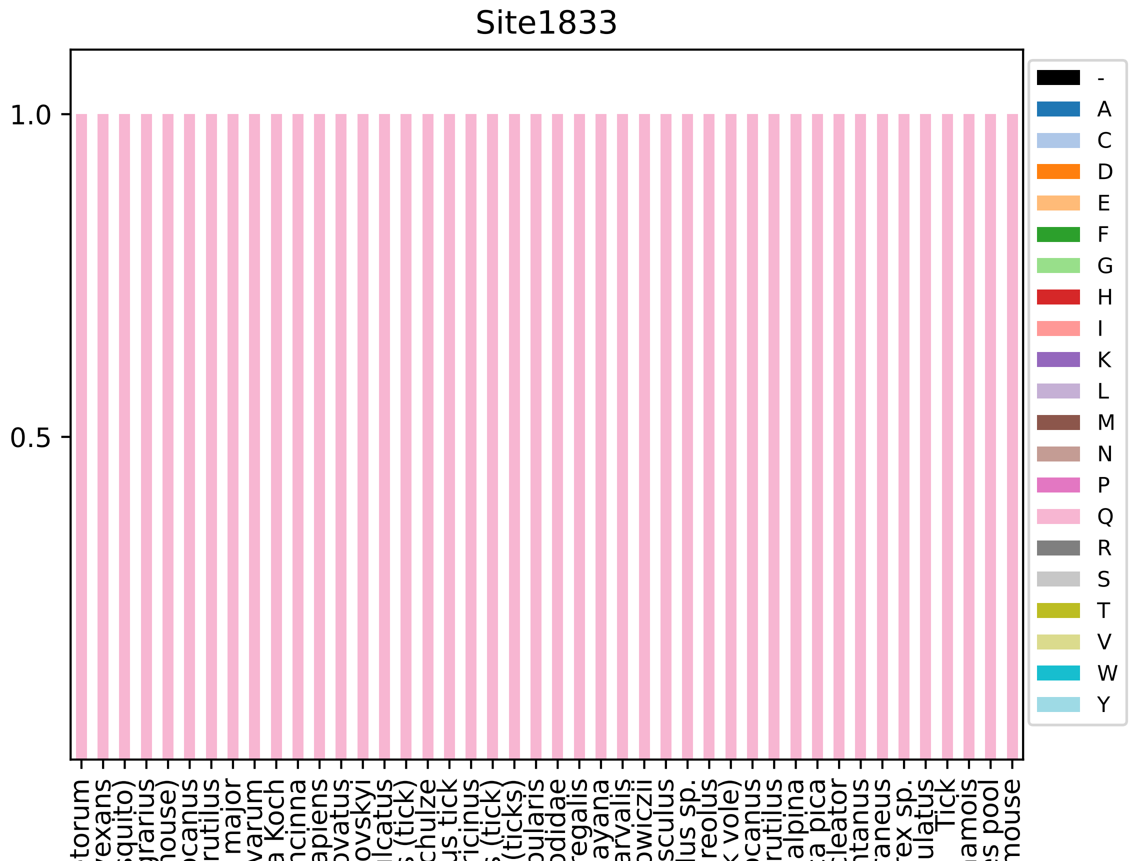

Site1833
1.0
0.5
-
A
C
D
E
F
G
H
I
K
L
M
N
P
Q
R
S
T
V
W
Y

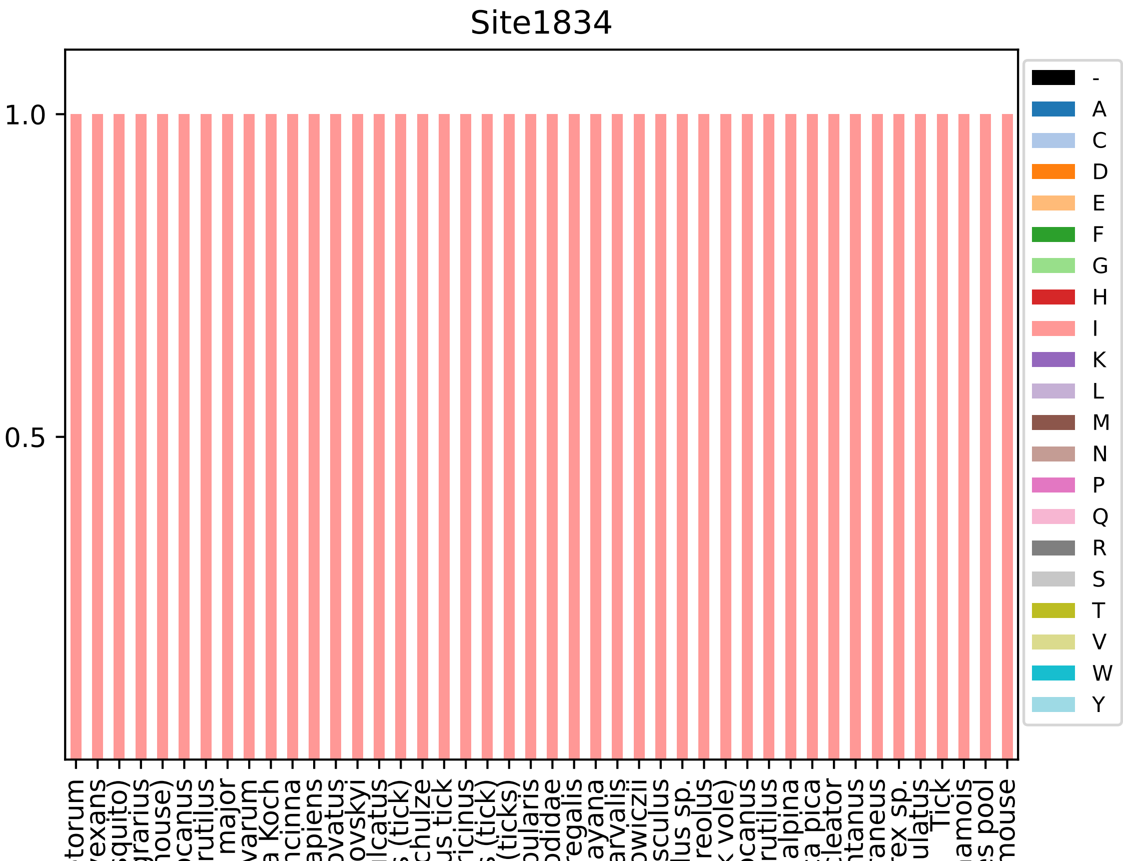
Site1834
1.0
0.5
-
A
C
D
E
F
G
H
I
K
L
M
N
P
Q
R
S
T
V
W
Y

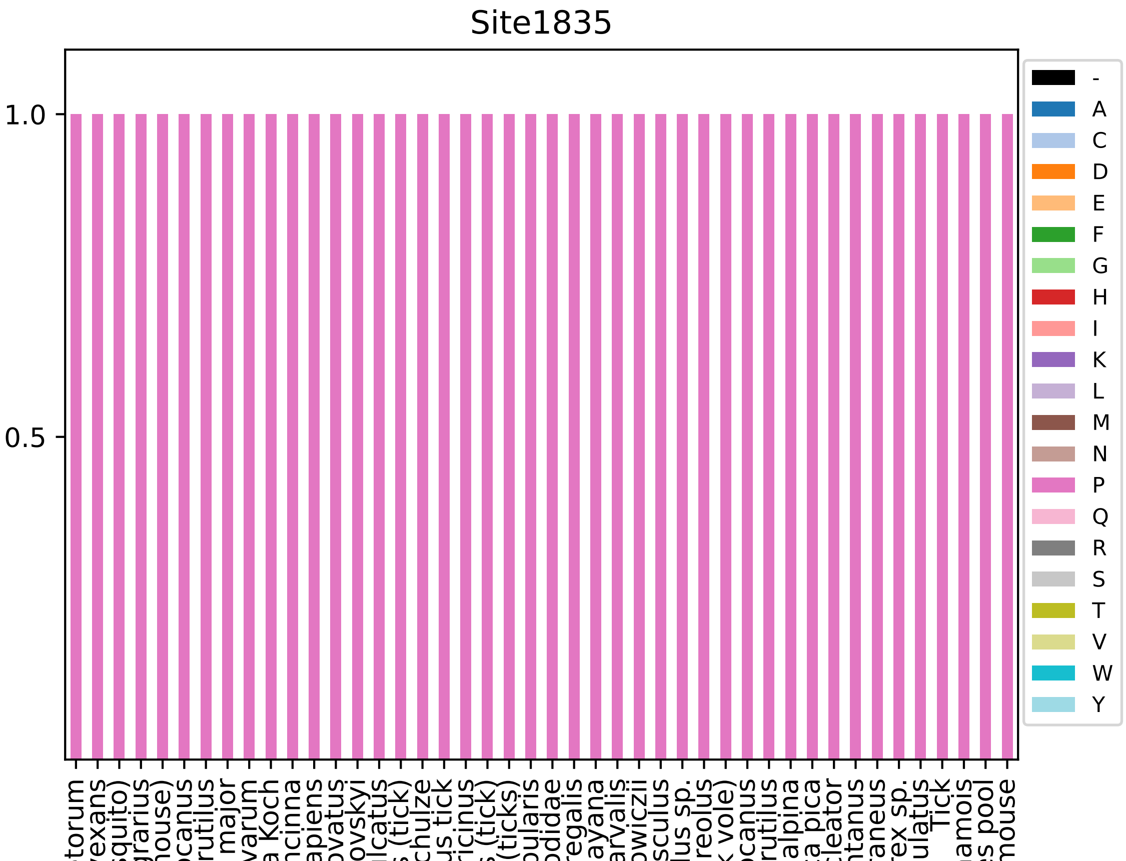

Site1835
1.0
0.5
-
A
C
D
E
F
G
H
I
K
L
M
N
P
Q
R
S
T
V
W
Y

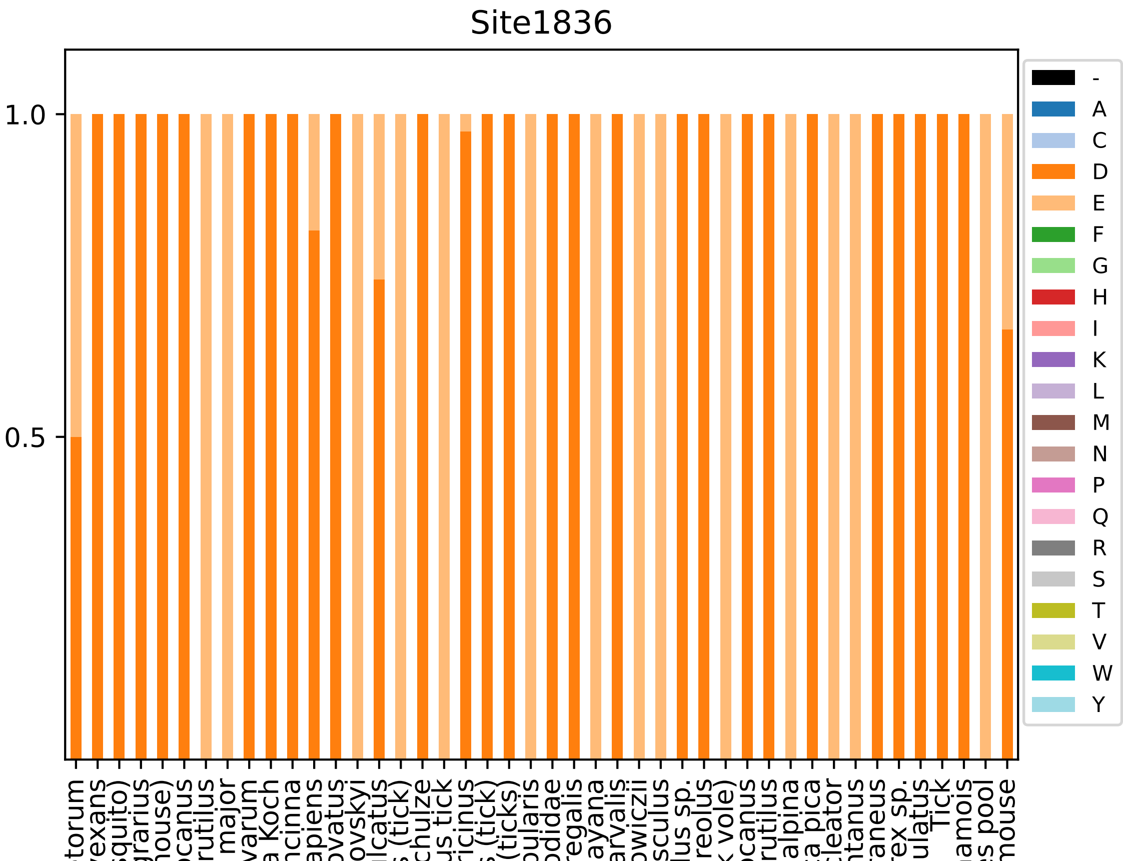
Site1836
1.0
0.5
-
A
C
D
E
F
G
H
I
K
L
M
N
P
Q
R
S
T
V
W
Y

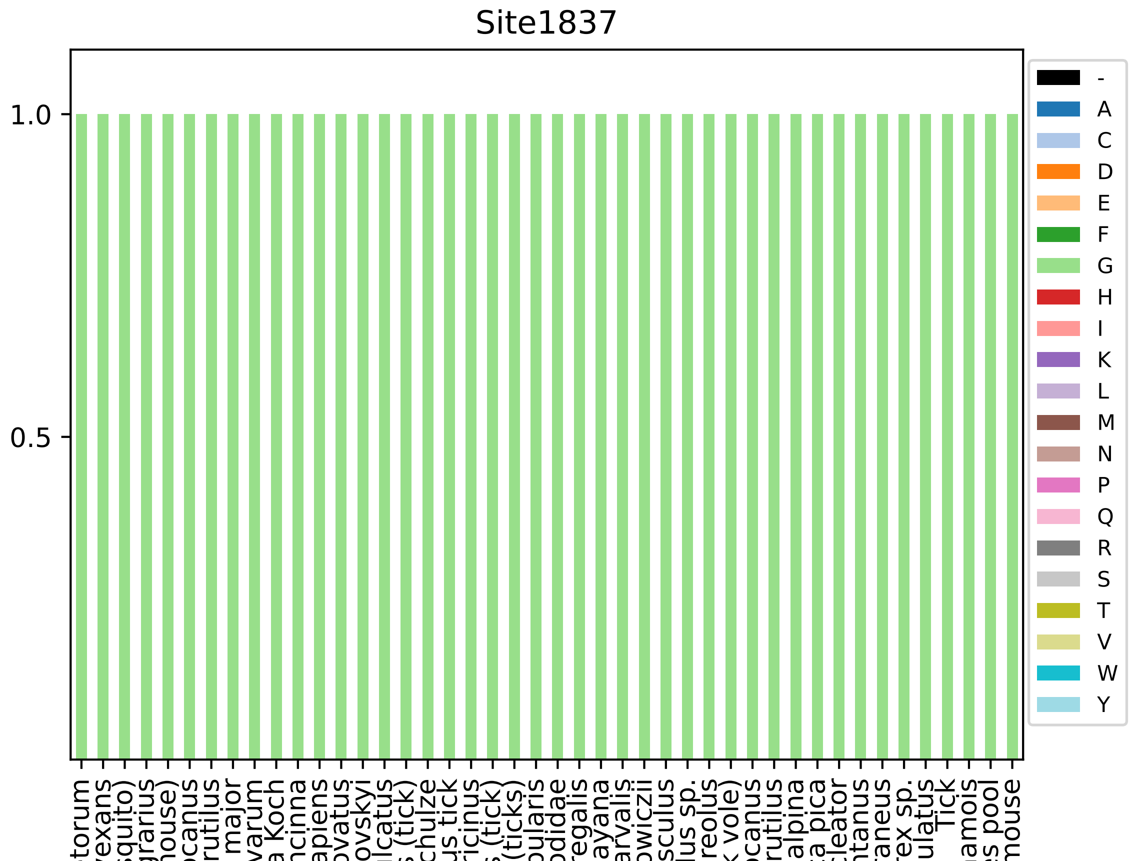
Site1837
1.0
0.5
-
A
C
D
E
F
G
H
I
K
L
M
N
P
Q
R
S
T
V
W
Y

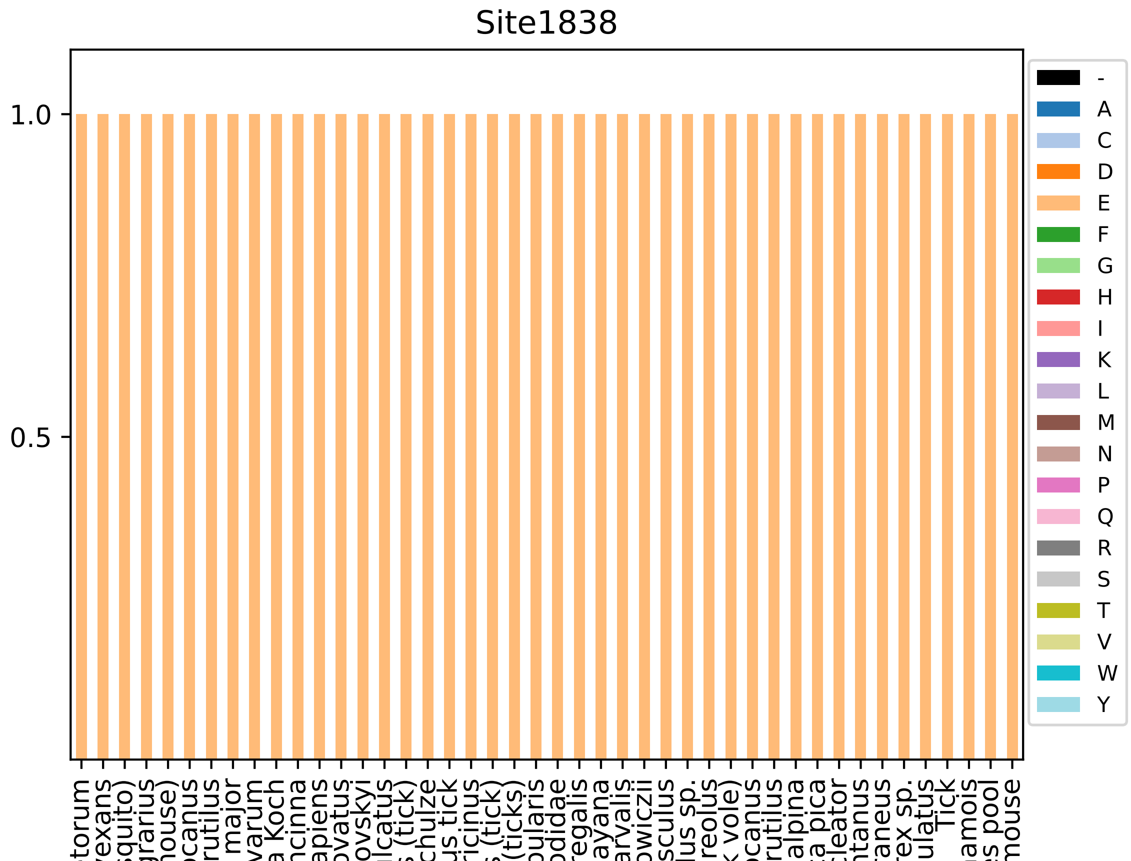
Site1838
1.0
0.5
-
A
C
D
E
F
G
H
I
K
L
M
N
P
Q
R
S
T
V
W
Y

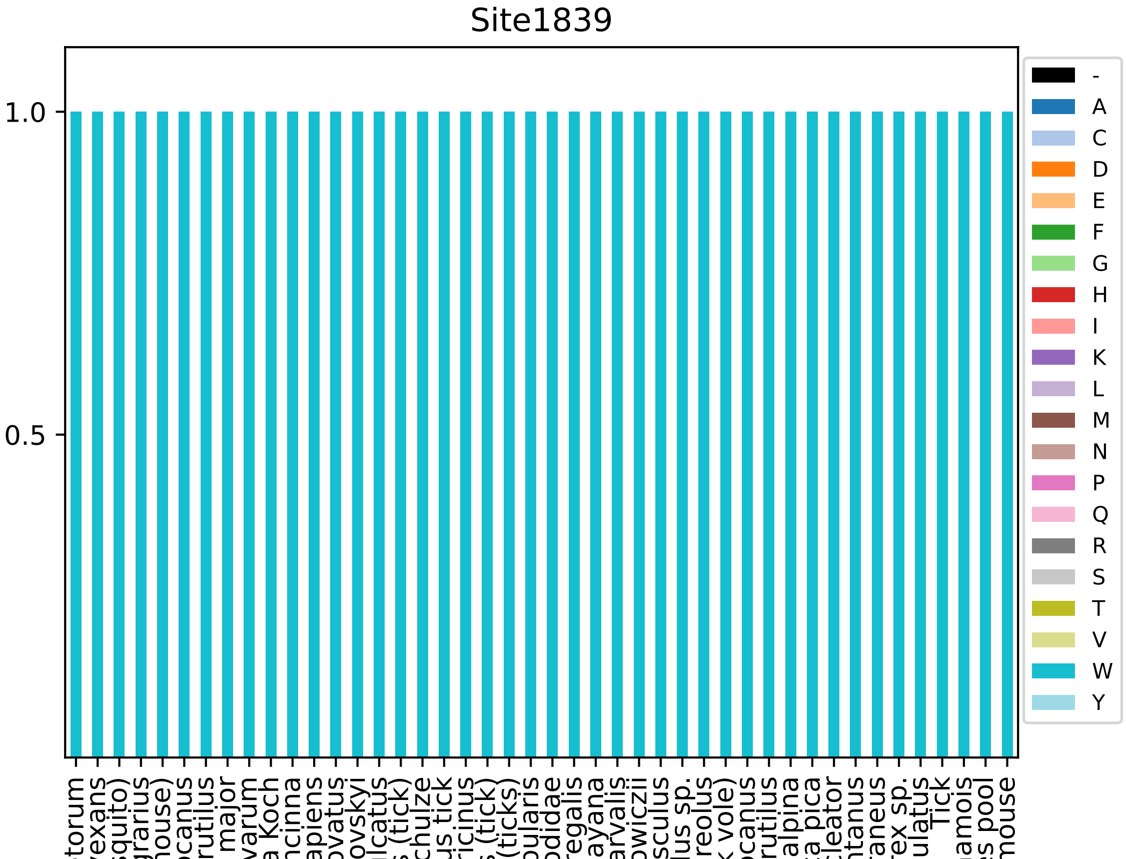
Site1839
1.0
0.5
-
A
C
D
E
F
G
H
I
K
L
M
N
P
Q
R
S
T
V
W
Y
torum
vexans
squito)
garius
nouse)
ocanus
rutilus
major
varum
a Koch
ncinna
apiens
ovatus
ovskyi
ulcatus
s (tick)
chulze
us tick
ricinus
s (tick)
(ticks)
pularis
podidae
regalis
ayana
arvalis
owiczii
sculus
lus sp.
reolus
x vole)
ocanus
rutilus
alpina
a pica
leator
ntanus
raneus
rex sp.
ulatus
Tick
amois
es pool
mouse

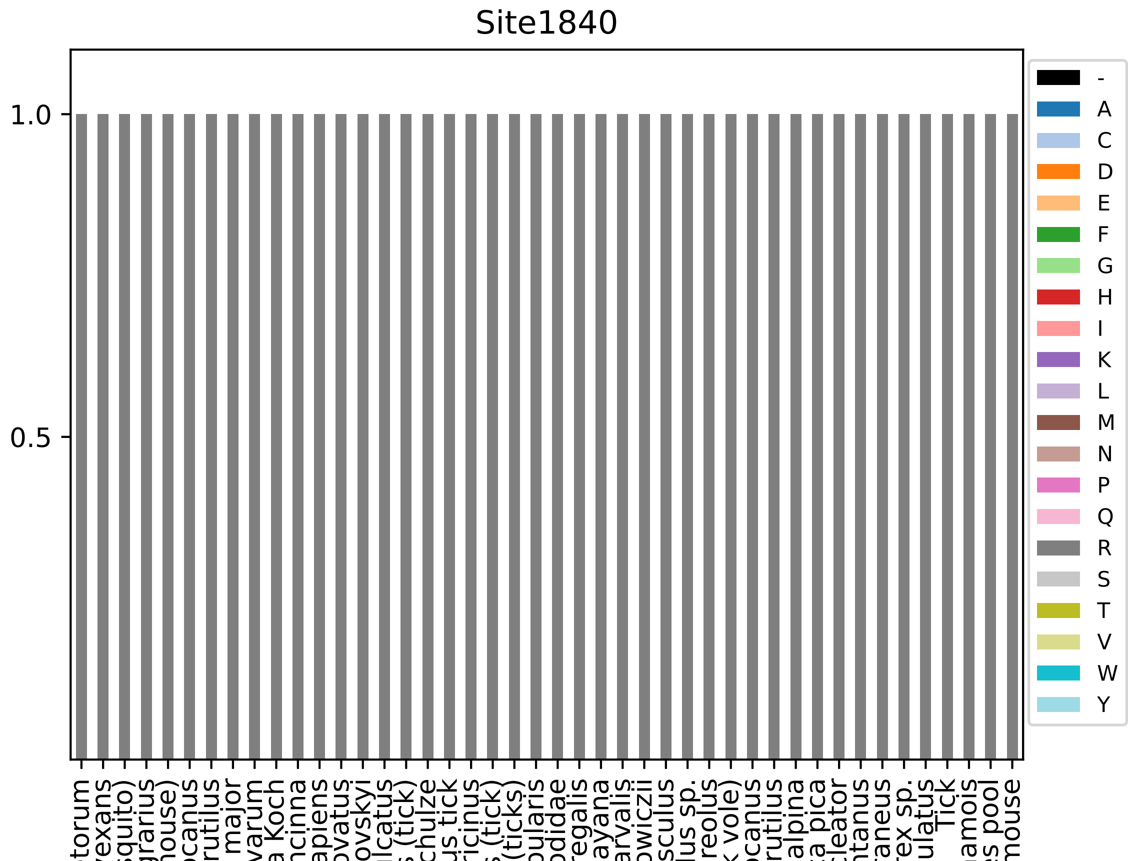
Site1840
1.0
0.5
-
A
C
D
E
F
G
H
I
K
L
M
N
P
Q
R
S
T
V
W
Y

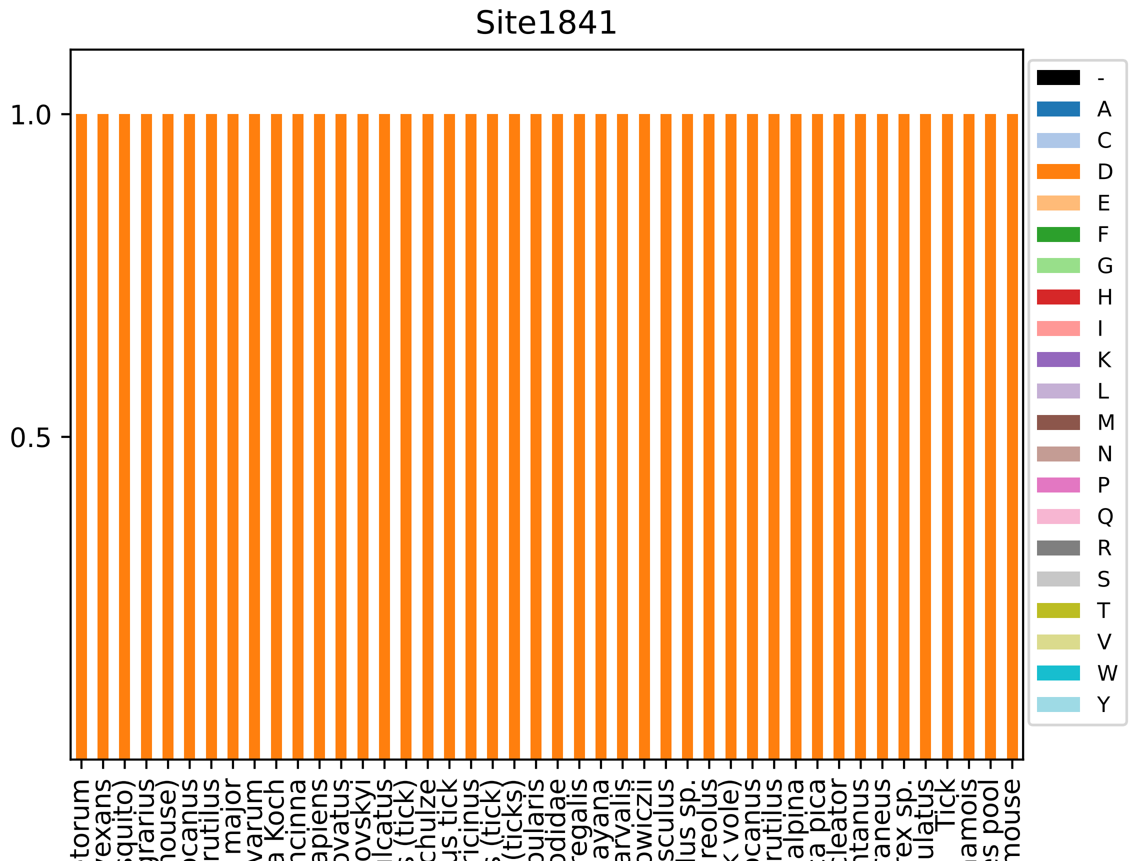
Site1841
1.0
0.5
-
A
C
D
E
F
G
H
I
K
L
M
N
P
Q
R
S
T
V
W
Y

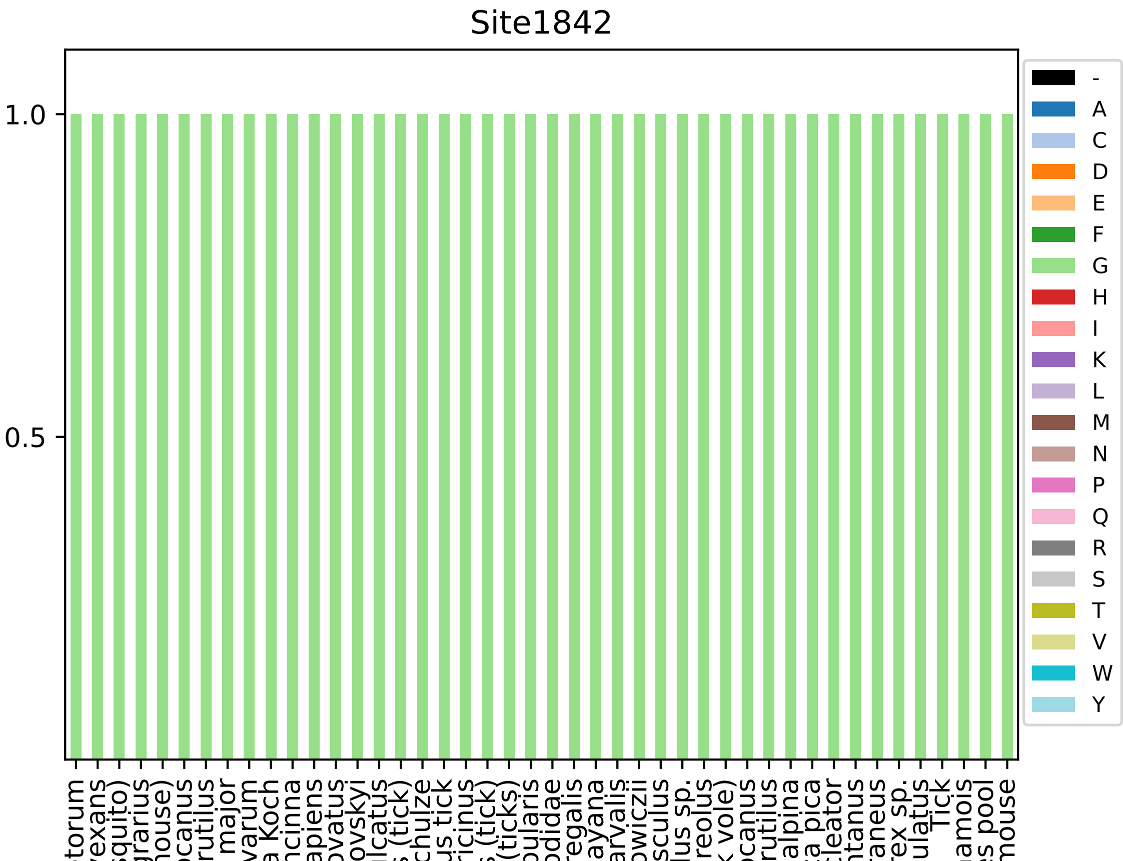
Site1842
1.0
0.5
-
A
C
D
E
F
G
H
I
K
L
M
N
P
Q
R
S
T
V
W
Y

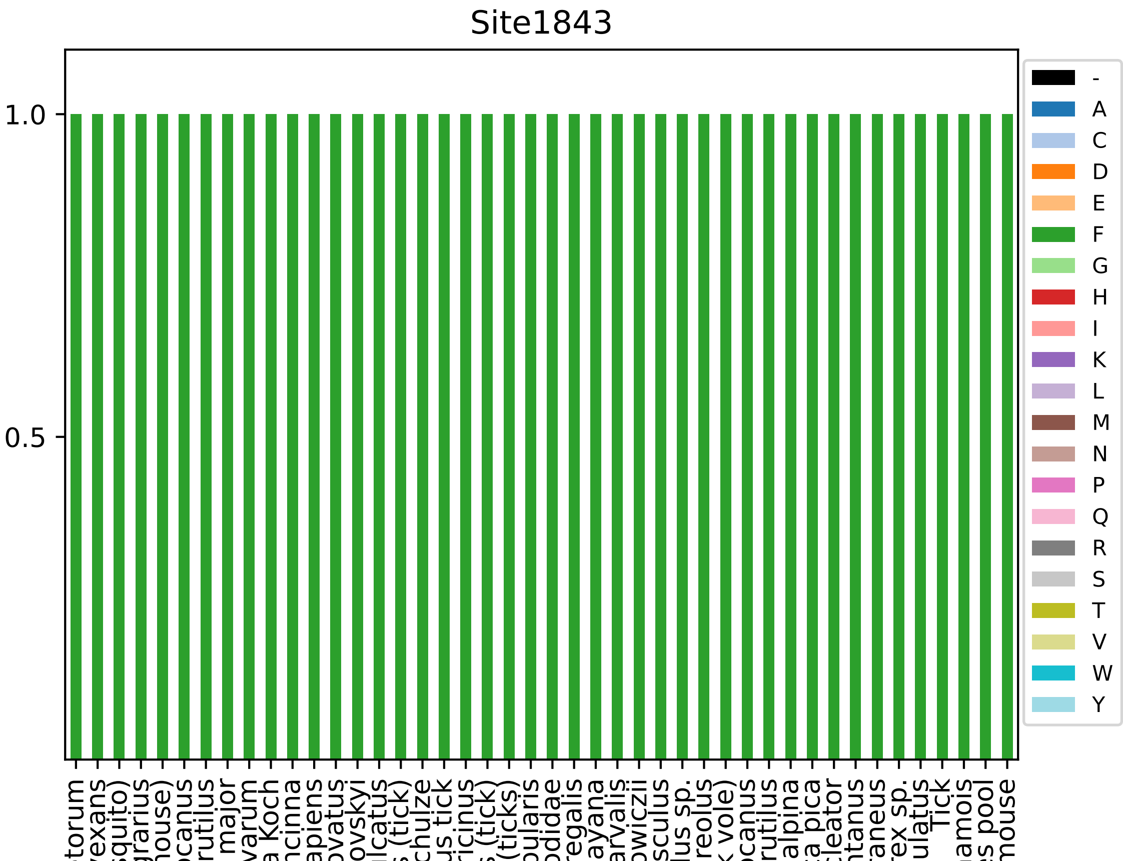
Site1843
1.0
0.5
-
A
C
D
E
F
G
H
I
K
L
M
N
P
Q
R
S
T
V
W
Y

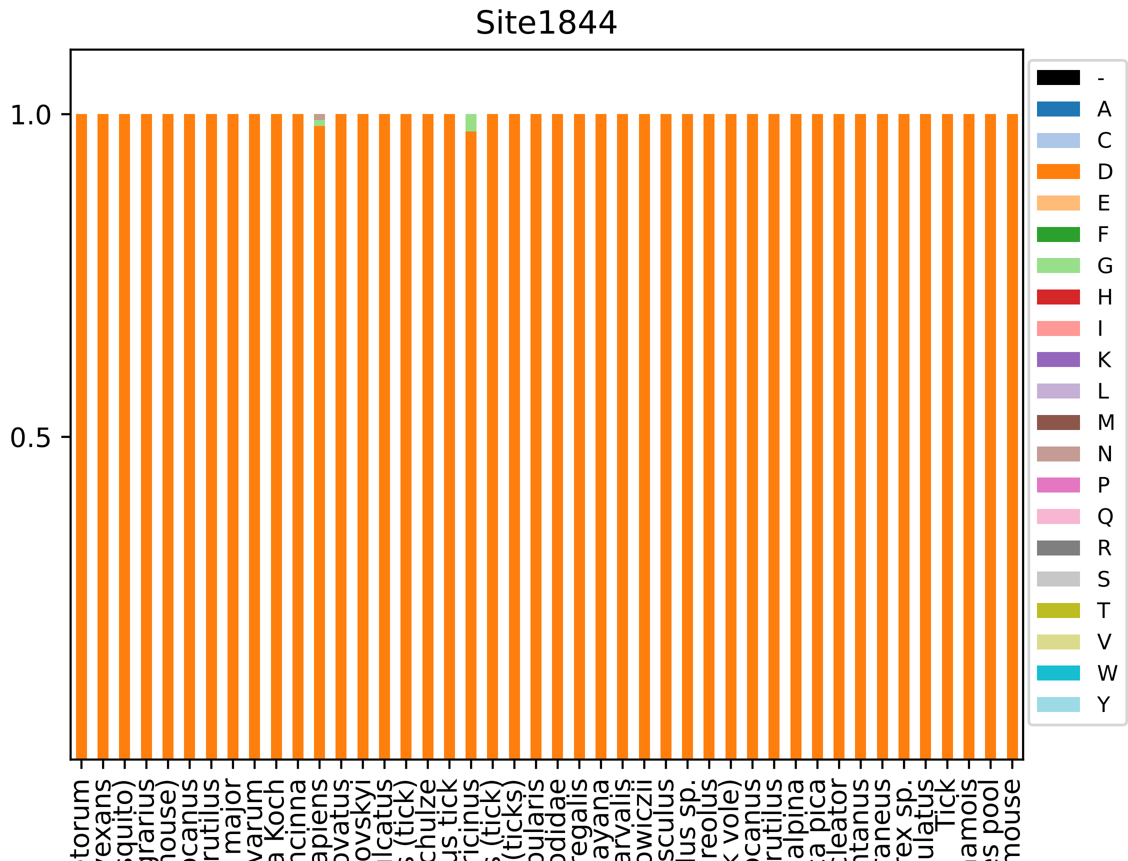

Site1844
1.0
0.5
-
A
C
D
E
F
G
H
I
K
L
M
N
P
Q
R
S
T
V
W
Y

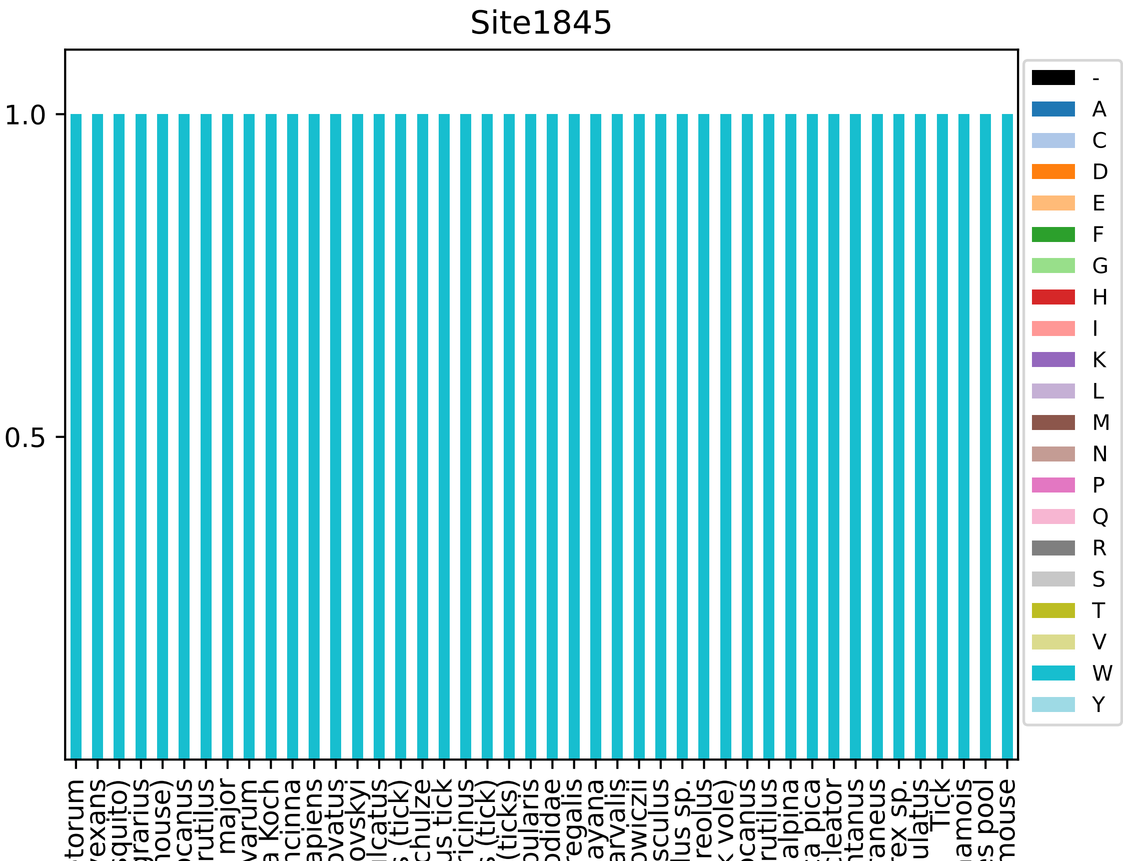
Site1845
1.0
0.5
-
A
C
D
E
F
G
H
I
K
L
M
N
P
Q
R
S
T
V
W
Y

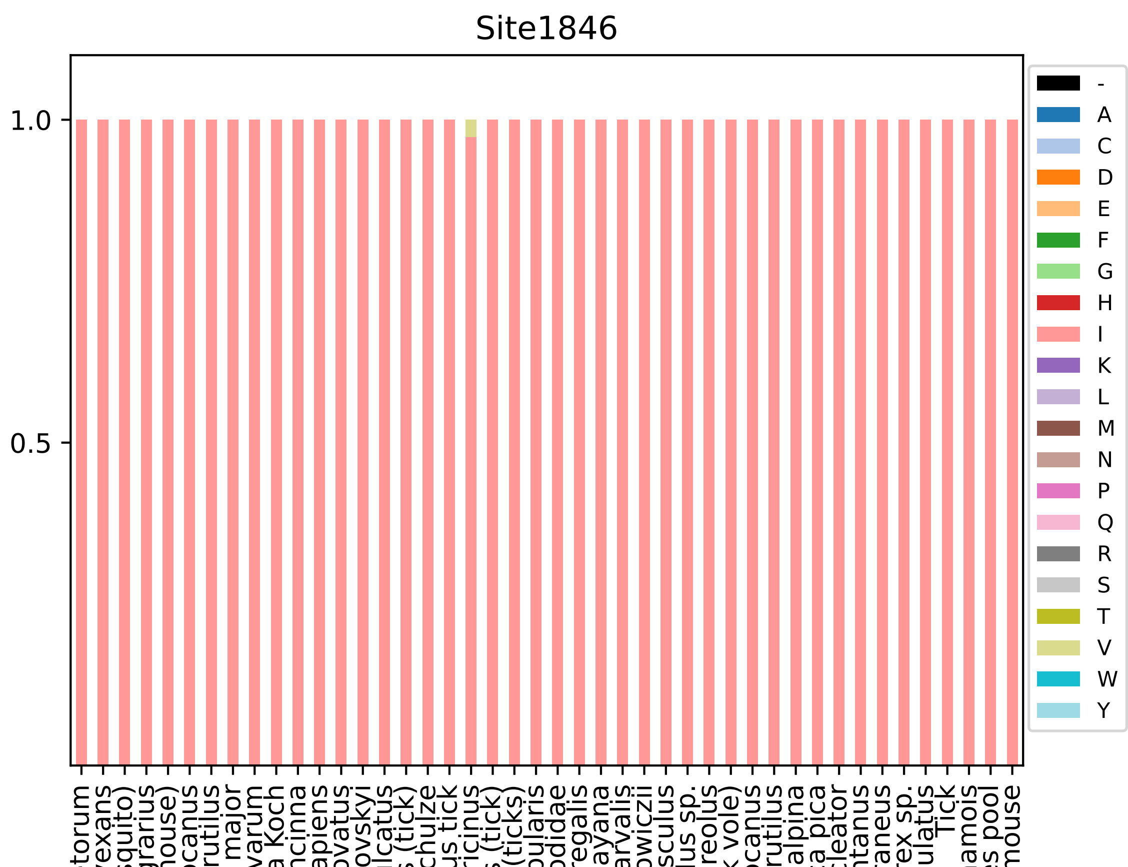

Site1846
1.0
0.5
-
A
C
D
E
F
G
H
I
K
L
M
N
P
Q
R
S
T
V
W
Y

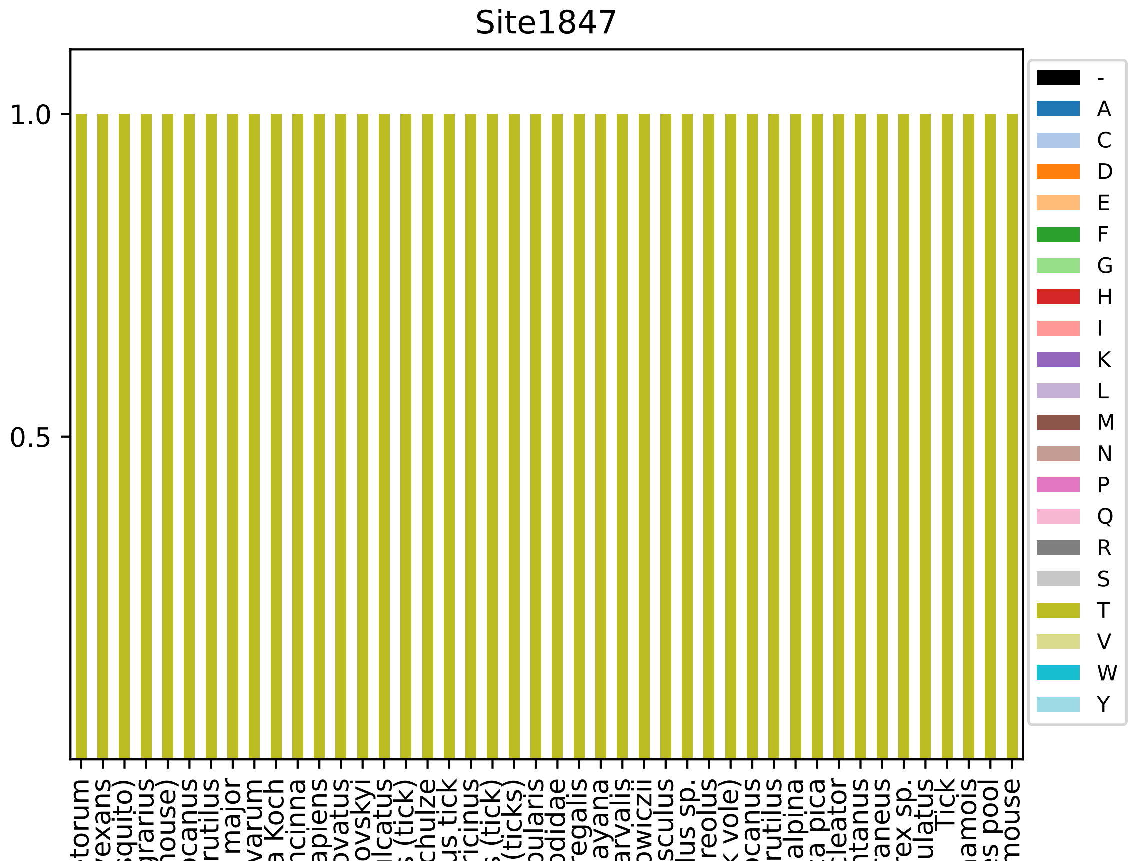

Site1847
1.0
0.5
-
A
C
D
E
F
G
H
I
K
L
M
N
P
Q
R
S
T
V
W
Y
Tick

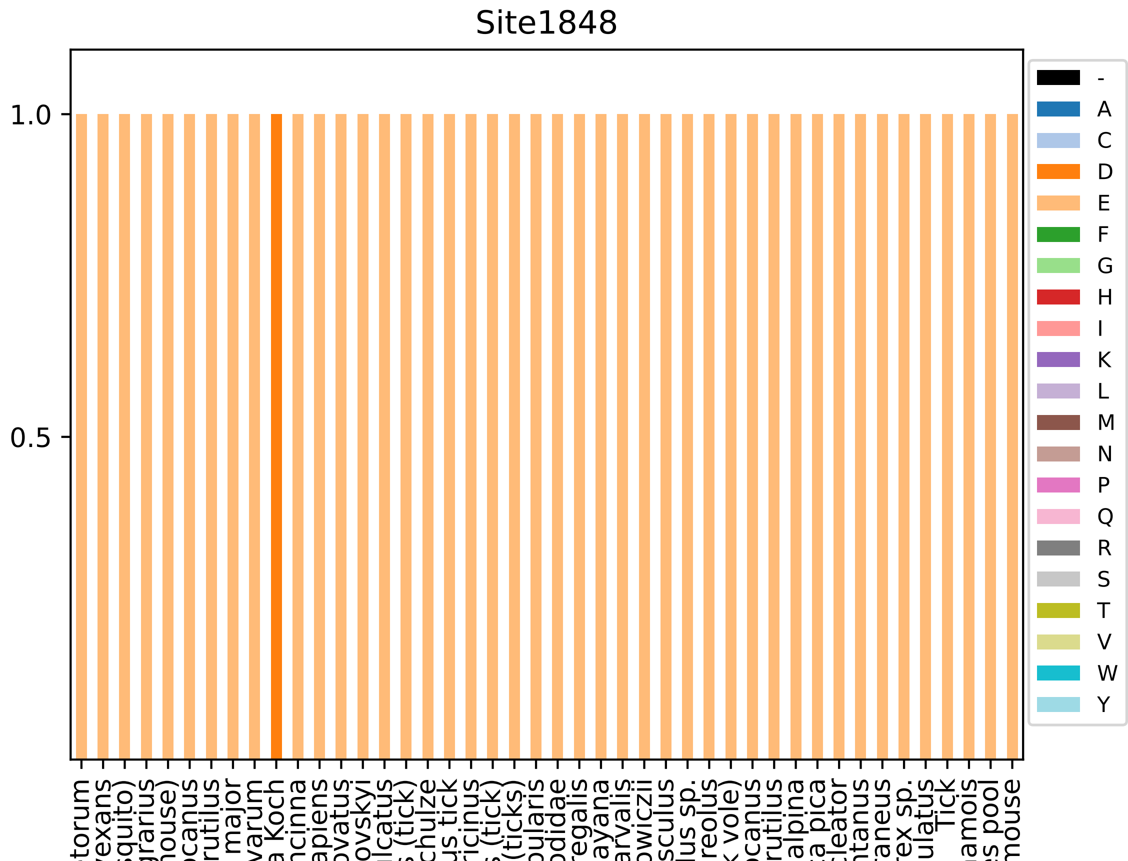
Site1848
1.0
0.5
-
A
C
D
E
F
G
H
I
K
L
M
N
P
Q
R
S
T
V
W
Y

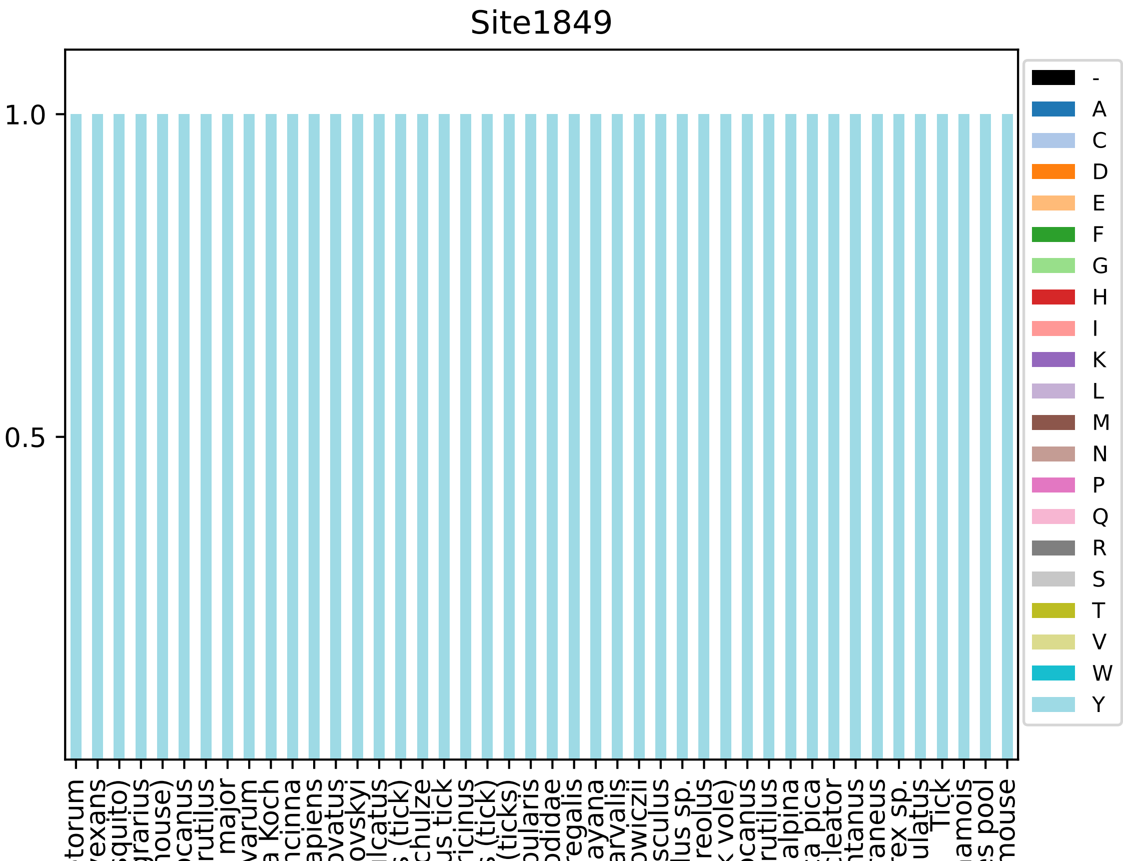
Site1849
1.0
0.5
-
A
C
D
E
F
G
H
I
K
L
M
N
P
Q
R
S
T
V
W
Y

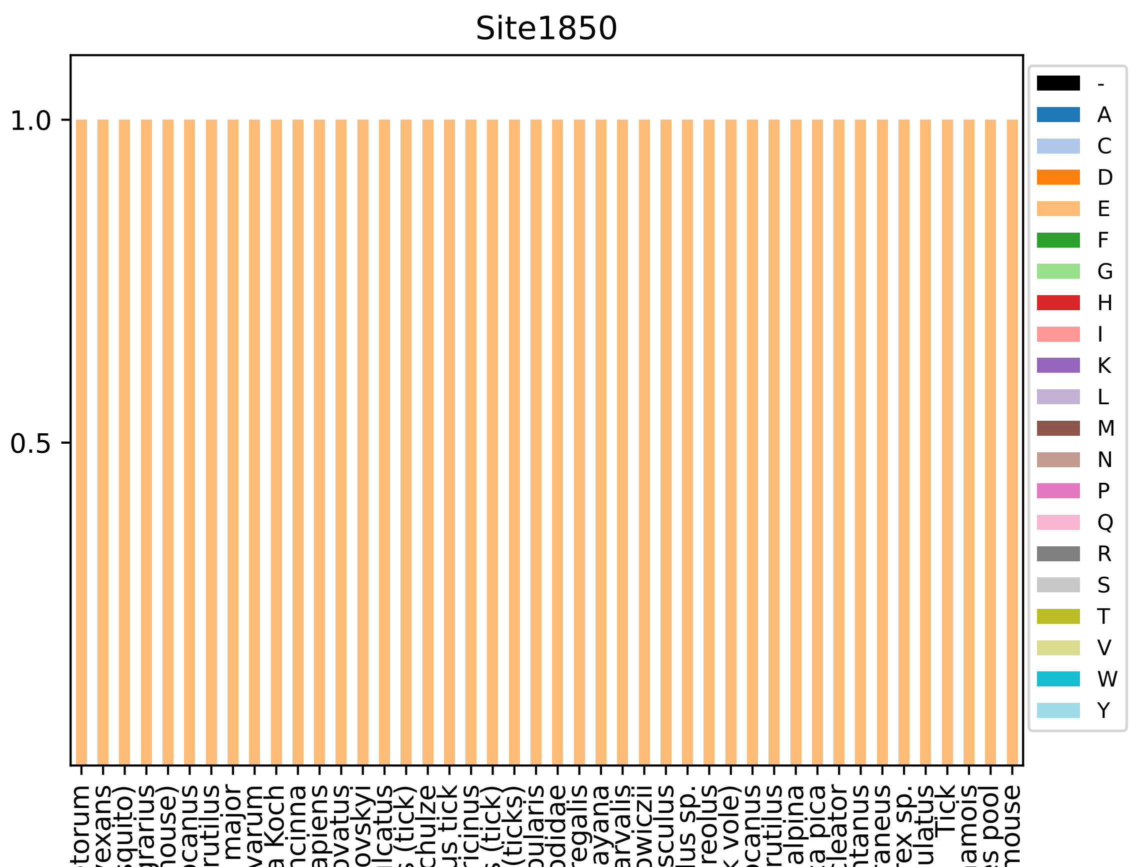
Site1850
1.0
0.5
-
A
C
D
E
F
G
H
I
K
L
M
N
P
Q
R
S
T
V
W
Y

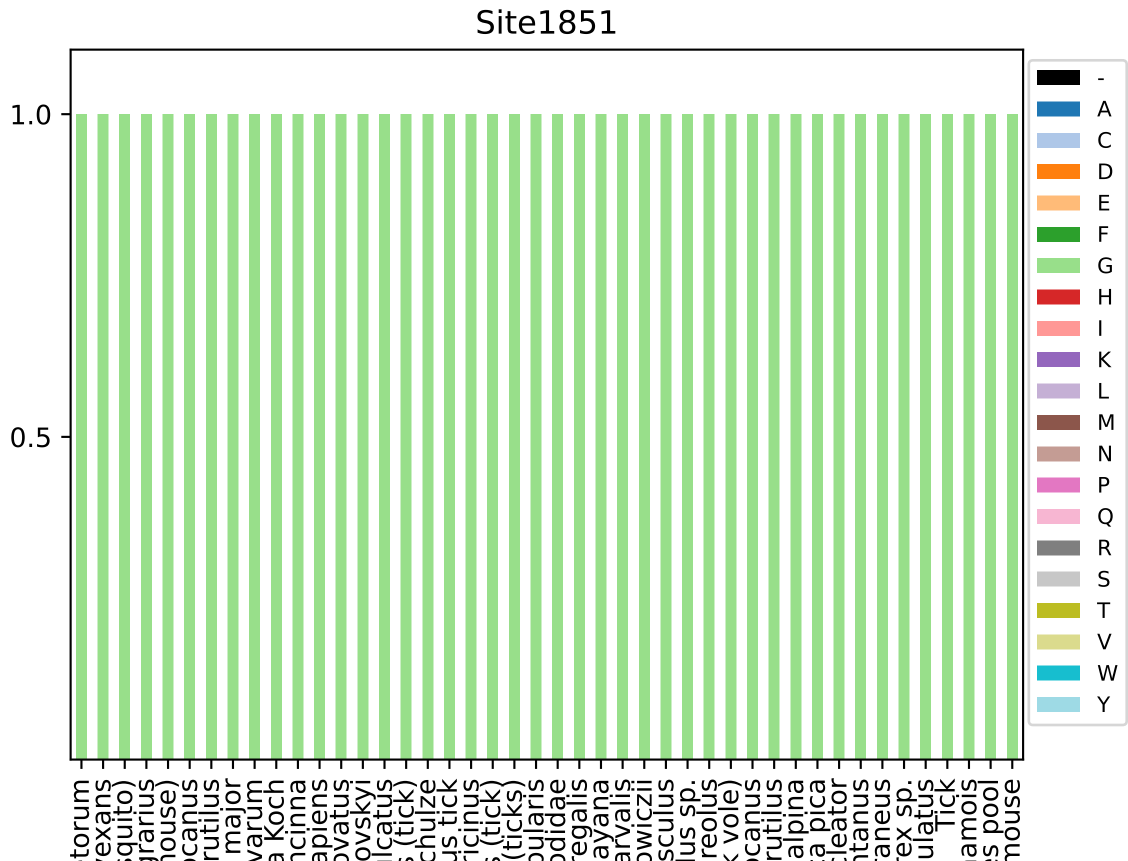
Site1851
1.0
0.5
-
A
C
D
E
F
G
H
I
K
L
M
N
P
Q
R
S
T
V
W
Y

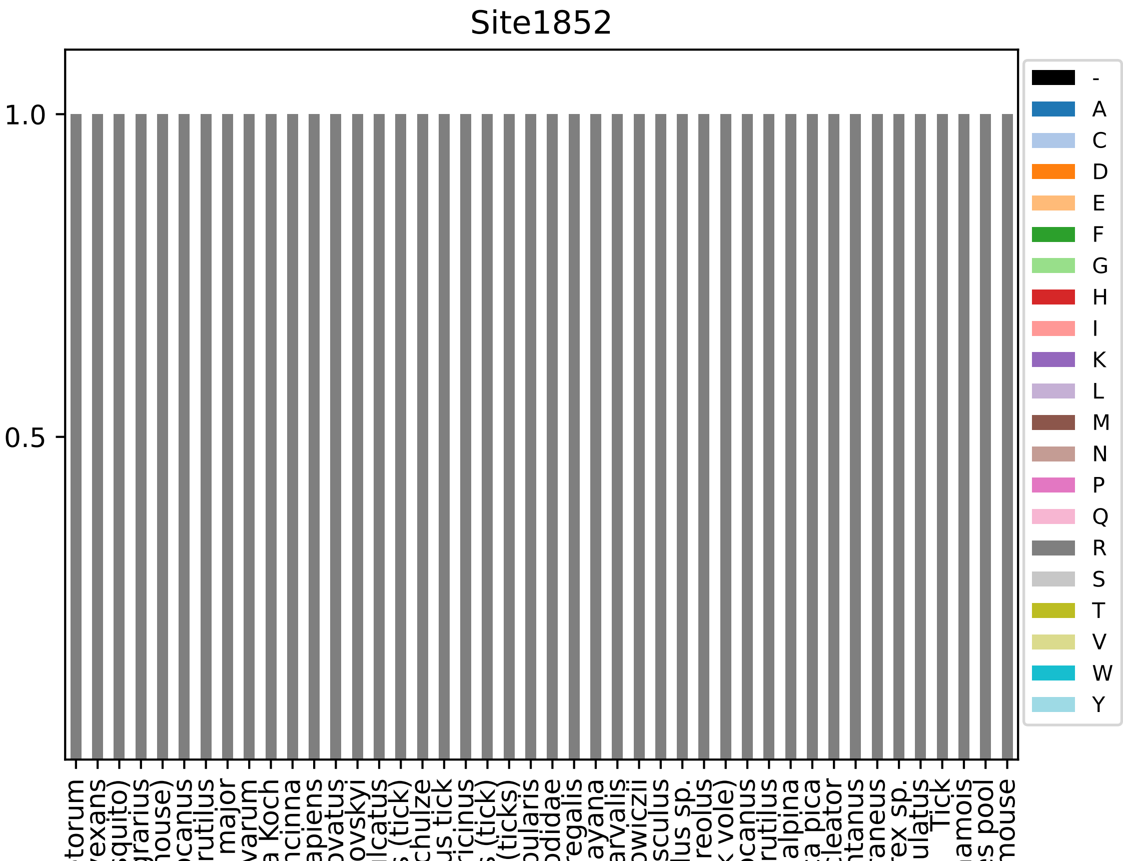
Site1852
1.0
0.5
-
A
C
D
E
F
G
H
I
K
L
M
N
P
Q
R
S
T
V
W
Y

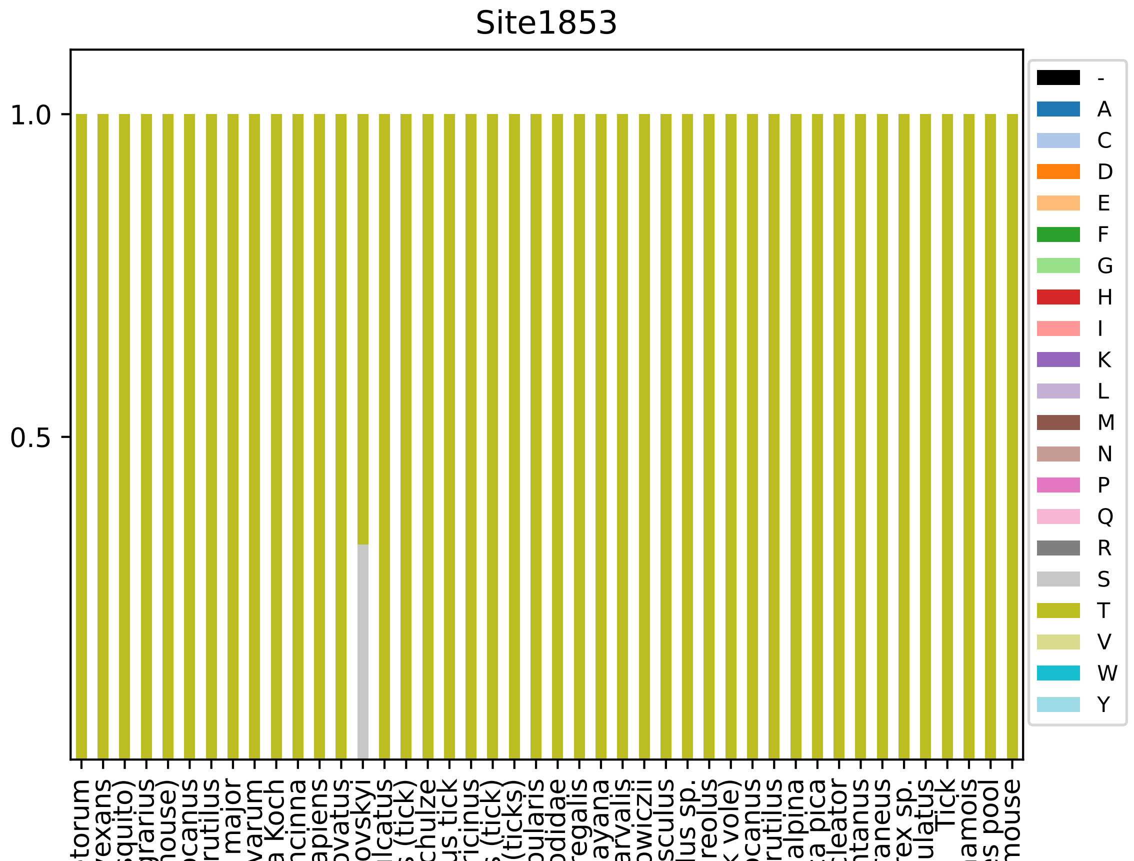
Site1853
1.0
0.5
-
A
C
D
E
F
G
H
I
K
L
M
N
P
Q
R
S
T
V
W
Y

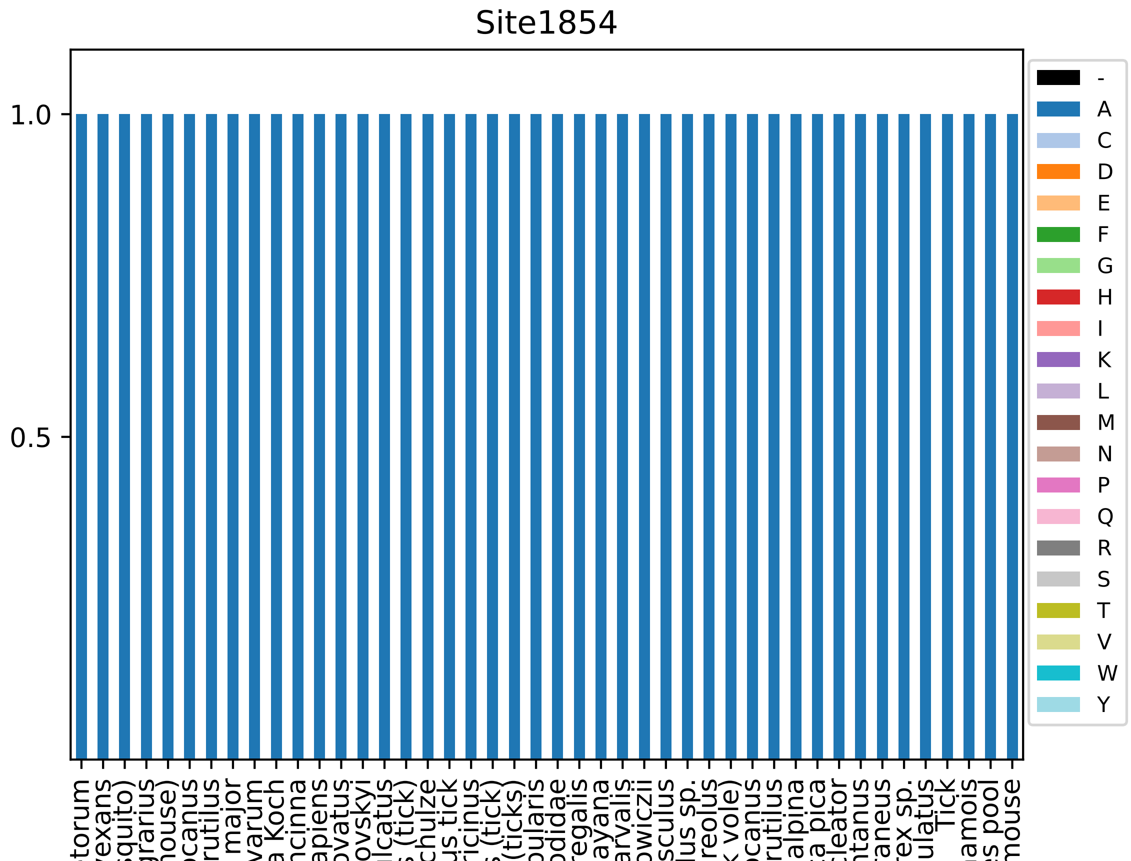
Site1854
1.0
0.5
-
A
C
D
E
F
G
H
I
K
L
M
N
P
Q
R
S
T
V
W
Y

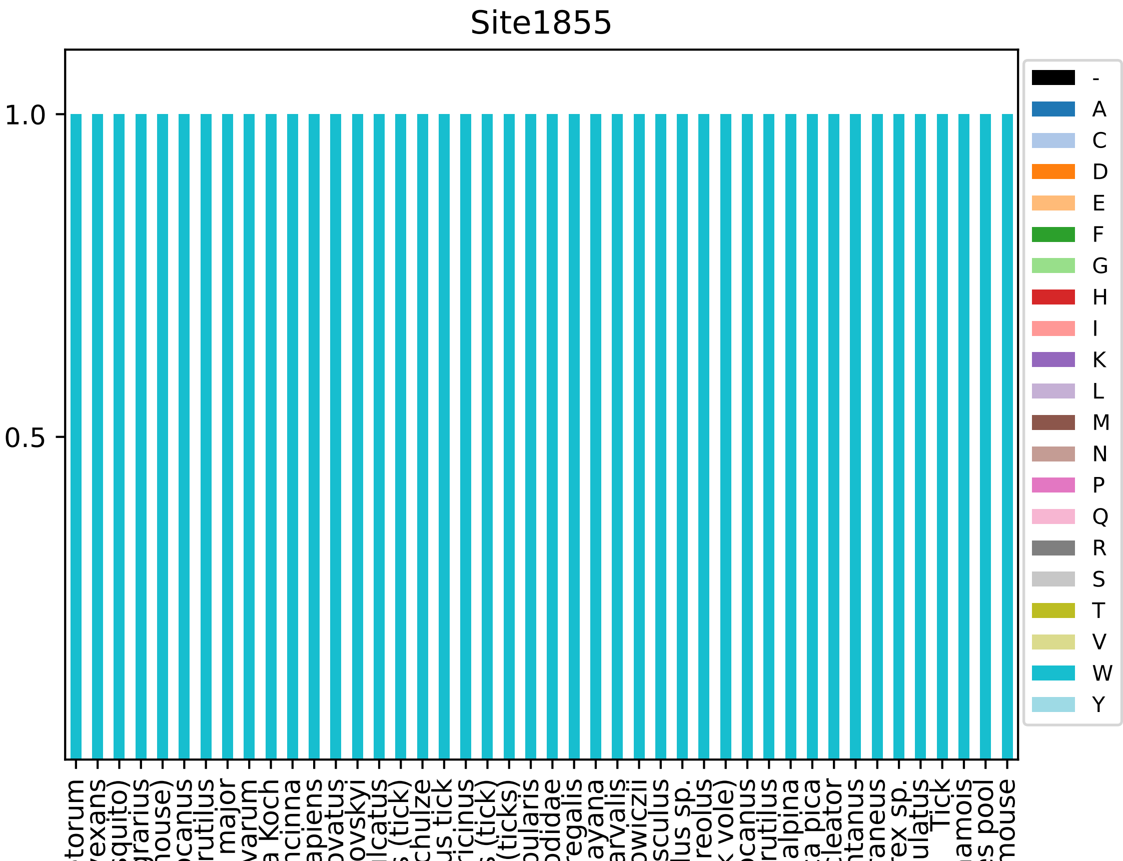
Site1855
1.0
0.5
-
A
C
D
E
F
G
H
I
K
L
M
N
P
Q
R
S
T
V
W
Y

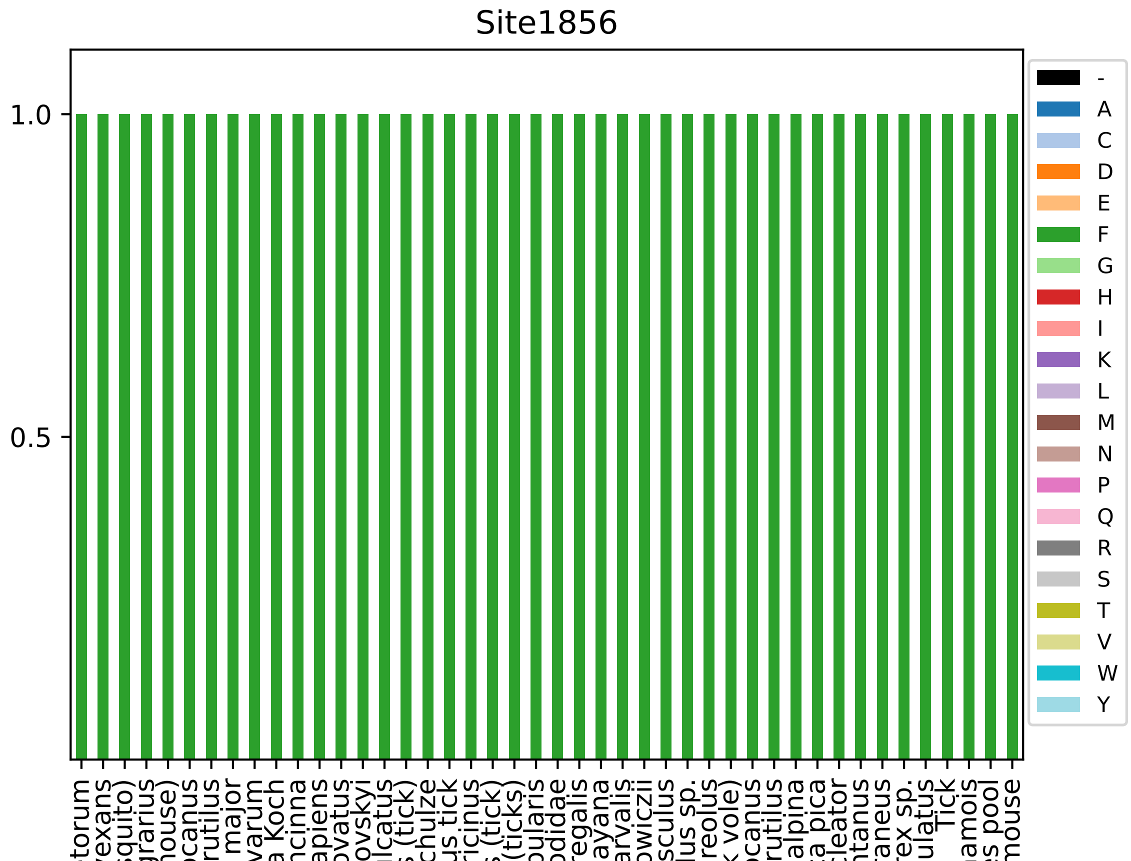
Site1856
1.0
0.5
-
A
C
D
E
F
G
H
I
K
L
M
N
P
Q
R
S
T
V
W
Y

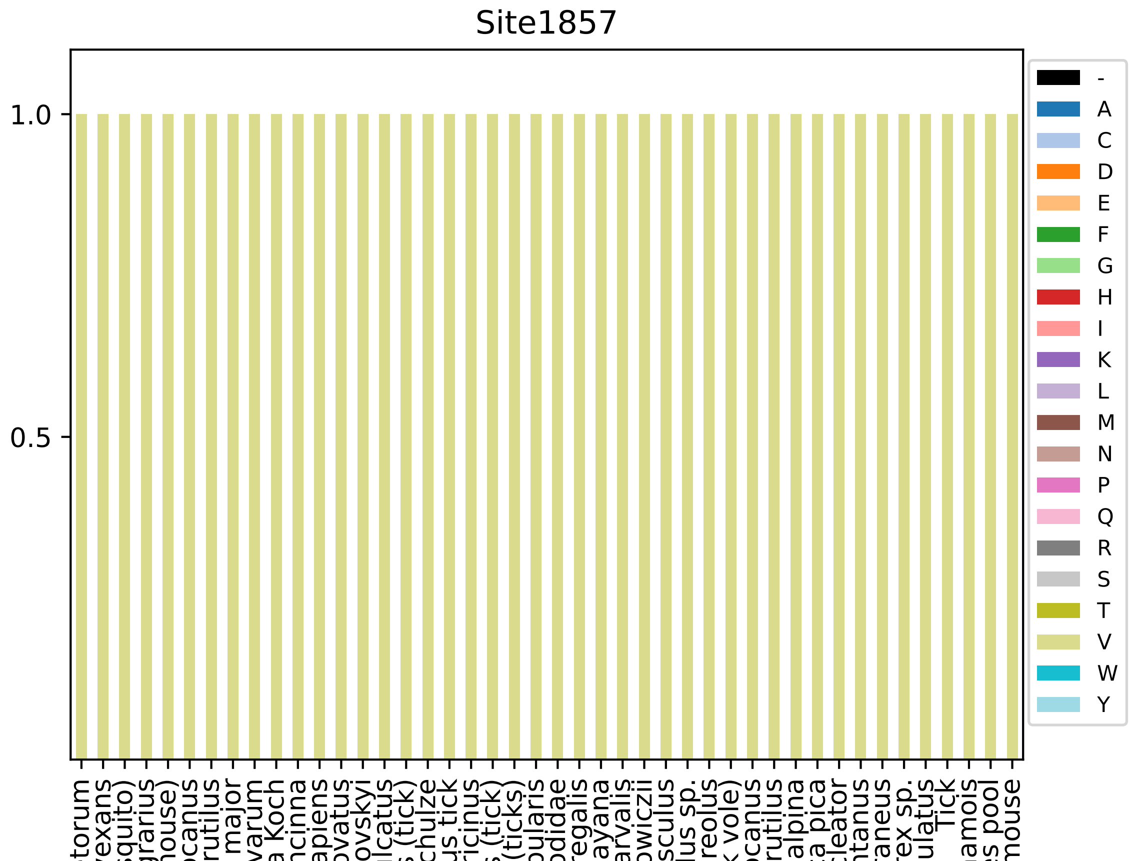
Site1857
1.0
0.5
-
A
C
D
E
F
G
H
I
K
L
M
N
P
Q
R
S
T
V
W
Y

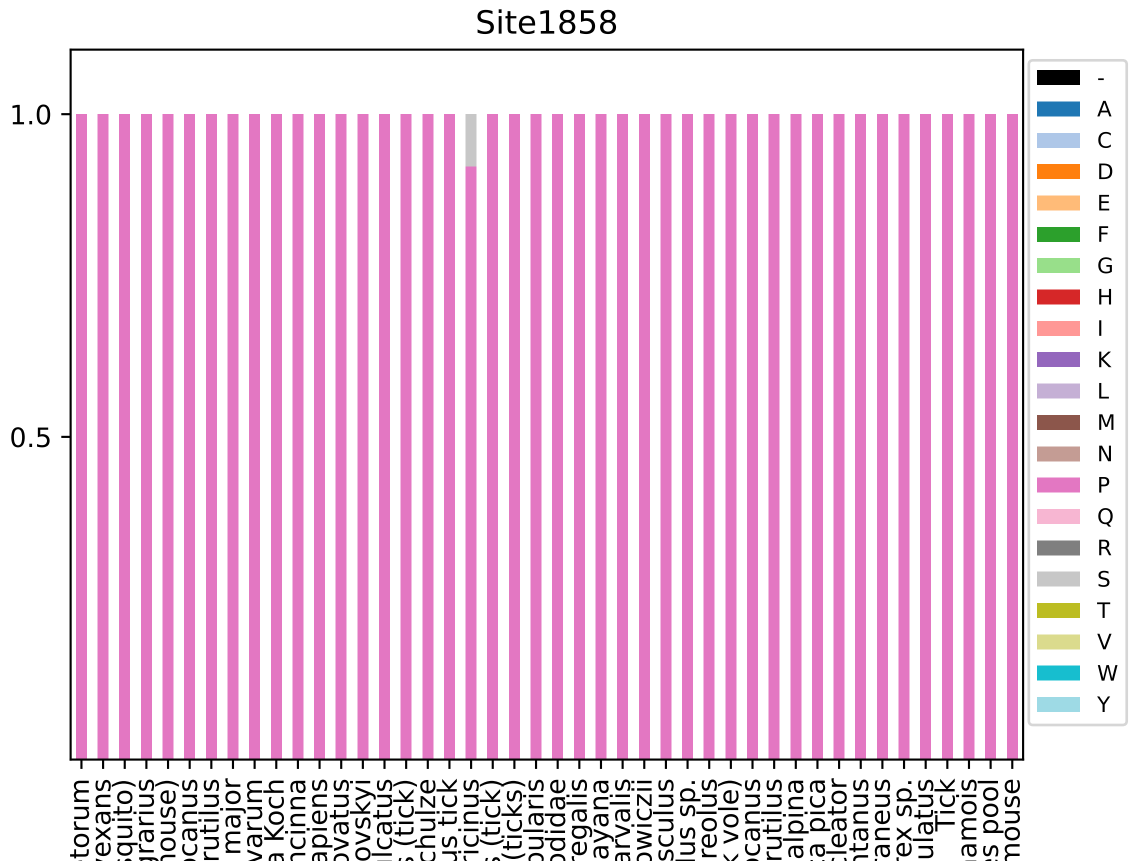

Site1858
1.0
0.5
-
A
C
D
E
F
G
H
I
K
L
M
N
P
Q
R
S
T
V
W
Y

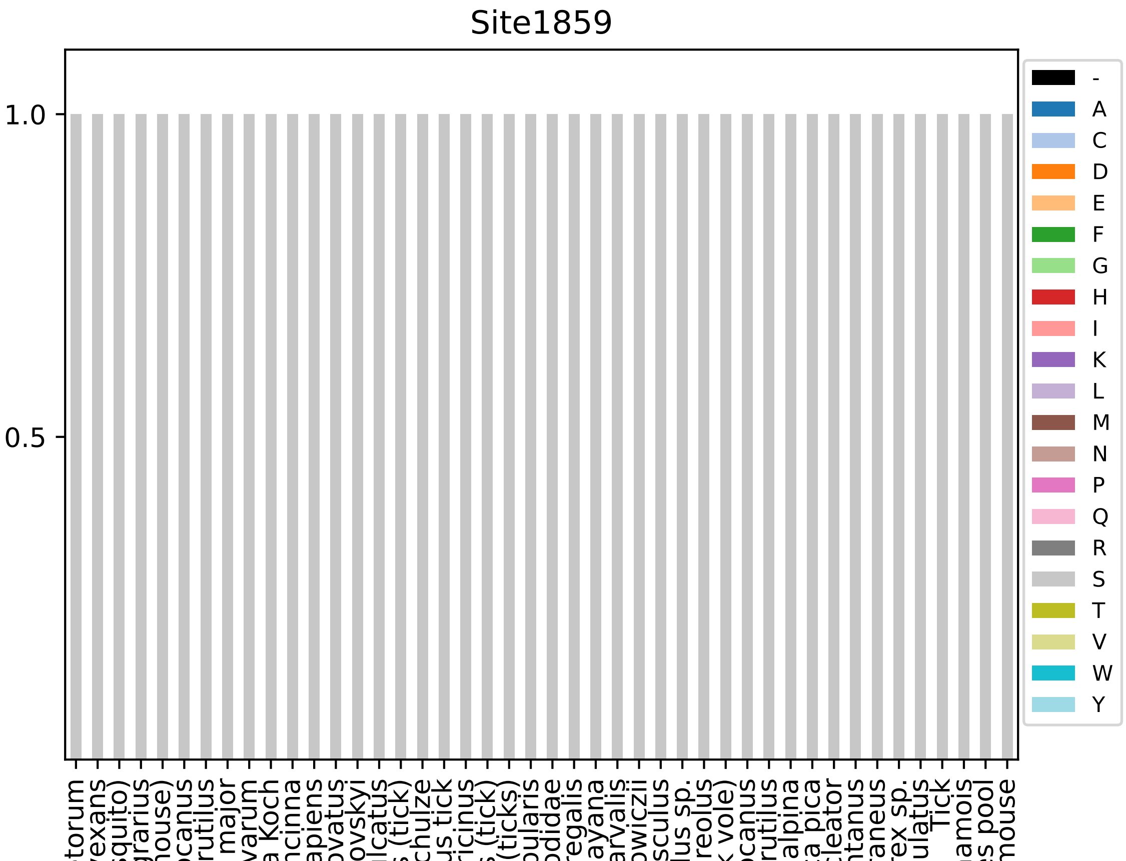
Site1859
1.0
0.5
-
A
C
D
E
F
G
H
I
K
L
M
N
P
Q
R
S
T
V
W
Y

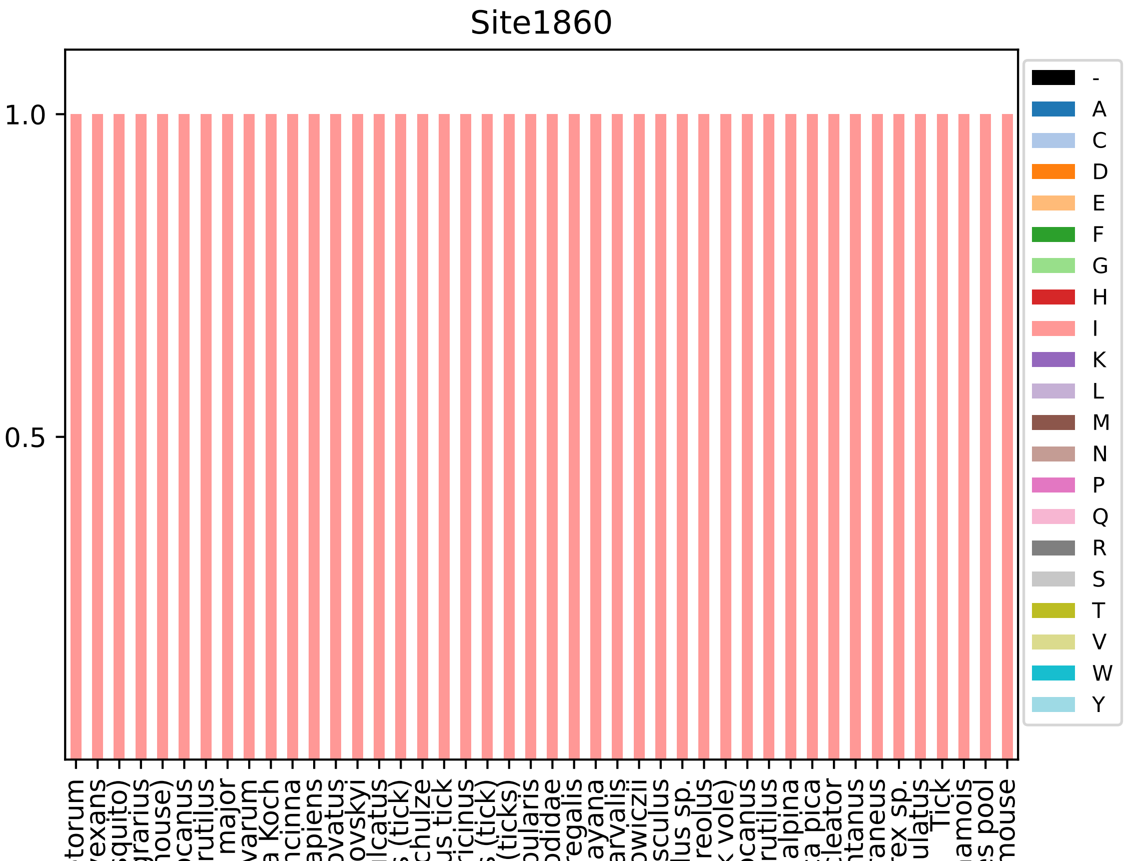

Site1860
1.0
0.5
-
A
C
D
E
F
G
H
I
K
L
M
N
P
Q
R
S
T
V
W
Y
Tick

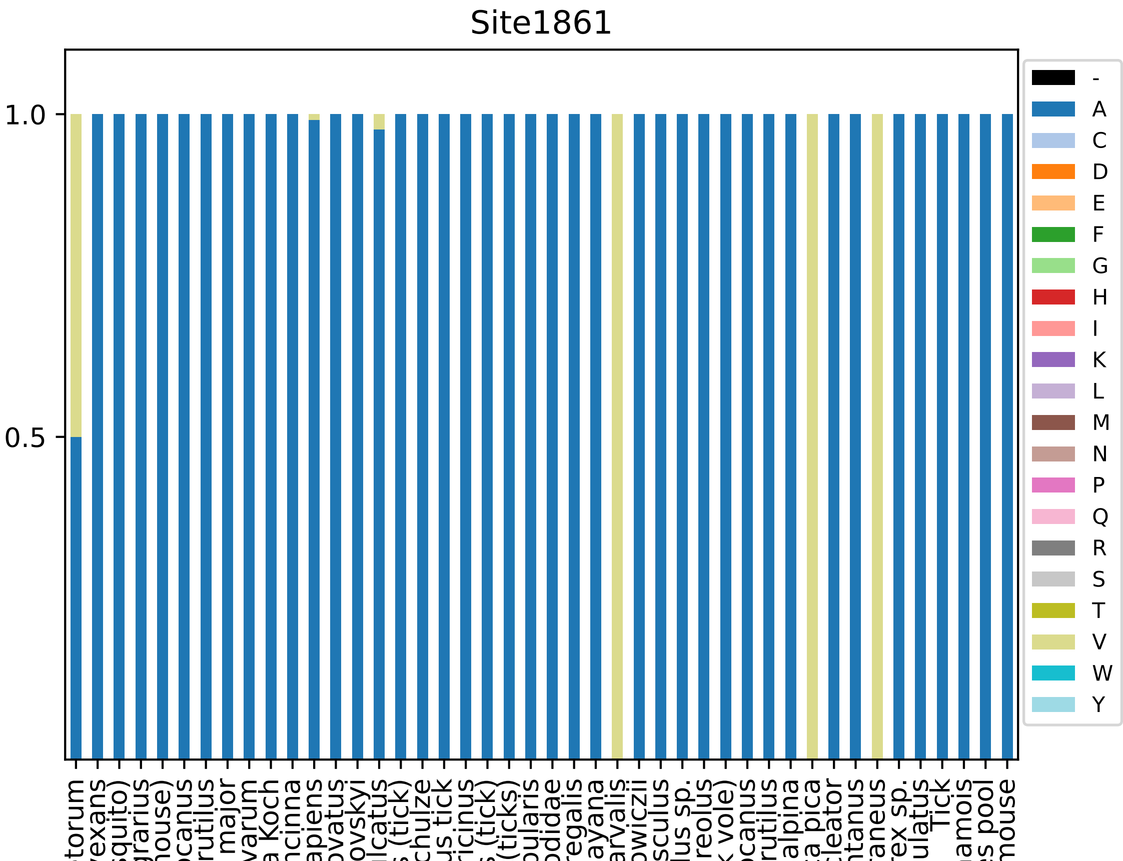
Site1861
1.0
0.5
-
A
C
D
E
F
G
H
I
K
L
M
N
P
Q
R
S
T
V
W
Y

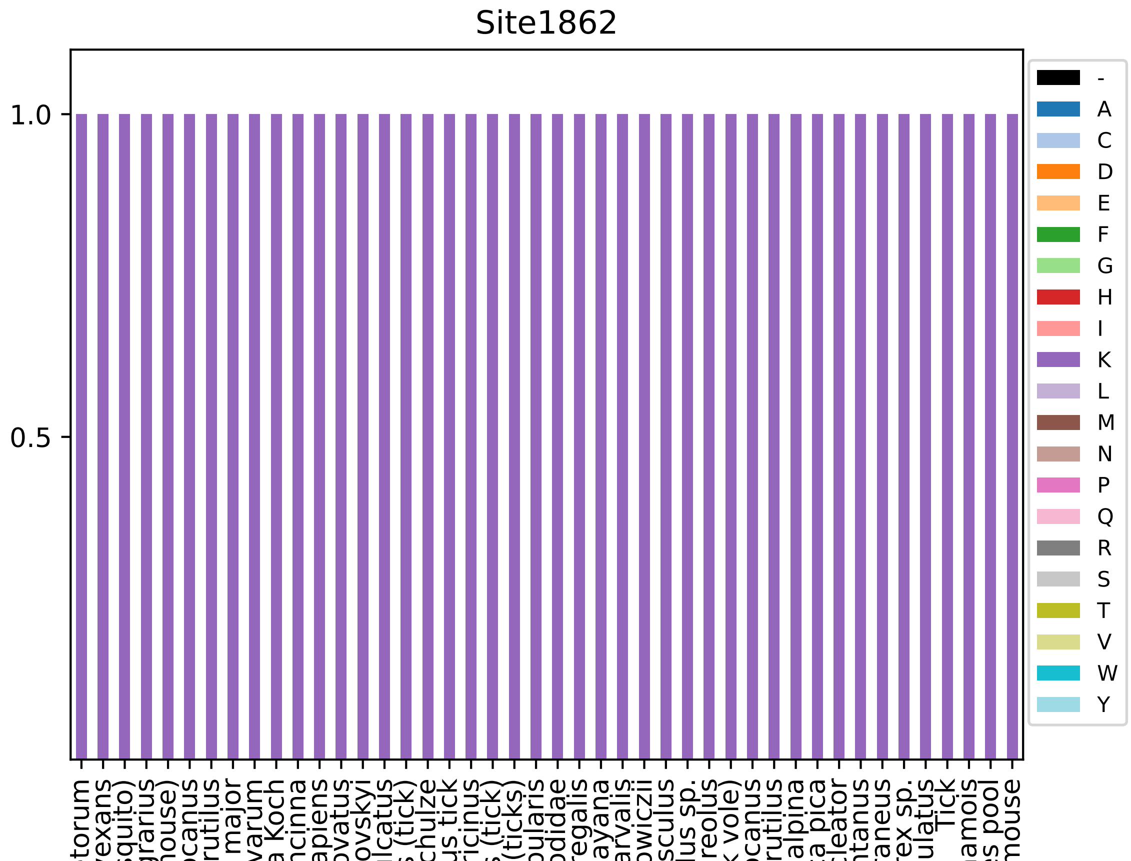
Site1862
1.0
0.5
-
A
C
D
E
F
G
H
I
K
L
M
N
P
Q
R
S
T
V
W
Y

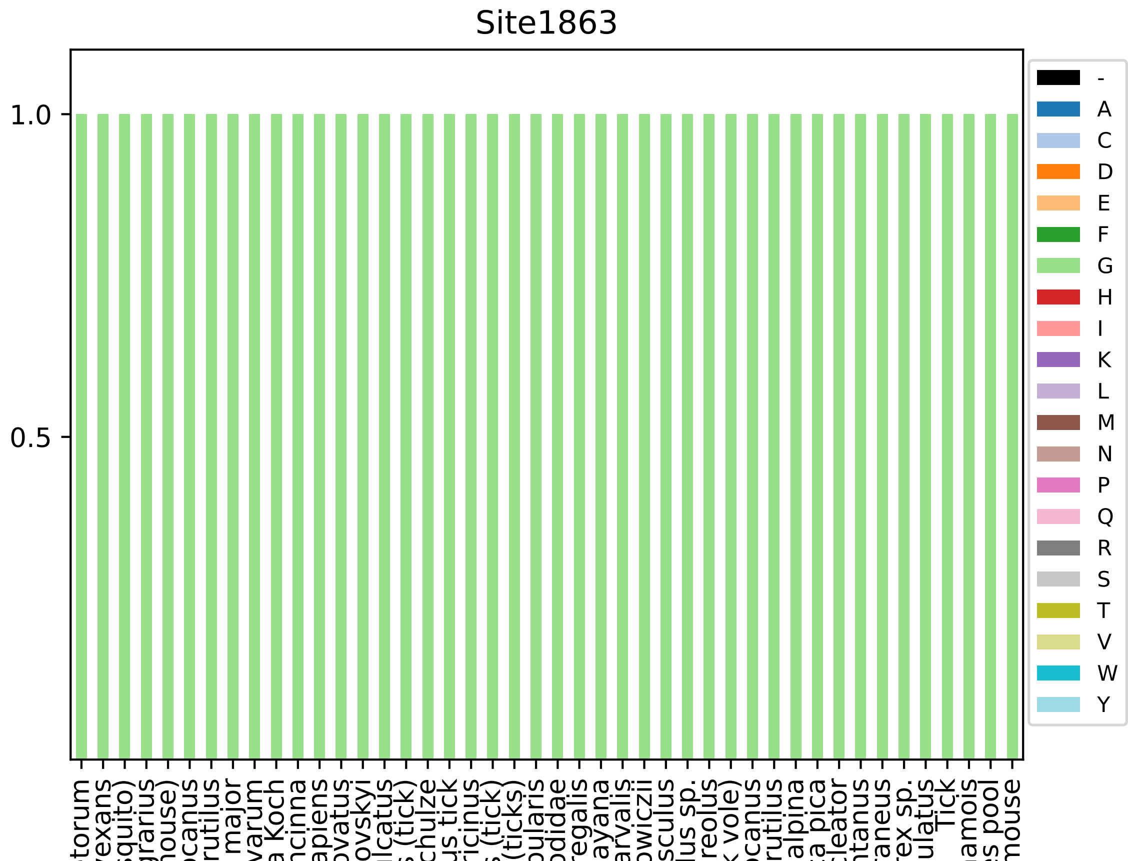
Site1863
1.0
0.5
-
A
C
D
E
F
G
H
I
K
L
M
N
P
Q
R
S
T
V
W
Y

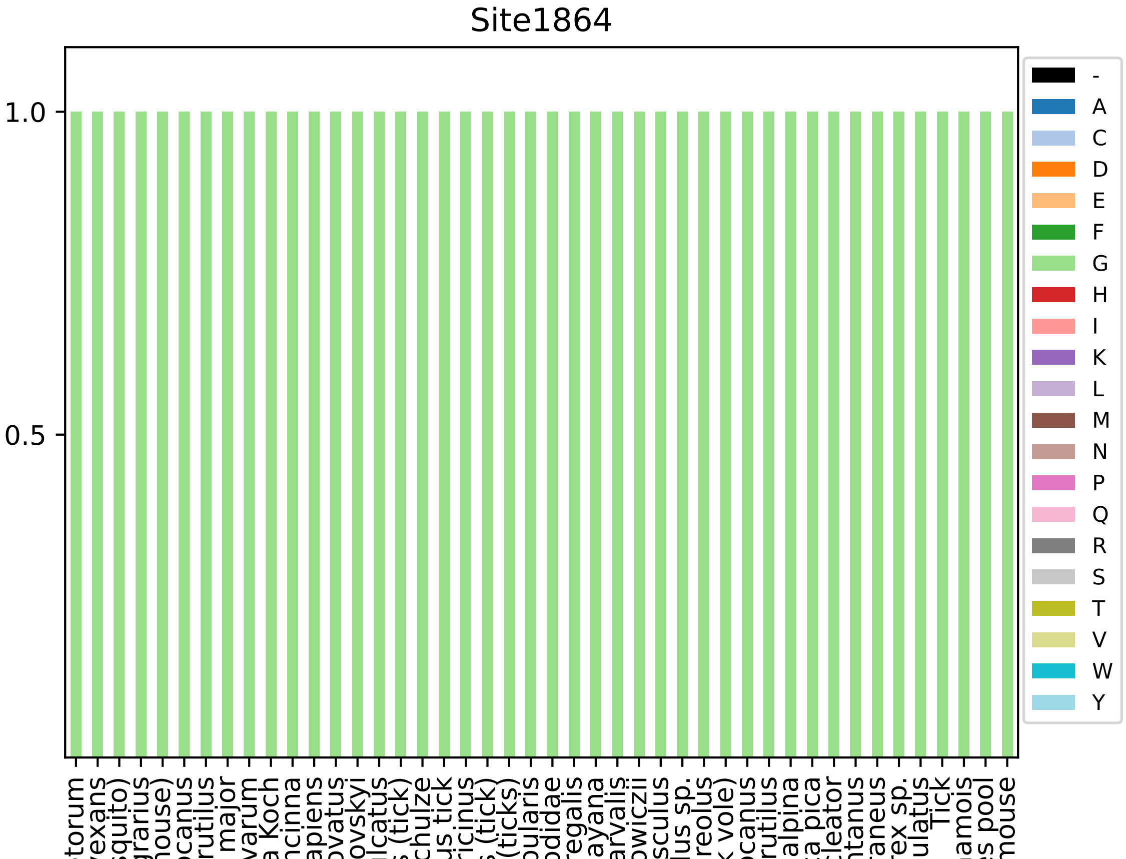

Site1864
1.0
0.5
-
A
C
D
E
F
G
H
I
K
L
M
N
P
Q
R
S
T
V
W
Y

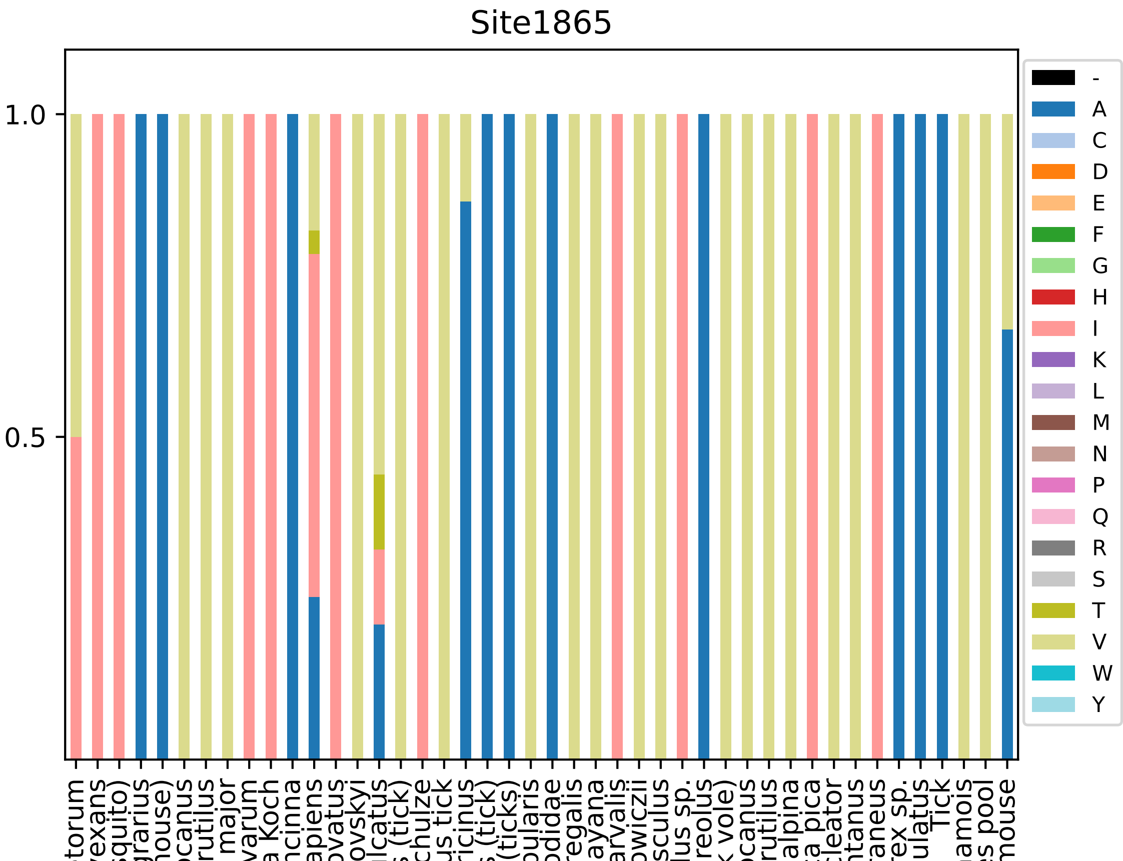
Site1865
1.0
0.5
-
A
C
D
E
F
G
H
I
K
L
M
N
P
Q
R
S
T
V
W
Y

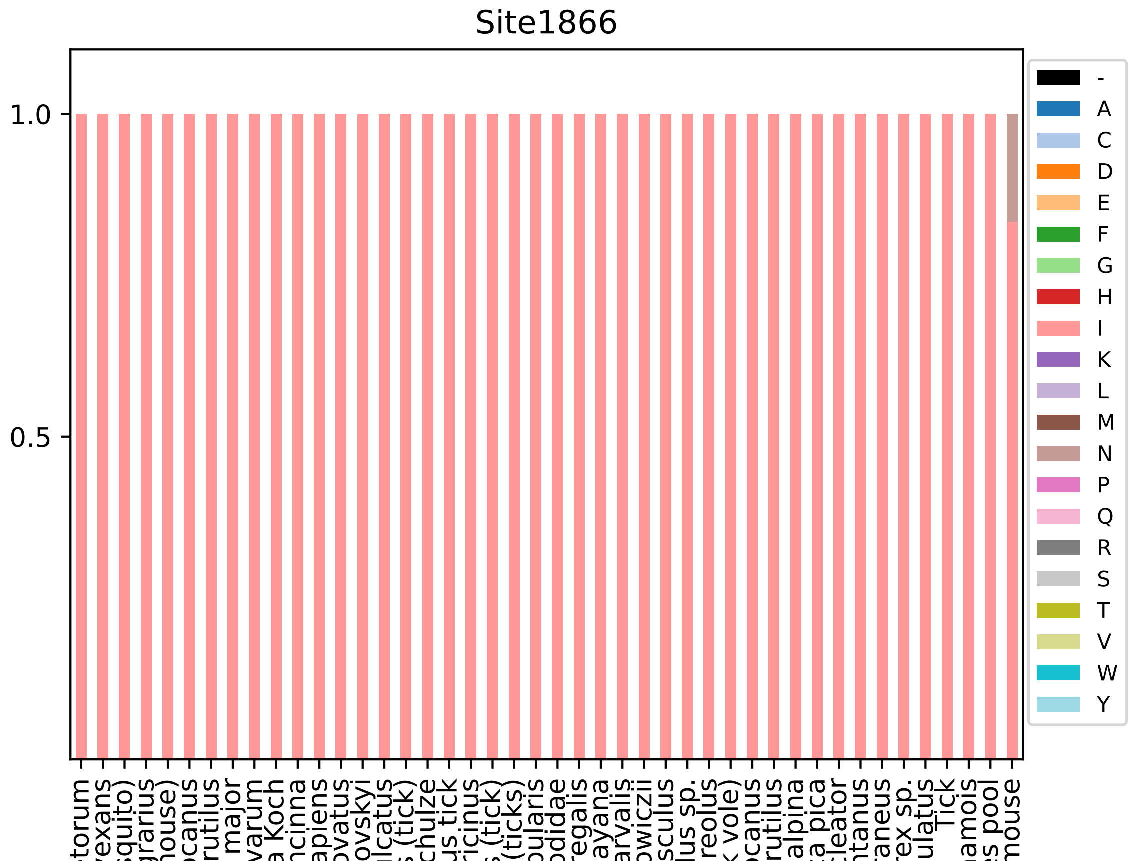

Site1866
1.0
0.5
-
A
C
D
E
F
G
H
I
K
L
M
N
P
Q
R
S
T
V
W
Y

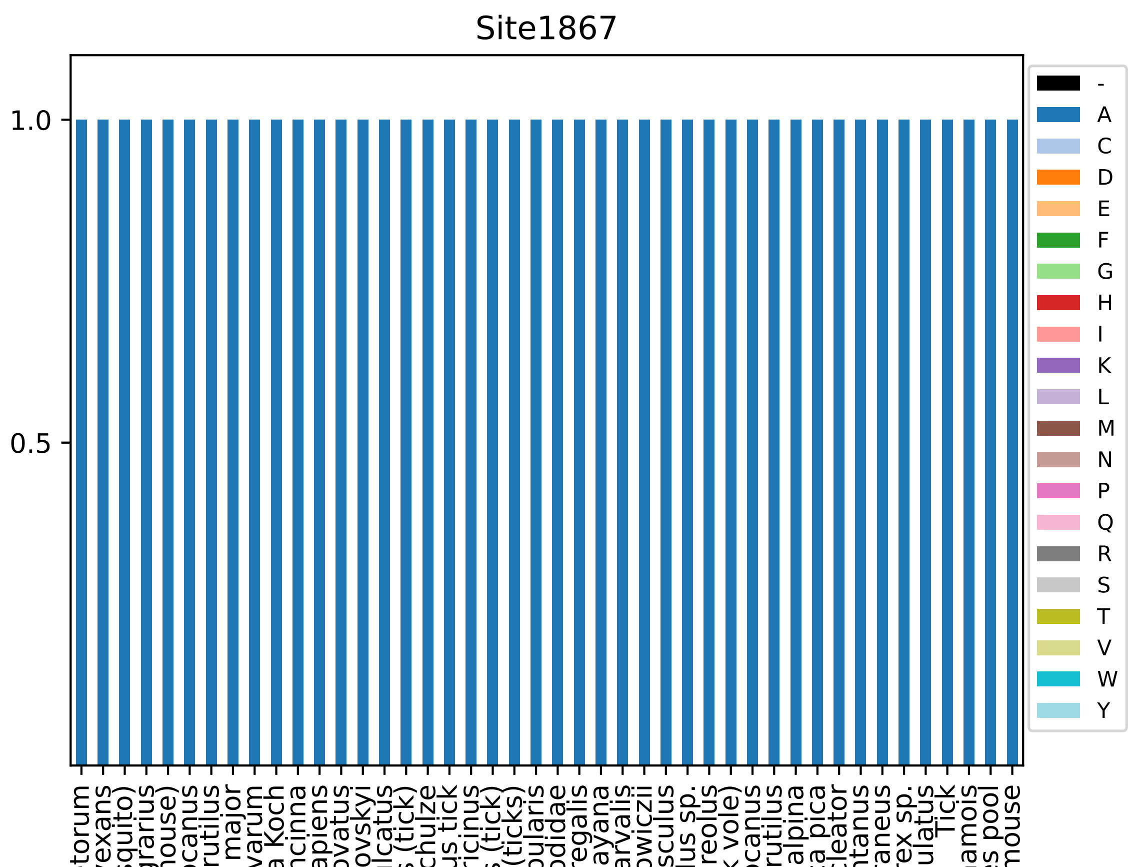
Site1867
1.0
0.5
-
A
C
D
E
F
G
H
I
K
L
M
N
P
Q
R
S
T
V
W
Y

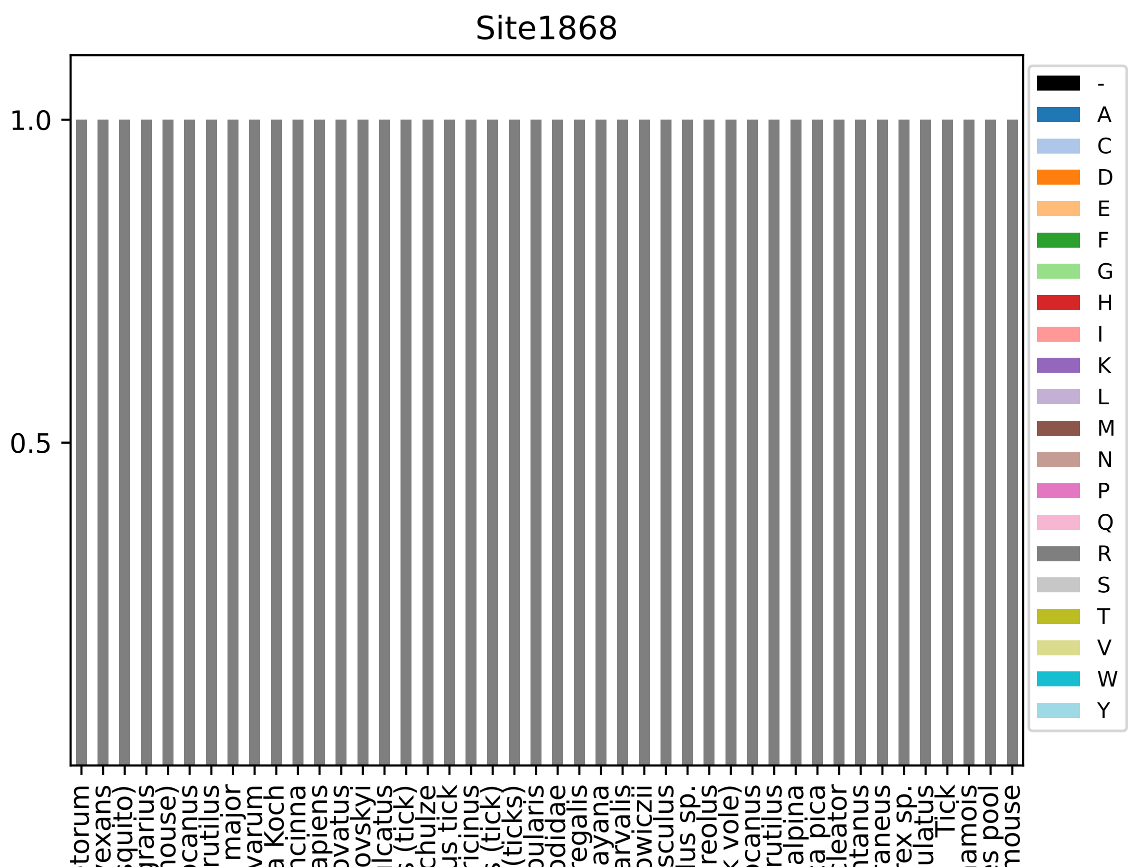
Site1868
1.0
0.5
-
A
C
D
E
F
G
H
I
K
L
M
N
P
Q
R
S
T
V
W
Y

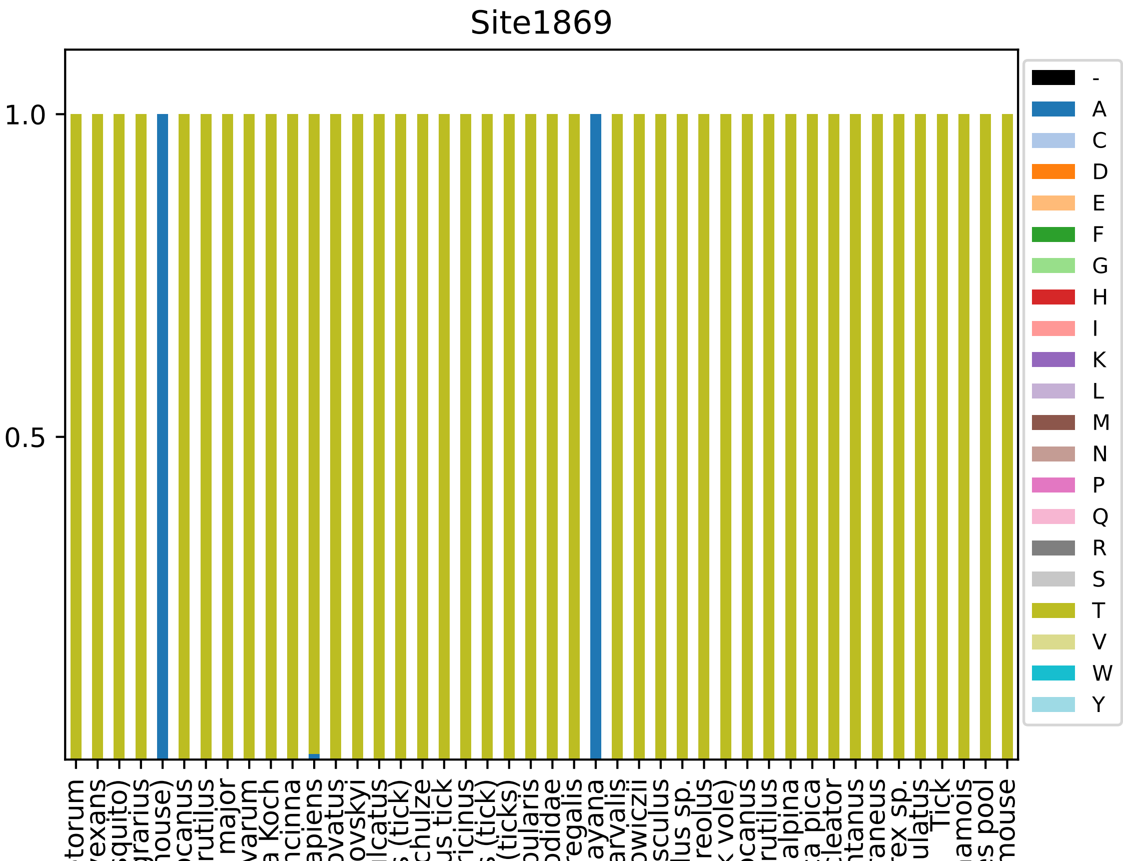
Site1869
1.0
0.5
-
A
C
D
E
F
G
H
I
K
L
M
N
P
Q
R
S
T
V
W
Y

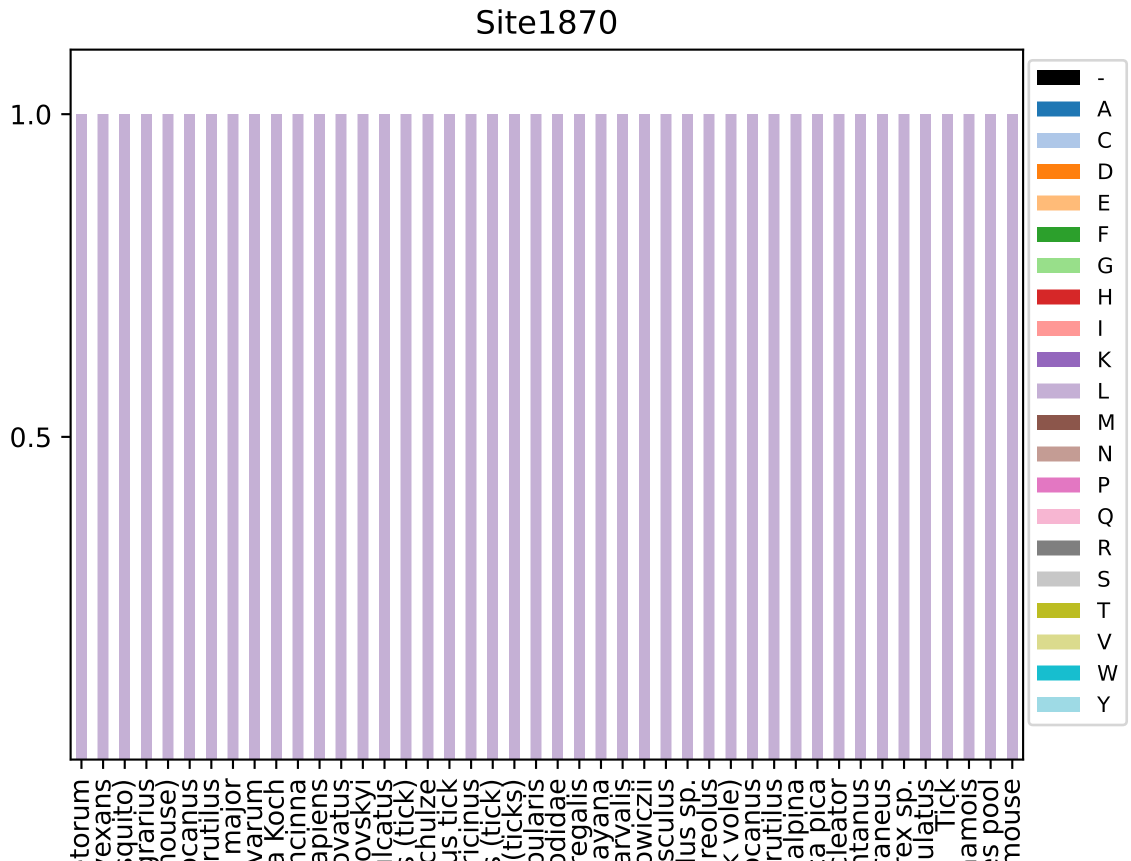

Site1870
1.0
0.5
-
A
C
D
E
F
G
H
I
K
L
M
N
P
Q
R
S
T
V
W
Y

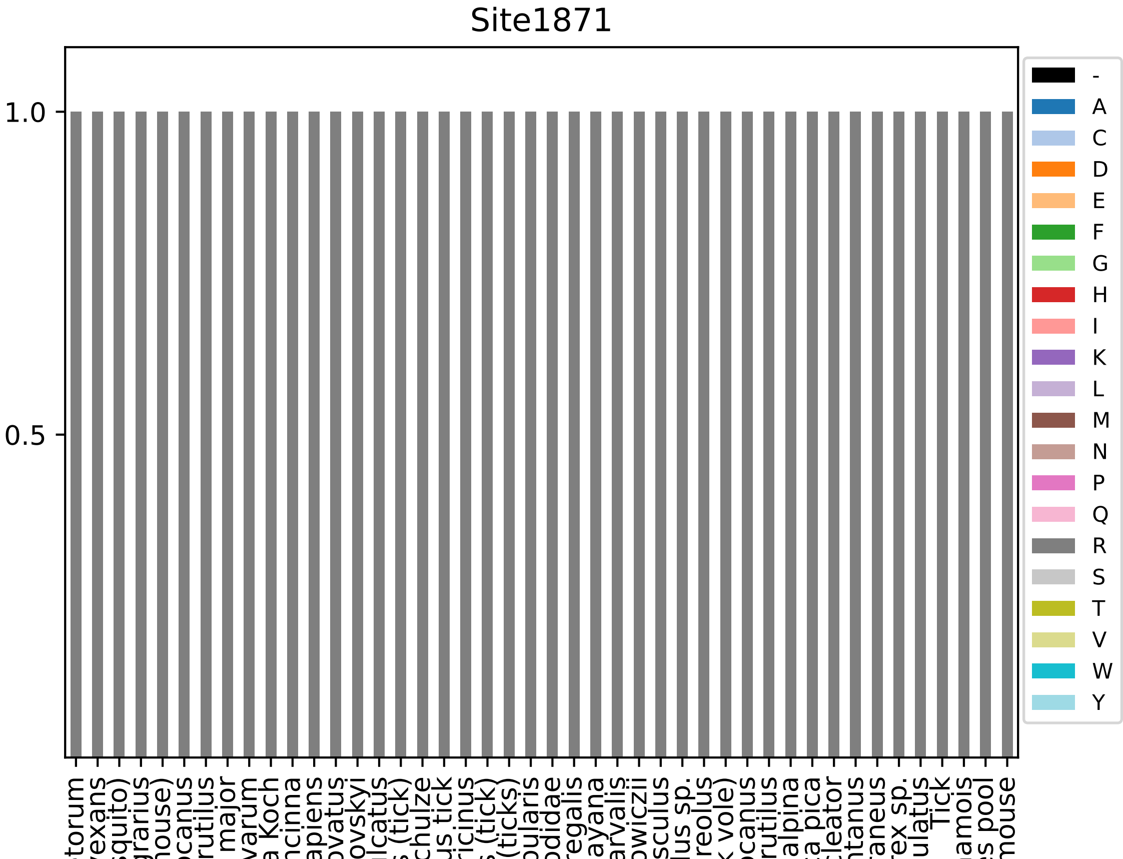
Site1871
1.0
0.5
-
A
C
D
E
F
G
H
I
K
L
M
N
P
Q
R
S
T
V
W
Y

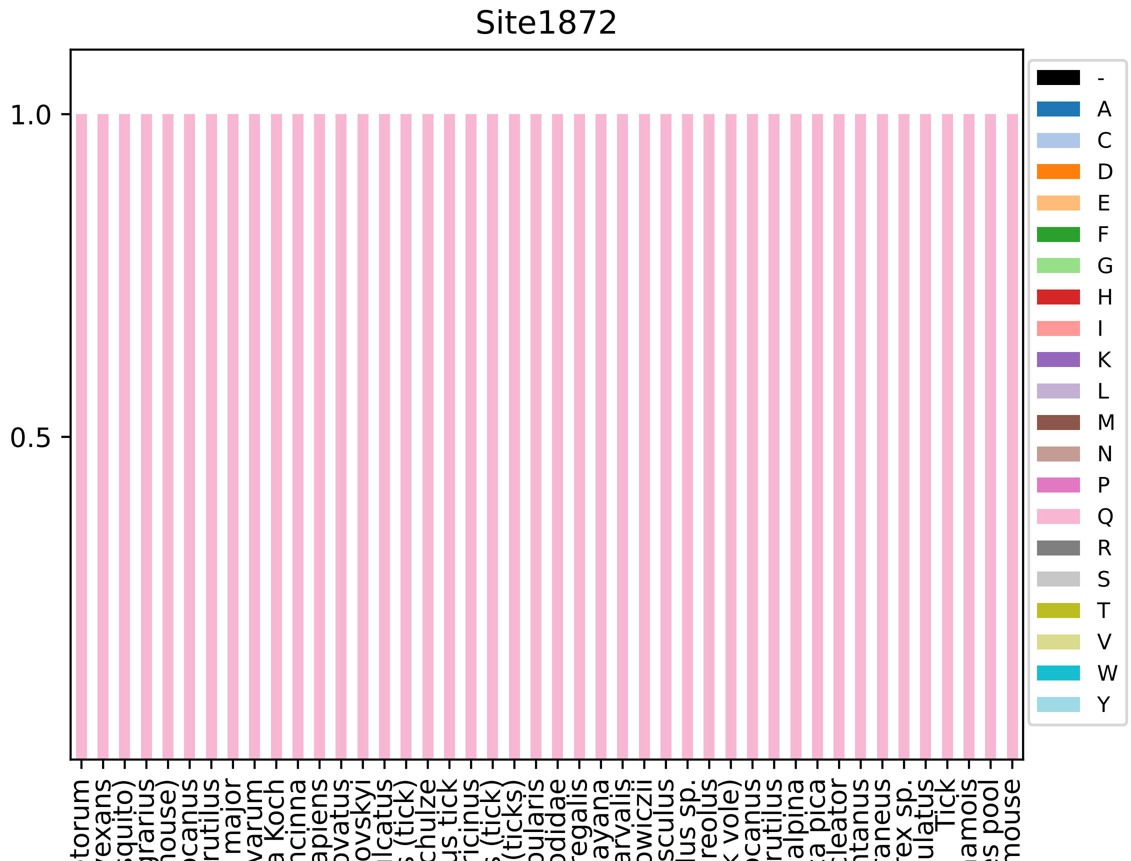
Site1872
1.0
0.5
-
A
C
D
E
F
G
H
I
K
L
M
N
P
Q
R
S
T
V
W
Y

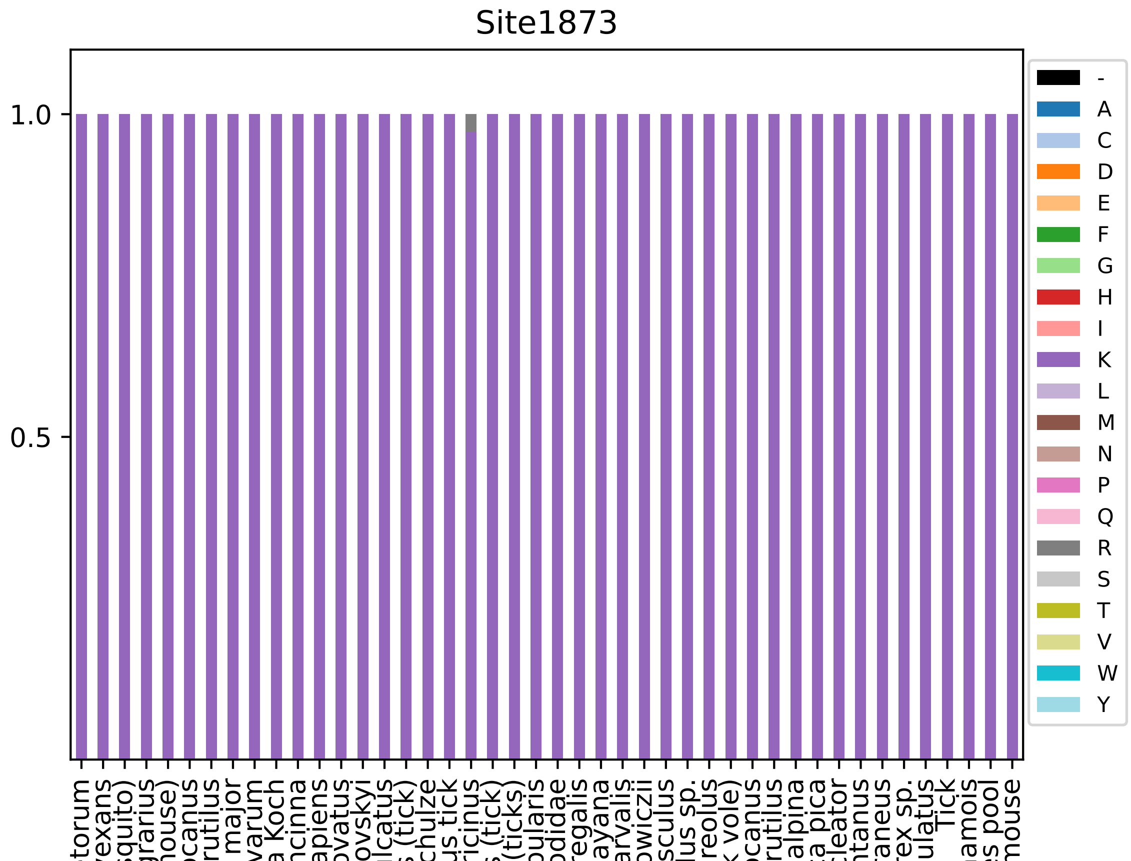
Site1873
1.0
0.5
-
A
C
D
E
F
G
H
I
K
L
M
N
P
Q
R
S
T
V
W
Y

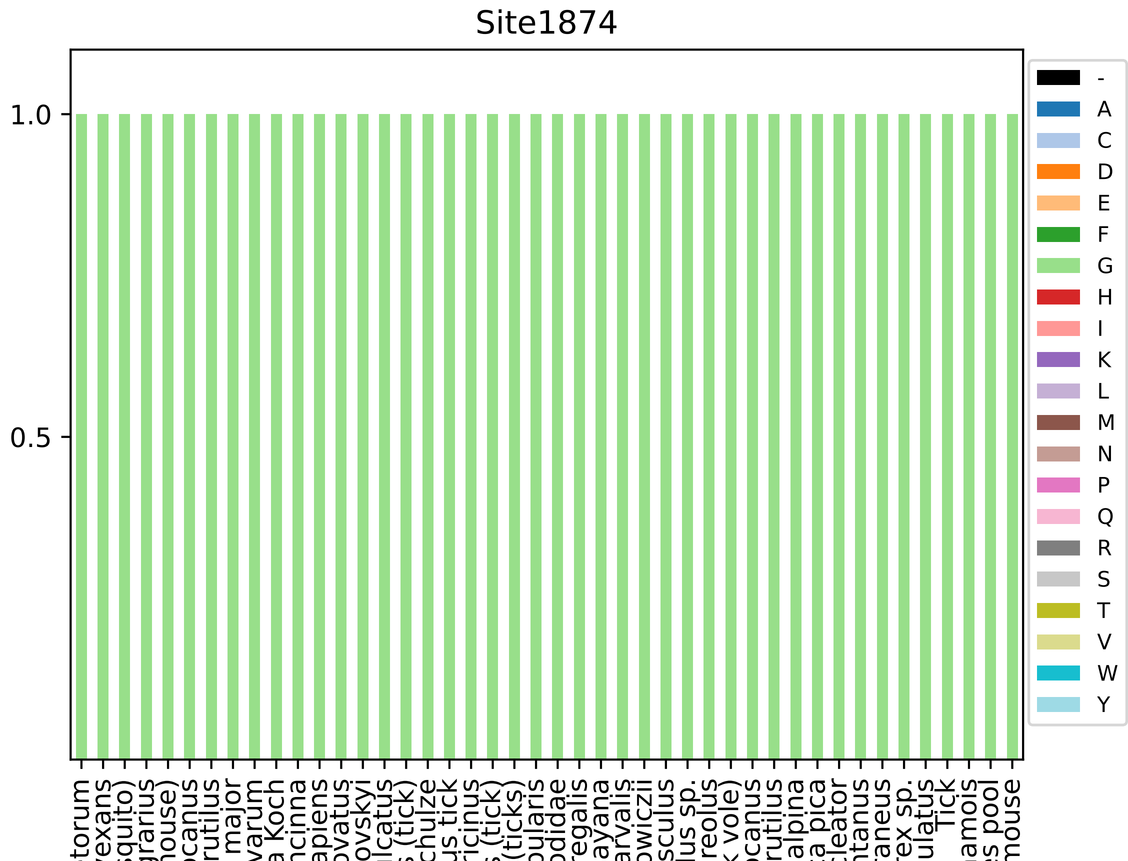
Site1874
1.0
0.5
-
A
C
D
E
F
G
H
I
K
L
M
N
P
Q
R
S
T
V
W
Y
Tick

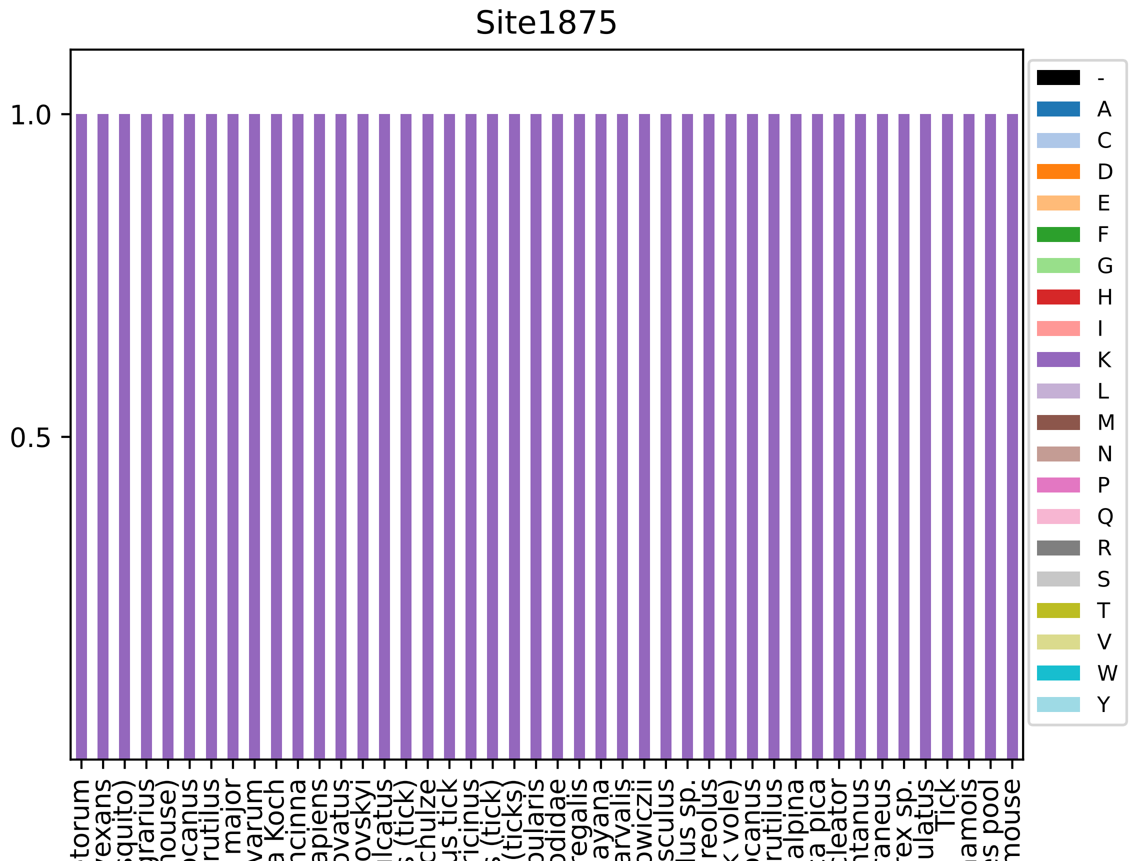

Site1875
1.0
0.5
-
A
C
D
E
F
G
H
I
K
L
M
N
P
Q
R
S
T
V
W
Y
torum
vexans
squito)
grarius
nouse)
ocanus
rutilus
major
varum
a Koch
ncinna
apiens
ovatus
ovskyi
ulcatus
s (tick)
chulze
us tick
ricinus
s (tick)
(ticks)
pularis
podidae
regalis
ayana
arvalis
owiczii
sculus
lus sp.
reolus
k vole)
ocanus
rutilus
alpina
a pica
cleator
ntanus
aneus
rex sp.
ulatus
Tick
amois
es pool
mouse

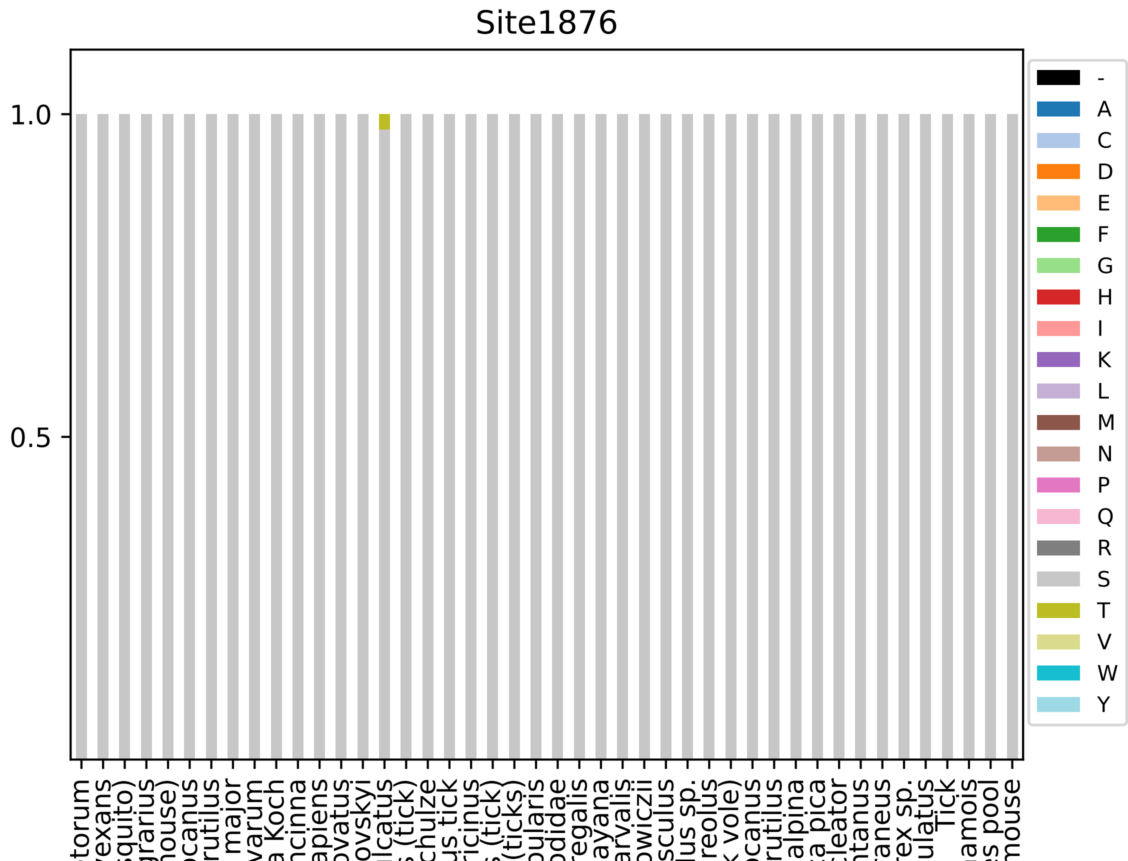
Site1876
1.0
0.5
-
A
C
D
E
F
G
H
I
K
L
M
N
P
Q
R
S
T
V
W
Y

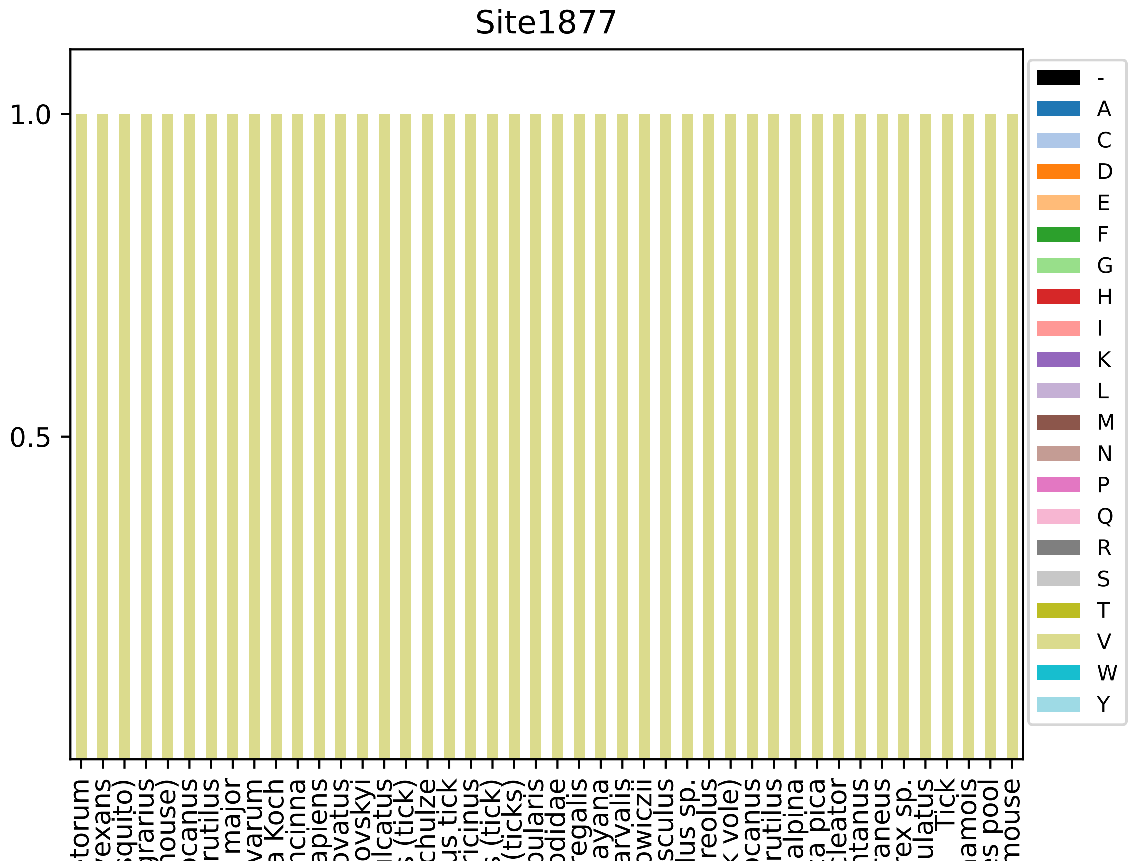
Site1877
1.0
0.5
-
A
C
D
E
F
G
H
I
K
L
M
N
P
Q
R
S
T
V
W
Y

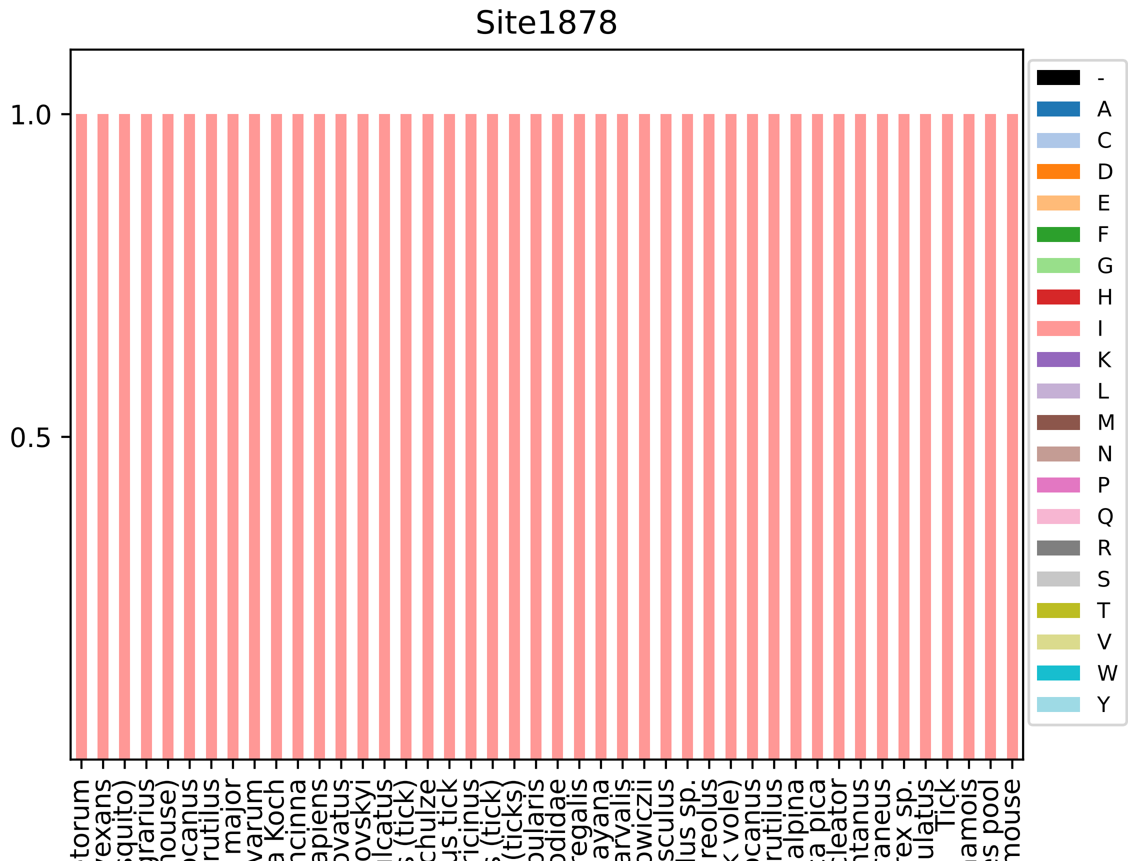
Site1878
1.0
0.5
-
A
C
D
E
F
G
H
I
K
L
M
N
P
Q
R
S
T
V
W
Y

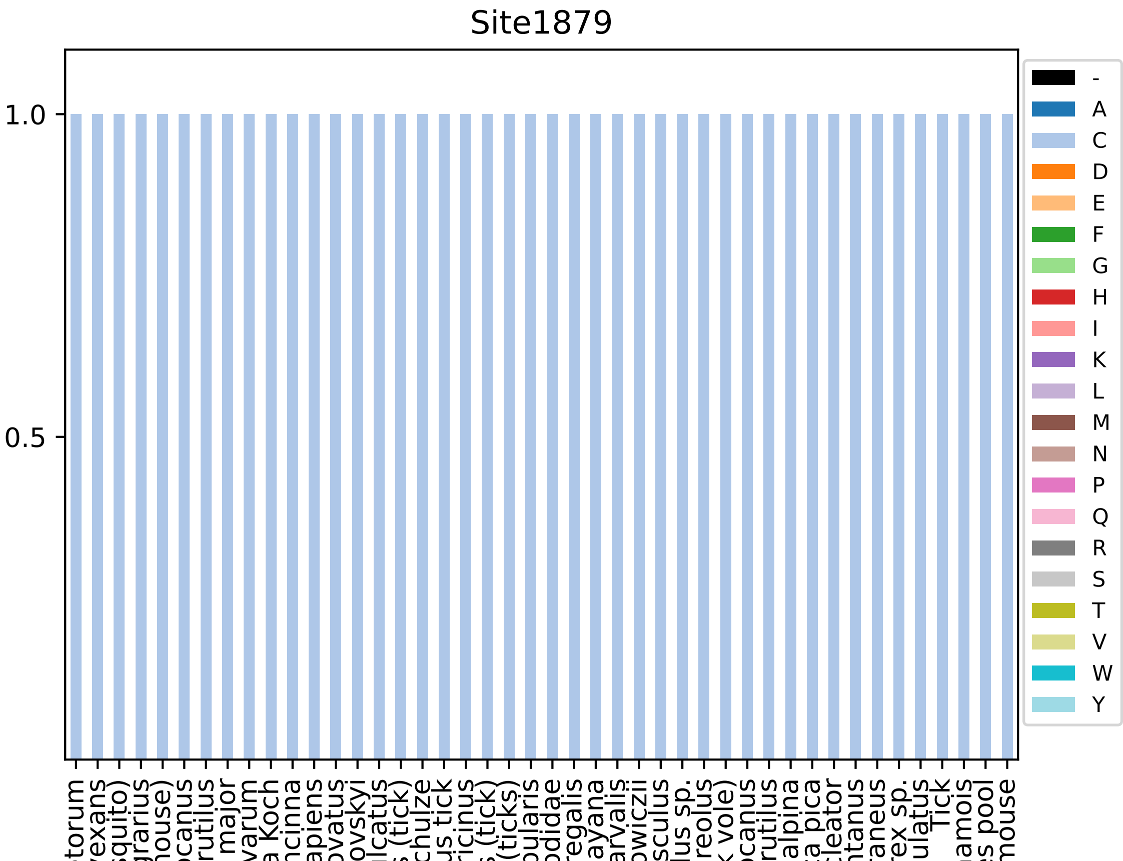

Site1879
1.0
0.5
-
A
C
D
E
F
G
H
I
K
L
M
N
P
Q
R
S
T
V
W
Y

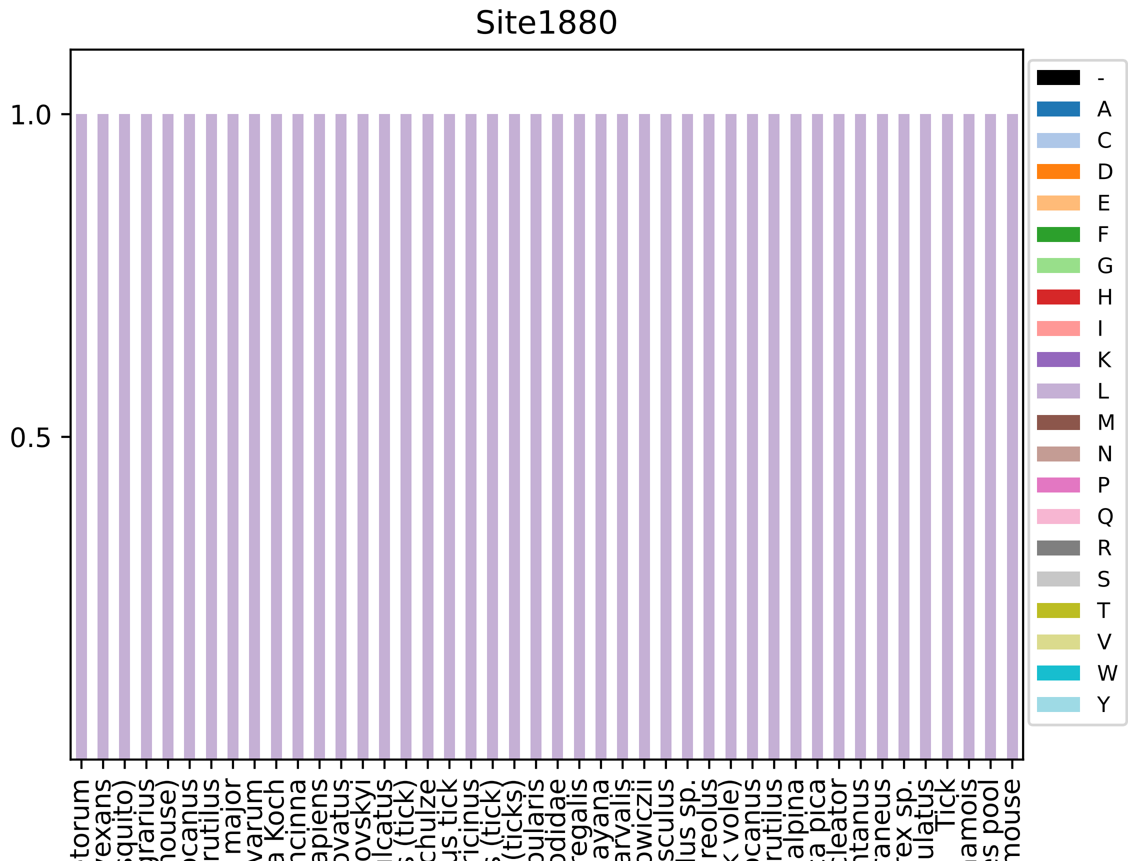
Site1880
1.0
0.5
-
A
C
D
E
F
G
H
I
K
L
M
N
P
Q
R
S
T
V
W
Y

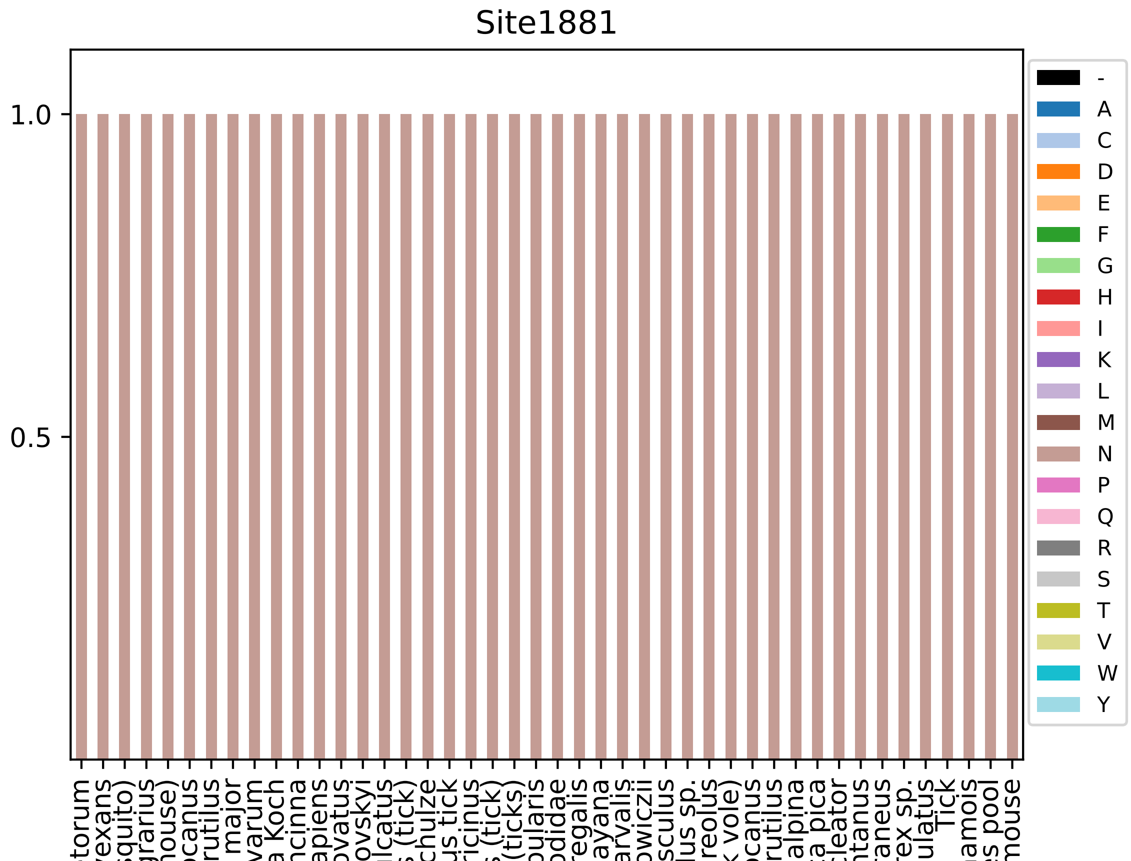
Site1881
1.0
0.5
-
A
C
D
E
F
G
H
I
K
L
M
N
P
Q
R
S
T
V
W
Y

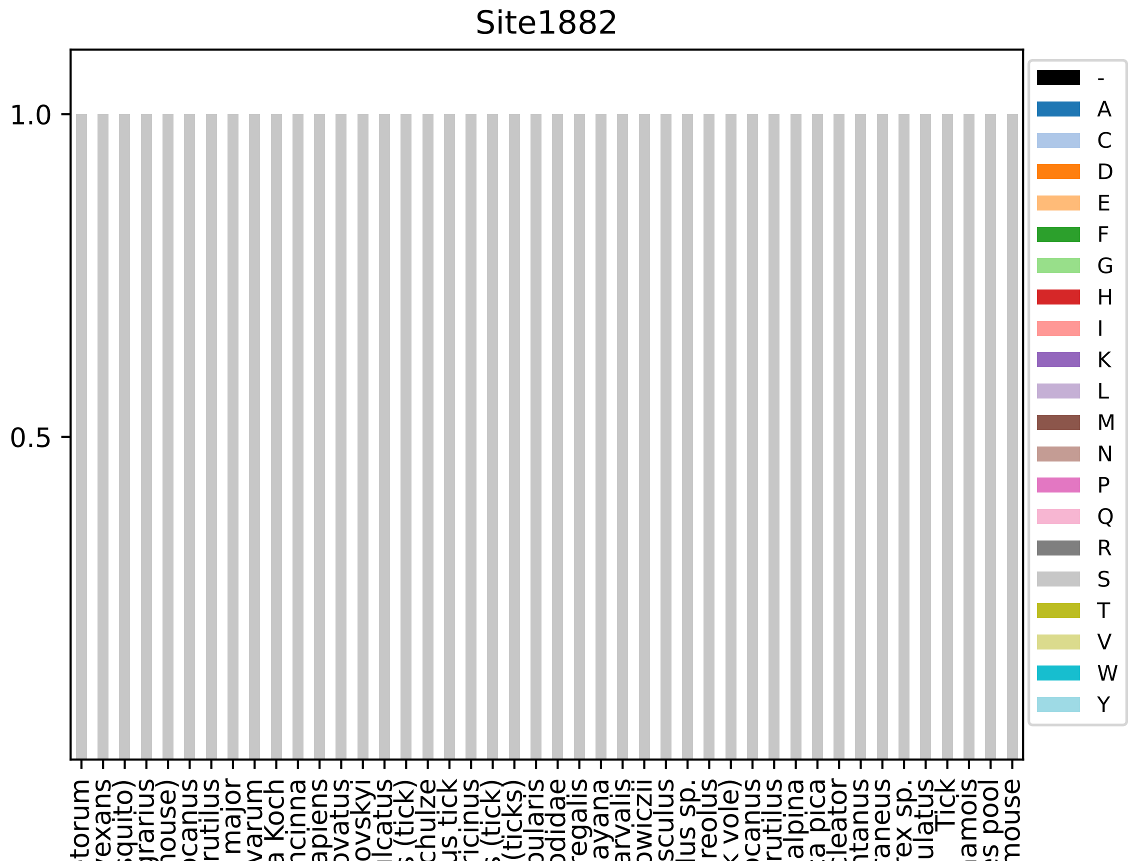
Site1882
1.0
0.5
-
A
C
D
E
F
G
H
I
K
L
M
N
P
Q
R
S
T
V
W
Y

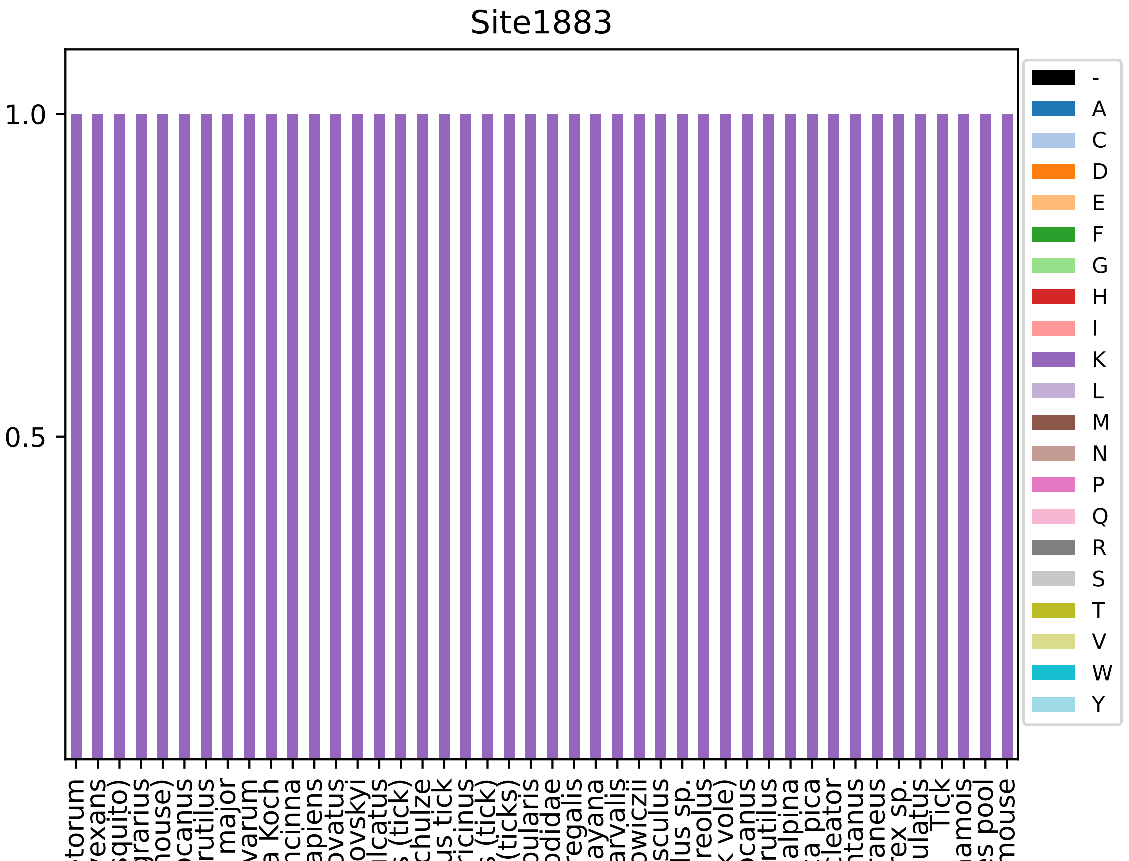
Site1883
1.0
0.5
-
A
C
D
E
F
G
H
I
K
L
M
N
P
Q
R
S
T
V
W
Y

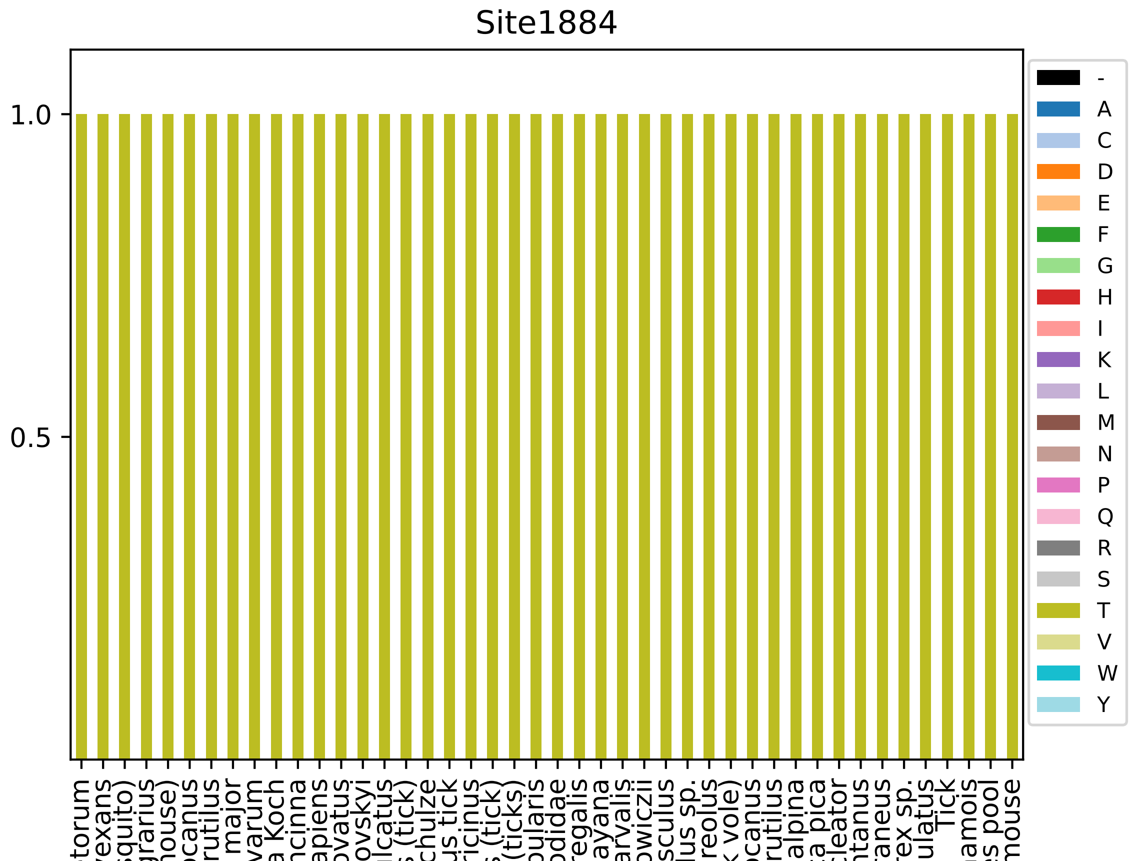
Site1884
1.0
0.5
-
A
C
D
E
F
G
H
I
K
L
M
N
P
Q
R
S
T
V
W
Y

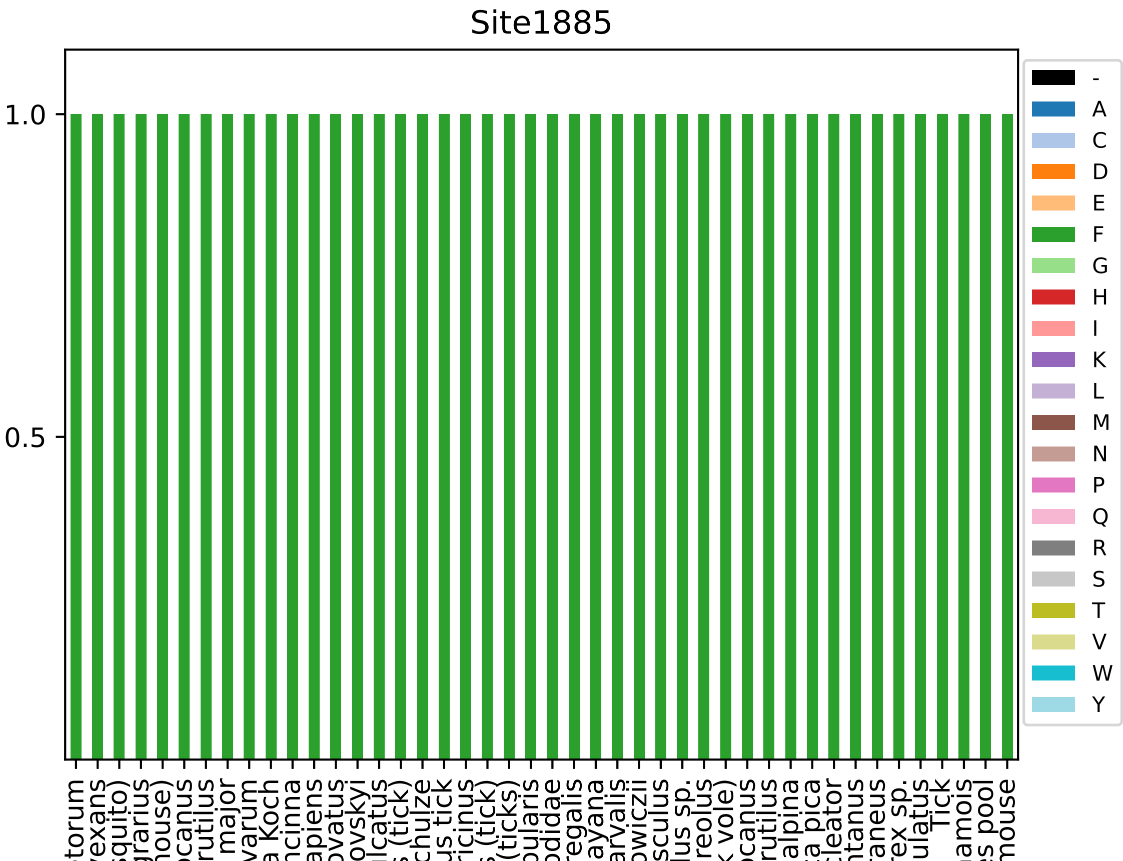
Site1885
1.0
0.5
-
A
C
D
E
F
G
H
I
K
L
M
N
P
Q
R
S
T
V
W
Y

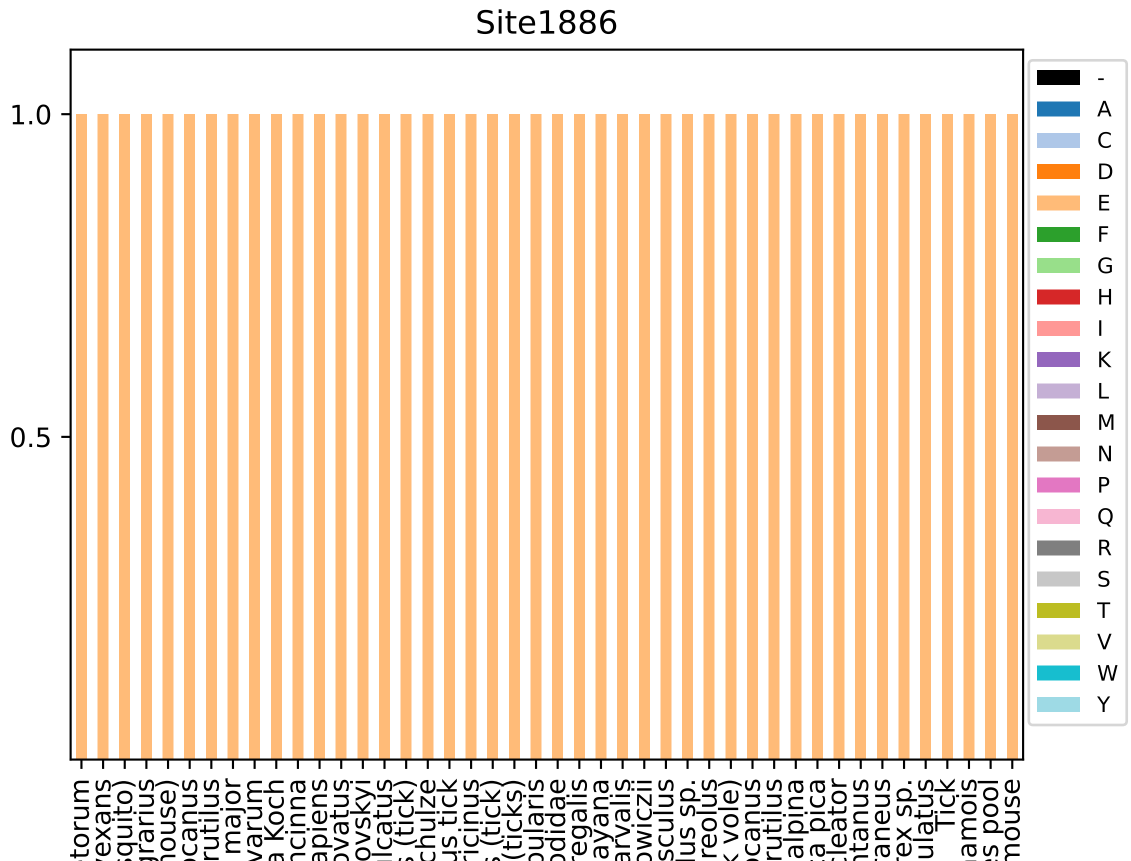
Site1886
1.0
0.5
-
A
C
D
E
F
G
H
I
K
L
M
N
P
Q
R
S
T
V
W
Y

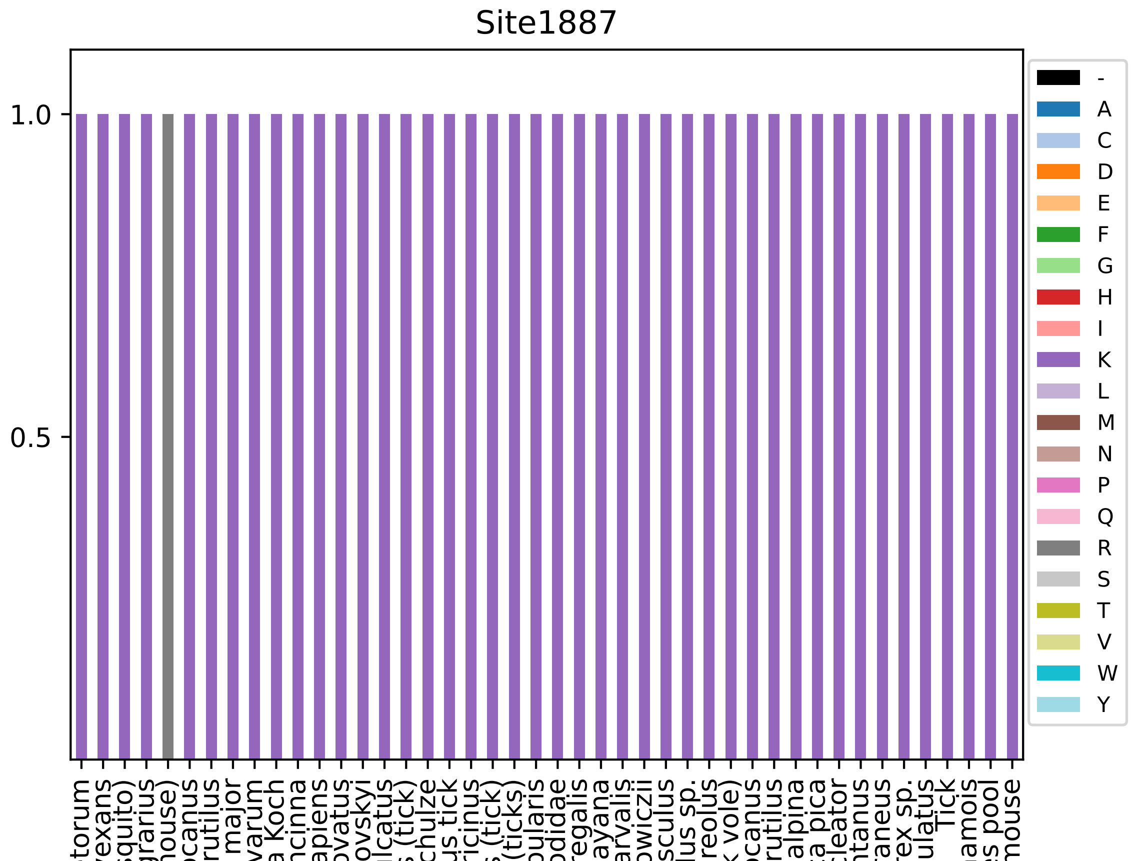
Site1887
1.0
0.5
-
A
C
D
E
F
G
H
I
K
L
M
N
P
Q
R
S
T
V
W
Y

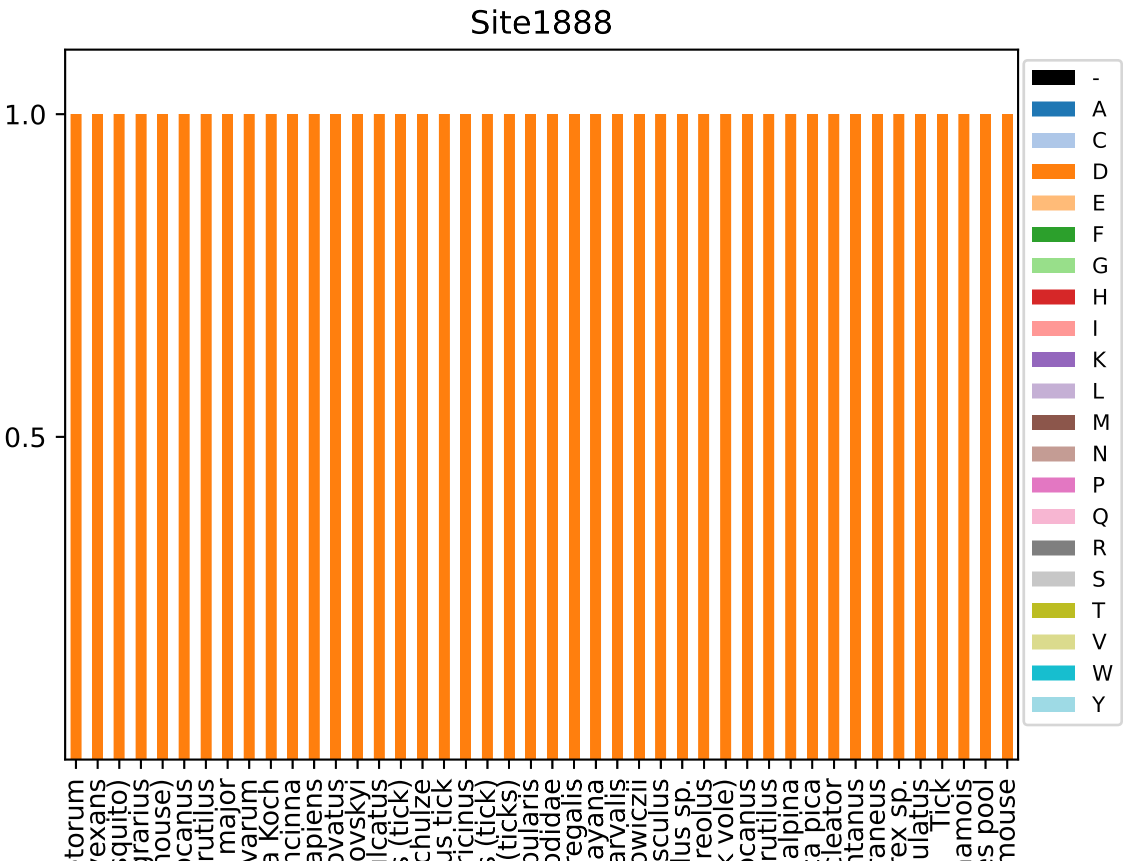
Site1888
1.0
0.5
-
A
C
D
E
F
G
H
I
K
L
M
N
P
Q
R
S
T
V
W
Y

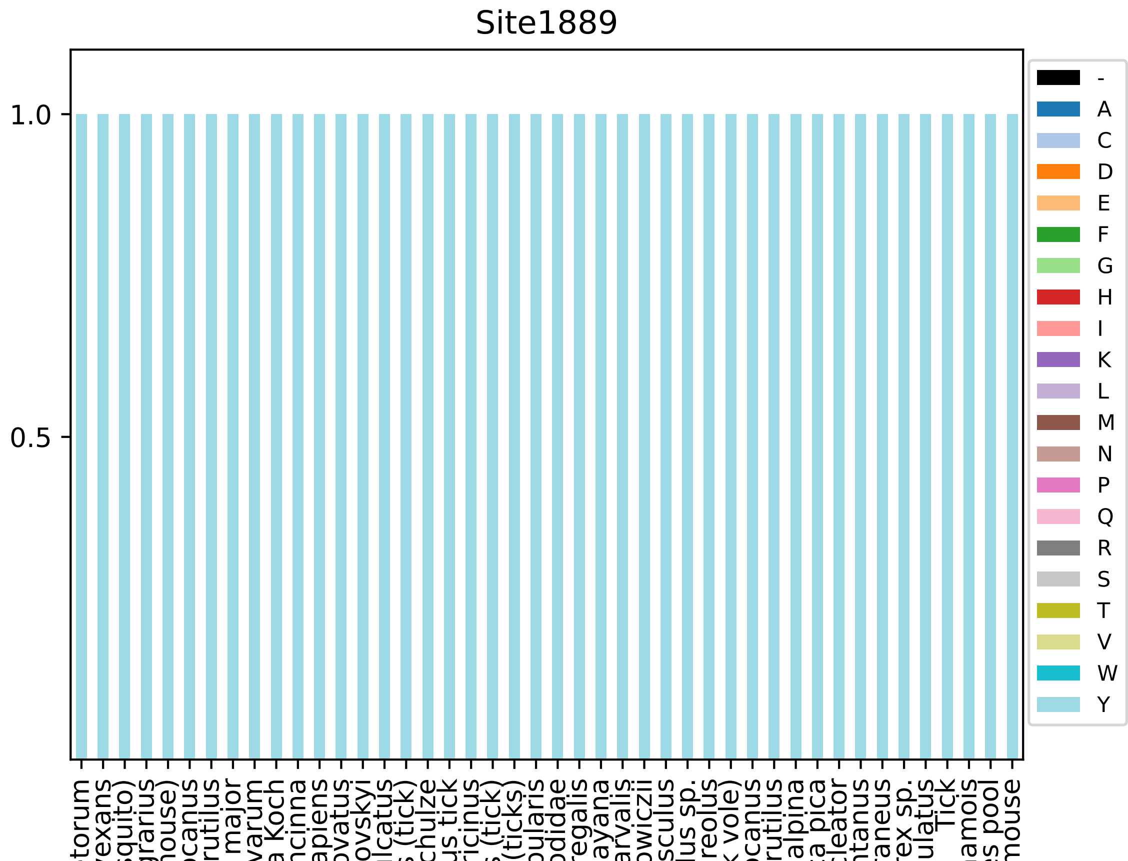
Site1889
1.0
0.5
-
A
C
D
E
F
G
H
I
K
L
M
N
P
Q
R
S
T
V
W
Y

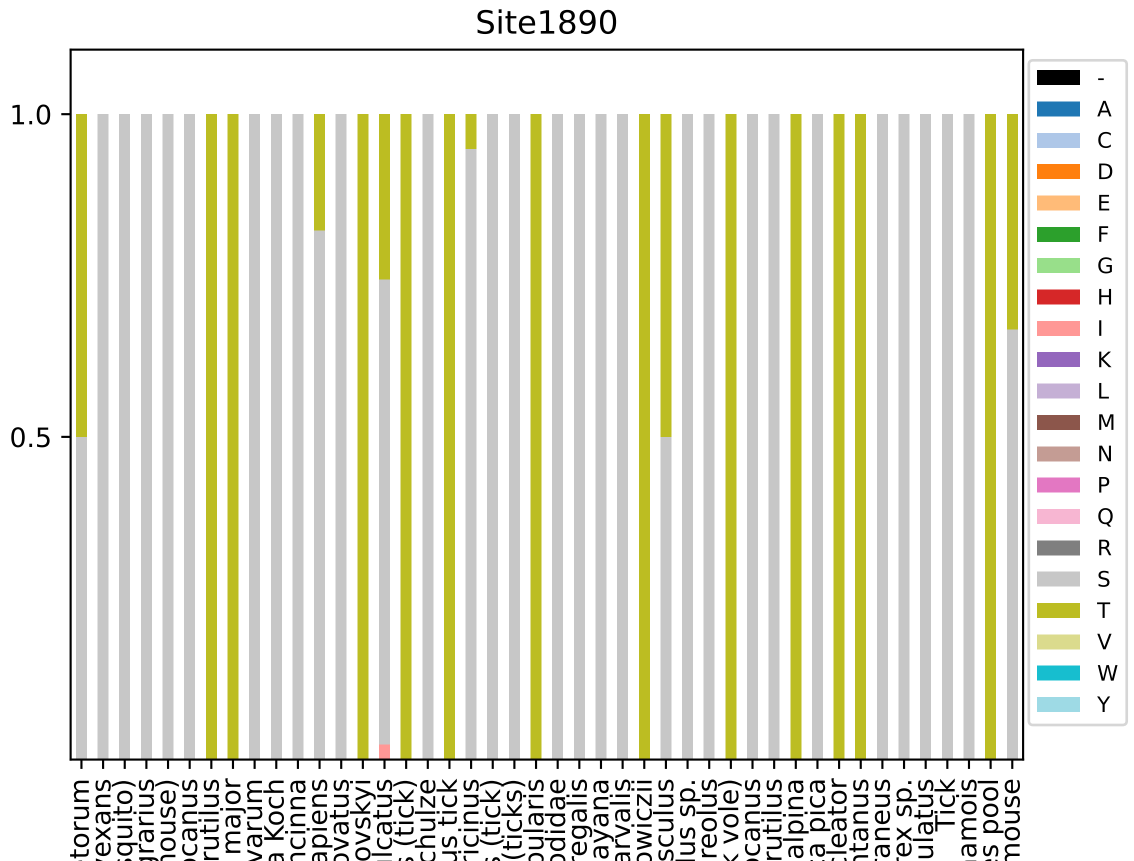
Site1890
1.0
0.5
-
A
C
D
E
F
G
H
I
K
L
M
N
P
Q
R
S
T
V
W
Y

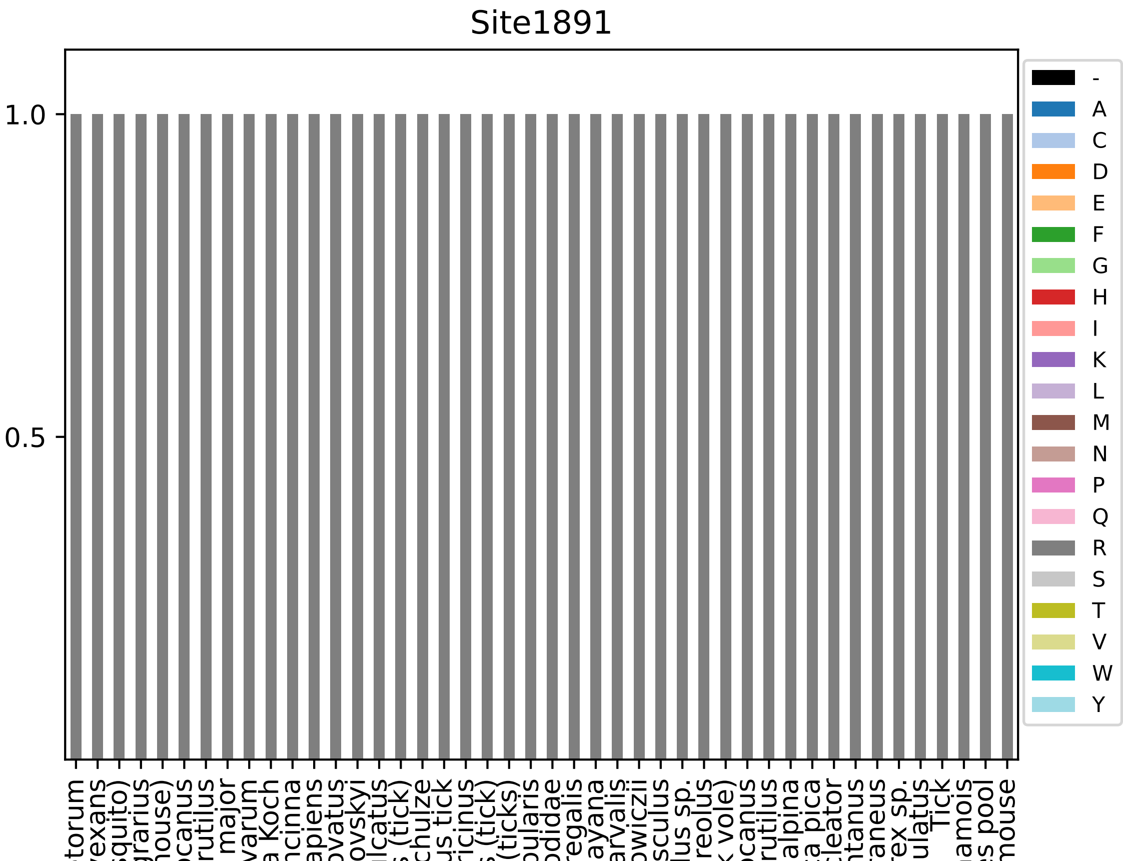
Site1891
1.0
0.5
-
A
C
D
E
F
G
H
I
K
L
M
N
P
Q
R
S
T
V
W
Y

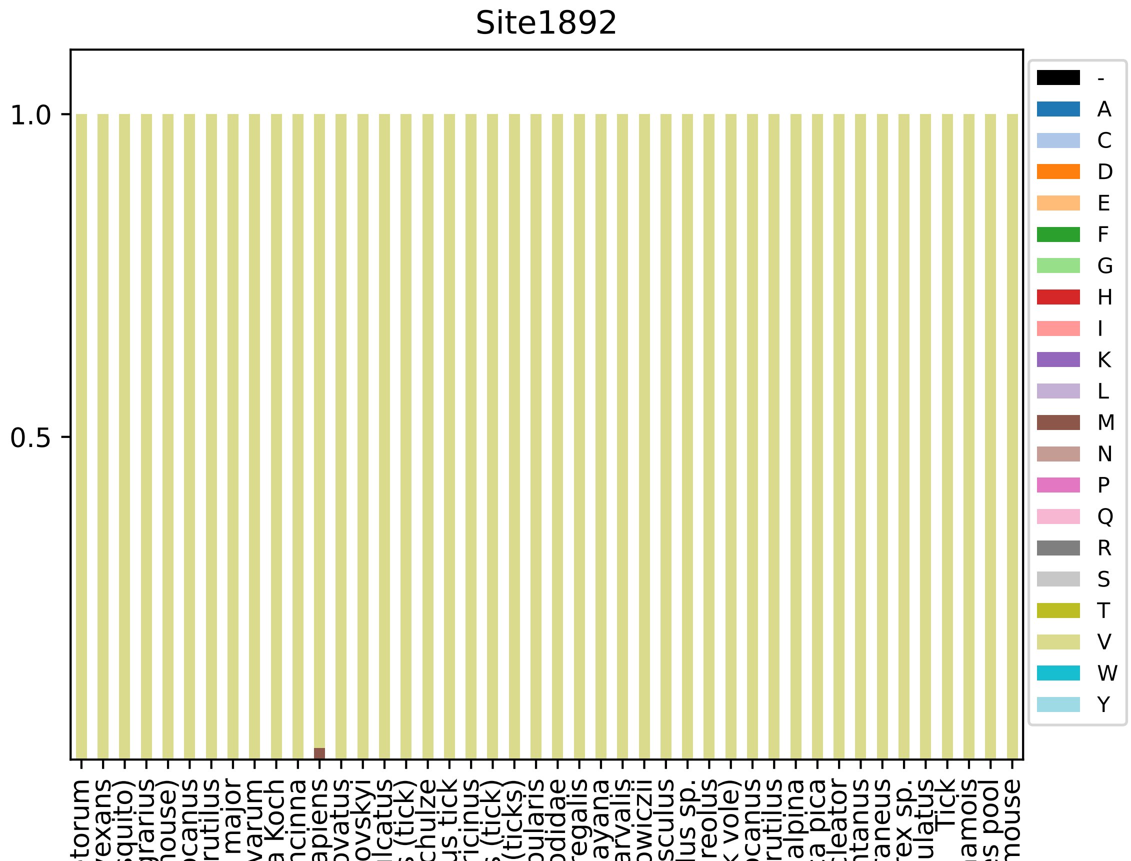
Site1892
1.0
0.5
-
A
C
D
E
F
G
H
I
K
L
M
N
P
Q
R
S
T
V
W
Y
Tick

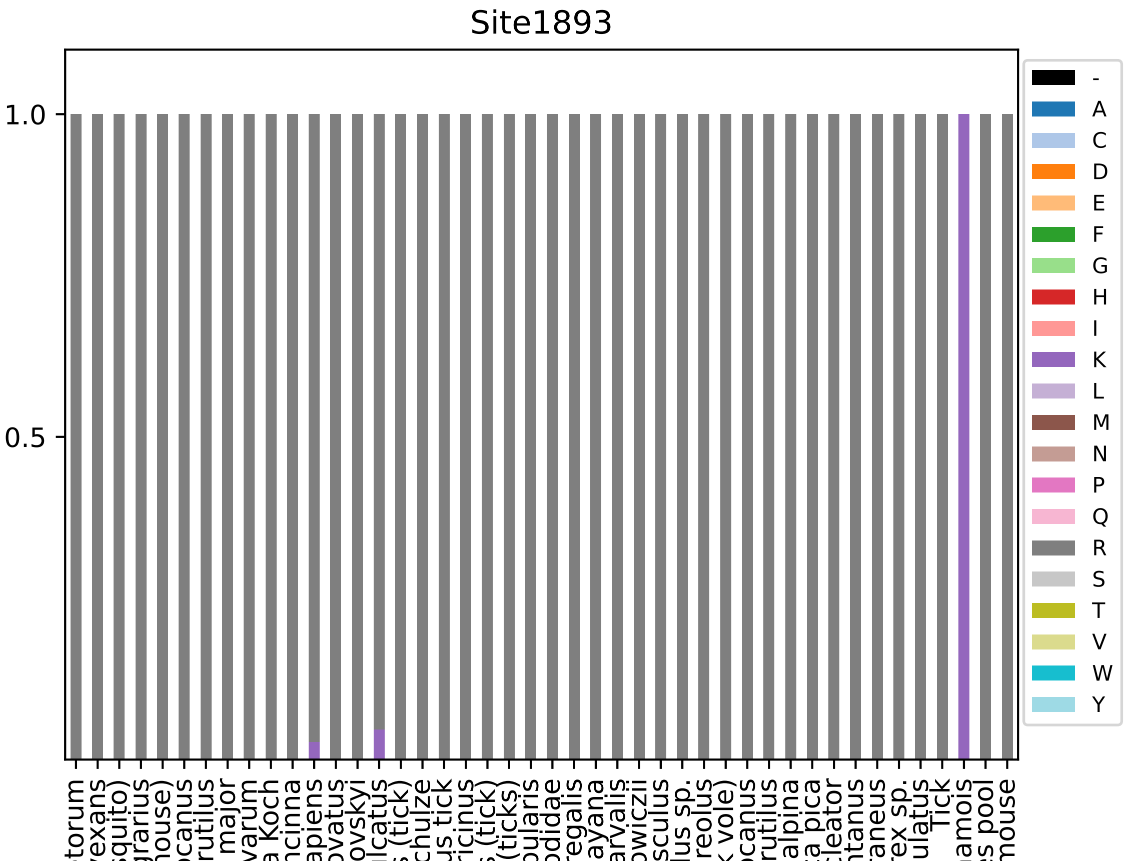
Site1893
1.0
0.5
-
A
C
D
E
F
G
H
I
K
L
M
N
P
Q
R
S
T
V
W
Y

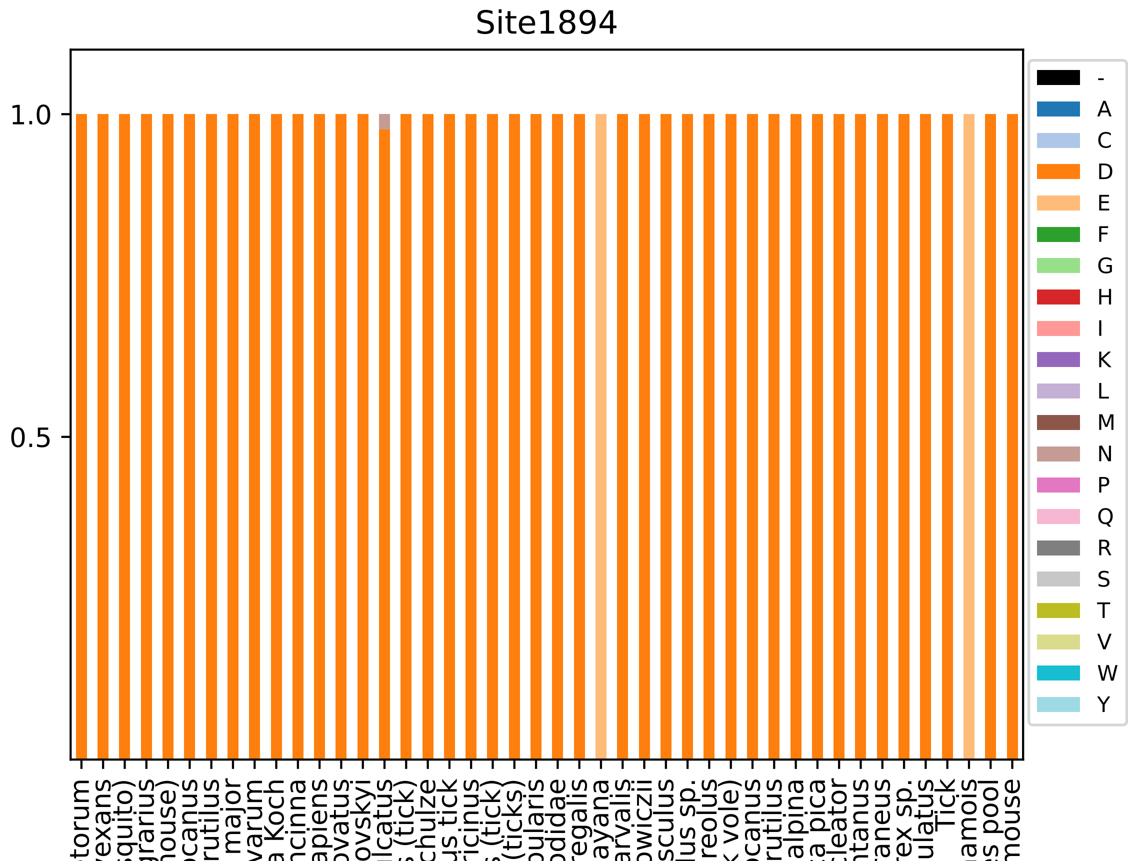
Site1894
1.0
0.5
-
A
C
D
E
F
G
H
I
K
L
M
N
P
Q
R
S
T
V
W
Y

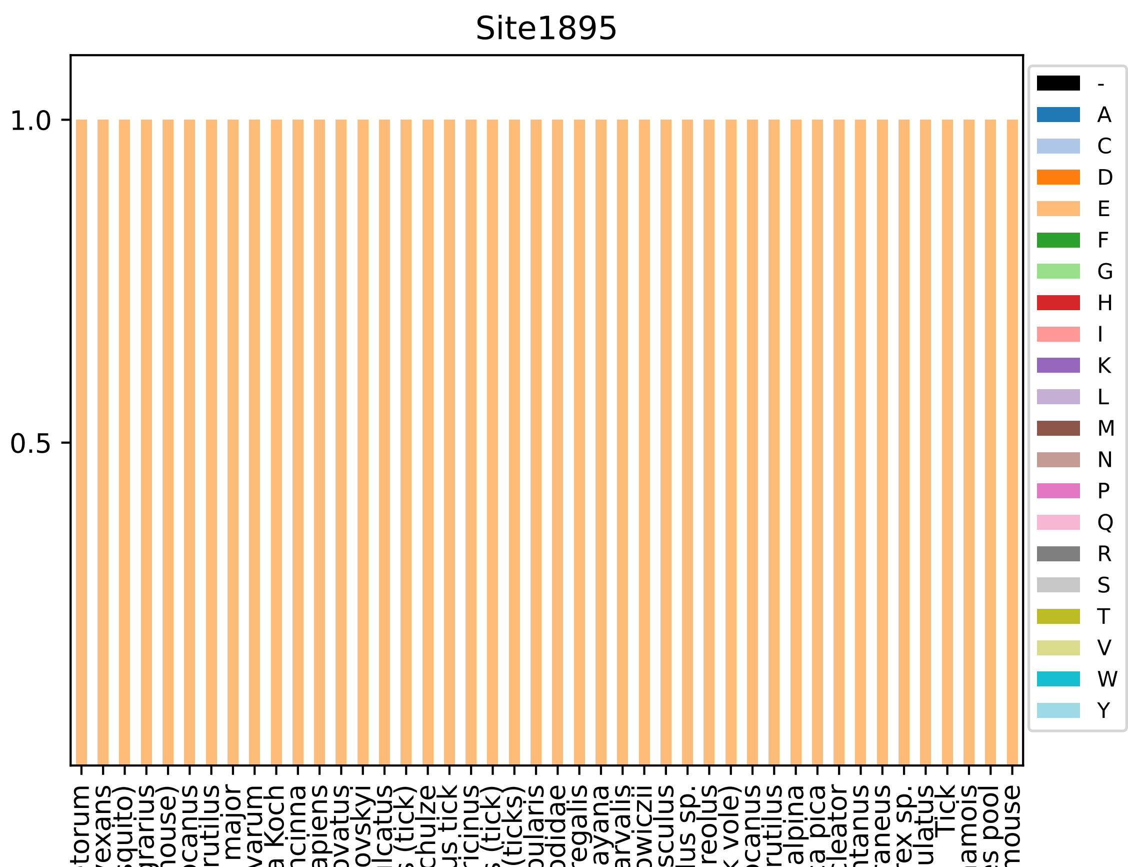
Site1895
1.0
0.5
-
A
C
D
E
F
G
H
I
K
L
M
N
P
Q
R
S
T
V
W
Y

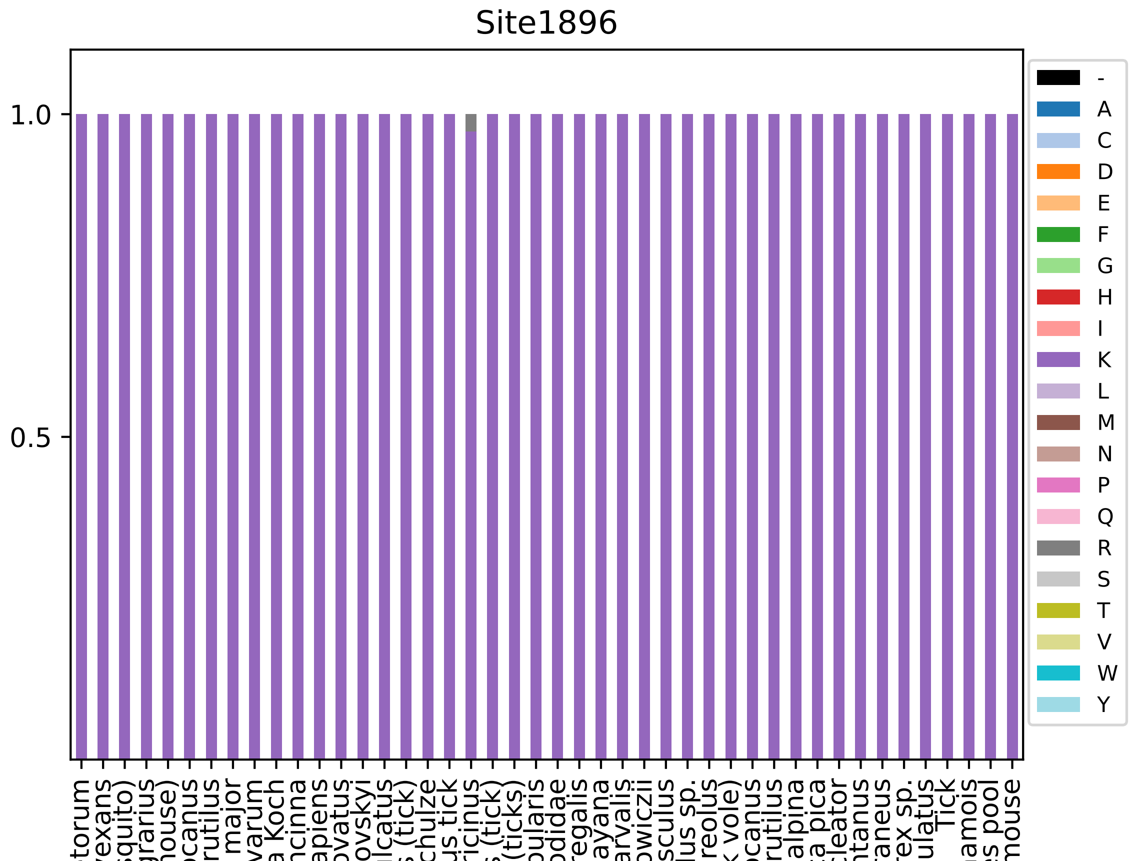
Site1896
1.0
0.5
-
A
C
D
E
F
G
H
I
K
L
M
N
P
Q
R
S
T
V
W
Y

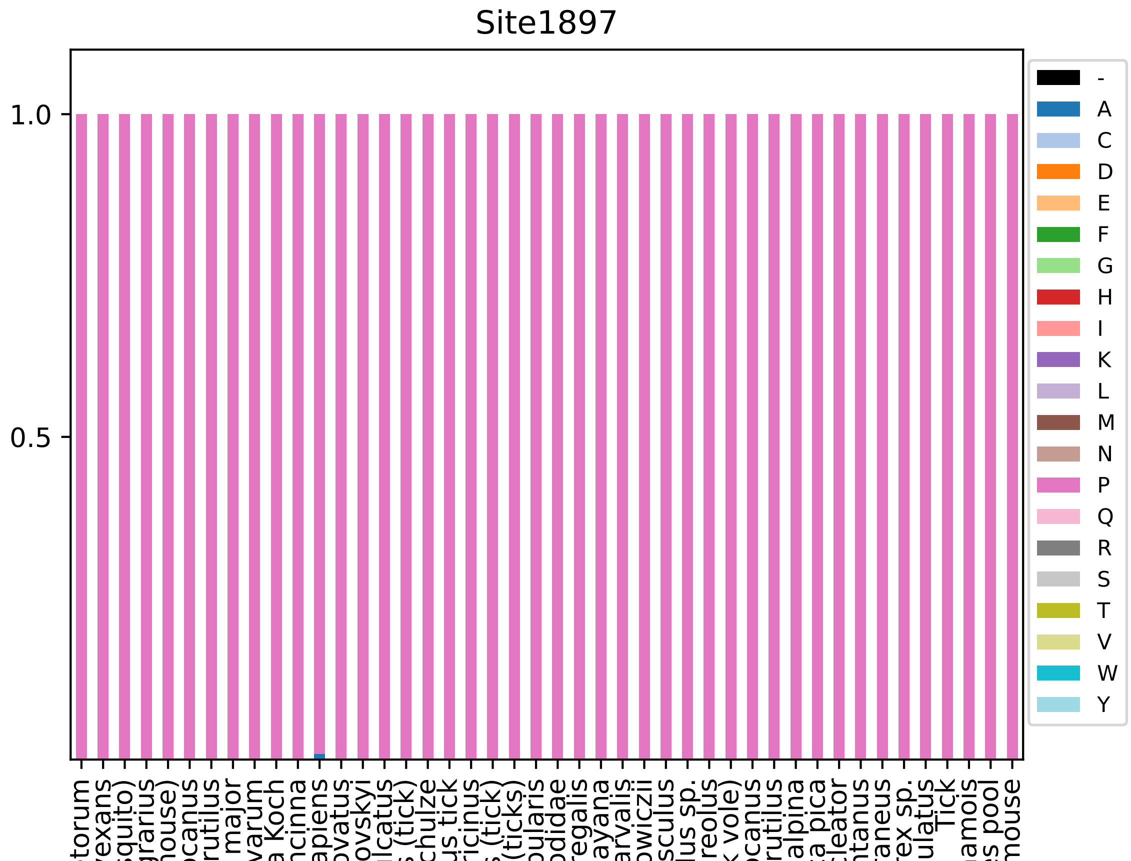
Site1897
1.0
0.5
-
A
C
D
E
F
G
H
I
K
L
M
N
P
Q
R
S
T
V
W
Y

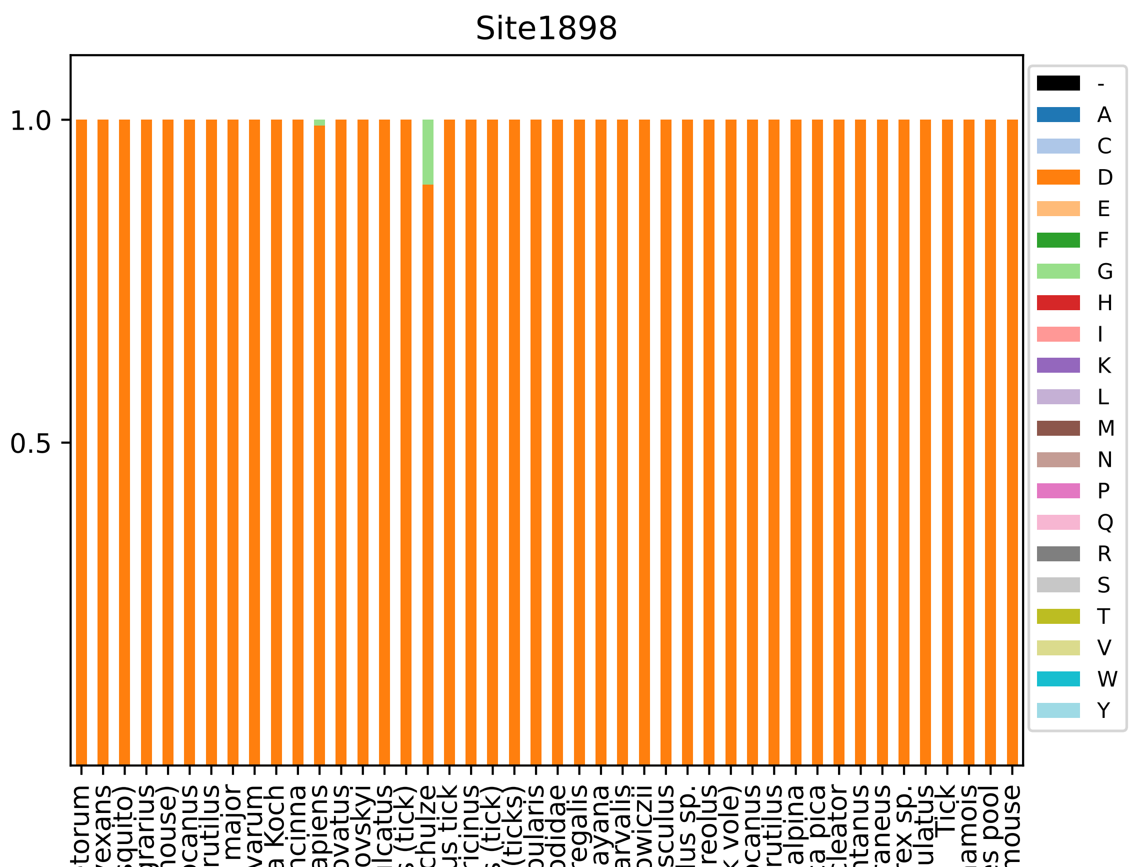
Site1898
1.0
0.5
-
A
C
D
E
F
G
H
I
K
L
M
N
P
Q
R
S
T
V
W
Y

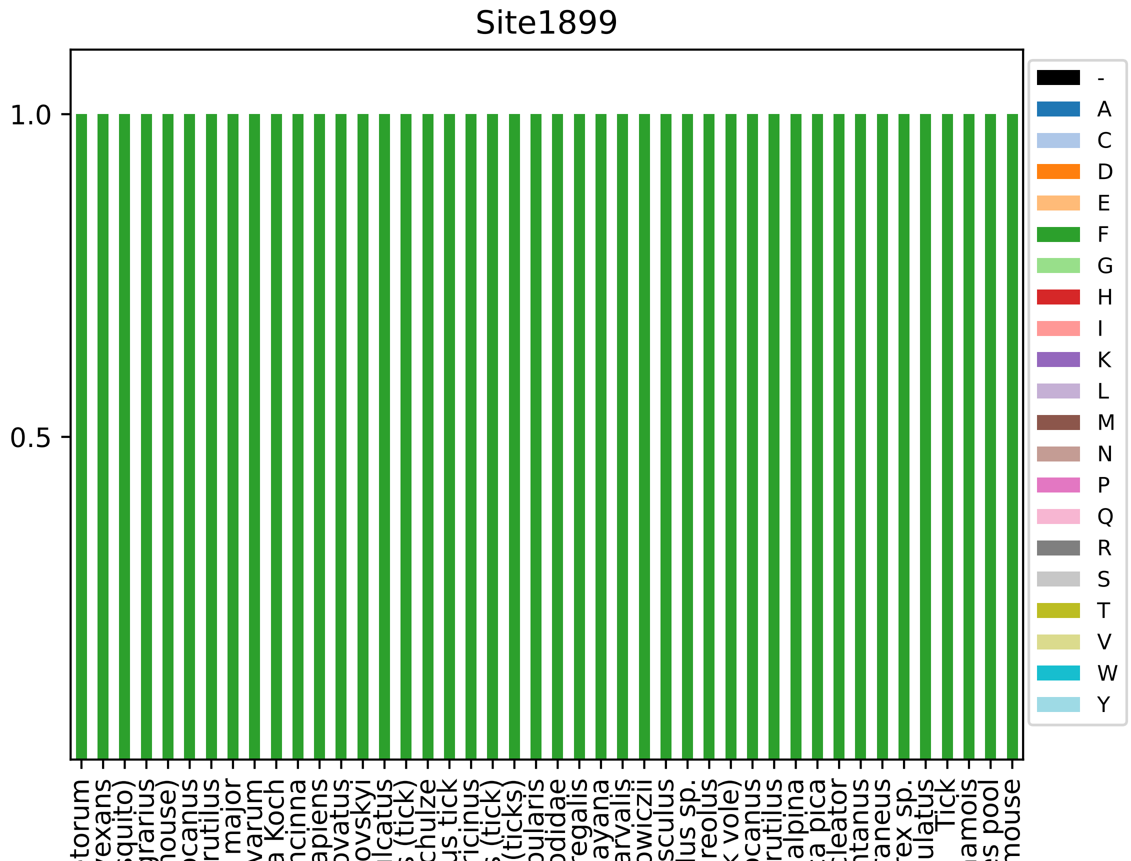
Site1899
1.0
0.5
-
A
C
D
E
F
G
H
I
K
L
M
N
P
Q
R
S
T
V
W
Y

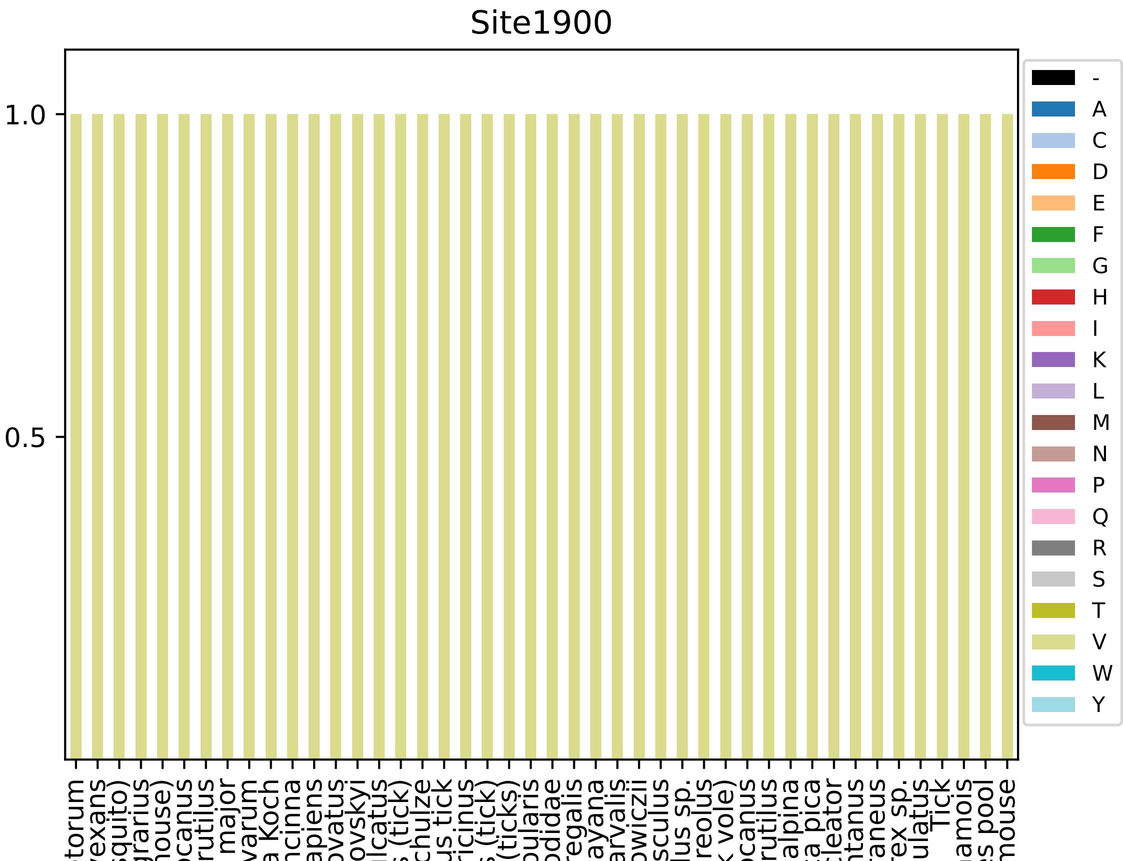
Site1900
1.0
0.5
-
A
C
D
E
F
G
H
I
K
L
M
N
P
Q
R
S
T
V
W
Y

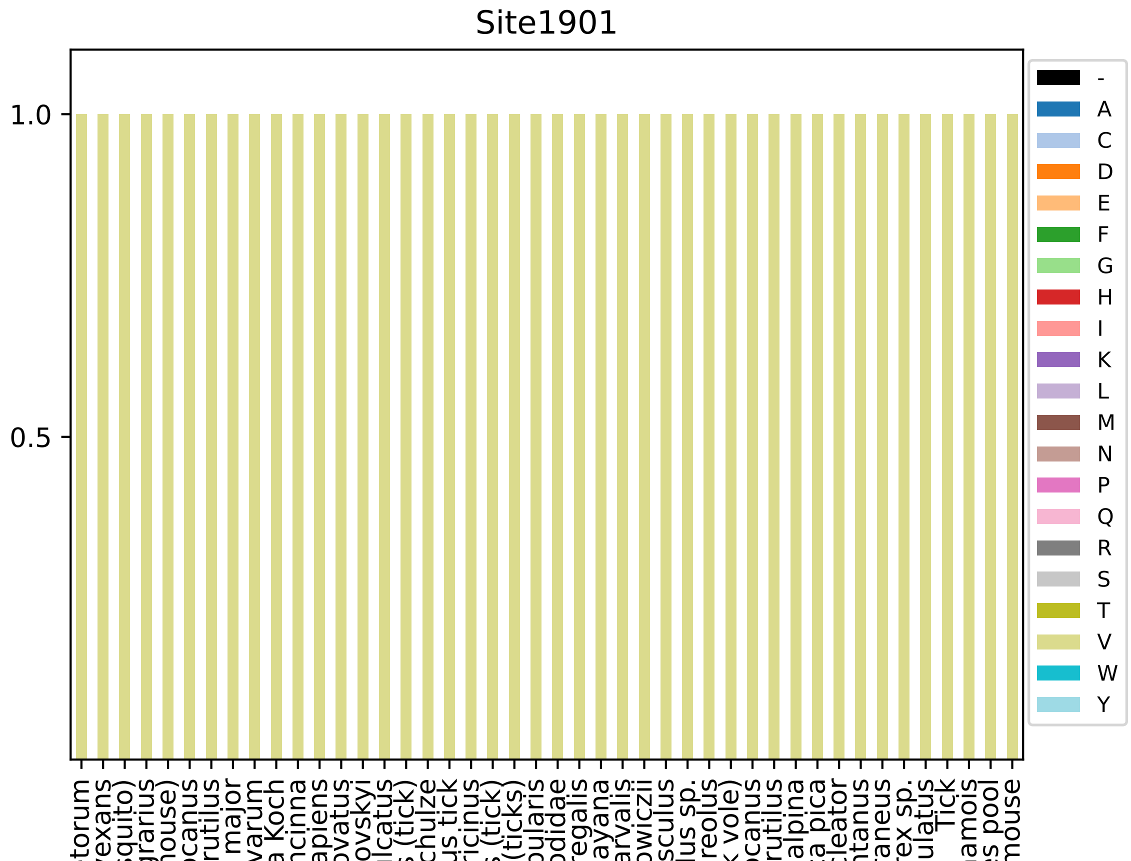
Site1901
1.0
0.5
-
A
C
D
E
F
G
H
I
K
L
M
N
P
Q
R
S
T
V
W
Y

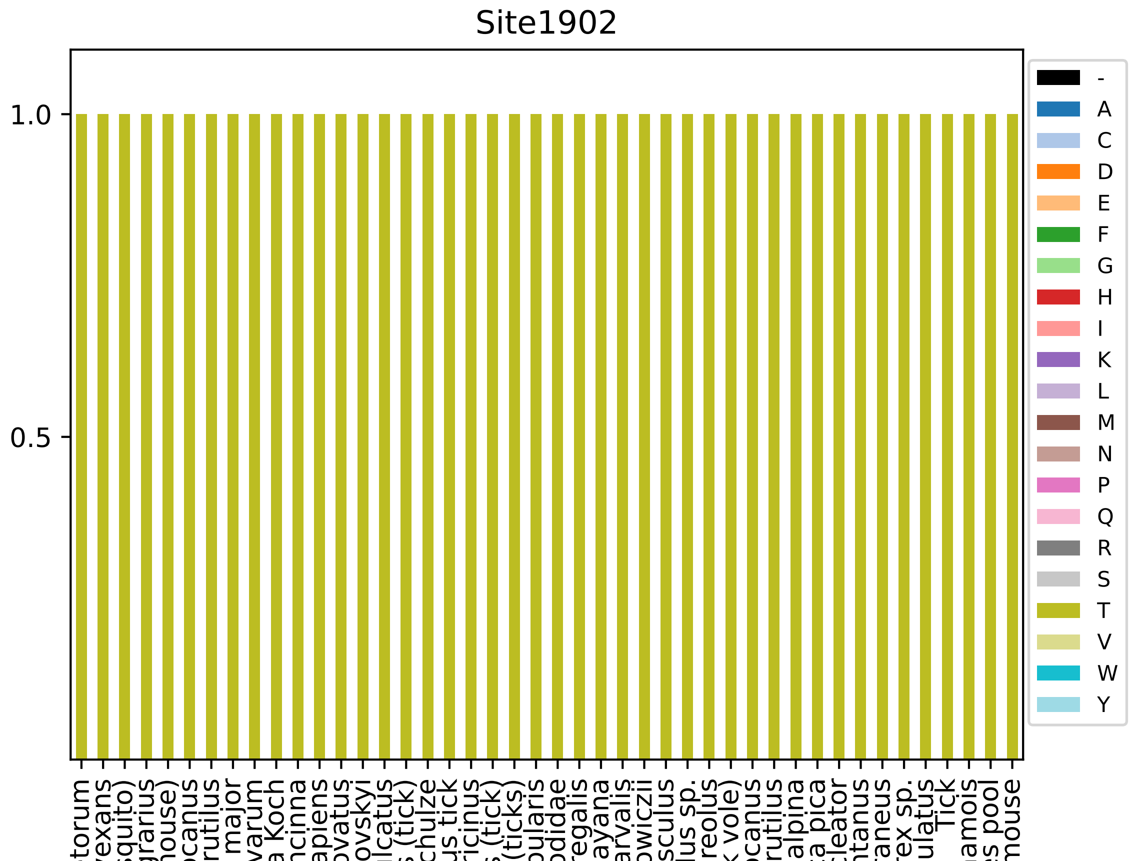
Site1902
1.0
0.5
-
A
C
D
E
F
G
H
I
K
L
M
N
P
Q
R
S
T
V
W
Y

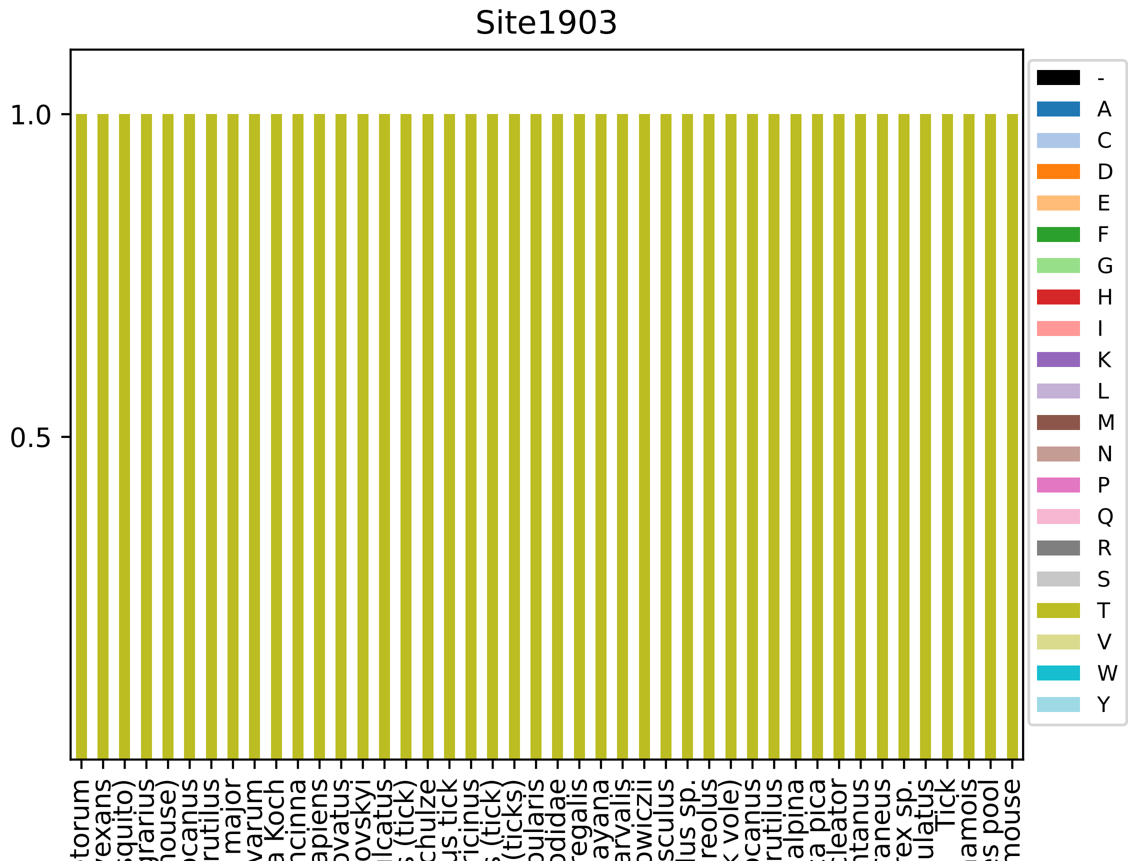

Site1903
1.0
0.5
-
A
C
D
E
F
G
H
I
K
L
M
N
P
Q
R
S
T
V
W
Y

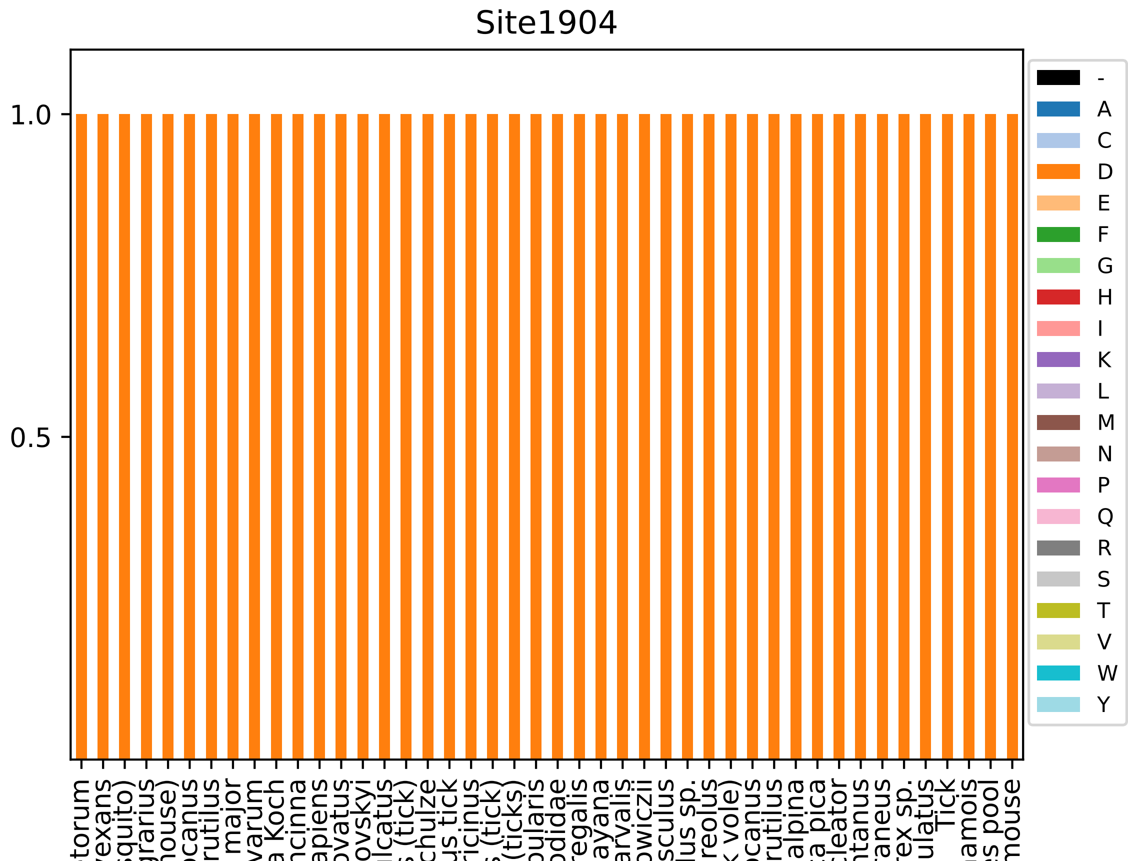
Site1904
1.0
0.5
-
A
C
D
E
F
G
H
I
K
L
M
N
P
Q
R
S
T
V
W
Y
Tick

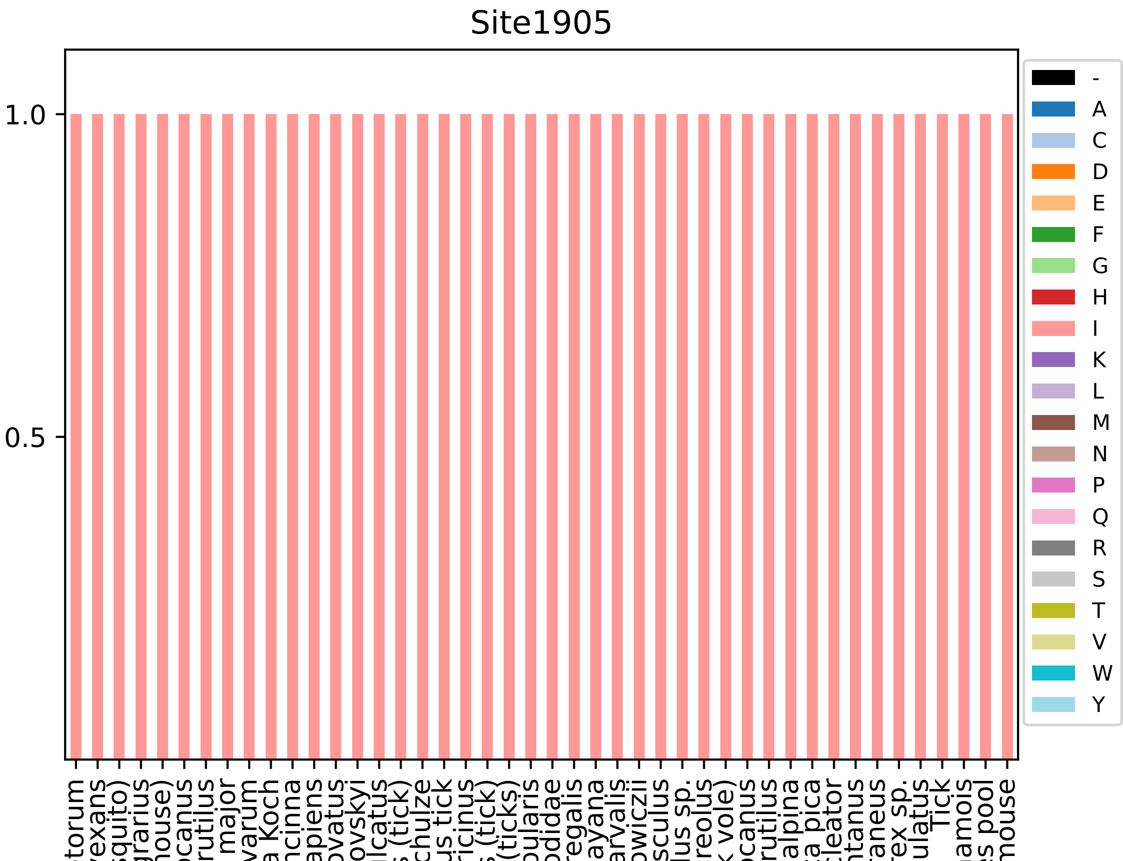
Site1905
1.0
0.5
-
A
C
D
E
F
G
H
I
K
L
M
N
P
Q
R
S
T
V
W
Y

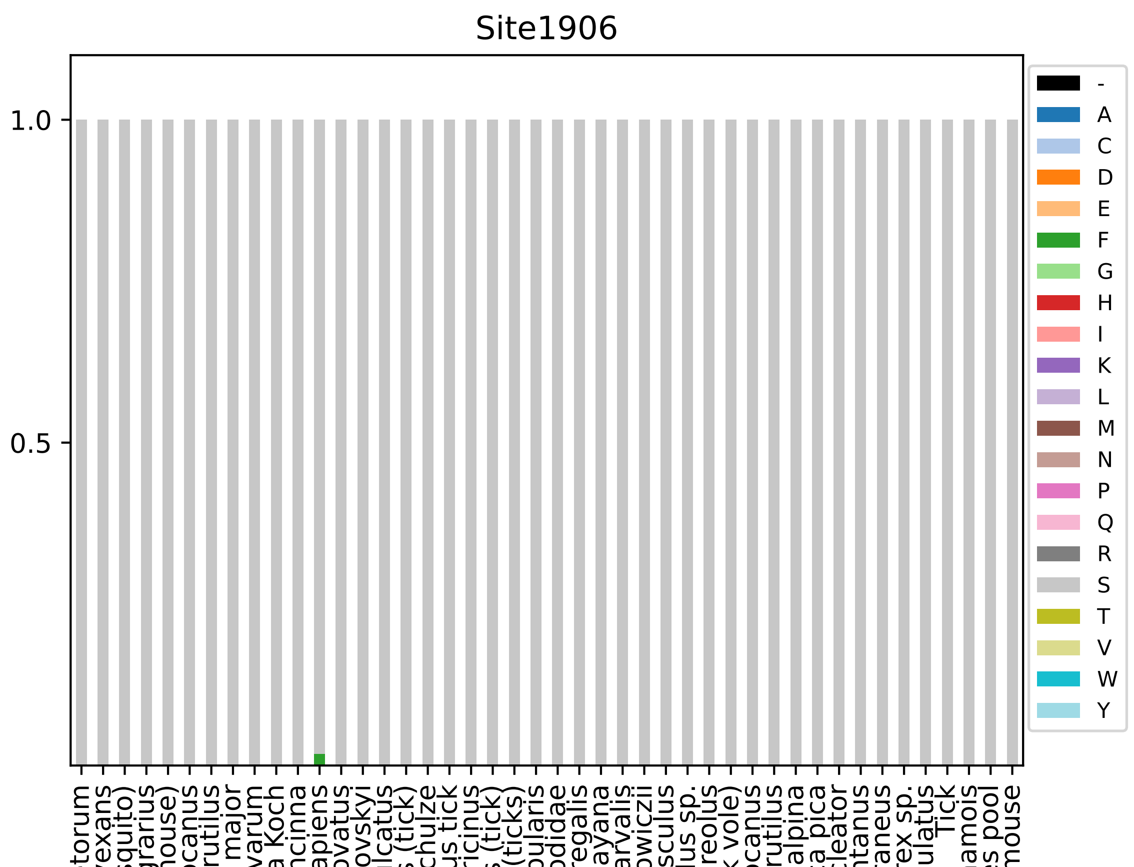
Site1906
1.0
0.5
-
A
C
D
E
F
G
H
I
K
L
M
N
P
Q
R
S
T
V
W
Y

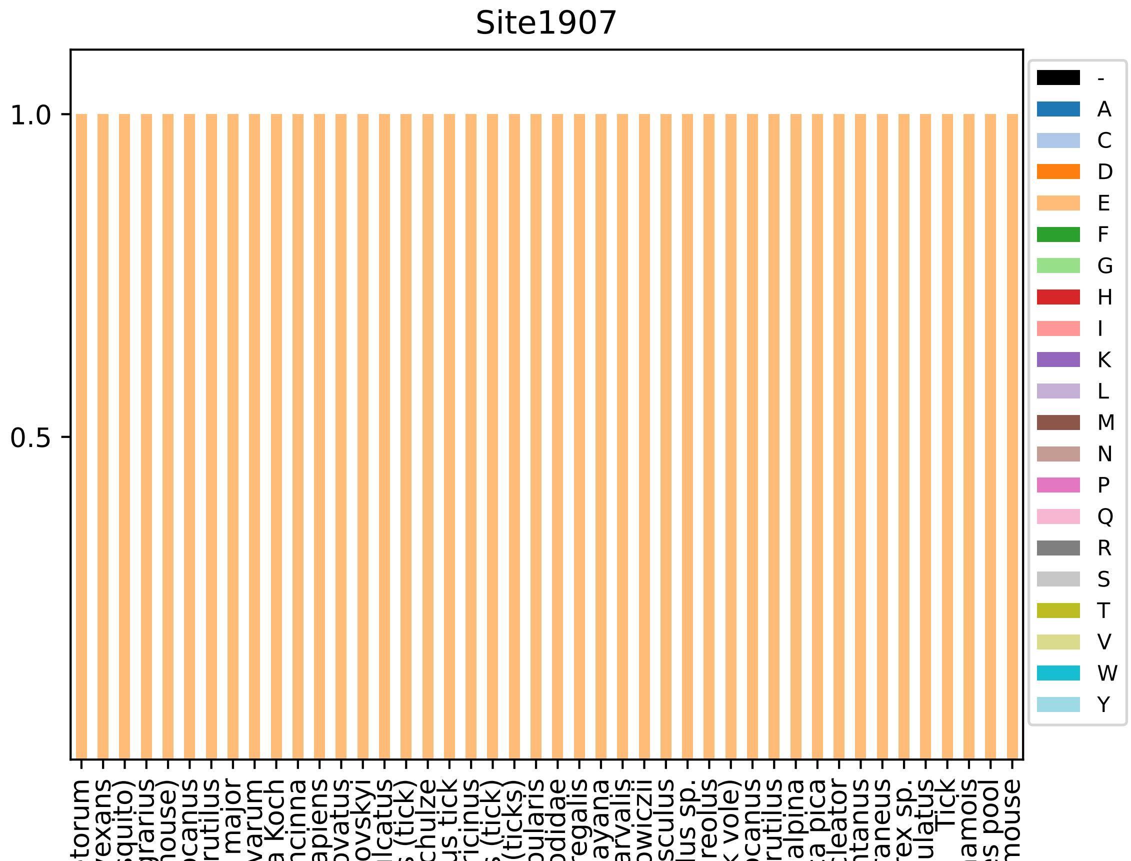
Site1907
1.0
0.5
-
A
C
D
E
F
G
H
I
K
L
M
N
P
Q
R
S
T
V
W
Y

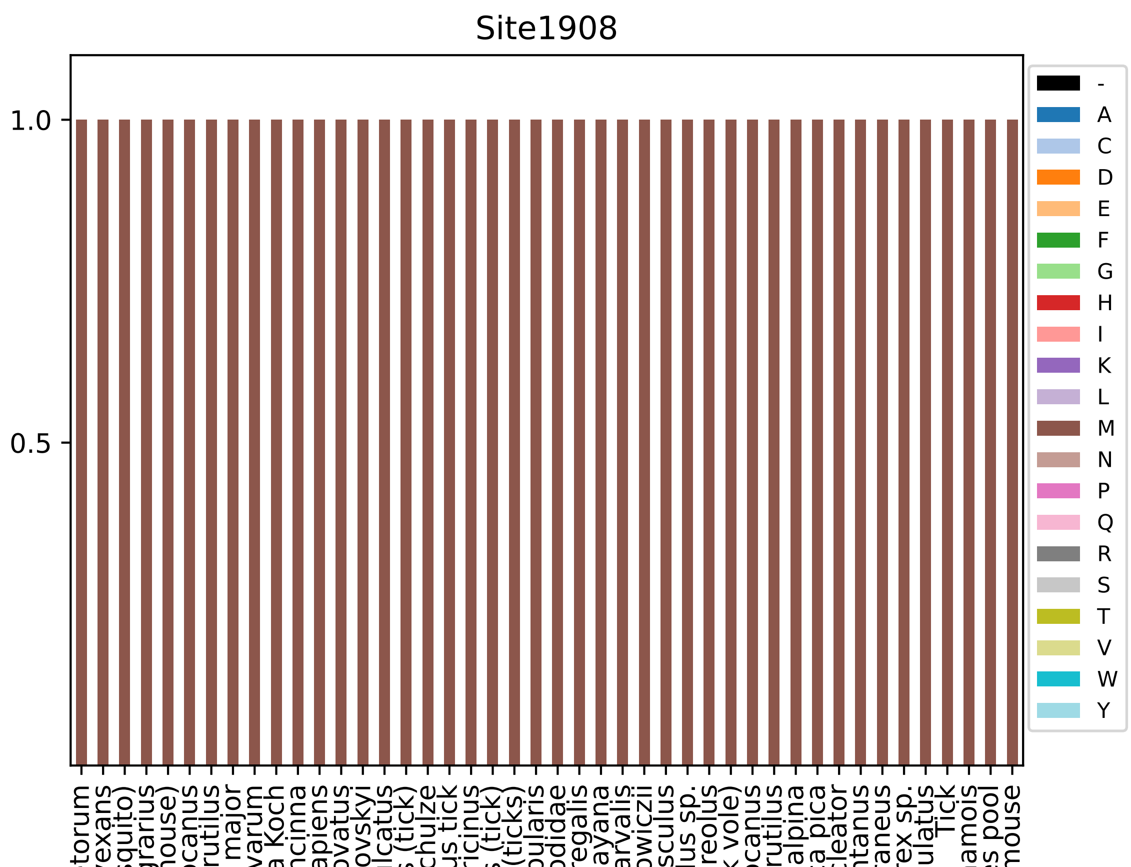
Site1908
1.0
0.5
-
A
C
D
E
F
G
H
I
K
L
M
N
P
Q
R
S
T
V
W
Y
Tick

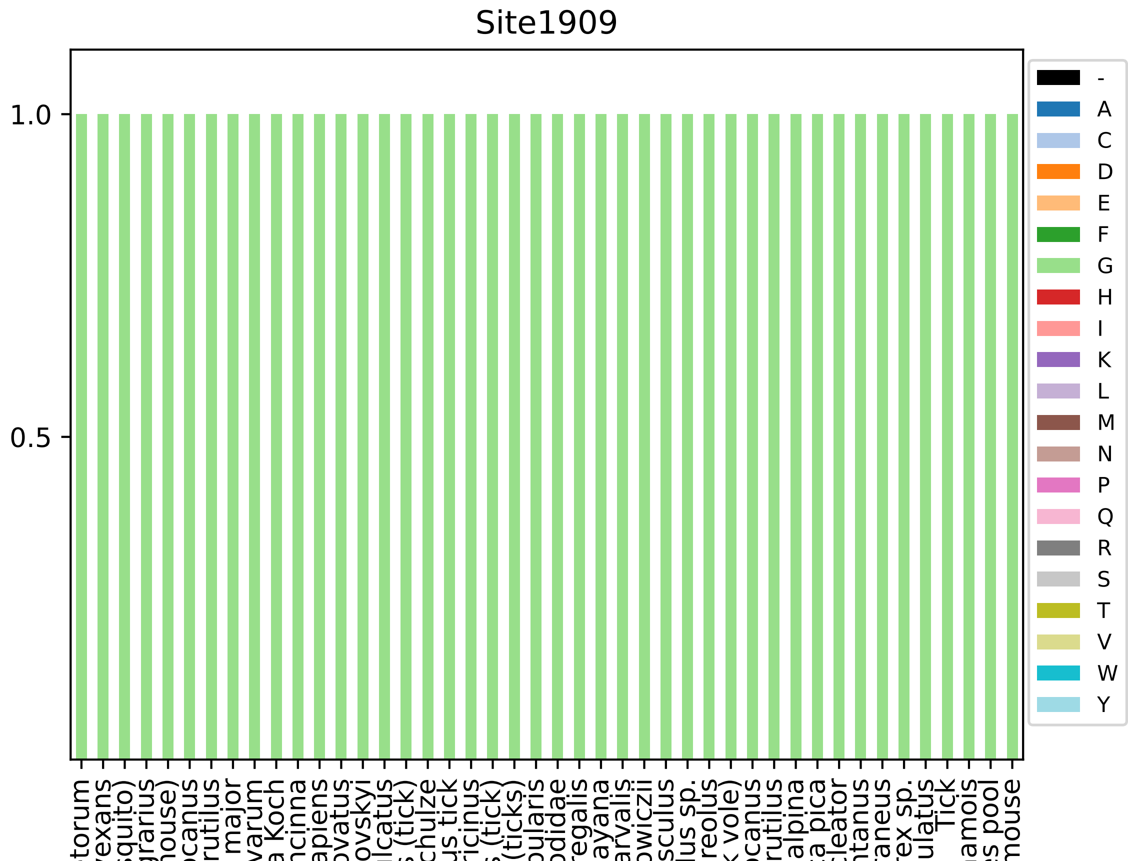
Site1909
1.0
0.5
-
A
C
D
E
F
G
H
I
K
L
M
N
P
Q
R
S
T
V
W
Y

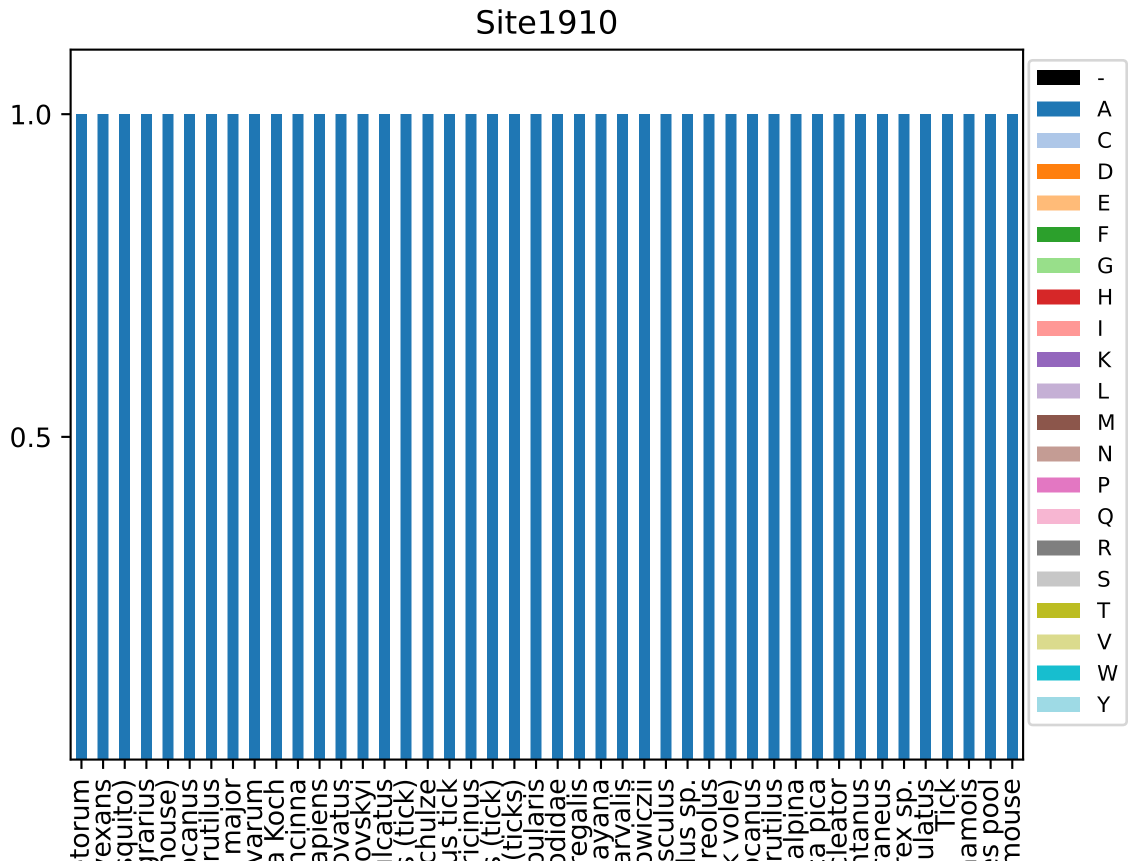
Site1910
1.0
0.5
-
A
C
D
E
F
G
H
I
K
L
M
N
P
Q
R
S
T
V
W
Y

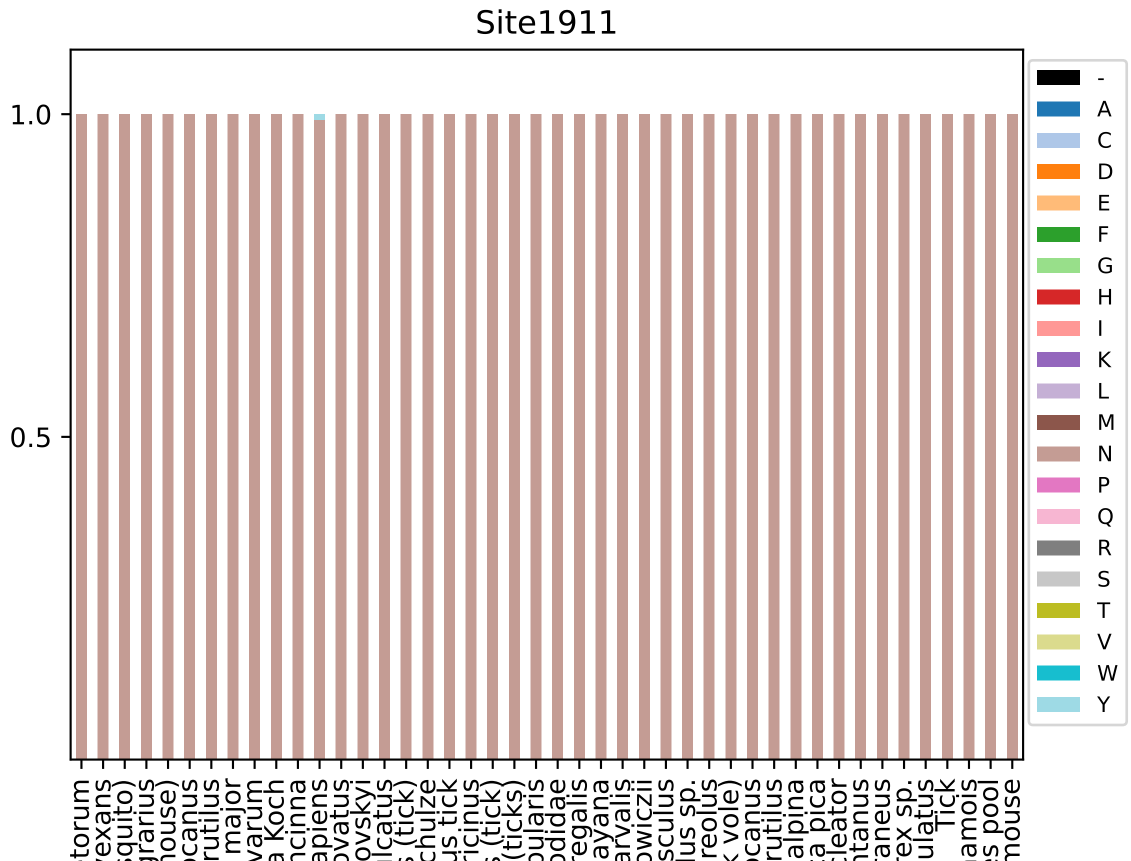
Site1911
1.0
0.5
-
A
C
D
E
F
G
H
I
K
L
M
N
P
Q
R
S
T
V
W
Y

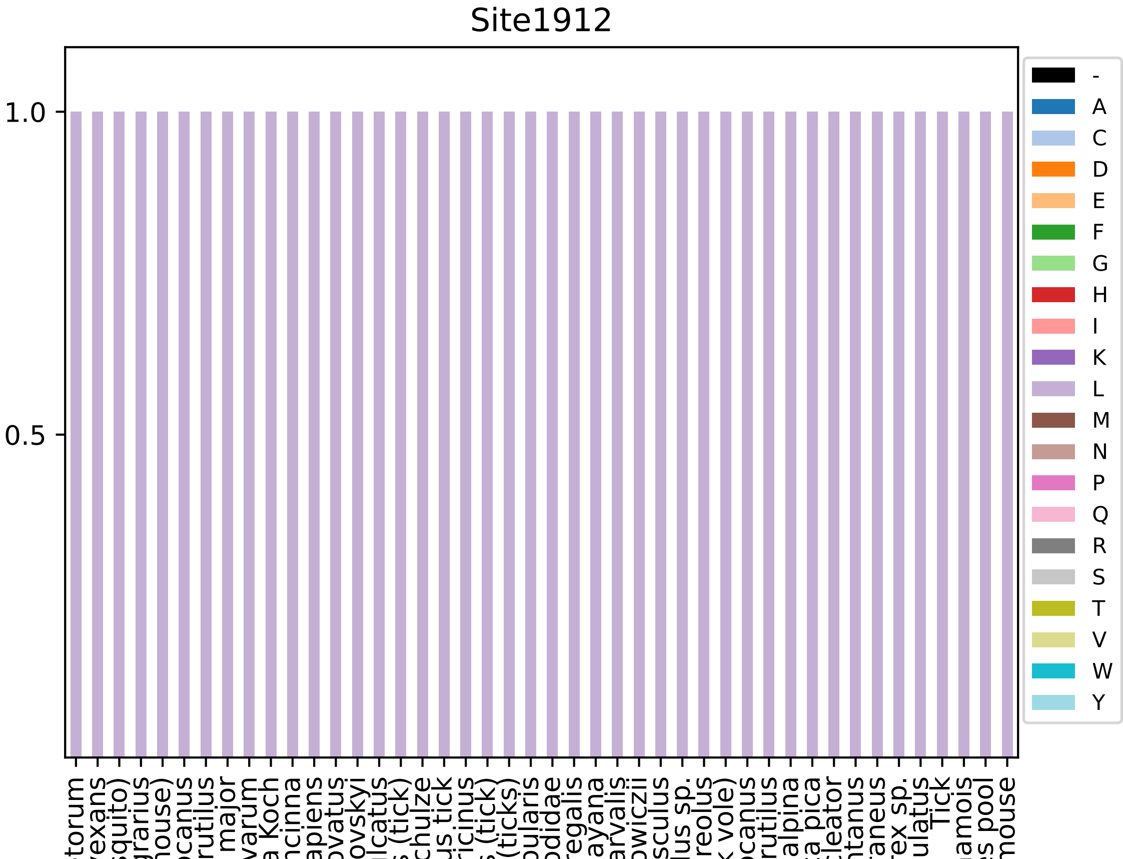
Site1912
1.0
0.5
-
A
C
D
E
F
G
H
I
K
L
M
N
P
Q
R
S
T
V
W
Y

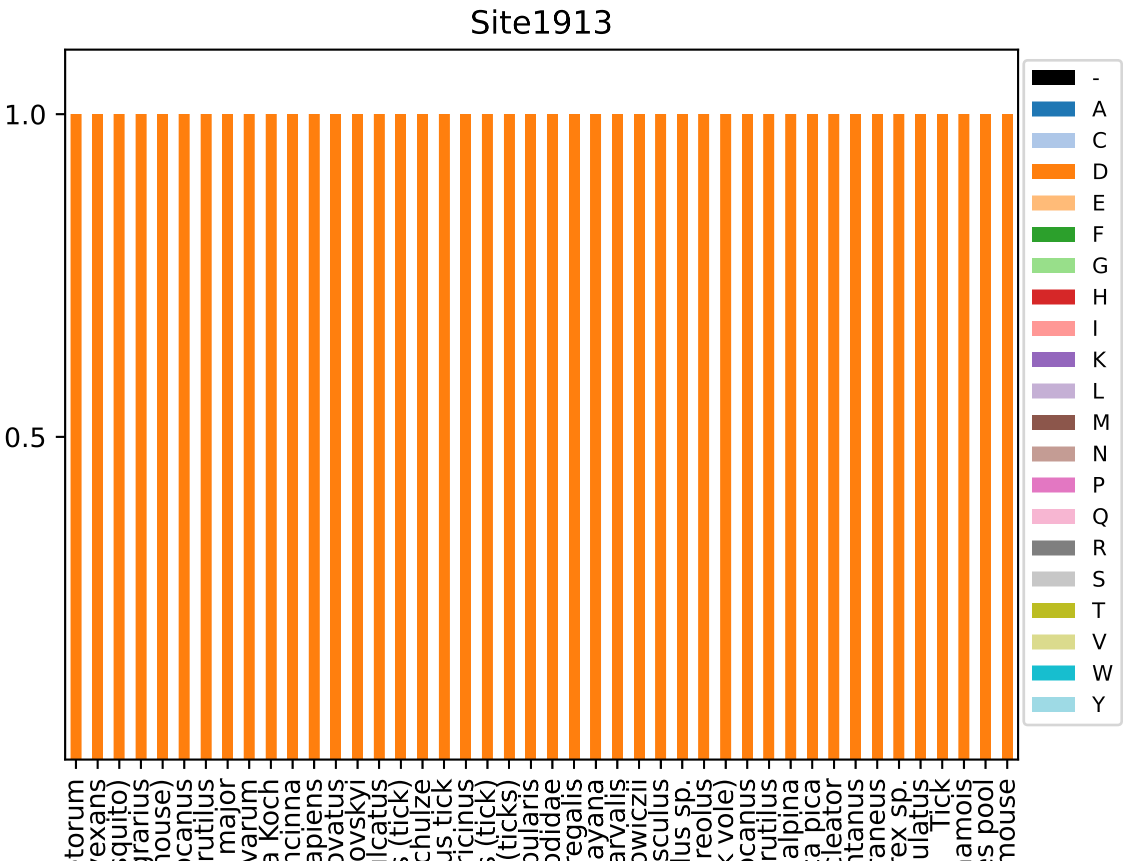
Site1913
1.0
0.5
-
A
C
D
E
F
G
H
I
K
L
M
N
P
Q
R
S
T
V
W
Y

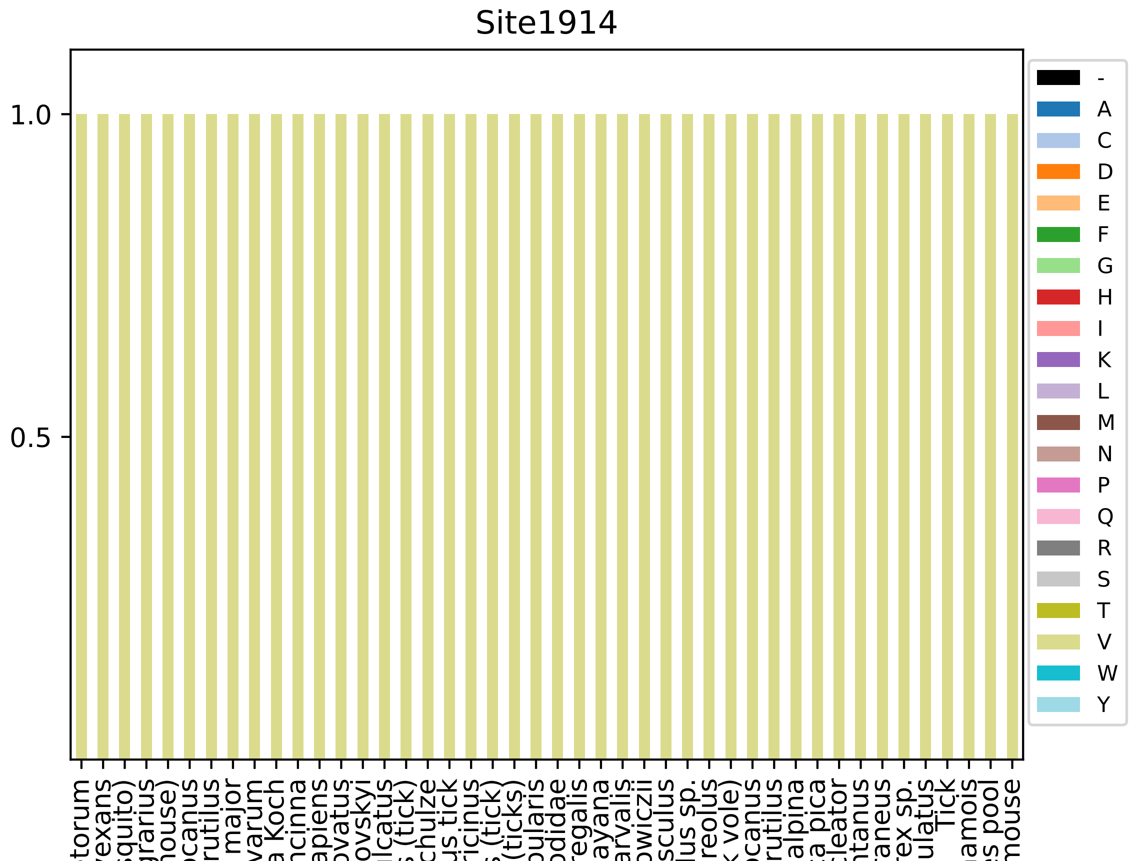

Site1914
1.0
0.5
-
A
C
D
E
F
G
H
I
K
L
M
N
P
Q
R
S
T
V
W
Y

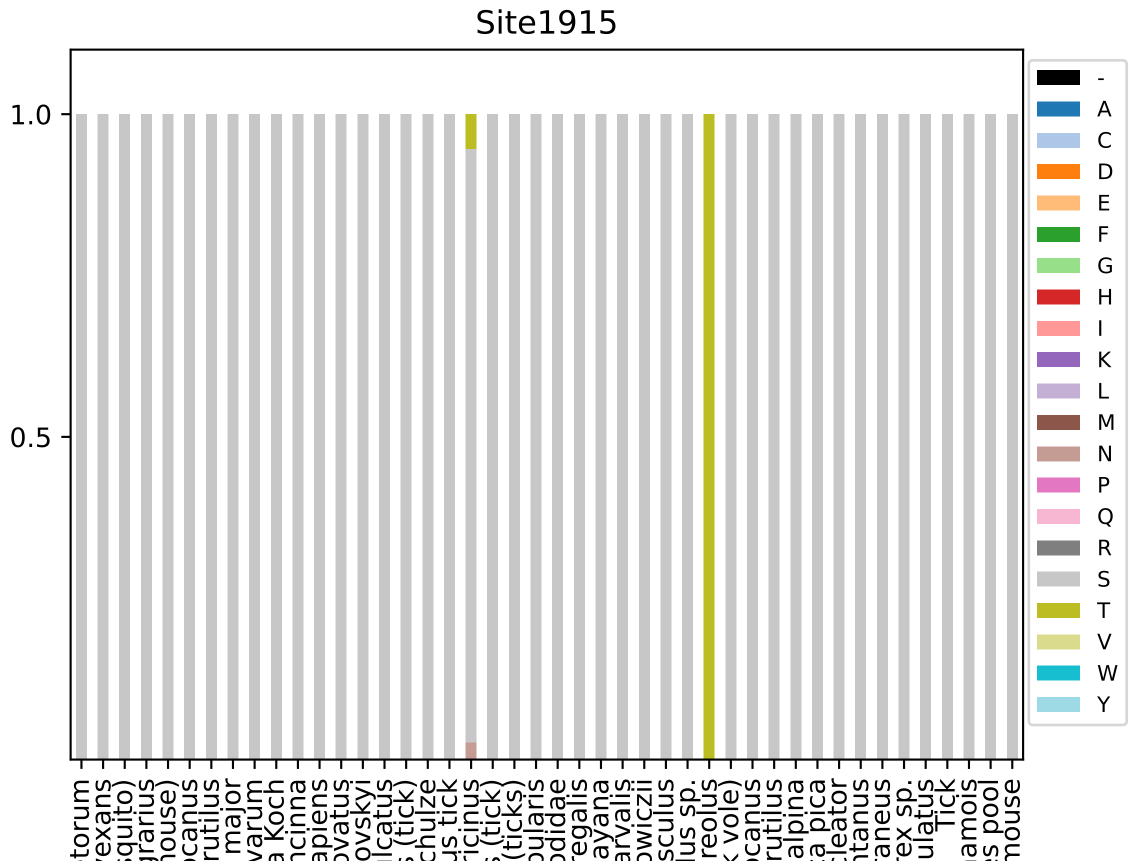
Site1915
1.0
0.5
-
A
C
D
E
F
G
H
I
K
L
M
N
P
Q
R
S
T
V
W
Y

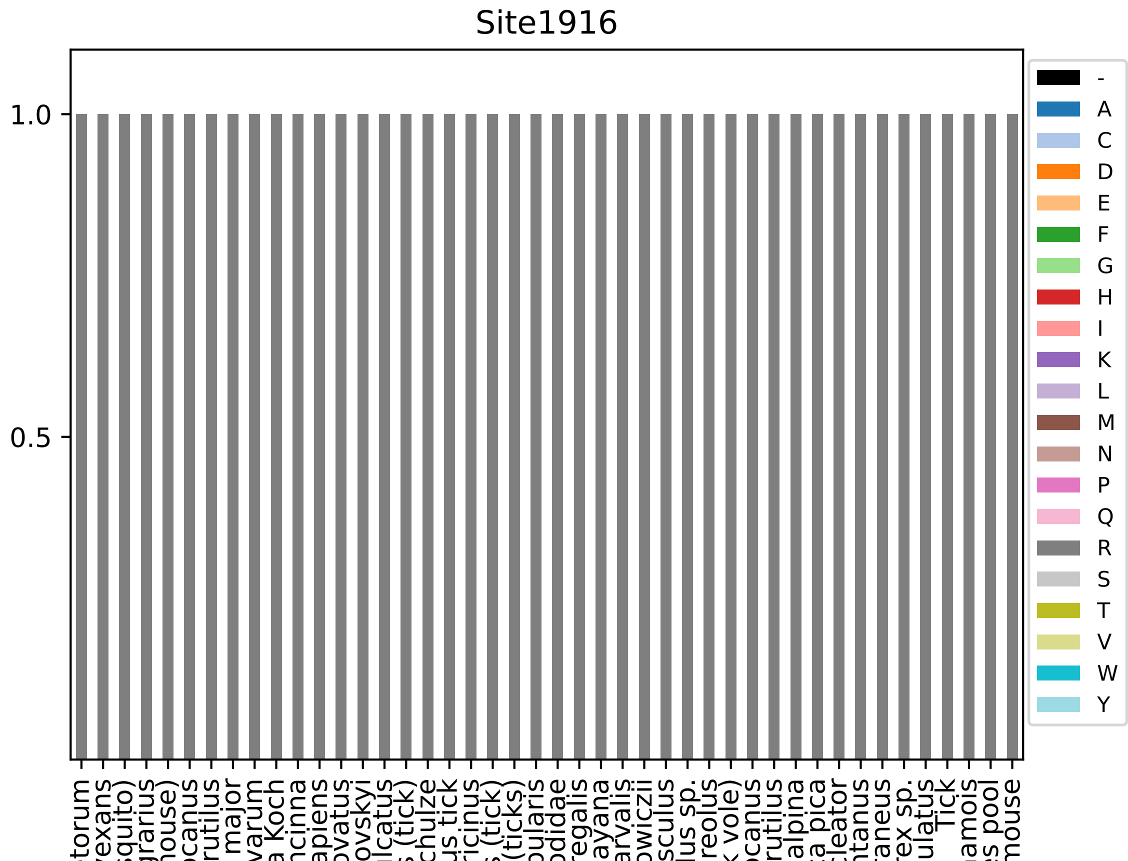
Site1916
1.0
0.5
-
A
C
D
E
F
G
H
I
K
L
M
N
P
Q
R
S
T
V
W
Y

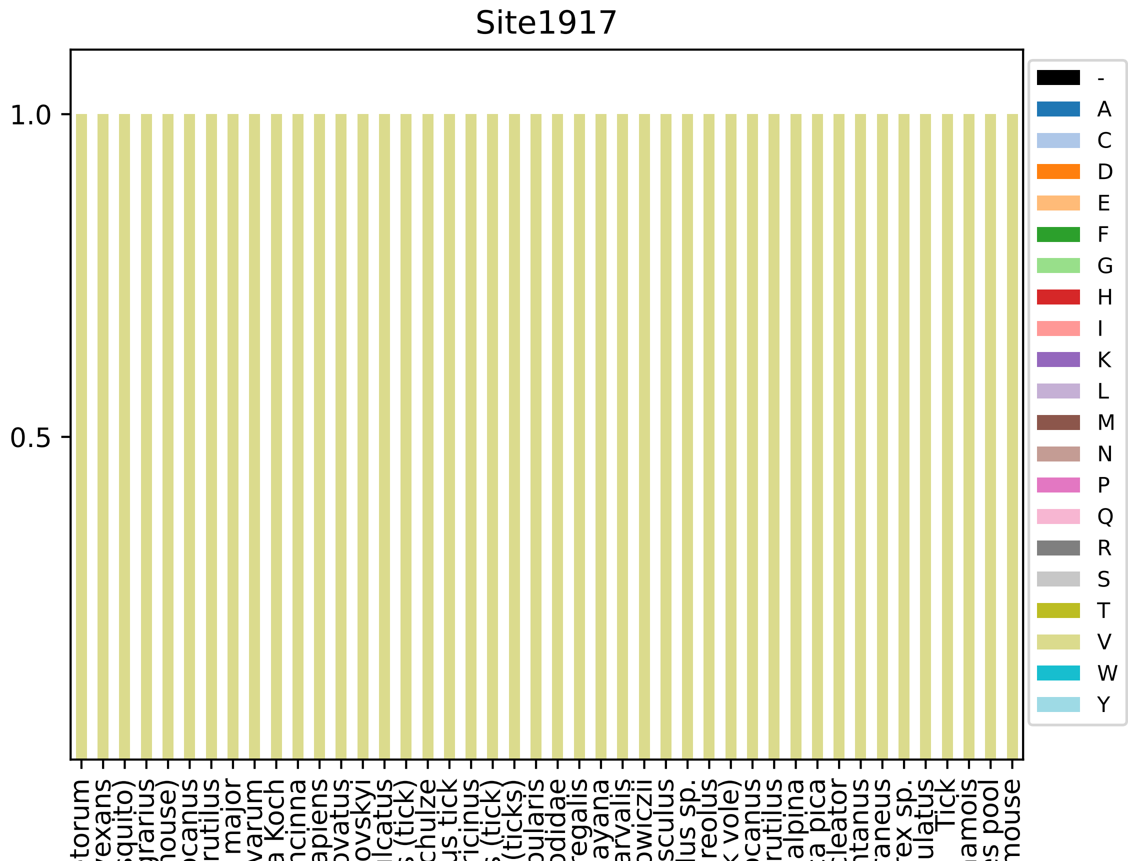
Site1917
1.0
0.5
-
A
C
D
E
F
G
H
I
K
L
M
N
P
Q
R
S
T
V
W
Y

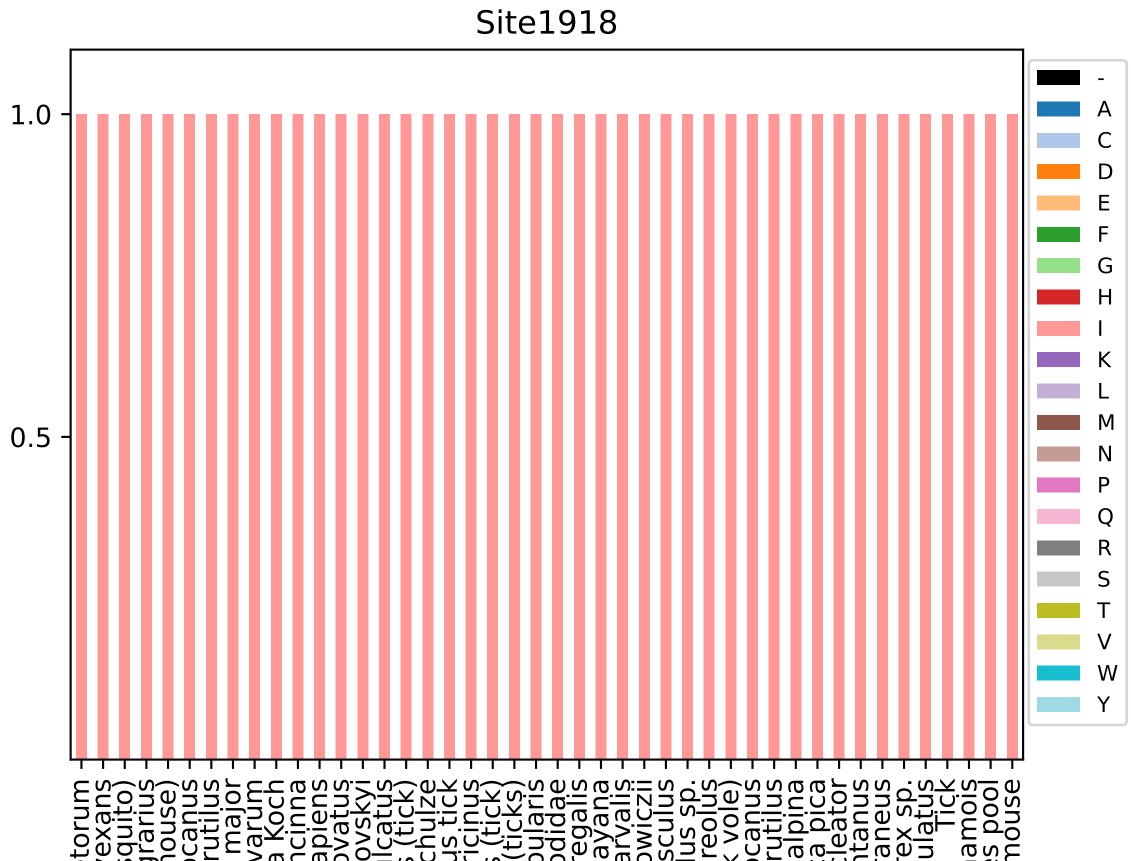
Site1918
1.0
0.5
-
A
C
D
E
F
G
H
I
K
L
M
N
P
Q
R
S
T
V
W
Y

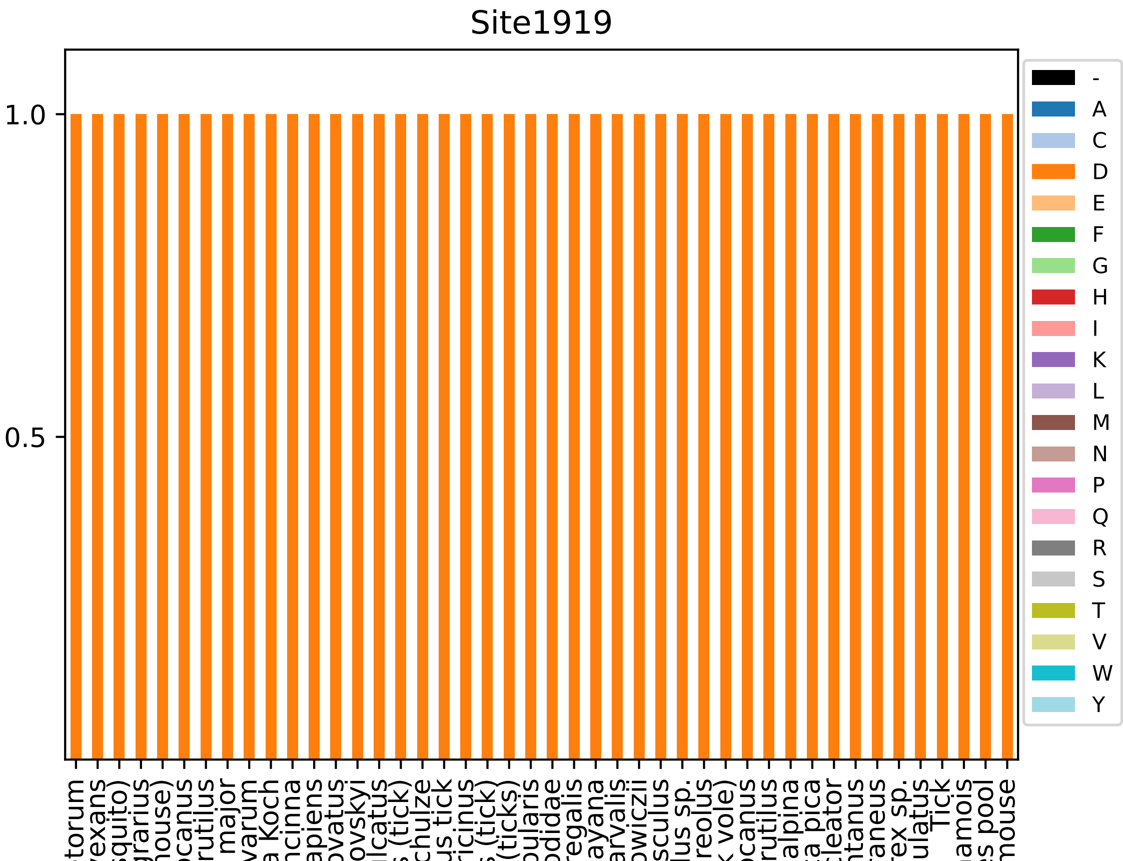

Site1919
1.0
0.5
-
A
C
D
E
F
G
H
I
K
L
M
N
P
Q
R
S
T
V
W
Y
torum
vexans
squito)
garius
nouse)
ocanus
rutilus
major
varum
a Koch
ncinna
apiens
ovatus
ovskyi
ulcatus
s (tick)
chulze
us tick
ricinus
s (tick)
(ticks)
pularis
odidae
regalis
ayana
arvalis
owiczii
sculus
lus sp.
reolus
k vole)
ocanus
rutilus
alpina
a pica
leator
ntanus
aneus
rex sp.
ulatus
Tick
amois
s pool
mouse

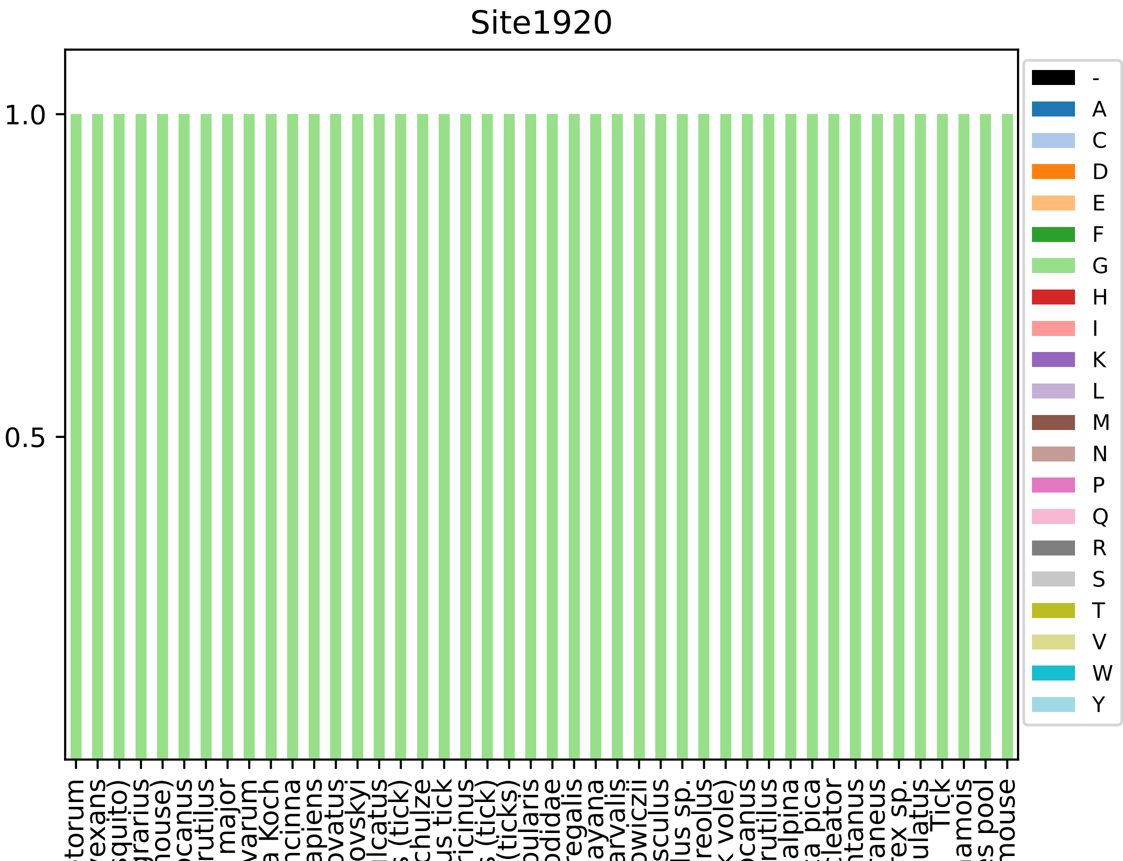
Site1920
1.0
0.5
-
A
C
D
E
F
G
H
I
K
L
M
N
P
Q
R
S
T
V
W
Y

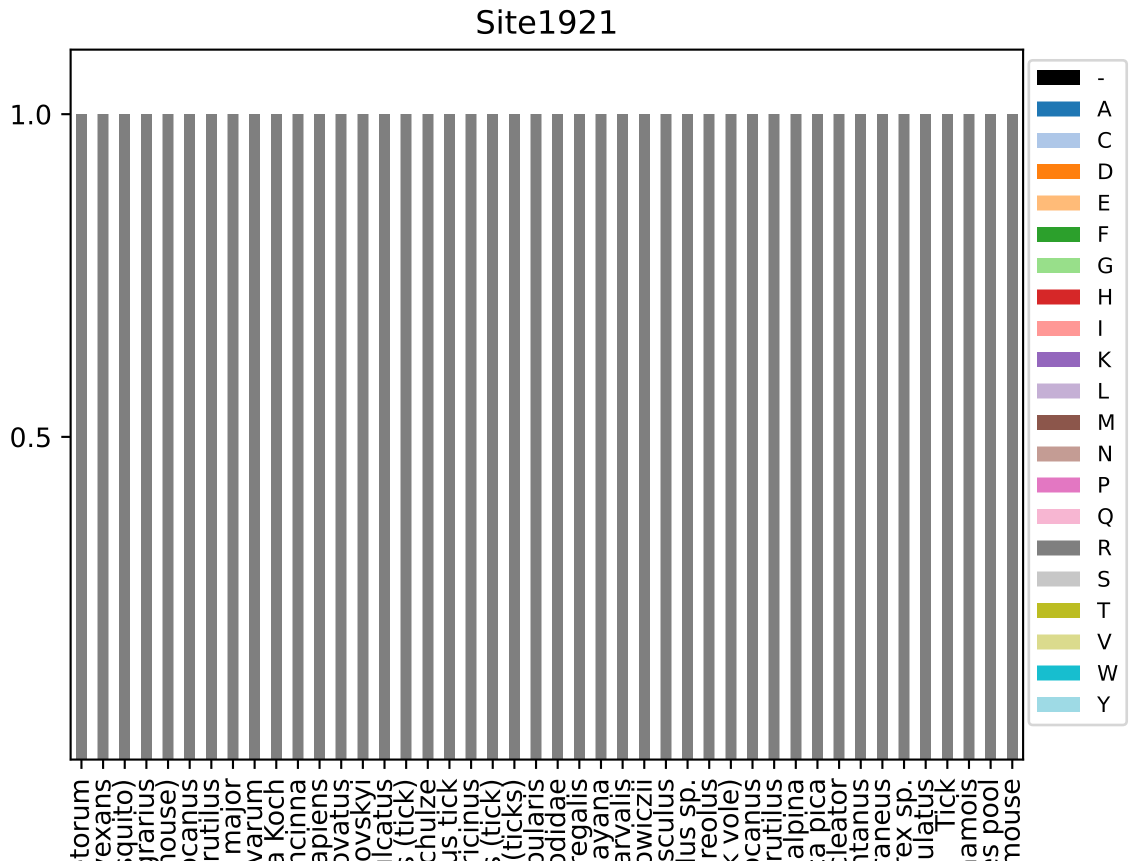
Site1921
1.0
0.5
-
A
C
D
E
F
G
H
I
K
L
M
N
P
Q
R
S
T
V
W
Y

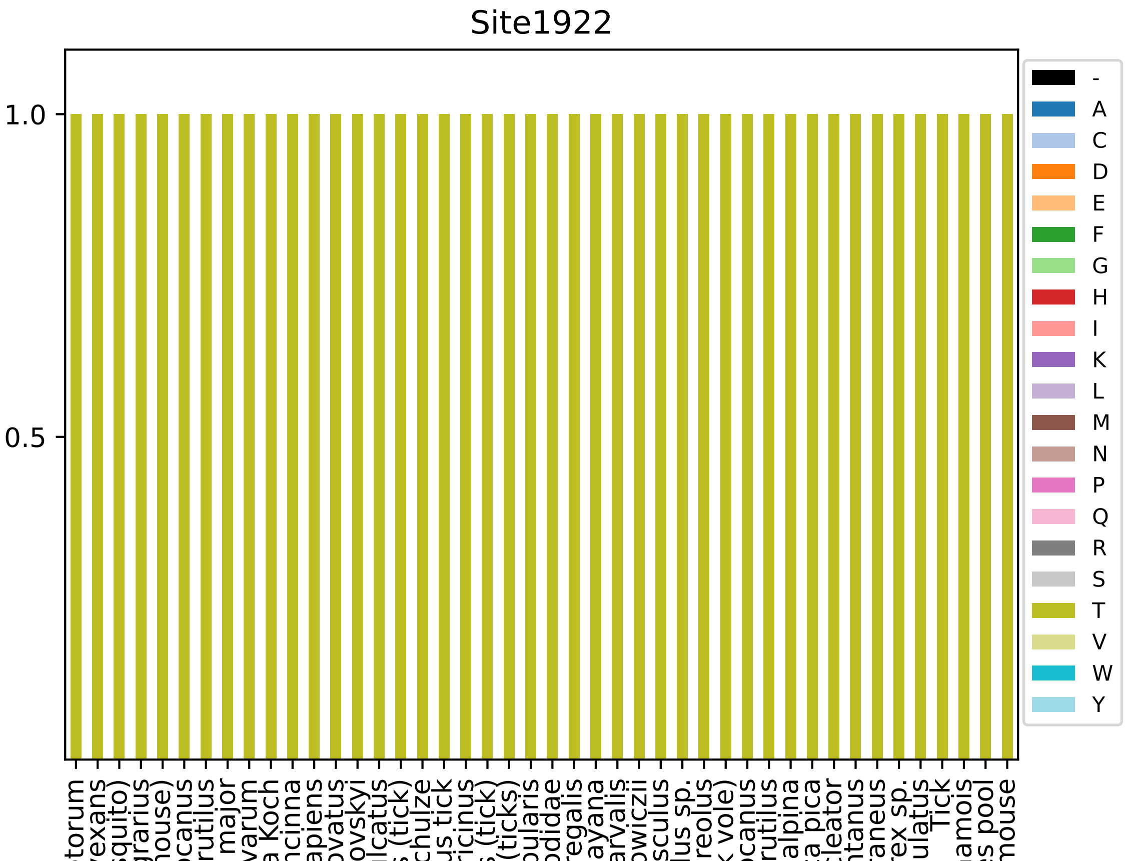
Site1922
1.0
0.5
-
A
C
D
E
F
G
H
I
K
L
M
N
P
Q
R
S
T
V
W
Y

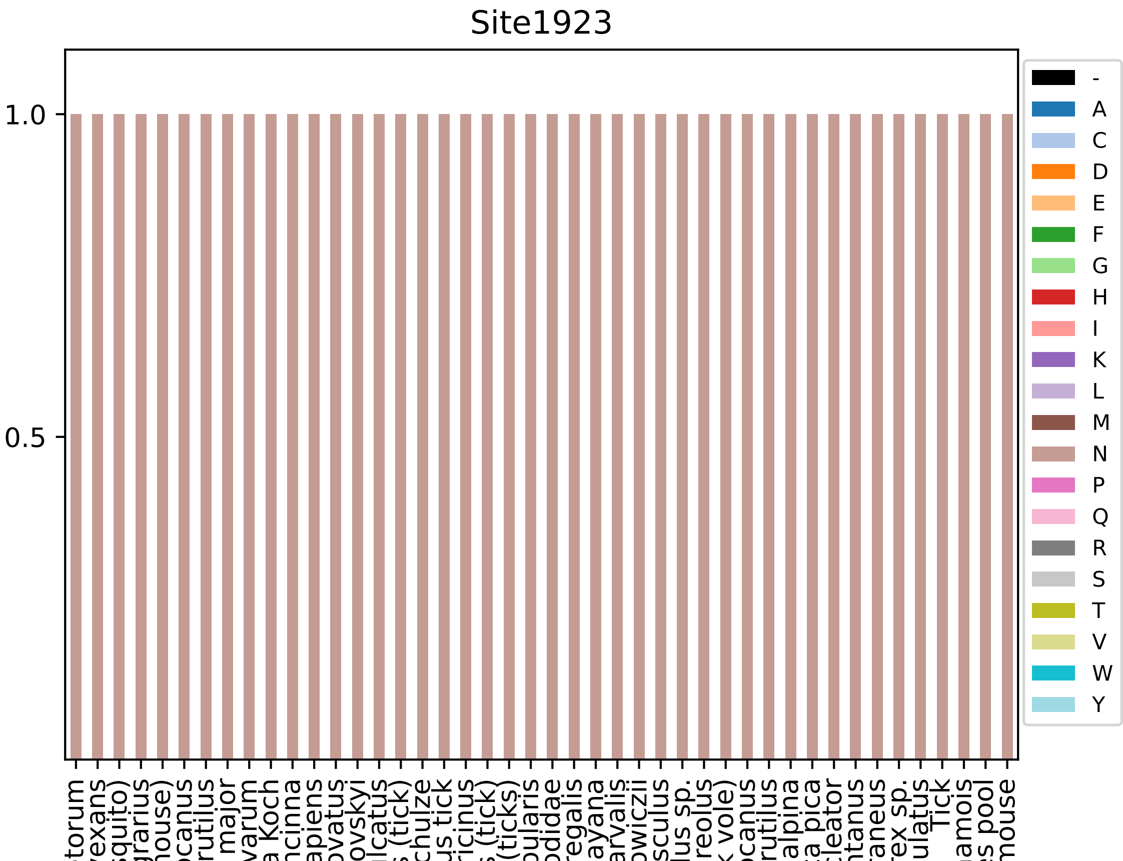
Site1923
1.0
0.5
-
A
C
D
E
F
G
H
I
K
L
M
N
P
Q
R
S
T
V
W
Y

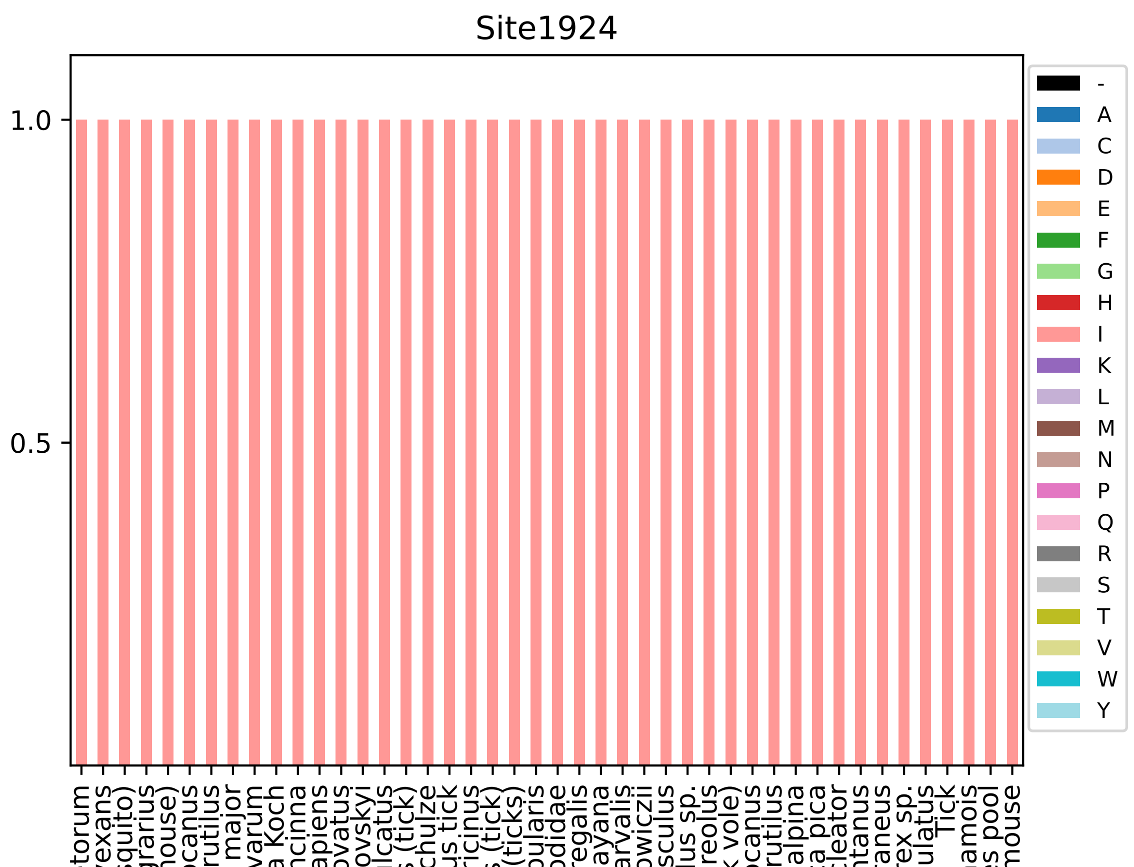
Site1924
1.0
0.5
-
A
C
D
E
F
G
H
I
K
L
M
N
P
Q
R
S
T
V
W
Y

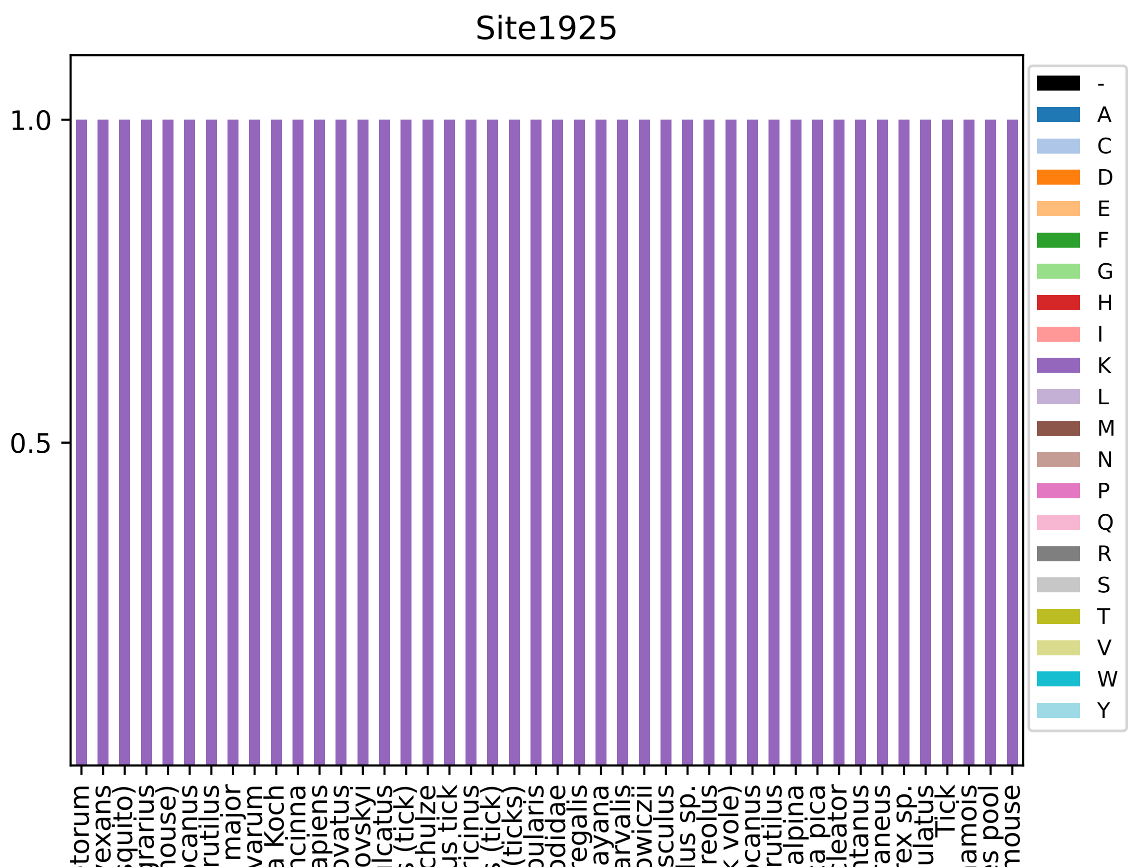

Site1925
1.0
0.5
-
A
C
D
E
F
G
H
I
K
L
M
N
P
Q
R
S
T
V
W
Y

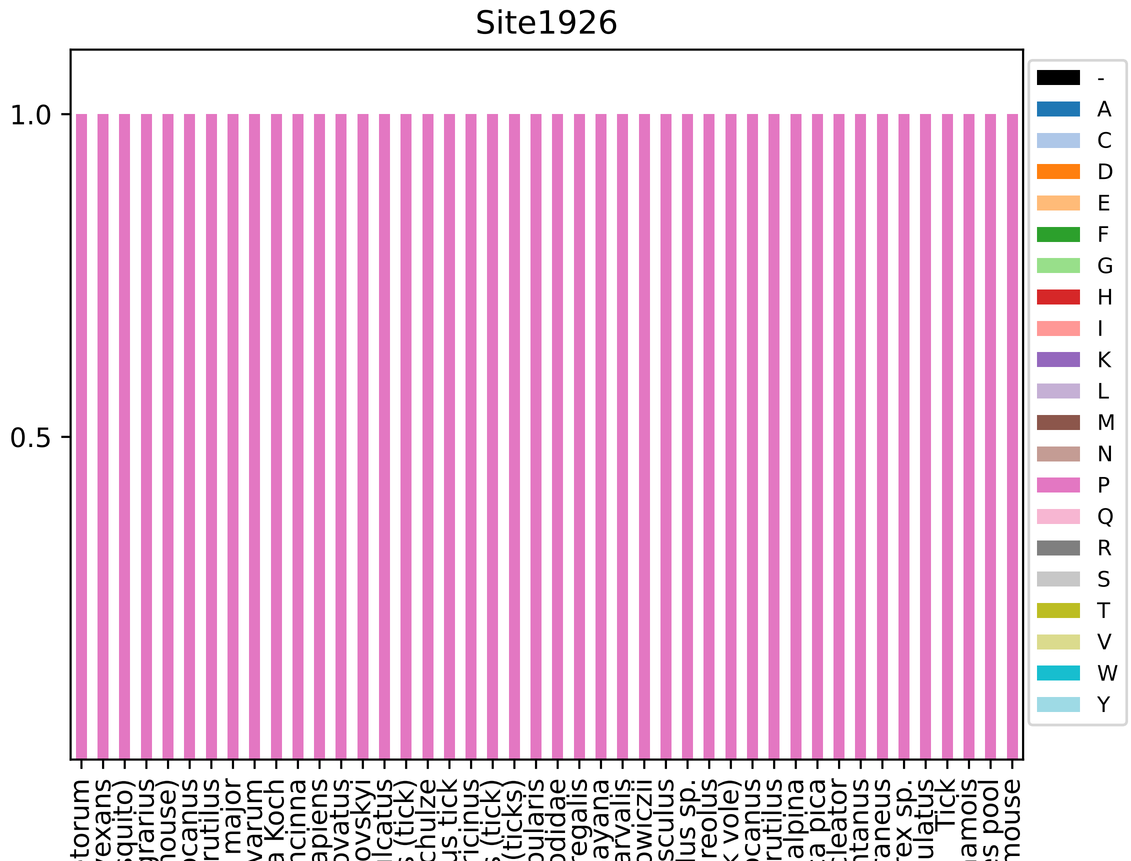
Site1926
1.0
0.5
-
A
C
D
E
F
G
H
I
K
L
M
N
P
Q
R
S
T
V
W
Y

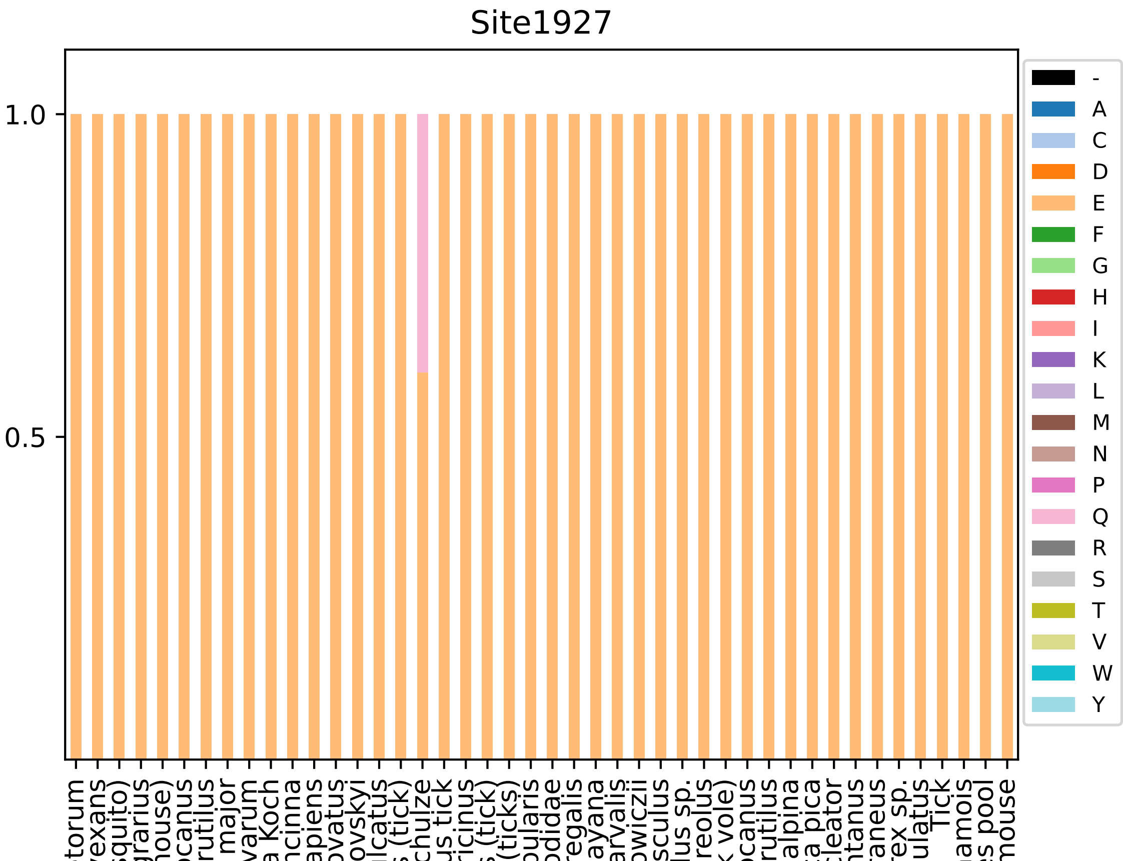
Site1927
1.0
0.5
-
A
C
D
E
F
G
H
I
K
L
M
N
P
Q
R
S
T
V
W
Y

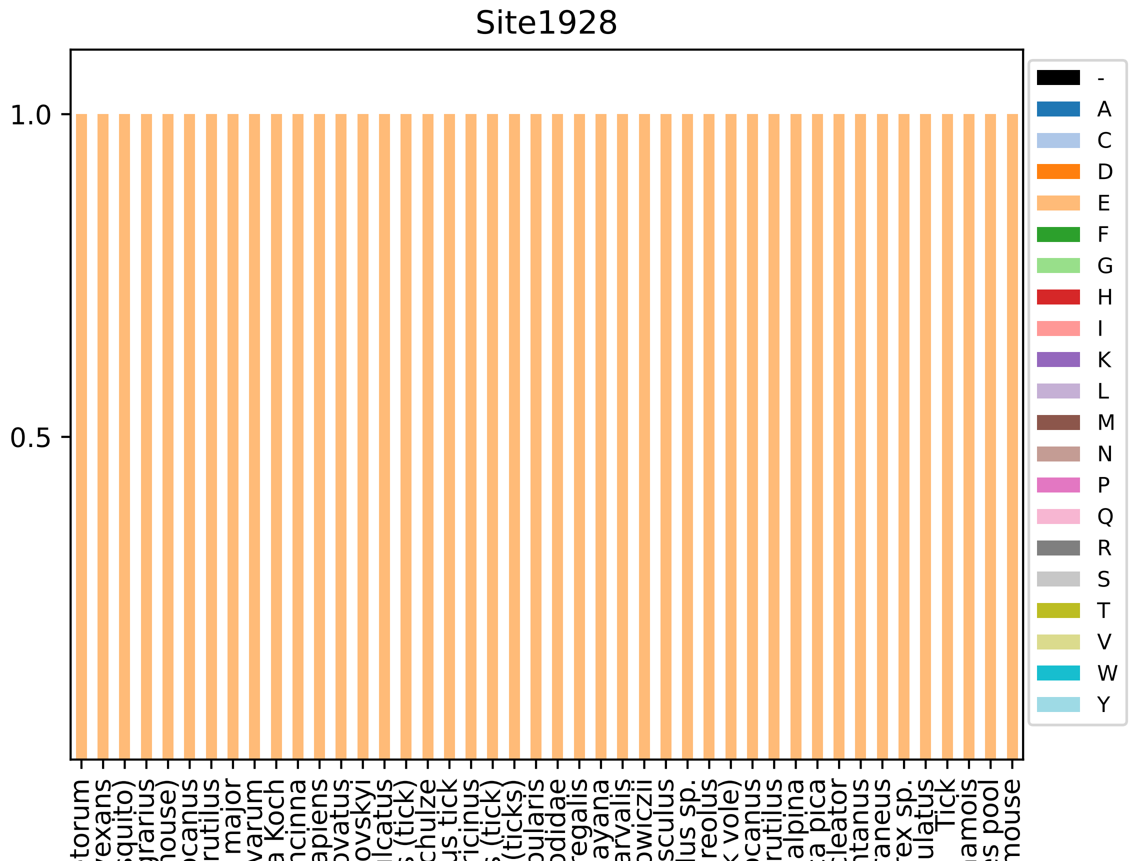
Site1928
1.0
0.5
-
A
C
D
E
F
G
H
I
K
L
M
N
P
Q
R
S
T
V
W
Y

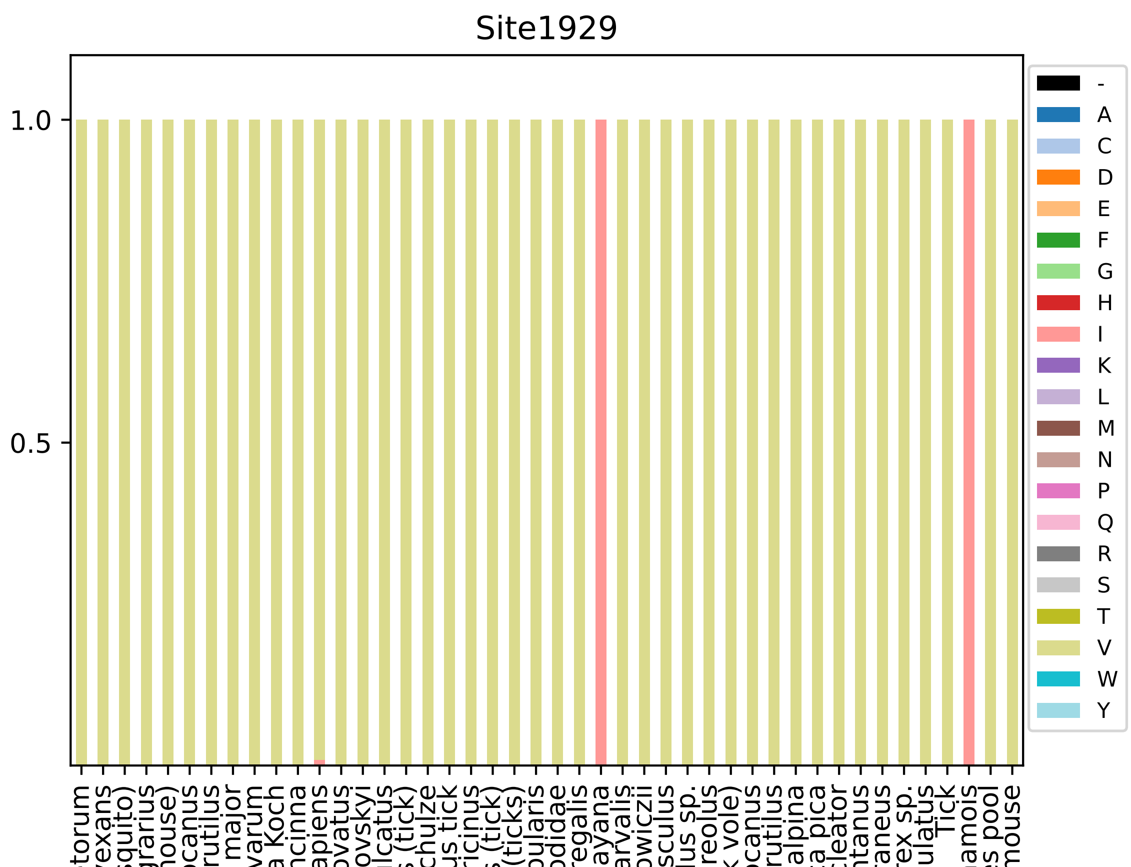

Site1929
1.0
0.5
-
A
C
D
E
F
G
H
I
K
L
M
N
P
Q
R
S
T
V
W
Y

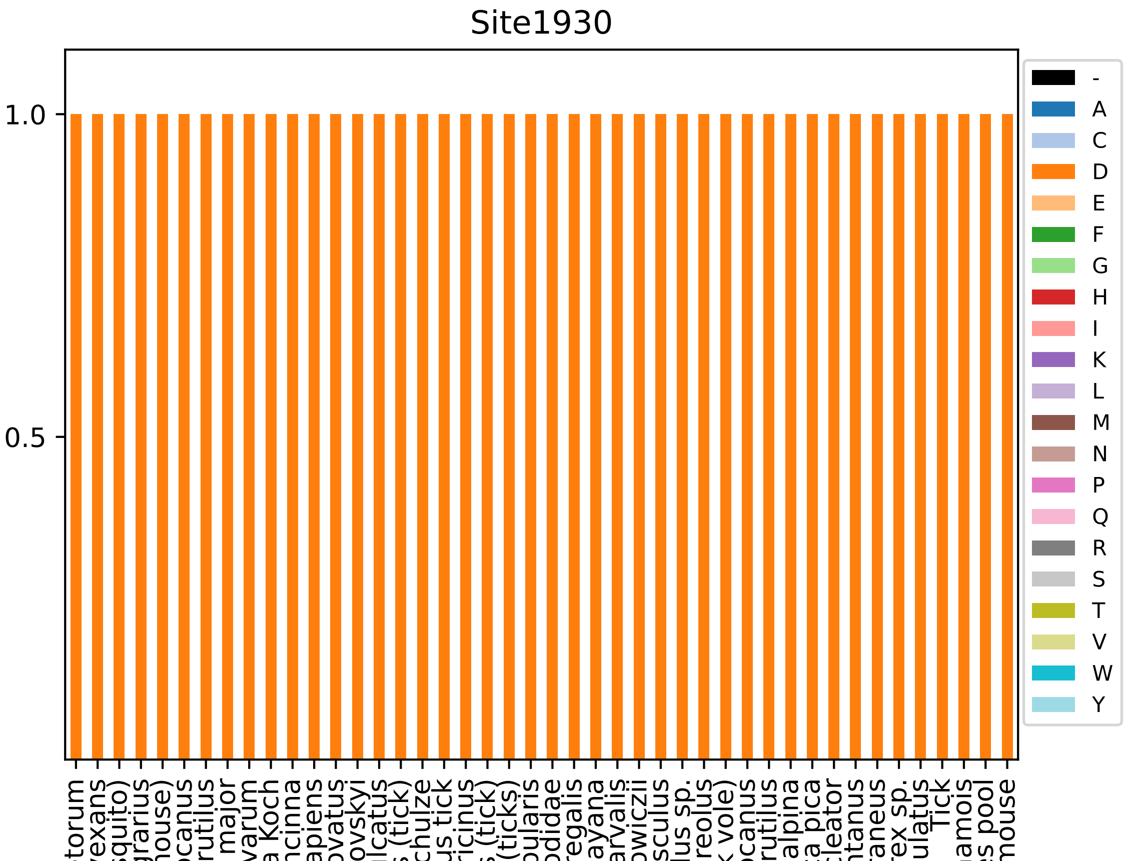

Site1930
1.0
0.5
-
A
C
D
E
F
G
H
I
K
L
M
N
P
Q
R
S
T
V
W
Y

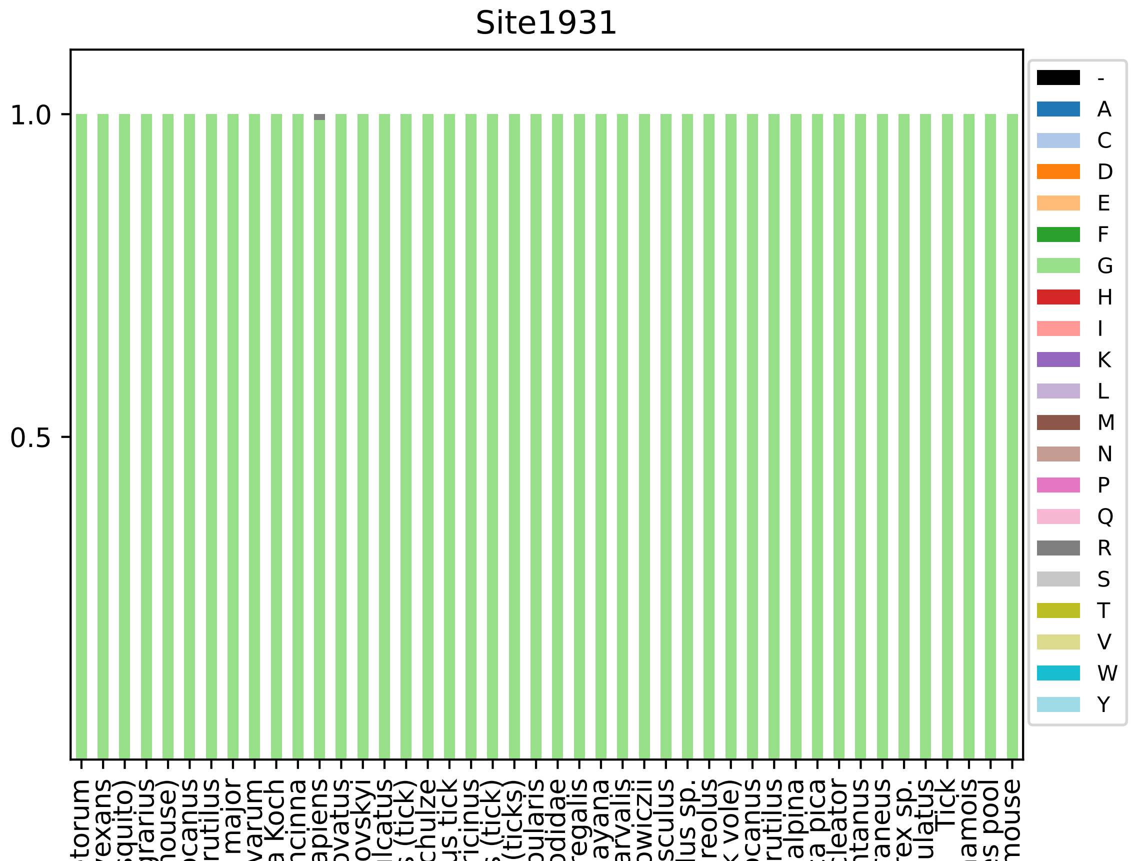
Site1931
1.0
0.5
-
A
C
D
E
F
G
H
I
K
L
M
N
P
Q
R
S
T
V
W
Y

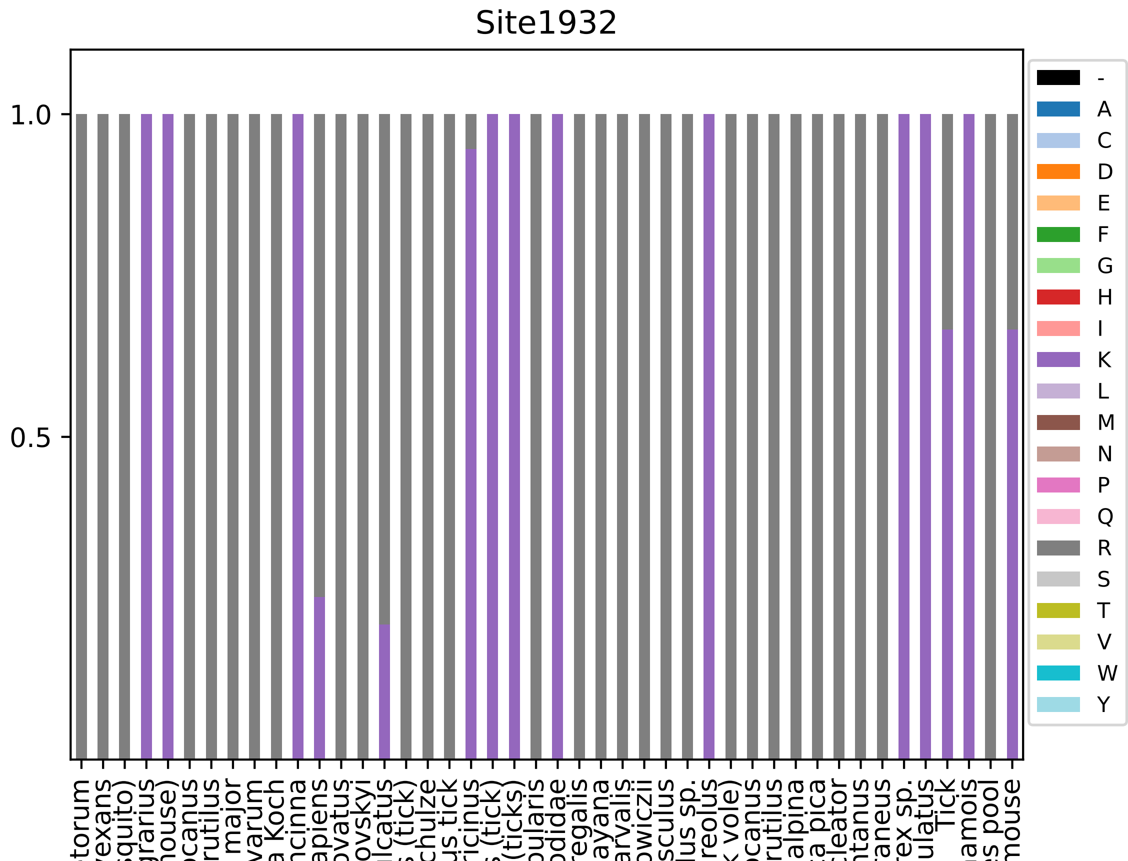

Site1932
1.0
0.5
-
A
C
D
E
F
G
H
I
K
L
M
N
P
Q
R
S
T
V
W
Y

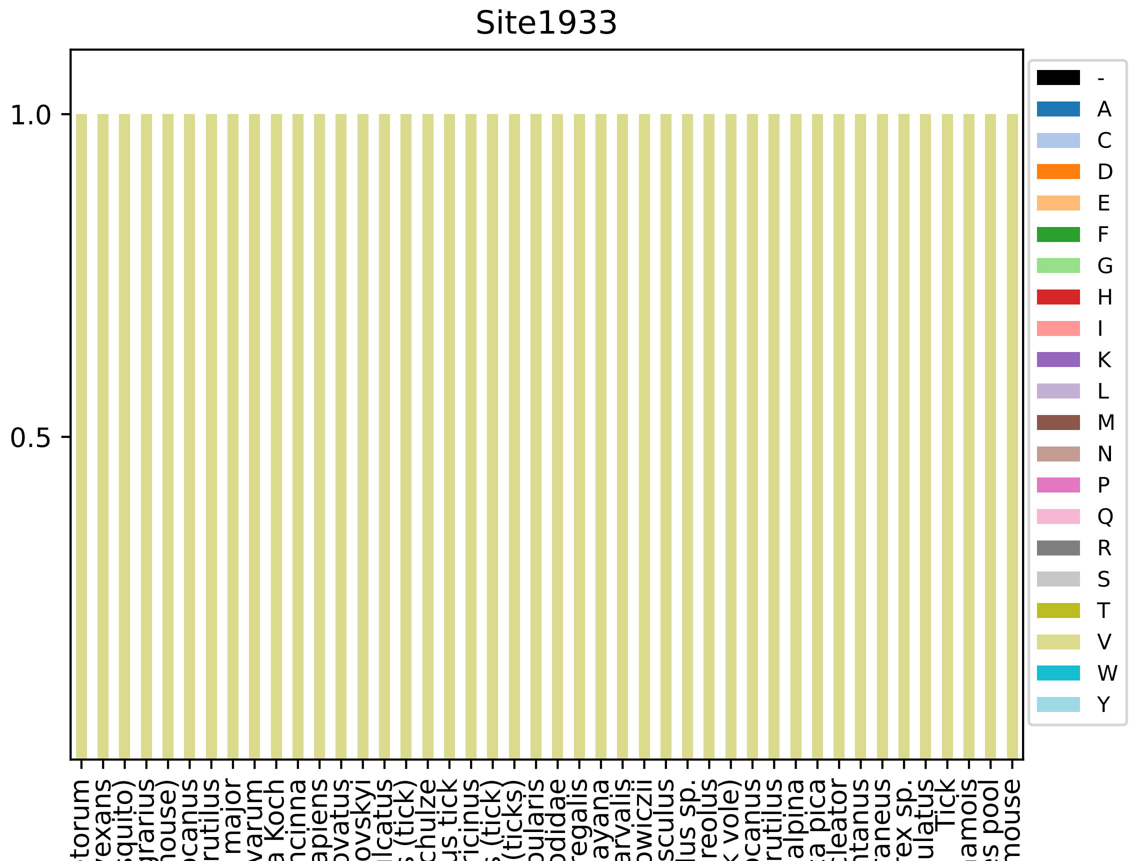
Site1933
1.0
0.5
-
A
C
D
E
F
G
H
I
K
L
M
N
P
Q
R
S
T
V
W
Y

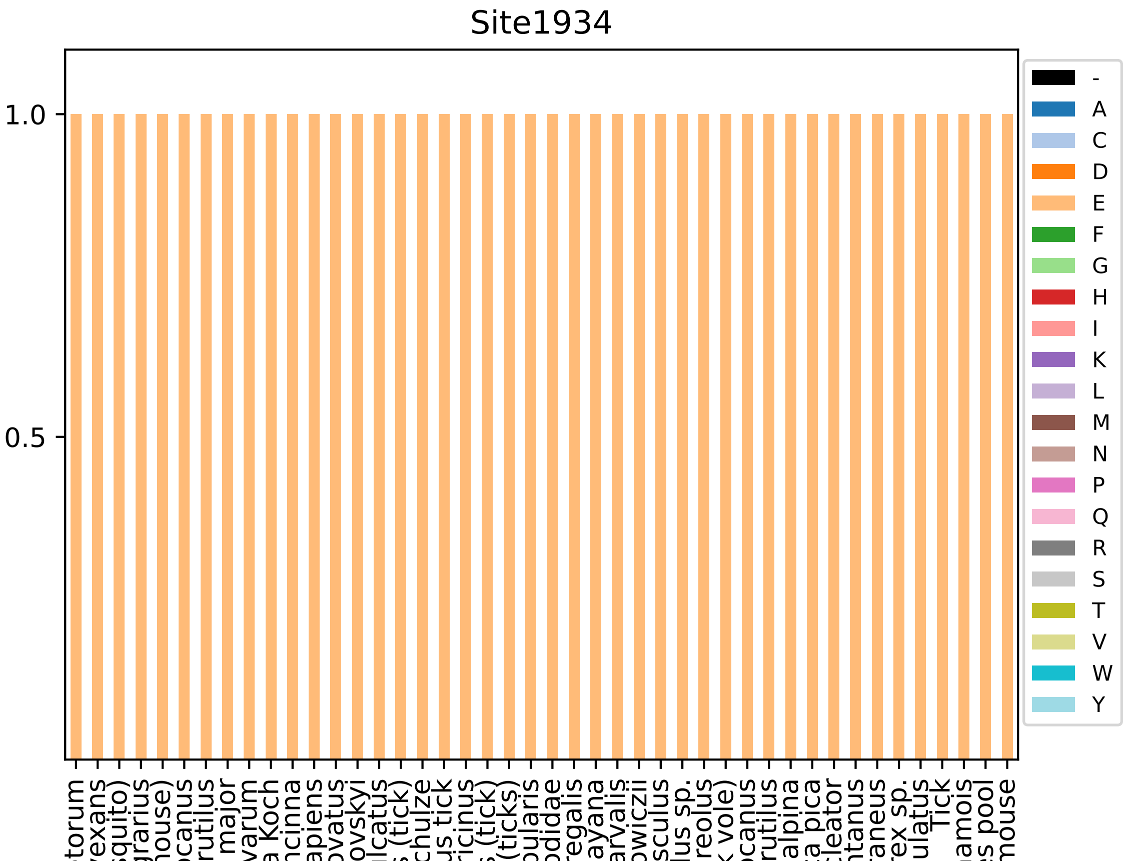
Site1934
1.0
0.5
-
A
C
D
E
F
G
H
I
K
L
M
N
P
Q
R
S
T
V
W
Y

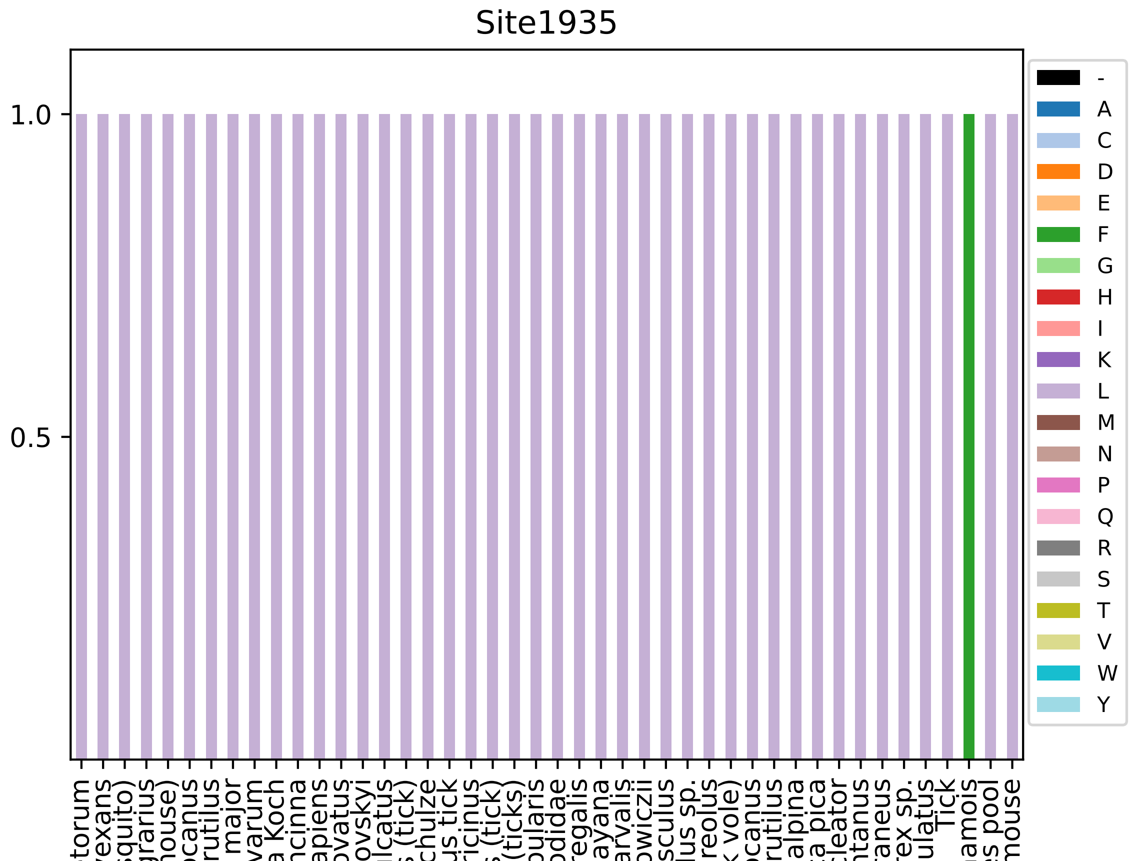
Site1935
1.0
0.5
-
A
C
D
E
F
G
H
I
K
L
M
N
P
Q
R
S
T
V
W
Y

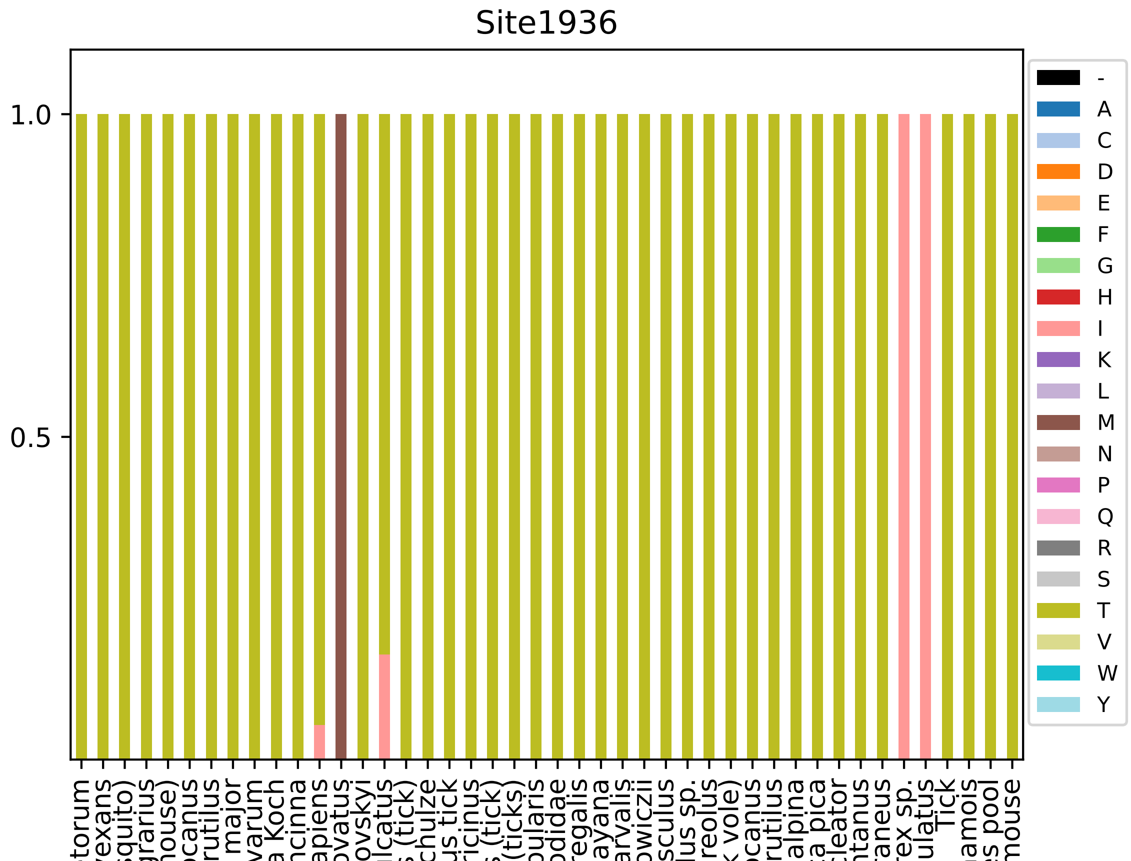
Site1936
1.0
0.5
-
A
C
D
E
F
G
H
I
K
L
M
N
P
Q
R
S
T
V
W
Y
Tick

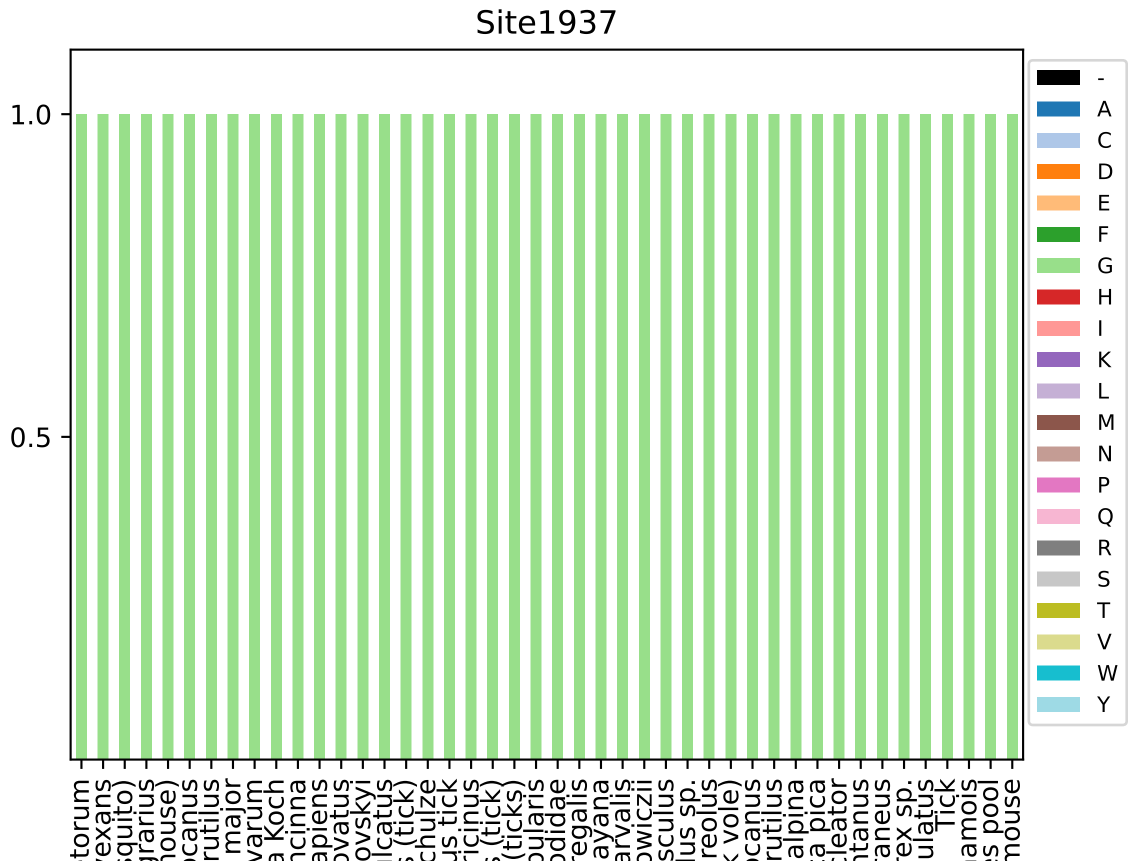
Site1937
1.0
0.5
-
A
C
D
E
F
G
H
I
K
L
M
N
P
Q
R
S
T
V
W
Y

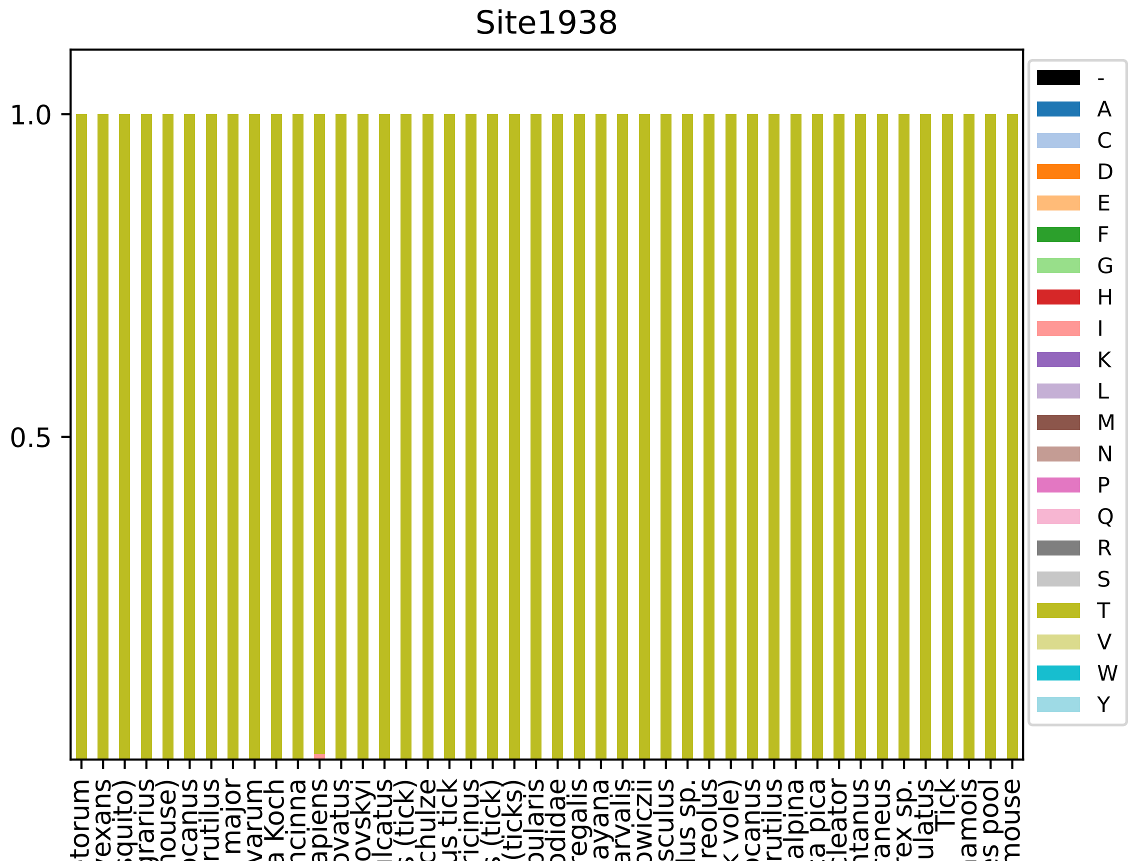
Site1938
1.0
0.5
-
A
C
D
E
F
G
H
I
K
L
M
N
P
Q
R
S
T
V
W
Y
Tick

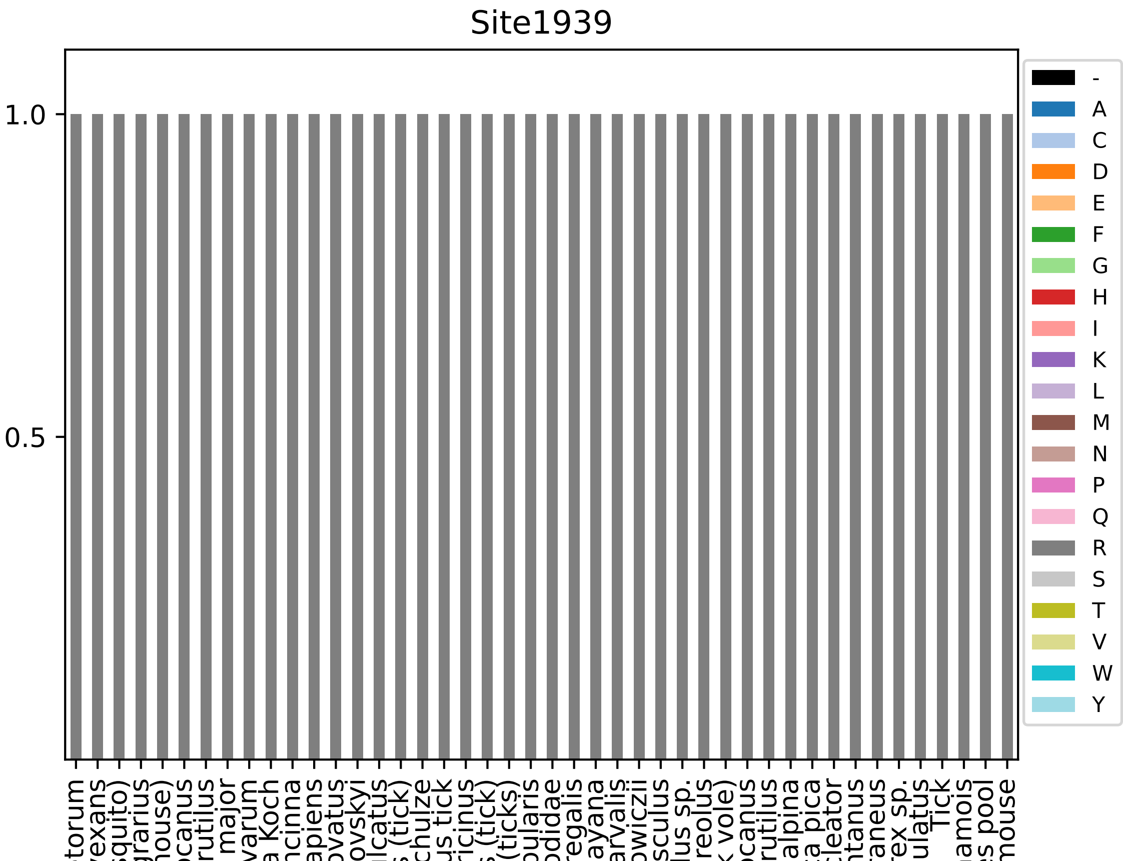
Site1939
1.0
0.5
-
A
C
D
E
F
G
H
I
K
L
M
N
P
Q
R
S
T
V
W
Y
Tick

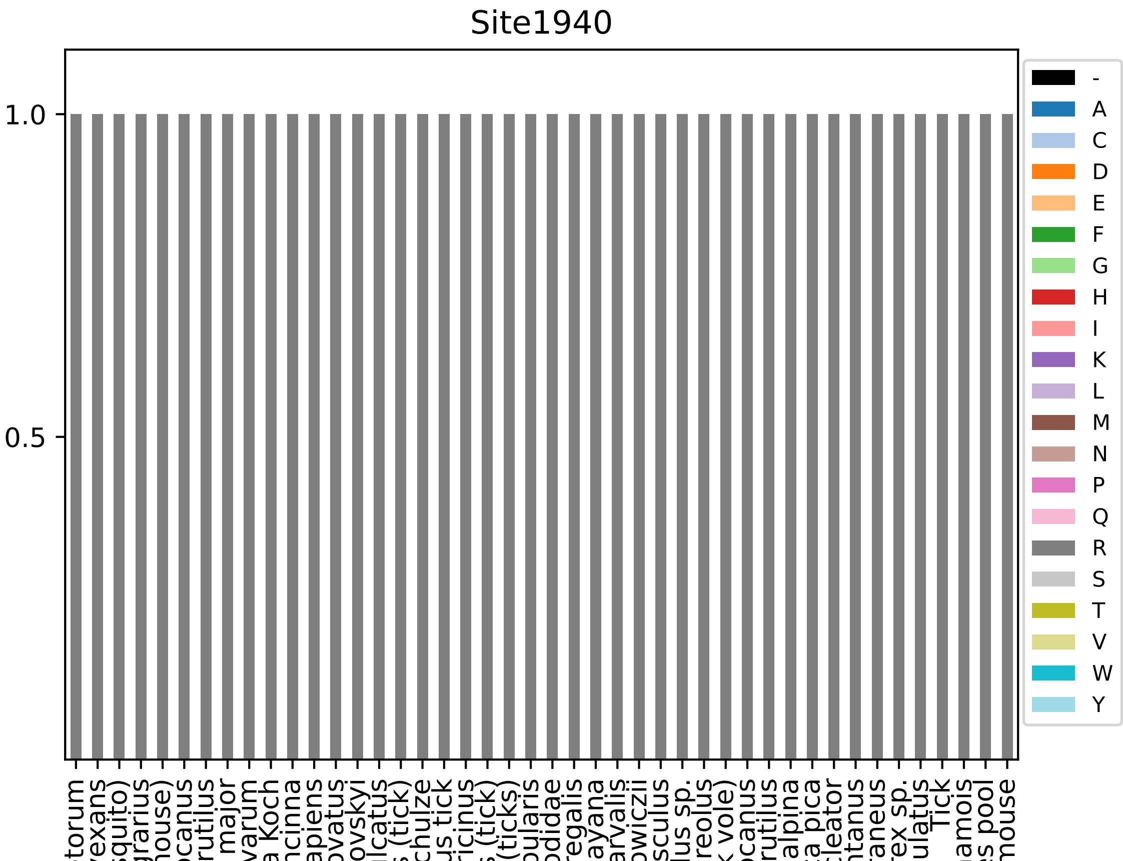
Site1940
1.0
0.5
-
A
C
D
E
F
G
H
I
K
L
M
N
P
Q
R
S
T
V
W
Y

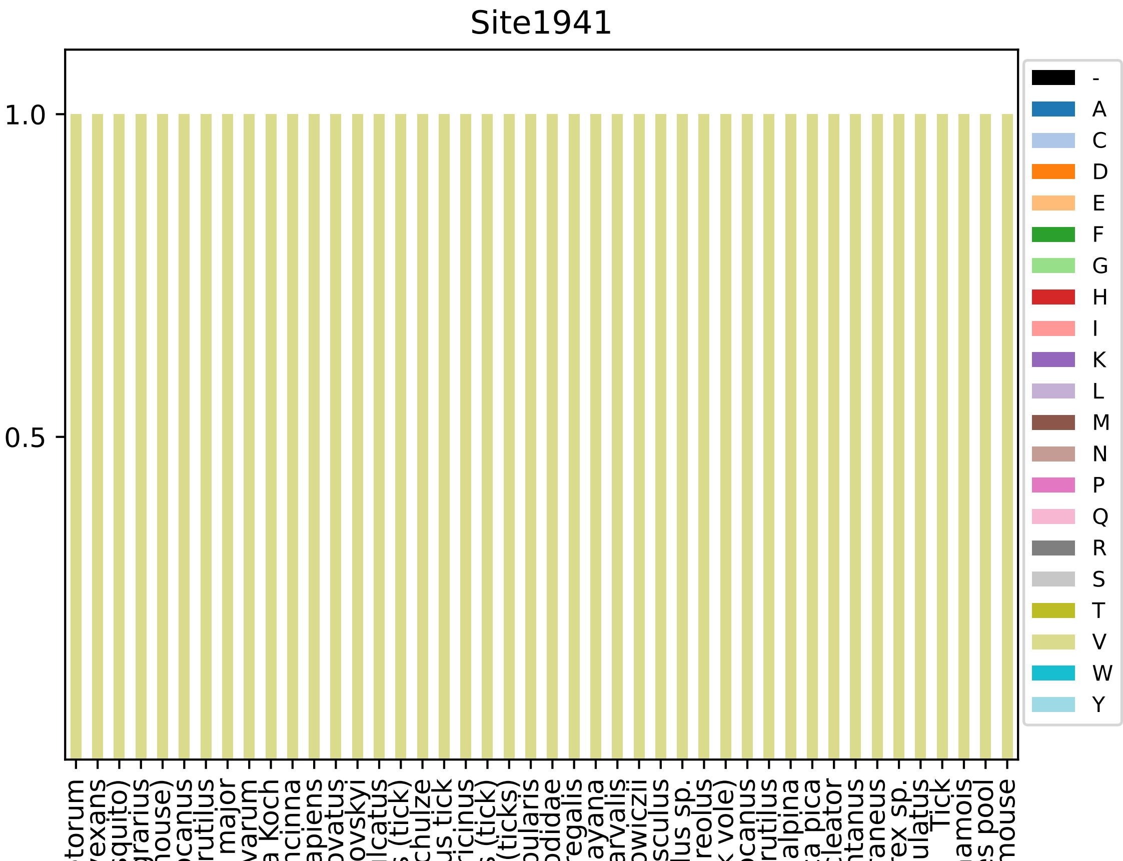

Site1941
1.0
0.5
-
A
C
D
E
F
G
H
I
K
L
M
N
P
Q
R
S
T
V
W
Y

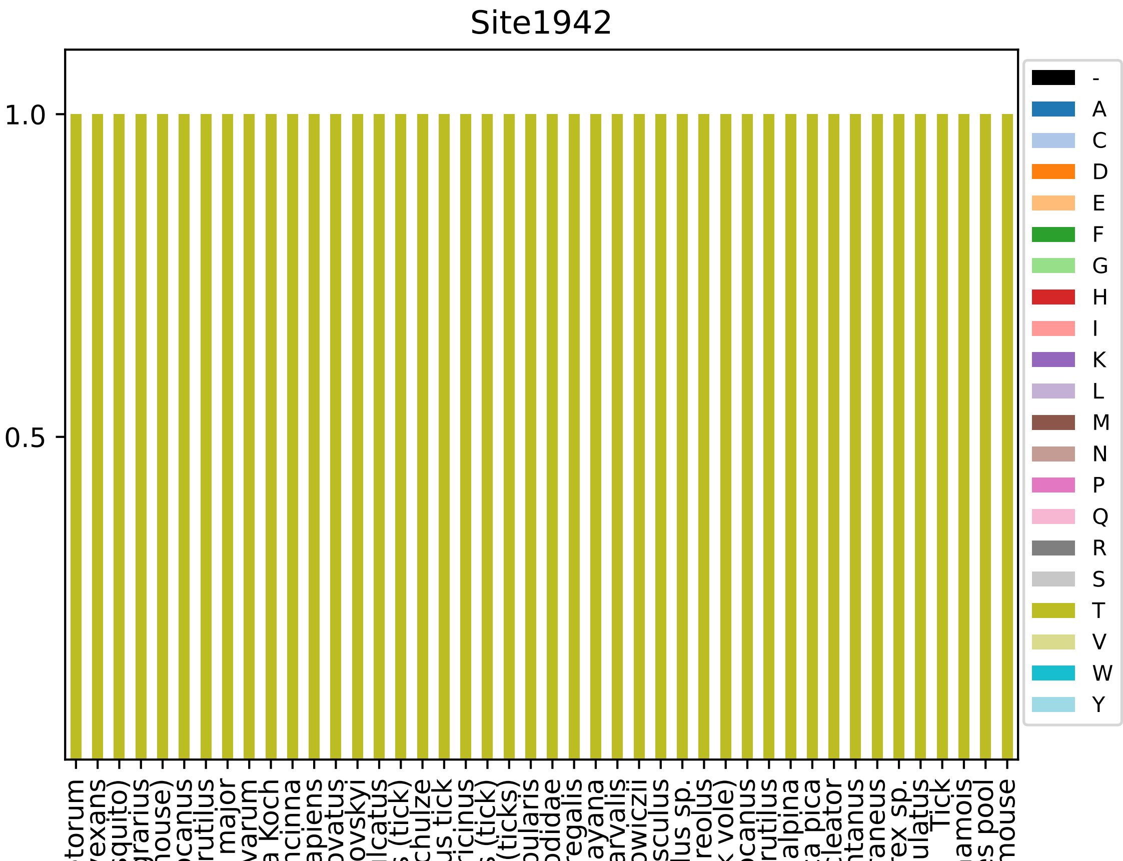
Site1942
1.0
0.5
-
A
C
D
E
F
G
H
I
K
L
M
N
P
Q
R
S
T
V
W
Y

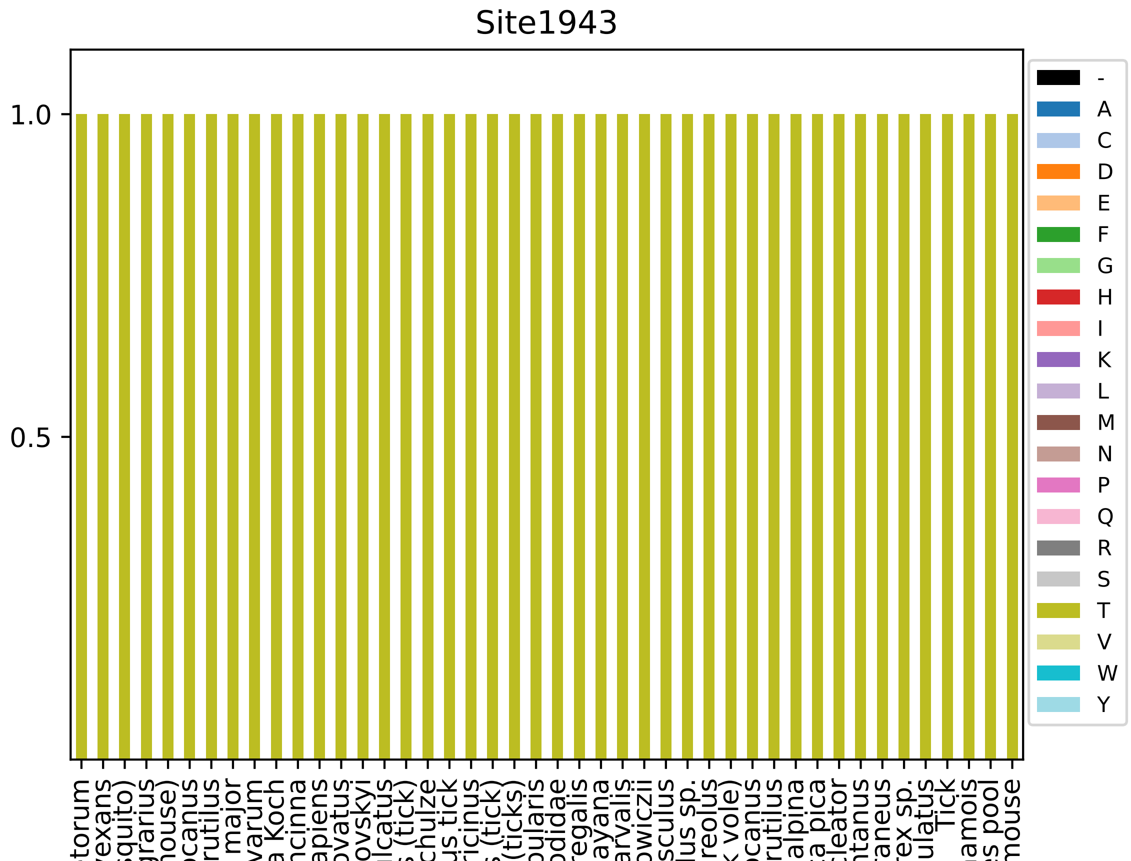
Site1943
1.0
0.5
-
A
C
D
E
F
G
H
I
K
L
M
N
P
Q
R
S
T
V
W
Y

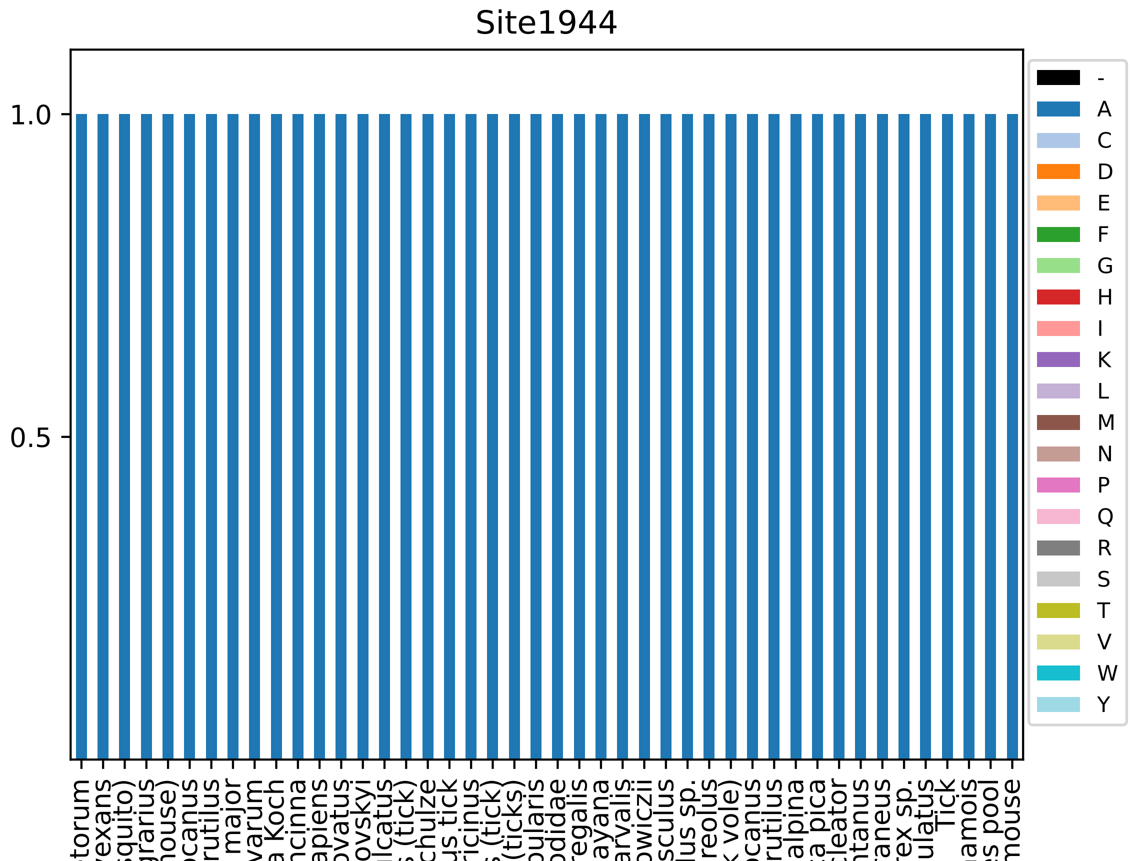
Site1944
1.0
0.5
-
A
C
D
E
F
G
H
I
K
L
M
N
P
Q
R
S
T
V
W
Y

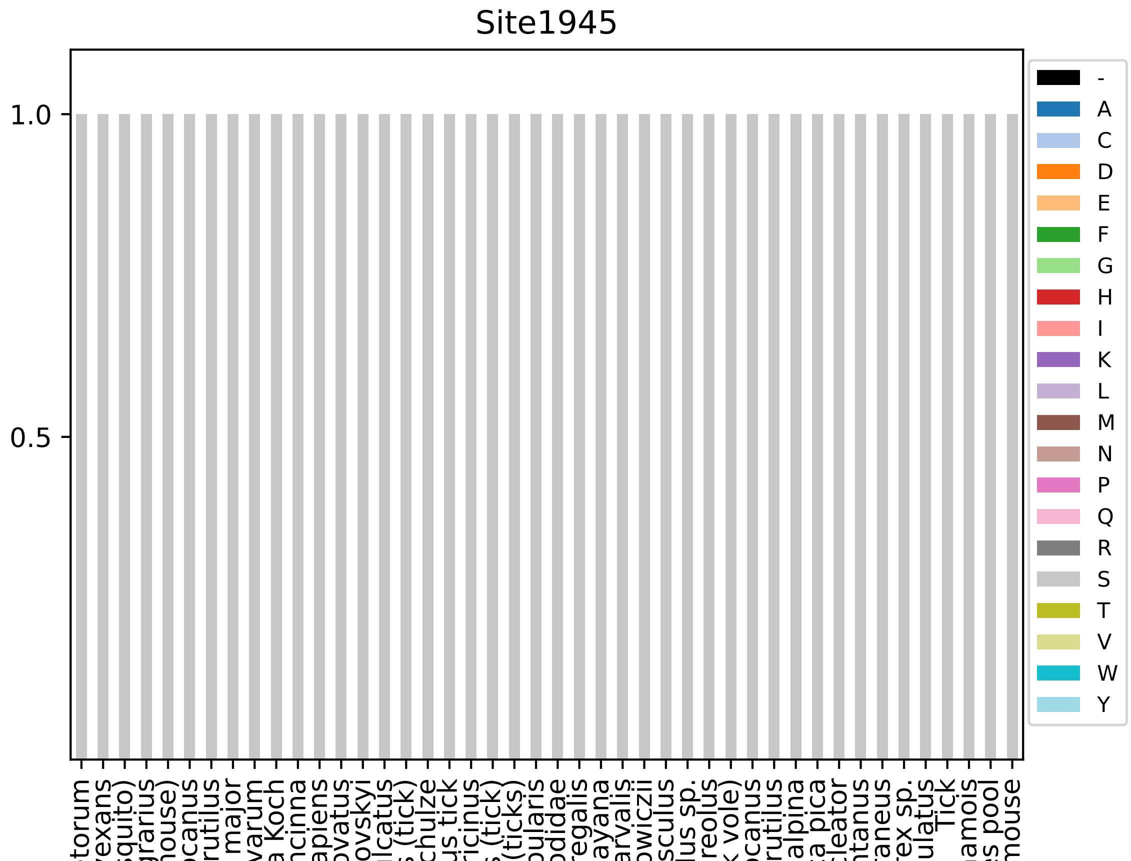
Site1945
1.0
0.5
-
A
C
D
E
F
G
H
I
K
L
M
N
P
Q
R
S
T
V
W
Y

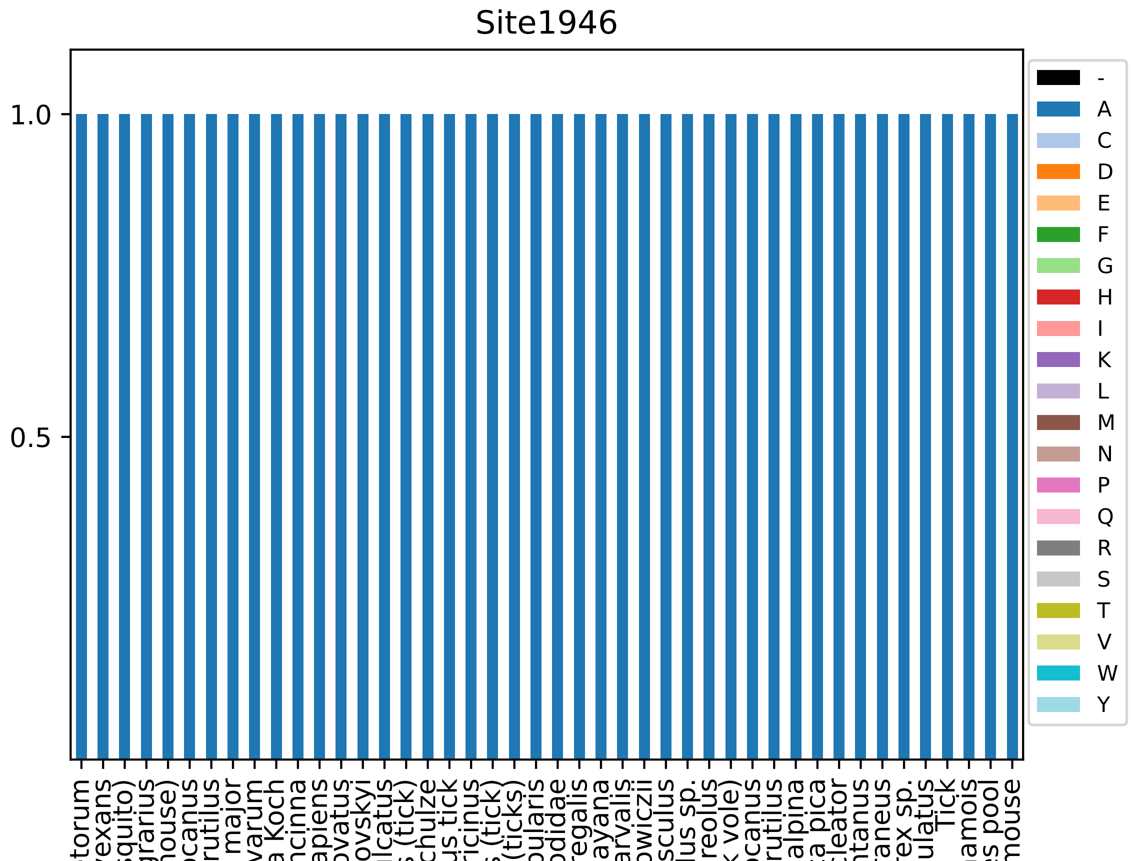
Site1946
1.0
0.5
-
A
C
D
E
F
G
H
I
K
L
M
N
P
Q
R
S
T
V
W
Y

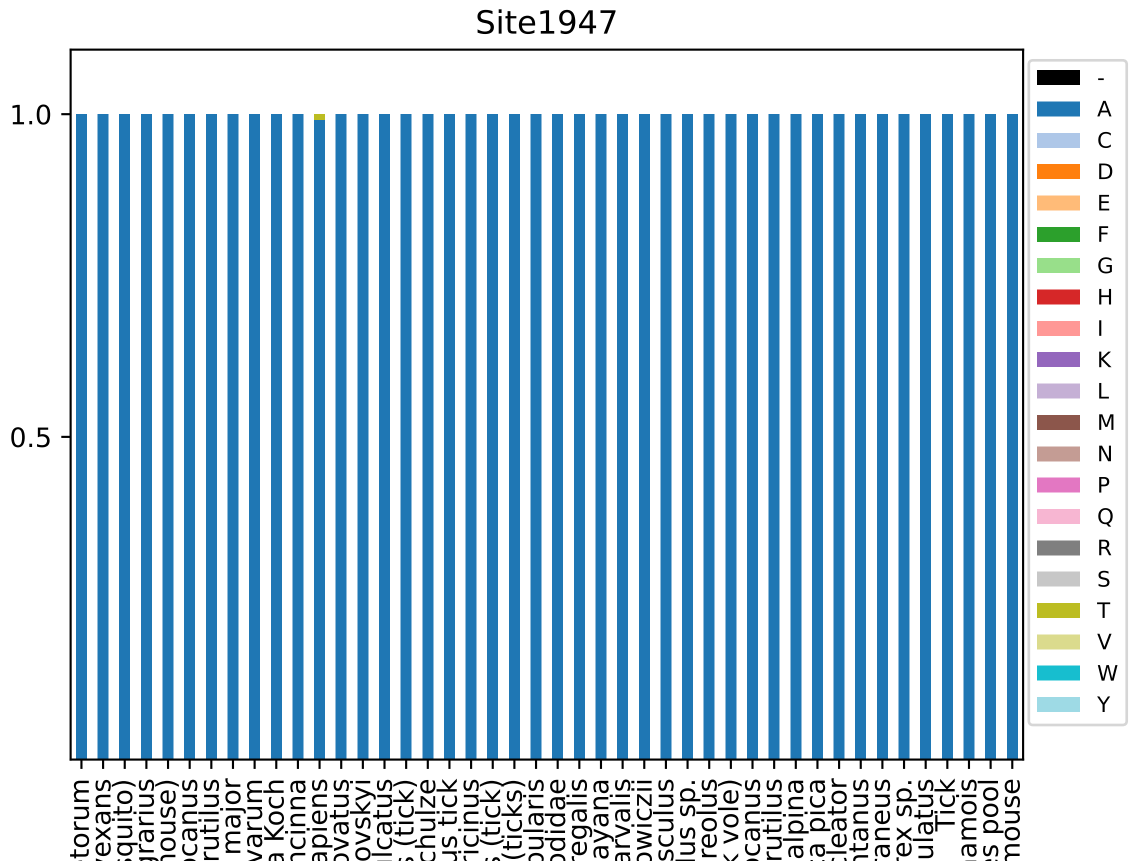

Site1947
1.0
0.5
-
A
C
D
E
F
G
H
I
K
L
M
N
P
Q
R
S
T
V
W
Y

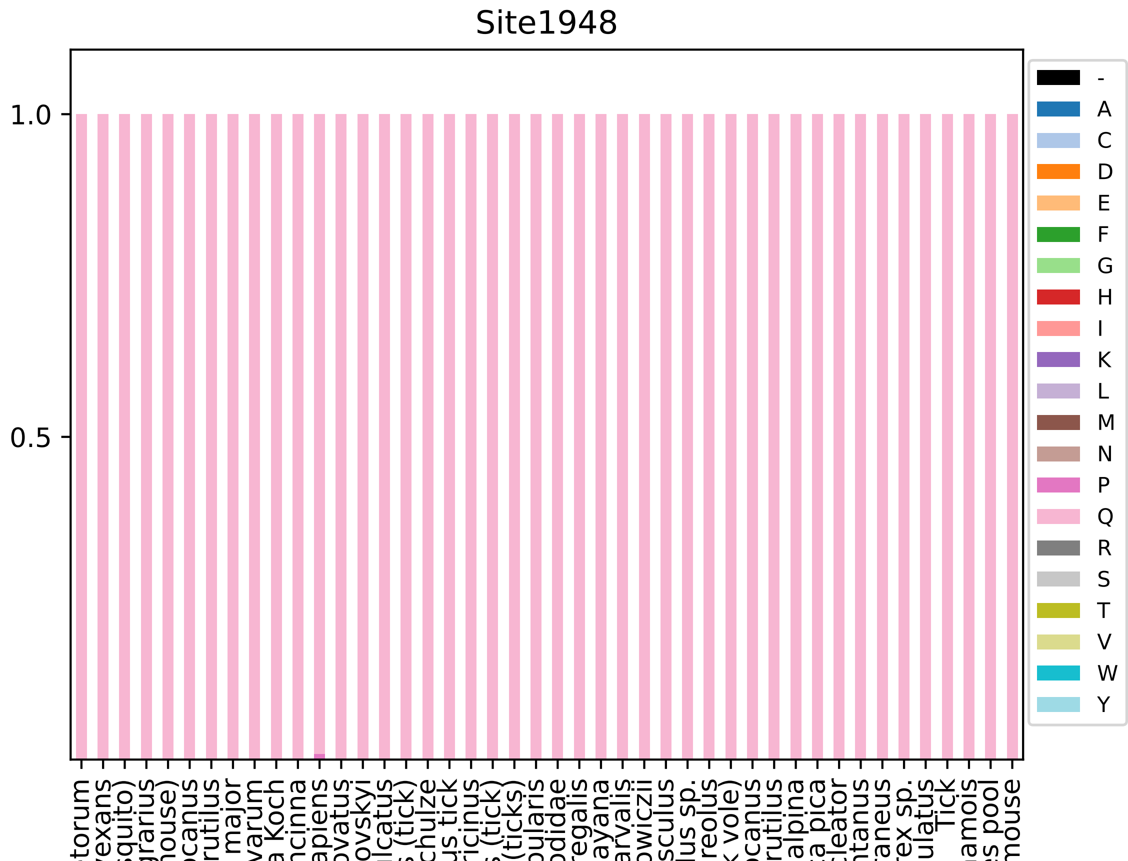

Site1948
1.0
0.5
-
A
C
D
E
F
G
H
I
K
L
M
N
P
Q
R
S
T
V
W
Y

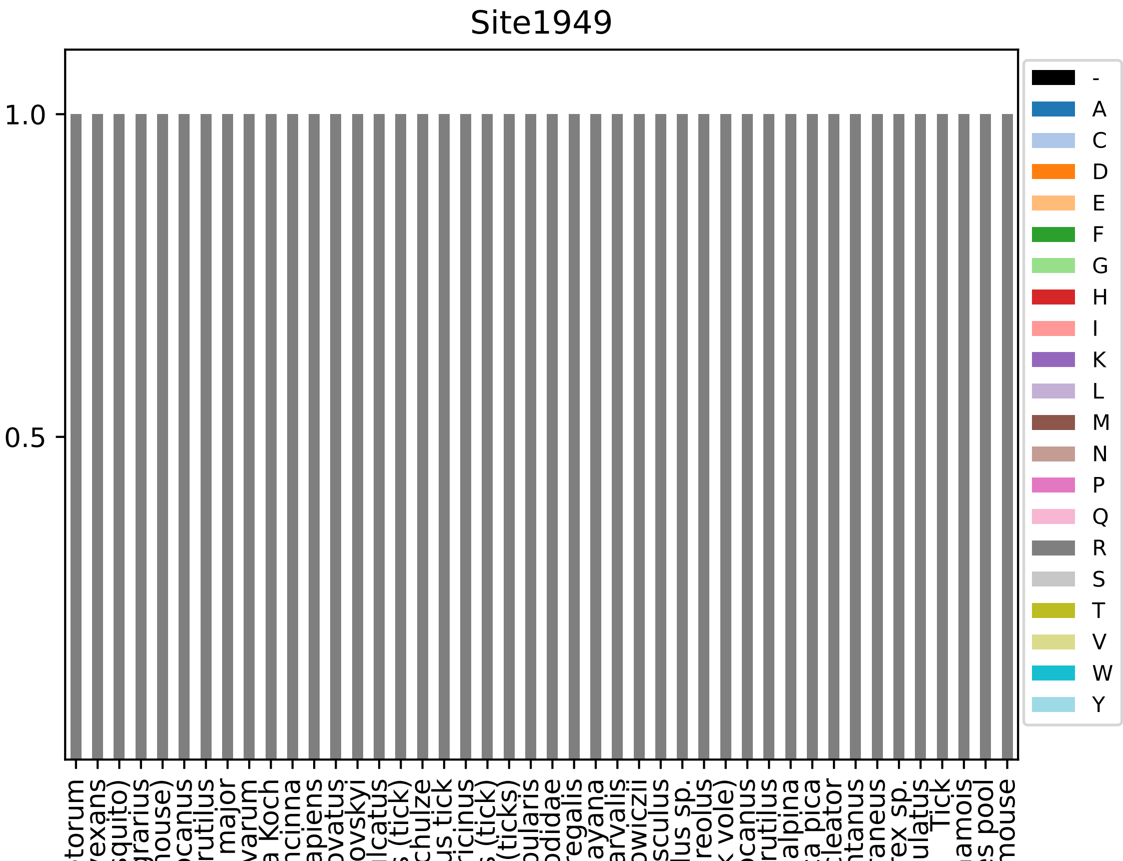
Site1949
1.0
0.5
-
A
C
D
E
F
G
H
I
K
L
M
N
P
Q
R
S
T
V
W
Y

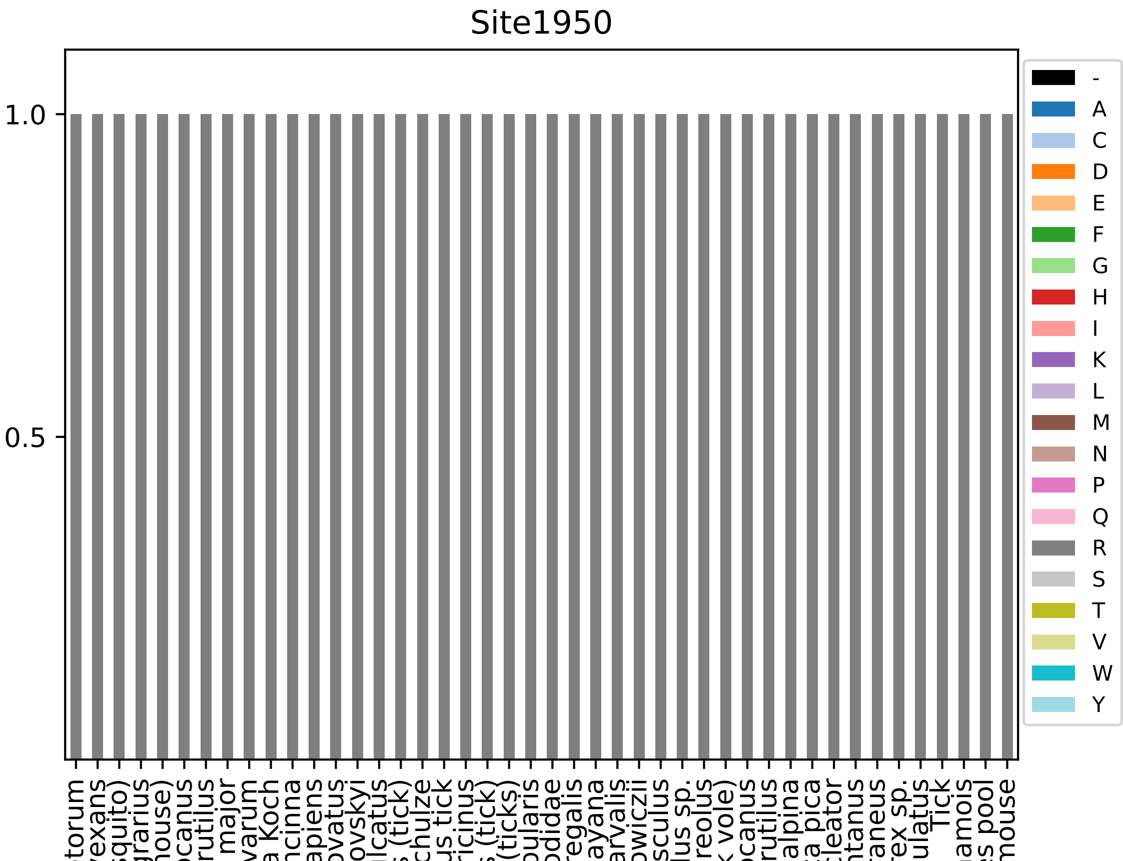
Site1950
1.0
0.5
-
A
C
D
E
F
G
H
I
K
L
M
N
P
Q
R
S
T
V
W
Y

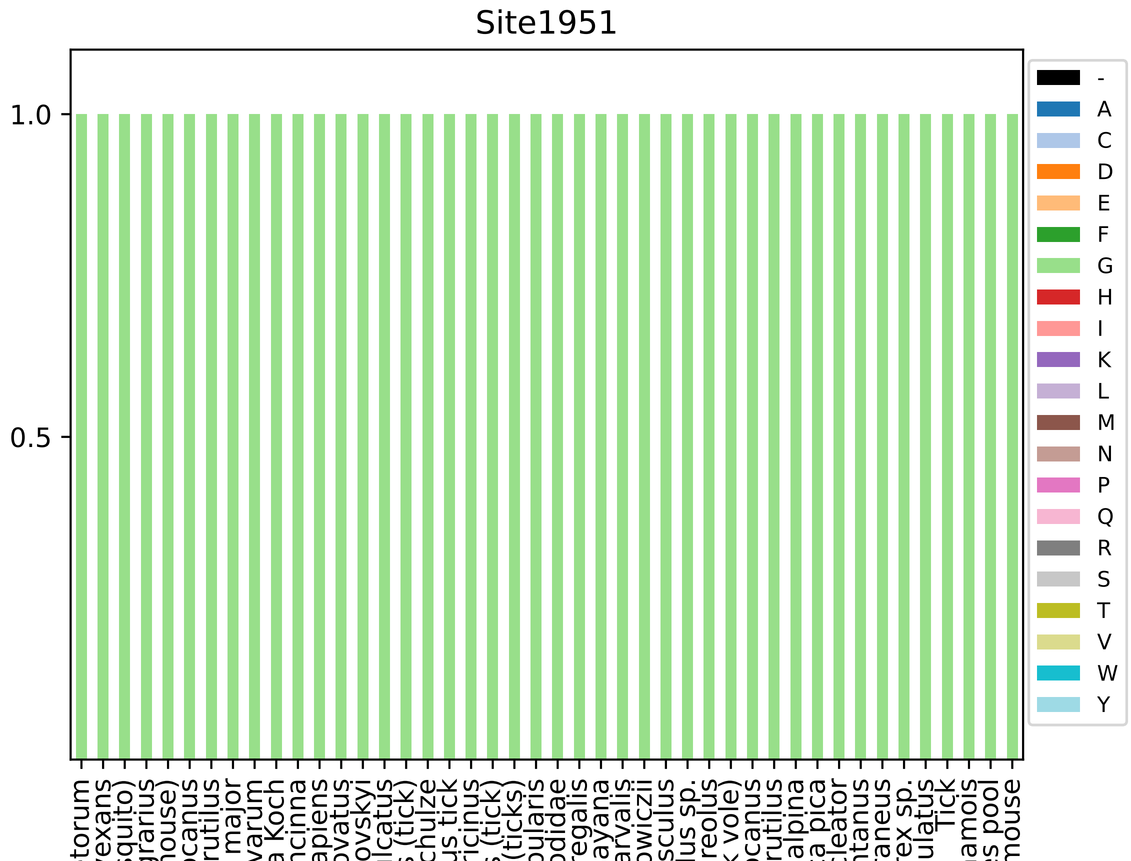
Site1951
1.0
0.5
-
A
C
D
E
F
G
H
I
K
L
M
N
P
Q
R
S
T
V
W
Y
Tick

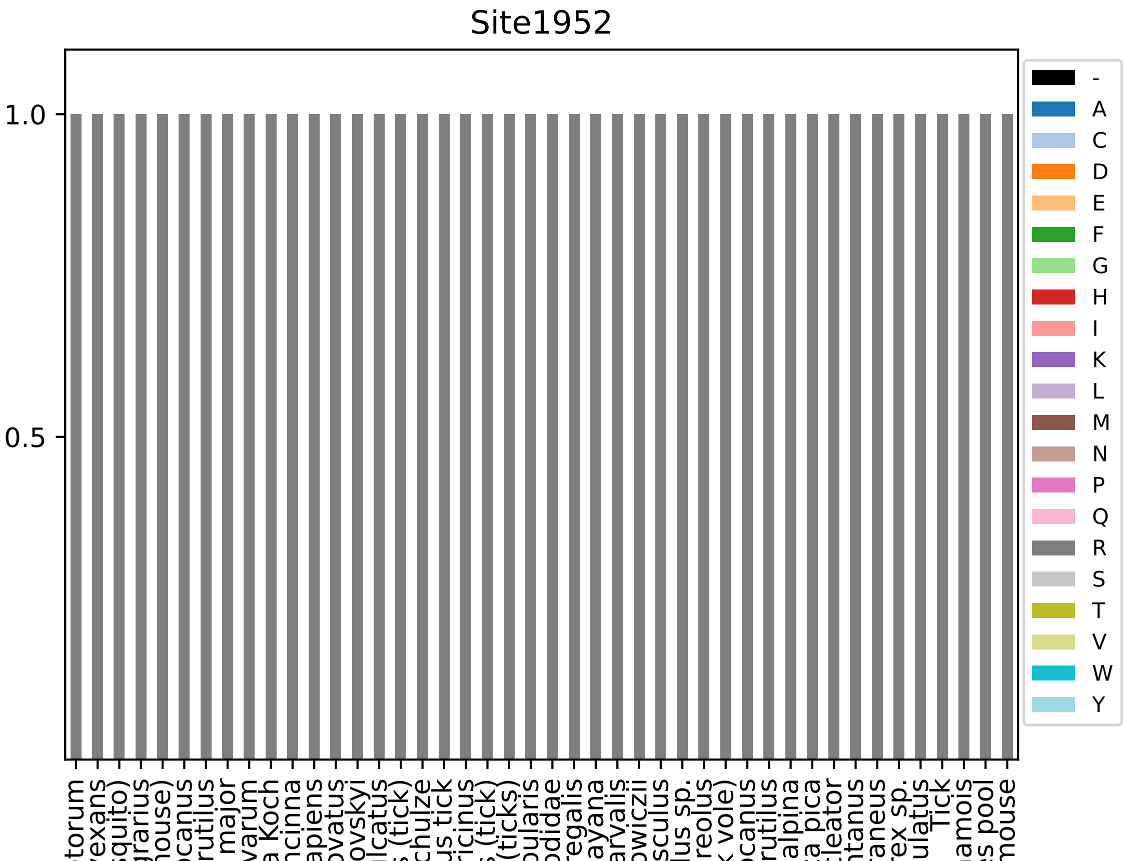
Site1952
1.0
0.5
-
A
C
D
E
F
G
H
I
K
L
M
N
P
Q
R
S
T
V
W
Y
Tick

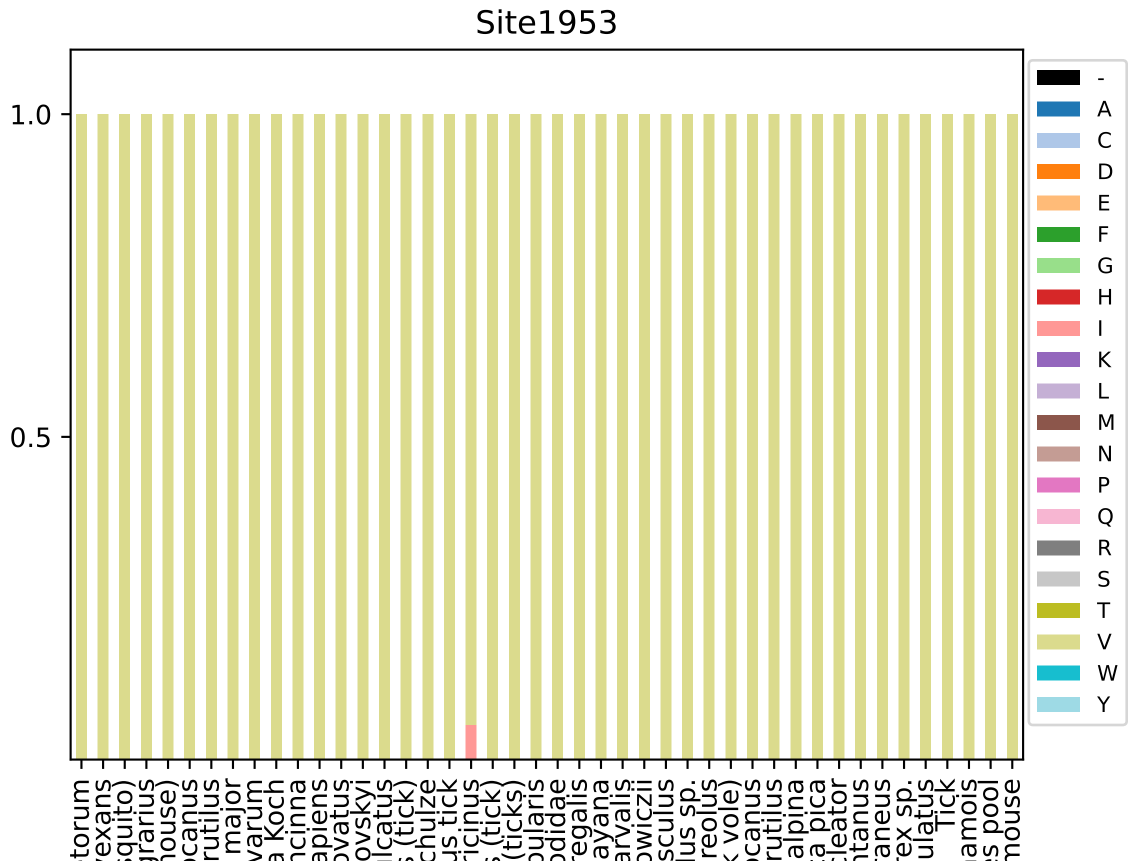
Site1953
1.0
0.5
-
A
C
D
E
F
G
H
I
K
L
M
N
P
Q
R
S
T
V
W
Y

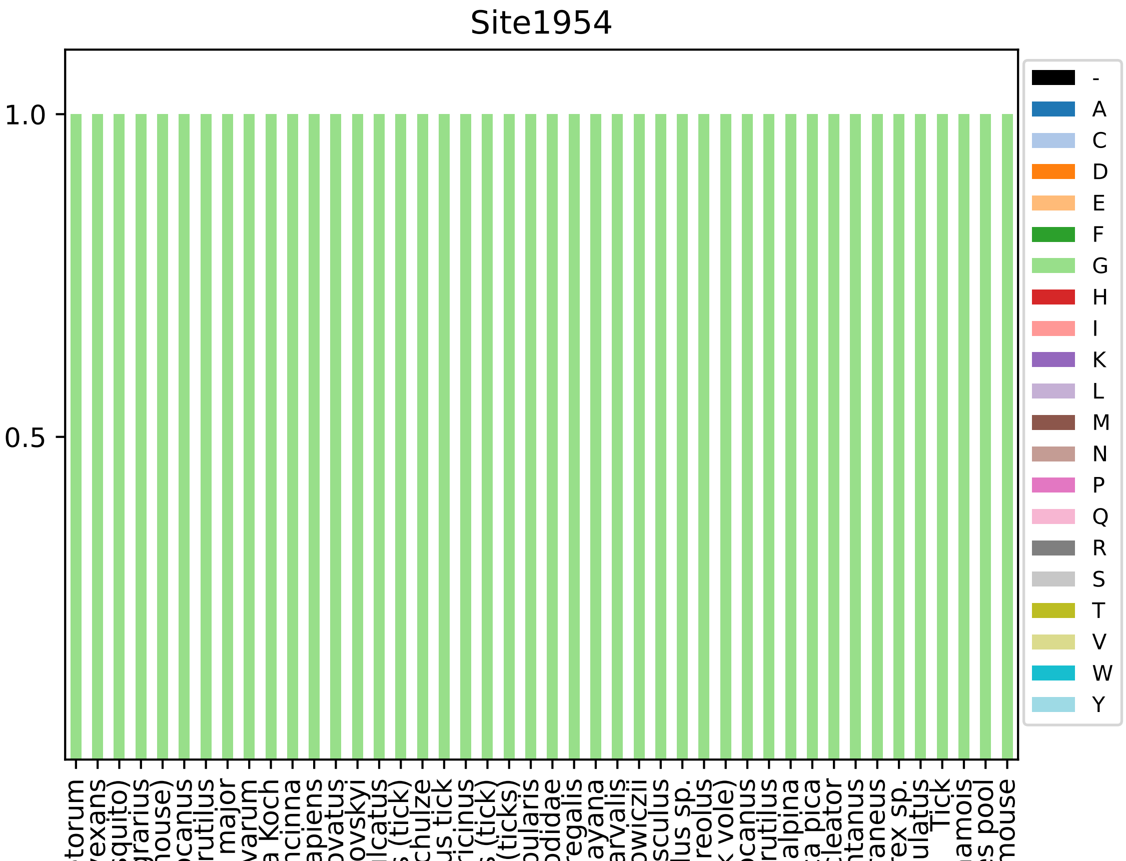
Site1954
1.0
0.5
-
A
C
D
E
F
G
H
I
K
L
M
N
P
Q
R
S
T
V
W
Y

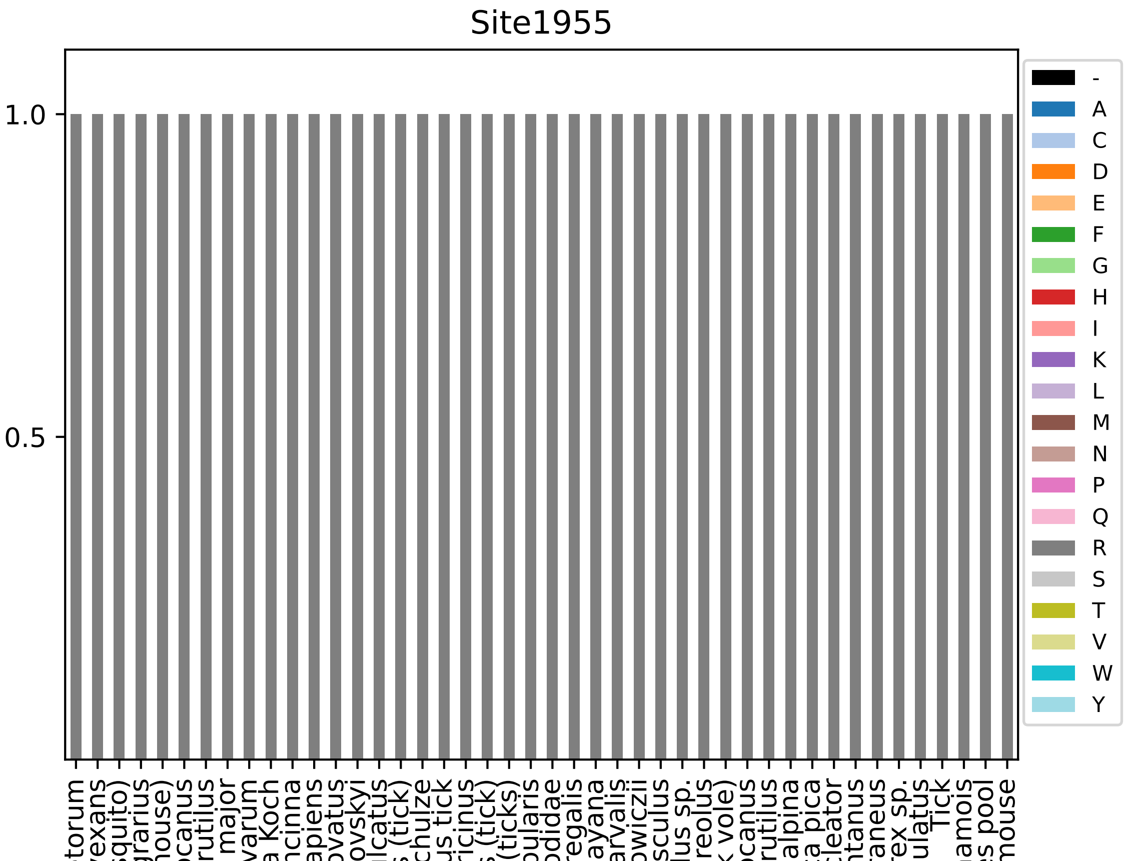
Site1955
1.0
0.5
-
A
C
D
E
F
G
H
I
K
L
M
N
P
Q
R
S
T
V
W
Y

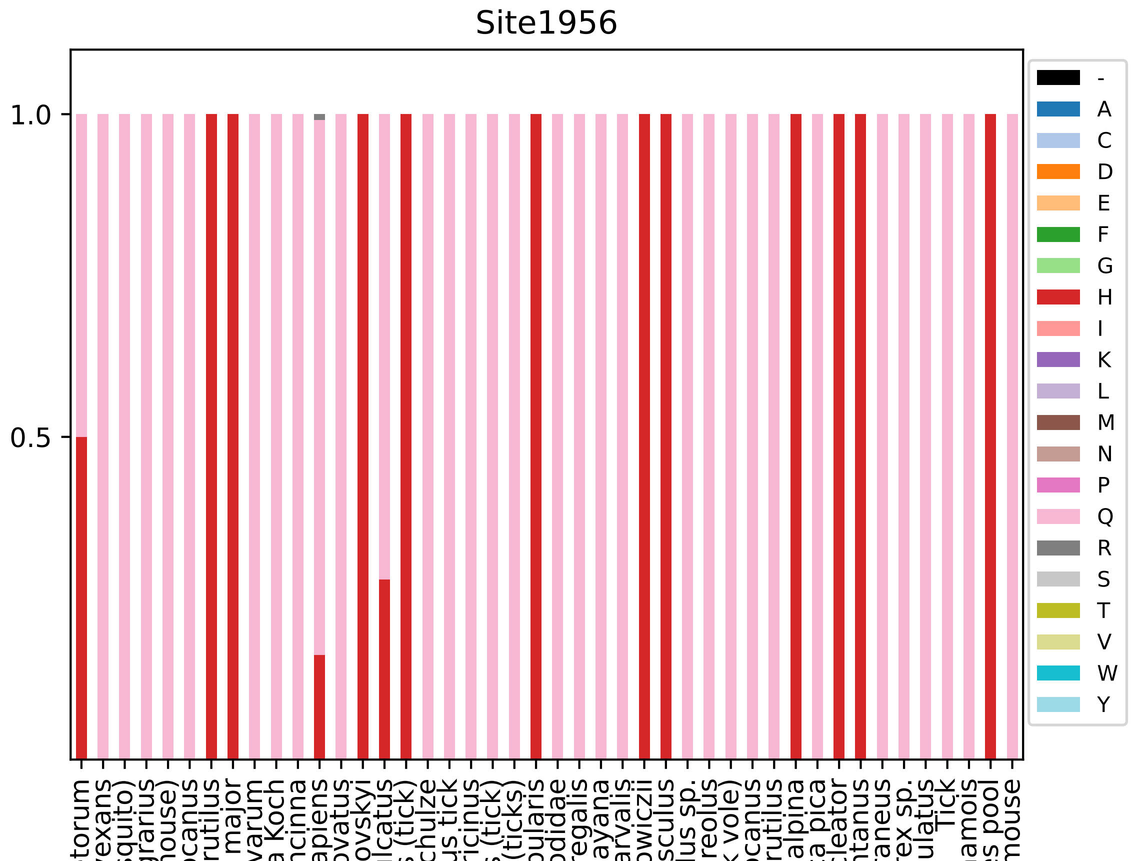
Site1956
1.0
0.5
-
A
C
D
E
F
G
H
I
K
L
M
N
P
Q
R
S
T
V
W
Y

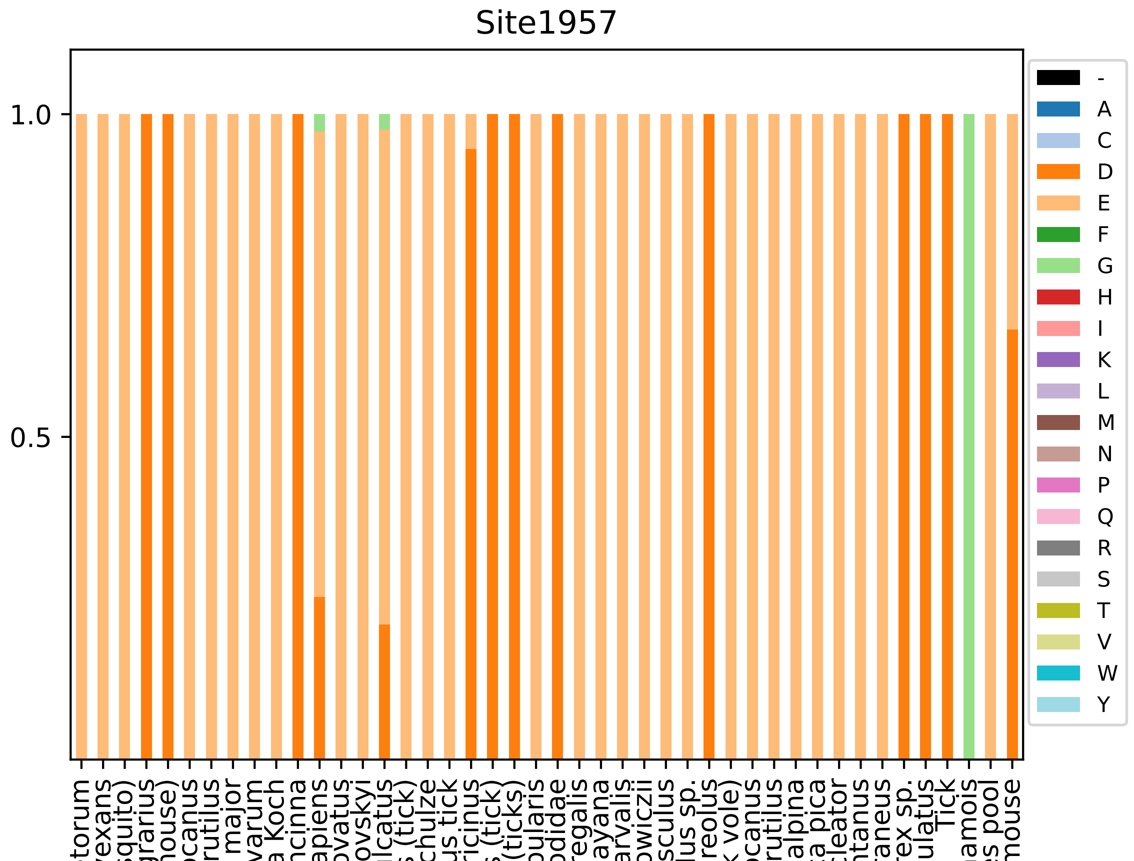

Site1957
1.0
0.5
-
A
C
D
E
F
G
H
I
K
L
M
N
P
Q
R
S
T
V
W
Y

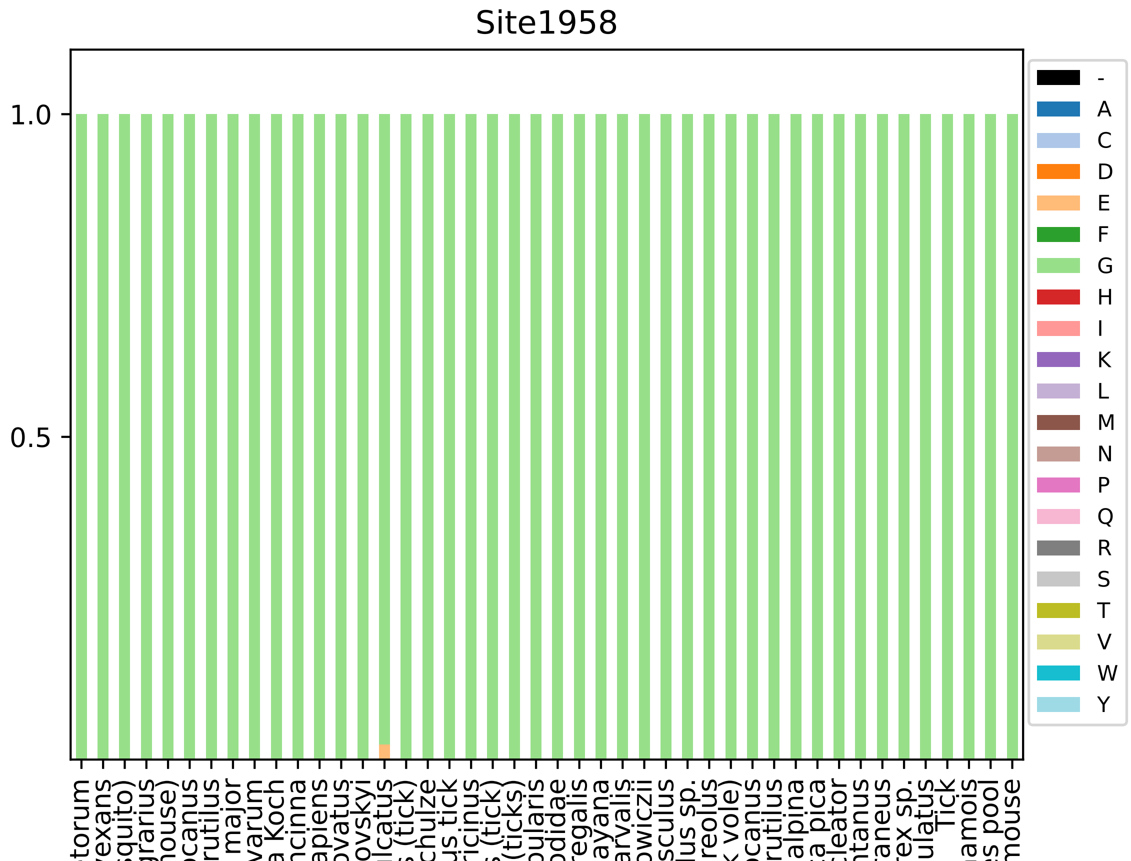

Site1958
1.0
0.5
-
A
C
D
E
F
G
H
I
K
L
M
N
P
Q
R
S
T
V
W
Y

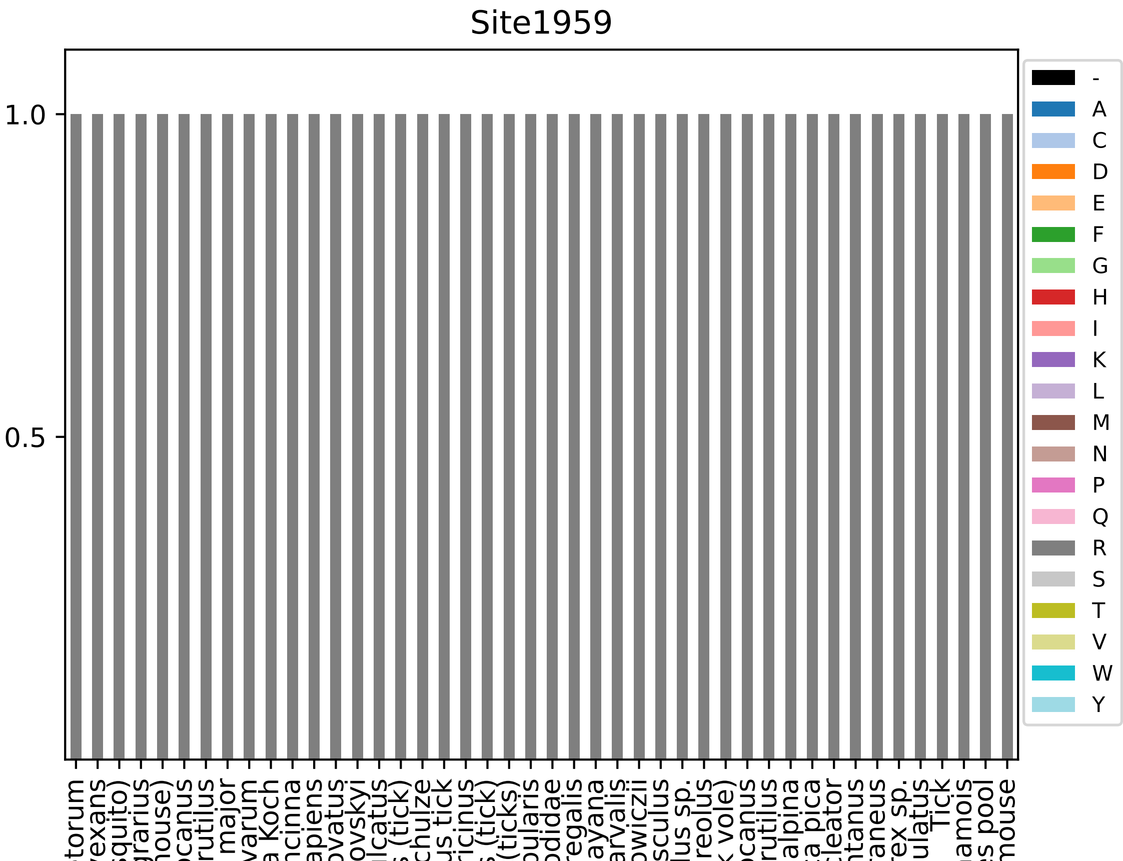

Site1959
1.0
0.5
-
A
C
D
E
F
G
H
I
K
L
M
N
P
Q
R
S
T
V
W
Y

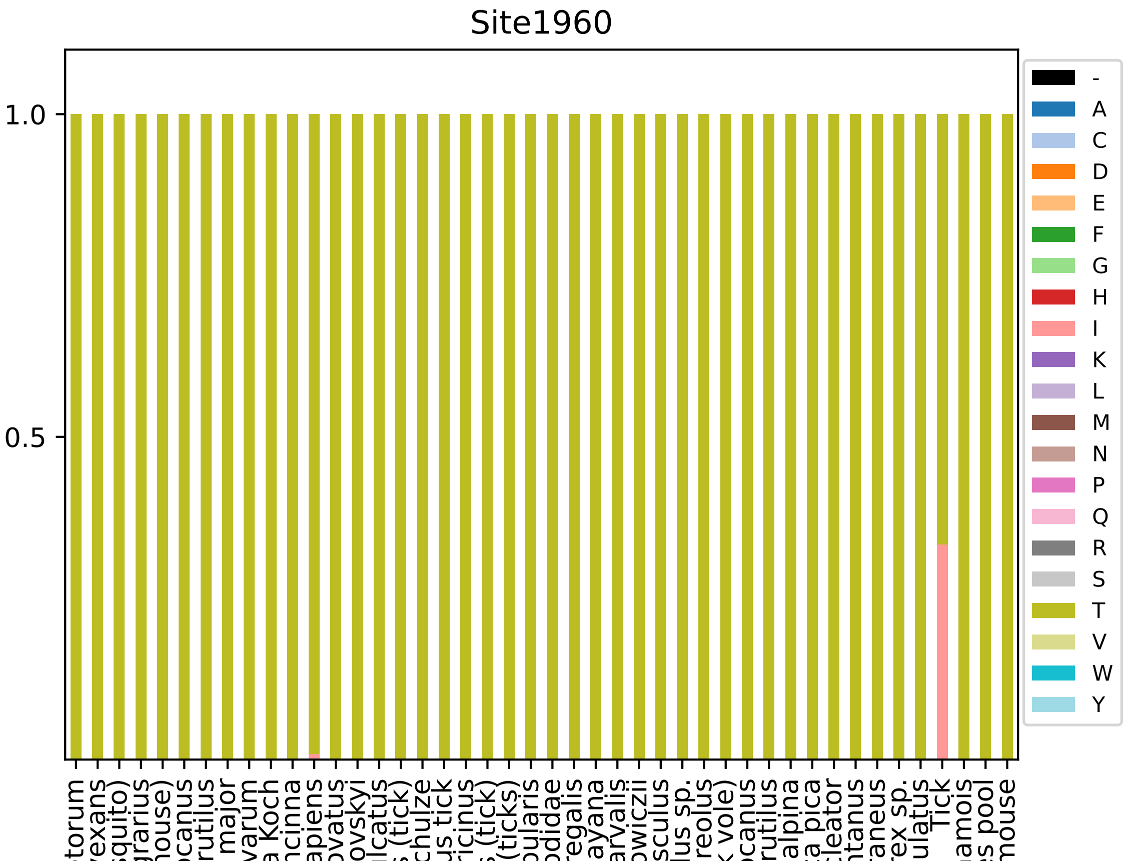

Site1960
1.0
0.5
-
A
C
D
E
F
G
H
I
K
L
M
N
P
Q
R
S
T
V
W
Y
Tick

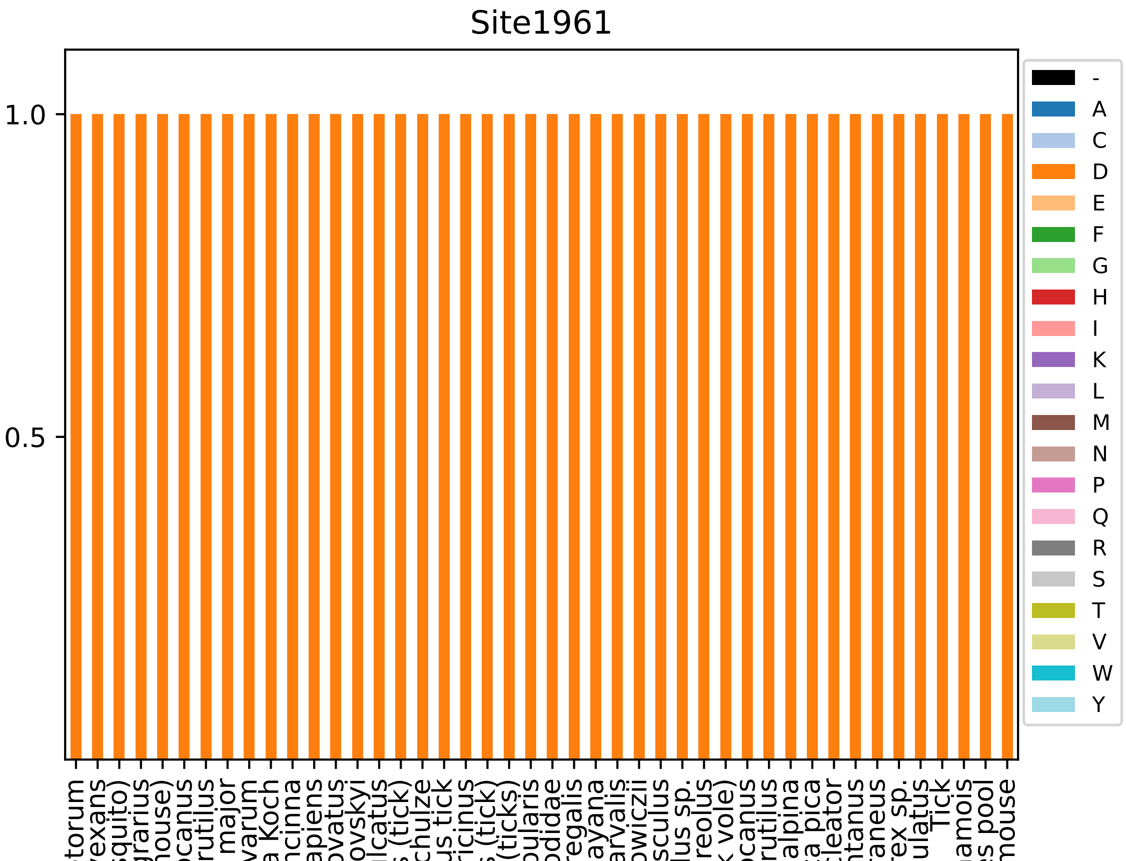
Site1961
1.0
0.5
-
A
C
D
E
F
G
H
I
K
L
M
N
P
Q
R
S
T
V
W
Y

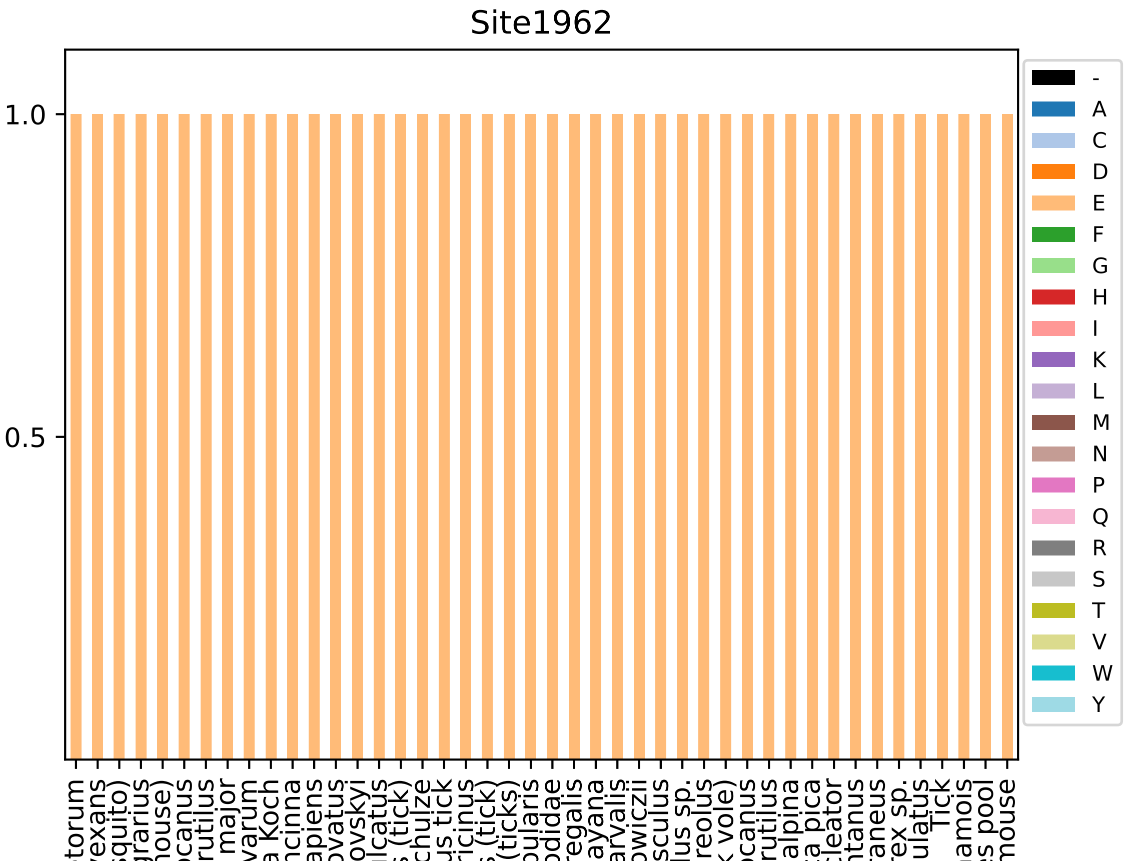
Site1962
1.0
0.5
-
A
C
D
E
F
G
H
I
K
L
M
N
P
Q
R
S
T
V
W
Y

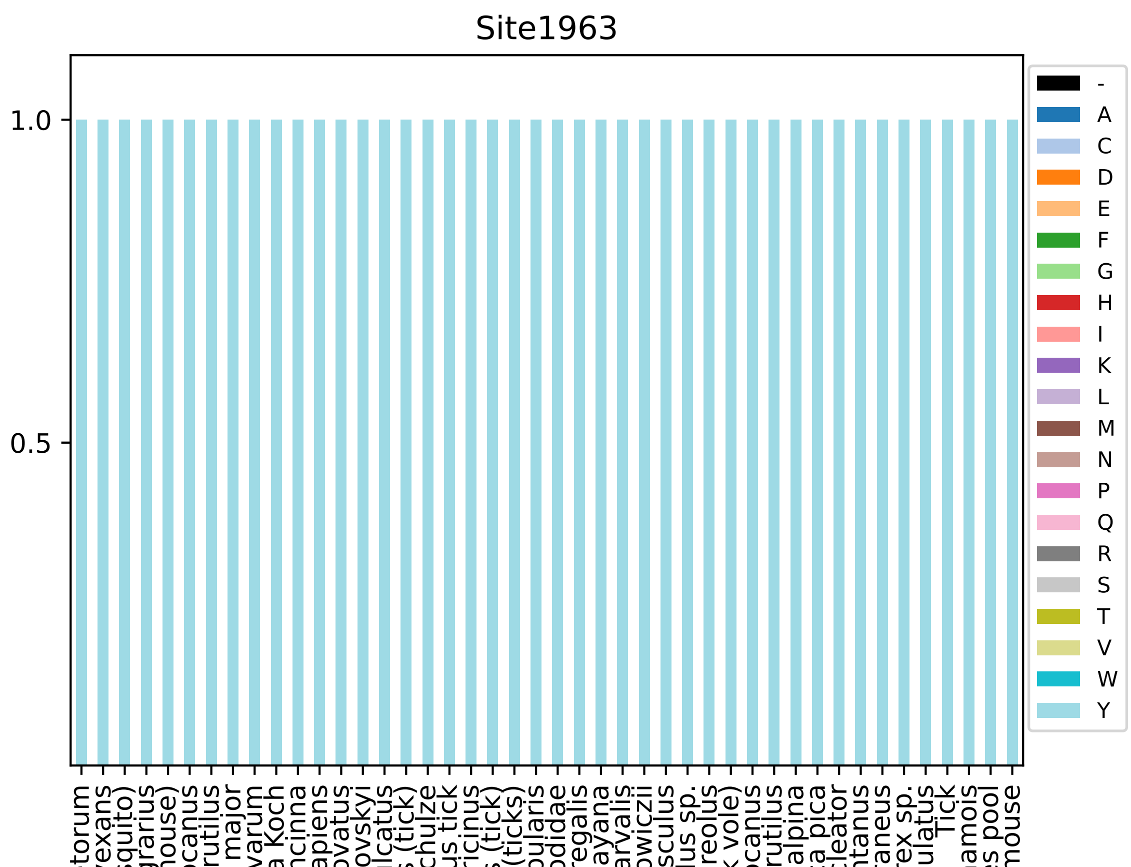
Site1963
1.0
0.5
-
A
C
D
E
F
G
H
I
K
L
M
N
P
Q
R
S
T
V
W
Y

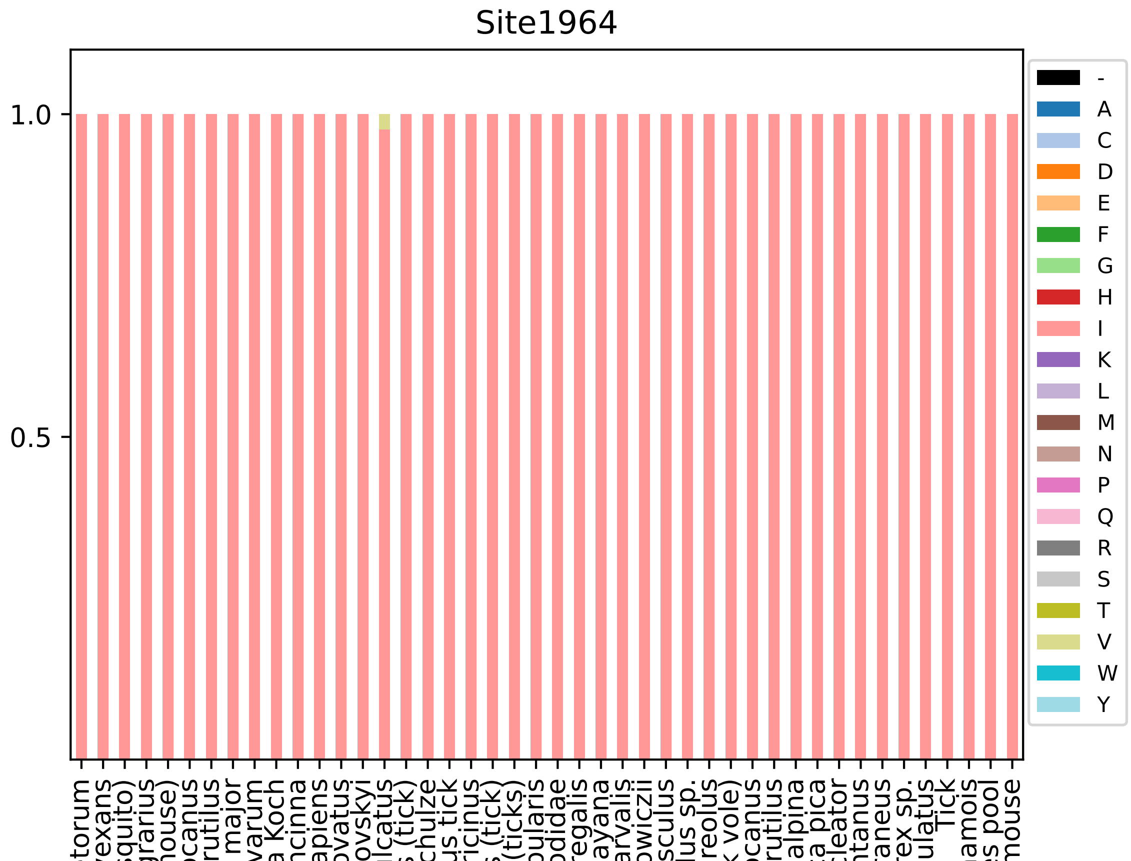
Site1964
1.0
0.5
-
A
C
D
E
F
G
H
I
K
L
M
N
P
Q
R
S
T
V
W
Y

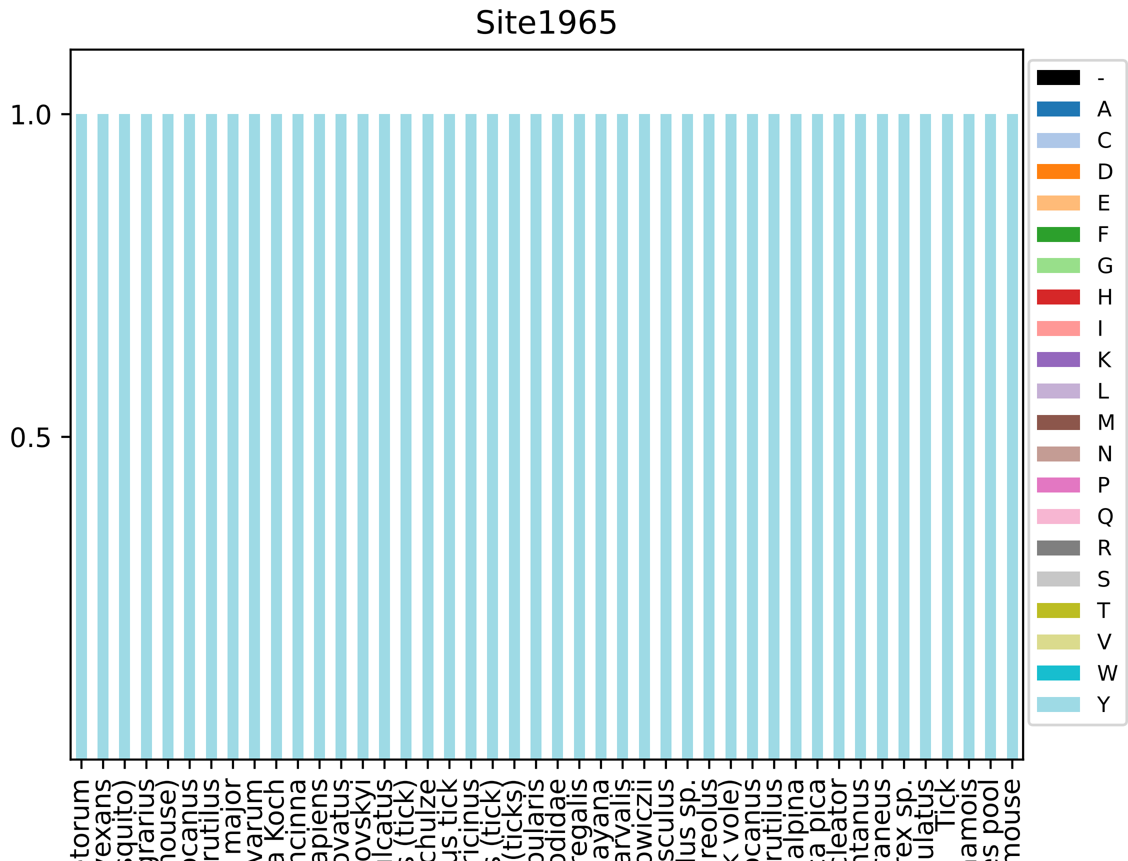

Site1965
1.0
0.5
-
A
C
D
E
F
G
H
I
K
L
M
N
P
Q
R
S
T
V
W
Y

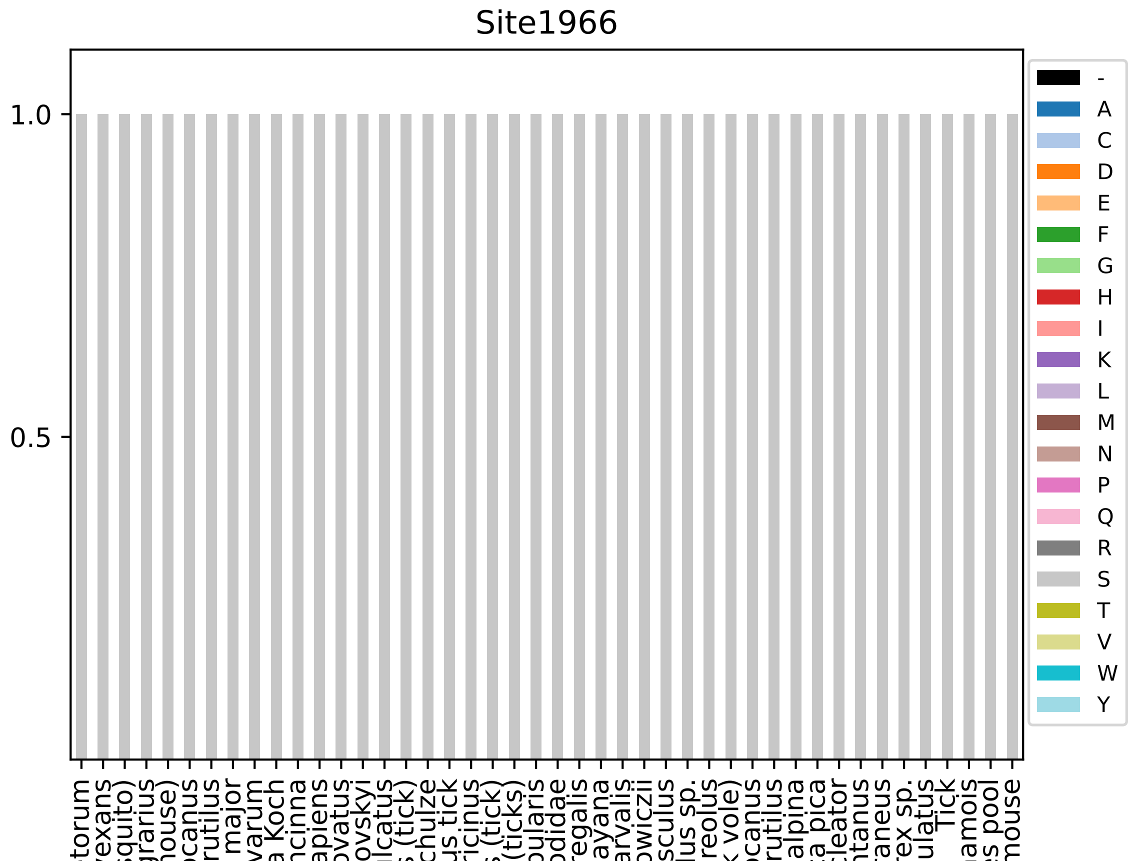
Site1966
1.0
0.5
-
A
C
D
E
F
G
H
I
K
L
M
N
P
Q
R
S
T
V
W
Y
Tick

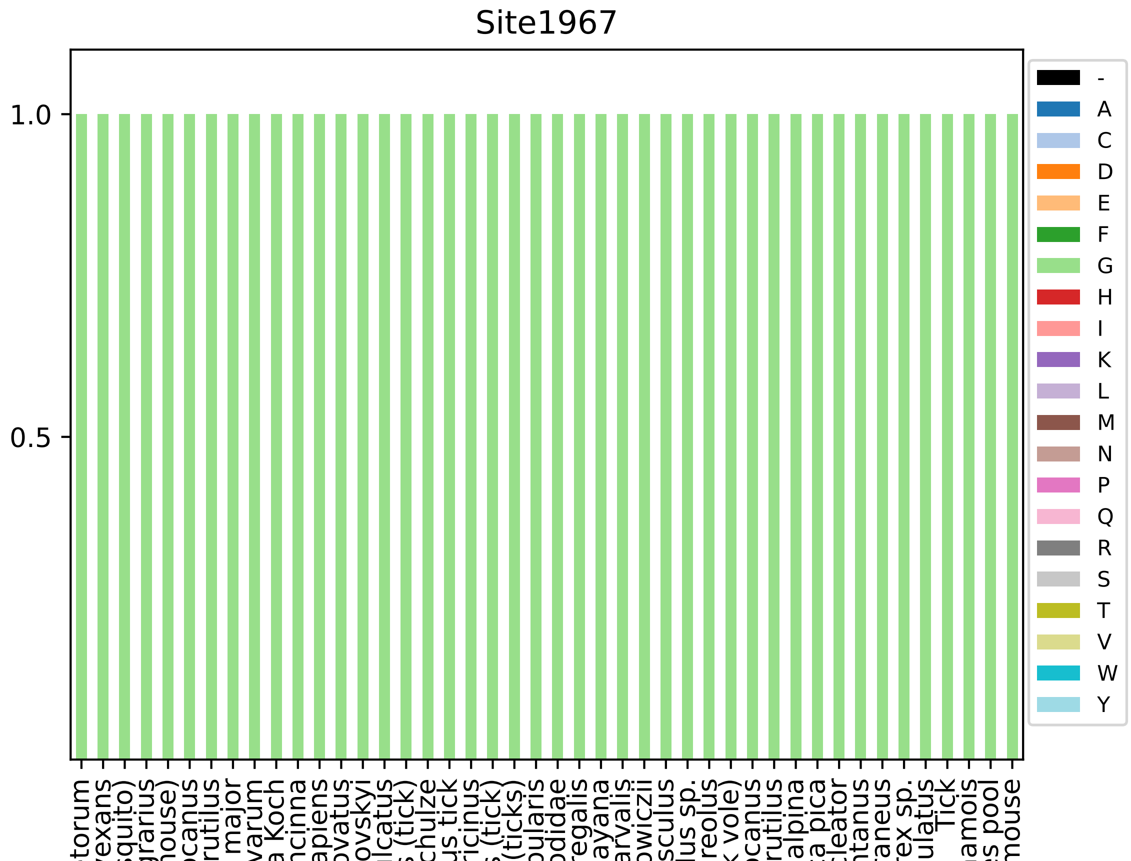

Site1967
1.0
0.5
-
A
C
D
E
F
G
H
I
K
L
M
N
P
Q
R
S
T
V
W
Y

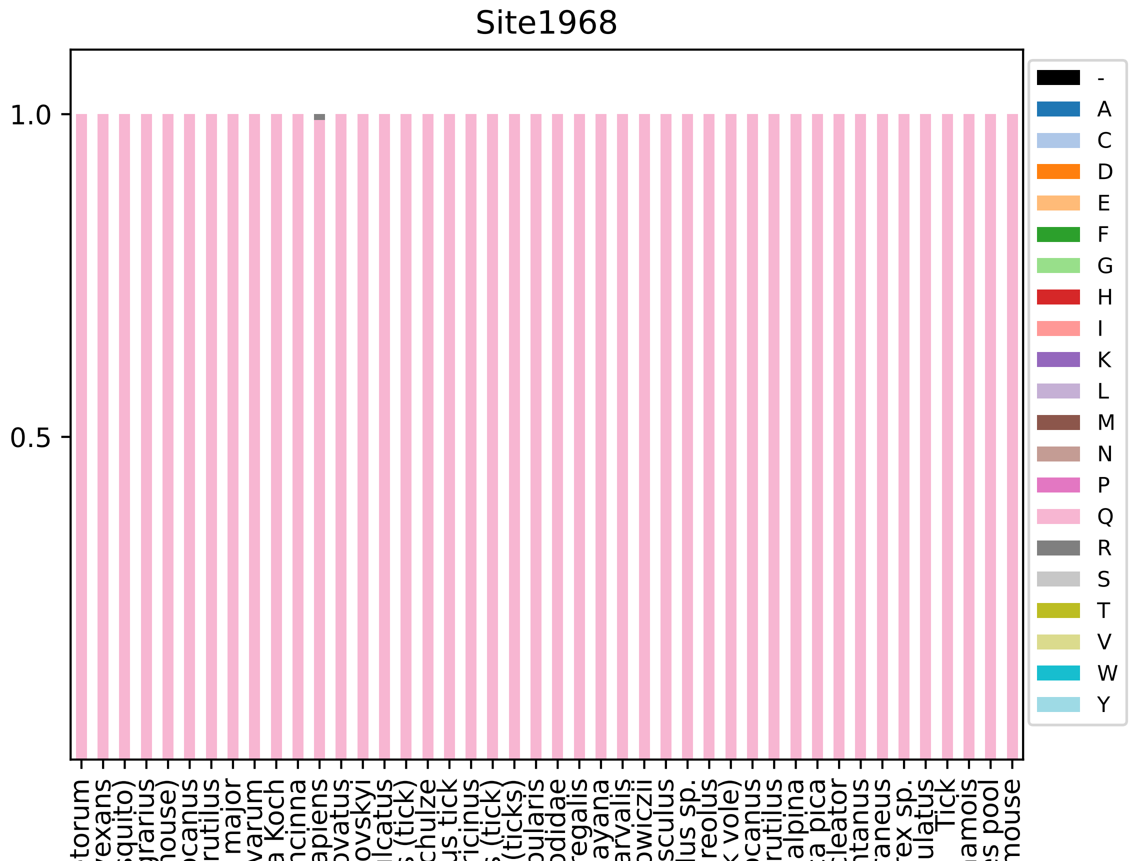

Site1968
1.0
0.5
-
A
C
D
E
F
G
H
I
K
L
M
N
P
Q
R
S
T
V
W
Y
Tick

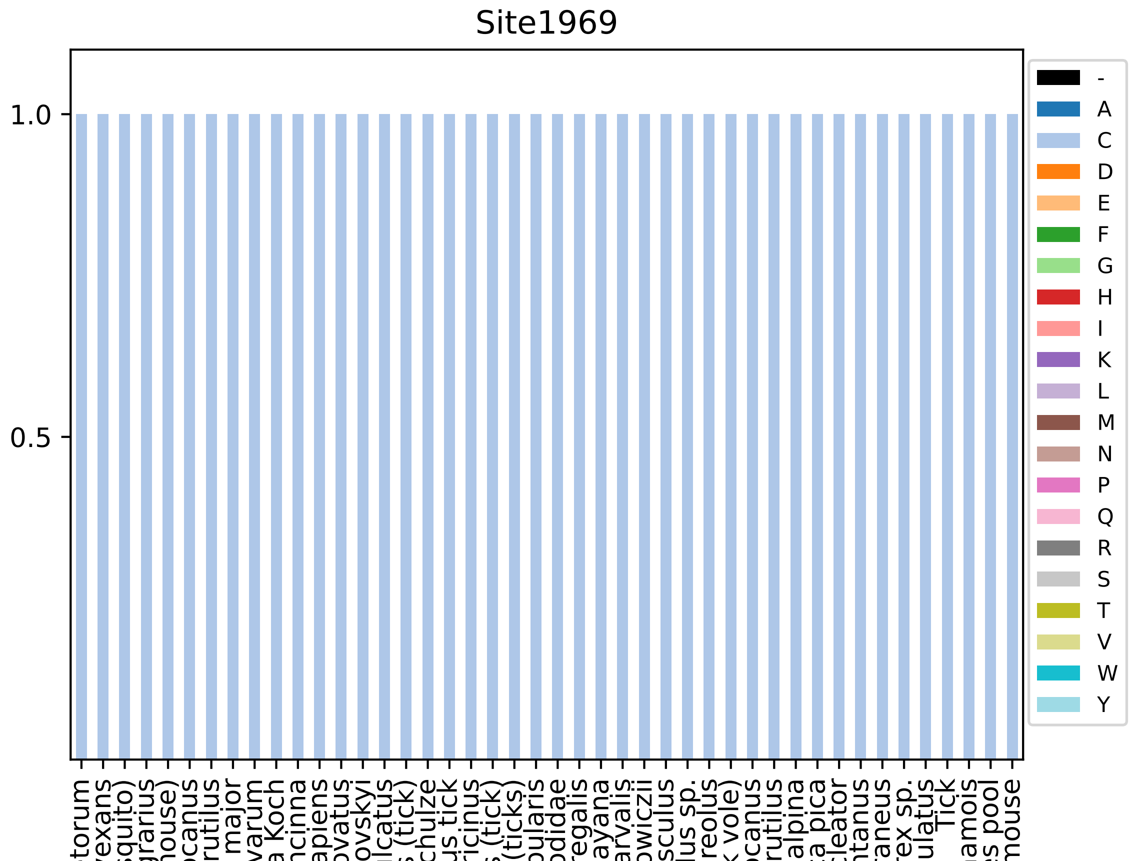
Site1969
1.0
0.5
-
A
C
D
E
F
G
H
I
K
L
M
N
P
Q
R
S
T
V
W
Y

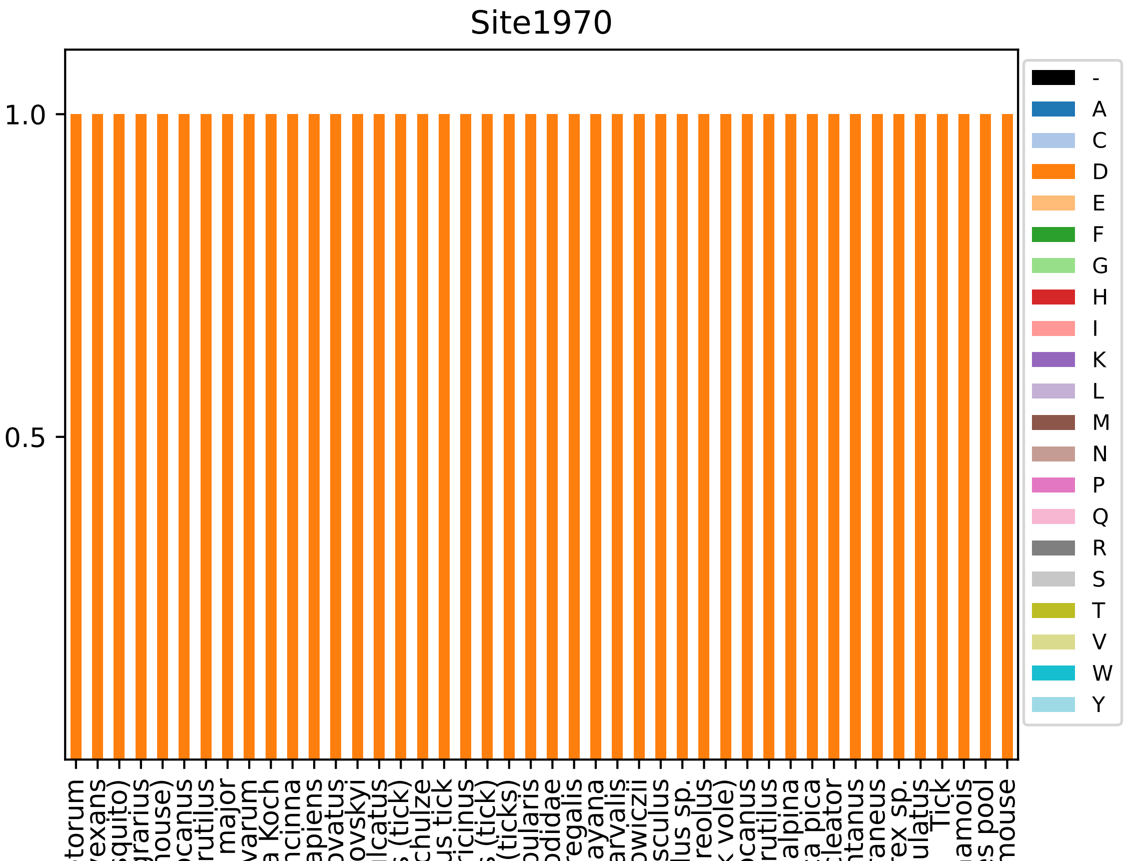
Site1970
1.0
0.5
-
A
C
D
E
F
G
H
I
K
L
M
N
P
Q
R
S
T
V
W
Y

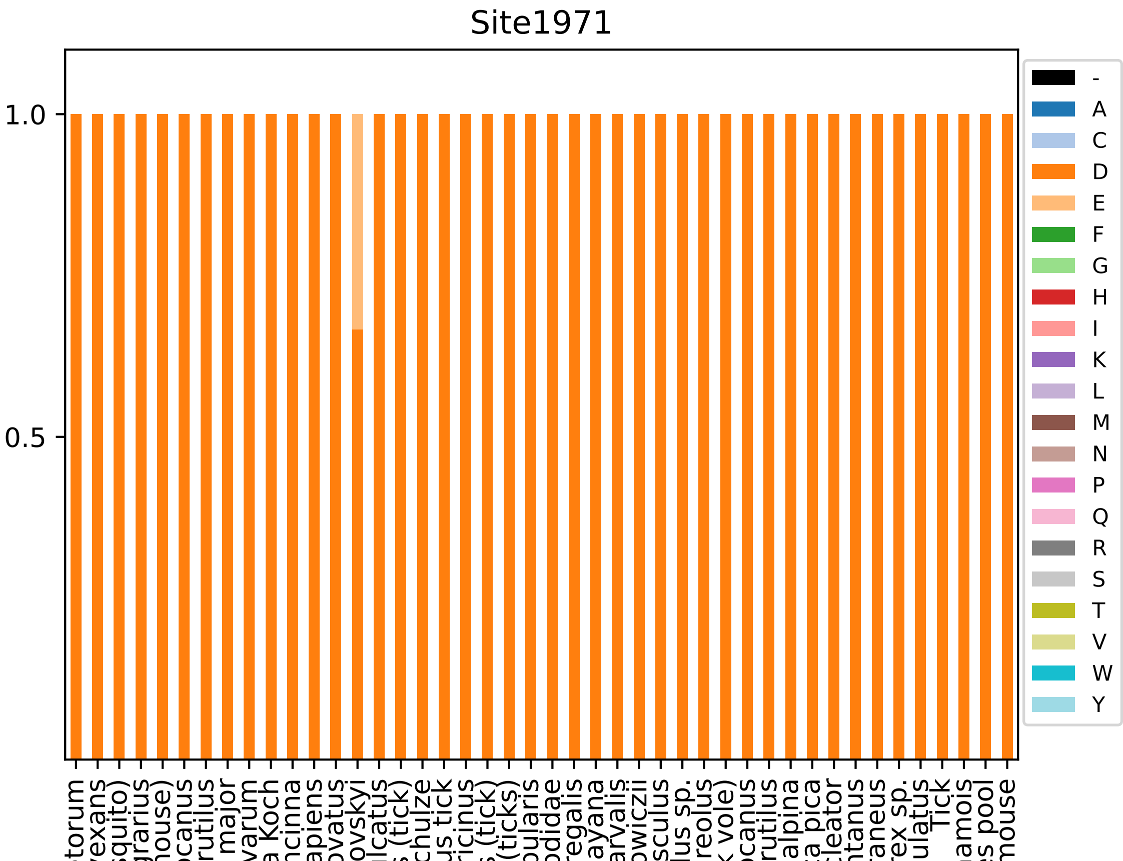
Site1971
1.0
0.5
-
A
C
D
E
F
G
H
I
K
L
M
N
P
Q
R
S
T
V
W
Y

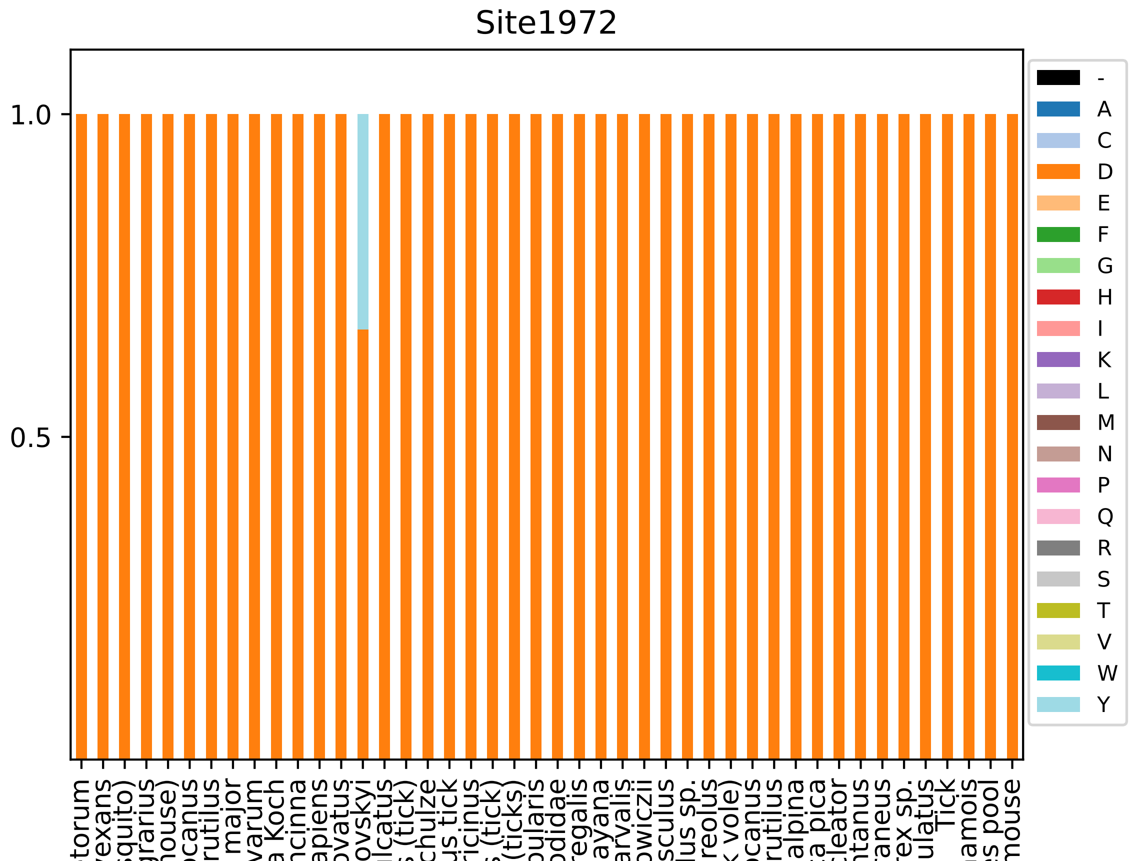
Site1972
1.0
0.5
-
A
C
D
E
F
G
H
I
K
L
M
N
P
Q
R
S
T
V
W
Y

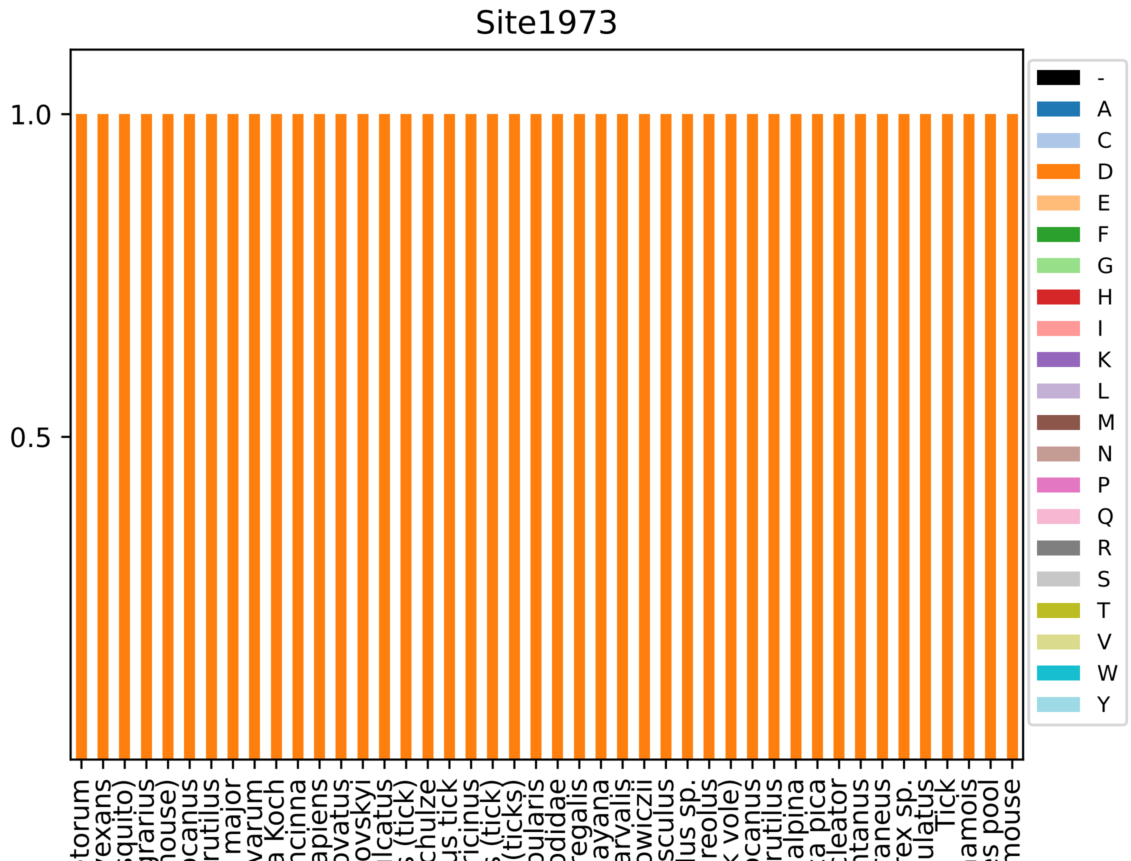
Site1973
1.0
0.5
-
A
C
D
E
F
G
H
I
K
L
M
N
P
Q
R
S
T
V
W
Y
Tick

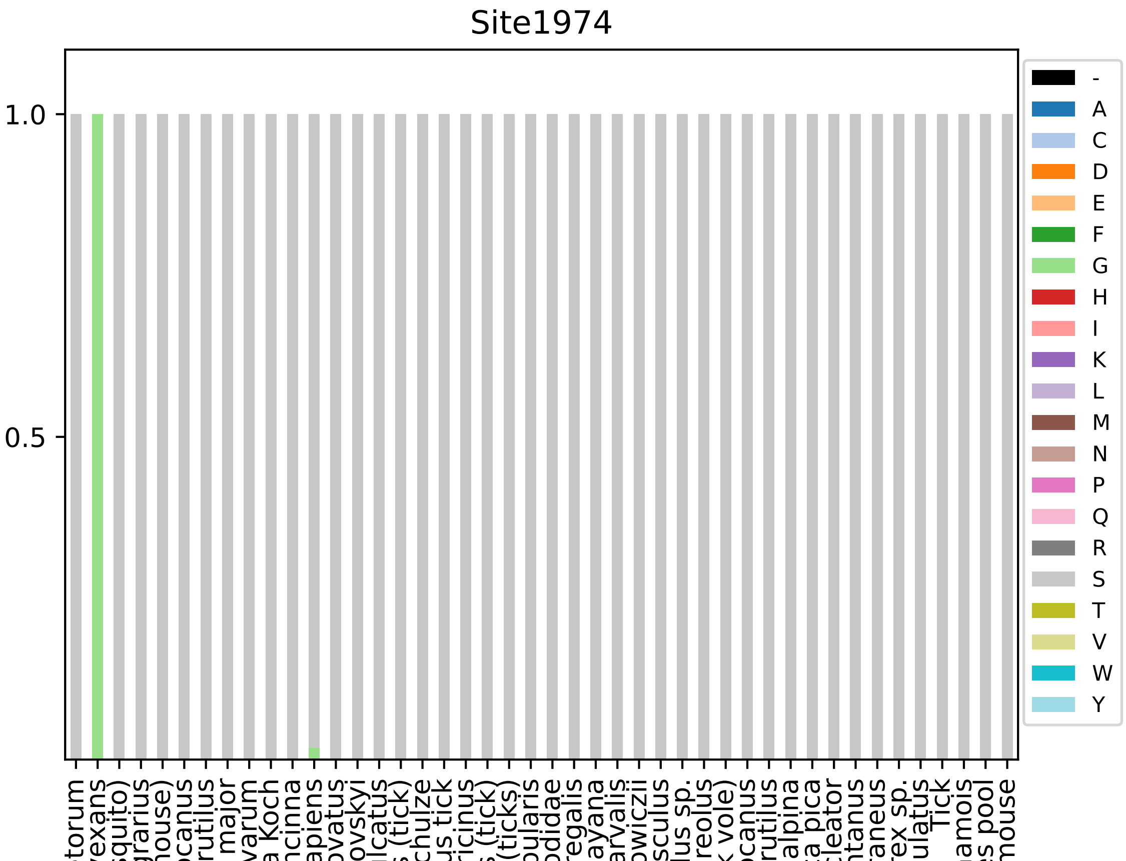
Site1974
1.0
0.5
-
A
C
D
E
F
G
H
I
K
L
M
N
P
Q
R
S
T
V
W
Y

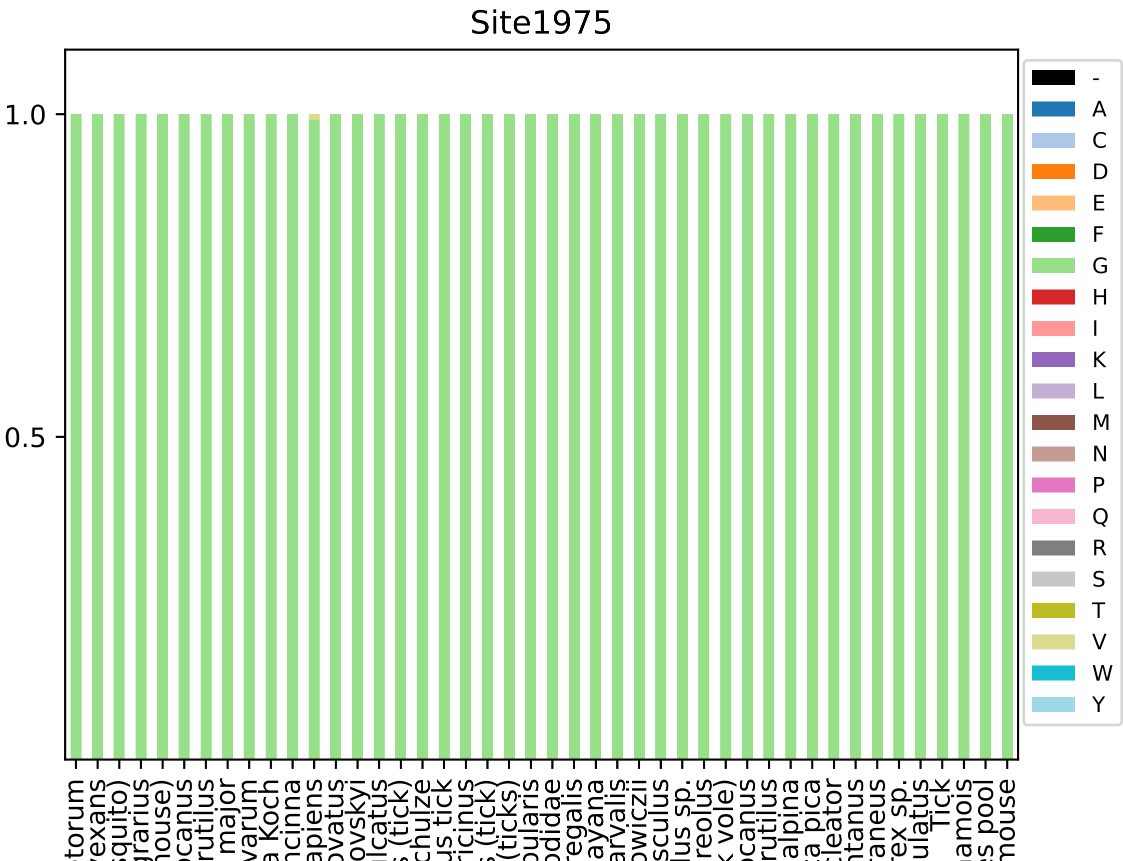
Site1975
1.0
0.5
-
A
C
D
E
F
G
H
I
K
L
M
N
P
Q
R
S
T
V
W
Y

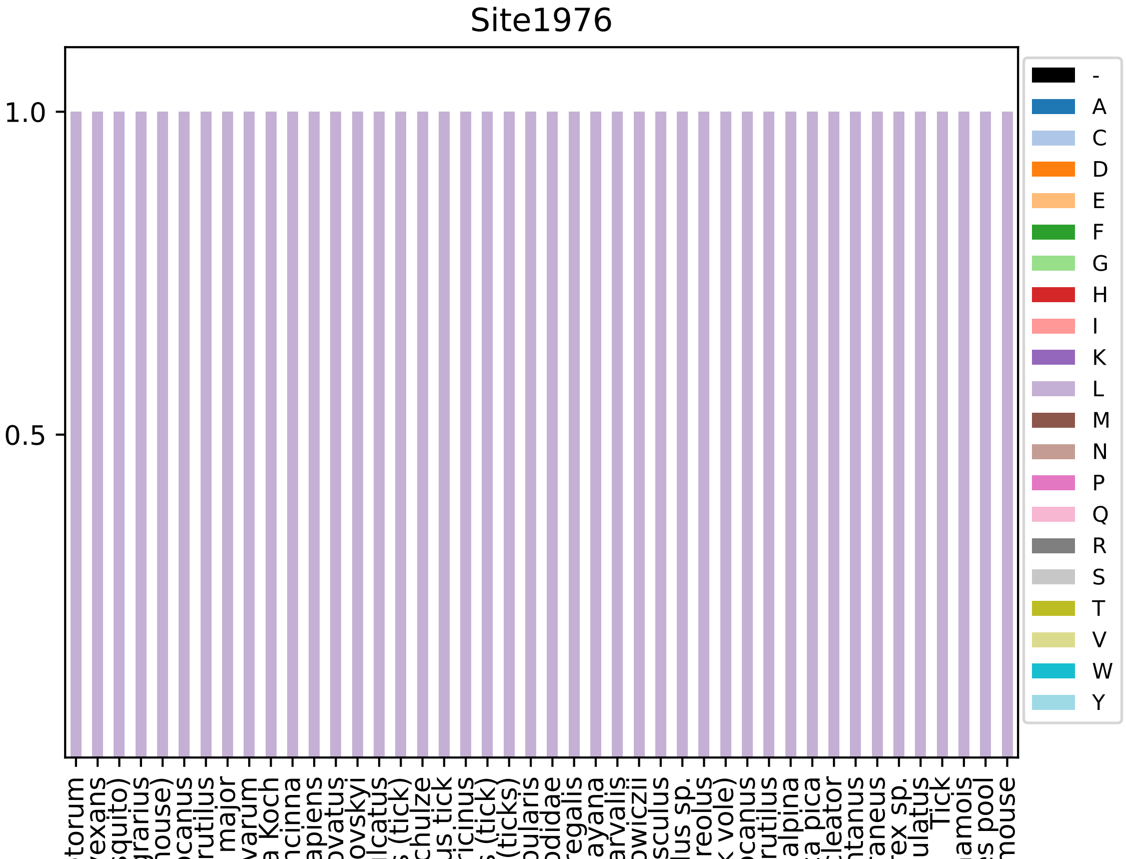
Site1976
1.0
0.5
-
A
C
D
E
F
G
H
I
K
L
M
N
P
Q
R
S
T
V
W
Y

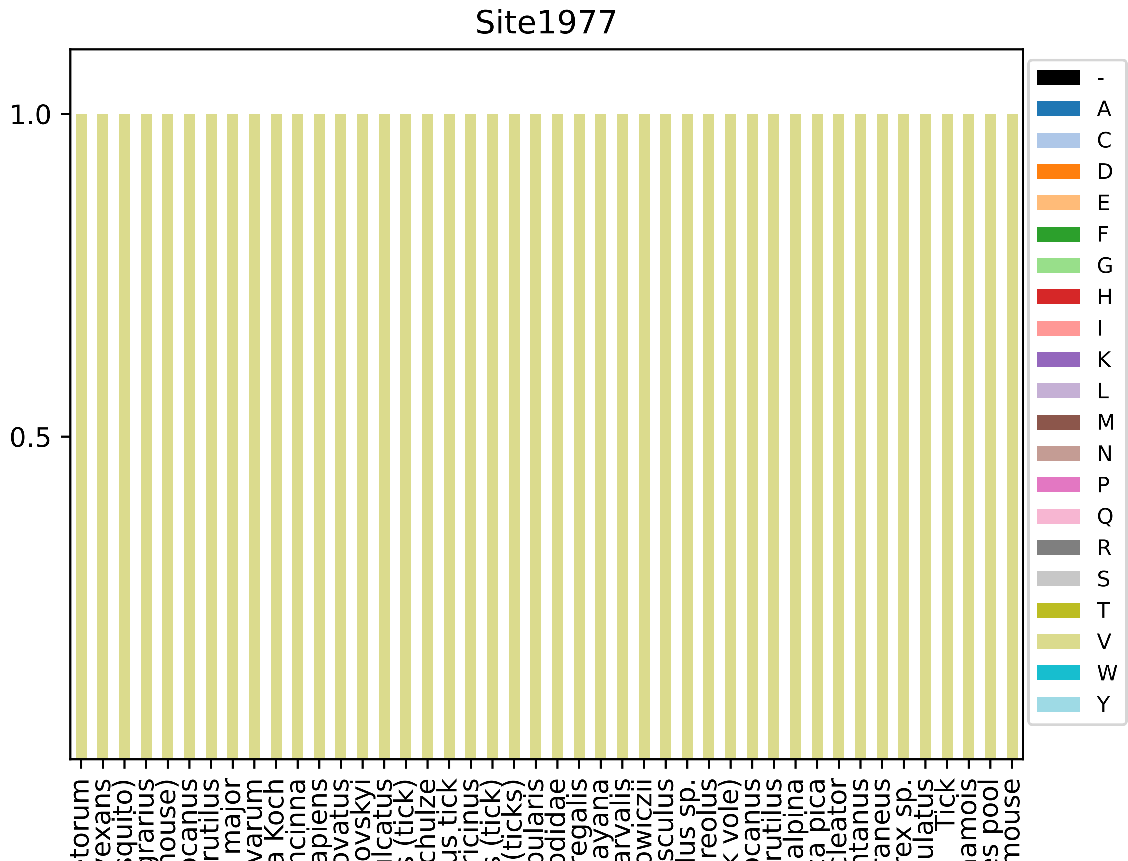

Site1977
1.0
0.5
-
A
C
D
E
F
G
H
I
K
L
M
N
P
Q
R
S
T
V
W
Y

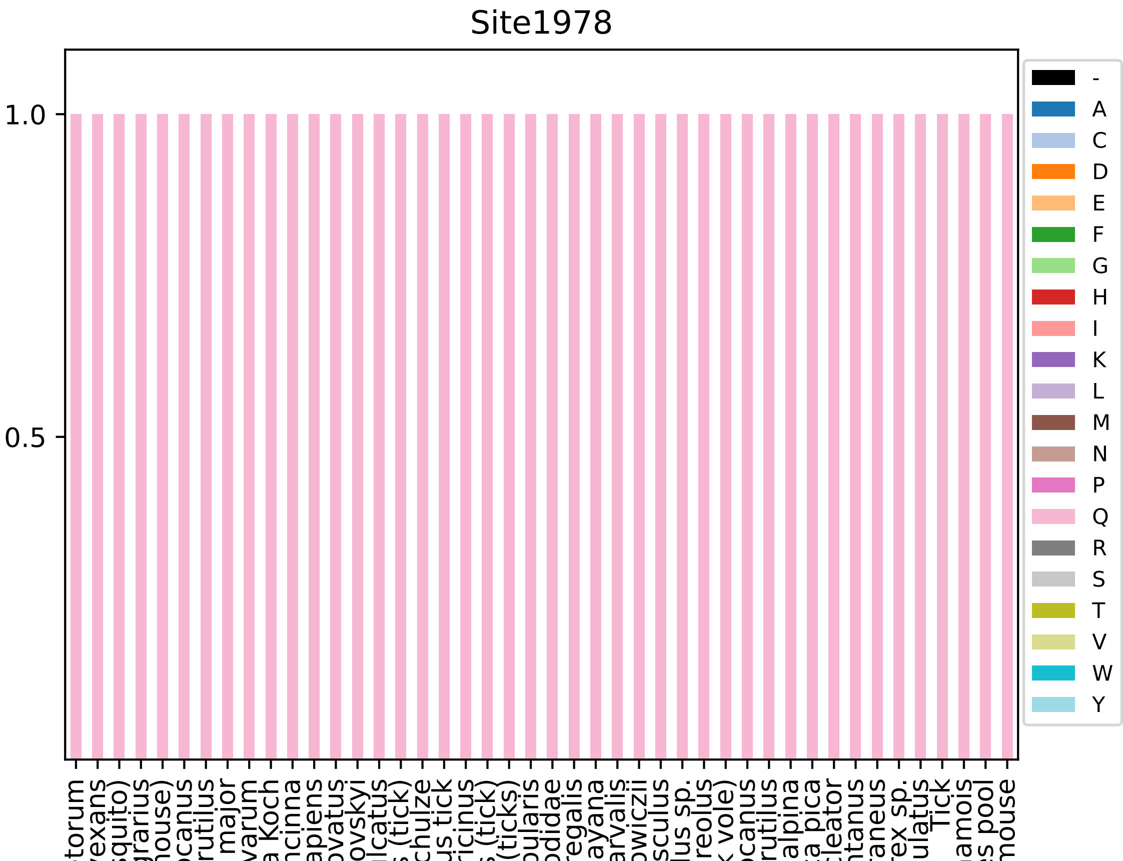

Site1978
1.0
0.5
-
A
C
D
E
F
G
H
I
K
L
M
N
P
Q
R
S
T
V
W
Y

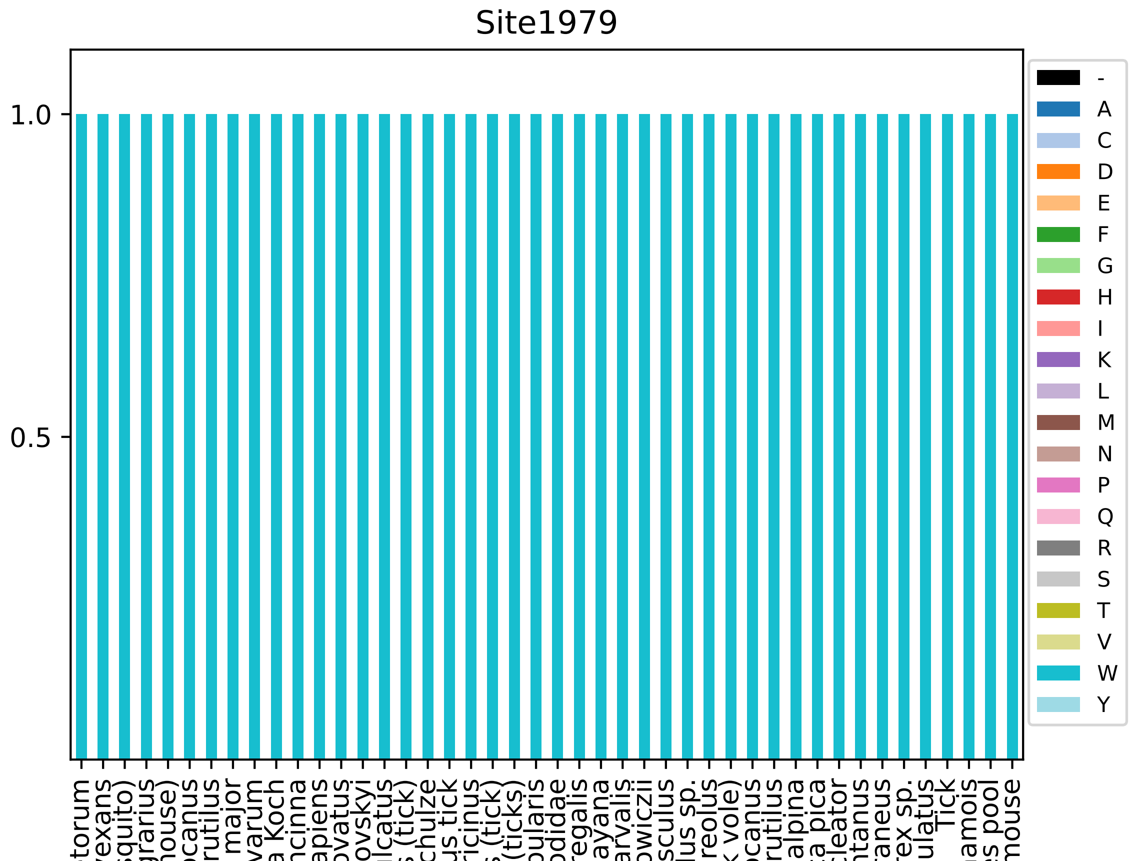
Site1979
1.0
0.5
-
A
C
D
E
F
G
H
I
K
L
M
N
P
Q
R
S
T
V
W
Y

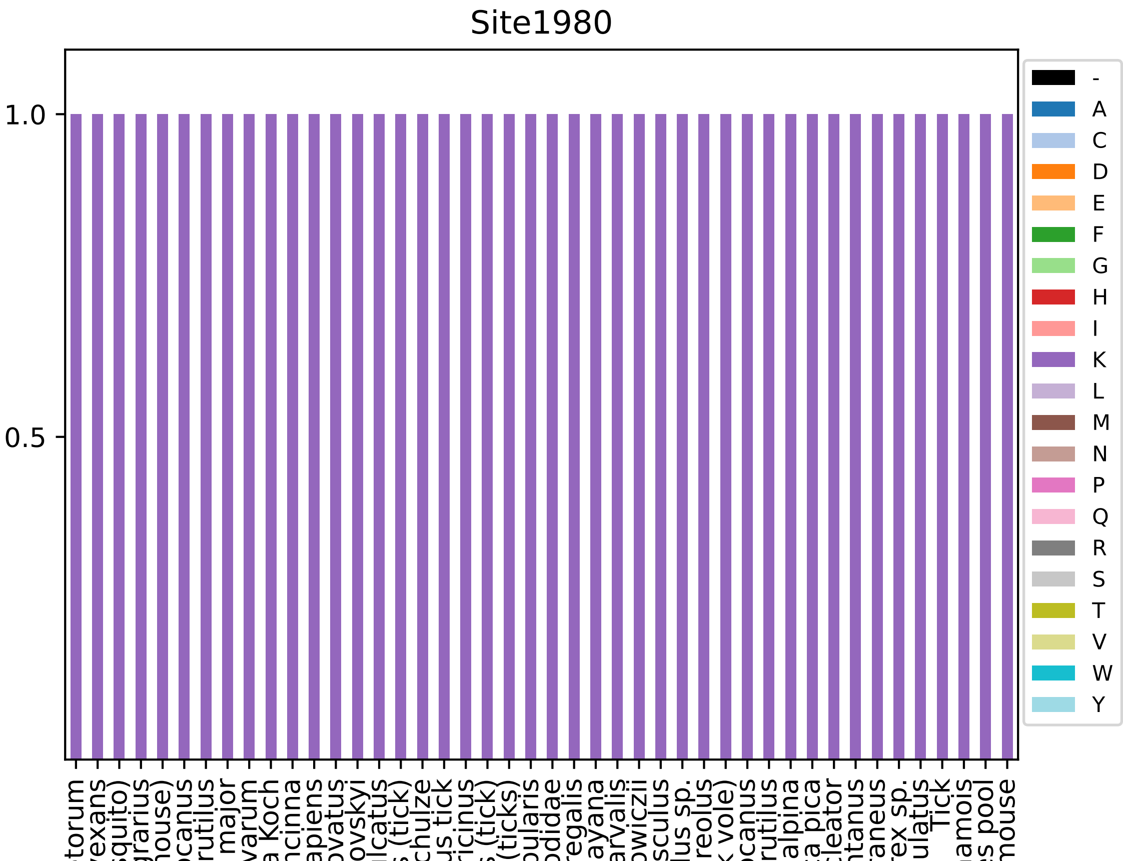
Site1980
1.0
0.5
-
A
C
D
E
F
G
H
I
K
L
M
N
P
Q
R
S
T
V
W
Y

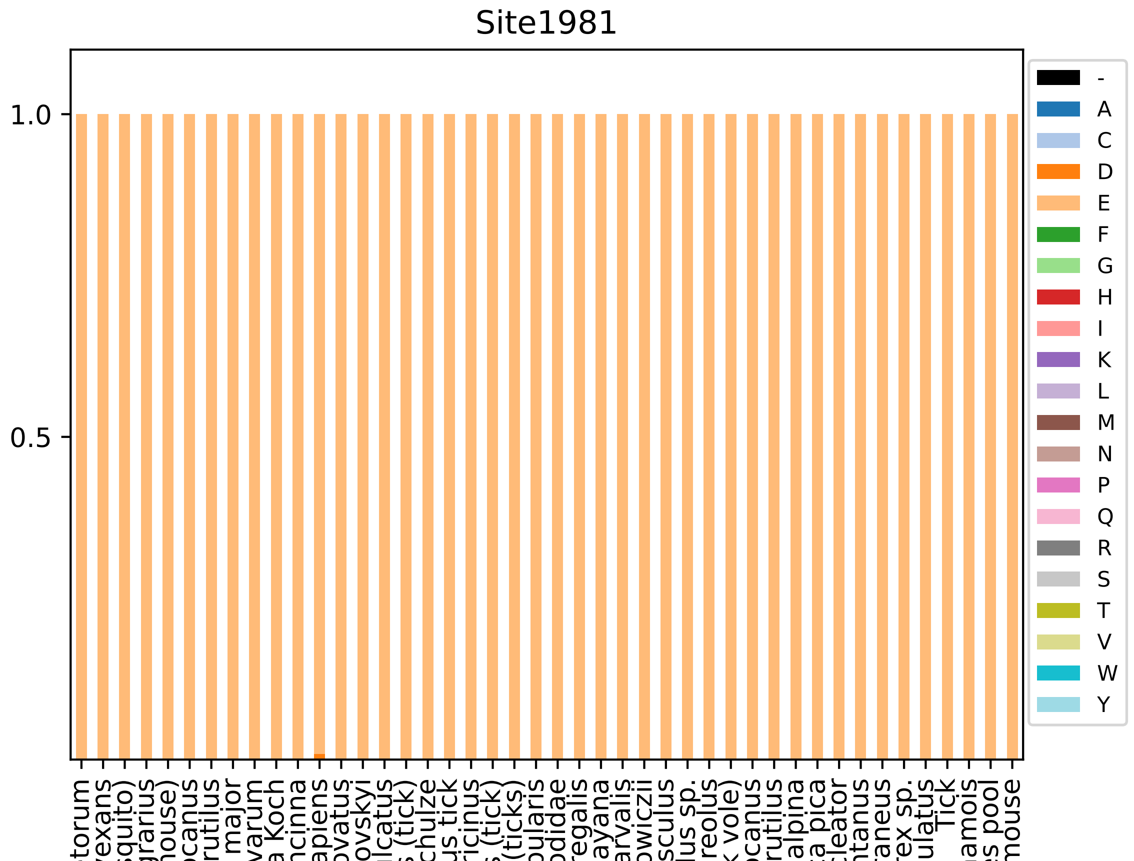

Site1981
1.0
0.5
-
A
C
D
E
F
G
H
I
K
L
M
N
P
Q
R
S
T
V
W
Y

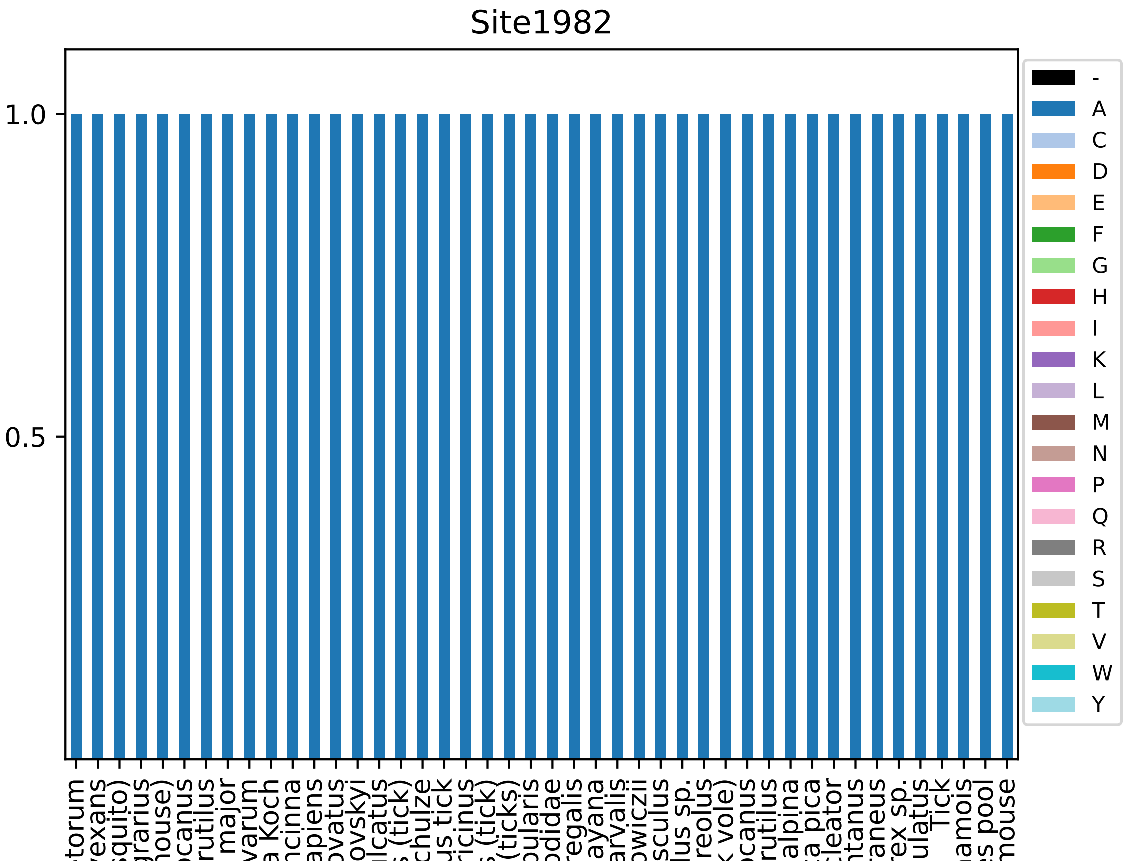
Site1982
1.0
0.5
-
A
C
D
E
F
G
H
I
K
L
M
N
P
Q
R
S
T
V
W
Y

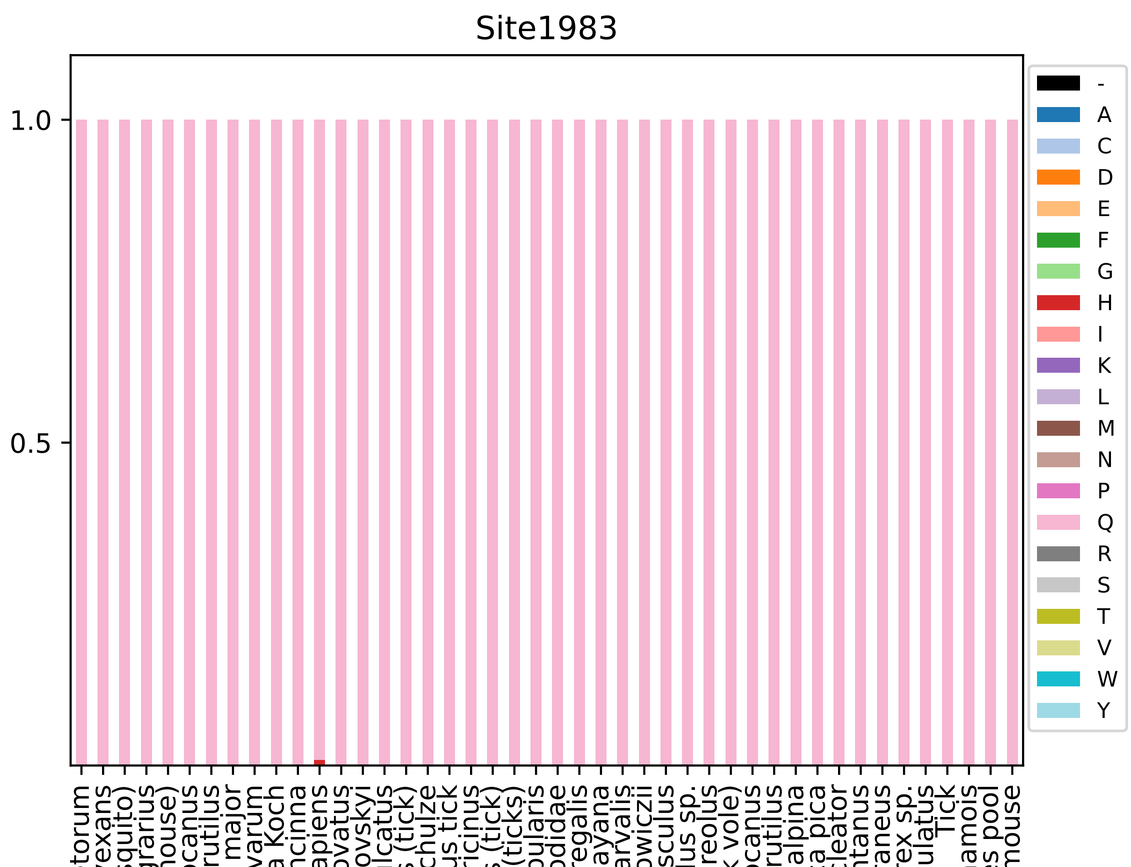
Site1983
1.0
0.5
-
A
C
D
E
F
G
H
I
K
L
M
N
P
Q
R
S
T
V
W
Y
Tick

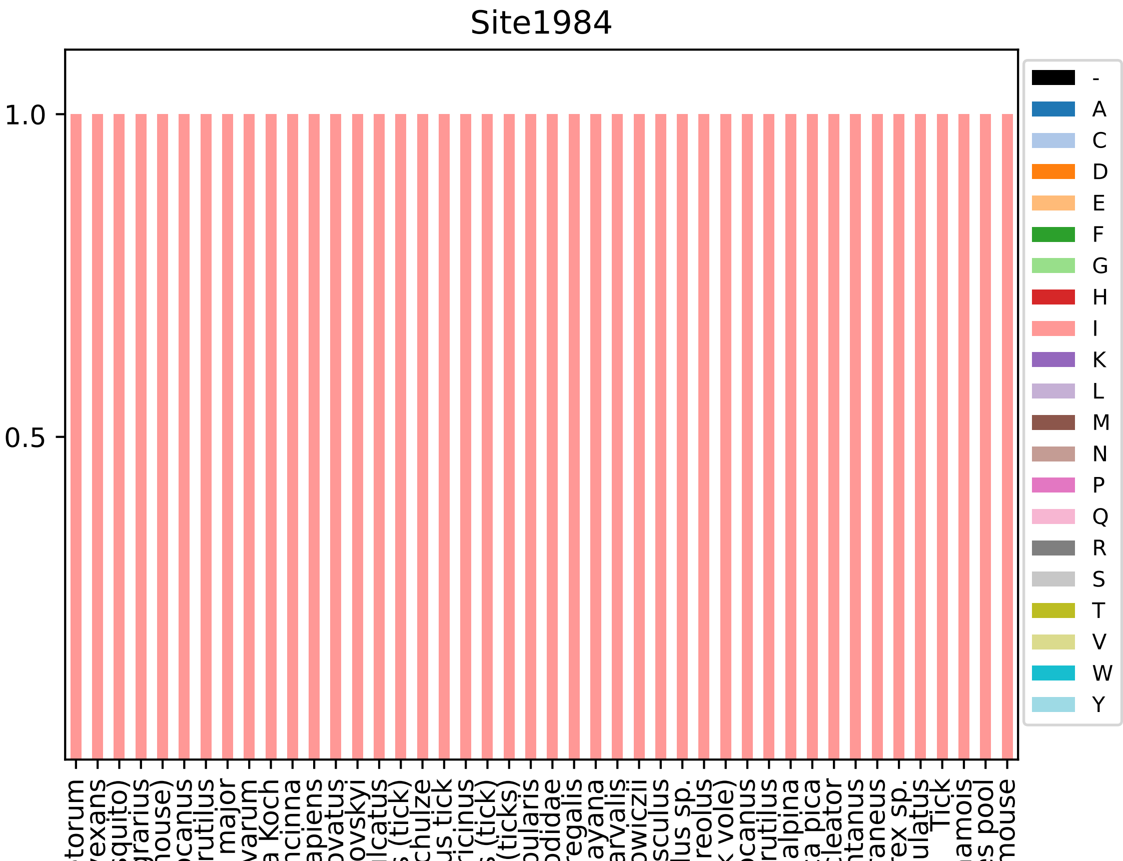
Site1984
1.0
0.5
-
A
C
D
E
F
G
H
I
K
L
M
N
P
Q
R
S
T
V
W
Y

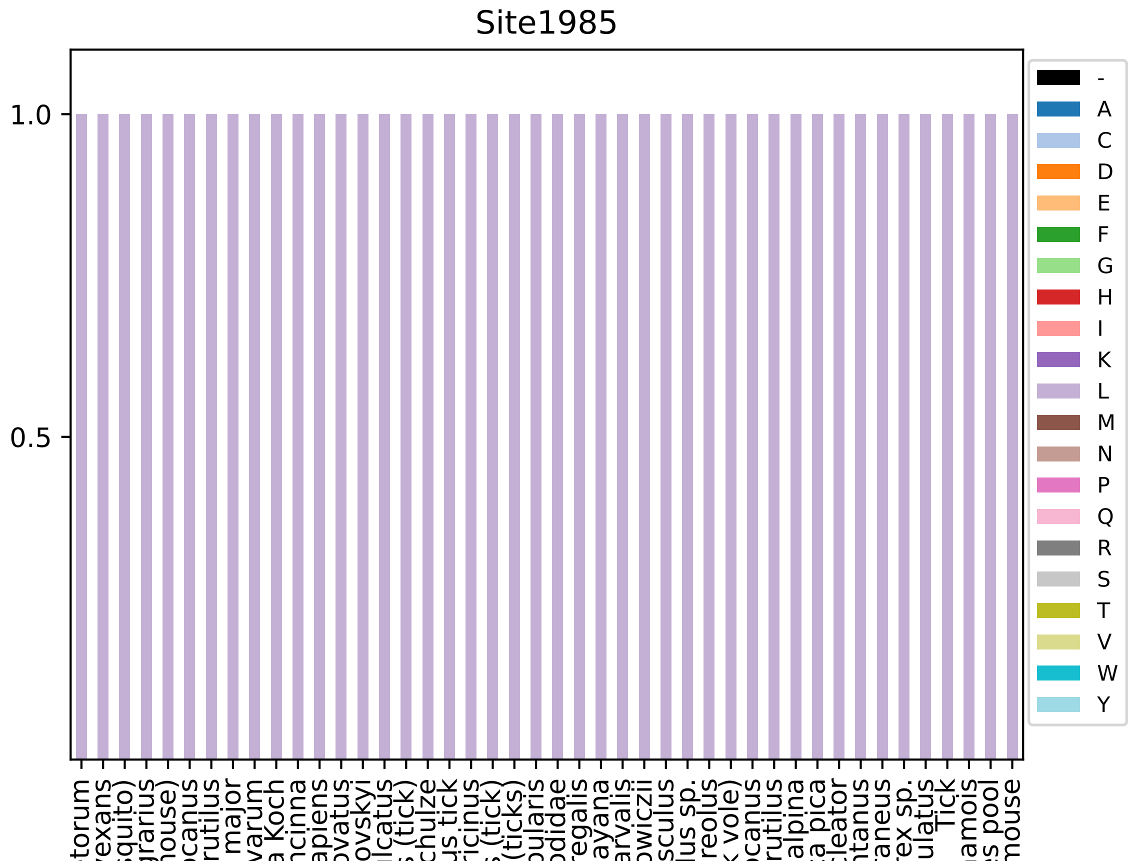
Site1985
1.0
0.5
-
A
C
D
E
F
G
H
I
K
L
M
N
P
Q
R
S
T
V
W
Y

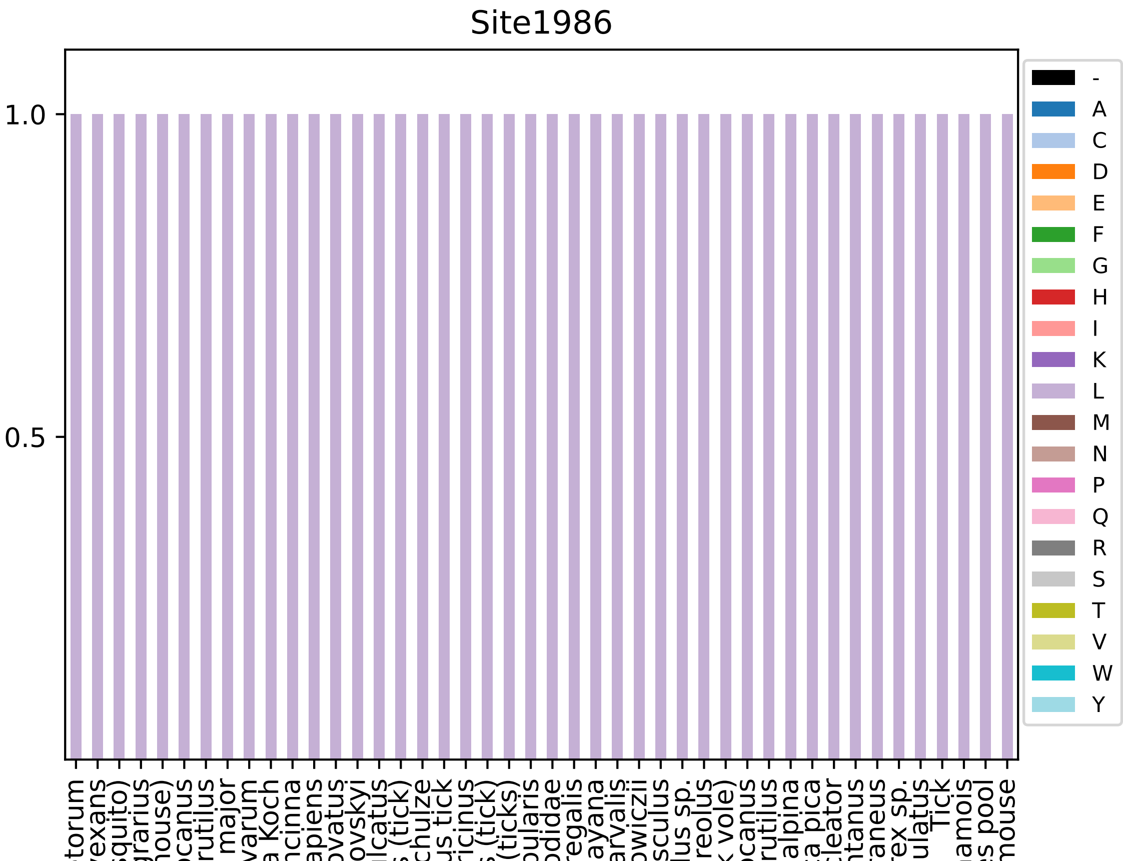
Site1986
1.0
0.5
-
A
C
D
E
F
G
H
I
K
L
M
N
P
Q
R
S
T
V
W
Y
Tick

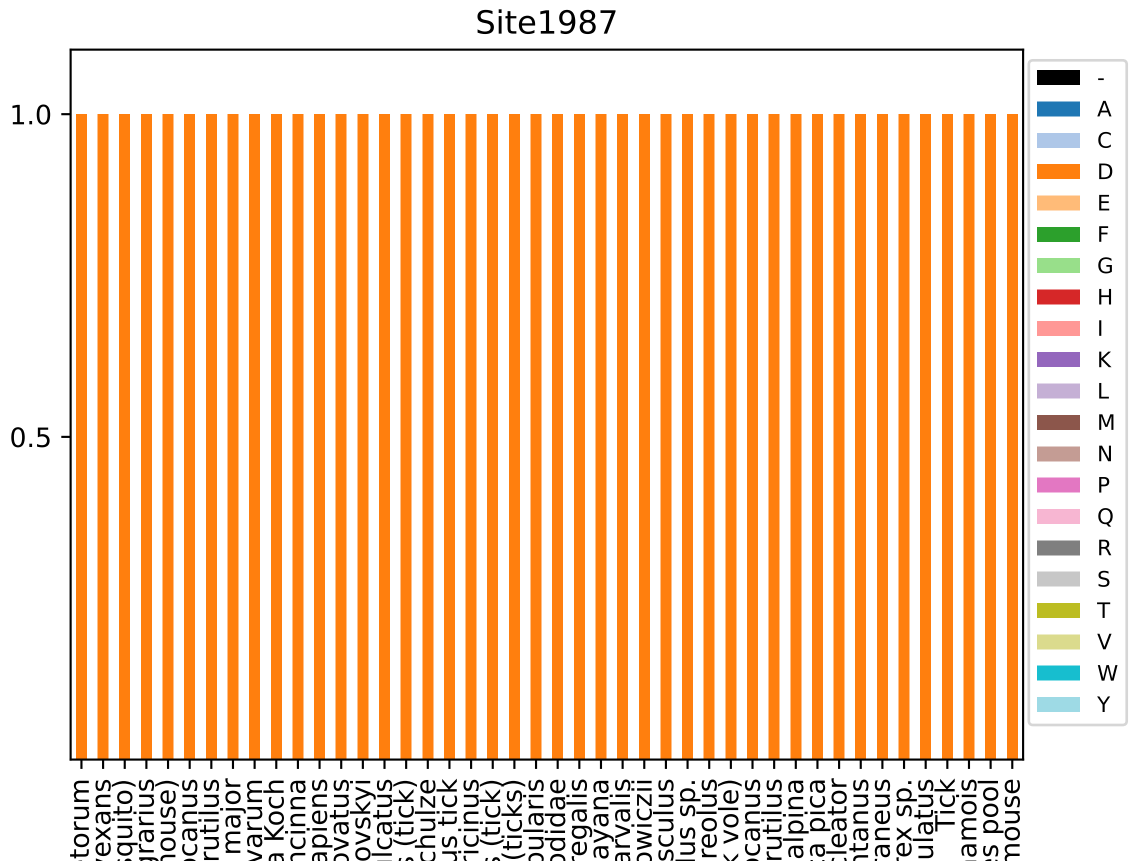
Site1987
1.0
0.5
-
A
C
D
E
F
G
H
I
K
L
M
N
P
Q
R
S
T
V
W
Y

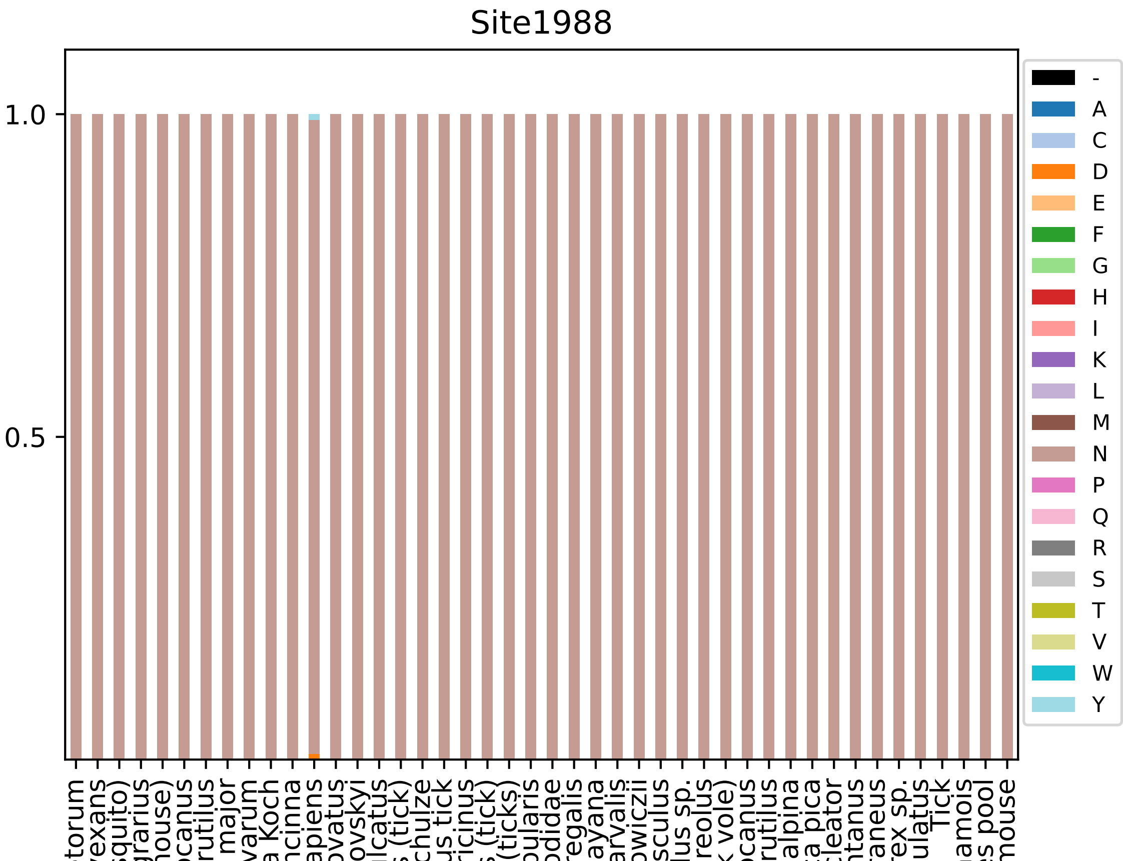
Site1988
1.0
0.5
-
A
C
D
E
F
G
H
I
K
L
M
N
P
Q
R
S
T
V
W
Y

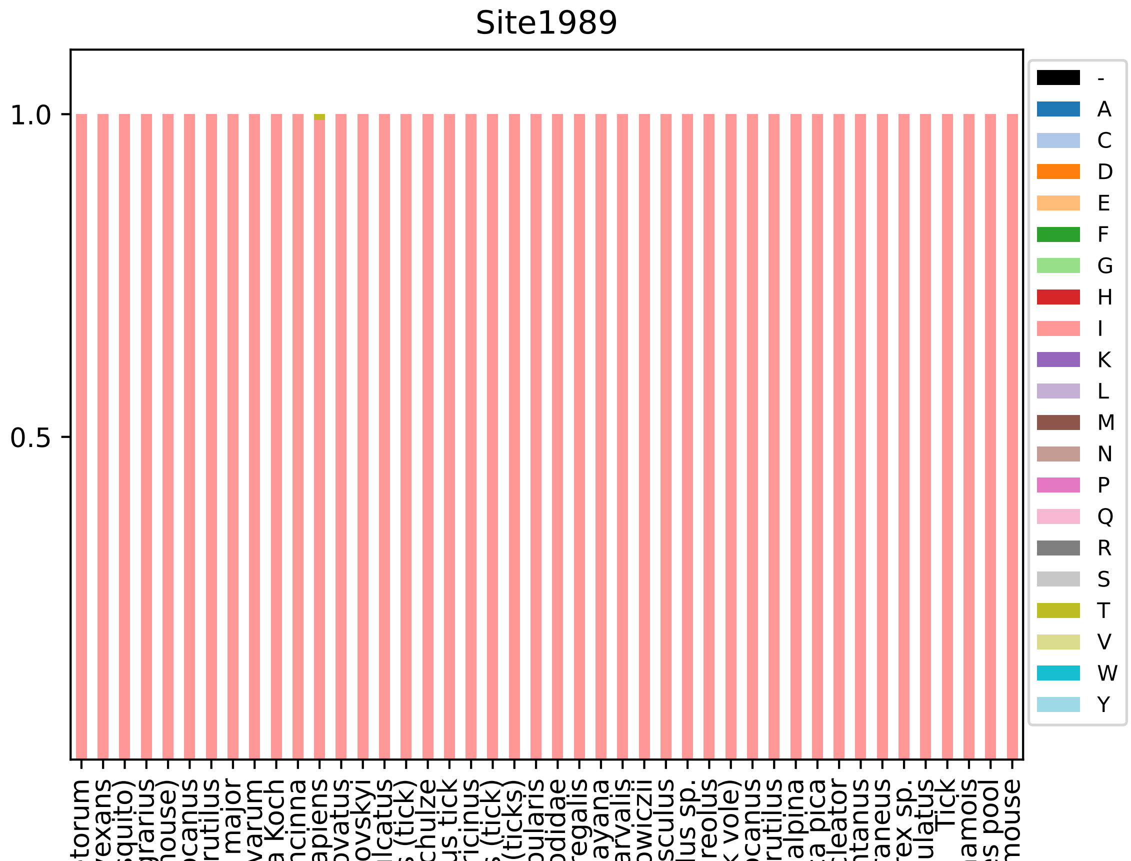

Site1989
1.0
0.5
-
A
C
D
E
F
G
H
I
K
L
M
N
P
Q
R
S
T
V
W
Y

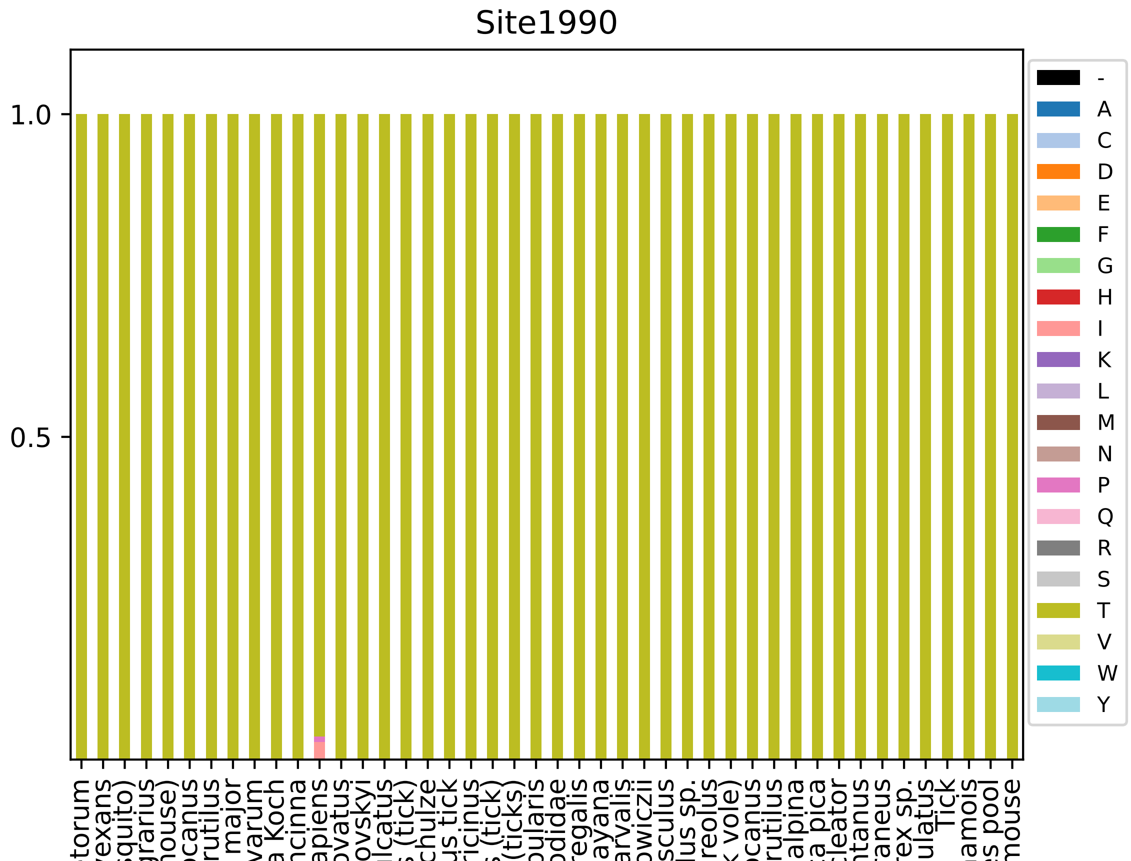
Site1990
1.0
0.5
-
A
C
D
E
F
G
H
I
K
L
M
N
P
Q
R
S
T
V
W
Y

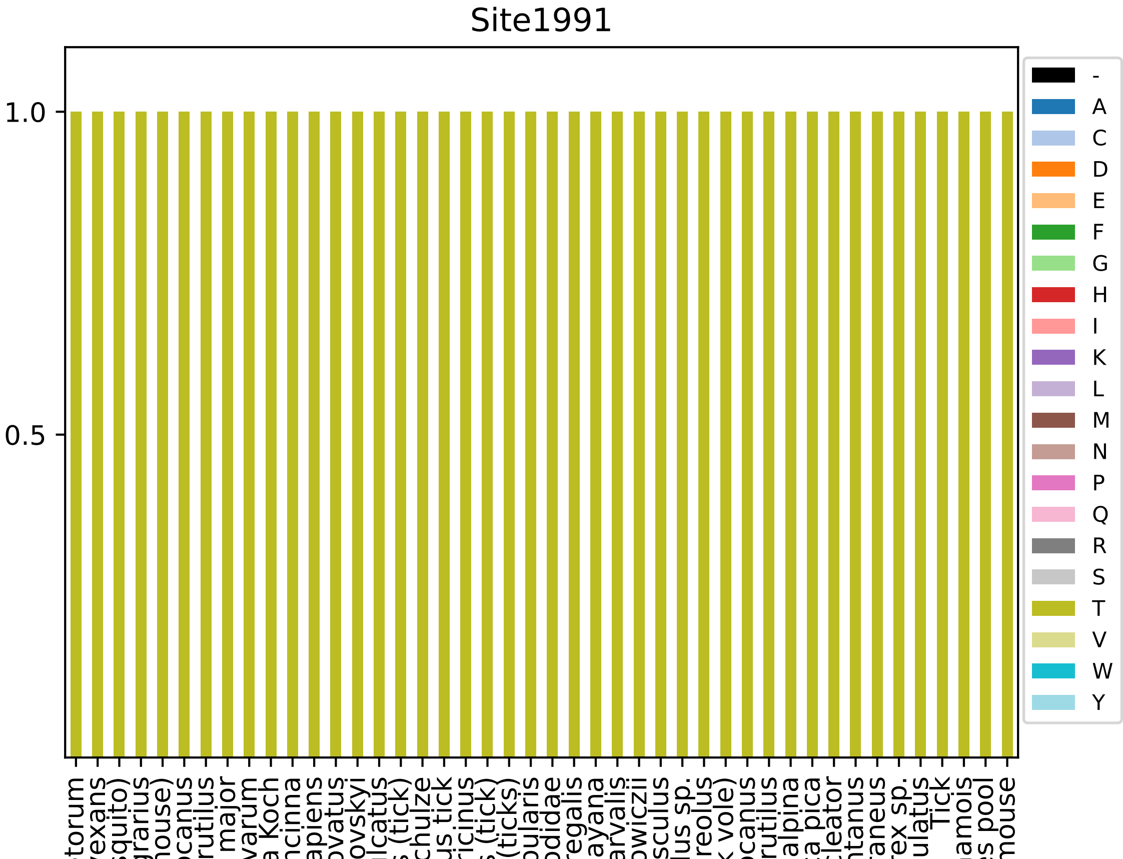

Site1991
1.0
0.5
-
A
C
D
E
F
G
H
I
K
L
M
N
P
Q
R
S
T
V
W
Y

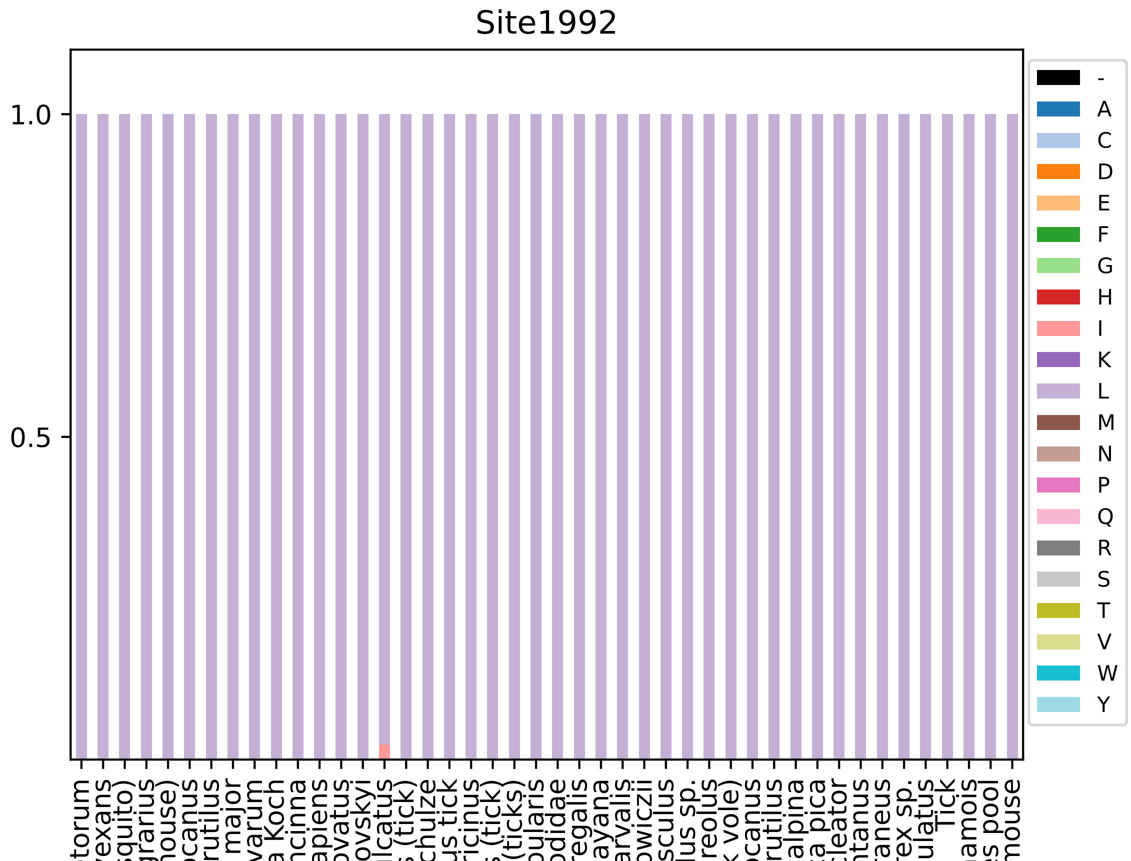
Site1992
1.0
0.5
-
A
C
D
E
F
G
H
I
K
L
M
N
P
Q
R
S
T
V
W
Y

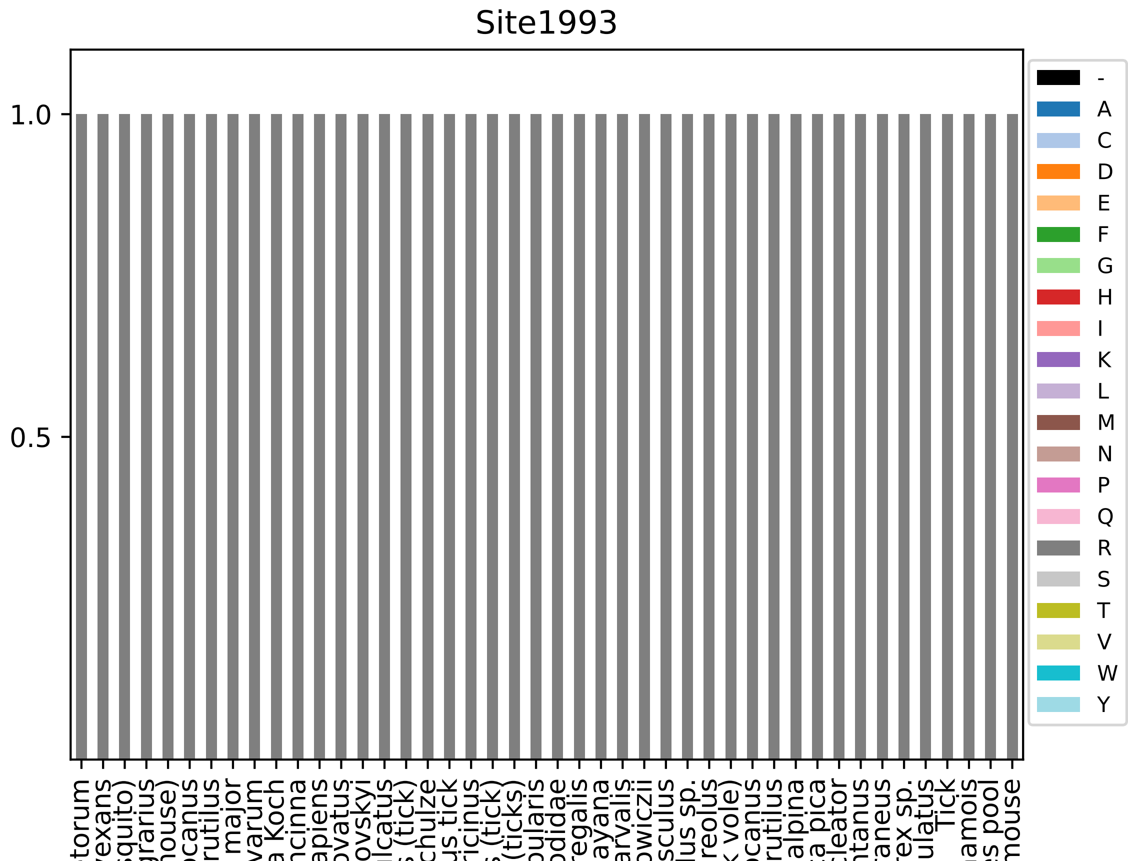
Site1993
1.0
0.5
-
A
C
D
E
F
G
H
I
K
L
M
N
P
Q
R
S
T
V
W
Y

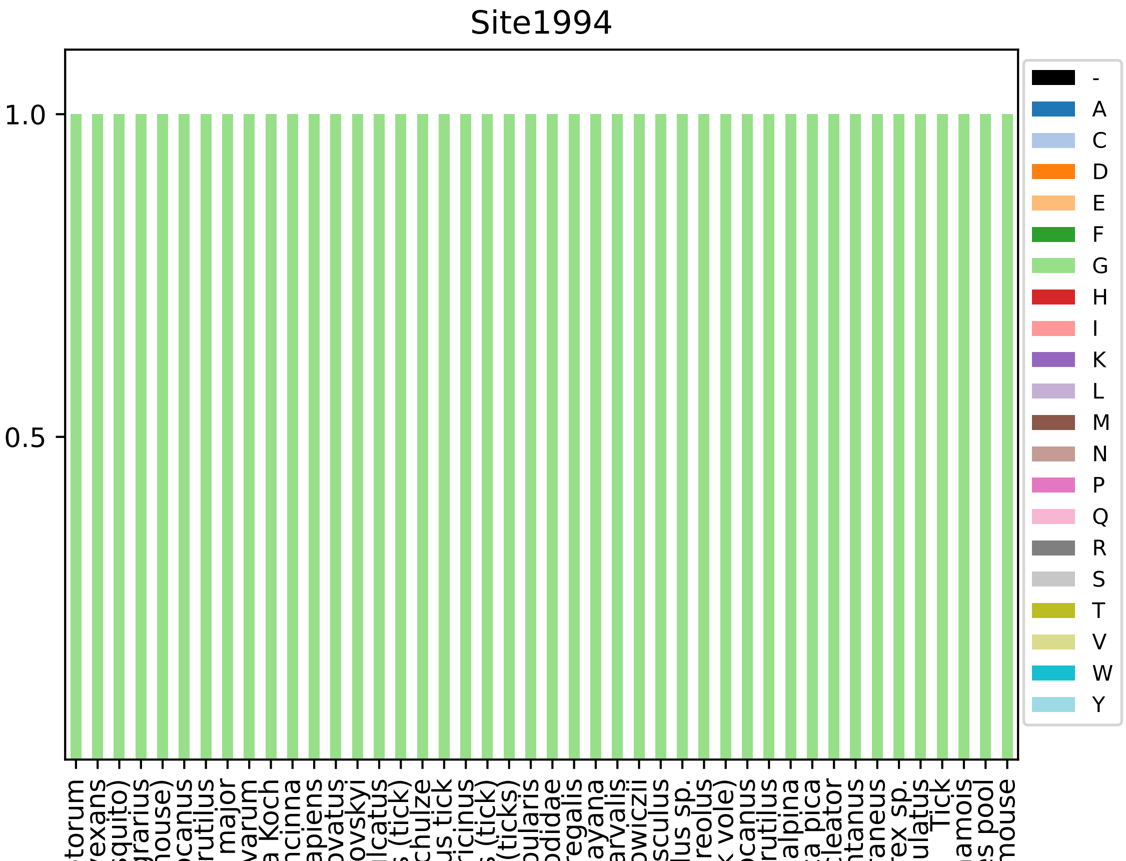
Site1994
1.0
0.5
-
A
C
D
E
F
G
H
I
K
L
M
N
P
Q
R
S
T
V
W
Y
Tick

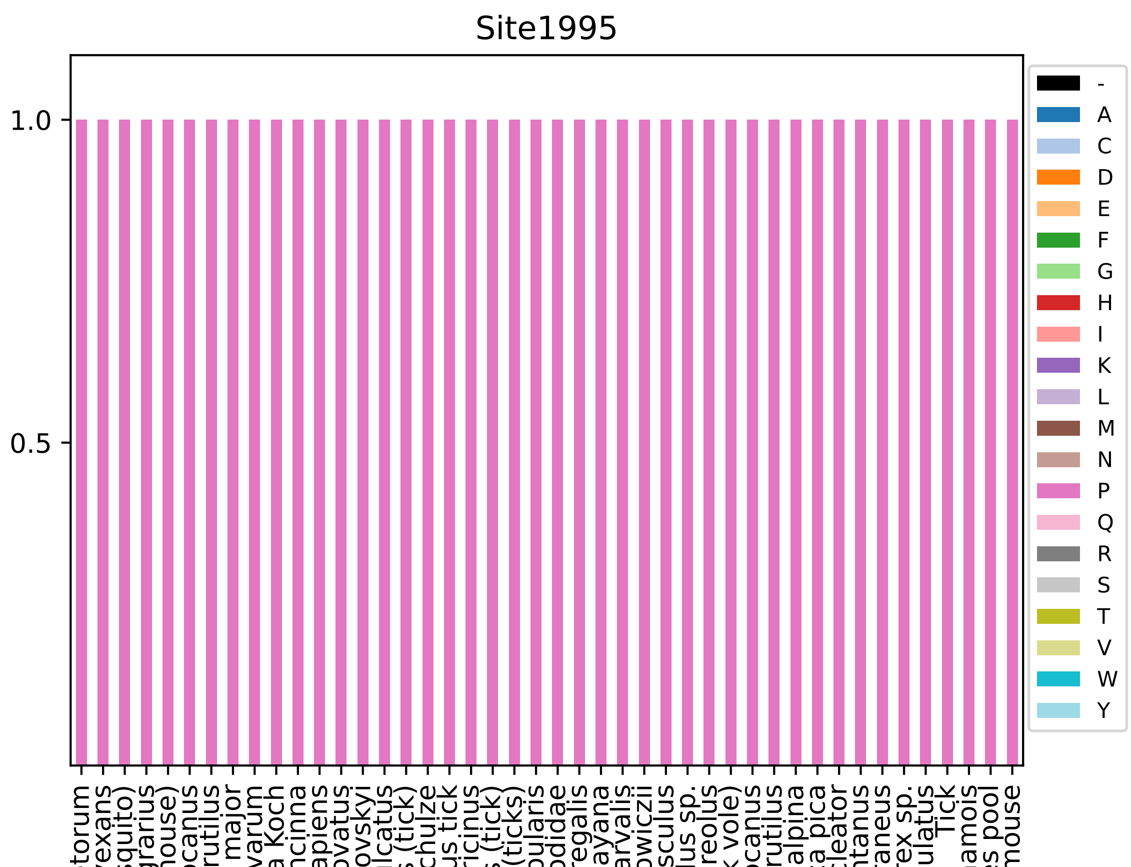
Site1995
1.0
0.5
-
A
C
D
E
F
G
H
I
K
L
M
N
P
Q
R
S
T
V
W
Y

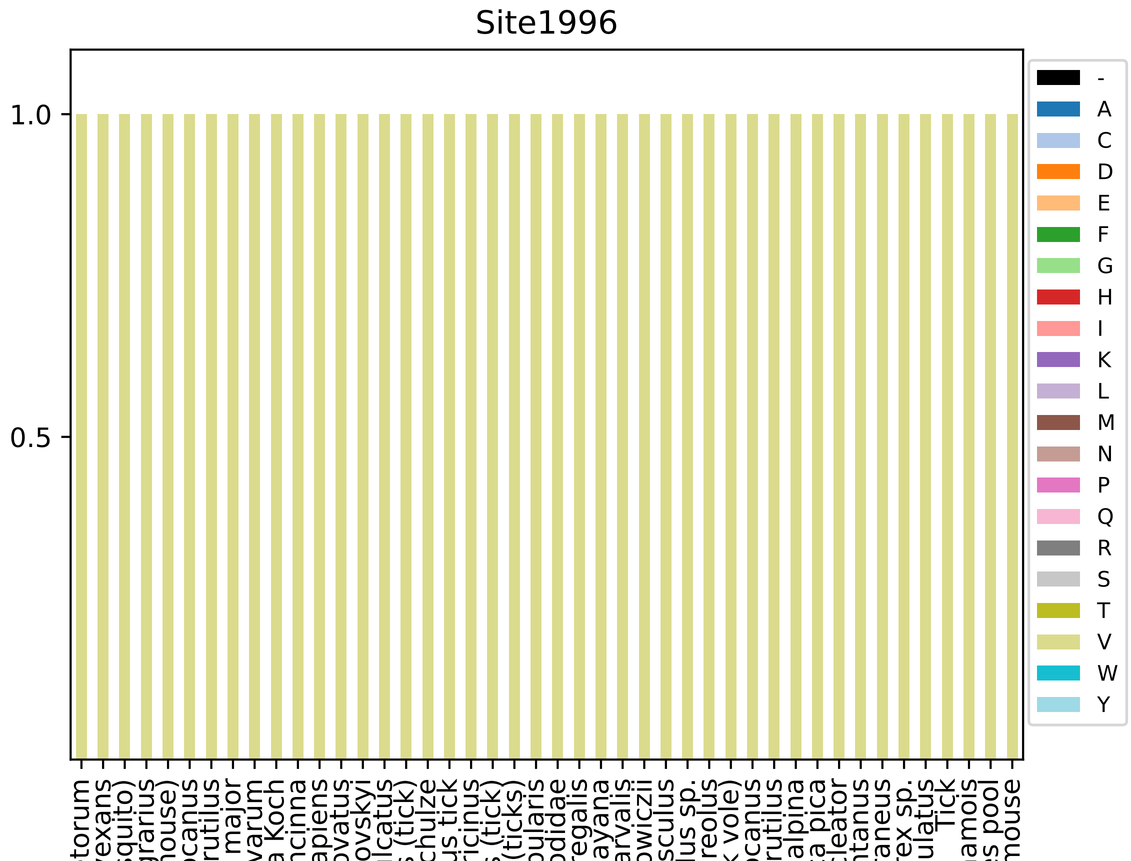

Site1996
1.0
0.5
-
A
C
D
E
F
G
H
I
K
L
M
N
P
Q
R
S
T
V
W
Y

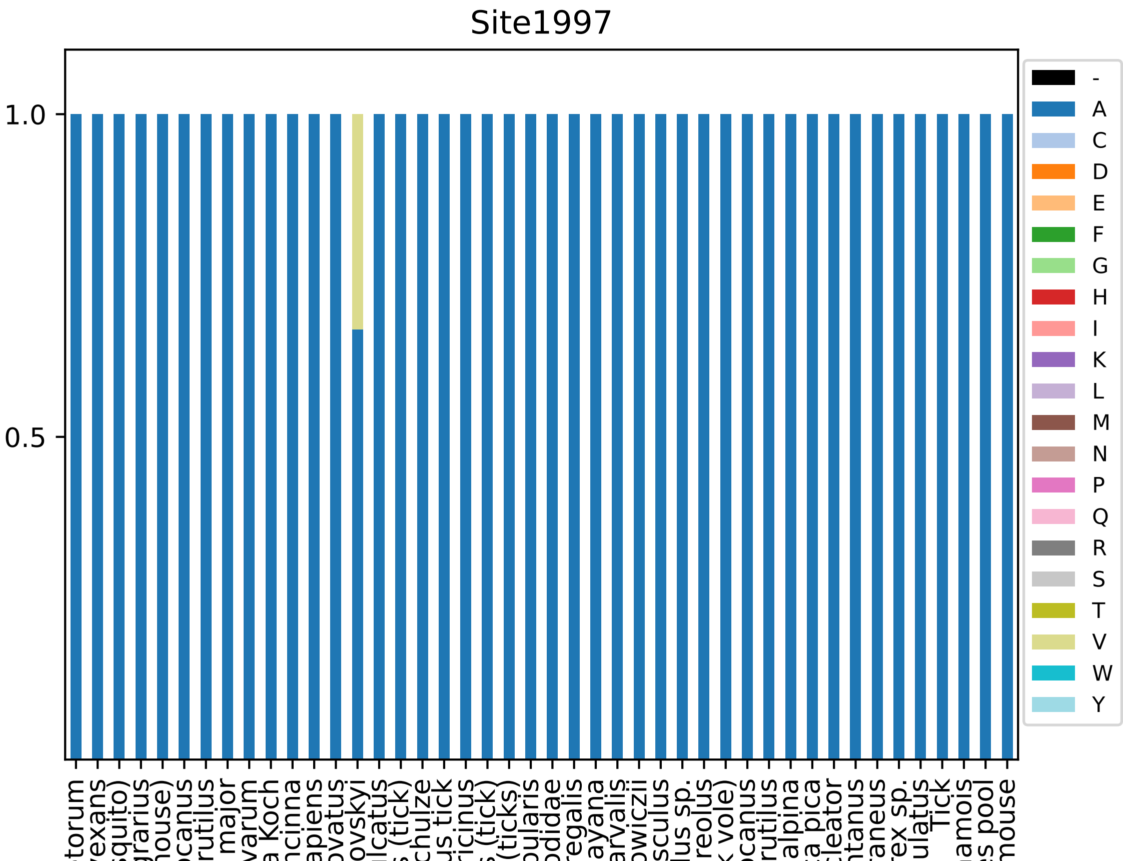

Site1997
1.0
0.5
-
A
C
D
E
F
G
H
I
K
L
M
N
P
Q
R
S
T
V
W
Y

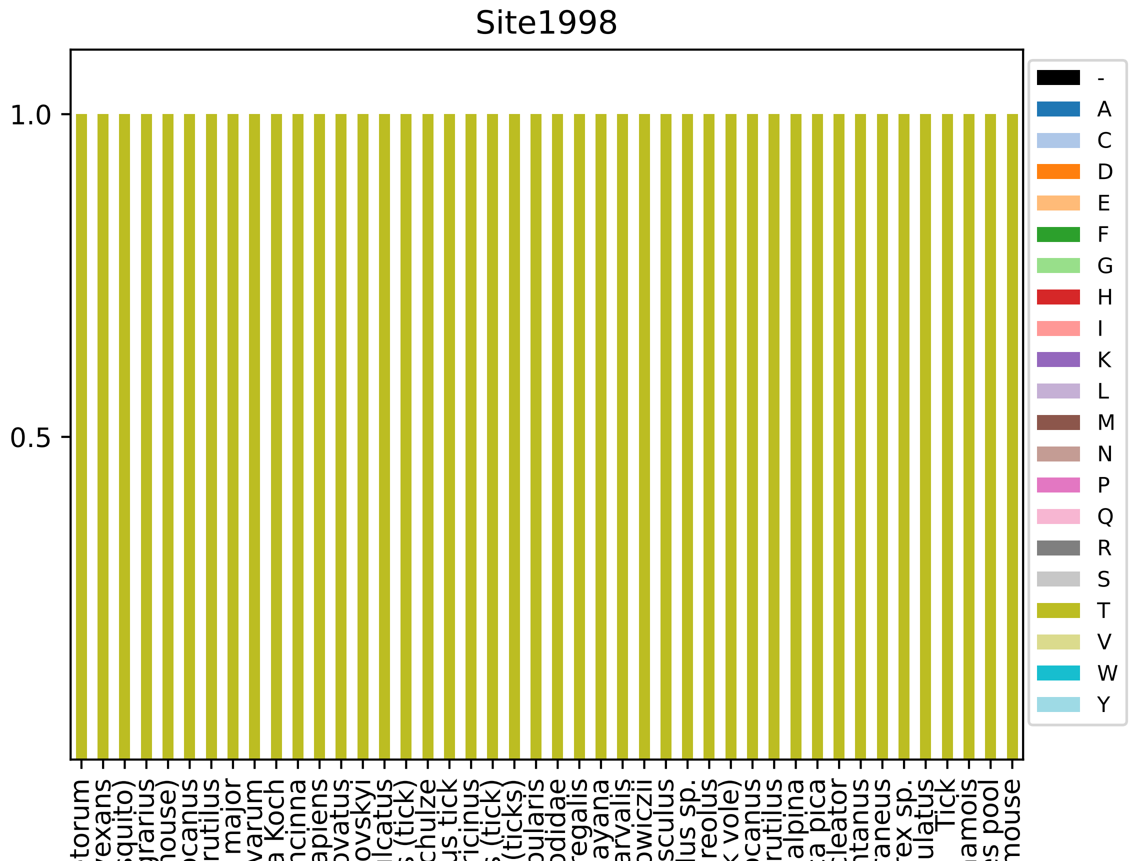

Site1998
1.0
0.5
-
A
C
D
E
F
G
H
I
K
L
M
N
P
Q
R
S
T
V
W
Y
Tick

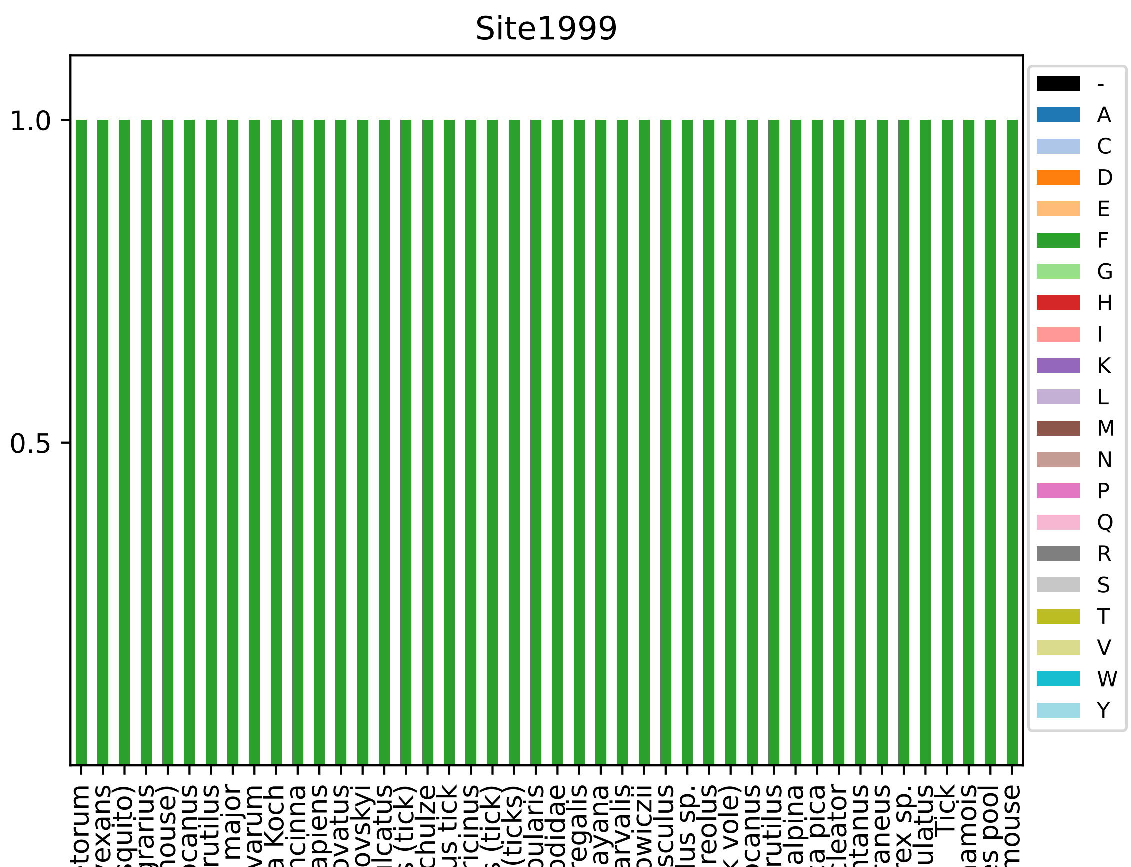
Site1999
1.0
0.5
-
A
C
D
E
F
G
H
I
K
L
M
N
P
Q
R
S
T
V
W
Y

Site2000

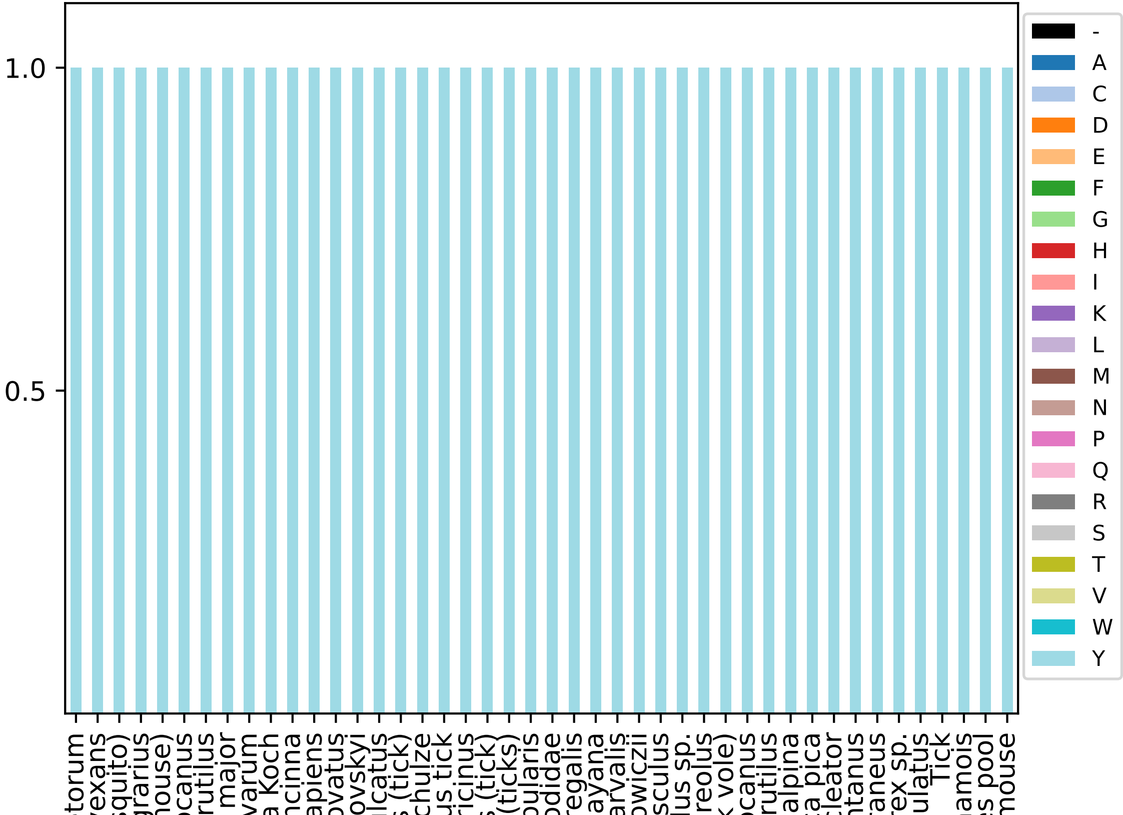

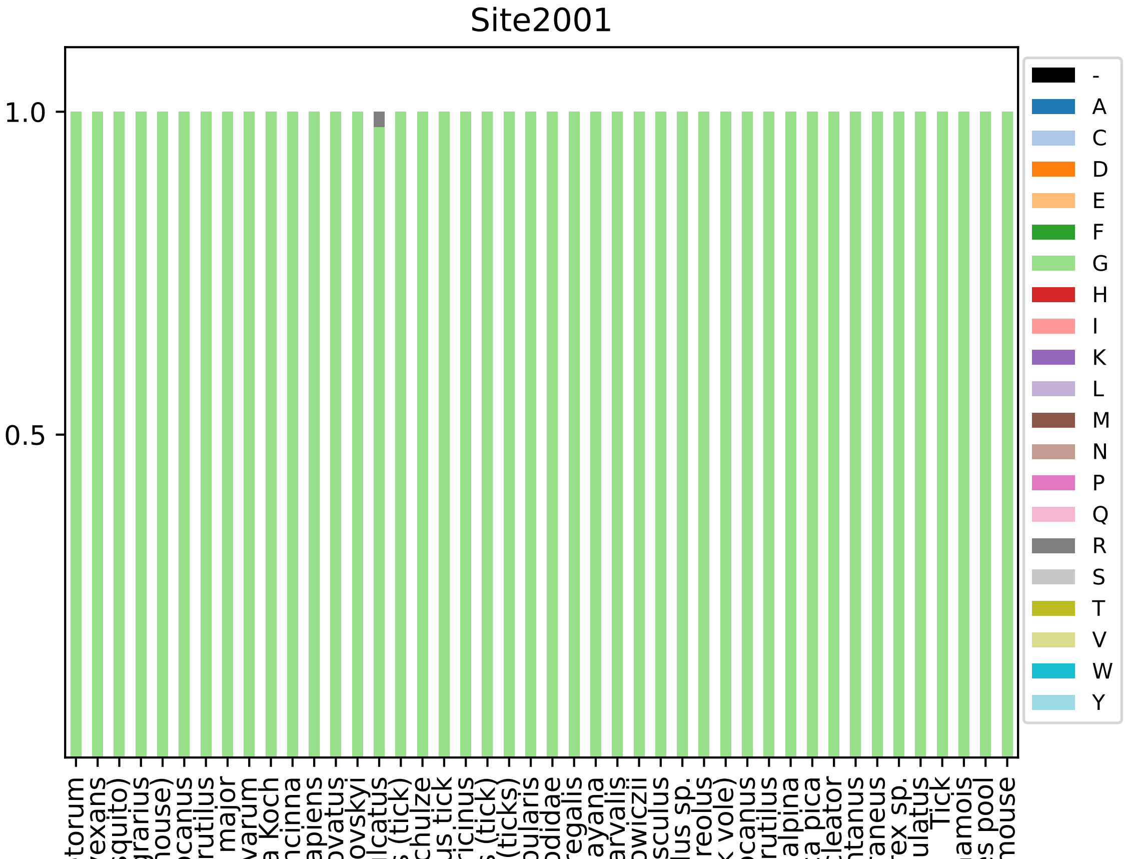

Site2001
1.0
0.5
-
A
C
D
E
F
G
H
I
K
L
M
N
P
Q
R
S
T
V
W
Y

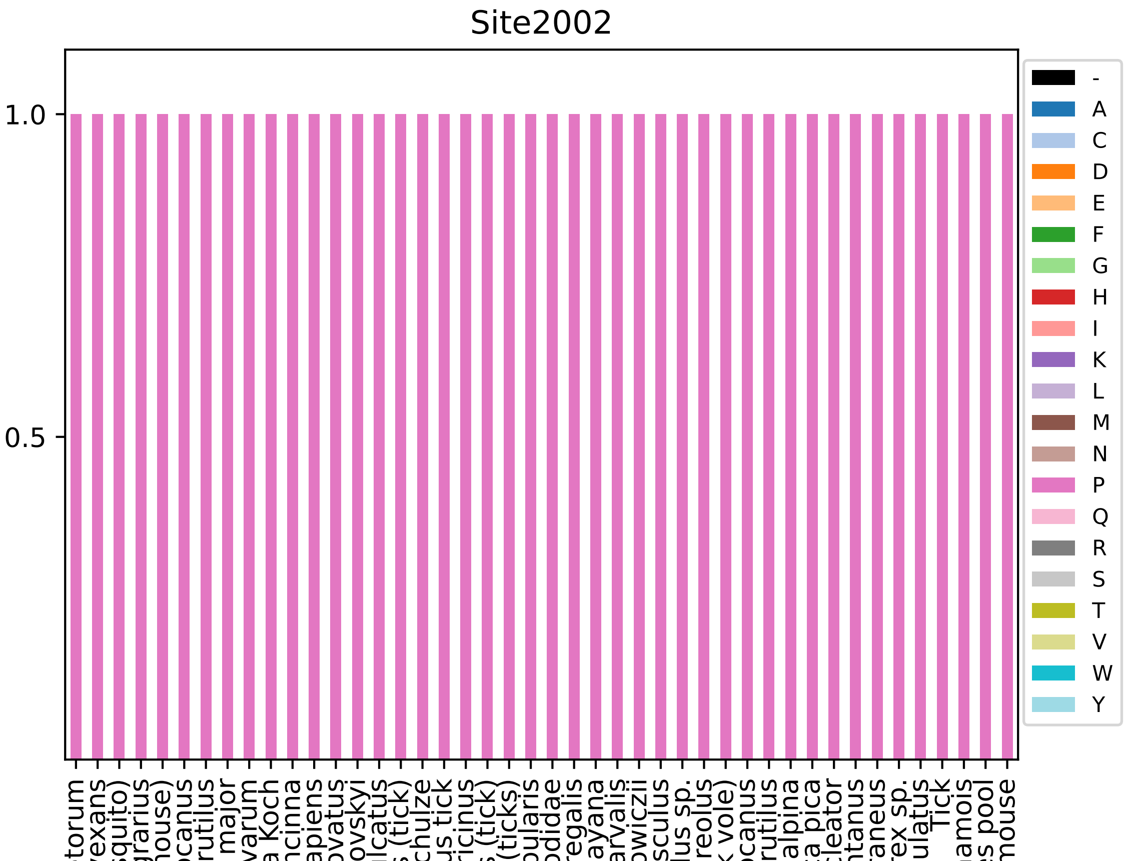
Site2002
1.0
0.5
-
A
C
D
E
F
G
H
I
K
L
M
N
P
Q
R
S
T
V
W
Y
torum
vexans
squito)
grarius
nouse)
ocanus
rutilus
major
varum
a Koch
ncinna
apiens
ovatus
ovskyi
ulcatus
s (tick)
chulze
us tick
ricinus
s (tick)
(ticks)
pularis
odidae
regalis
ayana
arvalis
owiczii
sculus
lus sp.
reolus
x vole)
ocanus
rutilus
alpina
a pica
leator
ntanus
raneus
rex sp.
ulatus
Tick
amois
es pool
mouse

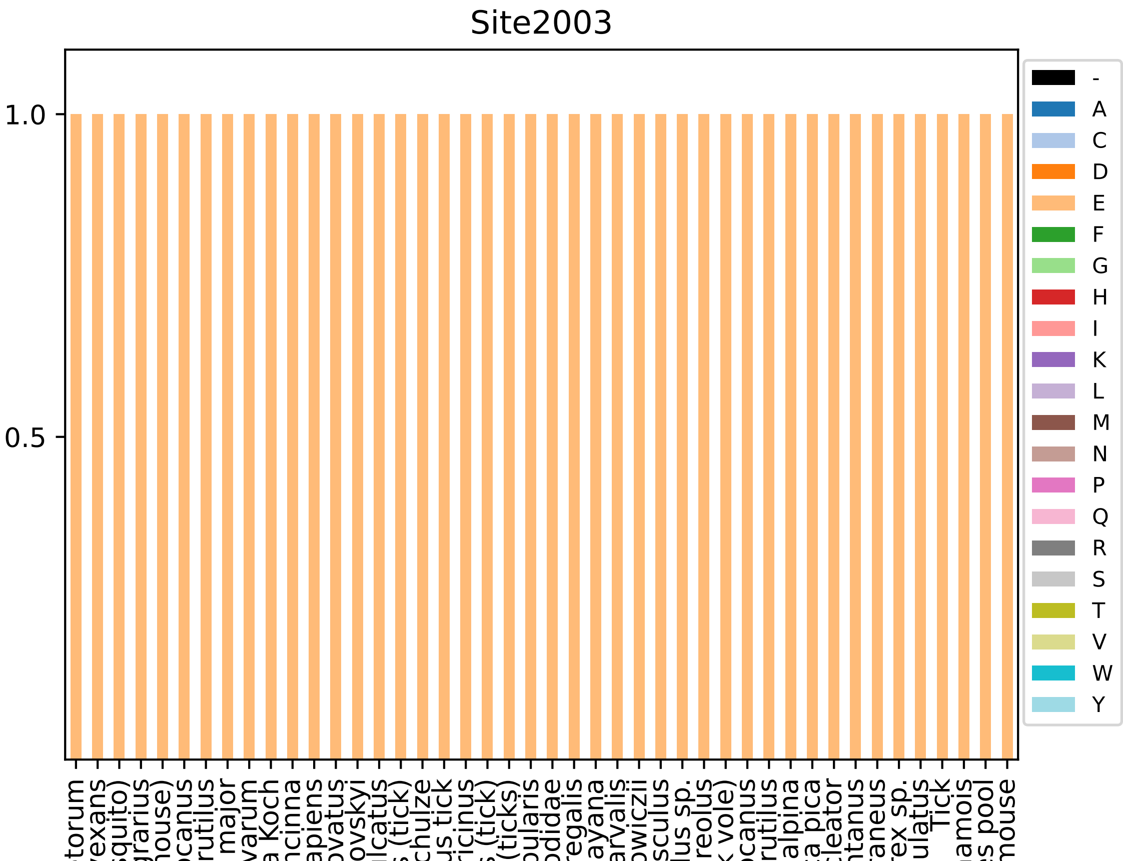

Site2003
1.0
0.5
-
A
C
D
E
F
G
H
I
K
L
M
N
P
Q
R
S
T
V
W
Y

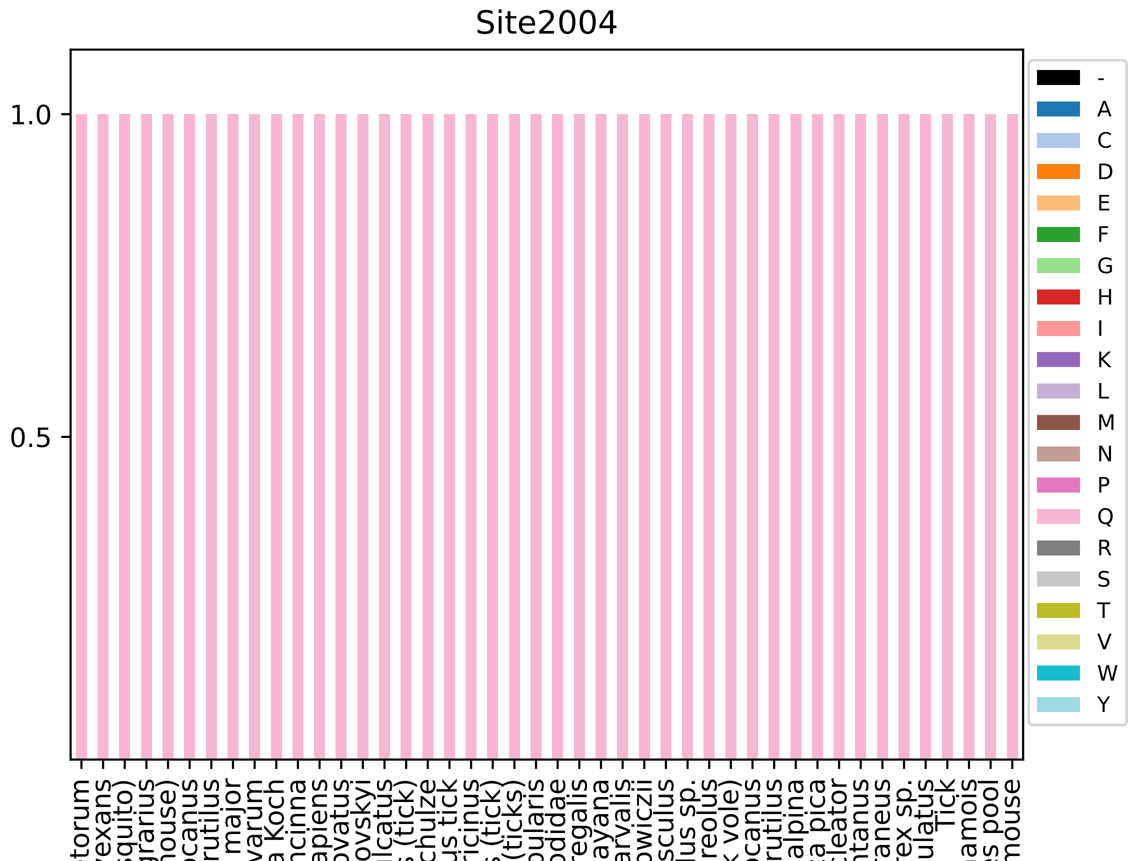
Site2004
1.0
0.5
-
A
C
D
E
F
G
H
I
K
L
M
N
P
Q
R
S
T
V
W
Y

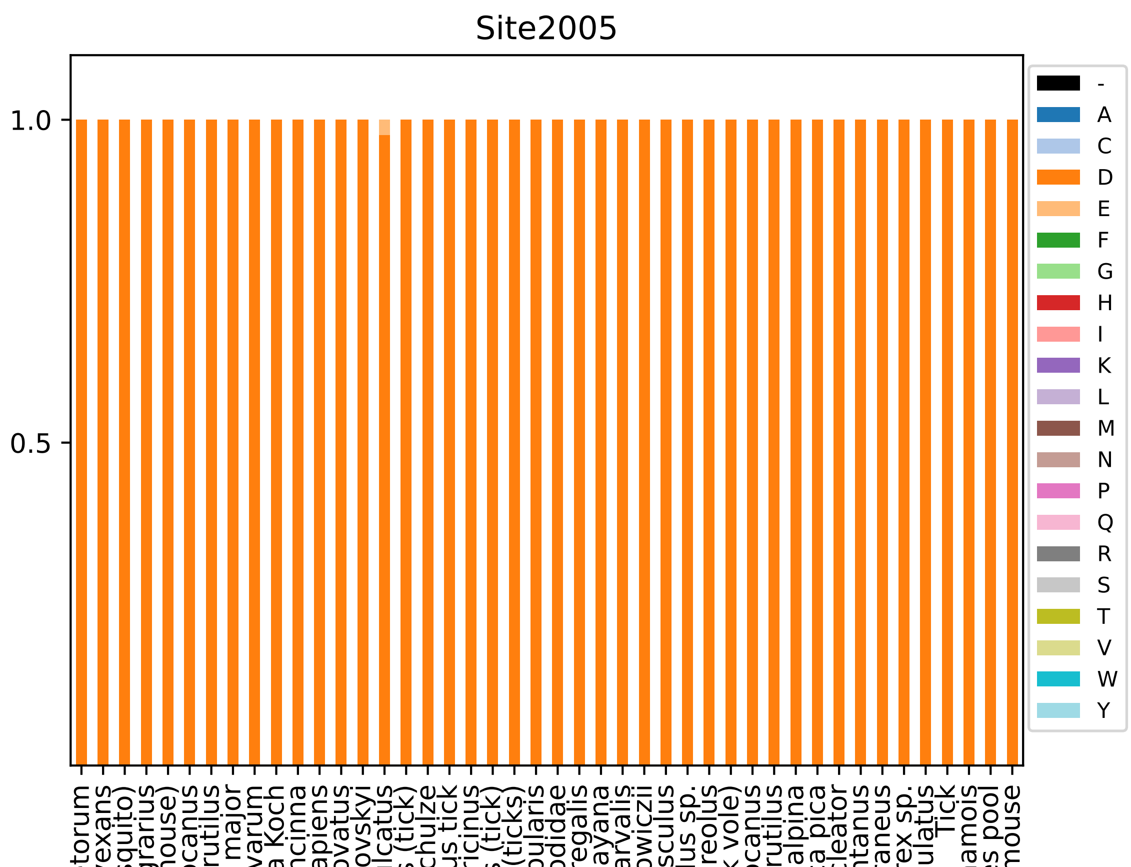

Site2005
1.0
0.5
-
A
C
D
E
F
G
H
I
K
L
M
N
P
Q
R
S
T
V
W
Y

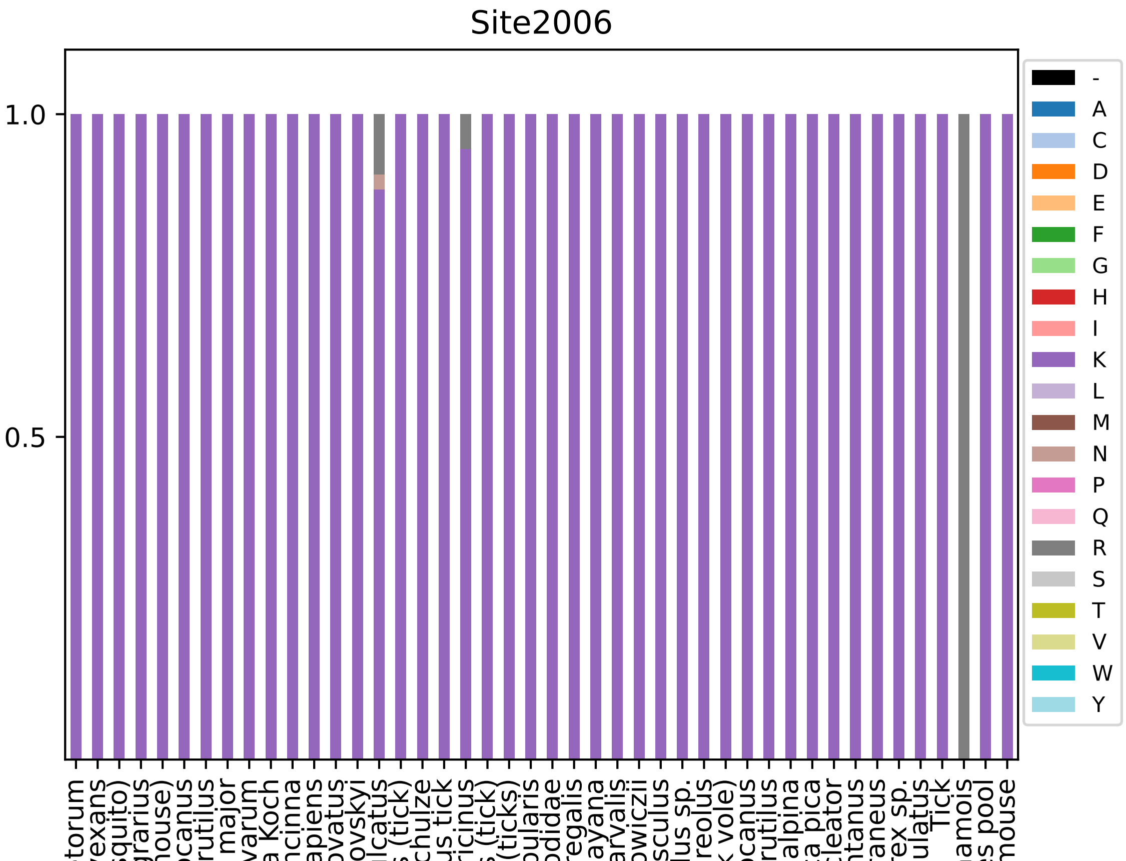

Site2006
1.0
0.5
-
A
C
D
E
F
G
H
I
K
L
M
N
P
Q
R
S
T
V
W
Y

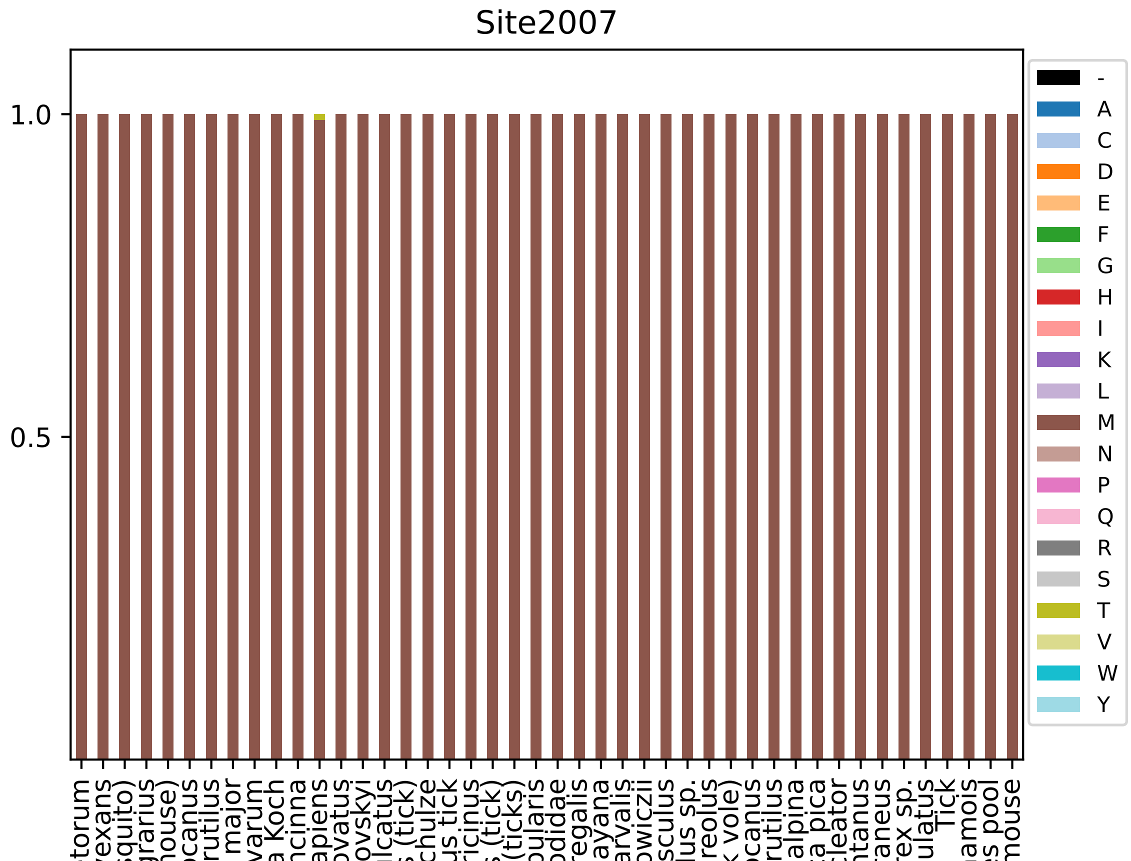
Site2007
1.0
0.5
-
A
C
D
E
F
G
H
I
K
L
M
N
P
Q
R
S
T
V
W
Y

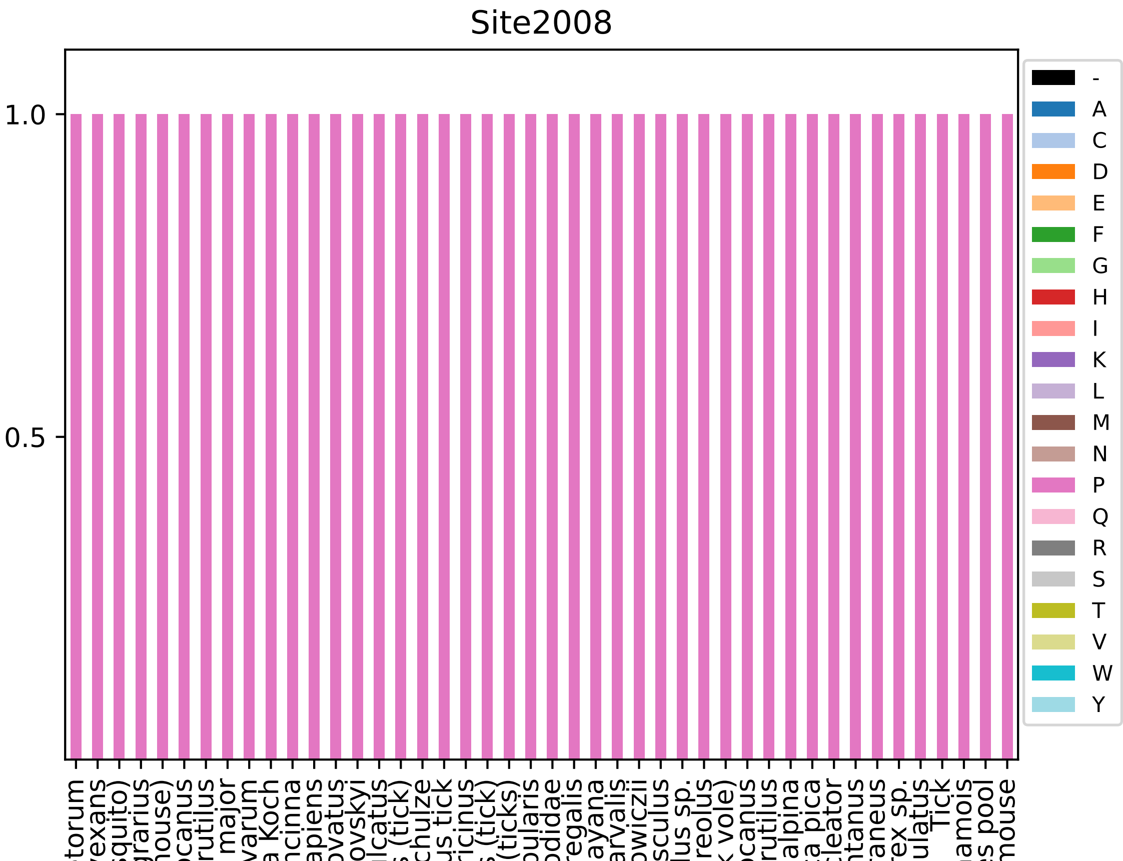
Site2008
1.0
0.5
-
A
C
D
E
F
G
H
I
K
L
M
N
P
Q
R
S
T
V
W
Y

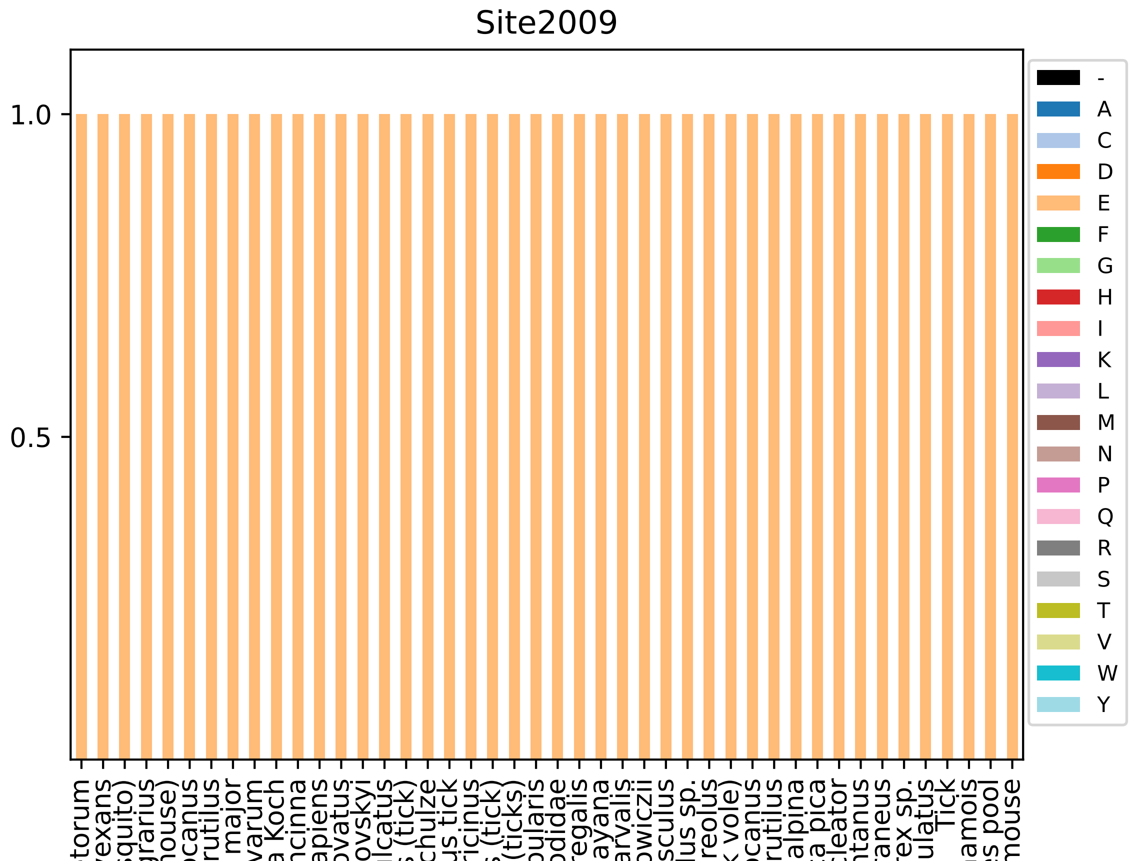
Site2009
1.0
0.5
-
A
C
D
E
F
G
H
I
K
L
M
N
P
Q
R
S
T
V
W
Y
Tick

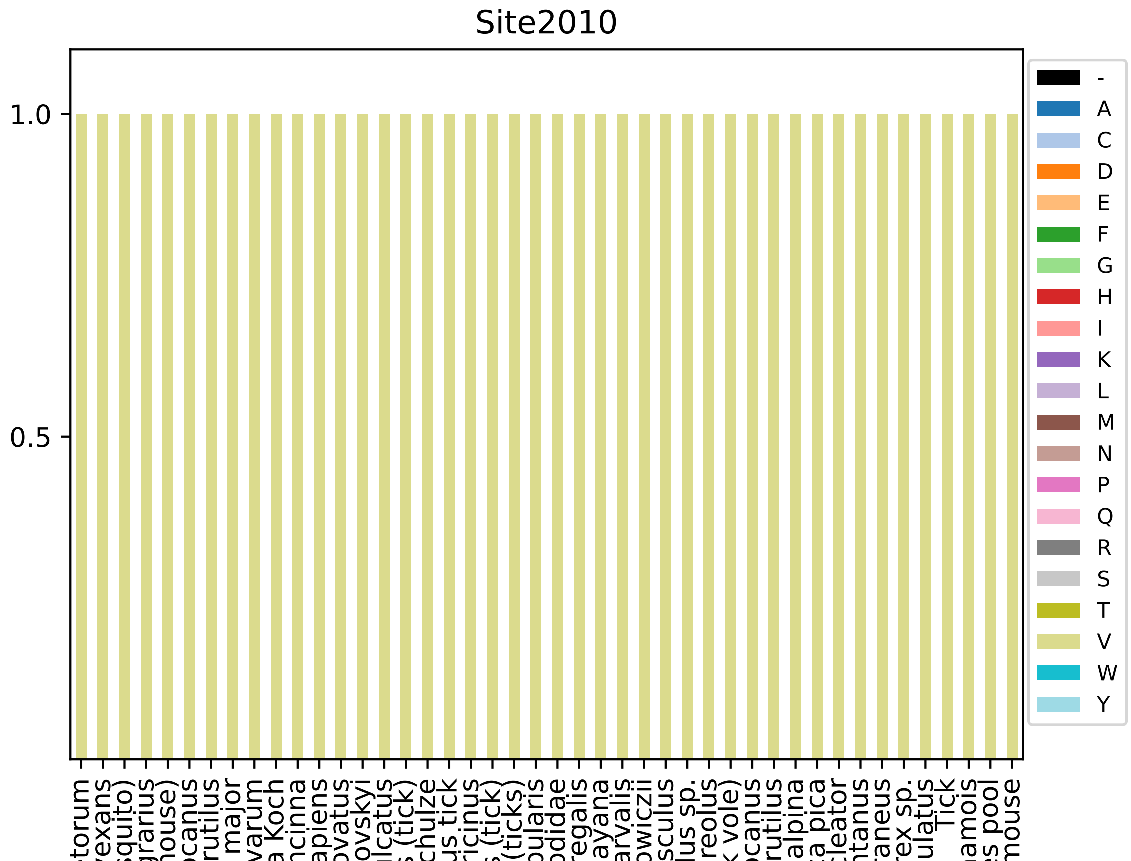

Site2010
1.0
0.5
-
A
C
D
E
F
G
H
I
K
L
M
N
P
Q
R
S
T
V
W
Y

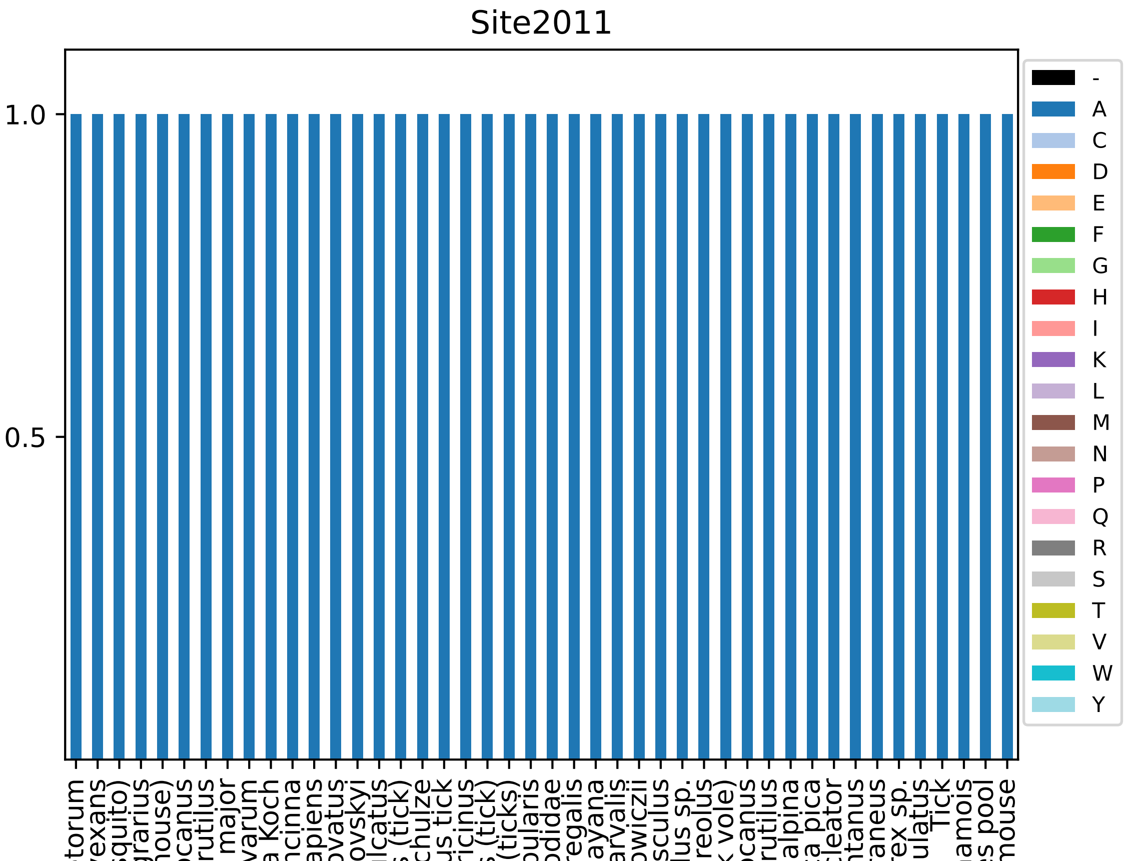
Site2011
1.0
0.5
-
A
C
D
E
F
G
H
I
K
L
M
N
P
Q
R
S
T
V
W
Y
Tick

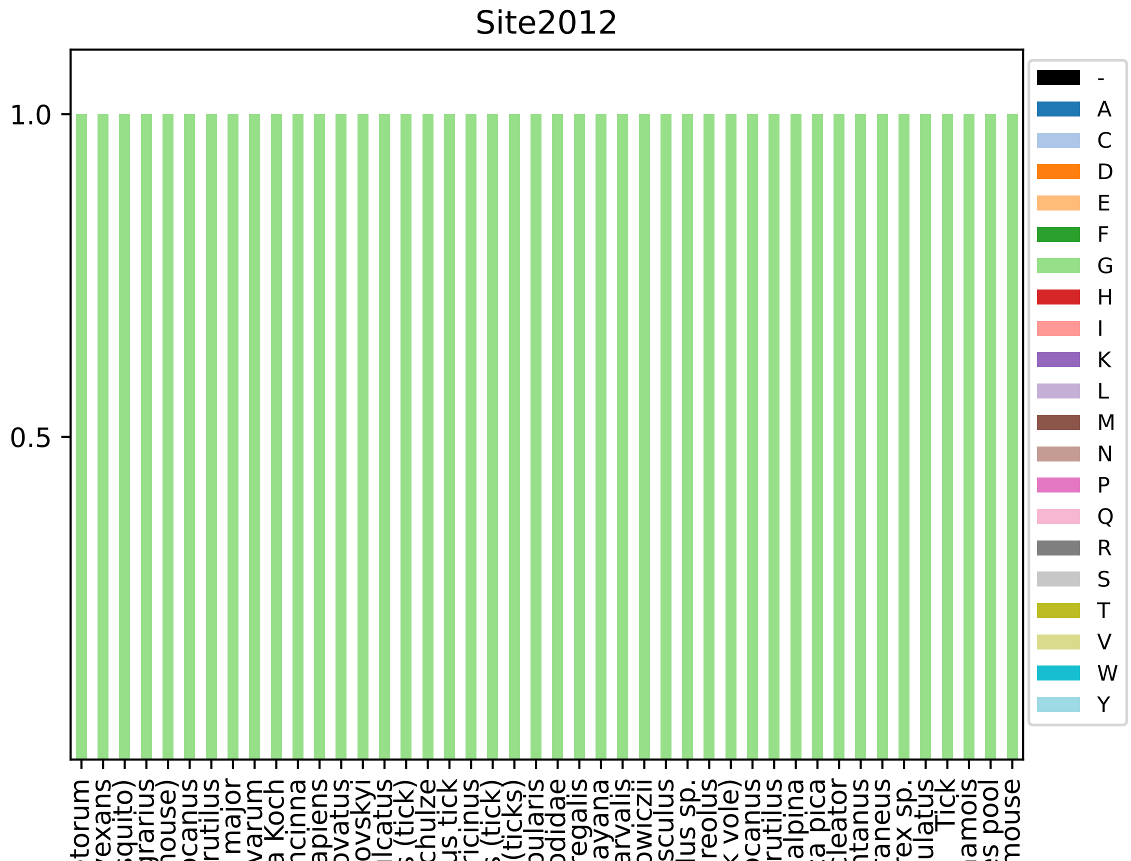
Site2012
1.0
0.5
-
A
C
D
E
F
G
H
I
K
L
M
N
P
Q
R
S
T
V
W
Y
Tick

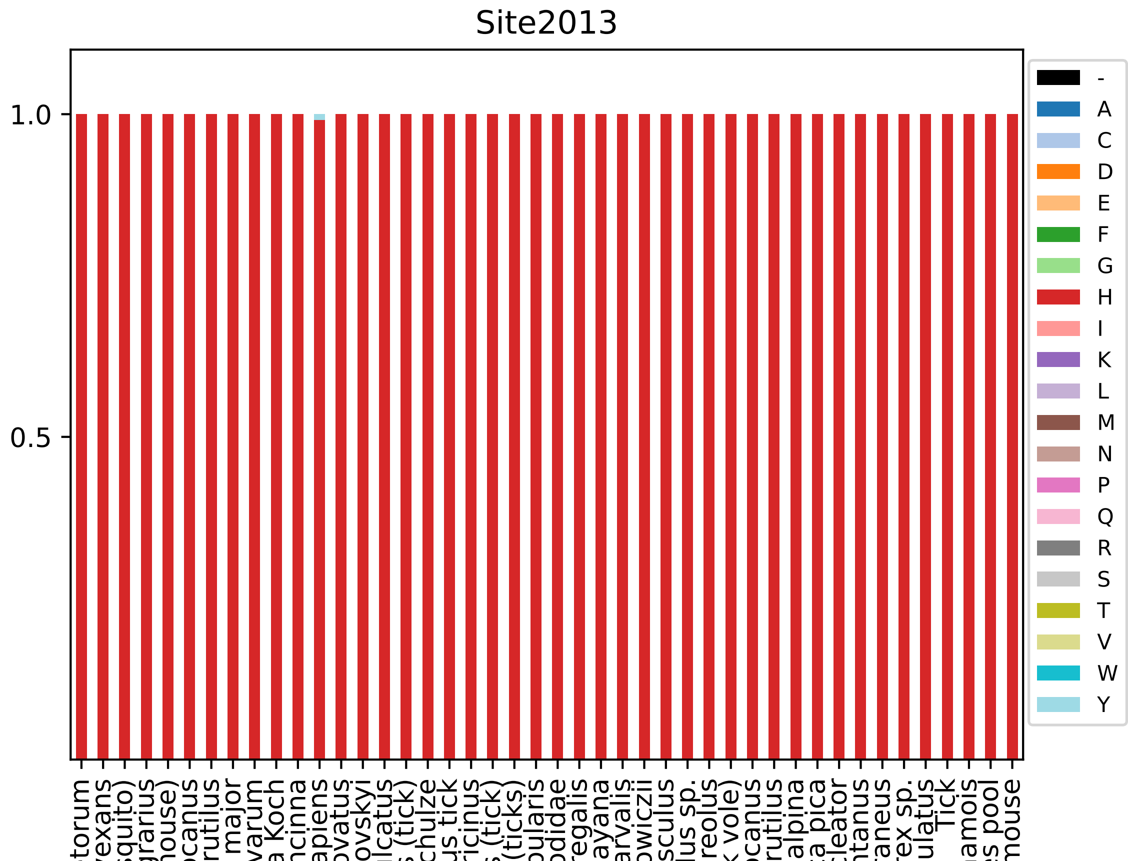

Site2013
1.0
0.5
-
A
C
D
E
F
G
H
I
K
L
M
N
P
Q
R
S
T
V
W
Y

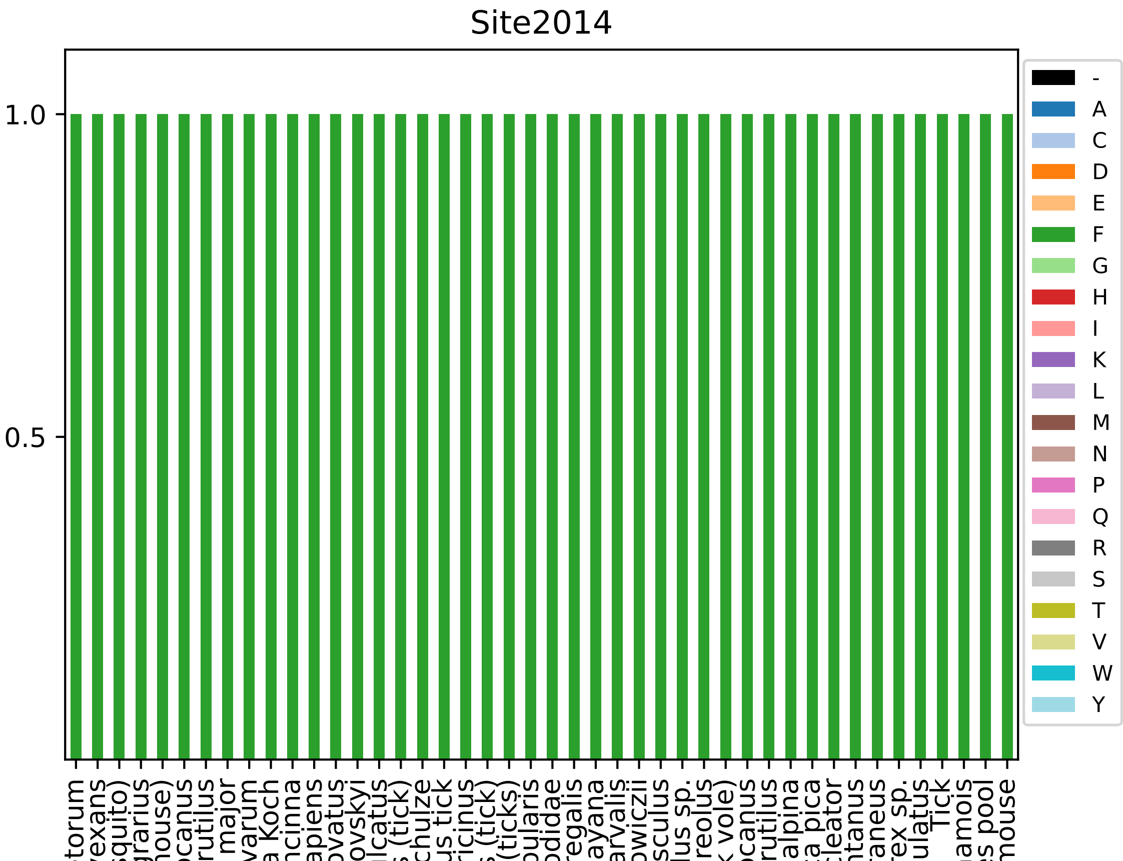
Site2014
1.0
0.5
-
A
C
D
E
F
G
H
I
K
L
M
N
P
Q
R
S
T
V
W
Y

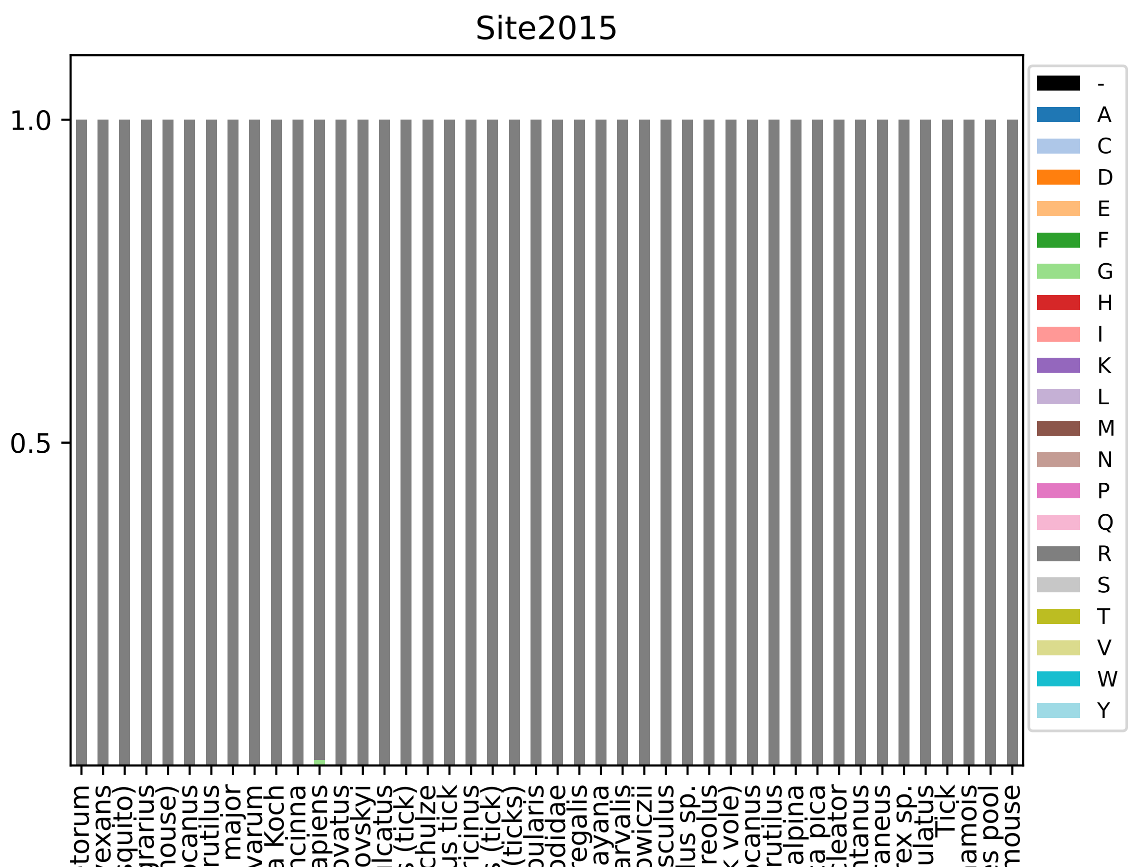
Site2015
1.0
0.5
-
A
C
D
E
F
G
H
I
K
L
M
N
P
Q
R
S
T
V
W
Y

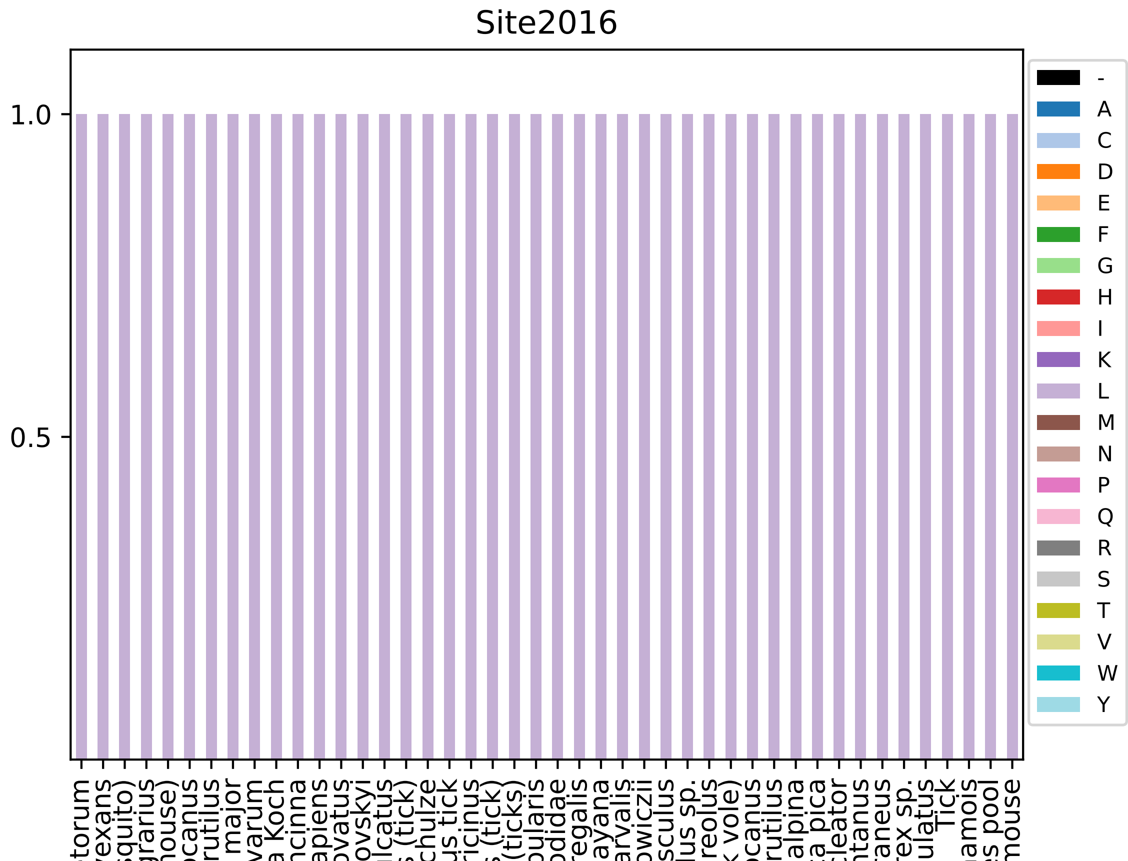
Site2016
1.0
0.5
-
A
C
D
E
F
G
H
I
K
L
M
N
P
Q
R
S
T
V
W
Y
Tick

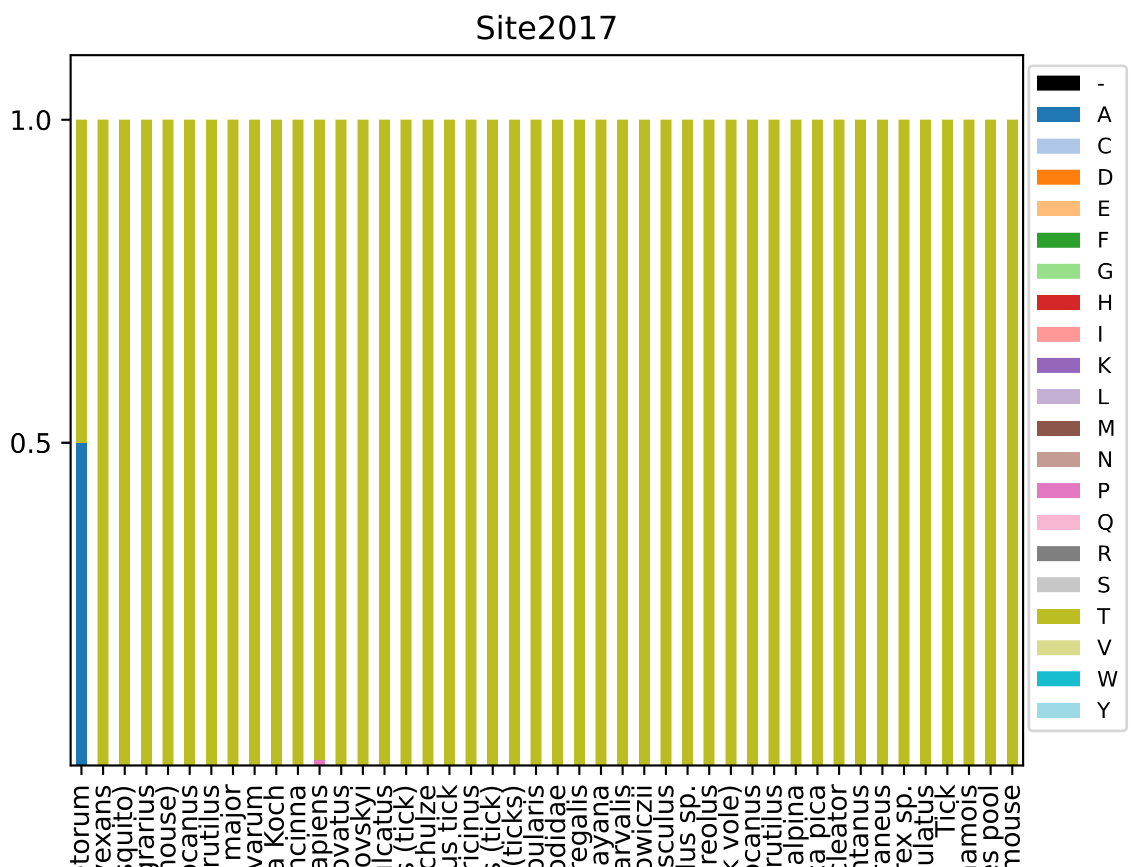
Site2017
1.0
0.5
-
A
C
D
E
F
G
H
I
K
L
M
N
P
Q
R
S
T
V
W
Y

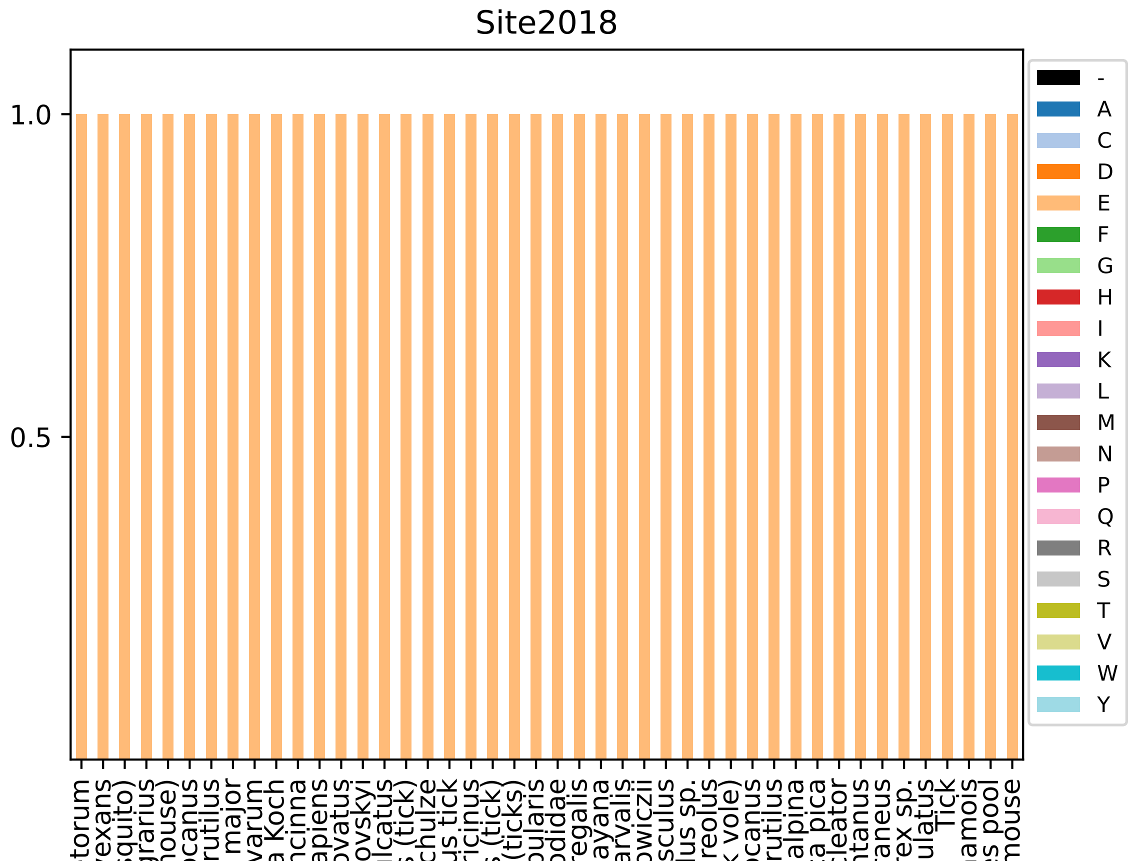

Site2018
1.0
0.5
-
A
C
D
E
F
G
H
I
K
L
M
N
P
Q
R
S
T
V
W
Y

Site2019

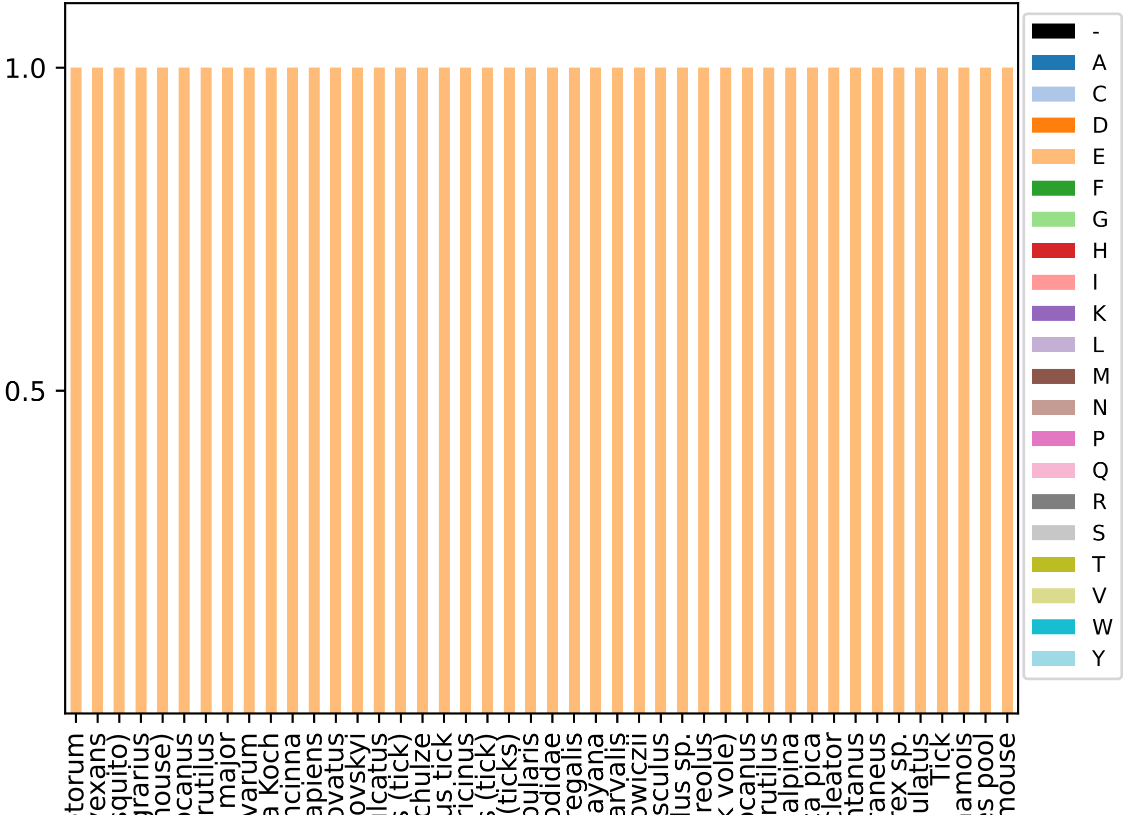

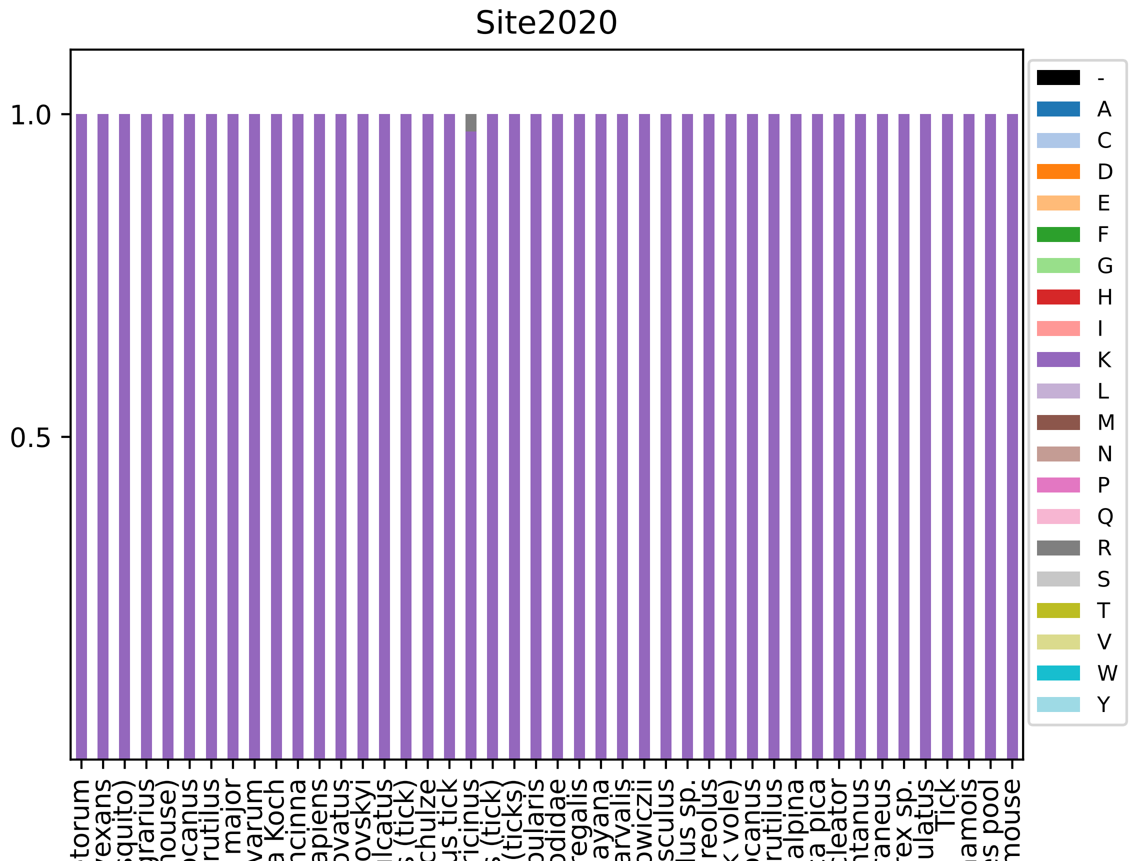
Site2020
1.0
0.5
-
A
C
D
E
F
G
H
I
K
L
M
N
P
Q
R
S
T
V
W
Y
Tick

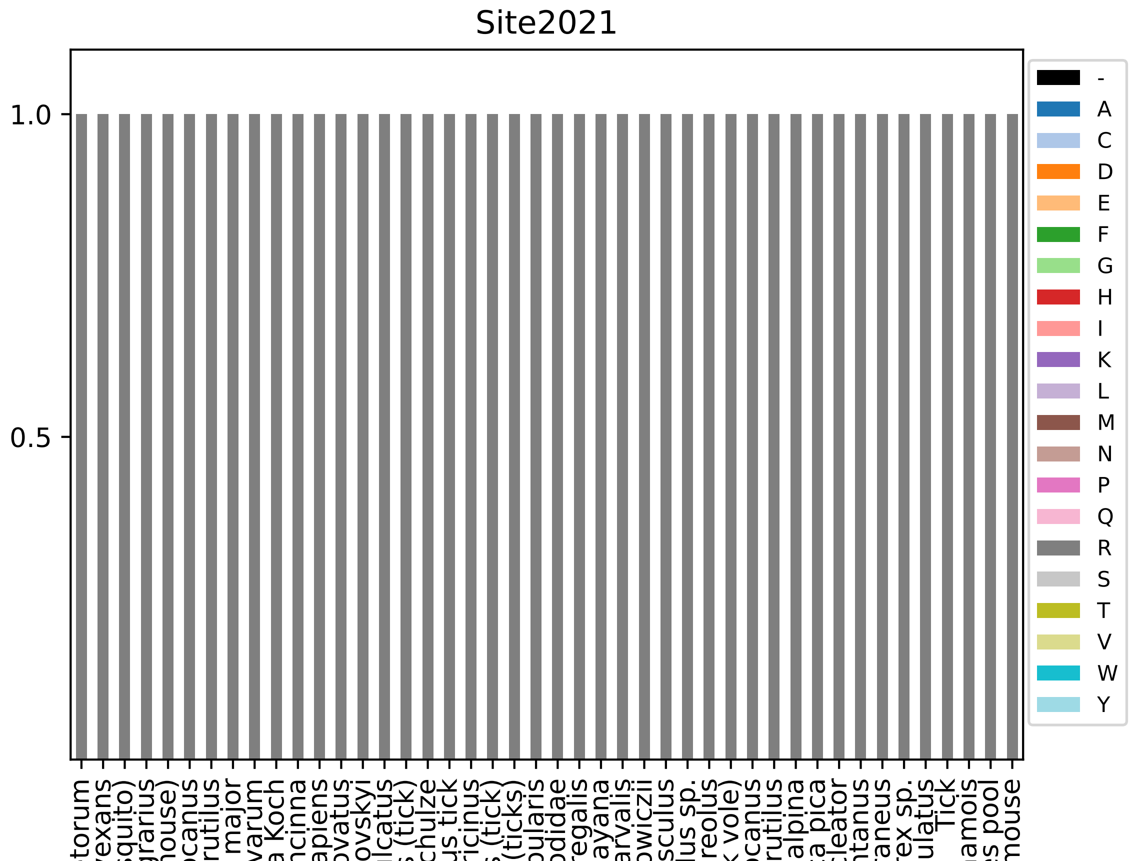
Site2021
1.0
0.5
-
A
C
D
E
F
G
H
I
K
L
M
N
P
Q
R
S
T
V
W
Y

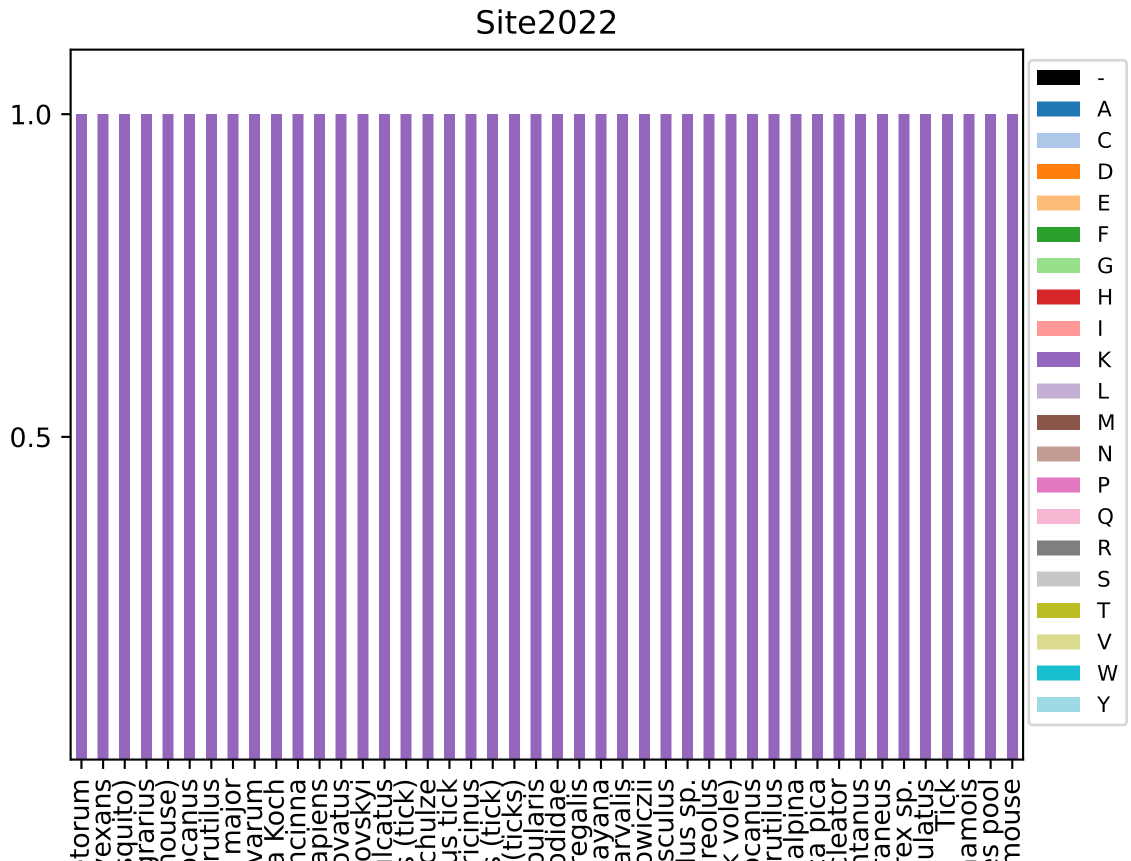
Site2022
1.0
0.5
-
A
C
D
E
F
G
H
I
K
L
M
N
P
Q
R
S
T
V
W
Y
Tick

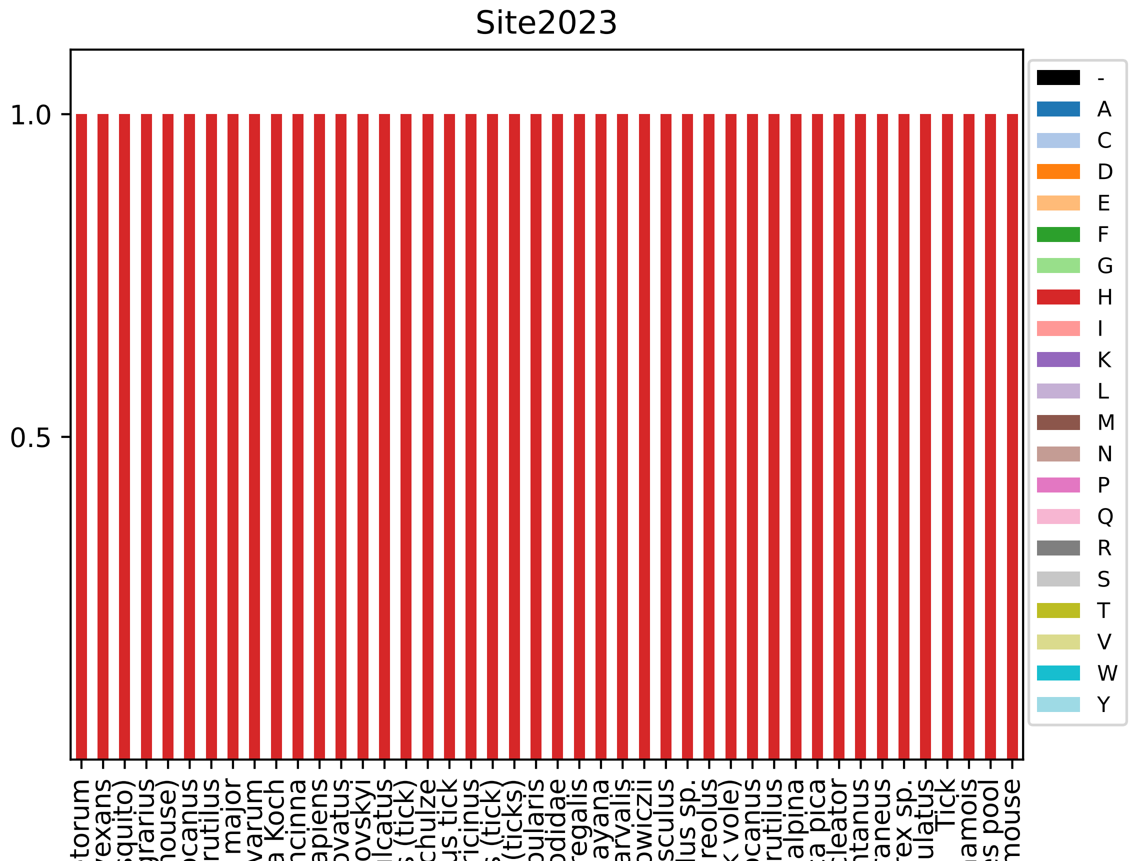

Site2023
1.0
0.5
-
A
C
D
E
F
G
H
I
K
L
M
N
P
Q
R
S
T
V
W
Y

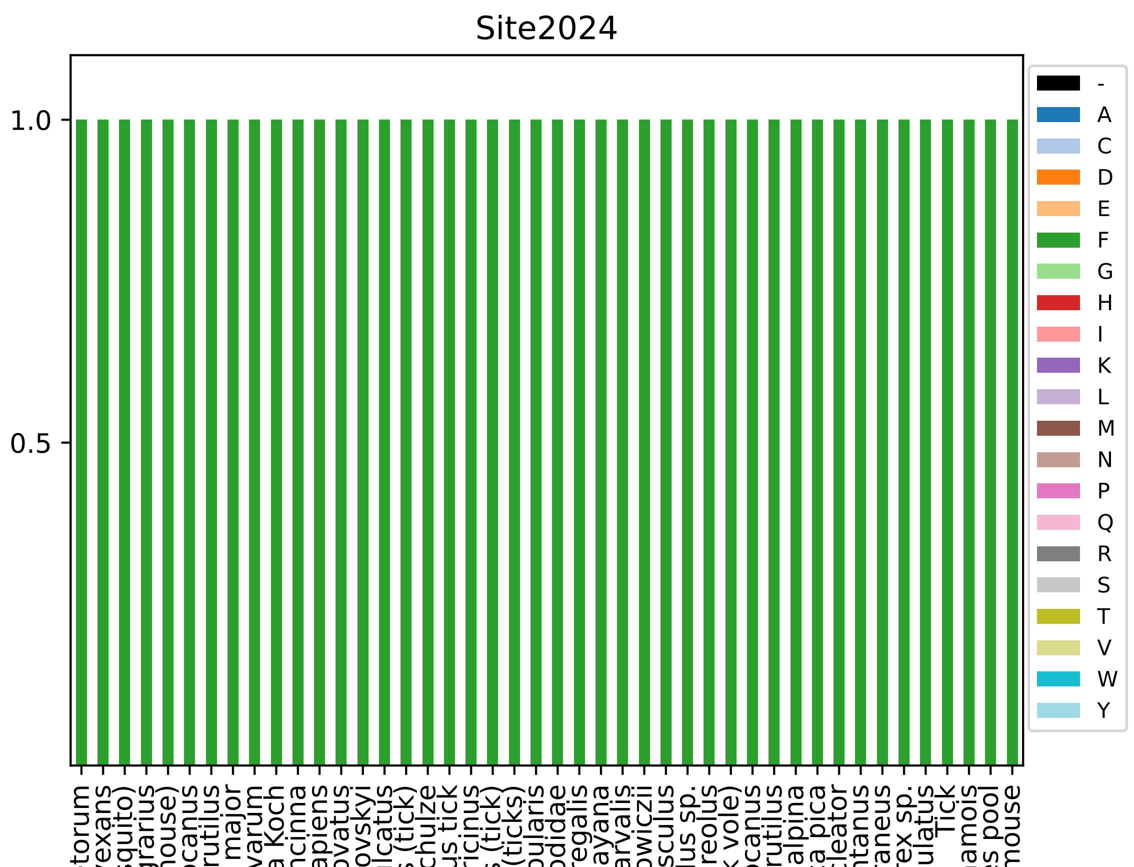
Site2024
1.0
0.5
-
A
C
D
E
F
G
H
I
K
L
M
N
P
Q
R
S
T
V
W
Y
Tick

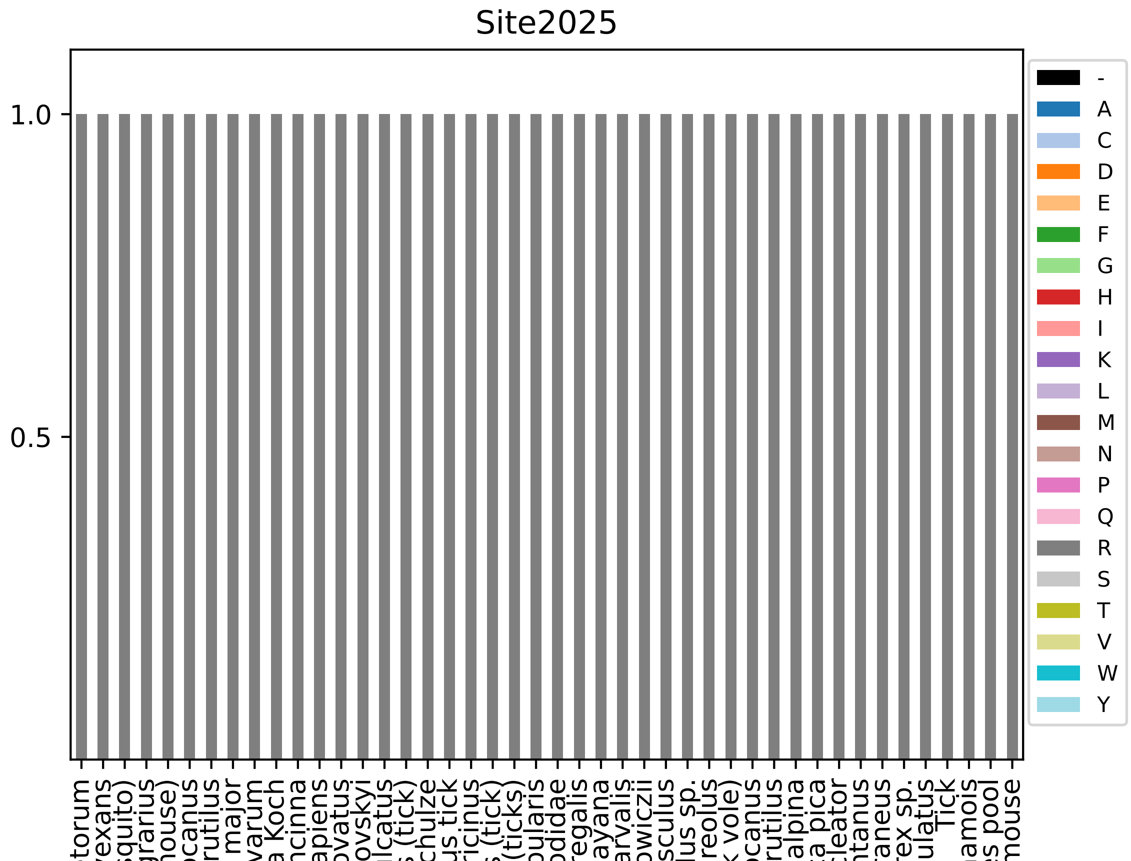
Site2025
1.0
0.5
-
A
C
D
E
F
G
H
I
K
L
M
N
P
Q
R
S
T
V
W
Y

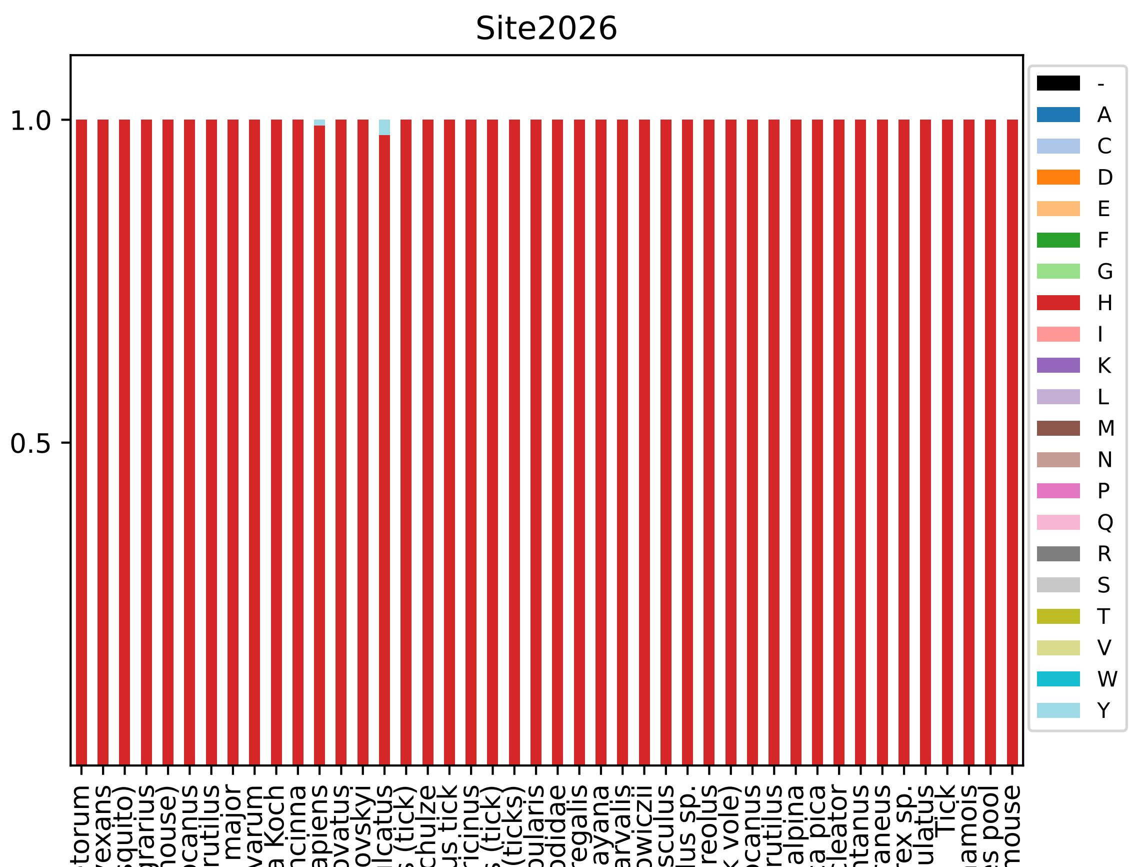
Site2026
1.0
0.5
-
A
C
D
E
F
G
H
I
K
L
M
N
P
Q
R
S
T
V
W
Y

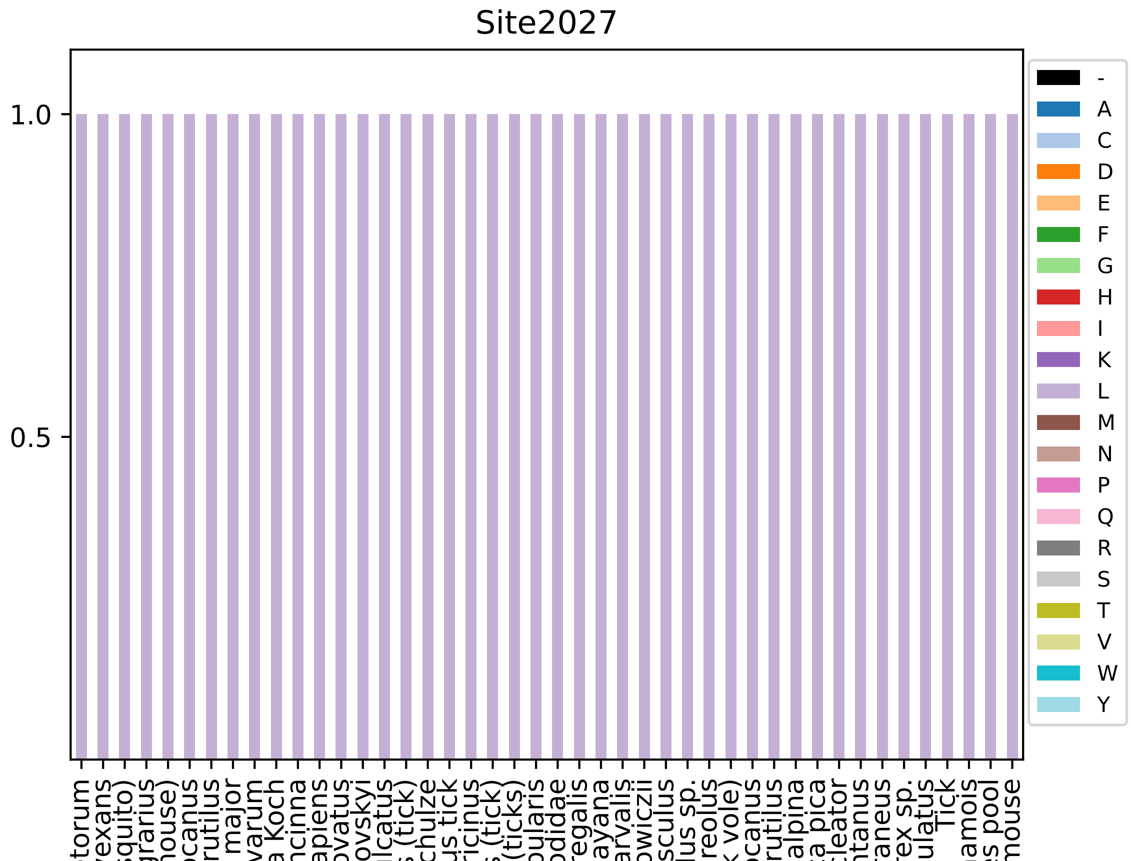
Site2027
1.0
0.5
-
A
C
D
E
F
G
H
I
K
L
M
N
P
Q
R
S
T
V
W
Y

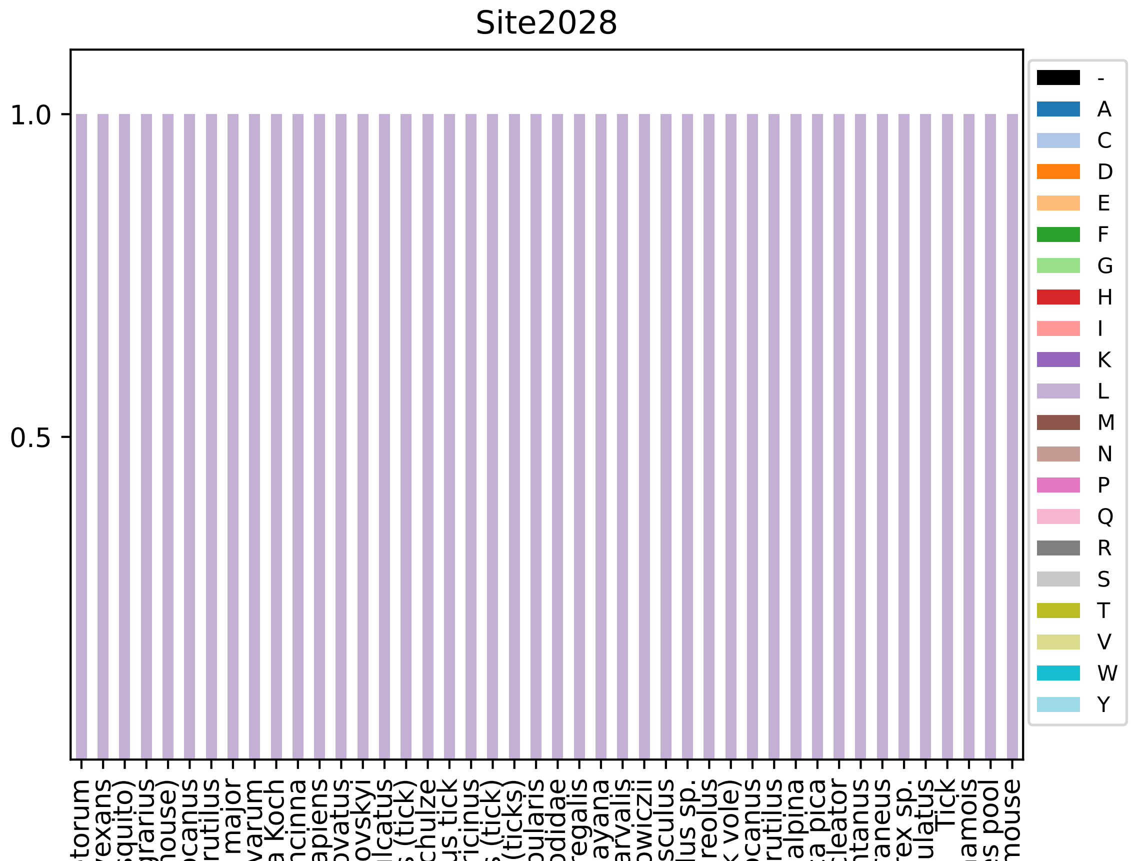
Site2028
1.0
0.5
-
A
C
D
E
F
G
H
I
K
L
M
N
P
Q
R
S
T
V
W
Y

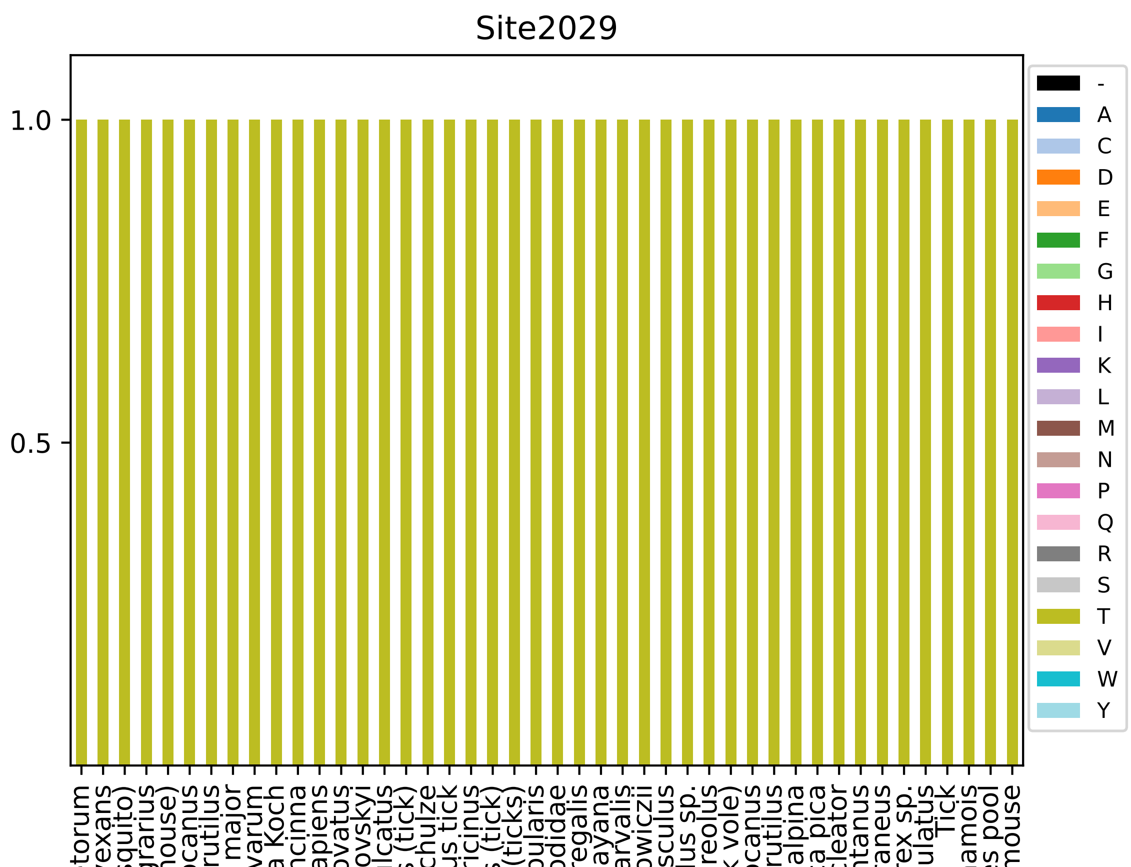

Site2029
1.0
0.5
-
A
C
D
E
F
G
H
I
K
L
M
N
P
Q
R
S
T
V
W
Y

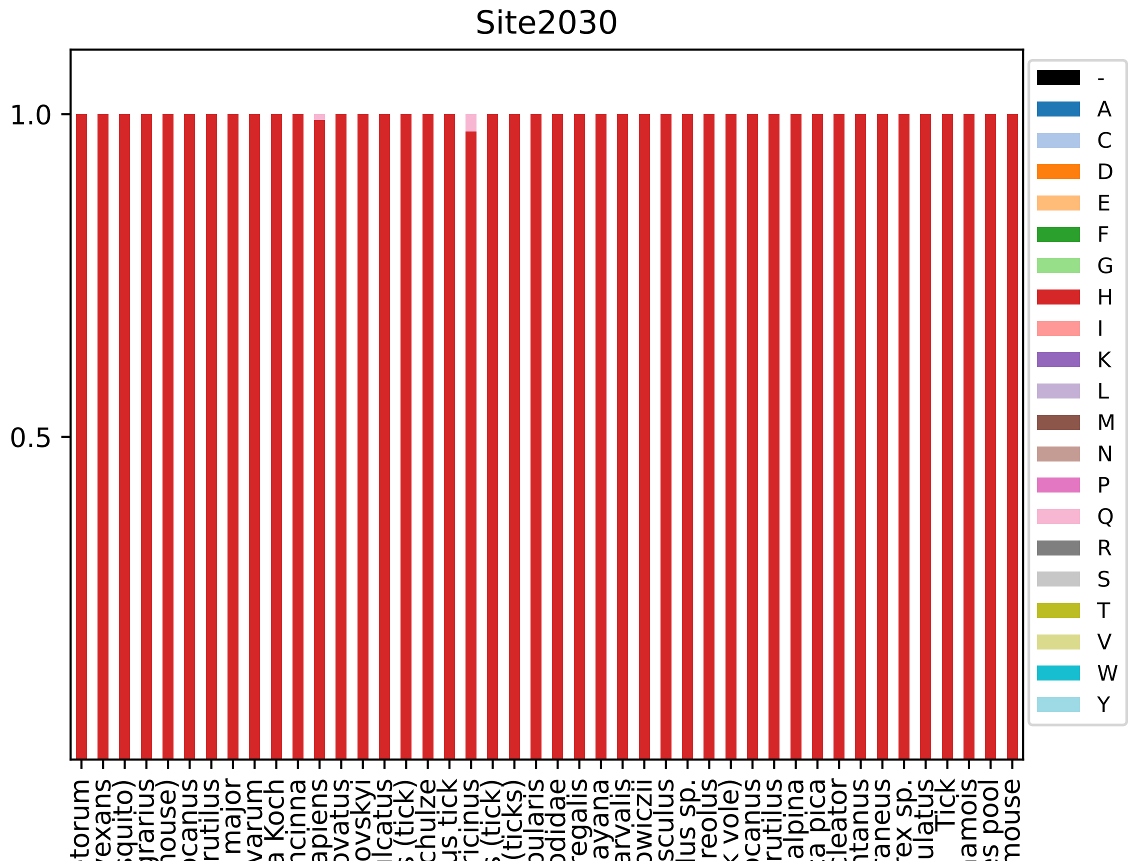
Site2030
1.0
0.5
-
A
C
D
E
F
G
H
I
K
L
M
N
P
Q
R
S
T
V
W
Y

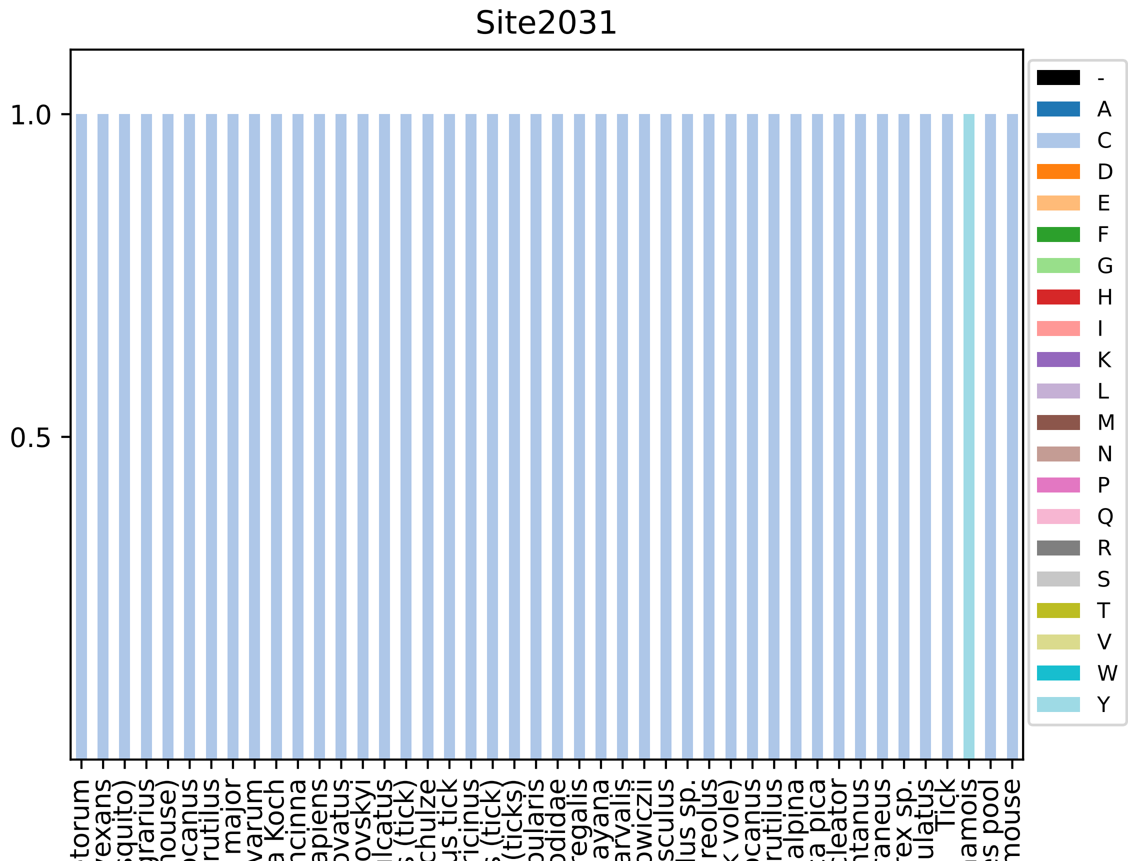

Site2031
1.0
0.5
-
A
C
D
E
F
G
H
I
K
L
M
N
P
Q
R
S
T
V
W
Y

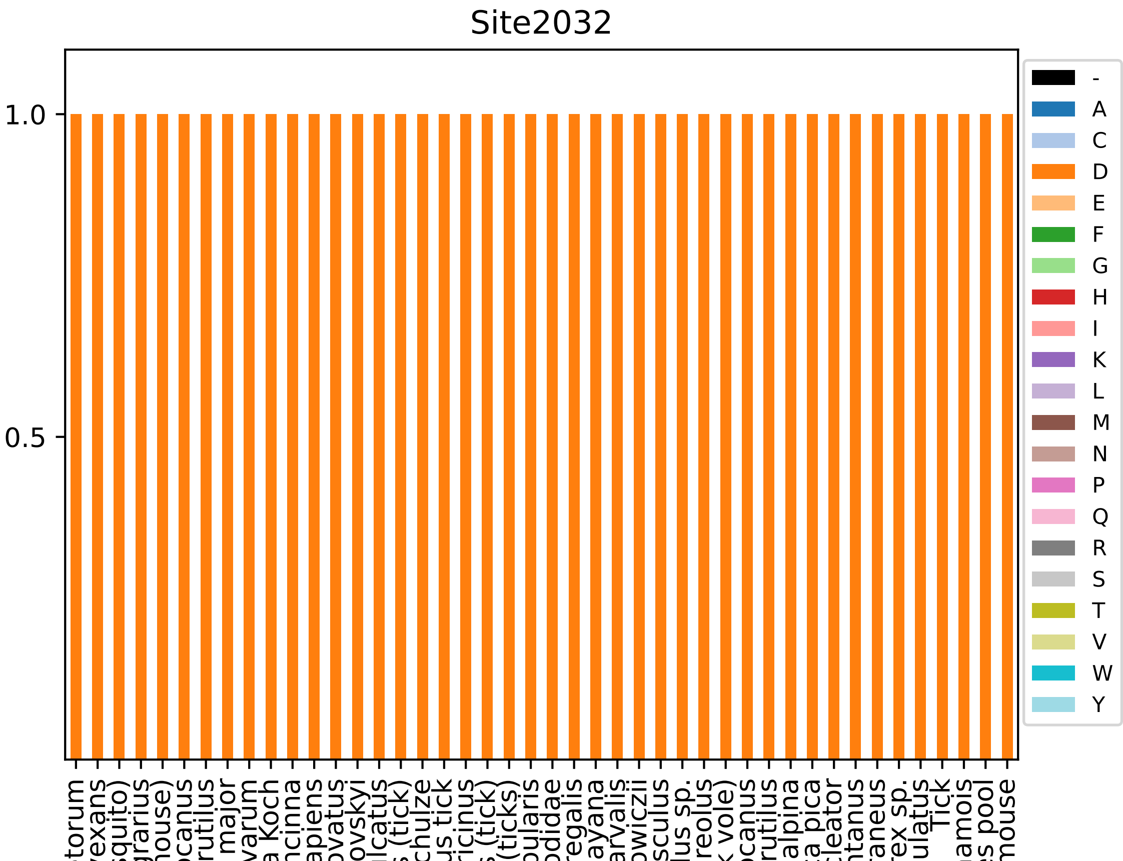
Site2032
1.0
0.5
-
A
C
D
E
F
G
H
I
K
L
M
N
P
Q
R
S
T
V
W
Y

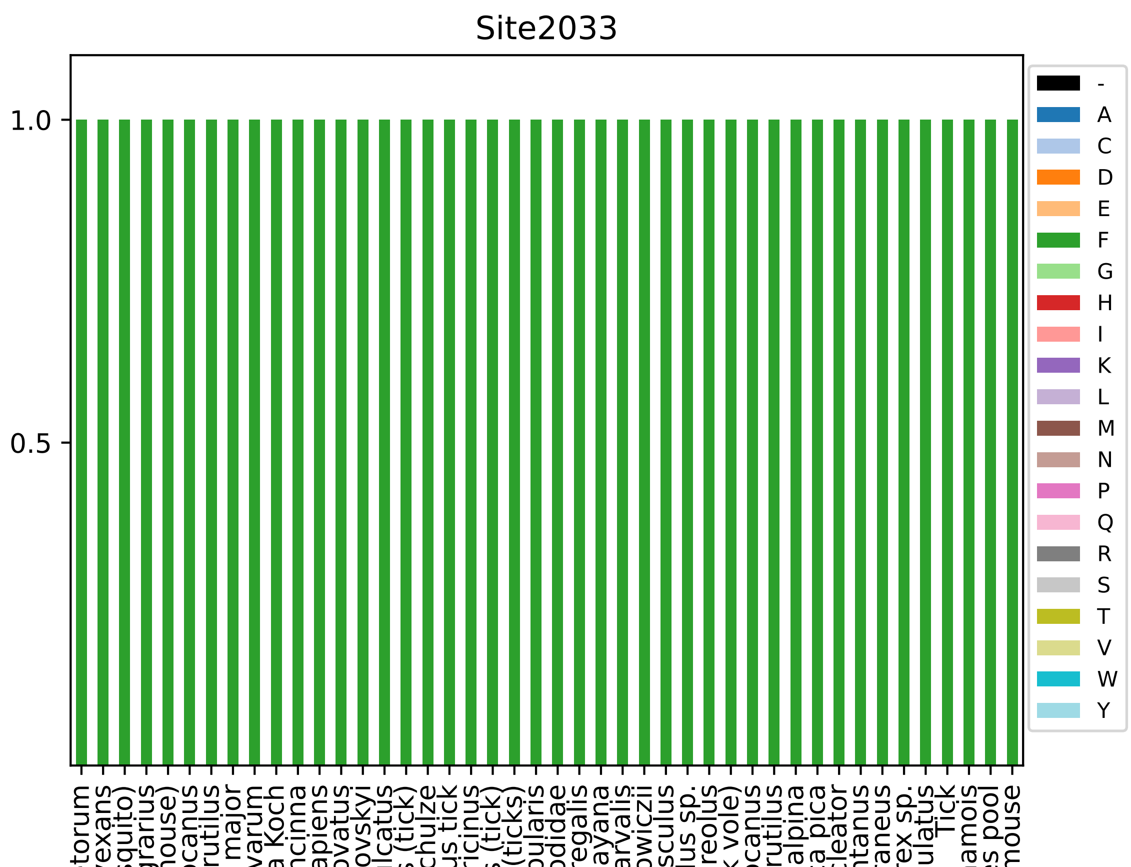

Site2033
1.0
0.5
-
A
C
D
E
F
G
H
I
K
L
M
N
P
Q
R
S
T
V
W
Y

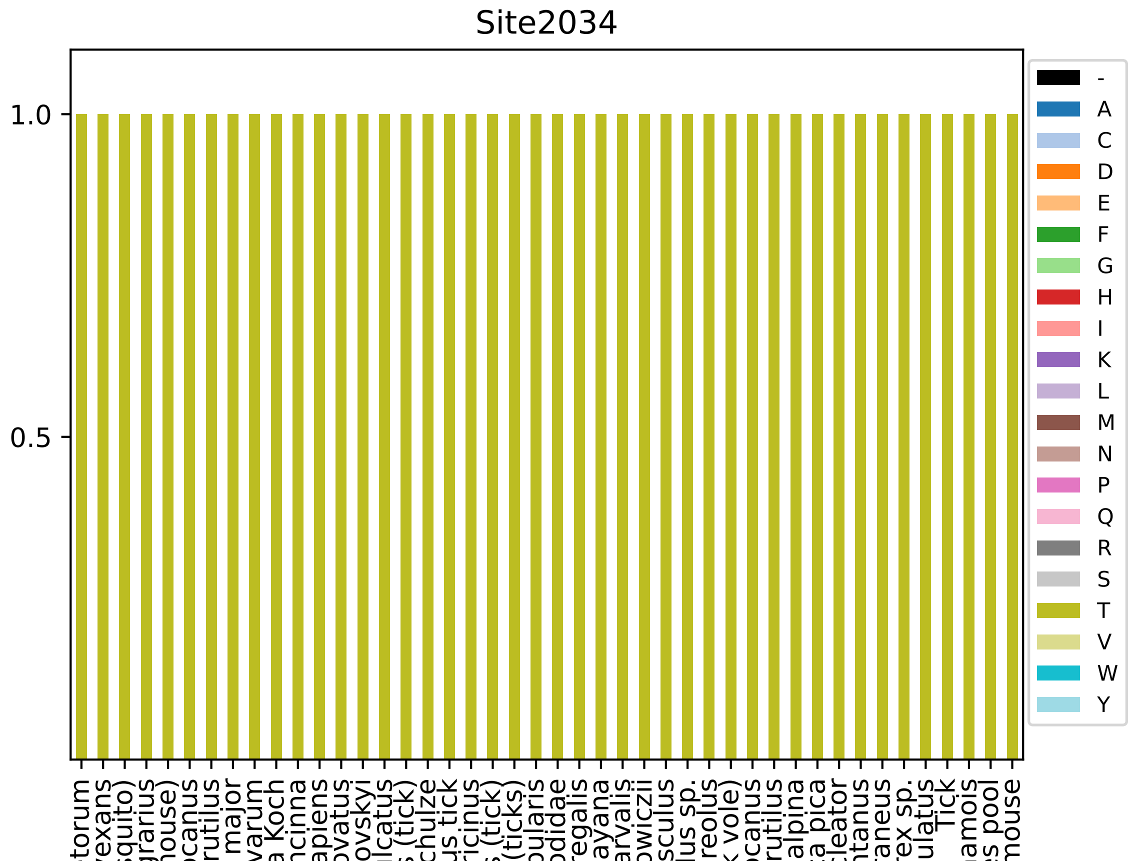
Site2034
1.0
0.5
-
A
C
D
E
F
G
H
I
K
L
M
N
P
Q
R
S
T
V
W
Y

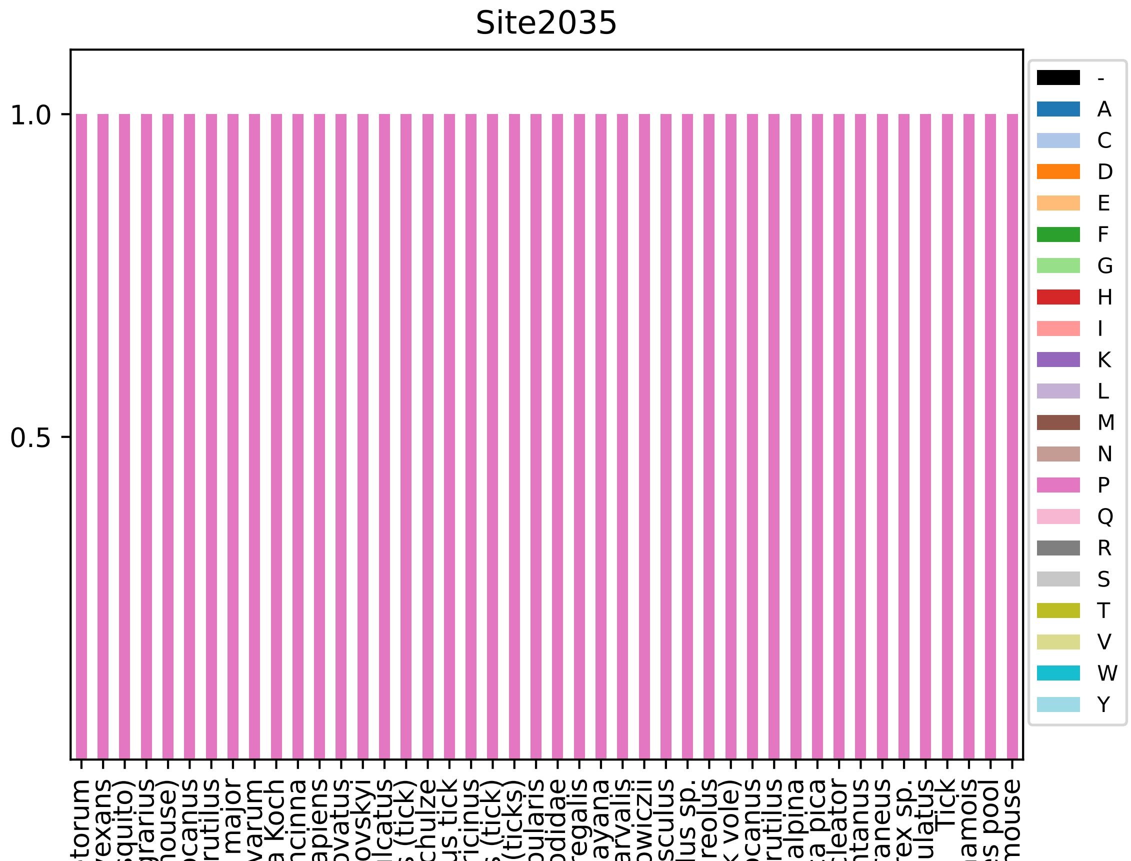
Site2035
1.0
0.5
-
A
C
D
E
F
G
H
I
K
L
M
N
P
Q
R
S
T
V
W
Y

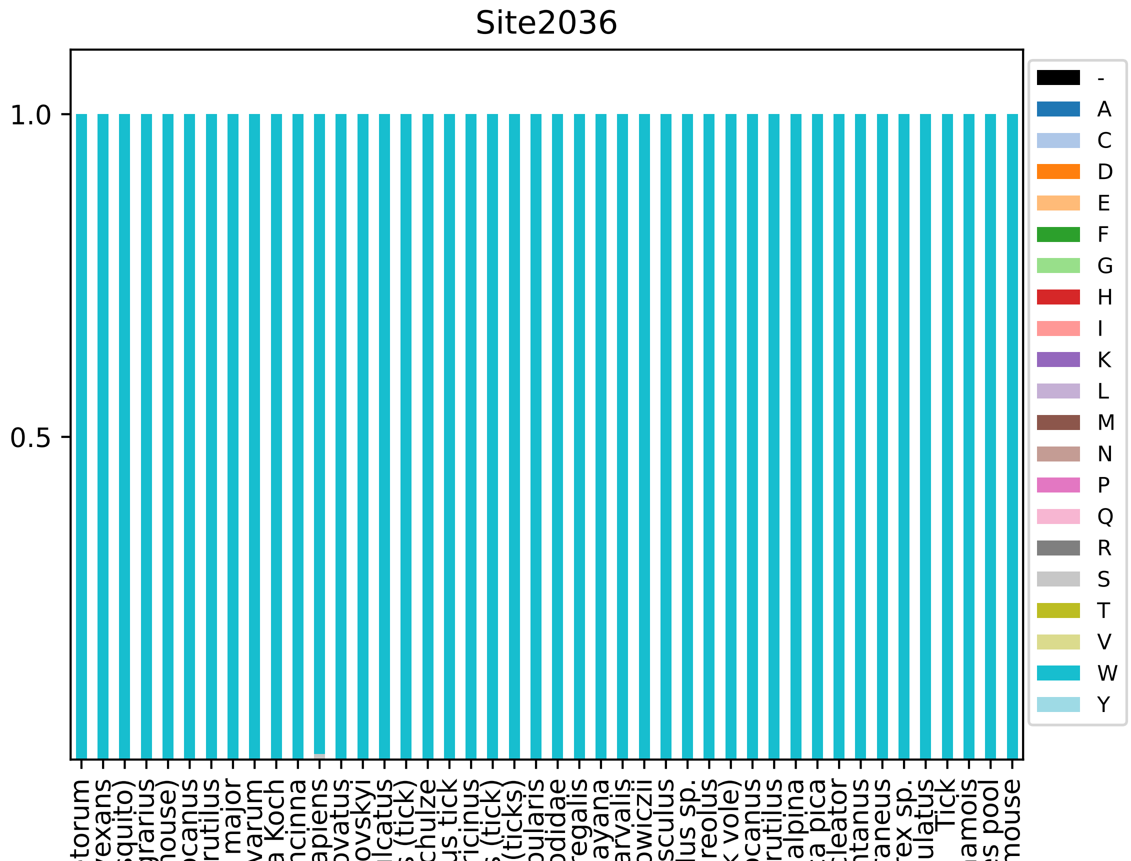
Site2036
1.0
0.5
-
A
C
D
E
F
G
H
I
K
L
M
N
P
Q
R
S
T
V
W
Y

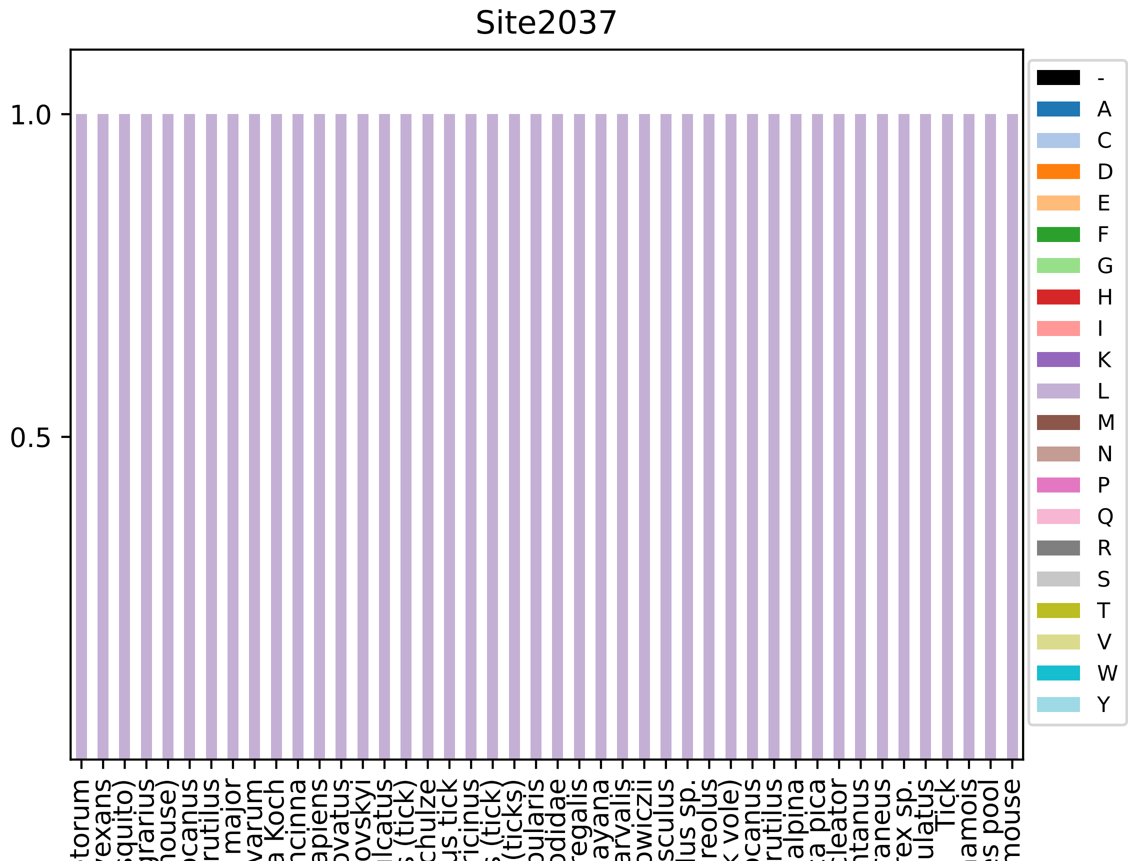

Site2037
1.0
0.5
-
A
C
D
E
F
G
H
I
K
L
M
N
P
Q
R
S
T
V
W
Y

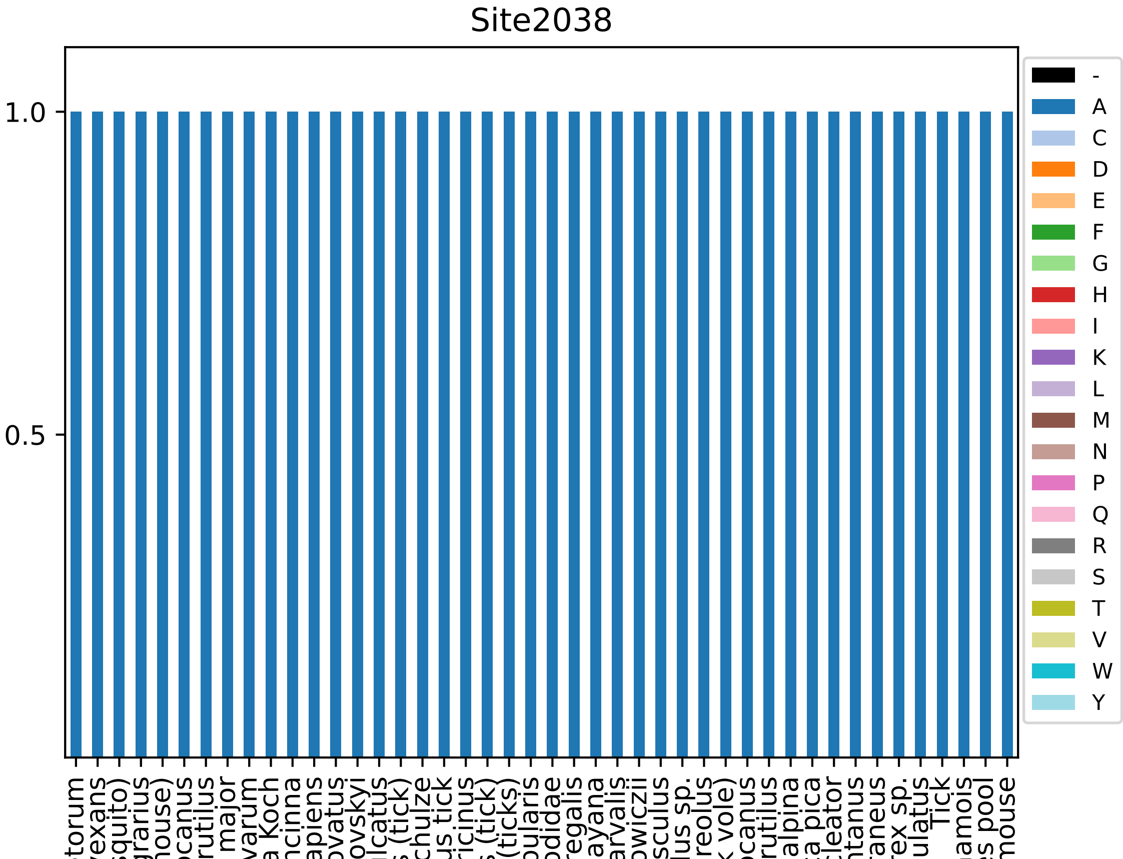
Site2038
1.0
0.5
-
A
C
D
E
F
G
H
I
K
L
M
N
P
Q
R
S
T
V
W
Y
Tick

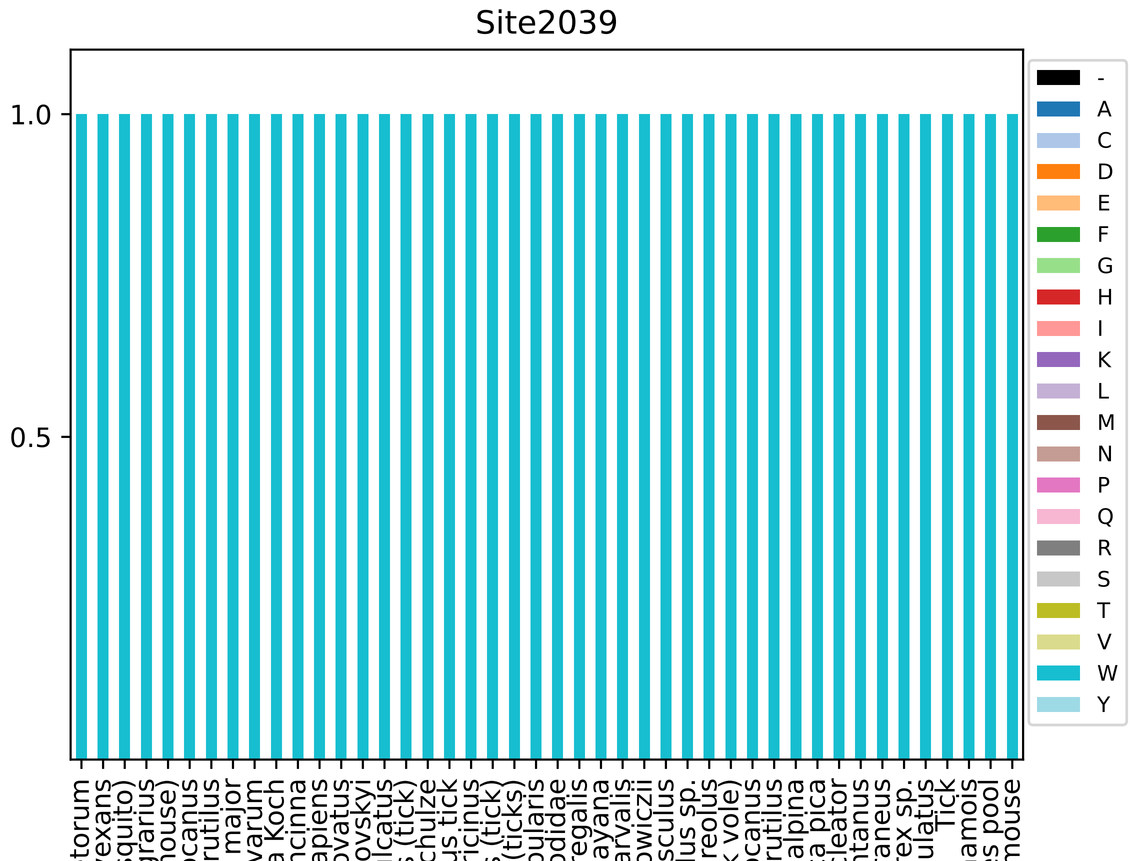
Site2039
1.0
0.5
-
A
C
D
E
F
G
H
I
K
L
M
N
P
Q
R
S
T
V
W
Y

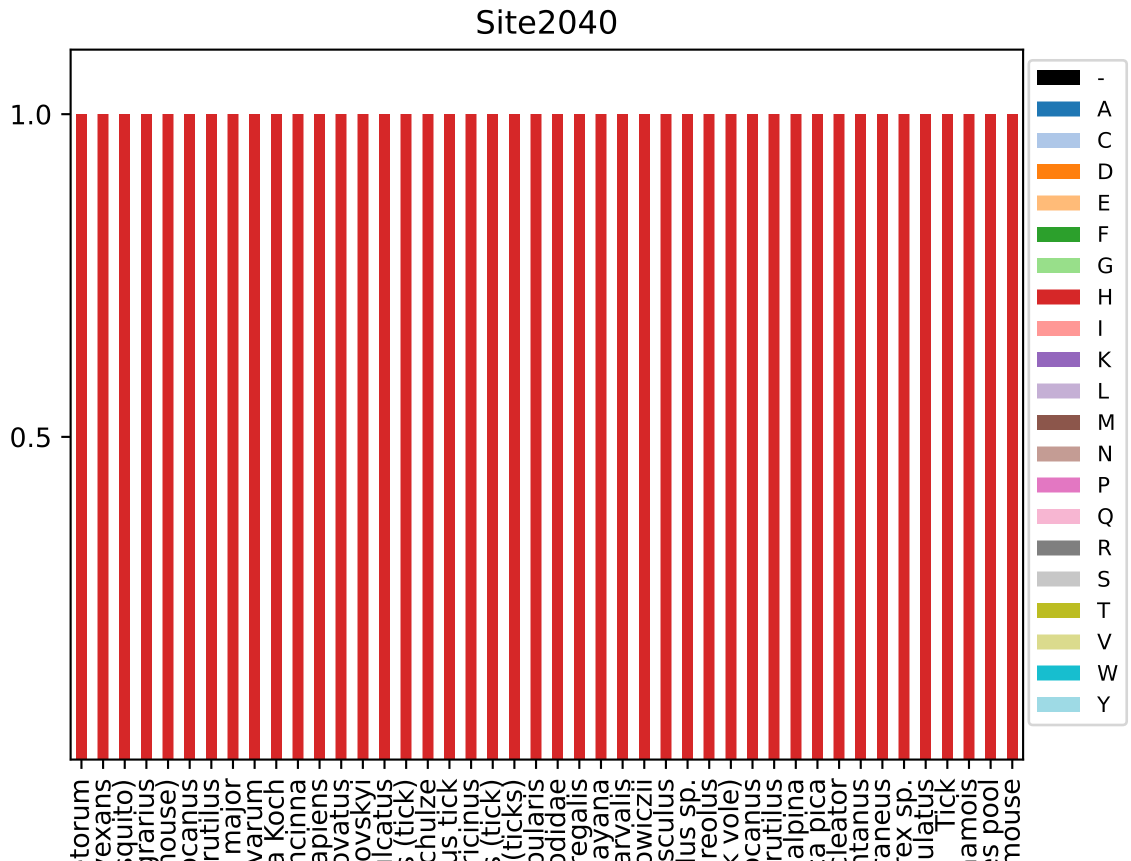
Site2040
1.0
0.5
-
A
C
D
E
F
G
H
I
K
L
M
N
P
Q
R
S
T
V
W
Y
Tick

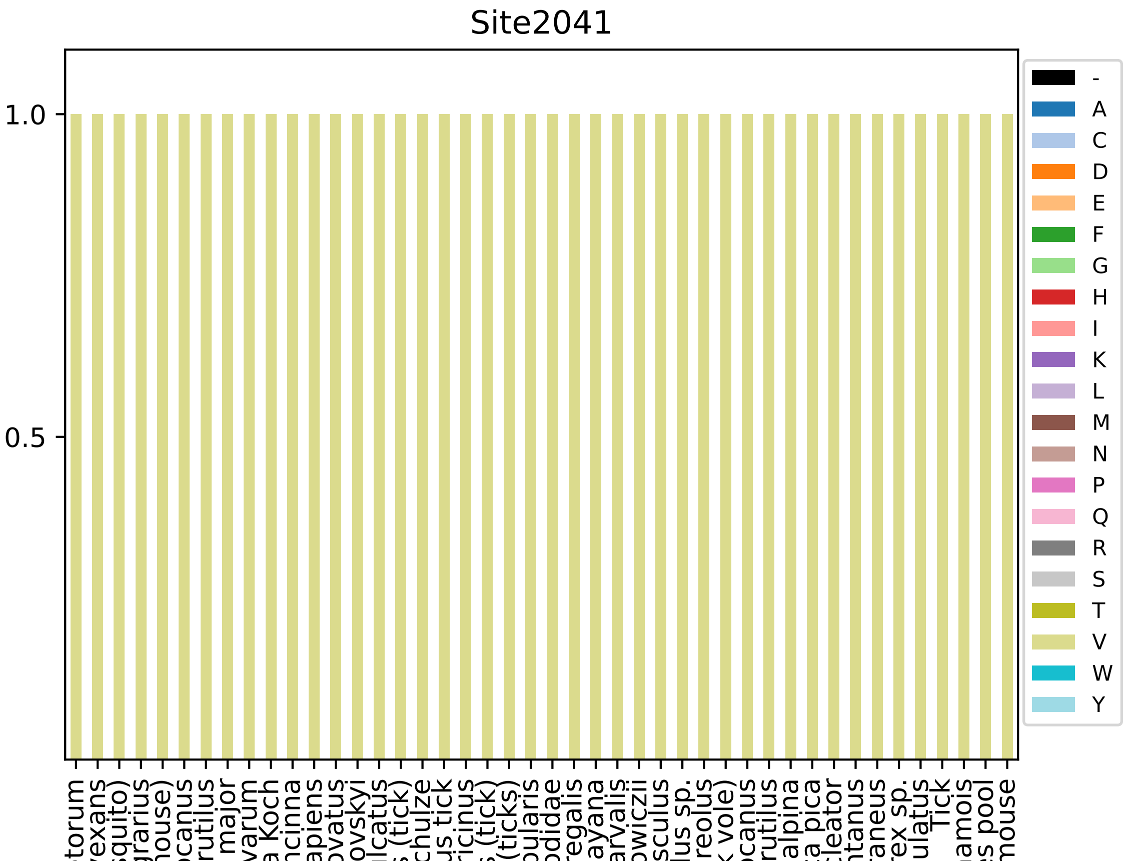
Site2041
1.0
0.5
-
A
C
D
E
F
G
H
I
K
L
M
N
P
Q
R
S
T
V
W
Y

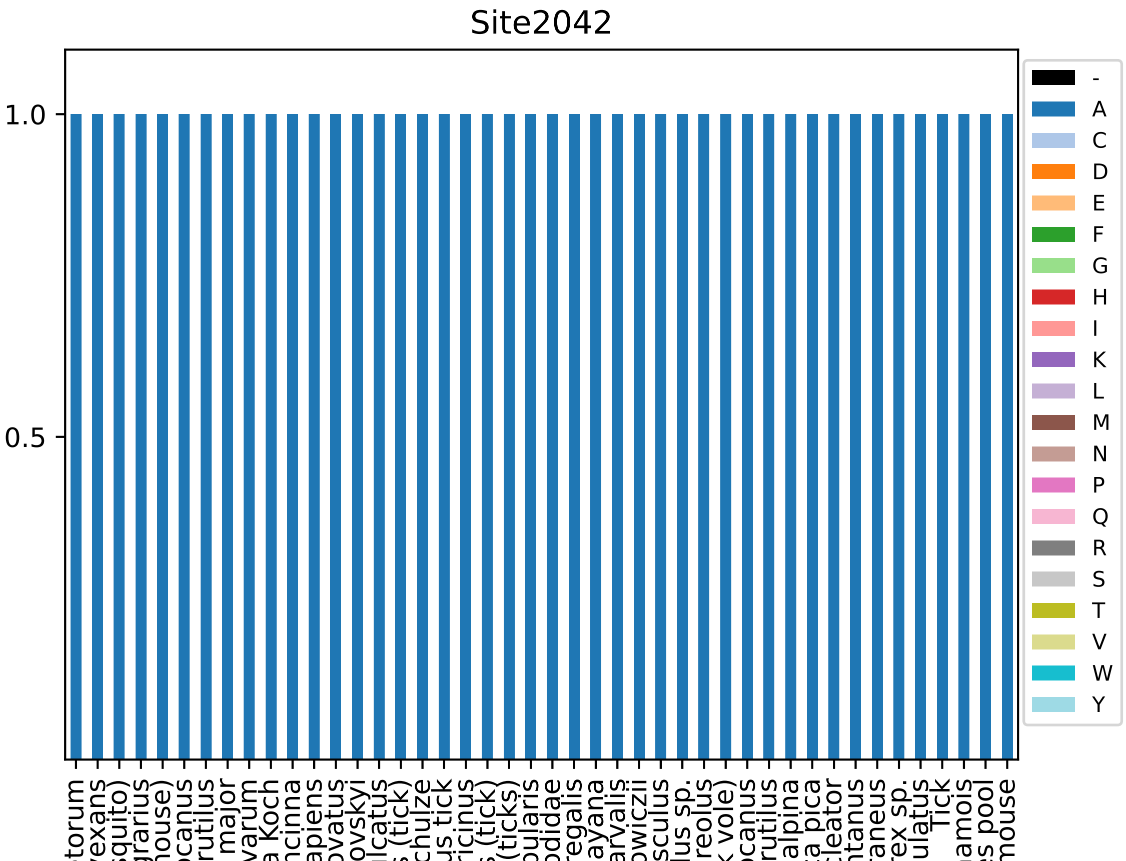

Site2042
1.0
0.5
-
A
C
D
E
F
G
H
I
K
L
M
N
P
Q
R
S
T
V
W
Y

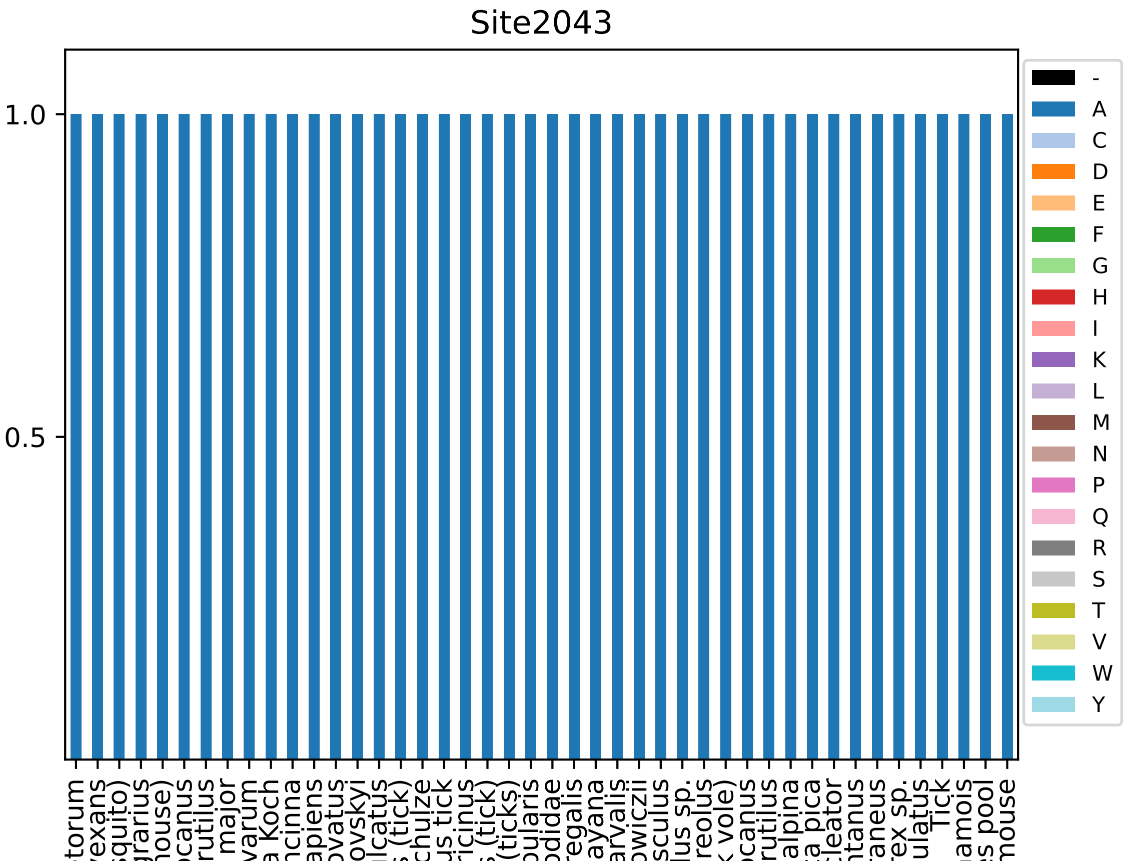
Site2043
1.0
0.5
-
A
C
D
E
F
G
H
I
K
L
M
N
P
Q
R
S
T
V
W
Y
Tick

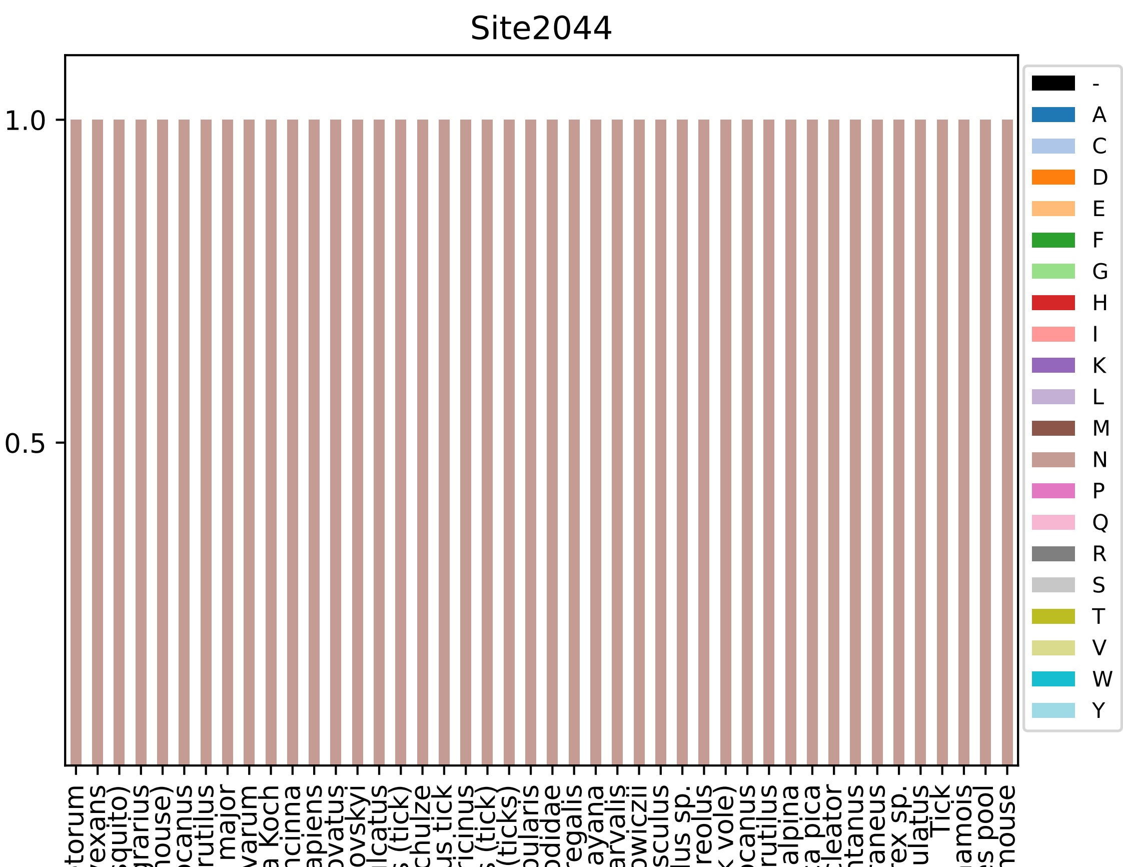
Site2044
1.0
0.5
-
A
C
D
E
F
G
H
I
K
L
M
N
P
Q
R
S
T
V
W
Y

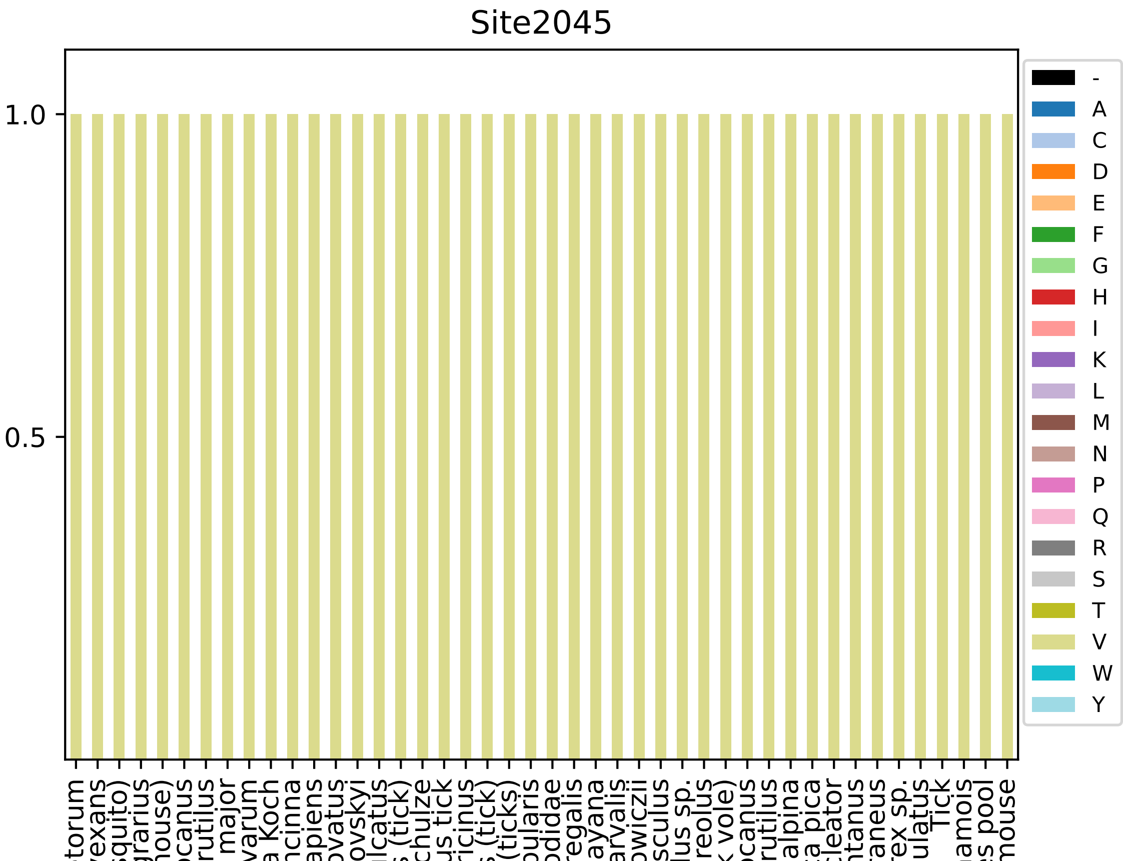
Site2045
1.0
0.5
-
A
C
D
E
F
G
H
I
K
L
M
N
P
Q
R
S
T
V
W
Y

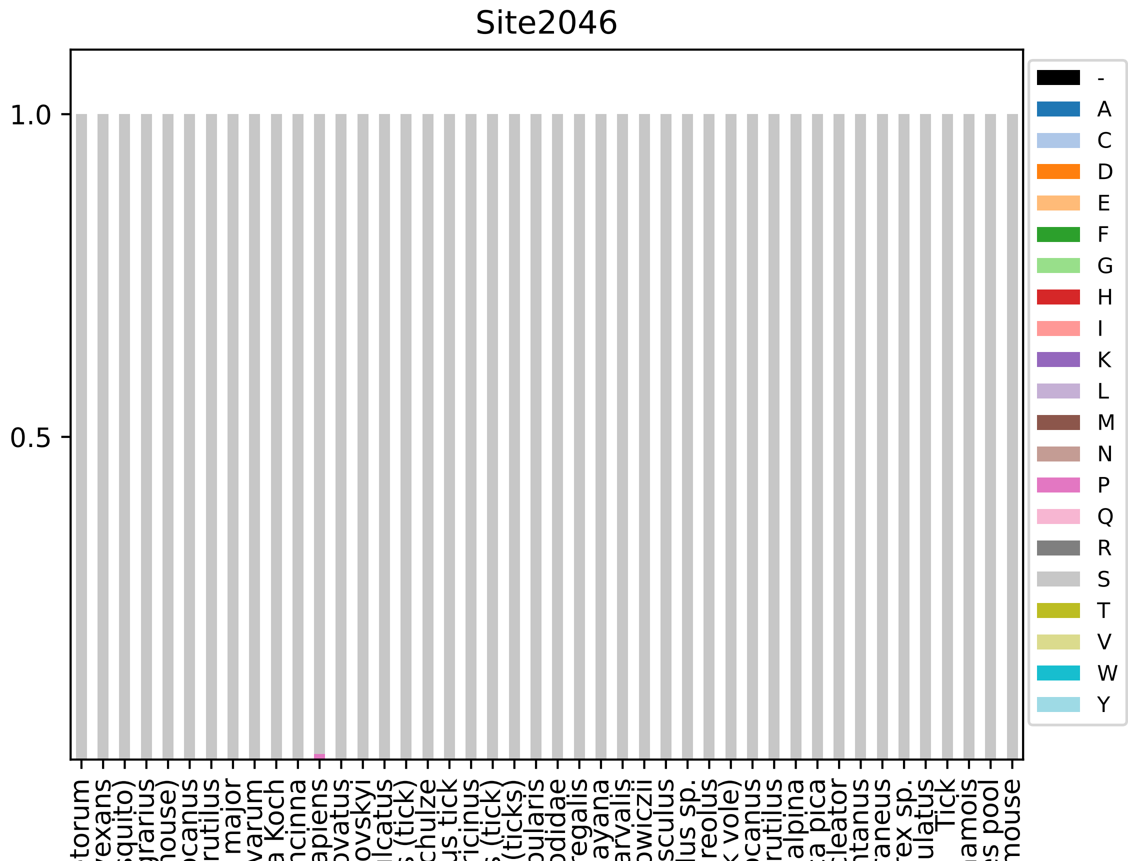

Site2046
1.0
0.5
-
A
C
D
E
F
G
H
I
K
L
M
N
P
Q
R
S
T
V
W
Y

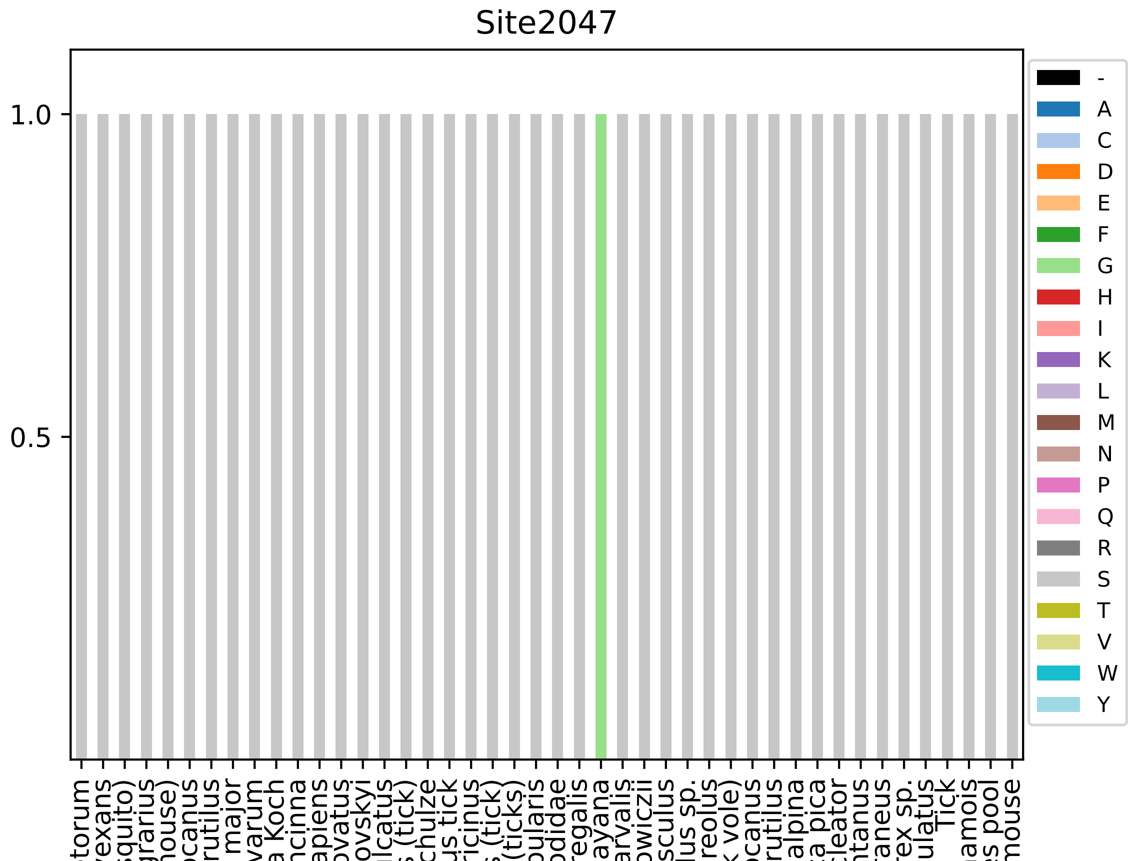
Site2047
1.0
0.5
-
A
C
D
E
F
G
H
I
K
L
M
N
P
Q
R
S
T
V
W
Y

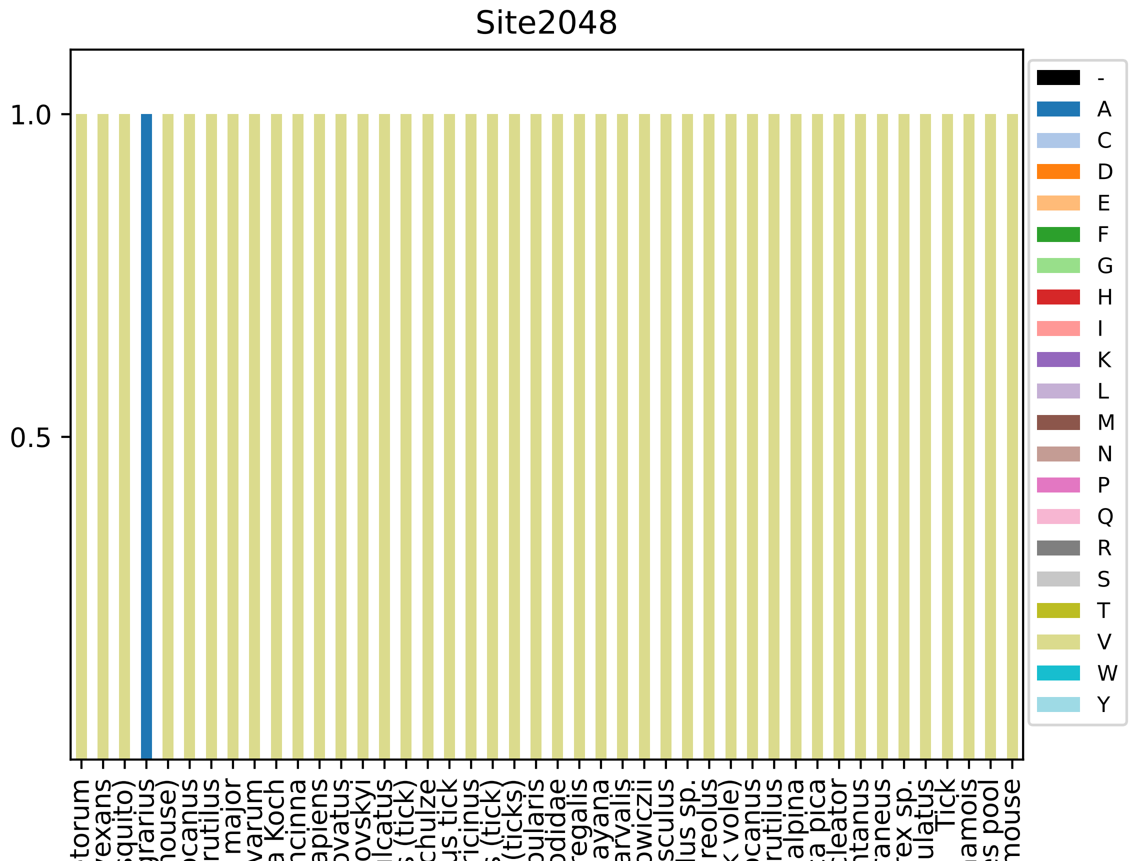
Site2048
1.0
0.5
-
A
C
D
E
F
G
H
I
K
L
M
N
P
Q
R
S
T
V
W
Y

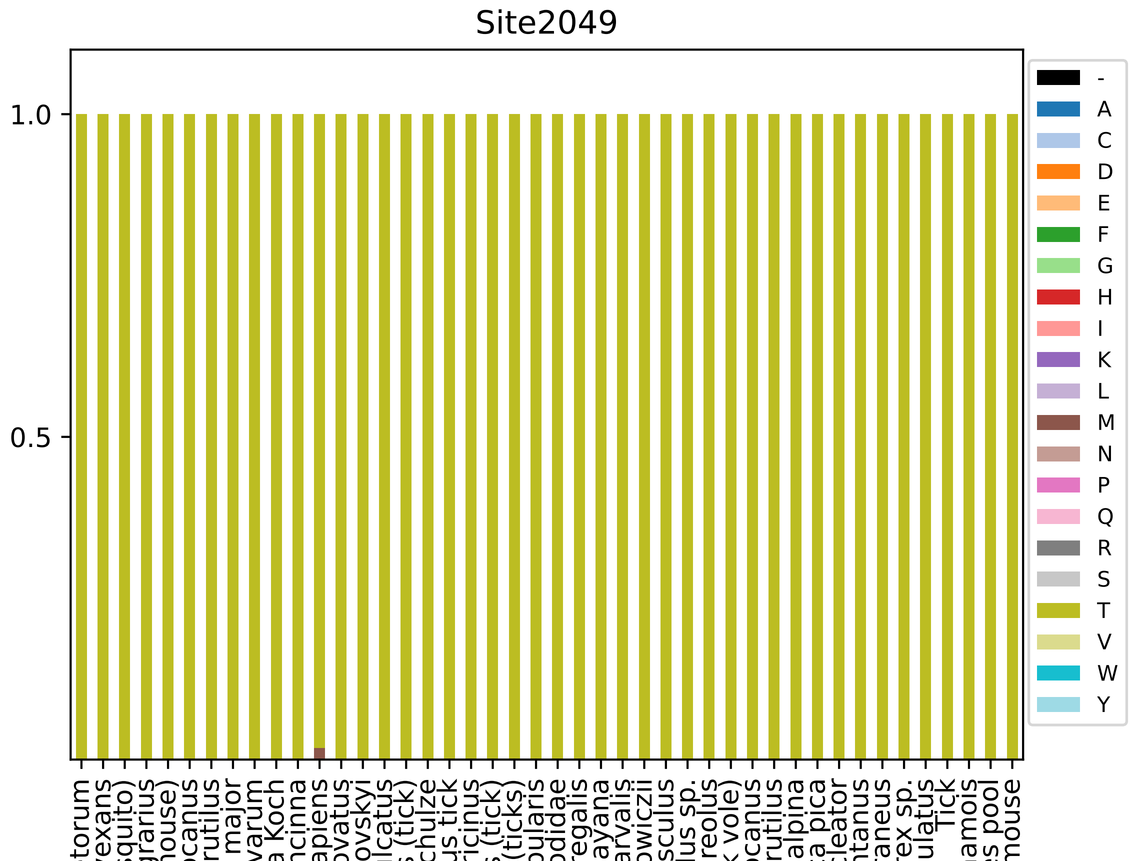
Site2049
1.0
0.5
-
A
C
D
E
F
G
H
I
K
L
M
N
P
Q
R
S
T
V
W
Y

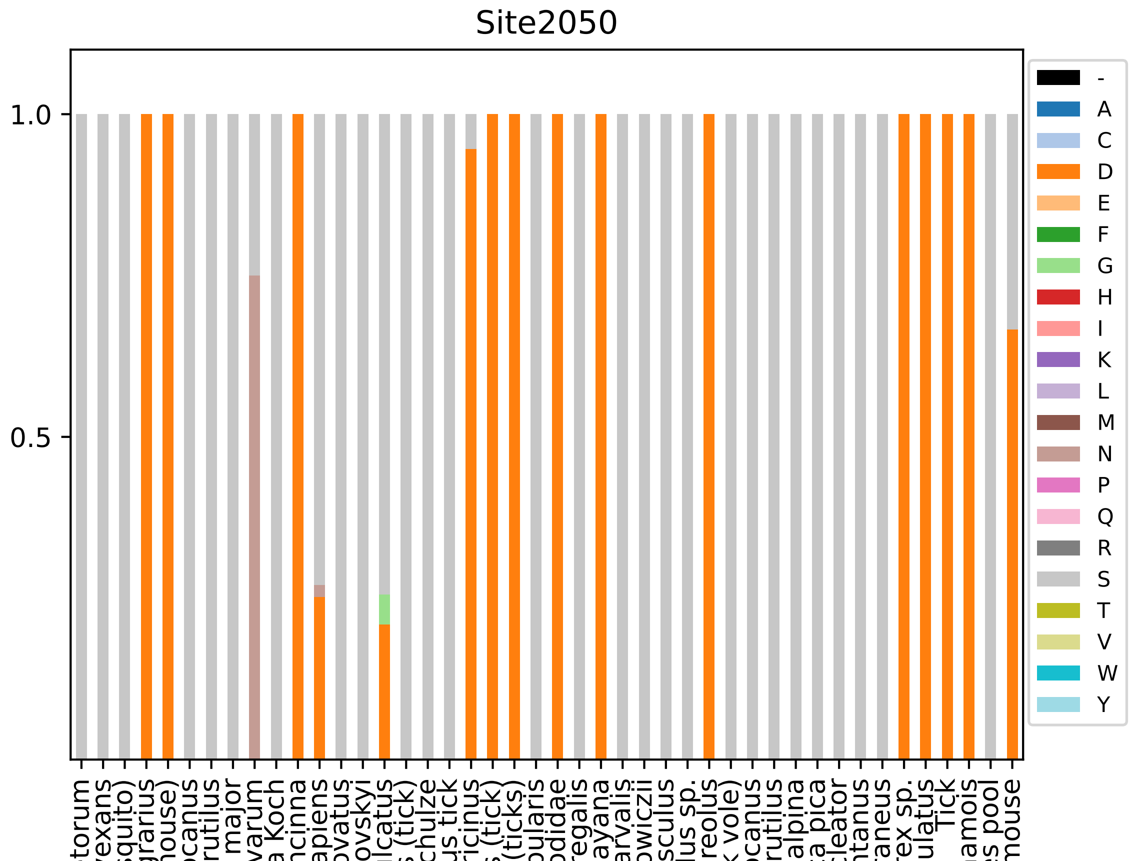
Site2050
1.0
0.5
-
A
C
D
E
F
G
H
I
K
L
M
N
P
Q
R
S
T
V
W
Y

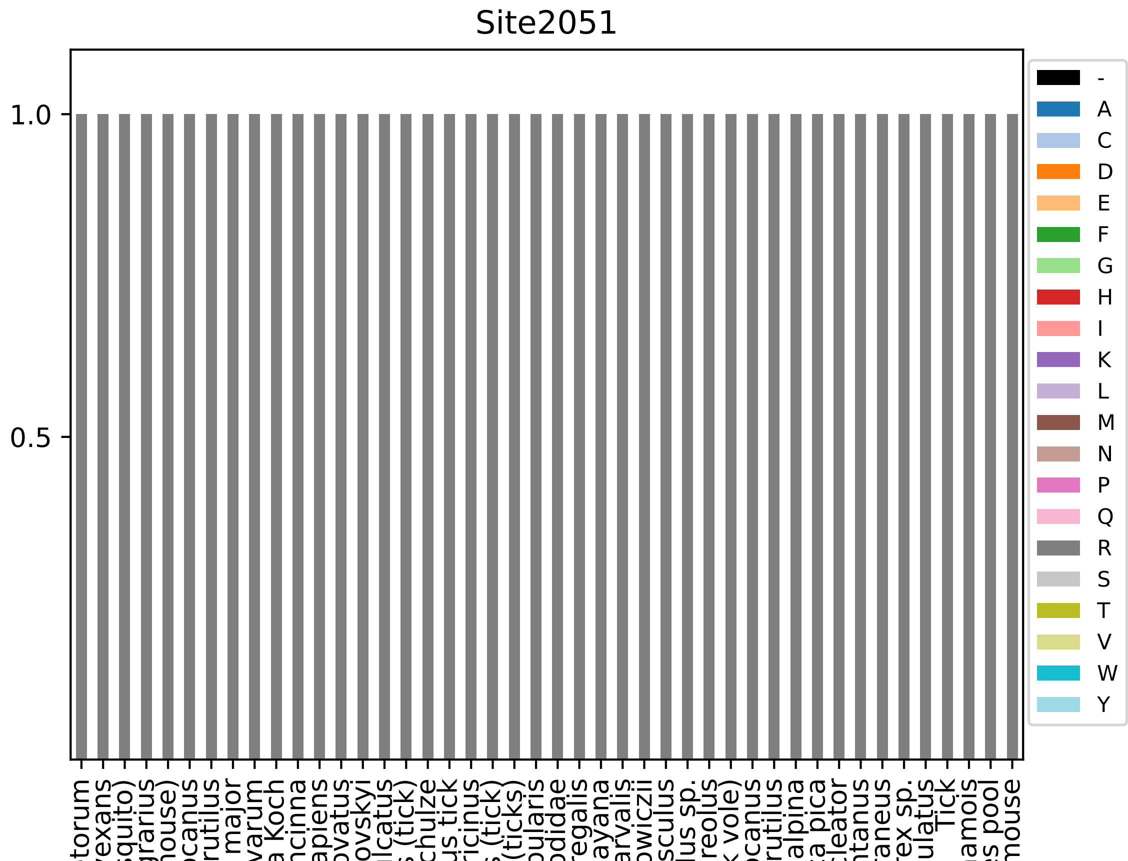

Site2051
1.0
0.5
-
A
C
D
E
F
G
H
I
K
L
M
N
P
Q
R
S
T
V
W
Y

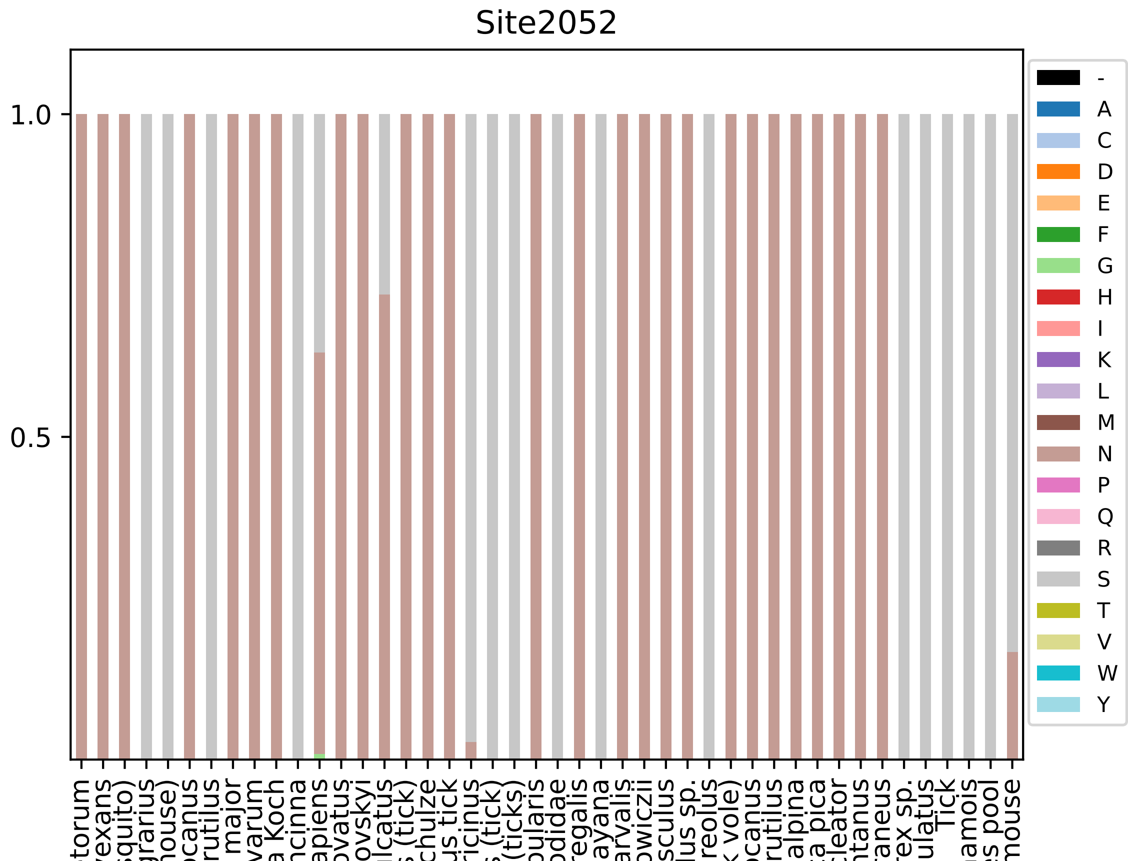

Site2052
1.0
0.5
-
A
C
D
E
F
G
H
I
K
L
M
N
P
Q
R
S
T
V
W
Y
torum
vexans
squito)
grarius
nouse)
ocanus
rutilus
major
varum
a Koch
ncinna
apiens
ovatus
ovskyi
ulcatus
s (tick)
chulze
us tick
ricinus
s (tick)
(ticks)
pularis
podidae
regalis
ayana
arvalis
owiczii
sculus
lus sp.
reolus
x vole)
ocanus
rutilus
alpina
a pica
cleator
ntanus
raneus
ex sp.
ulatus
Tick
amois
es pool
mouse

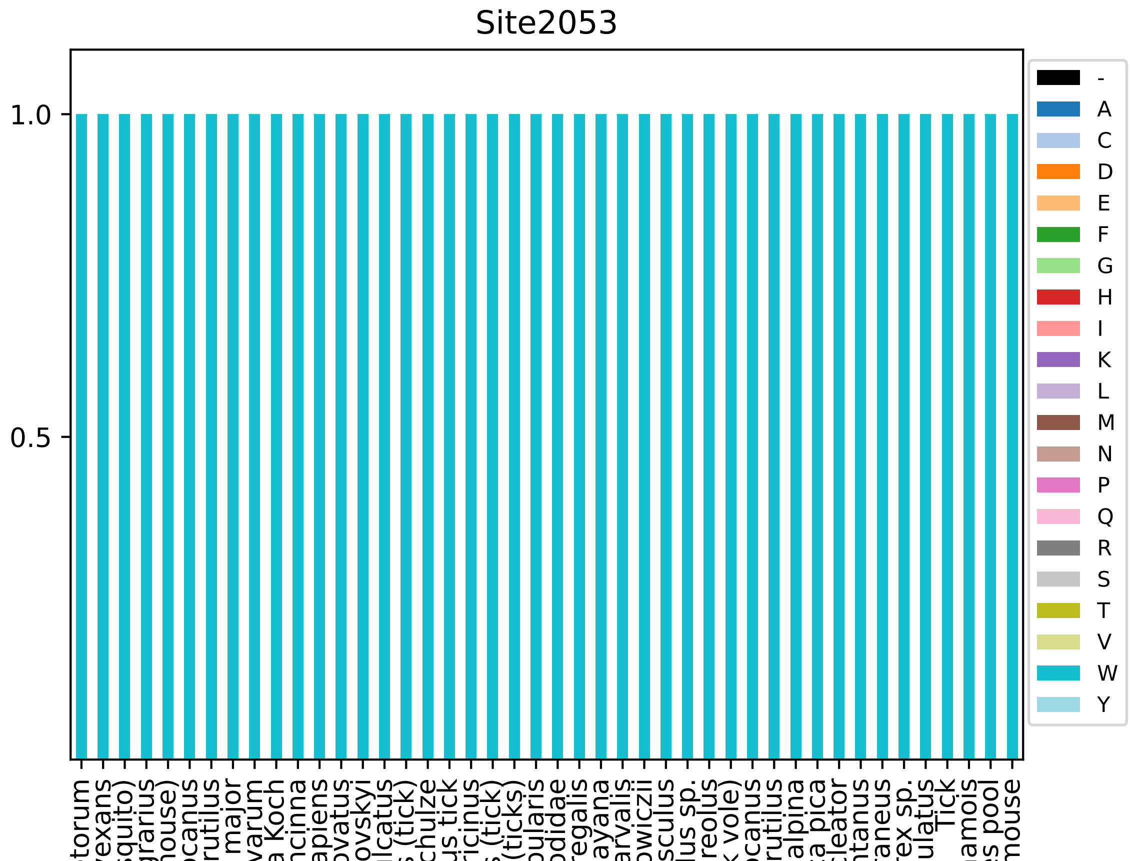

Site2053
1.0
0.5
-
A
C
D
E
F
G
H
I
K
L
M
N
P
Q
R
S
T
V
W
Y

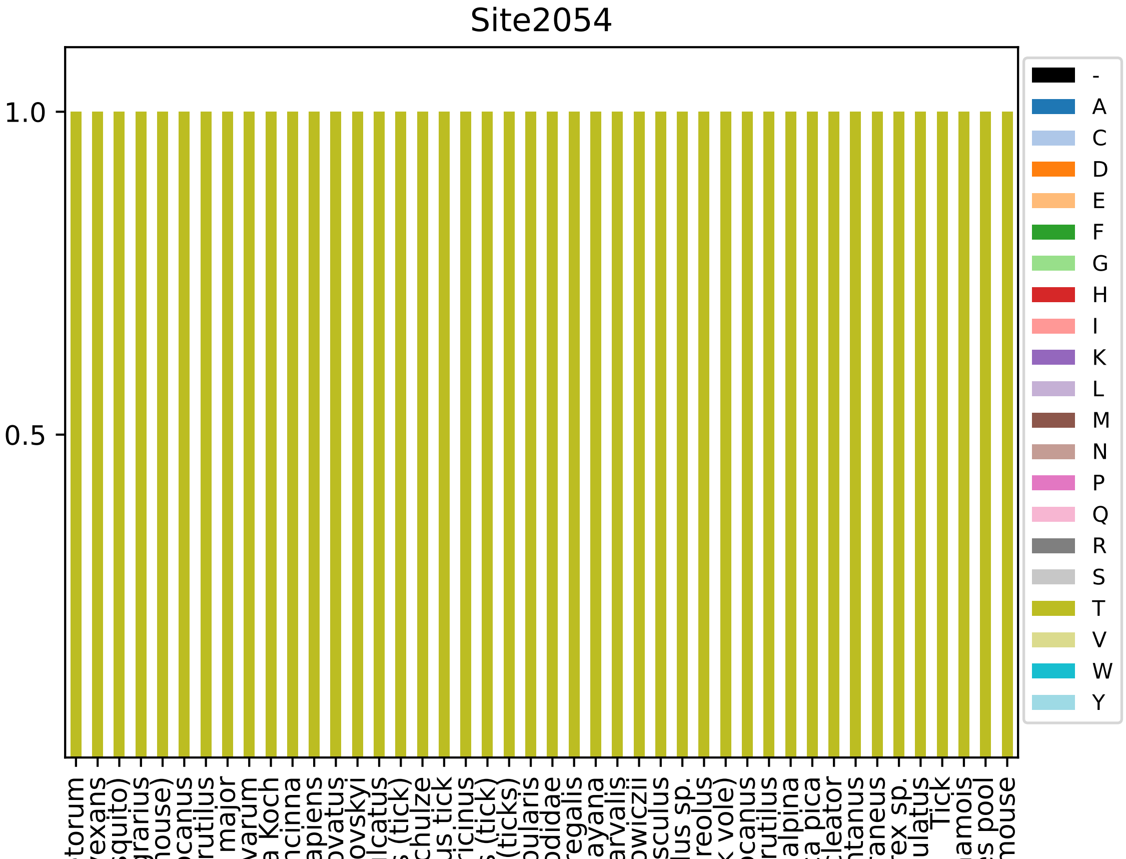
Site2054
1.0
0.5
-
A
C
D
E
F
G
H
I
K
L
M
N
P
Q
R
S
T
V
W
Y

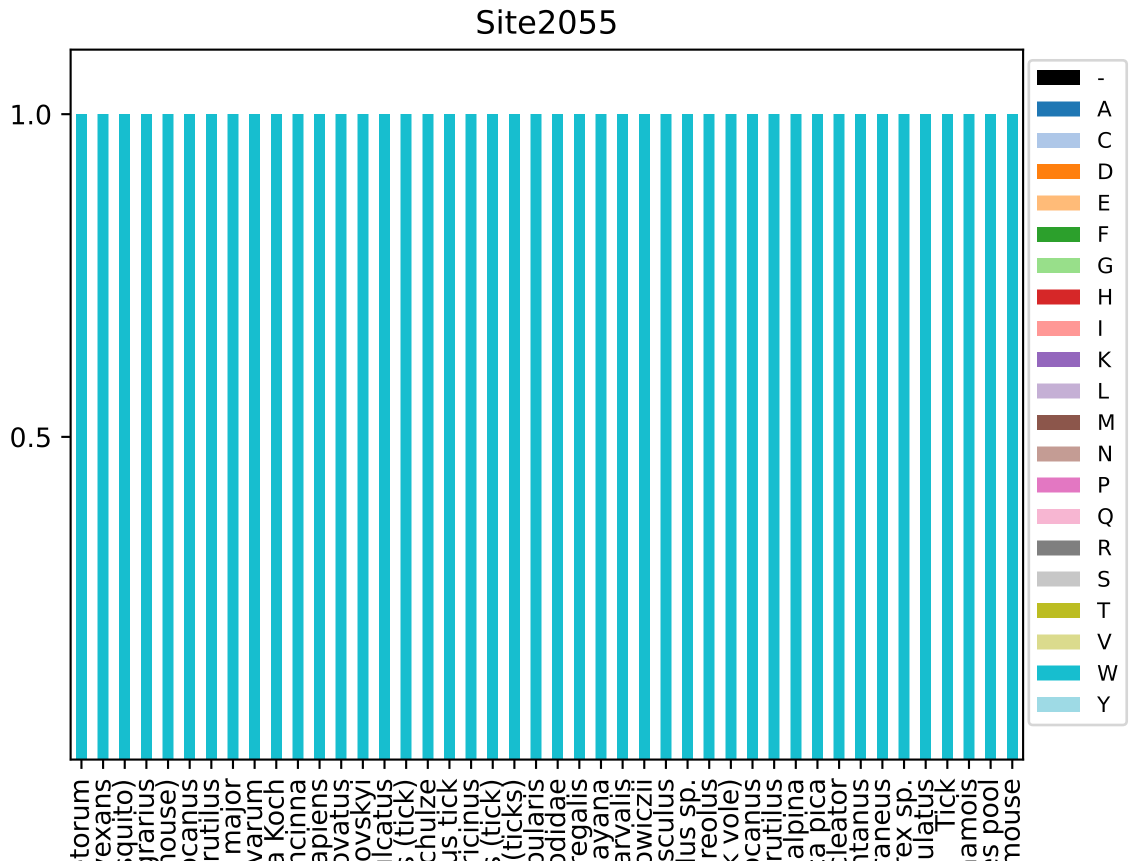
Site2055
1.0
0.5
-
A
C
D
E
F
G
H
I
K
L
M
N
P
Q
R
S
T
V
W
Y

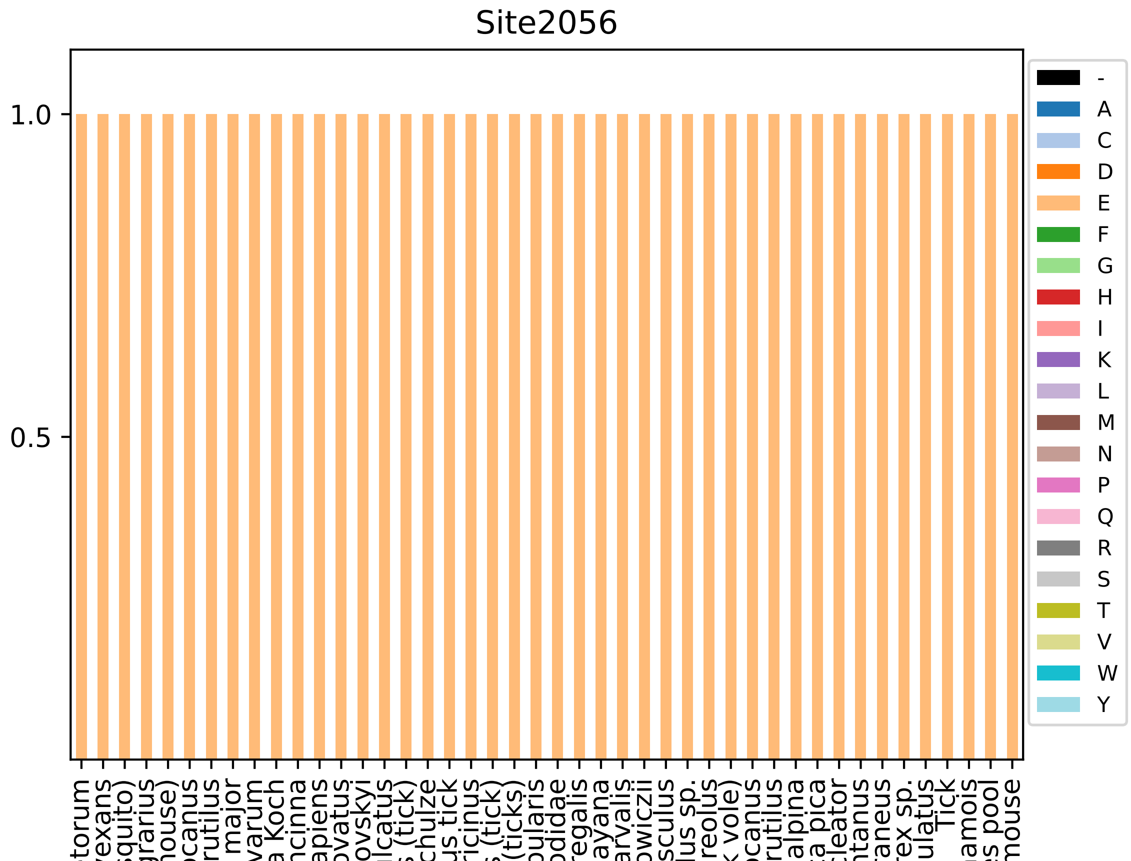
Site2056
1.0
0.5
-
A
C
D
E
F
G
H
I
K
L
M
N
P
Q
R
S
T
V
W
Y

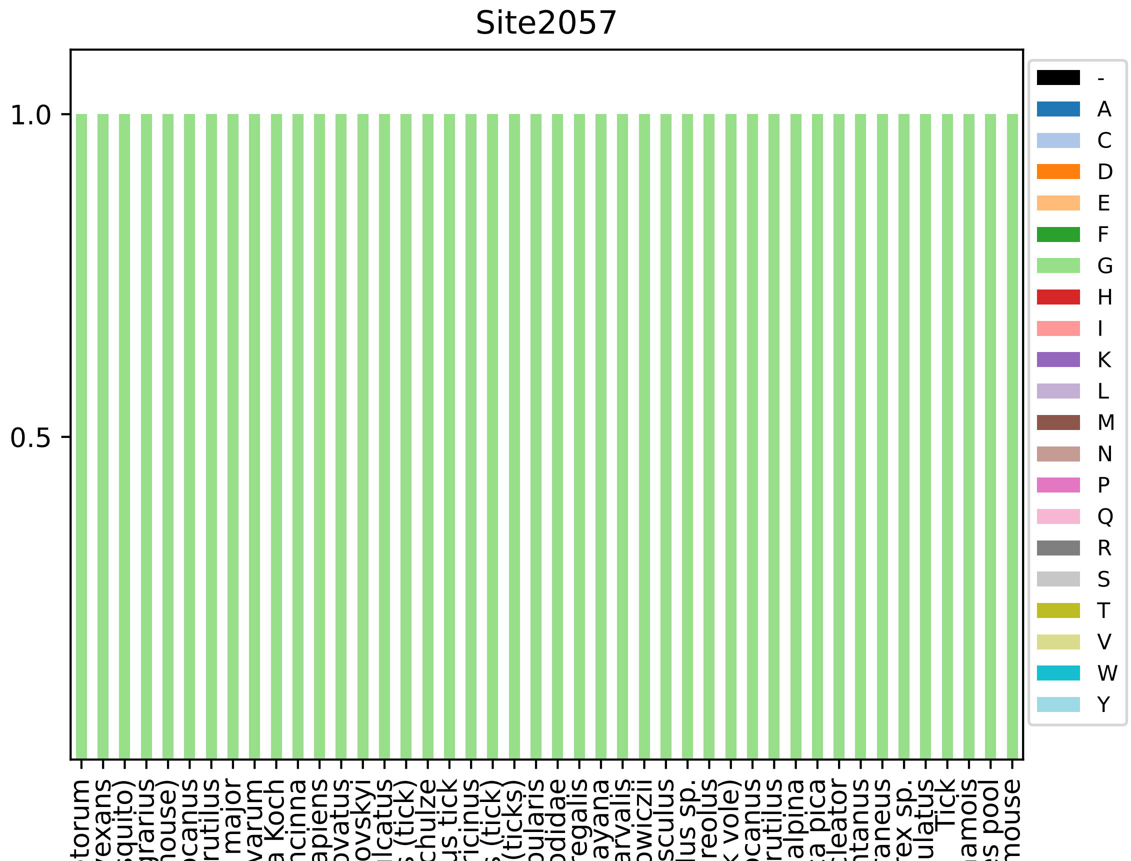
Site2057
1.0
0.5
-
A
C
D
E
F
G
H
I
K
L
M
N
P
Q
R
S
T
V
W
Y

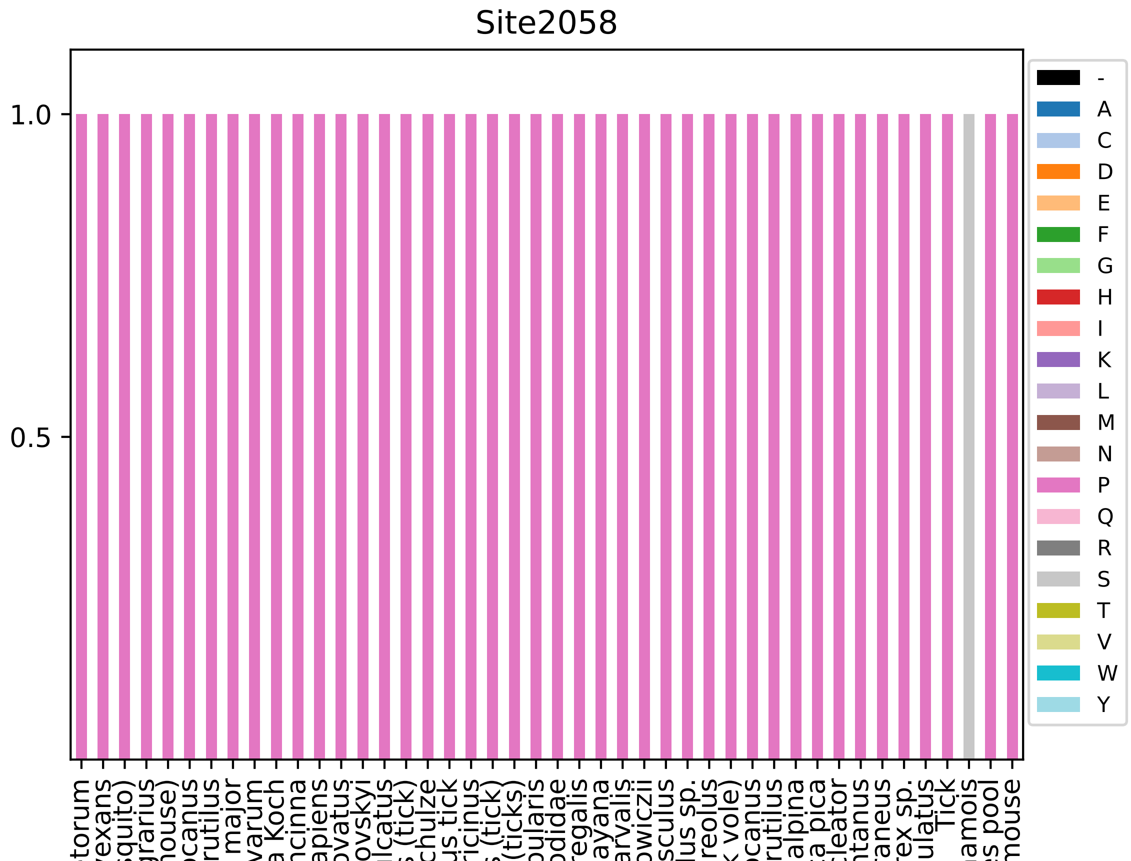
Site2058
1.0
0.5
-
A
C
D
E
F
G
H
I
K
L
M
N
P
Q
R
S
T
V
W
Y
Tick

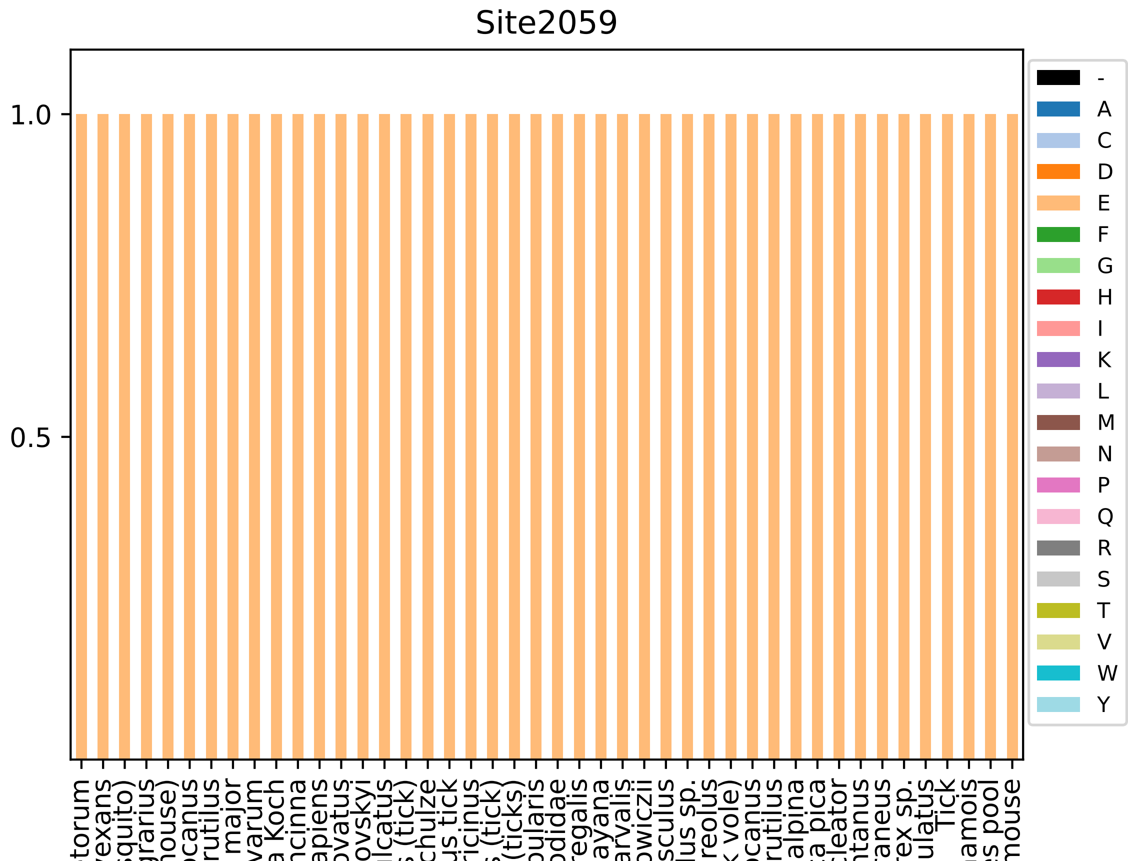
Site2059
1.0
0.5
-
A
C
D
E
F
G
H
I
K
L
M
N
P
Q
R
S
T
V
W
Y

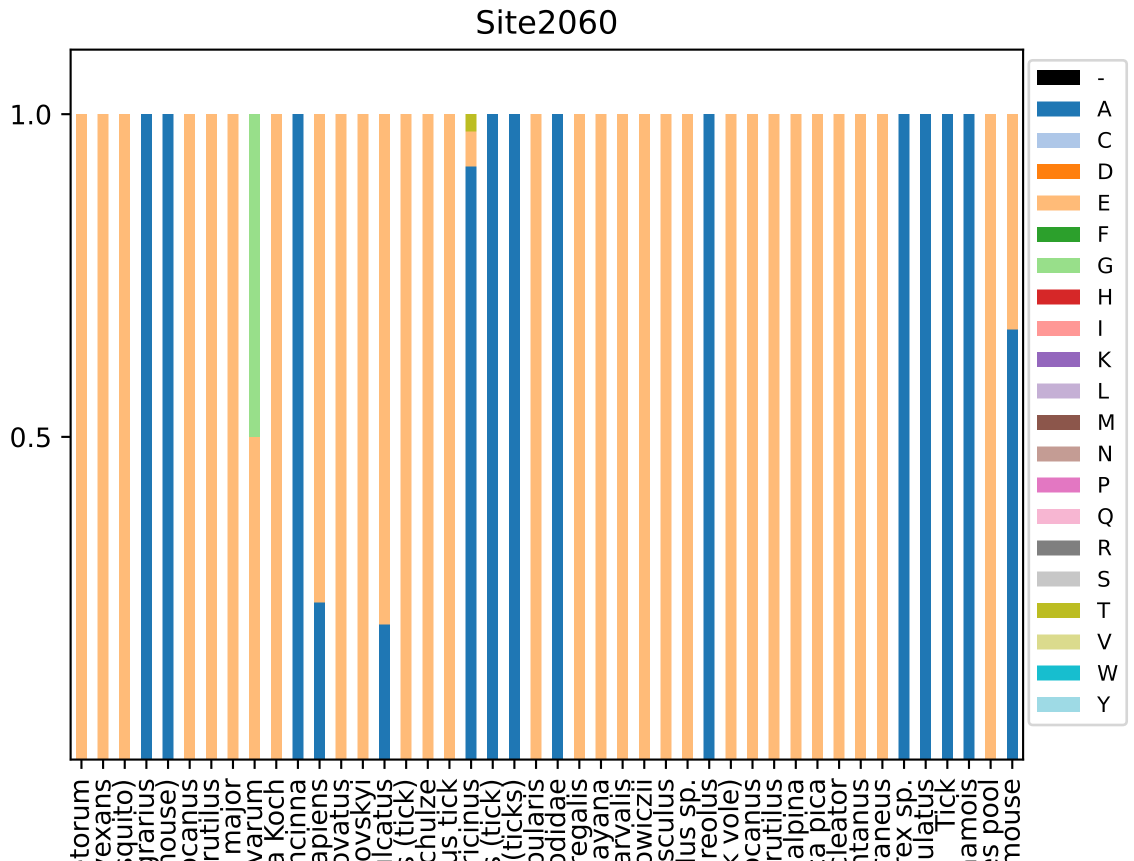

Site2060
1.0
0.5
-
A
C
D
E
F
G
H
I
K
L
M
N
P
Q
R
S
T
V
W
Y

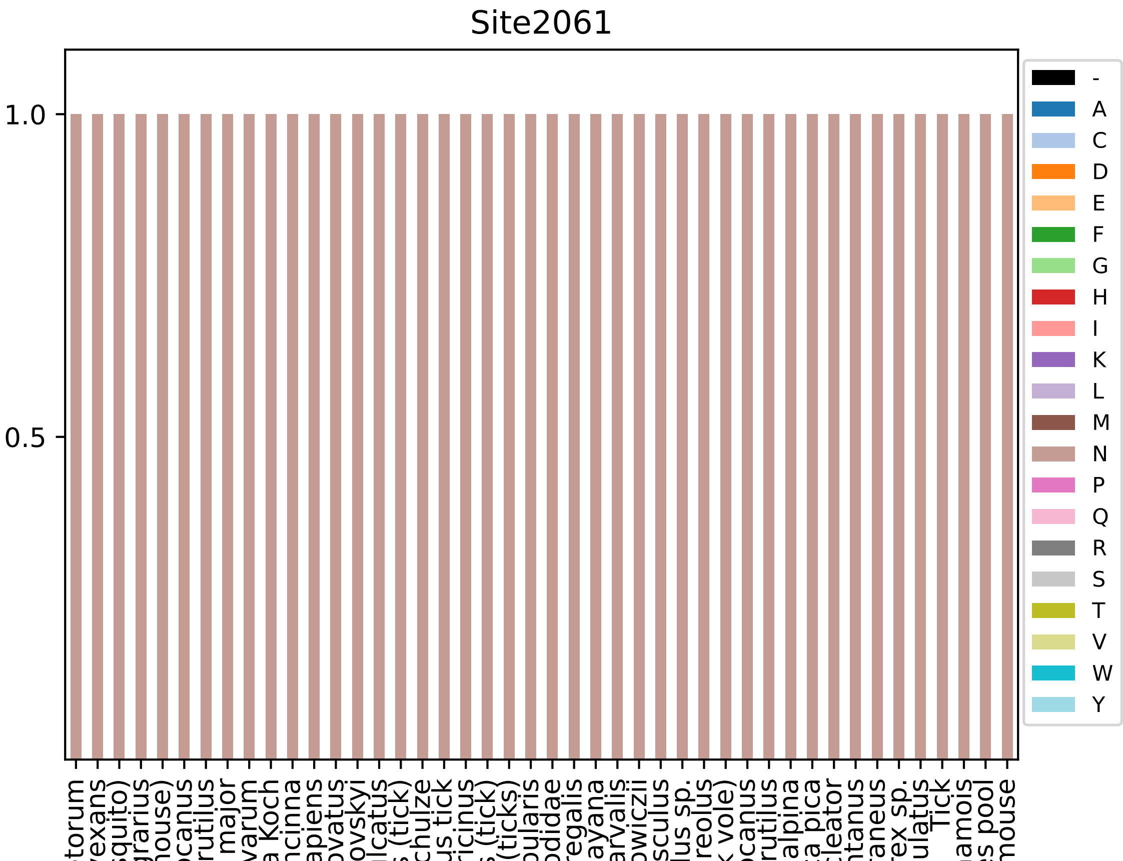
Site2061
1.0
0.5
-
A
C
D
E
F
G
H
I
K
L
M
N
P
Q
R
S
T
V
W
Y

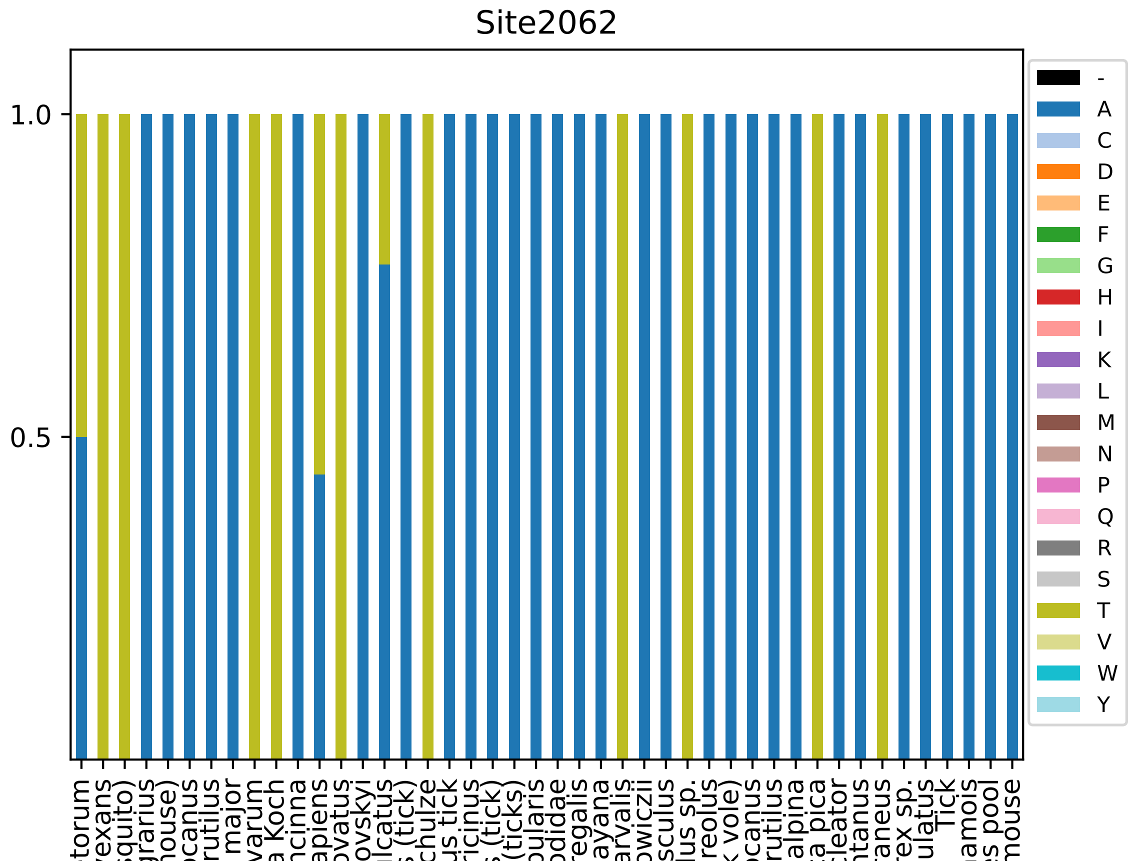

Site2062
1.0
0.5
-
A
C
D
E
F
G
H
I
K
L
M
N
P
Q
R
S
T
V
W
Y

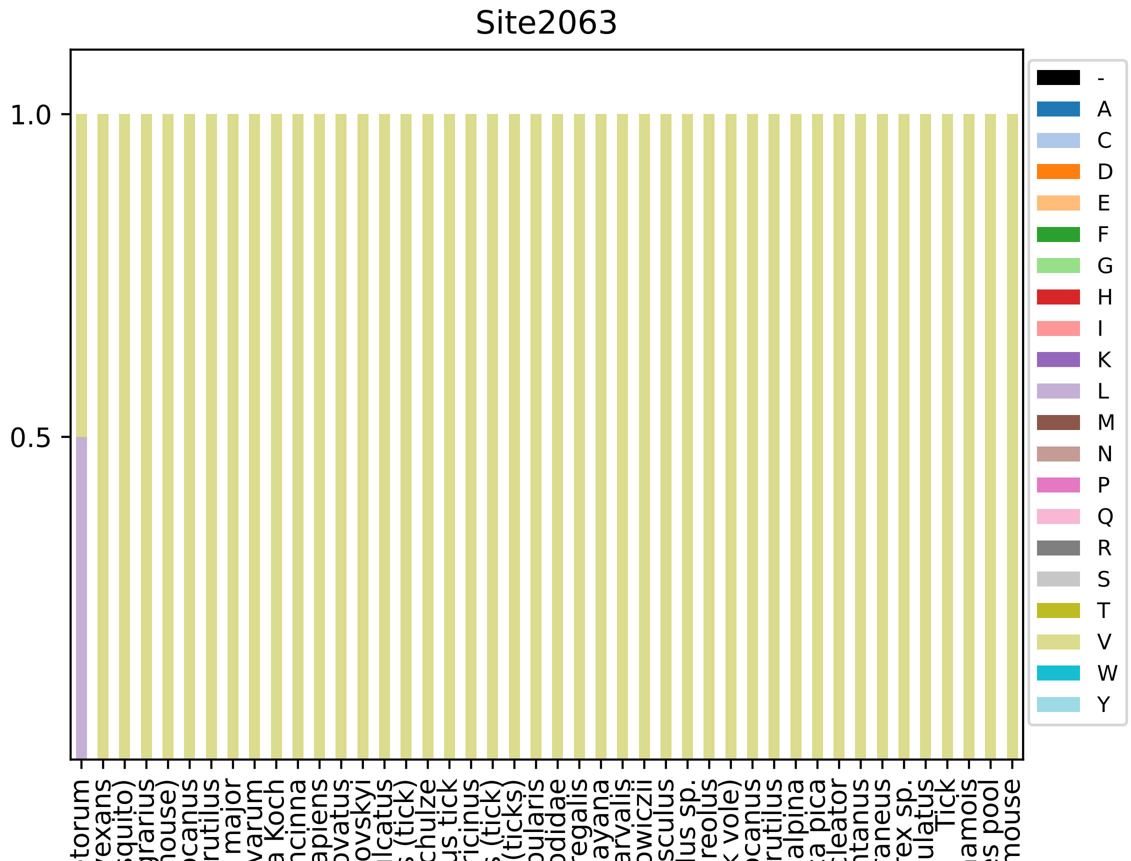
Site2063
1.0
0.5
-
A
C
D
E
F
G
H
I
K
L
M
N
P
Q
R
S
T
V
W
Y

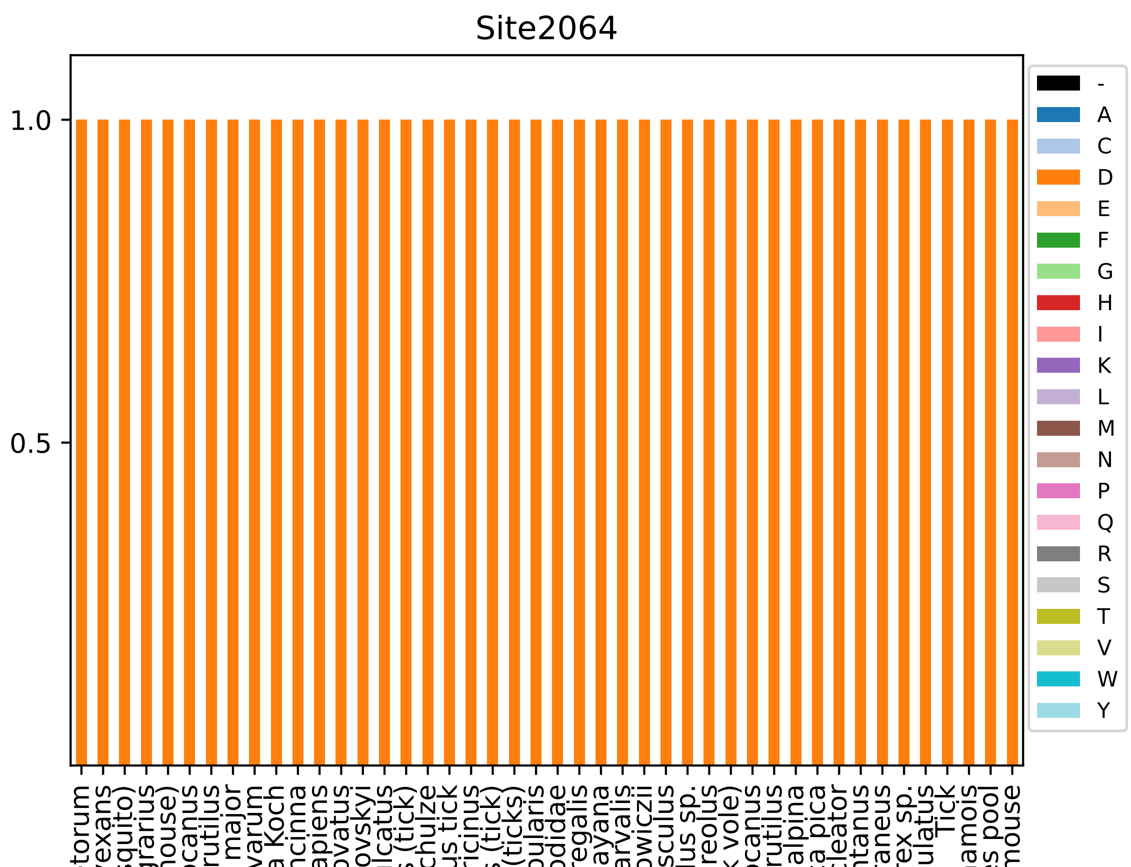
Site2064
1.0
0.5
-
A
C
D
E
F
G
H
I
K
L
M
N
P
Q
R
S
T
V
W
Y

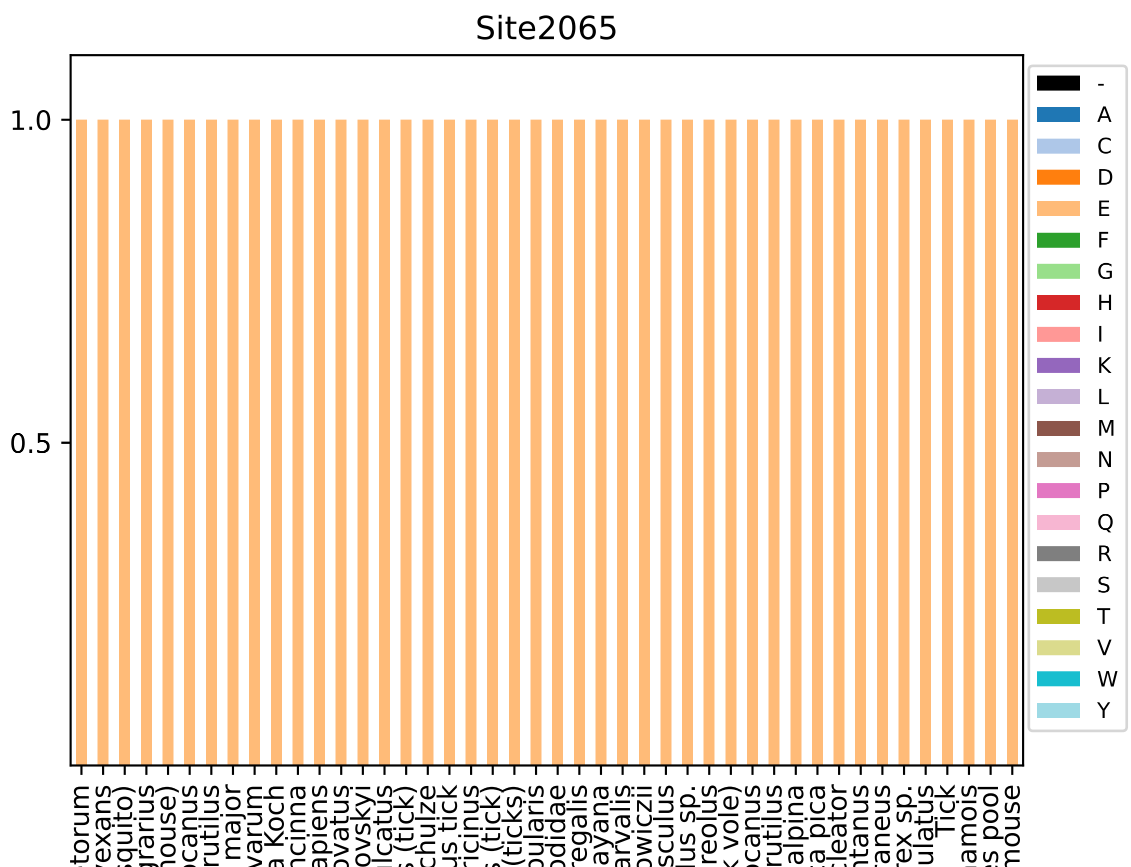
Site2065
1.0
0.5
-
A
C
D
E
F
G
H
I
K
L
M
N
P
Q
R
S
T
V
W
Y

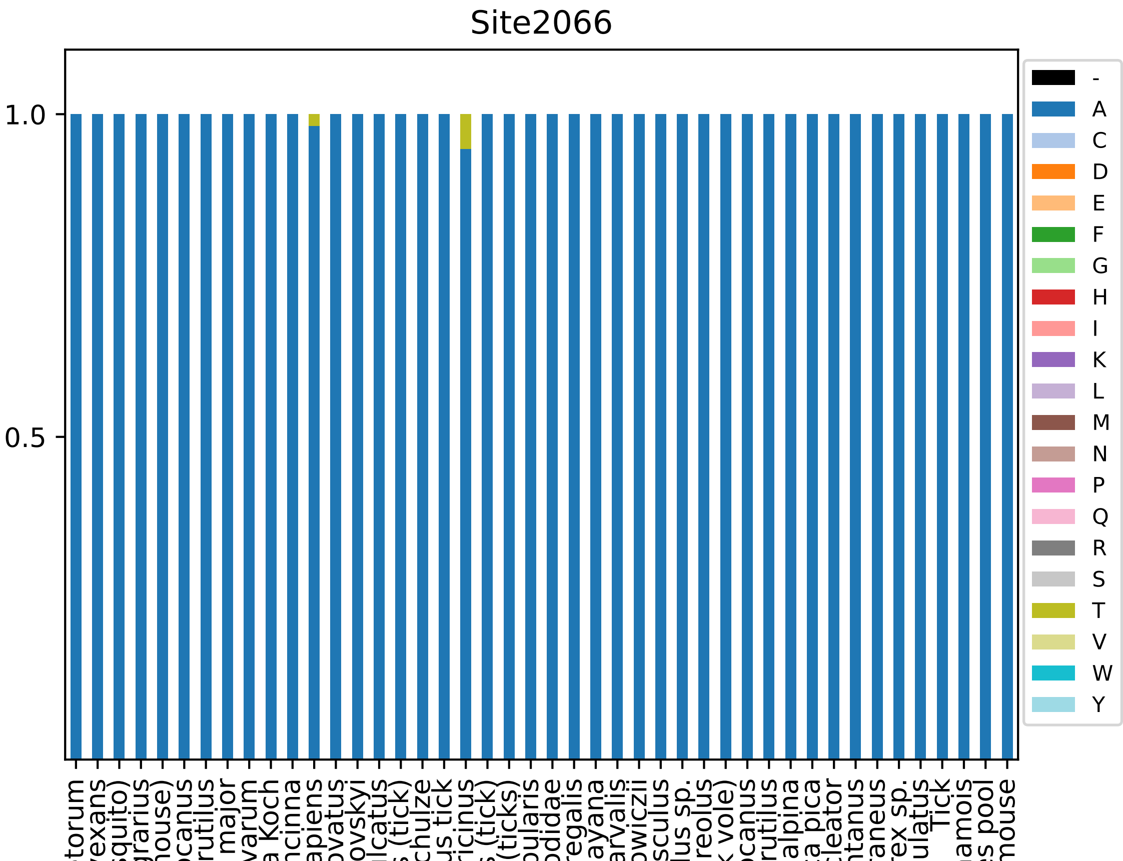

Site2066
1.0
0.5
-
A
C
D
E
F
G
H
I
K
L
M
N
P
Q
R
S
T
V
W
Y

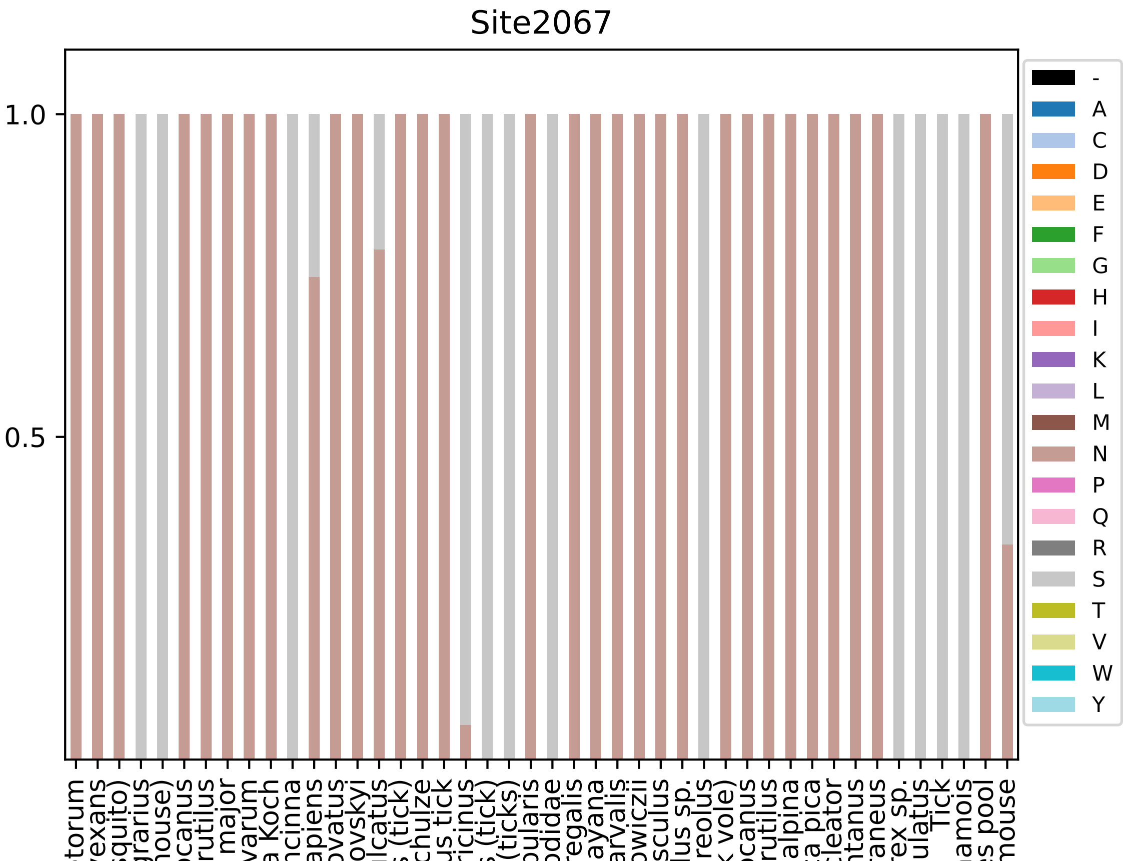
Site2067
1.0
0.5
-
A
C
D
E
F
G
H
I
K
L
M
N
P
Q
R
S
T
V
W
Y

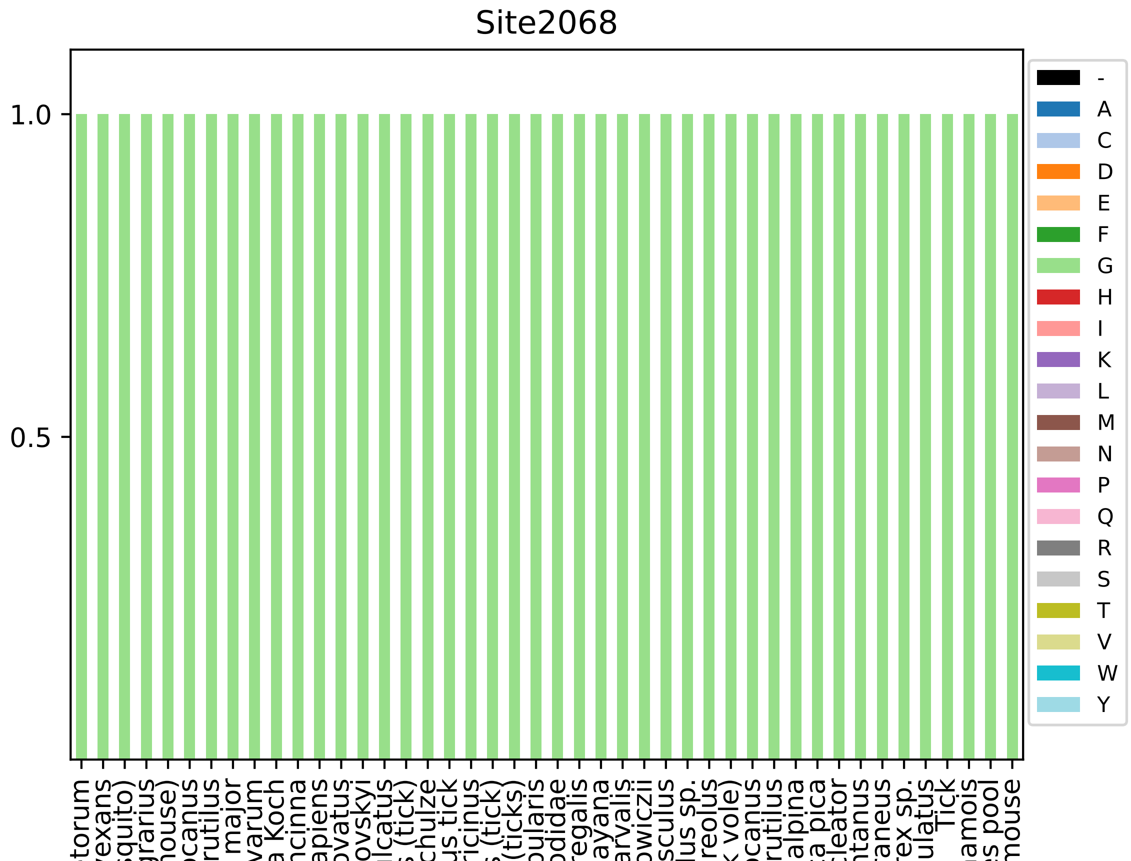
Site2068
1.0
0.5
-
A
C
D
E
F
G
H
I
K
L
M
N
P
Q
R
S
T
V
W
Y

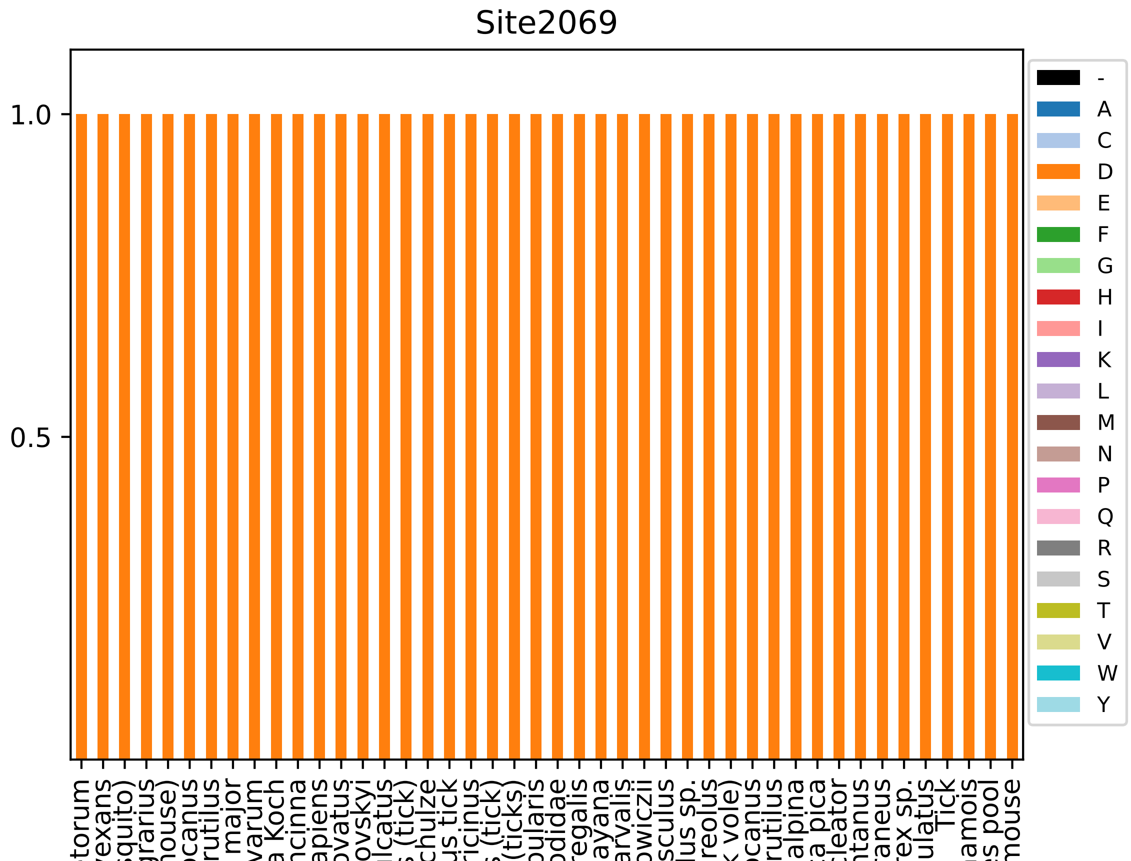

Site2069
1.0
0.5
-
A
C
D
E
F
G
H
I
K
L
M
N
P
Q
R
S
T
V
W
Y

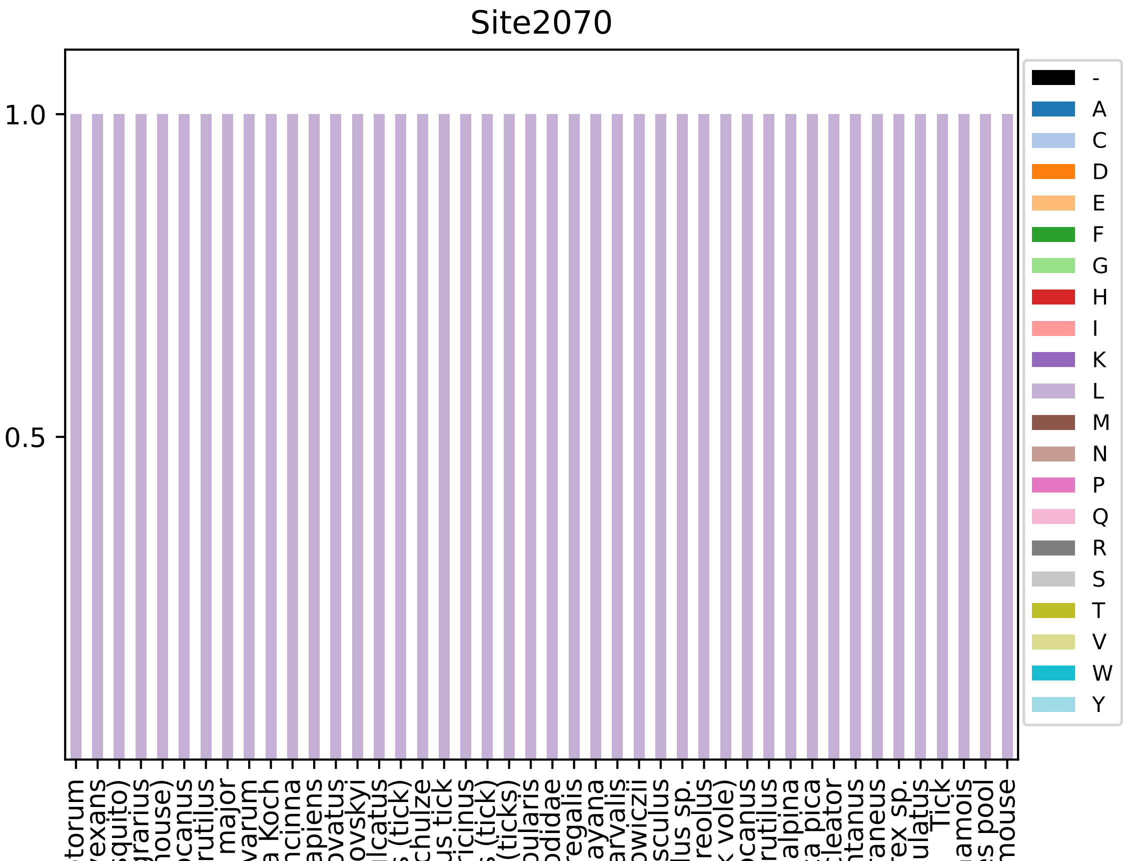
Site2070
1.0
0.5
-
A
C
D
E
F
G
H
I
K
L
M
N
P
Q
R
S
T
V
W
Y

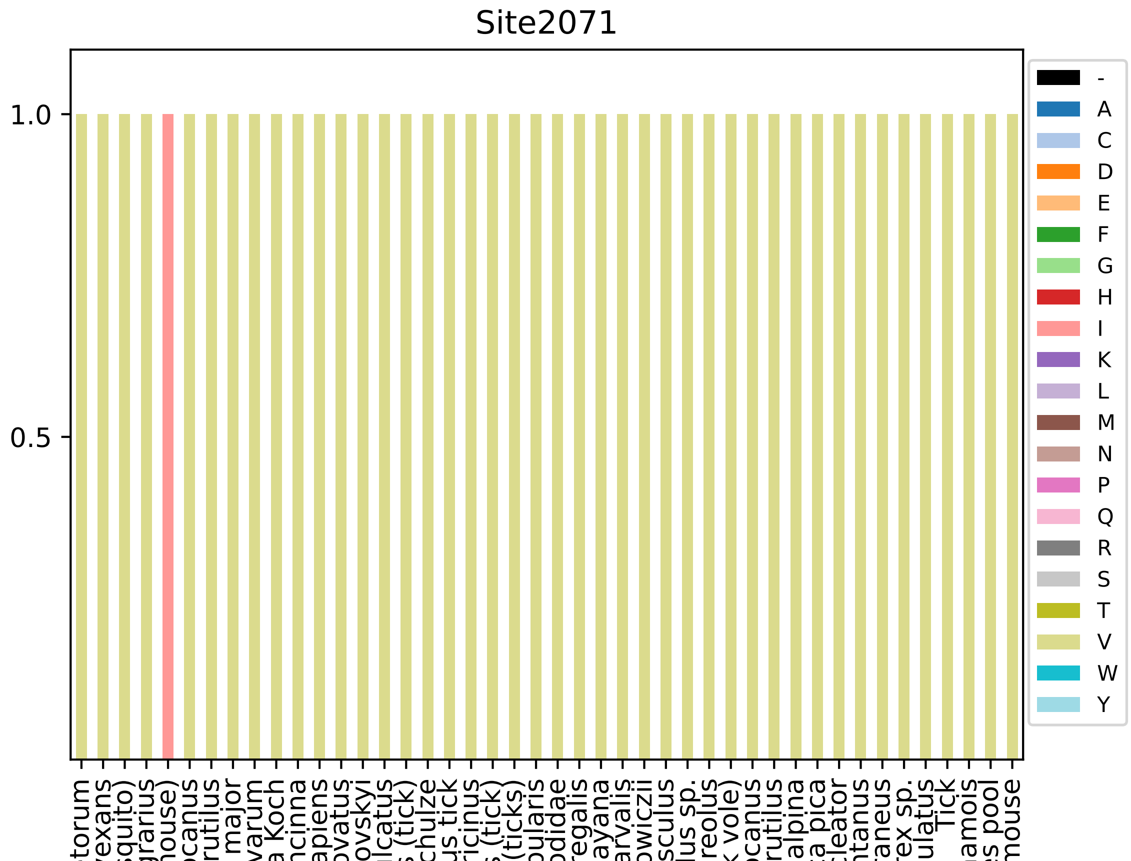
Site2071
1.0
0.5
-
A
C
D
E
F
G
H
I
K
L
M
N
P
Q
R
S
T
V
W
Y

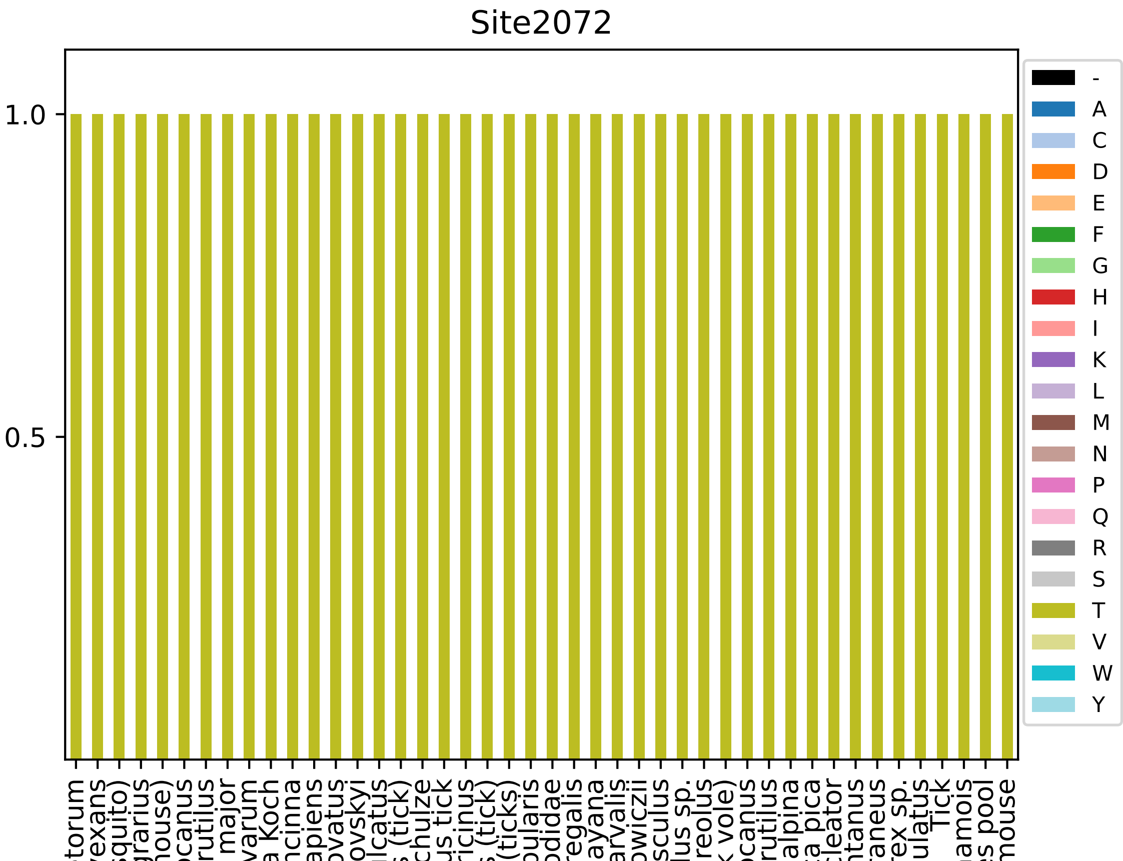

Site2072
1.0
0.5
-
A
C
D
E
F
G
H
I
K
L
M
N
P
Q
R
S
T
V
W
Y

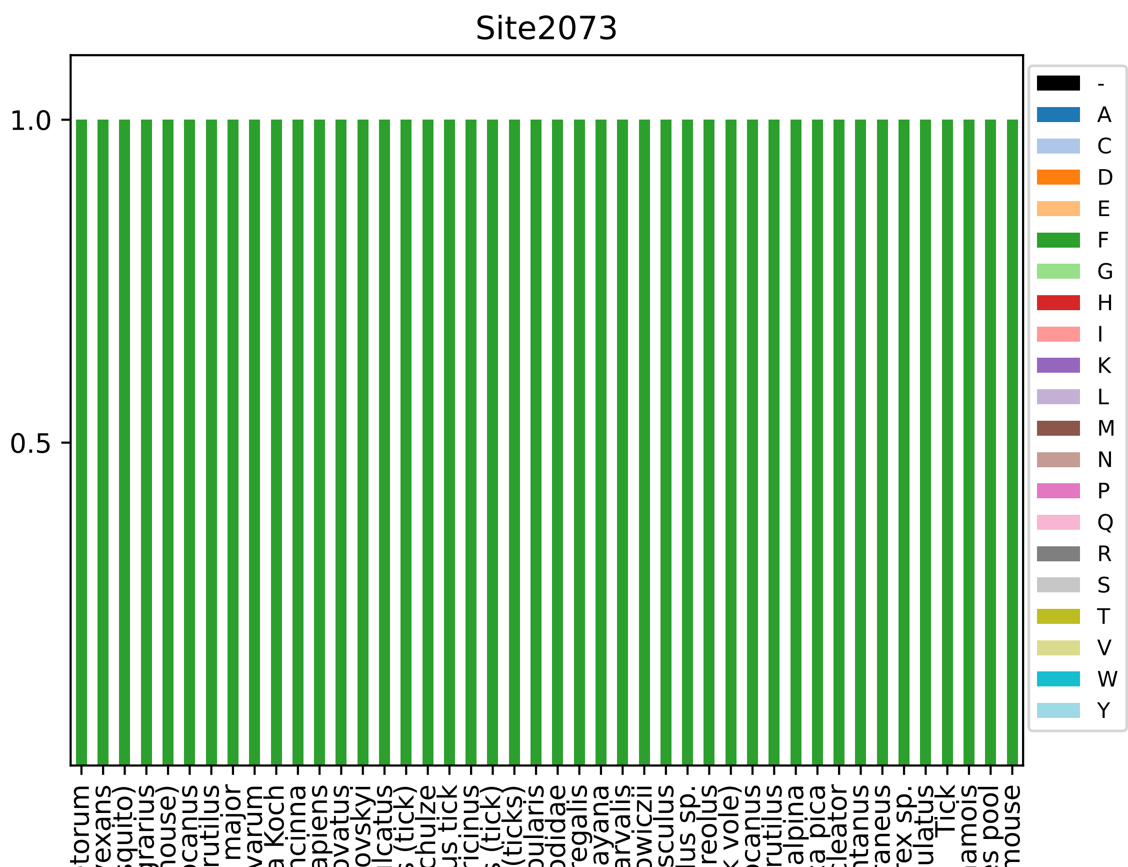
Site2073
1.0
0.5
-
A
C
D
E
F
G
H
I
K
L
M
N
P
Q
R
S
T
V
W
Y

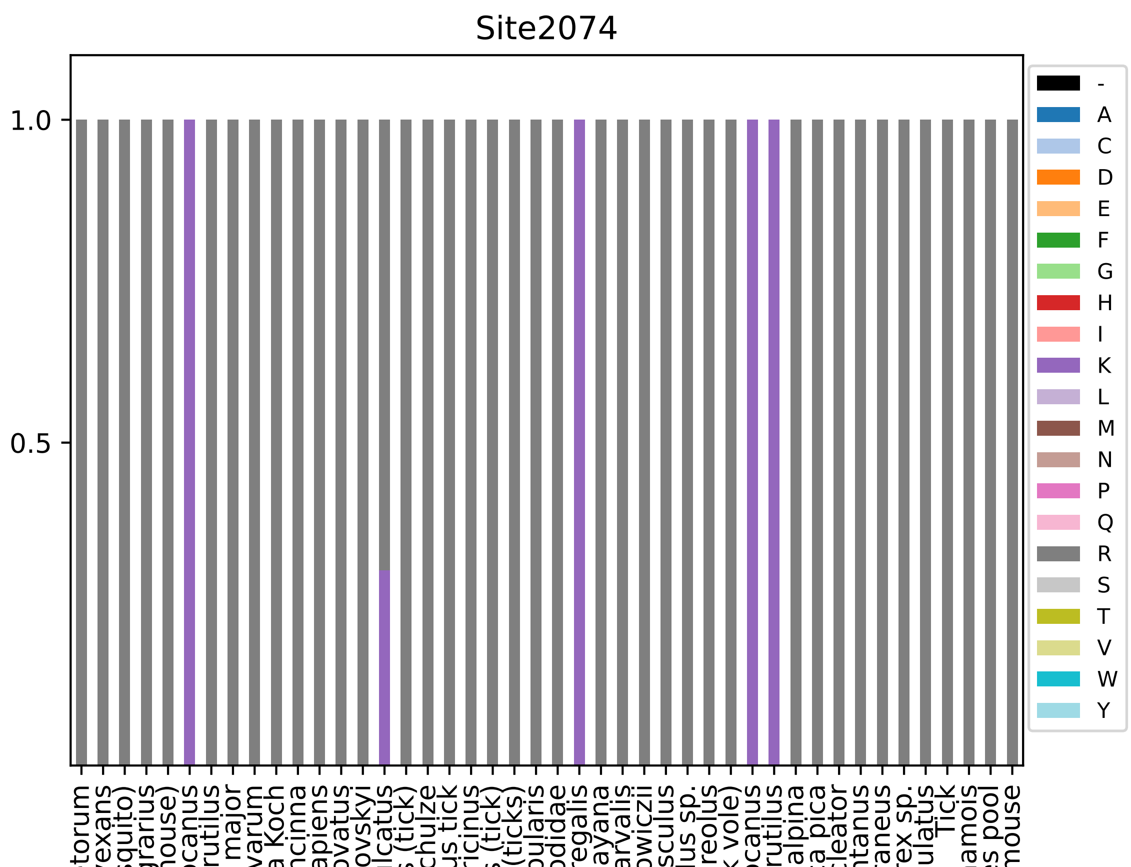
Site2074
1.0
0.5
-
A
C
D
E
F
G
H
I
K
L
M
N
P
Q
R
S
T
V
W
Y

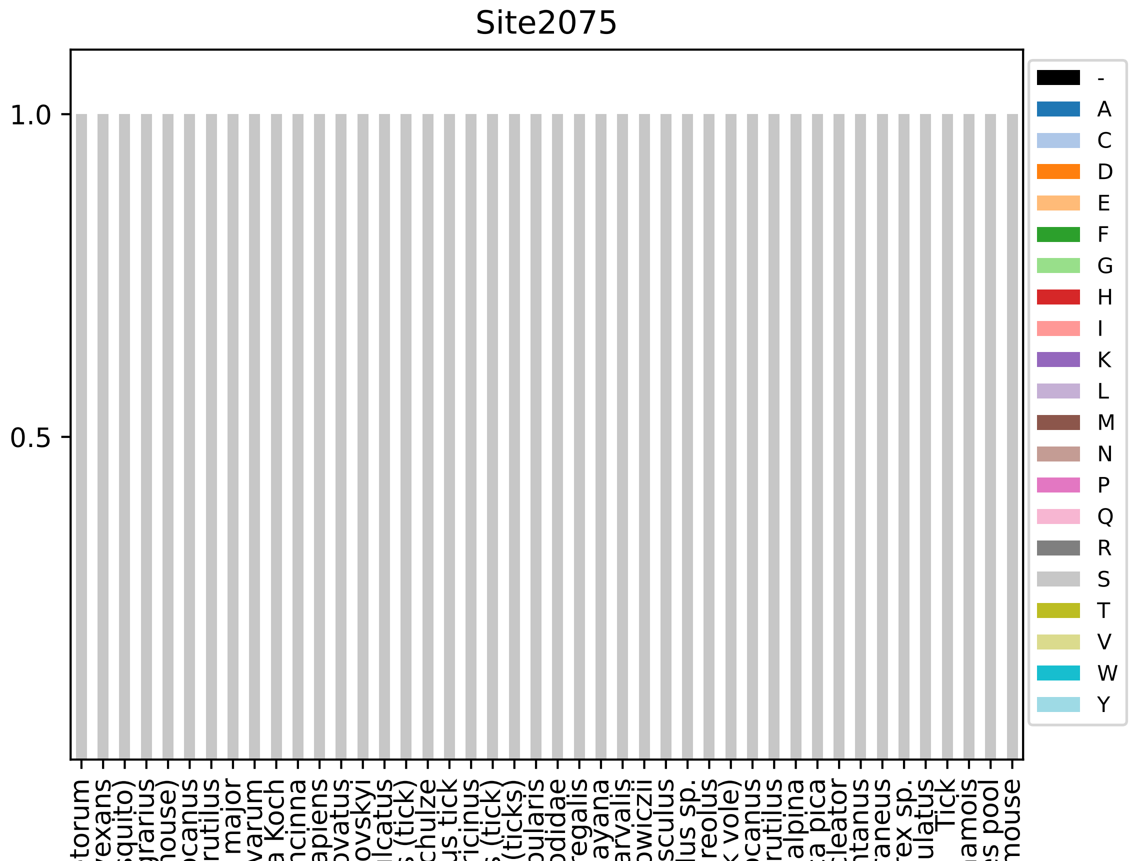
Site2075
1.0
0.5
-
A
C
D
E
F
G
H
I
K
L
M
N
P
Q
R
S
T
V
W
Y

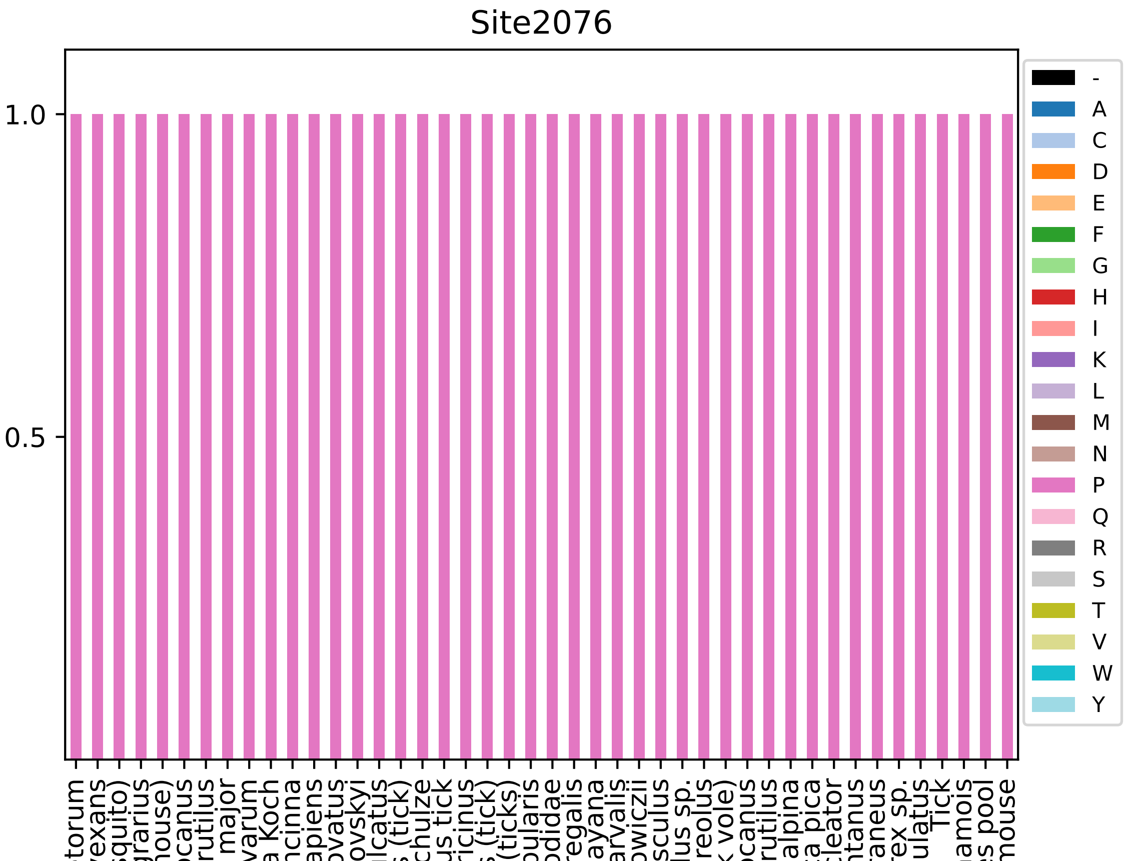
Site2076
1.0
0.5
-
A
C
D
E
F
G
H
I
K
L
M
N
P
Q
R
S
T
V
W
Y

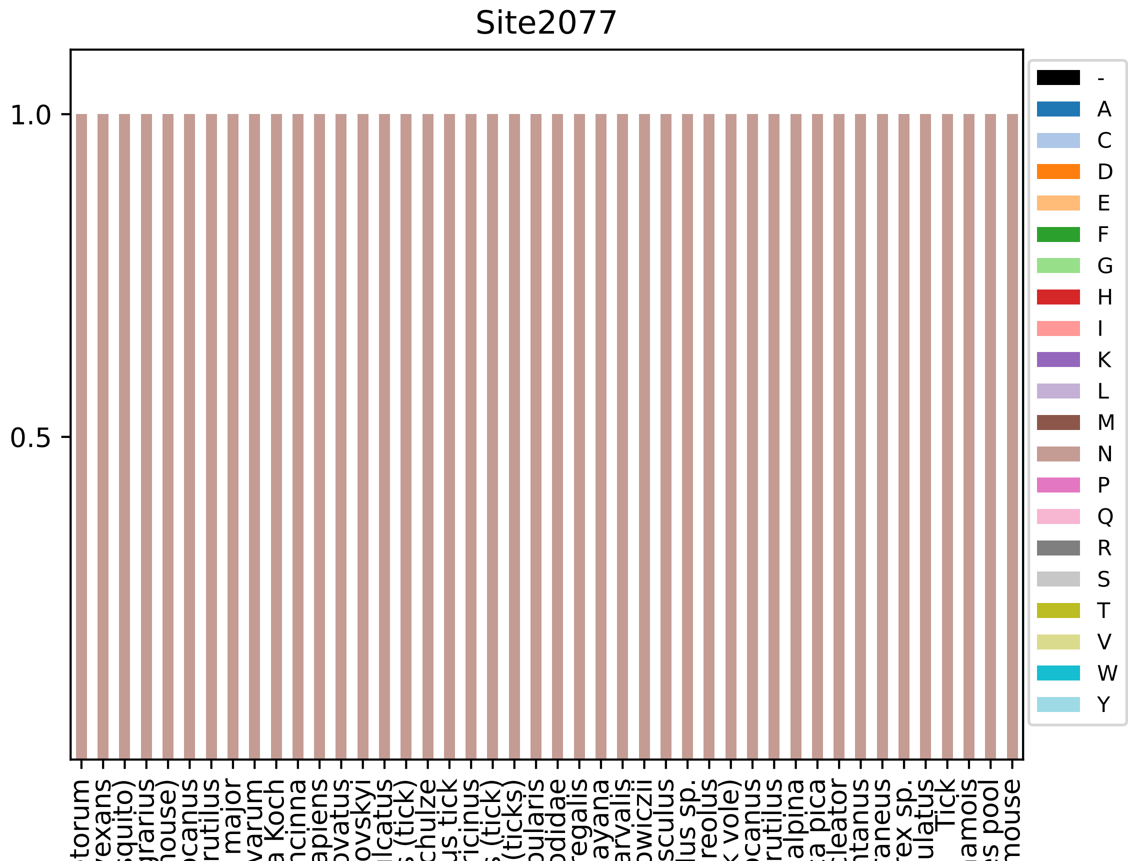
Site2077
1.0
0.5
-
A
C
D
E
F
G
H
I
K
L
M
N
P
Q
R
S
T
V
W
Y

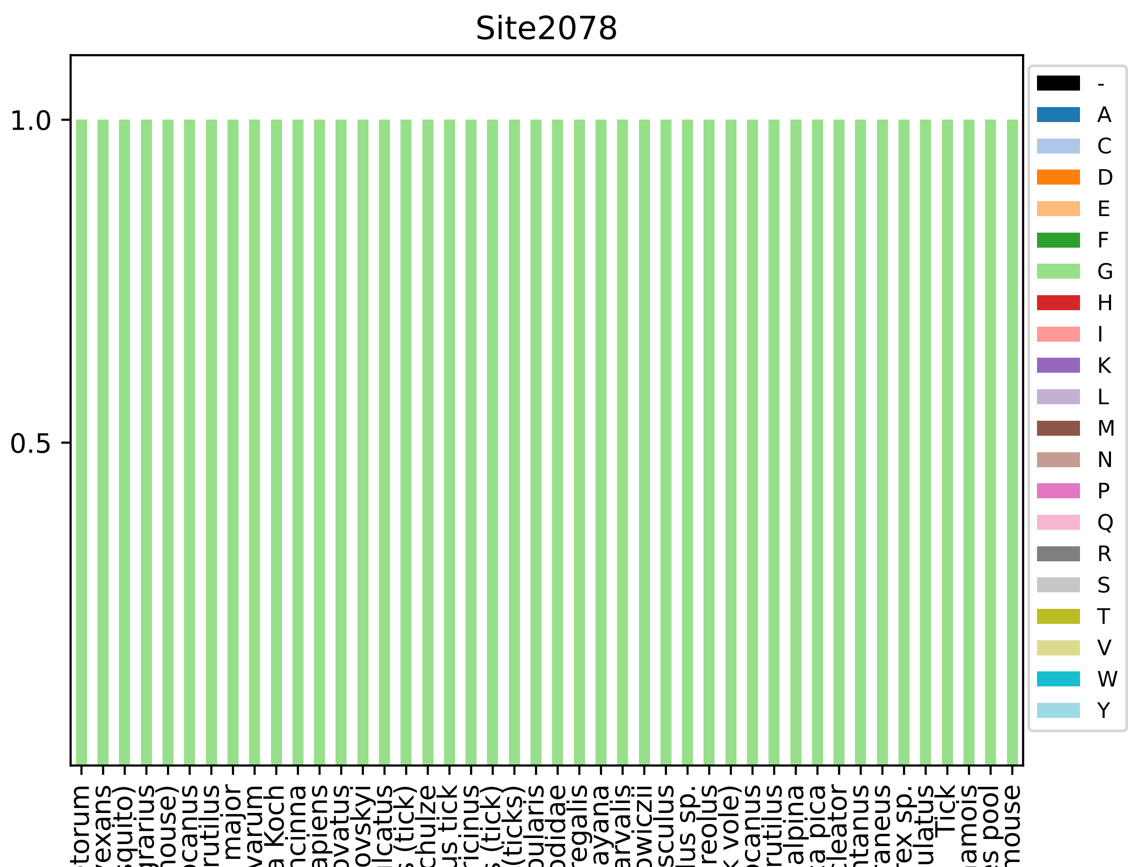
Site2078
1.0
0.5
-
A
C
D
E
F
G
H
I
K
L
M
N
P
Q
R
S
T
V
W
Y

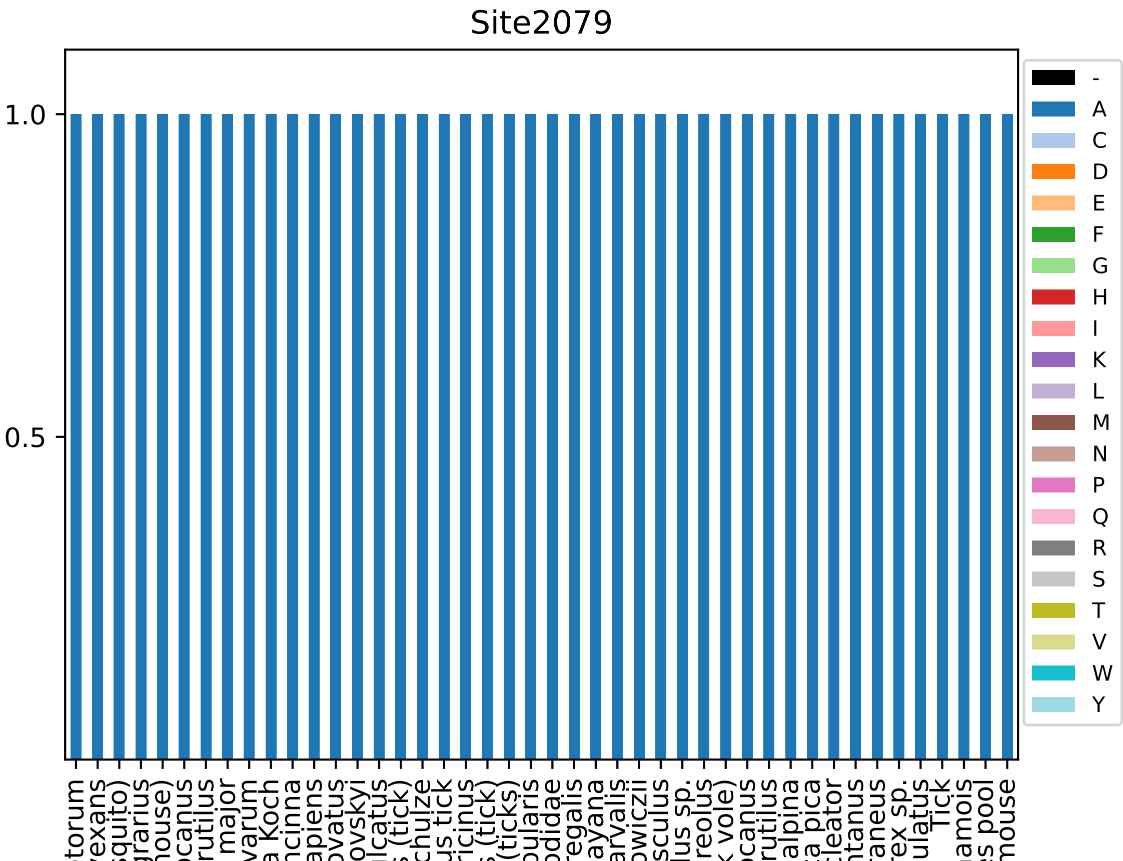
Site2079
1.0
0.5
-
A
C
D
E
F
G
H
I
K
L
M
N
P
Q
R
S
T
V
W
Y
Tick

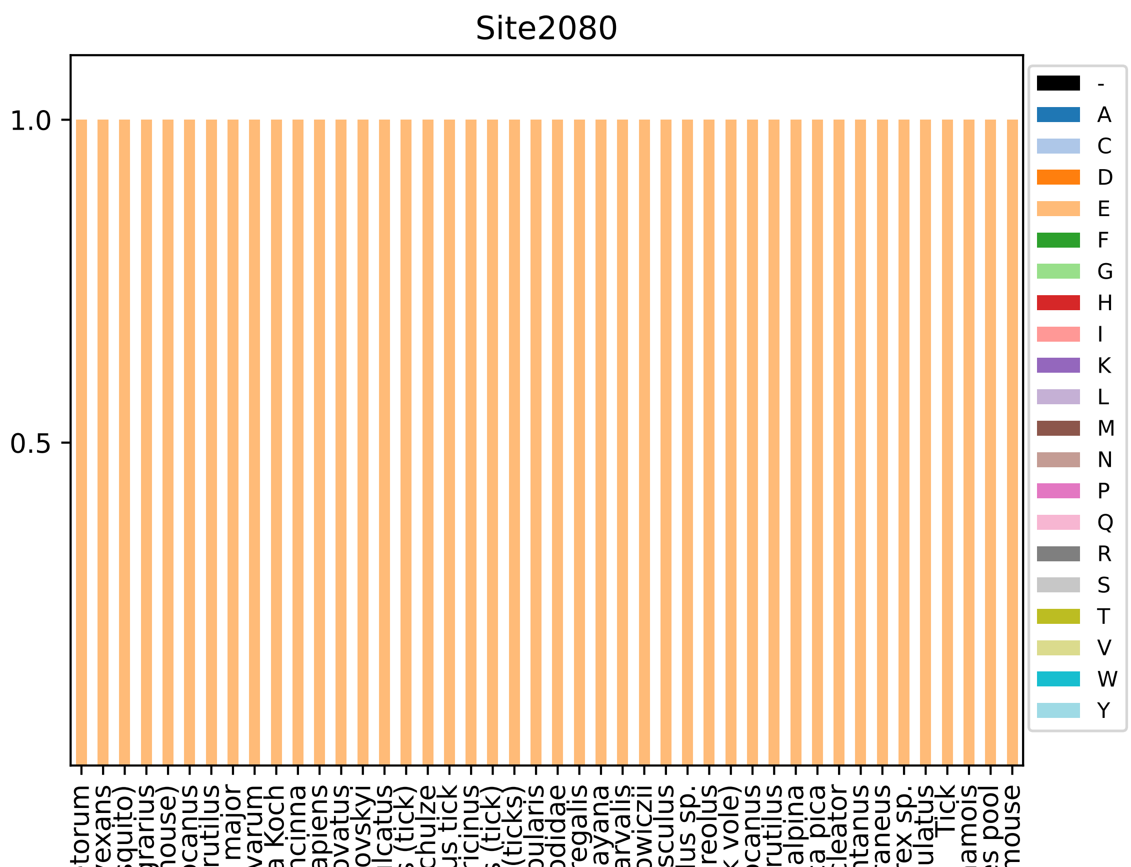
Site2080
1.0
0.5
-
A
C
D
E
F
G
H
I
K
L
M
N
P
Q
R
S
T
V
W
Y

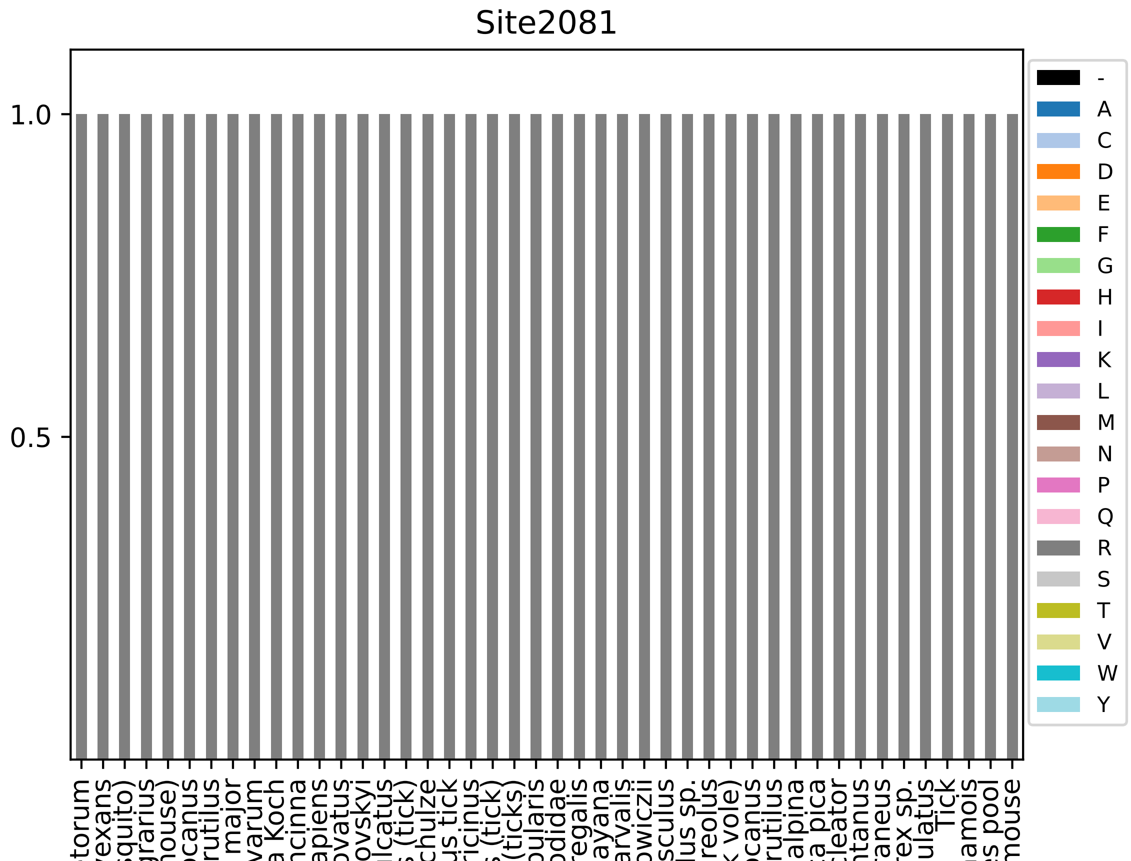
Site2081
1.0
0.5
-
A
C
D
E
F
G
H
I
K
L
M
N
P
Q
R
S
T
V
W
Y

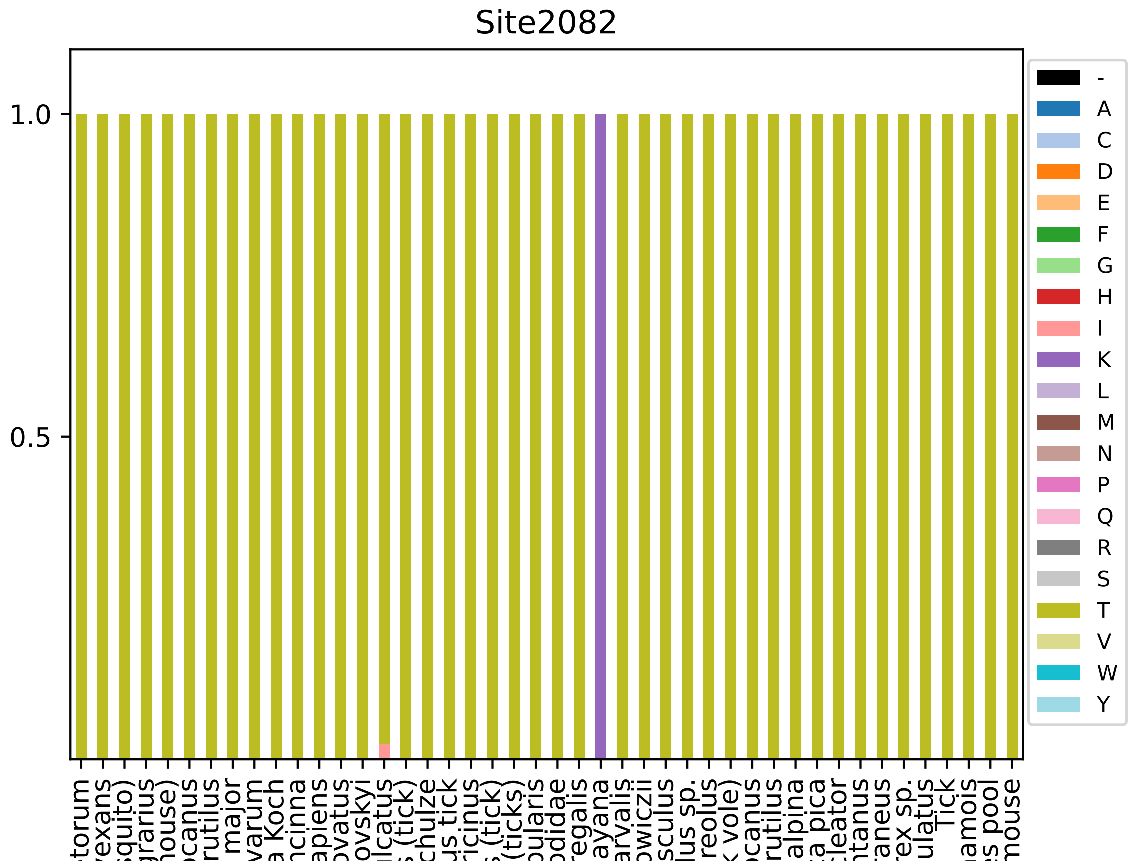
Site2082
1.0
0.5
-
A
C
D
E
F
G
H
I
K
L
M
N
P
Q
R
S
T
V
W
Y

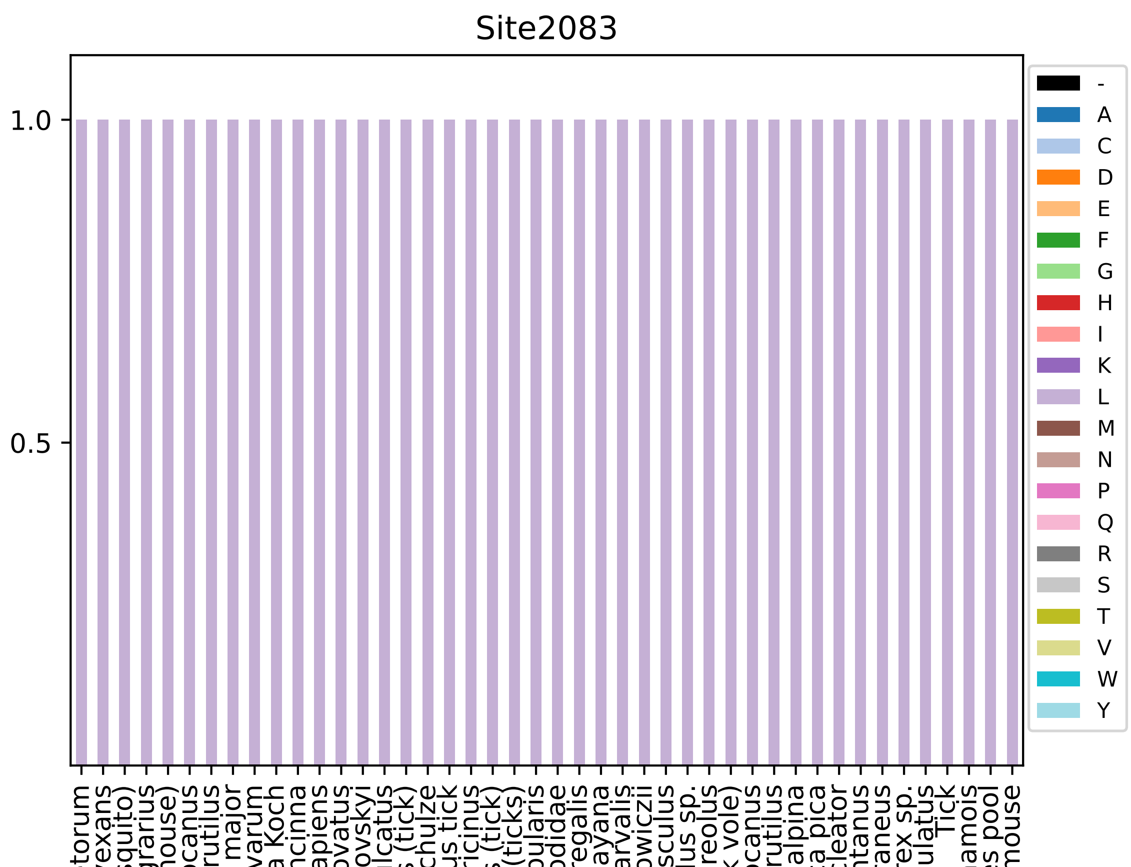
Site2083
1.0
0.5
-
A
C
D
E
F
G
H
I
K
L
M
N
P
Q
R
S
T
V
W
Y

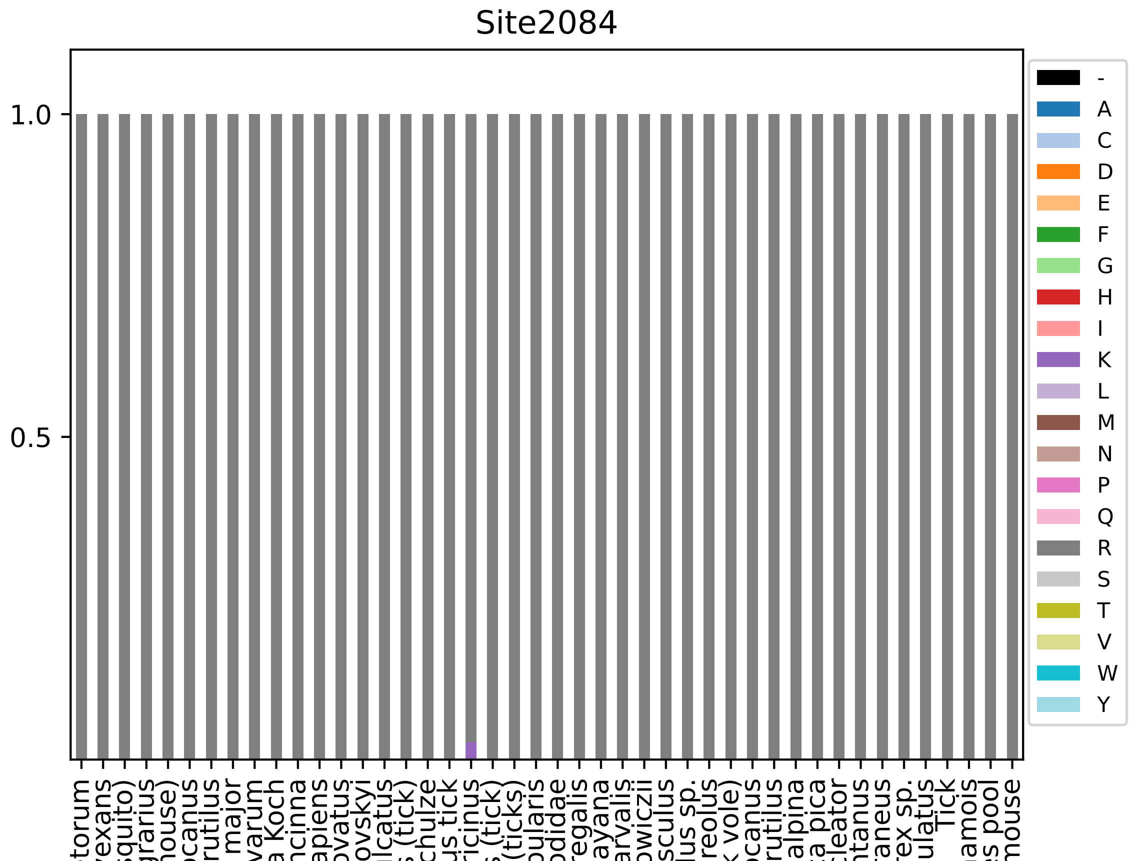
Site2084
1.0
0.5
-
A
C
D
E
F
G
H
I
K
L
M
N
P
Q
R
S
T
V
W
Y

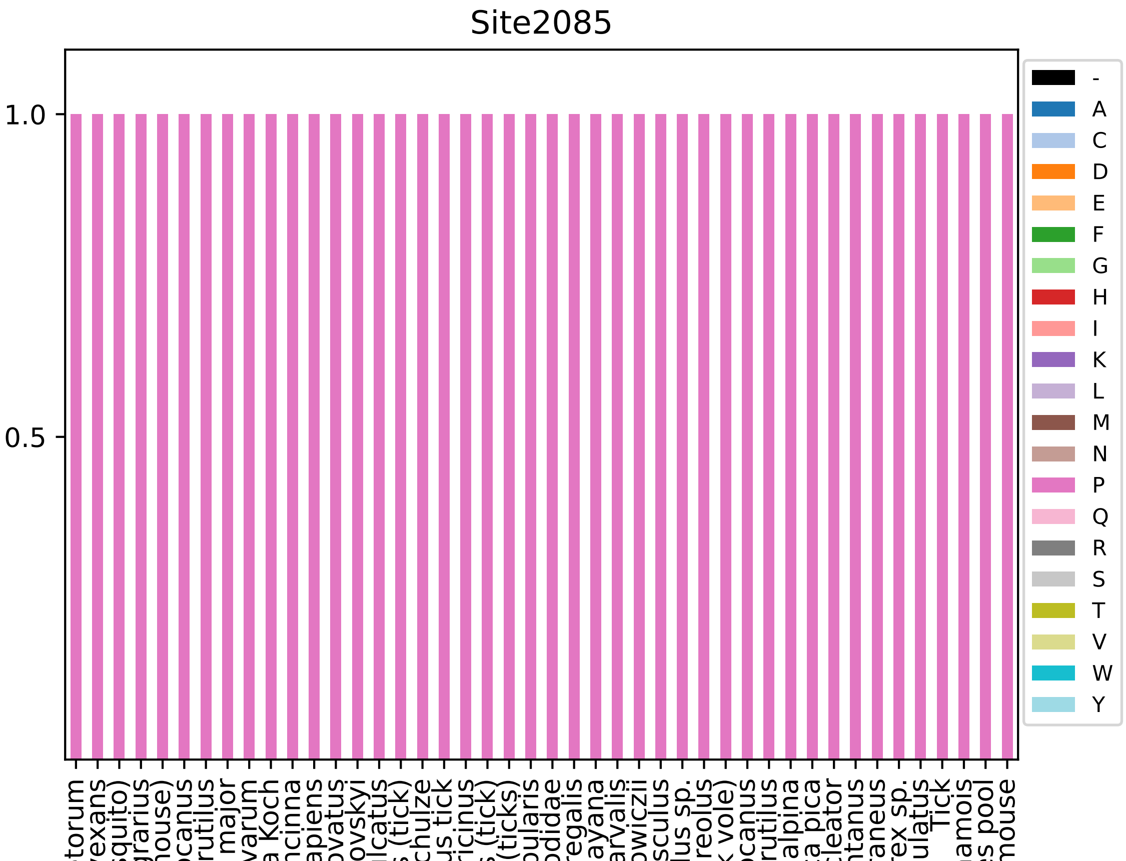

Site2085
1.0
0.5
-
A
C
D
E
F
G
H
I
K
L
M
N
P
Q
R
S
T
V
W
Y

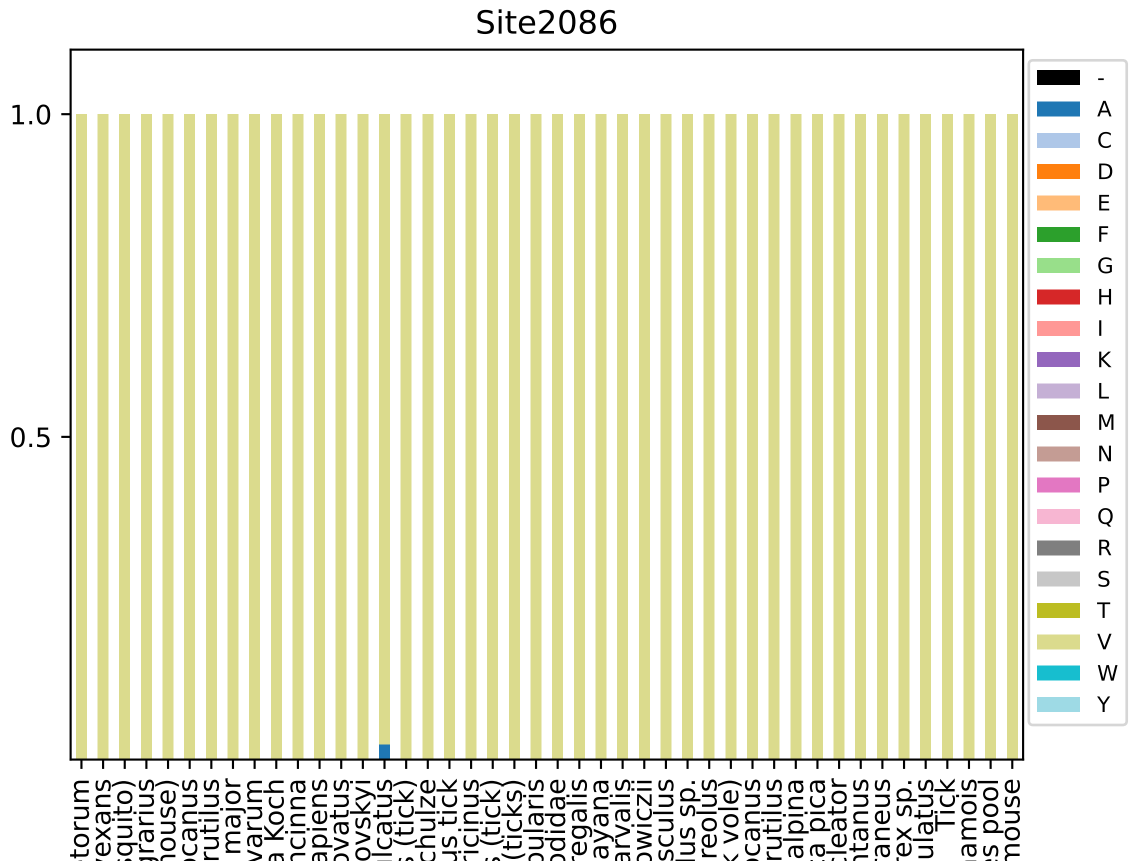

Site2086
1.0
0.5
-
A
C
D
E
F
G
H
I
K
L
M
N
P
Q
R
S
T
V
W
Y

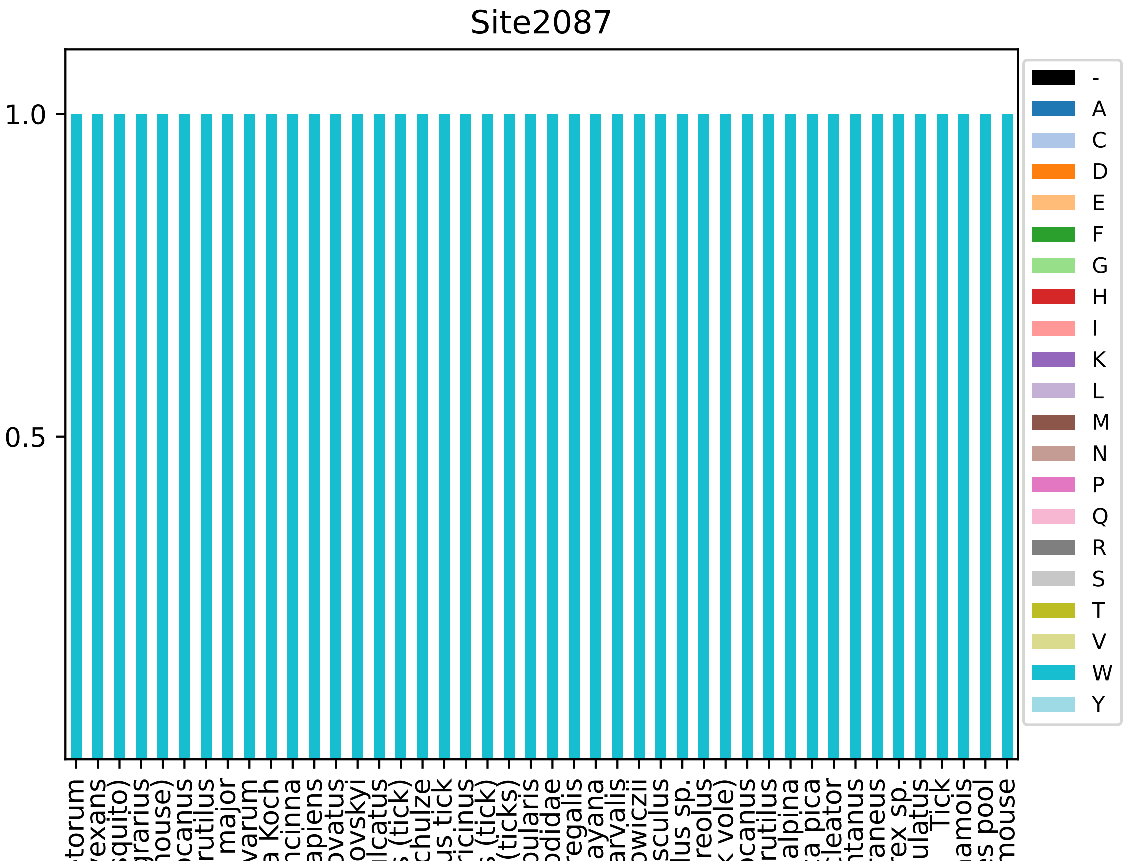
Site2087
1.0
0.5
-
A
C
D
E
F
G
H
I
K
L
M
N
P
Q
R
S
T
V
W
Y

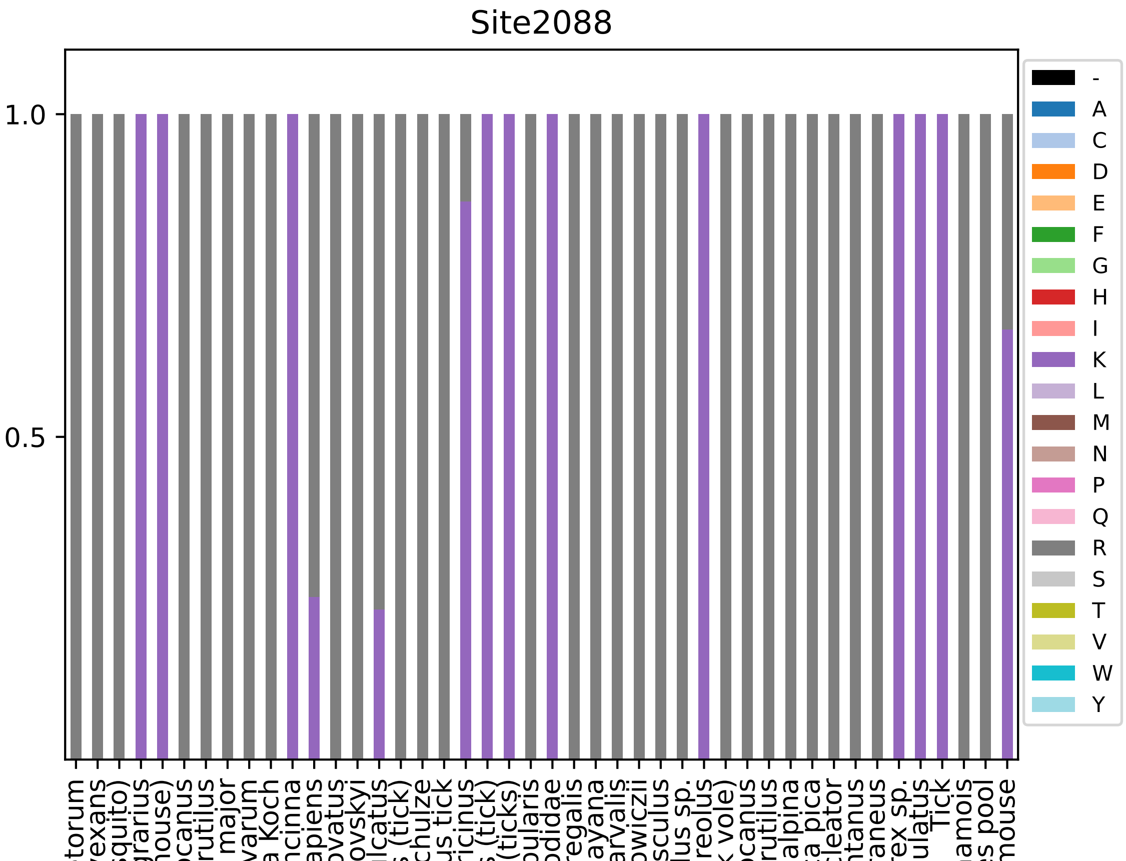
Site2088
1.0
0.5
-
A
C
D
E
F
G
H
I
K
L
M
N
P
Q
R
S
T
V
W
Y

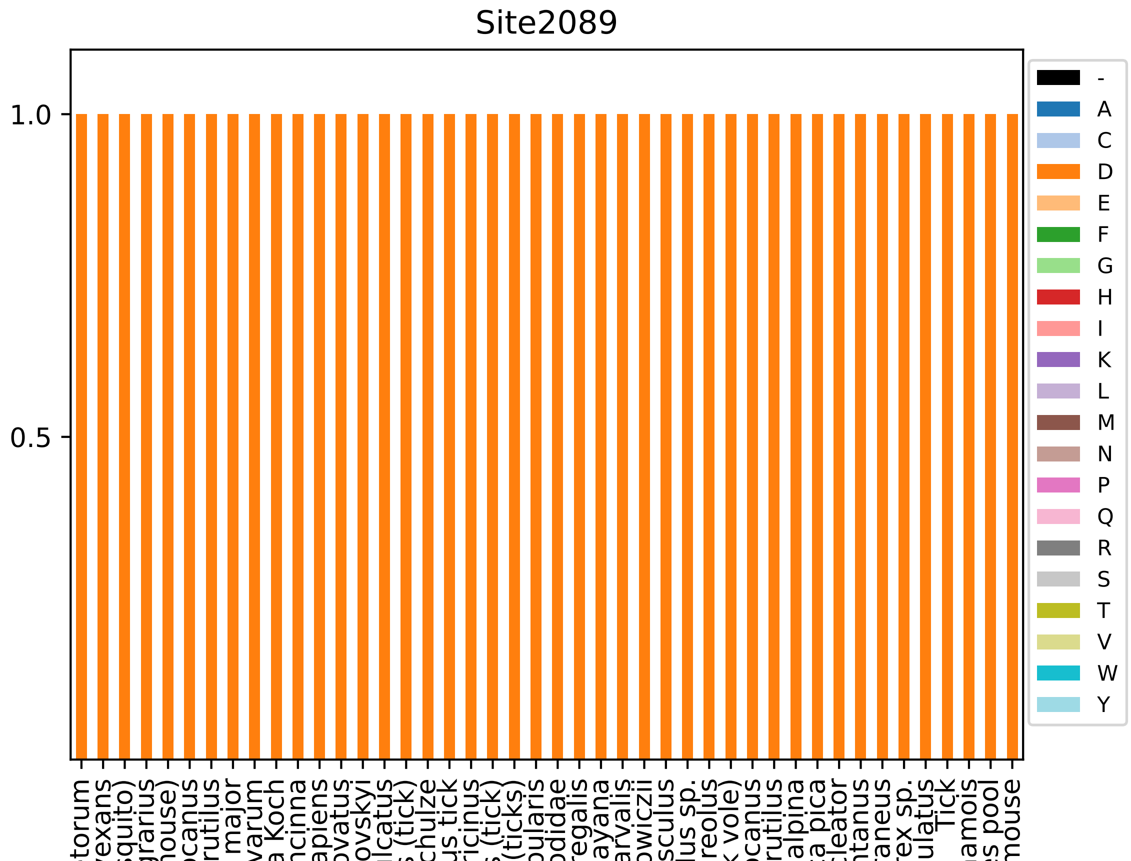
Site2089
1.0
0.5
-
A
C
D
E
F
G
H
I
K
L
M
N
P
Q
R
S
T
V
W
Y

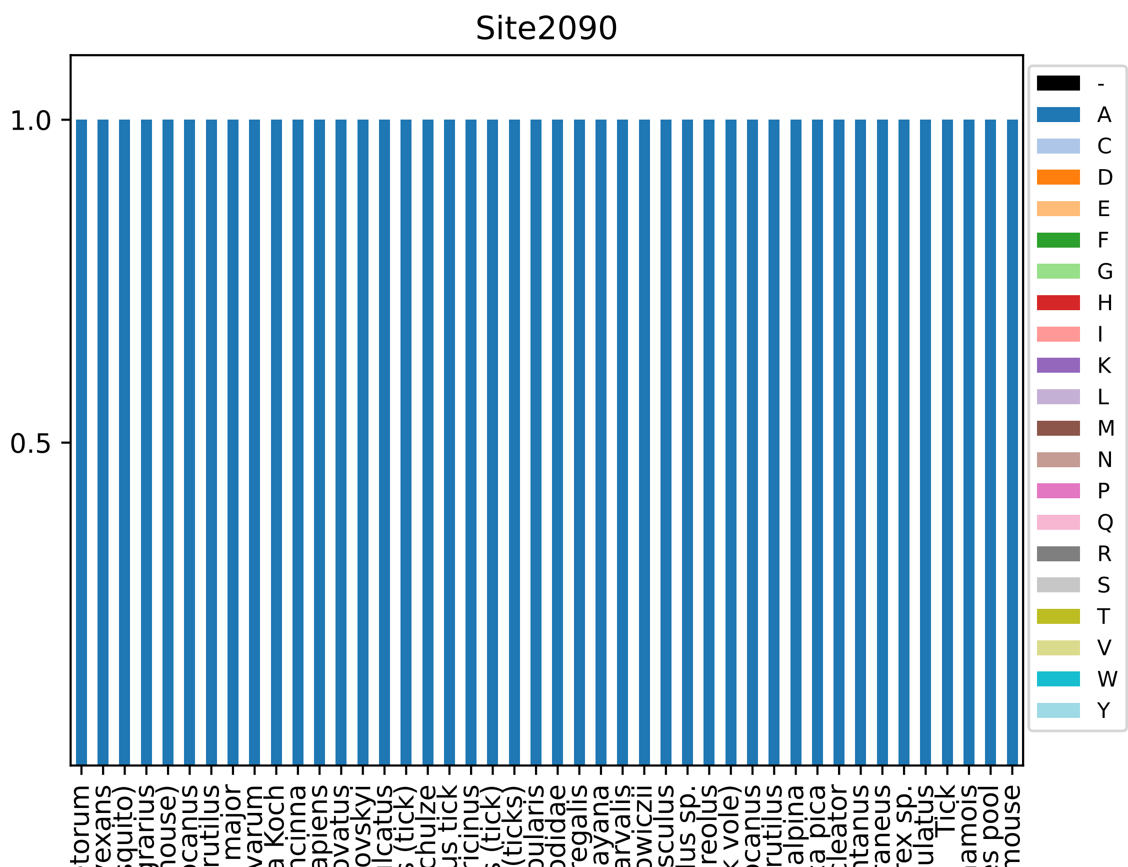

Site2090
1.0
0.5
-
A
C
D
E
F
G
H
I
K
L
M
N
P
Q
R
S
T
V
W
Y

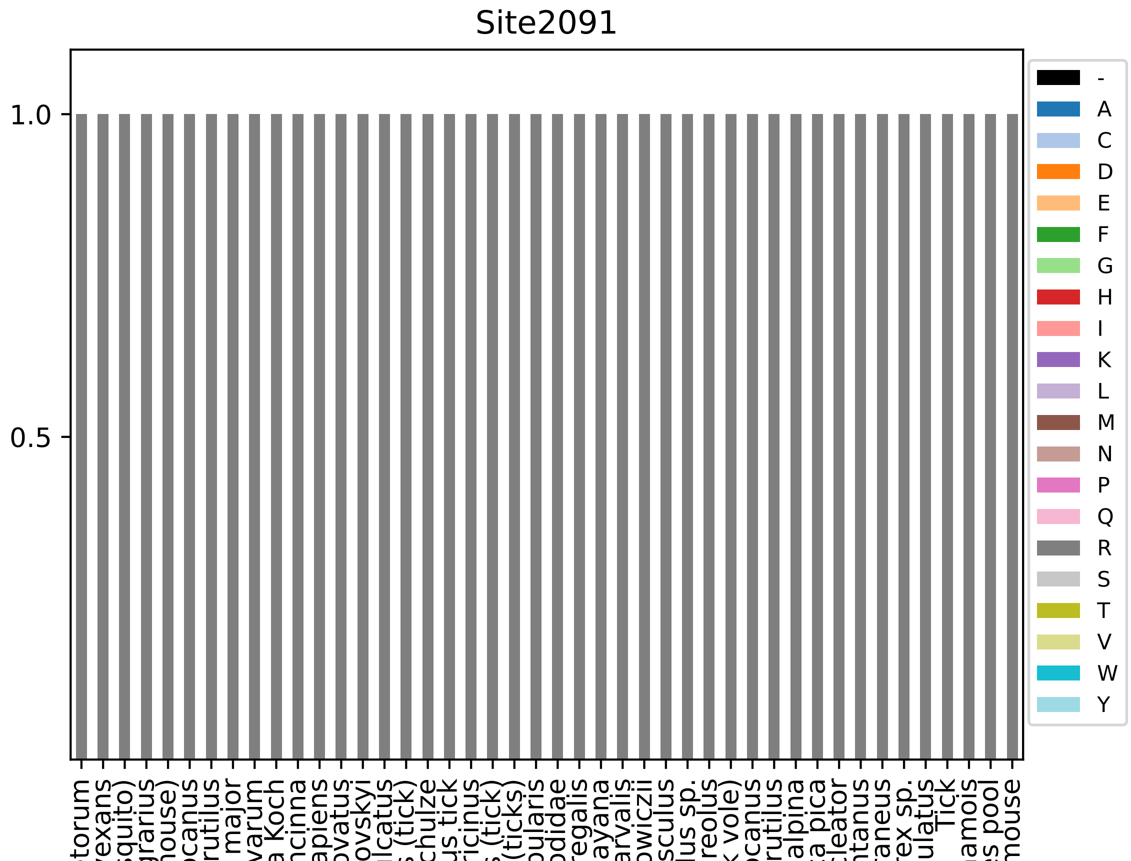
Site2091
1.0
0.5
-
A
C
D
E
F
G
H
I
K
L
M
N
P
Q
R
S
T
V
W
Y

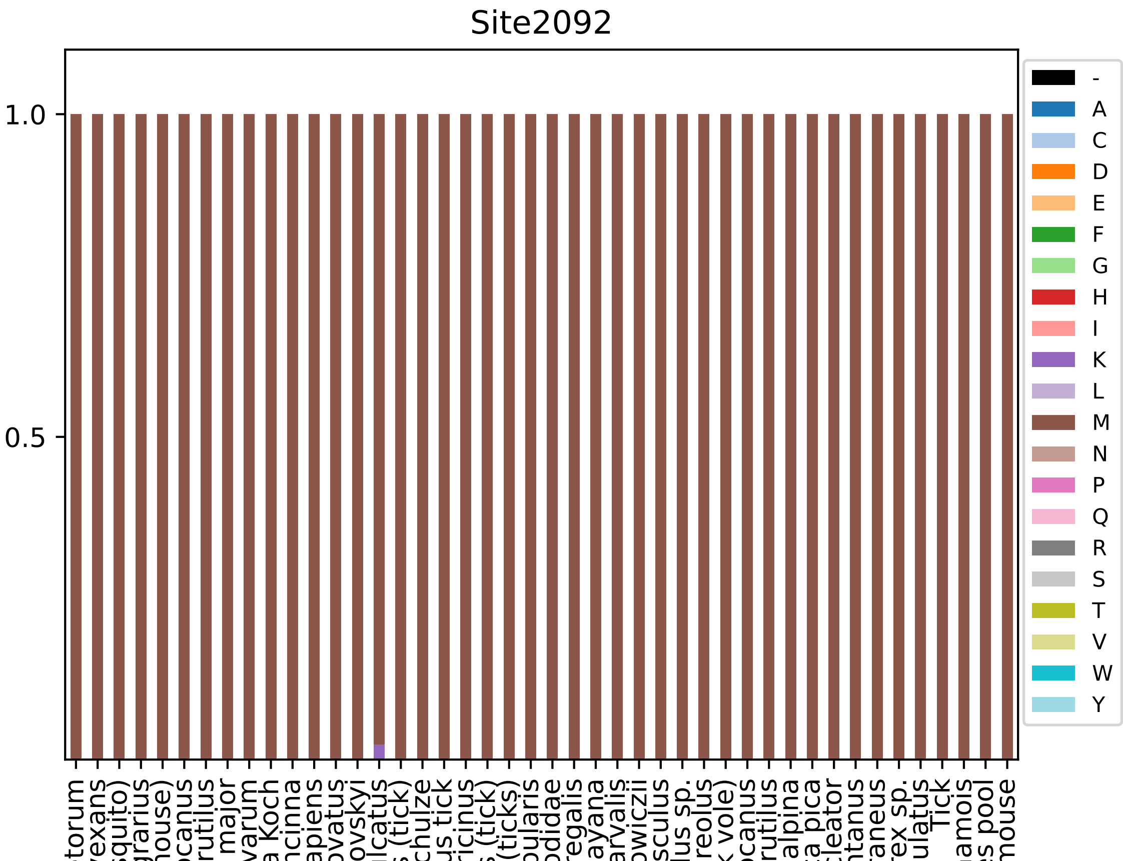
Site2092
1.0
0.5
-
A
C
D
E
F
G
H
I
K
L
M
N
P
Q
R
S
T
V
W
Y

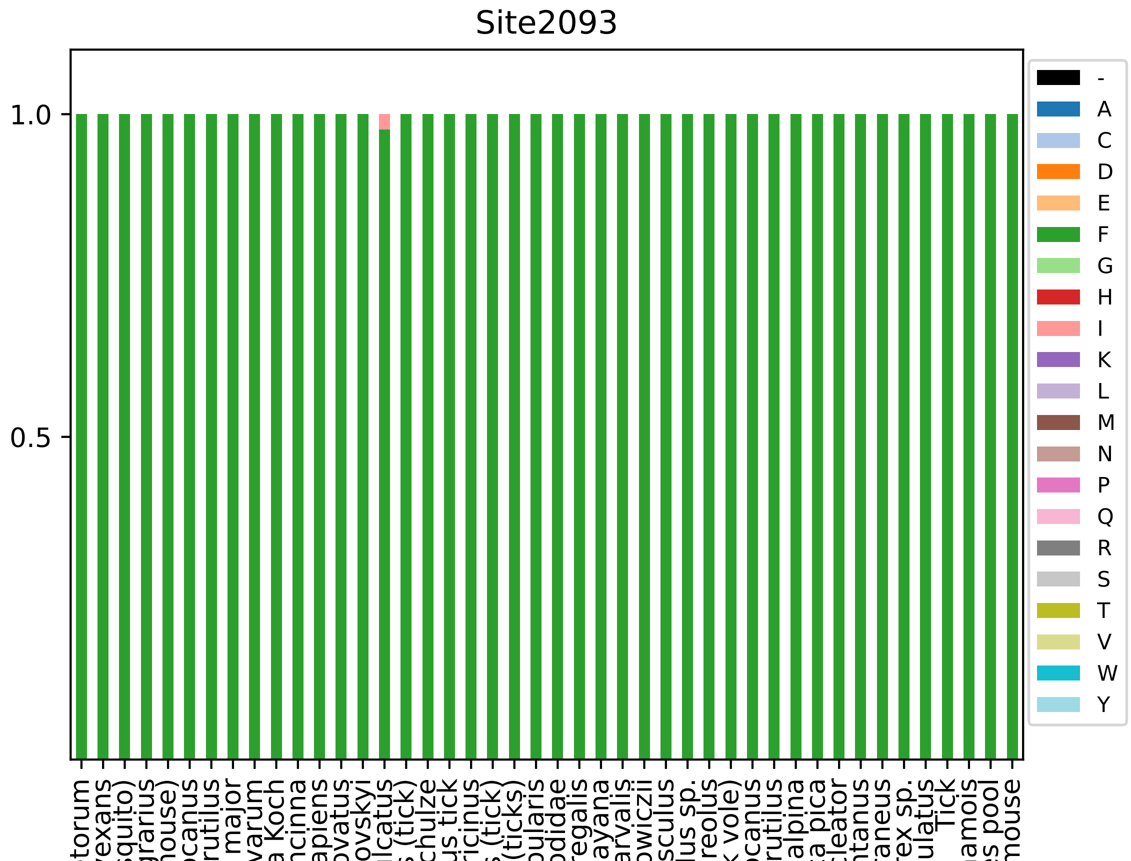

Site2093
1.0
0.5
-
A
C
D
E
F
G
H
I
K
L
M
N
P
Q
R
S
T
V
W
Y

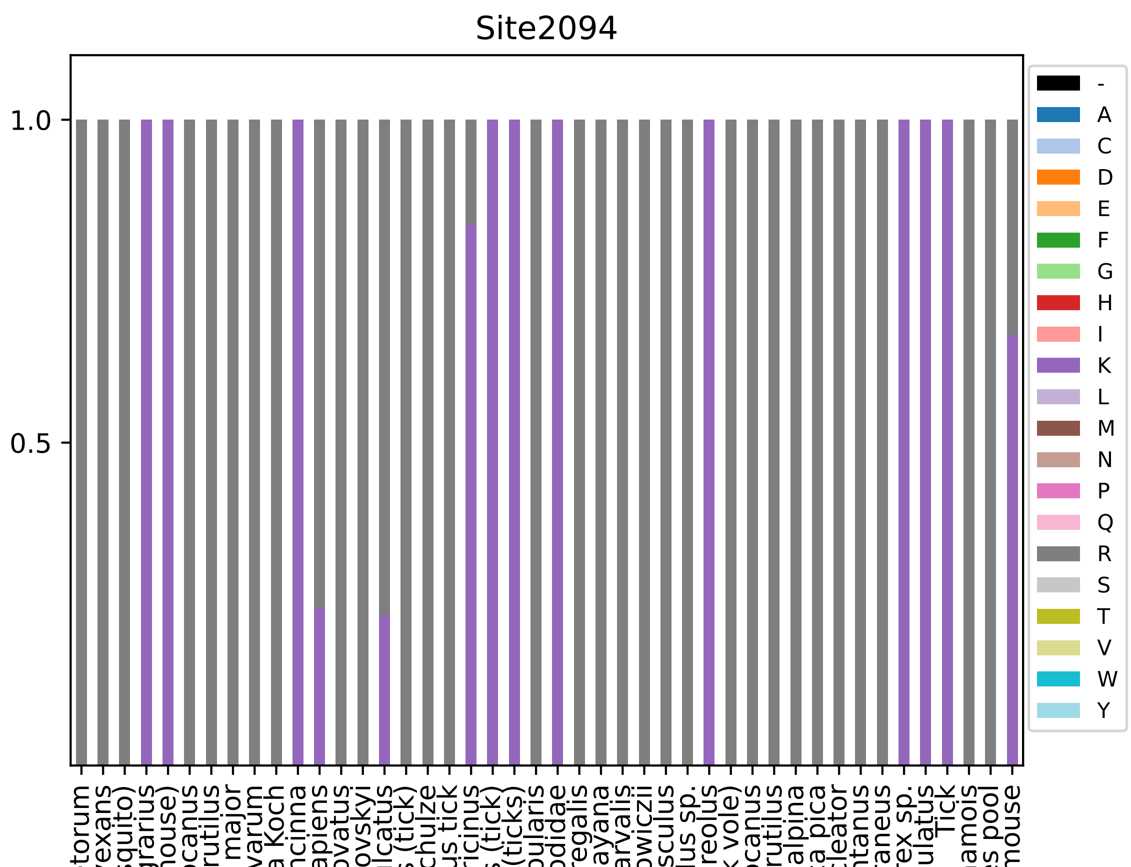
Site2094
1.0
0.5
-
A
C
D
E
F
G
H
I
K
L
M
N
P
Q
R
S
T
V
W
Y

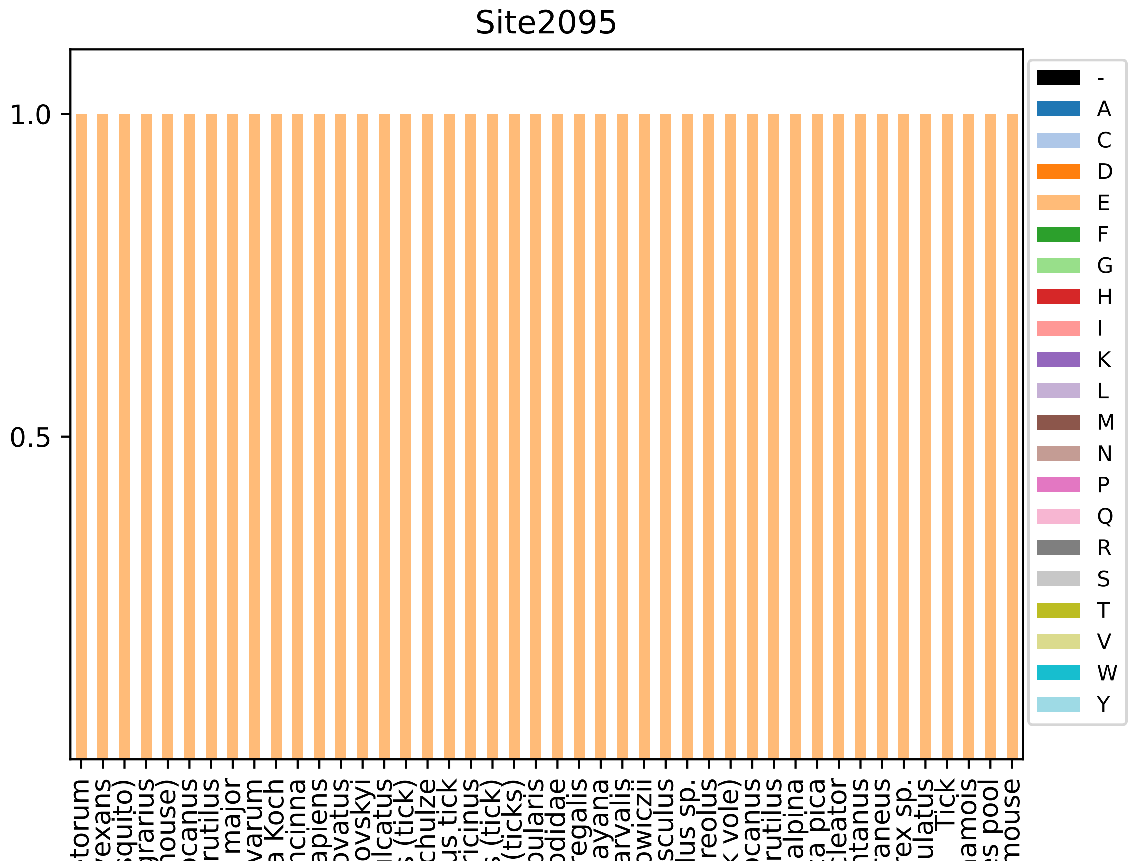
Site2095
1.0
0.5
-
A
C
D
E
F
G
H
I
K
L
M
N
P
Q
R
S
T
V
W
Y

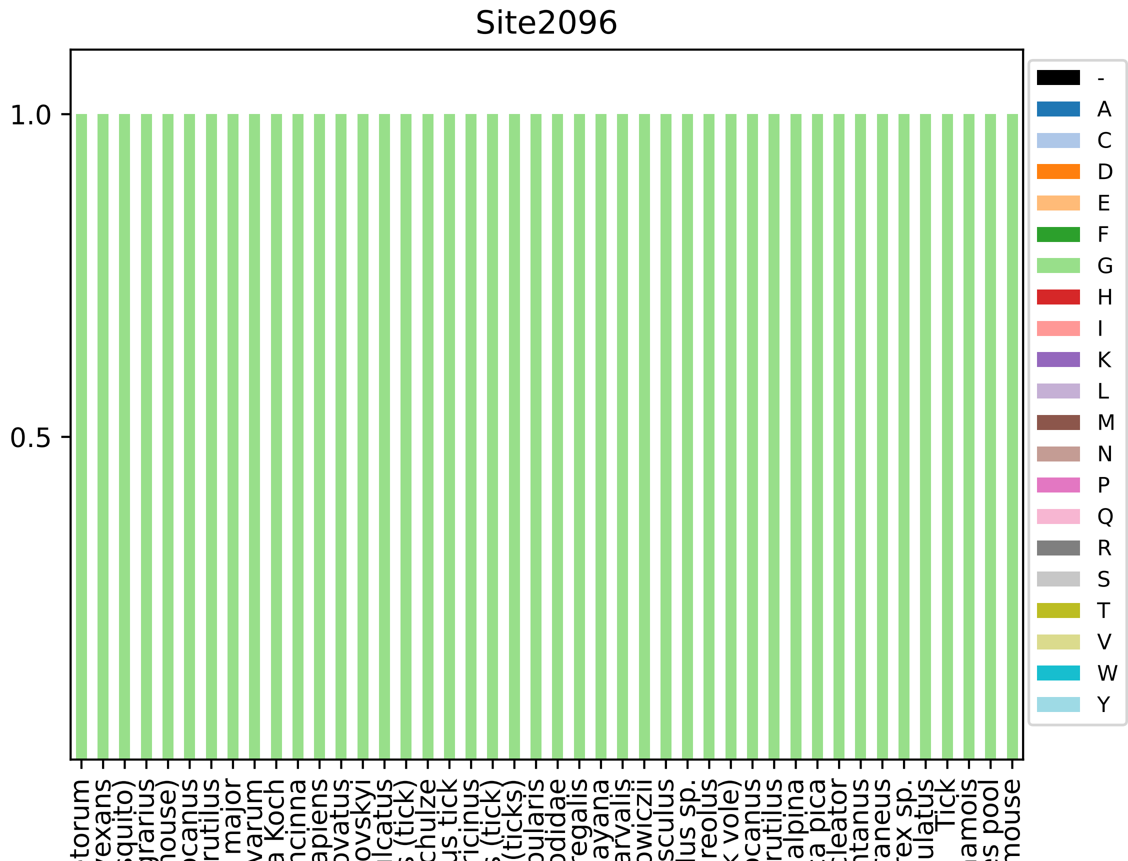
Site2096
1.0
0.5
-
A
C
D
E
F
G
H
I
K
L
M
N
P
Q
R
S
T
V
W
Y

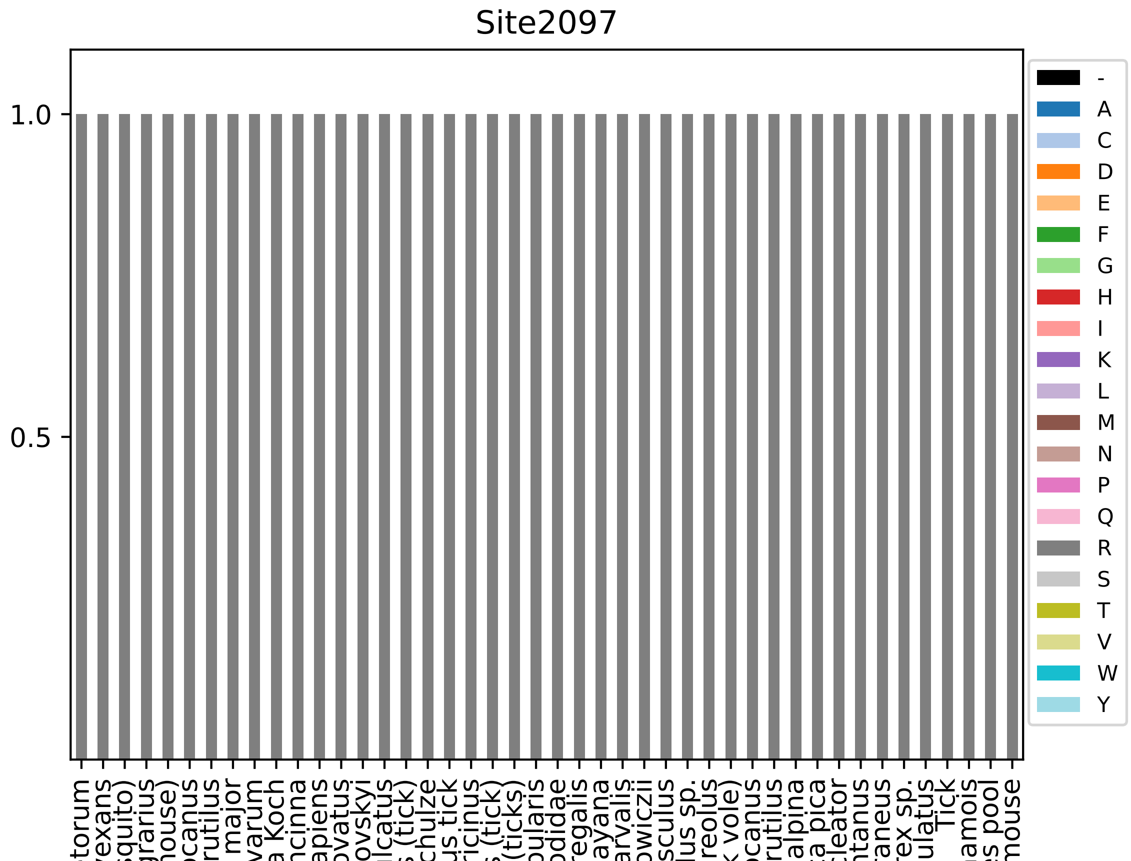
Site2097
1.0
0.5
-
A
C
D
E
F
G
H
I
K
L
M
N
P
Q
R
S
T
V
W
Y

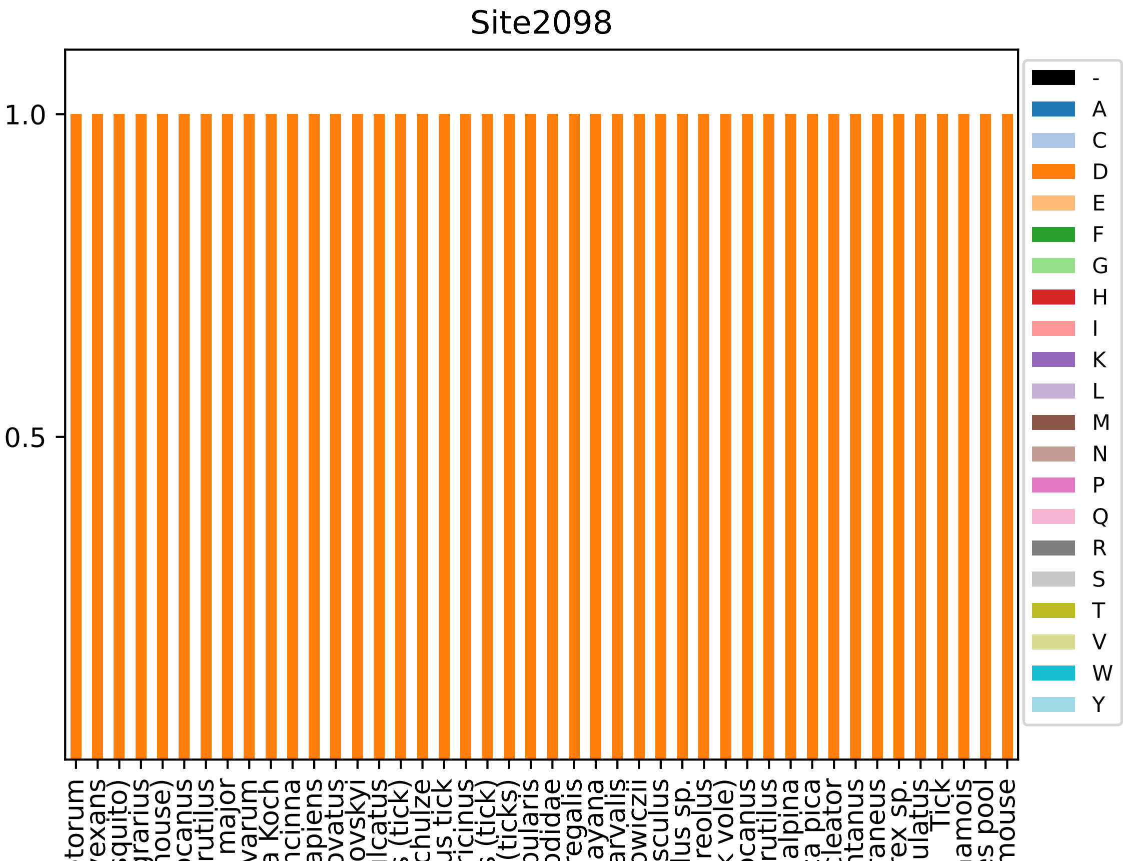
Site2098
1.0
0.5
-
A
C
D
E
F
G
H
I
K
L
M
N
P
Q
R
S
T
V
W
Y

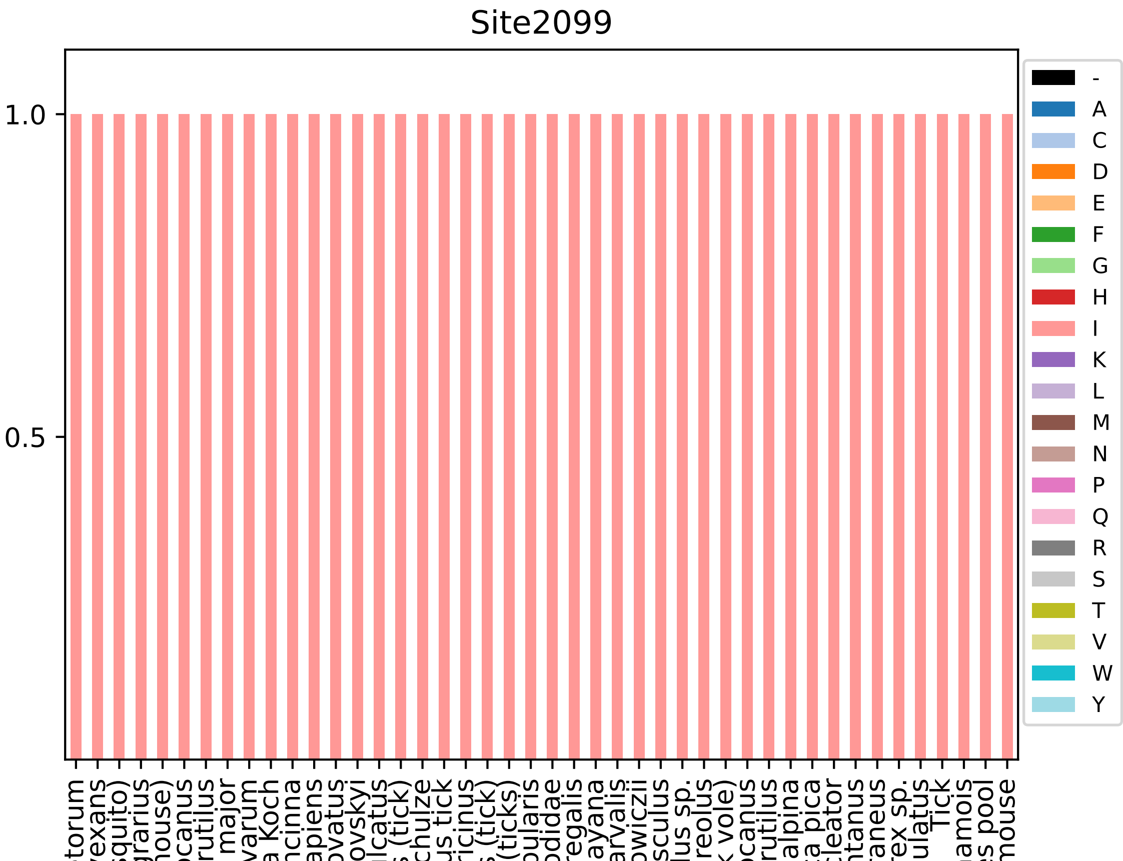
Site2099
1.0
0.5
-
A
C
D
E
F
G
H
I
K
L
M
N
P
Q
R
S
T
V
W
Y

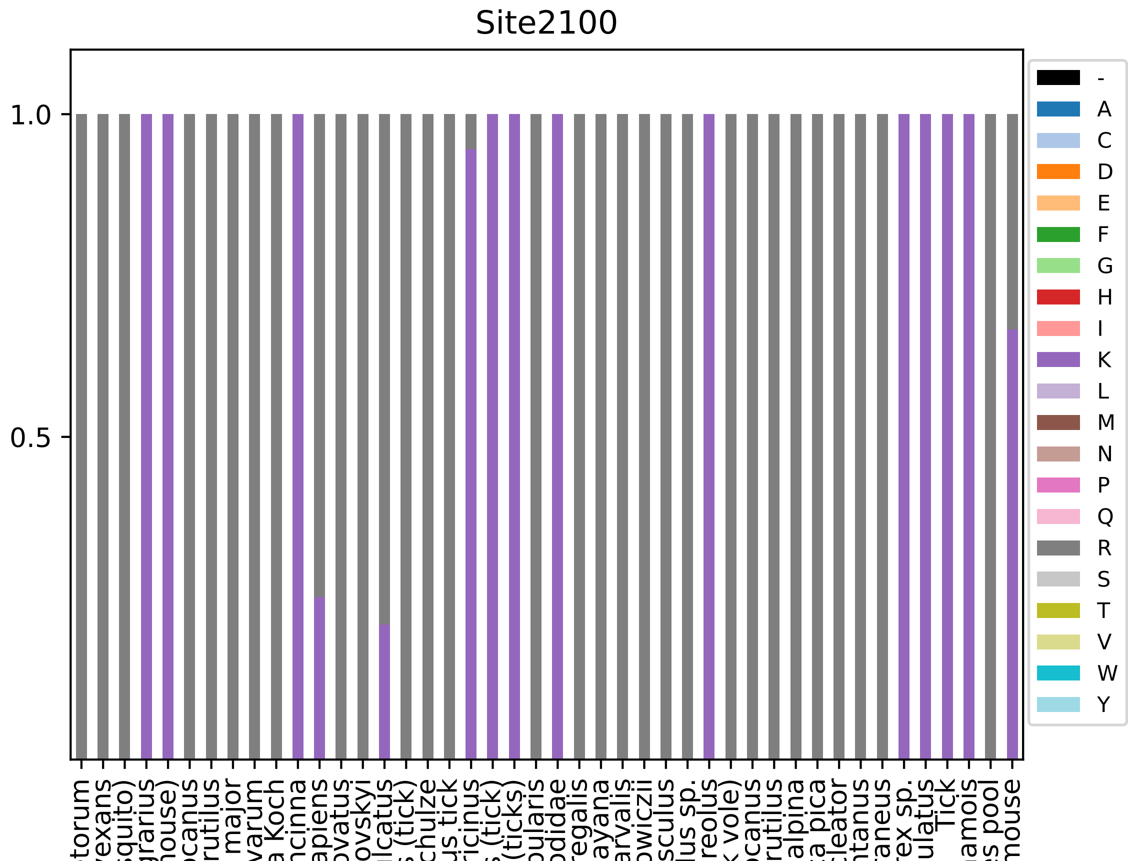
Site2100
1.0
0.5
-
A
C
D
E
F
G
H
I
K
L
M
N
P
Q
R
S
T
V
W
Y

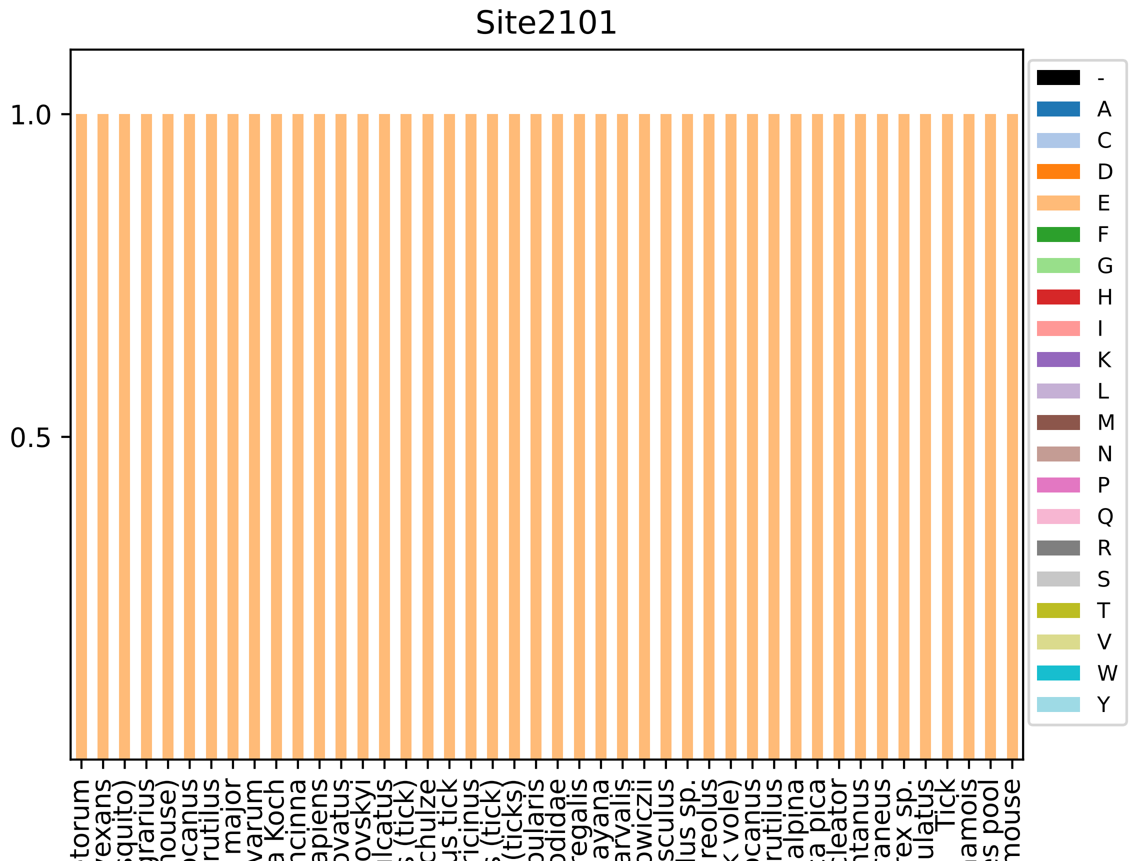

Site2101
1.0
0.5
-
A
C
D
E
F
G
H
I
K
L
M
N
P
Q
R
S
T
V
W
Y

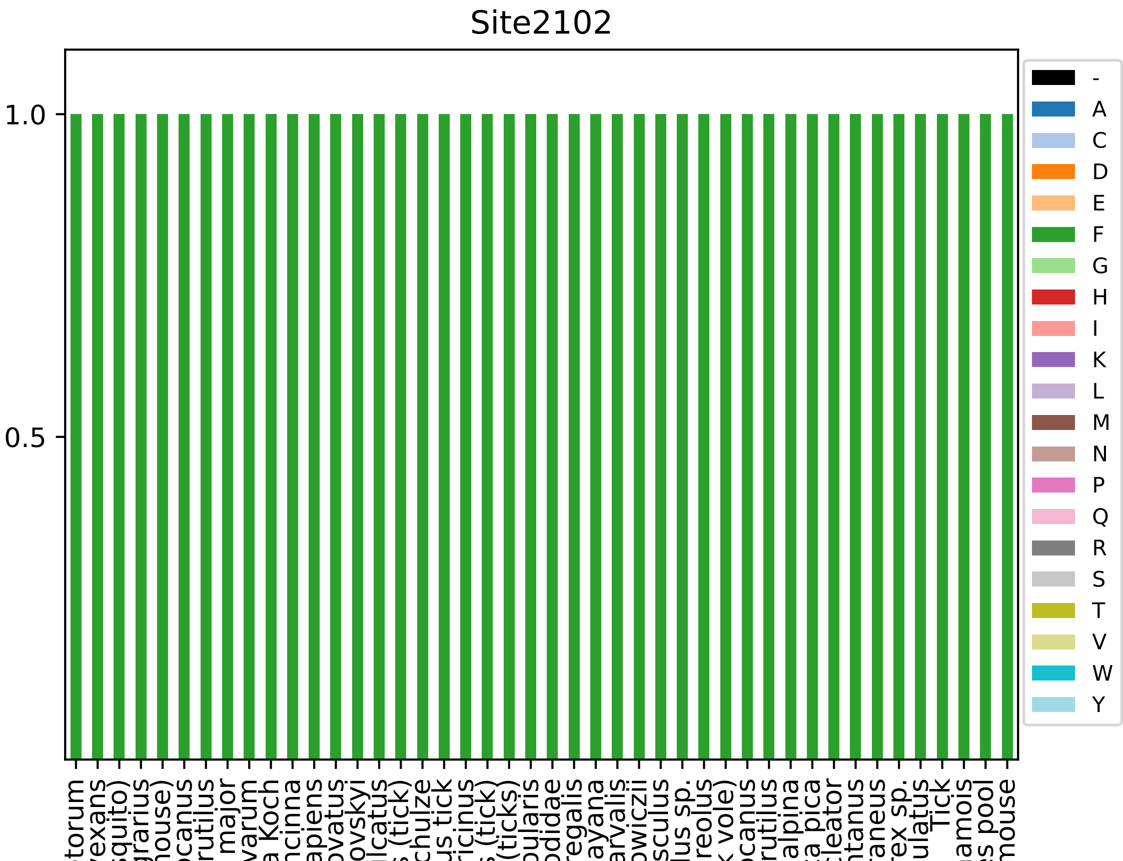
Site2102
1.0
0.5
-
A
C
D
E
F
G
H
I
K
L
M
N
P
Q
R
S
T
V
W
Y

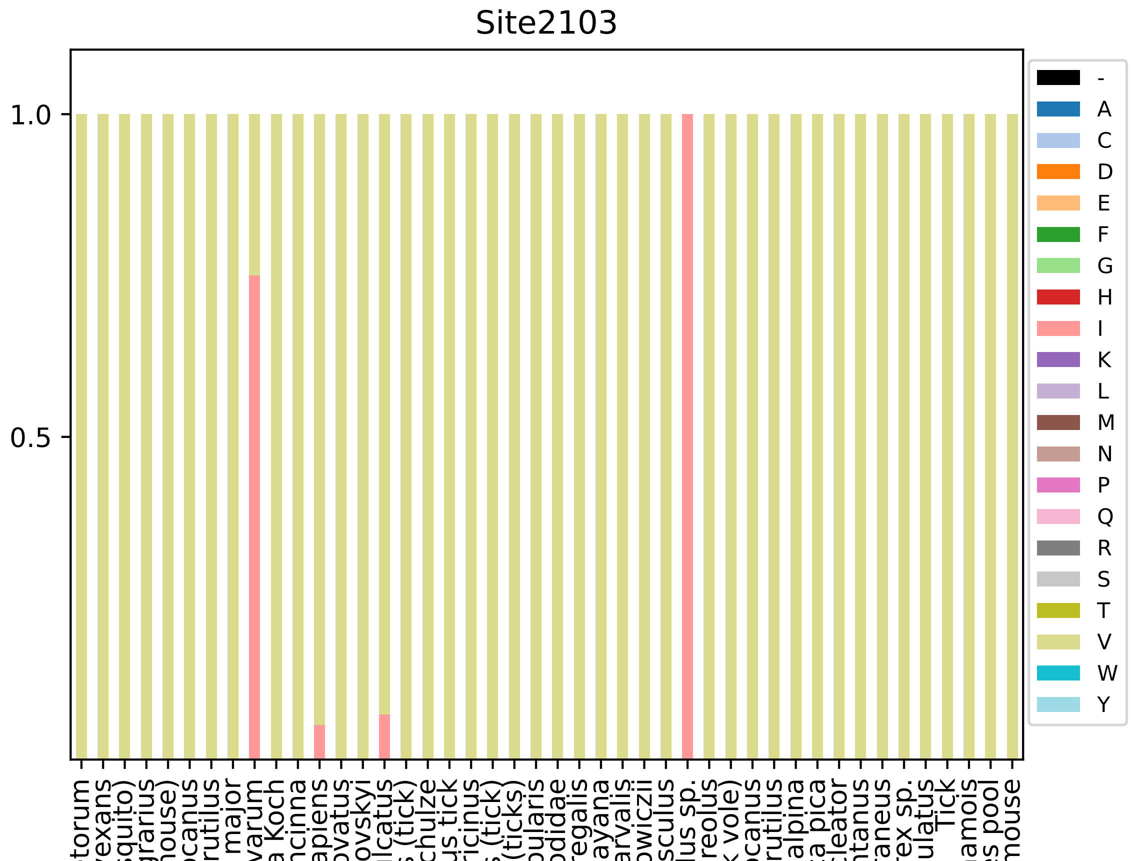
Site2103
1.0
0.5
-
A
C
D
E
F
G
H
I
K
L
M
N
P
Q
R
S
T
V
W
Y

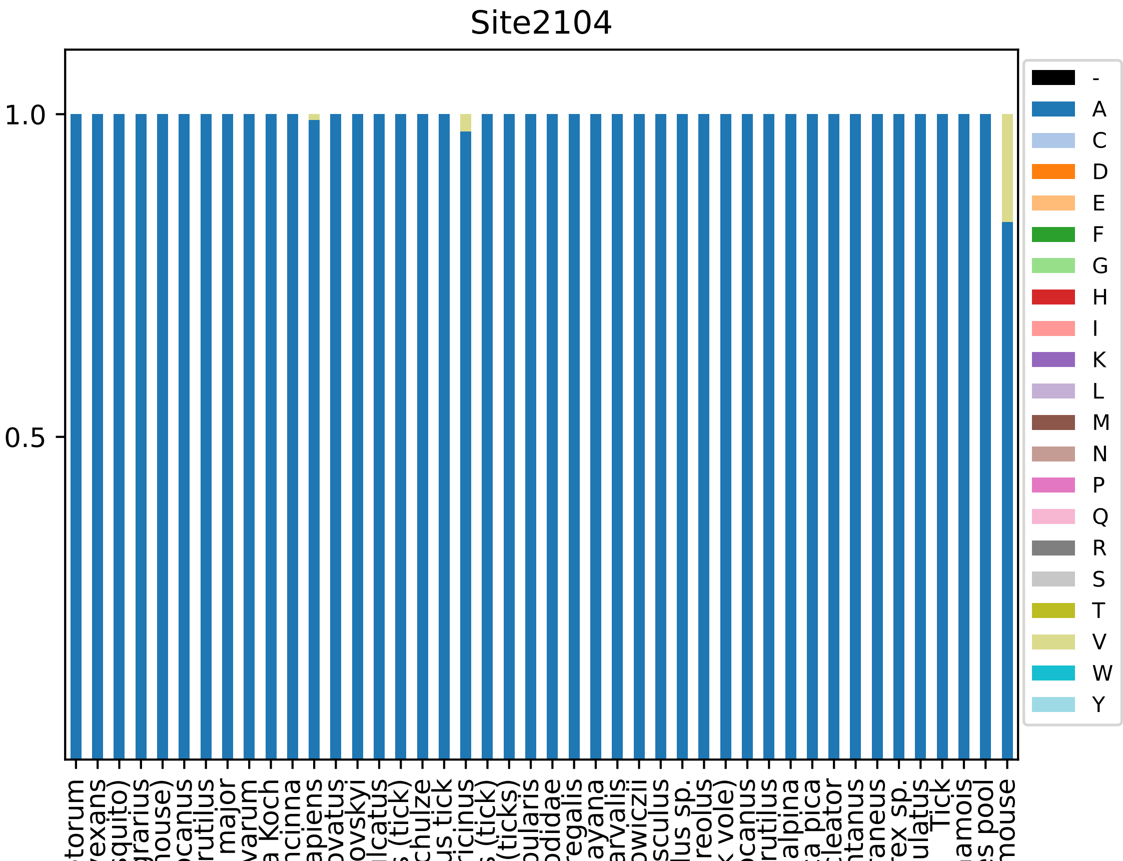
Site2104
1.0
0.5
-
A
C
D
E
F
G
H
I
K
L
M
N
P
Q
R
S
T
V
W
Y

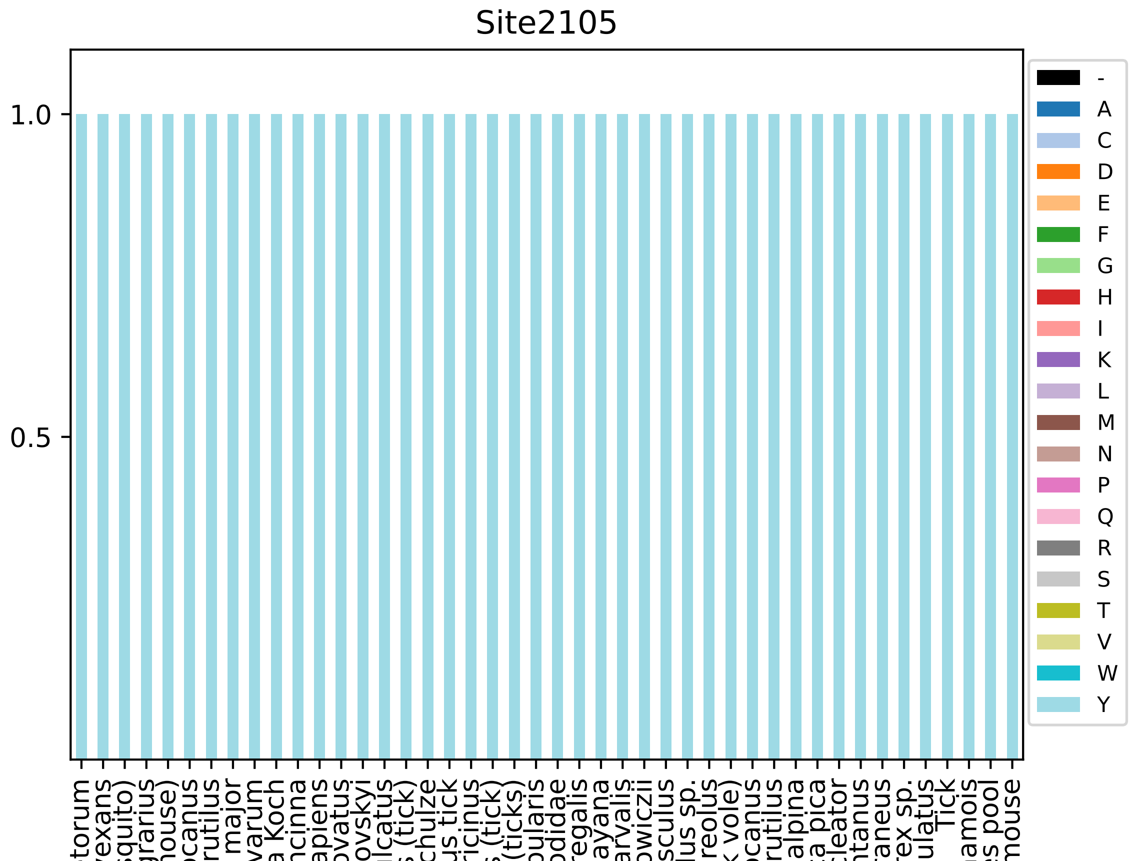

Site2105
1.0
0.5
-
A
C
D
E
F
G
H
I
K
L
M
N
P
Q
R
S
T
V
W
Y

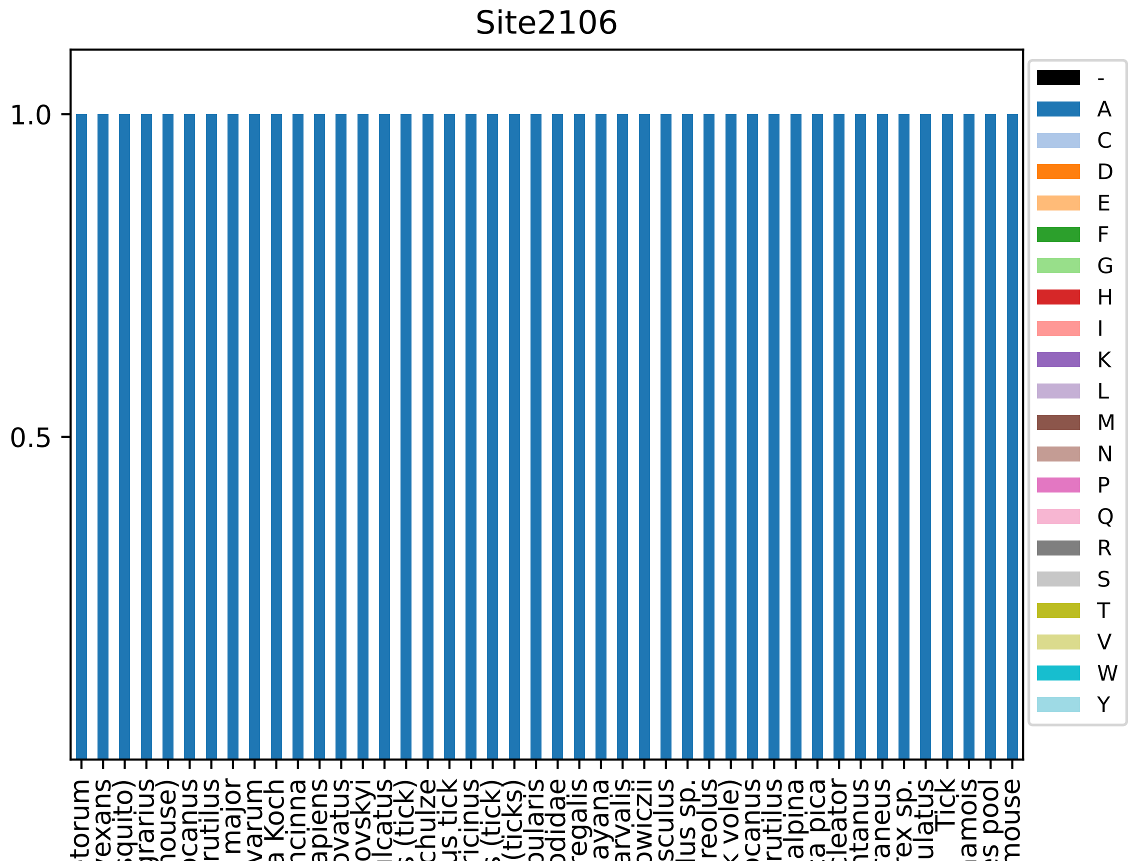

Site2106
1.0
0.5
-
A
C
D
E
F
G
H
I
K
L
M
N
P
Q
R
S
T
V
W
Y
Tick

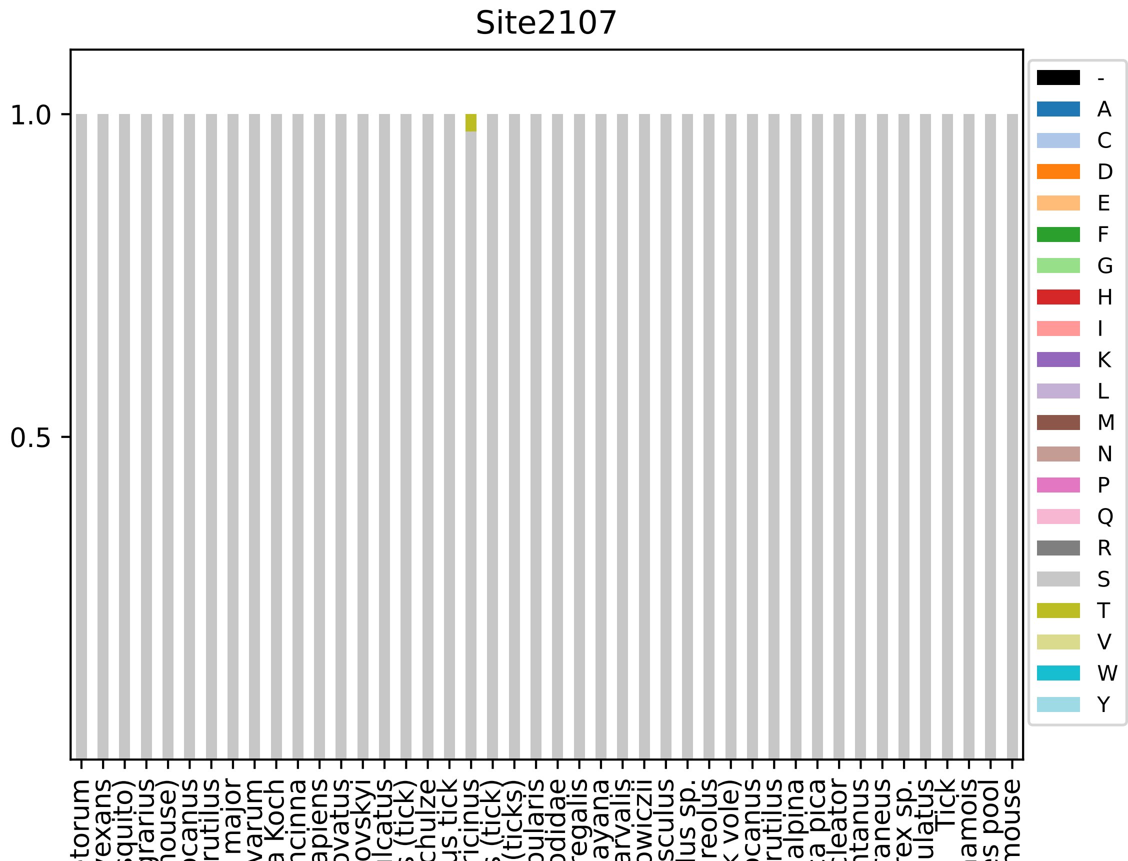
Site2107
1.0
0.5
-
A
C
D
E
F
G
H
I
K
L
M
N
P
Q
R
S
T
V
W
Y

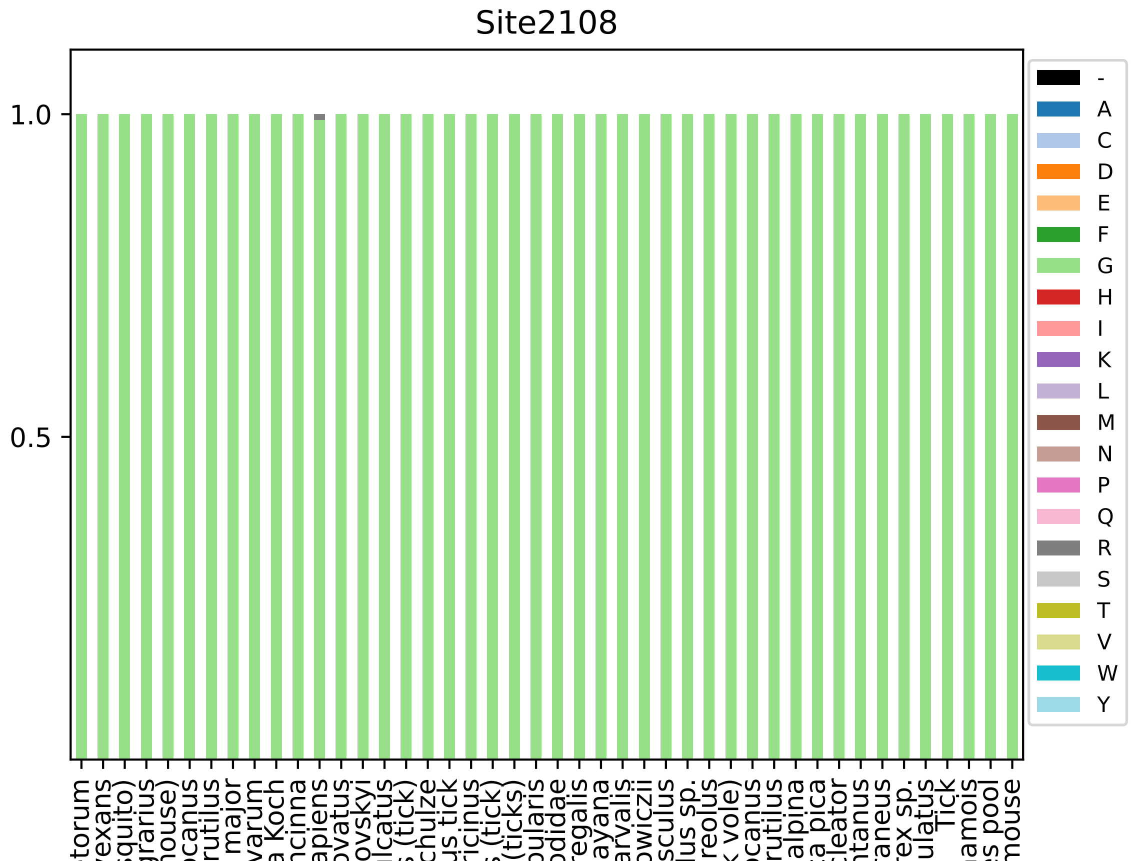
Site2108
1.0
0.5
-
A
C
D
E
F
G
H
I
K
L
M
N
P
Q
R
S
T
V
W
Y

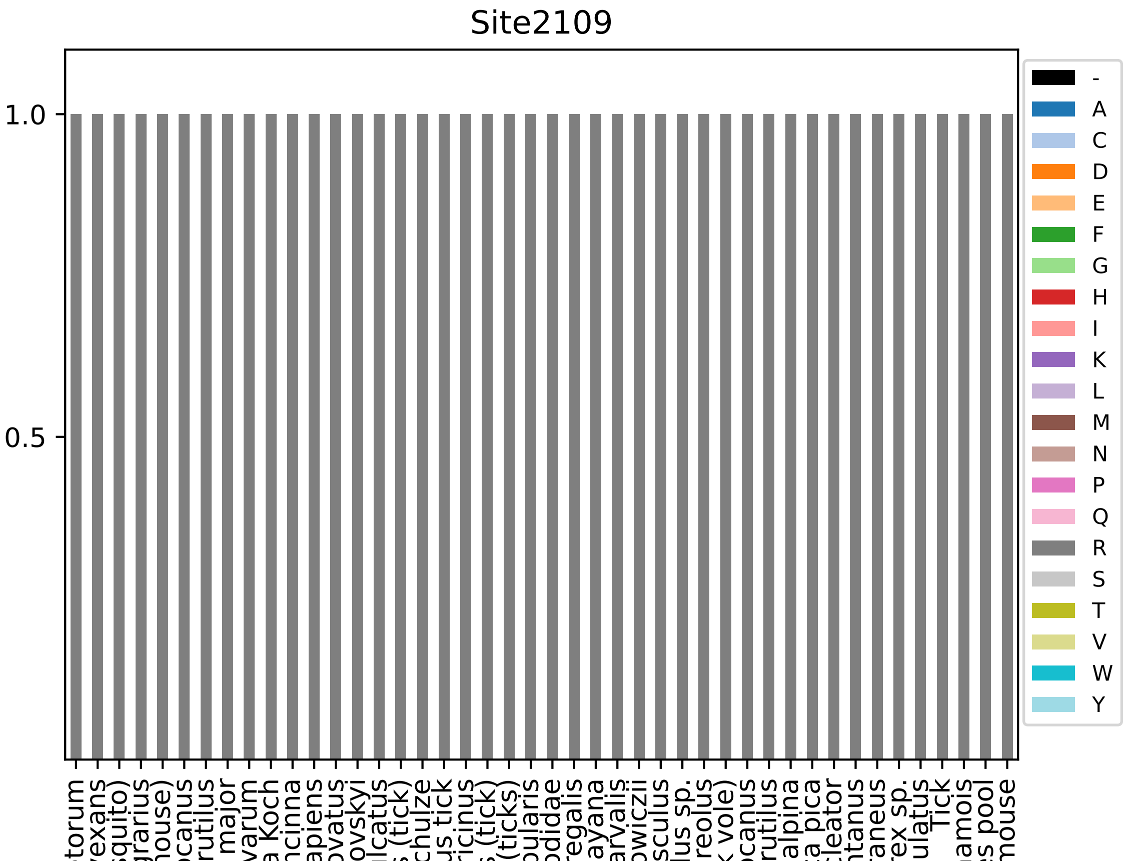
Site2109
1.0
0.5
-
A
C
D
E
F
G
H
I
K
L
M
N
P
Q
R
S
T
V
W
Y

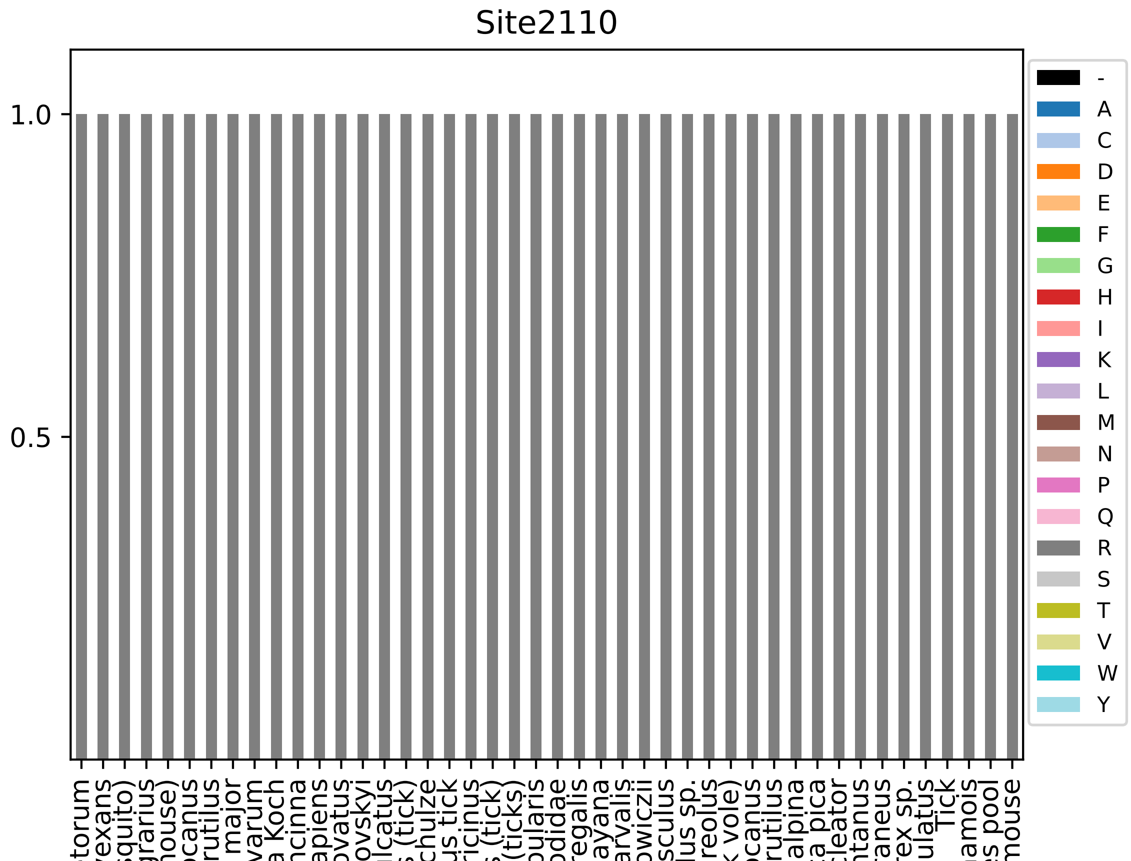

Site2110
1.0
0.5
-
A
C
D
E
F
G
H
I
K
L
M
N
P
Q
R
S
T
V
W
Y

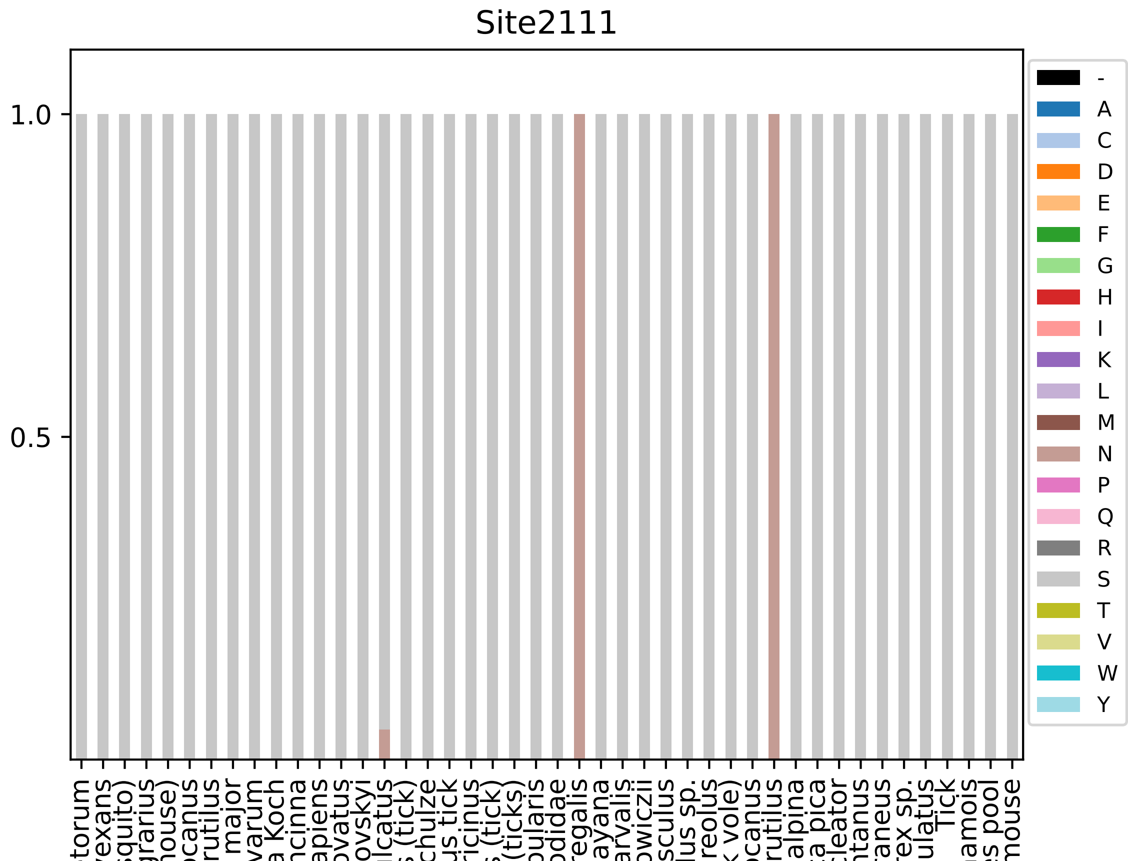
Site2111
1.0
0.5
-
A
C
D
E
F
G
H
I
K
L
M
N
P
Q
R
S
T
V
W
Y

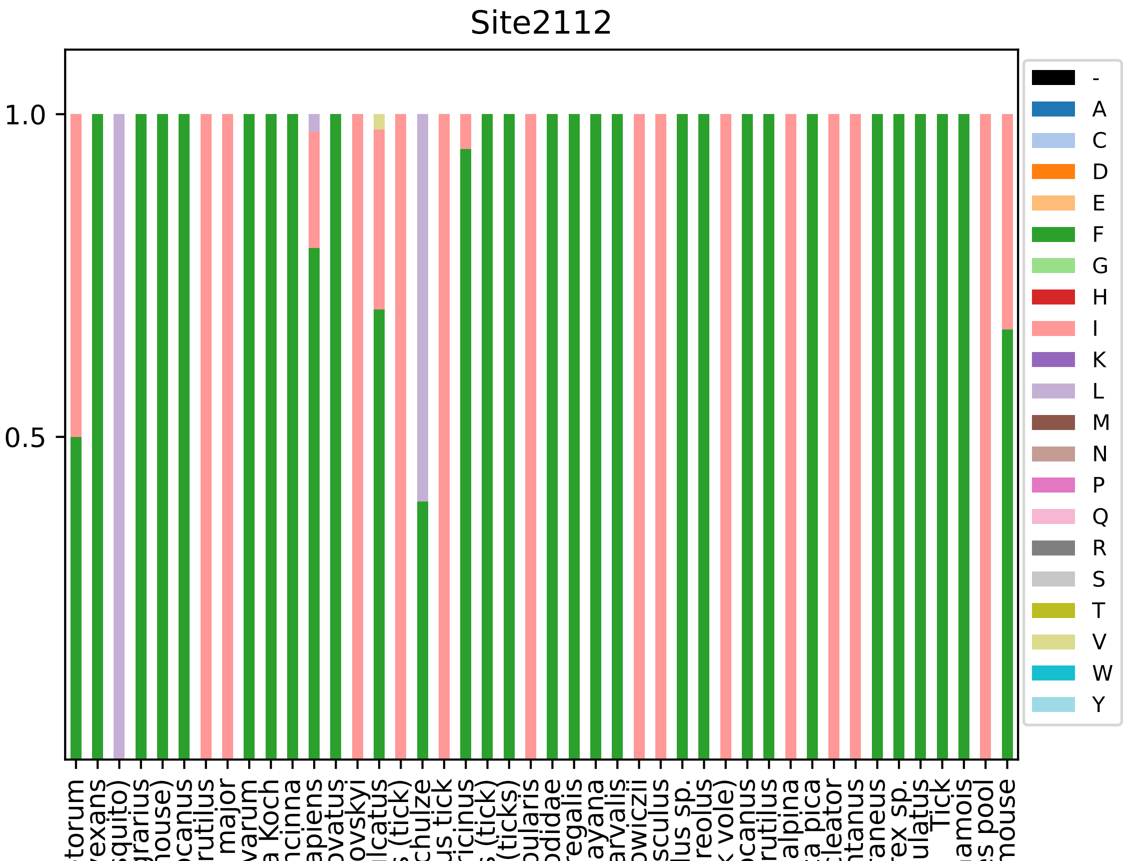
Site2112
1.0
0.5
-
A
C
D
E
F
G
H
I
K
L
M
N
P
Q
R
S
T
V
W
Y

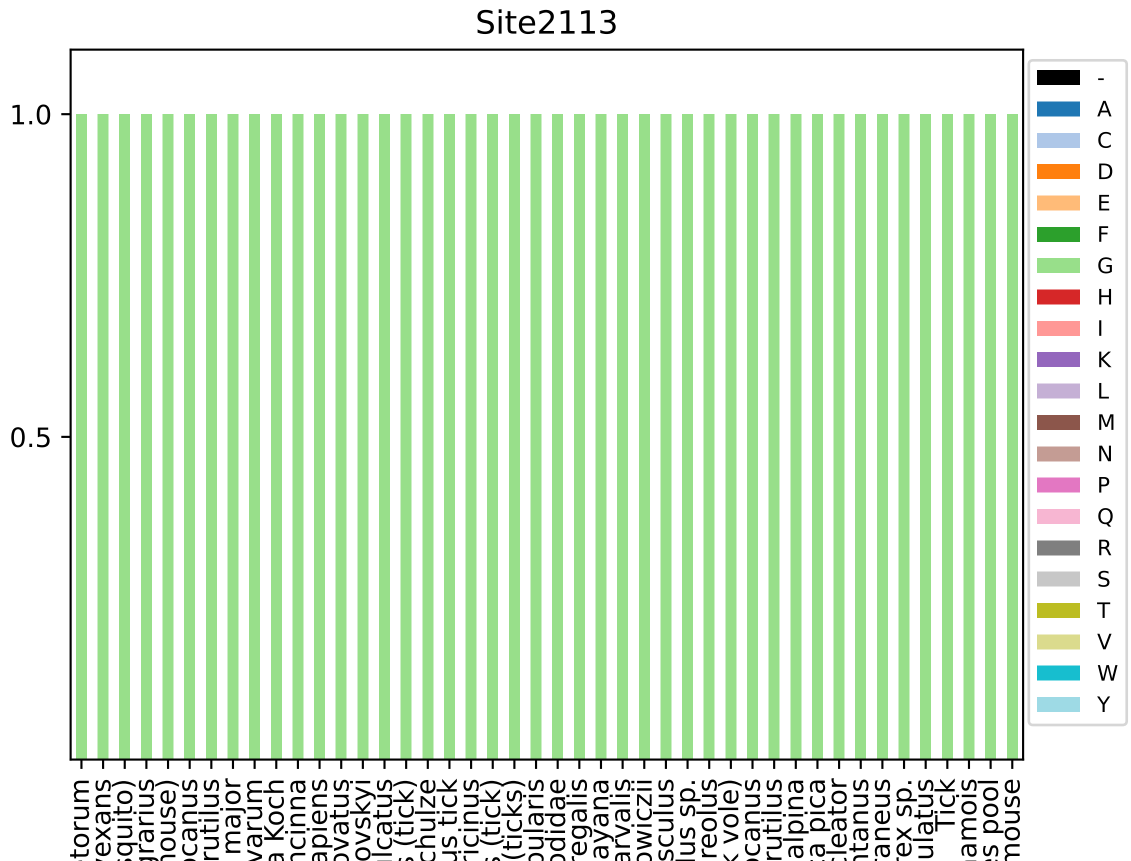
Site2113
1.0
0.5
-
A
C
D
E
F
G
H
I
K
L
M
N
P
Q
R
S
T
V
W
Y

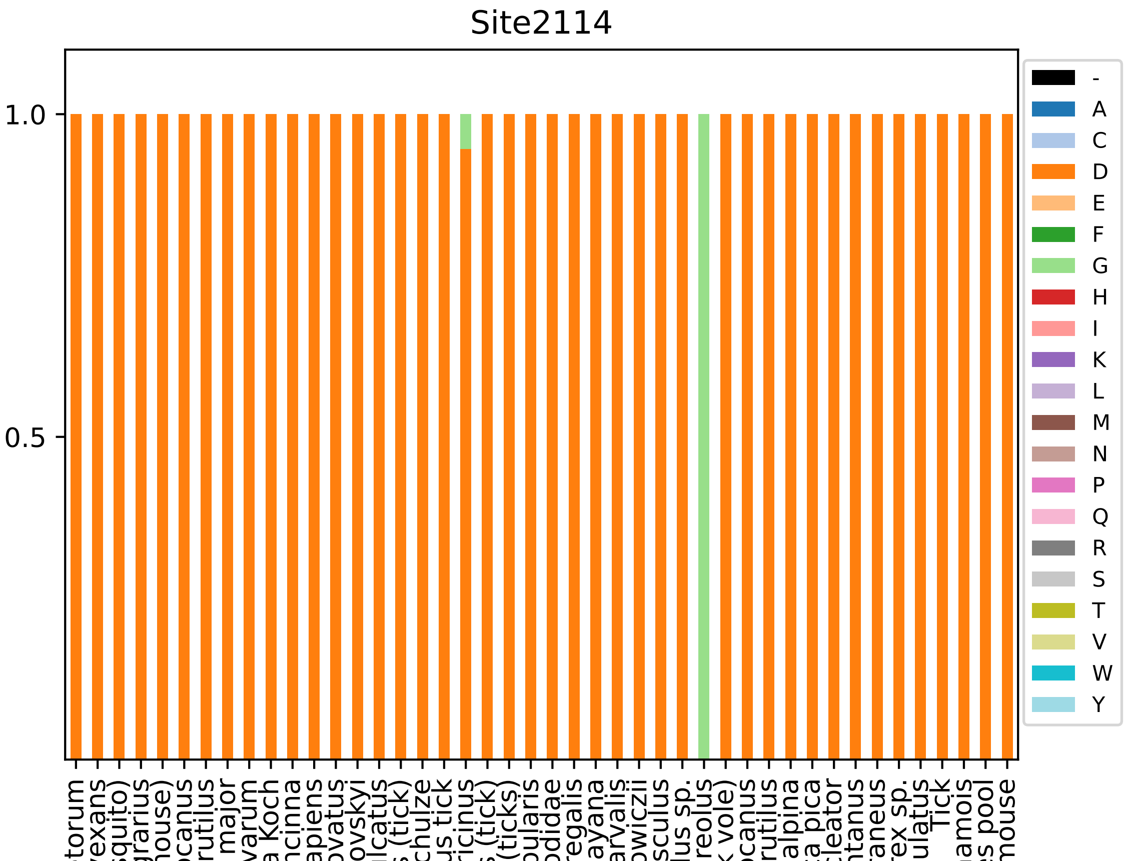
Site2114
1.0
0.5
-
A
C
D
E
F
G
H
I
K
L
M
N
P
Q
R
S
T
V
W
Y

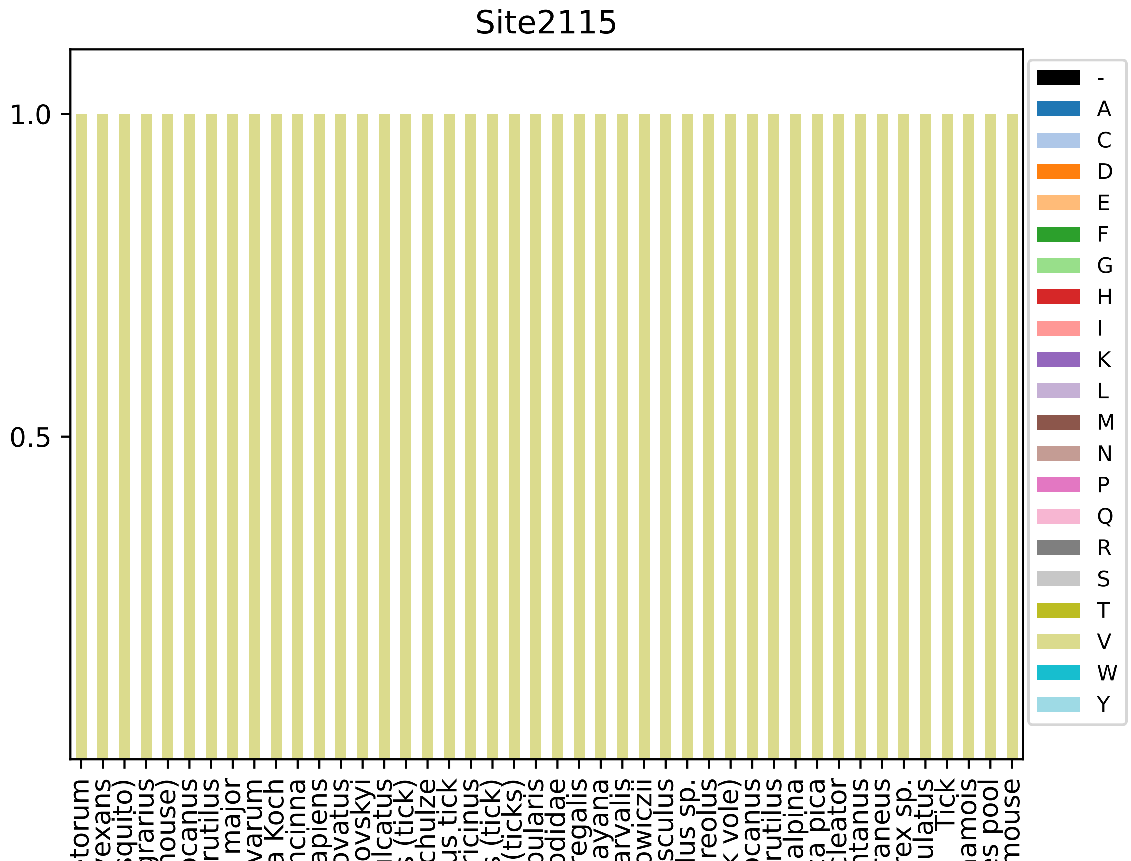
Site2115
1.0
0.5
-
A
C
D
E
F
G
H
I
K
L
M
N
P
Q
R
S
T
V
W
Y

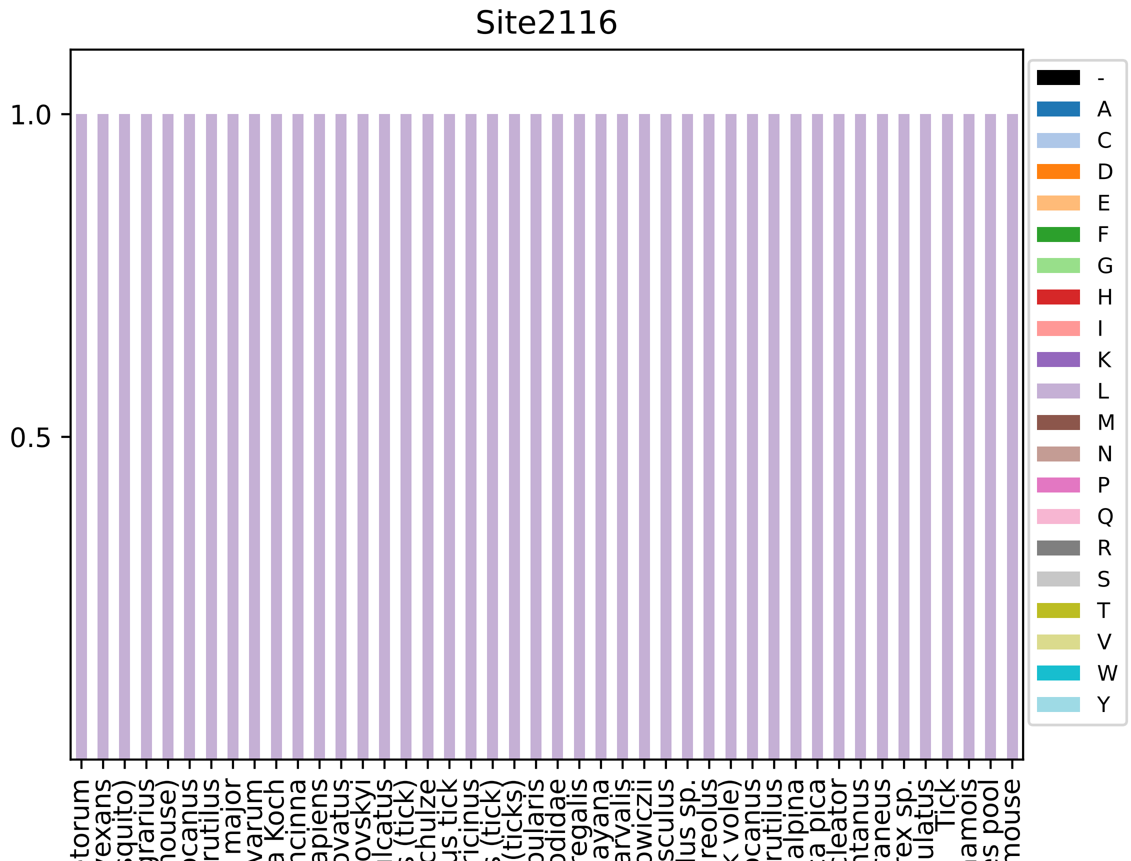

Site2116
1.0
0.5
-
A
C
D
E
F
G
H
I
K
L
M
N
P
Q
R
S
T
V
W
Y

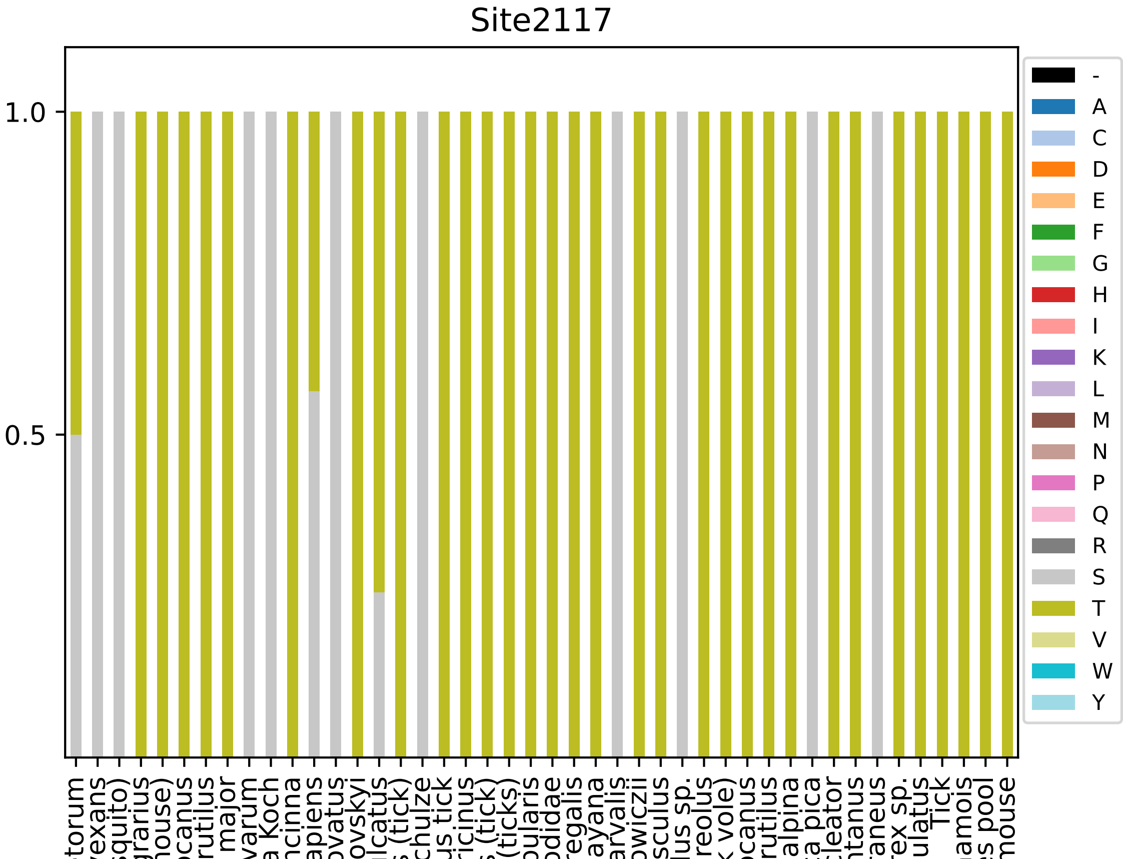

Site2117
1.0
0.5
-
A
C
D
E
F
G
H
I
K
L
M
N
P
Q
R
S
T
V
W
Y

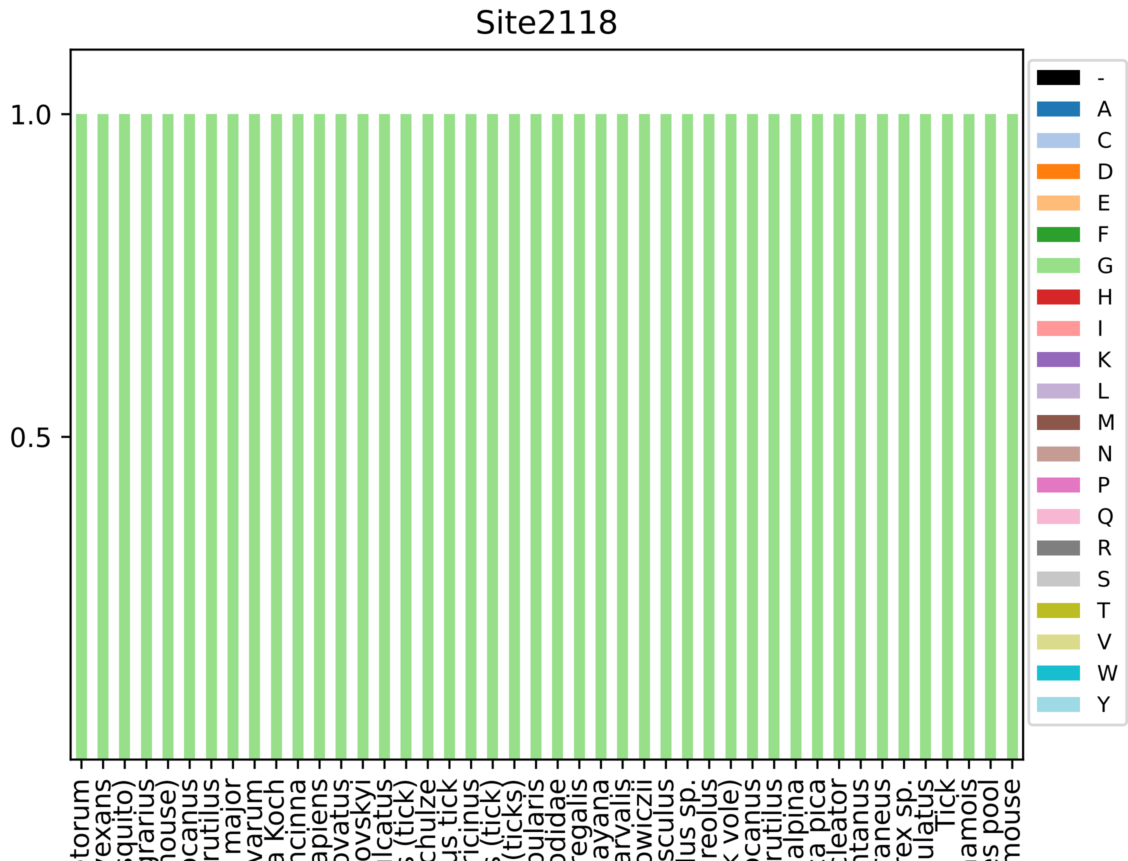

Site2118
1.0
0.5
-
A
C
D
E
F
G
H
I
K
L
M
N
P
Q
R
S
T
V
W
Y

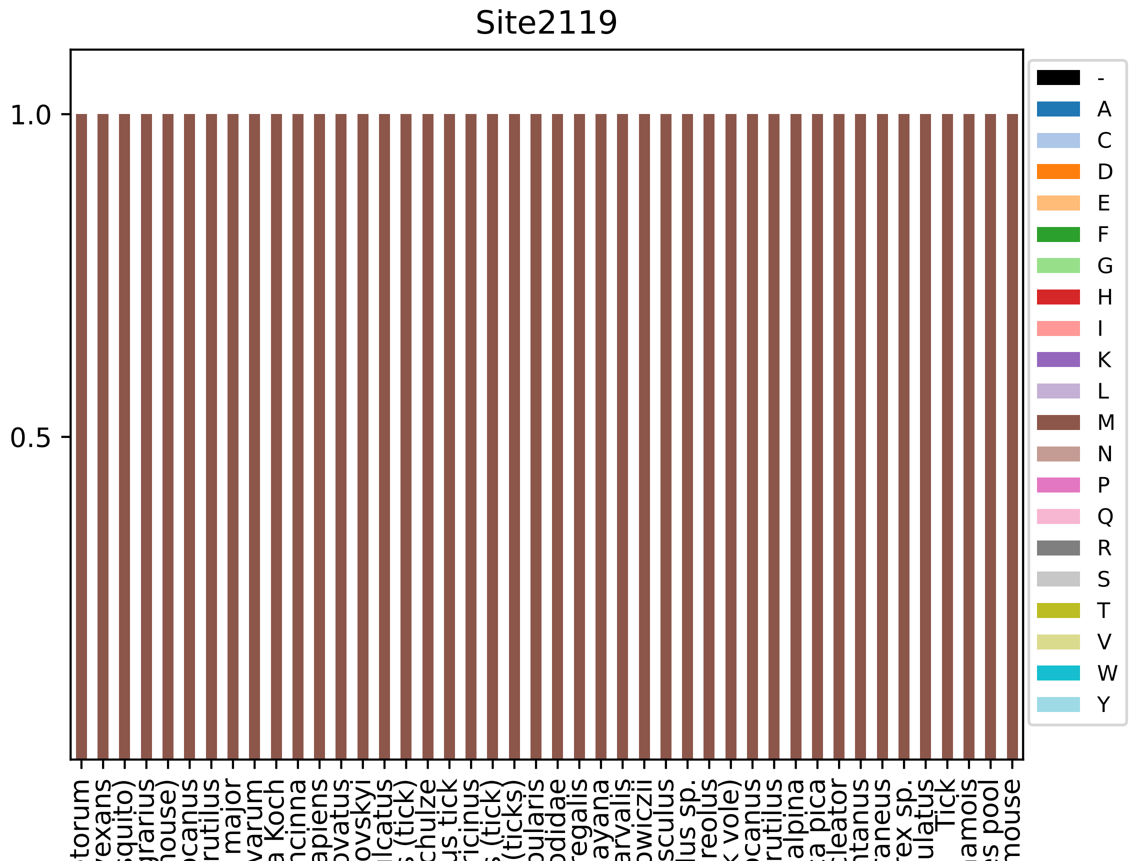
Site2119
1.0
0.5
-
A
C
D
E
F
G
H
I
K
L
M
N
P
Q
R
S
T
V
W
Y

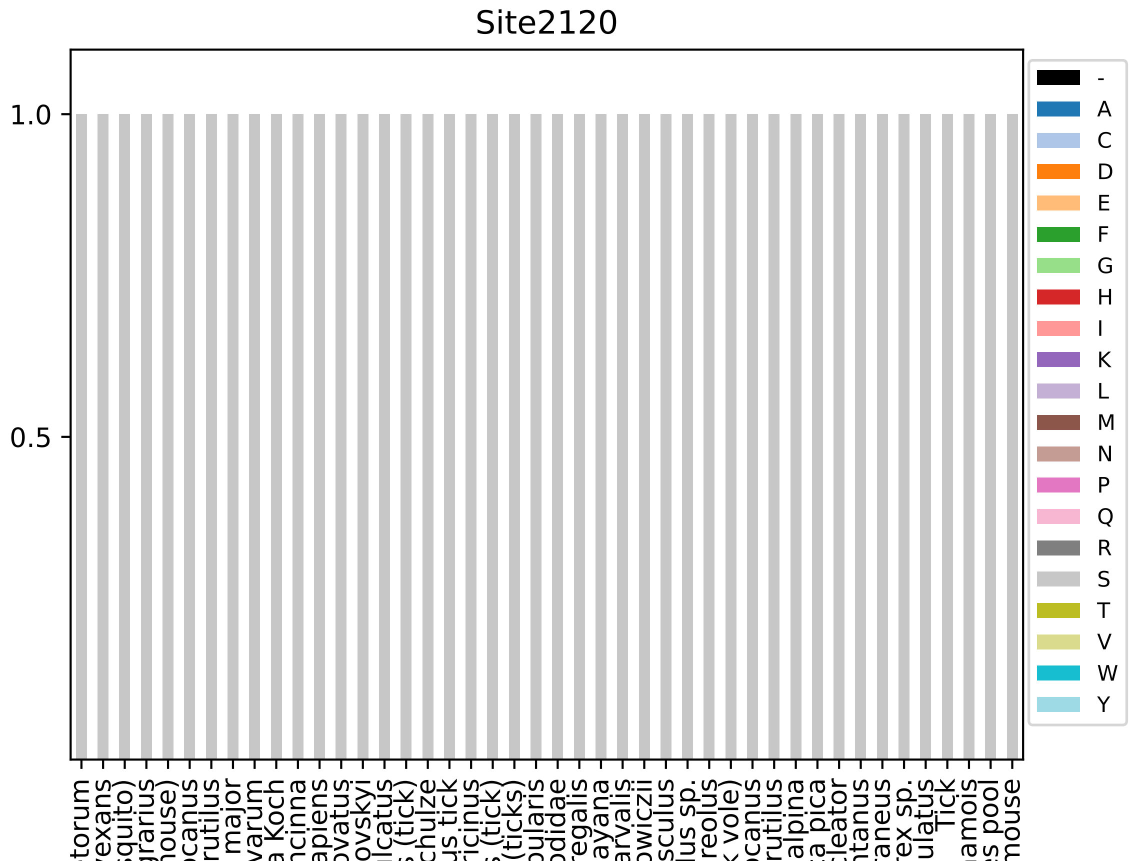

Site2120
1.0
0.5
-
A
C
D
E
F
G
H
I
K
L
M
N
P
Q
R
S
T
V
W
Y

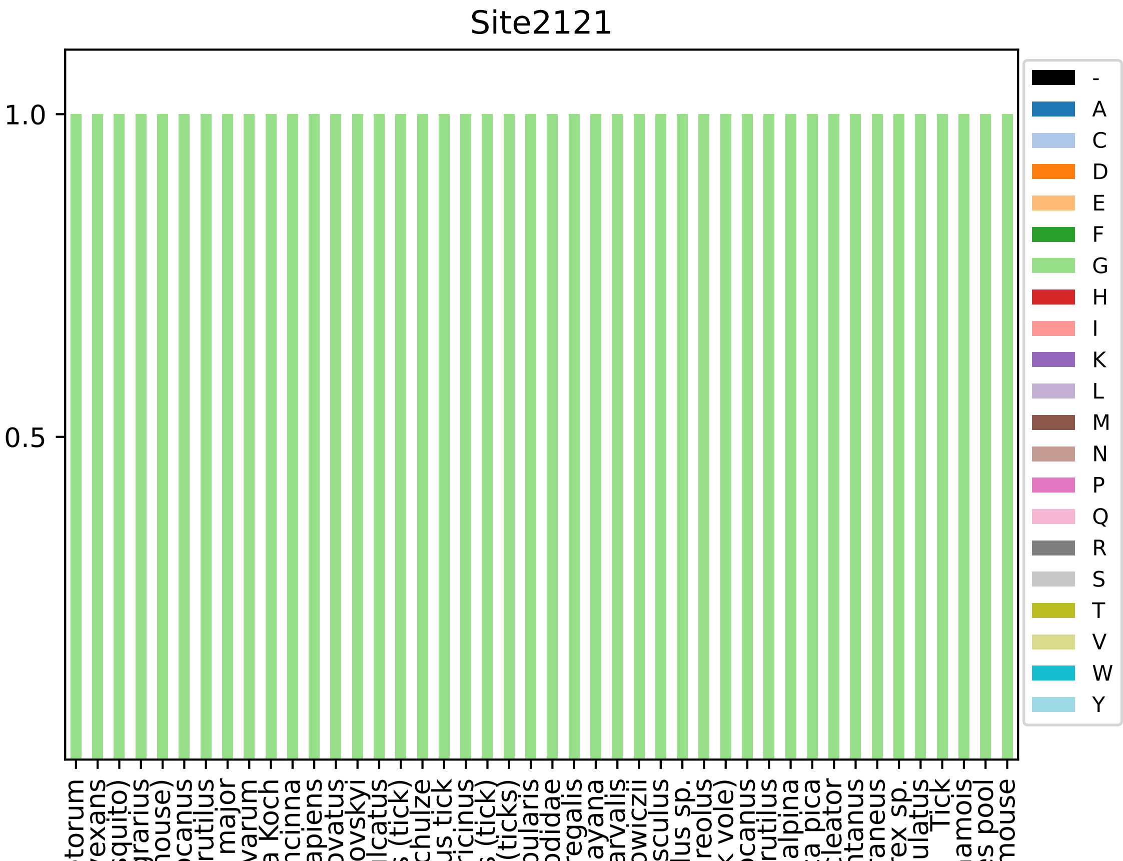

Site2121
1.0
0.5
-
A
C
D
E
F
G
H
I
K
L
M
N
P
Q
R
S
T
V
W
Y

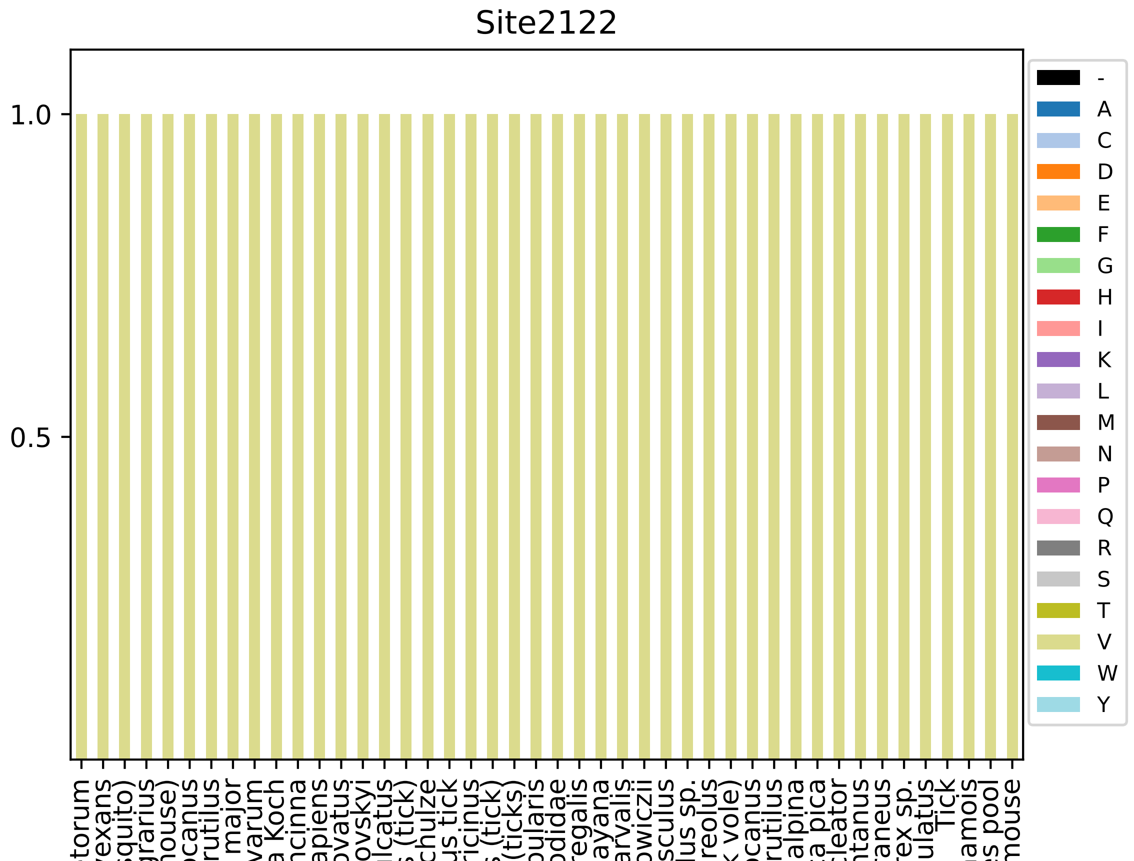
Site2122
1.0
0.5
-
A
C
D
E
F
G
H
I
K
L
M
N
P
Q
R
S
T
V
W
Y

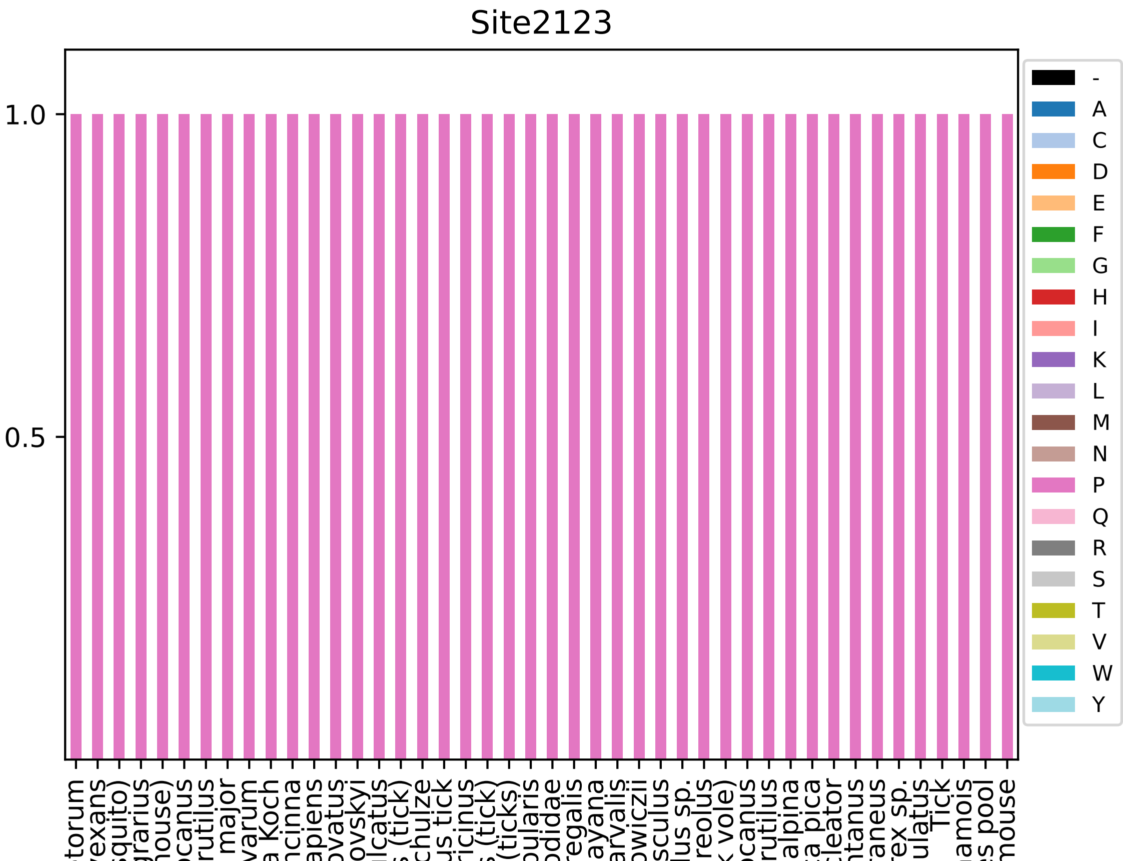

Site2123
1.0
0.5
-
A
C
D
E
F
G
H
I
K
L
M
N
P
Q
R
S
T
V
W
Y

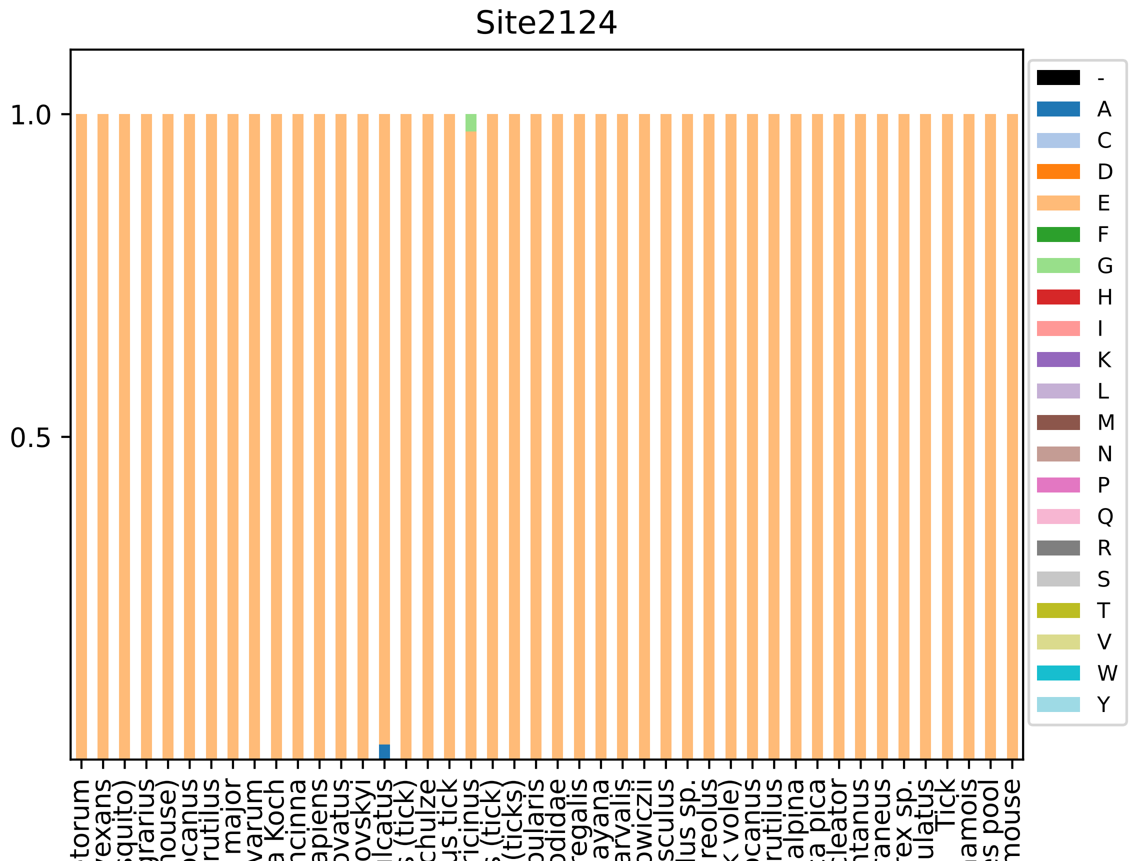
Site2124
1.0
0.5
-
A
C
D
E
F
G
H
I
K
L
M
N
P
Q
R
S
T
V
W
Y

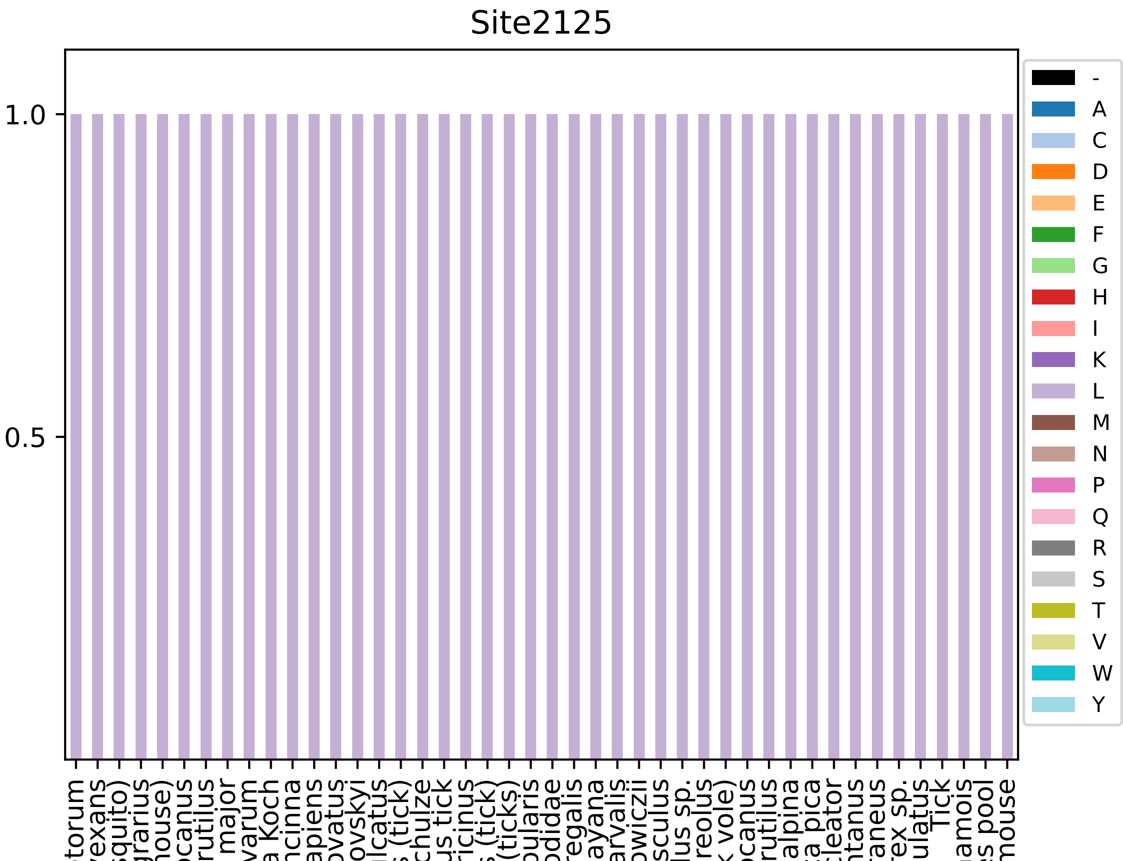
Site2125
1.0
0.5
-
A
C
D
E
F
G
H
I
K
L
M
N
P
Q
R
S
T
V
W
Y

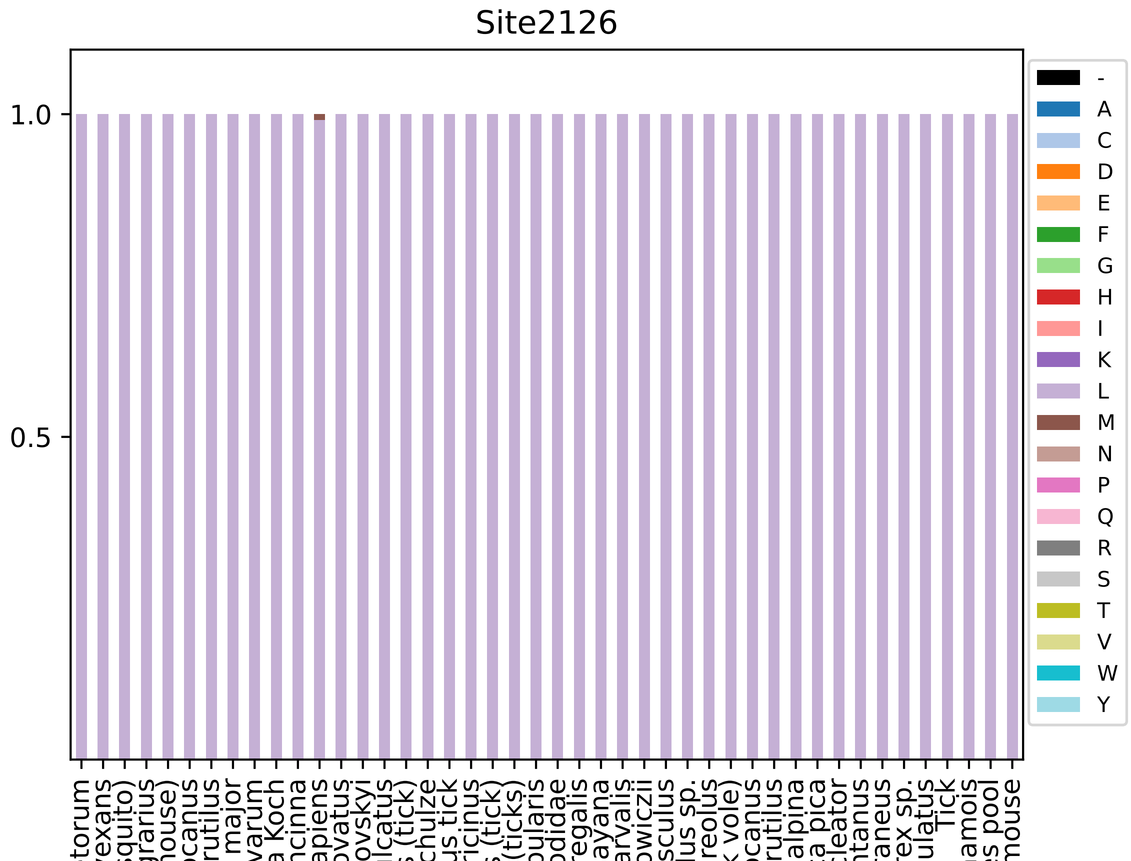
Site2126
1.0
0.5
-
A
C
D
E
F
G
H
I
K
L
M
N
P
Q
R
S
T
V
W
Y

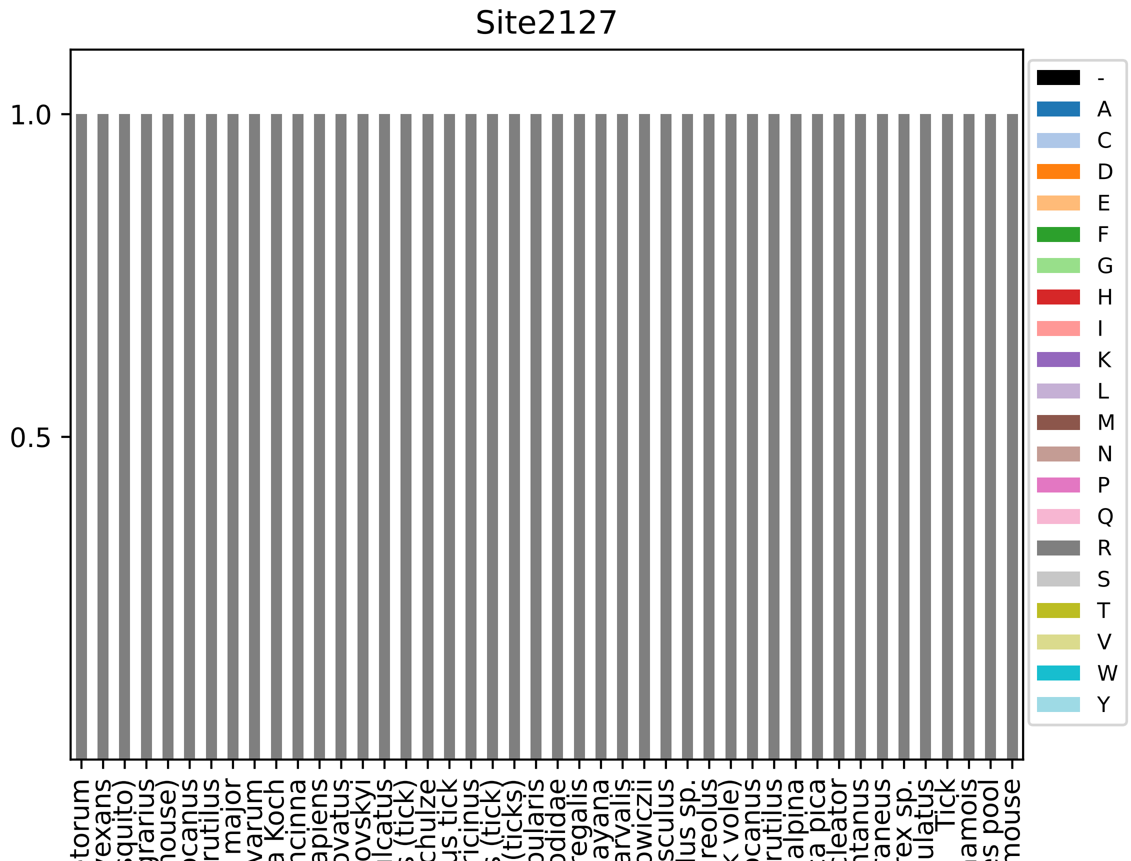
Site2127
1.0
0.5
-
A
C
D
E
F
G
H
I
K
L
M
N
P
Q
R
S
T
V
W
Y

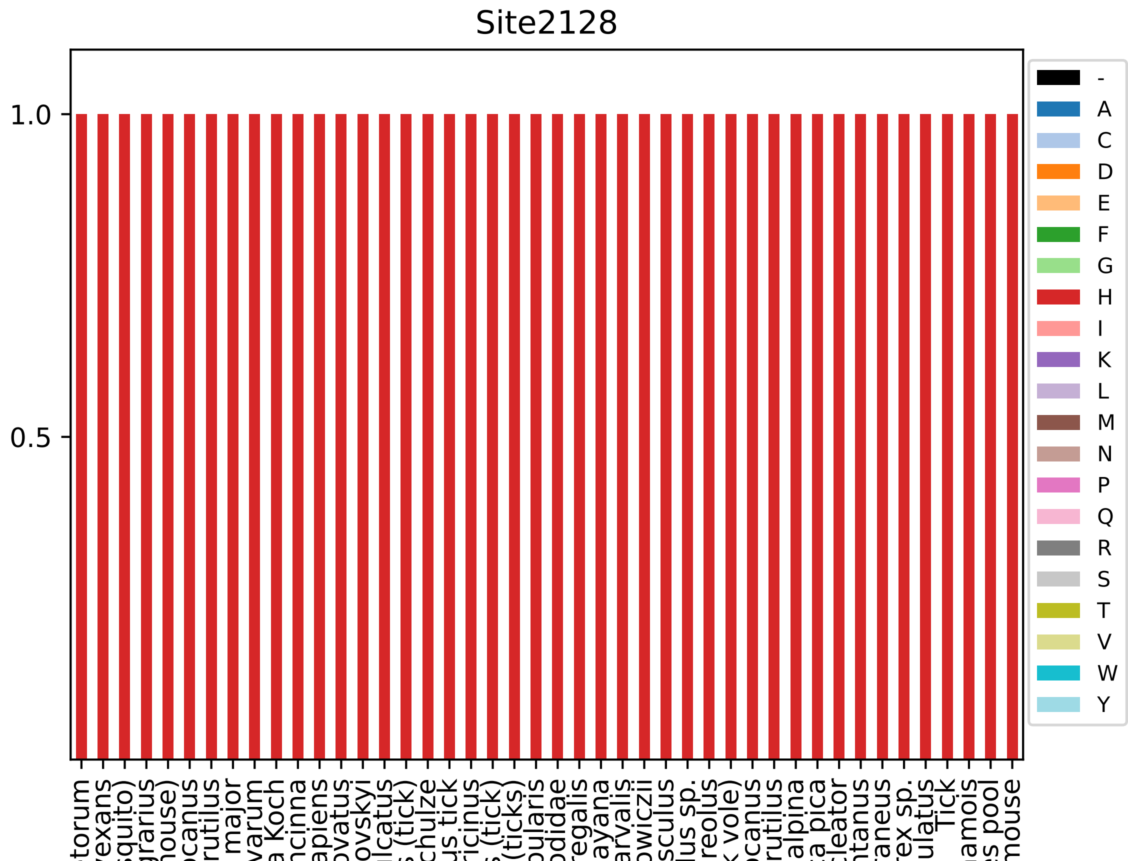
Site2128
1.0
0.5
-
A
C
D
E
F
G
H
I
K
L
M
N
P
Q
R
S
T
V
W
Y

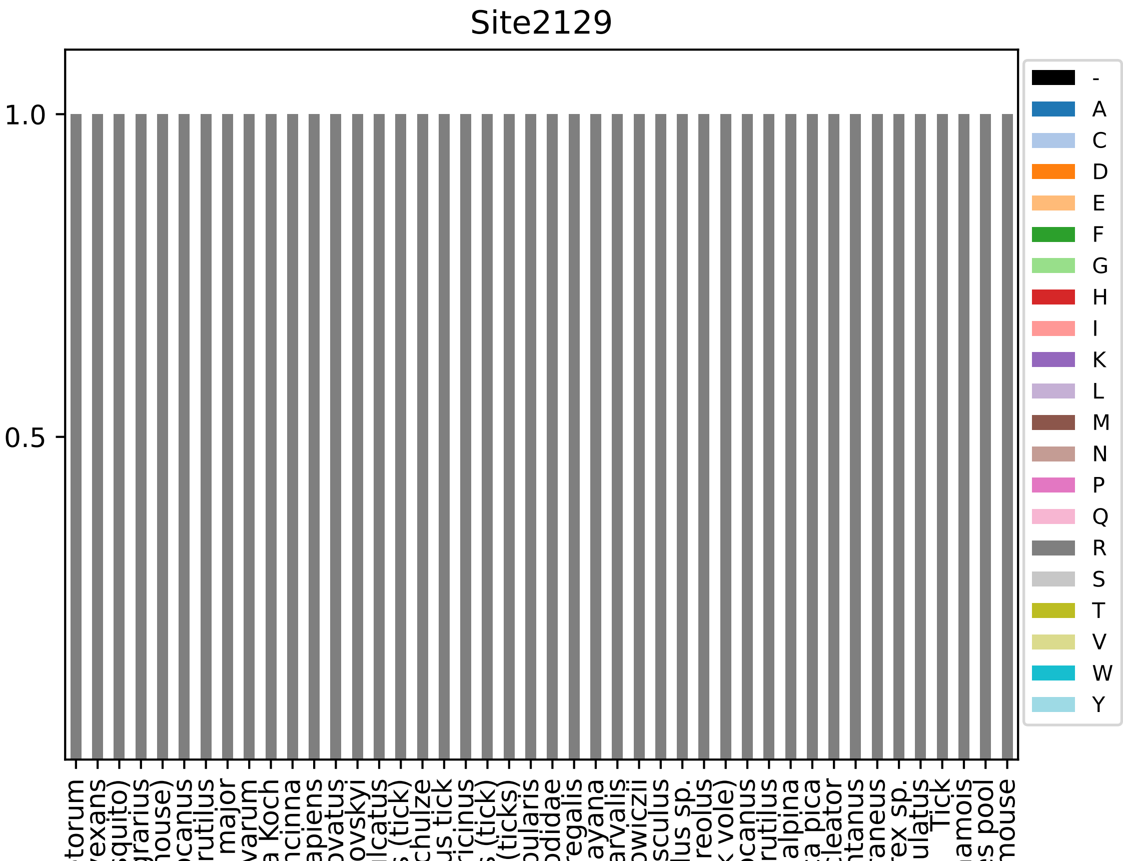
Site2129
1.0
0.5
-
A
C
D
E
F
G
H
I
K
L
M
N
P
Q
R
S
T
V
W
Y

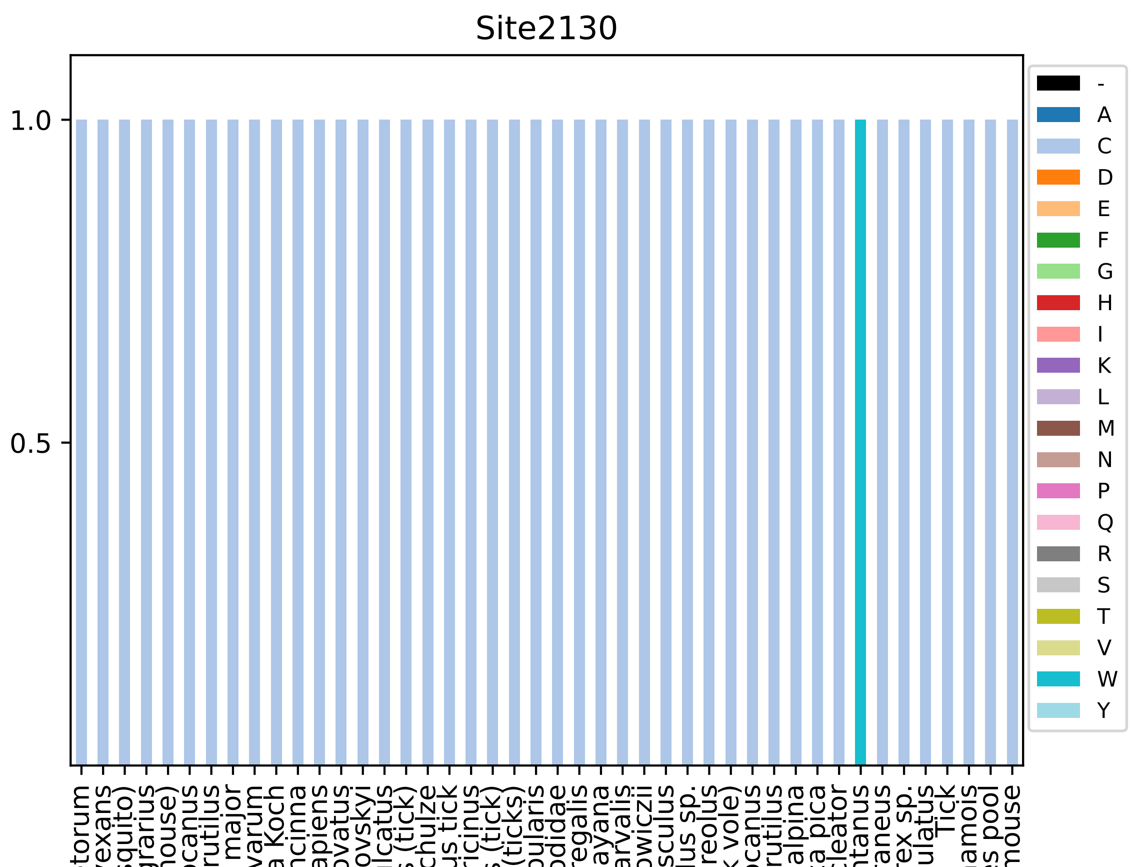
Site2130
1.0
0.5
-
A
C
D
E
F
G
H
I
K
L
M
N
P
Q
R
S
T
V
W
Y

Site2131

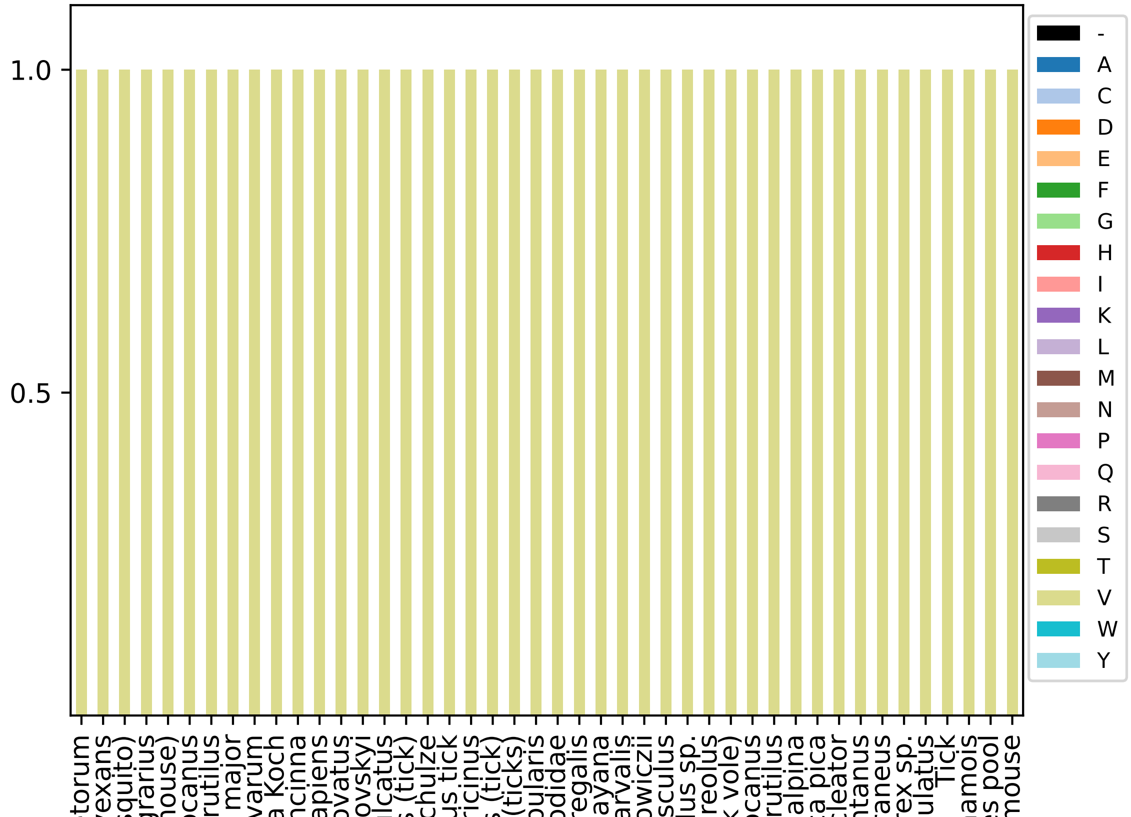

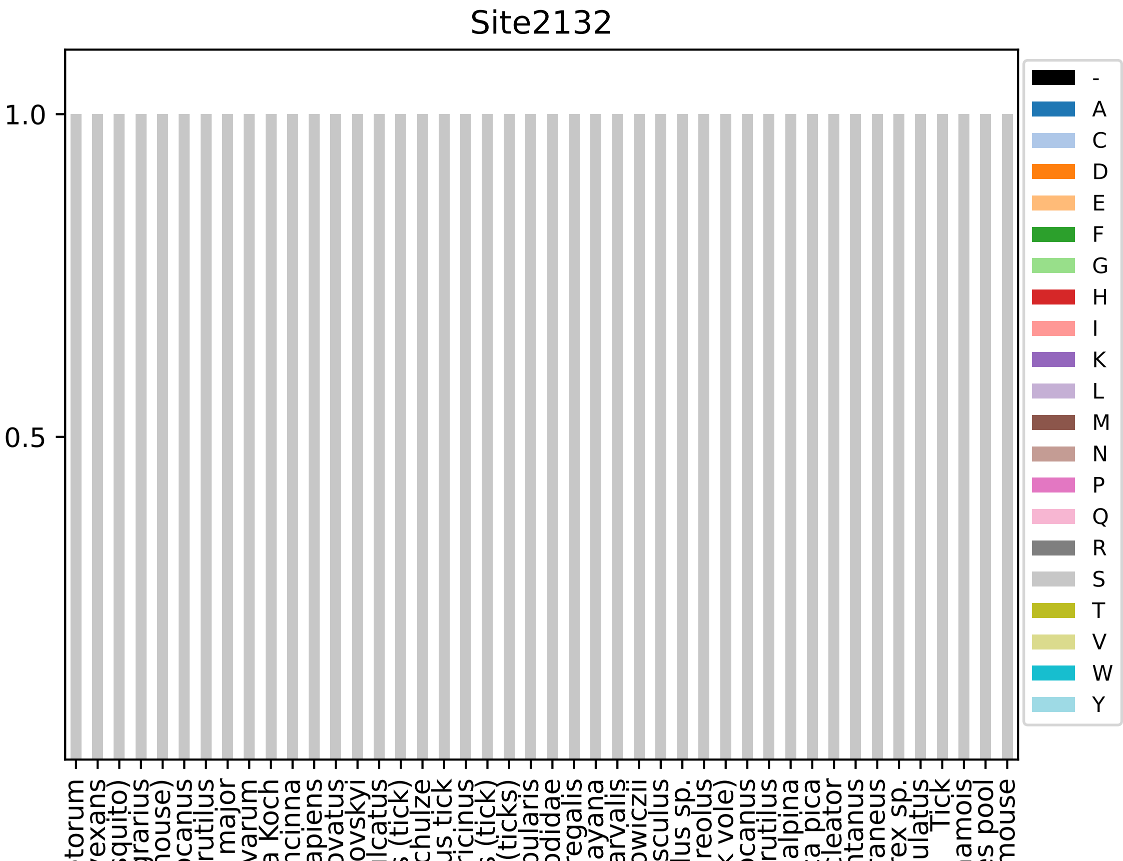
Site2132
1.0
0.5
-
A
C
D
E
F
G
H
I
K
L
M
N
P
Q
R
S
T
V
W
Y

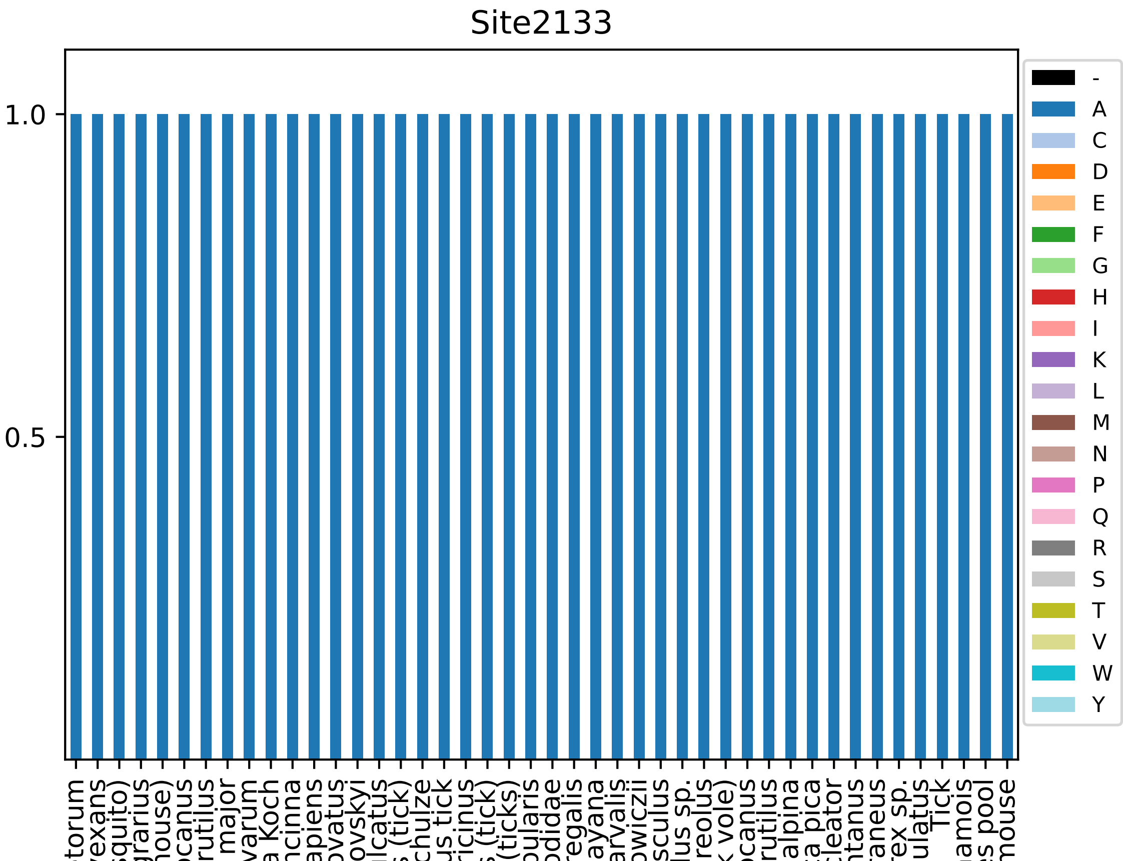
Site2133
1.0
0.5
-
A
C
D
E
F
G
H
I
K
L
M
N
P
Q
R
S
T
V
W
Y
torum
exans
squito)
garius
house)
ocanus
rutilus
major
varum
a Koch
ncinna
apiens
ovatus
ovskyi
lcatus
s (tick)
chulze
us tick
ricinus
s (tick)
(ticks)
pularis
odidae
regalis
ayana
arvalis
owiczii
sculus
lus sp.
reolus
x vole)
ocanus
rutilus
alpina
a pica
leator
ntanus
raneus
rex sp.
ulatus
Tick
amois
es pool
mouse

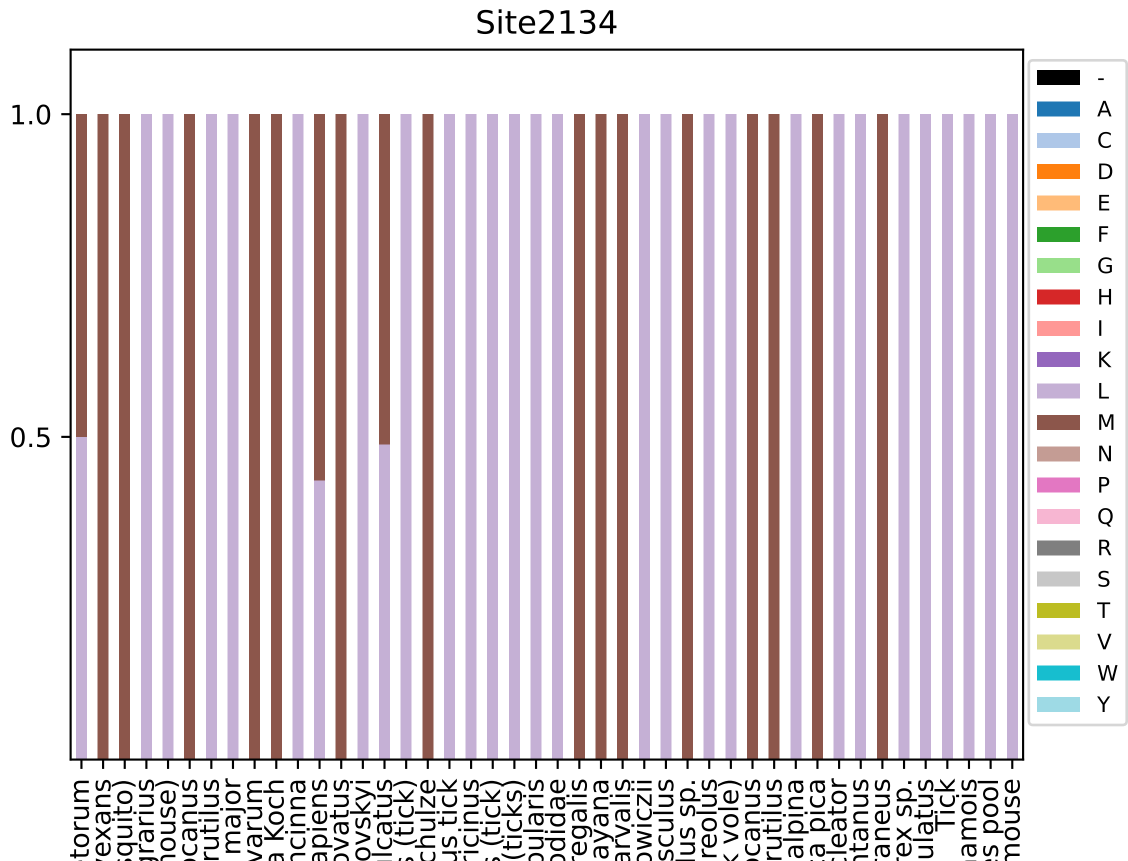

Site2134
1.0
0.5
-
A
C
D
E
F
G
H
I
K
L
M
N
P
Q
R
S
T
V
W
Y

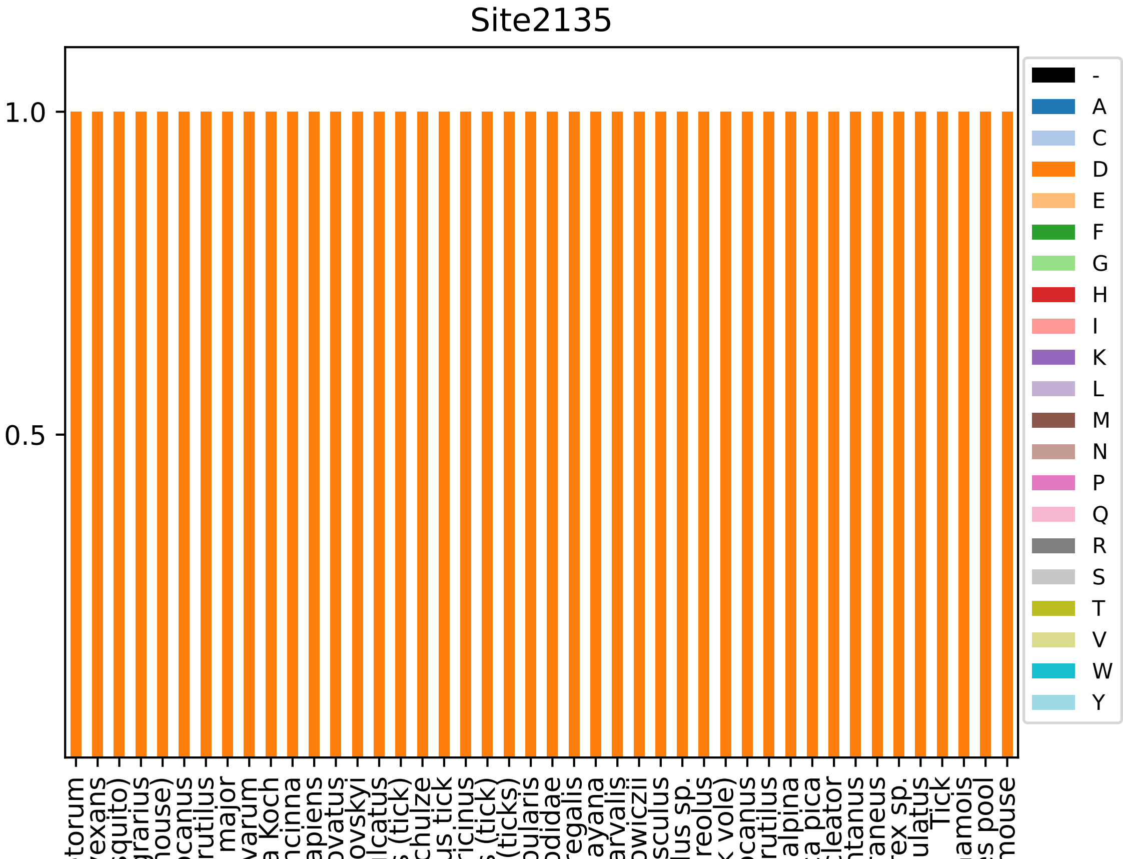
Site2135
1.0
0.5
-
A
C
D
E
F
G
H
I
K
L
M
N
P
Q
R
S
T
V
W
Y

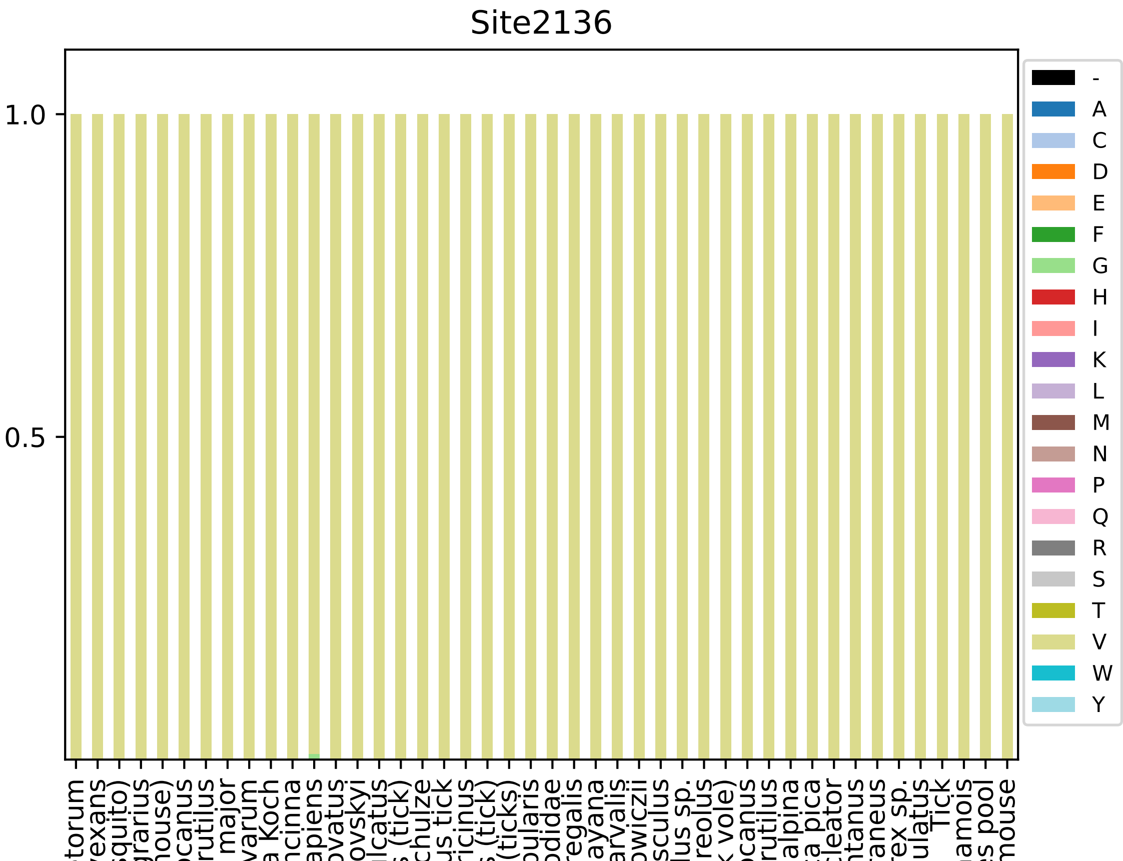
Site2136
1.0
0.5
-
A
C
D
E
F
G
H
I
K
L
M
N
P
Q
R
S
T
V
W
Y

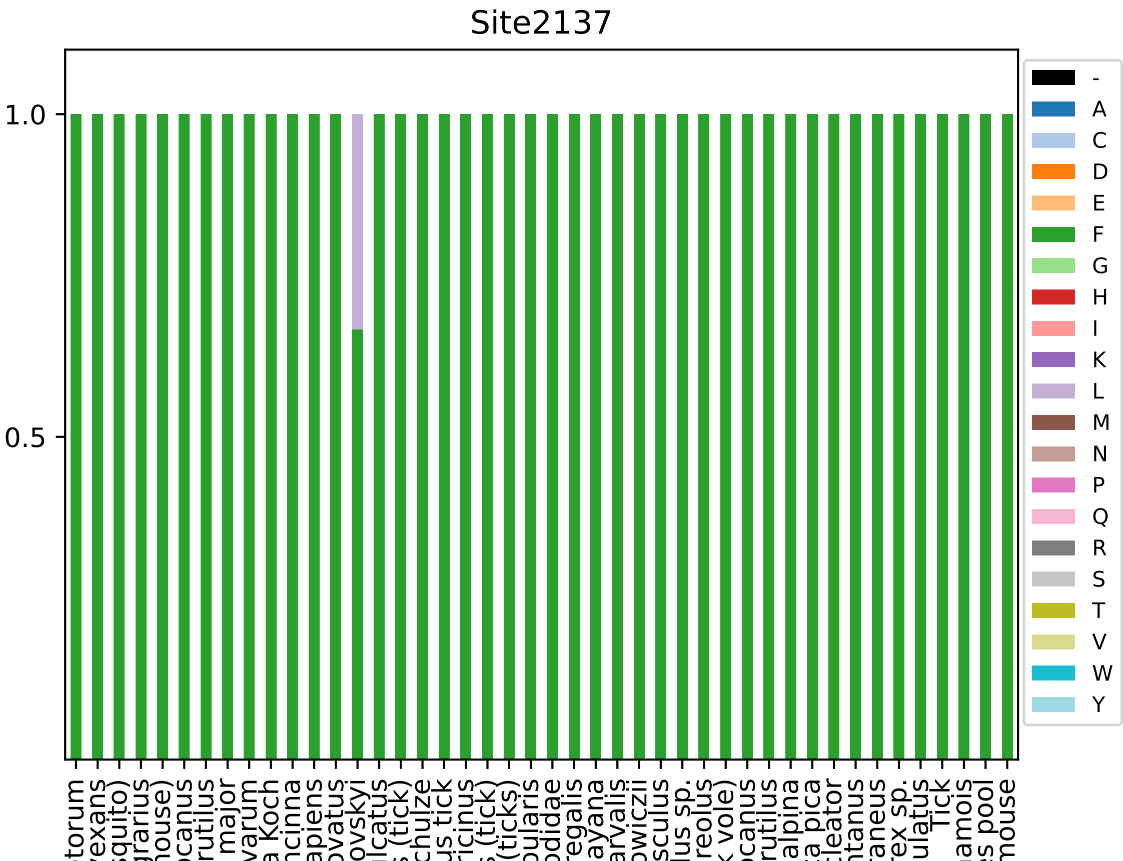
Site2137
1.0
0.5
-
A
C
D
E
F
G
H
I
K
L
M
N
P
Q
R
S
T
V
W
Y
Tick

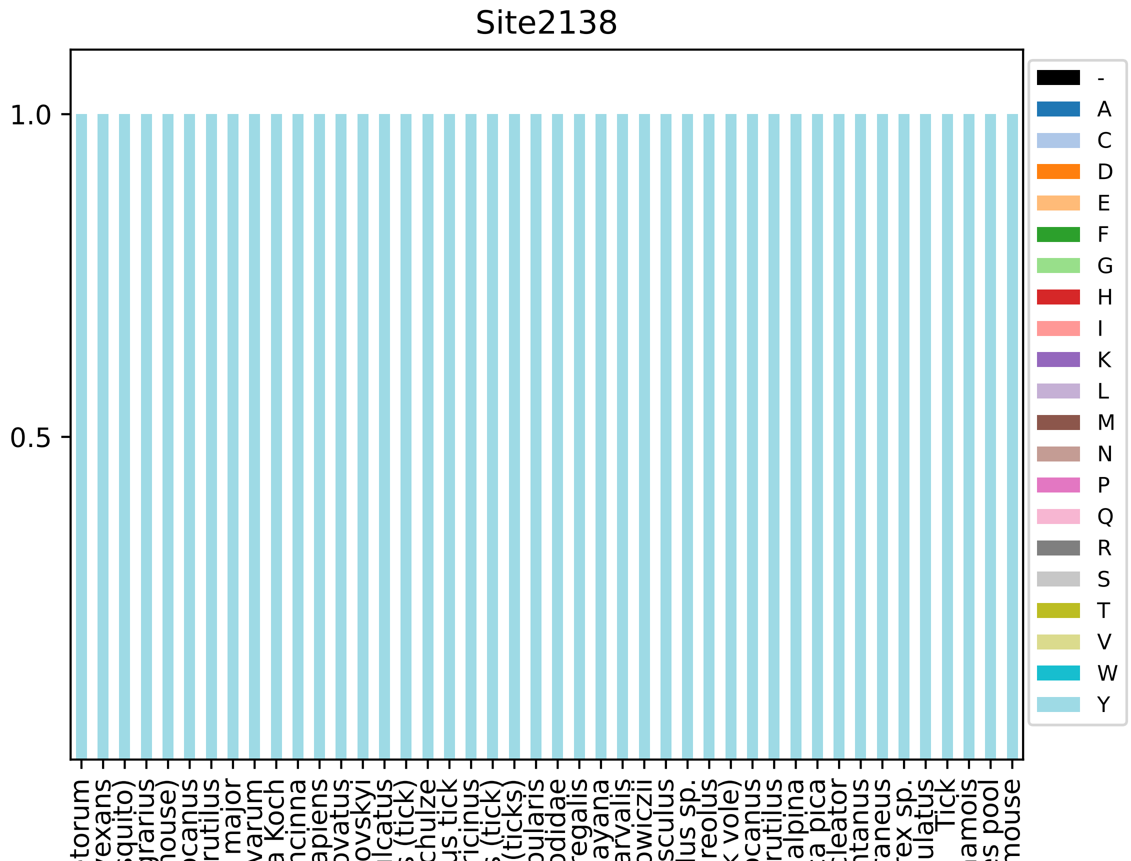
Site2138
1.0
0.5
-
A
C
D
E
F
G
H
I
K
L
M
N
P
Q
R
S
T
V
W
Y
Tick

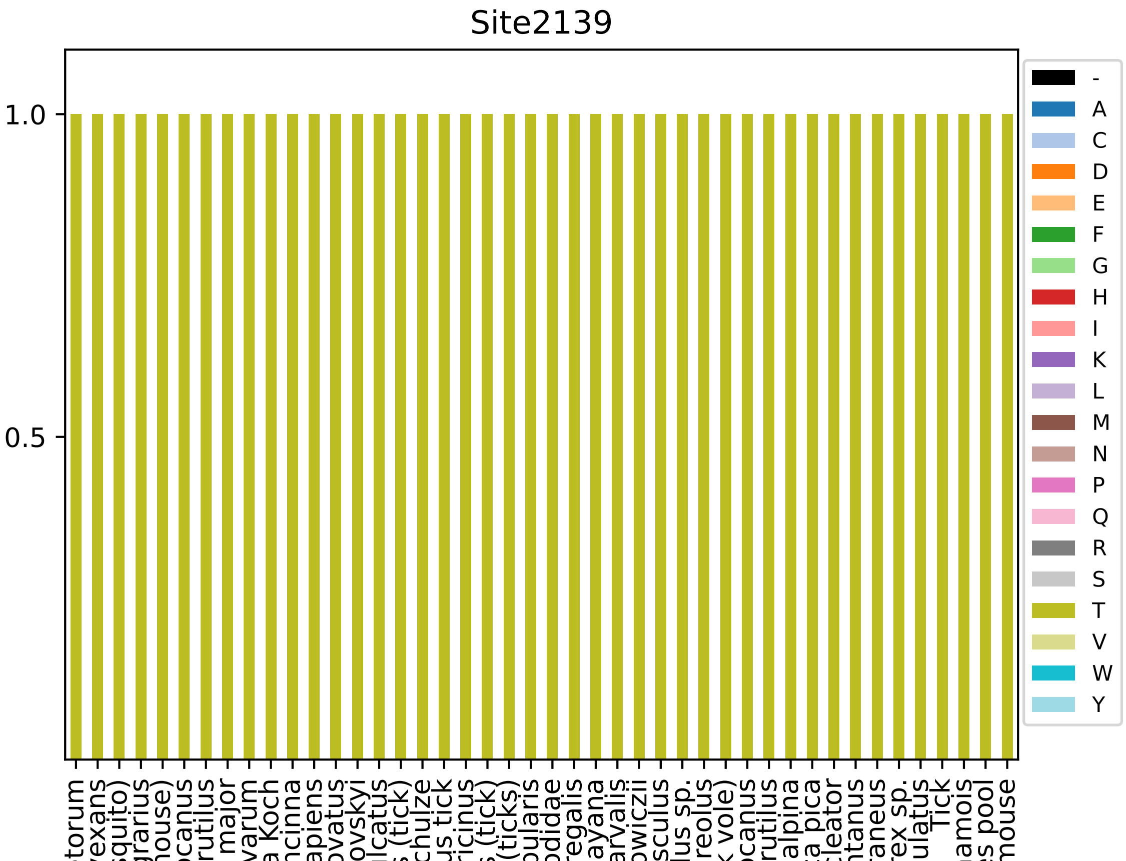
Site2139
1.0
0.5
-
A
C
D
E
F
G
H
I
K
L
M
N
P
Q
R
S
T
V
W
Y

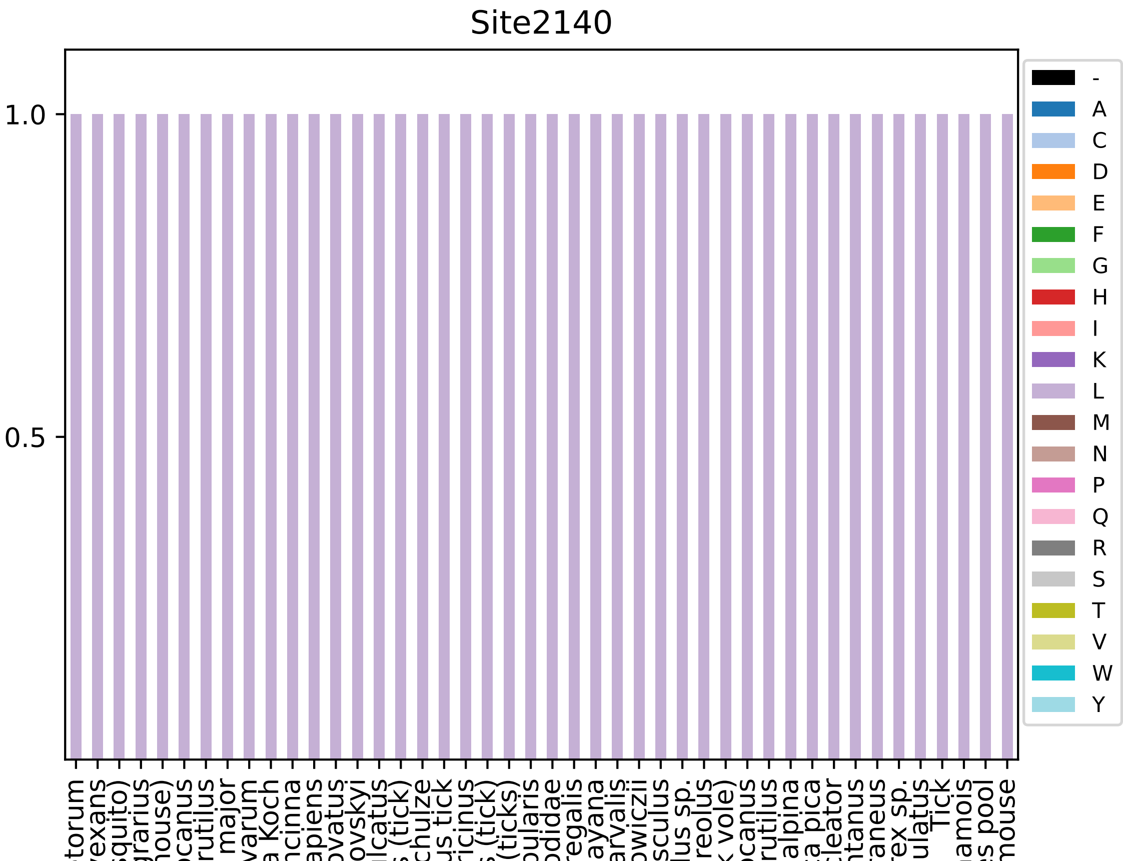
Site2140
1.0
0.5
-
A
C
D
E
F
G
H
I
K
L
M
N
P
Q
R
S
T
V
W
Y

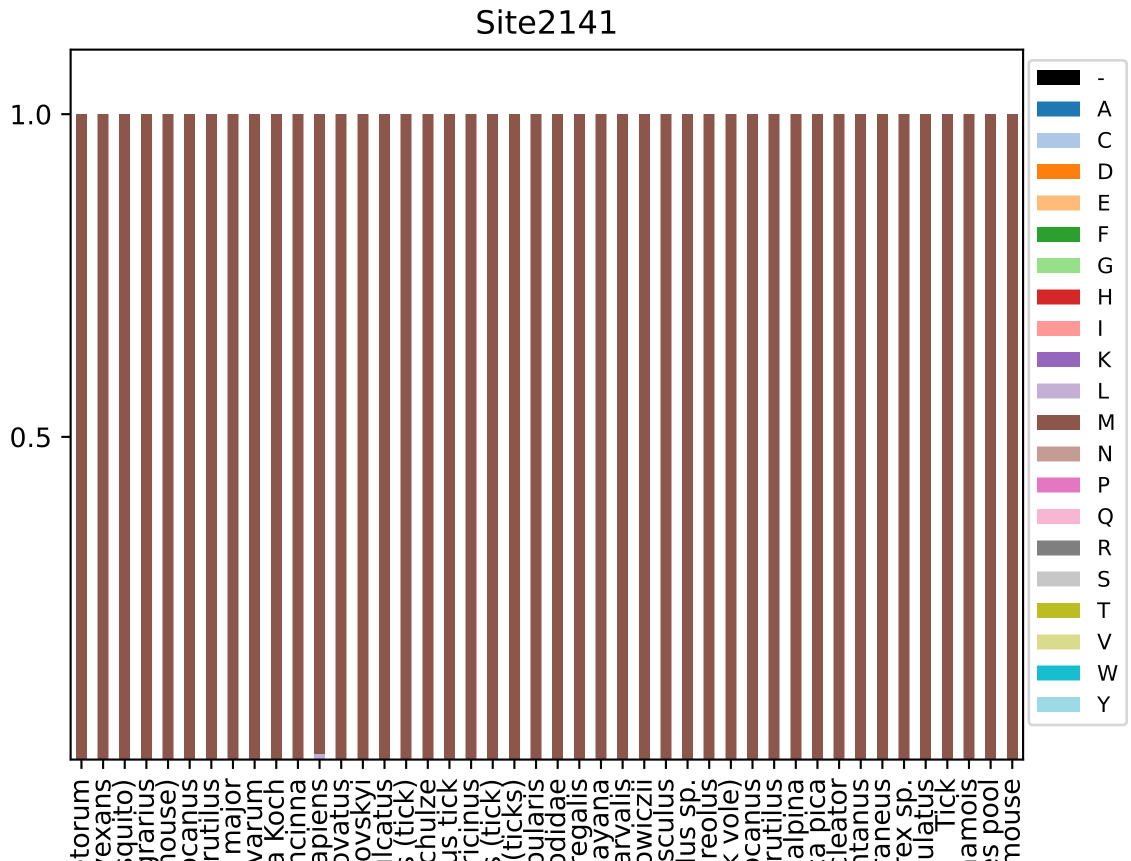
Site2141
1.0
0.5
-
A
C
D
E
F
G
H
I
K
L
M
N
P
Q
R
S
T
V
W
Y

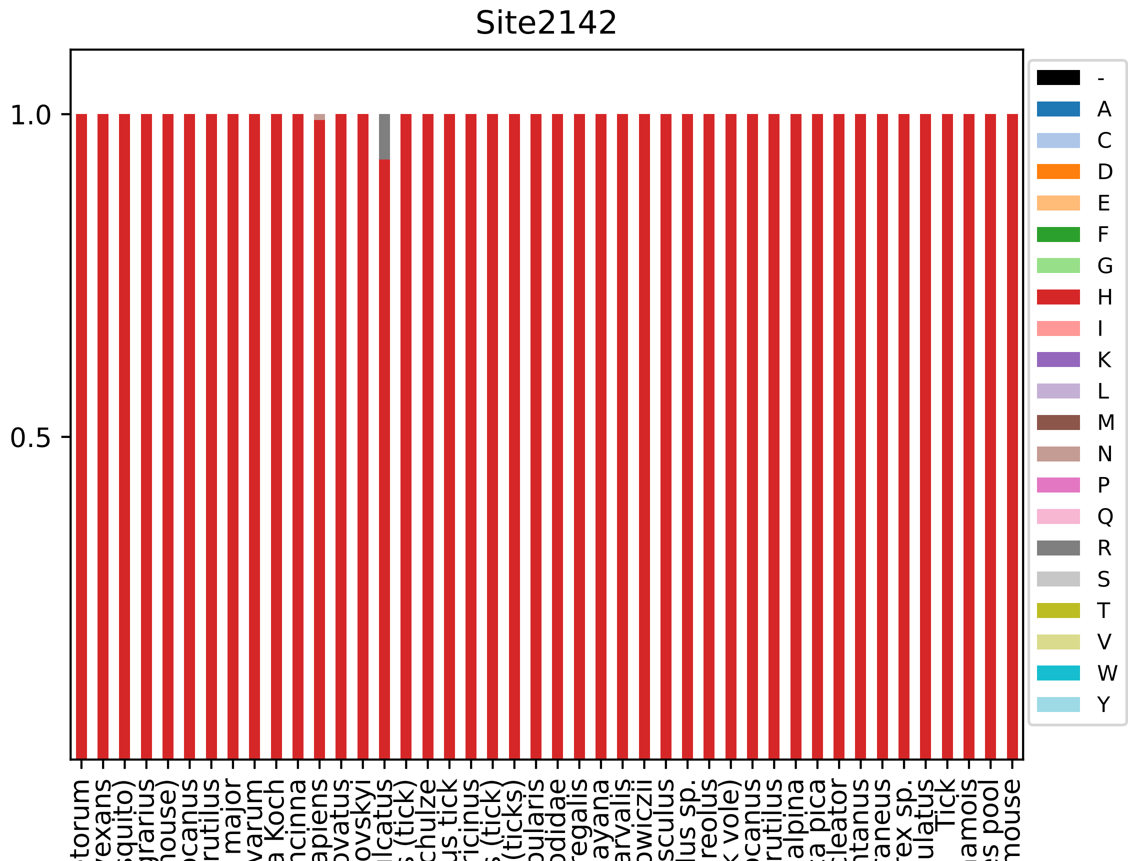

Site2142
1.0
0.5
-
A
C
D
E
F
G
H
I
K
L
M
N
P
Q
R
S
T
V
W
Y

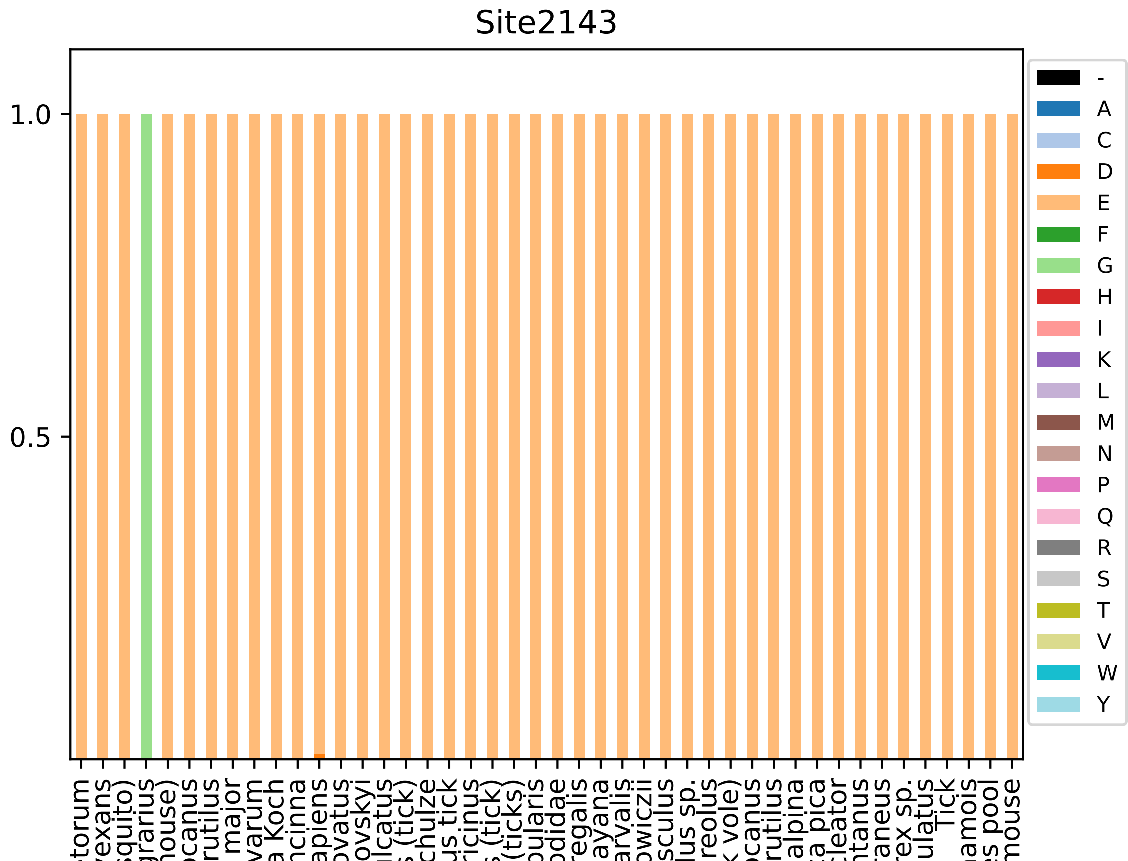
Site2143
1.0
0.5
-
A
C
D
E
F
G
H
I
K
L
M
N
P
Q
R
S
T
V
W
Y
Tick

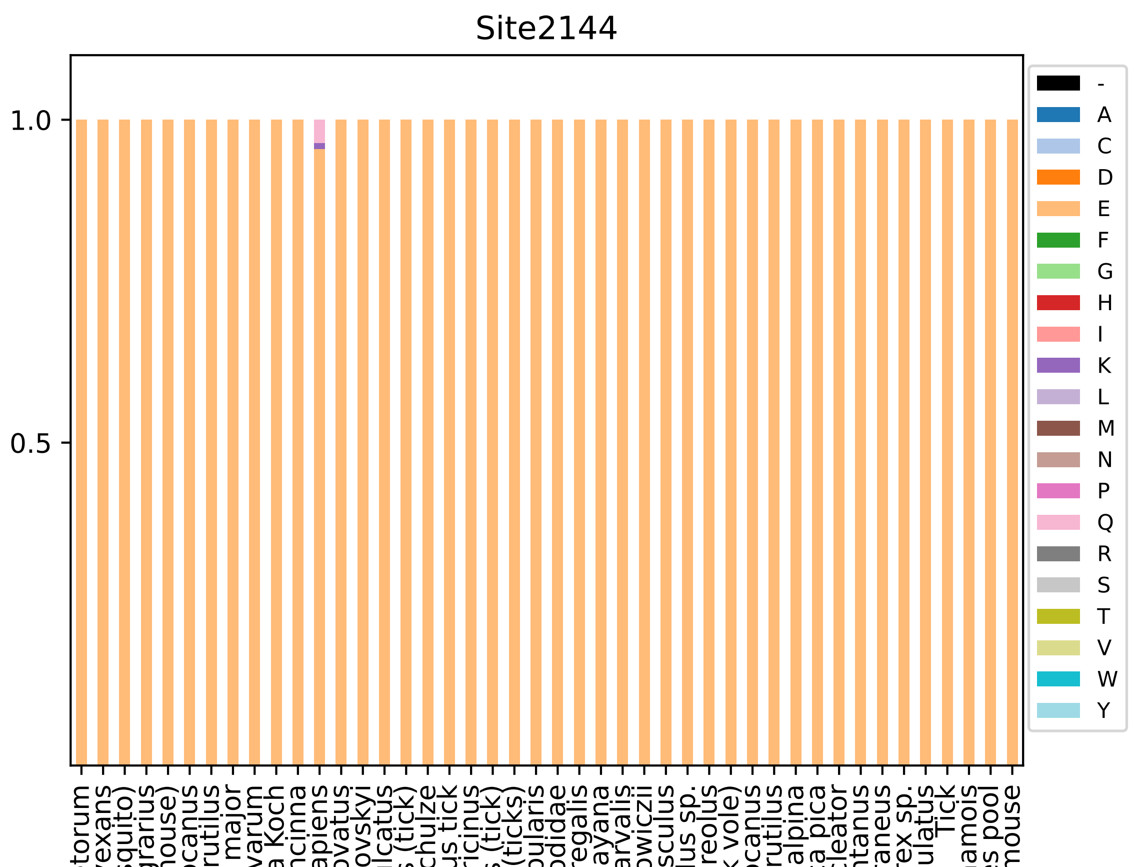
Site2144
1.0
0.5
-
A
C
D
E
F
G
H
I
K
L
M
N
P
Q
R
S
T
V
W
Y

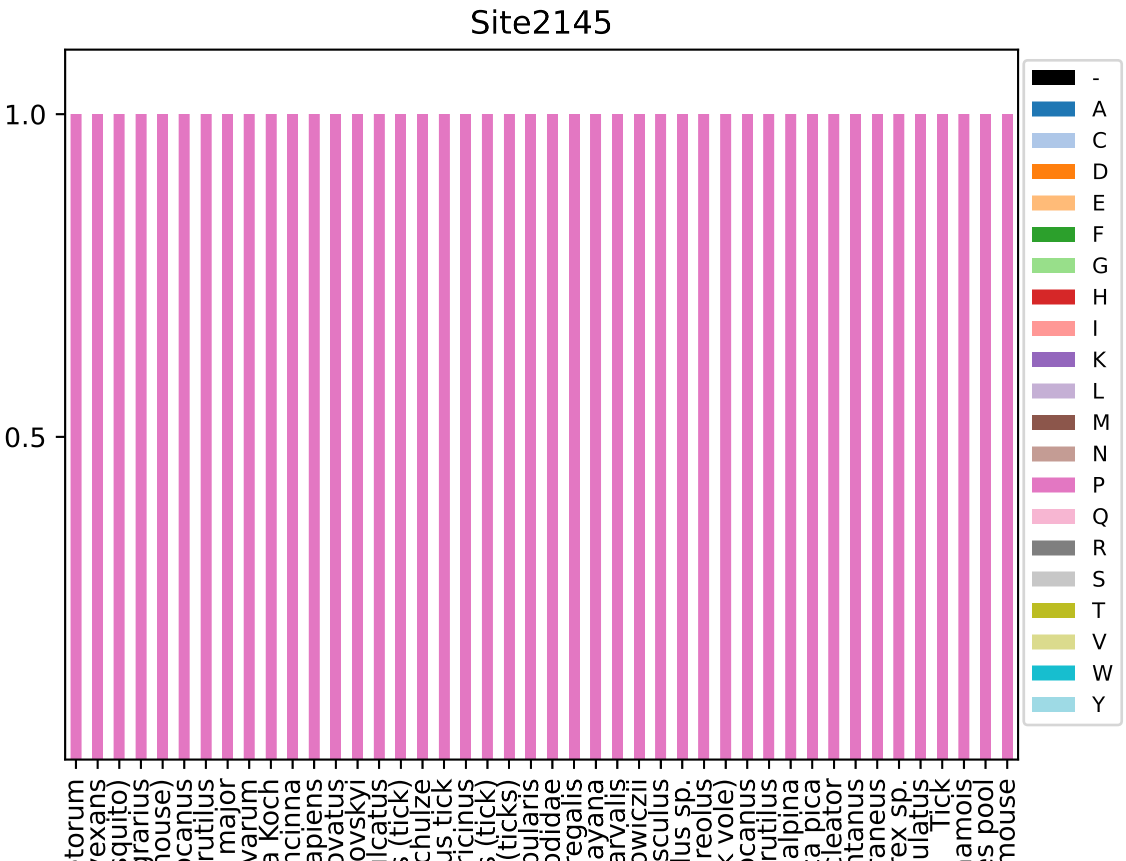

Site2145
1.0
0.5
-
A
C
D
E
F
G
H
I
K
L
M
N
P
Q
R
S
T
V
W
Y

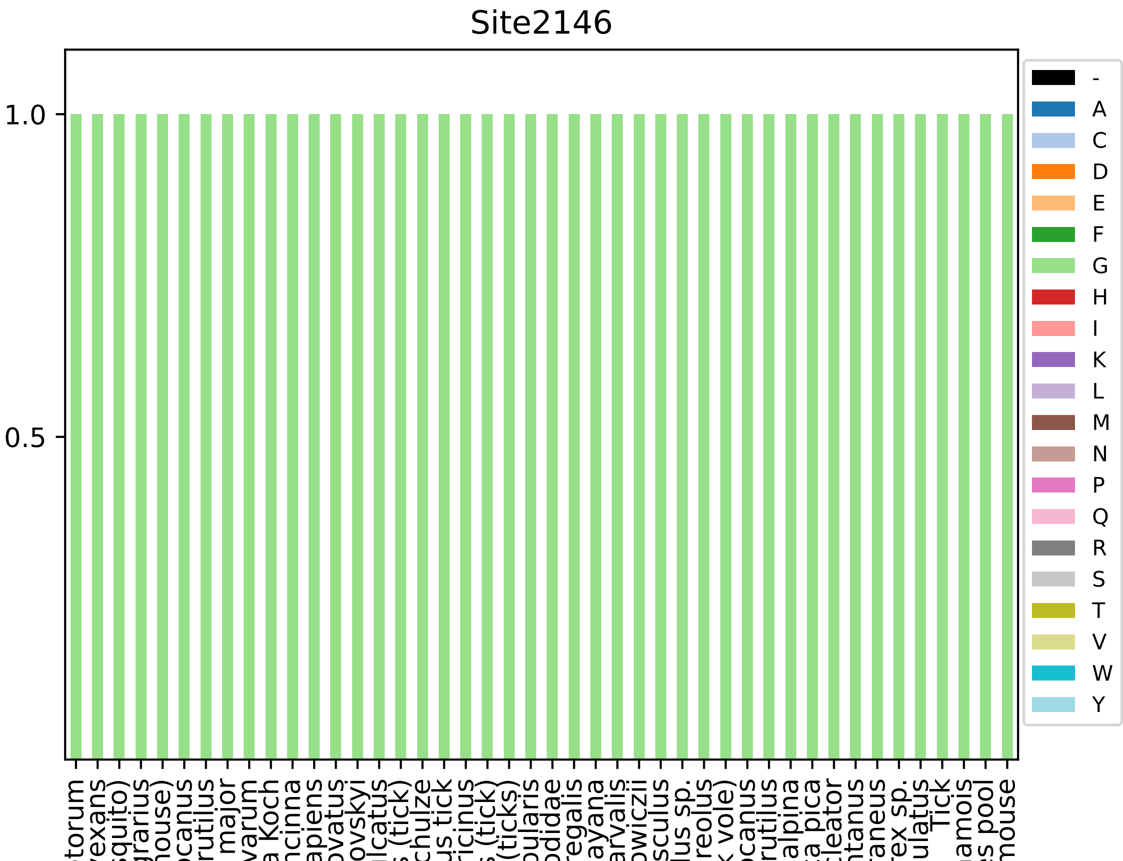
Site2146
1.0
0.5
-
A
C
D
E
F
G
H
I
K
L
M
N
P
Q
R
S
T
V
W
Y

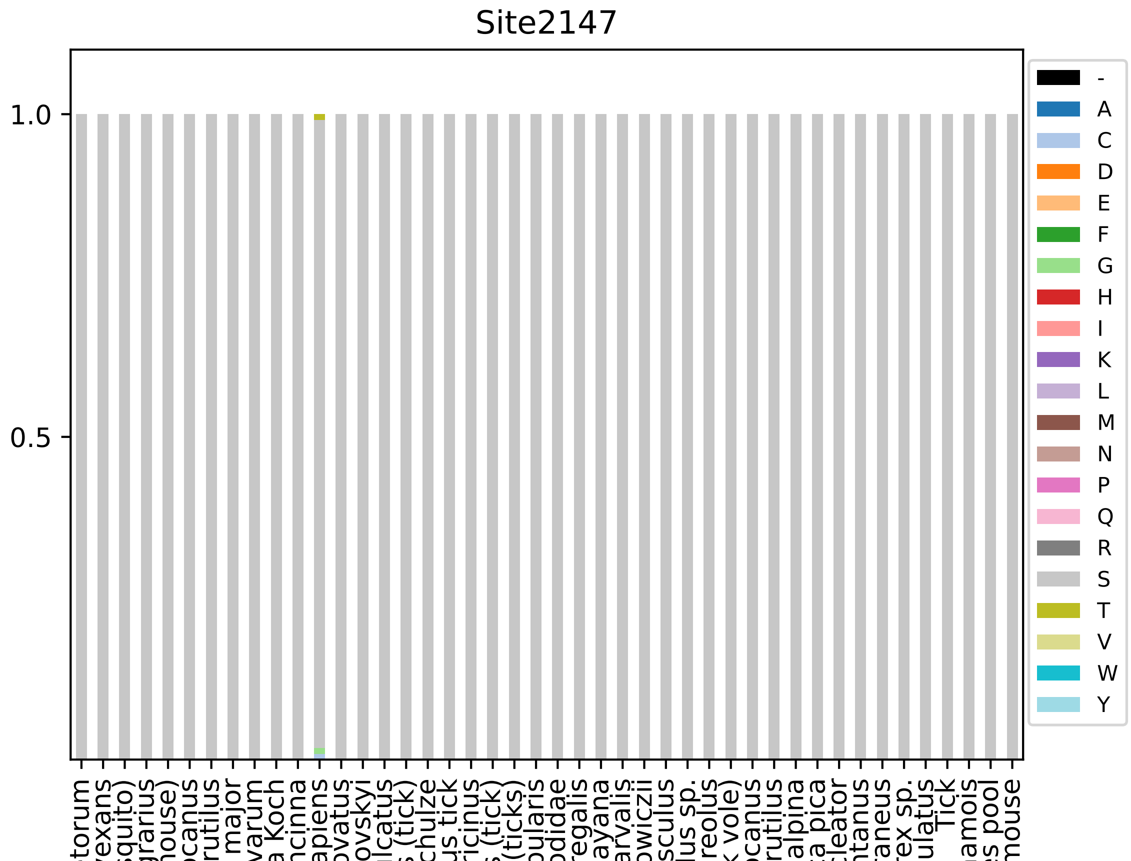
Site2147
1.0
0.5
-
A
C
D
E
F
G
H
I
K
L
M
N
P
Q
R
S
T
V
W
Y

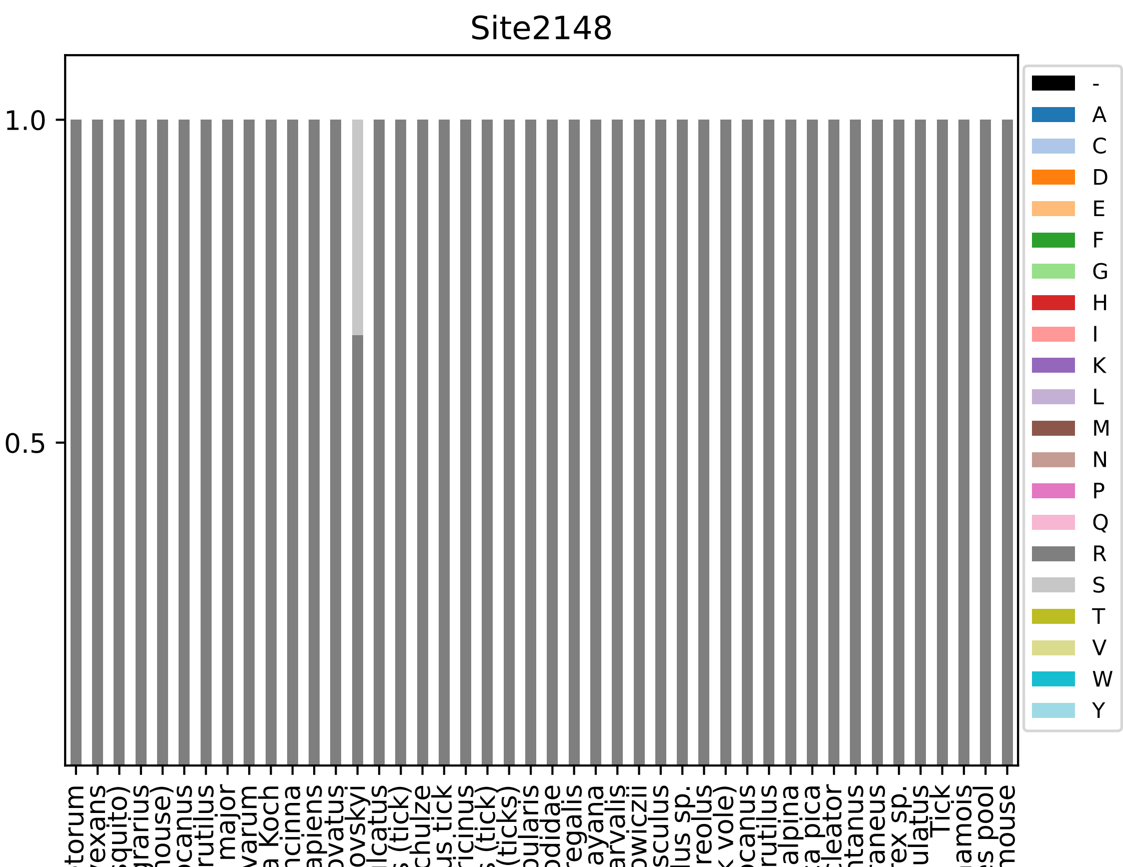

Site2148
1.0
0.5
-
A
C
D
E
F
G
H
I
K
L
M
N
P
Q
R
S
T
V
W
Y
torum
vexans
squito)
garius
house)
canus
rutilus
major
varum
a Koch
ncinna
apiens
ovatus
ovskyi
lcatus
(tick)
chulze
us tick
ricinus
(tick)
(ticks)
pularis
podidae
regalis
ayana
arvalis
owiczii
sculus
lus sp.
reolus
k vole)
canus
rutilus
alpina
a pica
cleator
ntanus
raneus
rex sp.
ulatus
Tick
amois
es pool
mouse

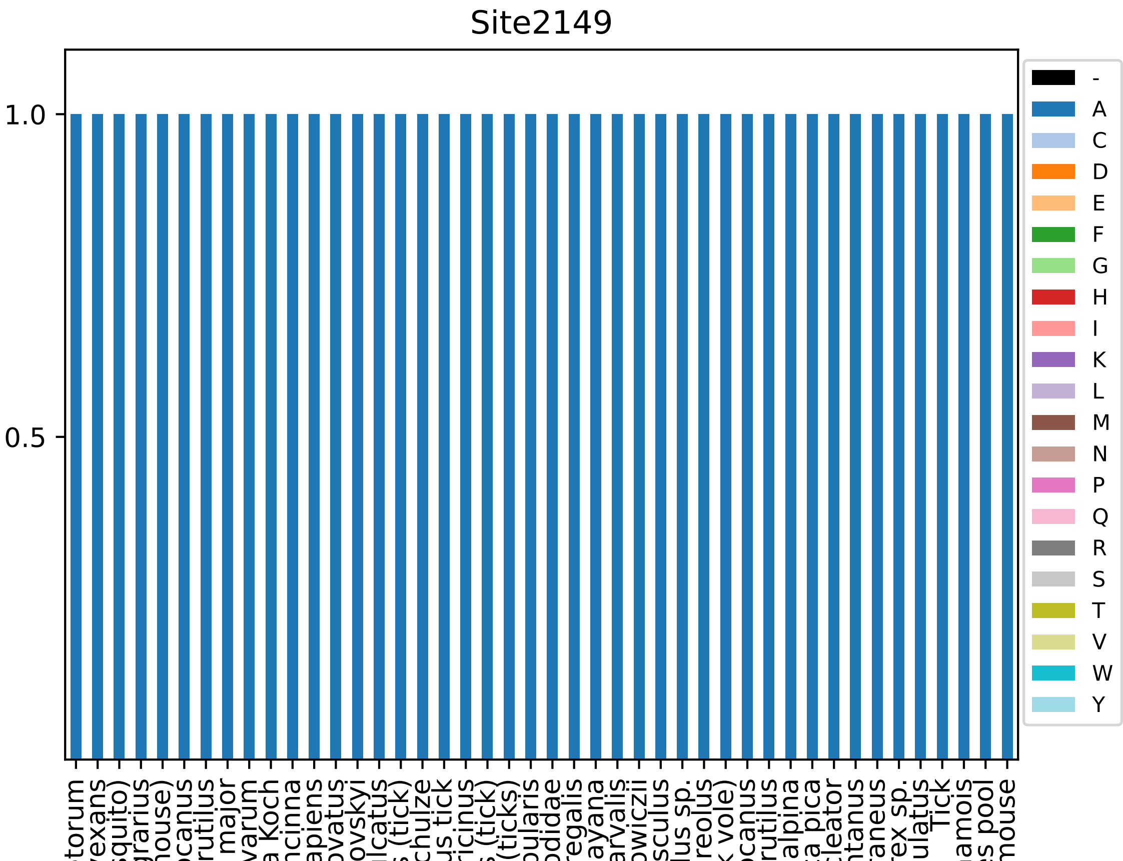
Site2149
1.0
0.5
-
A
C
D
E
F
G
H
I
K
L
M
N
P
Q
R
S
T
V
W
Y

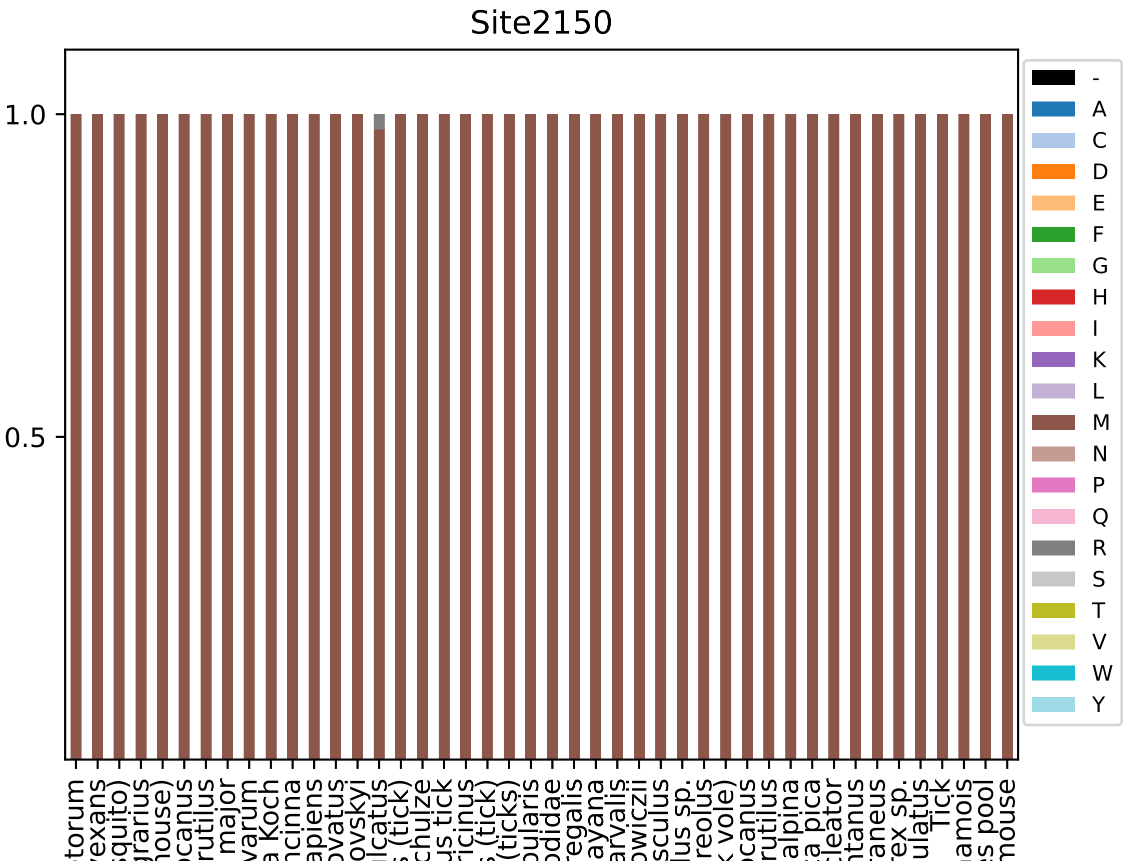
Site2150
1.0
0.5
-
A
C
D
E
F
G
H
I
K
L
M
N
P
Q
R
S
T
V
W
Y

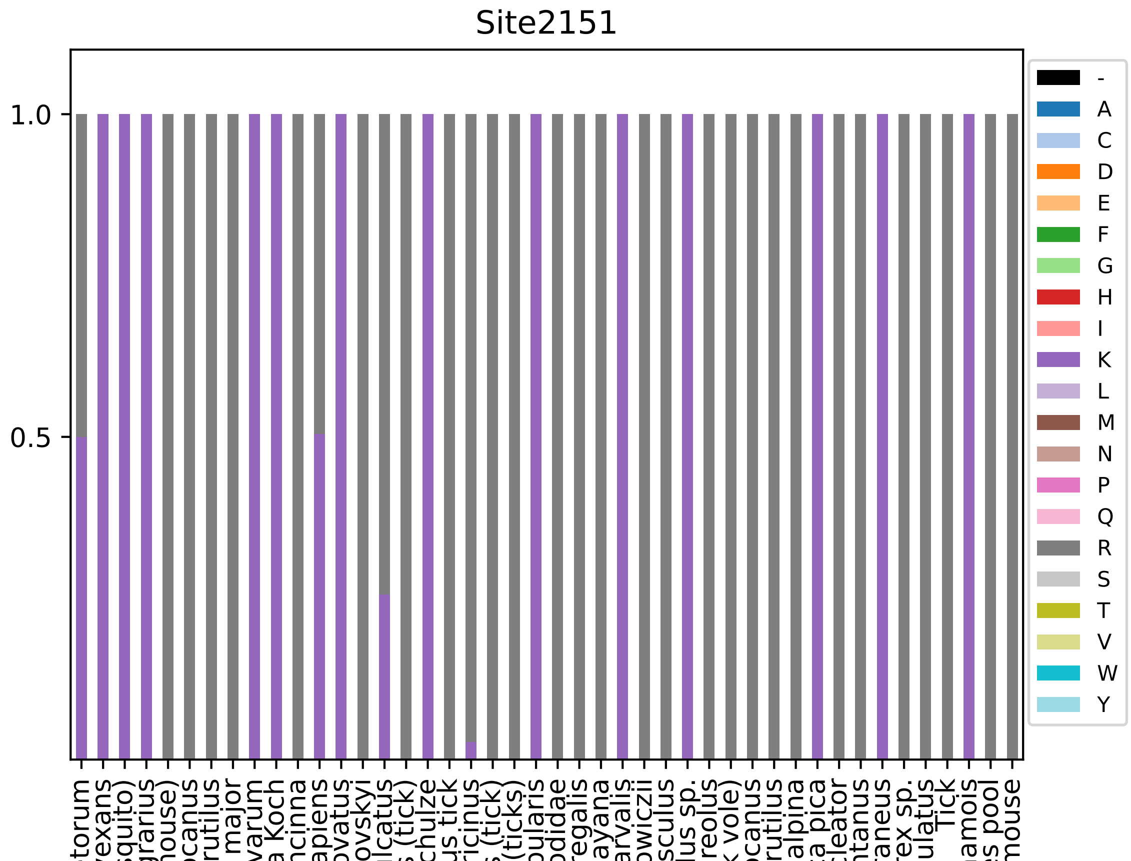
Site2151
1.0
0.5
-
A
C
D
E
F
G
H
I
K
L
M
N
P
Q
R
S
T
V
W
Y

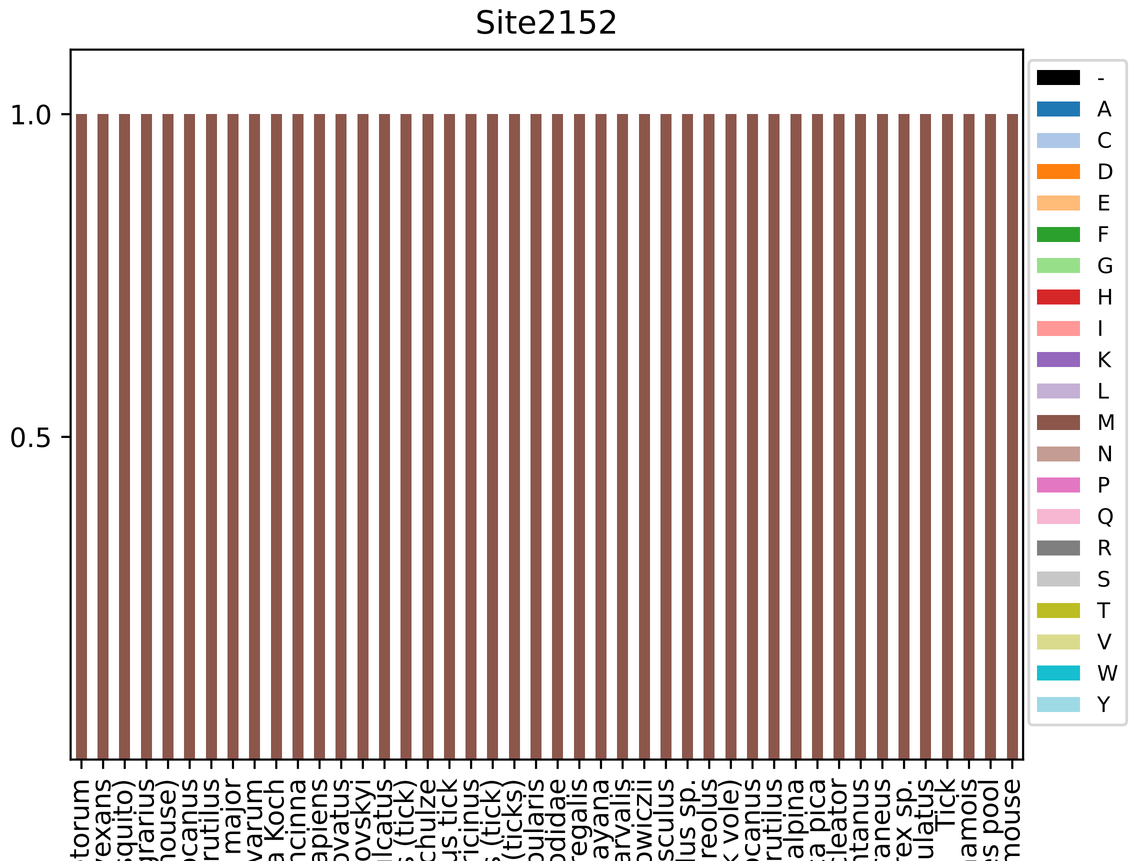
Site2152
1.0
0.5
-
A
C
D
E
F
G
H
I
K
L
M
N
P
Q
R
S
T
V
W
Y

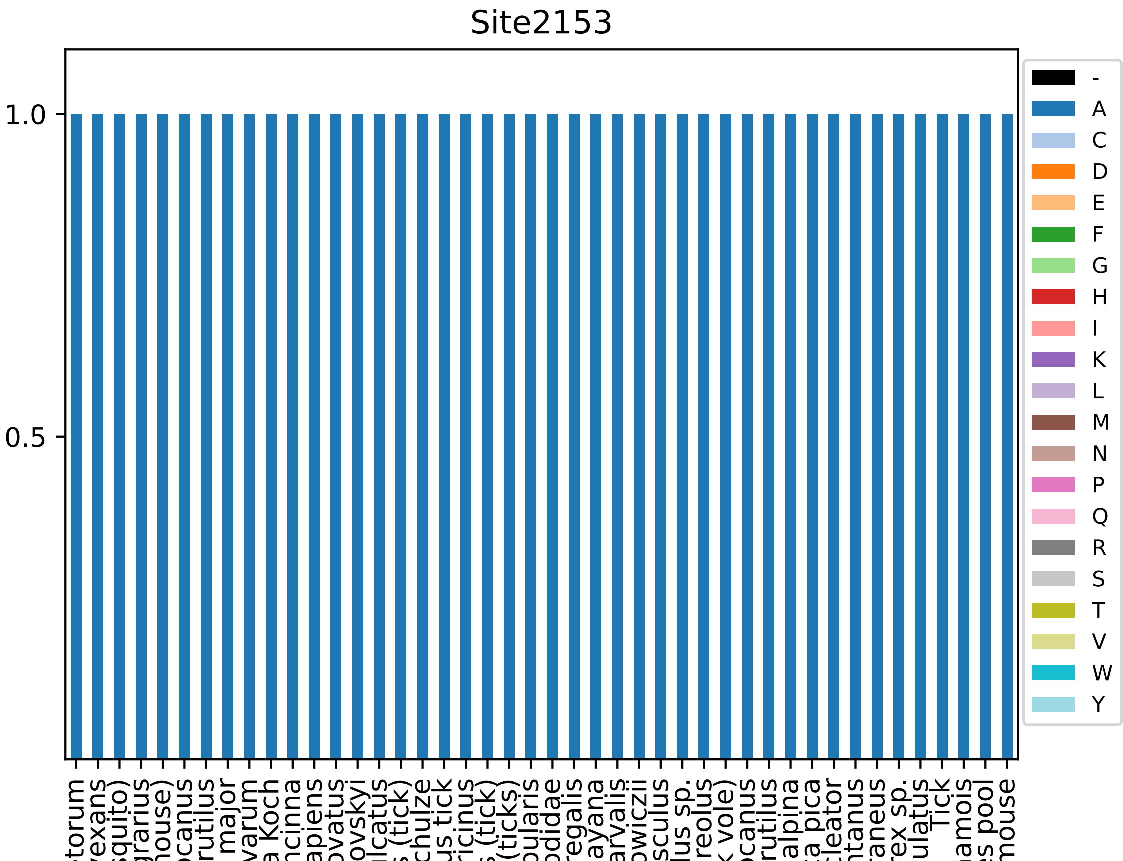
Site2153
1.0
0.5
-
A
C
D
E
F
G
H
I
K
L
M
N
P
Q
R
S
T
V
W
Y
Tick

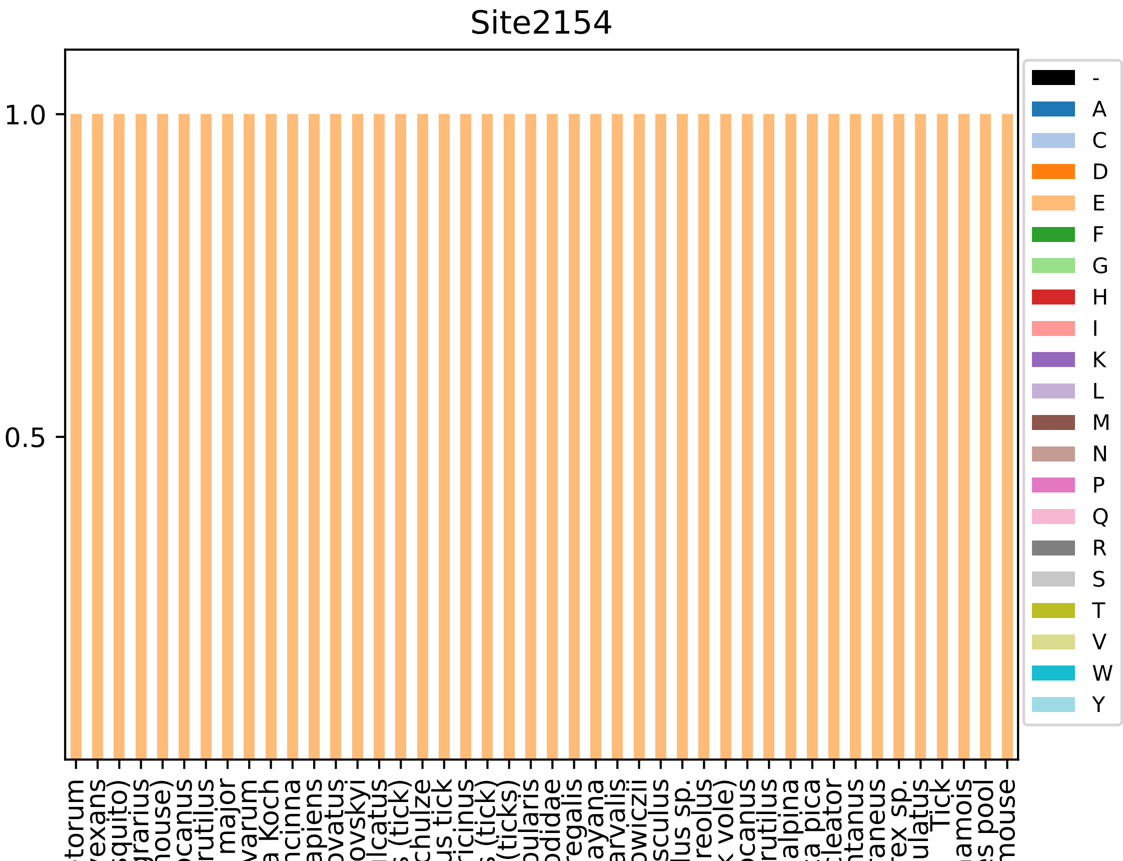

Site2154
1.0
0.5
-
A
C
D
E
F
G
H
I
K
L
M
N
P
Q
R
S
T
V
W
Y

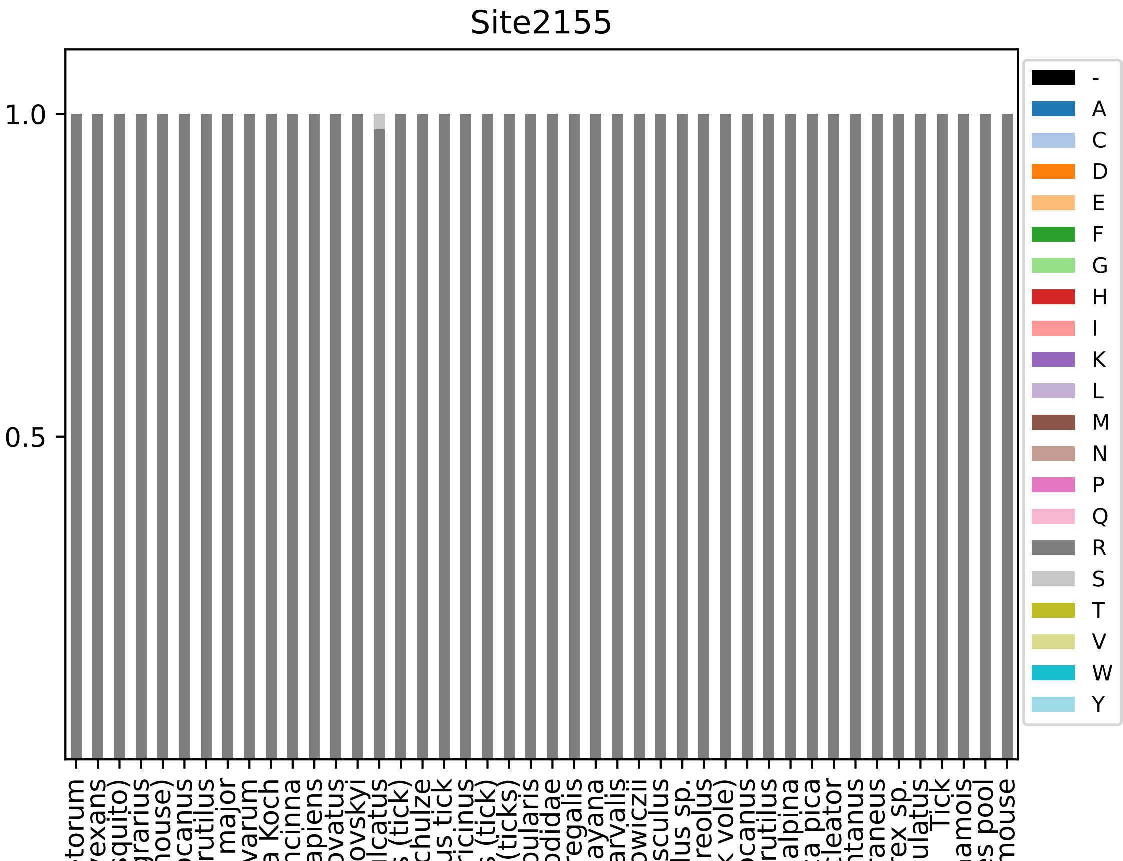
Site2155
1.0
0.5
-
A
C
D
E
F
G
H
I
K
L
M
N
P
Q
R
S
T
V
W
Y

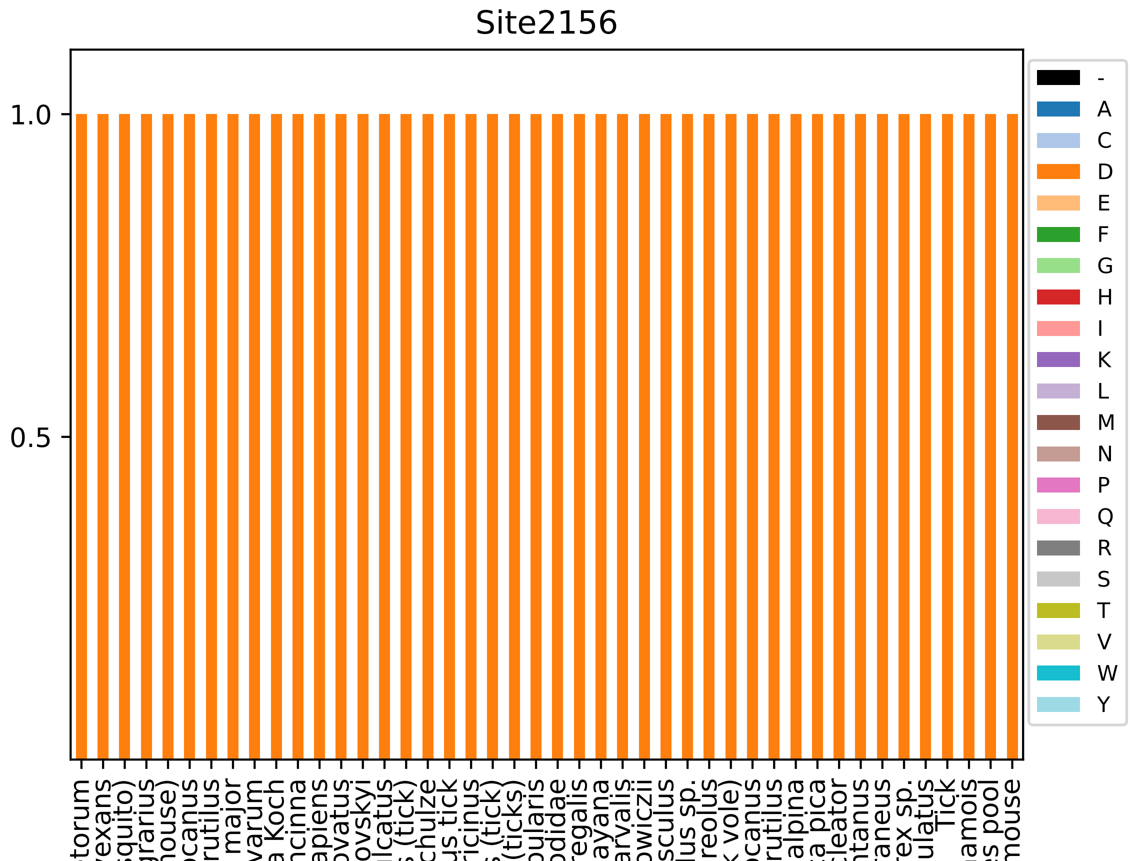
Site2156
1.0
0.5
-
A
C
D
E
F
G
H
I
K
L
M
N
P
Q
R
S
T
V
W
Y

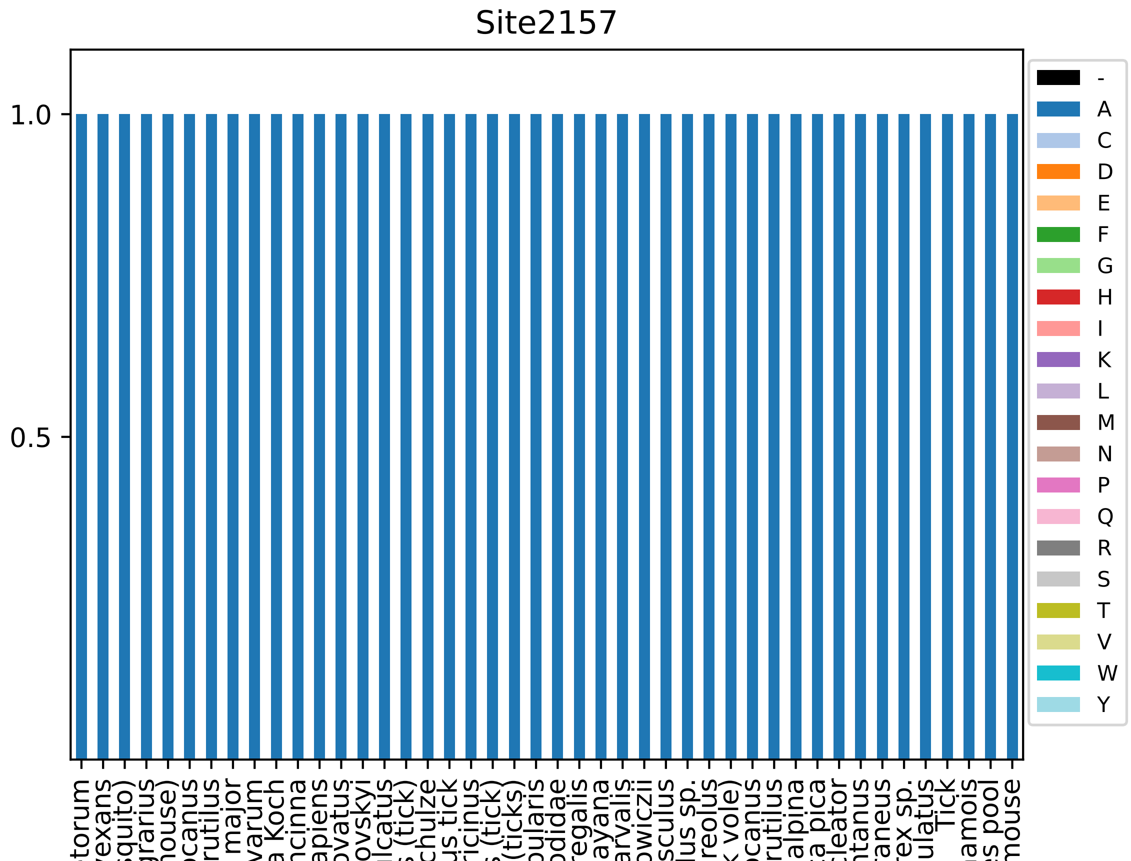

Site2157
1.0
0.5
-
A
C
D
E
F
G
H
I
K
L
M
N
P
Q
R
S
T
V
W
Y

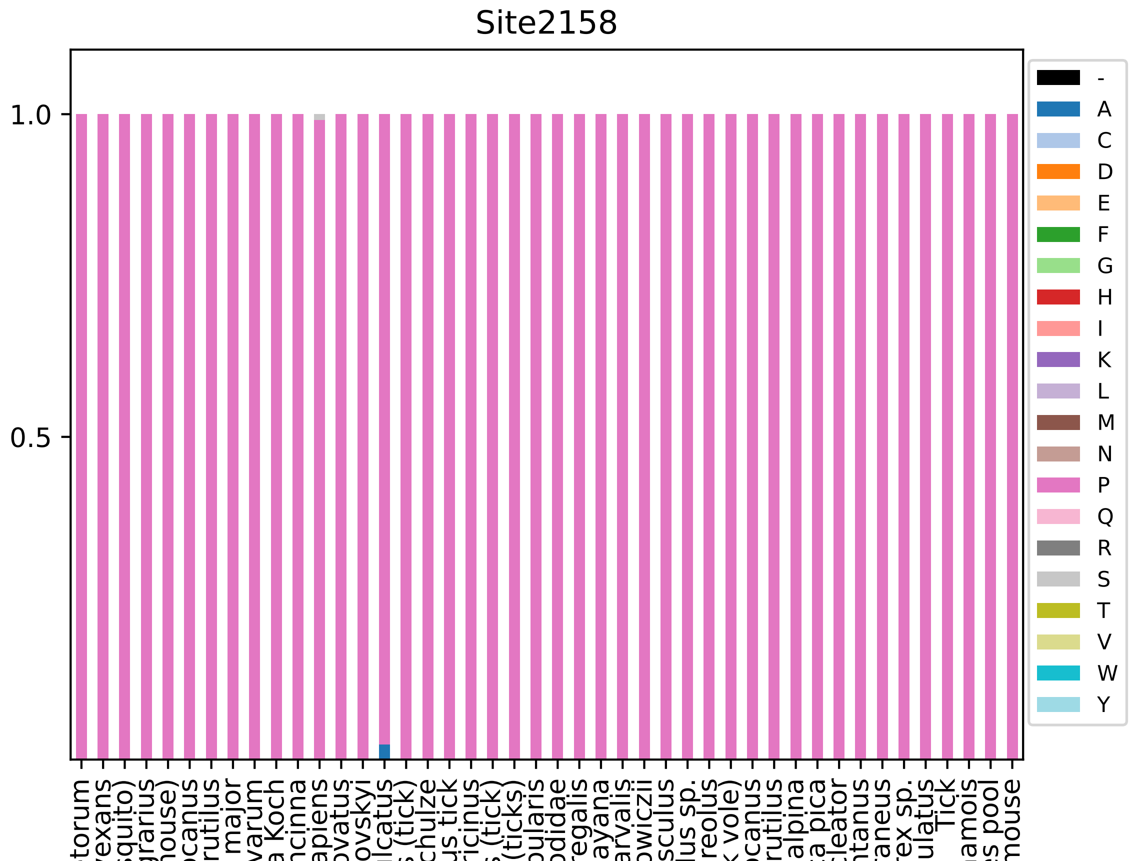
Site2158
1.0
0.5
-
A
C
D
E
F
G
H
I
K
L
M
N
P
Q
R
S
T
V
W
Y

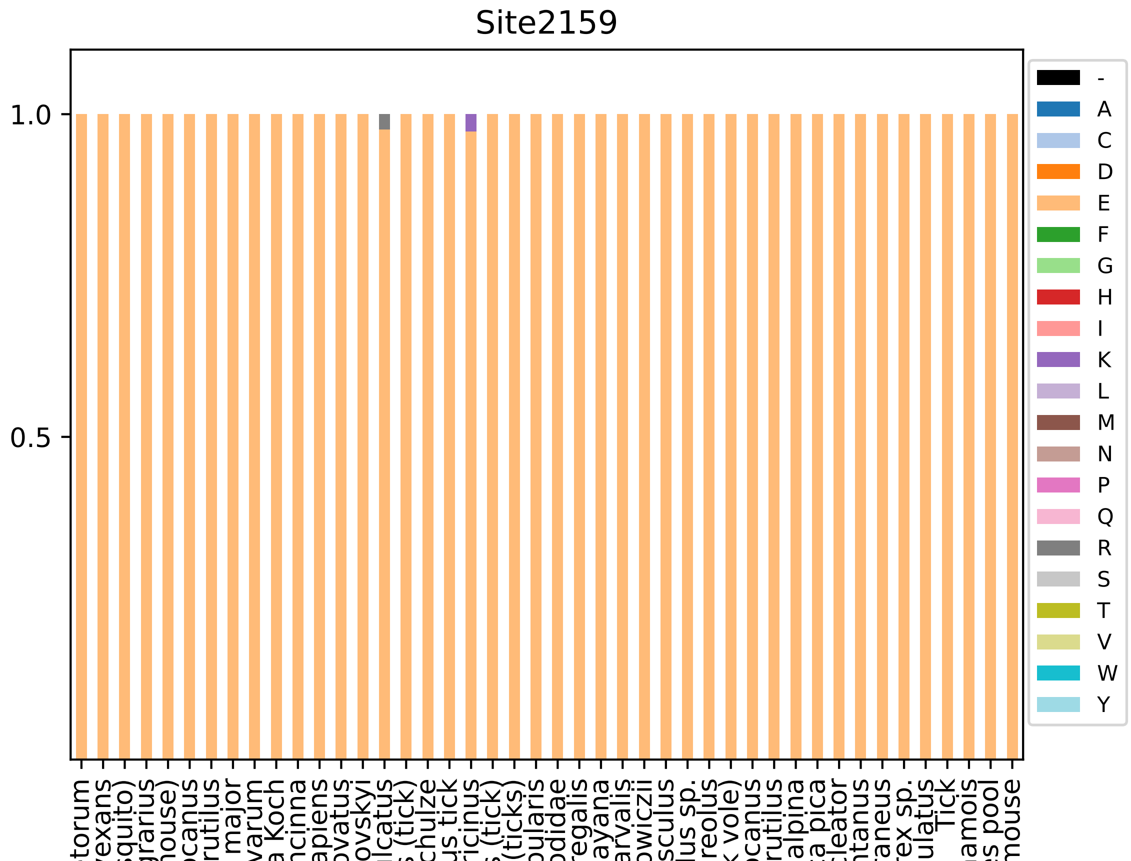
Site2159
1.0
0.5
-
A
C
D
E
F
G
H
I
K
L
M
N
P
Q
R
S
T
V
W
Y
Tick

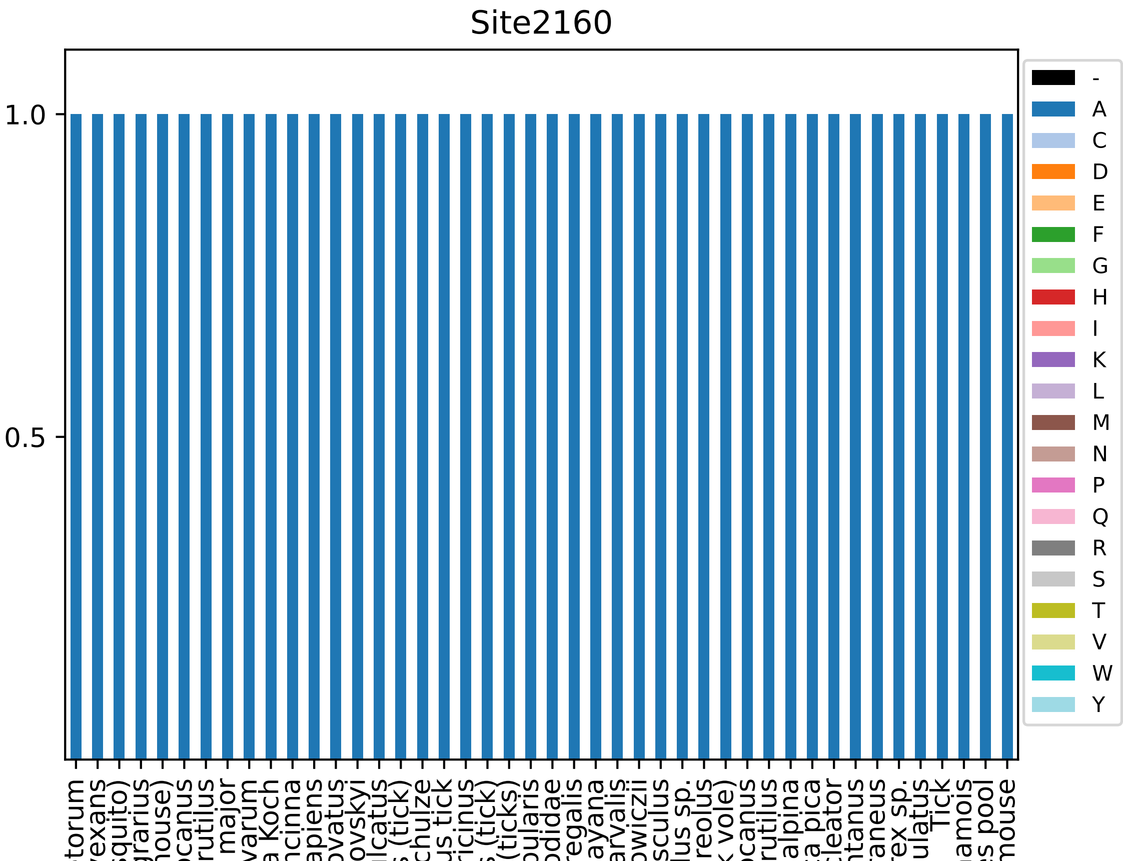
Site2160
1.0
0.5
-
A
C
D
E
F
G
H
I
K
L
M
N
P
Q
R
S
T
V
W
Y

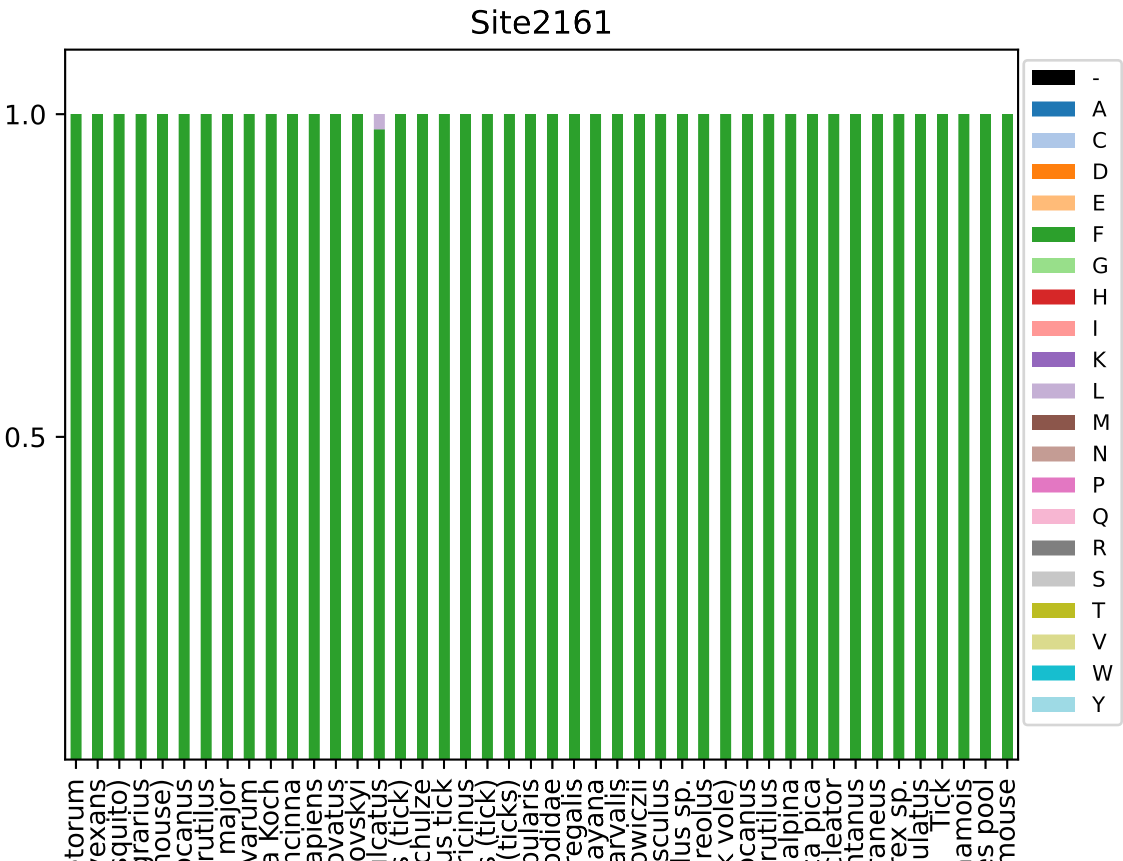
Site2161
1.0
0.5
-
A
C
D
E
F
G
H
I
K
L
M
N
P
Q
R
S
T
V
W
Y

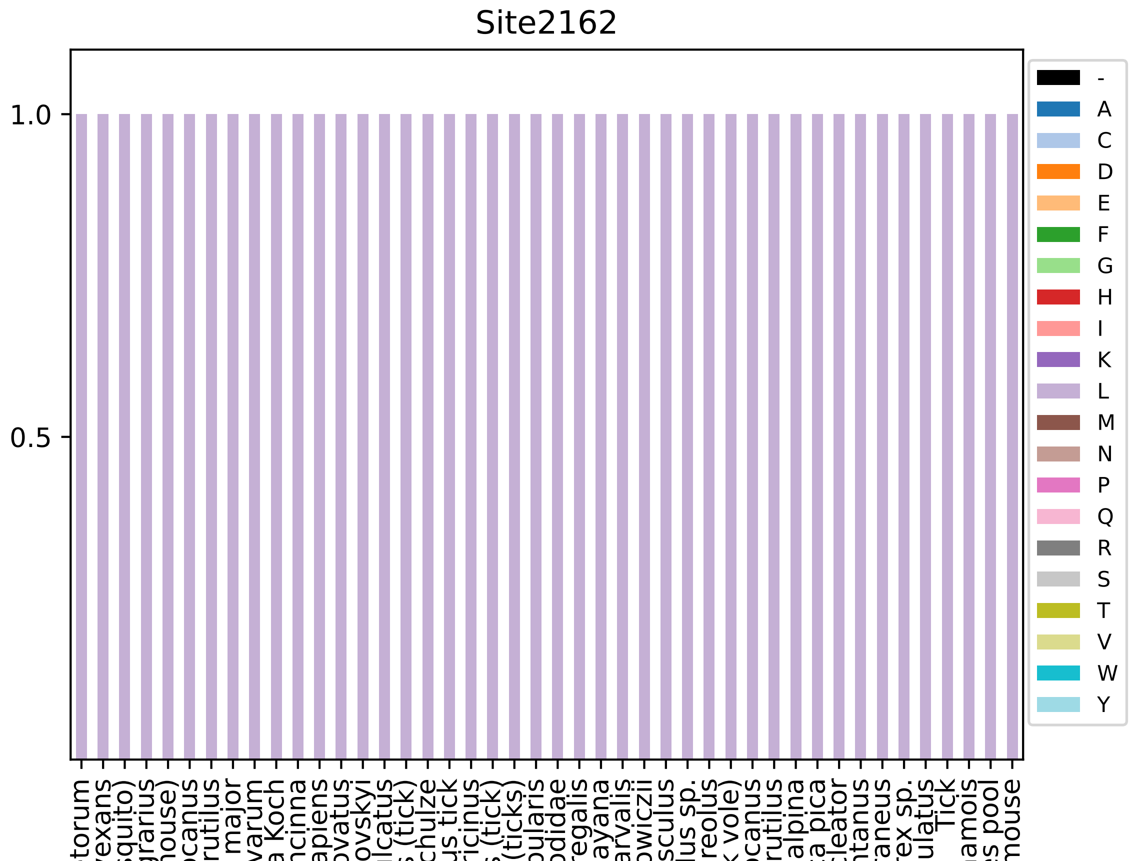
Site2162
1.0
0.5
-
A
C
D
E
F
G
H
I
K
L
M
N
P
Q
R
S
T
V
W
Y

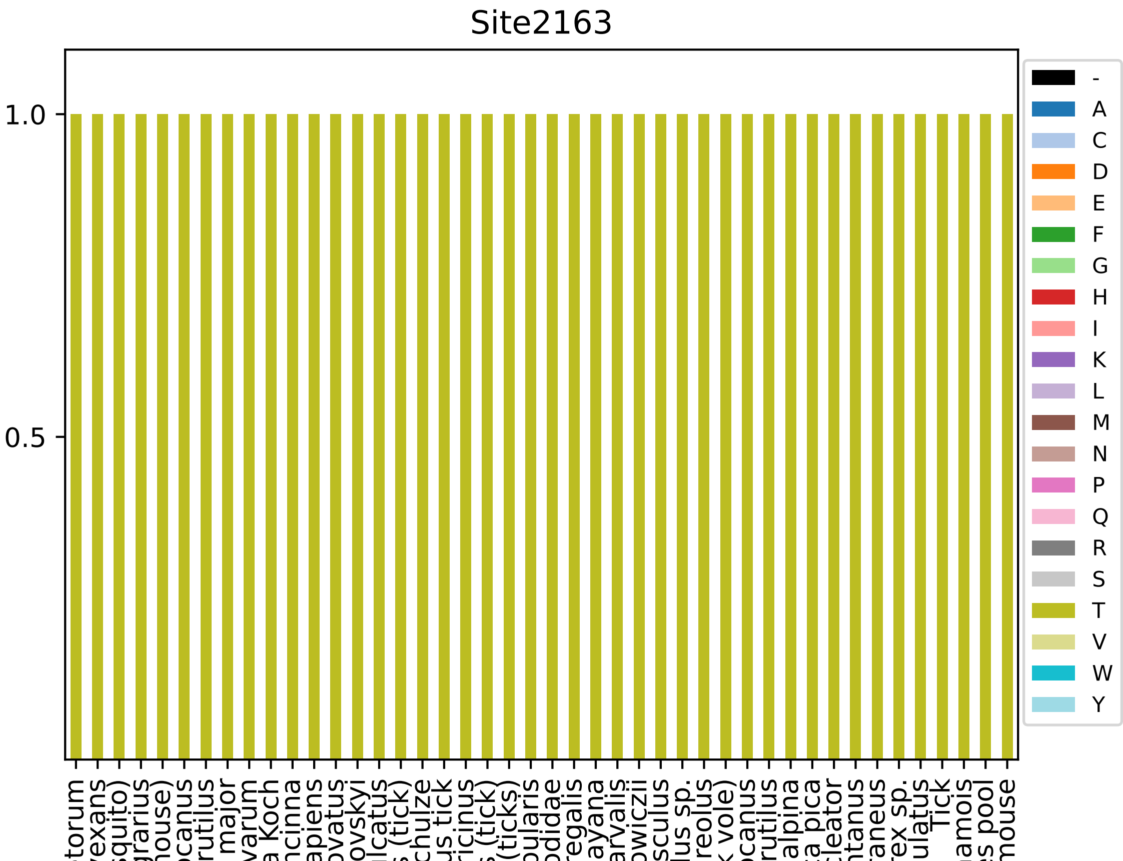
Site2163
1.0
0.5
-
A
C
D
E
F
G
H
I
K
L
M
N
P
Q
R
S
T
V
W
Y

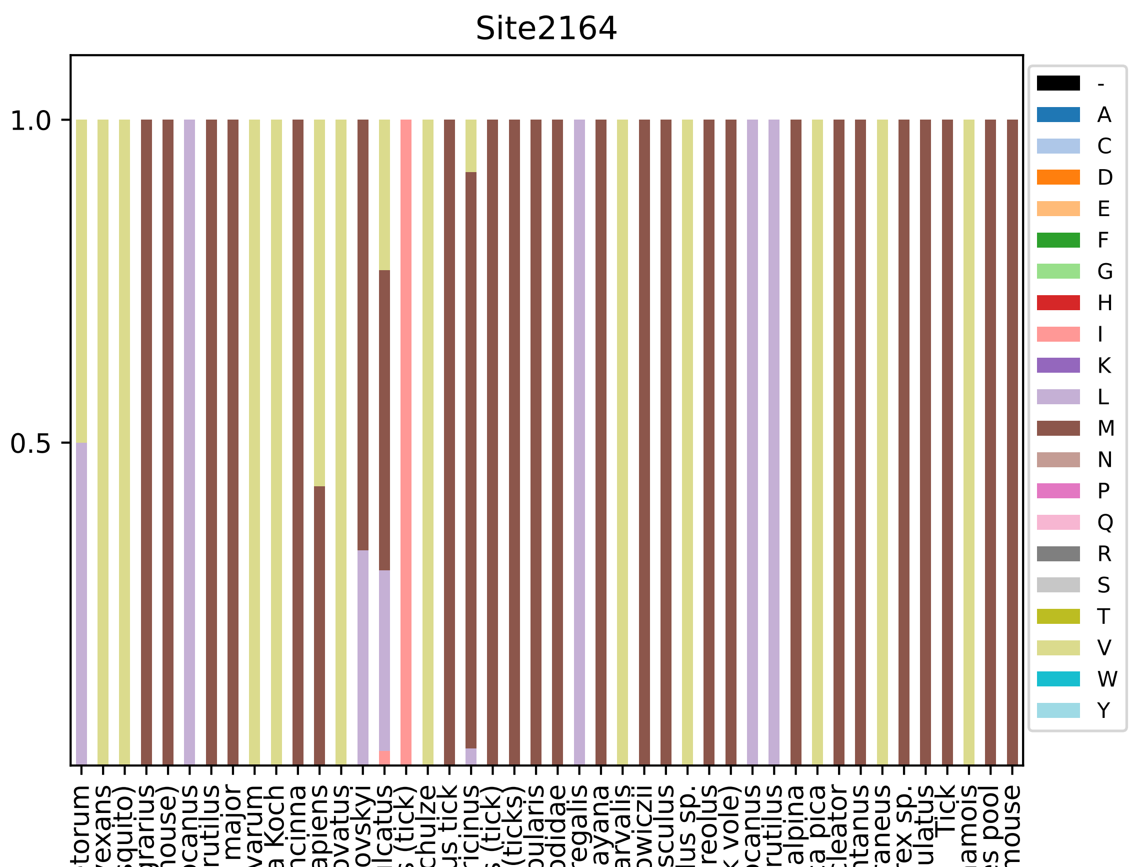

Site2164
1.0
0.5
-
A
C
D
E
F
G
H
I
K
L
M
N
P
Q
R
S
T
V
W
Y
torum
vexans
squito)
grarius
nouse)
ocanus
rutilus
major
varum
a Koch
ncinna
apiens
ovatus
ovskyi
ulcatus
s (tick)
chulze
us tick
ricinus
s (tick)
(ticks)
pularis
odidae
regalis
ayana
arvalis
owiczii
sculus
lus sp.
reolus
k vole)
ocanus
rutilus
alpina
a pica
cleator
ntanus
raneus
rex sp.
ulatus
Tick
amois
es pool
mouse

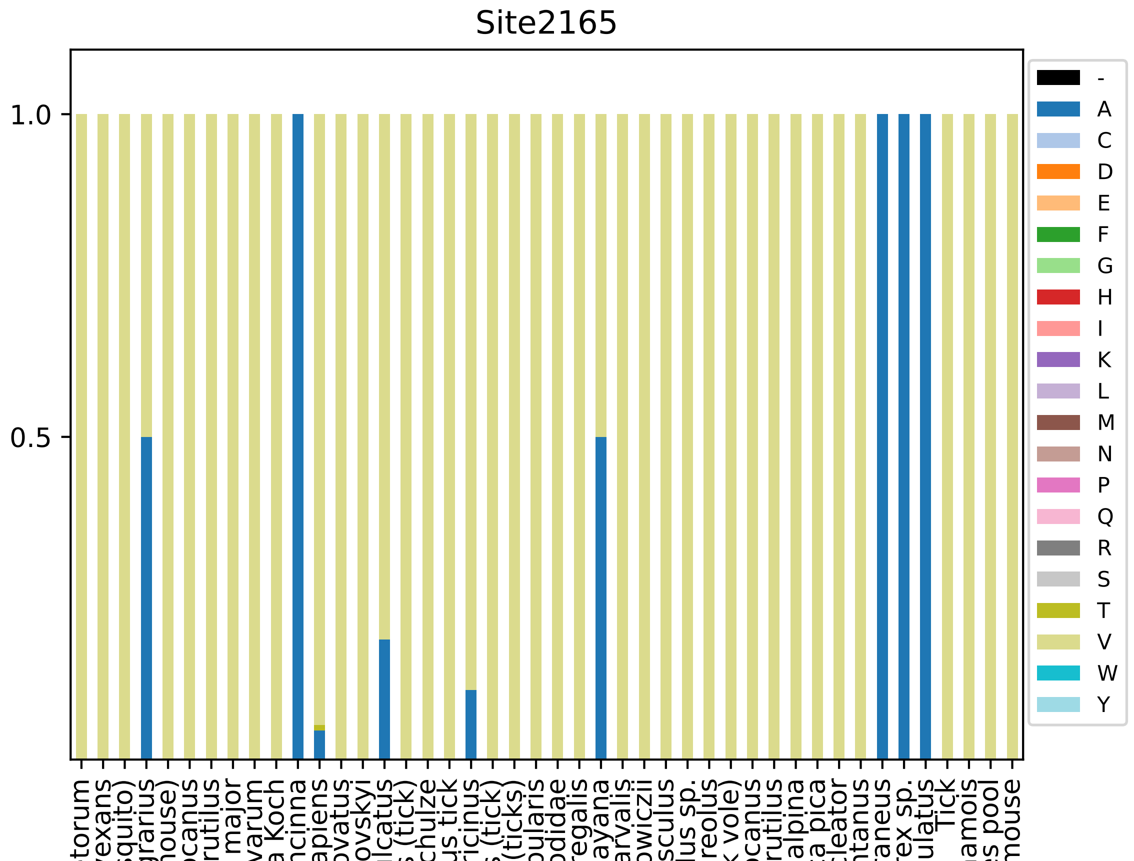
Site2165
1.0
0.5
-
A
C
D
E
F
G
H
I
K
L
M
N
P
Q
R
S
T
V
W
Y

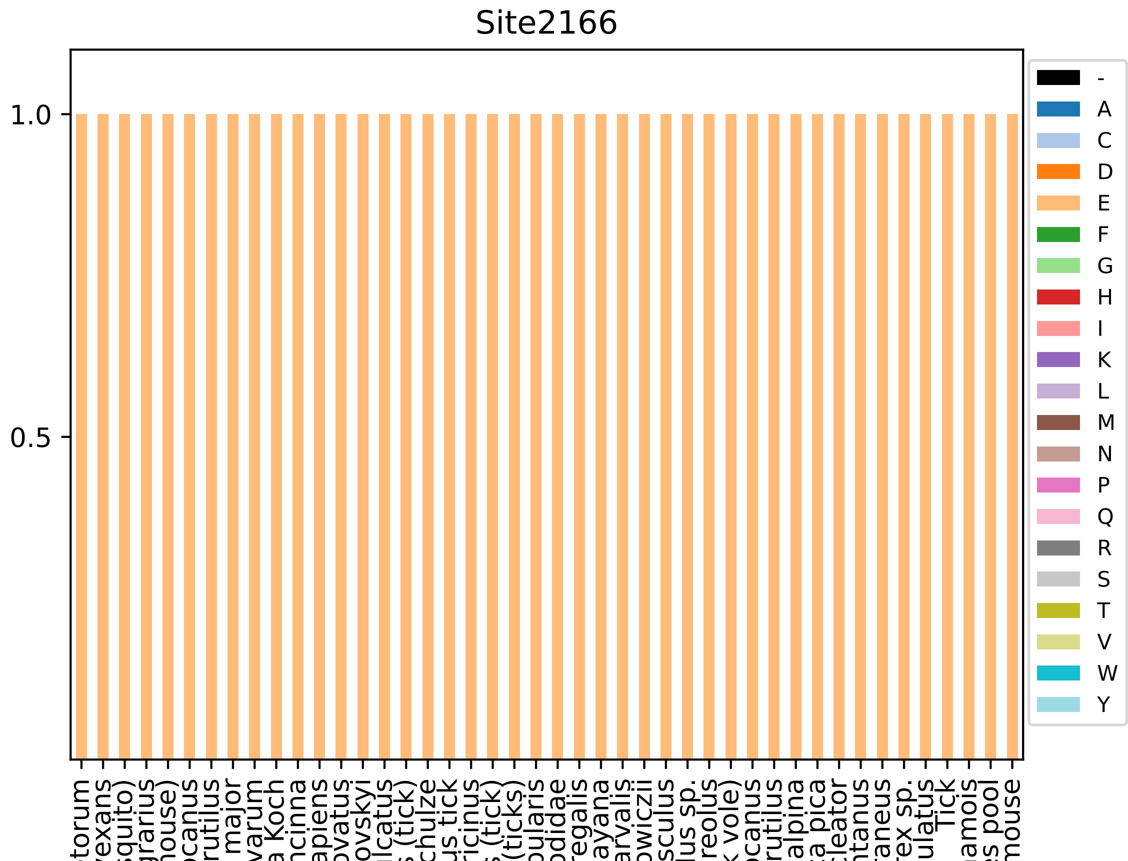
Site2166
1.0
0.5
-
A
C
D
E
F
G
H
I
K
L
M
N
P
Q
R
S
T
V
W
Y

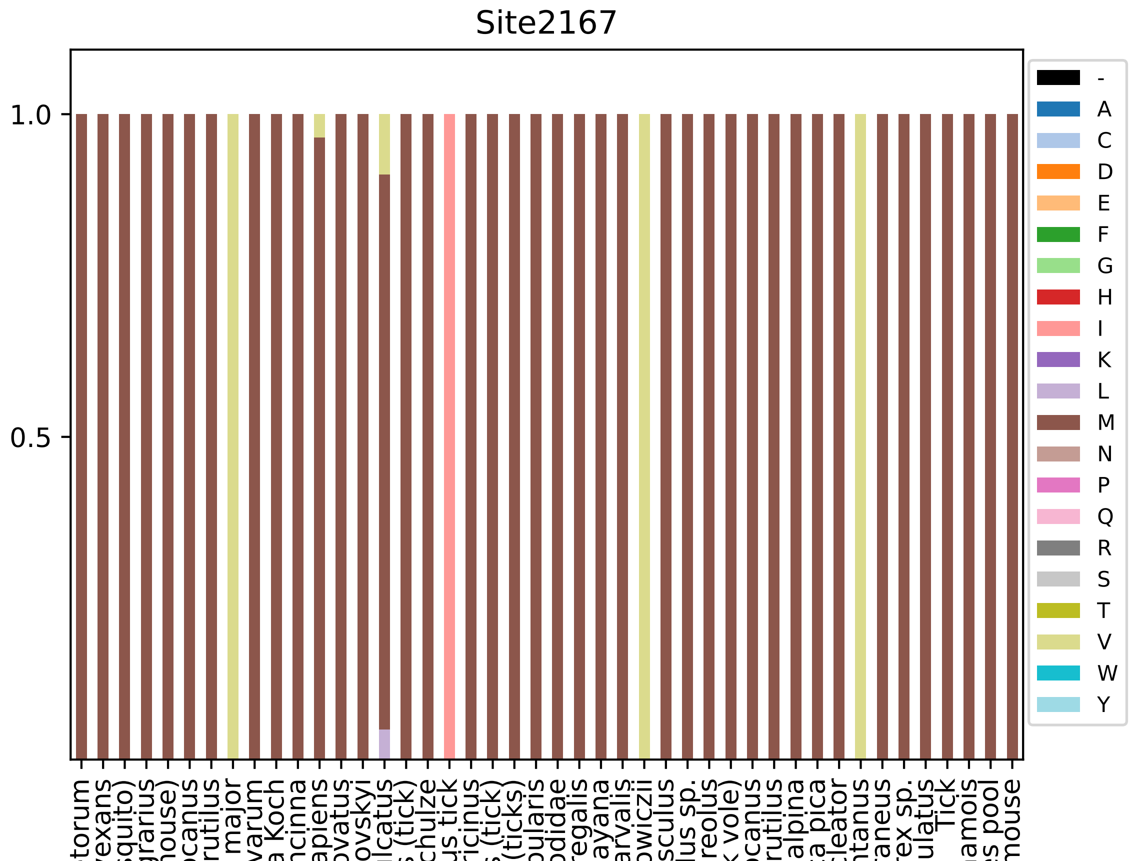
Site2167
1.0
0.5
-
A
C
D
E
F
G
H
I
K
L
M
N
P
Q
R
S
T
V
W
Y

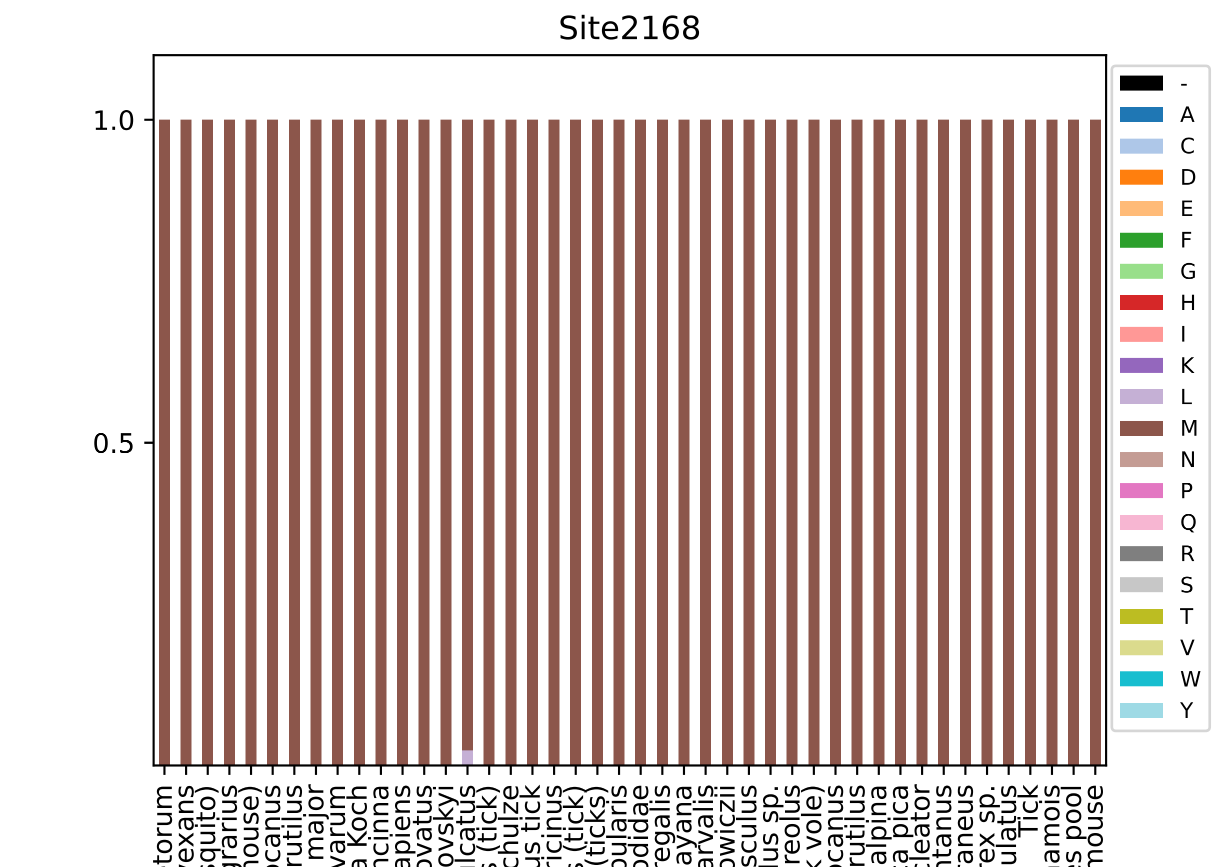

Site2168
1.0
0.5
-
A
C
D
E
F
G
H
I
K
L
M
N
P
Q
R
S
T
V
W
Y

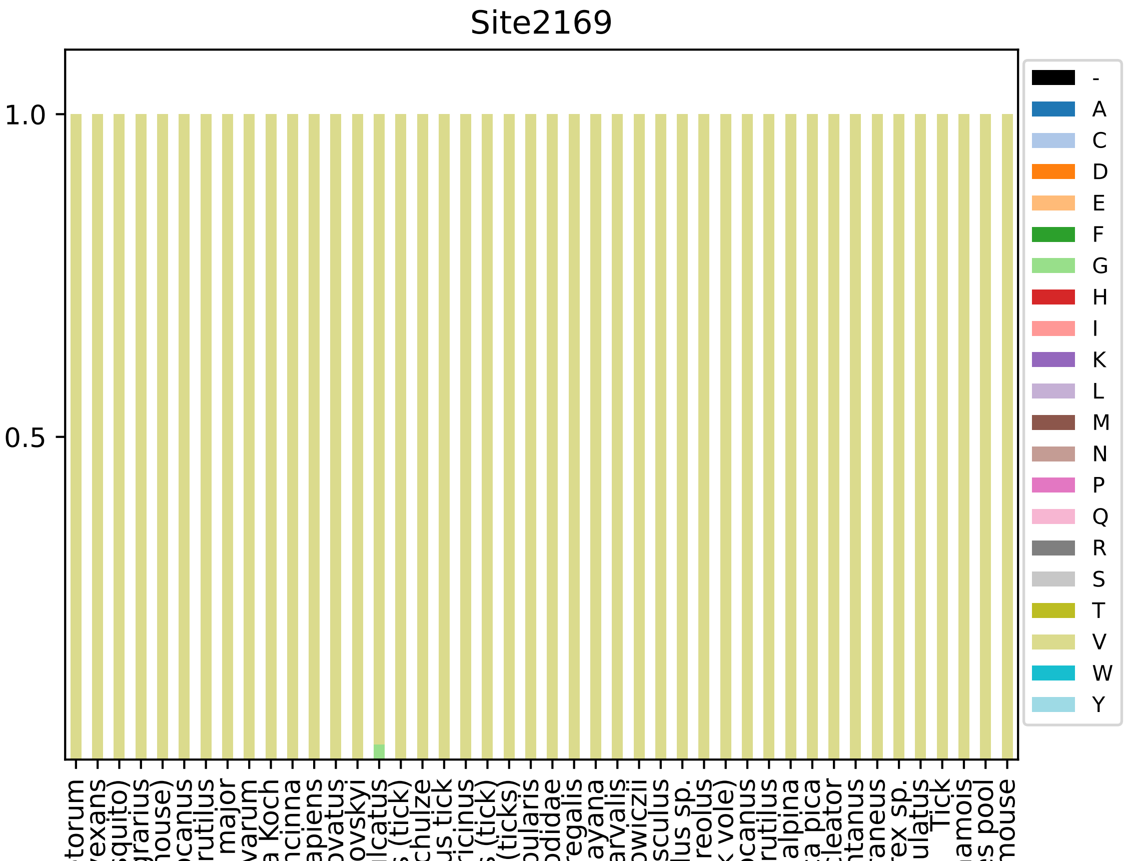
Site2169
1.0
0.5
-
A
C
D
E
F
G
H
I
K
L
M
N
P
Q
R
S
T
V
W
Y

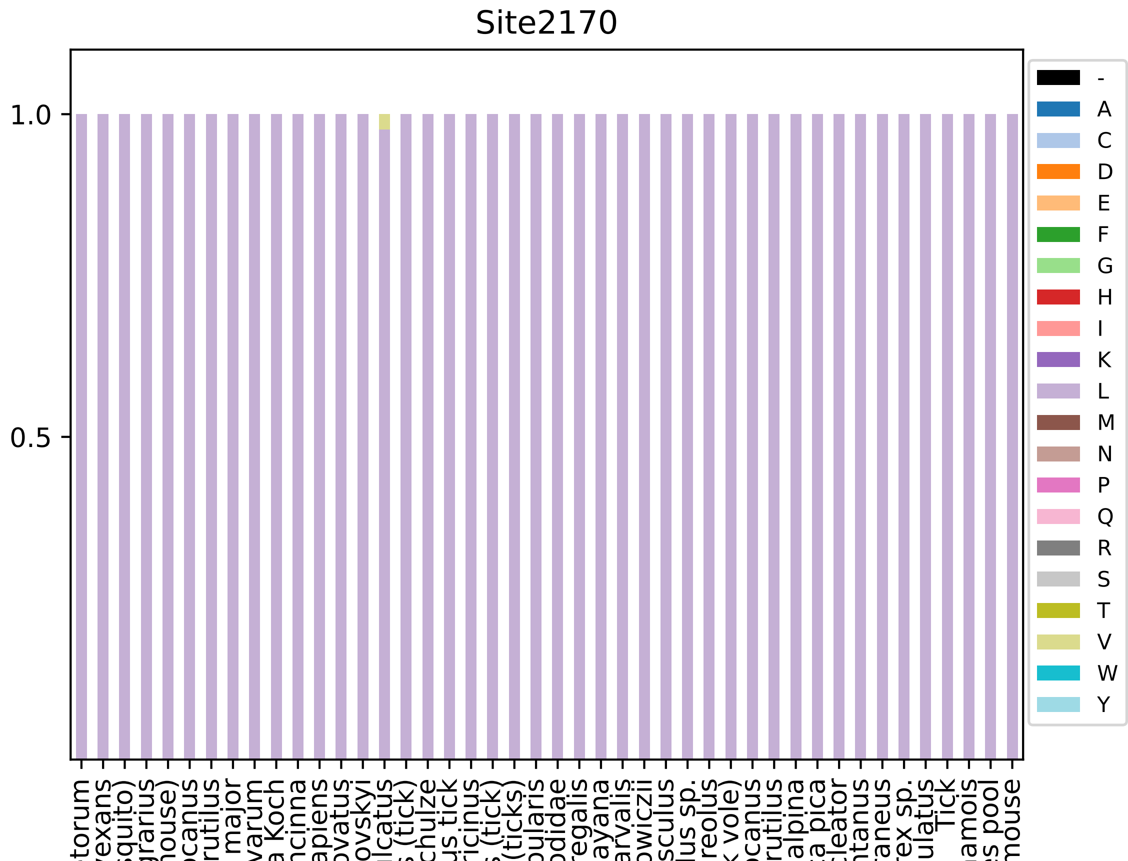
Site2170
1.0
0.5
-
A
C
D
E
F
G
H
I
K
L
M
N
P
Q
R
S
T
V
W
Y

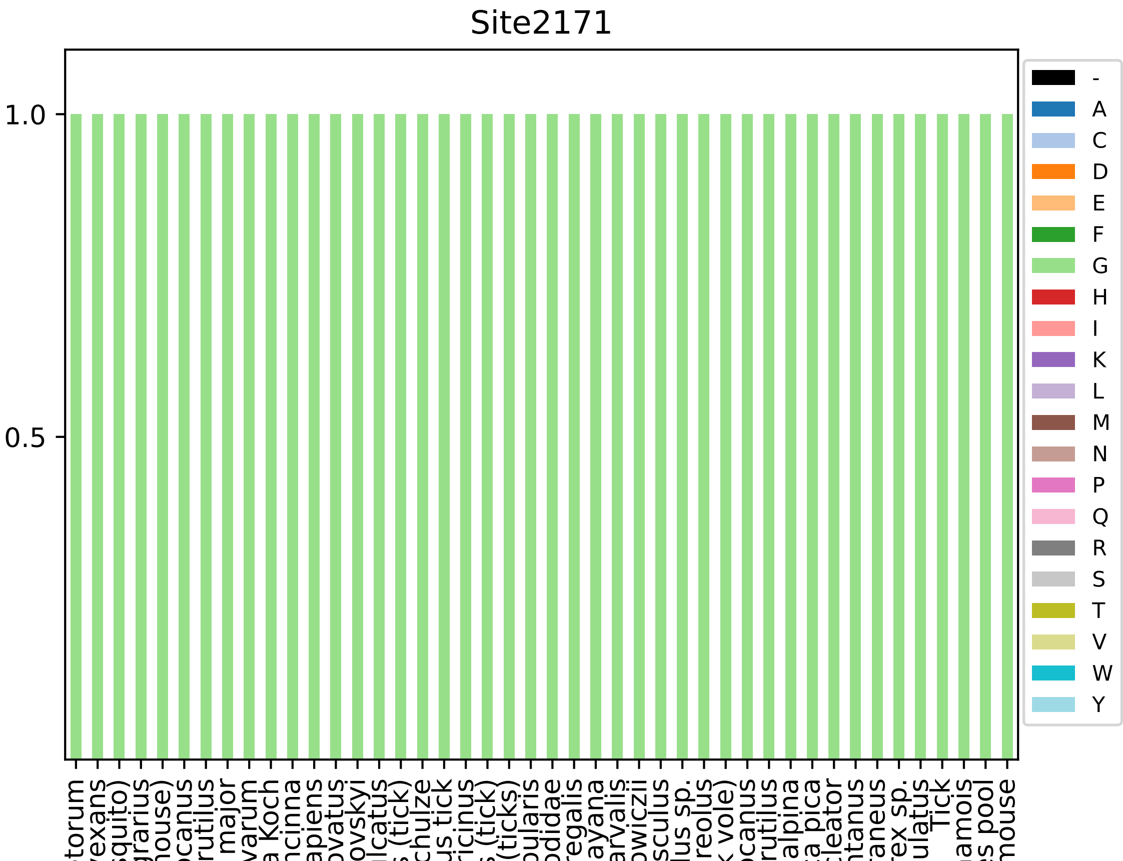
Site2171
1.0
0.5
-
A
C
D
E
F
G
H
I
K
L
M
N
P
Q
R
S
T
V
W
Y

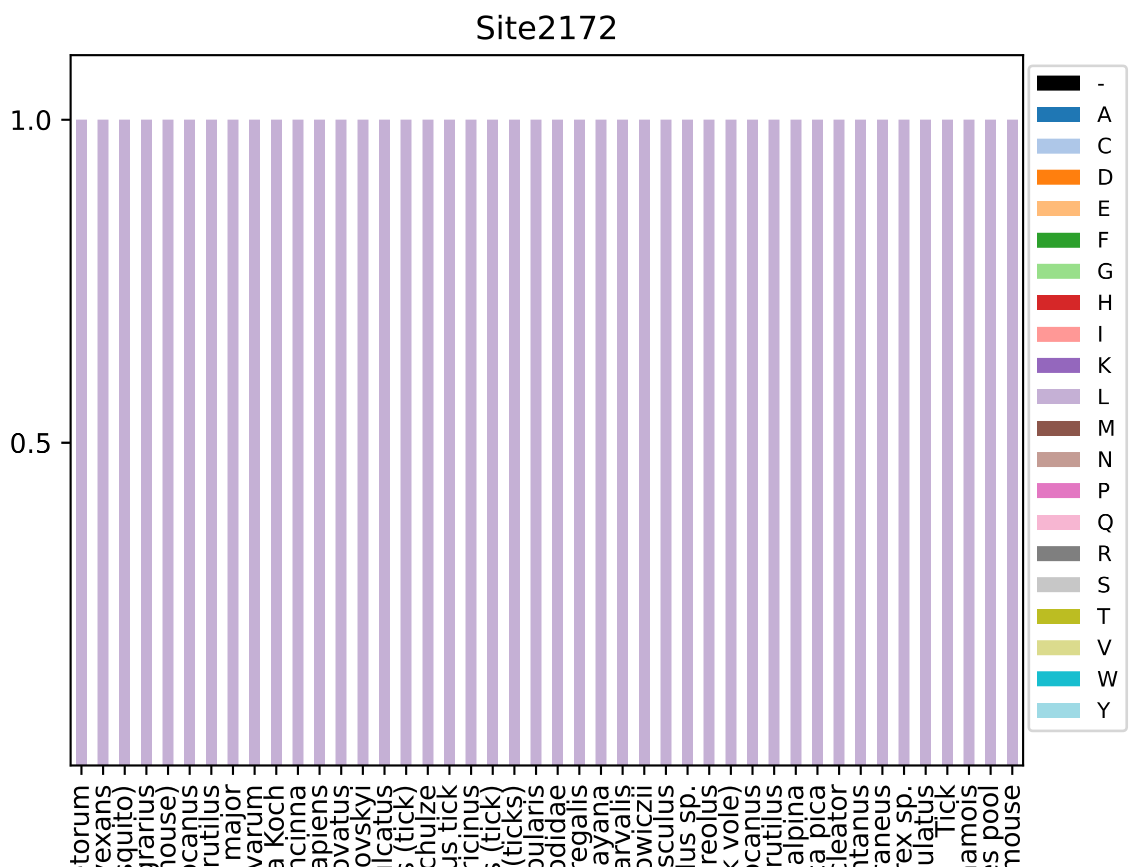
Site2172
1.0
0.5
-
A
C
D
E
F
G
H
I
K
L
M
N
P
Q
R
S
T
V
W
Y

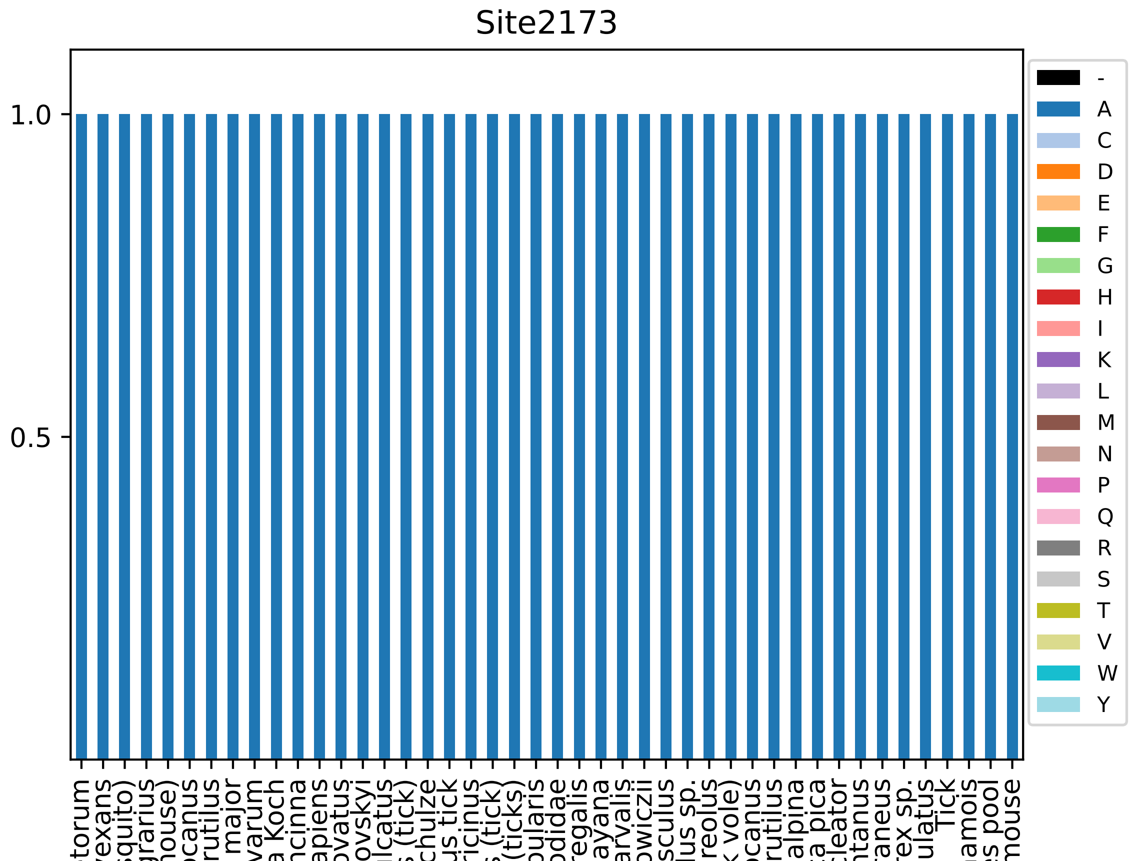
Site2173
1.0
0.5
-
A
C
D
E
F
G
H
I
K
L
M
N
P
Q
R
S
T
V
W
Y

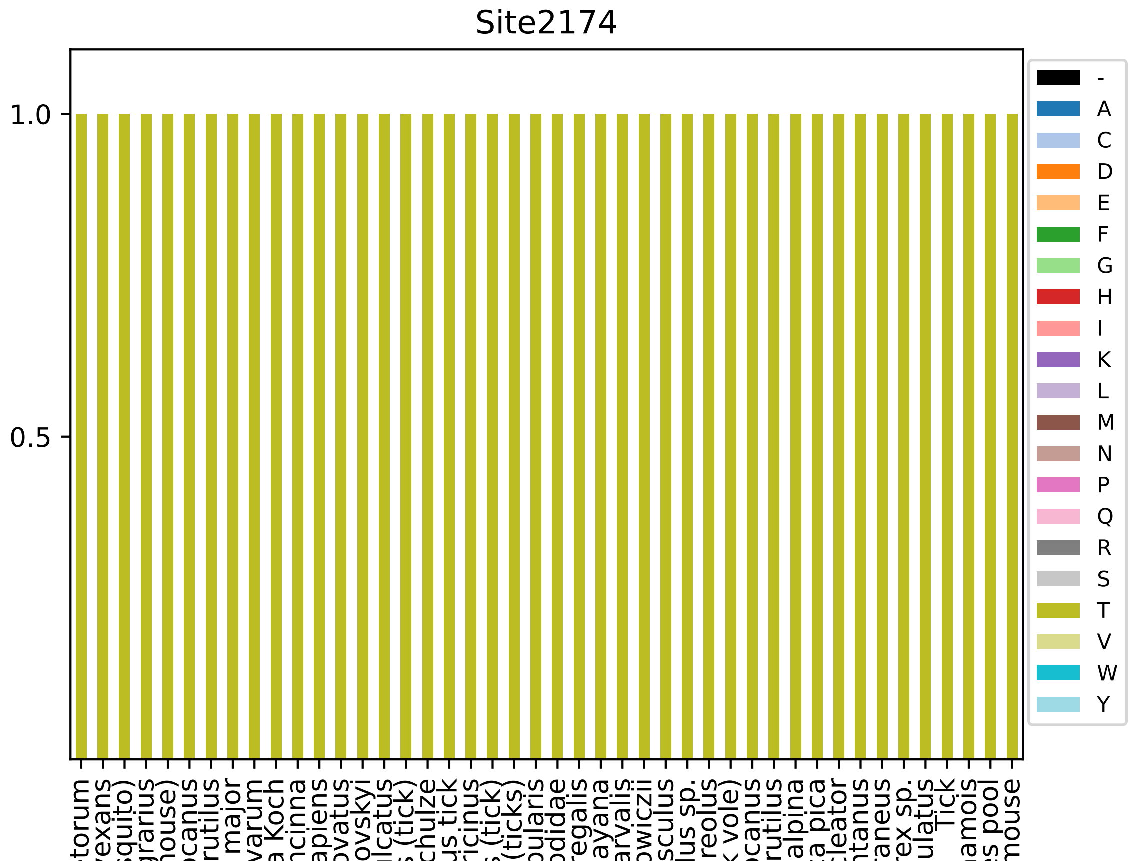
Site2174
1.0
0.5
-
A
C
D
E
F
G
H
I
K
L
M
N
P
Q
R
S
T
V
W
Y

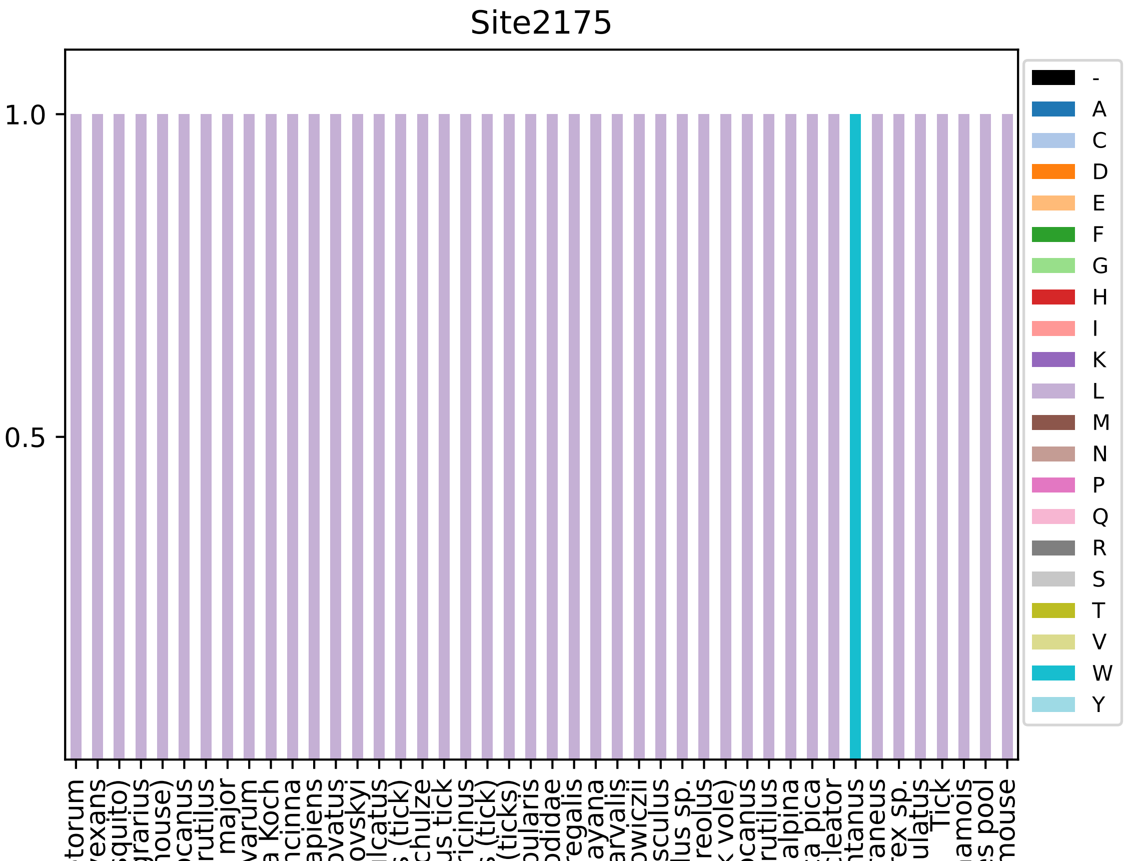
Site2175
1.0
0.5
-
A
C
D
E
F
G
H
I
K
L
M
N
P
Q
R
S
T
V
W
Y

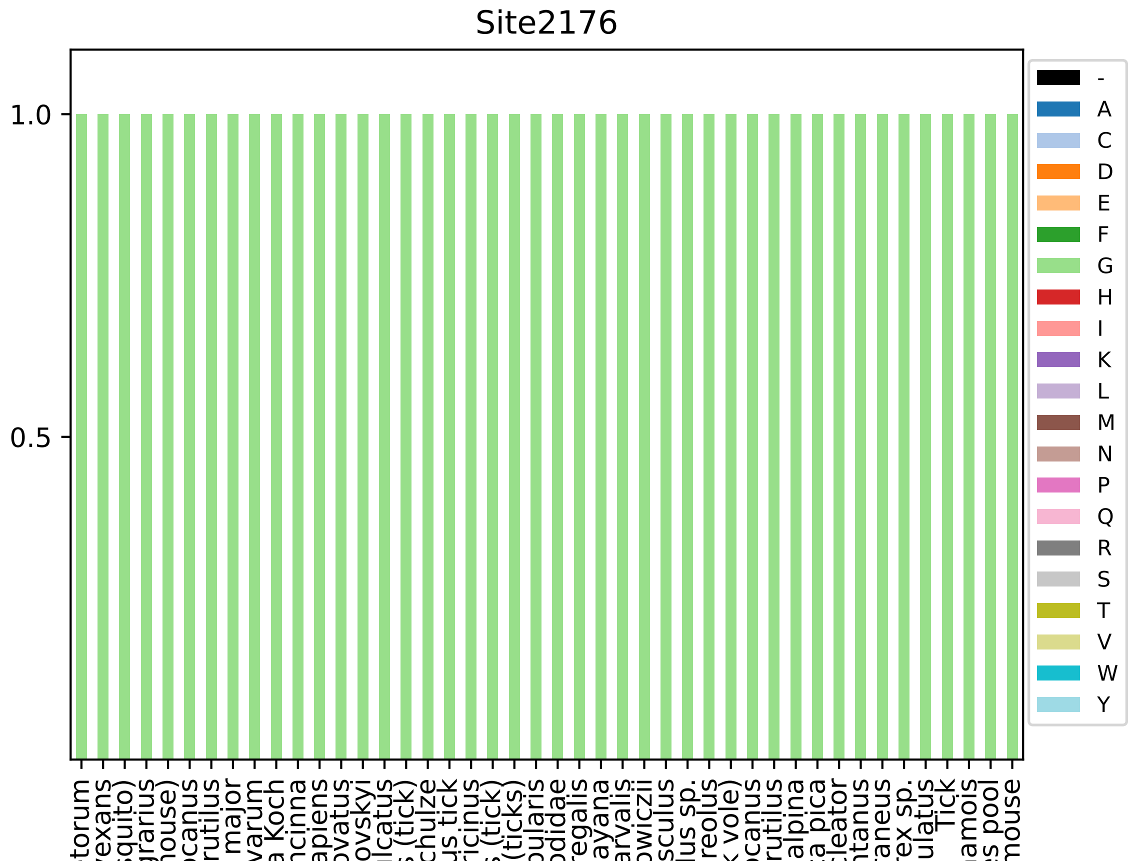
Site2176
1.0
0.5
-
A
C
D
E
F
G
H
I
K
L
M
N
P
Q
R
S
T
V
W
Y

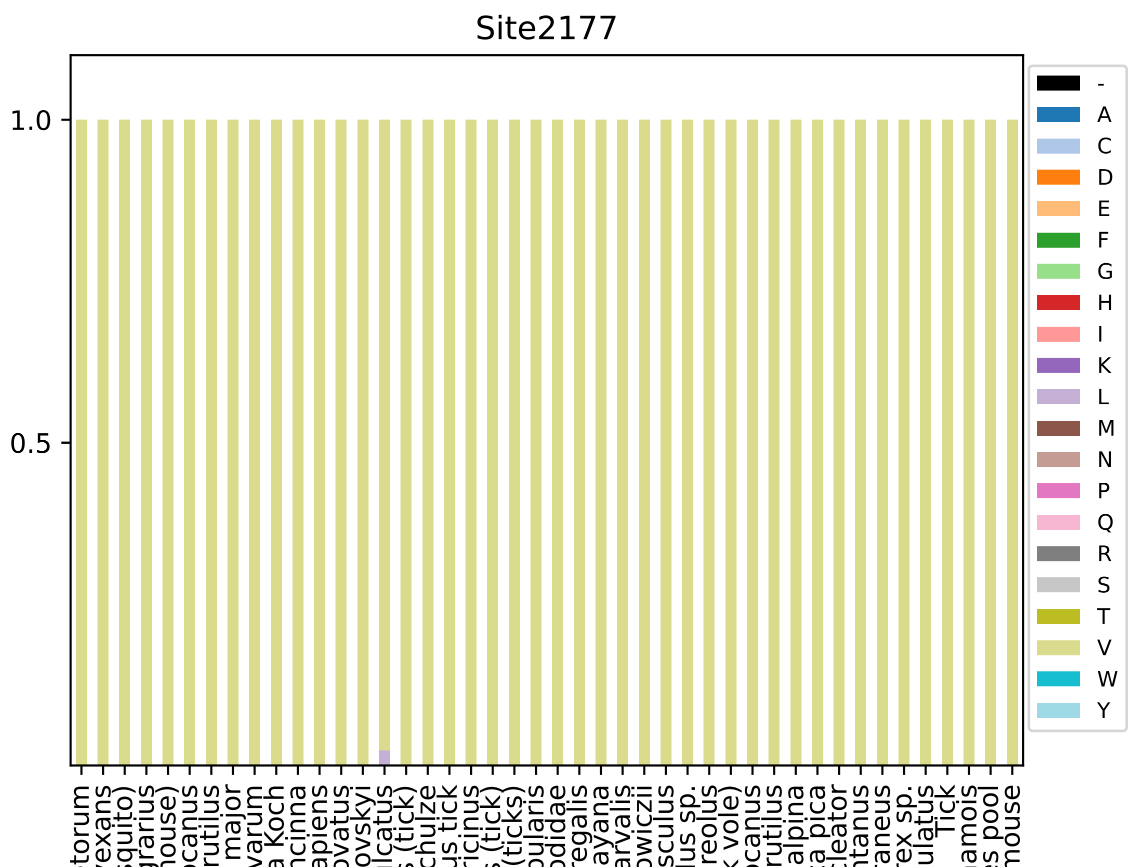
Site2177
1.0
0.5
-
A
C
D
E
F
G
H
I
K
L
M
N
P
Q
R
S
T
V
W
Y

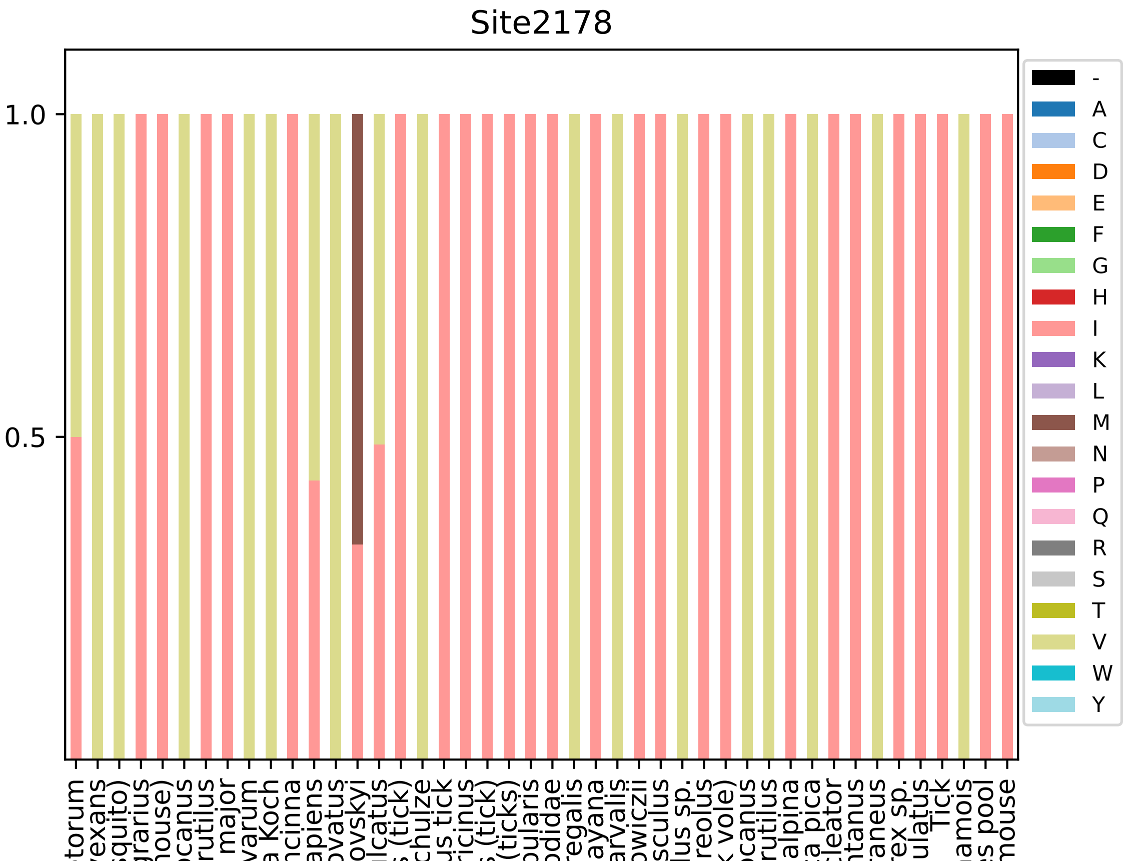
Site2178
1.0
0.5
-
A
C
D
E
F
G
H
I
K
L
M
N
P
Q
R
S
T
V
W
Y

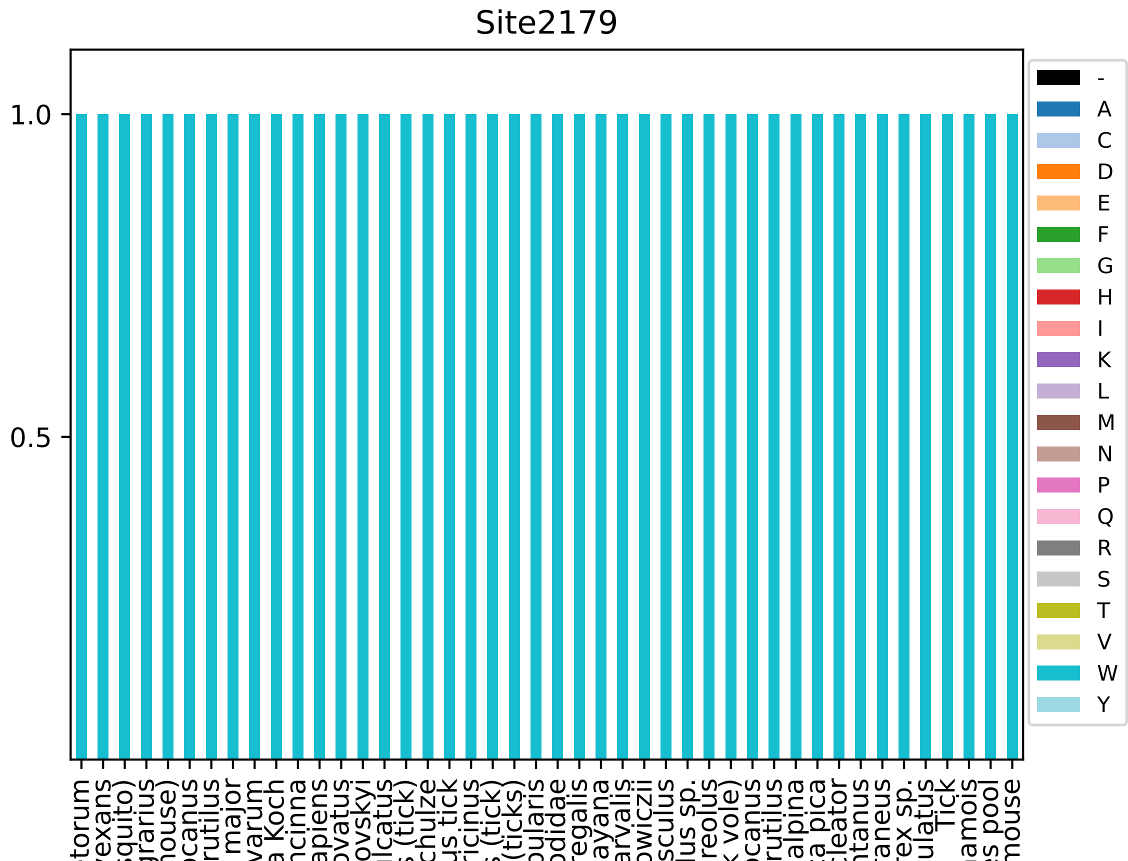
Site2179
1.0
0.5
-
A
C
D
E
F
G
H
I
K
L
M
N
P
Q
R
S
T
V
W
Y

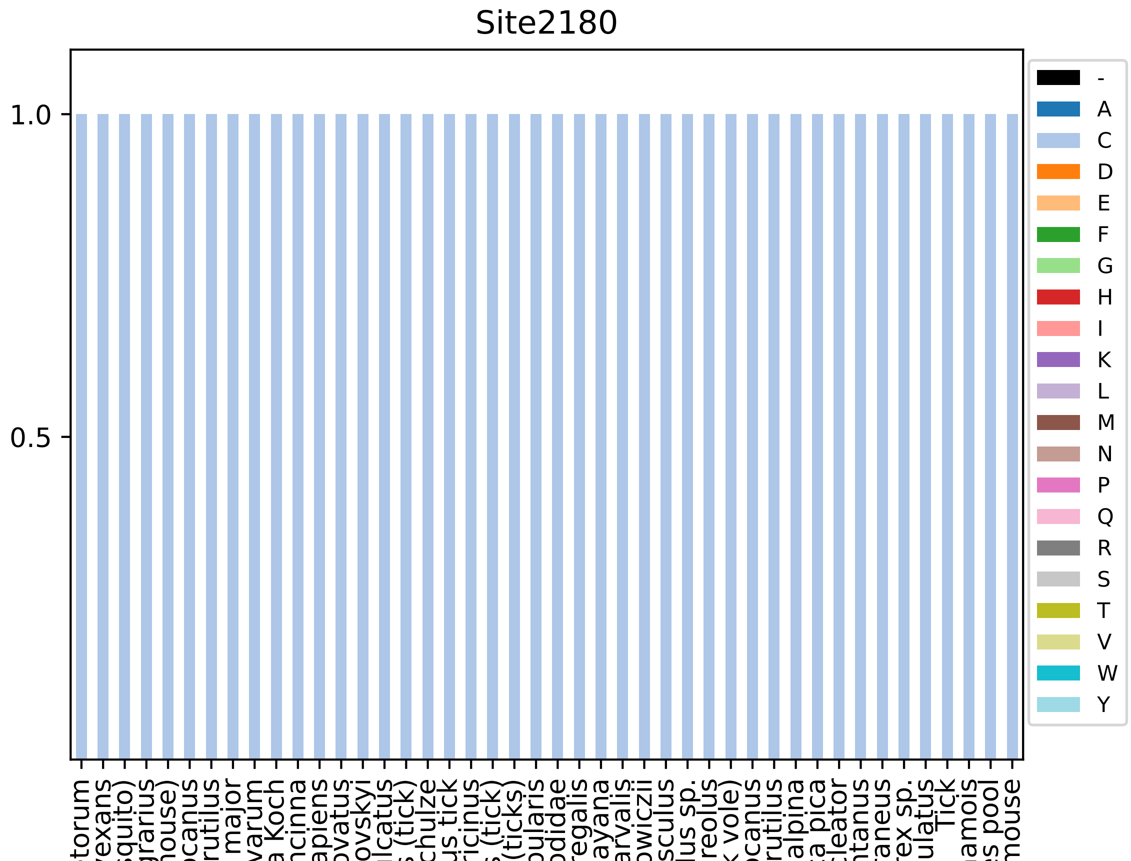
Site2180
1.0
0.5
-
A
C
D
E
F
G
H
I
K
L
M
N
P
Q
R
S
T
V
W
Y
torum
vexans
squito)
grarius
house)
ocanus
rutilus
major
varum
a Koch
ncinna
apiens
ovatus
ovskyi
ulcatus
s (tick)
chulze
us tick
ricinus
s (tick)
(ticks)
pularis
podidae
regalis
ayana
arvalis
owiczii
sculus
lus sp.
reolus
k vole)
ocanus
rutilus
alpina
a pica
leator
ntanus
raneus
rex sp.
ulatus
Tick
amois
s pool
mouse

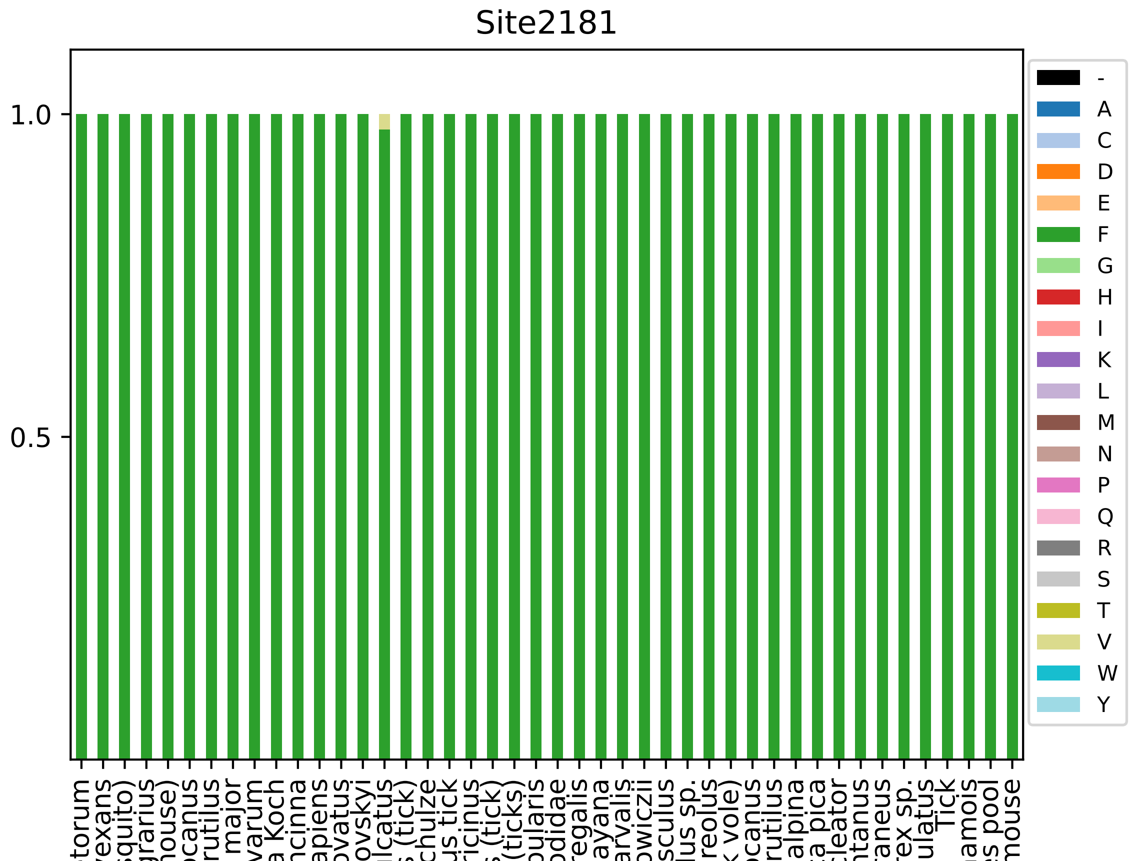
Site2181
1.0
0.5
-
A
C
D
E
F
G
H
I
K
L
M
N
P
Q
R
S
T
V
W
Y
Tick

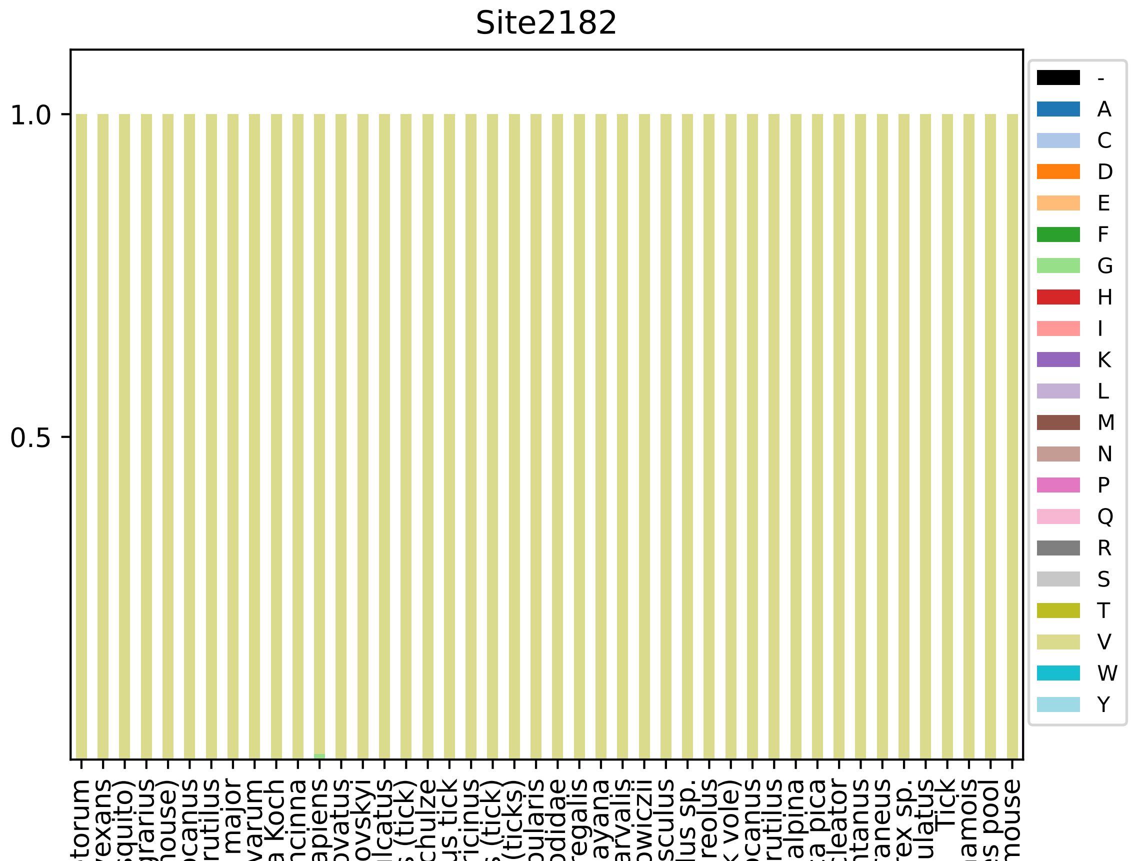
Site2182
1.0
0.5
-
A
C
D
E
F
G
H
I
K
L
M
N
P
Q
R
S
T
V
W
Y

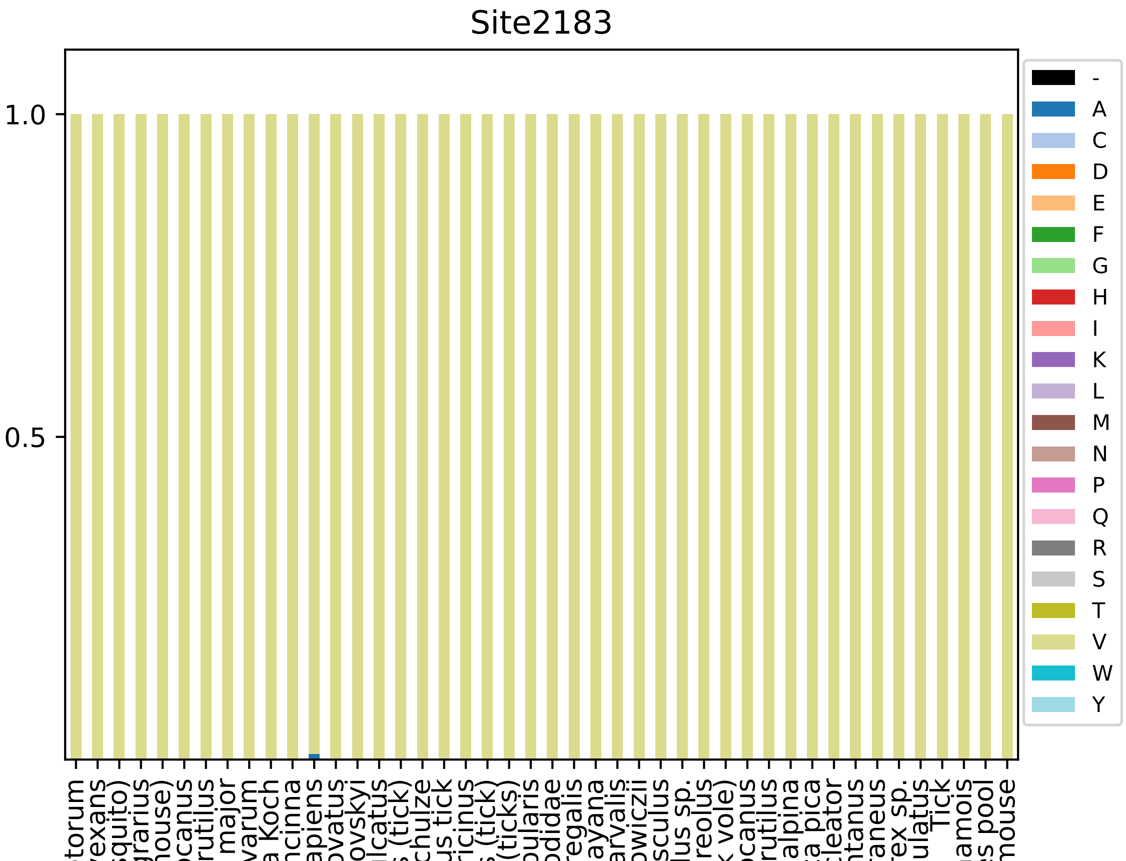

Site2183
1.0
0.5
-
A
C
D
E
F
G
H
I
K
L
M
N
P
Q
R
S
T
V
W
Y

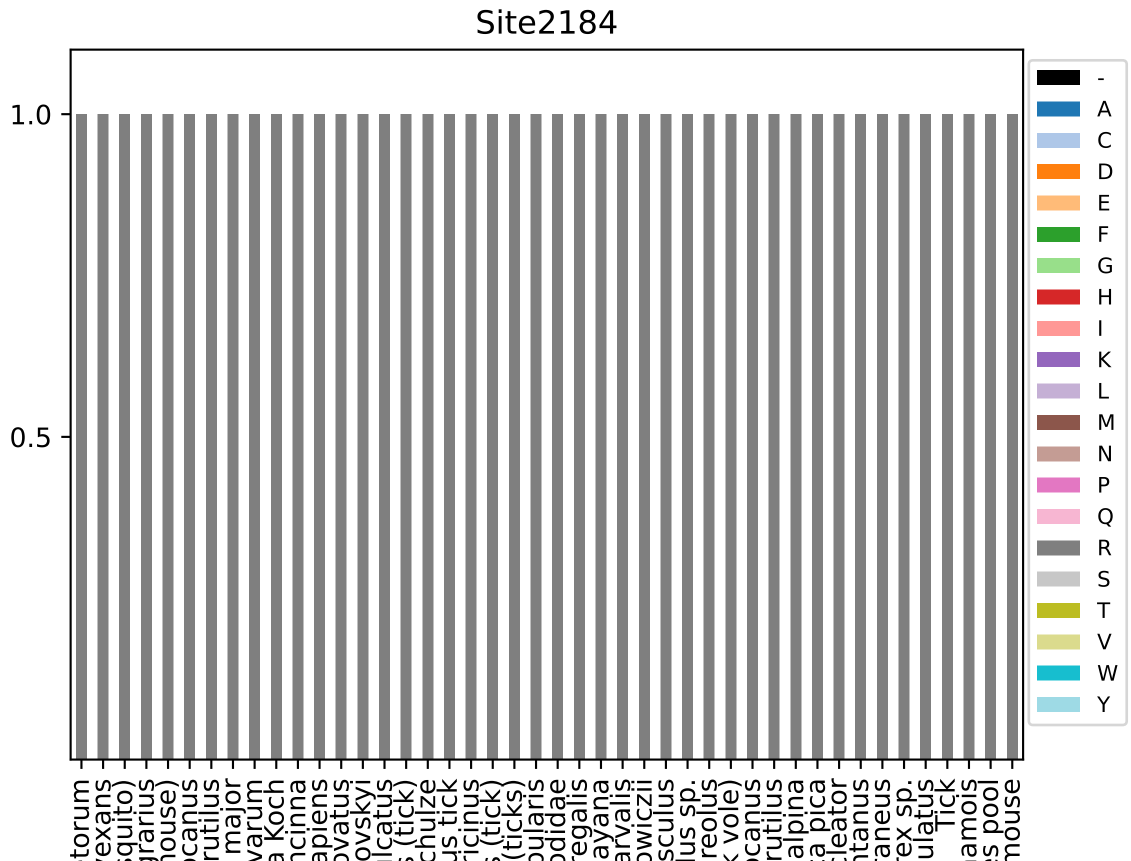
Site2184
1.0
0.5
-
A
C
D
E
F
G
H
I
K
L
M
N
P
Q
R
S
T
V
W
Y

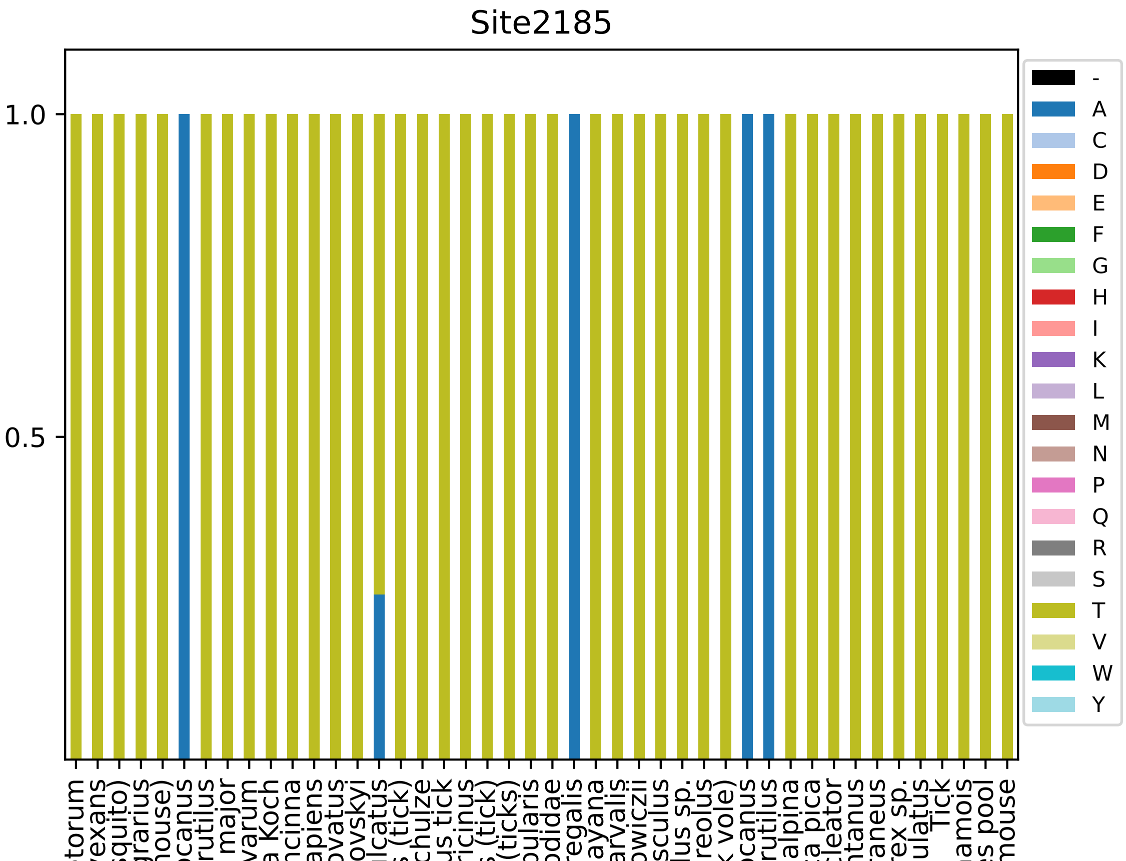

Site2185
1.0
0.5
-
A
C
D
E
F
G
H
I
K
L
M
N
P
Q
R
S
T
V
W
Y

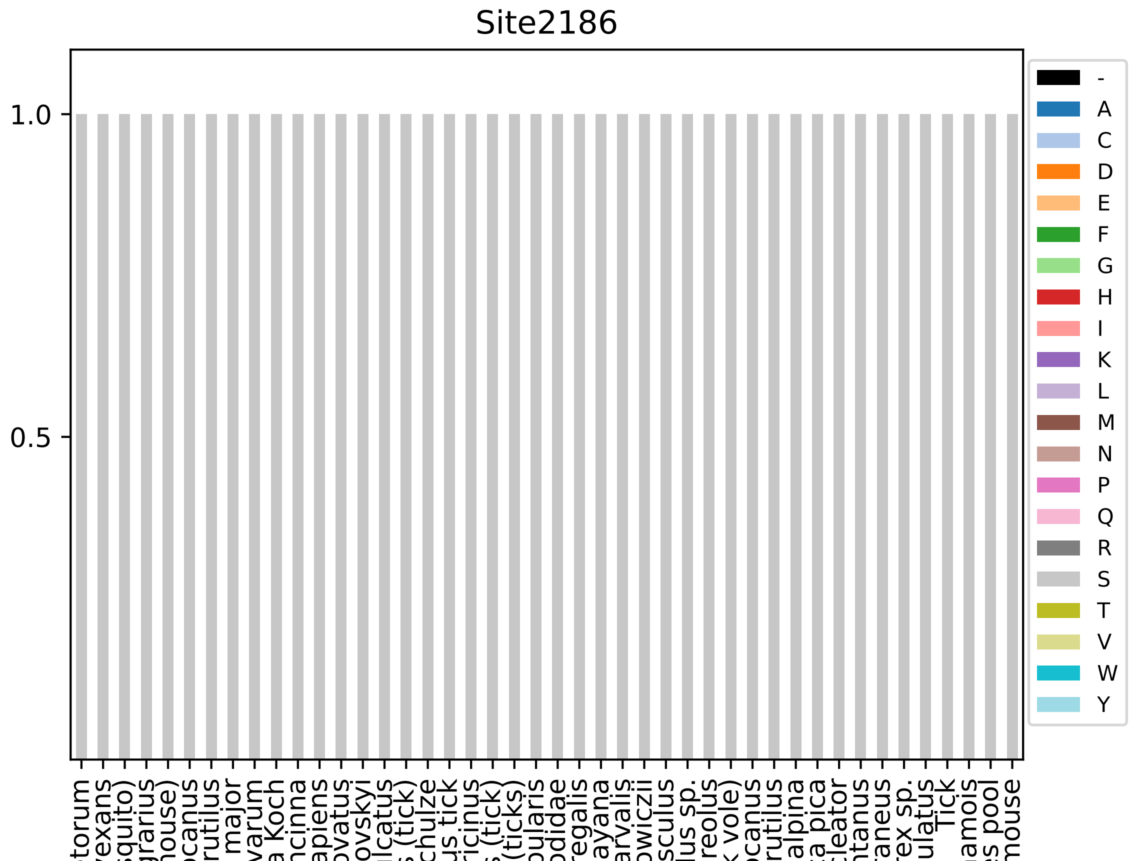

Site2186
1.0
0.5
-
A
C
D
E
F
G
H
I
K
L
M
N
P
Q
R
S
T
V
W
Y

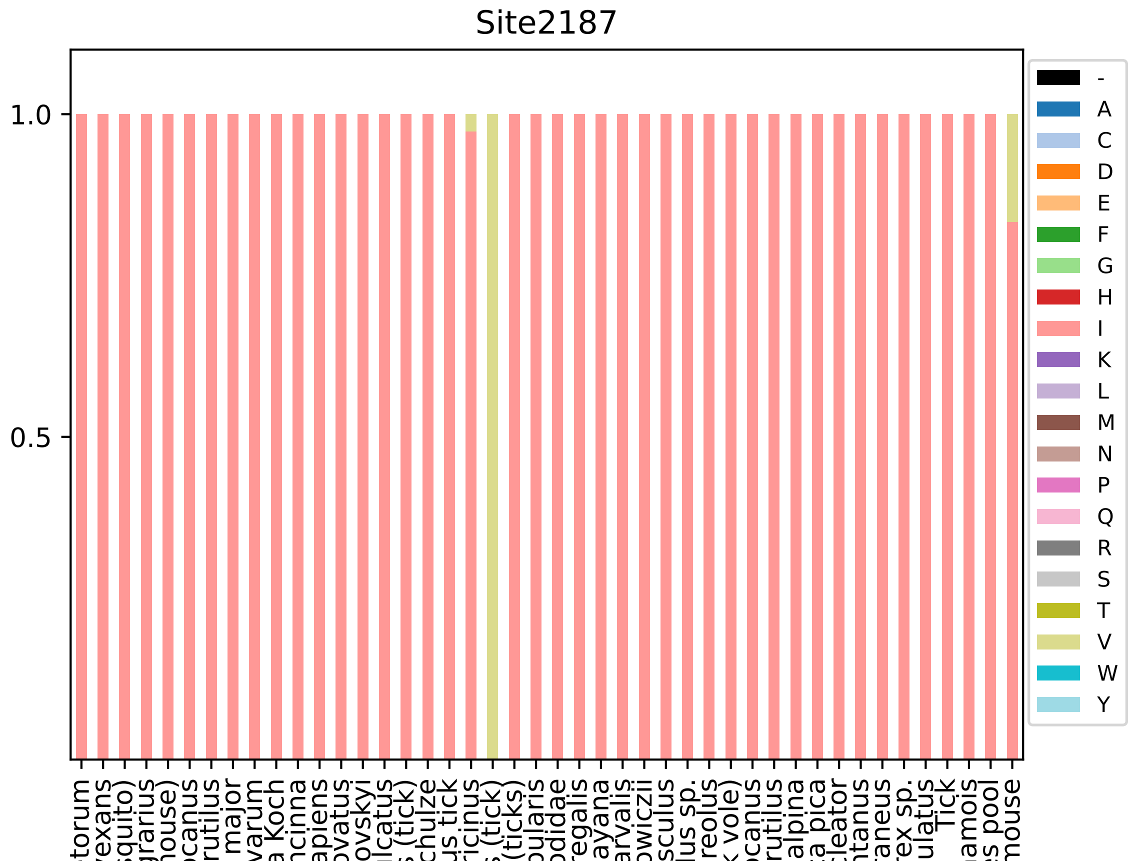

Site2187
1.0
0.5
-
A
C
D
E
F
G
H
I
K
L
M
N
P
Q
R
S
T
V
W
Y

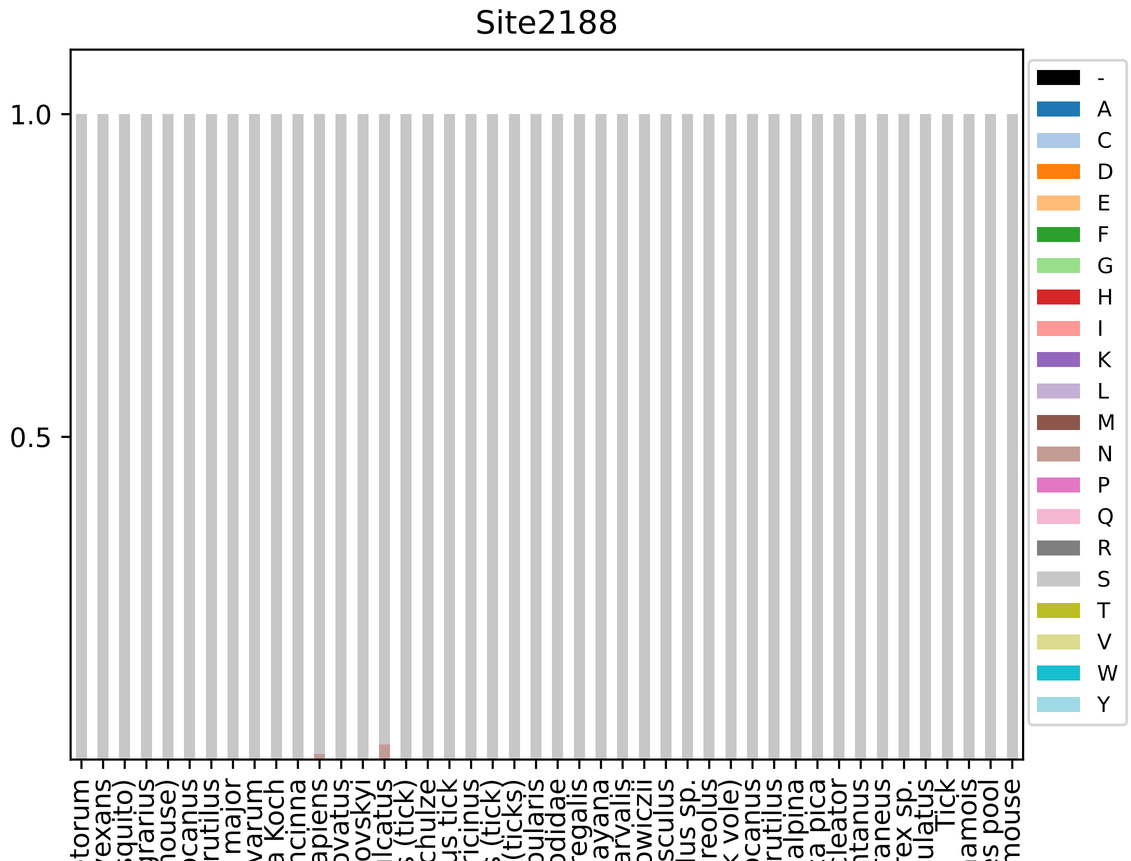
Site2188
1.0
0.5
-
A
C
D
E
F
G
H
I
K
L
M
N
P
Q
R
S
T
V
W
Y

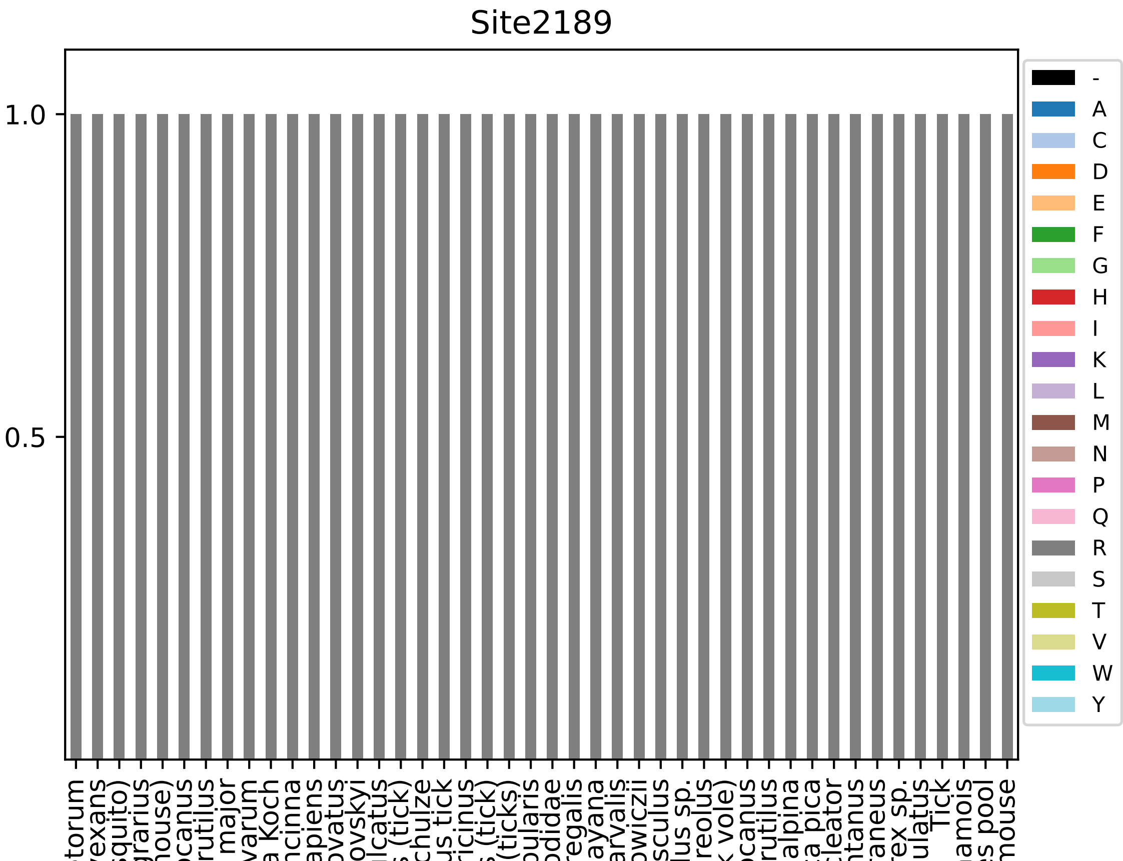
Site2189
1.0
0.5
-
A
C
D
E
F
G
H
I
K
L
M
N
P
Q
R
S
T
V
W
Y

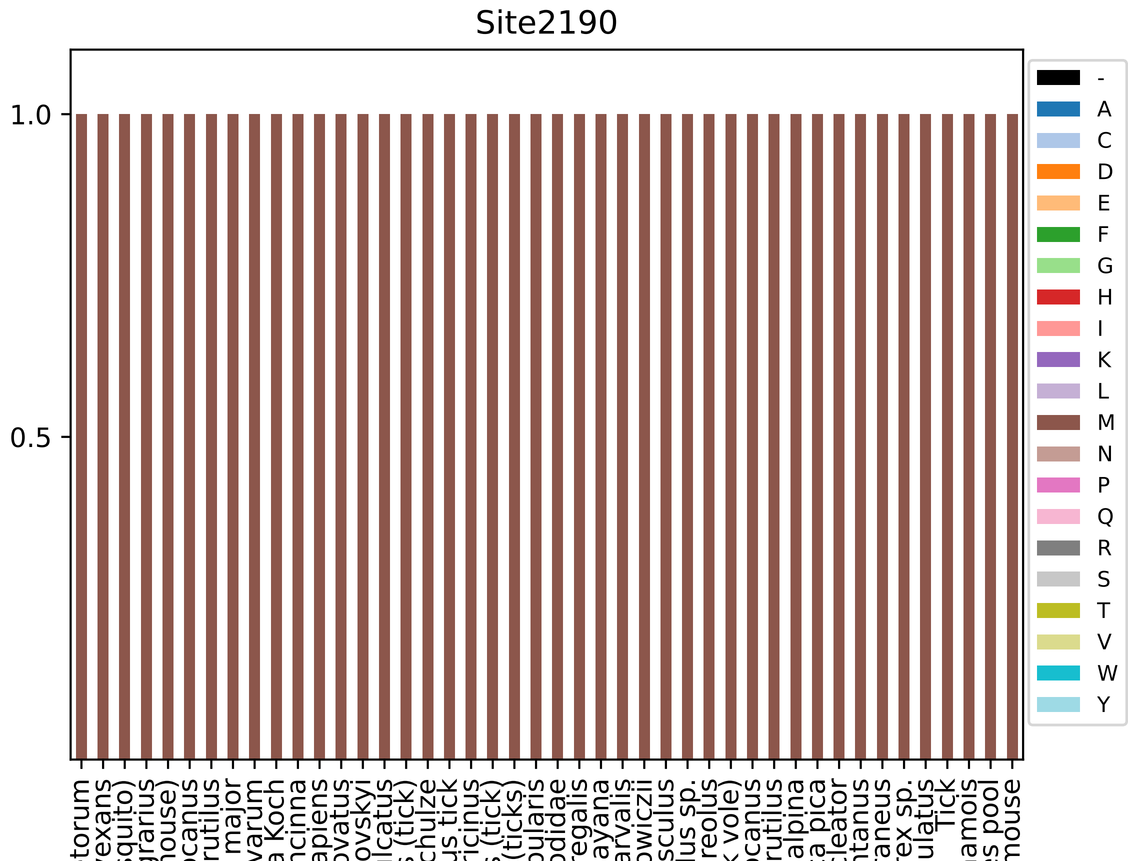
Site2190
1.0
0.5
-
A
C
D
E
F
G
H
I
K
L
M
N
P
Q
R
S
T
V
W
Y

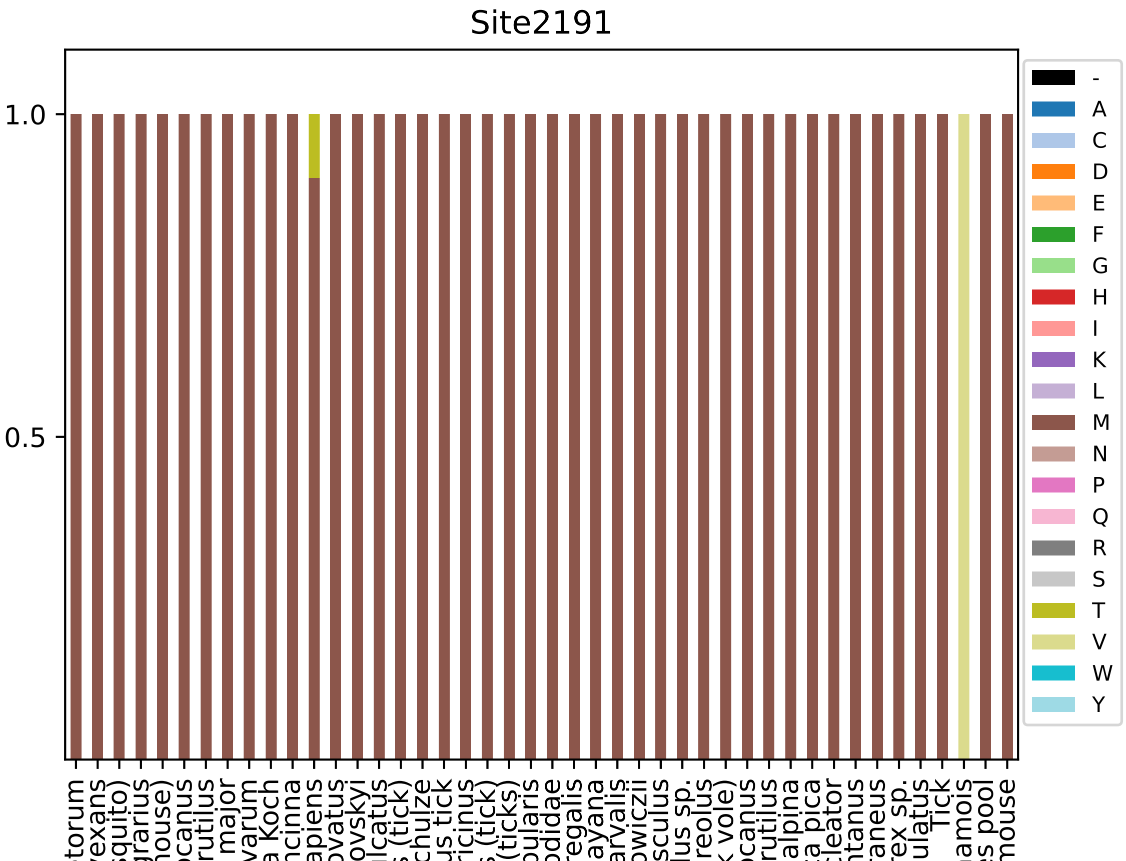
Site2191
1.0
0.5
-
A
C
D
E
F
G
H
I
K
L
M
N
P
Q
R
S
T
V
W
Y
Tick

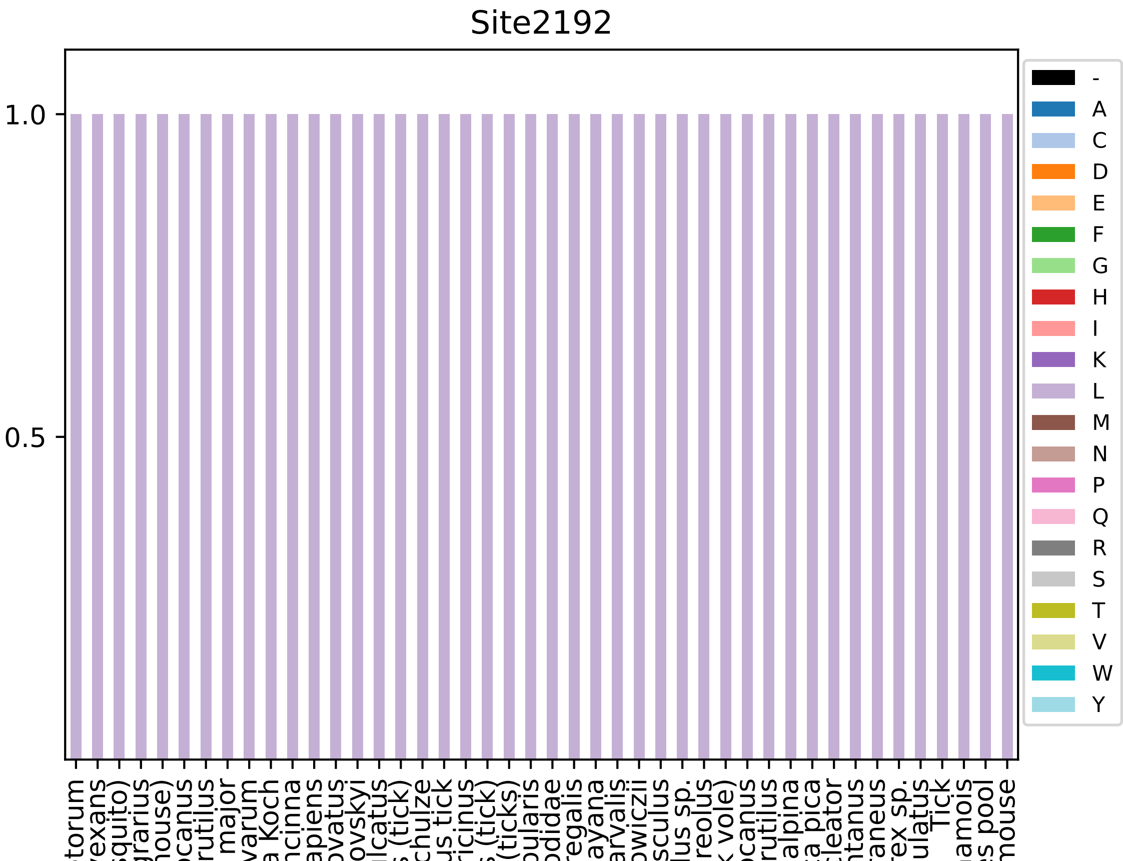
Site2192
1.0
0.5
-
A
C
D
E
F
G
H
I
K
L
M
N
P
Q
R
S
T
V
W
Y

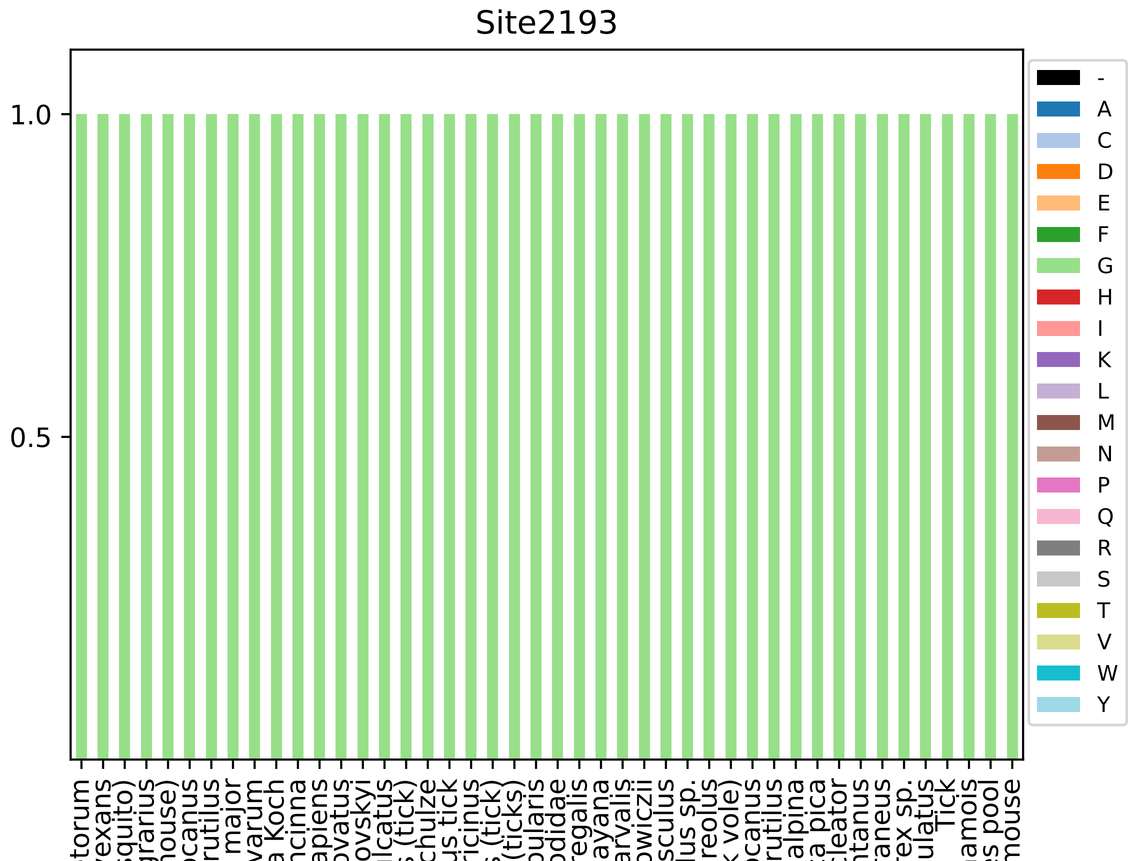
Site2193
1.0
0.5
-
A
C
D
E
F
G
H
I
K
L
M
N
P
Q
R
S
T
V
W
Y
Tick

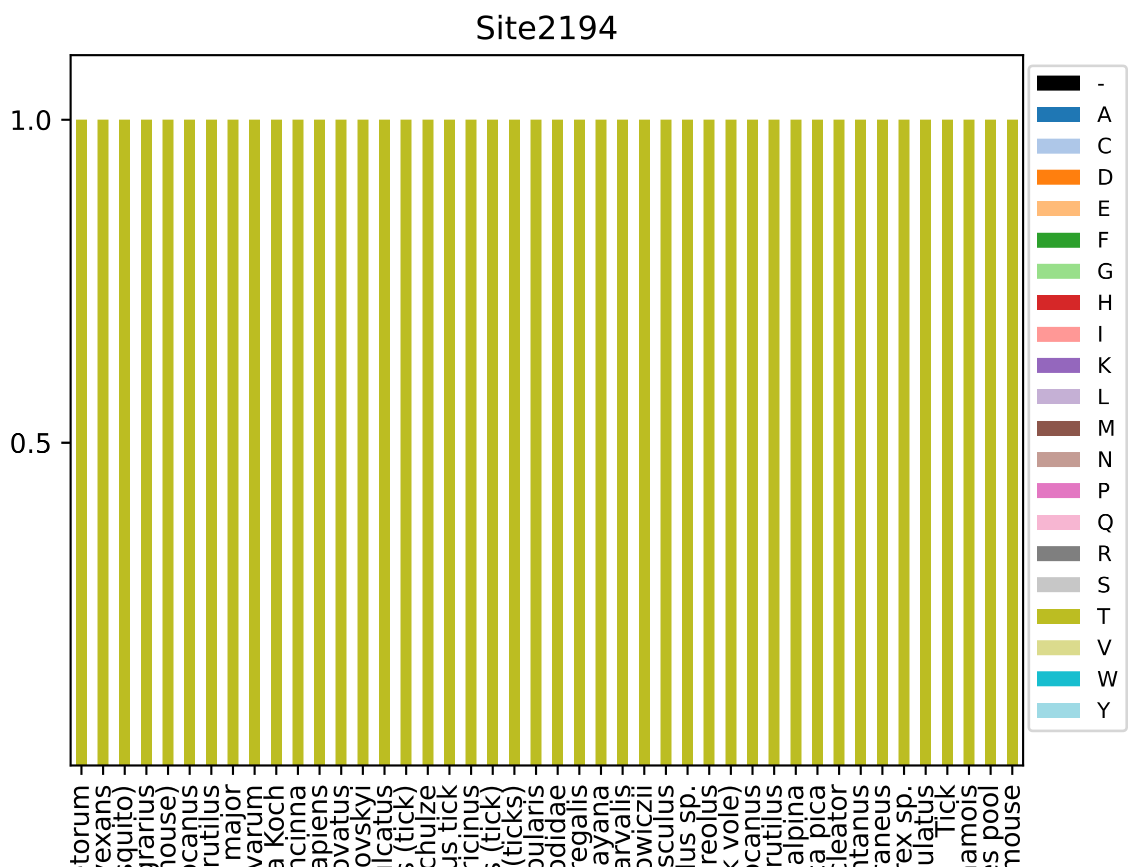
Site2194
1.0
0.5
-
A
C
D
E
F
G
H
I
K
L
M
N
P
Q
R
S
T
V
W
Y

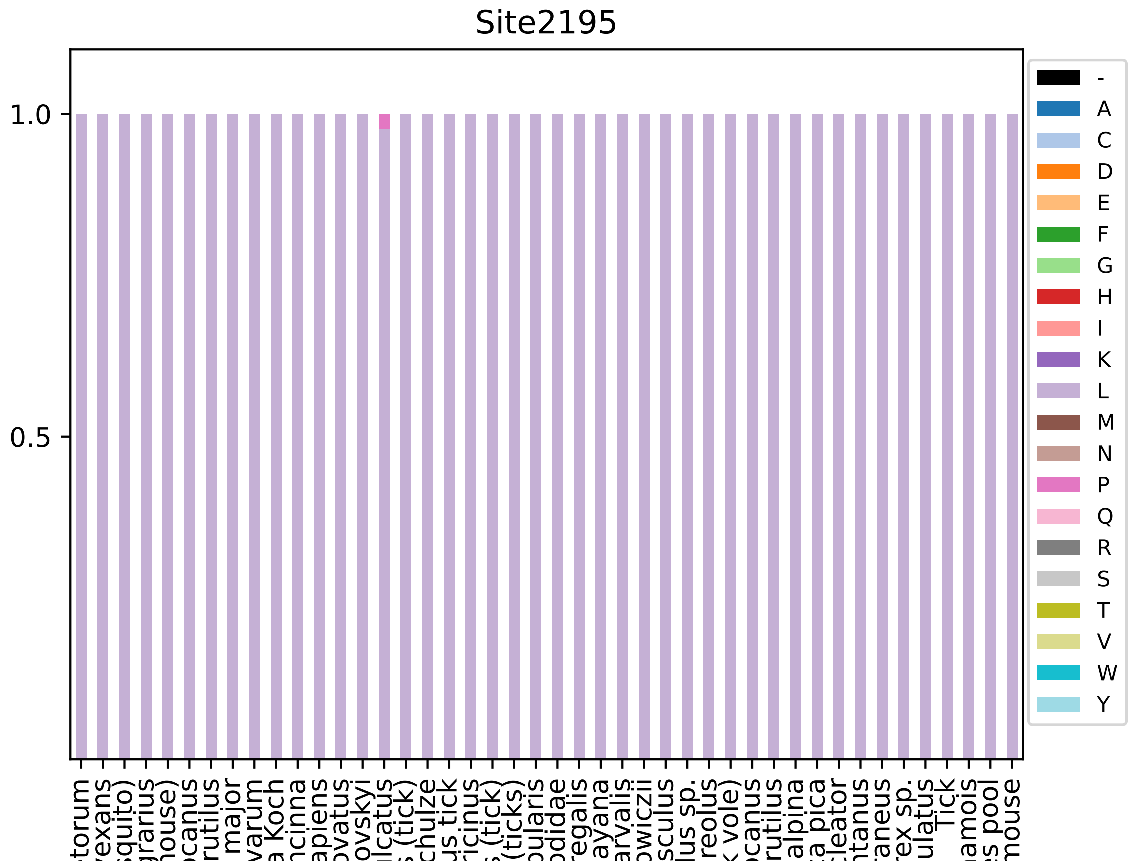

Site2195
1.0
0.5
-
A
C
D
E
F
G
H
I
K
L
M
N
P
Q
R
S
T
V
W
Y

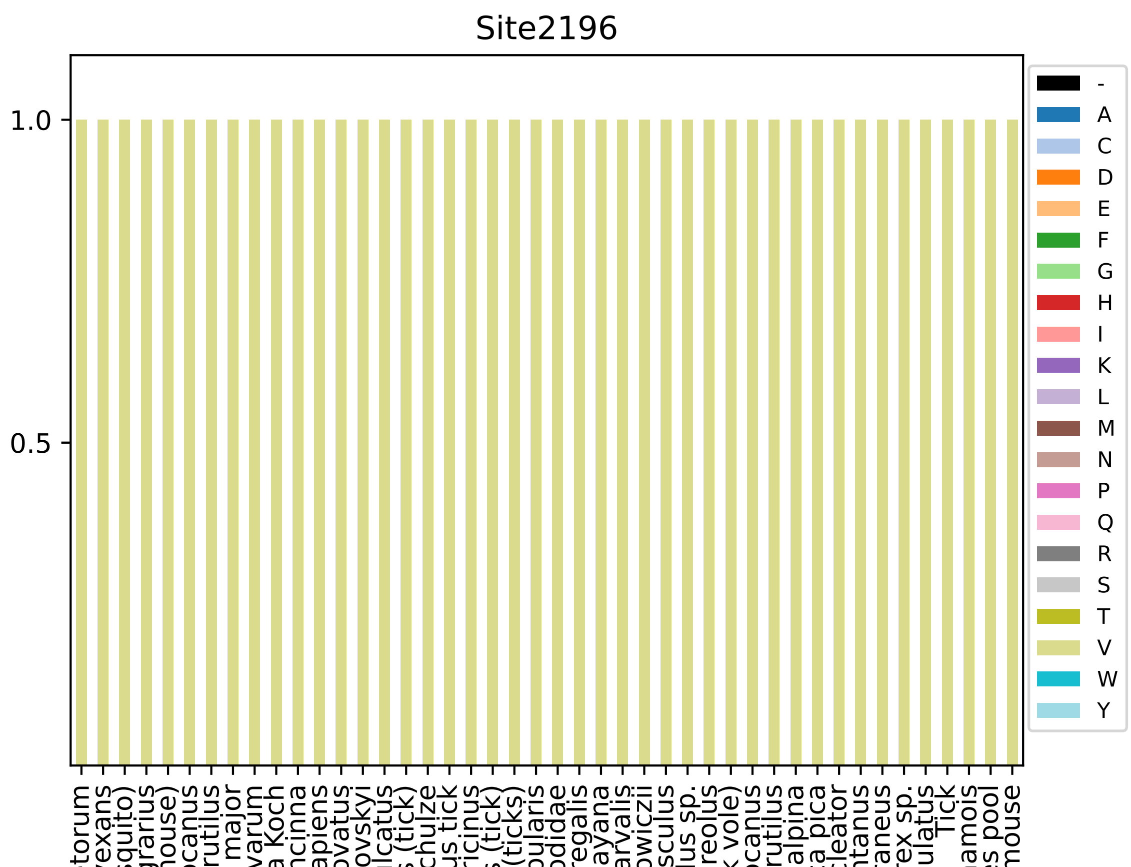
Site2196
1.0
0.5
-
A
C
D
E
F
G
H
I
K
L
M
N
P
Q
R
S
T
V
W
Y

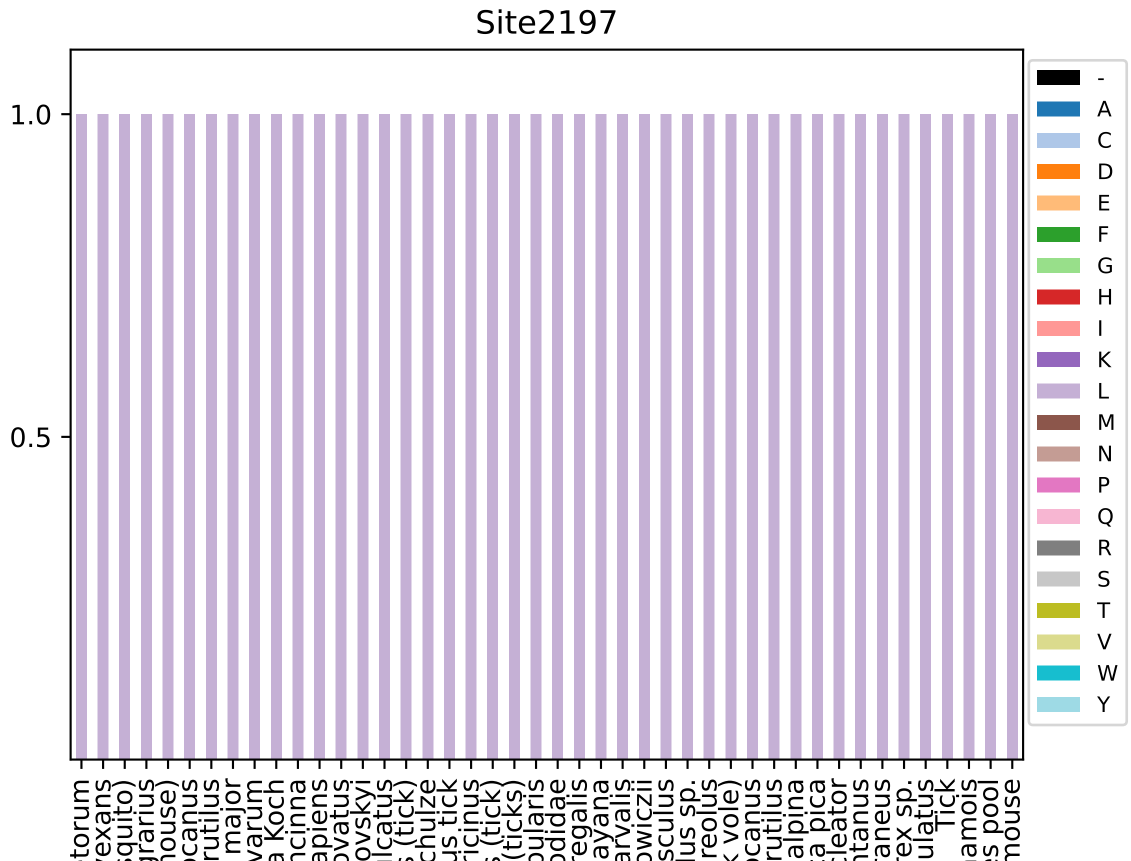
Site2197
1.0
0.5
-
A
C
D
E
F
G
H
I
K
L
M
N
P
Q
R
S
T
V
W
Y

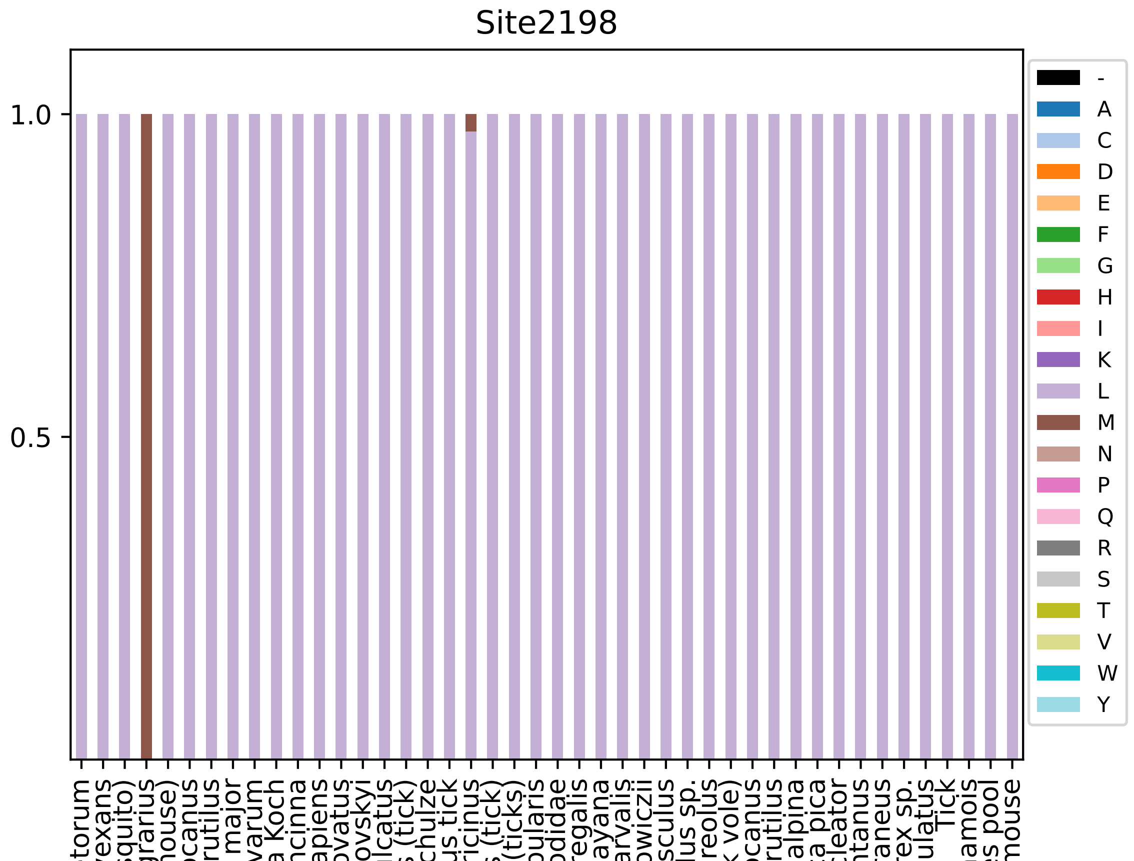
Site2198
1.0
0.5
-
A
C
D
E
F
G
H
I
K
L
M
N
P
Q
R
S
T
V
W
Y

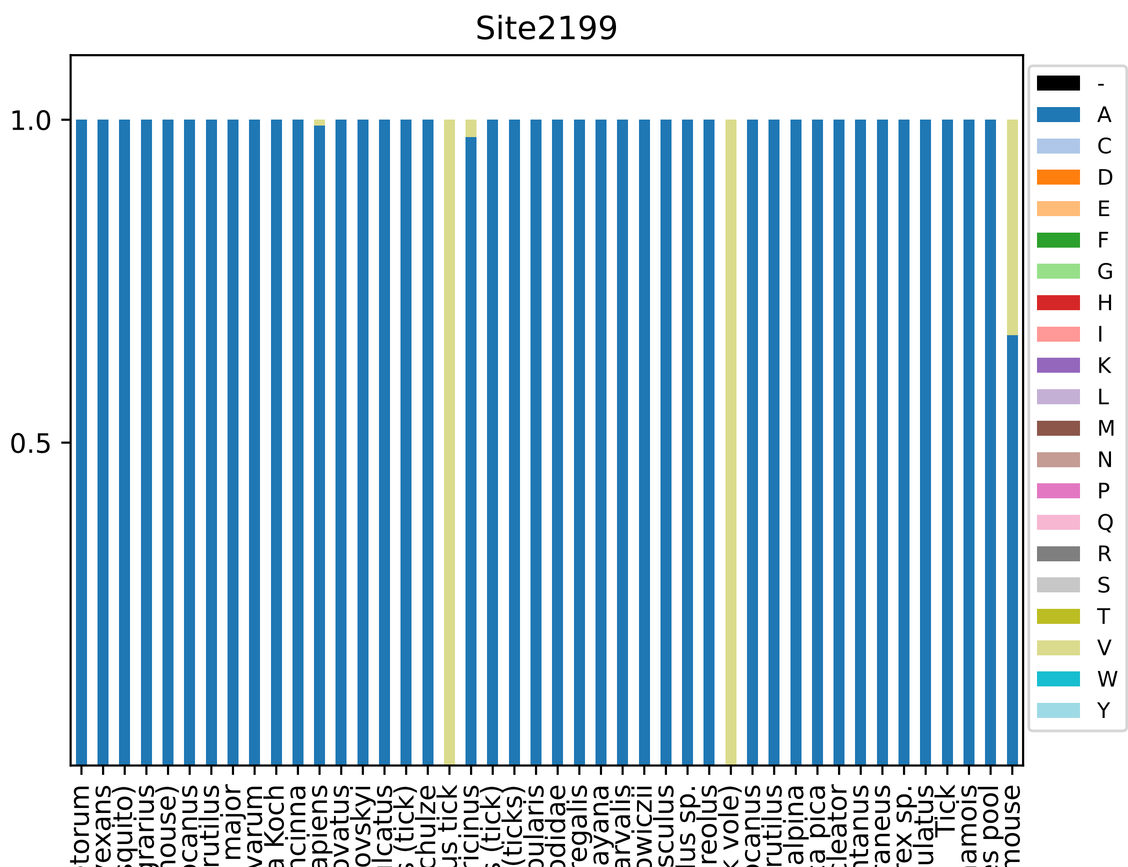
Site2199
1.0
0.5
-
A
C
D
E
F
G
H
I
K
L
M
N
P
Q
R
S
T
V
W
Y

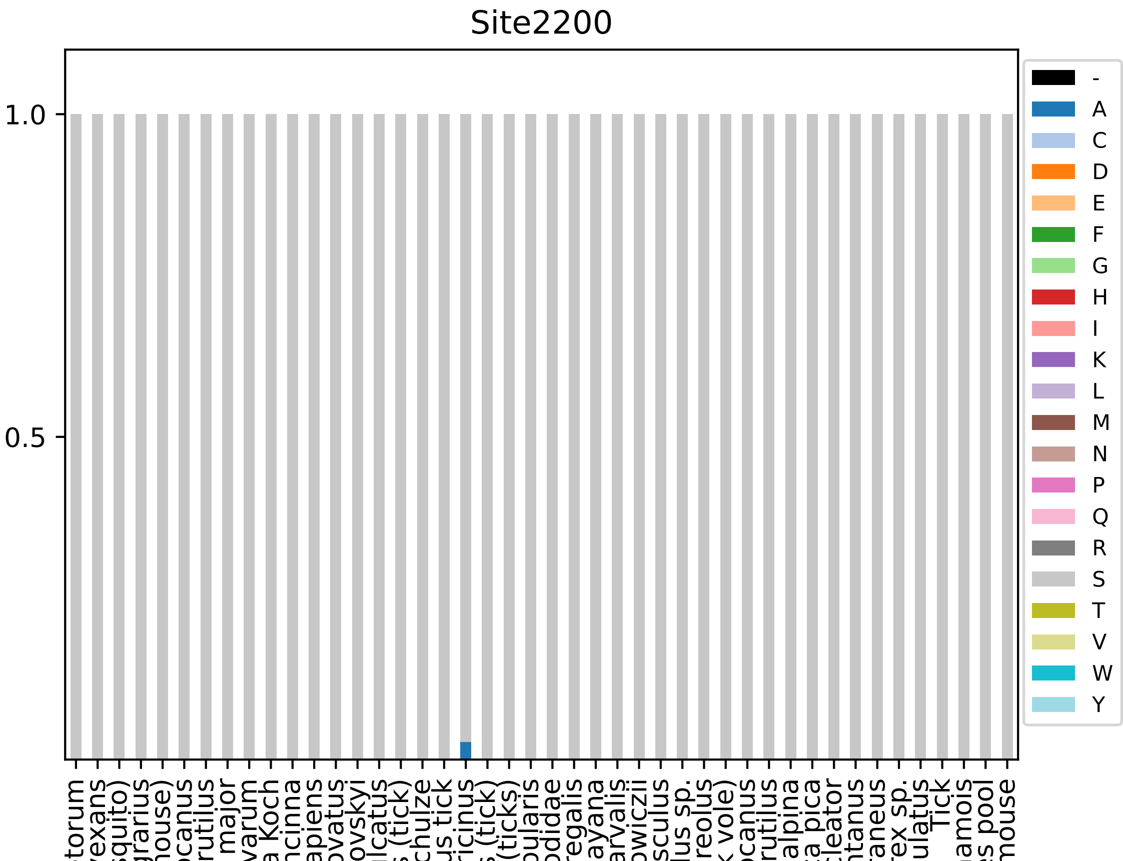
Site2200
1.0
0.5
-
A
C
D
E
F
G
H
I
K
L
M
N
P
Q
R
S
T
V
W
Y
Tick

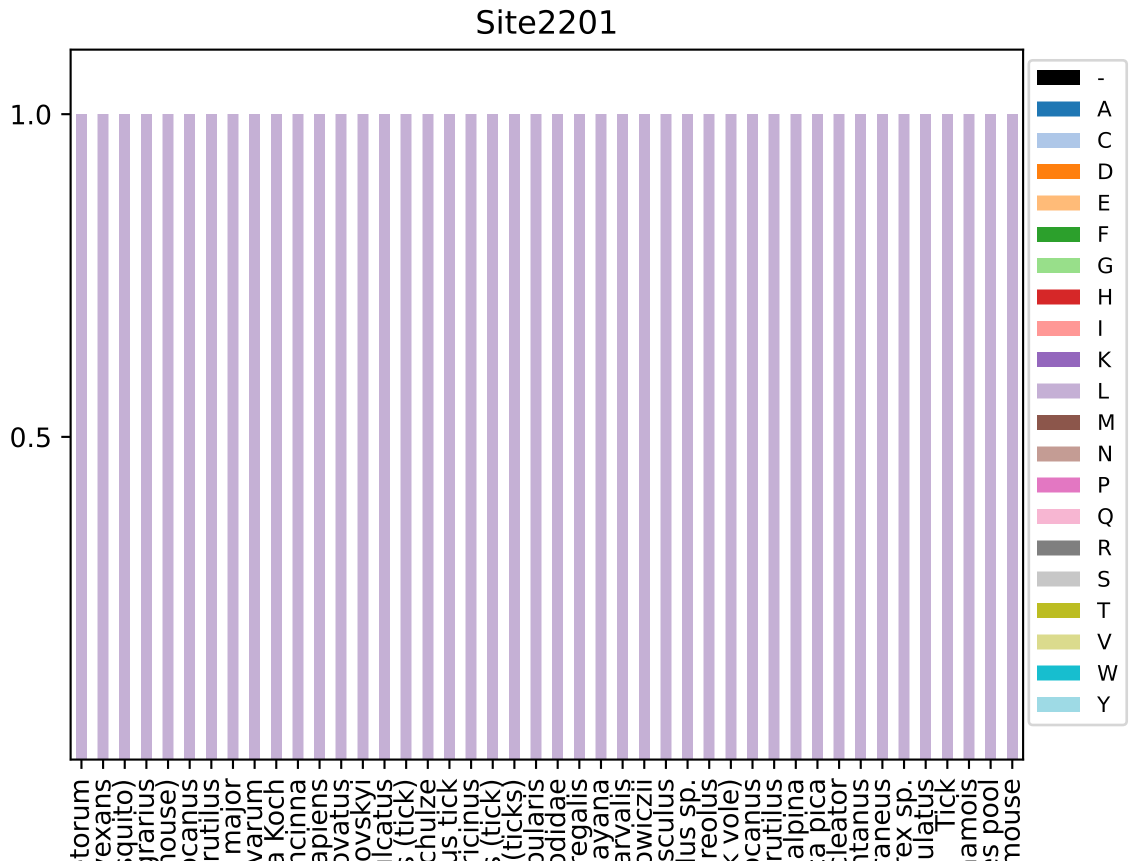
Site2201
1.0
0.5
-
A
C
D
E
F
G
H
I
K
L
M
N
P
Q
R
S
T
V
W
Y

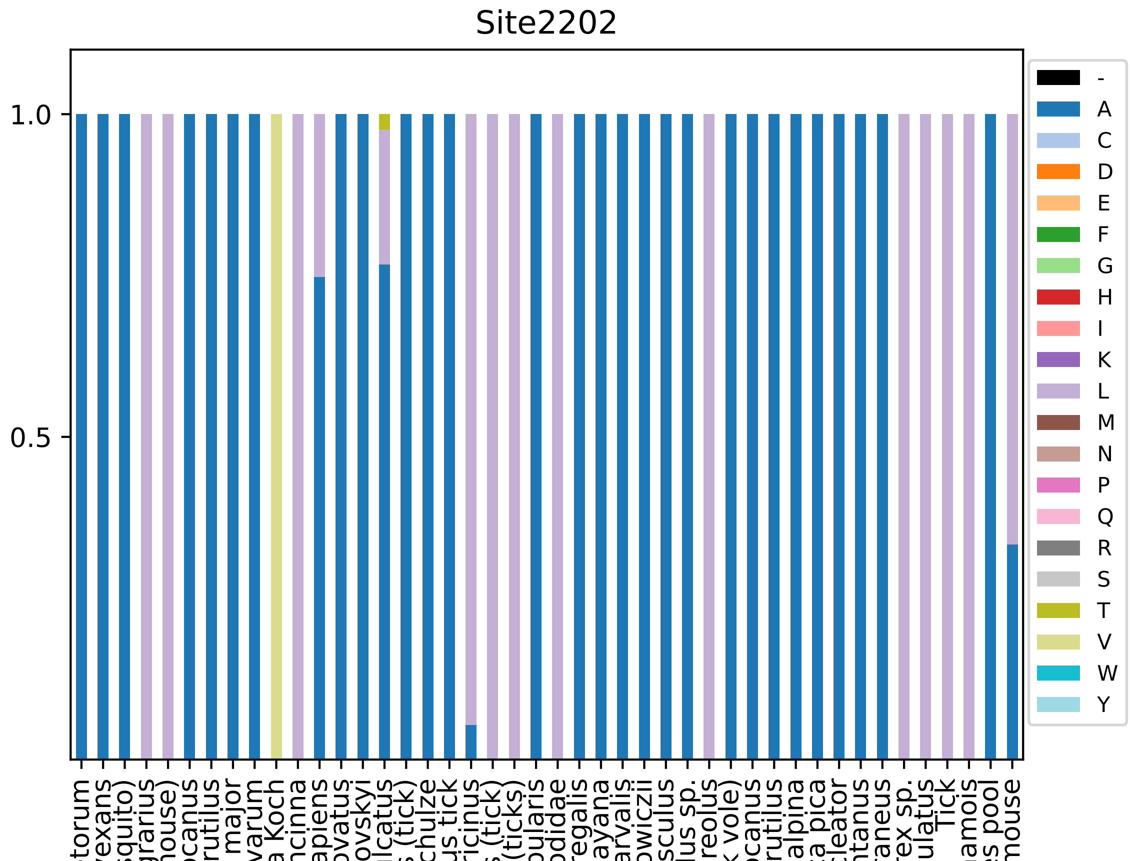

Site2202
1.0
0.5
-
A
C
D
E
F
G
H
I
K
L
M
N
P
Q
R
S
T
V
W
Y

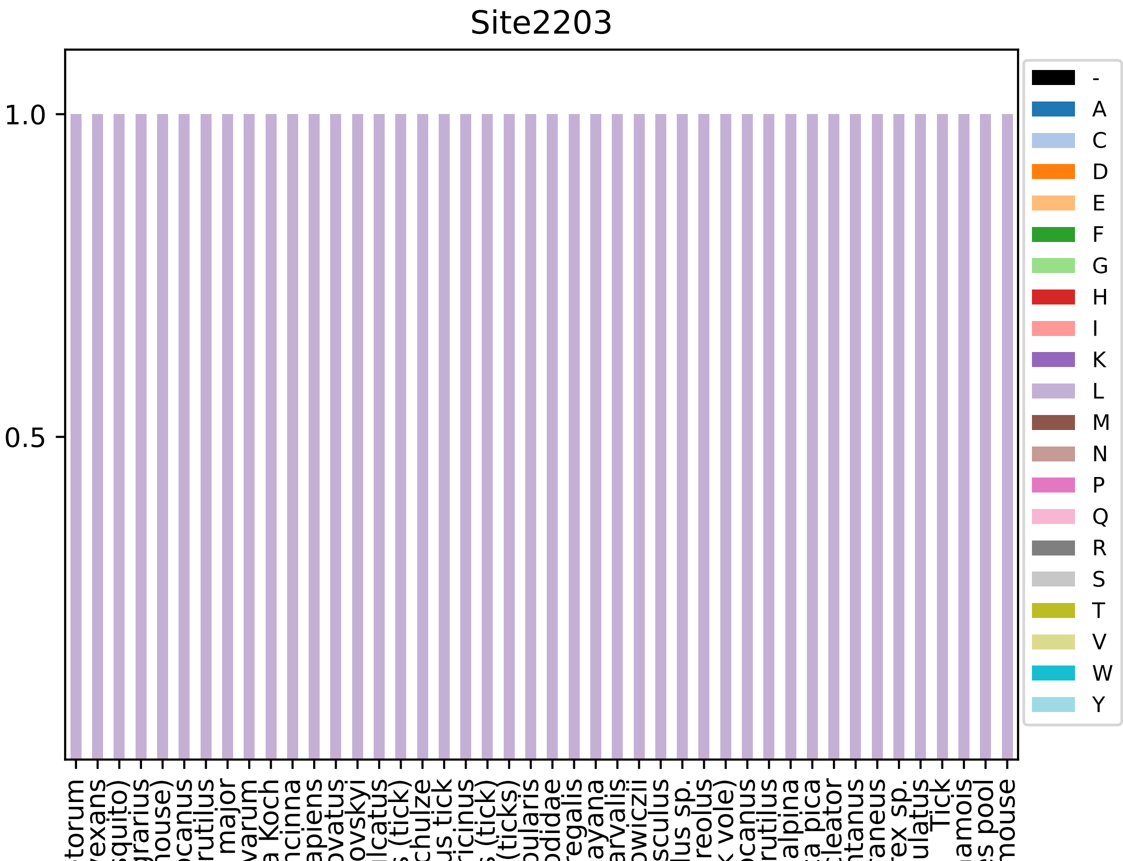
Site2203
1.0
0.5
-
A
C
D
E
F
G
H
I
K
L
M
N
P
Q
R
S
T
V
W
Y

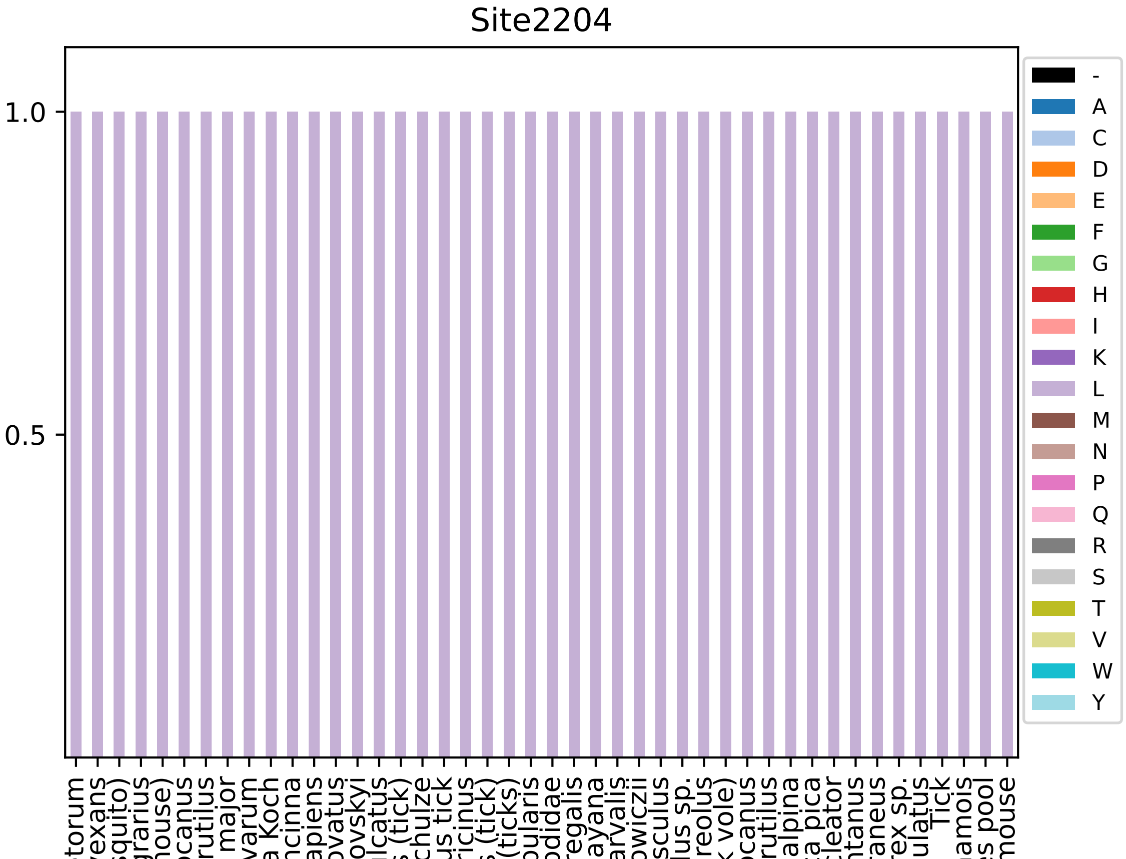

Site2204
1.0
0.5
-
A
C
D
E
F
G
H
I
K
L
M
N
P
Q
R
S
T
V
W
Y
Tick

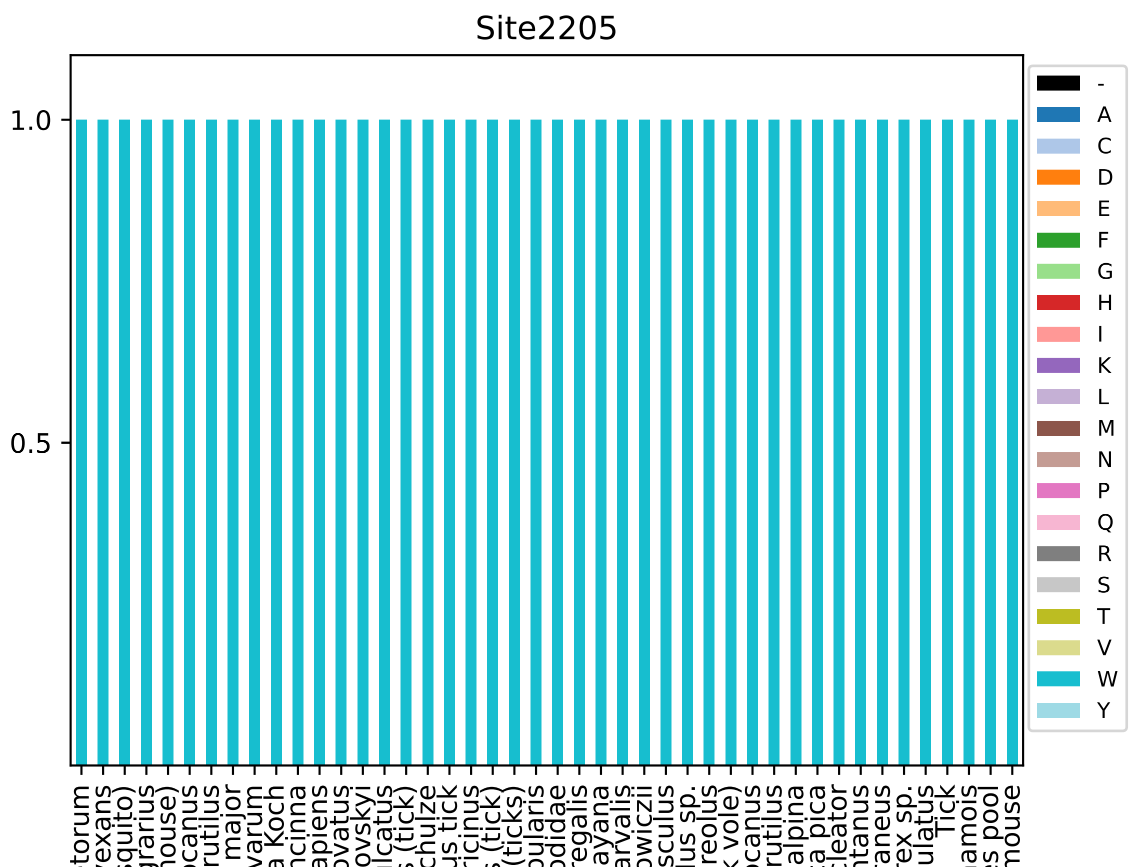
Site2205
1.0
0.5
-
A
C
D
E
F
G
H
I
K
L
M
N
P
Q
R
S
T
V
W
Y

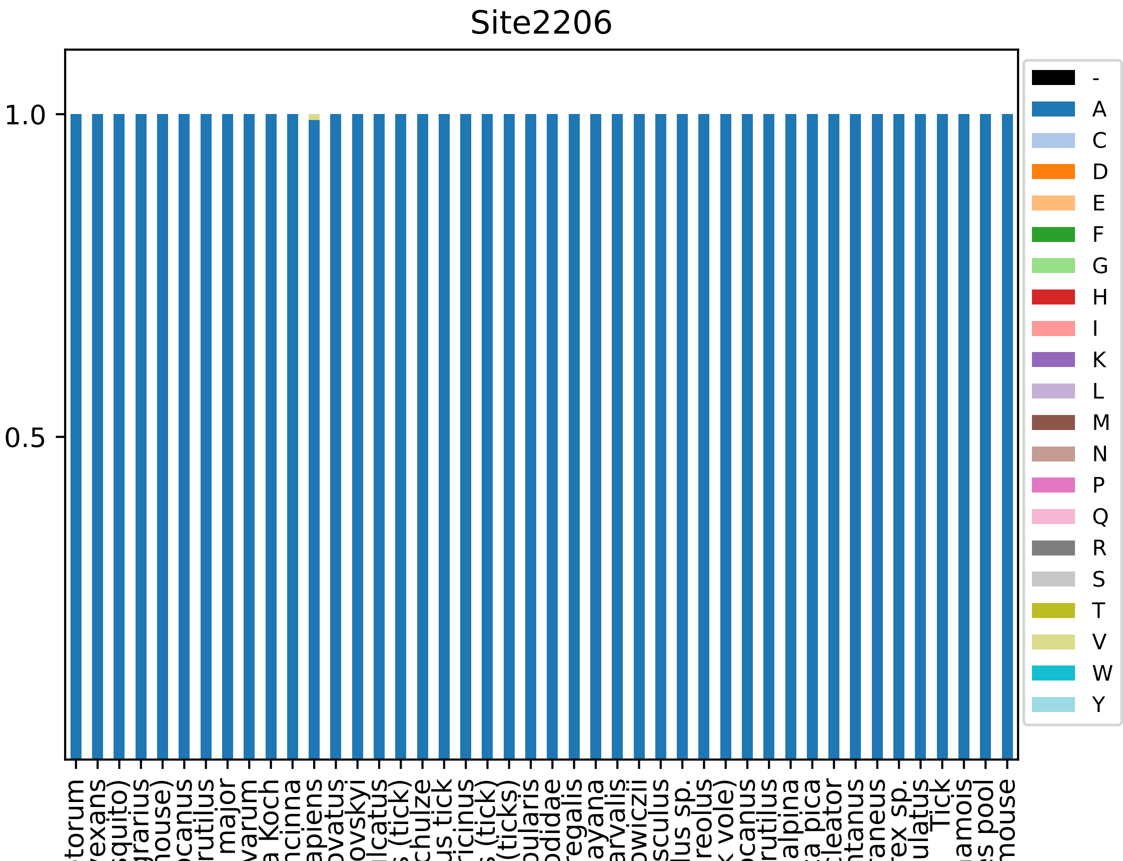
Site2206
1.0
0.5
-
A
C
D
E
F
G
H
I
K
L
M
N
P
Q
R
S
T
V
W
Y

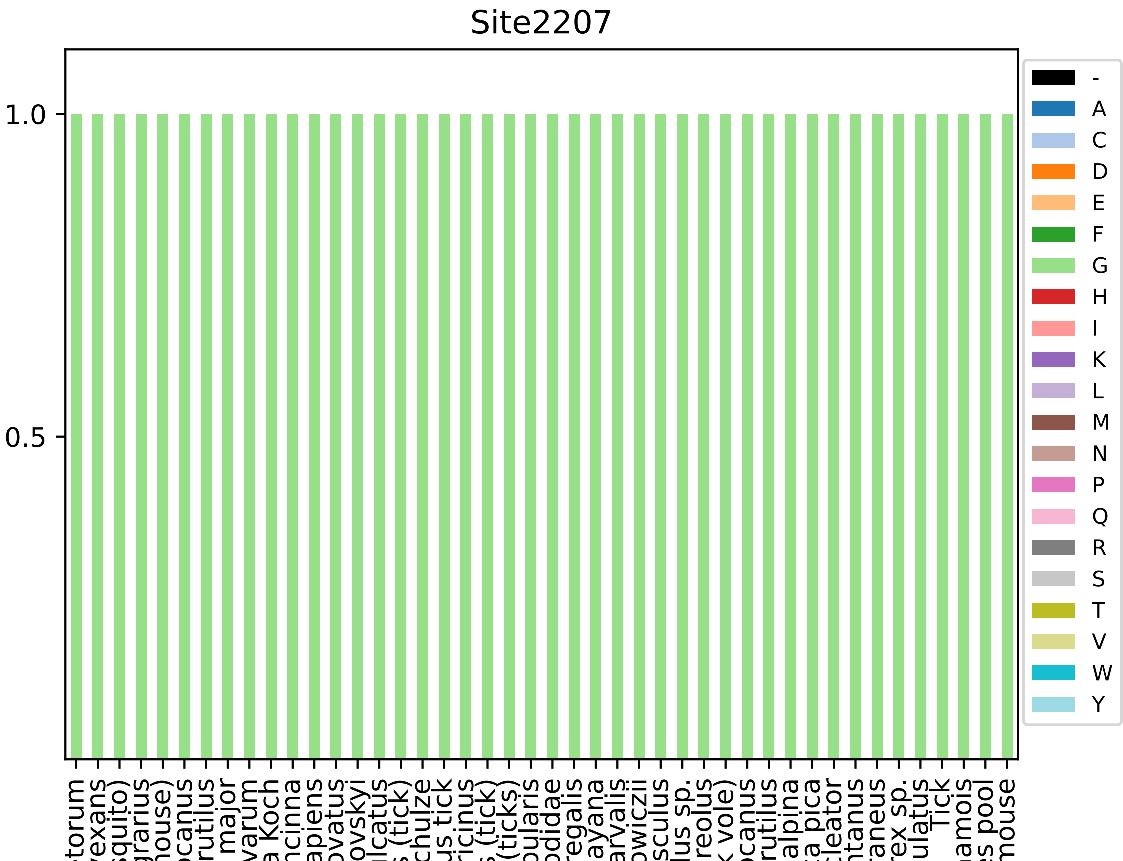
Site2207
1.0
0.5
-
A
C
D
E
F
G
H
I
K
L
M
N
P
Q
R
S
T
V
W
Y

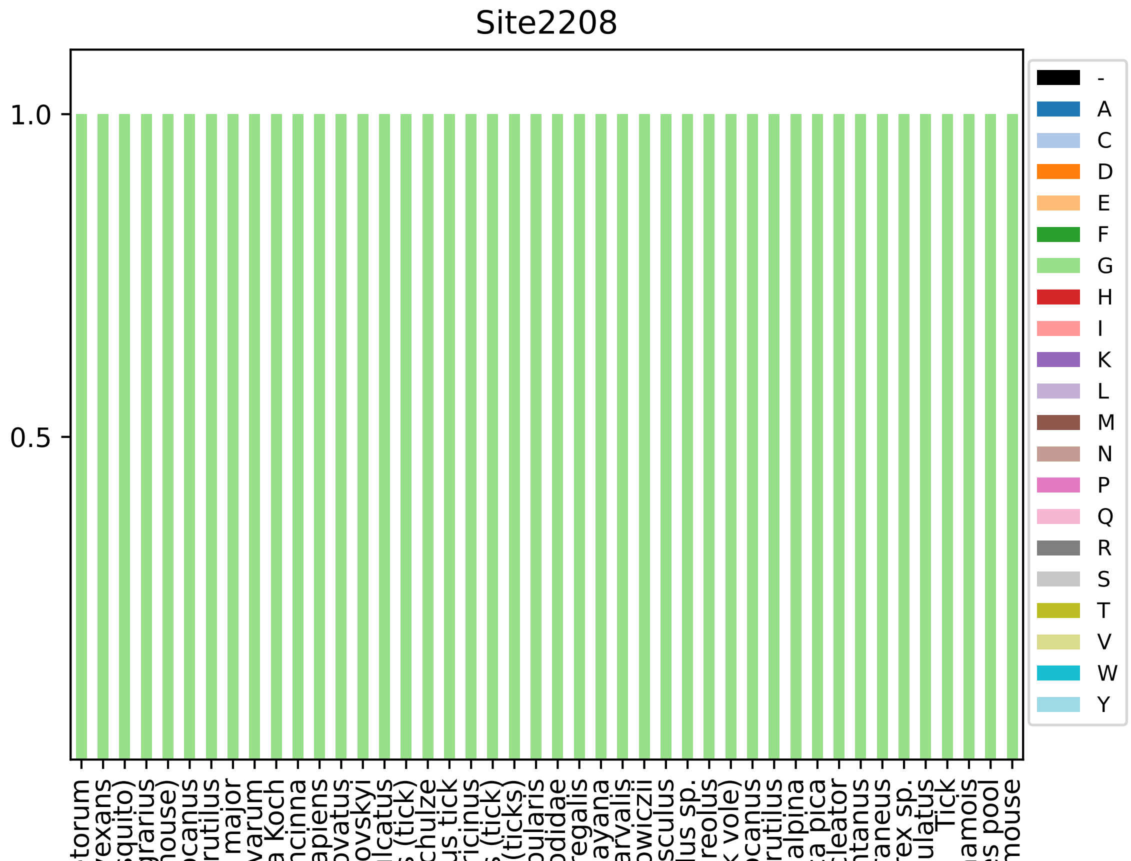
Site2208
1.0
0.5
-
A
C
D
E
F
G
H
I
K
L
M
N
P
Q
R
S
T
V
W
Y

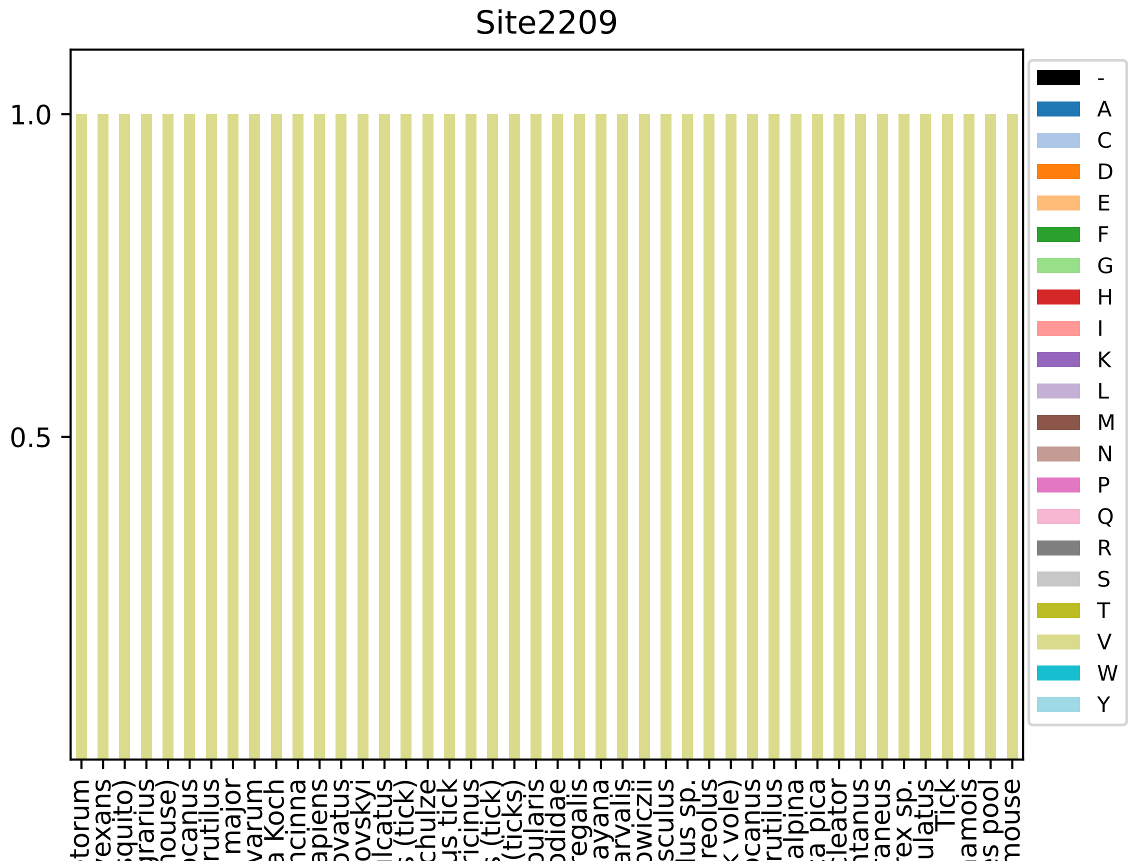
Site2209
1.0
0.5
-
A
C
D
E
F
G
H
I
K
L
M
N
P
Q
R
S
T
V
W
Y

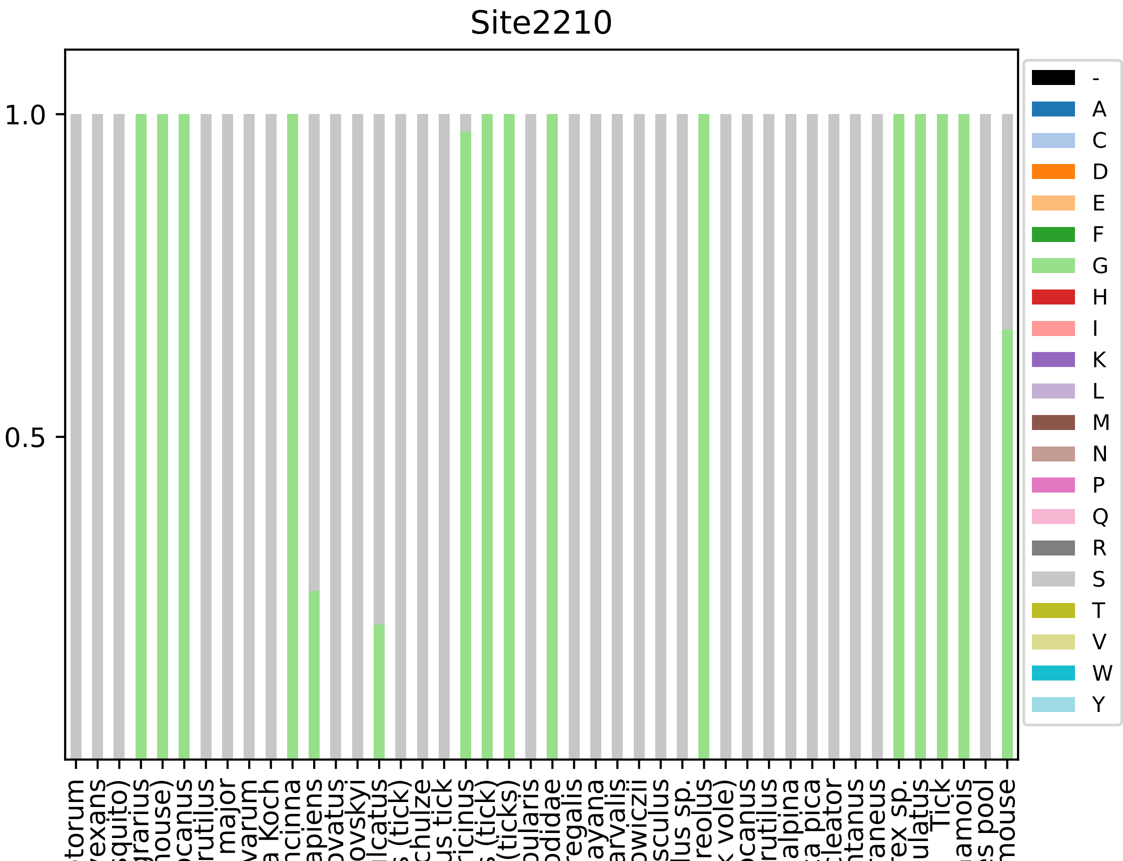

Site2210
1.0
0.5
-
A
C
D
E
F
G
H
I
K
L
M
N
P
Q
R
S
T
V
W
Y

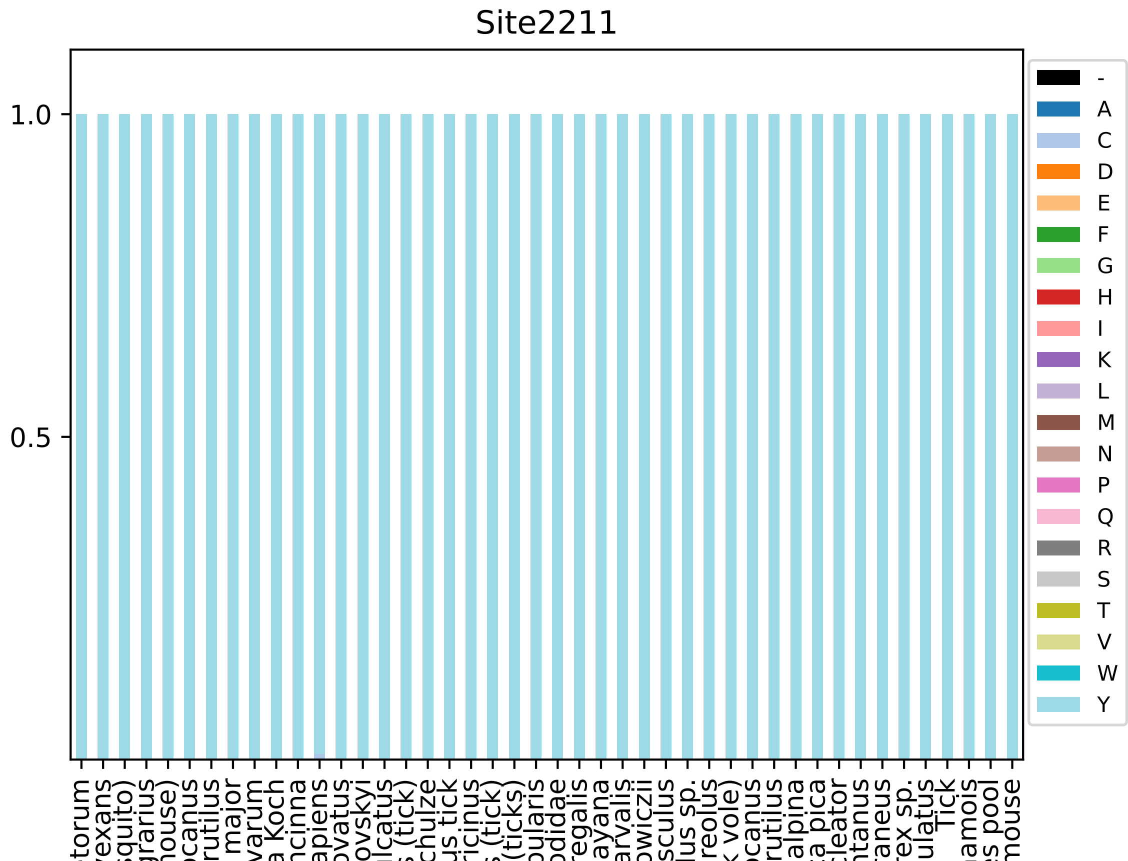
Site2211
1.0
0.5
-
A
C
D
E
F
G
H
I
K
L
M
N
P
Q
R
S
T
V
W
Y

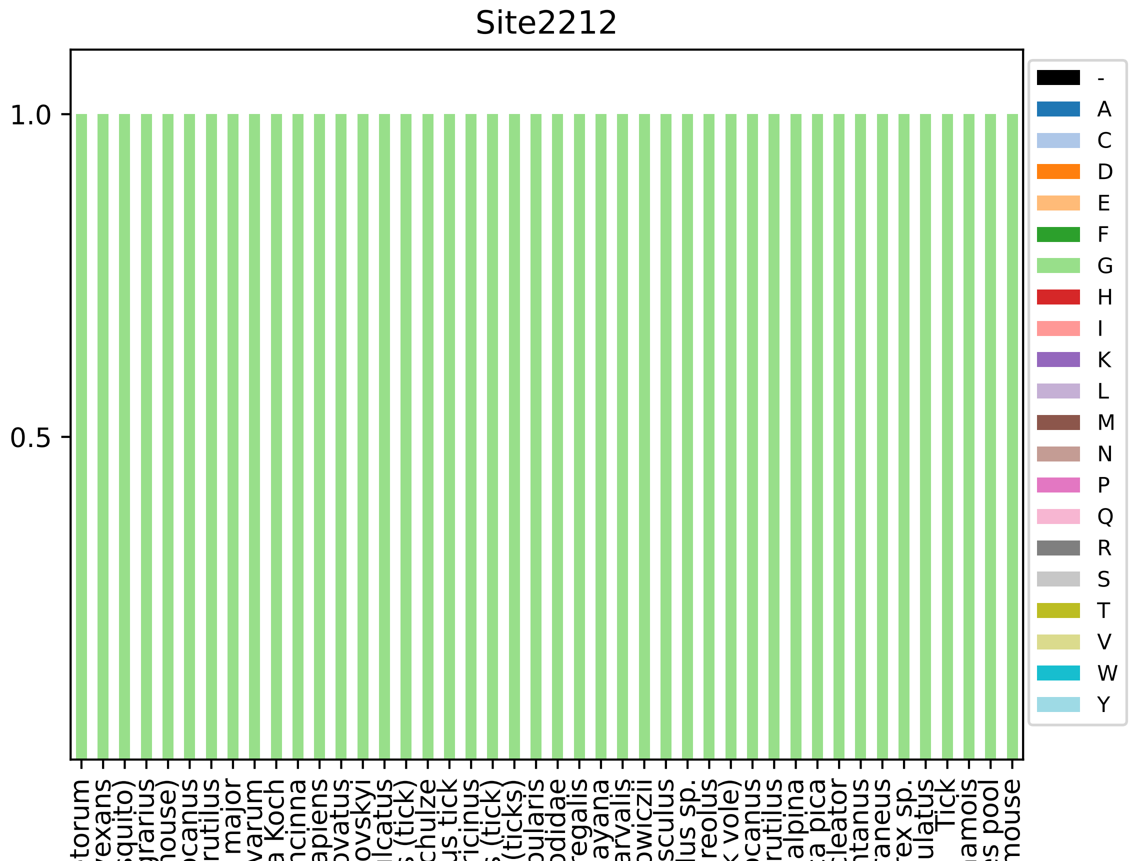
Site2212
1.0
0.5
-
A
C
D
E
F
G
H
I
K
L
M
N
P
Q
R
S
T
V
W
Y
torum
vexans
squito)
garius
nouse)
ocanus
rutilus
major
varum
a Koch
ncinna
apiens
ovatus
ovskyi
ulcatus
s (tick)
chulze
us tick
ricinus
s (tick)
(ticks)
pularis
podidae
regalis
ayana
arvalis
owiczii
sculus
lus sp.
reolus
k vole)
ocanus
rutilus
alpina
a pica
cleator
ntanus
raneus
rex sp.
ulatus
Tick
amois
es pool
mouse

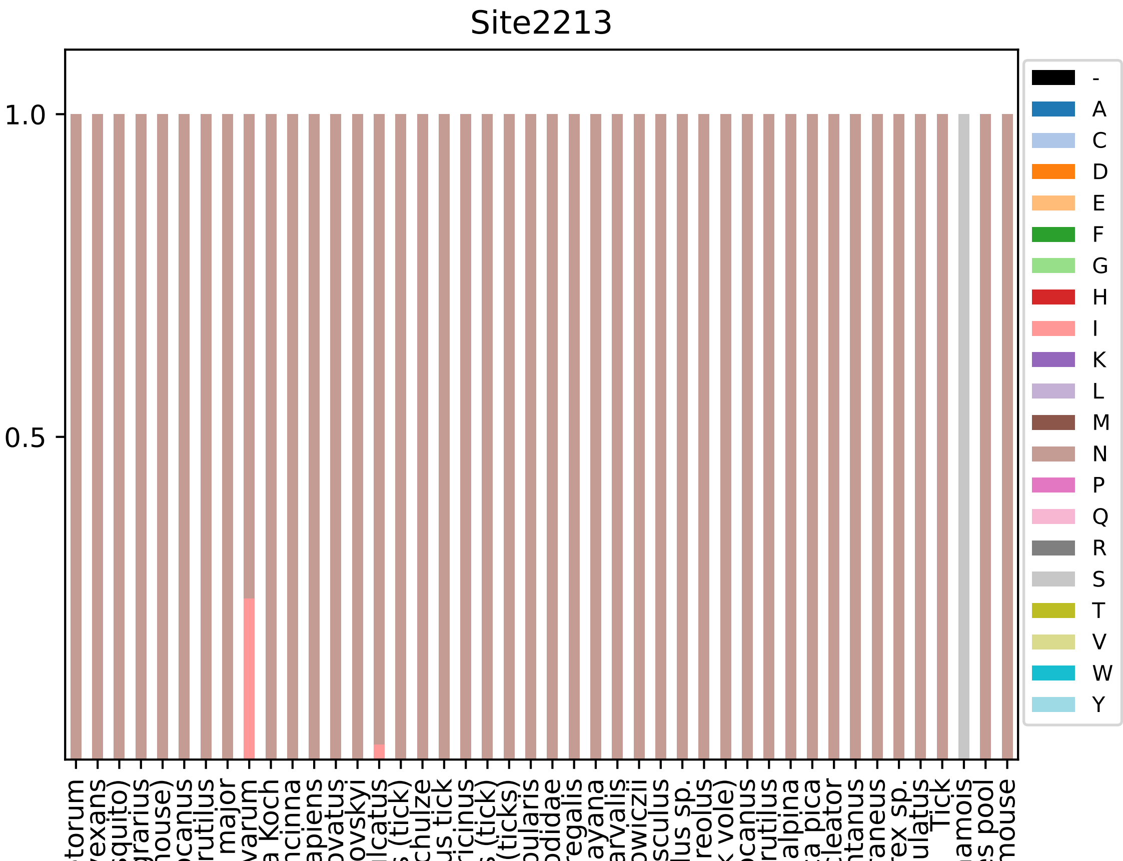

Site2213
1.0
0.5
-
A
C
D
E
F
G
H
I
K
L
M
N
P
Q
R
S
T
V
W
Y

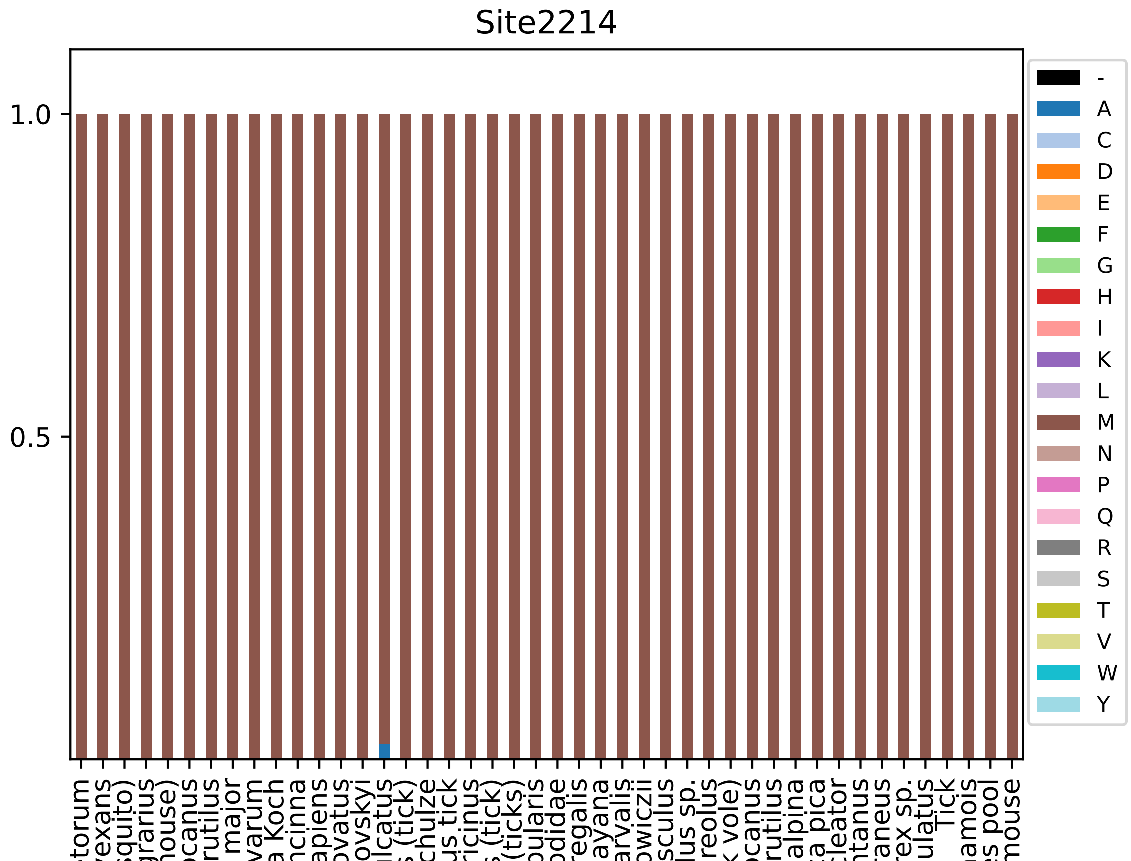
Site2214
1.0
0.5
-
A
C
D
E
F
G
H
I
K
L
M
N
P
Q
R
S
T
V
W
Y

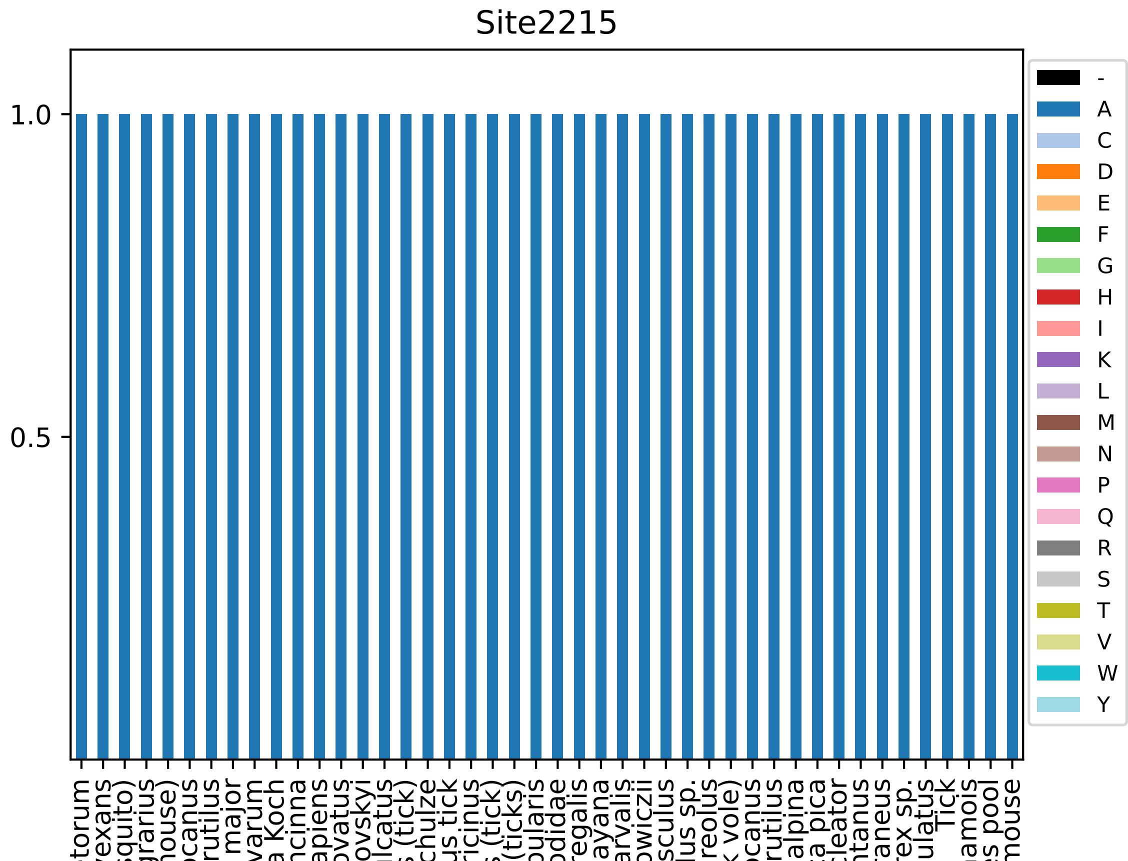
Site2215
1.0
0.5
-
A
C
D
E
F
G
H
I
K
L
M
N
P
Q
R
S
T
V
W
Y
Tick

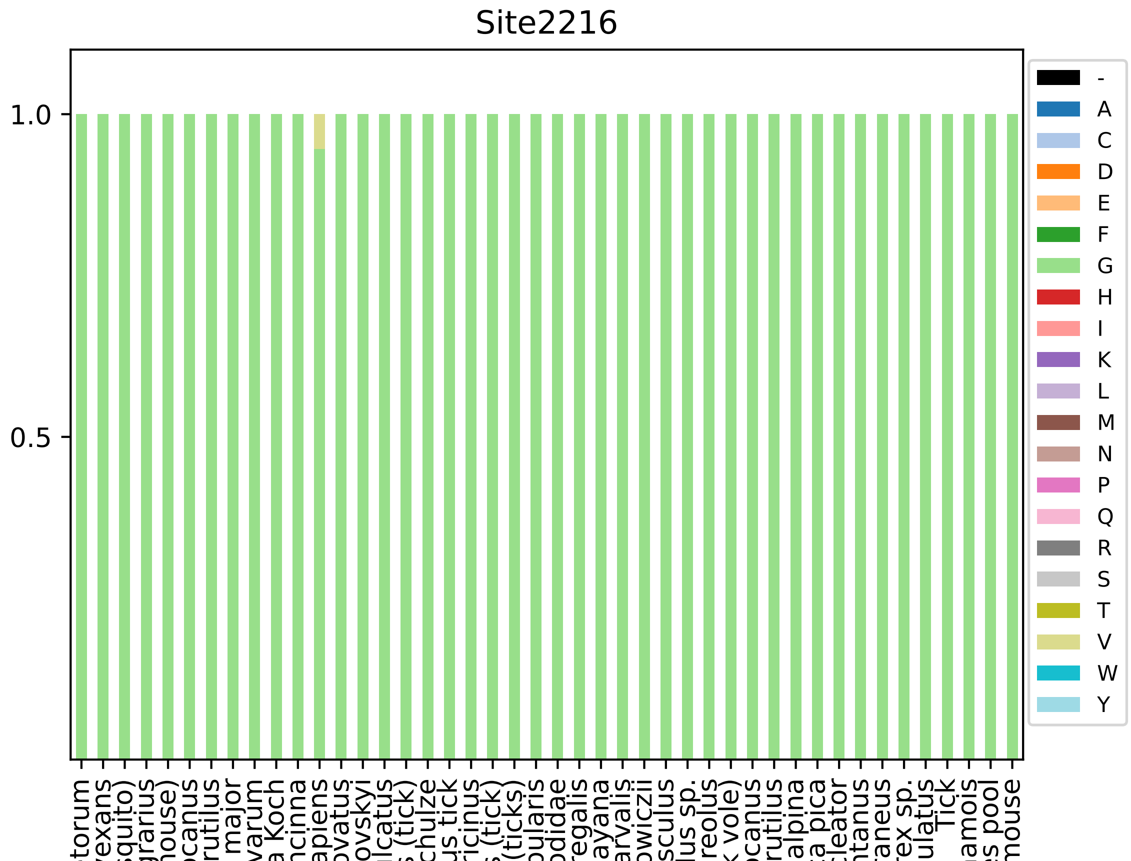
Site2216
1.0
0.5
-
A
C
D
E
F
G
H
I
K
L
M
N
P
Q
R
S
T
V
W
Y

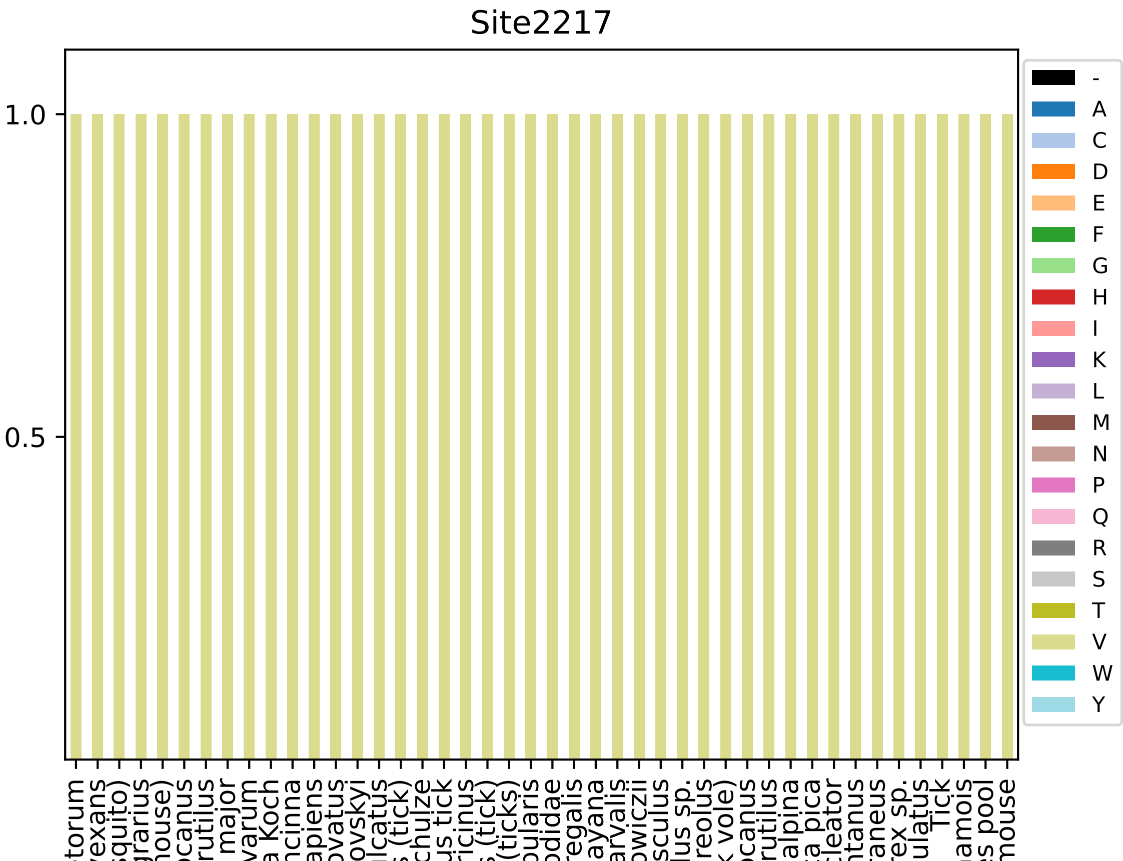
Site2217
1.0
0.5
-
A
C
D
E
F
G
H
I
K
L
M
N
P
Q
R
S
T
V
W
Y

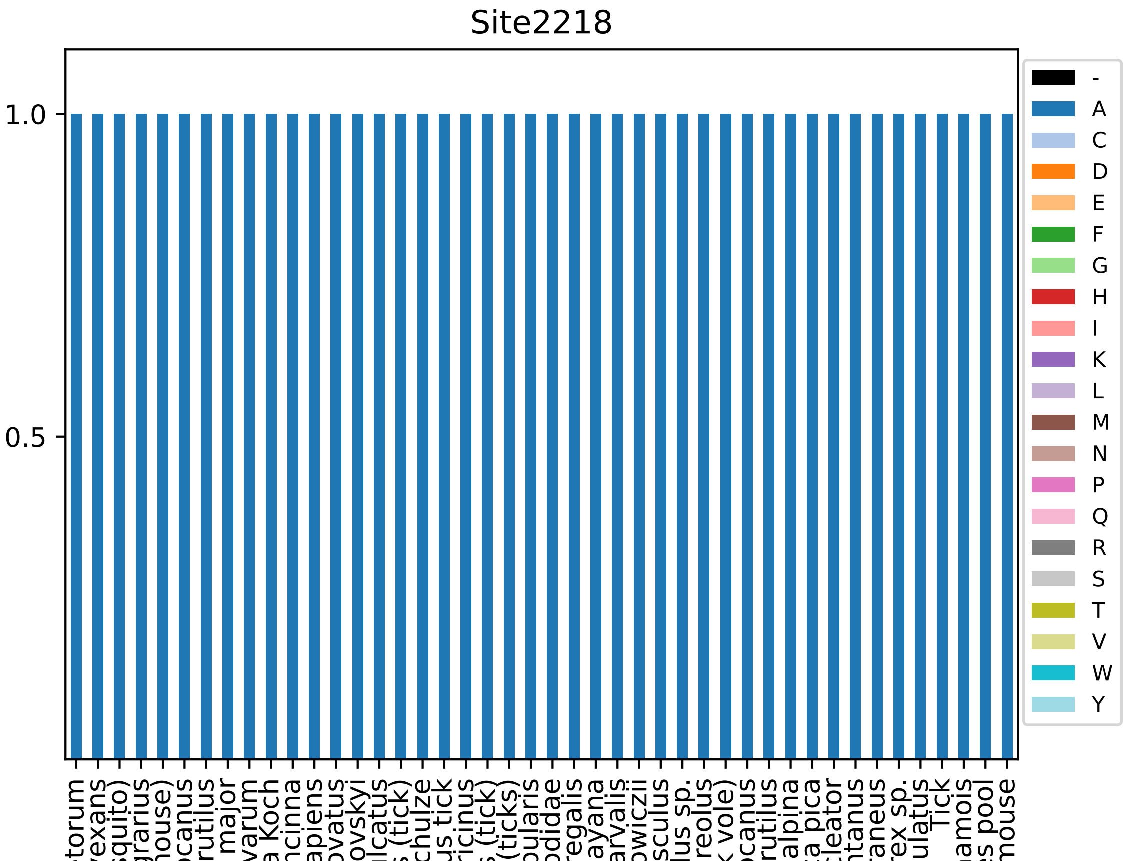

Site2218
1.0
0.5
-
A
C
D
E
F
G
H
I
K
L
M
N
P
Q
R
S
T
V
W
Y

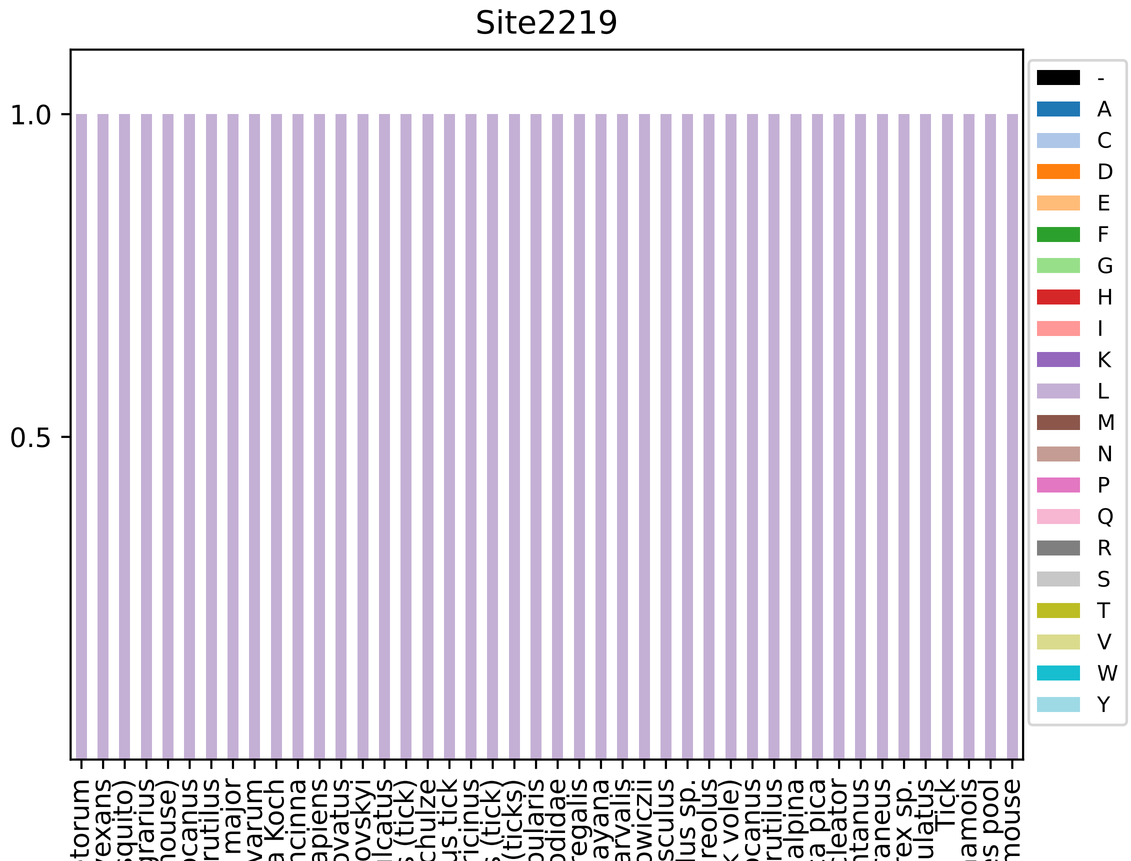
Site2219
1.0
0.5
-
A
C
D
E
F
G
H
I
K
L
M
N
P
Q
R
S
T
V
W
Y

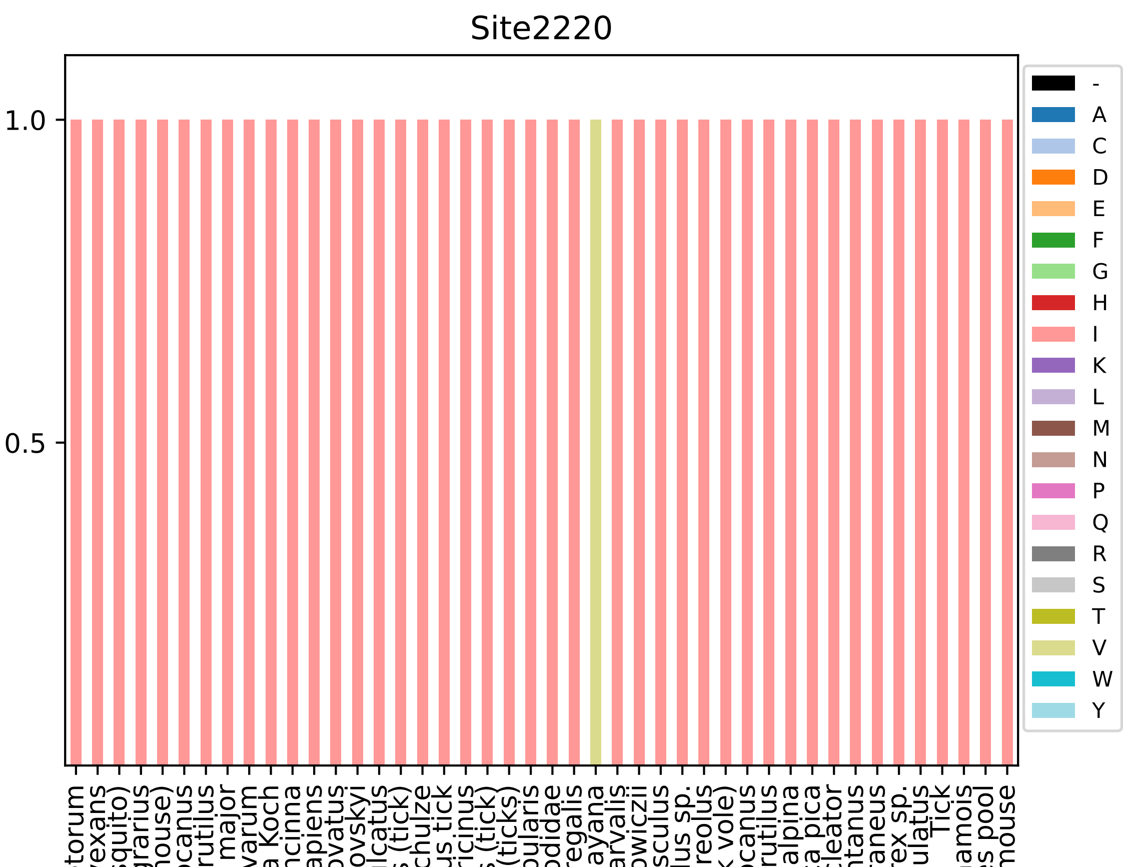

Site2220
1.0
0.5
-
A
C
D
E
F
G
H
I
K
L
M
N
P
Q
R
S
T
V
W
Y

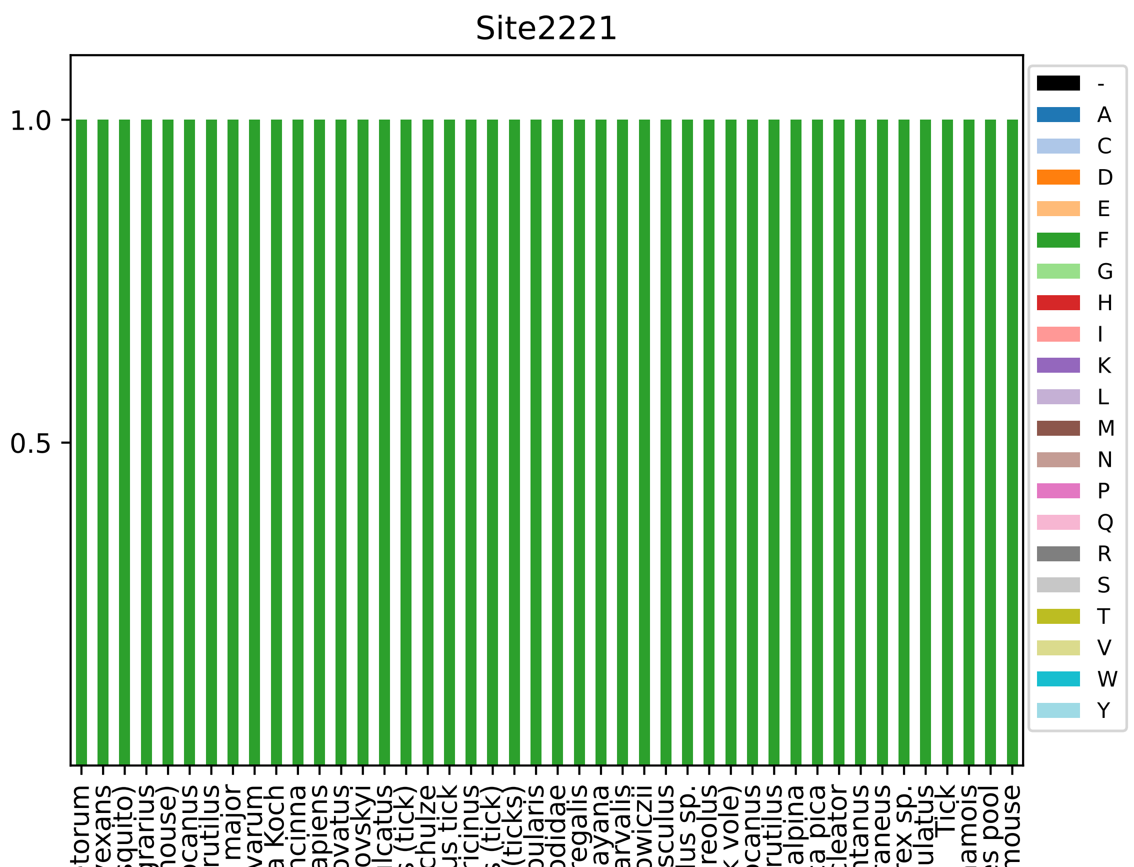
Site2221
1.0
0.5
-
A
C
D
E
F
G
H
I
K
L
M
N
P
Q
R
S
T
V
W
Y

Site2222

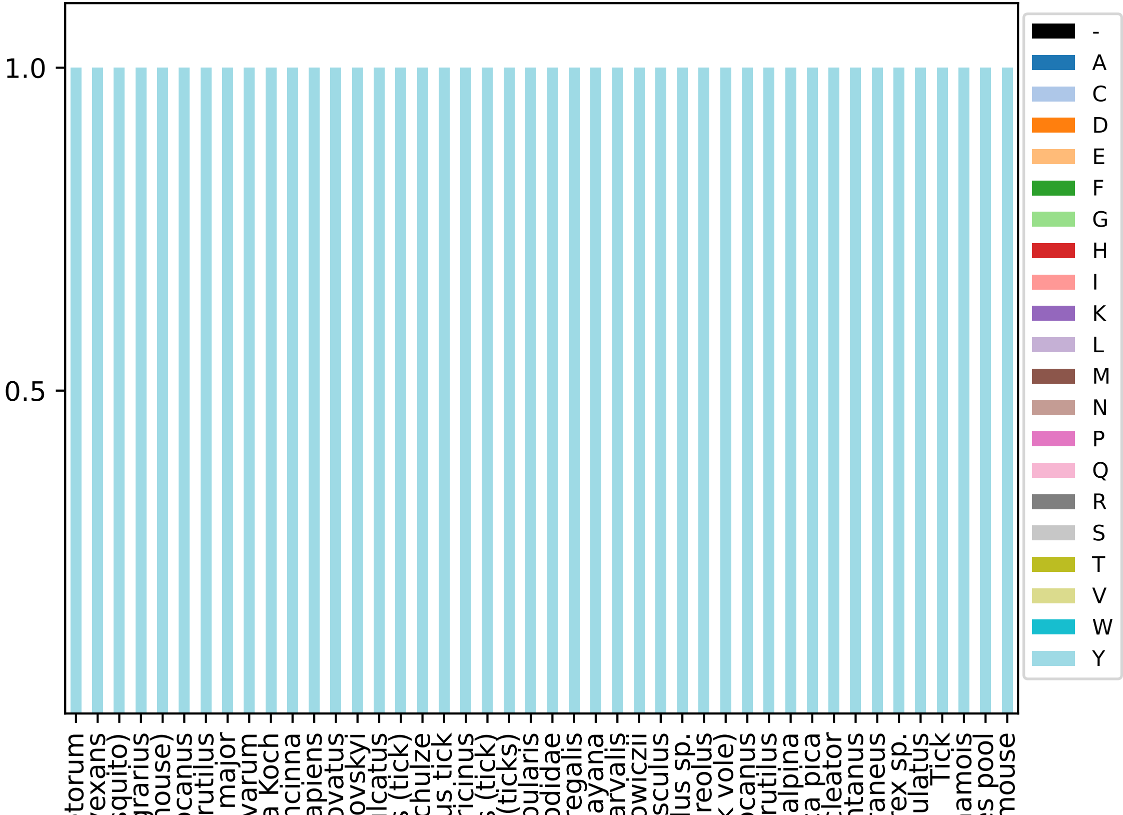

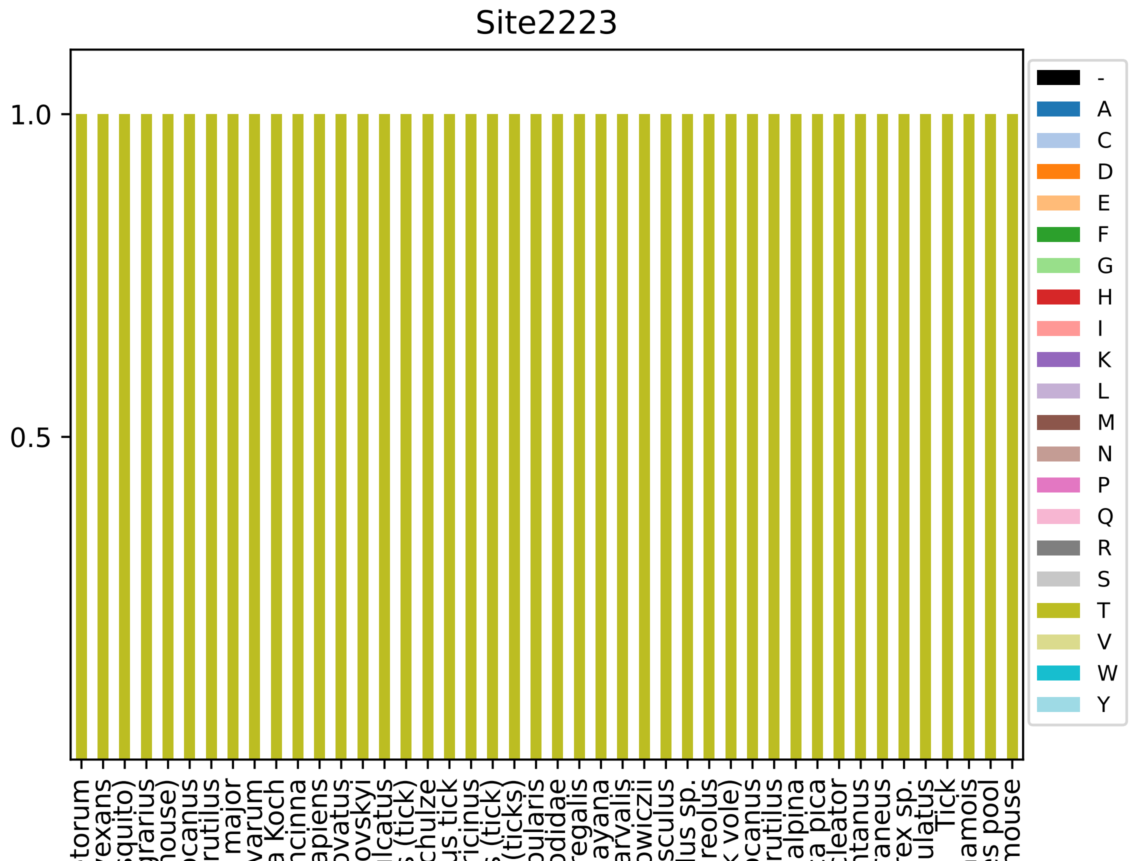
Site2223
1.0
0.5
-
A
C
D
E
F
G
H
I
K
L
M
N
P
Q
R
S
T
V
W
Y

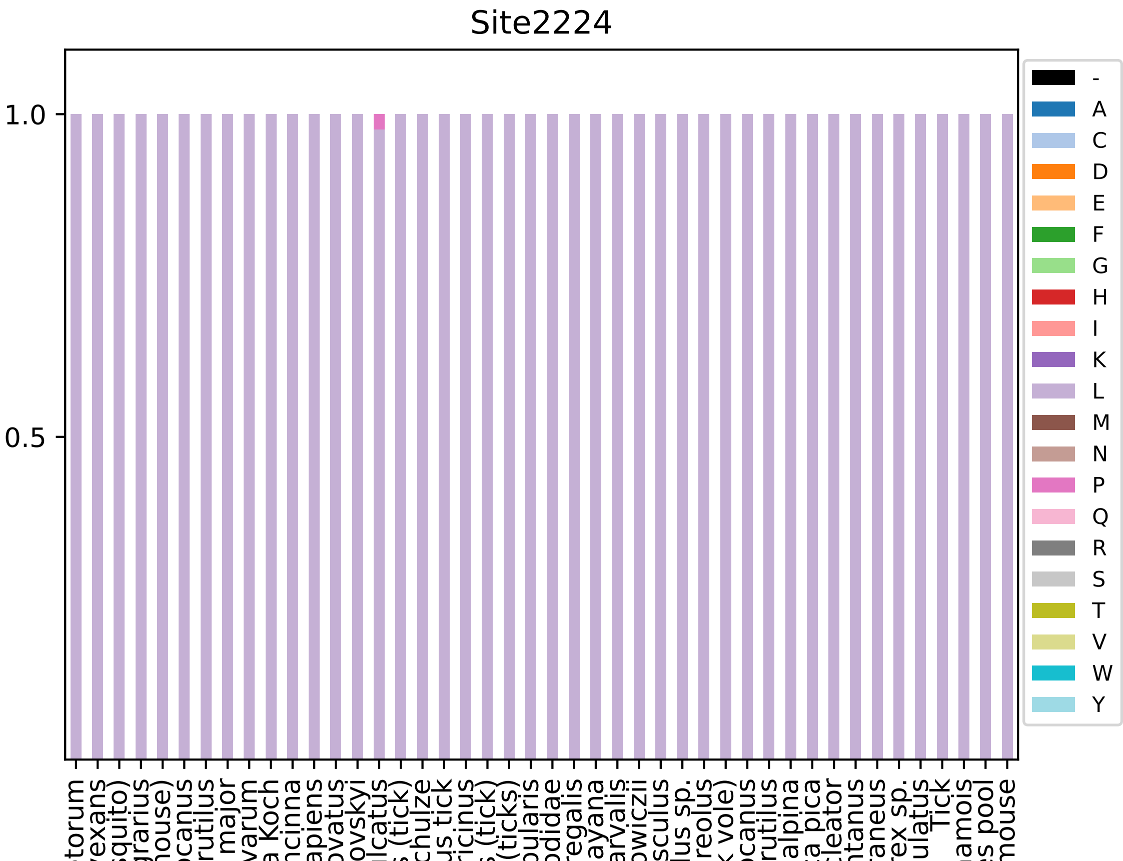
Site2224
1.0
0.5
-
A
C
D
E
F
G
H
I
K
L
M
N
P
Q
R
S
T
V
W
Y

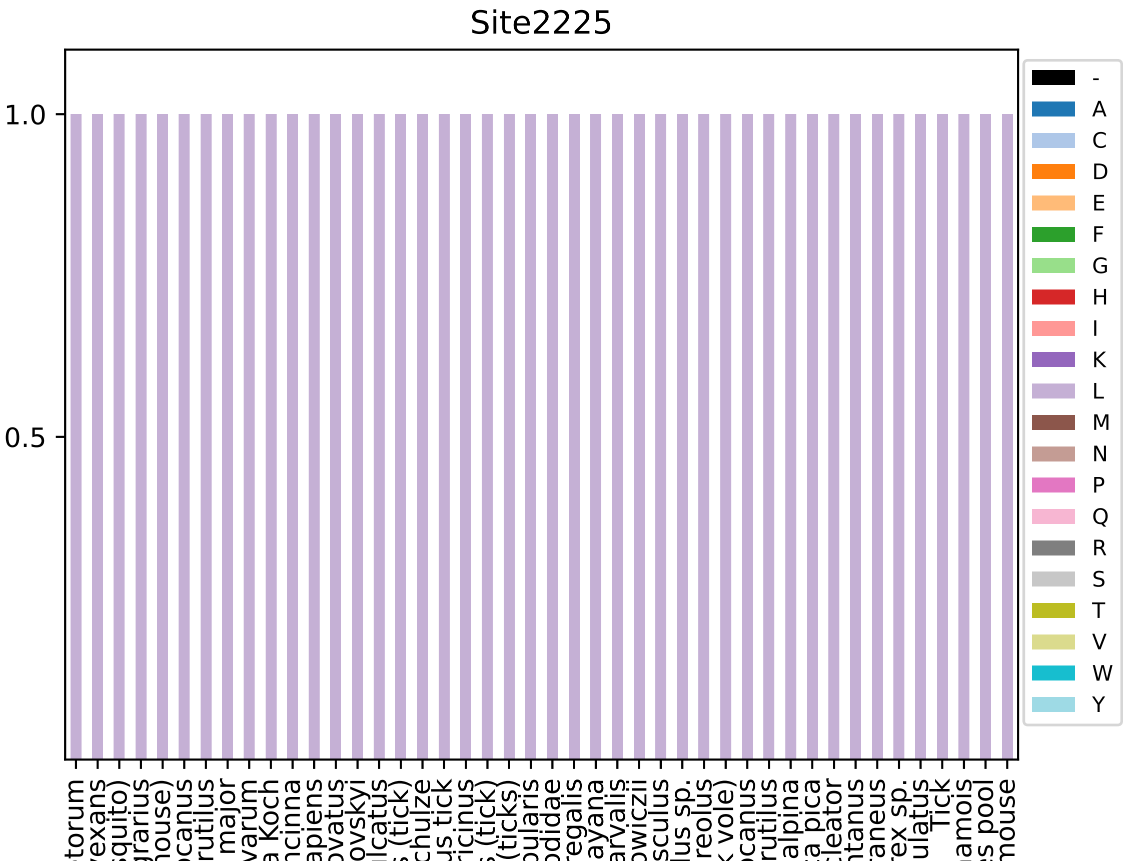
Site2225
1.0
0.5
-
A
C
D
E
F
G
H
I
K
L
M
N
P
Q
R
S
T
V
W
Y

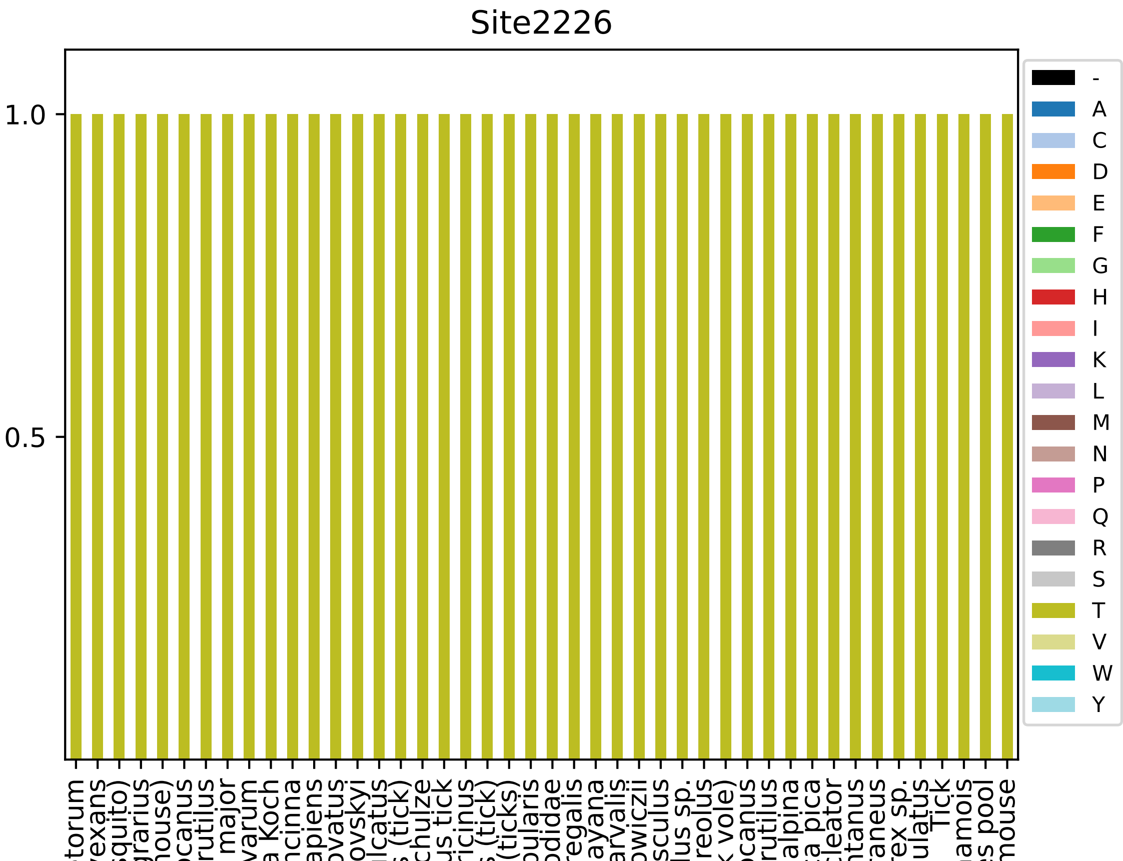
Site2226
1.0
0.5
-
A
C
D
E
F
G
H
I
K
L
M
N
P
Q
R
S
T
V
W
Y

Site2227

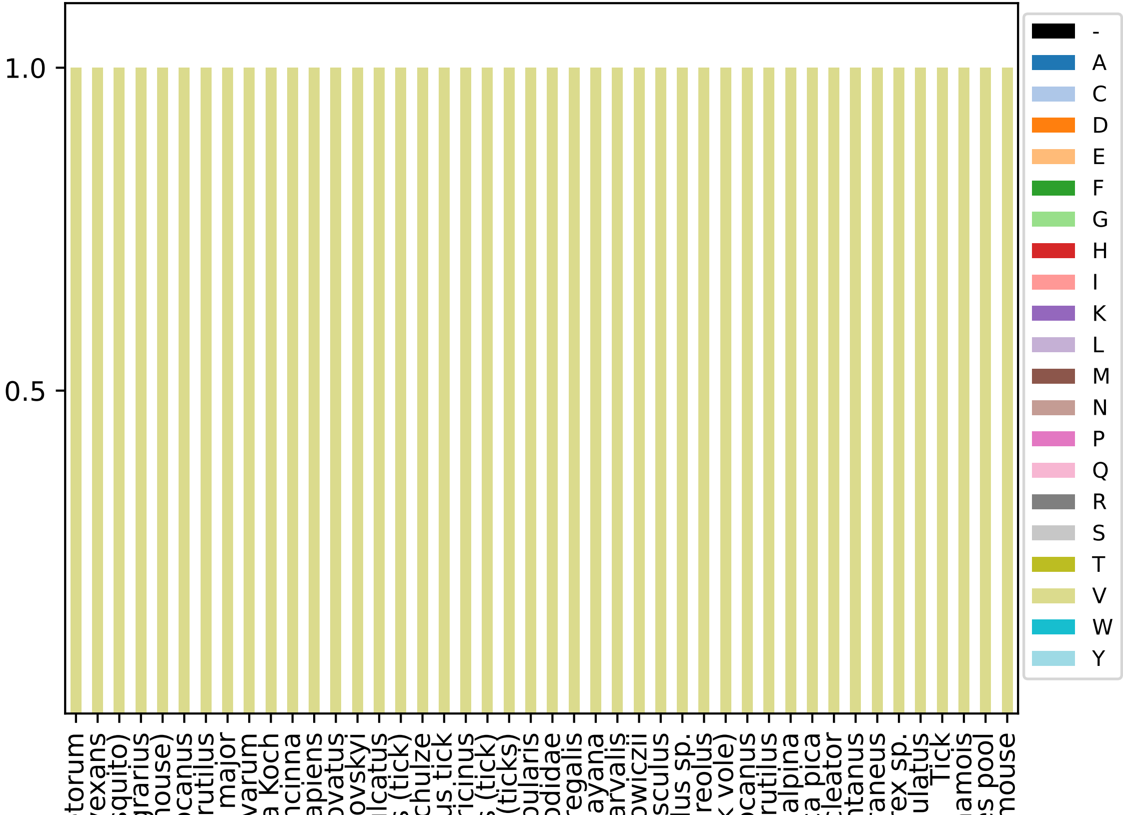

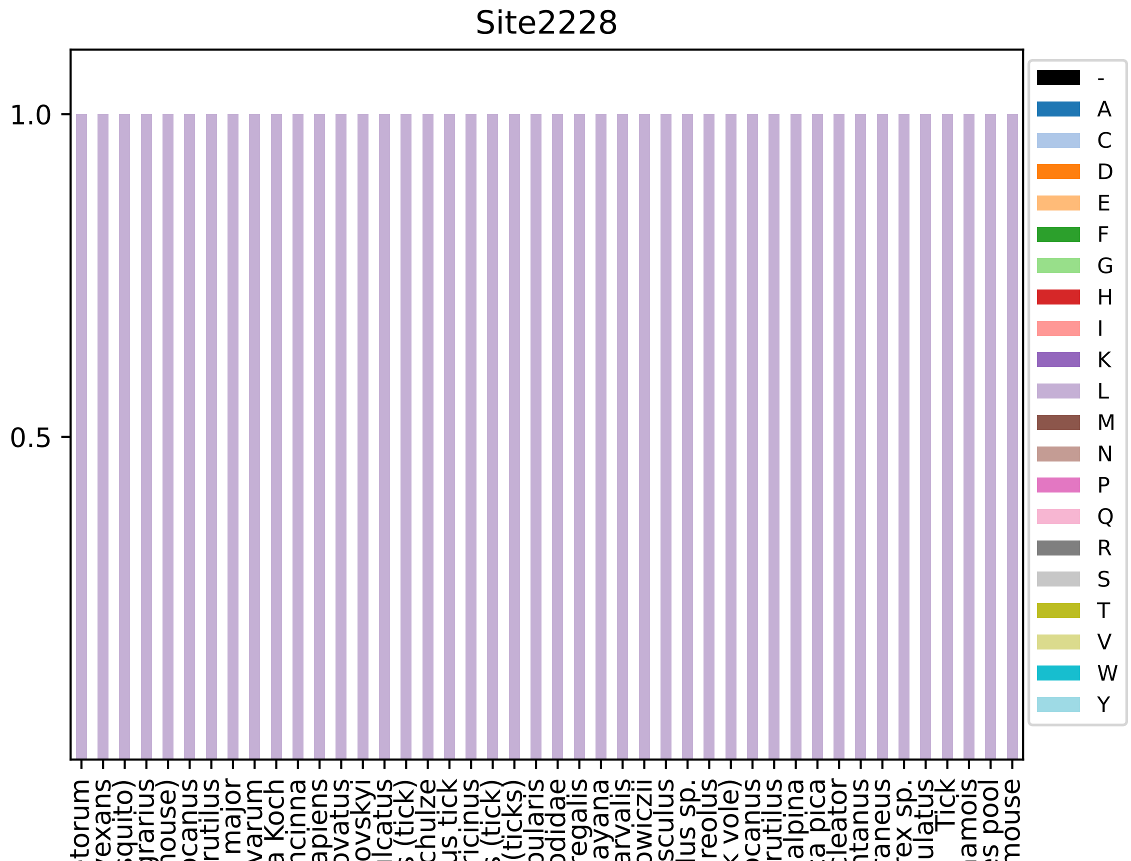
Site2228
1.0
0.5
-
A
C
D
E
F
G
H
I
K
L
M
N
P
Q
R
S
T
V
W
Y

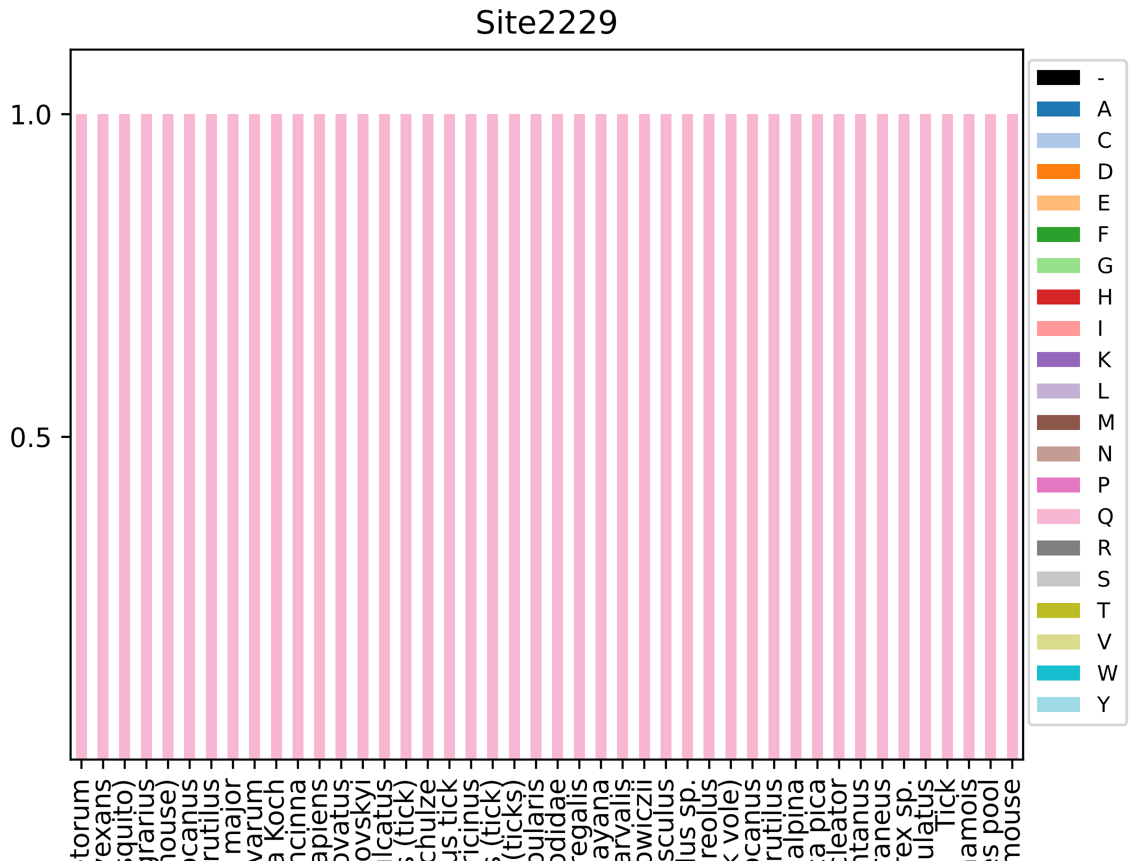
Site2229
1.0
0.5
-
A
C
D
E
F
G
H
I
K
L
M
N
P
Q
R
S
T
V
W
Y

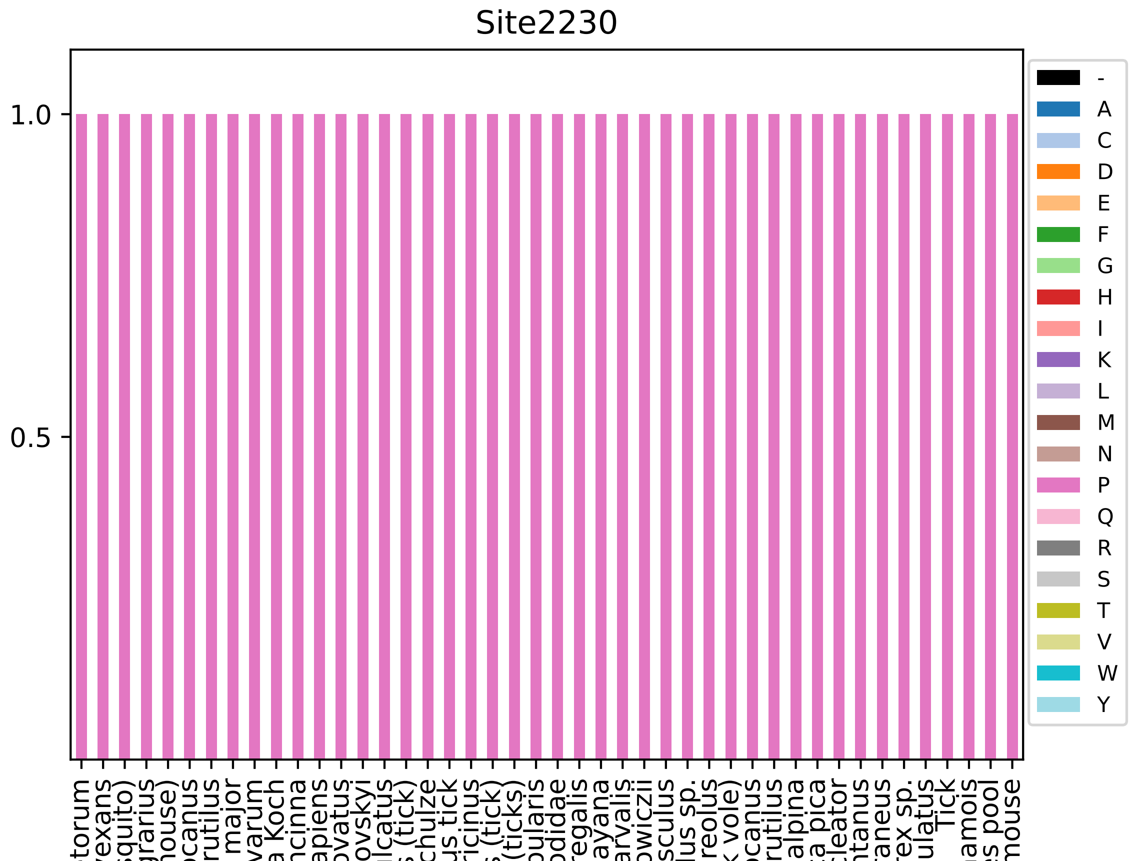
Site2230
1.0
0.5
-
A
C
D
E
F
G
H
I
K
L
M
N
P
Q
R
S
T
V
W
Y

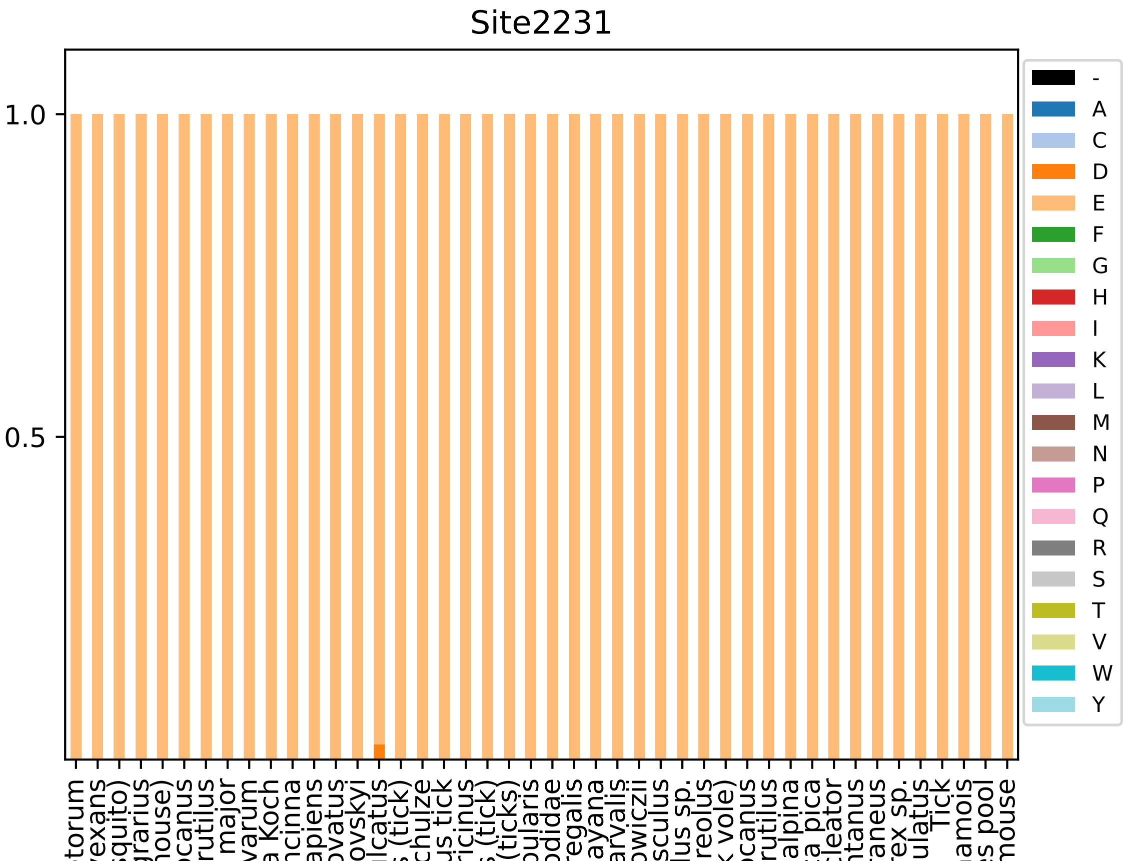

Site2231
1.0
0.5
-
A
C
D
E
F
G
H
I
K
L
M
N
P
Q
R
S
T
V
W
Y
torum
vexans
squito)
grarius
house)
ocanus
rutilus
major
varum
a Koch
ncinna
apiens
ovatus
ovskyi
ulcatus
(tick)
chulze
us tick
ricinus
(tick)
(ticks)
pularis
odidae
regalis
ayana
arvalis
owiczii
sculus
lus sp.
reolus
vole)
ocanus
rutilus
alpina
a pica
leator
ntanus
raneus
rex sp.
ulatus
Tick
amois
es pool
mouse

Site2232

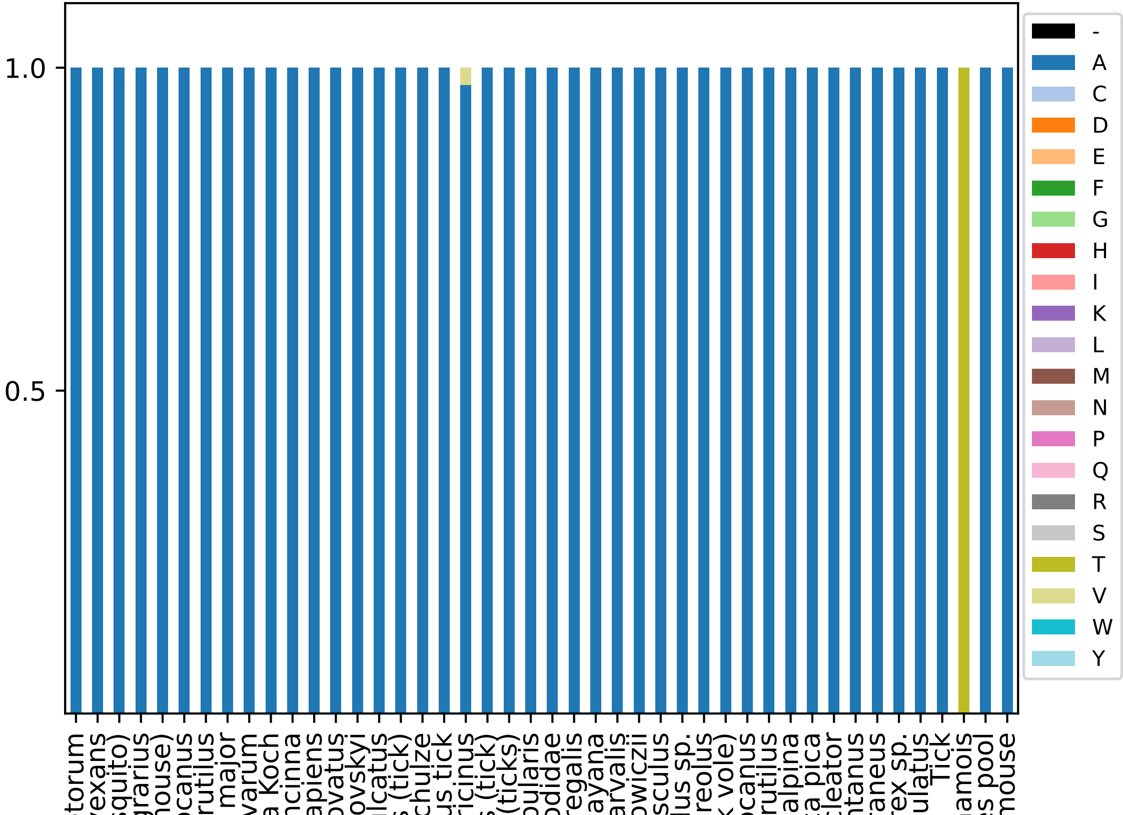

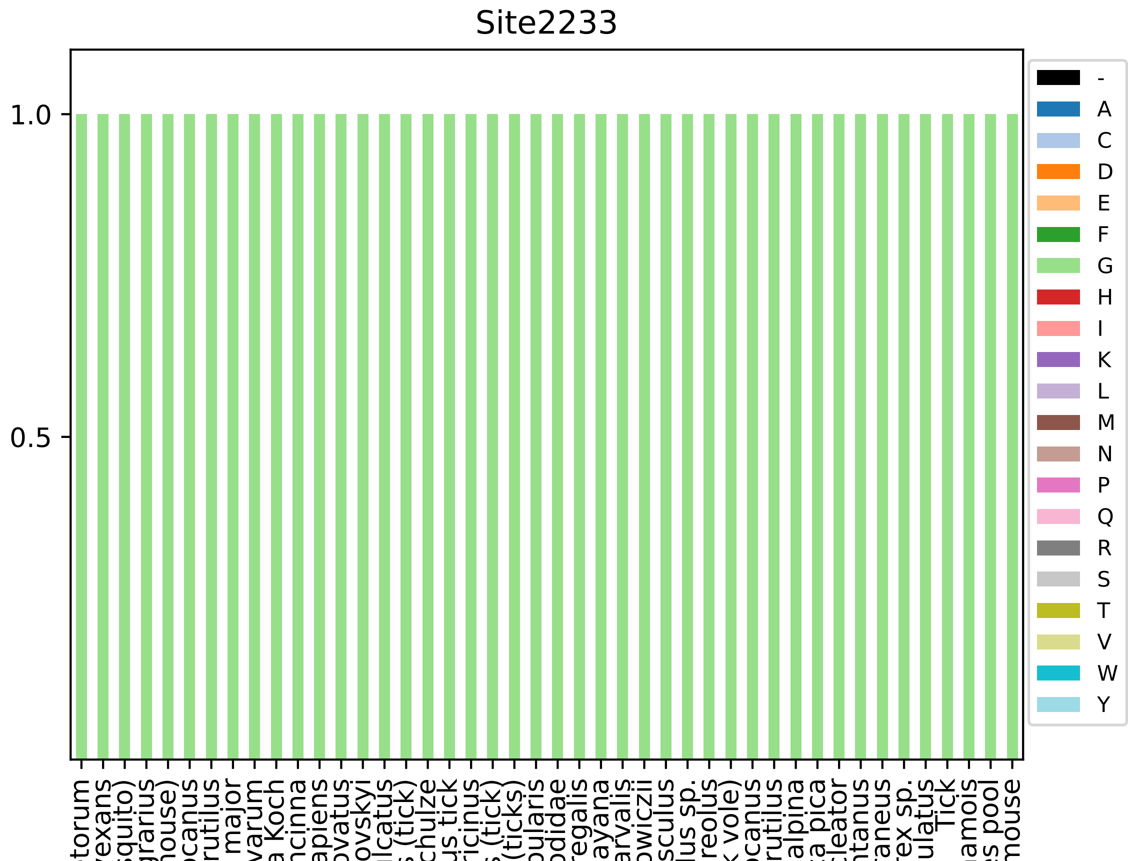

Site2233
1.0
0.5
-
A
C
D
E
F
G
H
I
K
L
M
N
P
Q
R
S
T
V
W
Y

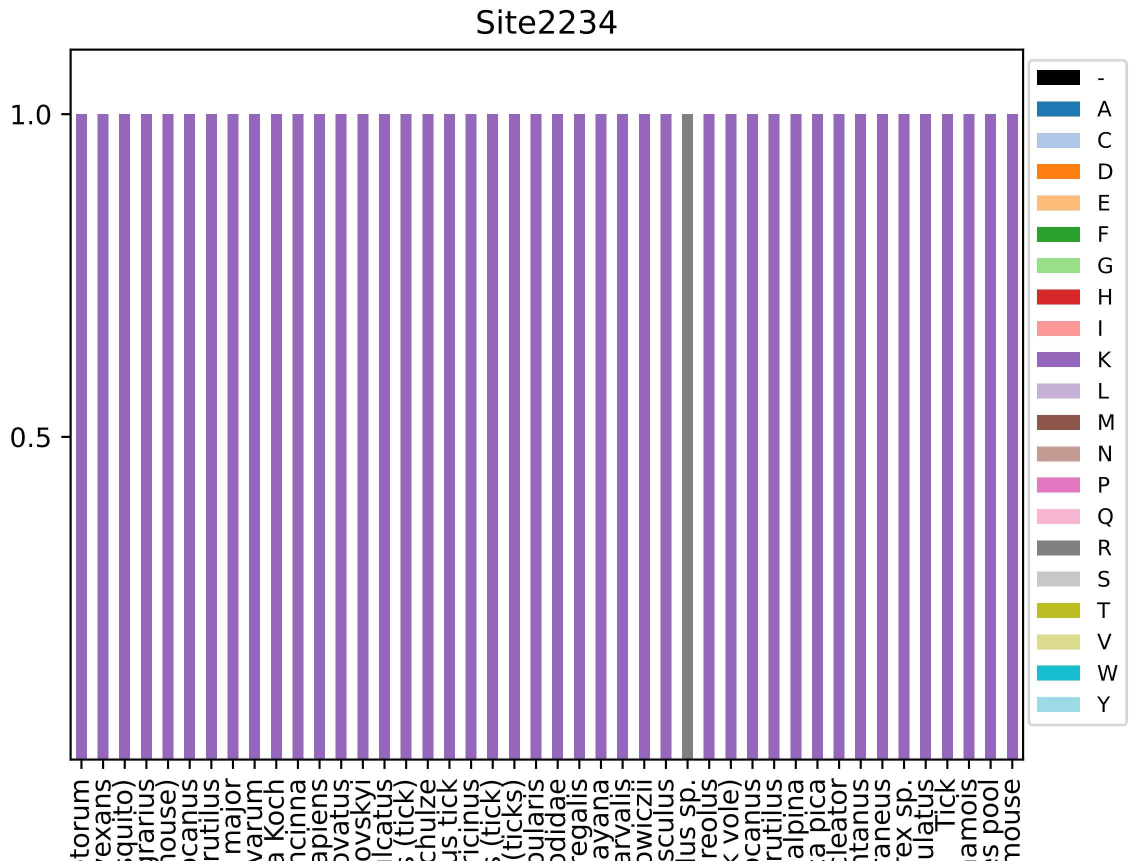

Site2234
1.0
0.5
-
A
C
D
E
F
G
H
I
K
L
M
N
P
Q
R
S
T
V
W
Y

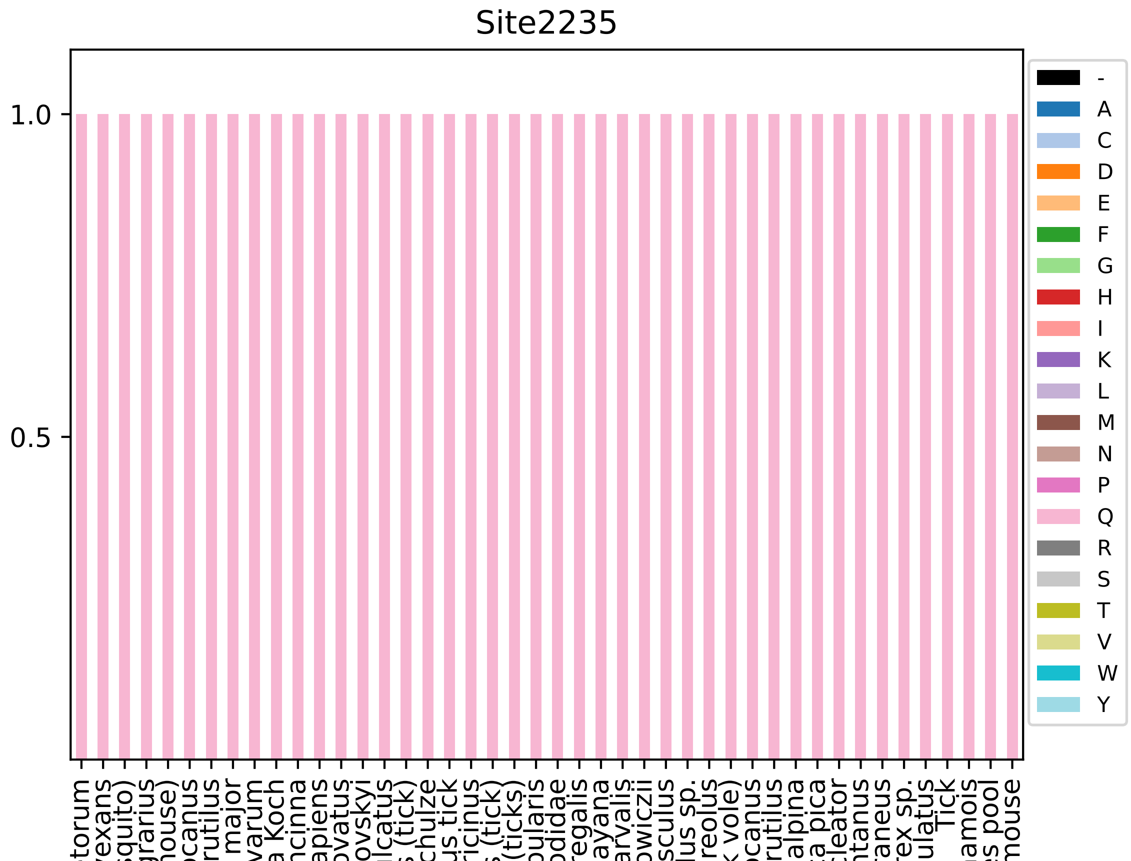
Site2235
1.0
0.5
-
A
C
D
E
F
G
H
I
K
L
M
N
P
Q
R
S
T
V
W
Y

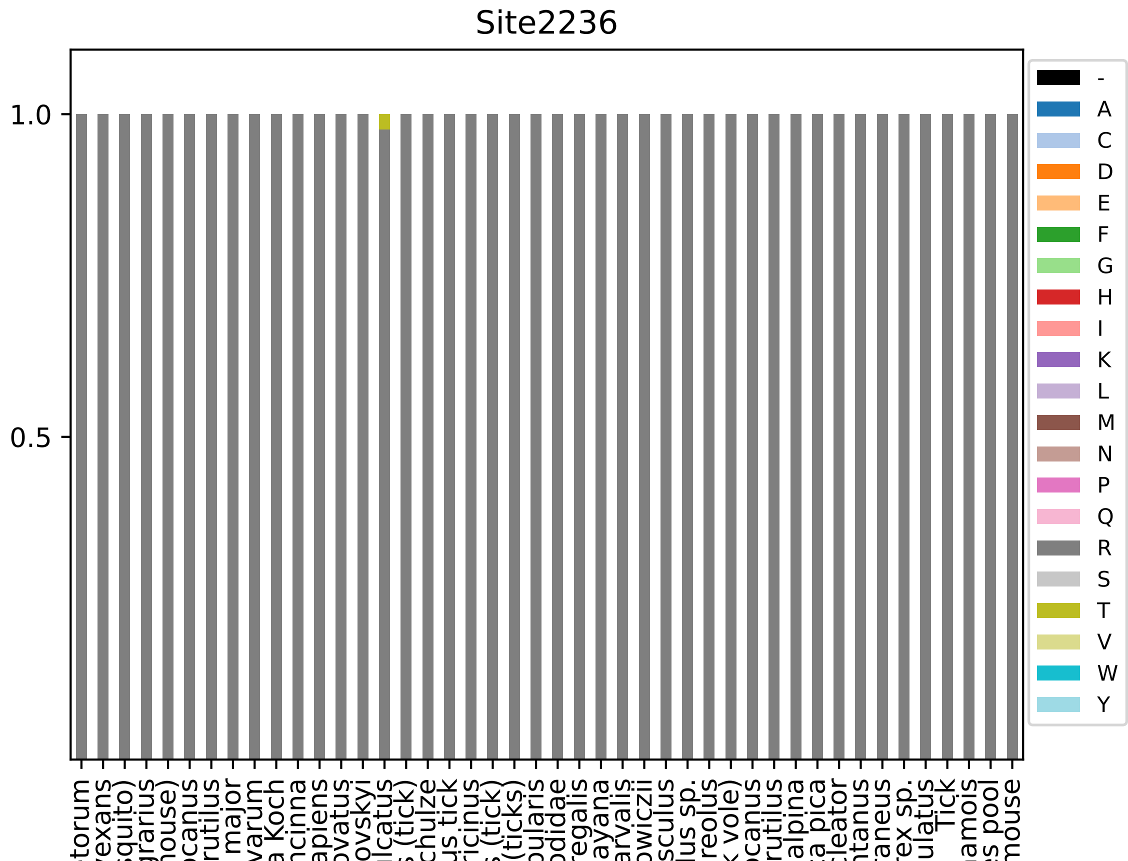
Site2236
1.0
0.5
-
A
C
D
E
F
G
H
I
K
L
M
N
P
Q
R
S
T
V
W
Y

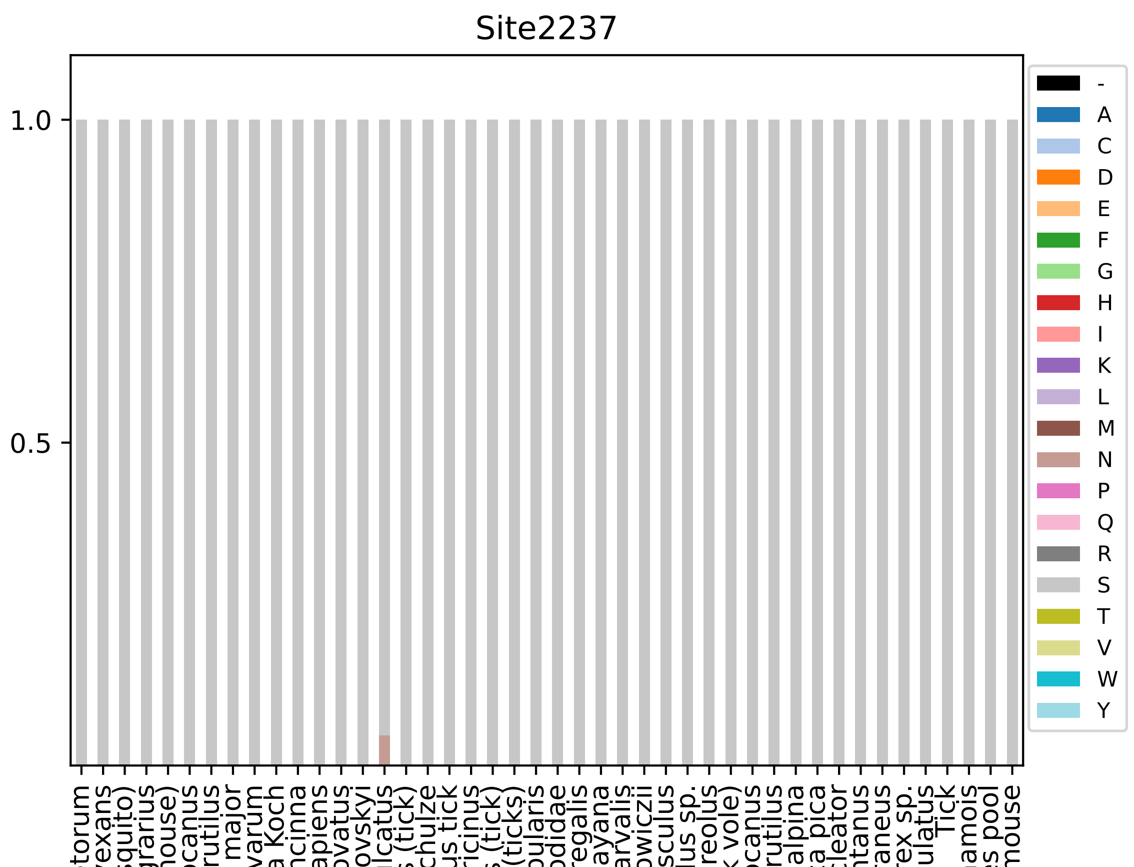
Site2237
1.0
0.5
-
A
C
D
E
F
G
H
I
K
L
M
N
P
Q
R
S
T
V
W
Y

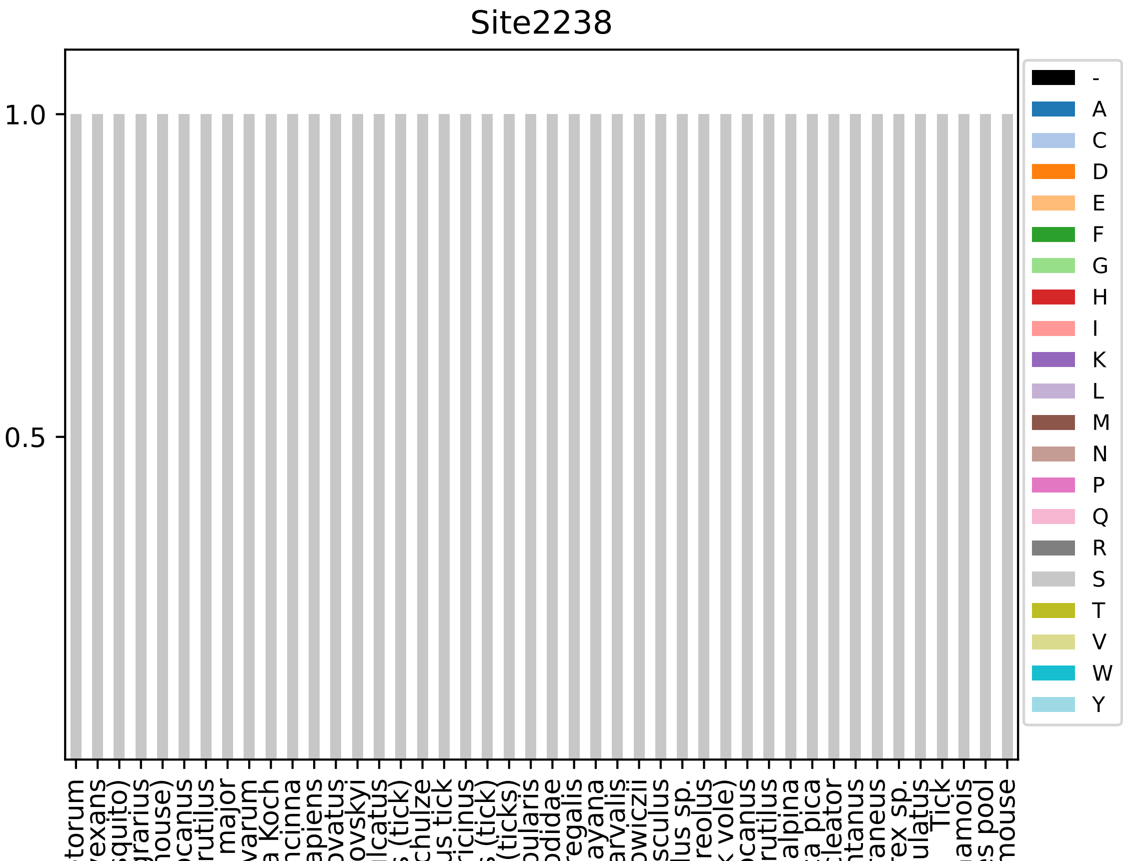
Site2238
1.0
0.5
-
A
C
D
E
F
G
H
I
K
L
M
N
P
Q
R
S
T
V
W
Y

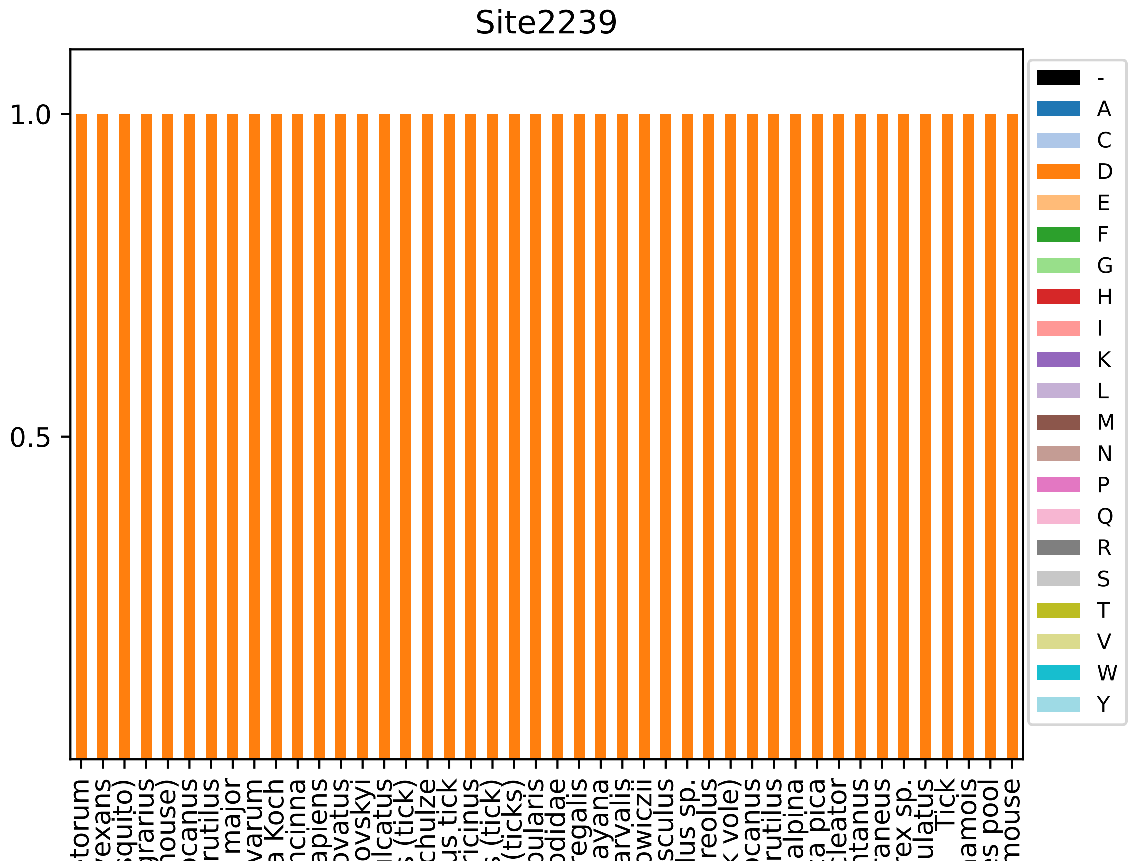

Site2239
1.0
0.5
-
A
C
D
E
F
G
H
I
K
L
M
N
P
Q
R
S
T
V
W
Y

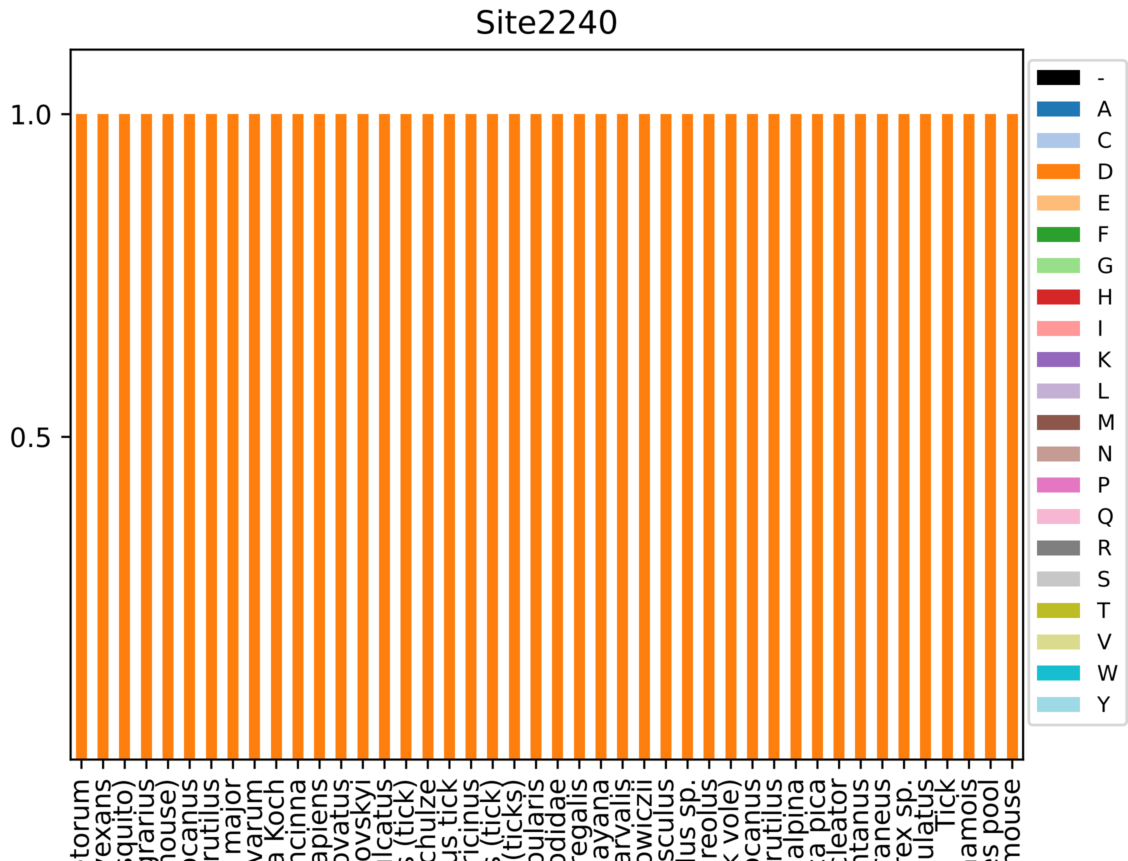

Site2240
1.0
0.5
-
A
C
D
E
F
G
H
I
K
L
M
N
P
Q
R
S
T
V
W
Y
Tick

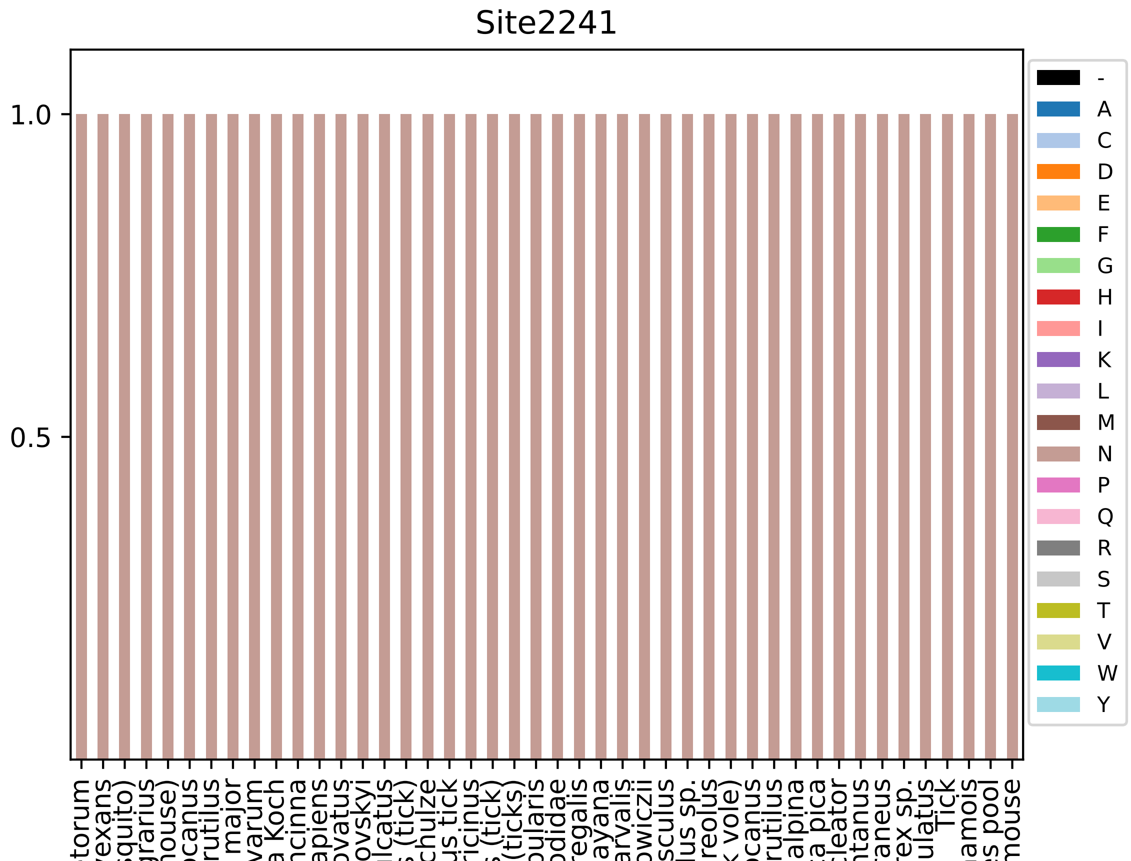
Site2241
1.0
0.5
-
A
C
D
E
F
G
H
I
K
L
M
N
P
Q
R
S
T
V
W
Y

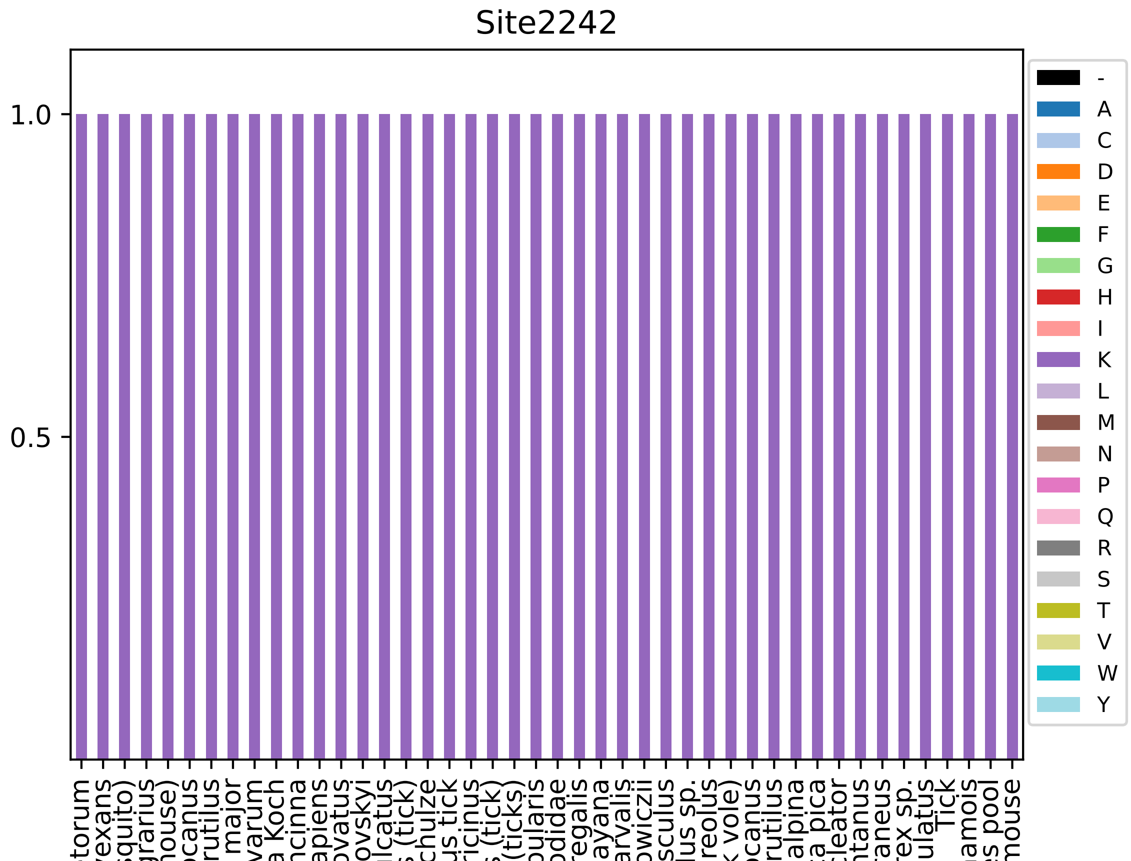
Site2242
1.0
0.5
-
A
C
D
E
F
G
H
I
K
L
M
N
P
Q
R
S
T
V
W
Y

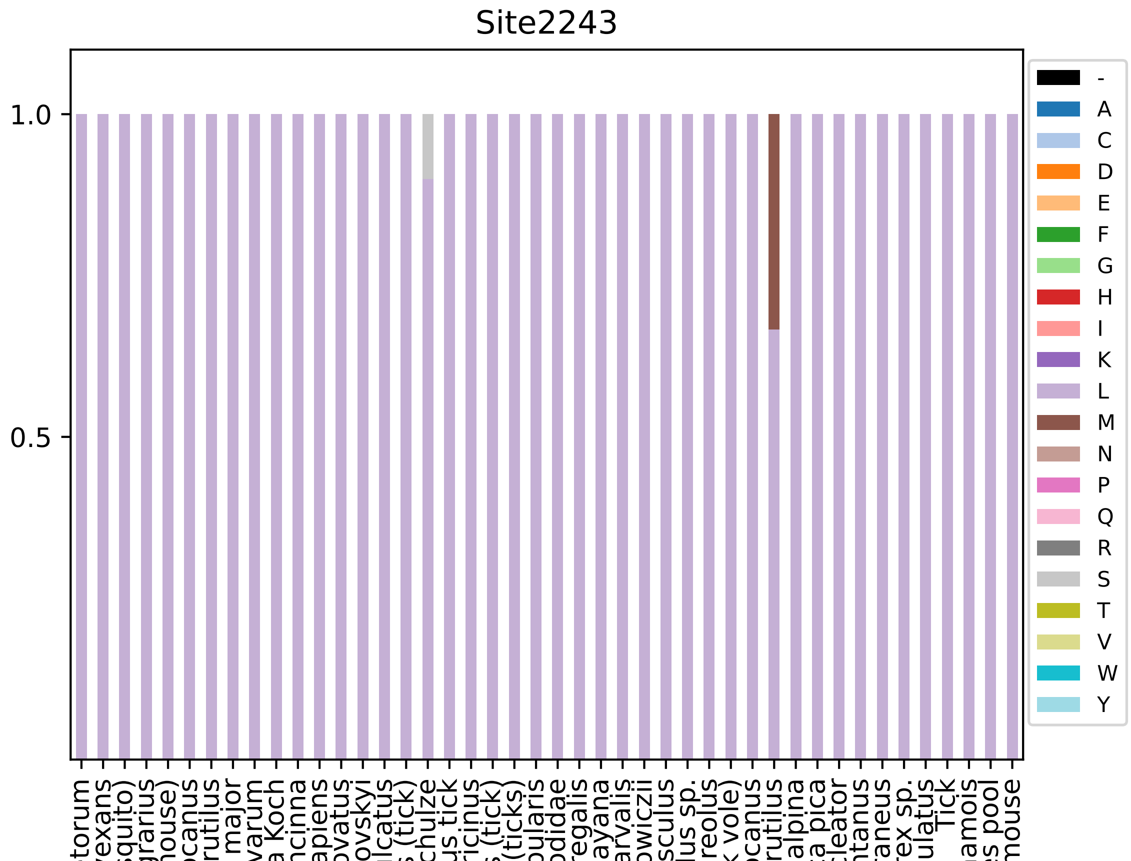
Site2243
1.0
0.5
-
A
C
D
E
F
G
H
I
K
L
M
N
P
Q
R
S
T
V
W
Y

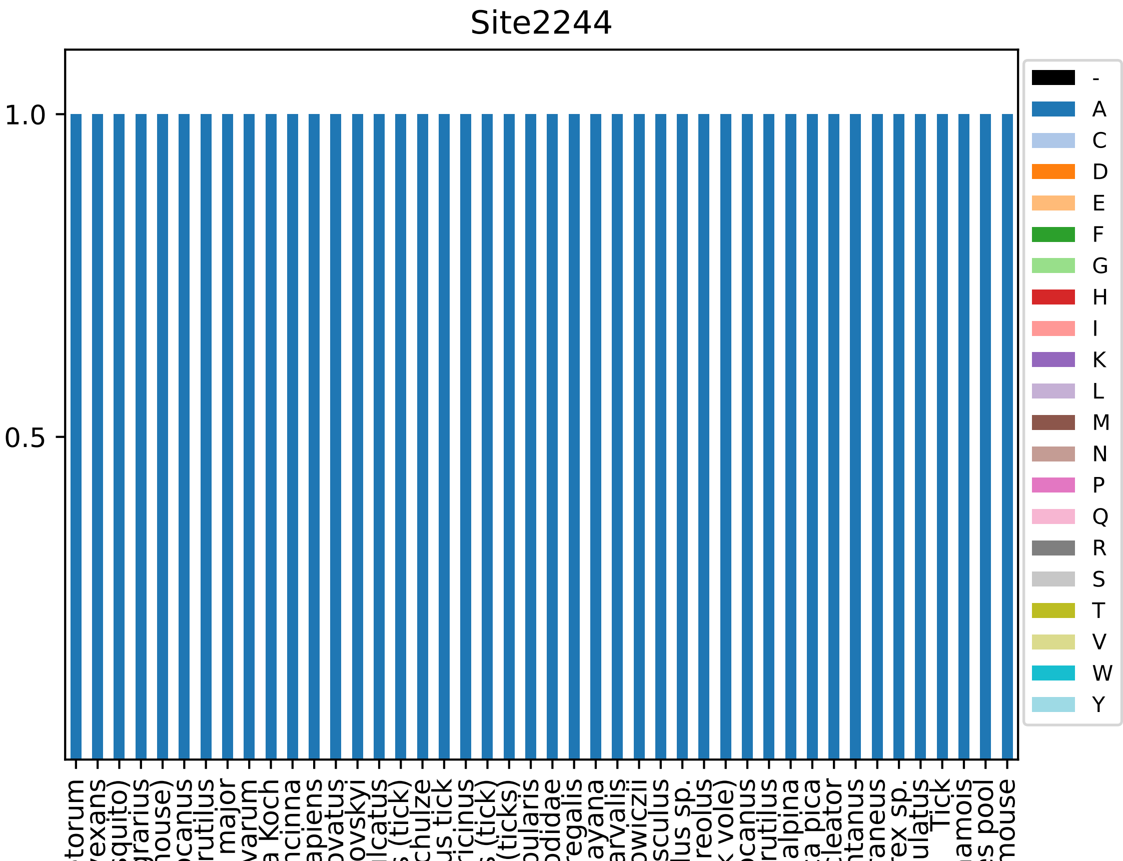
Site2244
1.0
0.5
-
A
C
D
E
F
G
H
I
K
L
M
N
P
Q
R
S
T
V
W
Y

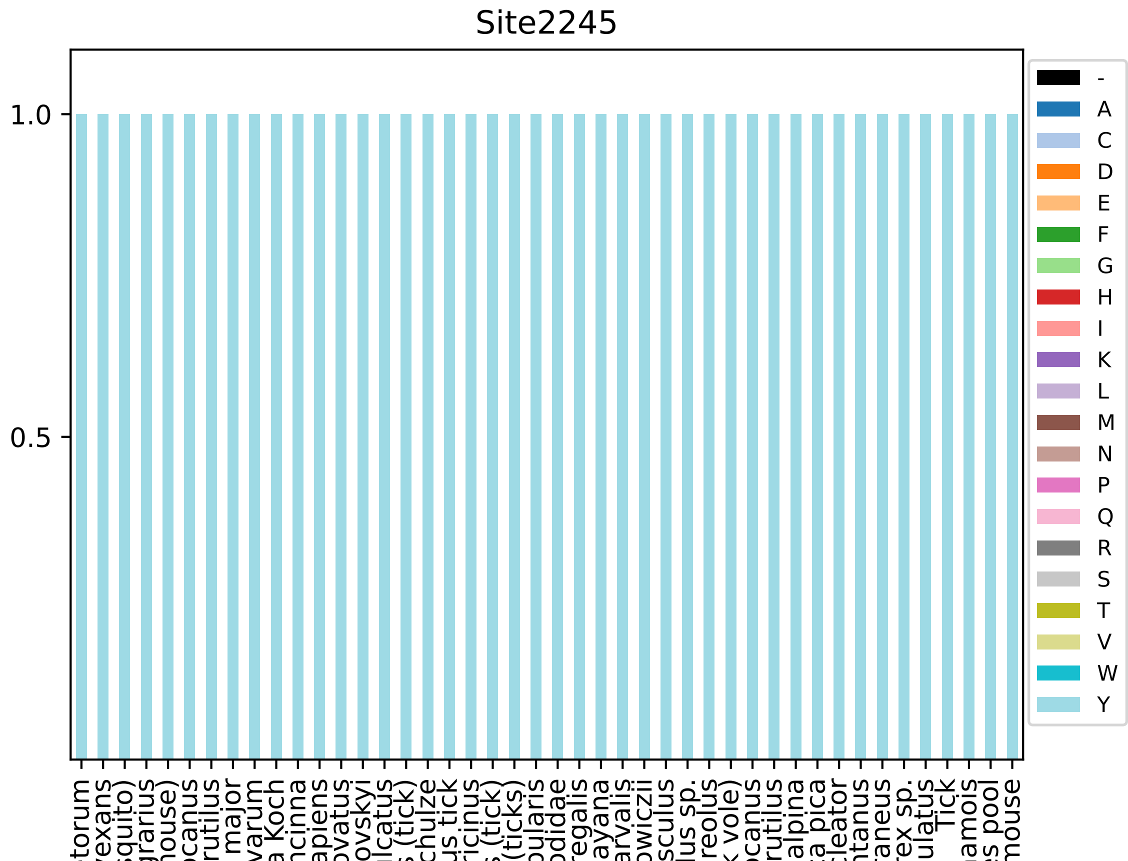
Site2245
1.0
0.5
-
A
C
D
E
F
G
H
I
K
L
M
N
P
Q
R
S
T
V
W
Y

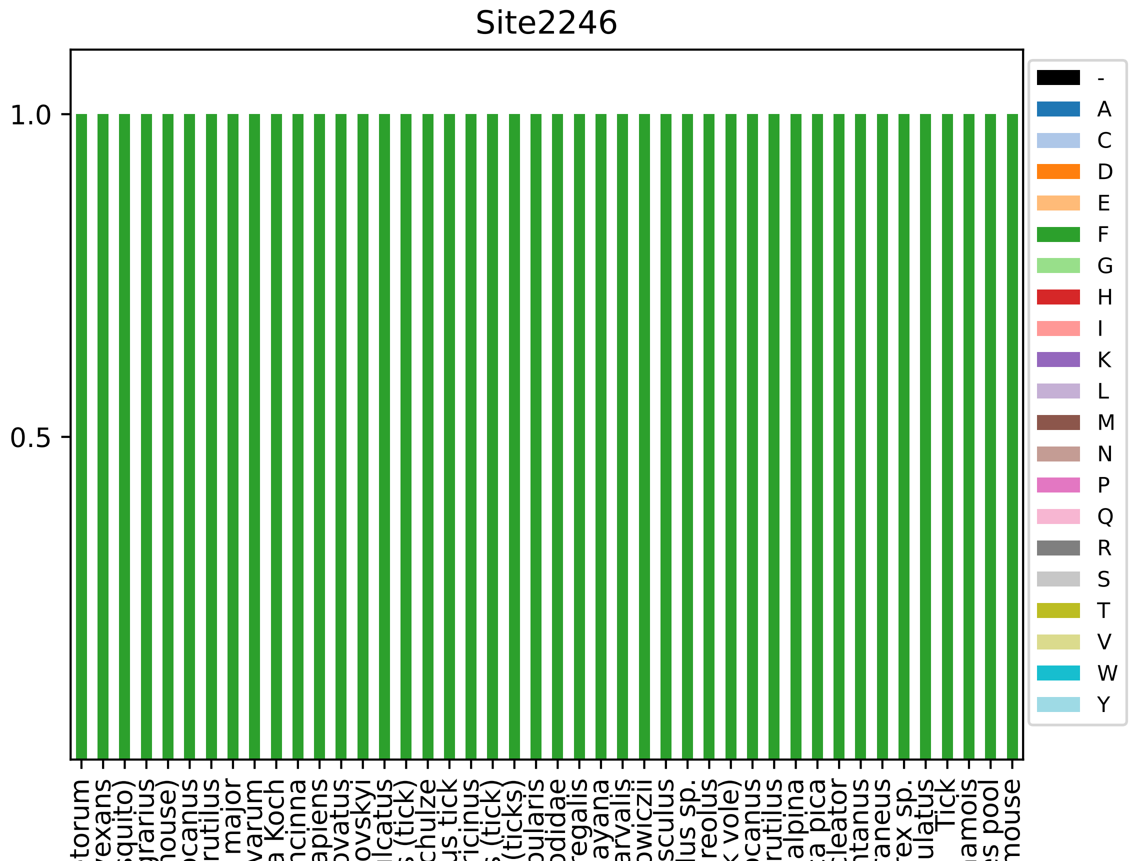
Site2246
1.0
0.5
-
A
C
D
E
F
G
H
I
K
L
M
N
P
Q
R
S
T
V
W
Y

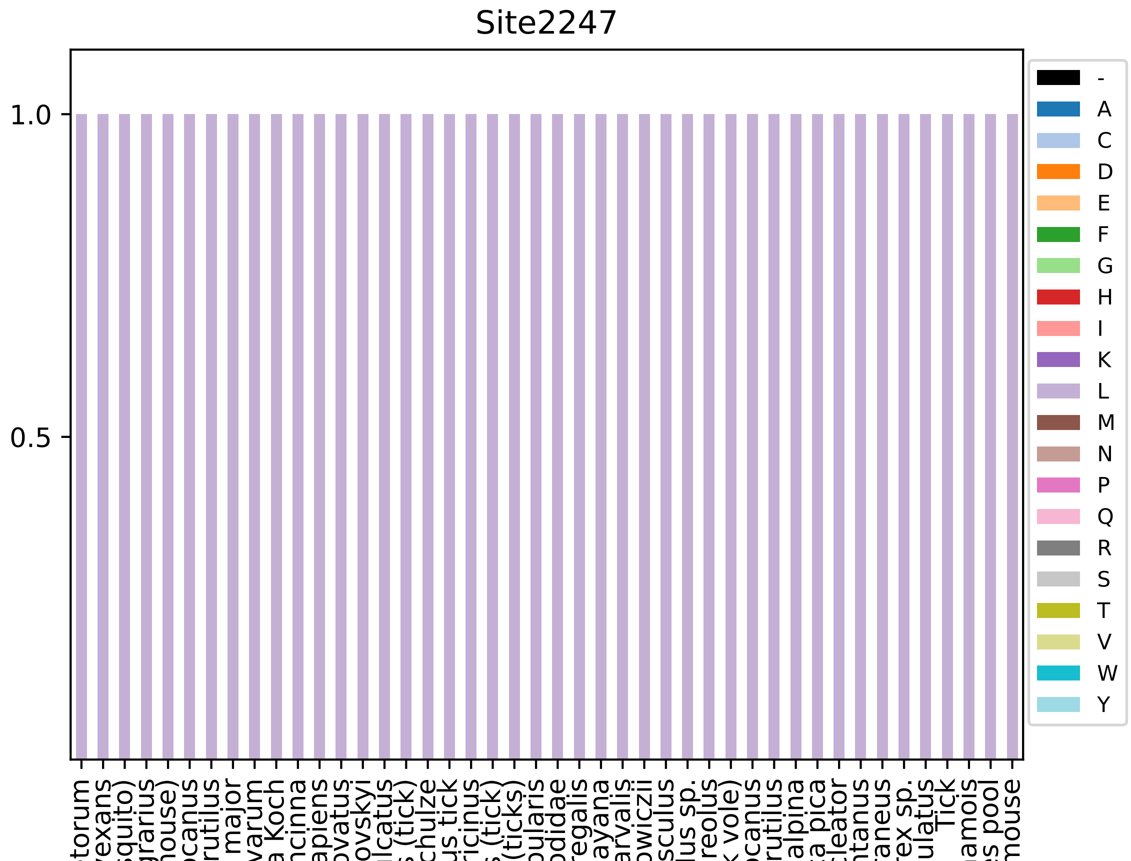
Site2247
1.0
0.5
-
A
C
D
E
F
G
H
I
K
L
M
N
P
Q
R
S
T
V
W
Y

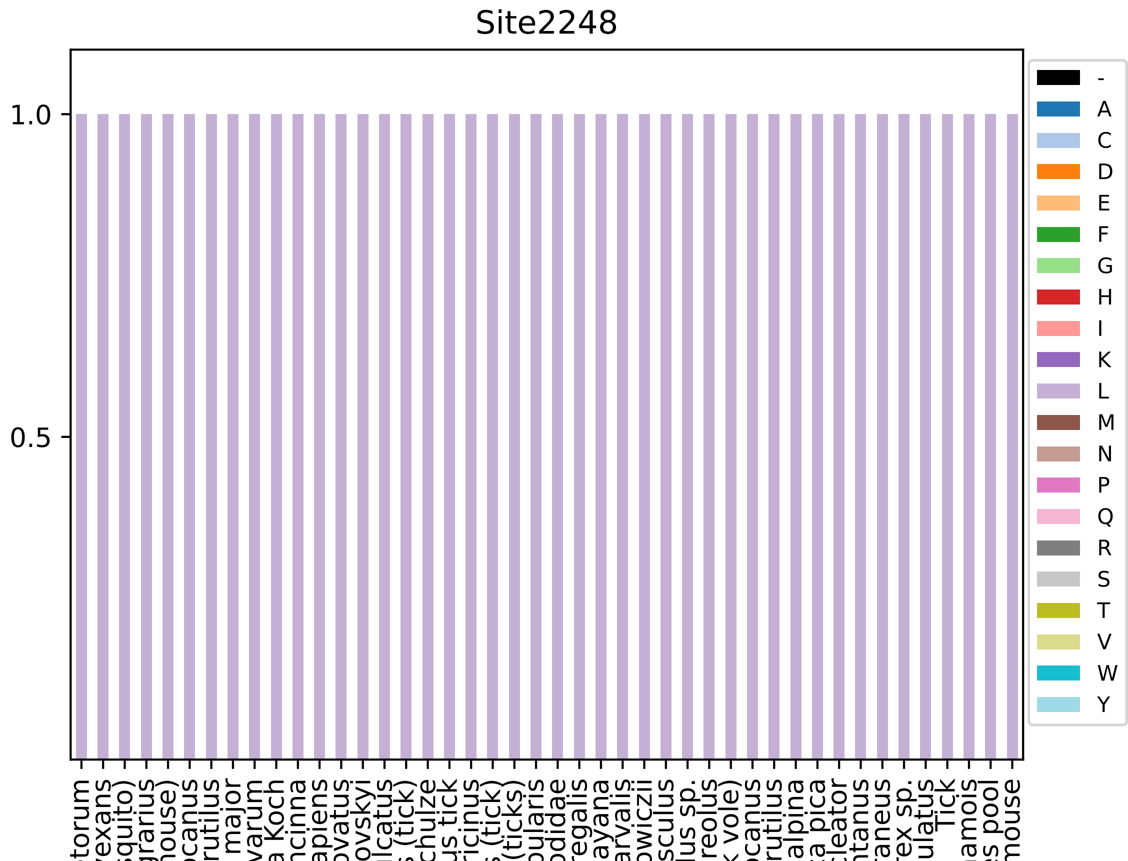
Site2248
1.0
0.5
-
A
C
D
E
F
G
H
I
K
L
M
N
P
Q
R
S
T
V
W
Y

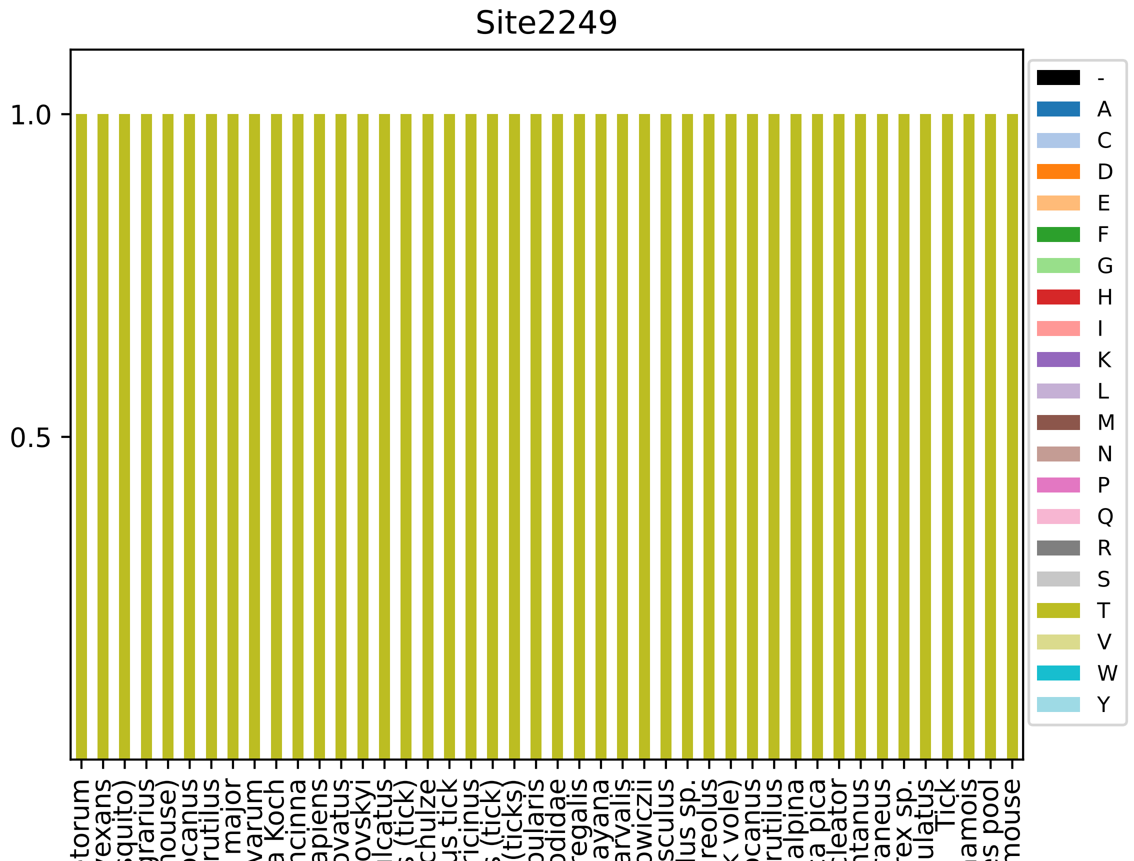

Site2249
1.0
0.5
-
A
C
D
E
F
G
H
I
K
L
M
N
P
Q
R
S
T
V
W
Y

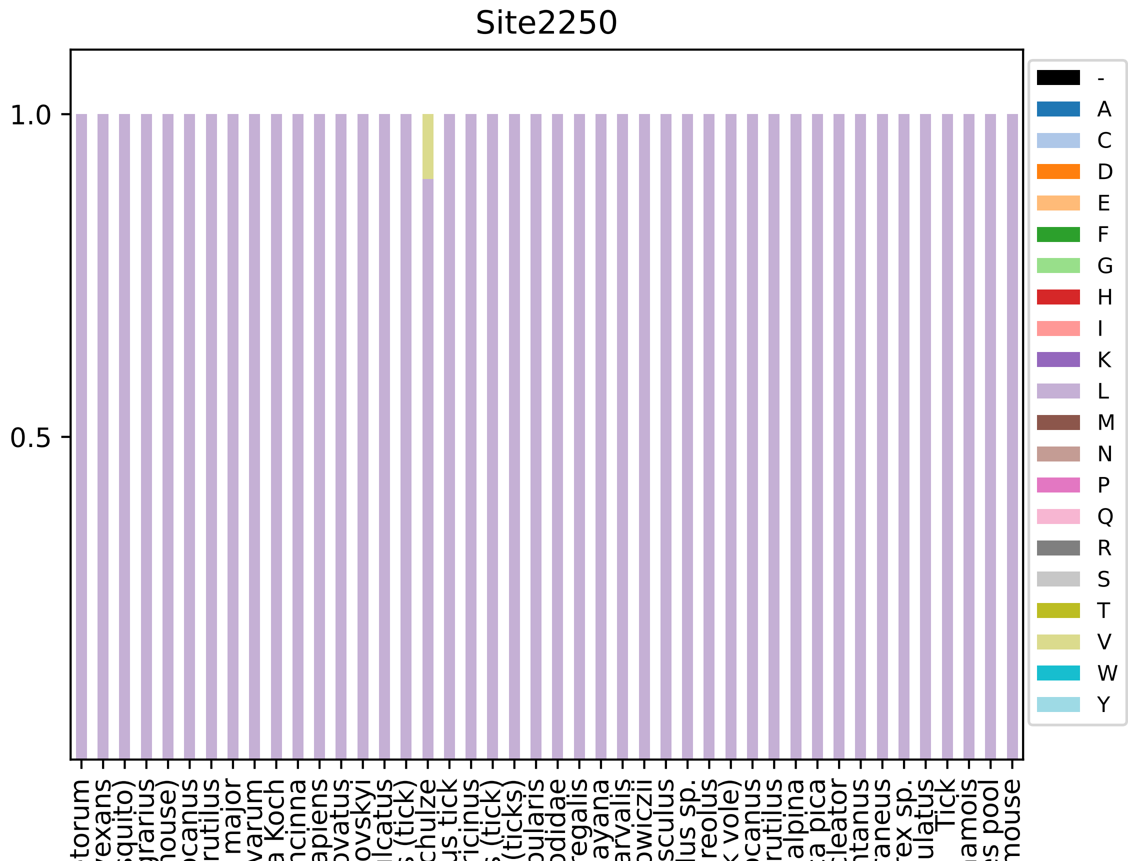
Site2250
1.0
0.5
-
A
C
D
E
F
G
H
I
K
L
M
N
P
Q
R
S
T
V
W
Y
Tick

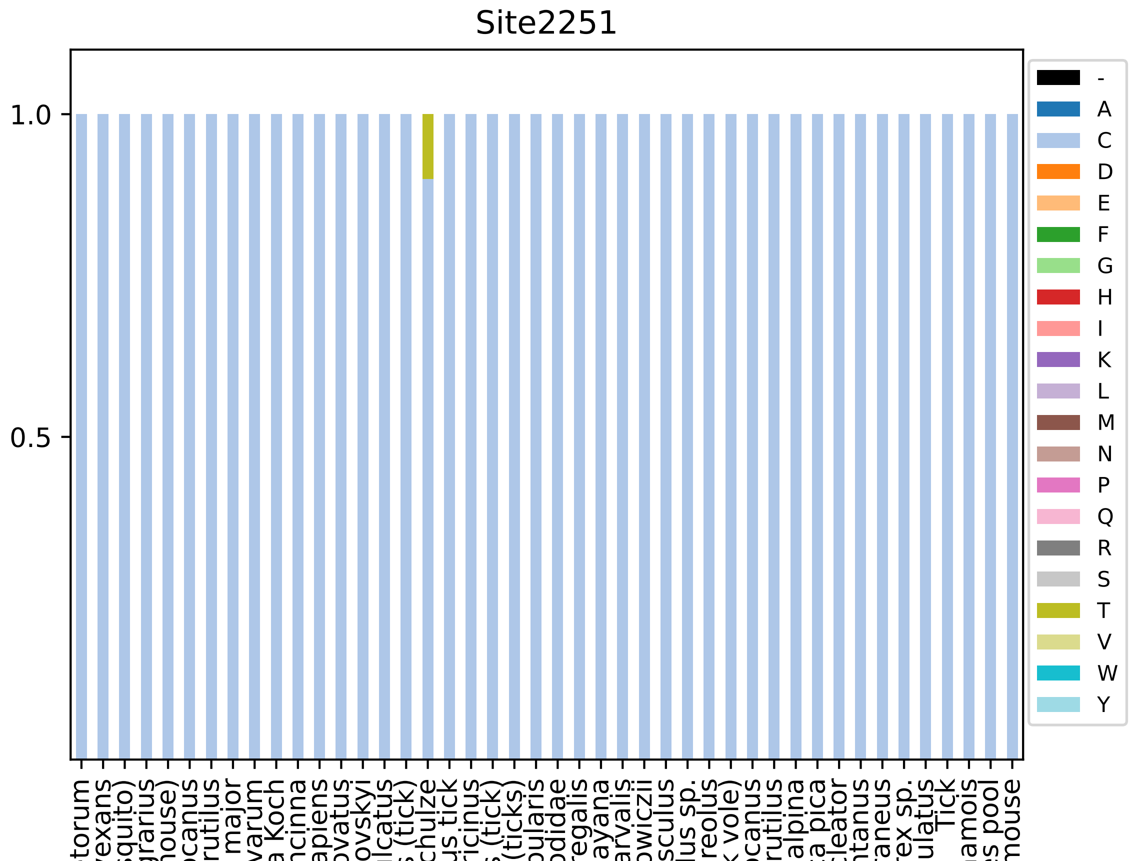
Site2251
1.0
0.5
-
A
C
D
E
F
G
H
I
K
L
M
N
P
Q
R
S
T
V
W
Y

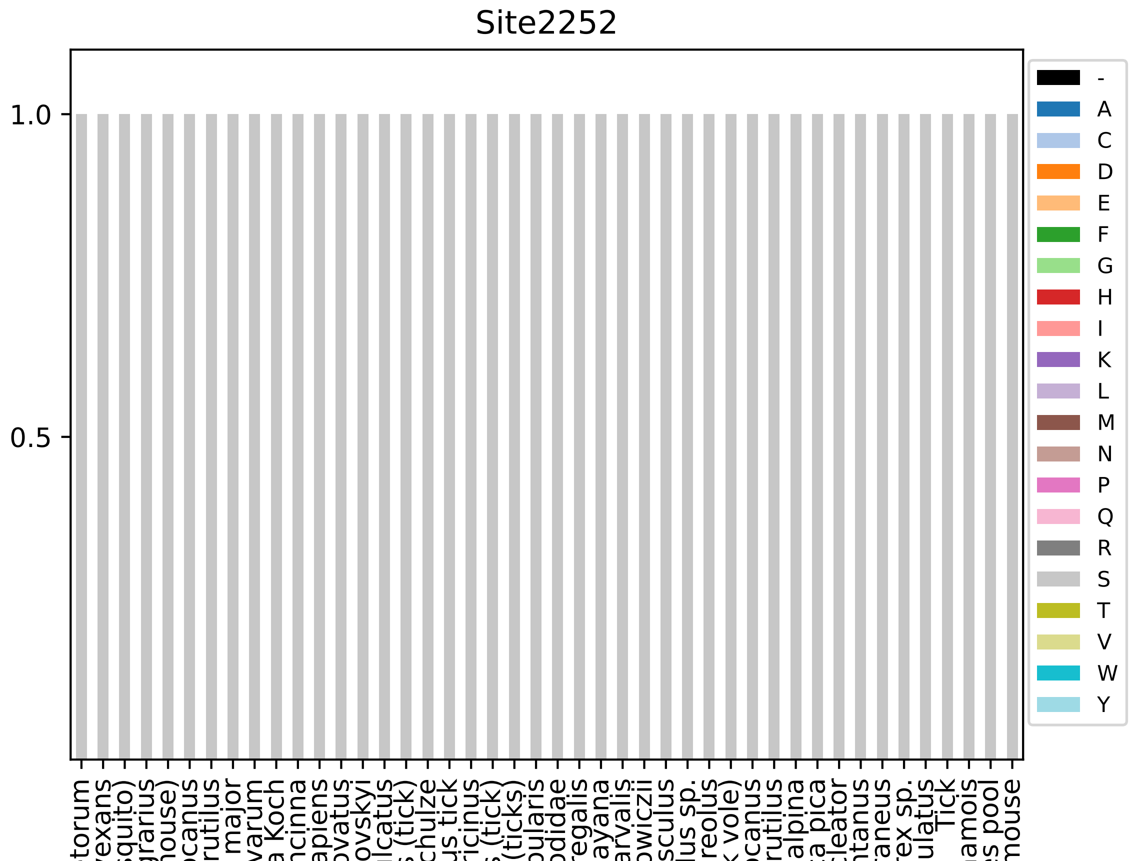
Site2252
1.0
0.5
-
A
C
D
E
F
G
H
I
K
L
M
N
P
Q
R
S
T
V
W
Y

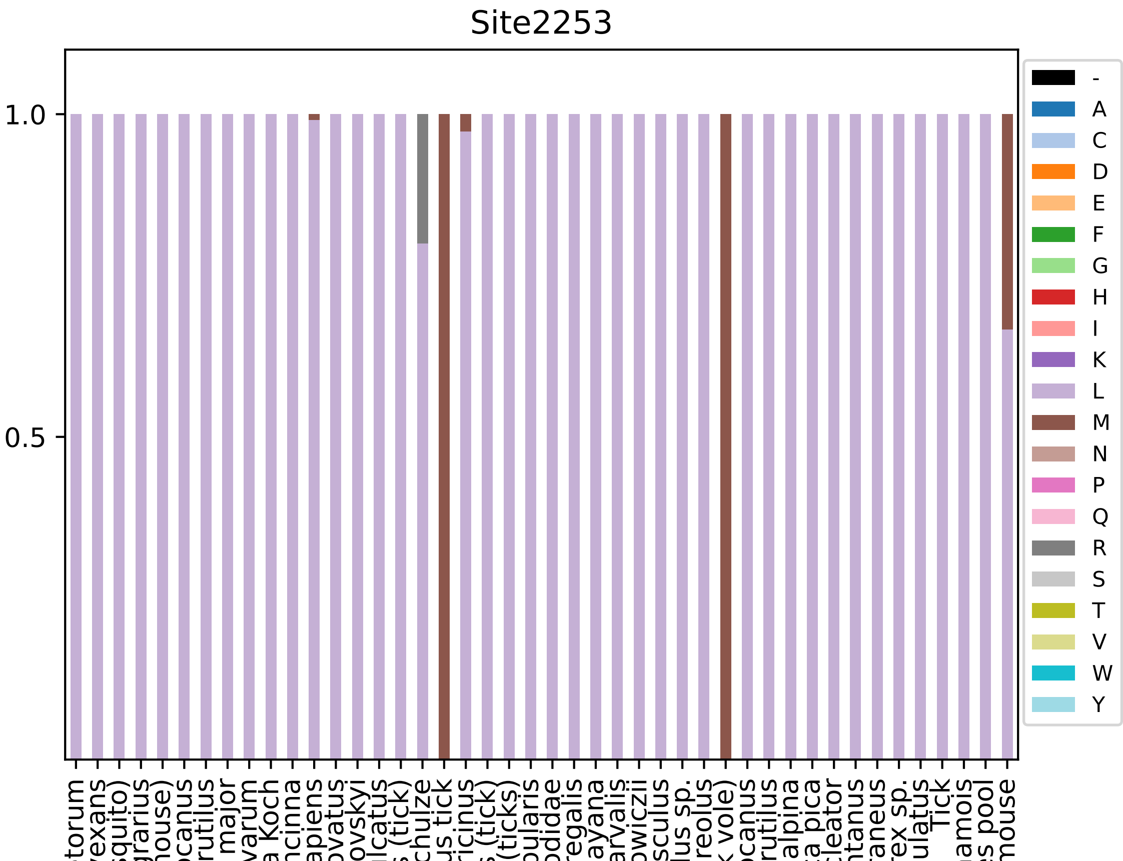
Site2253
1.0
0.5
-
A
C
D
E
F
G
H
I
K
L
M
N
P
Q
R
S
T
V
W
Y

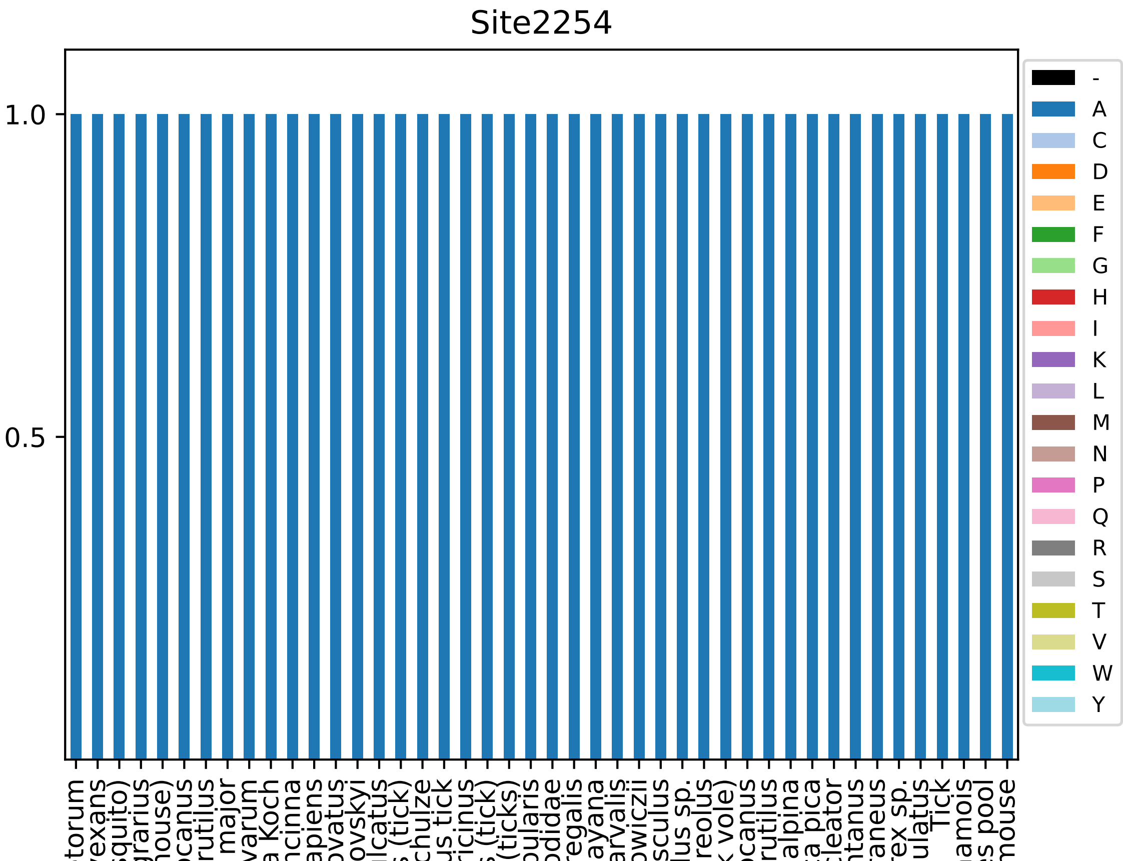
Site2254
1.0
0.5
-
A
C
D
E
F
G
H
I
K
L
M
N
P
Q
R
S
T
V
W
Y
Tick

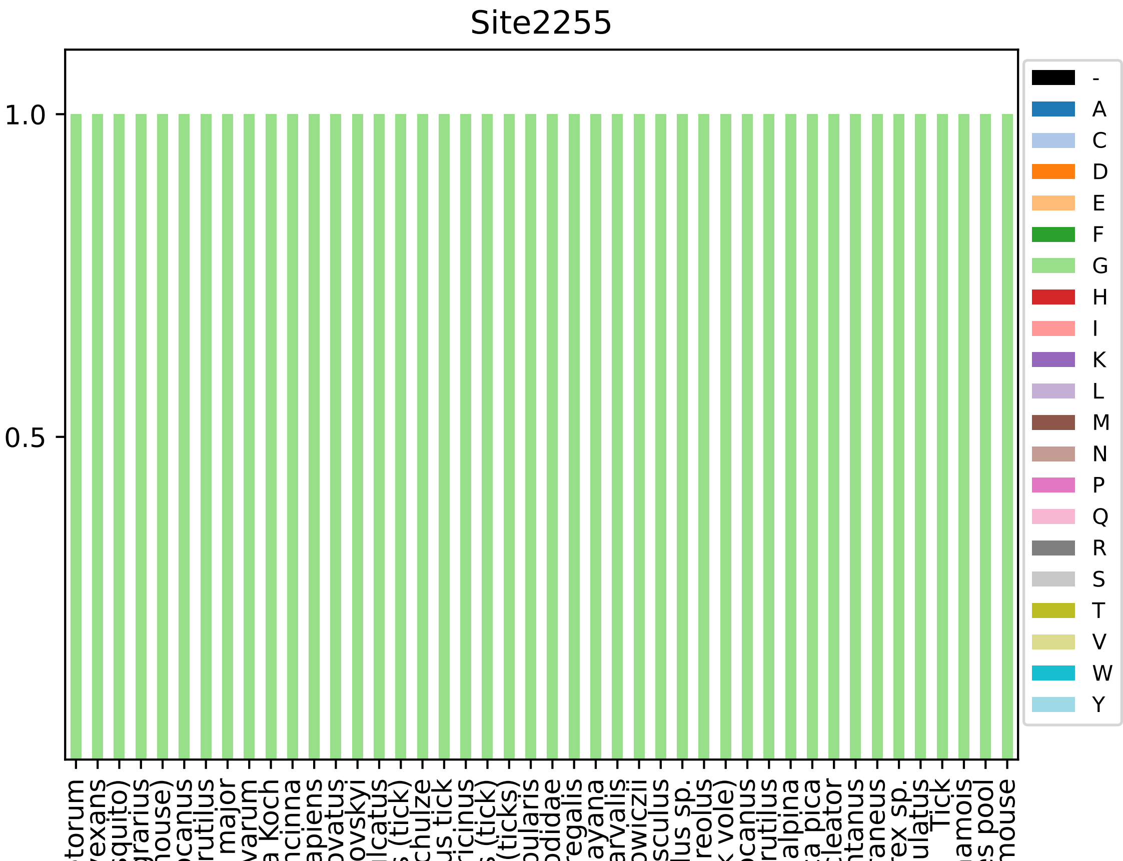
Site2255
1.0
0.5
-
A
C
D
E
F
G
H
I
K
L
M
N
P
Q
R
S
T
V
W
Y

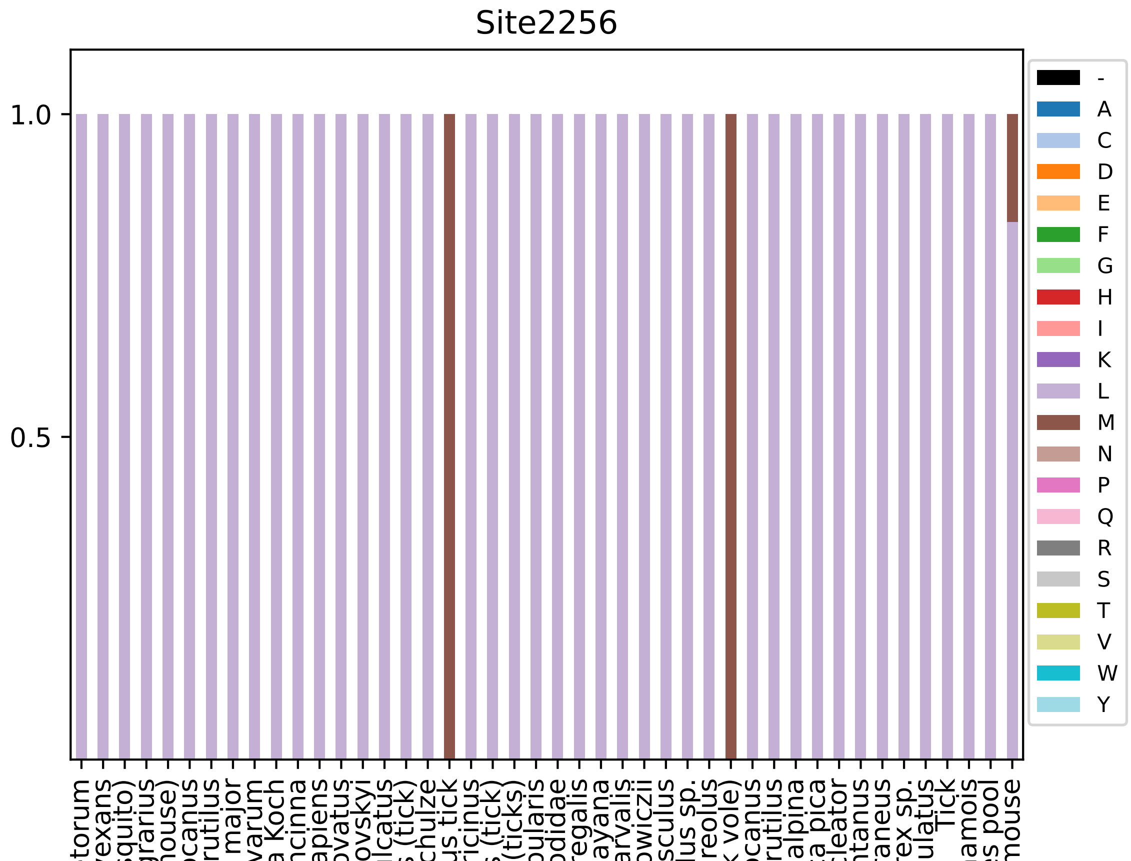
Site2256
1.0
0.5
-
A
C
D
E
F
G
H
I
K
L
M
N
P
Q
R
S
T
V
W
Y
Tick

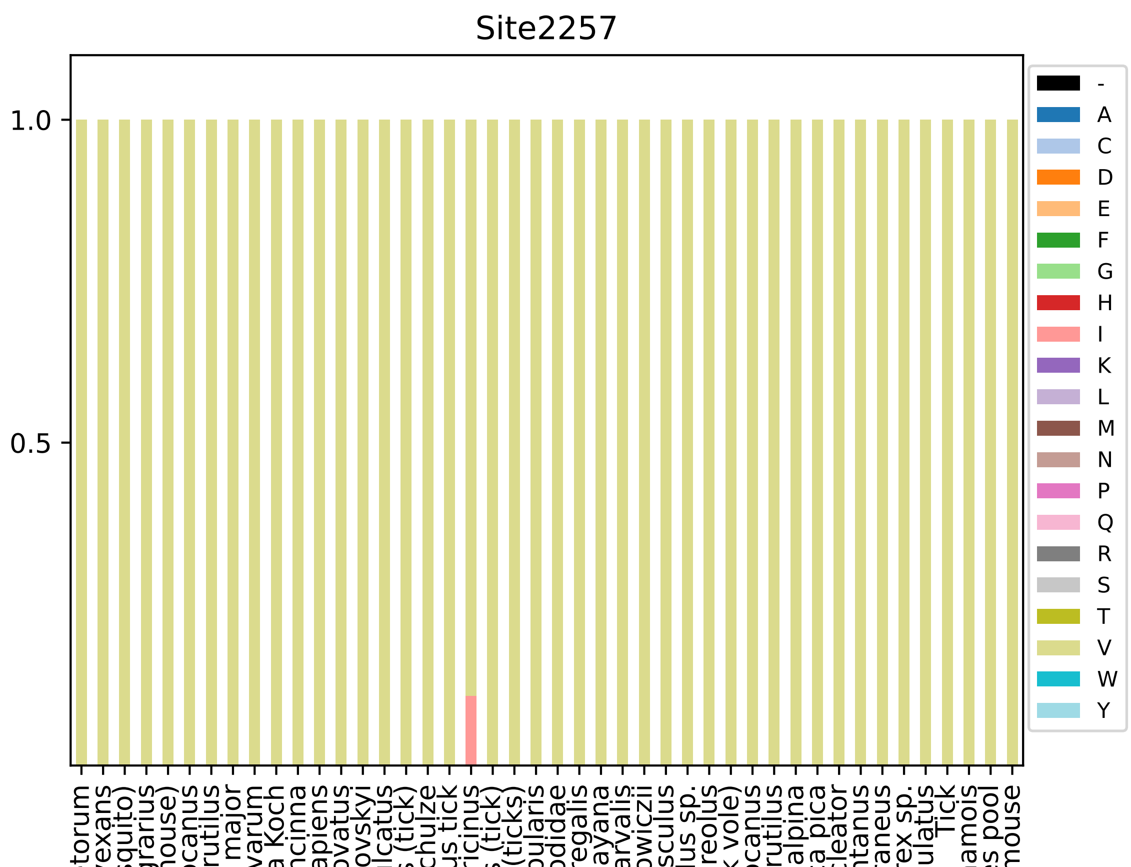
Site2257
1.0
0.5
-
A
C
D
E
F
G
H
I
K
L
M
N
P
Q
R
S
T
V
W
Y
torum
vexans
squito)
garius
house)
ocanus
rutilus
major
varum
a Koch
ncinna
apiens
ovatus
ovskyi
lcatus
s (tick)
chulze
us tick
ricinus
s (tick)
(ticks)
pularis
podidae
regalis
ayana
arvalis
owiczii
sculus
lus sp.
reolus
x vole)
ocanus
rutilus
alpina
a pica
cleator
ntanus
raneus
rex sp.
ulatus
Tick
amois
es pool
mouse

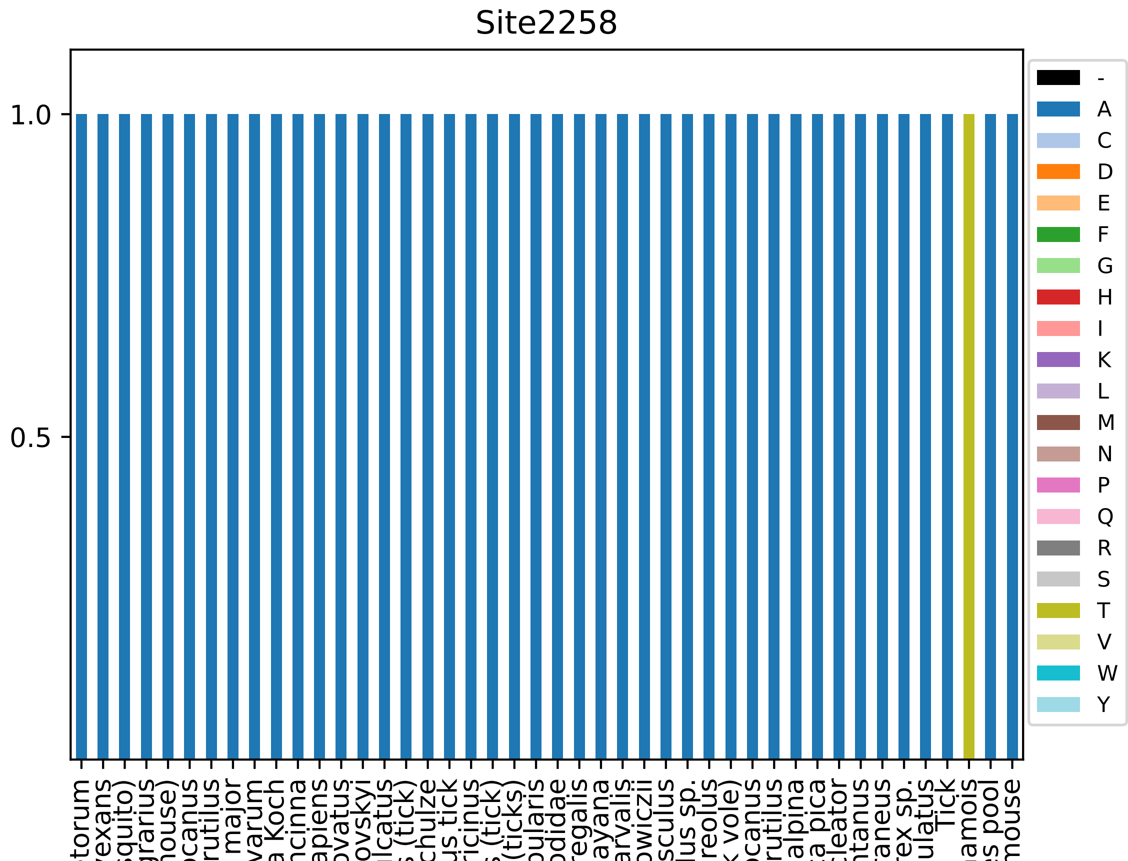
Site2258
1.0
0.5
-
A
C
D
E
F
G
H
I
K
L
M
N
P
Q
R
S
T
V
W
Y

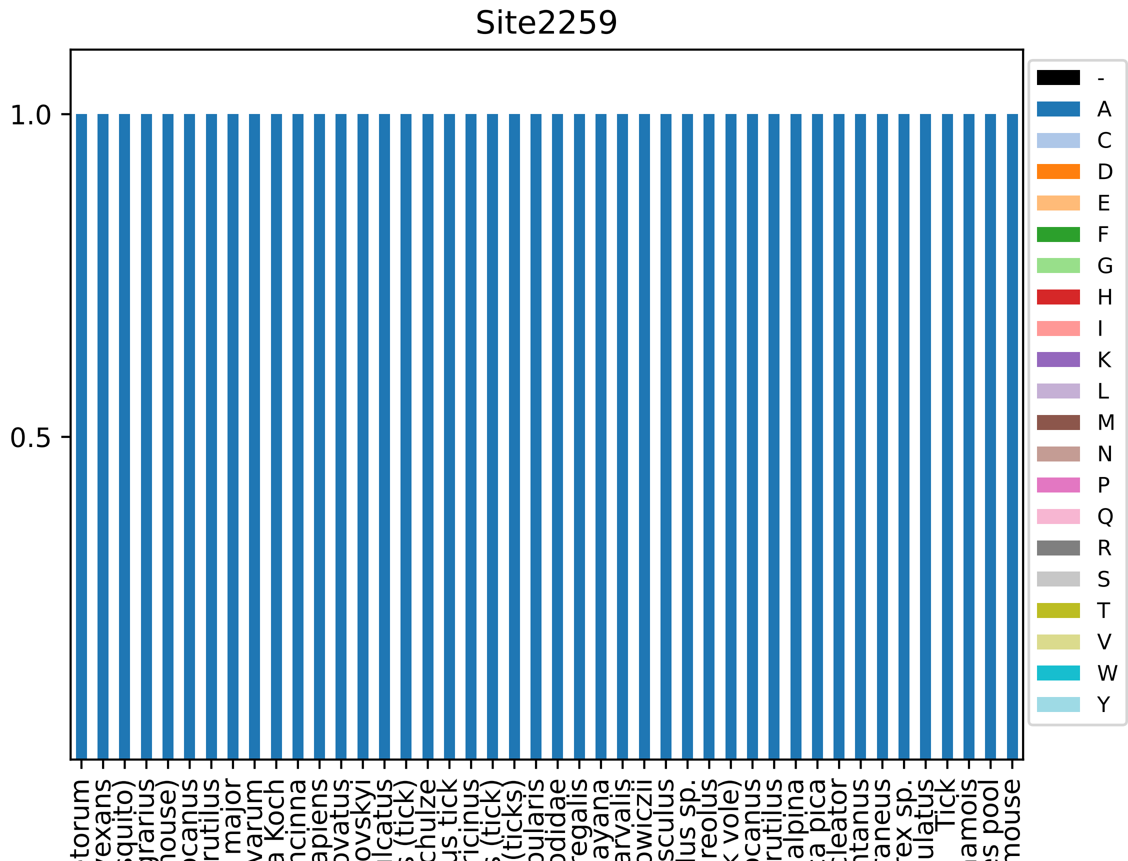
Site2259
1.0
0.5
-
A
C
D
E
F
G
H
I
K
L
M
N
P
Q
R
S
T
V
W
Y
Tick

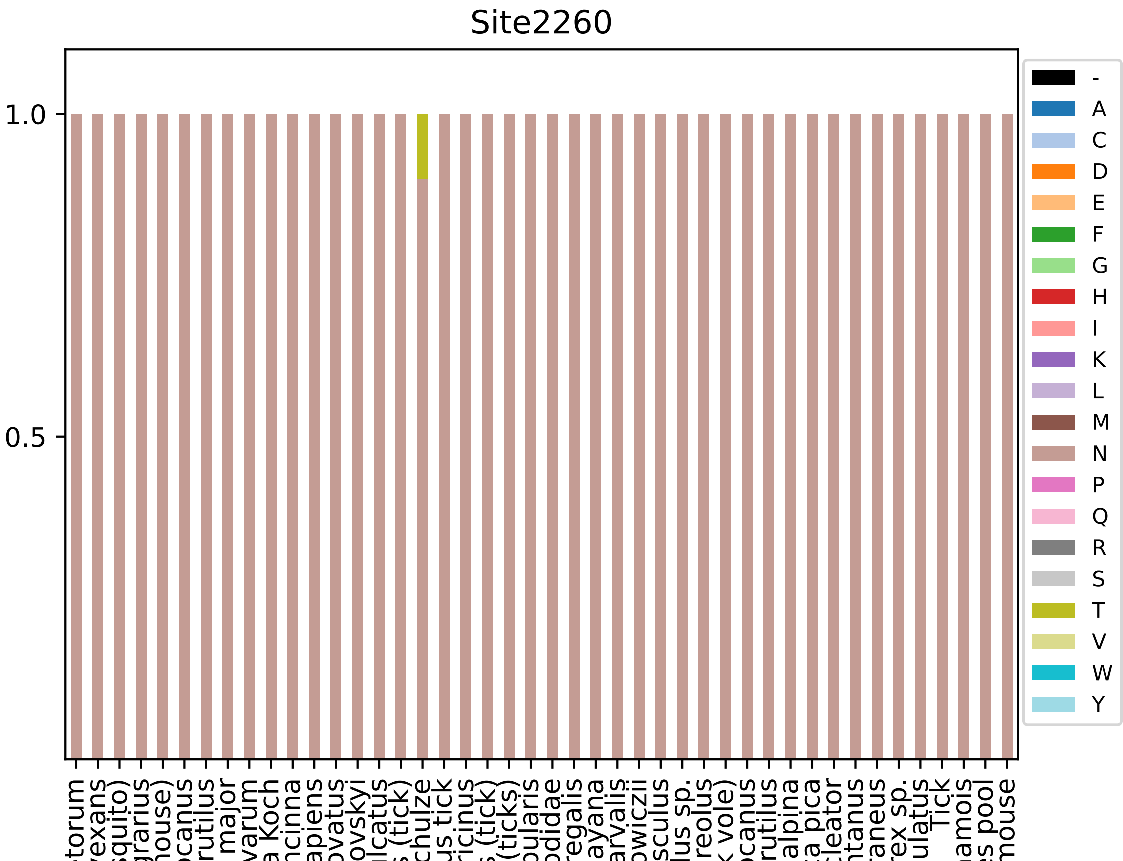
Site2260
1.0
0.5
-
A
C
D
E
F
G
H
I
K
L
M
N
P
Q
R
S
T
V
W
Y

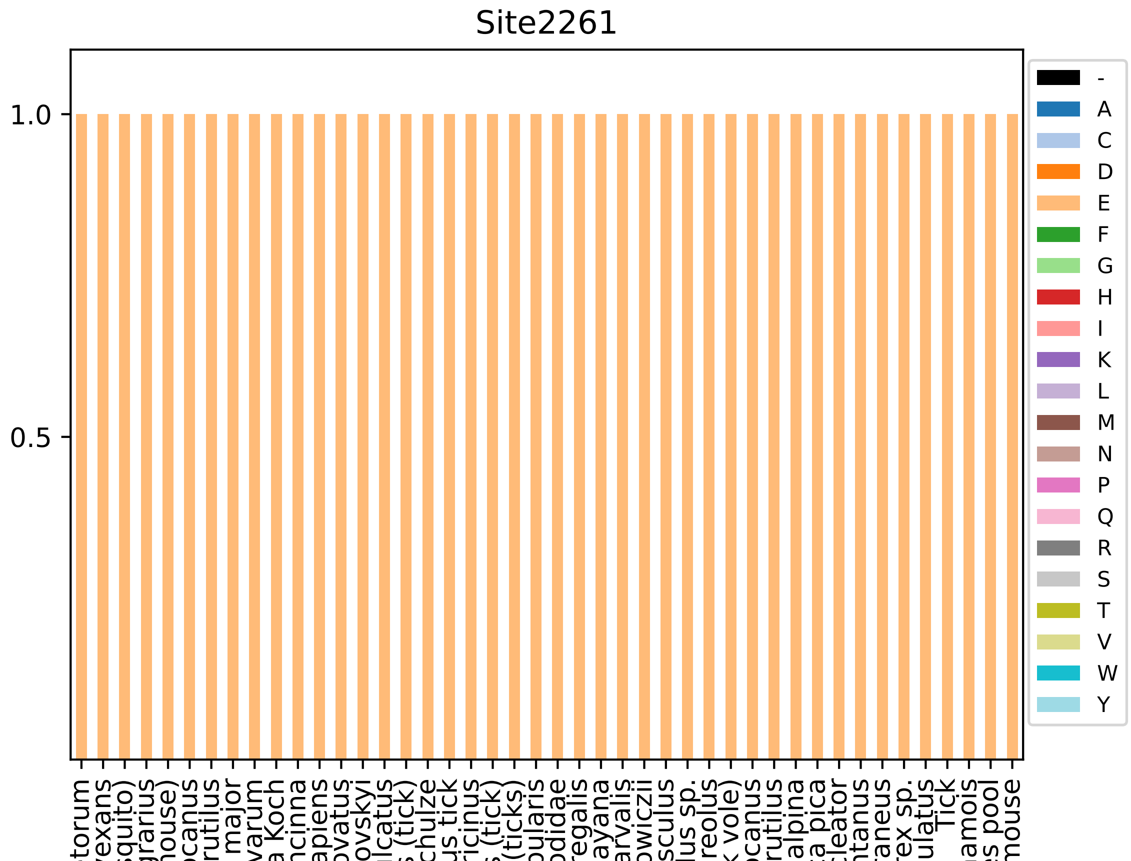
Site2261
1.0
0.5
-
A
C
D
E
F
G
H
I
K
L
M
N
P
Q
R
S
T
V
W
Y

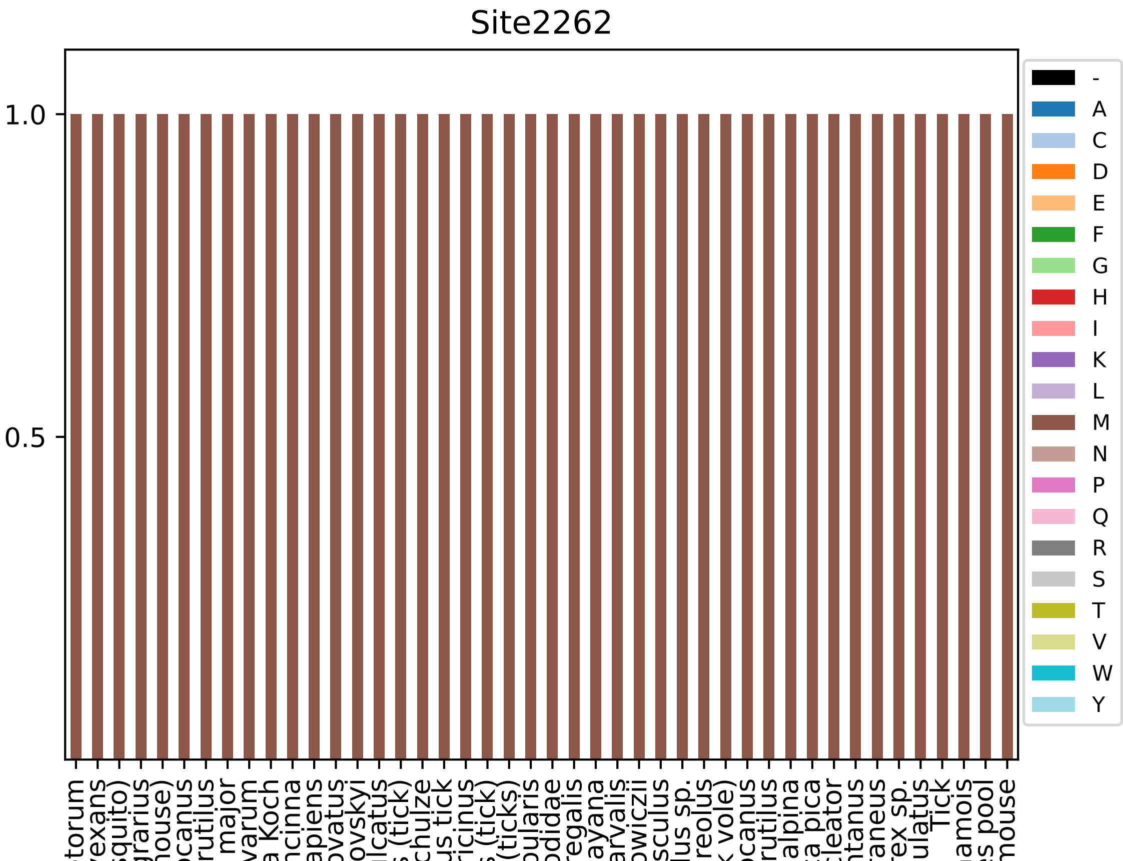
Site2262
1.0
0.5
-
A
C
D
E
F
G
H
I
K
L
M
N
P
Q
R
S
T
V
W
Y

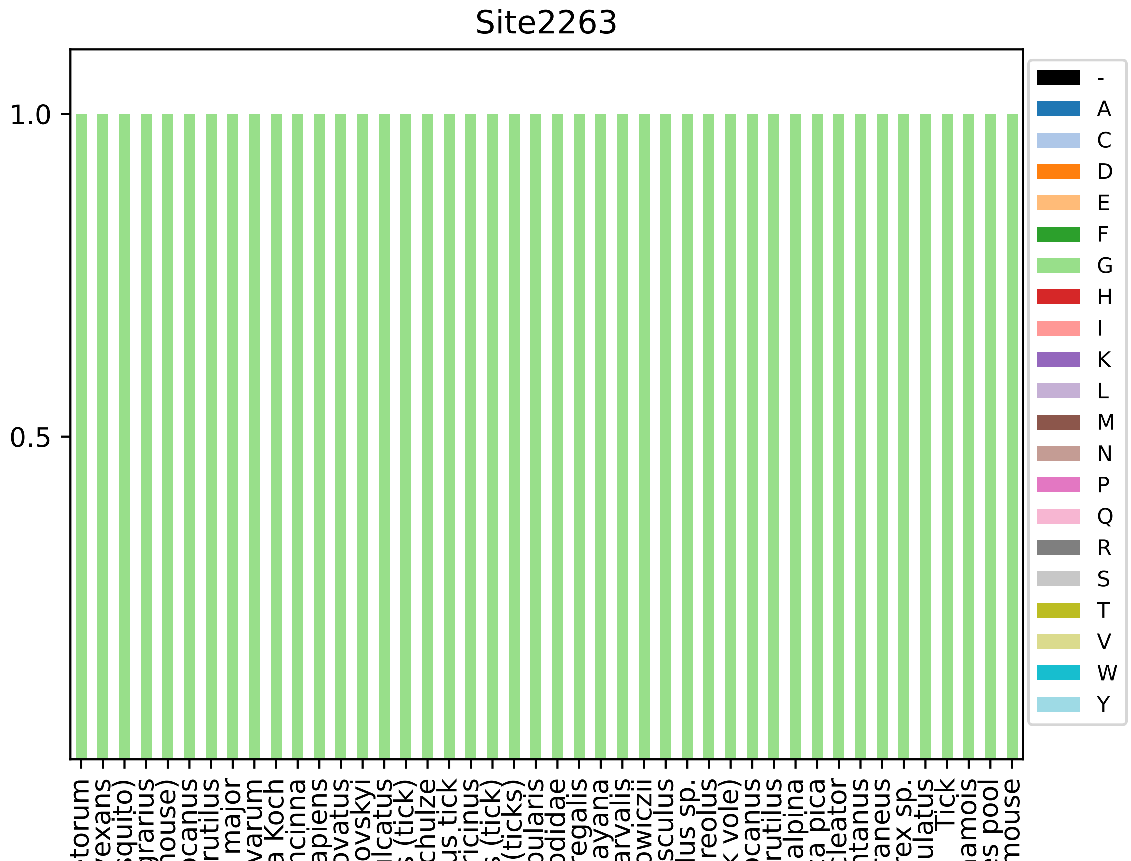

Site2263
1.0
0.5
-
A
C
D
E
F
G
H
I
K
L
M
N
P
Q
R
S
T
V
W
Y

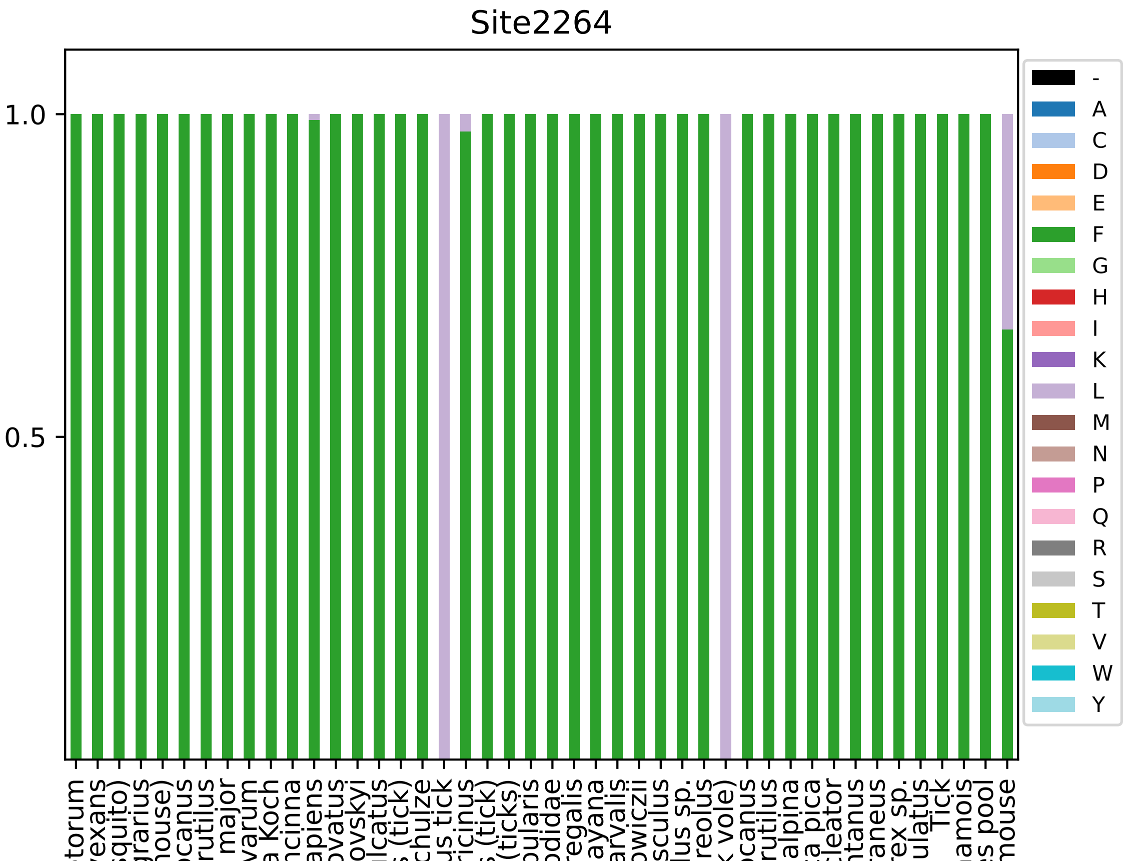
Site2264
1.0
0.5
-
A
C
D
E
F
G
H
I
K
L
M
N
P
Q
R
S
T
V
W
Y

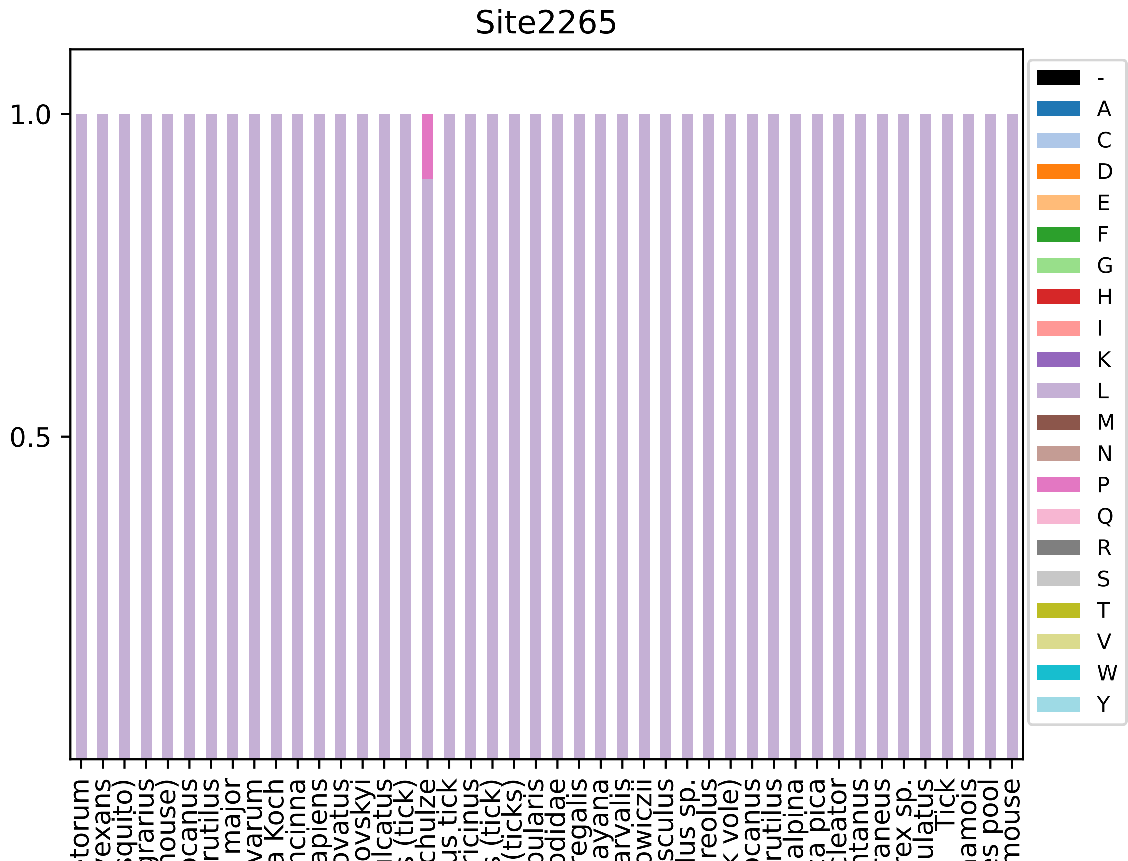
Site2265
1.0
0.5
-
A
C
D
E
F
G
H
I
K
L
M
N
P
Q
R
S
T
V
W
Y

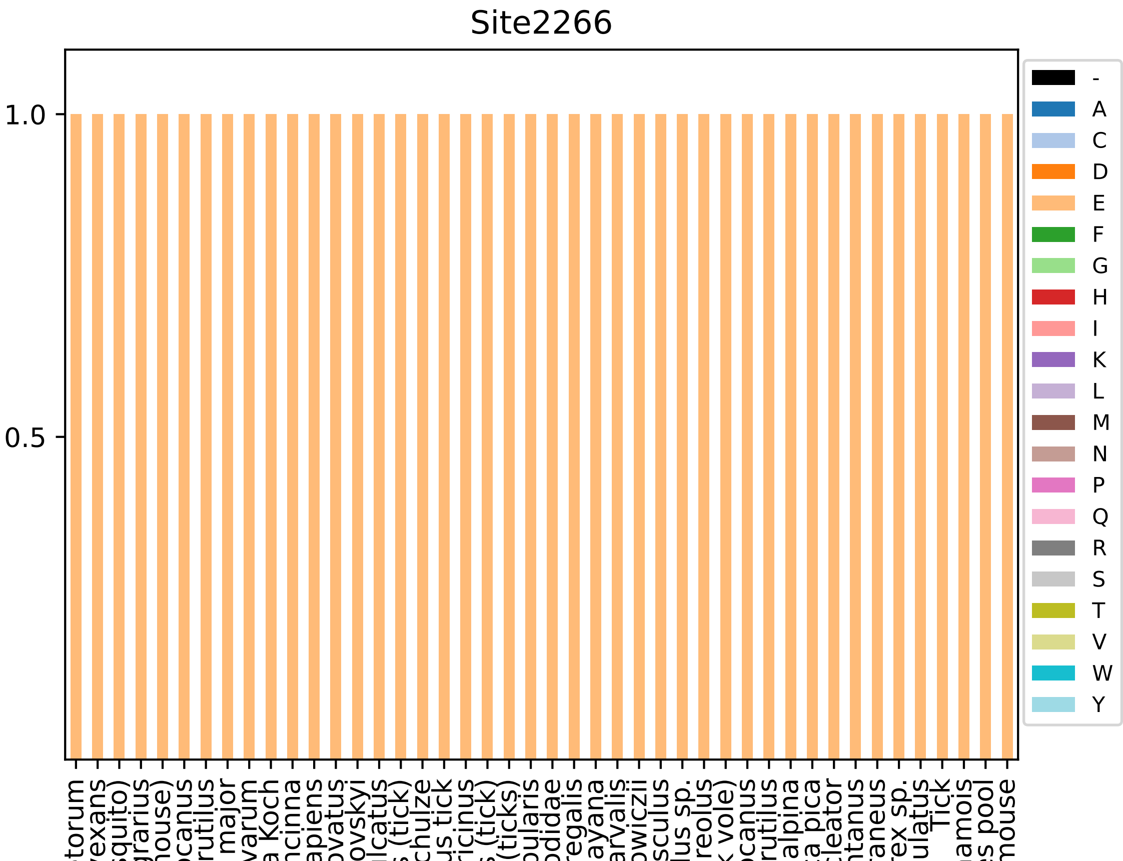
Site2266
1.0
0.5
-
A
C
D
E
F
G
H
I
K
L
M
N
P
Q
R
S
T
V
W
Y

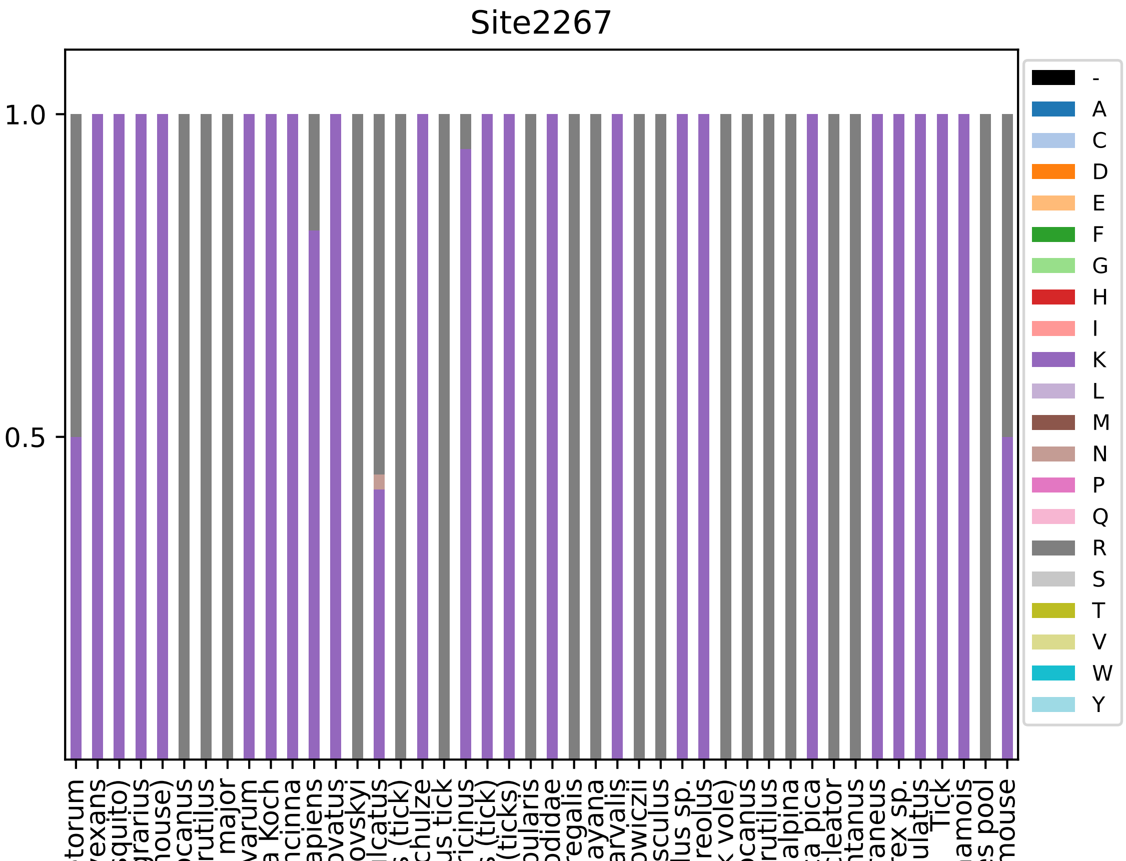
Site2267
1.0
0.5
-
A
C
D
E
F
G
H
I
K
L
M
N
P
Q
R
S
T
V
W
Y

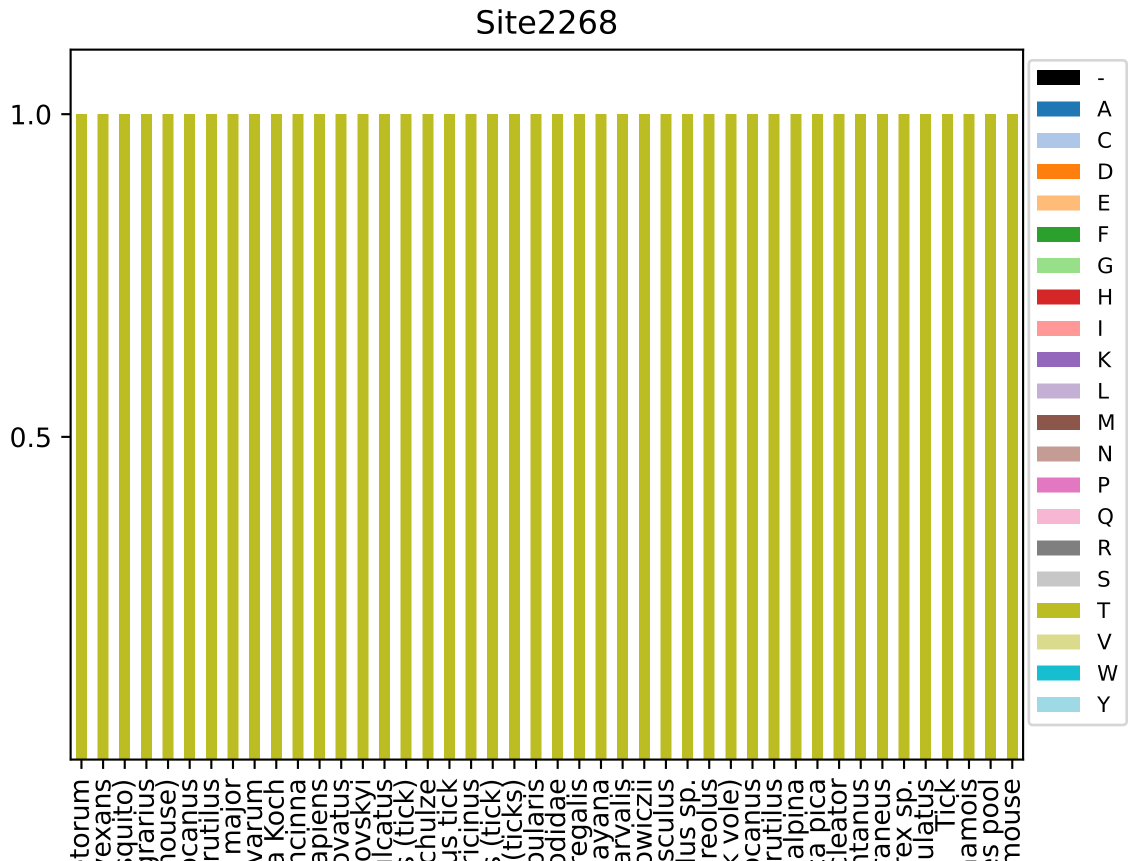
Site2268
1.0
0.5
-
A
C
D
E
F
G
H
I
K
L
M
N
P
Q
R
S
T
V
W
Y
torum
vexans
squito)
grarius
nouse)
ocanus
rutilus
major
varum
a Koch
ncinna
apiens
ovatus
ovskyi
ulcatus
s (tick)
chulze
us tick
ricinus
s (tick)
(ticks)
pularis
odidae
regalis
ayana
arvalis
owiczii
sculus
lus sp.
reolus
x vole)
ocanus
rutilus
alpina
a pica
cleator
ntanus
raneus
rex sp.
ulatus
Tick
amois
es pool
mouse

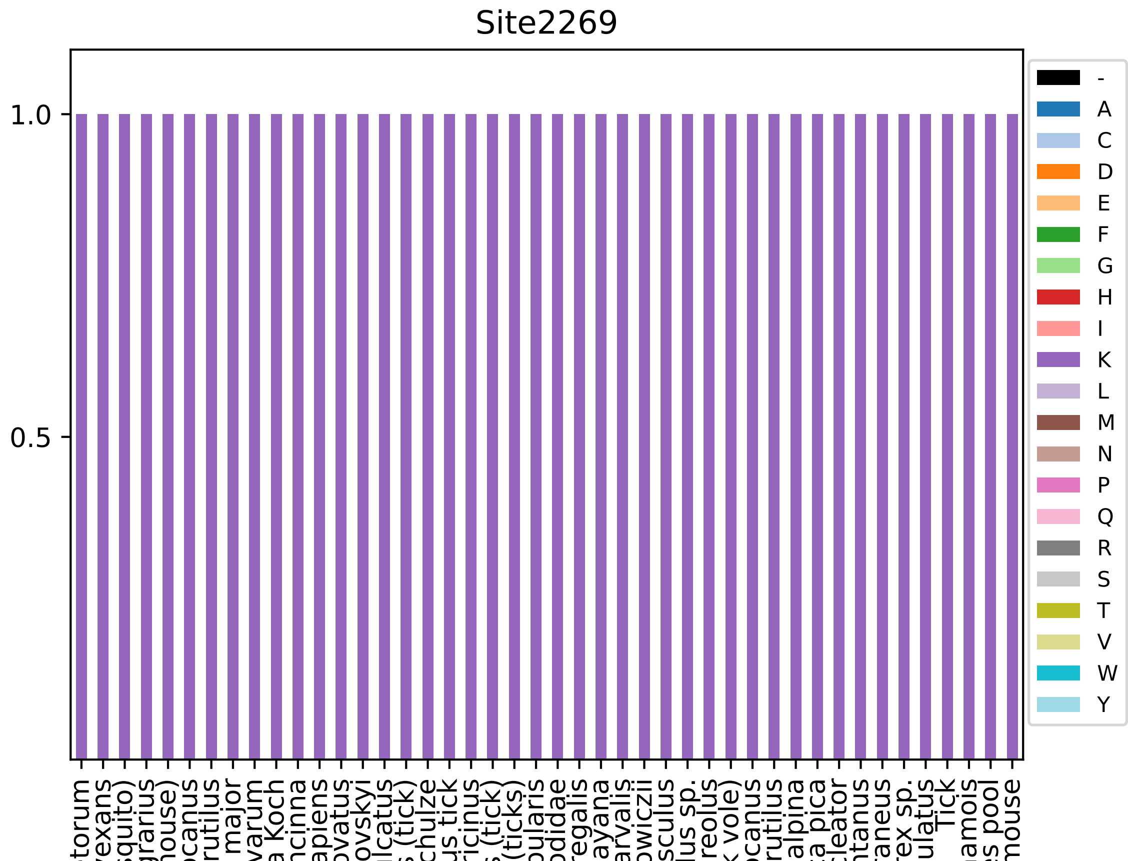

Site2269
1.0
0.5
-
A
C
D
E
F
G
H
I
K
L
M
N
P
Q
R
S
T
V
W
Y
torum
exans
squito)
garius
house)
ocanus
rutilus
major
varum
a Koch
ncinna
apiens
ovatus
ovskyi
lcatus
(tick)
chulze
us tick
ricinus
(tick)
(ticks)
pularis
podidae
regalis
ayana
arvalis
owiczii
sculus
lus sp.
reolus
k vole)
ocanus
rutilus
alpina
a pica
cleator
ntanus
aneus
ex sp.
ulatus
Tick
amois
es pool
mouse

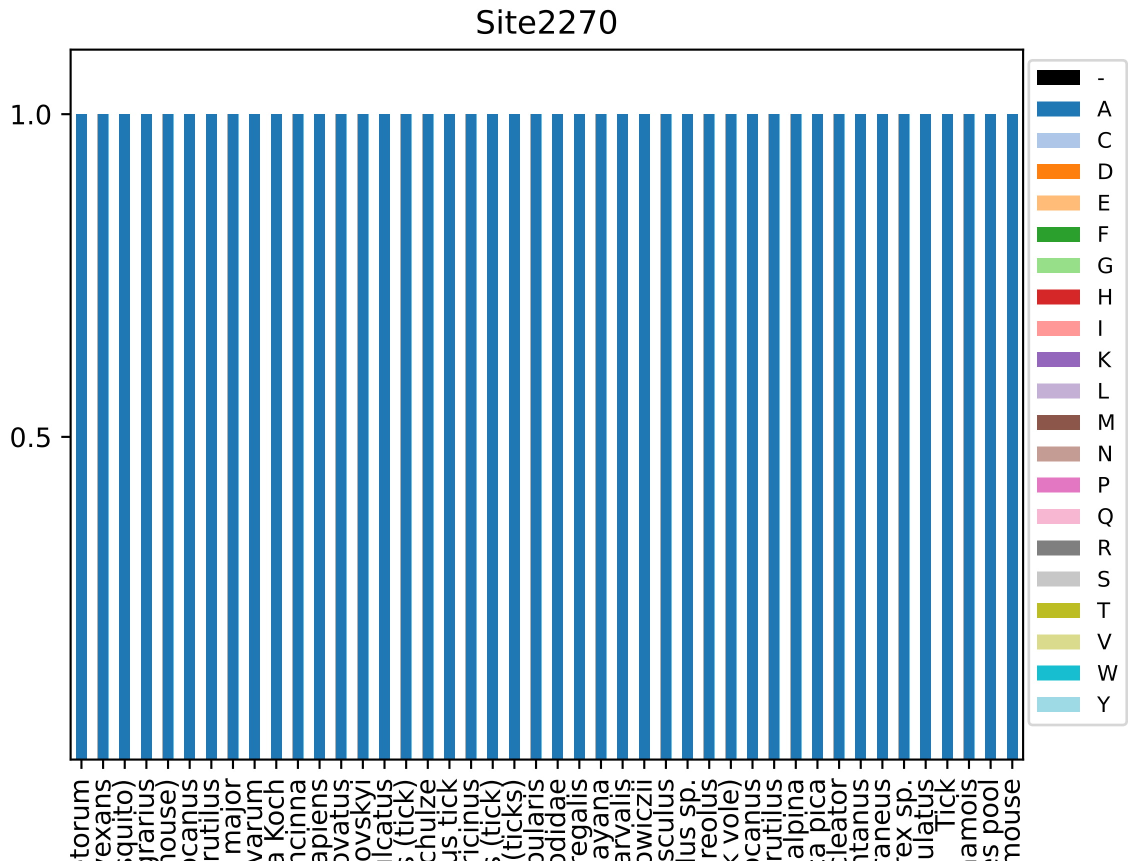

Site2270
1.0
0.5
-
A
C
D
E
F
G
H
I
K
L
M
N
P
Q
R
S
T
V
W
Y

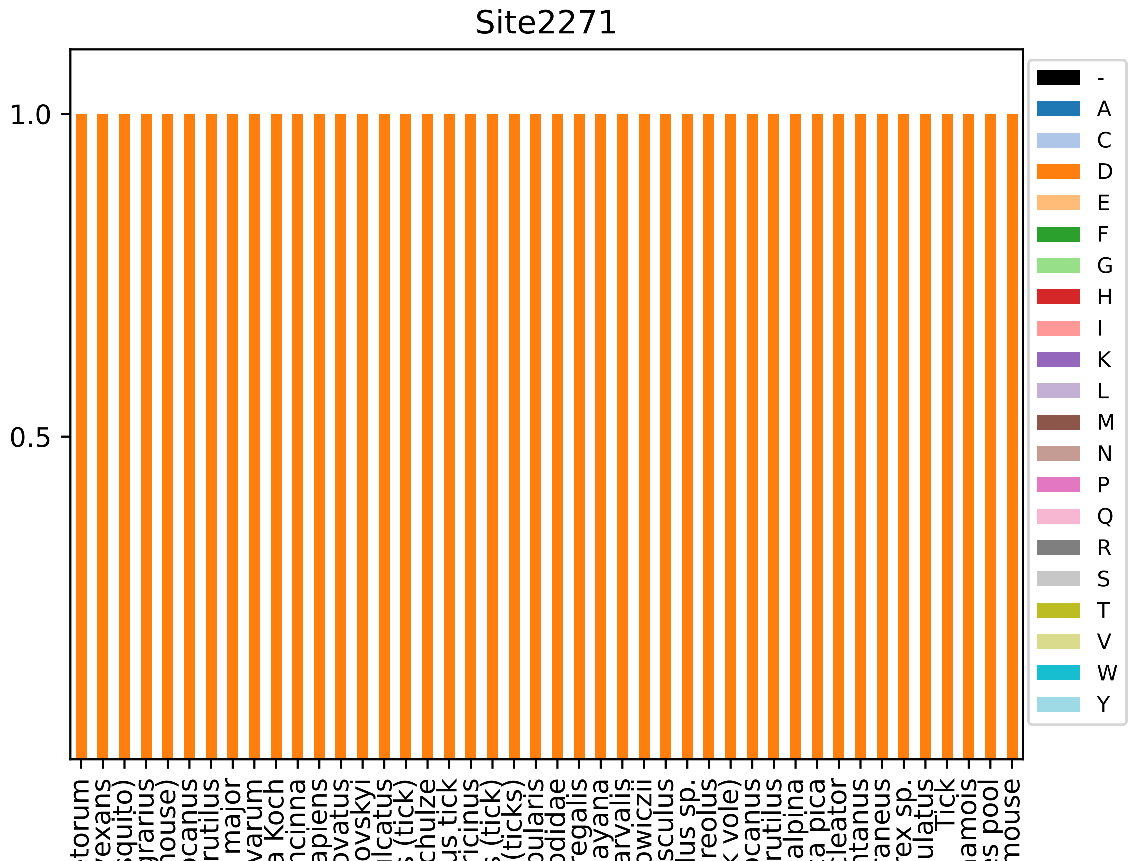
Site2271
1.0
0.5
-
A
C
D
E
F
G
H
I
K
L
M
N
P
Q
R
S
T
V
W
Y

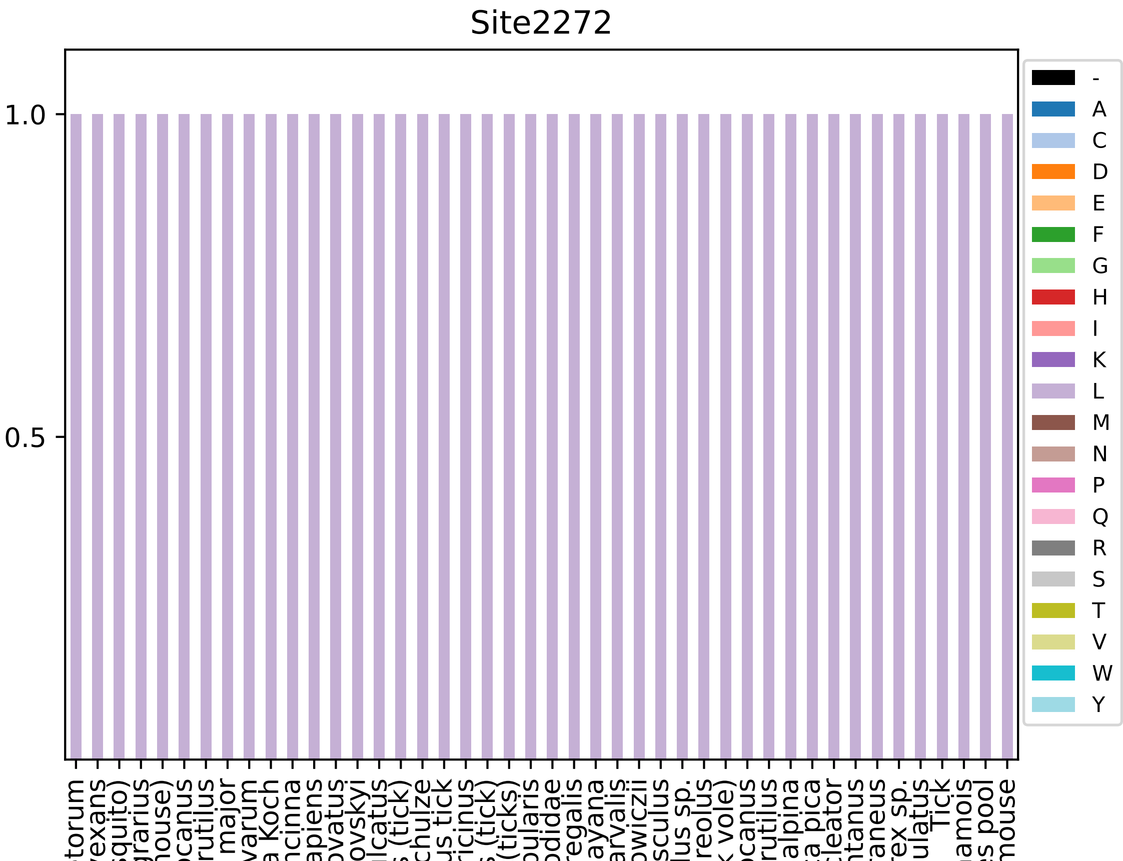

Site2272
1.0
0.5
-
A
C
D
E
F
G
H
I
K
L
M
N
P
Q
R
S
T
V
W
Y
Tick

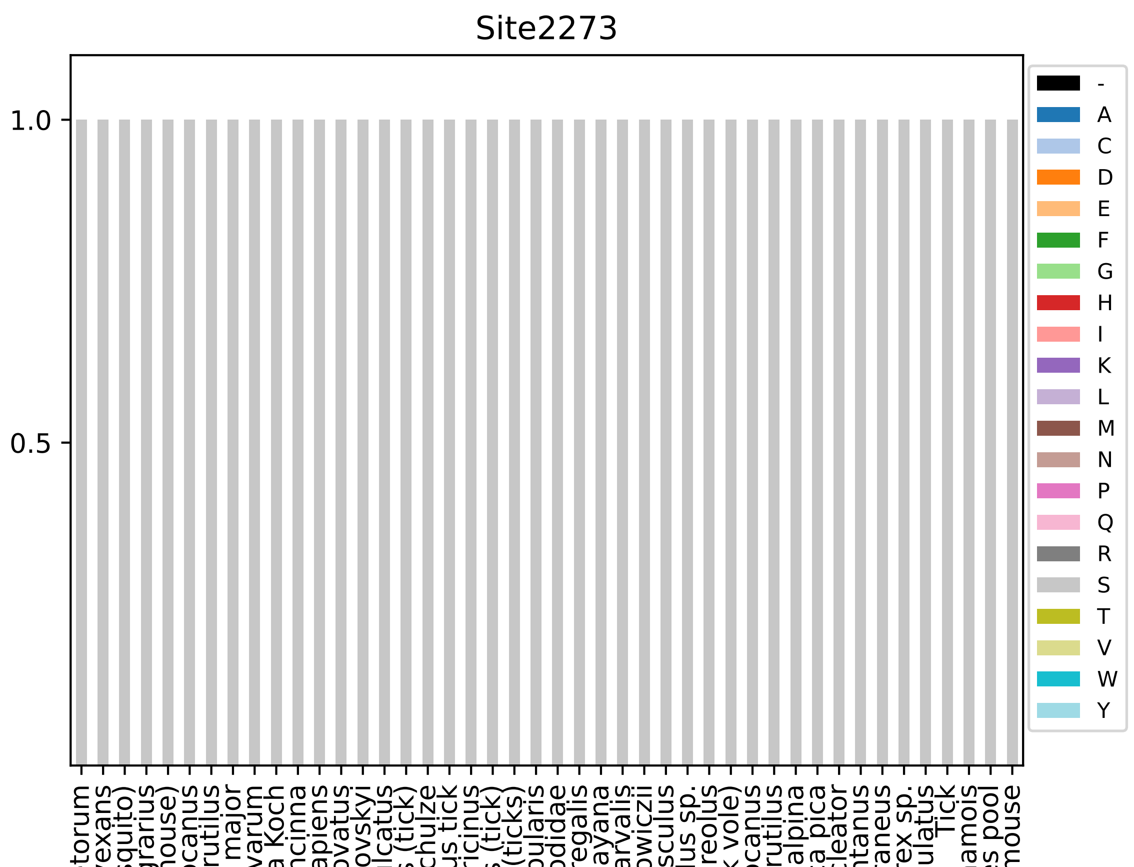
Site2273
1.0
0.5
-
A
C
D
E
F
G
H
I
K
L
M
N
P
Q
R
S
T
V
W
Y

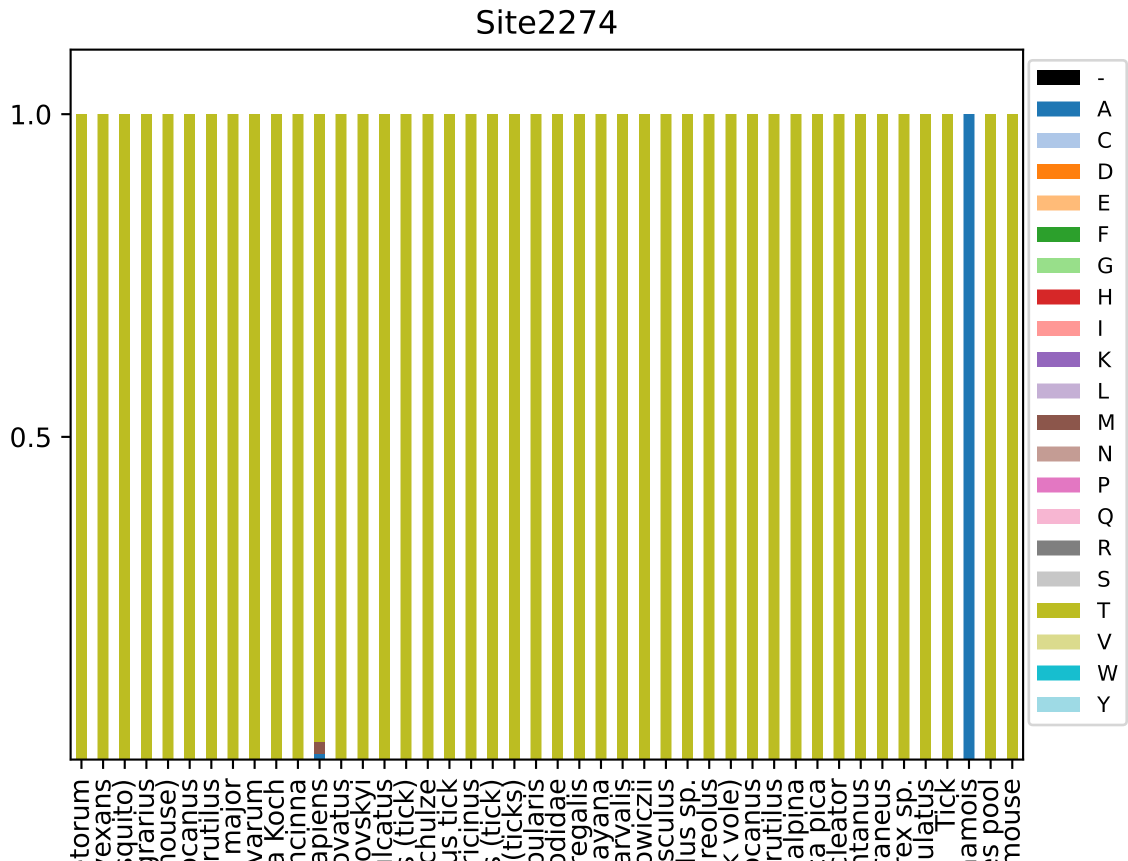
Site2274
1.0
0.5
-
A
C
D
E
F
G
H
I
K
L
M
N
P
Q
R
S
T
V
W
Y
Tick

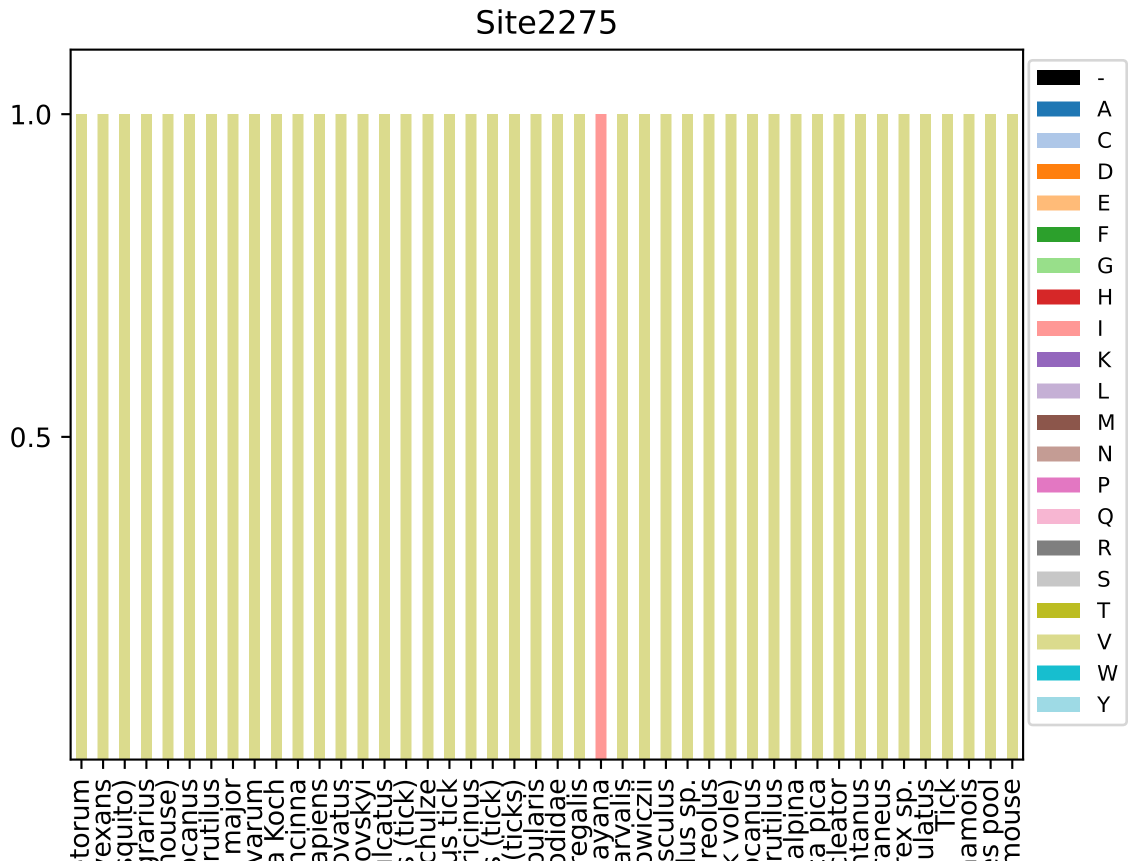
Site2275
1.0
0.5
-
A
C
D
E
F
G
H
I
K
L
M
N
P
Q
R
S
T
V
W
Y

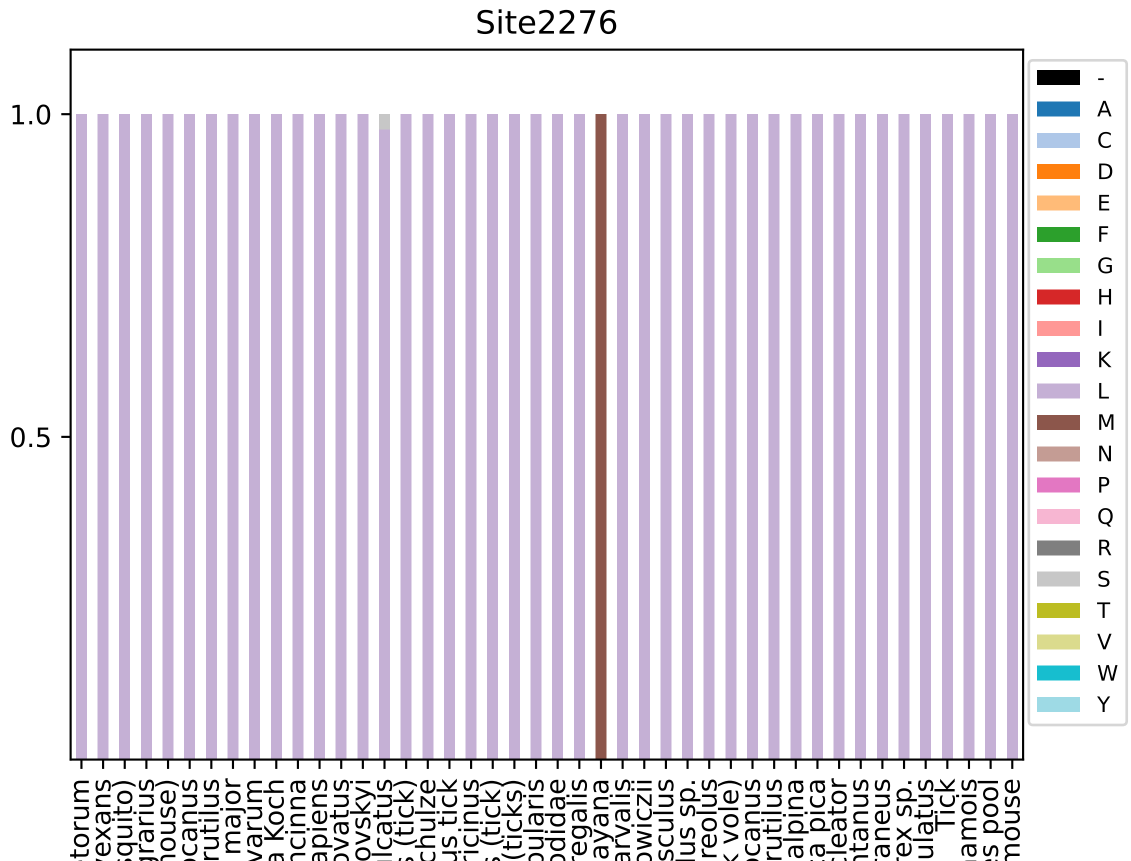
Site2276
1.0
0.5
-
A
C
D
E
F
G
H
I
K
L
M
N
P
Q
R
S
T
V
W
Y

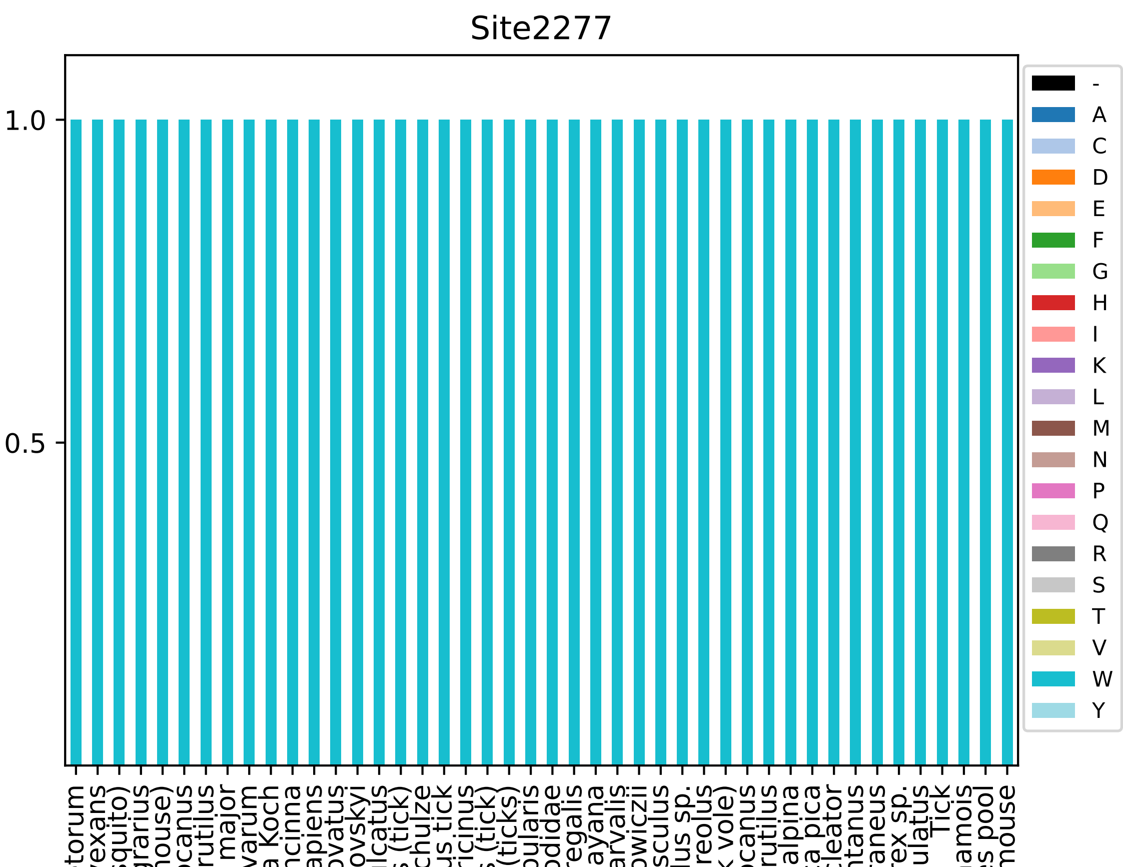
Site2277
1.0
0.5
-
A
C
D
E
F
G
H
I
K
L
M
N
P
Q
R
S
T
V
W
Y

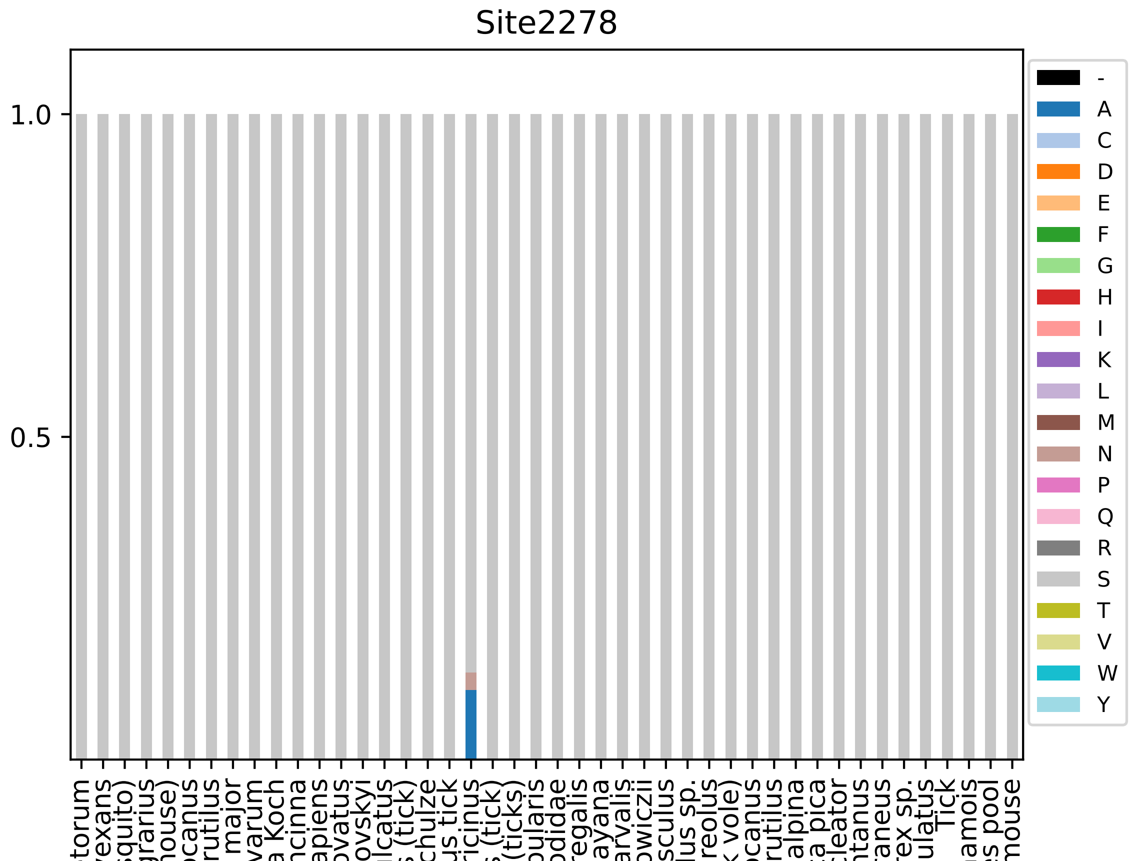

Site2278
1.0
0.5
-
A
C
D
E
F
G
H
I
K
L
M
N
P
Q
R
S
T
V
W
Y

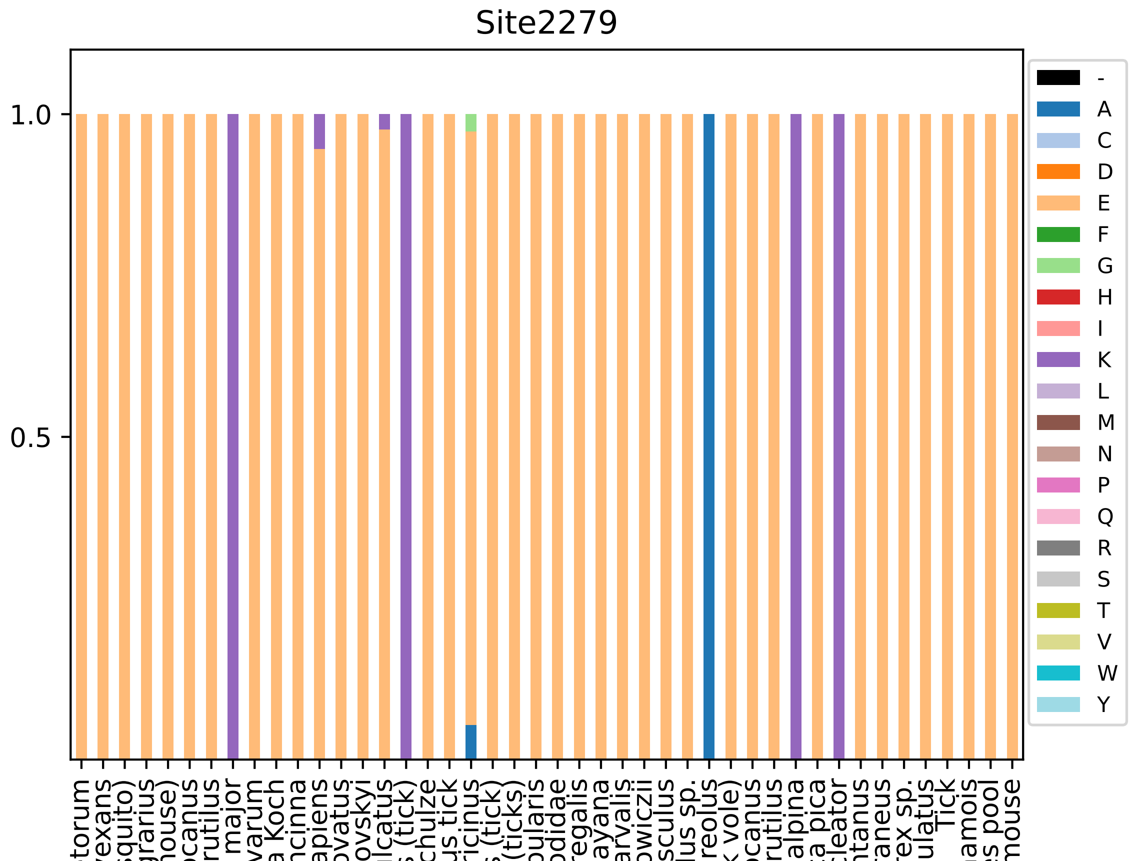
Site2279
1.0
0.5
-
A
C
D
E
F
G
H
I
K
L
M
N
P
Q
R
S
T
V
W
Y

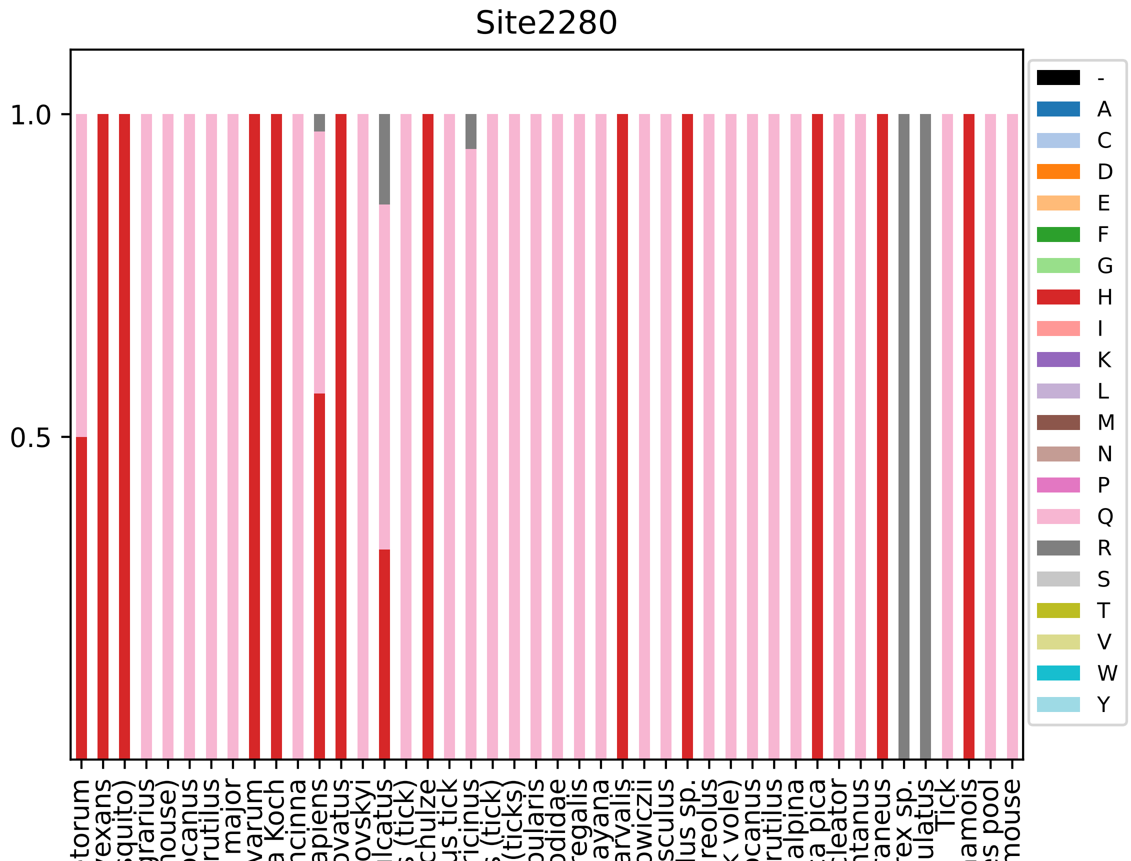
Site2280
1.0
0.5
-
A
C
D
E
F
G
H
I
K
L
M
N
P
Q
R
S
T
V
W
Y
torum
vexans
squito)
grarius
house)
ocanus
rutilus
major
varum
a Koch
ncinna
apiens
ovatus
ovskyi
lcatus
(tick)
chulze
us tick
ricinus
(tick)
(ticks)
pularis
odidae
regalis
ayana
arvalis
owiczii
sculus
lus sp.
reolus
k vole)
ocanus
rutilus
alpina
a pica
leator
ntanus
aneus
ex sp.
ulatus
Tick
amois
es pool
mouse

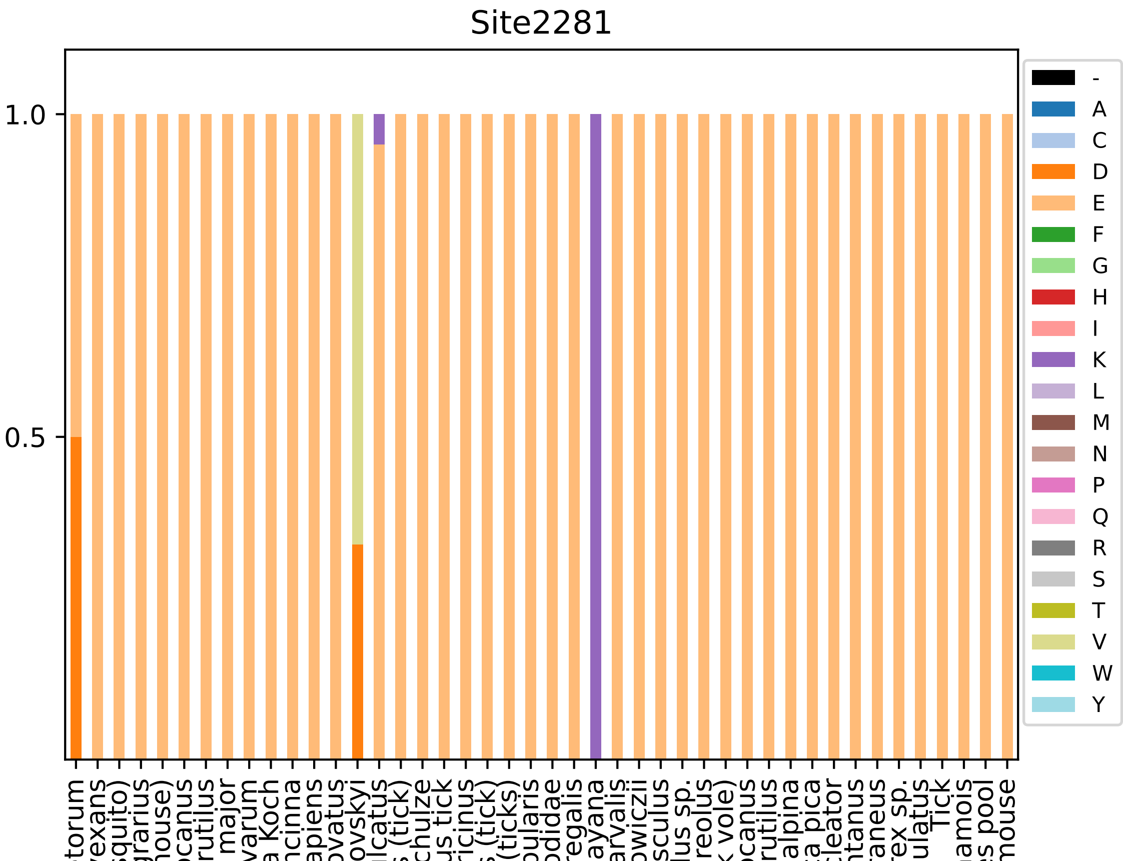
Site2281
1.0
0.5
-
A
C
D
E
F
G
H
I
K
L
M
N
P
Q
R
S
T
V
W
Y

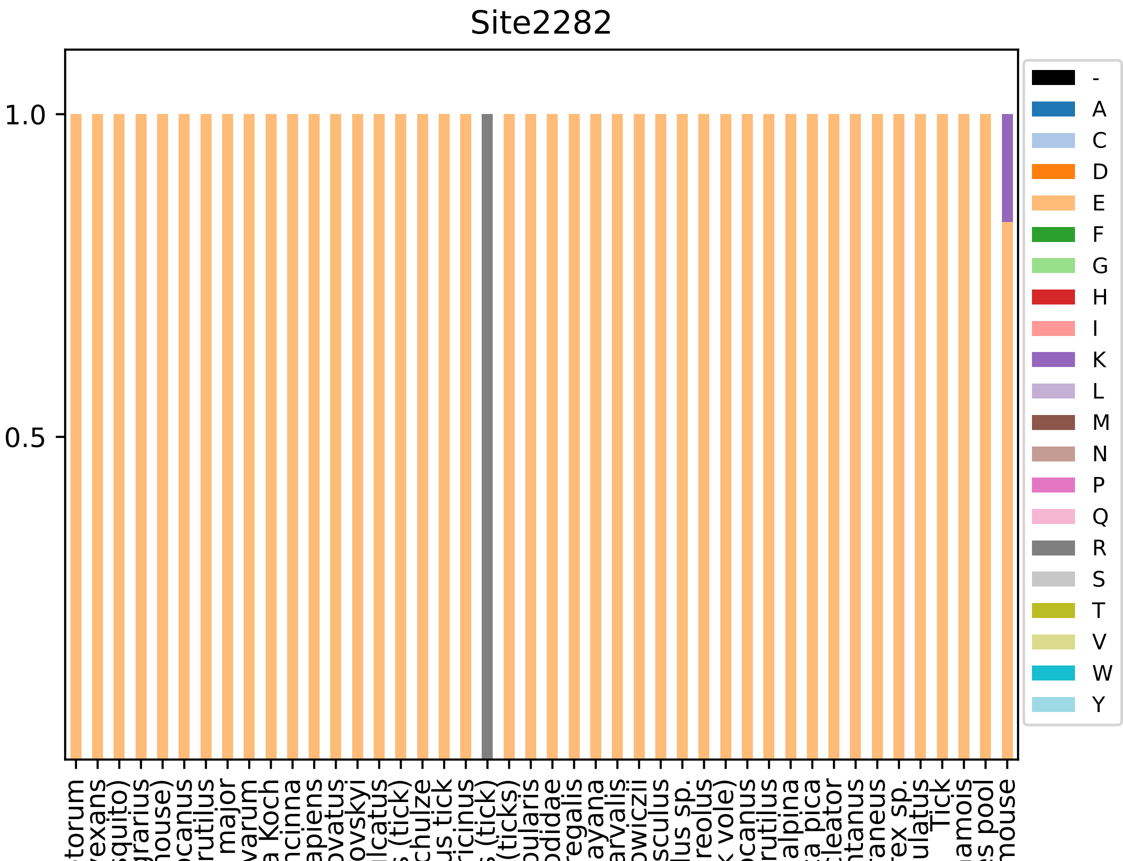
Site2282
1.0
0.5
-
A
C
D
E
F
G
H
I
K
L
M
N
P
Q
R
S
T
V
W
Y

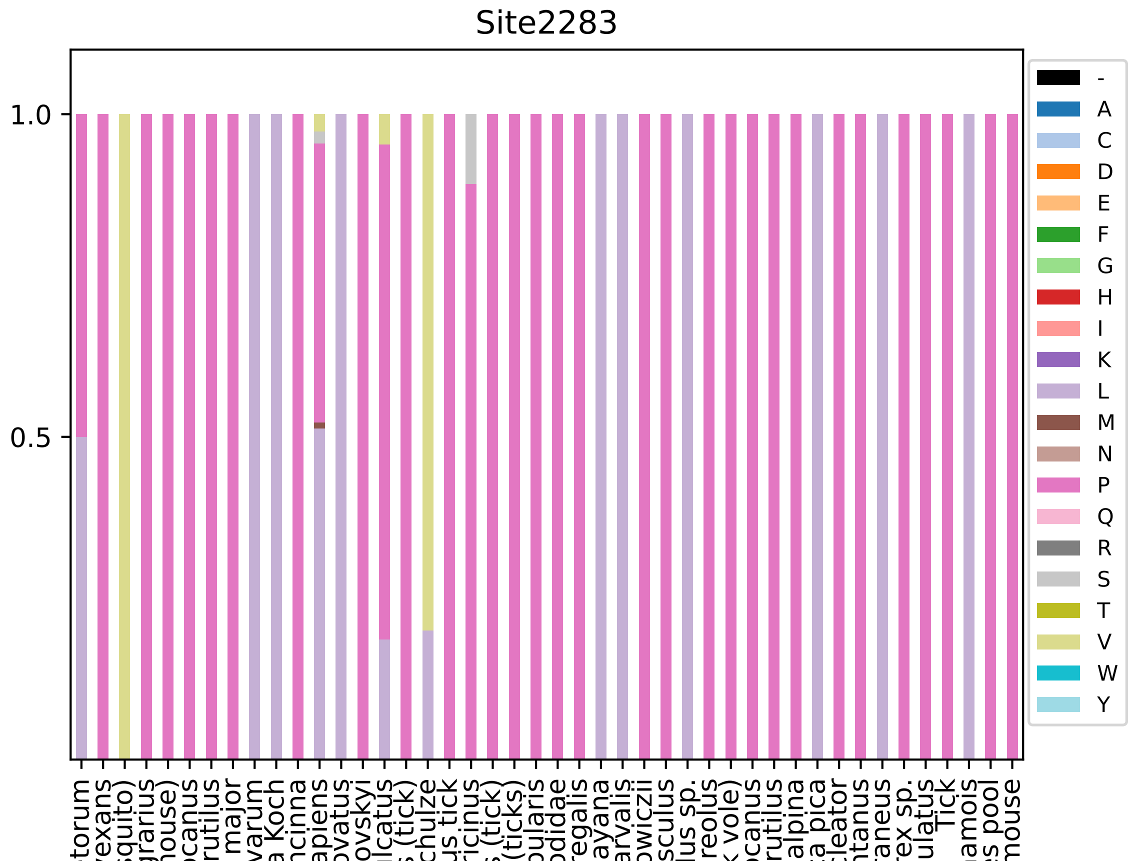
Site2283
1.0
0.5
-
A
C
D
E
F
G
H
I
K
L
M
N
P
Q
R
S
T
V
W
Y

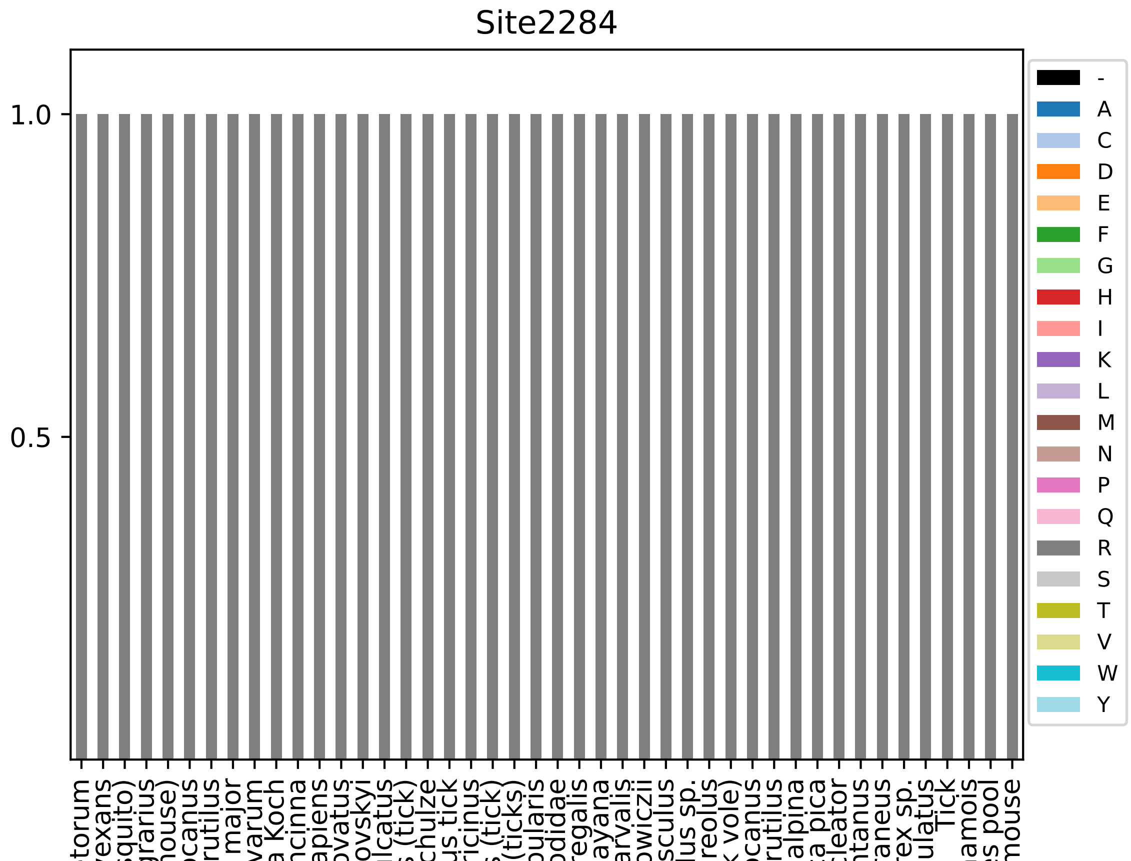

Site2284
1.0
0.5
-
A
C
D
E
F
G
H
I
K
L
M
N
P
Q
R
S
T
V
W
Y

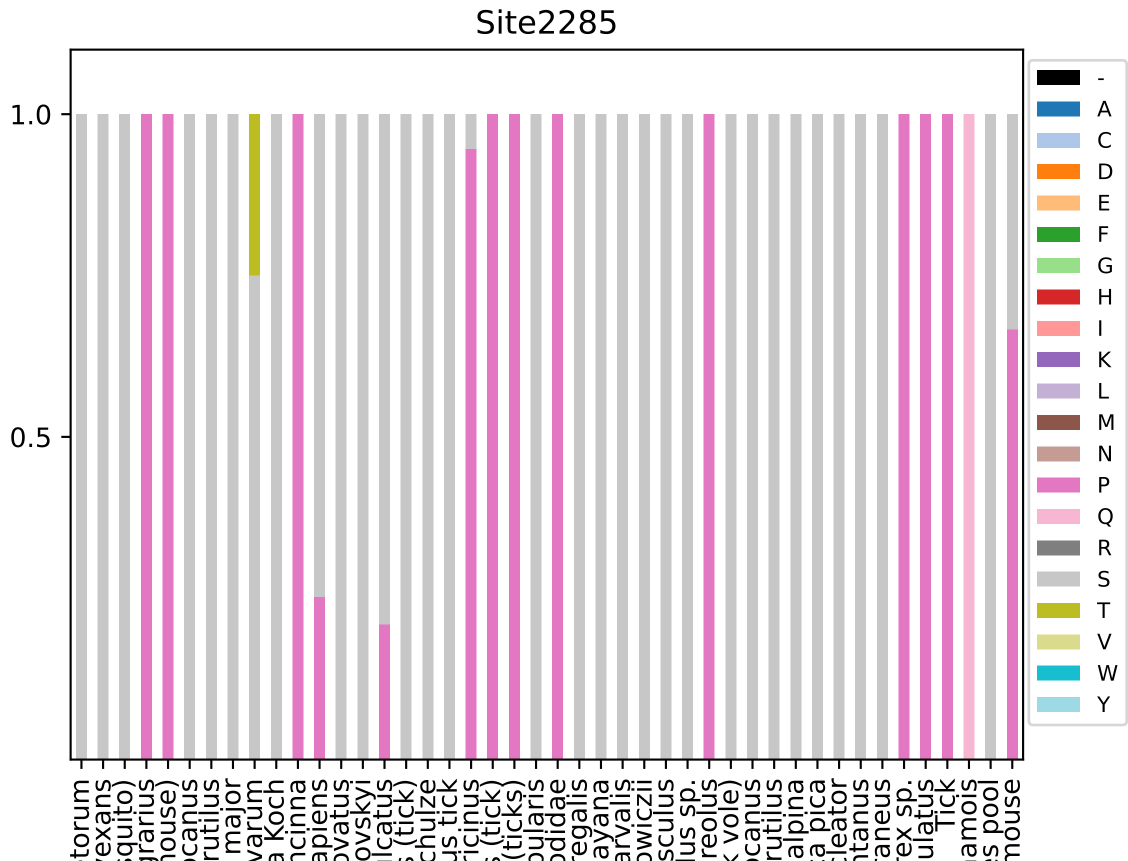
Site2285
1.0
0.5
-
A
C
D
E
F
G
H
I
K
L
M
N
P
Q
R
S
T
V
W
Y

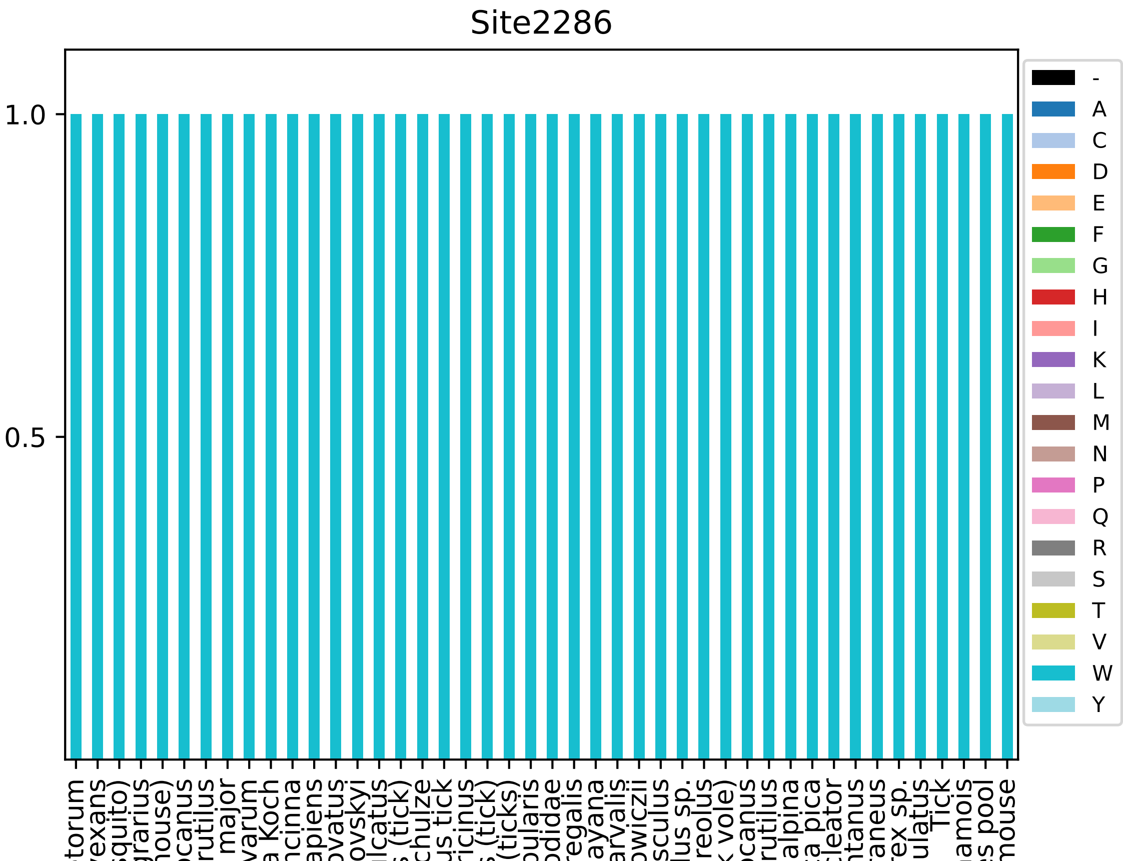

Site2286
1.0
0.5
-
A
C
D
E
F
G
H
I
K
L
M
N
P
Q
R
S
T
V
W
Y

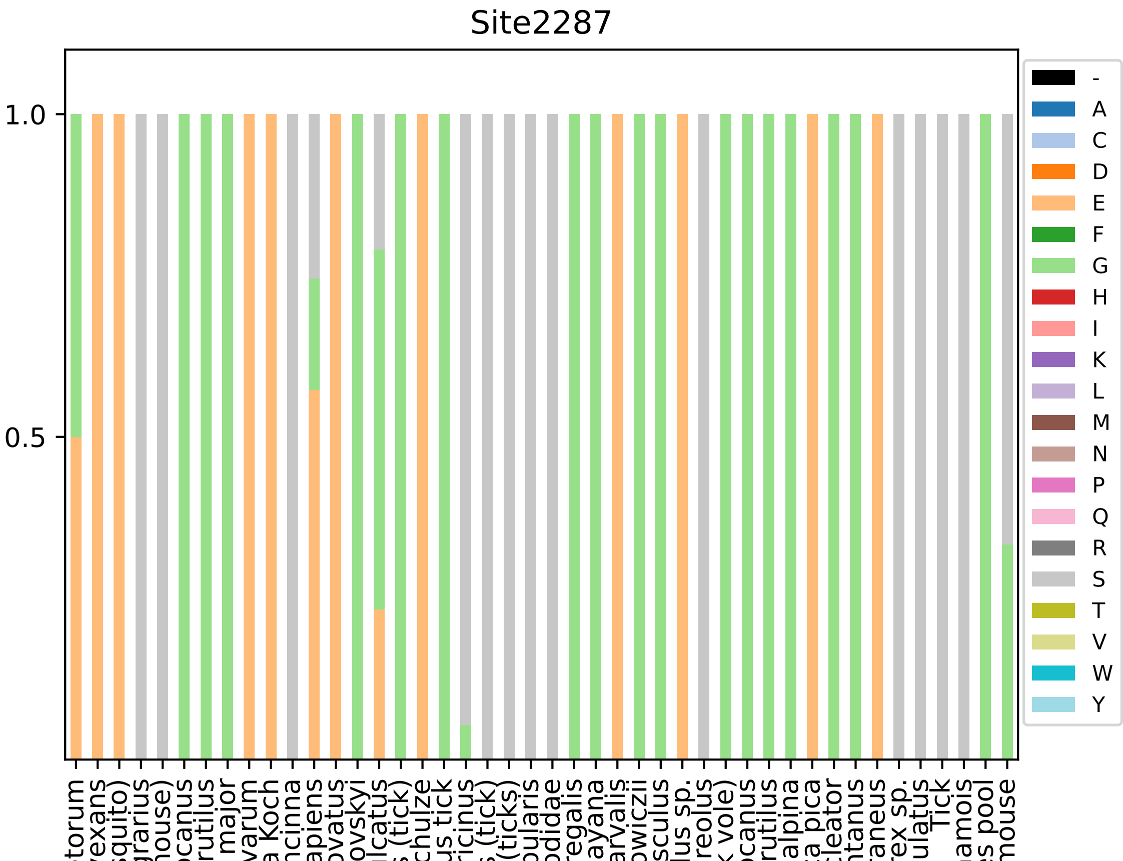
Site2287
1.0
0.5
-
A
C
D
E
F
G
H
I
K
L
M
N
P
Q
R
S
T
V
W
Y

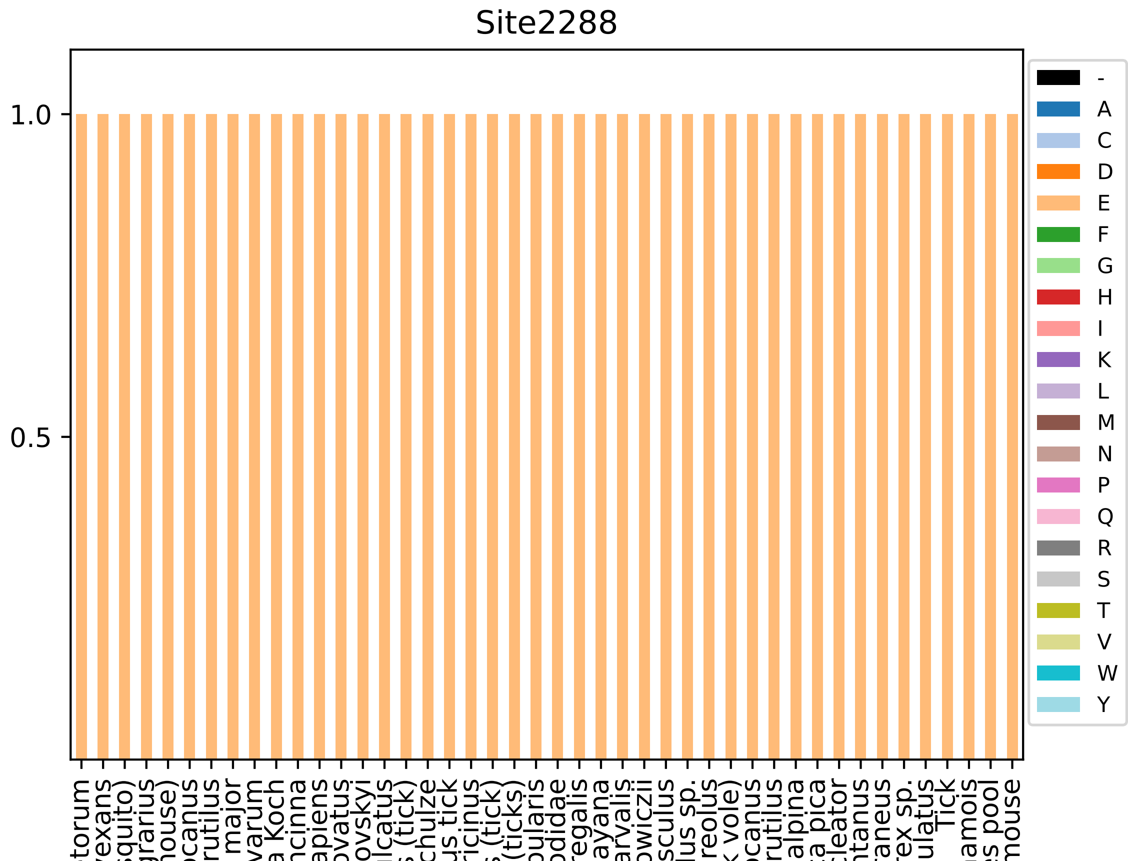
Site2288
1.0
0.5
-
A
C
D
E
F
G
H
I
K
L
M
N
P
Q
R
S
T
V
W
Y

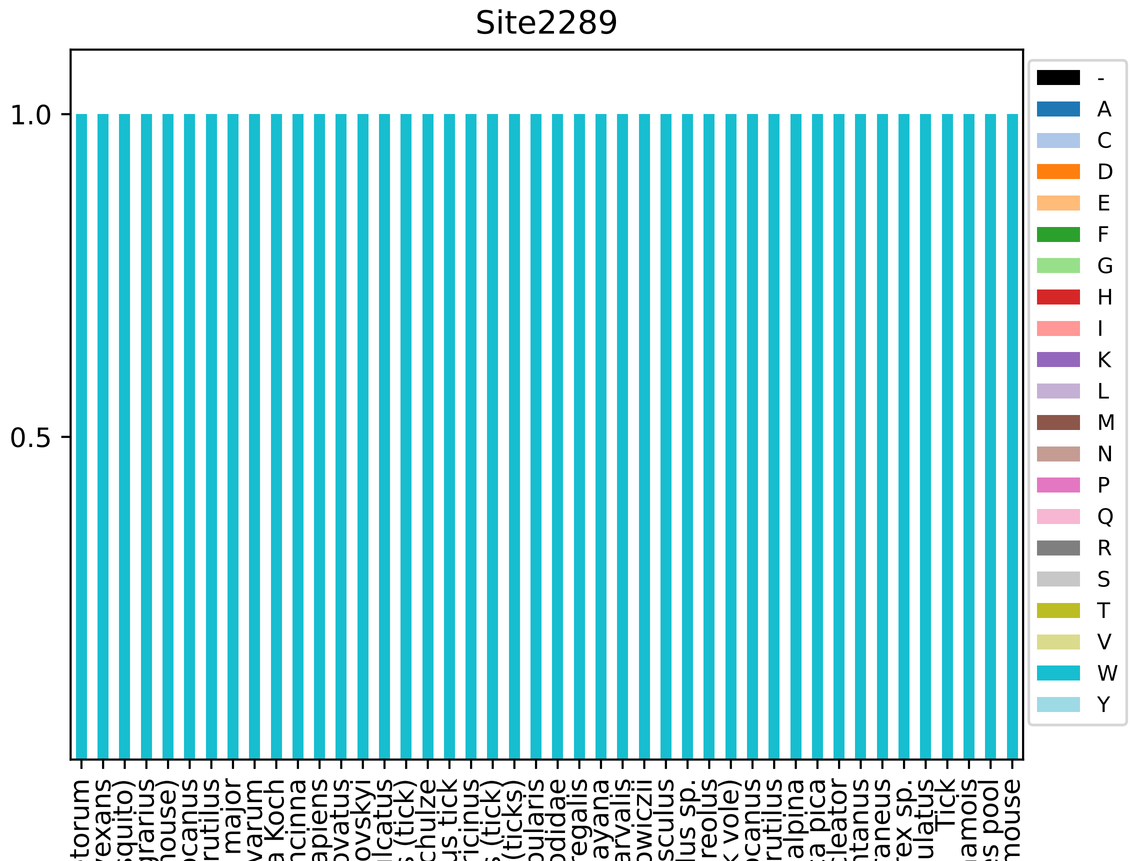
Site2289
1.0
0.5
-
A
C
D
E
F
G
H
I
K
L
M
N
P
Q
R
S
T
V
W
Y

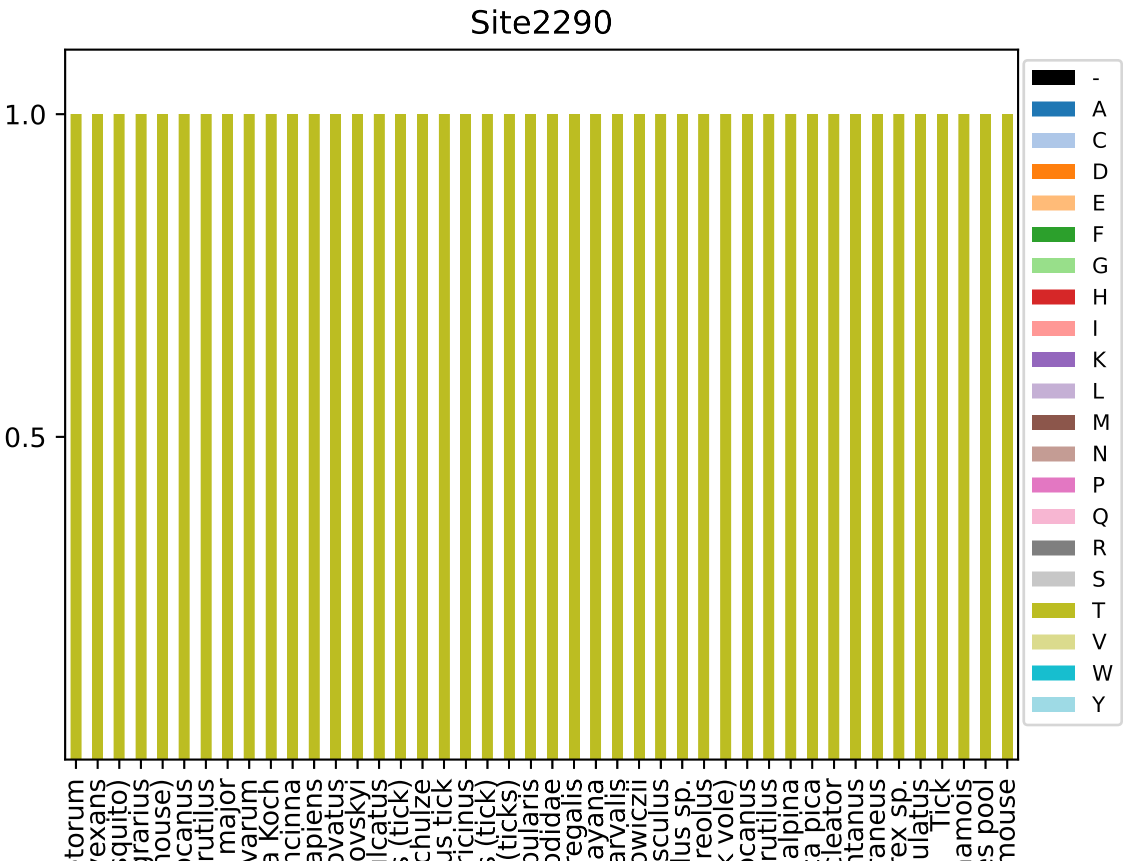
Site2290
1.0
0.5
-
A
C
D
E
F
G
H
I
K
L
M
N
P
Q
R
S
T
V
W
Y

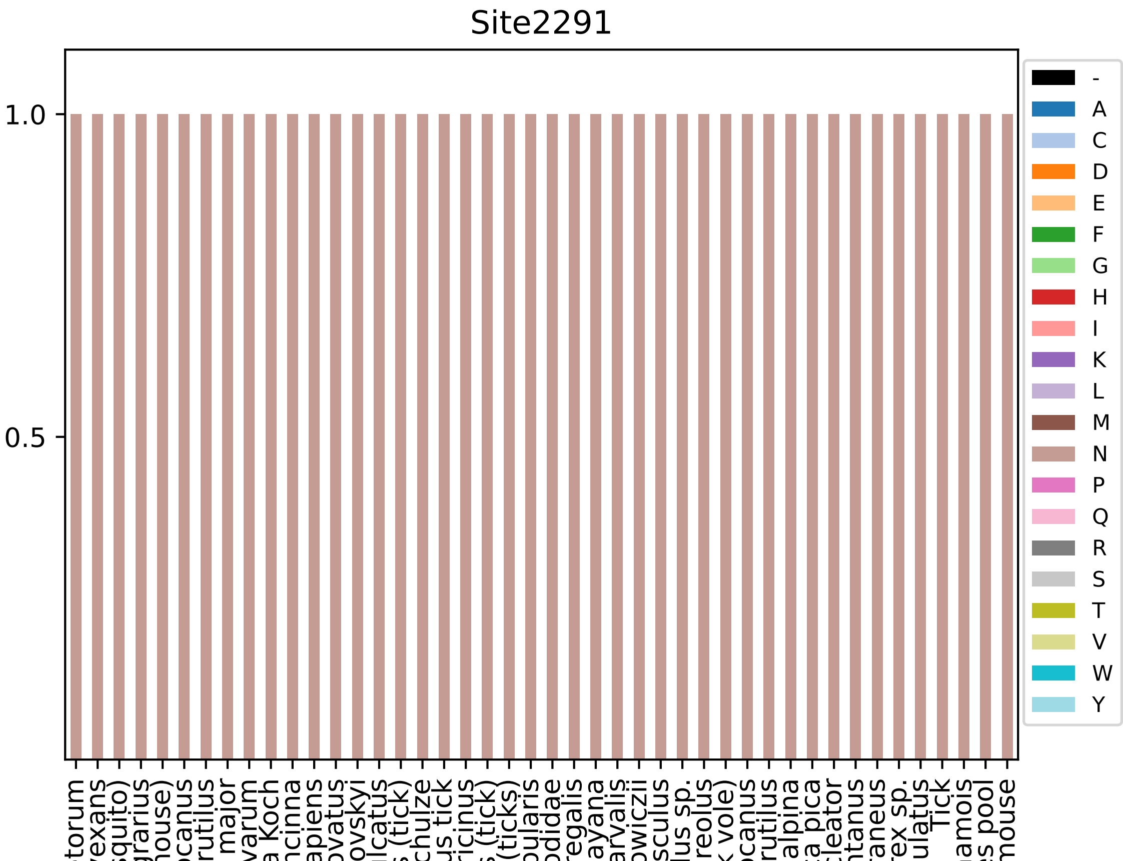
Site2291
1.0
0.5
-
A
C
D
E
F
G
H
I
K
L
M
N
P
Q
R
S
T
V
W
Y

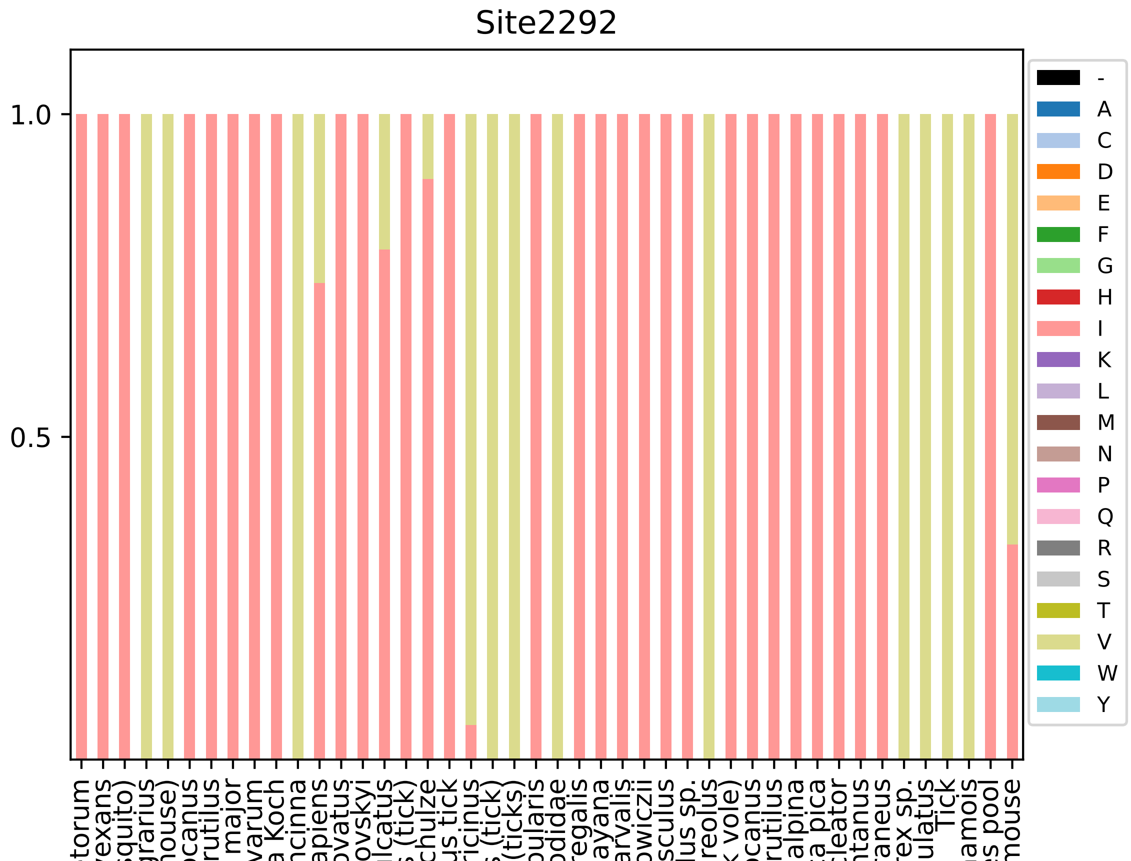
Site2292
1.0
0.5
-
A
C
D
E
F
G
H
I
K
L
M
N
P
Q
R
S
T
V
W
Y

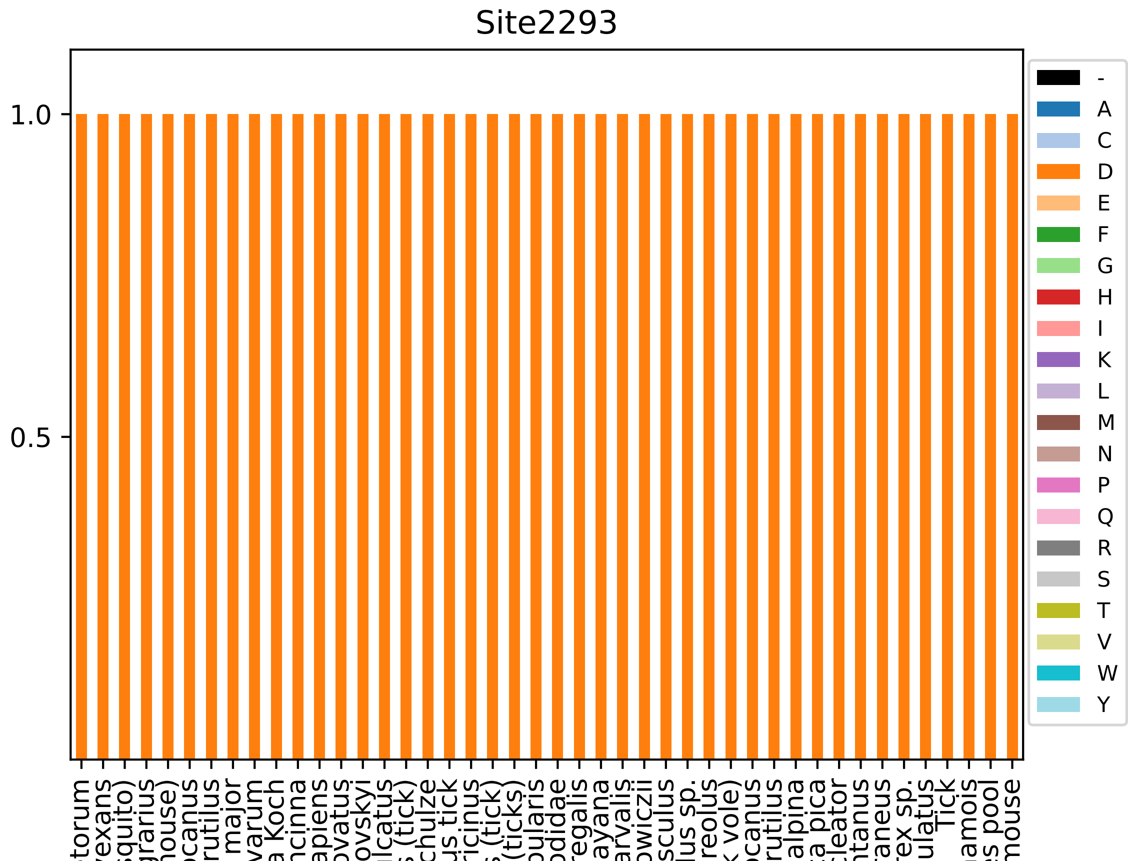
Site2293
1.0
0.5
-
A
C
D
E
F
G
H
I
K
L
M
N
P
Q
R
S
T
V
W
Y

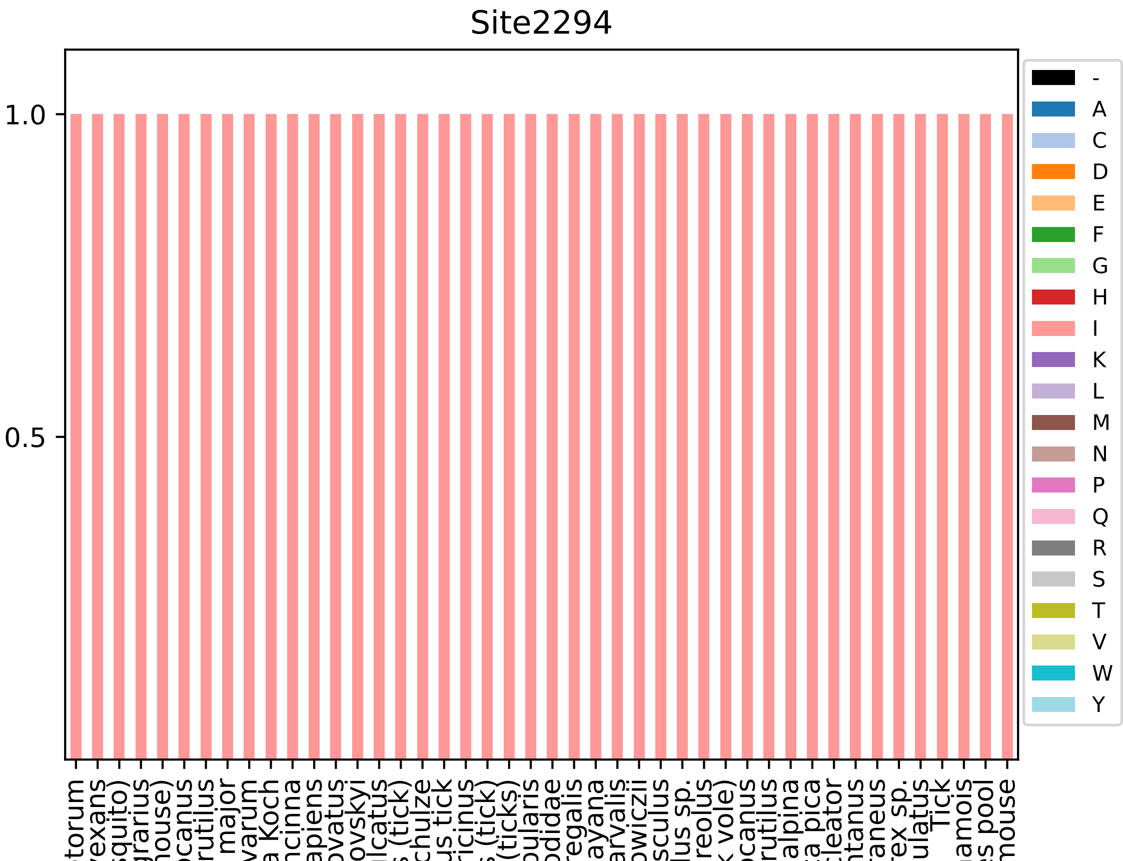
Site2294
1.0
0.5
-
A
C
D
E
F
G
H
I
K
L
M
N
P
Q
R
S
T
V
W
Y

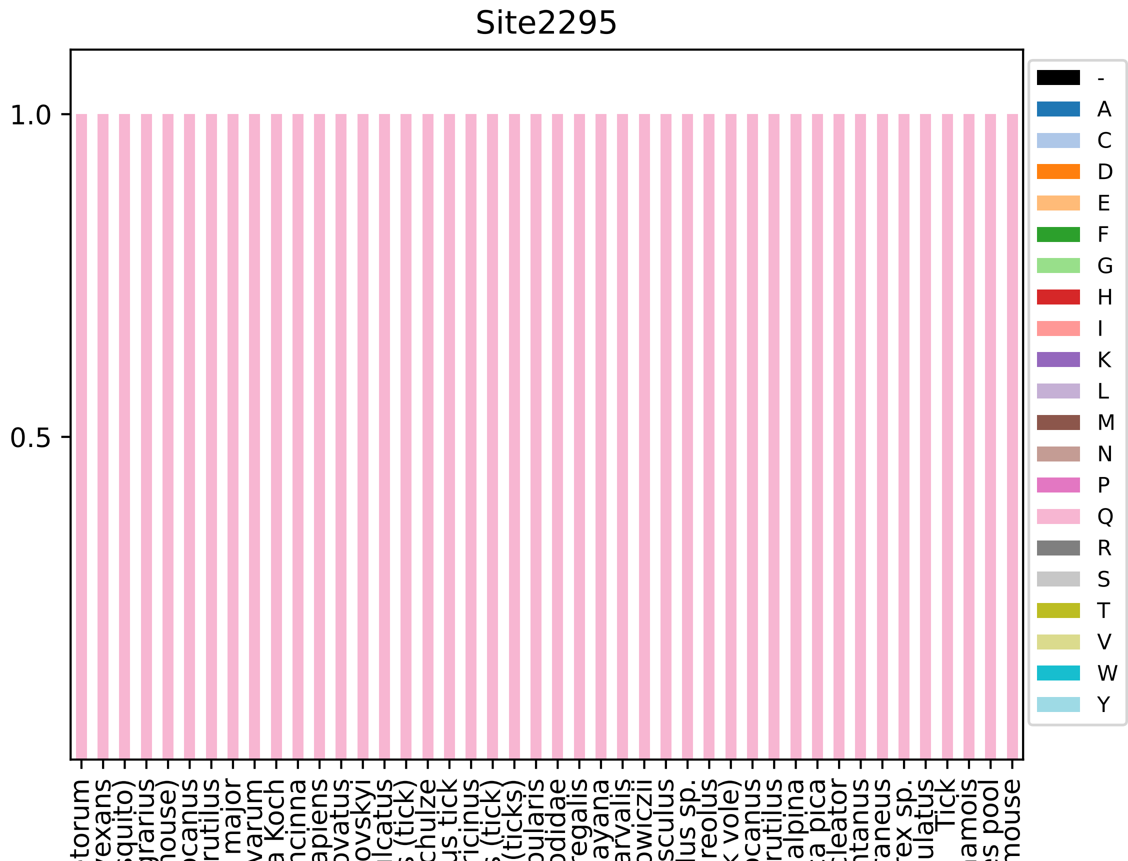
Site2295
1.0
0.5
-
A
C
D
E
F
G
H
I
K
L
M
N
P
Q
R
S
T
V
W
Y

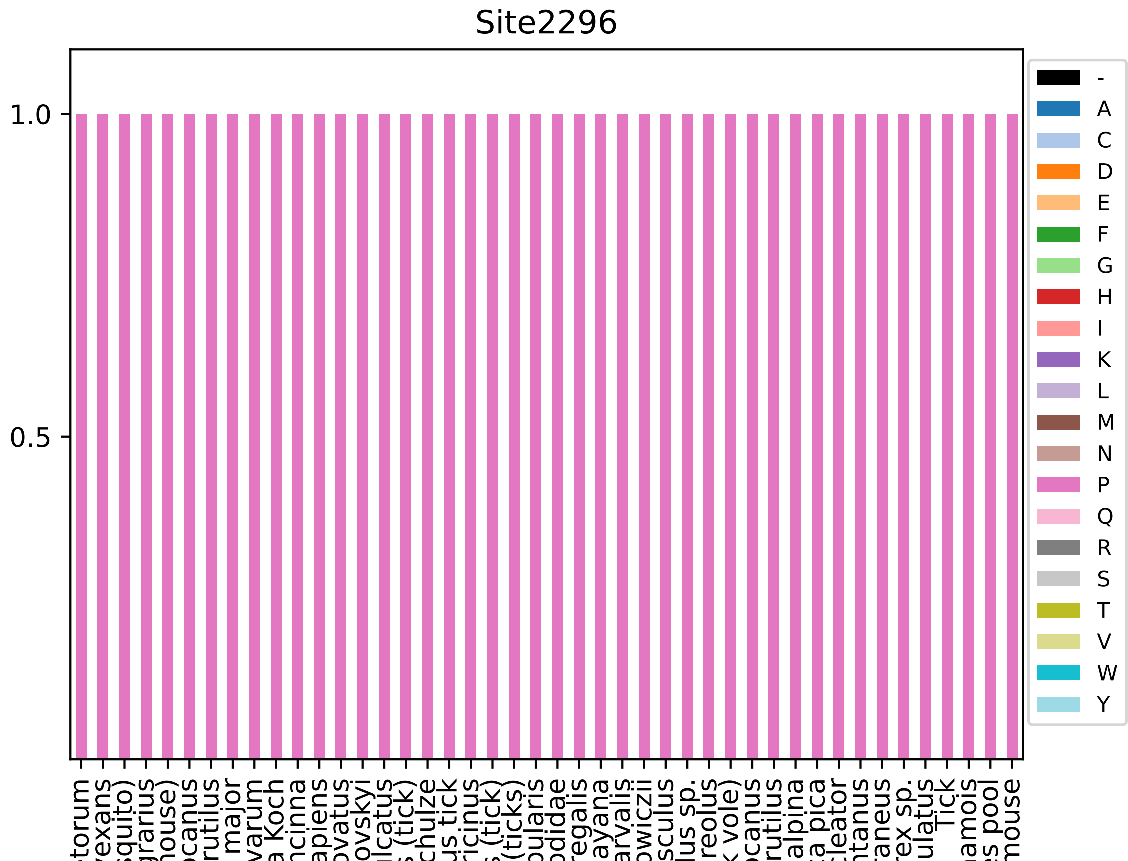
Site2296
1.0
0.5
-
A
C
D
E
F
G
H
I
K
L
M
N
P
Q
R
S
T
V
W
Y

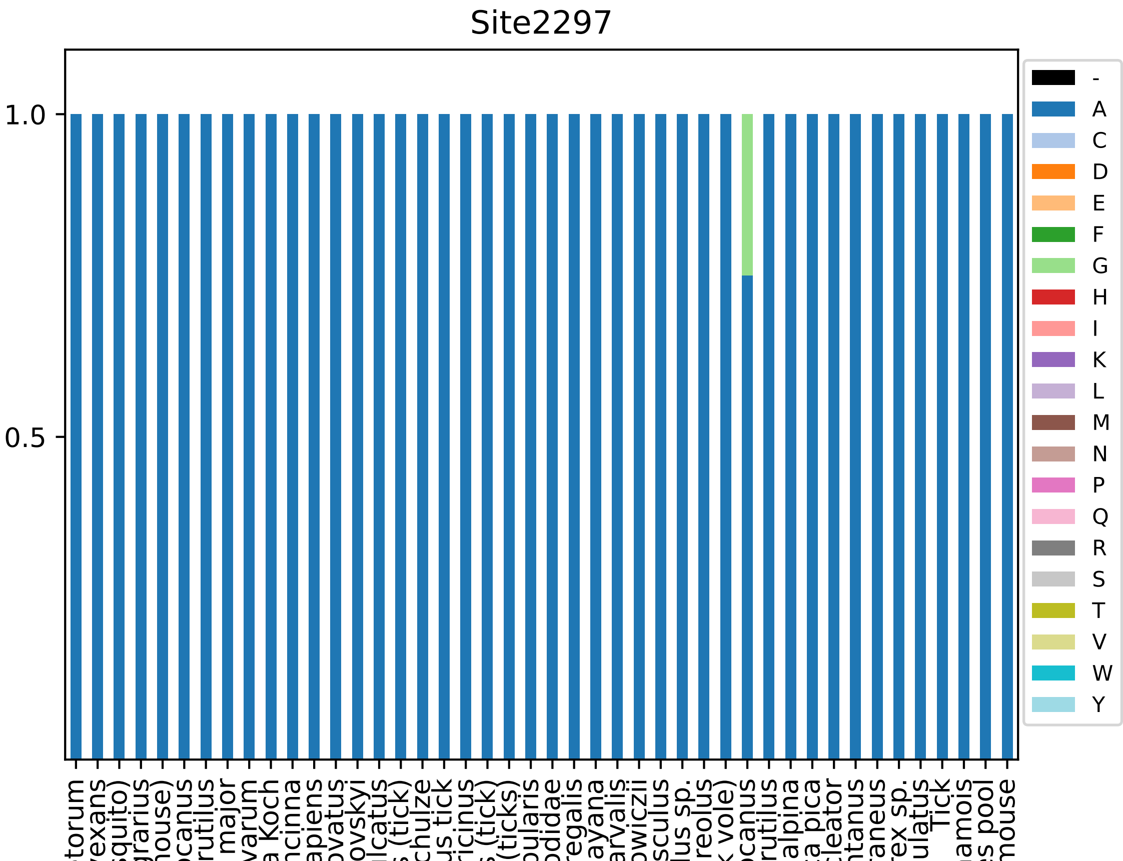

Site2297
1.0
0.5
-
A
C
D
E
F
G
H
I
K
L
M
N
P
Q
R
S
T
V
W
Y

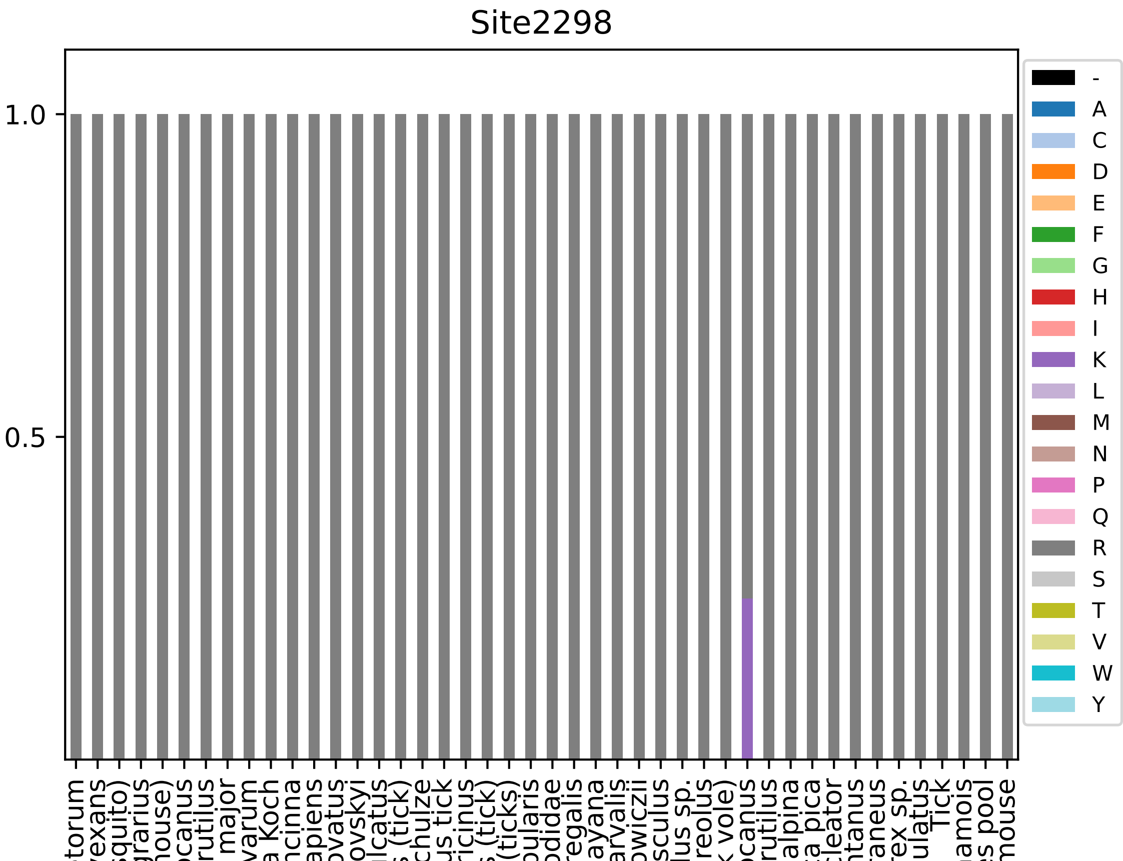
Site2298
1.0
0.5
-
A
C
D
E
F
G
H
I
K
L
M
N
P
Q
R
S
T
V
W
Y

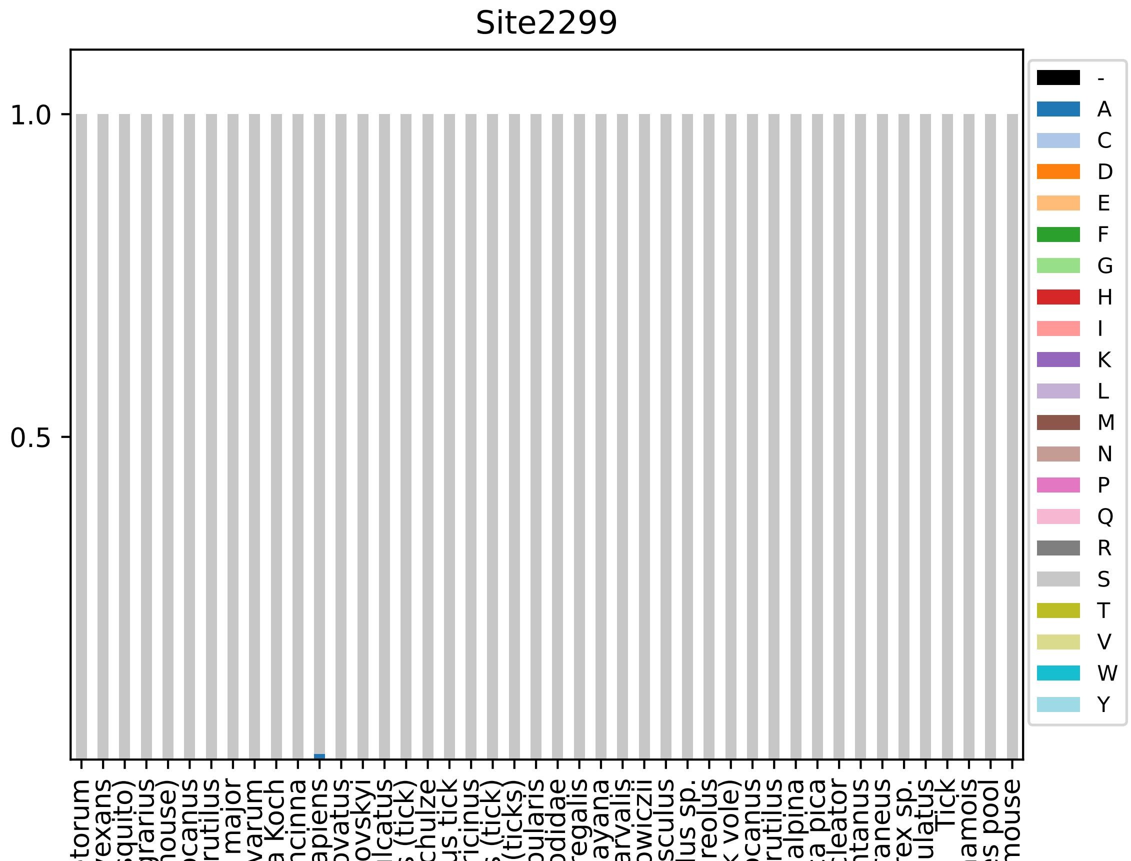

Site2299
1.0
0.5
-
A
C
D
E
F
G
H
I
K
L
M
N
P
Q
R
S
T
V
W
Y

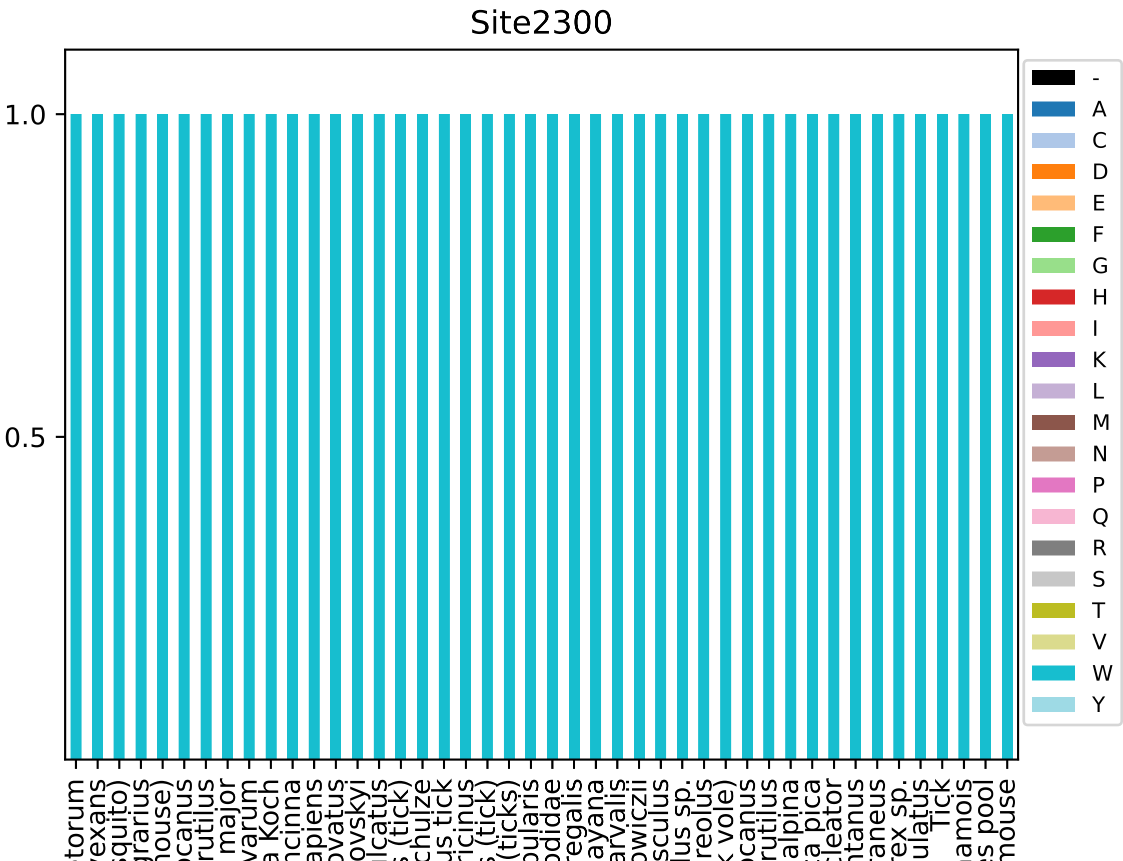
Site2300
1.0
0.5
-
A
C
D
E
F
G
H
I
K
L
M
N
P
Q
R
S
T
V
W
Y

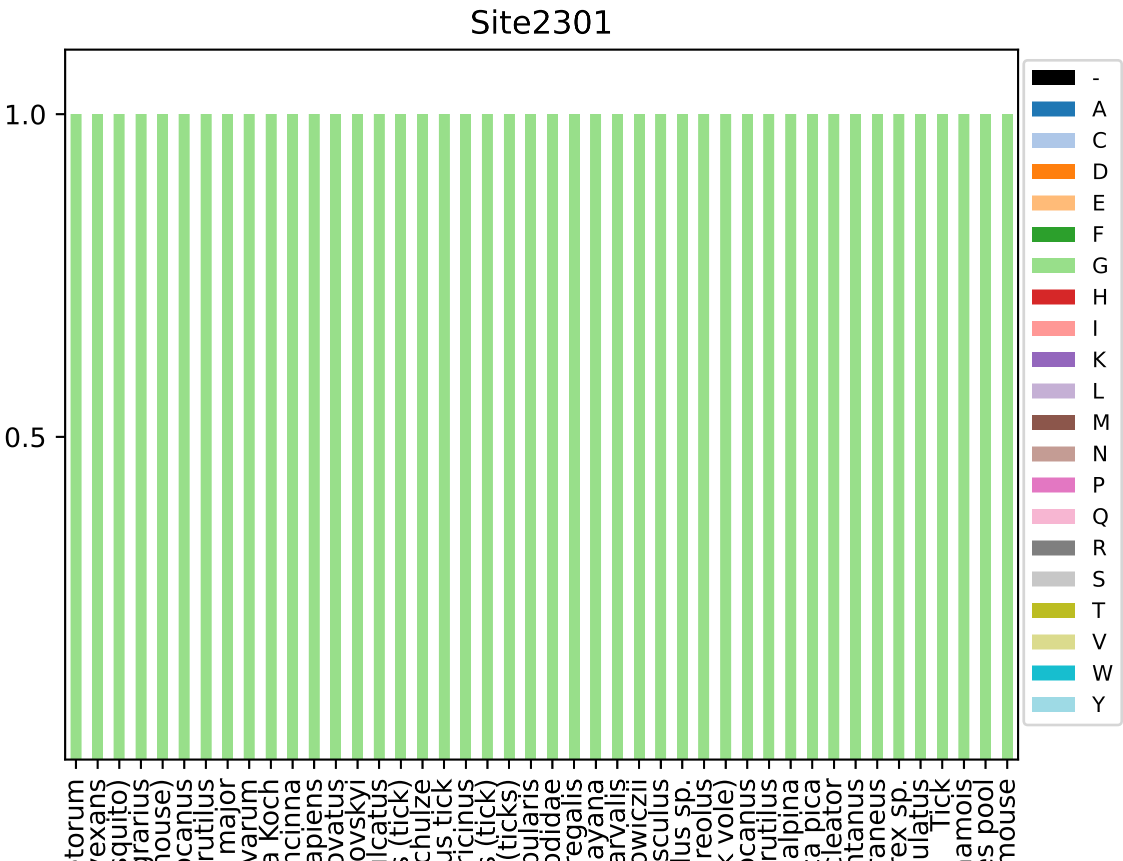
Site2301
1.0
0.5
-
A
C
D
E
F
G
H
I
K
L
M
N
P
Q
R
S
T
V
W
Y

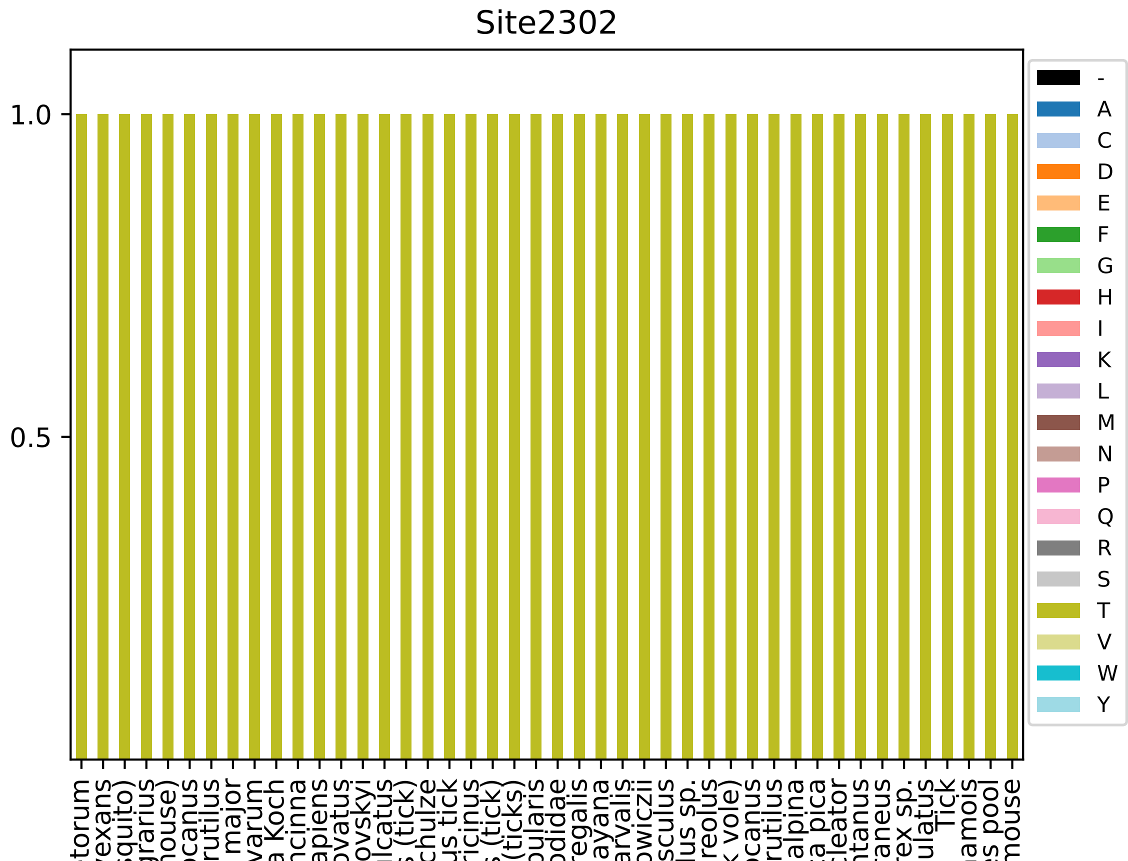
Site2302
1.0
0.5
-
A
C
D
E
F
G
H
I
K
L
M
N
P
Q
R
S
T
V
W
Y

Site2303

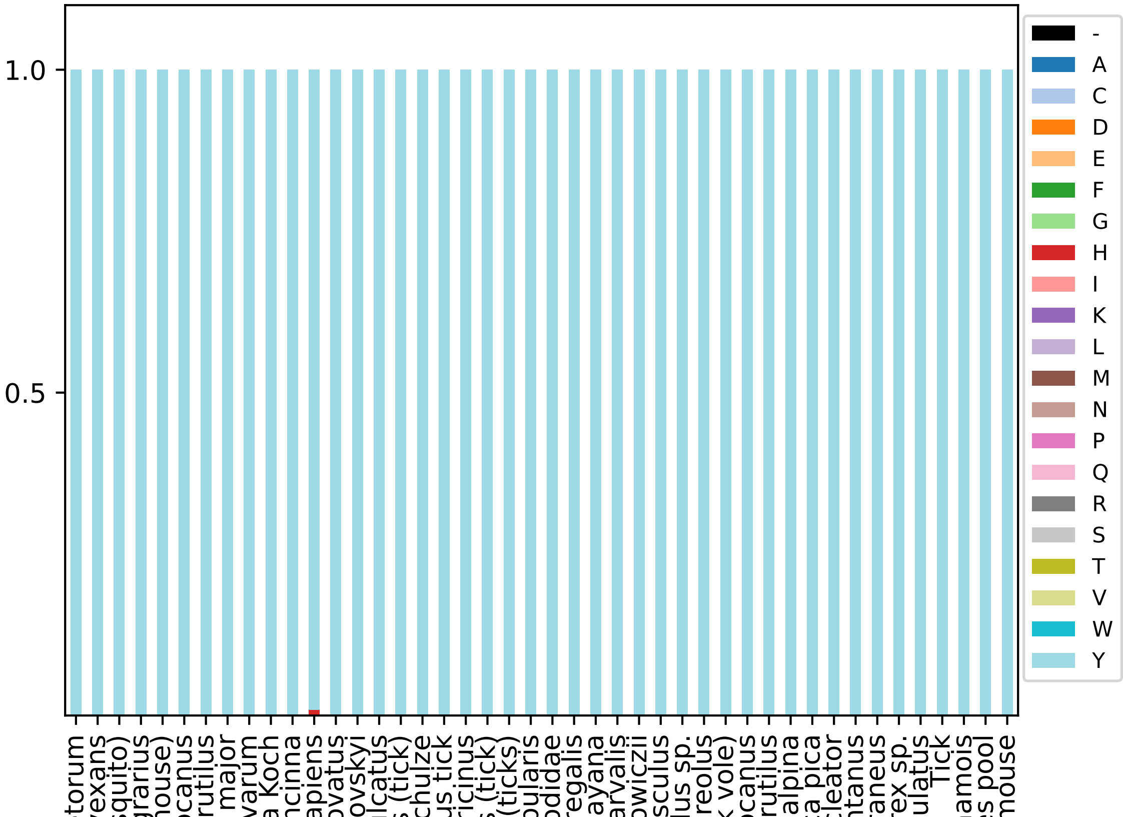

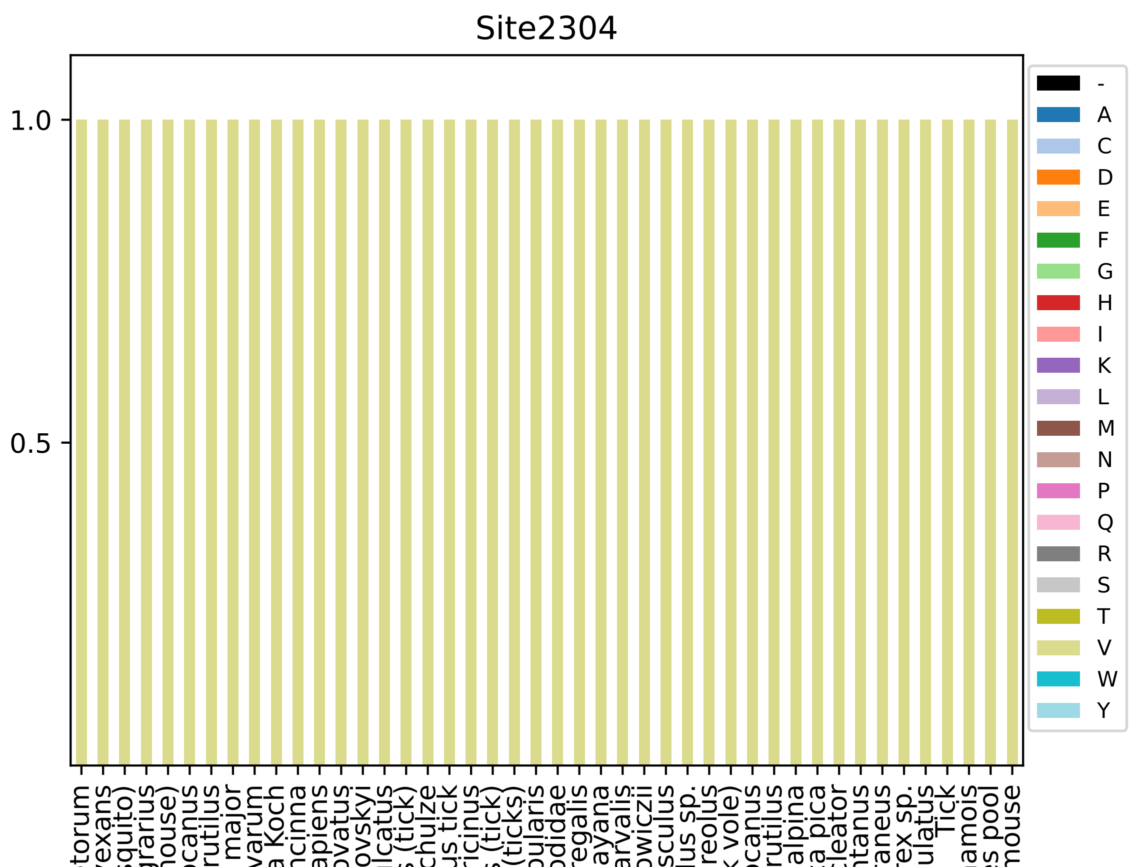

Site2304
1.0
0.5
-
A
C
D
E
F
G
H
I
K
L
M
N
P
Q
R
S
T
V
W
Y

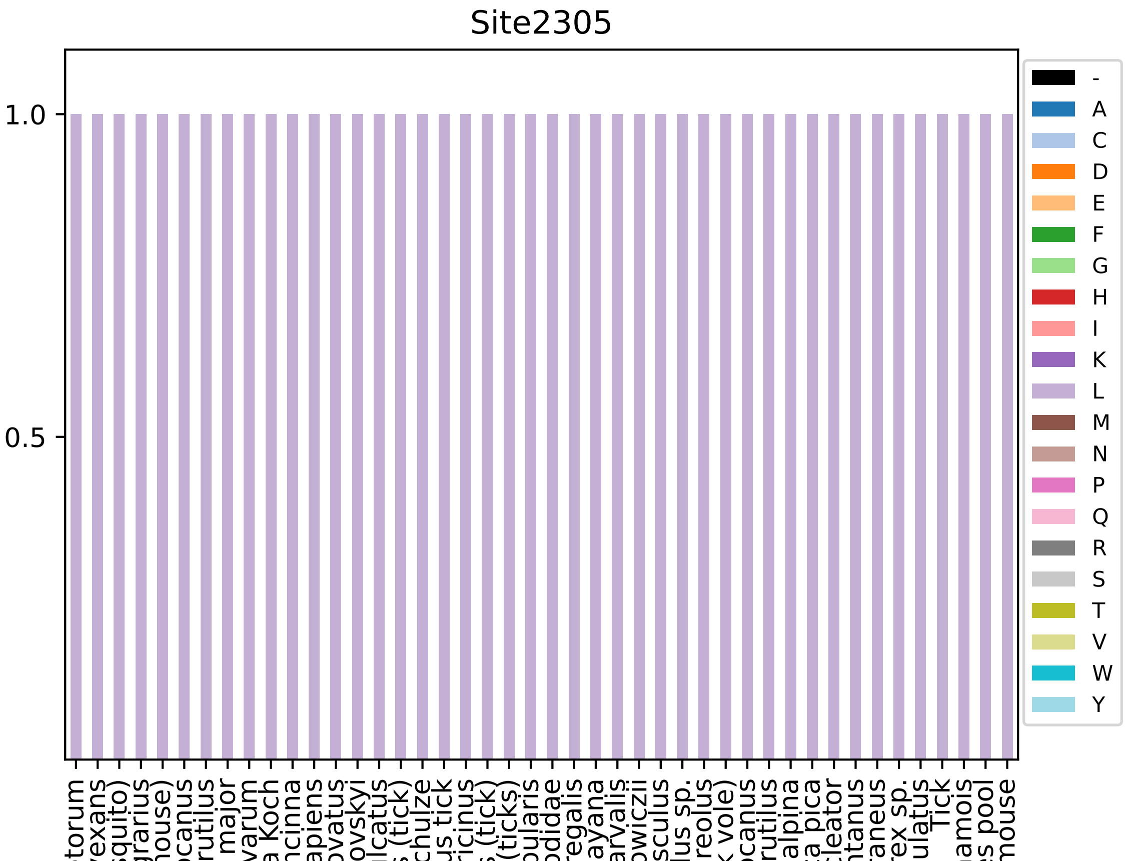
Site2305
1.0
0.5
-
A
C
D
E
F
G
H
I
K
L
M
N
P
Q
R
S
T
V
W
Y

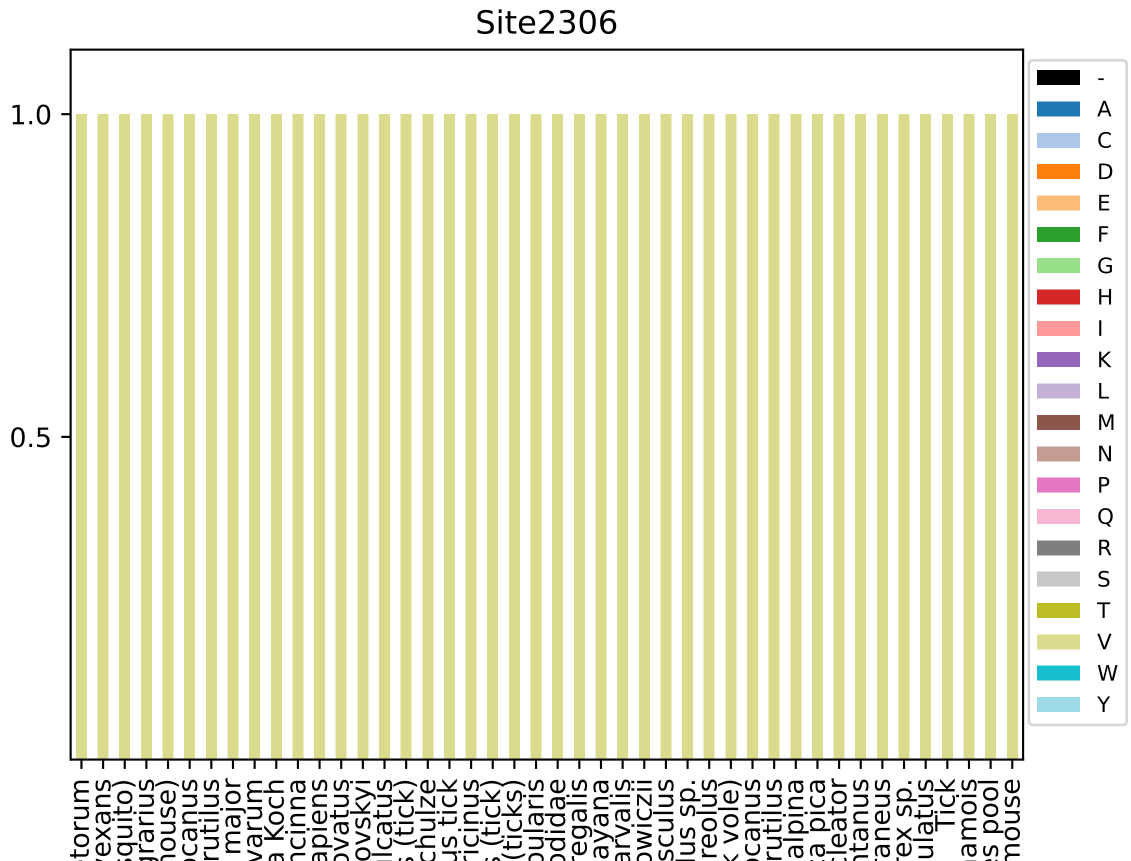
Site2306
1.0
0.5
-
A
C
D
E
F
G
H
I
K
L
M
N
P
Q
R
S
T
V
W
Y

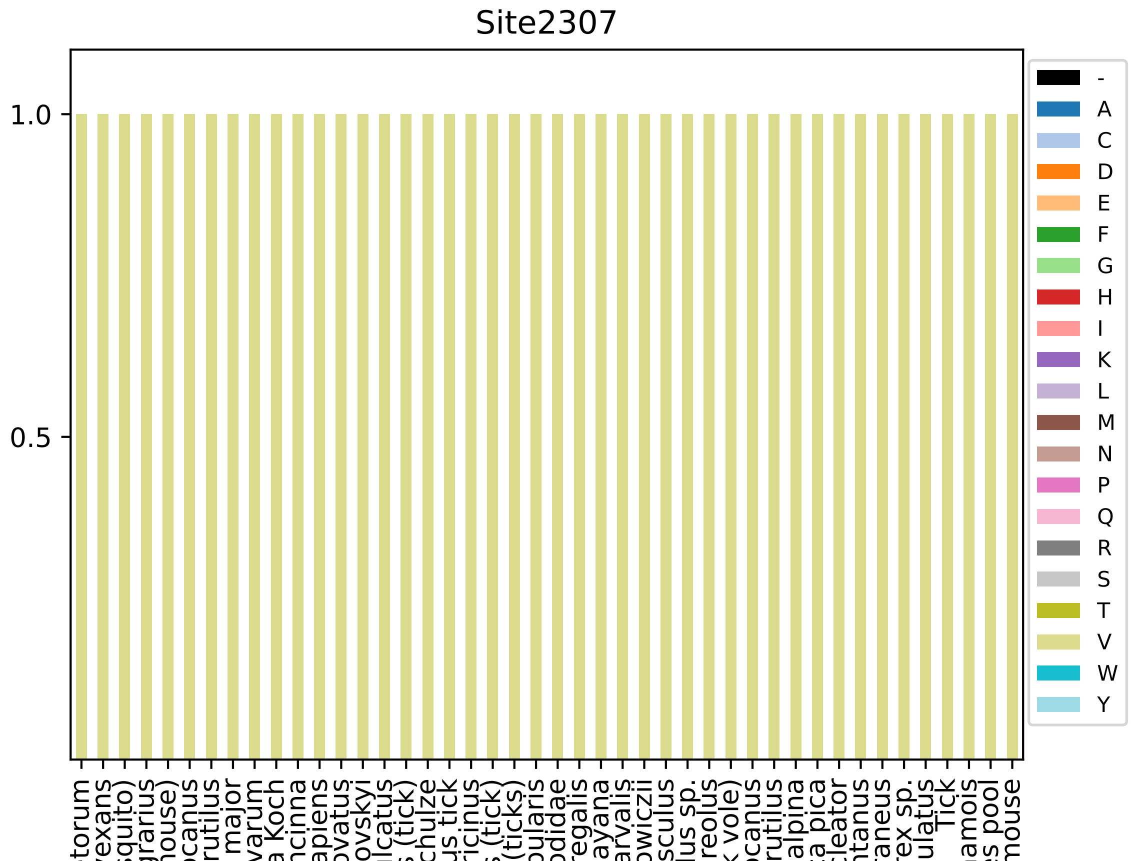
Site2307
1.0
0.5
-
A
C
D
E
F
G
H
I
K
L
M
N
P
Q
R
S
T
V
W
Y

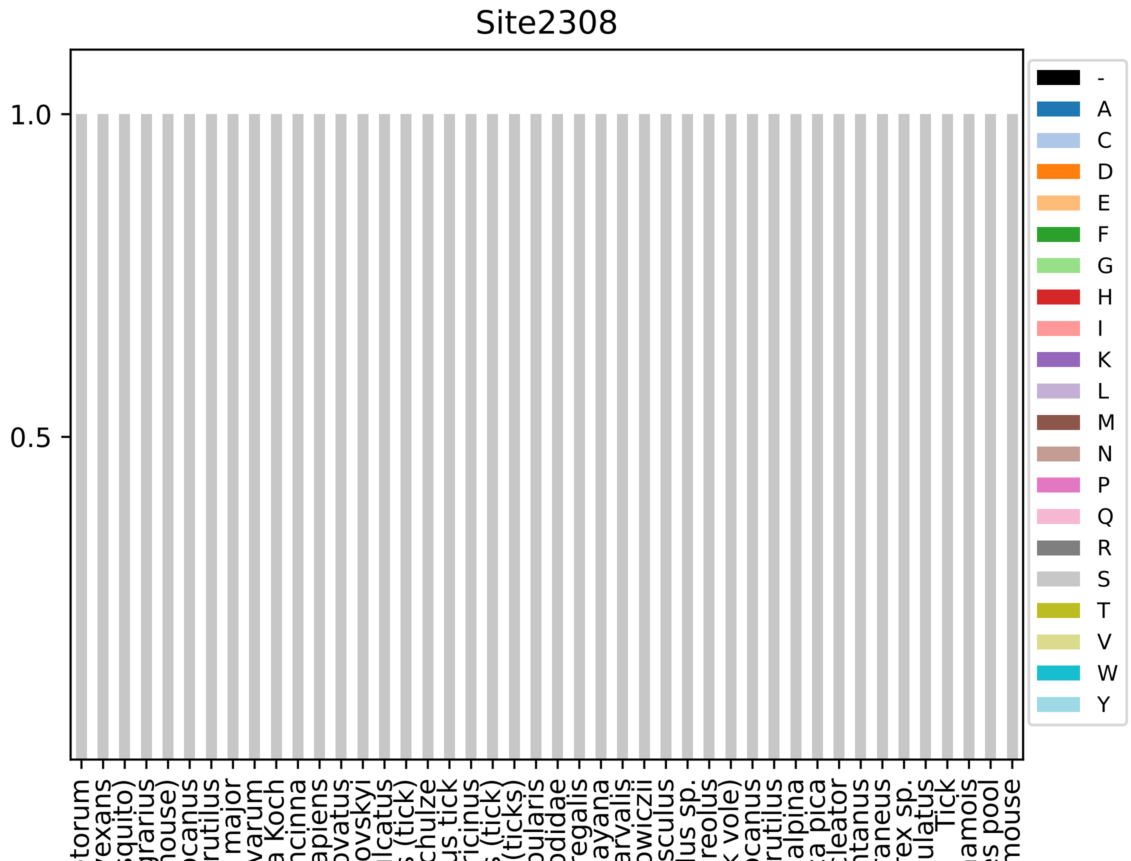
Site2308
1.0
0.5
-
A
C
D
E
F
G
H
I
K
L
M
N
P
Q
R
S
T
V
W
Y

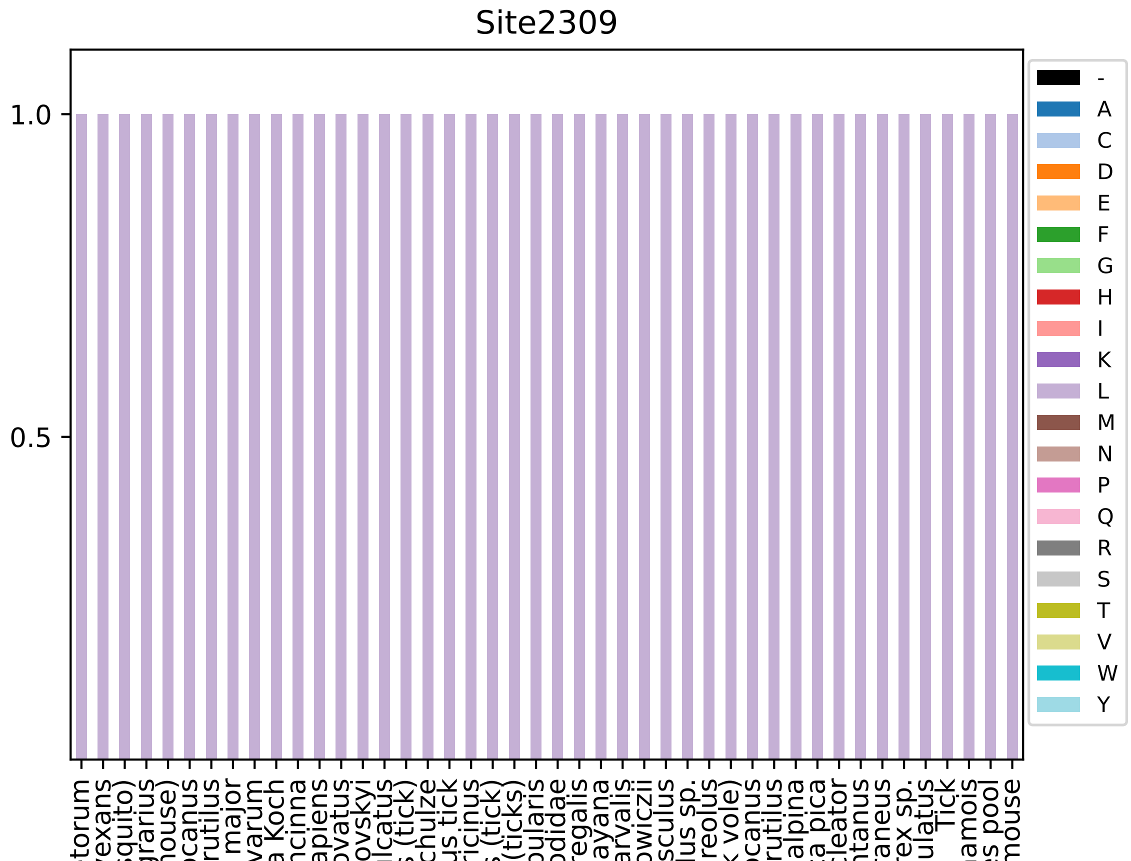

Site2309
1.0
0.5
-
A
C
D
E
F
G
H
I
K
L
M
N
P
Q
R
S
T
V
W
Y

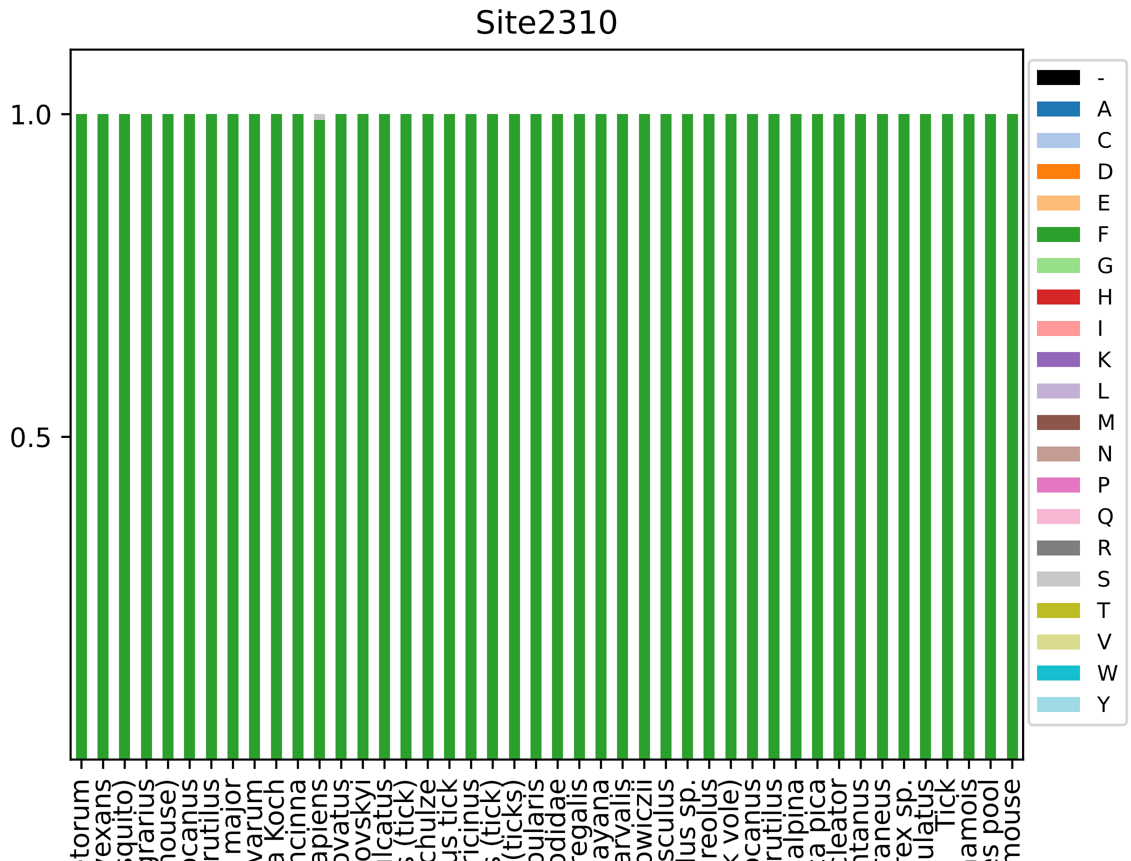

Site2310
1.0
0.5
-
A
C
D
E
F
G
H
I
K
L
M
N
P
Q
R
S
T
V
W
Y

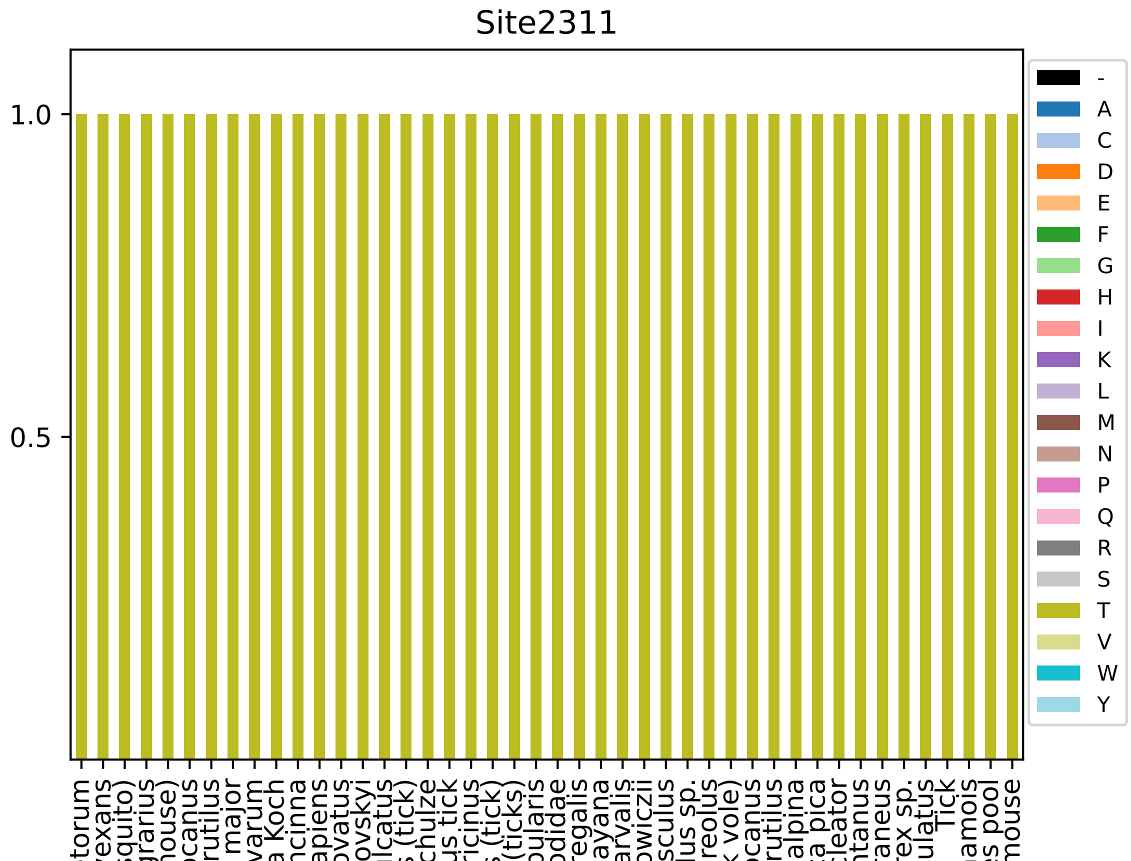
Site2311
1.0
0.5
-
A
C
D
E
F
G
H
I
K
L
M
N
P
Q
R
S
T
V
W
Y

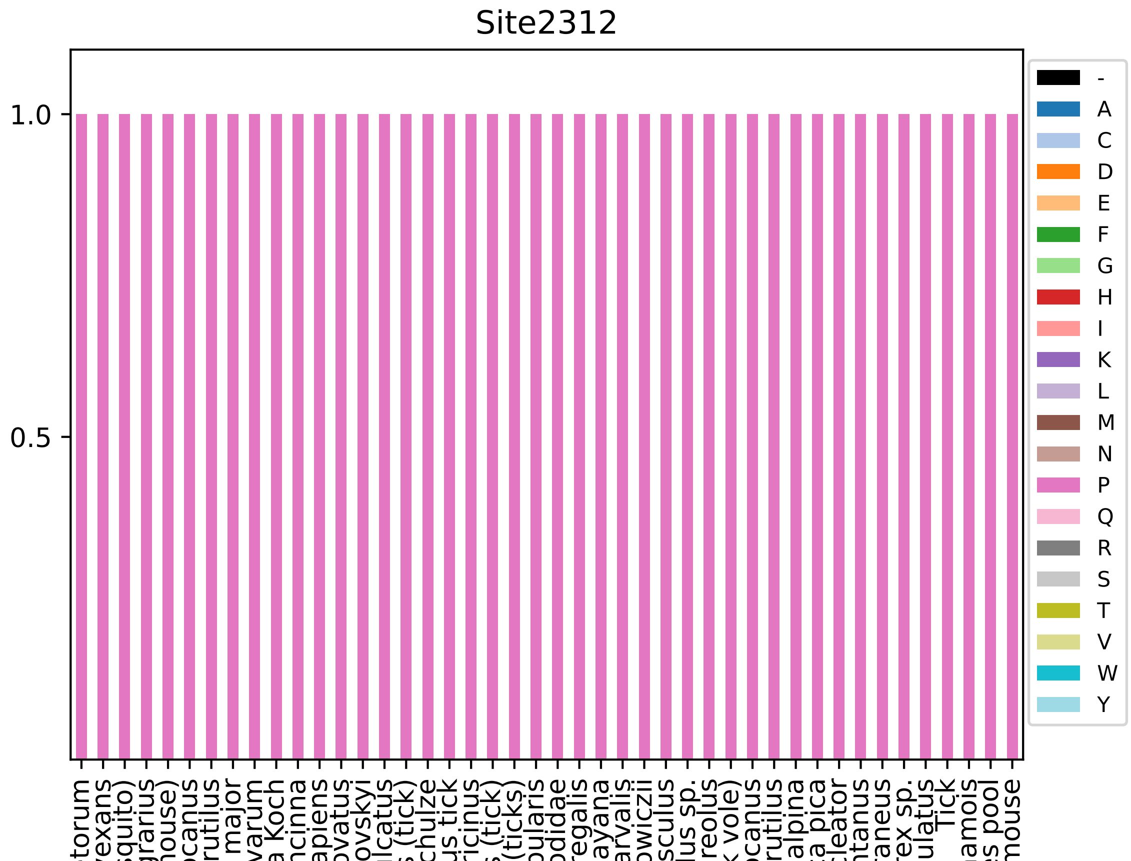
Site2312
1.0
0.5
-
A
C
D
E
F
G
H
I
K
L
M
N
P
Q
R
S
T
V
W
Y

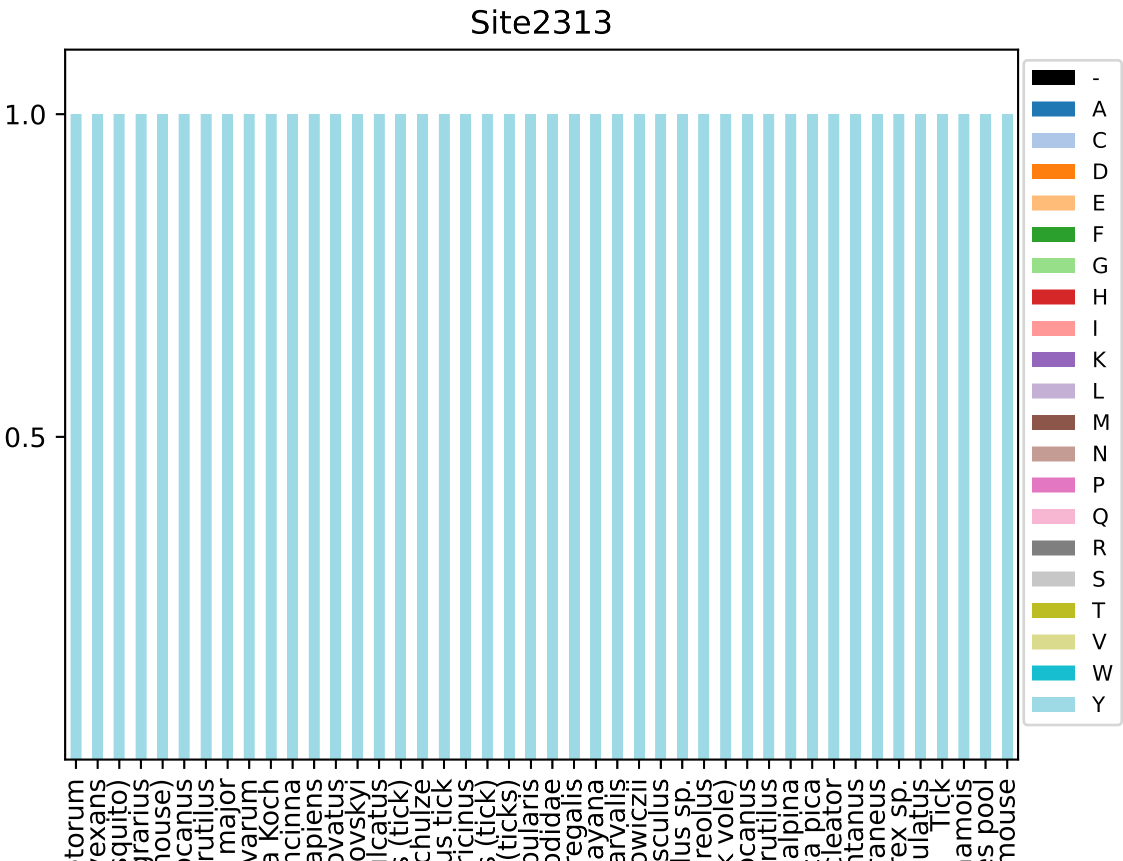
Site2313
1.0
0.5
-
A
C
D
E
F
G
H
I
K
L
M
N
P
Q
R
S
T
V
W
Y

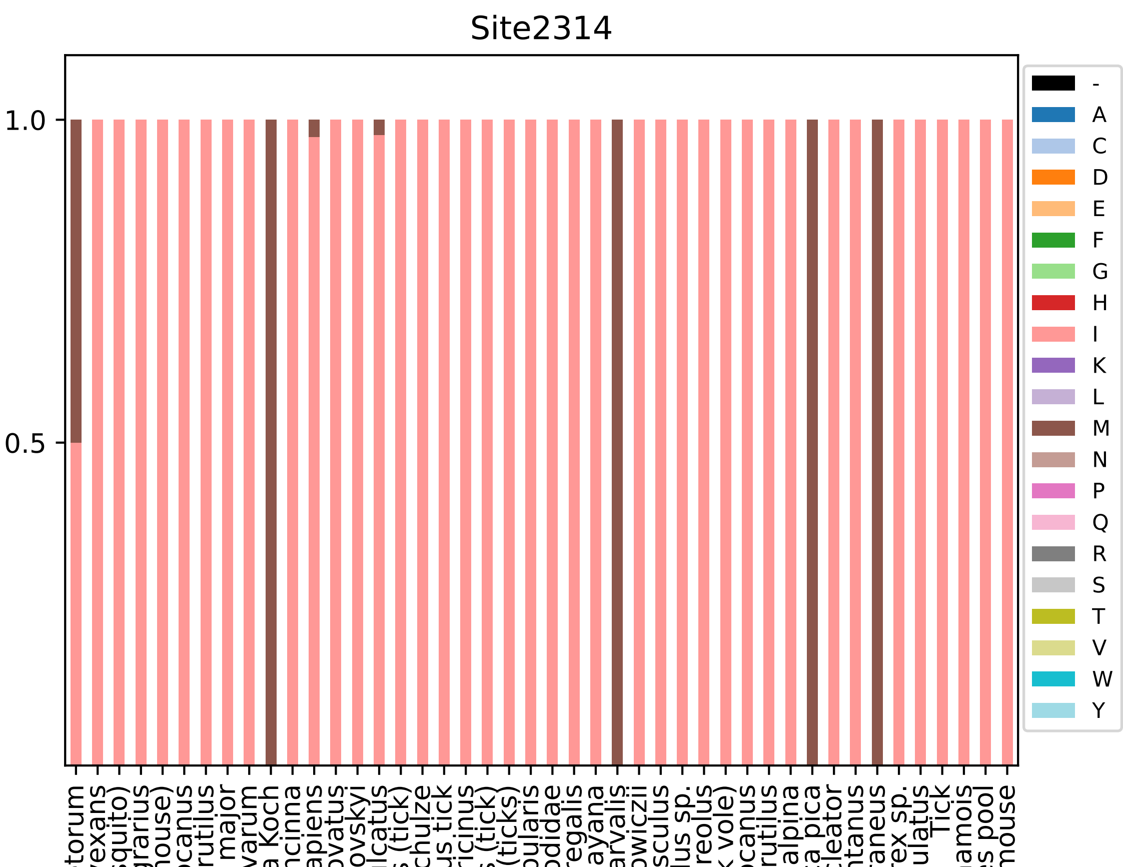
Site2314
1.0
0.5
-
A
C
D
E
F
G
H
I
K
L
M
N
P
Q
R
S
T
V
W
Y

Site2315

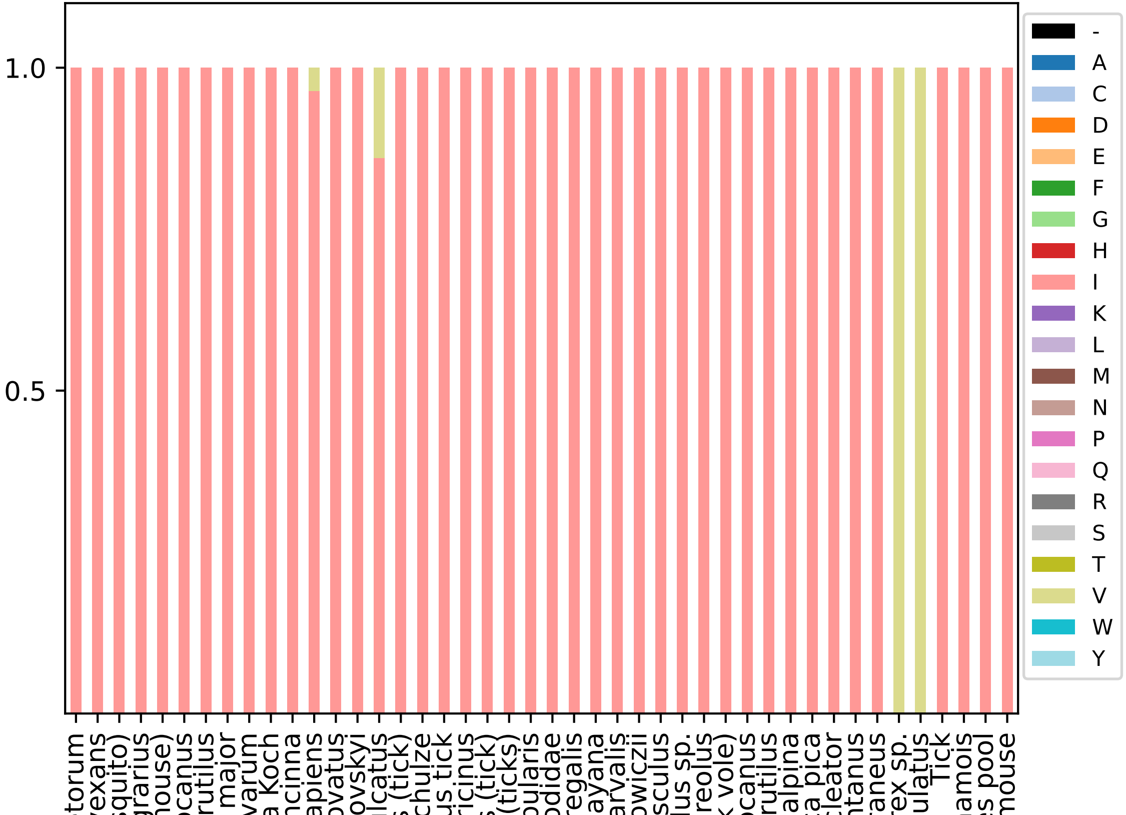

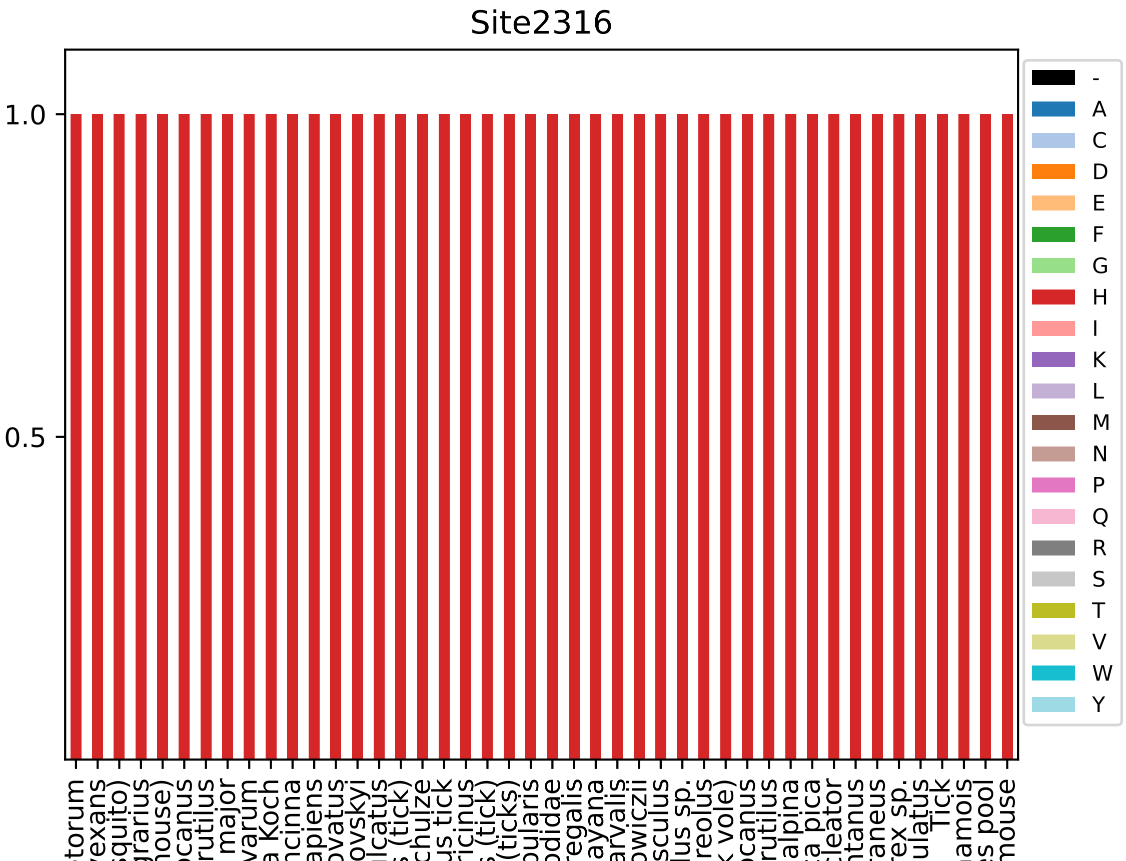
Site2316
1.0
0.5
-
A
C
D
E
F
G
H
I
K
L
M
N
P
Q
R
S
T
V
W
Y

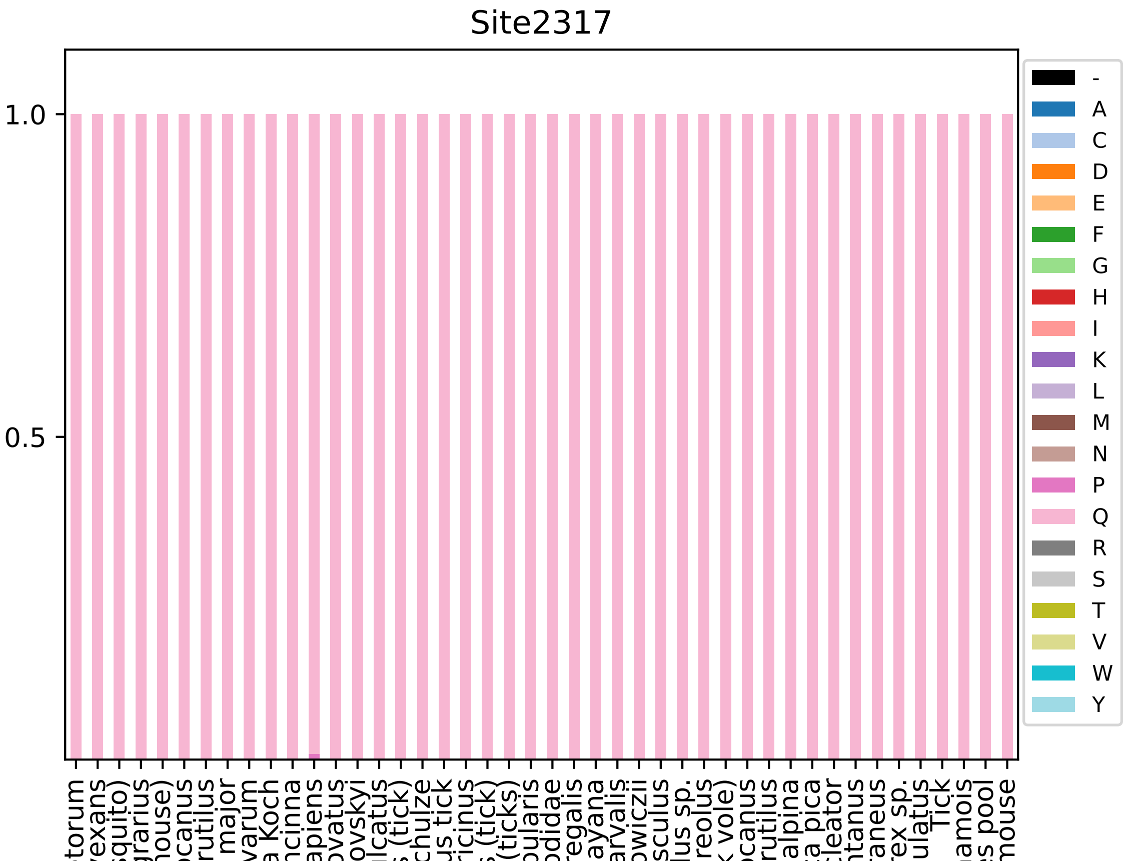
Site2317
1.0
0.5
-
A
C
D
E
F
G
H
I
K
L
M
N
P
Q
R
S
T
V
W
Y
Tick

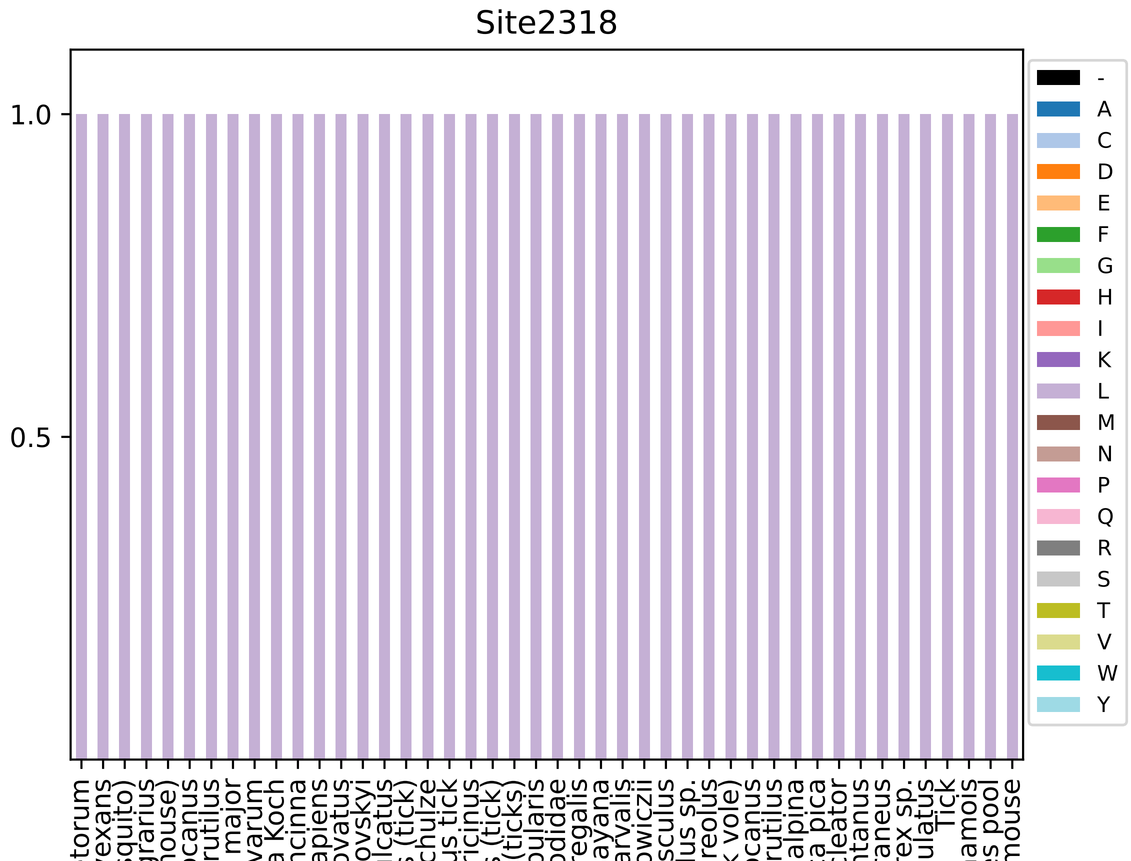

Site2318
1.0
0.5
-
A
C
D
E
F
G
H
I
K
L
M
N
P
Q
R
S
T
V
W
Y

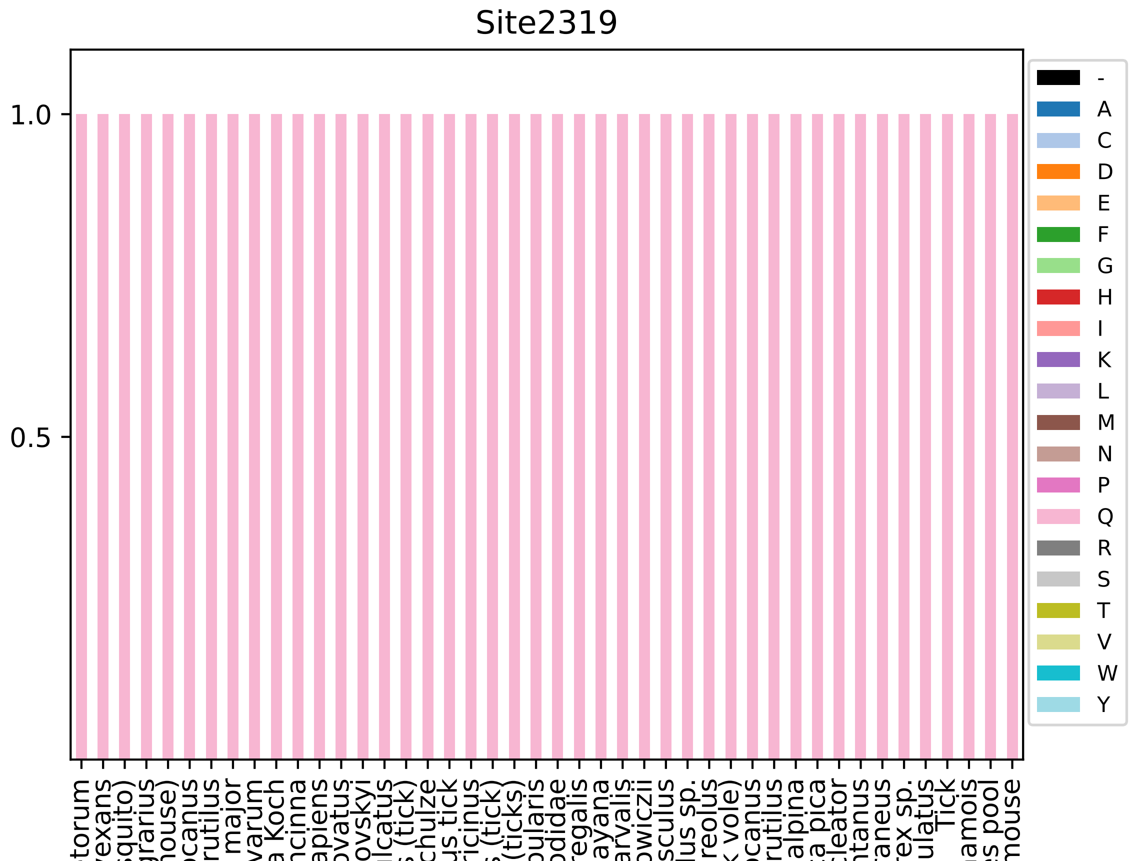
Site2319
1.0
0.5
-
A
C
D
E
F
G
H
I
K
L
M
N
P
Q
R
S
T
V
W
Y

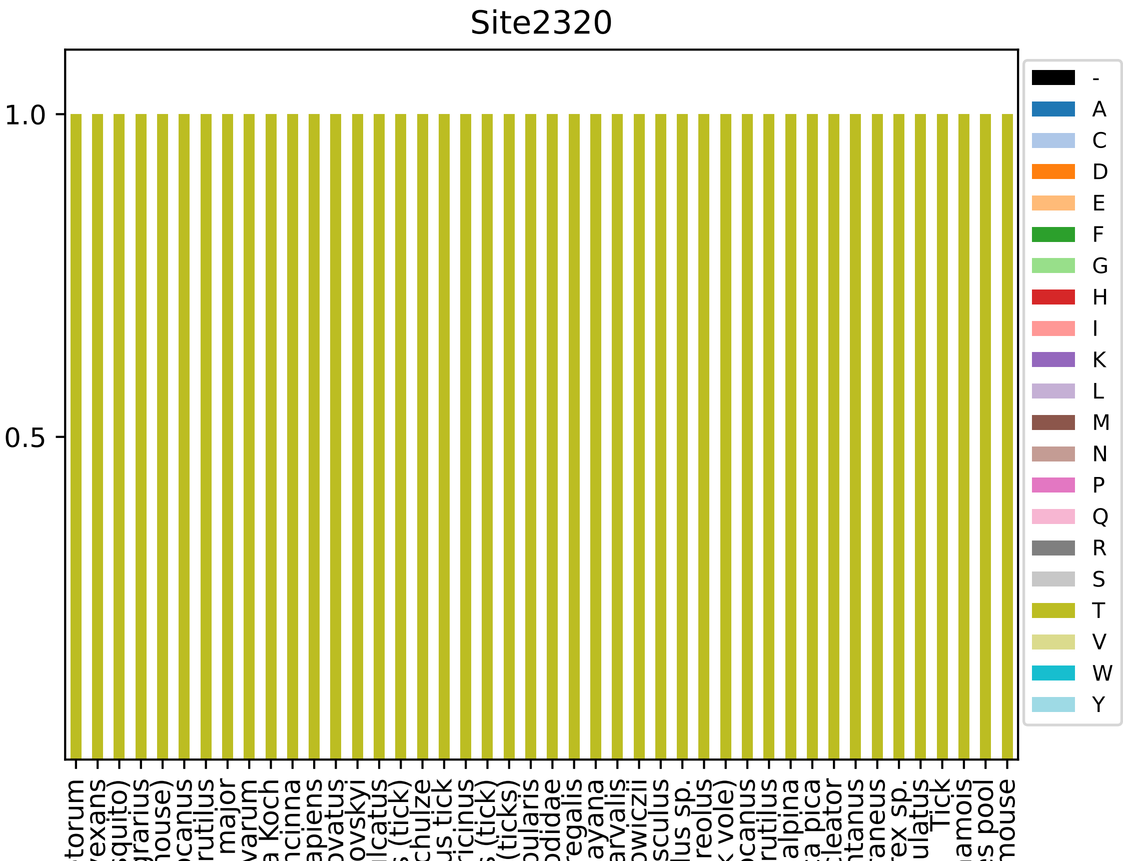
Site2320
1.0
0.5
-
A
C
D
E
F
G
H
I
K
L
M
N
P
Q
R
S
T
V
W
Y

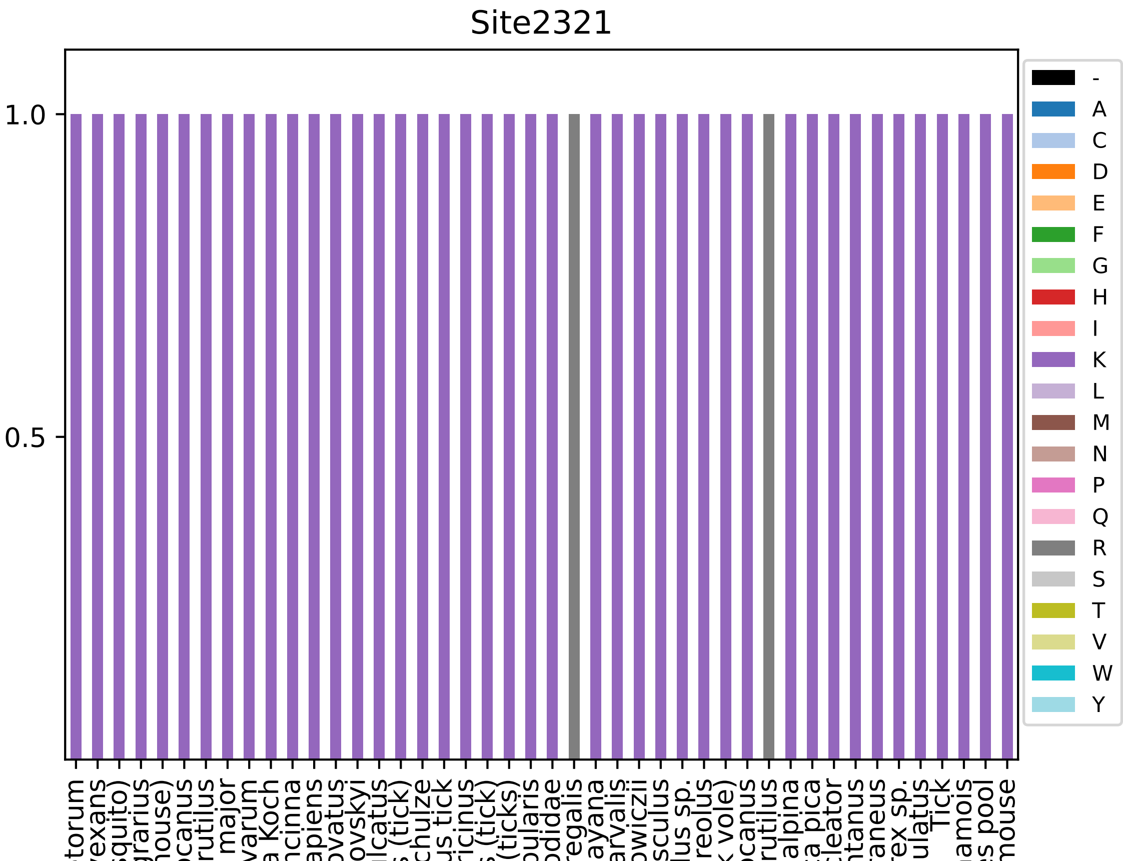
Site2321
1.0
0.5
-
A
C
D
E
F
G
H
I
K
L
M
N
P
Q
R
S
T
V
W
Y
torum
vexans
squito)
grarius
house)
ocanus
rutilus
major
varum
a Koch
ncinna
apiens
ovatus
ovskyi
ulcatus
s (tick)
chulze
us tick
ricinus
s (tick)
(ticks)
pularis
podidae
regalis
ayana
arvalis
owiczii
sculus
lus sp.
reolus
k vole)
ocanus
rutilus
alpina
a pica
cleator
ntanus
raneus
rex sp.
ulatus
Tick
amois
es pool
mouse

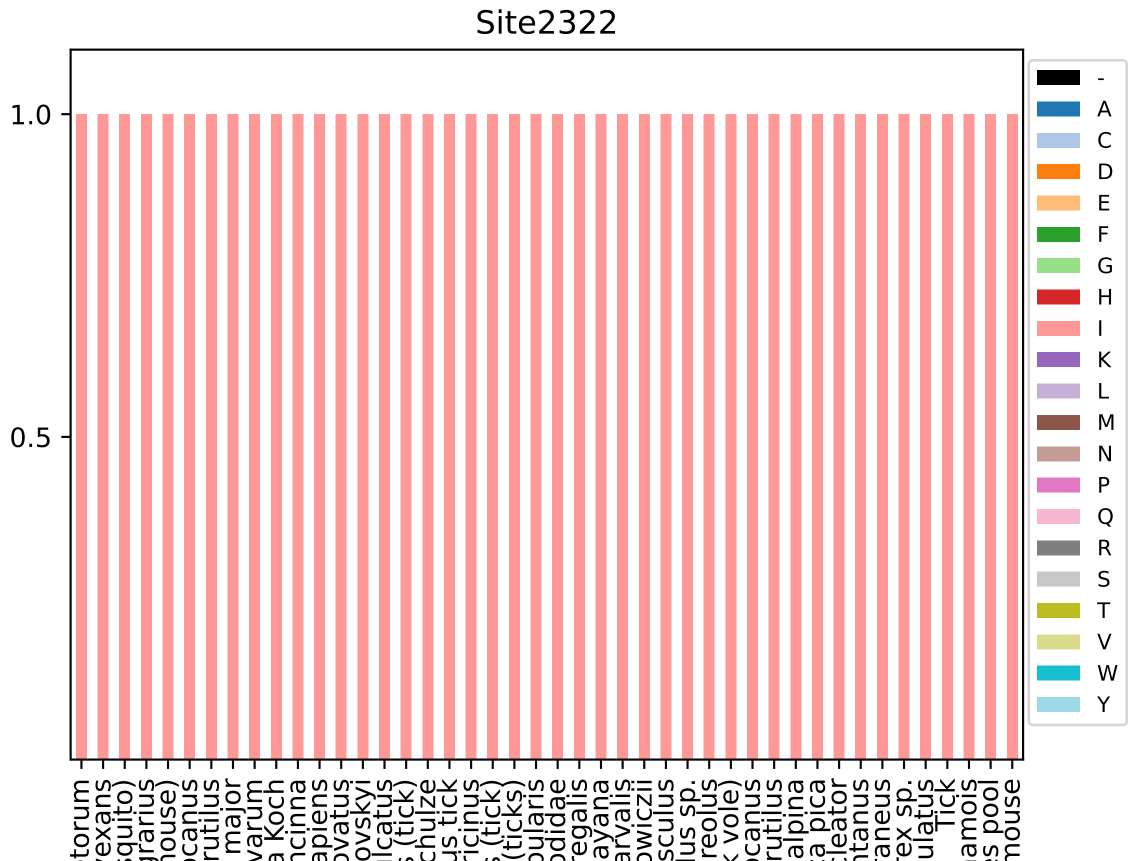

Site2322
1.0
0.5
-
A
C
D
E
F
G
H
I
K
L
M
N
P
Q
R
S
T
V
W
Y

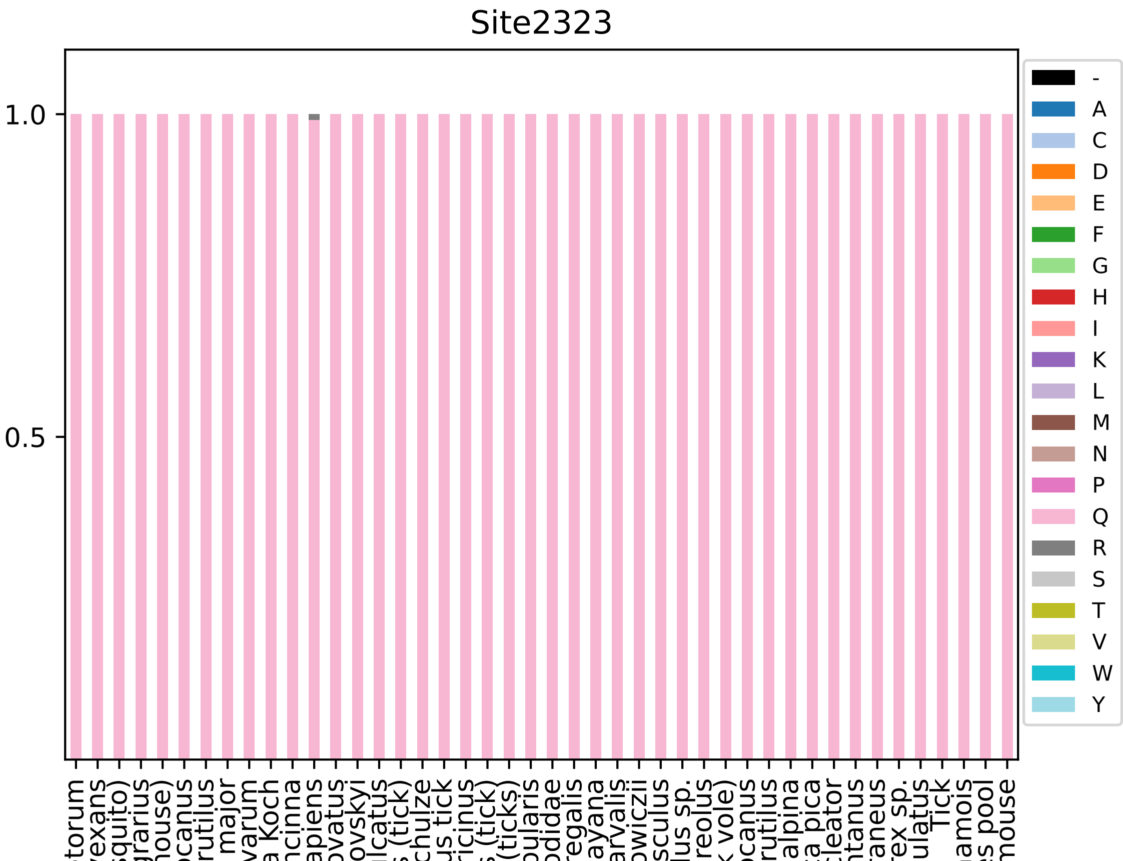

Site2323
1.0
0.5
-
A
C
D
E
F
G
H
I
K
L
M
N
P
Q
R
S
T
V
W
Y

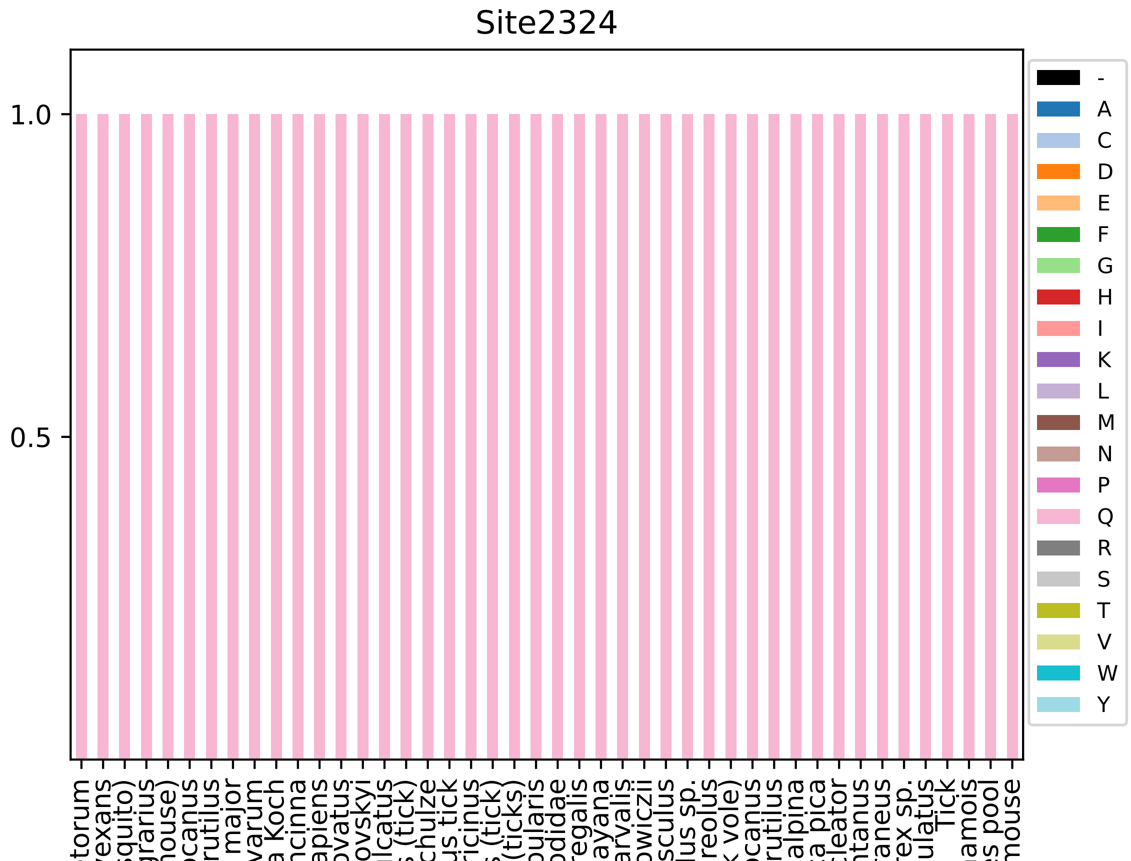
Site2324
1.0
0.5
-
A
C
D
E
F
G
H
I
K
L
M
N
P
Q
R
S
T
V
W
Y

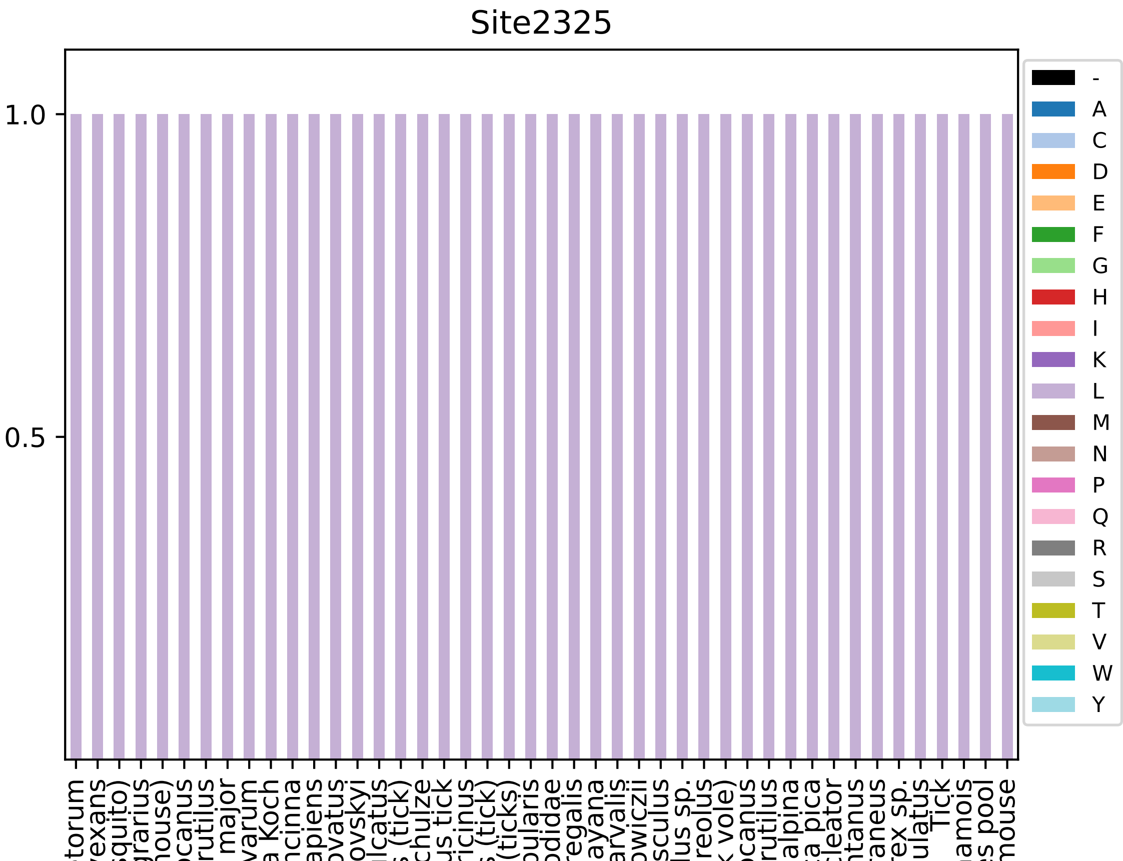
Site2325
1.0
0.5
-
A
C
D
E
F
G
H
I
K
L
M
N
P
Q
R
S
T
V
W
Y

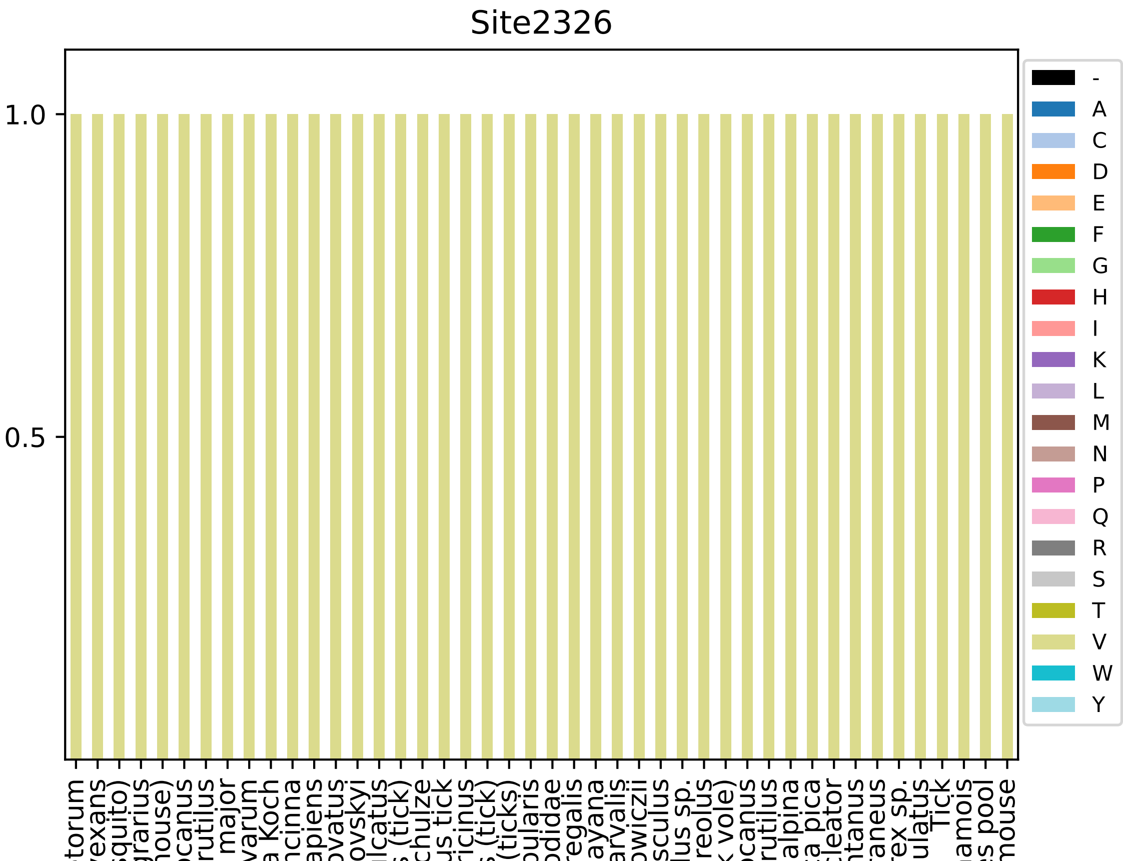
Site2326
1.0
0.5
-
A
C
D
E
F
G
H
I
K
L
M
N
P
Q
R
S
T
V
W
Y

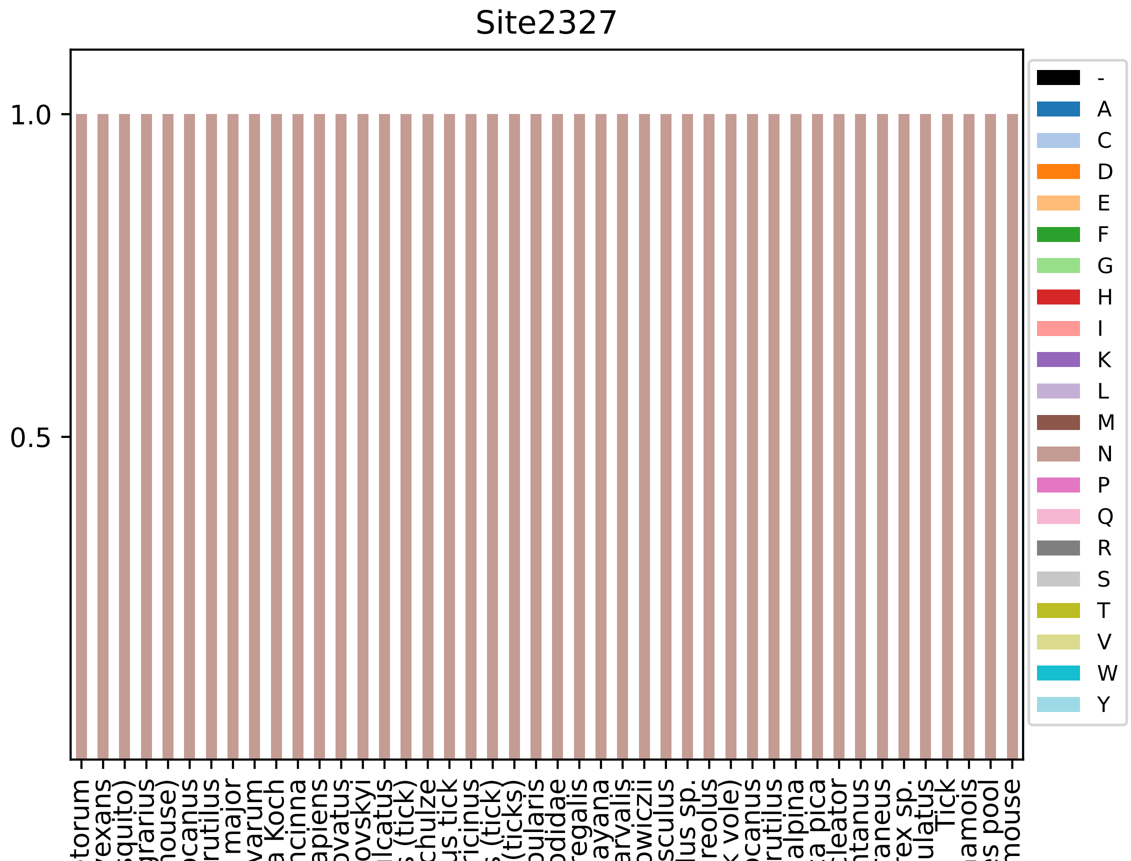
Site2327
1.0
0.5
-
A
C
D
E
F
G
H
I
K
L
M
N
P
Q
R
S
T
V
W
Y
Tick

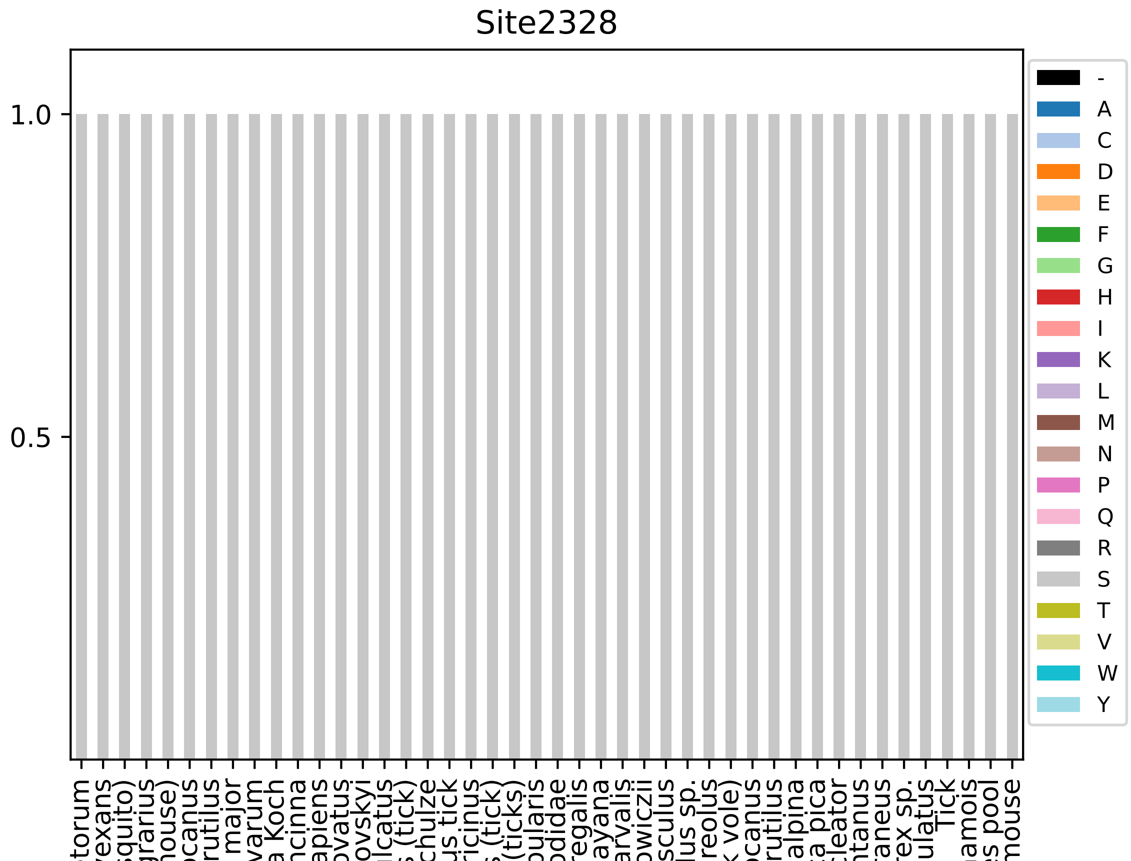

Site2328
1.0
0.5
-
A
C
D
E
F
G
H
I
K
L
M
N
P
Q
R
S
T
V
W
Y

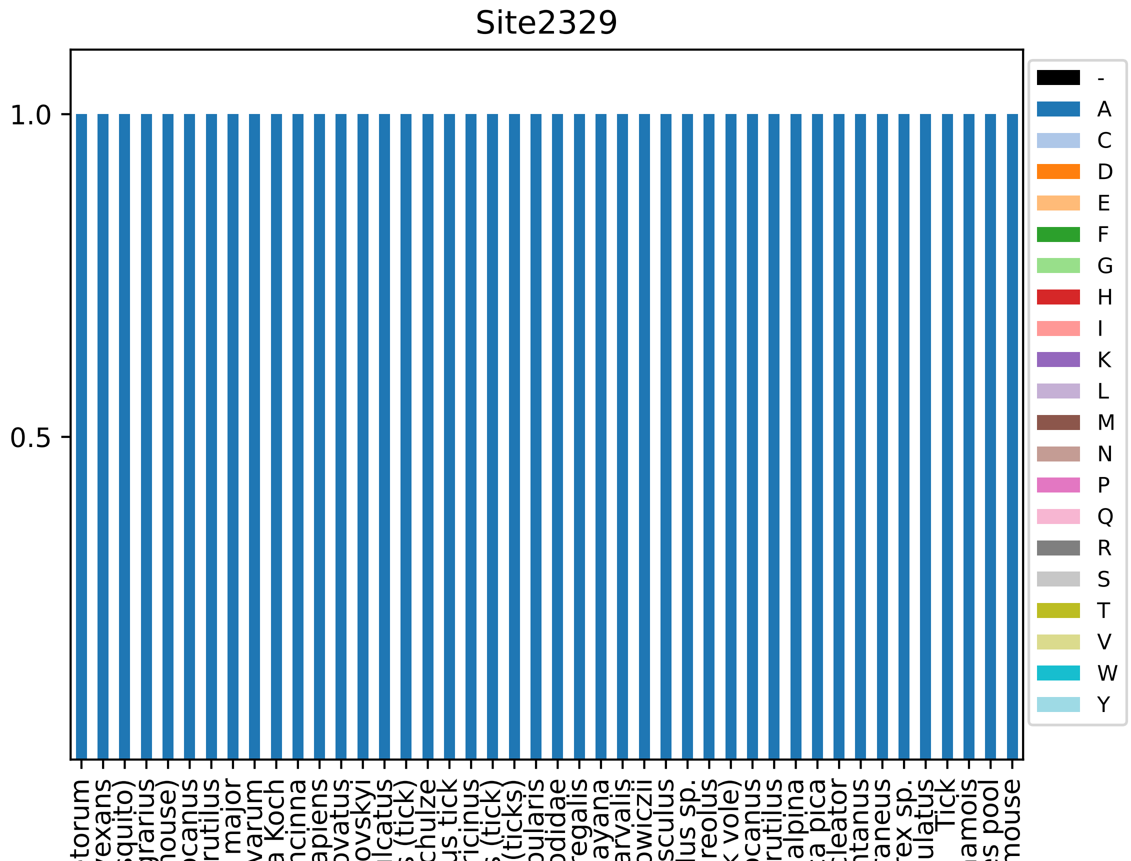
Site2329
1.0
0.5
-
A
C
D
E
F
G
H
I
K
L
M
N
P
Q
R
S
T
V
W
Y

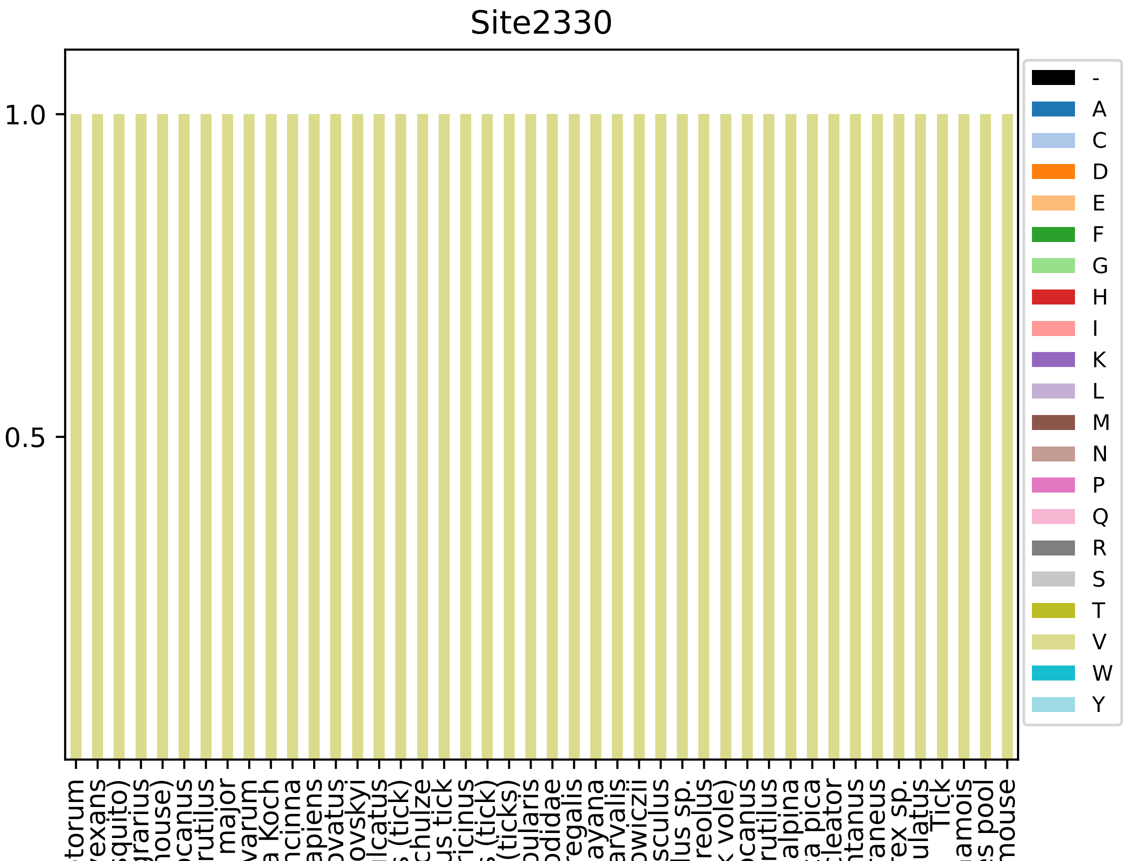
Site2330
1.0
0.5
-
A
C
D
E
F
G
H
I
K
L
M
N
P
Q
R
S
T
V
W
Y

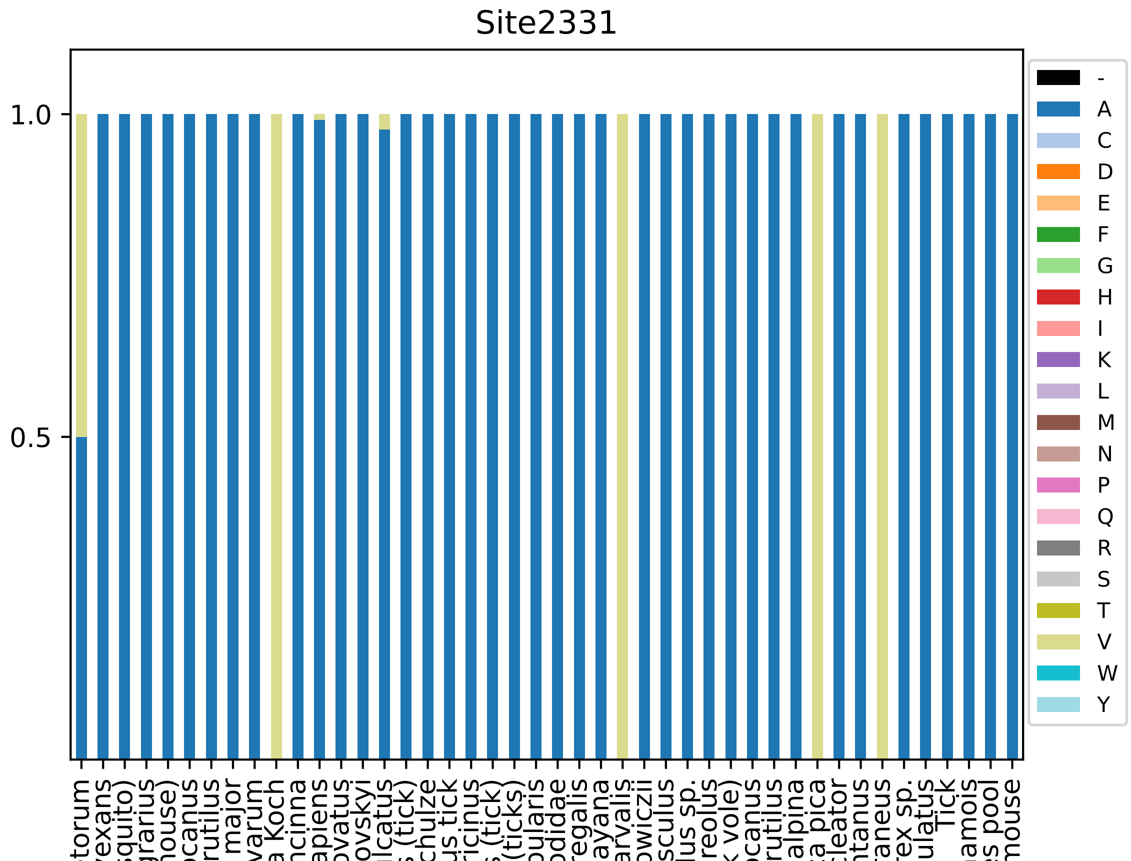

Site2331
1.0
0.5
-
A
C
D
E
F
G
H
I
K
L
M
N
P
Q
R
S
T
V
W
Y

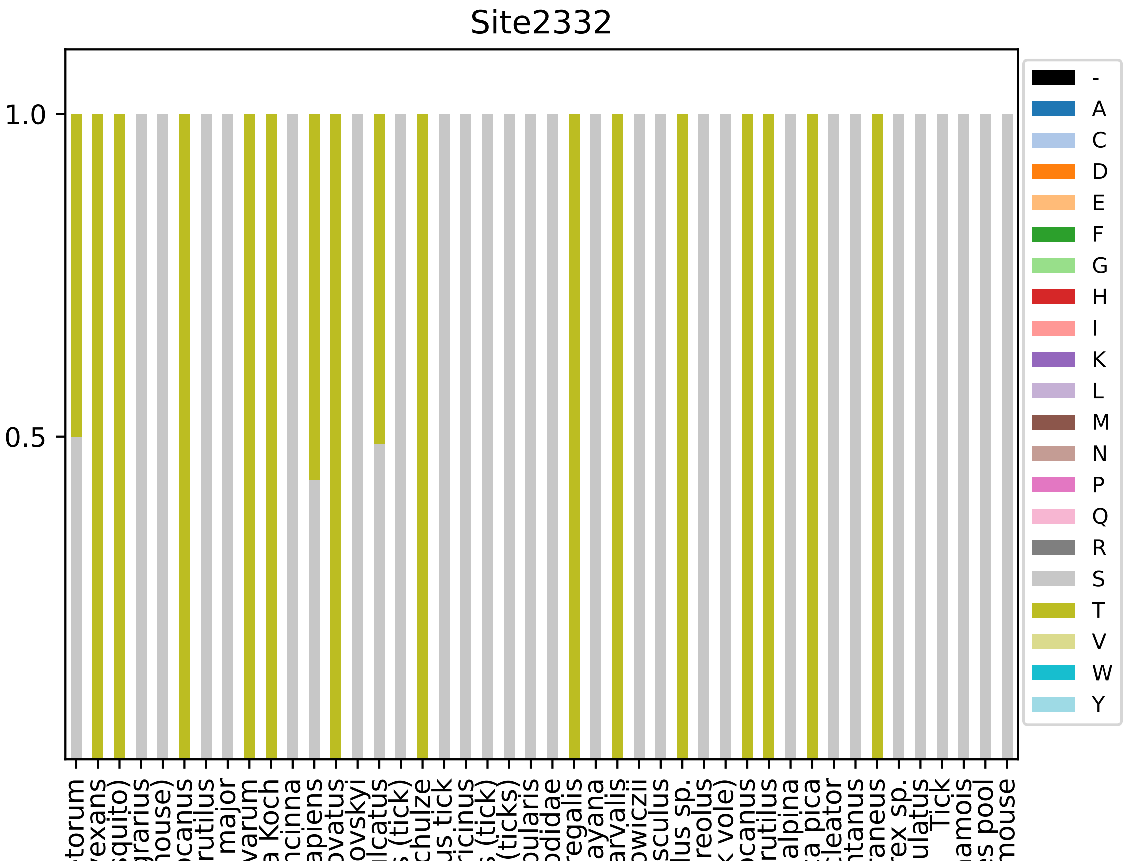
Site2332
1.0
0.5
-
A
C
D
E
F
G
H
I
K
L
M
N
P
Q
R
S
T
V
W
Y

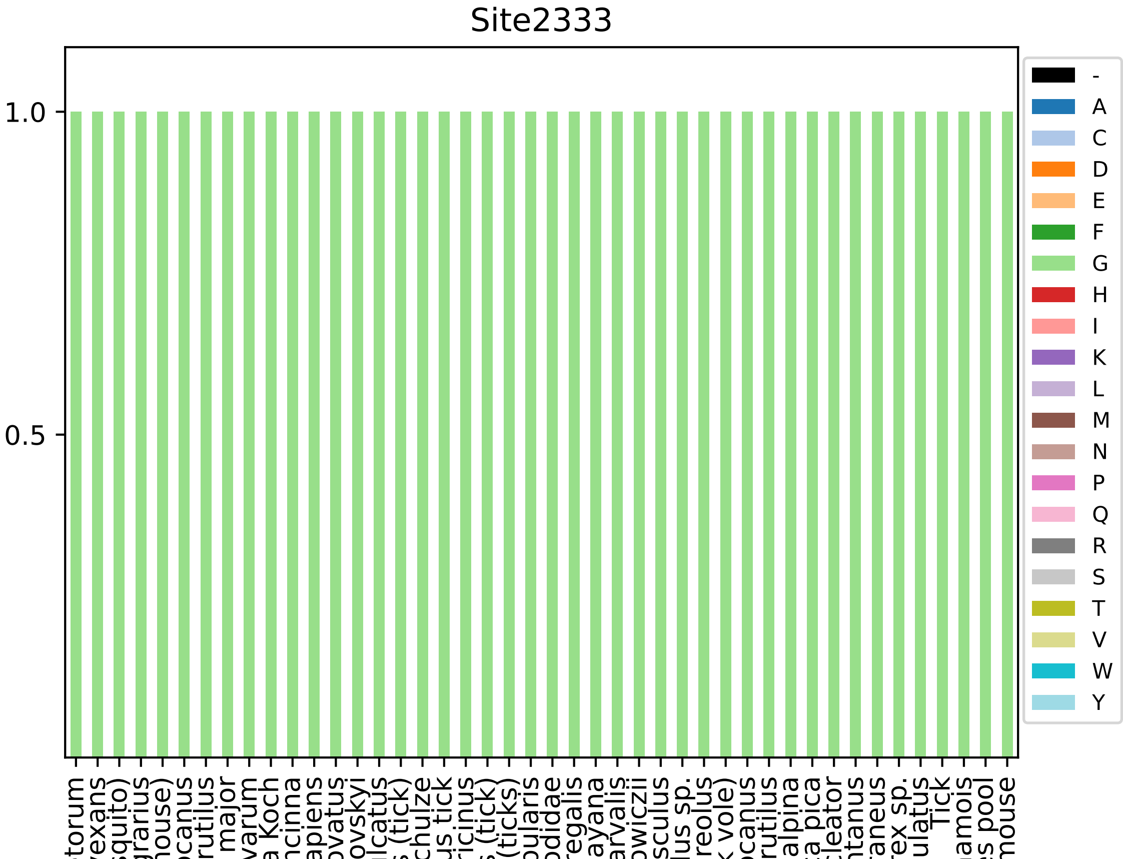
Site2333
1.0
0.5
-
A
C
D
E
F
G
H
I
K
L
M
N
P
Q
R
S
T
V
W
Y

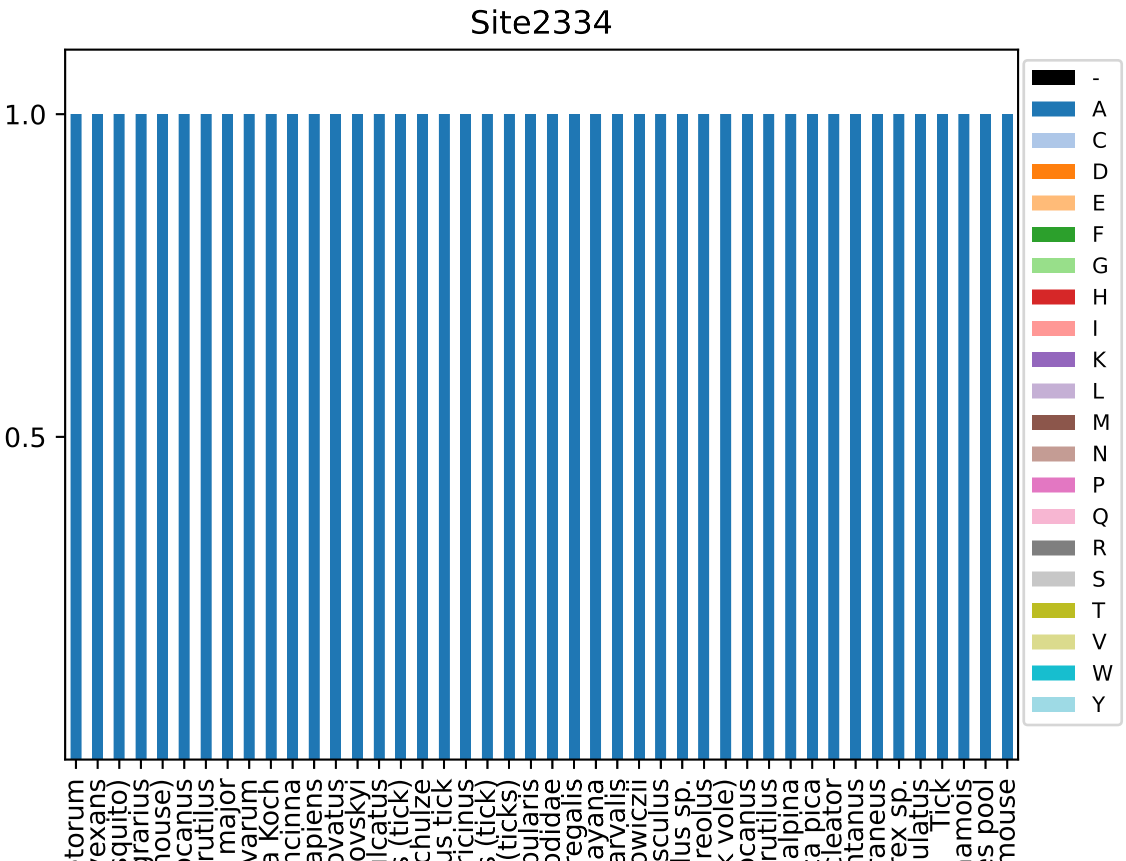
Site2334
1.0
0.5
-
A
C
D
E
F
G
H
I
K
L
M
N
P
Q
R
S
T
V
W
Y
torum
vexans
squito)
grarius
nouse)
ocanus
rutilus
major
varum
a Koch
ncinna
apiens
ovatus
ovskyi
ulcatus
s (tick)
chulze
us tick
ricinus
s (tick)
(ticks)
pularis
odidae
regalis
ayana
arvalis
owiczii
sculus
lus sp.
reolus
k vole)
ocanus
rutilus
alpina
a pica
cleator
ntanus
raneus
rex sp.
ulatus
Tick
amois
es pool
mouse

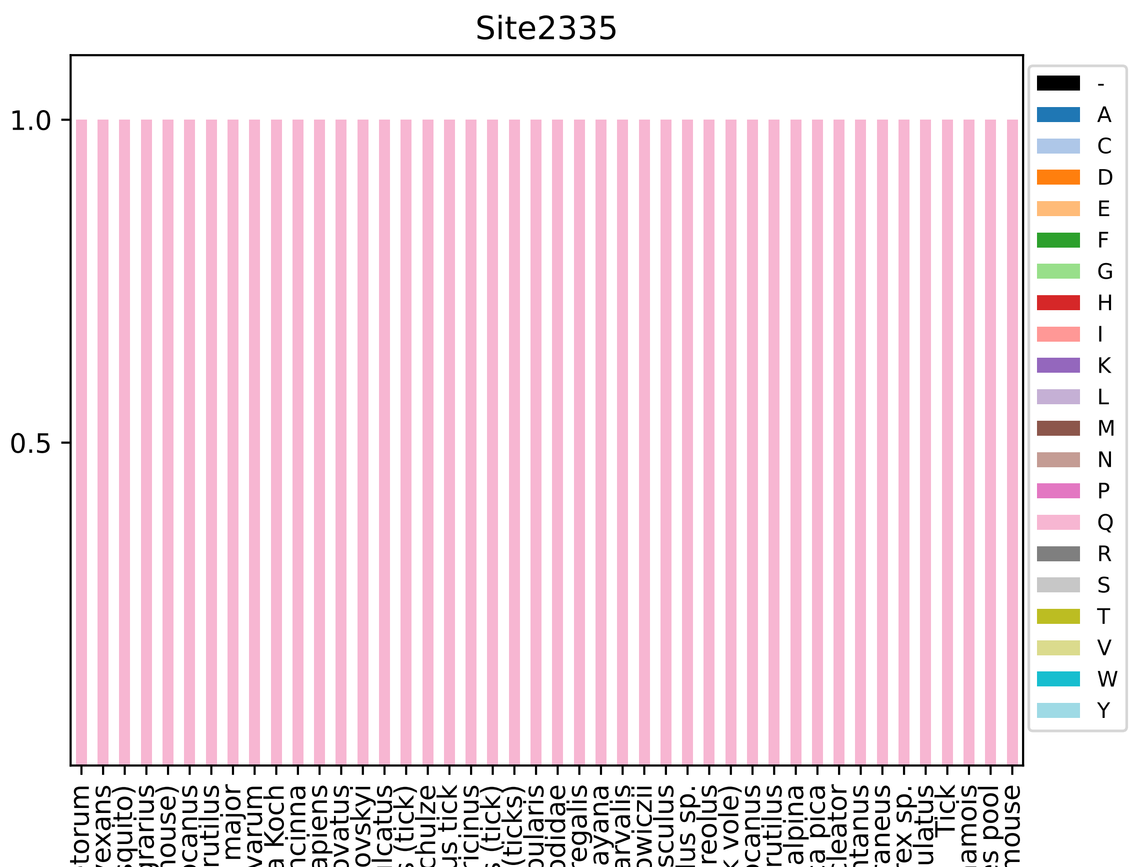
Site2335
1.0
0.5
-
A
C
D
E
F
G
H
I
K
L
M
N
P
Q
R
S
T
V
W
Y
Tick

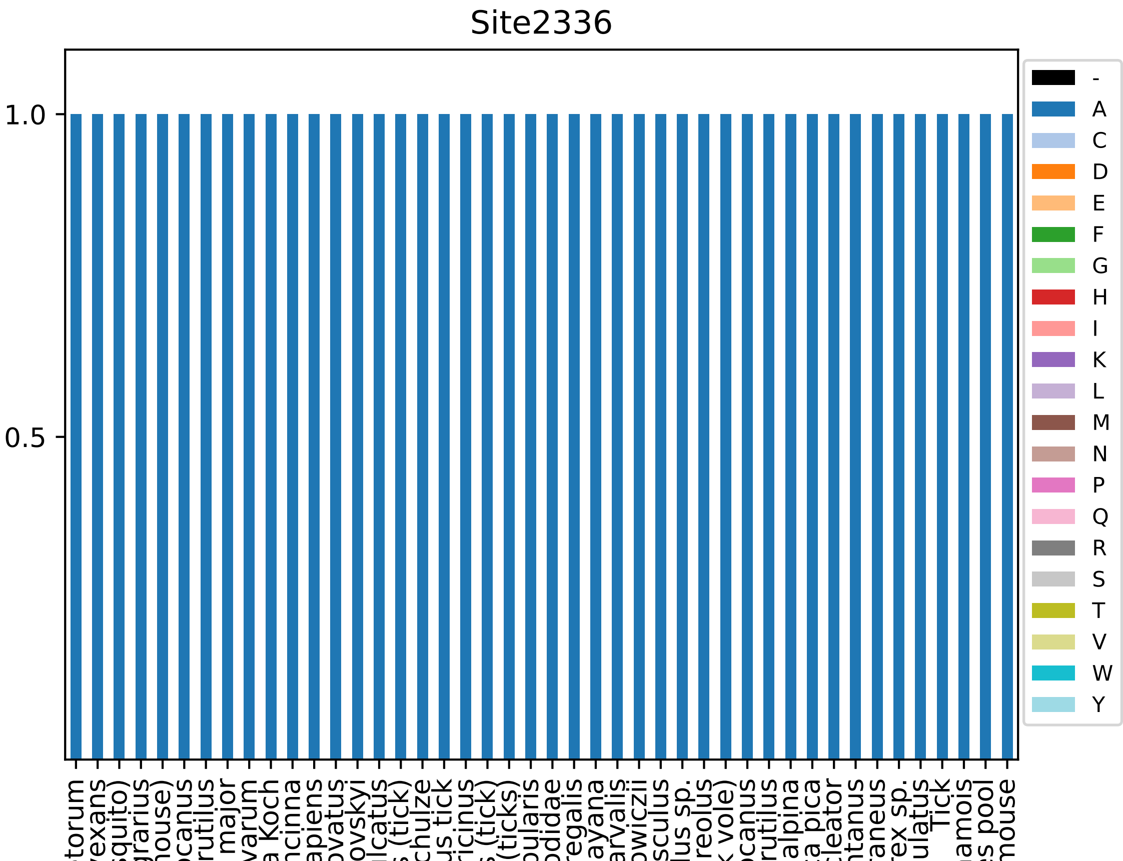

Site2336
1.0
0.5
-
A
C
D
E
F
G
H
I
K
L
M
N
P
Q
R
S
T
V
W
Y

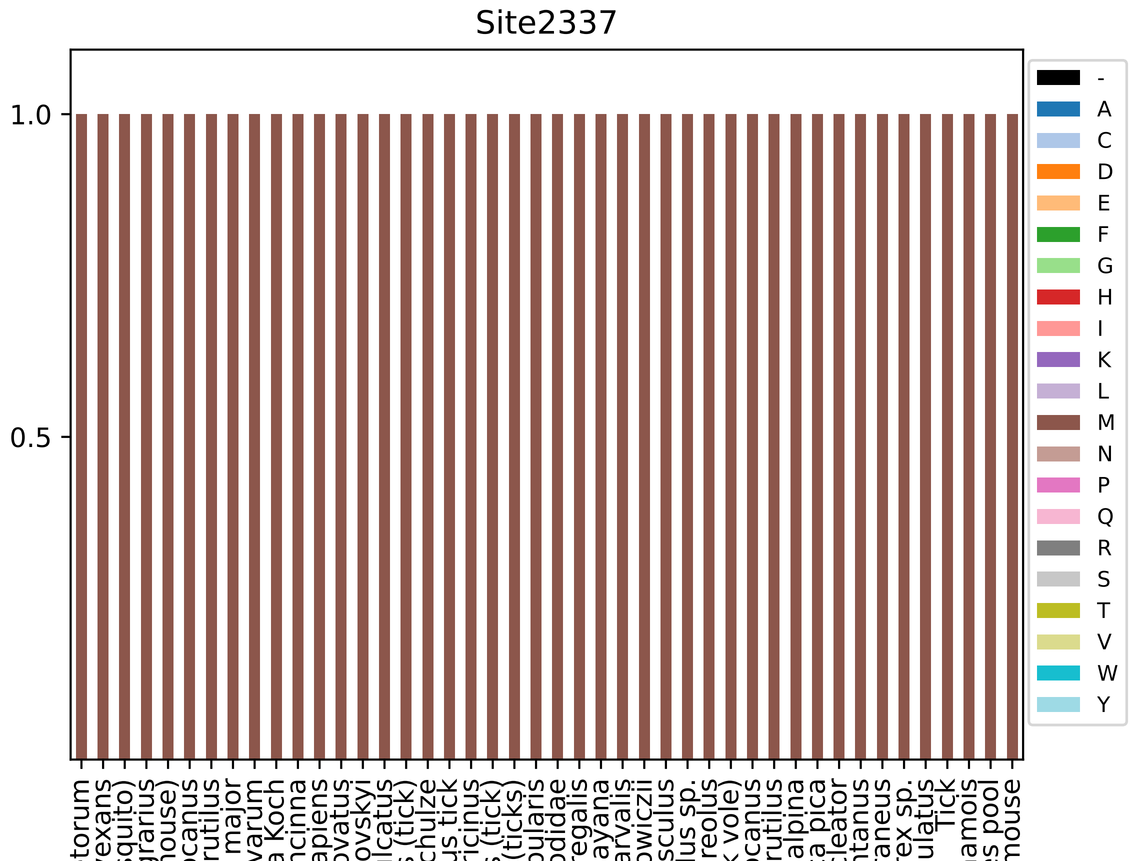
Site2337
1.0
0.5
-
A
C
D
E
F
G
H
I
K
L
M
N
P
Q
R
S
T
V
W
Y

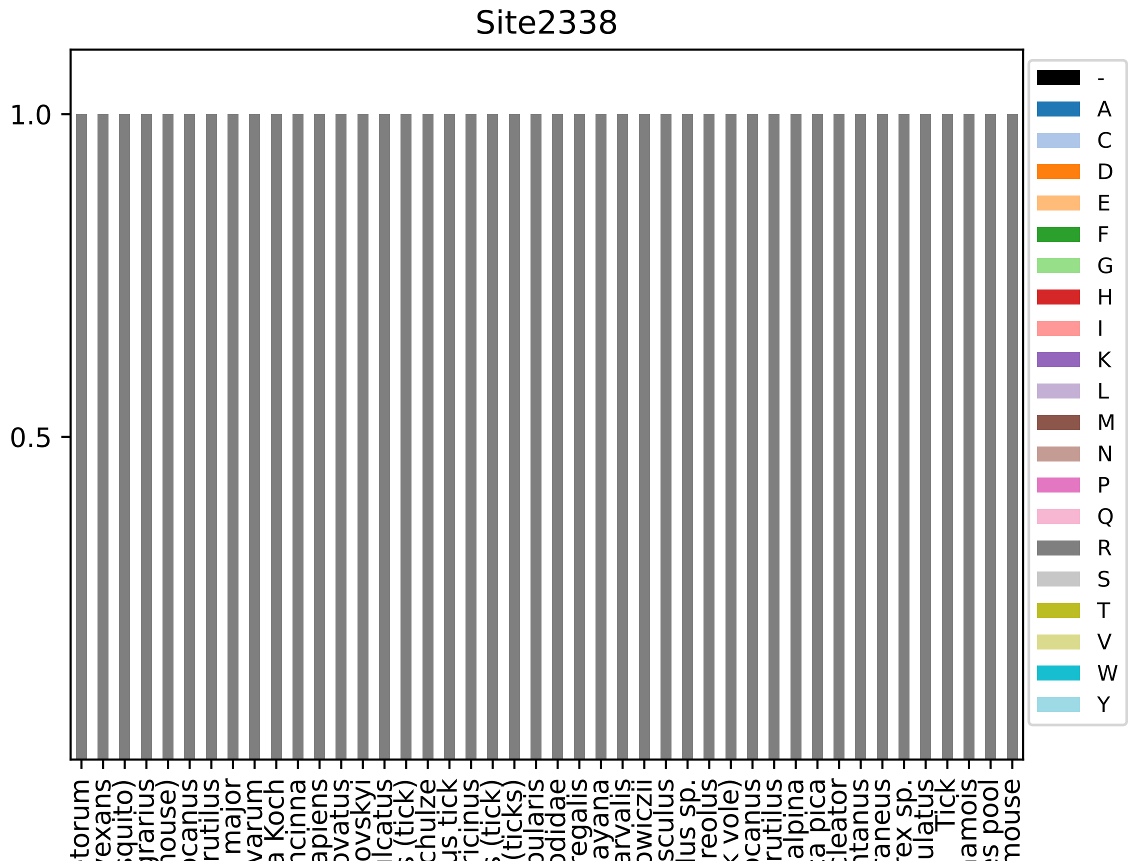
Site2338
1.0
0.5
-
A
C
D
E
F
G
H
I
K
L
M
N
P
Q
R
S
T
V
W
Y

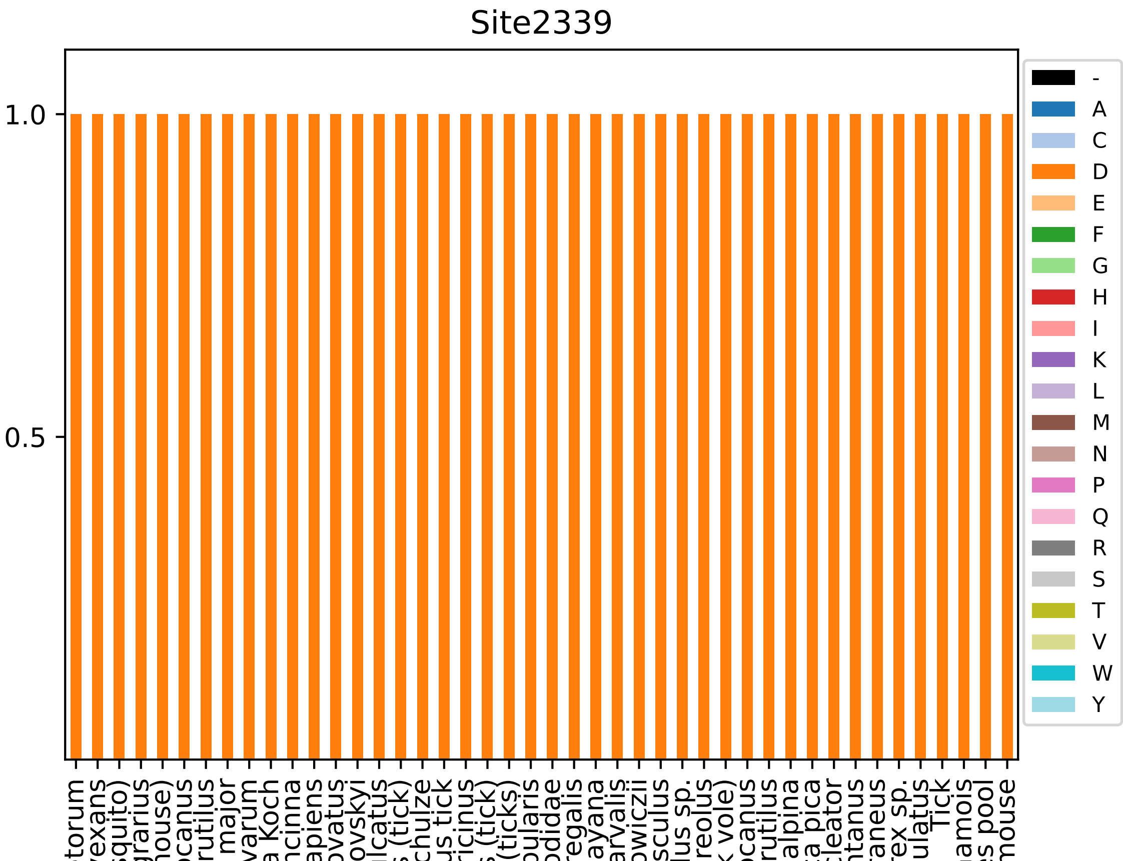
Site2339
1.0
0.5
-
A
C
D
E
F
G
H
I
K
L
M
N
P
Q
R
S
T
V
W
Y

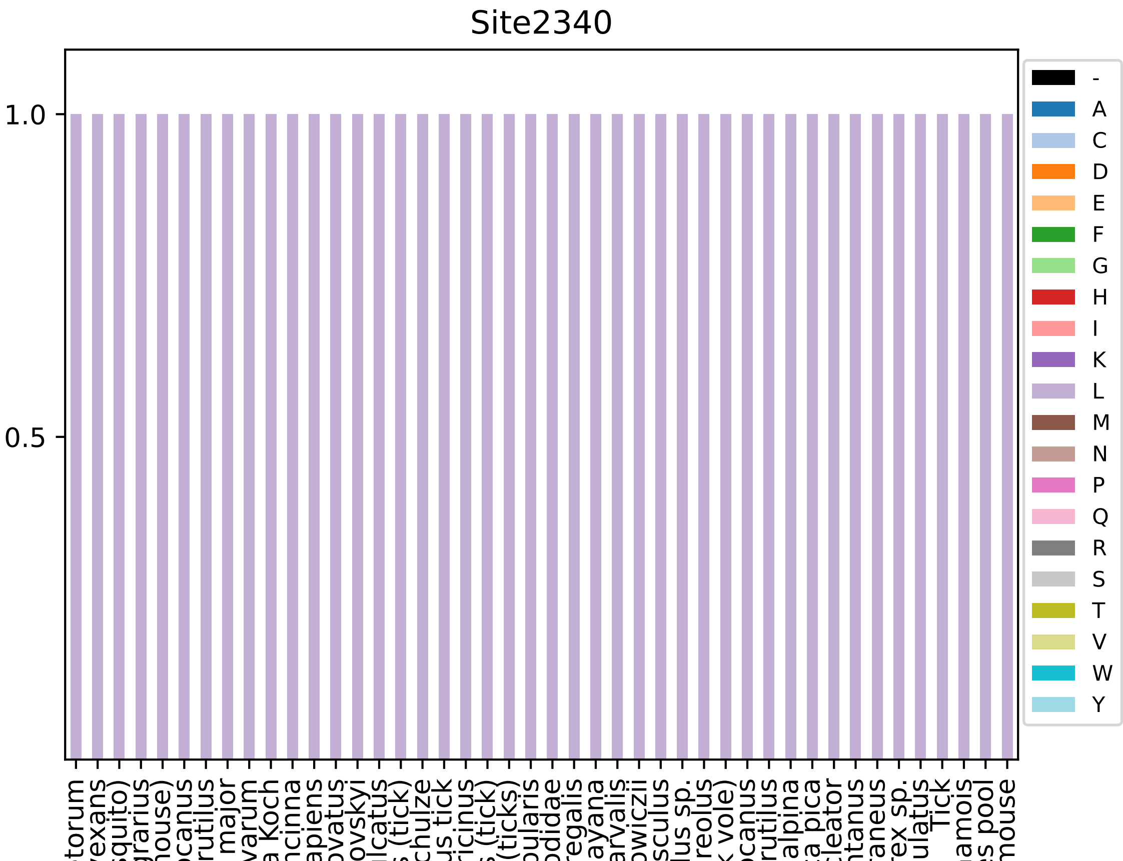
Site2340
1.0
0.5
-
A
C
D
E
F
G
H
I
K
L
M
N
P
Q
R
S
T
V
W
Y

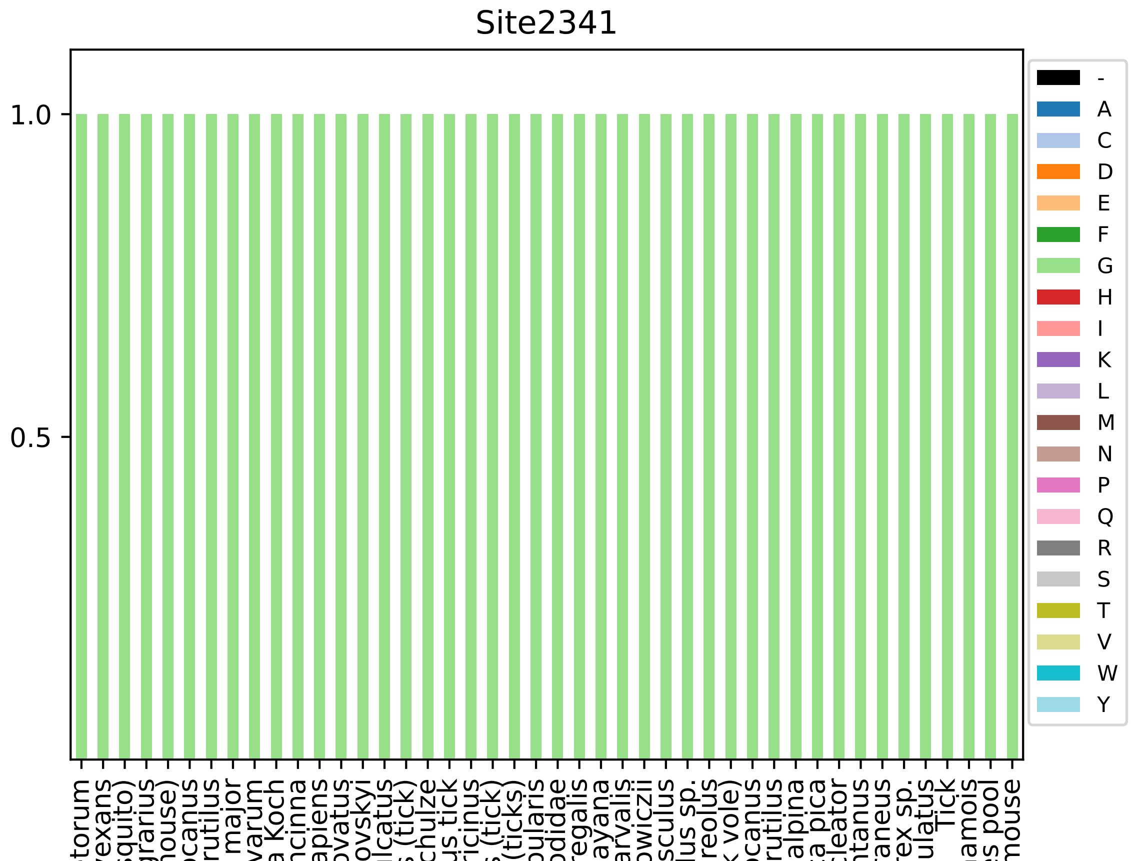
Site2341
1.0
0.5
-
A
C
D
E
F
G
H
I
K
L
M
N
P
Q
R
S
T
V
W
Y

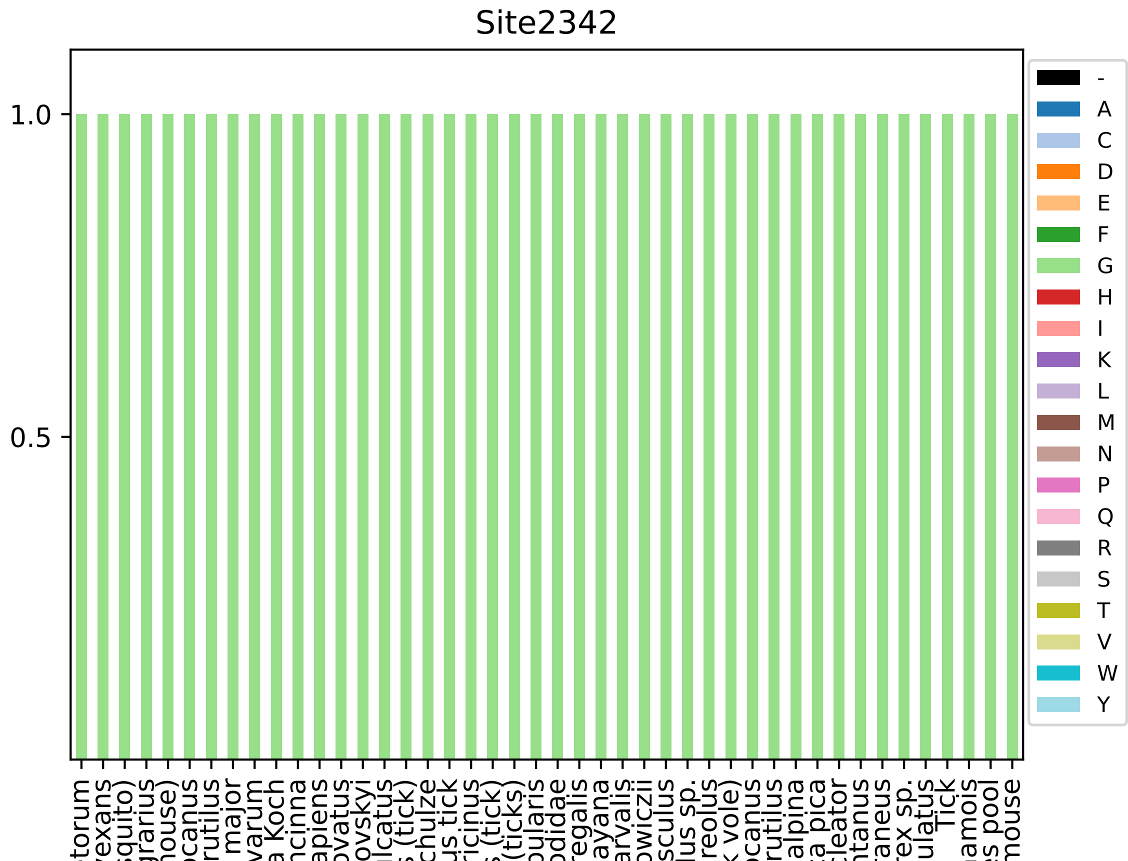
Site2342
1.0
0.5
-
A
C
D
E
F
G
H
I
K
L
M
N
P
Q
R
S
T
V
W
Y

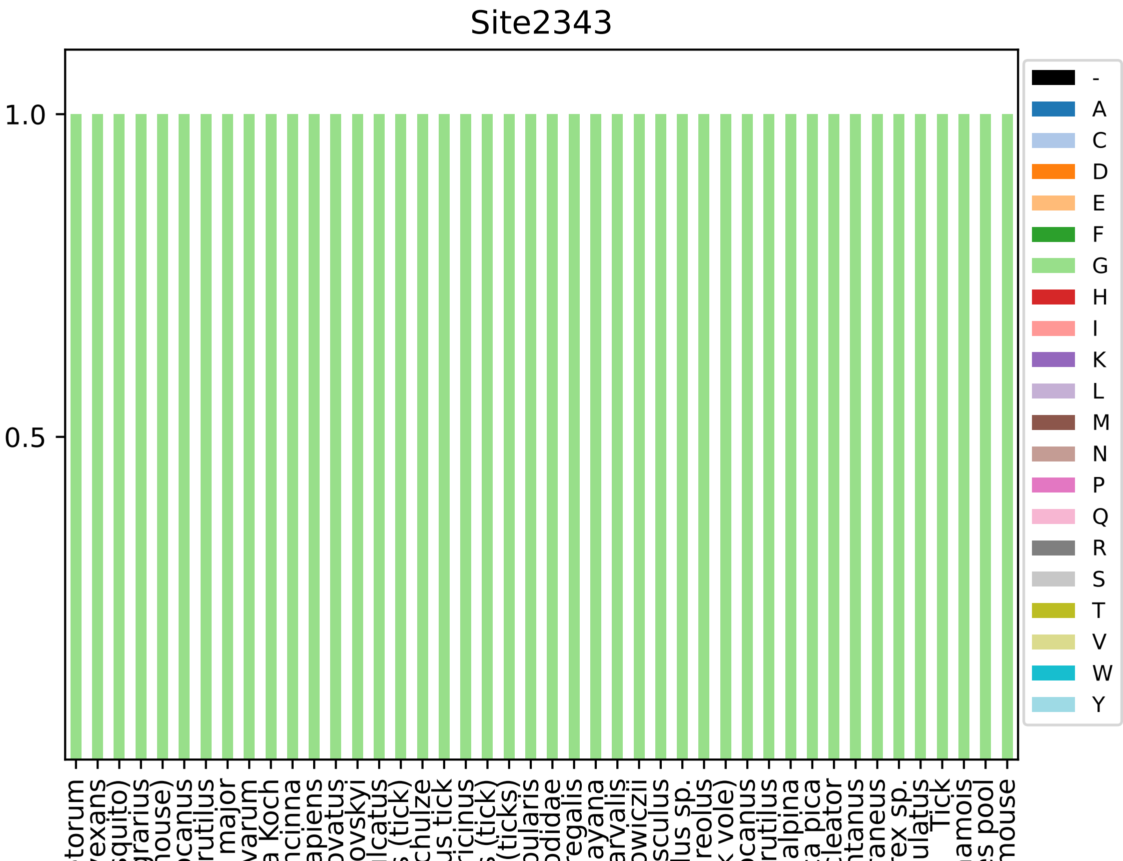
Site2343
1.0
0.5
-
A
C
D
E
F
G
H
I
K
L
M
N
P
Q
R
S
T
V
W
Y

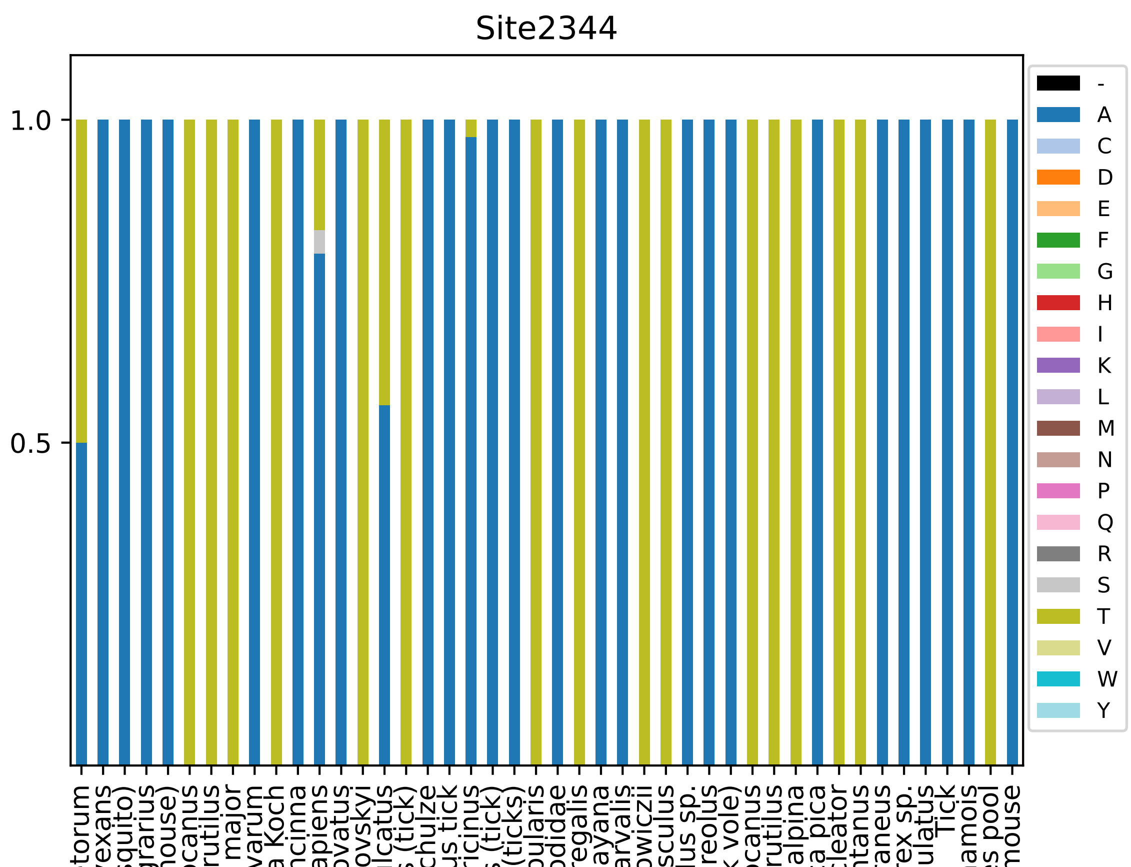
Site2344
1.0
0.5
-
A
C
D
E
F
G
H
I
K
L
M
N
P
Q
R
S
T
V
W
Y

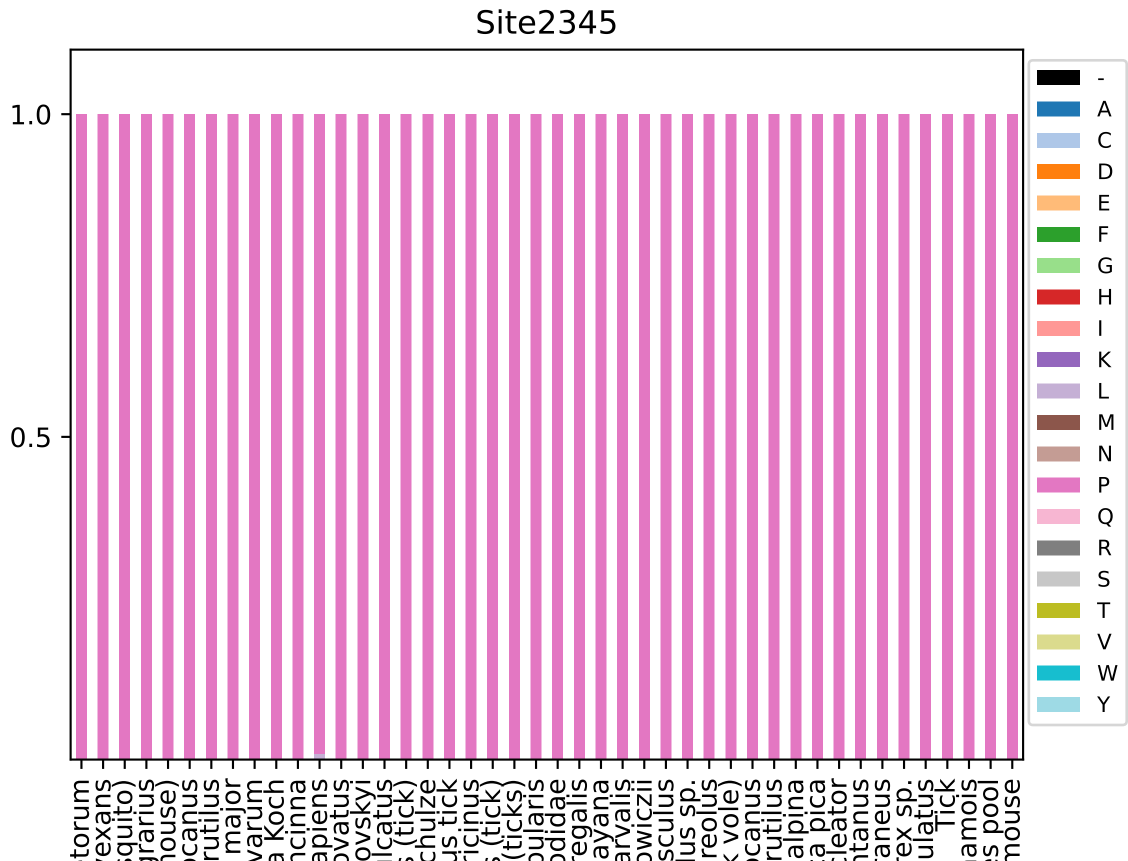
Site2345
1.0
0.5
-
A
C
D
E
F
G
H
I
K
L
M
N
P
Q
R
S
T
V
W
Y

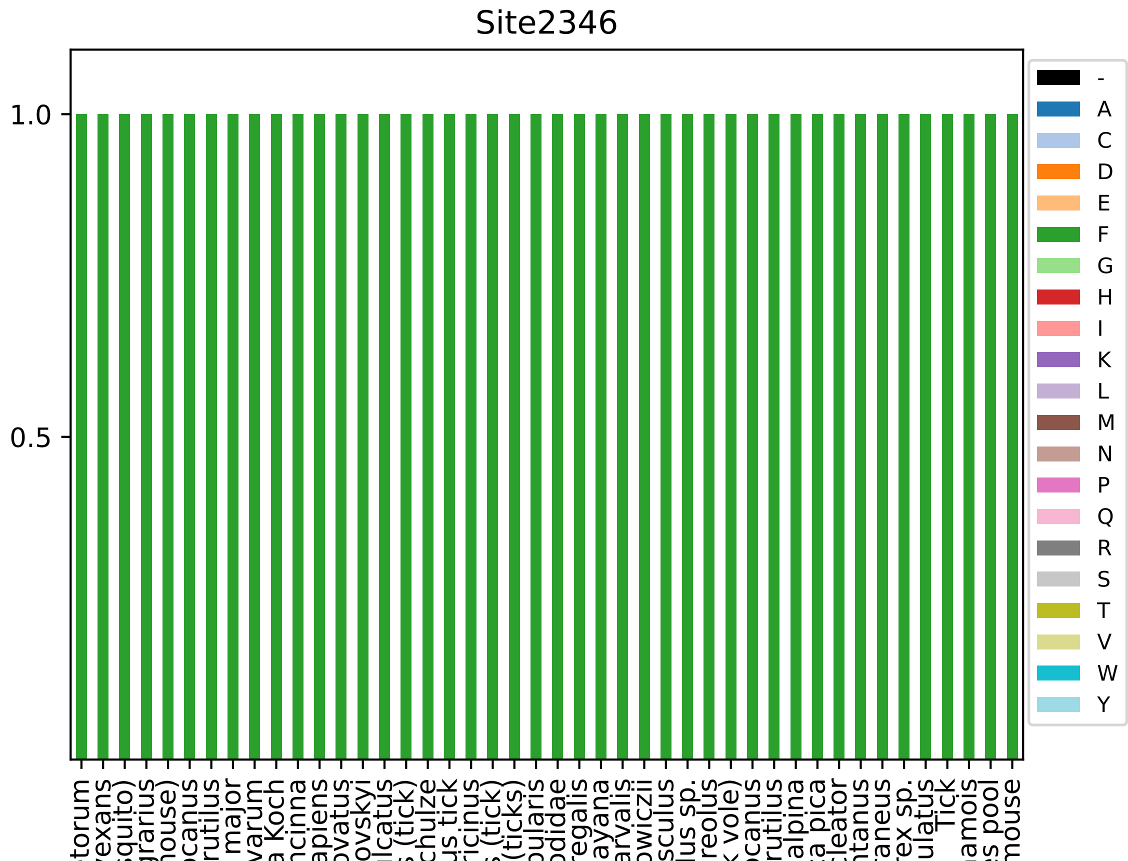
Site2346
1.0
0.5
-
A
C
D
E
F
G
H
I
K
L
M
N
P
Q
R
S
T
V
W
Y

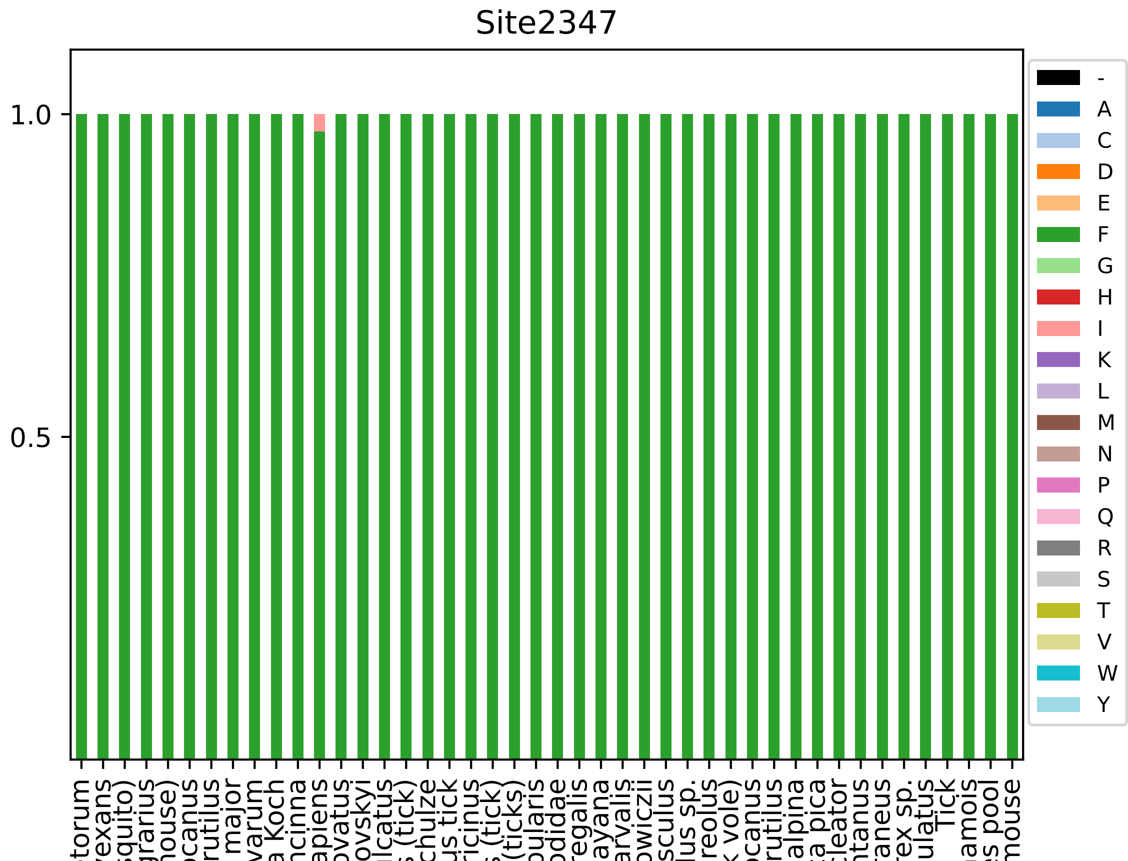

Site2347
1.0
0.5
-
A
C
D
E
F
G
H
I
K
L
M
N
P
Q
R
S
T
V
W
Y

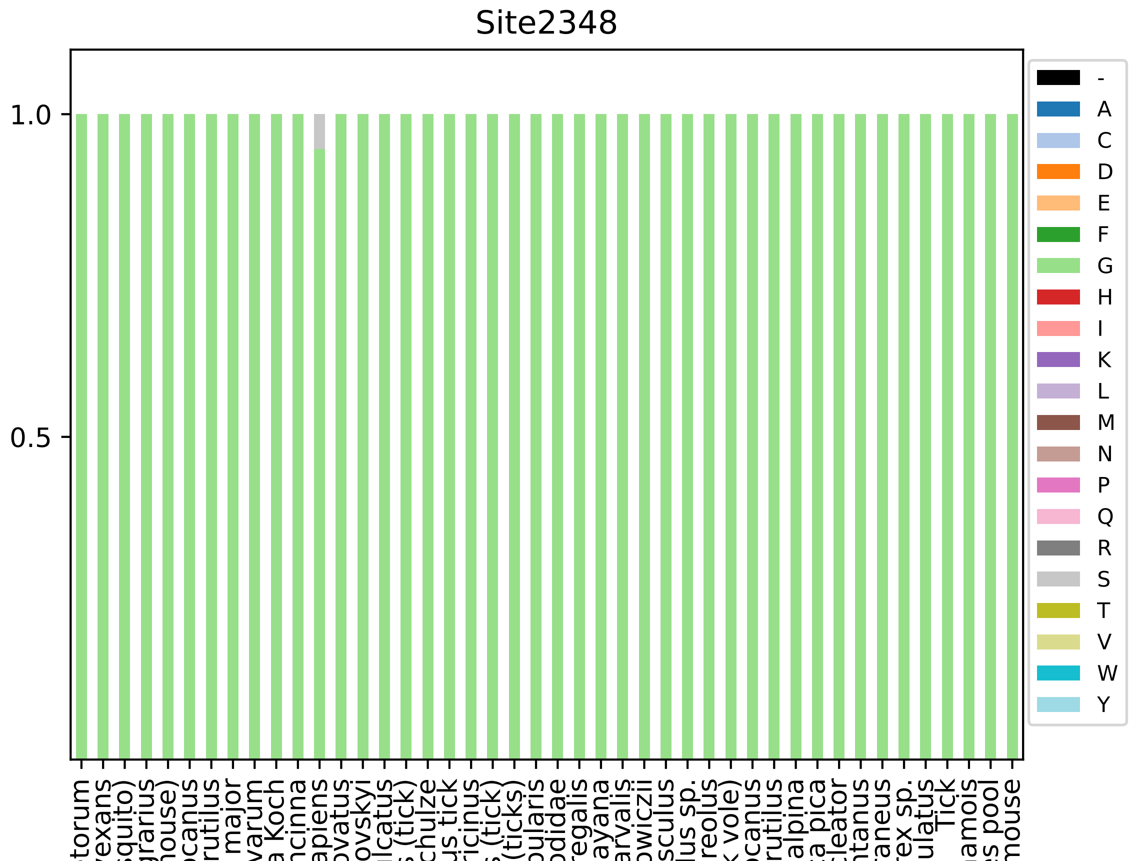
Site2348
1.0
0.5
-
A
C
D
E
F
G
H
I
K
L
M
N
P
Q
R
S
T
V
W
Y
Tick

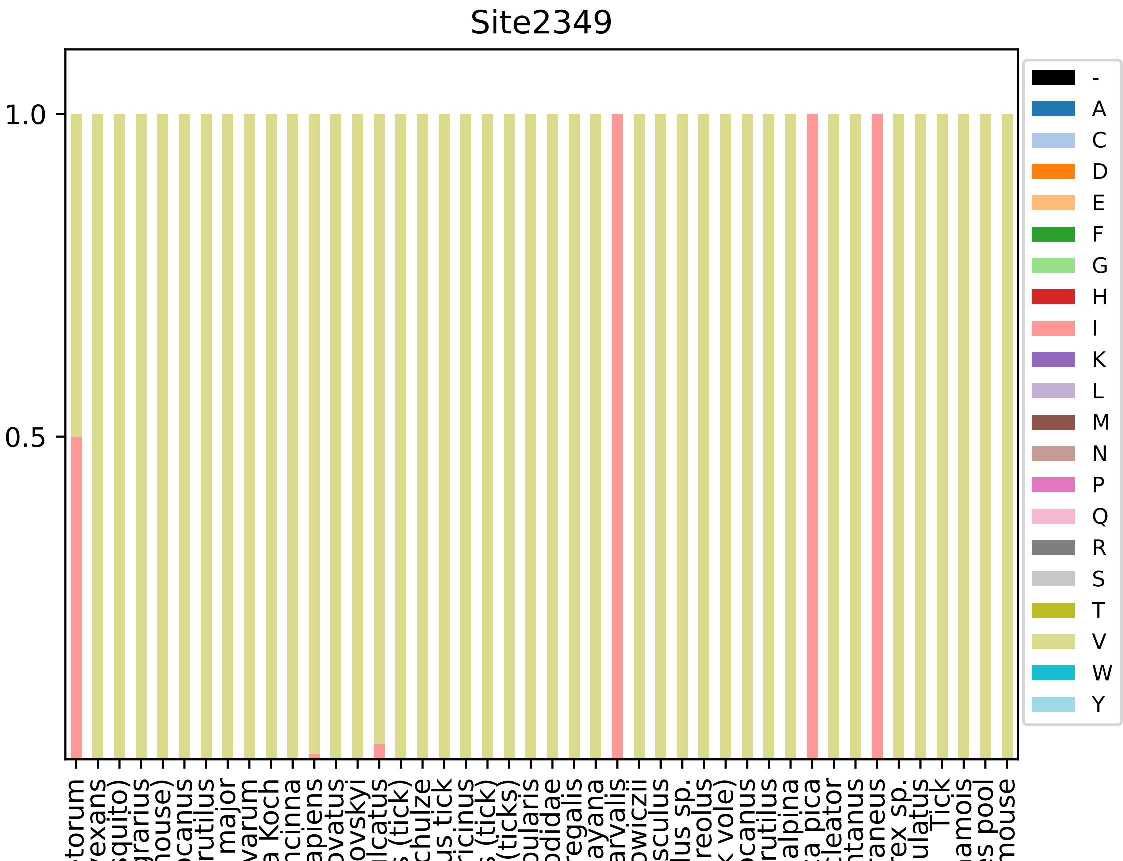
Site2349
1.0
0.5
-
A
C
D
E
F
G
H
I
K
L
M
N
P
Q
R
S
T
V
W
Y

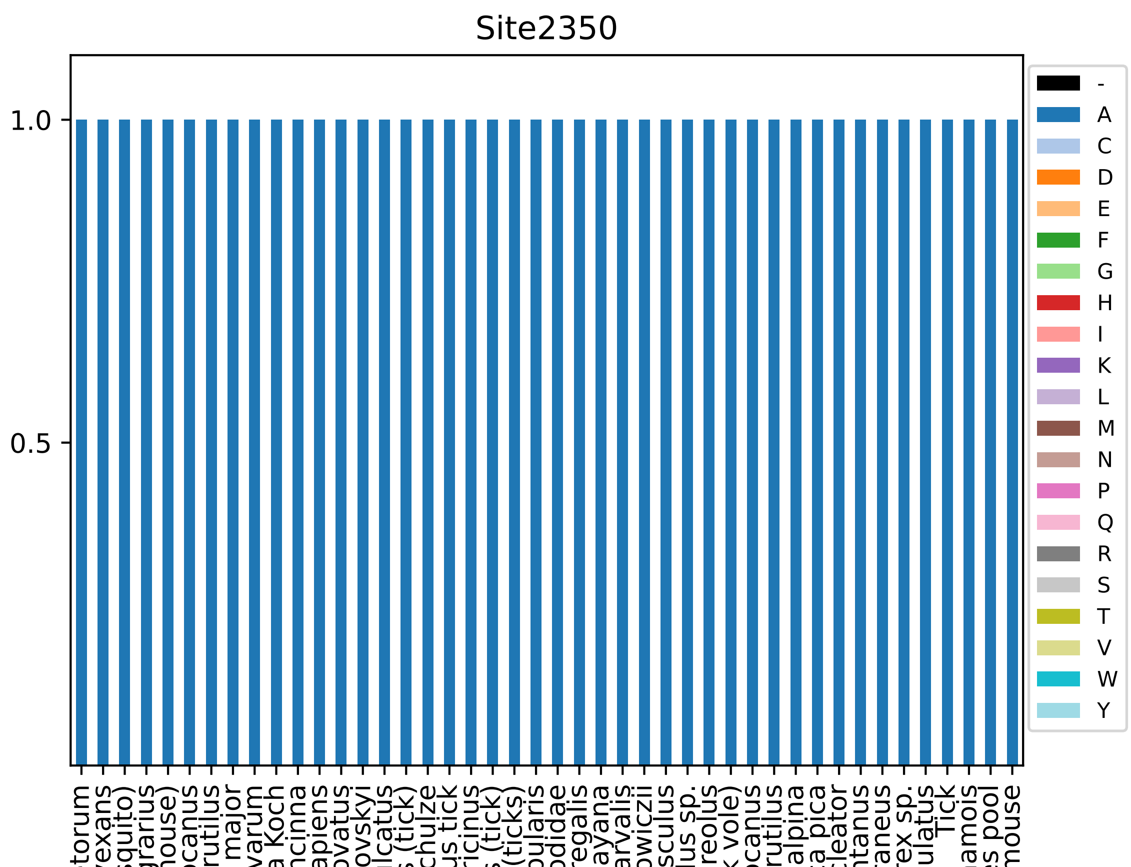
Site2350
1.0
0.5
-
A
C
D
E
F
G
H
I
K
L
M
N
P
Q
R
S
T
V
W
Y

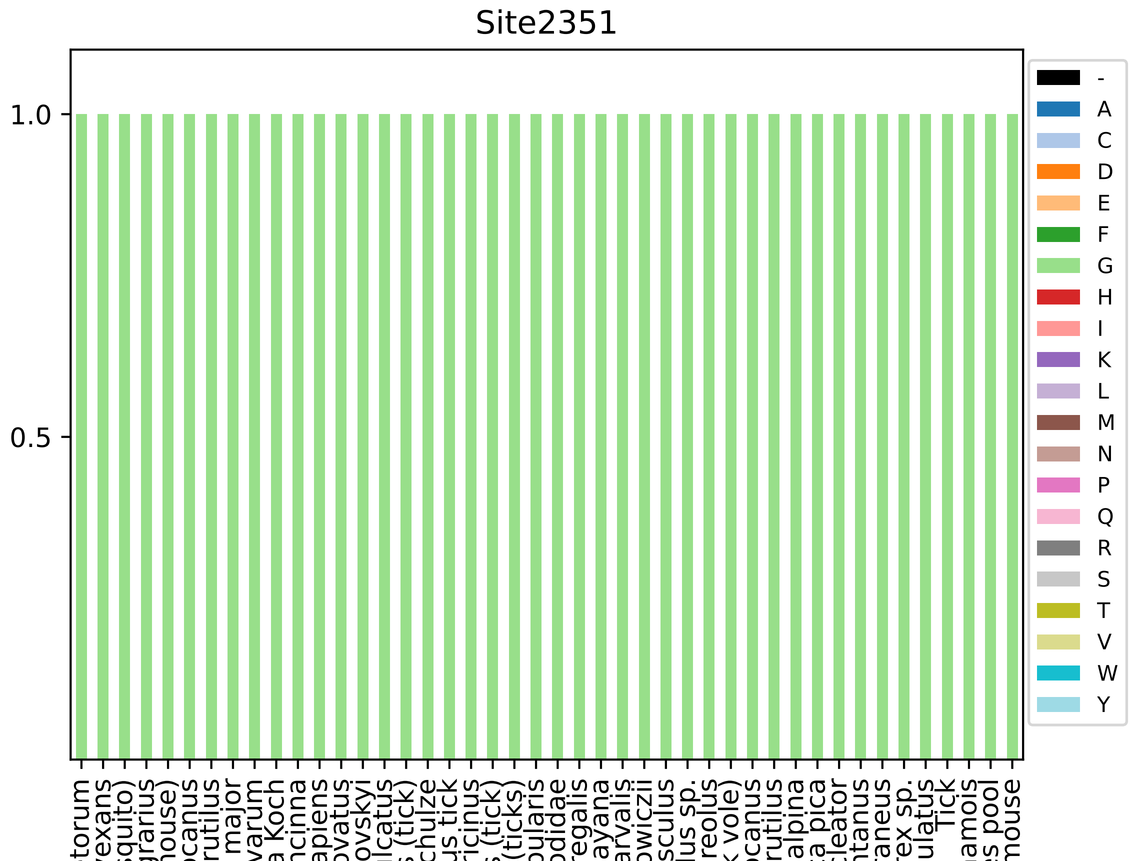
Site2351
1.0
0.5
-
A
C
D
E
F
G
H
I
K
L
M
N
P
Q
R
S
T
V
W
Y
Tick

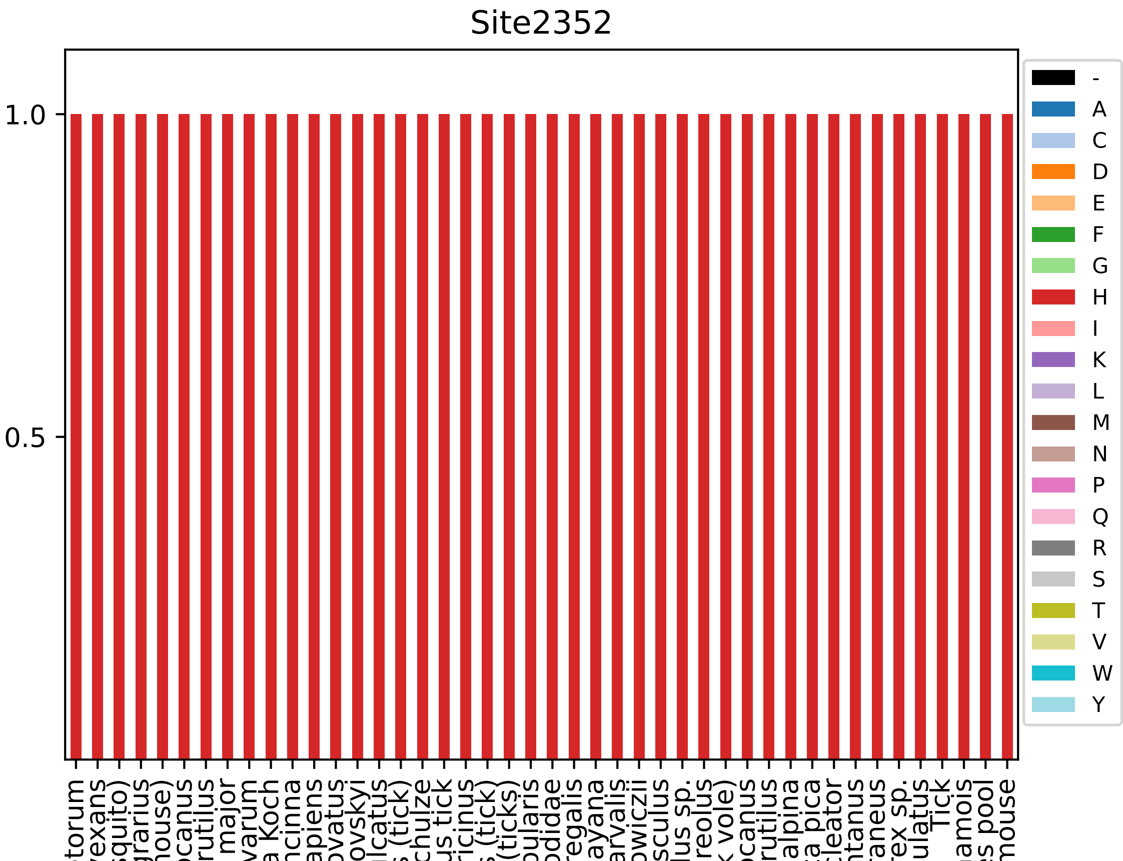
Site2352
1.0
0.5
-
A
C
D
E
F
G
H
I
K
L
M
N
P
Q
R
S
T
V
W
Y

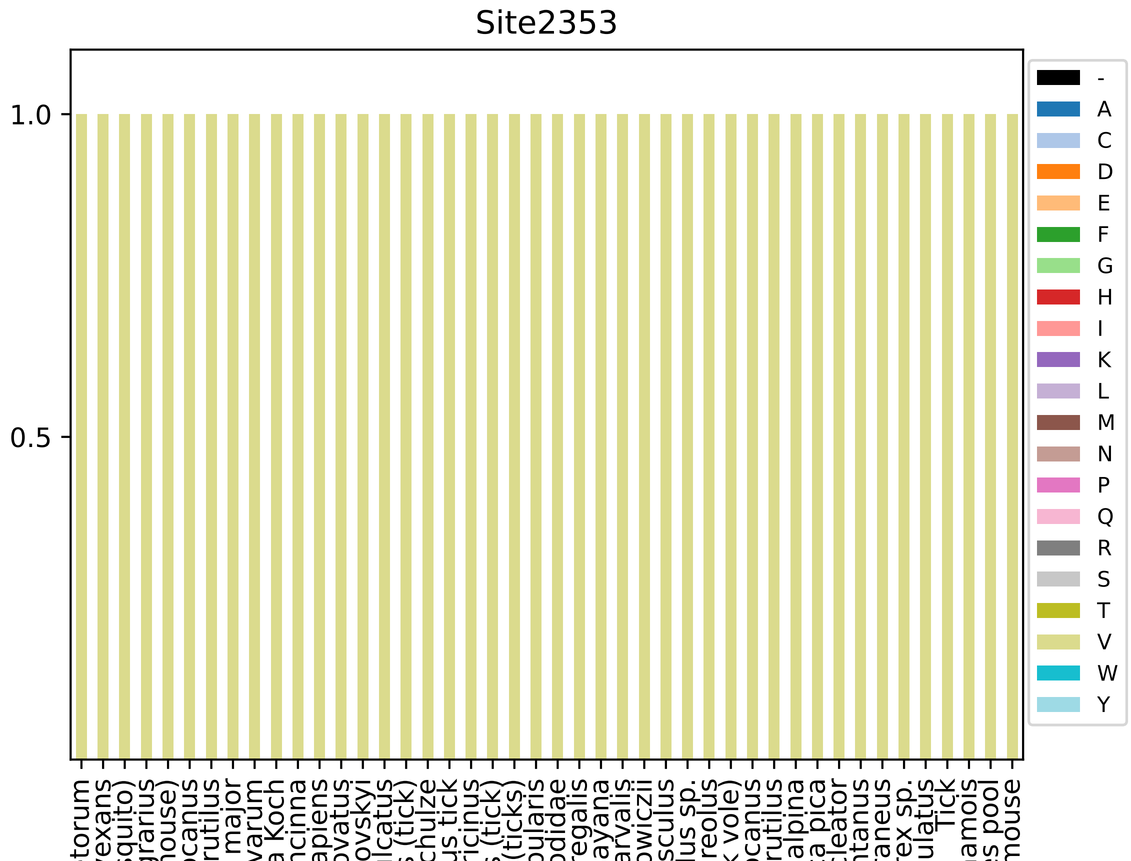

Site2353
1.0
0.5
-
A
C
D
E
F
G
H
I
K
L
M
N
P
Q
R
S
T
V
W
Y

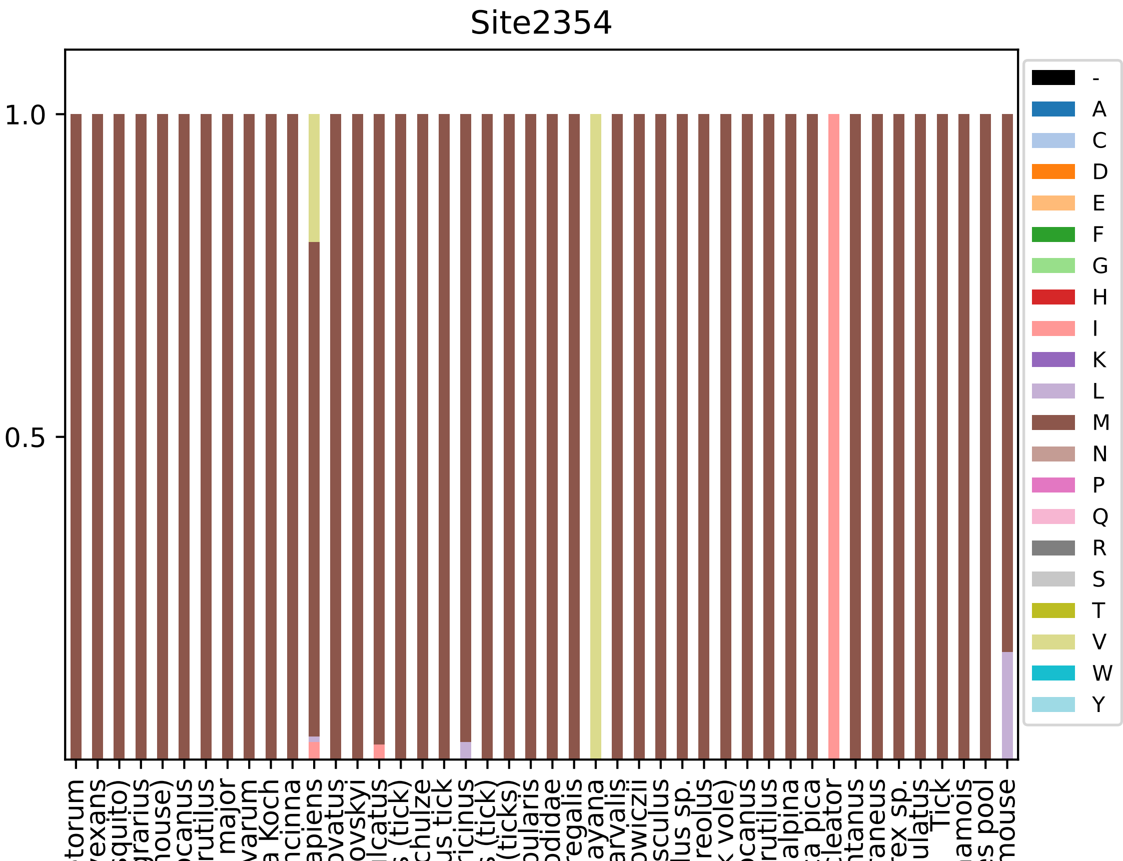

Site2354
1.0
0.5
-
A
C
D
E
F
G
H
I
K
L
M
N
P
Q
R
S
T
V
W
Y

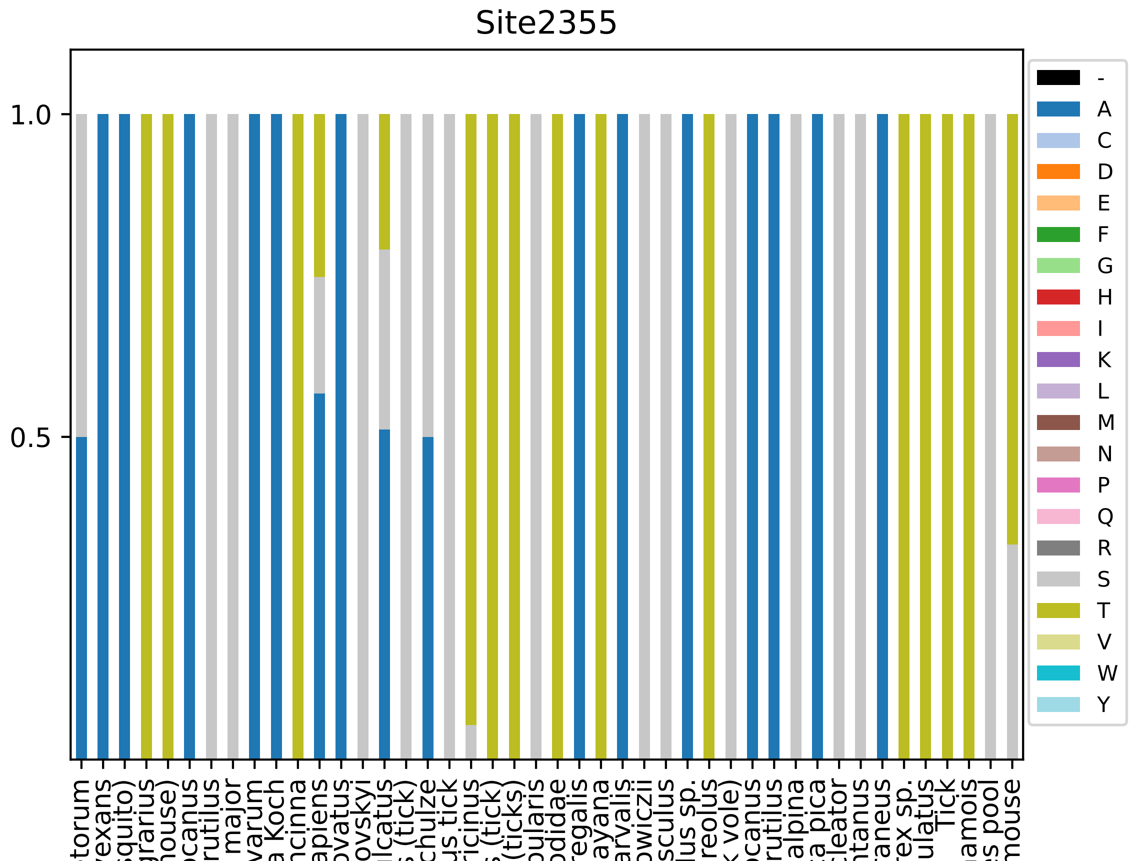
Site2355
1.0
0.5
-
A
C
D
E
F
G
H
I
K
L
M
N
P
Q
R
S
T
V
W
Y
torum
vexans
squito)
grarius
house)
ocanus
rutilus
major
varum
a Koch
ncinna
apiens
ovatus
ovskyi
ulcatus
s (tick)
chulze
us tick
ricinus
s (tick)
(ticks)
pularis
odidae
regalis
ayana
arvalis
owiczii
sculus
lus sp.
reolus
k vole)
ocanus
rutilus
alpina
a pica
cleator
ntanus
raneus
rex sp.
ulatus
Tick
amois
es pool
mouse

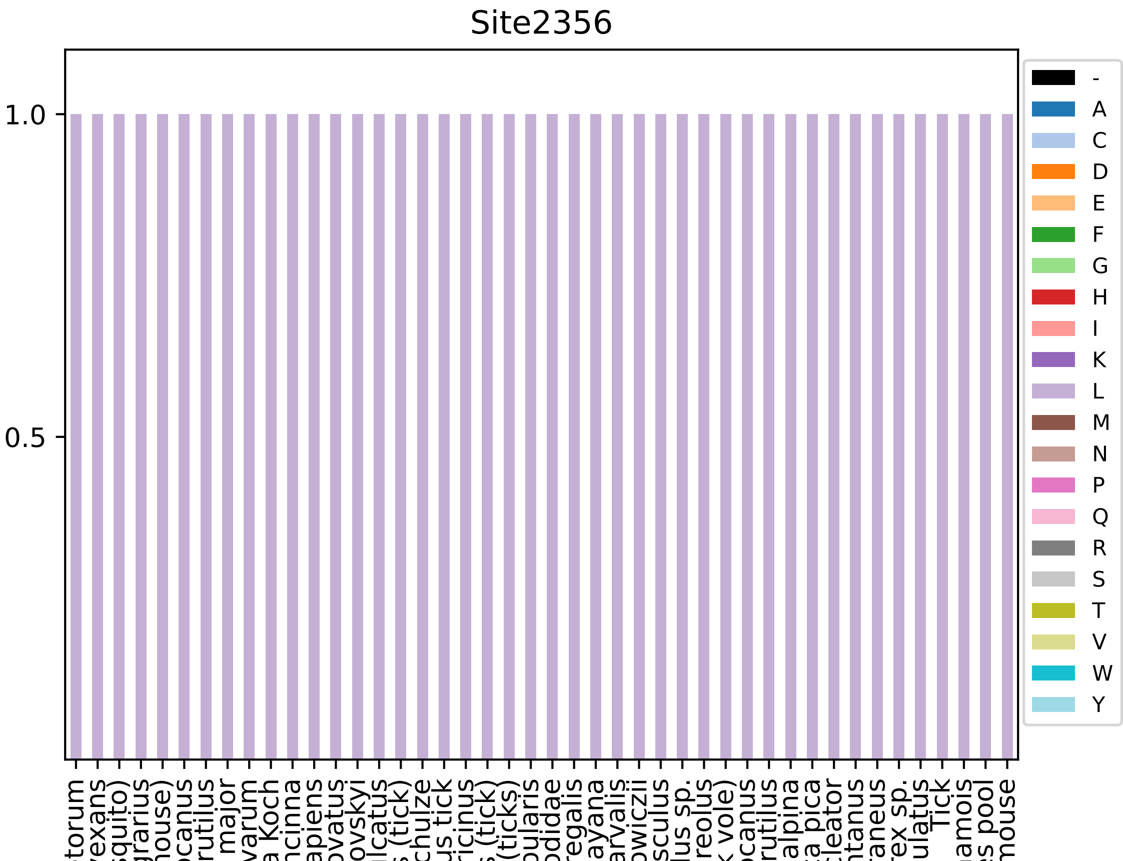
Site2356
1.0
0.5
-
A
C
D
E
F
G
H
I
K
L
M
N
P
Q
R
S
T
V
W
Y
torum
vexans
squito)
grarius
nouse)
ocanus
rutilus
major
varum
a Koch
ncinna
apiens
ovatus
ovskyi
lcatus
(tick)
chulze
us tick
ricinus
(tick)
(ticks)
pularis
podidae
regalis
ayana
arvalis
owiczii
sculus
lus sp.
reolus
k vole)
ocanus
rutilus
alpina
a pica
leator
ntanus
raneus
rex sp.
ulatus
Tick
amois
es pool
mouse

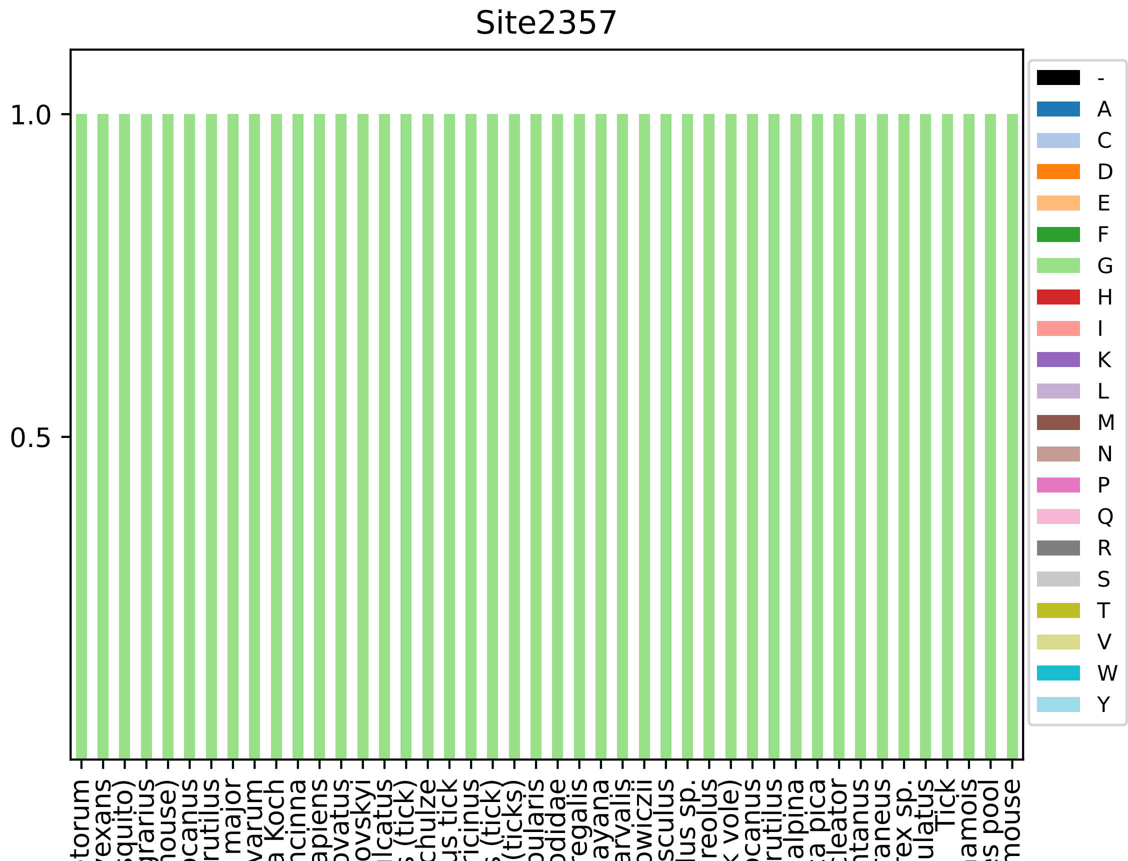
Site2357
1.0
0.5
-
A
C
D
E
F
G
H
I
K
L
M
N
P
Q
R
S
T
V
W
Y

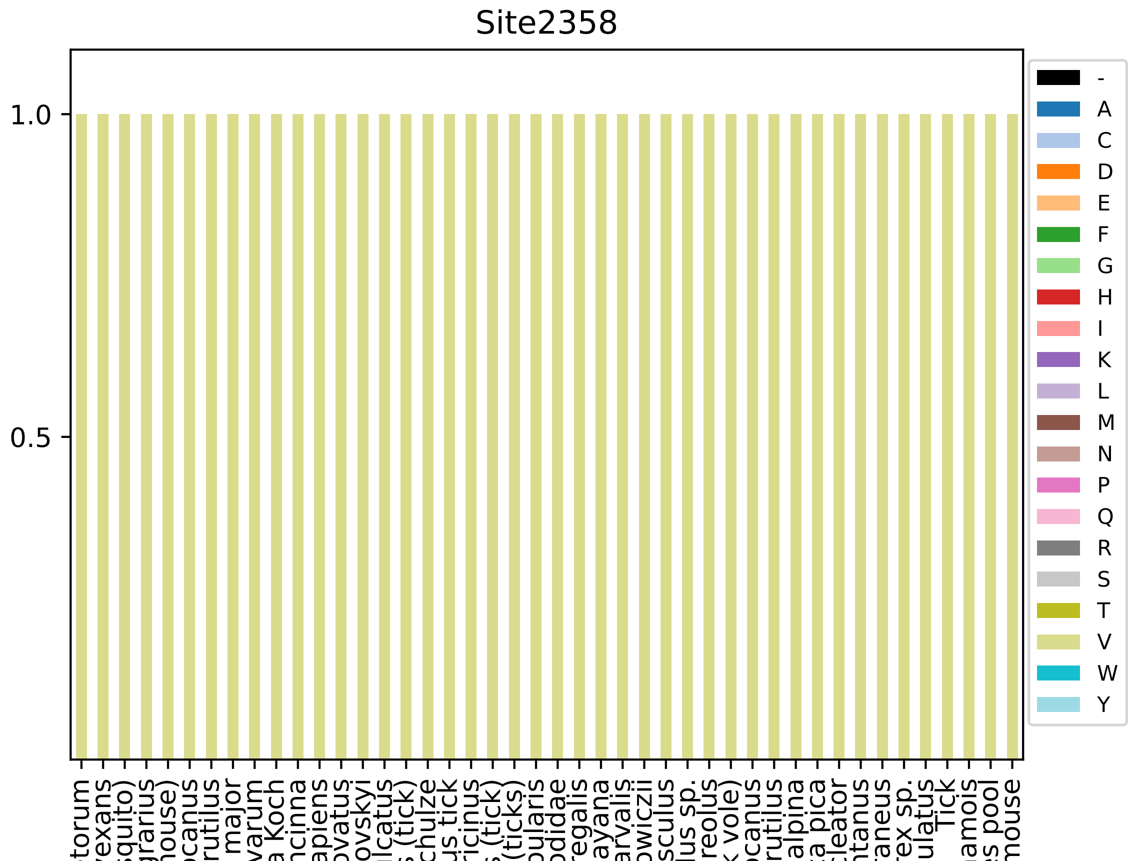
Site2358
1.0
0.5
-
A
C
D
E
F
G
H
I
K
L
M
N
P
Q
R
S
T
V
W
Y

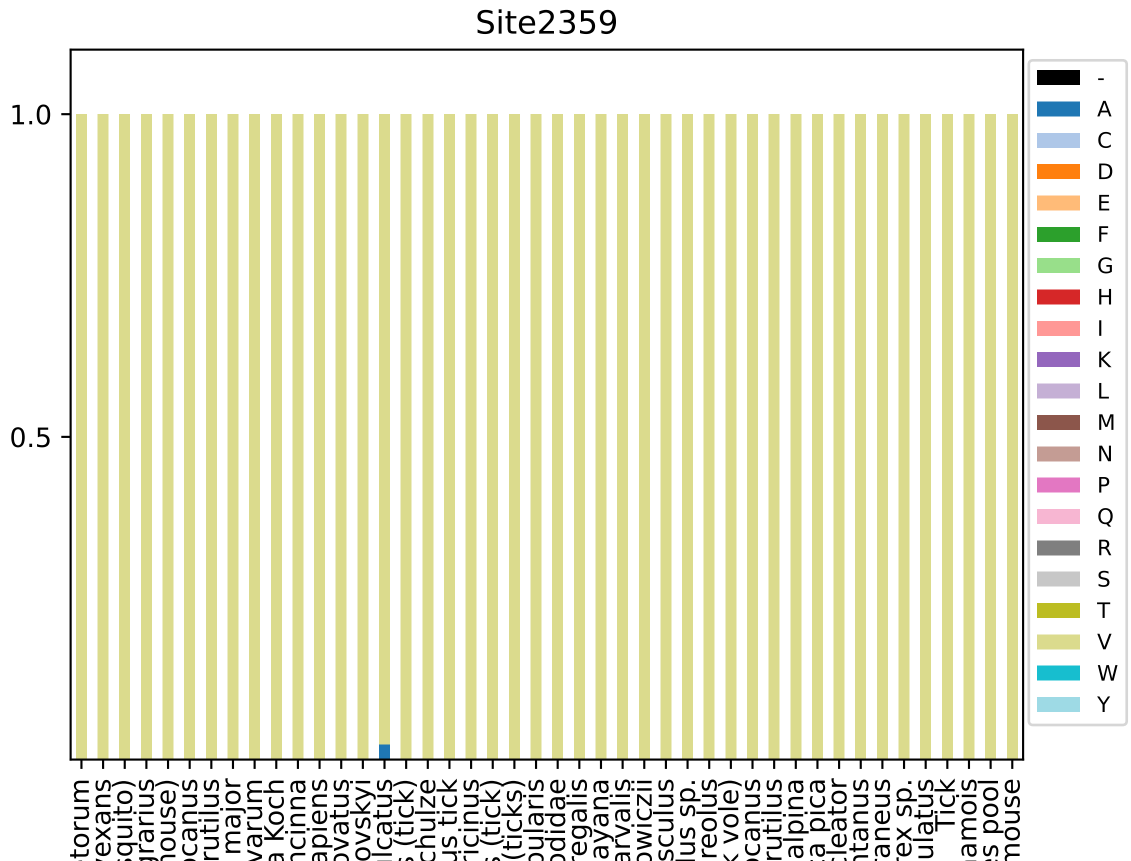
Site2359
1.0
0.5
-
A
C
D
E
F
G
H
I
K
L
M
N
P
Q
R
S
T
V
W
Y

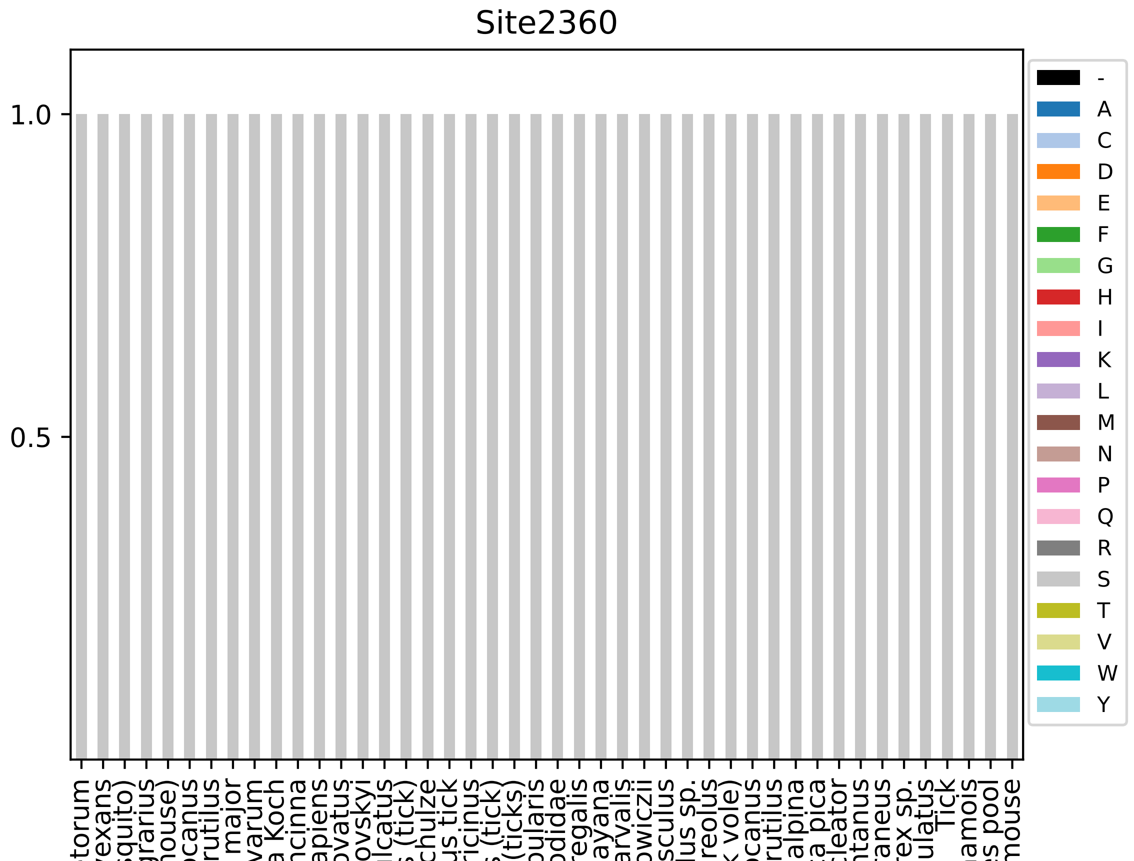
Site2360
1.0
0.5
-
A
C
D
E
F
G
H
I
K
L
M
N
P
Q
R
S
T
V
W
Y

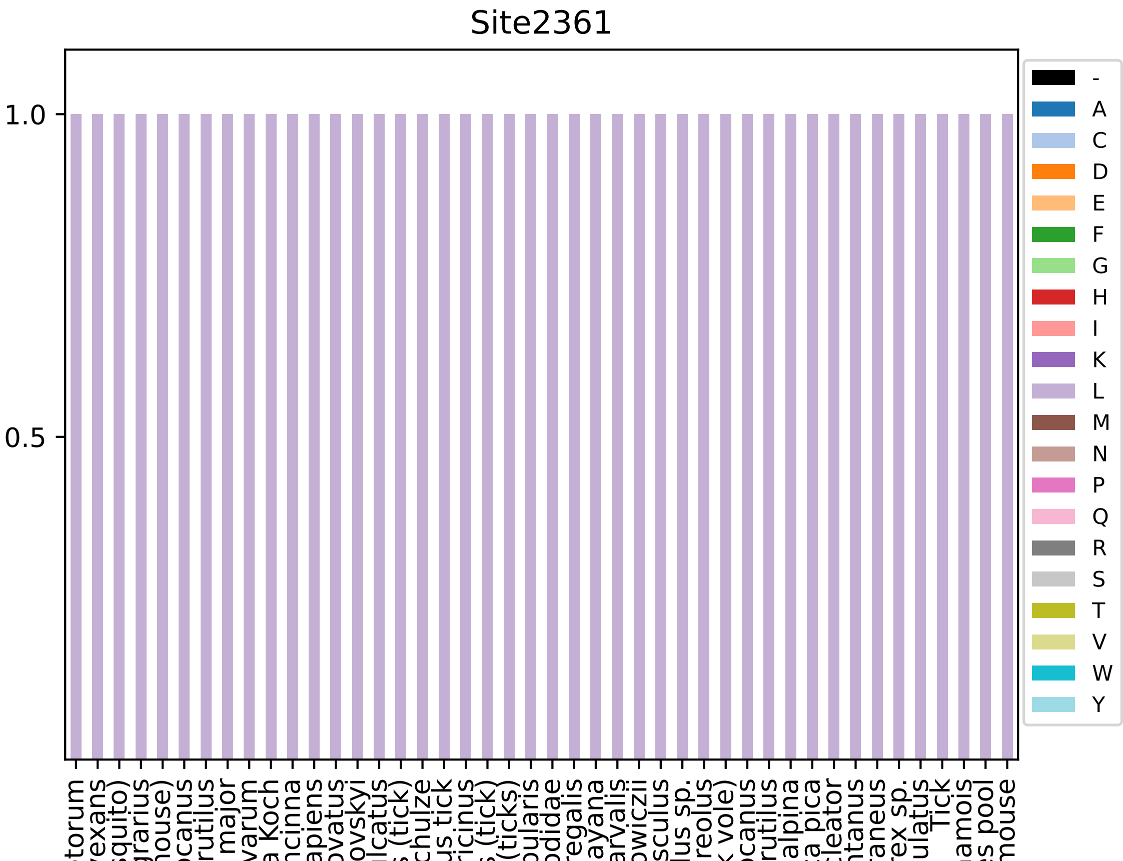
Site2361
1.0
0.5
-
A
C
D
E
F
G
H
I
K
L
M
N
P
Q
R
S
T
V
W
Y

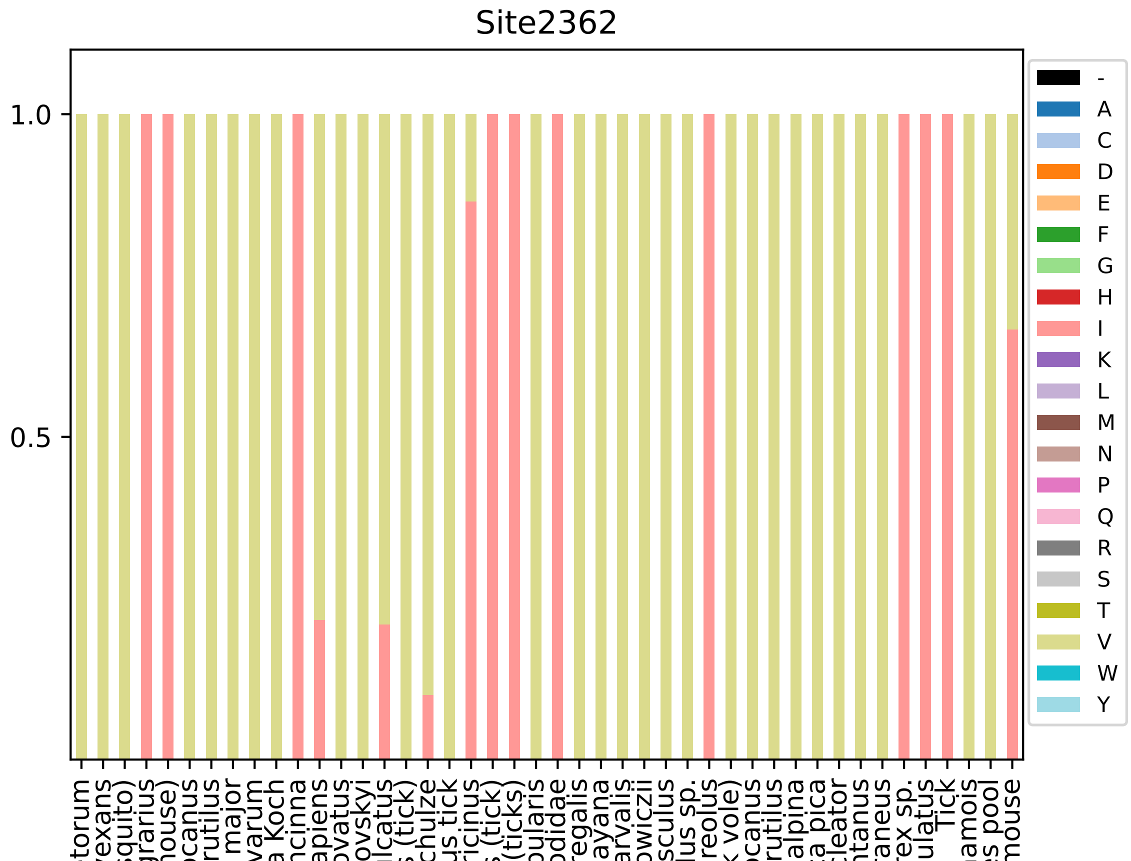

Site2362
1.0
0.5
-
A
C
D
E
F
G
H
I
K
L
M
N
P
Q
R
S
T
V
W
Y

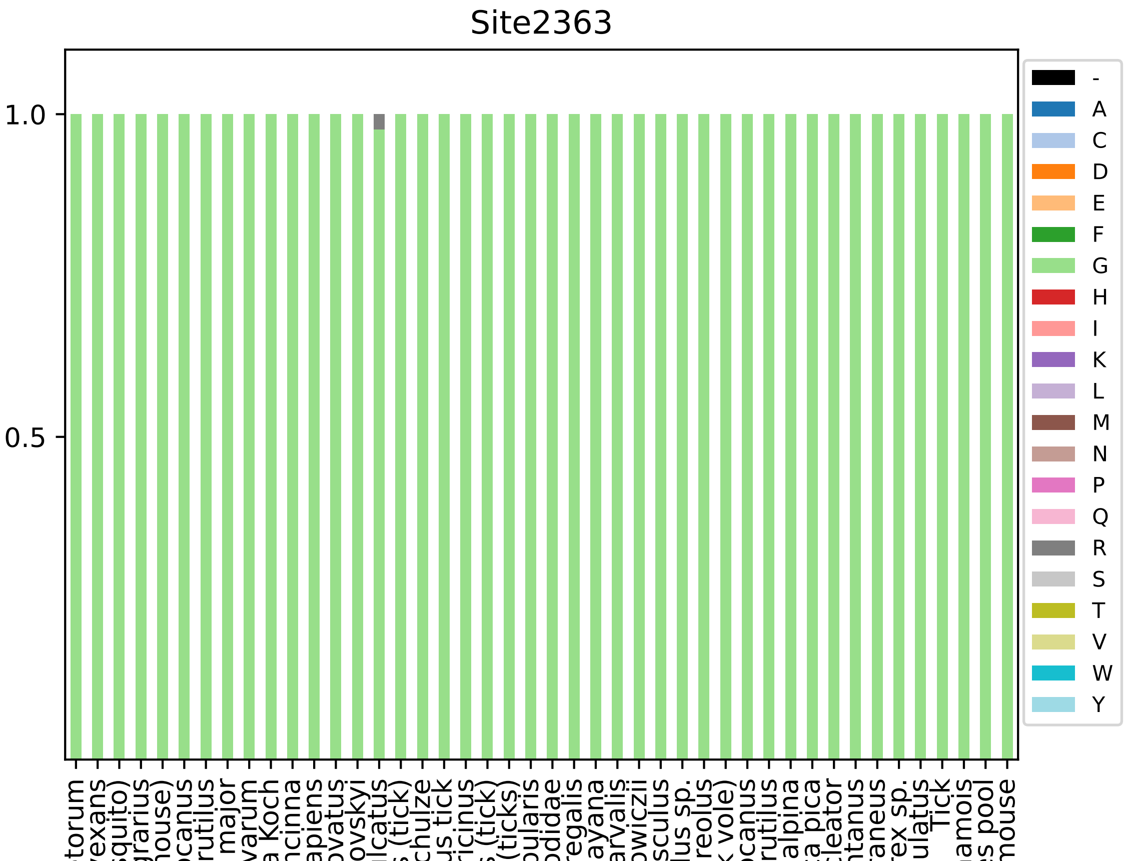
Site2363
1.0
0.5
-
A
C
D
E
F
G
H
I
K
L
M
N
P
Q
R
S
T
V
W
Y

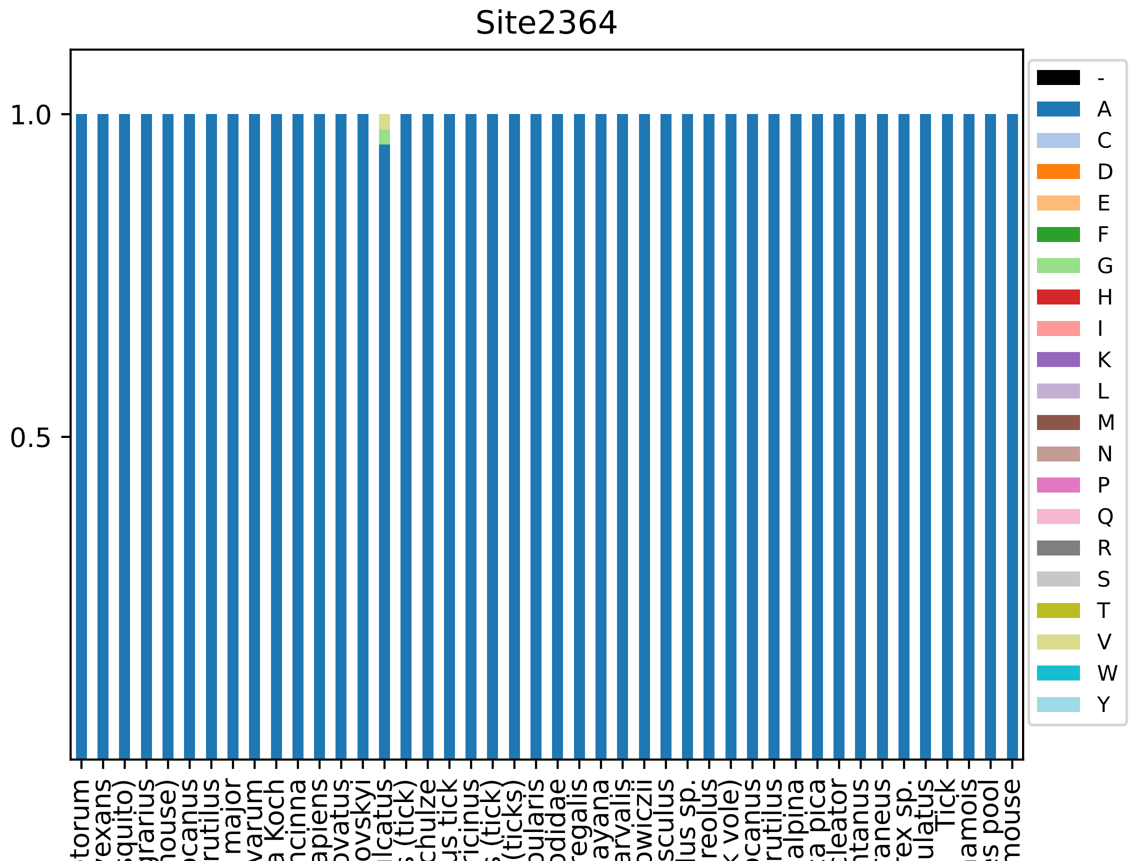
Site2364
1.0
0.5
-
A
C
D
E
F
G
H
I
K
L
M
N
P
Q
R
S
T
V
W
Y

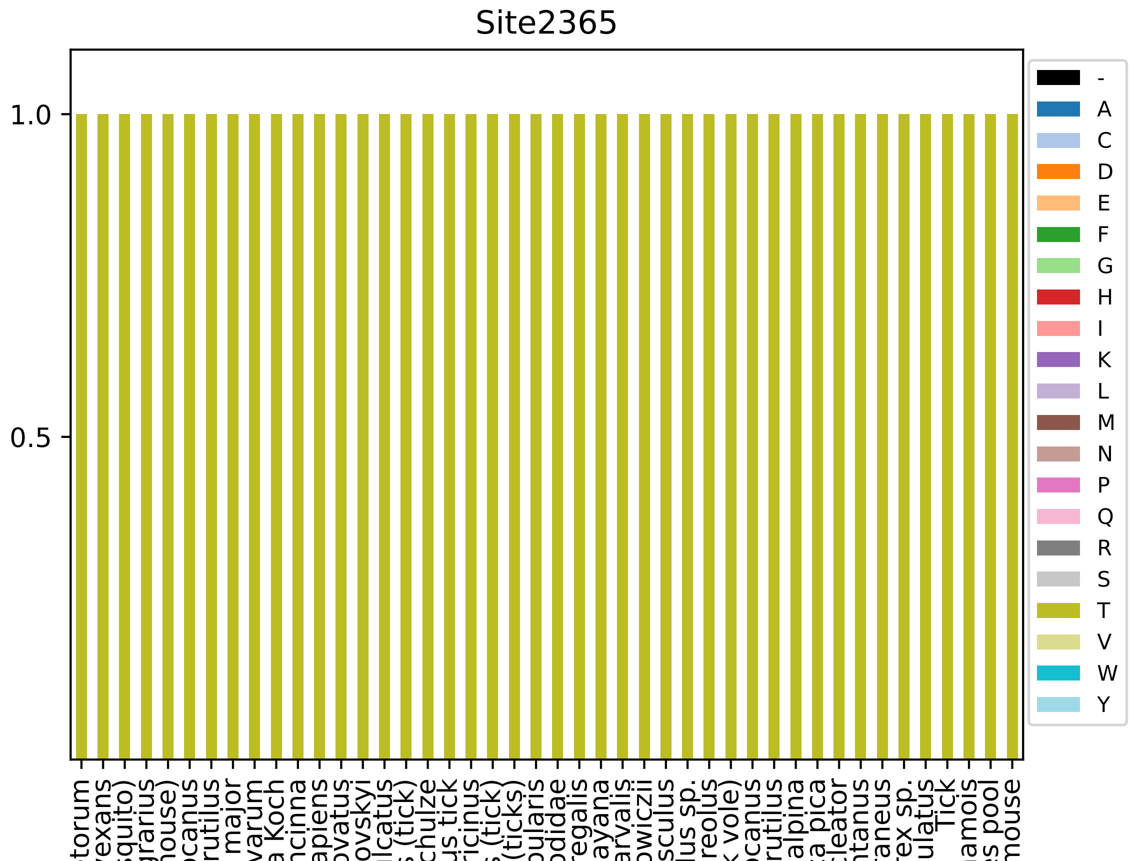
Site2365
1.0
0.5
-
A
C
D
E
F
G
H
I
K
L
M
N
P
Q
R
S
T
V
W
Y
Tick

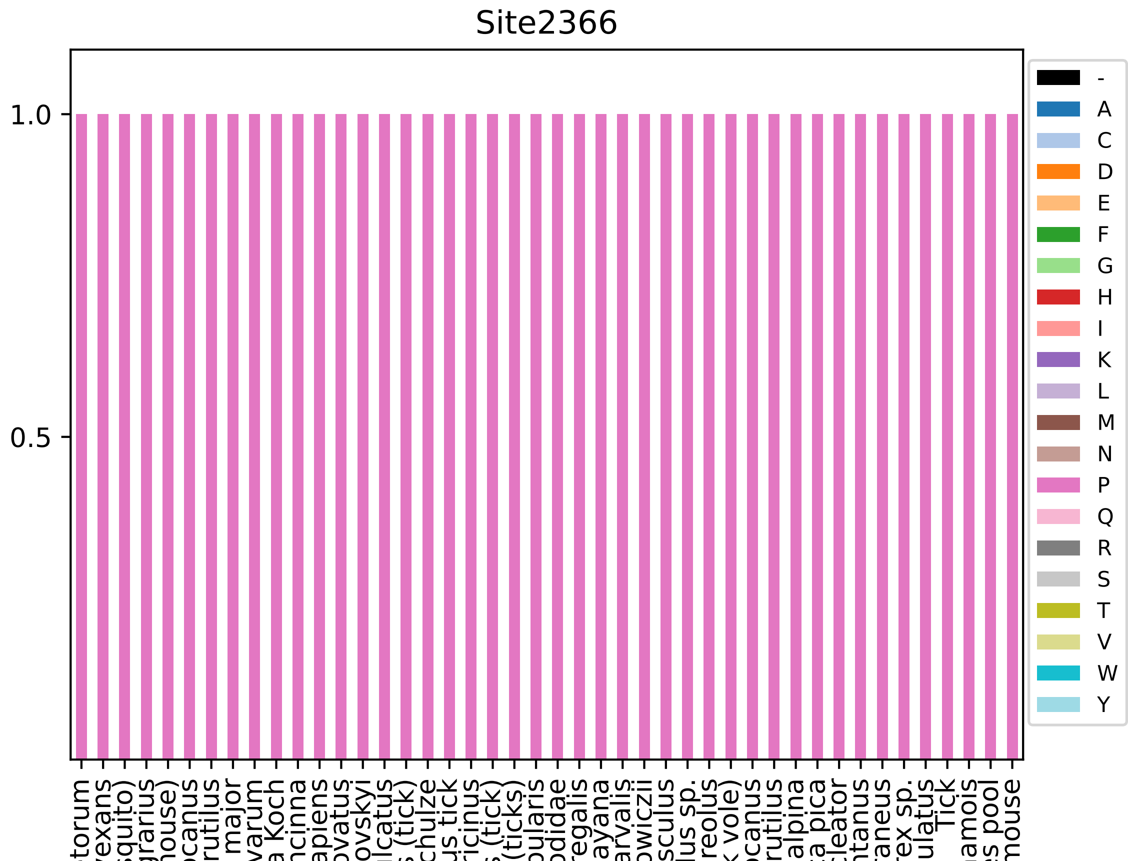

Site2366
1.0
0.5
-
A
C
D
E
F
G
H
I
K
L
M
N
P
Q
R
S
T
V
W
Y

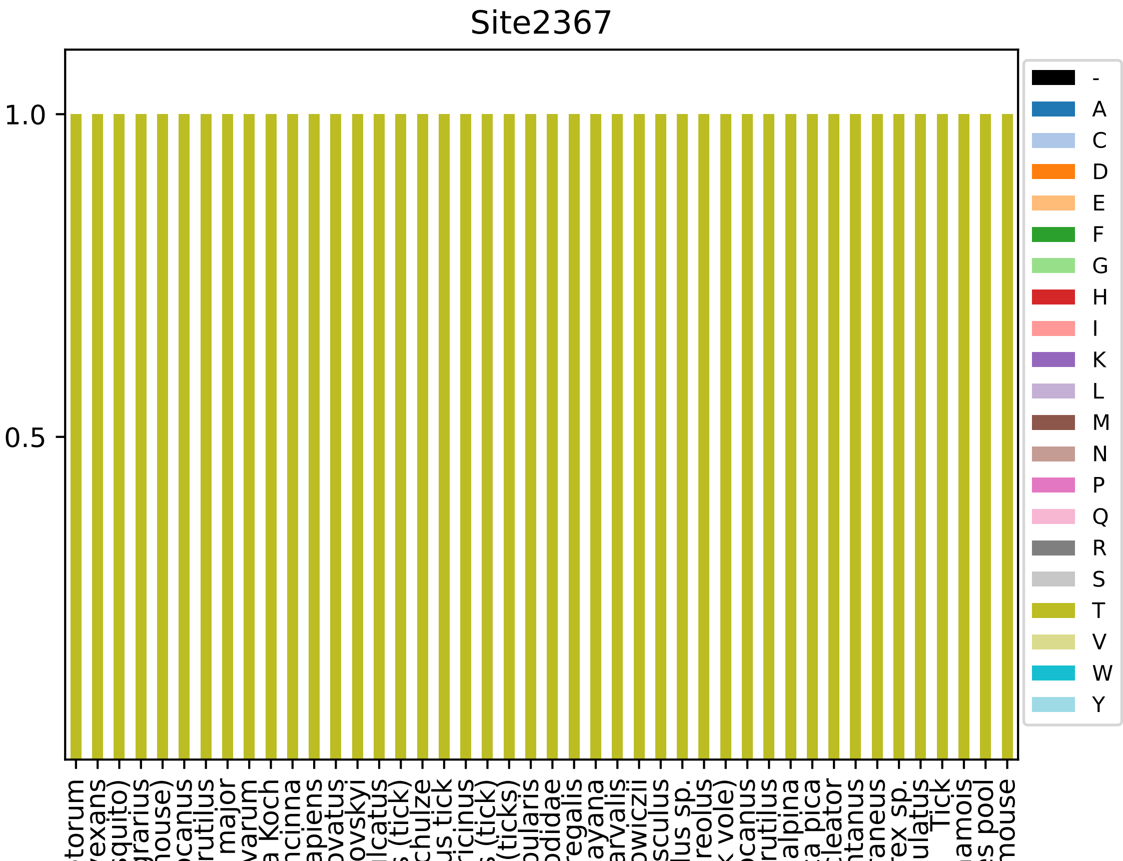
Site2367
1.0
0.5
-
A
C
D
E
F
G
H
I
K
L
M
N
P
Q
R
S
T
V
W
Y

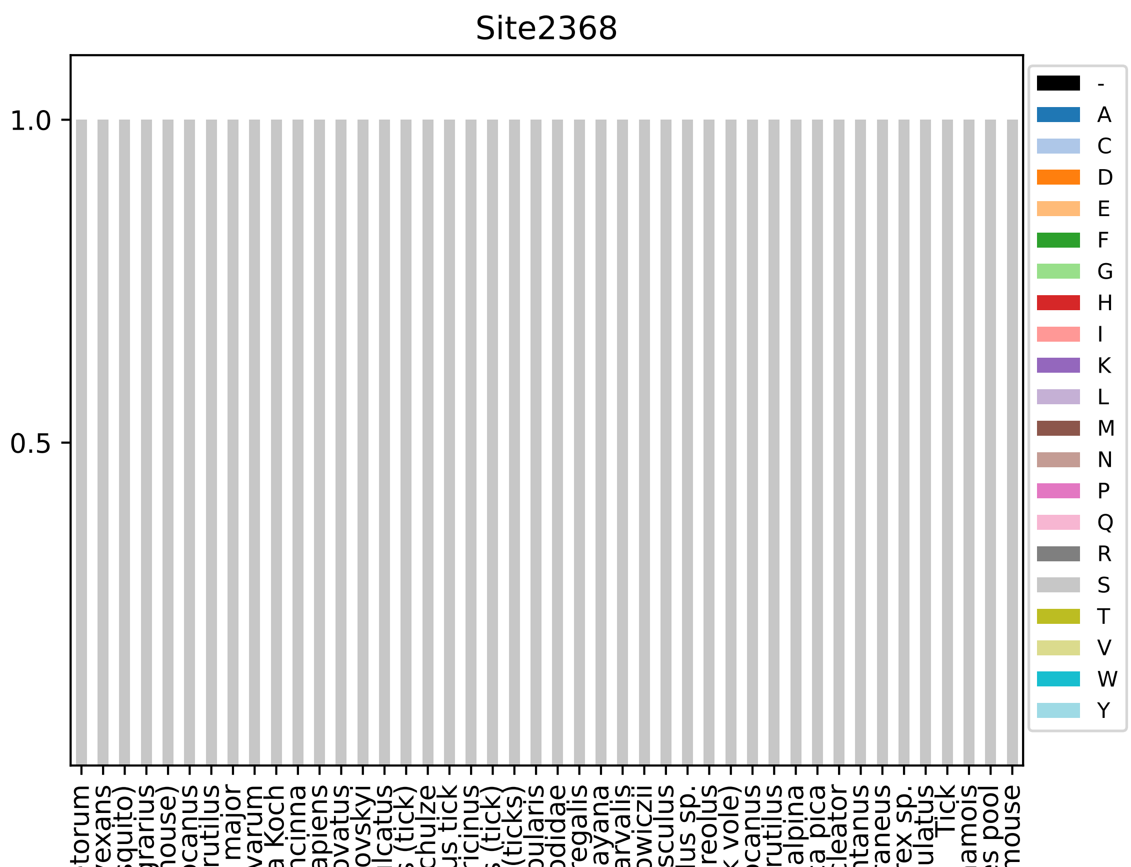
Site2368
1.0
0.5
-
A
C
D
E
F
G
H
I
K
L
M
N
P
Q
R
S
T
V
W
Y
torum
vexans
squito)
grarius
nouse)
ocanus
rutilus
major
varum
a Koch
ncinna
apiens
ovatus
ovskyi
ılcatus
s (tick)
chulze
us tick
ricinus
s (tick)
(ticks)
pularis
odidae
regalis
ayana
arvalis
owiczii
sculus
lus sp.
reolus
x vole)
ocanus
rutilus
alpina
a pica
cleator
ntanus
raneus
rex sp.
ulatus
Tick
amois
es pool
mouse

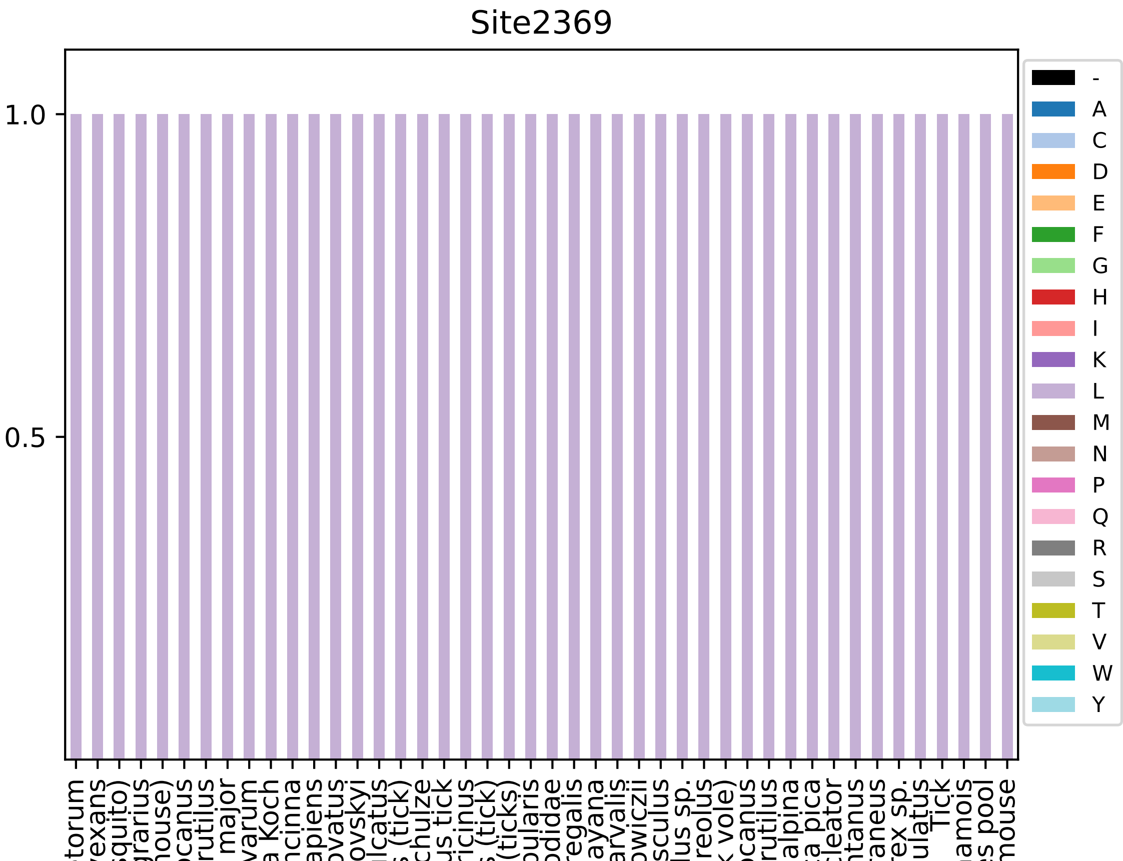
Site2369
1.0
0.5
-
A
C
D
E
F
G
H
I
K
L
M
N
P
Q
R
S
T
V
W
Y

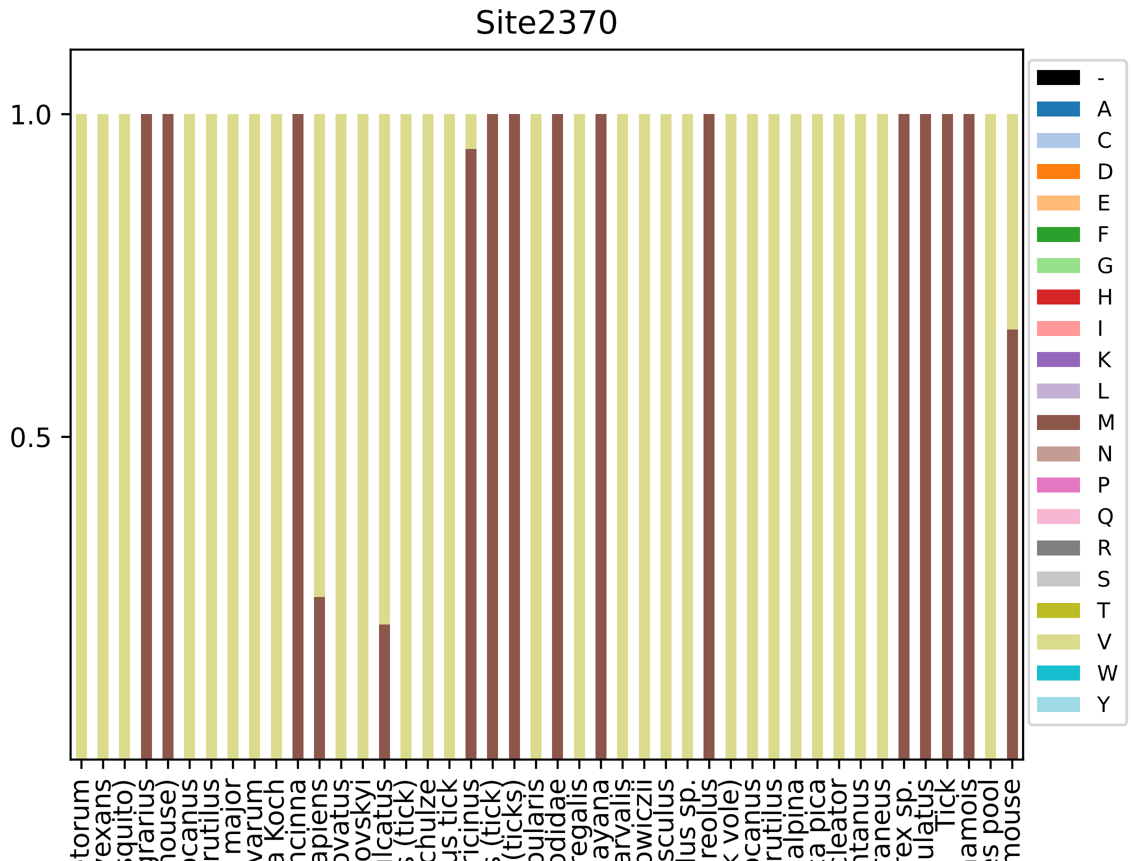
Site2370
1.0
0.5
-
A
C
D
E
F
G
H
I
K
L
M
N
P
Q
R
S
T
V
W
Y

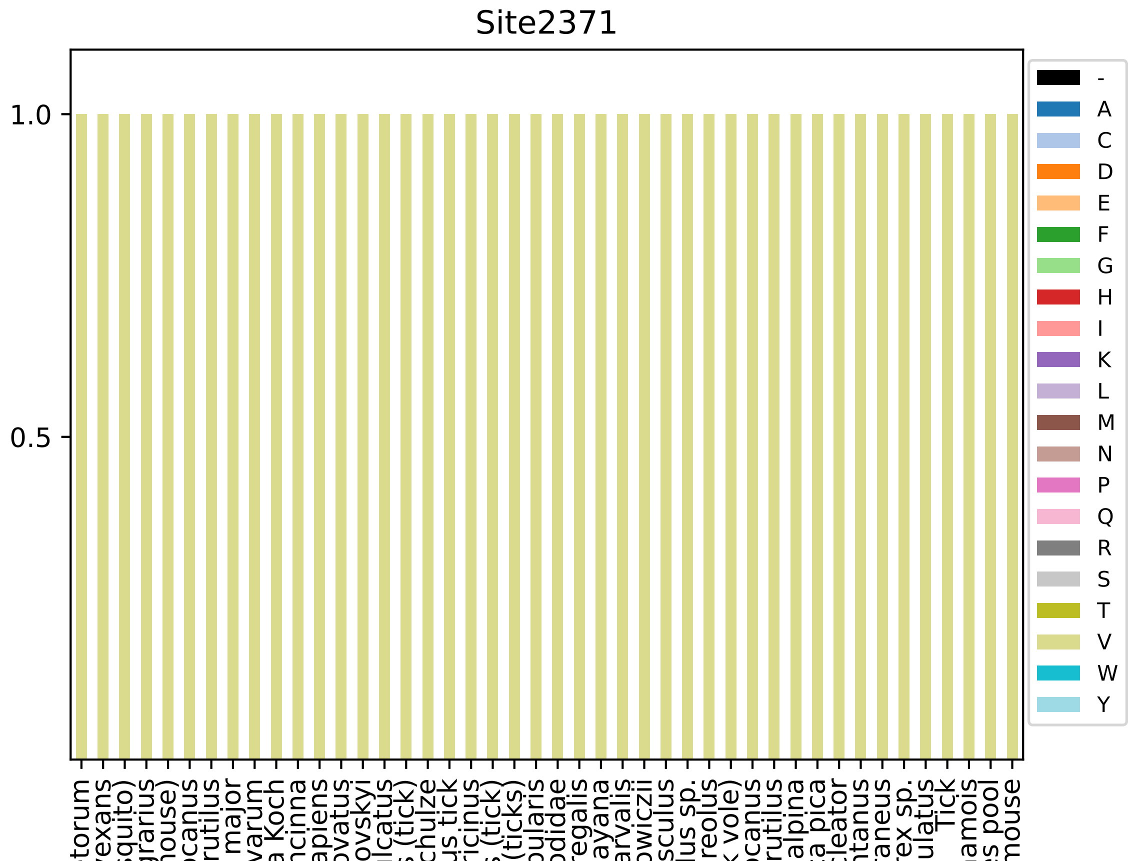
Site2371
1.0
0.5
-
A
C
D
E
F
G
H
I
K
L
M
N
P
Q
R
S
T
V
W
Y

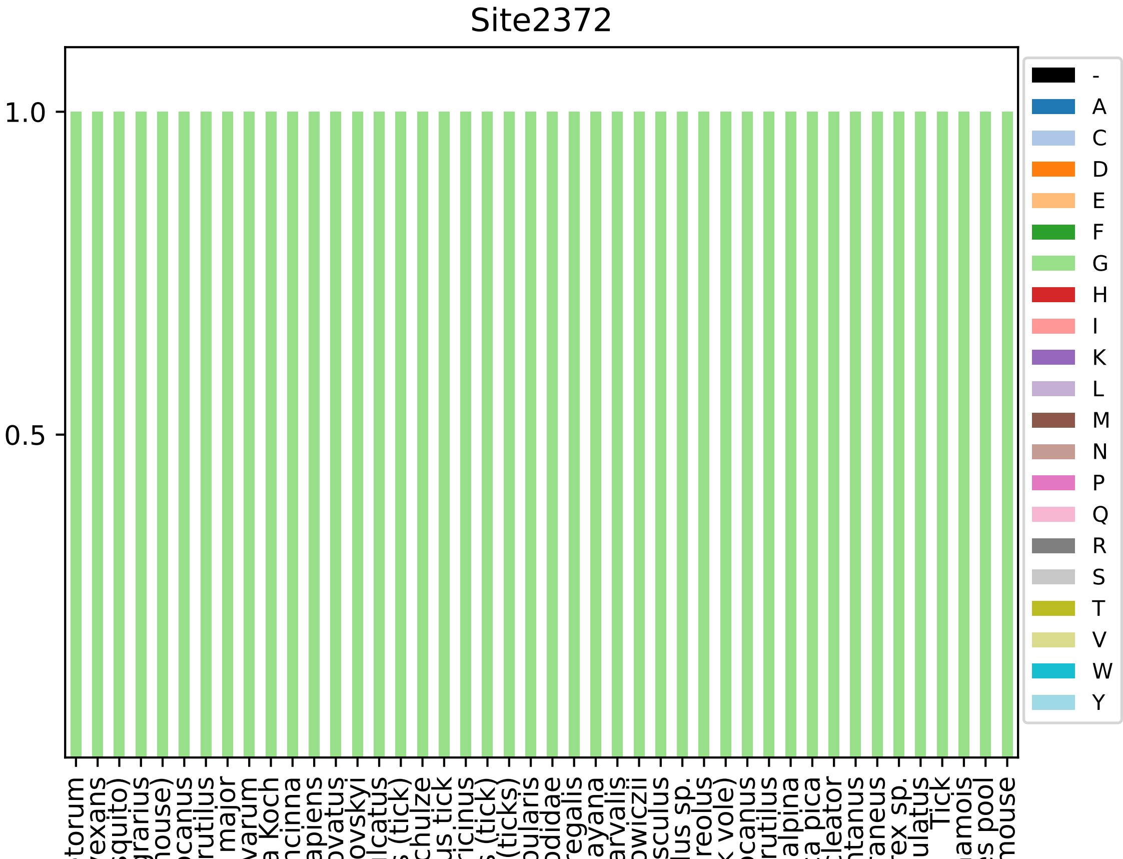

Site2372
1.0
0.5
-
A
C
D
E
F
G
H
I
K
L
M
N
P
Q
R
S
T
V
W
Y

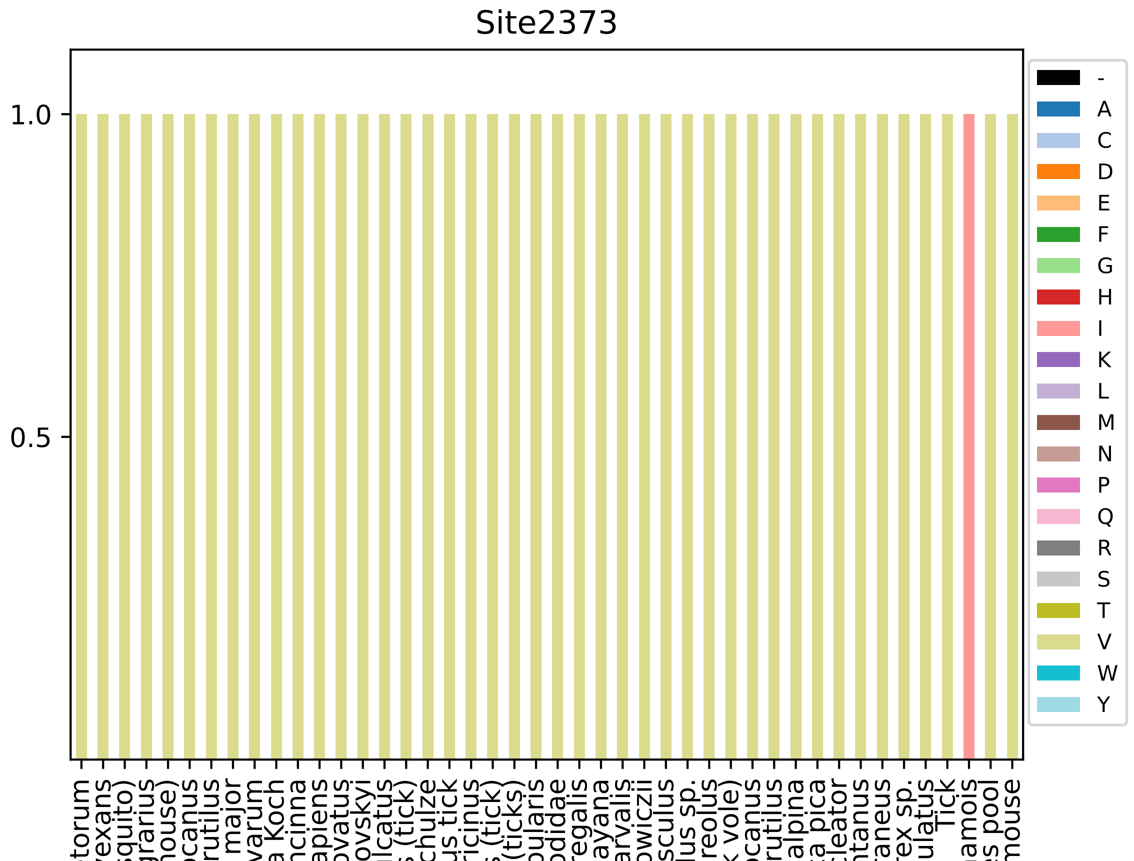

Site2373
1.0
0.5
-
A
C
D
E
F
G
H
I
K
L
M
N
P
Q
R
S
T
V
W
Y

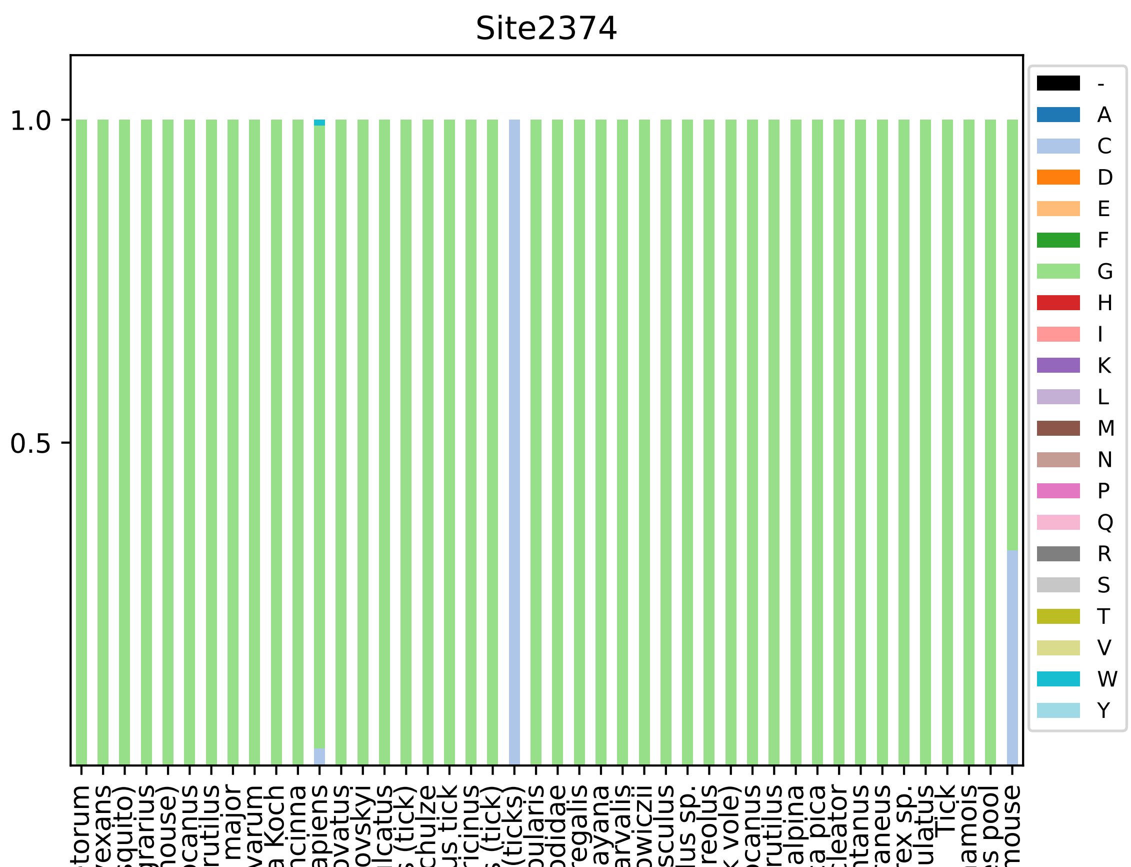
Site2374
1.0
0.5
-
A
C
D
E
F
G
H
I
K
L
M
N
P
Q
R
S
T
V
W
Y

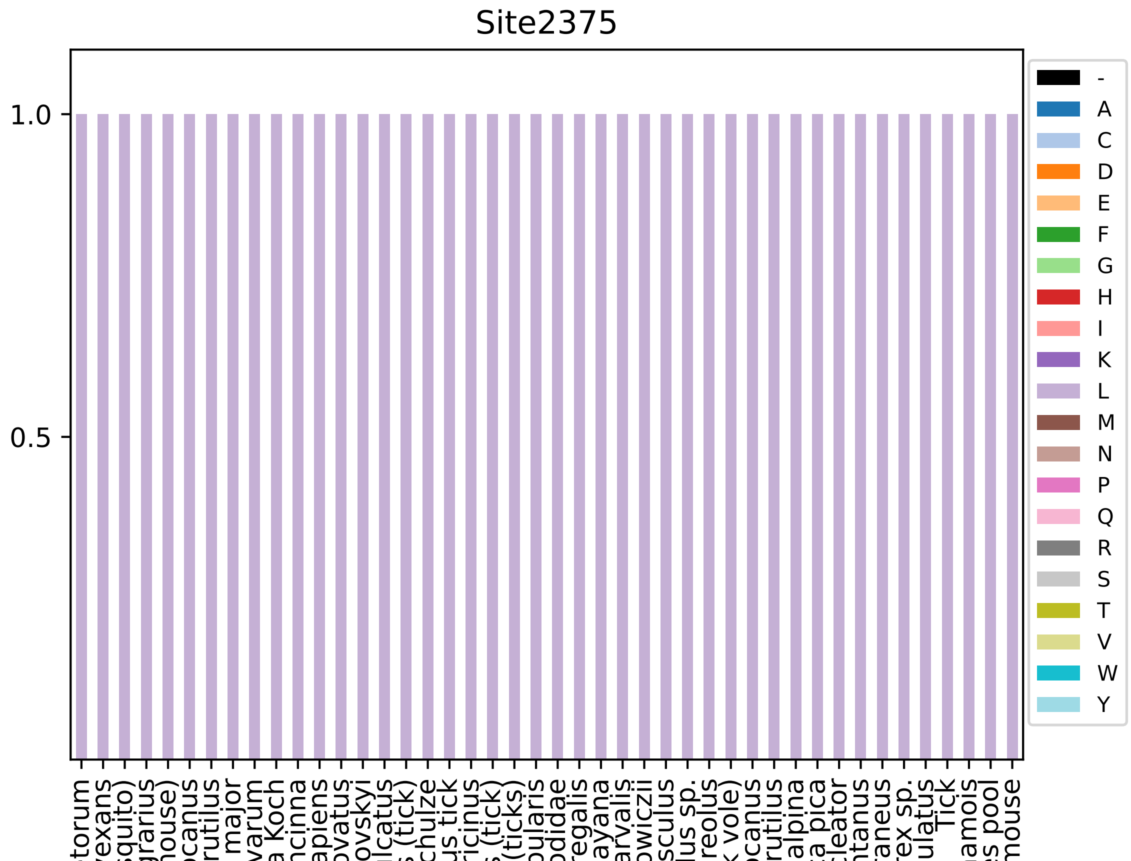
Site2375
1.0
0.5
-
A
C
D
E
F
G
H
I
K
L
M
N
P
Q
R
S
T
V
W
Y

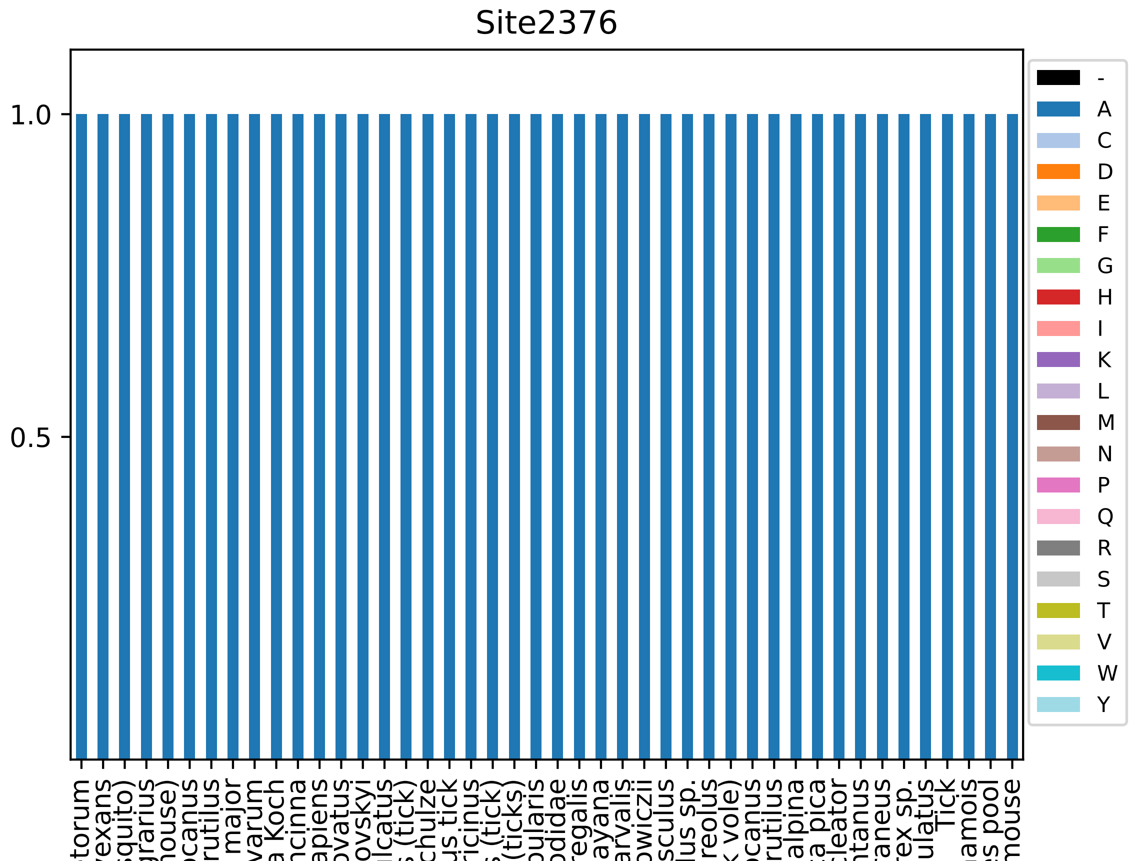
Site2376
1.0
0.5
-
A
C
D
E
F
G
H
I
K
L
M
N
P
Q
R
S
T
V
W
Y

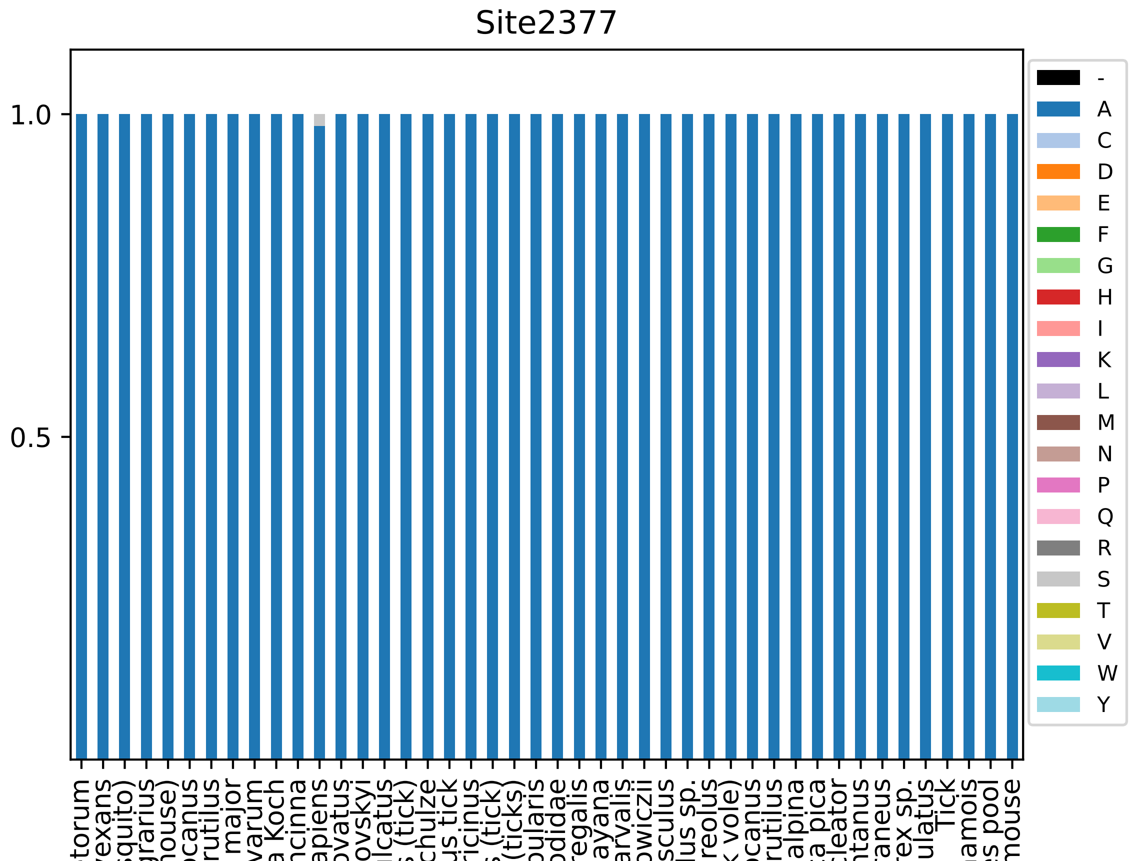

Site2377
1.0
0.5
-
A
C
D
E
F
G
H
I
K
L
M
N
P
Q
R
S
T
V
W
Y

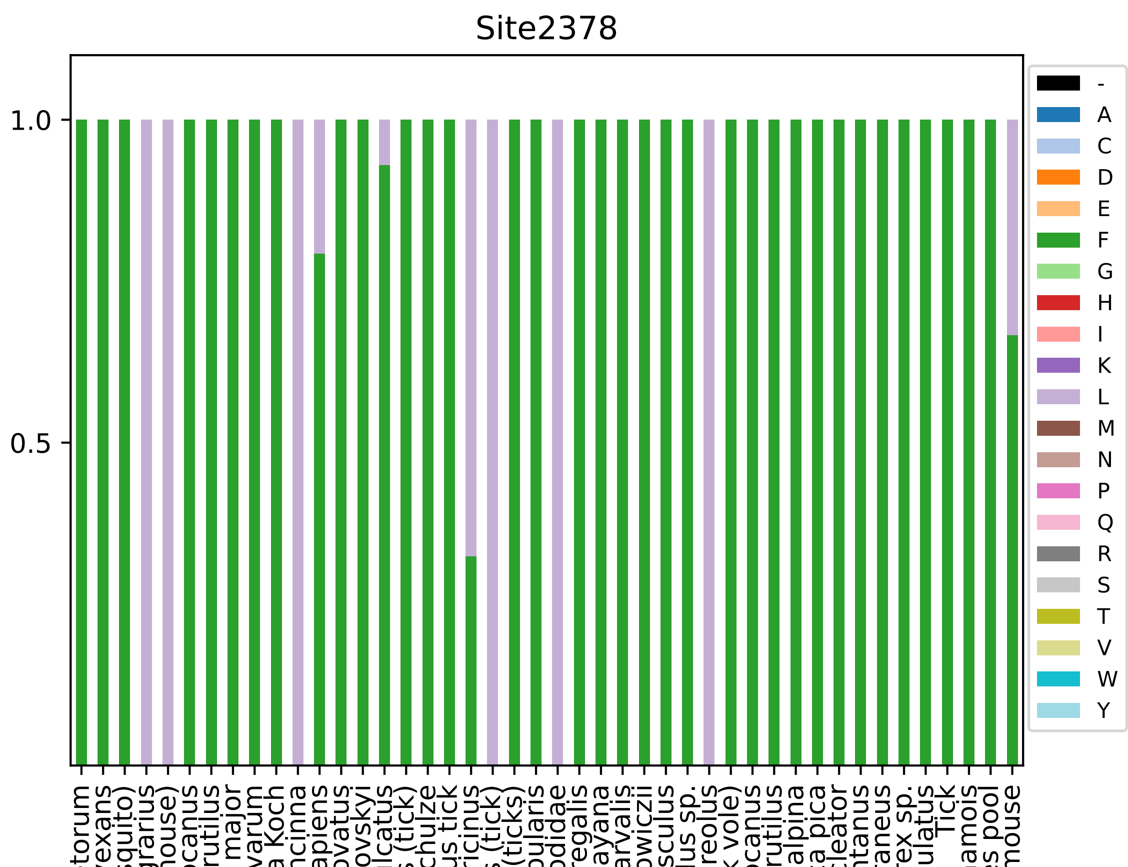
Site2378
1.0
0.5
torum
vexans
squito)
grarius
nouse)
ocanus
rutilus
major
varum
a Koch
ncinna
apiens
ovatus
ovskyi
ulcatus
s (tick)
chulze
us tick
ricinus
s (tick)
(ticks)
pularis
podidae
regalis
ayana
arvalis
owiczii
sculus
lus sp.
reolus
k vole)
ocanus
rutilus
alpina
a pica
cleator
ntanus
raneus
rex sp.
ulatus
Tick
amois
es pool
mouse
-
A
C
D
E
F
G
H
I
K
L
M
N
P
Q
R
S
T
V
W
Y

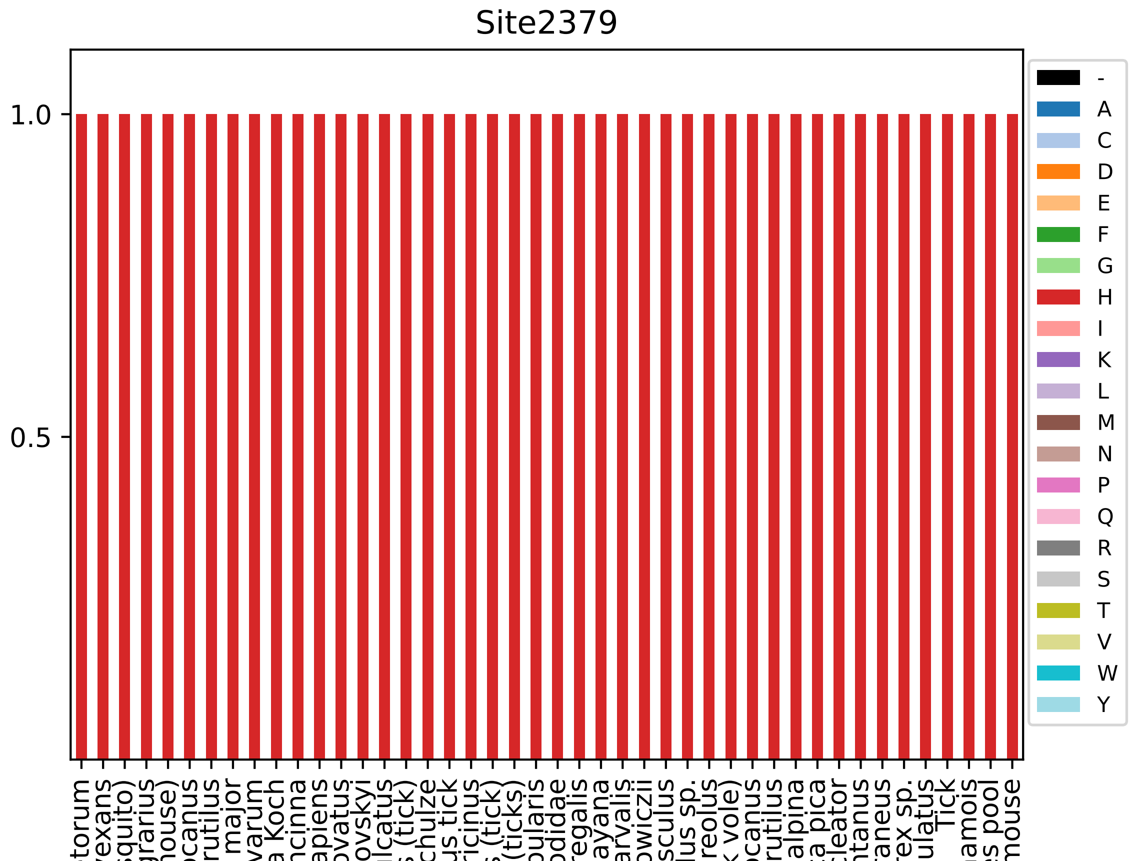

Site2379
1.0
0.5
-
A
C
D
E
F
G
H
I
K
L
M
N
P
Q
R
S
T
V
W
Y

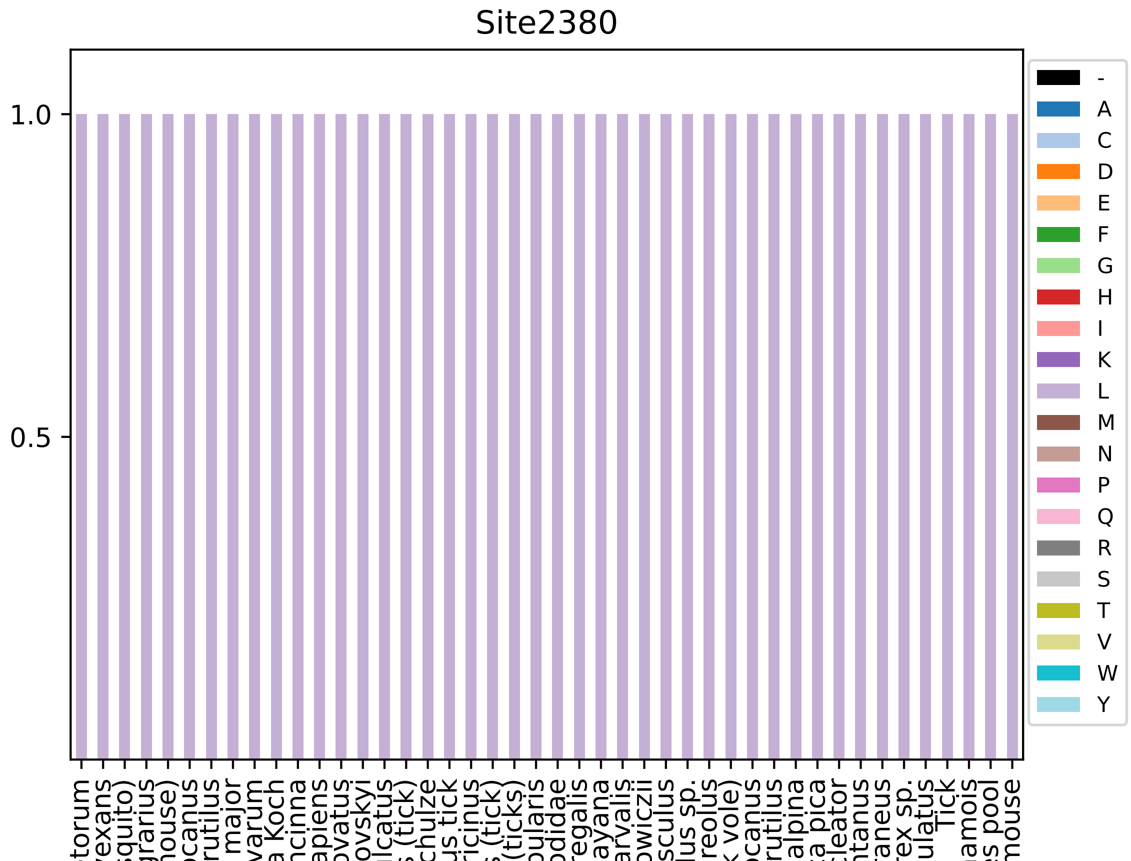
Site2380
1.0
0.5
-
A
C
D
E
F
G
H
I
K
L
M
N
P
Q
R
S
T
V
W
Y

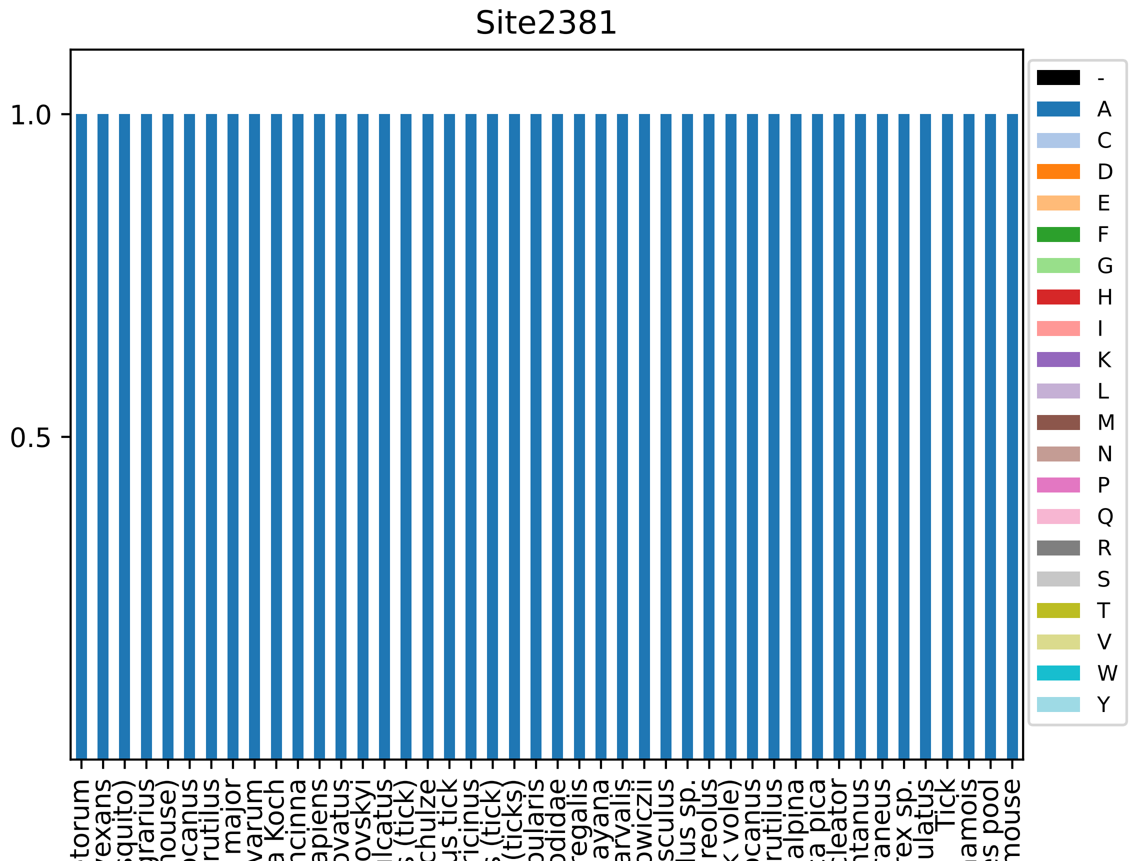
Site2381
1.0
0.5
-
A
C
D
E
F
G
H
I
K
L
M
N
P
Q
R
S
T
V
W
Y

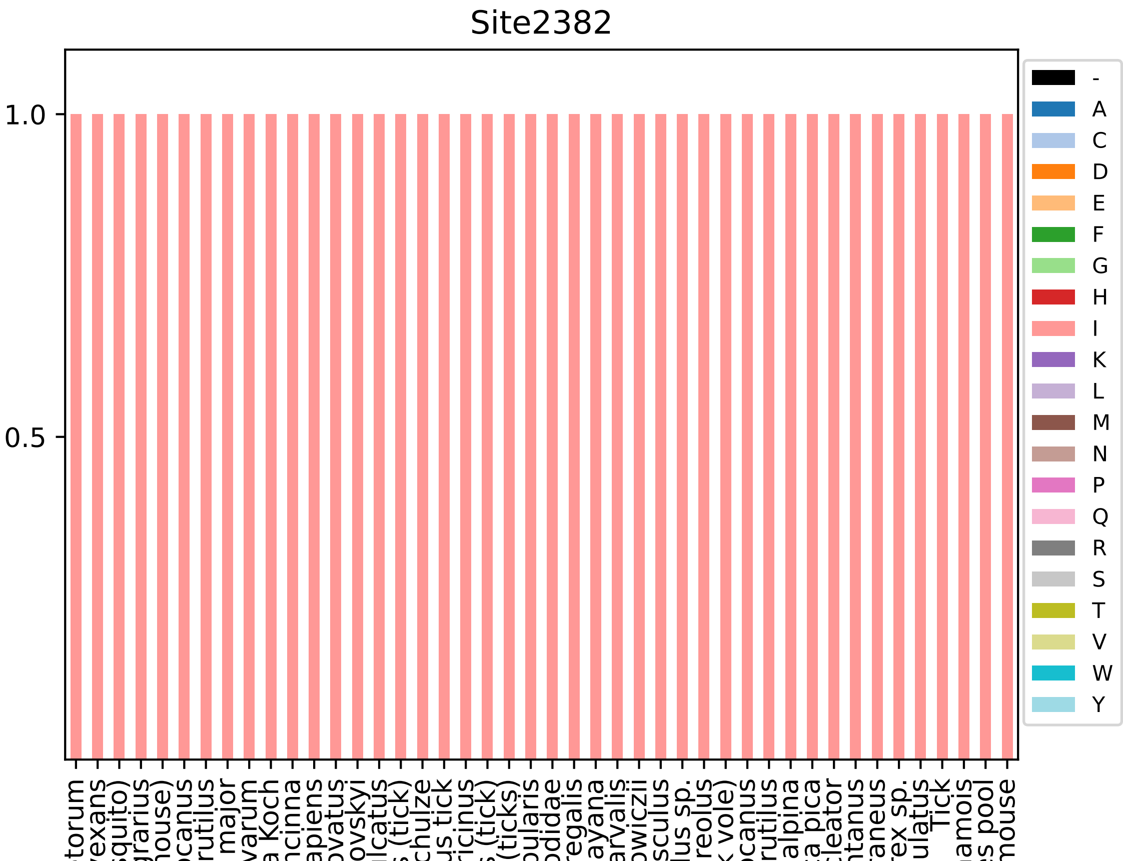
Site2382
1.0
0.5
-
A
C
D
E
F
G
H
I
K
L
M
N
P
Q
R
S
T
V
W
Y

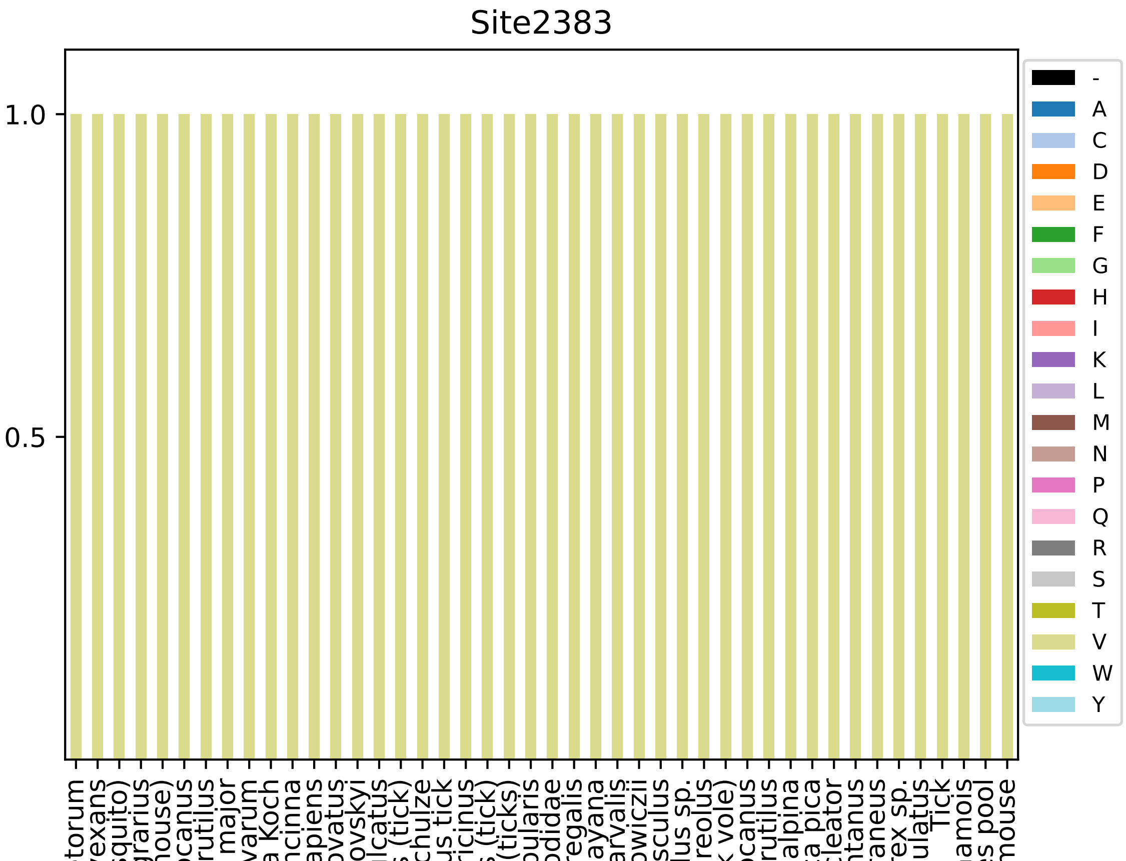
Site2383
1.0
0.5
-
A
C
D
E
F
G
H
I
K
L
M
N
P
Q
R
S
T
V
W
Y

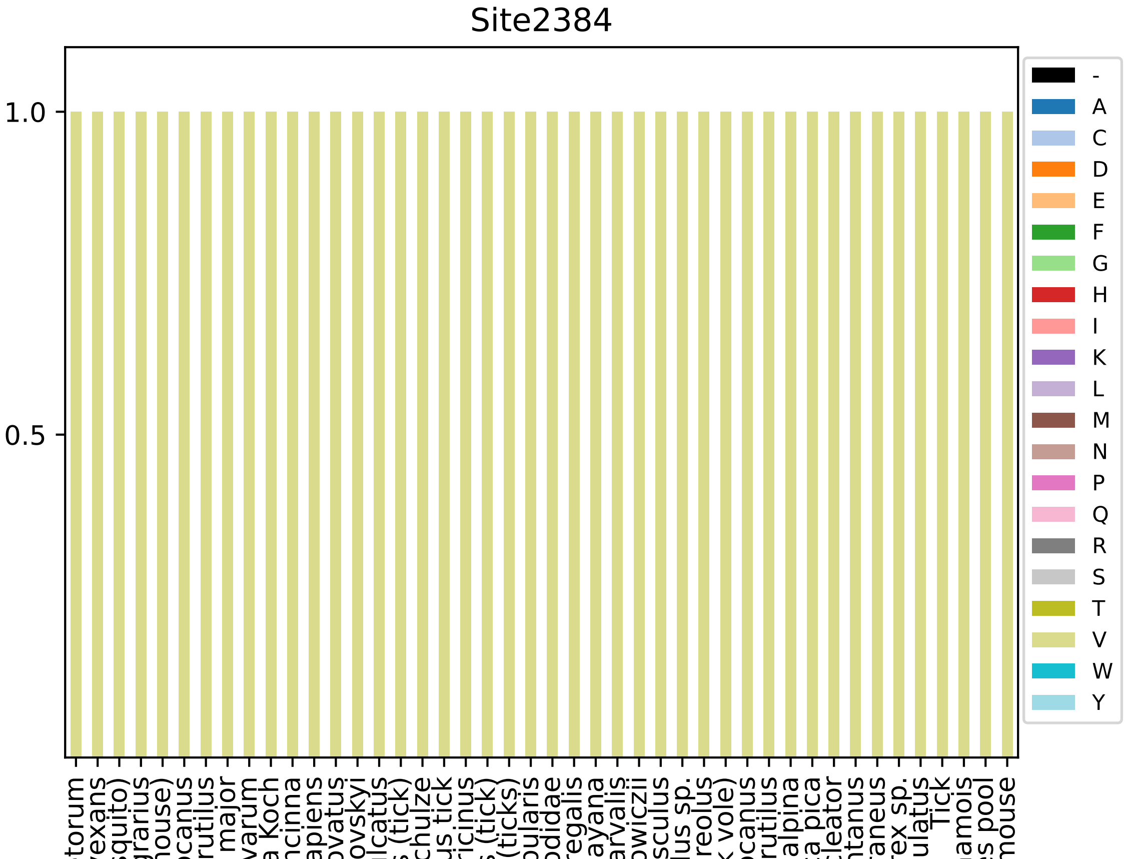
Site2384
1.0
0.5
-
A
C
D
E
F
G
H
I
K
L
M
N
P
Q
R
S
T
V
W
Y
Tick

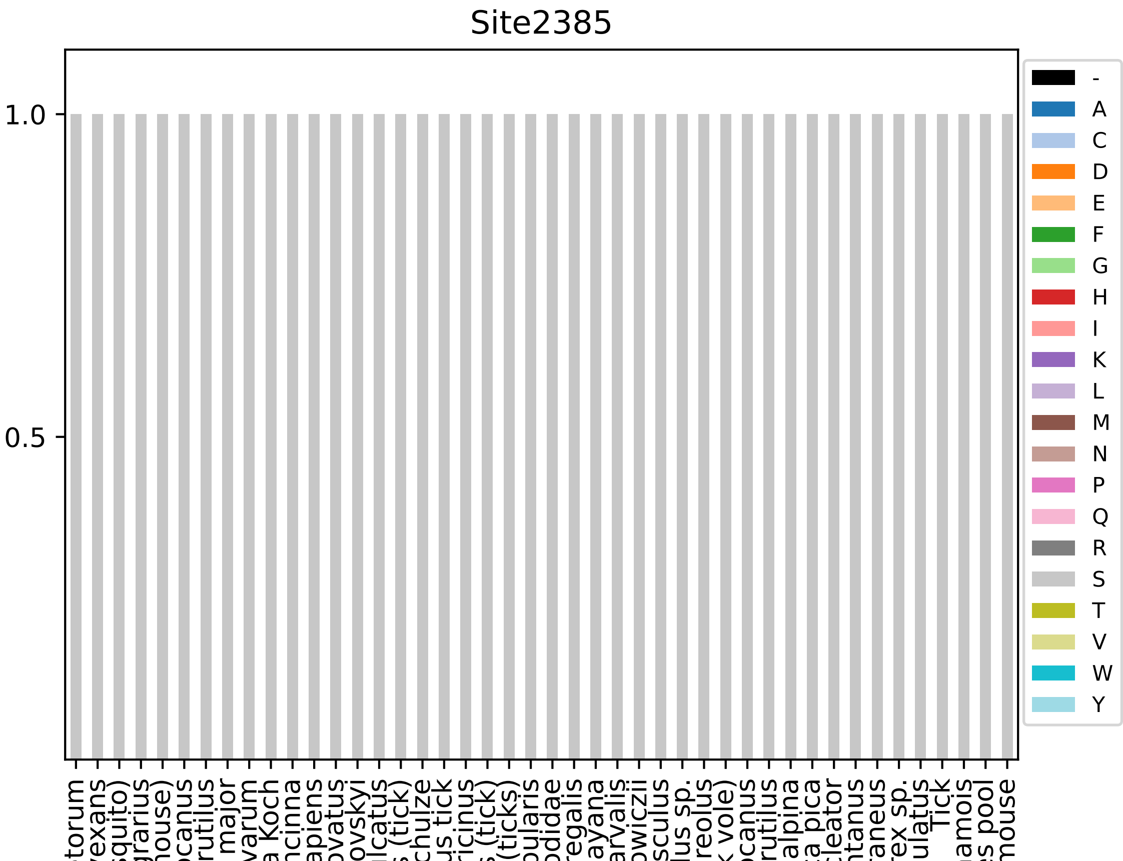
Site2385
1.0
0.5
-
A
C
D
E
F
G
H
I
K
L
M
N
P
Q
R
S
T
V
W
Y

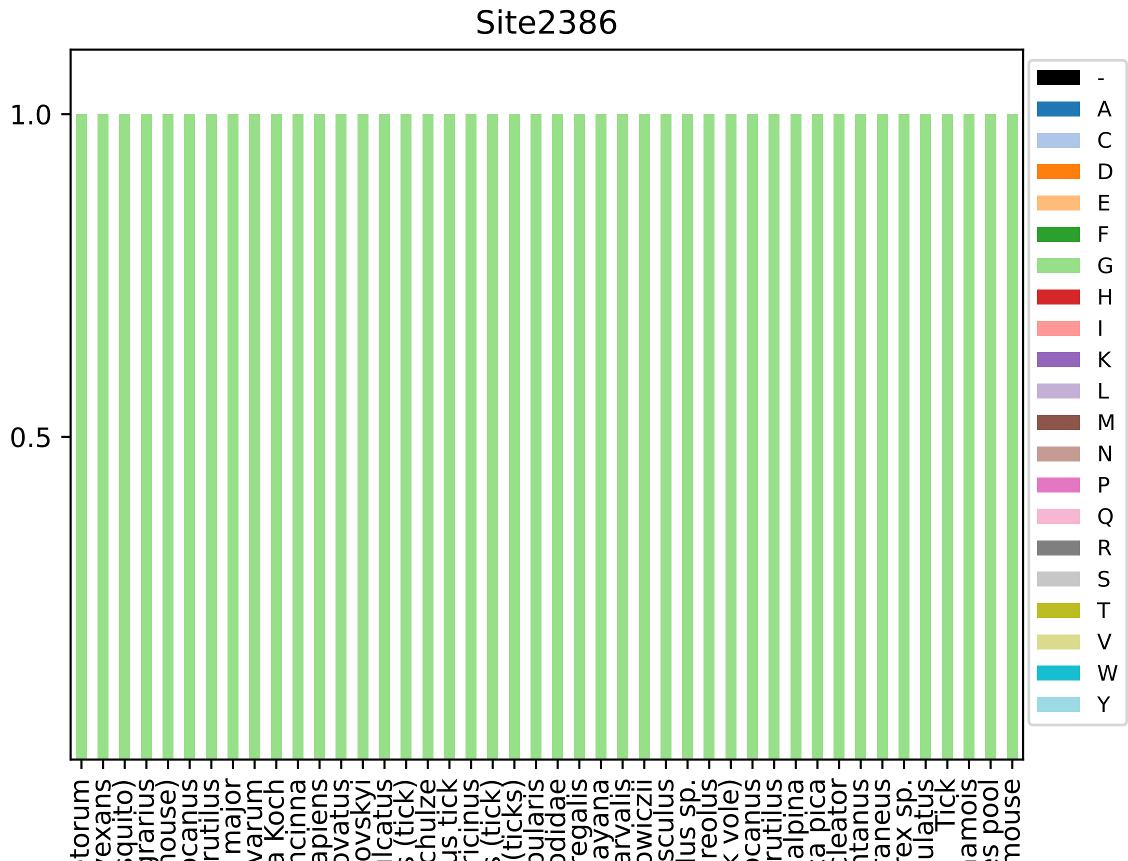
Site2386
1.0
0.5
-
A
C
D
E
F
G
H
I
K
L
M
N
P
Q
R
S
T
V
W
Y

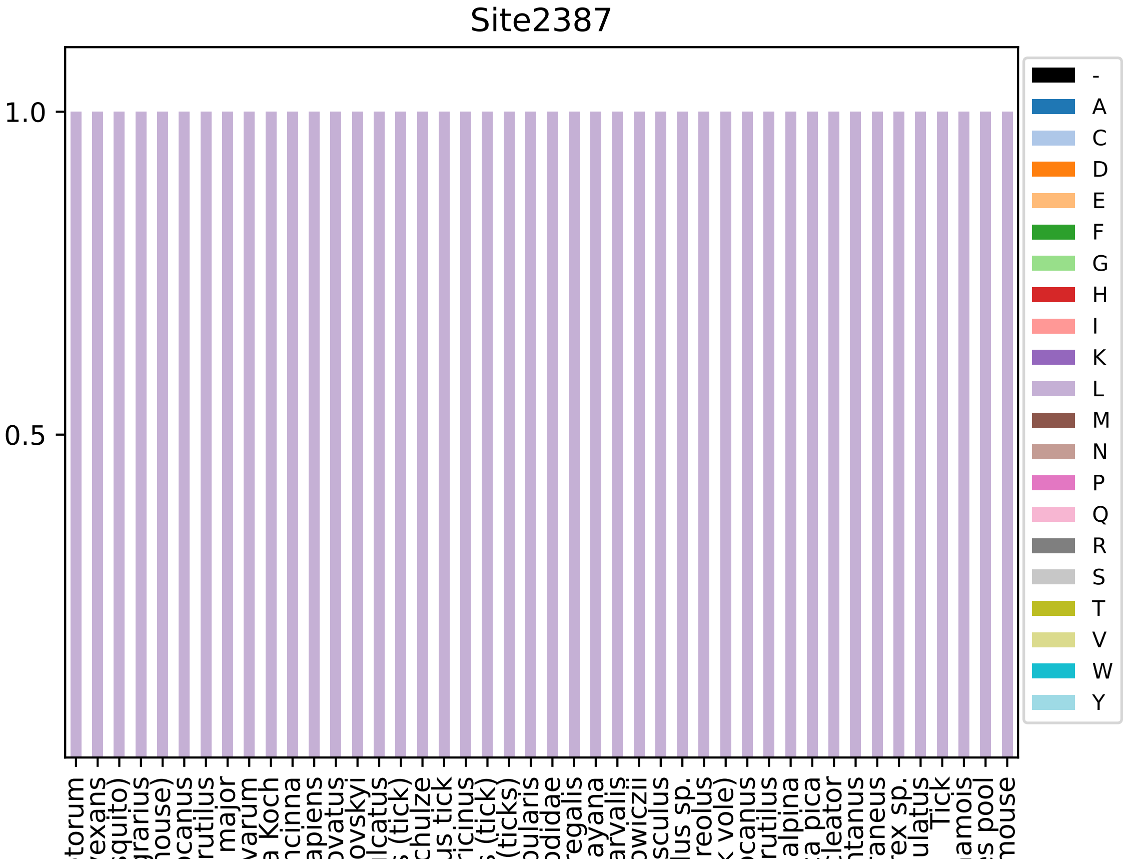
Site2387
1.0
0.5
-
A
C
D
E
F
G
H
I
K
L
M
N
P
Q
R
S
T
V
W
Y

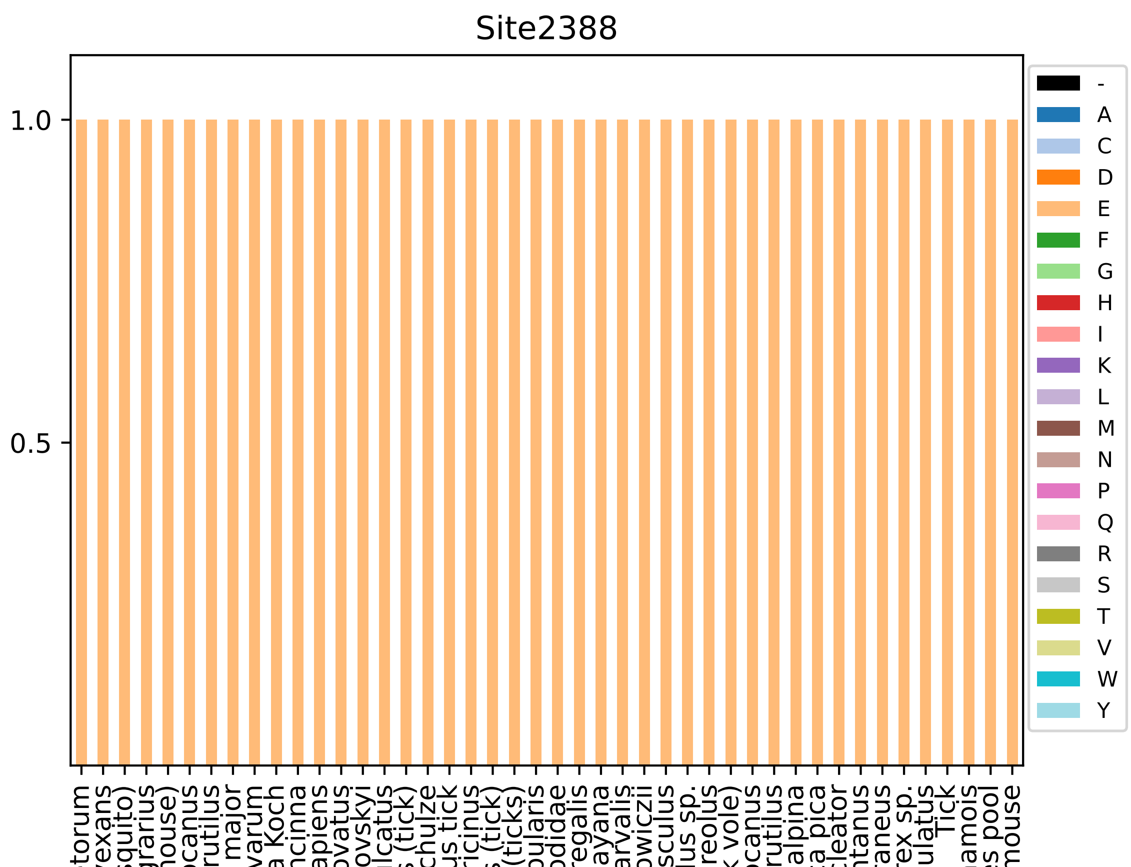
Site2388
1.0
0.5
-
A
C
D
E
F
G
H
I
K
L
M
N
P
Q
R
S
T
V
W
Y

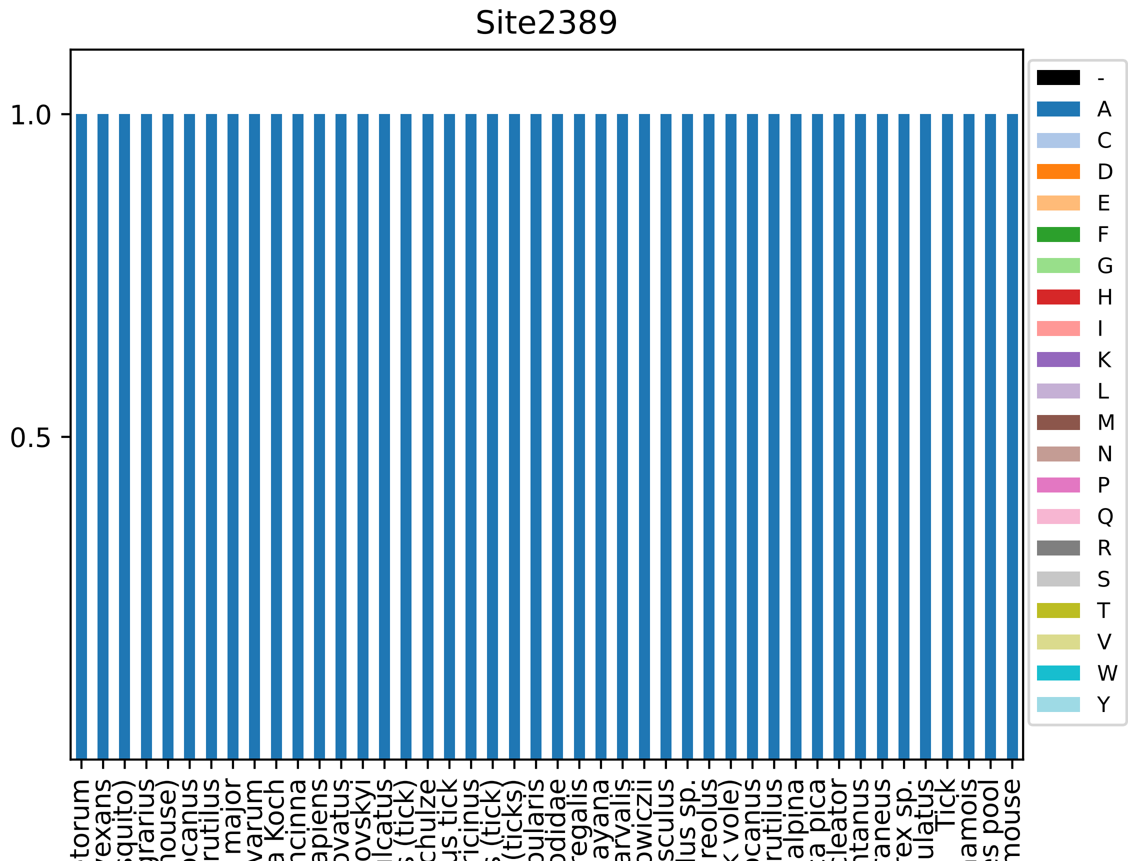
Site2389
1.0
0.5
-
A
C
D
E
F
G
H
I
K
L
M
N
P
Q
R
S
T
V
W
Y

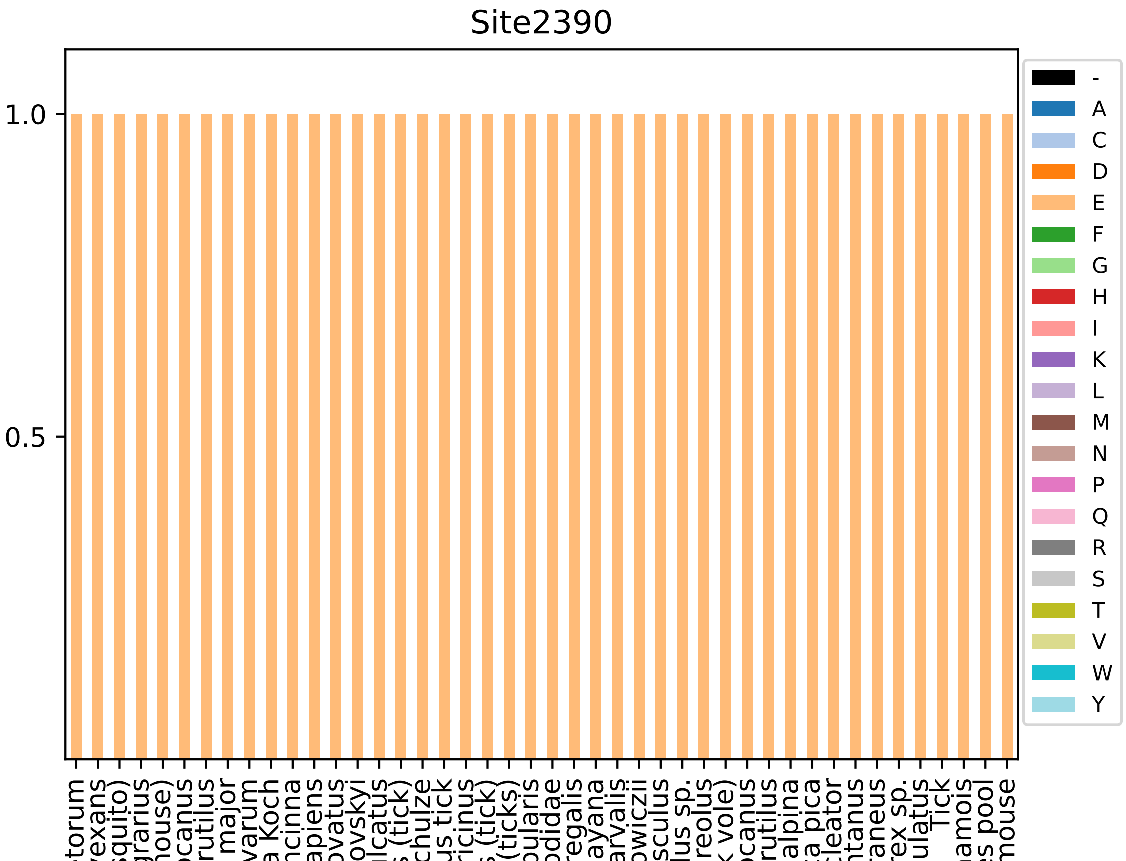

Site2390
1.0
0.5
-
A
C
D
E
F
G
H
I
K
L
M
N
P
Q
R
S
T
V
W
Y

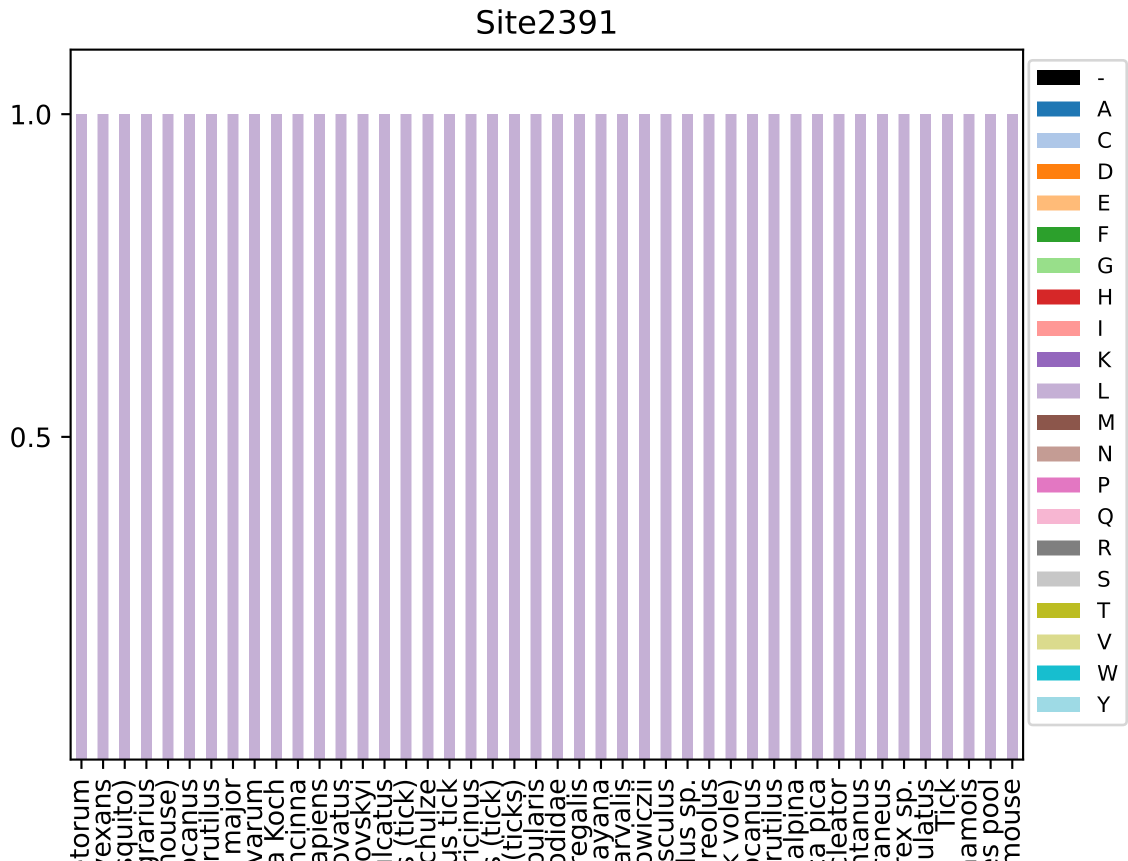

Site2391
1.0
0.5
-
A
C
D
E
F
G
H
I
K
L
M
N
P
Q
R
S
T
V
W
Y
torum
vexans
squito)
grarius
nouse)
ocanus
rutilus
major
varum
a Koch
ncinna
apiens
ovatus
ovskyi
ulcatus
(tick)
chulze
us tick
ricinus
(tick)
(ticks)
pularis
podidae
regalis
ayana
arvalis
owiczii
sculus
lus sp.
reolus
k vole)
ocanus
rutilus
alpina
a pica
cleator
ntanus
raneus
rex sp.
ulatus
Tick
amois
es pool
mouse

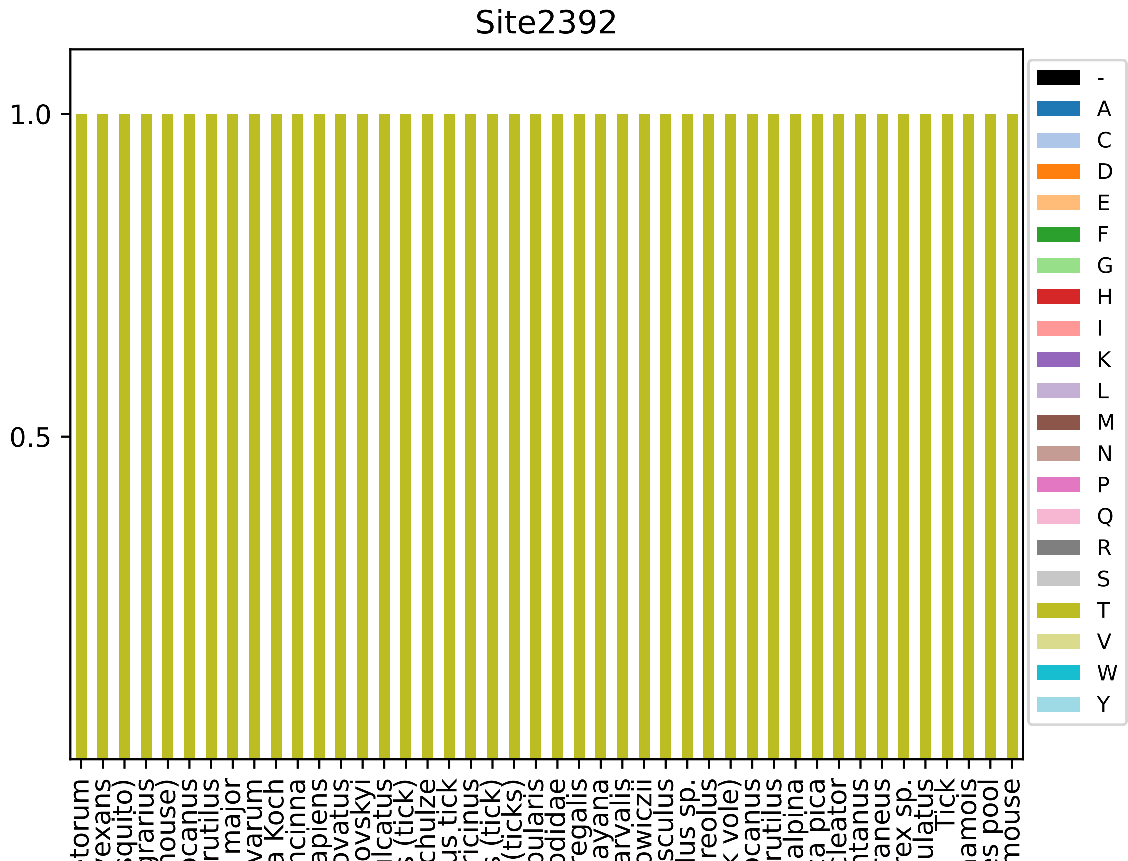

Site2392
1.0
0.5
-
A
C
D
E
F
G
H
I
K
L
M
N
P
Q
R
S
T
V
W
Y

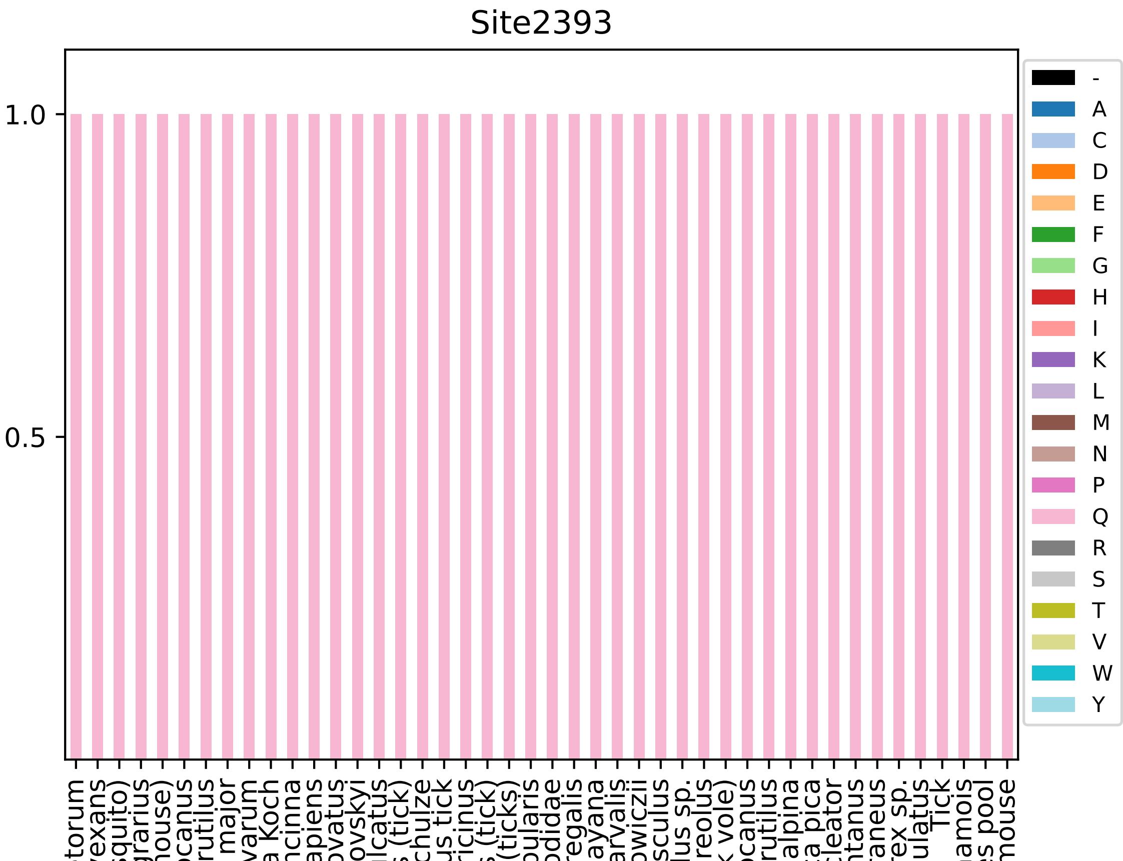
Site2393
1.0
0.5
-
A
C
D
E
F
G
H
I
K
L
M
N
P
Q
R
S
T
V
W
Y

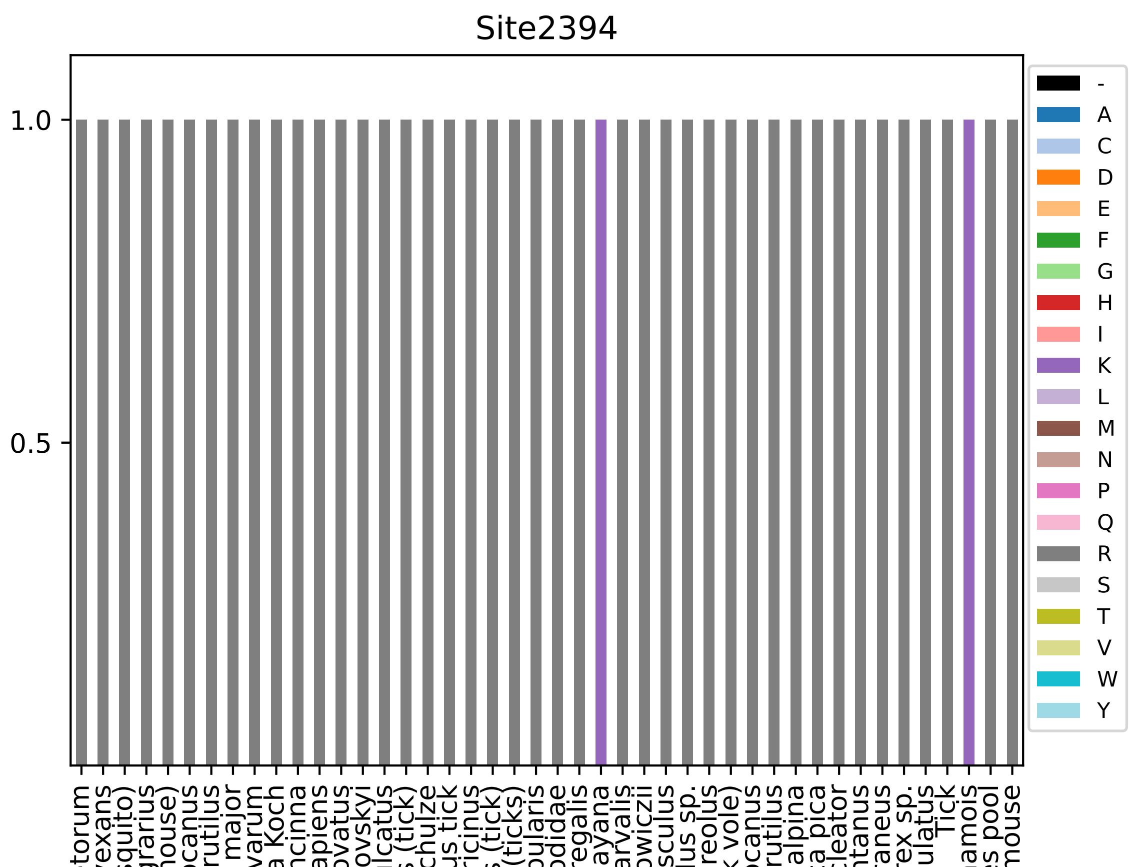
Site2394
1.0
0.5
-
A
C
D
E
F
G
H
I
K
L
M
N
P
Q
R
S
T
V
W
Y
Tick

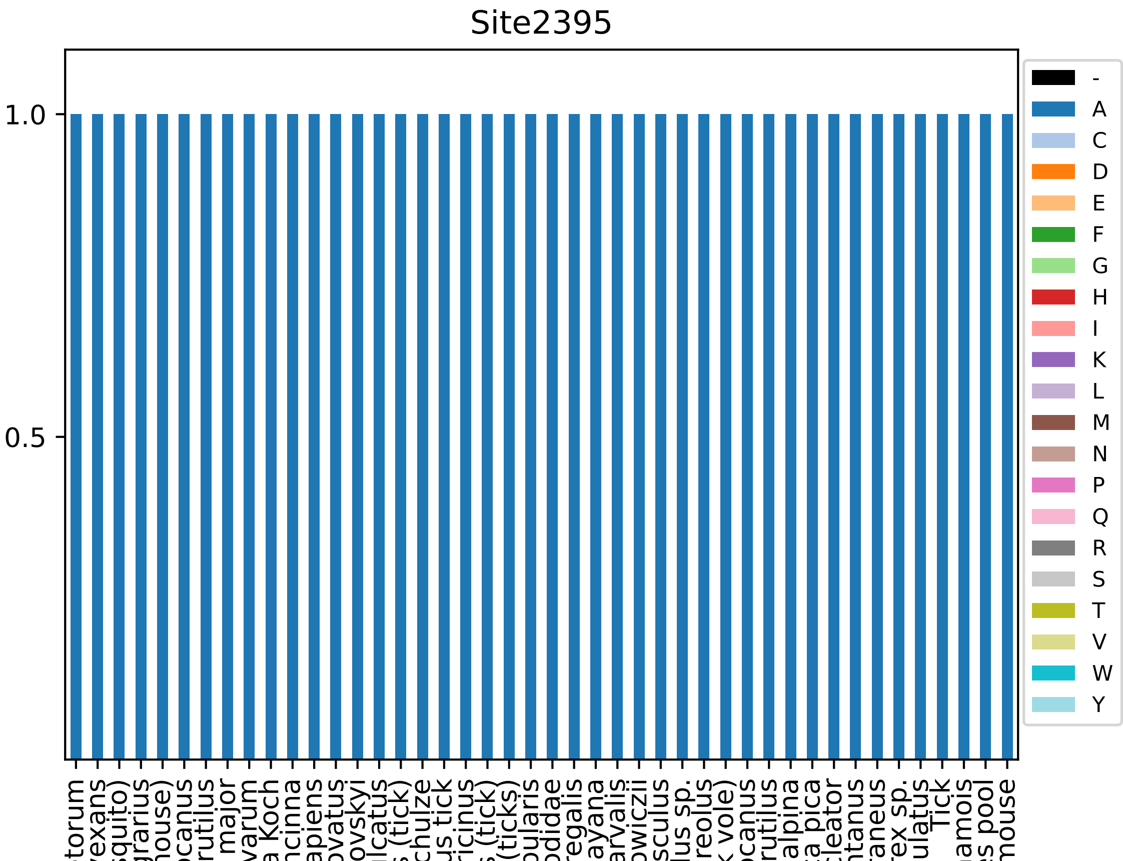

Site2395
1.0
0.5
-
A
C
D
E
F
G
H
I
K
L
M
N
P
Q
R
S
T
V
W
Y

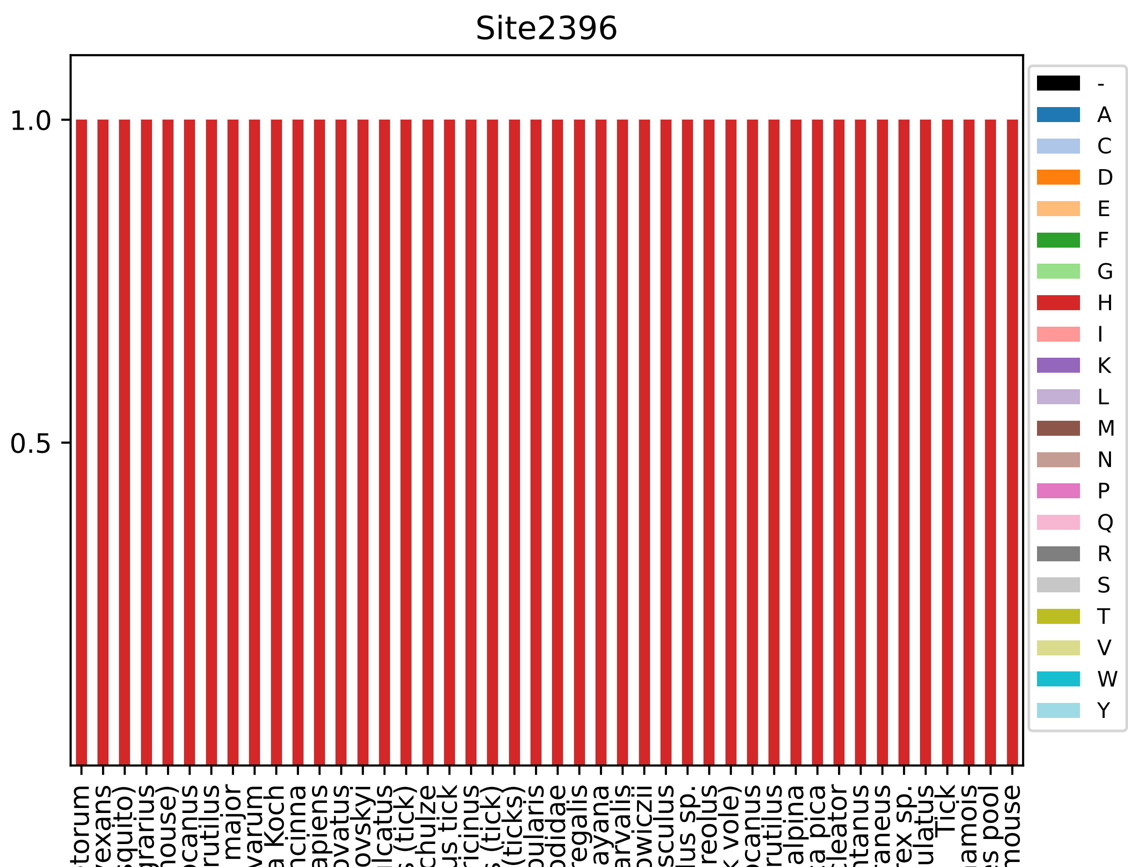
Site2396
1.0
0.5
-
A
C
D
E
F
G
H
I
K
L
M
N
P
Q
R
S
T
V
W
Y

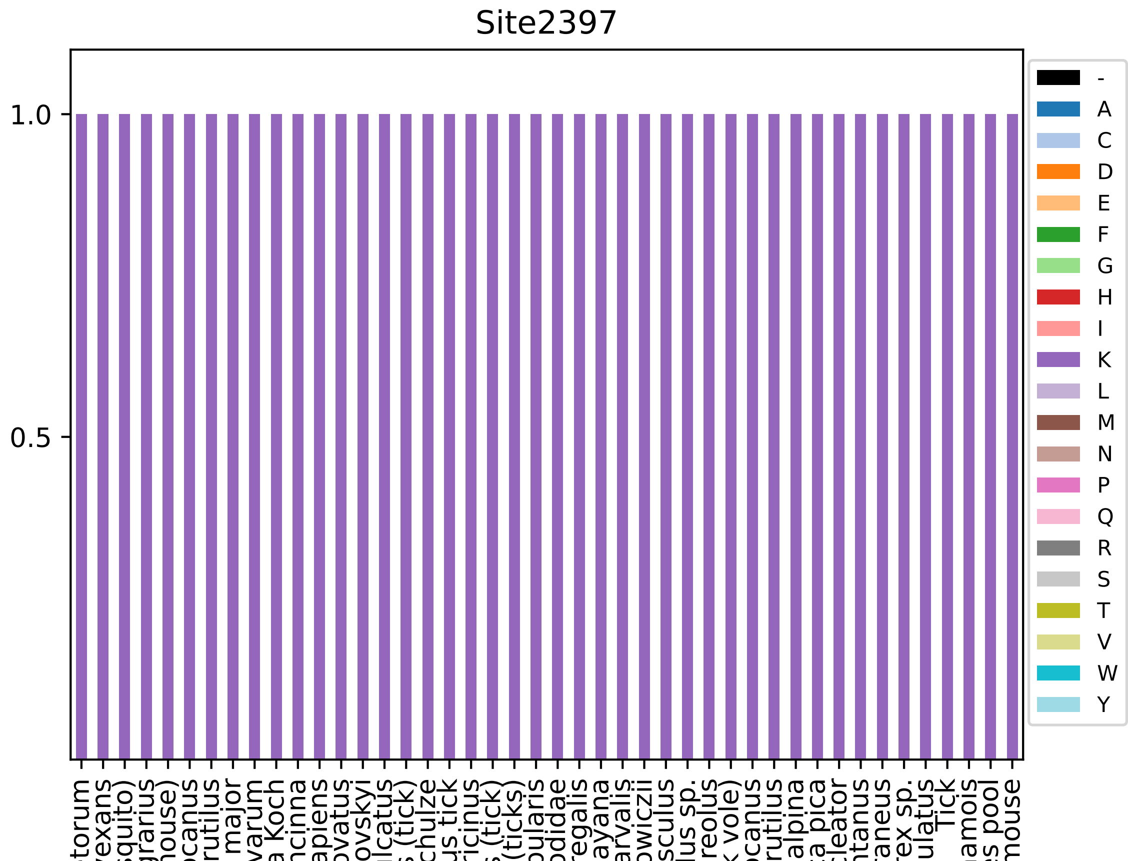
Site2397
1.0
0.5
-
A
C
D
E
F
G
H
I
K
L
M
N
P
Q
R
S
T
V
W
Y
Tick

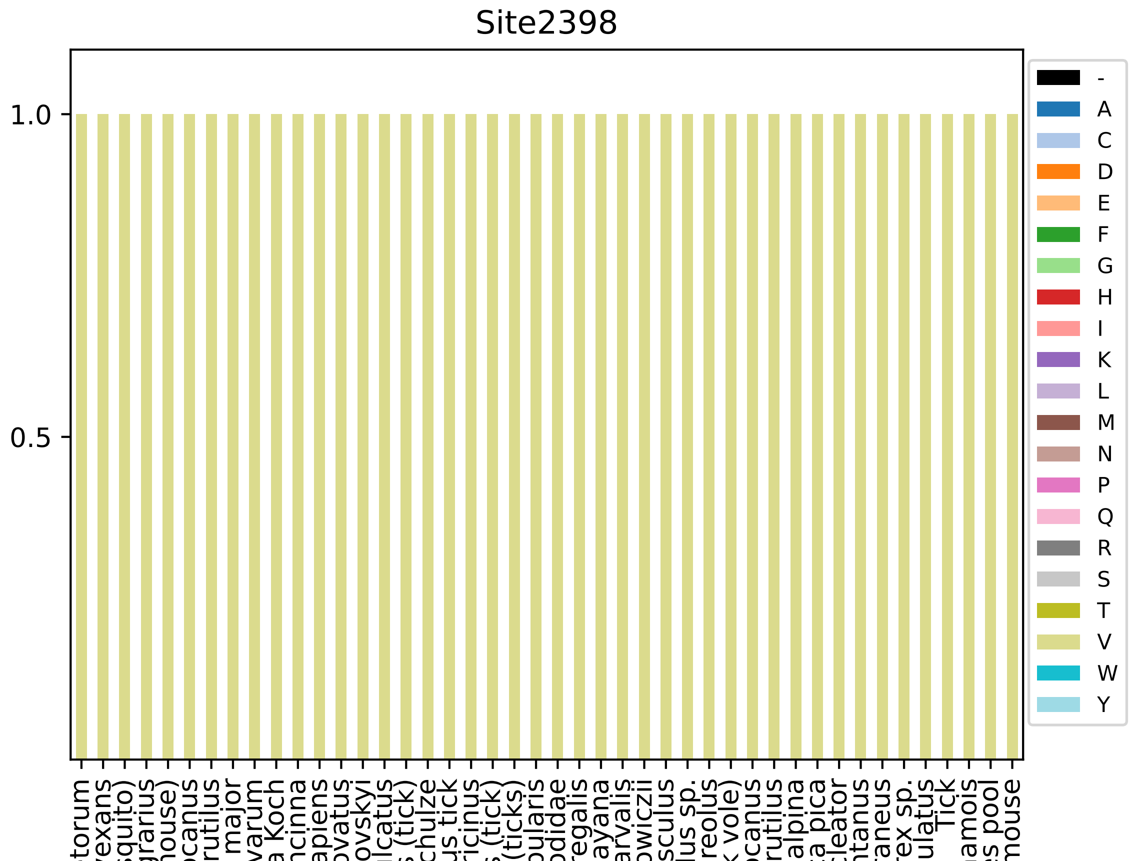
Site2398
1.0
0.5
-
A
C
D
E
F
G
H
I
K
L
M
N
P
Q
R
S
T
V
W
Y

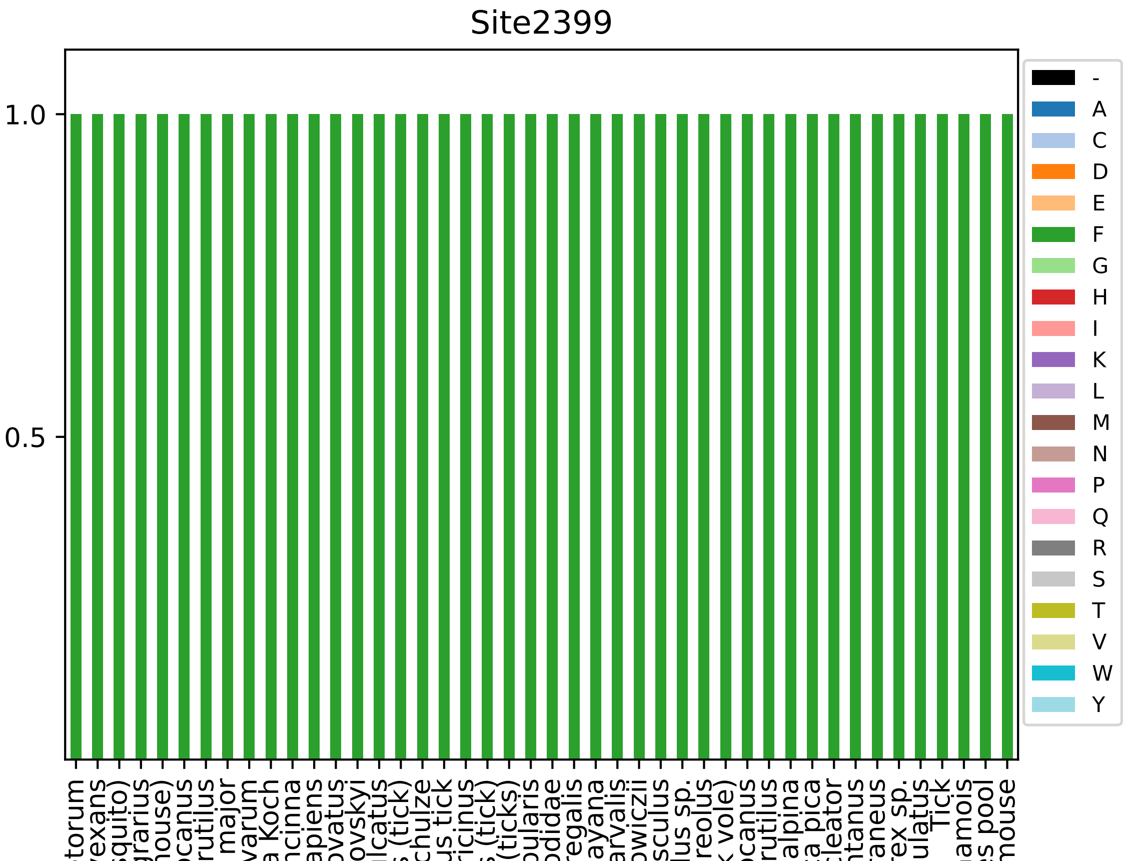
Site2399
1.0
0.5
-
A
C
D
E
F
G
H
I
K
L
M
N
P
Q
R
S
T
V
W
Y

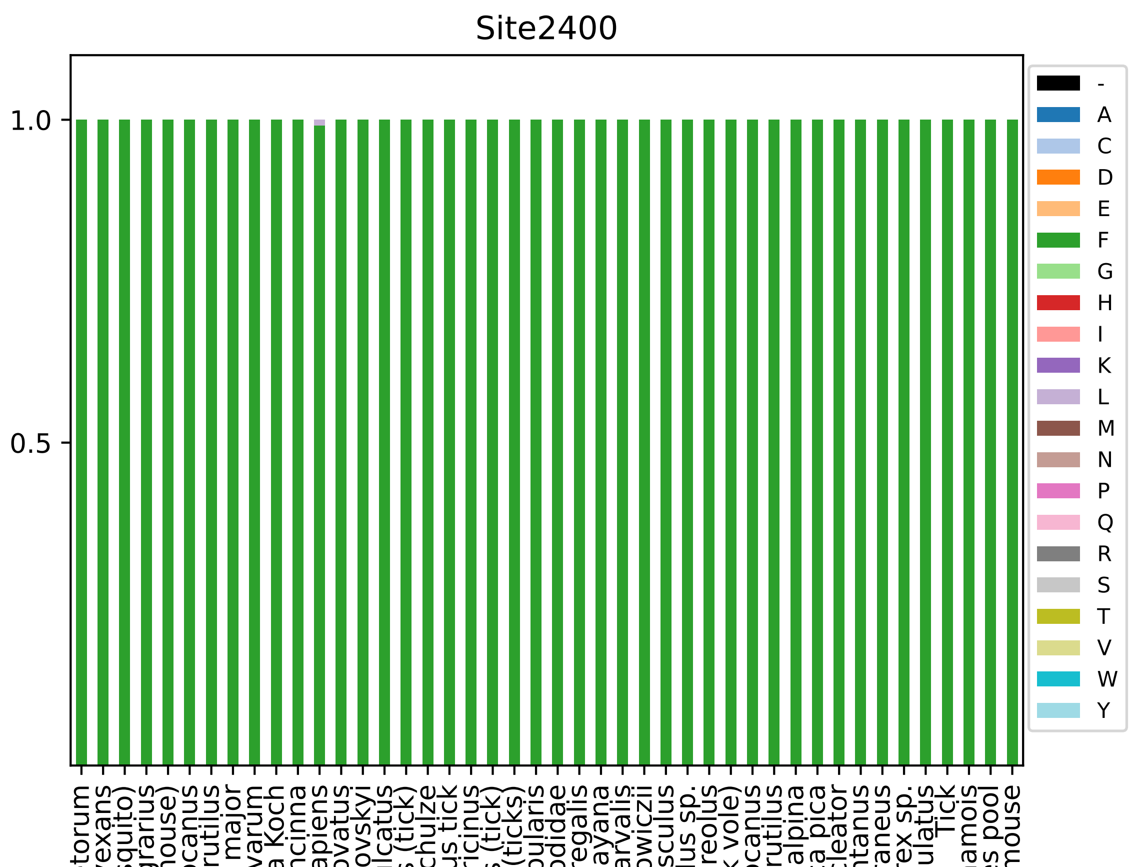
Site2400
1.0
0.5
-
A
C
D
E
F
G
H
I
K
L
M
N
P
Q
R
S
T
V
W
Y

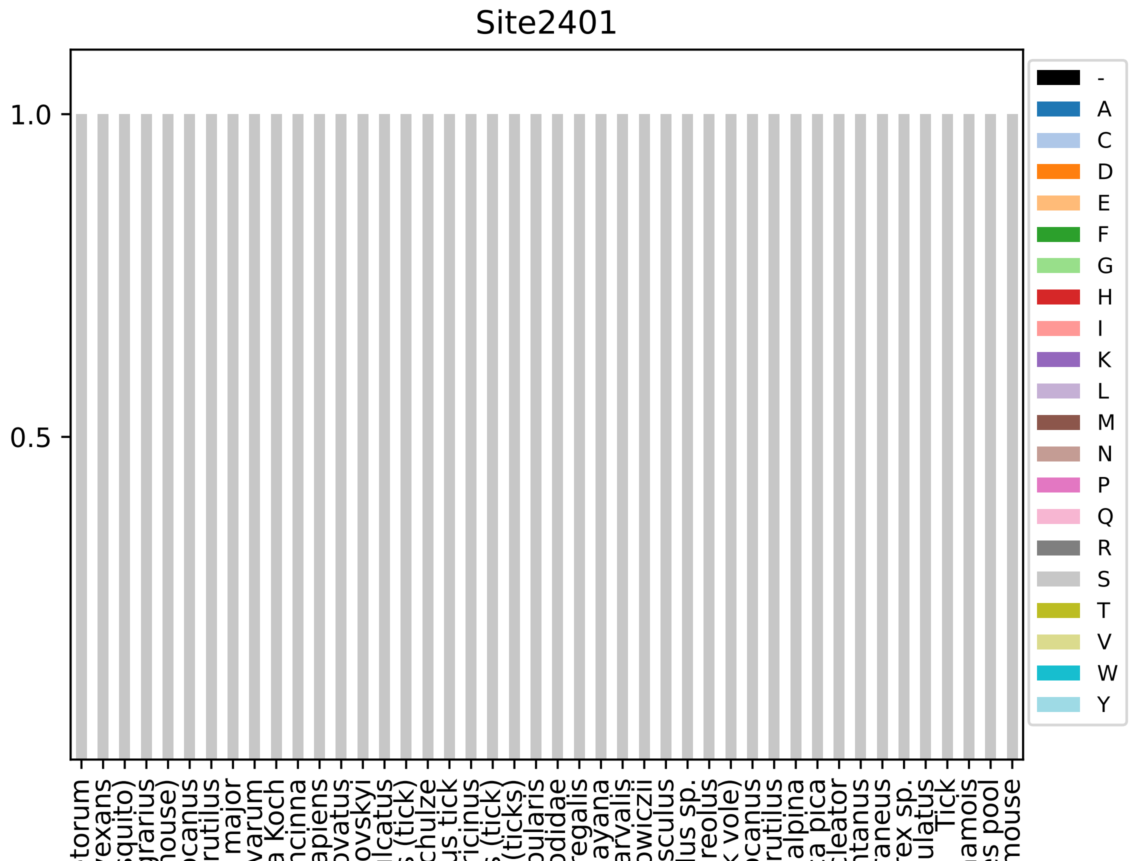
Site2401
1.0
0.5
-
A
C
D
E
F
G
H
I
K
L
M
N
P
Q
R
S
T
V
W
Y

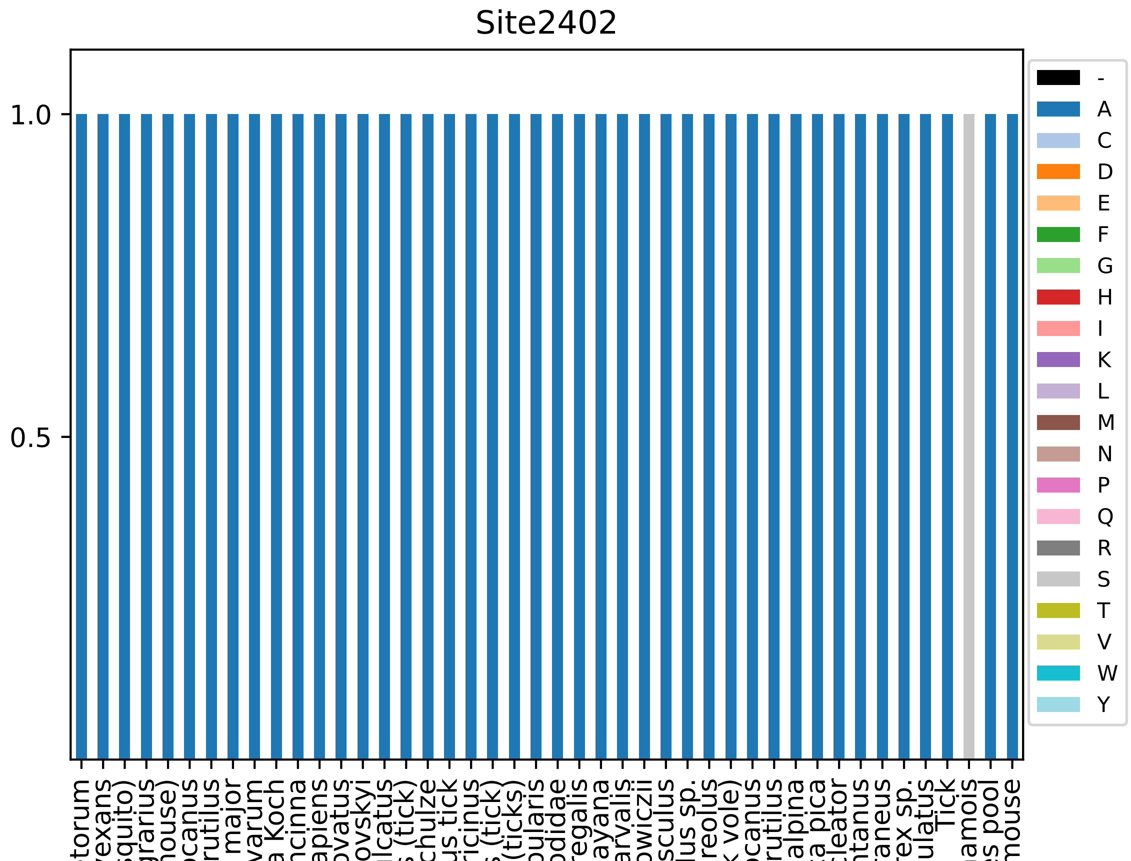
Site2402
1.0
0.5
-
A
C
D
E
F
G
H
I
K
L
M
N
P
Q
R
S
T
V
W
Y

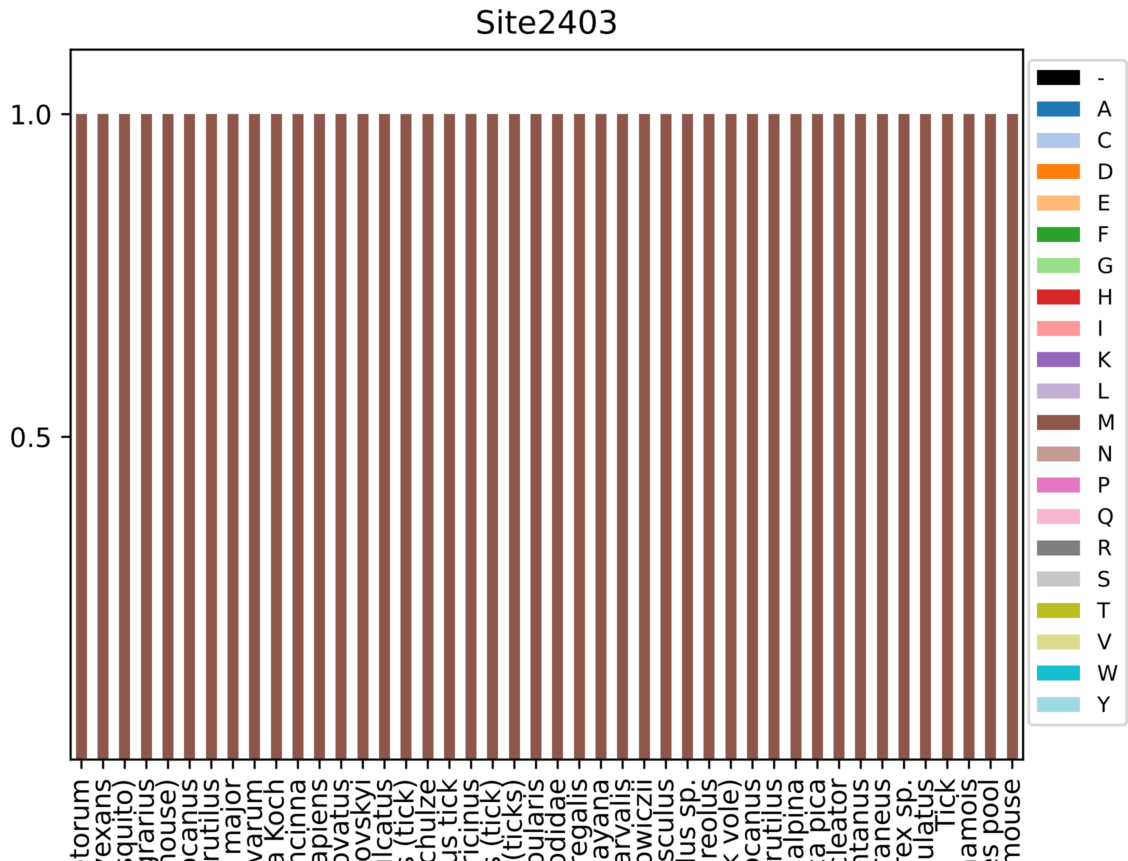
Site2403
1.0
0.5
-
A
C
D
E
F
G
H
I
K
L
M
N
P
Q
R
S
T
V
W
Y

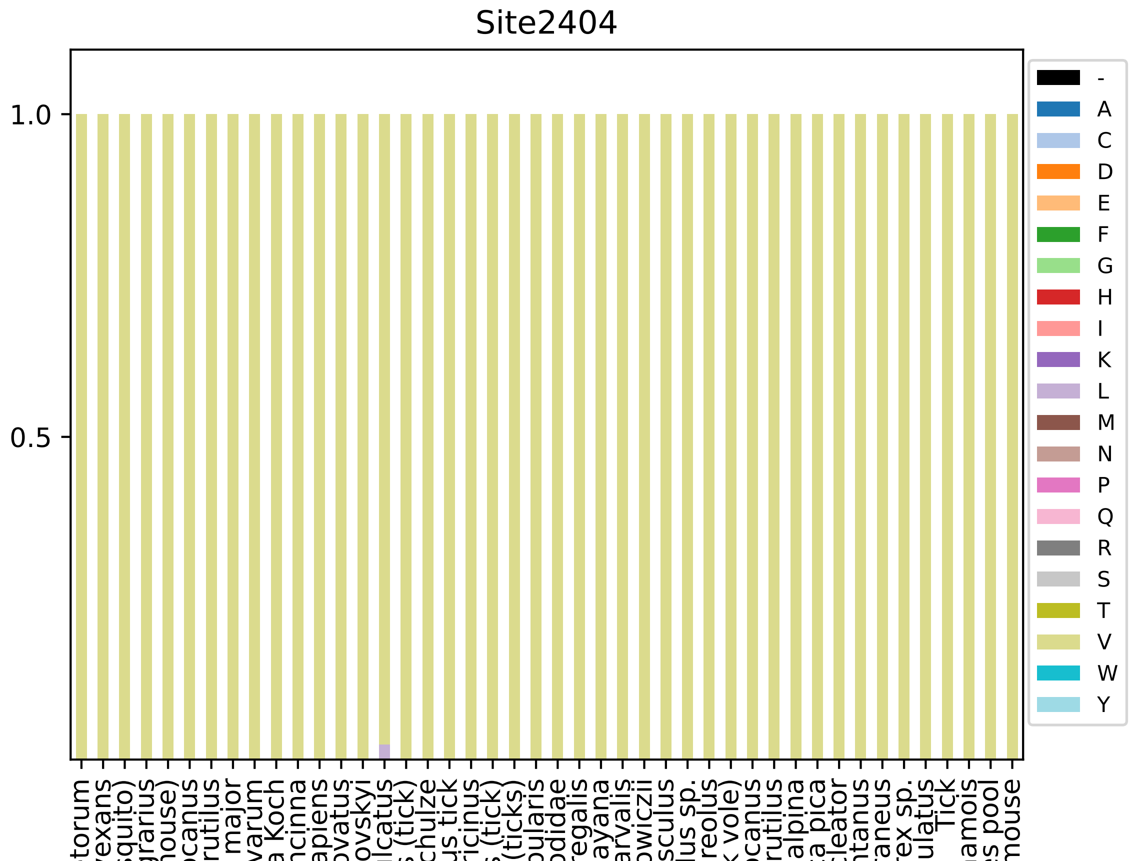
Site2404
1.0
0.5
-
A
C
D
E
F
G
H
I
K
L
M
N
P
Q
R
S
T
V
W
Y

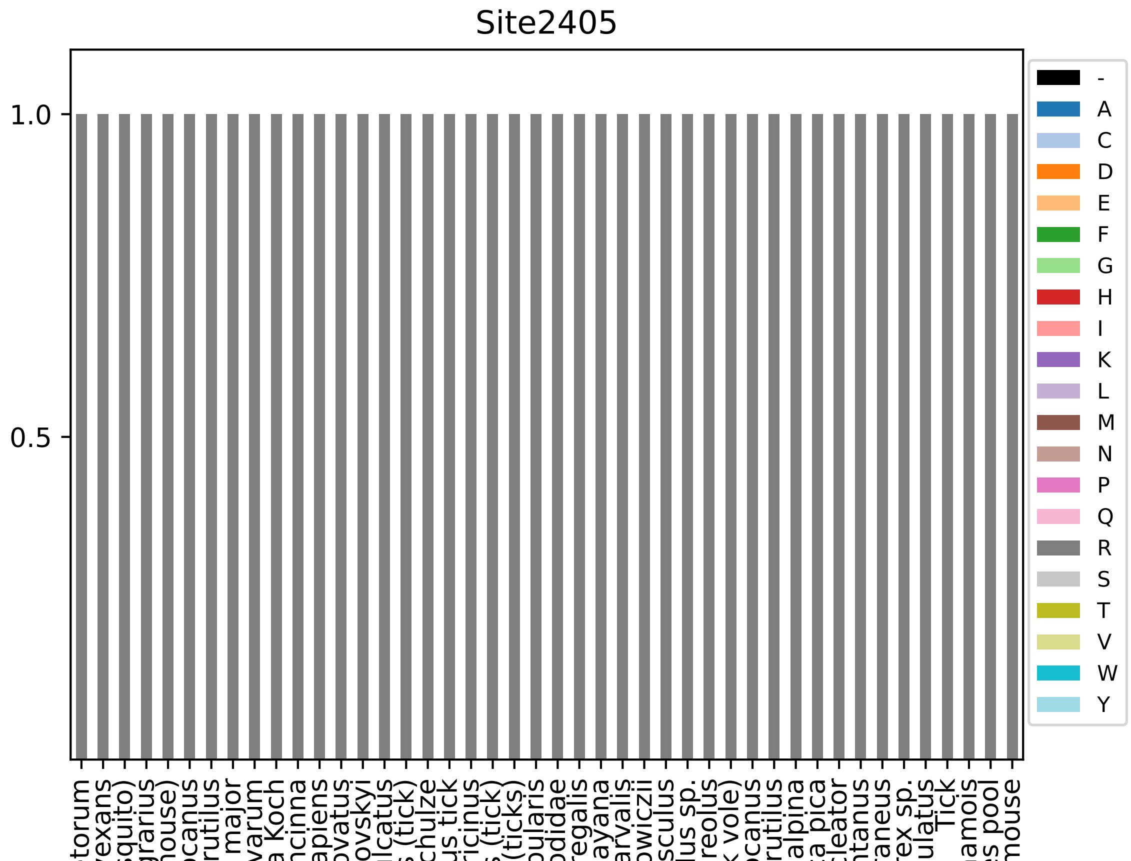
Site2405
1.0
0.5
-
A
C
D
E
F
G
H
I
K
L
M
N
P
Q
R
S
T
V
W
Y

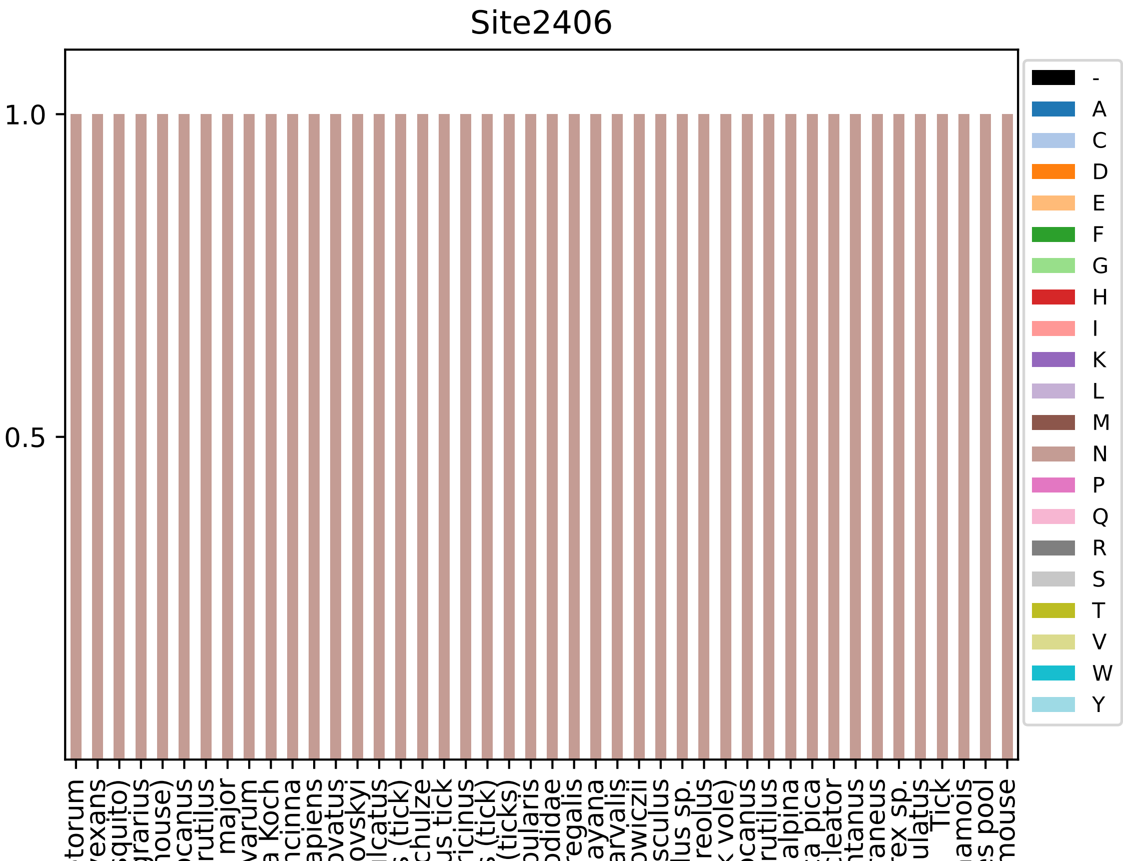
Site2406
1.0
0.5
-
A
C
D
E
F
G
H
I
K
L
M
N
P
Q
R
S
T
V
W
Y

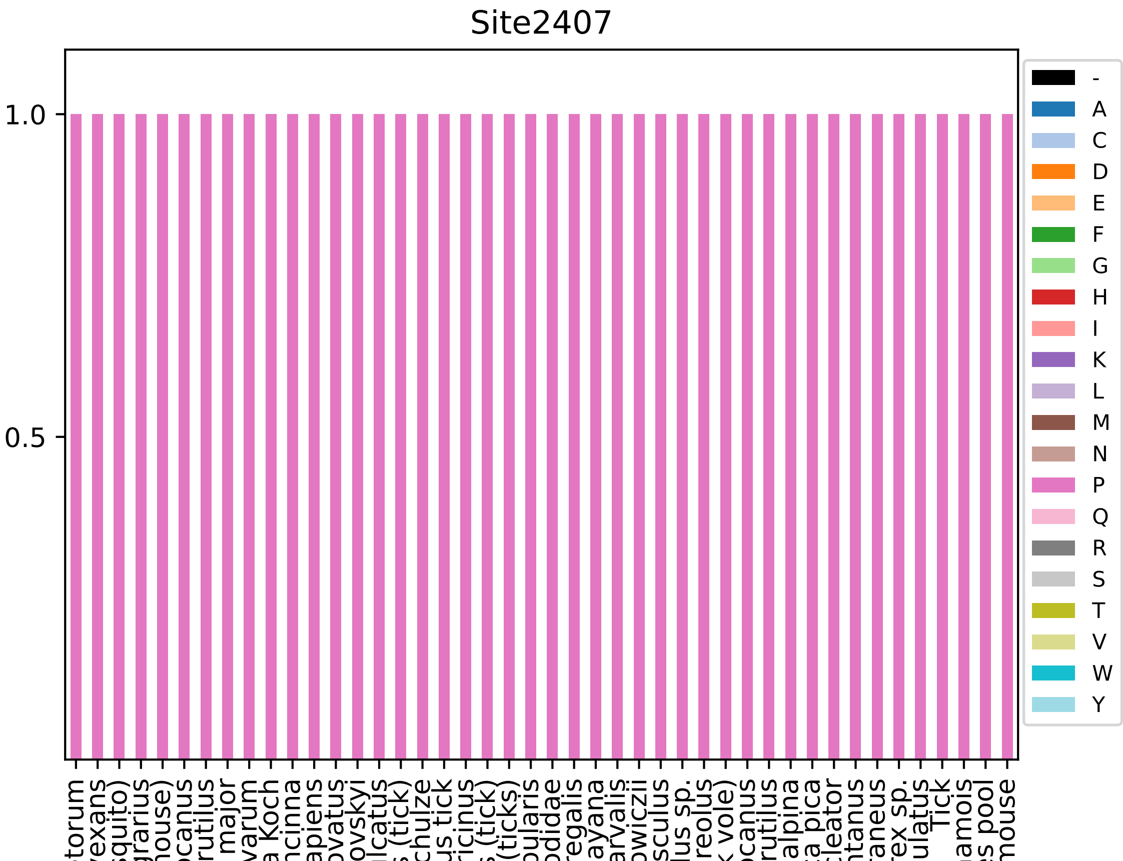

Site2407
1.0
0.5
-
A
C
D
E
F
G
H
I
K
L
M
N
P
Q
R
S
T
V
W
Y
Tick

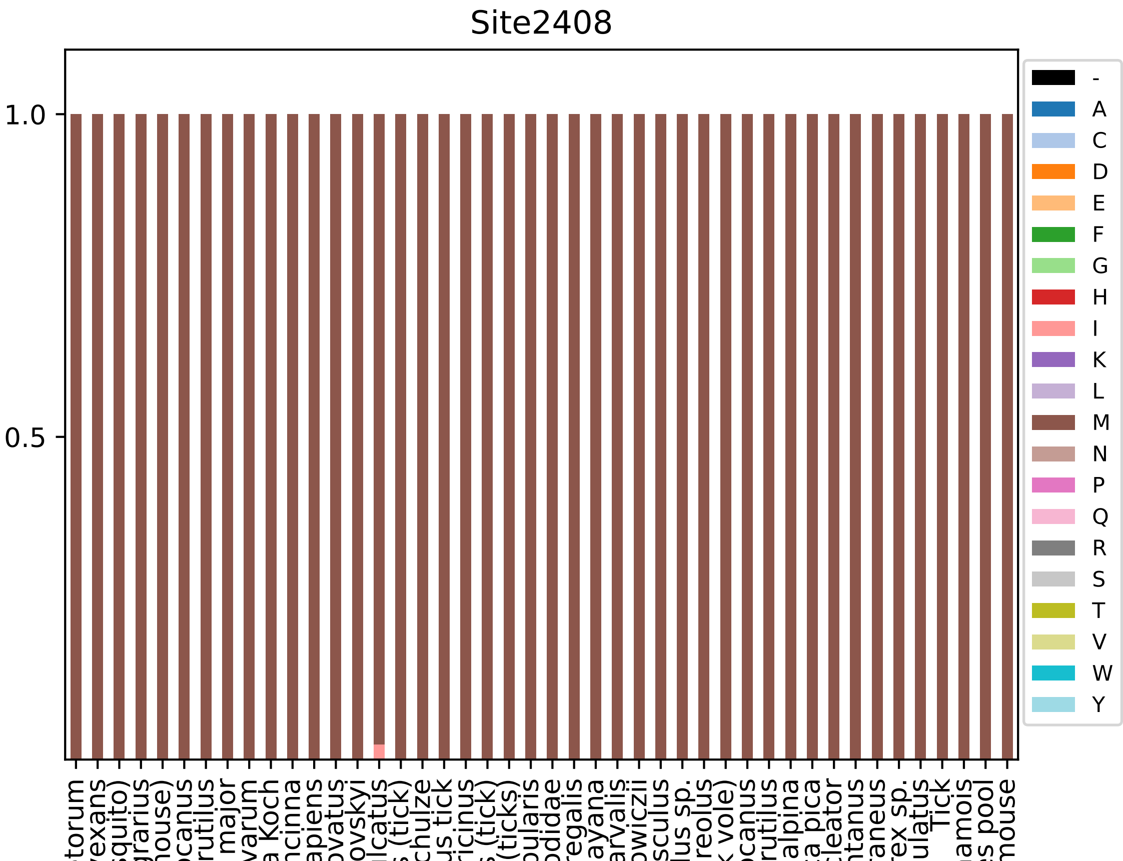
Site2408
1.0
0.5
-
A
C
D
E
F
G
H
I
K
L
M
N
P
Q
R
S
T
V
W
Y

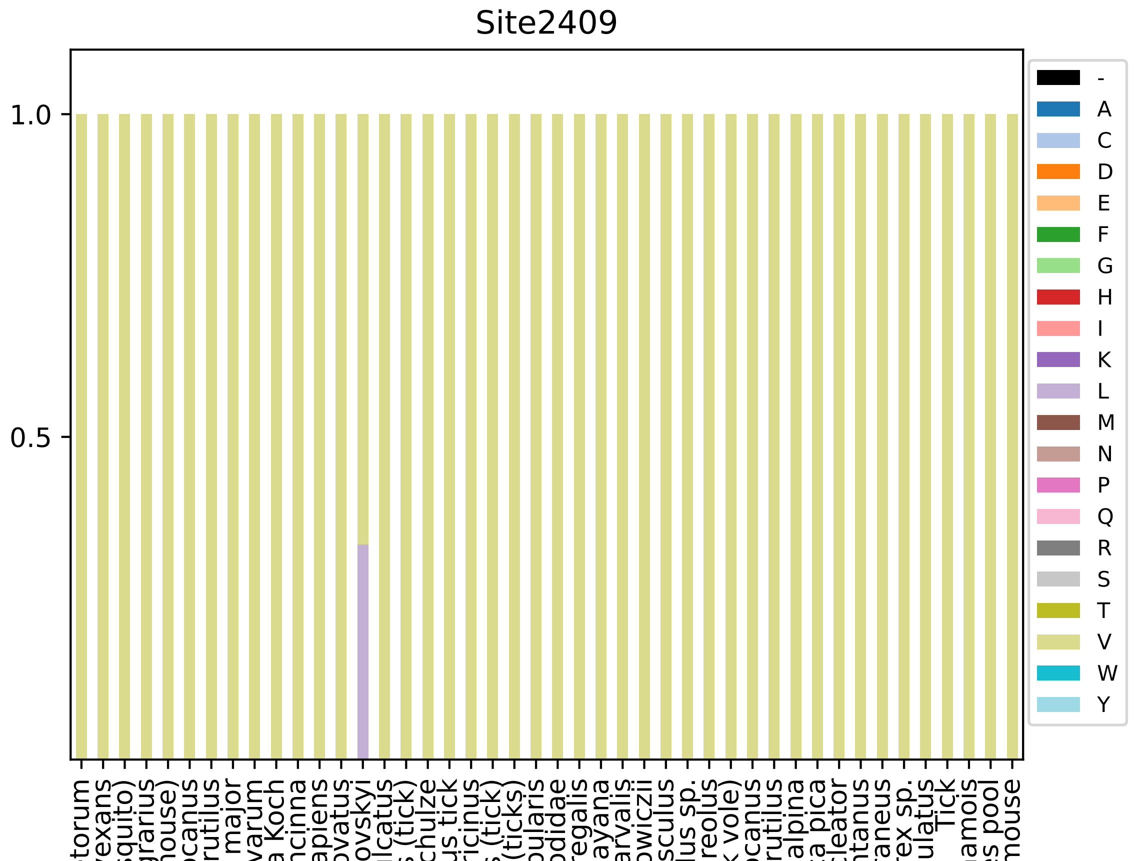
Site2409
1.0
0.5
-
A
C
D
E
F
G
H
I
K
L
M
N
P
Q
R
S
T
V
W
Y

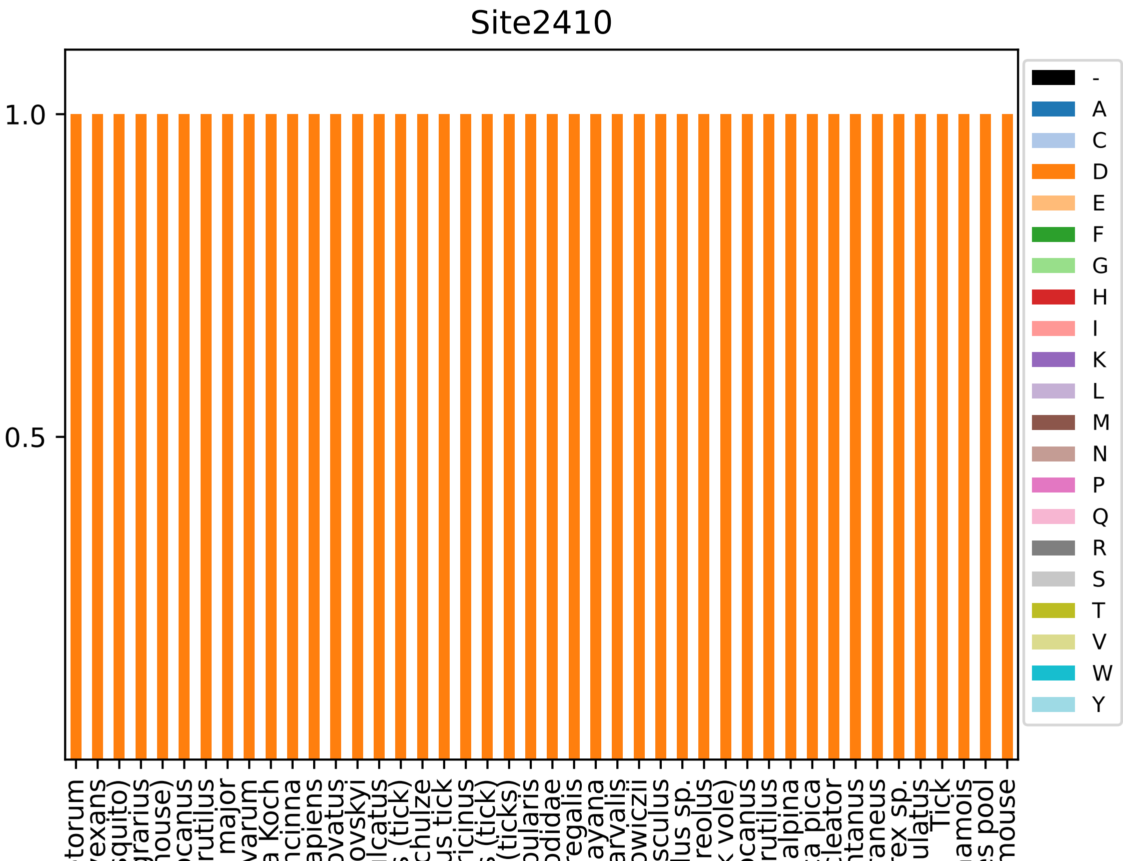
Site2410
1.0
0.5
-
A
C
D
E
F
G
H
I
K
L
M
N
P
Q
R
S
T
V
W
Y

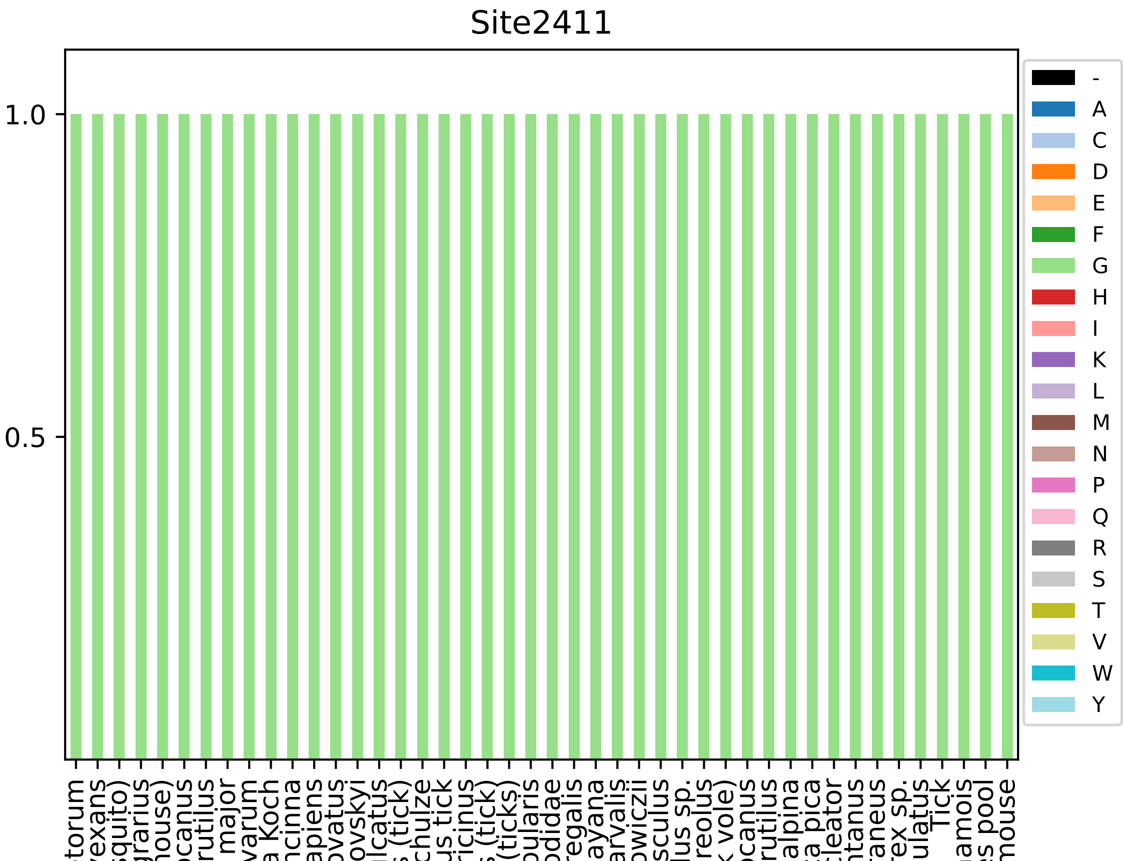
Site2411
1.0
0.5
-
A
C
D
E
F
G
H
I
K
L
M
N
P
Q
R
S
T
V
W
Y

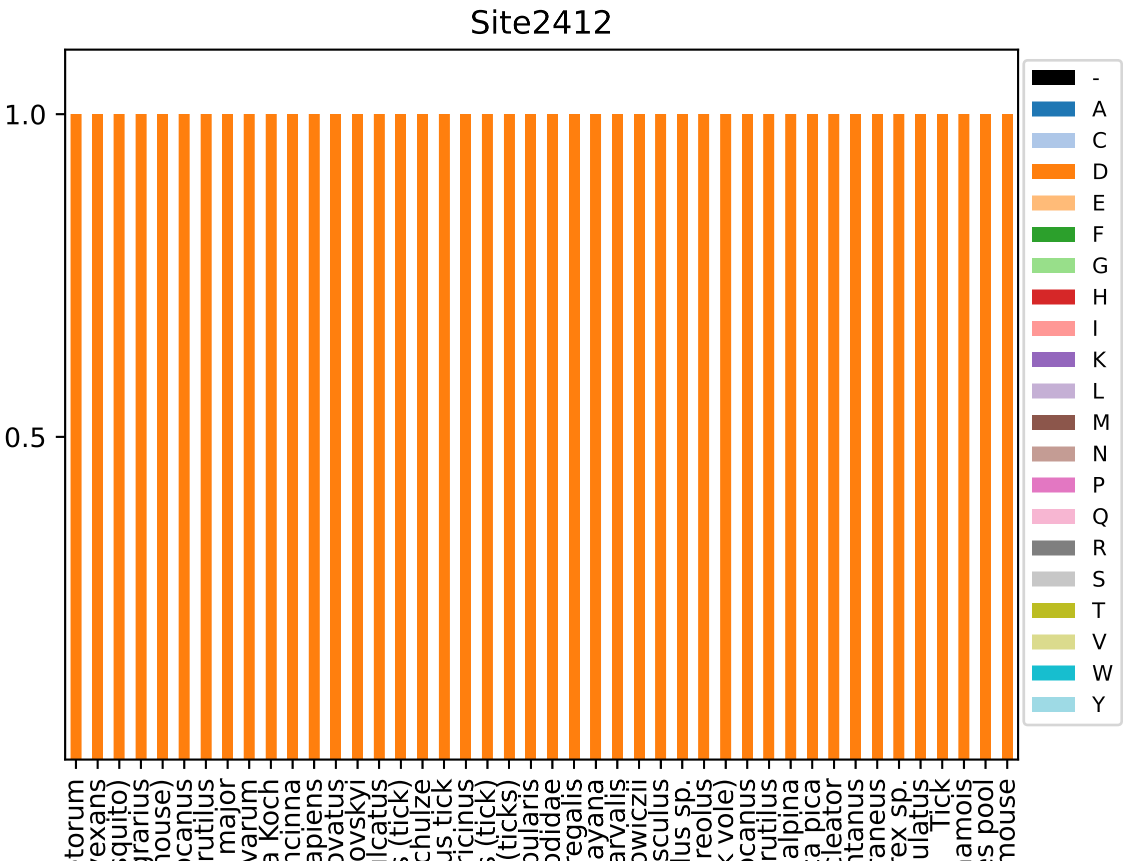
Site2412
1.0
0.5
-
A
C
D
E
F
G
H
I
K
L
M
N
P
Q
R
S
T
V
W
Y

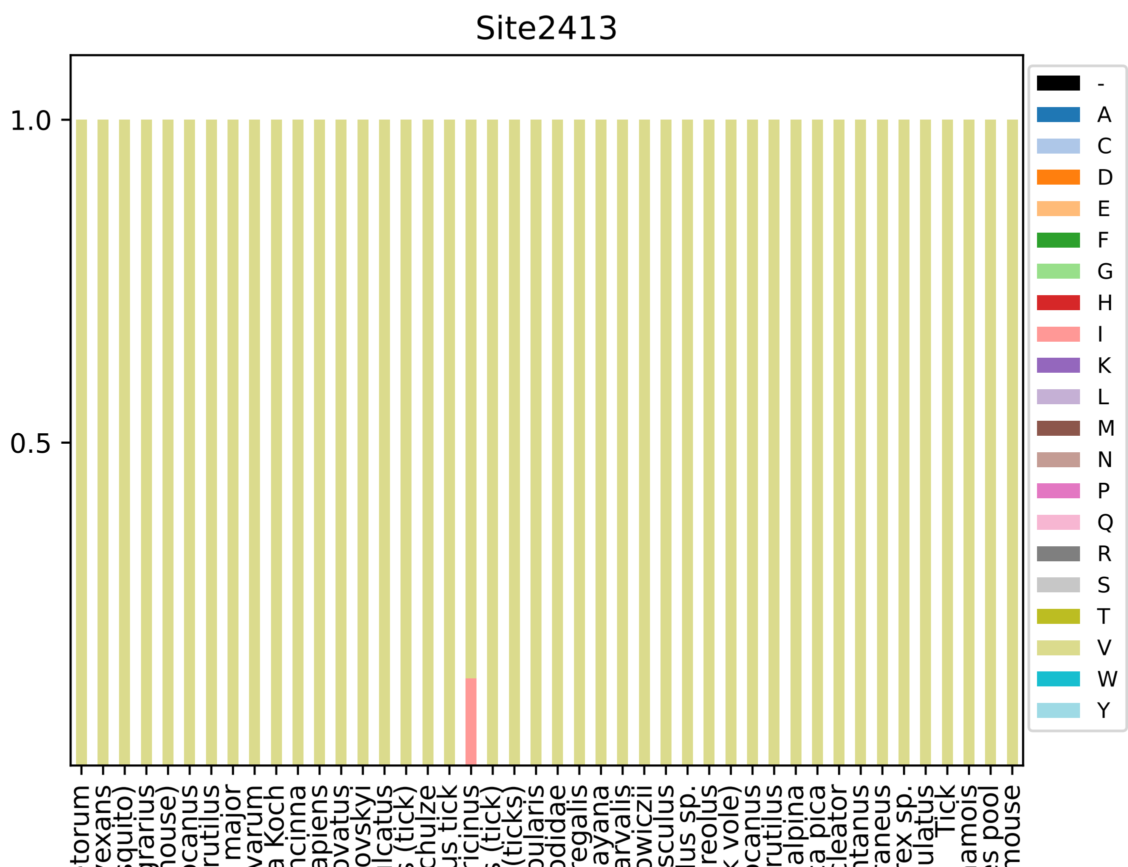
Site2413
1.0
0.5
-
A
C
D
E
F
G
H
I
K
L
M
N
P
Q
R
S
T
V
W
Y

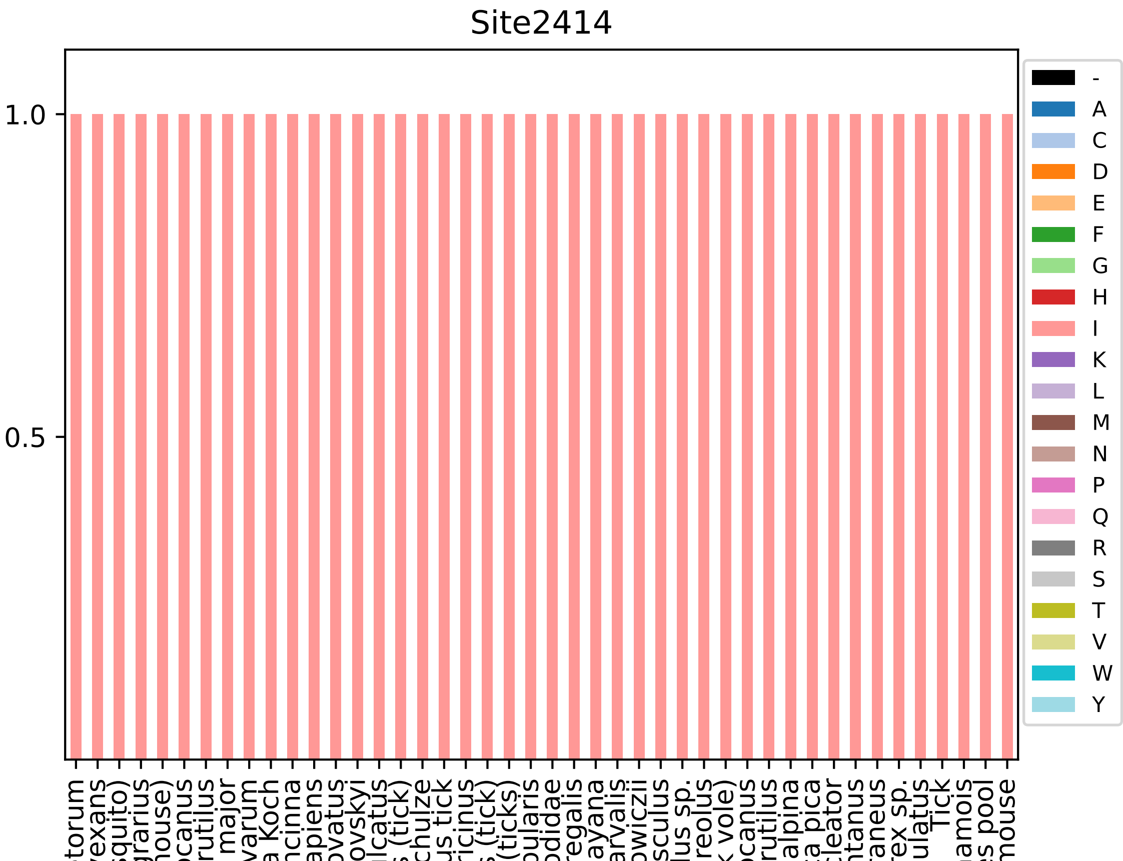
Site2414
1.0
0.5
-
A
C
D
E
F
G
H
I
K
L
M
N
P
Q
R
S
T
V
W
Y

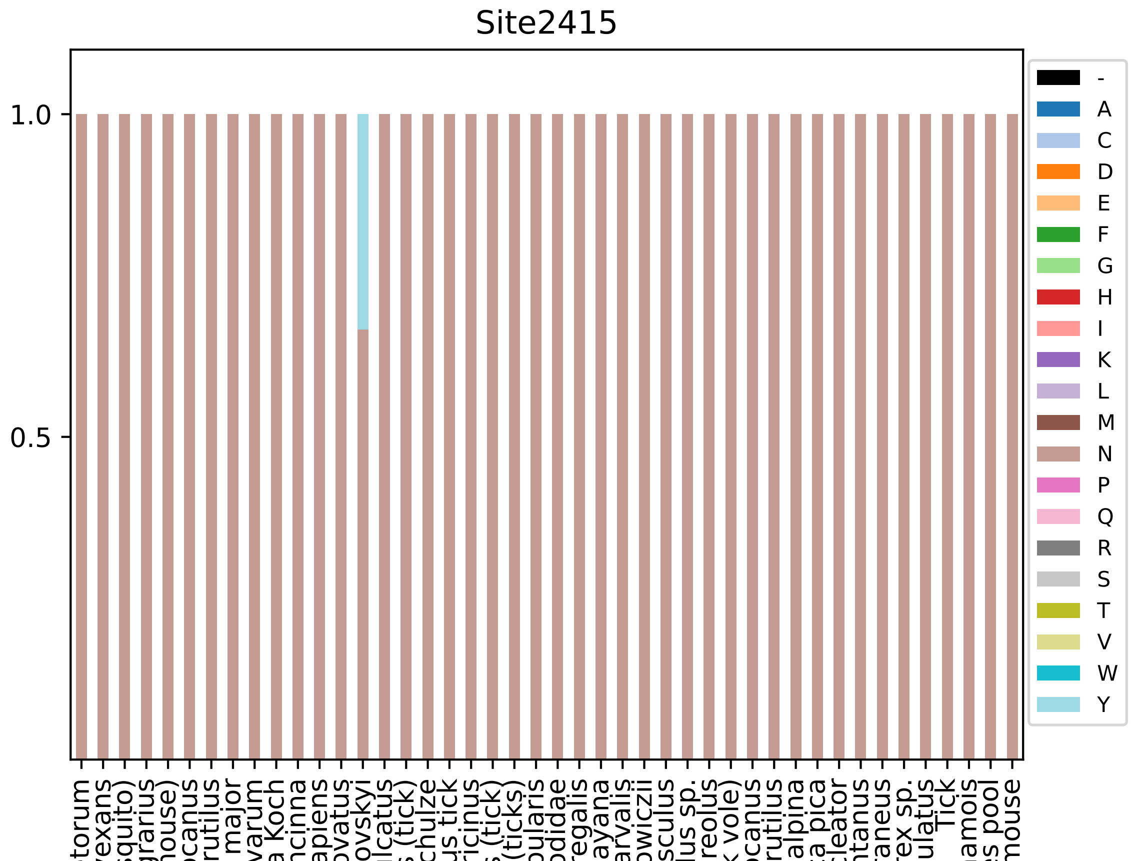
Site2415
1.0
0.5
-
A
C
D
E
F
G
H
I
K
L
M
N
P
Q
R
S
T
V
W
Y

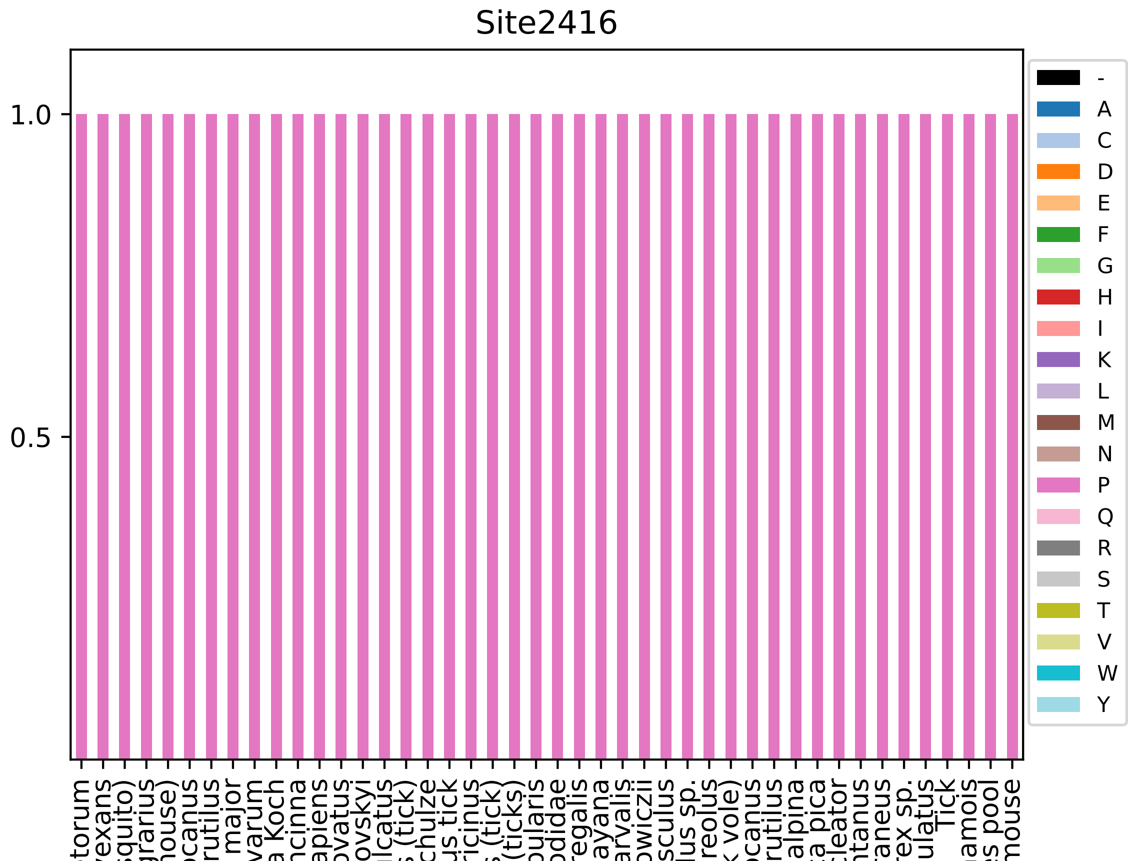

Site2416
1.0
0.5
-
A
C
D
E
F
G
H
I
K
L
M
N
P
Q
R
S
T
V
W
Y

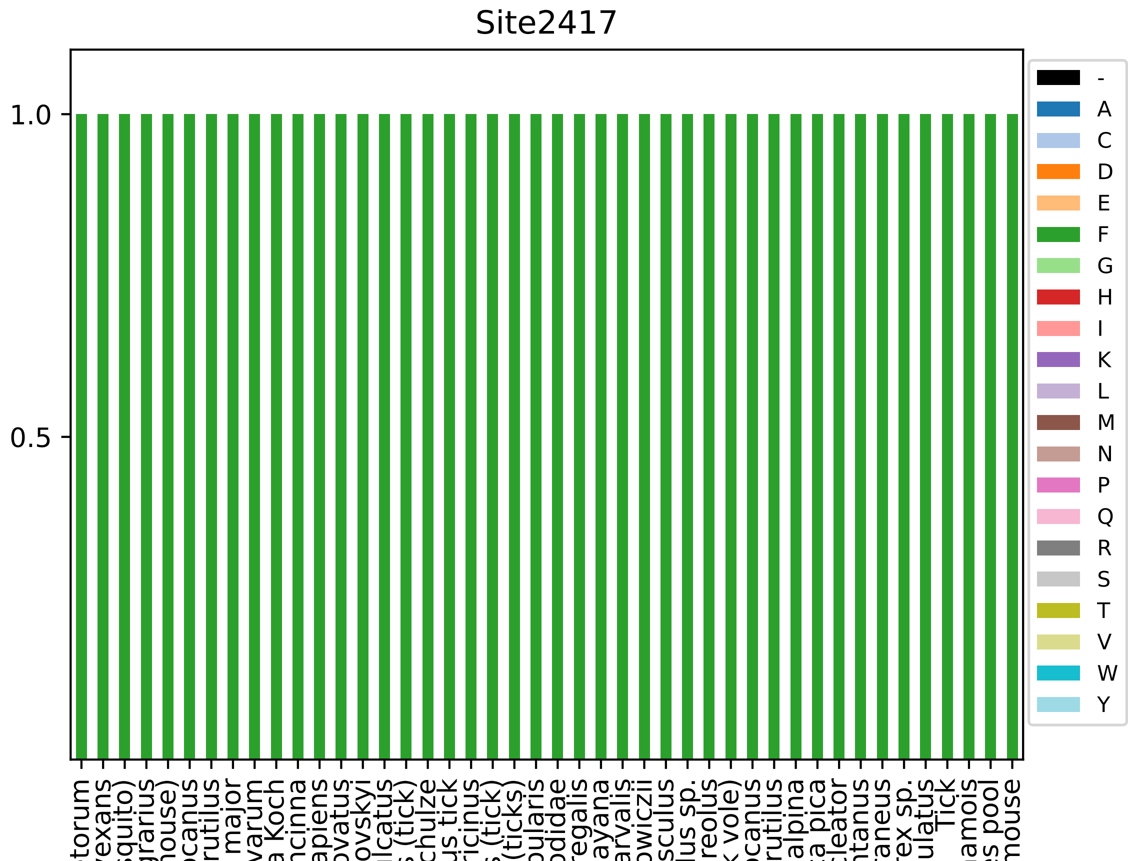
Site2417
1.0
0.5
-
A
C
D
E
F
G
H
I
K
L
M
N
P
Q
R
S
T
V
W
Y

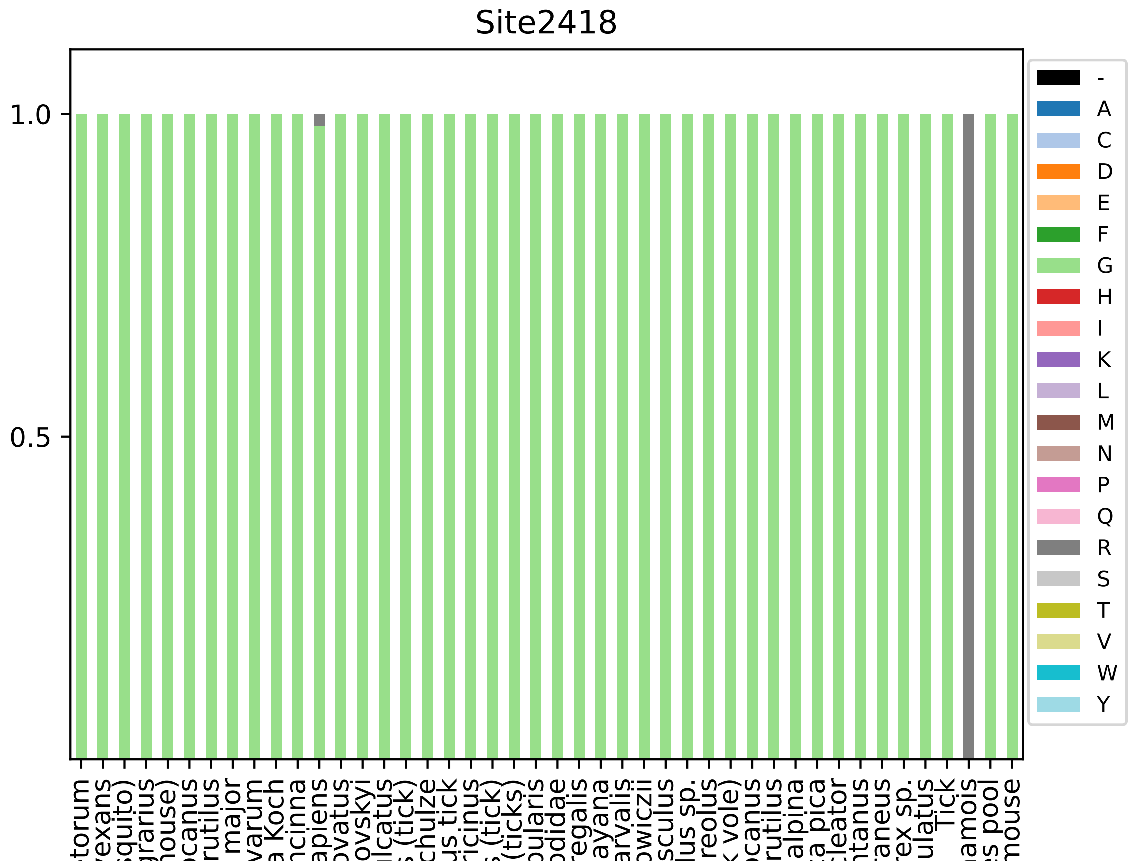
Site2418
1.0
0.5
-
A
C
D
E
F
G
H
I
K
L
M
N
P
Q
R
S
T
V
W
Y

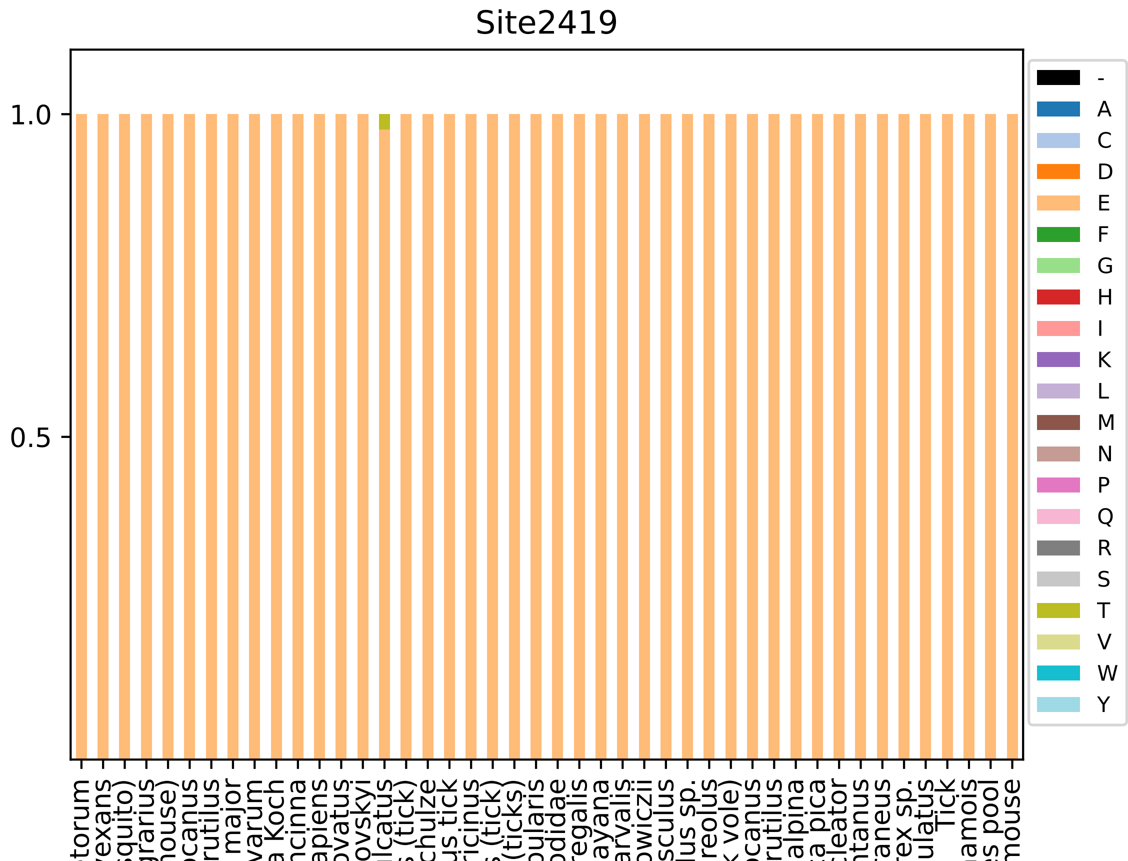
Site2419
1.0
0.5
-
A
C
D
E
F
G
H
I
K
L
M
N
P
Q
R
S
T
V
W
Y

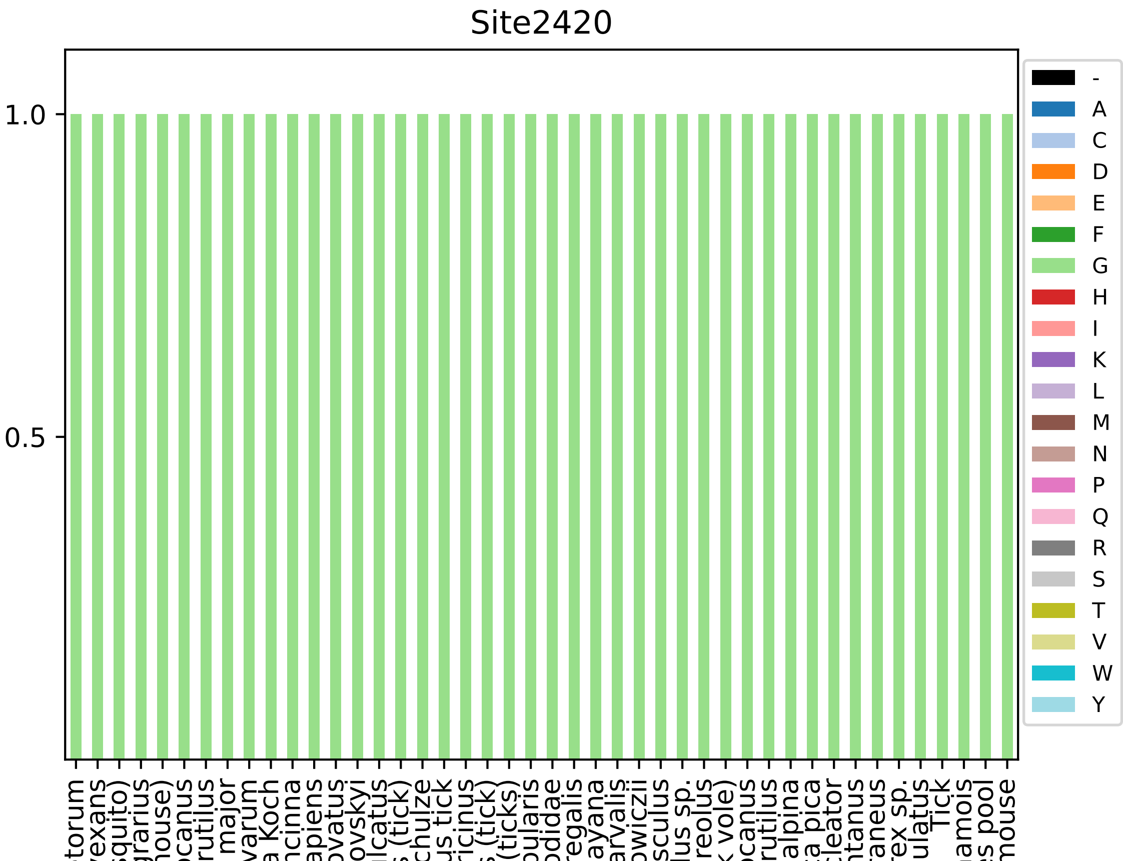

Site2420
1.0
0.5
-
A
C
D
E
F
G
H
I
K
L
M
N
P
Q
R
S
T
V
W
Y

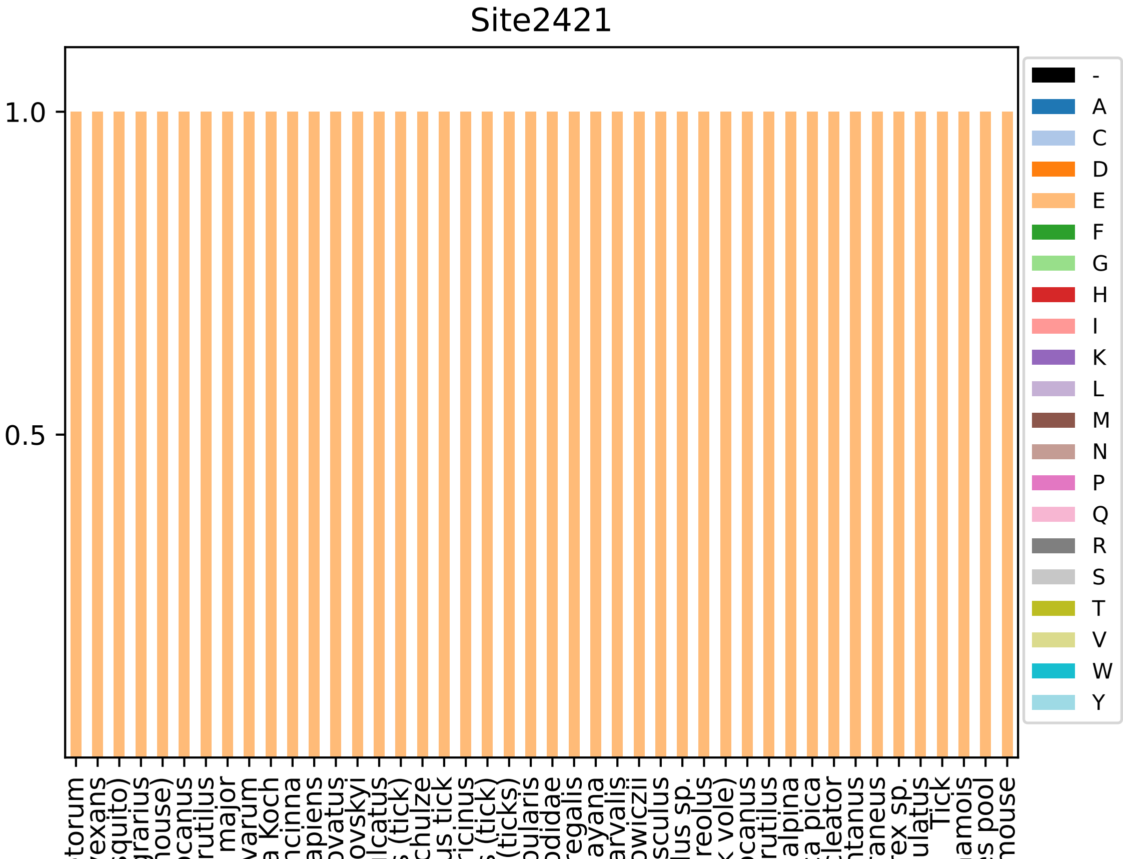
Site2421
1.0
0.5
-
A
C
D
E
F
G
H
I
K
L
M
N
P
Q
R
S
T
V
W
Y

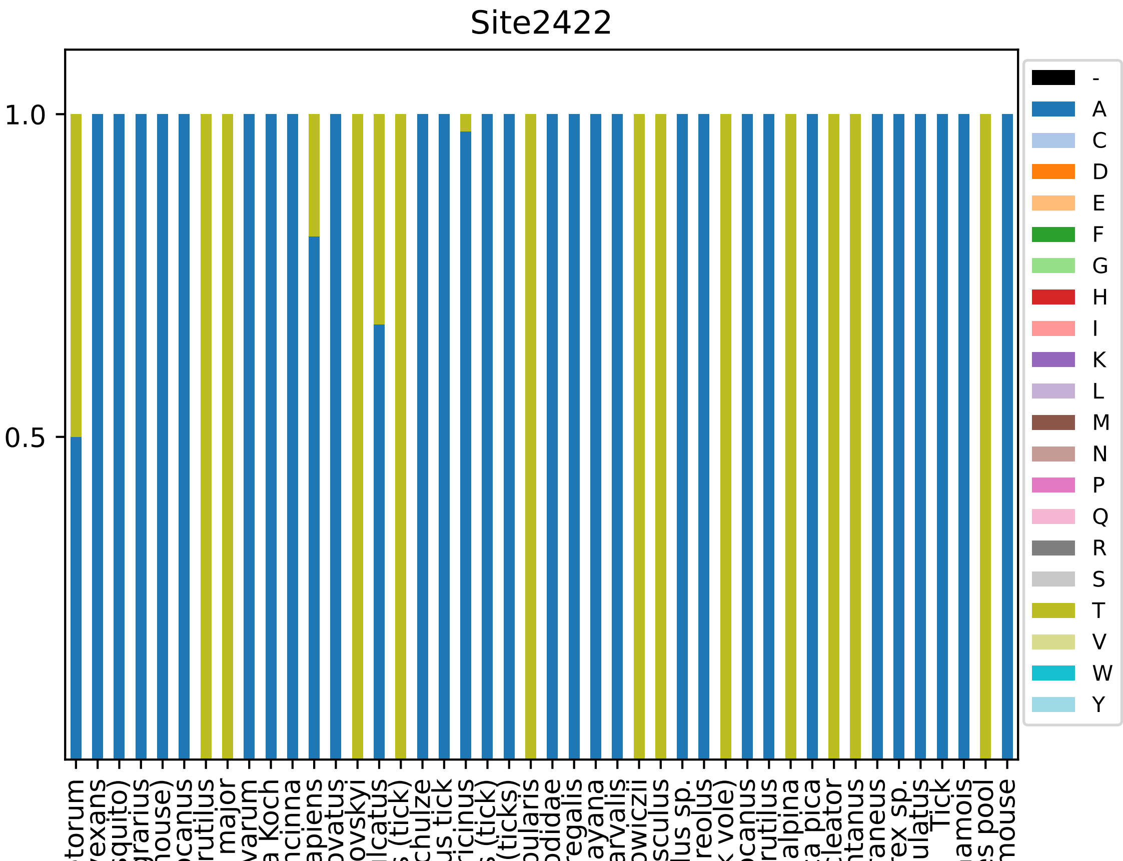
Site2422
1.0
0.5
-
A
C
D
E
F
G
H
I
K
L
M
N
P
Q
R
S
T
V
W
Y

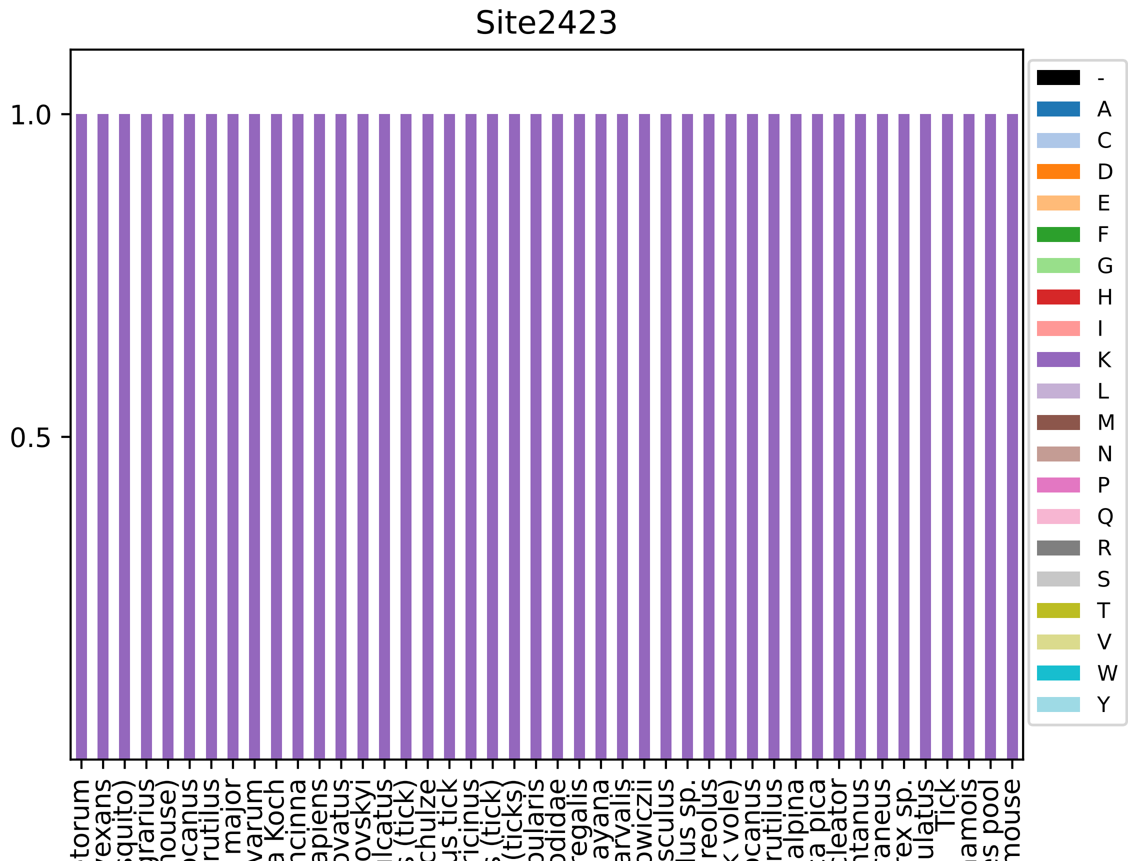
Site2423
1.0
0.5
-
A
C
D
E
F
G
H
I
K
L
M
N
P
Q
R
S
T
V
W
Y

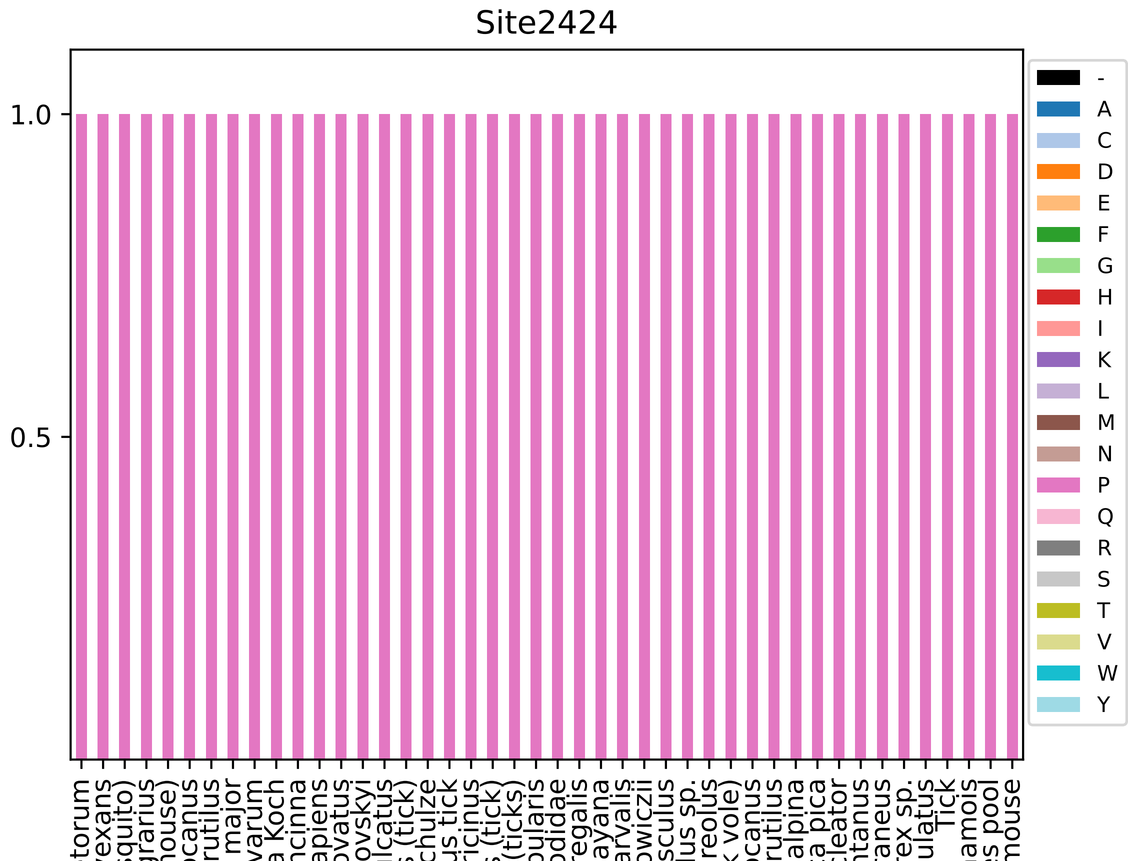
Site2424
1.0
0.5
-
A
C
D
E
F
G
H
I
K
L
M
N
P
Q
R
S
T
V
W
Y
major
Tick

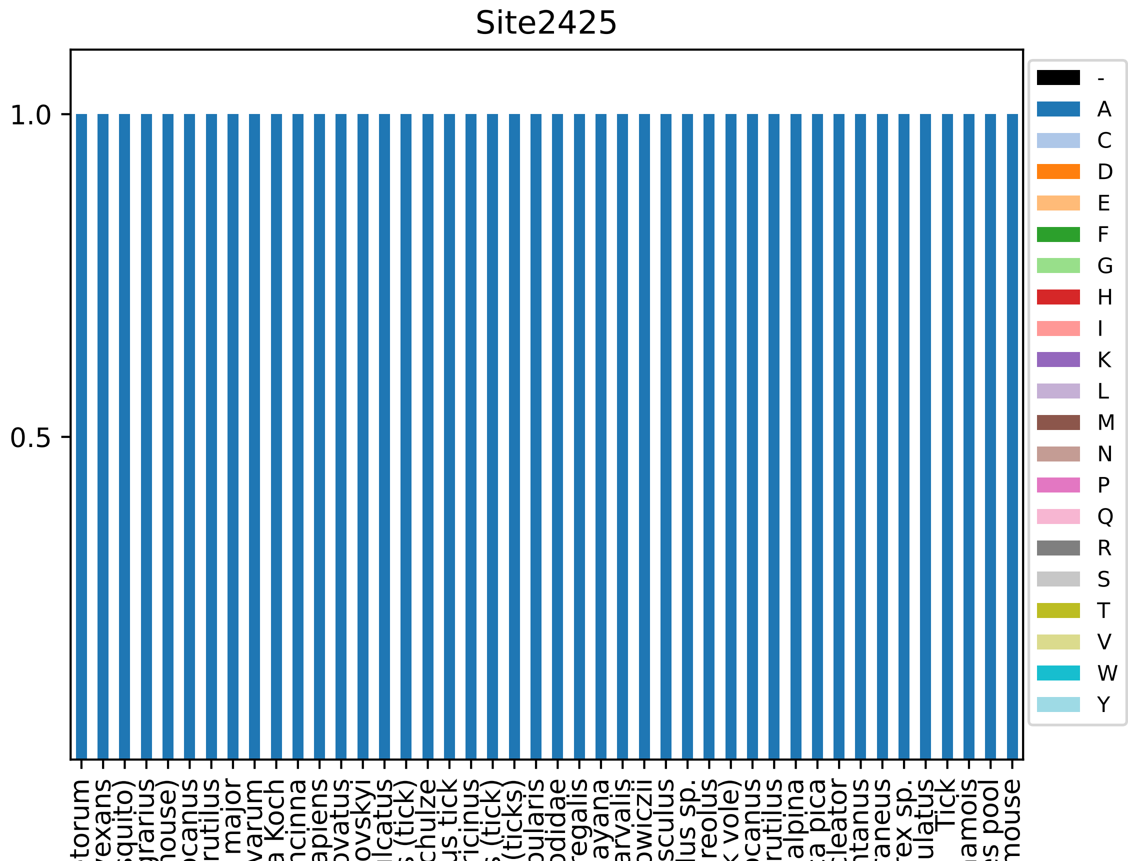
Site2425
1.0
0.5
-
A
C
D
E
F
G
H
I
K
L
M
N
P
Q
R
S
T
V
W
Y

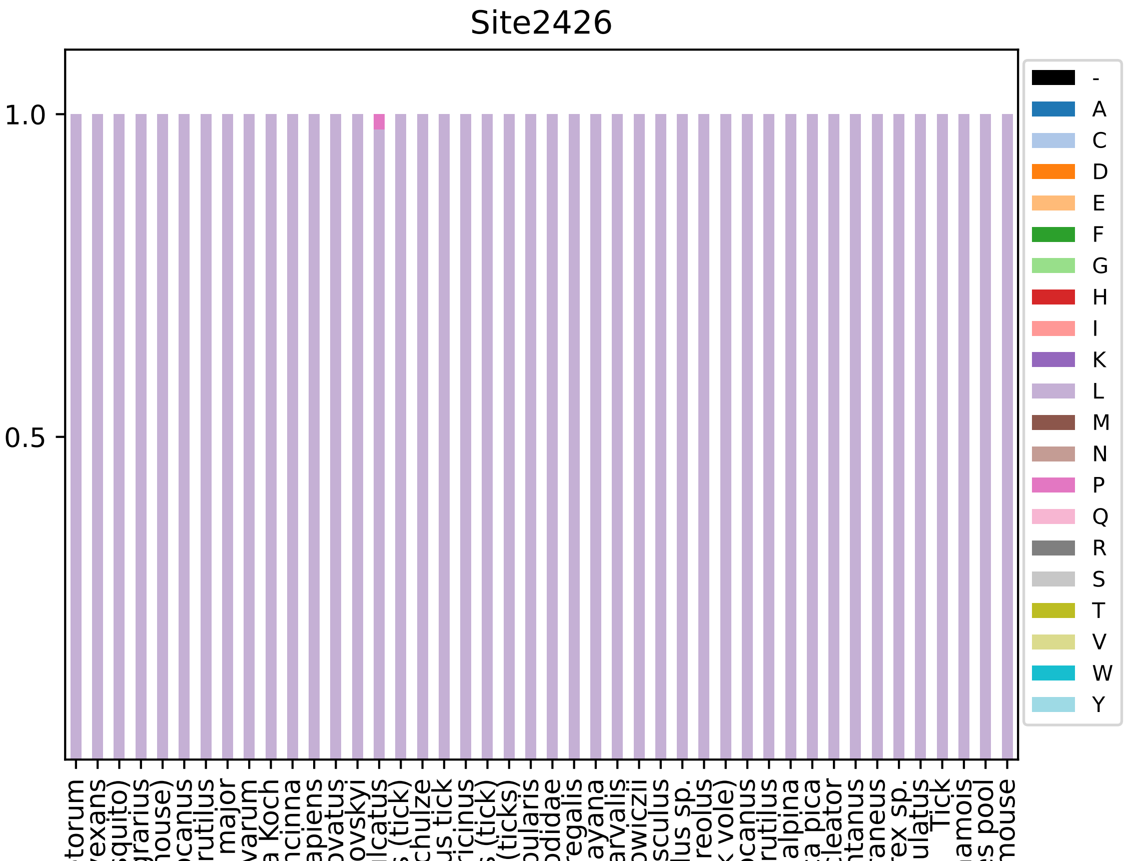
Site2426
1.0
0.5
-
A
C
D
E
F
G
H
I
K
L
M
N
P
Q
R
S
T
V
W
Y

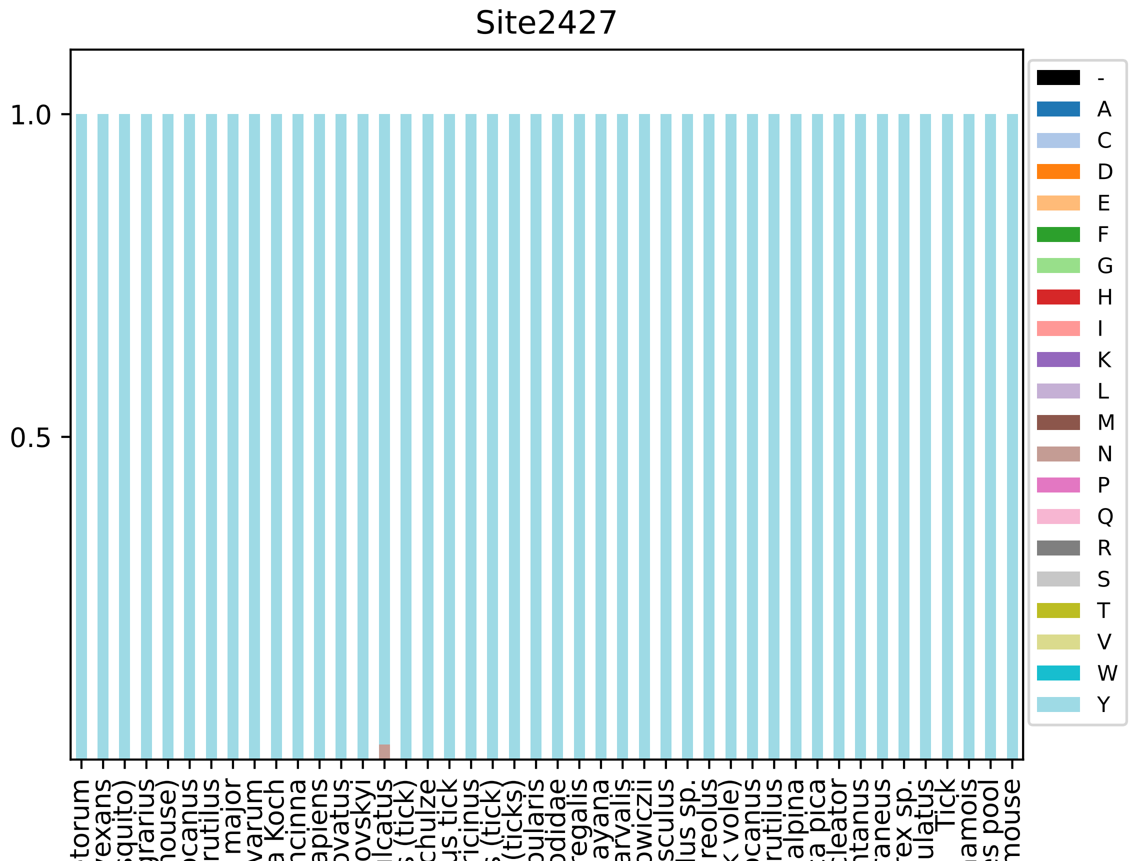
Site2427
1.0
0.5
-
A
C
D
E
F
G
H
I
K
L
M
N
P
Q
R
S
T
V
W
Y

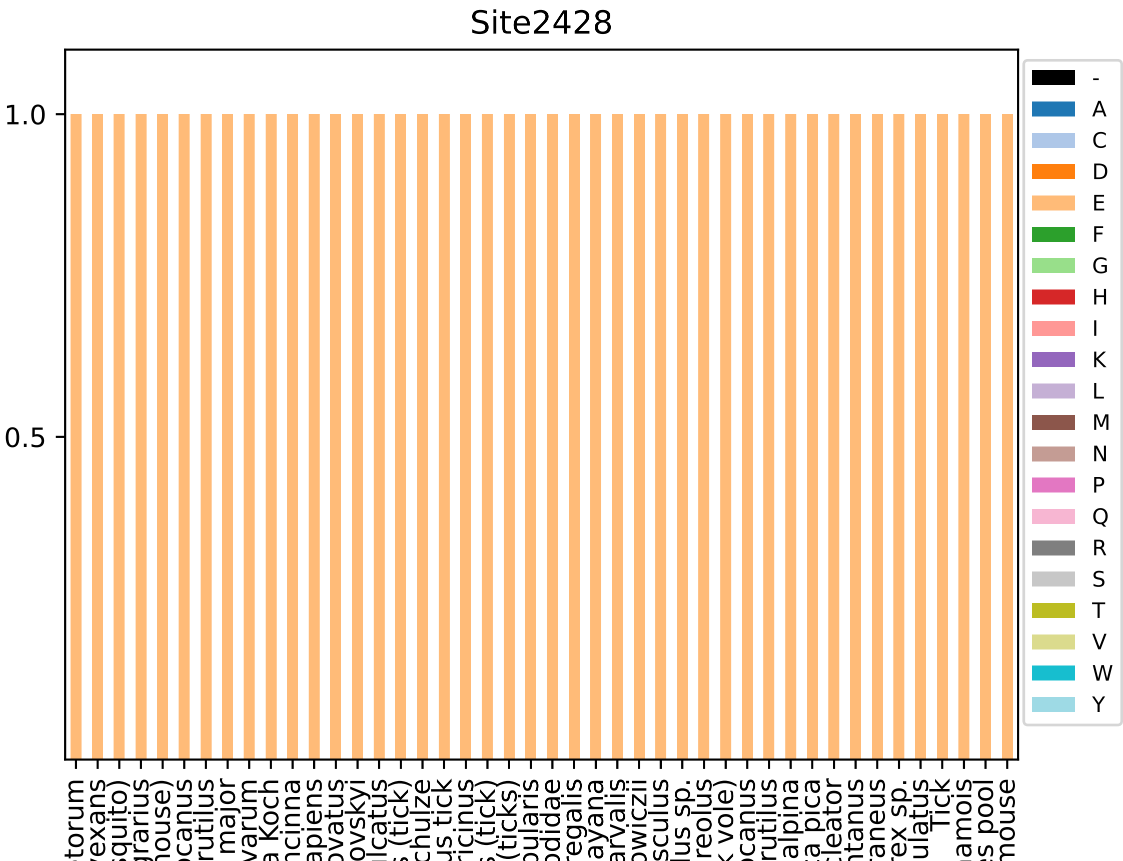
Site2428
1.0
0.5
-
A
C
D
E
F
G
H
I
K
L
M
N
P
Q
R
S
T
V
W
Y

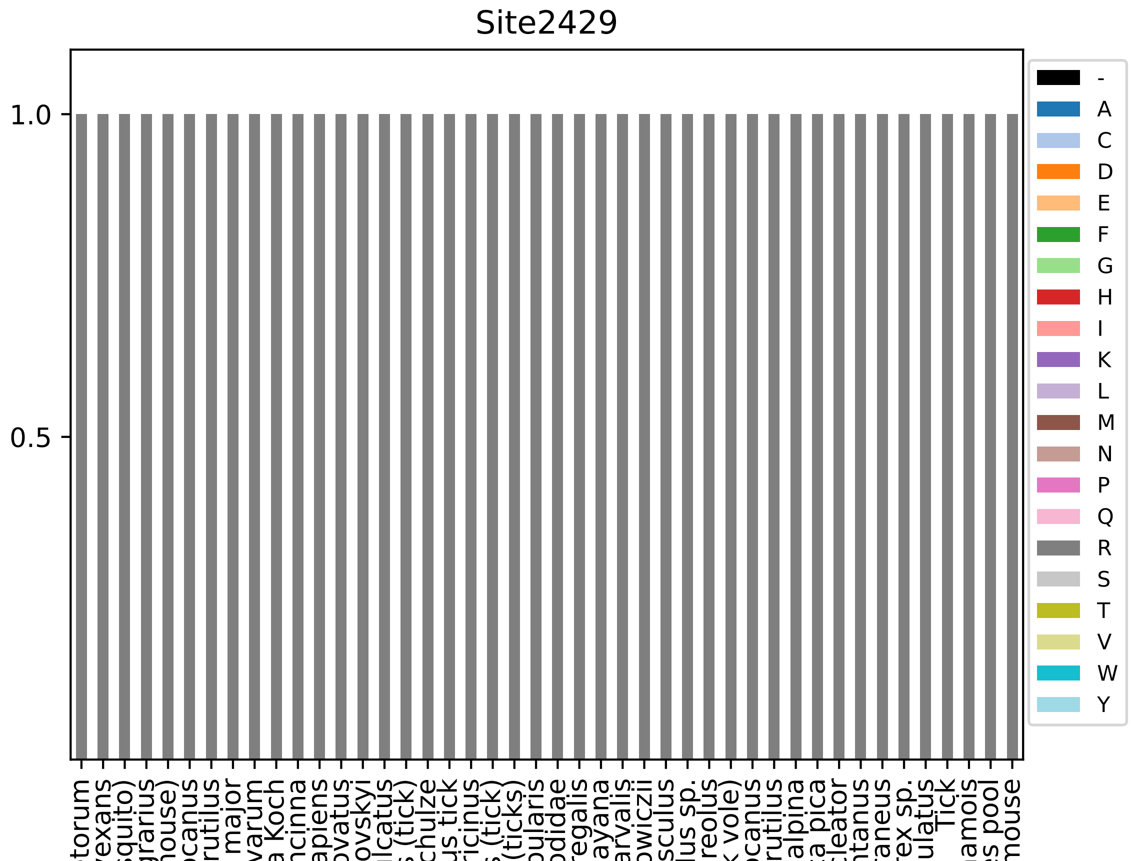
Site2429
1.0
0.5
-
A
C
D
E
F
G
H
I
K
L
M
N
P
Q
R
S
T
V
W
Y
Tick

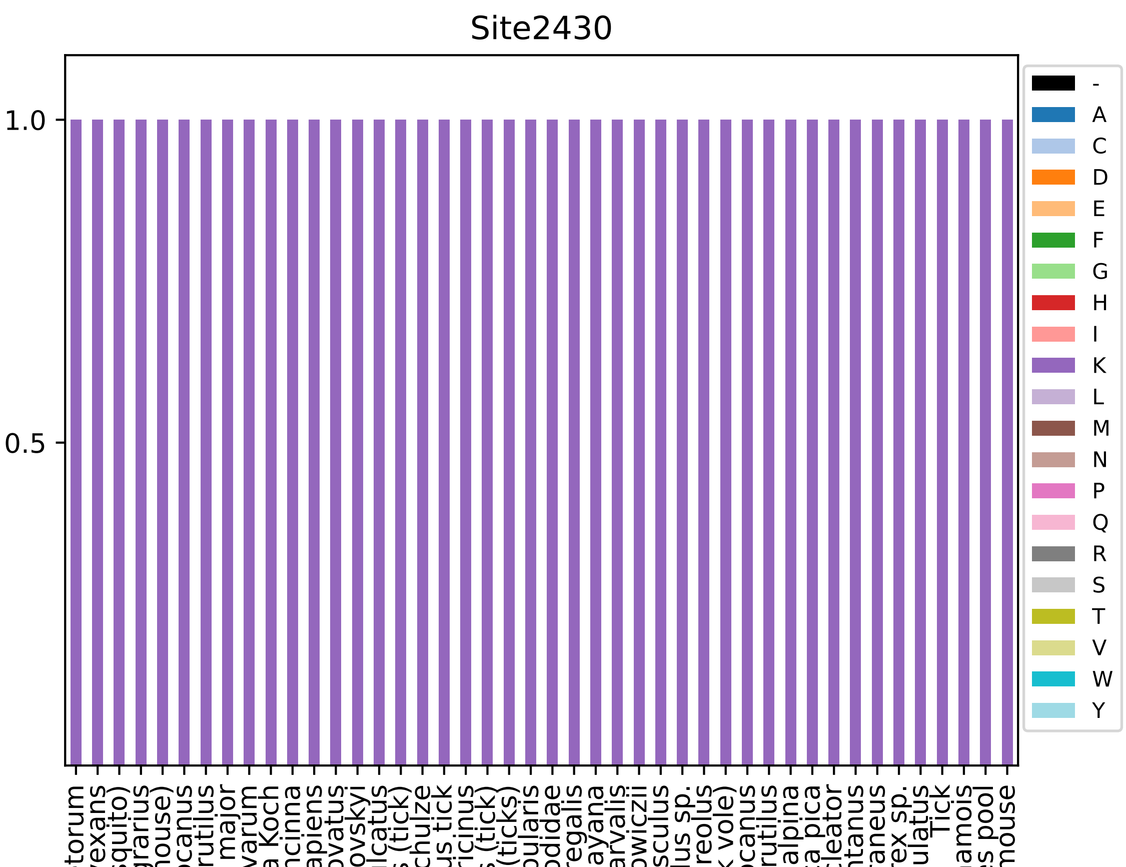
Site2430
1.0
0.5
-
A
C
D
E
F
G
H
I
K
L
M
N
P
Q
R
S
T
V
W
Y
torum
vexans
squito)
grarius
nouse)
ocanus
rutilus
major
varum
a Koch
ncinna
apiens
ovatus
ovskyi
ilcatus
s (tick)
chulze
us tick
ricinus
s (tick)
(ticks)
pularis
podidae
regalis
ayana
arvalis
owiczii
sculus
lus sp.
reolus
k vole)
ocanus
rutilus
alpina
a pica
cleator
ntanus
aneus
rex sp.
ulatus
Tick
amois
s pool
mouse

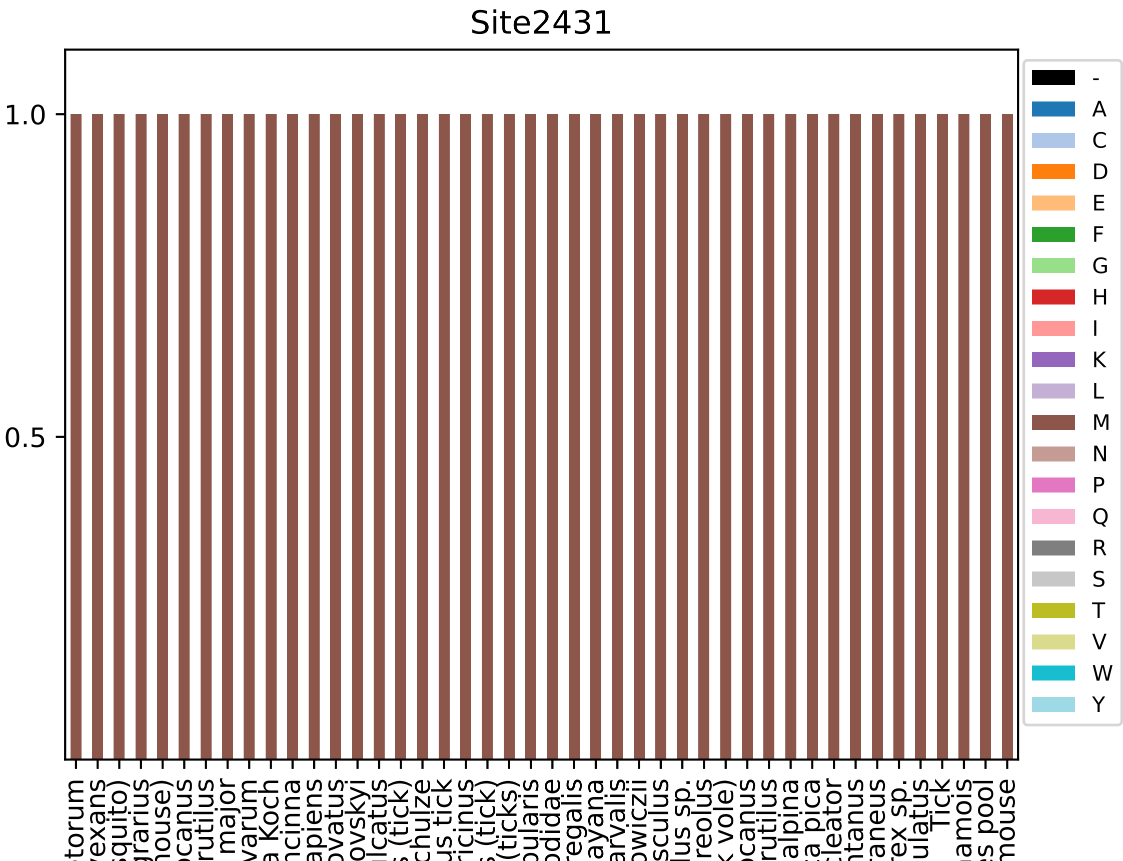
Site2431
1.0
0.5
-
A
C
D
E
F
G
H
I
K
L
M
N
P
Q
R
S
T
V
W
Y

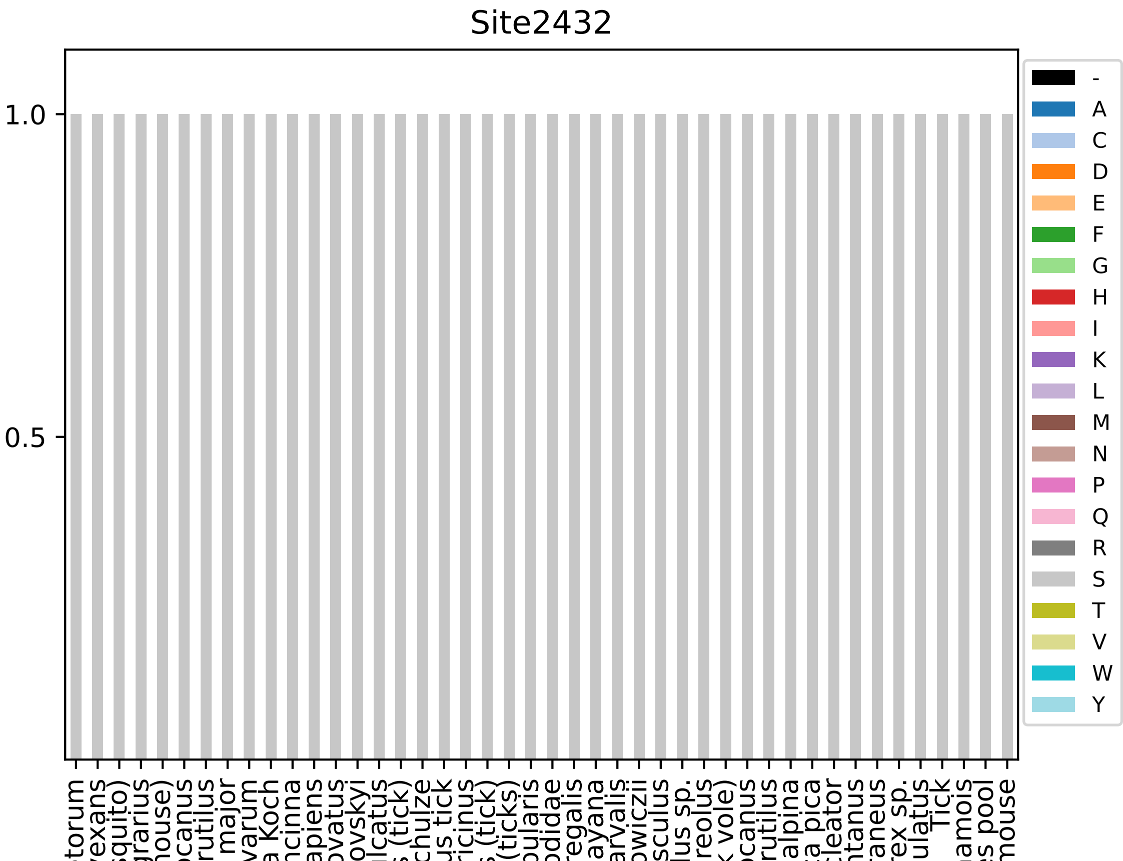

Site2432
1.0
0.5
-
A
C
D
E
F
G
H
I
K
L
M
N
P
Q
R
S
T
V
W
Y

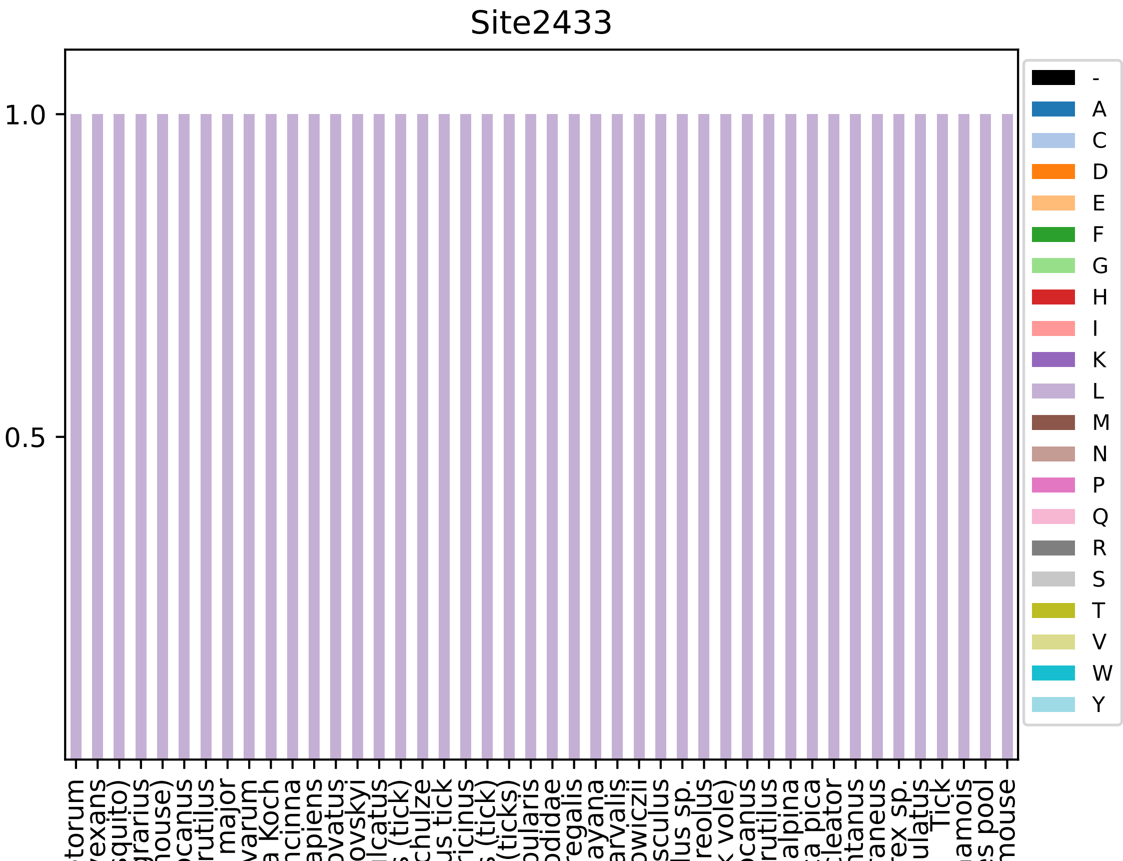

Site2433
1.0
0.5
-
A
C
D
E
F
G
H
I
K
L
M
N
P
Q
R
S
T
V
W
Y
torum
vexans
squito)
grarius
nouse)
ocanus
rutilus
major
varum
a Koch
ncinna
apiens
ovatus
ovskyi
ulcatus
s (tick)
chulze
us tick
ricinus
s (tick)
(ticks)
pularis
odidae
regalis
ayana
arvalis
owiczii
sculus
lus sp.
reolus
k vole)
ocanus
rutilus
alpina
a pica
leator
ntanus
raneus
rex sp.
ulatus
Tick
amois
es pool
mouse

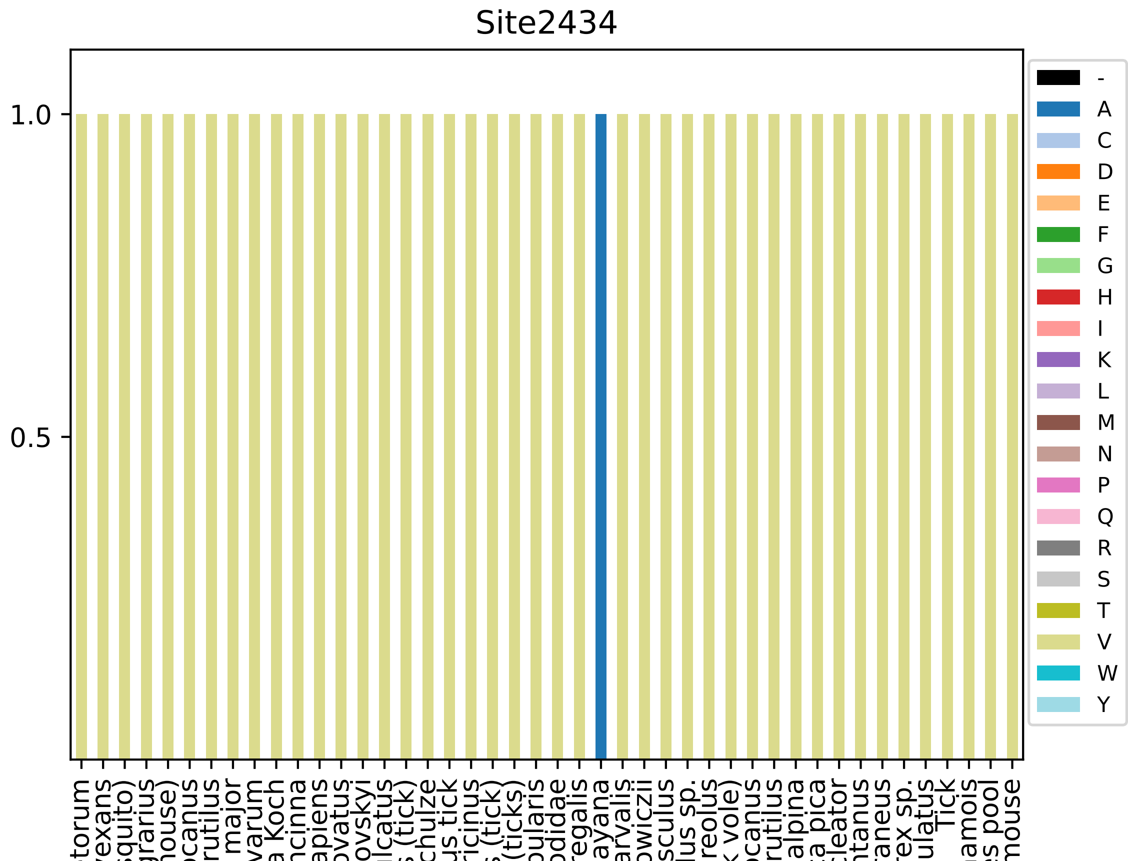
Site2434
1.0
0.5
-
A
C
D
E
F
G
H
I
K
L
M
N
P
Q
R
S
T
V
W
Y

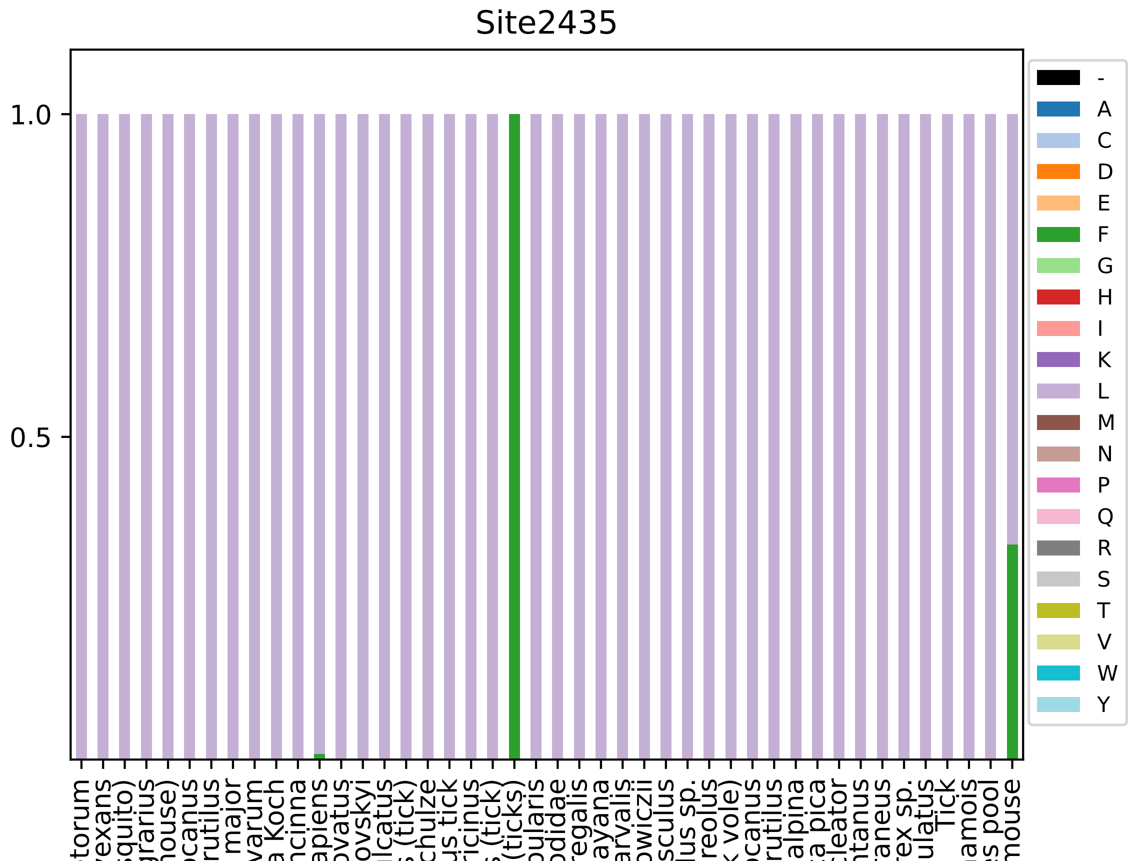

Site2435
1.0
0.5
-
A
C
D
E
F
G
H
I
K
L
M
N
P
Q
R
S
T
V
W
Y

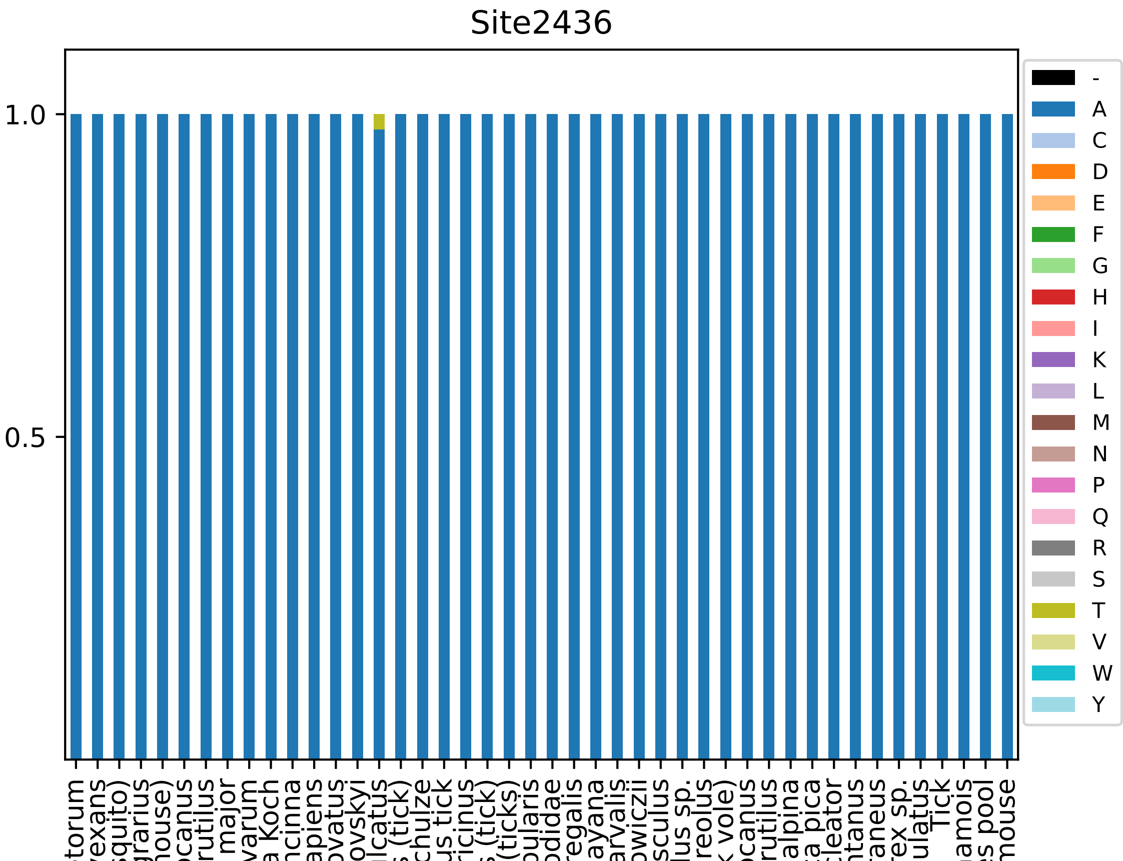
Site2436
1.0
0.5
-
A
C
D
E
F
G
H
I
K
L
M
N
P
Q
R
S
T
V
W
Y

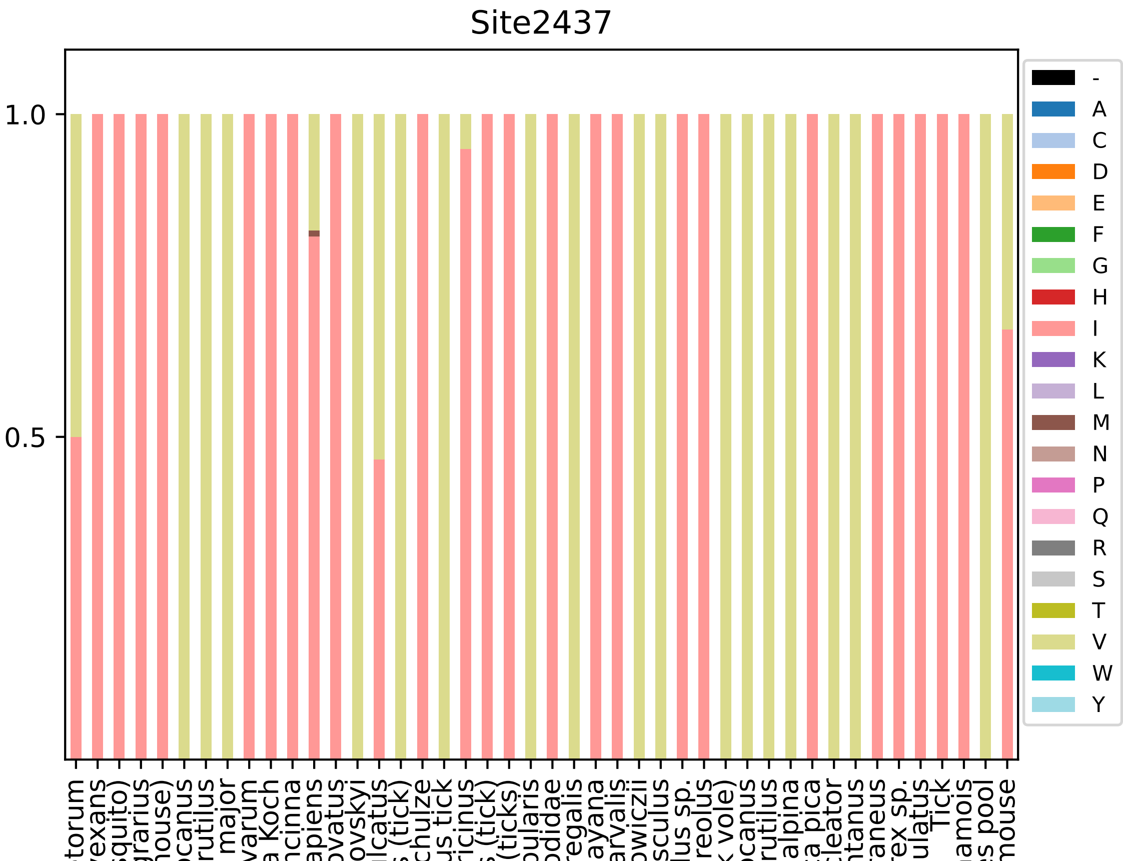
Site2437
1.0
0.5
-
A
C
D
E
F
G
H
I
K
L
M
N
P
Q
R
S
T
V
W
Y

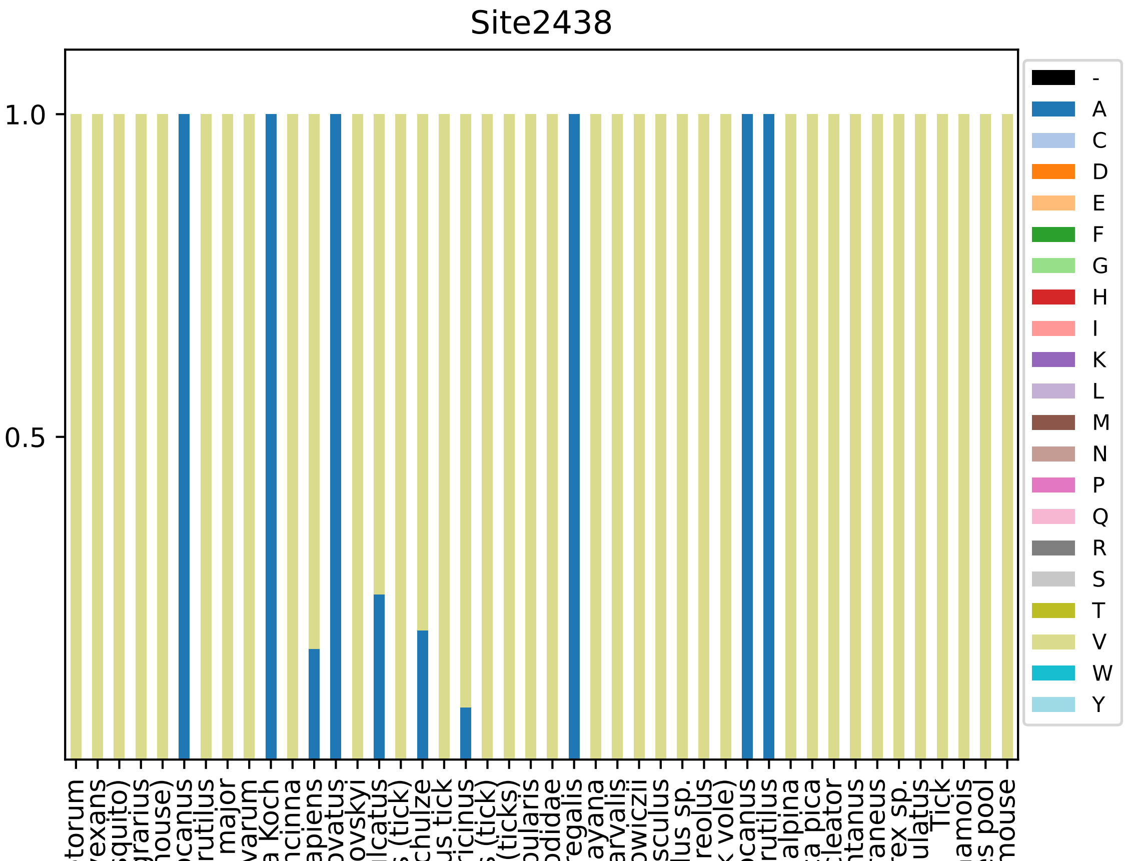
Site2438
1.0
0.5
-
A
C
D
E
F
G
H
I
K
L
M
N
P
Q
R
S
T
V
W
Y

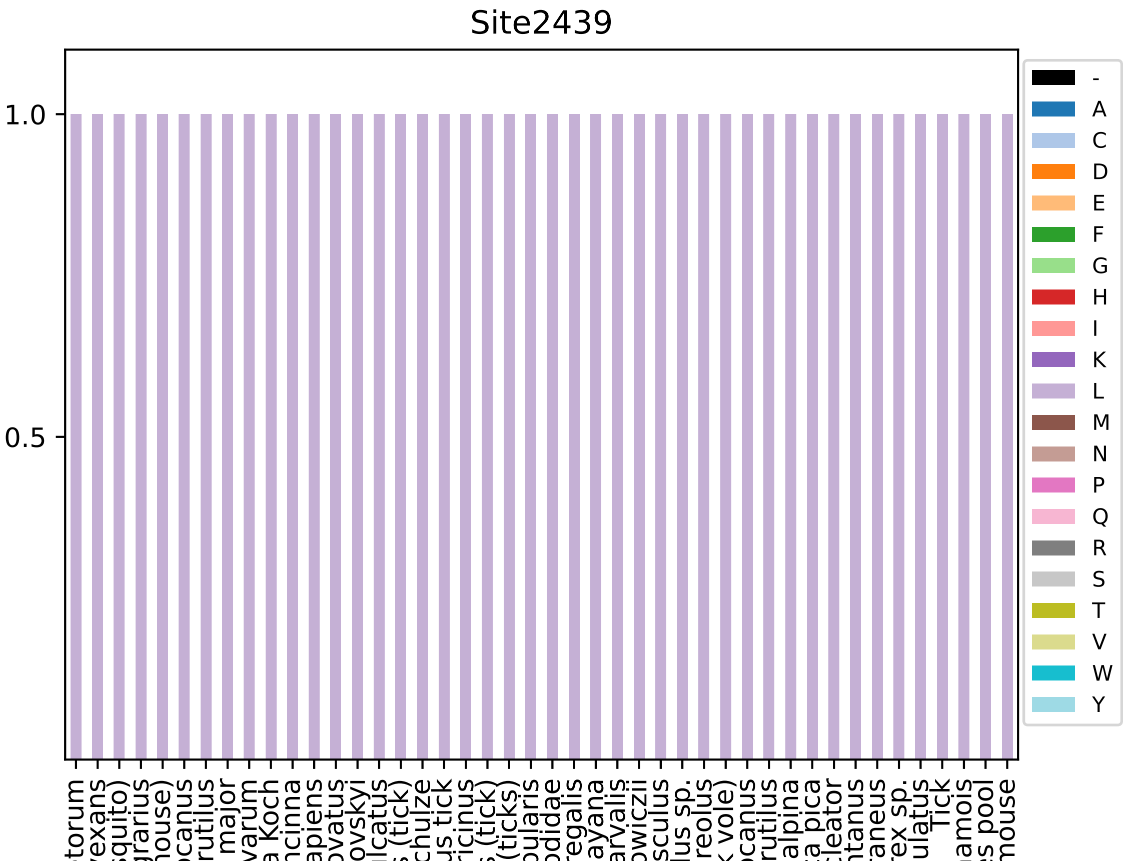
Site2439
1.0
0.5
-
A
C
D
E
F
G
H
I
K
L
M
N
P
Q
R
S
T
V
W
Y

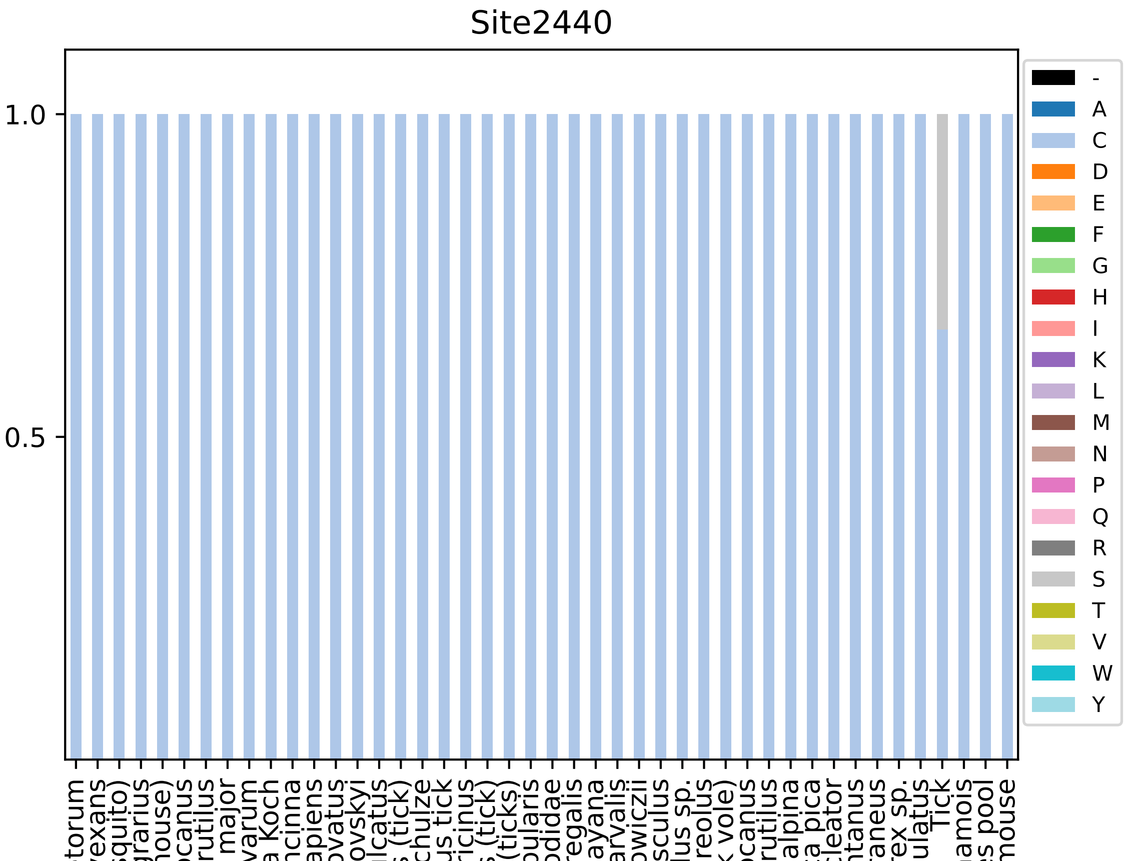
Site2440
1.0
0.5
-
A
C
D
E
F
G
H
I
K
L
M
N
P
Q
R
S
T
V
W
Y

Site2441

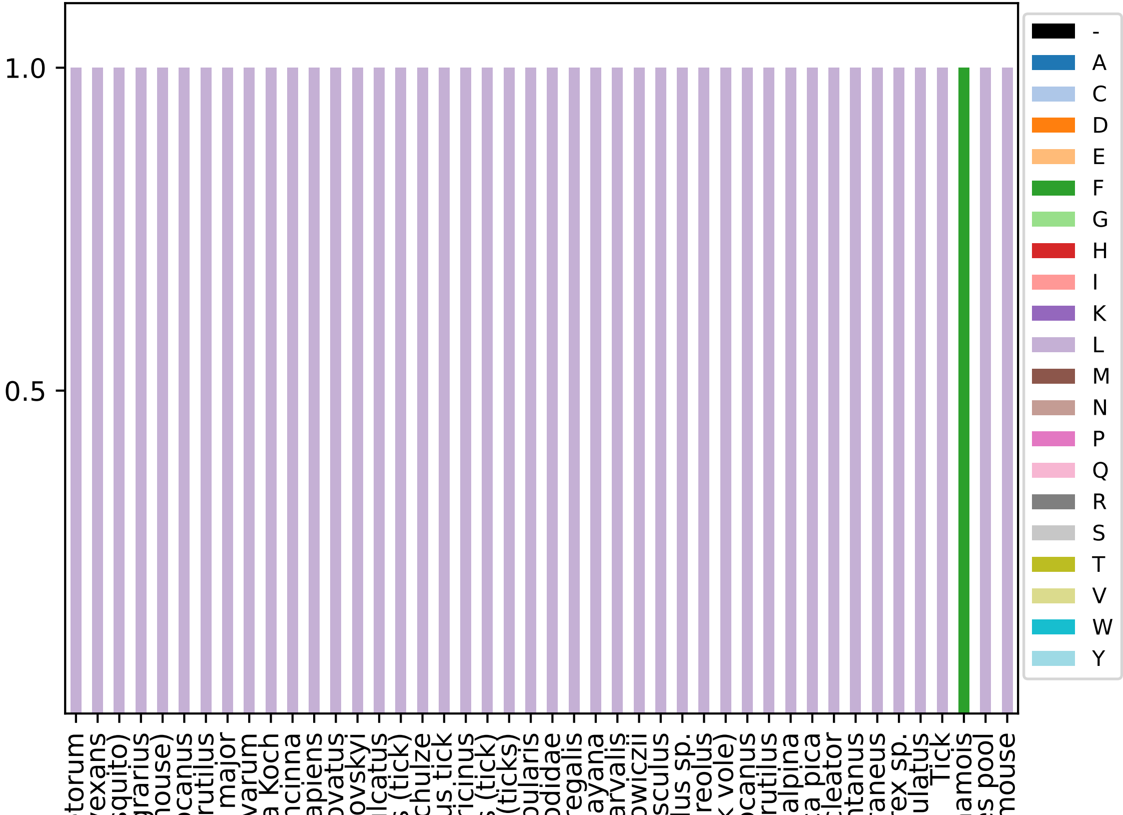

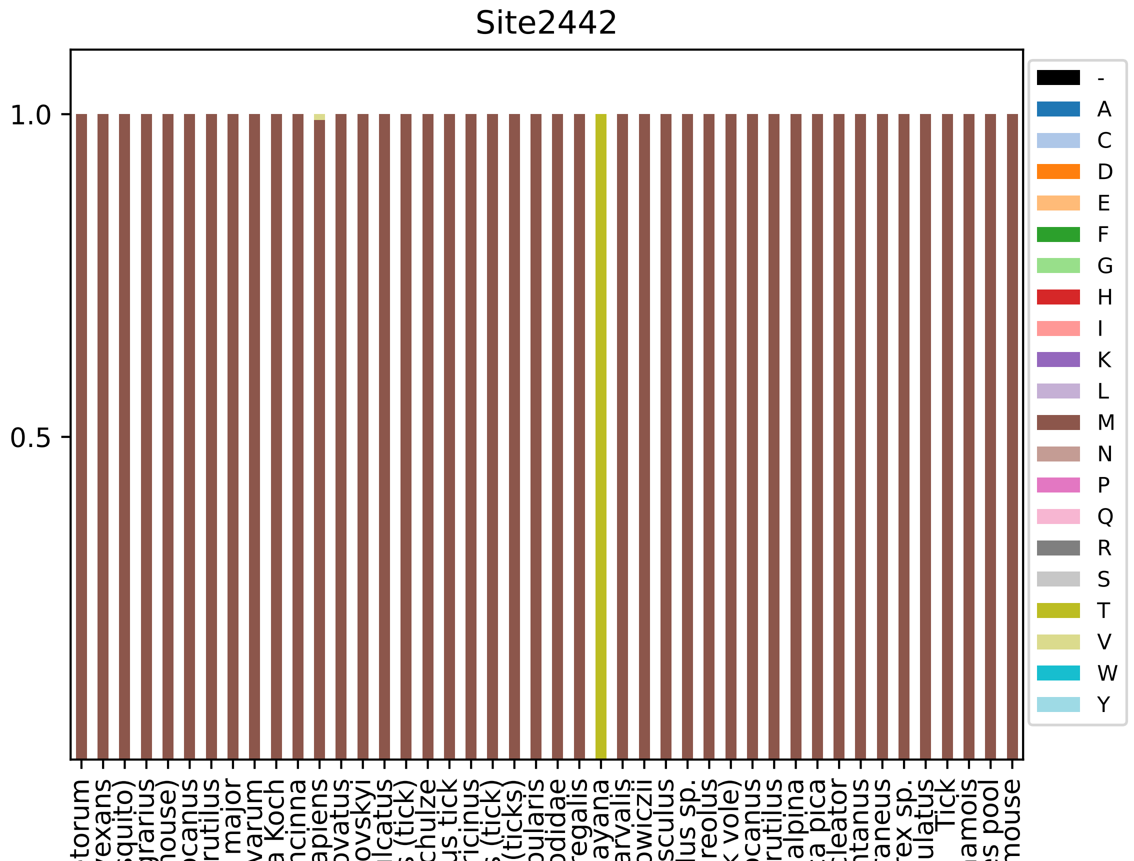
Site2442
1.0
0.5
-
A
C
D
E
F
G
H
I
K
L
M
N
P
Q
R
S
T
V
W
Y

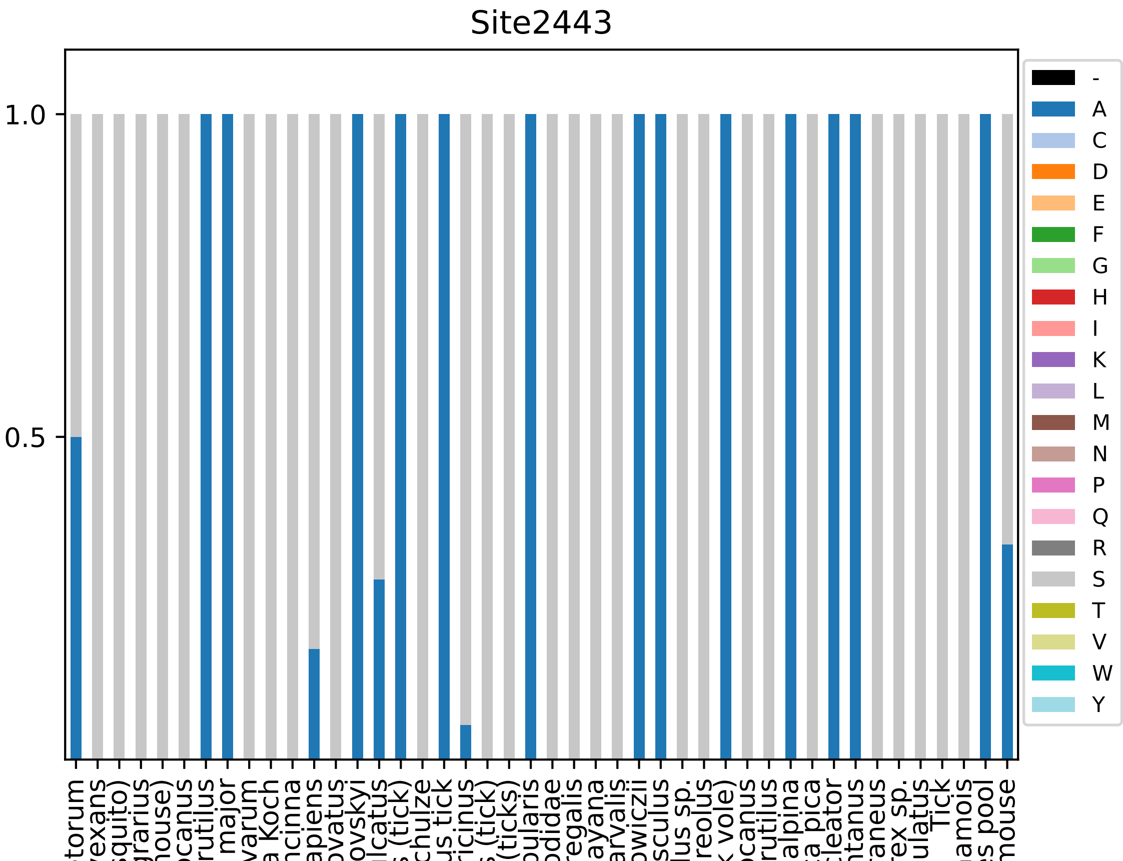

Site2443
1.0
0.5
-
A
C
D
E
F
G
H
I
K
L
M
N
P
Q
R
S
T
V
W
Y

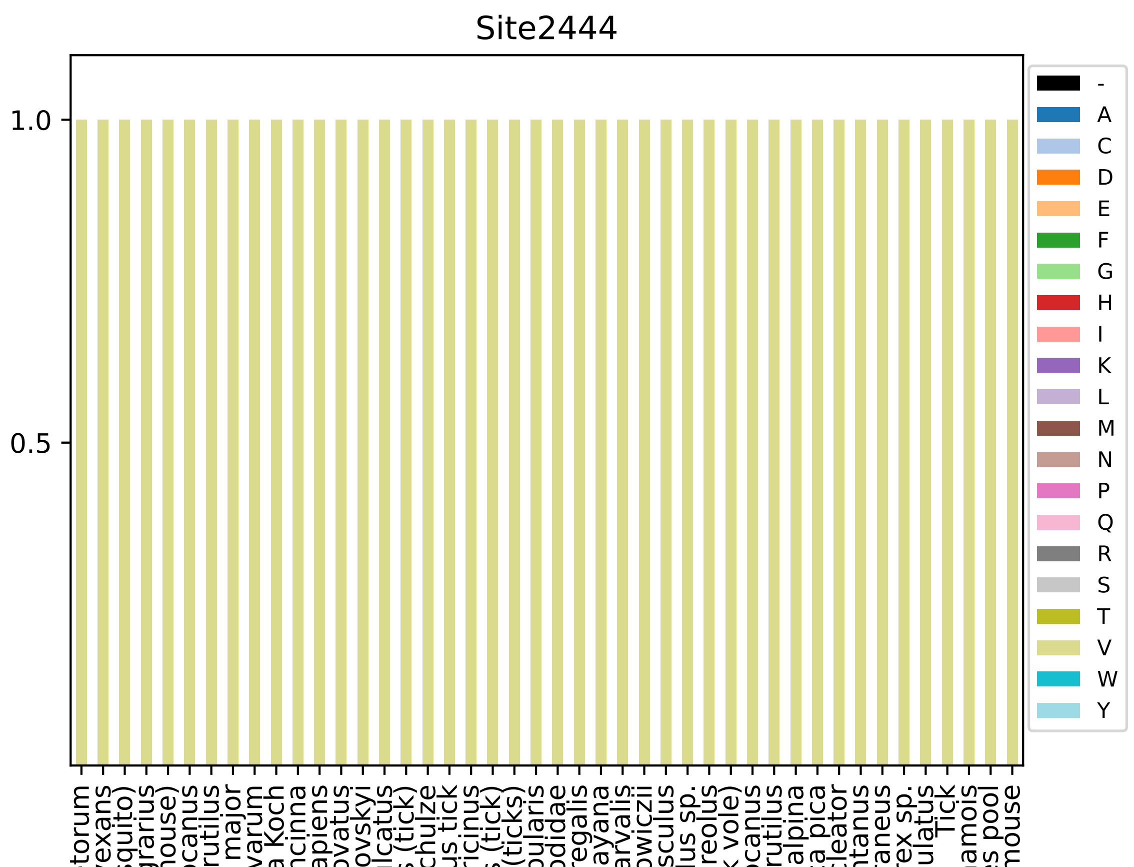
Site2444
1.0
0.5
-
A
C
D
E
F
G
H
I
K
L
M
N
P
Q
R
S
T
V
W
Y

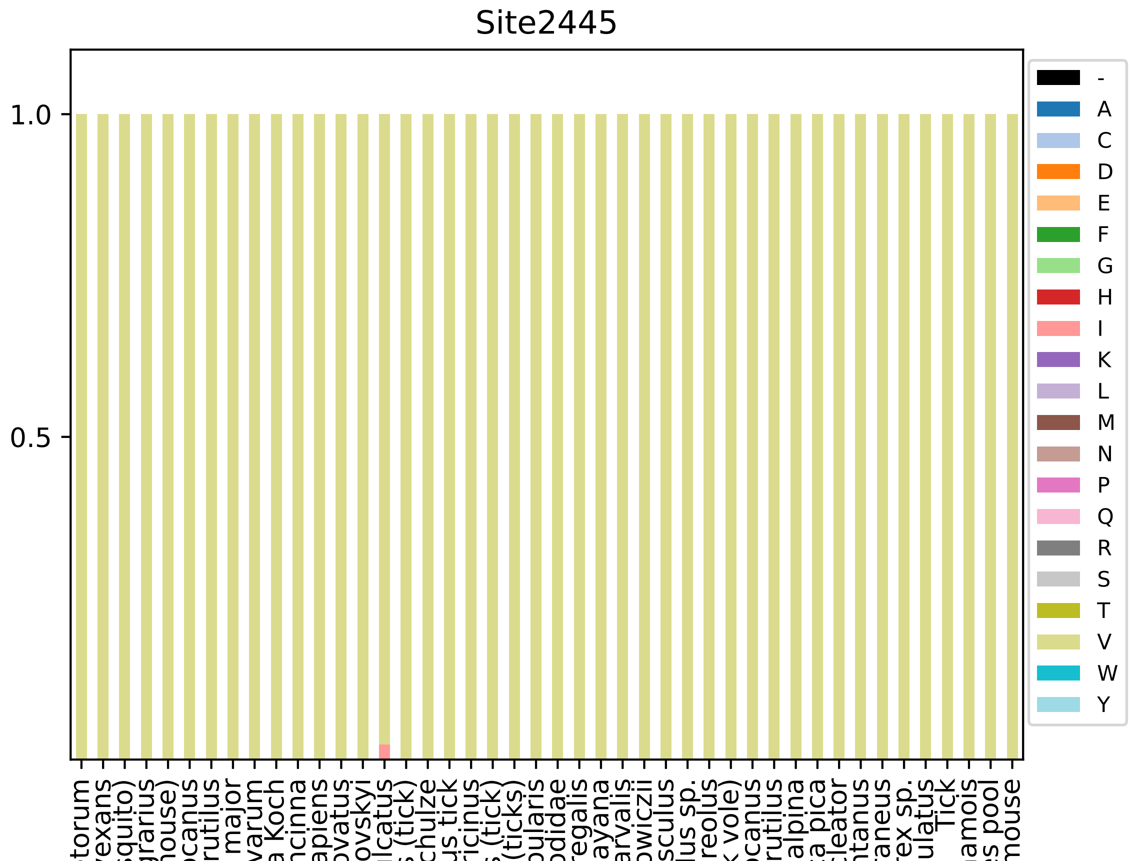
Site2445
1.0
0.5
-
A
C
D
E
F
G
H
I
K
L
M
N
P
Q
R
S
T
V
W
Y

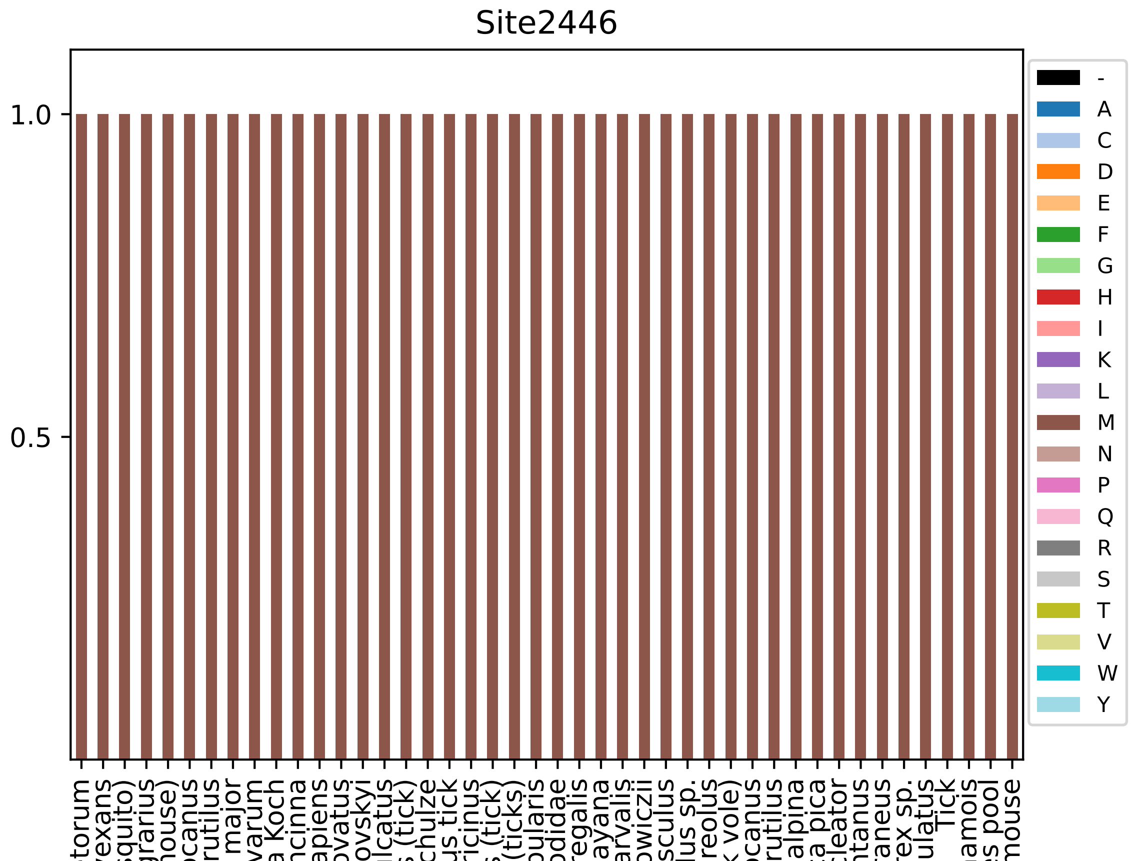
Site2446
1.0
0.5
-
A
C
D
E
F
G
H
I
K
L
M
N
P
Q
R
S
T
V
W
Y
Tick

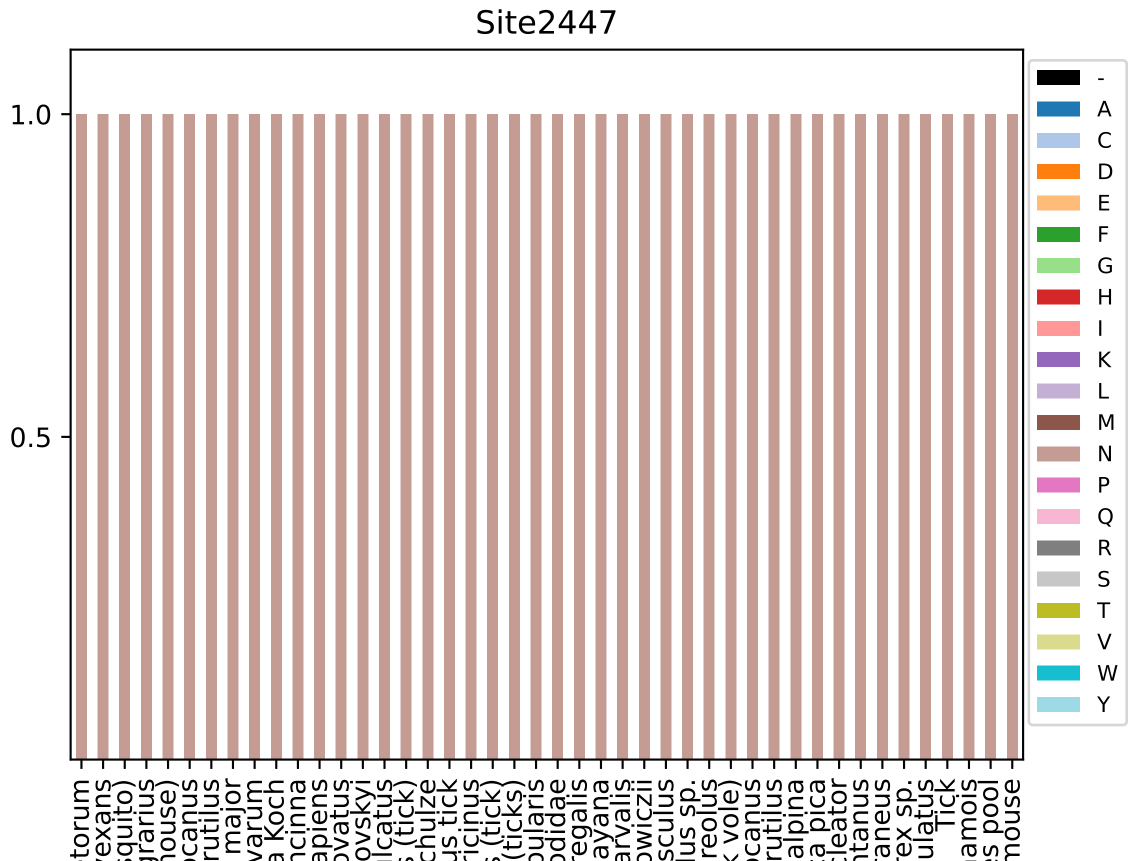
Site2447
1.0
0.5
-
A
C
D
E
F
G
H
I
K
L
M
N
P
Q
R
S
T
V
W
Y

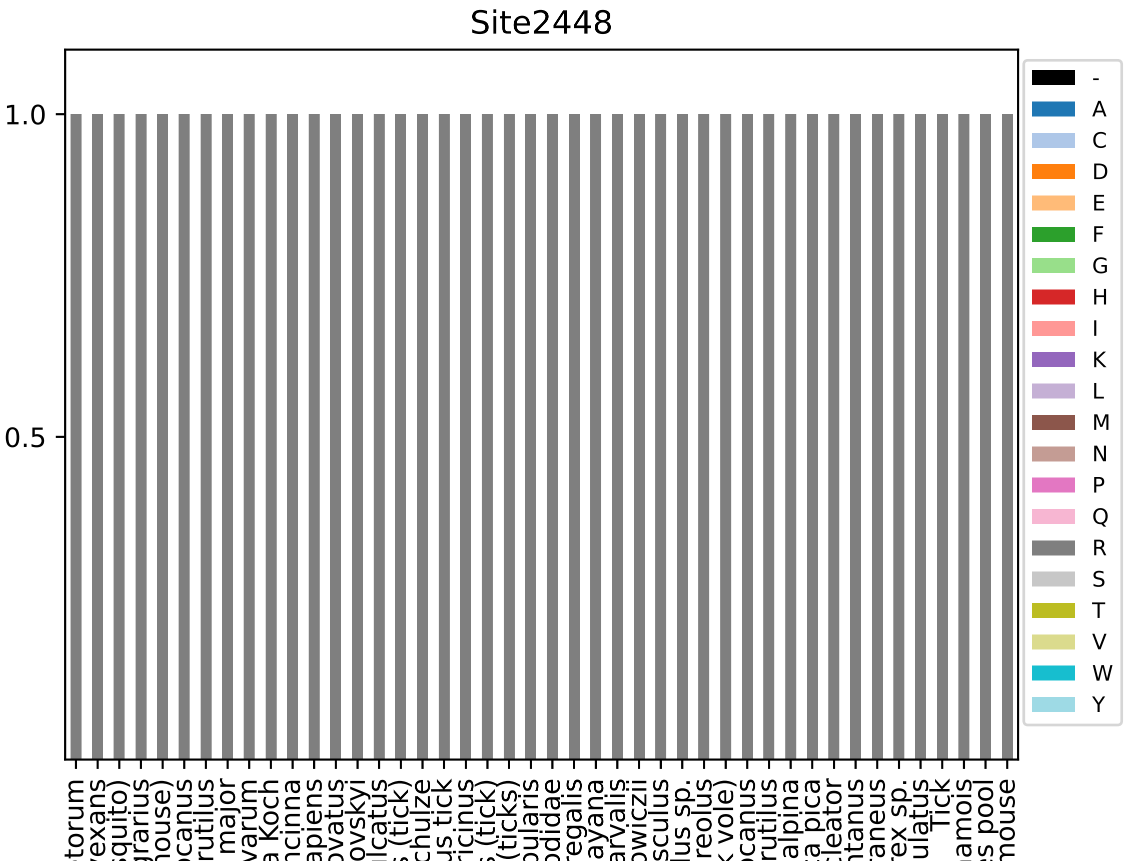

Site2448
1.0
0.5
-
A
C
D
E
F
G
H
I
K
L
M
N
P
Q
R
S
T
V
W
Y

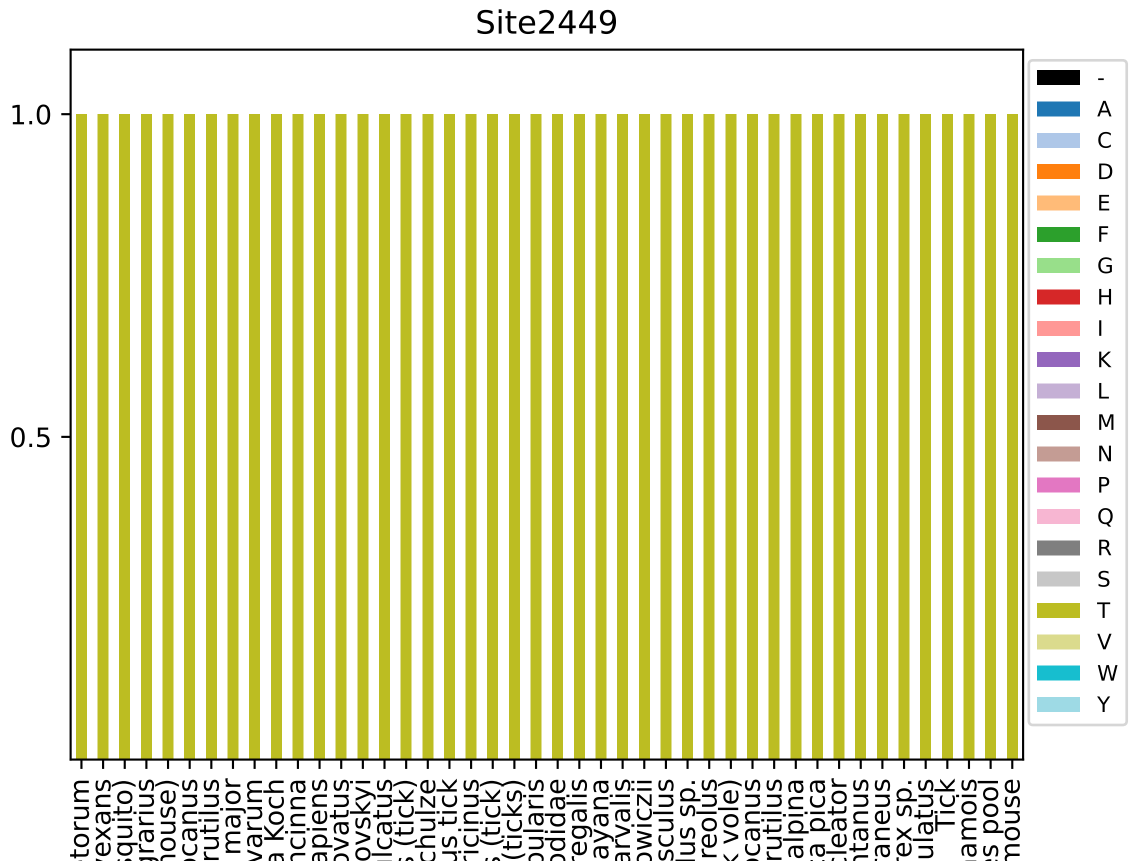

Site2449
1.0
0.5
-
A
C
D
E
F
G
H
I
K
L
M
N
P
Q
R
S
T
V
W
Y

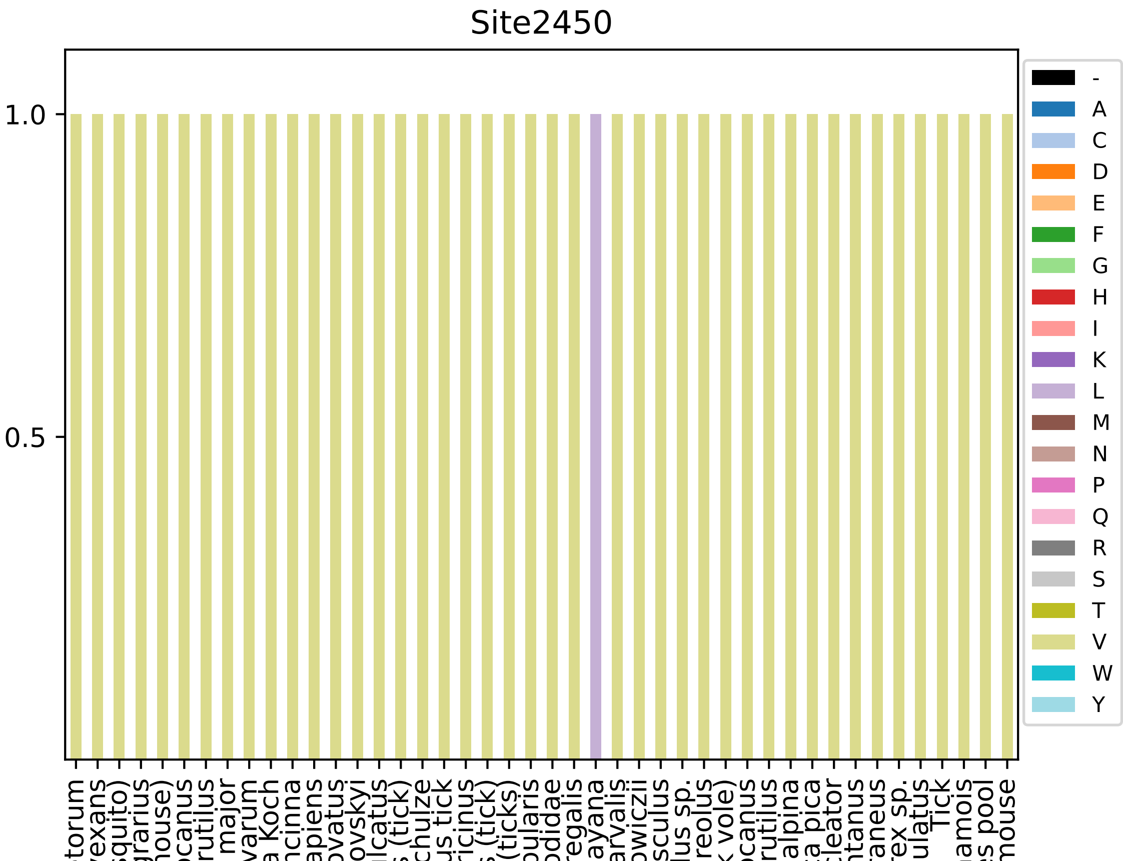

Site2450
1.0
0.5
-
A
C
D
E
F
G
H
I
K
L
M
N
P
Q
R
S
T
V
W
Y

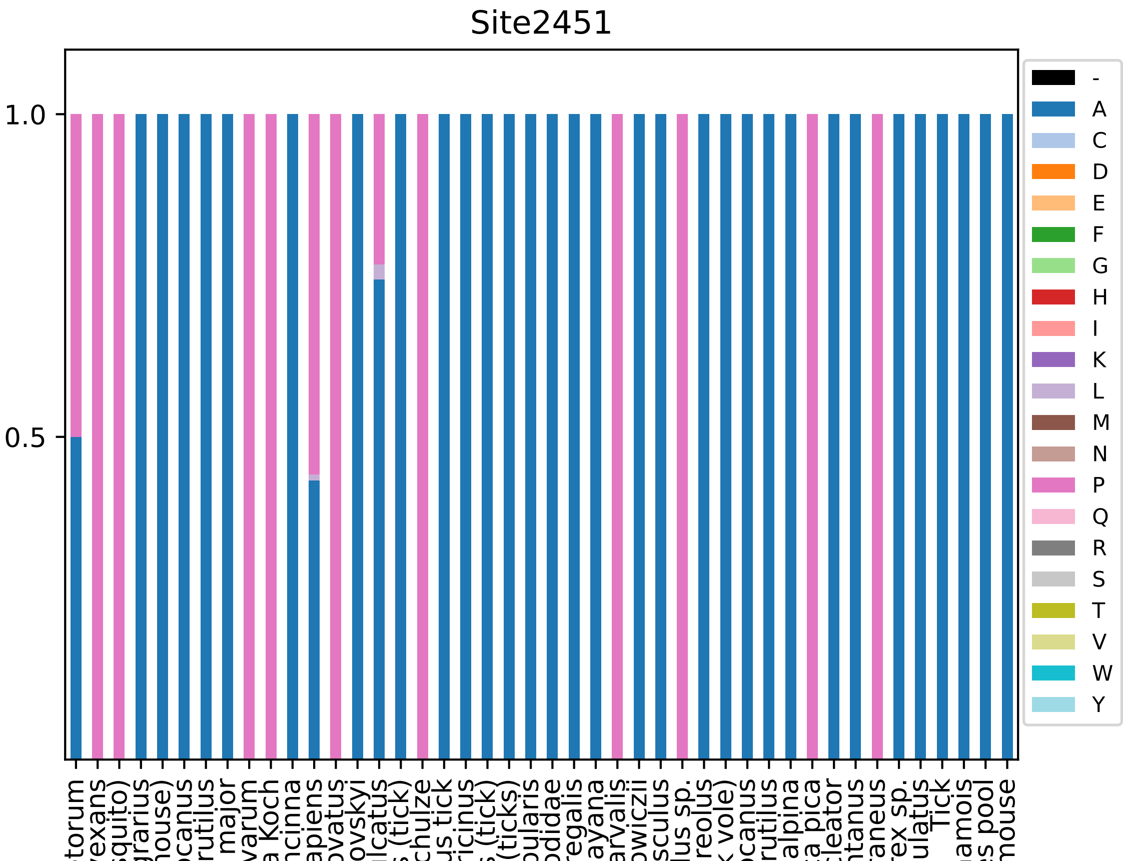
Site2451
1.0
0.5
-
A
C
D
E
F
G
H
I
K
L
M
N
P
Q
R
S
T
V
W
Y

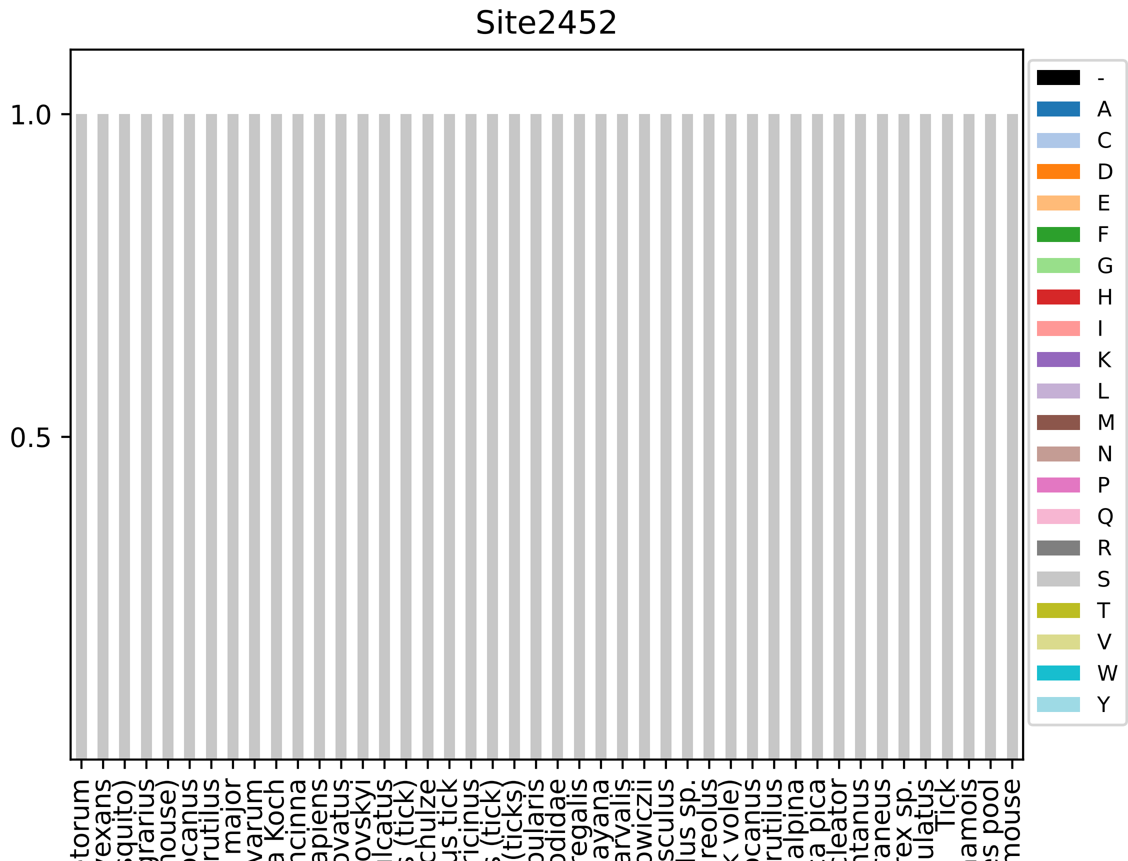
Site2452
1.0
0.5
-
A
C
D
E
F
G
H
I
K
L
M
N
P
Q
R
S
T
V
W
Y

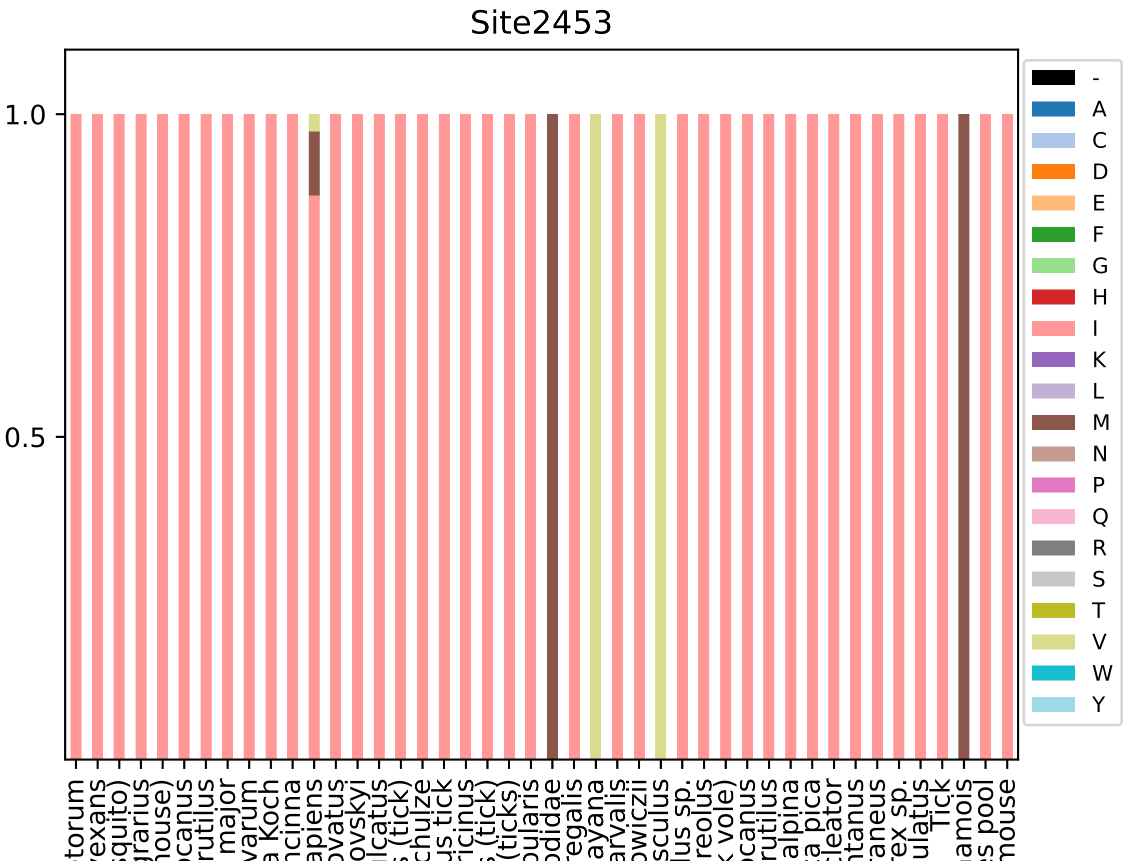
Site2453
1.0
0.5
-
A
C
D
E
F
G
H
I
K
L
M
N
P
Q
R
S
T
V
W
Y

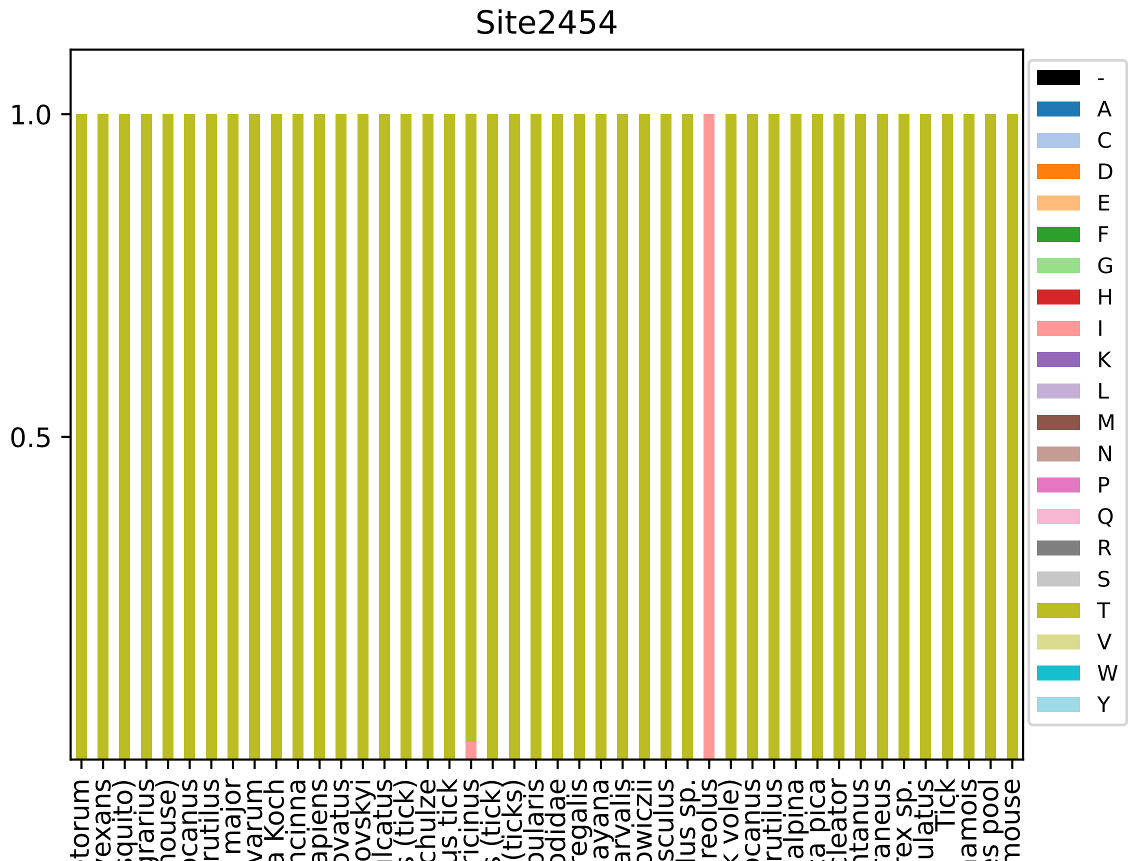

Site2454
1.0
0.5
-
A
C
D
E
F
G
H
I
K
L
M
N
P
Q
R
S
T
V
W
Y

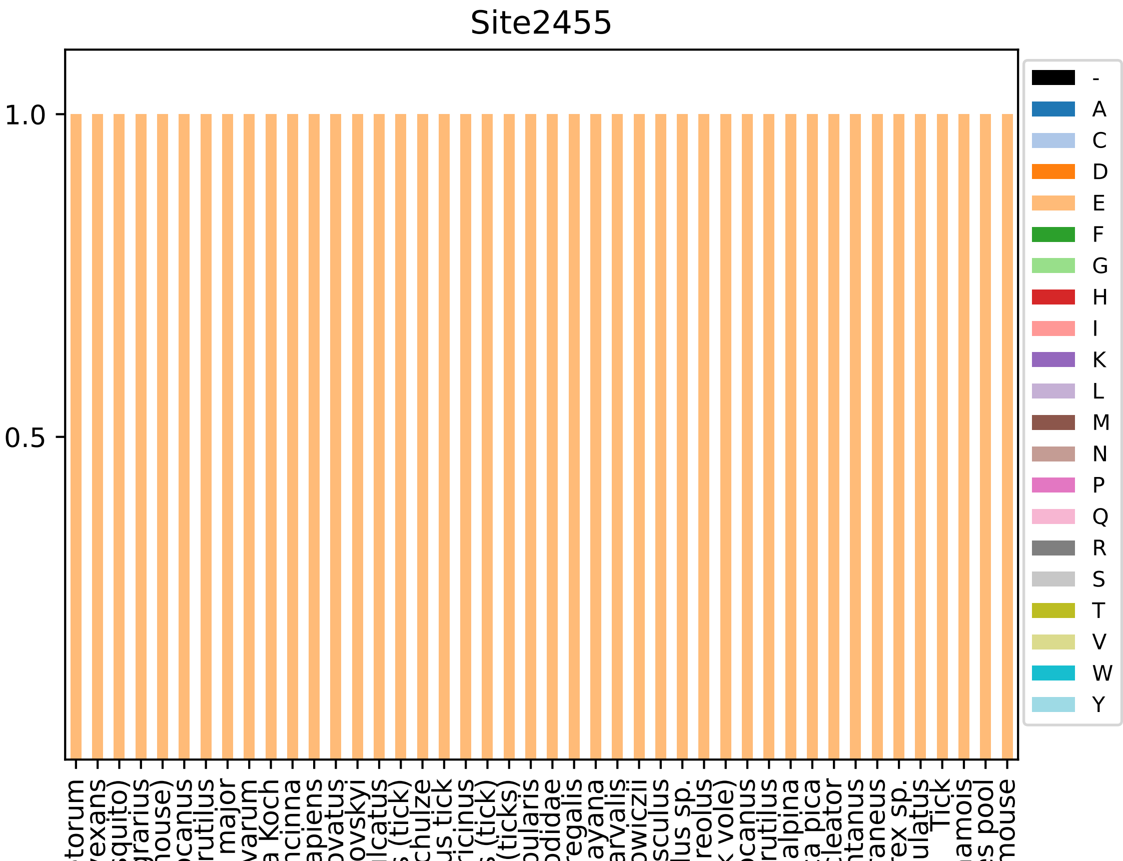

Site2455
1.0
0.5
-
A
C
D
E
F
G
H
I
K
L
M
N
P
Q
R
S
T
V
W
Y

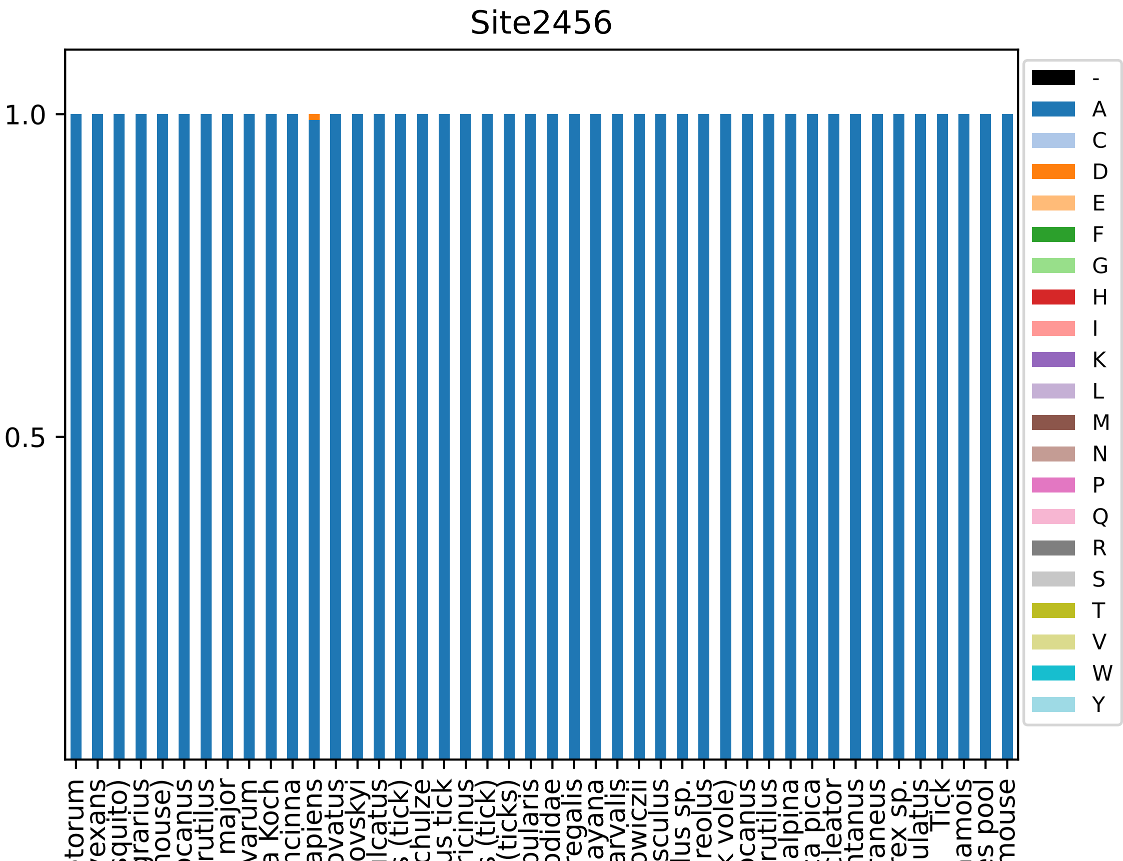

Site2456
1.0
0.5
-
A
C
D
E
F
G
H
I
K
L
M
N
P
Q
R
S
T
V
W
Y

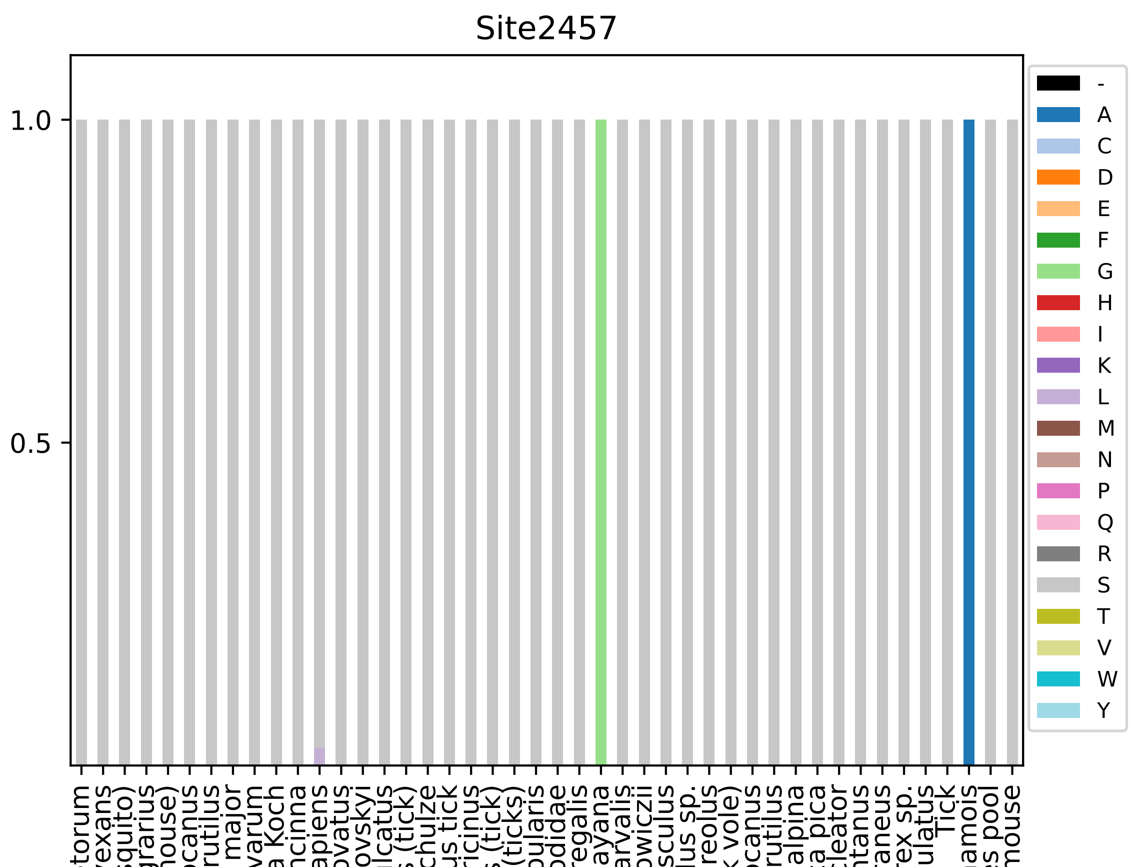
Site2457
1.0
0.5
-
A
C
D
E
F
G
H
I
K
L
M
N
P
Q
R
S
T
V
W
Y
torum
vexans
squito)
grarius
nouse)
ocanus
rutilus
major
varum
a Koch
ncinna
apiens
ovatus
ovskyi
ulcatus
s (tick)
chulze
us tick
ricinus
s (tick)
(ticks)
pularis
odidae
regalis
ayana
arvalis
owiczii
sculus
lus sp.
reolus
k vole)
ocanus
rutilus
alpina
a pica
cleator
ntanus
raneus
rex sp.
ulatus
Tick
amois
es pool
mouse

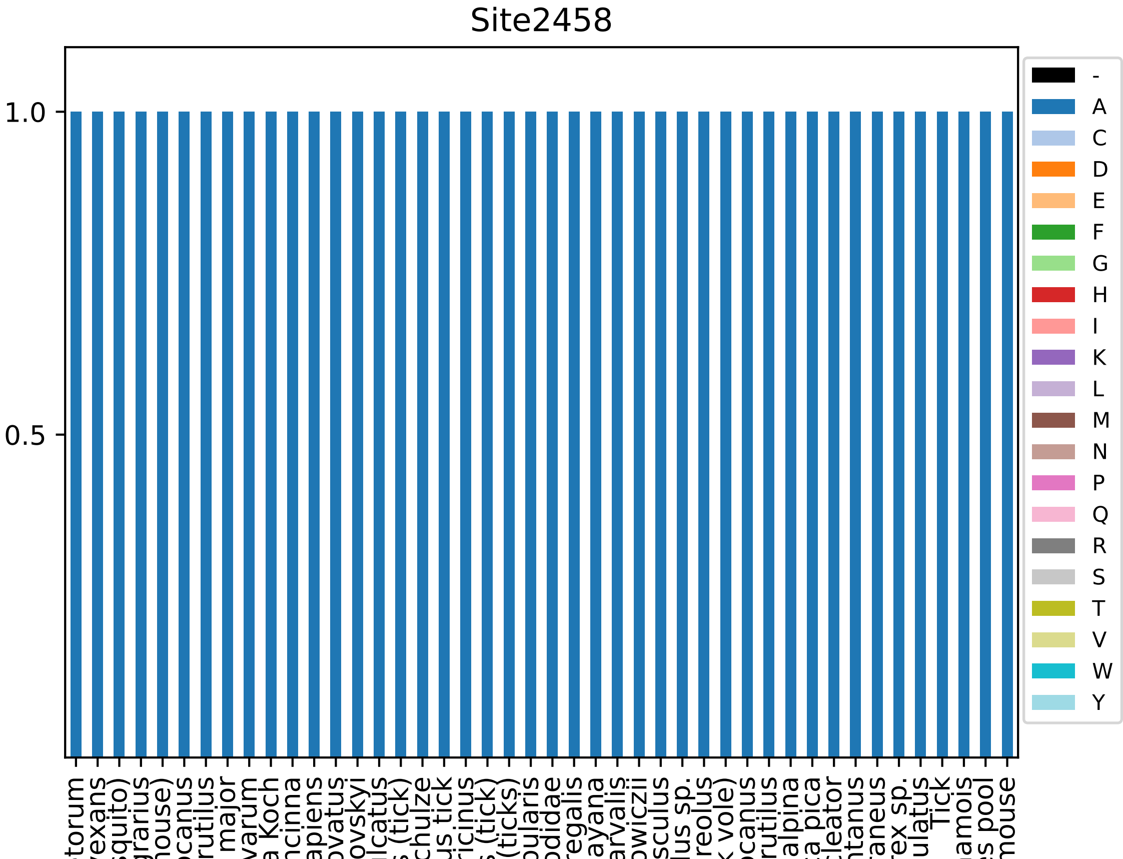
Site2458
1.0
0.5
-
A
C
D
E
F
G
H
I
K
L
M
N
P
Q
R
S
T
V
W
Y

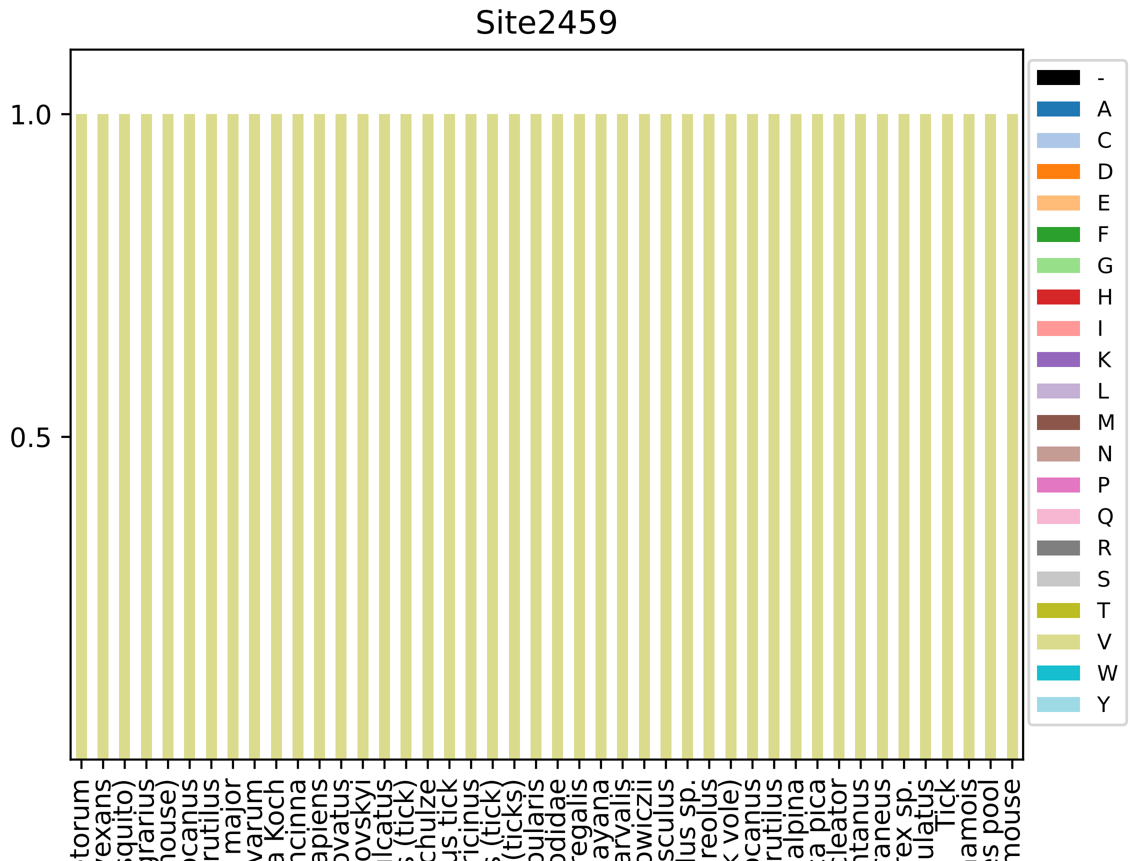
Site2459
1.0
0.5
-
A
C
D
E
F
G
H
I
K
L
M
N
P
Q
R
S
T
V
W
Y

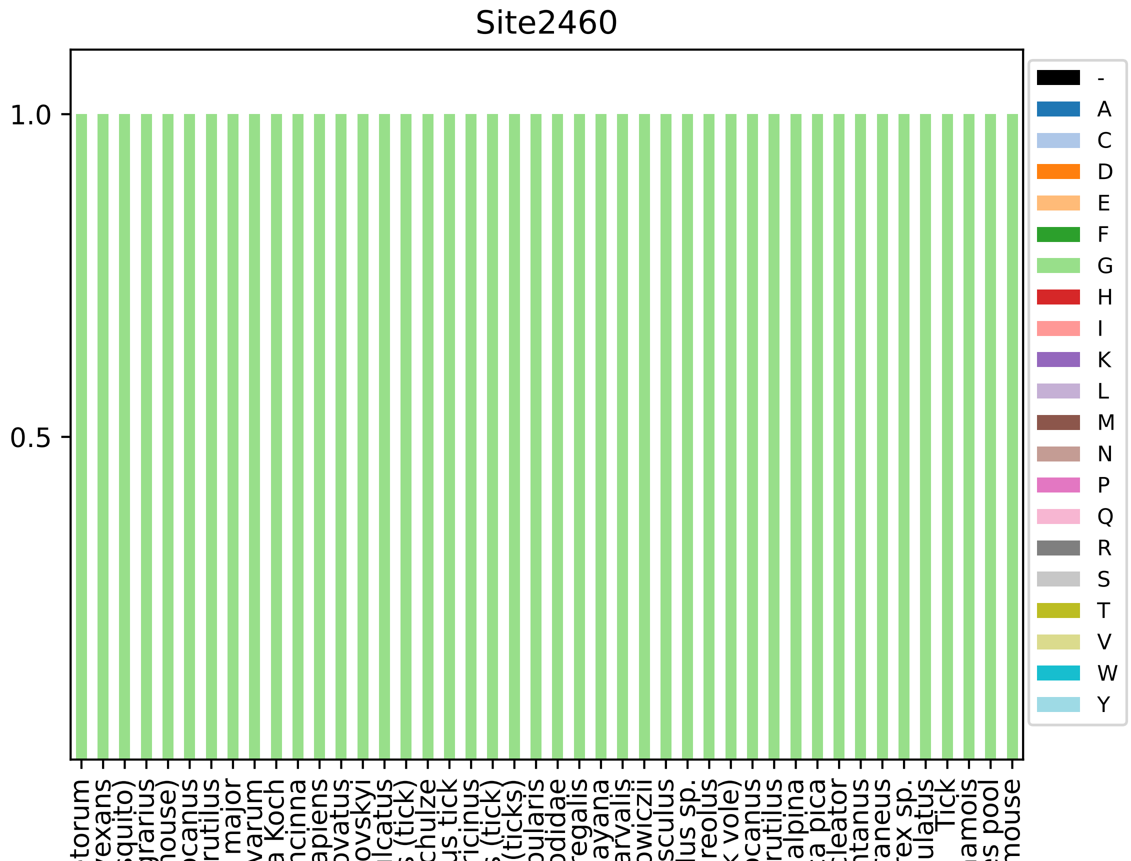
Site2460
1.0
0.5
-
A
C
D
E
F
G
H
I
K
L
M
N
P
Q
R
S
T
V
W
Y

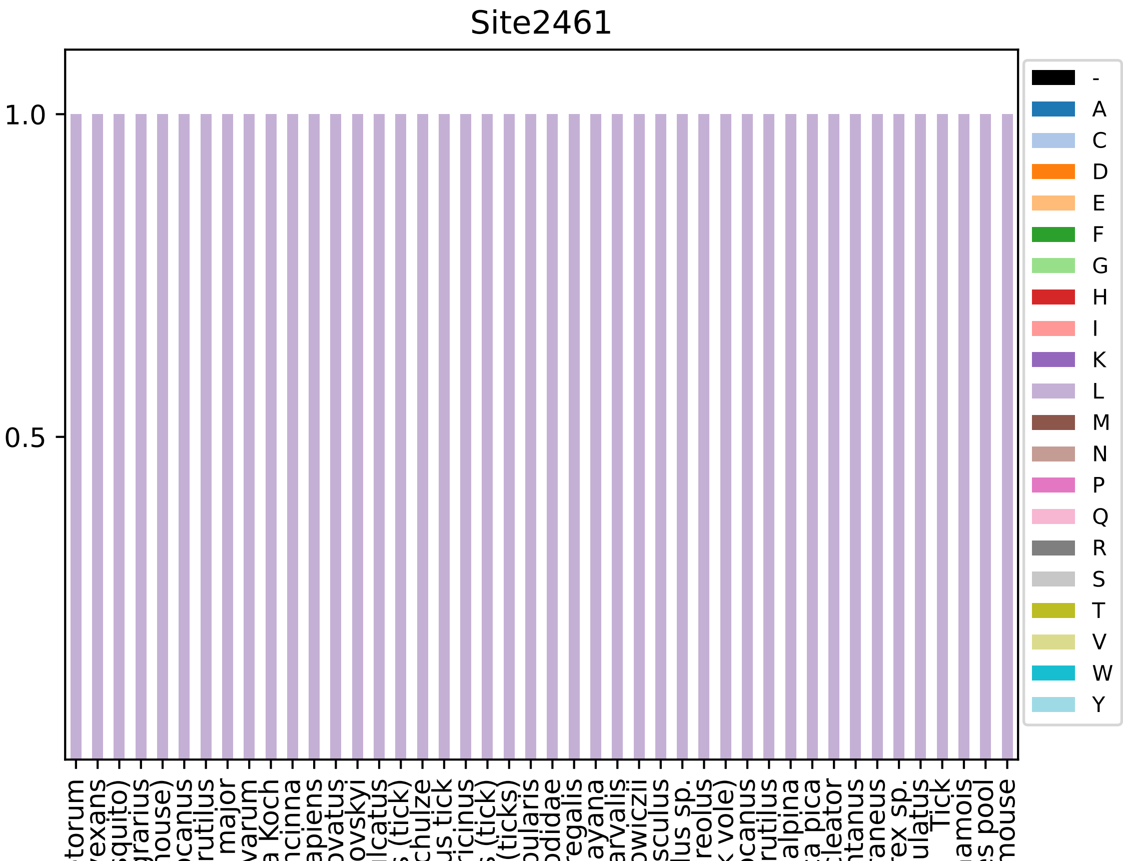

Site2461
1.0
0.5
-
A
C
D
E
F
G
H
I
K
L
M
N
P
Q
R
S
T
V
W
Y

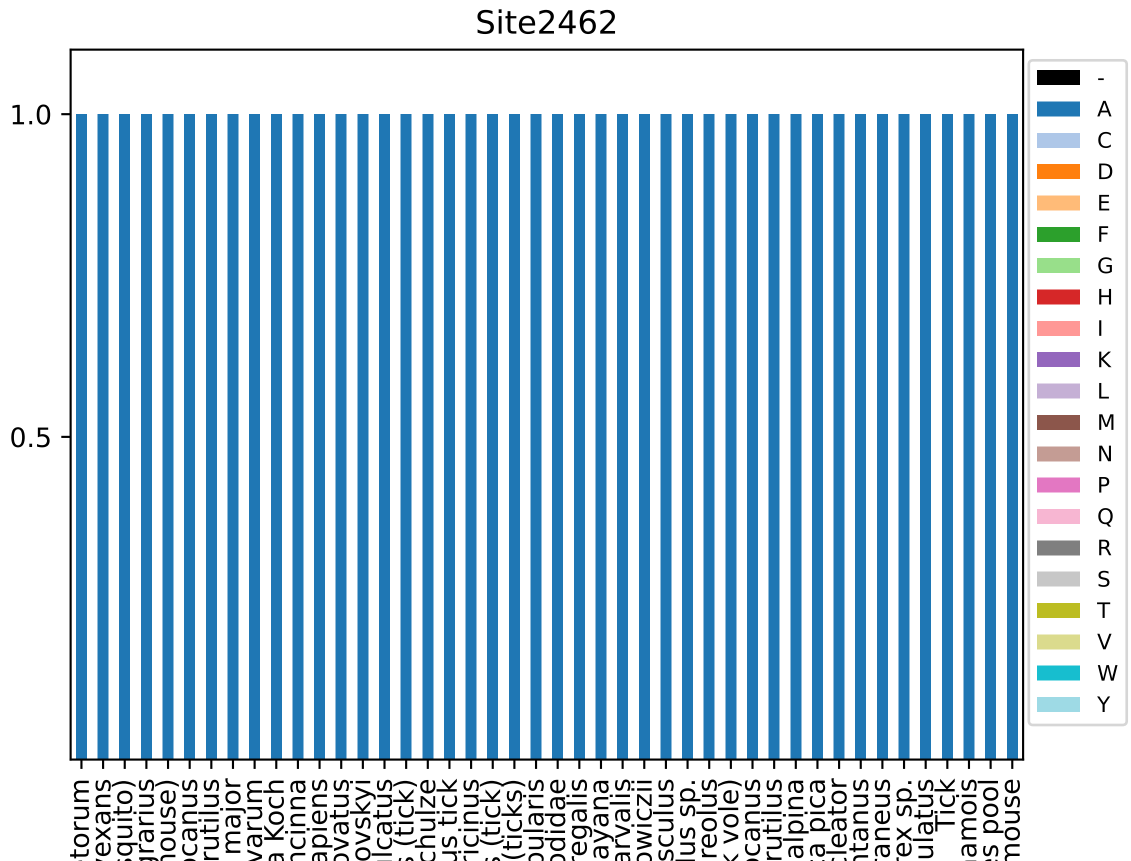
Site2462
1.0
0.5
-
A
C
D
E
F
G
H
I
K
L
M
N
P
Q
R
S
T
V
W
Y

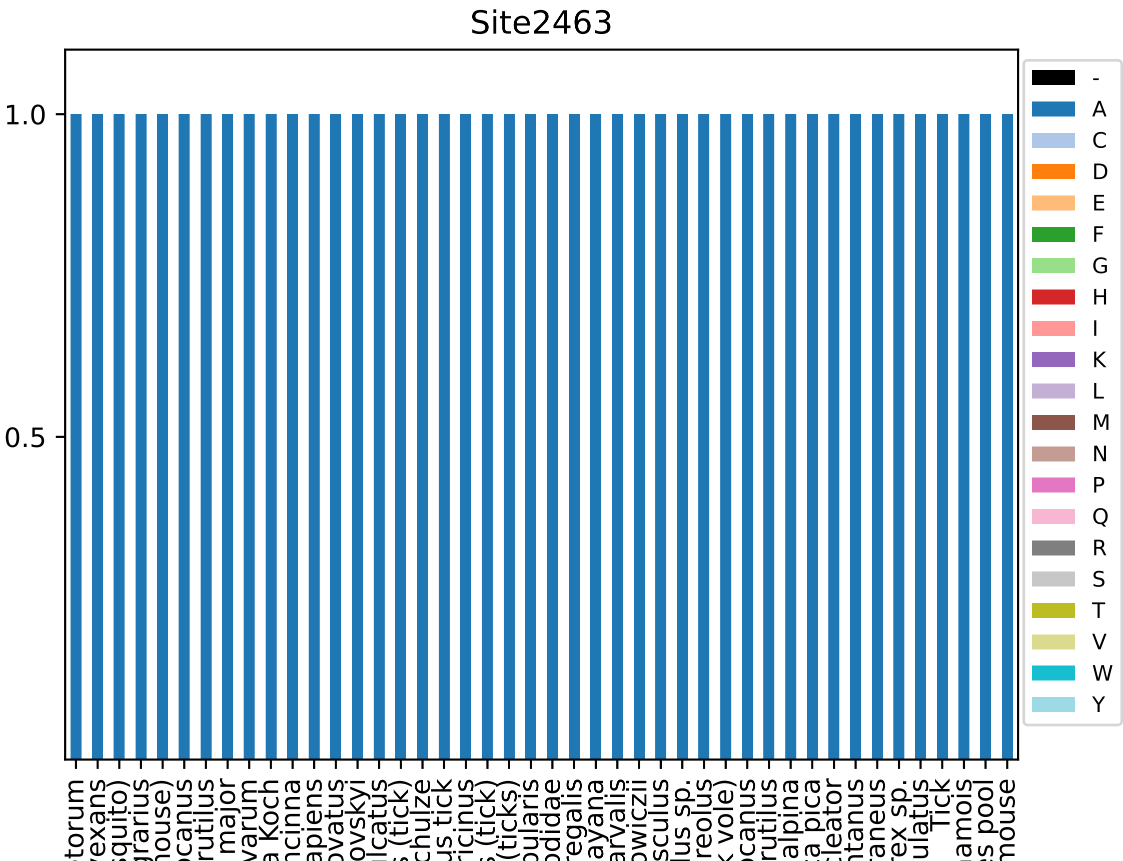

Site2463
1.0
0.5
-
A
C
D
E
F
G
H
I
K
L
M
N
P
Q
R
S
T
V
W
Y

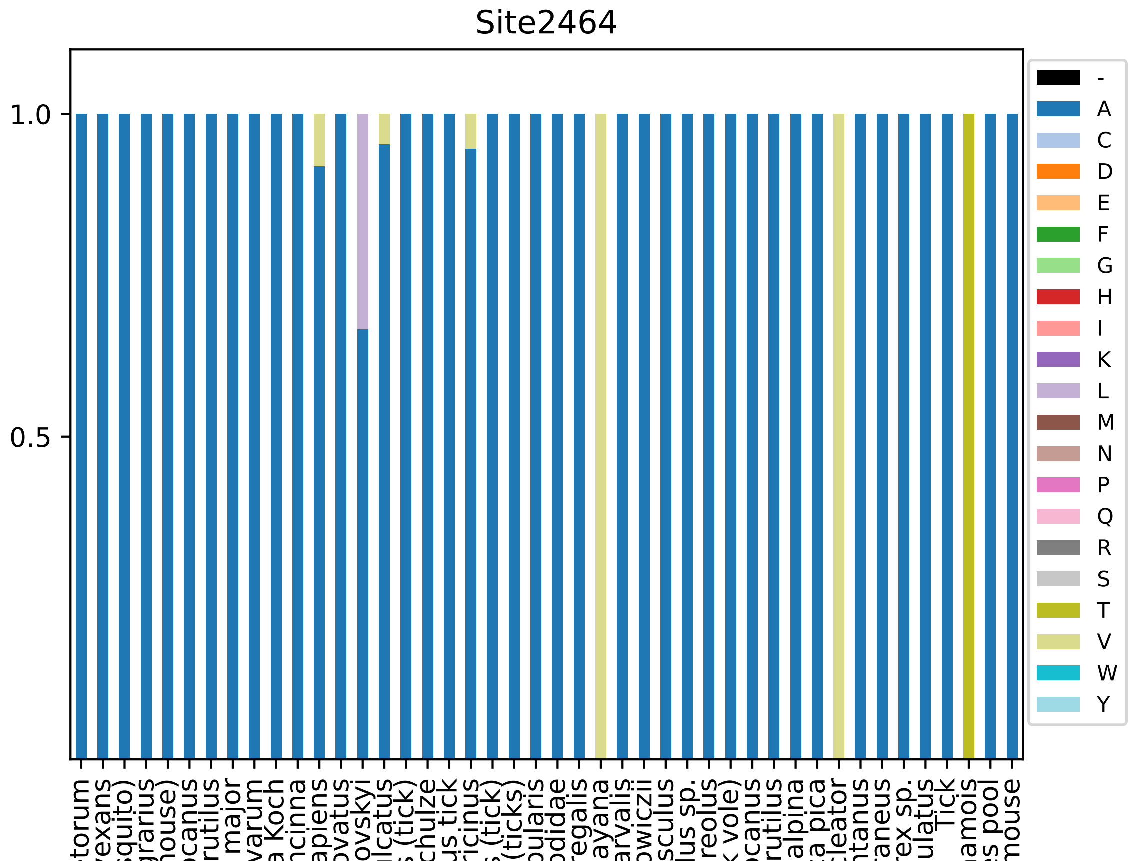
Site2464
1.0
0.5
-
A
C
D
E
F
G
H
I
K
L
M
N
P
Q
R
S
T
V
W
Y

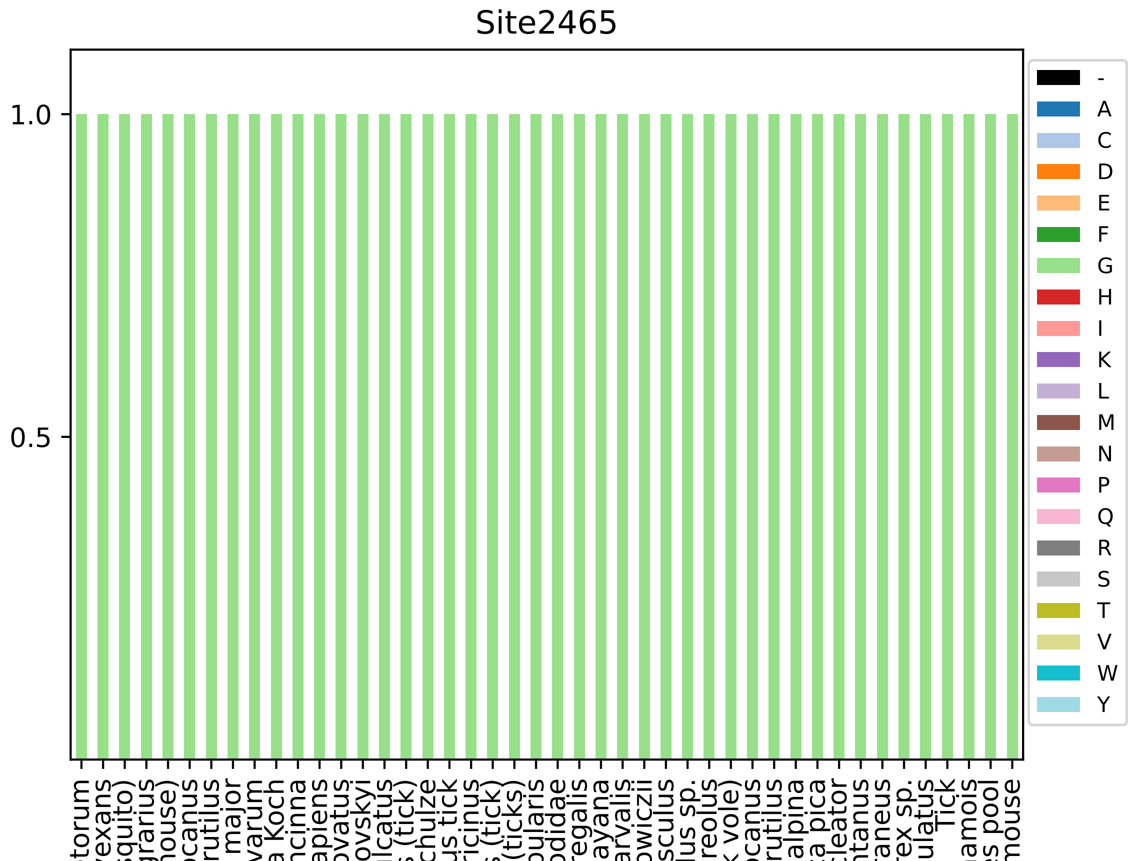
Site2465
1.0
0.5
-
A
C
D
E
F
G
H
I
K
L
M
N
P
Q
R
S
T
V
W
Y

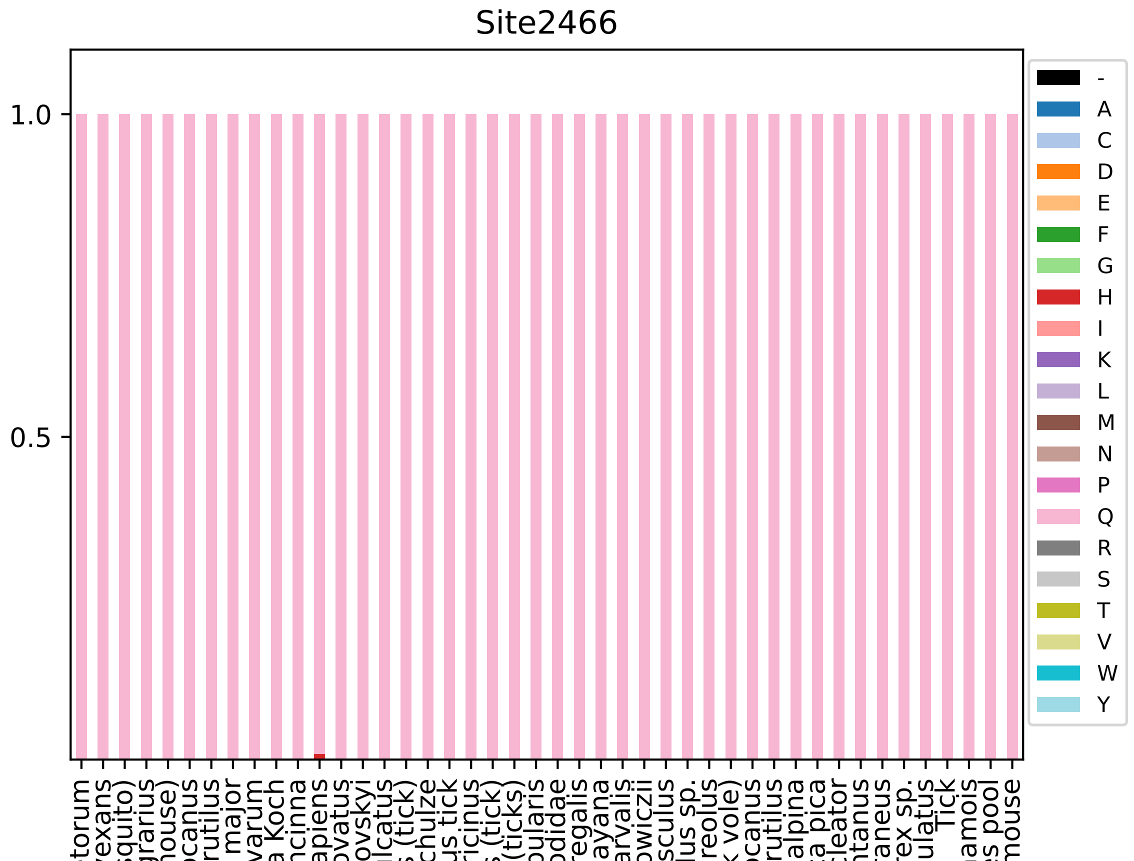

Site2466
1.0
0.5
-
A
C
D
E
F
G
H
I
K
L
M
N
P
Q
R
S
T
V
W
Y

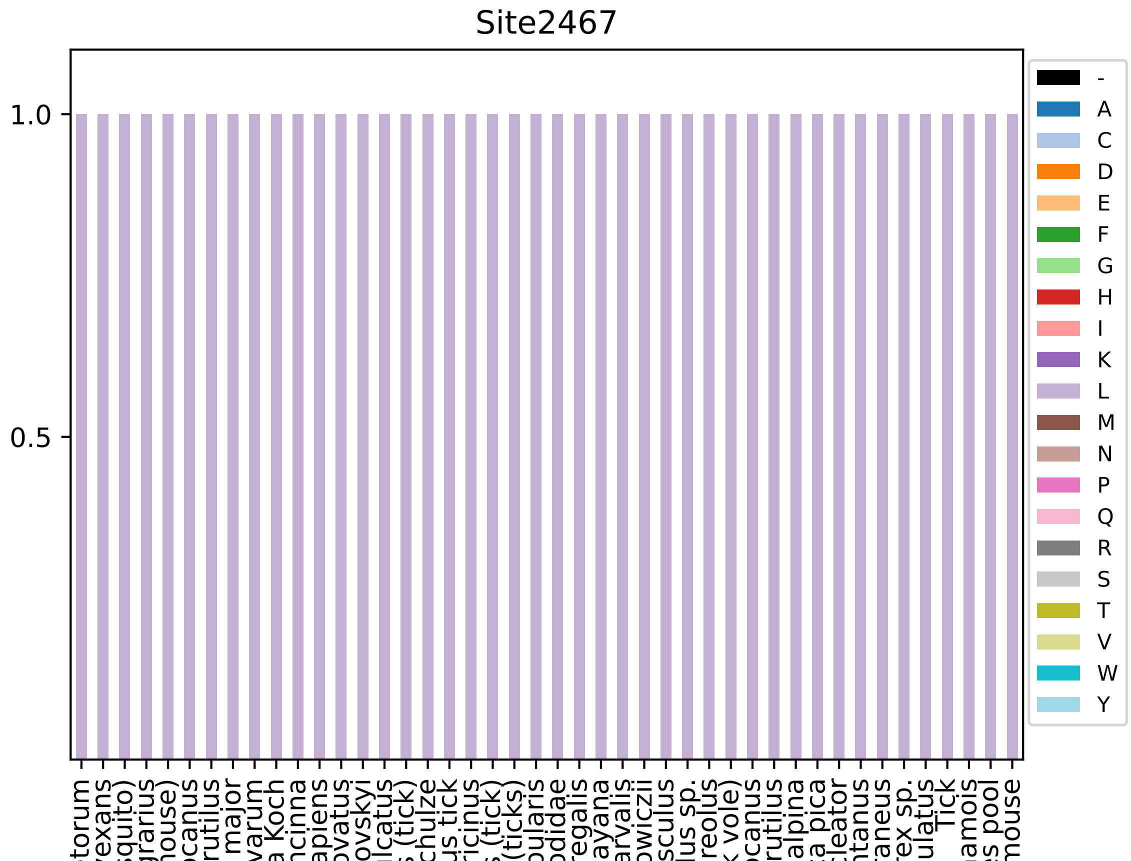

Site2467
1.0
0.5
-
A
C
D
E
F
G
H
I
K
L
M
N
P
Q
R
S
T
V
W
Y

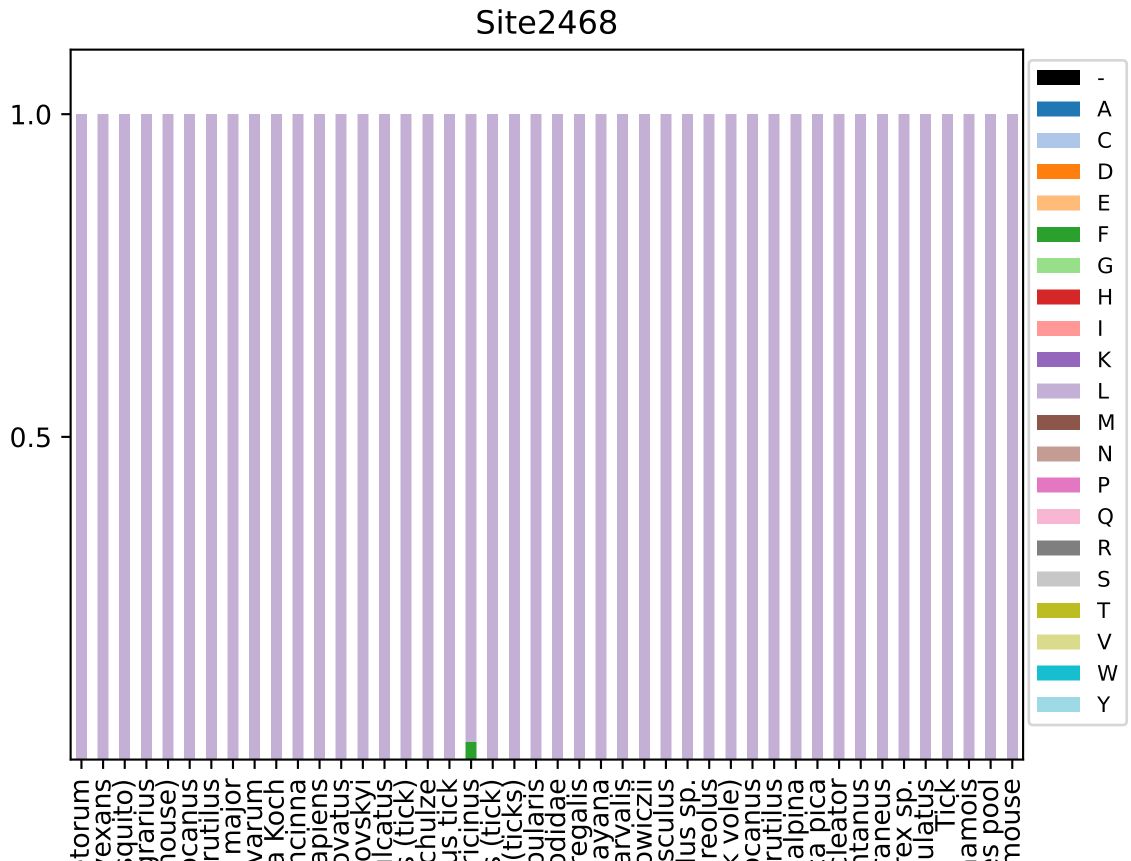
Site2468
1.0
0.5
-
A
C
D
E
F
G
H
I
K
L
M
N
P
Q
R
S
T
V
W
Y

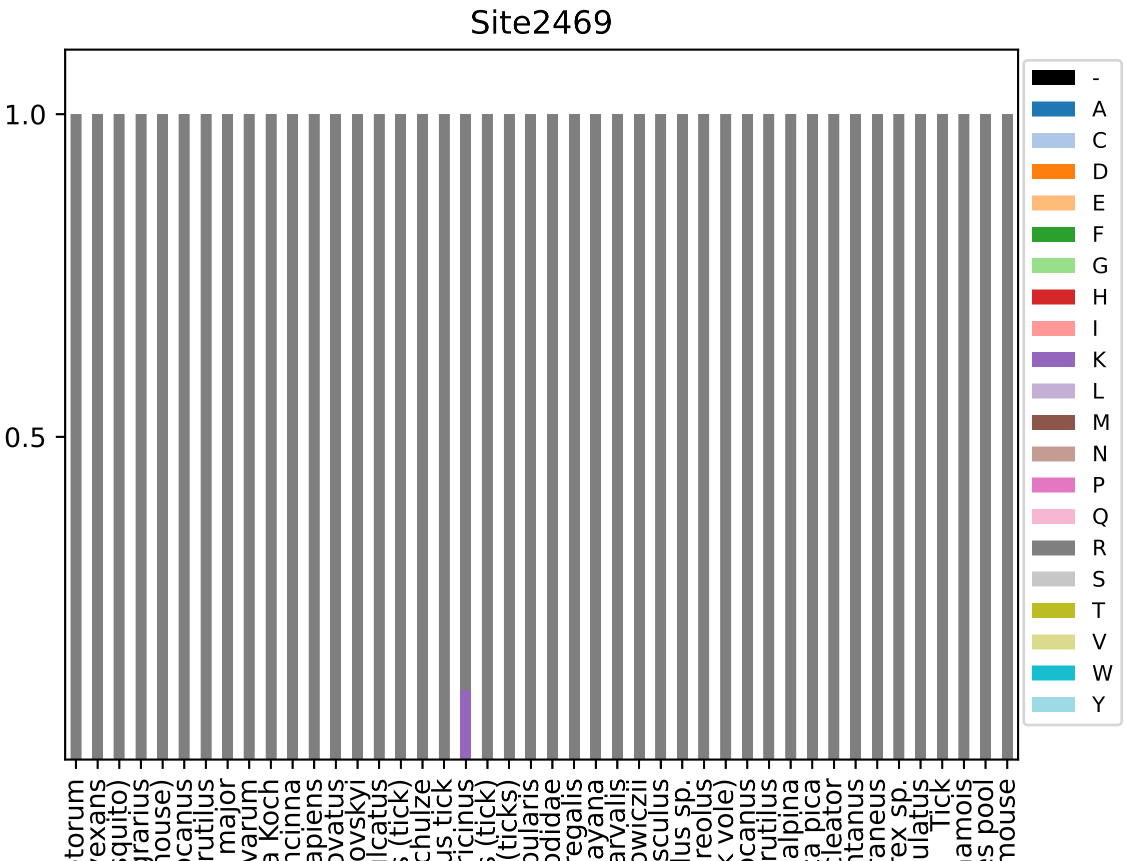
Site2469
1.0
0.5
-
A
C
D
E
F
G
H
I
K
L
M
N
P
Q
R
S
T
V
W
Y

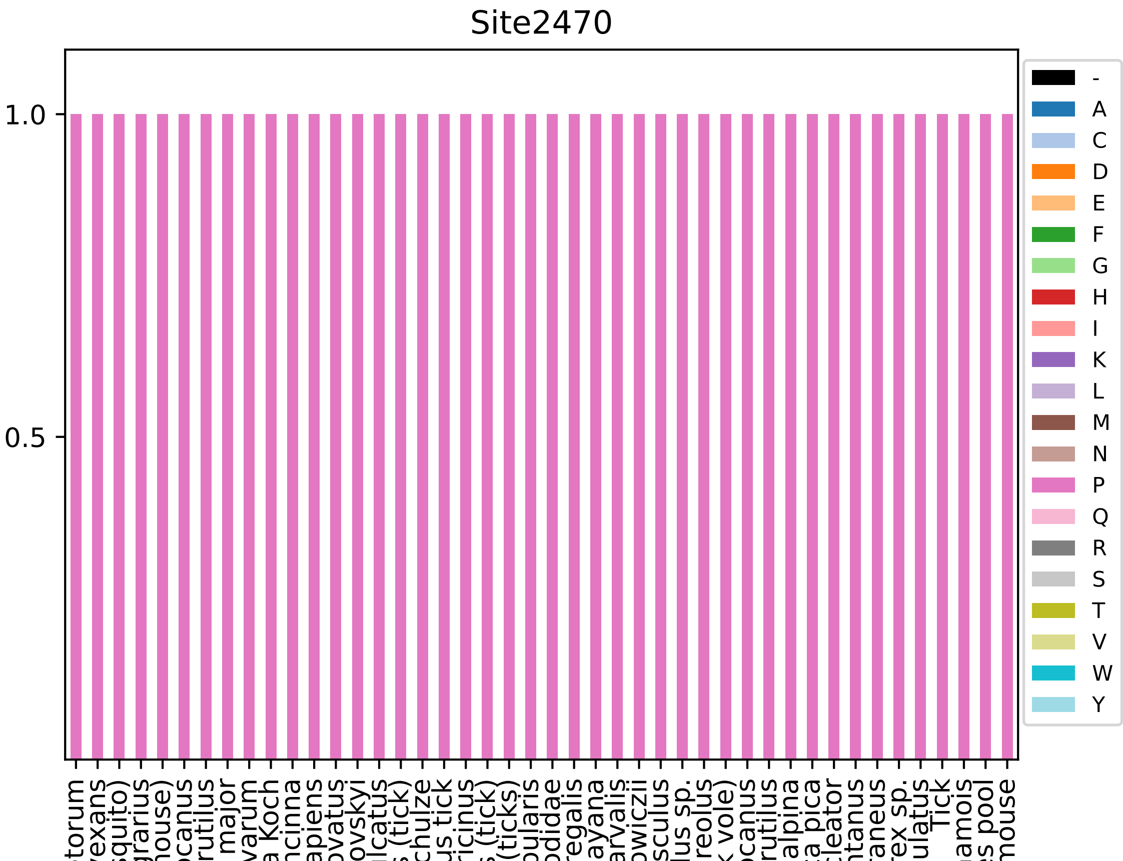

Site2470
1.0
0.5
-
A
C
D
E
F
G
H
I
K
L
M
N
P
Q
R
S
T
V
W
Y

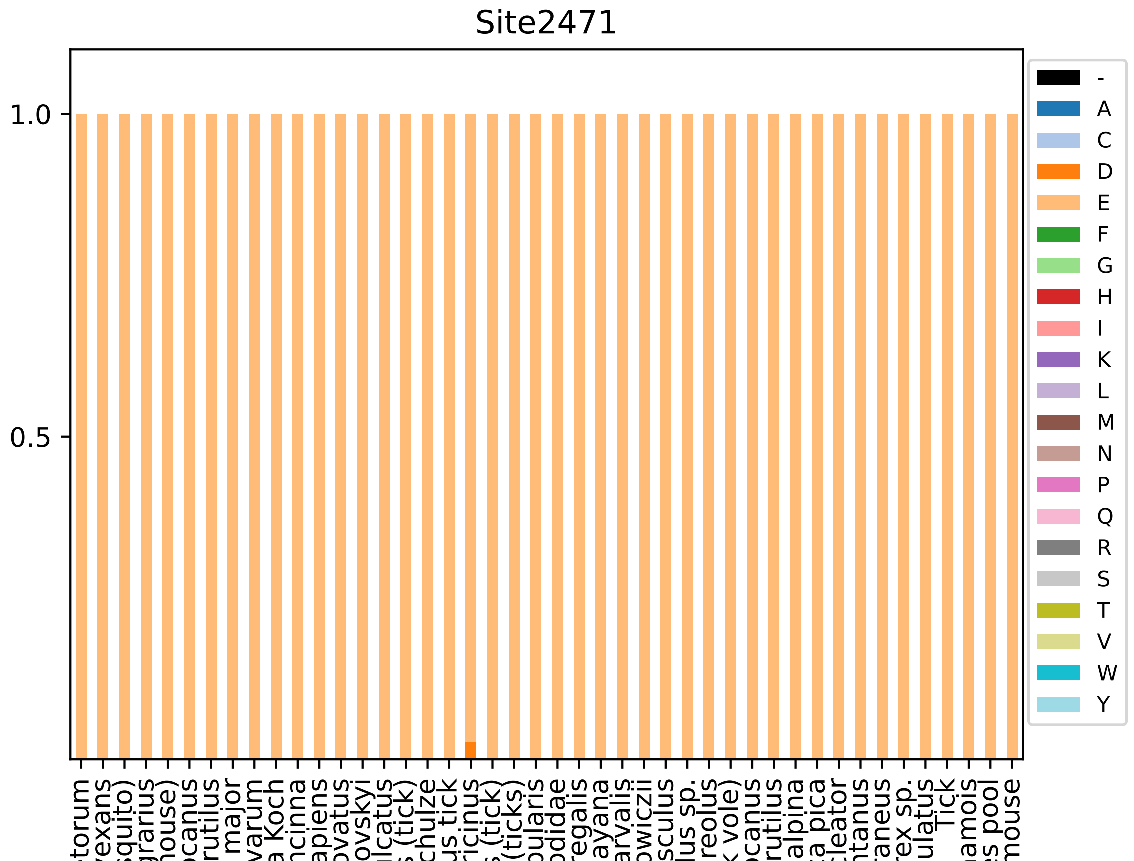
Site2471
1.0
0.5
-
A
C
D
E
F
G
H
I
K
L
M
N
P
Q
R
S
T
V
W
Y
torum
vexans
squito)
grarius
house)
ocanus
rutilus
major
varum
a Koch
ncinna
apiens
ovatus
ovskyi
ulcatus
s (tick)
chulze
us tick
ricinus
s (tick)
(ticks)
pularis
odidae
regalis
ayana
arvalis
owiczii
sculus
lus sp.
reolus
k vole)
ocanus
rutilus
alpina
a pica
cleator
ntanus
aneus
rex sp.
ulatus
Tick
amois
es pool
mouse

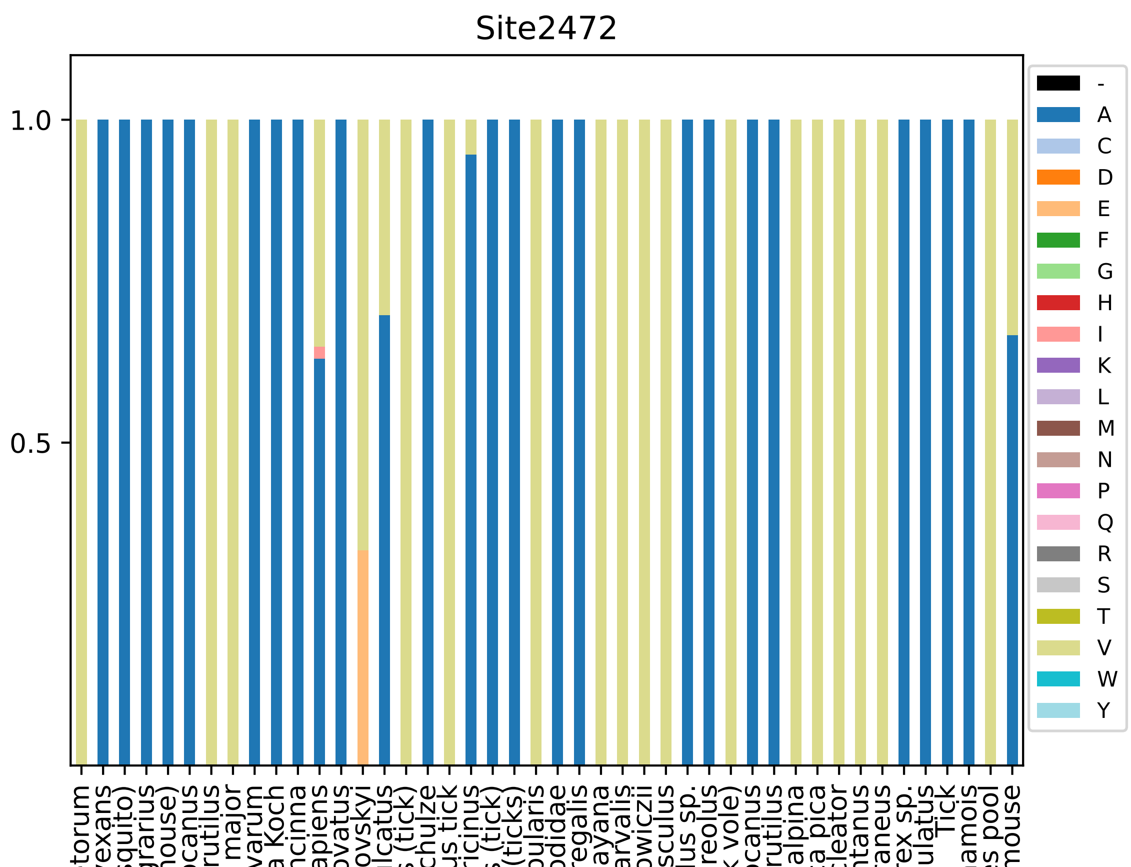

Site2472
1.0
0.5
-
A
C
D
E
F
G
H
I
K
L
M
N
P
Q
R
S
T
V
W
Y

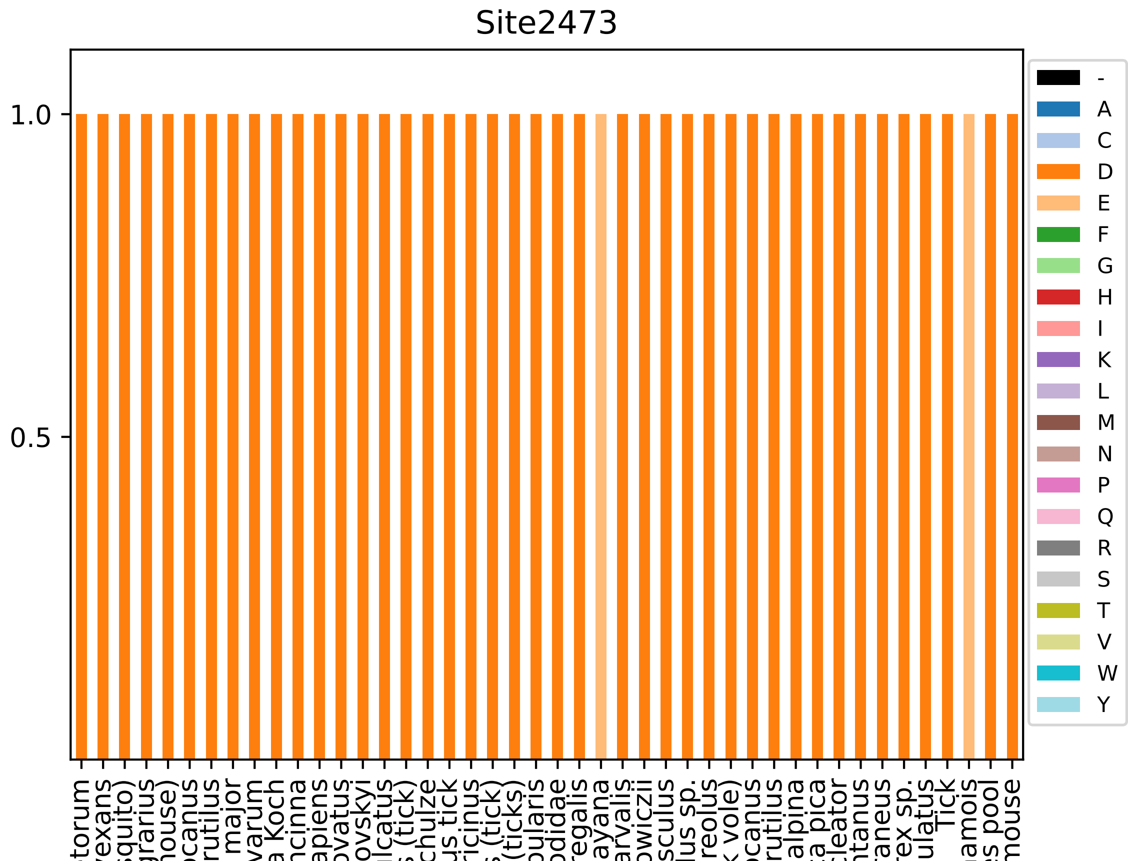
Site2473
1.0
0.5
-
A
C
D
E
F
G
H
I
K
L
M
N
P
Q
R
S
T
V
W
Y

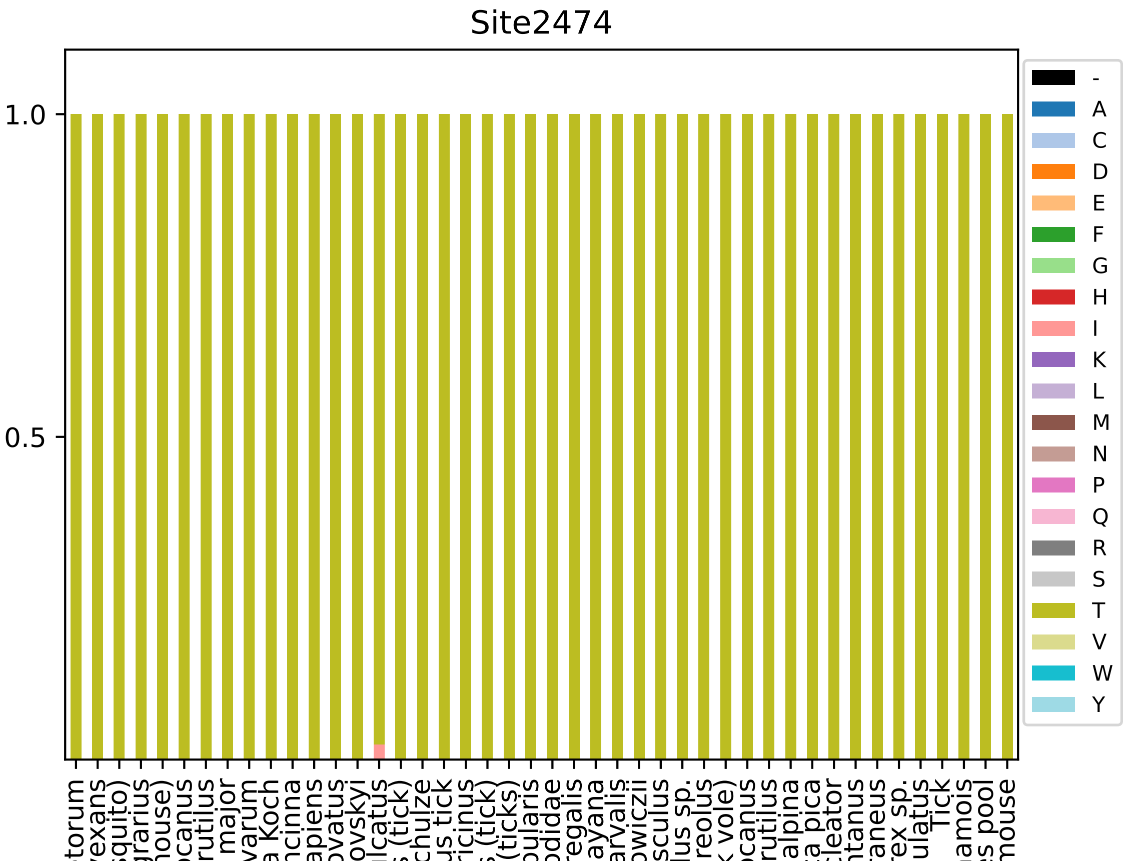
Site2474
1.0
0.5
-
A
C
D
E
F
G
H
I
K
L
M
N
P
Q
R
S
T
V
W
Y

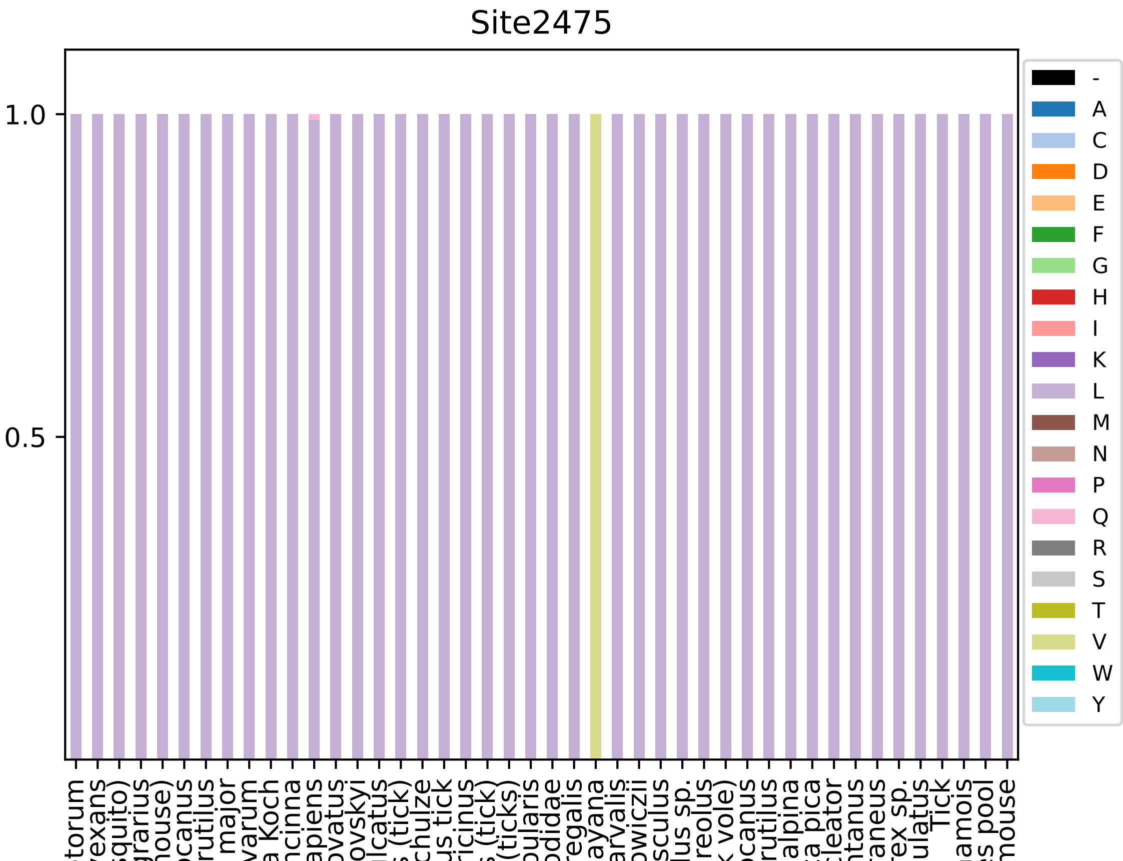

Site2475
1.0
0.5
-
A
C
D
E
F
G
H
I
K
L
M
N
P
Q
R
S
T
V
W
Y

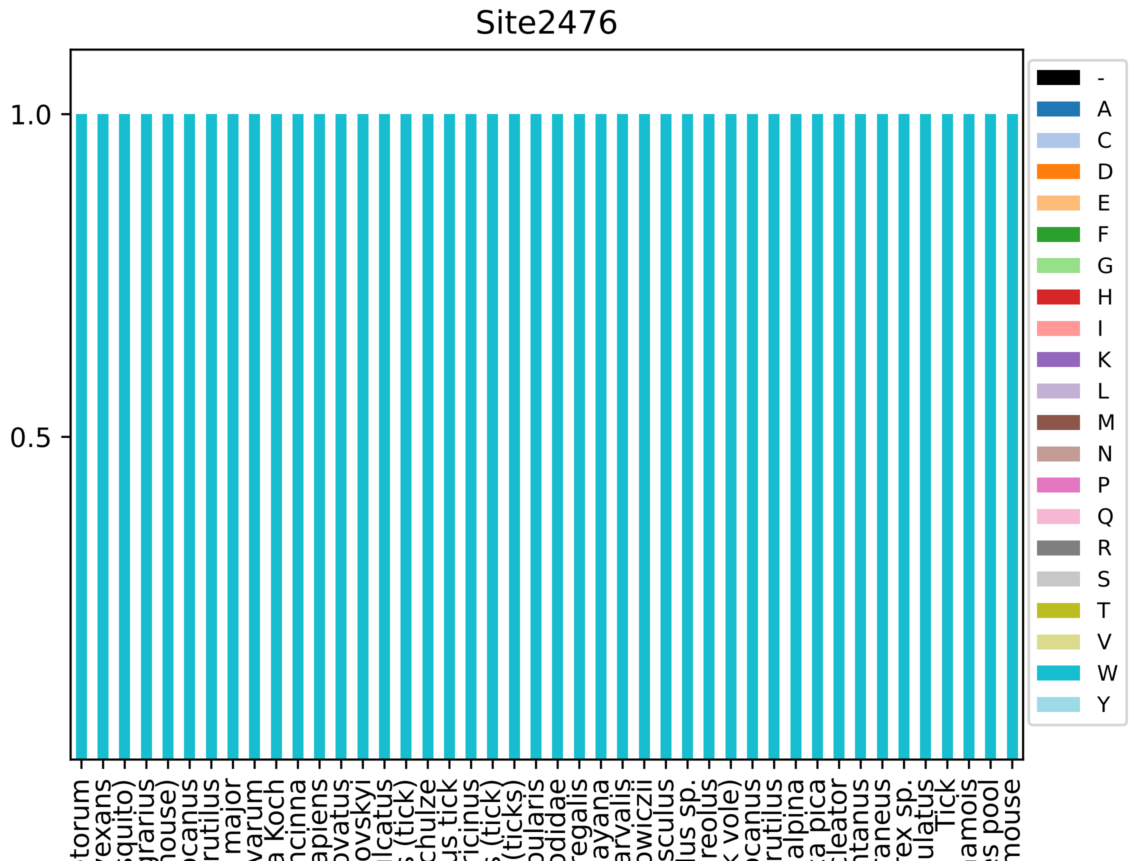

Site2476
1.0
0.5
-
A
C
D
E
F
G
H
I
K
L
M
N
P
Q
R
S
T
V
W
Y

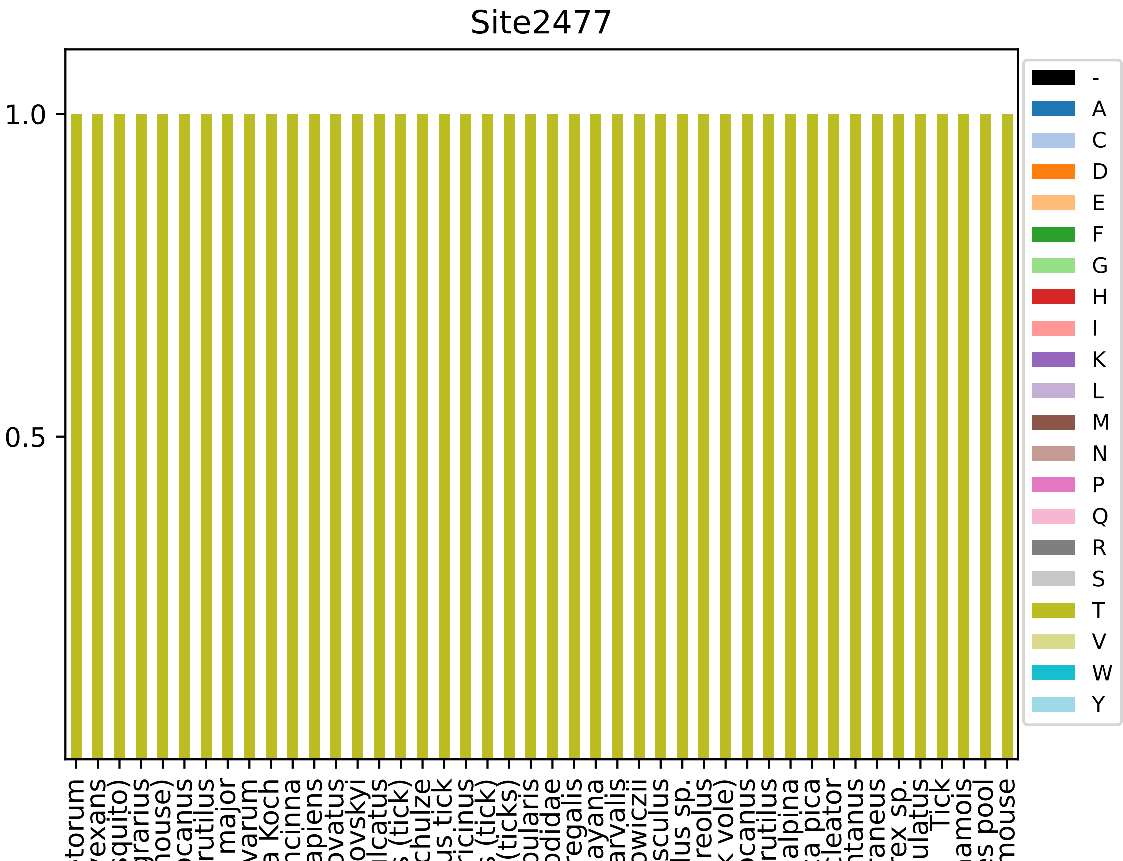

Site2477
1.0
0.5
-
A
C
D
E
F
G
H
I
K
L
M
N
P
Q
R
S
T
V
W
Y

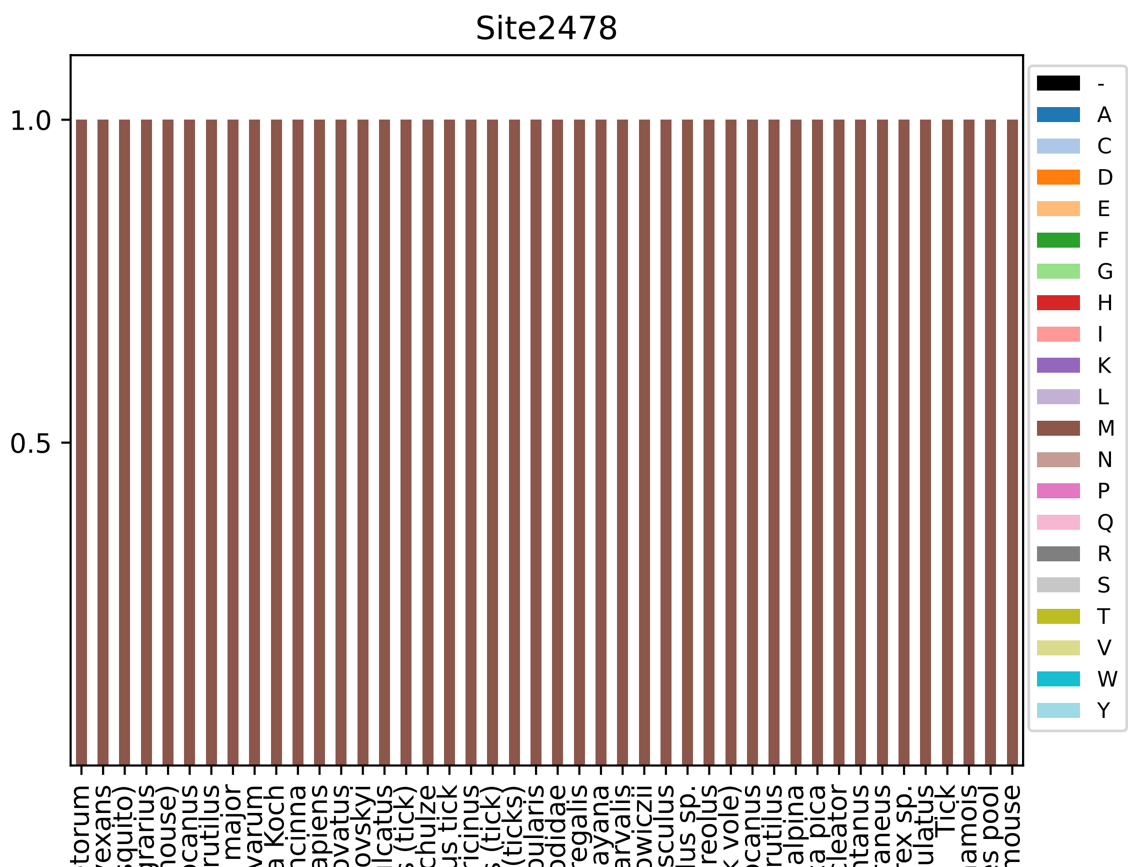

Site2478
1.0
0.5
-
A
C
D
E
F
G
H
I
K
L
M
N
P
Q
R
S
T
V
W
Y

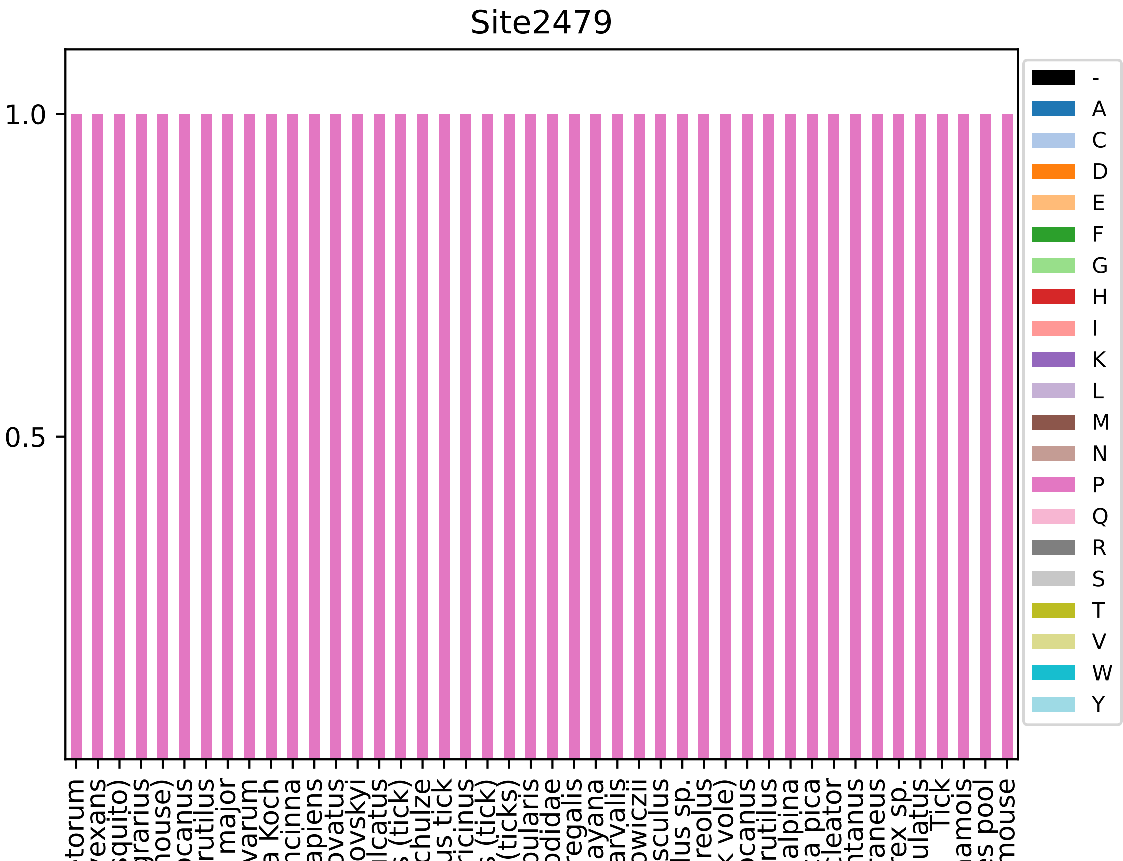
Site2479
1.0
0.5
-
A
C
D
E
F
G
H
I
K
L
M
N
P
Q
R
S
T
V
W
Y

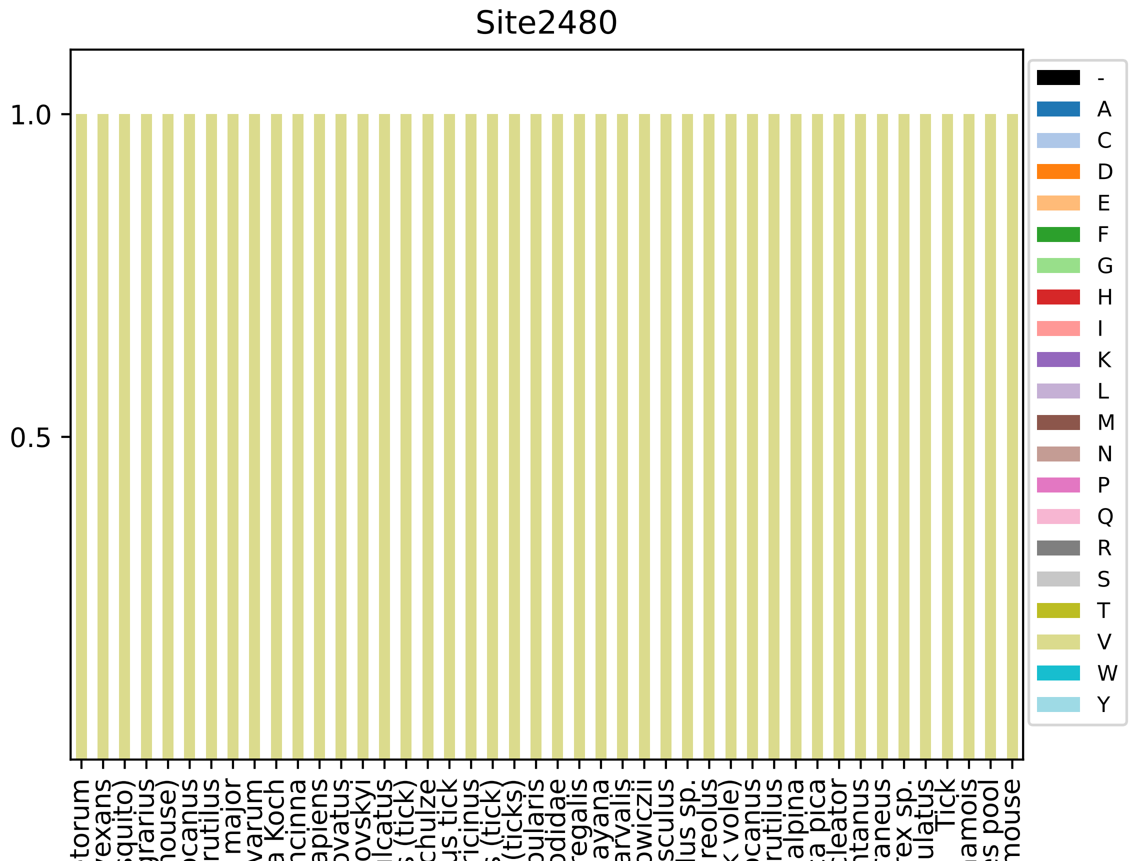

Site2480
1.0
0.5
-
A
C
D
E
F
G
H
I
K
L
M
N
P
Q
R
S
T
V
W
Y

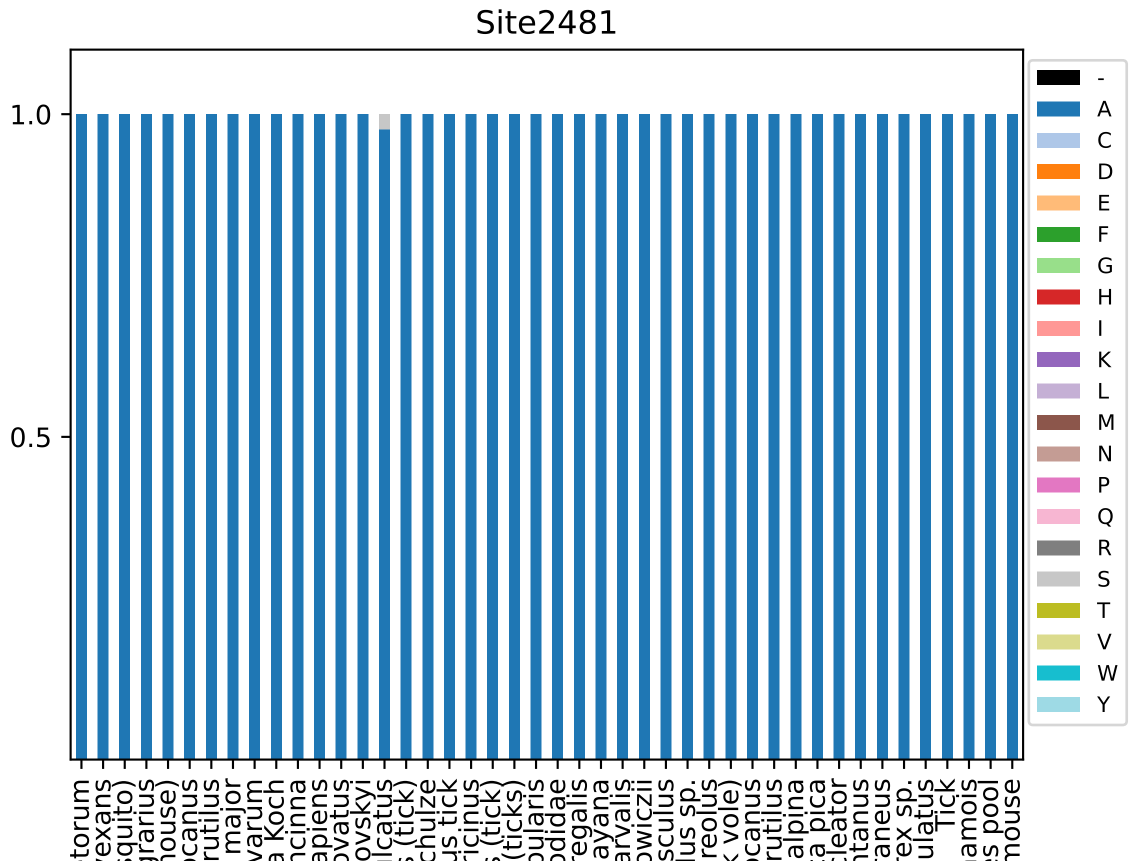

Site2481
1.0
0.5
-
A
C
D
E
F
G
H
I
K
L
M
N
P
Q
R
S
T
V
W
Y

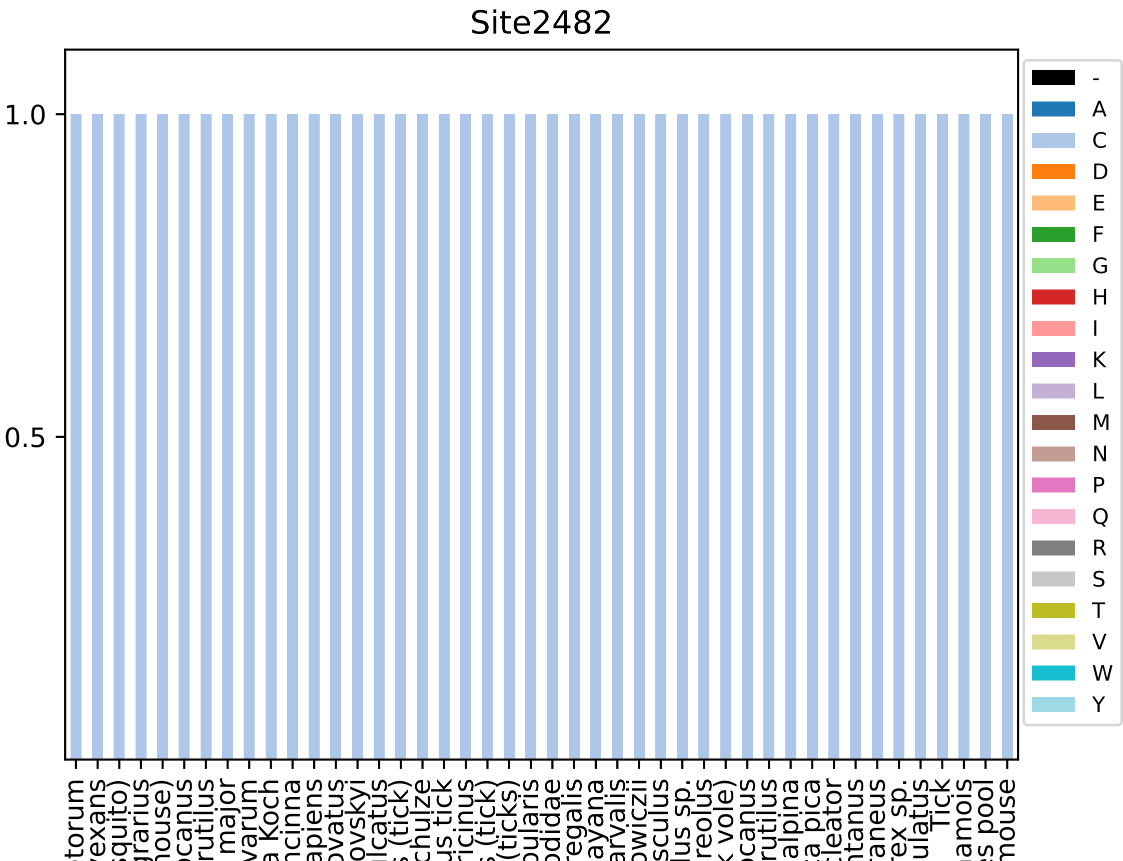

Site2482
1.0
0.5
-
A
C
D
E
F
G
H
I
K
L
M
N
P
Q
R
S
T
V
W
Y

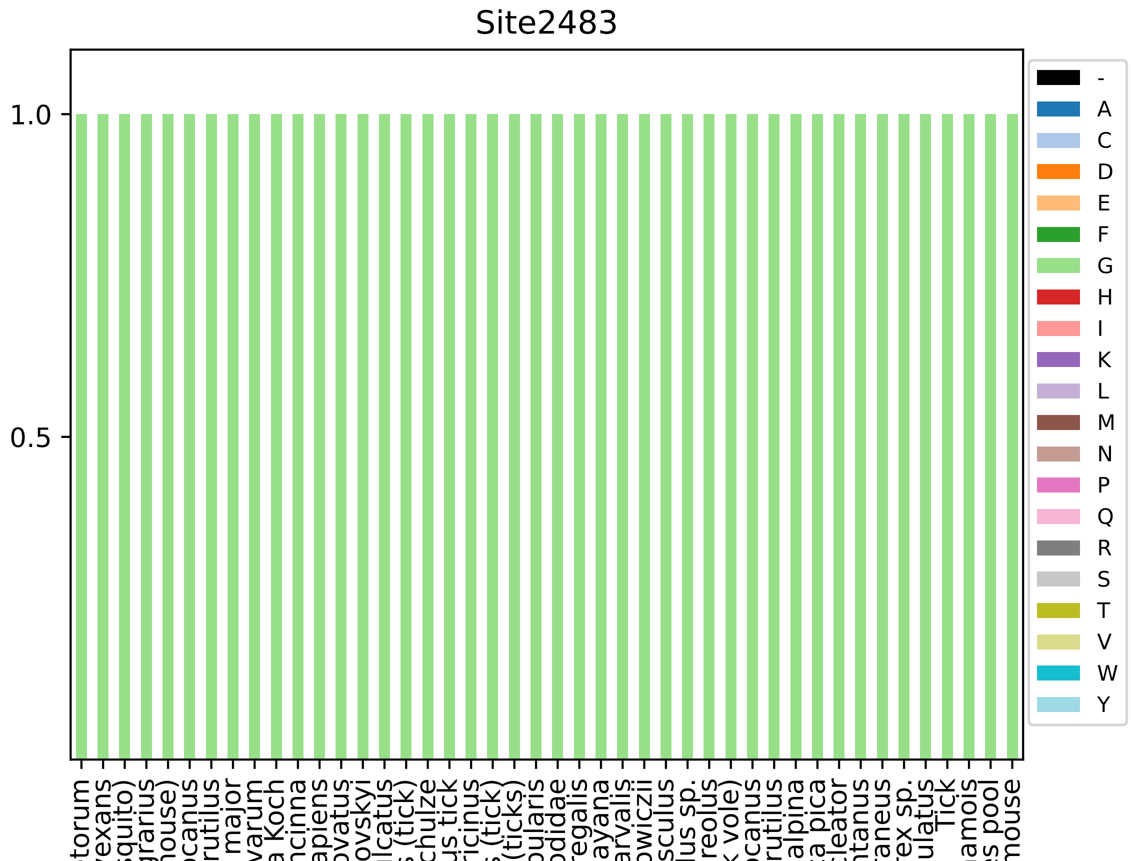
Site2483
1.0
0.5
-
A
C
D
E
F
G
H
I
K
L
M
N
P
Q
R
S
T
V
W
Y

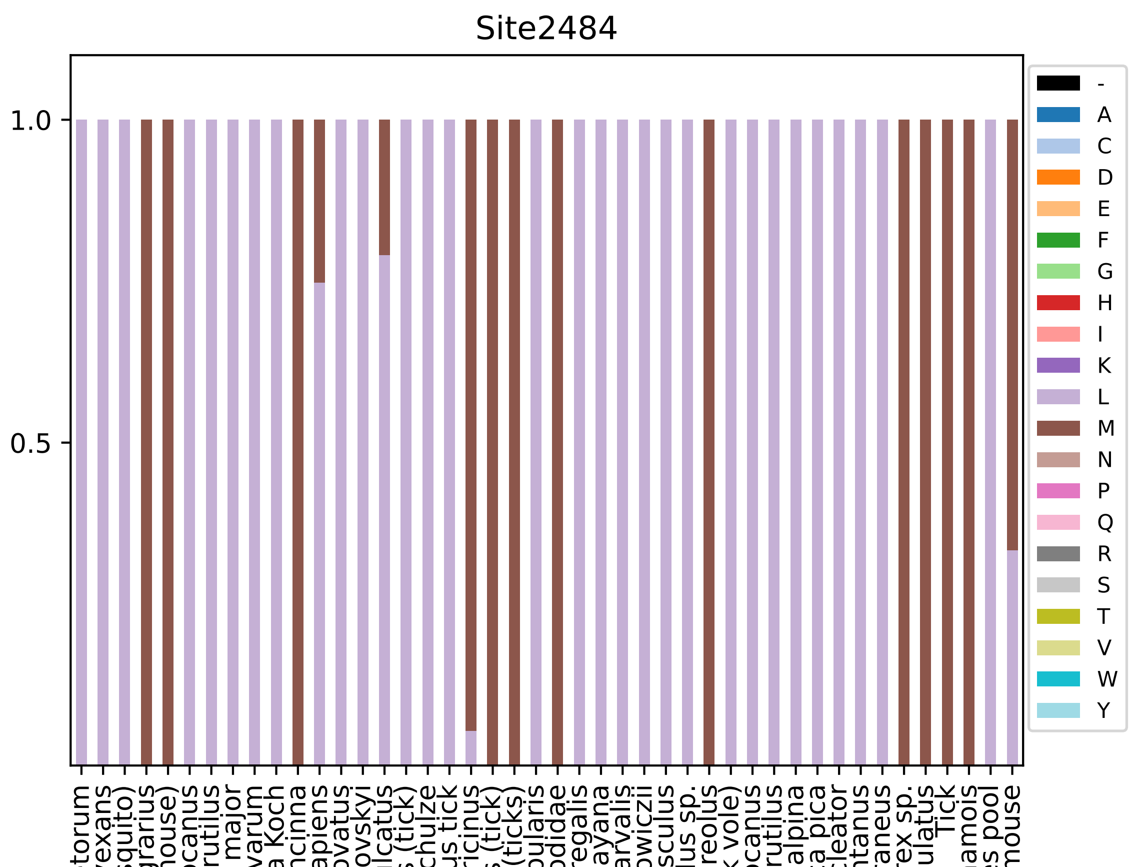
Site2484
1.0
0.5
-
A
C
D
E
F
G
H
I
K
L
M
N
P
Q
R
S
T
V
W
Y
Tick

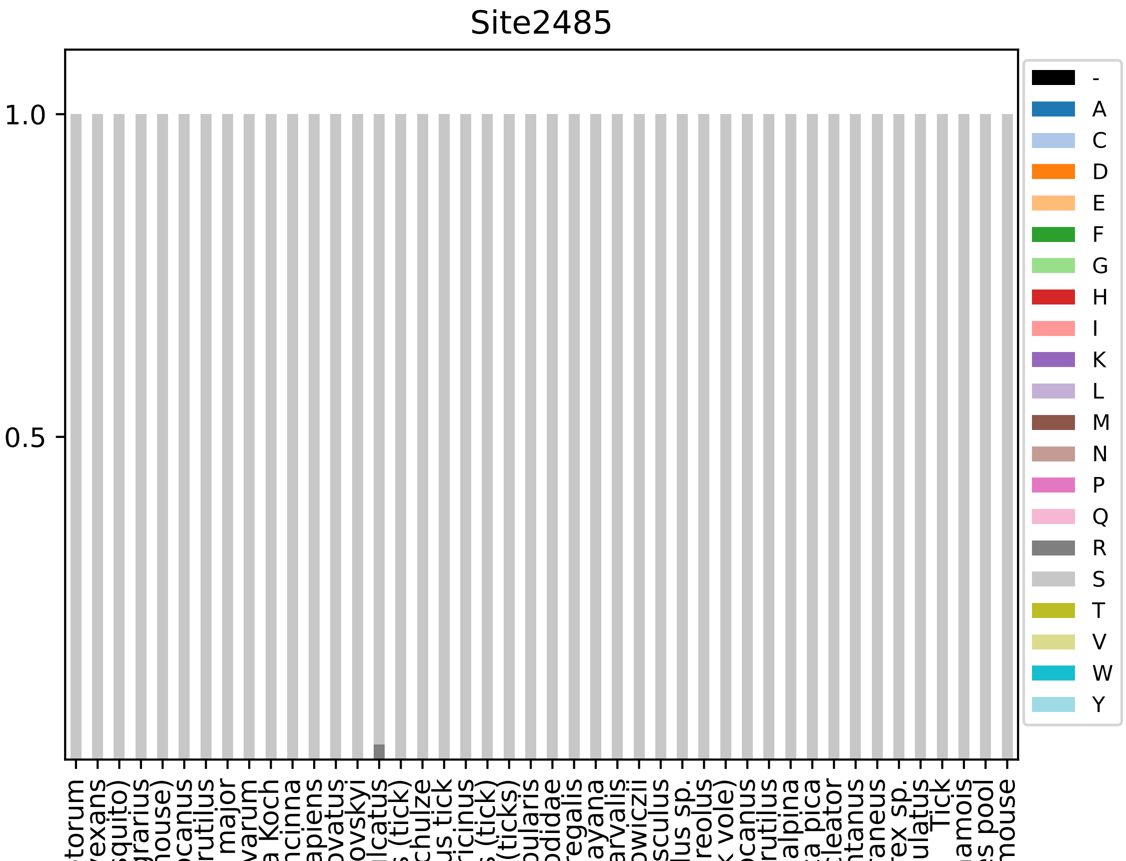
Site2485
1.0
0.5
-
A
C
D
E
F
G
H
I
K
L
M
N
P
Q
R
S
T
V
W
Y

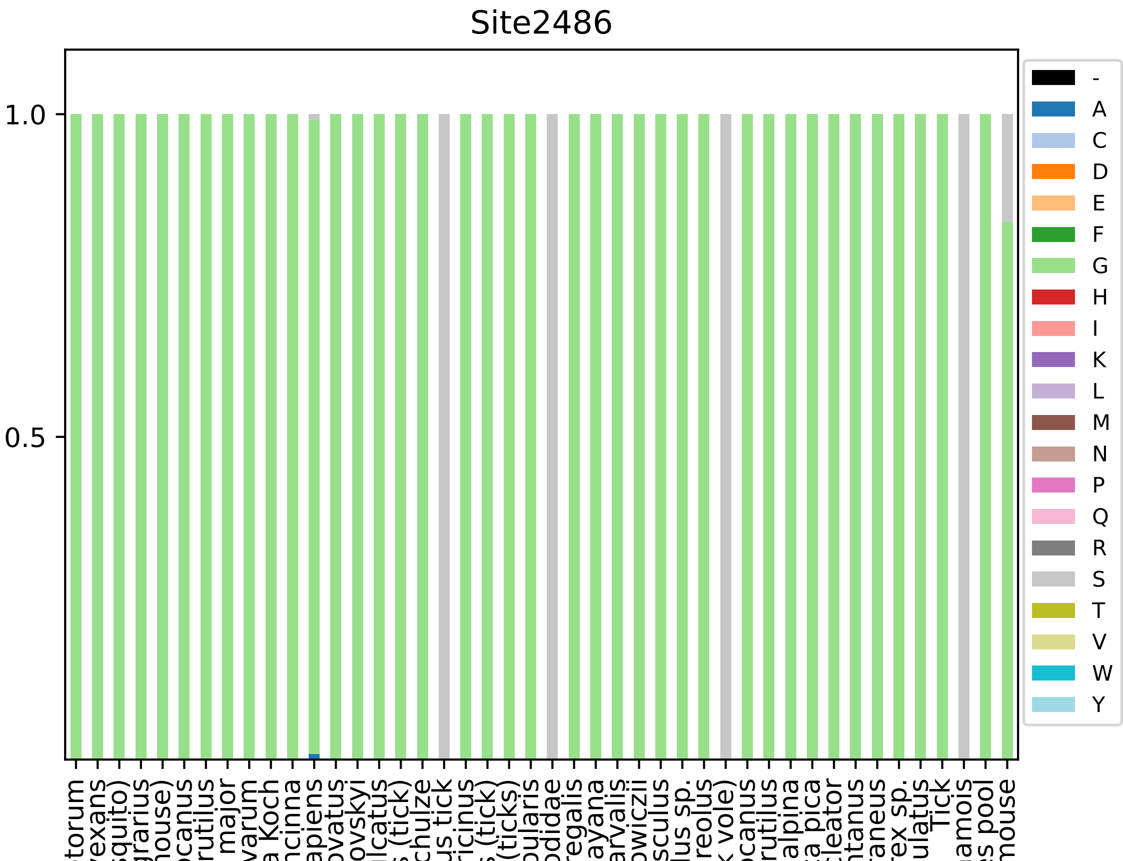

Site2486
1.0
0.5
-
A
C
D
E
F
G
H
I
K
L
M
N
P
Q
R
S
T
V
W
Y

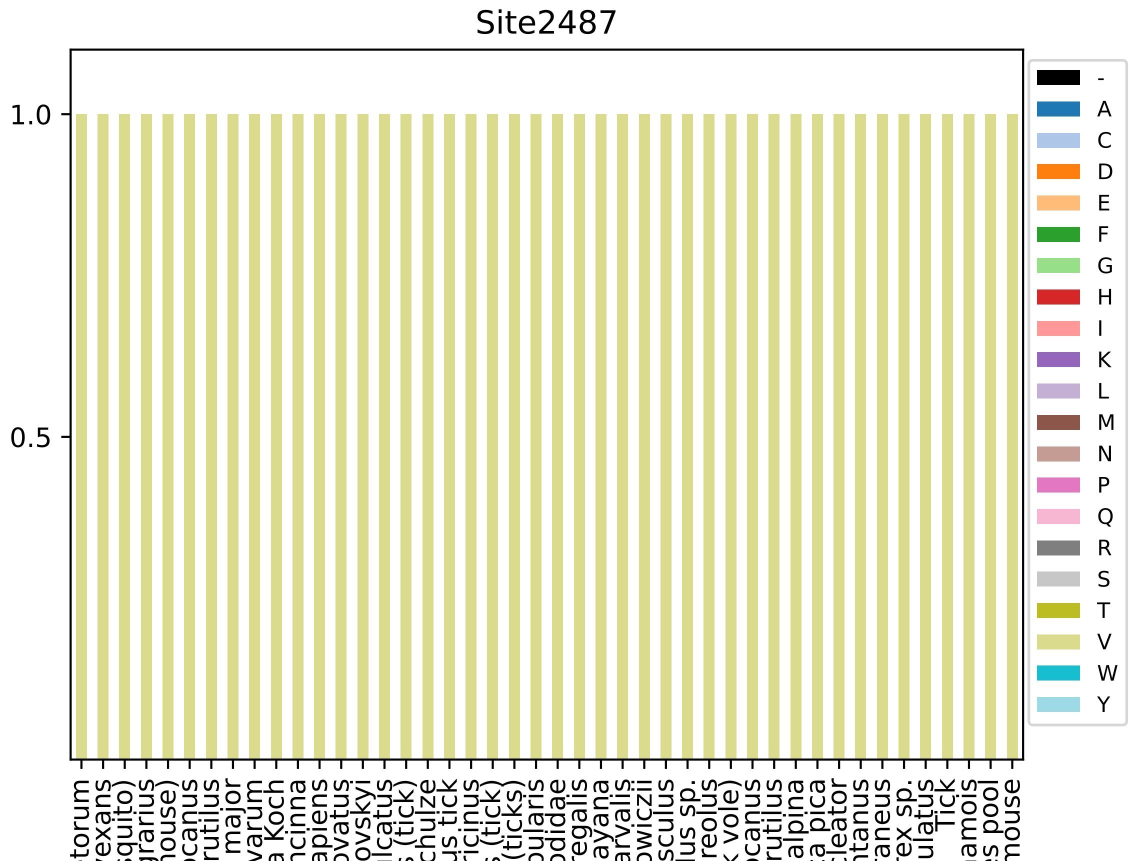

Site2487
1.0
0.5
-
A
C
D
E
F
G
H
I
K
L
M
N
P
Q
R
S
T
V
W
Y

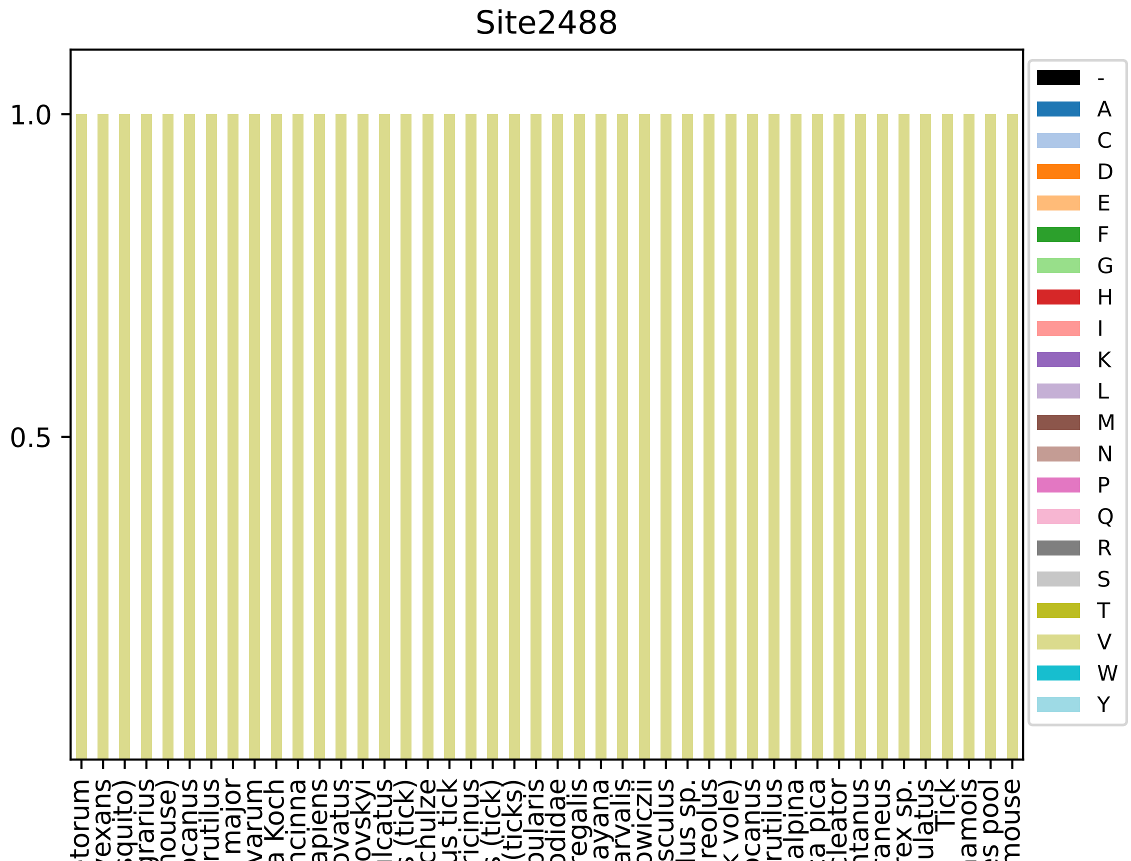
Site2488
1.0
0.5
-
A
C
D
E
F
G
H
I
K
L
M
N
P
Q
R
S
T
V
W
Y

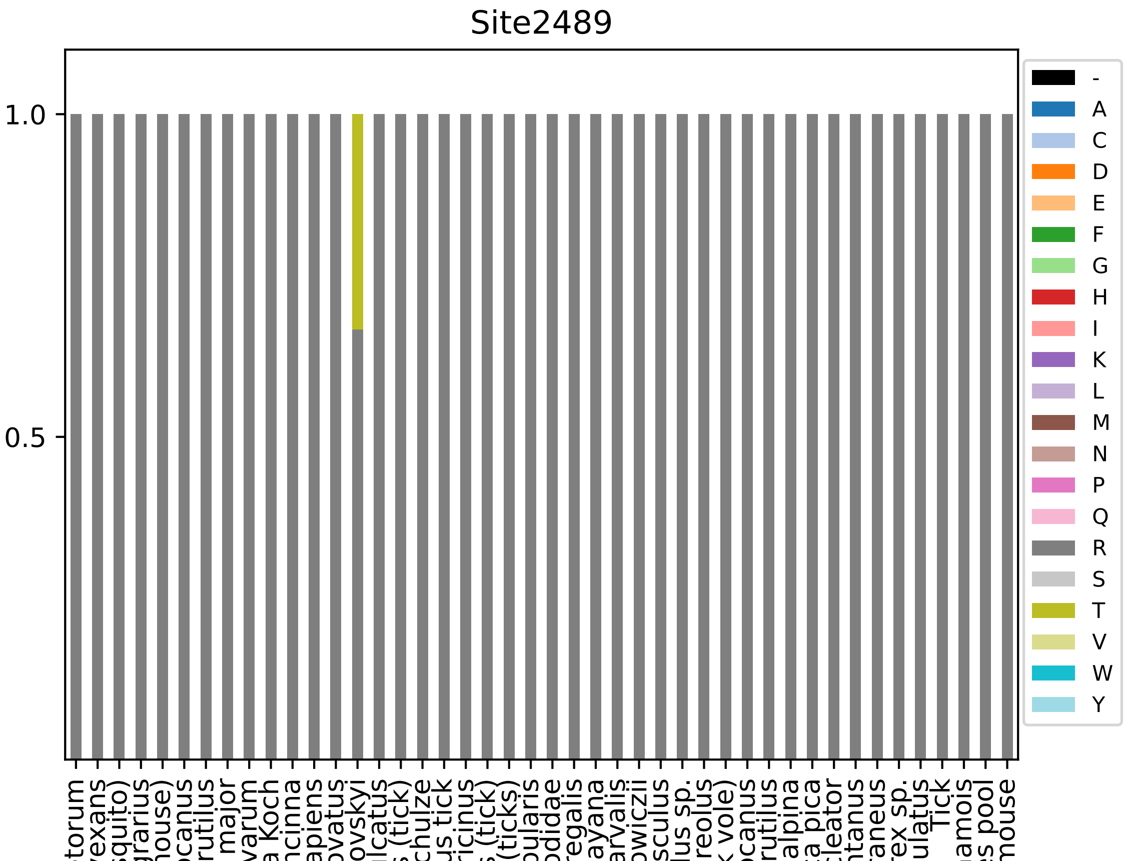
Site2489
1.0
0.5
-
A
C
D
E
F
G
H
I
K
L
M
N
P
Q
R
S
T
V
W
Y
Tick

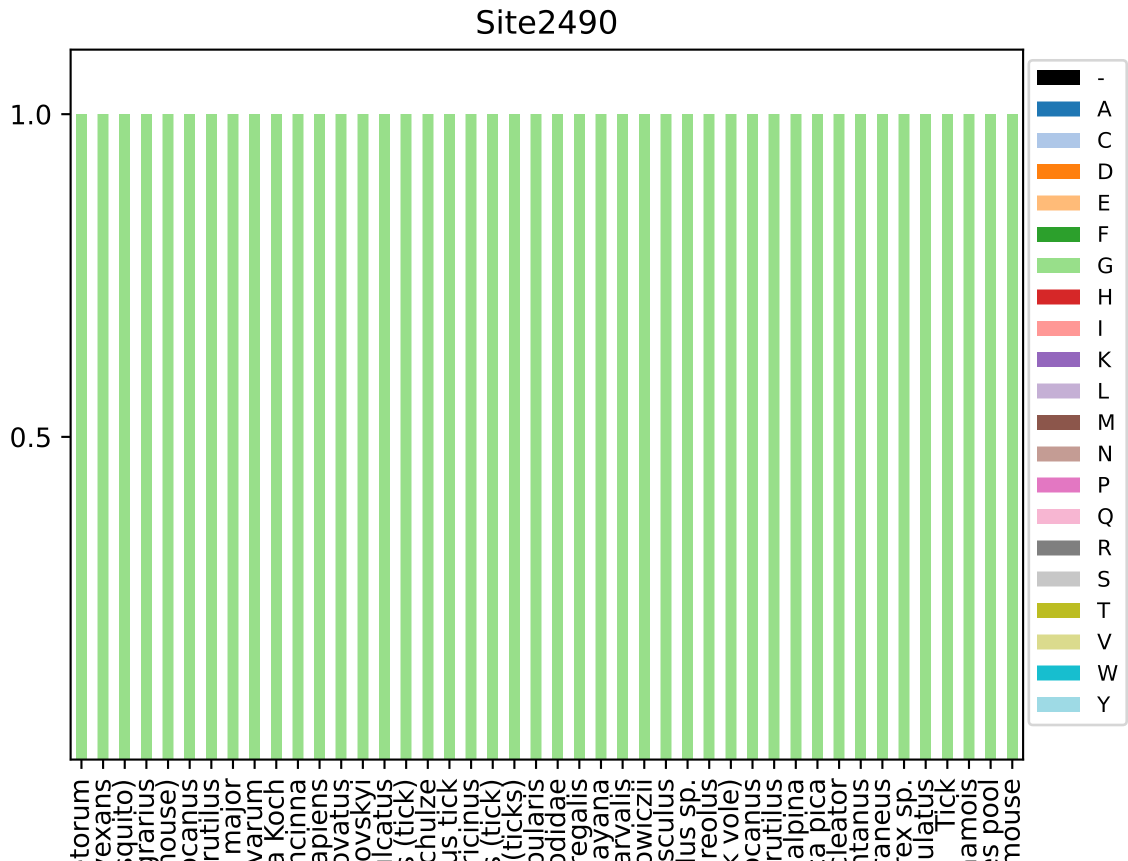

Site2490
1.0
0.5
-
A
C
D
E
F
G
H
I
K
L
M
N
P
Q
R
S
T
V
W
Y

Site2491

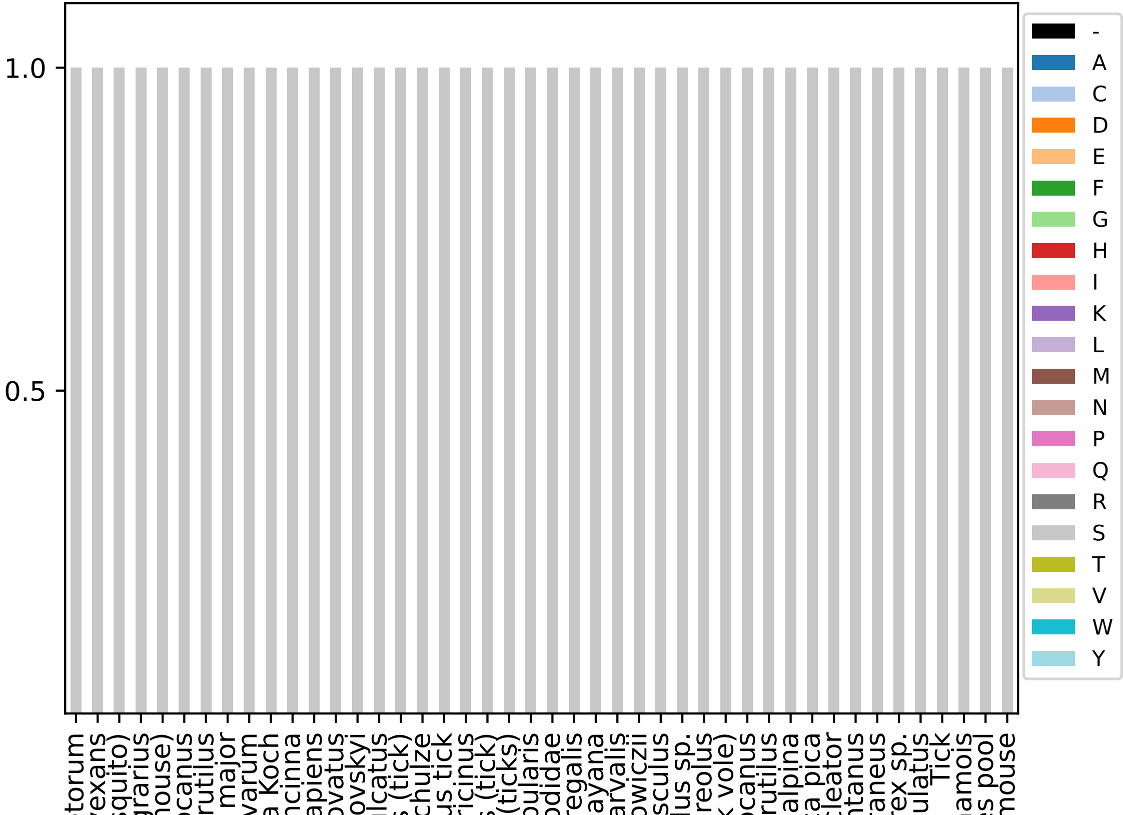

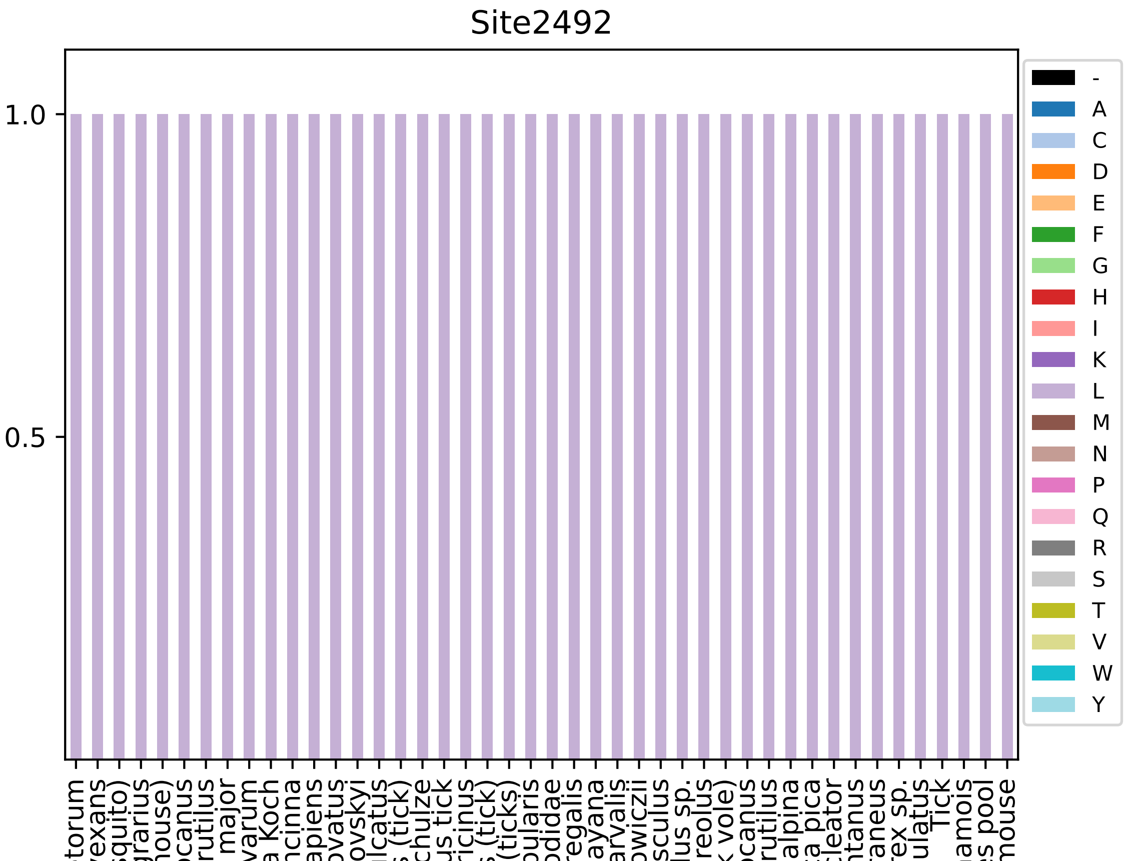
Site2492
1.0
0.5
-
A
C
D
E
F
G
H
I
K
L
M
N
P
Q
R
S
T
V
W
Y

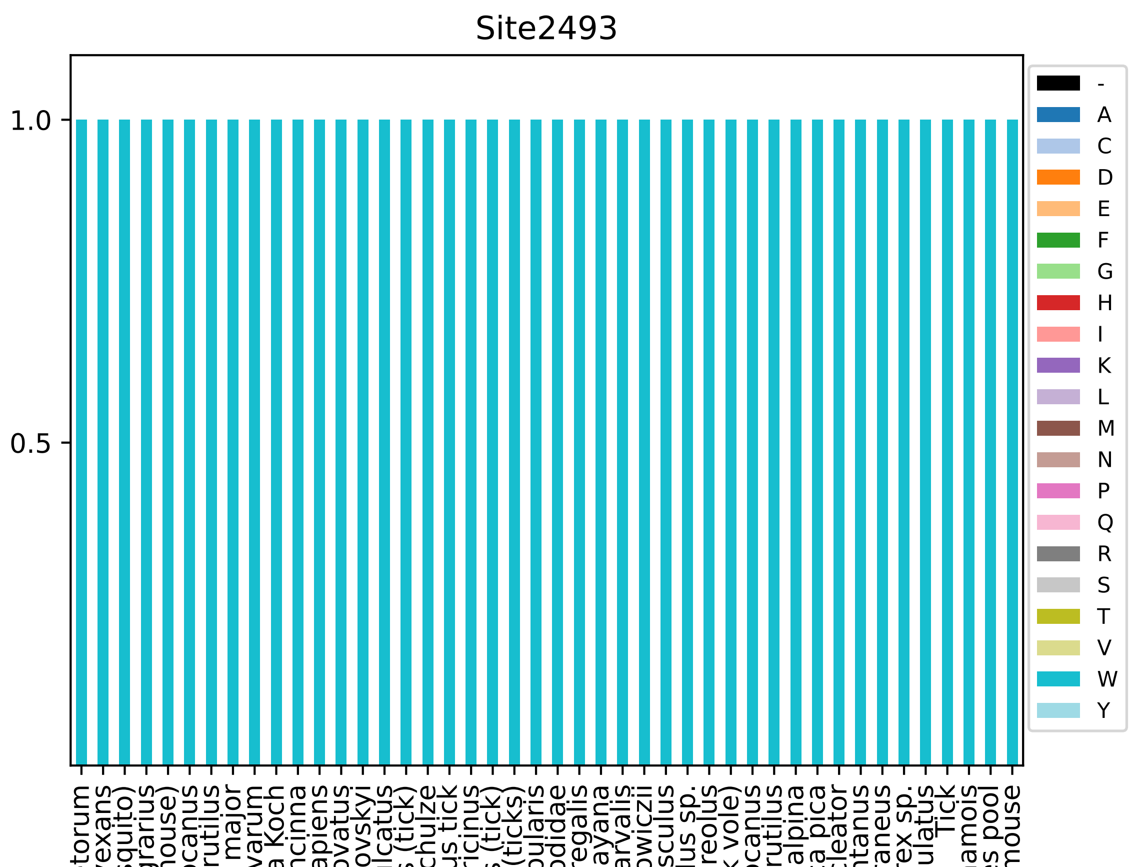

Site2493
1.0
0.5
-
A
C
D
E
F
G
H
I
K
L
M
N
P
Q
R
S
T
V
W
Y

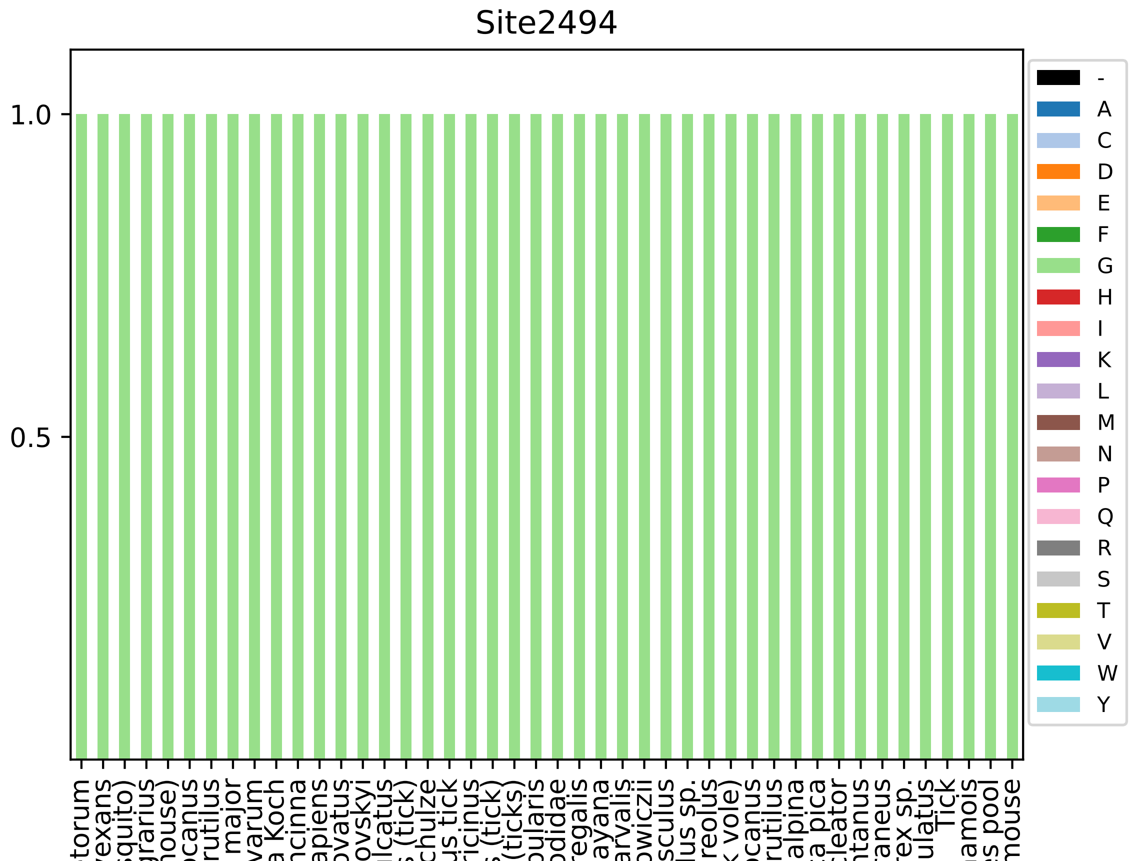
Site2494
1.0
0.5
-
A
C
D
E
F
G
H
I
K
L
M
N
P
Q
R
S
T
V
W
Y

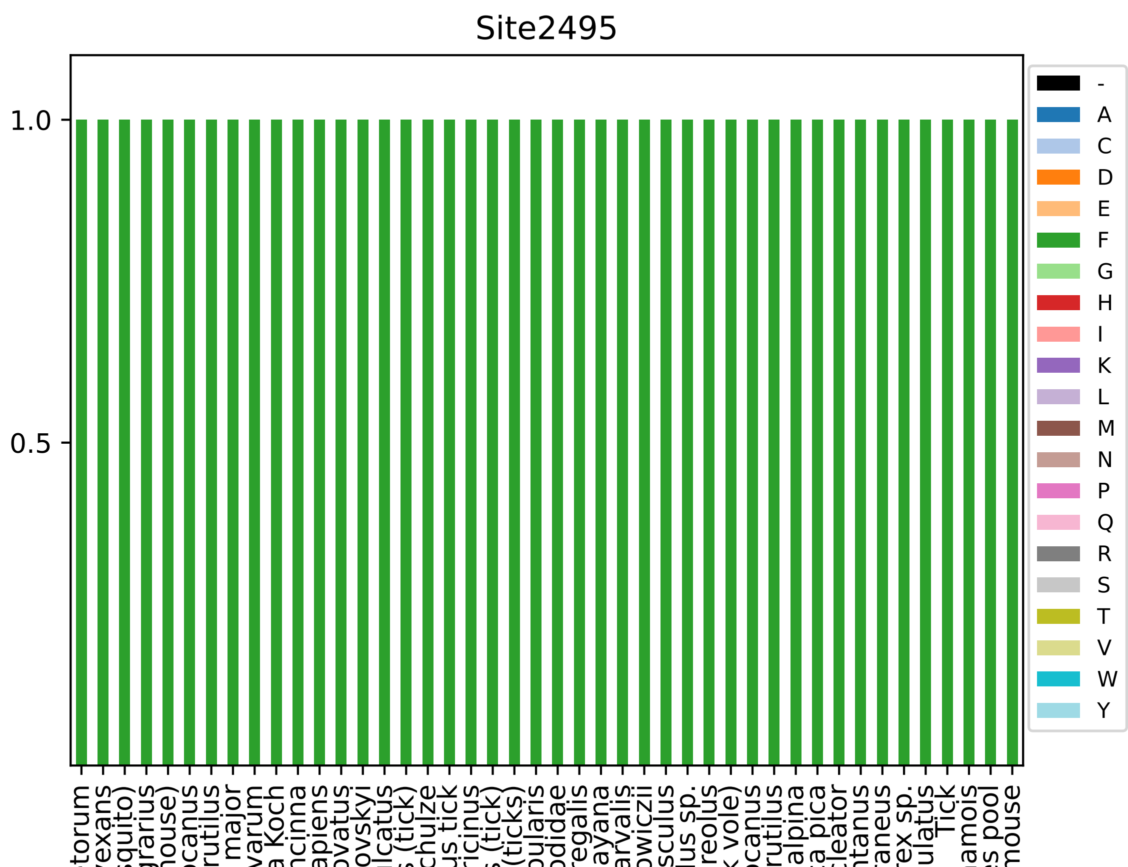
Site2495
1.0
0.5
-
A
C
D
E
F
G
H
I
K
L
M
N
P
Q
R
S
T
V
W
Y

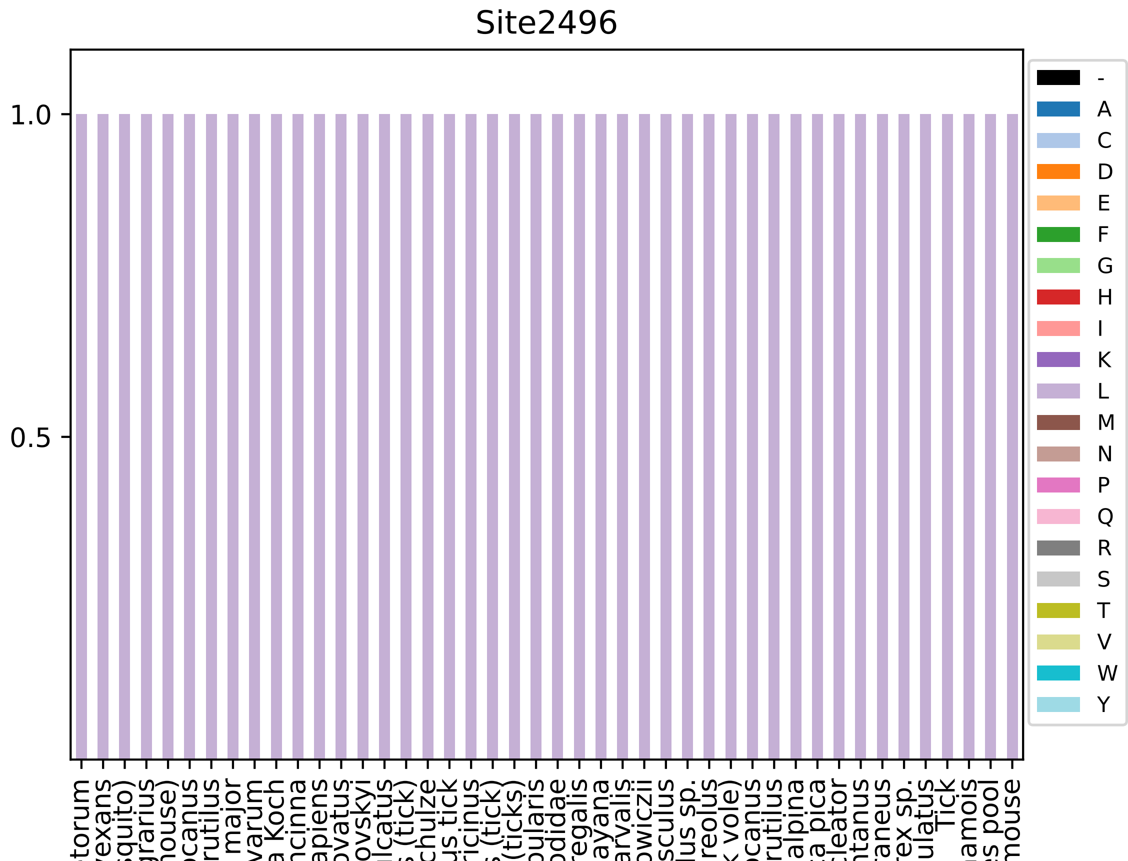

Site2496
1.0
0.5
-
A
C
D
E
F
G
H
I
K
L
M
N
P
Q
R
S
T
V
W
Y

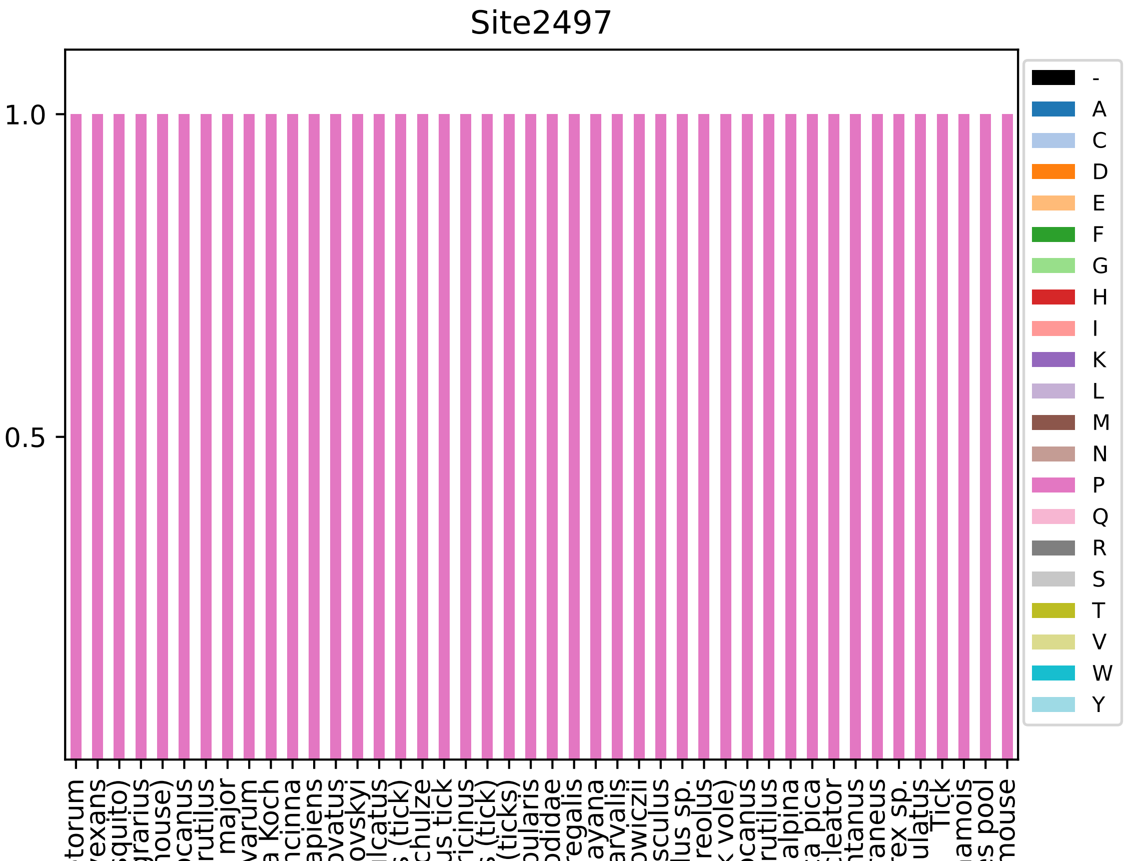

Site2497
1.0
0.5
-
A
C
D
E
F
G
H
I
K
L
M
N
P
Q
R
S
T
V
W
Y

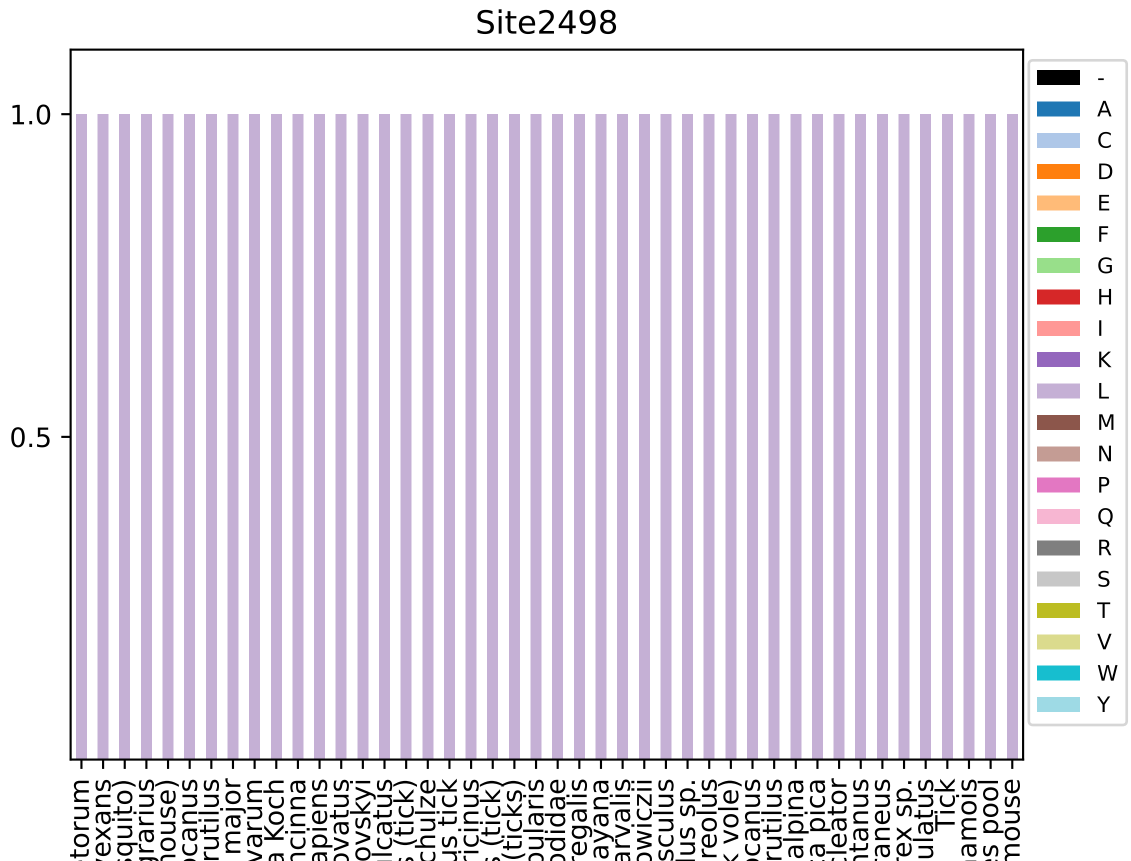
Site2498
1.0
0.5
-
A
C
D
E
F
G
H
I
K
L
M
N
P
Q
R
S
T
V
W
Y

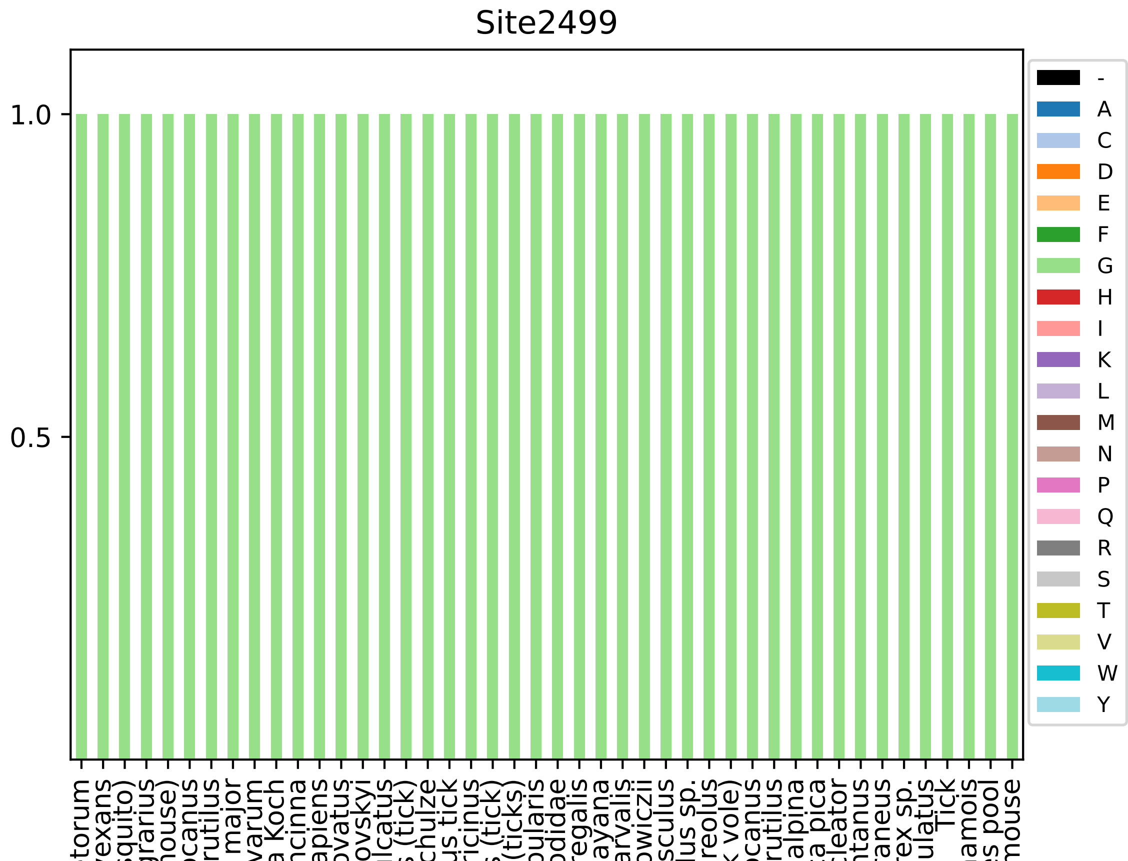
Site2499
1.0
0.5
-
A
C
D
E
F
G
H
I
K
L
M
N
P
Q
R
S
T
V
W
Y

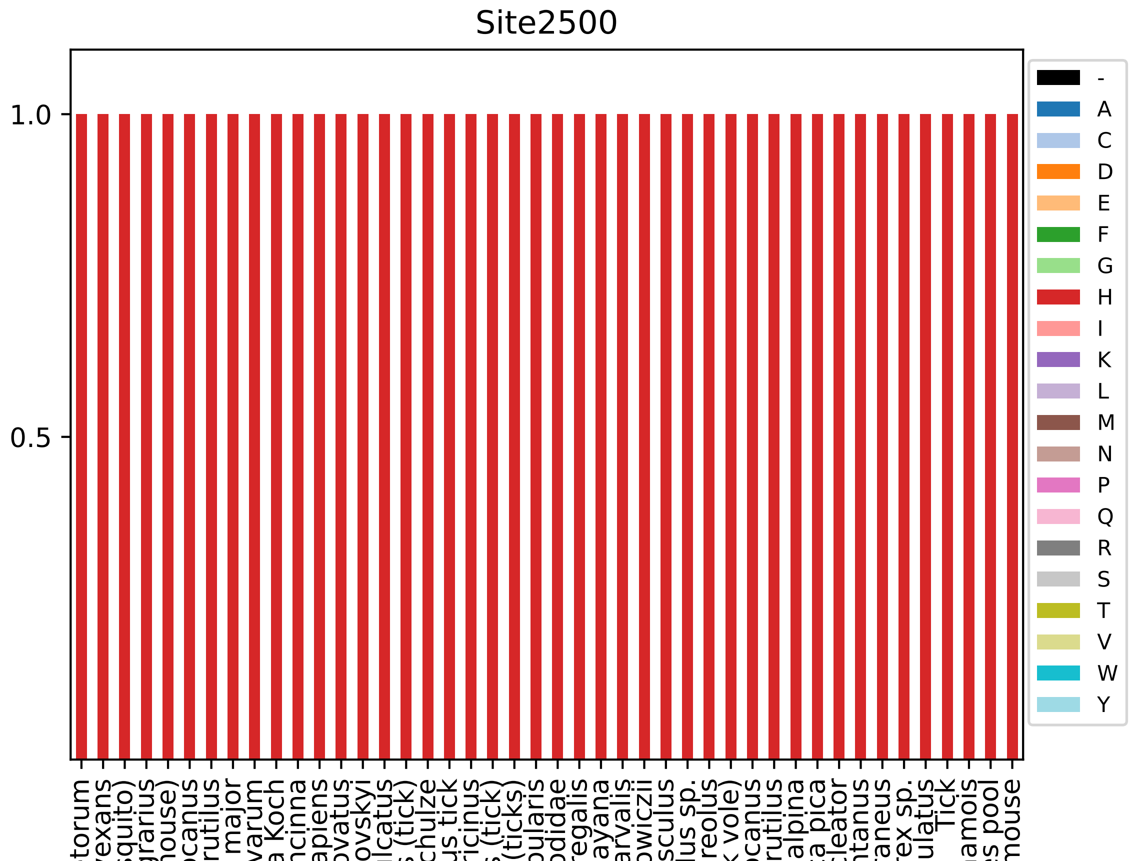

Site2500
1.0
0.5
-
A
C
D
E
F
G
H
I
K
L
M
N
P
Q
R
S
T
V
W
Y

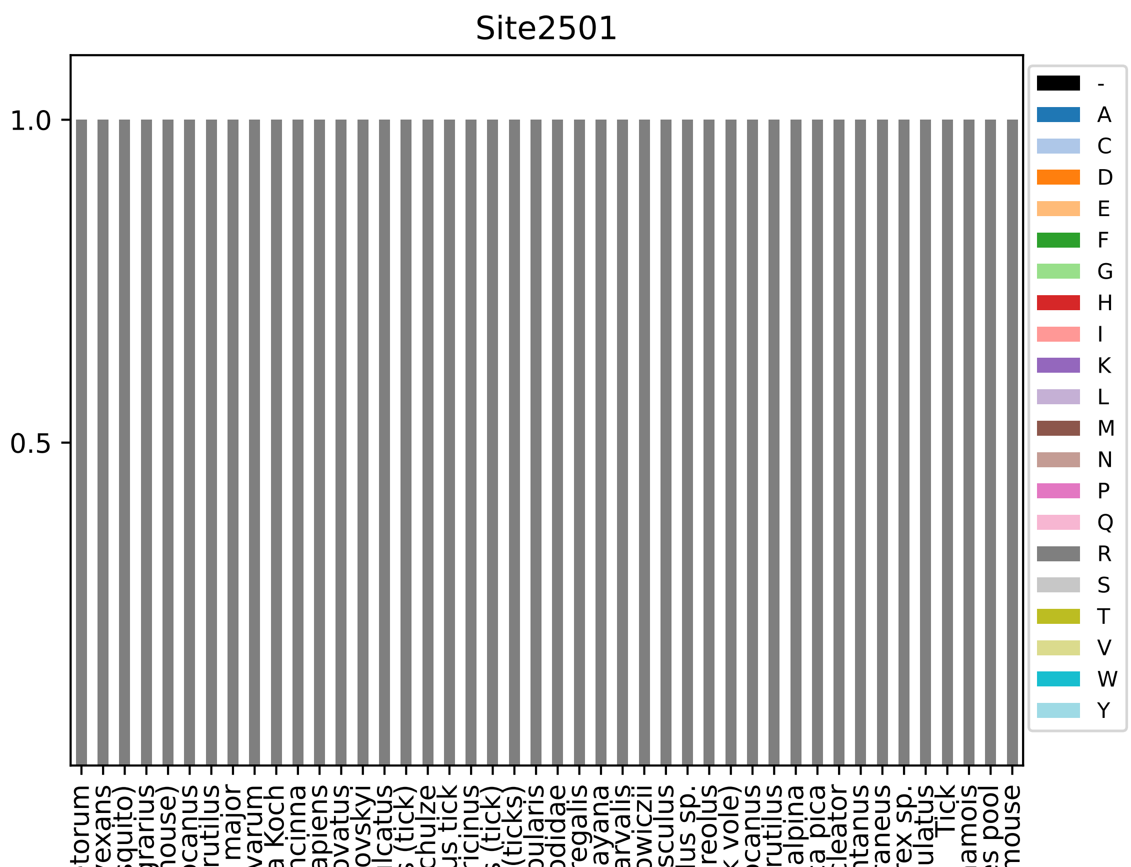

Site2501
1.0
0.5
-
A
C
D
E
F
G
H
I
K
L
M
N
P
Q
R
S
T
V
W
Y

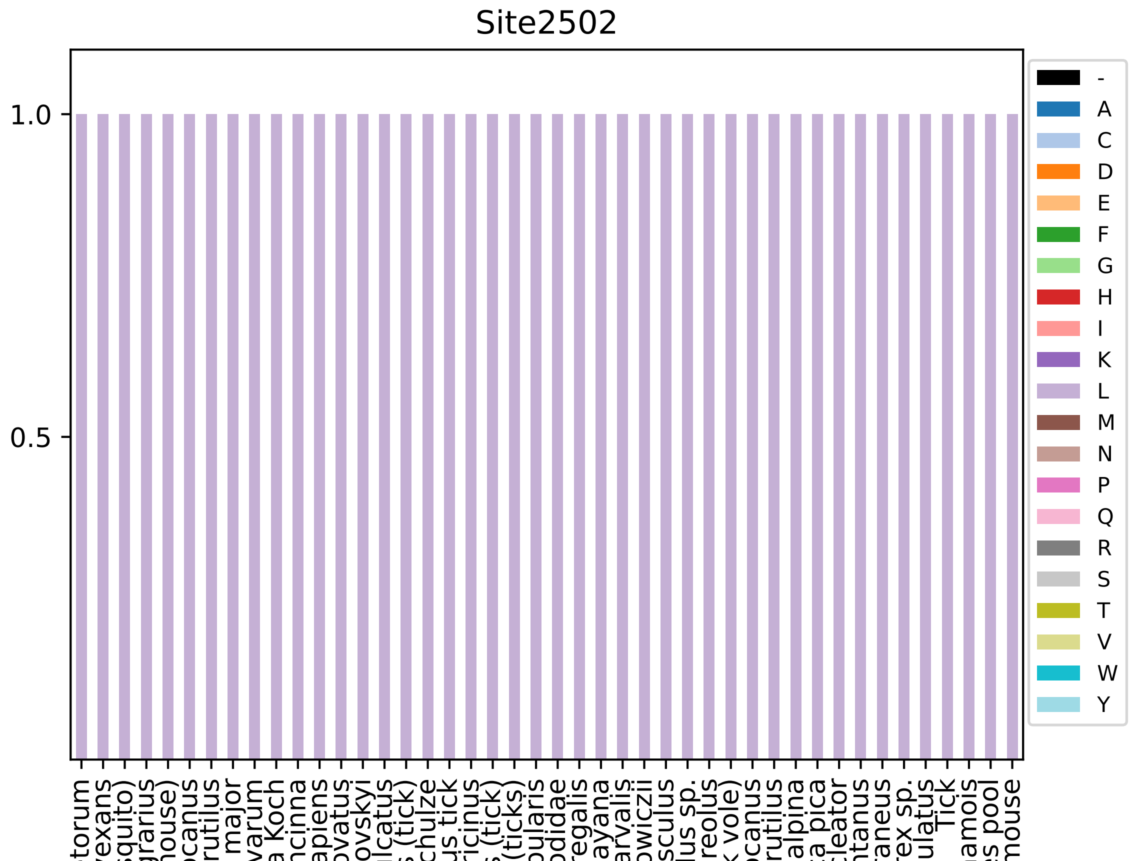
Site2502
1.0
0.5
-
A
C
D
E
F
G
H
I
K
L
M
N
P
Q
R
S
T
V
W
Y

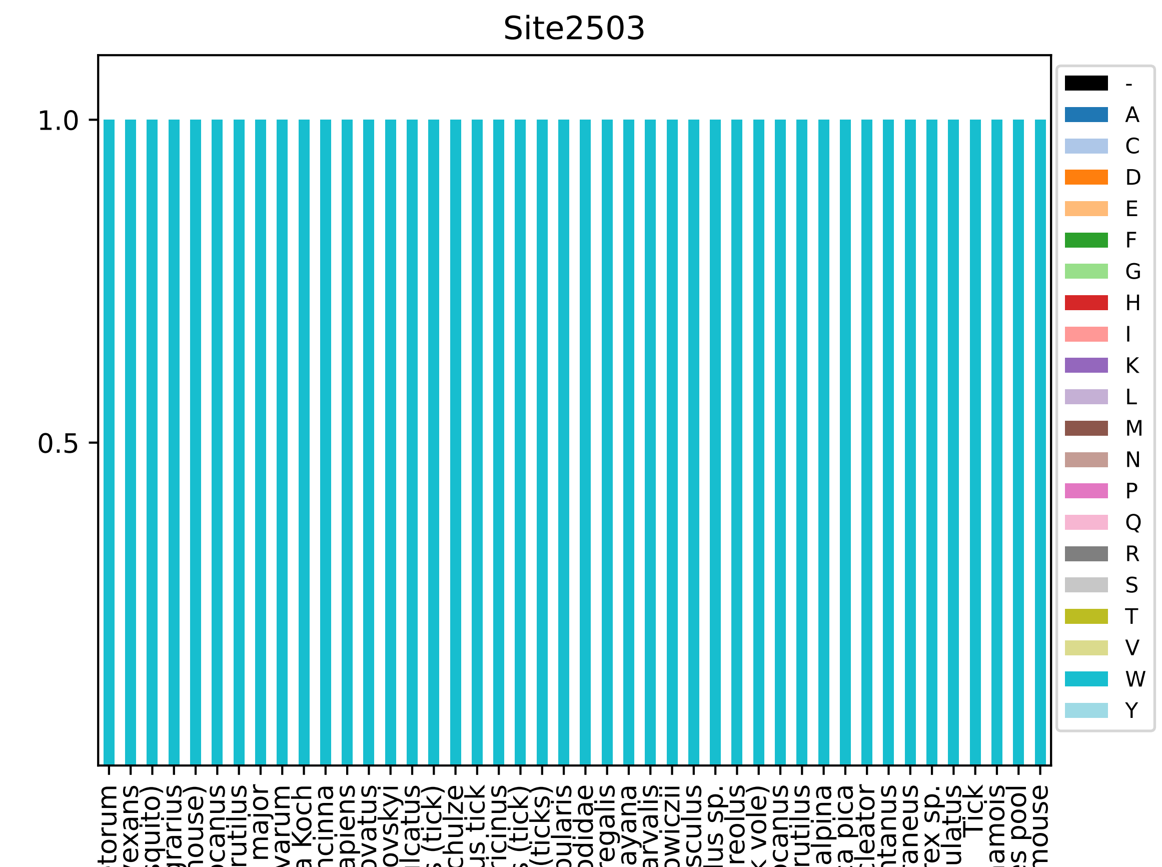
Site2503
1.0
0.5
-
A
C
D
E
F
G
H
I
K
L
M
N
P
Q
R
S
T
V
W
Y

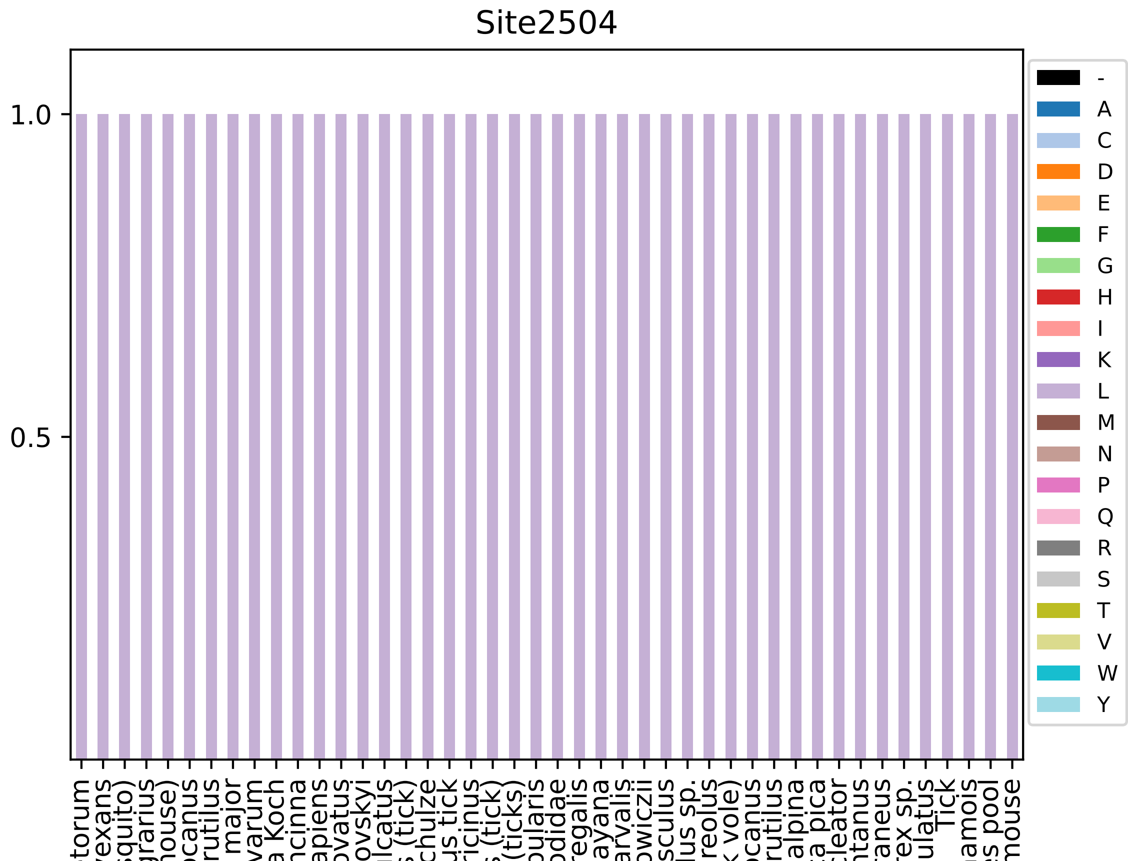

Site2504
1.0
0.5
-
A
C
D
E
F
G
H
I
K
L
M
N
P
Q
R
S
T
V
W
Y

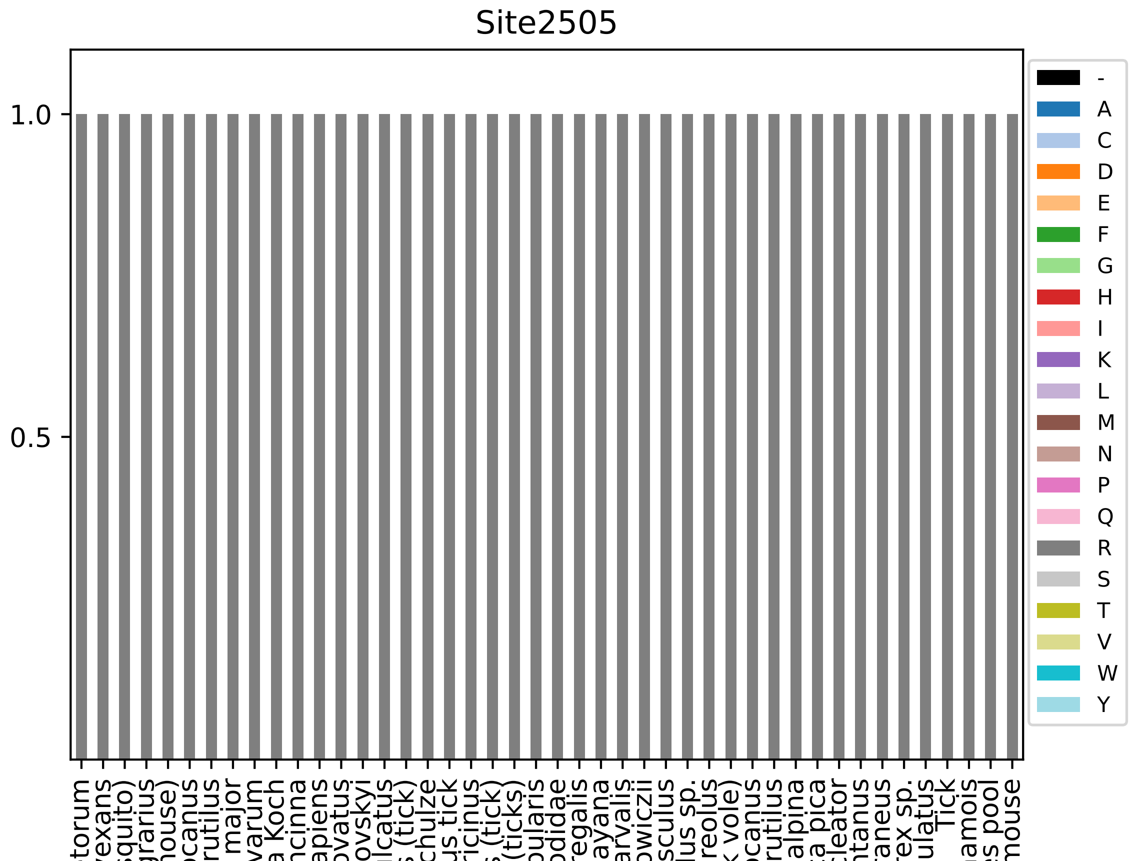
Site2505
1.0
0.5
-
A
C
D
E
F
G
H
I
K
L
M
N
P
Q
R
S
T
V
W
Y

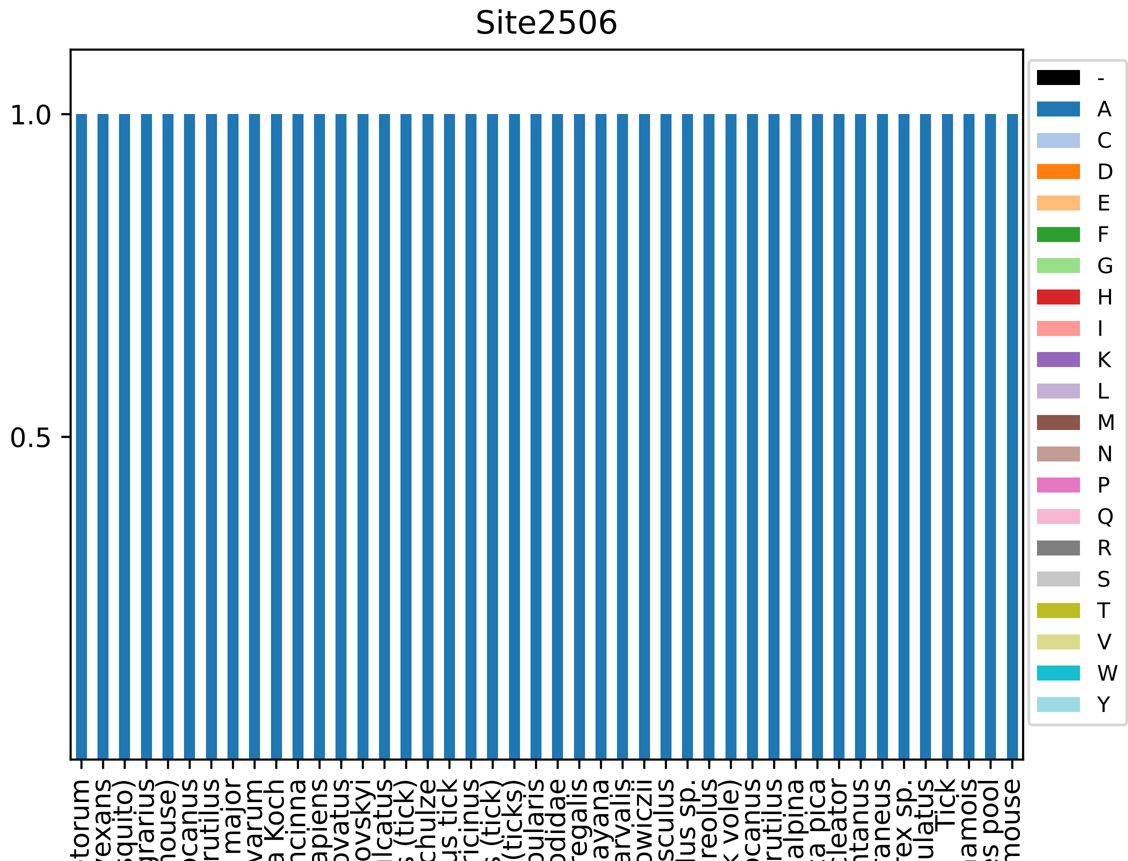
Site2506
1.0
0.5
-
A
C
D
E
F
G
H
I
K
L
M
N
P
Q
R
S
T
V
W
Y

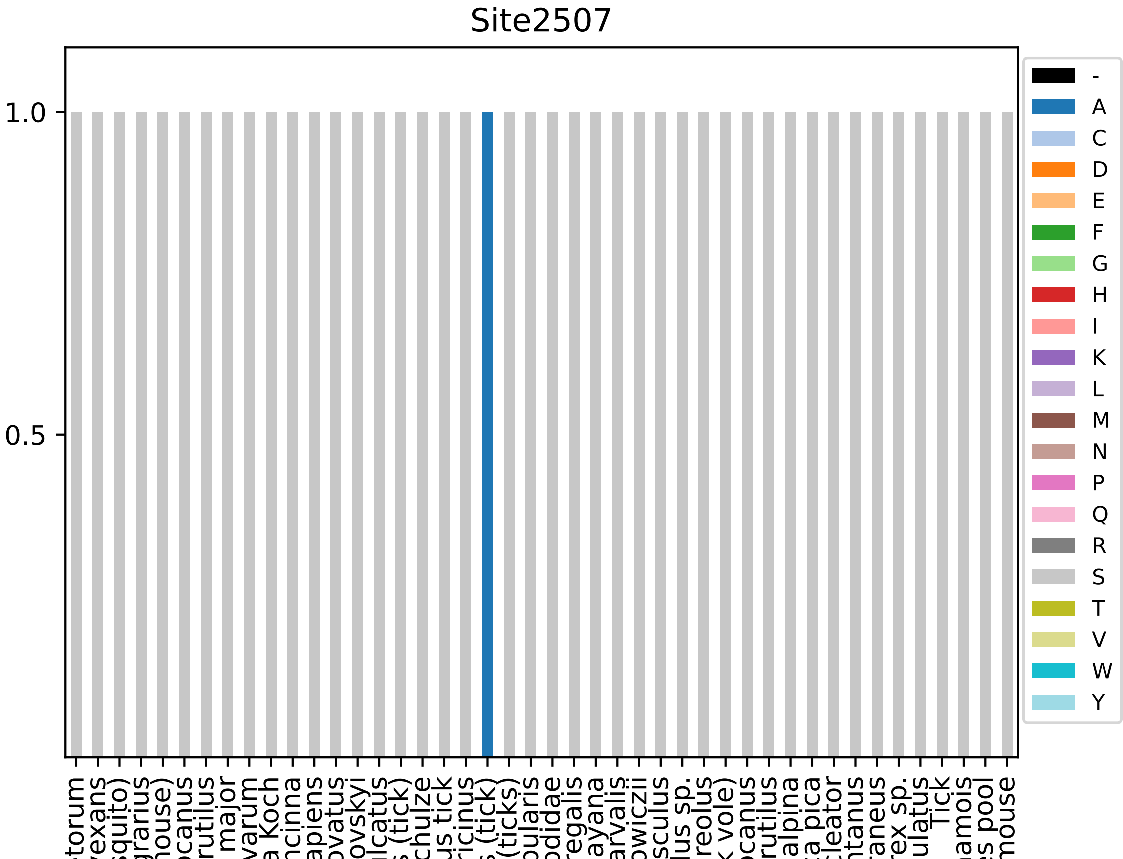

Site2507
1.0
0.5
-
A
C
D
E
F
G
H
I
K
L
M
N
P
Q
R
S
T
V
W
Y

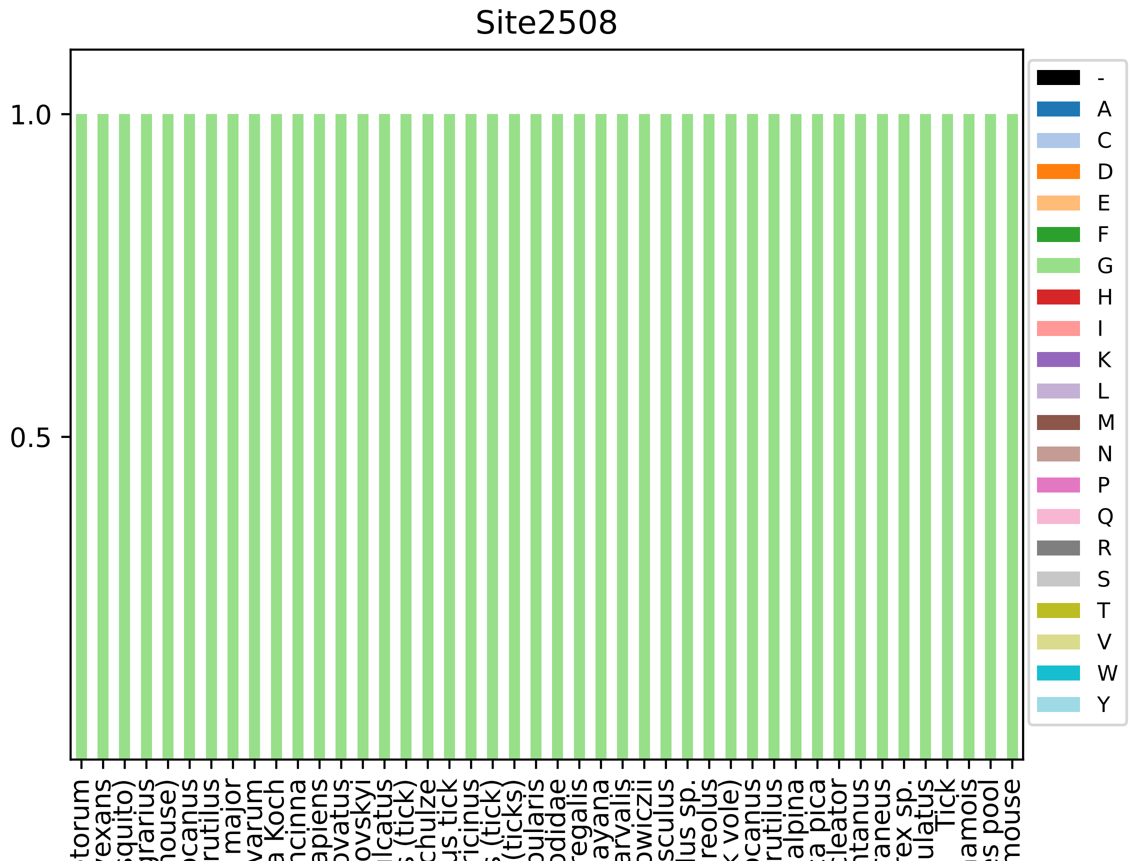
Site2508
1.0
0.5
-
A
C
D
E
F
G
H
I
K
L
M
N
P
Q
R
S
T
V
W
Y

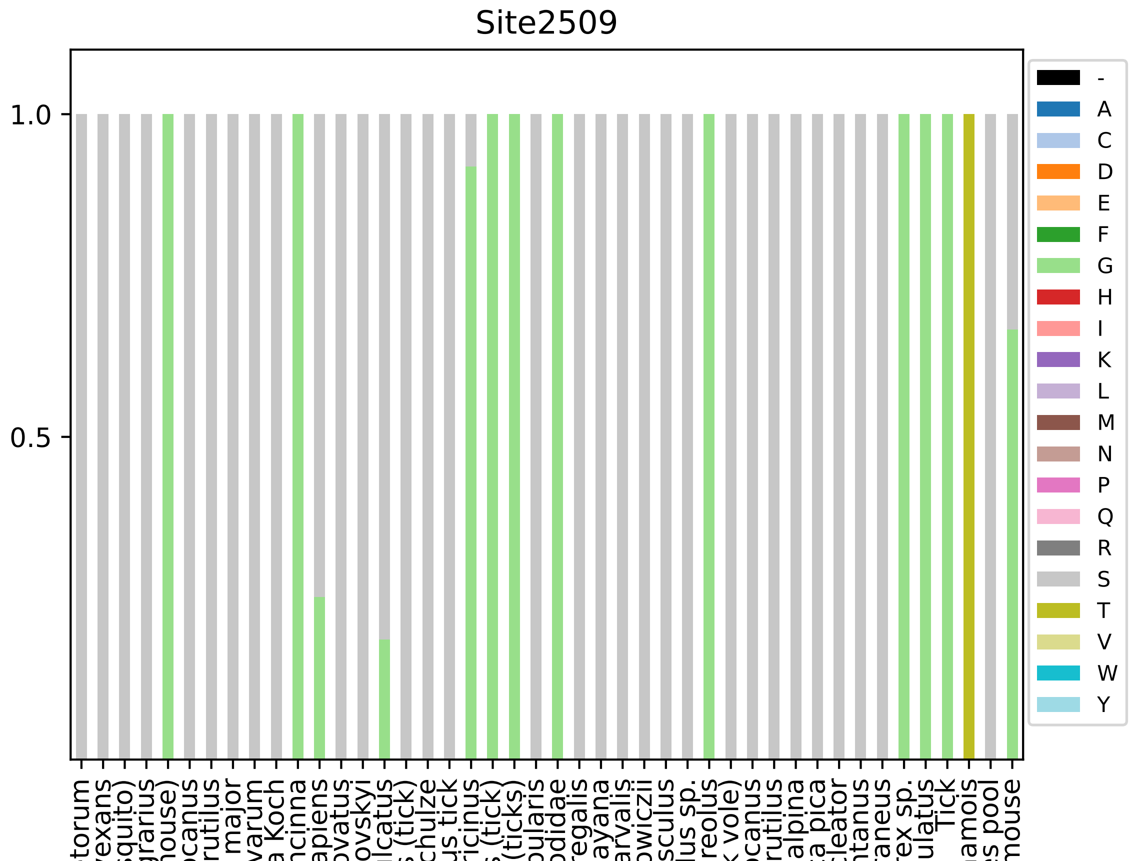
Site2509
1.0
0.5
-
A
C
D
E
F
G
H
I
K
L
M
N
P
Q
R
S
T
V
W
Y

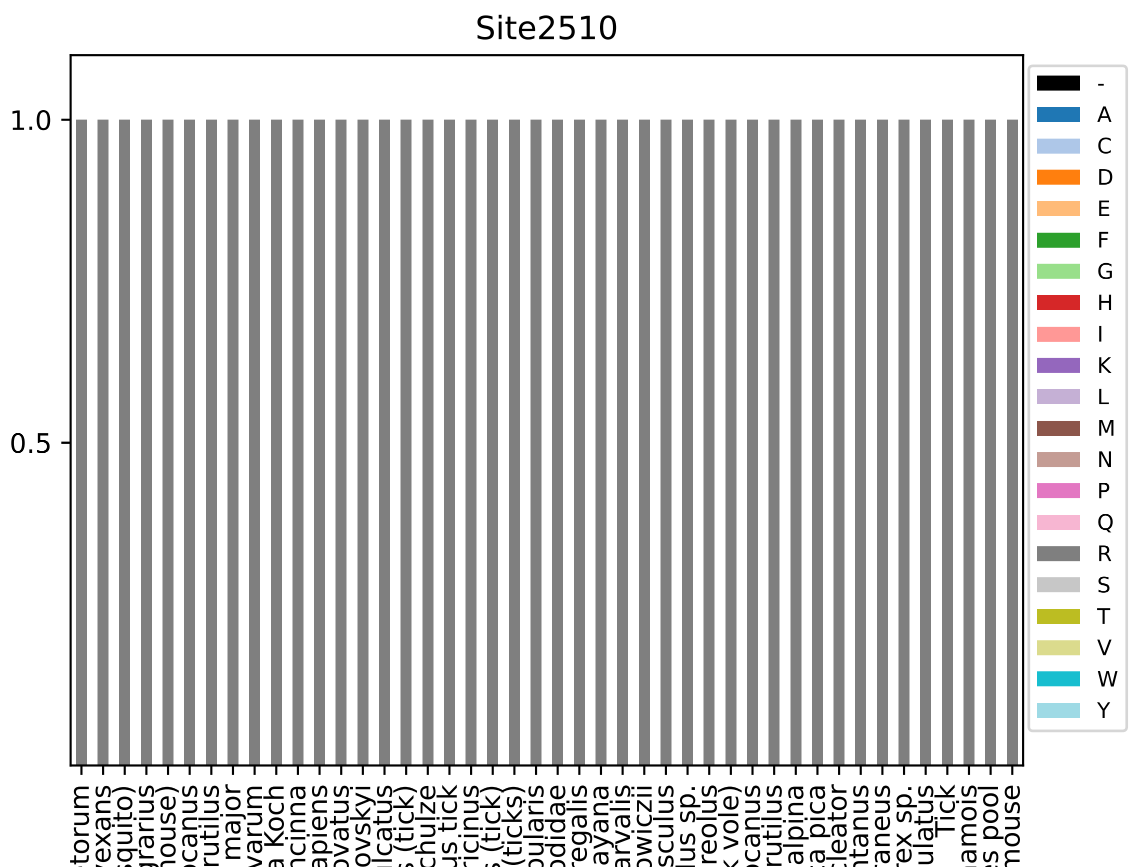
Site2510
1.0
0.5
-
A
C
D
E
F
G
H
I
K
L
M
N
P
Q
R
S
T
V
W
Y
Tick

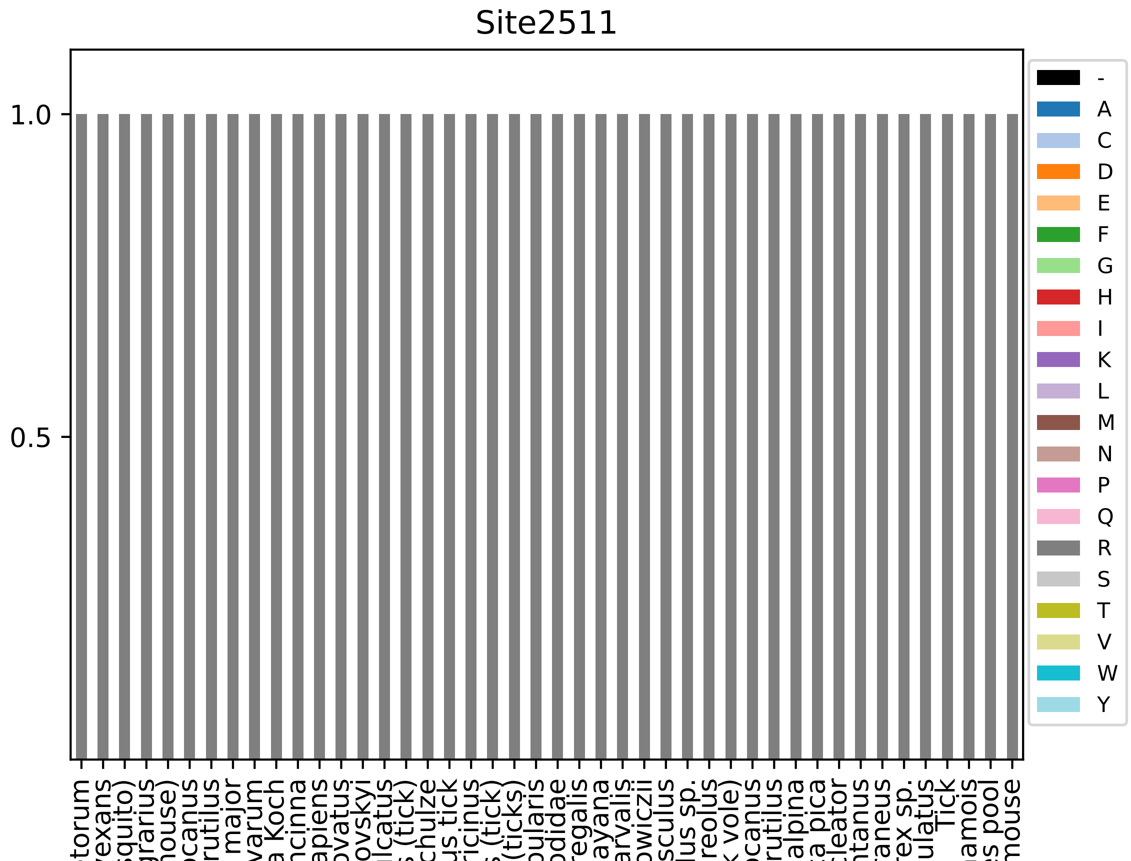
Site2511
1.0
0.5
-
A
C
D
E
F
G
H
I
K
L
M
N
P
Q
R
S
T
V
W
Y
Tick

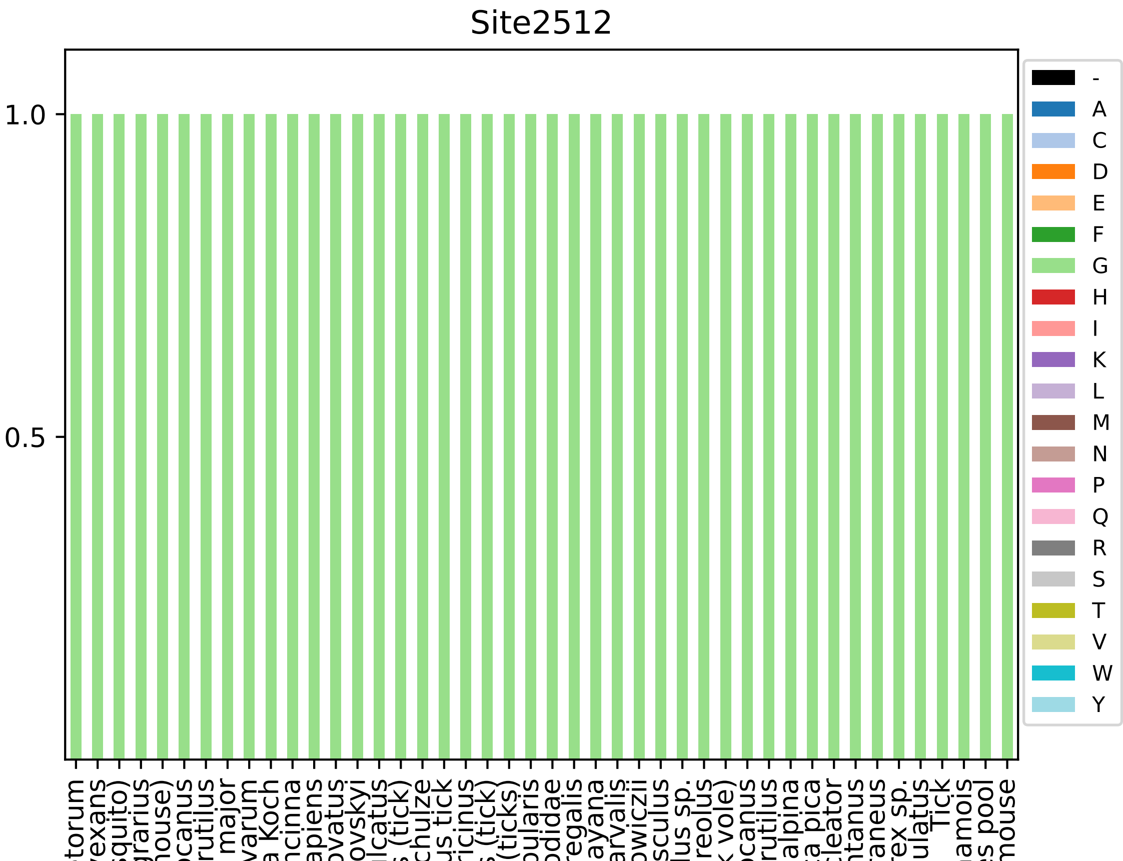
Site2512
1.0
0.5
-
A
C
D
E
F
G
H
I
K
L
M
N
P
Q
R
S
T
V
W
Y

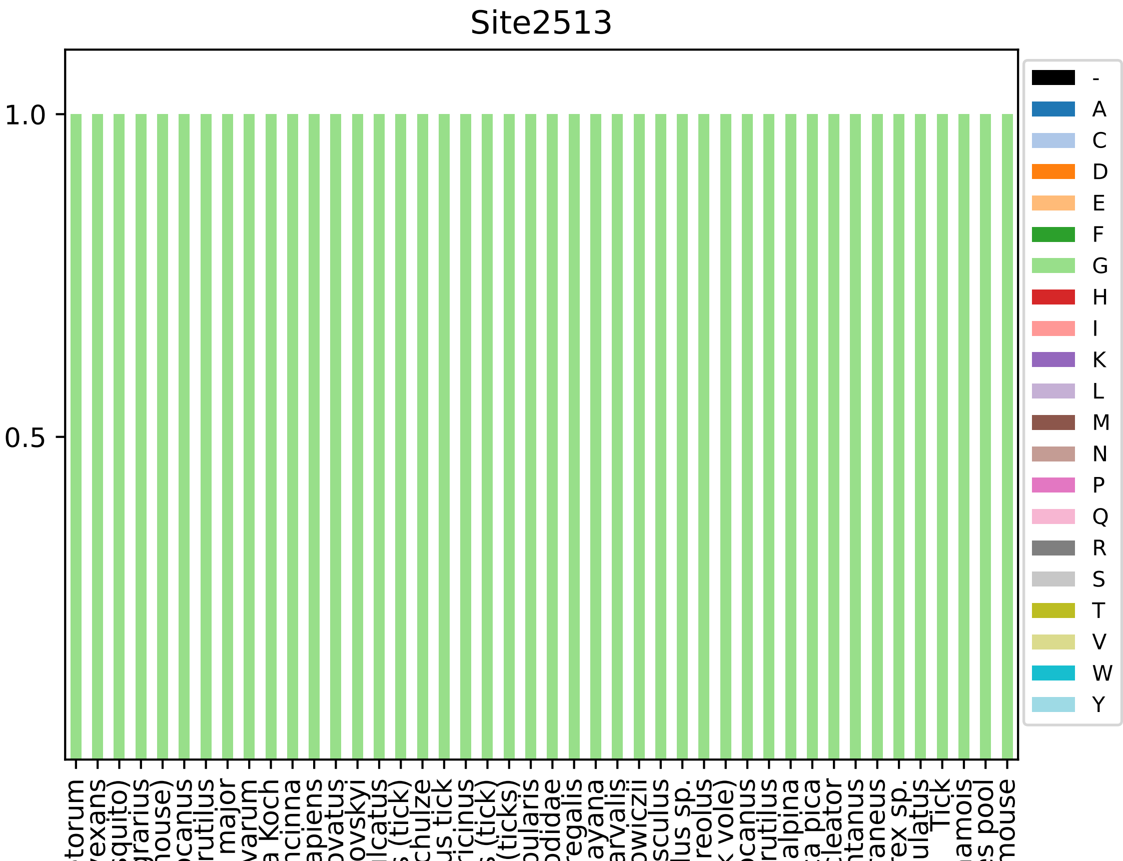
Site2513
1.0
0.5
-
A
C
D
E
F
G
H
I
K
L
M
N
P
Q
R
S
T
V
W
Y

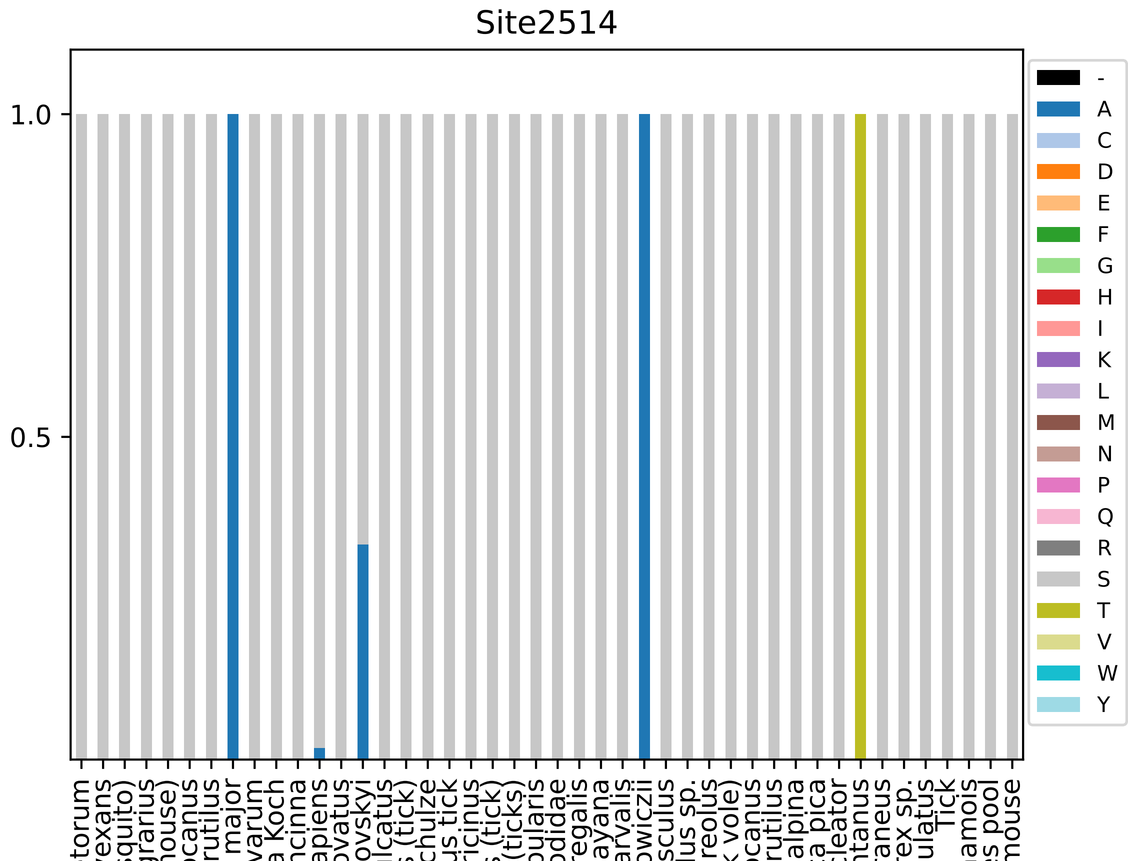
Site2514
1.0
0.5
-
A
C
D
E
F
G
H
I
K
L
M
N
P
Q
R
S
T
V
W
Y

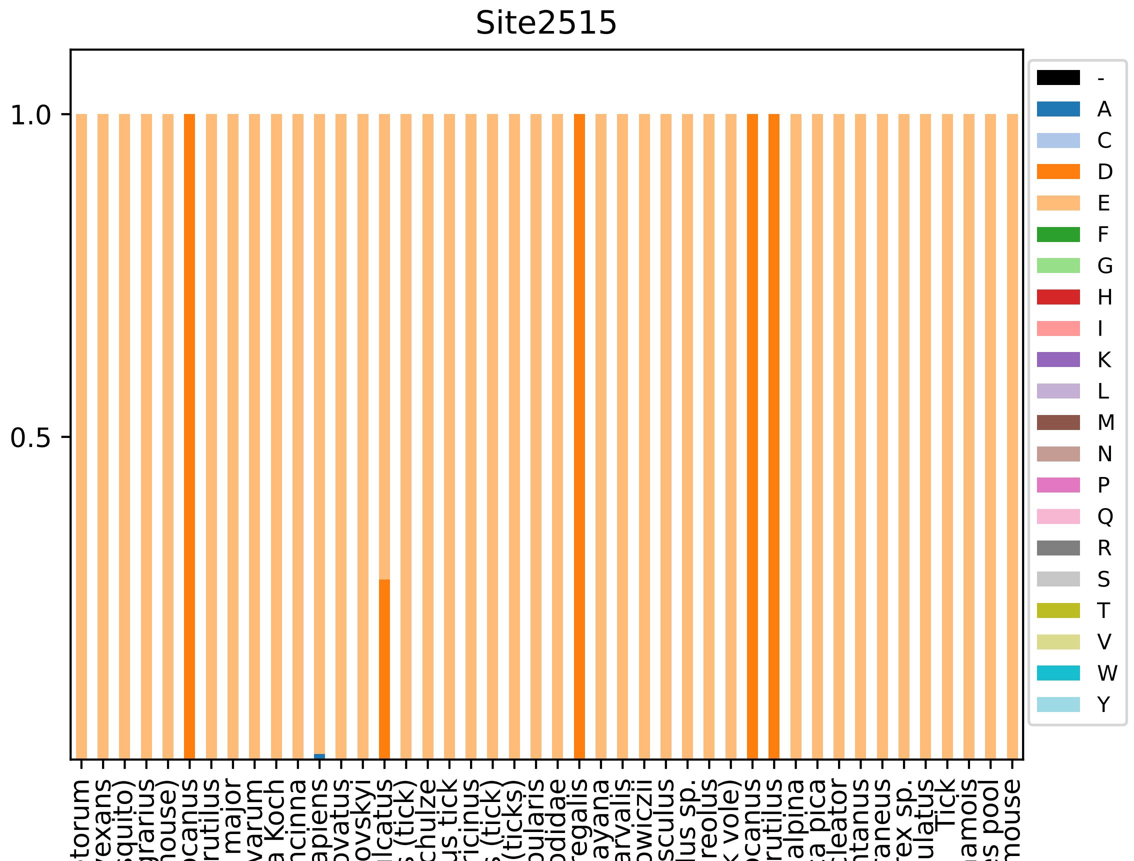
Site2515
1.0
0.5
-
A
C
D
E
F
G
H
I
K
L
M
N
P
Q
R
S
T
V
W
Y

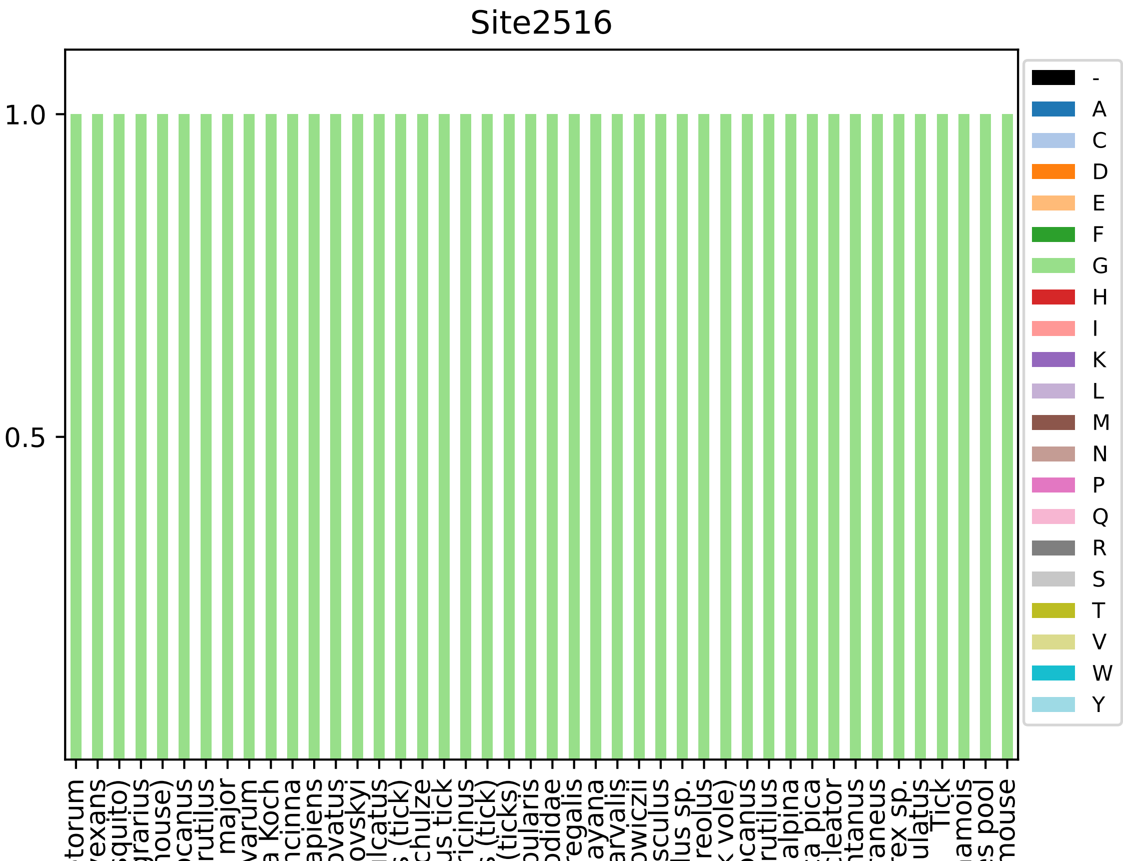

Site2516
1.0
0.5
-
A
C
D
E
F
G
H
I
K
L
M
N
P
Q
R
S
T
V
W
Y

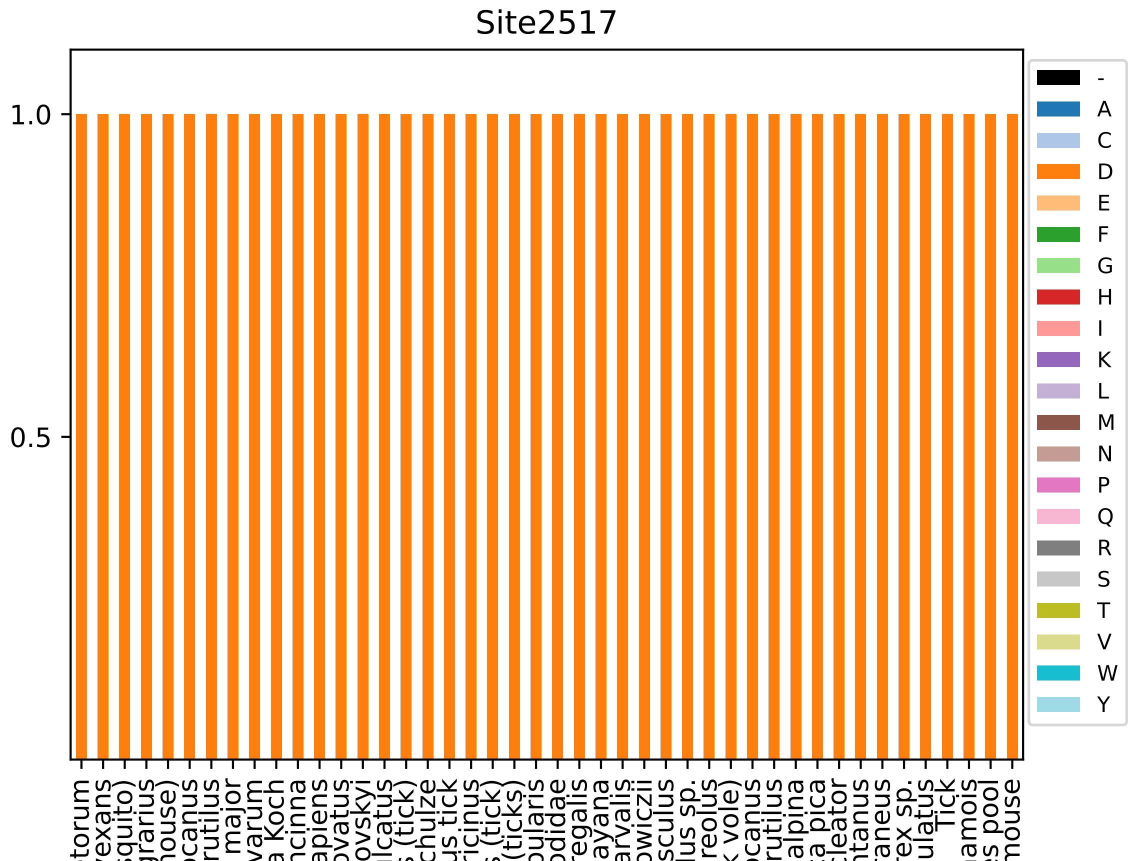
Site2517
1.0
0.5
-
A
C
D
E
F
G
H
I
K
L
M
N
P
Q
R
S
T
V
W
Y

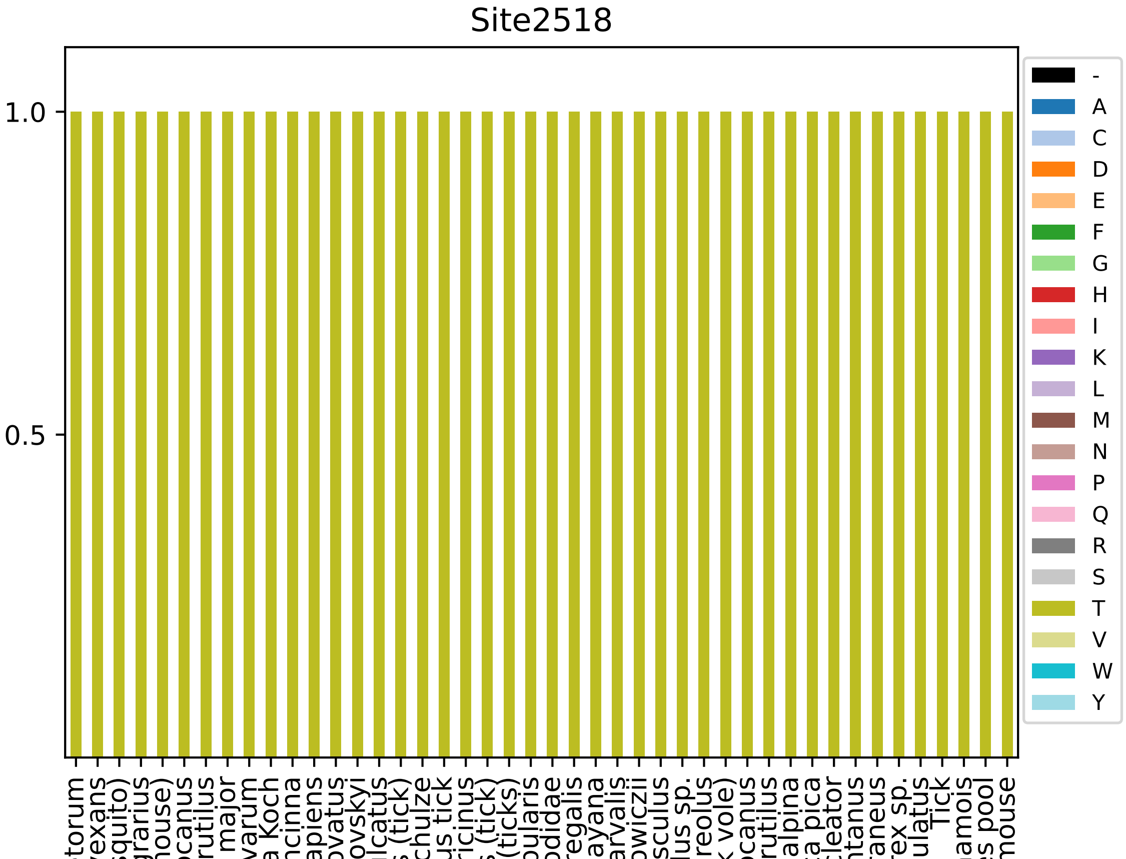
Site2518
1.0
0.5
-
A
C
D
E
F
G
H
I
K
L
M
N
P
Q
R
S
T
V
W
Y

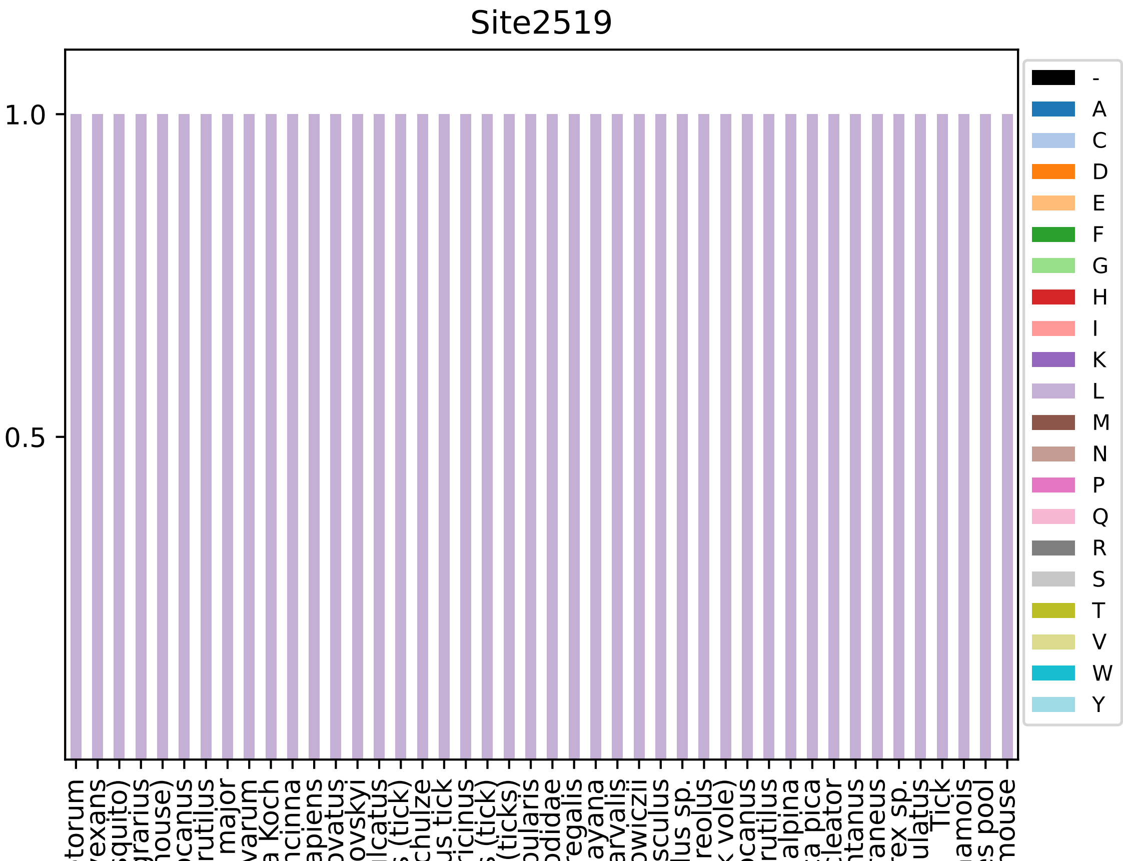
Site2519
1.0
0.5
-
A
C
D
E
F
G
H
I
K
L
M
N
P
Q
R
S
T
V
W
Y

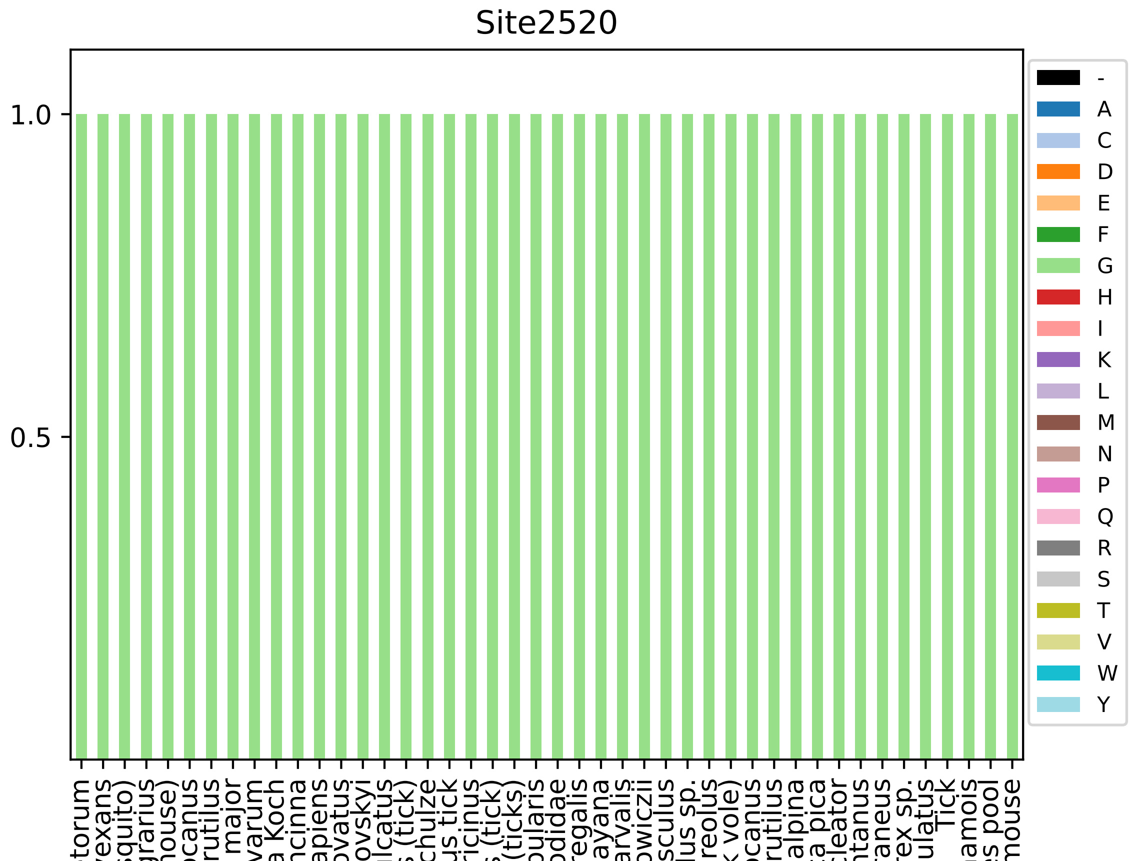
Site2520
1.0
0.5
-
A
C
D
E
F
G
H
I
K
L
M
N
P
Q
R
S
T
V
W
Y

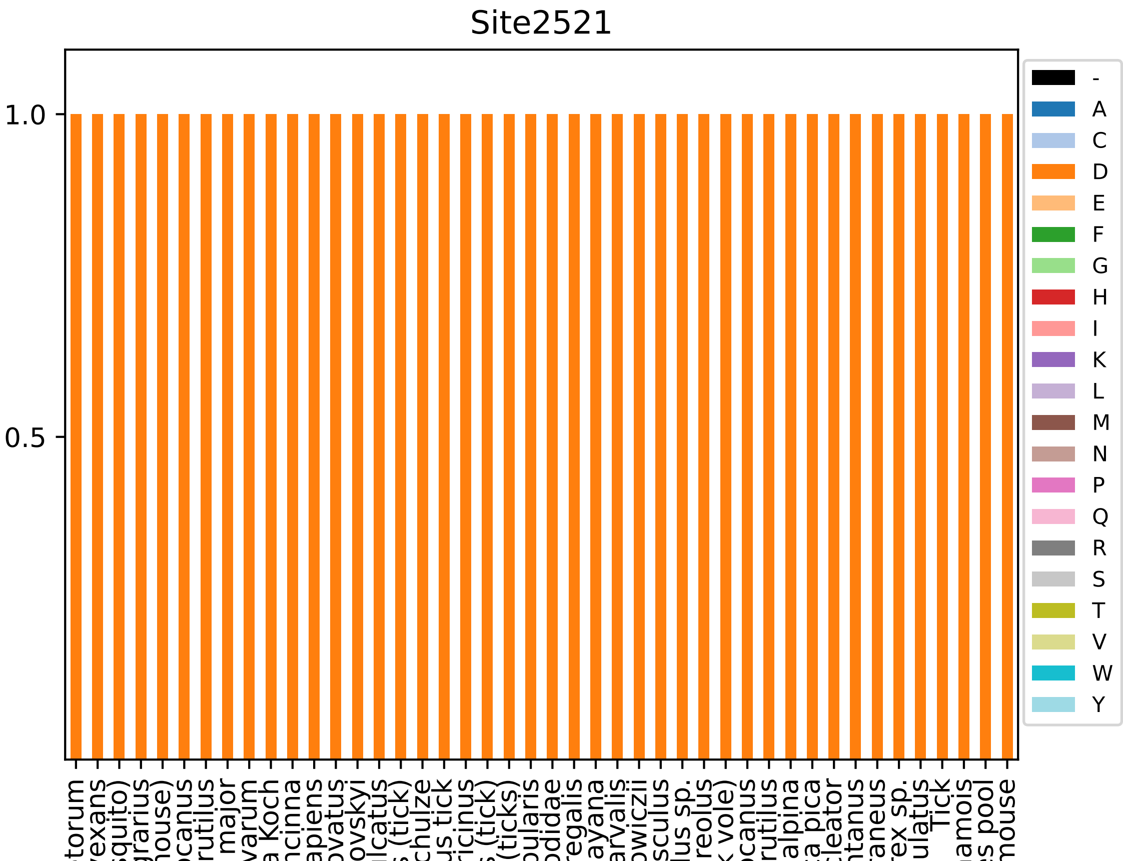
Site2521
1.0
0.5
-
A
C
D
E
F
G
H
I
K
L
M
N
P
Q
R
S
T
V
W
Y

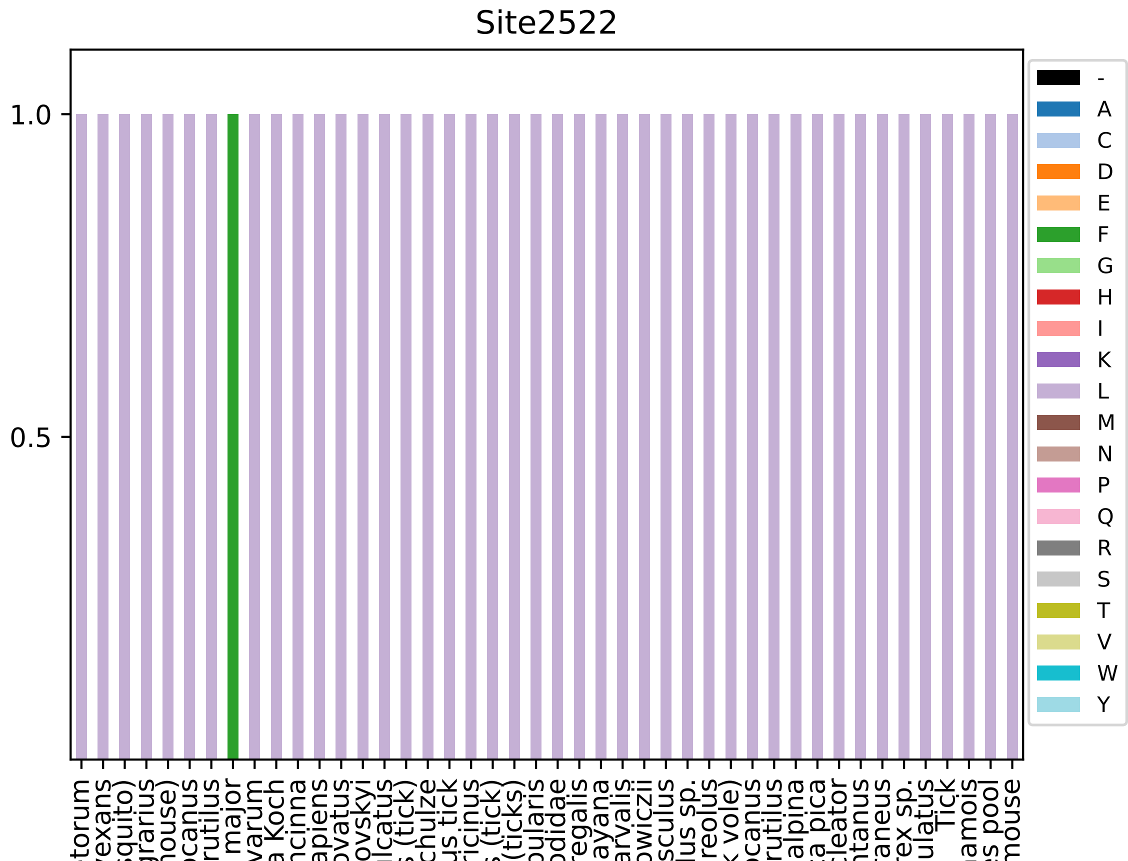

Site2522
1.0
0.5
-
A
C
D
E
F
G
H
I
K
L
M
N
P
Q
R
S
T
V
W
Y

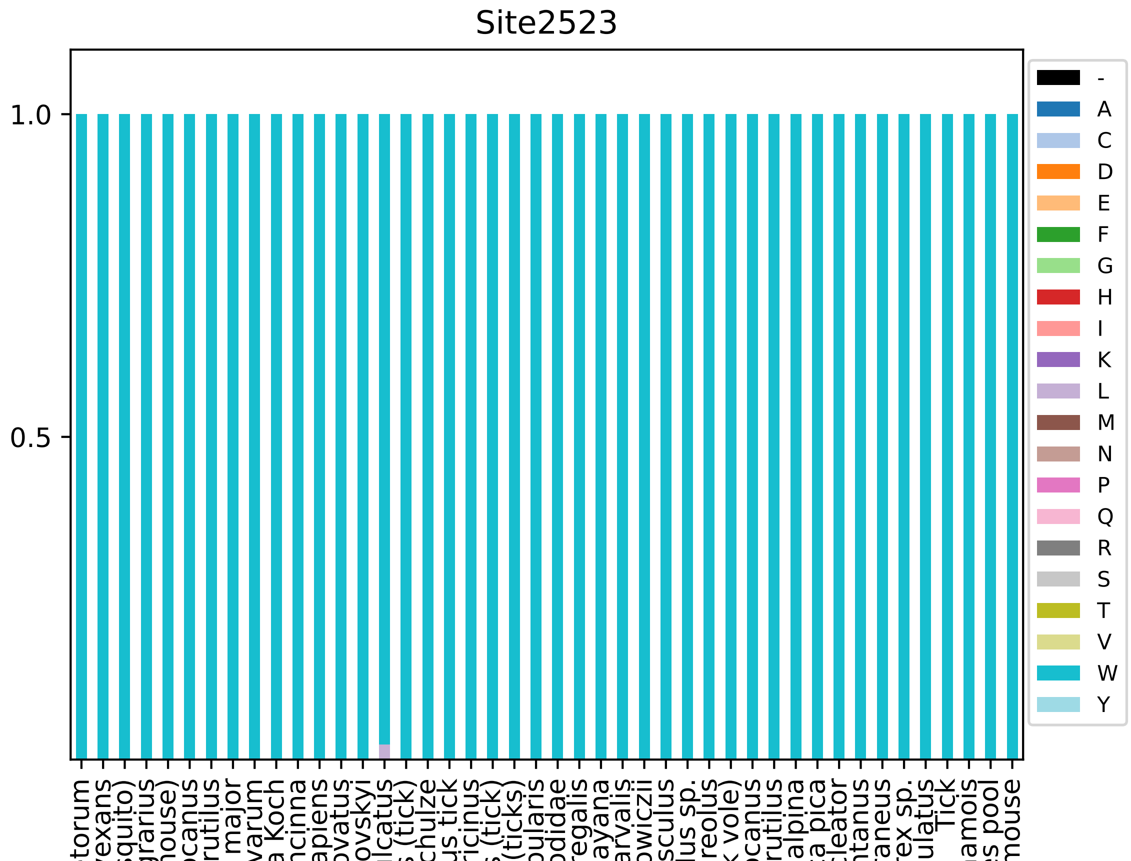
Site2523
1.0
0.5
-
A
C
D
E
F
G
H
I
K
L
M
N
P
Q
R
S
T
V
W
Y

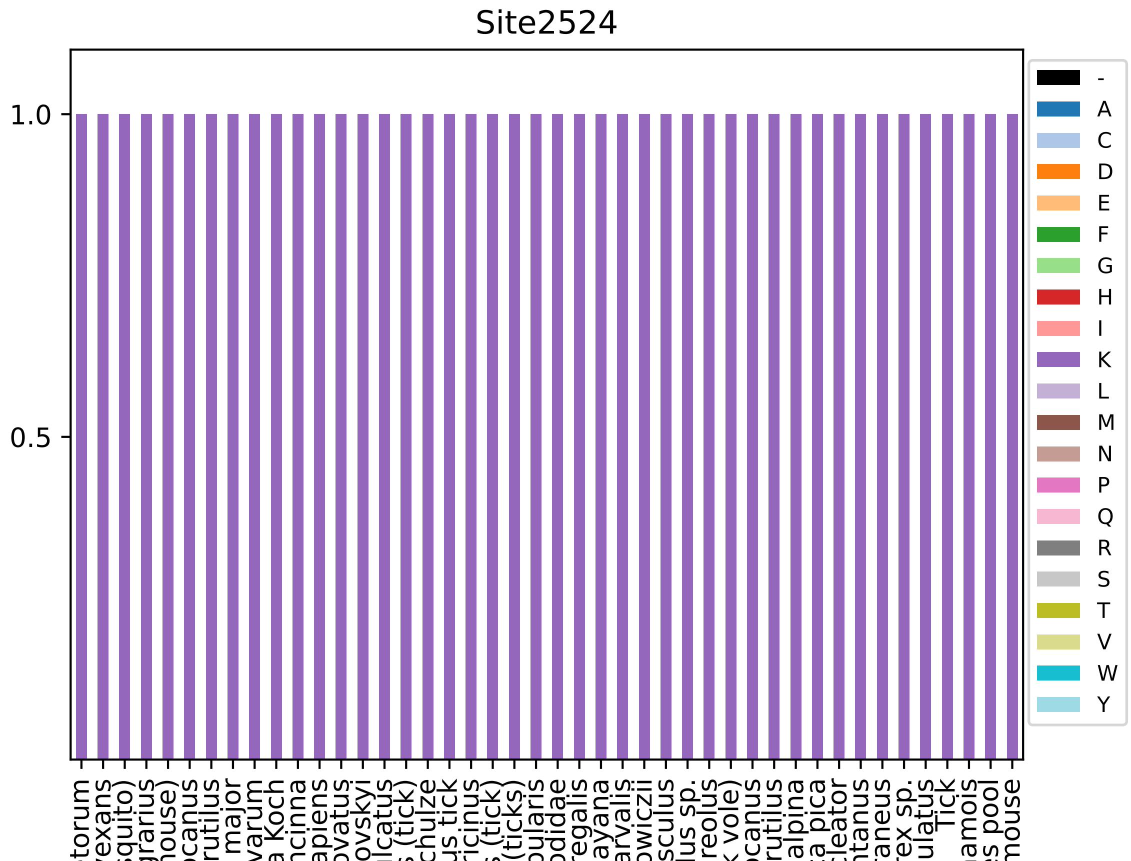

Site2524
1.0
0.5
-
A
C
D
E
F
G
H
I
K
L
M
N
P
Q
R
S
T
V
W
Y

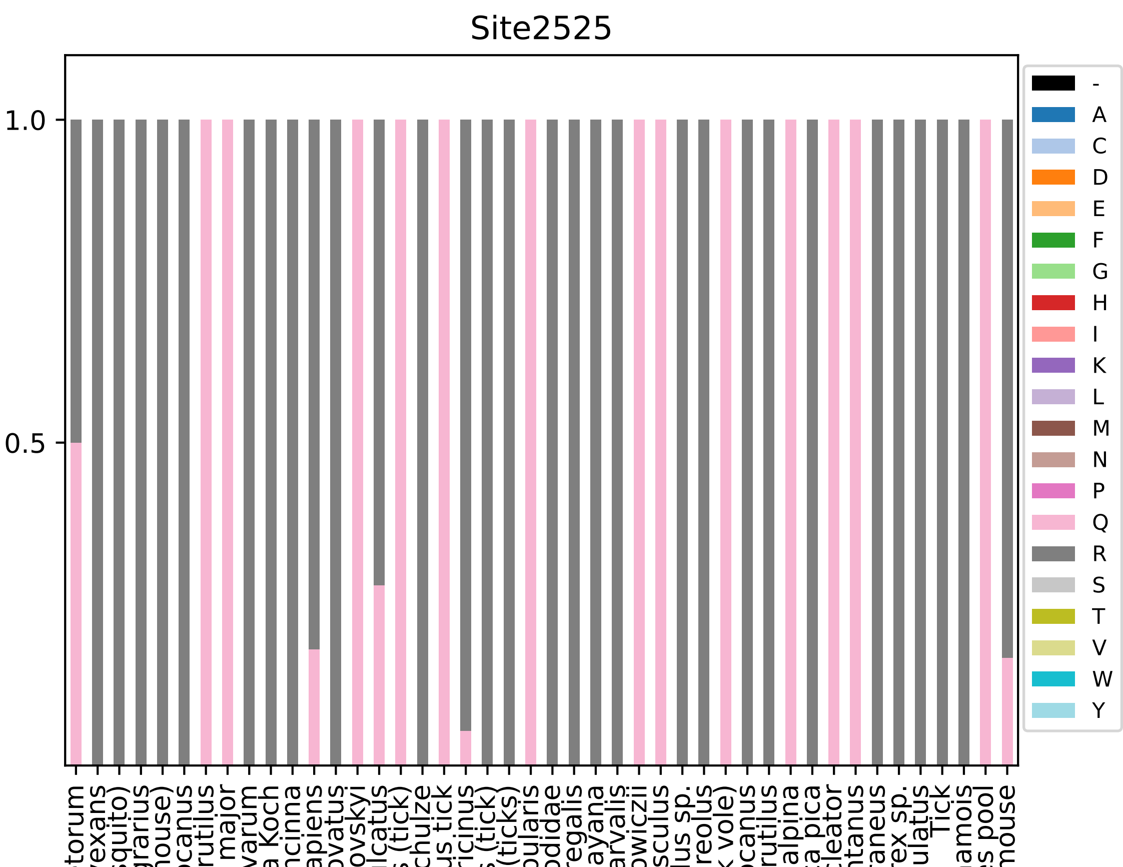

Site2525
1.0
0.5
-
A
C
D
E
F
G
H
I
K
L
M
N
P
Q
R
S
T
V
W
Y
torum
vexans
squito)
grarius
nouse)
ocanus
rutilus
major
varum
a Koch
ncinna
apiens
ovatus
ovskyi
ulcatus
s (tick)
chulze
us tick
ricinus
s (ticks)
ularis
podidae
regalis
ayana
arvalis
owiczii
sculus
lus sp.
reolus
k vole)
ocanus
rutilus
alpina
a pica
cleator
ntanus
raneus
rex sp.
ulatus
Tick
amois
es pool
mouse

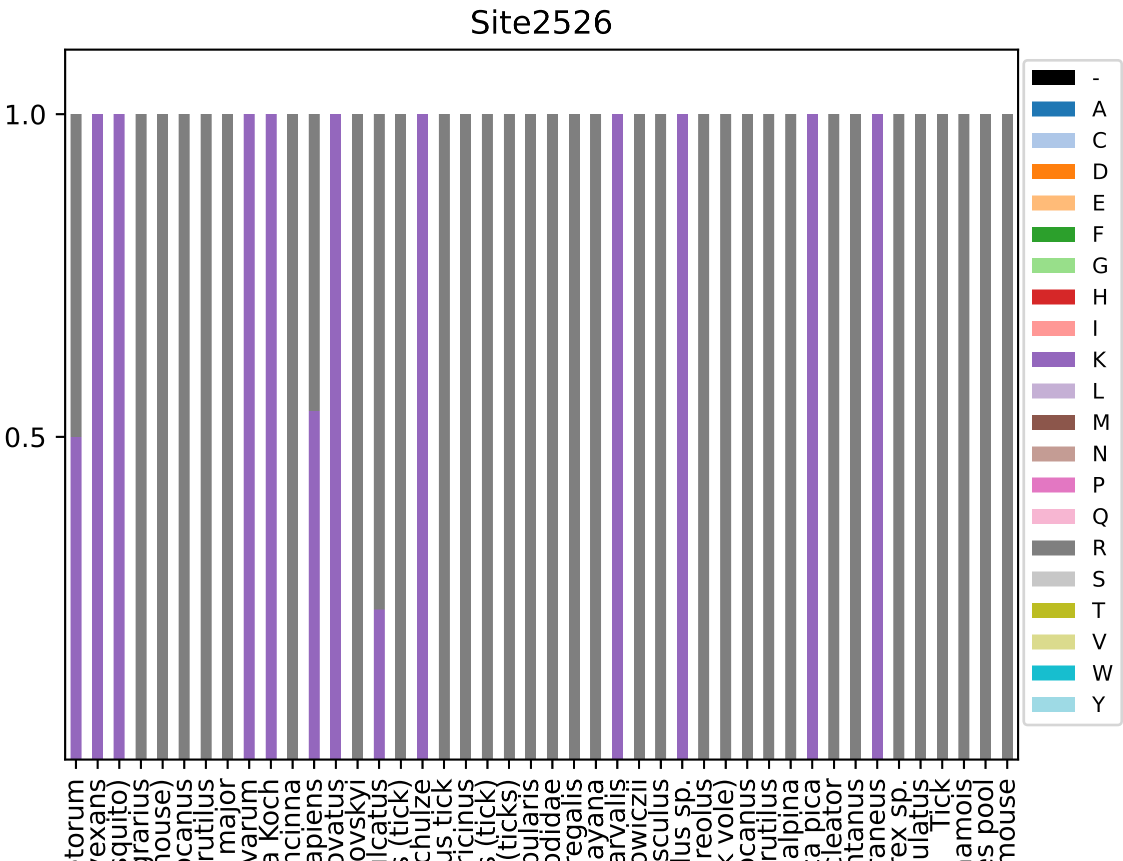
Site2526
1.0
0.5
-
A
C
D
E
F
G
H
I
K
L
M
N
P
Q
R
S
T
V
W
Y

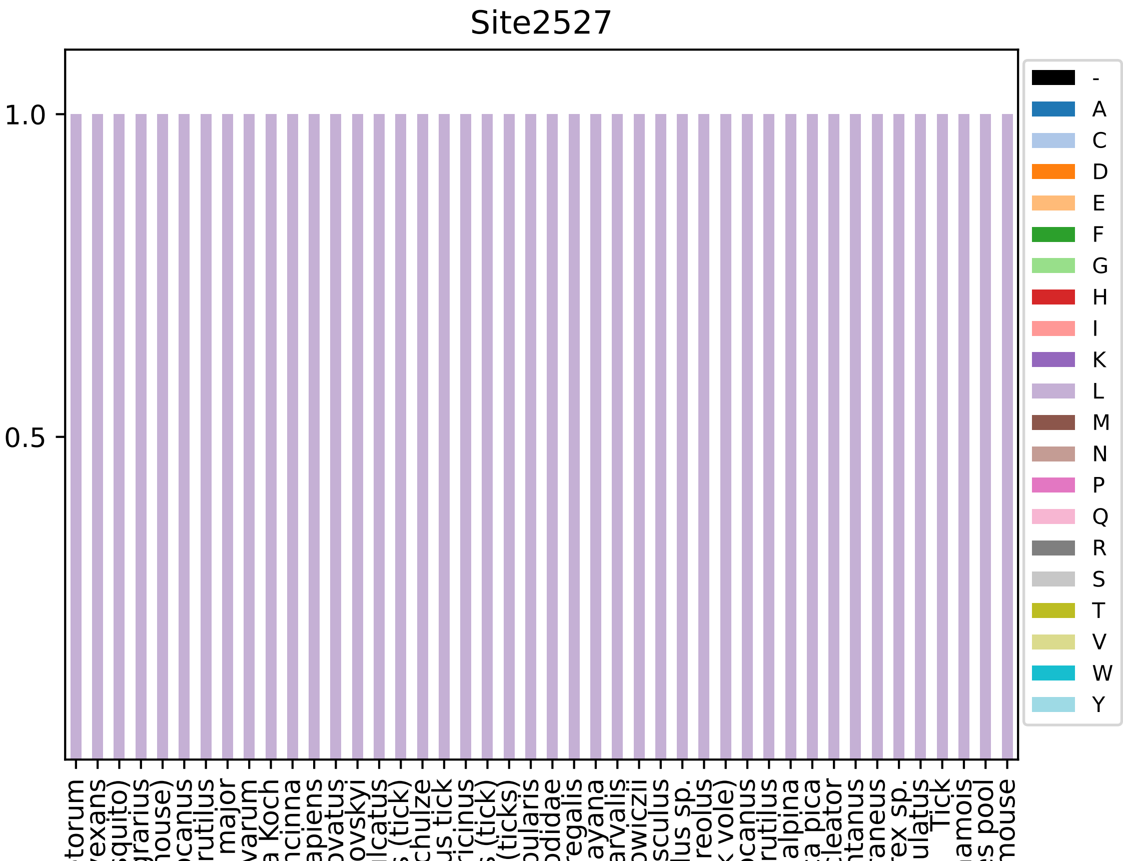

Site2527
1.0
0.5
-
A
C
D
E
F
G
H
I
K
L
M
N
P
Q
R
S
T
V
W
Y

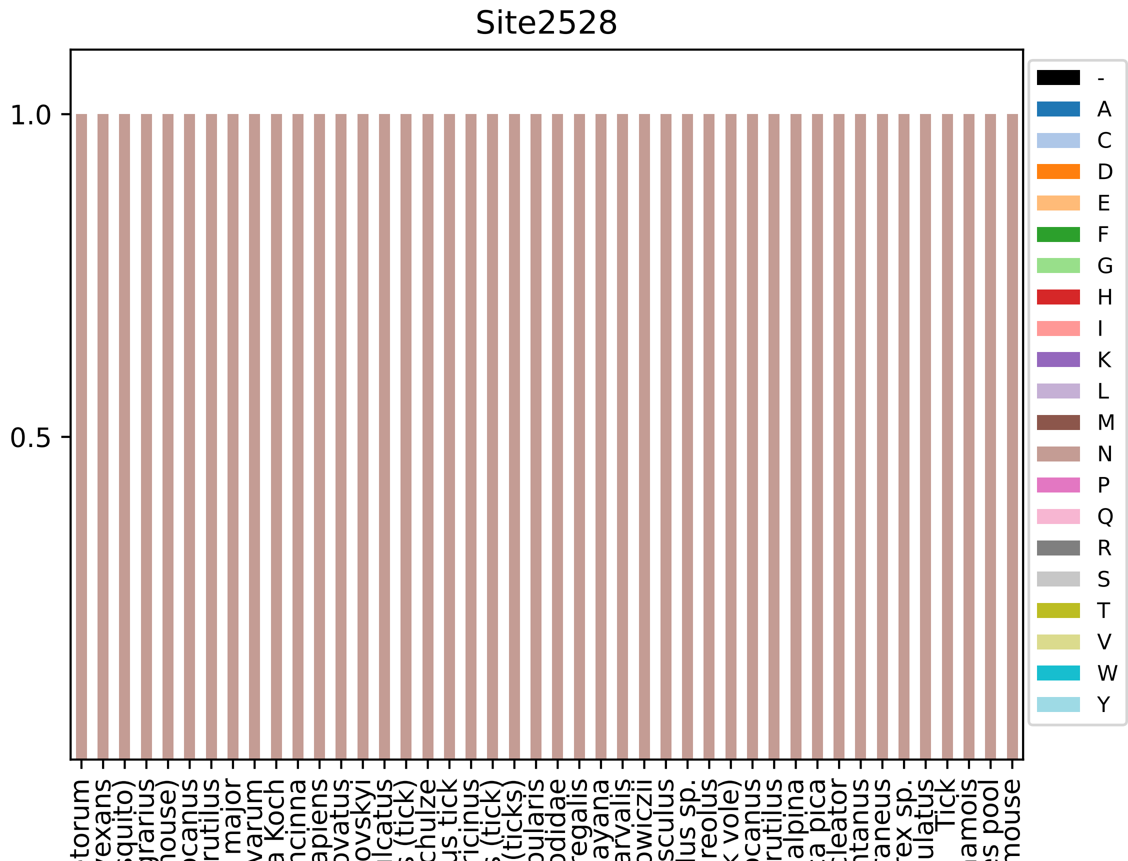
Site2528
1.0
0.5
-
A
C
D
E
F
G
H
I
K
L
M
N
P
Q
R
S
T
V
W
Y

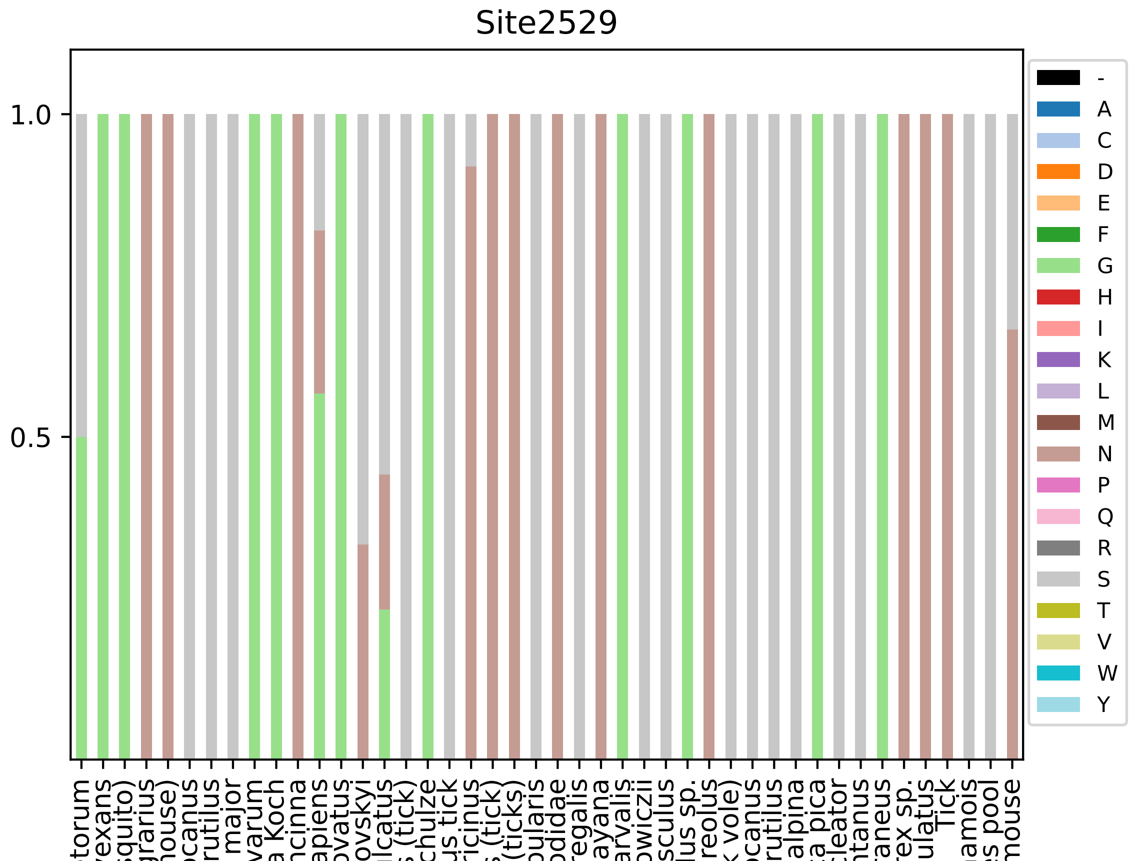
Site2529
1.0
0.5
-
A
C
D
E
F
G
H
I
K
L
M
N
P
Q
R
S
T
V
W
Y

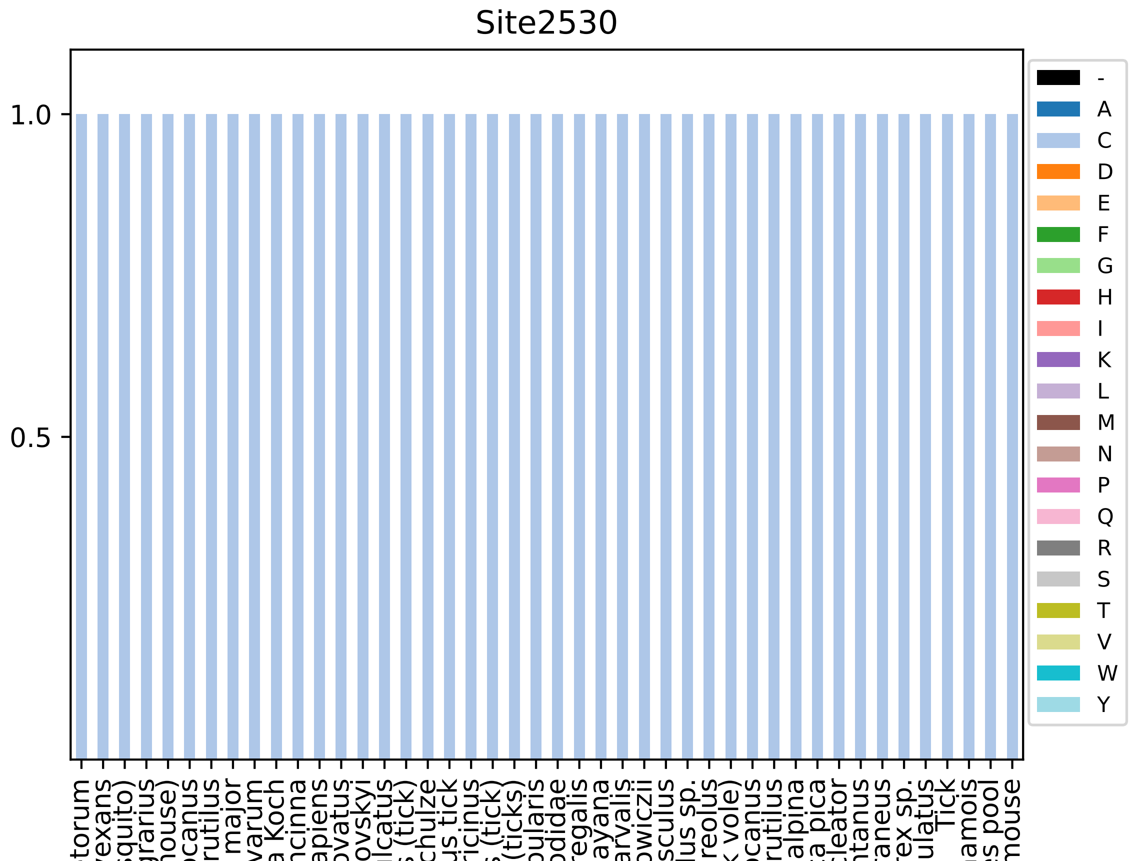
Site2530
1.0
0.5
-
A
C
D
E
F
G
H
I
K
L
M
N
P
Q
R
S
T
V
W
Y

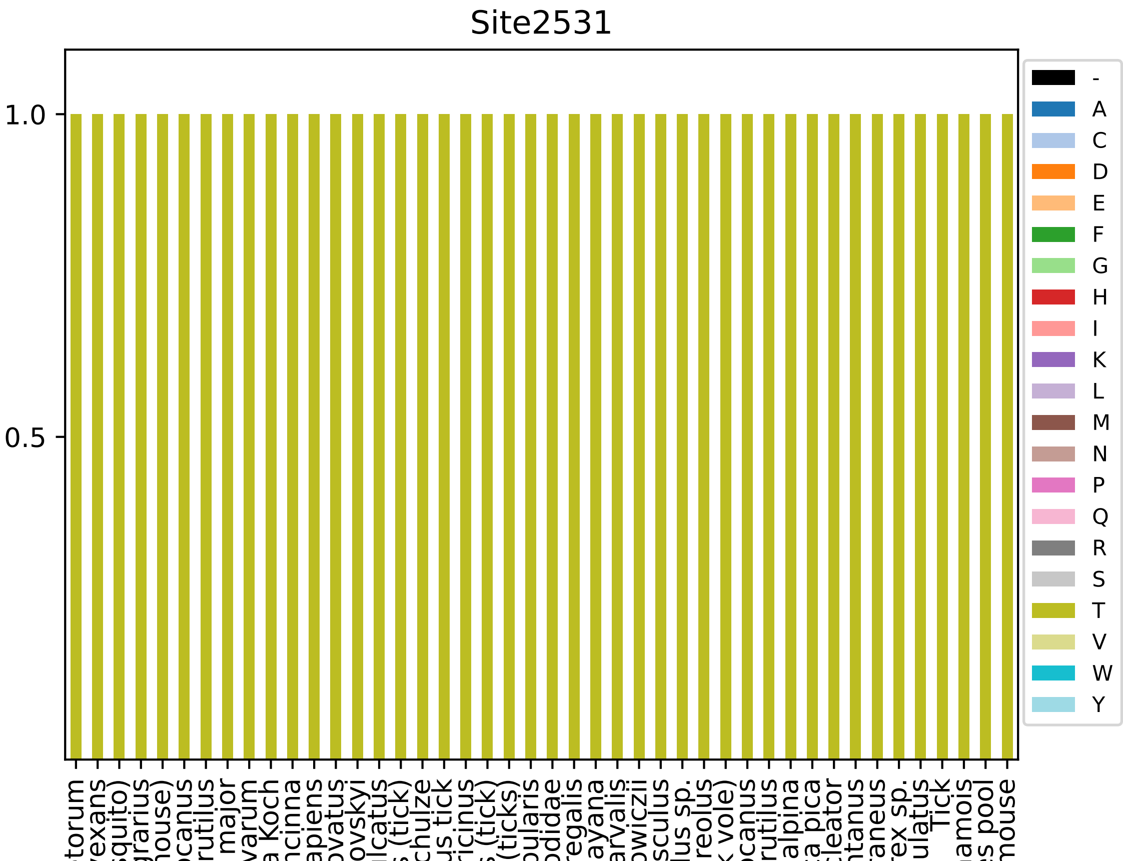

Site2531
1.0
0.5
-
A
C
D
E
F
G
H
I
K
L
M
N
P
Q
R
S
T
V
W
Y

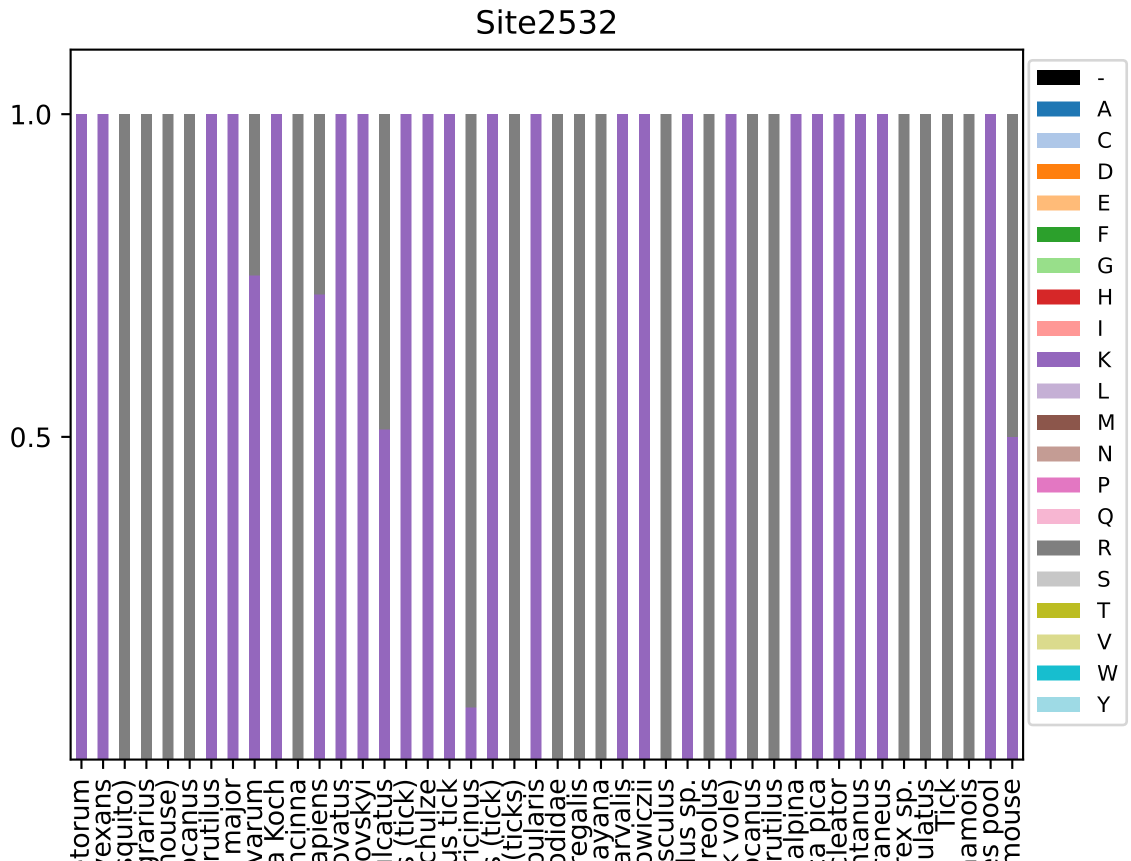

Site2532
1.0
0.5
-
A
C
D
E
F
G
H
I
K
L
M
N
P
Q
R
S
T
V
W
Y

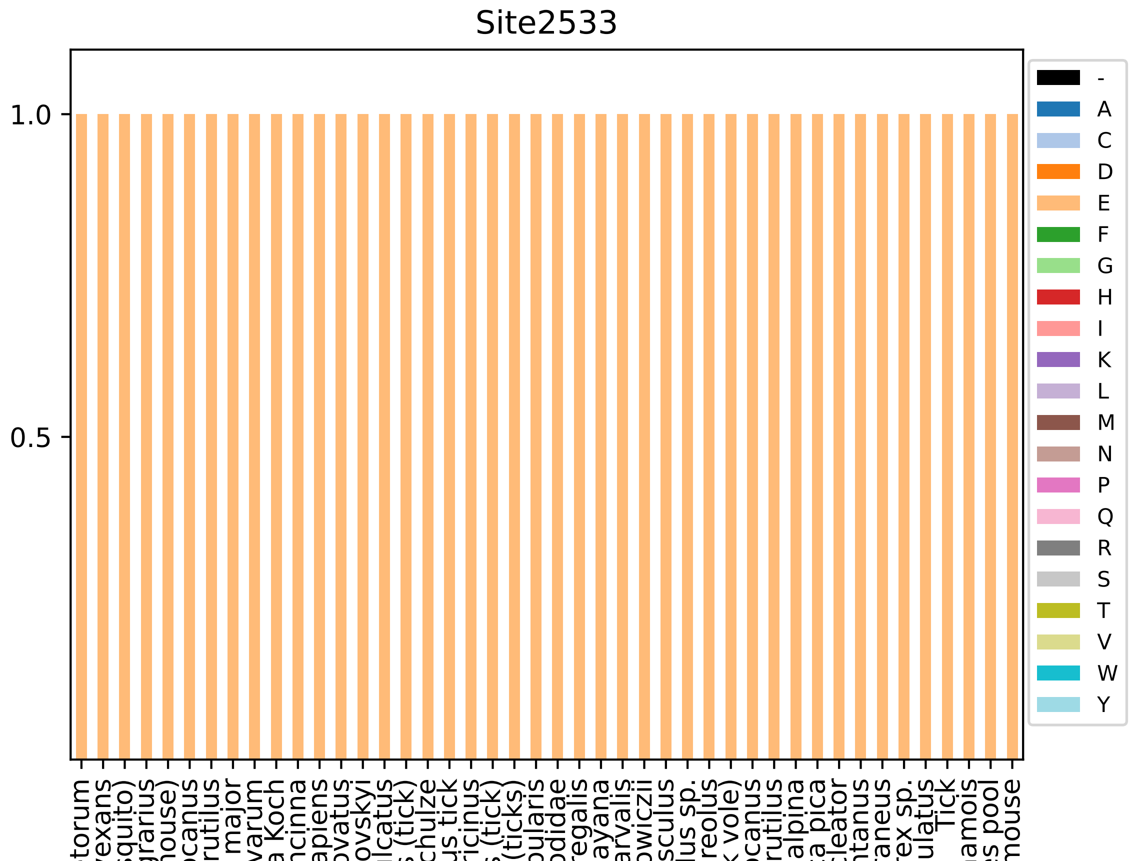

Site2533
1.0
0.5
-
A
C
D
E
F
G
H
I
K
L
M
N
P
Q
R
S
T
V
W
Y

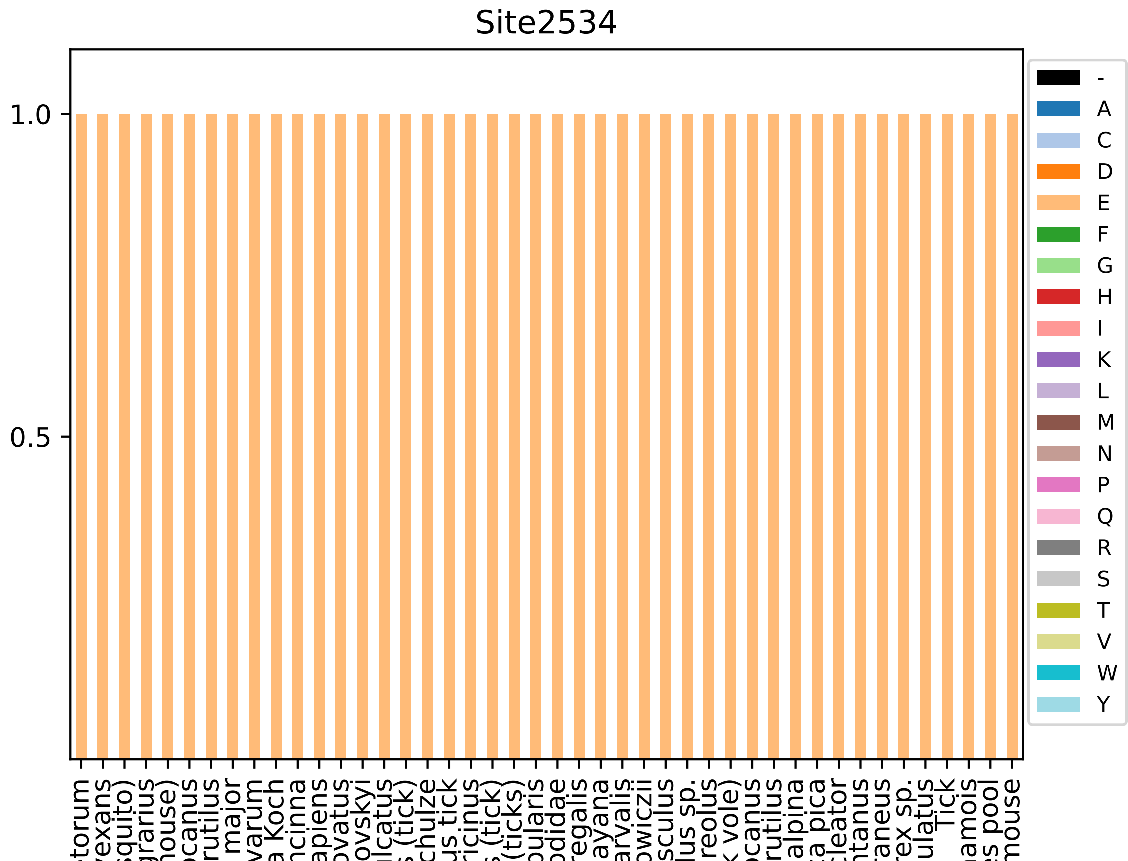
Site2534
1.0
0.5
-
A
C
D
E
F
G
H
I
K
L
M
N
P
Q
R
S
T
V
W
Y

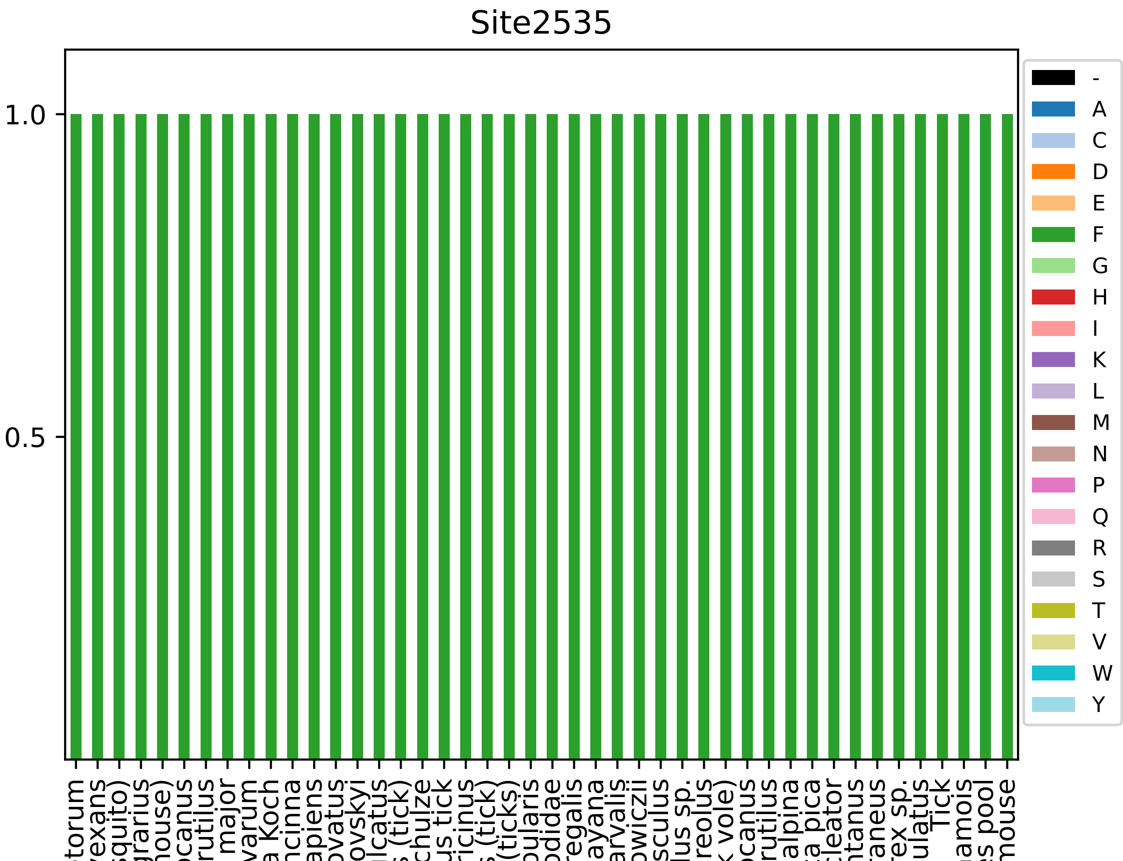
Site2535
1.0
0.5
-
A
C
D
E
F
G
H
I
K
L
M
N
P
Q
R
S
T
V
W
Y

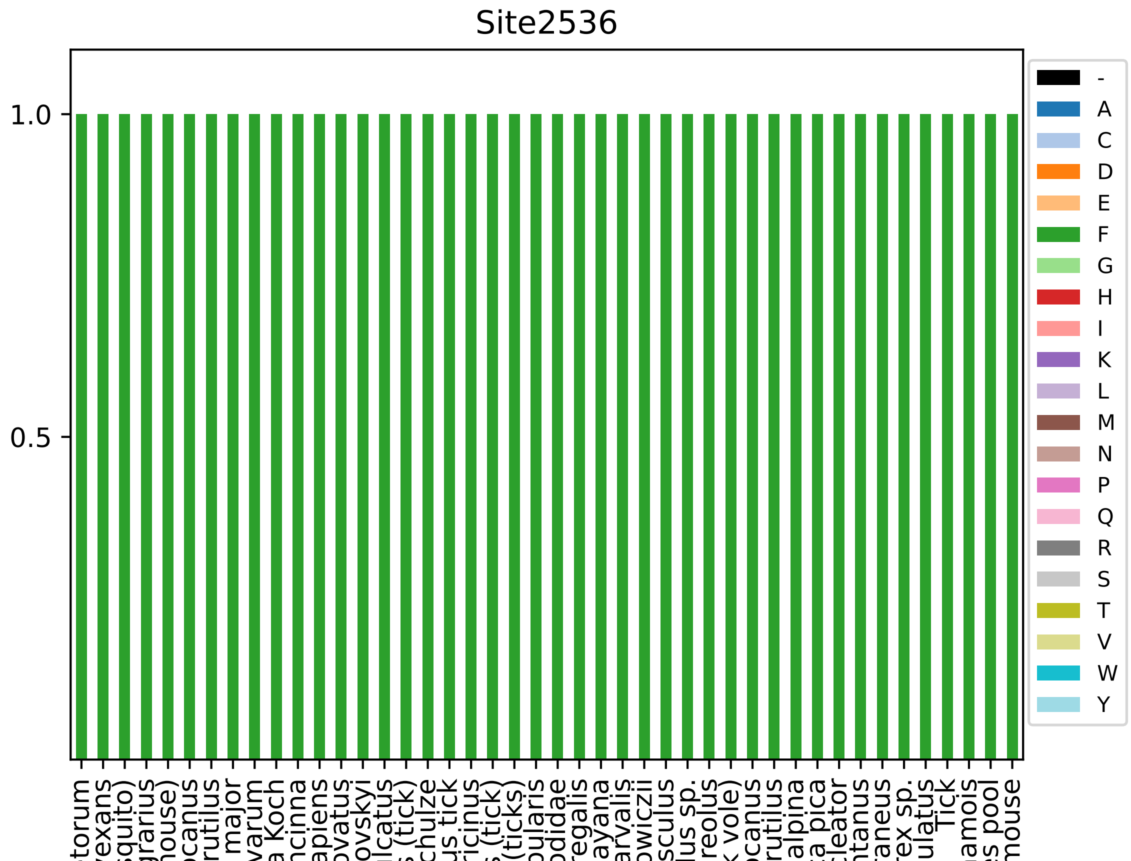
Site2536
1.0
0.5
-
A
C
D
E
F
G
H
I
K
L
M
N
P
Q
R
S
T
V
W
Y

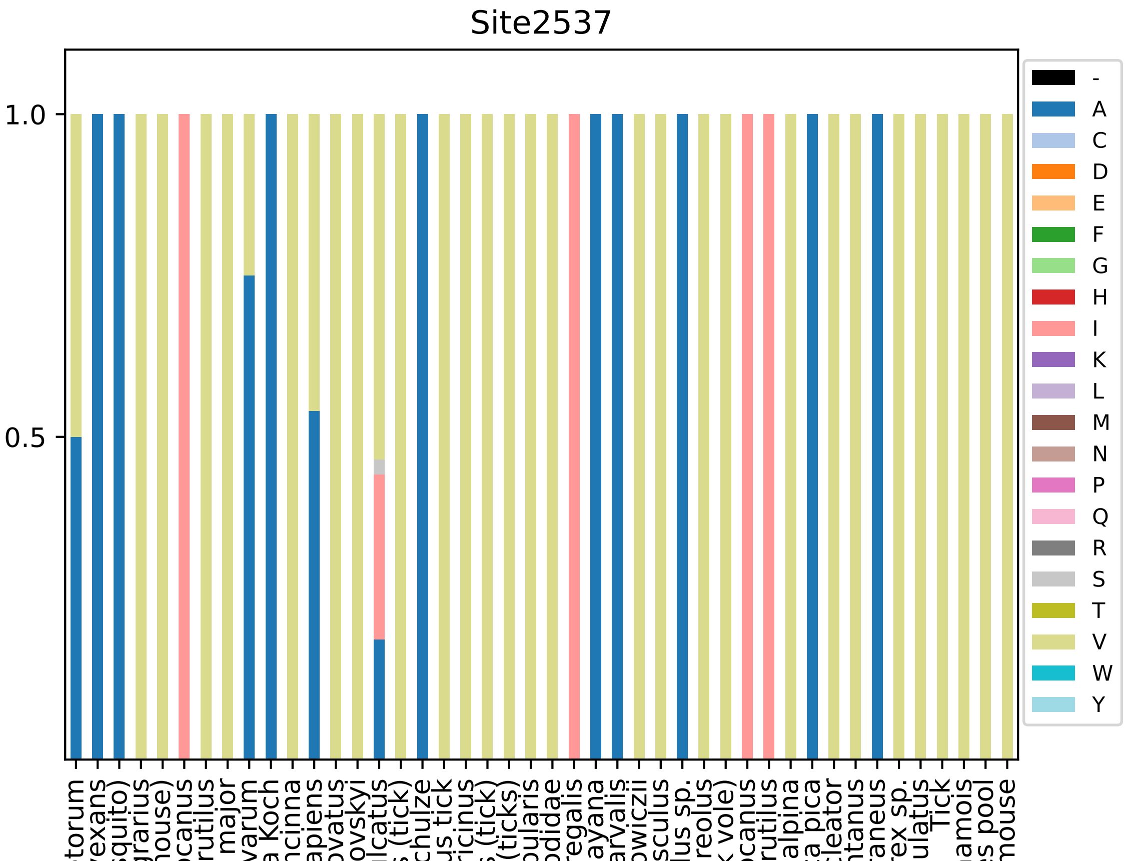
Site2537
1.0
0.5
-
A
C
D
E
F
G
H
I
K
L
M
N
P
Q
R
S
T
V
W
Y

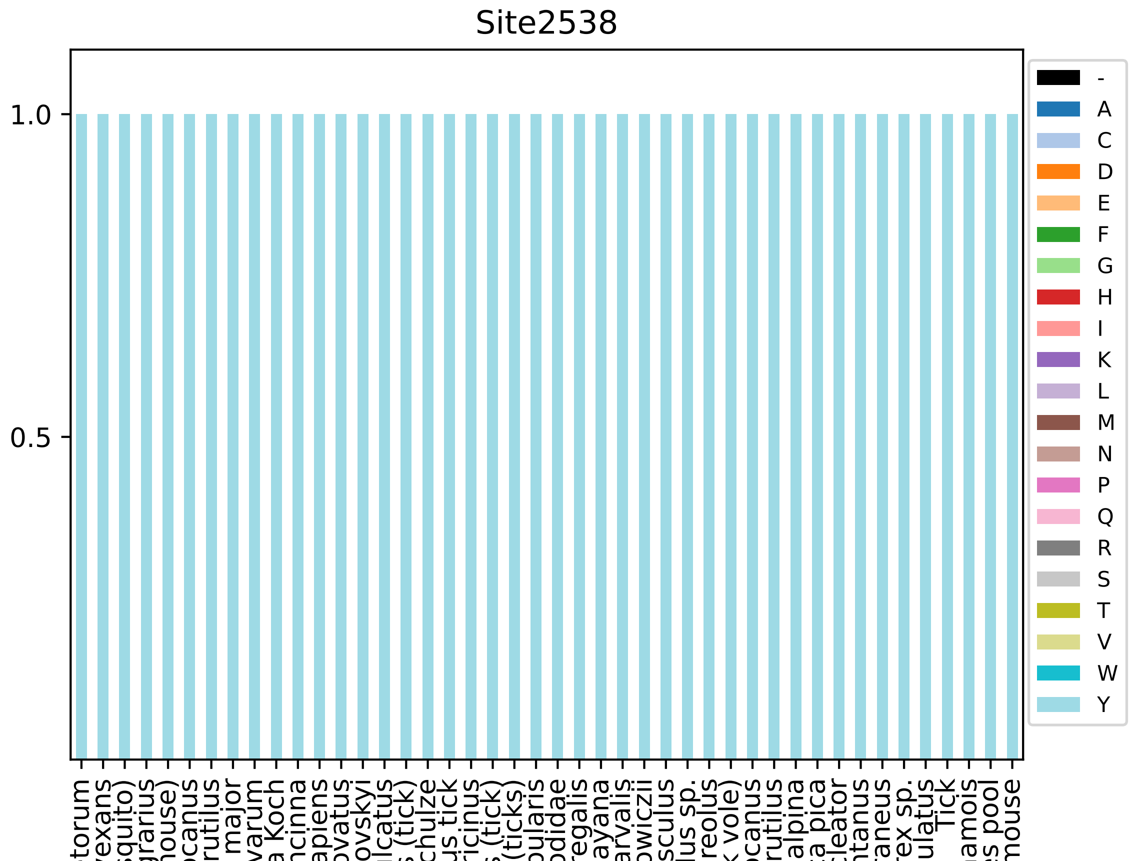

Site2538
1.0
0.5
-
A
C
D
E
F
G
H
I
K
L
M
N
P
Q
R
S
T
V
W
Y

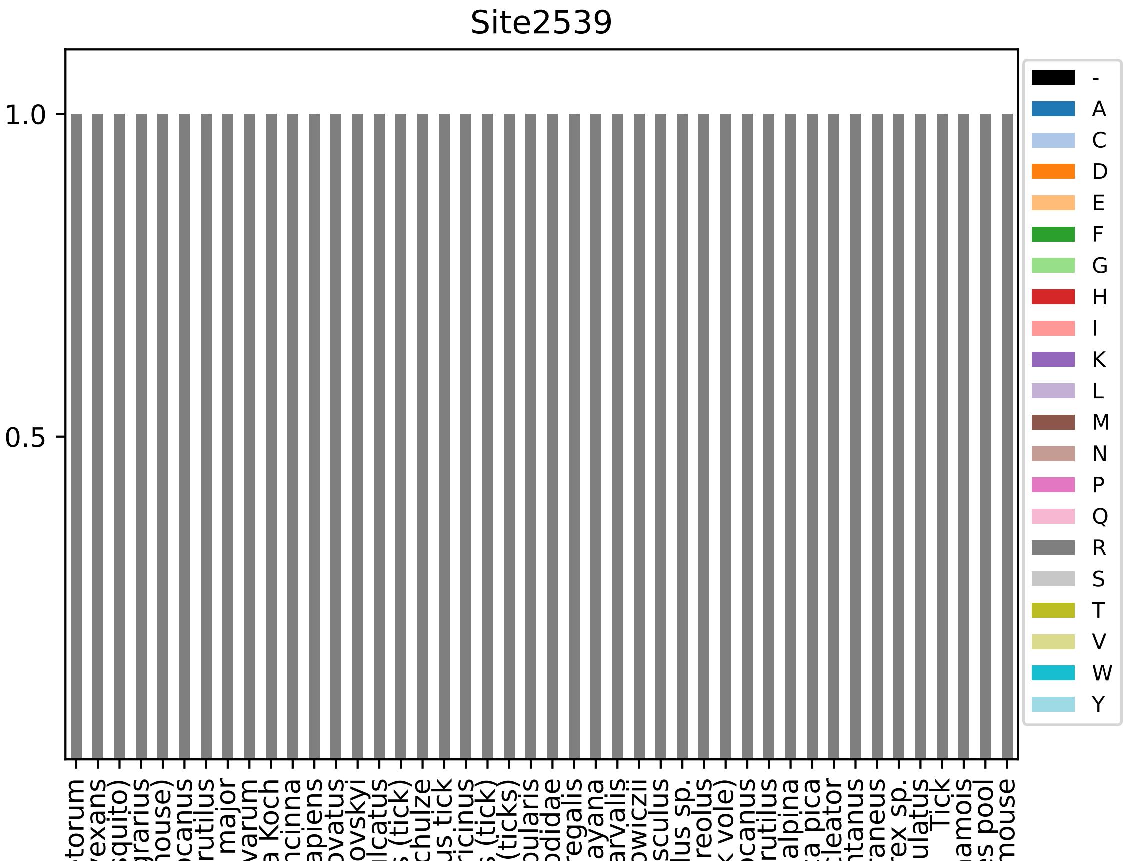
Site2539
1.0
0.5
-
A
C
D
E
F
G
H
I
K
L
M
N
P
Q
R
S
T
V
W
Y

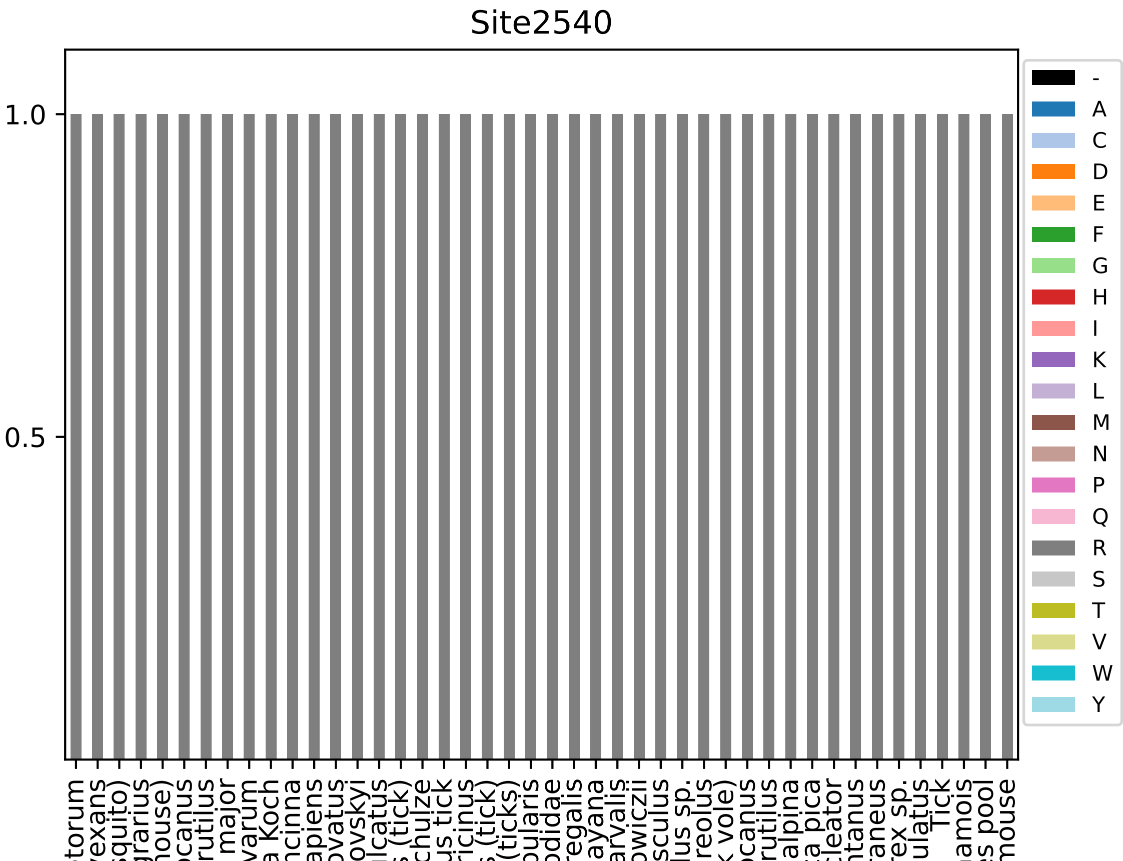
Site2540
1.0
0.5
-
A
C
D
E
F
G
H
I
K
L
M
N
P
Q
R
S
T
V
W
Y

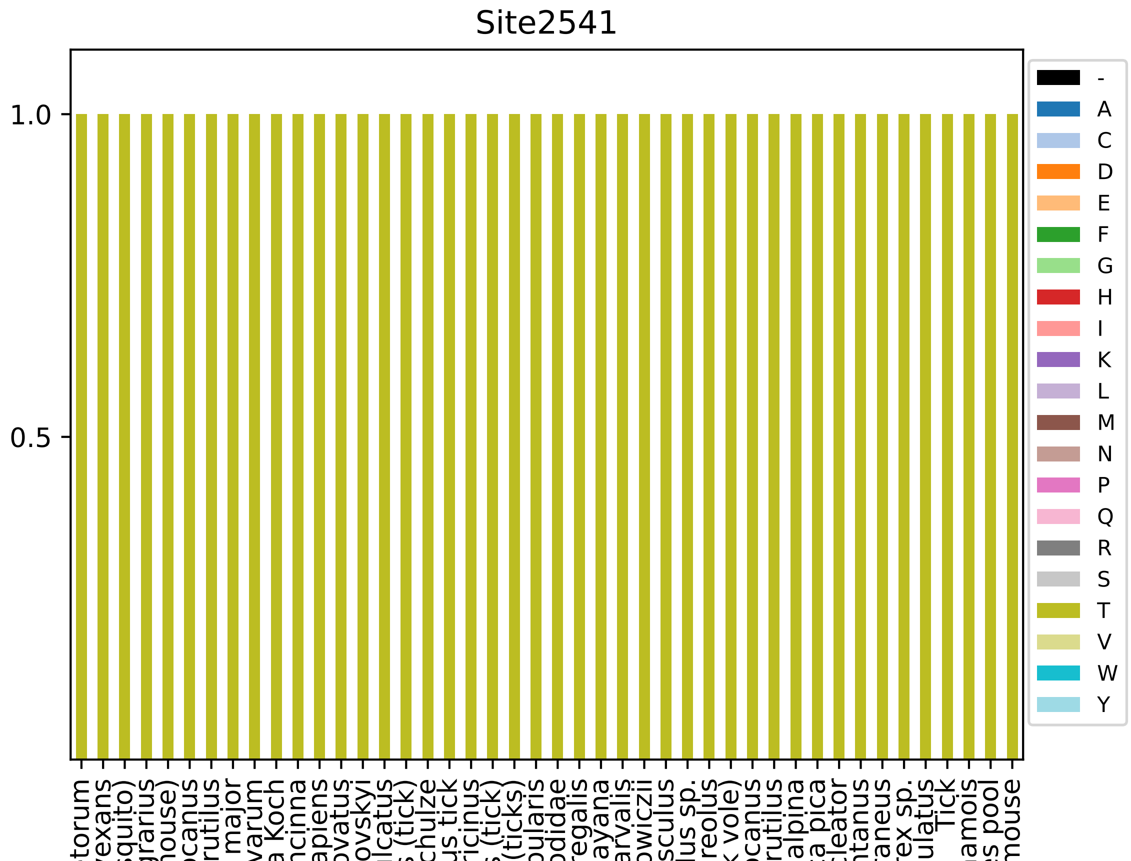
Site2541
1.0
0.5
-
A
C
D
E
F
G
H
I
K
L
M
N
P
Q
R
S
T
V
W
Y

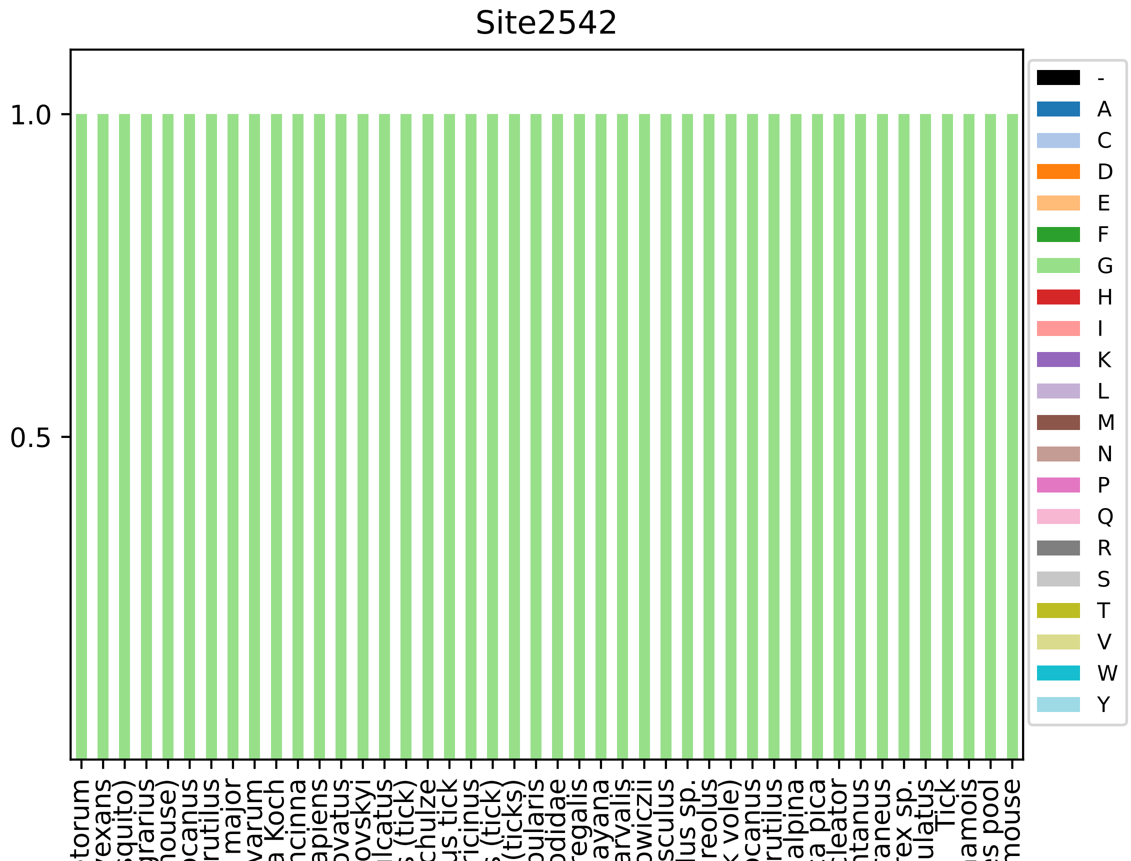
Site2542
1.0
0.5
-
A
C
D
E
F
G
H
I
K
L
M
N
P
Q
R
S
T
V
W
Y

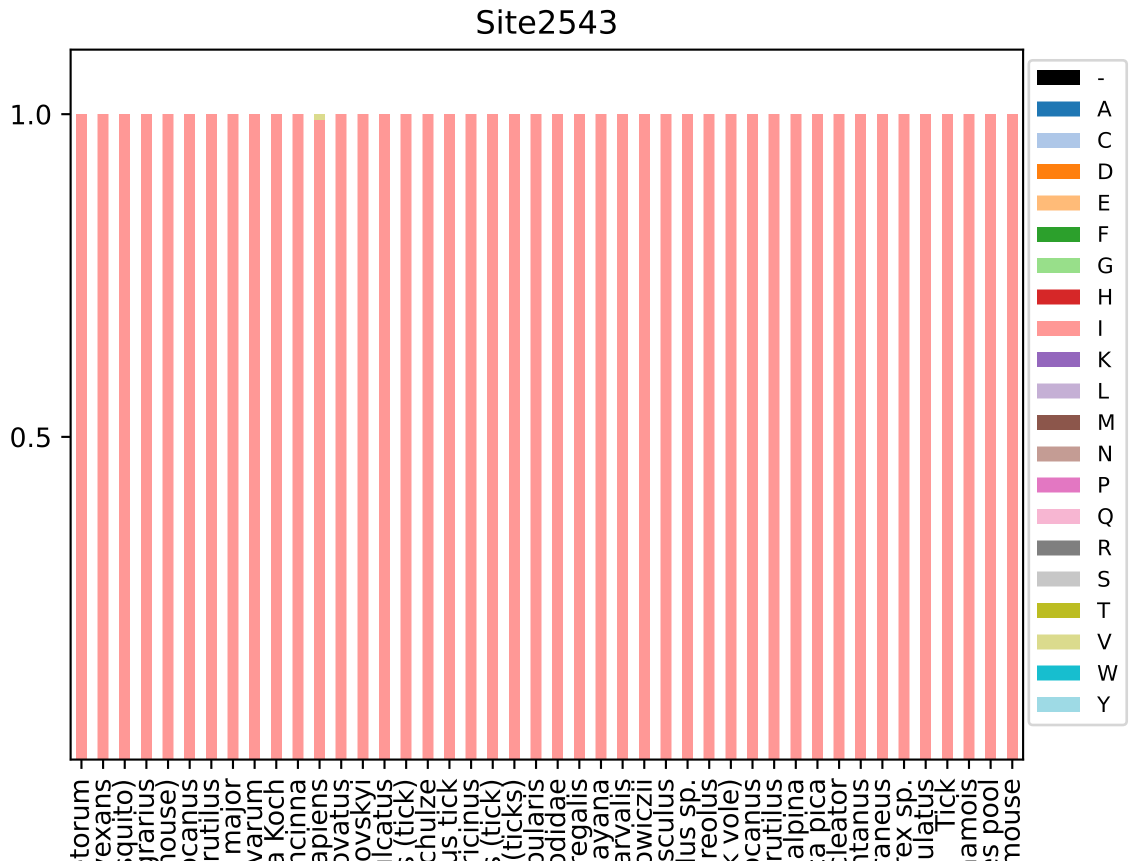

Site2543
1.0
0.5
-
A
C
D
E
F
G
H
I
K
L
M
N
P
Q
R
S
T
V
W
Y

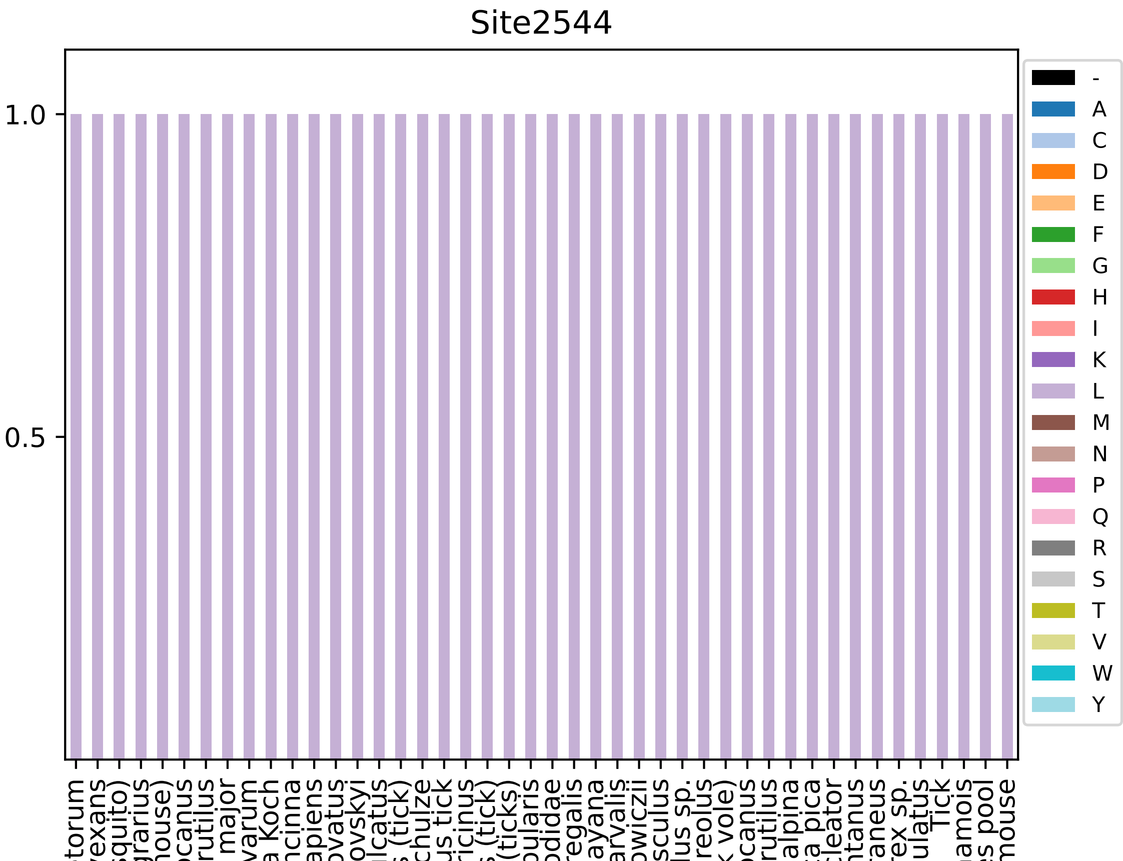

Site2544
1.0
0.5
-
A
C
D
E
F
G
H
I
K
L
M
N
P
Q
R
S
T
V
W
Y

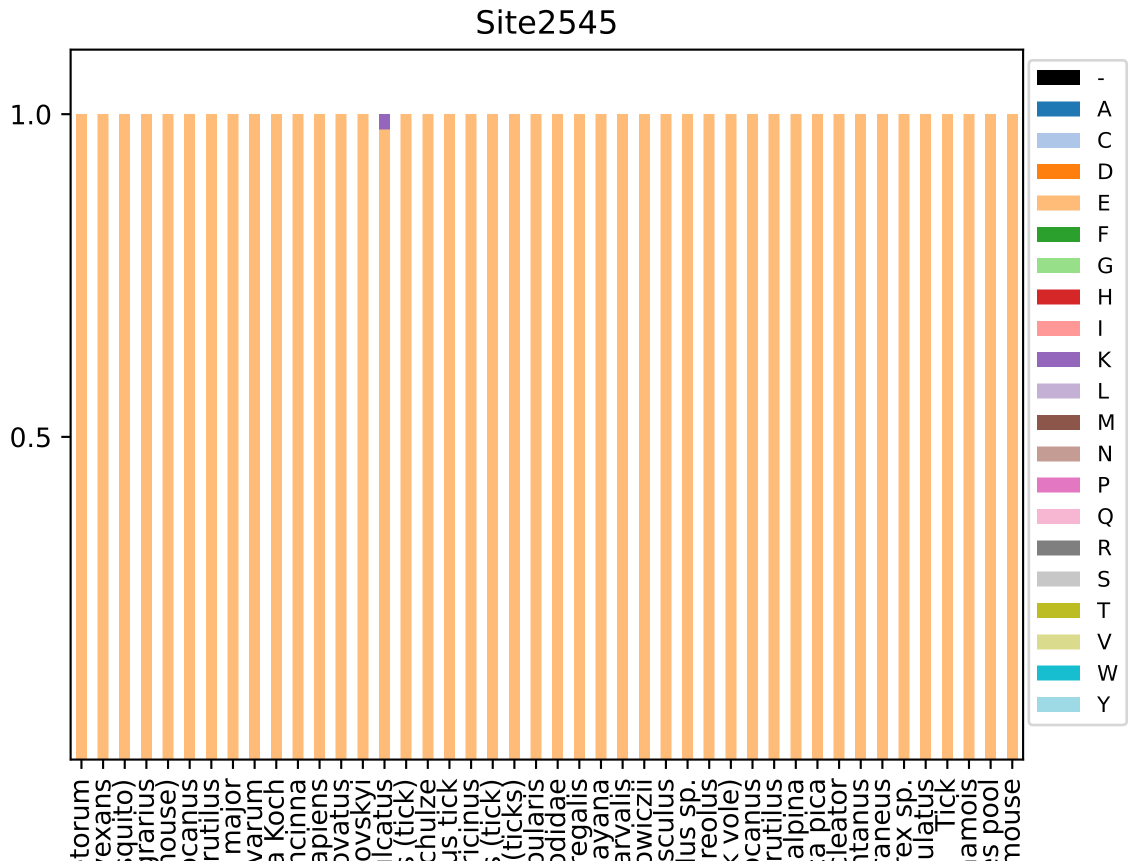
Site2545
1.0
0.5
-
A
C
D
E
F
G
H
I
K
L
M
N
P
Q
R
S
T
V
W
Y

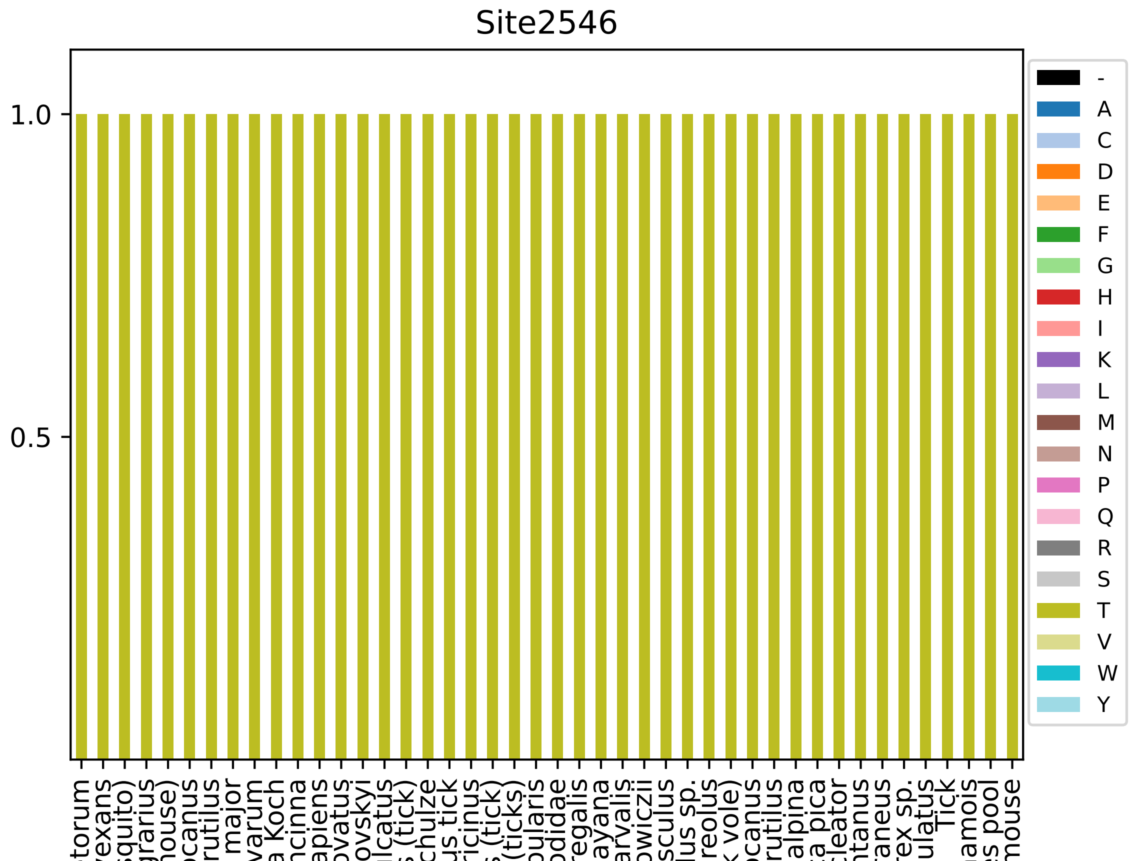
Site2546
1.0
0.5
-
A
C
D
E
F
G
H
I
K
L
M
N
P
Q
R
S
T
V
W
Y

Site2547

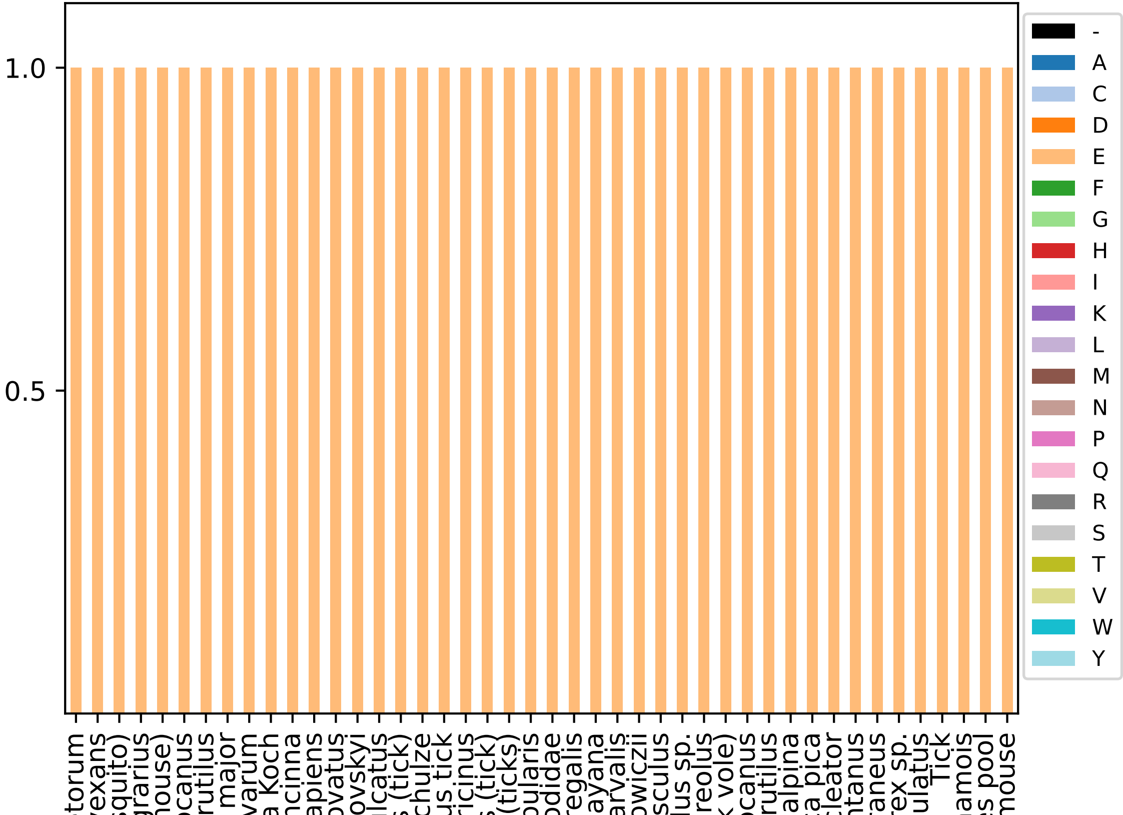

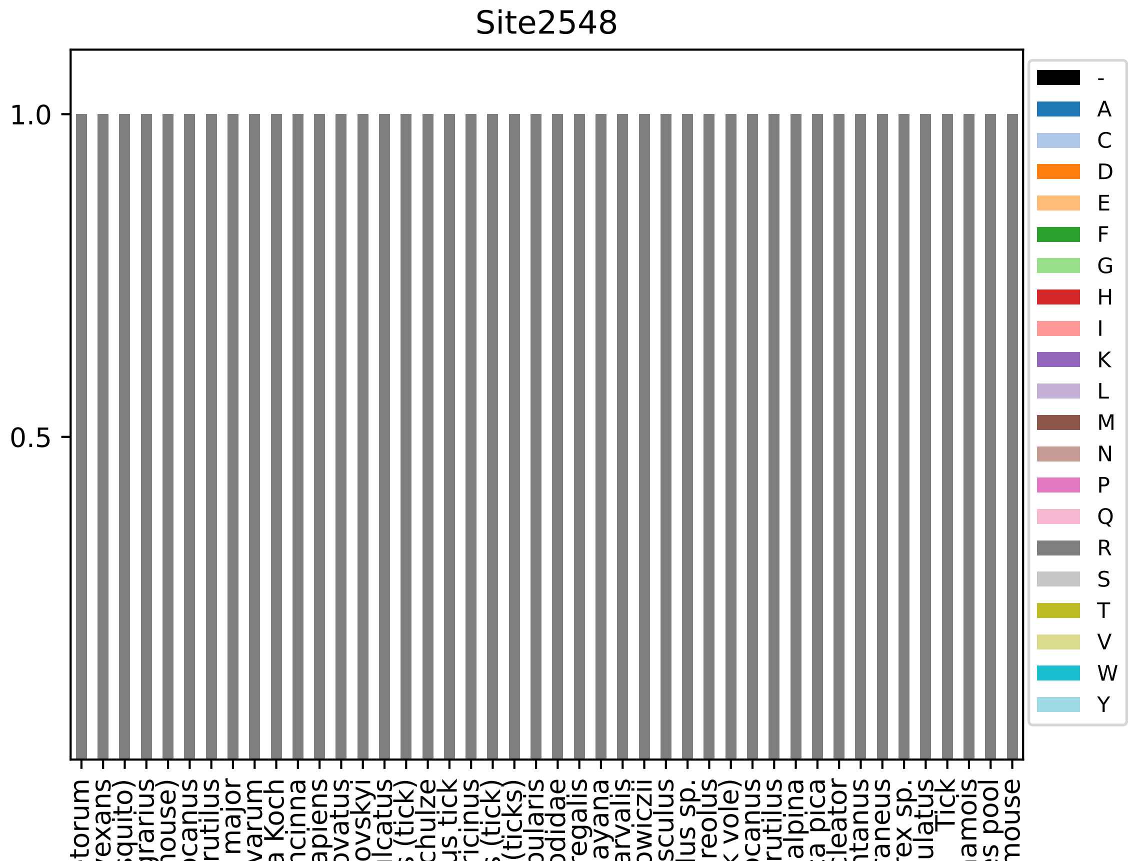
Site2548
1.0
0.5
-
A
C
D
E
F
G
H
I
K
L
M
N
P
Q
R
S
T
V
W
Y

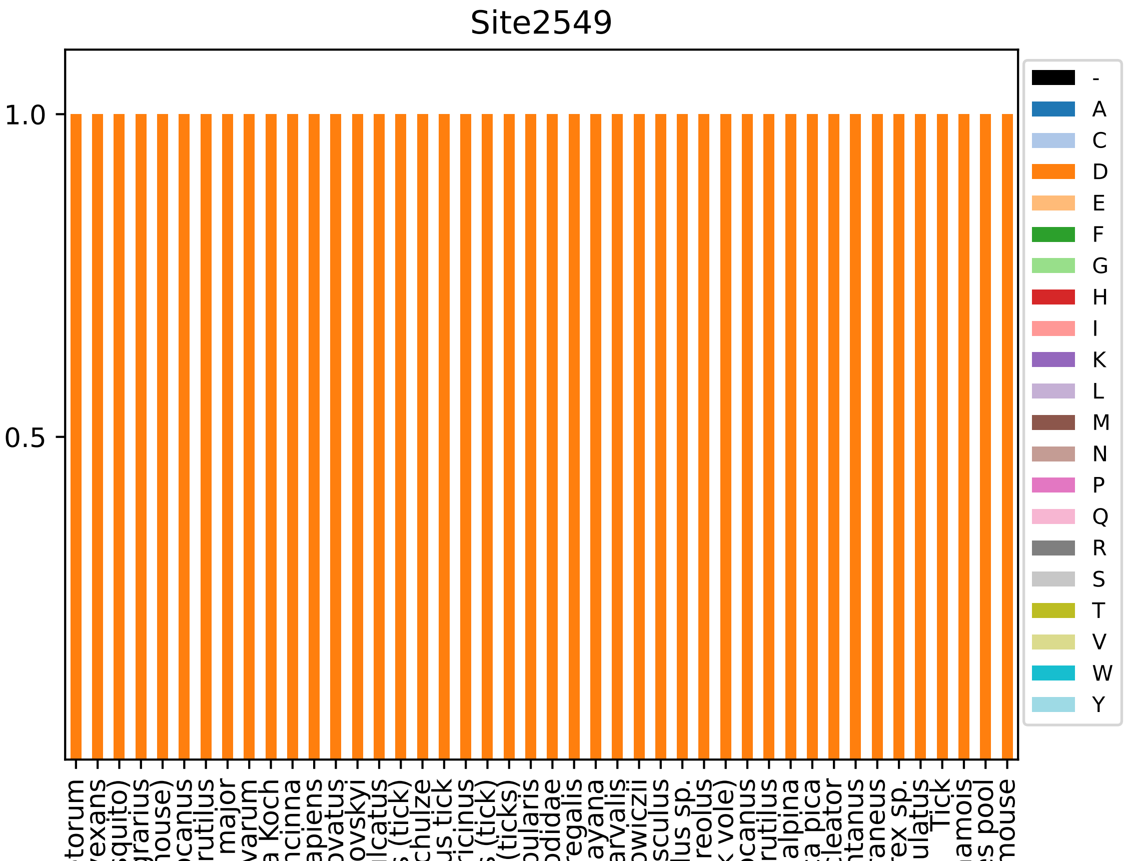

Site2549
1.0
0.5
-
A
C
D
E
F
G
H
I
K
L
M
N
P
Q
R
S
T
V
W
Y

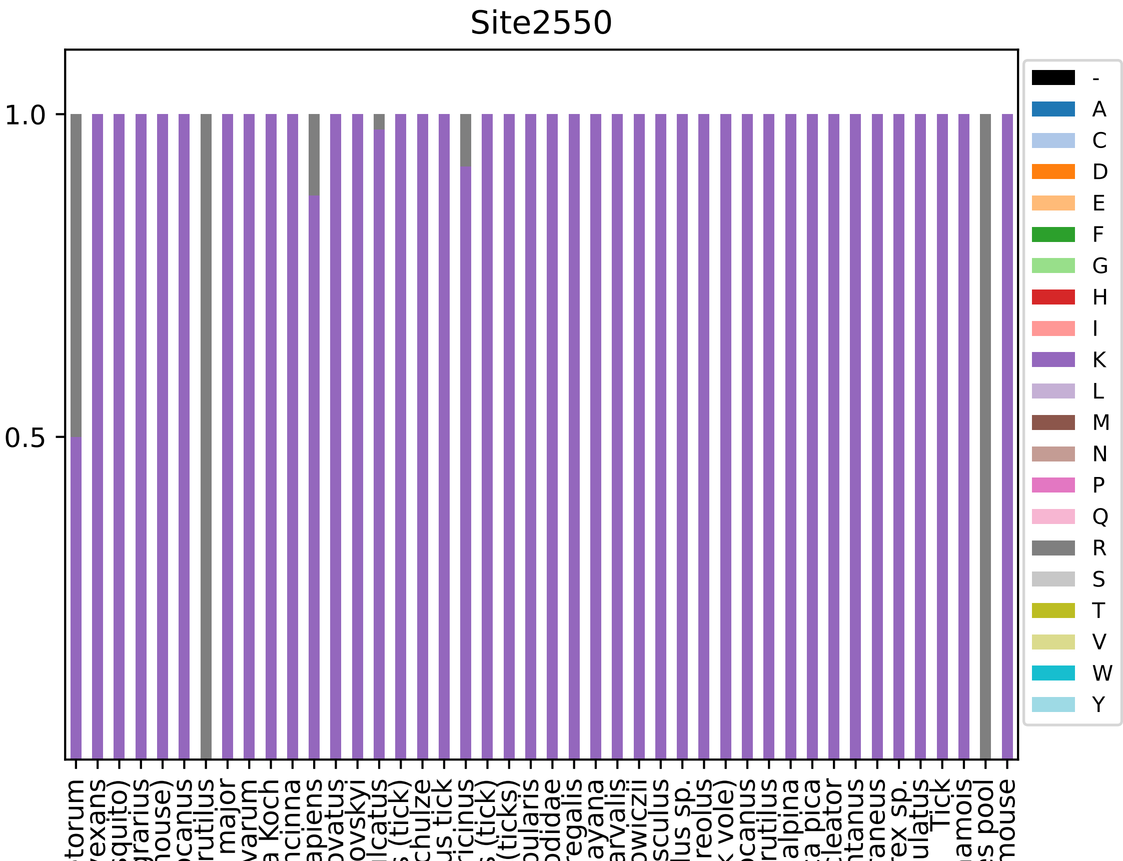
Site2550
1.0
0.5
-
A
C
D
E
F
G
H
I
K
L
M
N
P
Q
R
S
T
V
W
Y

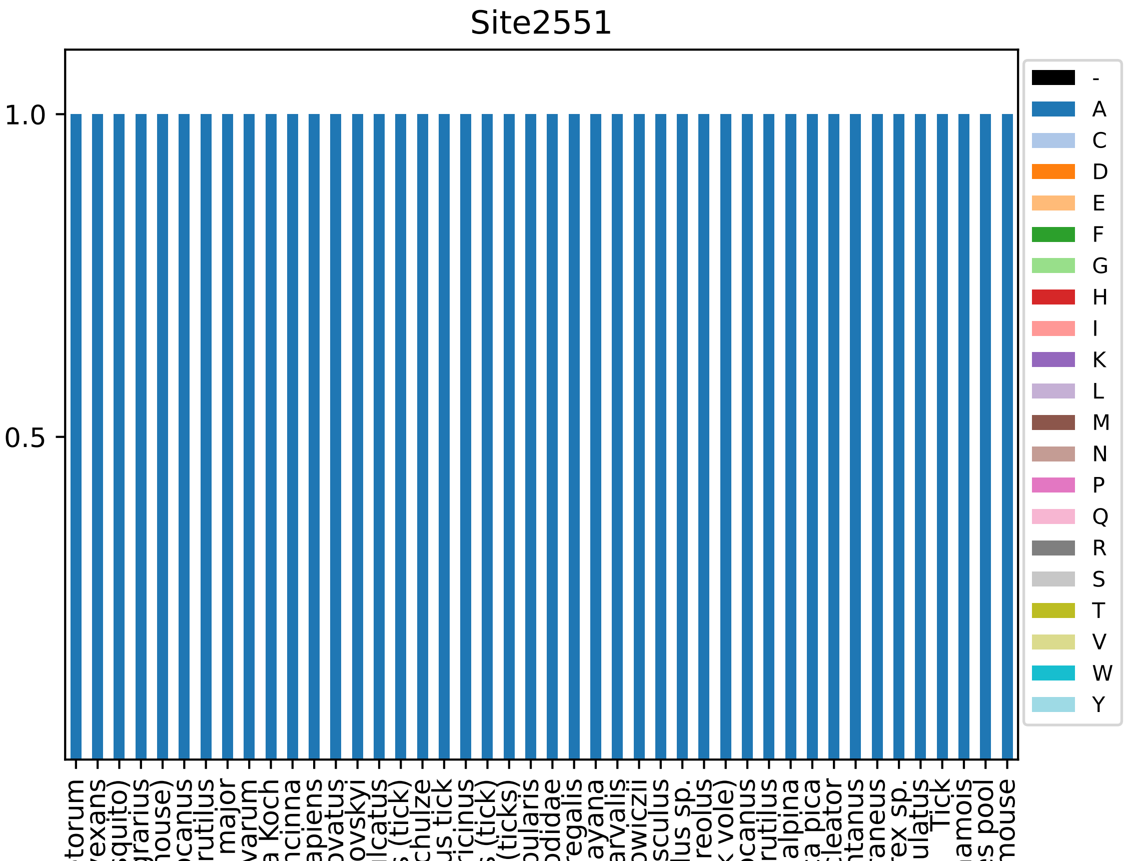
Site2551
1.0
0.5
-
A
C
D
E
F
G
H
I
K
L
M
N
P
Q
R
S
T
V
W
Y

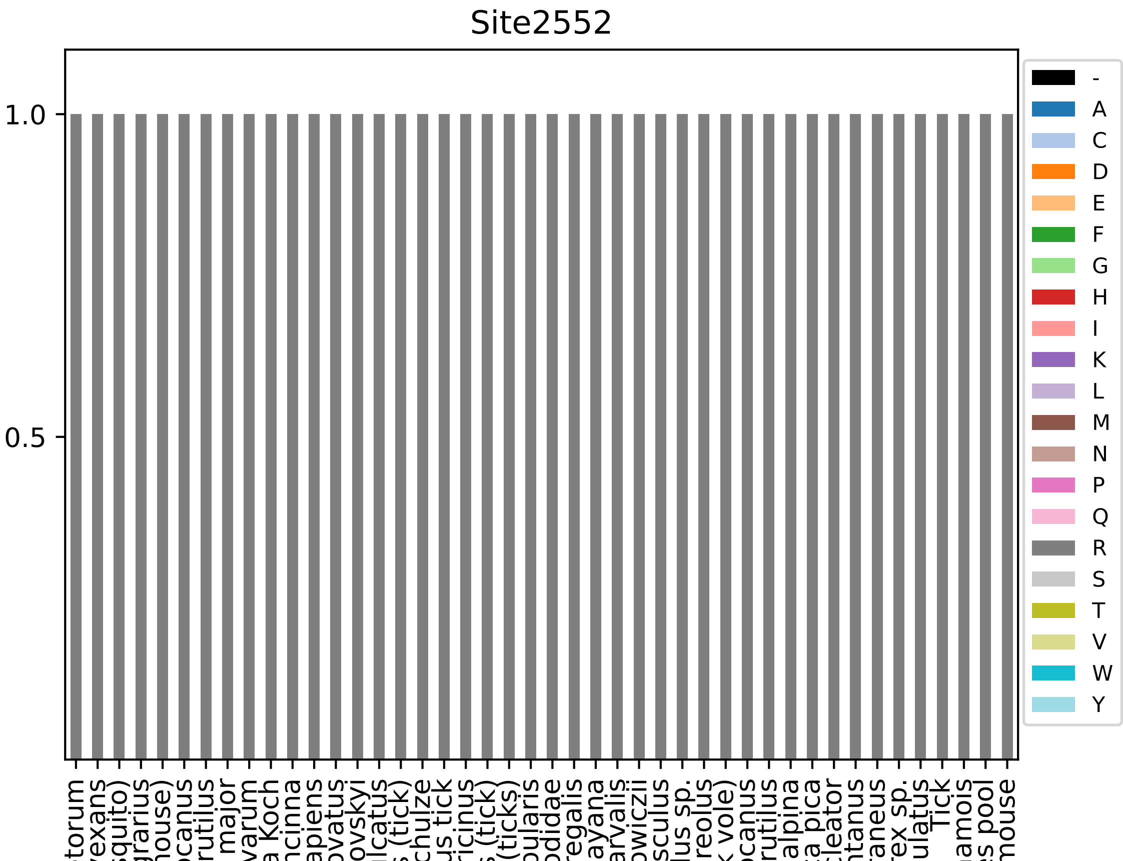
Site2552
1.0
0.5
-
A
C
D
E
F
G
H
I
K
L
M
N
P
Q
R
S
T
V
W
Y

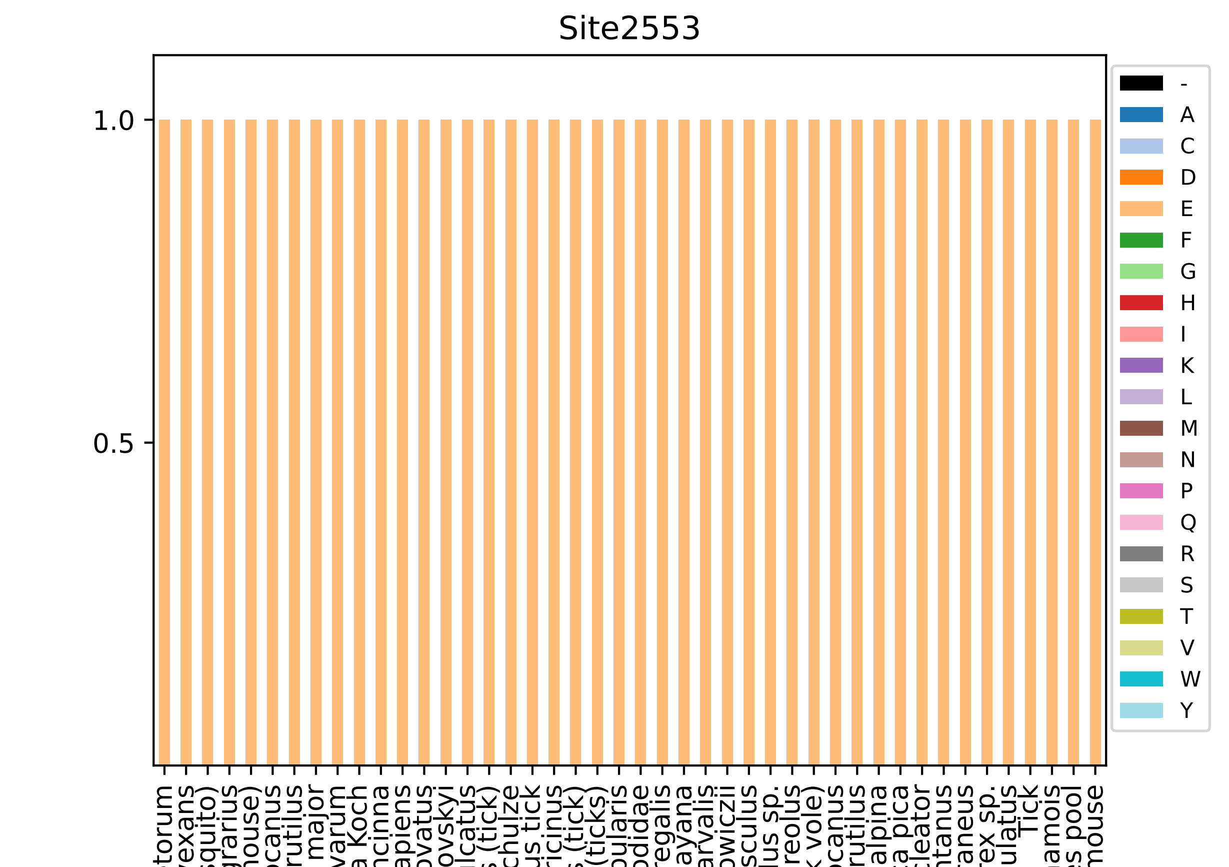
Site2553
1.0
0.5
-
A
C
D
E
F
G
H
I
K
L
M
N
P
Q
R
S
T
V
W
Y

Site2554

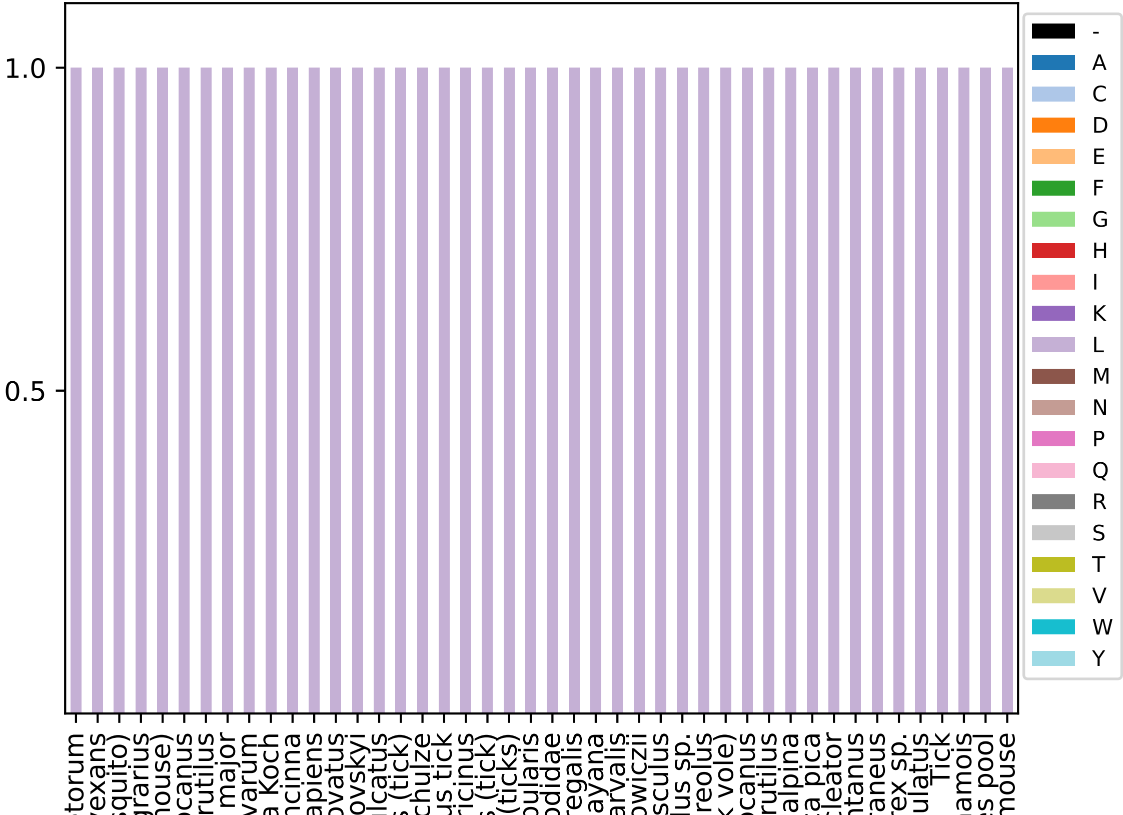

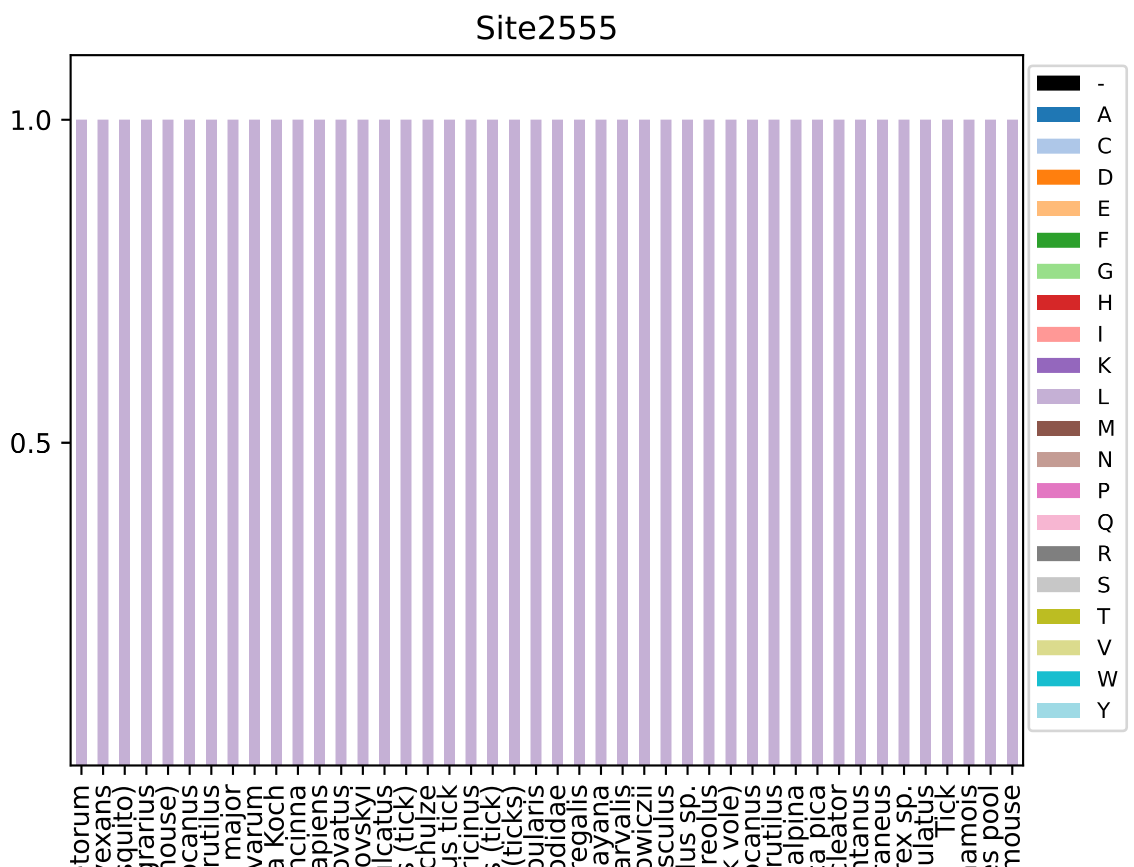
Site2555
1.0
0.5
-
A
C
D
E
F
G
H
I
K
L
M
N
P
Q
R
S
T
V
W
Y

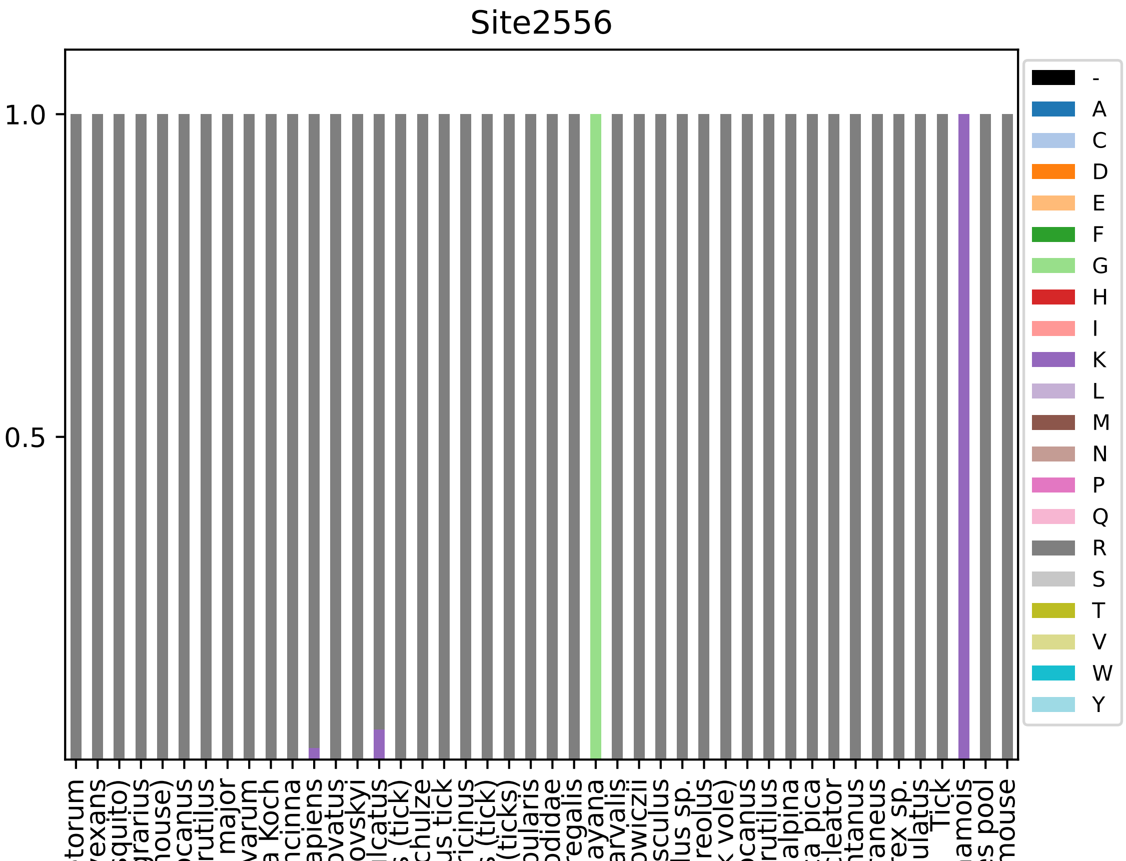
Site2556
1.0
0.5
-
A
C
D
E
F
G
H
I
K
L
M
N
P
Q
R
S
T
V
W
Y

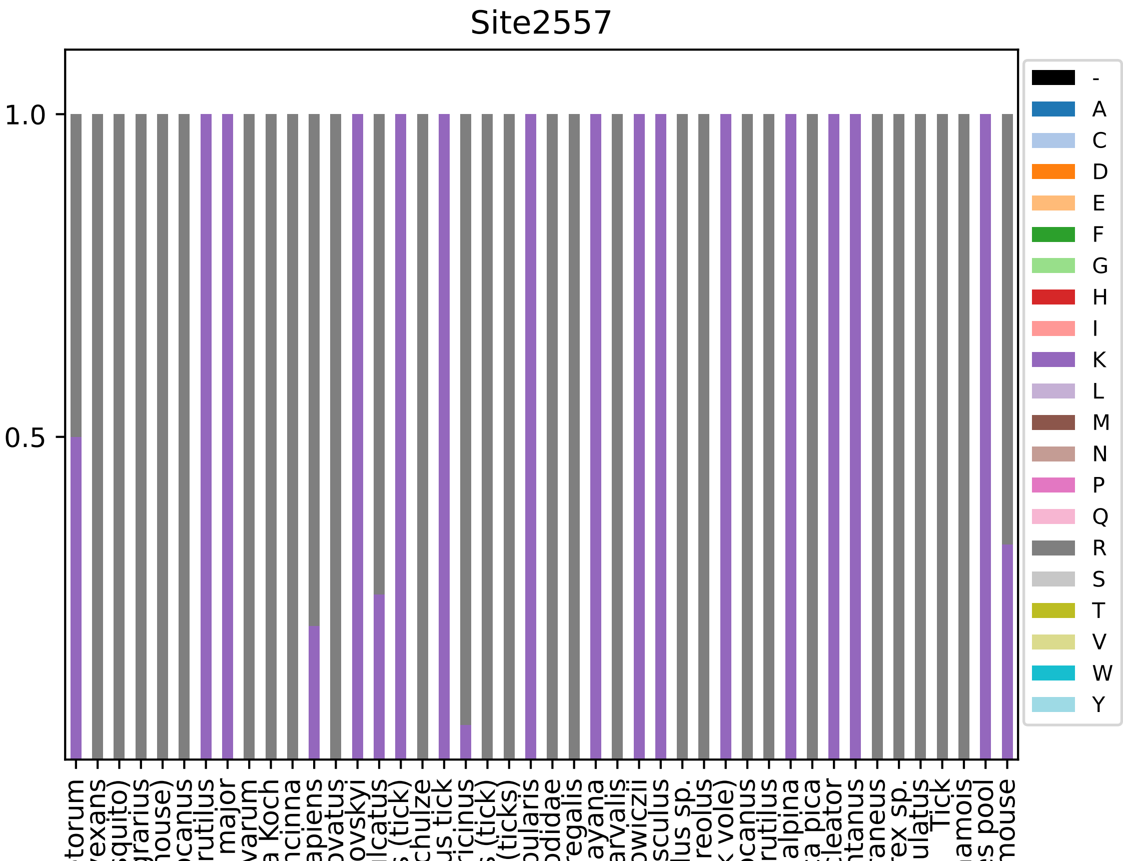
Site2557
1.0
0.5
-
A
C
D
E
F
G
H
I
K
L
M
N
P
Q
R
S
T
V
W
Y

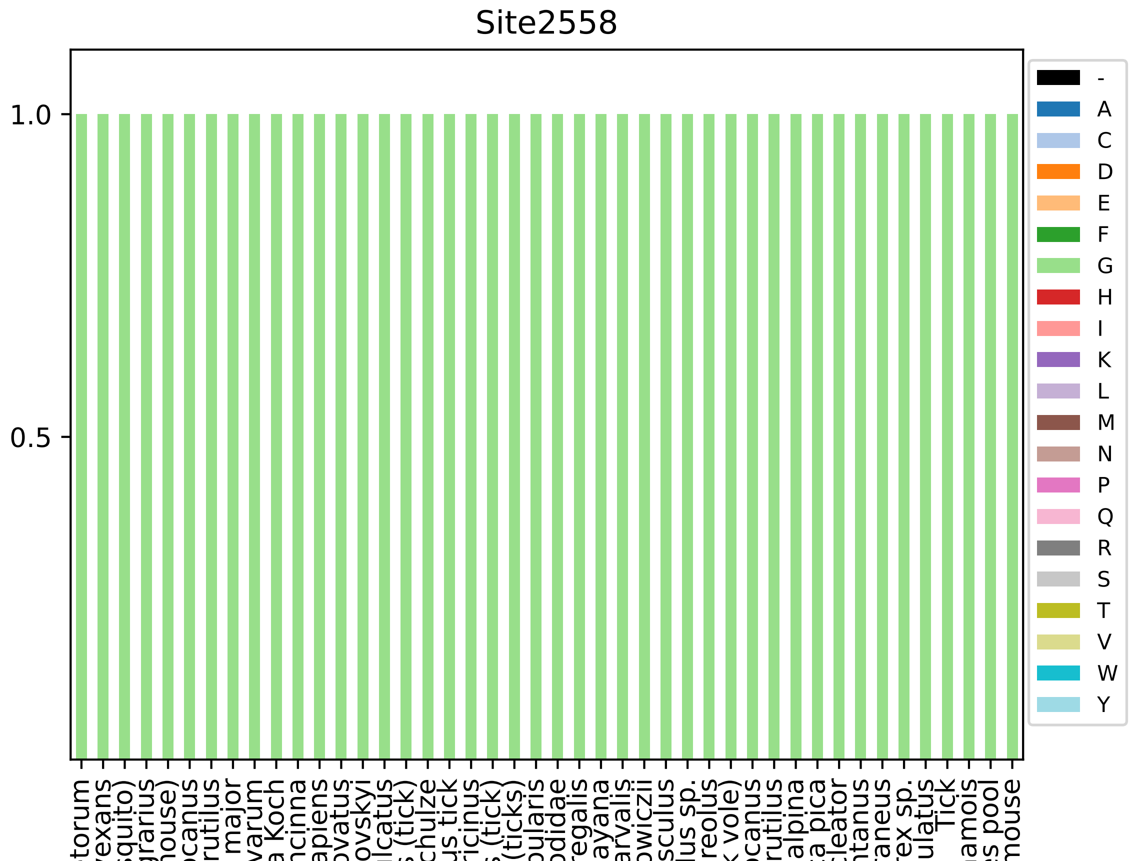

Site2558
1.0
0.5
-
A
C
D
E
F
G
H
I
K
L
M
N
P
Q
R
S
T
V
W
Y

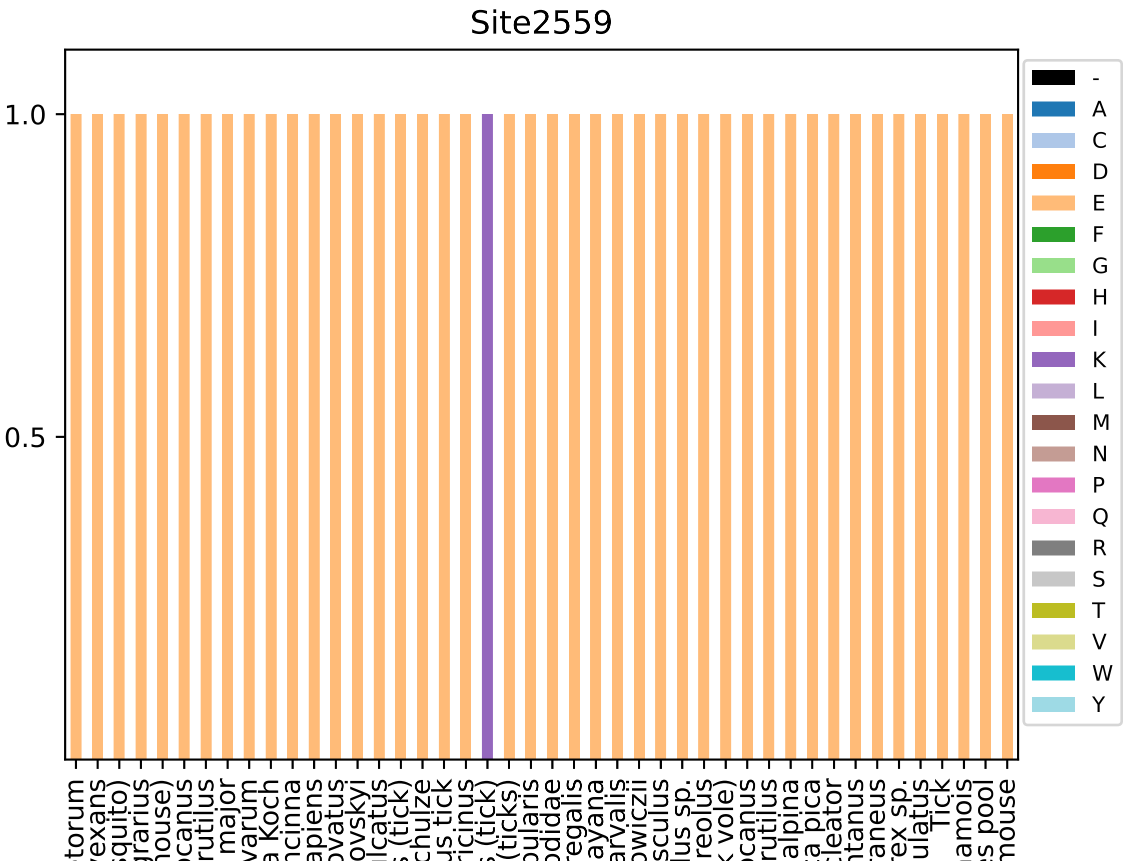
Site2559
1.0
0.5
-
A
C
D
E
F
G
H
I
K
L
M
N
P
Q
R
S
T
V
W
Y

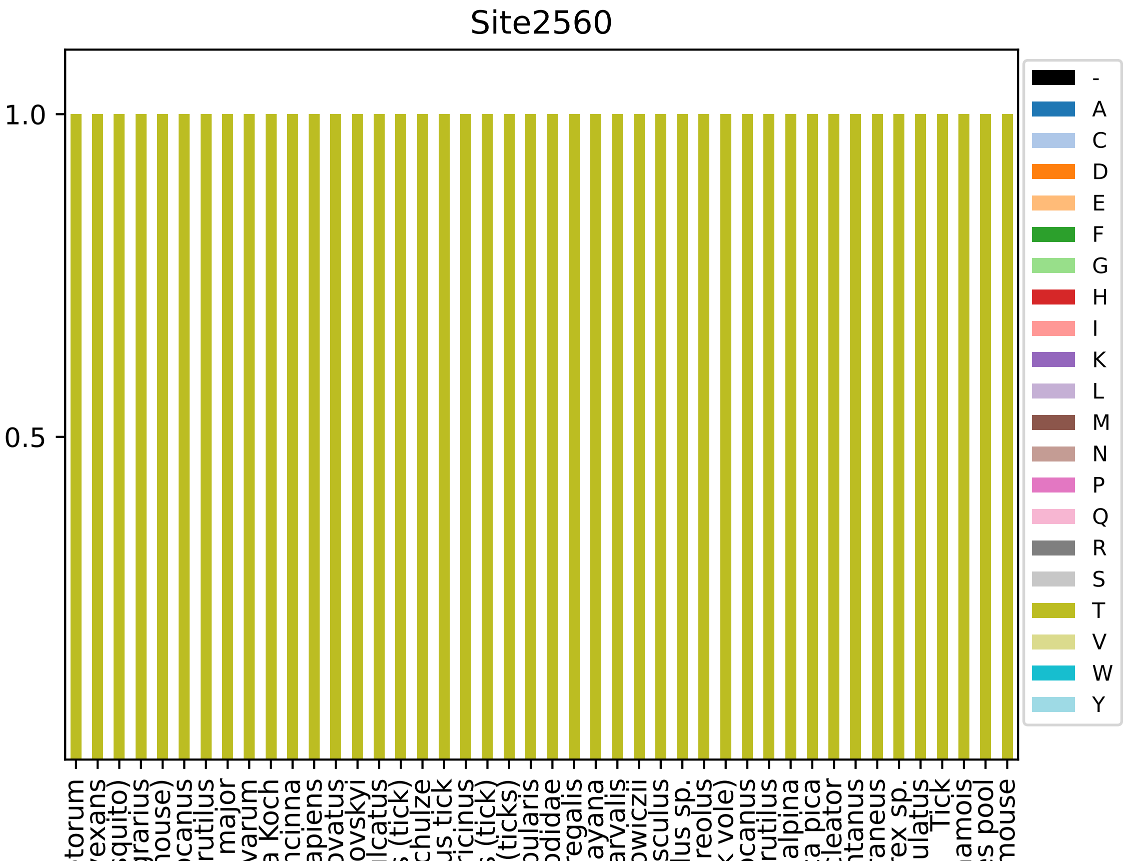

Site2560
1.0
0.5
-
A
C
D
E
F
G
H
I
K
L
M
N
P
Q
R
S
T
V
W
Y

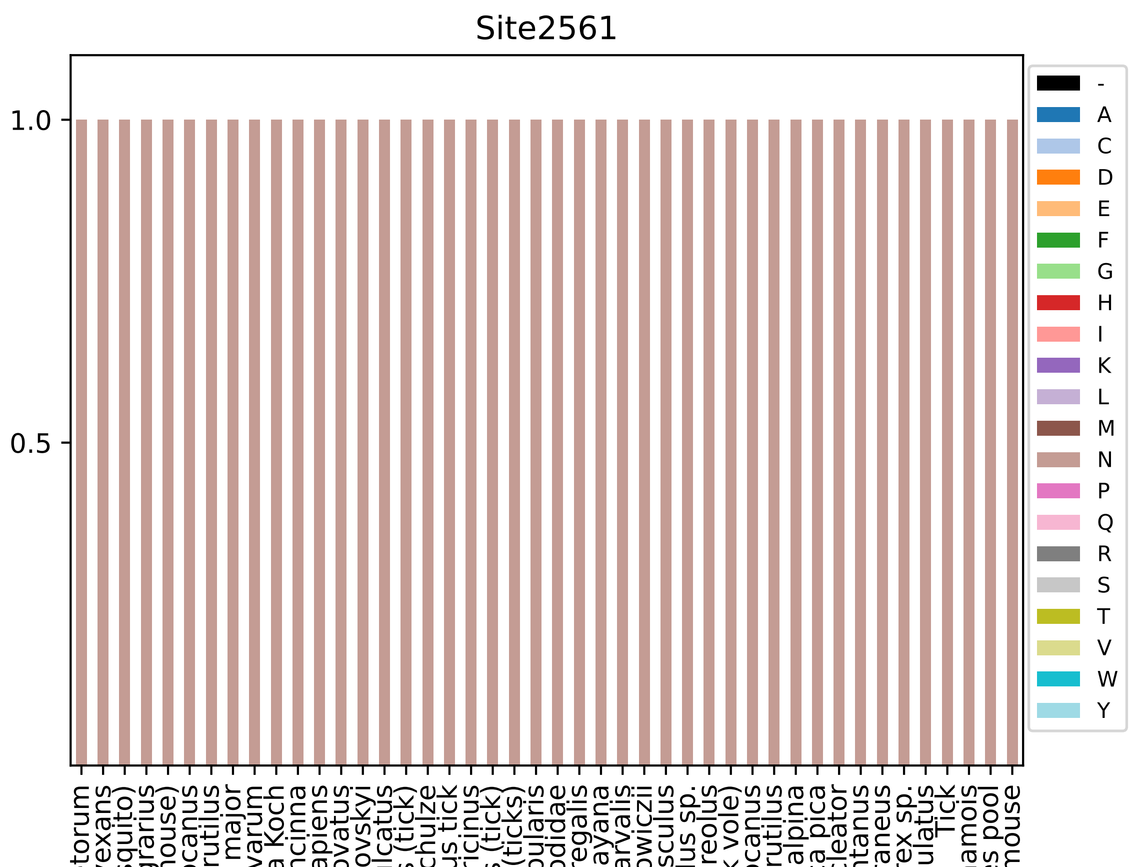
Site2561
1.0
0.5
-
A
C
D
E
F
G
H
I
K
L
M
N
P
Q
R
S
T
V
W
Y

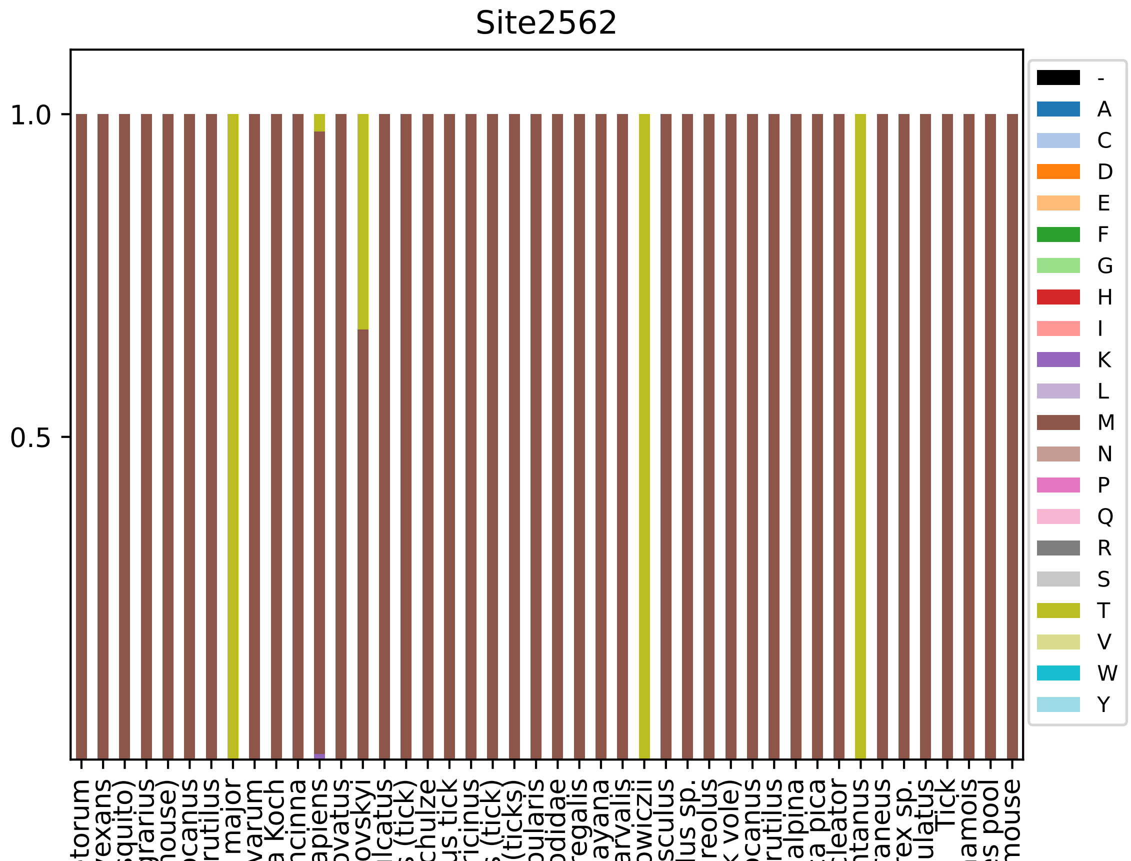

Site2562
1.0
0.5
-
A
C
D
E
F
G
H
I
K
L
M
N
P
Q
R
S
T
V
W
Y

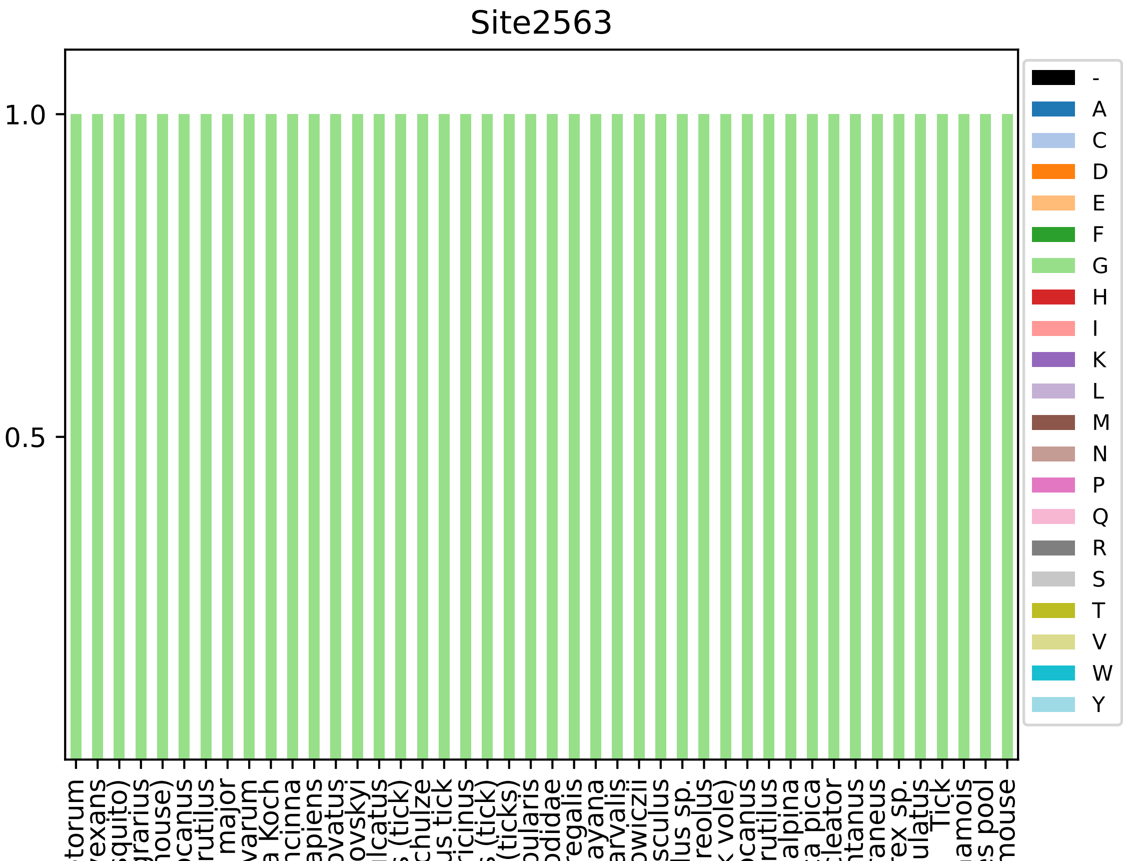
Site2563
1.0
0.5
-
A
C
D
E
F
G
H
I
K
L
M
N
P
Q
R
S
T
V
W
Y

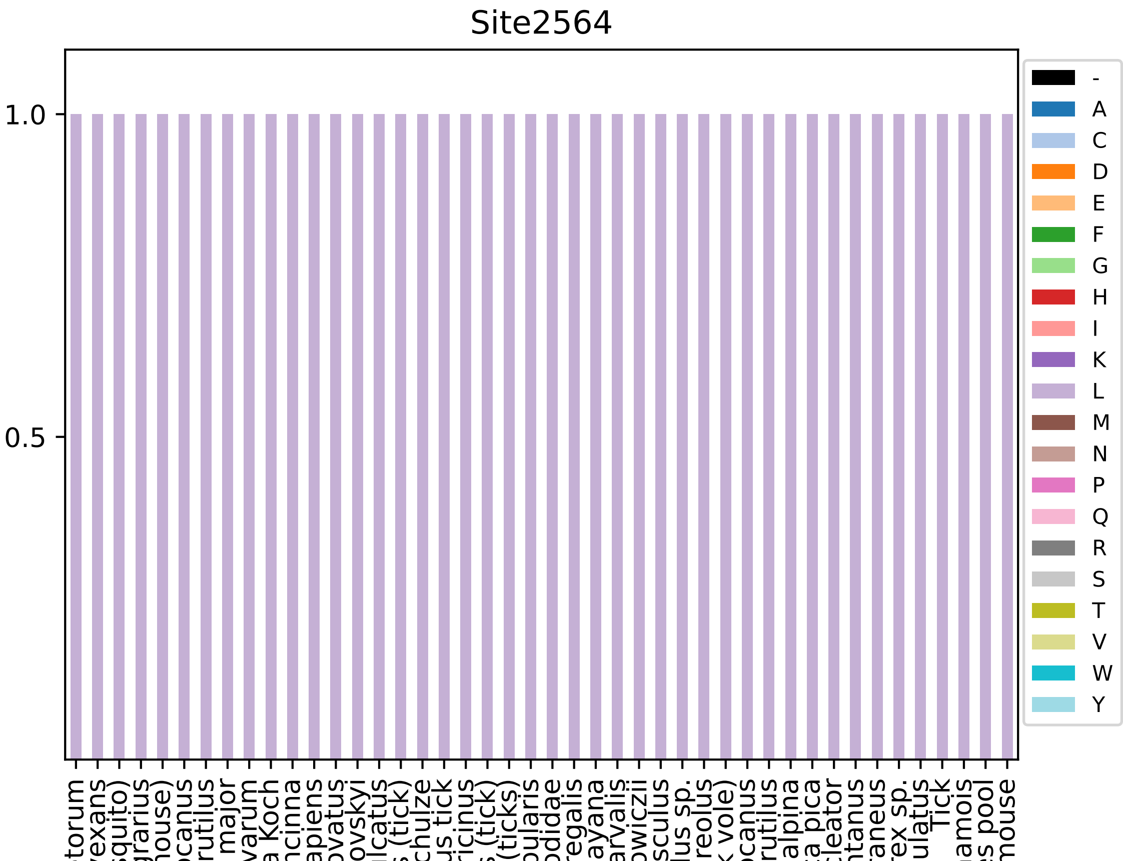
Site2564
1.0
0.5
-
A
C
D
E
F
G
H
I
K
L
M
N
P
Q
R
S
T
V
W
Y

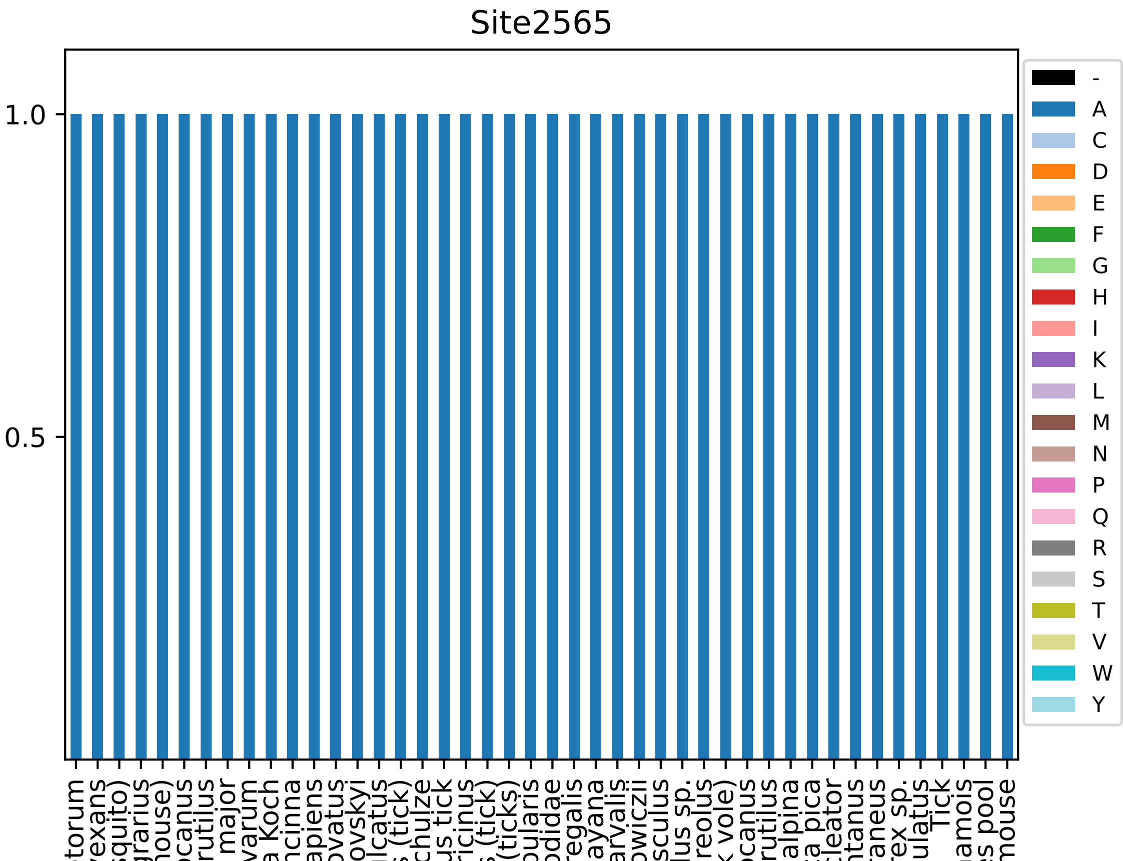
Site2565
1.0
0.5
-
A
C
D
E
F
G
H
I
K
L
M
N
P
Q
R
S
T
V
W
Y

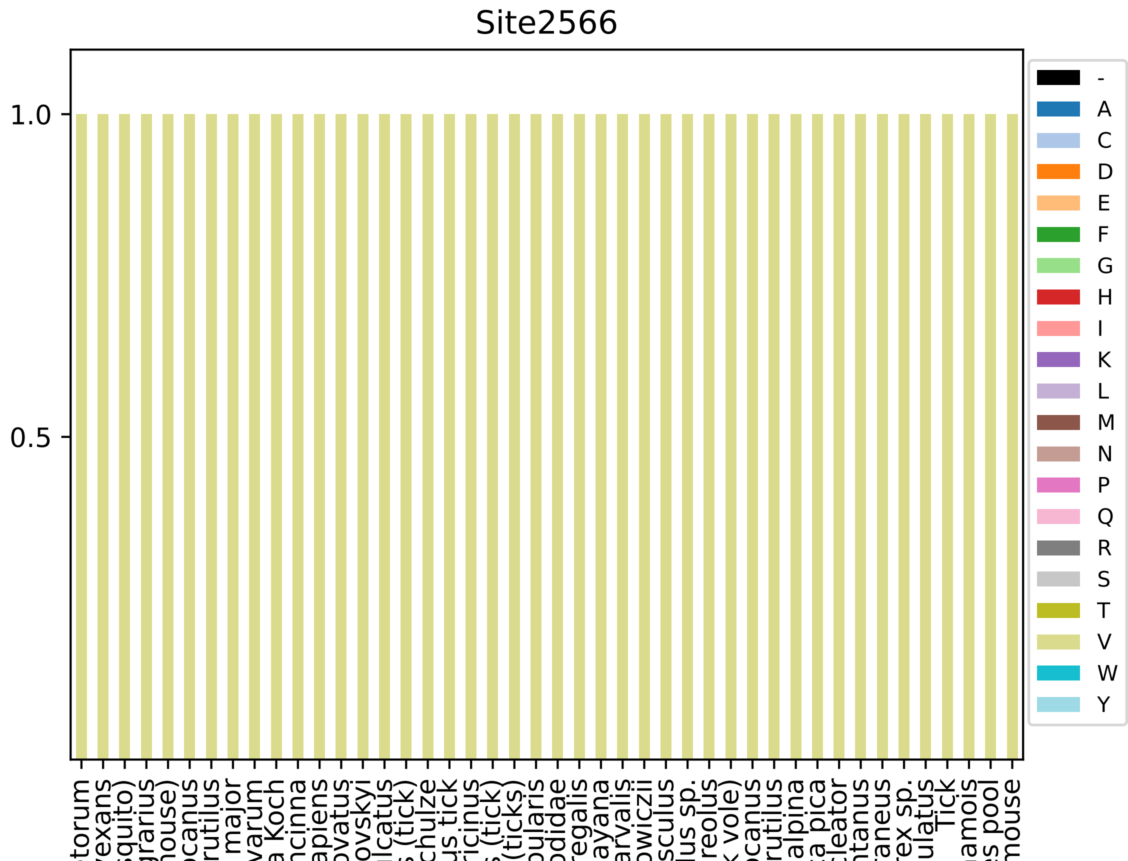
Site2566
1.0
0.5
-
A
C
D
E
F
G
H
I
K
L
M
N
P
Q
R
S
T
V
W
Y
torum
vexans
squito)
garius
nouse)
ocanus
rutilus
major
varum
a Koch
ncinna
apiens
ovatus
ovskyi
lcatus
s (tick)
chulze
us tick
ricinus
s (tick)
(ticks)
pularis
podidae
regalis
ayana
arvalis
owiczii
sculus
lus sp.
reolus
k vole)
ocanus
rutilus
alpina
a pica
leator
ntanus
raneus
rex sp.
ulatus
Tick
amois
es pool
mouse

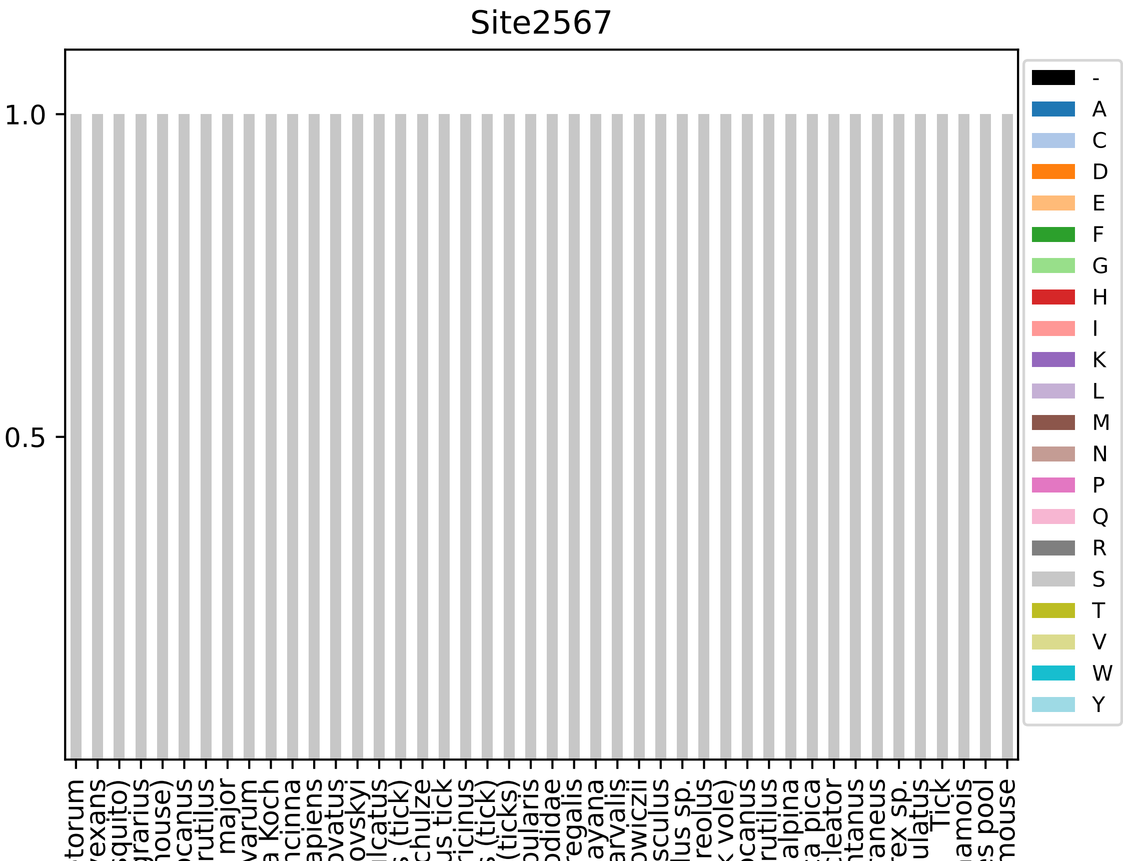

Site2567
1.0
0.5
-
A
C
D
E
F
G
H
I
K
L
M
N
P
Q
R
S
T
V
W
Y

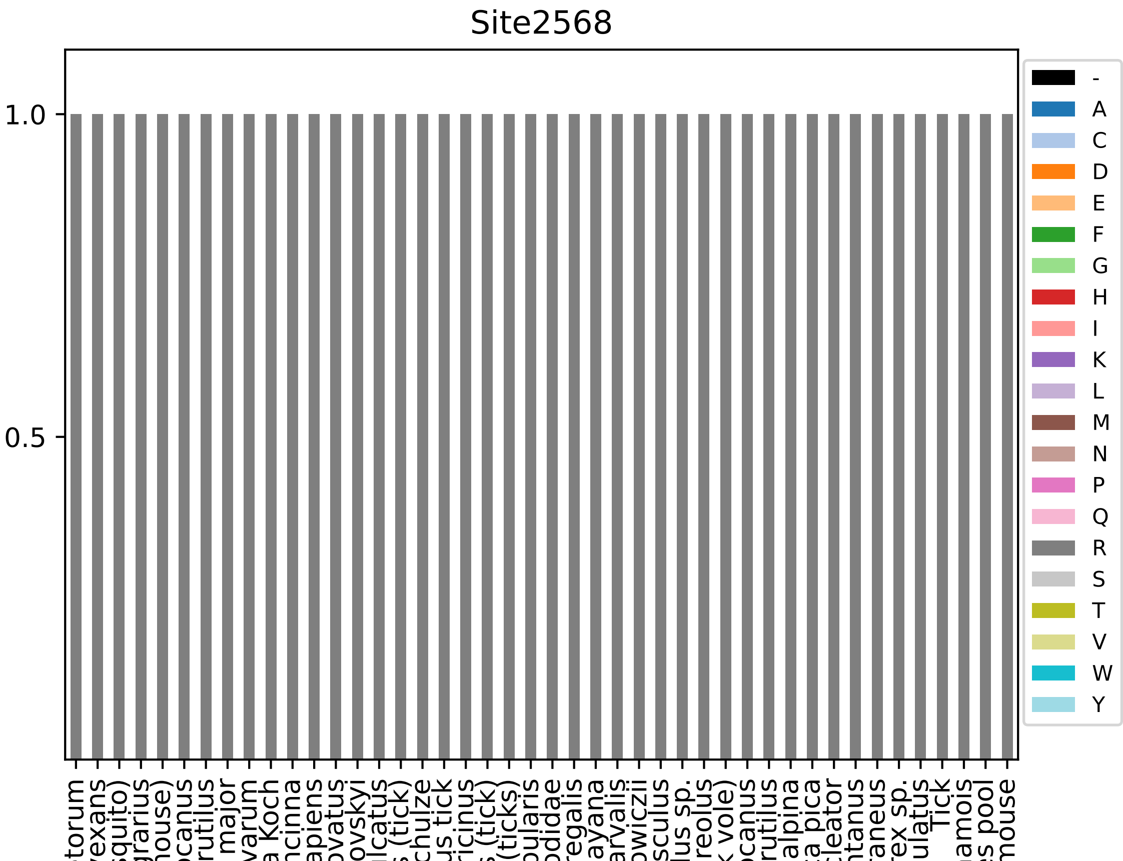
Site2568
1.0
0.5
-
A
C
D
E
F
G
H
I
K
L
M
N
P
Q
R
S
T
V
W
Y

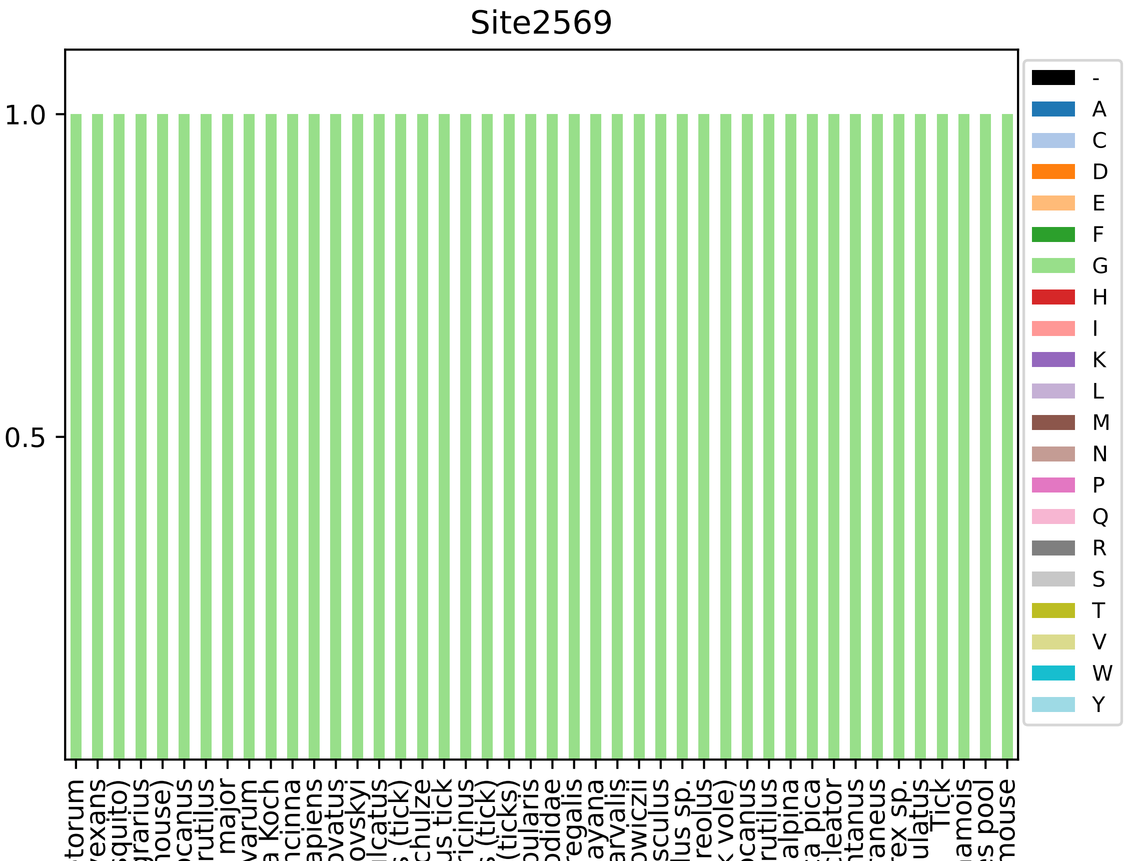
Site2569
1.0
0.5
-
A
C
D
E
F
G
H
I
K
L
M
N
P
Q
R
S
T
V
W
Y

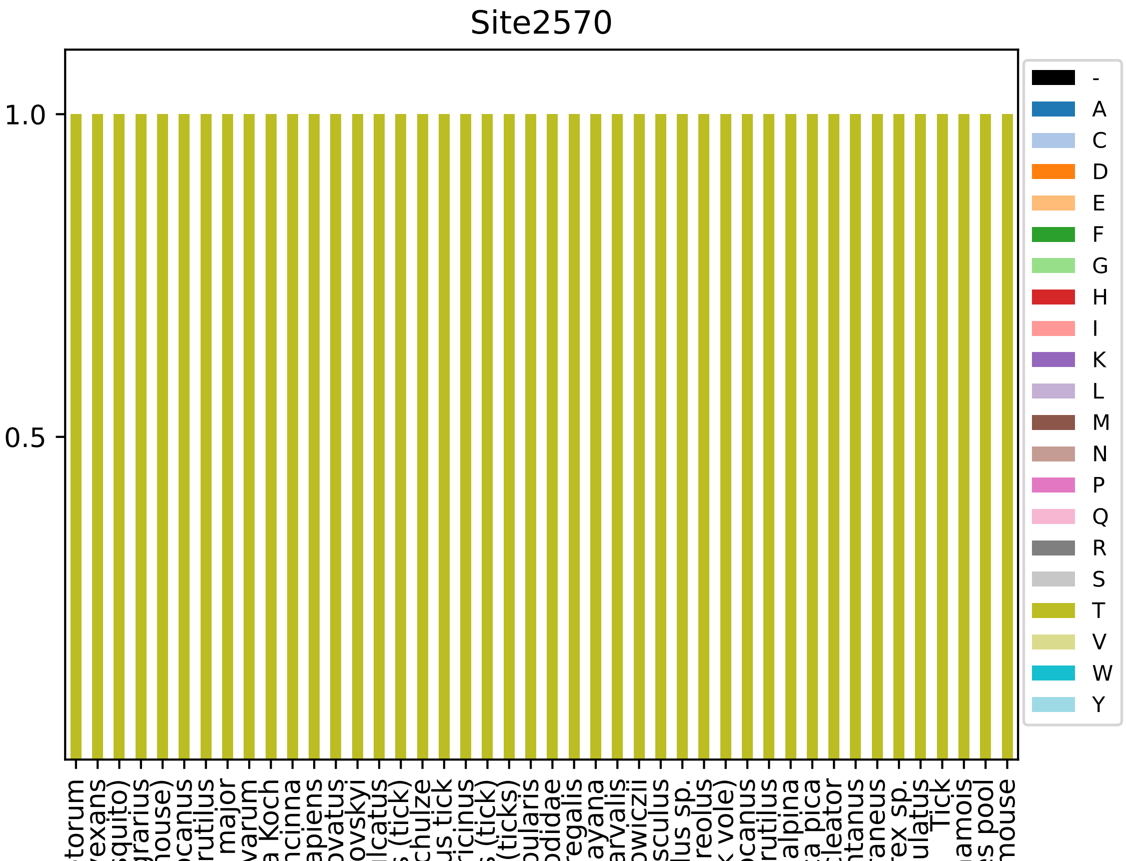

Site2570
1.0
0.5
-
A
C
D
E
F
G
H
I
K
L
M
N
P
Q
R
S
T
V
W
Y

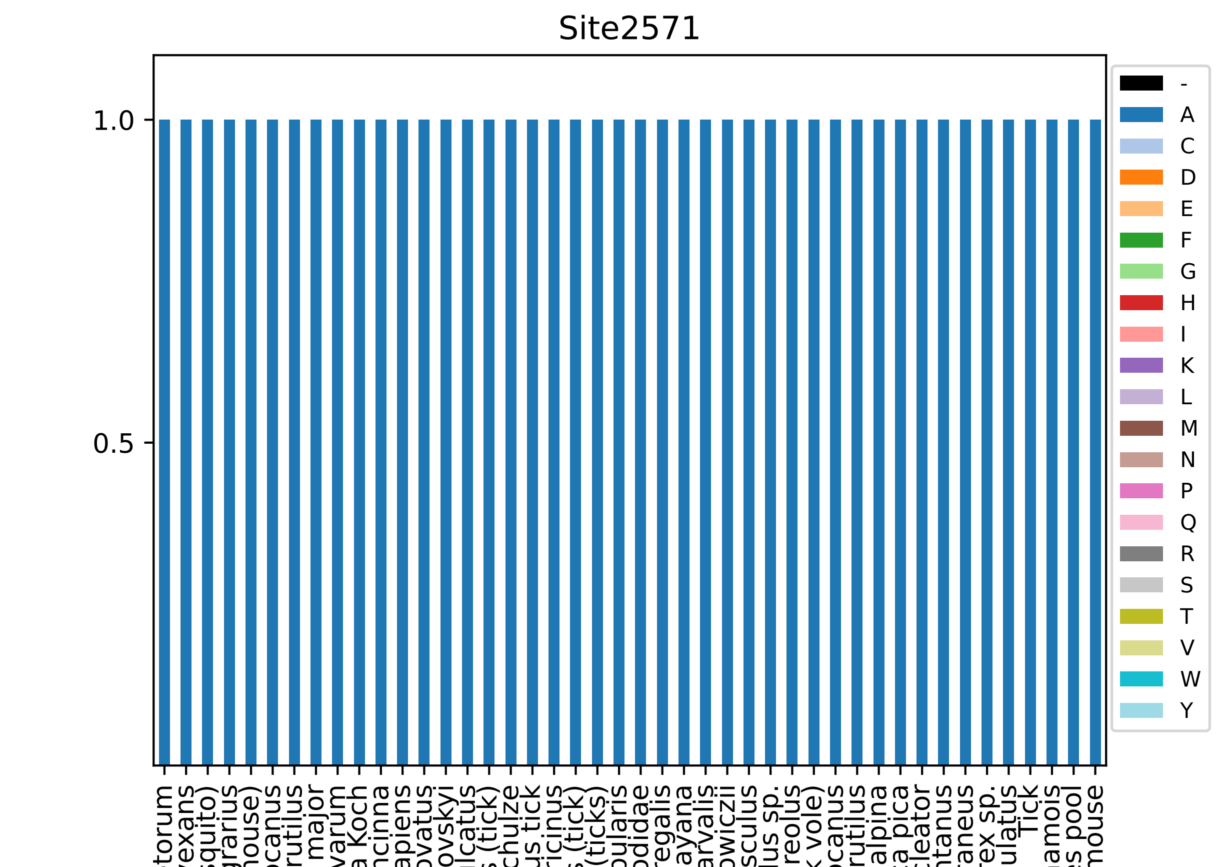
Site2571
1.0
0.5
-
A
C
D
E
F
G
H
I
K
L
M
N
P
Q
R
S
T
V
W
Y

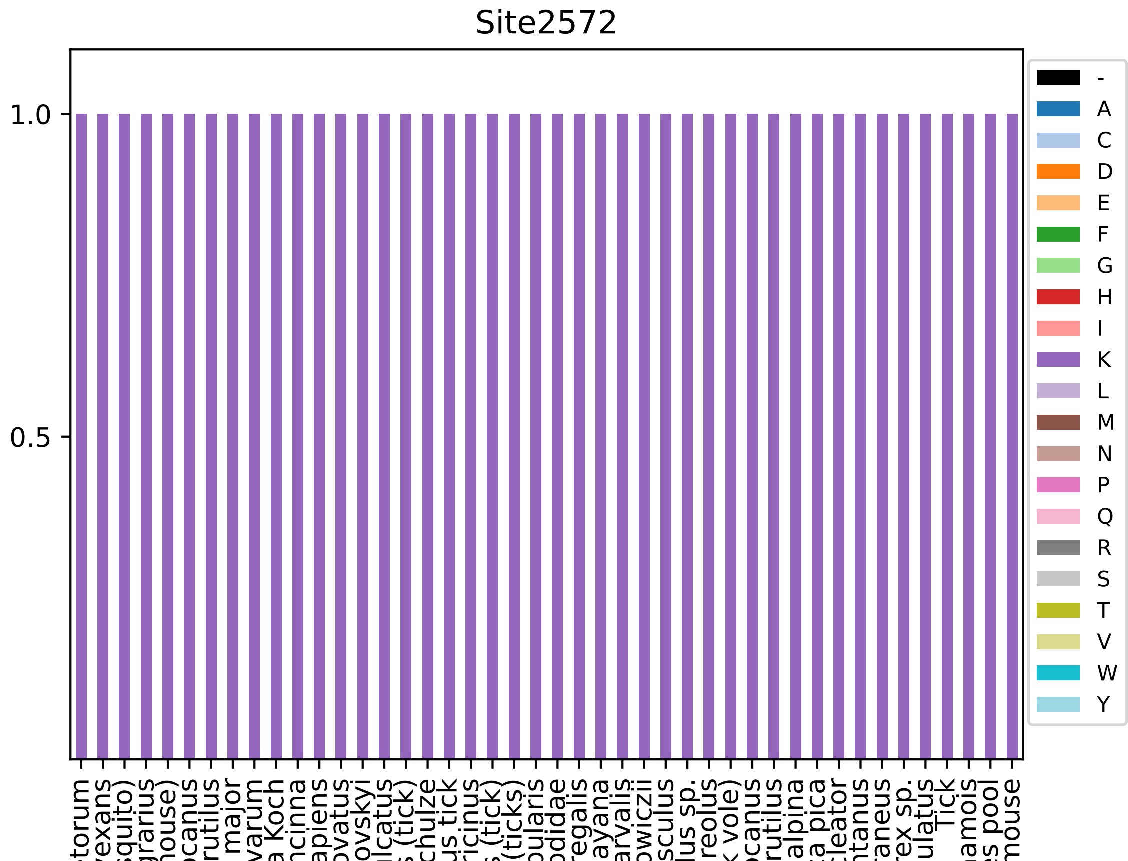
Site2572
1.0
0.5
-
A
C
D
E
F
G
H
I
K
L
M
N
P
Q
R
S
T
V
W
Y

Site2573

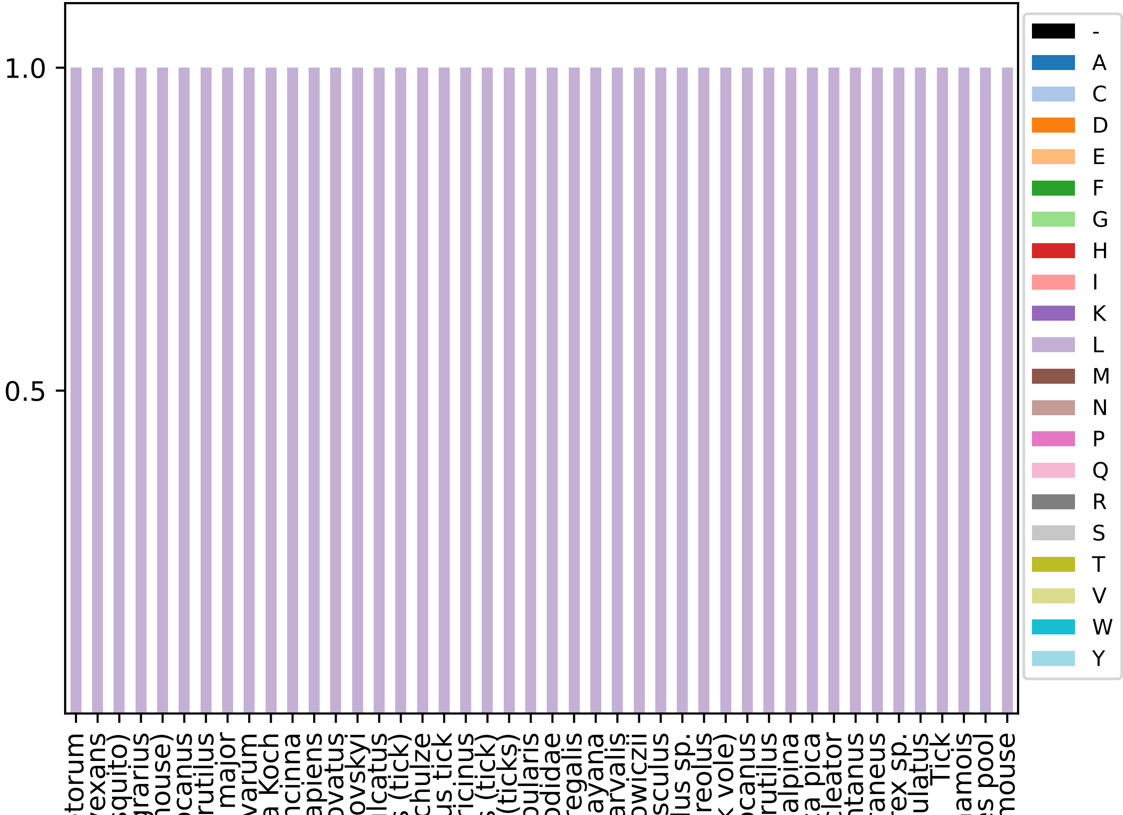

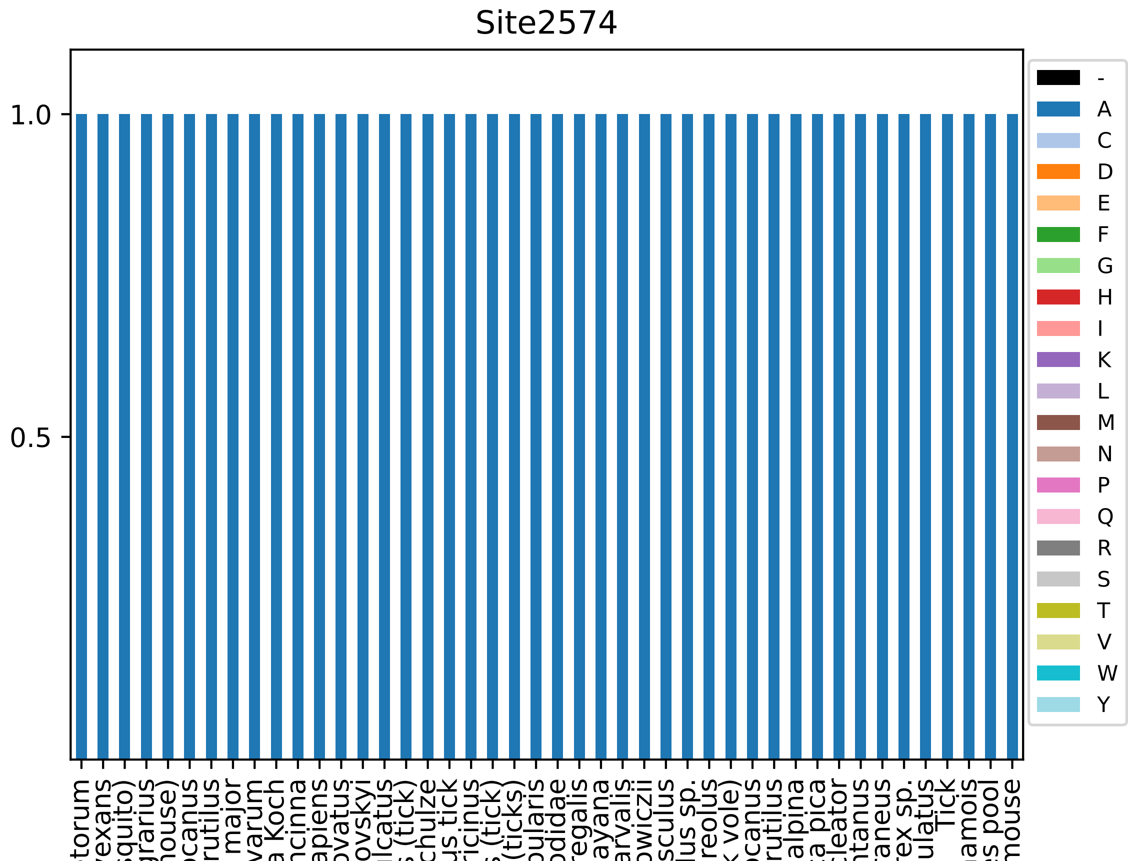

Site2574
1.0
0.5
-
A
C
D
E
F
G
H
I
K
L
M
N
P
Q
R
S
T
V
W
Y

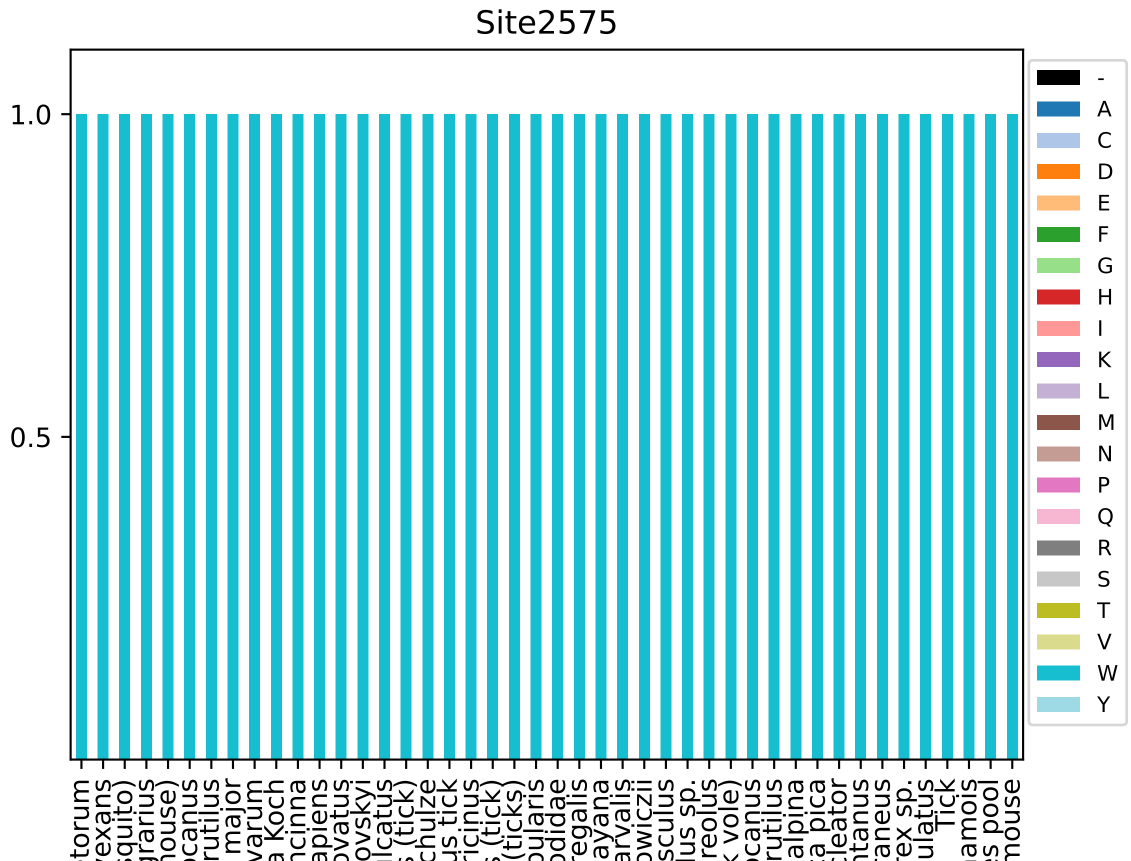

Site2575
1.0
0.5
-
A
C
D
E
F
G
H
I
K
L
M
N
P
Q
R
S
T
V
W
Y

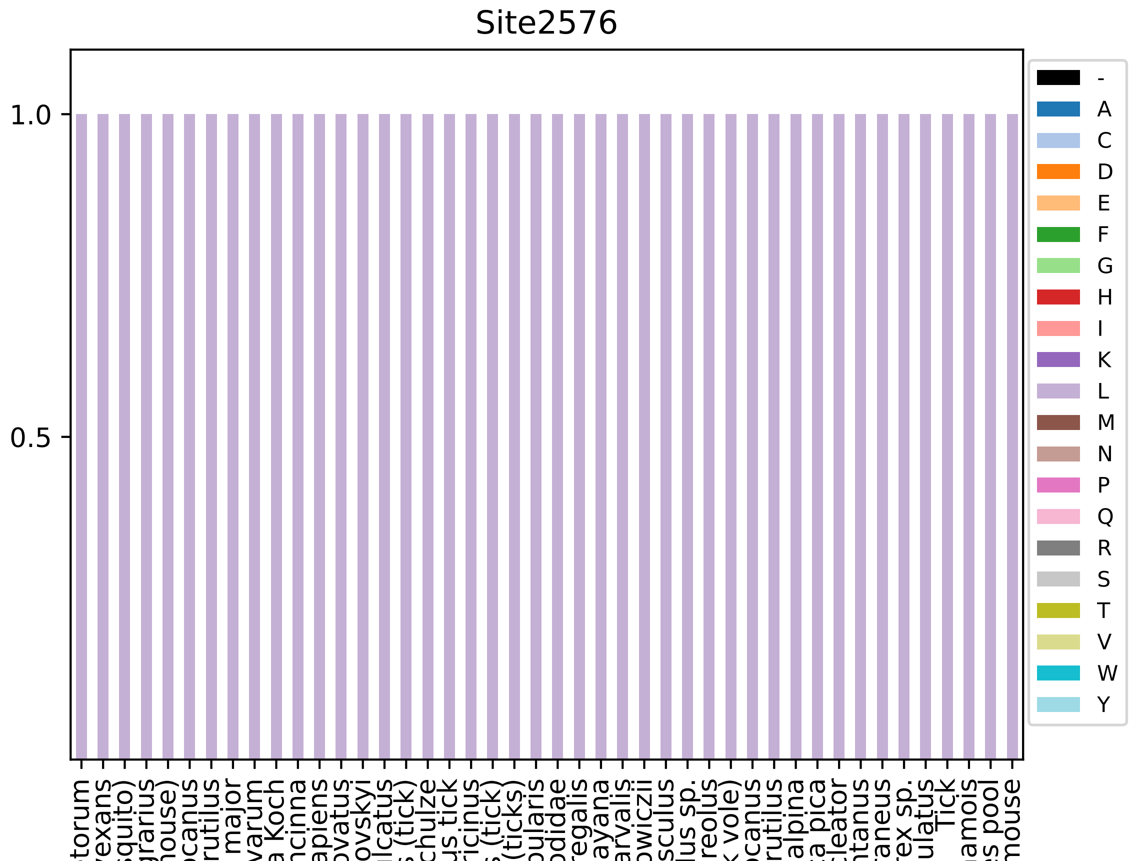
Site2576
1.0
0.5
-
A
C
D
E
F
G
H
I
K
L
M
N
P
Q
R
S
T
V
W
Y
Tick

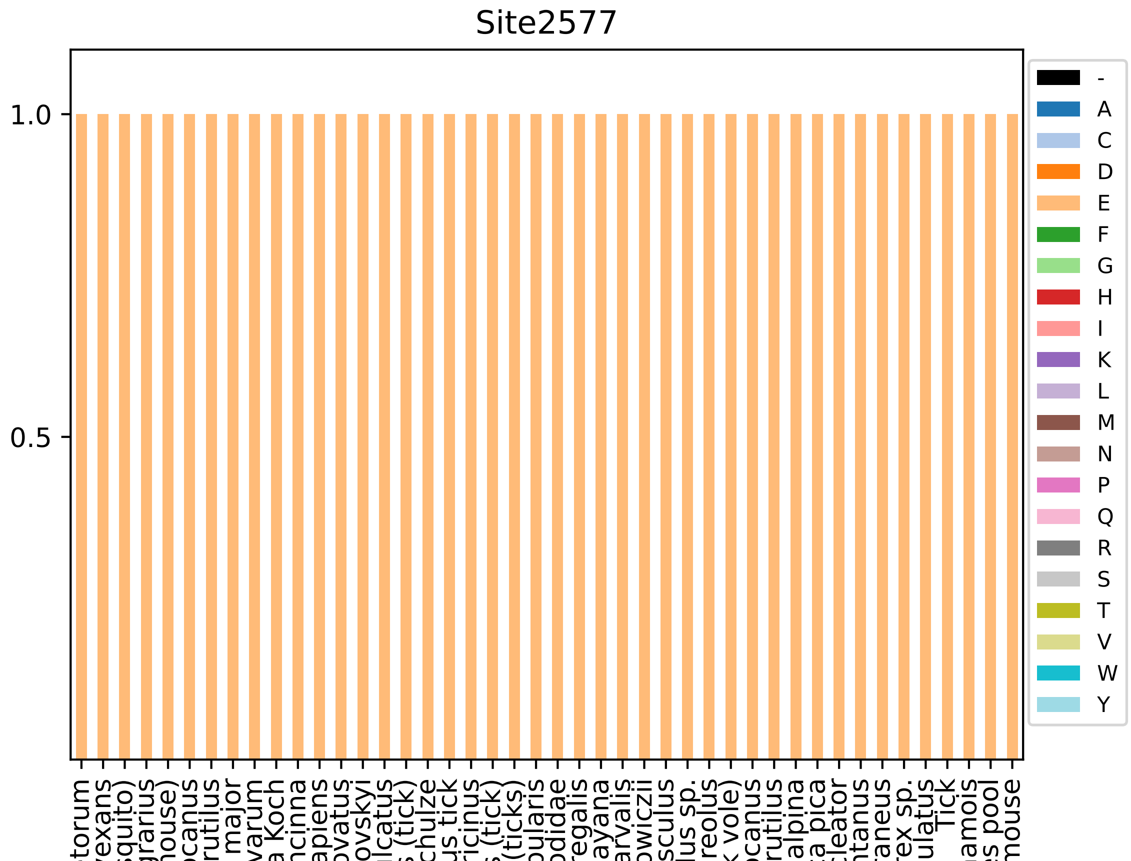
Site2577
1.0
0.5
-
A
C
D
E
F
G
H
I
K
L
M
N
P
Q
R
S
T
V
W
Y

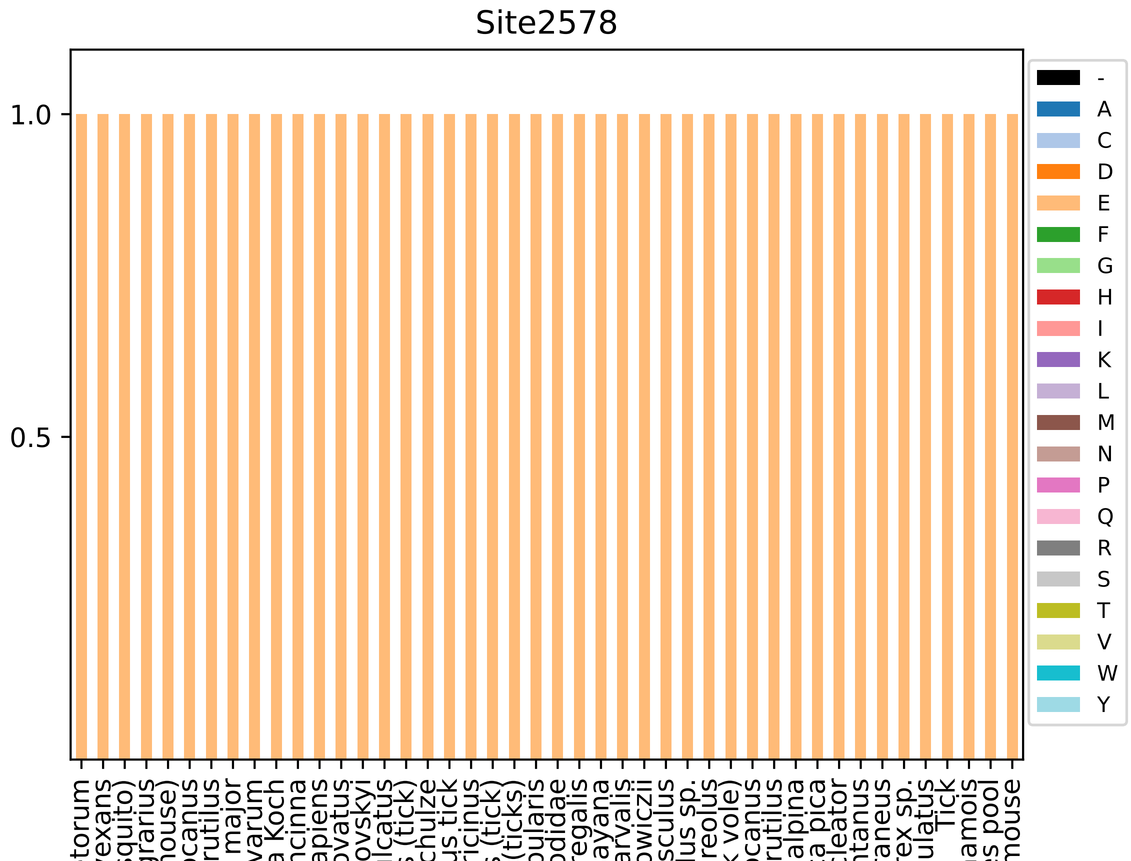
Site2578
1.0
0.5
-
A
C
D
E
F
G
H
I
K
L
M
N
P
Q
R
S
T
V
W
Y
Tick

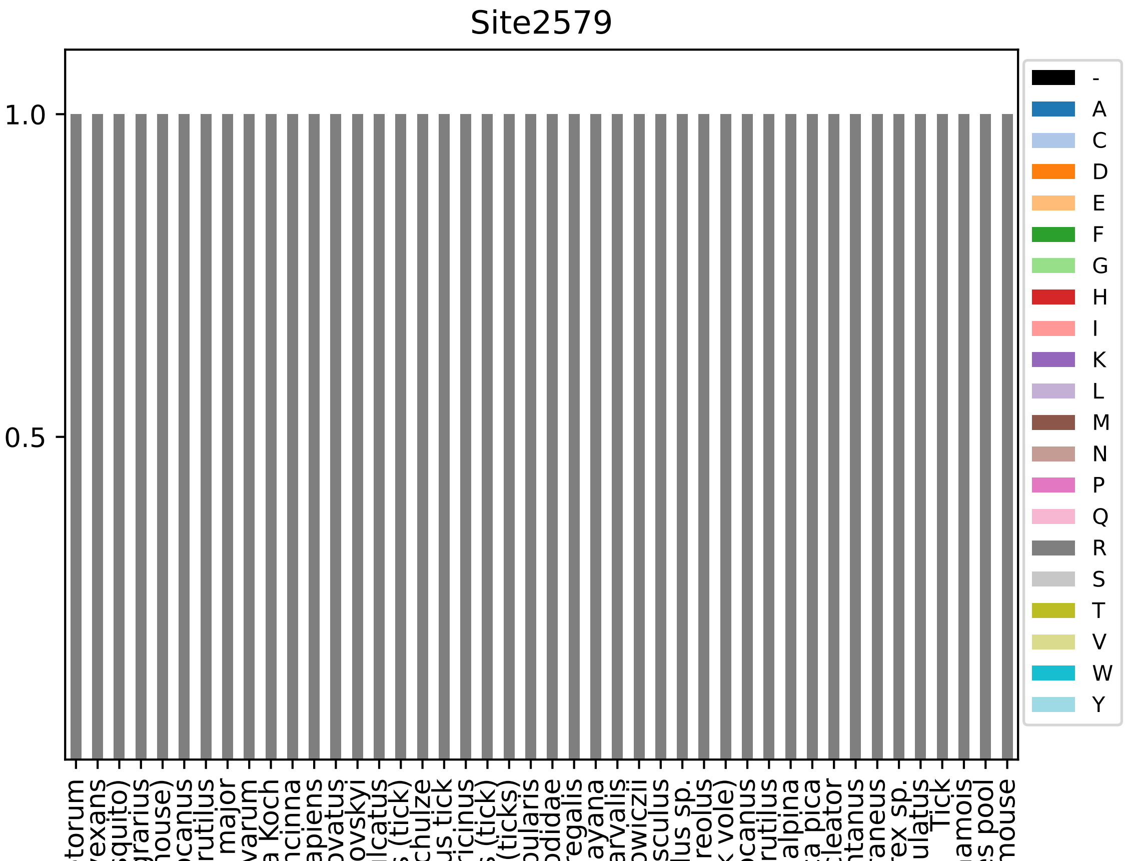

Site2579
1.0
0.5
-
A
C
D
E
F
G
H
I
K
L
M
N
P
Q
R
S
T
V
W
Y

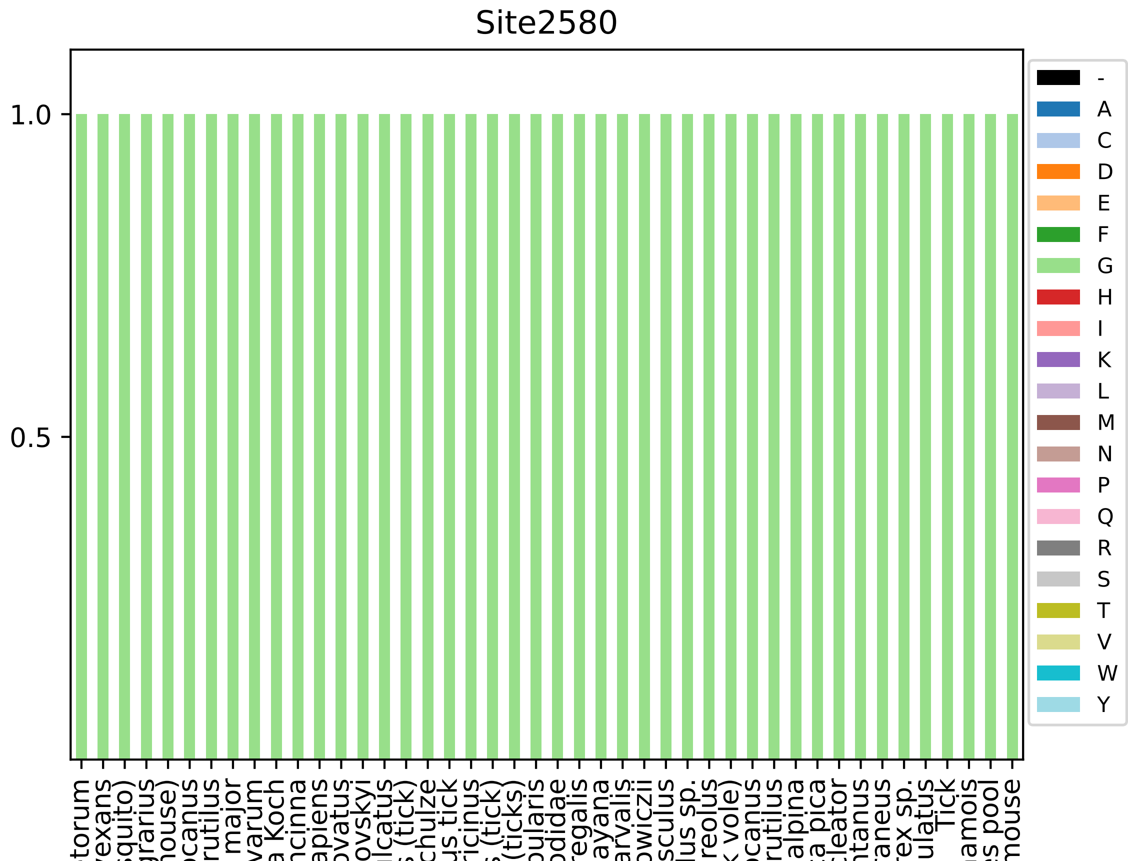

Site2580
1.0
0.5
-
A
C
D
E
F
G
H
I
K
L
M
N
P
Q
R
S
T
V
W
Y

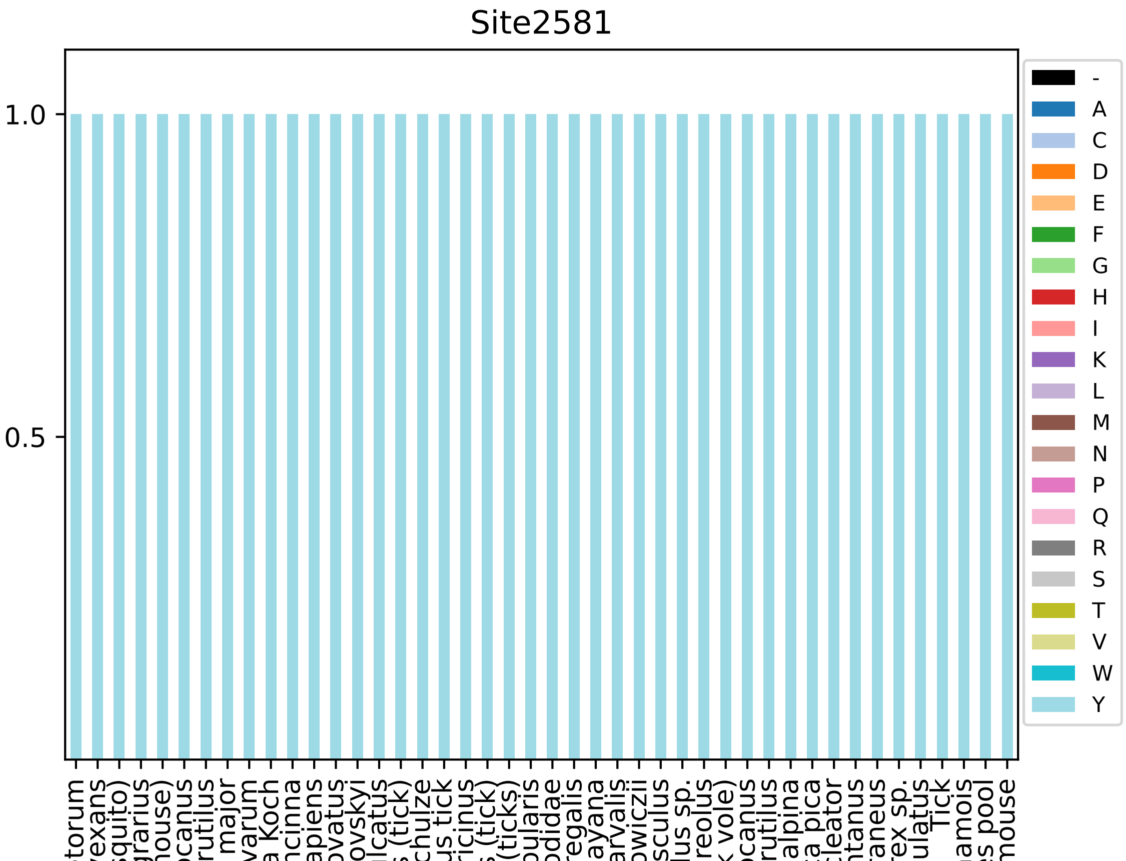
Site2581
1.0
0.5
-
A
C
D
E
F
G
H
I
K
L
M
N
P
Q
R
S
T
V
W
Y

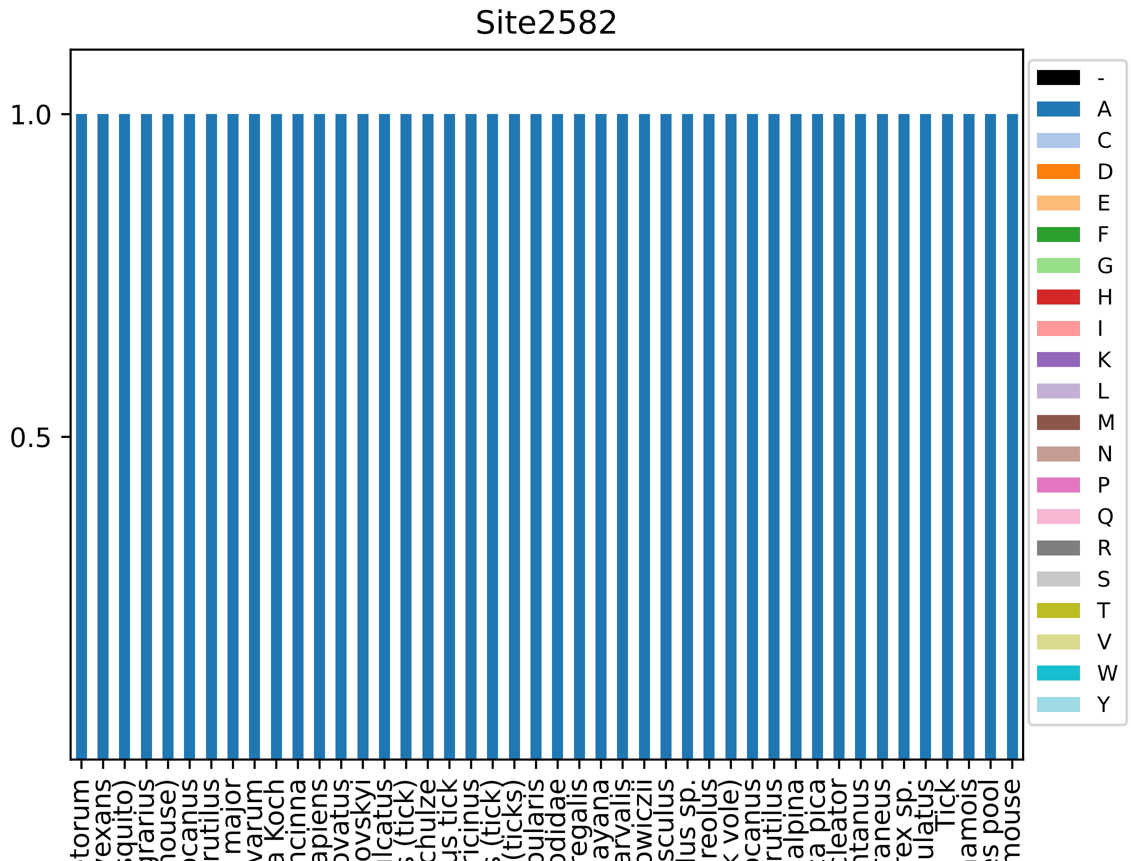
Site2582
1.0
0.5
-
A
C
D
E
F
G
H
I
K
L
M
N
P
Q
R
S
T
V
W
Y
Tick

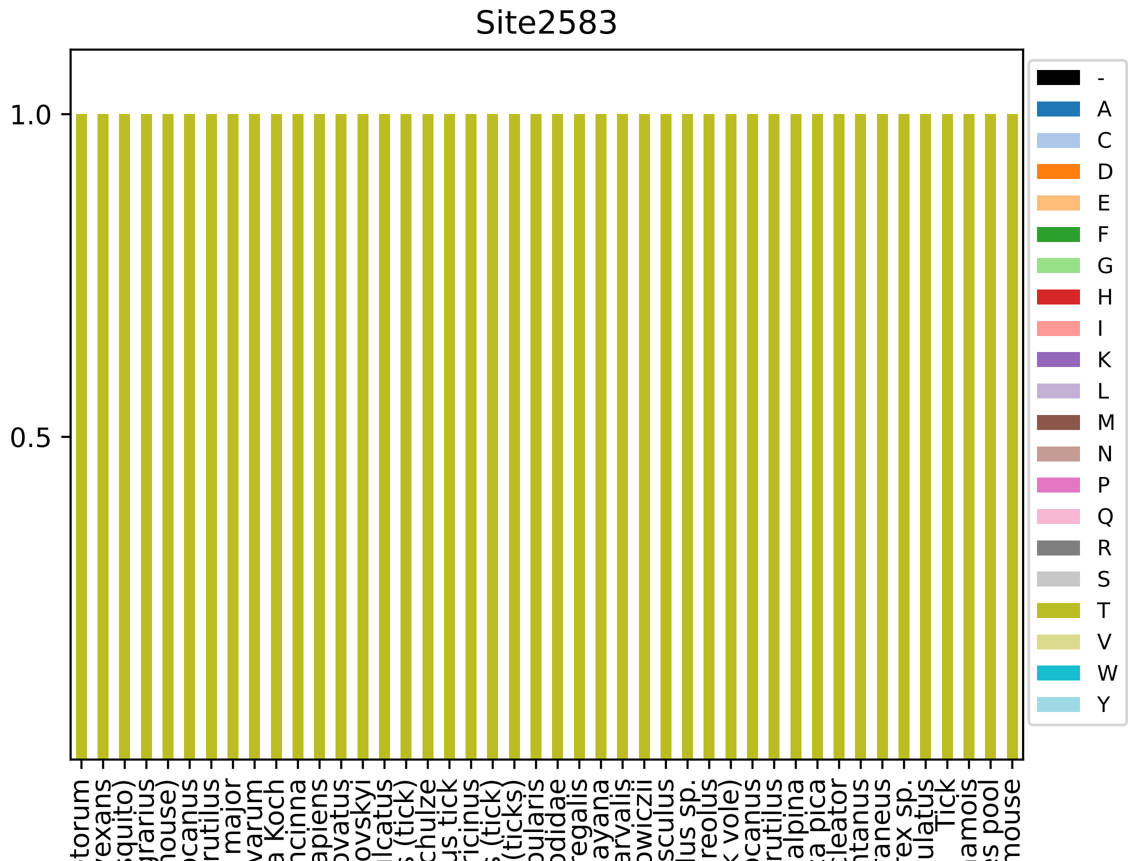
Site2583
1.0
0.5
-
A
C
D
E
F
G
H
I
K
L
M
N
P
Q
R
S
T
V
W
Y

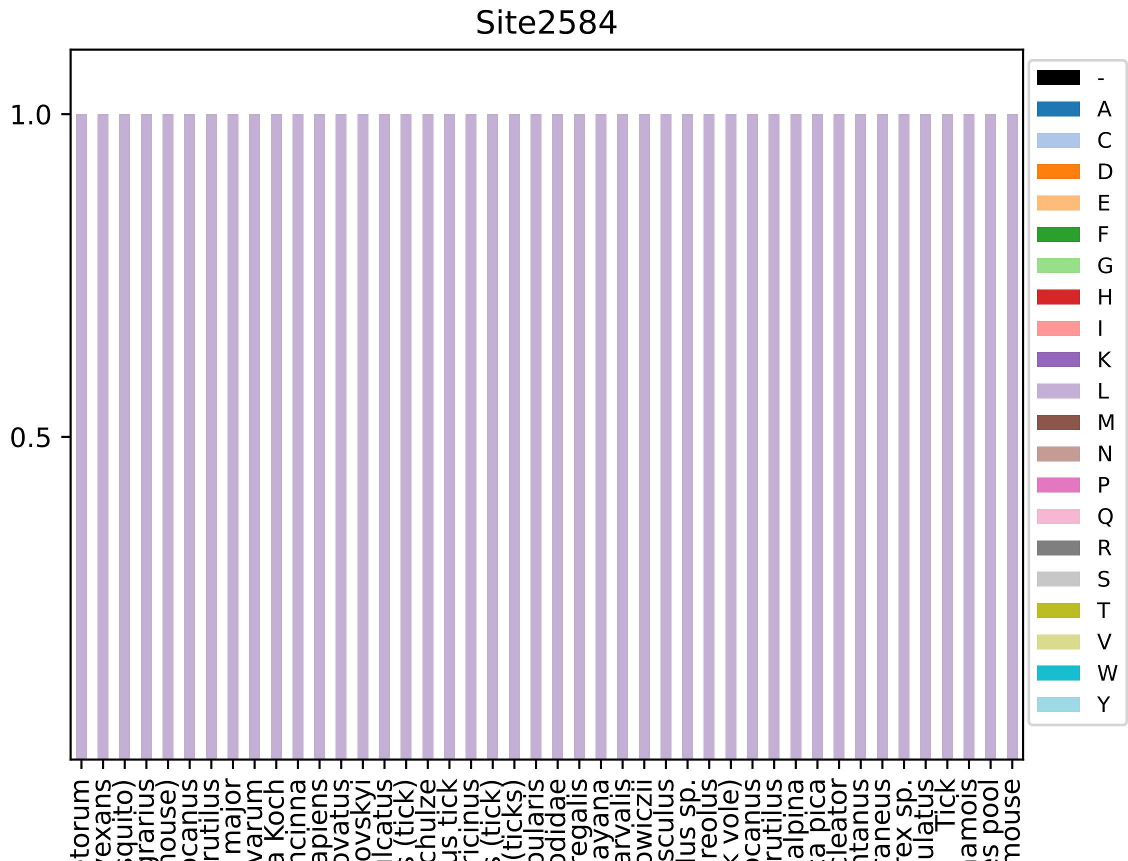
Site2584
1.0
0.5
-
A
C
D
E
F
G
H
I
K
L
M
N
P
Q
R
S
T
V
W
Y

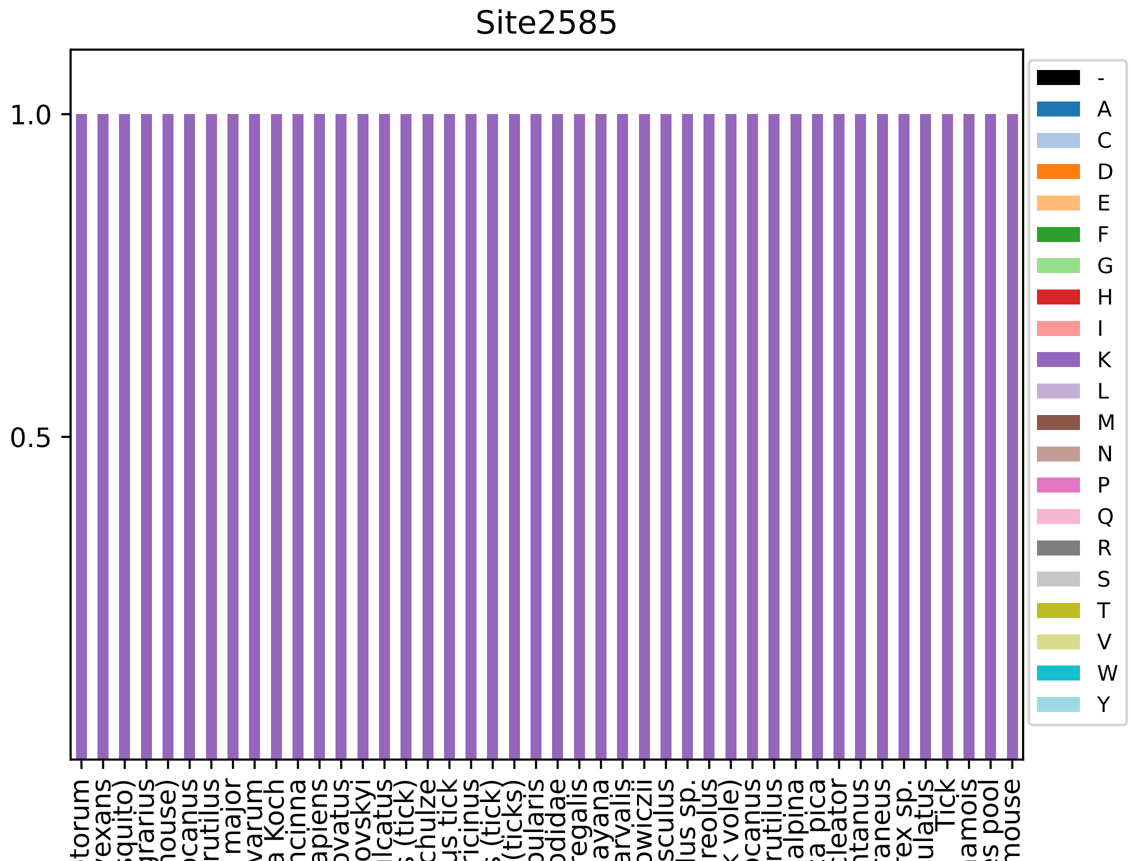

Site2585
1.0
0.5
-
A
C
D
E
F
G
H
I
K
L
M
N
P
Q
R
S
T
V
W
Y

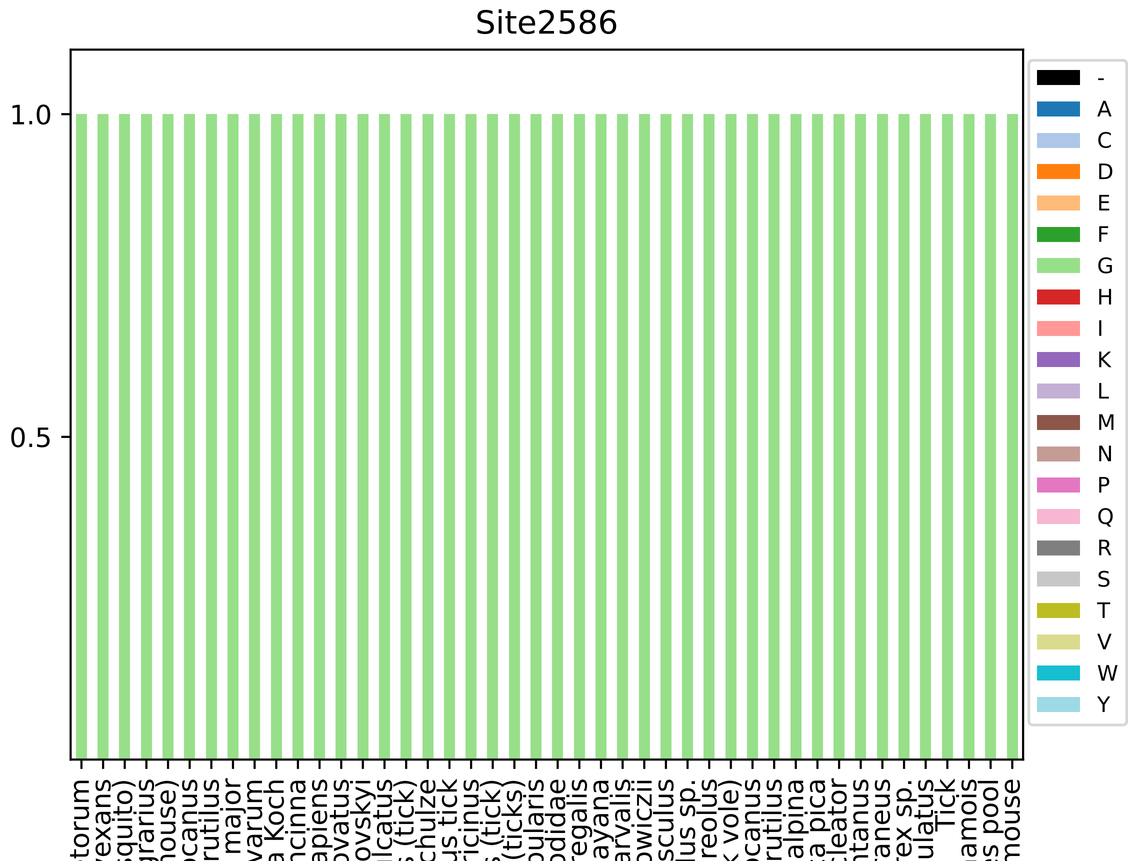
Site2586
1.0
0.5
-
A
C
D
E
F
G
H
I
K
L
M
N
P
Q
R
S
T
V
W
Y
Tick

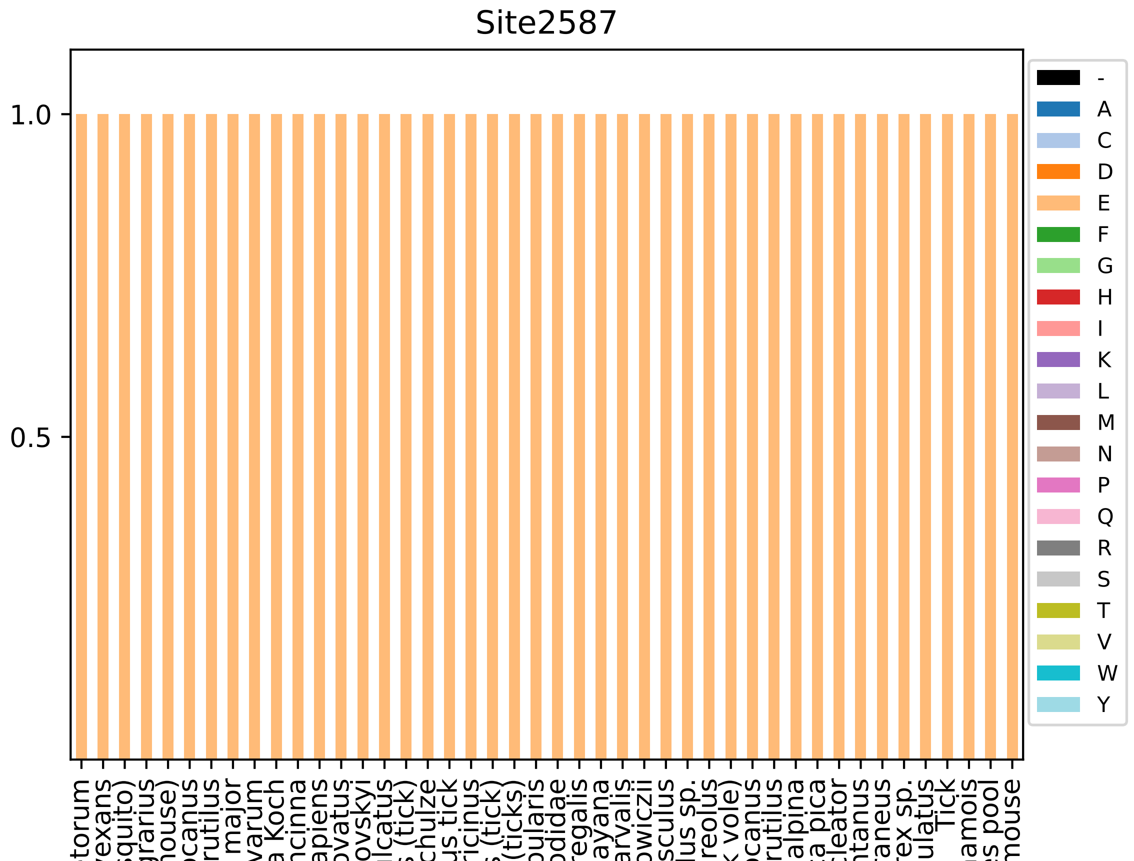
Site2587
1.0
0.5
-
A
C
D
E
F
G
H
I
K
L
M
N
P
Q
R
S
T
V
W
Y

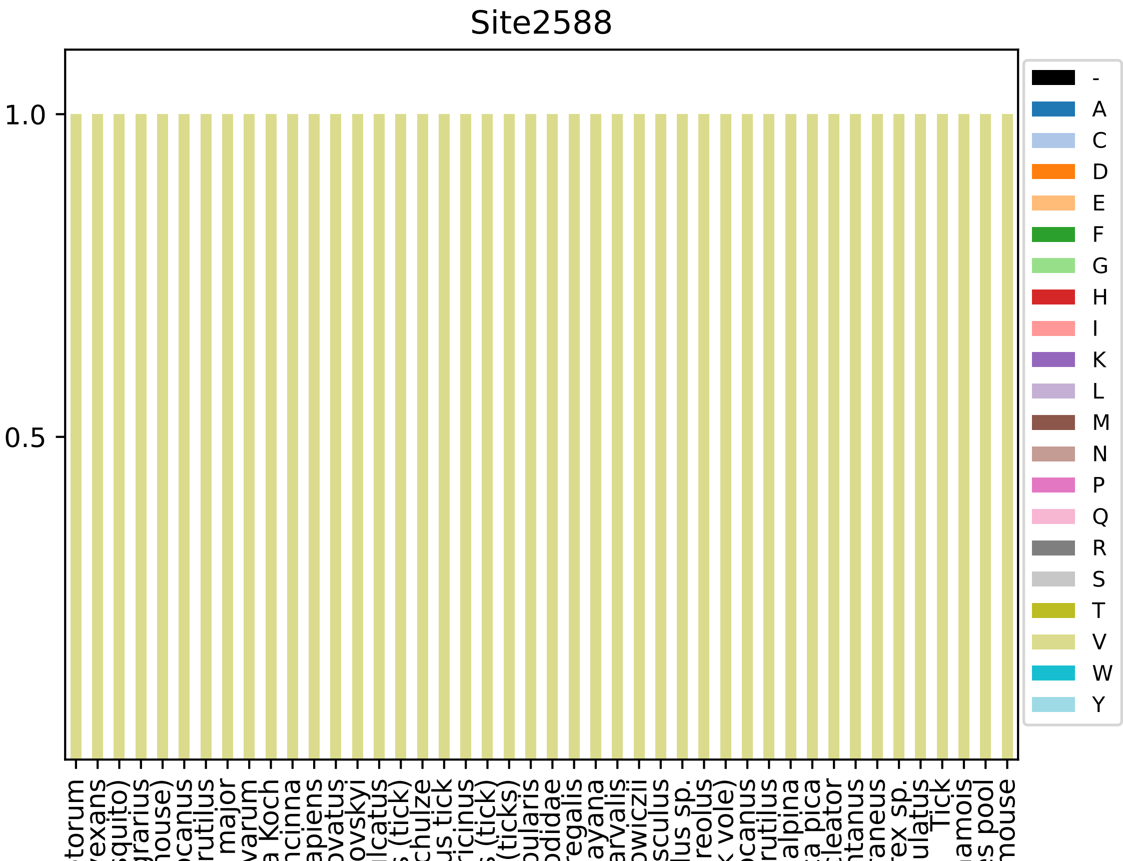

Site2588
1.0
0.5
-
A
C
D
E
F
G
H
I
K
L
M
N
P
Q
R
S
T
V
W
Y

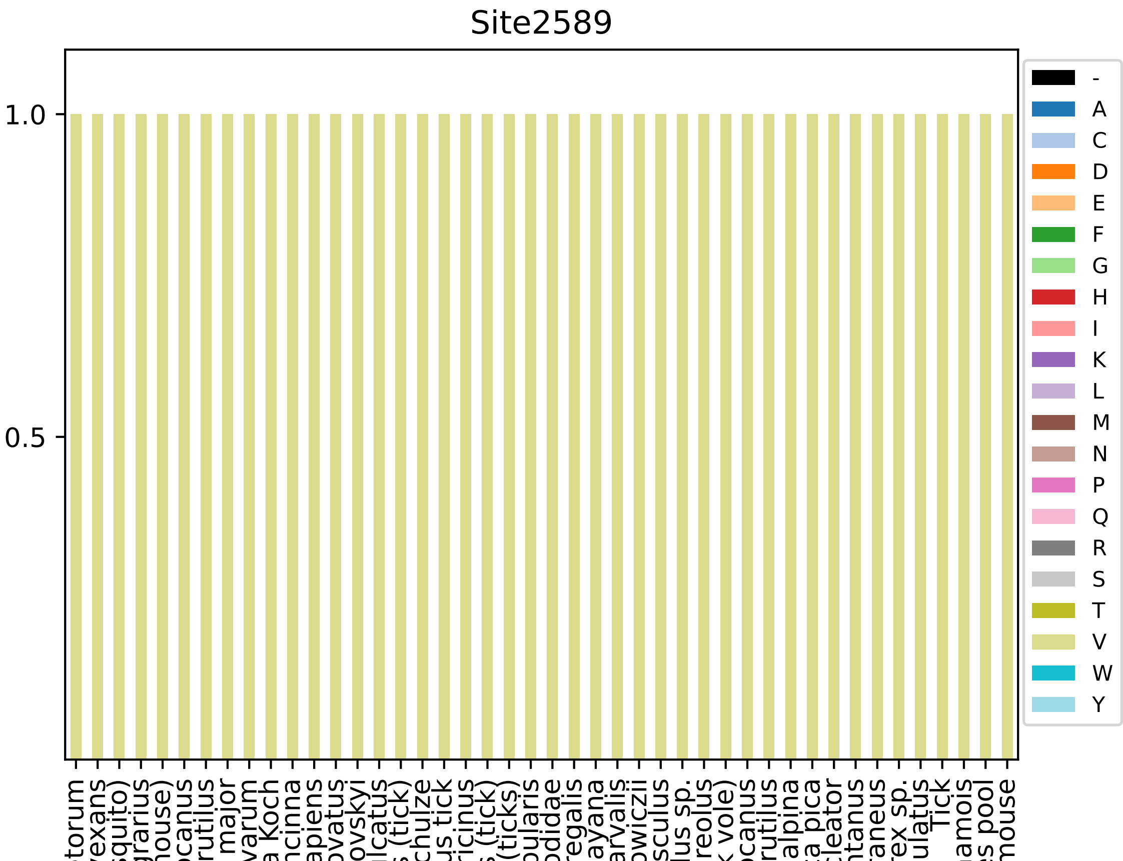
Site2589
1.0
0.5
-
A
C
D
E
F
G
H
I
K
L
M
N
P
Q
R
S
T
V
W
Y

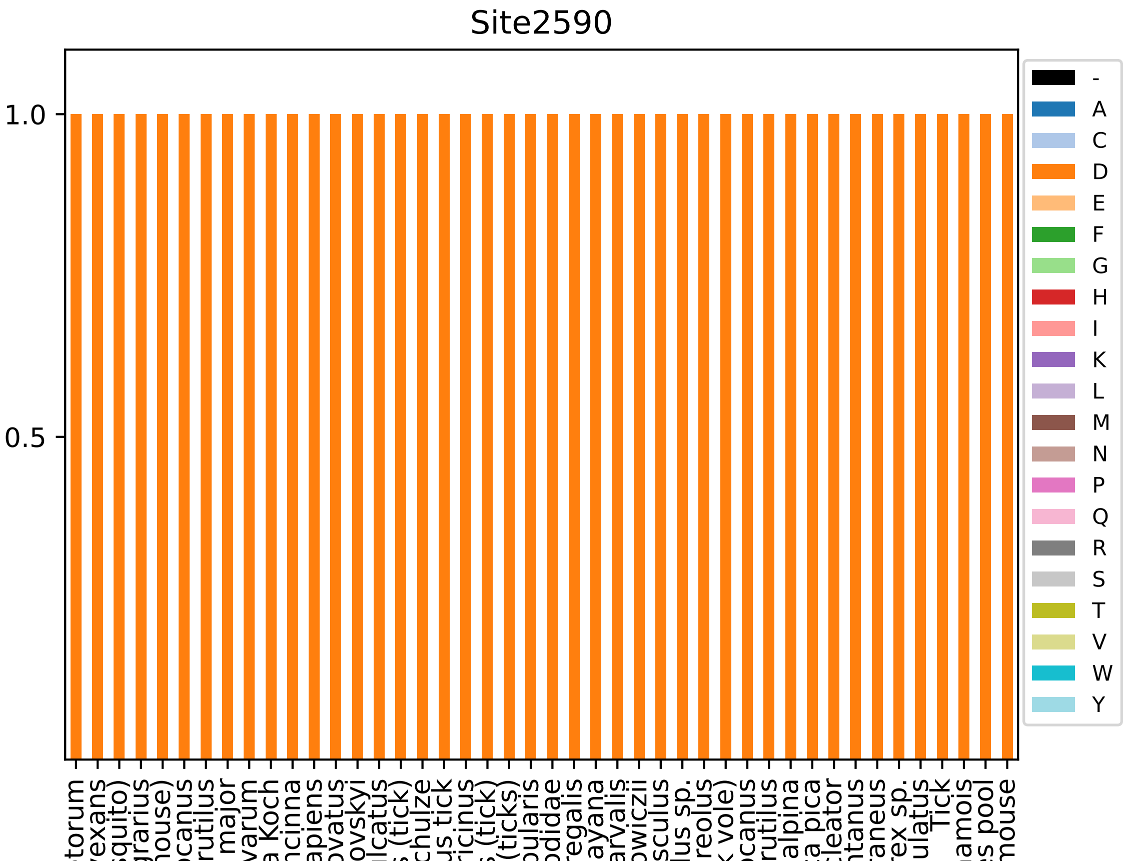
Site2590
1.0
0.5
-
A
C
D
E
F
G
H
I
K
L
M
N
P
Q
R
S
T
V
W
Y

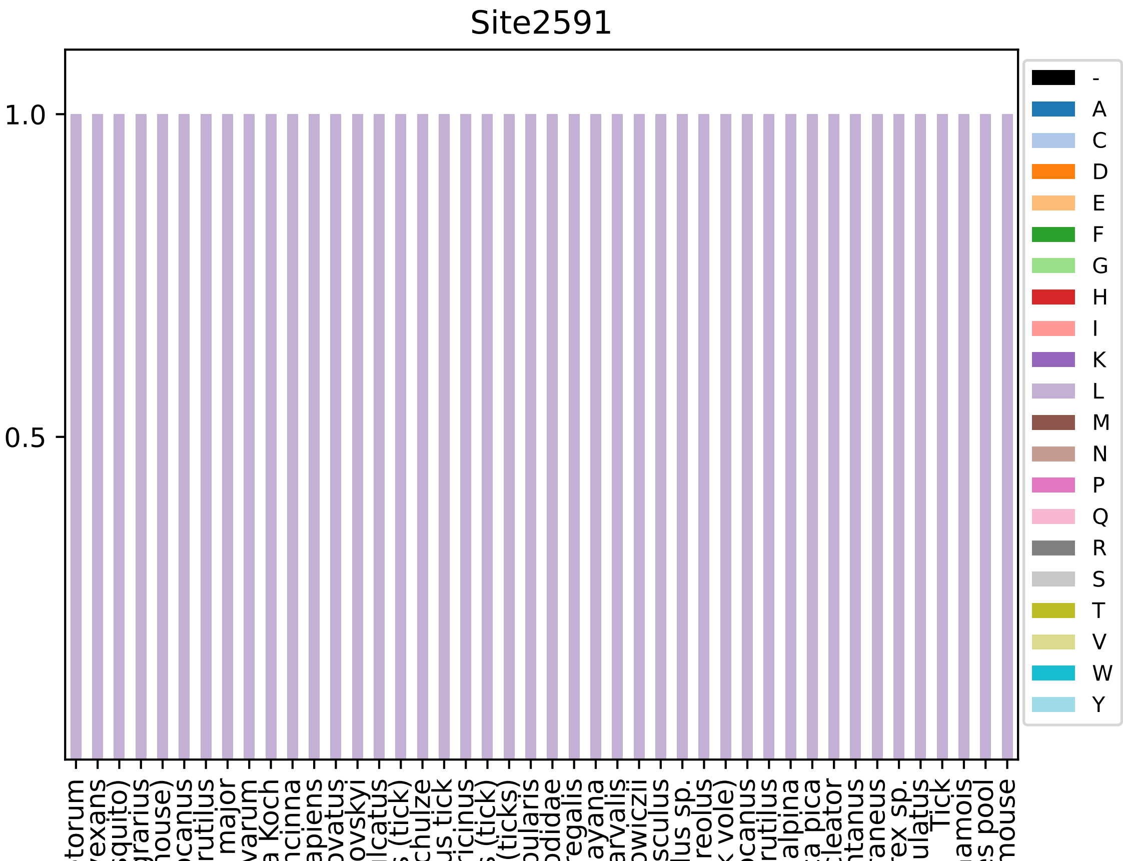
Site2591
1.0
0.5
-
A
C
D
E
F
G
H
I
K
L
M
N
P
Q
R
S
T
V
W
Y

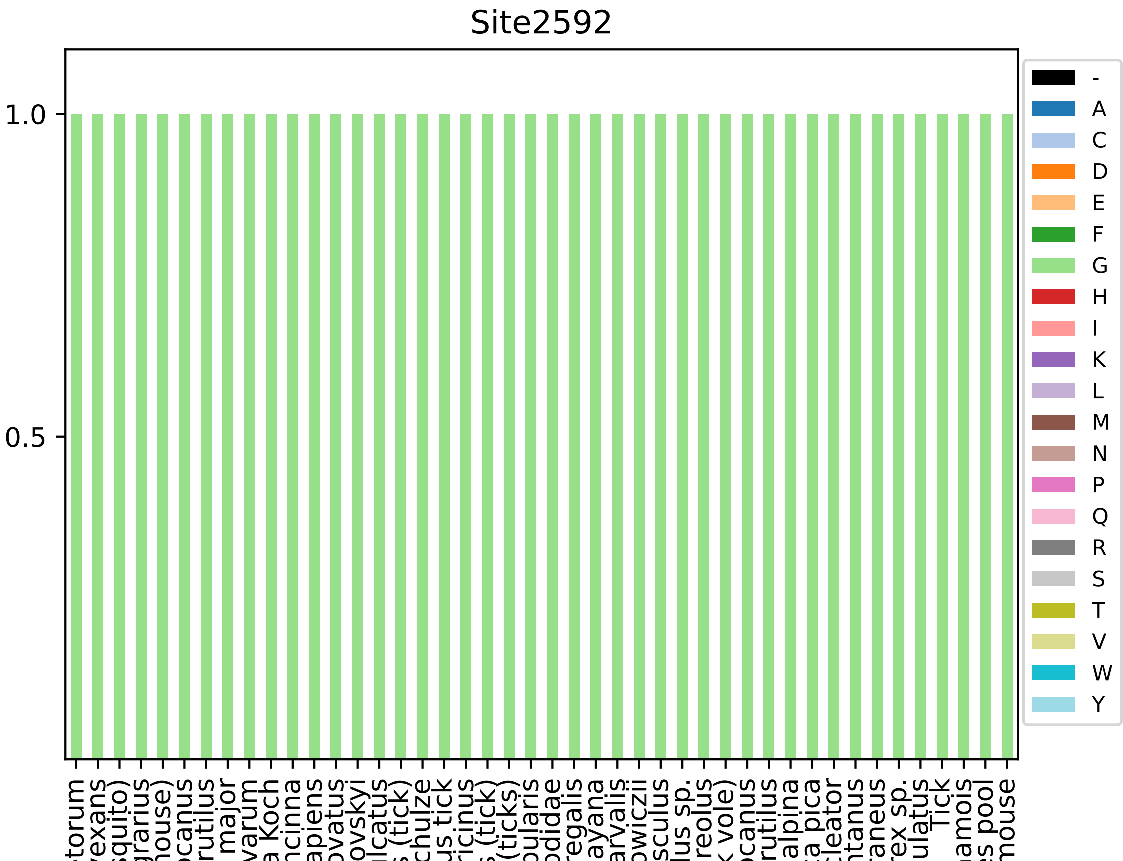
Site2592
1.0
0.5
-
A
C
D
E
F
G
H
I
K
L
M
N
P
Q
R
S
T
V
W
Y

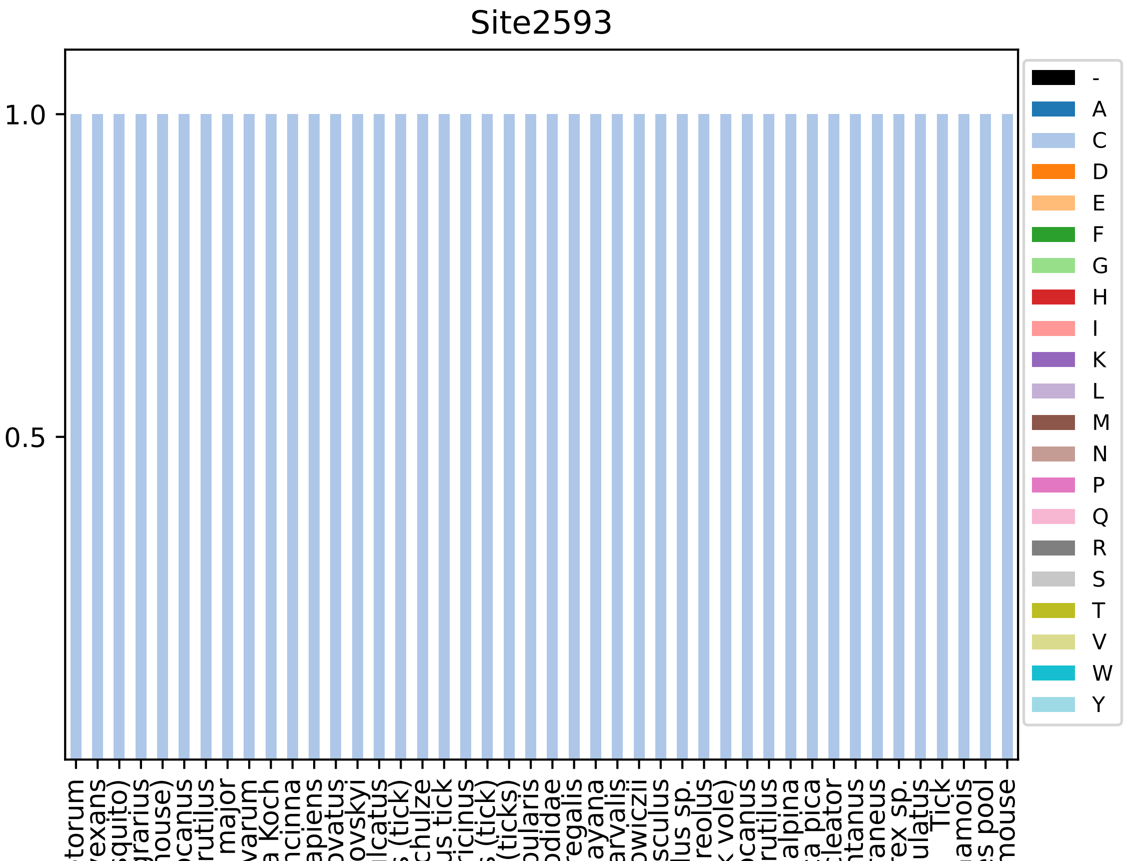

Site2593
1.0
0.5
-
A
C
D
E
F
G
H
I
K
L
M
N
P
Q
R
S
T
V
W
Y

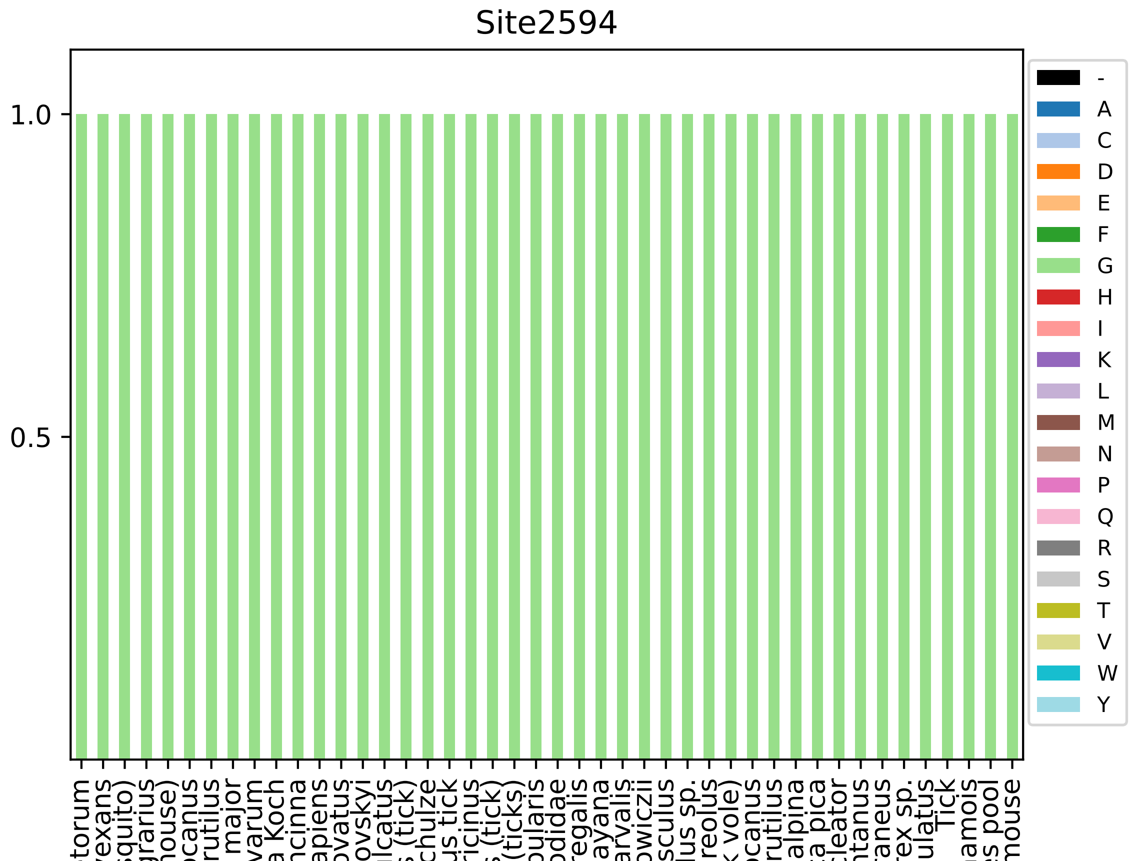
Site2594
1.0
0.5
-
A
C
D
E
F
G
H
I
K
L
M
N
P
Q
R
S
T
V
W
Y
Tick

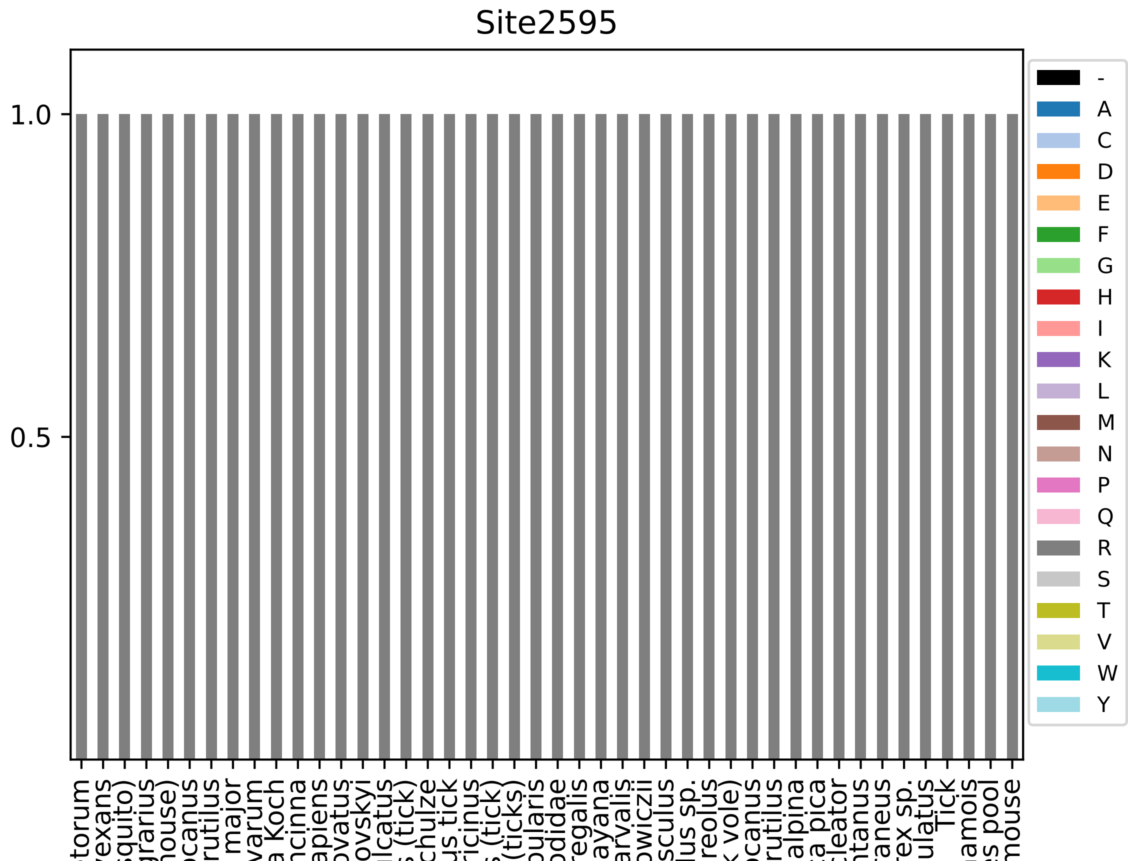

Site2595
1.0
0.5
-
A
C
D
E
F
G
H
I
K
L
M
N
P
Q
R
S
T
V
W
Y

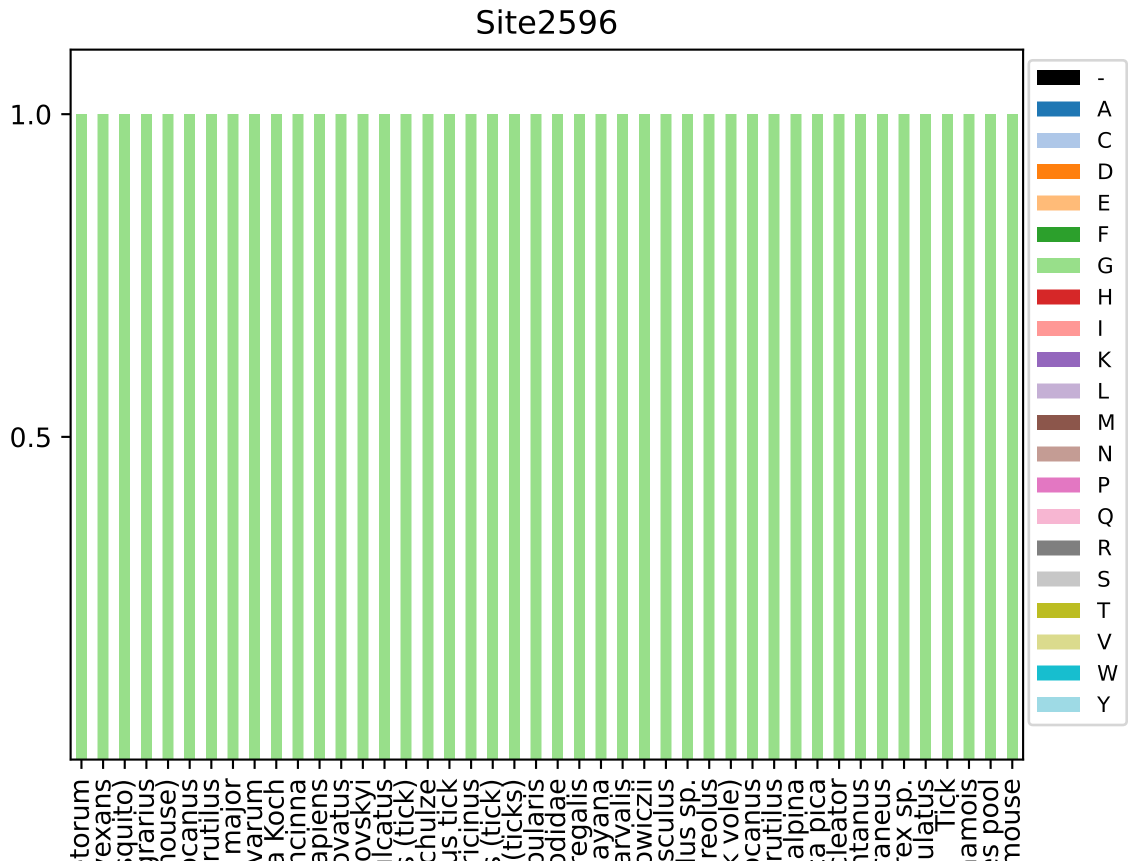
Site2596
1.0
0.5
-
A
C
D
E
F
G
H
I
K
L
M
N
P
Q
R
S
T
V
W
Y

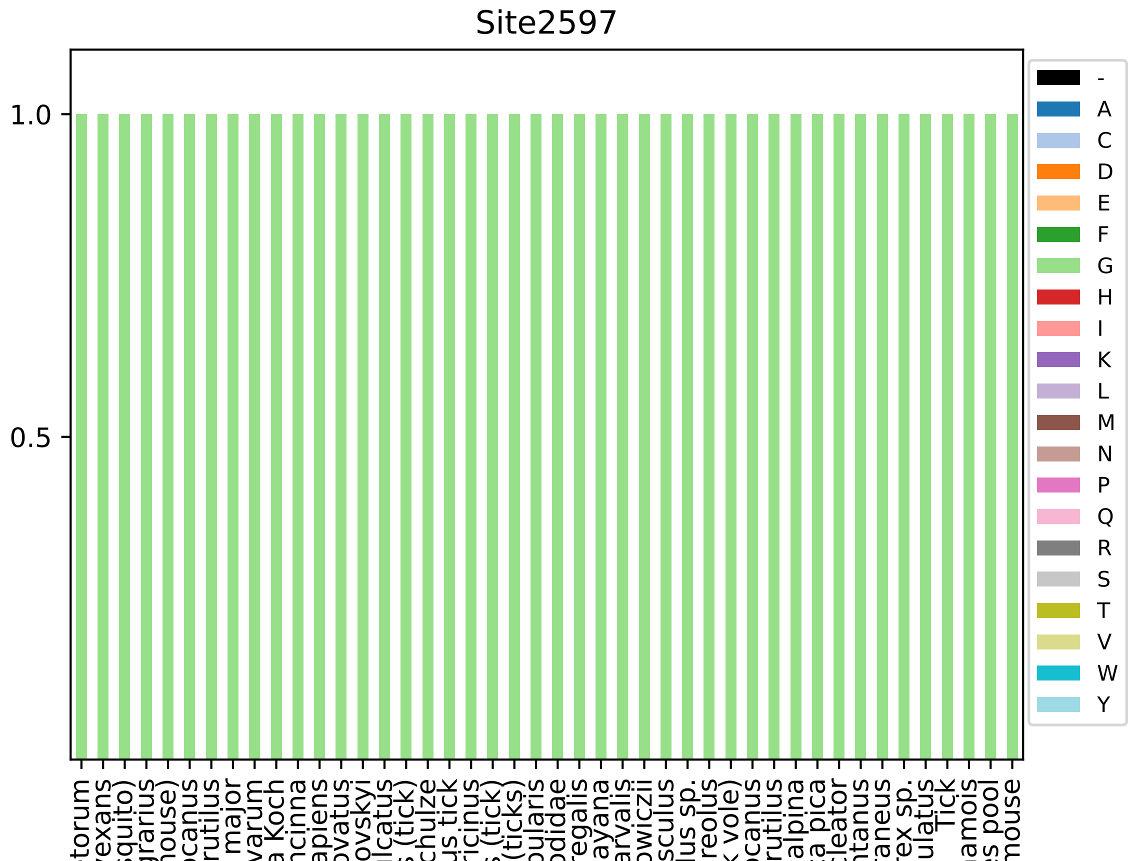
Site2597
1.0
0.5
-
A
C
D
E
F
G
H
I
K
L
M
N
P
Q
R
S
T
V
W
Y

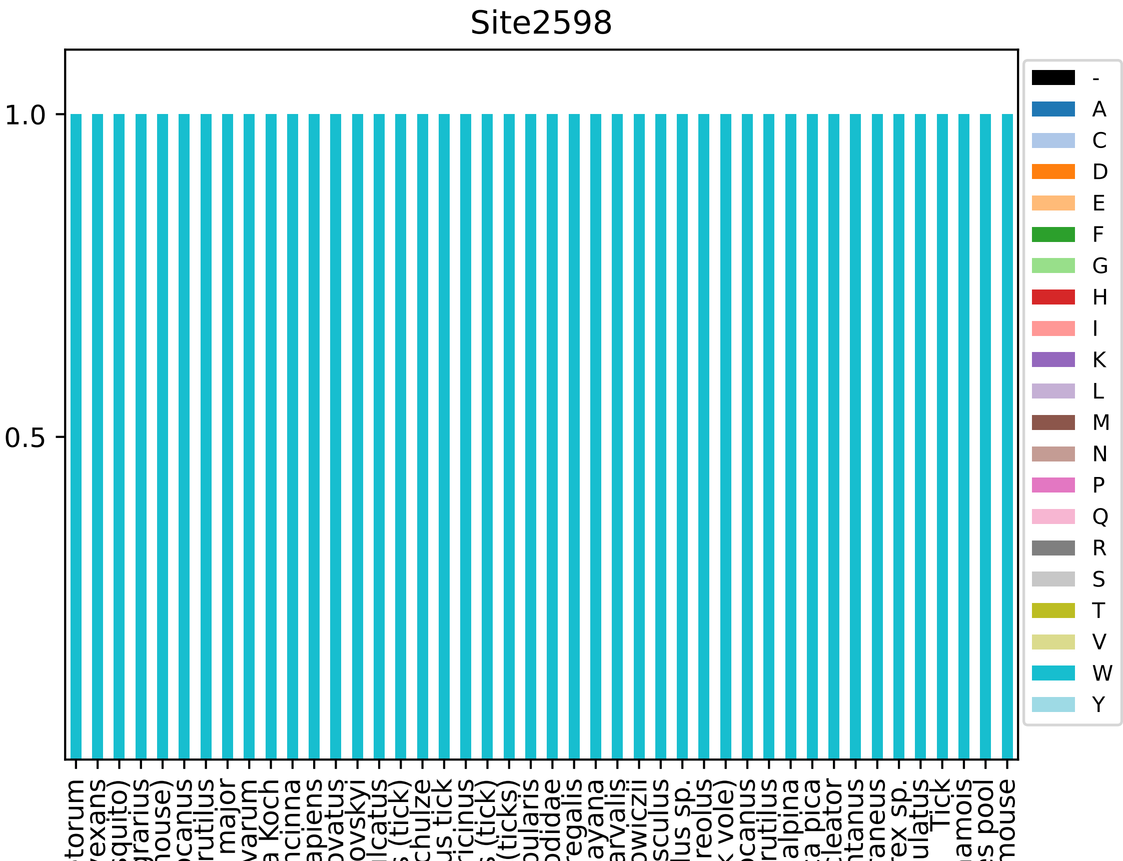
Site2598
1.0
0.5
-
A
C
D
E
F
G
H
I
K
L
M
N
P
Q
R
S
T
V
W
Y

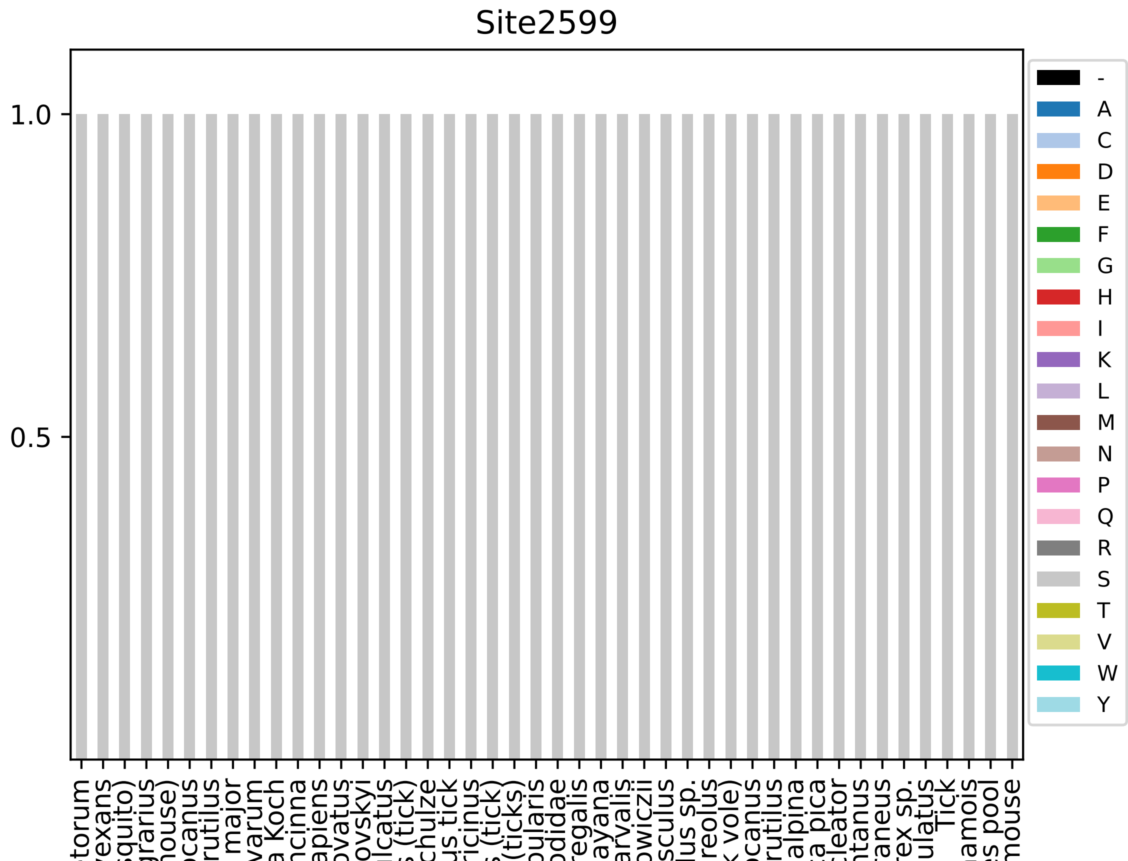
Site2599
1.0
0.5
-
A
C
D
E
F
G
H
I
K
L
M
N
P
Q
R
S
T
V
W
Y

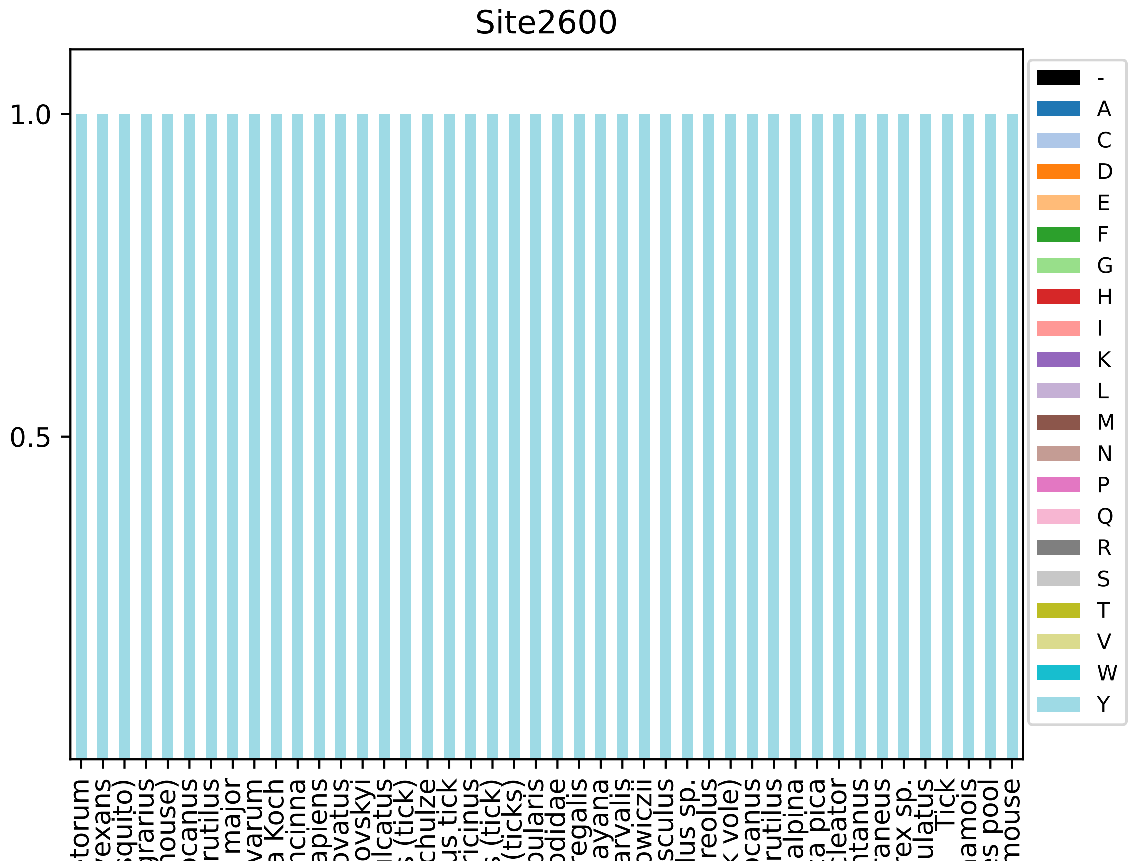
Site2600
1.0
0.5
-
A
C
D
E
F
G
H
I
K
L
M
N
P
Q
R
S
T
V
W
Y

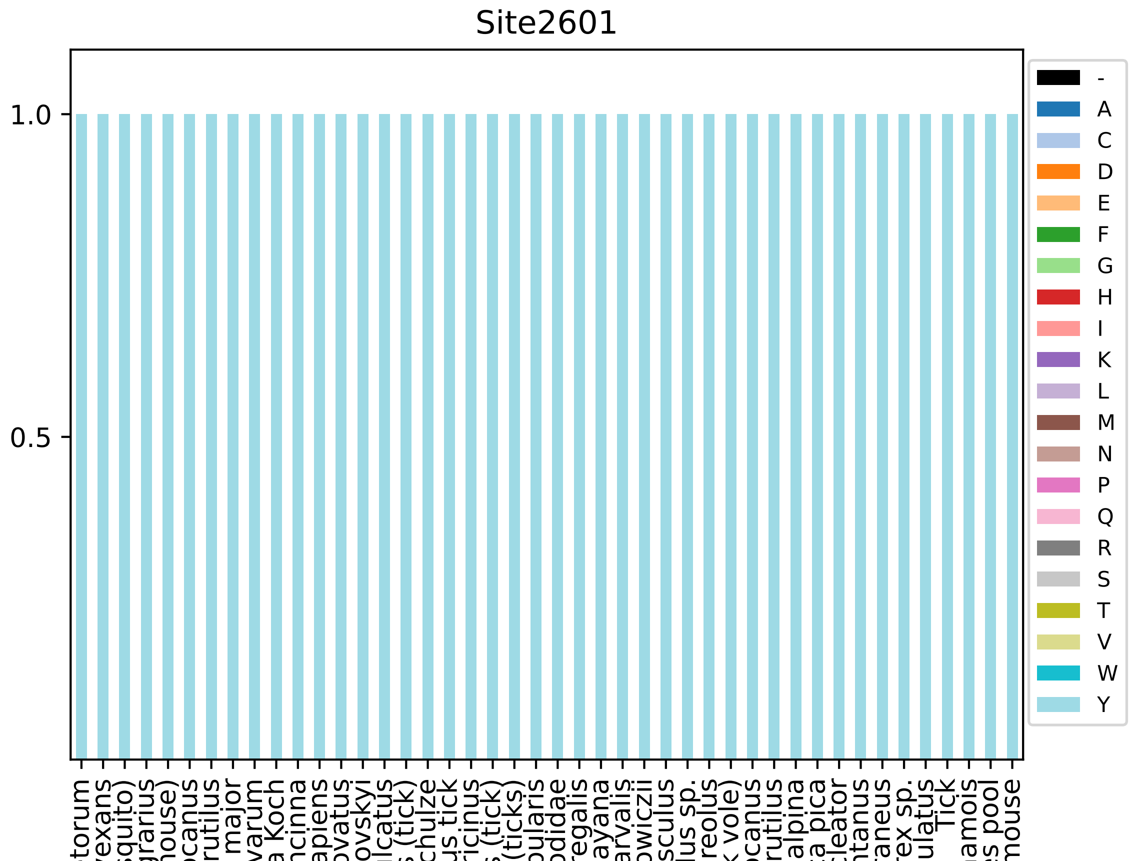
Site2601
1.0
0.5
-
A
C
D
E
F
G
H
I
K
L
M
N
P
Q
R
S
T
V
W
Y

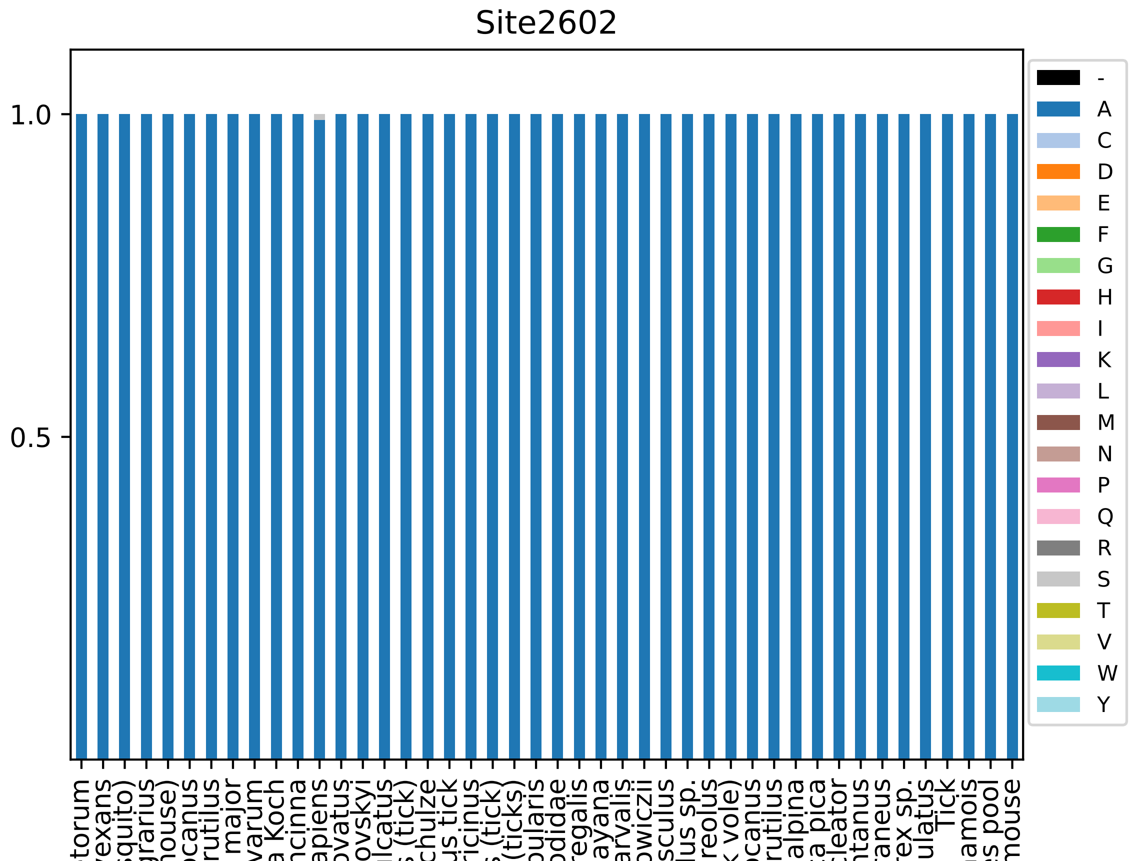
Site2602
1.0
0.5
-
A
C
D
E
F
G
H
I
K
L
M
N
P
Q
R
S
T
V
W
Y

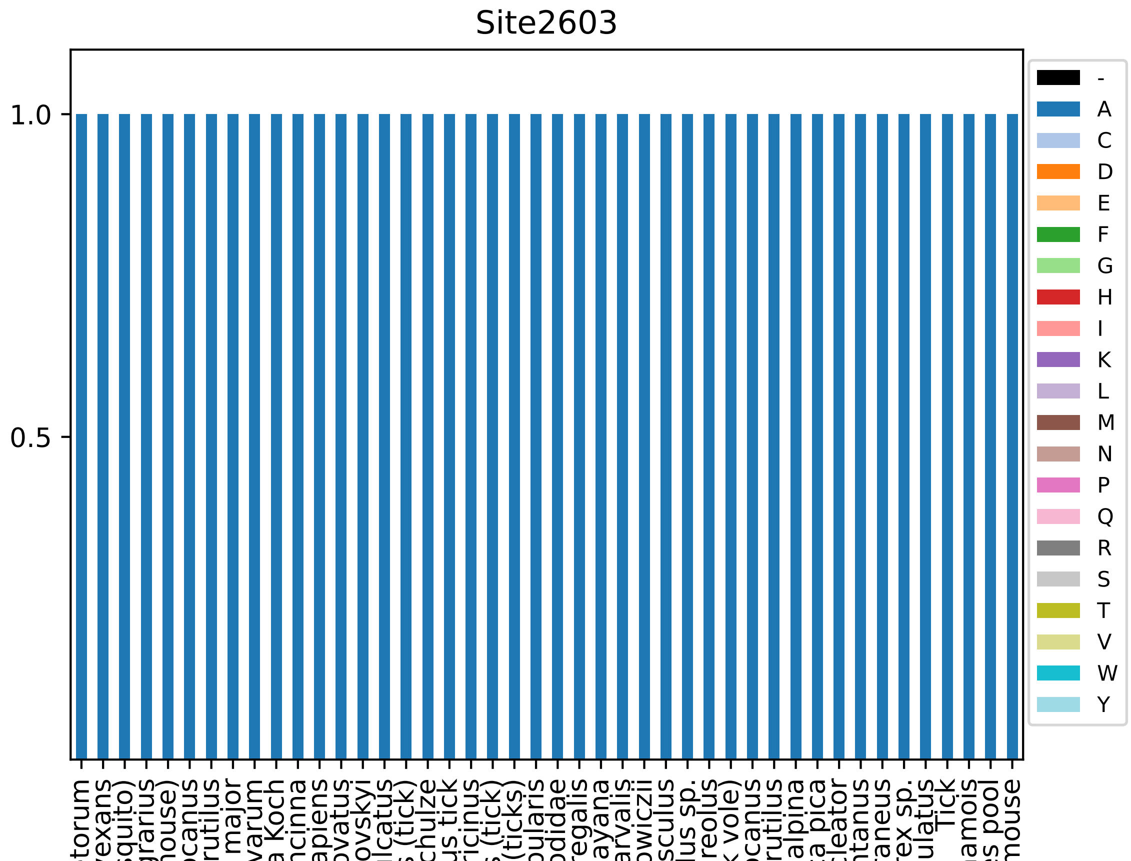
Site2603
1.0
0.5
-
A
C
D
E
F
G
H
I
K
L
M
N
P
Q
R
S
T
V
W
Y

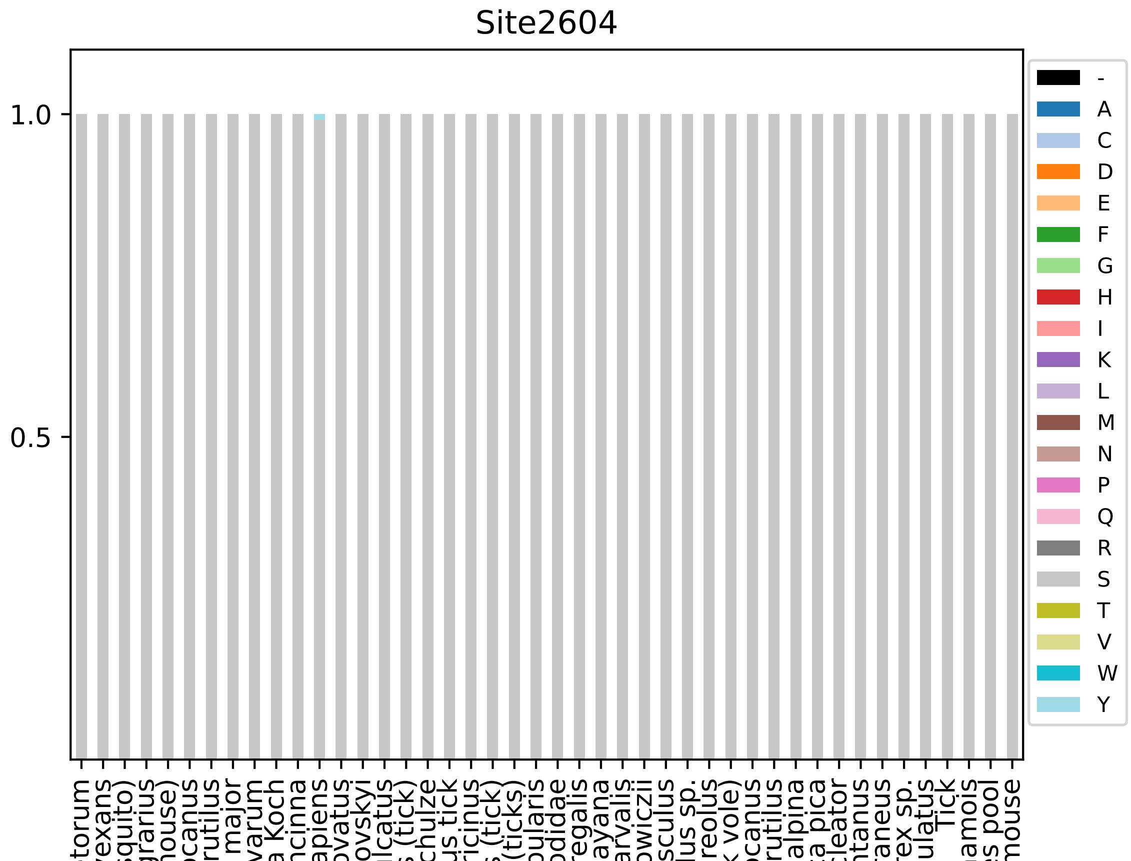

Site2604
1.0
0.5
-
A
C
D
E
F
G
H
I
K
L
M
N
P
Q
R
S
T
V
W
Y

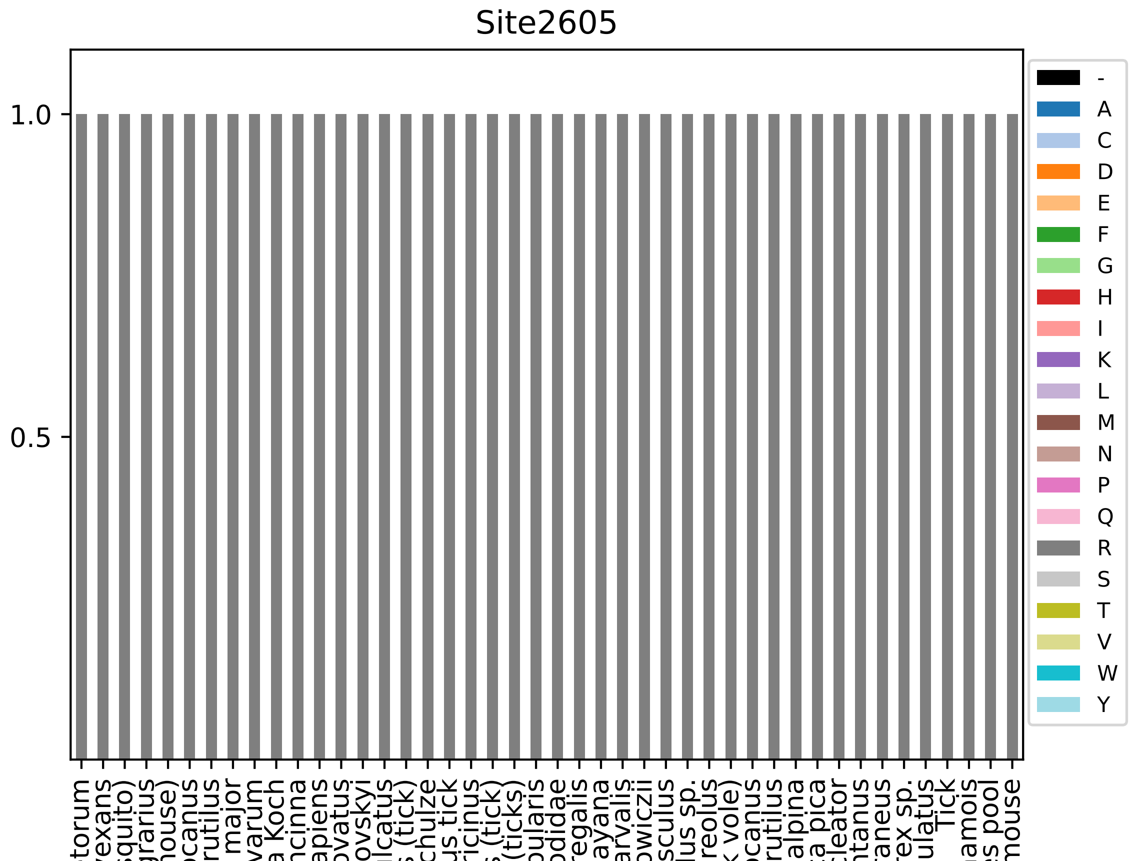
Site2605
1.0
0.5
-
A
C
D
E
F
G
H
I
K
L
M
N
P
Q
R
S
T
V
W
Y

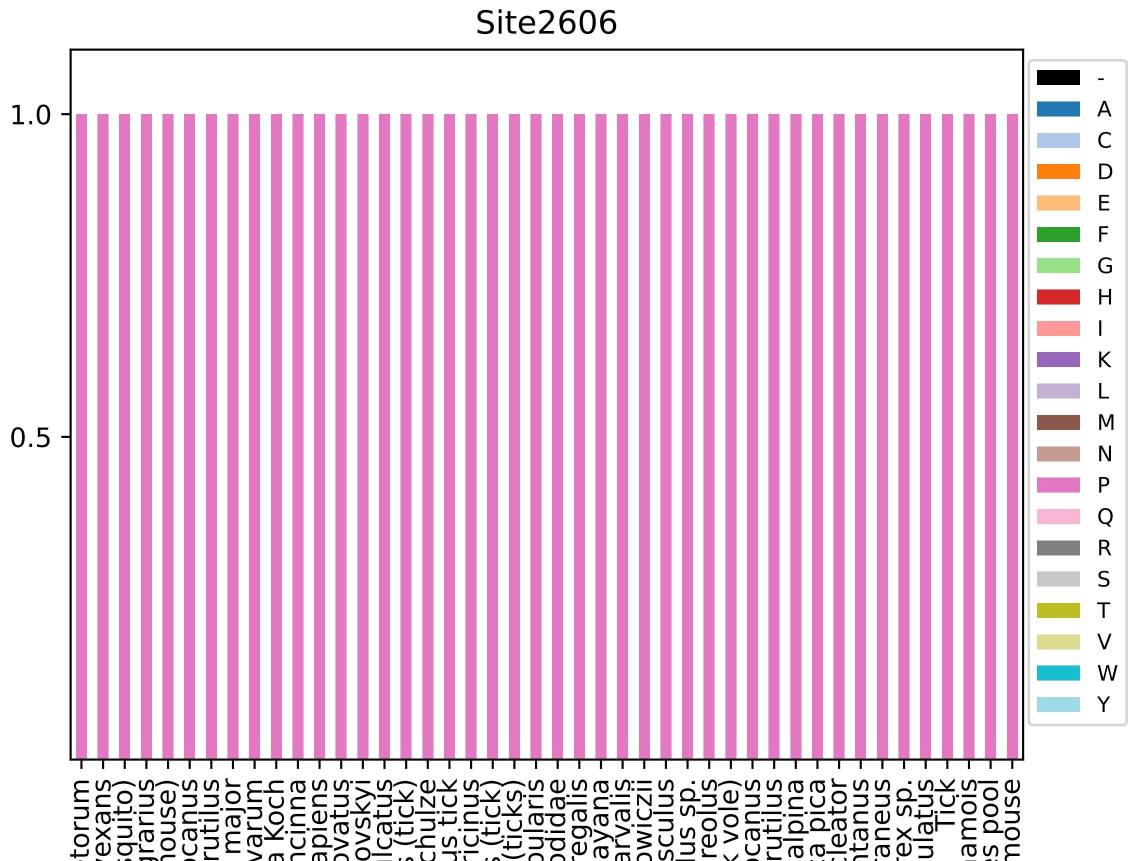

Site2606
1.0
0.5
-
A
C
D
E
F
G
H
I
K
L
M
N
P
Q
R
S
T
V
W
Y

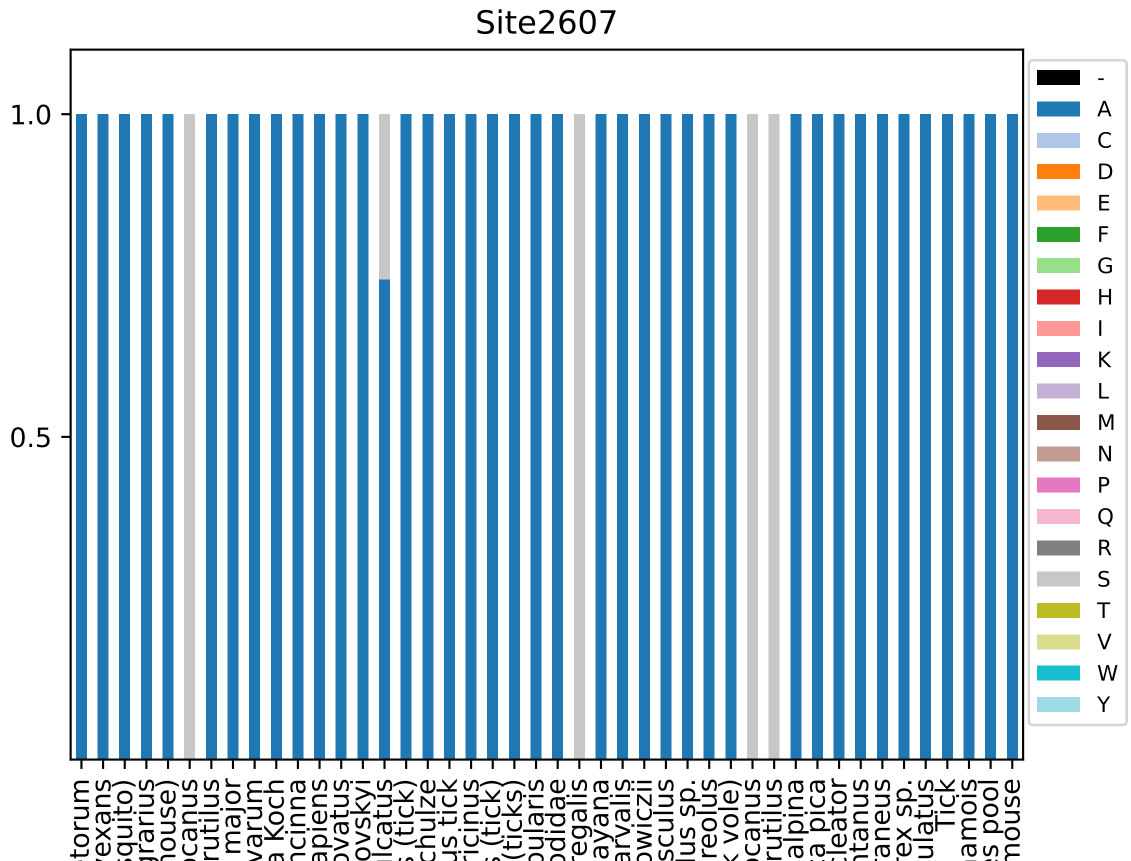
Site2607
1.0
0.5
-
A
C
D
E
F
G
H
I
K
L
M
N
P
Q
R
S
T
V
W
Y

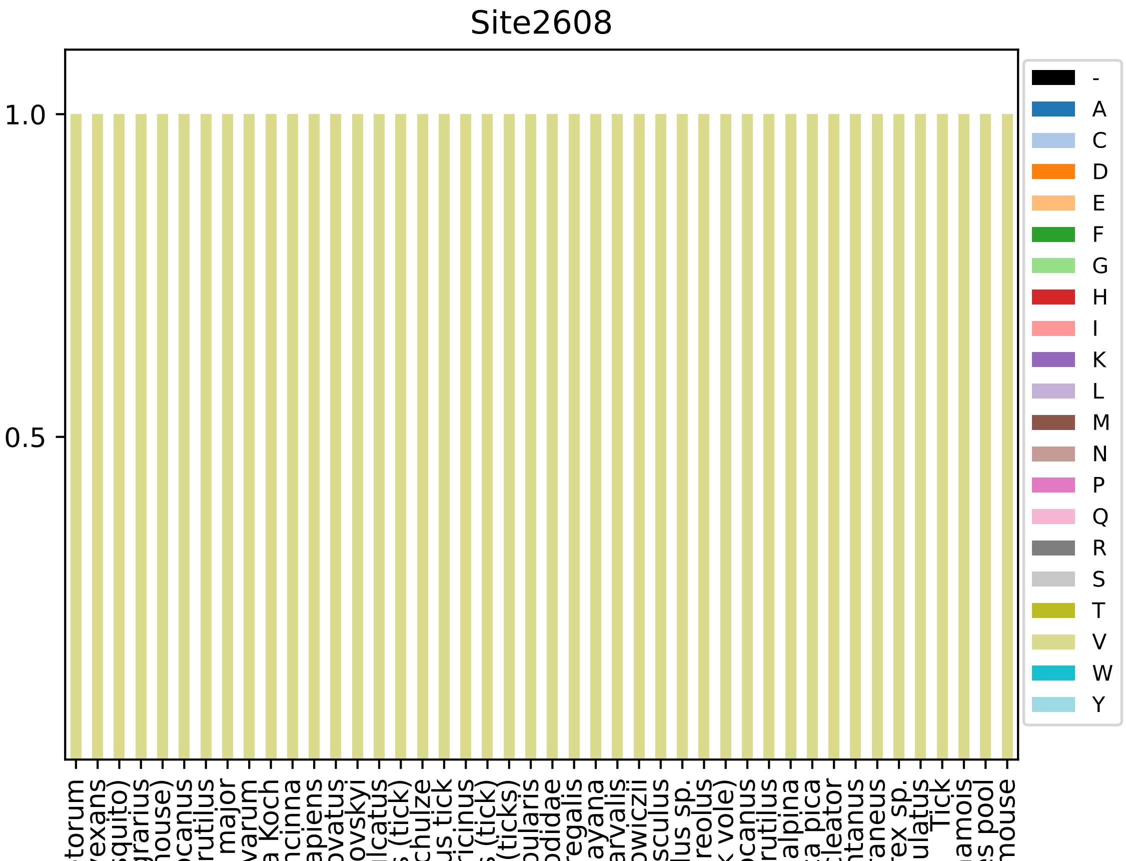

Site2608
1.0
0.5
-
A
C
D
E
F
G
H
I
K
L
M
N
P
Q
R
S
T
V
W
Y
Tick

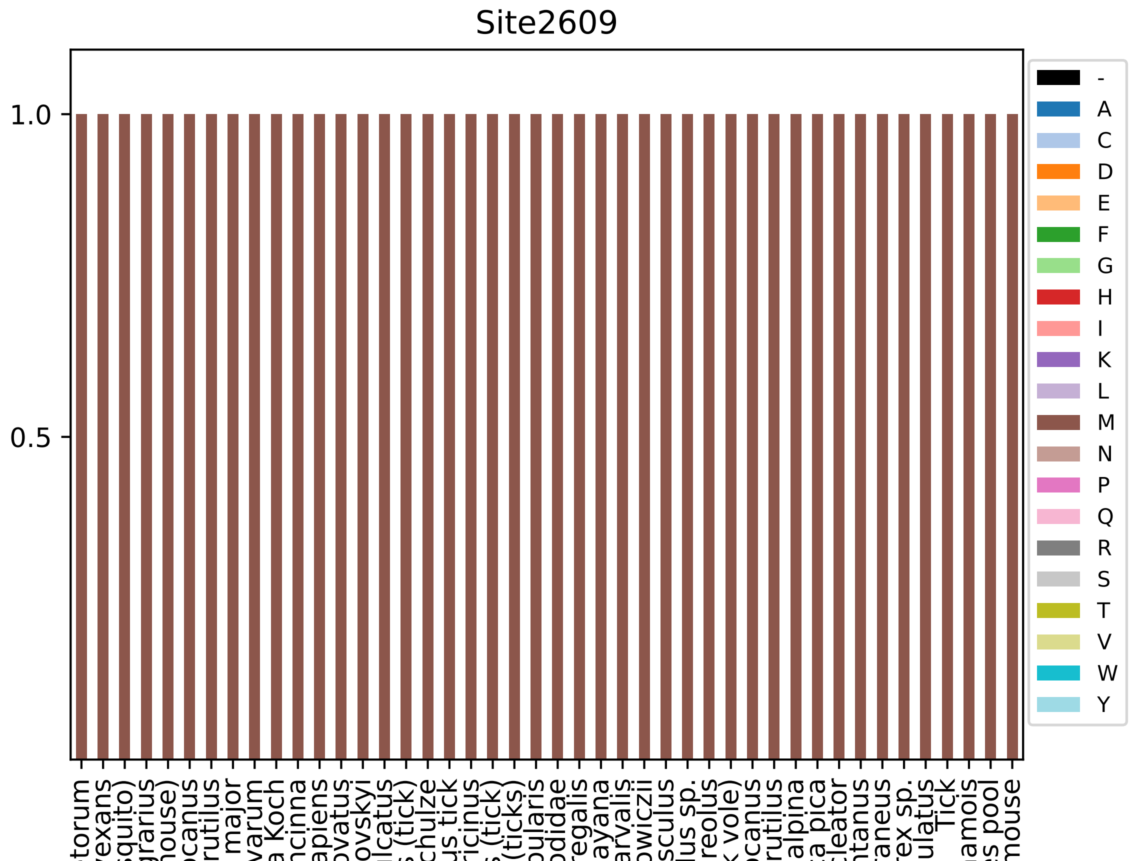
Site2609
1.0
0.5
-
A
C
D
E
F
G
H
I
K
L
M
N
P
Q
R
S
T
V
W
Y

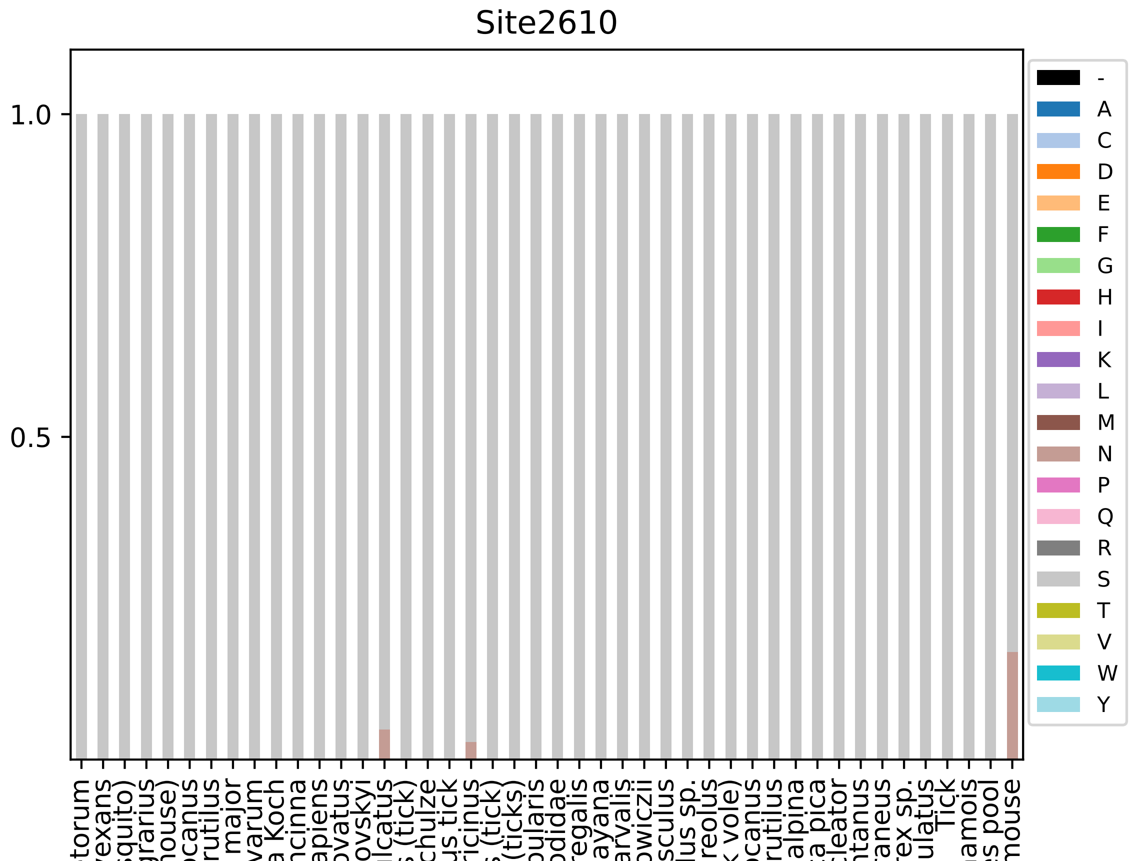
Site2610
1.0
0.5
-
A
C
D
E
F
G
H
I
K
L
M
N
P
Q
R
S
T
V
W
Y

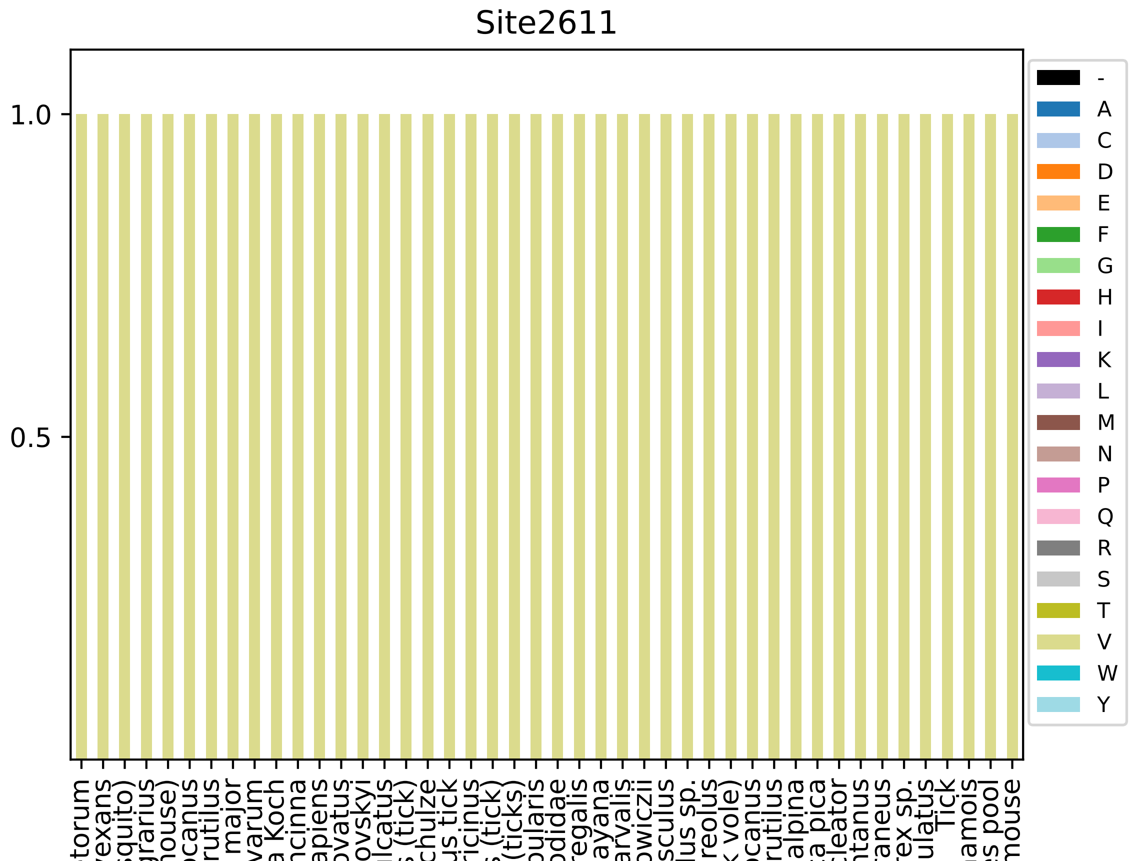
Site2611
1.0
0.5
-
A
C
D
E
F
G
H
I
K
L
M
N
P
Q
R
S
T
V
W
Y

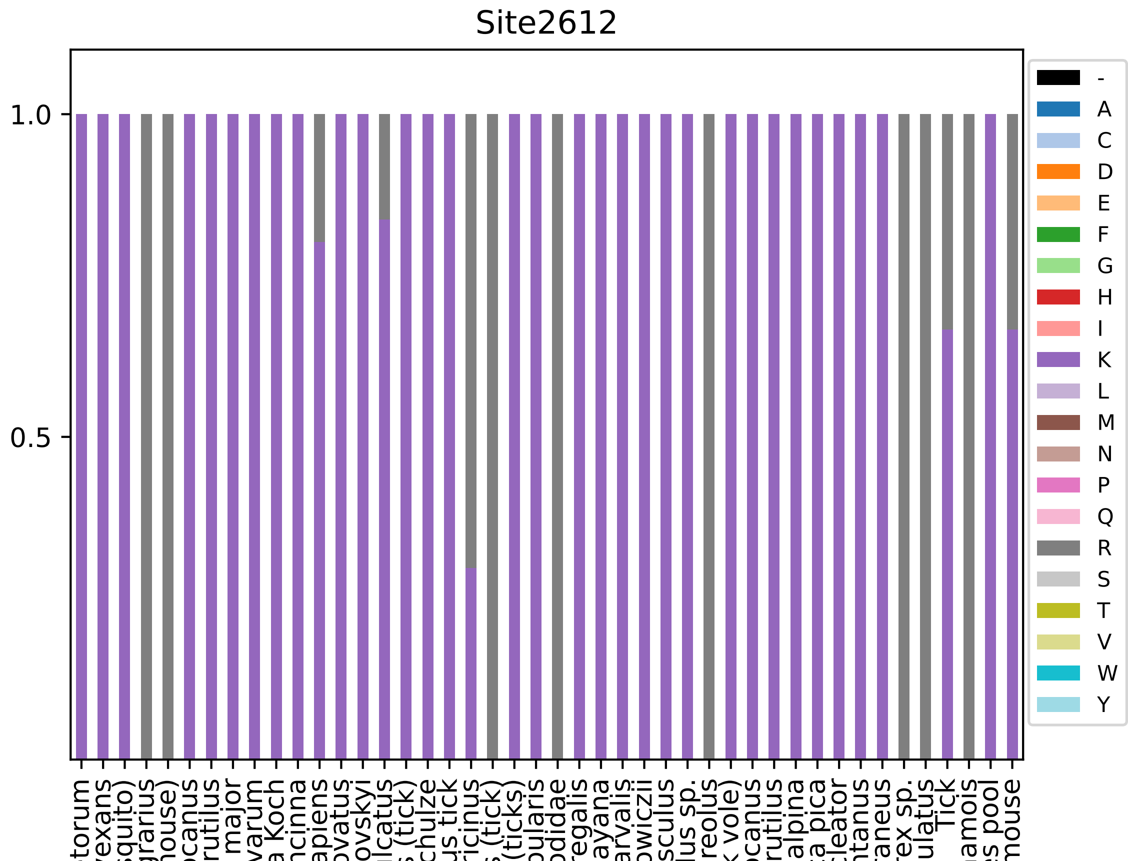

Site2612
1.0
0.5
-
A
C
D
E
F
G
H
I
K
L
M
N
P
Q
R
S
T
V
W
Y

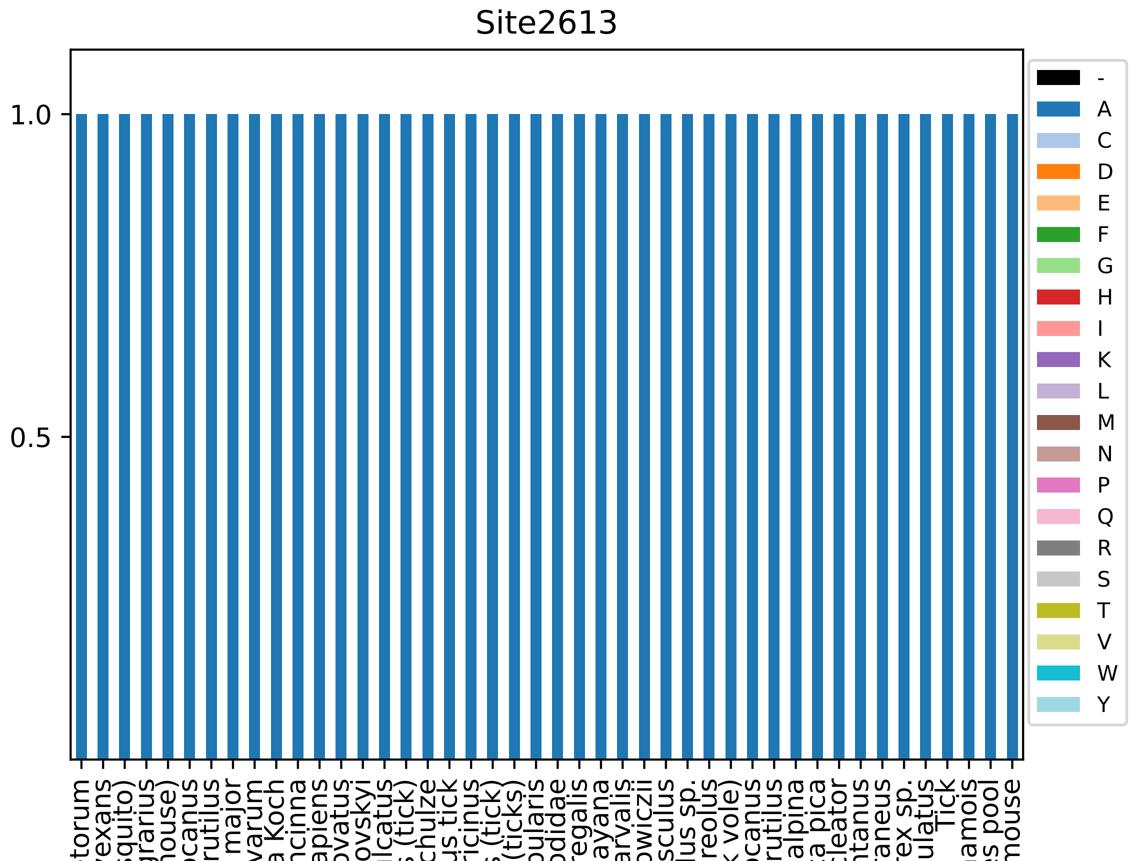
Site2613
1.0
0.5
-
A
C
D
E
F
G
H
I
K
L
M
N
P
Q
R
S
T
V
W
Y

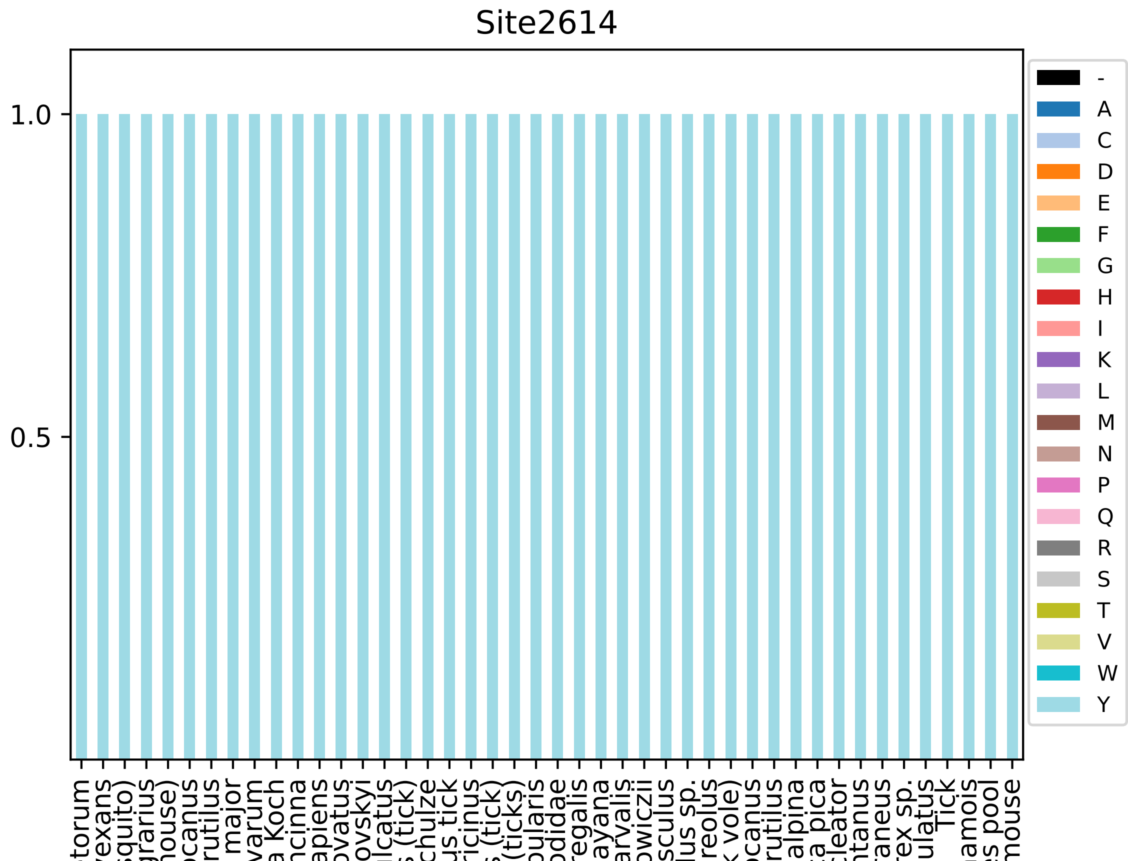
Site2614
1.0
0.5
-
A
C
D
E
F
G
H
I
K
L
M
N
P
Q
R
S
T
V
W
Y
Tick

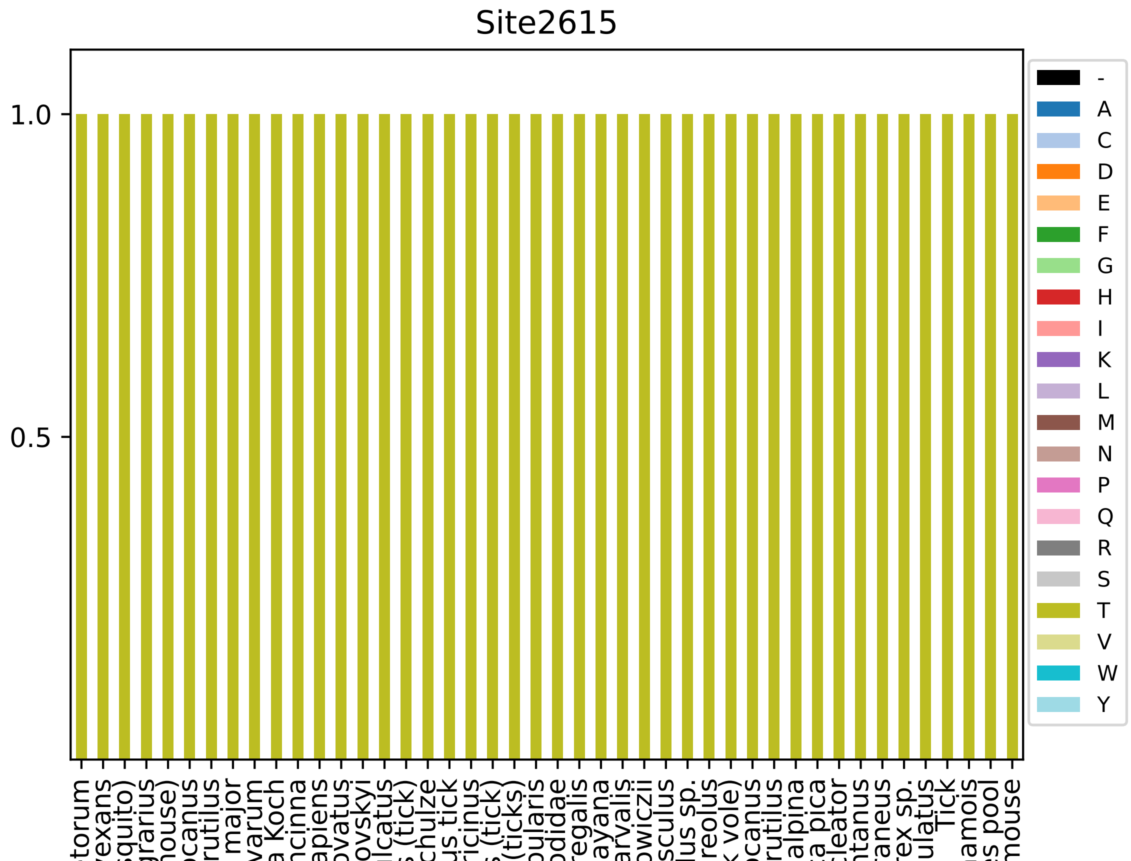
Site2615
1.0
0.5
-
A
C
D
E
F
G
H
I
K
L
M
N
P
Q
R
S
T
V
W
Y

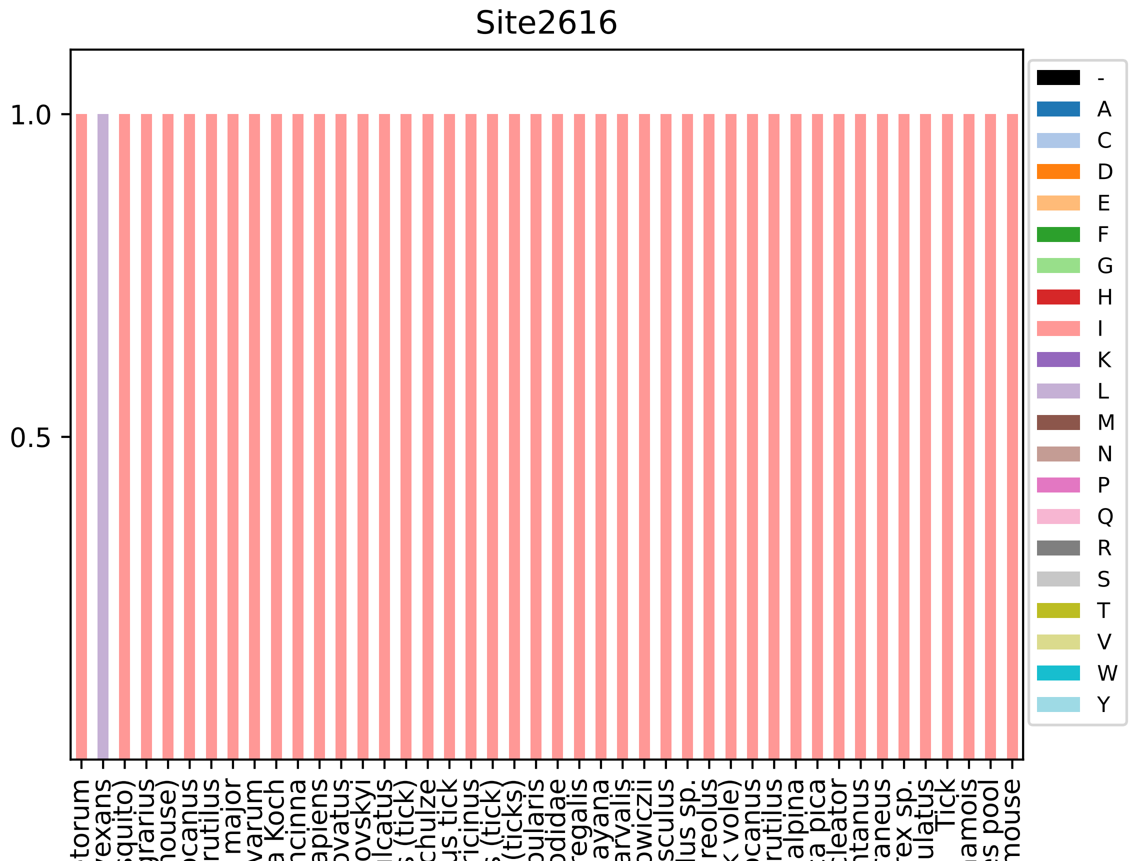
Site2616
1.0
0.5
-
A
C
D
E
F
G
H
I
K
L
M
N
P
Q
R
S
T
V
W
Y

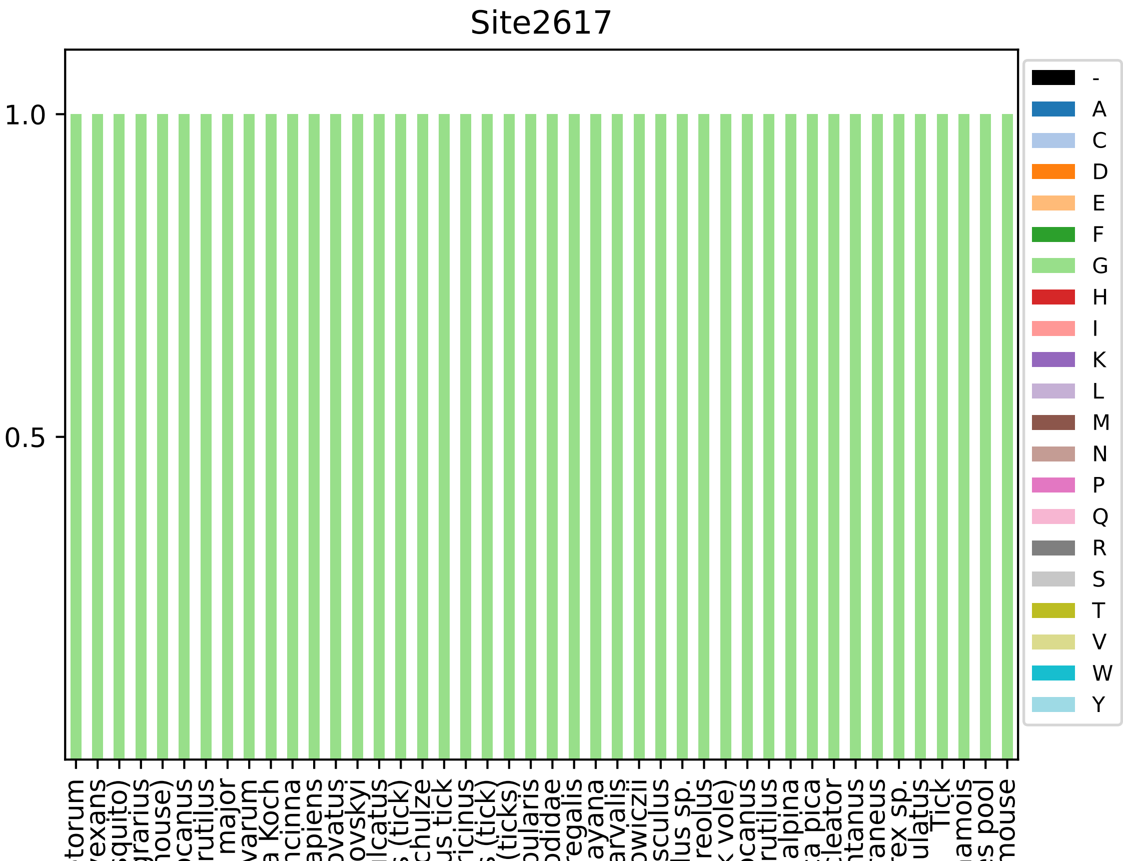

Site2617
1.0
0.5
-
A
C
D
E
F
G
H
I
K
L
M
N
P
Q
R
S
T
V
W
Y
torum
vexans
squito)
grarius
house)
ocanus
rutilus
major
varum
a Koch
ncinna
apiens
ovatus
ovskyi
ulcatus
s (tick)
chulze
us tick
ricinus
s (tick)
(ticks)
pularis
odidae
regalis
ayana
arvalis
owiczii
sculus
lus sp.
reolus
k vole)
ocanus
rutilus
alpina
a pica
cleator
ntanus
raneus
rex sp.
ulatus
Tick
amois
es pool
mouse

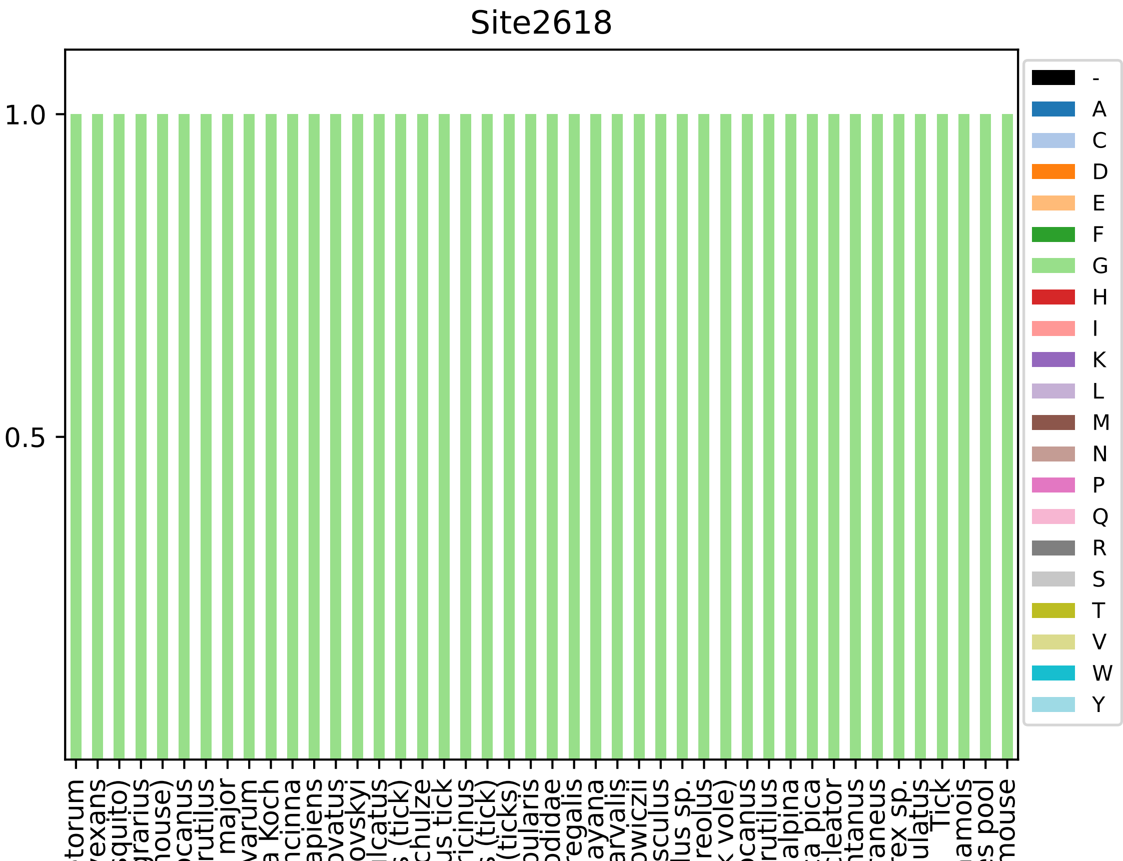

Site2618
1.0
0.5
-
A
C
D
E
F
G
H
I
K
L
M
N
P
Q
R
S
T
V
W
Y

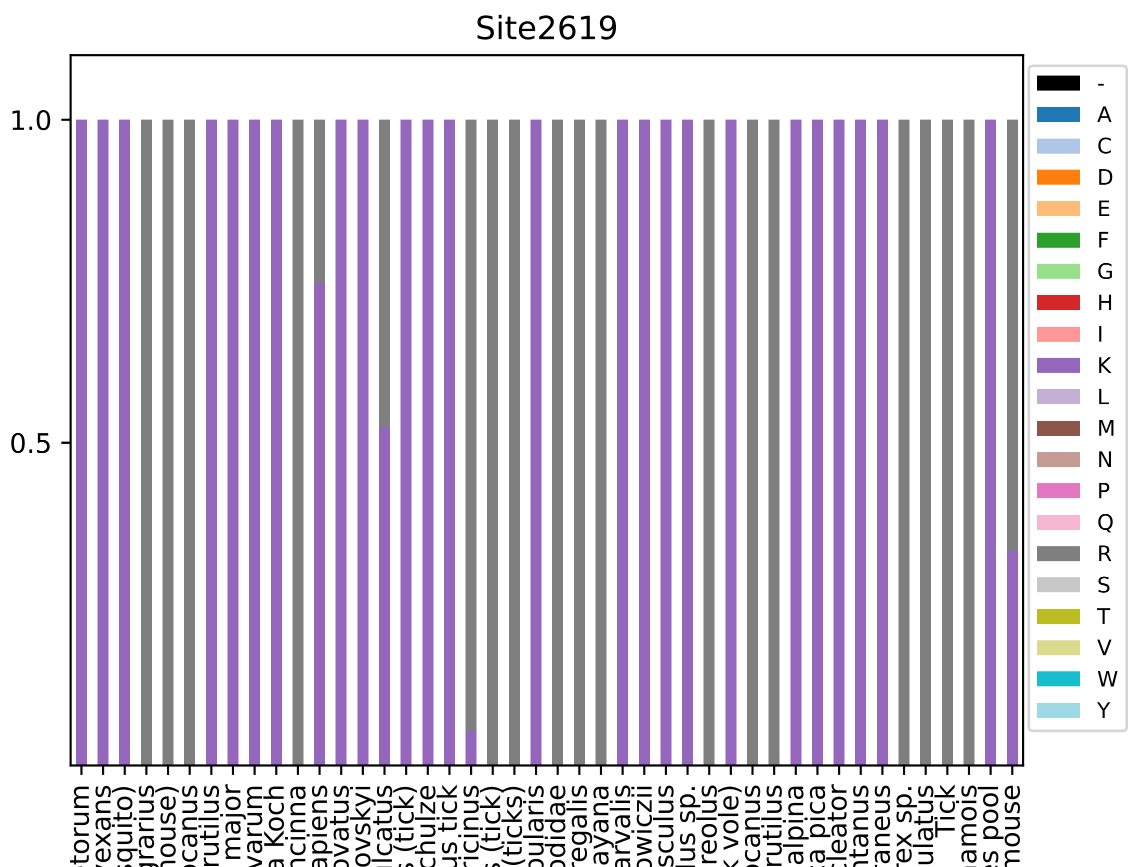
Site2619
1.0
0.5
-
A
C
D
E
F
G
H
I
K
L
M
N
P
Q
R
S
T
V
W
Y
Tick

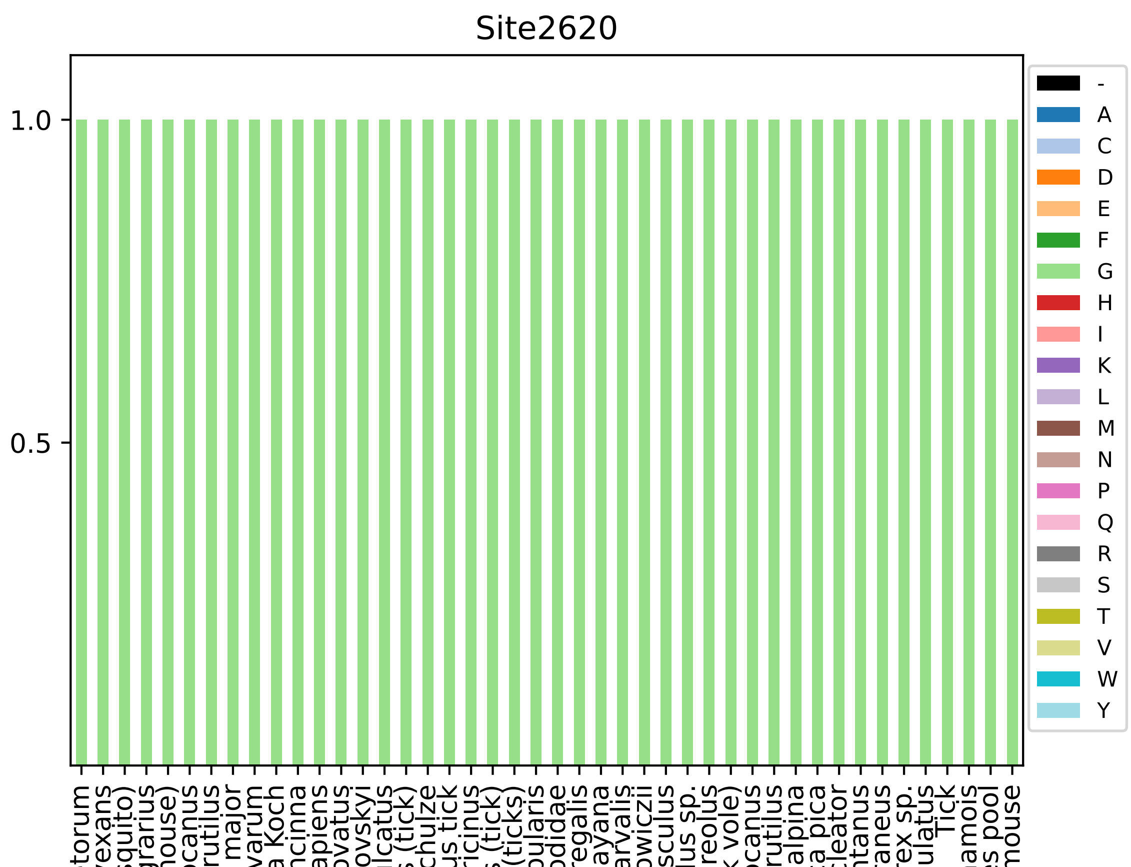

Site2620
1.0
0.5
-
A
C
D
E
F
G
H
I
K
L
M
N
P
Q
R
S
T
V
W
Y

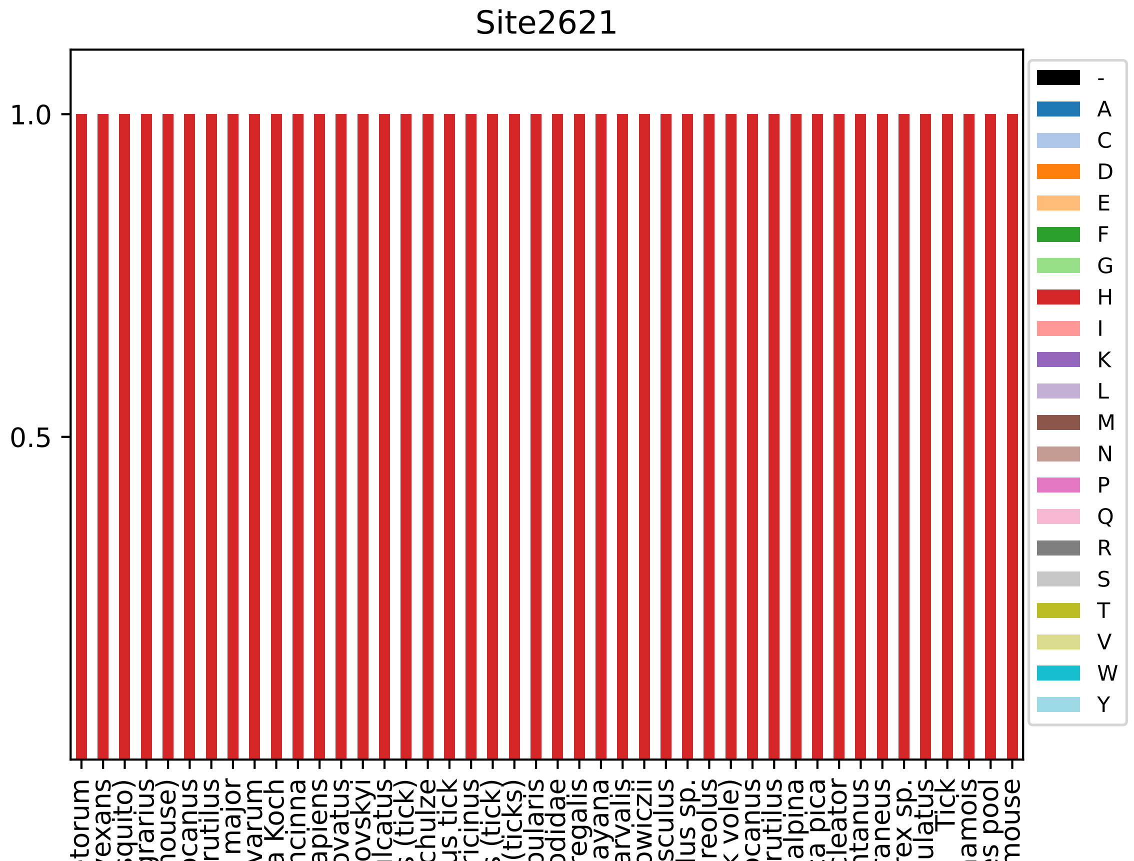
Site2621
1.0
0.5
-
A
C
D
E
F
G
H
I
K
L
M
N
P
Q
R
S
T
V
W
Y
torum
vexans
squito)
grarius
nouse)
ocanus
rutilus
major
varum
a Koch
ncinna
apiens
ovatus
ovskyi
ulcatus
s (tick)
chulze
us tick
ricinus
s (tick)
(ticks)
pularis
odidae
regalis
ayana
arvalis
owiczii
sculus
lus sp.
reolus
k vole)
ocanus
rutilus
alpina
a pica
leator
ntanus
raneus
rex sp.
ulatus
Tick
amois
es pool
mouse

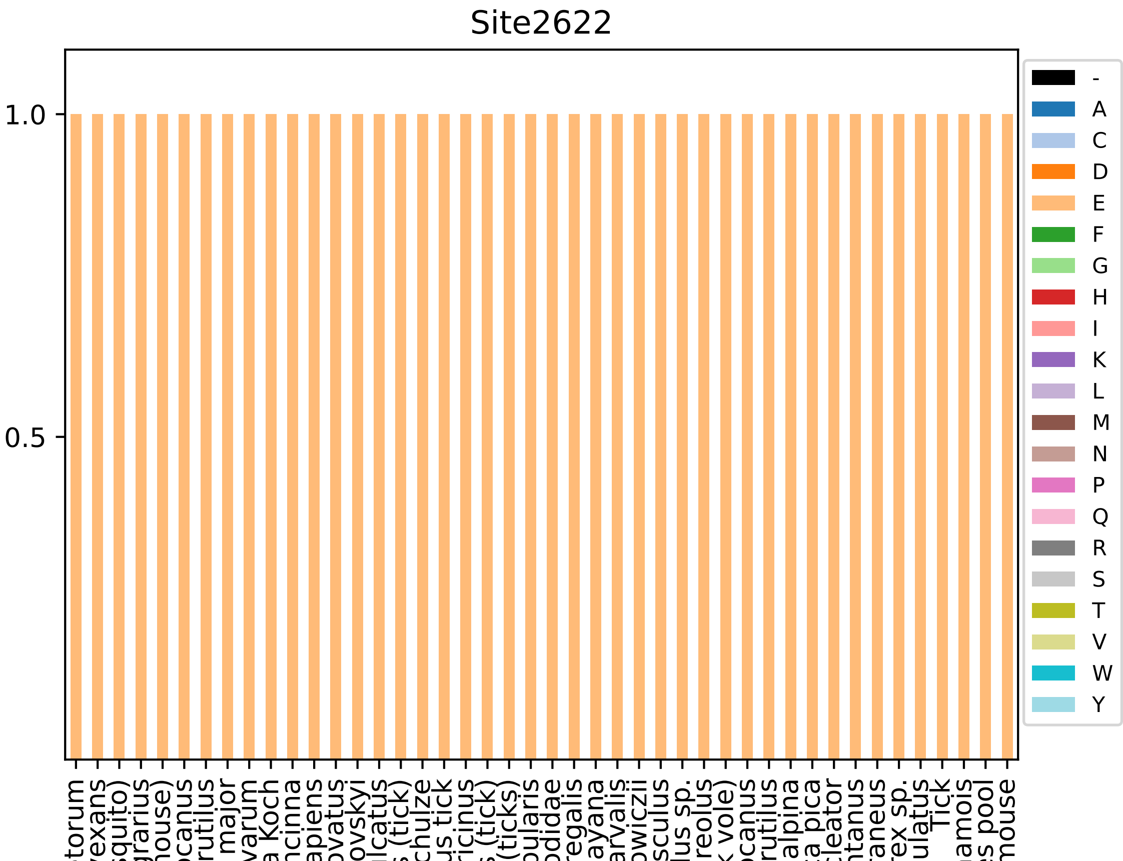

Site2622
1.0
0.5
-
A
C
D
E
F
G
H
I
K
L
M
N
P
Q
R
S
T
V
W
Y

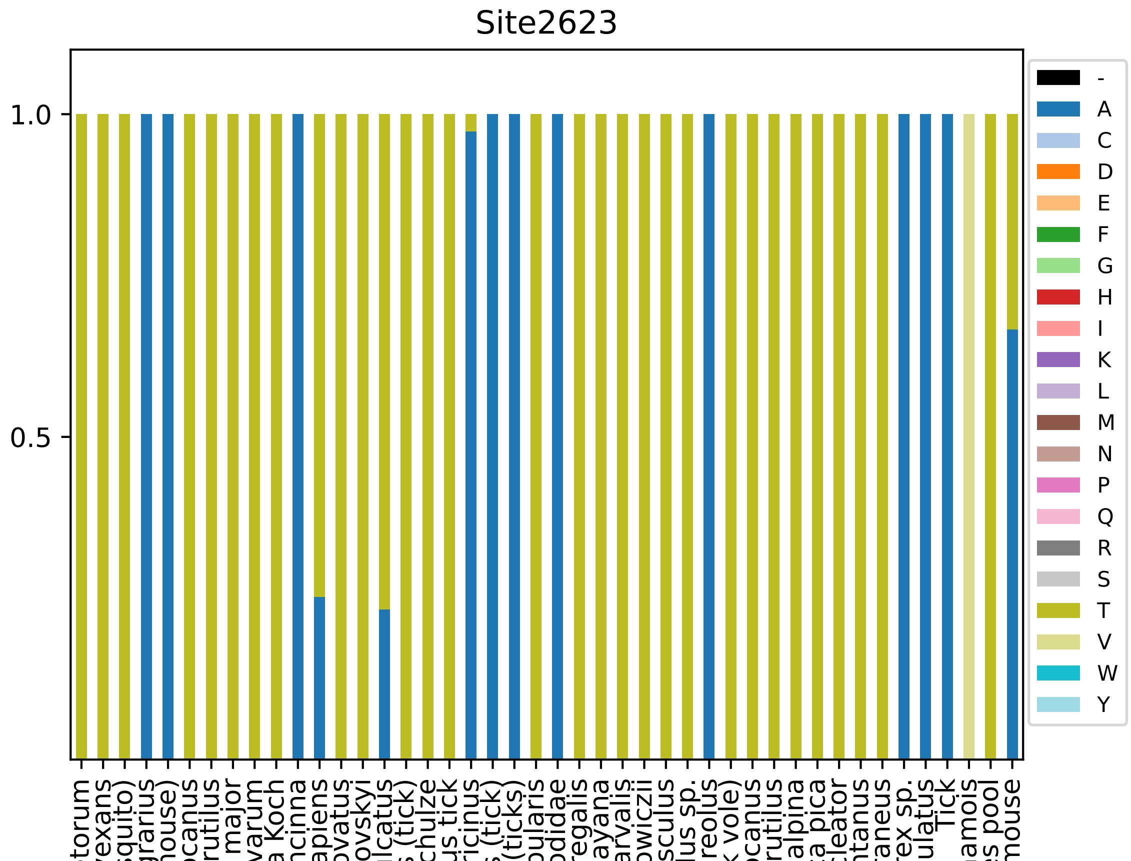
Site2623
1.0
0.5
-
A
C
D
E
F
G
H
I
K
L
M
N
P
Q
R
S
T
V
W
Y

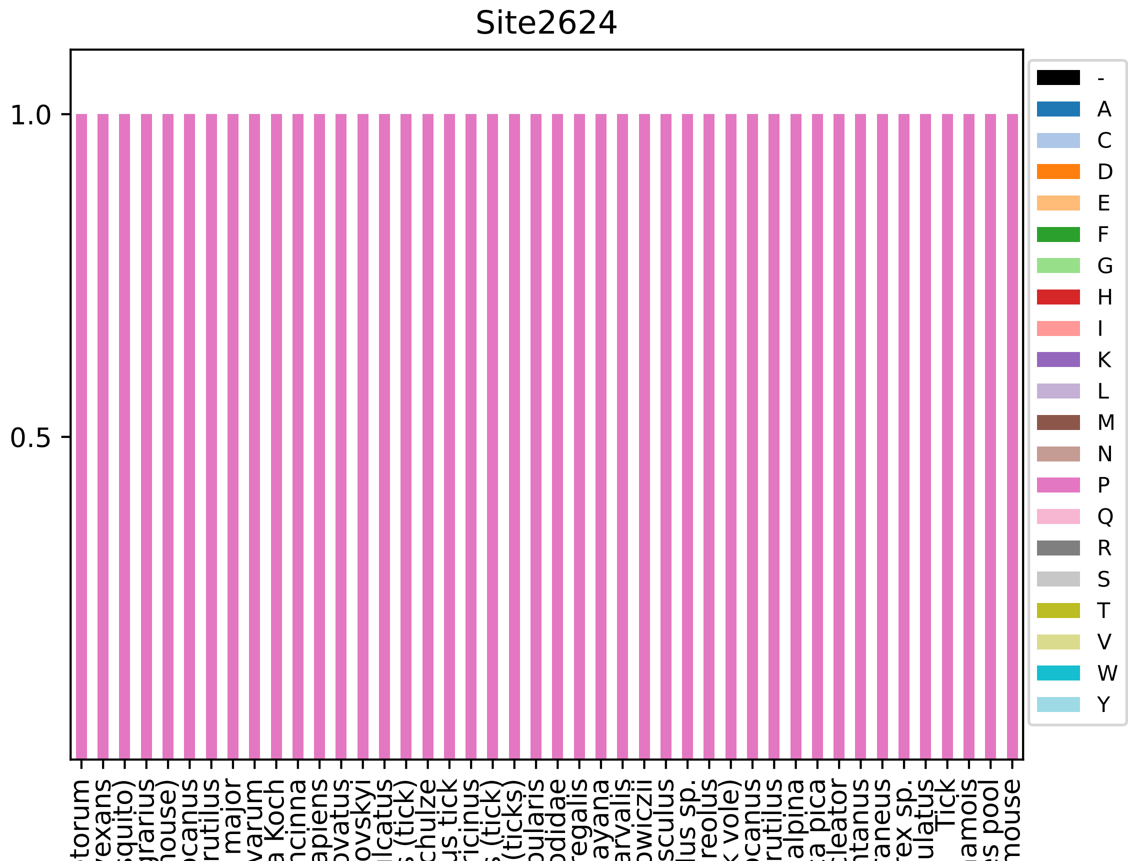

Site2624
1.0
0.5
-
A
C
D
E
F
G
H
I
K
L
M
N
P
Q
R
S
T
V
W
Y

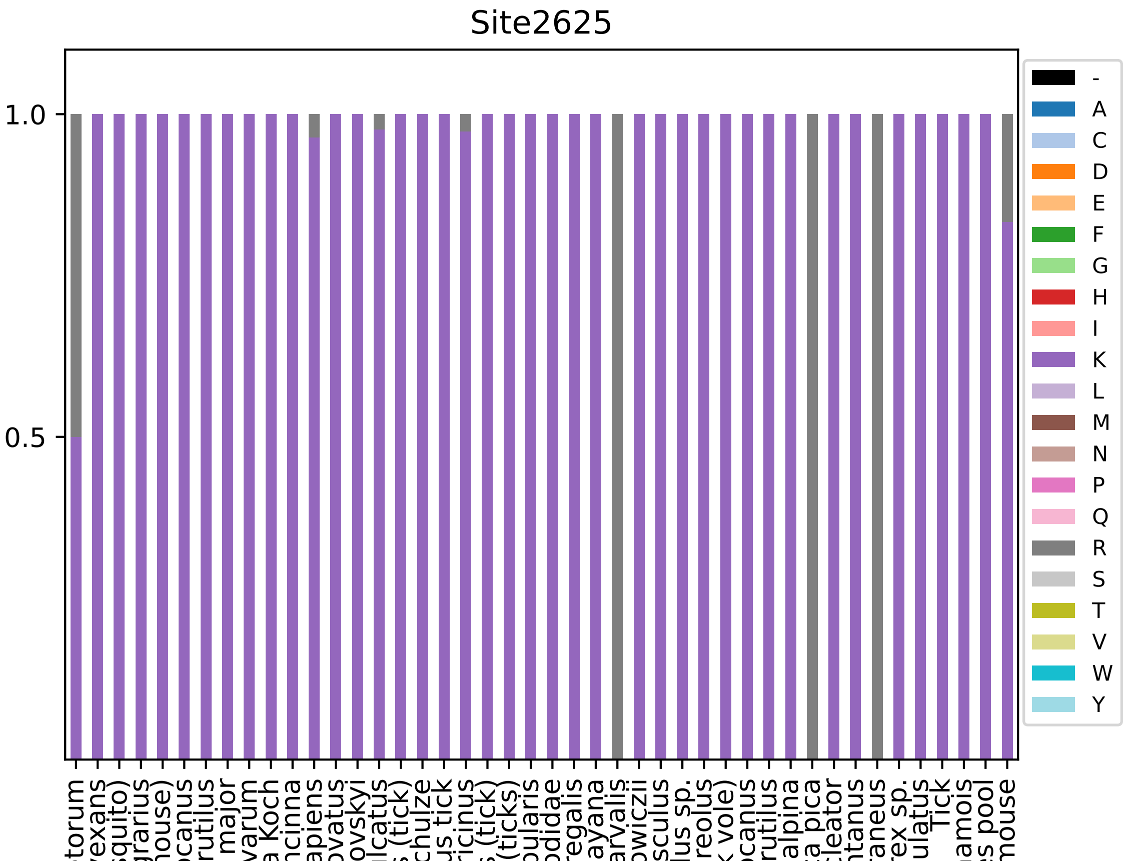
Site2625
1.0
0.5
-
A
C
D
E
F
G
H
I
K
L
M
N
P
Q
R
S
T
V
W
Y

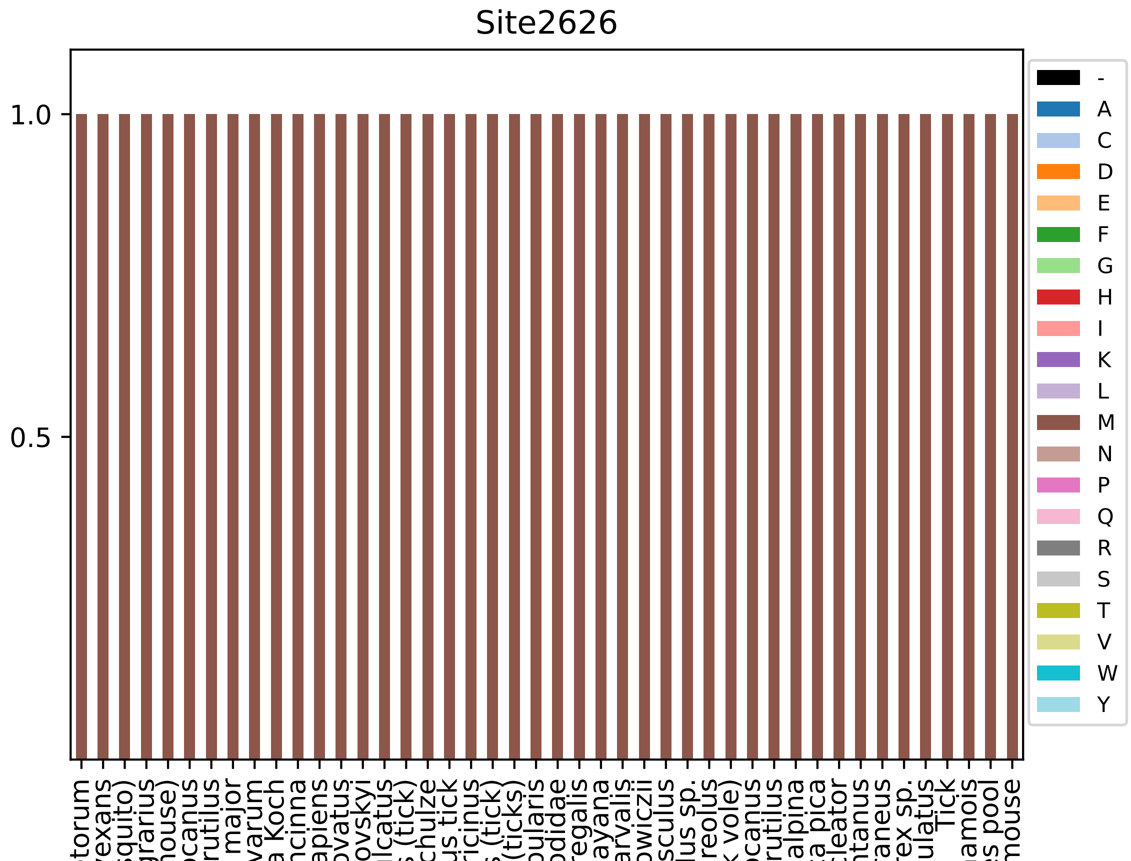

Site2626
1.0
0.5
-
A
C
D
E
F
G
H
I
K
L
M
N
P
Q
R
S
T
V
W
Y

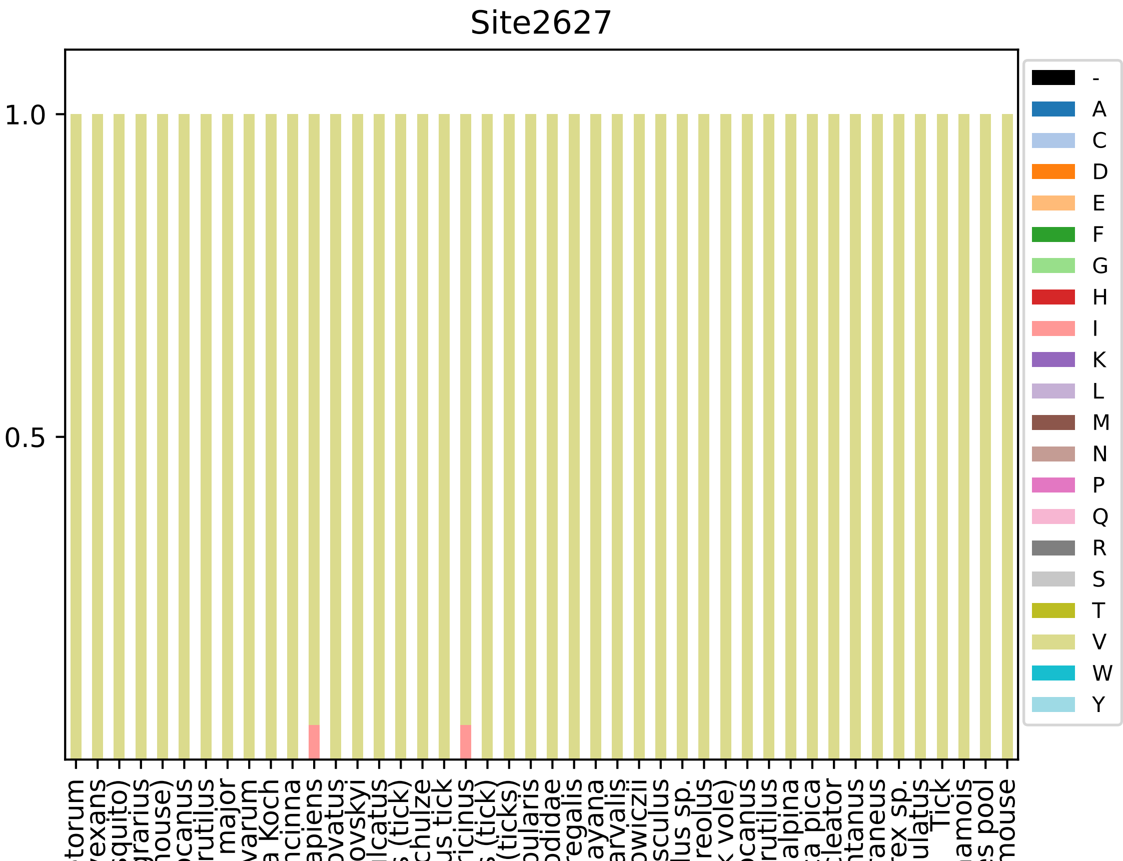
Site2627
1.0
0.5
-
A
C
D
E
F
G
H
I
K
L
M
N
P
Q
R
S
T
V
W
Y

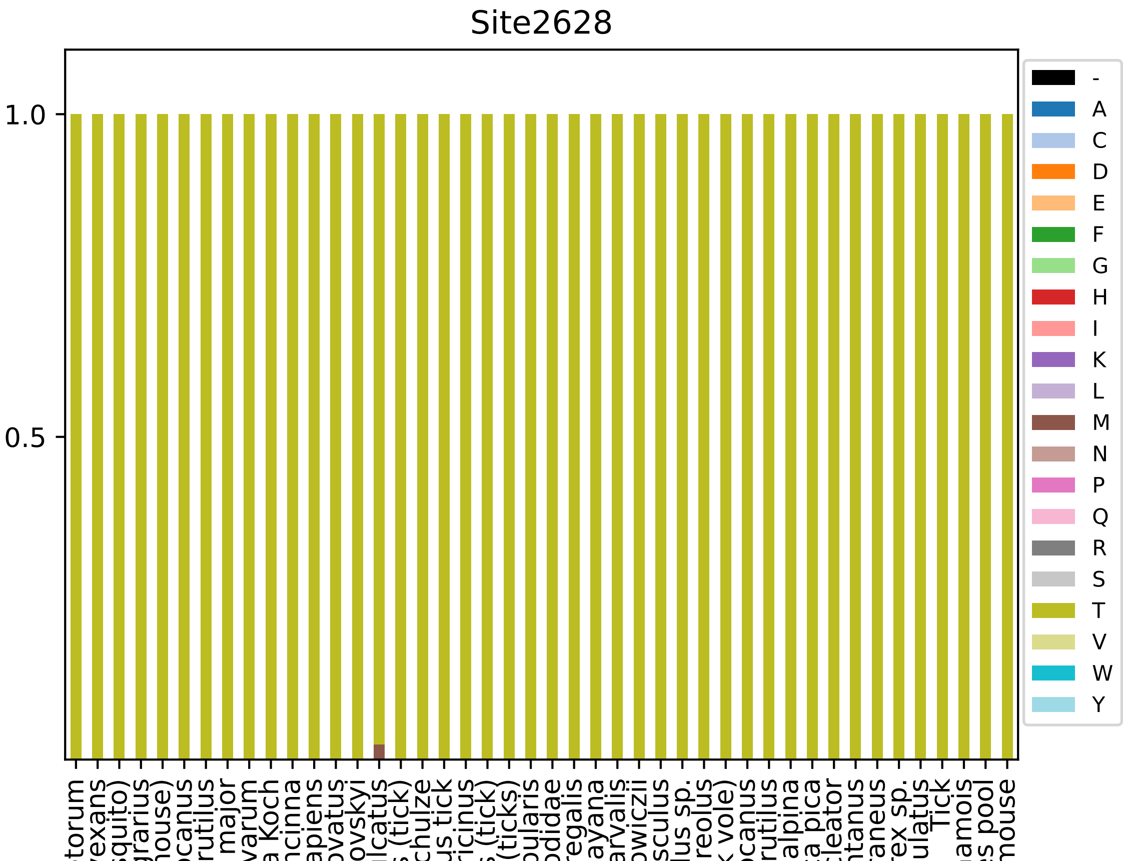
Site2628
1.0
0.5
-
A
C
D
E
F
G
H
I
K
L
M
N
P
Q
R
S
T
V
W
Y

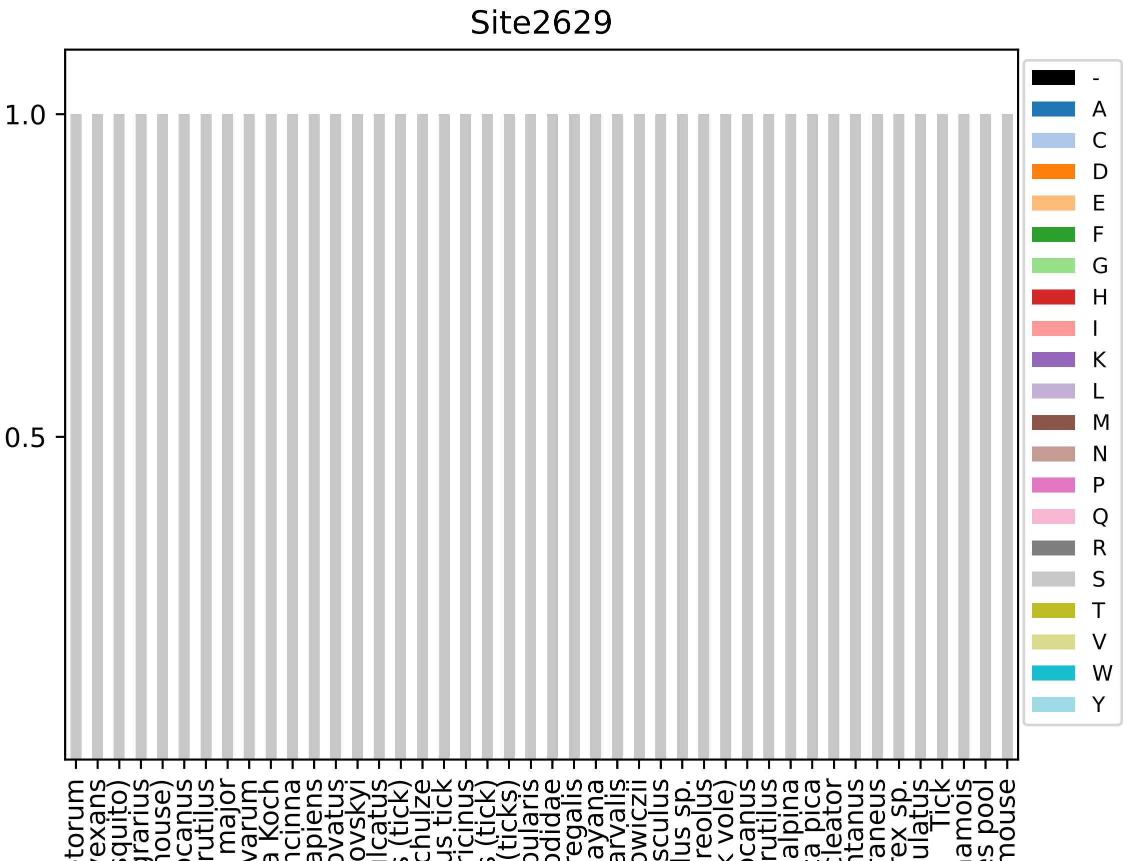
Site2629
1.0
0.5
-
A
C
D
E
F
G
H
I
K
L
M
N
P
Q
R
S
T
V
W
Y

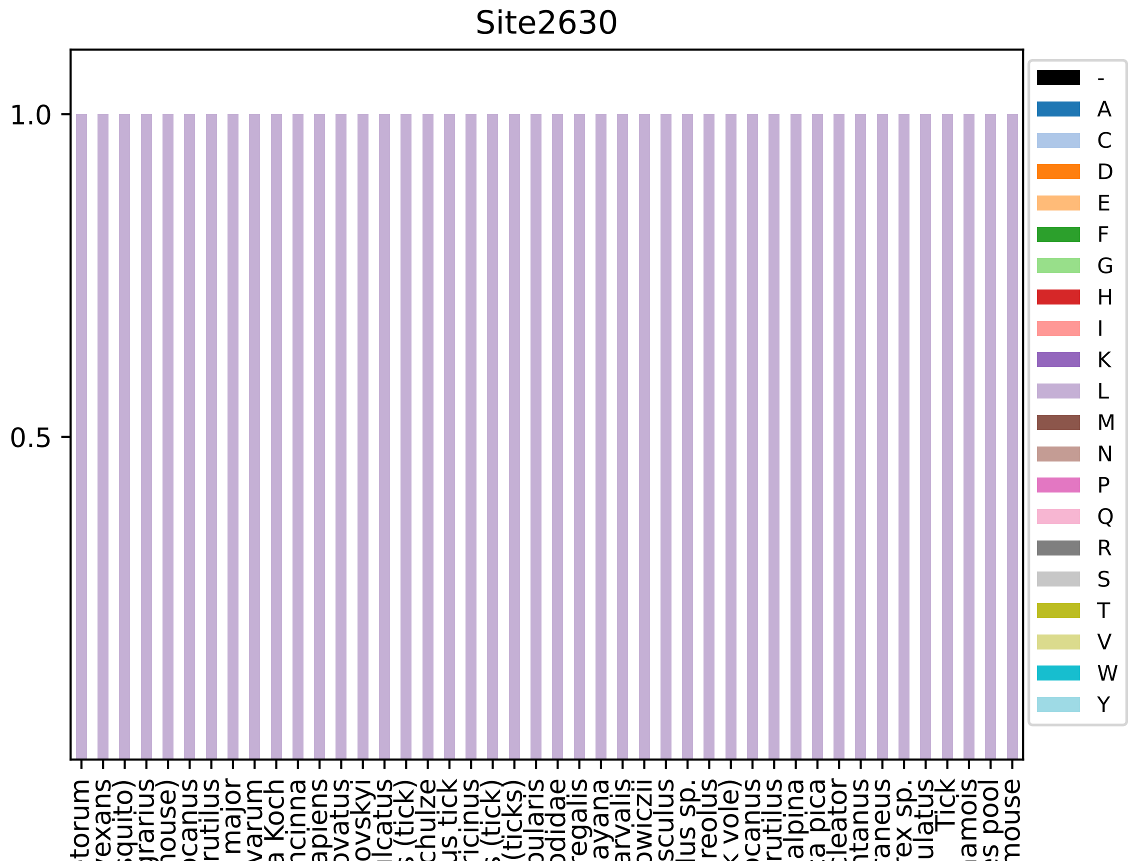
Site2630
1.0
0.5
-
A
C
D
E
F
G
H
I
K
L
M
N
P
Q
R
S
T
V
W
Y

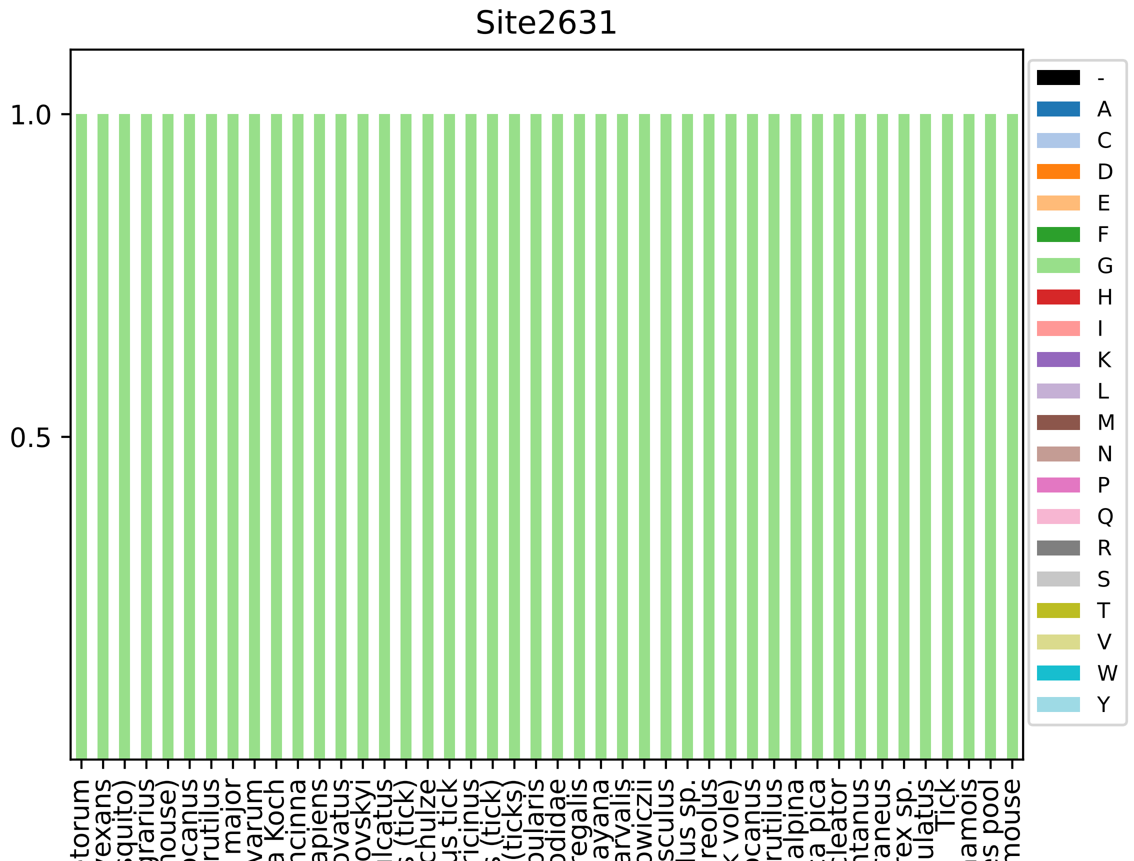
Site2631
1.0
0.5
-
A
C
D
E
F
G
H
I
K
L
M
N
P
Q
R
S
T
V
W
Y

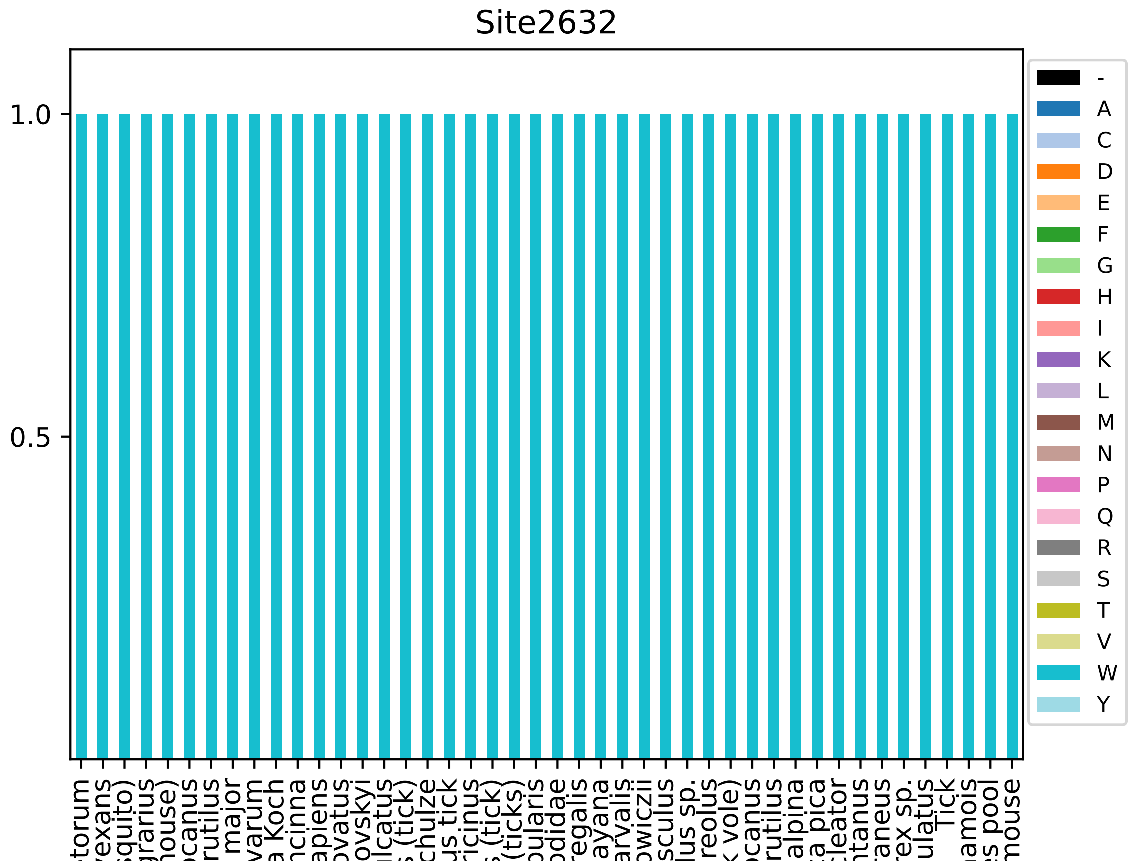
Site2632
1.0
0.5
-
A
C
D
E
F
G
H
I
K
L
M
N
P
Q
R
S
T
V
W
Y

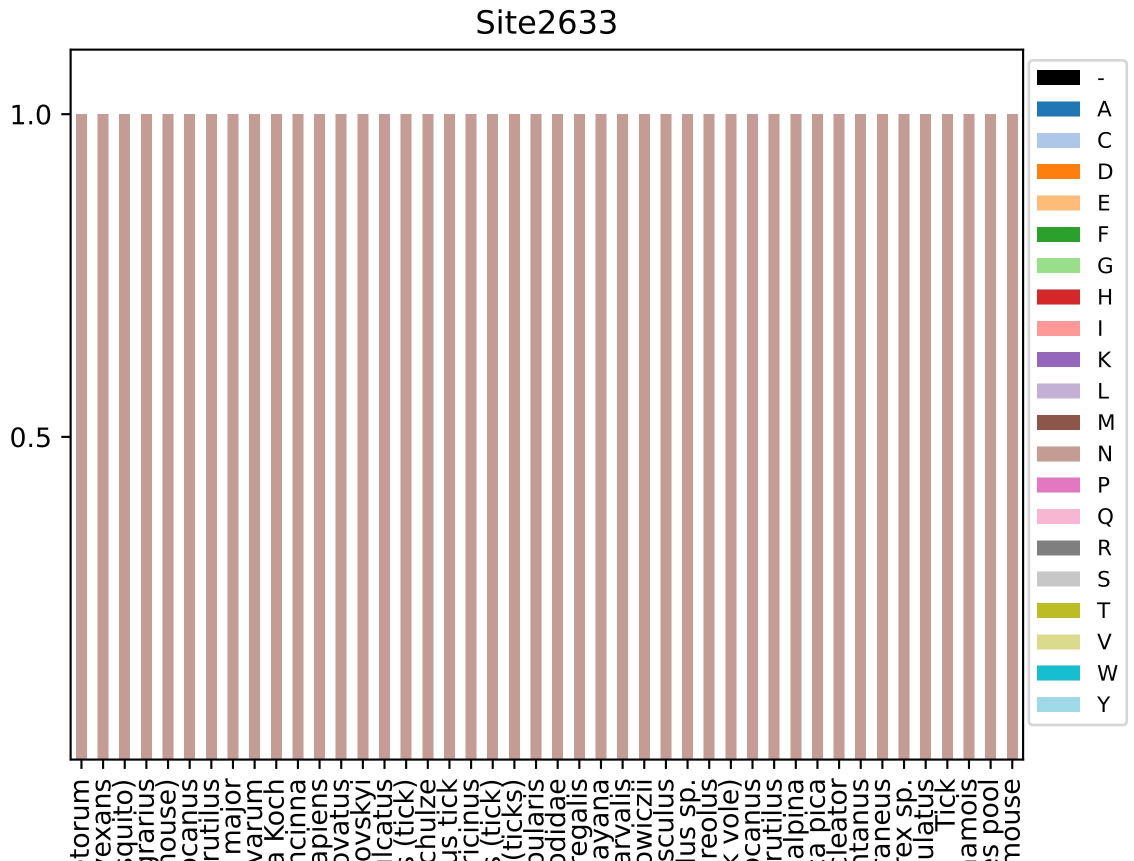
Site2633
1.0
0.5
-
A
C
D
E
F
G
H
I
K
L
M
N
P
Q
R
S
T
V
W
Y

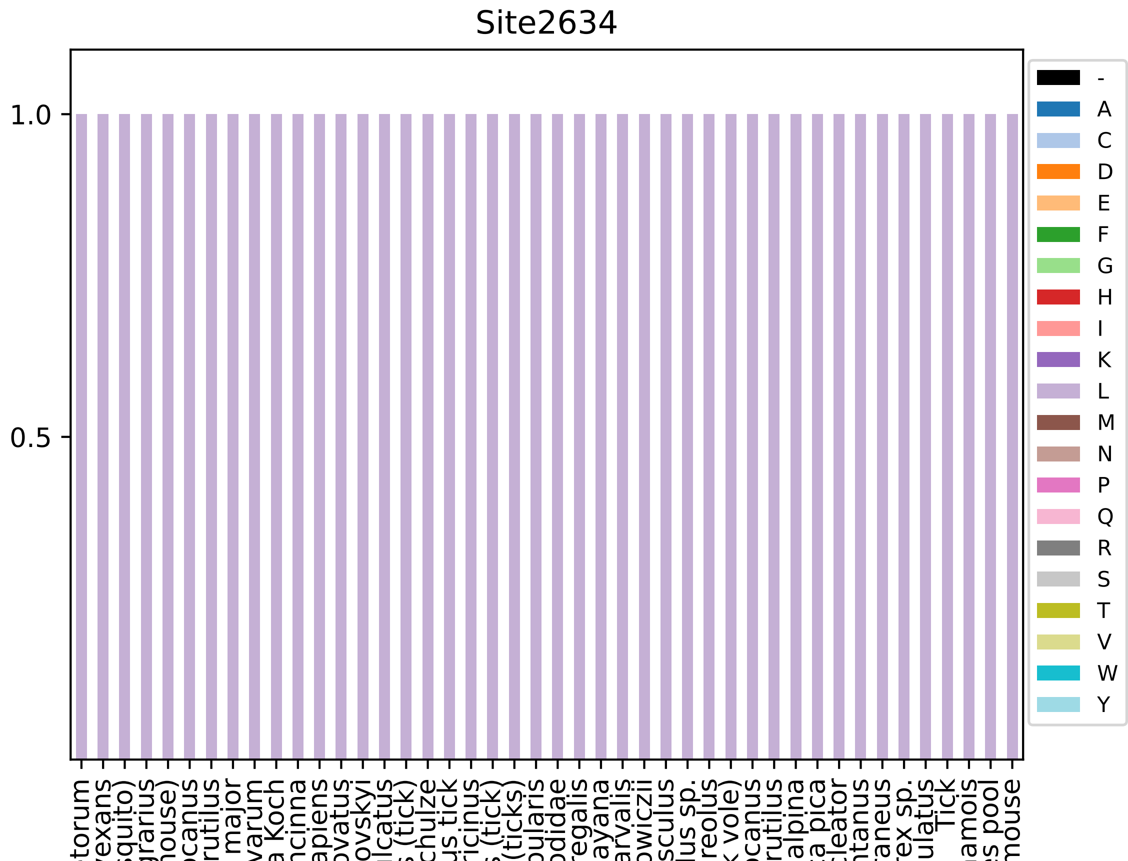

Site2634
1.0
0.5
-
A
C
D
E
F
G
H
I
K
L
M
N
P
Q
R
S
T
V
W
Y
torum
vexans
squito)
grarius
nouse)
ocanus
rutilus
major
varum
a Koch
ncinna
apiens
ovatus
ovskyi
ulcatus
s (tick)
chulze
us tick
ricinus
s (tick)
(ticks)
pularis
podidae
regalis
ayana
arvalis
owiczii
sculus
lus sp.
reolus
k vole)
ocanus
rutilus
alpina
a pica
cleator
ntanus
raneus
rex sp.
ulatus
Tick
amois
es pool
mouse

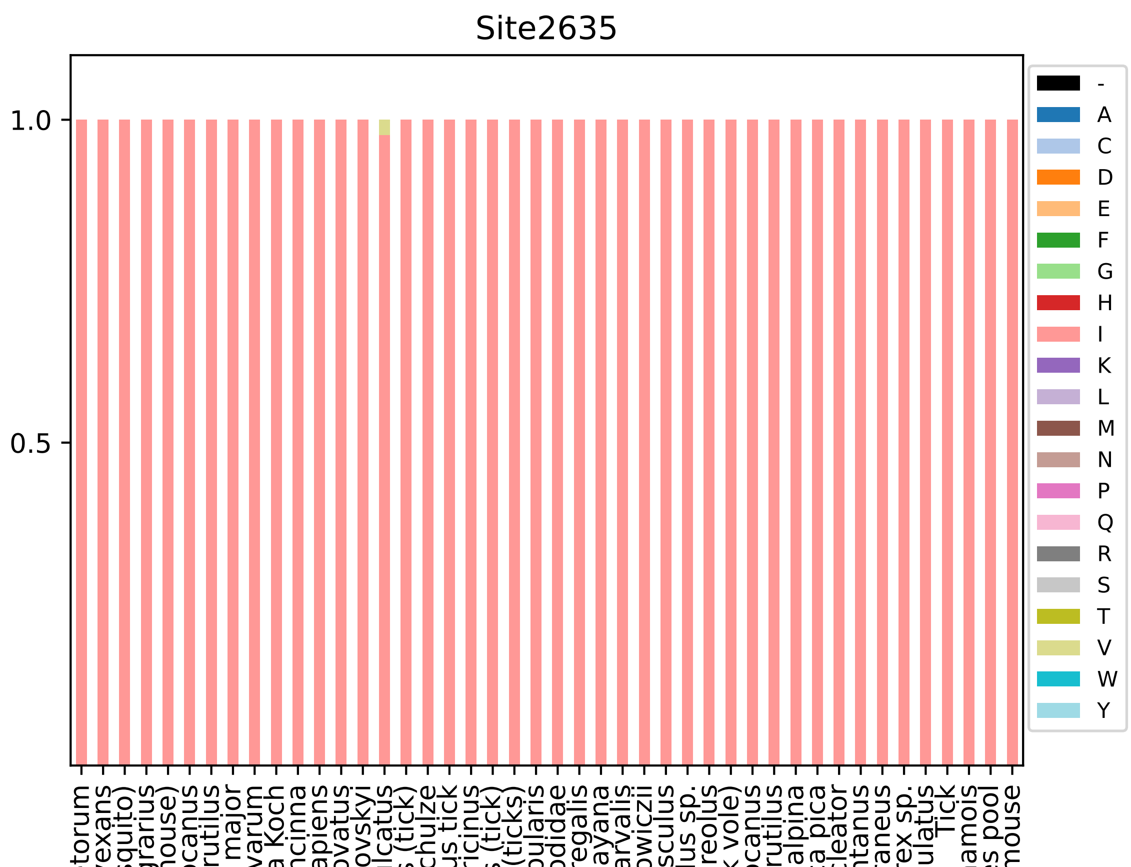
Site2635
1.0
0.5
-
A
C
D
E
F
G
H
I
K
L
M
N
P
Q
R
S
T
V
W
Y

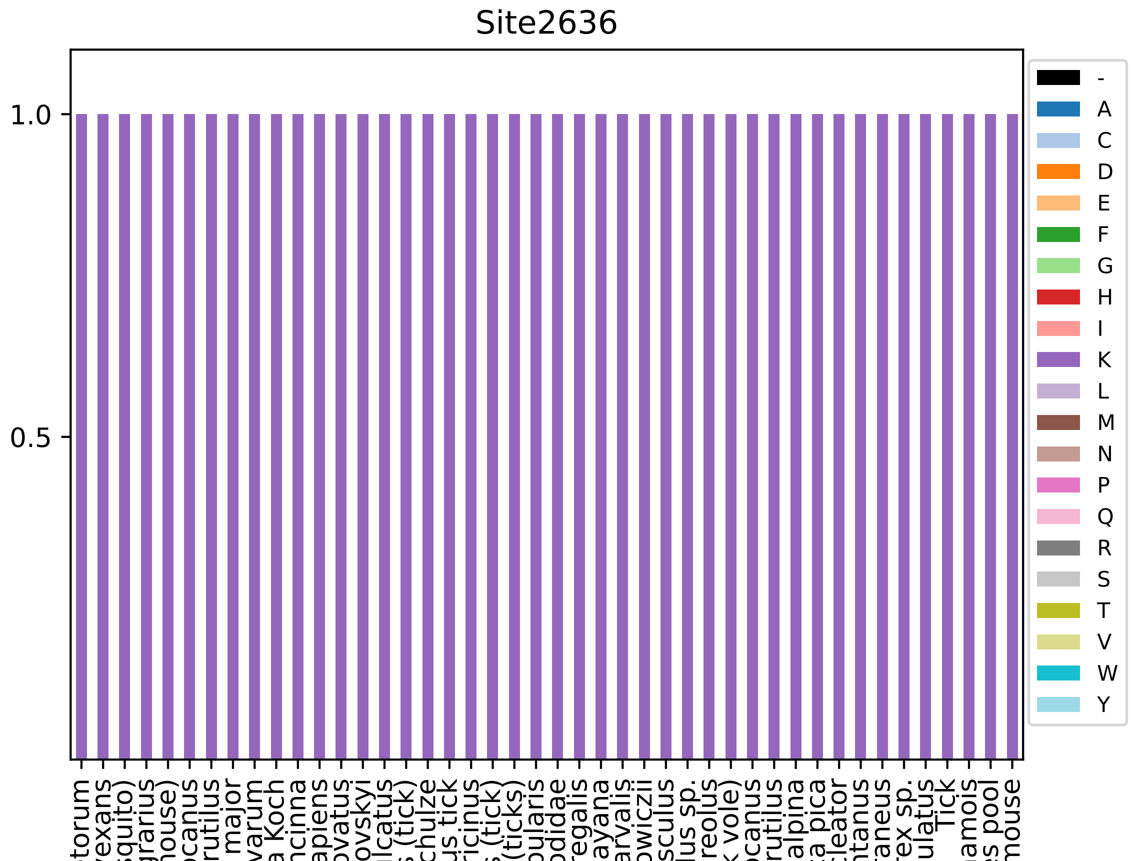
Site2636
1.0
0.5
-
A
C
D
E
F
G
H
I
K
L
M
N
P
Q
R
S
T
V
W
Y

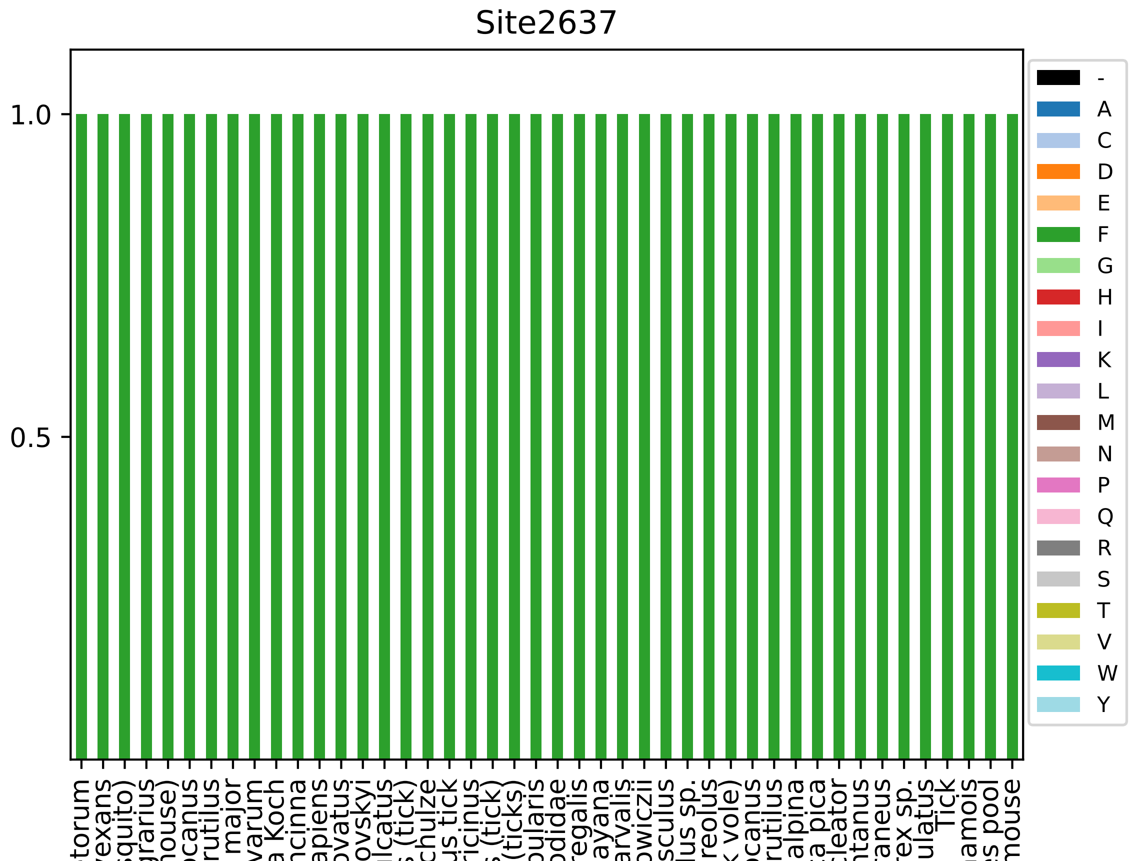
Site2637
1.0
0.5
-
A
C
D
E
F
G
H
I
K
L
M
N
P
Q
R
S
T
V
W
Y

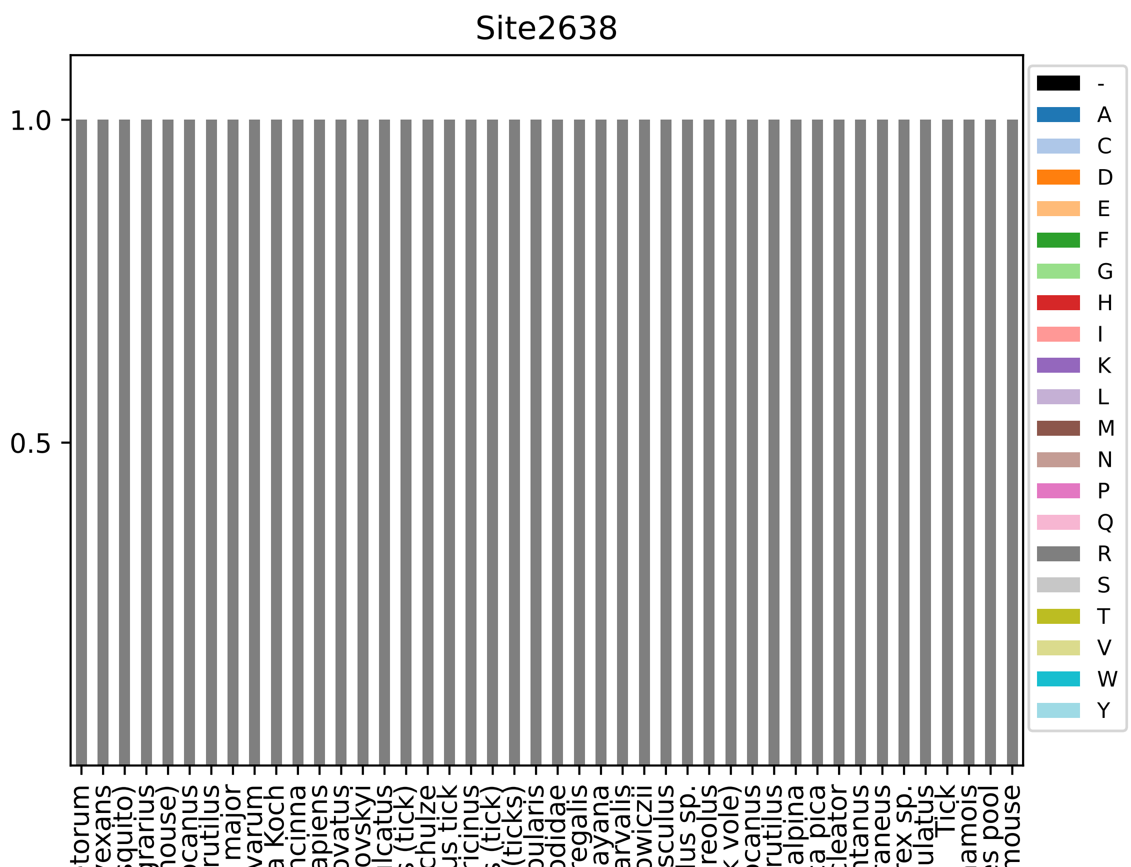
Site2638
1.0
0.5
-
A
C
D
E
F
G
H
I
K
L
M
N
P
Q
R
S
T
V
W
Y

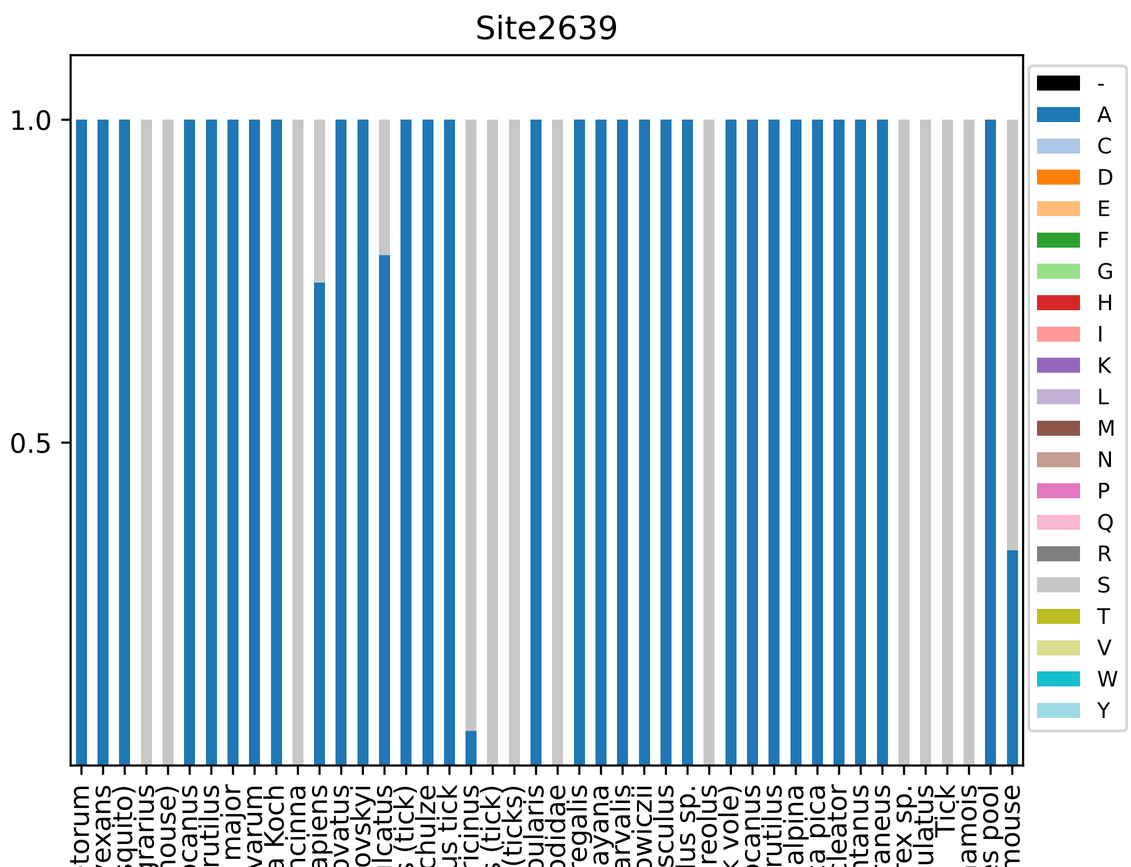
Site2639
1.0
0.5
-
A
C
D
E
F
G
H
I
K
L
M
N
P
Q
R
S
T
V
W
Y

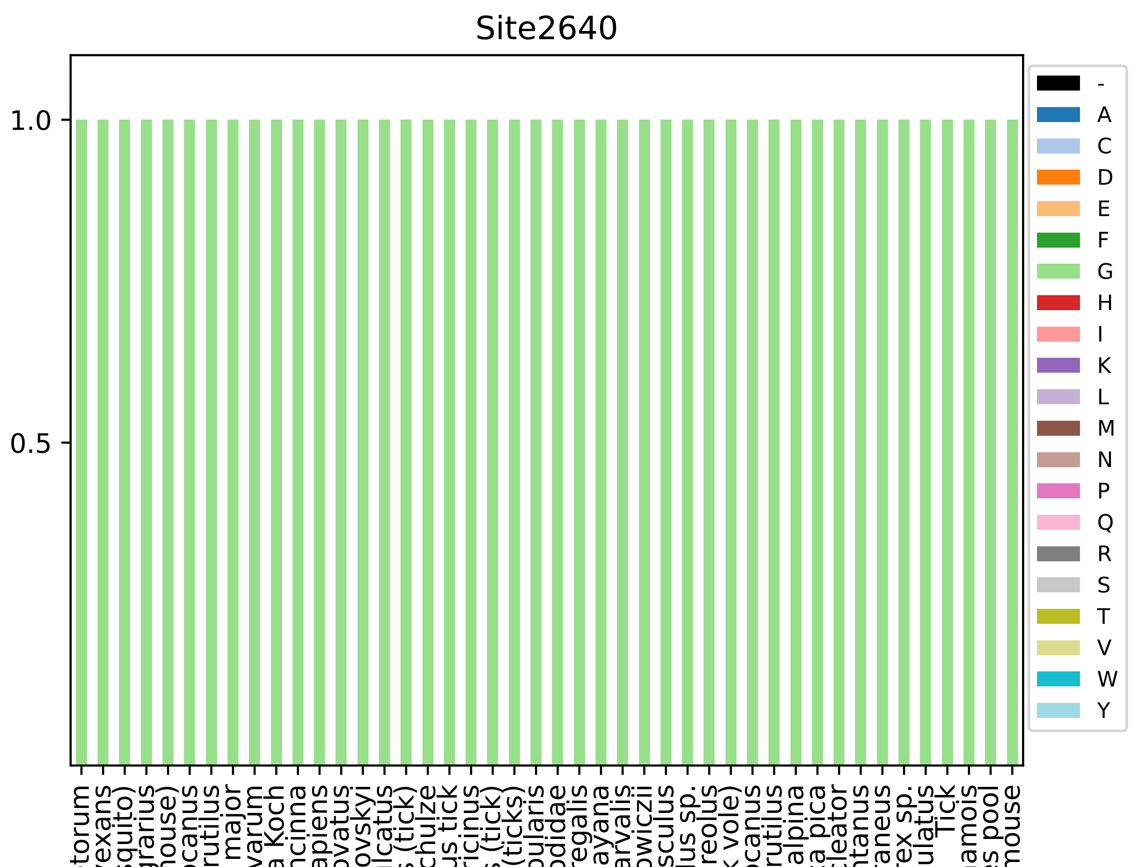
Site2640
1.0
0.5
-
A
C
D
E
F
G
H
I
K
L
M
N
P
Q
R
S
T
V
W
Y

Site2641

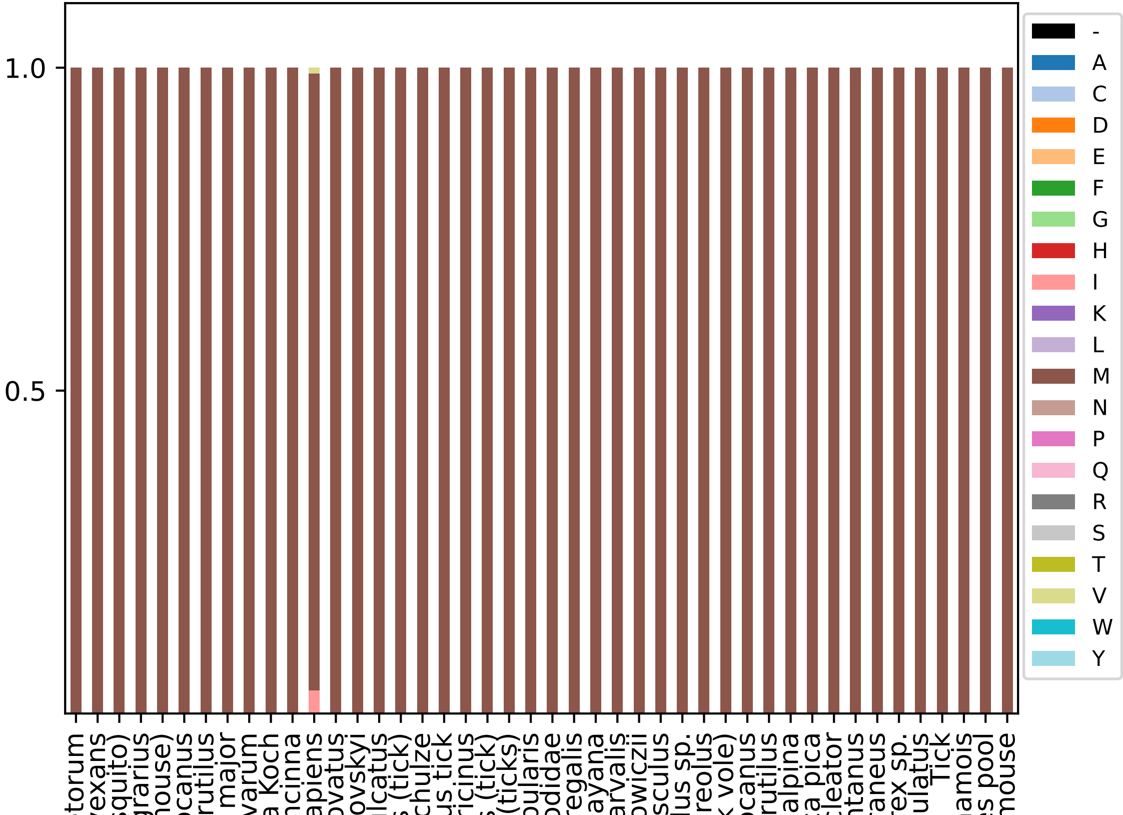

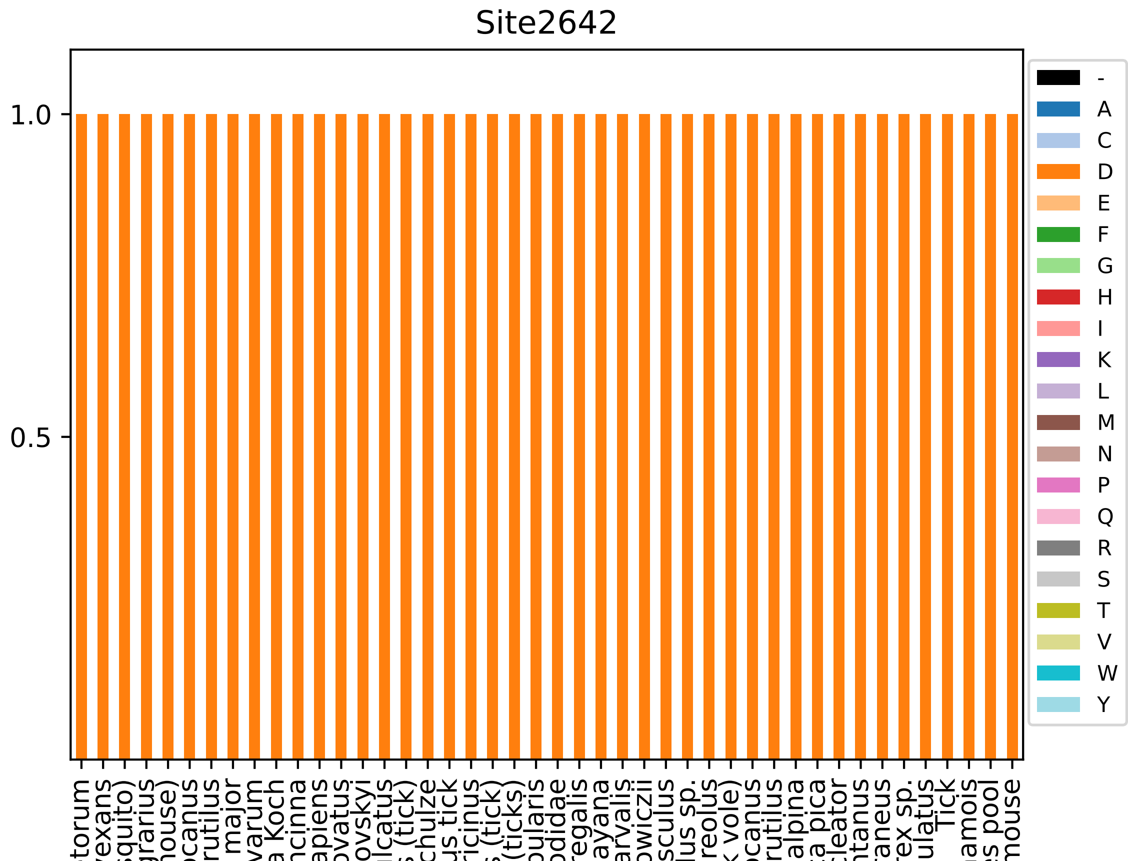

Site2642
1.0
0.5
-
A
C
D
E
F
G
H
I
K
L
M
N
P
Q
R
S
T
V
W
Y

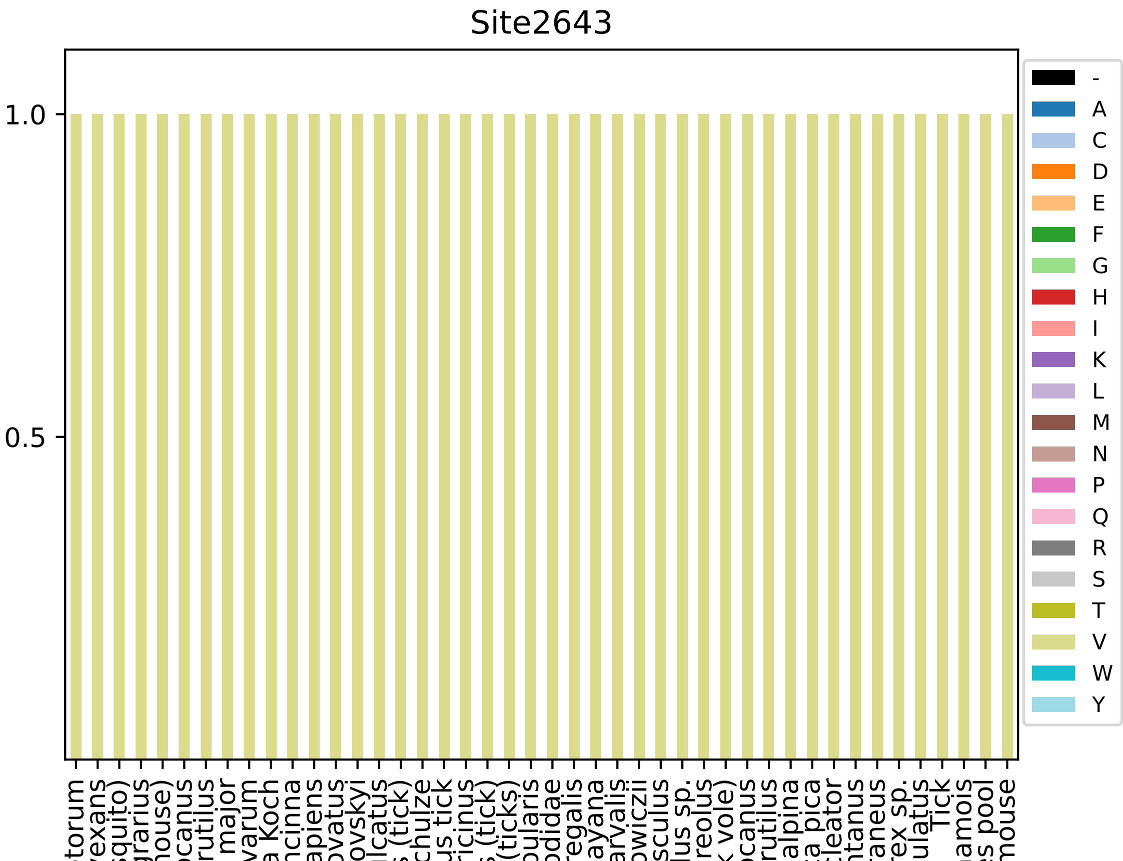
Site2643
1.0
0.5
-
A
C
D
E
F
G
H
I
K
L
M
N
P
Q
R
S
T
V
W
Y

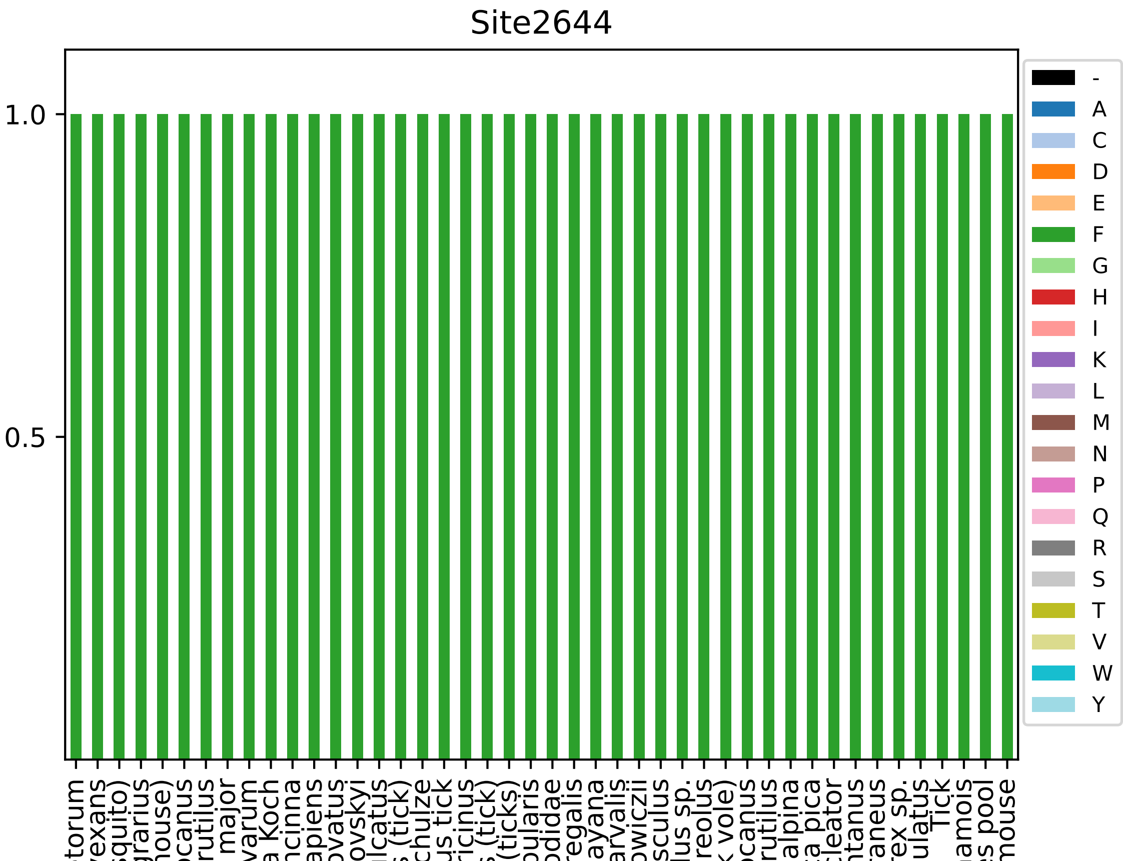

Site2644
1.0
0.5
-
A
C
D
E
F
G
H
I
K
L
M
N
P
Q
R
S
T
V
W
Y

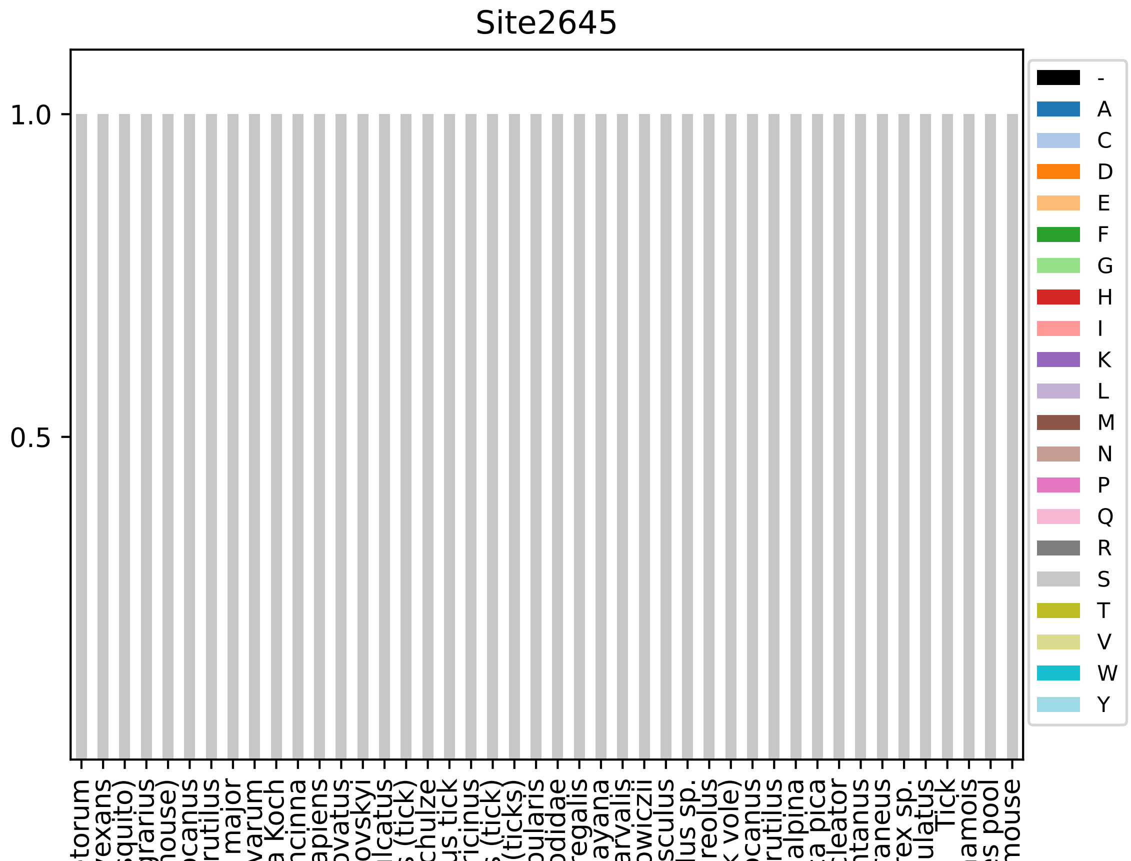
Site2645
1.0
0.5
-
A
C
D
E
F
G
H
I
K
L
M
N
P
Q
R
S
T
V
W
Y

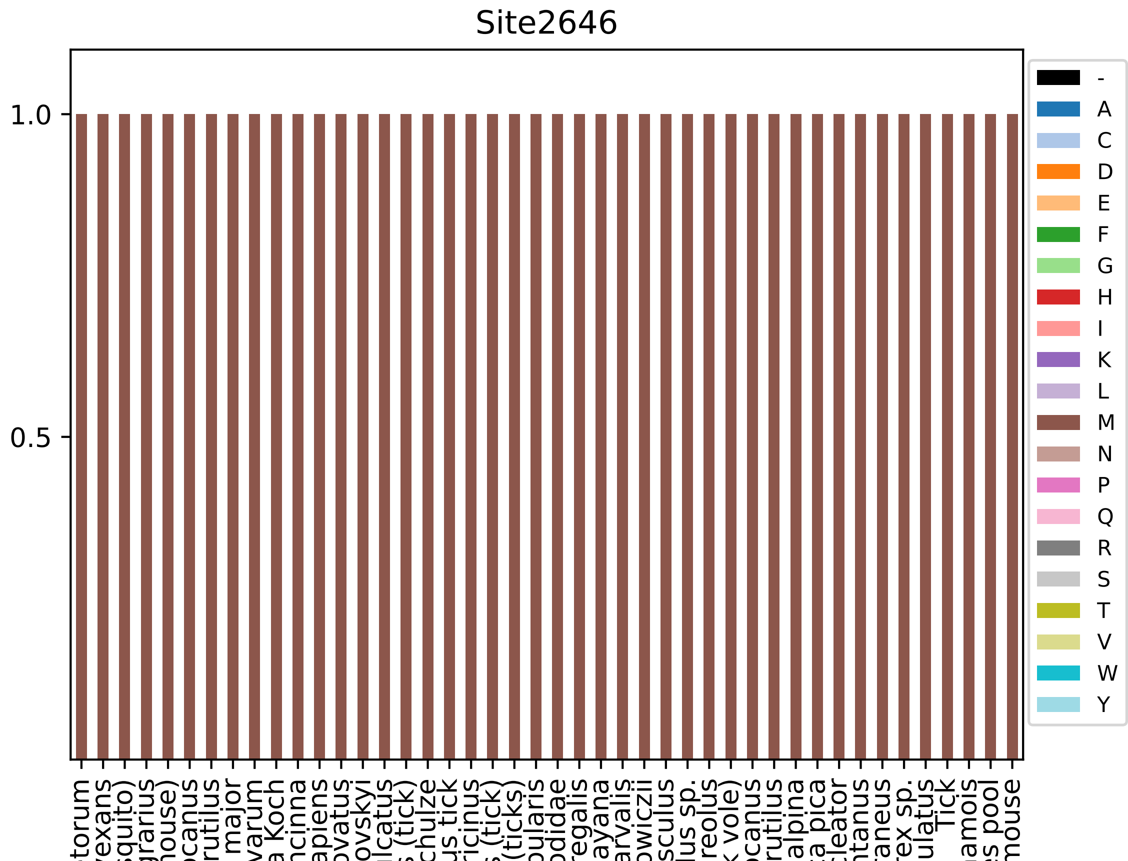
Site2646
1.0
0.5
-
A
C
D
E
F
G
H
I
K
L
M
N
P
Q
R
S
T
V
W
Y
torum
vexans
rarius
ocanus
rutilus
major
varum
a Koch
ncinna
apiens
ovatus
ovskyi
chulze
us tick
ricinus
(ticks)
pularis
podidae
regalis
ayana
arvalis
owiczii
sculus
lus sp.
reolus
ocanus
rutilus
alpina
a pica
cleator
ntanus
raneus
rex sp.
ulatus
Tick
amois
es pool
mouse

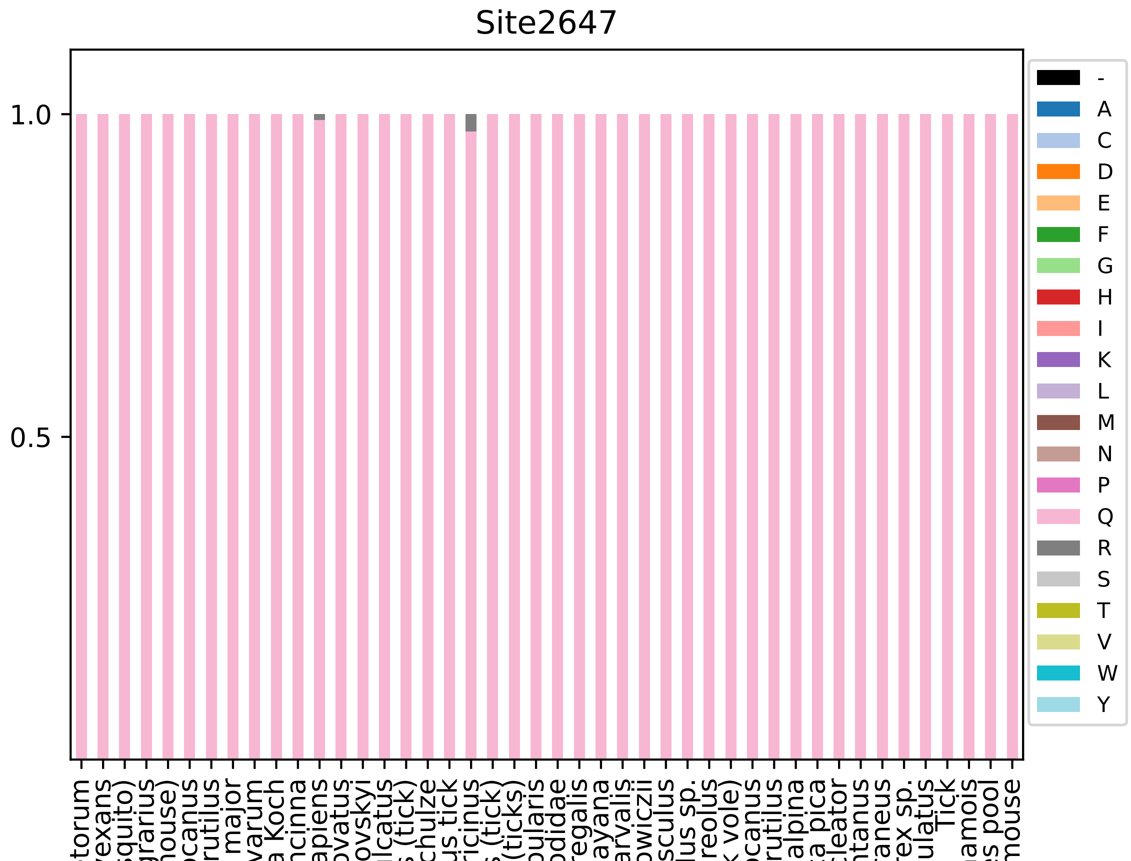
Site2647
1.0
0.5
-
A
C
D
E
F
G
H
I
K
L
M
N
P
Q
R
S
T
V
W
Y

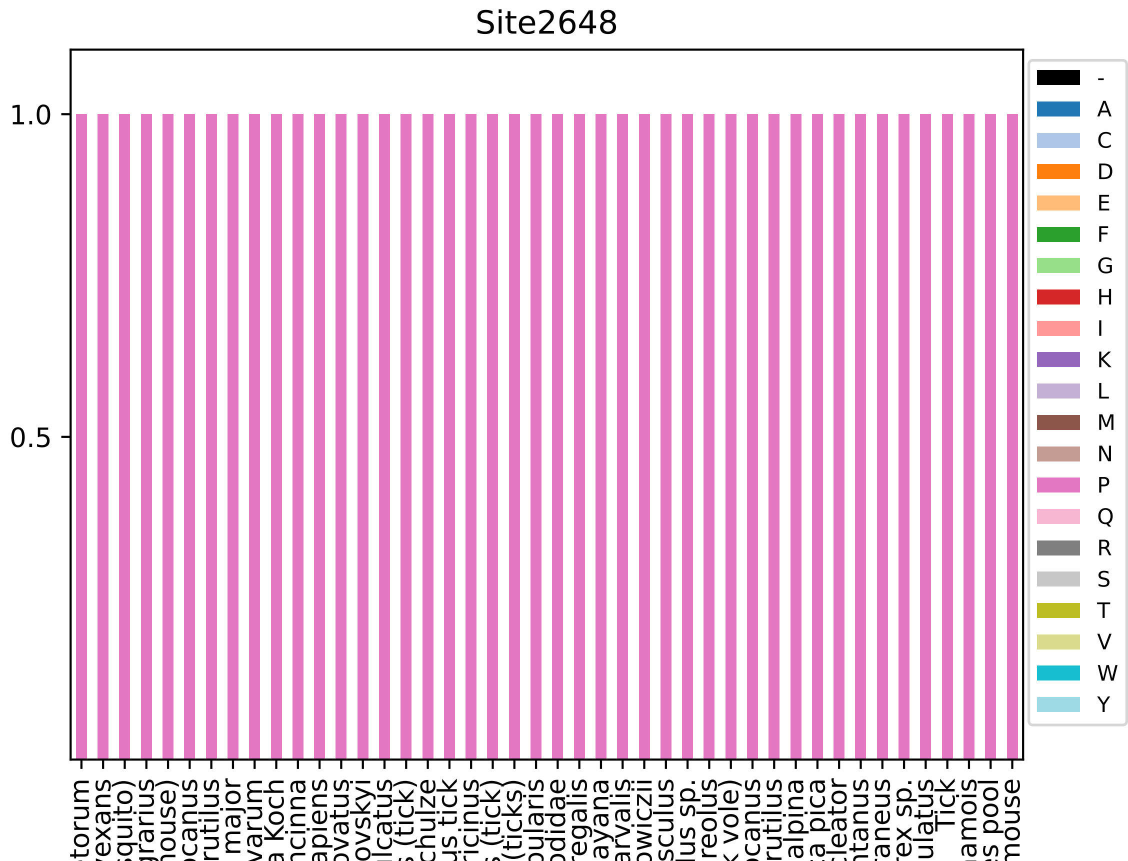
Site2648
1.0
0.5
-
A
C
D
E
F
G
H
I
K
L
M
N
P
Q
R
S
T
V
W
Y

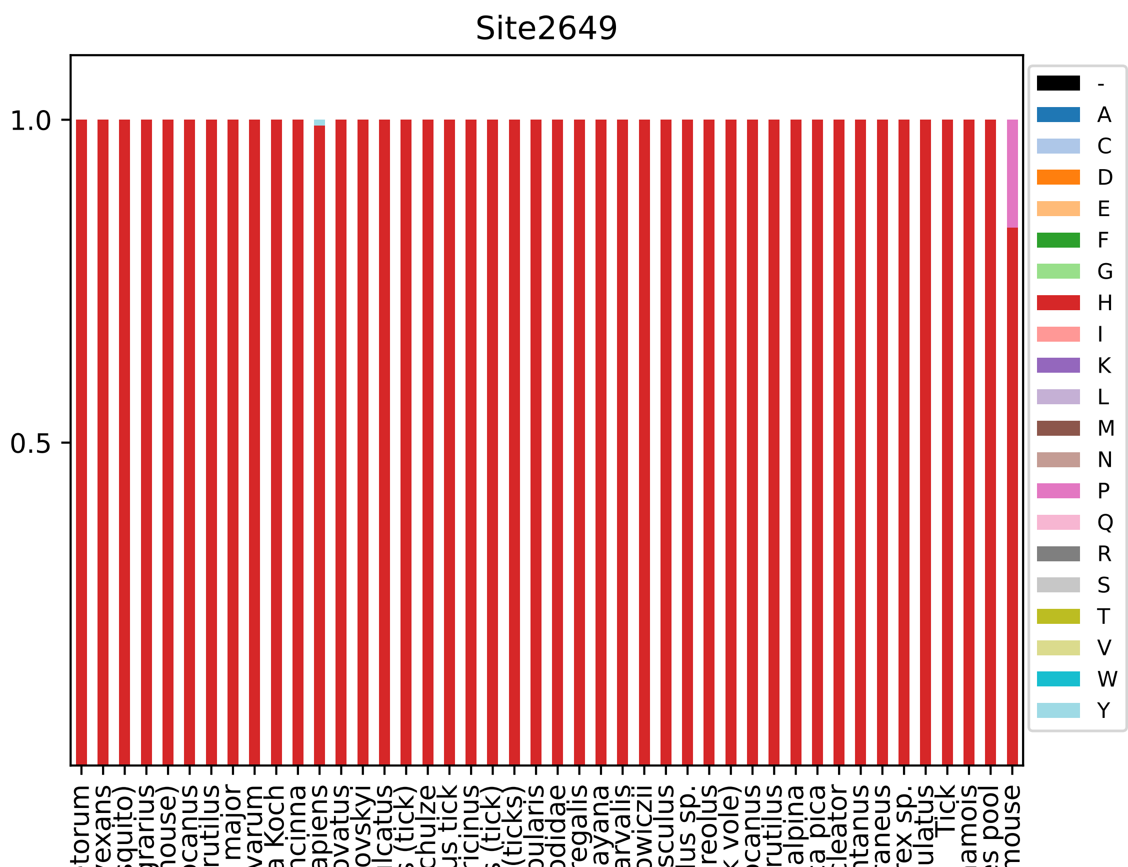
Site2649
1.0
0.5
-
A
C
D
E
F
G
H
I
K
L
M
N
P
Q
R
S
T
V
W
Y

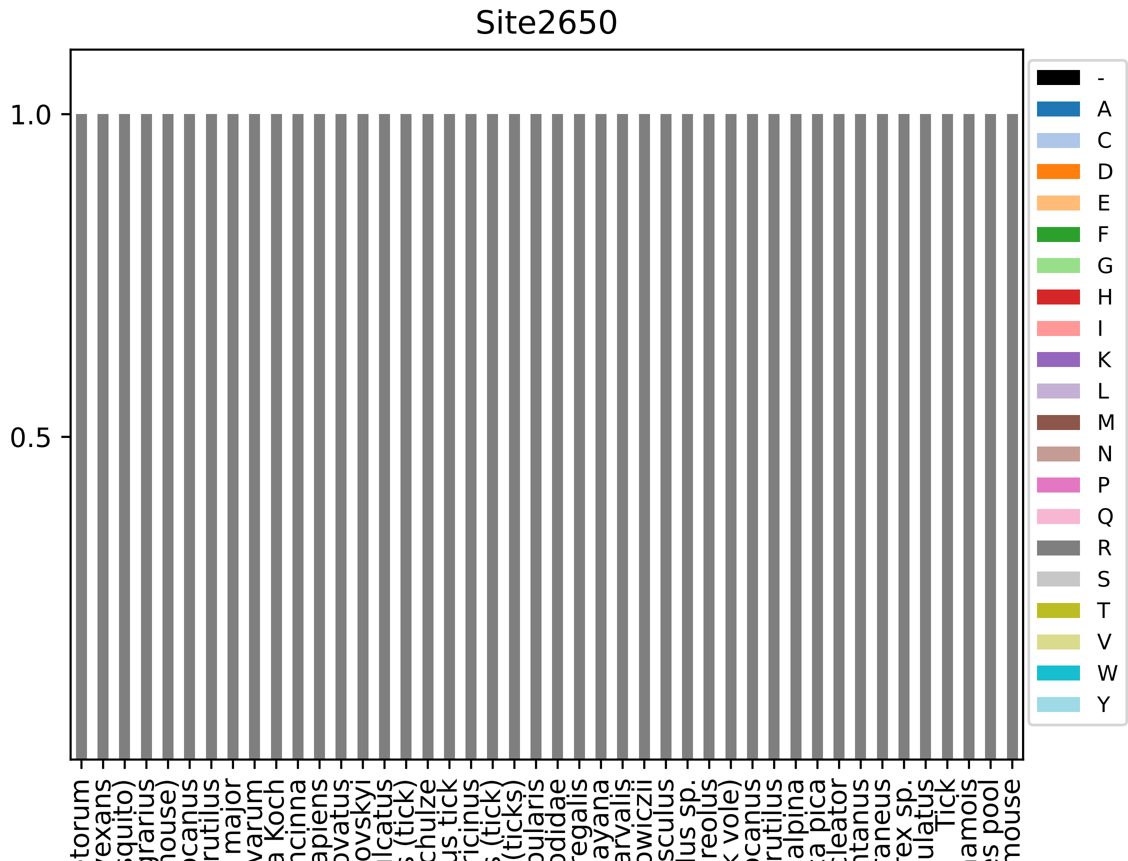
Site2650
1.0
0.5
-
A
C
D
E
F
G
H
I
K
L
M
N
P
Q
R
S
T
V
W
Y
torum
vexans
squito)
grarius
nouse)
ocanus
rutilus
major
varum
a Koch
ncinna
apiens
ovatus
ovskyi
ulcatus
s (tick)
chulze
us tick
ricinus
s (tick)
(ticks)
pularis
podidae
regalis
ayana
arvalis
owiczii
sculus
lus sp.
reolus
x vole)
ocanus
rutilus
alpina
a pica
cleator
ntanus
raneus
rex sp.
ulatus
Tick
amois
es pool
mouse

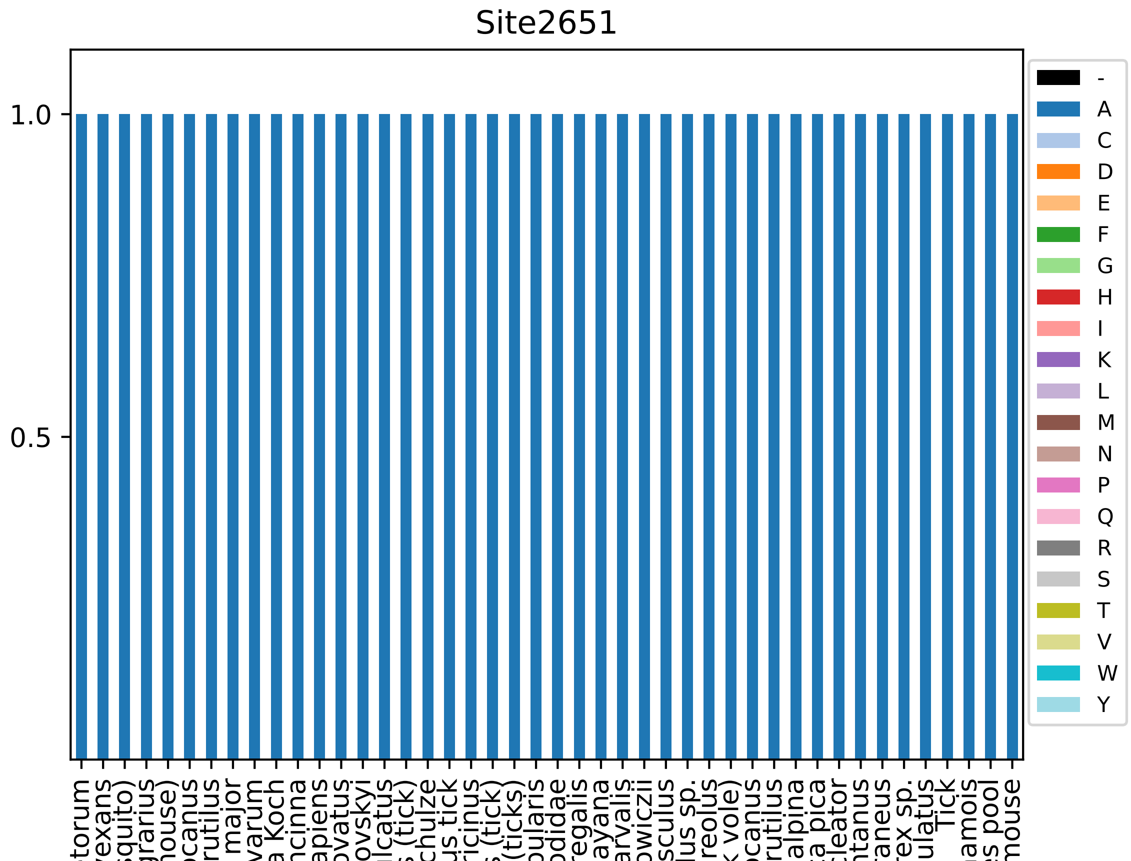
Site2651
1.0
0.5
-
A
C
D
E
F
G
H
I
K
L
M
N
P
Q
R
S
T
V
W
Y
torum
vexans
squito)
grarius
nouse)
ocanus
rutilus
major
varum
a Koch
ncinna
apiens
ovatus
ovskyi
ulcatus
(tick)
chulze
us tick
ricinus
(tick)
(ticks)
pularis
odidae
regalis
ayana
arvalis
owiczii
sculus
lus sp.
reolus
x vole)
ocanus
rutilus
alpina
a pica
cleator
ntanus
raneus
rex sp.
ulatus
Tick
amois
es pool
mouse

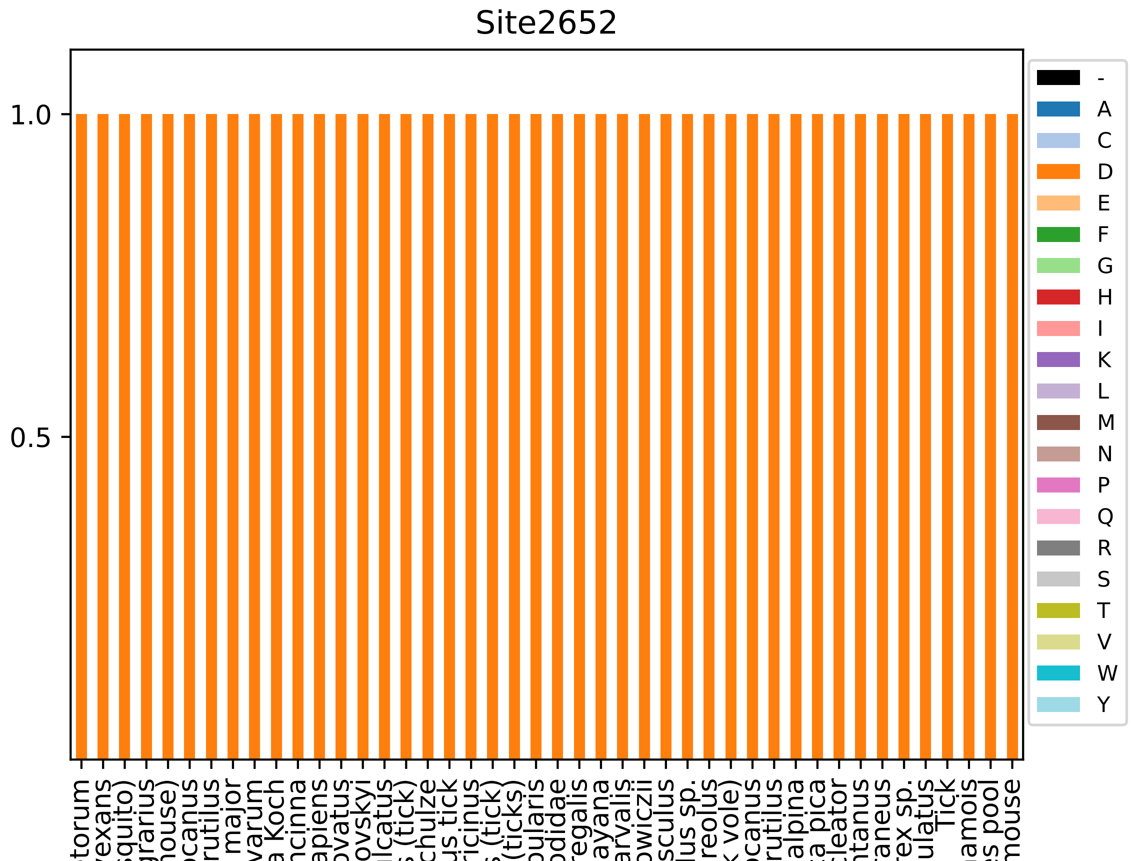

Site2652
1.0
0.5
-
A
C
D
E
F
G
H
I
K
L
M
N
P
Q
R
S
T
V
W
Y

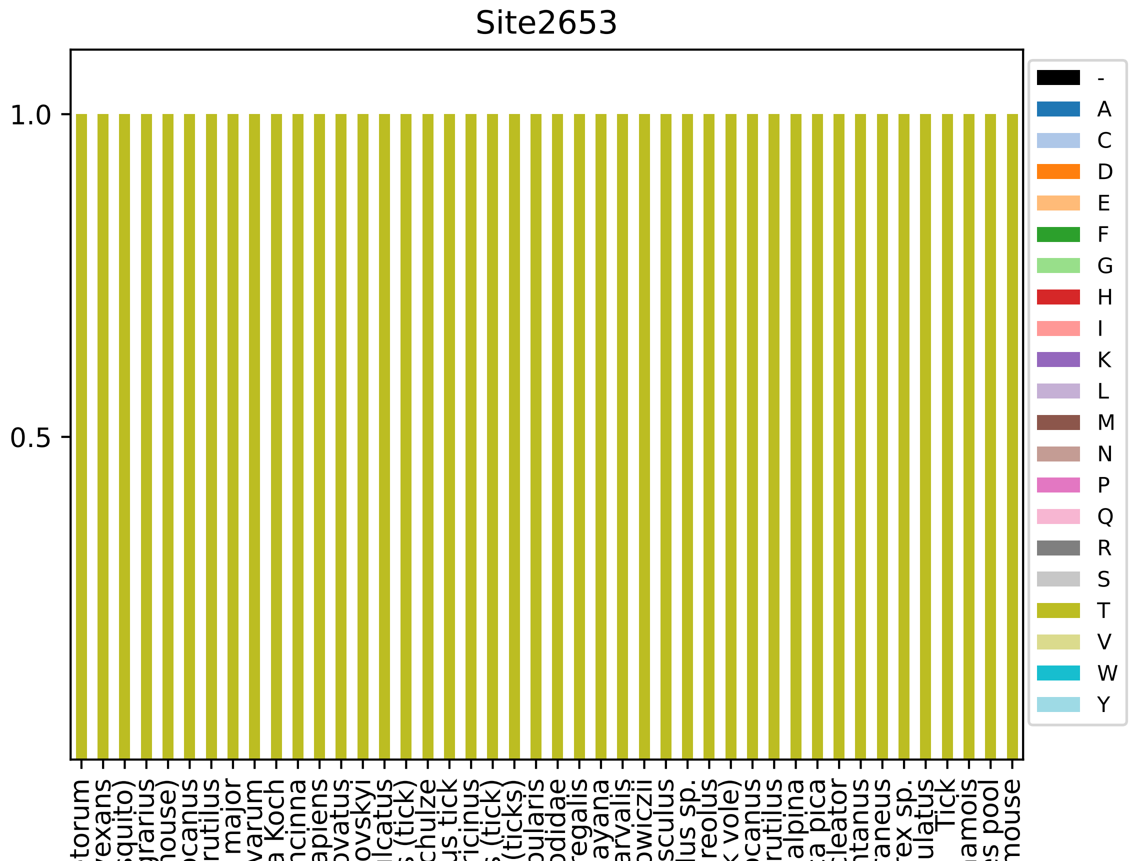
Site2653
1.0
0.5
-
A
C
D
E
F
G
H
I
K
L
M
N
P
Q
R
S
T
V
W
Y

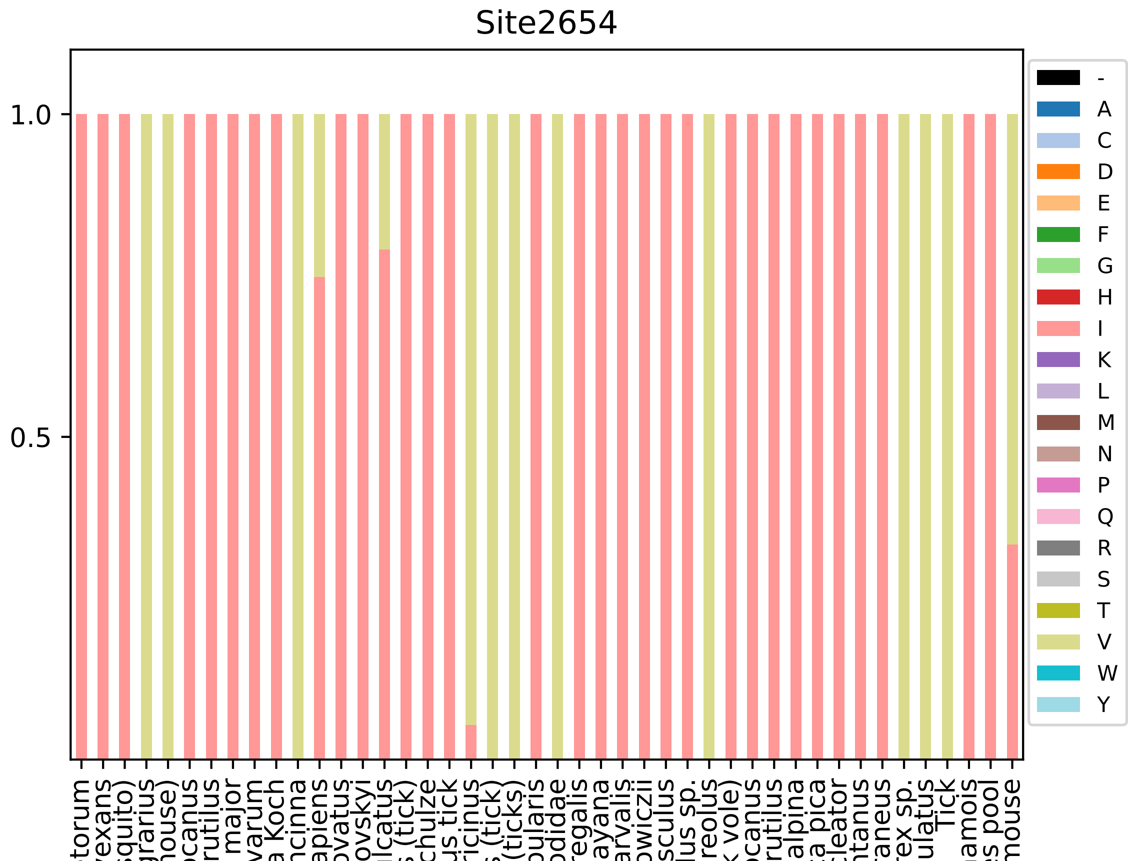

Site2654
1.0
0.5
-
A
C
D
E
F
G
H
I
K
L
M
N
P
Q
R
S
T
V
W
Y

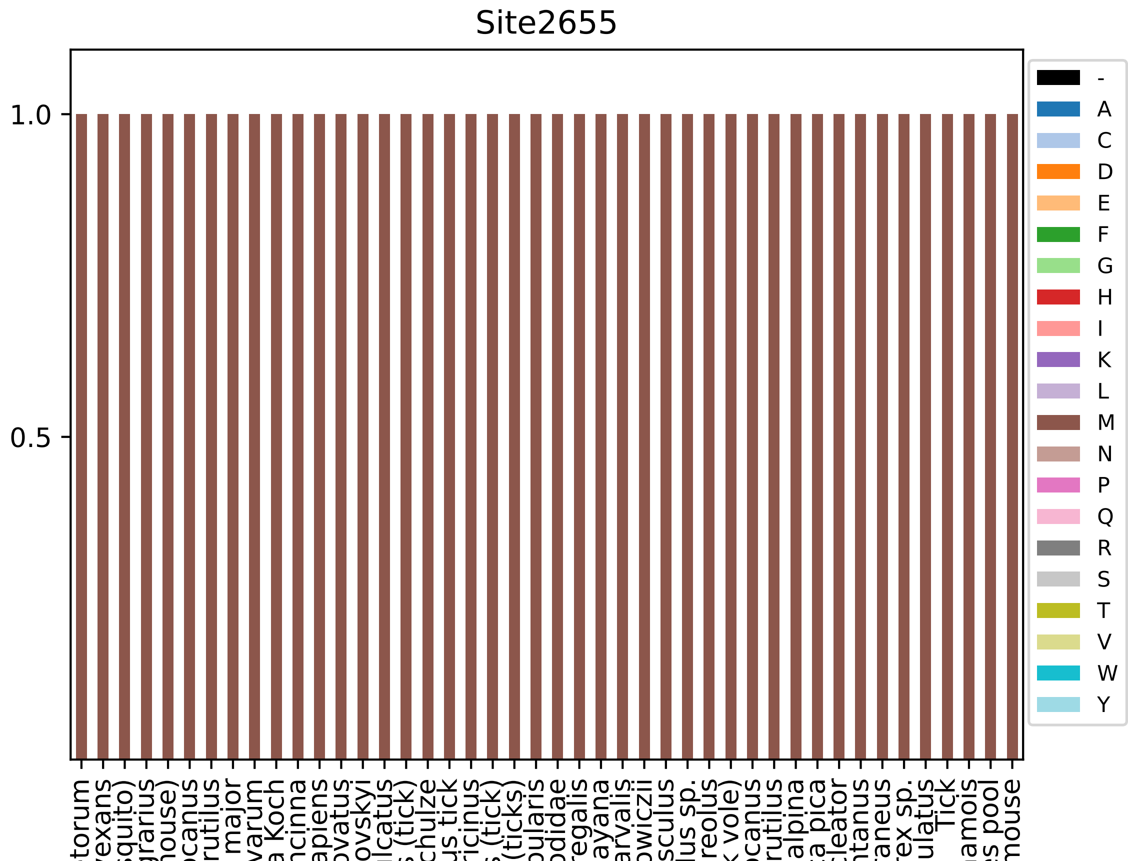

Site2655
1.0
0.5
-
A
C
D
E
F
G
H
I
K
L
M
N
P
Q
R
S
T
V
W
Y

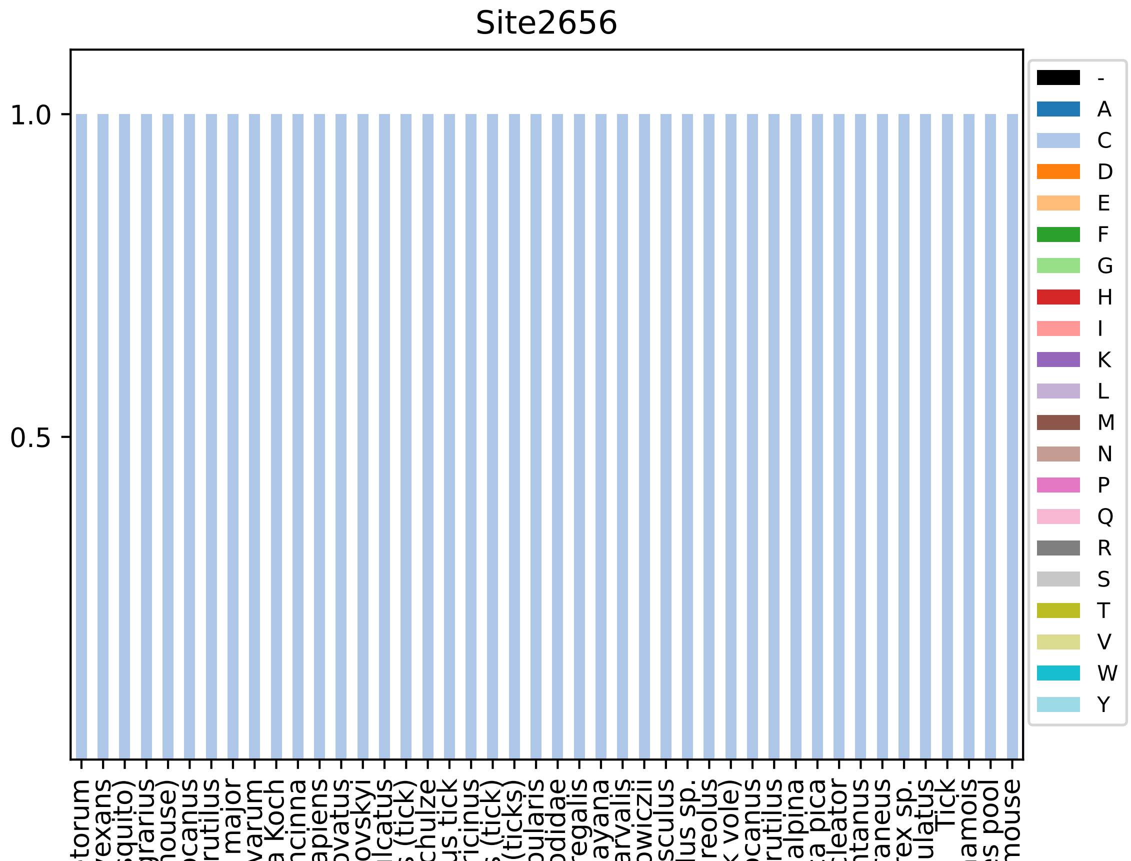

Site2656
1.0
0.5
-
A
C
D
E
F
G
H
I
K
L
M
N
P
Q
R
S
T
V
W
Y

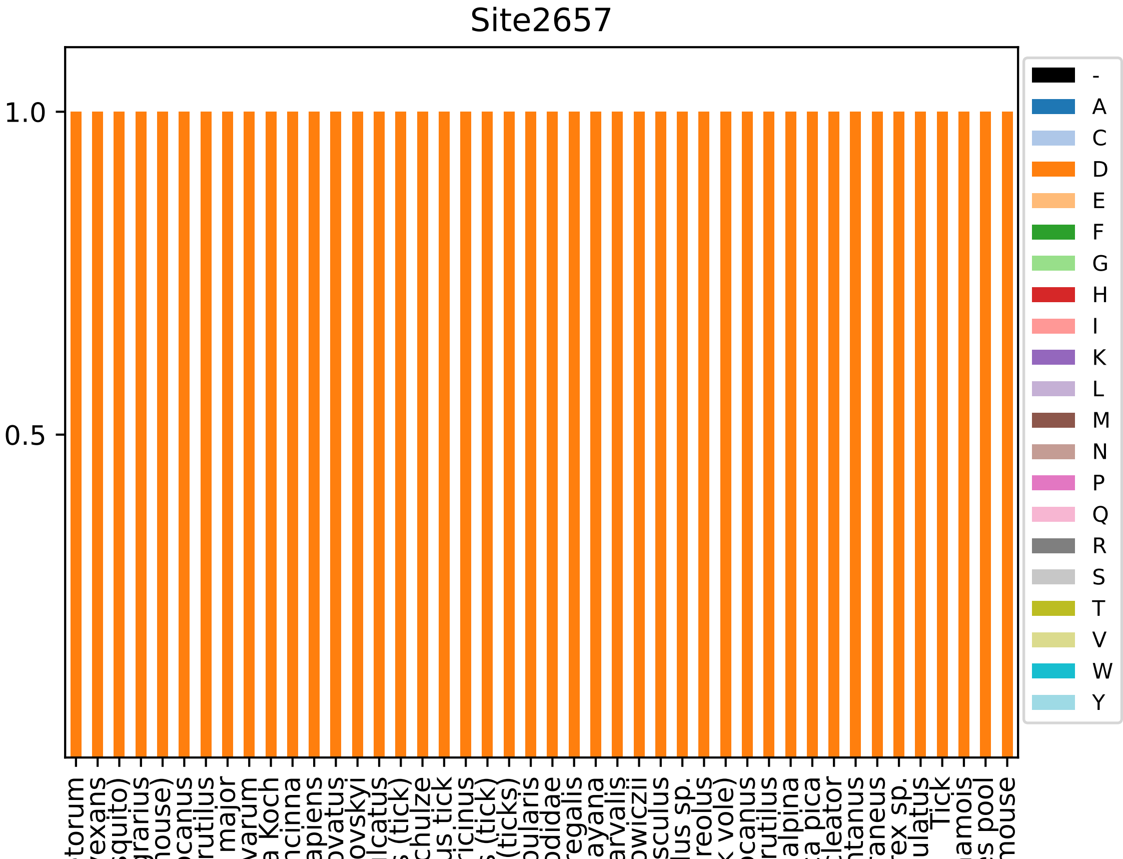
Site2657
1.0
0.5
-
A
C
D
E
F
G
H
I
K
L
M
N
P
Q
R
S
T
V
W
Y
torum
vexans
squito)
grarius
nouse)
ocanus
rutilus
major
varum
a Koch
ncinna
apiens
ovatus
ovskyi
lcatus
(tick)
chulze
us tick
ricinus
(tick)
(ticks)
pularis
odidae
regalis
ayana
arvalis
owiczii
sculus
lus sp.
reolus
x vole)
ocanus
rutilus
alpina
a pica
leator
ntanus
raneus
ex sp.
ulatus
Tick
amois
s pool
mouse

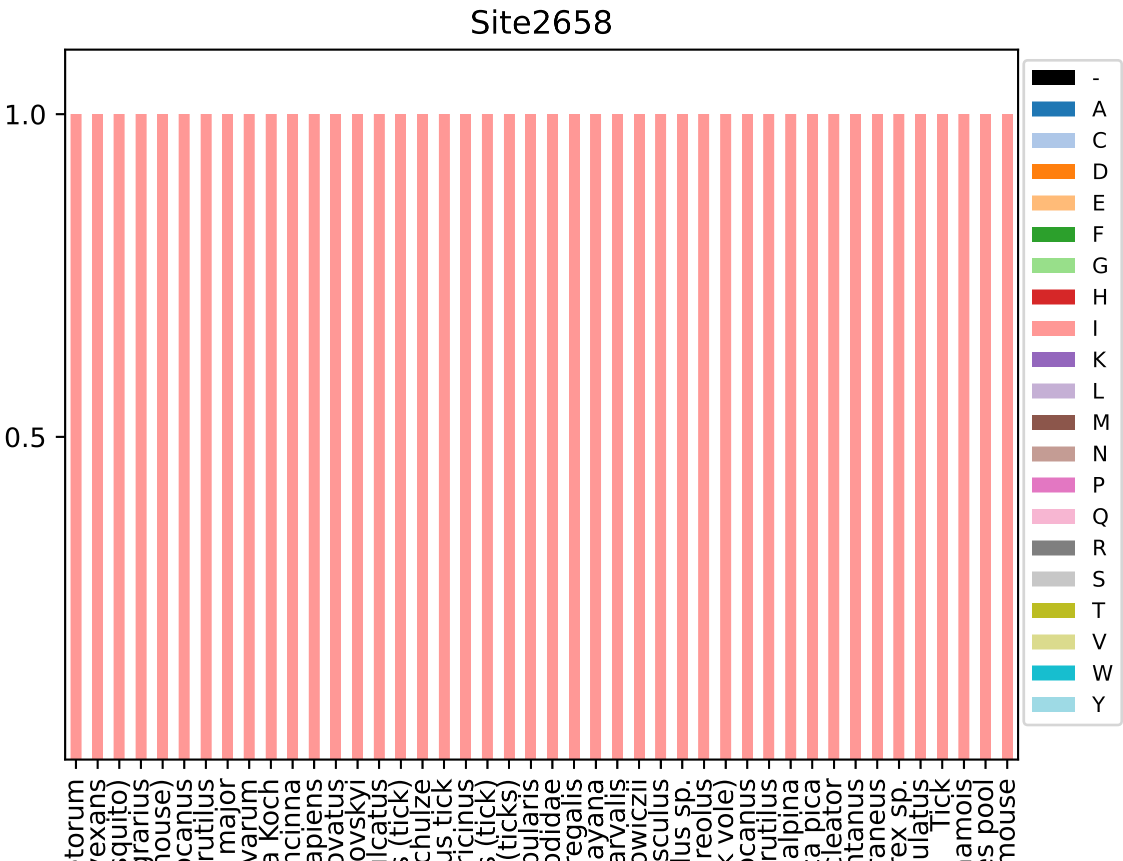
Site2658
1.0
0.5
-
A
C
D
E
F
G
H
I
K
L
M
N
P
Q
R
S
T
V
W
Y

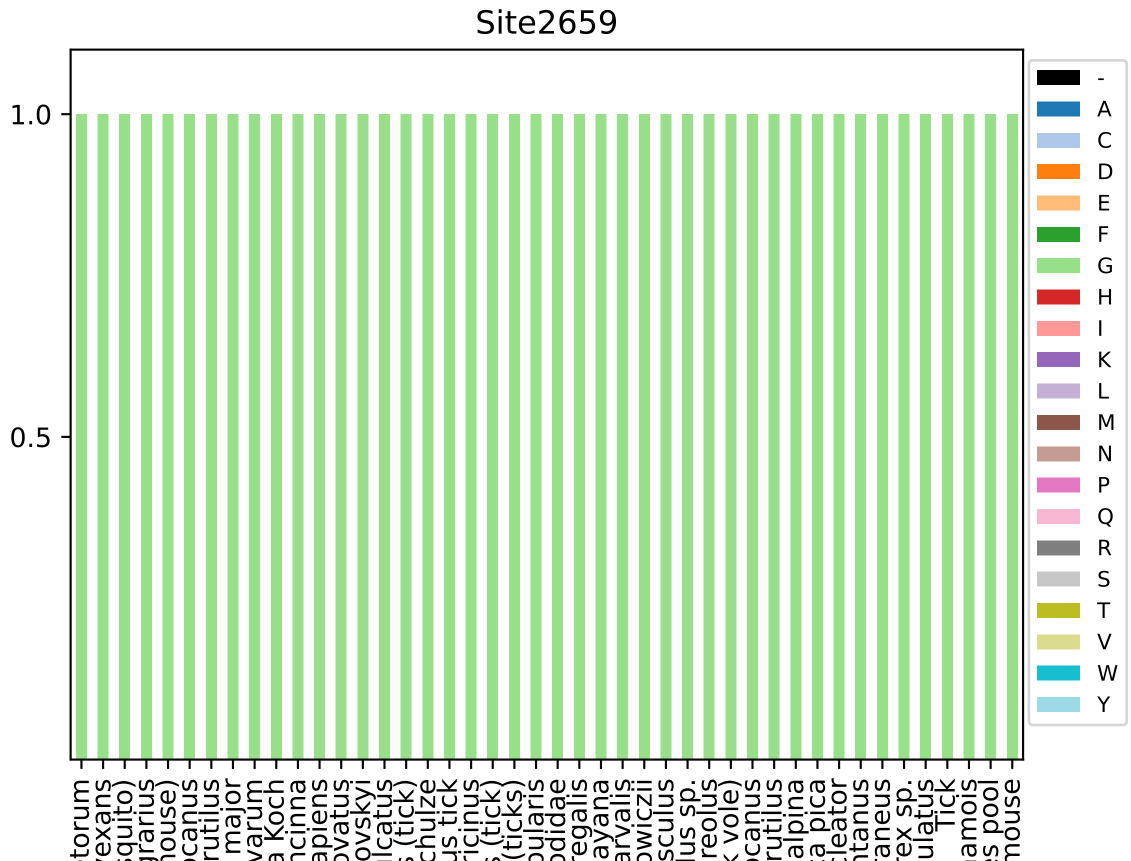
Site2659
1.0
0.5
-
A
C
D
E
F
G
H
I
K
L
M
N
P
Q
R
S
T
V
W
Y

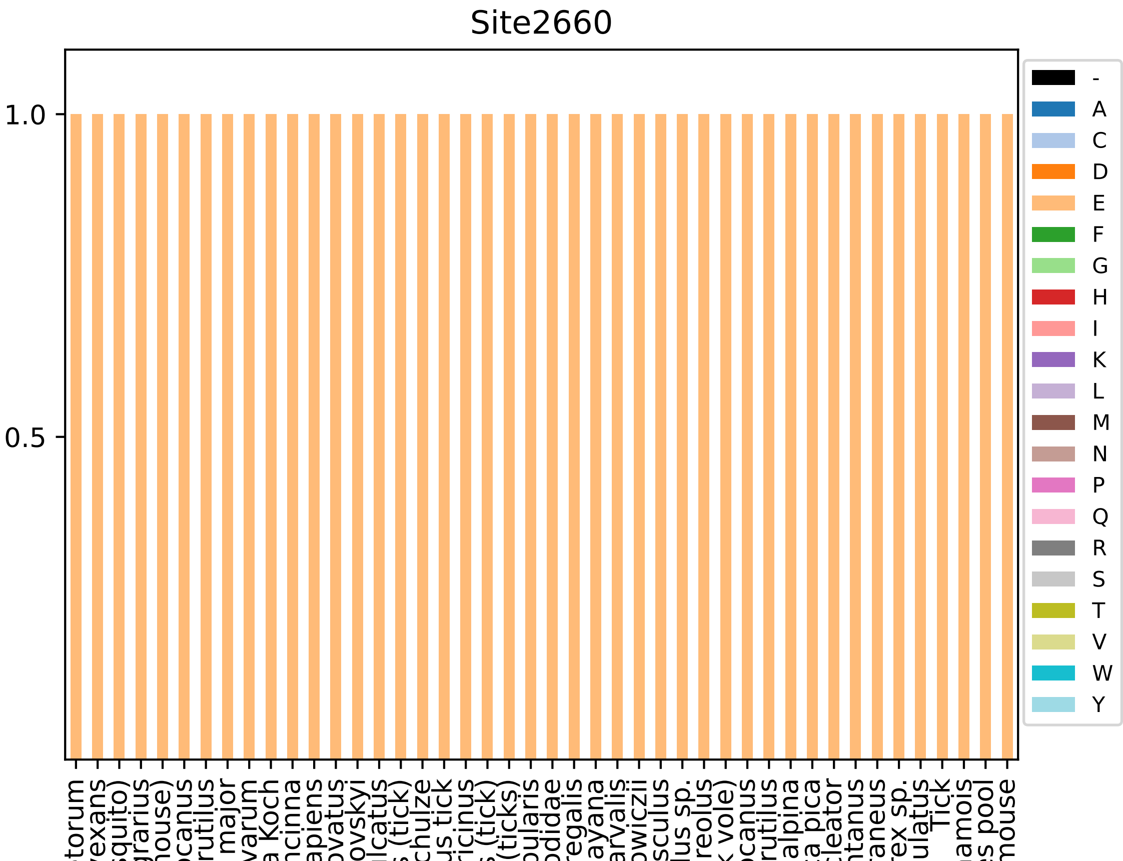

Site2660
1.0
0.5
-
A
C
D
E
F
G
H
I
K
L
M
N
P
Q
R
S
T
V
W
Y

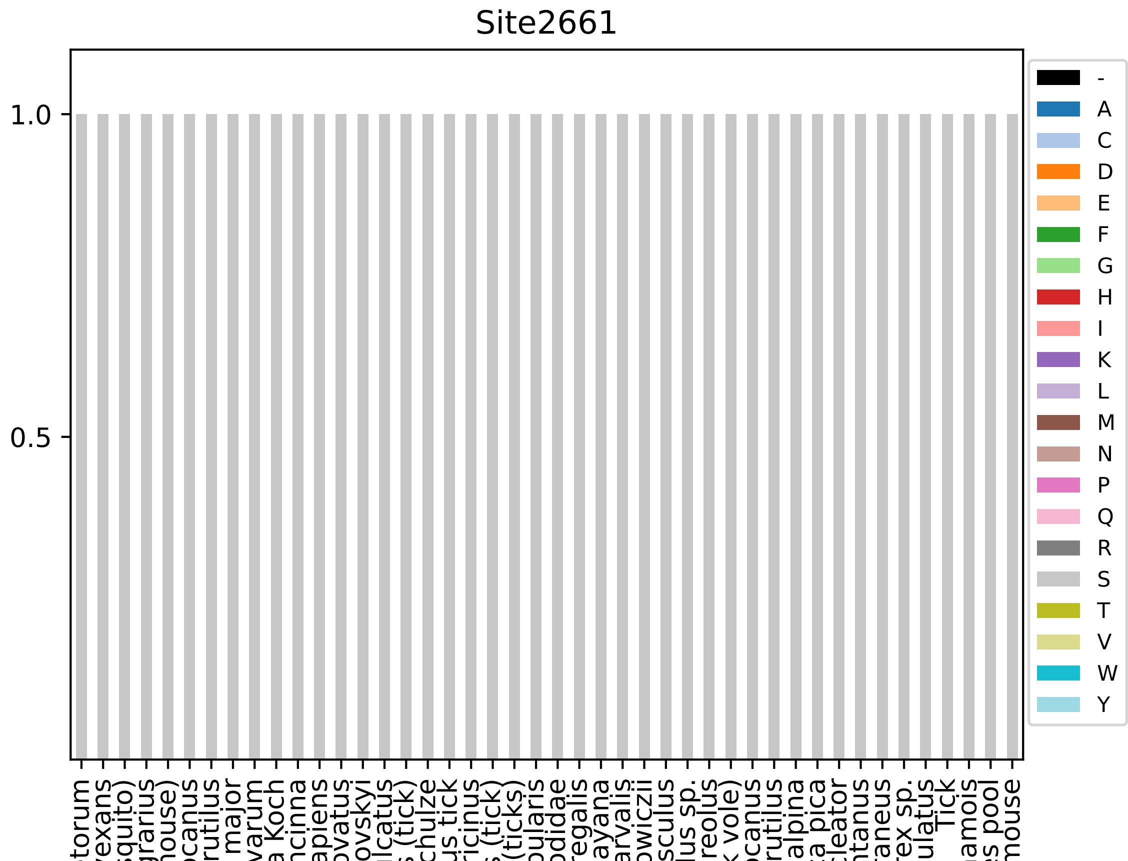

Site2661
1.0
0.5
-
A
C
D
E
F
G
H
I
K
L
M
N
P
Q
R
S
T
V
W
Y

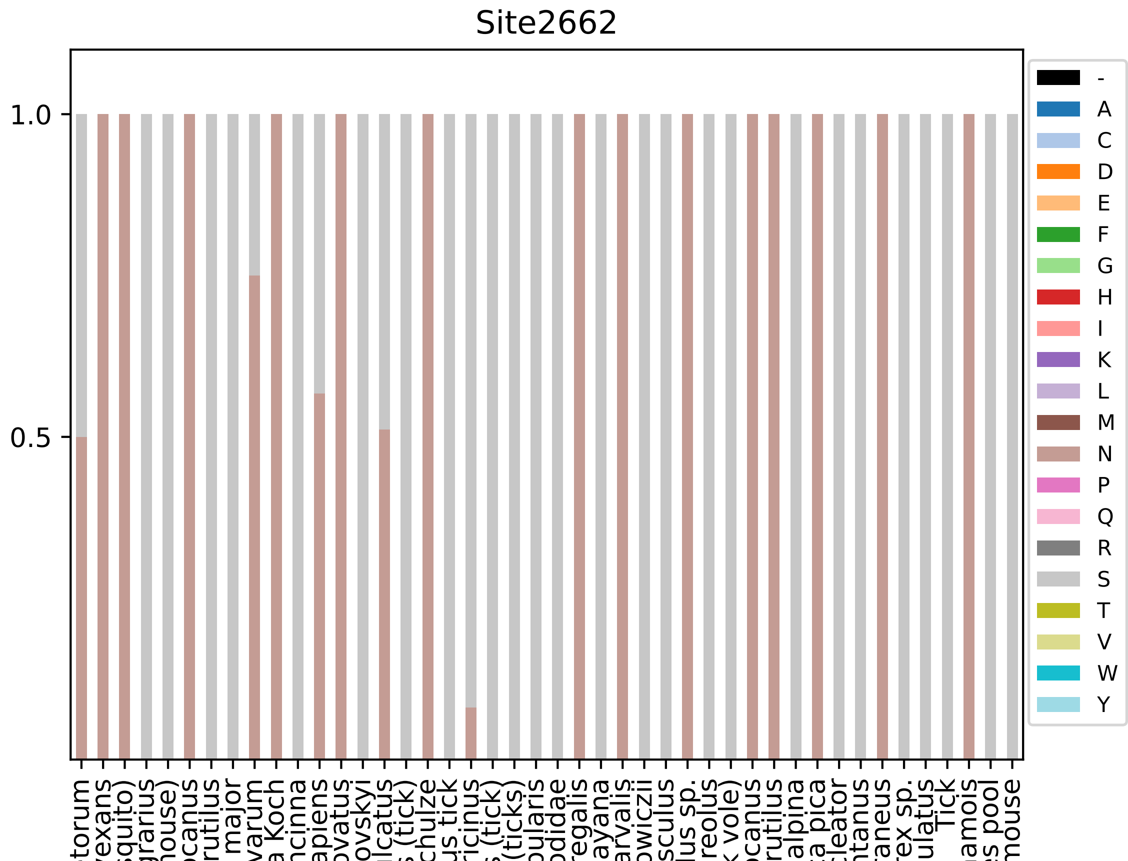

Site2662
1.0
0.5
-
A
C
D
E
F
G
H
I
K
L
M
N
P
Q
R
S
T
V
W
Y
Tick

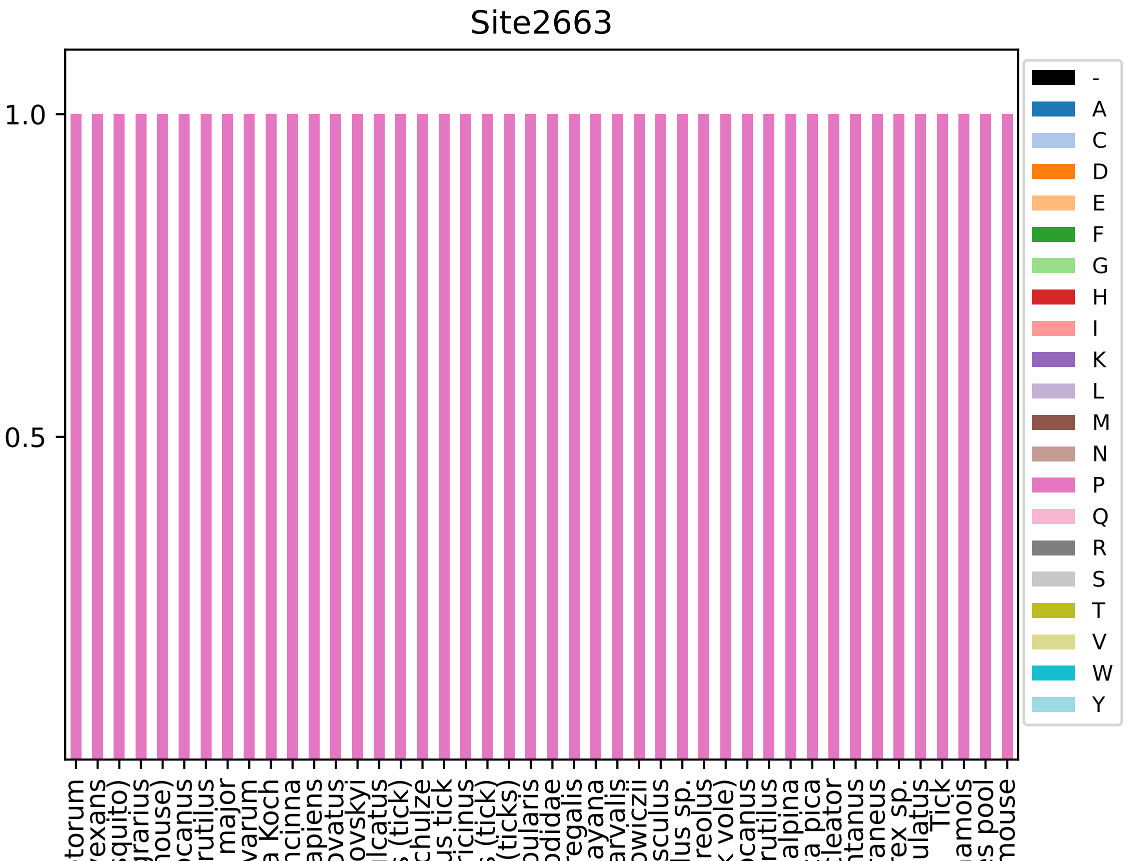
Site2663
1.0
0.5
-
A
C
D
E
F
G
H
I
K
L
M
N
P
Q
R
S
T
V
W
Y

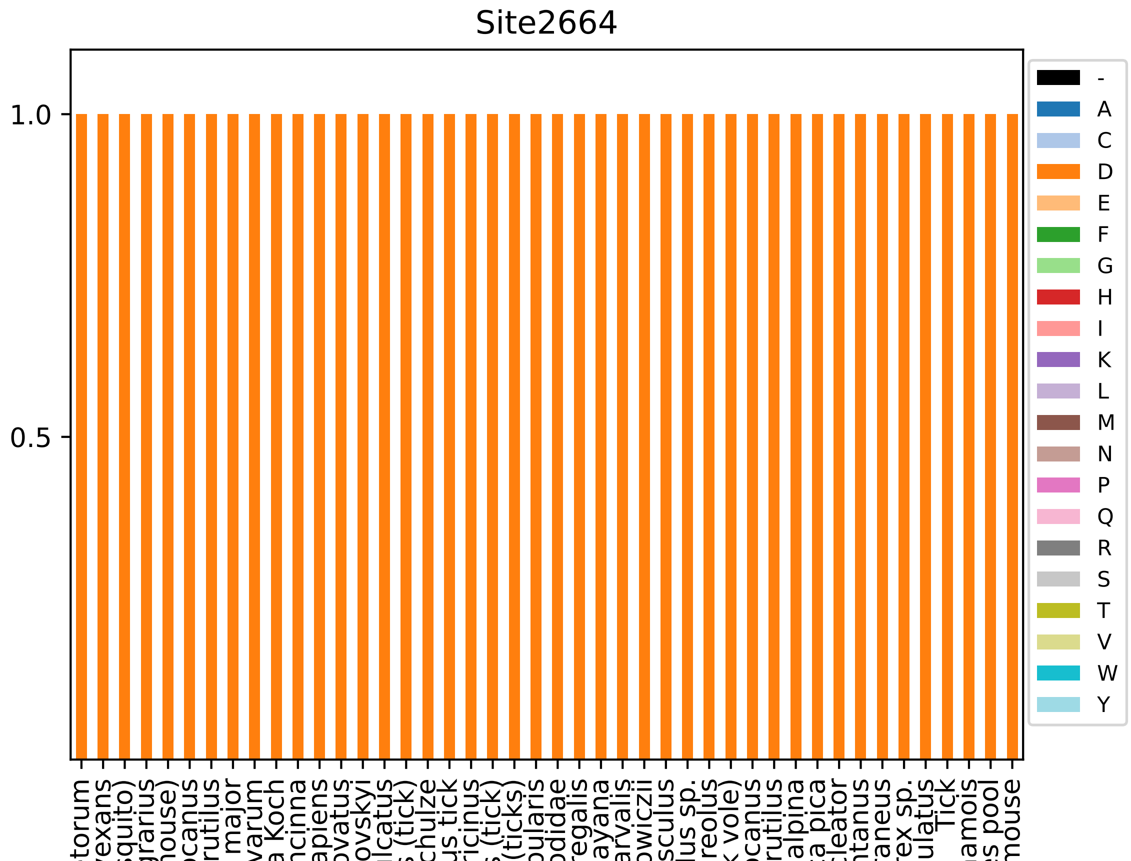
Site2664
1.0
0.5
-
A
C
D
E
F
G
H
I
K
L
M
N
P
Q
R
S
T
V
W
Y

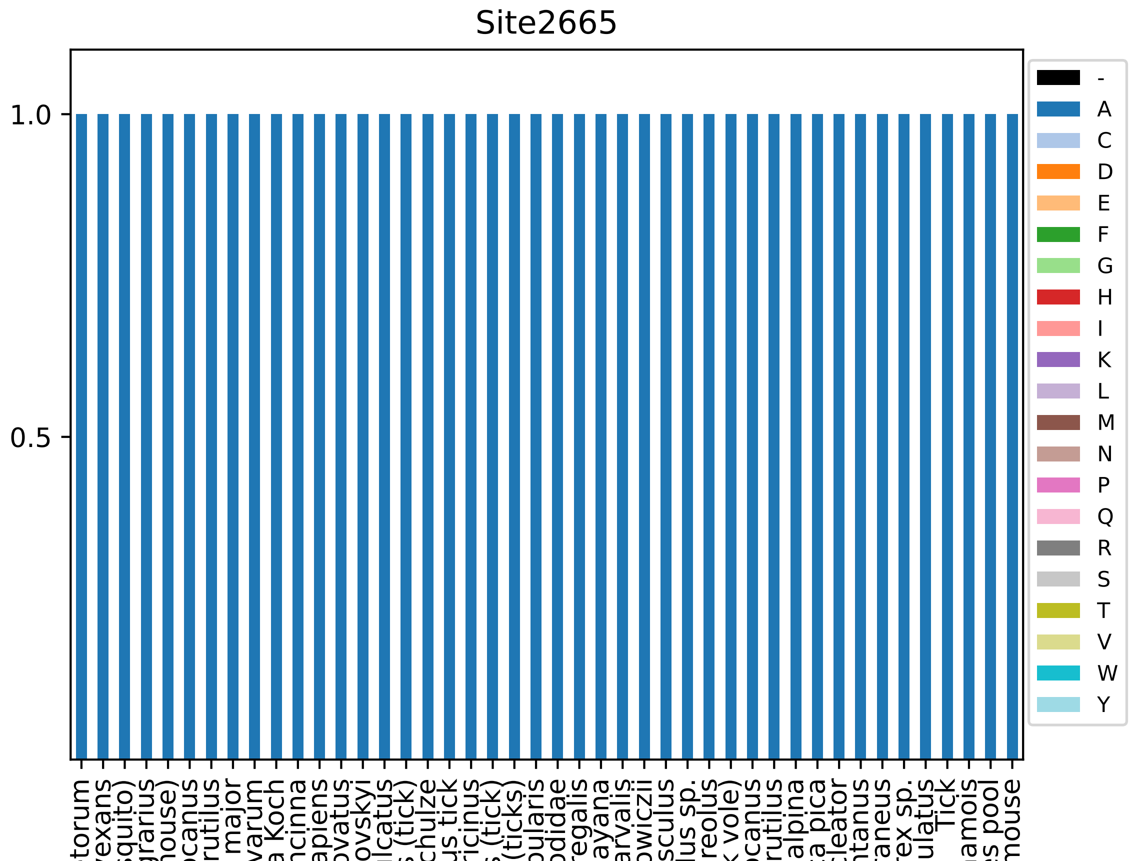
Site2665
1.0
0.5
-
A
C
D
E
F
G
H
I
K
L
M
N
P
Q
R
S
T
V
W
Y

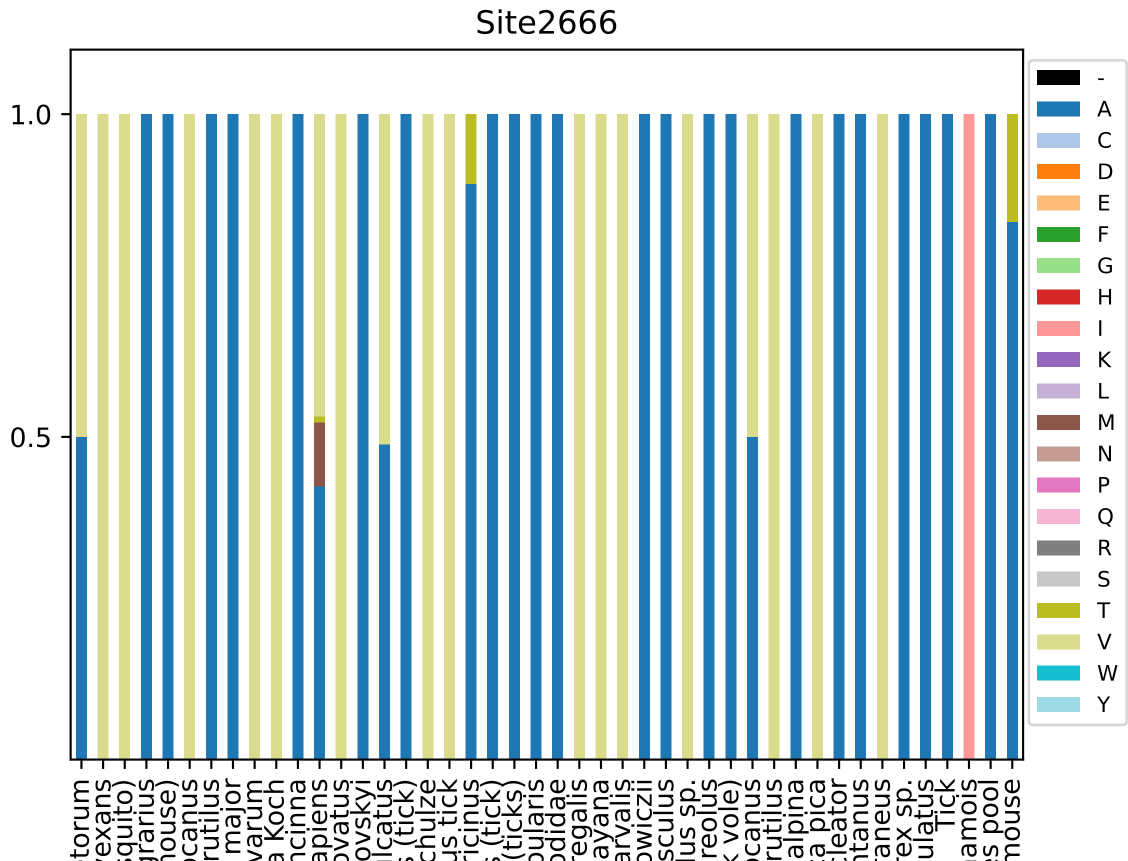
Site2666
1.0
0.5
-
A
C
D
E
F
G
H
I
K
L
M
N
P
Q
R
S
T
V
W
Y
Tick

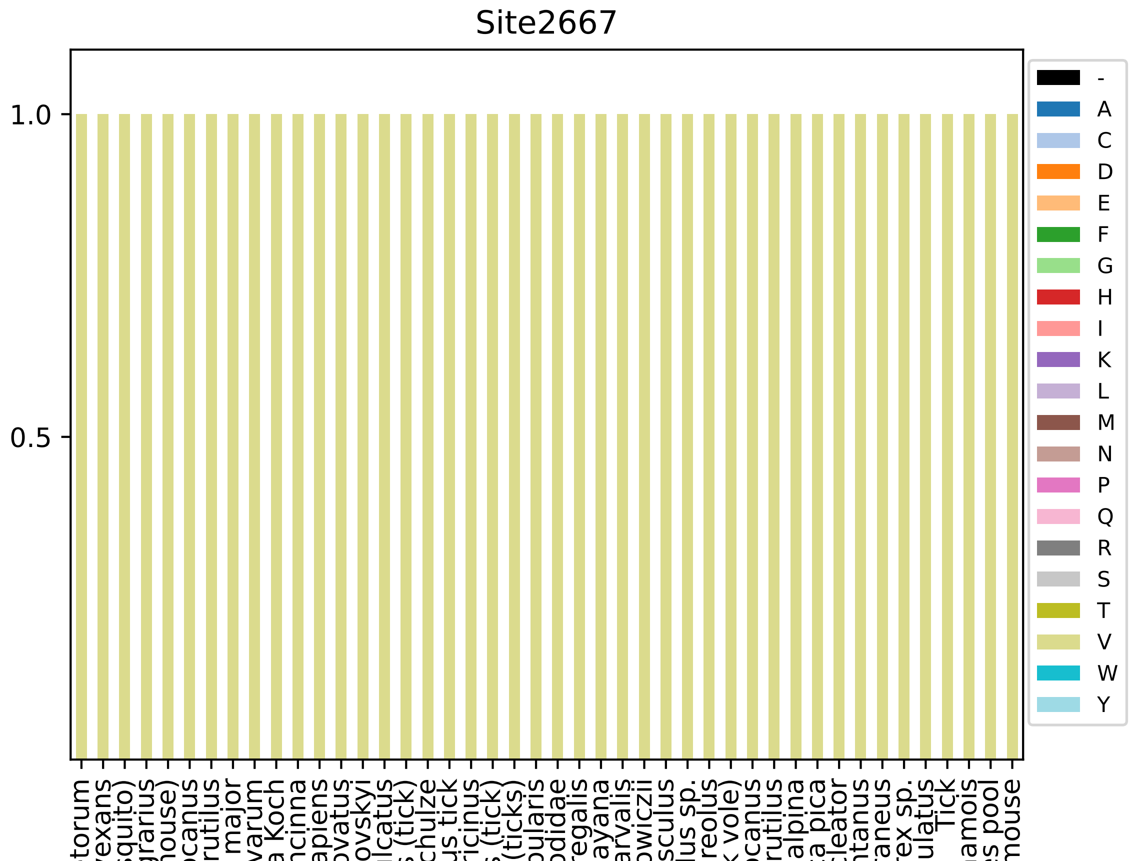

Site2667
1.0
0.5
-
A
C
D
E
F
G
H
I
K
L
M
N
P
Q
R
S
T
V
W
Y

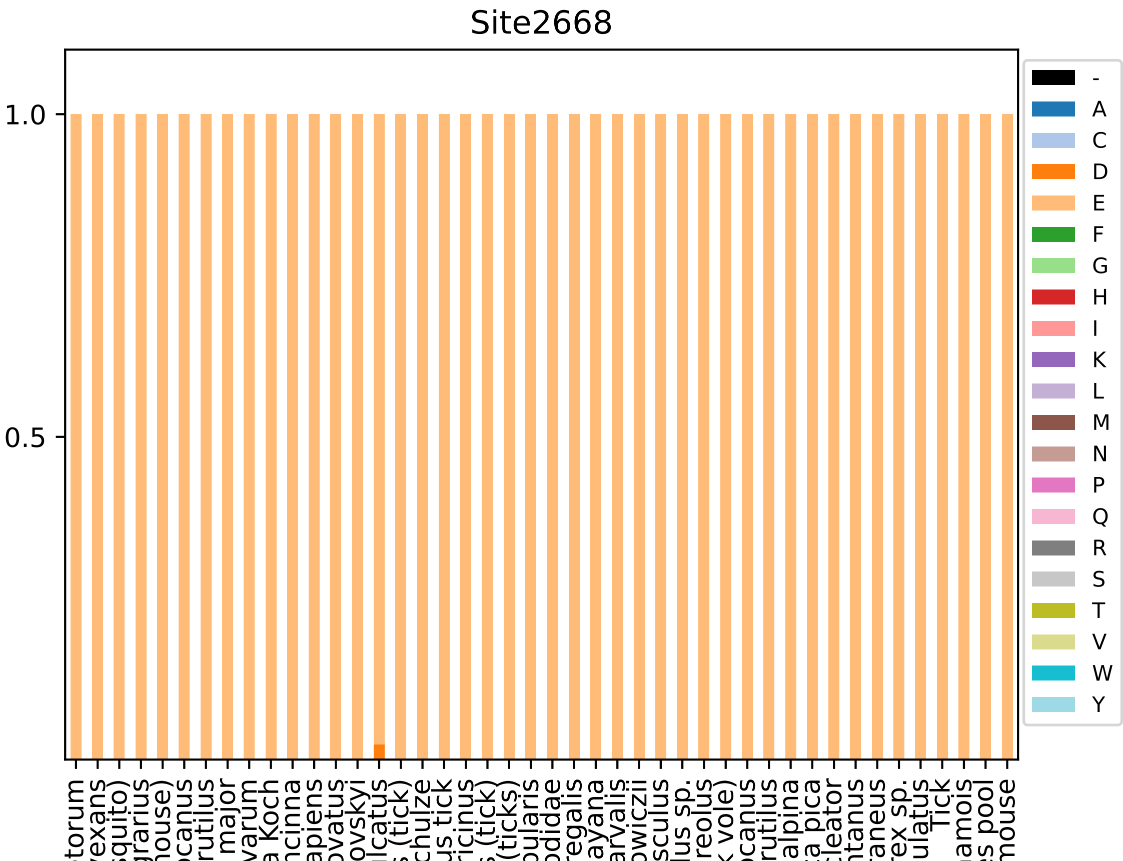
Site2668
1.0
0.5
-
A
C
D
E
F
G
H
I
K
L
M
N
P
Q
R
S
T
V
W
Y

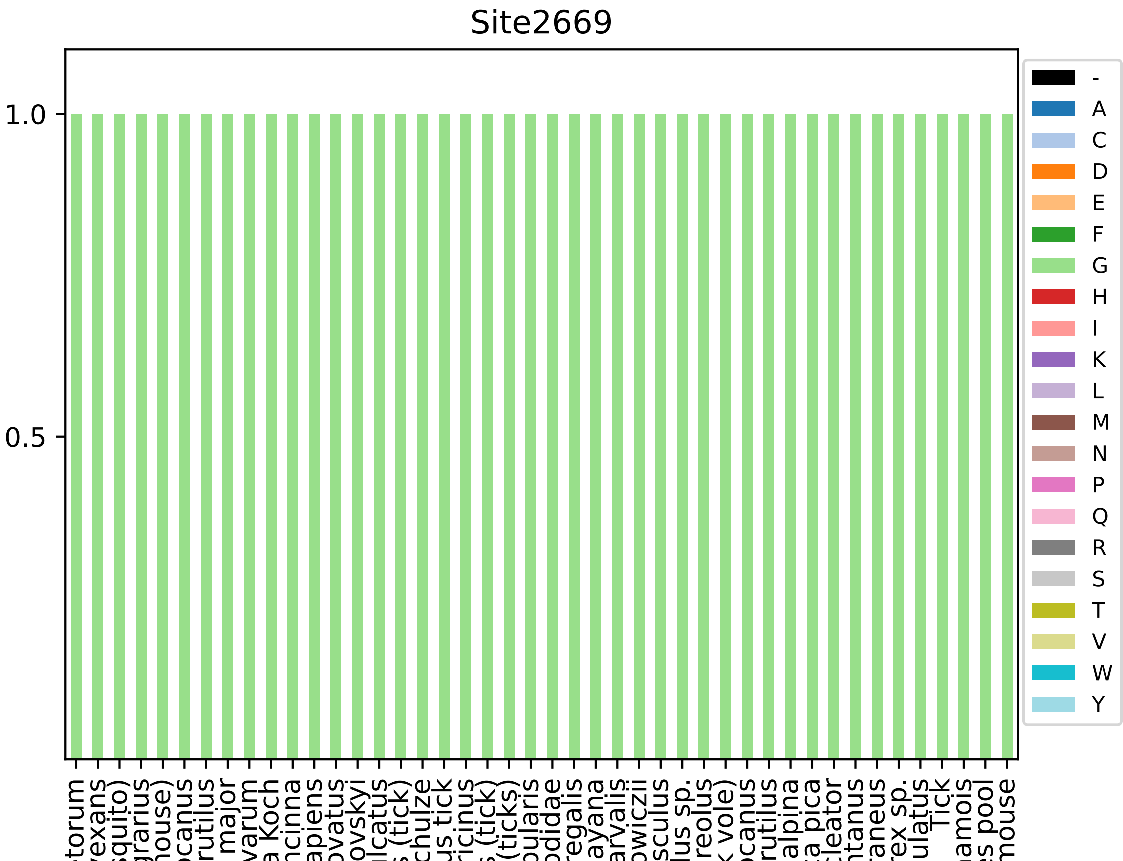
Site2669
1.0
0.5
-
A
C
D
E
F
G
H
I
K
L
M
N
P
Q
R
S
T
V
W
Y

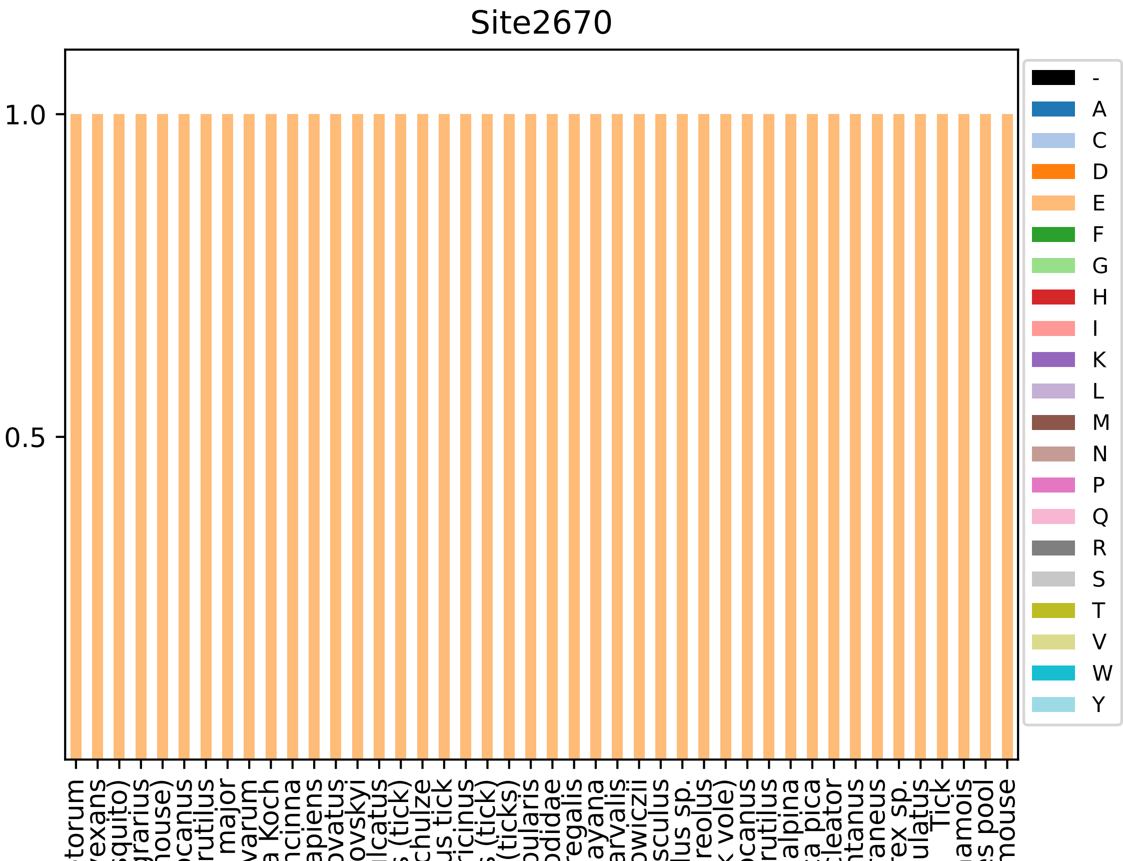
Site2670
1.0
0.5
-
A
C
D
E
F
G
H
I
K
L
M
N
P
Q
R
S
T
V
W
Y

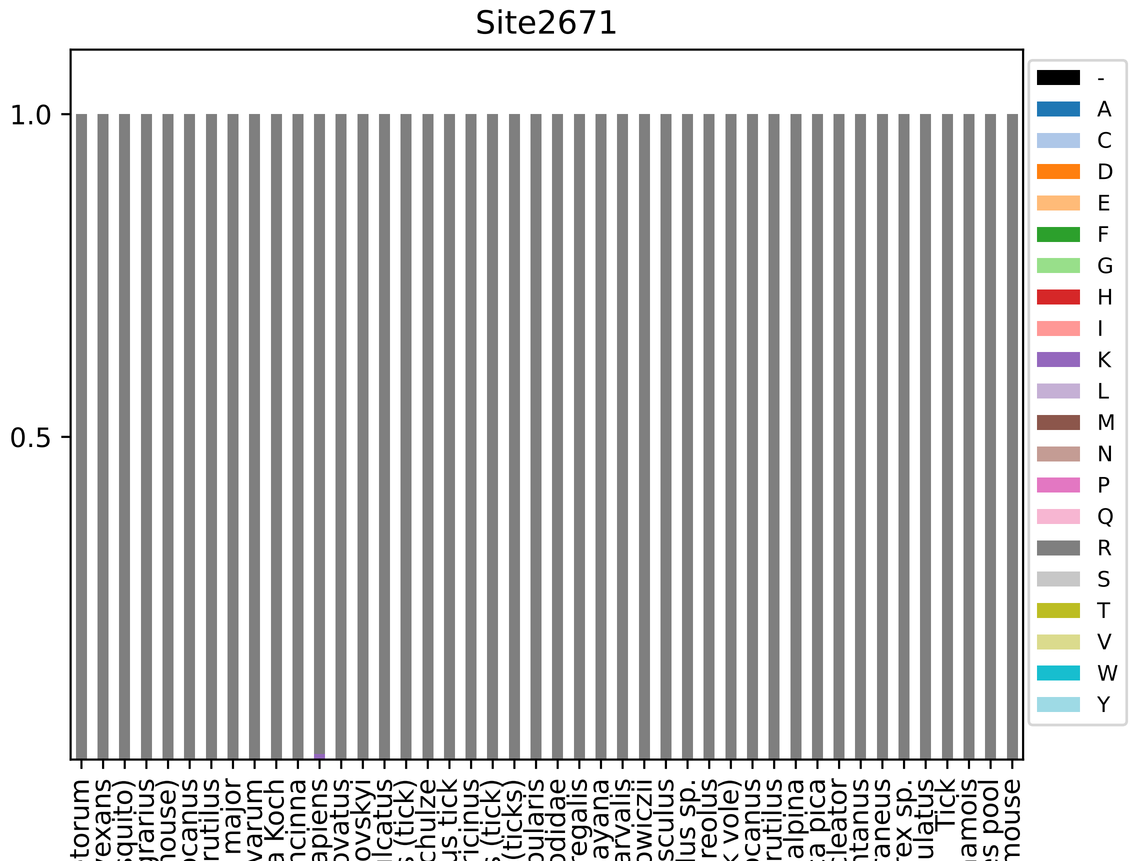
Site2671
1.0
0.5
-
A
C
D
E
F
G
H
I
K
L
M
N
P
Q
R
S
T
V
W
Y

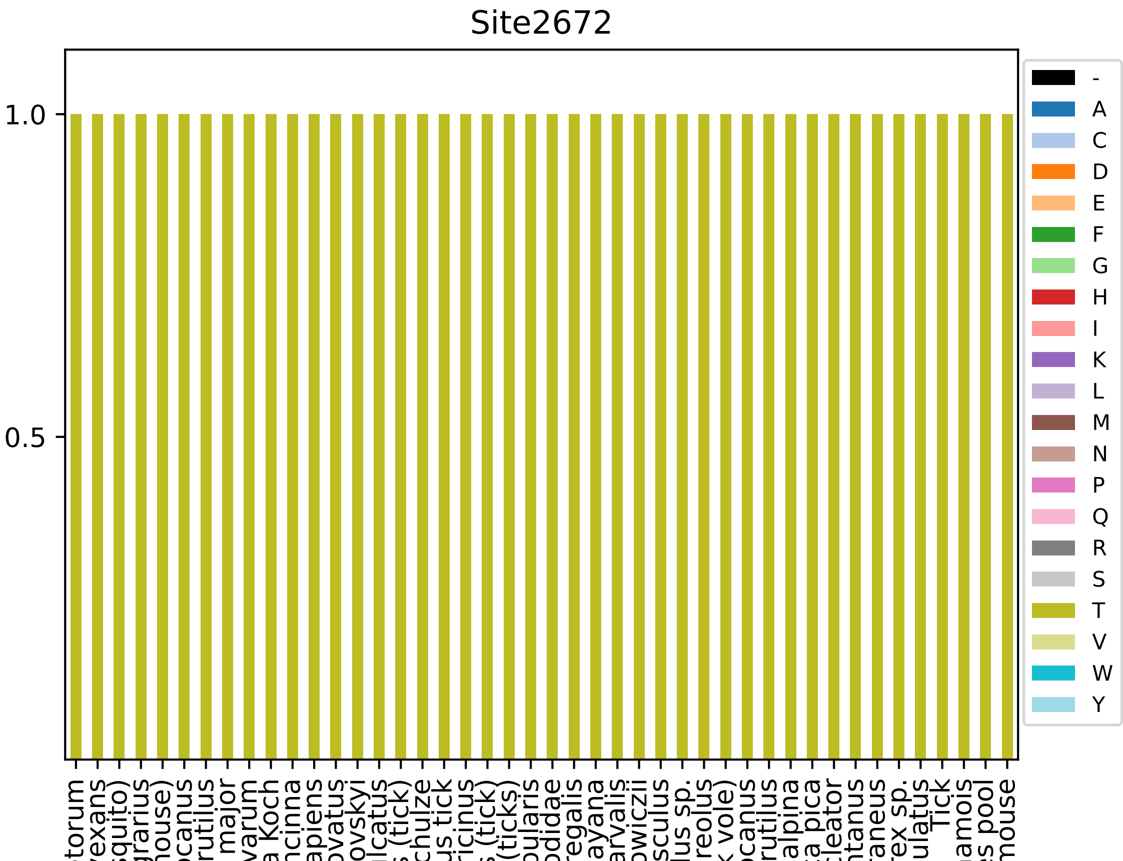
Site2672
1.0
0.5
-
A
C
D
E
F
G
H
I
K
L
M
N
P
Q
R
S
T
V
W
Y

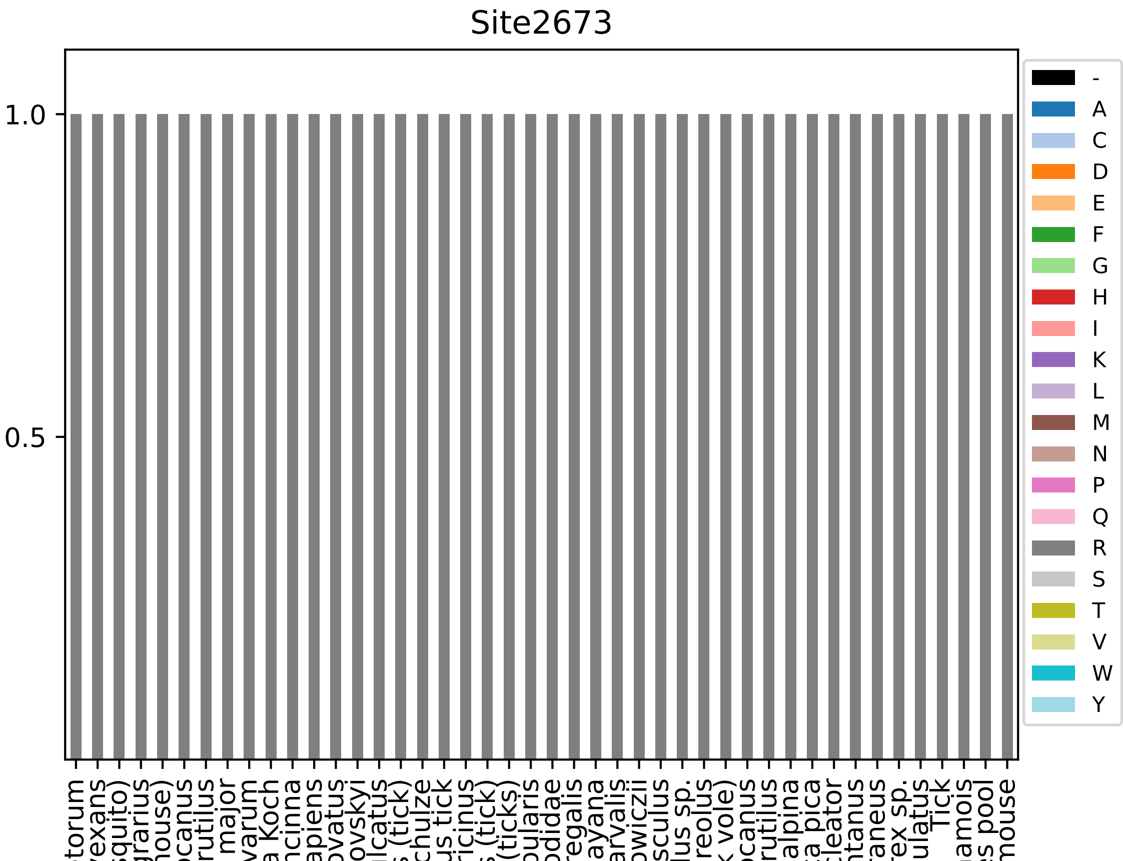
Site2673
1.0
0.5
-
A
C
D
E
F
G
H
I
K
L
M
N
P
Q
R
S
T
V
W
Y

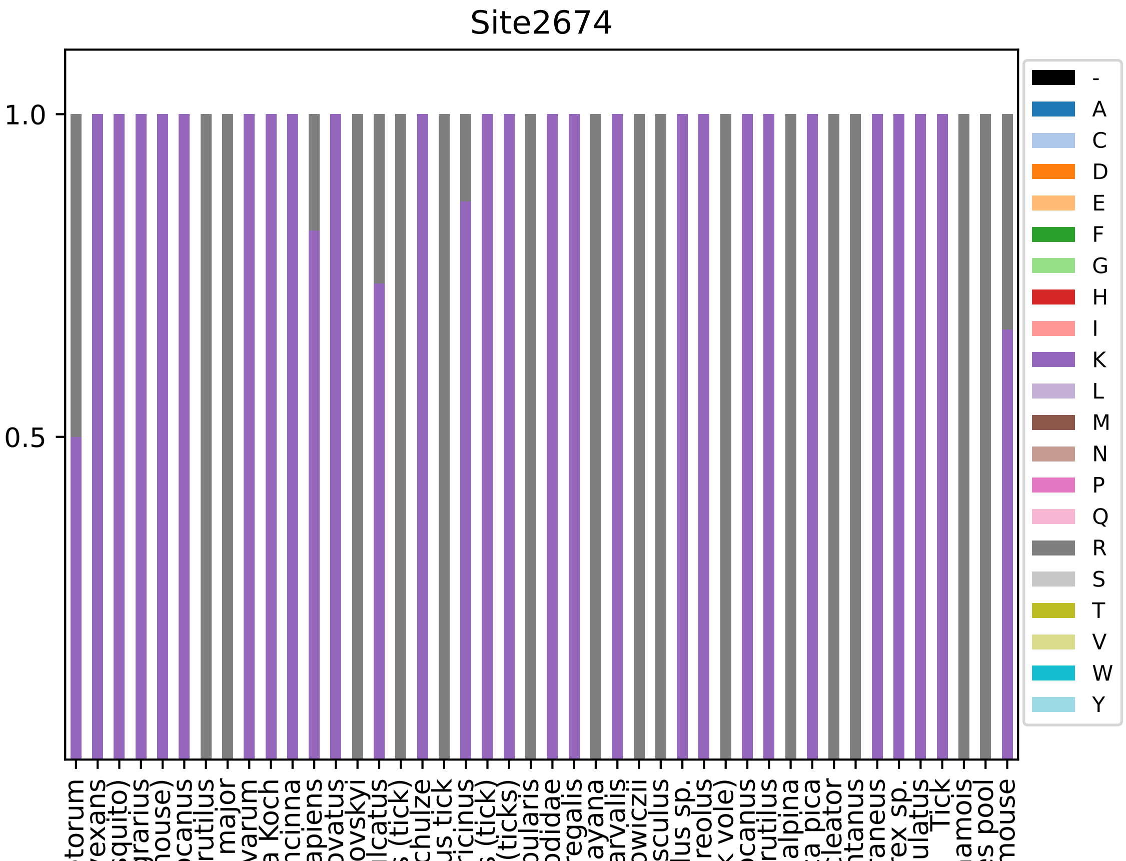

Site2674
1.0
0.5
-
A
C
D
E
F
G
H
I
K
L
M
N
P
Q
R
S
T
V
W
Y

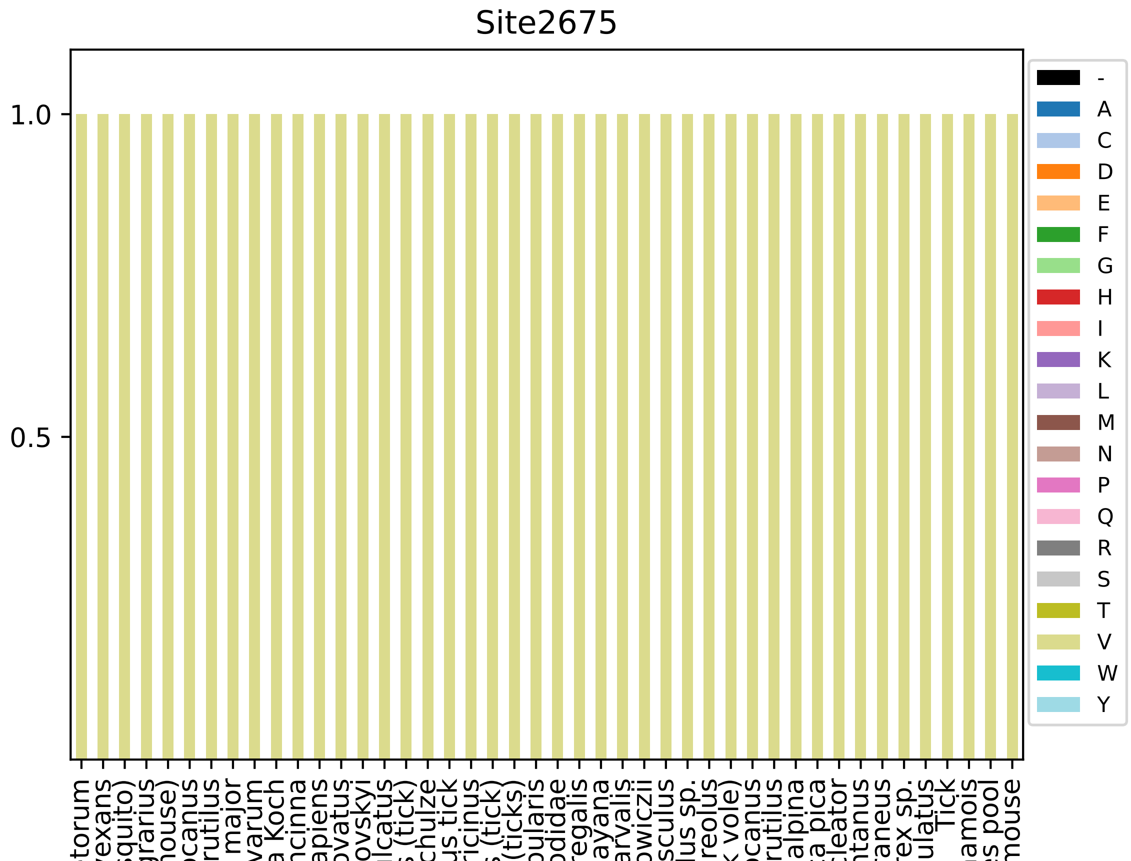

Site2675
1.0
0.5
-
A
C
D
E
F
G
H
I
K
L
M
N
P
Q
R
S
T
V
W
Y

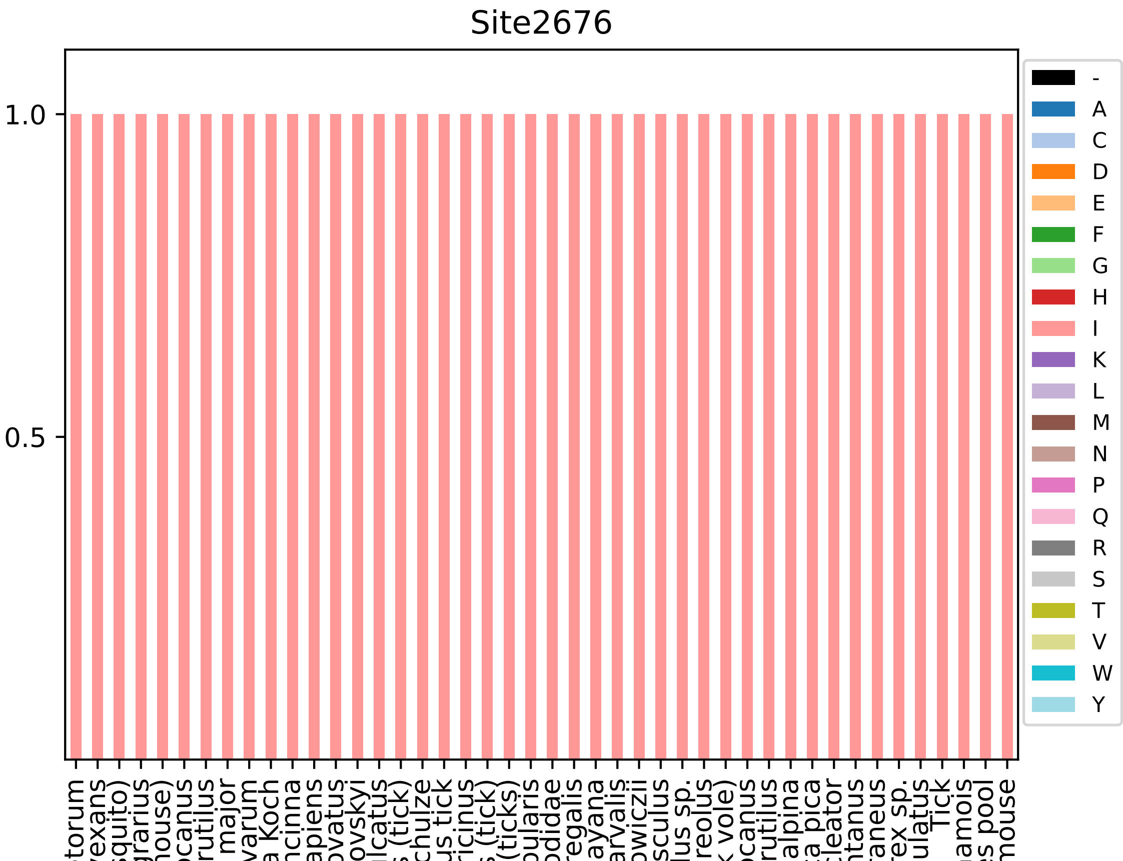
Site2676
1.0
0.5
-
A
C
D
E
F
G
H
I
K
L
M
N
P
Q
R
S
T
V
W
Y

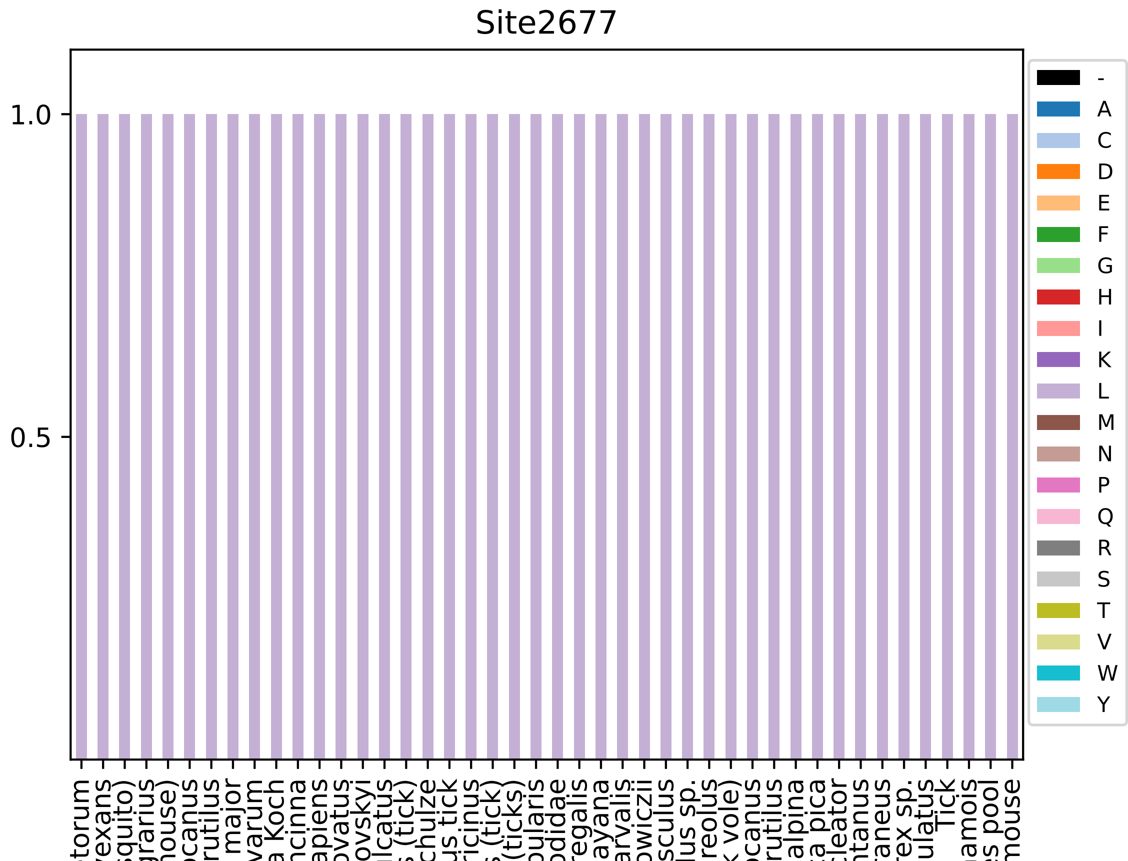
Site2677
1.0
0.5
-
A
C
D
E
F
G
H
I
K
L
M
N
P
Q
R
S
T
V
W
Y

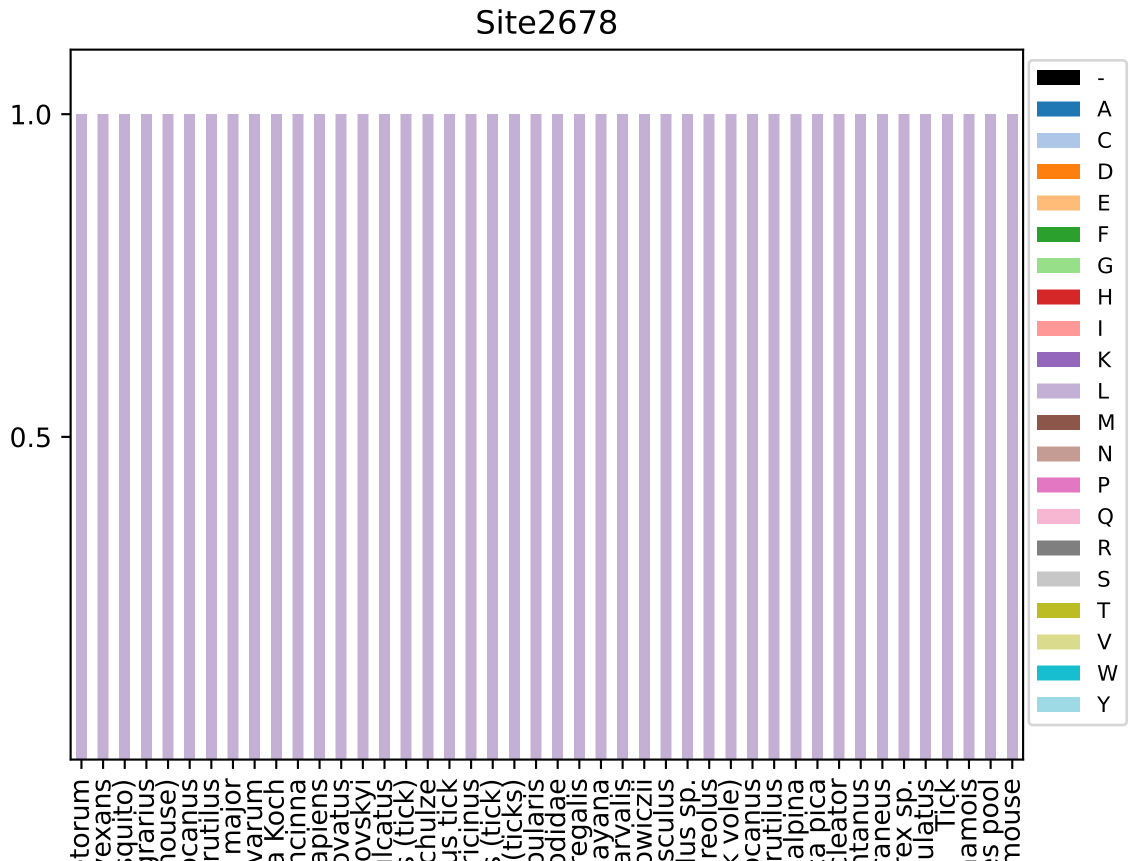
Site2678
1.0
0.5
-
A
C
D
E
F
G
H
I
K
L
M
N
P
Q
R
S
T
V
W
Y

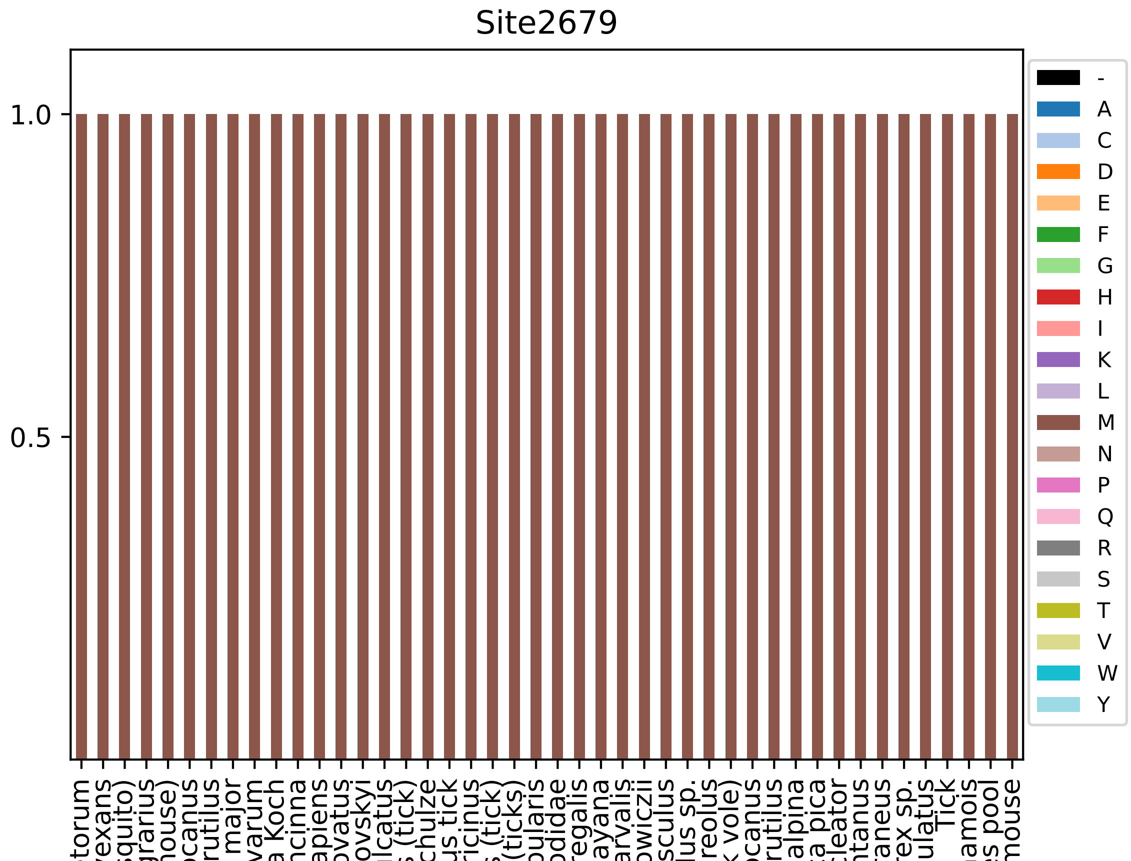

Site2679
1.0
0.5
-
A
C
D
E
F
G
H
I
K
L
M
N
P
Q
R
S
T
V
W
Y

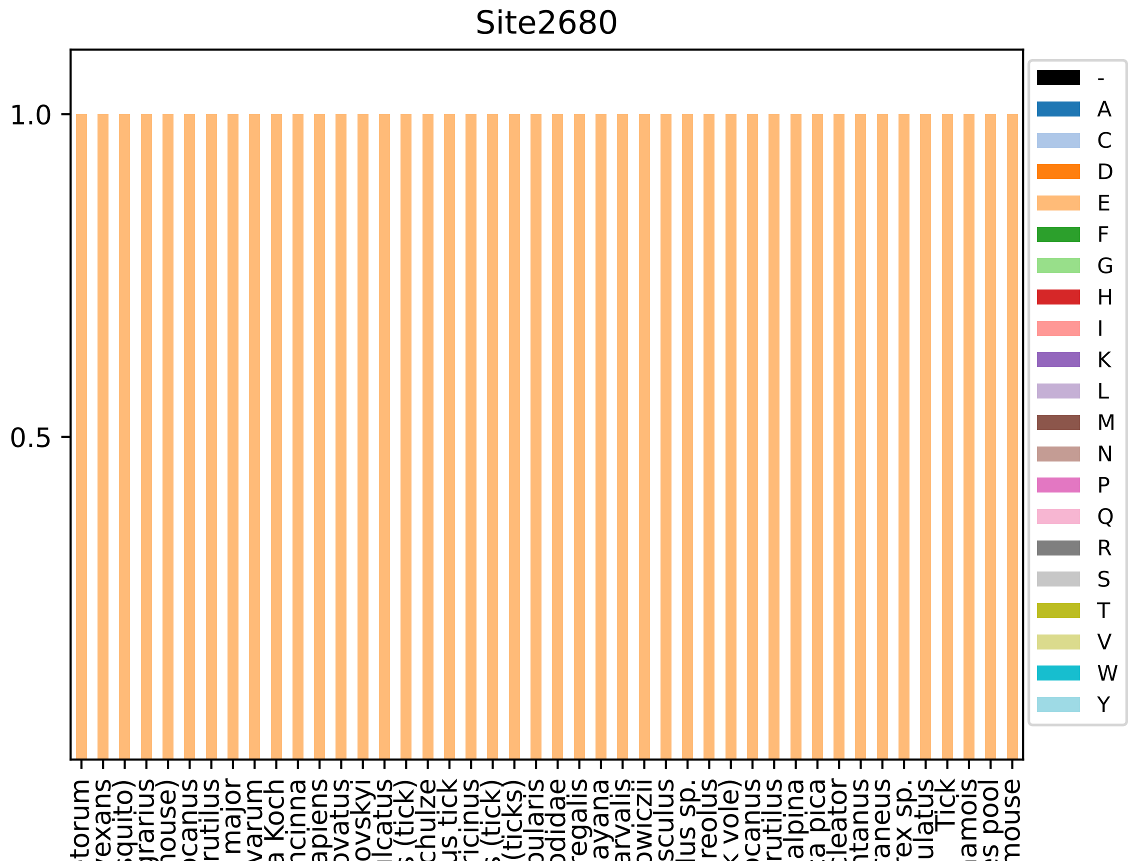
Site2680
1.0
0.5
-
A
C
D
E
F
G
H
I
K
L
M
N
P
Q
R
S
T
V
W
Y

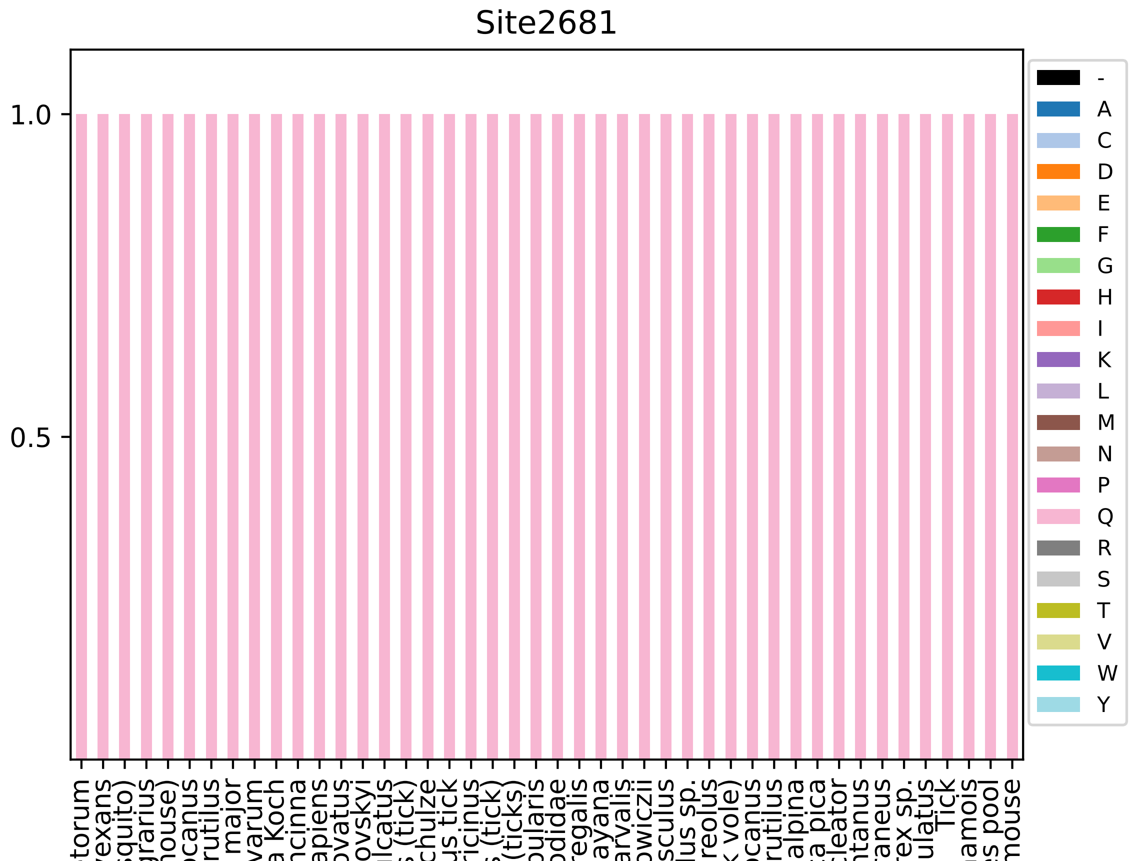

Site2681
1.0
0.5
-
A
C
D
E
F
G
H
I
K
L
M
N
P
Q
R
S
T
V
W
Y

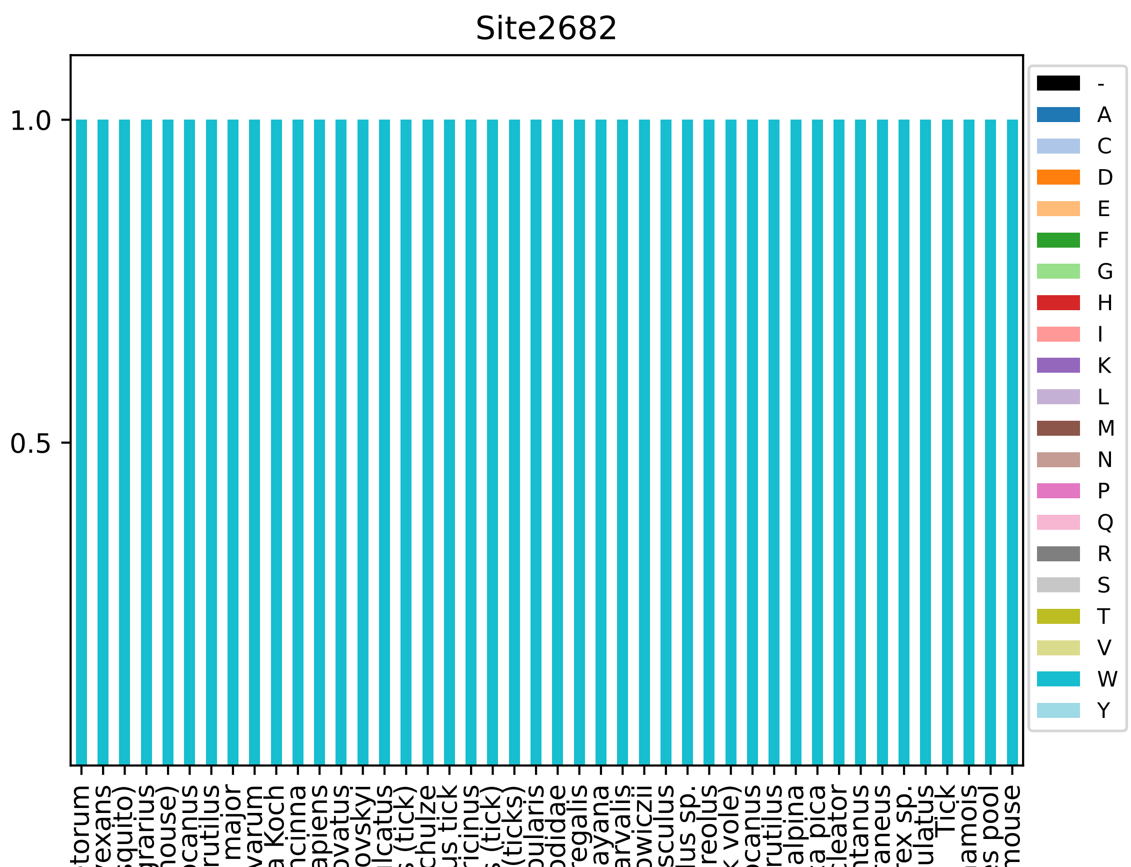

Site2682
1.0
0.5
-
A
C
D
E
F
G
H
I
K
L
M
N
P
Q
R
S
T
V
W
Y
torum
vexans
squito)
grarius
house)
ocanus
rutilus
major
varum
a Koch
ncinna
apiens
ovatus
ovskyi
lcatus
s (tick)
chulze
us tick
ricinus
s (tick)
(ticks)
pularis
podidae
regalis
ayana
arvalis
owiczii
sculus
lus sp.
reolus
k vole)
ocanus
rutilus
alpina
a pica
leator
ntanus
raneus
rex sp.
ulatus
Tick
amois
es pool
mouse

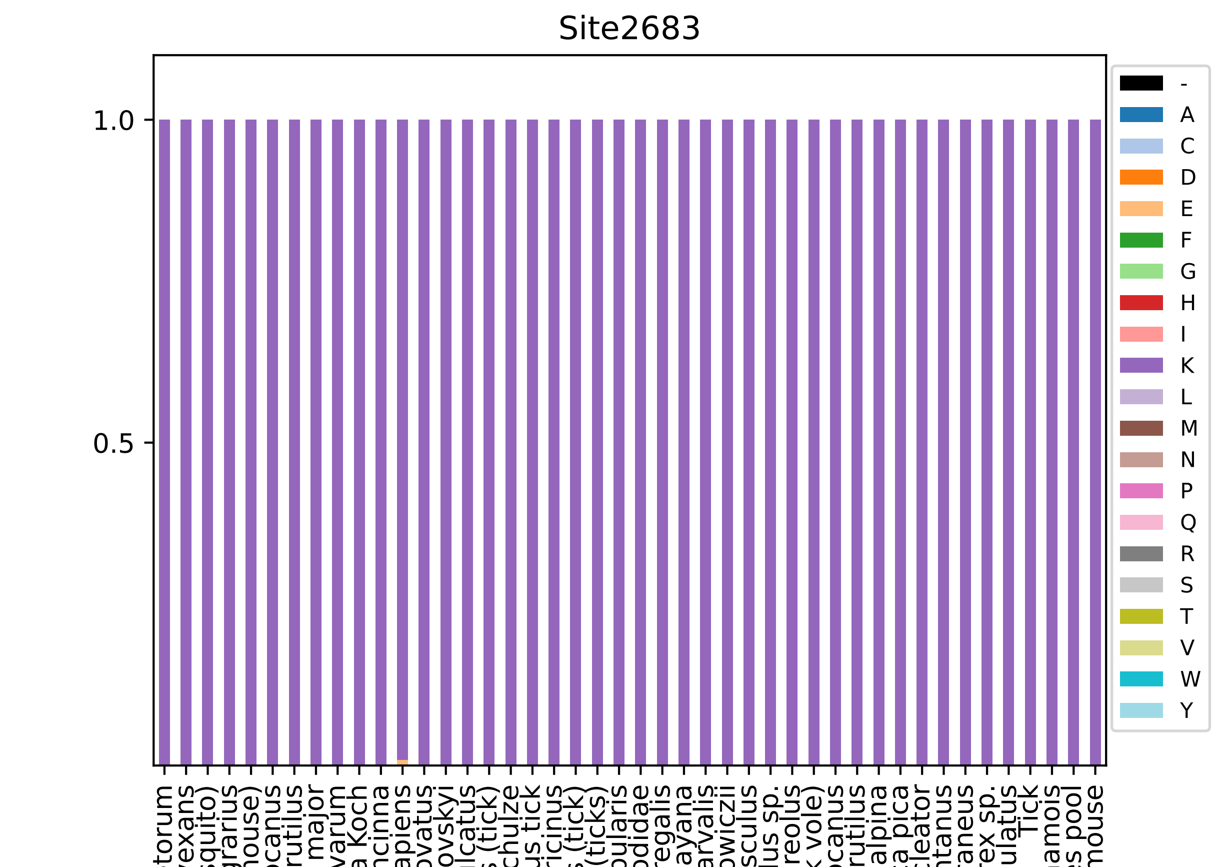
Site2683
1.0
0.5
-
A
C
D
E
F
G
H
I
K
L
M
N
P
Q
R
S
T
V
W
Y

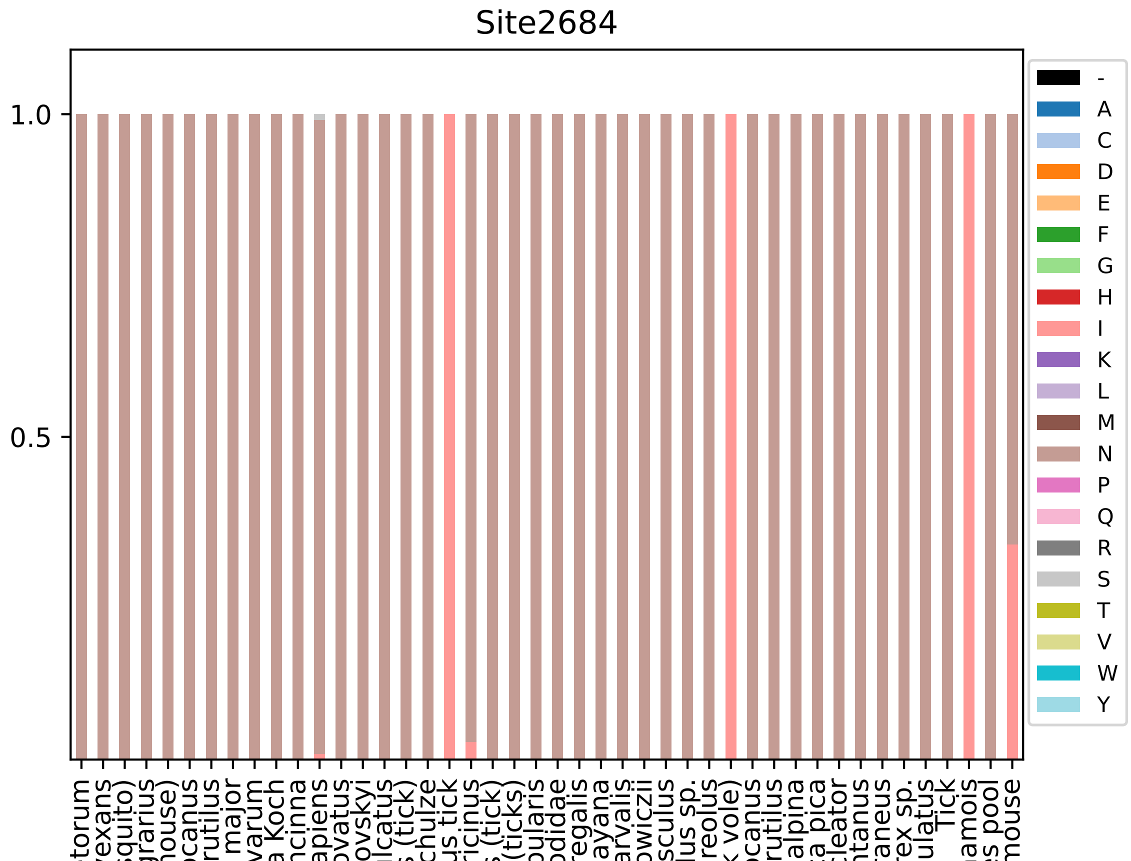

Site2684
1.0
0.5
-
A
C
D
E
F
G
H
I
K
L
M
N
P
Q
R
S
T
V
W
Y

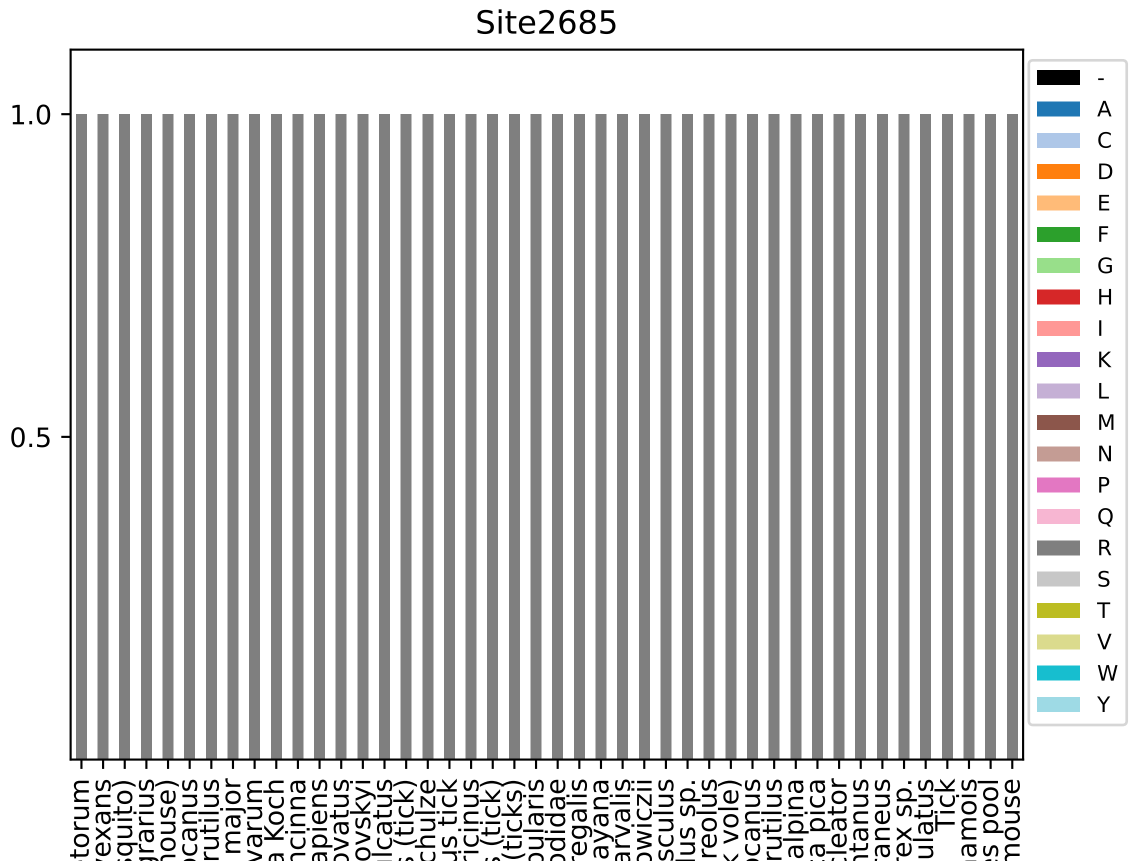
Site2685
1.0
0.5
-
A
C
D
E
F
G
H
I
K
L
M
N
P
Q
R
S
T
V
W
Y
torum
vexans
squito)
grarius
nouse)
canus
rutilus
major
varum
a Koch
ncinna
apiens
ovatus
ovskyi
lcatus
(tick)
chulze
us tick
ricinus
(tick)
(ticks)
pularis
podidae
regalis
ayana
arvalis
owiczii
sculus
us sp.
reolus
k vole)
canus
rutilus
alpina
a pica
cleator
ntanus
raneus
ex sp.
ulatus
Tick
amois
s pool
mouse

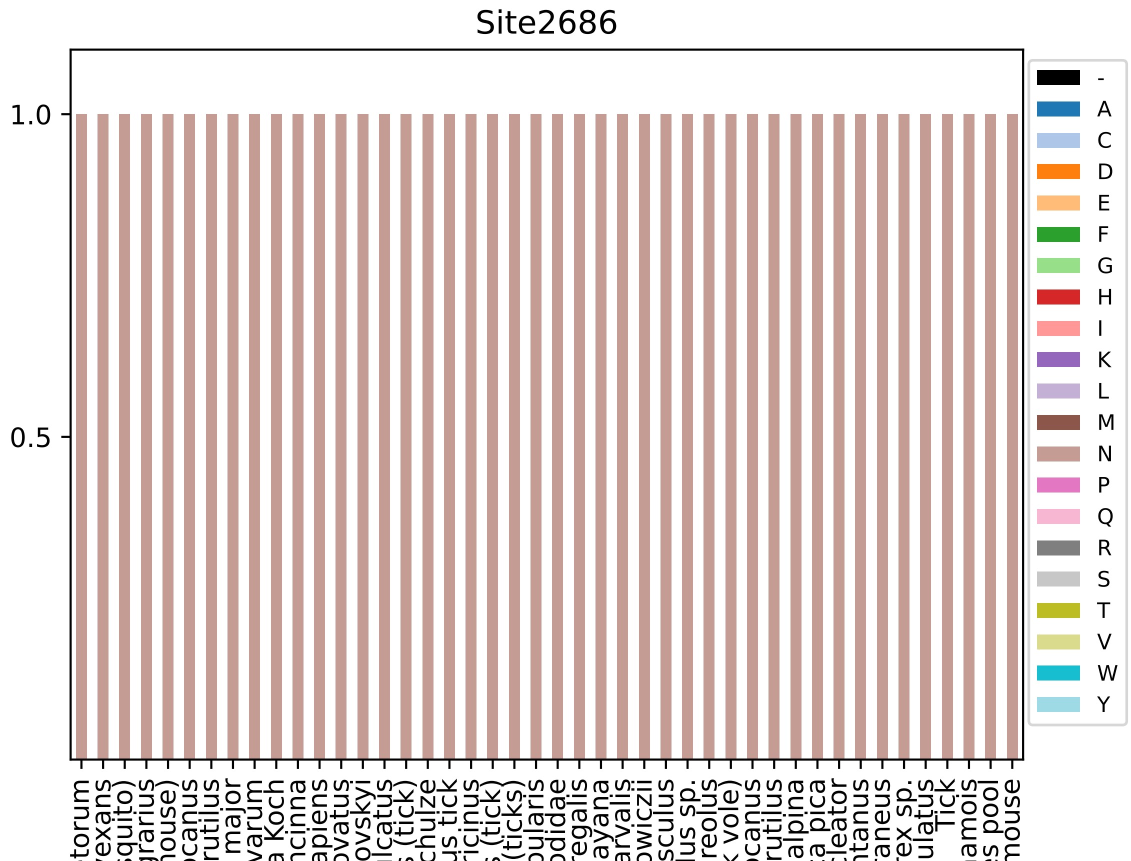
Site2686
1.0
0.5
-
A
C
D
E
F
G
H
I
K
L
M
N
P
Q
R
S
T
V
W
Y

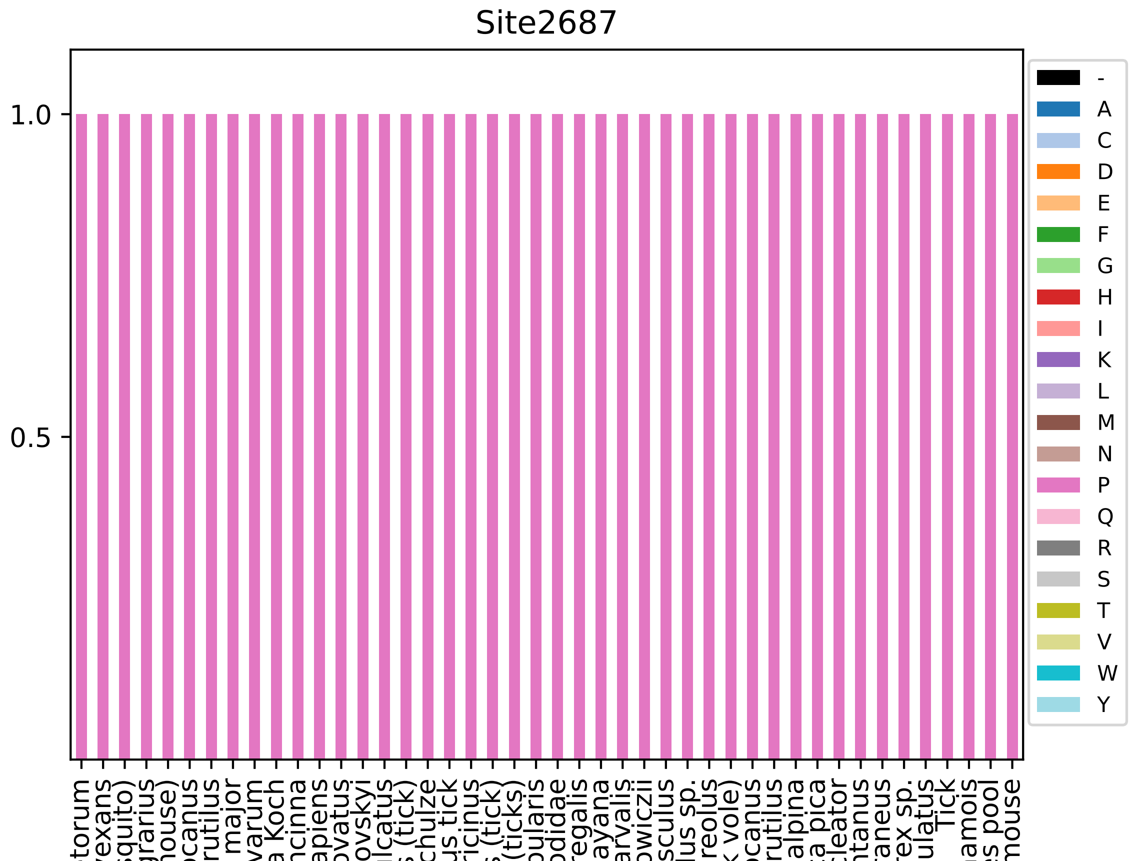

Site2687
1.0
0.5
-
A
C
D
E
F
G
H
I
K
L
M
N
P
Q
R
S
T
V
W
Y

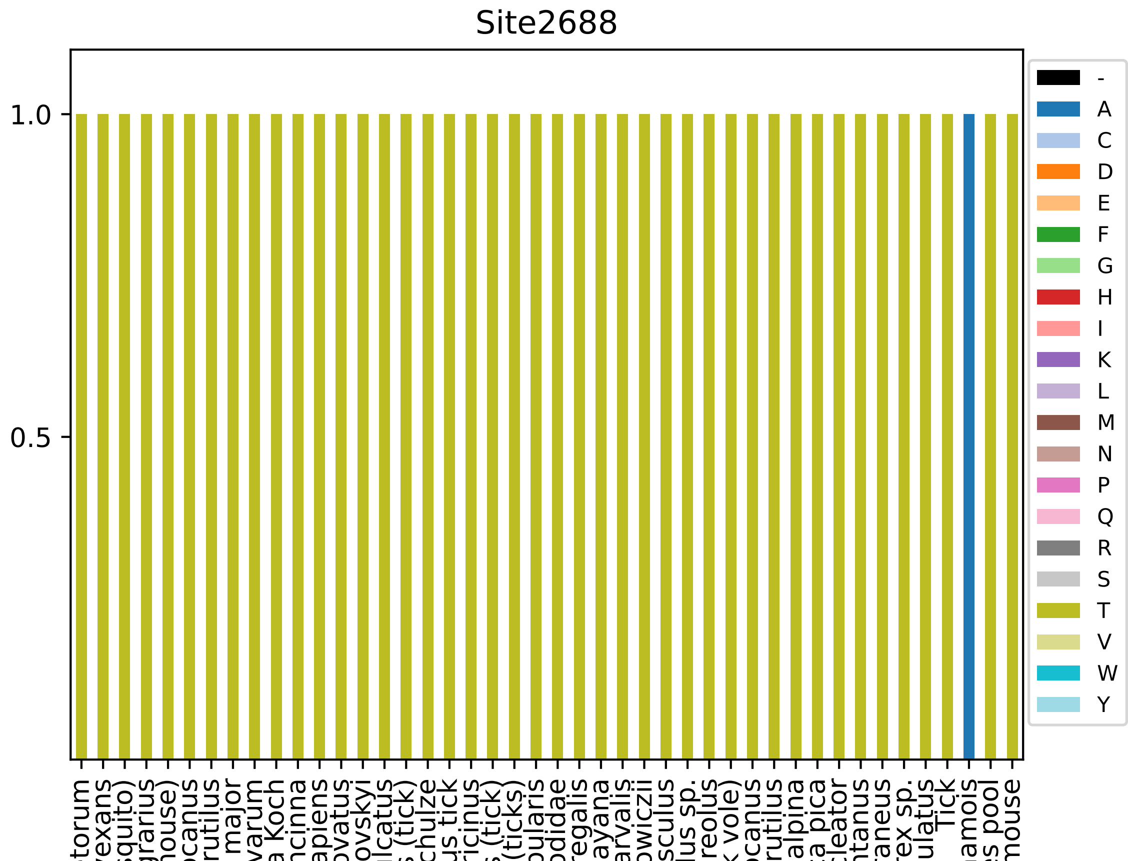

Site2688
1.0
0.5
-
A
C
D
E
F
G
H
I
K
L
M
N
P
Q
R
S
T
V
W
Y

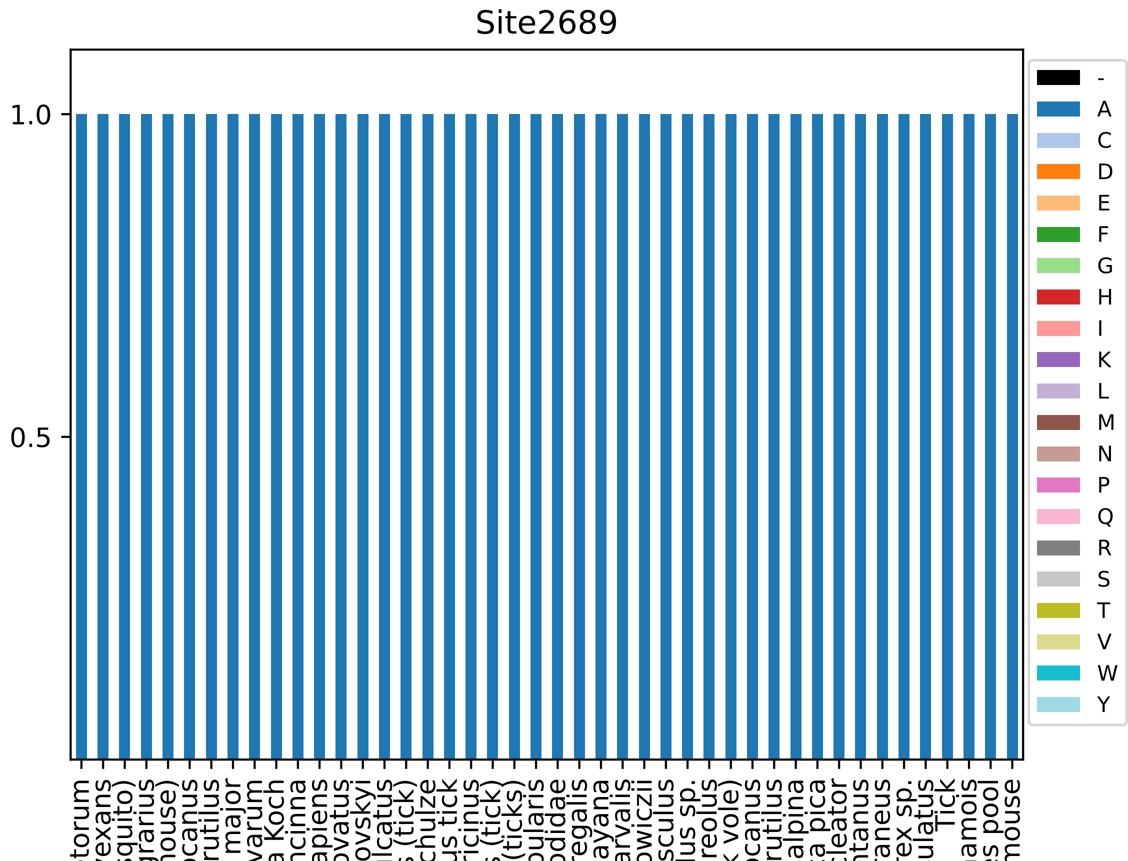
Site2689
1.0
0.5
-
A
C
D
E
F
G
H
I
K
L
M
N
P
Q
R
S
T
V
W
Y

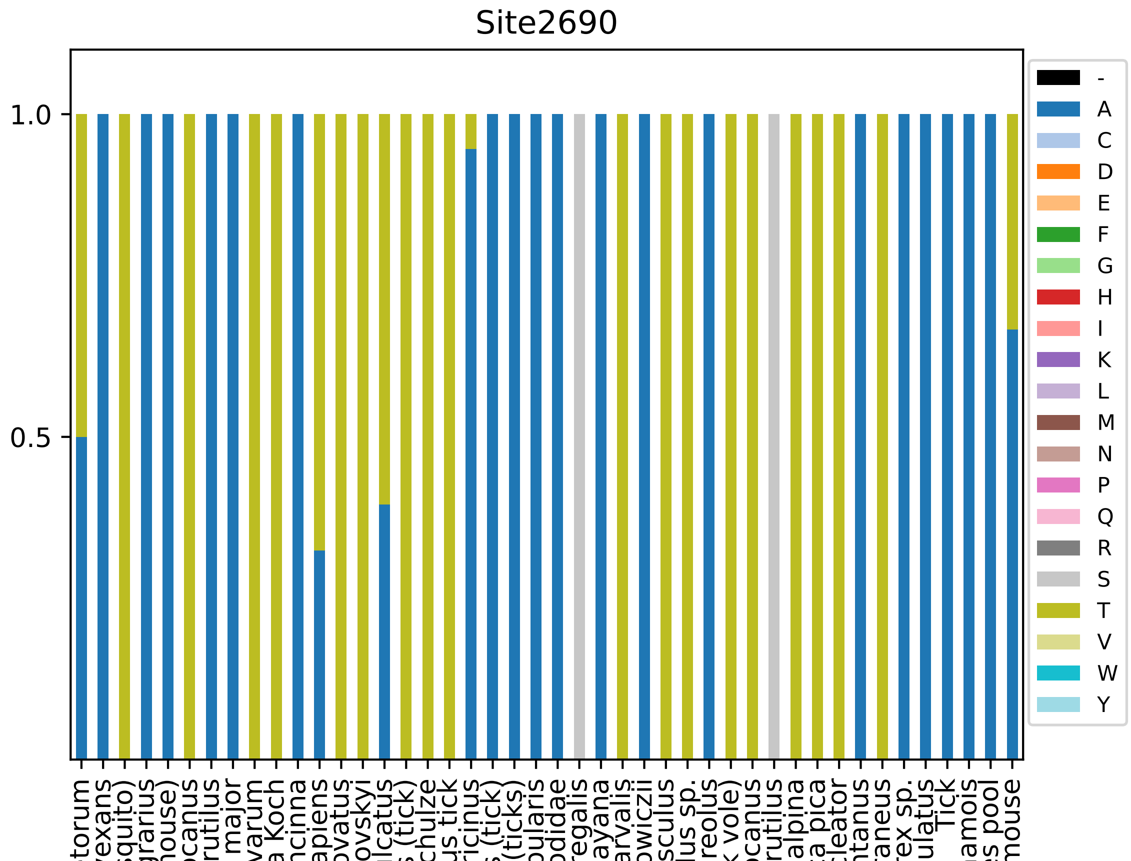
Site2690
1.0
0.5
-
A
C
D
E
F
G
H
I
K
L
M
N
P
Q
R
S
T
V
W
Y

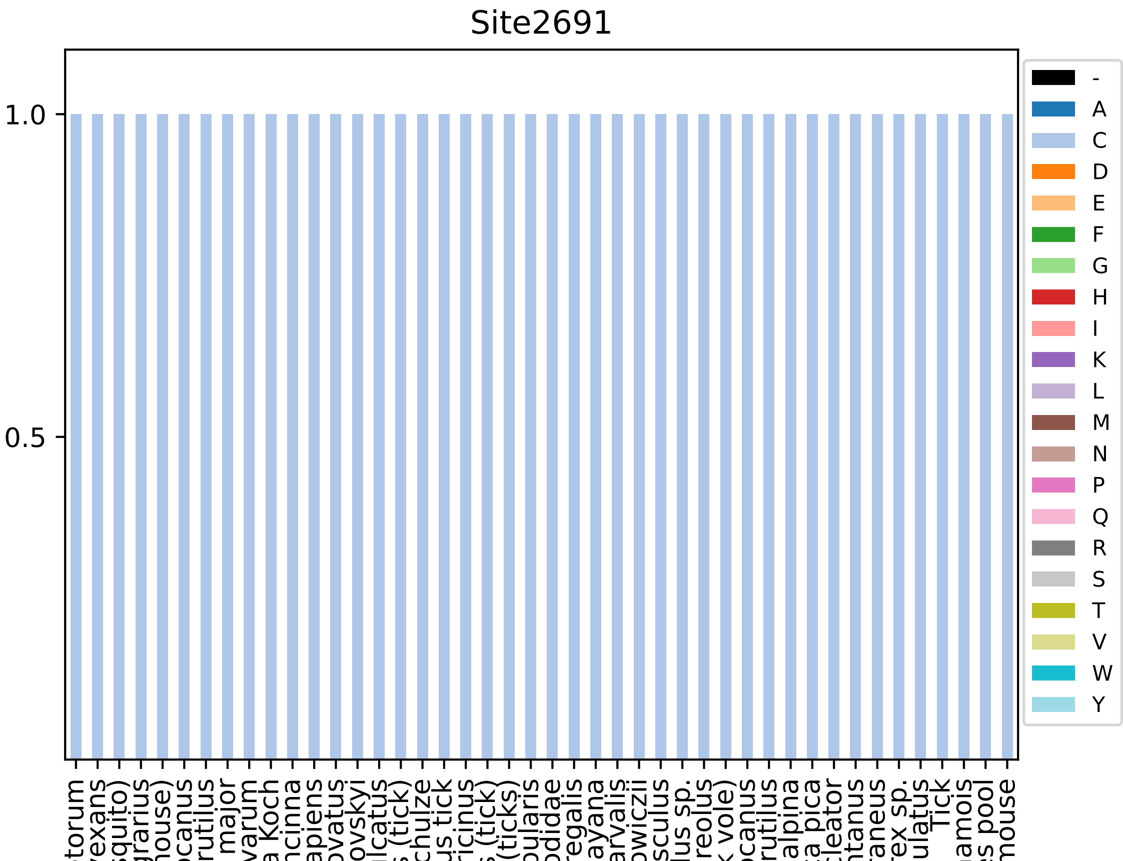
Site2691
1.0
0.5
-
A
C
D
E
F
G
H
I
K
L
M
N
P
Q
R
S
T
V
W
Y

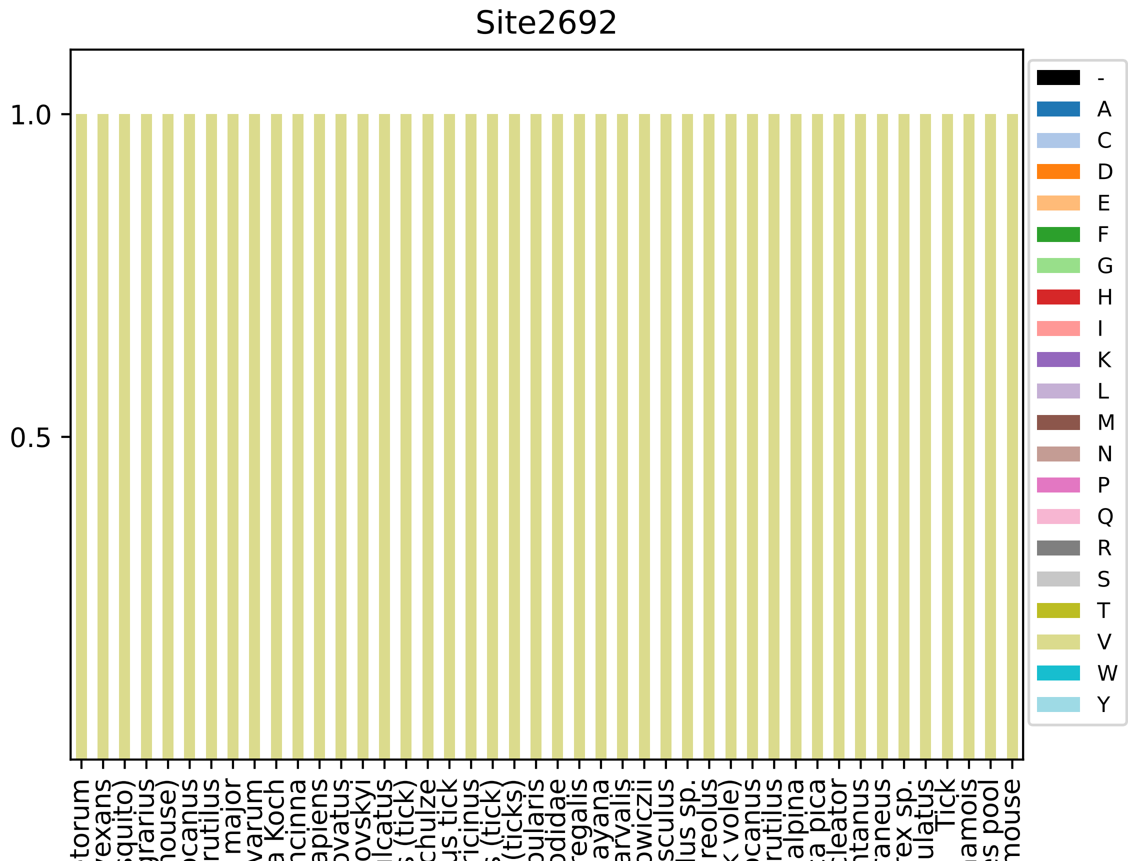
Site2692
1.0
0.5
-
A
C
D
E
F
G
H
I
K
L
M
N
P
Q
R
S
T
V
W
Y

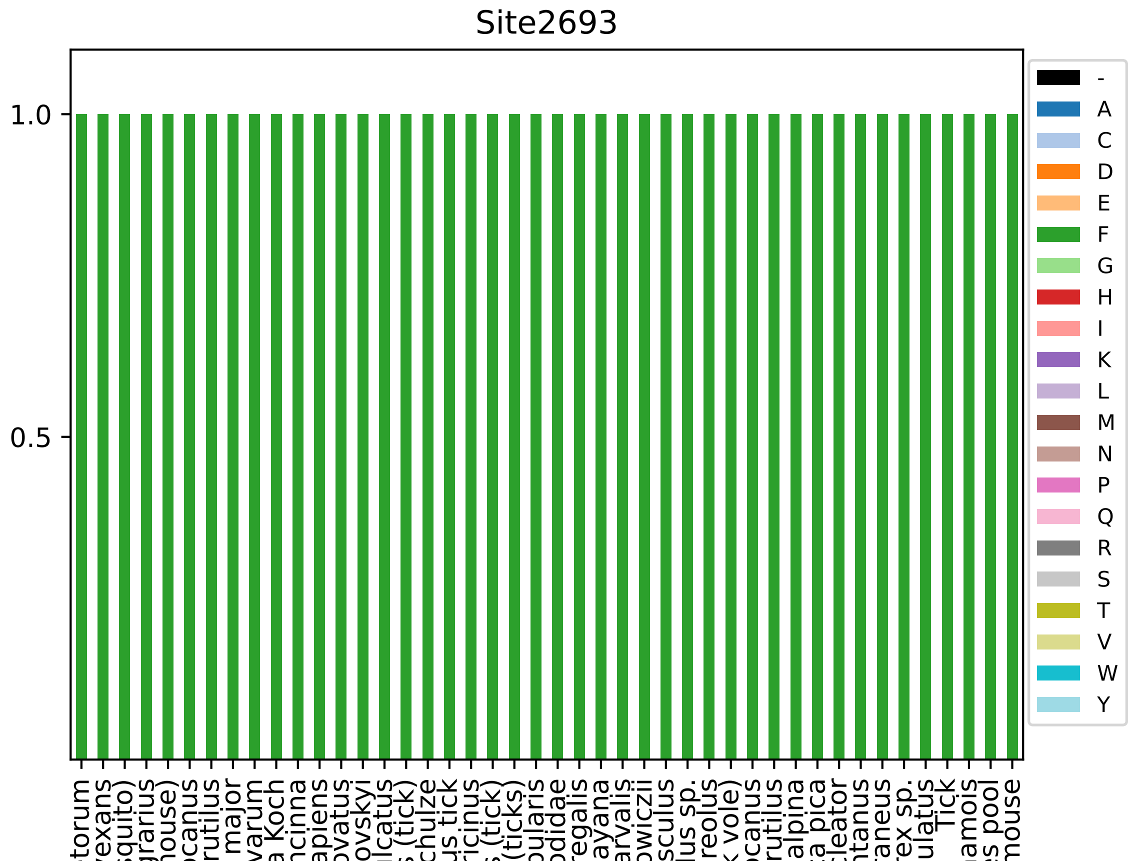
Site2693
1.0
0.5
-
A
C
D
E
F
G
H
I
K
L
M
N
P
Q
R
S
T
V
W
Y
Tick

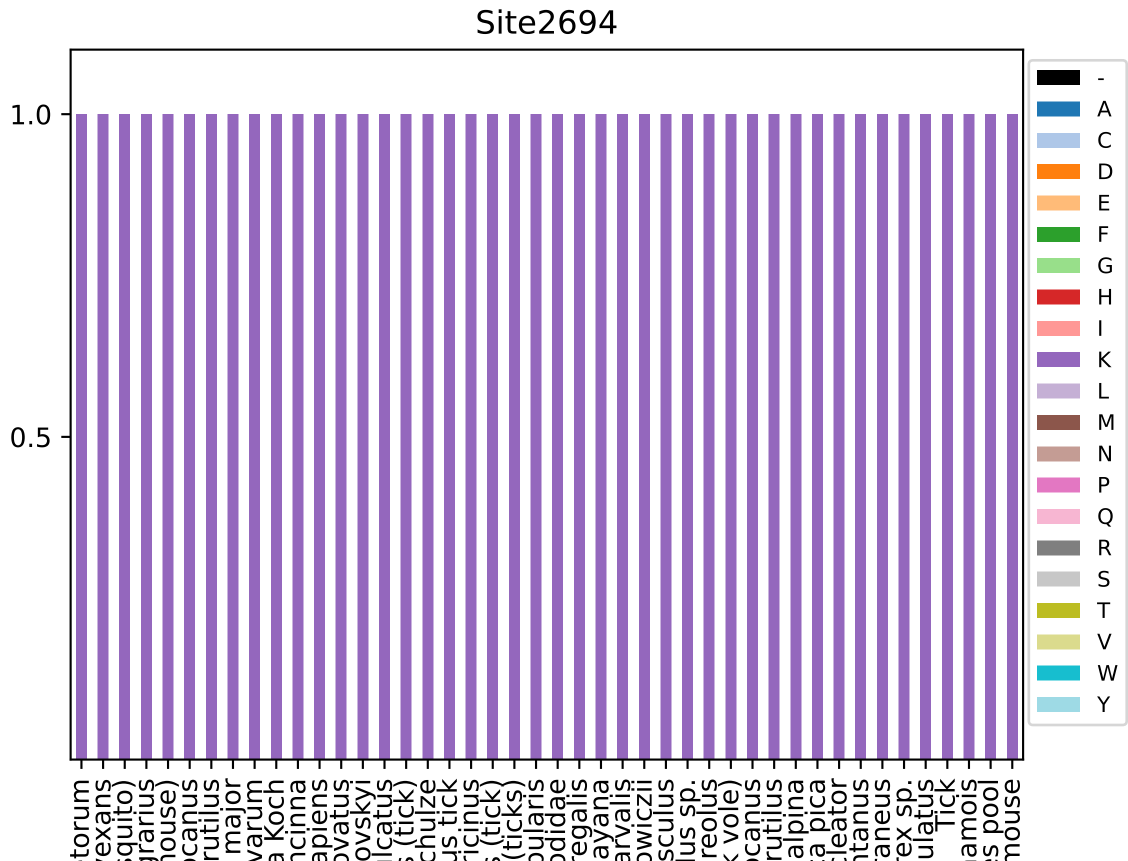
Site2694
1.0
0.5
-
A
C
D
E
F
G
H
I
K
L
M
N
P
Q
R
S
T
V
W
Y
Tick

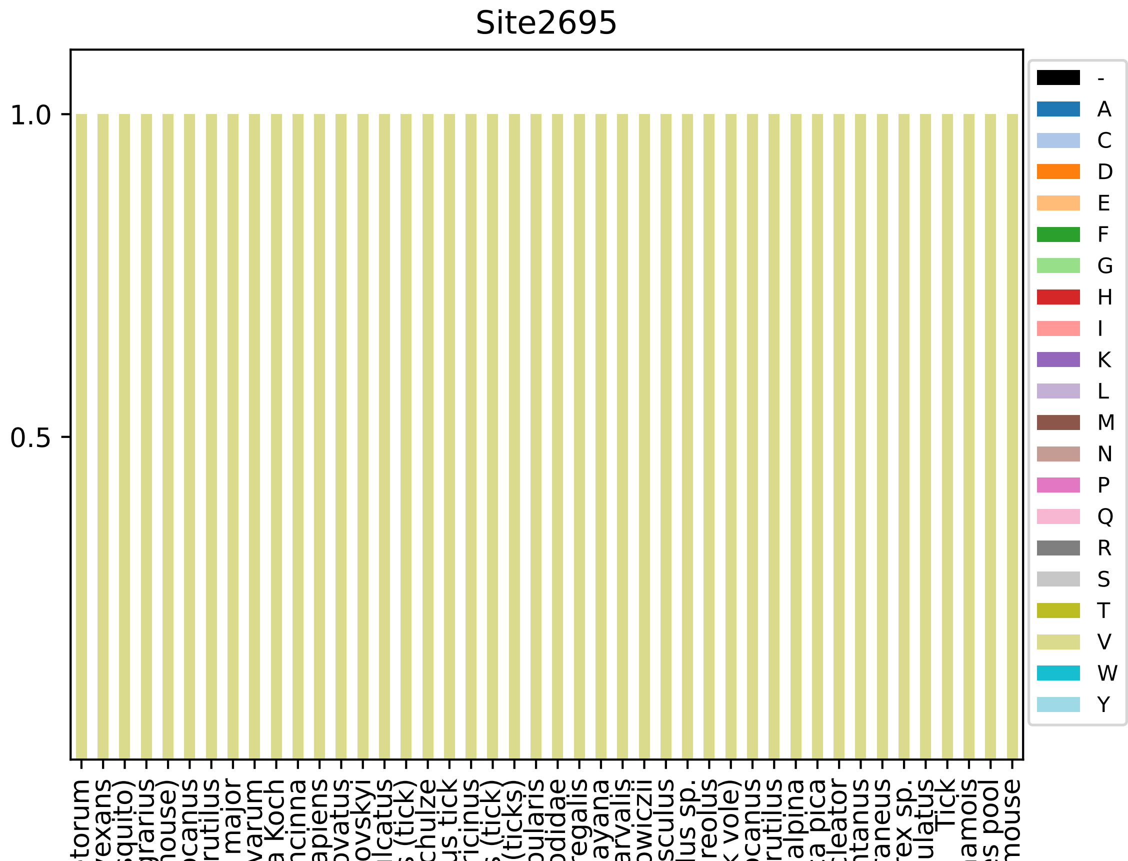
Site2695
1.0
0.5
-
A
C
D
E
F
G
H
I
K
L
M
N
P
Q
R
S
T
V
W
Y

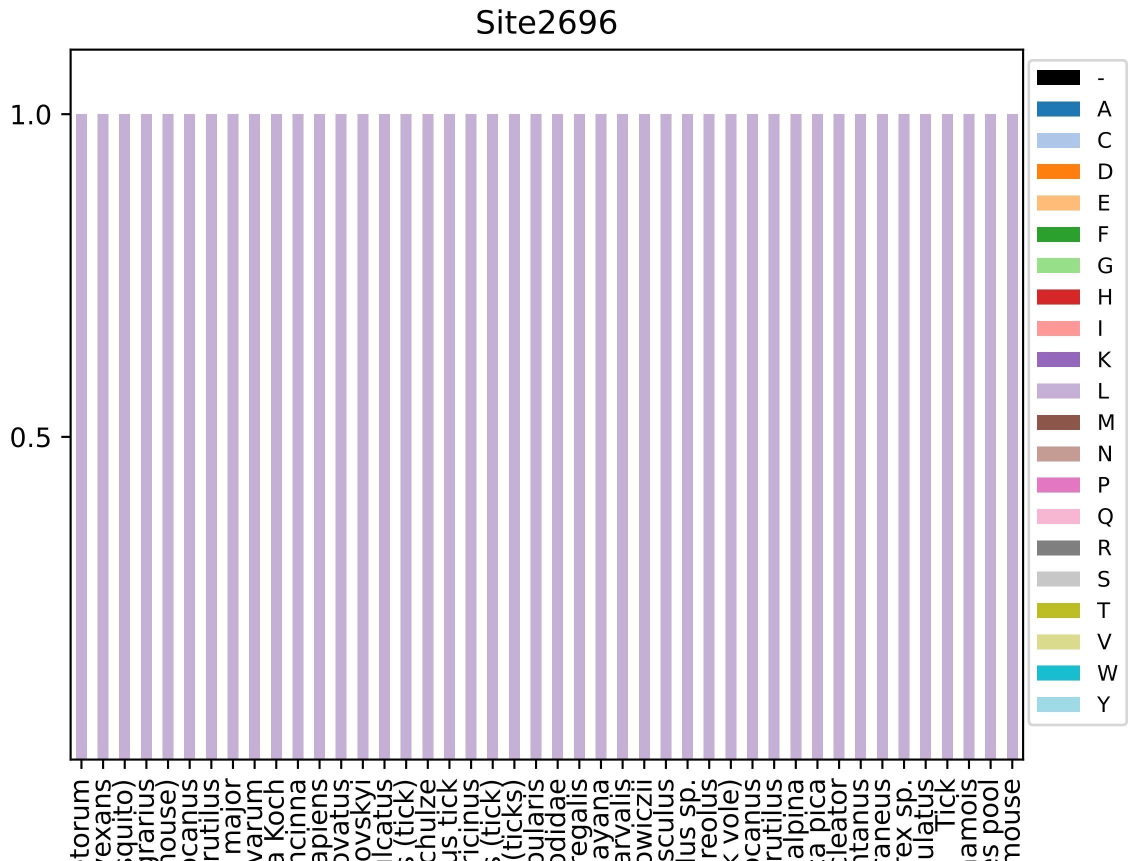

Site2696
1.0
0.5
-
A
C
D
E
F
G
H
I
K
L
M
N
P
Q
R
S
T
V
W
Y

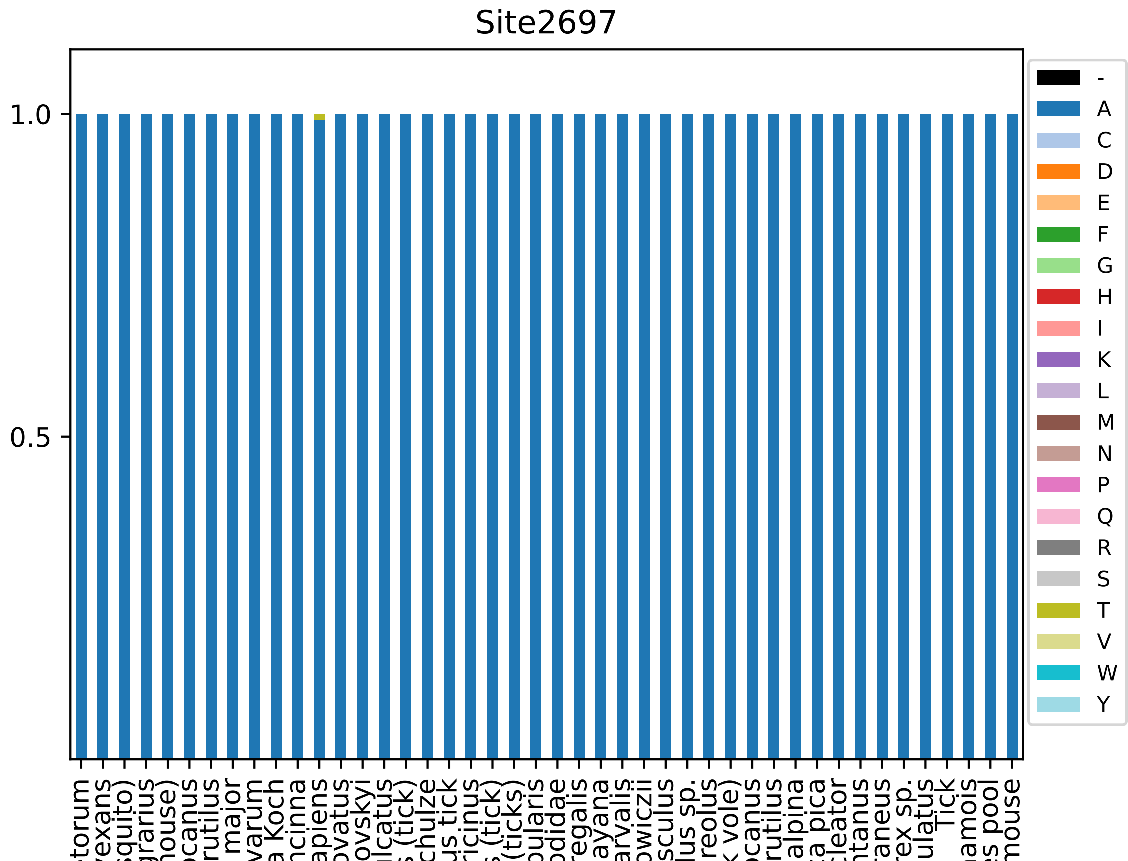
Site2697
1.0
0.5
-
A
C
D
E
F
G
H
I
K
L
M
N
P
Q
R
S
T
V
W
Y

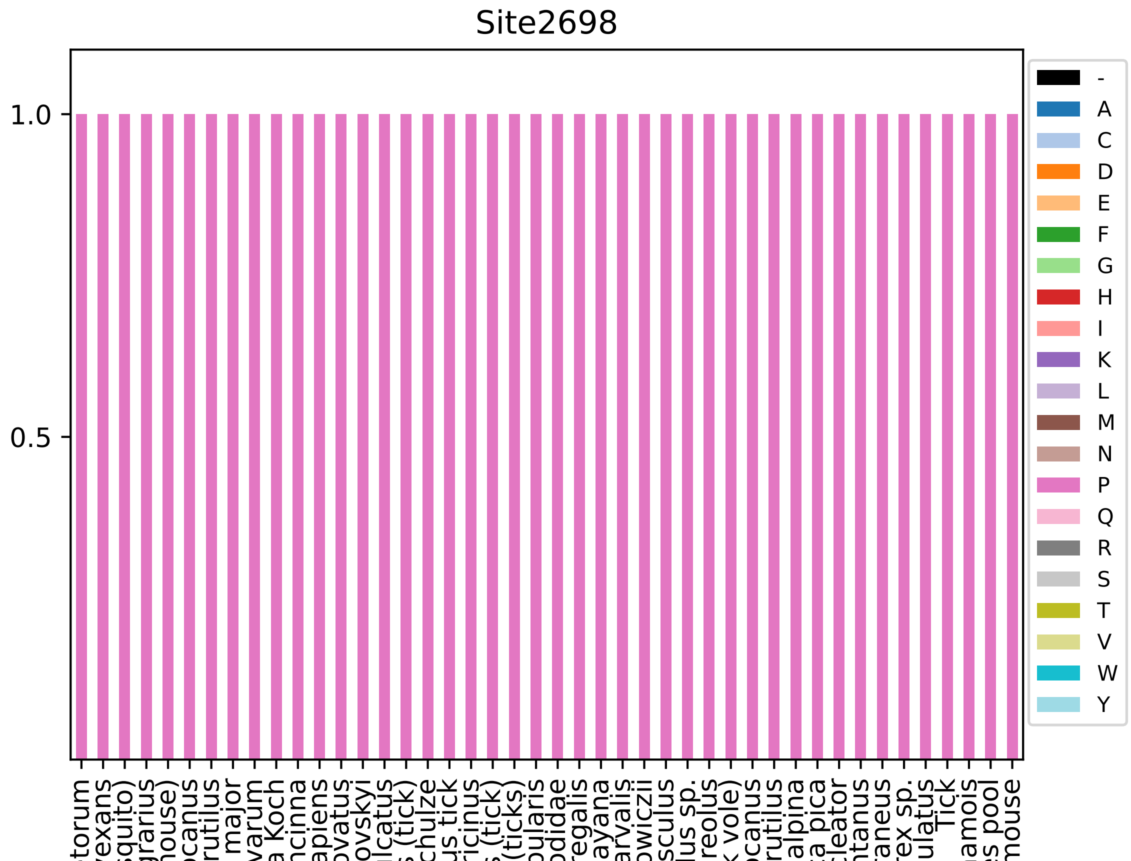

Site2698
1.0
0.5
-
A
C
D
E
F
G
H
I
K
L
M
N
P
Q
R
S
T
V
W
Y

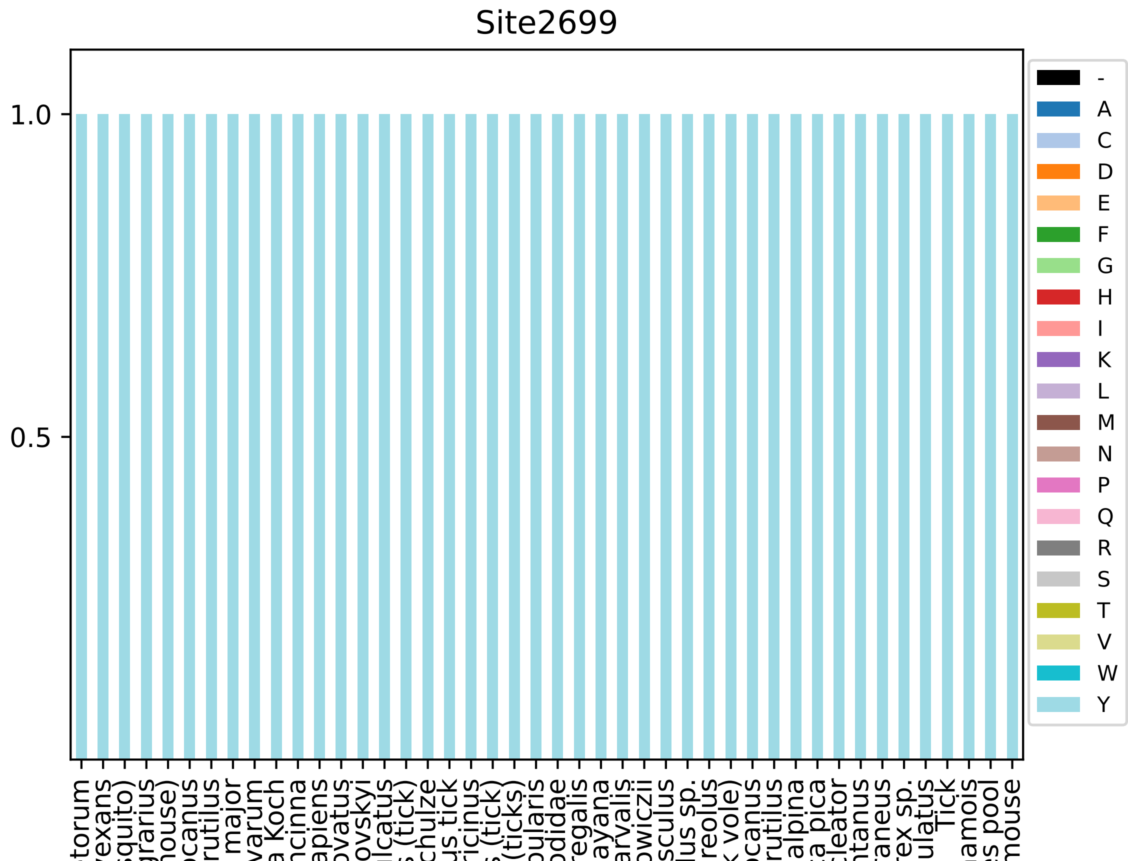
Site2699
1.0
0.5
-
A
C
D
E
F
G
H
I
K
L
M
N
P
Q
R
S
T
V
W
Y

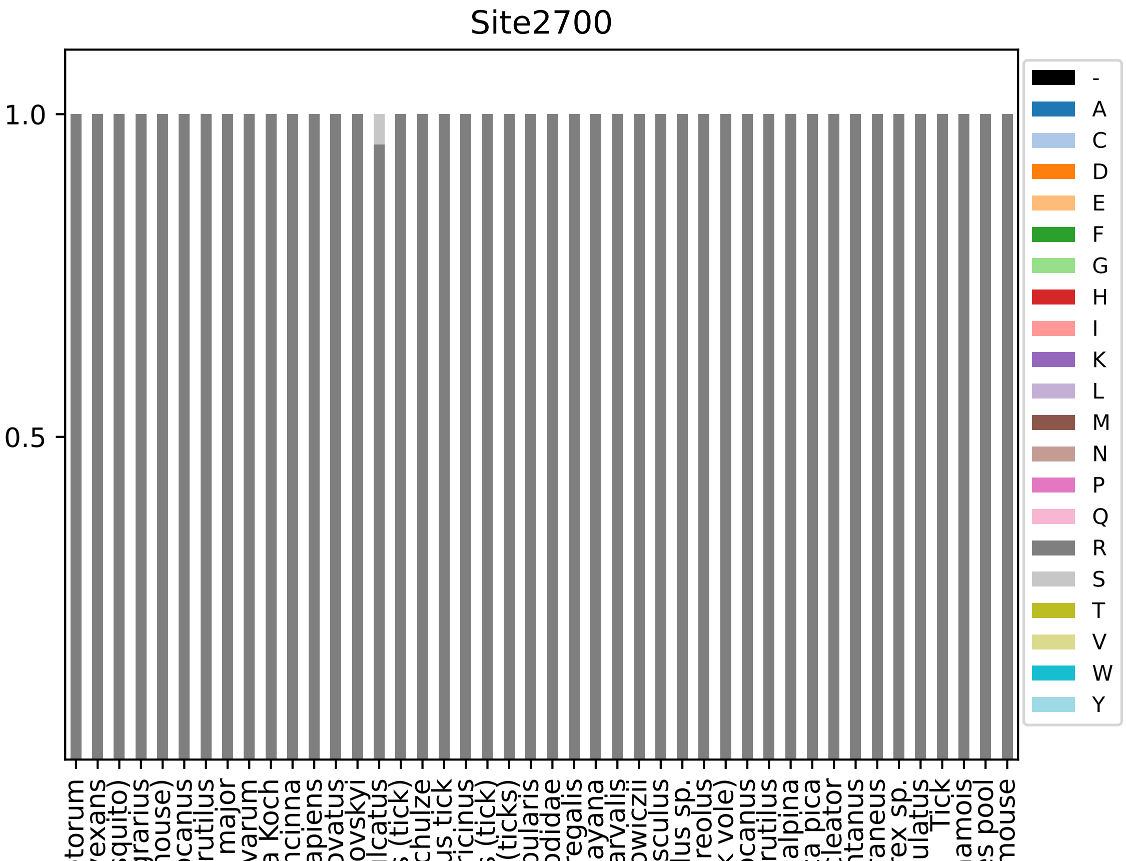

Site2700
1.0
0.5
-
A
C
D
E
F
G
H
I
K
L
M
N
P
Q
R
S
T
V
W
Y
Tick

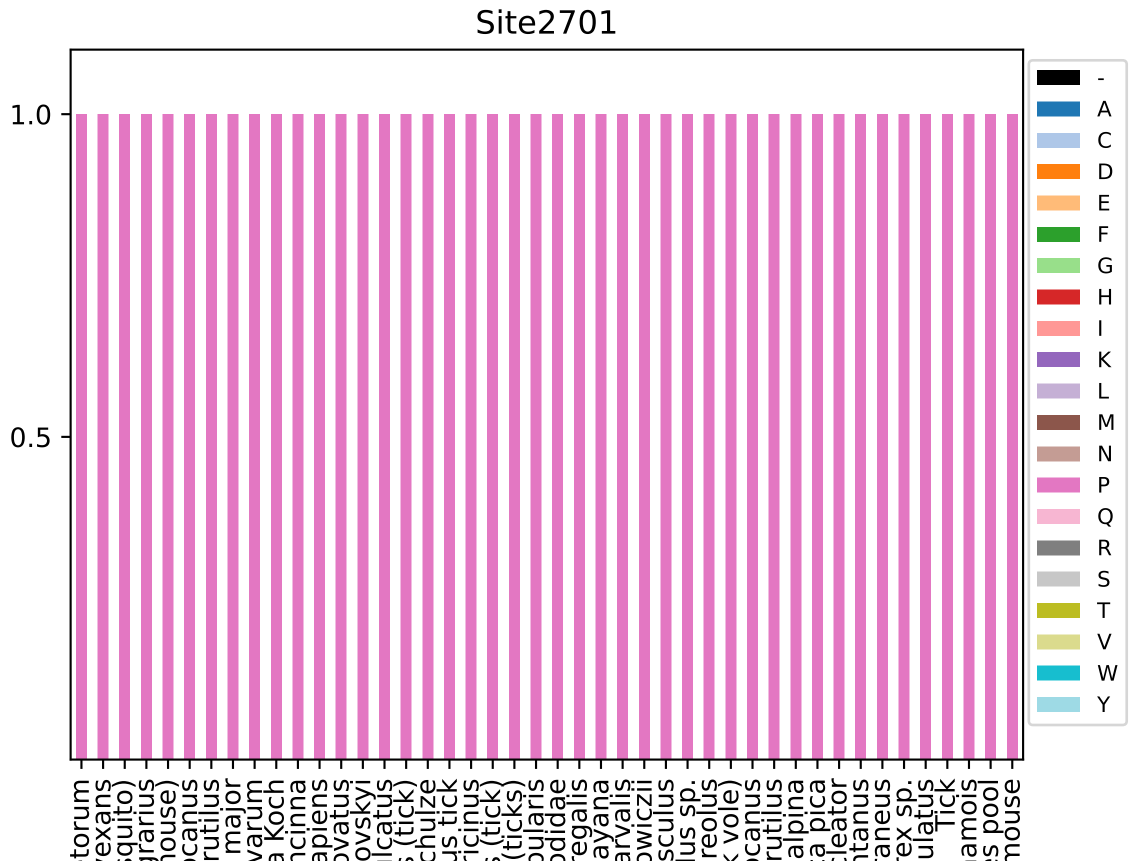

Site2701
1.0
0.5
-
A
C
D
E
F
G
H
I
K
L
M
N
P
Q
R
S
T
V
W
Y

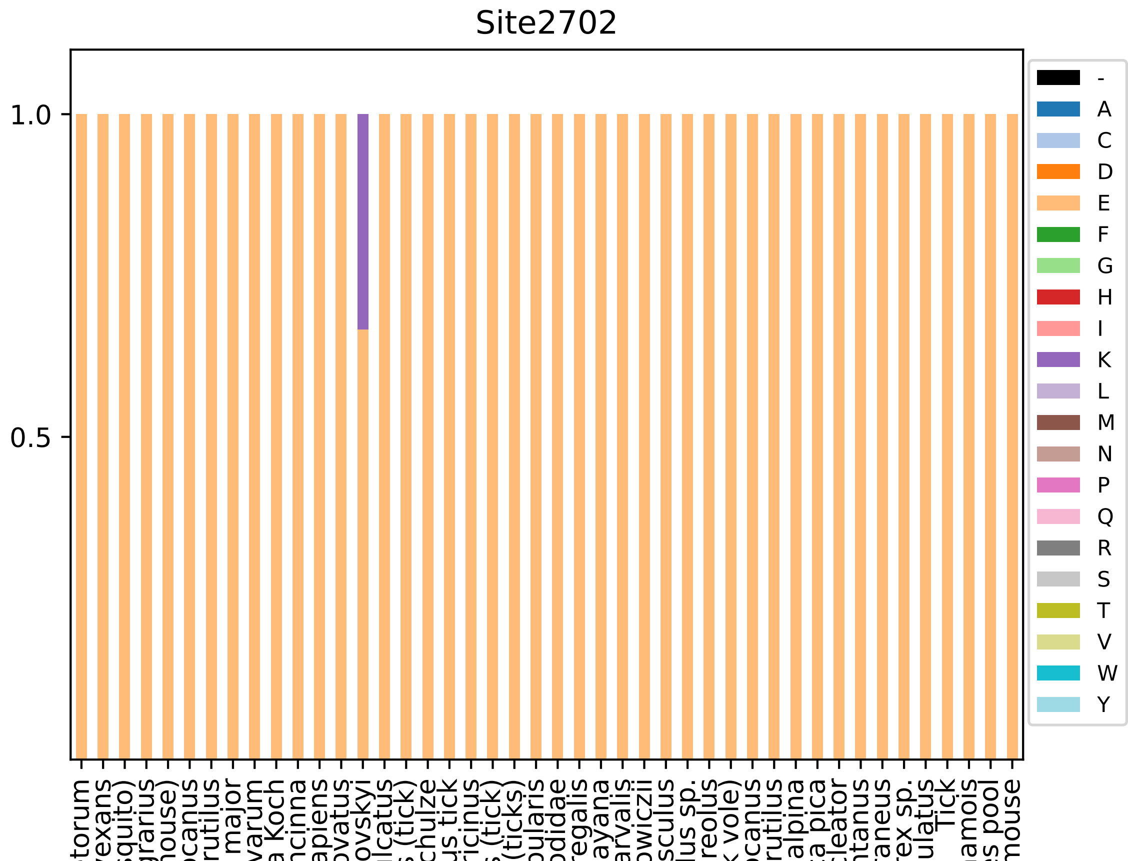
Site2702
1.0
0.5
-
A
C
D
E
F
G
H
I
K
L
M
N
P
Q
R
S
T
V
W
Y

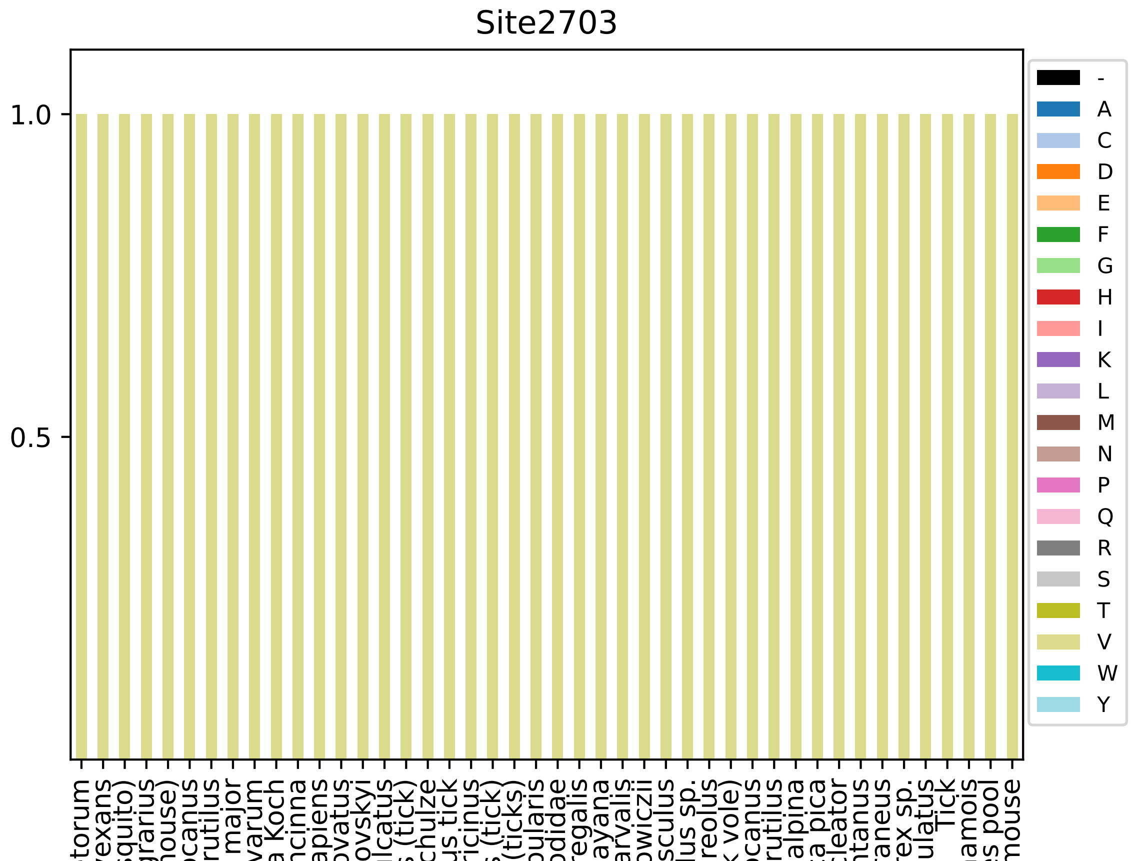
Site2703
1.0
0.5
-
A
C
D
E
F
G
H
I
K
L
M
N
P
Q
R
S
T
V
W
Y

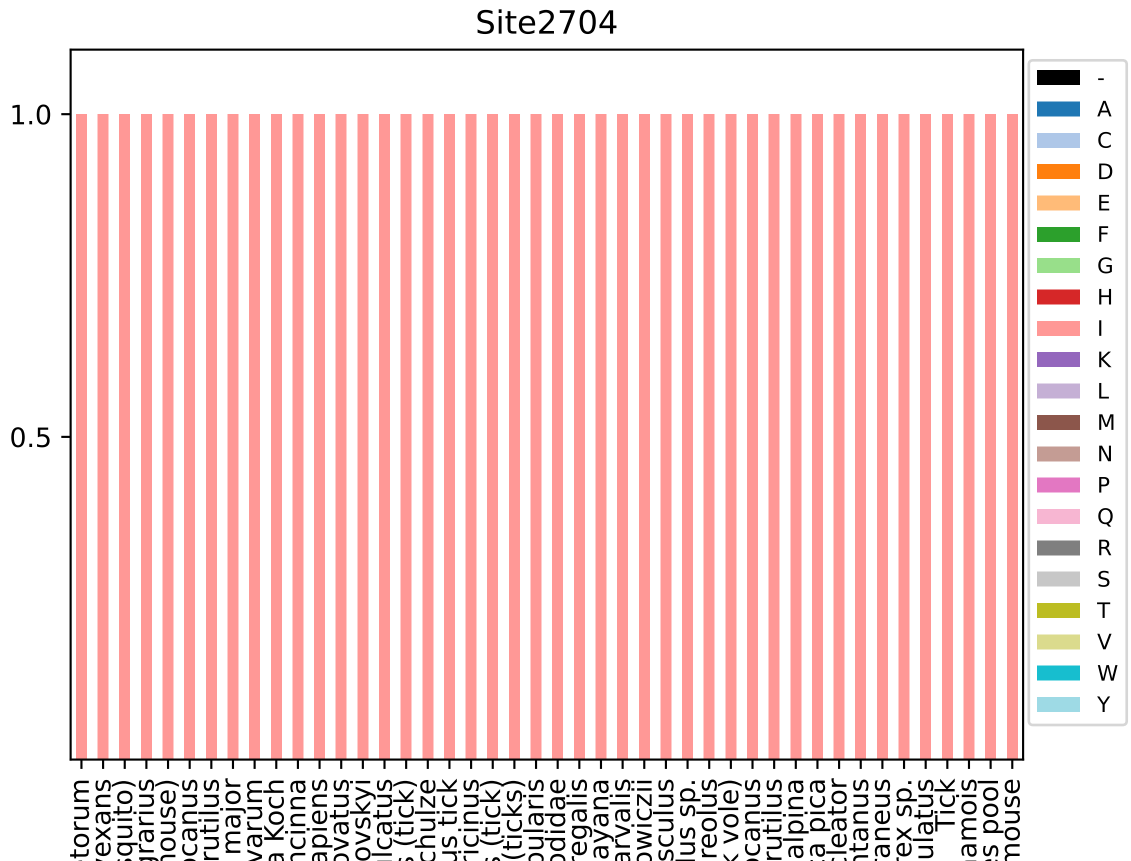
Site2704
1.0
0.5
-
A
C
D
E
F
G
H
I
K
L
M
N
P
Q
R
S
T
V
W
Y

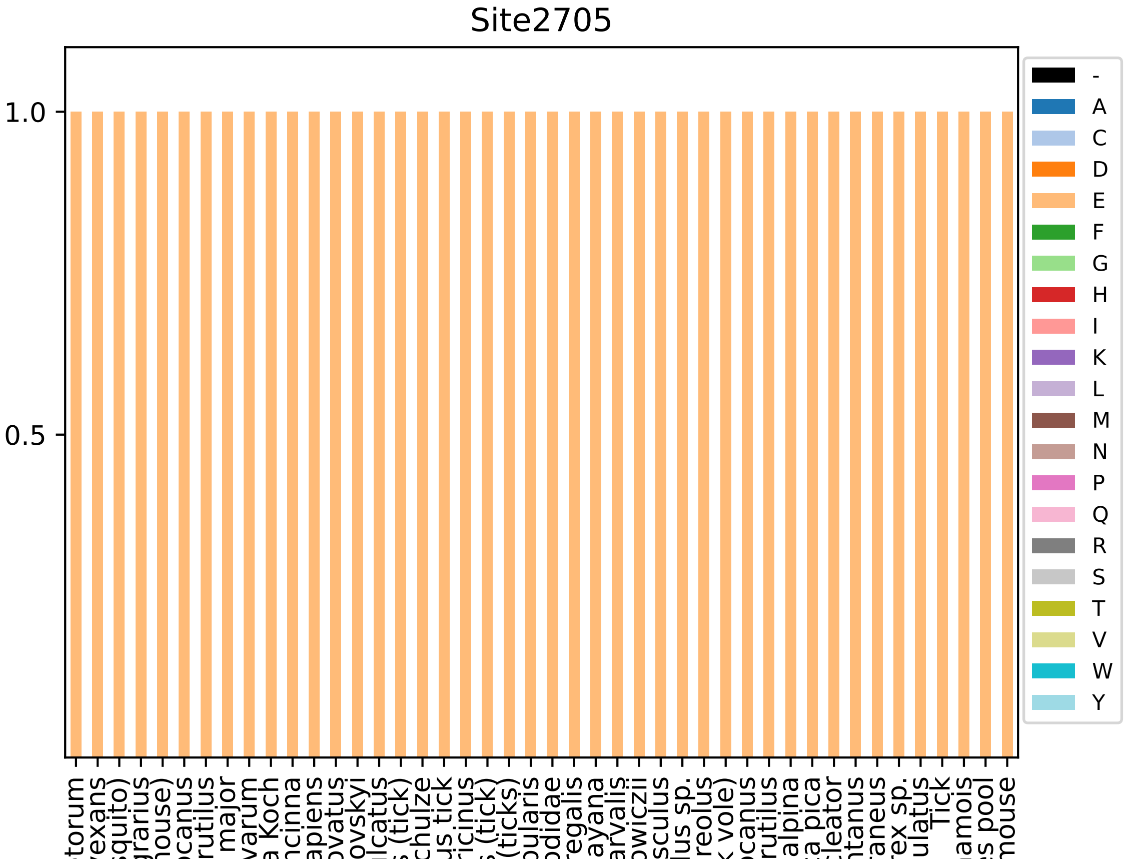
Site2705
1.0
0.5
-
A
C
D
E
F
G
H
I
K
L
M
N
P
Q
R
S
T
V
W
Y

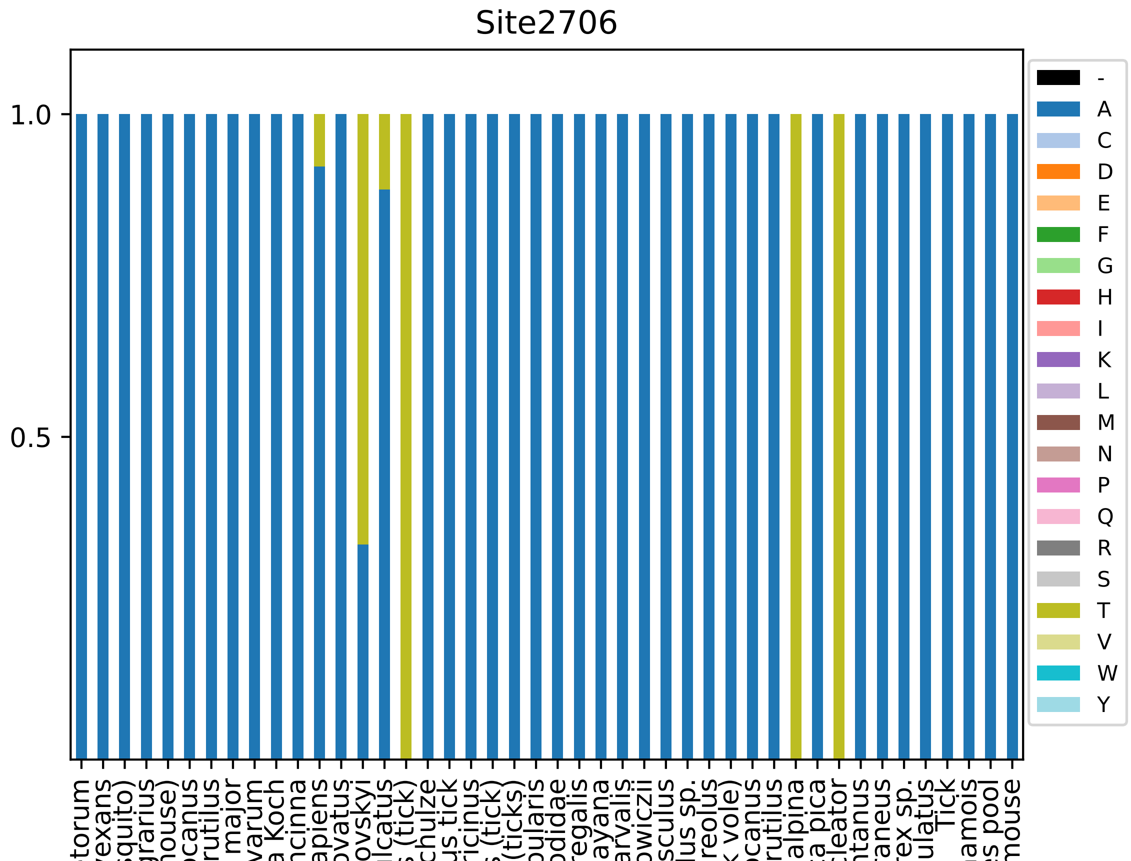
Site2706
1.0
0.5
-
A
C
D
E
F
G
H
I
K
L
M
N
P
Q
R
S
T
V
W
Y

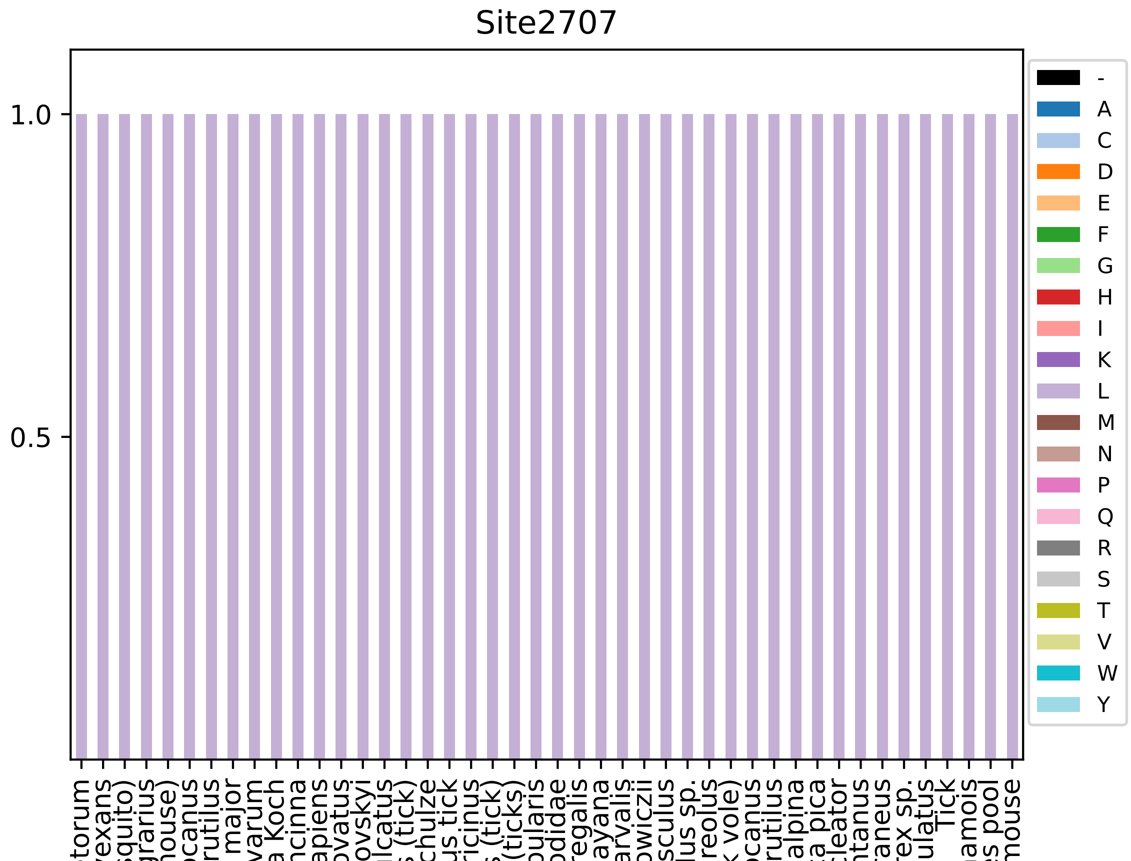
Site2707
1.0
0.5
-
A
C
D
E
F
G
H
I
K
L
M
N
P
Q
R
S
T
V
W
Y

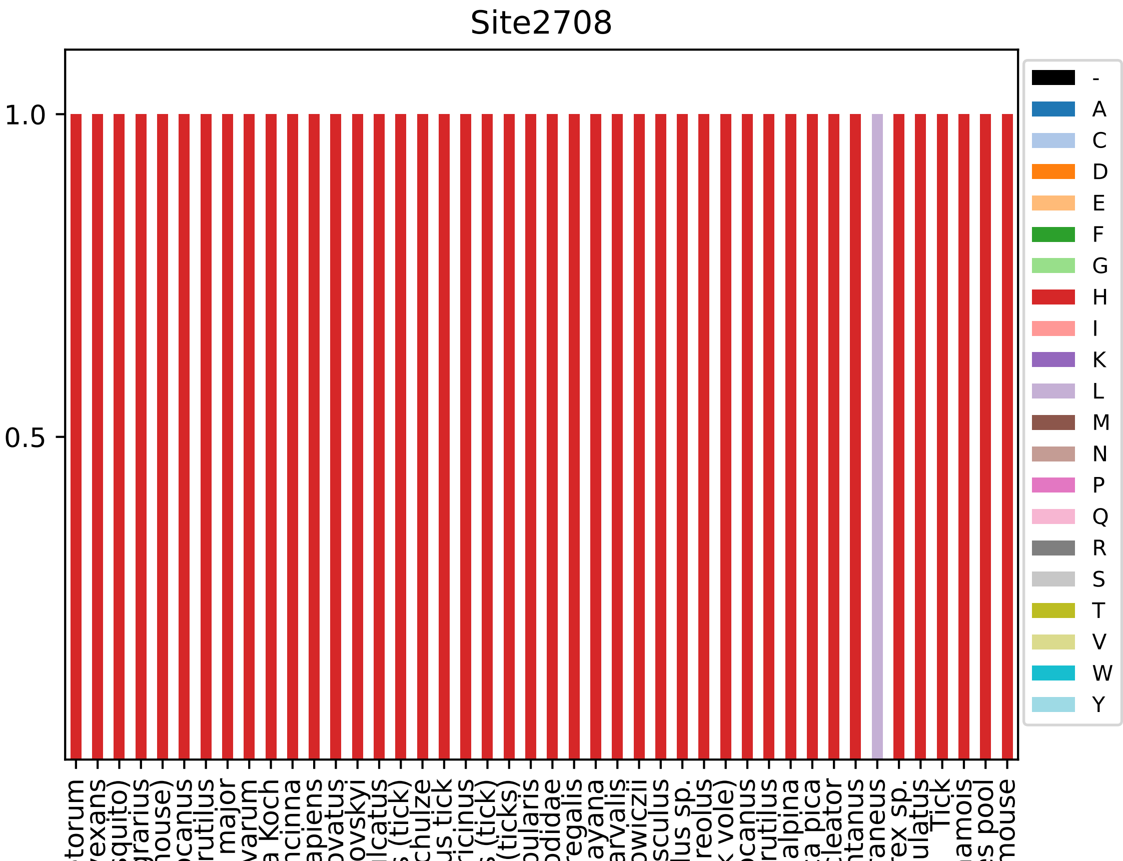
Site2708
1.0
0.5
-
A
C
D
E
F
G
H
I
K
L
M
N
P
Q
R
S
T
V
W
Y

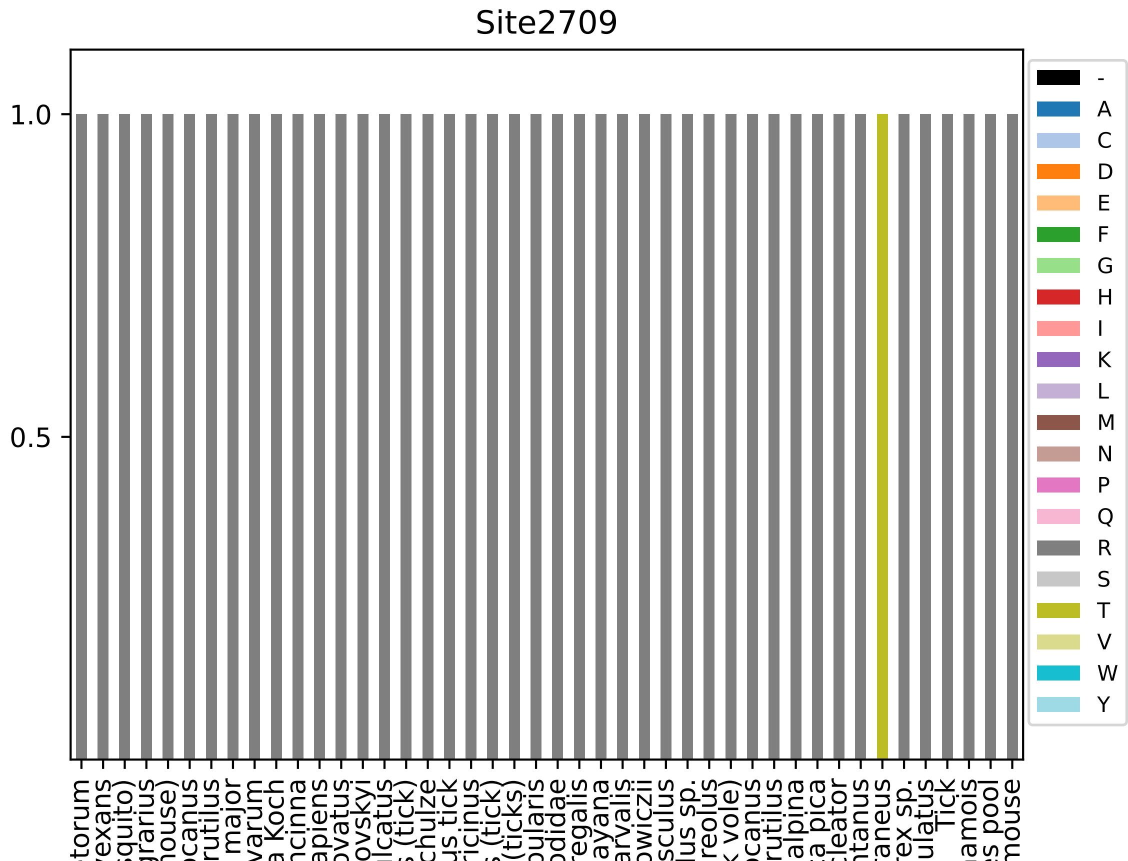

Site2709
1.0
0.5
-
A
C
D
E
F
G
H
I
K
L
M
N
P
Q
R
S
T
V
W
Y

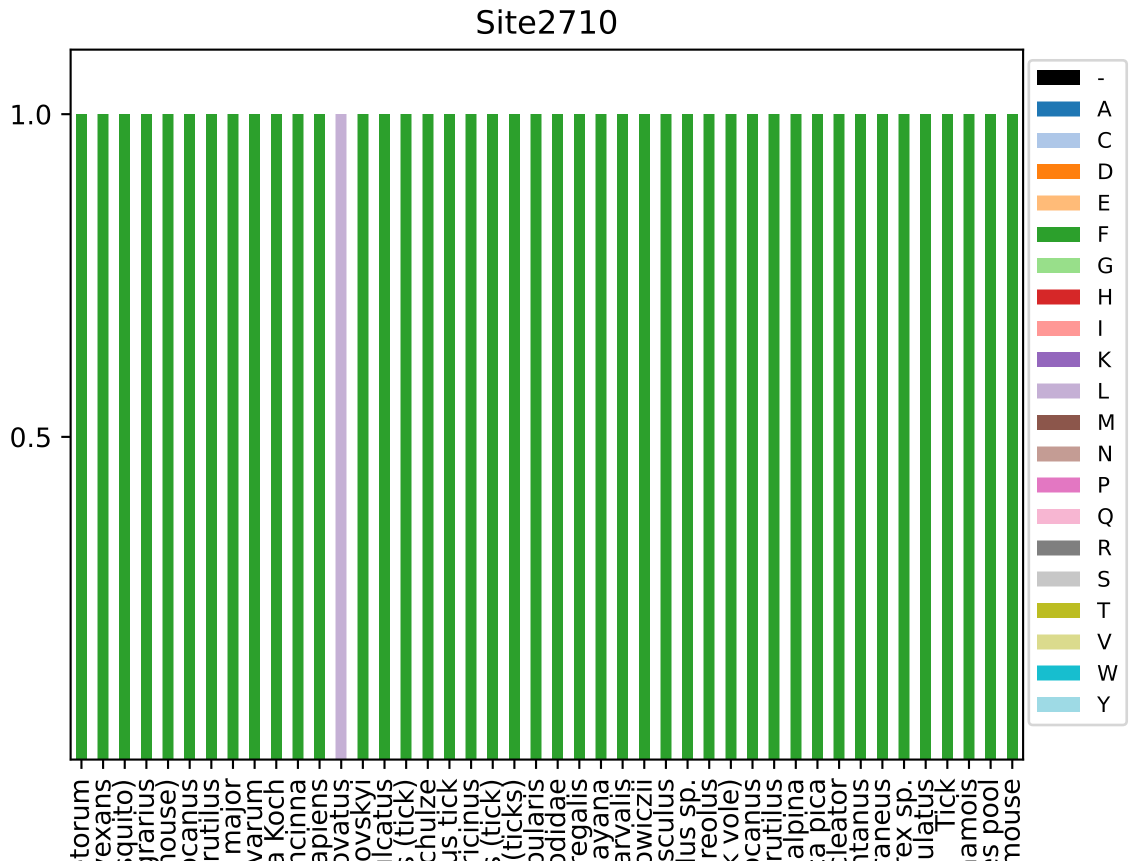

Site2710
1.0
0.5
-
A
C
D
E
F
G
H
I
K
L
M
N
P
Q
R
S
T
V
W
Y

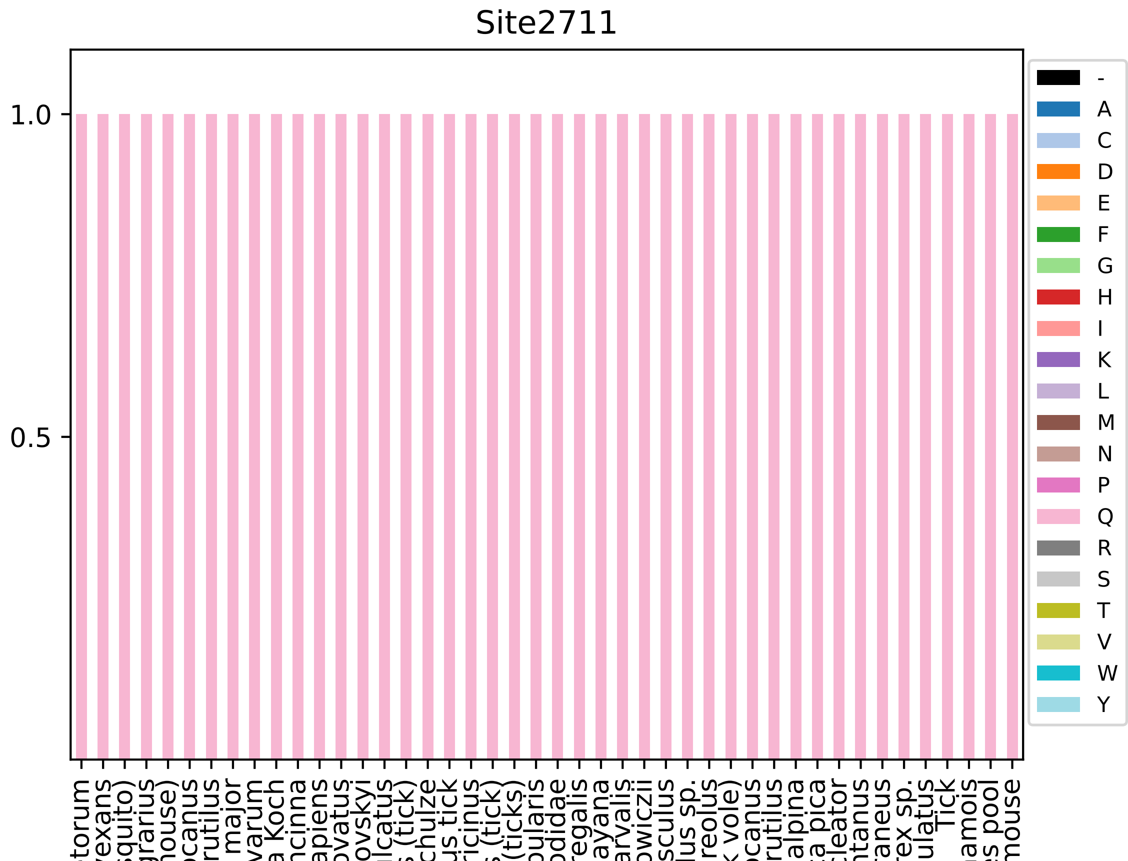
Site2711
1.0
0.5
-
A
C
D
E
F
G
H
I
K
L
M
N
P
Q
R
S
T
V
W
Y

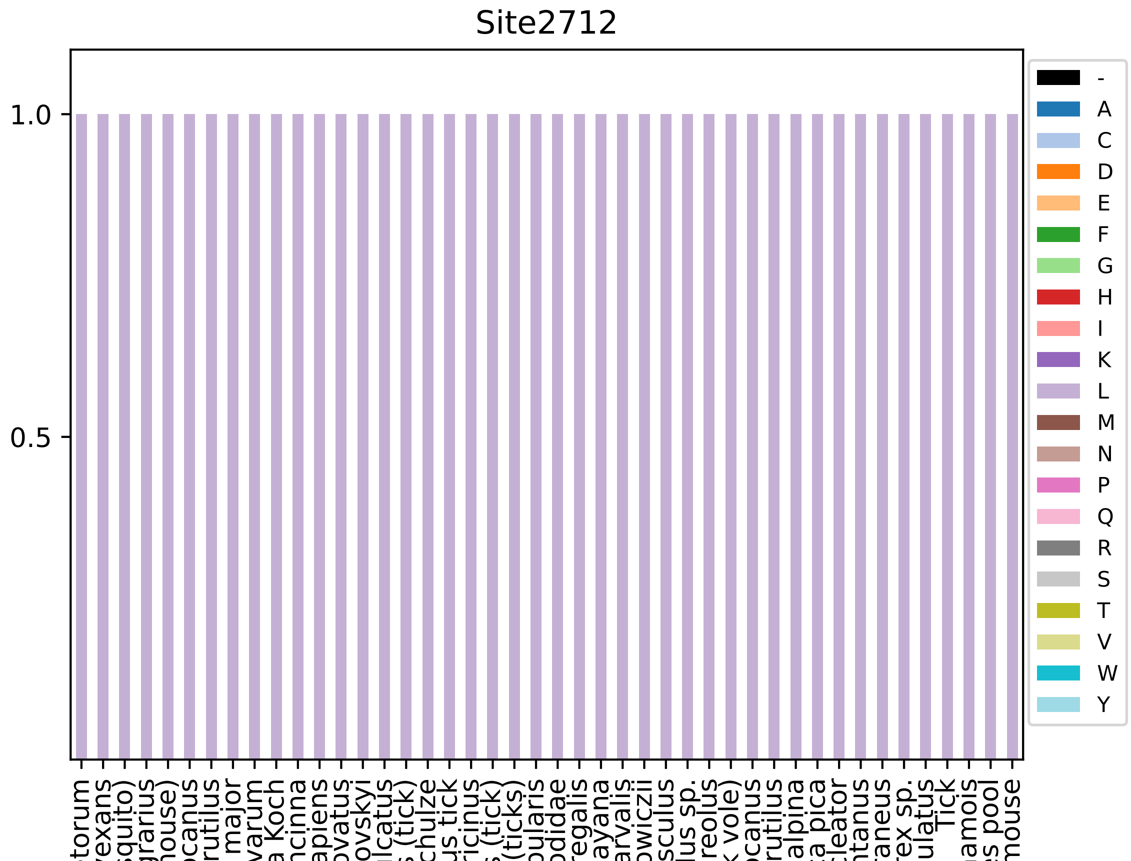
Site2712
1.0
0.5
-
A
C
D
E
F
G
H
I
K
L
M
N
P
Q
R
S
T
V
W
Y

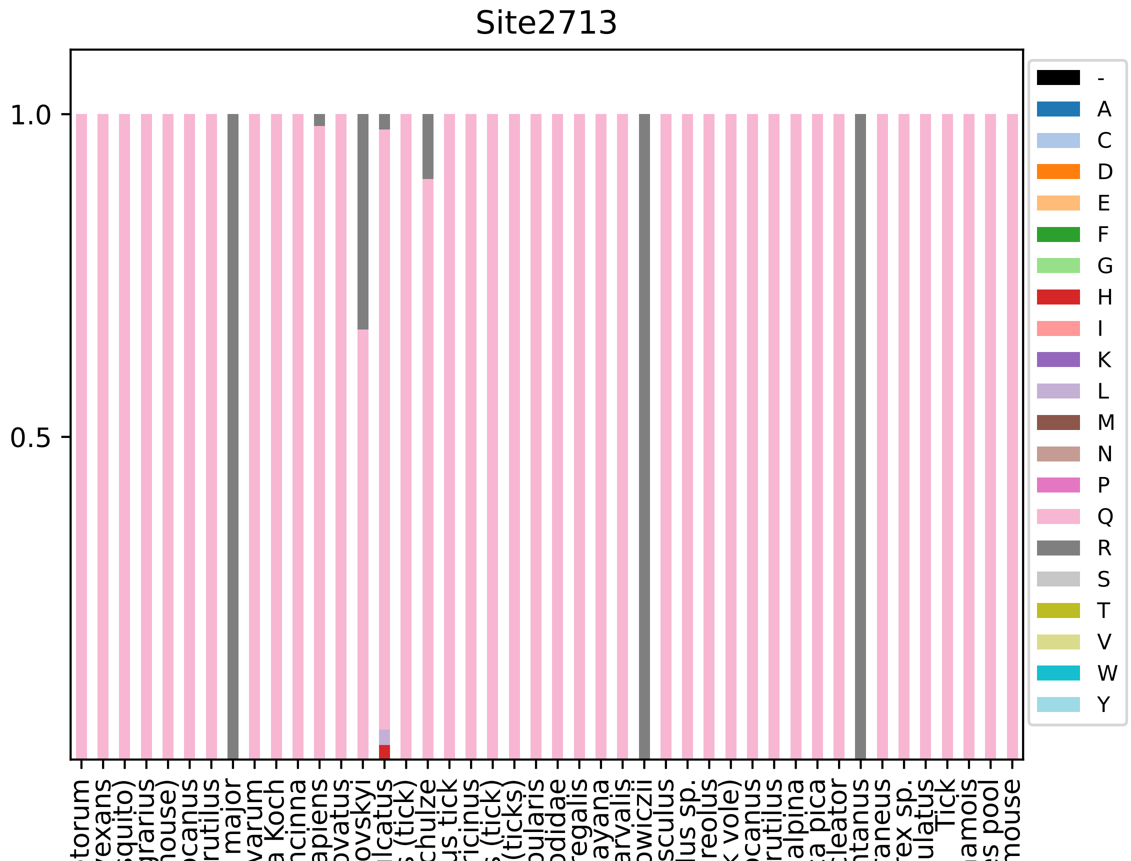
Site2713
1.0
0.5
-
A
C
D
E
F
G
H
I
K
L
M
N
P
Q
R
S
T
V
W
Y

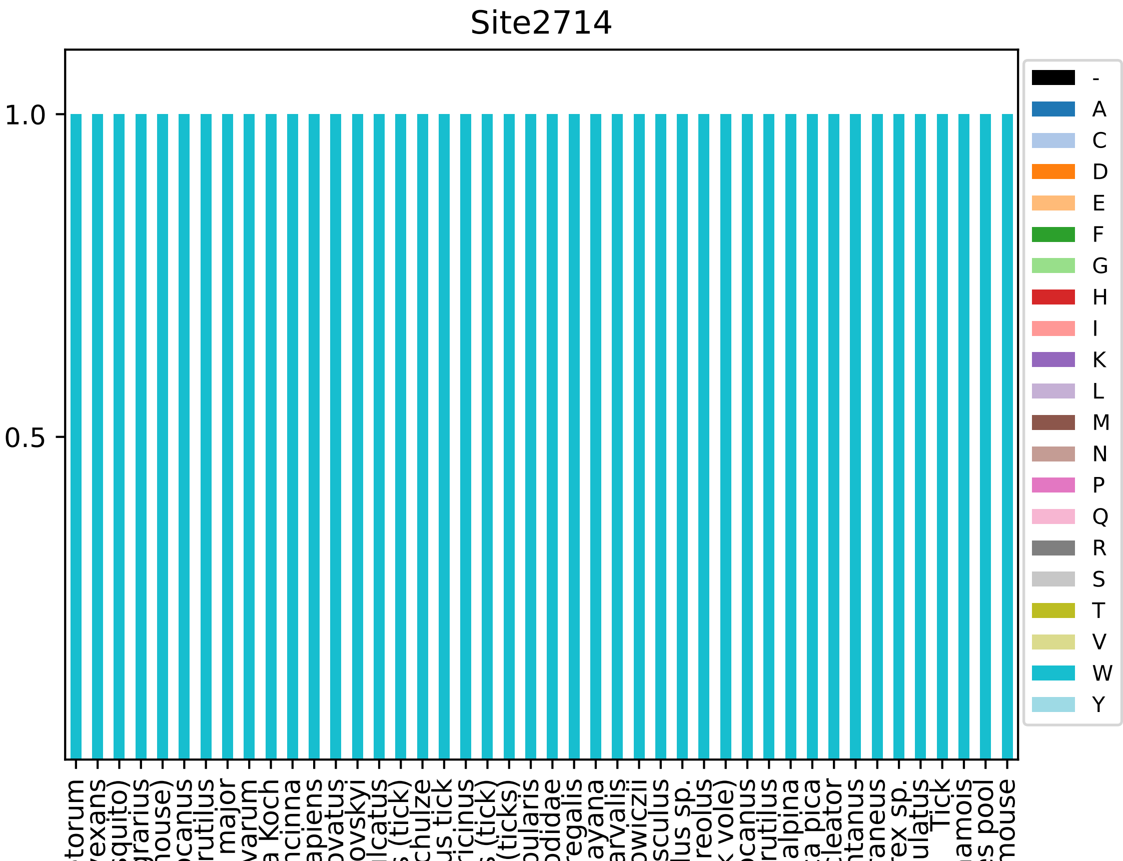
Site2714
1.0
0.5
-
A
C
D
E
F
G
H
I
K
L
M
N
P
Q
R
S
T
V
W
Y

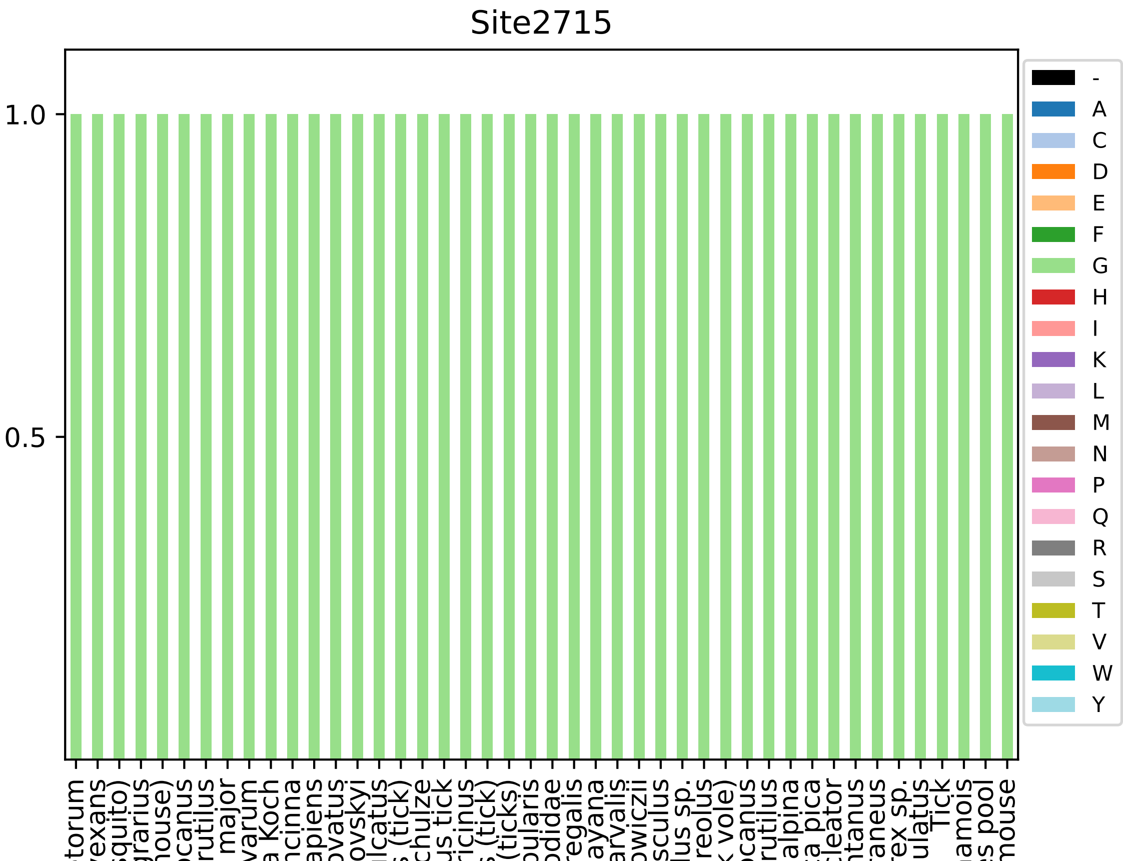
Site2715
1.0
0.5
-
A
C
D
E
F
G
H
I
K
L
M
N
P
Q
R
S
T
V
W
Y

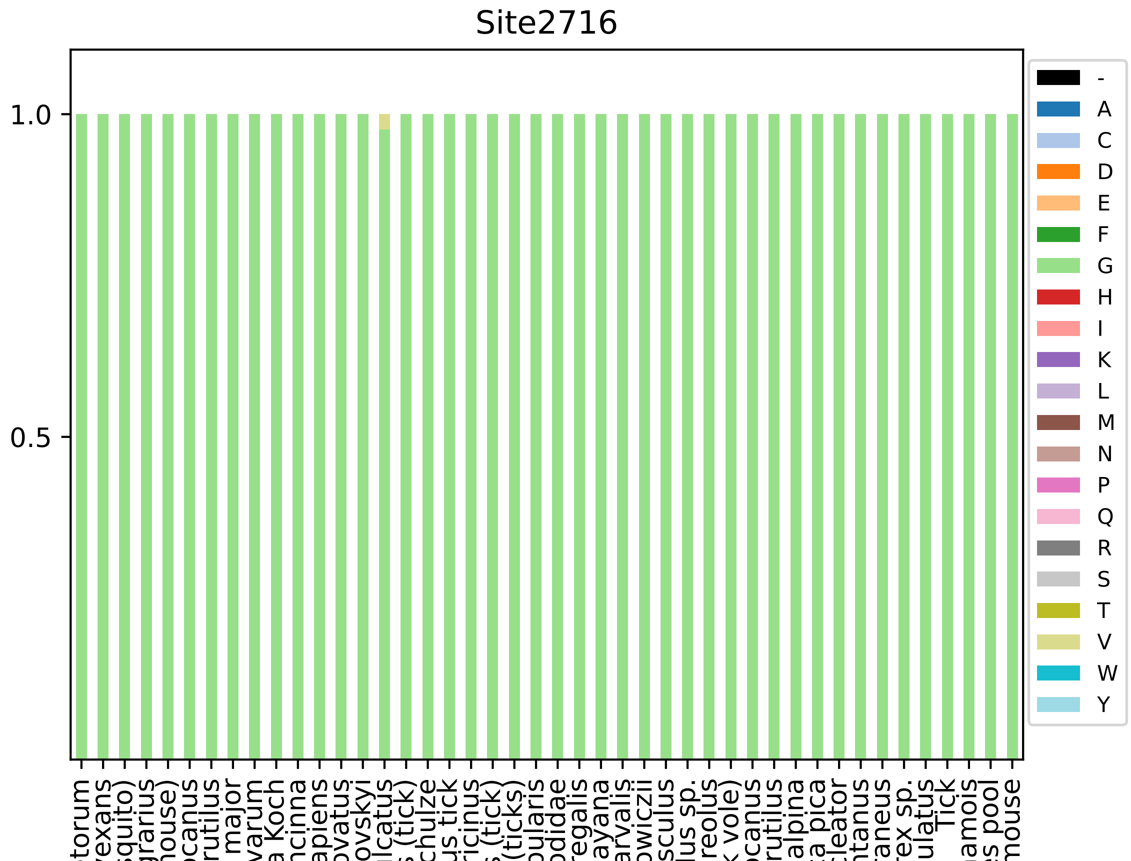
Site2716
1.0
0.5
-
A
C
D
E
F
G
H
I
K
L
M
N
P
Q
R
S
T
V
W
Y

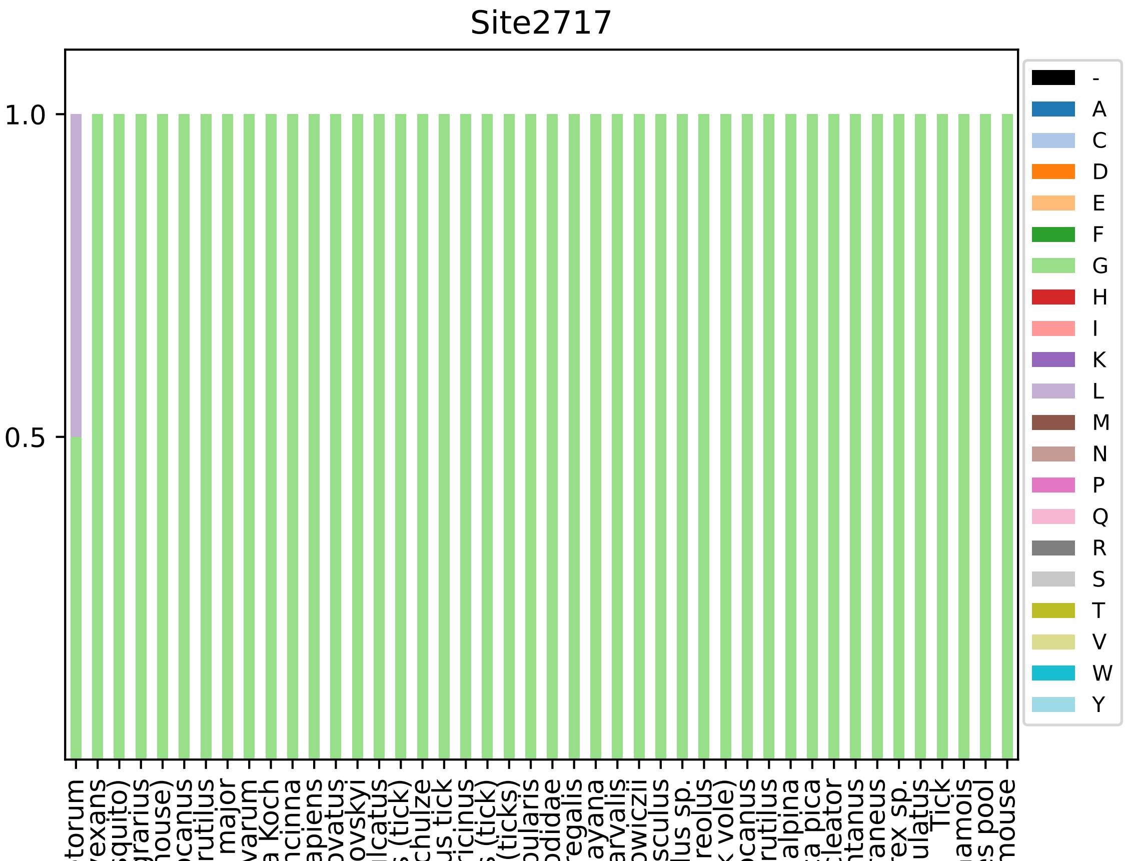
Site2717
1.0
0.5
-
A
C
D
E
F
G
H
I
K
L
M
N
P
Q
R
S
T
V
W
Y

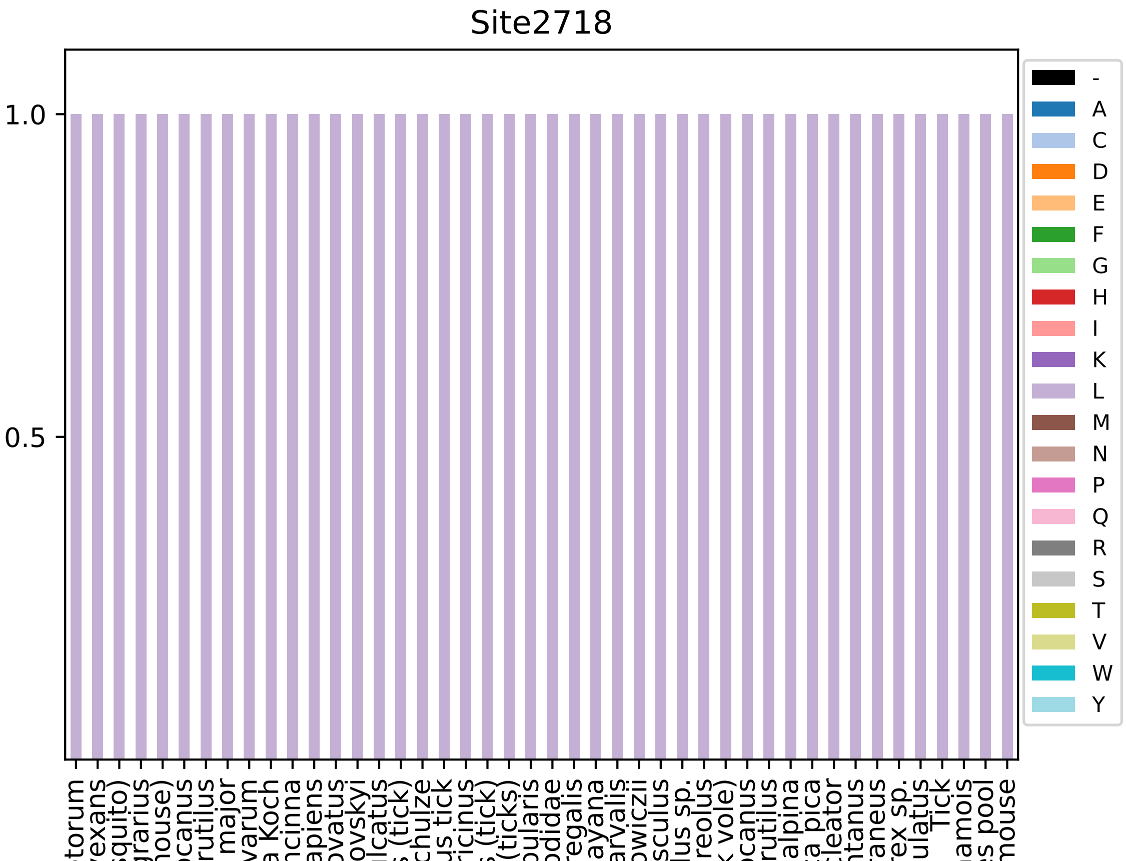
Site2718
1.0
0.5
-
A
C
D
E
F
G
H
I
K
L
M
N
P
Q
R
S
T
V
W
Y

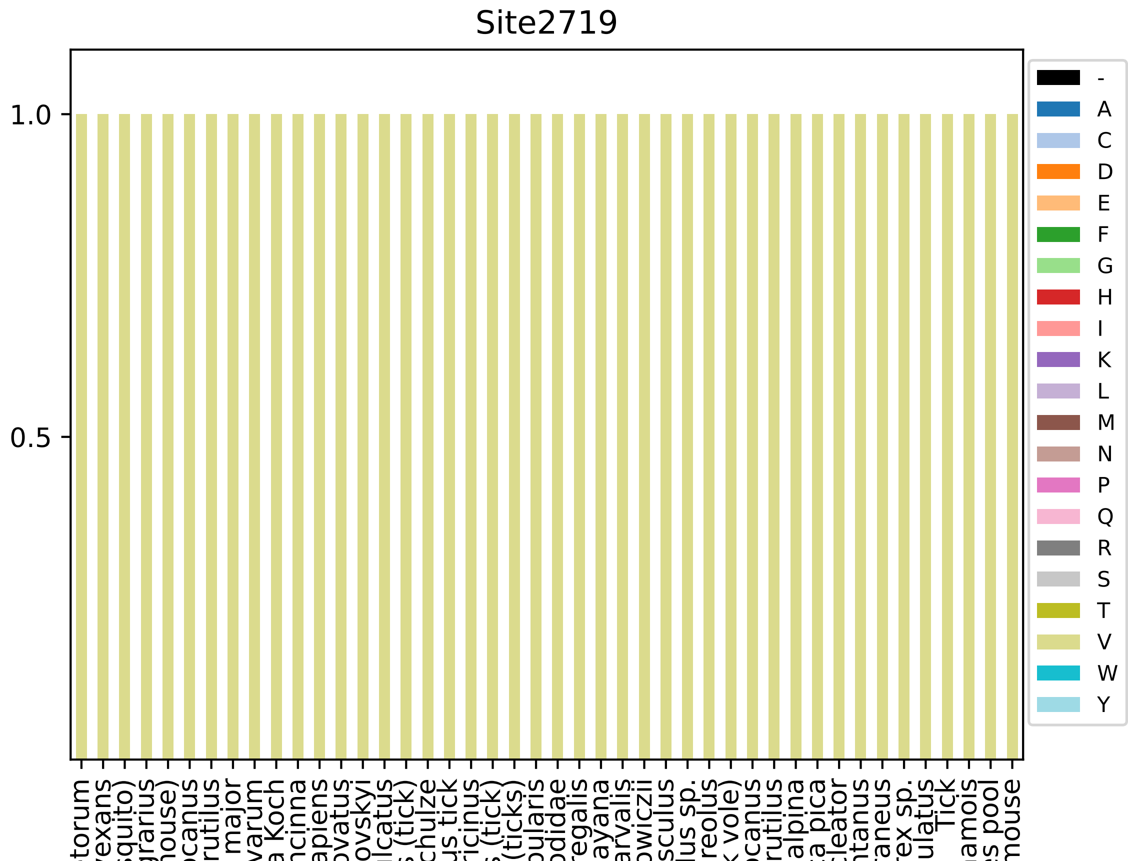
Site2719
1.0
0.5
-
A
C
D
E
F
G
H
I
K
L
M
N
P
Q
R
S
T
V
W
Y

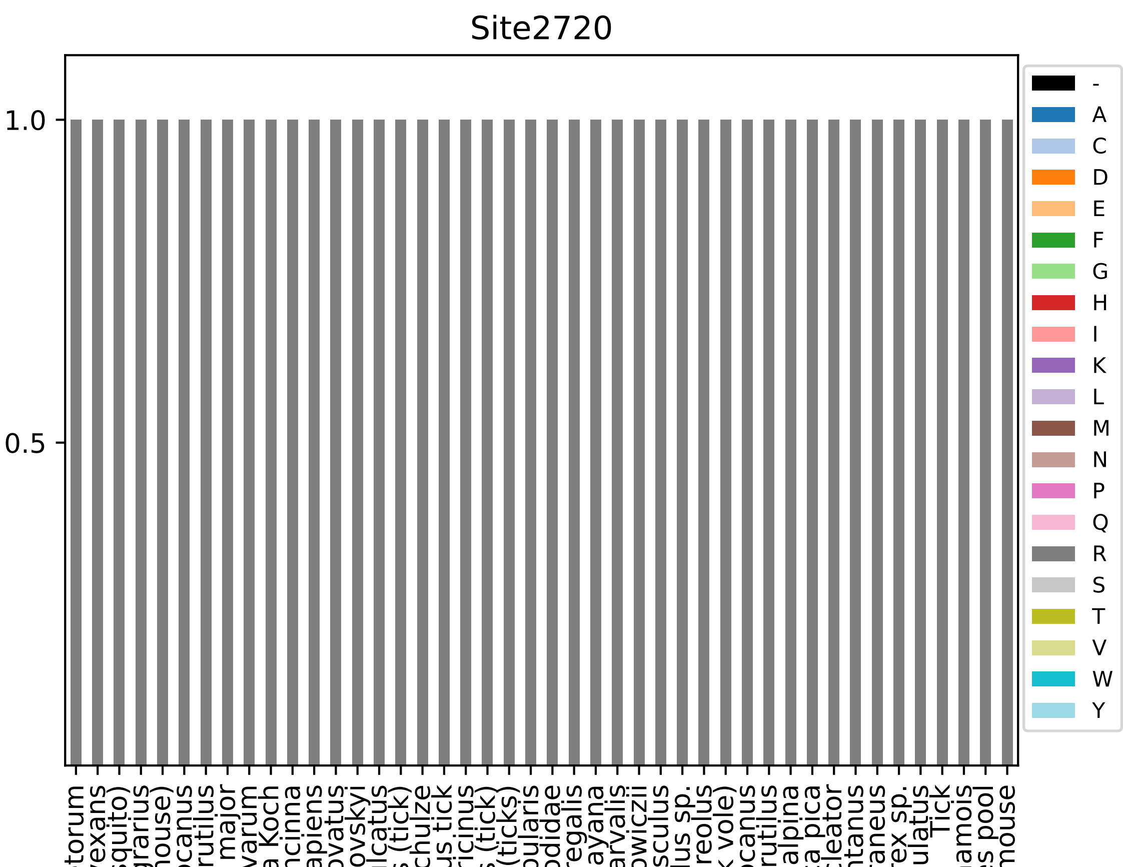
Site2720
1.0
0.5
-
A
C
D
E
F
G
H
I
K
L
M
N
P
Q
R
S
T
V
W
Y

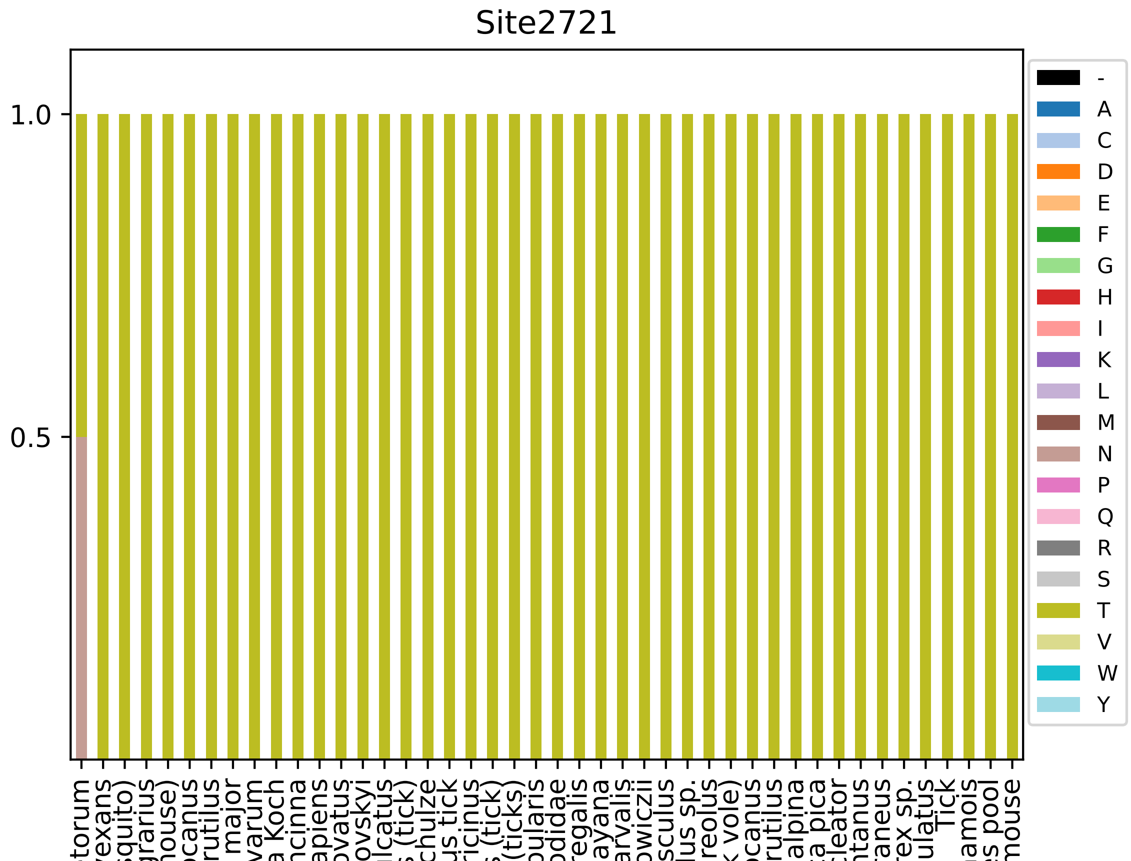

Site2721
1.0
0.5
-
A
C
D
E
F
G
H
I
K
L
M
N
P
Q
R
S
T
V
W
Y

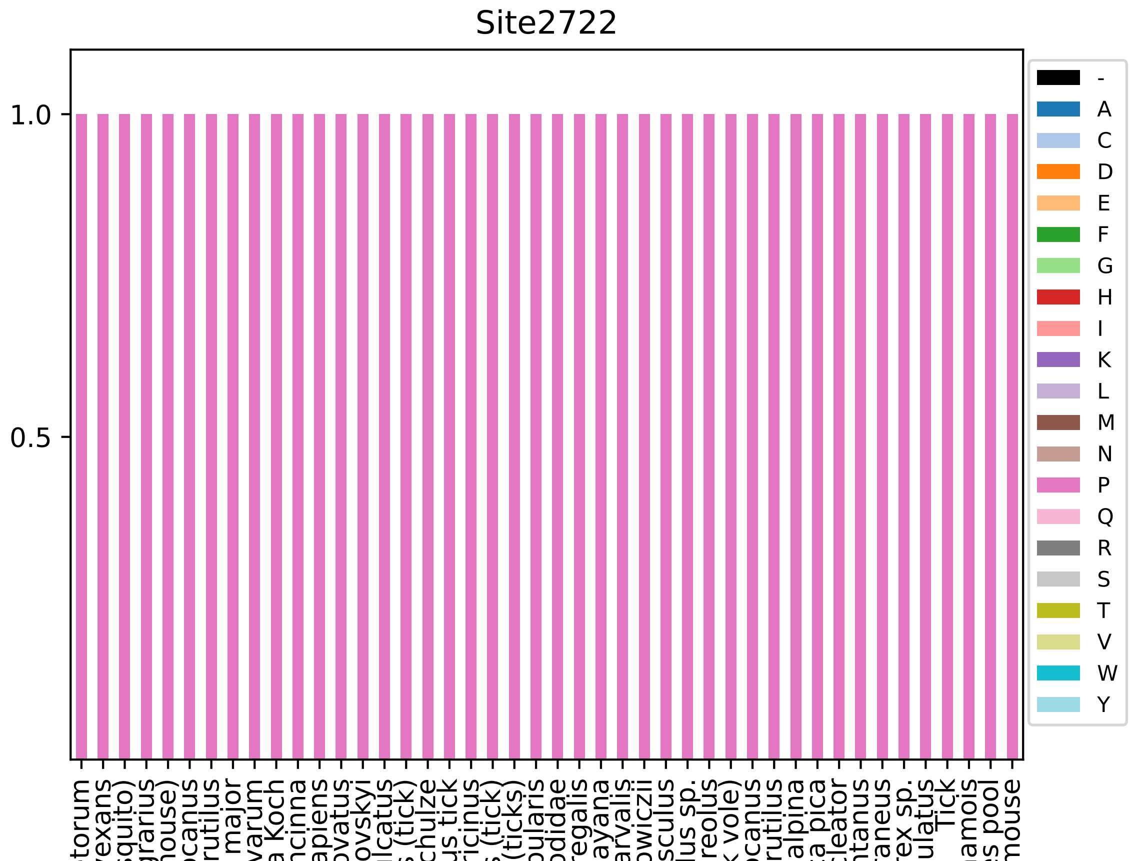
Site2722
1.0
0.5
-
A
C
D
E
F
G
H
I
K
L
M
N
P
Q
R
S
T
V
W
Y

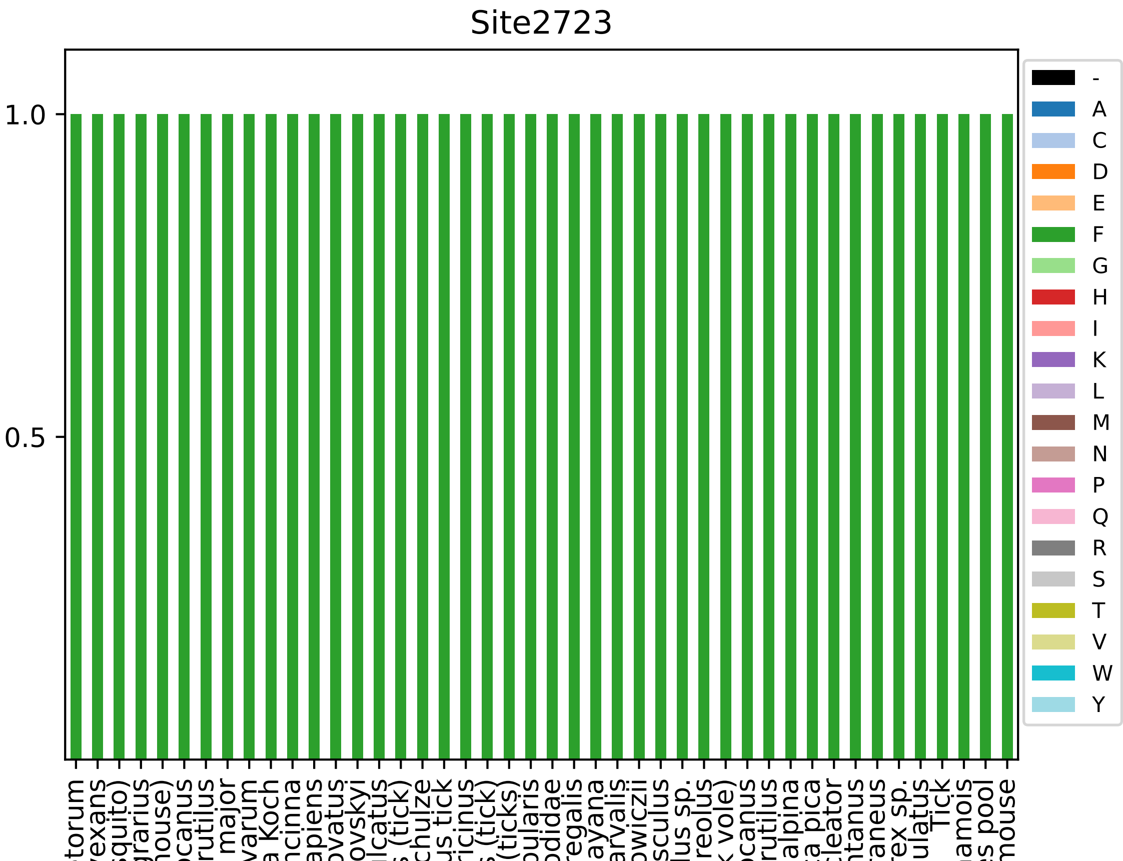

Site2723
1.0
0.5
-
A
C
D
E
F
G
H
I
K
L
M
N
P
Q
R
S
T
V
W
Y

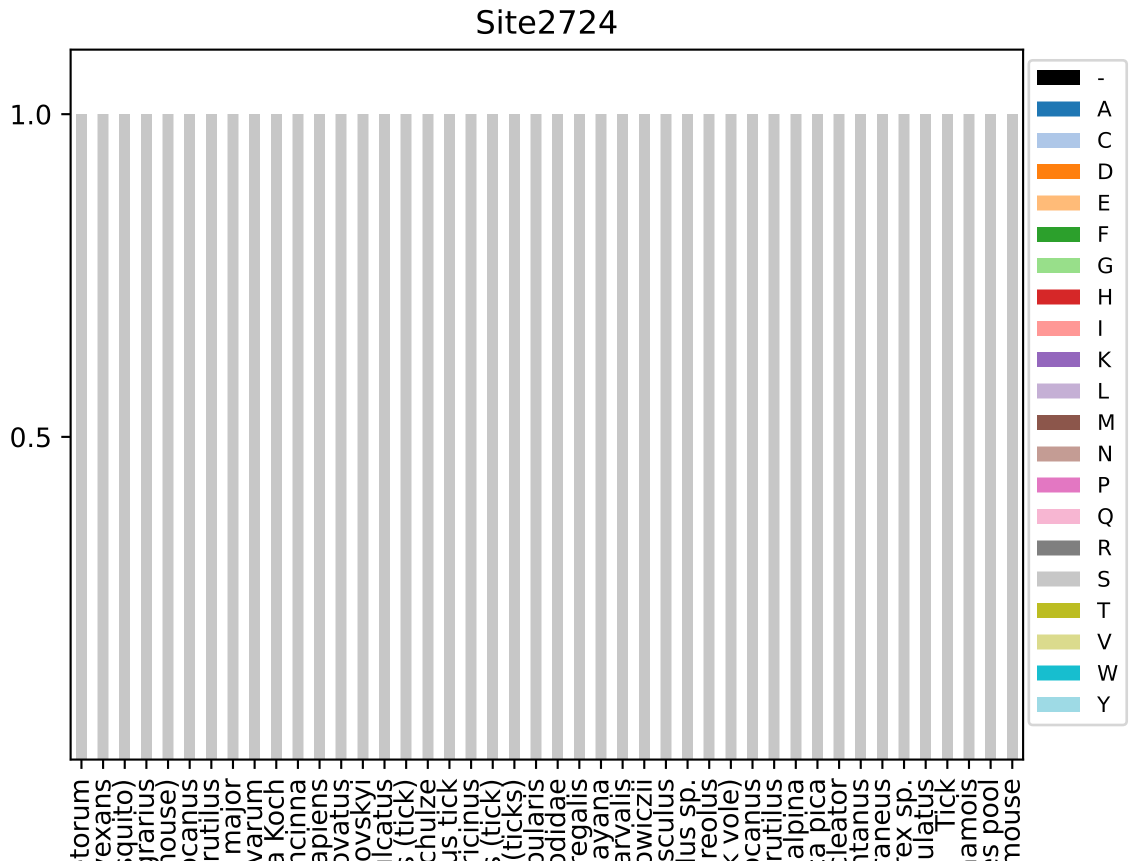

Site2724
1.0
0.5
-
A
C
D
E
F
G
H
I
K
L
M
N
P
Q
R
S
T
V
W
Y

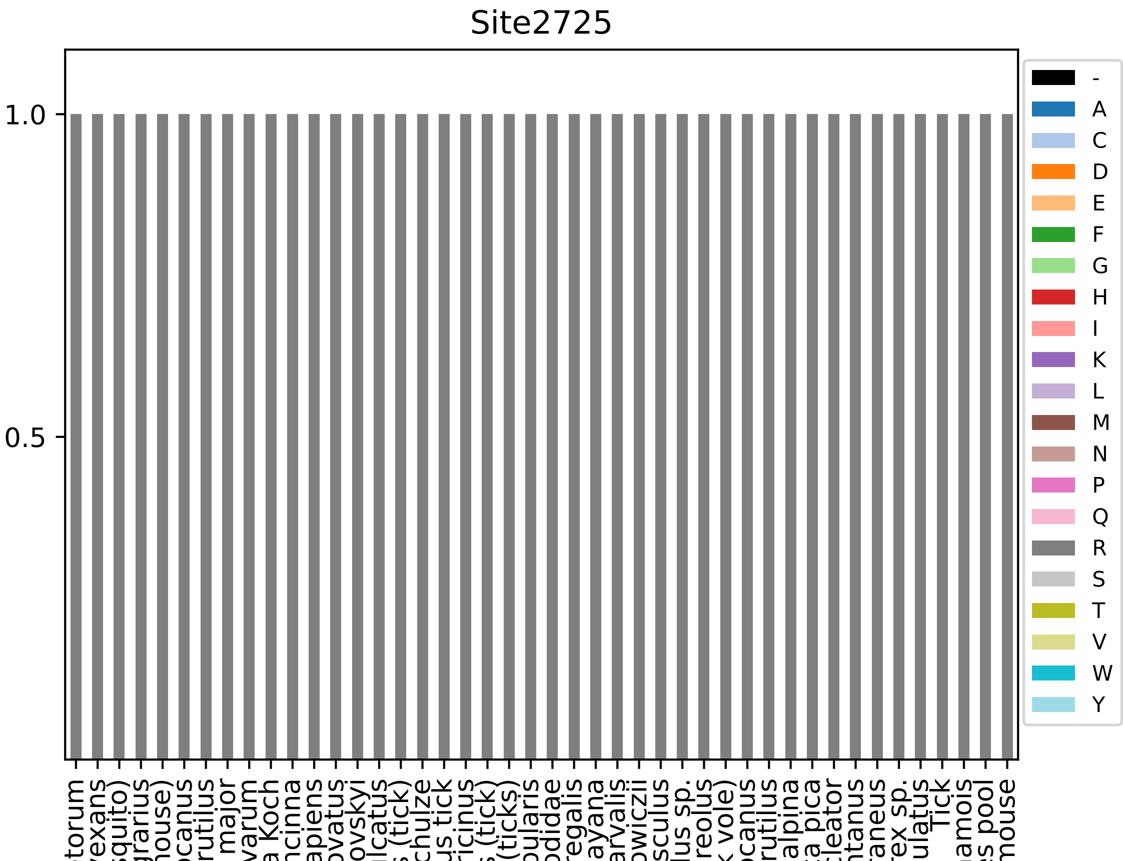
Site2725
1.0
0.5
-
A
C
D
E
F
G
H
I
K
L
M
N
P
Q
R
S
T
V
W
Y

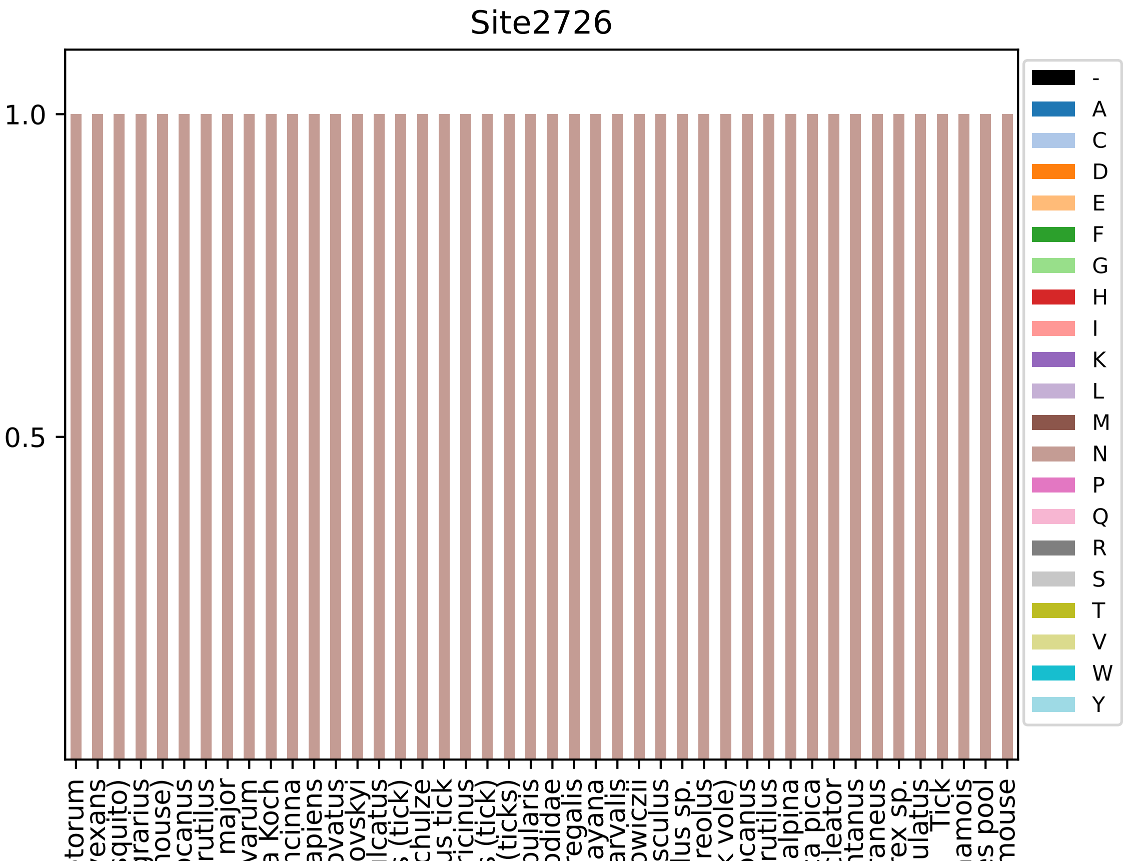
Site2726
1.0
0.5
-
A
C
D
E
F
G
H
I
K
L
M
N
P
Q
R
S
T
V
W
Y

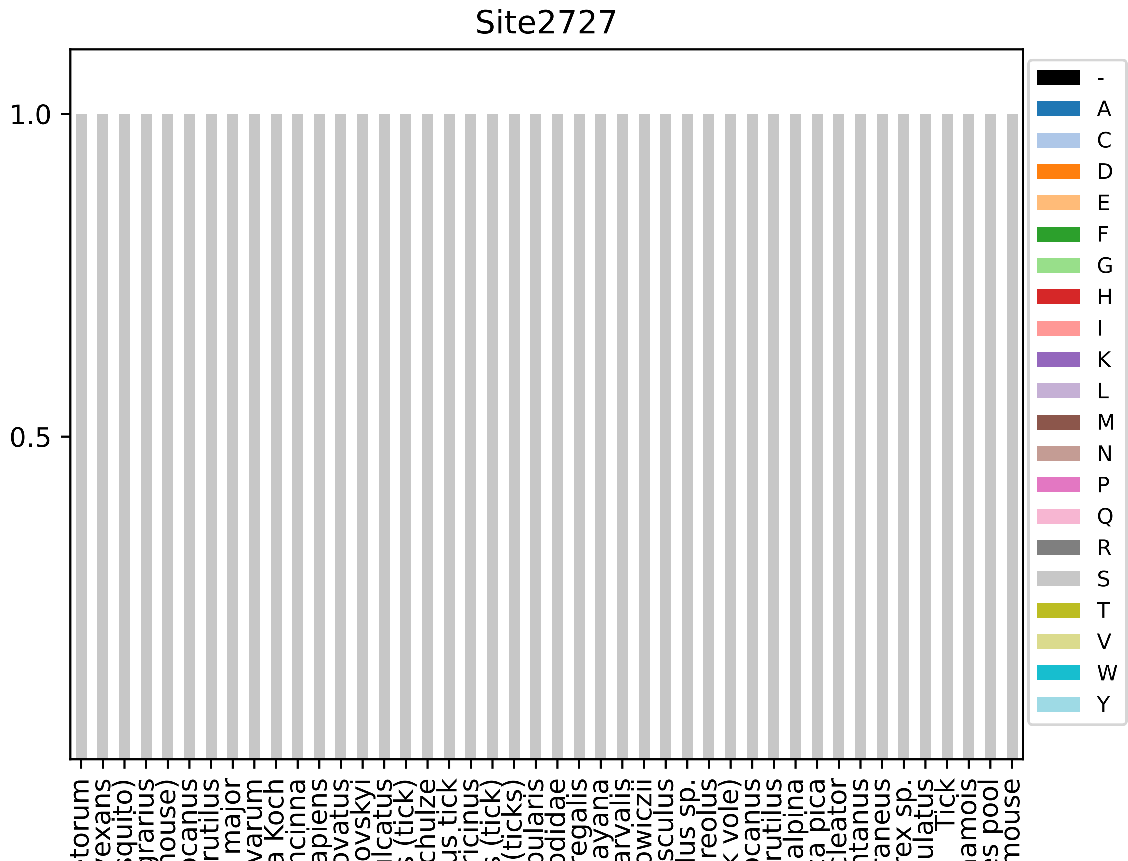

Site2727
1.0
0.5
-
A
C
D
E
F
G
H
I
K
L
M
N
P
Q
R
S
T
V
W
Y

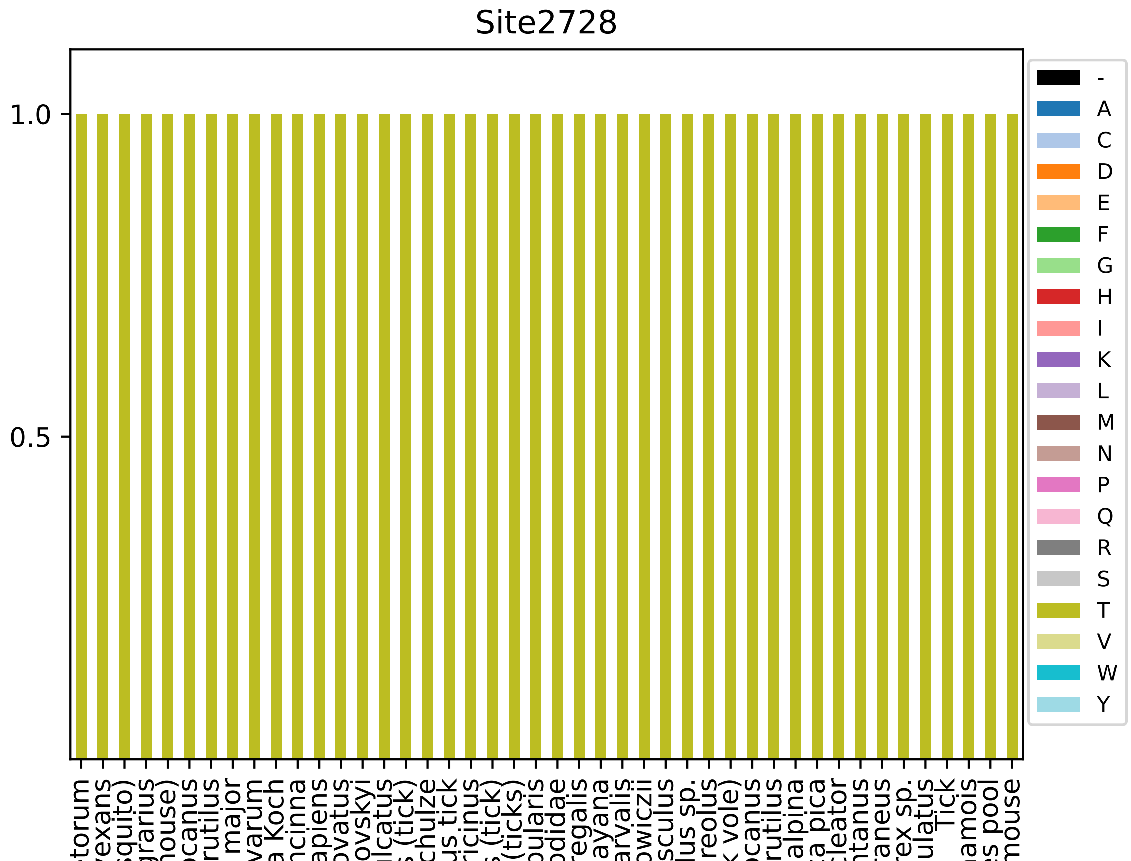

Site2728
1.0
0.5
-
A
C
D
E
F
G
H
I
K
L
M
N
P
Q
R
S
T
V
W
Y

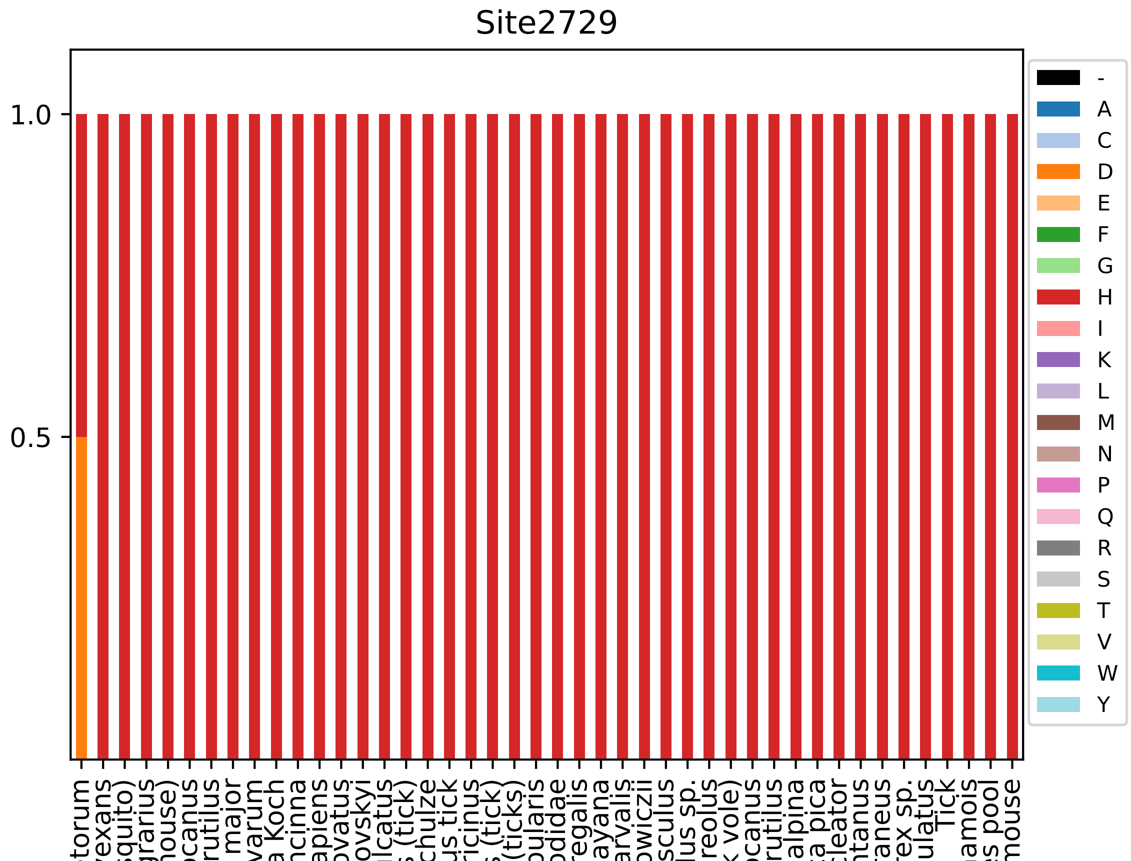
Site2729
1.0
0.5
-
A
C
D
E
F
G
H
I
K
L
M
N
P
Q
R
S
T
V
W
Y
major
Tick

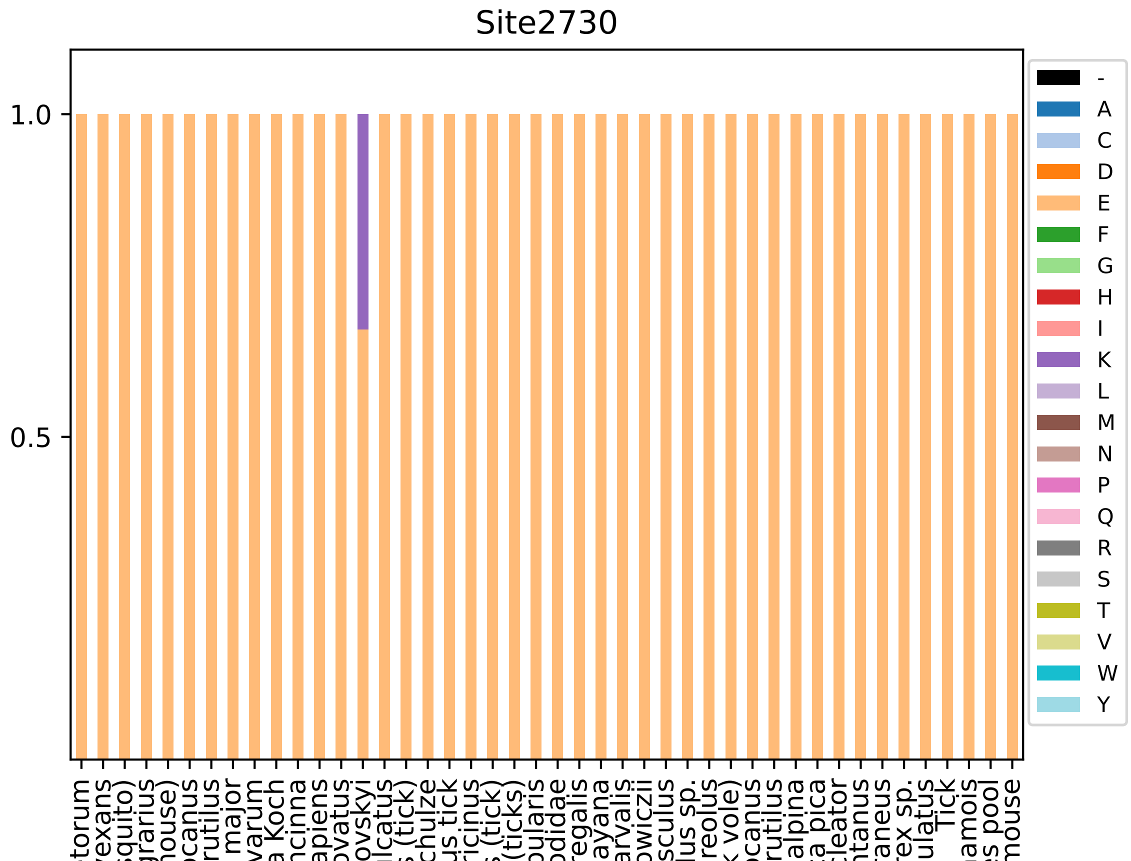
Site2730
1.0
0.5
-
A
C
D
E
F
G
H
I
K
L
M
N
P
Q
R
S
T
V
W
Y

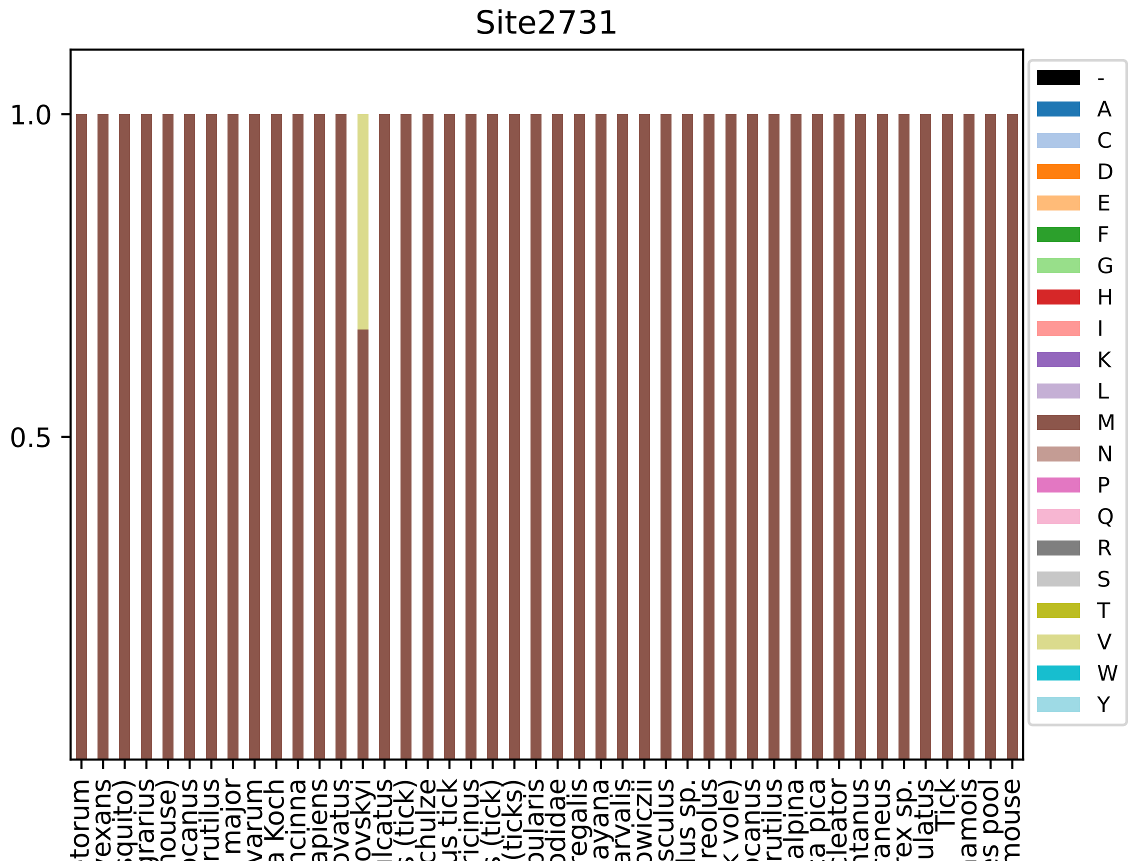
Site2731
1.0
0.5
-
A
C
D
E
F
G
H
I
K
L
M
N
P
Q
R
S
T
V
W
Y

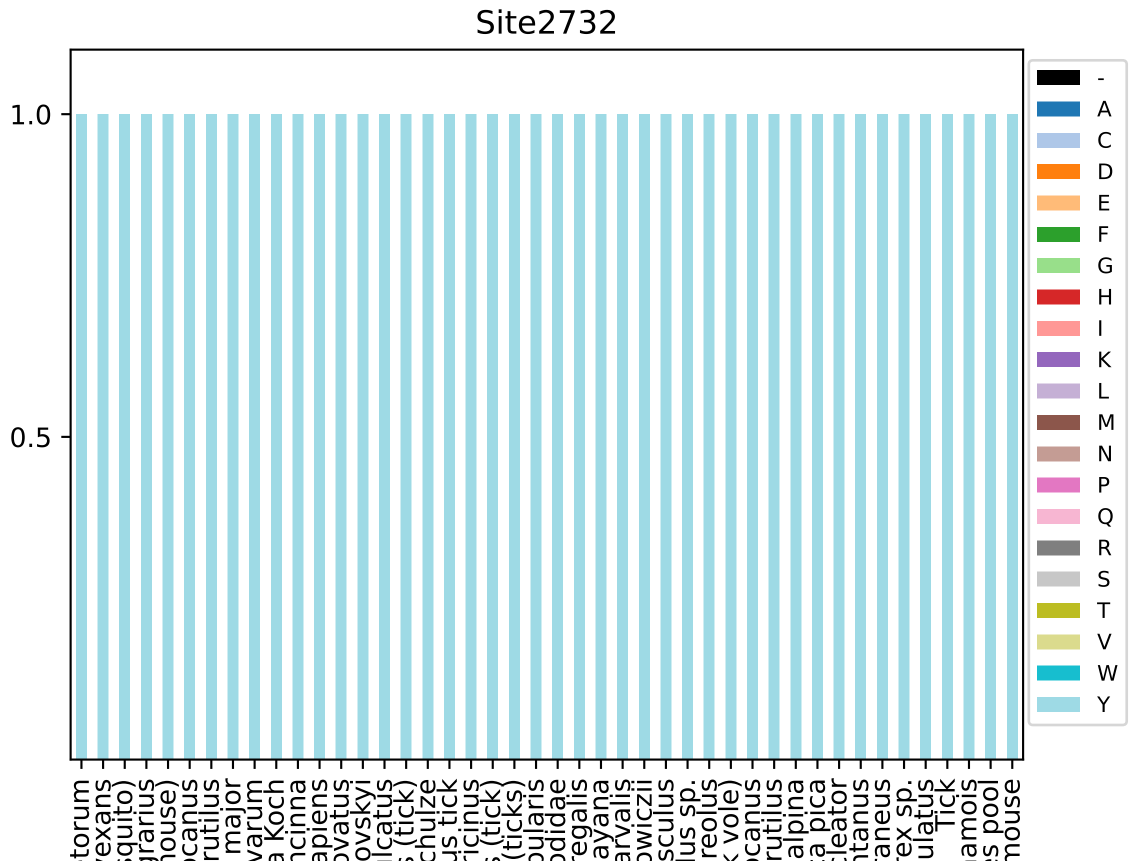
Site2732
1.0
0.5
-
A
C
D
E
F
G
H
I
K
L
M
N
P
Q
R
S
T
V
W
Y

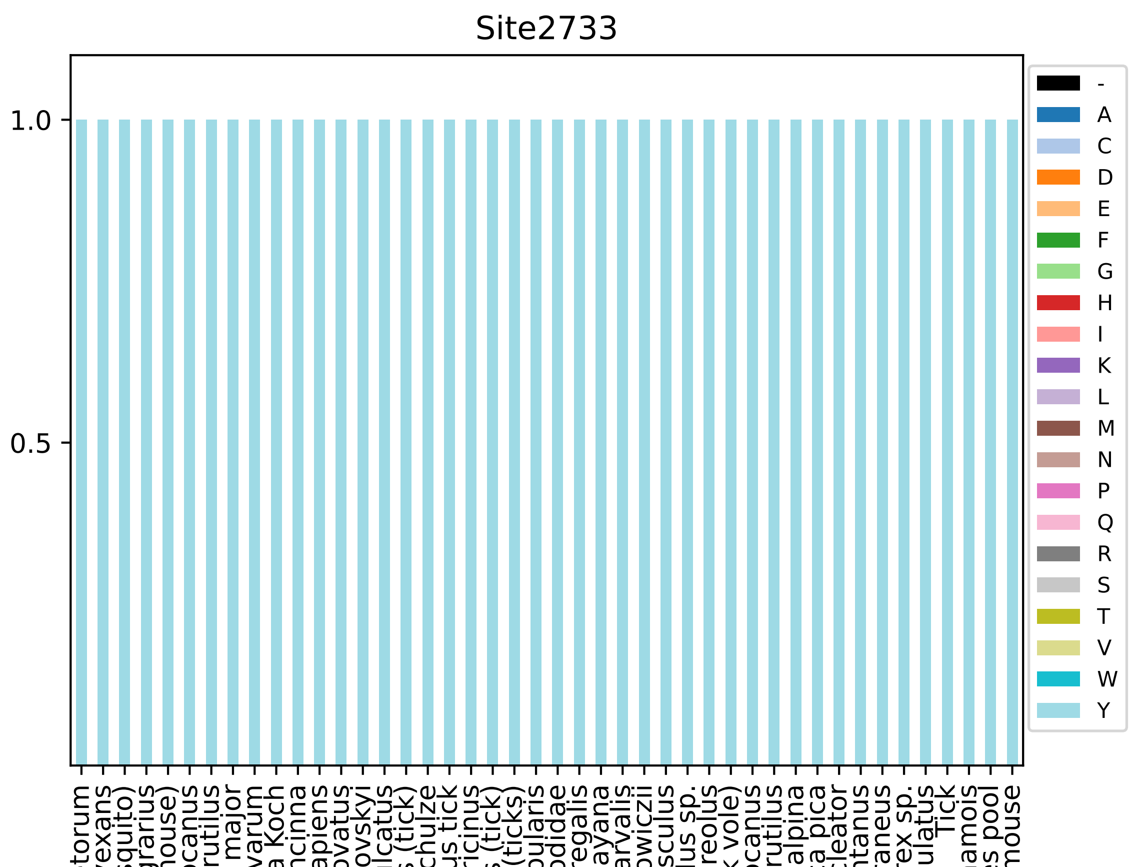
Site2733
1.0
0.5
-
A
C
D
E
F
G
H
I
K
L
M
N
P
Q
R
S
T
V
W
Y

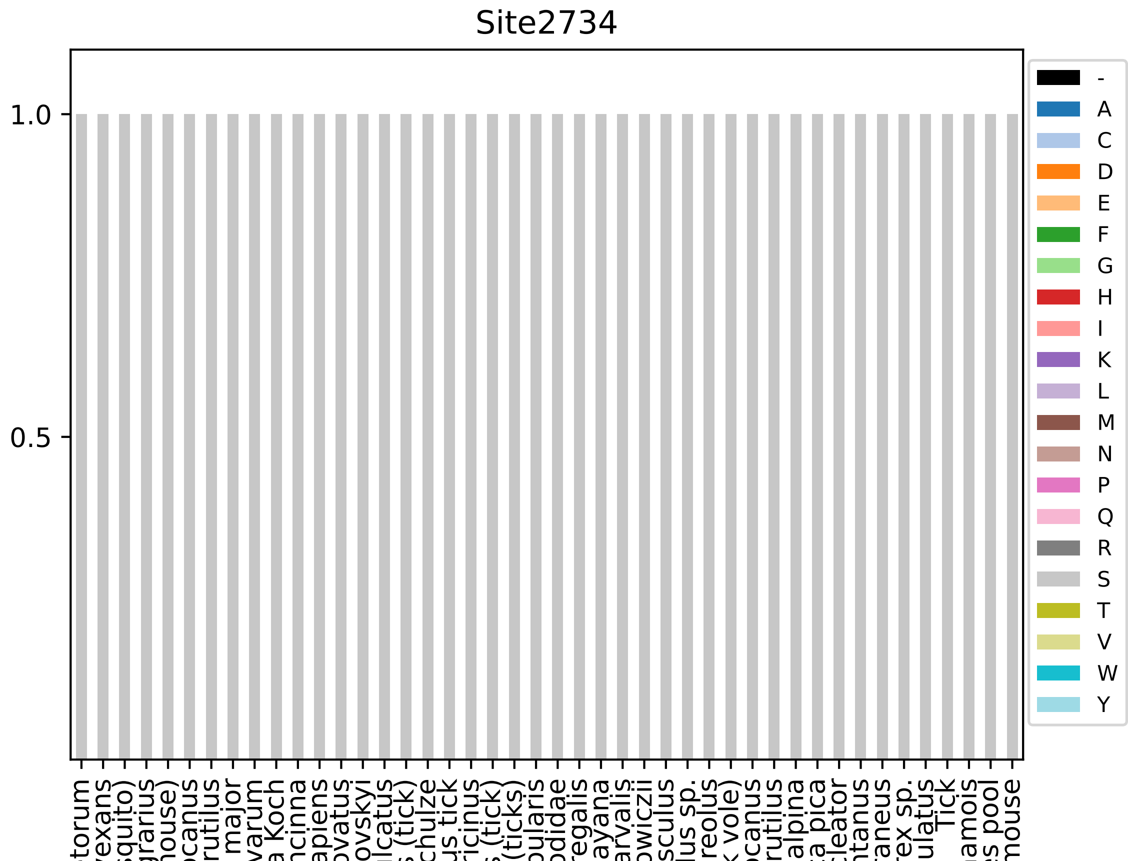

Site2734
1.0
0.5
-
A
C
D
E
F
G
H
I
K
L
M
N
P
Q
R
S
T
V
W
Y
Tick

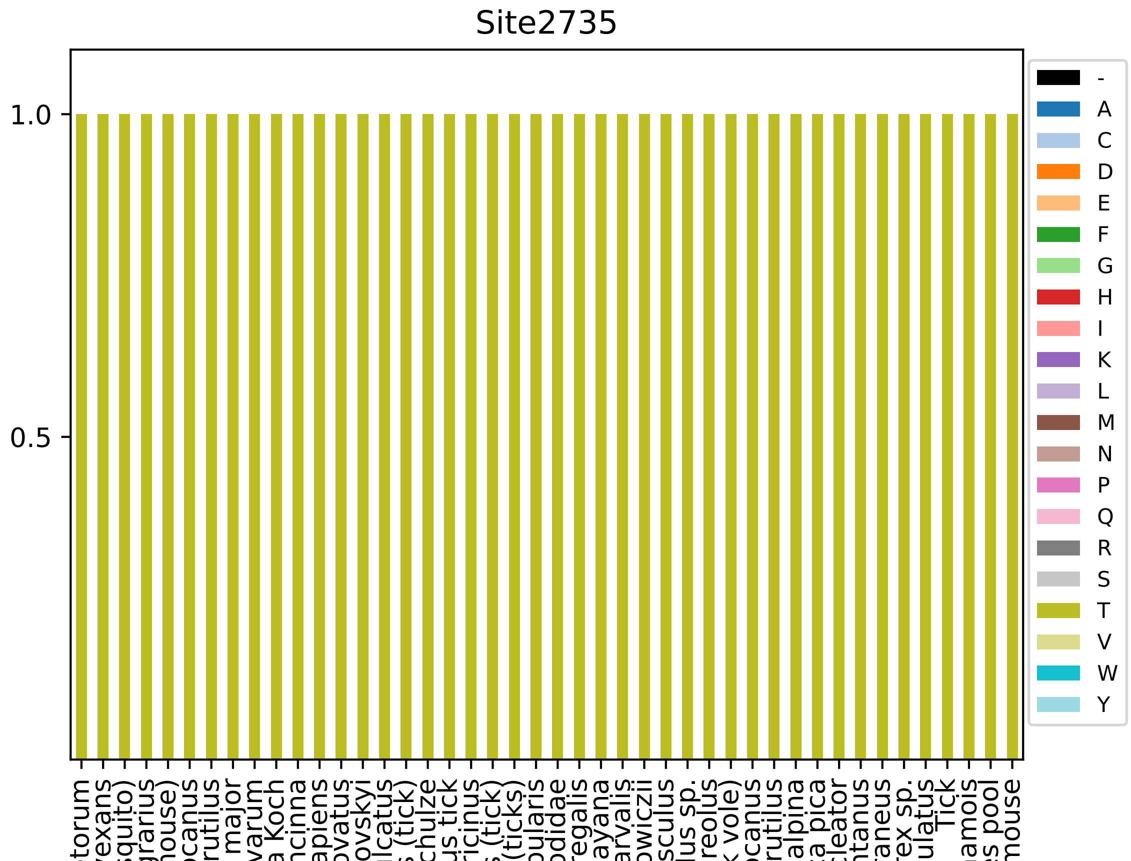
Site2735
1.0
0.5
-
A
C
D
E
F
G
H
I
K
L
M
N
P
Q
R
S
T
V
W
Y

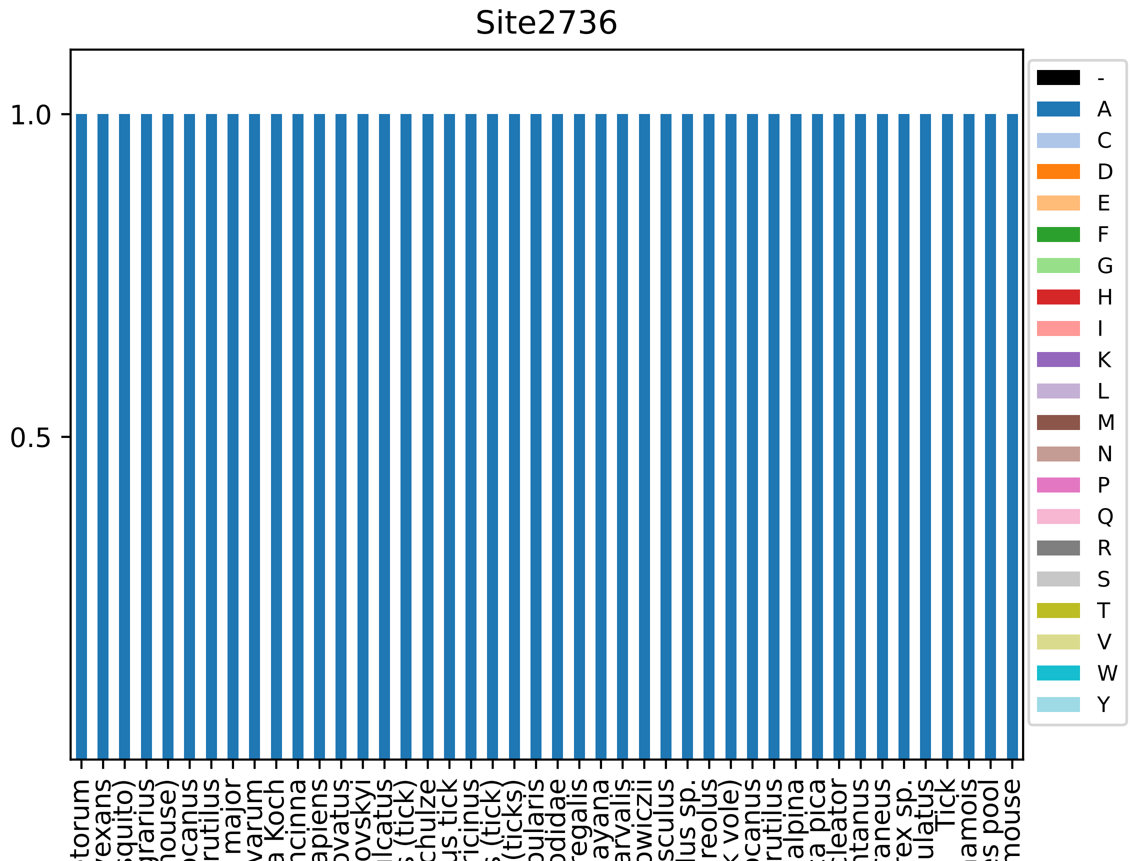

Site2736
1.0
0.5
-
A
C
D
E
F
G
H
I
K
L
M
N
P
Q
R
S
T
V
W
Y

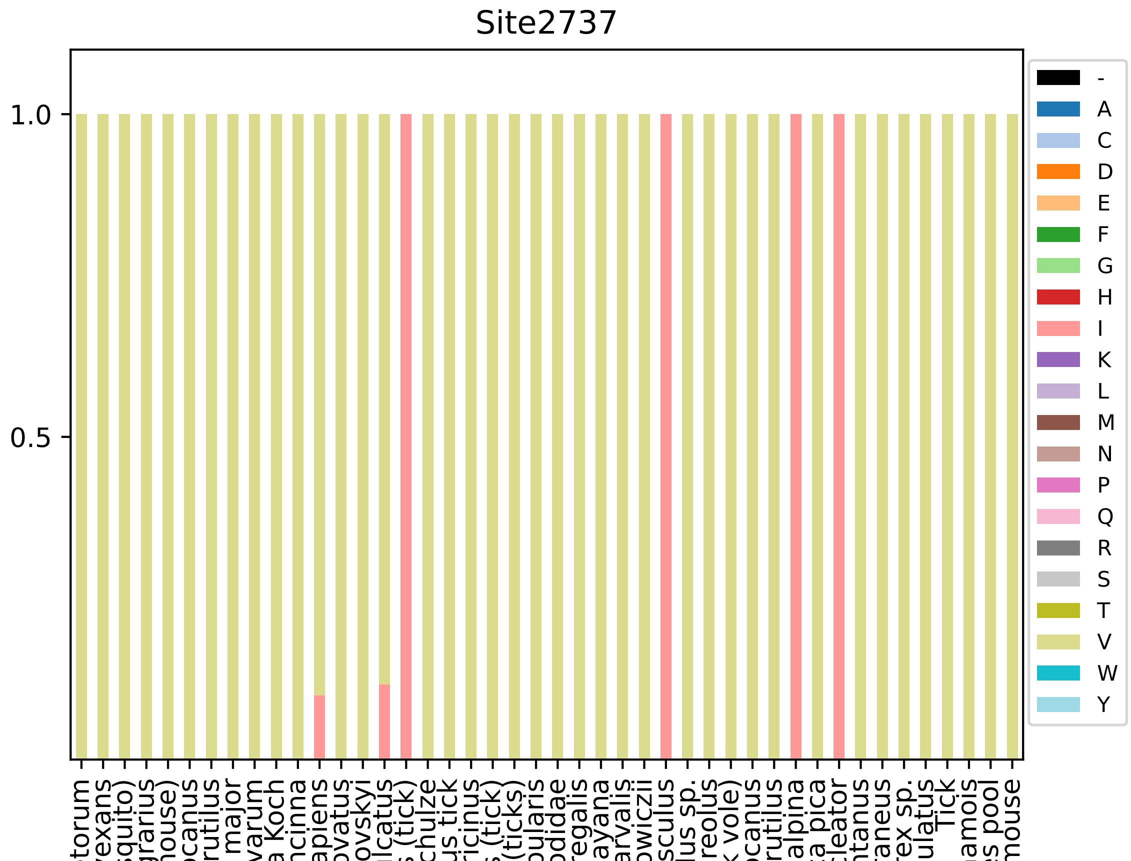
Site2737
1.0
0.5
-
A
C
D
E
F
G
H
I
K
L
M
N
P
Q
R
S
T
V
W
Y

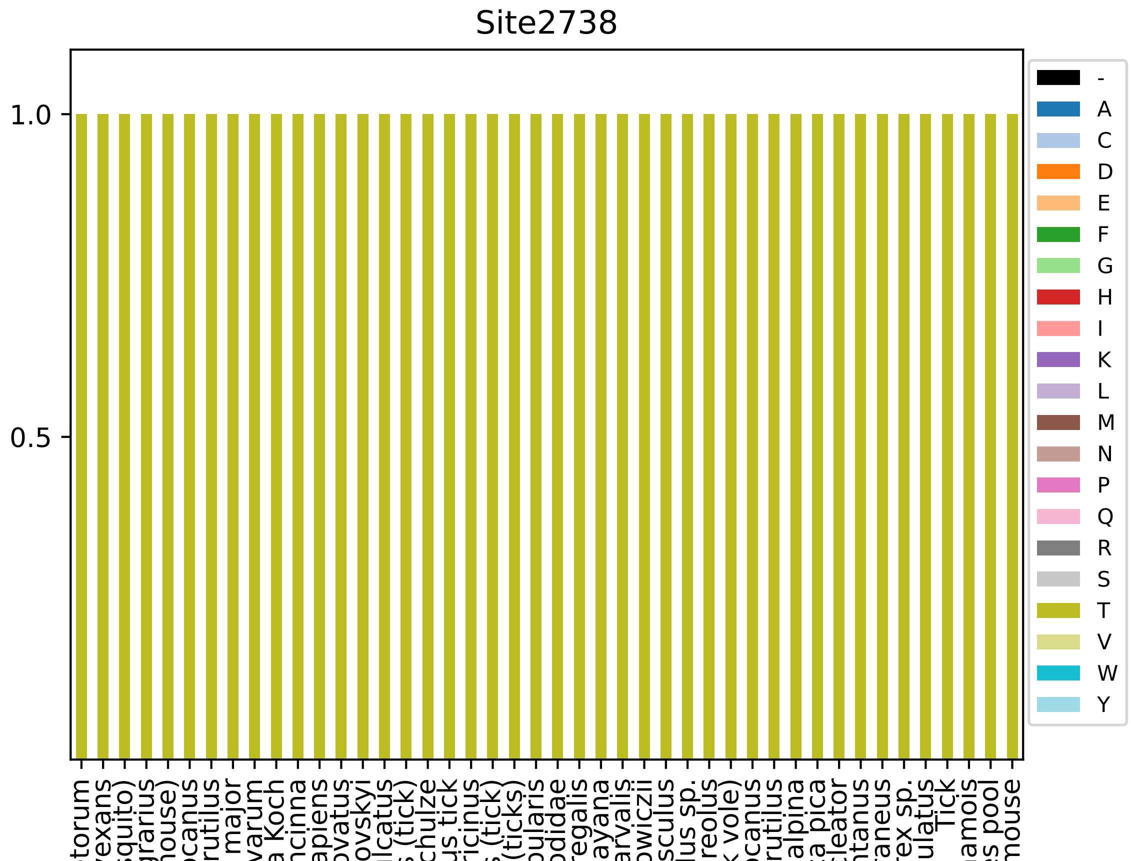
Site2738
1.0
0.5
-
A
C
D
E
F
G
H
I
K
L
M
N
P
Q
R
S
T
V
W
Y

Site2739

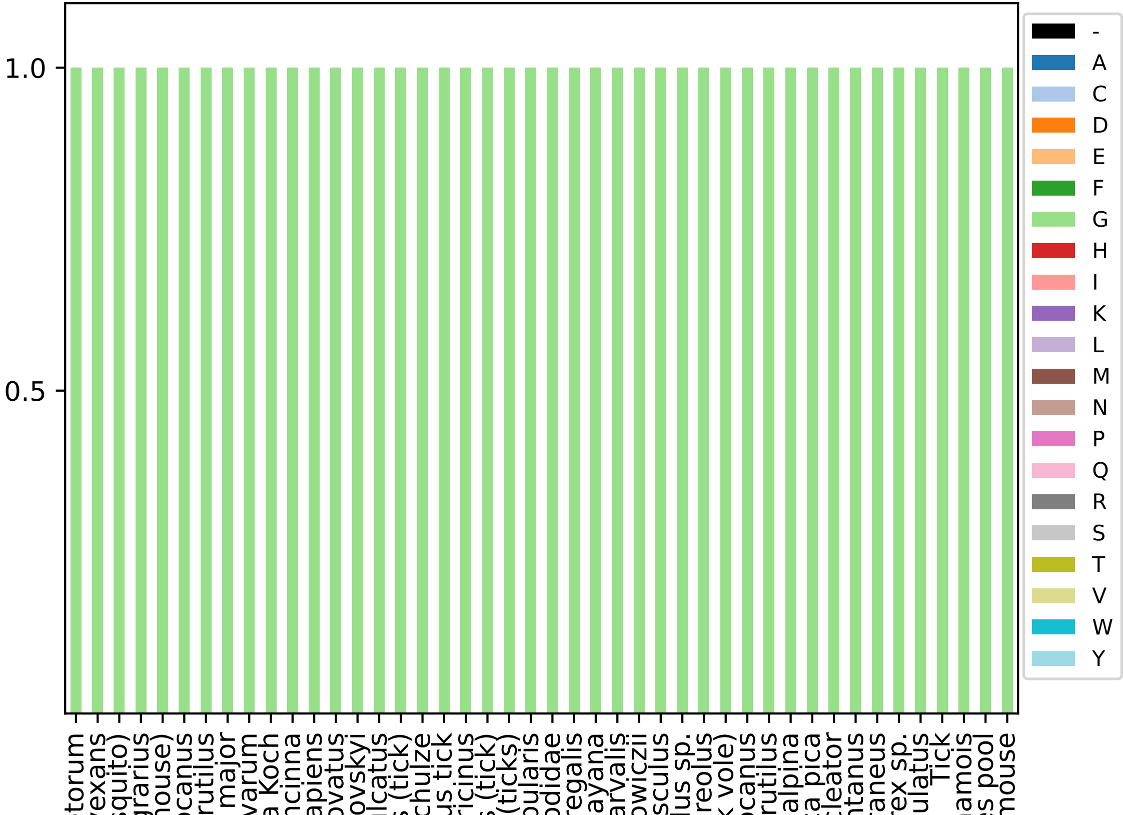

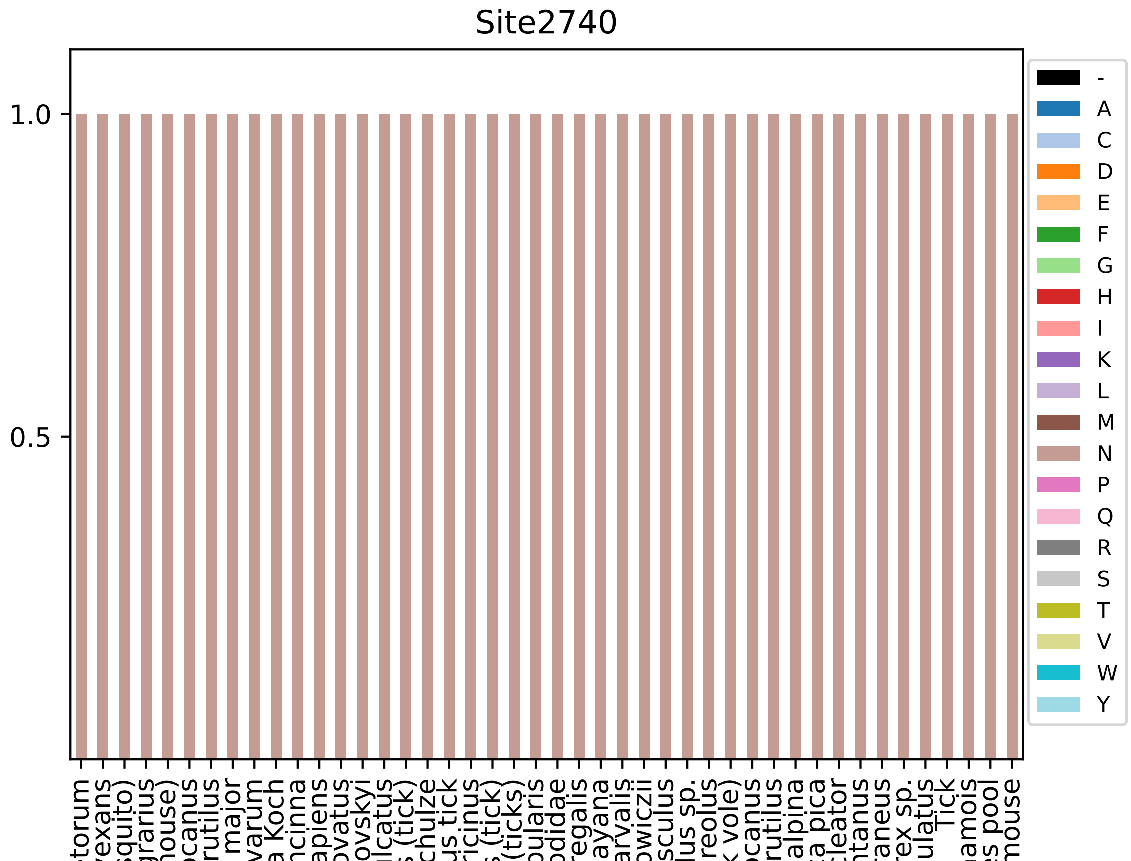

Site2740
1.0
0.5
-
A
C
D
E
F
G
H
I
K
L
M
N
P
Q
R
S
T
V
W
Y

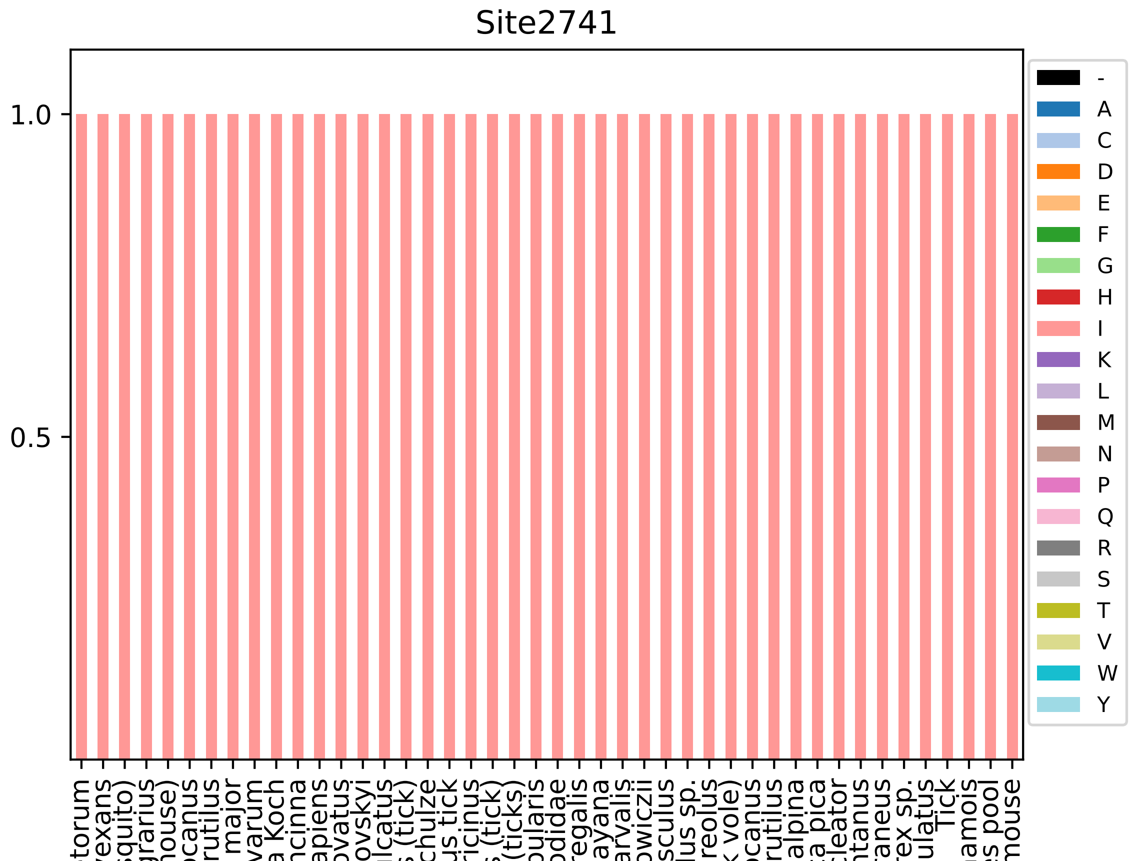

Site2741
1.0
0.5
-
A
C
D
E
F
G
H
I
K
L
M
N
P
Q
R
S
T
V
W
Y

Site2742

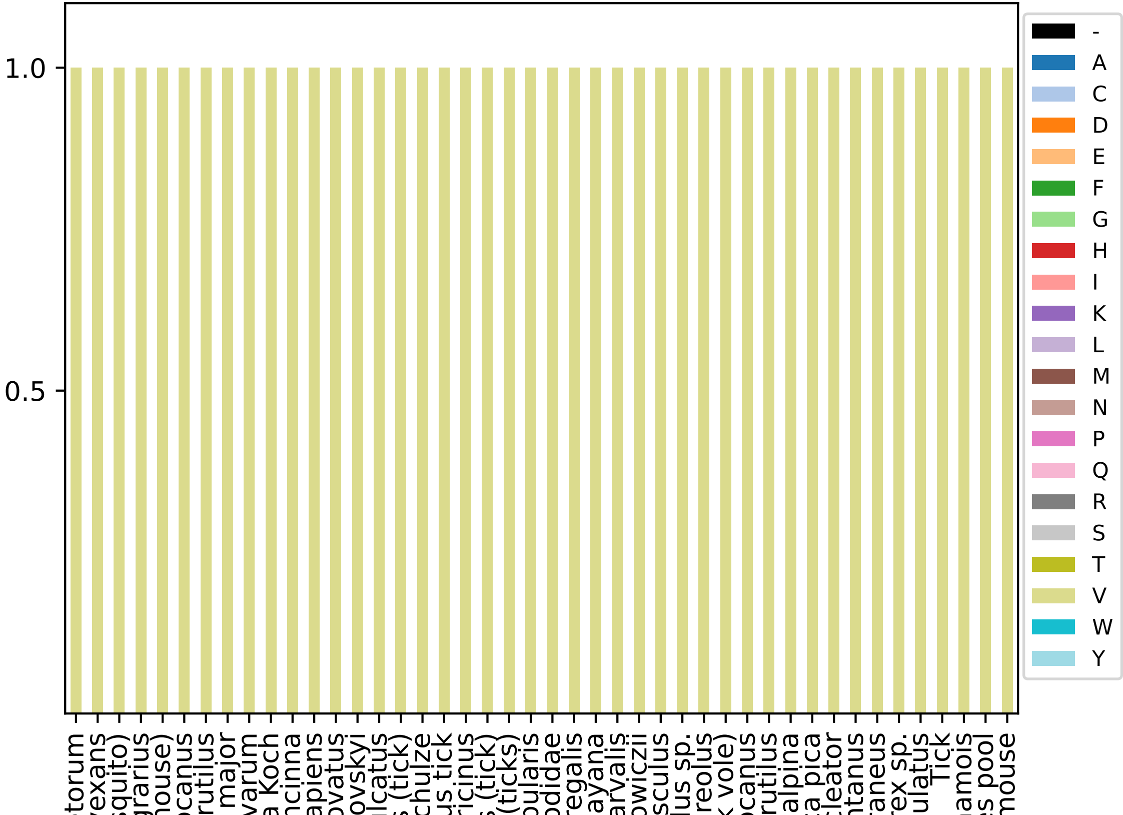

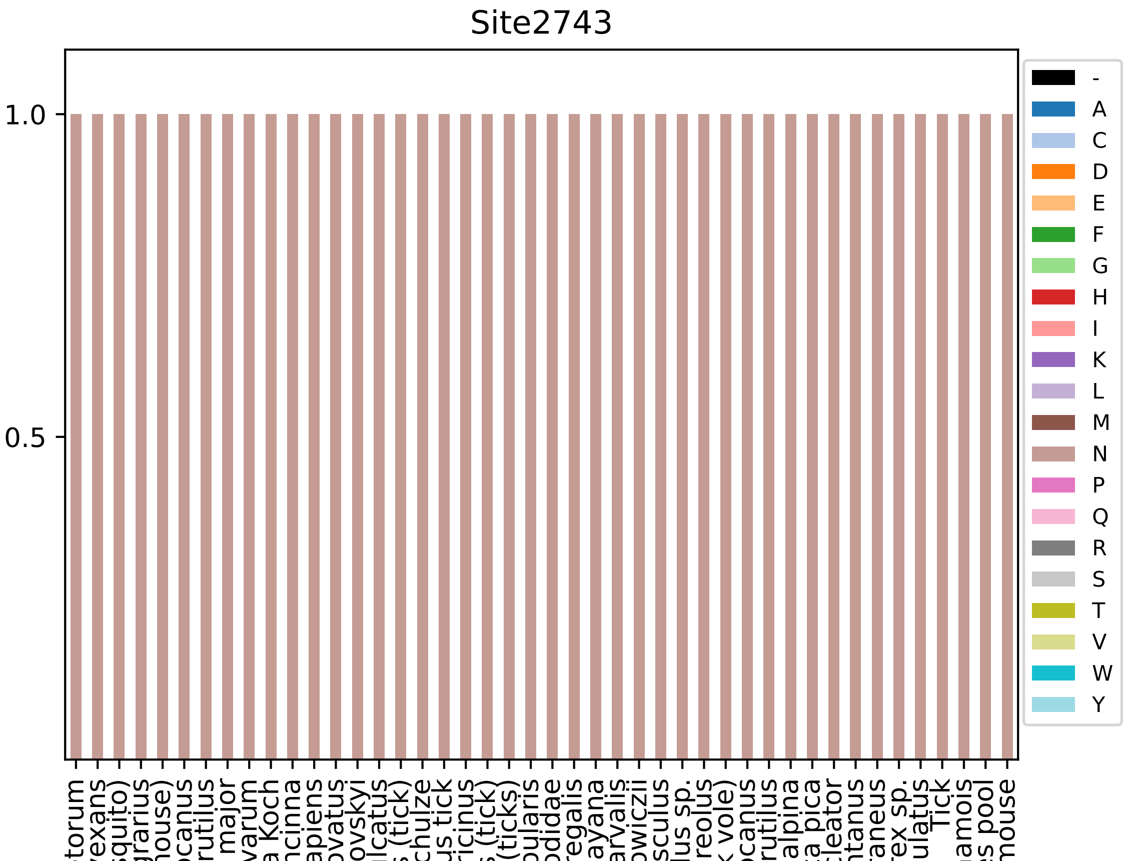

Site2743
1.0
0.5
-
A
C
D
E
F
G
H
I
K
L
M
N
P
Q
R
S
T
V
W
Y

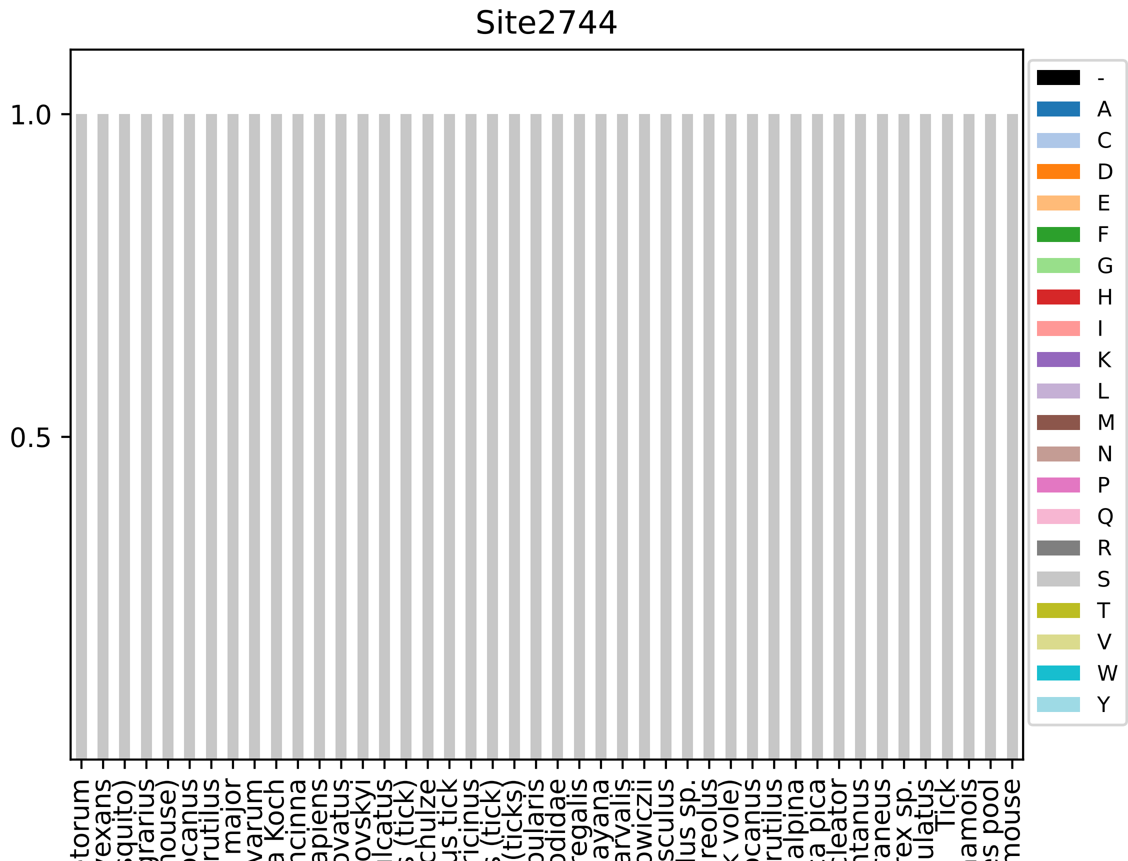
Site2744
1.0
0.5
-
A
C
D
E
F
G
H
I
K
L
M
N
P
Q
R
S
T
V
W
Y

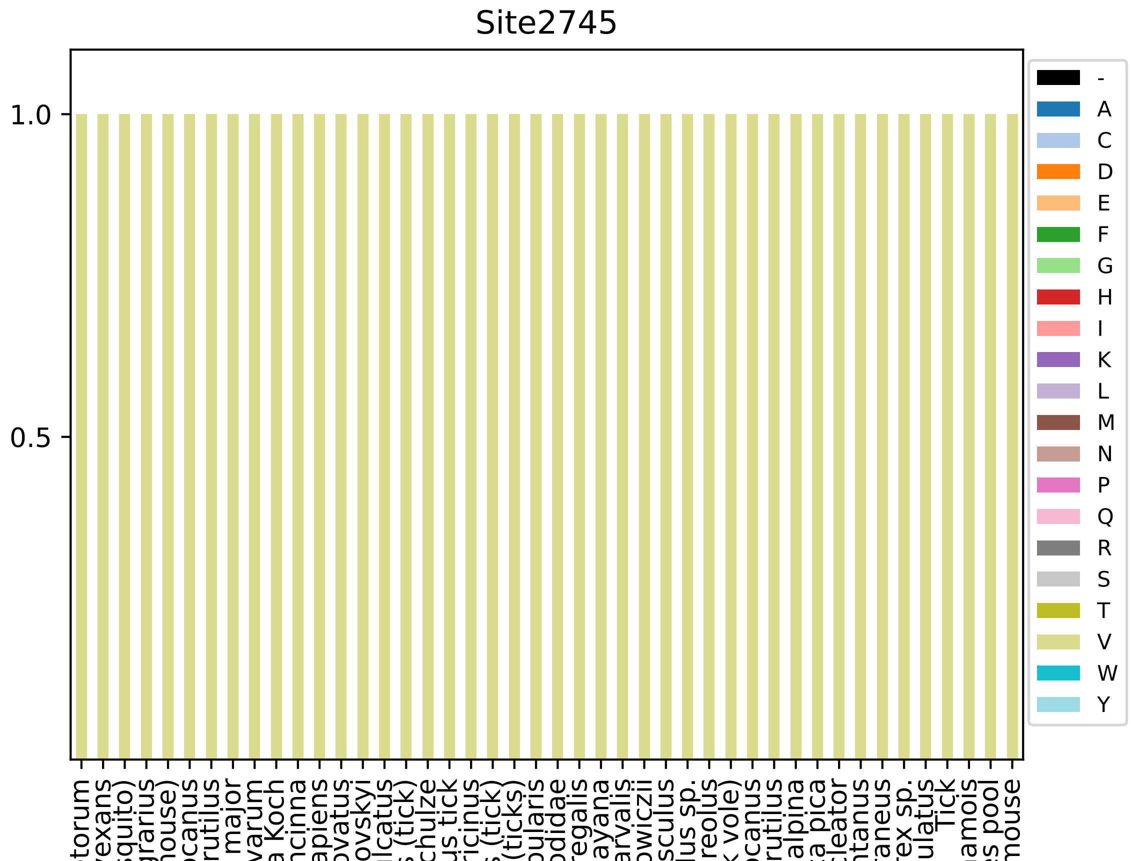
Site2745
1.0
0.5
-
A
C
D
E
F
G
H
I
K
L
M
N
P
Q
R
S
T
V
W
Y

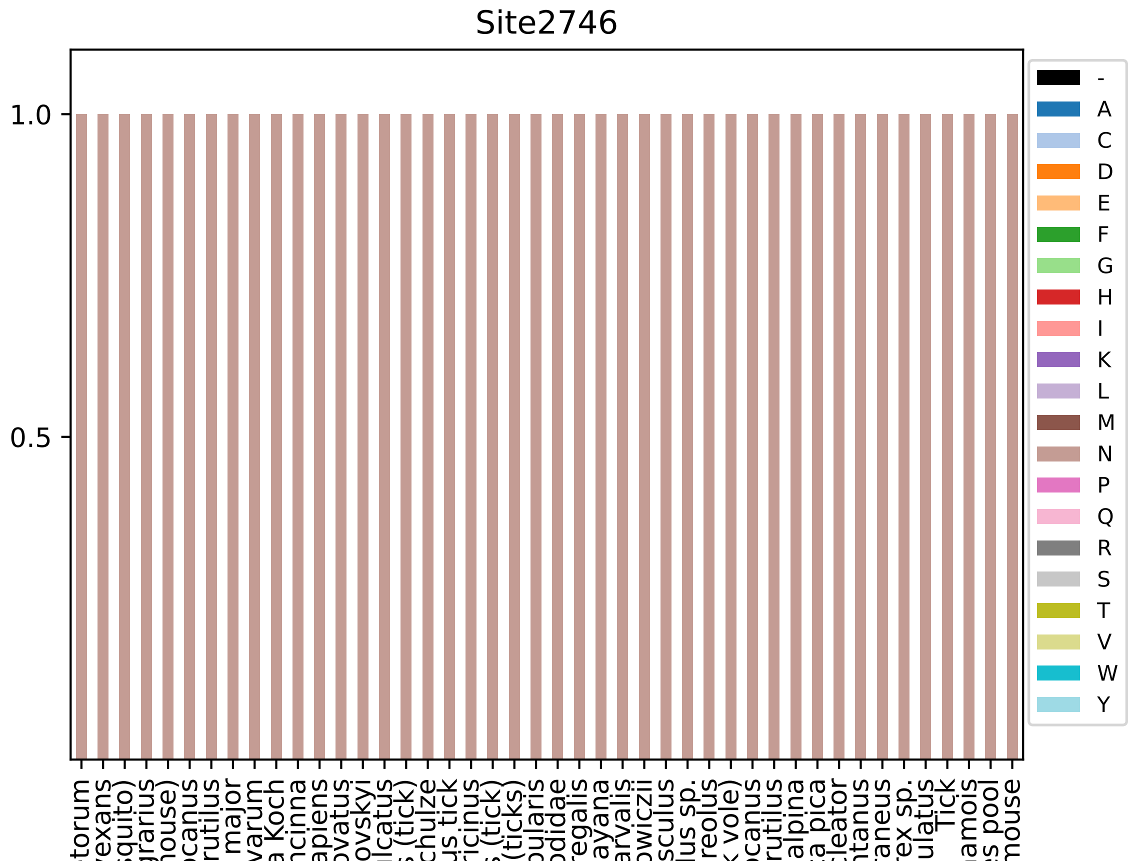
Site2746
1.0
0.5
-
A
C
D
E
F
G
H
I
K
L
M
N
P
Q
R
S
T
V
W
Y

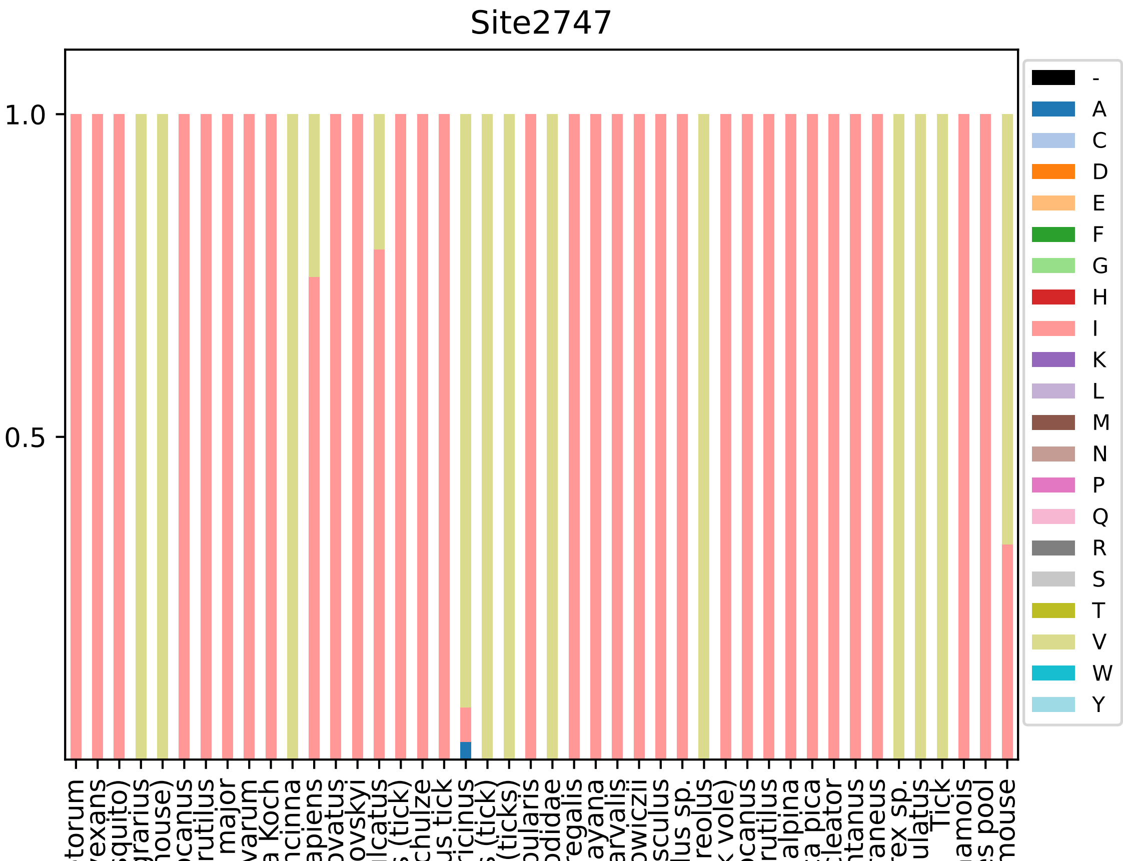
Site2747
1.0
0.5
-
A
C
D
E
F
G
H
I
K
L
M
N
P
Q
R
S
T
V
W
Y
Tick

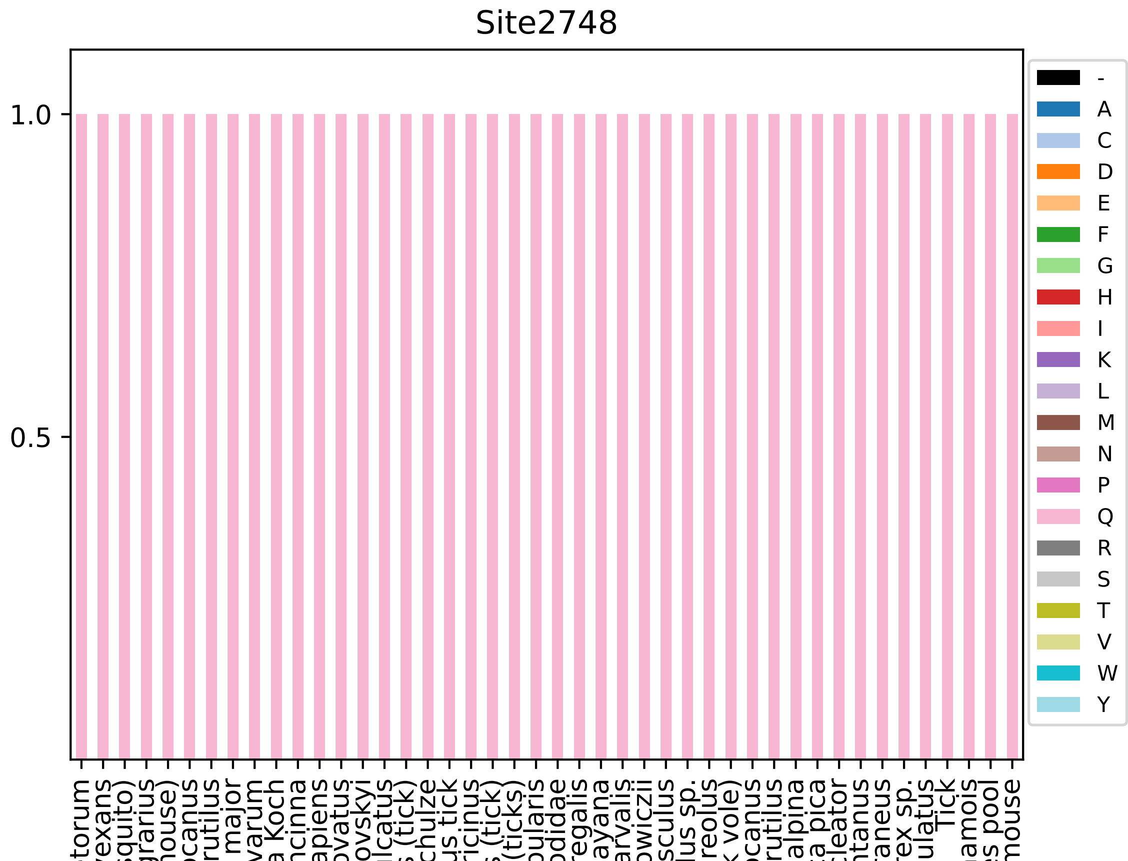
Site2748
1.0
0.5
-
A
C
D
E
F
G
H
I
K
L
M
N
P
Q
R
S
T
V
W
Y

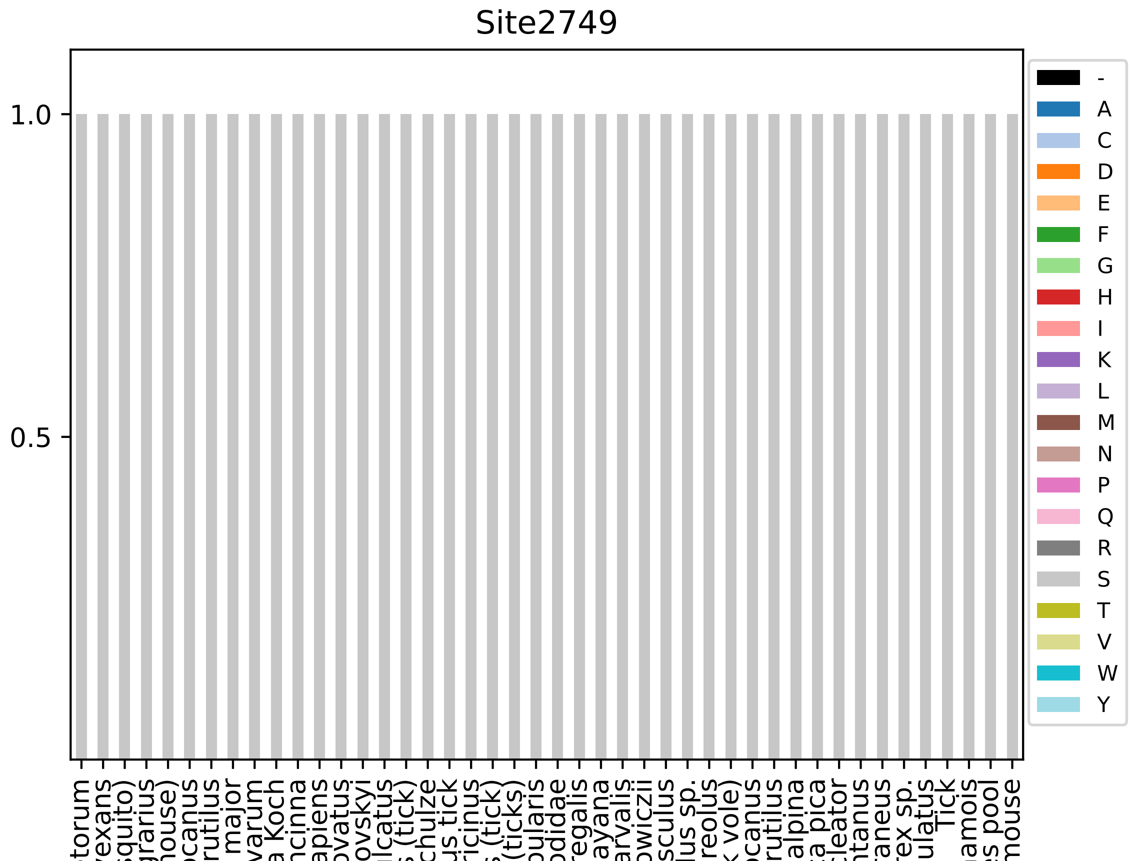
Site2749
1.0
0.5
-
A
C
D
E
F
G
H
I
K
L
M
N
P
Q
R
S
T
V
W
Y

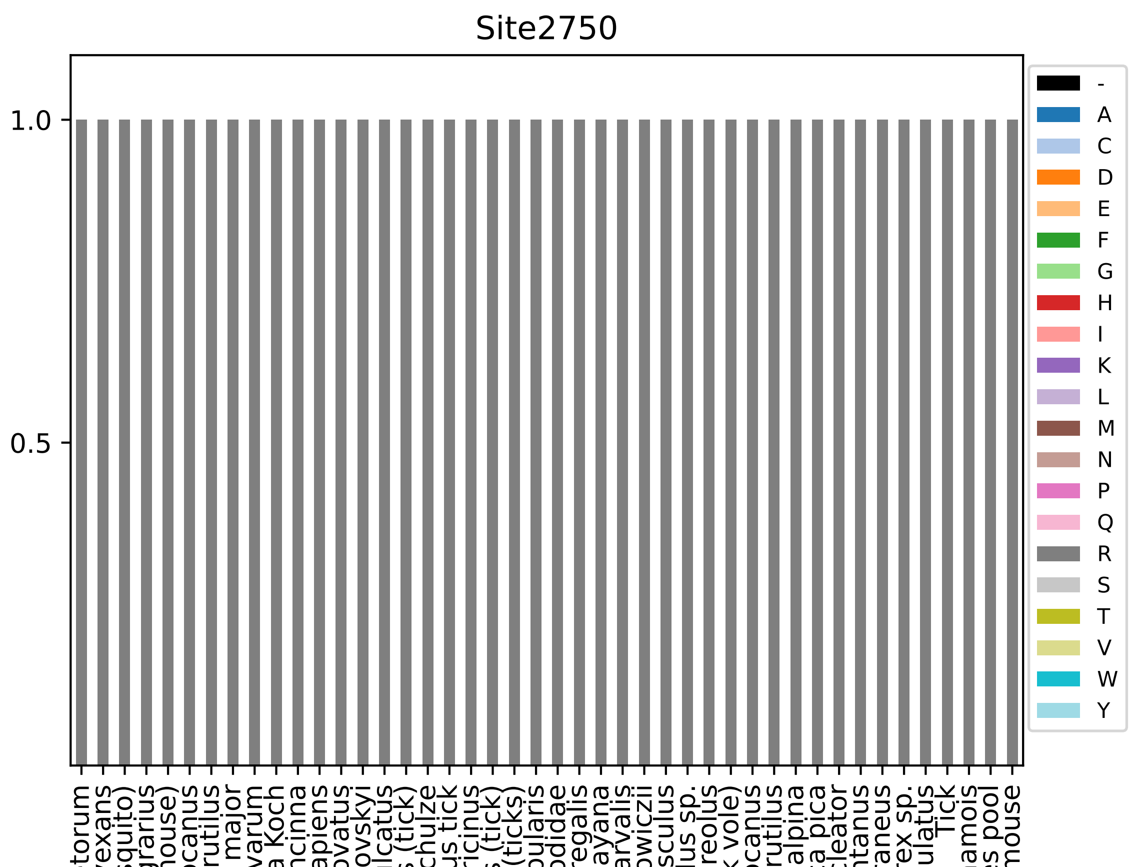
Site2750
1.0
0.5
-
A
C
D
E
F
G
H
I
K
L
M
N
P
Q
R
S
T
V
W
Y

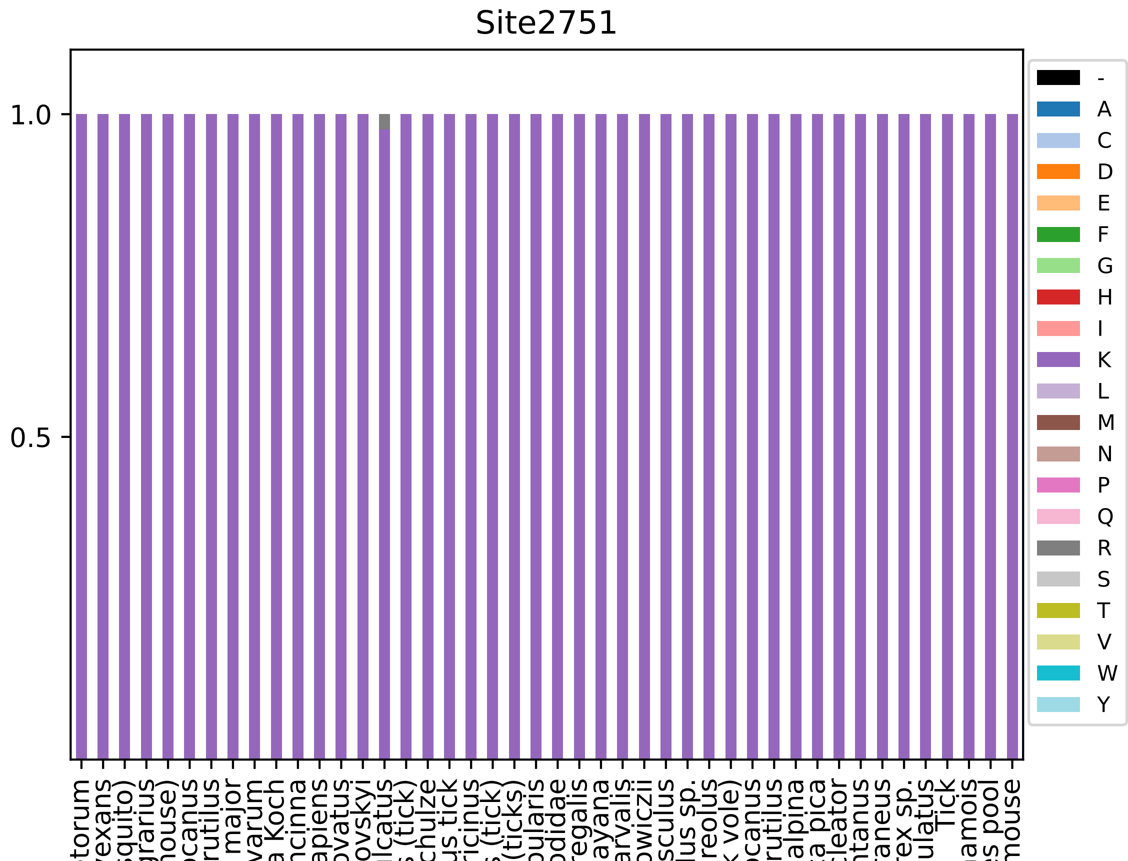
Site2751
1.0
0.5
-
A
C
D
E
F
G
H
I
K
L
M
N
P
Q
R
S
T
V
W
Y

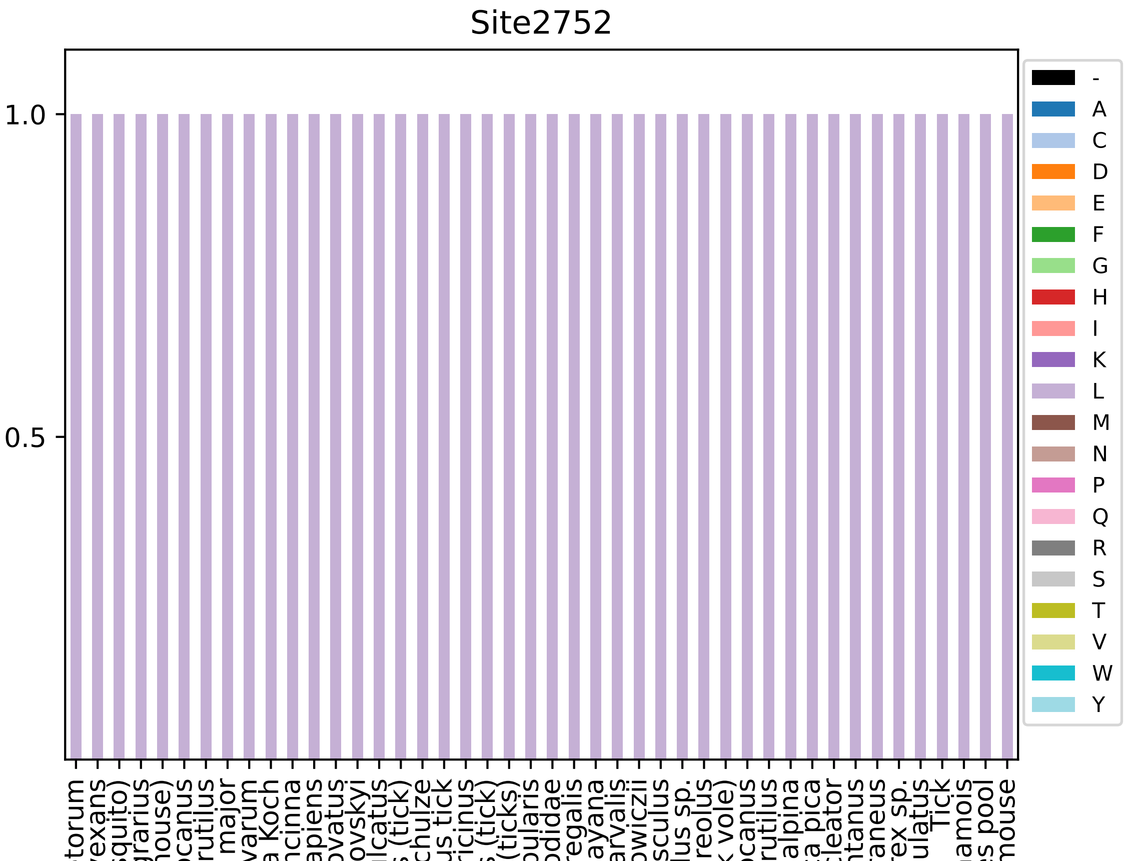
Site2752
1.0
0.5
-
A
C
D
E
F
G
H
I
K
L
M
N
P
Q
R
S
T
V
W
Y

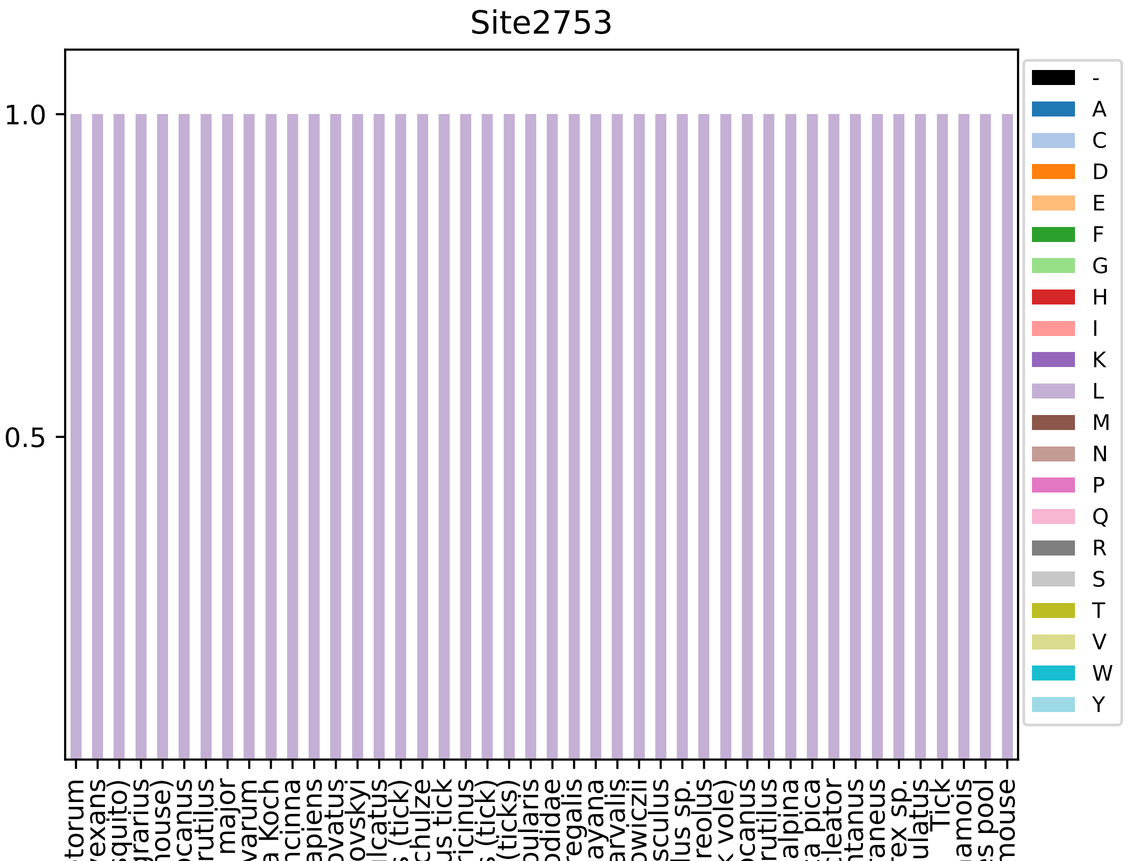
Site2753
1.0
0.5
-
A
C
D
E
F
G
H
I
K
L
M
N
P
Q
R
S
T
V
W
Y

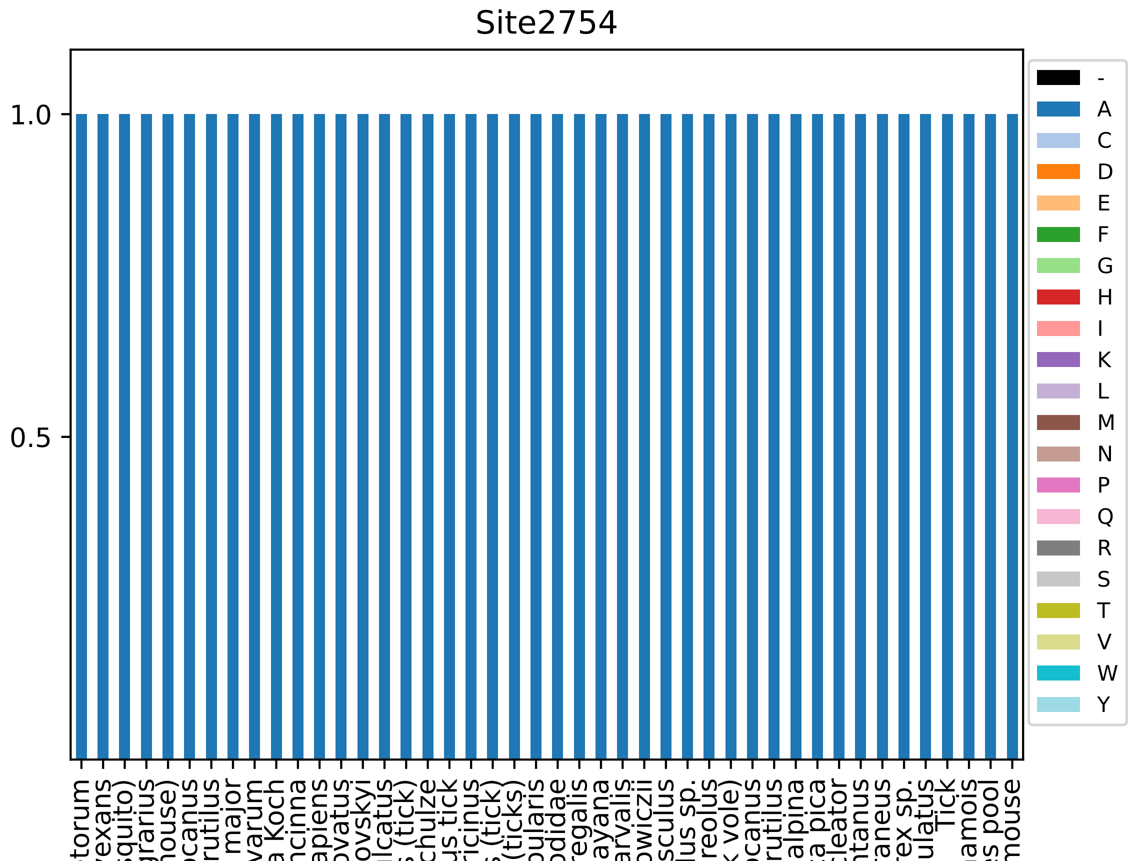
Site2754
1.0
0.5
-
A
C
D
E
F
G
H
I
K
L
M
N
P
Q
R
S
T
V
W
Y

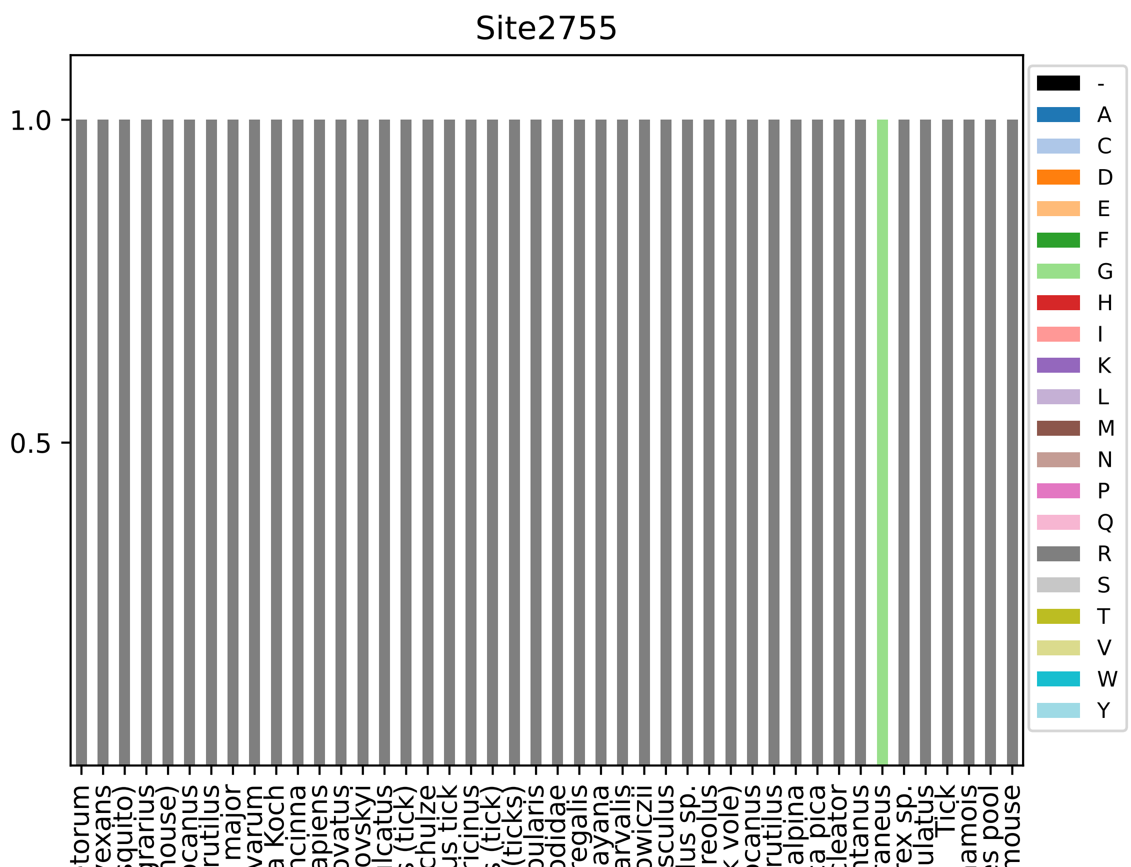
Site2755
1.0
0.5
-
A
C
D
E
F
G
H
I
K
L
M
N
P
Q
R
S
T
V
W
Y

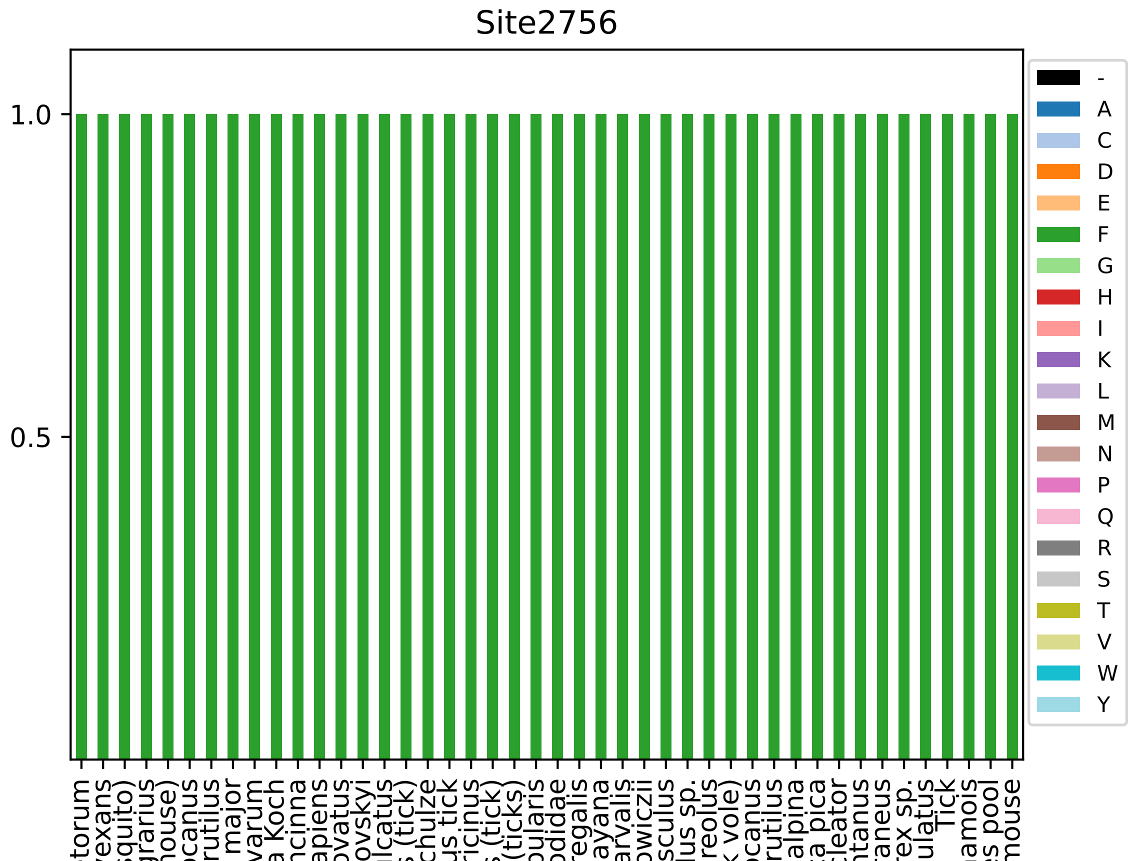
Site2756
1.0
0.5
-
A
C
D
E
F
G
H
I
K
L
M
N
P
Q
R
S
T
V
W
Y

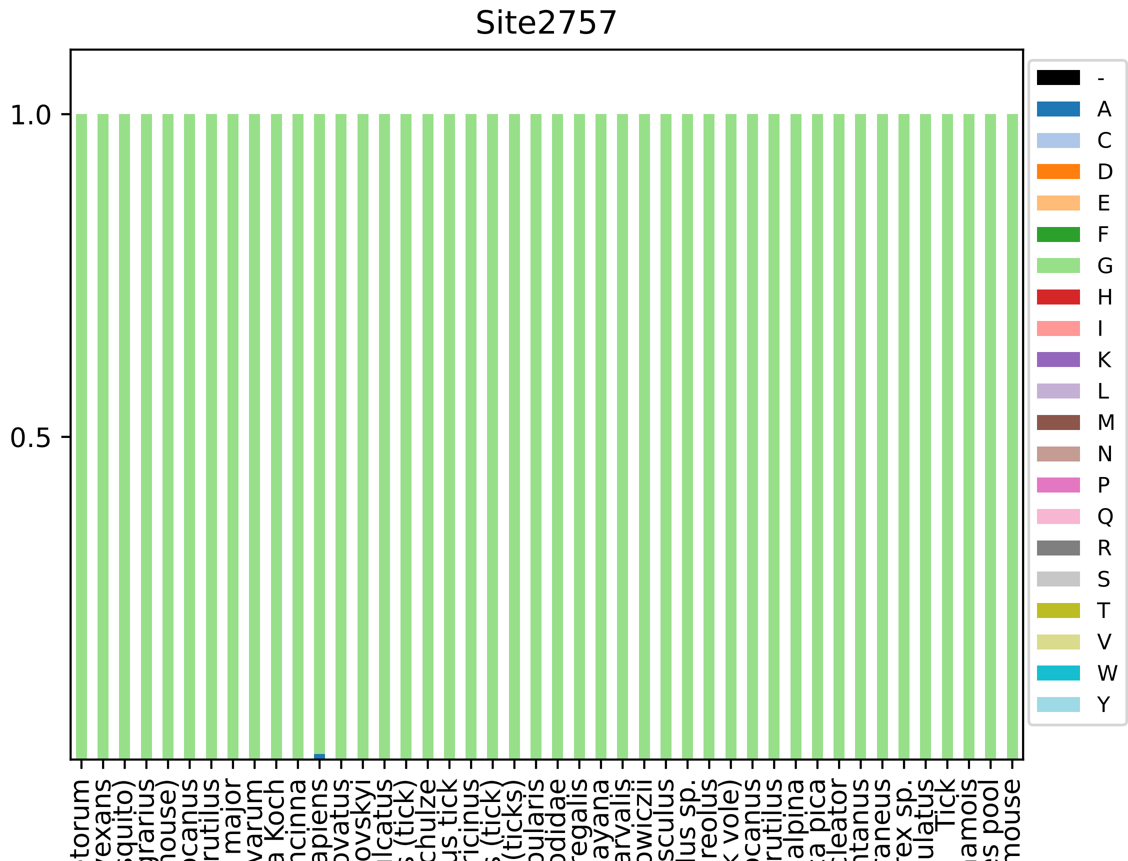
Site2757
1.0
0.5
-
A
C
D
E
F
G
H
I
K
L
M
N
P
Q
R
S
T
V
W
Y

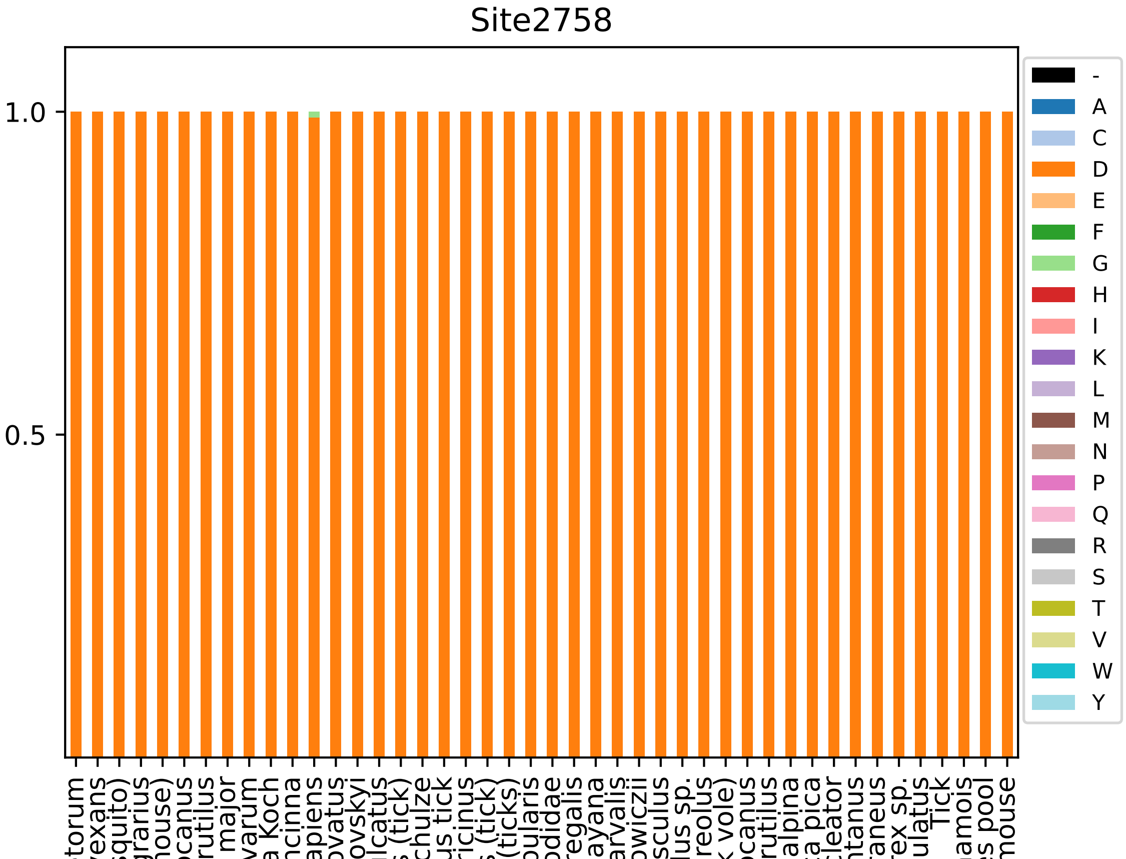

Site2758
1.0
0.5
-
A
C
D
E
F
G
H
I
K
L
M
N
P
Q
R
S
T
V
W
Y

Site2759

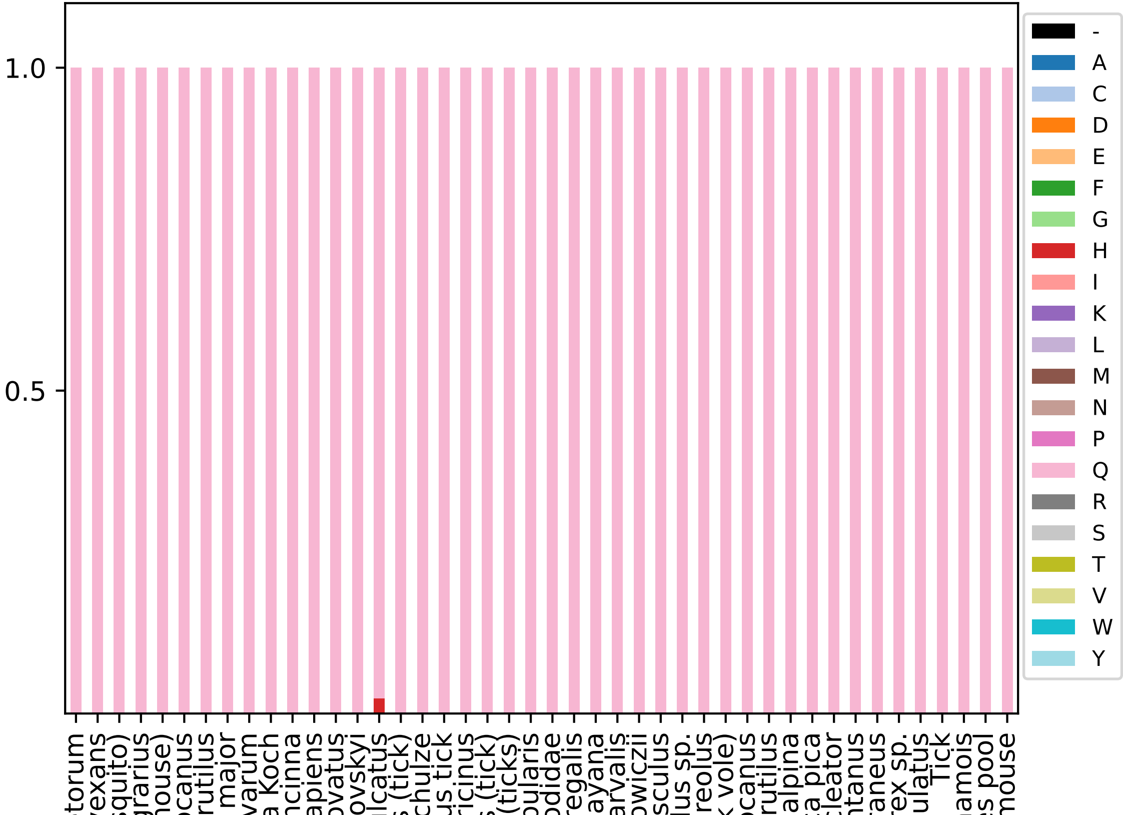

Site2760

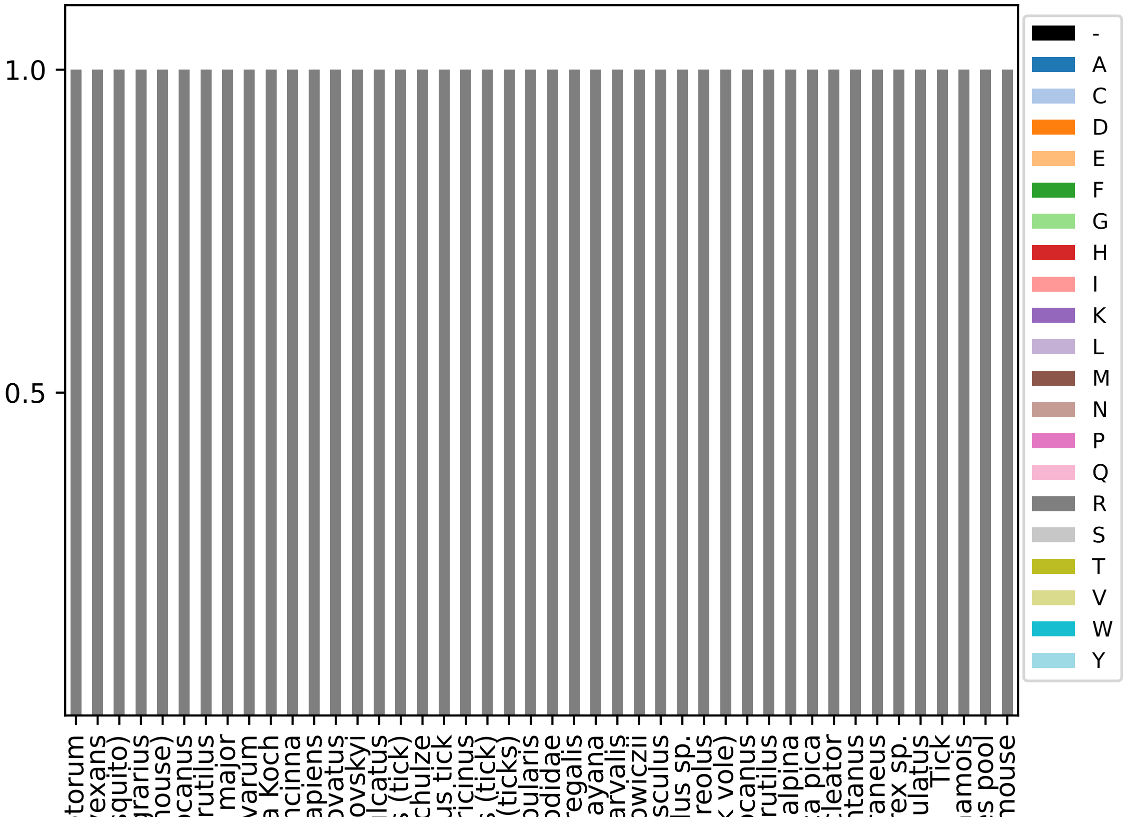

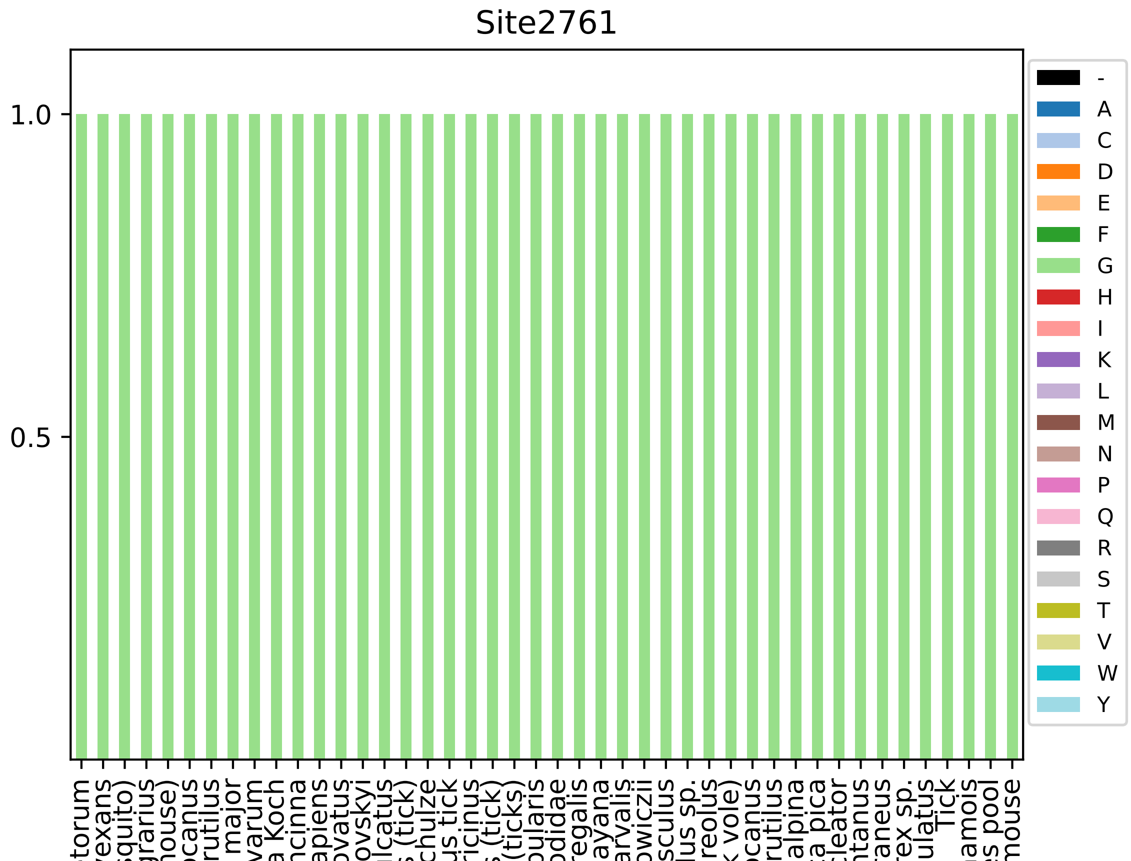

Site2761
1.0
0.5
-
A
C
D
E
F
G
H
I
K
L
M
N
P
Q
R
S
T
V
W
Y

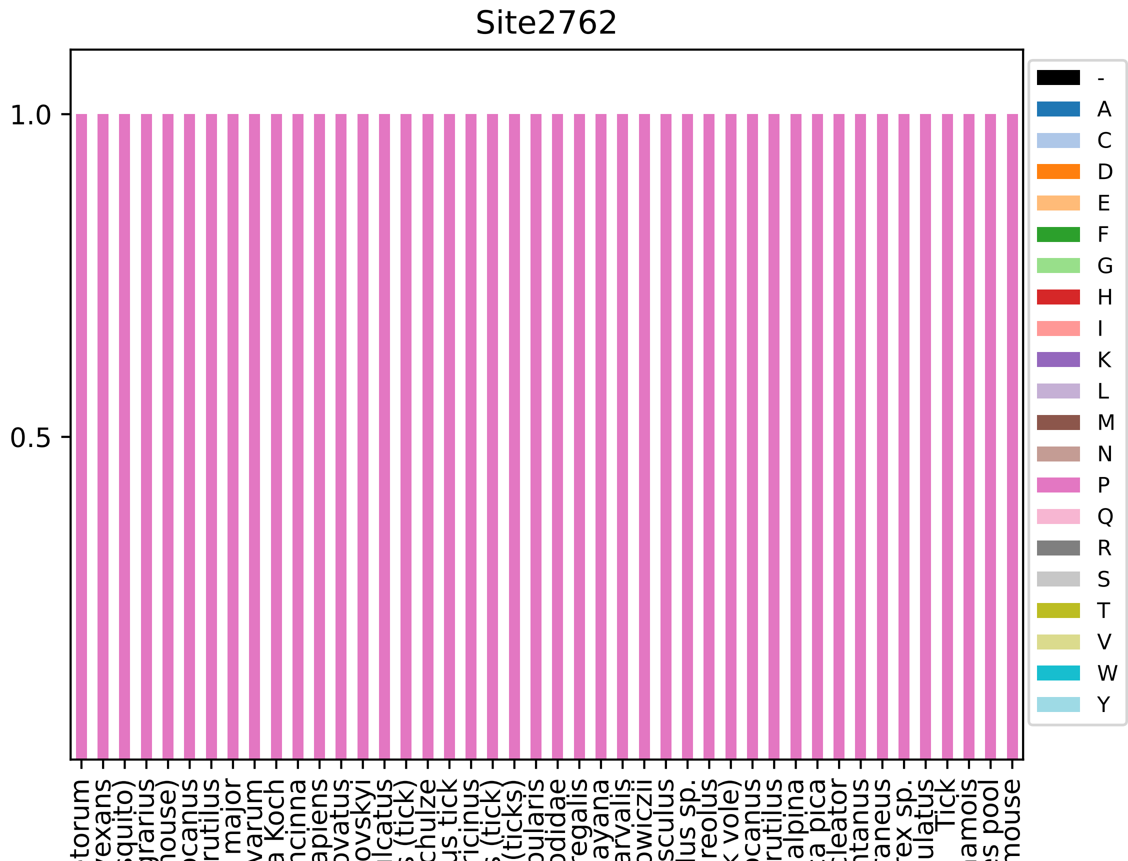
Site2762
1.0
0.5
-
A
C
D
E
F
G
H
I
K
L
M
N
P
Q
R
S
T
V
W
Y

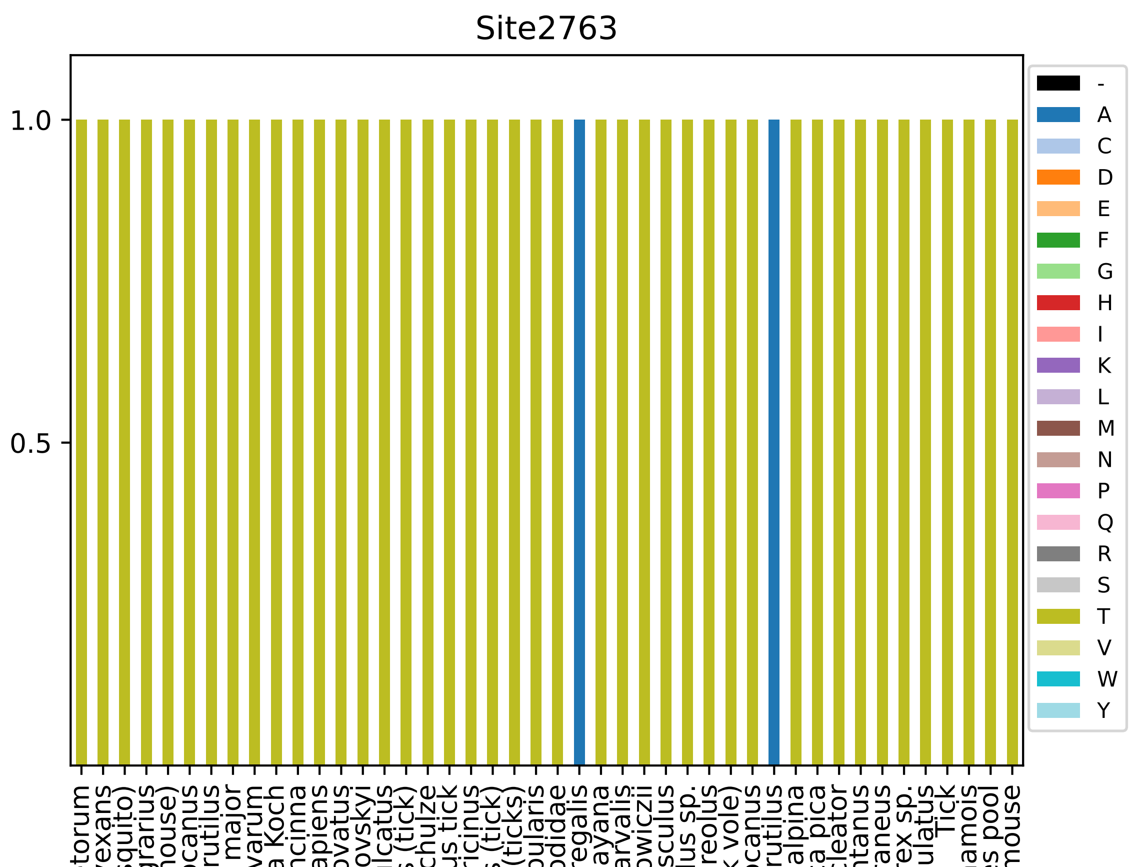
Site2763
1.0
0.5
-
A
C
D
E
F
G
H
I
K
L
M
N
P
Q
R
S
T
V
W
Y

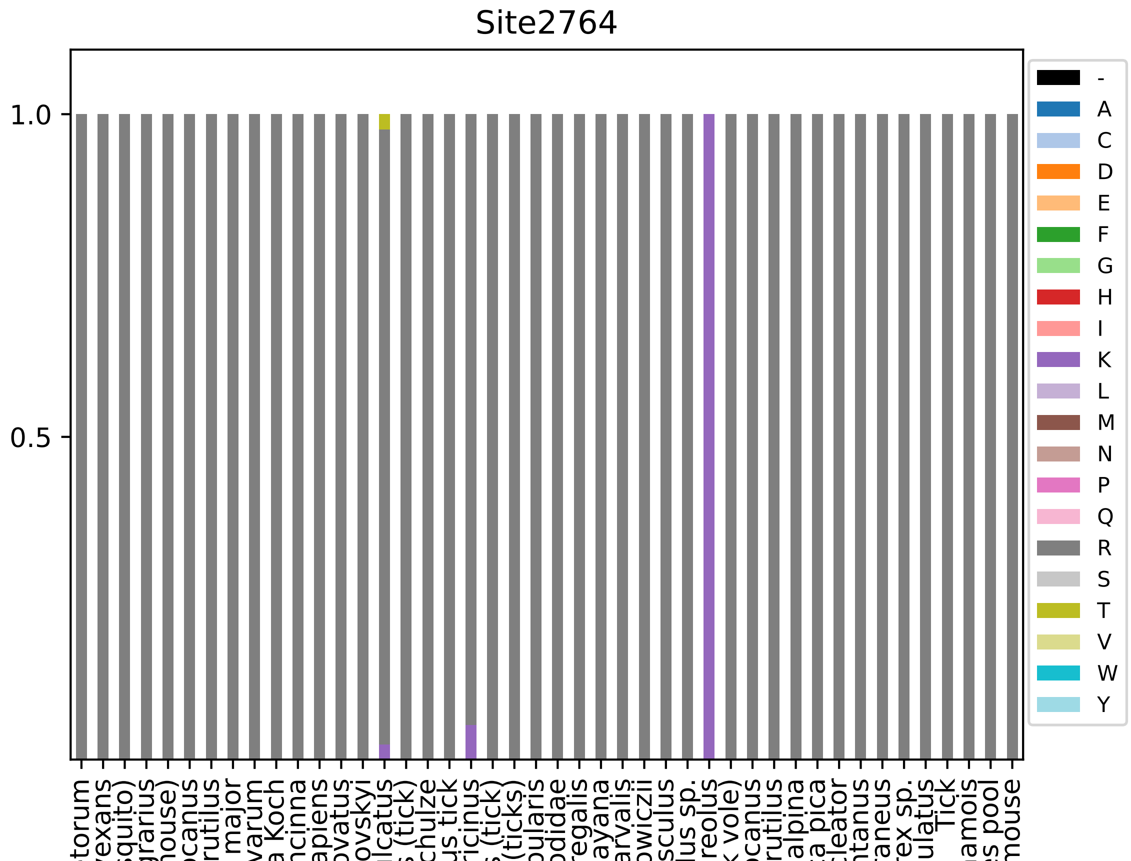
Site2764
1.0
0.5
-
A
C
D
E
F
G
H
I
K
L
M
N
P
Q
R
S
T
V
W
Y

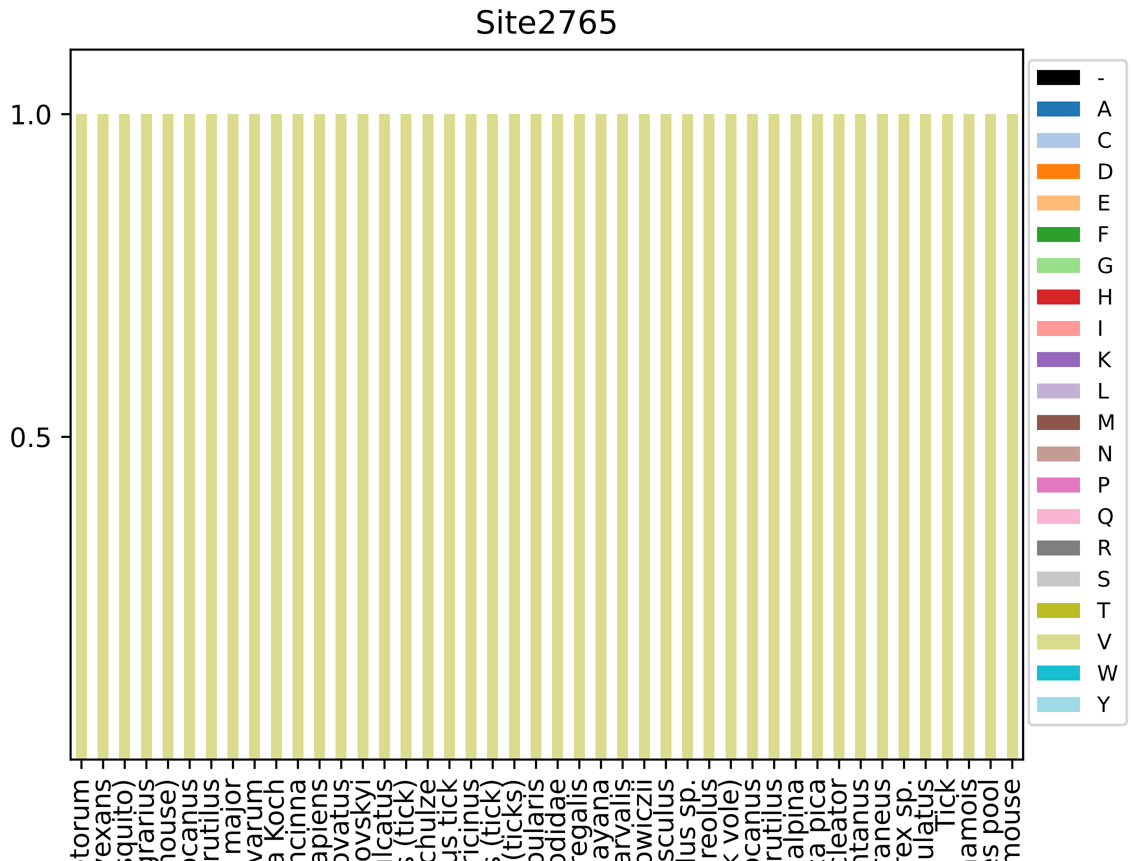
Site2765
1.0
0.5
-
A
C
D
E
F
G
H
I
K
L
M
N
P
Q
R
S
T
V
W
Y

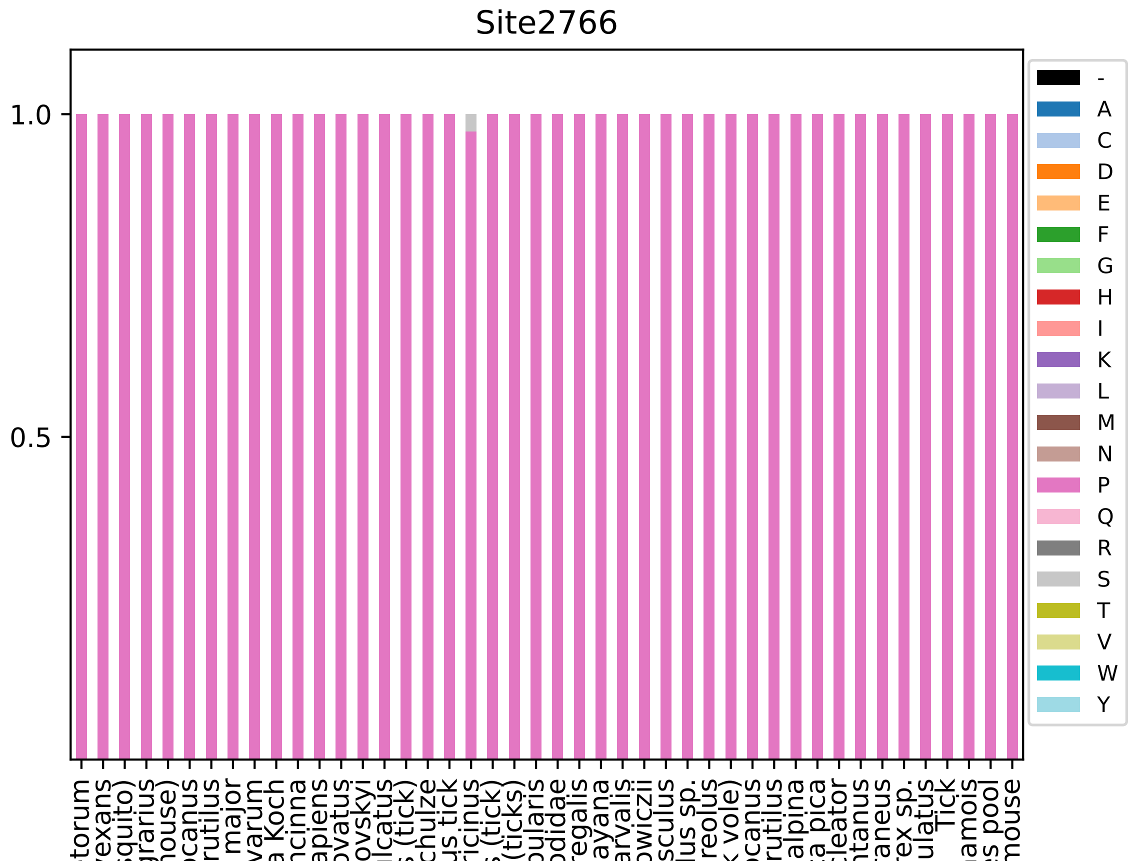

Site2766
1.0
0.5
-
A
C
D
E
F
G
H
I
K
L
M
N
P
Q
R
S
T
V
W
Y

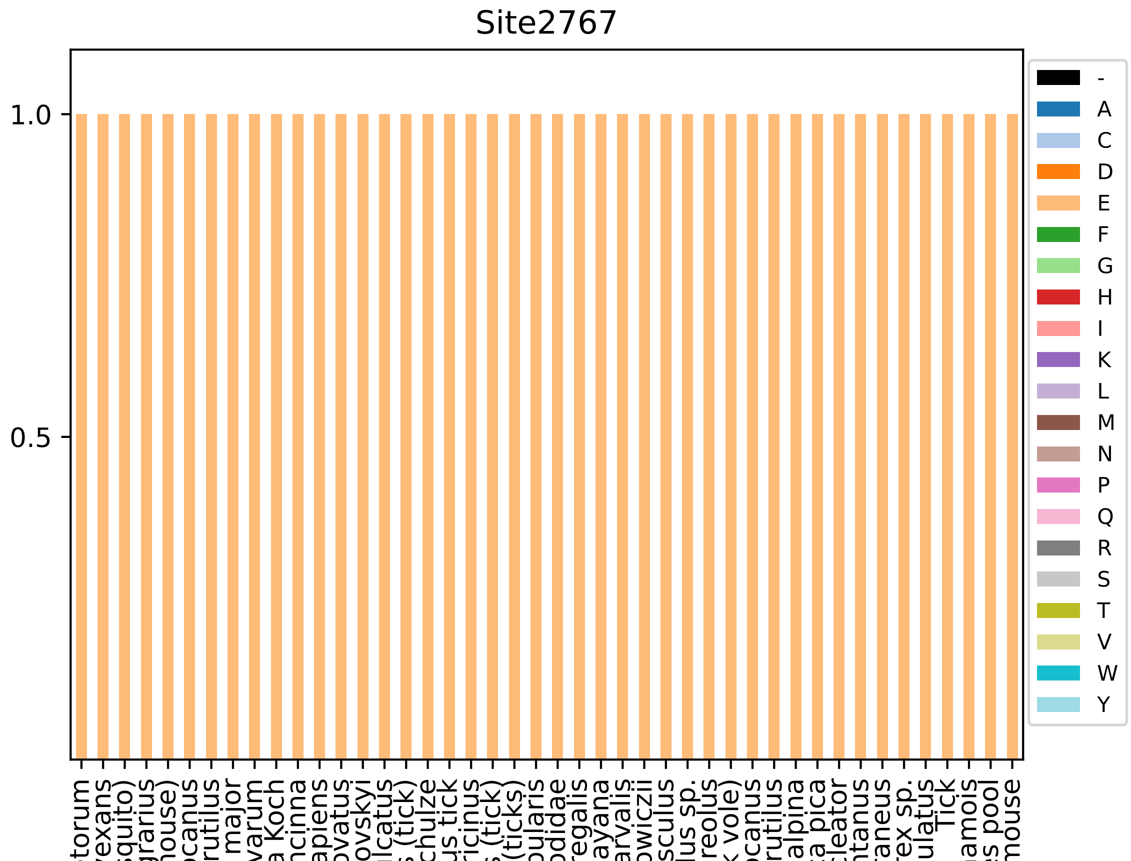
Site2767
1.0
0.5
-
A
C
D
E
F
G
H
I
K
L
M
N
P
Q
R
S
T
V
W
Y

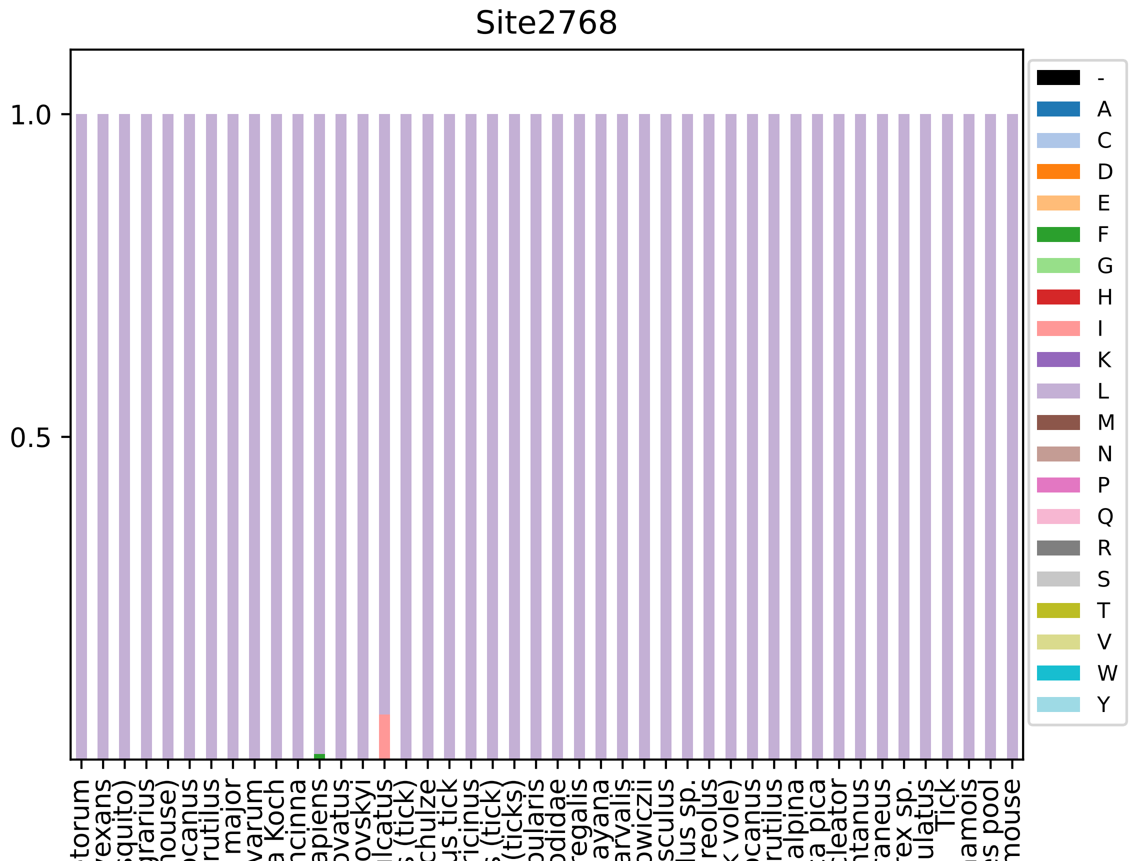

Site2768
1.0
0.5
-
A
C
D
E
F
G
H
I
K
L
M
N
P
Q
R
S
T
V
W
Y

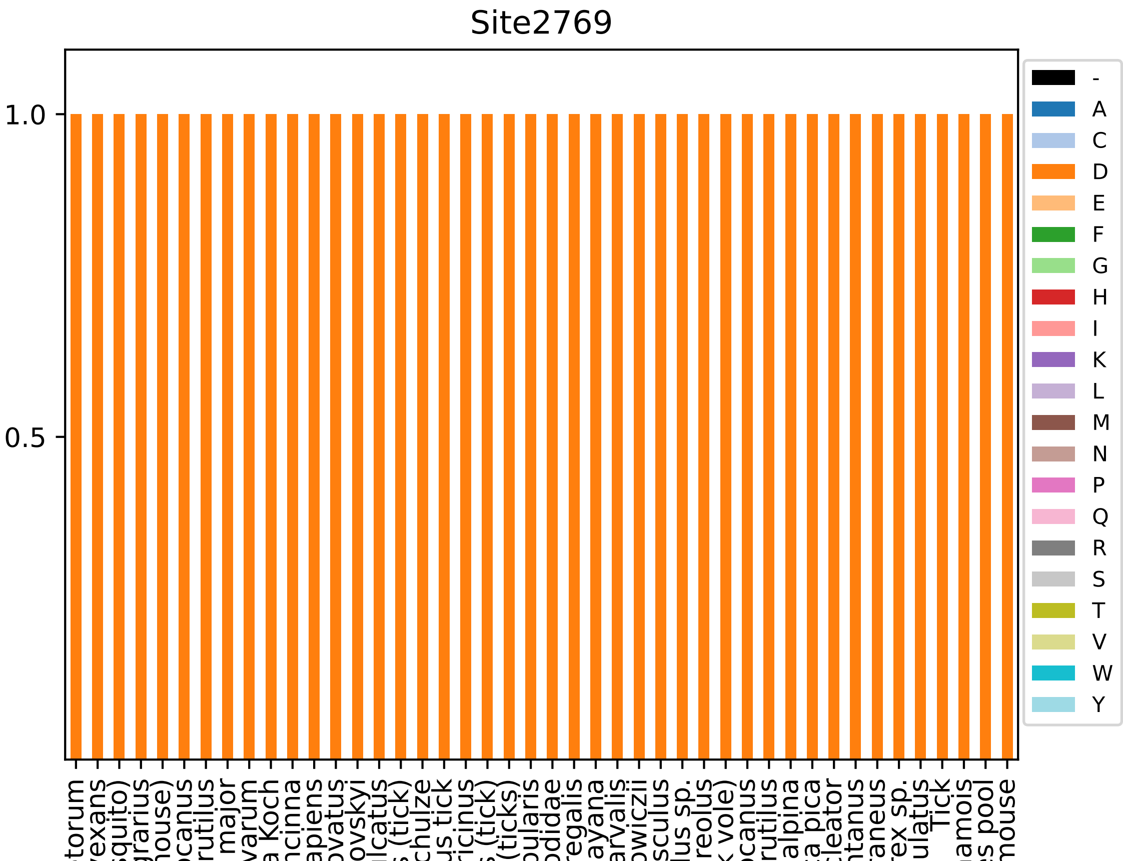
Site2769
1.0
0.5
-
A
C
D
E
F
G
H
I
K
L
M
N
P
Q
R
S
T
V
W
Y

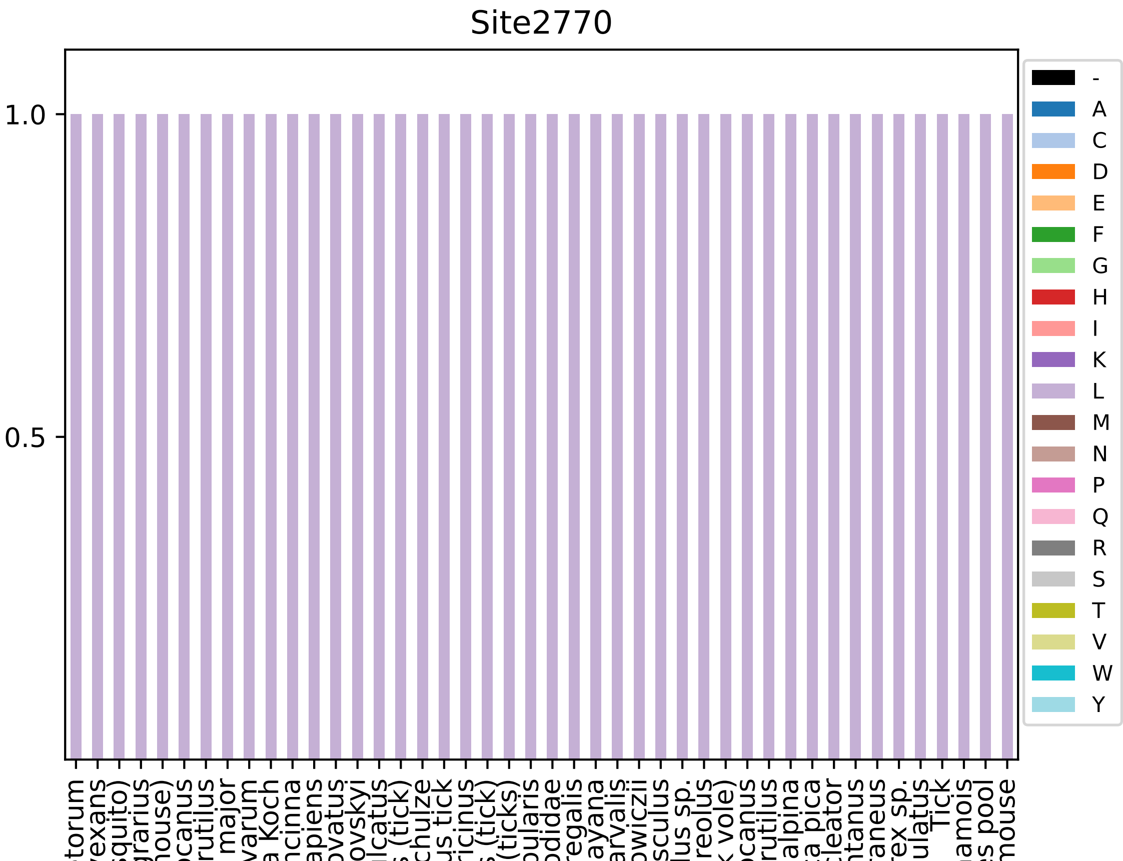

Site2770
1.0
0.5
-
A
C
D
E
F
G
H
I
K
L
M
N
P
Q
R
S
T
V
W
Y

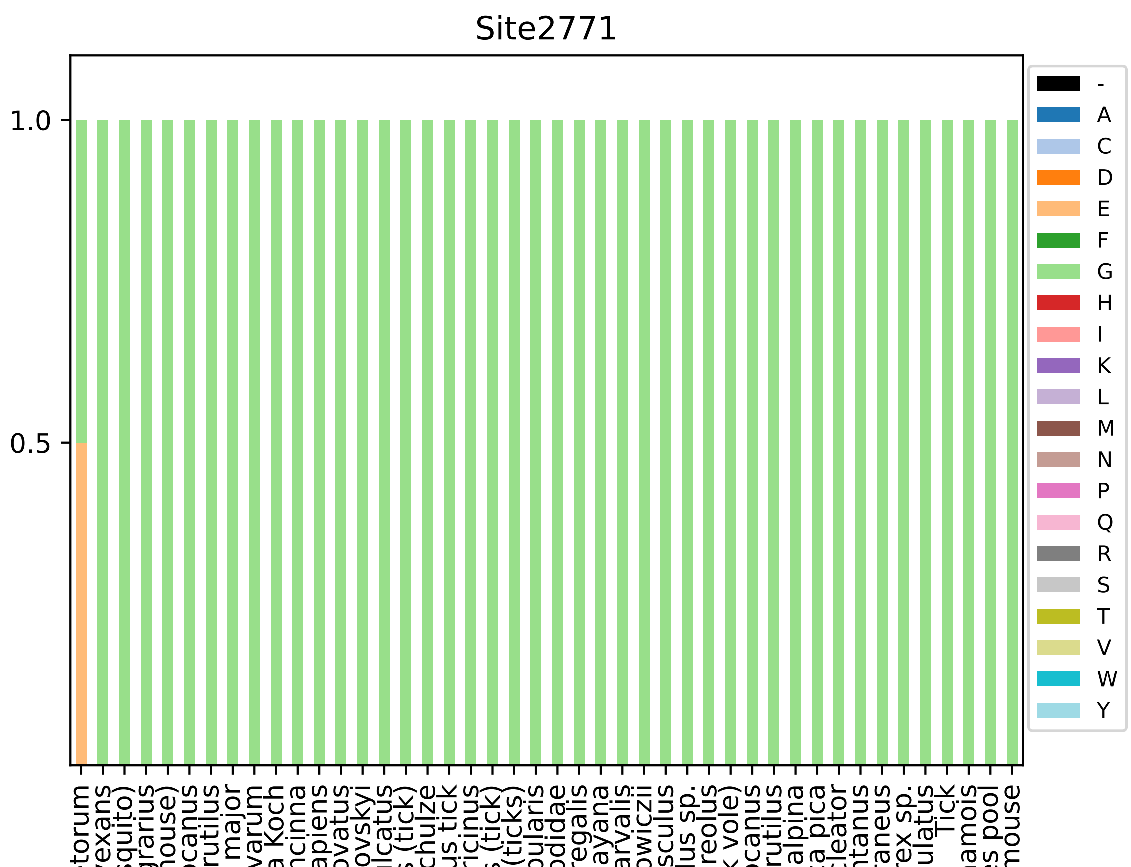
Site2771
1.0
0.5
-
A
C
D
E
F
G
H
I
K
L
M
N
P
Q
R
S
T
V
W
Y

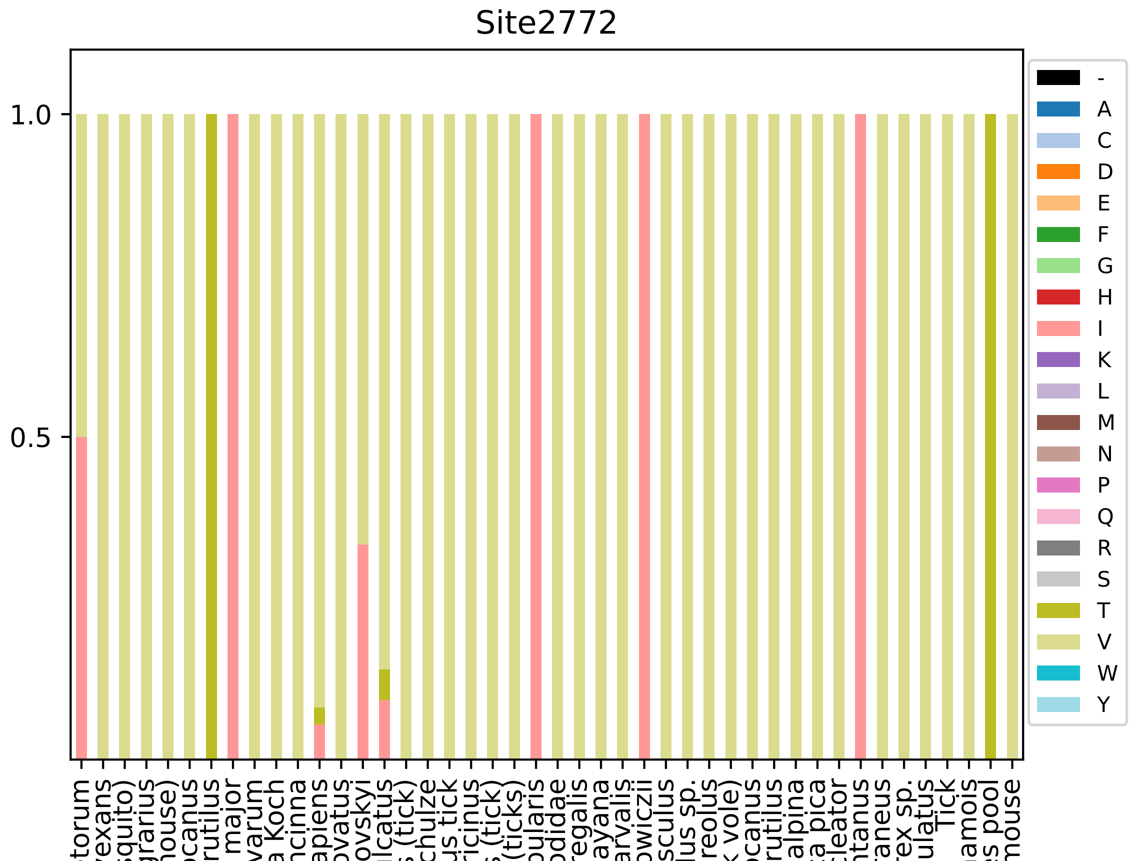
Site2772
1.0
0.5
-
A
C
D
E
F
G
H
I
K
L
M
N
P
Q
R
S
T
V
W
Y

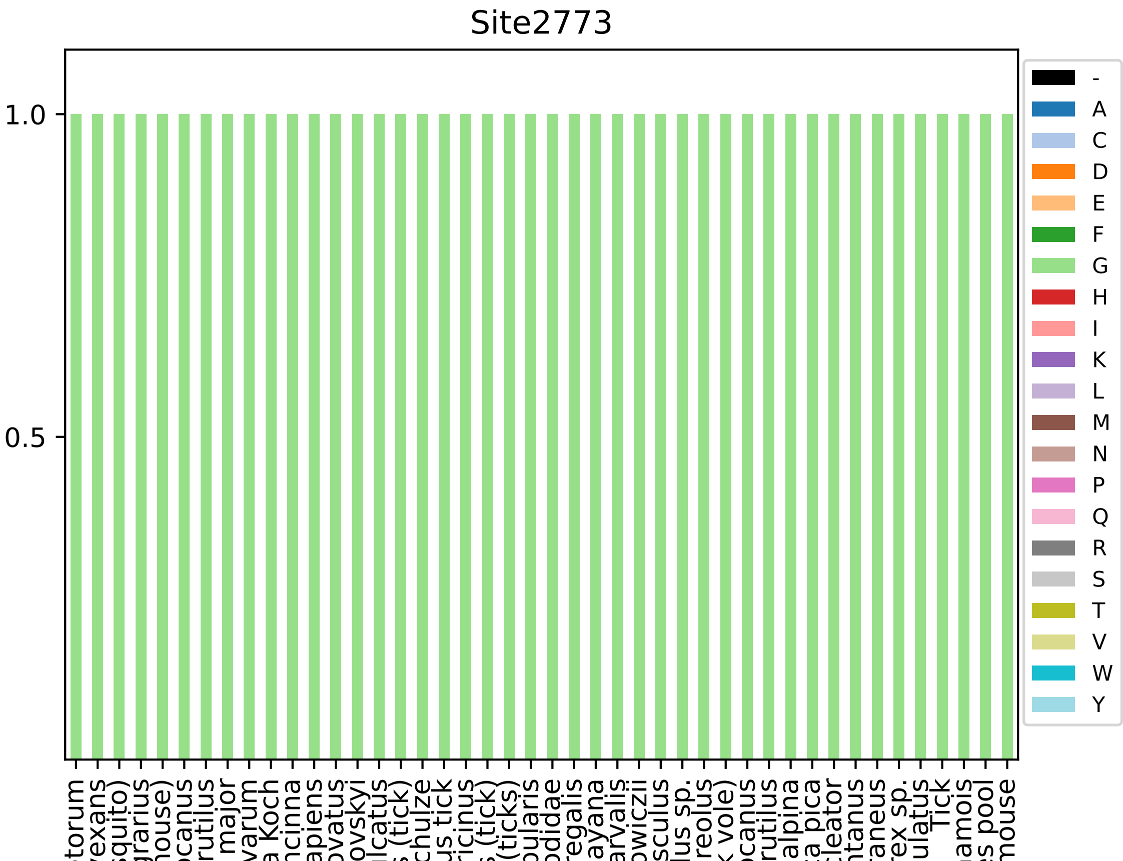
Site2773
1.0
0.5
-
A
C
D
E
F
G
H
I
K
L
M
N
P
Q
R
S
T
V
W
Y

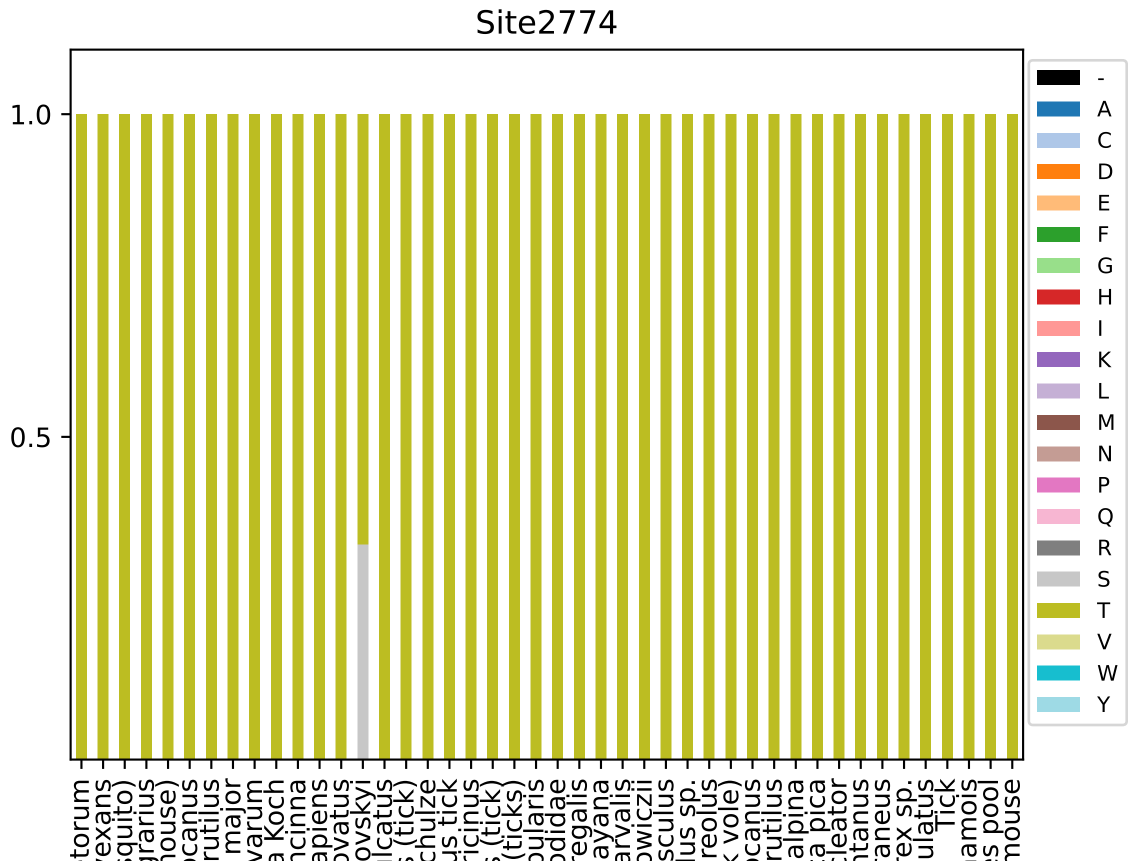

Site2774
1.0
0.5
-
A
C
D
E
F
G
H
I
K
L
M
N
P
Q
R
S
T
V
W
Y

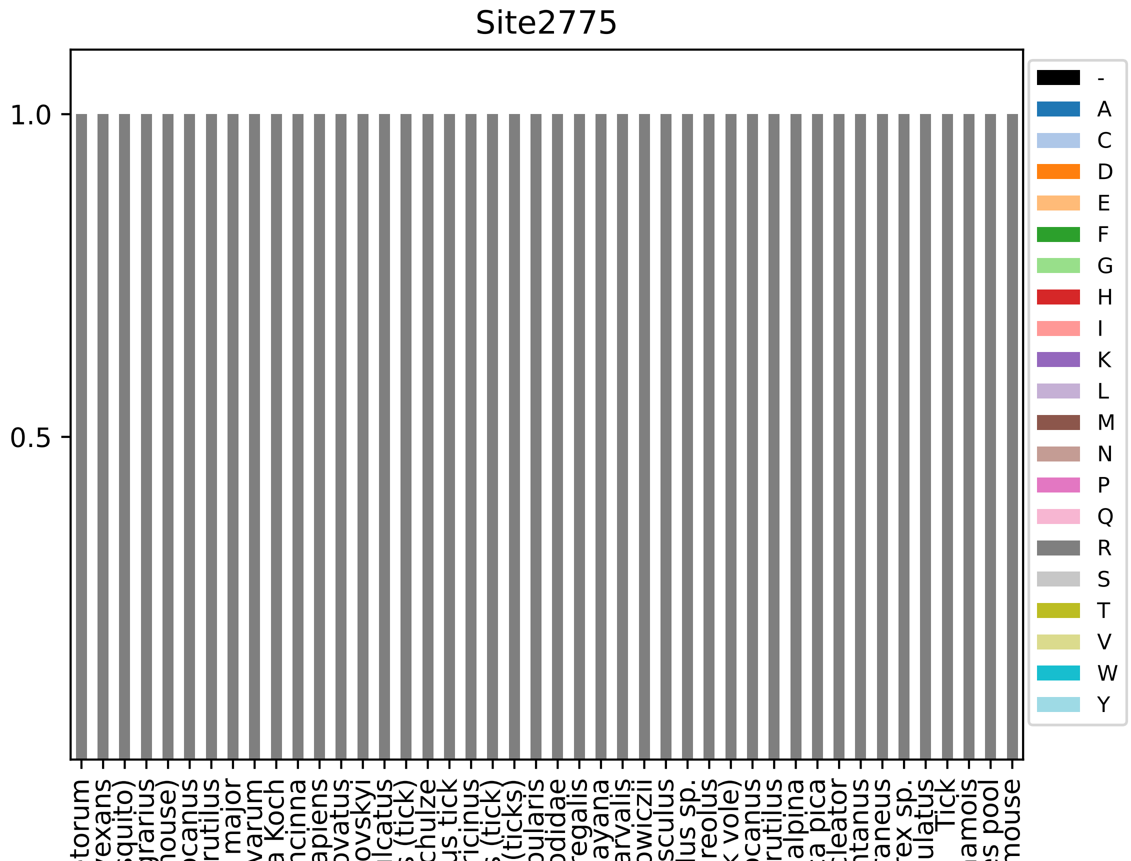

Site2775
1.0
0.5
-
A
C
D
E
F
G
H
I
K
L
M
N
P
Q
R
S
T
V
W
Y

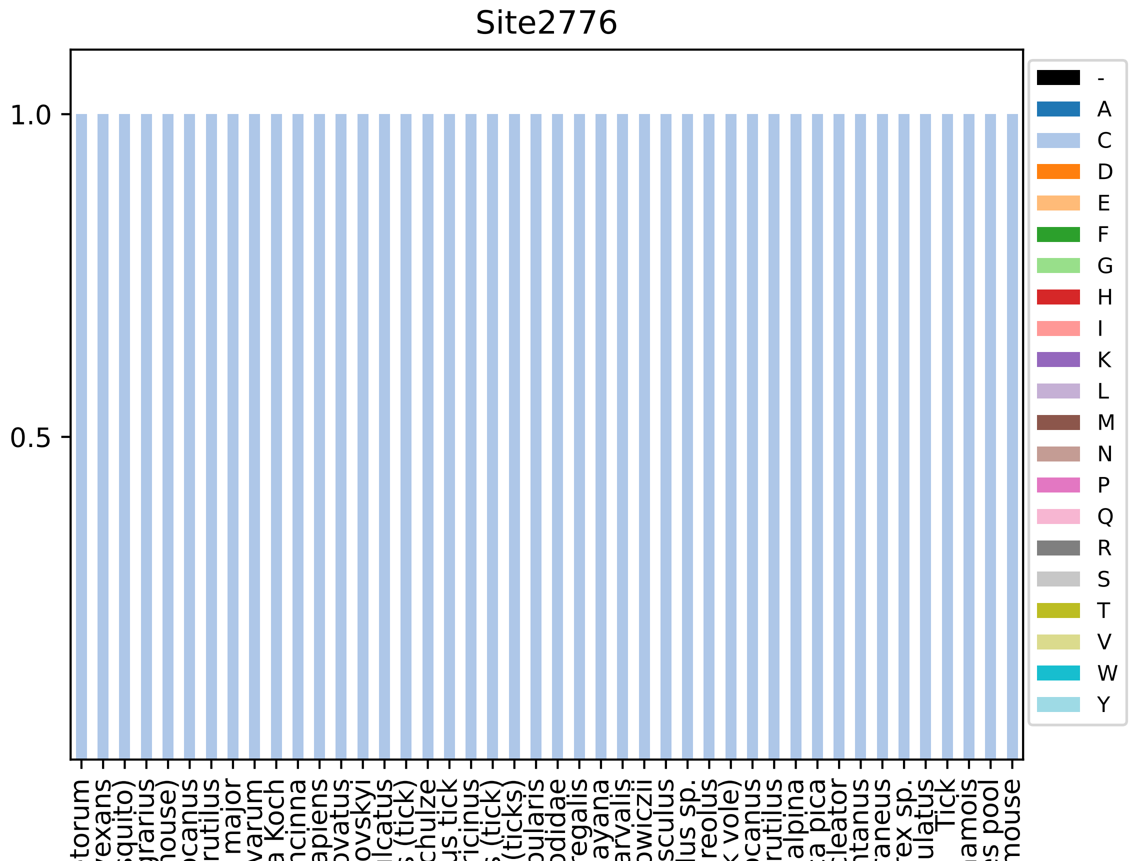
Site2776
1.0
0.5
-
A
C
D
E
F
G
H
I
K
L
M
N
P
Q
R
S
T
V
W
Y

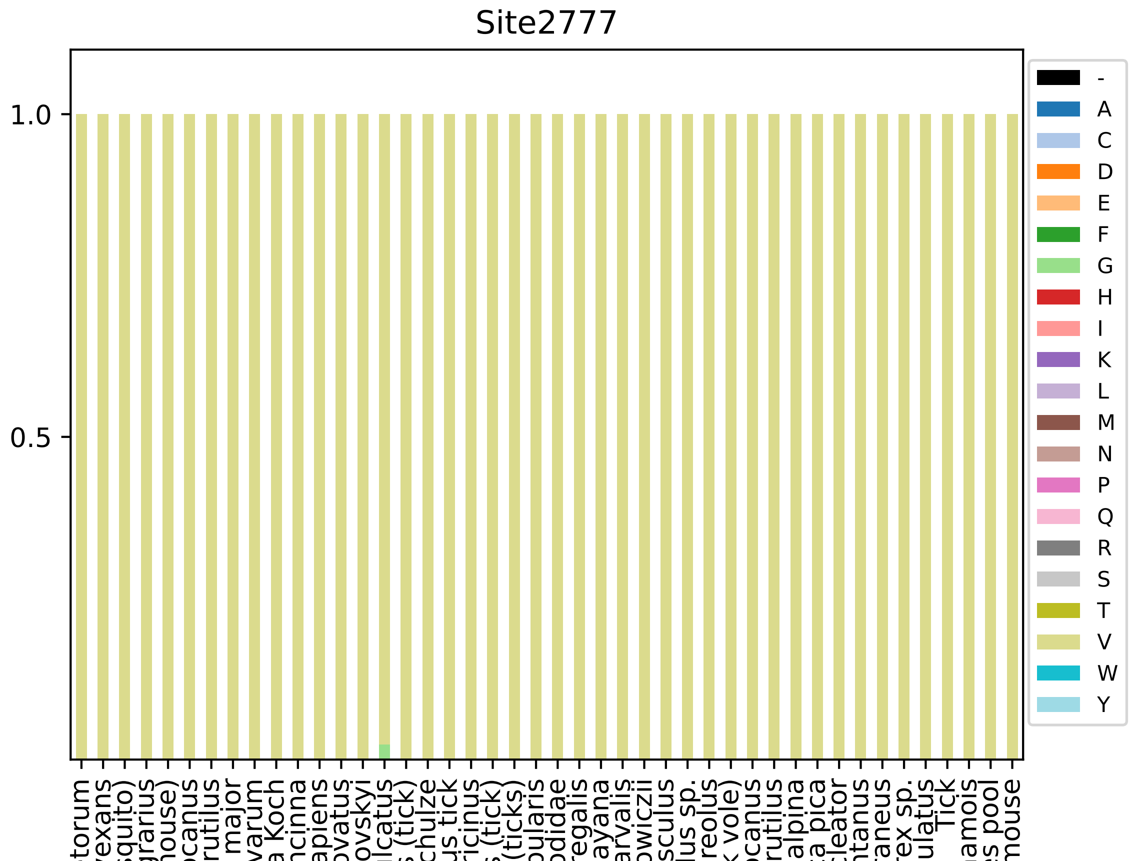
Site2777
1.0
0.5
-
A
C
D
E
F
G
H
I
K
L
M
N
P
Q
R
S
T
V
W
Y

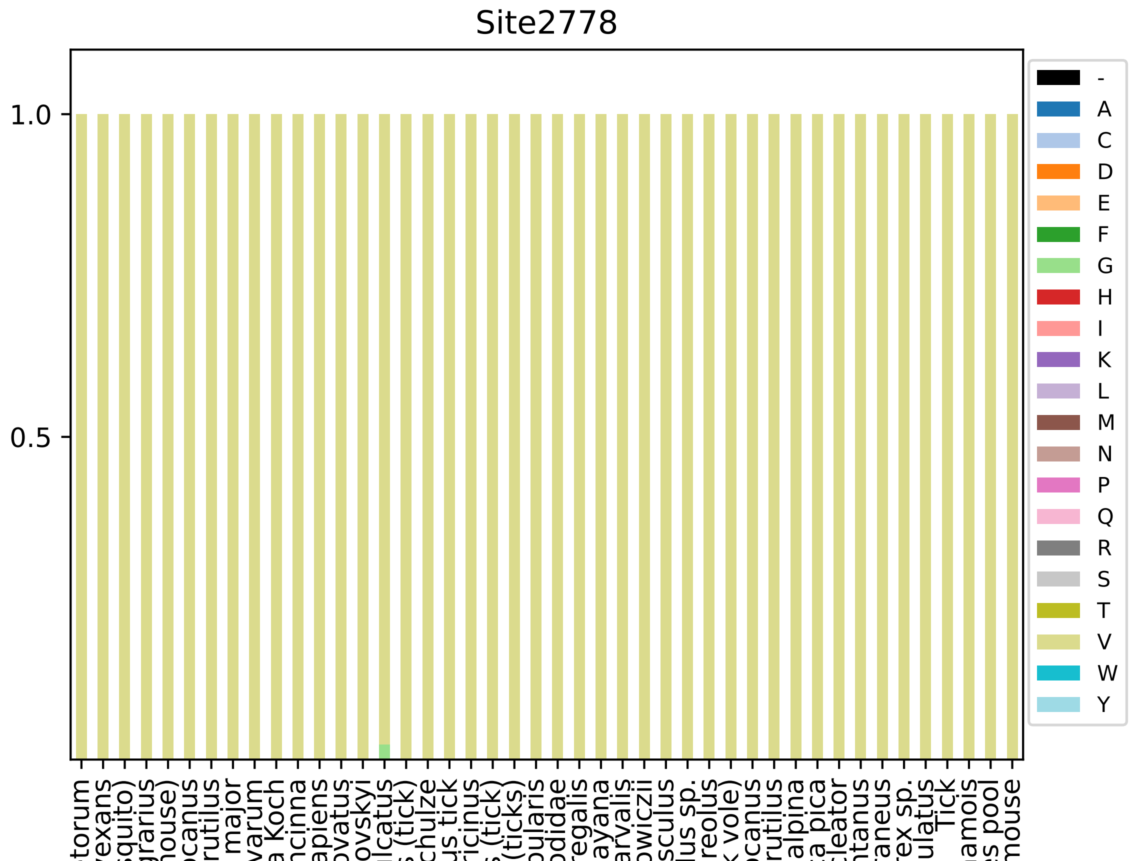
Site2778
1.0
0.5
-
A
C
D
E
F
G
H
I
K
L
M
N
P
Q
R
S
T
V
W
Y

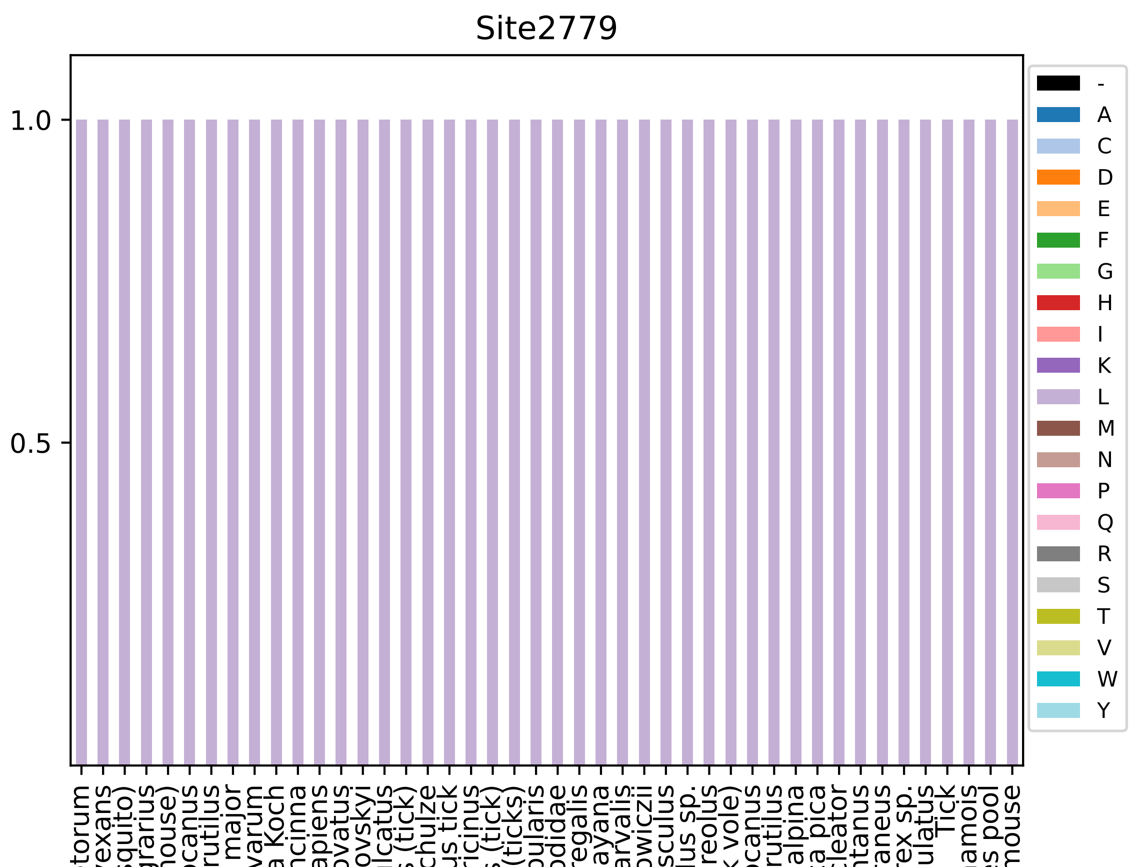
Site2779
1.0
0.5
-
A
C
D
E
F
G
H
I
K
L
M
N
P
Q
R
S
T
V
W
Y

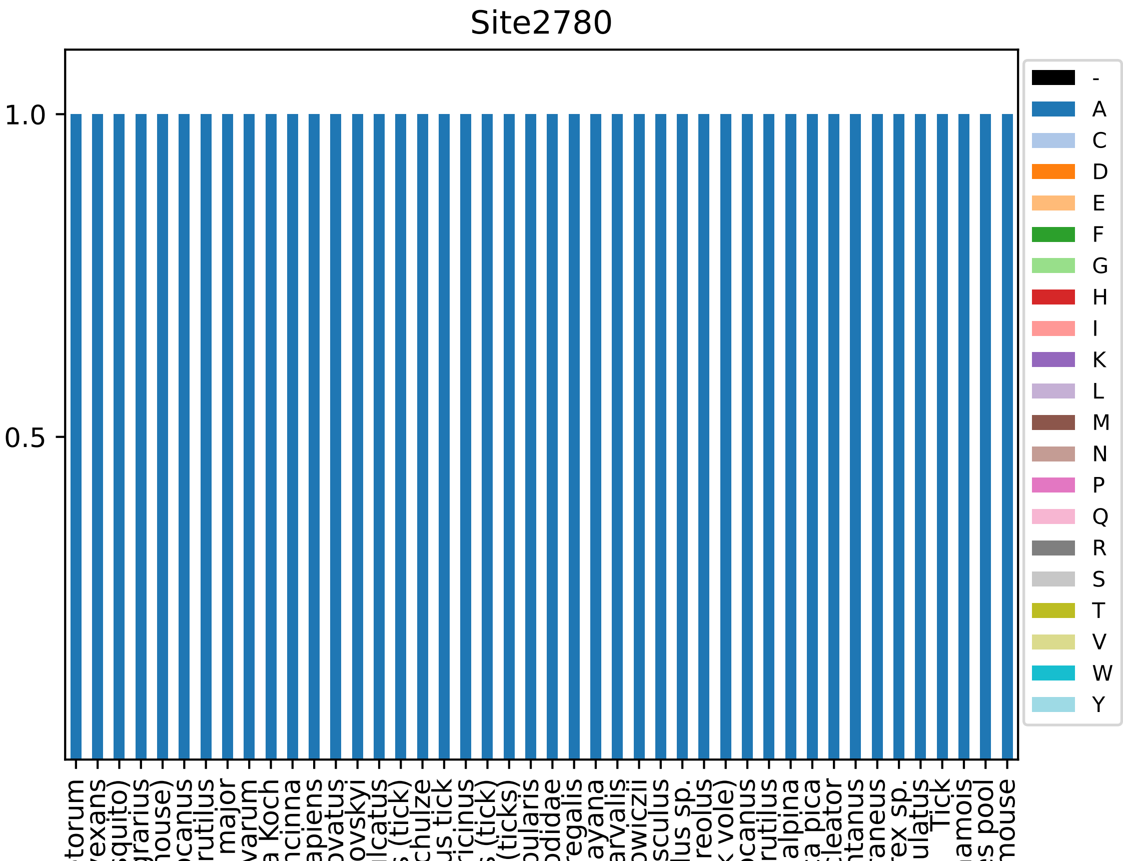
Site2780
1.0
0.5
-
A
C
D
E
F
G
H
I
K
L
M
N
P
Q
R
S
T
V
W
Y
Tick

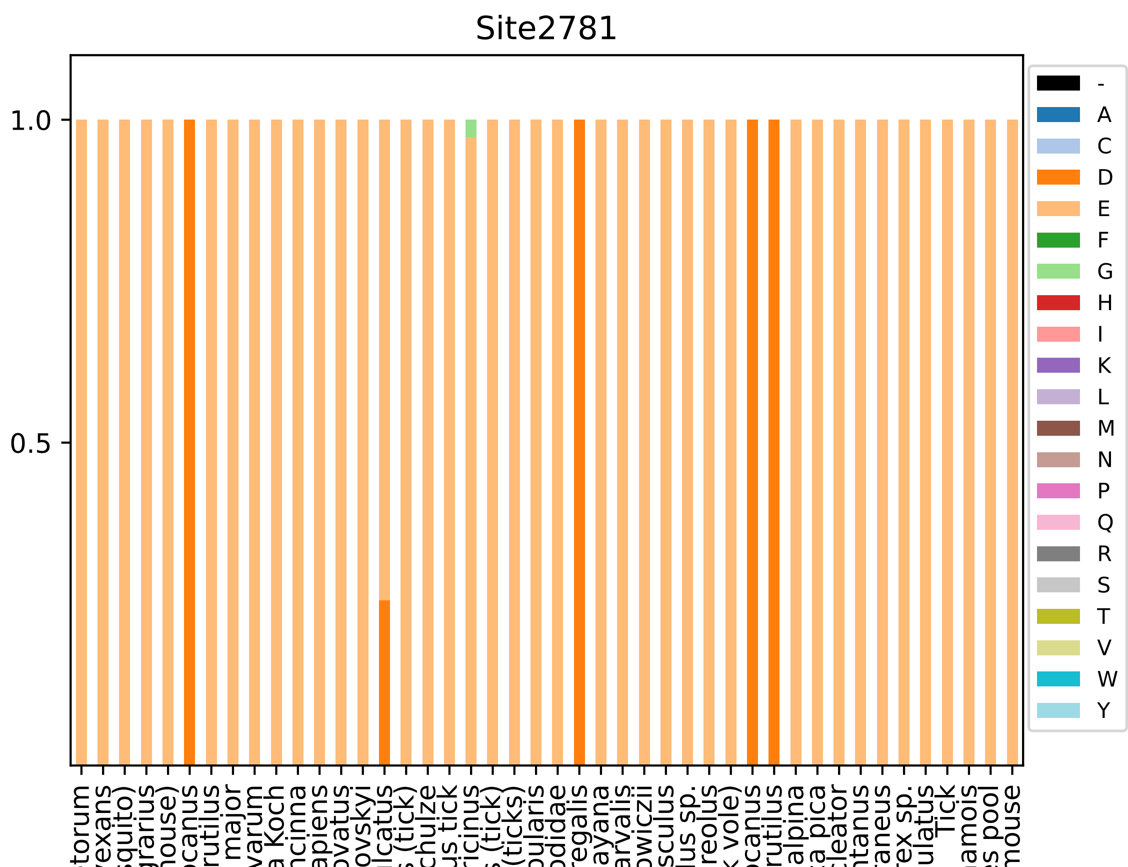

Site2781
1.0
0.5
-
A
C
D
E
F
G
H
I
K
L
M
N
P
Q
R
S
T
V
W
Y

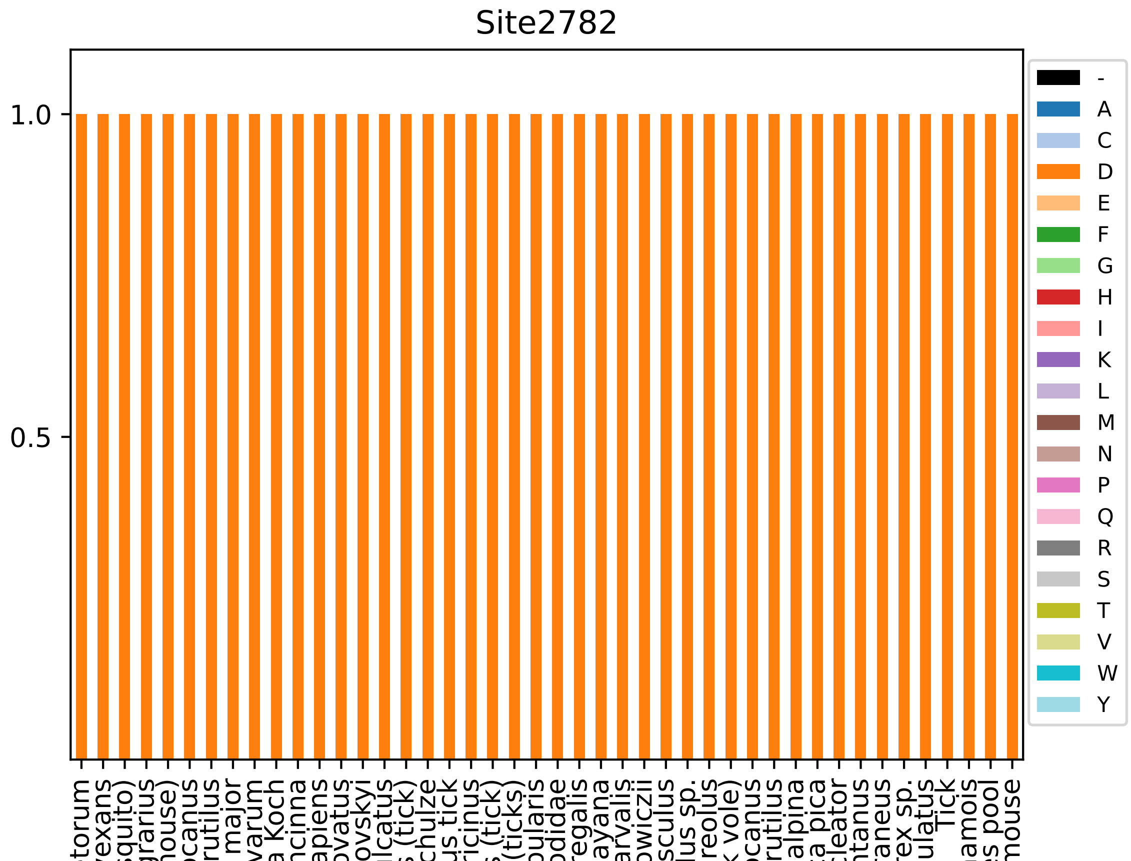
Site2782
1.0
0.5
-
A
C
D
E
F
G
H
I
K
L
M
N
P
Q
R
S
T
V
W
Y

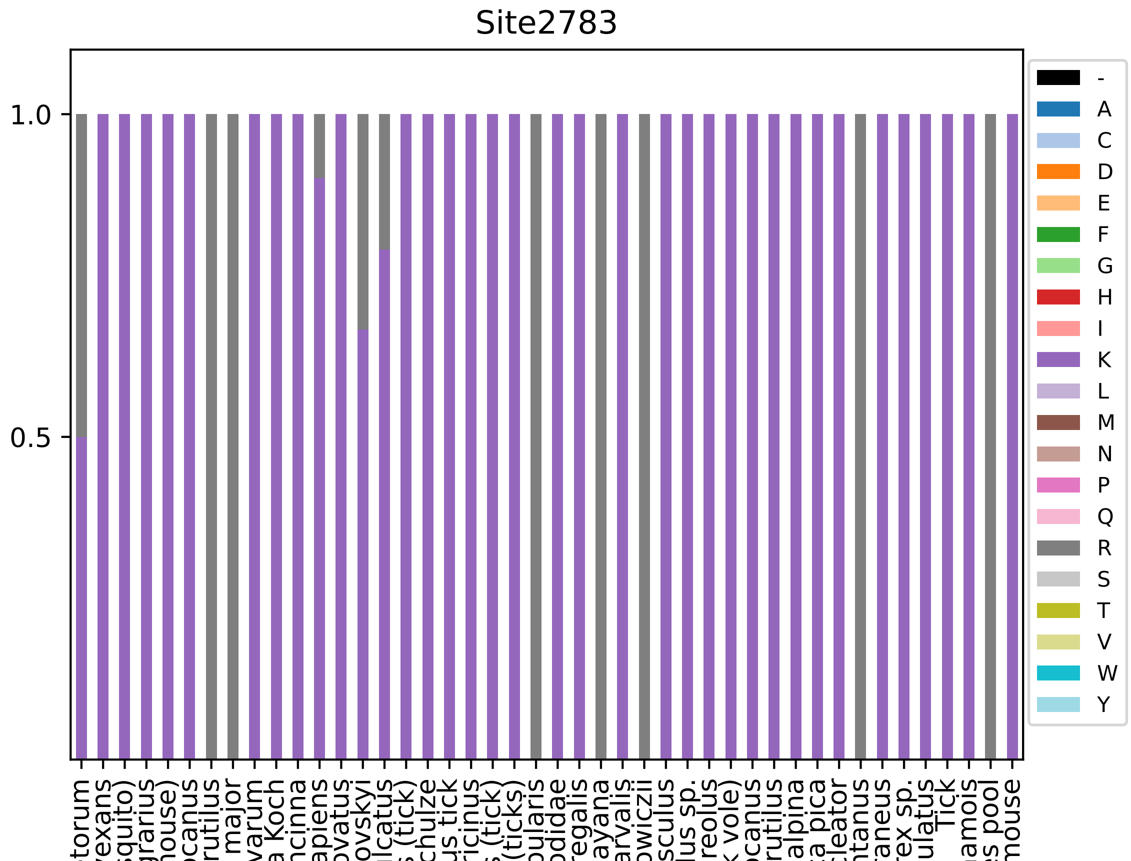

Site2783
1.0
0.5
-
A
C
D
E
F
G
H
I
K
L
M
N
P
Q
R
S
T
V
W
Y

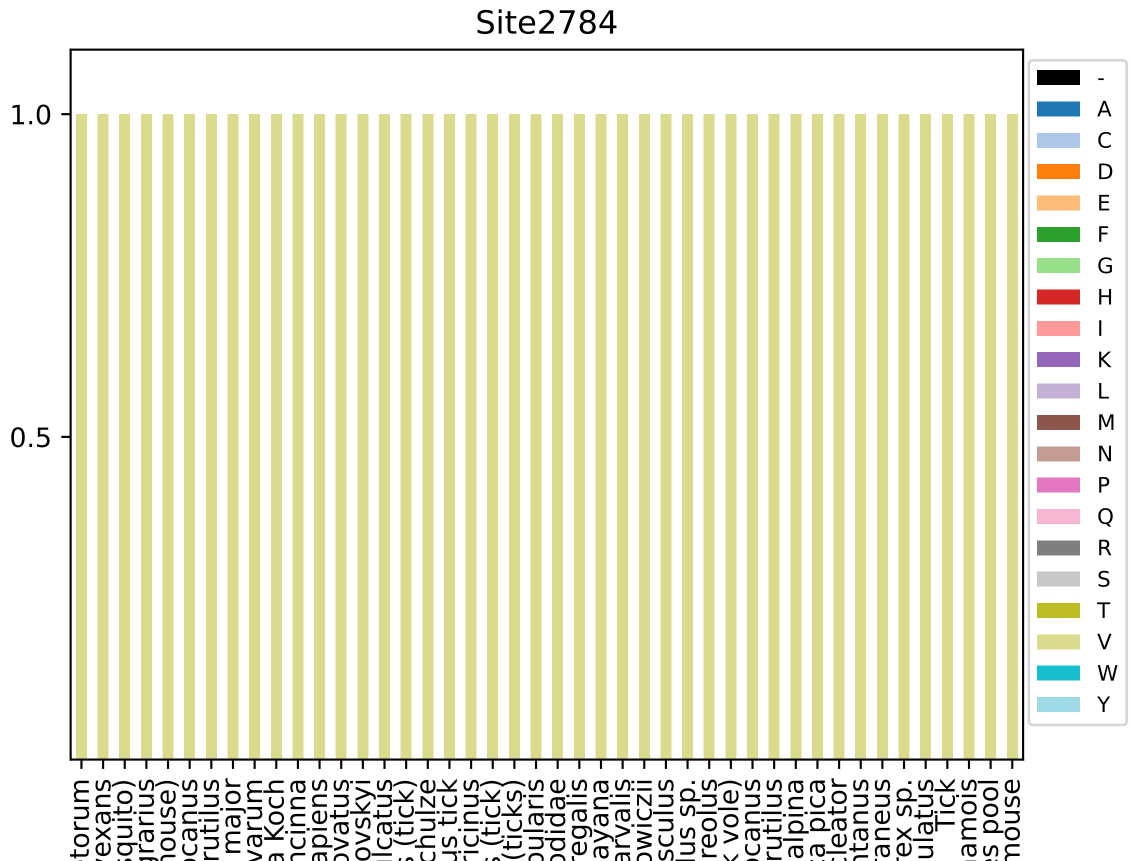
Site2784
1.0
0.5
-
A
C
D
E
F
G
H
I
K
L
M
N
P
Q
R
S
T
V
W
Y

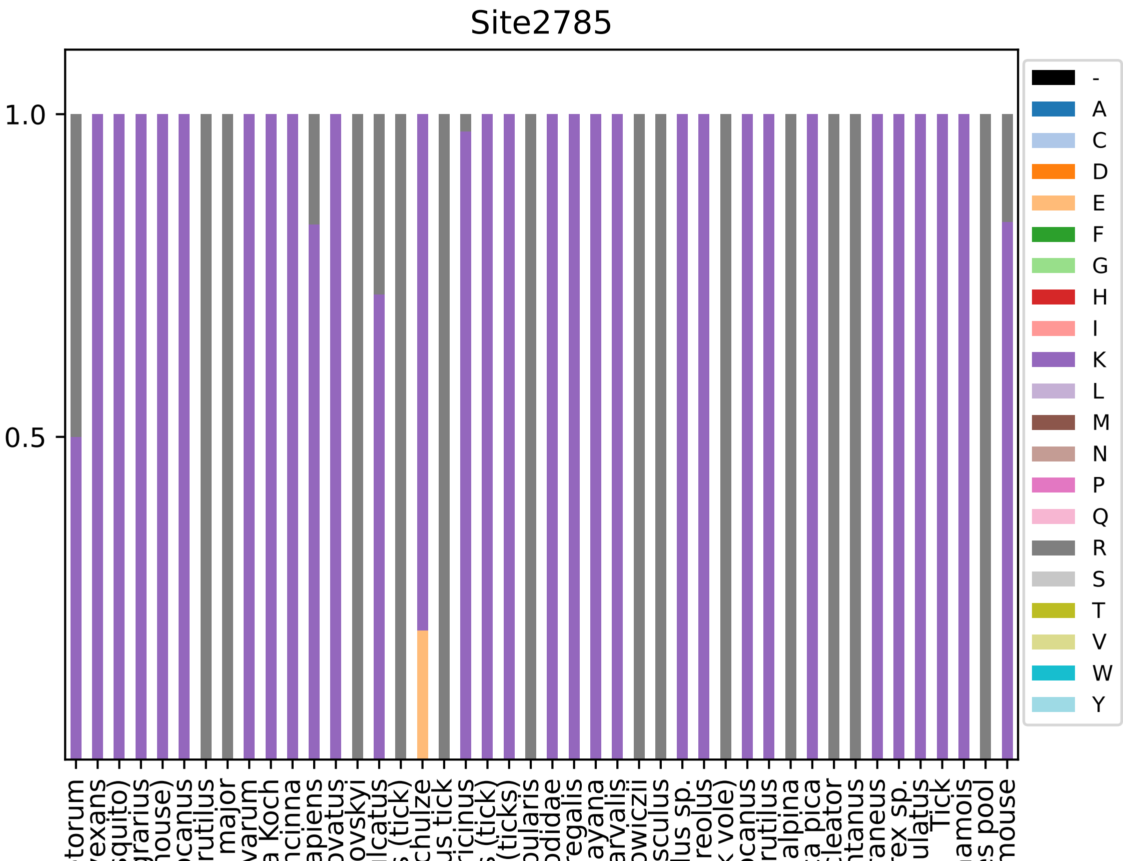

Site2785
1.0
0.5
-
A
C
D
E
F
G
H
I
K
L
M
N
P
Q
R
S
T
V
W
Y

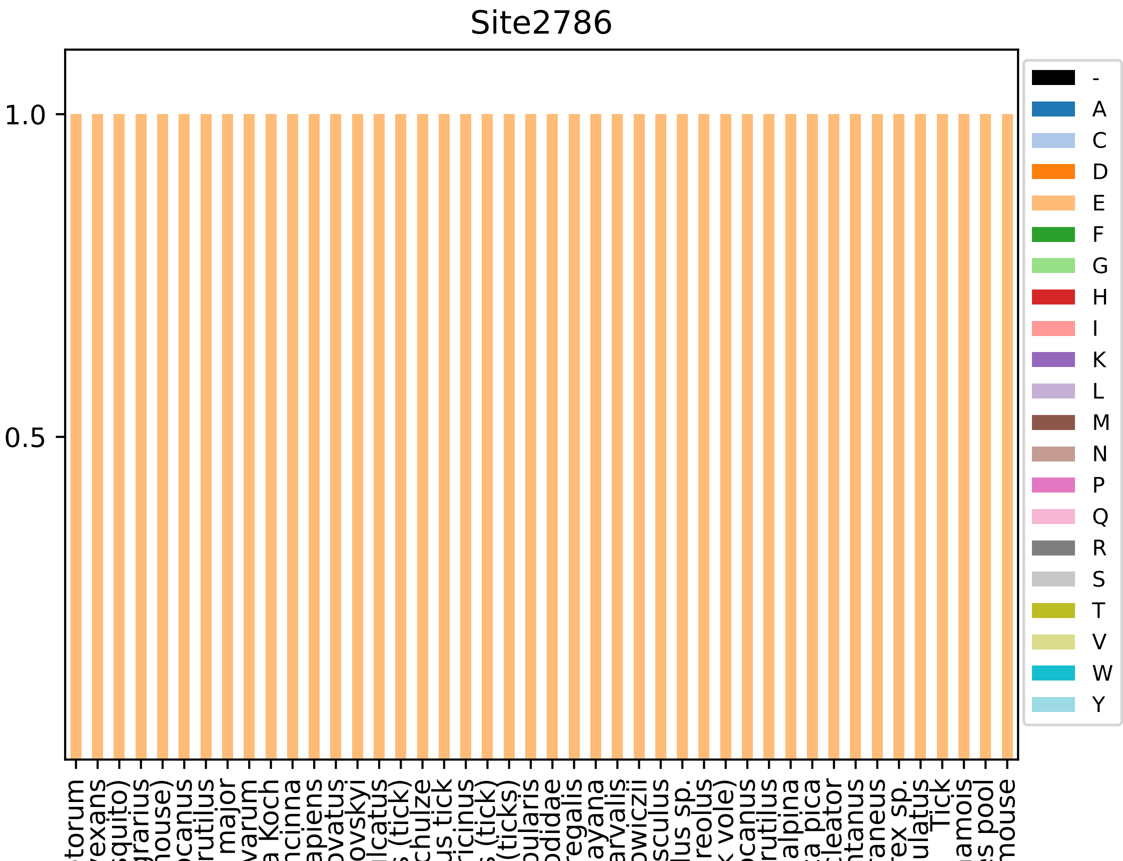

Site2786
1.0
0.5
-
A
C
D
E
F
G
H
I
K
L
M
N
P
Q
R
S
T
V
W
Y

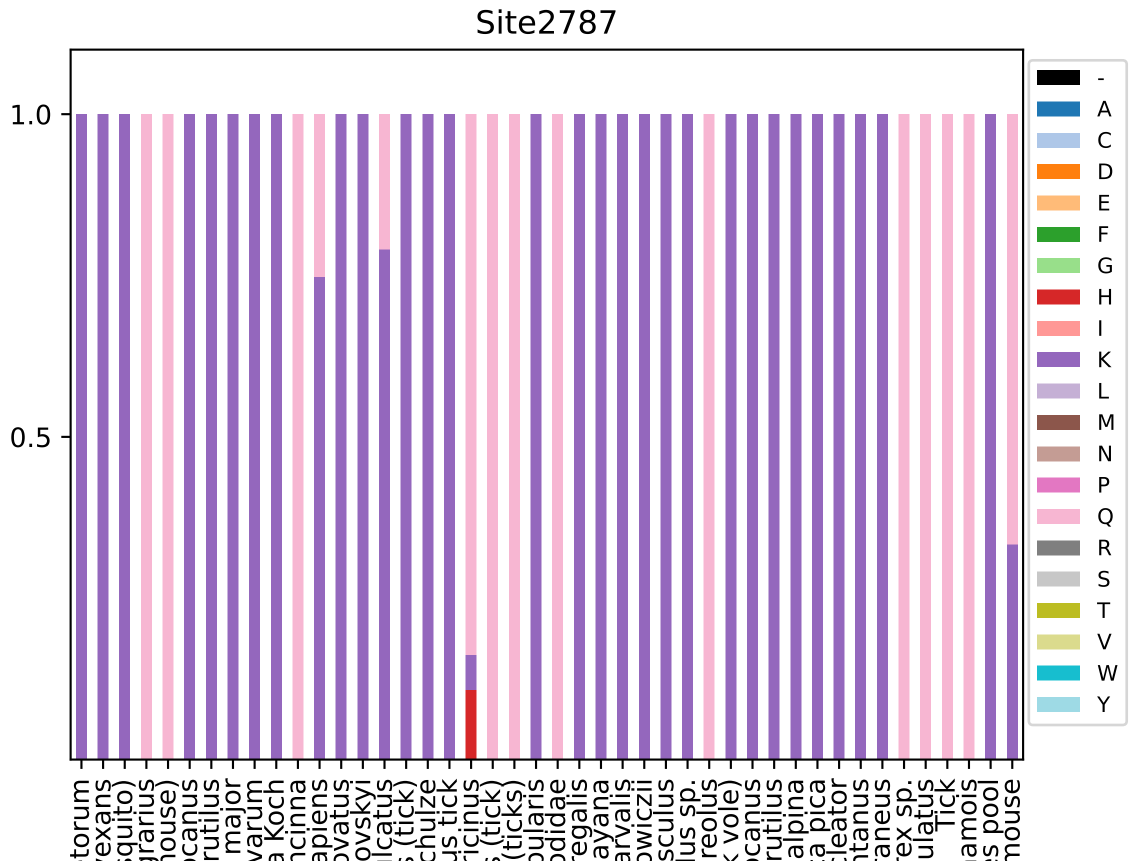
Site2787
1.0
0.5
-
A
C
D
E
F
G
H
I
K
L
M
N
P
Q
R
S
T
V
W
Y

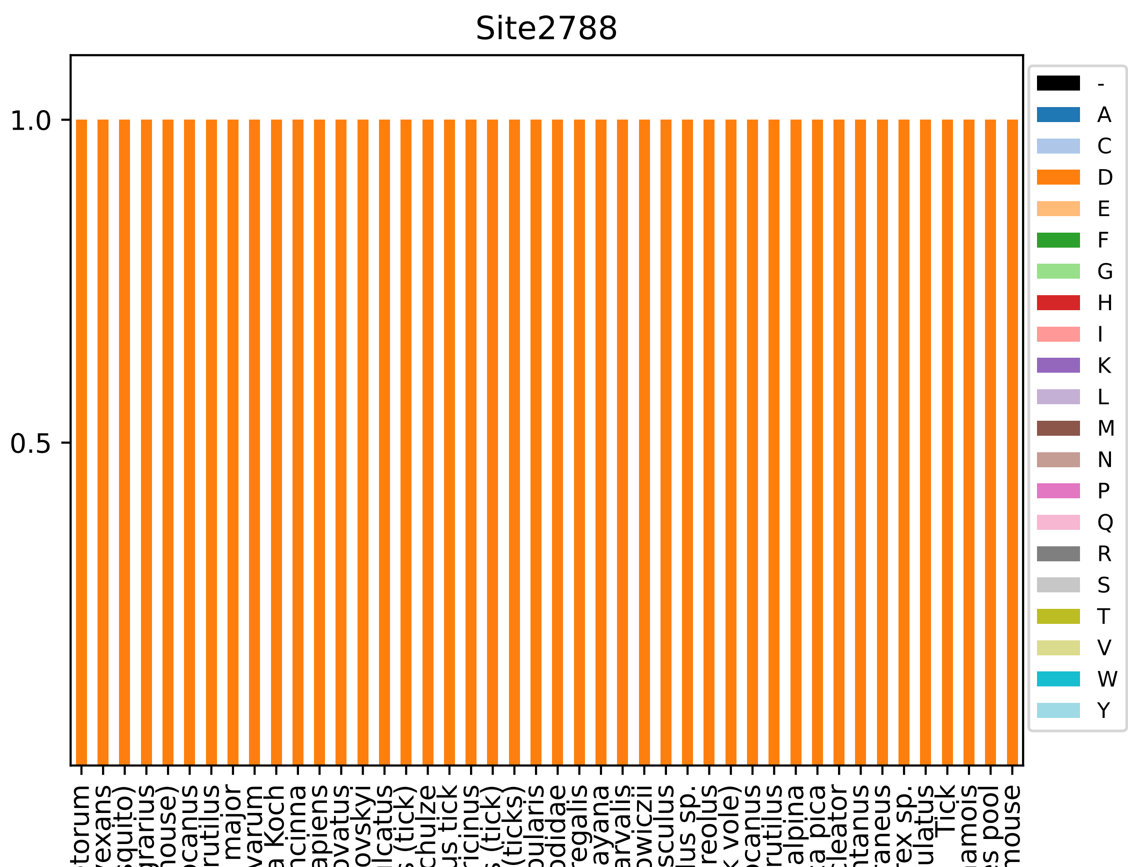

Site2788
1.0
0.5
-
A
C
D
E
F
G
H
I
K
L
M
N
P
Q
R
S
T
V
W
Y
Tick

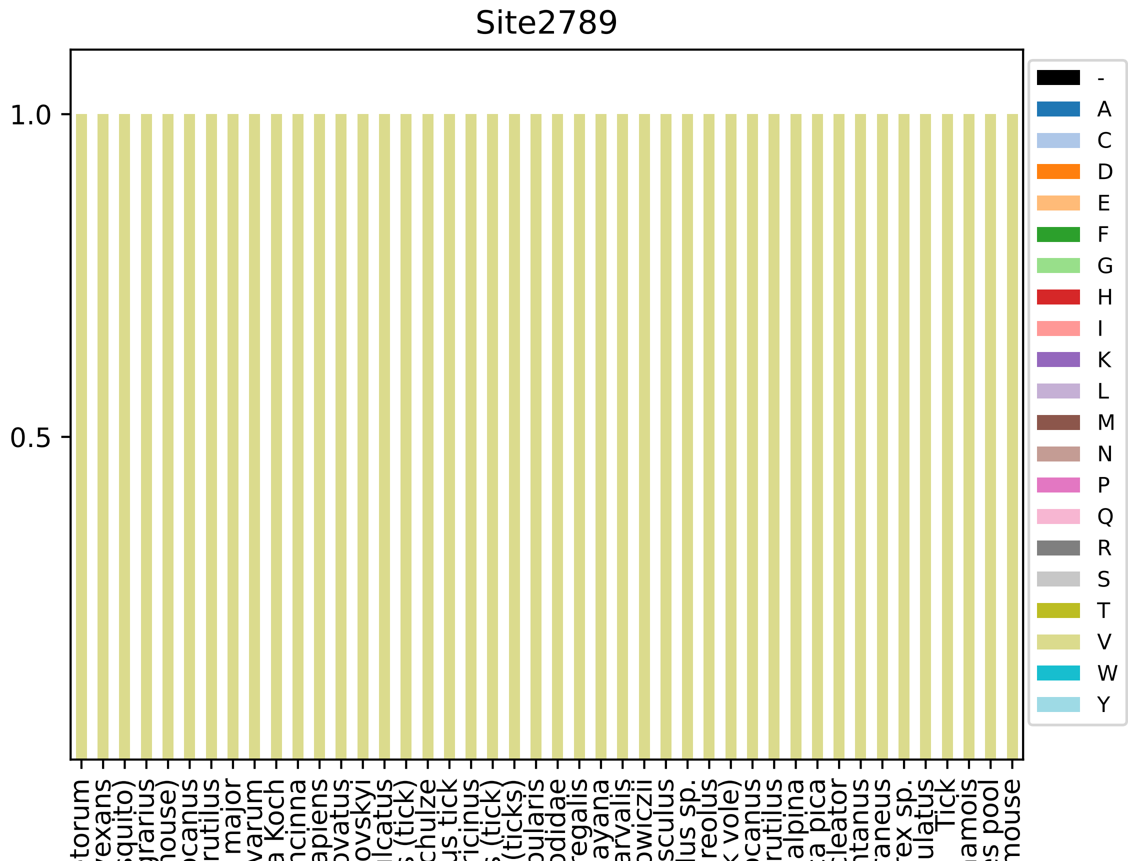
Site2789
1.0
0.5
-
A
C
D
E
F
G
H
I
K
L
M
N
P
Q
R
S
T
V
W
Y
torum
vexans
squito)
grarius
nouse)
ocanus
rutilus
major
varum
a Koch
ncinna
apiens
ovatus
ovskyi
ulcatus
s (tick)
chulze
us tick
ricinus
s (tick)
(ticks)
pularis
podidae
regalis
ayana
arvalis
owiczii
sculus
lus sp.
reolus
x vole)
ocanus
rutilus
alpina
a pica
tleator
ntanus
raneus
rex sp.
ulatus
Tick
amois
es pool
mouse

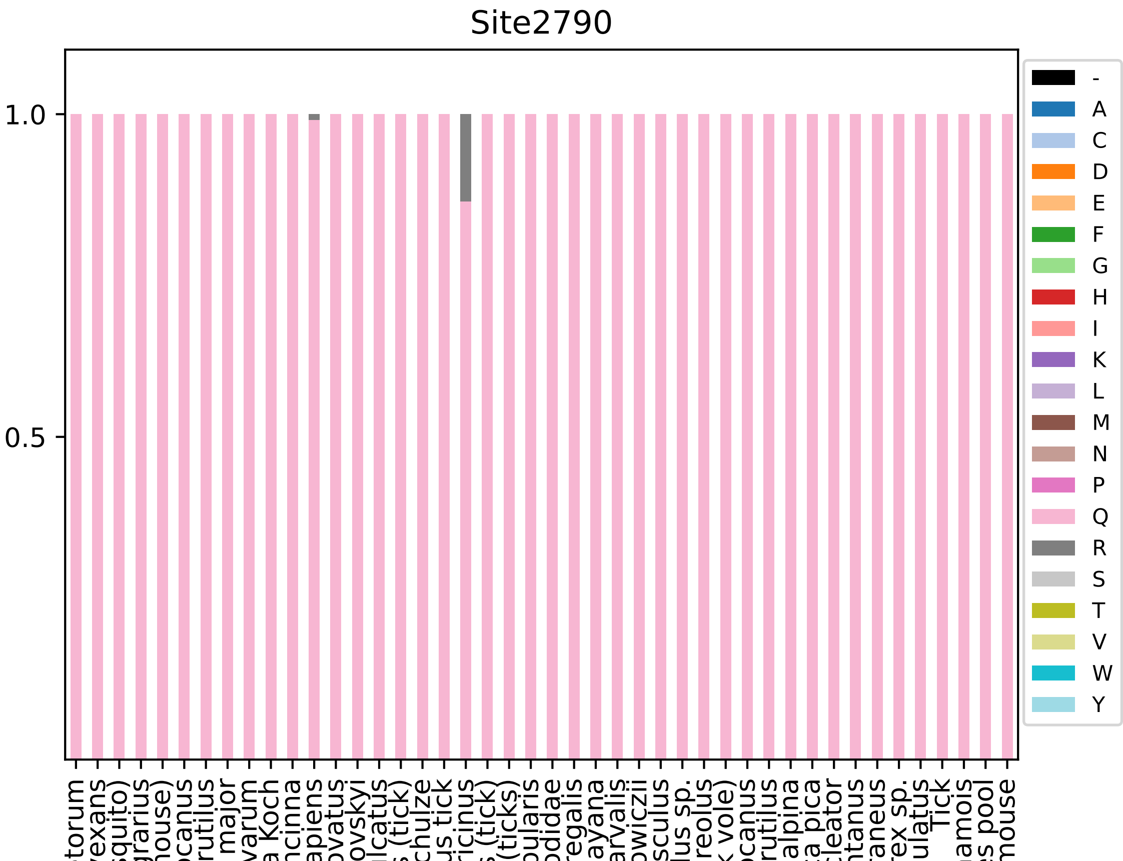
Site2790
1.0
0.5
-
A
C
D
E
F
G
H
I
K
L
M
N
P
Q
R
S
T
V
W
Y

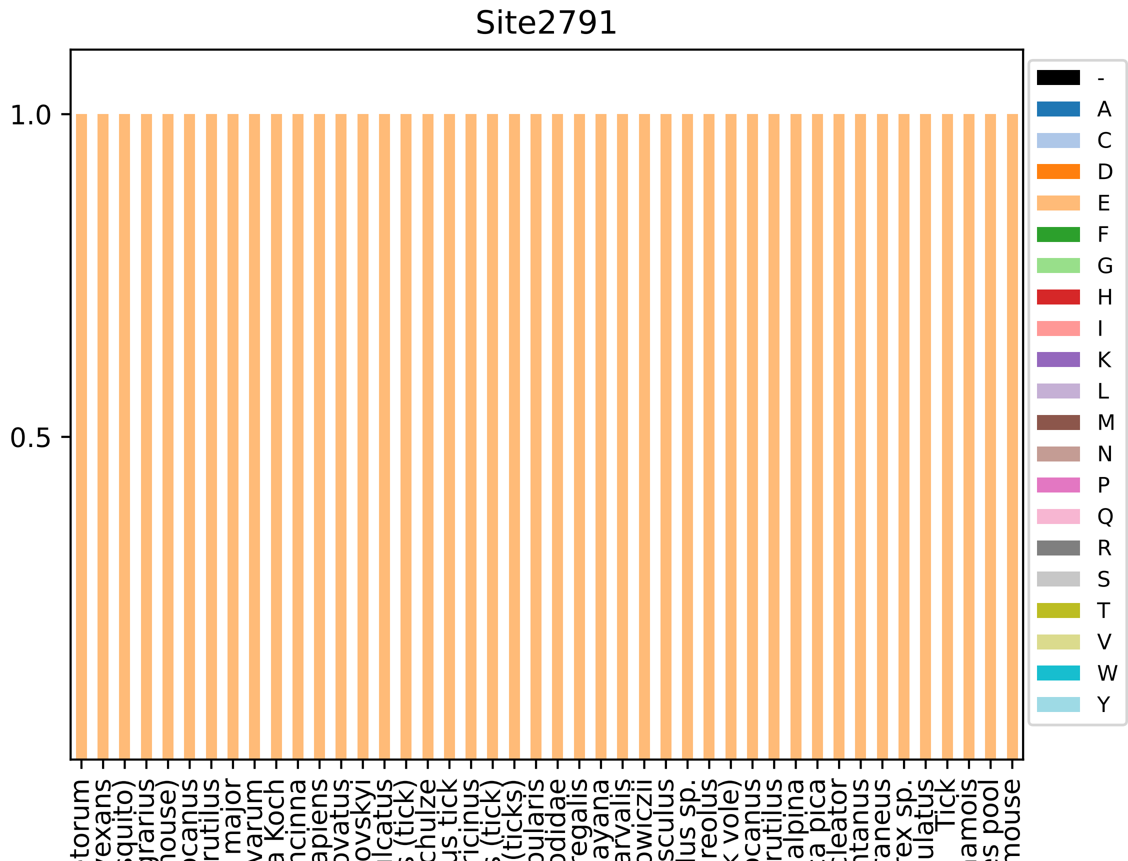
Site2791
1.0
0.5
-
A
C
D
E
F
G
H
I
K
L
M
N
P
Q
R
S
T
V
W
Y

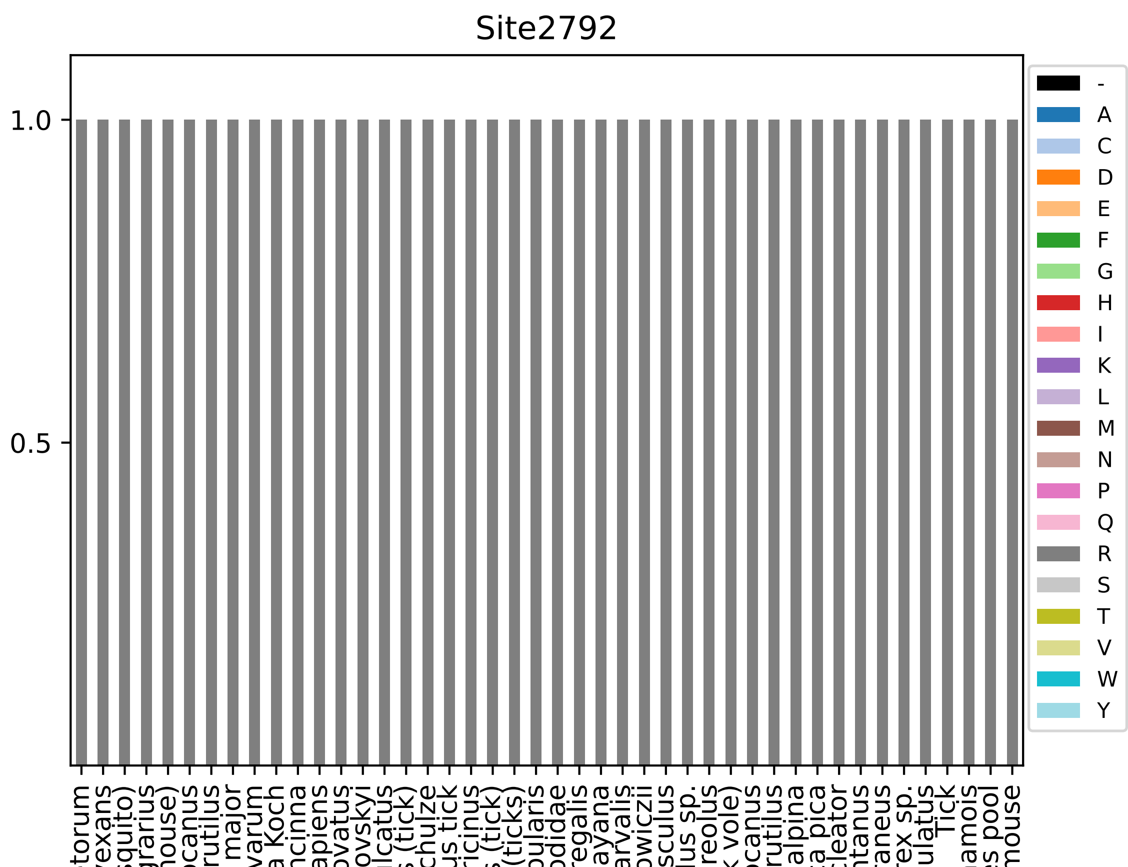

Site2792
1.0
0.5
-
A
C
D
E
F
G
H
I
K
L
M
N
P
Q
R
S
T
V
W
Y

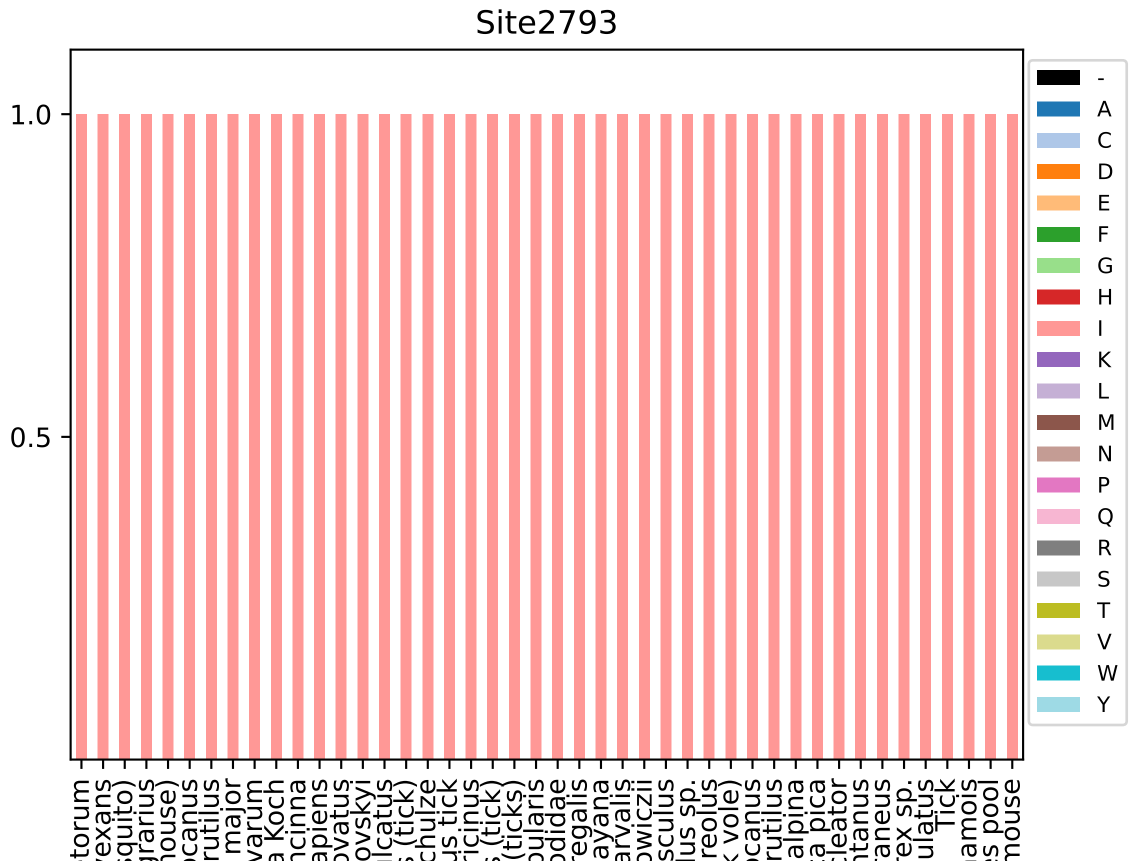
Site2793
1.0
0.5
-
A
C
D
E
F
G
H
I
K
L
M
N
P
Q
R
S
T
V
W
Y

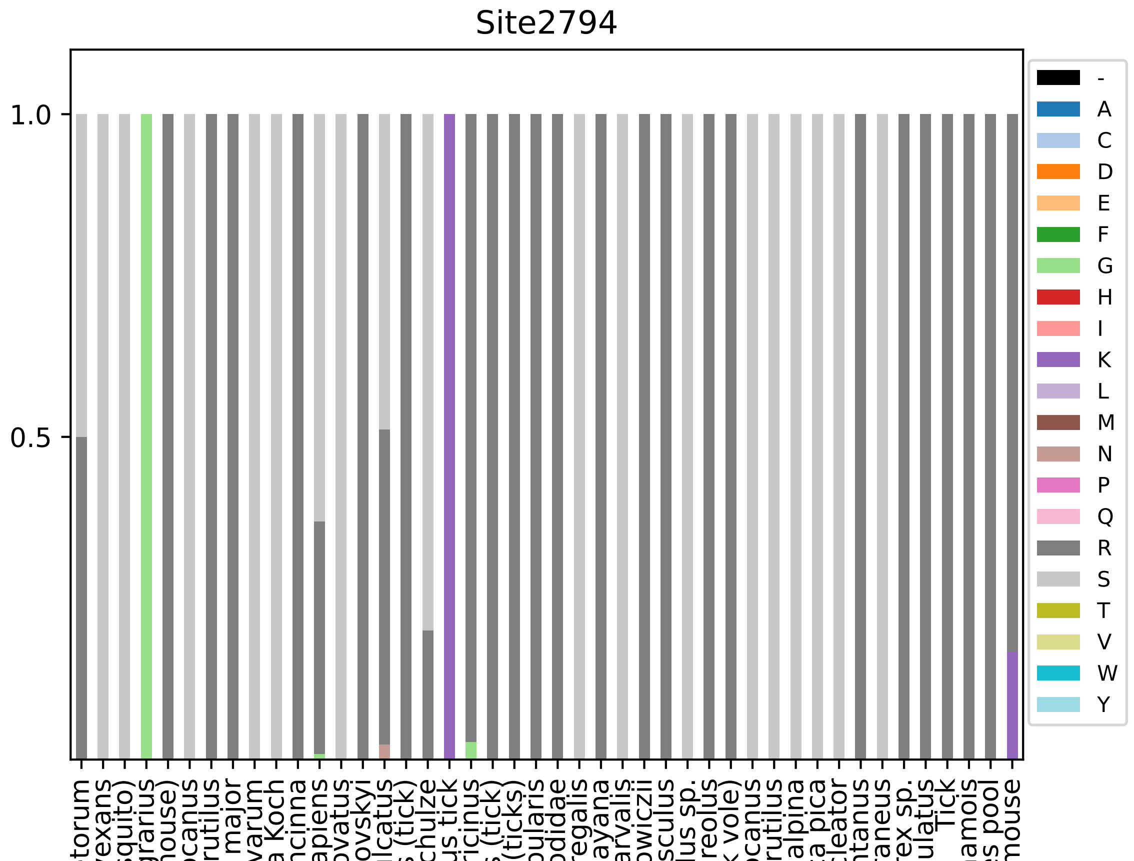

Site2794
1.0
0.5
-
A
C
D
E
F
G
H
I
K
L
M
N
P
Q
R
S
T
V
W
Y

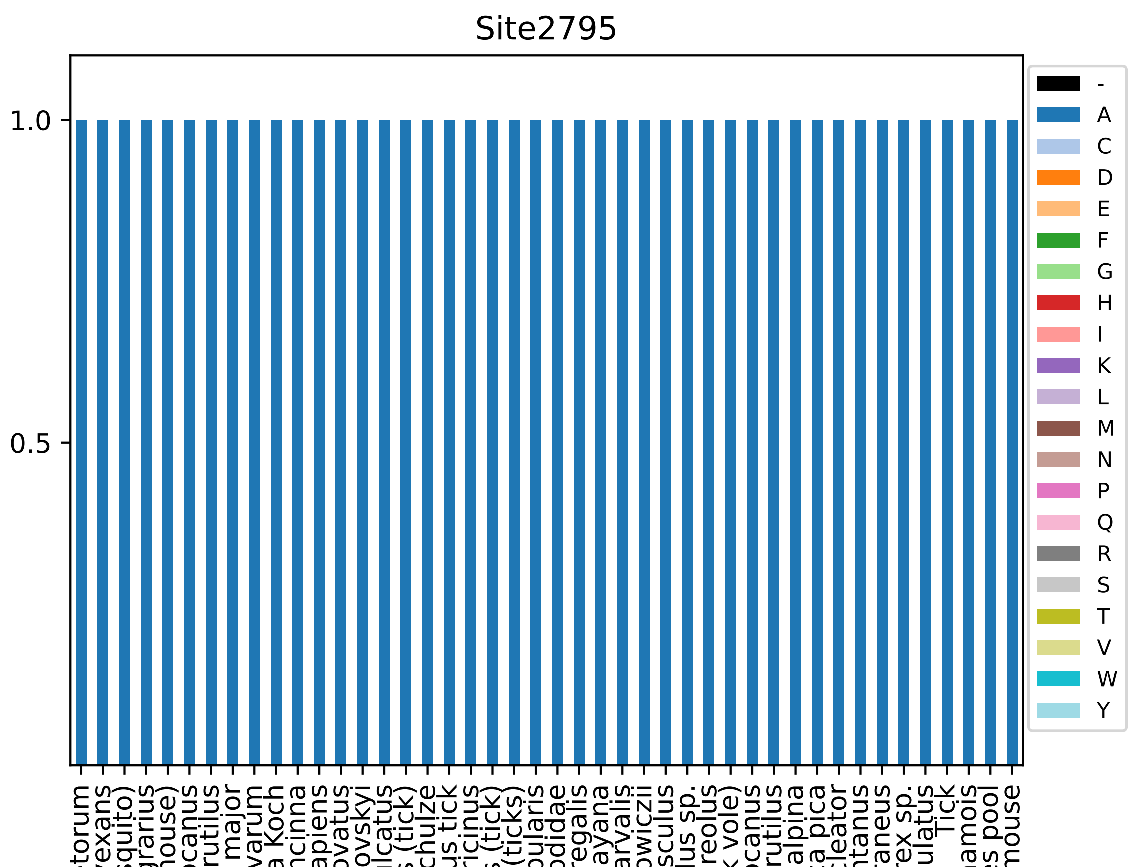
Site2795
1.0
0.5
-
A
C
D
E
F
G
H
I
K
L
M
N
P
Q
R
S
T
V
W
Y

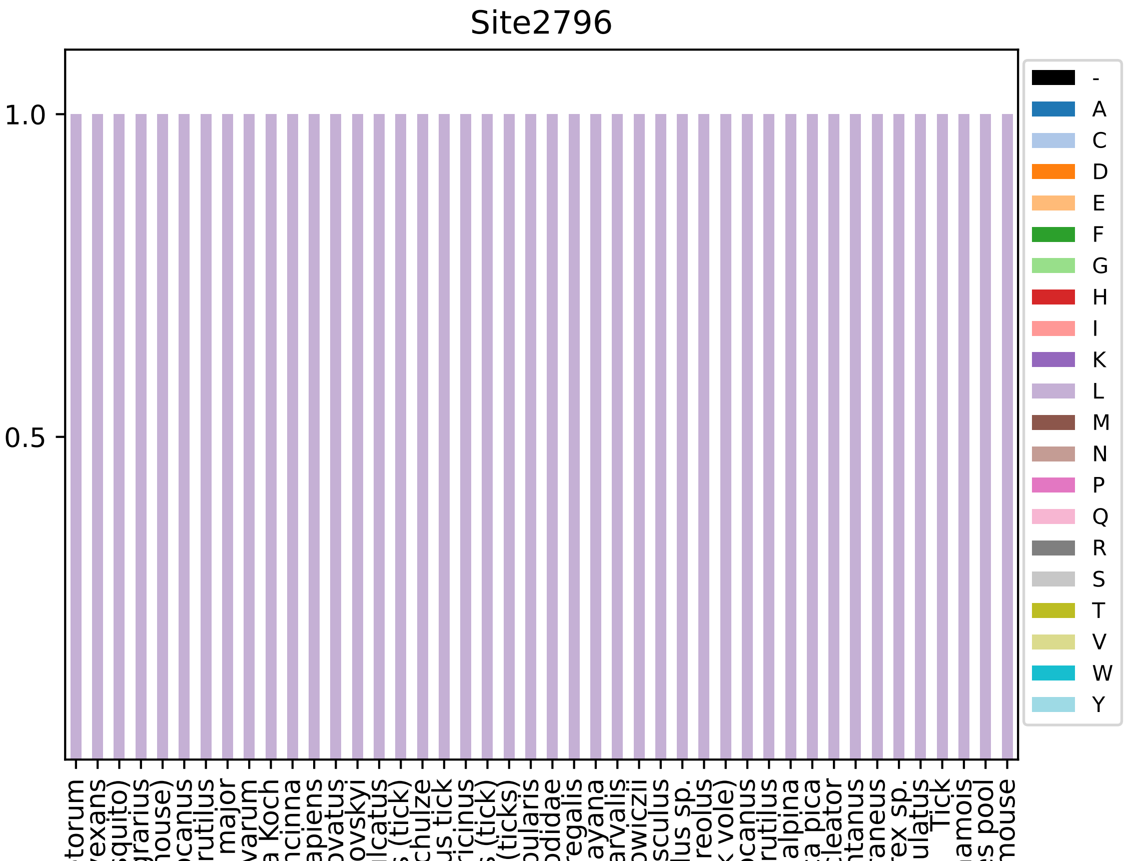
Site2796
1.0
0.5
-
A
C
D
E
F
G
H
I
K
L
M
N
P
Q
R
S
T
V
W
Y

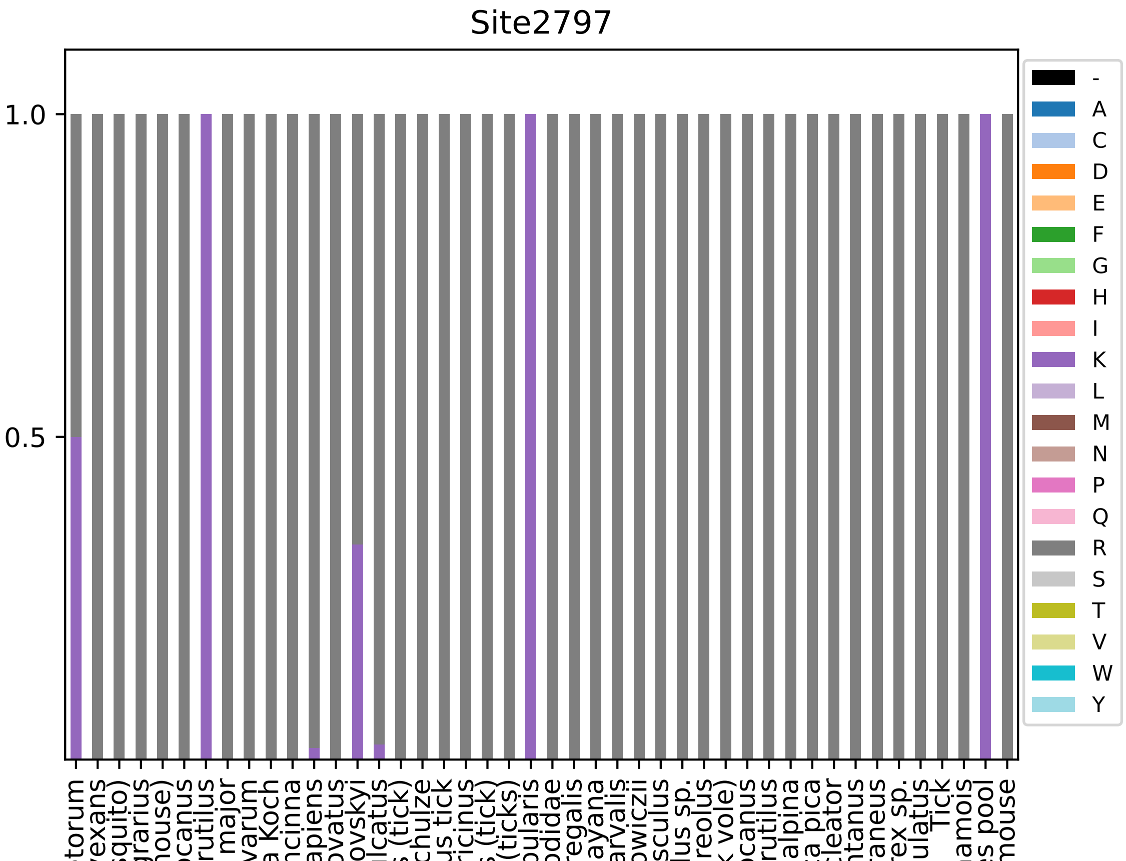
Site2797
1.0
0.5
-
A
C
D
E
F
G
H
I
K
L
M
N
P
Q
R
S
T
V
W
Y
Tick

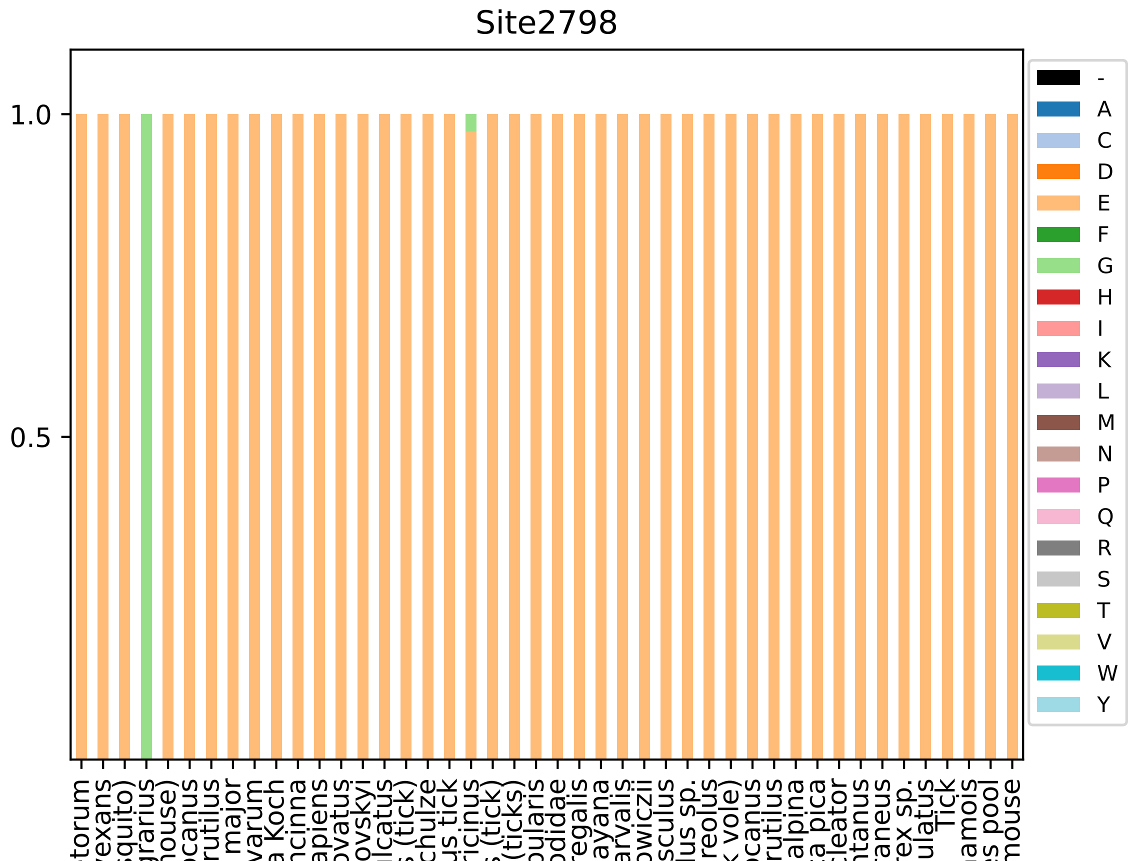
Site2798
1.0
0.5
-
A
C
D
E
F
G
H
I
K
L
M
N
P
Q
R
S
T
V
W
Y

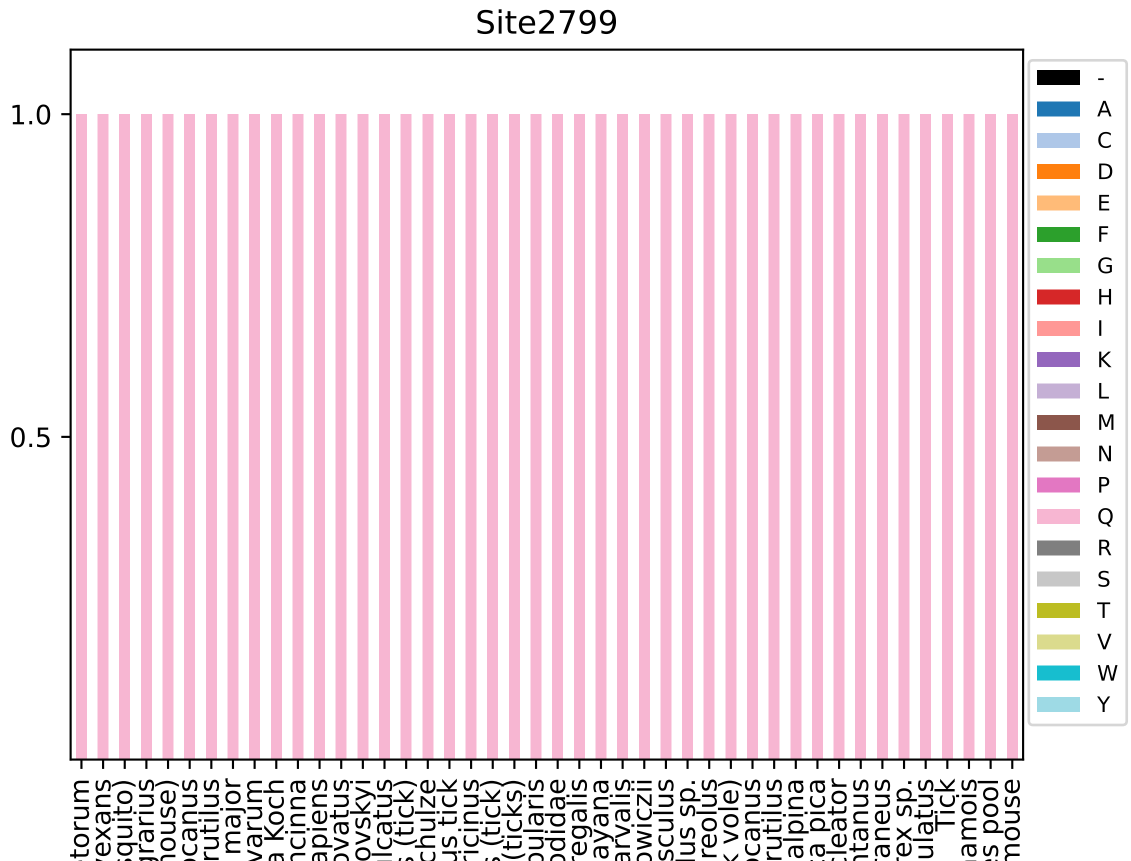
Site2799
1.0
0.5
-
A
C
D
E
F
G
H
I
K
L
M
N
P
Q
R
S
T
V
W
Y

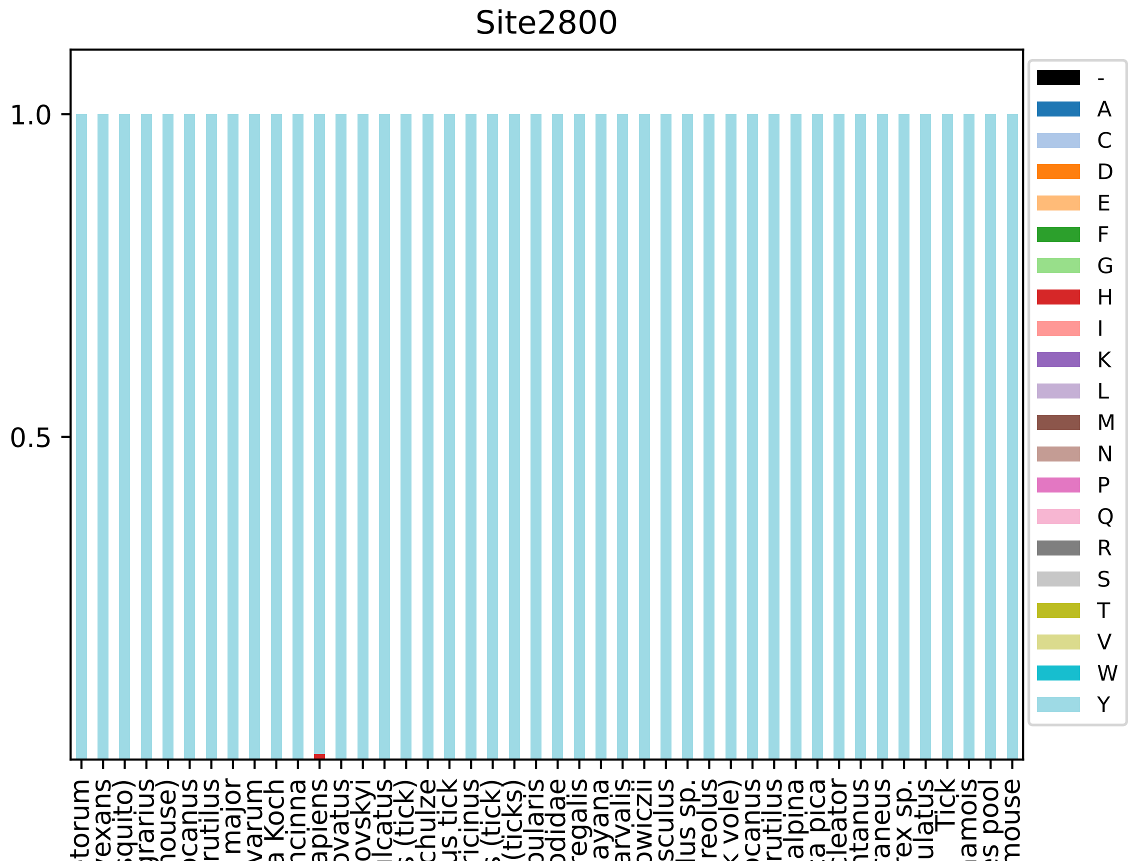
Site2800
1.0
0.5
-
A
C
D
E
F
G
H
I
K
L
M
N
P
Q
R
S
T
V
W
Y

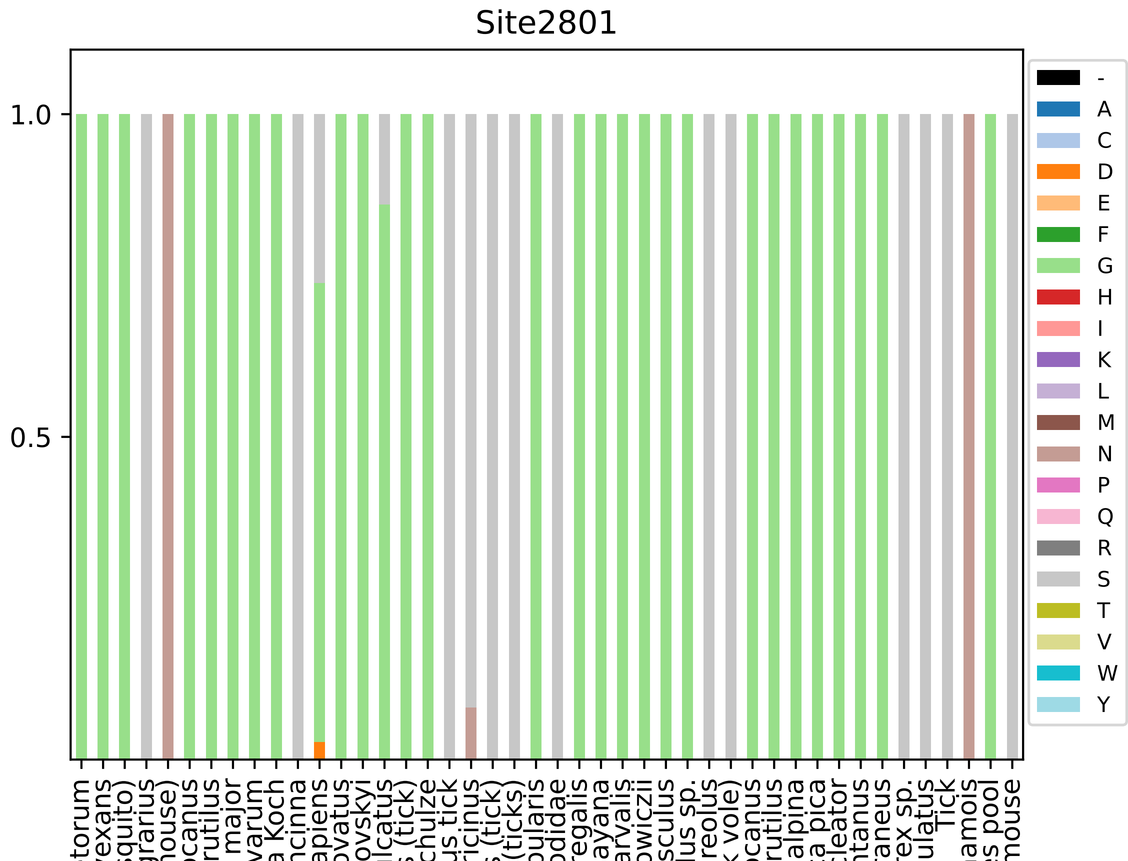
Site2801
1.0
0.5
-
A
C
D
E
F
G
H
I
K
L
M
N
P
Q
R
S
T
V
W
Y

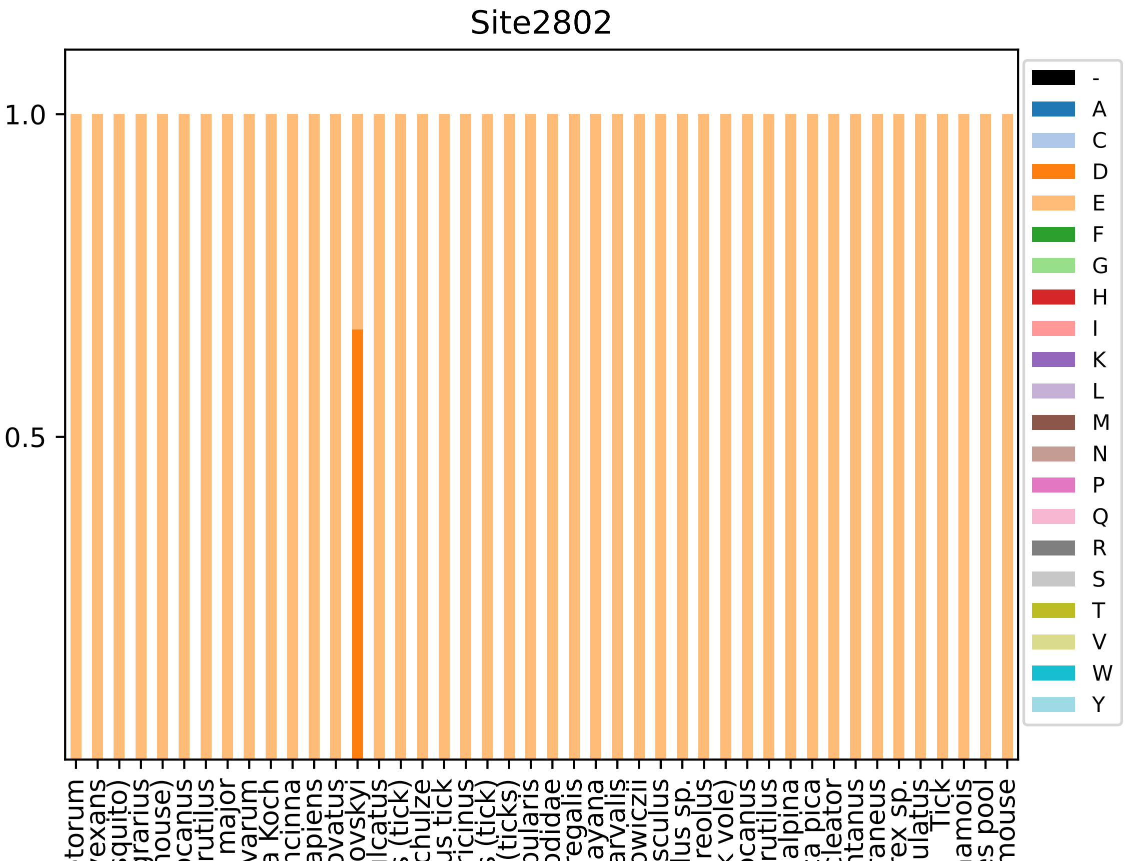
Site2802
1.0
0.5
-
A
C
D
E
F
G
H
I
K
L
M
N
P
Q
R
S
T
V
W
Y

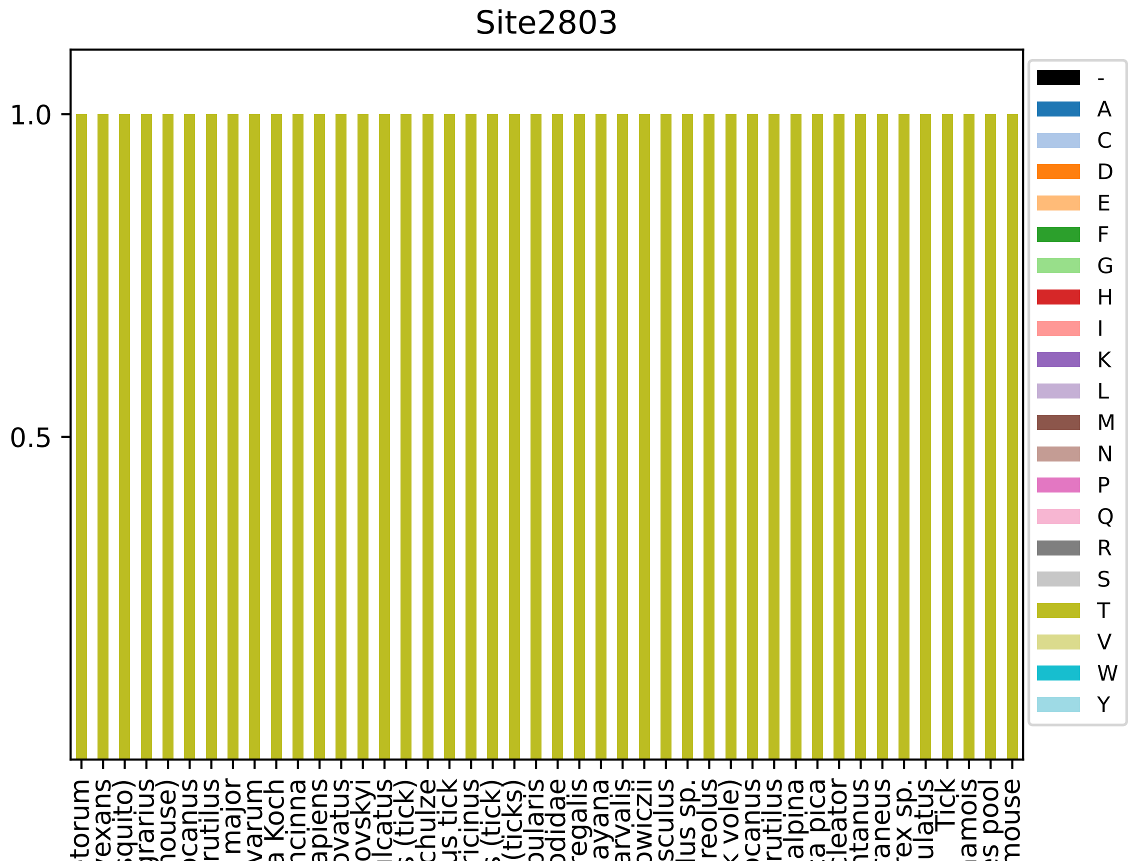

Site2803
1.0
0.5
-
A
C
D
E
F
G
H
I
K
L
M
N
P
Q
R
S
T
V
W
Y
Tick

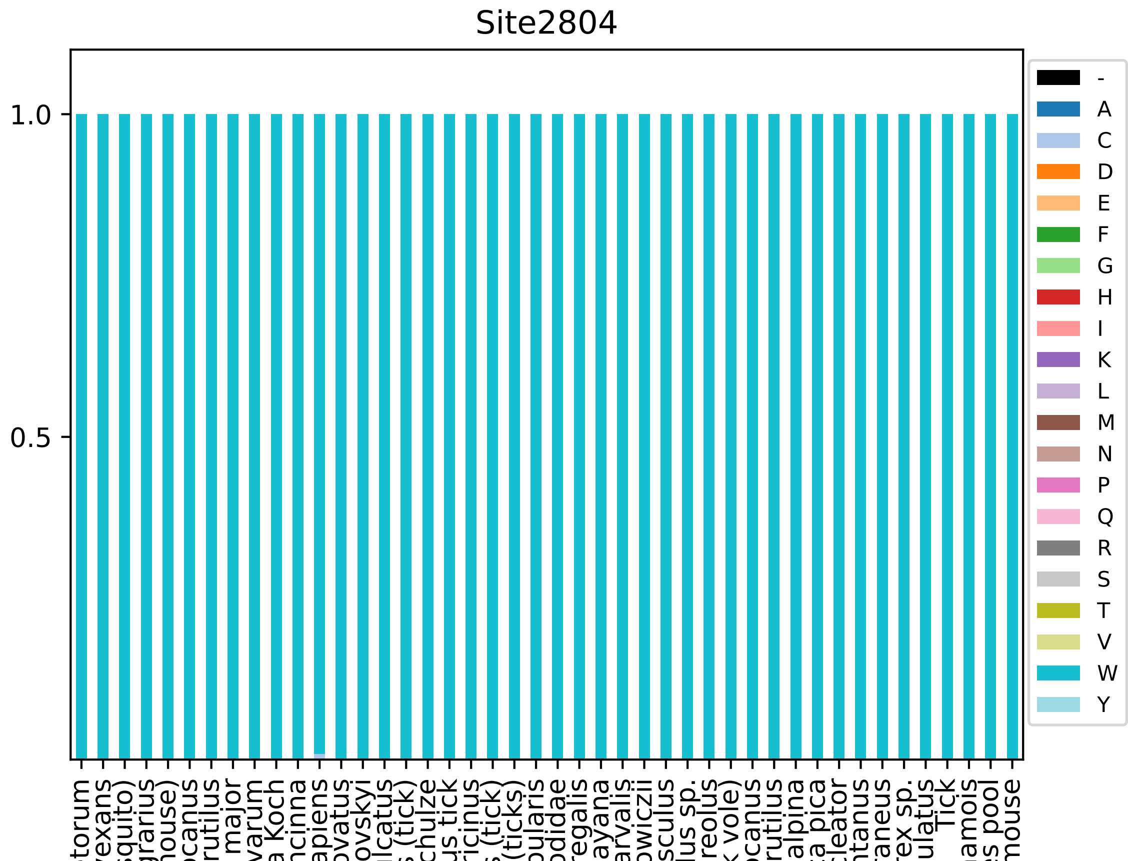
Site2804
1.0
0.5
-
A
C
D
E
F
G
H
I
K
L
M
N
P
Q
R
S
T
V
W
Y

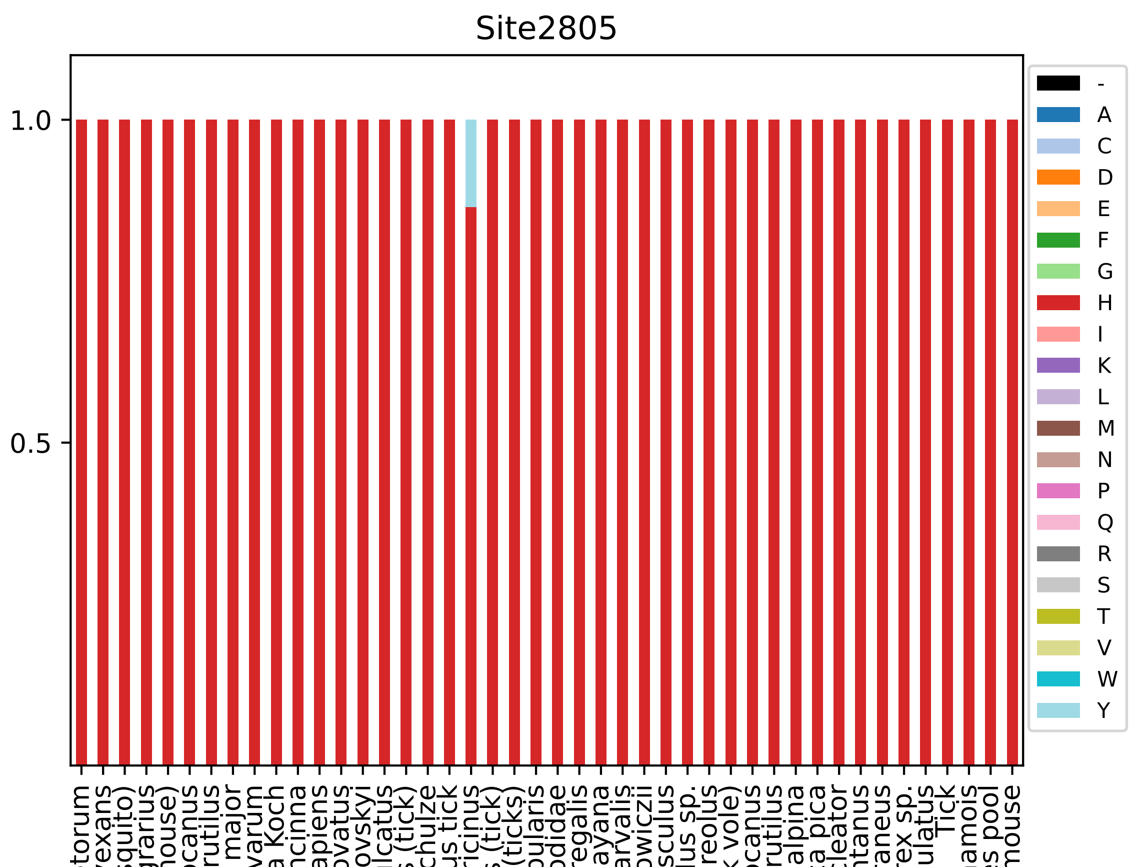
Site2805
1.0
0.5
-
A
C
D
E
F
G
H
I
K
L
M
N
P
Q
R
S
T
V
W
Y
torum
vexans
squito)
grarius
mouse)
ocanus
rutilus
major
varum
a Koch
ncinna
apiens
ovatus
ovskyi
ulcatus
s (tick)
chulze
us tick
ricinus
s (tick)
(ticks)
pularis
odidae
regalis
ayana
arvalis
owiczii
sculus
lus sp.
reolus
x vole)
ocanus
rutilus
alpina
a pica
leator
ntanus
raneus
rex sp.
ulatus
Tick
amois
s pool
mouse

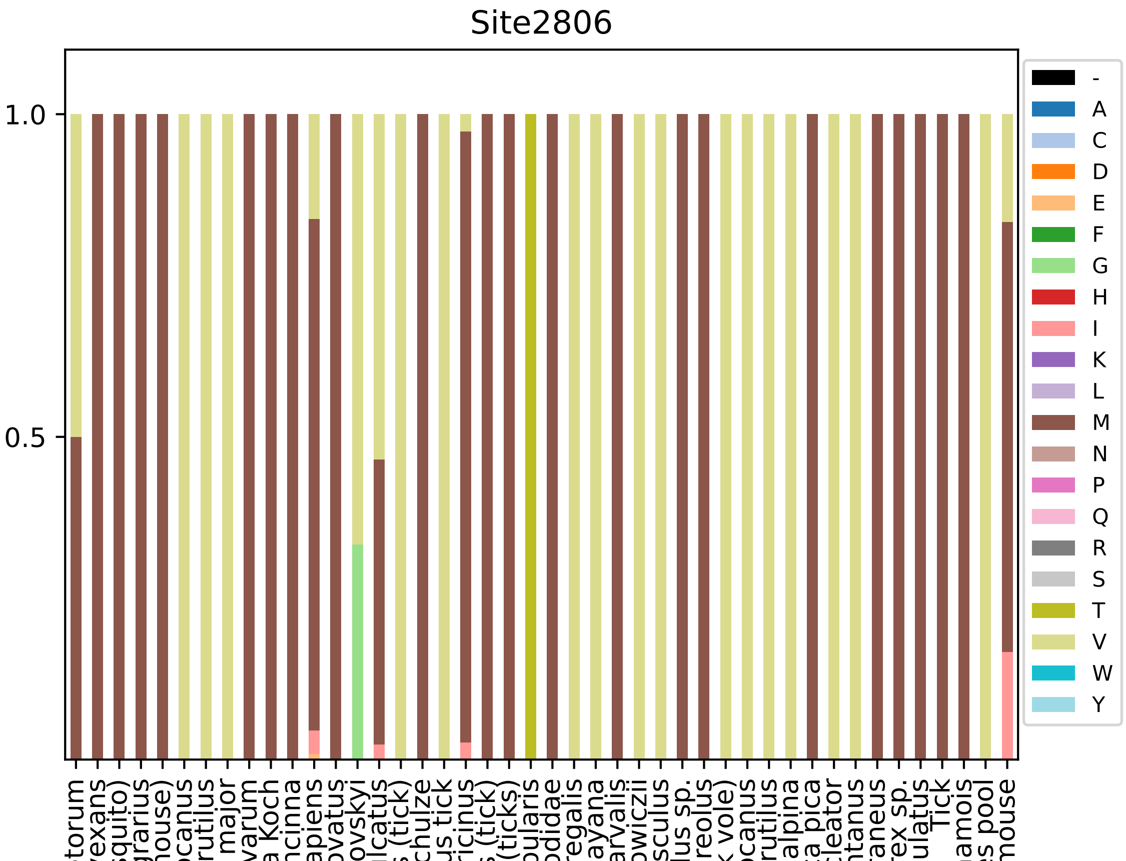
Site2806
1.0
0.5
-
A
C
D
E
F
G
H
I
K
L
M
N
P
Q
R
S
T
V
W
Y

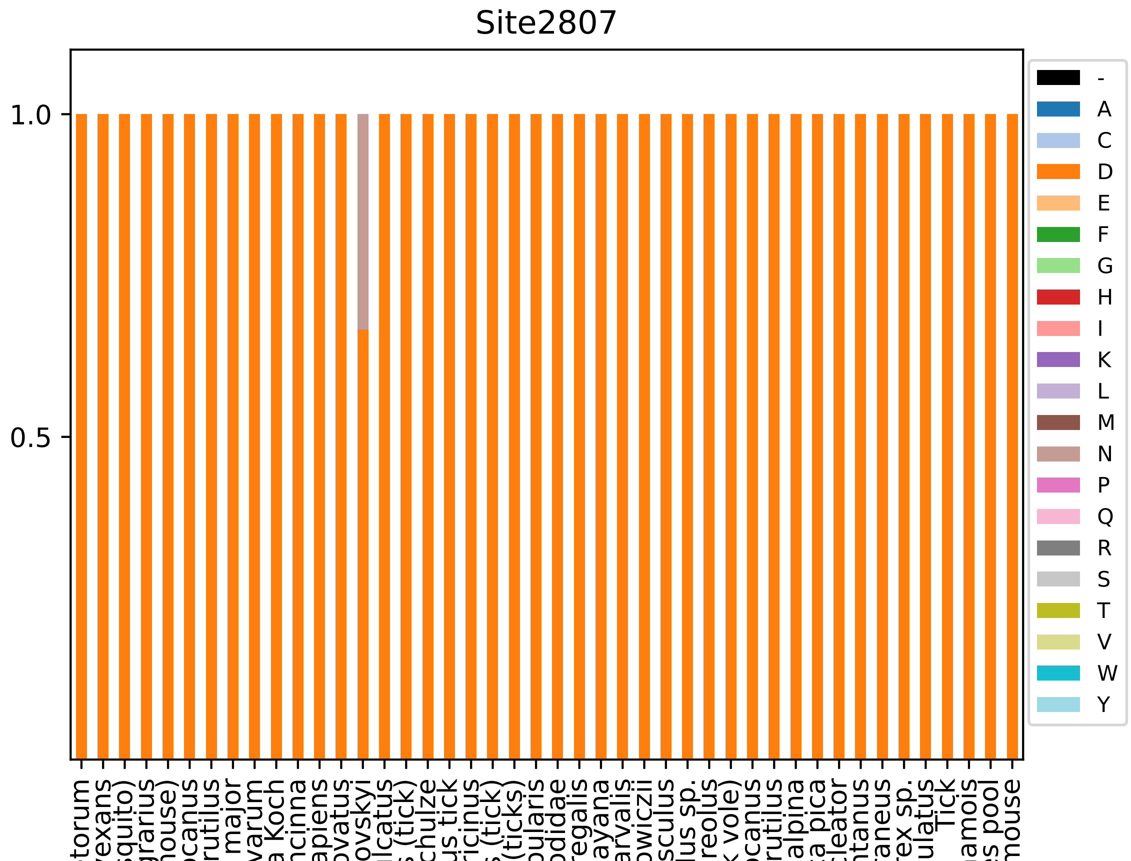
Site2807
1.0
0.5
-
A
C
D
E
F
G
H
I
K
L
M
N
P
Q
R
S
T
V
W
Y

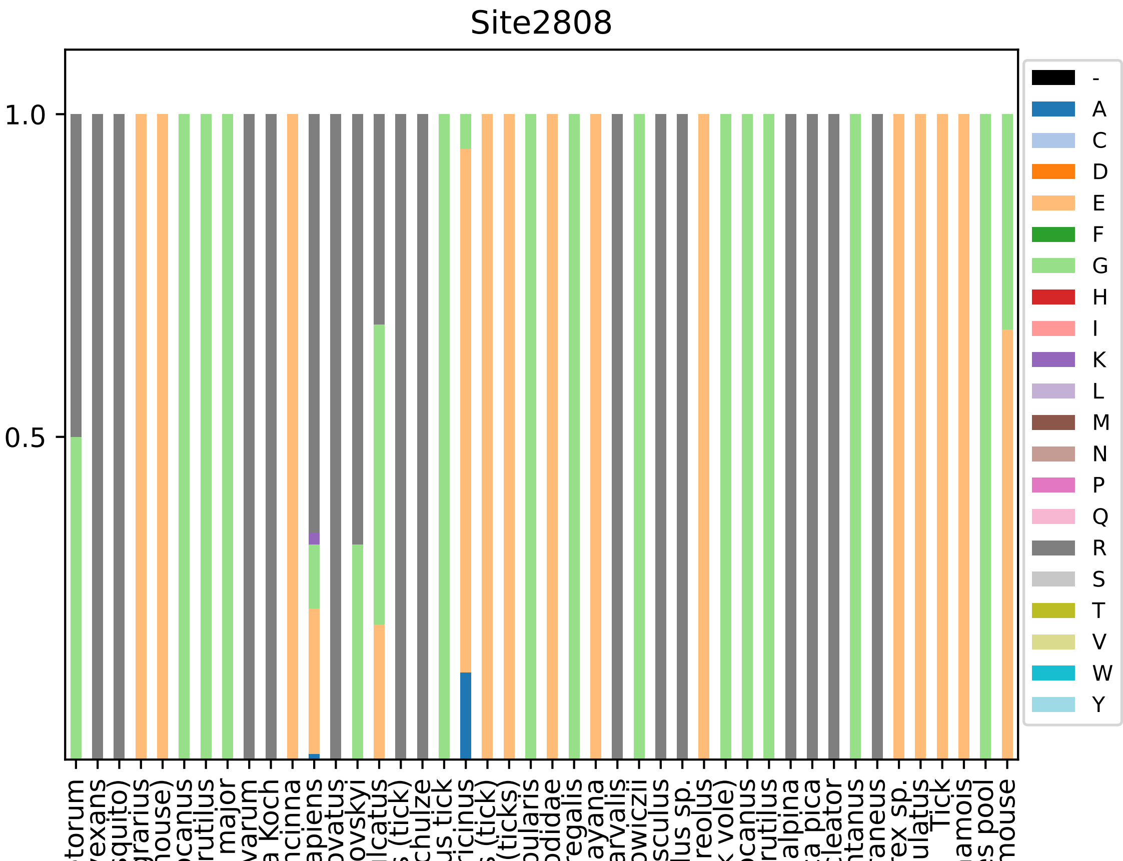
Site2808
1.0
0.5
-
A
C
D
E
F
G
H
I
K
L
M
N
P
Q
R
S
T
V
W
Y

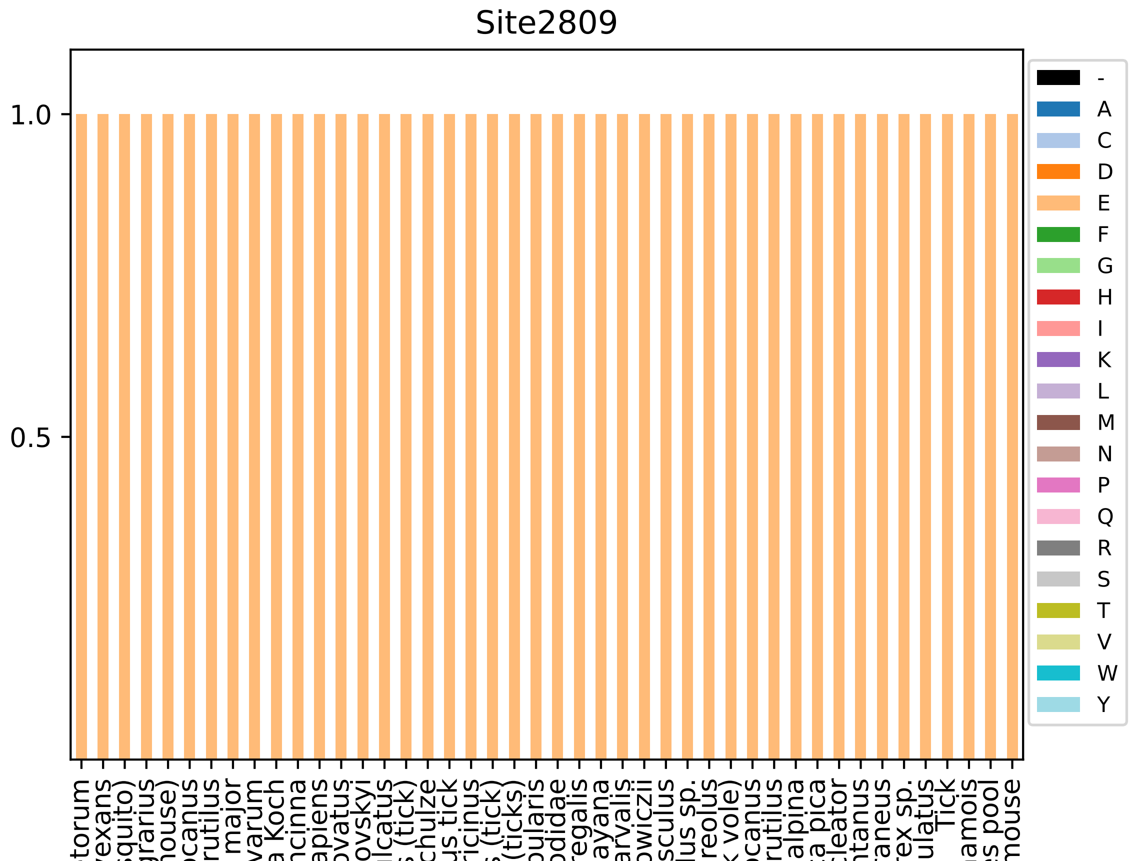

Site2809
1.0
0.5
-
A
C
D
E
F
G
H
I
K
L
M
N
P
Q
R
S
T
V
W
Y

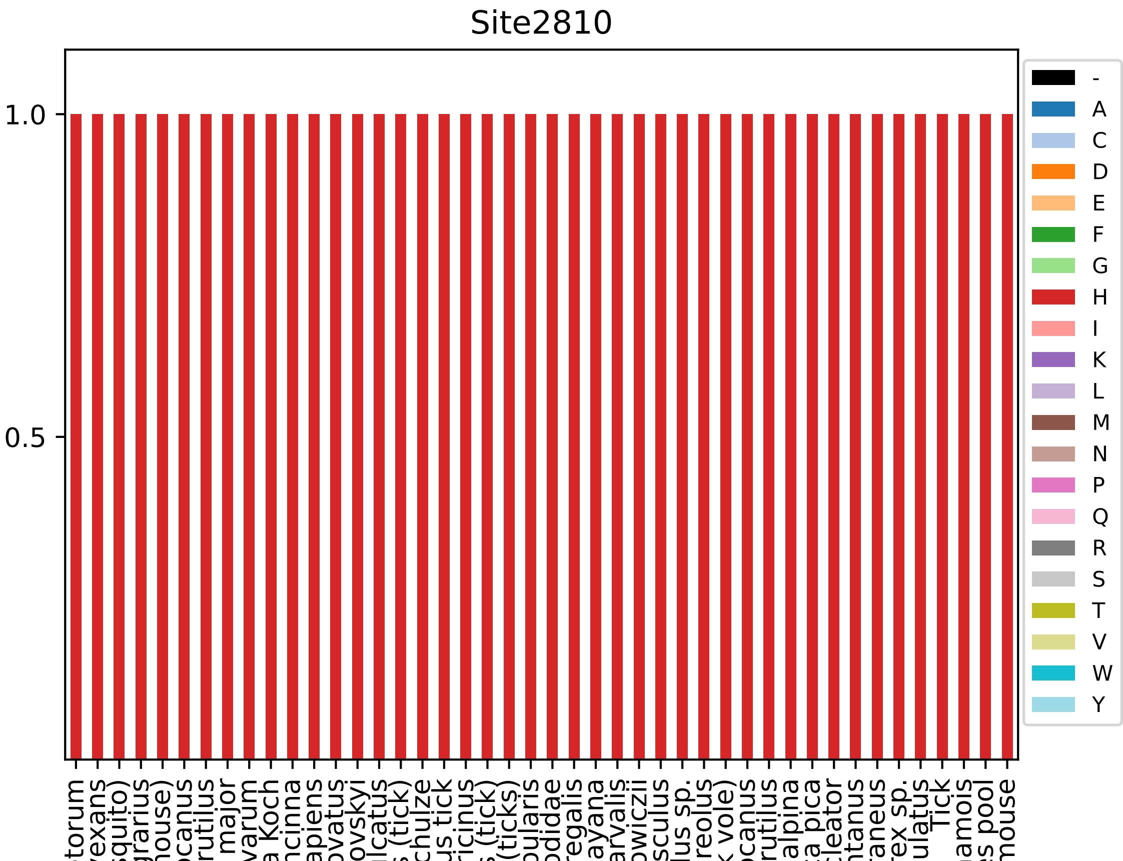
Site2810
1.0
0.5
-
A
C
D
E
F
G
H
I
K
L
M
N
P
Q
R
S
T
V
W
Y

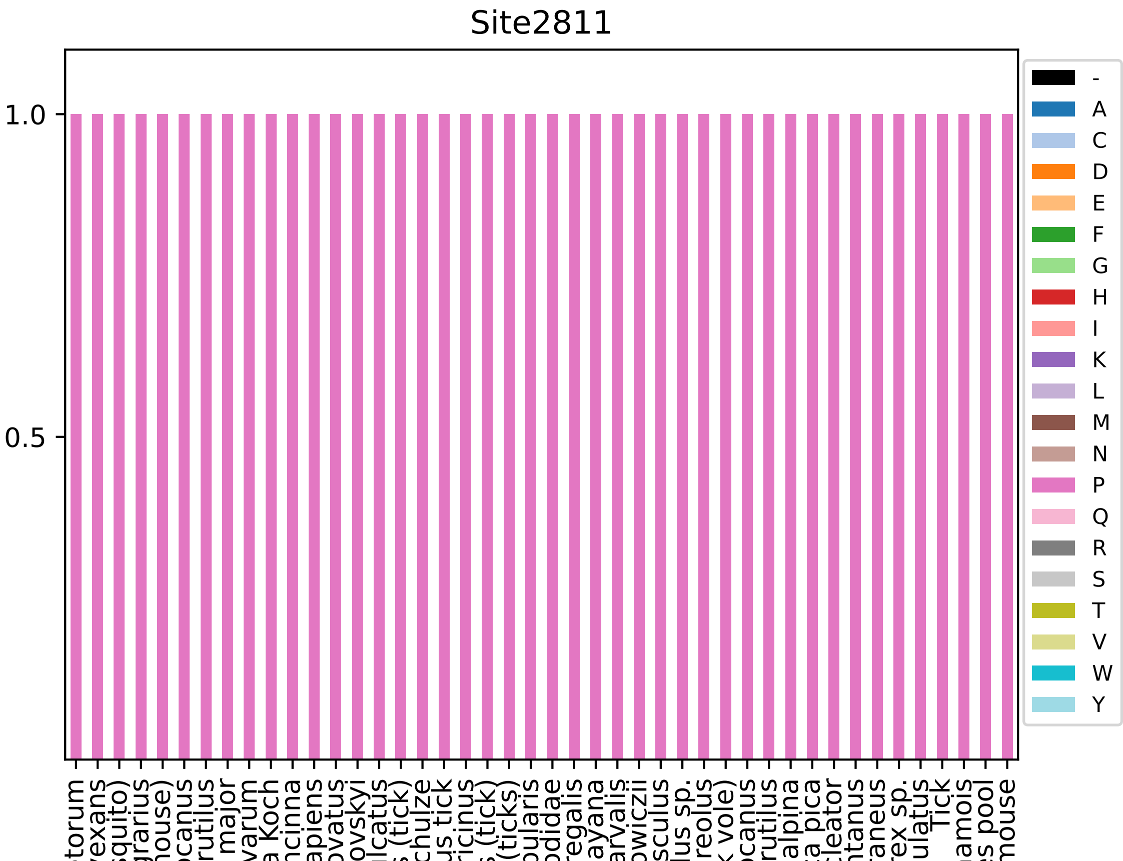
Site2811
1.0
0.5
-
A
C
D
E
F
G
H
I
K
L
M
N
P
Q
R
S
T
V
W
Y

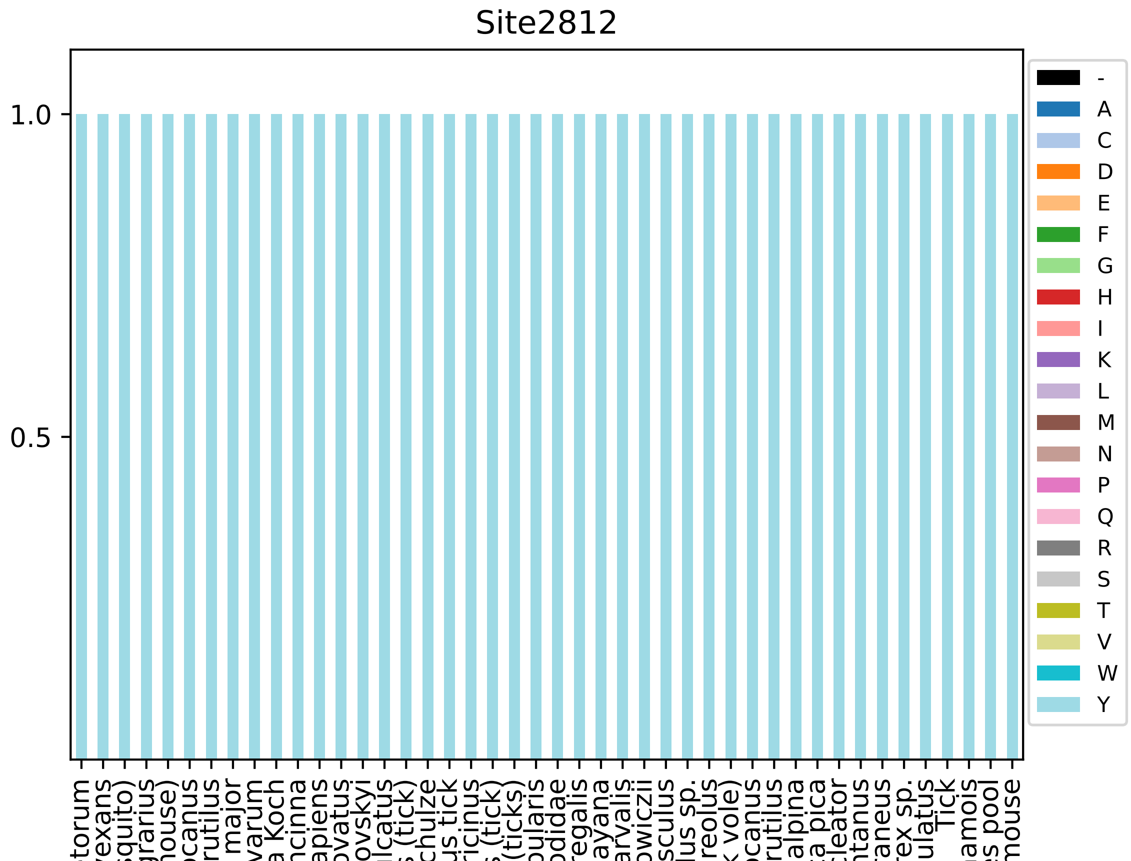
Site2812
1.0
0.5
-
A
C
D
E
F
G
H
I
K
L
M
N
P
Q
R
S
T
V
W
Y

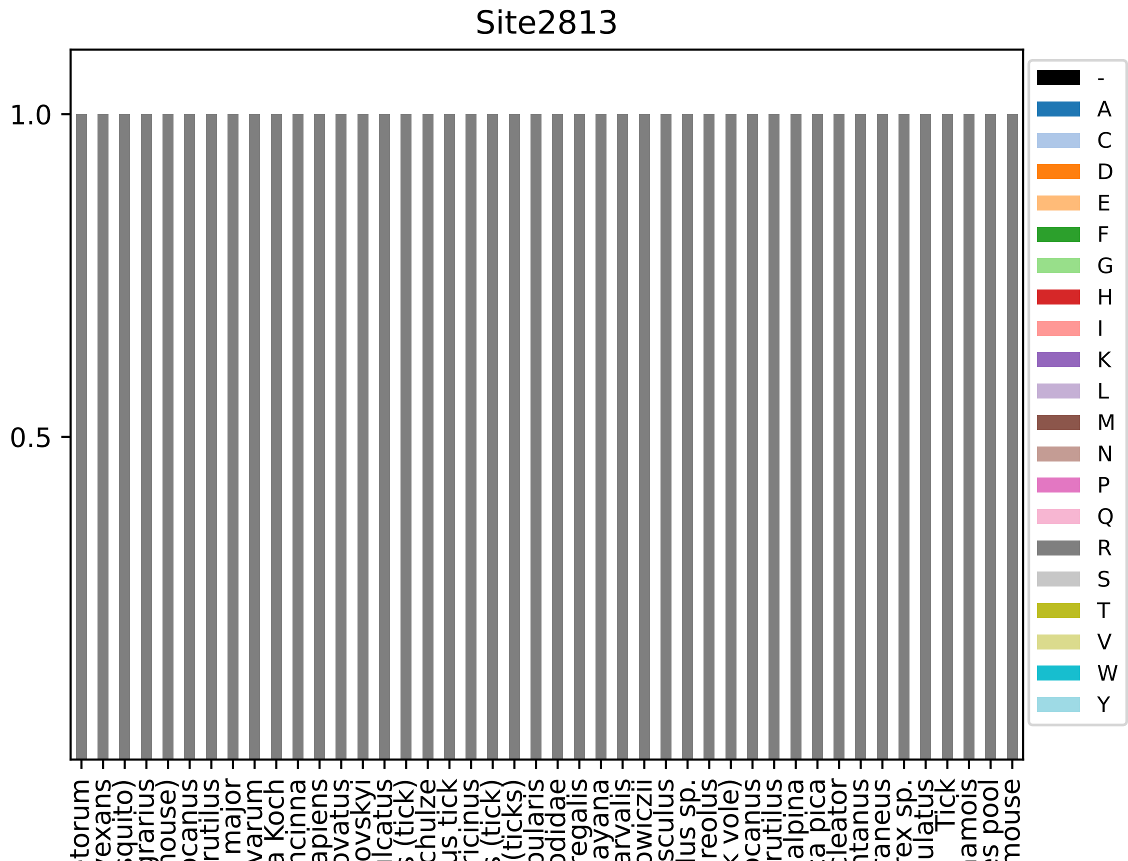
Site2813
1.0
0.5
-
A
C
D
E
F
G
H
I
K
L
M
N
P
Q
R
S
T
V
W
Y

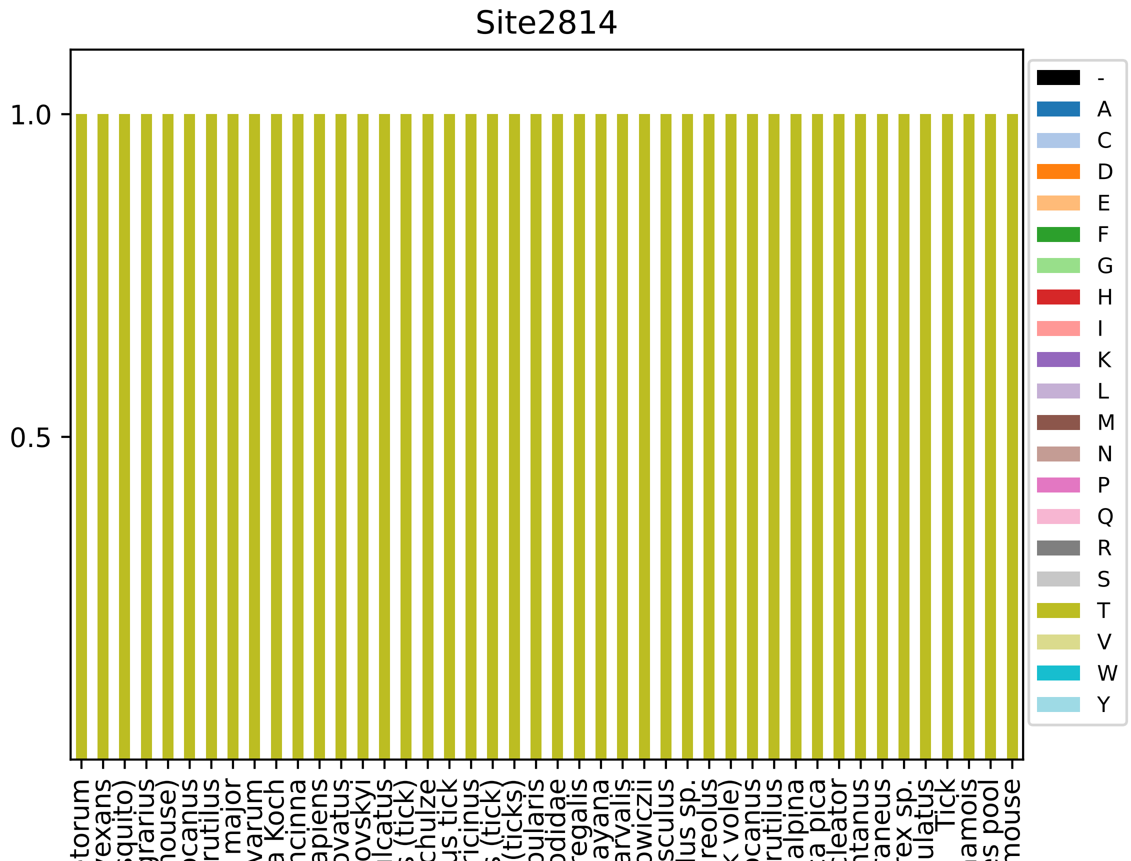

Site2814
1.0
0.5
-
A
C
D
E
F
G
H
I
K
L
M
N
P
Q
R
S
T
V
W
Y

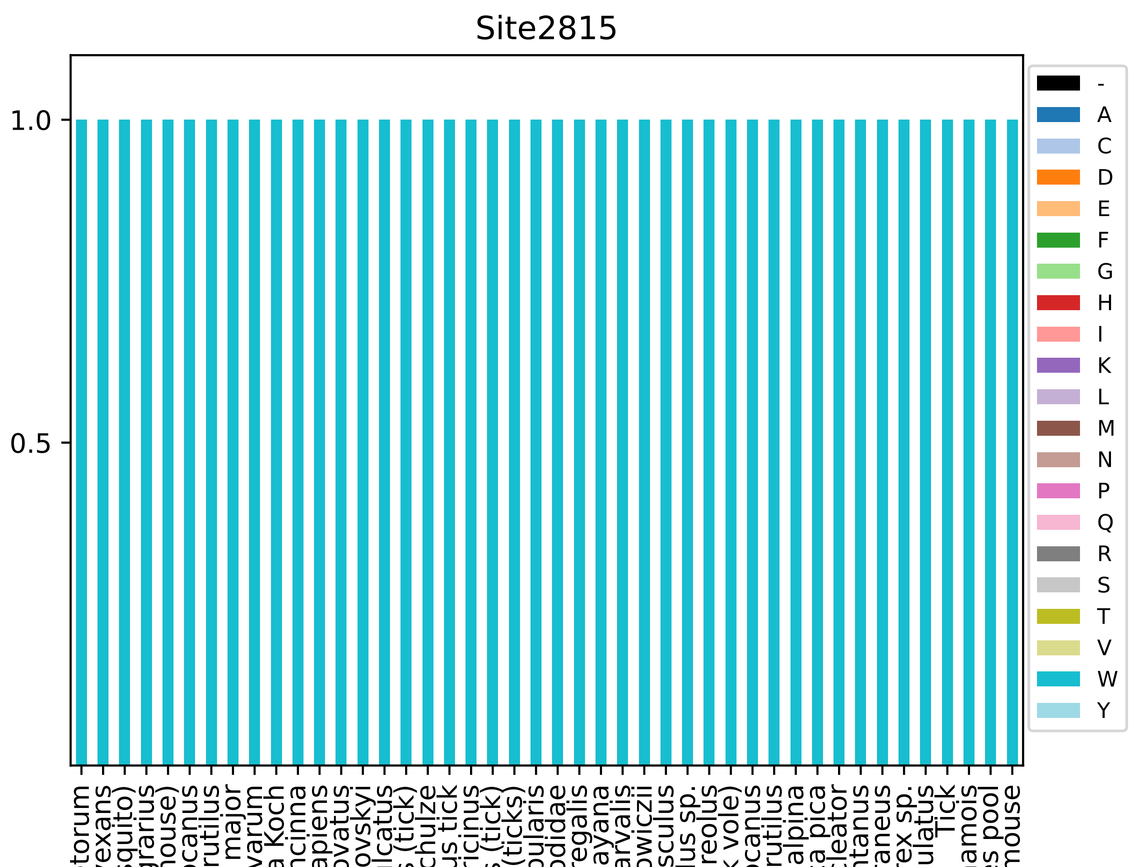
Site2815
1.0
0.5
-
A
C
D
E
F
G
H
I
K
L
M
N
P
Q
R
S
T
V
W
Y

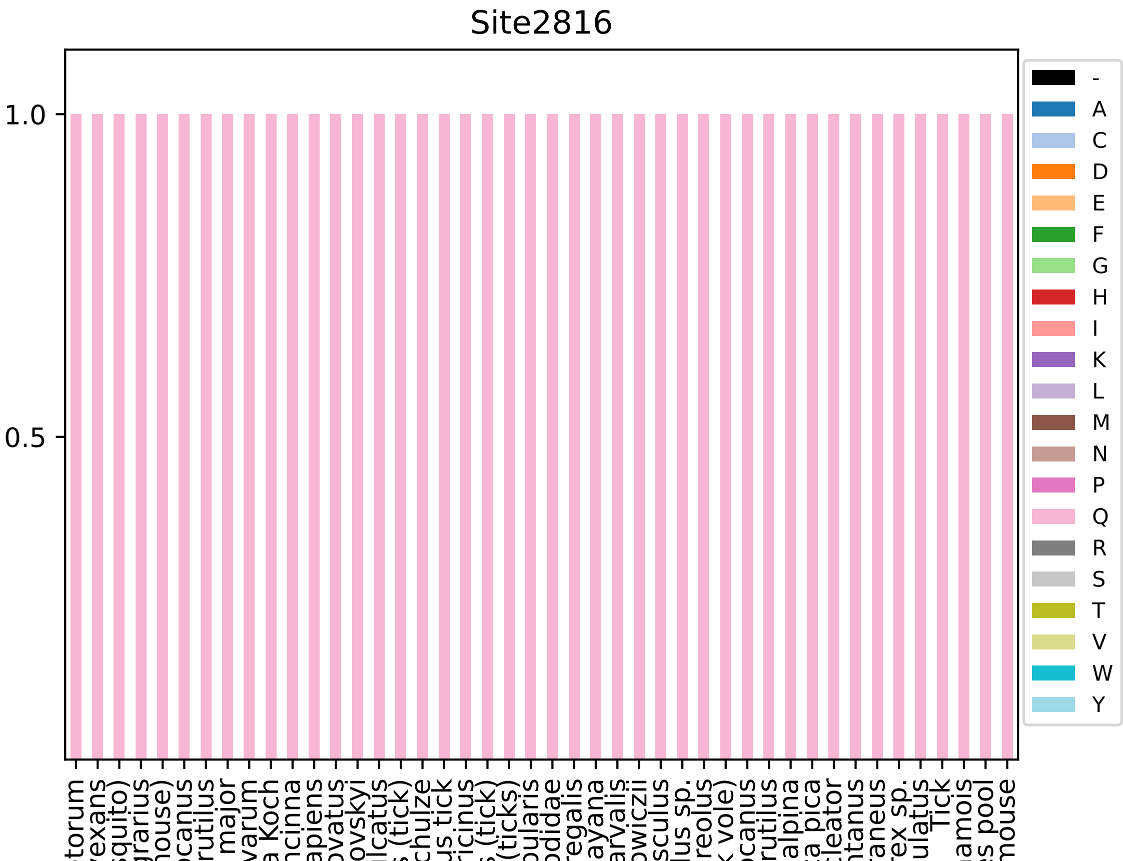

Site2816
1.0
0.5
-
A
C
D
E
F
G
H
I
K
L
M
N
P
Q
R
S
T
V
W
Y
torum
vexans
squito)
garius
nouse)
ocanus
rutilus
major
varum
a Koch
ncinna
apiens
ovatus
ovskyi
ulcatus
s (tick)
chulze
us tick
ricinus
s (tick)
(ticks)
pularis
odidae
regalis
ayana
arvalis
owiczii
sculus
lus sp.
reolus
k vole)
ocanus
rutilus
alpina
a pica
cleator
ntanus
raneus
rex sp.
ulatus
Tick
amois
es pool
mouse

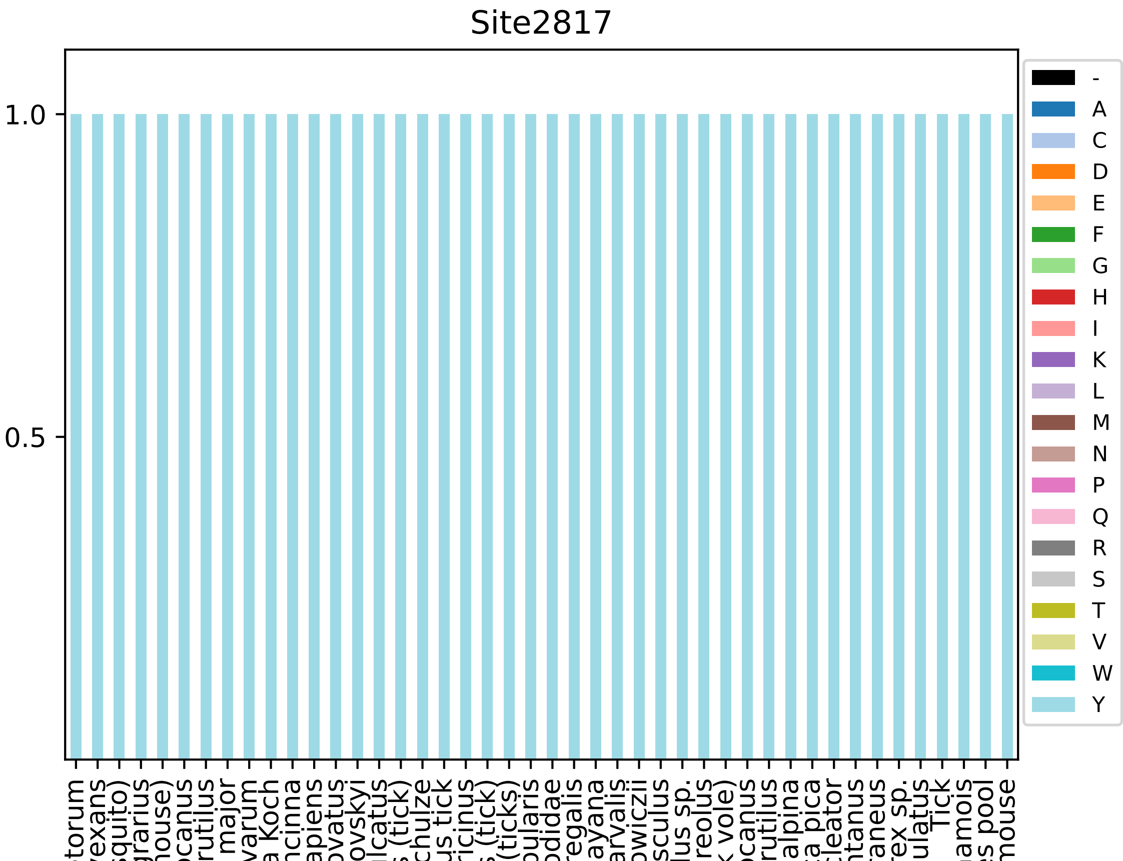
Site2817
1.0
0.5
-
A
C
D
E
F
G
H
I
K
L
M
N
P
Q
R
S
T
V
W
Y

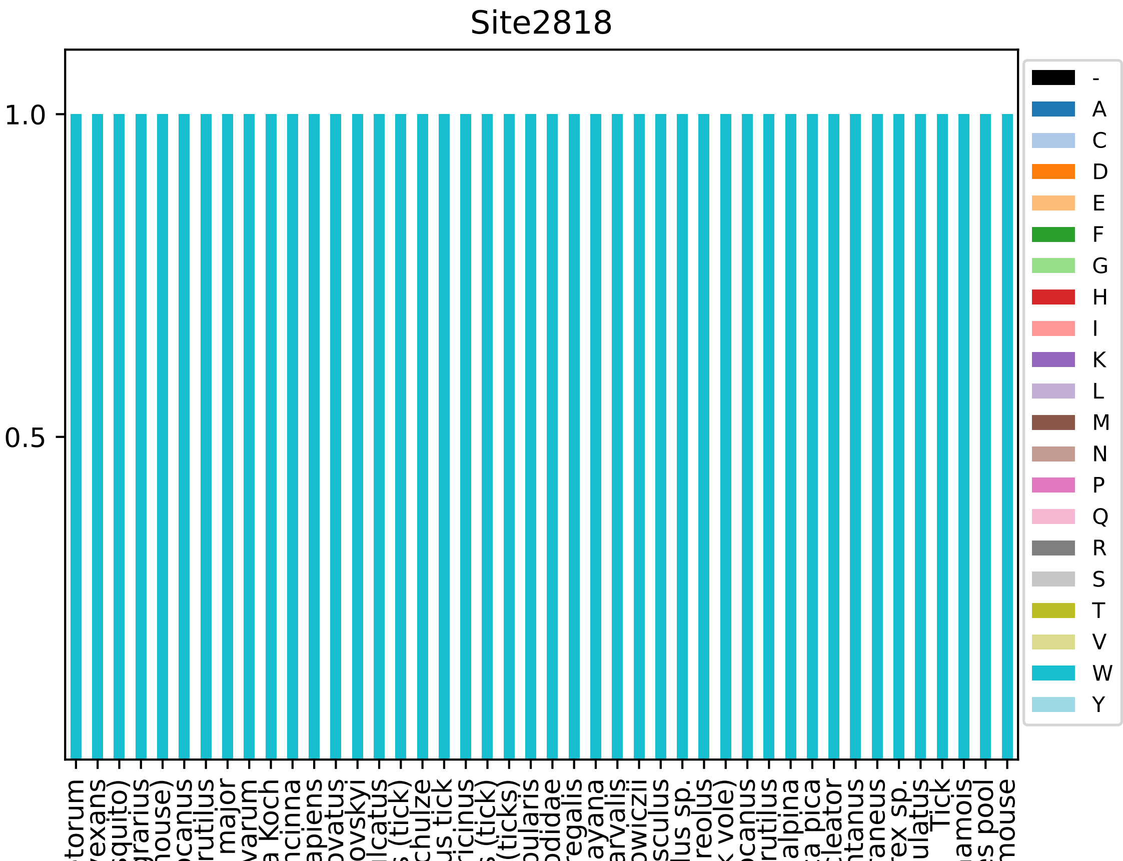
Site2818
1.0
0.5
-
A
C
D
E
F
G
H
I
K
L
M
N
P
Q
R
S
T
V
W
Y

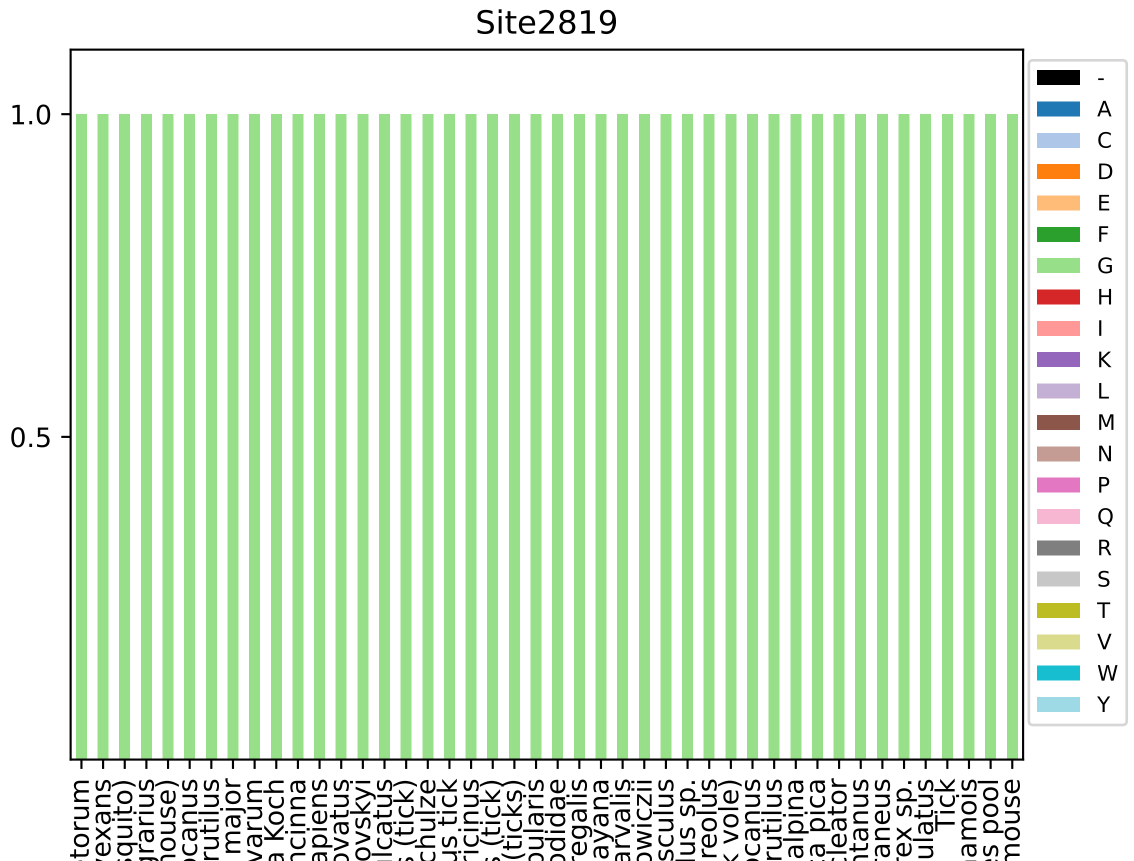
Site2819
1.0
0.5
-
A
C
D
E
F
G
H
I
K
L
M
N
P
Q
R
S
T
V
W
Y

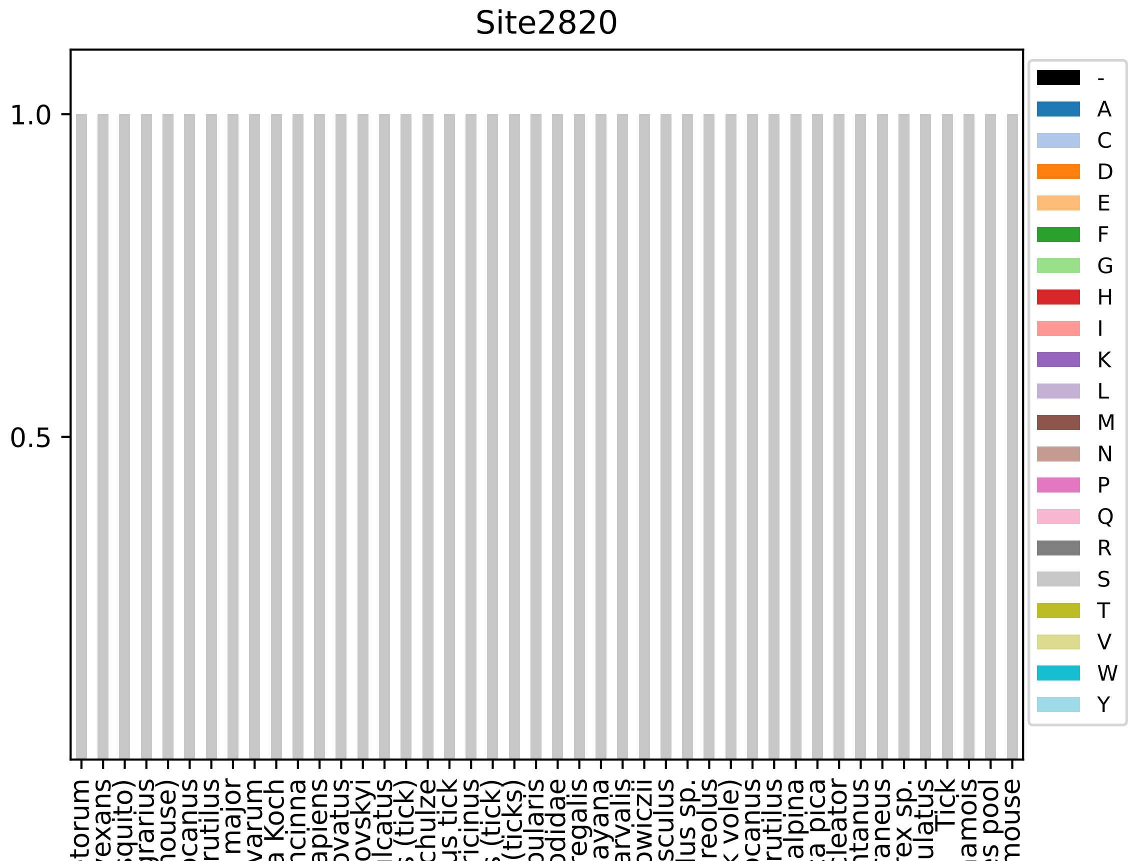

Site2820
1.0
0.5
-
A
C
D
E
F
G
H
I
K
L
M
N
P
Q
R
S
T
V
W
Y

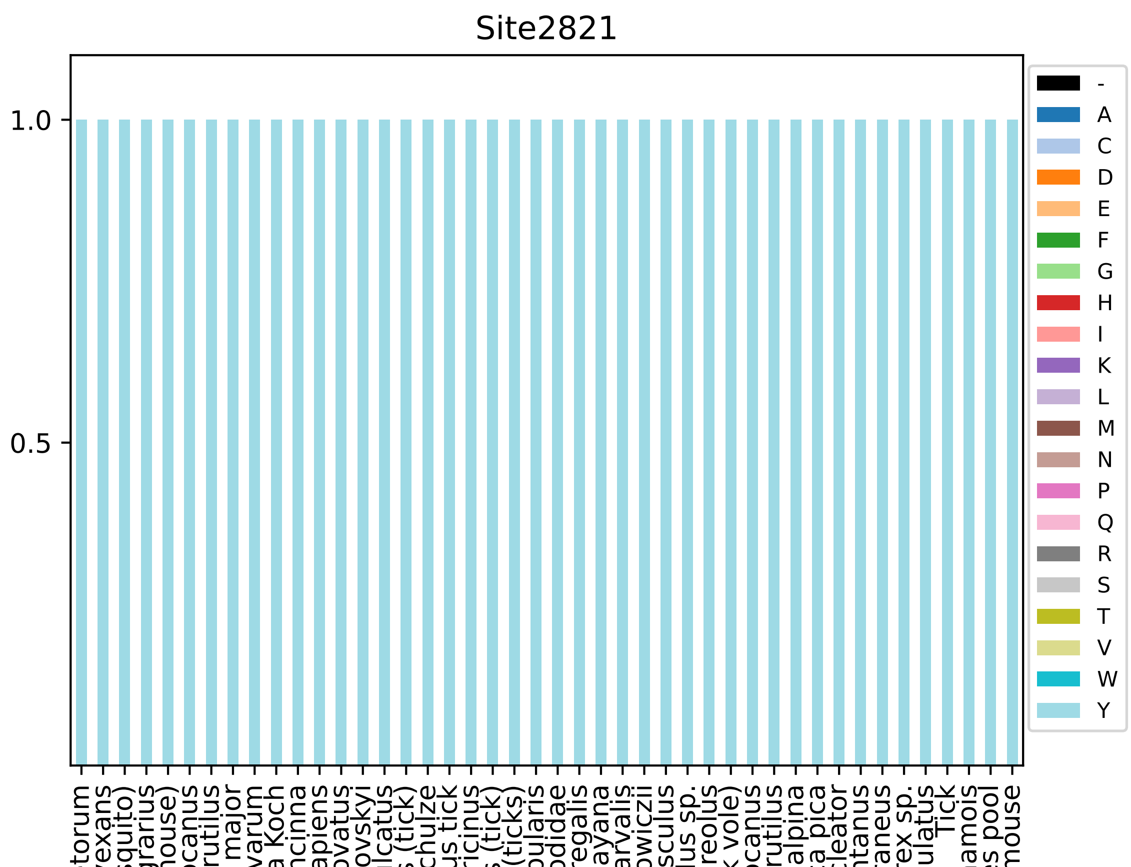
Site2821
1.0
0.5
-
A
C
D
E
F
G
H
I
K
L
M
N
P
Q
R
S
T
V
W
Y

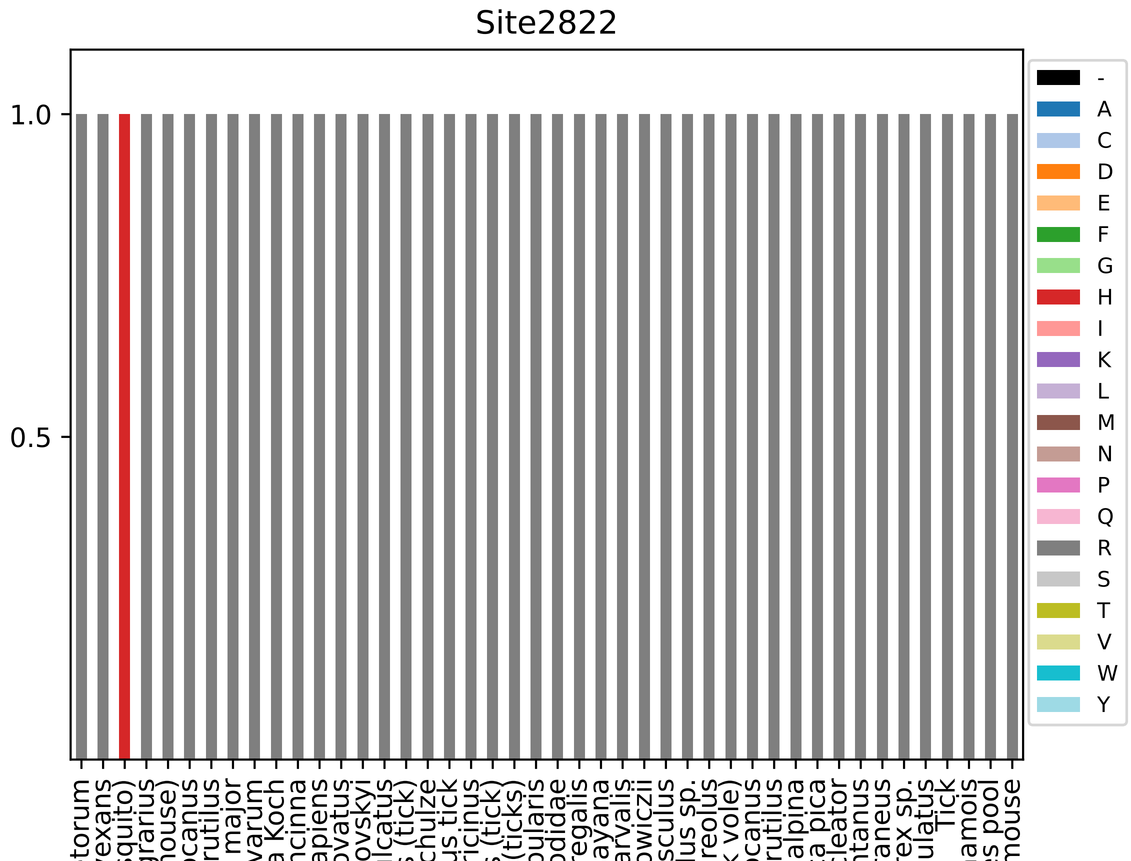

Site2822
1.0
0.5
-
A
C
D
E
F
G
H
I
K
L
M
N
P
Q
R
S
T
V
W
Y

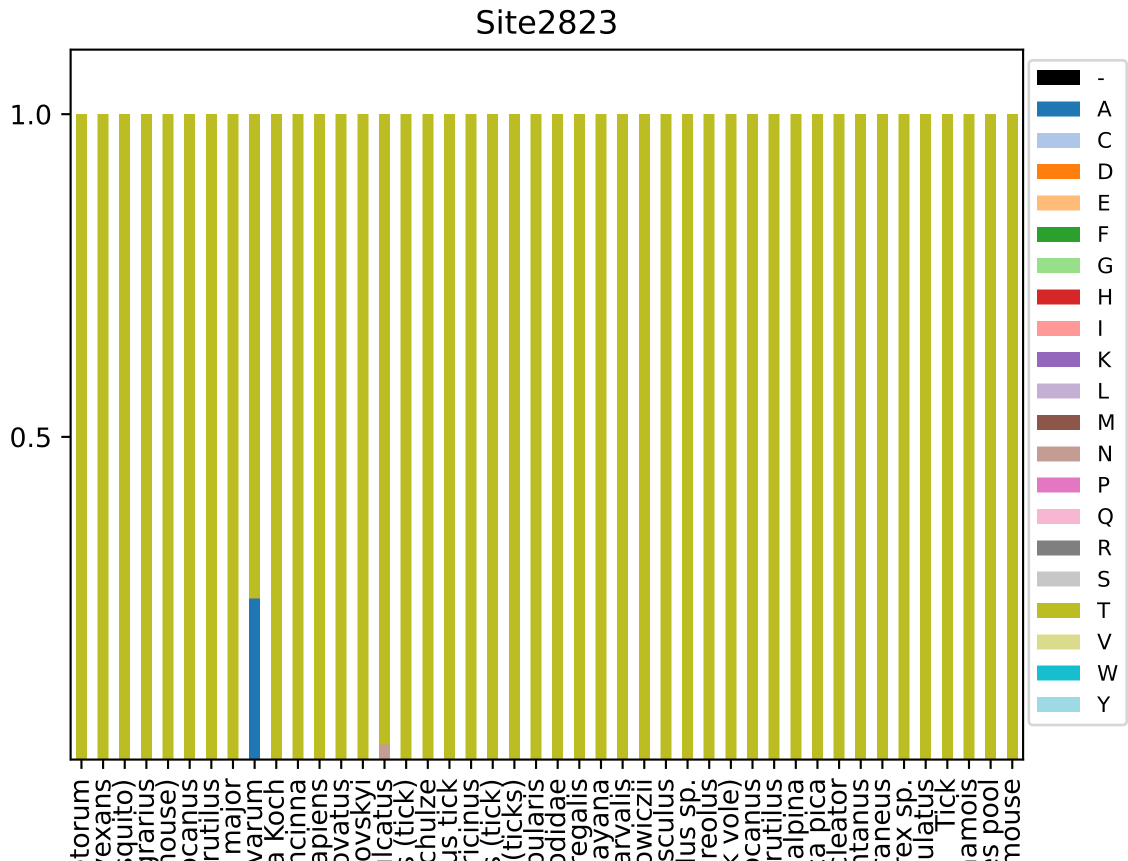
Site2823
1.0
0.5
-
A
C
D
E
F
G
H
I
K
L
M
N
P
Q
R
S
T
V
W
Y

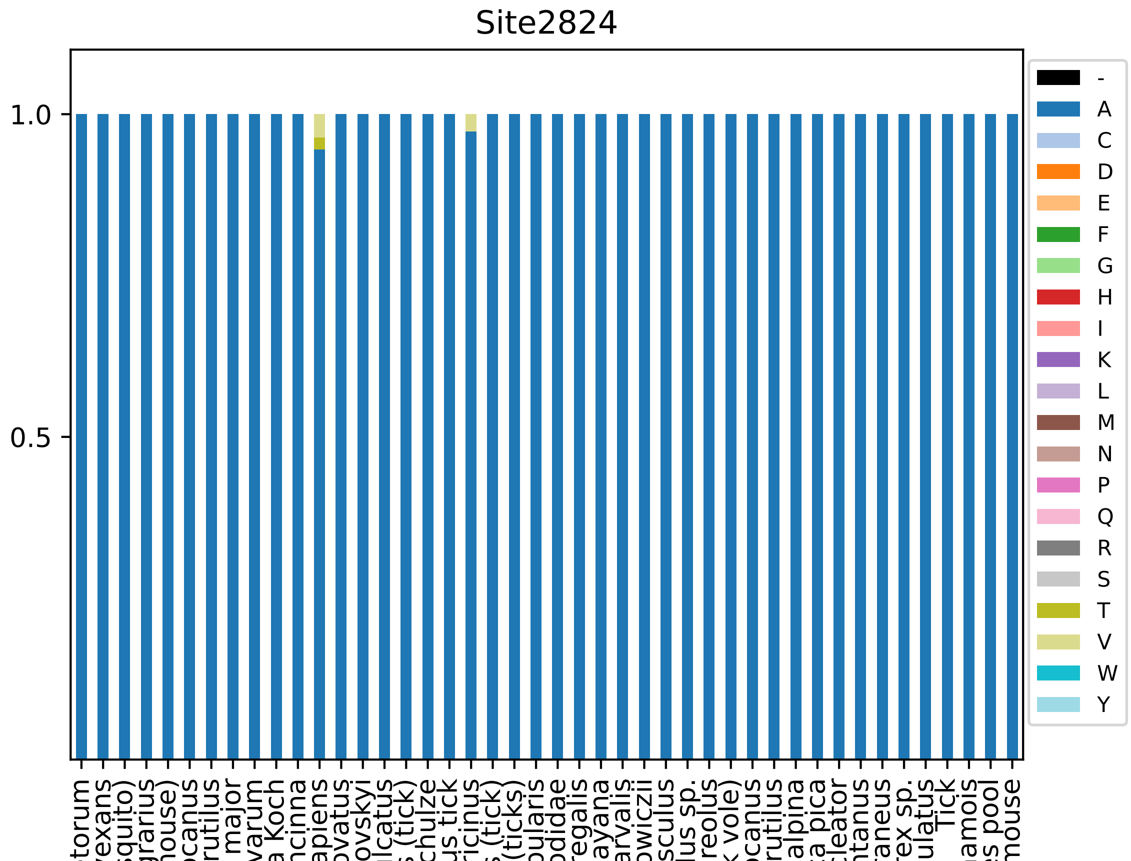
Site2824
1.0
0.5
-
A
C
D
E
F
G
H
I
K
L
M
N
P
Q
R
S
T
V
W
Y

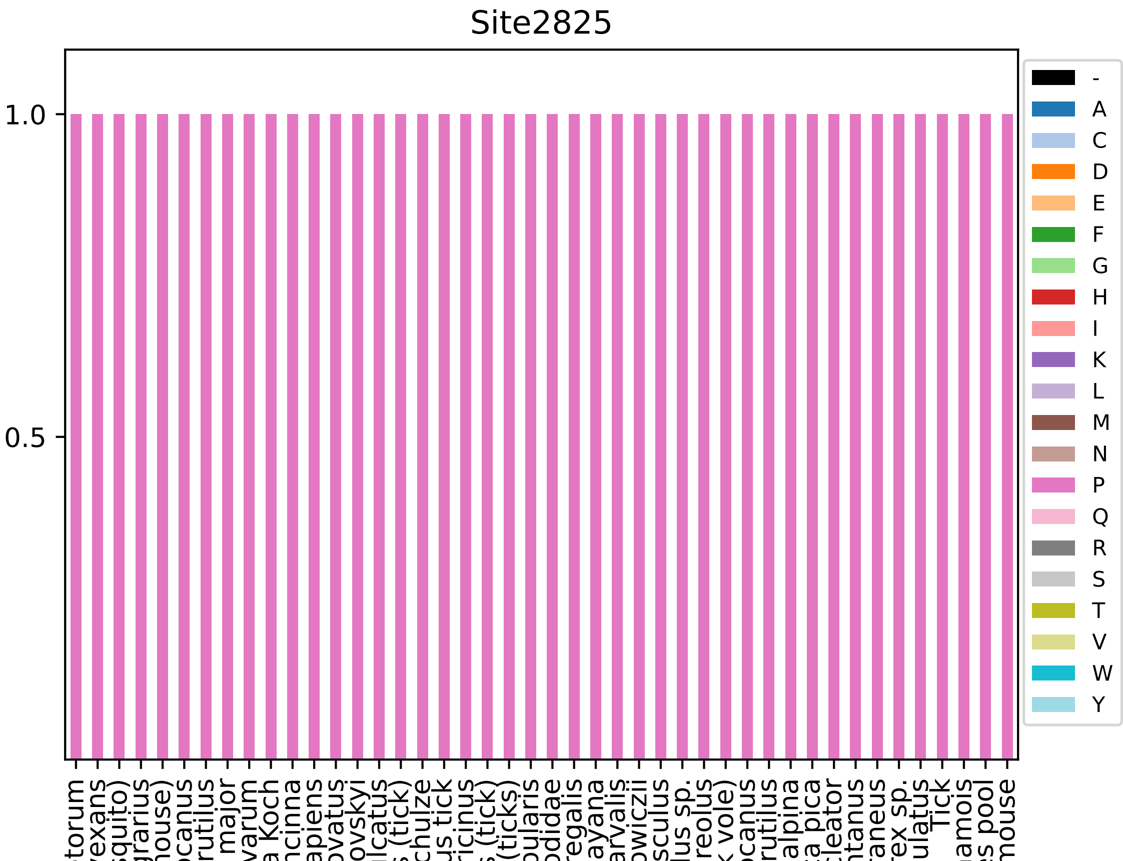
Site2825
1.0
0.5
-
A
C
D
E
F
G
H
I
K
L
M
N
P
Q
R
S
T
V
W
Y

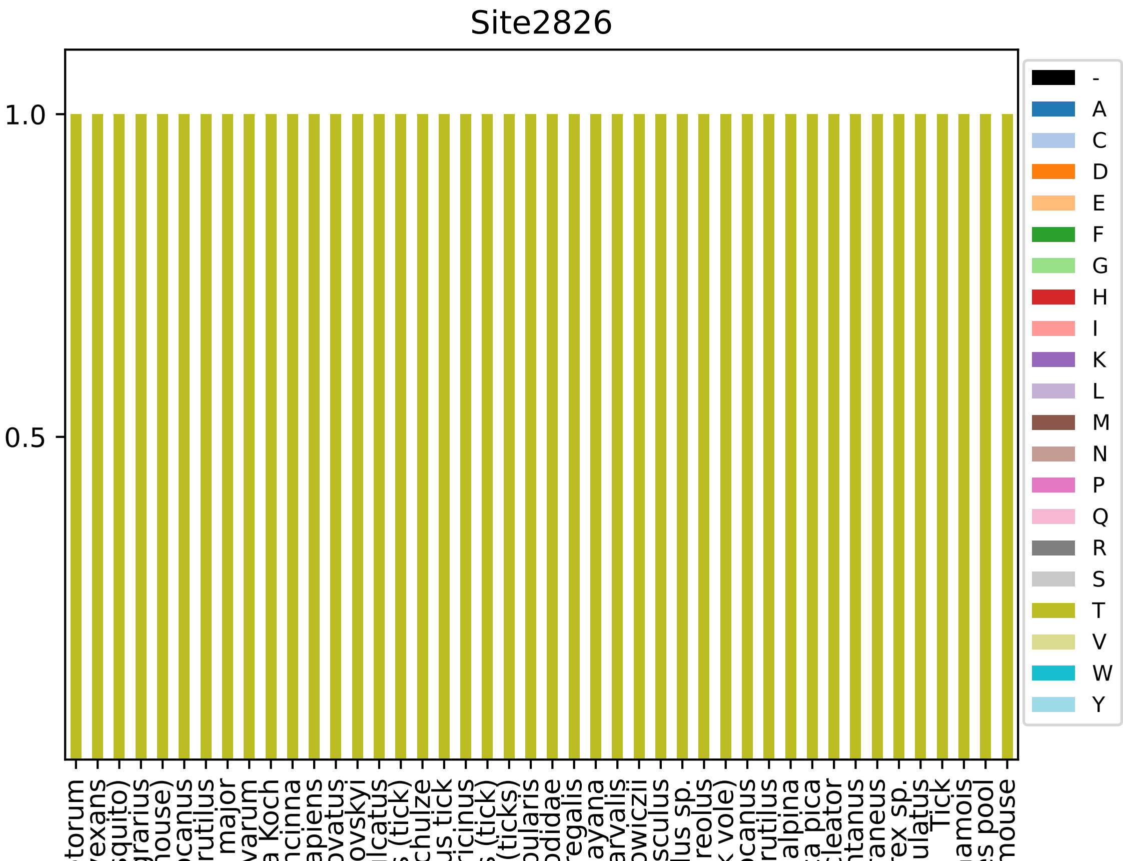
Site2826
1.0
0.5
-
A
C
D
E
F
G
H
I
K
L
M
N
P
Q
R
S
T
V
W
Y

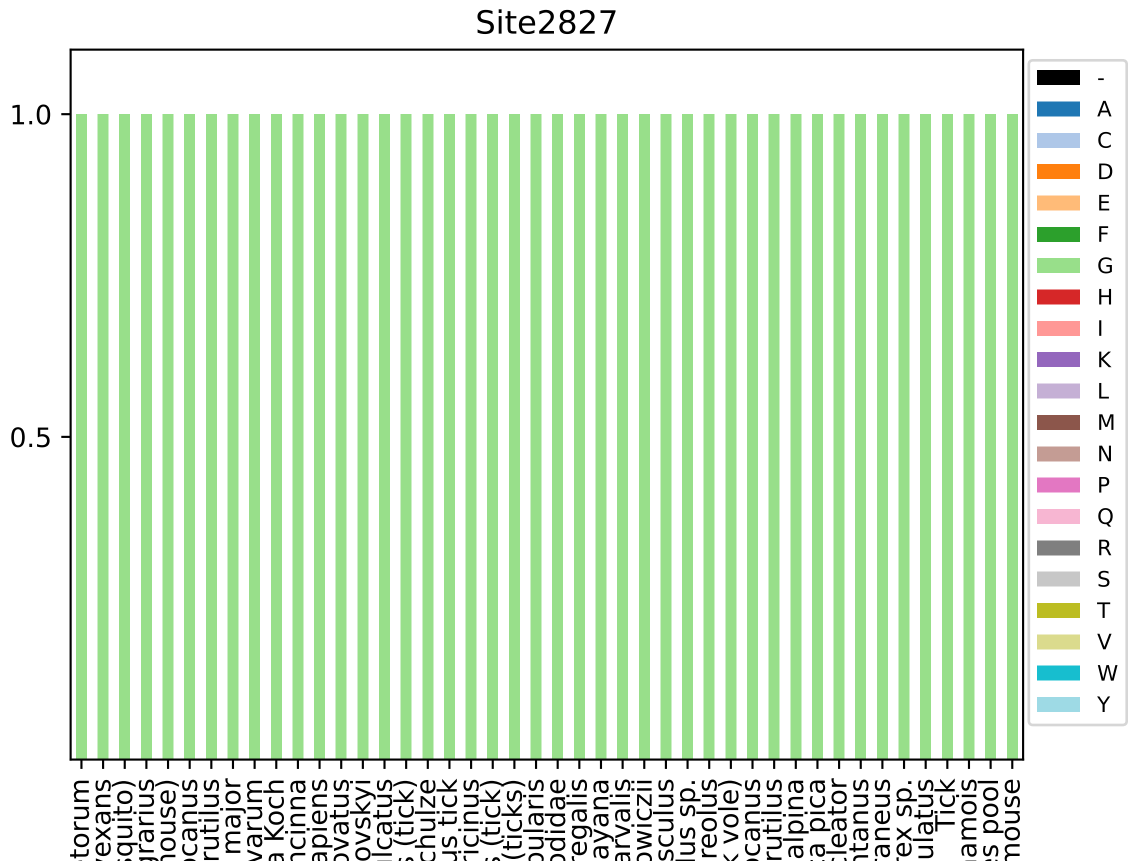
Site2827
1.0
0.5
-
A
C
D
E
F
G
H
I
K
L
M
N
P
Q
R
S
T
V
W
Y

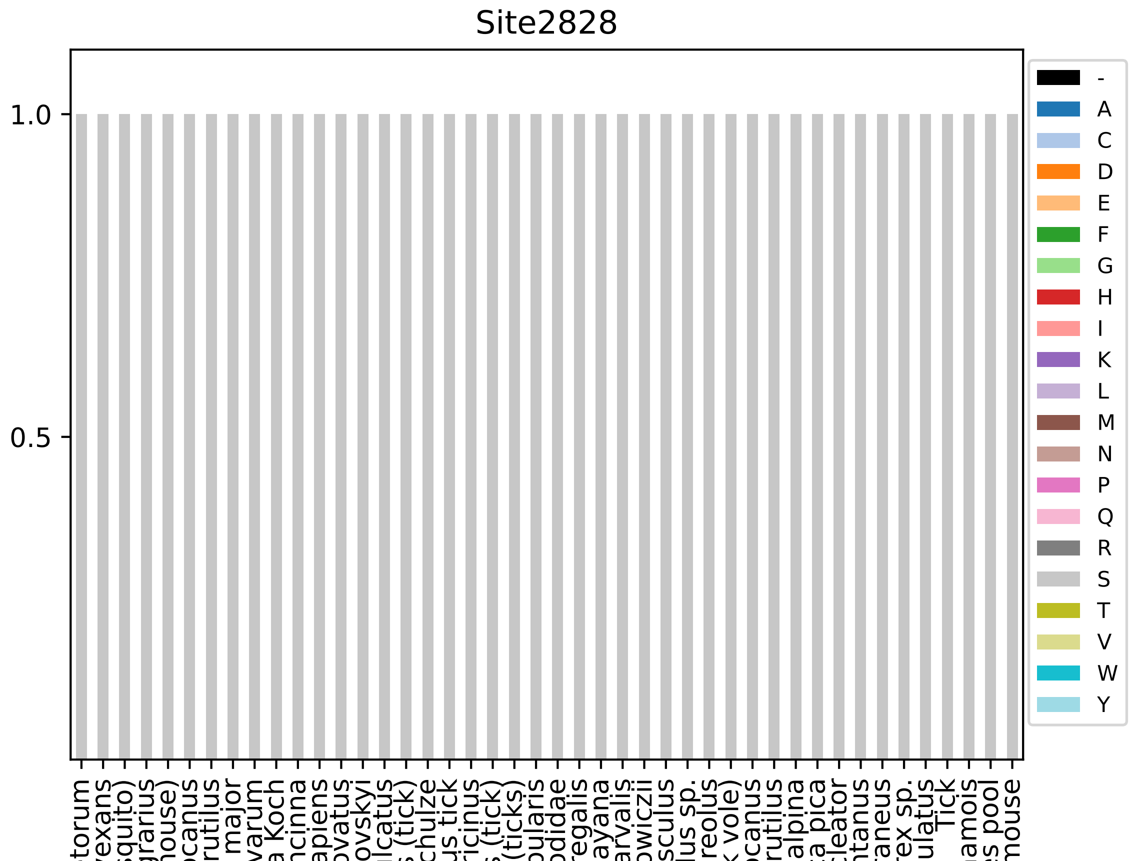
Site2828
1.0
0.5
-
A
C
D
E
F
G
H
I
K
L
M
N
P
Q
R
S
T
V
W
Y

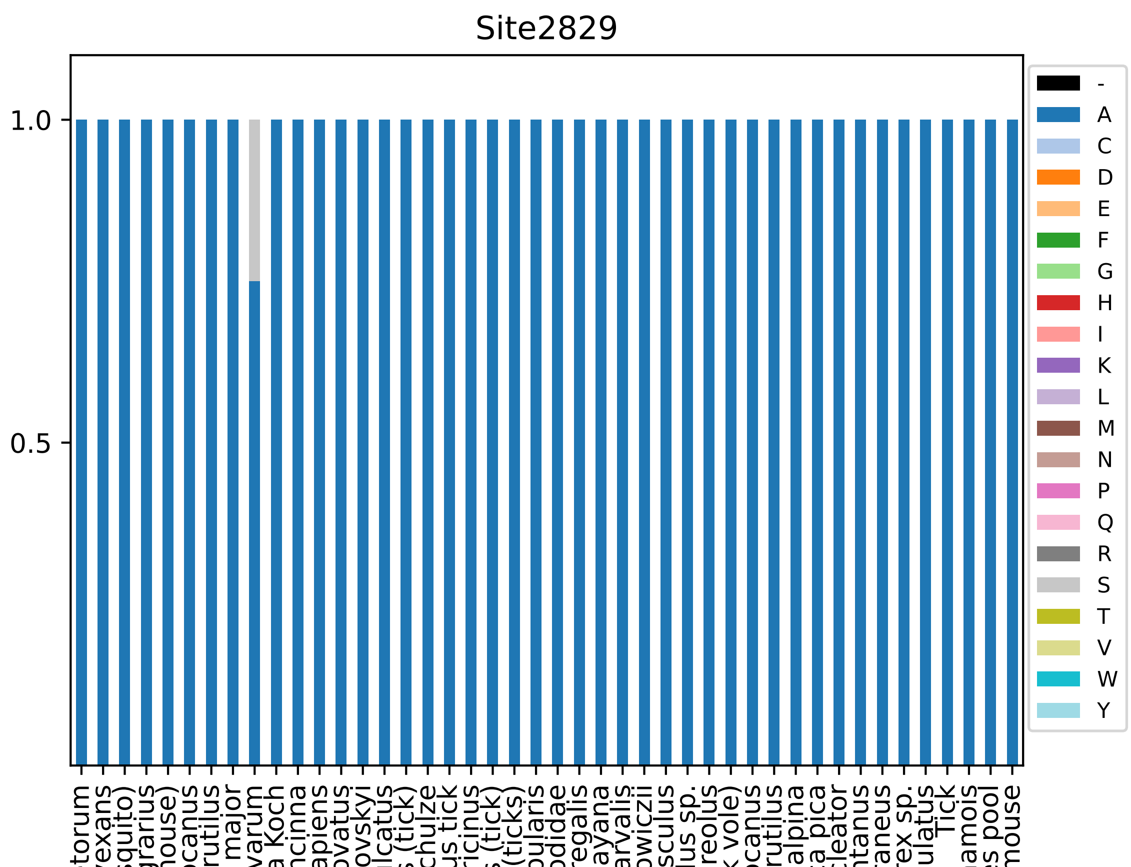
Site2829
1.0
0.5
-
A
C
D
E
F
G
H
I
K
L
M
N
P
Q
R
S
T
V
W
Y

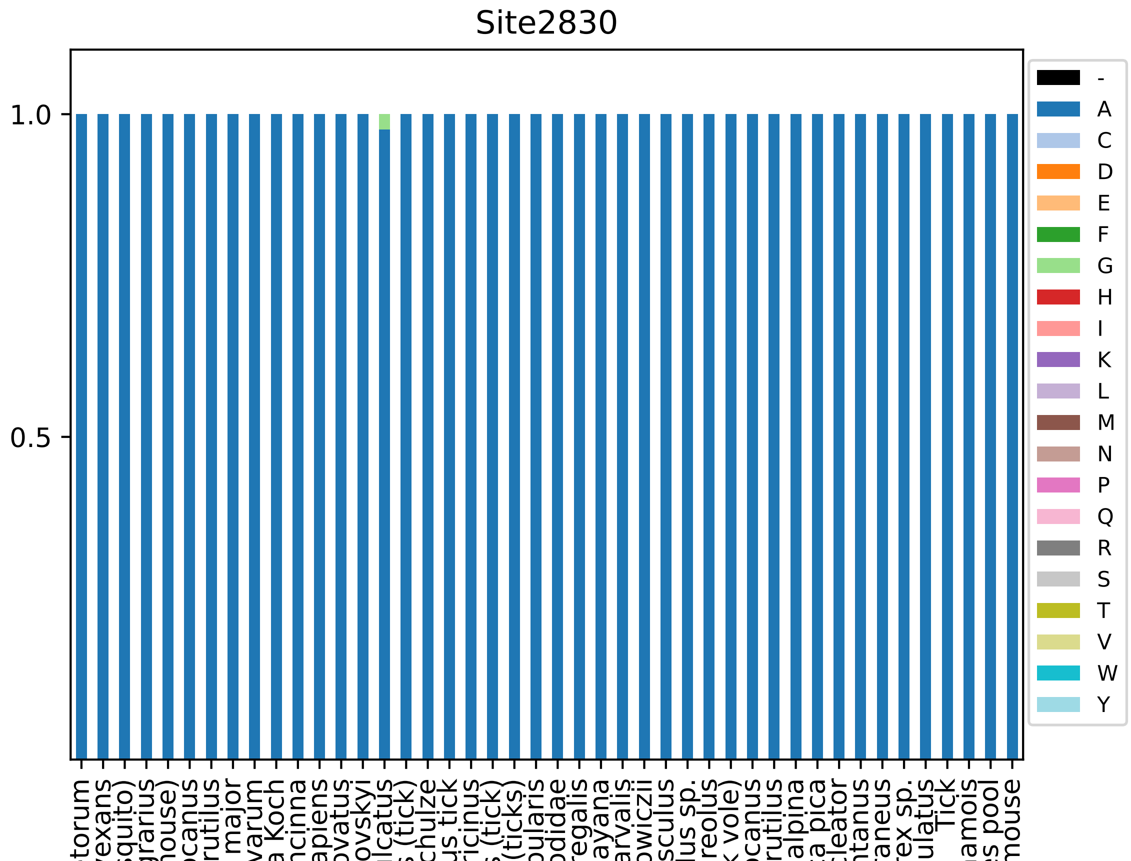
Site2830
1.0
0.5
-
A
C
D
E
F
G
H
I
K
L
M
N
P
Q
R
S
T
V
W
Y
Tick

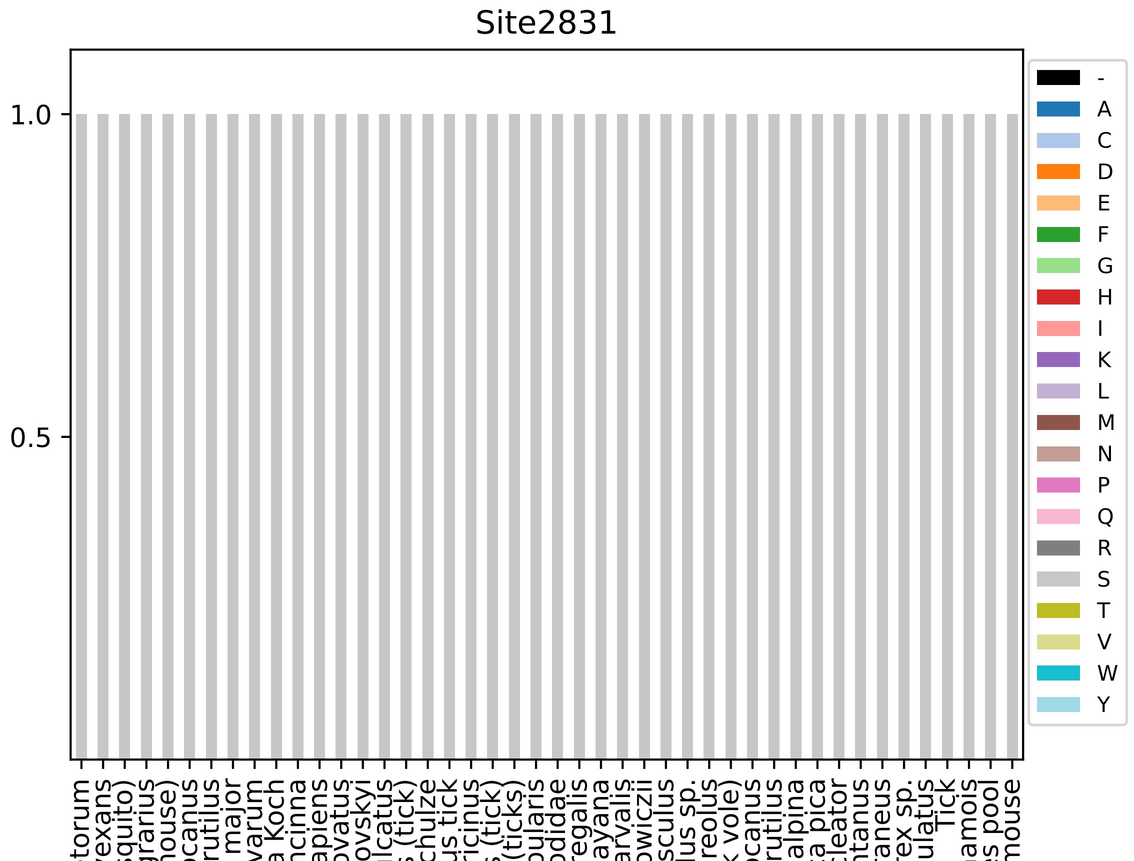
Site2831
1.0
0.5
-
A
C
D
E
F
G
H
I
K
L
M
N
P
Q
R
S
T
V
W
Y

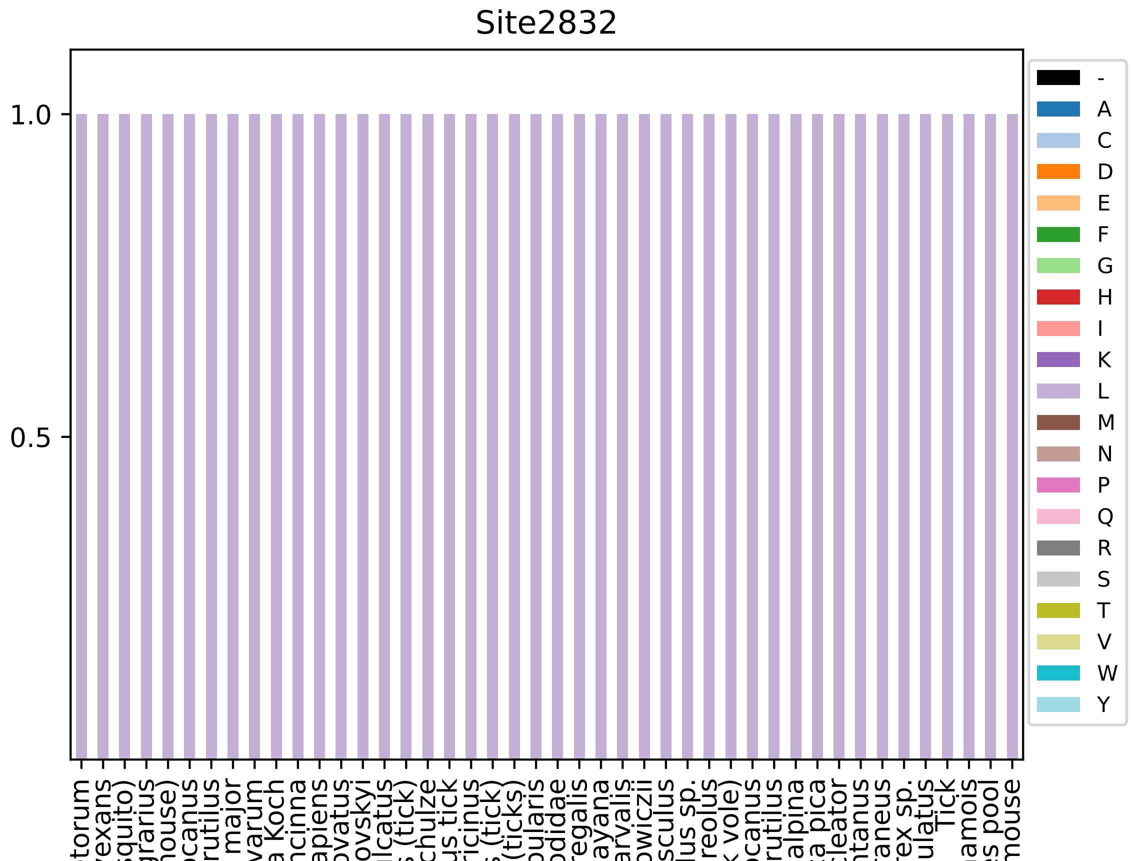
Site2832
1.0
0.5
-
A
C
D
E
F
G
H
I
K
L
M
N
P
Q
R
S
T
V
W
Y

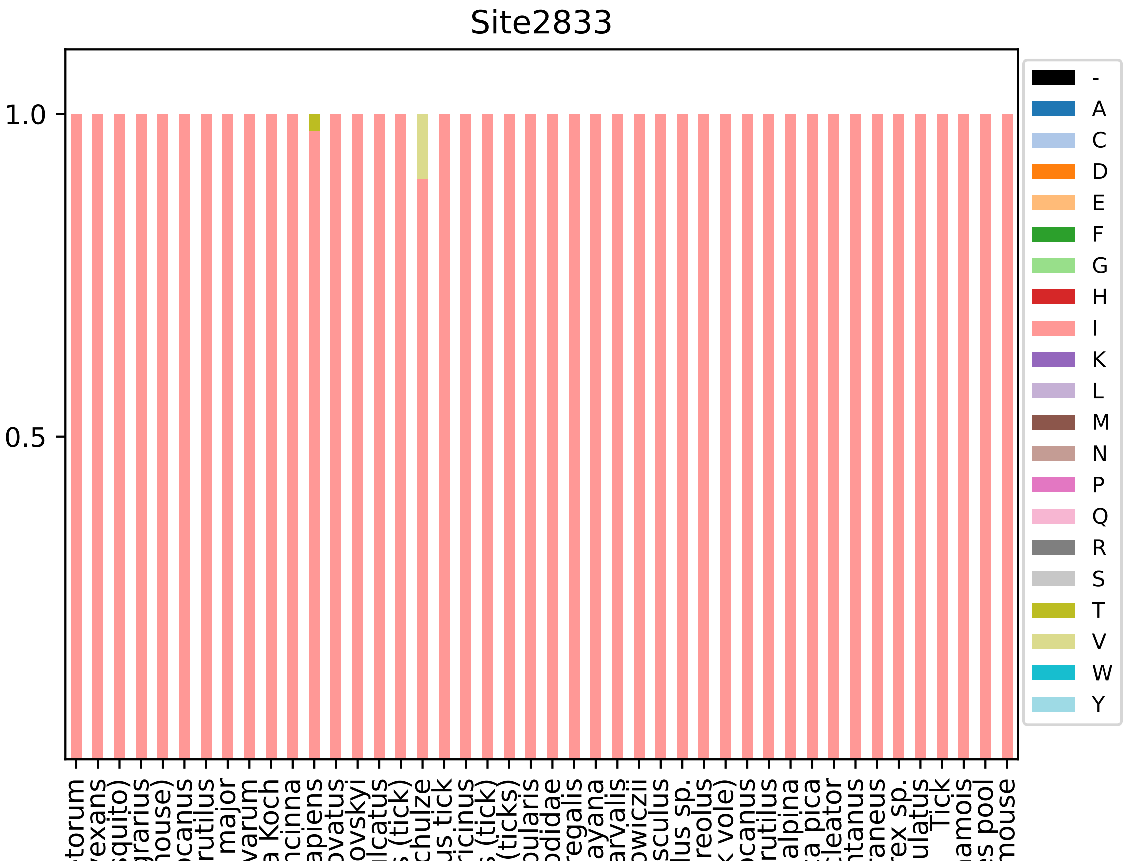

Site2833
1.0
0.5
-
A
C
D
E
F
G
H
I
K
L
M
N
P
Q
R
S
T
V
W
Y

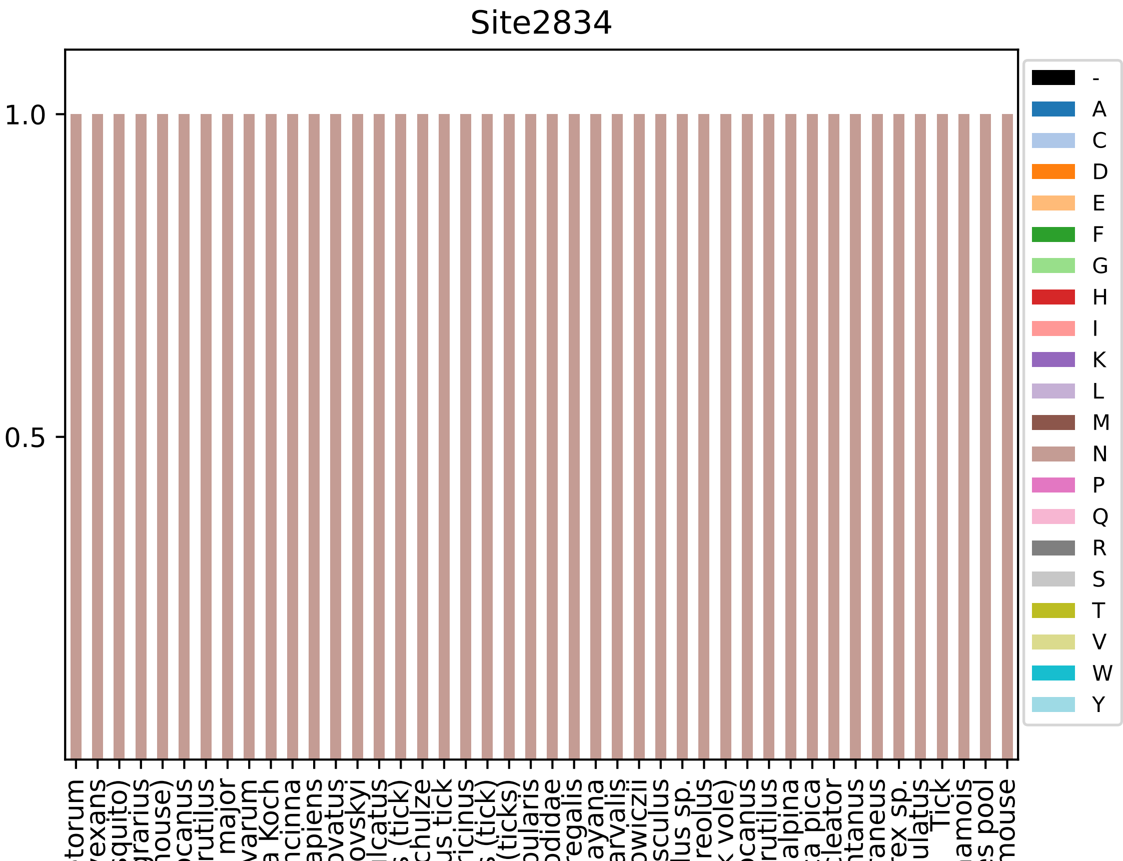

Site2834
1.0
0.5
-
A
C
D
E
F
G
H
I
K
L
M
N
P
Q
R
S
T
V
W
Y

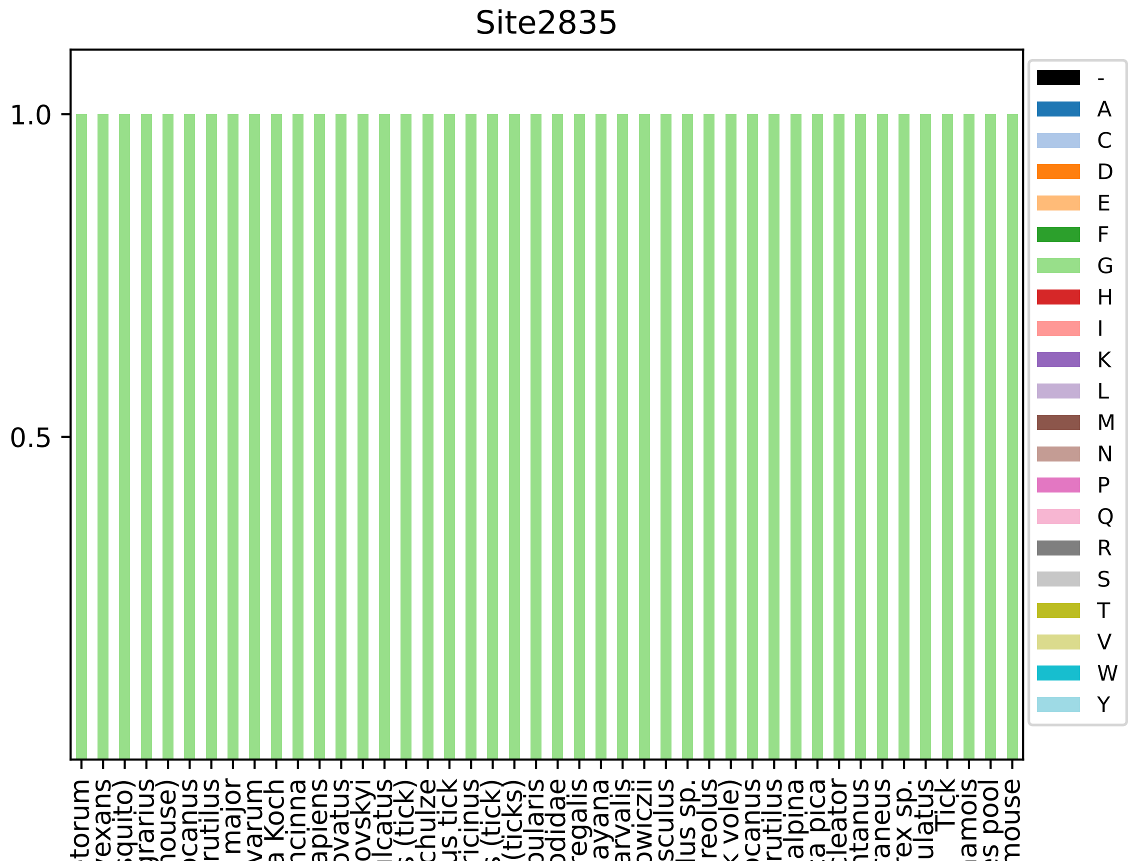

Site2835
1.0
0.5
-
A
C
D
E
F
G
H
I
K
L
M
N
P
Q
R
S
T
V
W
Y

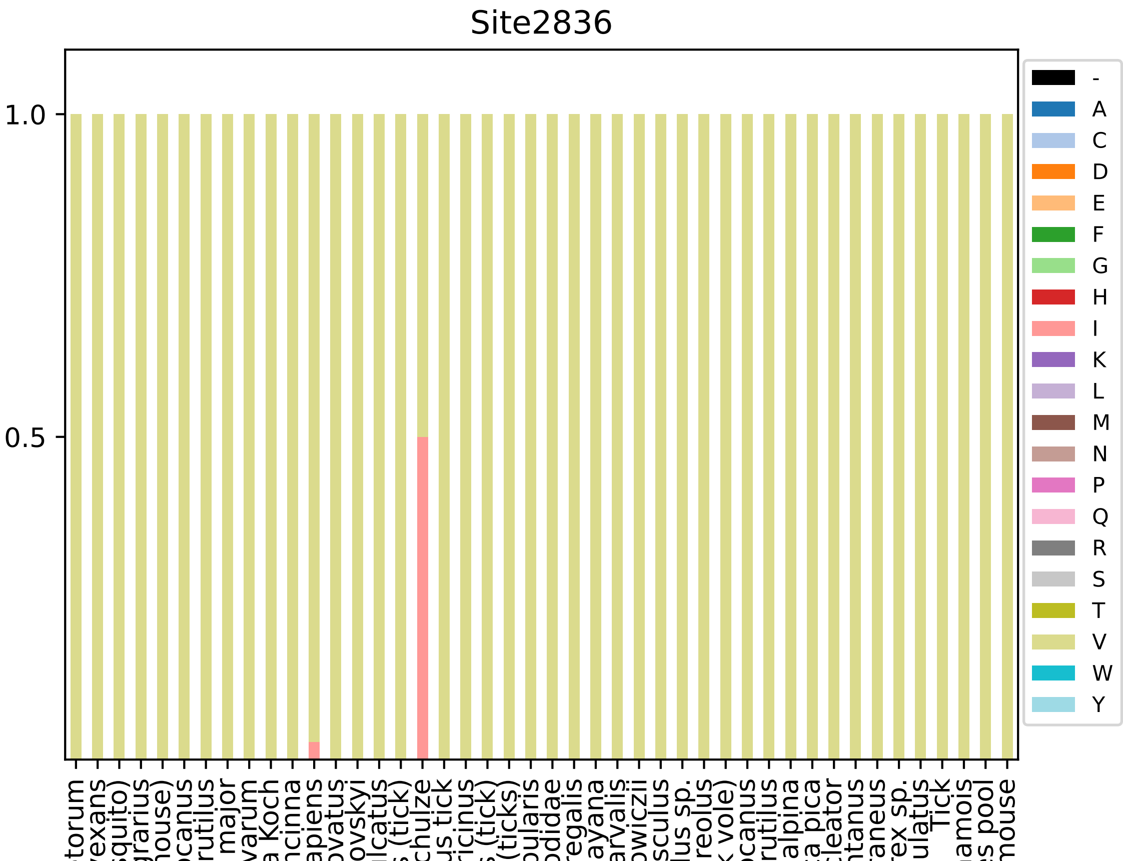
Site2836
1.0
0.5
-
A
C
D
E
F
G
H
I
K
L
M
N
P
Q
R
S
T
V
W
Y

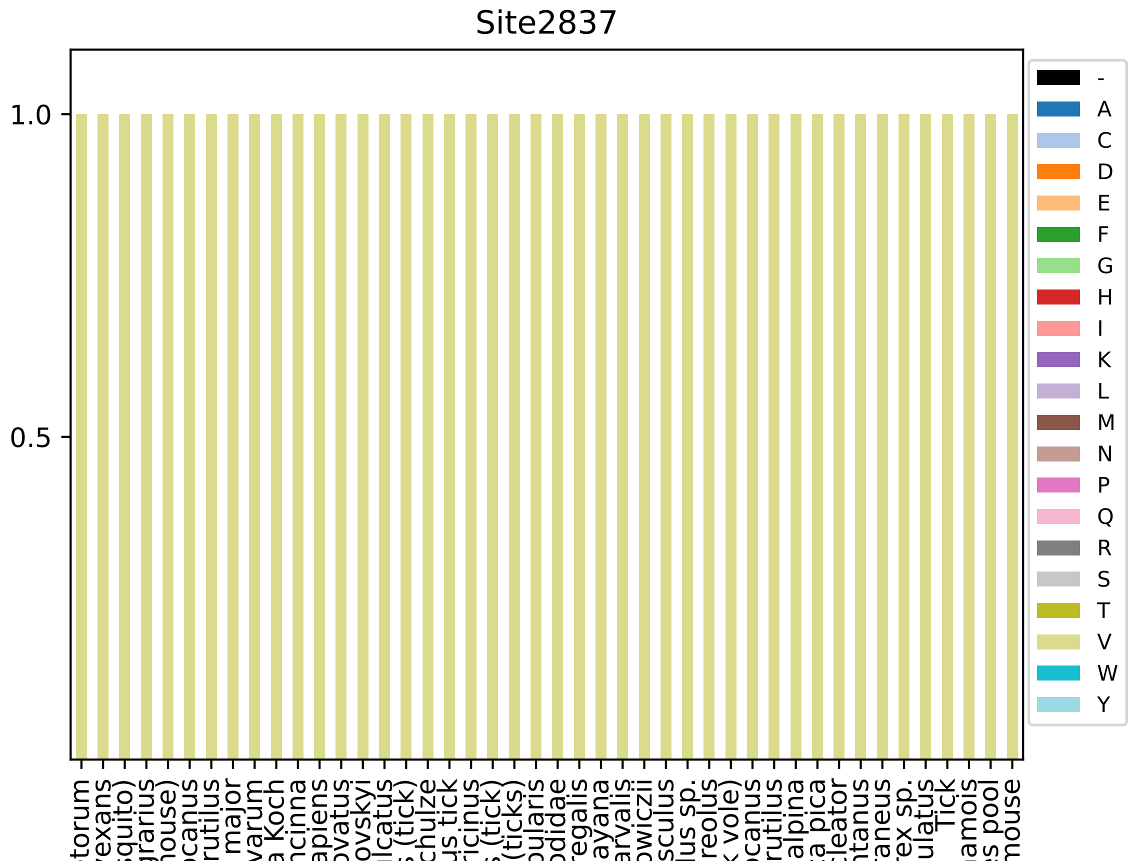
Site2837
1.0
0.5
-
A
C
D
E
F
G
H
I
K
L
M
N
P
Q
R
S
T
V
W
Y

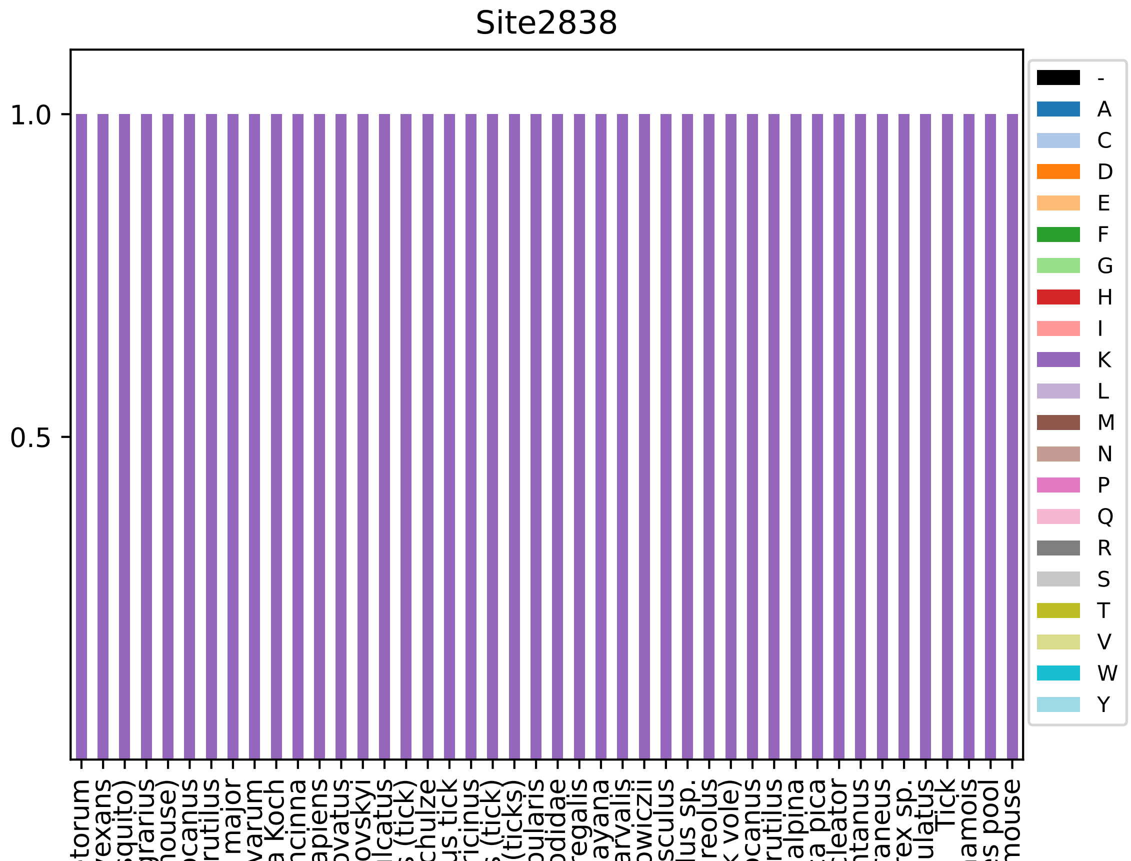
Site2838
1.0
0.5
-
A
C
D
E
F
G
H
I
K
L
M
N
P
Q
R
S
T
V
W
Y

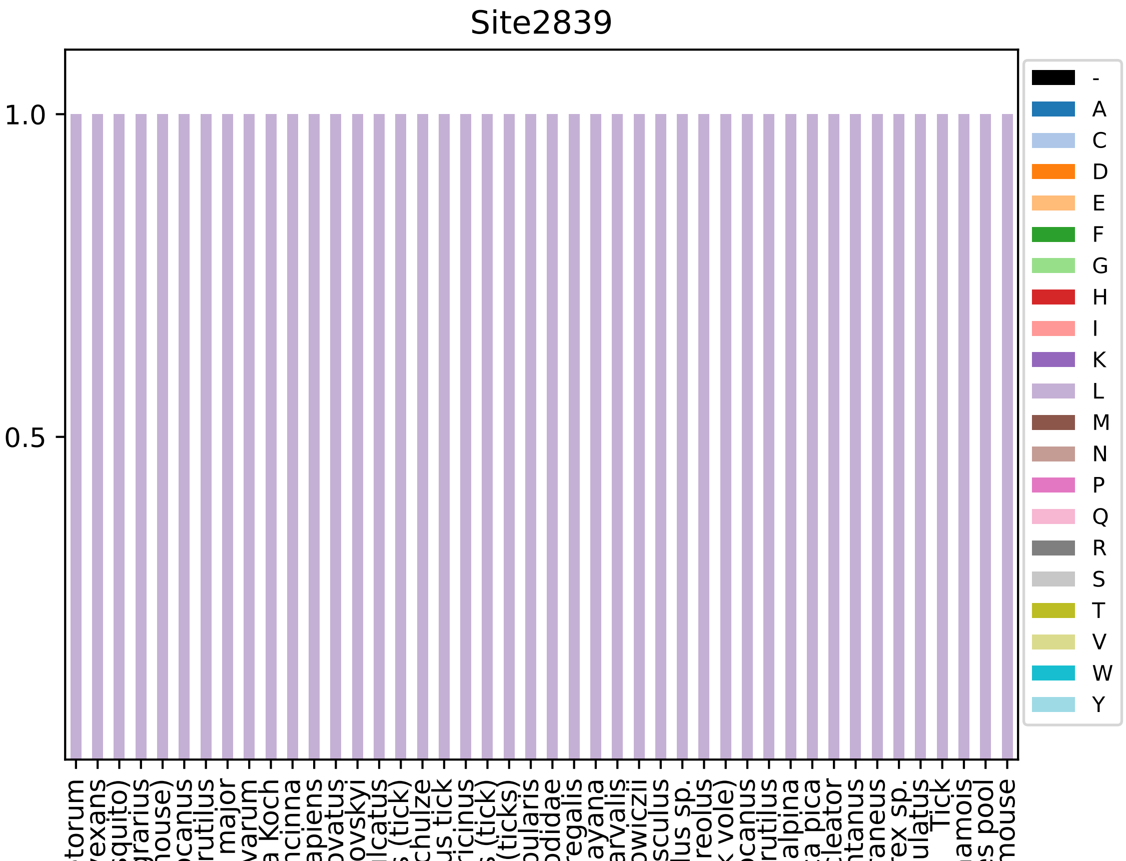
Site2839
1.0
0.5
-
A
C
D
E
F
G
H
I
K
L
M
N
P
Q
R
S
T
V
W
Y
torum
vexans
squito)
grarius
nouse)
ocanus
rutilus
major
varum
a Koch
ncinna
apiens
ovatus
ovskyi
lcatus
s (tick)
chulze
us tick
ricinus
s (tick)
(ticks)
pularis
odidae
regalis
ayana
arvalis
owiczii
sculus
lus sp.
reolus
k vole)
ocanus
rutilus
alpina
a pica
leator
ntanus
raneus
ex sp.
ulatus
Tick
amois
s pool
mouse

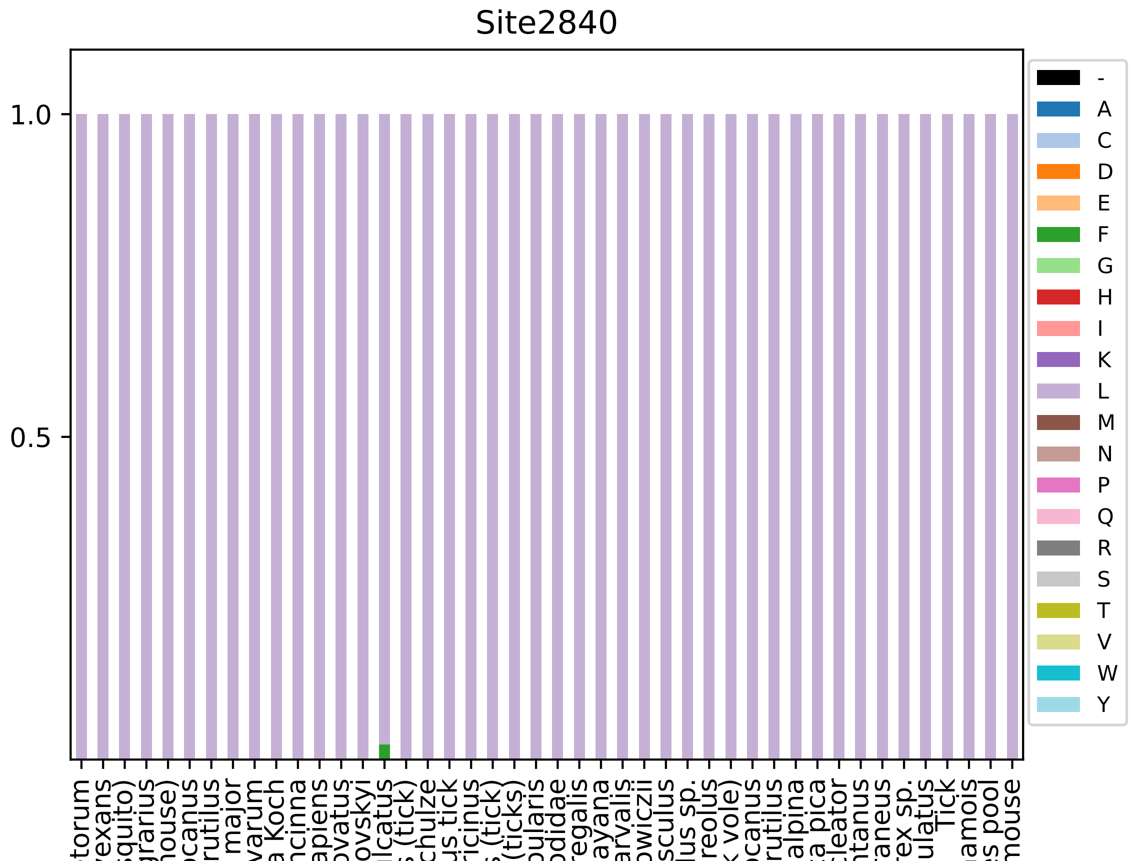
Site2840
1.0
0.5
-
A
C
D
E
F
G
H
I
K
L
M
N
P
Q
R
S
T
V
W
Y
Tick

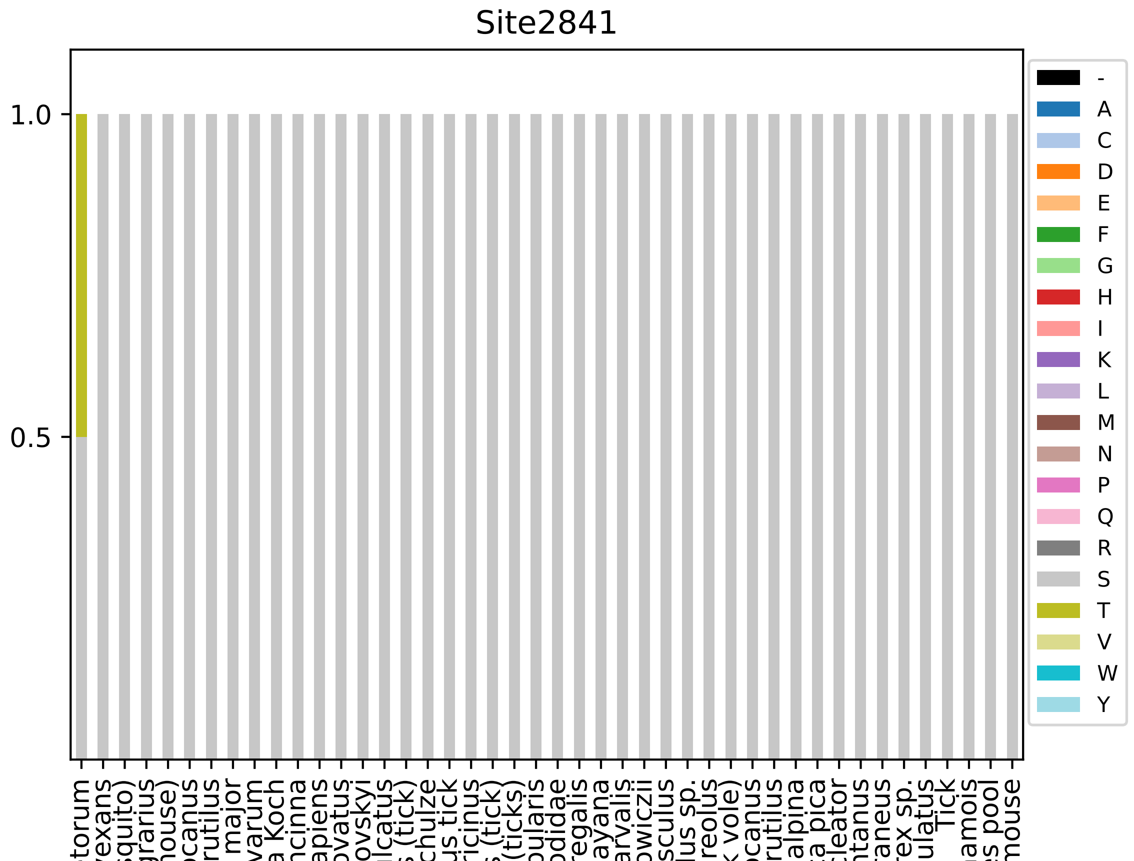

Site2841
1.0
0.5
-
A
C
D
E
F
G
H
I
K
L
M
N
P
Q
R
S
T
V
W
Y

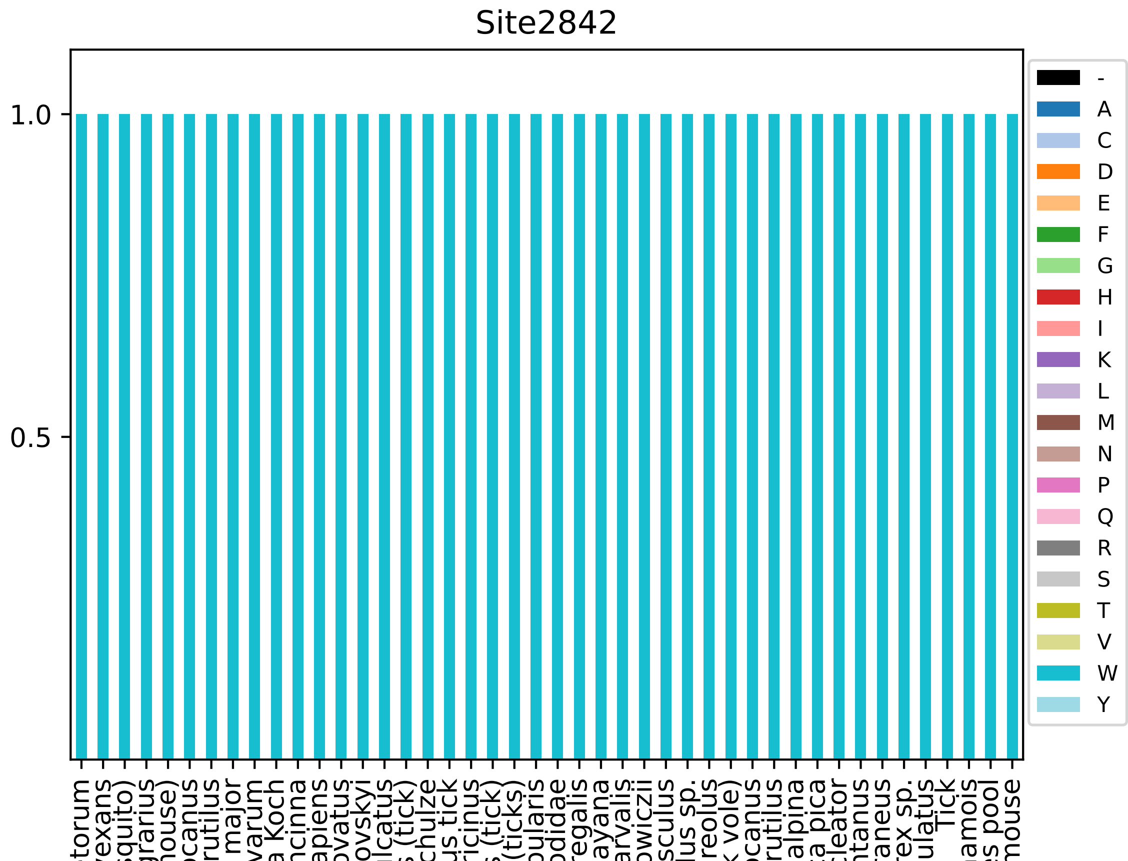
Site2842
1.0
0.5
-
A
C
D
E
F
G
H
I
K
L
M
N
P
Q
R
S
T
V
W
Y
Tick

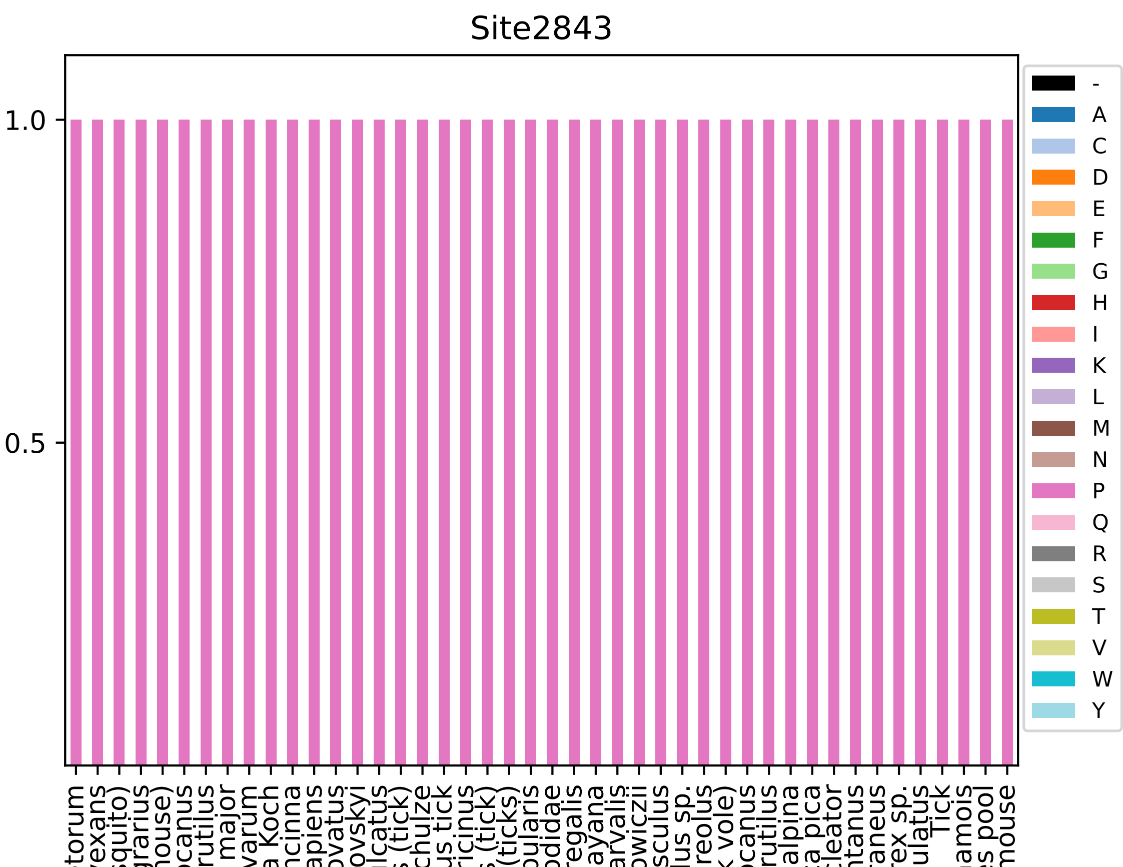
Site2843
1.0
0.5
-
A
C
D
E
F
G
H
I
K
L
M
N
P
Q
R
S
T
V
W
Y

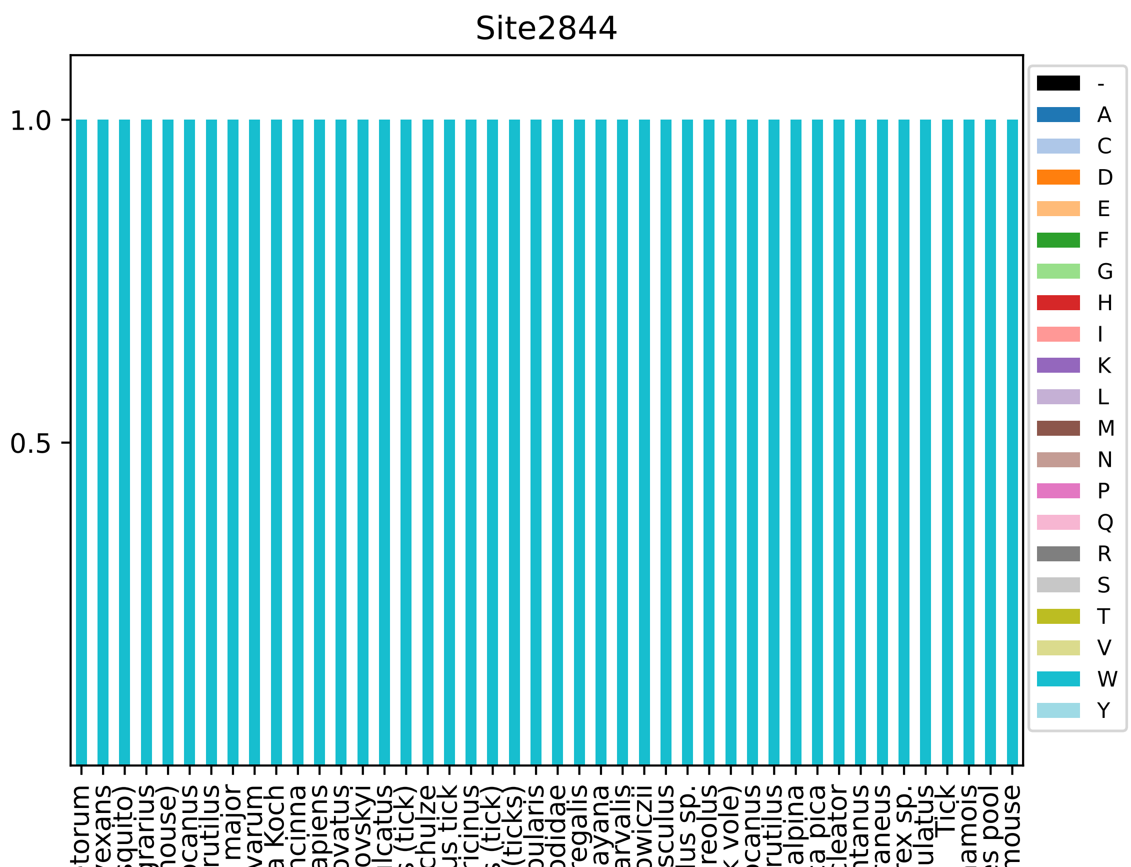
Site2844
1.0
0.5
-
A
C
D
E
F
G
H
I
K
L
M
N
P
Q
R
S
T
V
W
Y

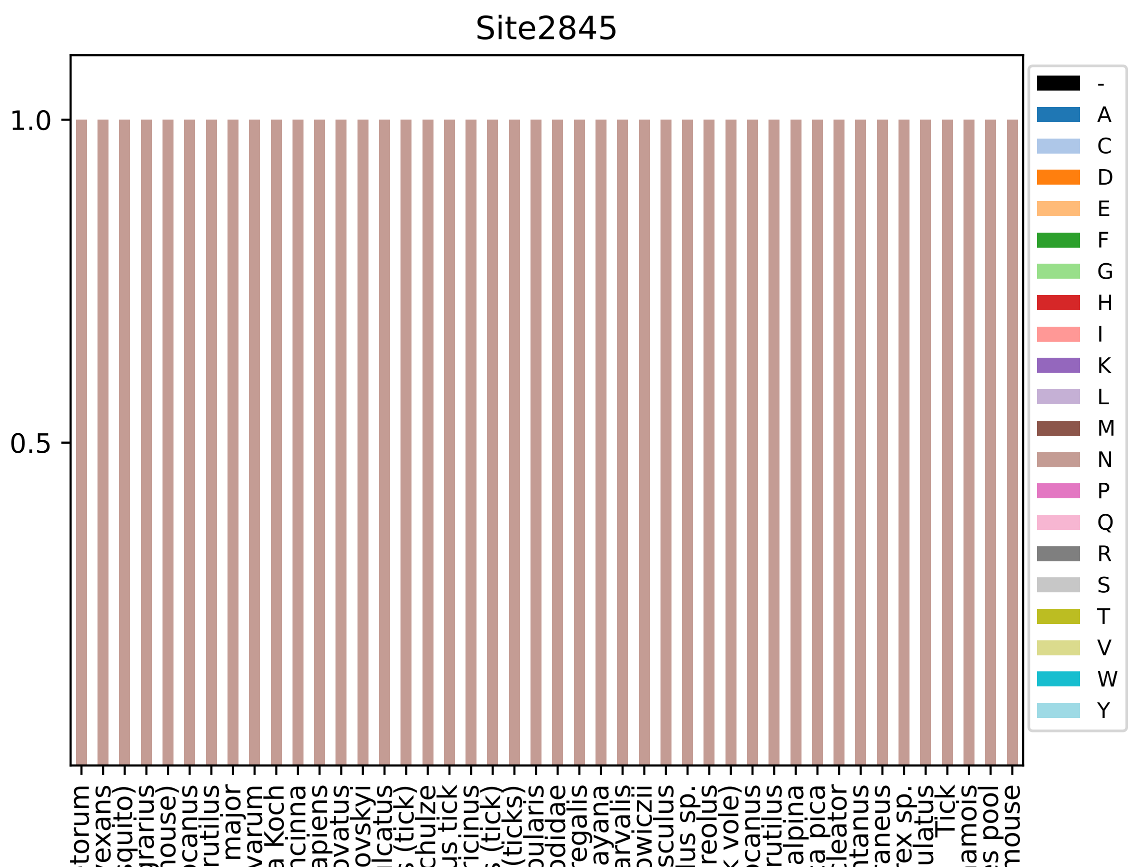
Site2845
1.0
0.5
-
A
C
D
E
F
G
H
I
K
L
M
N
P
Q
R
S
T
V
W
Y
Tick

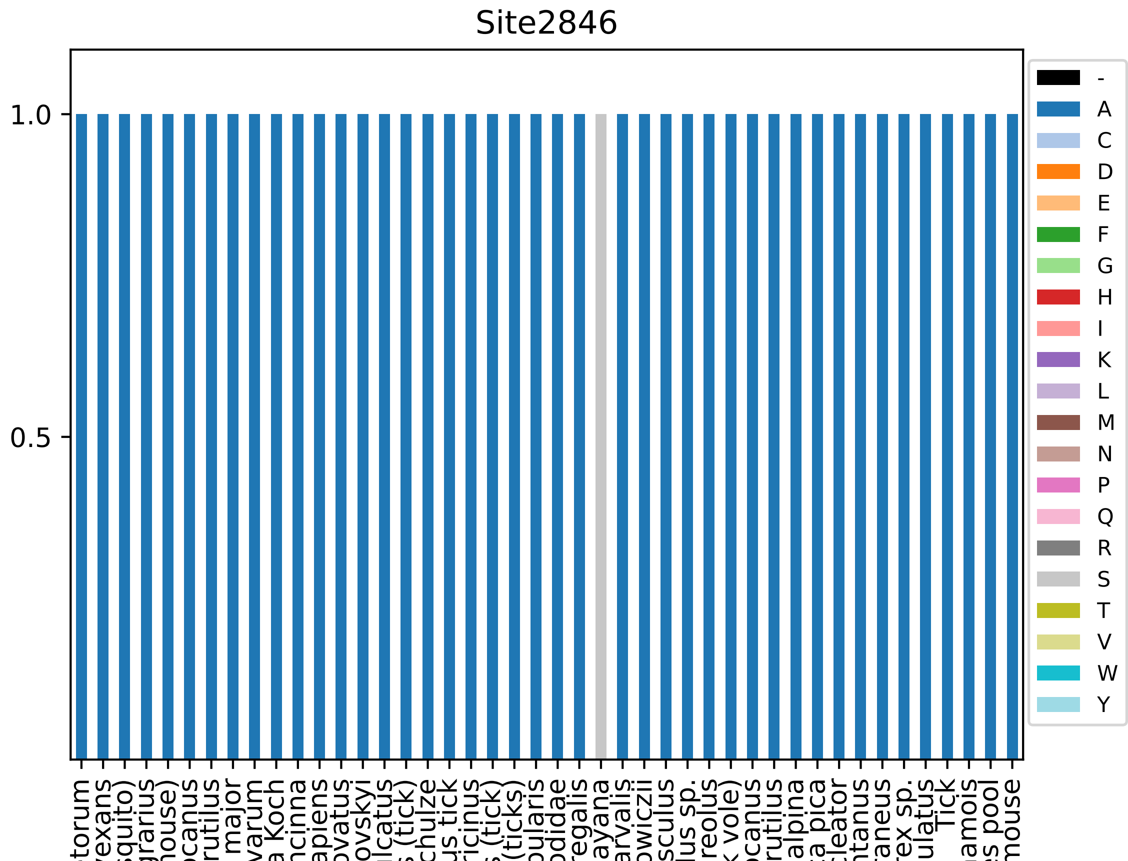
Site2846
1.0
0.5
-
A
C
D
E
F
G
H
I
K
L
M
N
P
Q
R
S
T
V
W
Y

Site2847

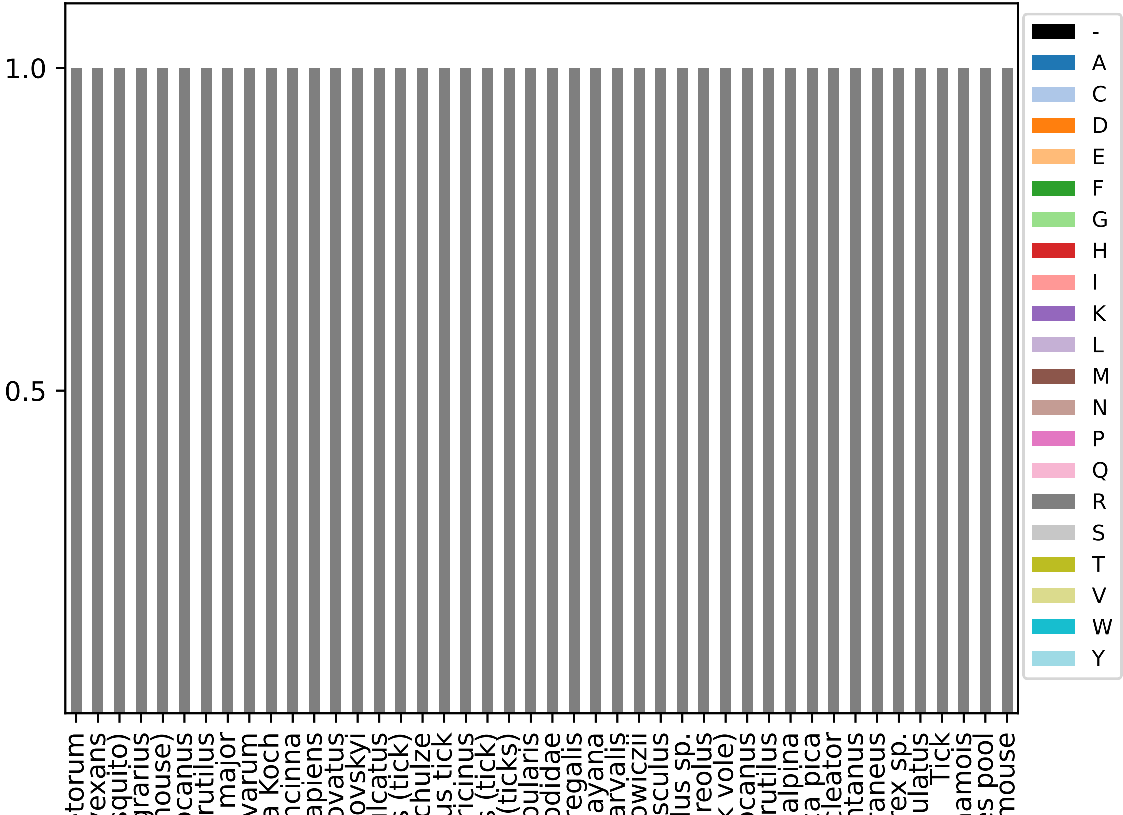

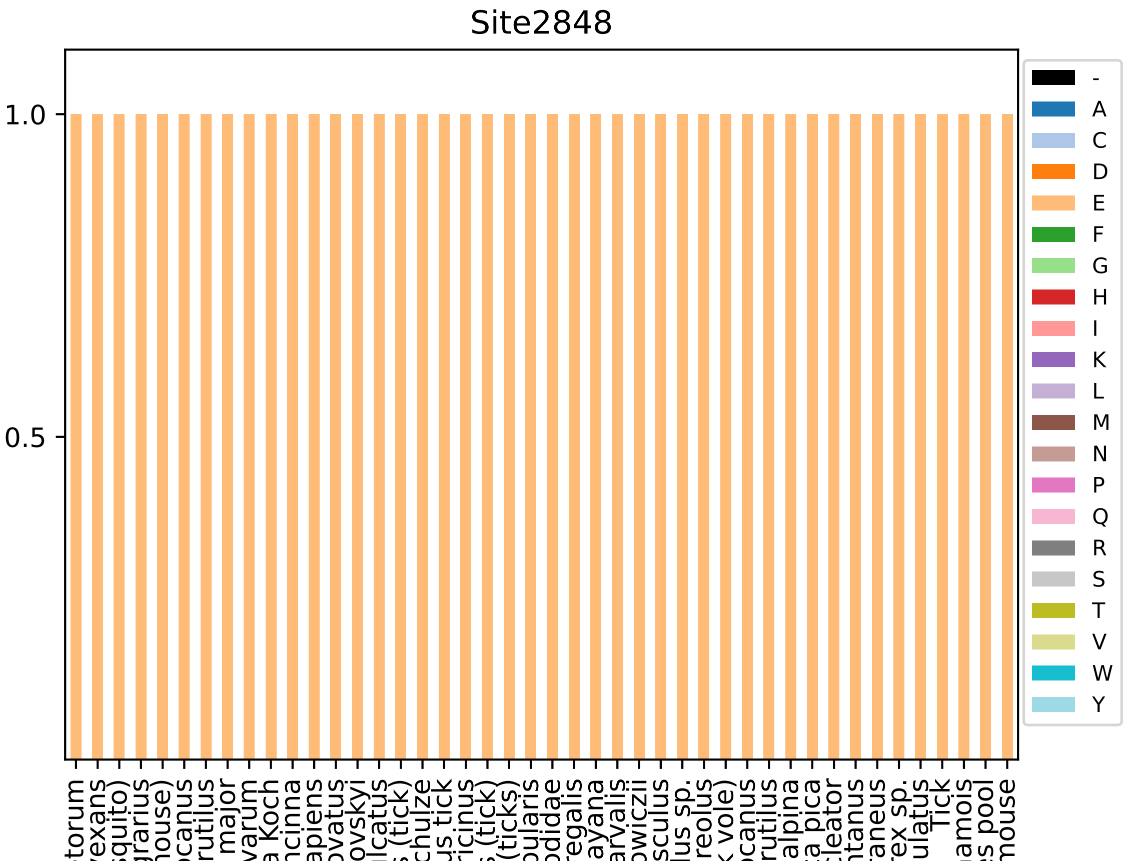
Site2848
1.0
0.5
-
A
C
D
E
F
G
H
I
K
L
M
N
P
Q
R
S
T
V
W
Y

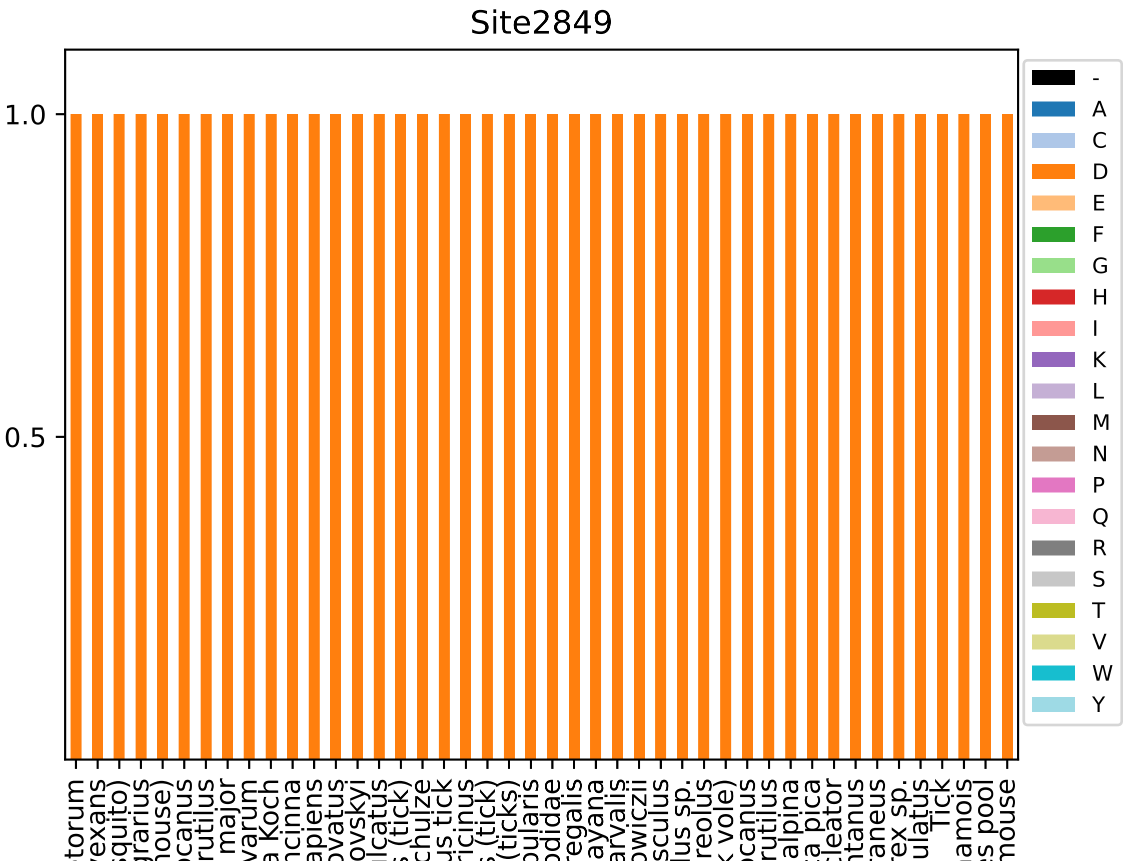

Site2849
1.0
0.5
-
A
C
D
E
F
G
H
I
K
L
M
N
P
Q
R
S
T
V
W
Y

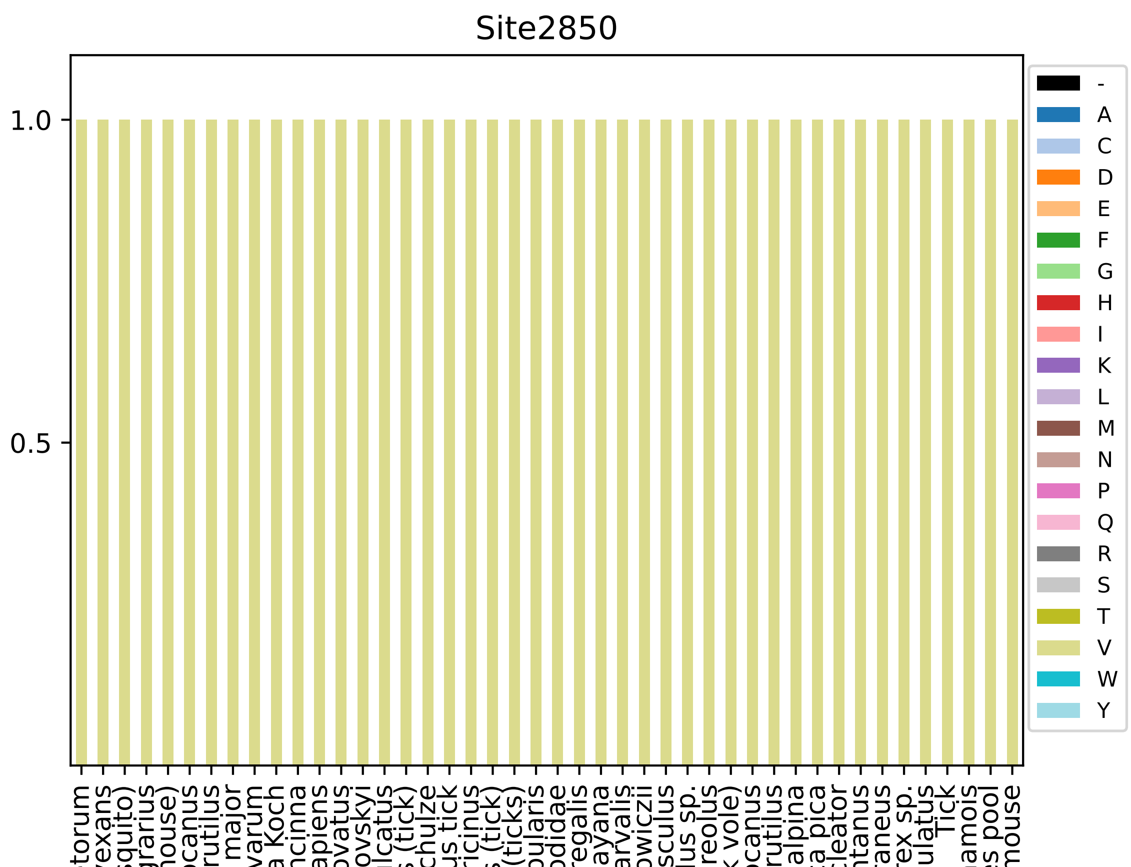
Site2850
1.0
0.5
-
A
C
D
E
F
G
H
I
K
L
M
N
P
Q
R
S
T
V
W
Y

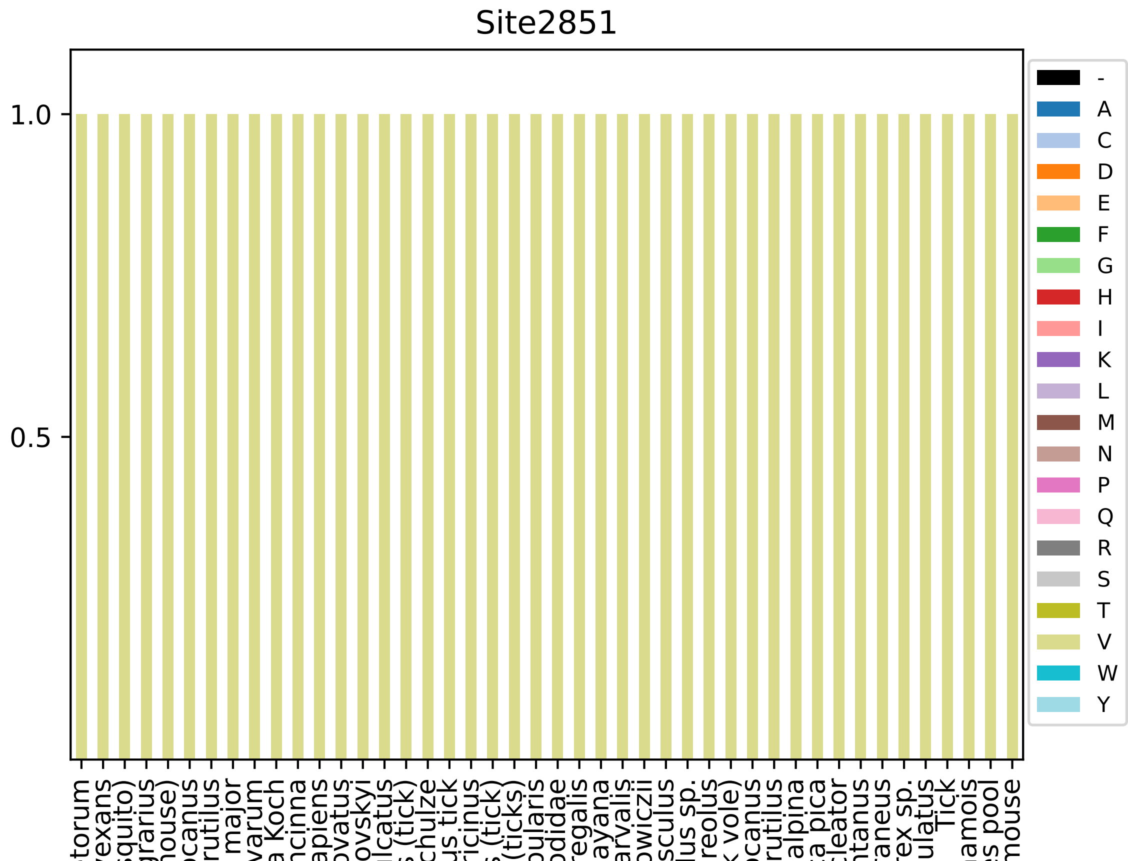
Site2851
1.0
0.5
-
A
C
D
E
F
G
H
I
K
L
M
N
P
Q
R
S
T
V
W
Y

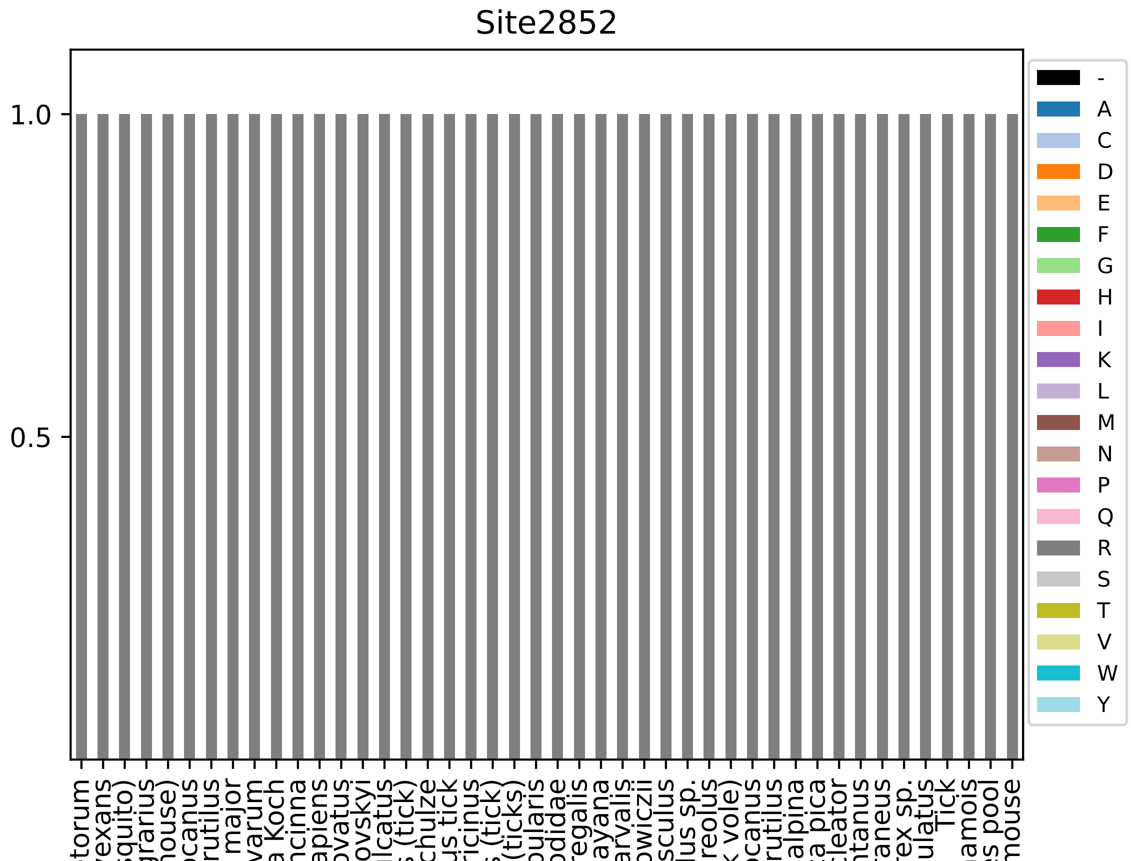
Site2852
1.0
0.5
-
A
C
D
E
F
G
H
I
K
L
M
N
P
Q
R
S
T
V
W
Y
Tick

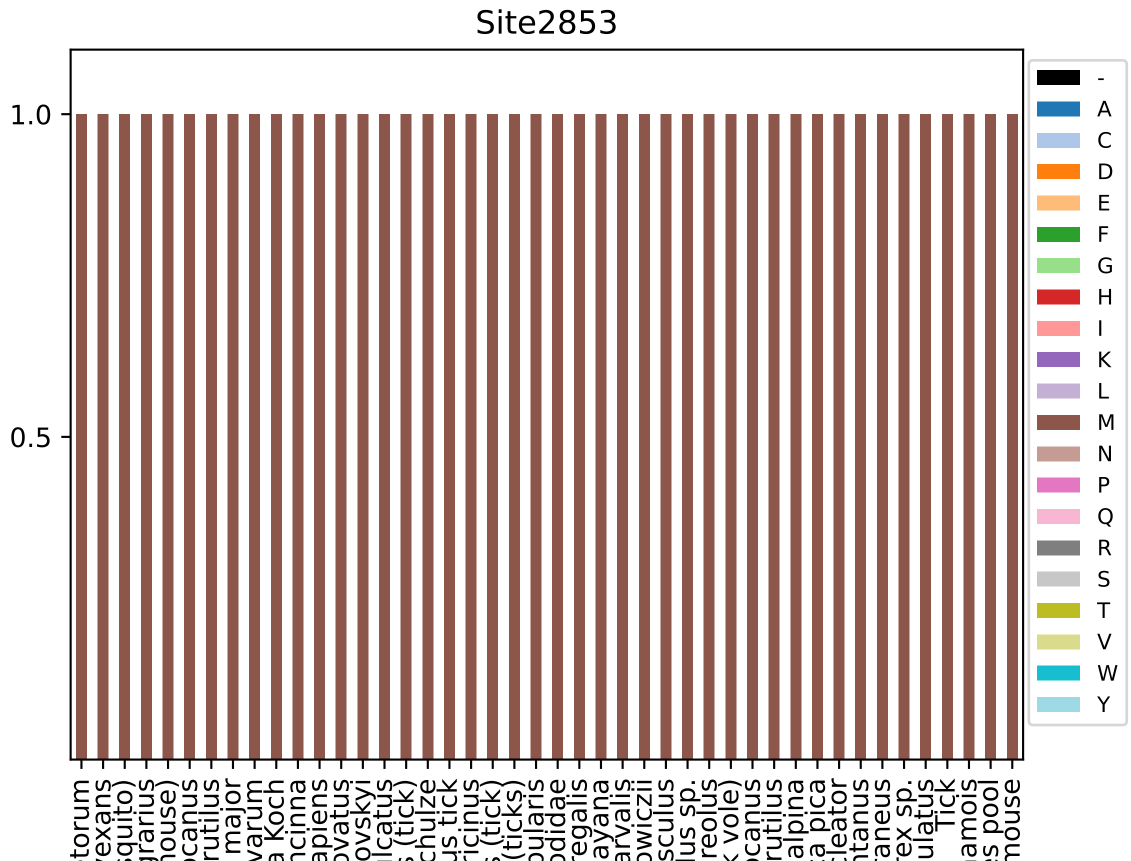
Site2853
1.0
0.5
-
A
C
D
E
F
G
H
I
K
L
M
N
P
Q
R
S
T
V
W
Y

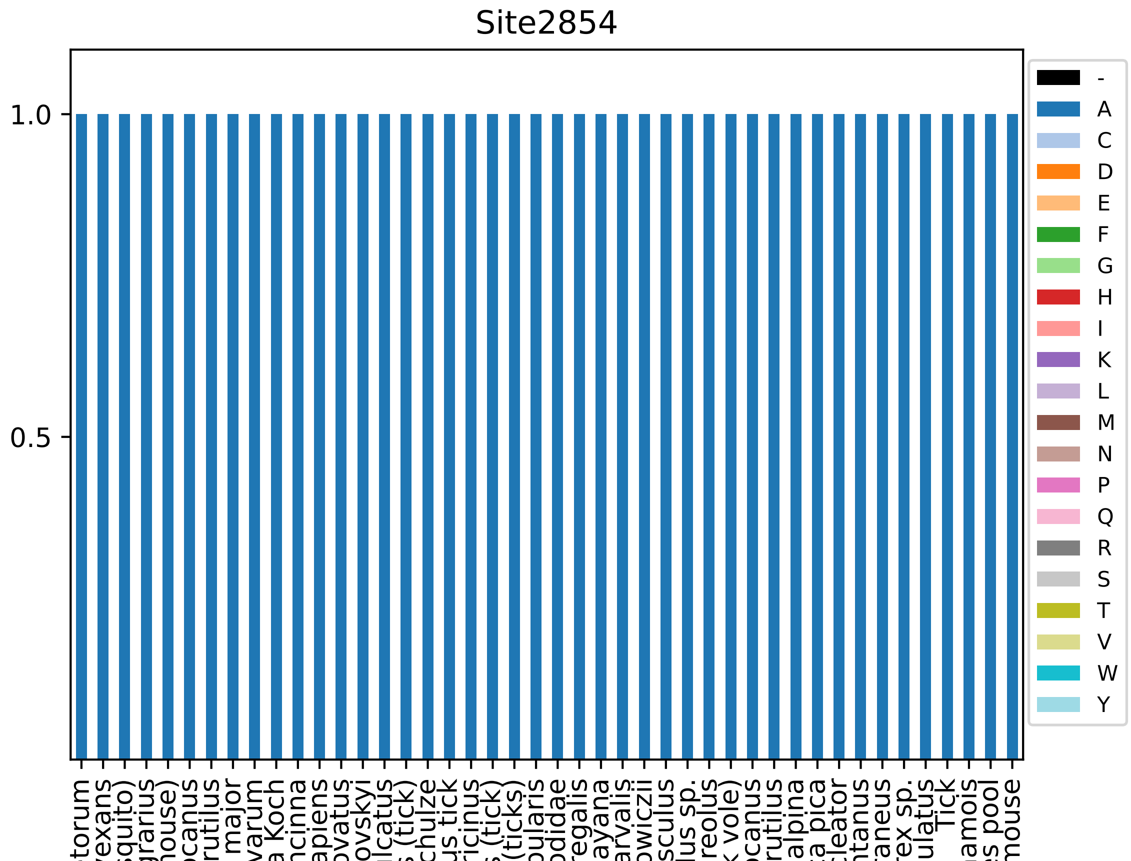

Site2854
1.0
0.5
-
A
C
D
E
F
G
H
I
K
L
M
N
P
Q
R
S
T
V
W
Y

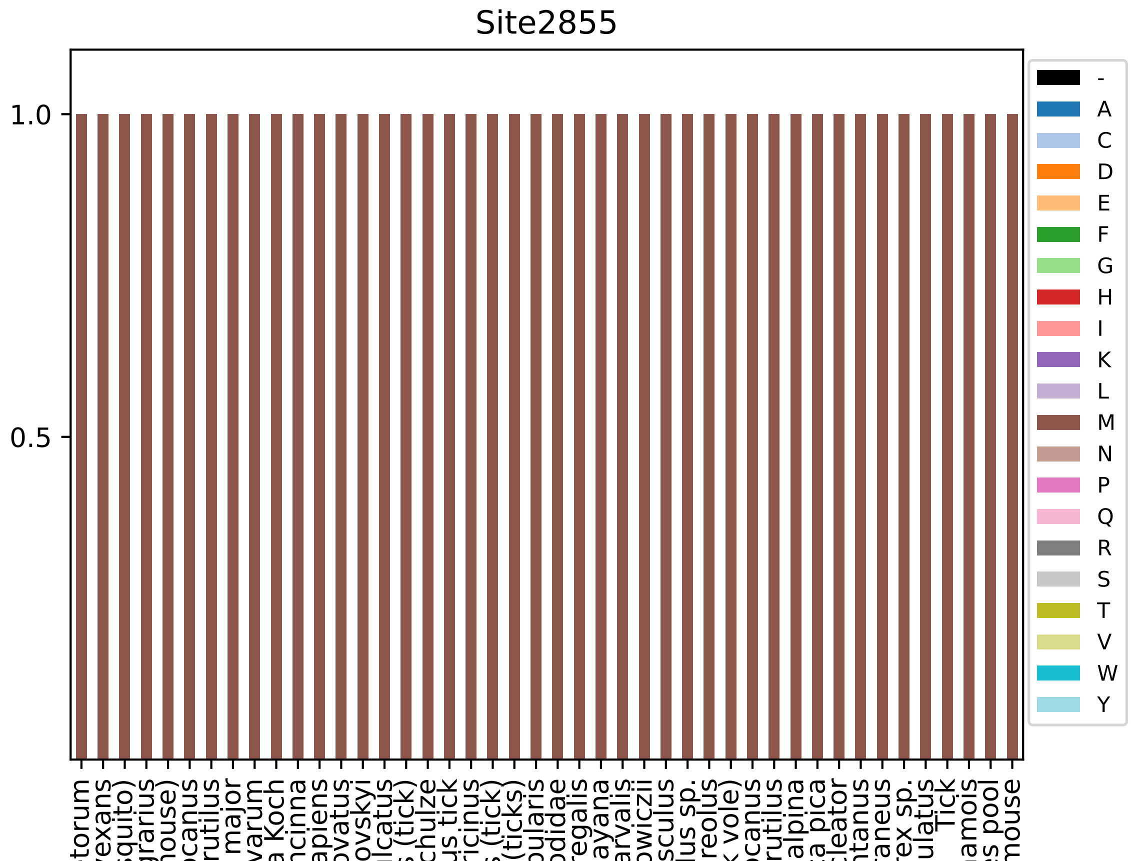
Site2855
1.0
0.5
-
A
C
D
E
F
G
H
I
K
L
M
N
P
Q
R
S
T
V
W
Y

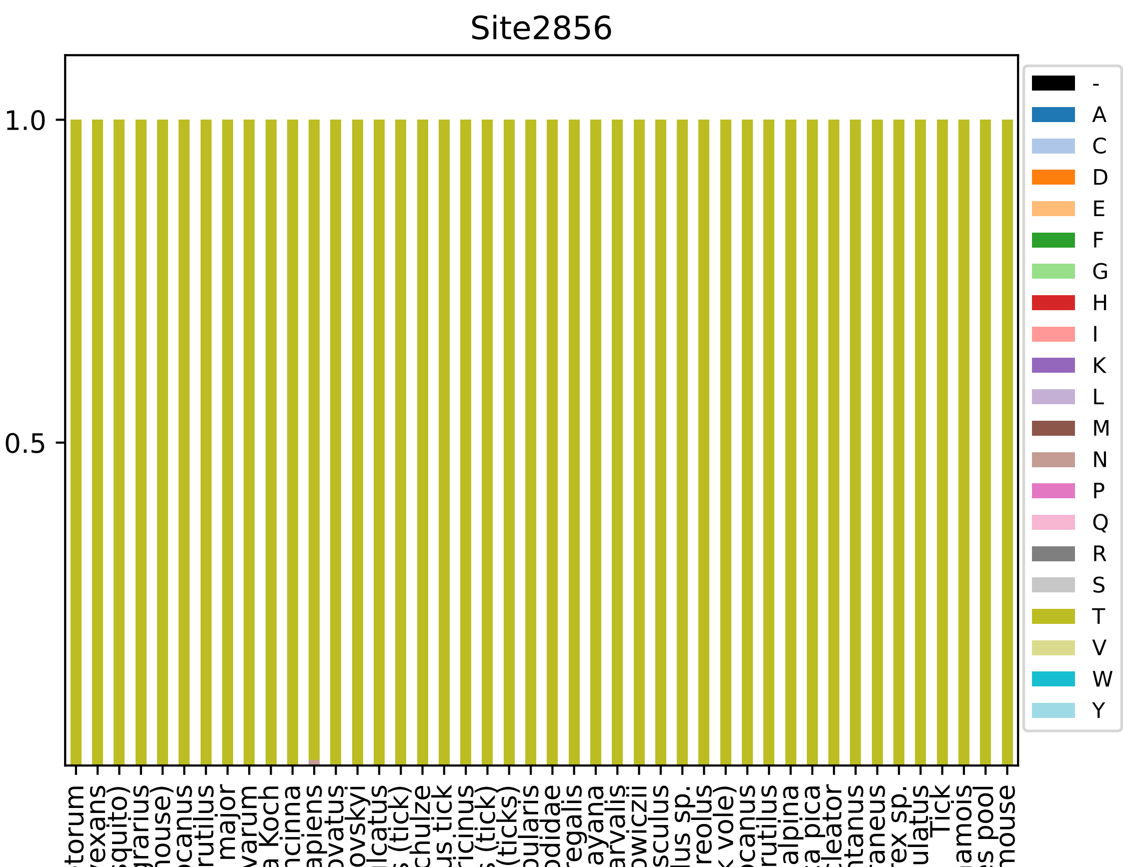

Site2856
1.0
0.5
-
A
C
D
E
F
G
H
I
K
L
M
N
P
Q
R
S
T
V
W
Y

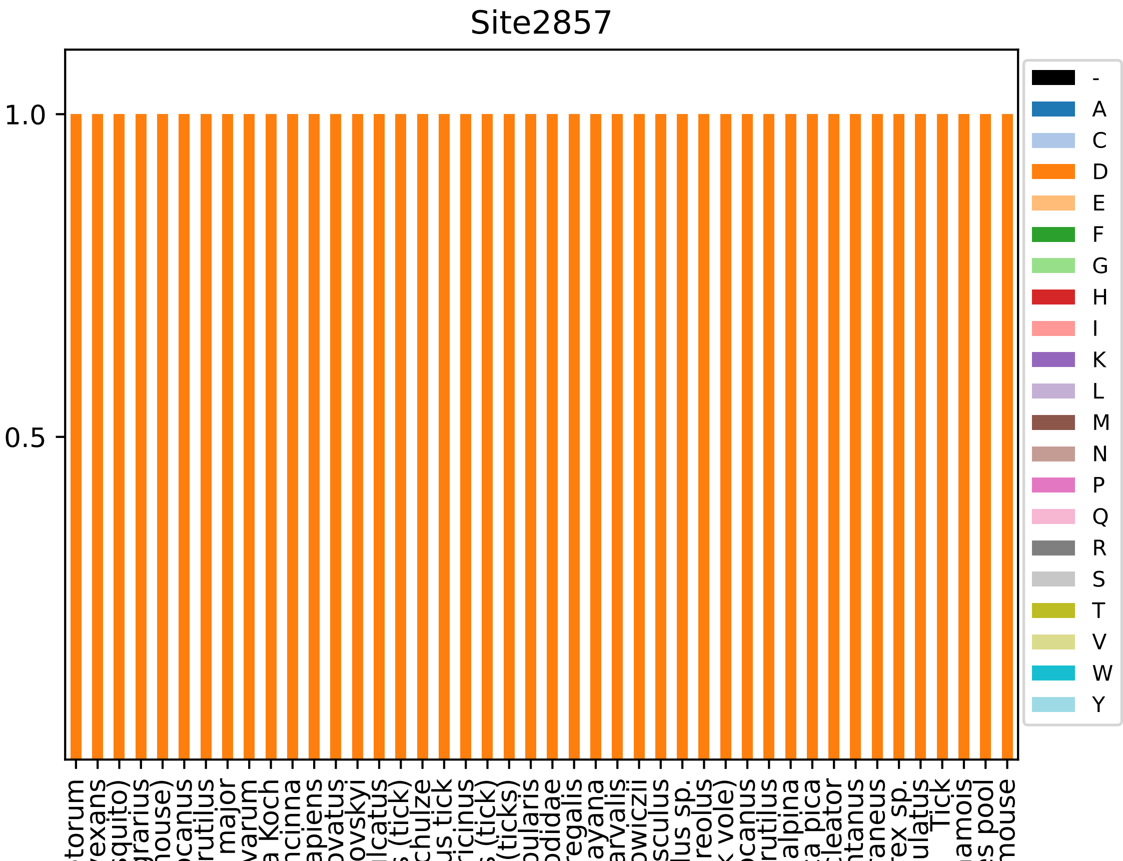
Site2857
1.0
0.5
-
A
C
D
E
F
G
H
I
K
L
M
N
P
Q
R
S
T
V
W
Y
torum
vexans
squito)
grarius
nouse)
ocanus
rutilus
major
varum
a Koch
ncinna
apiens
ovatus
ovskyi
ulcatus
s (tick)
chulze
us tick
ricinus
s (tick)
(ticks)
pularis
odidae
regalis
ayana
arvalis
owiczii
isculus
lus sp.
reolus
k vole)
ocanus
rutilus
alpina
a pica
cleator
ntanus
raneus
rex sp.
ulatus
Tick
amois
es pool
mouse

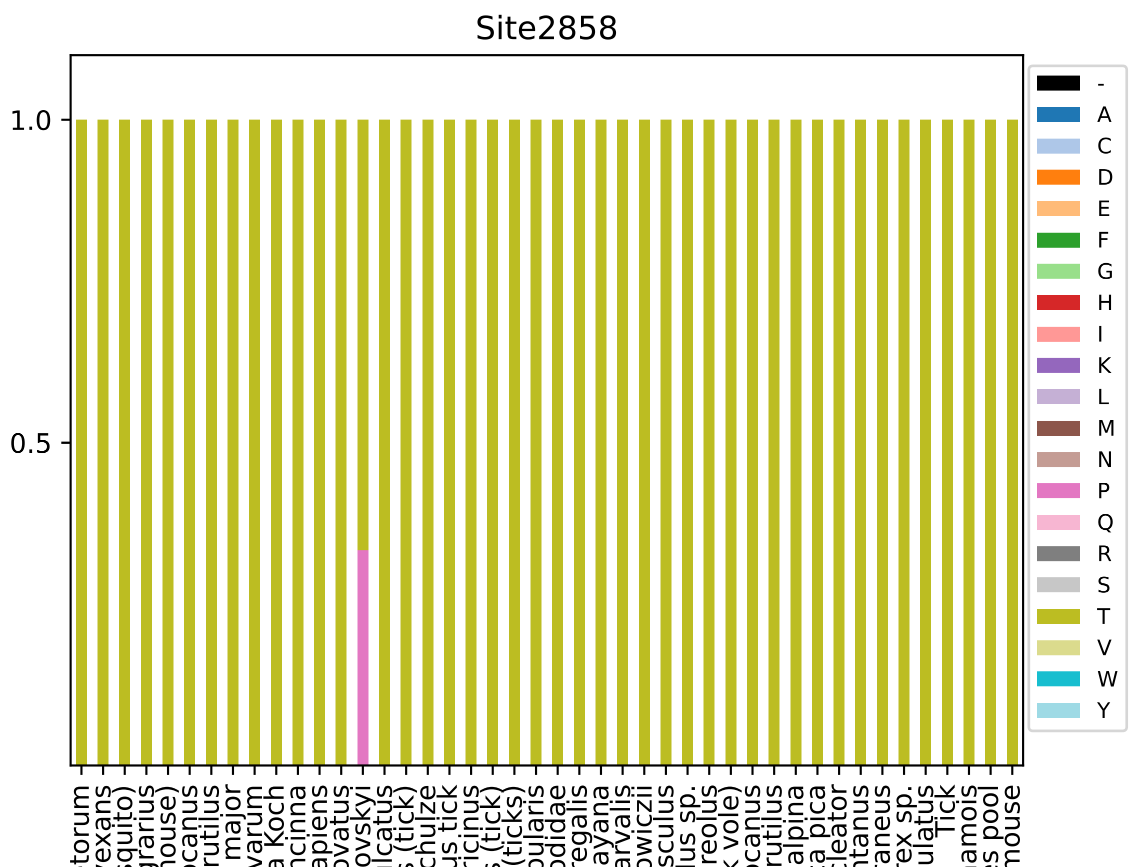
Site2858
1.0
0.5
-
A
C
D
E
F
G
H
I
K
L
M
N
P
Q
R
S
T
V
W
Y

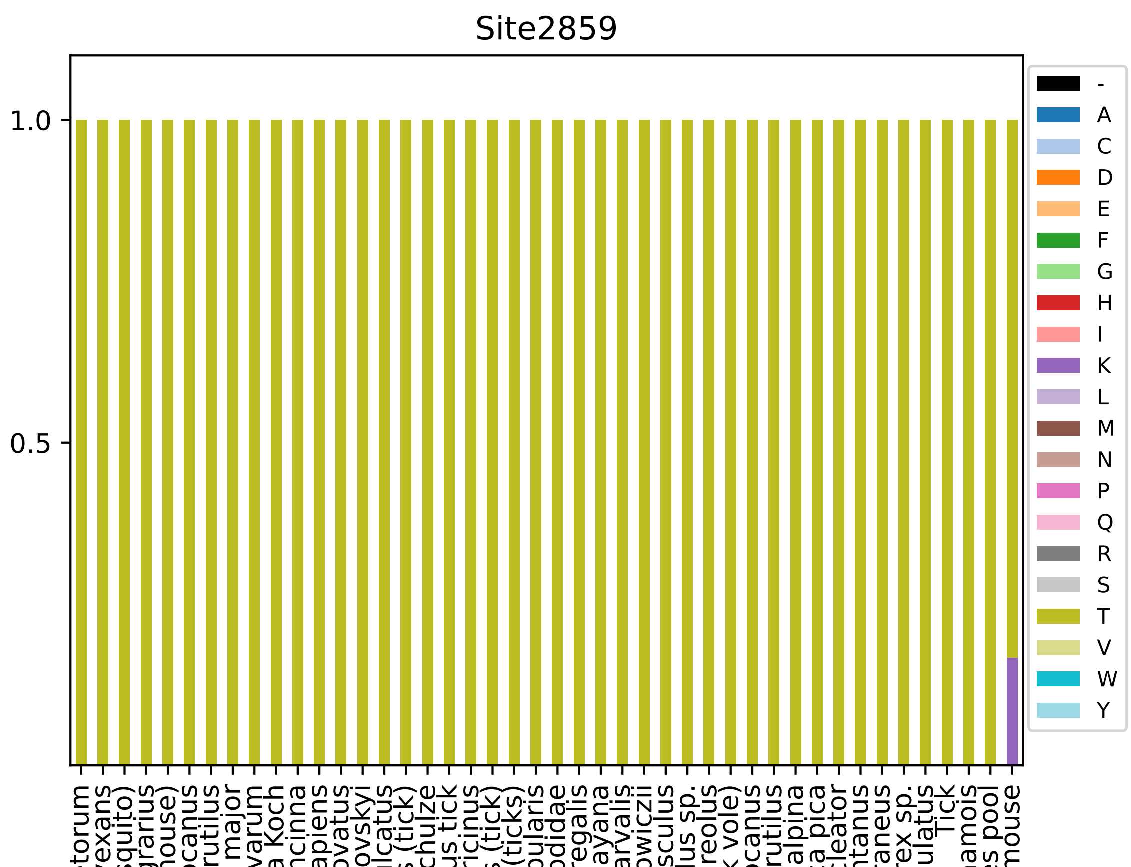
Site2859
1.0
0.5
-
A
C
D
E
F
G
H
I
K
L
M
N
P
Q
R
S
T
V
W
Y
torum
vexans
squito)
grarius
house)
ocanus
rutilus
major
varum
a Koch
ncinna
apiens
ovatus
ovskyi
ulcatus
s (tick)
chulze
us tick
ricinus
s (tick)
(ticks)
pularis
podidae
regalis
ayana
arvalis
owiczii
sculus
lus sp.
reolus
x vole)
ocanus
rutilus
alpina
a pica
cleator
ntanus
raneus
rex sp.
ulatus
Tick
amois
es pool
mouse

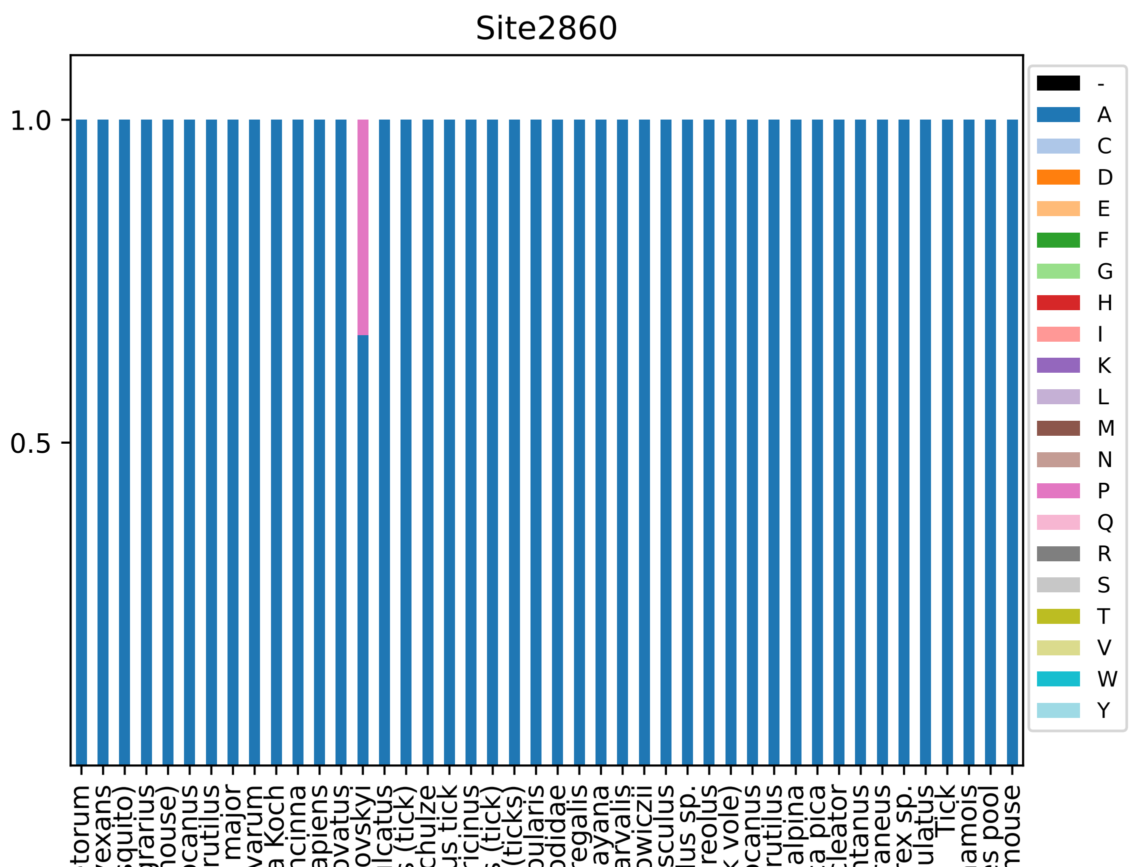

Site2860
1.0
0.5
-
A
C
D
E
F
G
H
I
K
L
M
N
P
Q
R
S
T
V
W
Y

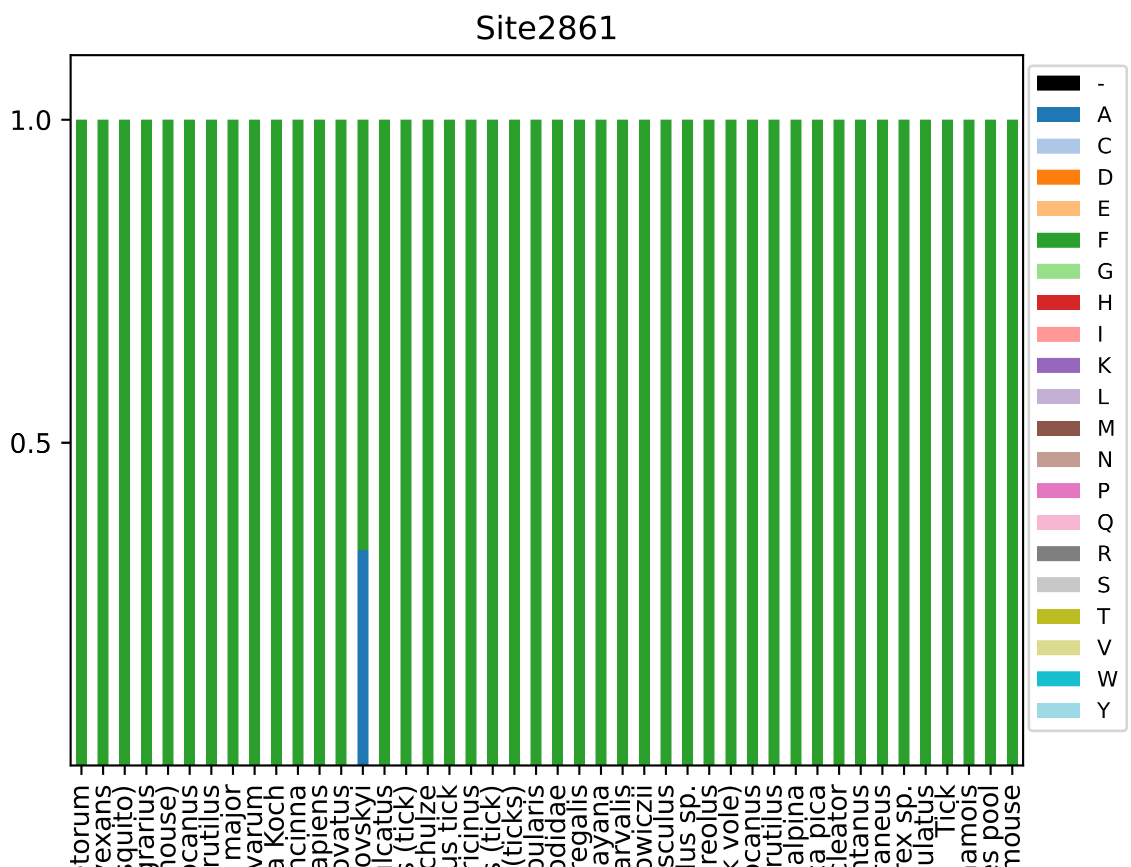
Site2861
1.0
0.5
-
A
C
D
E
F
G
H
I
K
L
M
N
P
Q
R
S
T
V
W
Y

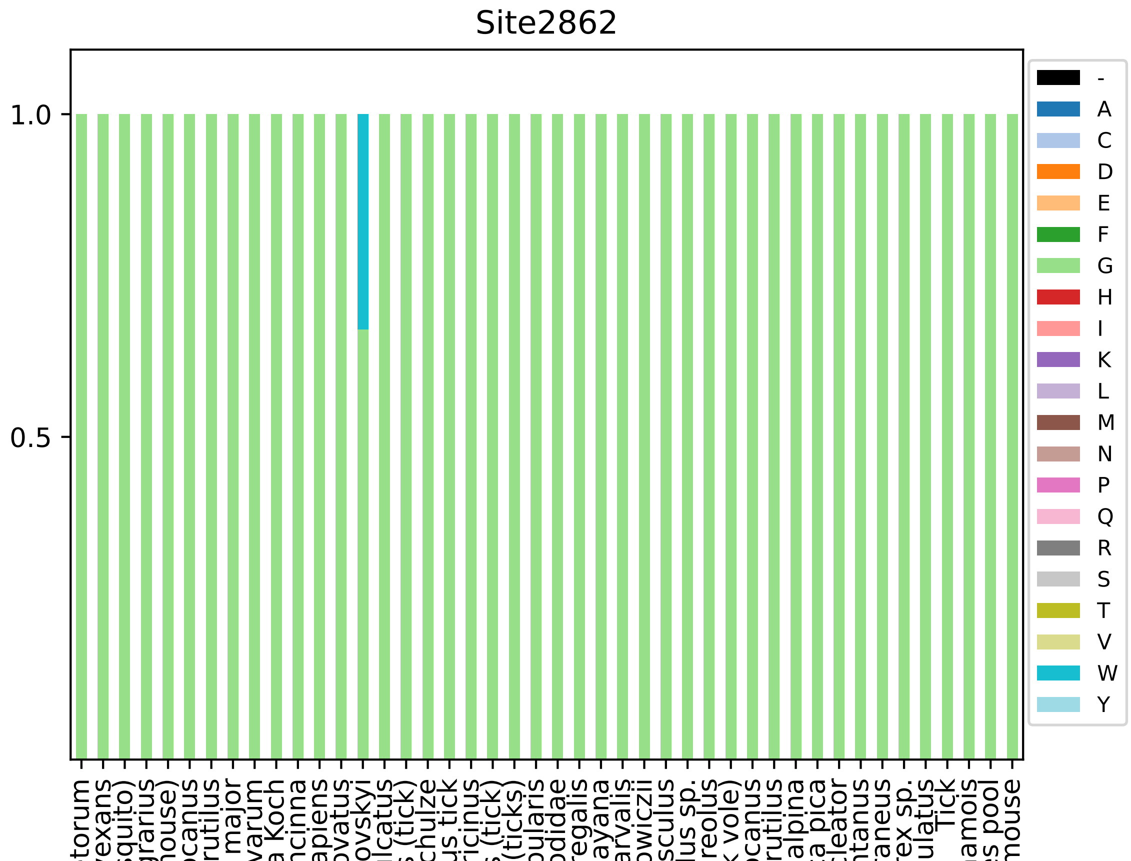

Site2862
1.0
0.5
-
A
C
D
E
F
G
H
I
K
L
M
N
P
Q
R
S
T
V
W
Y

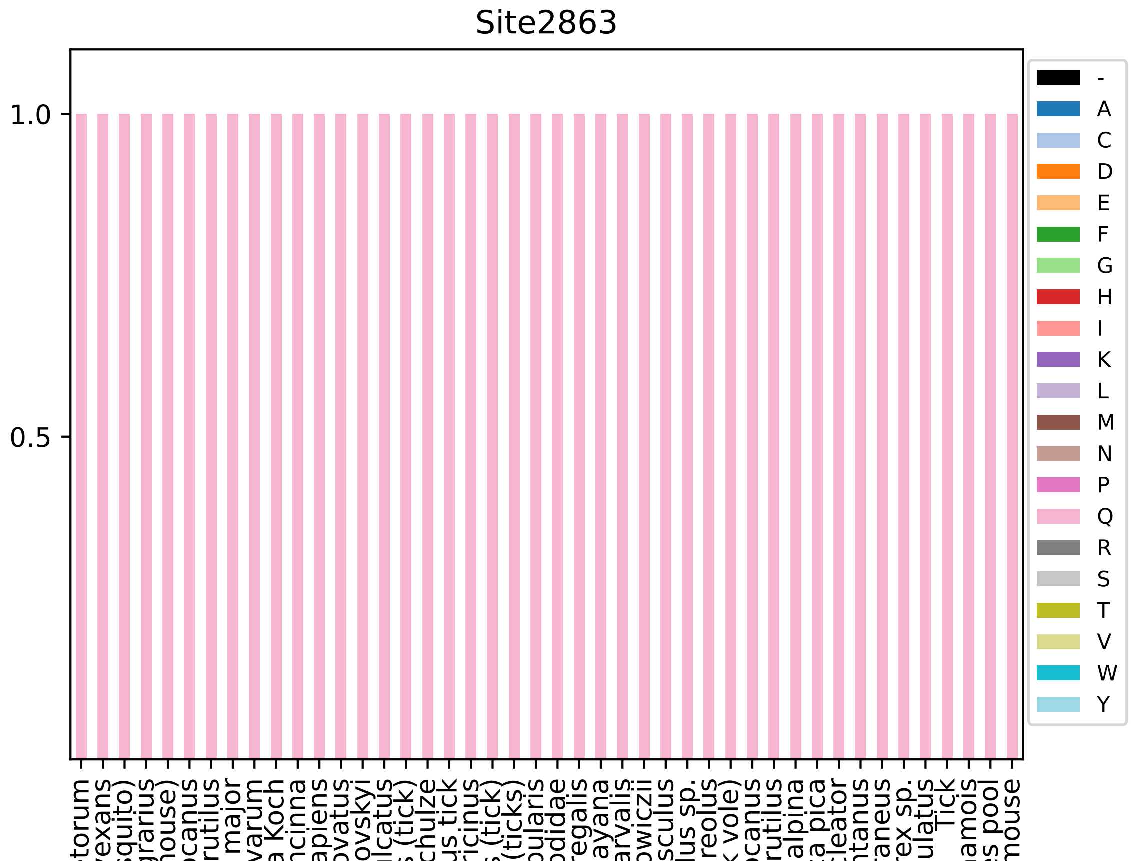
Site2863
1.0
0.5
-
A
C
D
E
F
G
H
I
K
L
M
N
P
Q
R
S
T
V
W
Y

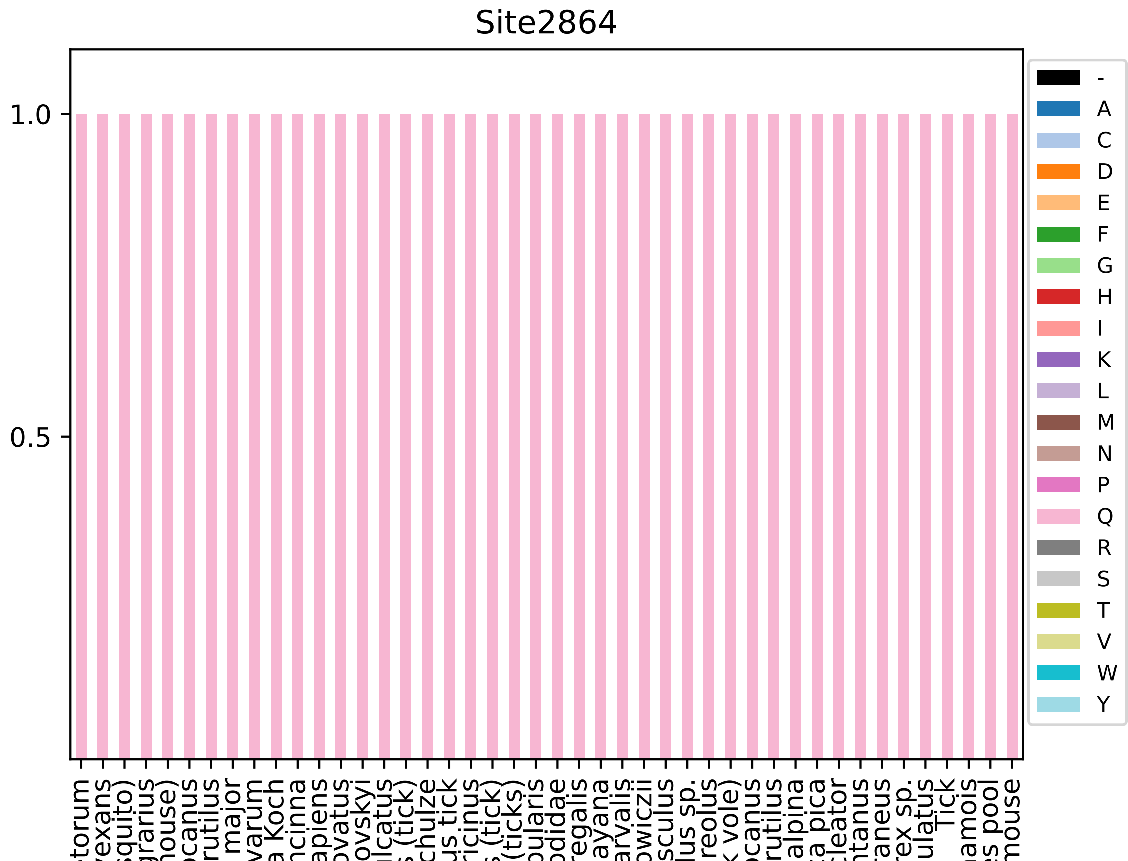
Site2864
1.0
0.5
-
A
C
D
E
F
G
H
I
K
L
M
N
P
Q
R
S
T
V
W
Y

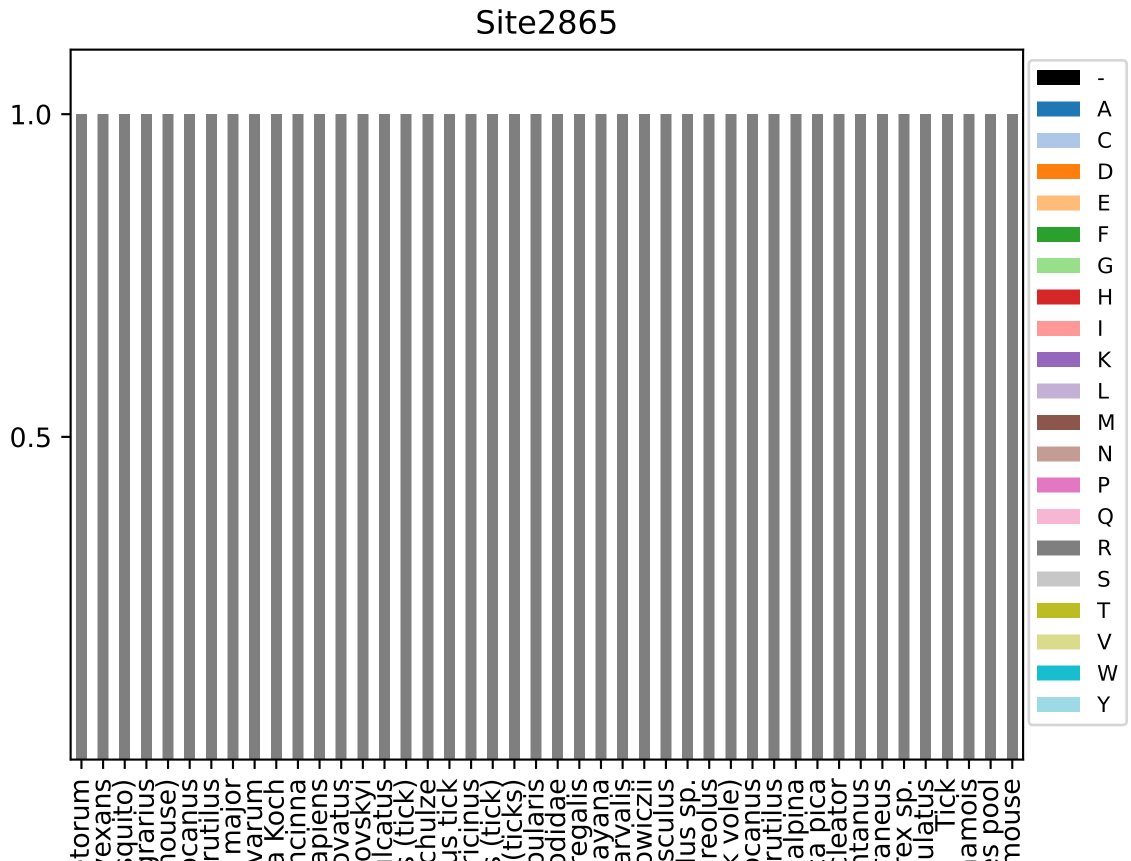
Site2865
1.0
0.5
-
A
C
D
E
F
G
H
I
K
L
M
N
P
Q
R
S
T
V
W
Y

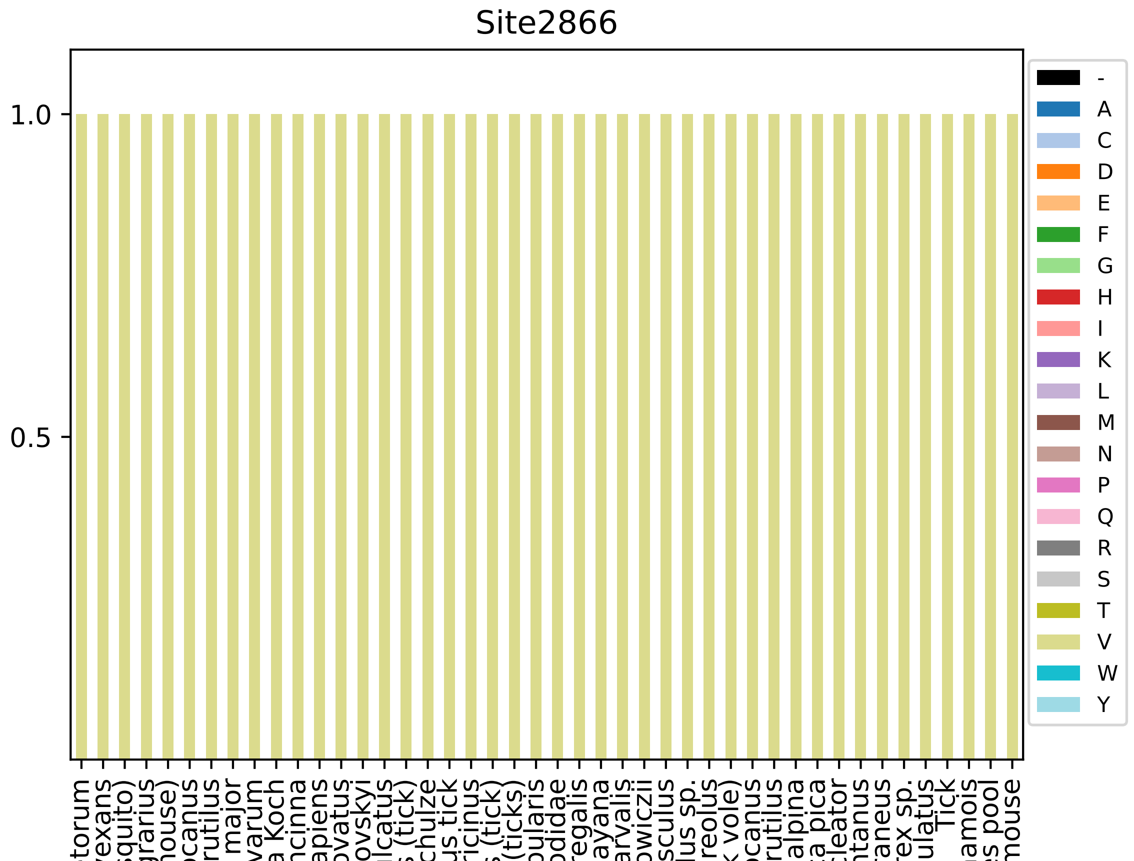

Site2866
1.0
0.5
-
A
C
D
E
F
G
H
I
K
L
M
N
P
Q
R
S
T
V
W
Y

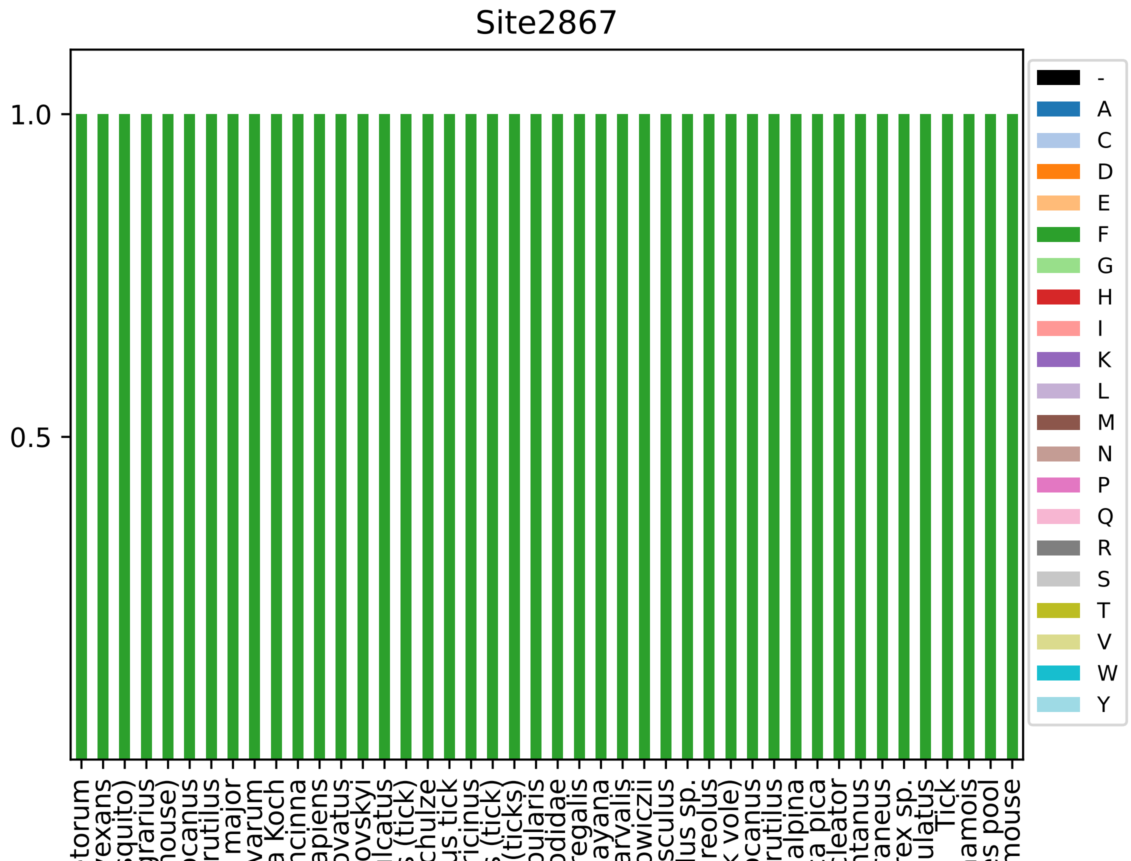
Site2867
1.0
0.5
-
A
C
D
E
F
G
H
I
K
L
M
N
P
Q
R
S
T
V
W
Y

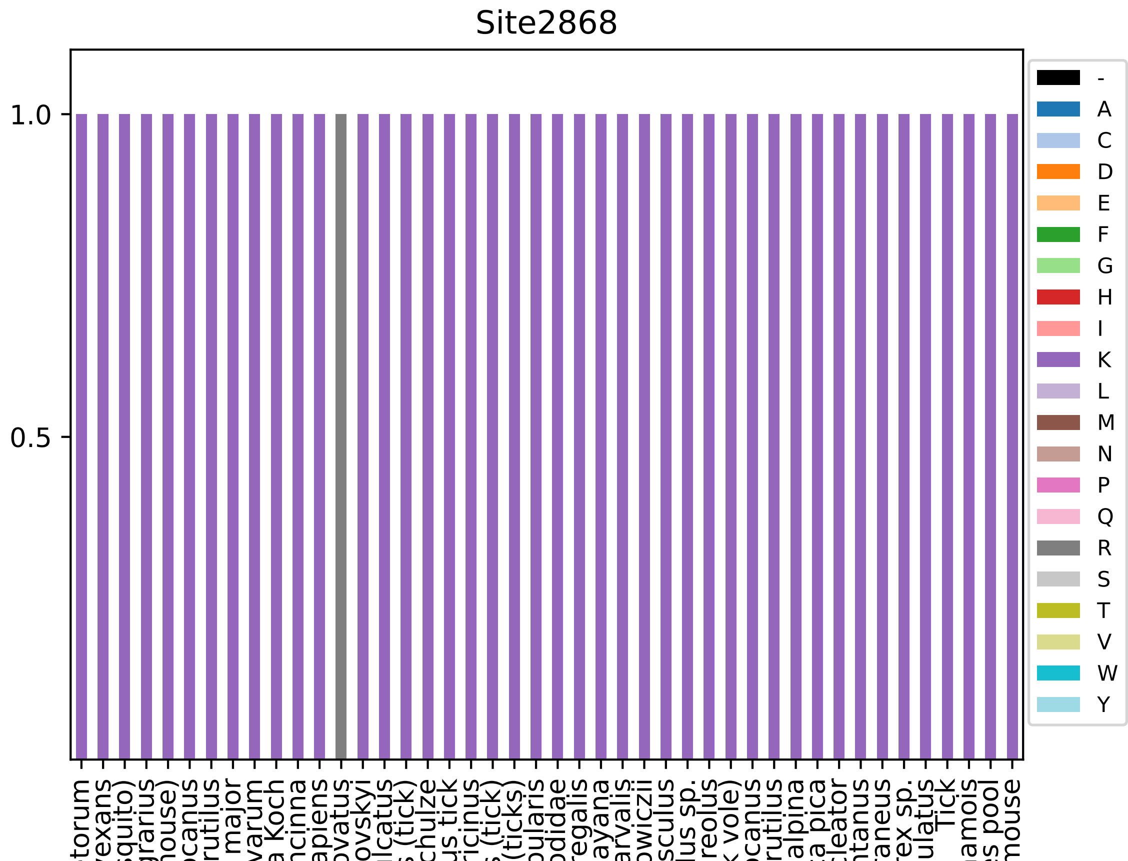
Site2868
1.0
0.5
-
A
C
D
E
F
G
H
I
K
L
M
N
P
Q
R
S
T
V
W
Y

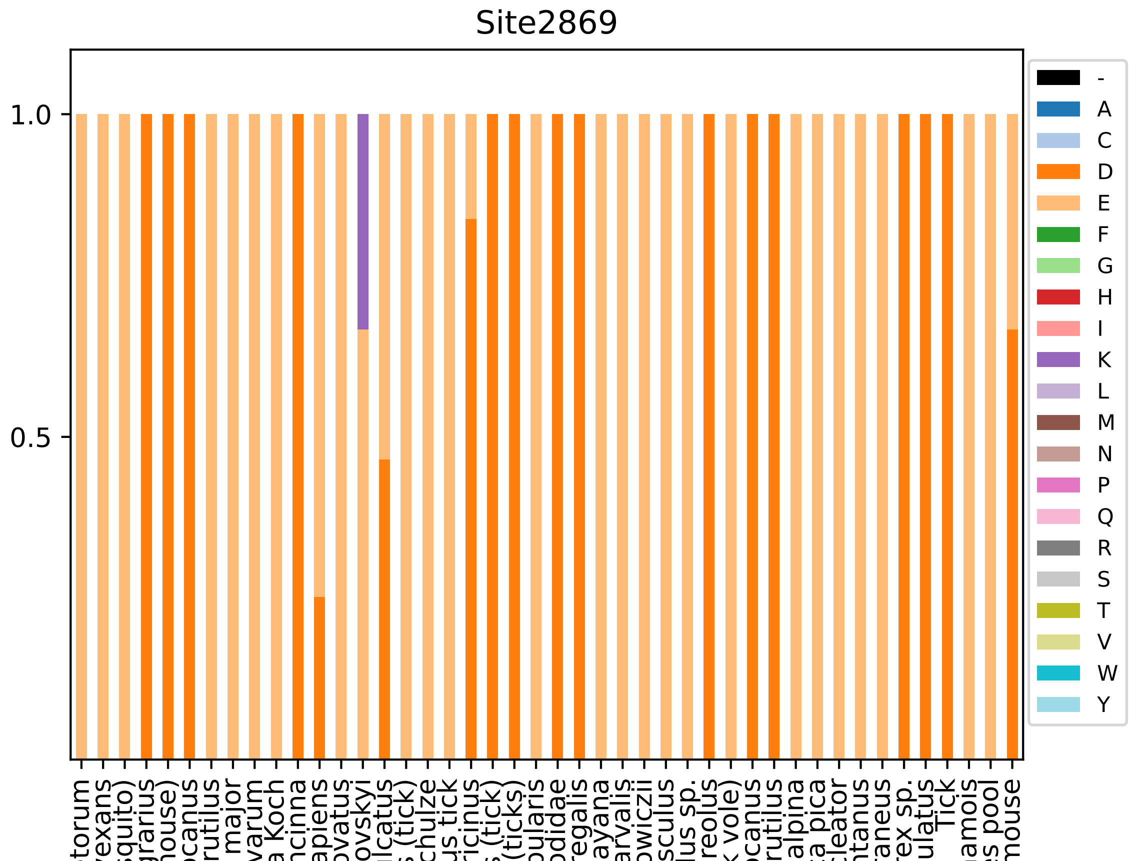
Site2869
1.0
0.5
-
A
C
D
E
F
G
H
I
K
L
M
N
P
Q
R
S
T
V
W
Y

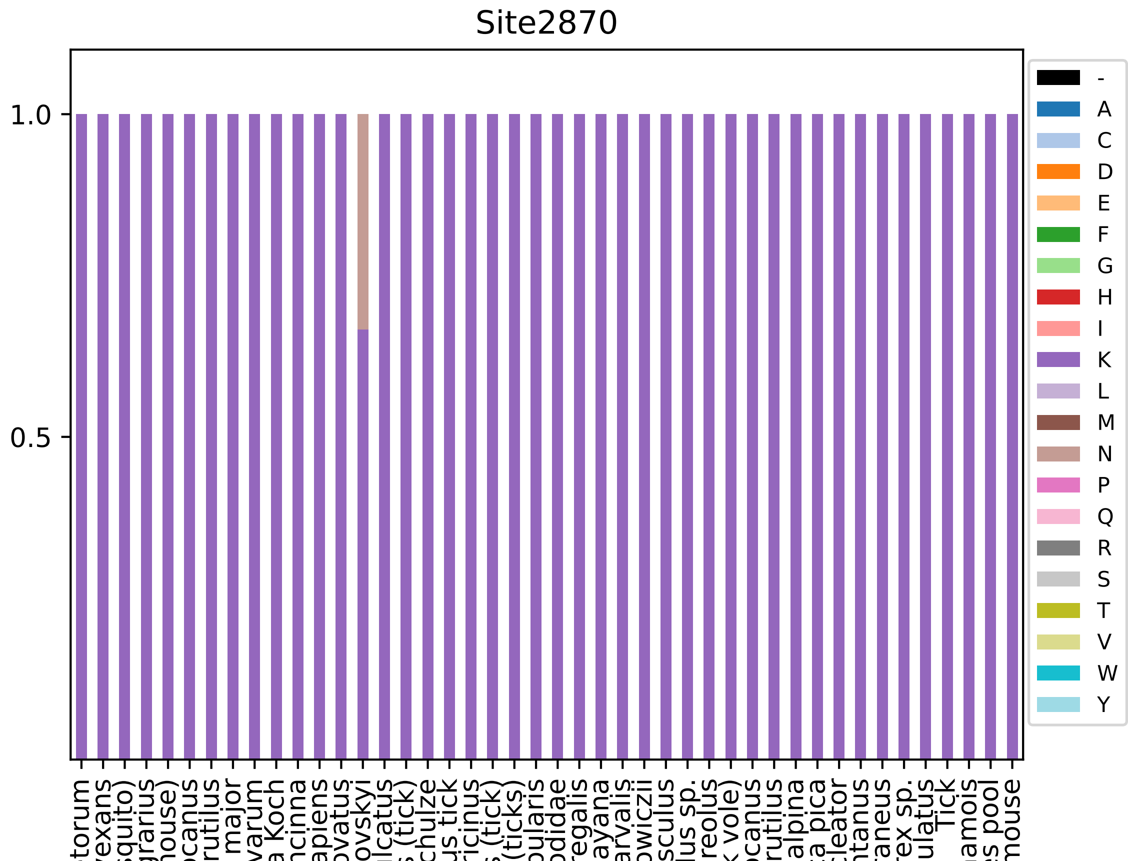

Site2870
1.0
0.5
-
A
C
D
E
F
G
H
I
K
L
M
N
P
Q
R
S
T
V
W
Y

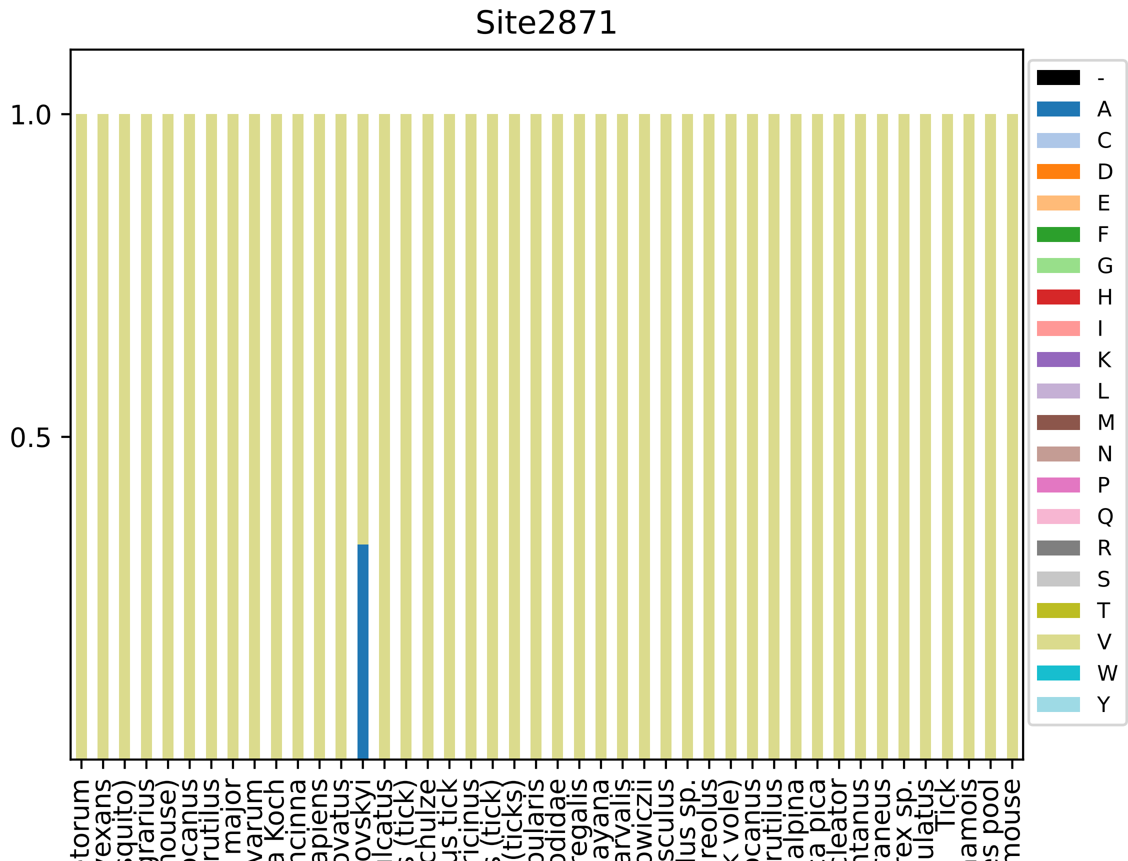
Site2871
1.0
0.5
-
A
C
D
E
F
G
H
I
K
L
M
N
P
Q
R
S
T
V
W
Y

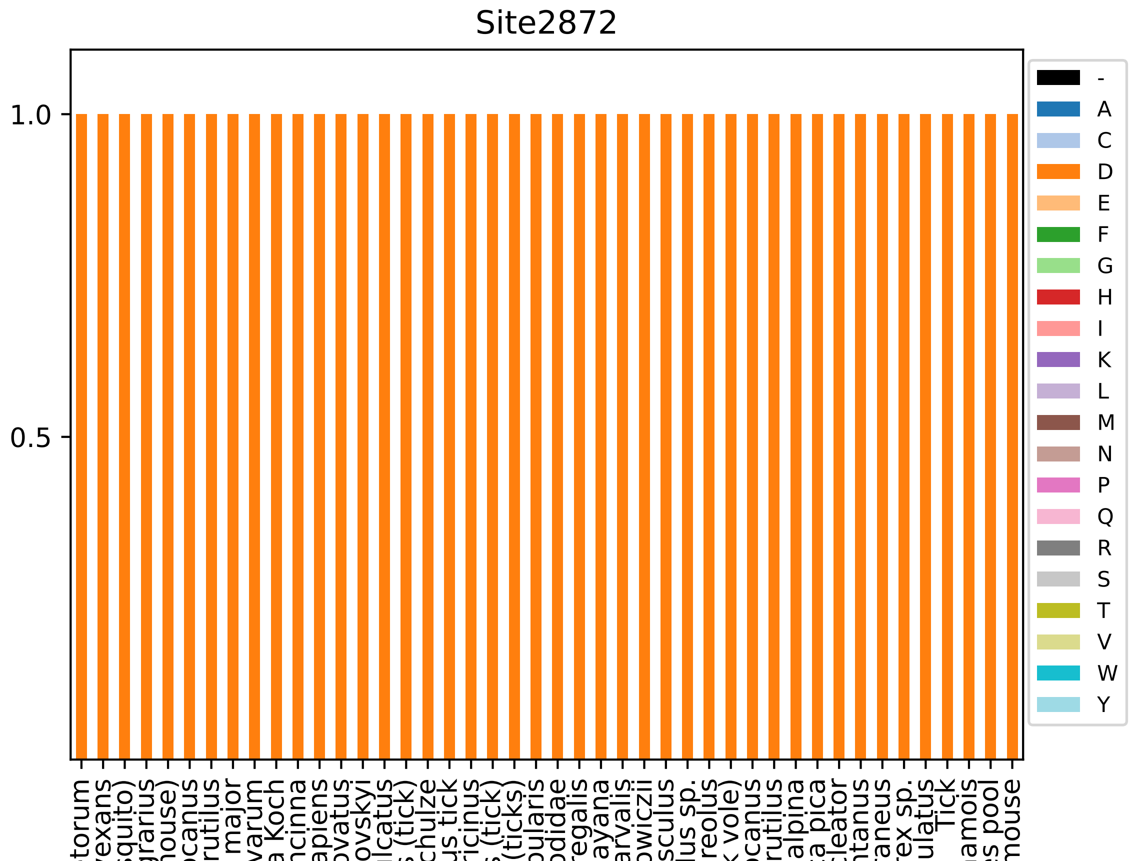
Site2872
1.0
0.5
-
A
C
D
E
F
G
H
I
K
L
M
N
P
Q
R
S
T
V
W
Y

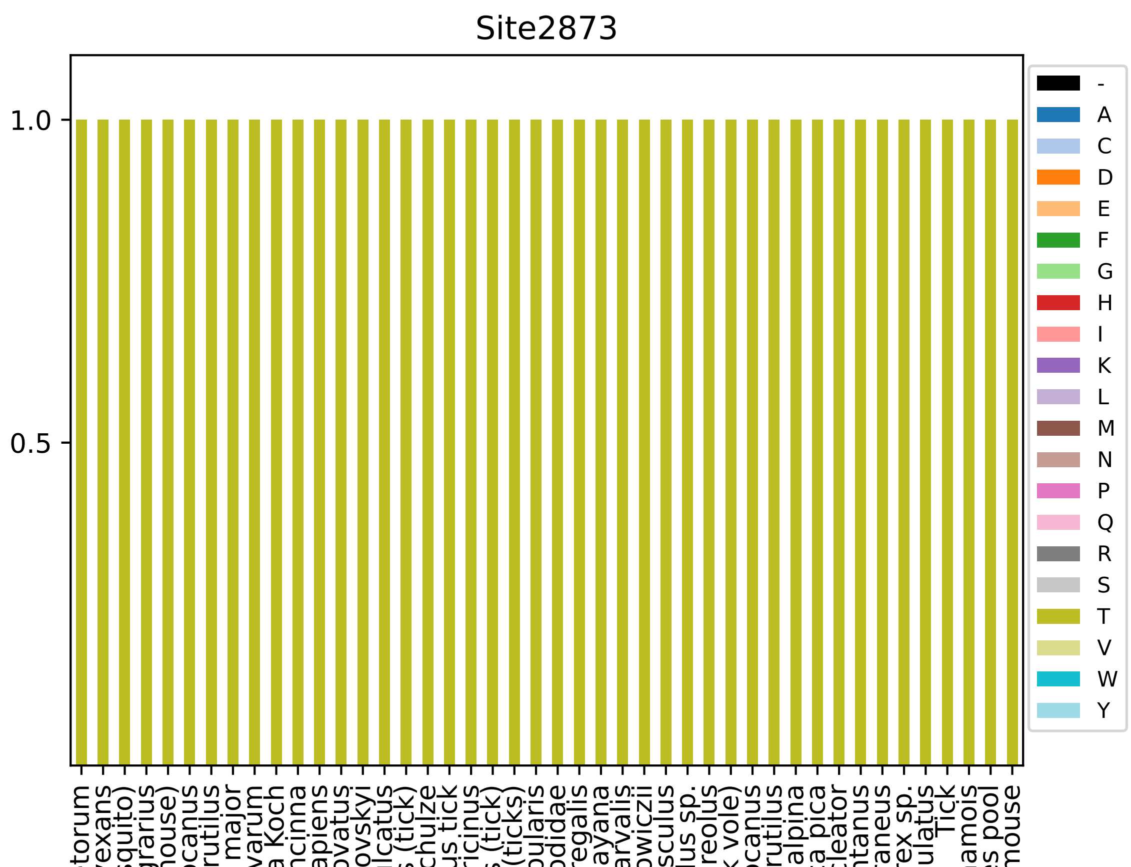

Site2873
1.0
0.5
-
A
C
D
E
F
G
H
I
K
L
M
N
P
Q
R
S
T
V
W
Y

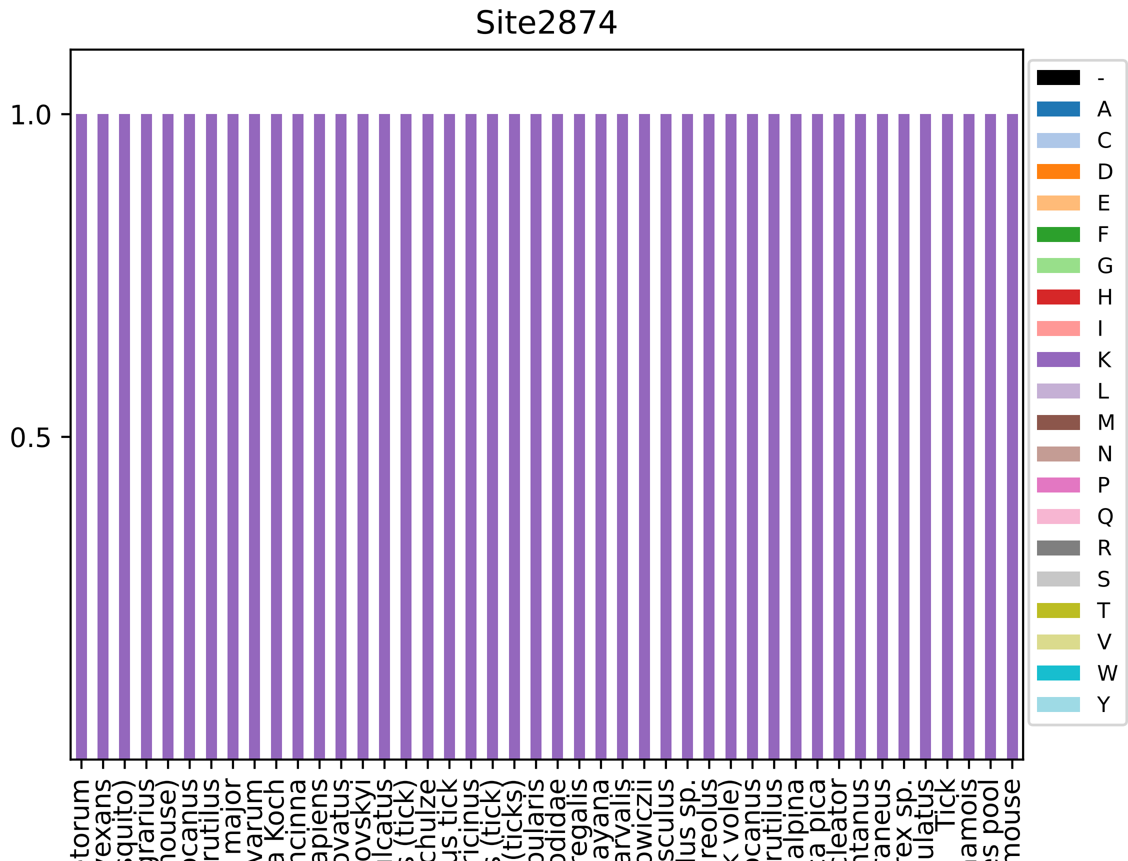
Site2874
1.0
0.5
-
A
C
D
E
F
G
H
I
K
L
M
N
P
Q
R
S
T
V
W
Y

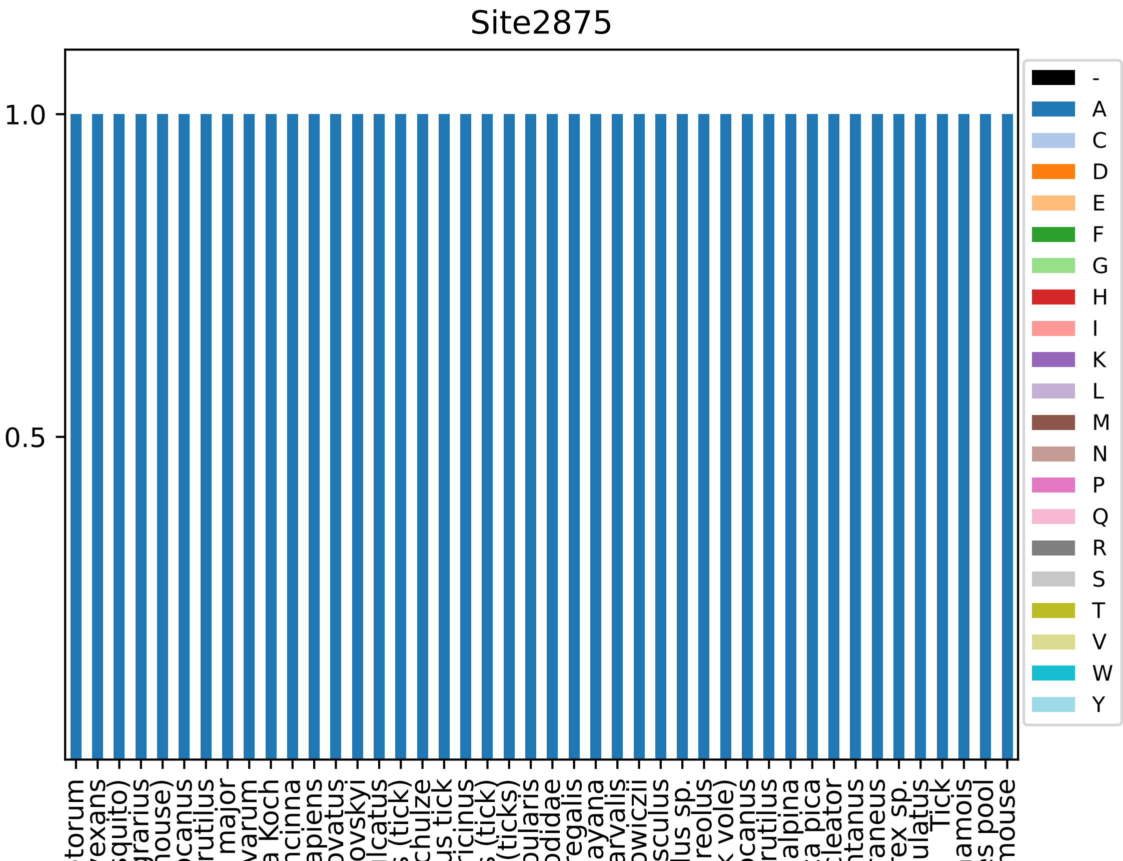
Site2875
1.0
0.5
-
A
C
D
E
F
G
H
I
K
L
M
N
P
Q
R
S
T
V
W
Y

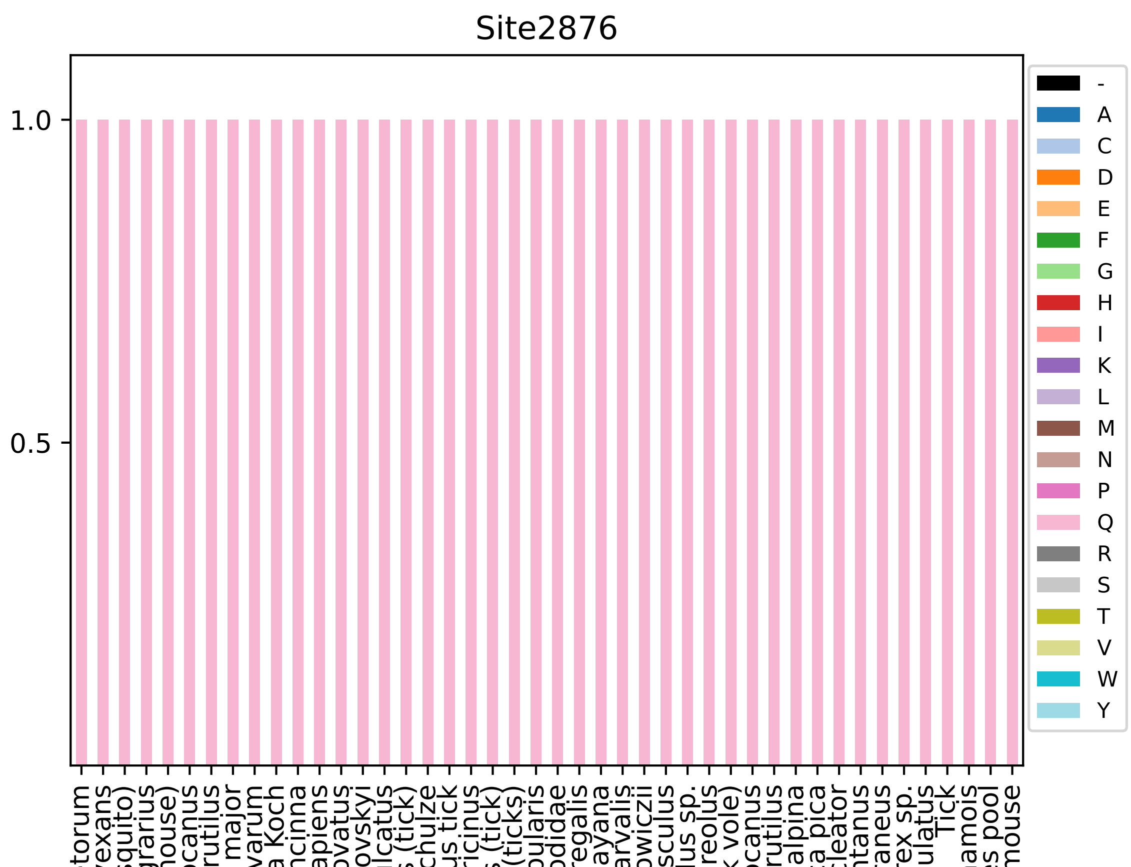
Site2876
1.0
0.5
-
A
C
D
E
F
G
H
I
K
L
M
N
P
Q
R
S
T
V
W
Y

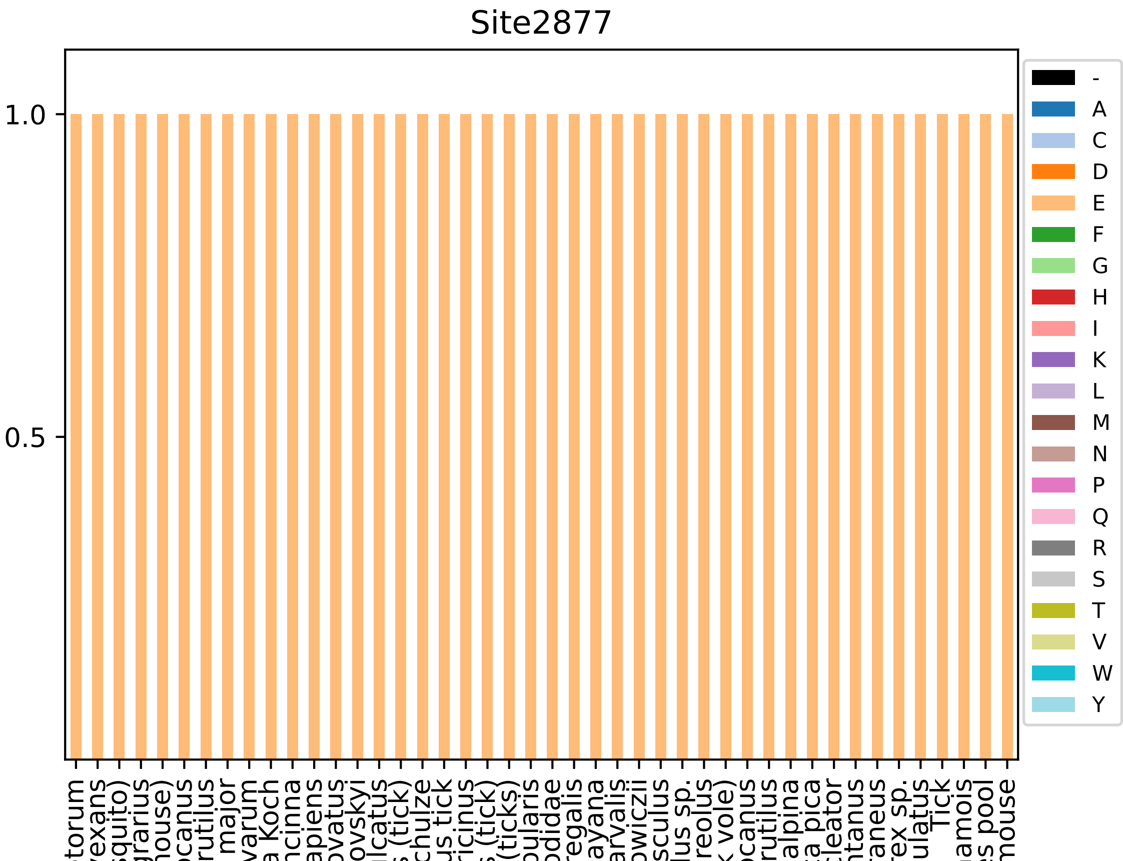

Site2877
1.0
0.5
-
A
C
D
E
F
G
H
I
K
L
M
N
P
Q
R
S
T
V
W
Y

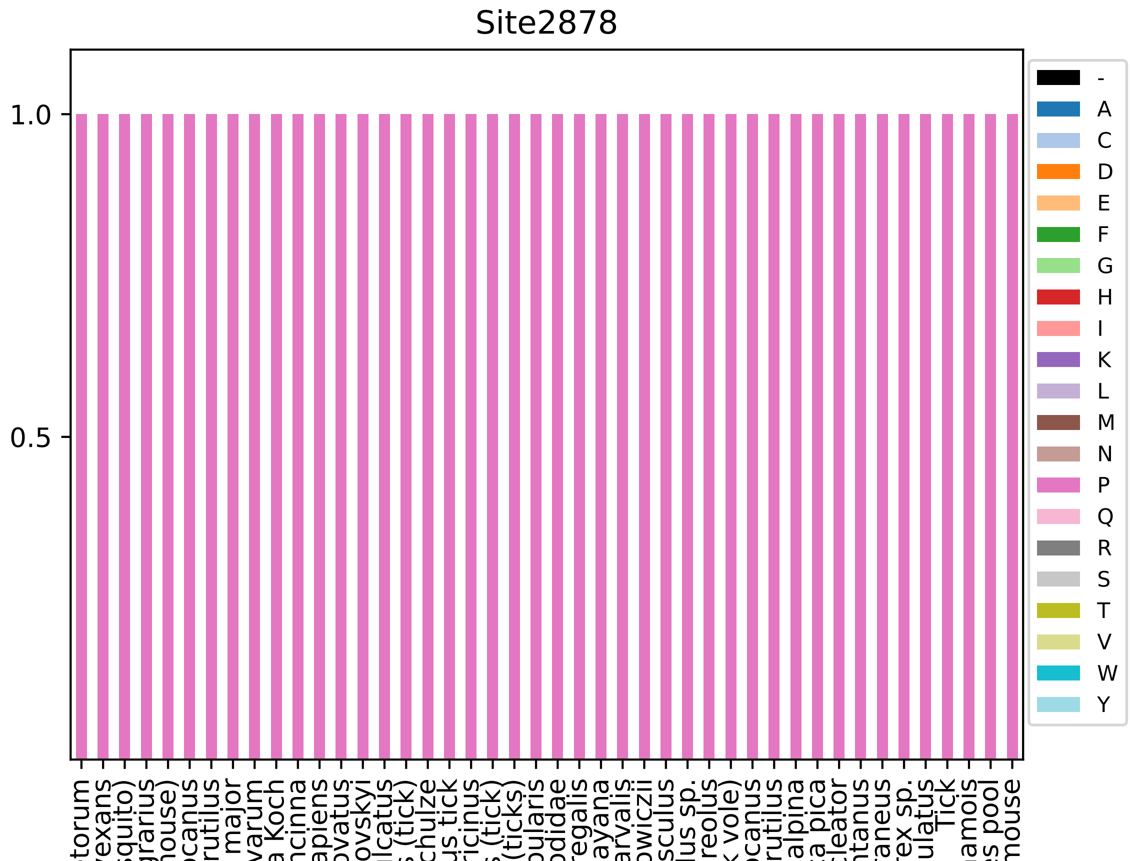
Site2878
1.0
0.5
-
A
C
D
E
F
G
H
I
K
L
M
N
P
Q
R
S
T
V
W
Y

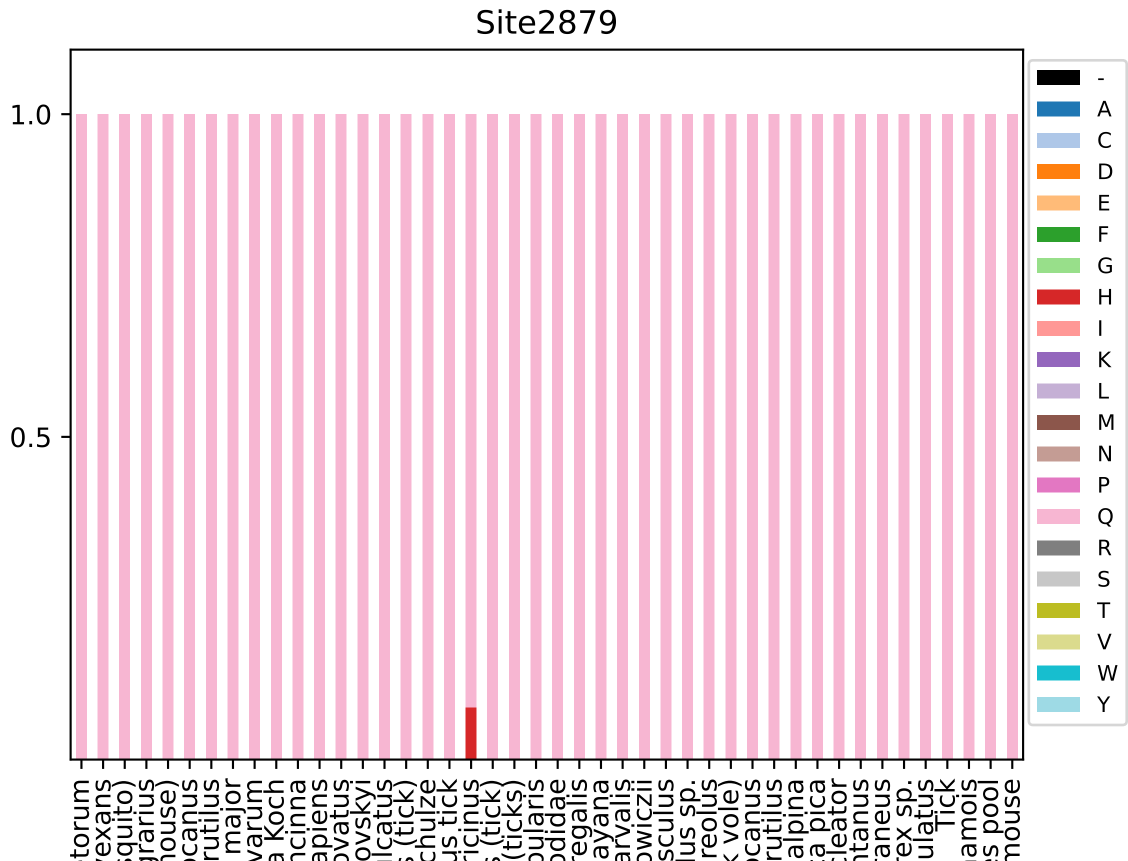

Site2879
1.0
0.5
-
A
C
D
E
F
G
H
I
K
L
M
N
P
Q
R
S
T
V
W
Y

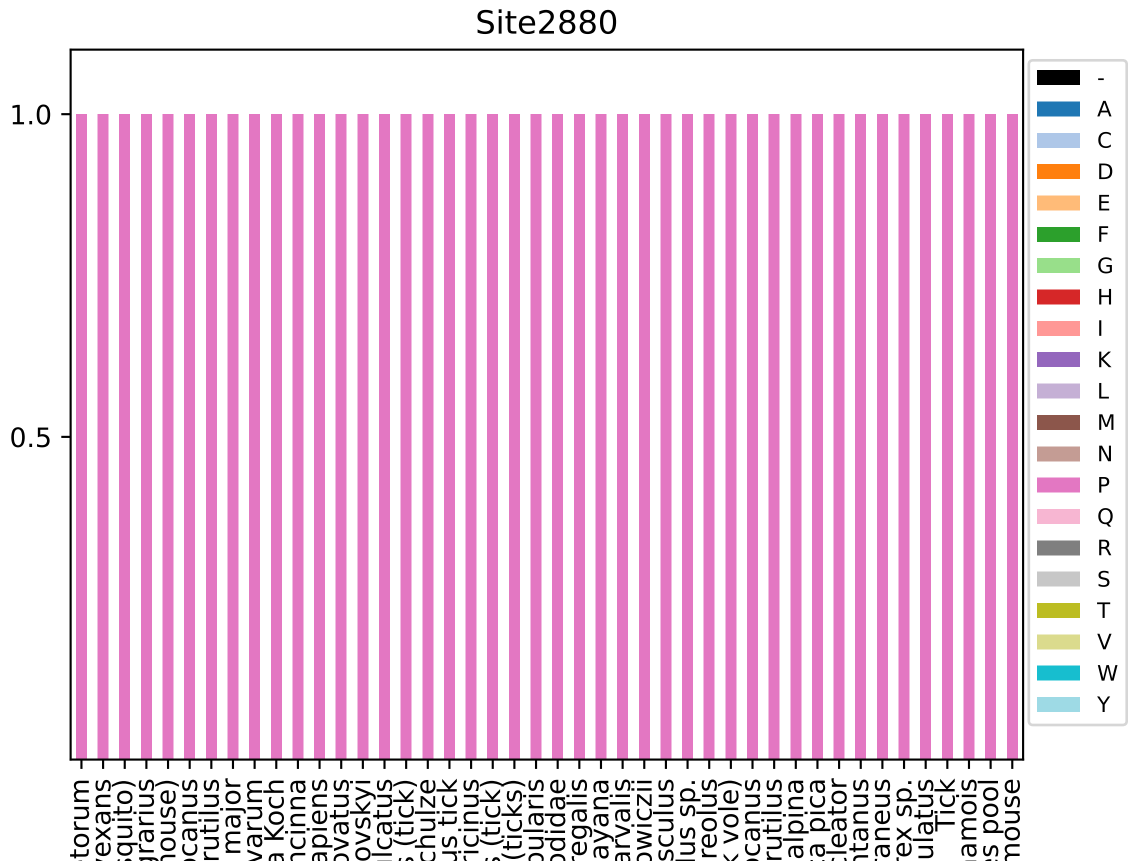
Site2880
1.0
0.5
-
A
C
D
E
F
G
H
I
K
L
M
N
P
Q
R
S
T
V
W
Y

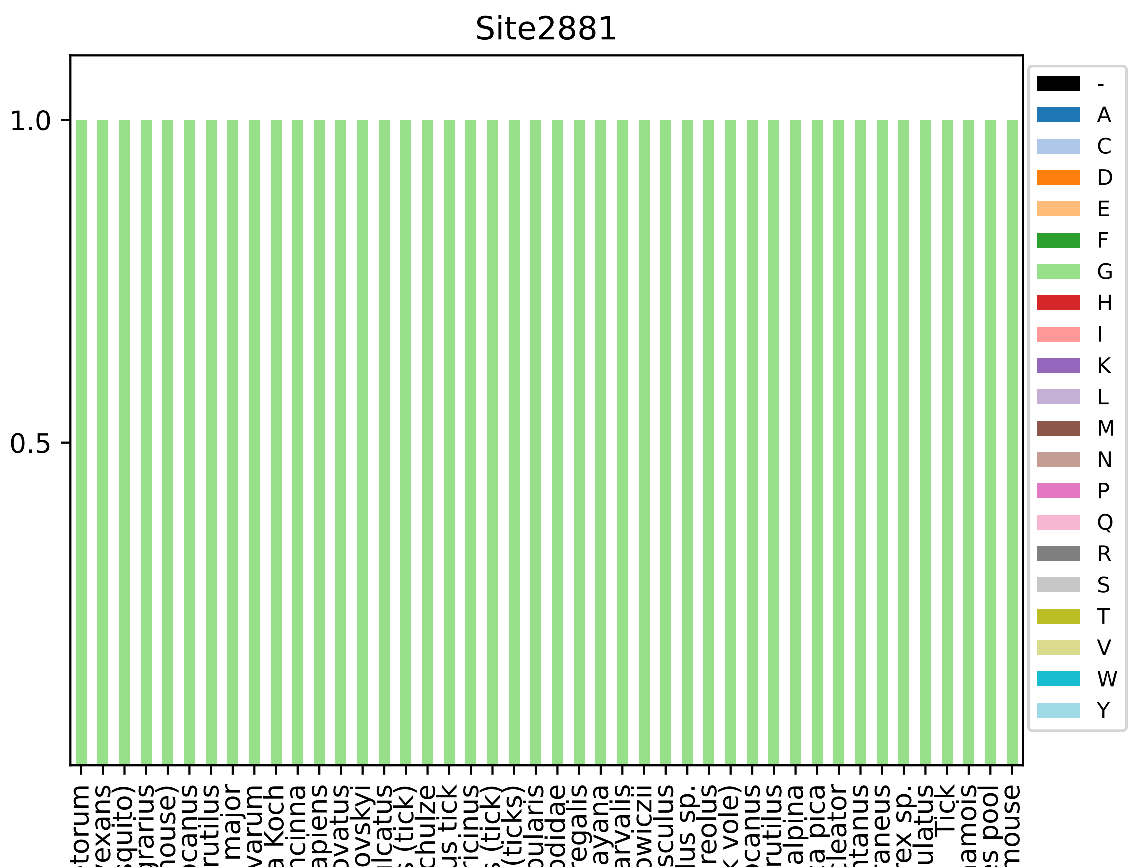

Site2881
1.0
0.5
-
A
C
D
E
F
G
H
I
K
L
M
N
P
Q
R
S
T
V
W
Y

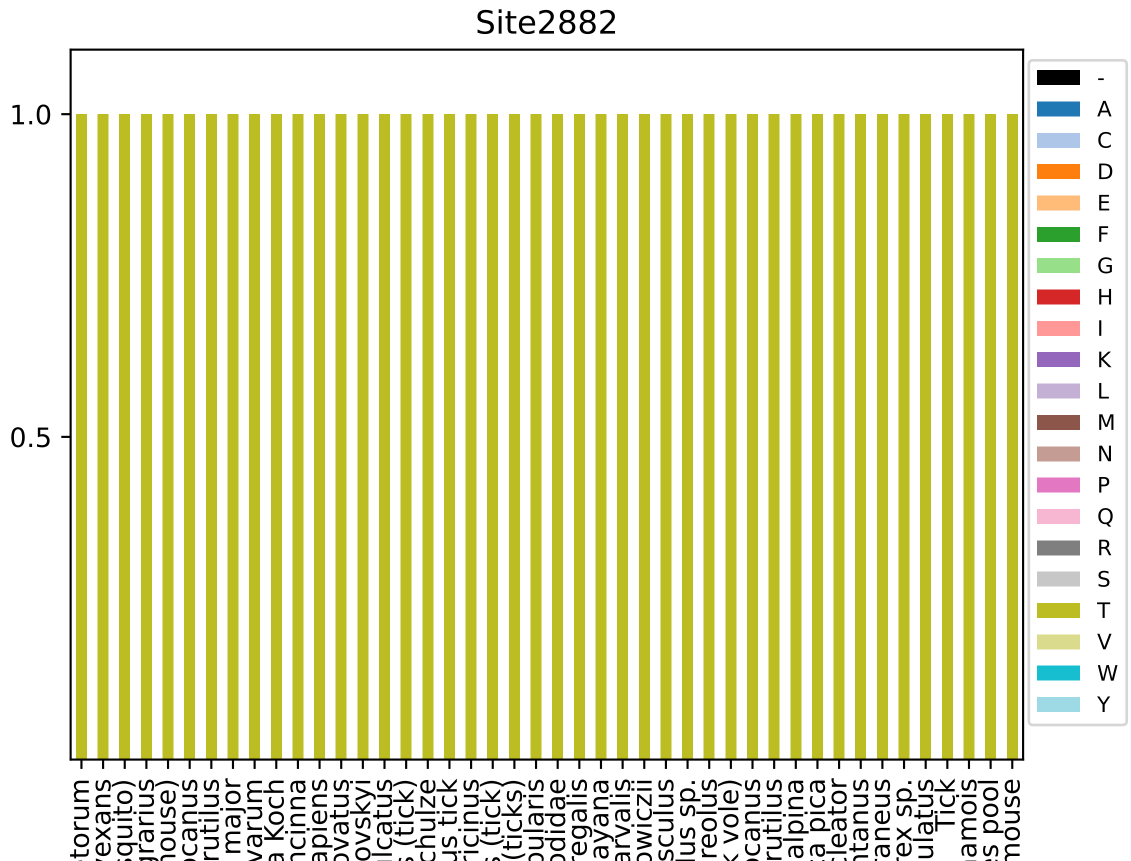

Site2882
1.0
0.5
-
A
C
D
E
F
G
H
I
K
L
M
N
P
Q
R
S
T
V
W
Y

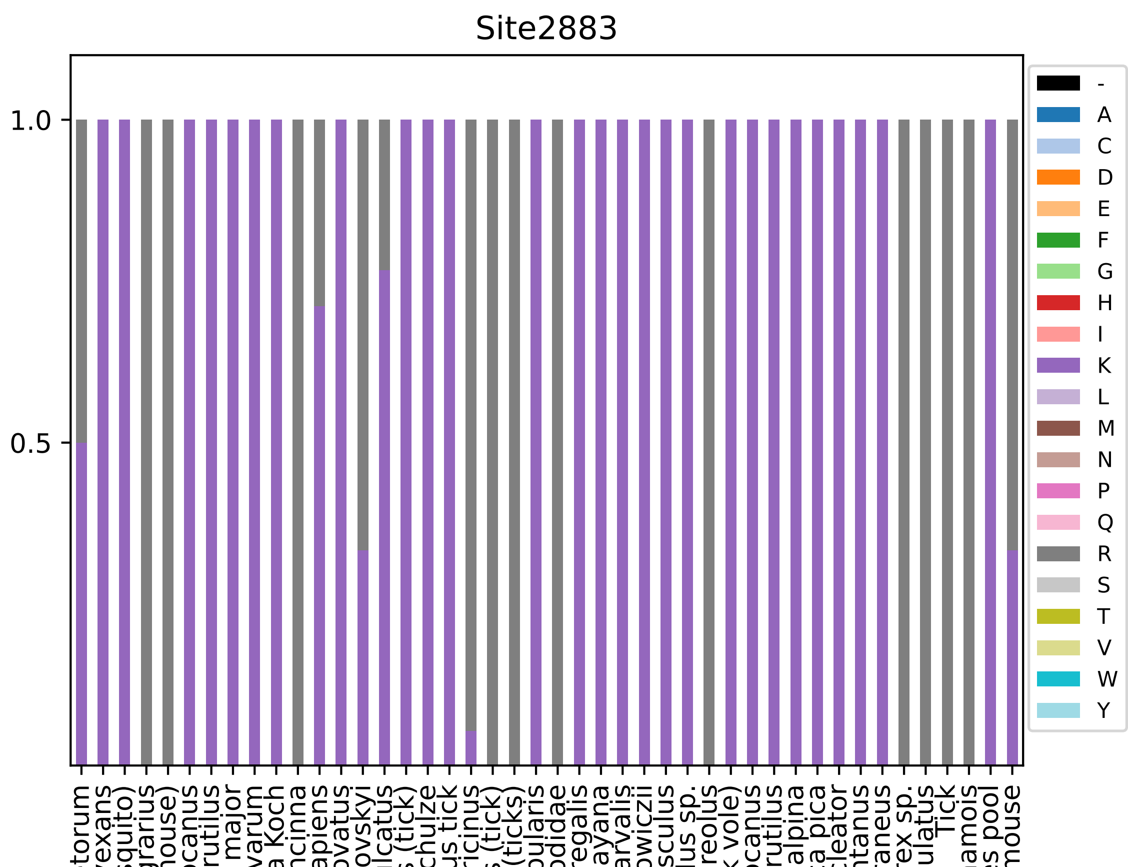
Site2883
1.0
0.5
-
A
C
D
E
F
G
H
I
K
L
M
N
P
Q
R
S
T
V
W
Y

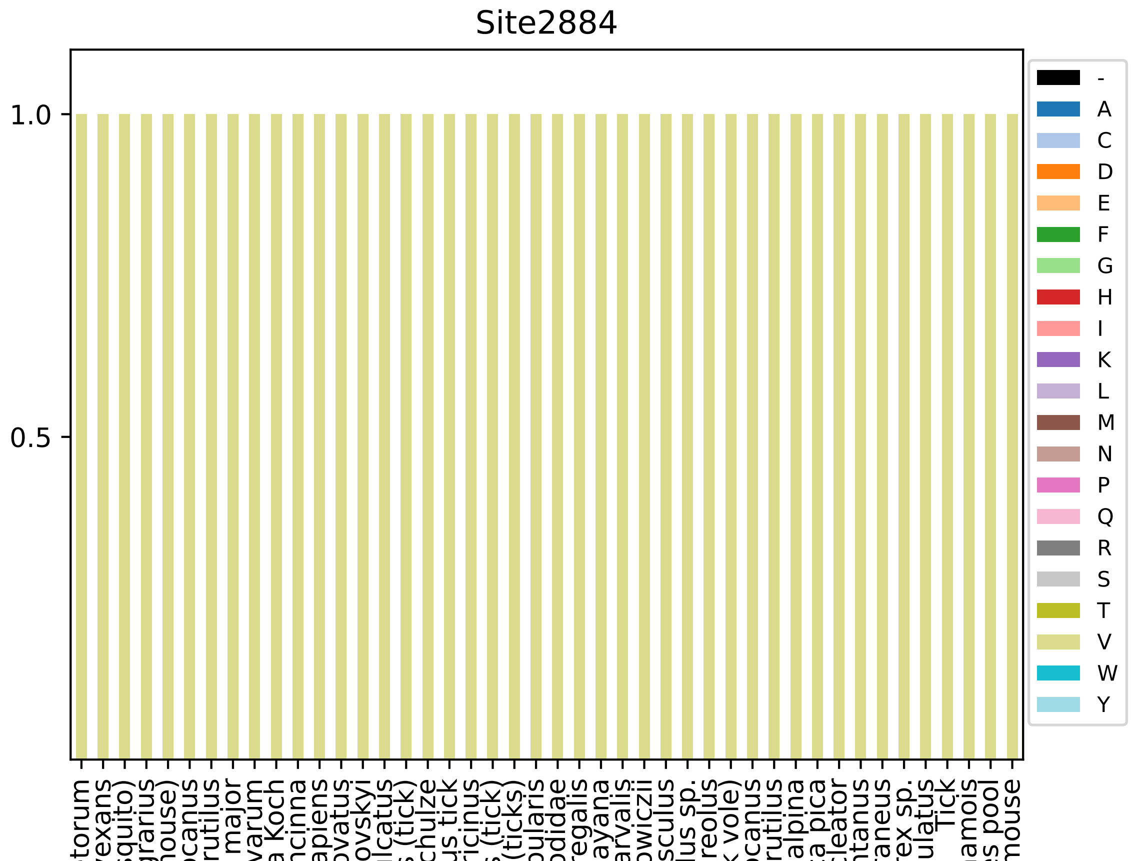
Site2884
1.0
0.5
-
A
C
D
E
F
G
H
I
K
L
M
N
P
Q
R
S
T
V
W
Y
Tick

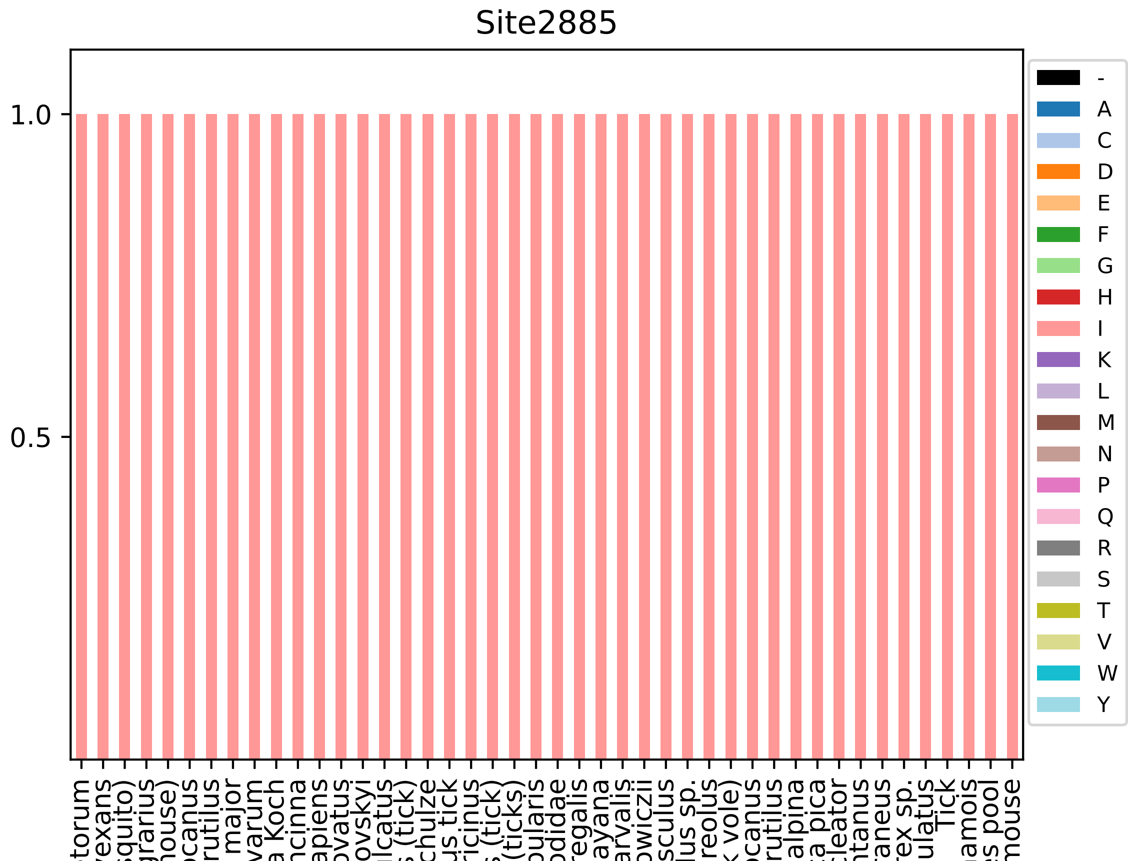

Site2885
1.0
0.5
-
A
C
D
E
F
G
H
I
K
L
M
N
P
Q
R
S
T
V
W
Y

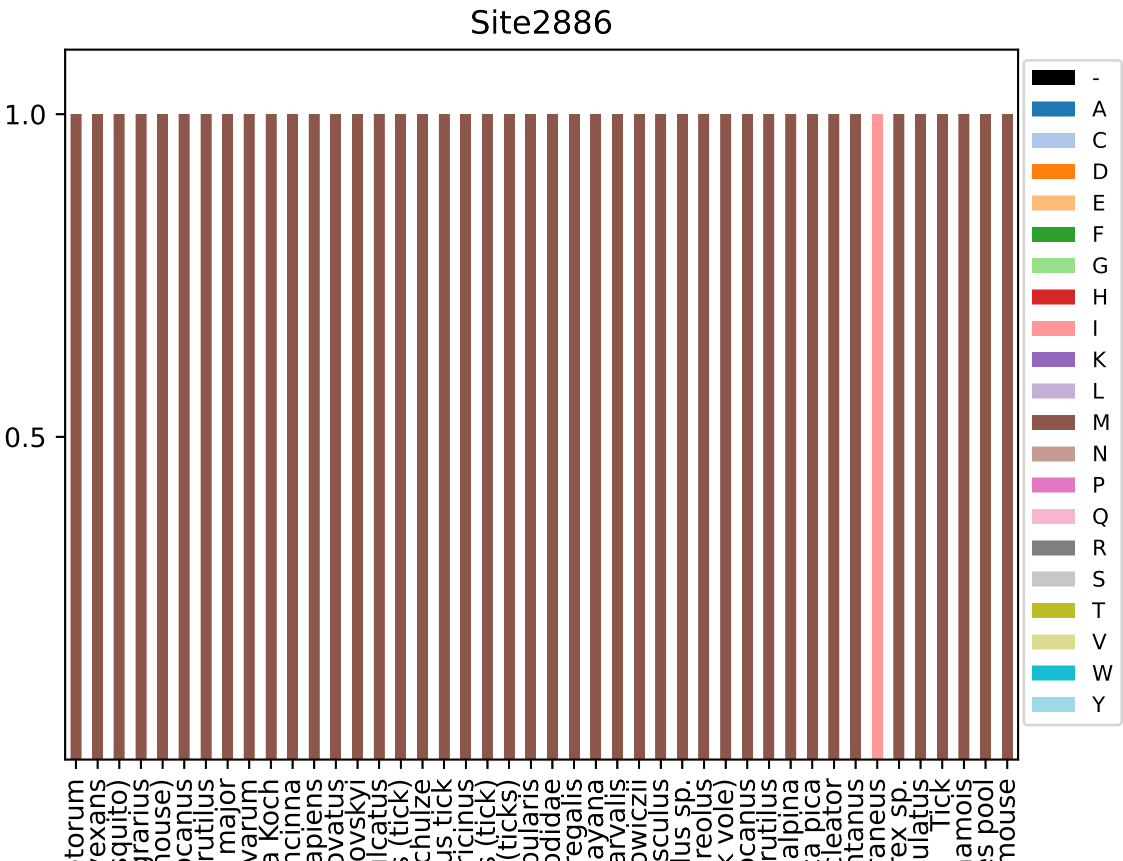
Site2886
1.0
0.5
-
A
C
D
E
F
G
H
I
K
L
M
N
P
Q
R
S
T
V
W
Y

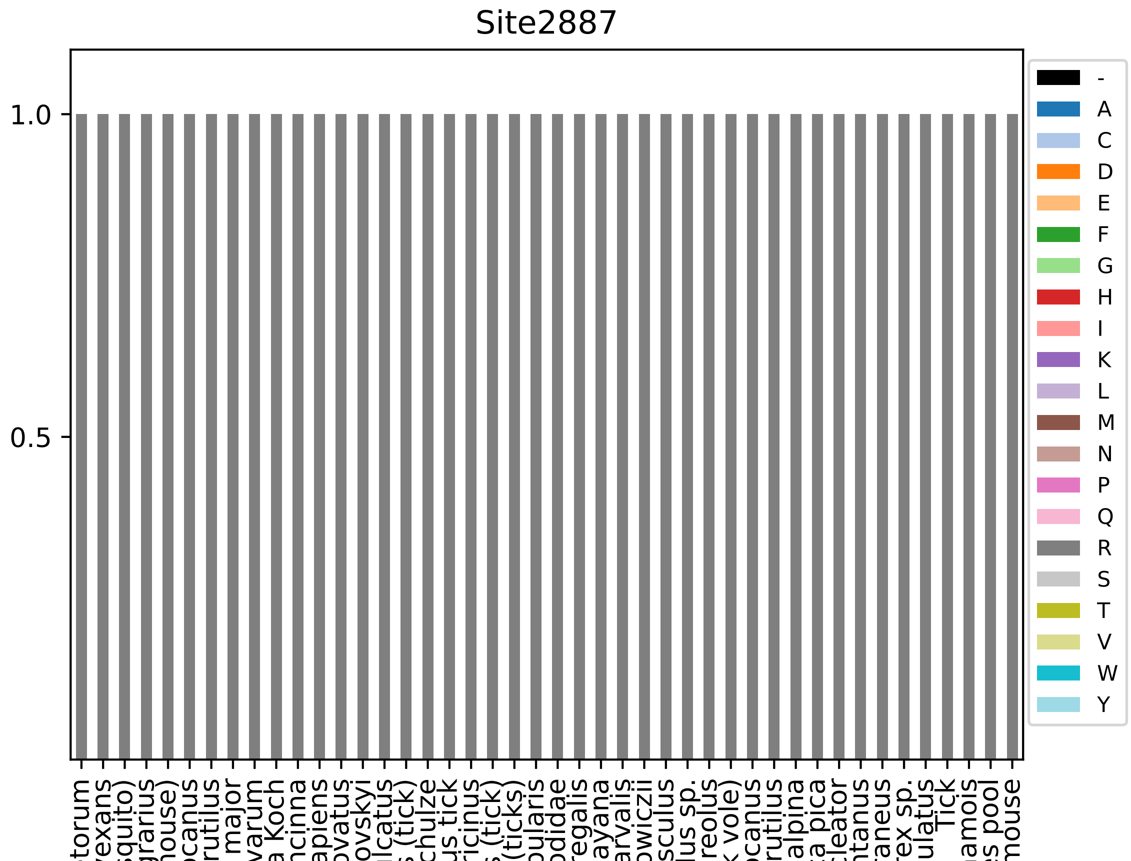
Site2887
1.0
0.5
-
A
C
D
E
F
G
H
I
K
L
M
N
P
Q
R
S
T
V
W
Y

Site2888

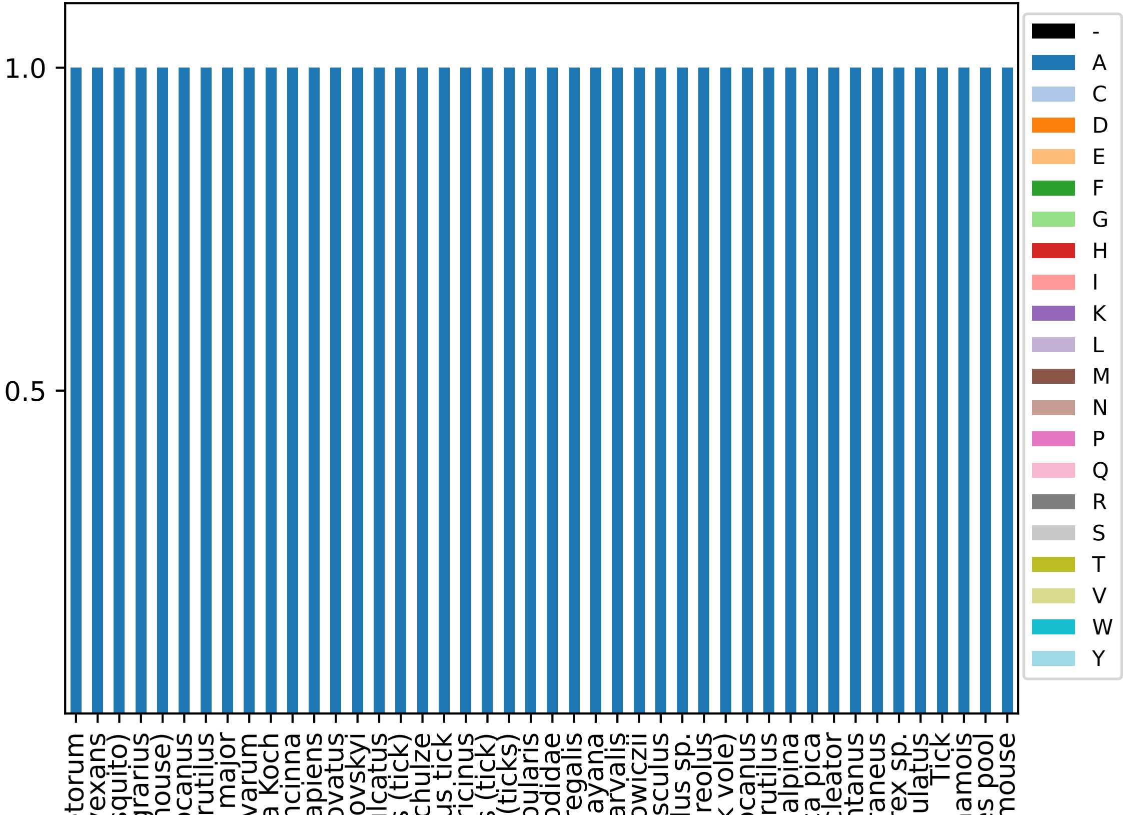

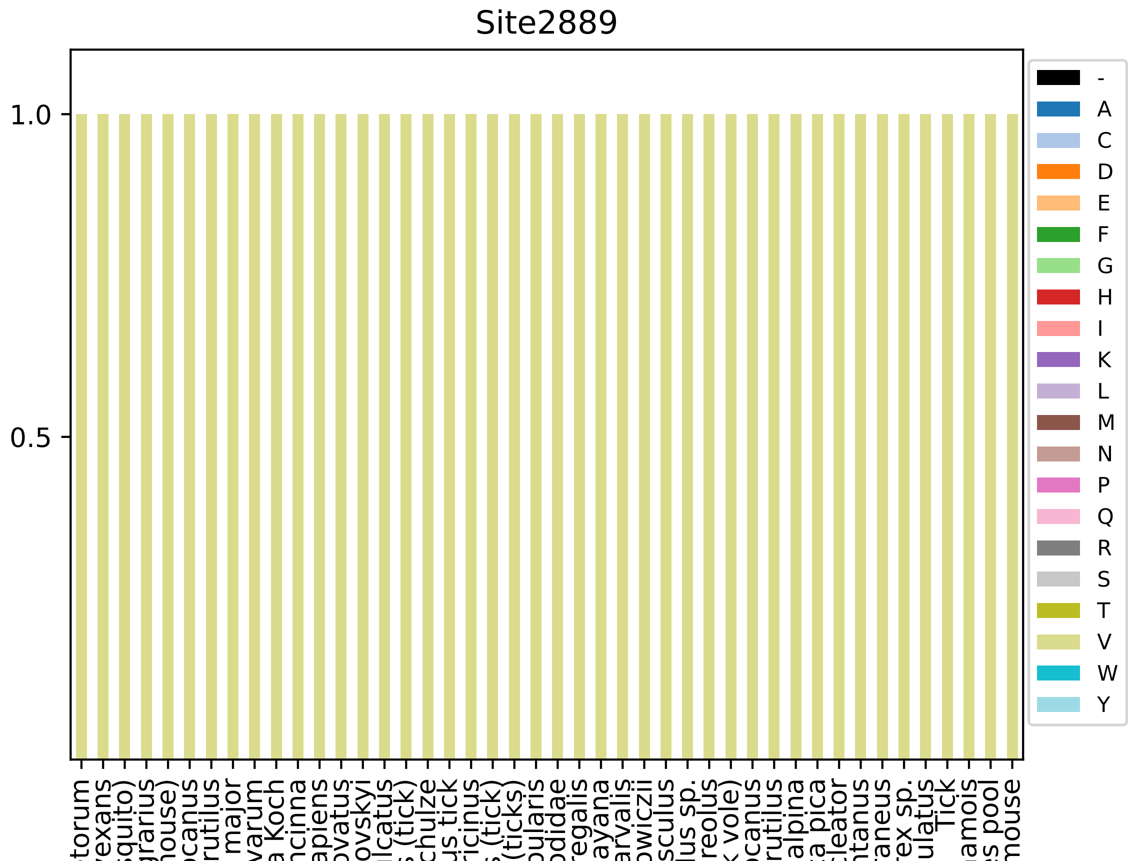
Site2889
1.0
0.5
-
A
C
D
E
F
G
H
I
K
L
M
N
P
Q
R
S
T
V
W
Y

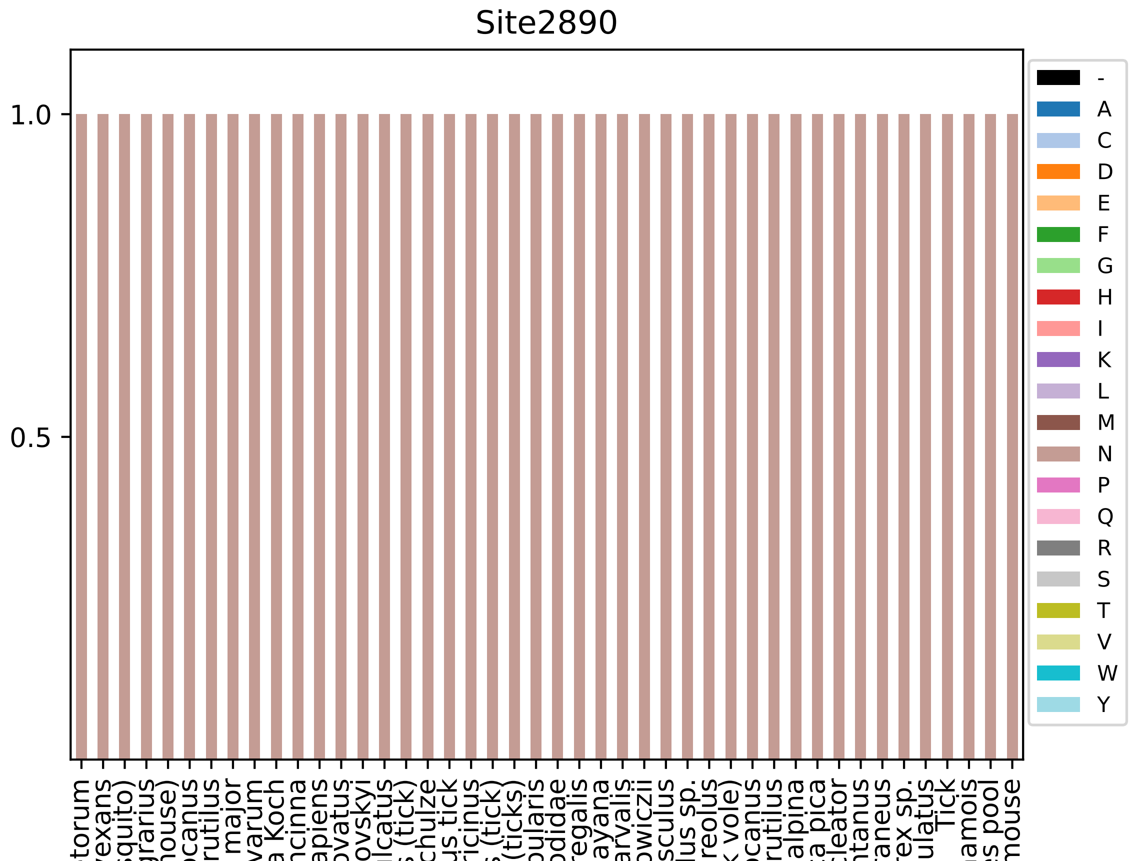

Site2890
1.0
0.5
-
A
C
D
E
F
G
H
I
K
L
M
N
P
Q
R
S
T
V
W
Y

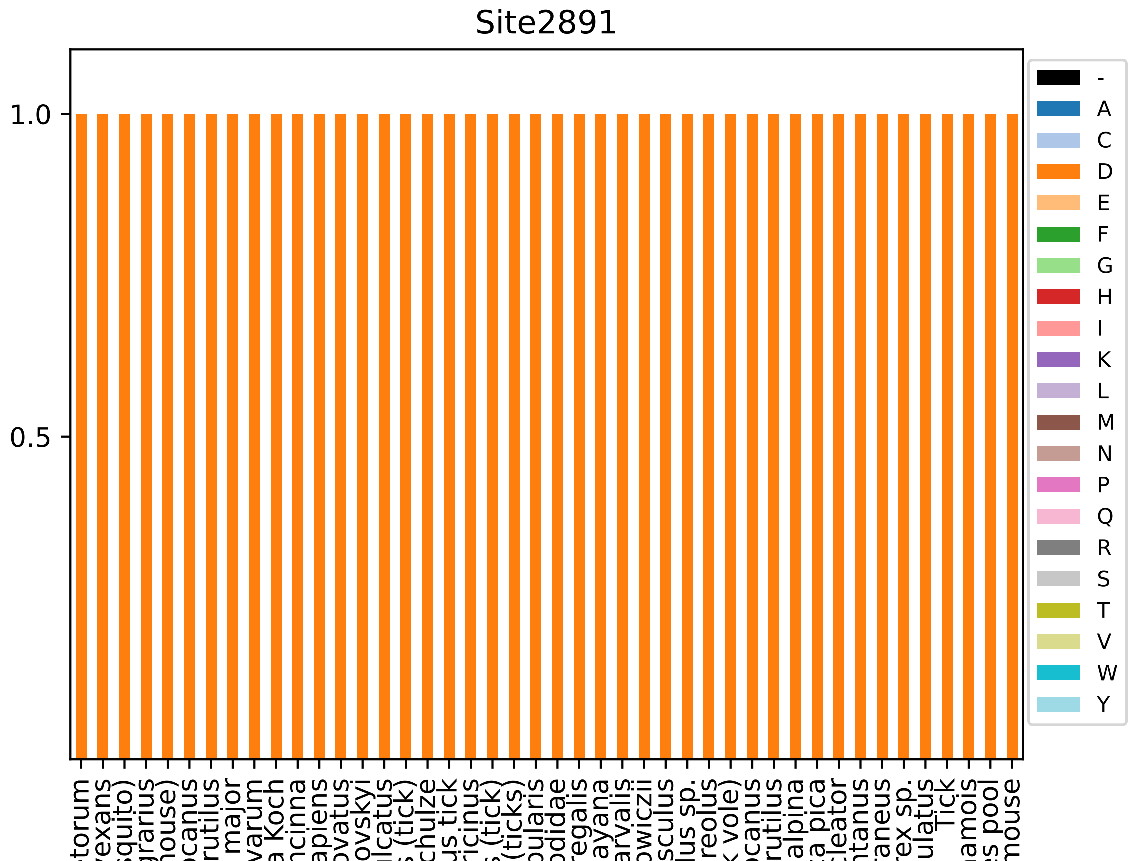
Site2891
1.0
0.5
-
A
C
D
E
F
G
H
I
K
L
M
N
P
Q
R
S
T
V
W
Y

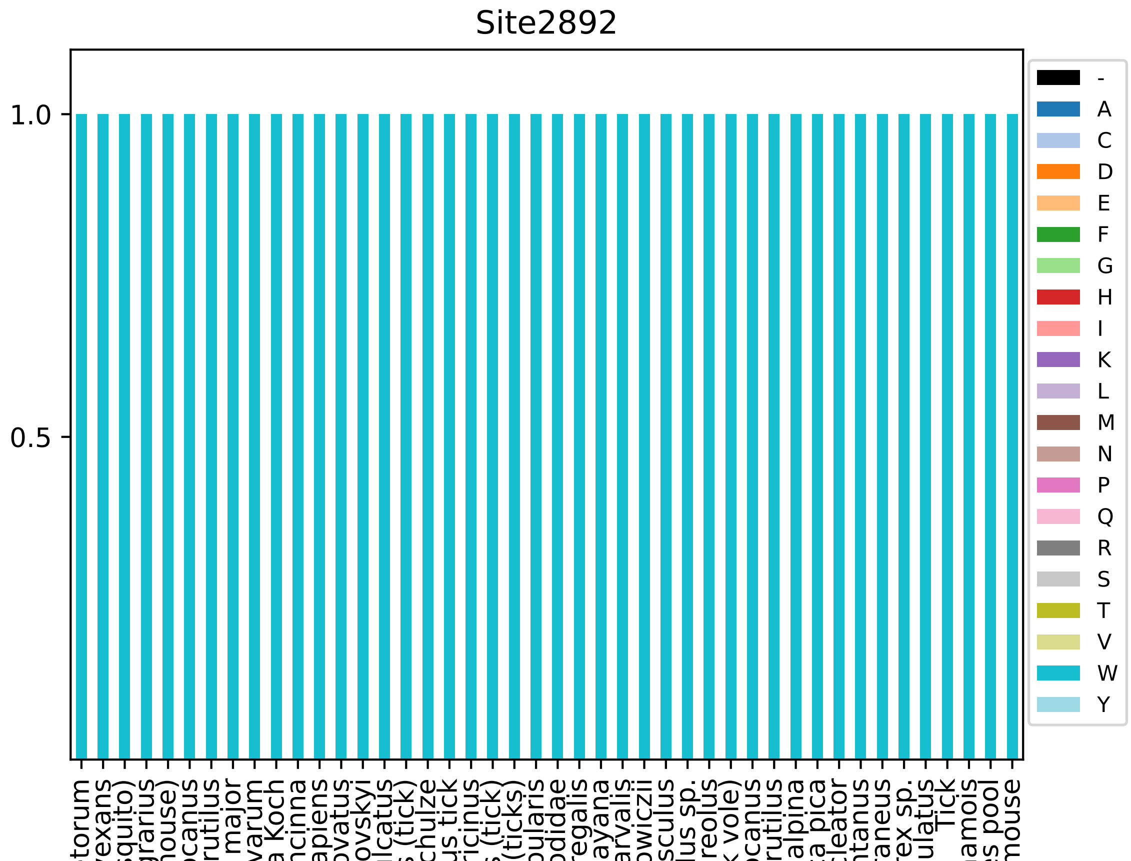
Site2892
1.0
0.5
-
A
C
D
E
F
G
H
I
K
L
M
N
P
Q
R
S
T
V
W
Y
torum
vexans
squito)
grarius
nouse)
ocanus
rutilus
major
varum
a Koch
ncinna
apiens
ovatus
ovskyi
ulcatus
s (tick)
chulze
us tick
ricinus
s (tick)
(ticks)
pularis
podidae
regalis
ayana
arvalis
owiczii
sculus
lus sp.
reolus
x vole)
ocanus
rutilus
alpina
a pica
leator
ntanus
raneus
rex sp.
ulatus
Tick
amois
es pool
mouse

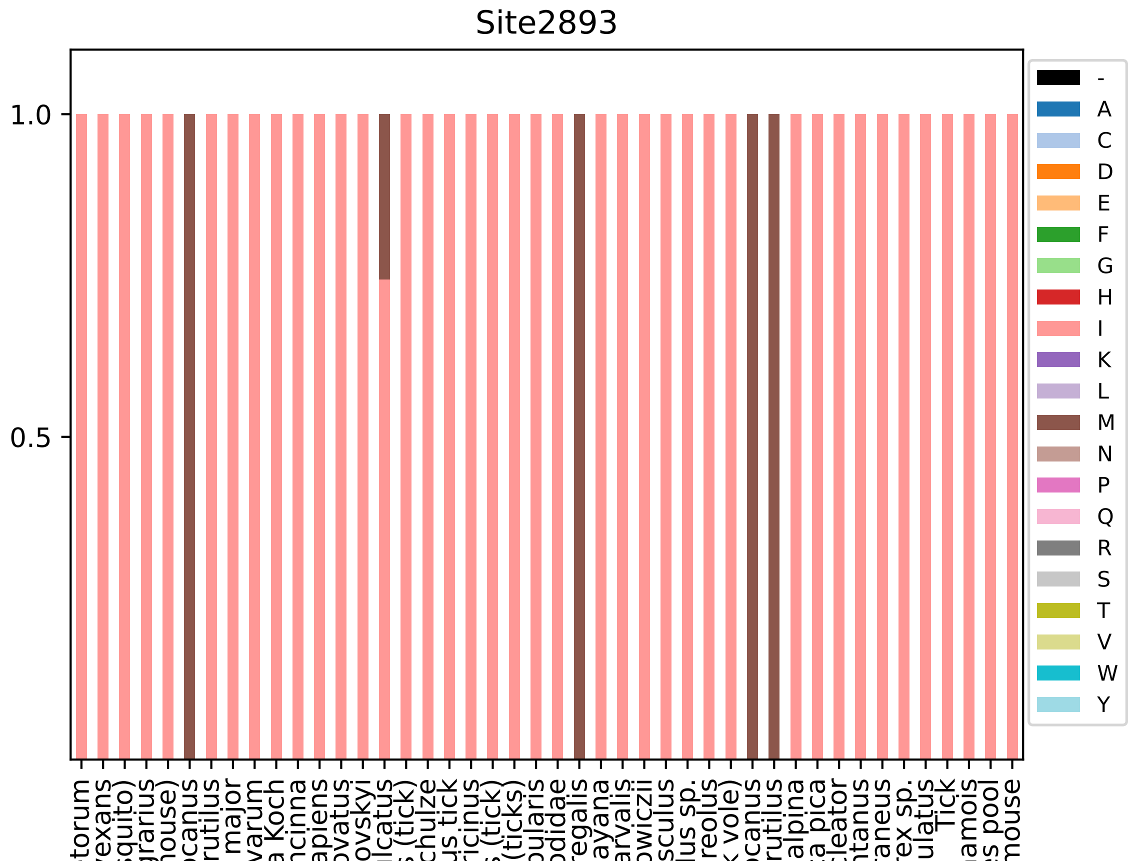

Site2893
1.0
0.5
-
A
C
D
E
F
G
H
I
K
L
M
N
P
Q
R
S
T
V
W
Y

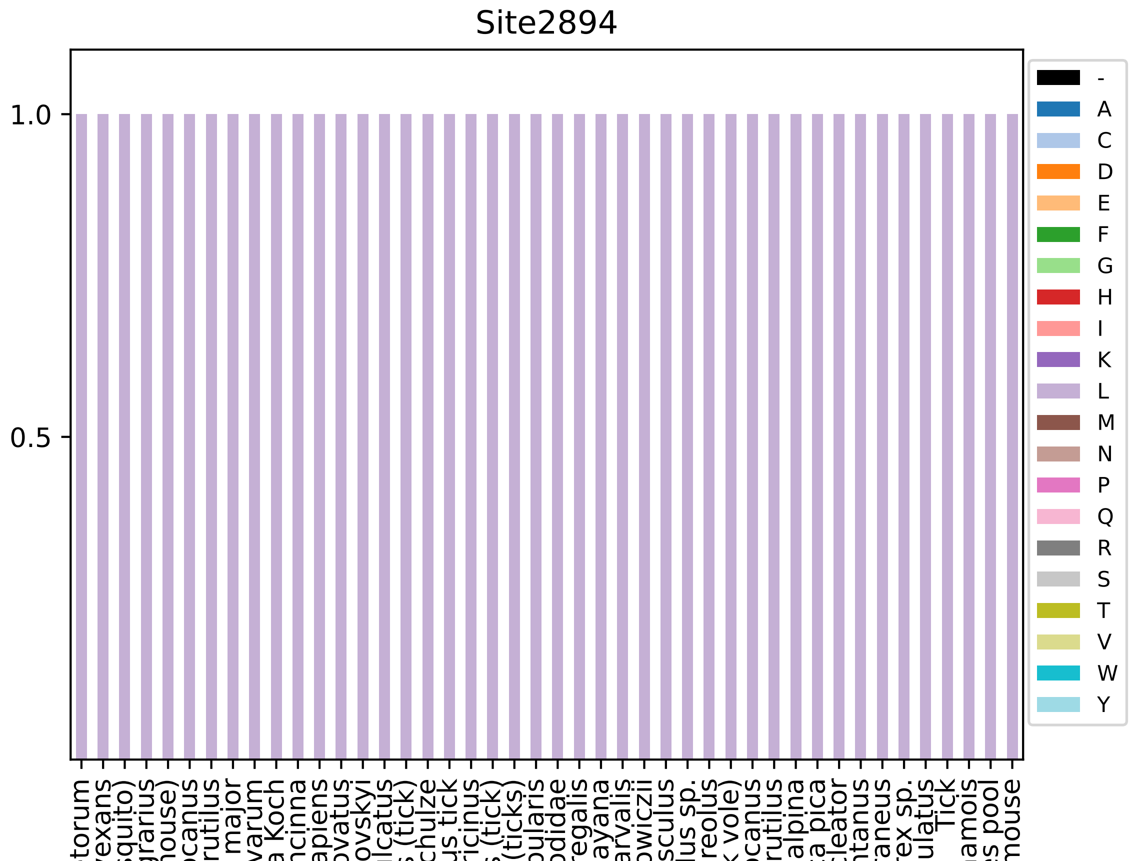

Site2894
1.0
0.5
-
A
C
D
E
F
G
H
I
K
L
M
N
P
Q
R
S
T
V
W
Y

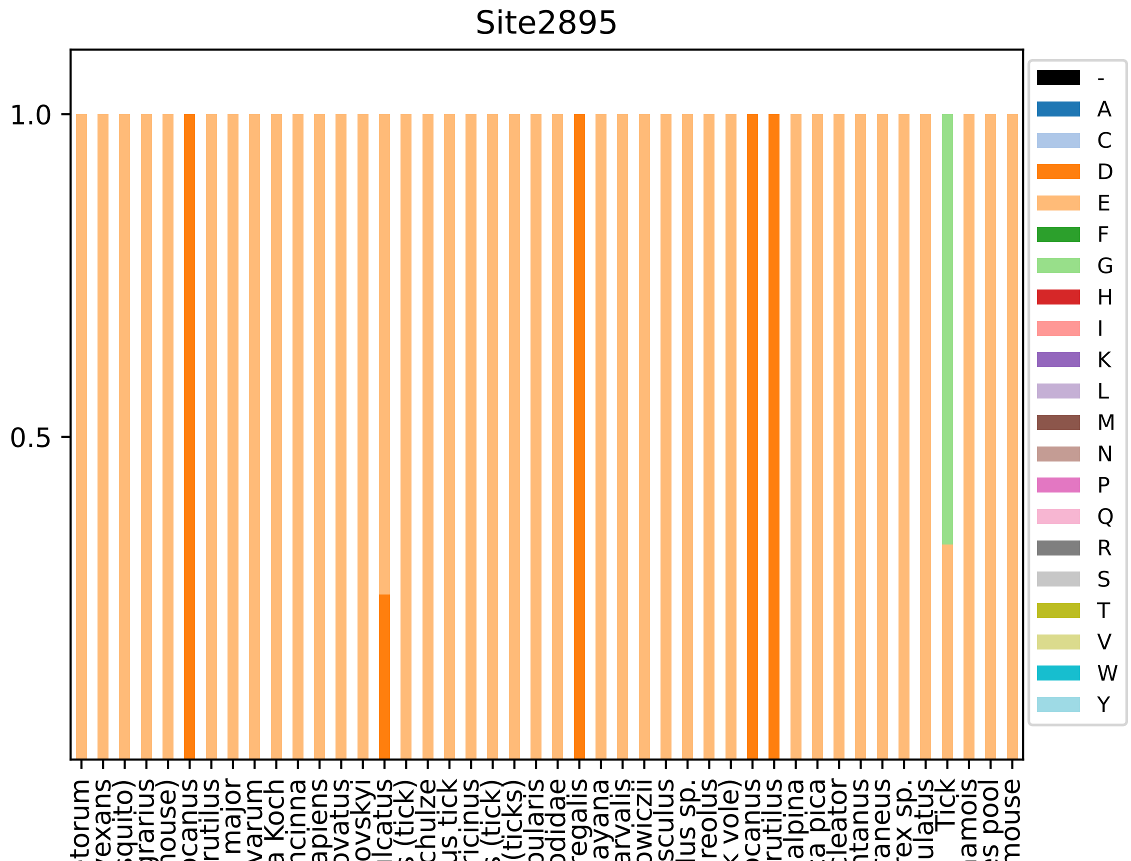
Site2895
1.0
0.5
-
A
C
D
E
F
G
H
I
K
L
M
N
P
Q
R
S
T
V
W
Y

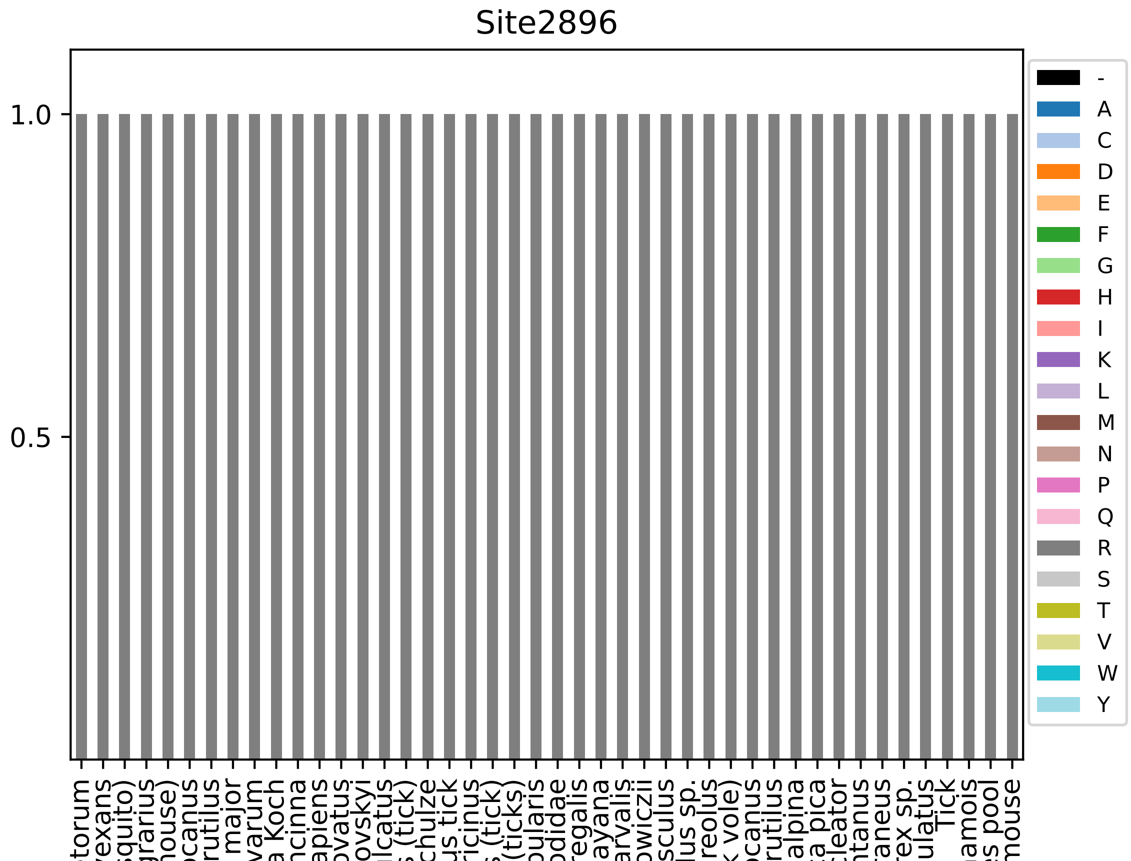

Site2896
1.0
0.5
-
A
C
D
E
F
G
H
I
K
L
M
N
P
Q
R
S
T
V
W
Y

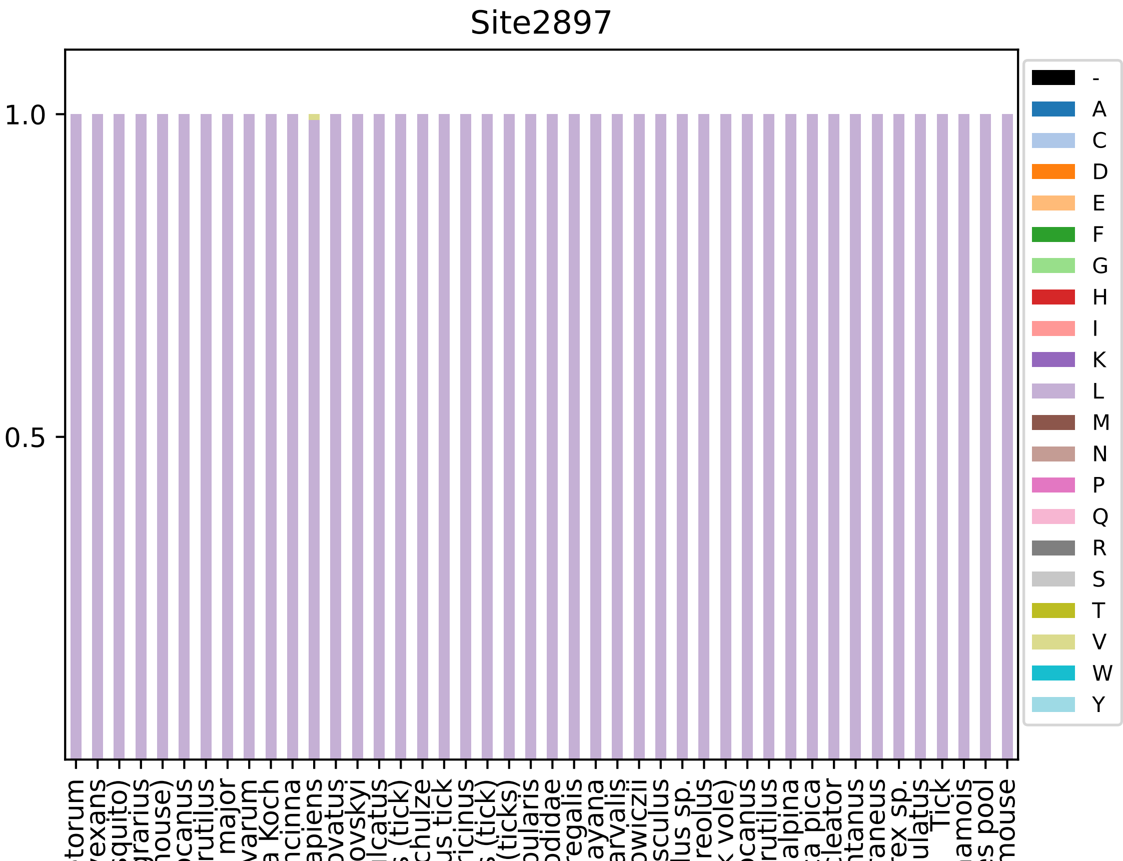
Site2897
1.0
0.5
-
A
C
D
E
F
G
H
I
K
L
M
N
P
Q
R
S
T
V
W
Y

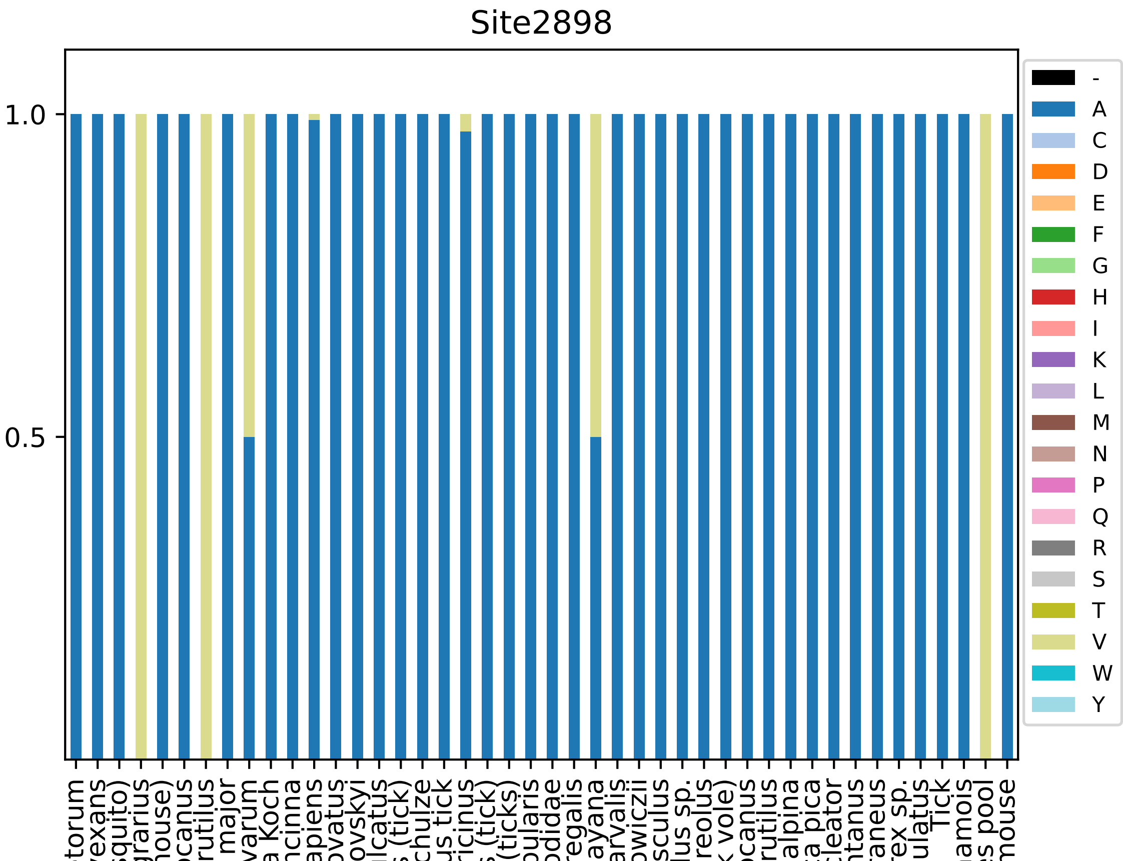
Site2898
1.0
0.5
-
A
C
D
E
F
G
H
I
K
L
M
N
P
Q
R
S
T
V
W
Y

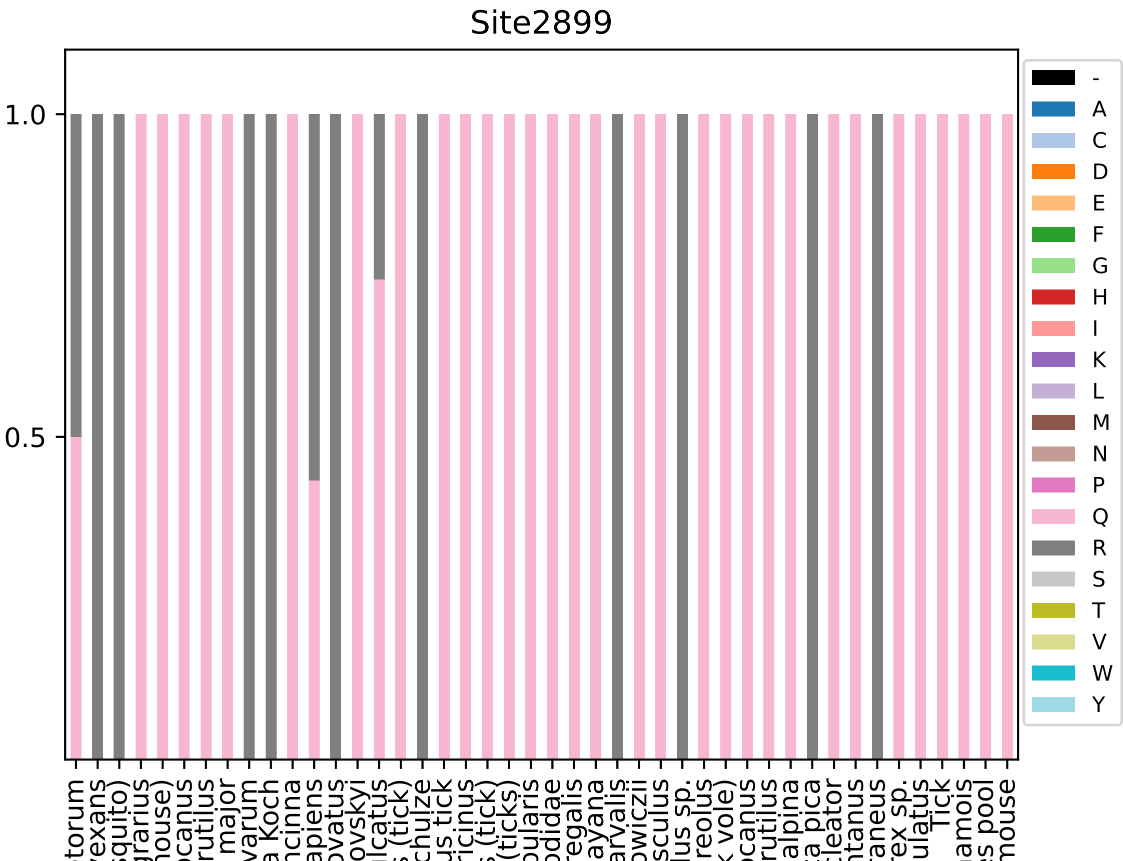

Site2899
1.0
0.5
-
A
C
D
E
F
G
H
I
K
L
M
N
P
Q
R
S
T
V
W
Y

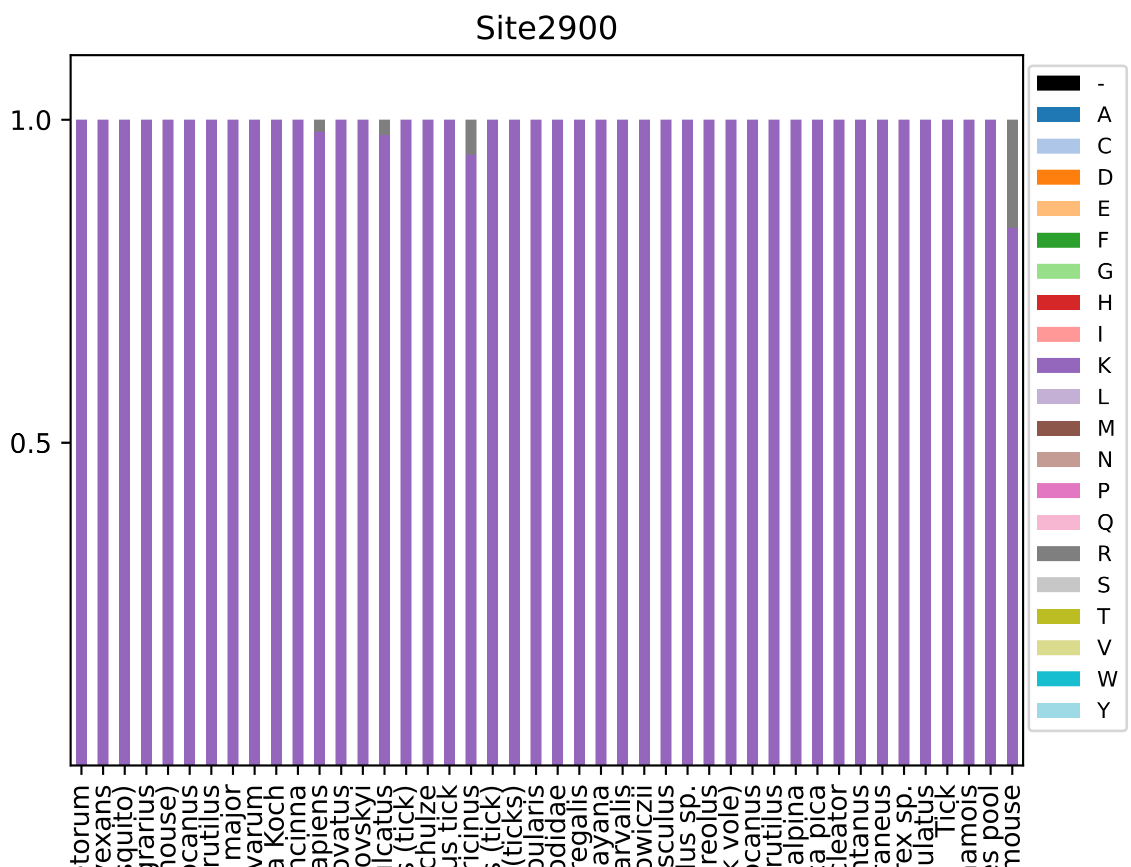

Site2900
1.0
0.5
-
A
C
D
E
F
G
H
I
K
L
M
N
P
Q
R
S
T
V
W
Y
Tick

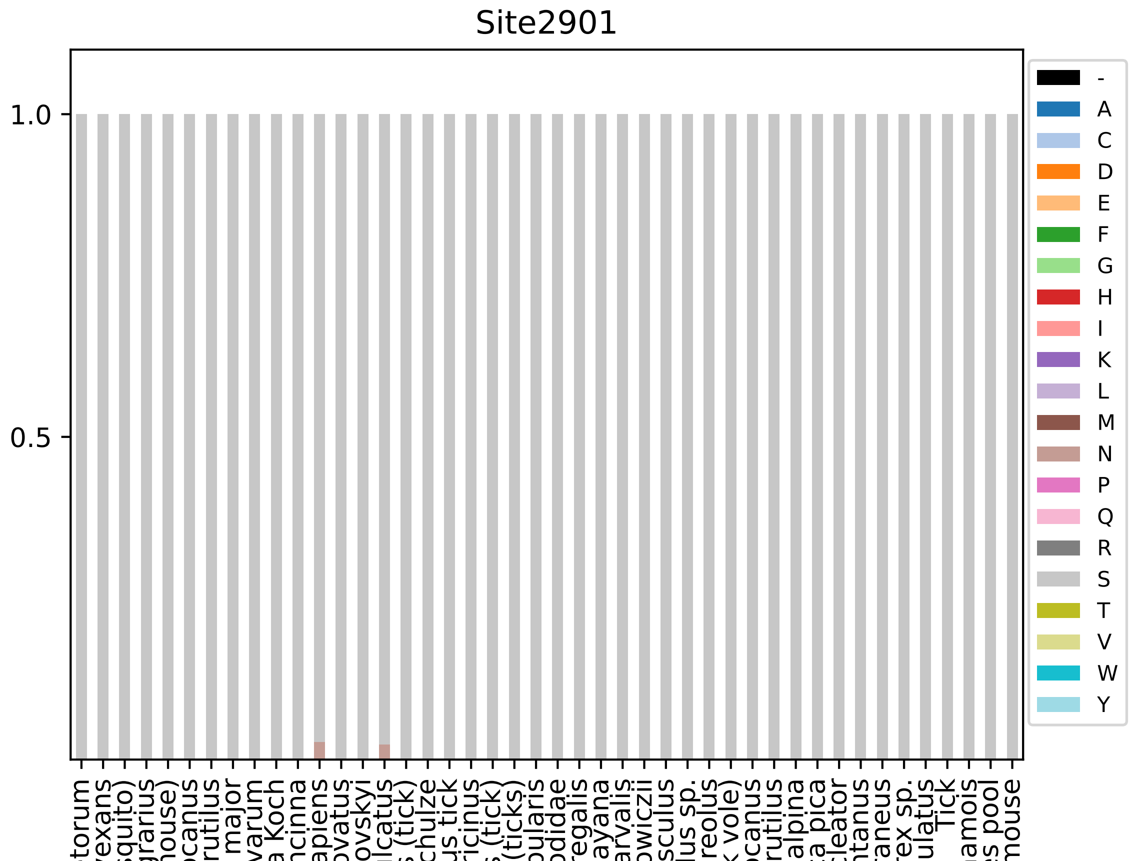

Site2901
1.0
0.5
-
A
C
D
E
F
G
H
I
K
L
M
N
P
Q
R
S
T
V
W
Y

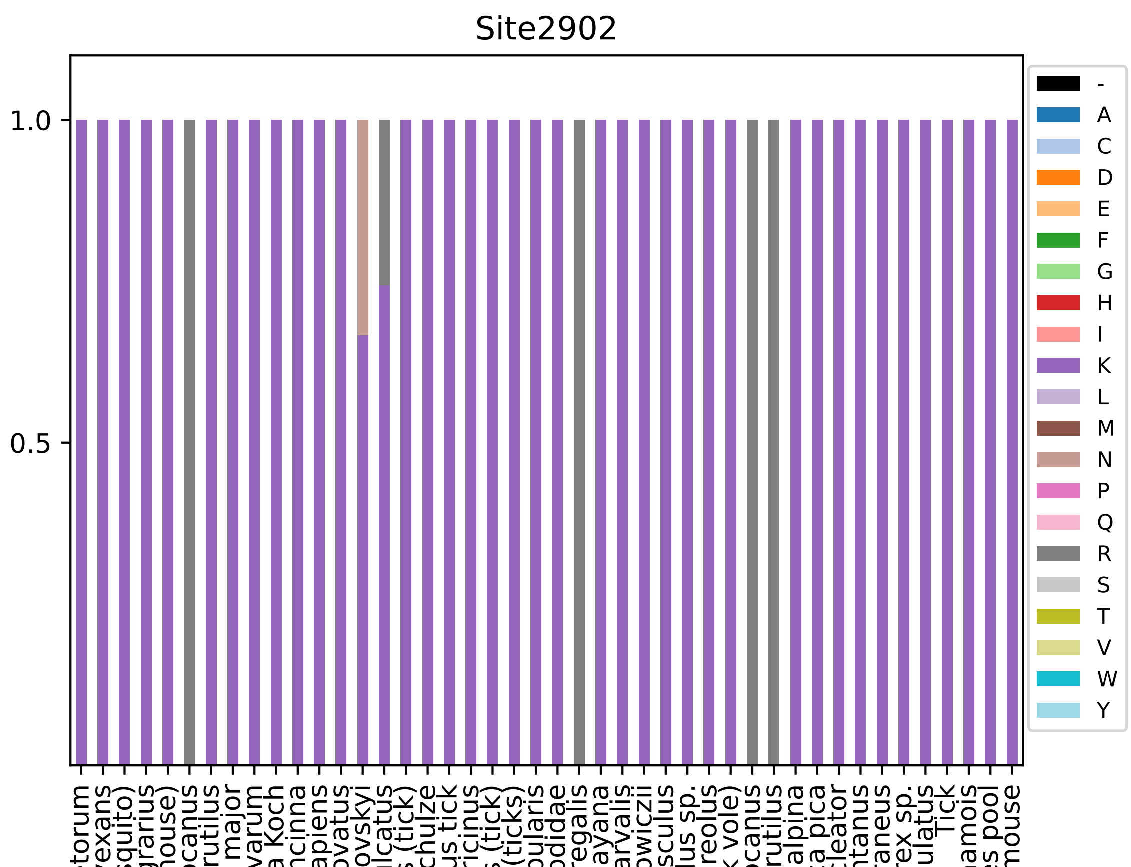

Site2902
1.0
0.5
-
A
C
D
E
F
G
H
I
K
L
M
N
P
Q
R
S
T
V
W
Y

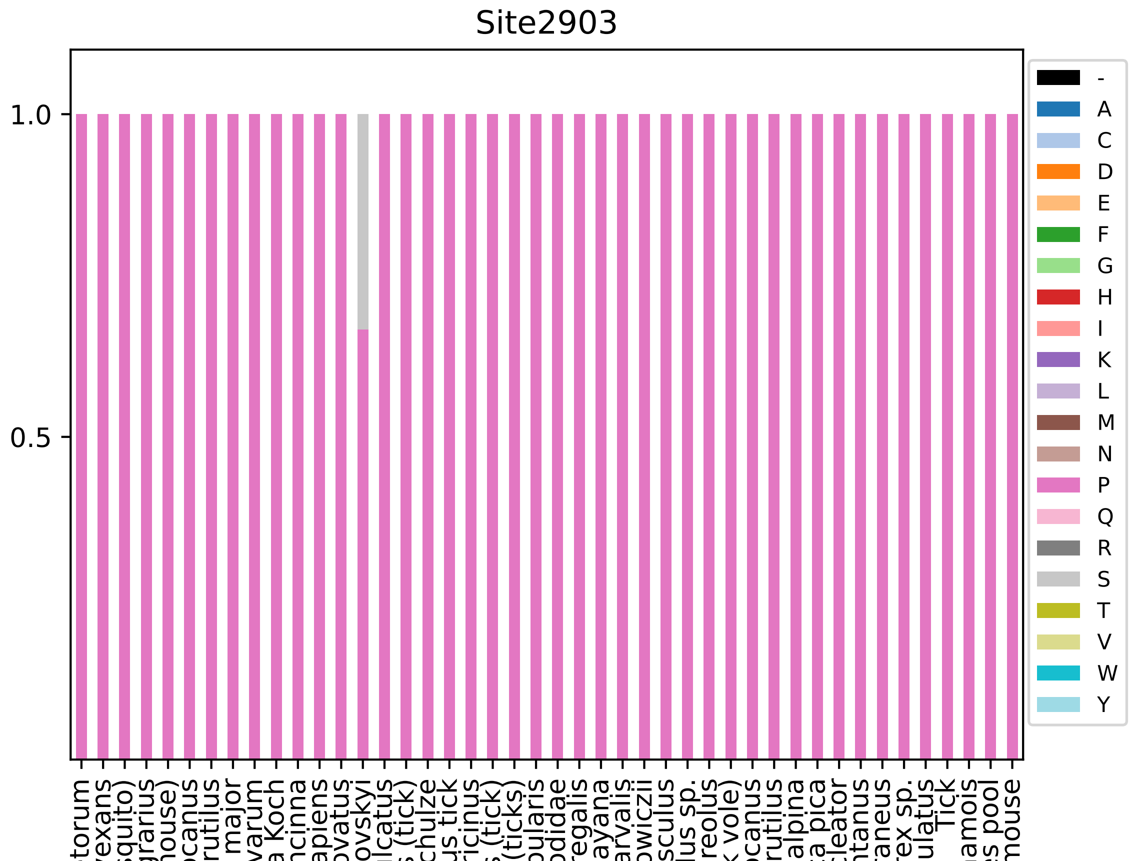
Site2903
1.0
0.5
-
A
C
D
E
F
G
H
I
K
L
M
N
P
Q
R
S
T
V
W
Y

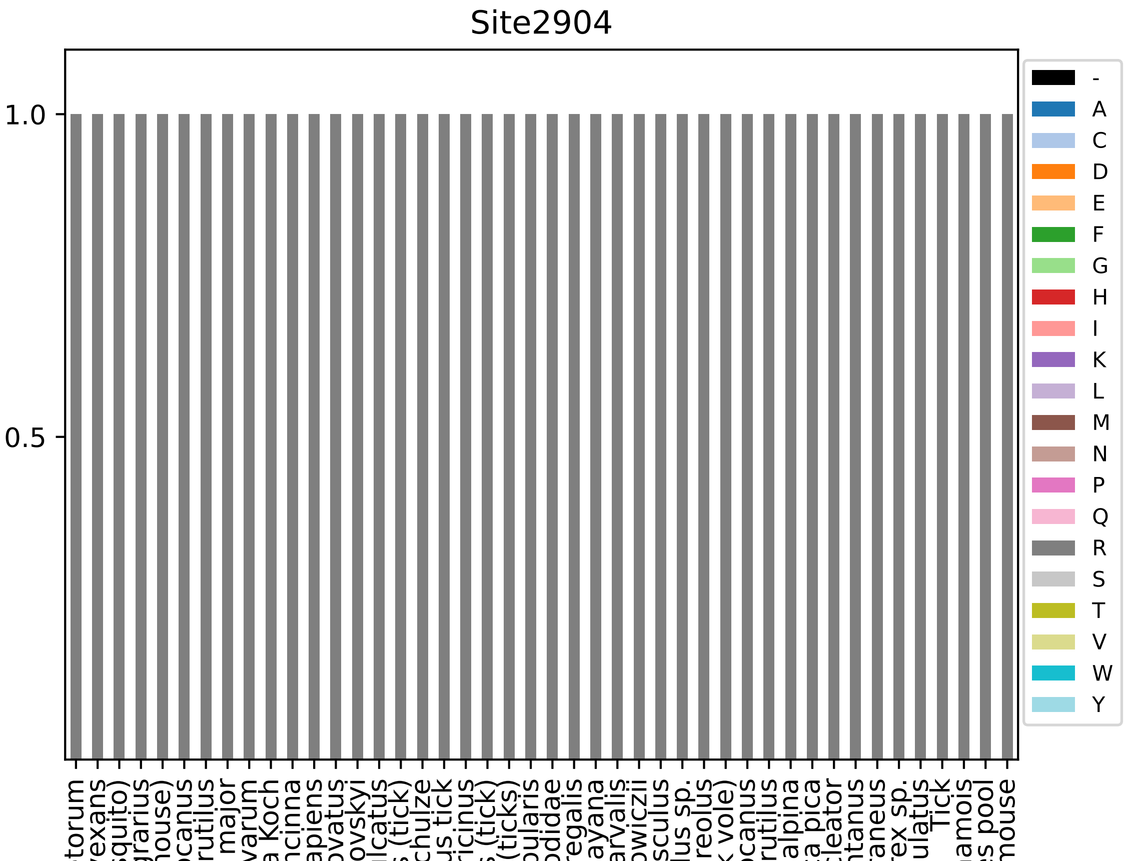
Site2904
1.0
0.5
-
A
C
D
E
F
G
H
I
K
L
M
N
P
Q
R
S
T
V
W
Y

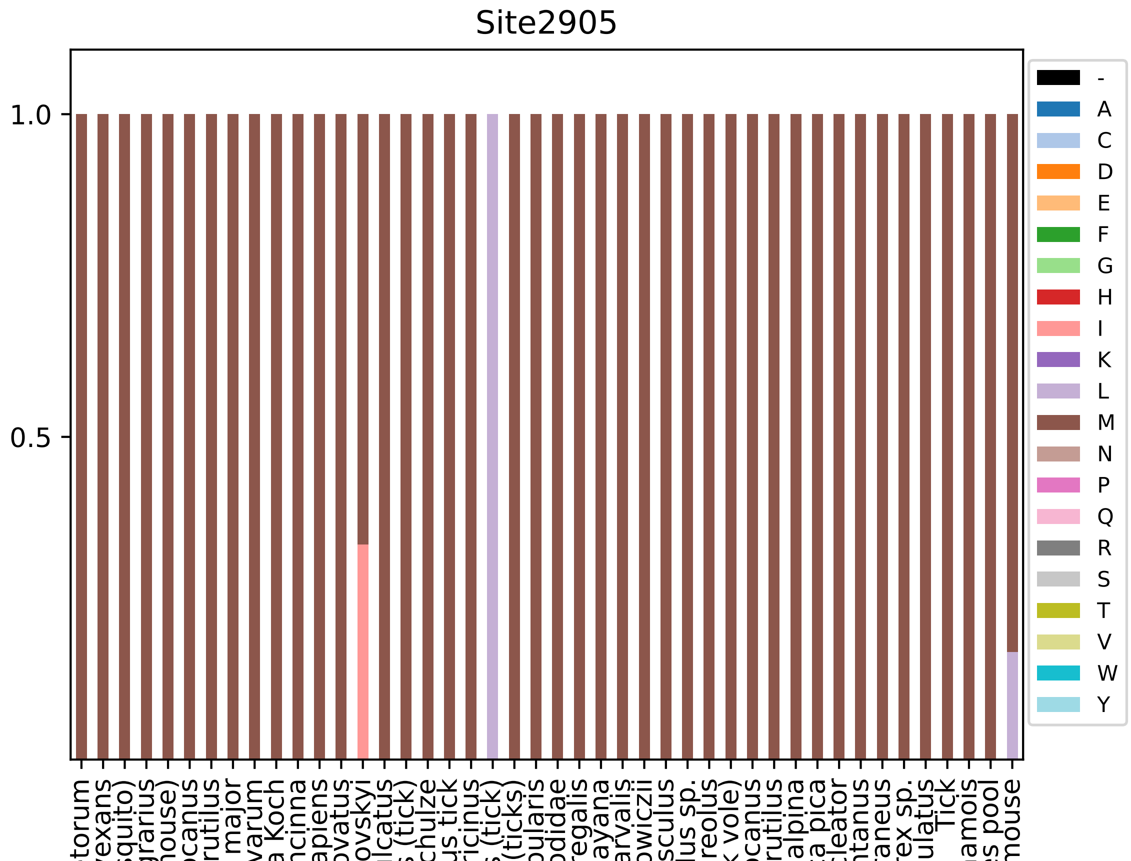

Site2905
1.0
0.5
-
A
C
D
E
F
G
H
I
K
L
M
N
P
Q
R
S
T
V
W
Y

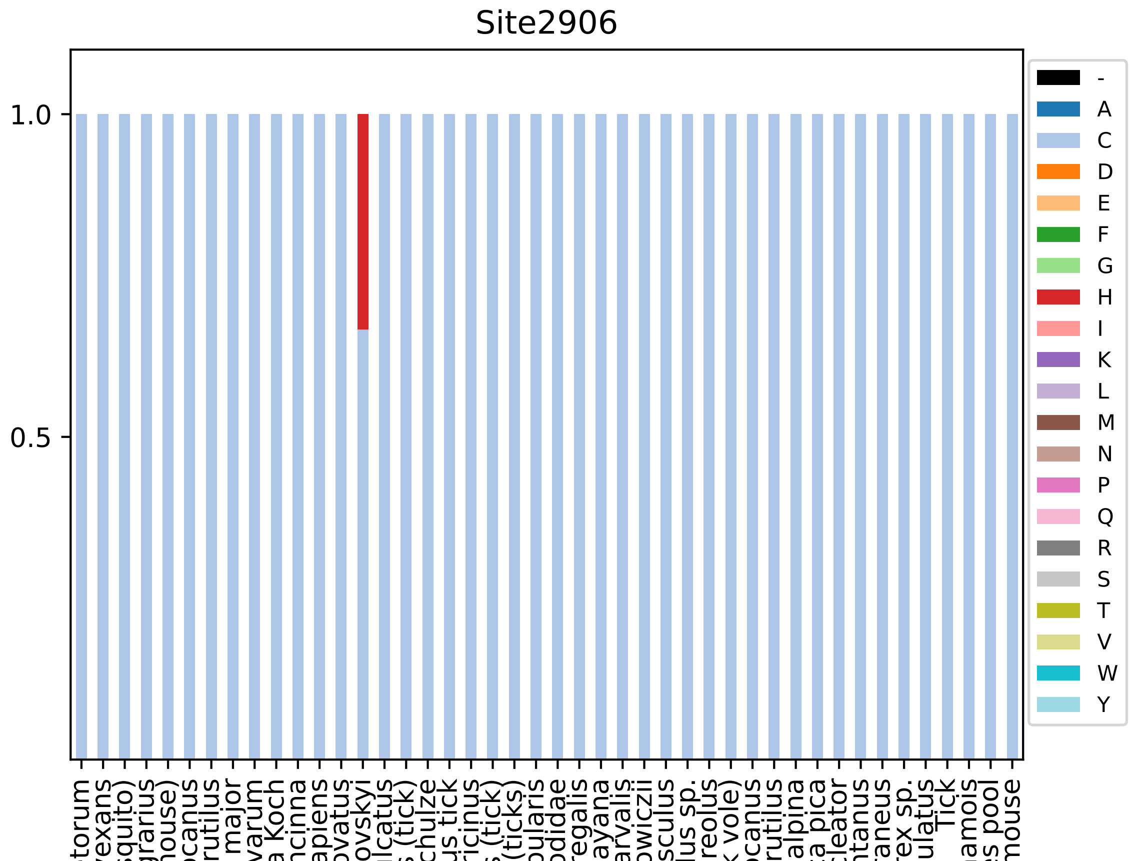
Site2906
1.0
0.5
-
A
C
D
E
F
G
H
I
K
L
M
N
P
Q
R
S
T
V
W
Y
Tick

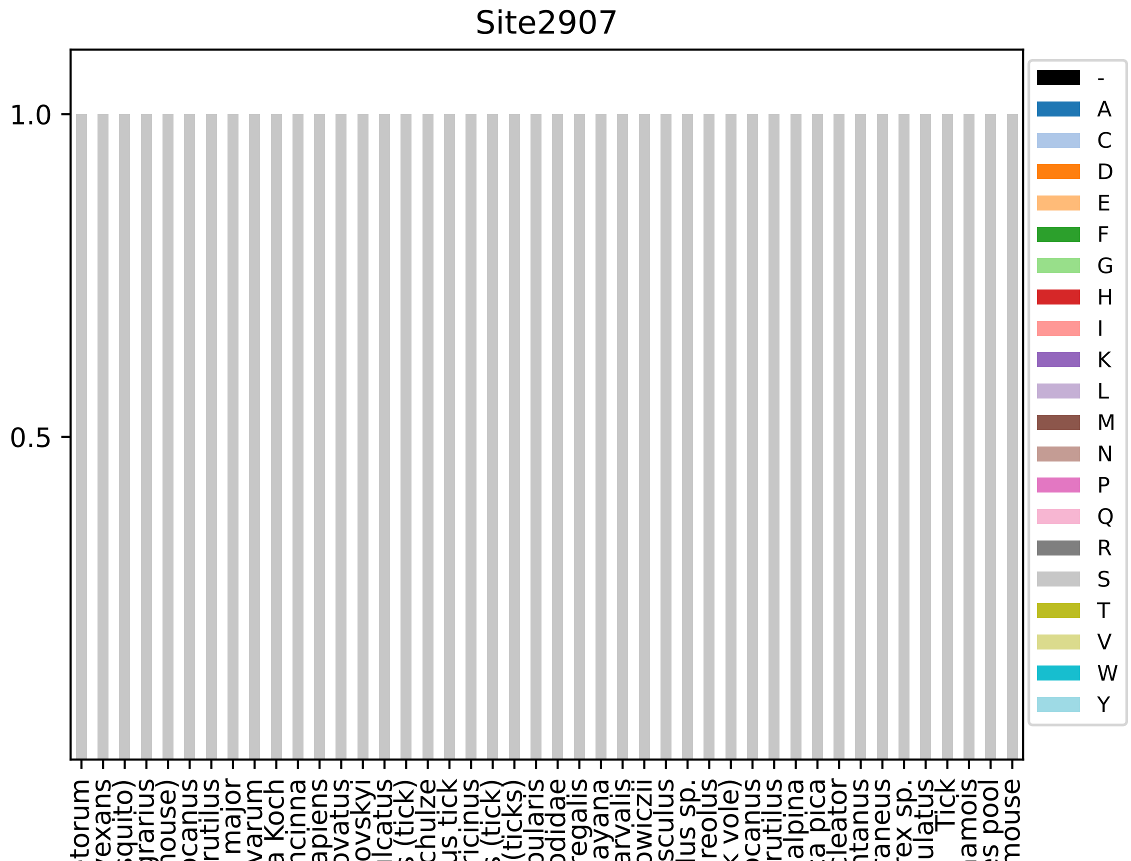
Site2907
1.0
0.5
-
A
C
D
E
F
G
H
I
K
L
M
N
P
Q
R
S
T
V
W
Y

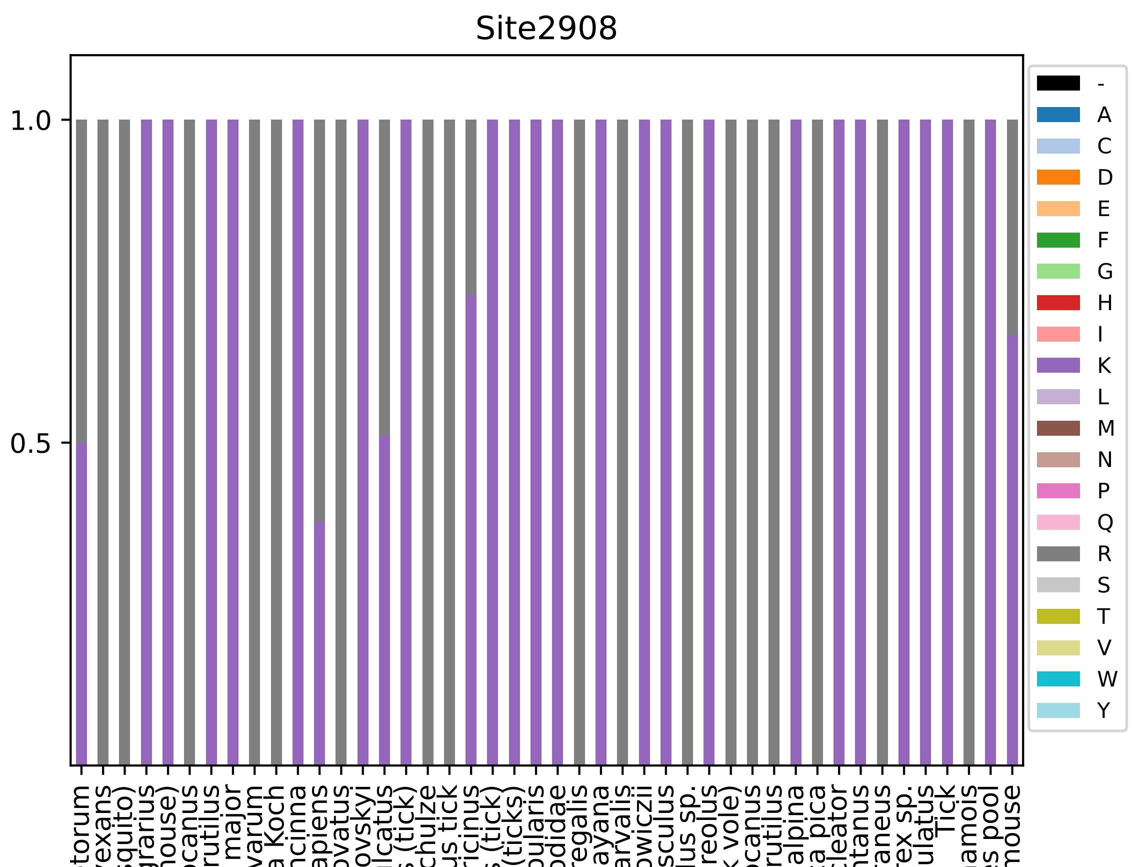
Site2908
1.0
0.5
-
A
C
D
E
F
G
H
I
K
L
M
N
P
Q
R
S
T
V
W
Y

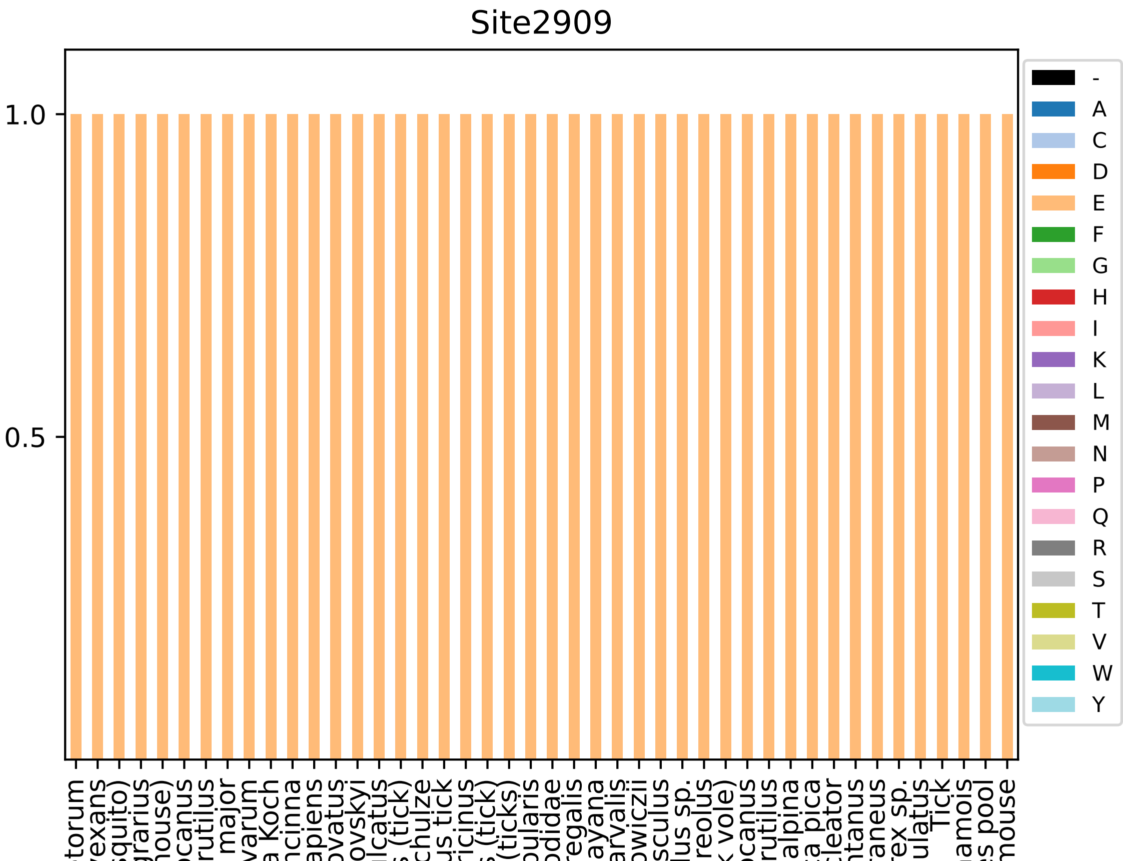

Site2909
1.0
0.5
-
A
C
D
E
F
G
H
I
K
L
M
N
P
Q
R
S
T
V
W
Y

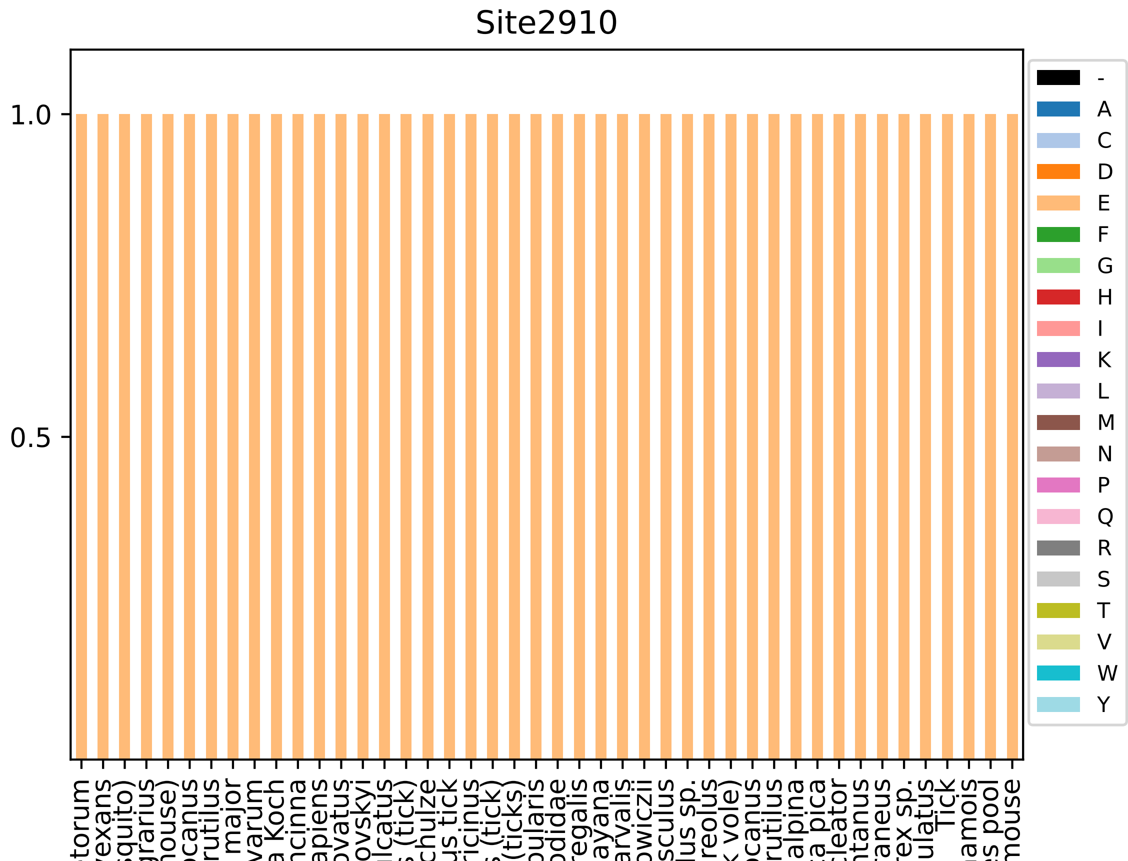
Site2910
1.0
0.5
-
A
C
D
E
F
G
H
I
K
L
M
N
P
Q
R
S
T
V
W
Y

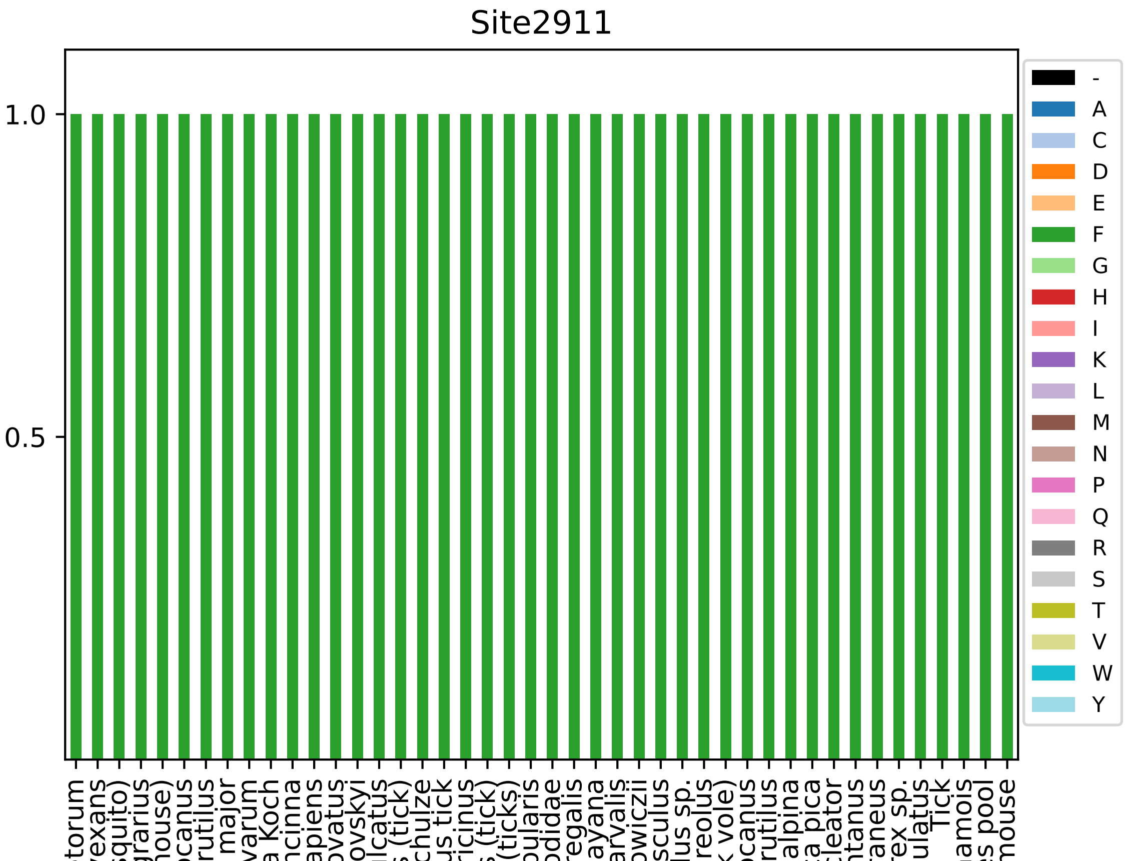

Site2911
1.0
0.5
-
A
C
D
E
F
G
H
I
K
L
M
N
P
Q
R
S
T
V
W
Y

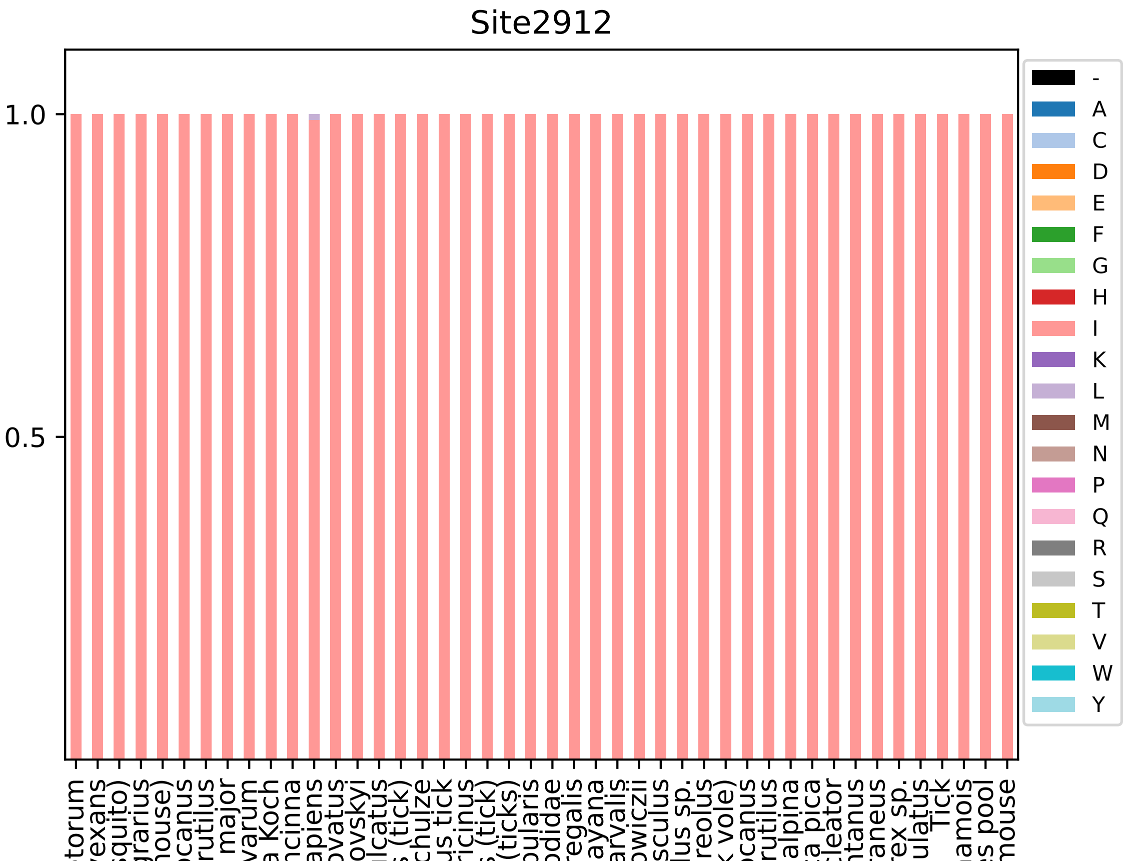
Site2912
1.0
0.5
-
A
C
D
E
F
G
H
I
K
L
M
N
P
Q
R
S
T
V
W
Y

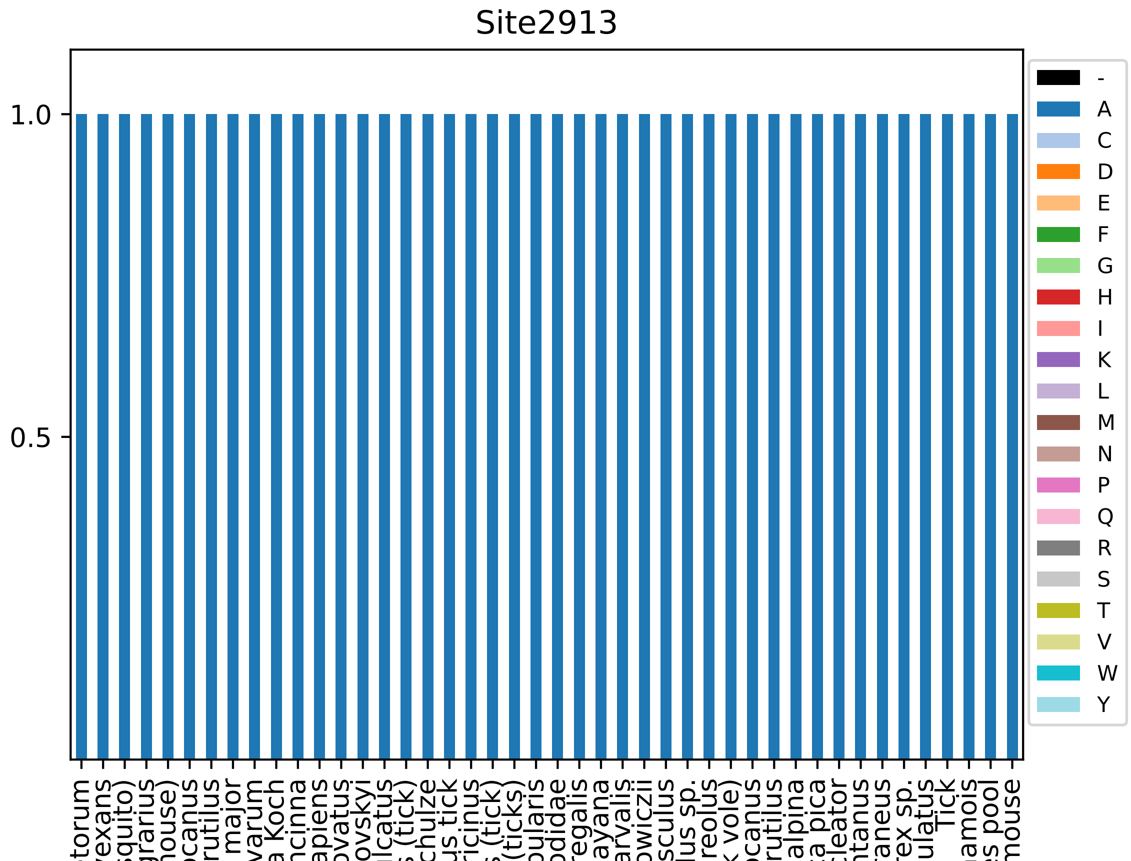
Site2913
1.0
0.5
-
A
C
D
E
F
G
H
I
K
L
M
N
P
Q
R
S
T
V
W
Y

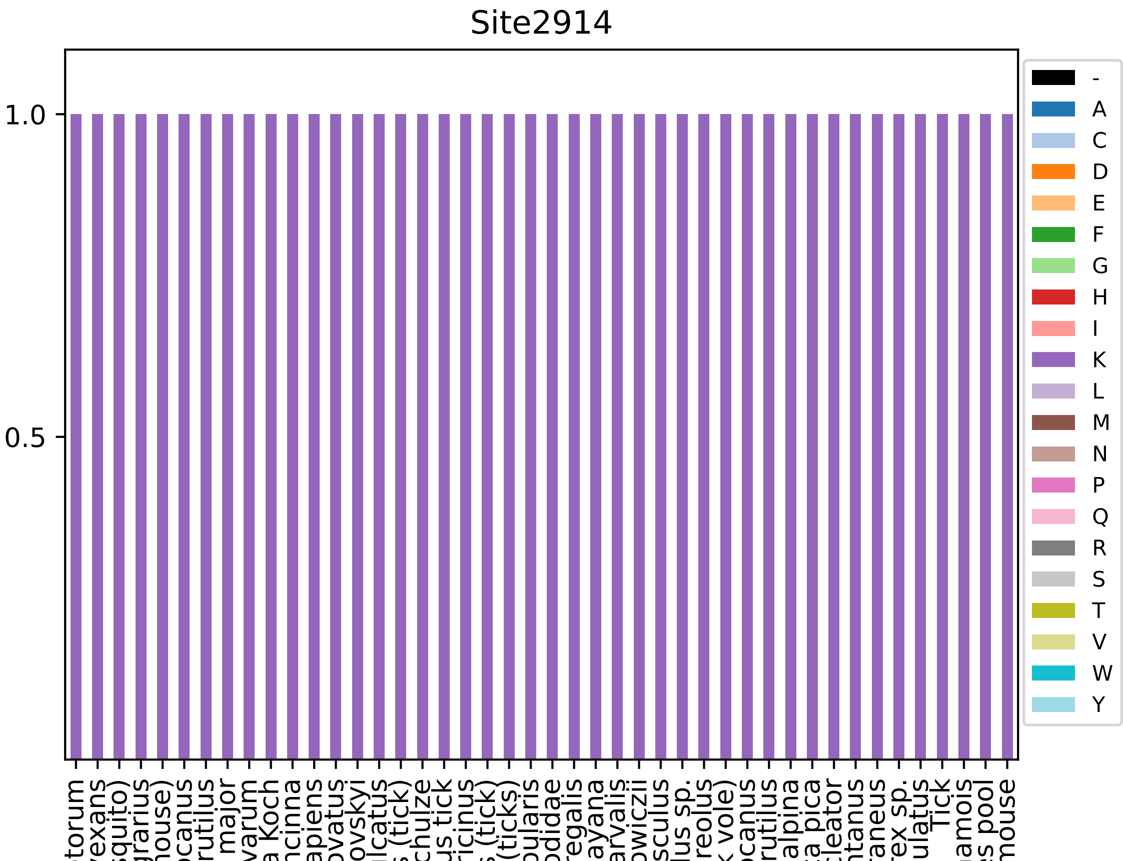
Site2914
1.0
0.5
-
A
C
D
E
F
G
H
I
K
L
M
N
P
Q
R
S
T
V
W
Y

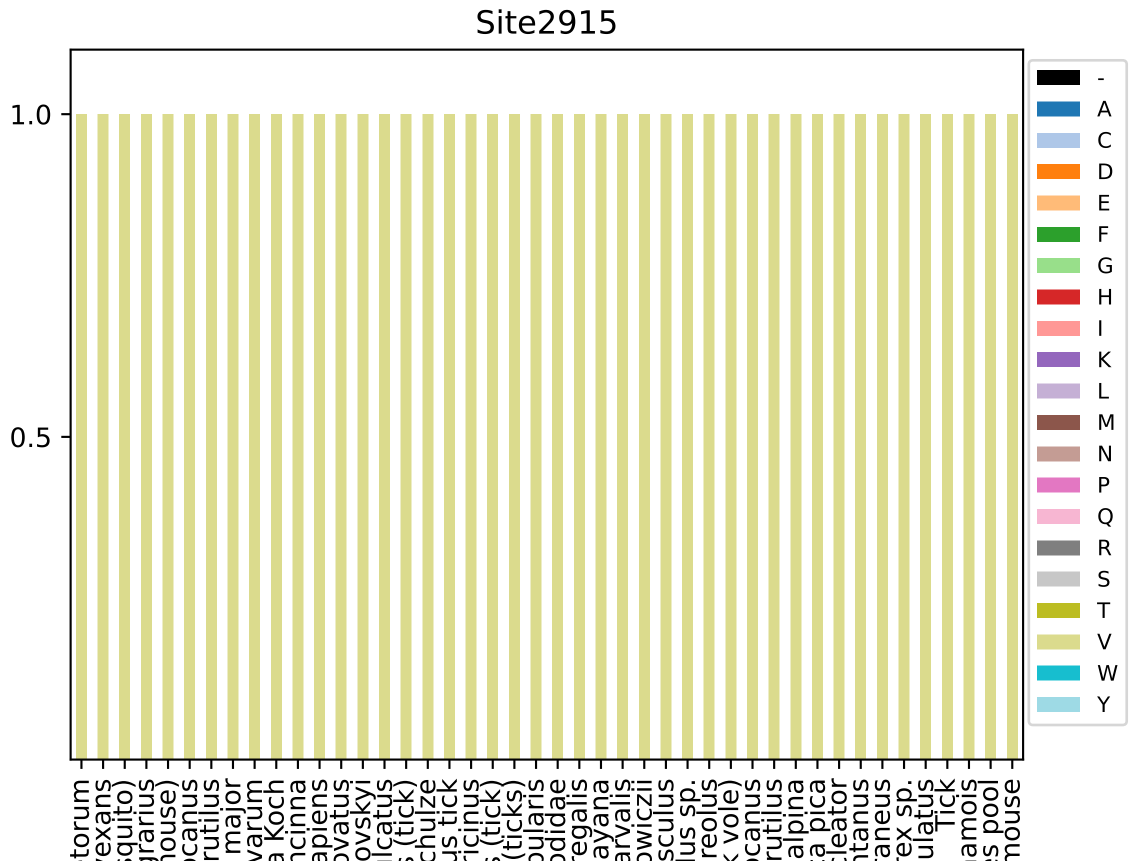
Site2915
1.0
0.5
-
A
C
D
E
F
G
H
I
K
L
M
N
P
Q
R
S
T
V
W
Y

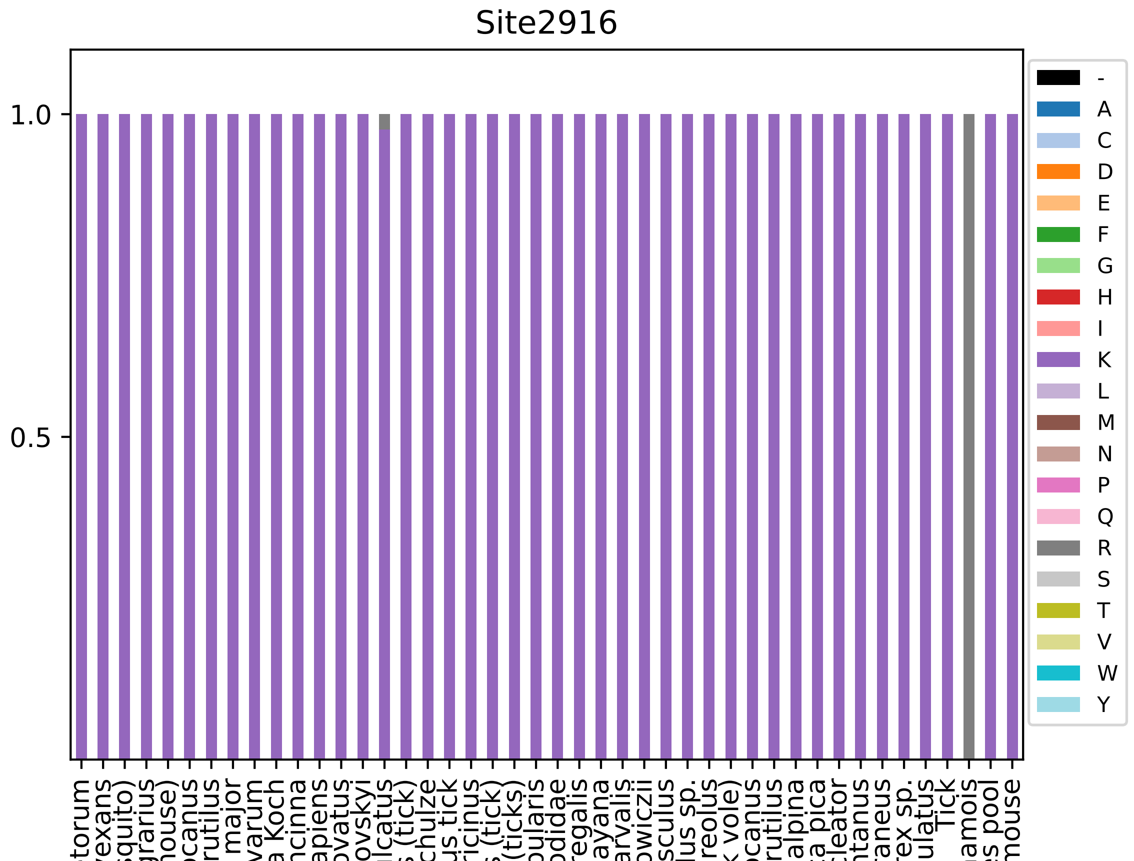
Site2916
1.0
0.5
-
A
C
D
E
F
G
H
I
K
L
M
N
P
Q
R
S
T
V
W
Y

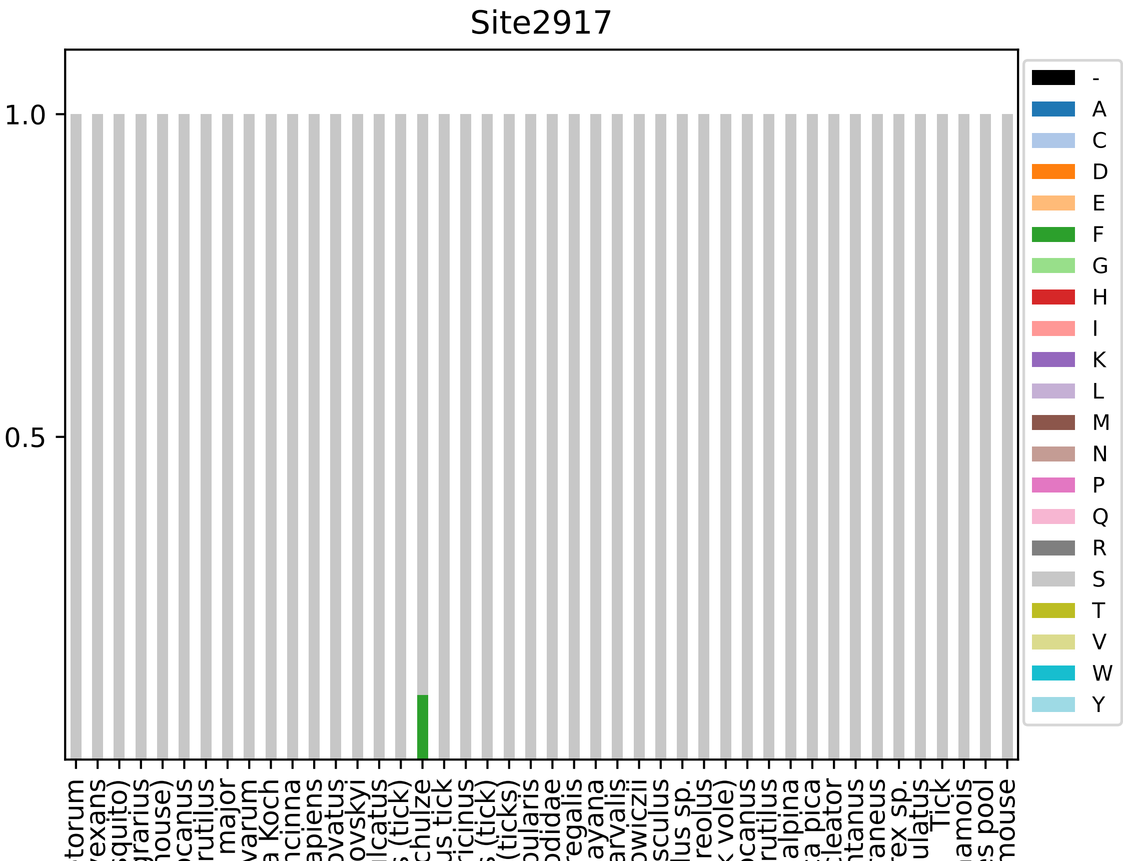

Site2917
1.0
0.5
-
A
C
D
E
F
G
H
I
K
L
M
N
P
Q
R
S
T
V
W
Y

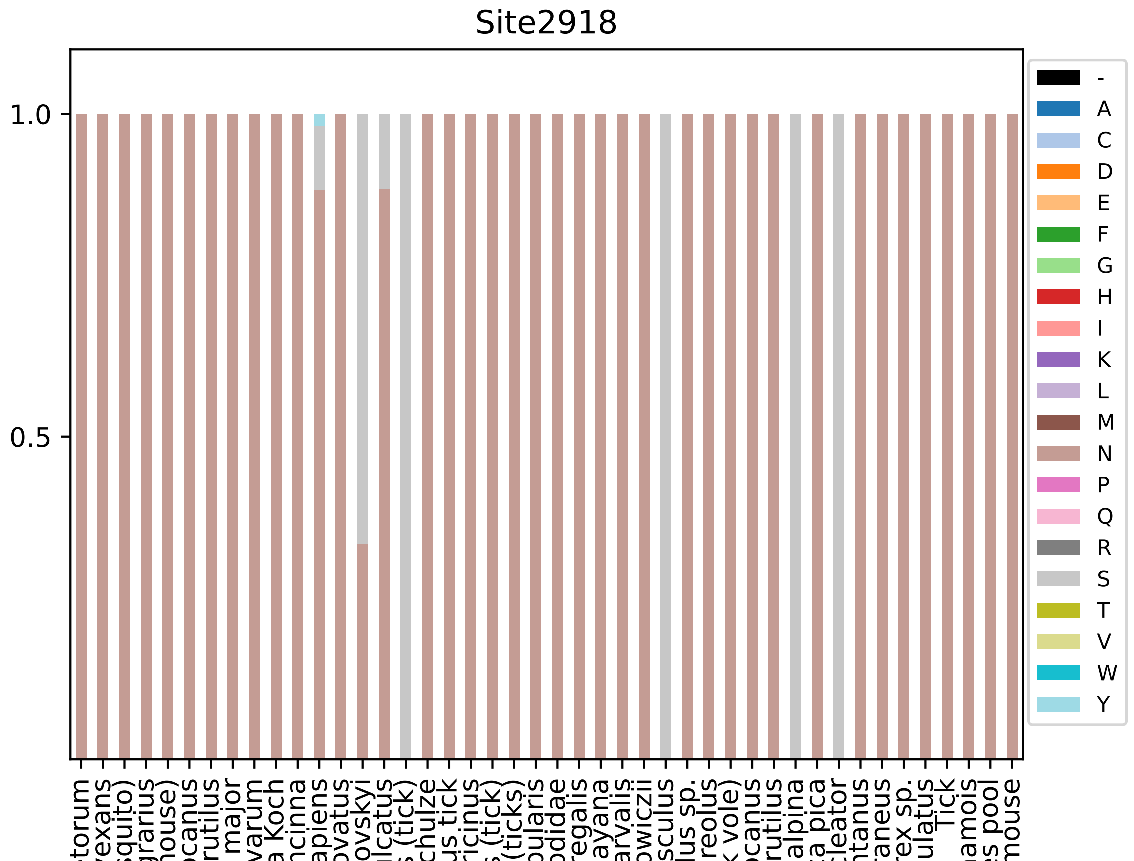
Site2918
1.0
0.5
-
A
C
D
E
F
G
H
I
K
L
M
N
P
Q
R
S
T
V
W
Y

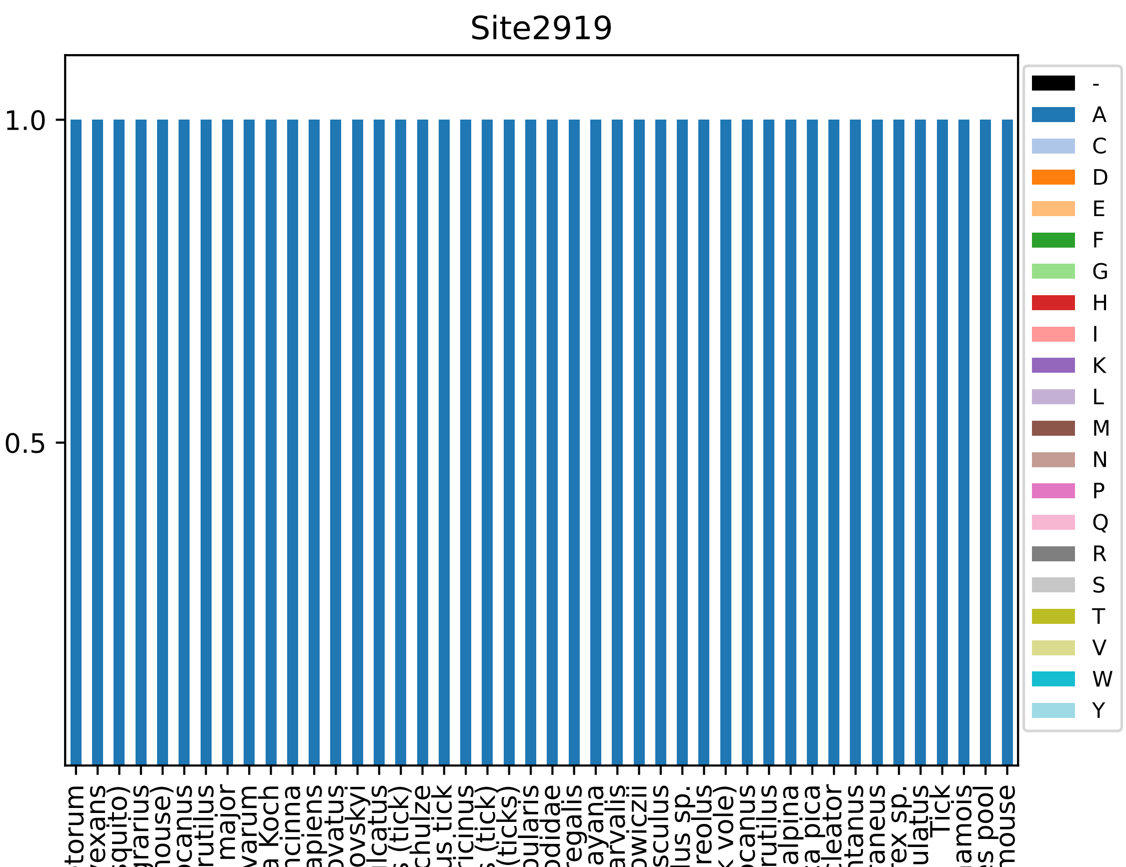
Site2919
1.0
0.5
-
A
C
D
E
F
G
H
I
K
L
M
N
P
Q
R
S
T
V
W
Y

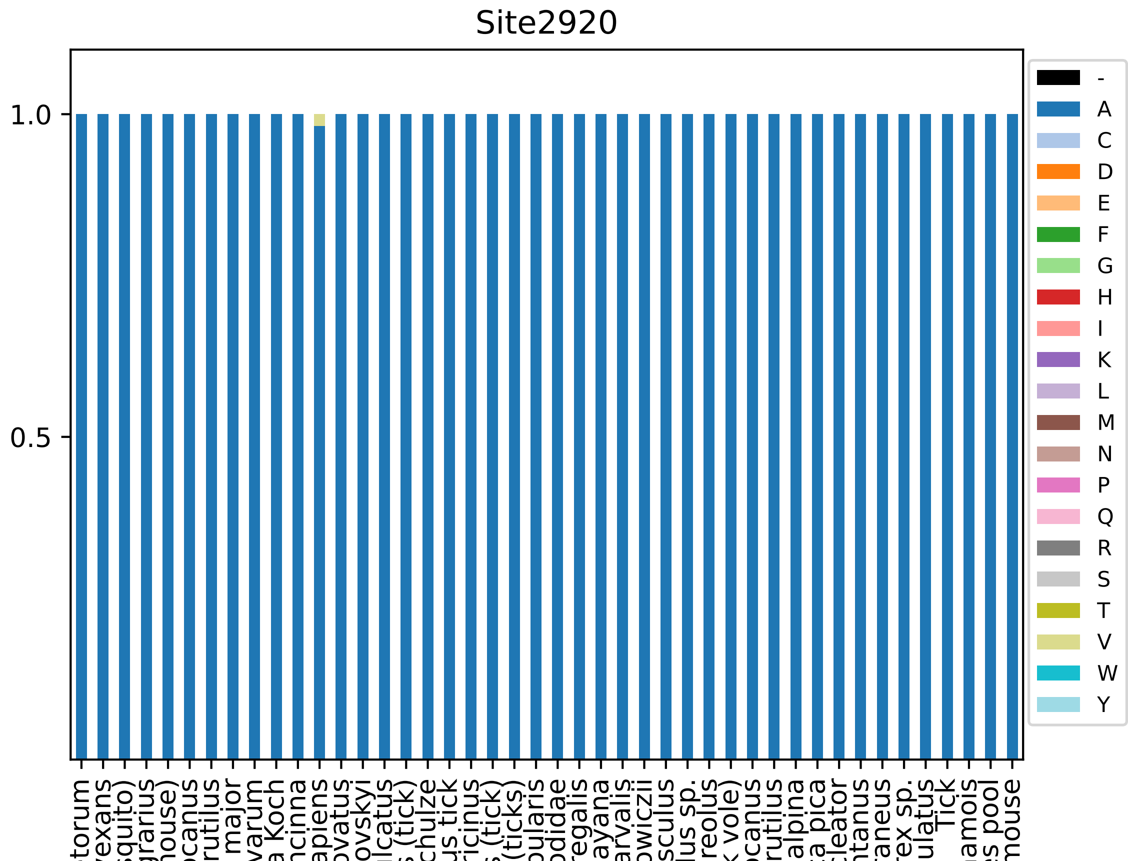
Site2920
1.0
0.5
-
A
C
D
E
F
G
H
I
K
L
M
N
P
Q
R
S
T
V
W
Y

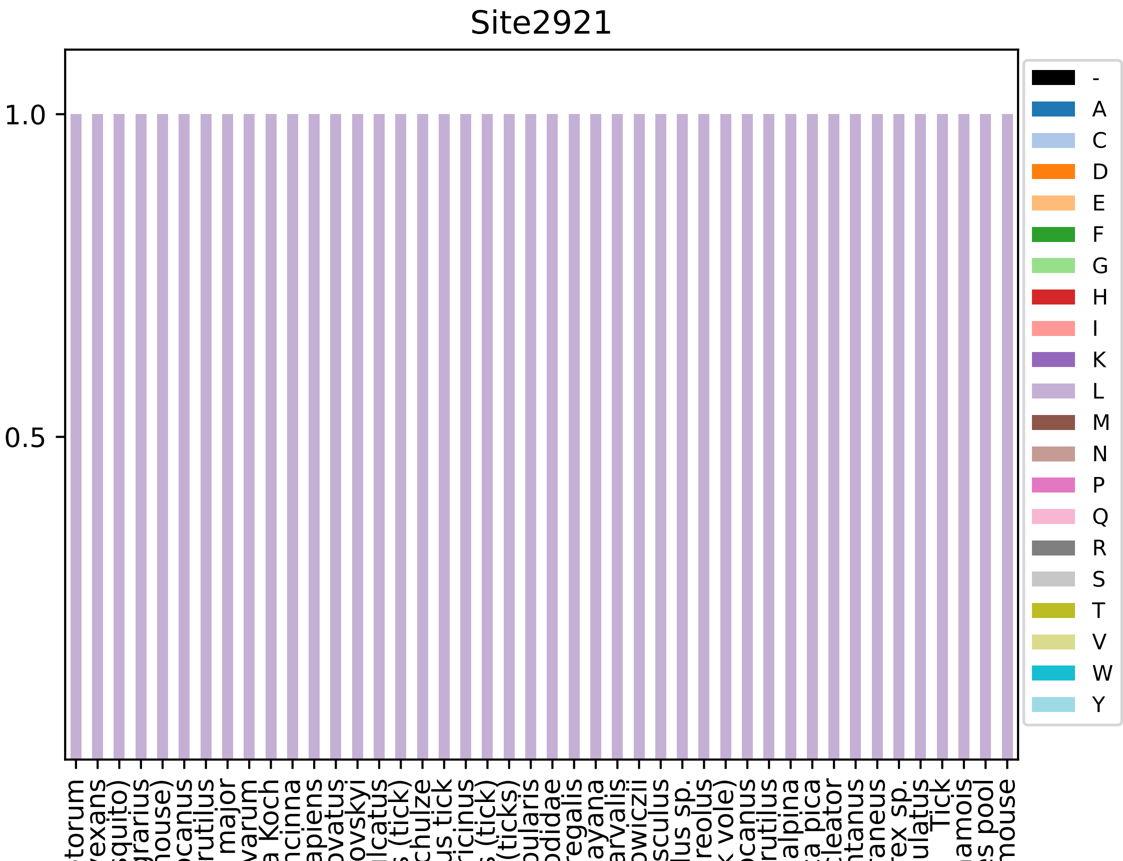
Site2921
1.0
0.5
-
A
C
D
E
F
G
H
I
K
L
M
N
P
Q
R
S
T
V
W
Y

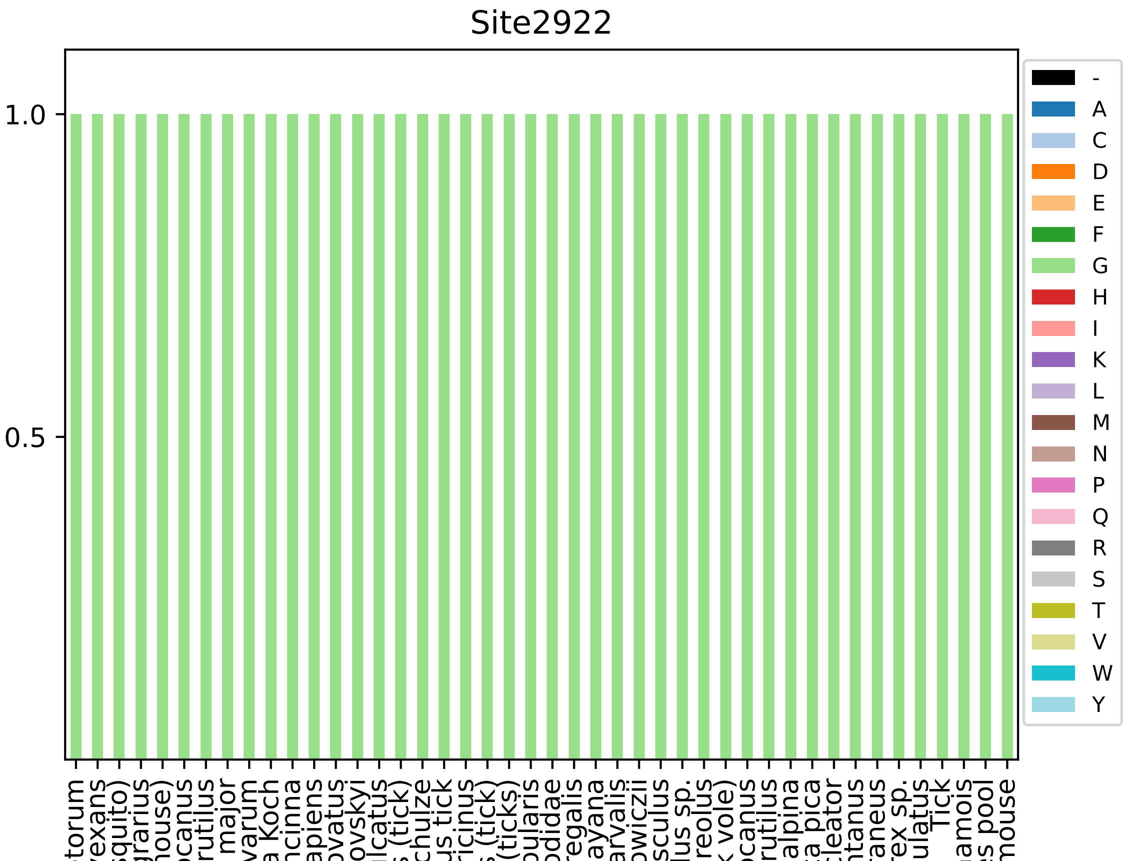
Site2922
1.0
0.5
-
A
C
D
E
F
G
H
I
K
L
M
N
P
Q
R
S
T
V
W
Y

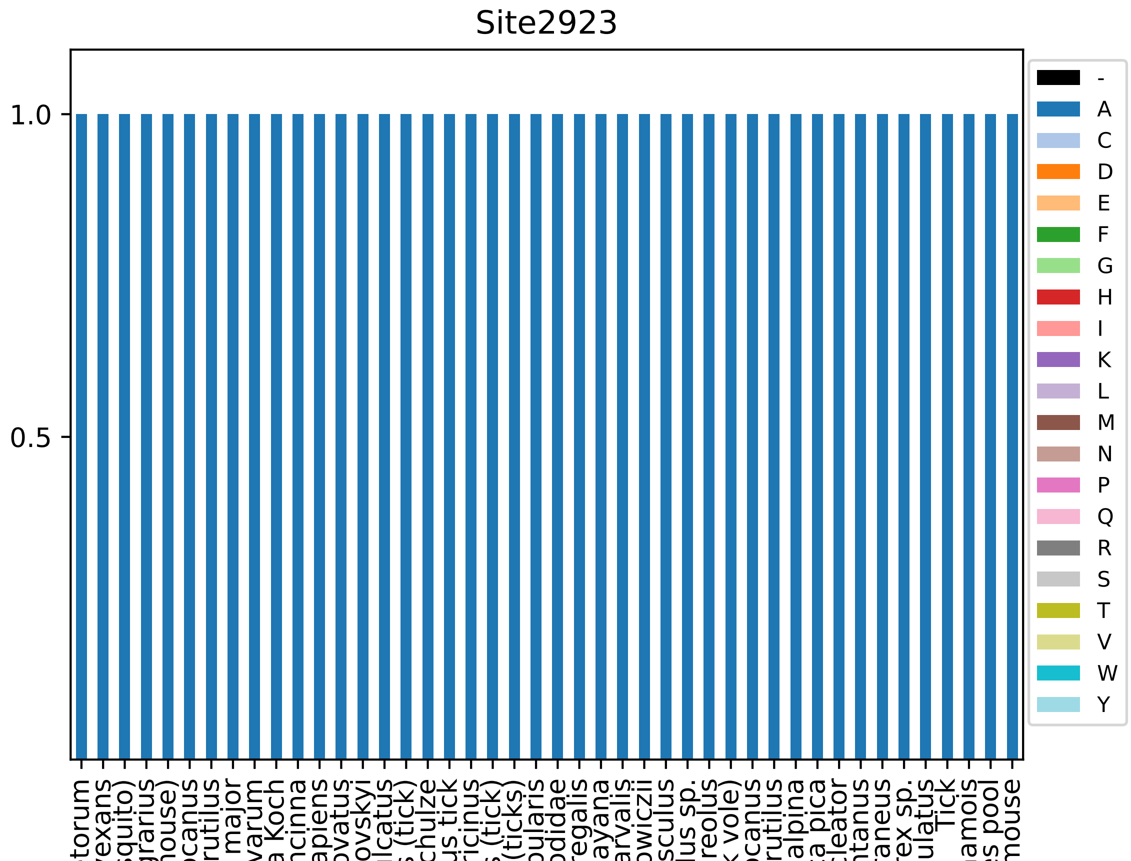

Site2923
1.0
0.5
-
A
C
D
E
F
G
H
I
K
L
M
N
P
Q
R
S
T
V
W
Y

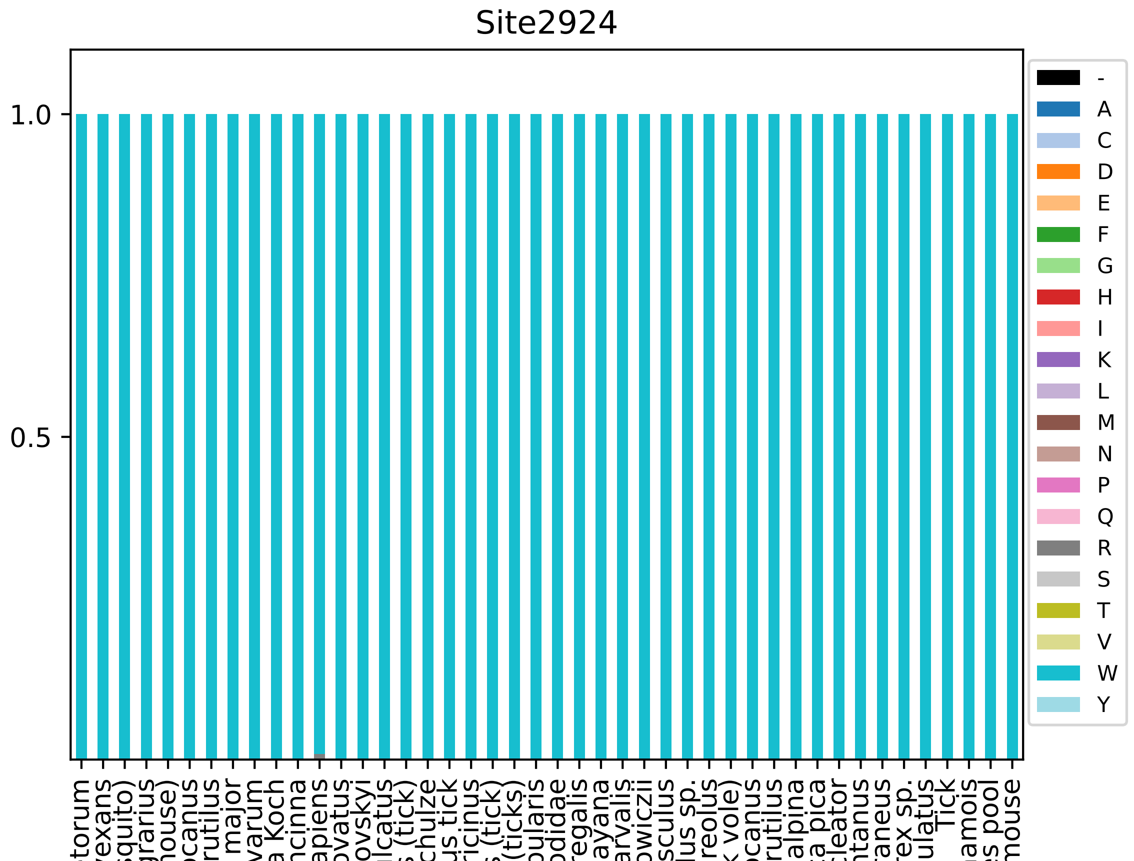
Site2924
1.0
0.5
-
A
C
D
E
F
G
H
I
K
L
M
N
P
Q
R
S
T
V
W
Y
Tick

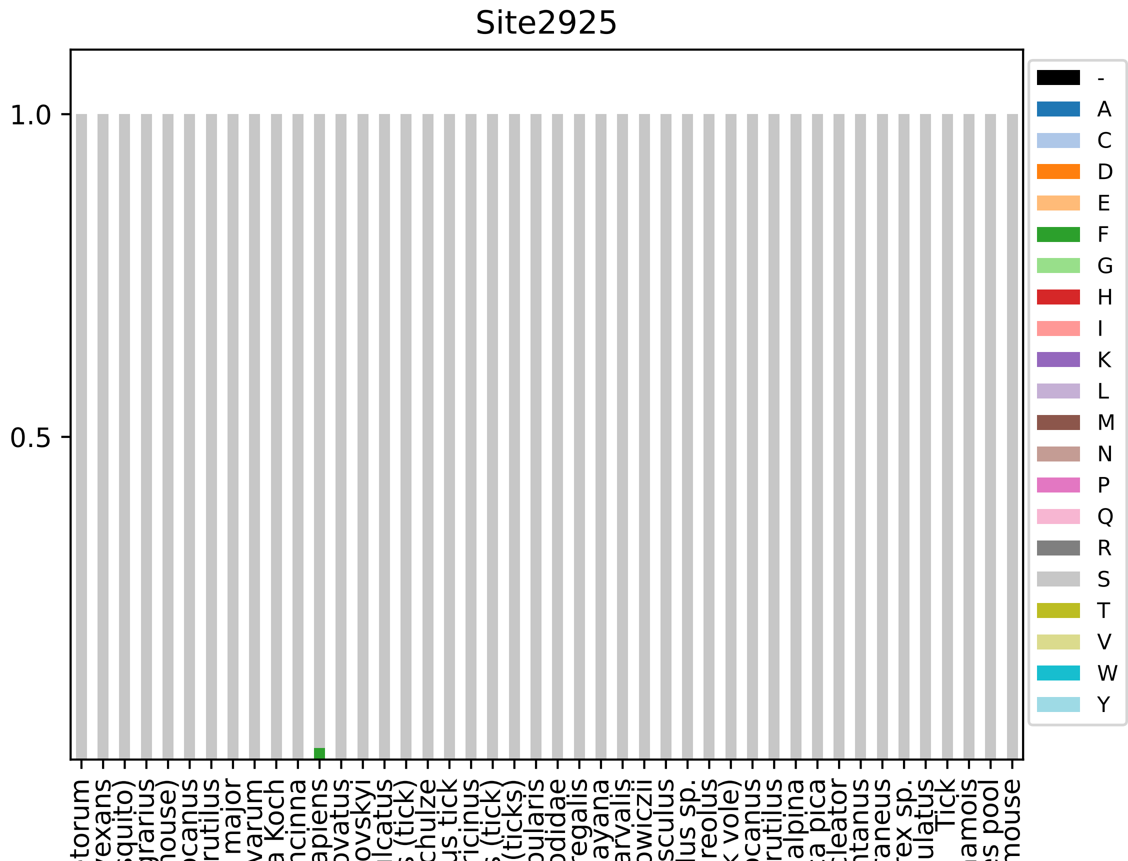
Site2925
1.0
0.5
-
A
C
D
E
F
G
H
I
K
L
M
N
P
Q
R
S
T
V
W
Y

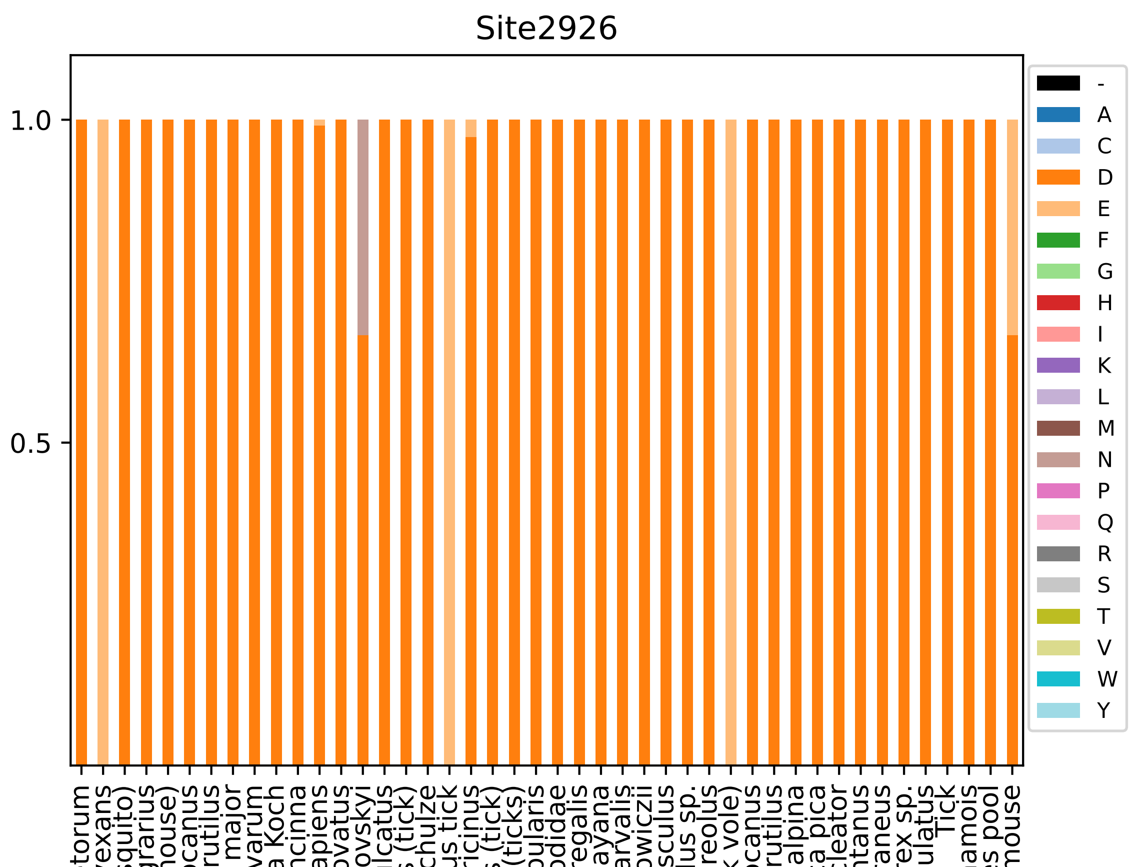
Site2926
1.0
0.5
-
A
C
D
E
F
G
H
I
K
L
M
N
P
Q
R
S
T
V
W
Y

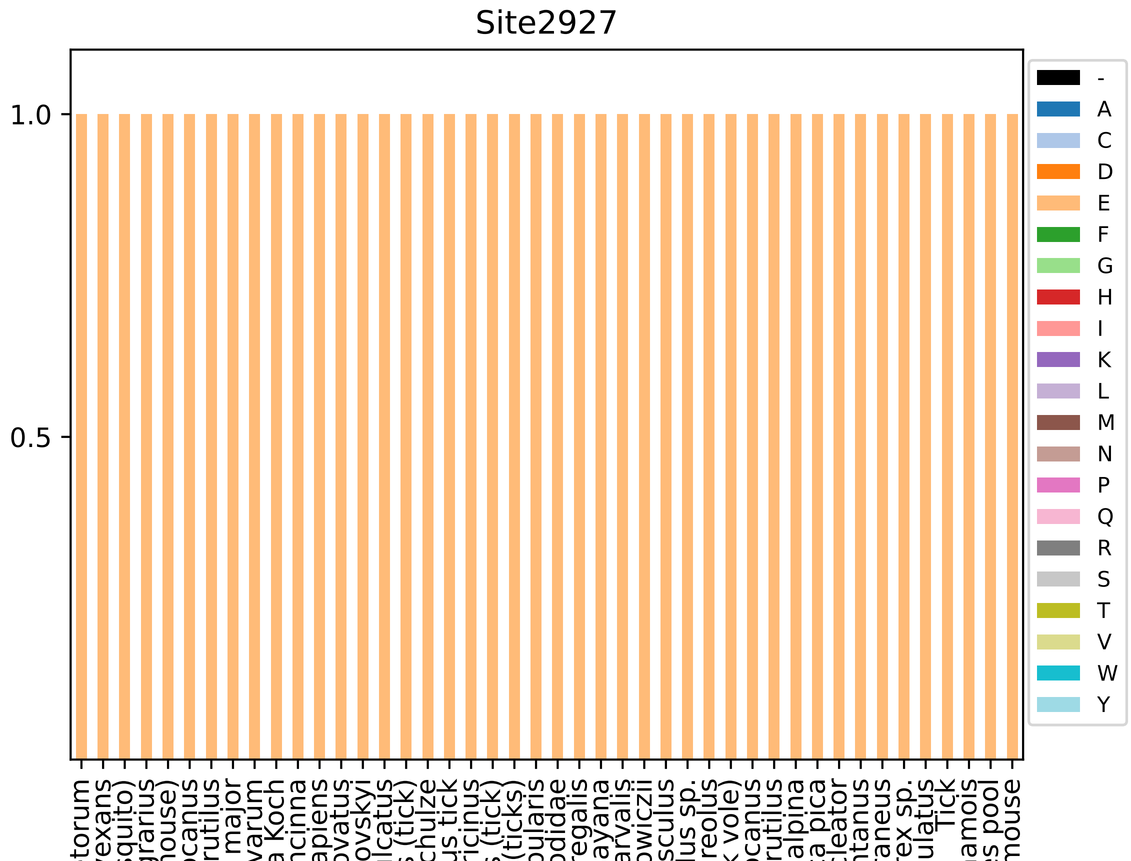

Site2927
1.0
0.5
-
A
C
D
E
F
G
H
I
K
L
M
N
P
Q
R
S
T
V
W
Y

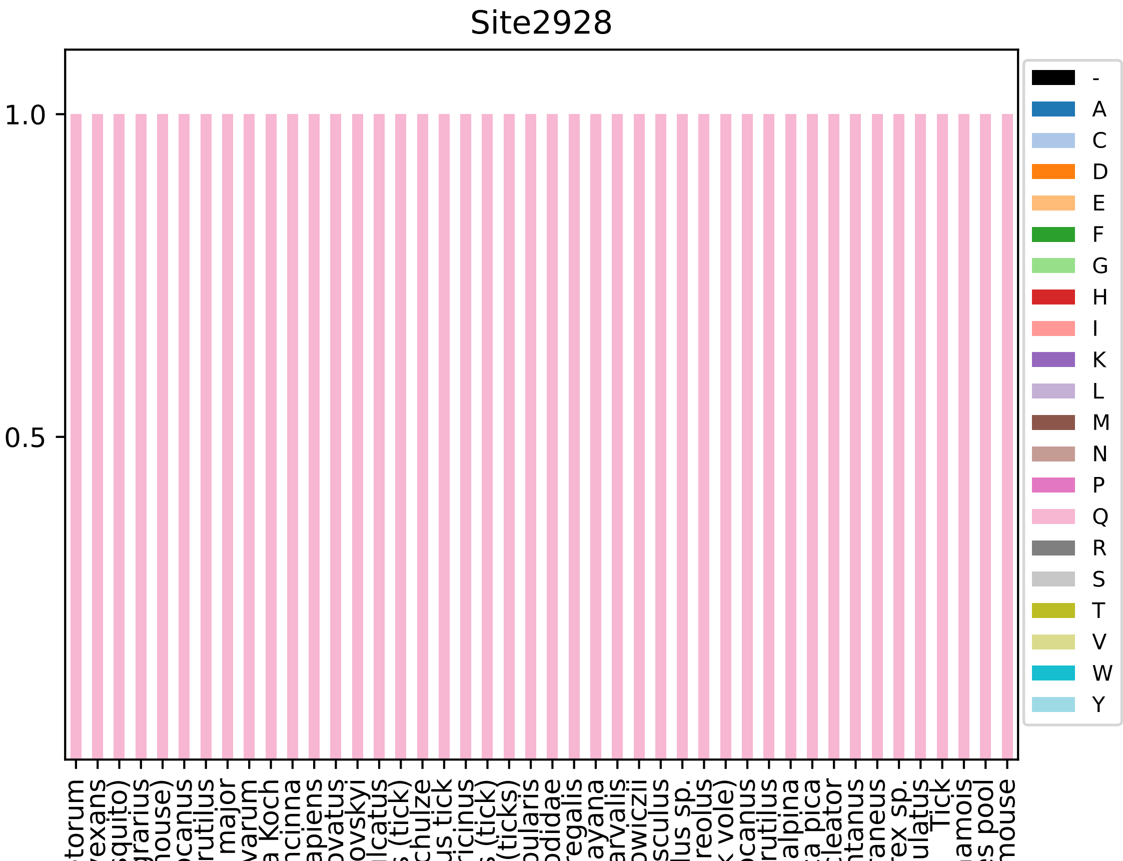

Site2928
1.0
0.5
-
A
C
D
E
F
G
H
I
K
L
M
N
P
Q
R
S
T
V
W
Y

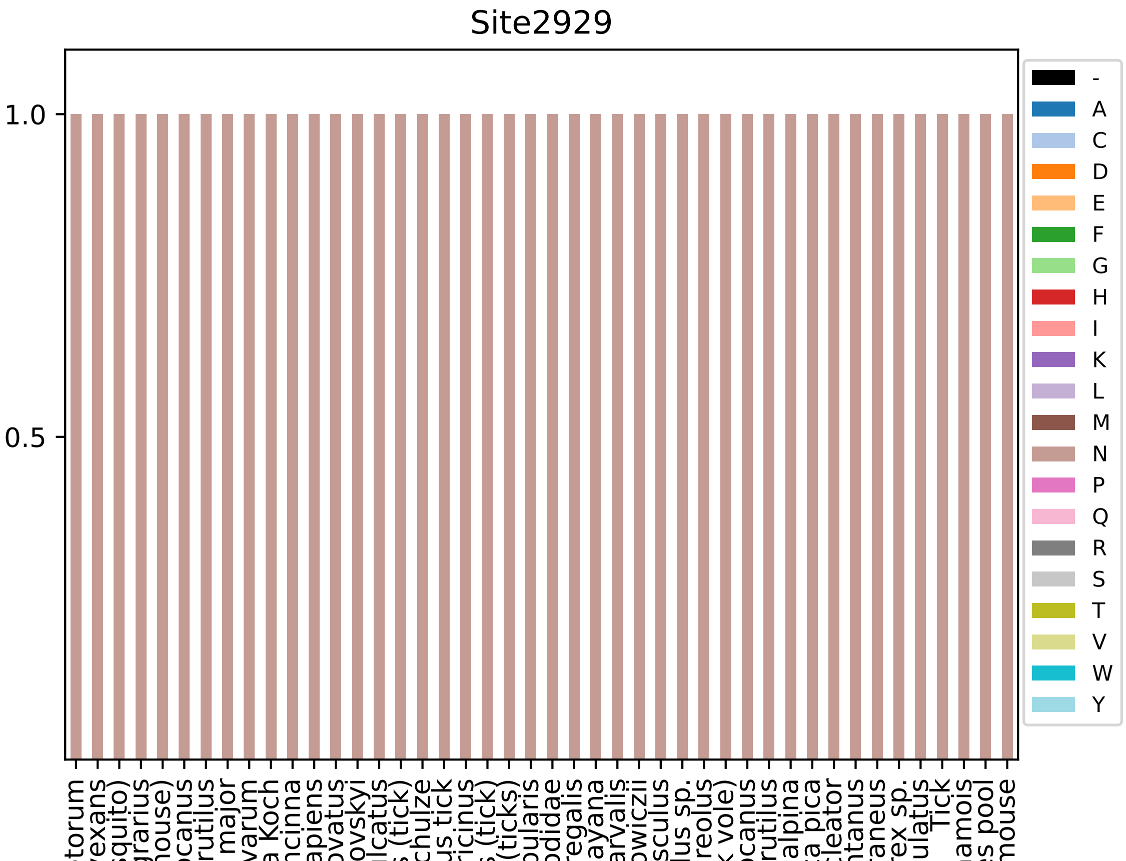
Site2929
1.0
0.5
-
A
C
D
E
F
G
H
I
K
L
M
N
P
Q
R
S
T
V
W
Y

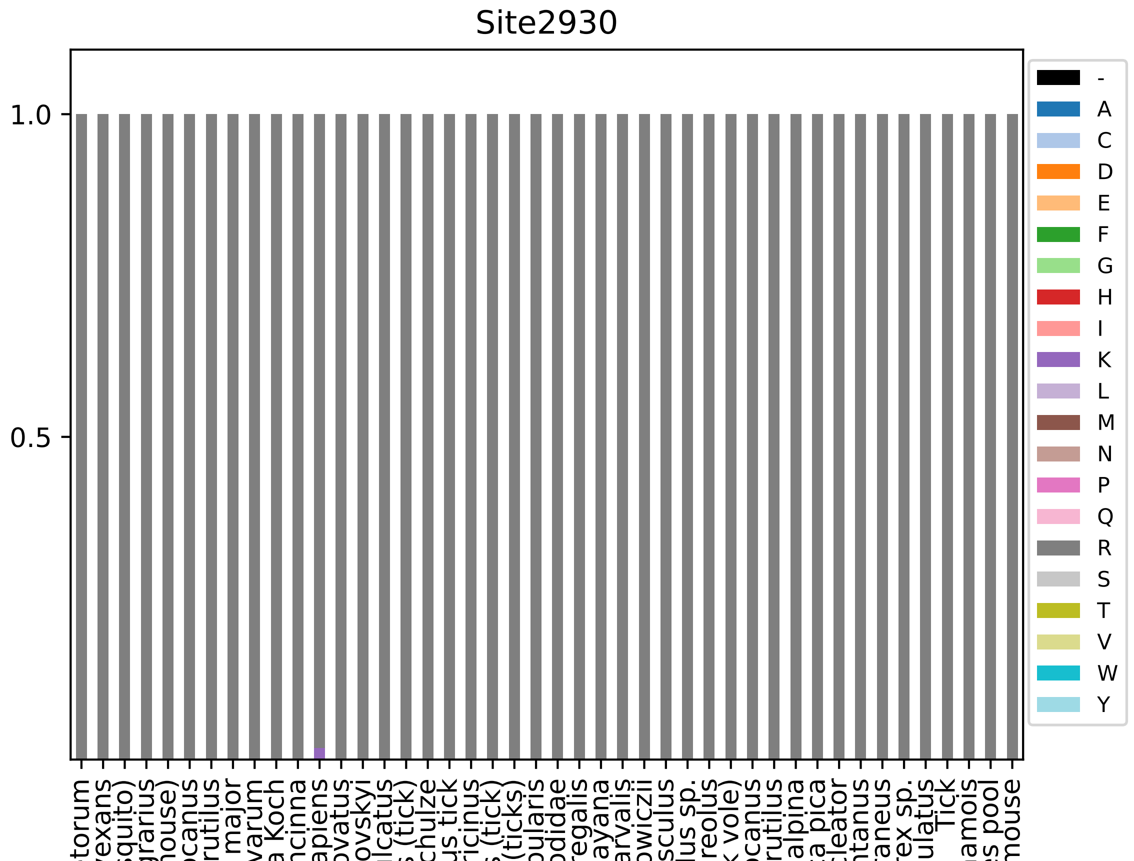

Site2930
1.0
0.5
-
A
C
D
E
F
G
H
I
K
L
M
N
P
Q
R
S
T
V
W
Y

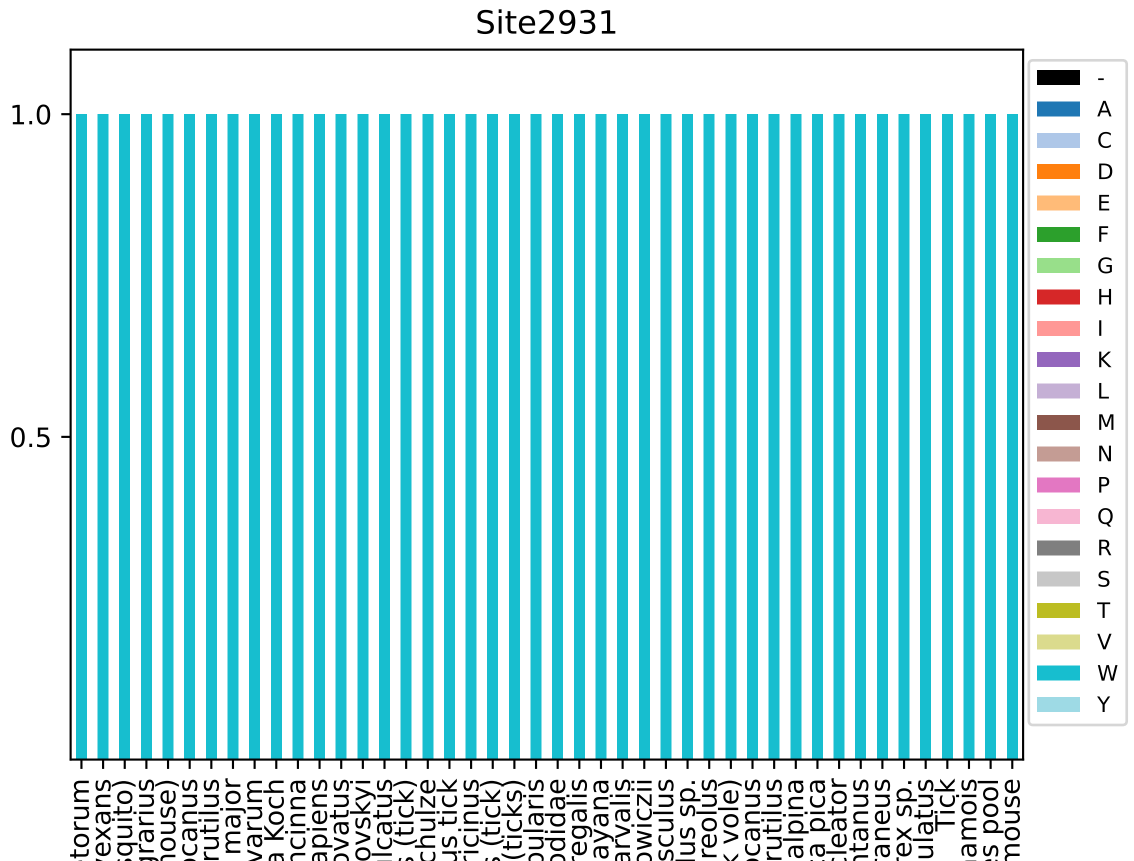
Site2931
1.0
0.5
-
A
C
D
E
F
G
H
I
K
L
M
N
P
Q
R
S
T
V
W
Y
Tick

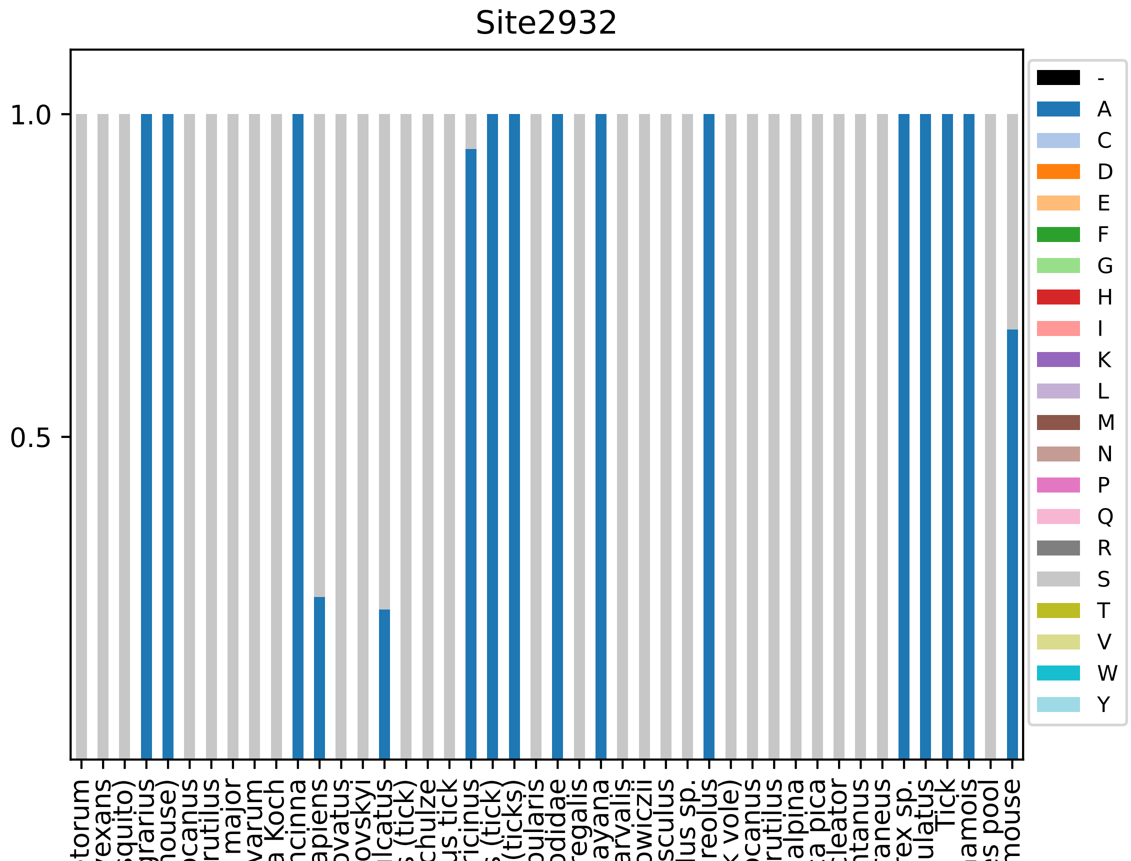
Site2932
1.0
0.5
-
A
C
D
E
F
G
H
I
K
L
M
N
P
Q
R
S
T
V
W
Y

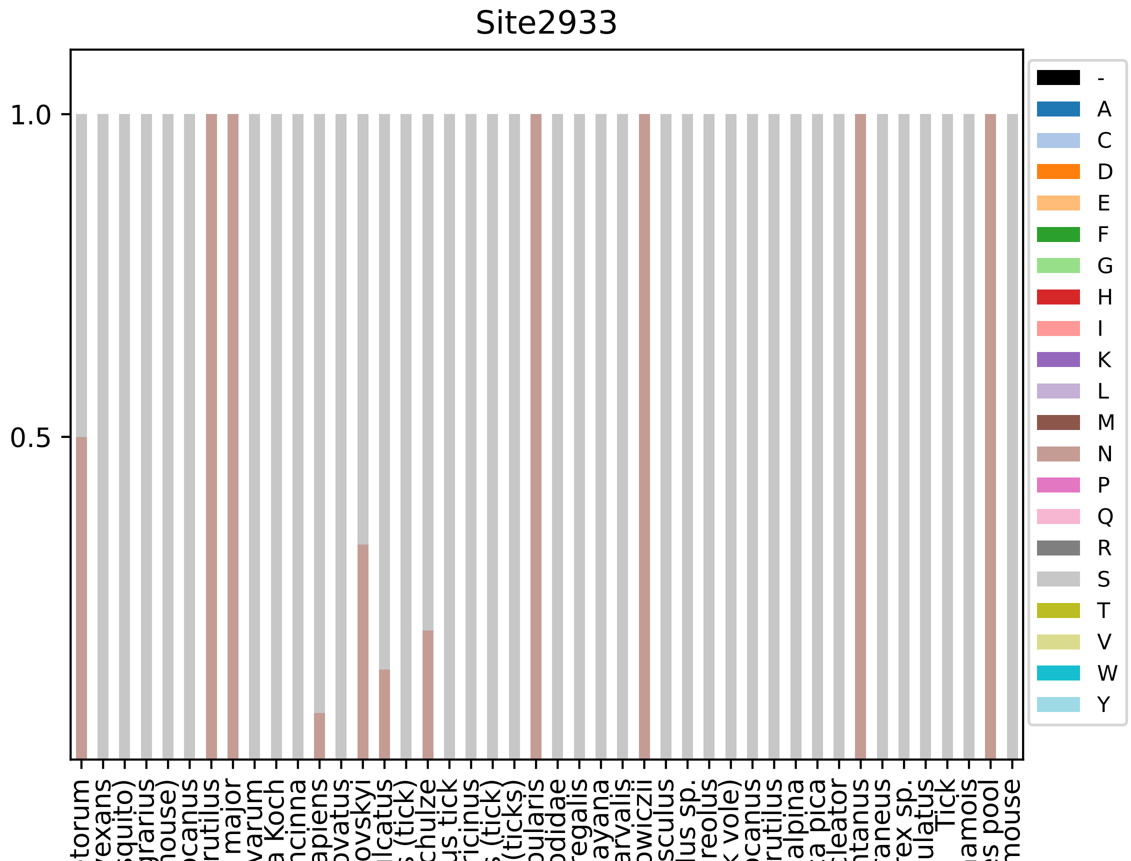
Site2933
1.0
0.5
-
A
C
D
E
F
G
H
I
K
L
M
N
P
Q
R
S
T
V
W
Y

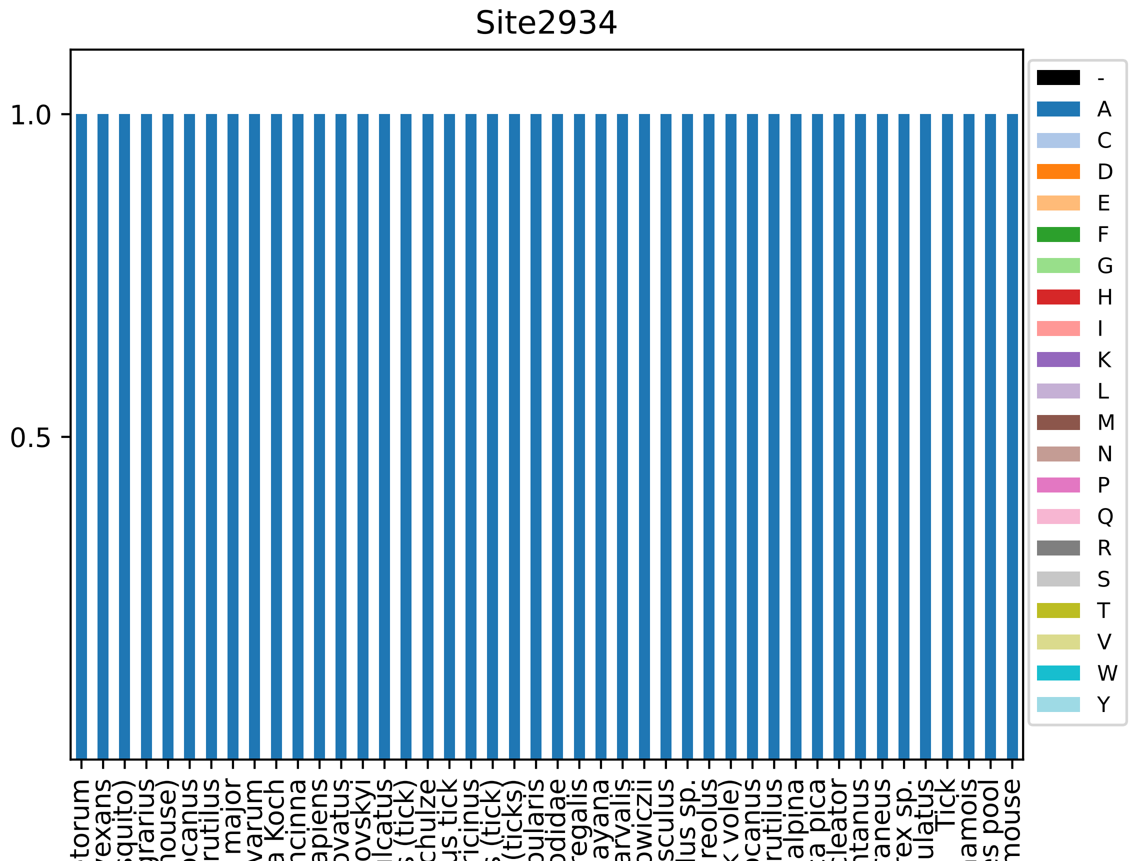

Site2934
1.0
0.5
-
A
C
D
E
F
G
H
I
K
L
M
N
P
Q
R
S
T
V
W
Y

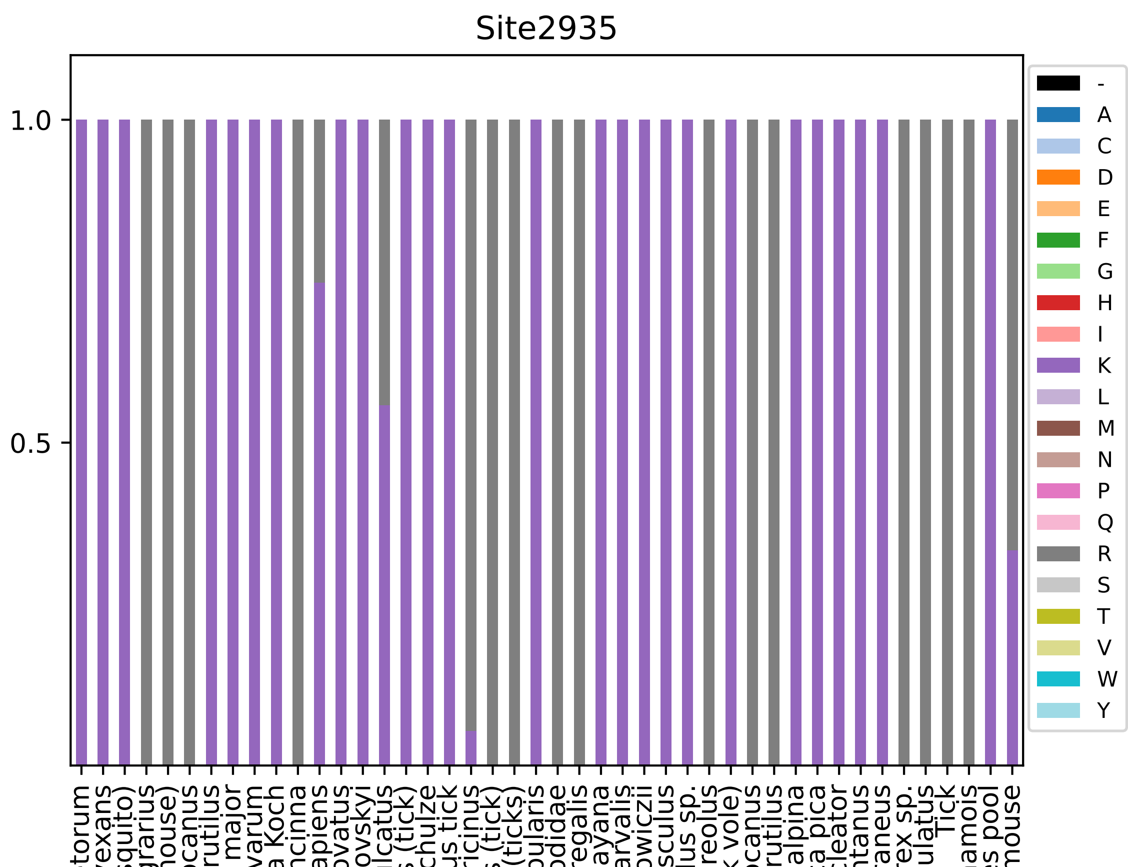

Site2935
1.0
0.5
-
A
C
D
E
F
G
H
I
K
L
M
N
P
Q
R
S
T
V
W
Y

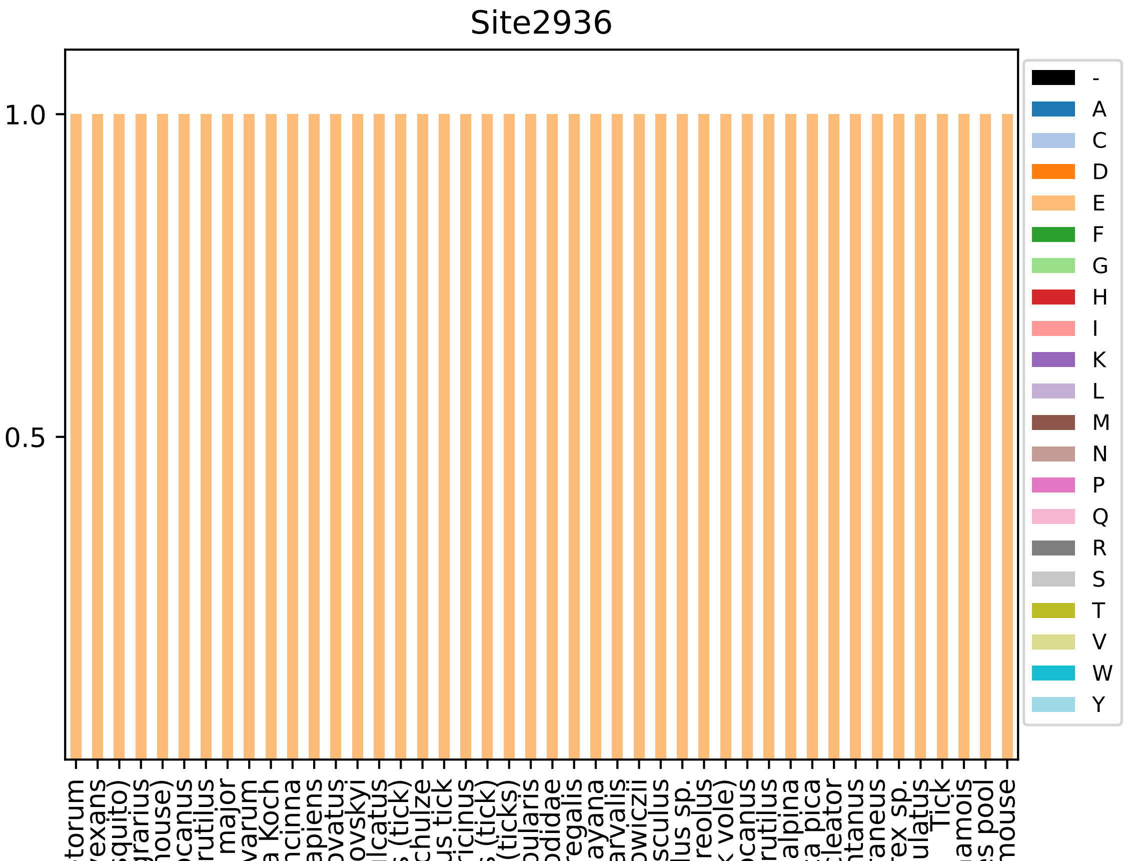
Site2936
1.0
0.5
-
A
C
D
E
F
G
H
I
K
L
M
N
P
Q
R
S
T
V
W
Y

Site2937

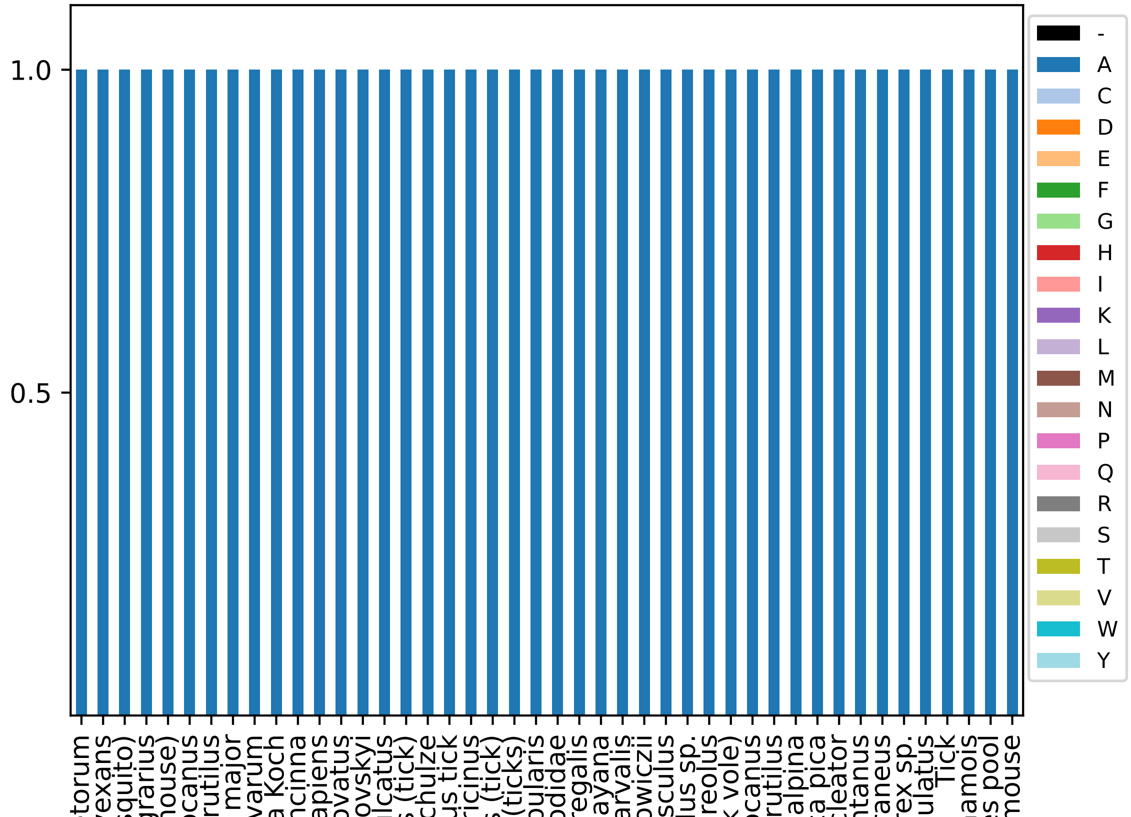
1.0
0.5
-
A
C
D
E
F
G
H
I
K
L
M
N
P
Q
R
S
T
V
W
Y
torum
vexans
squito)
garius
nouse)
ocanus
rutilus
major
varum
a Koch
ncinna
apiens
ovatus
ovskyi
ulcatus
s (tick)
chulze
us tick
ricinus
s (tick)
(ticks)
pularis
podidae
regalis
ayana
arvalis
owiczii
sculus
lus sp.
reolus
k vole)
ocanus
rutilus
alpina
a pica
cleator
ntanus
raneus
rex sp.
ulatus
Tick
amois
s pool
mouse

Site2938

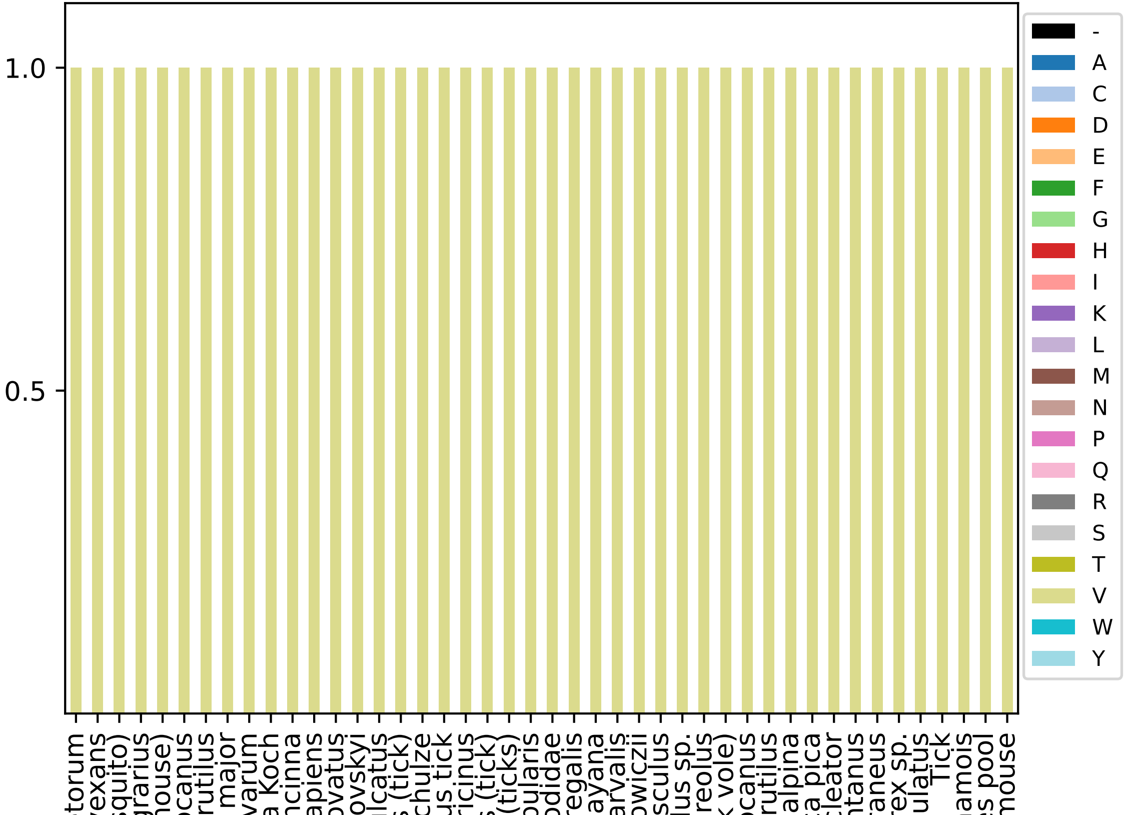

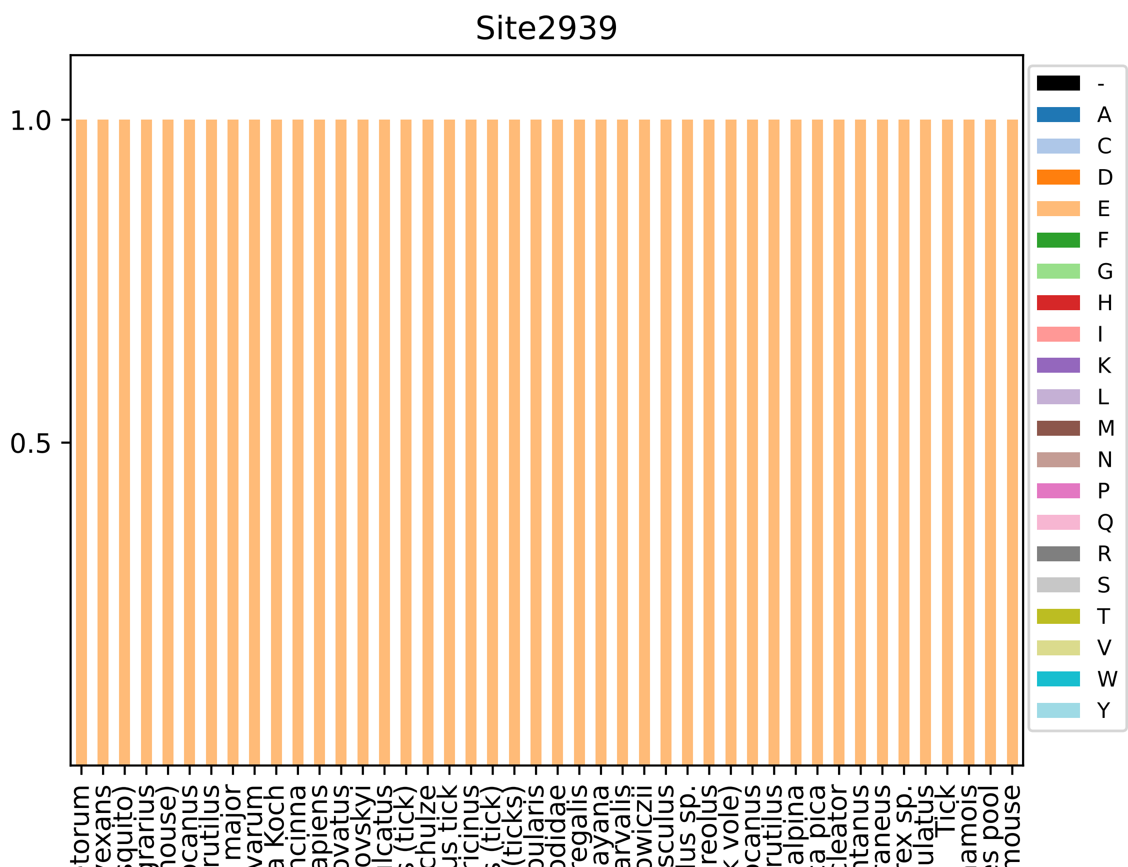
Site2939
1.0
0.5
-
A
C
D
E
F
G
H
I
K
L
M
N
P
Q
R
S
T
V
W
Y

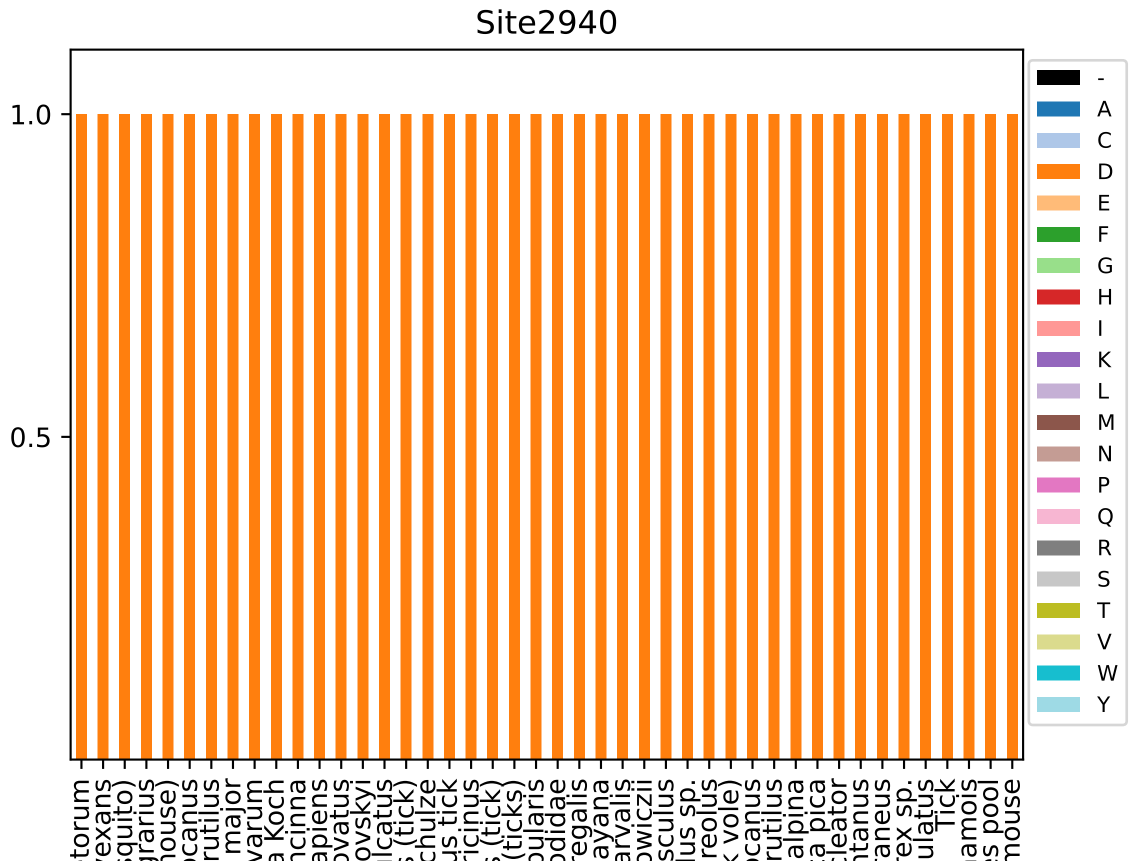
Site2940
1.0
0.5
-
A
C
D
E
F
G
H
I
K
L
M
N
P
Q
R
S
T
V
W
Y

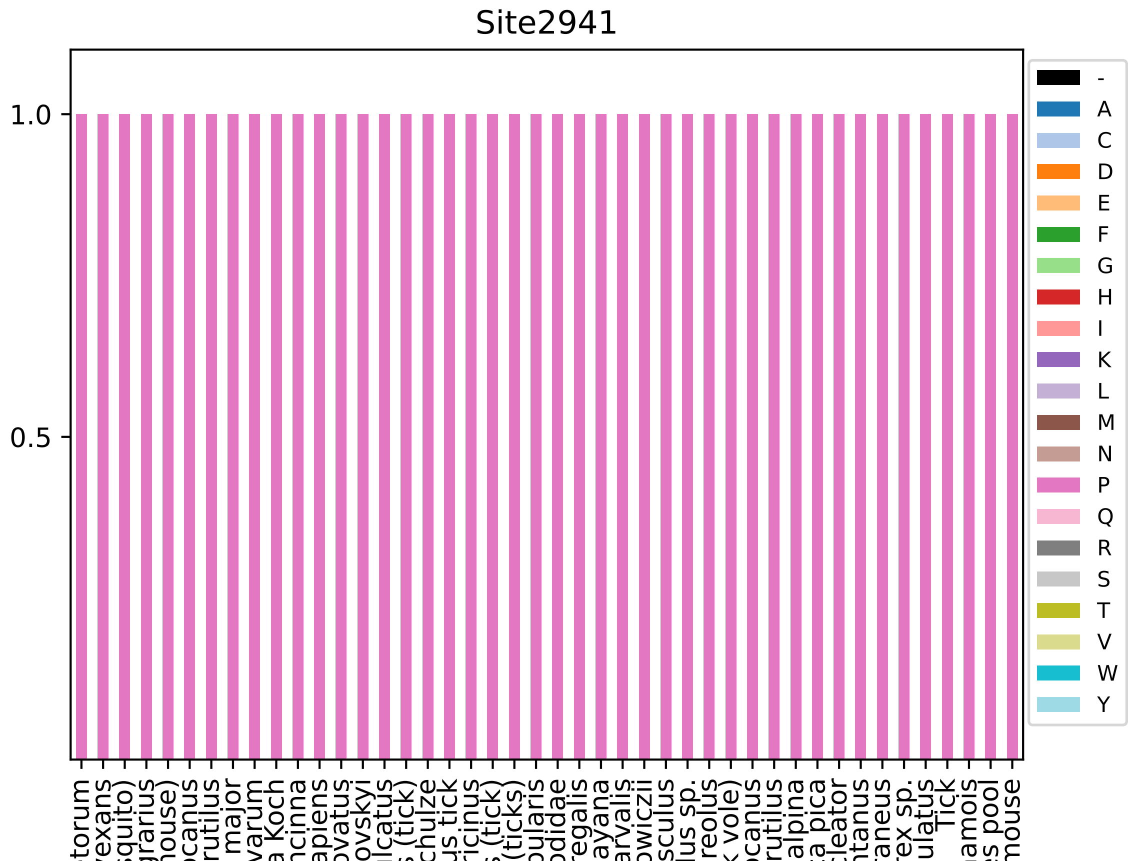
Site2941
1.0
0.5
-
A
C
D
E
F
G
H
I
K
L
M
N
P
Q
R
S
T
V
W
Y

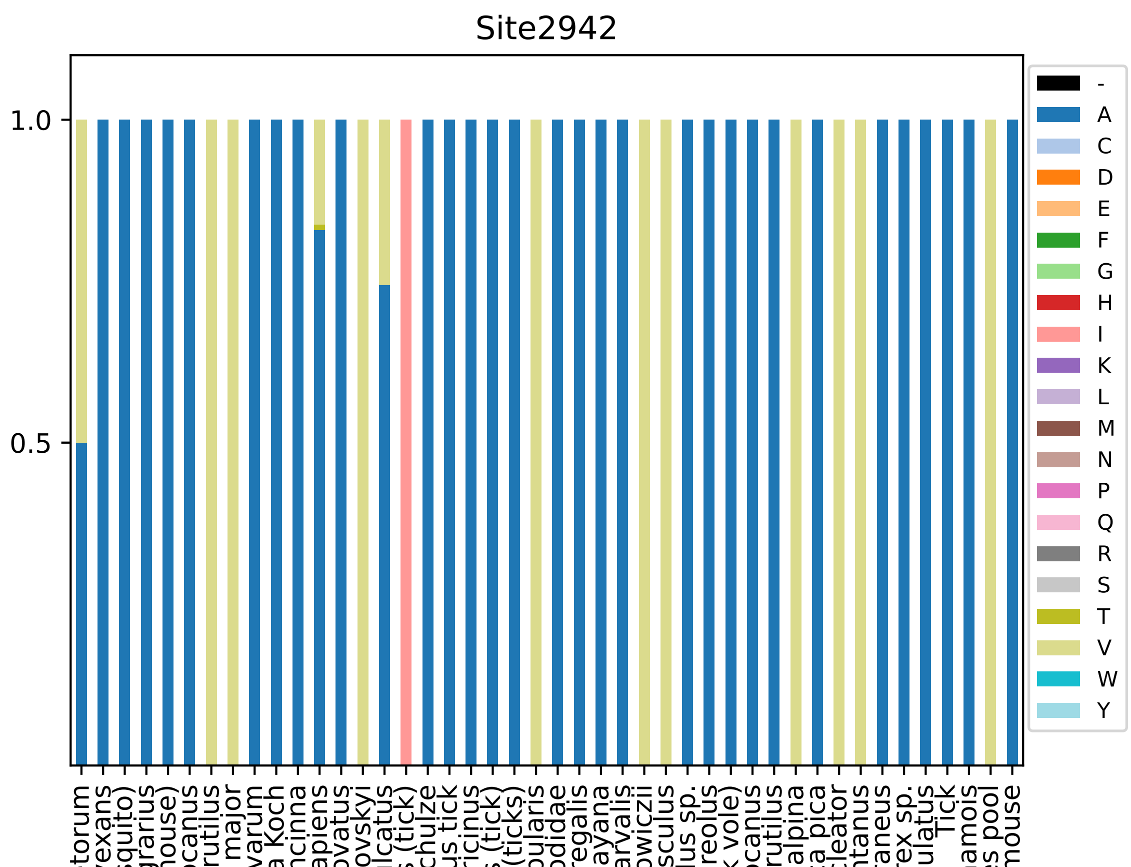
Site2942
1.0
0.5
-
A
C
D
E
F
G
H
I
K
L
M
N
P
Q
R
S
T
V
W
Y

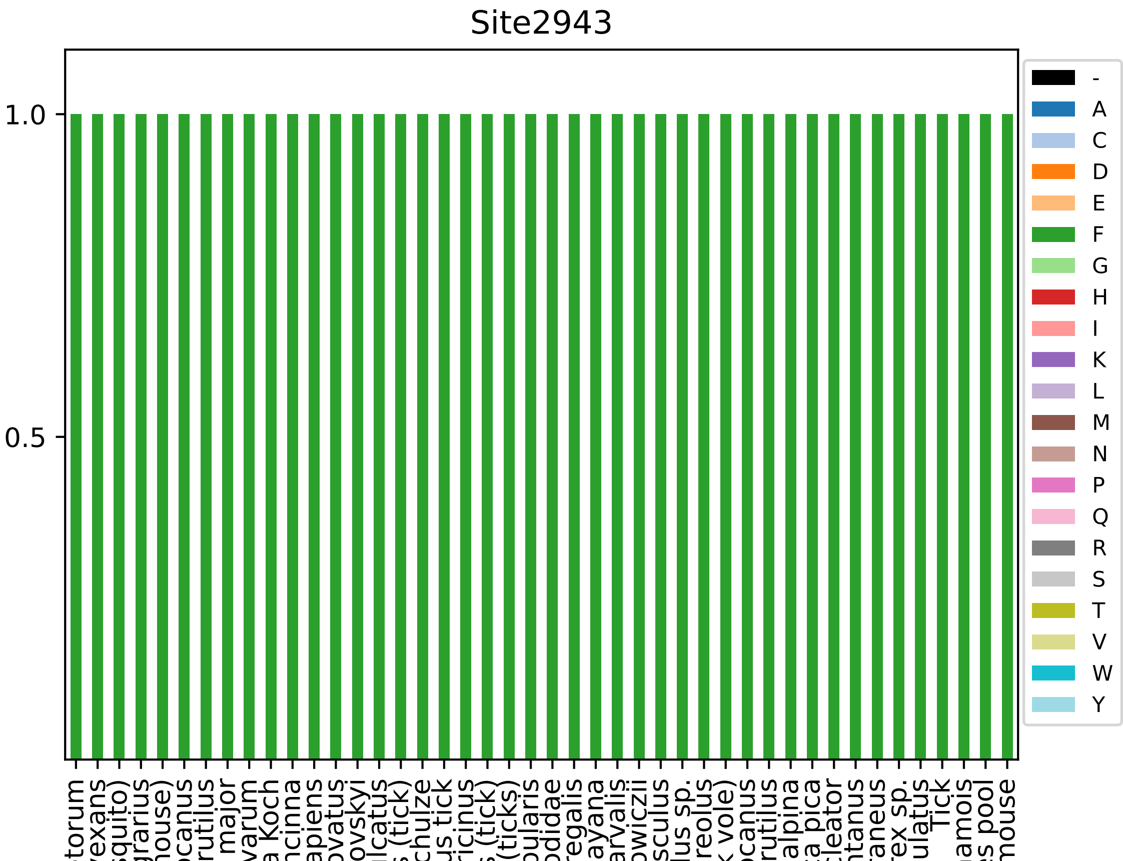

Site2943
1.0
0.5
-
A
C
D
E
F
G
H
I
K
L
M
N
P
Q
R
S
T
V
W
Y

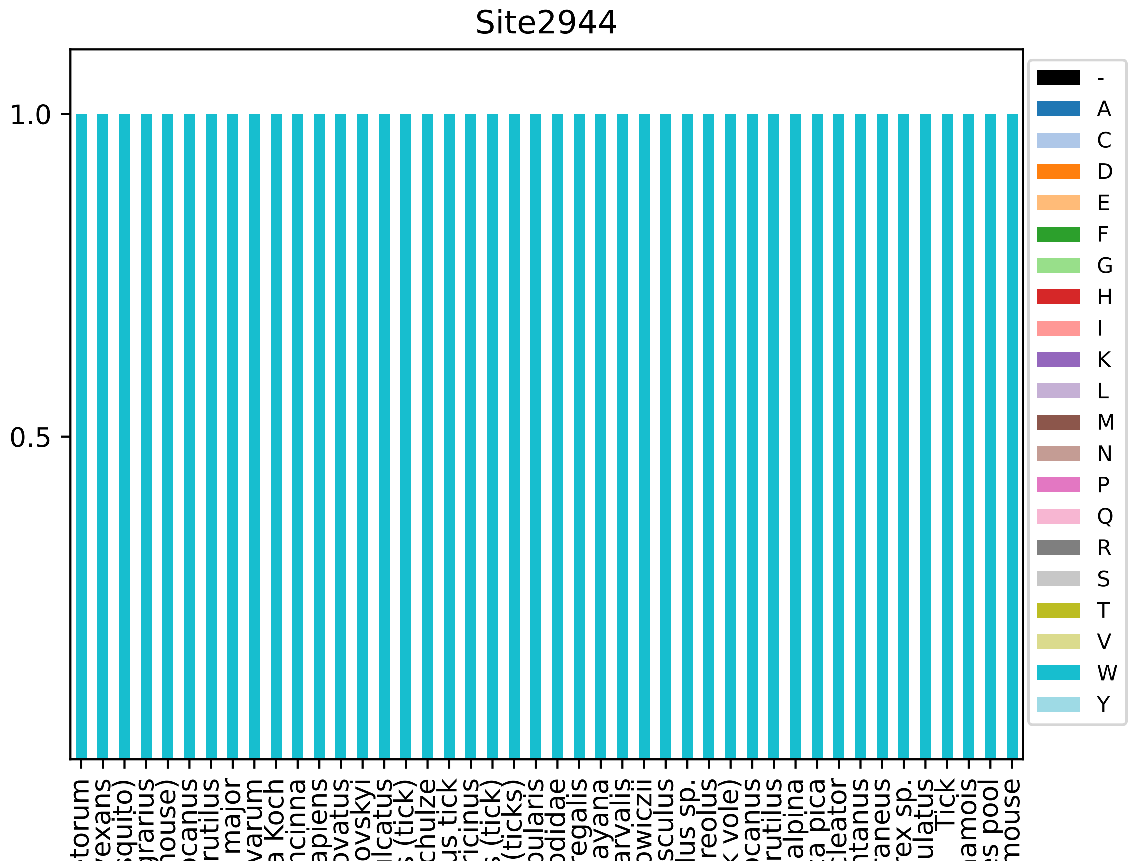

Site2944
1.0
0.5
-
A
C
D
E
F
G
H
I
K
L
M
N
P
Q
R
S
T
V
W
Y

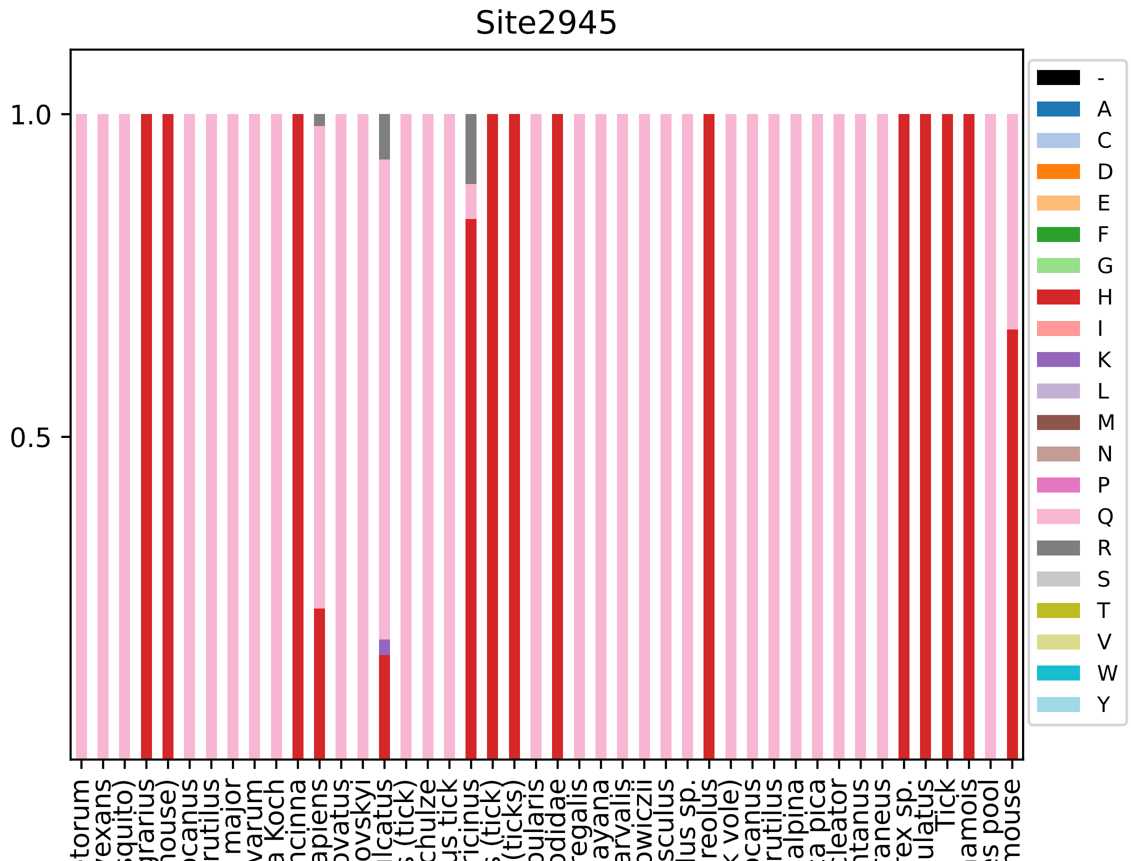

Site2945
1.0
0.5
-
A
C
D
E
F
G
H
I
K
L
M
N
P
Q
R
S
T
V
W
Y

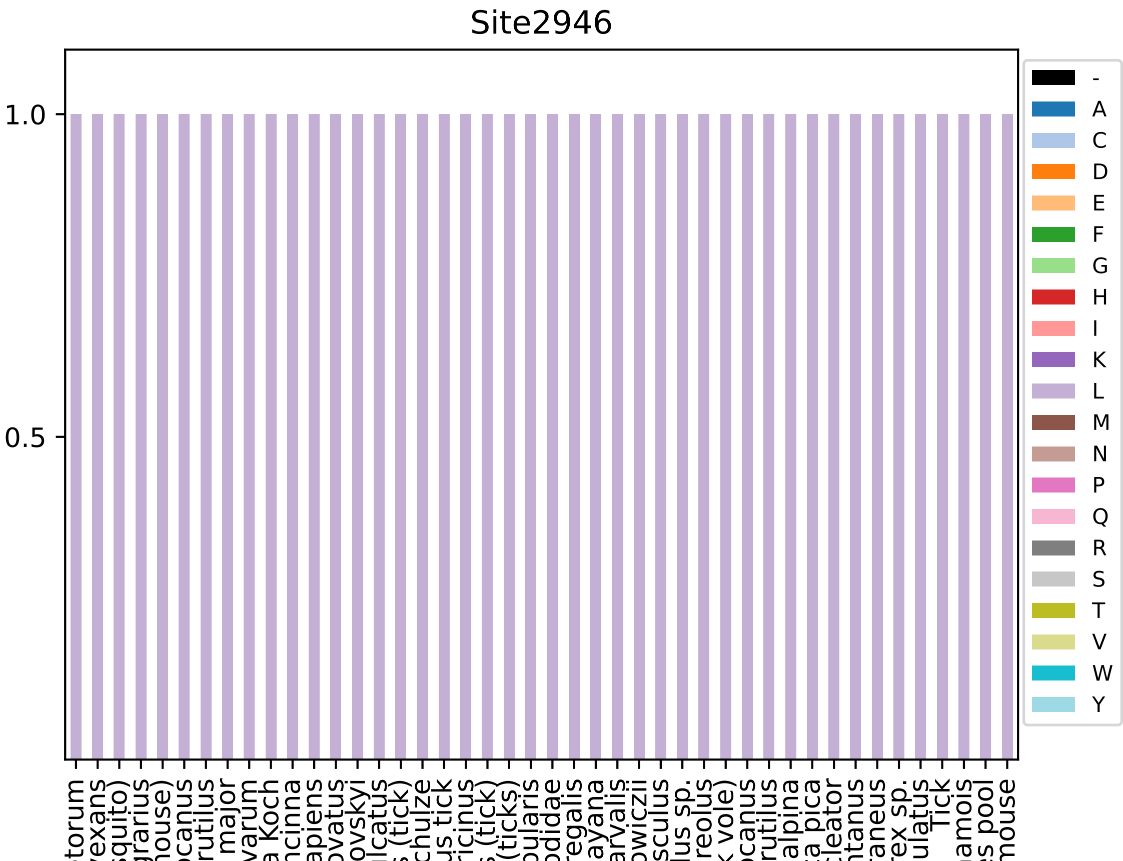
Site2946
1.0
0.5
-
A
C
D
E
F
G
H
I
K
L
M
N
P
Q
R
S
T
V
W
Y
torum
exans
squito)
grarius
nouse)
ocanus
rutilus
major
varum
a Koch
ncinna
apiens
ovatus
ovskyi
ulcatus
(tick)
chulze
us tick
ricinus
(tick)
(ticks)
pularis
podidae
regalis
ayana
arvalis
owiczii
sculus
lus sp.
reolus
k vole)
ocanus
rutilus
alpina
a pica
cleator
ntanus
raneus
rex sp.
ulatus
Tick
amois
s pool
mouse

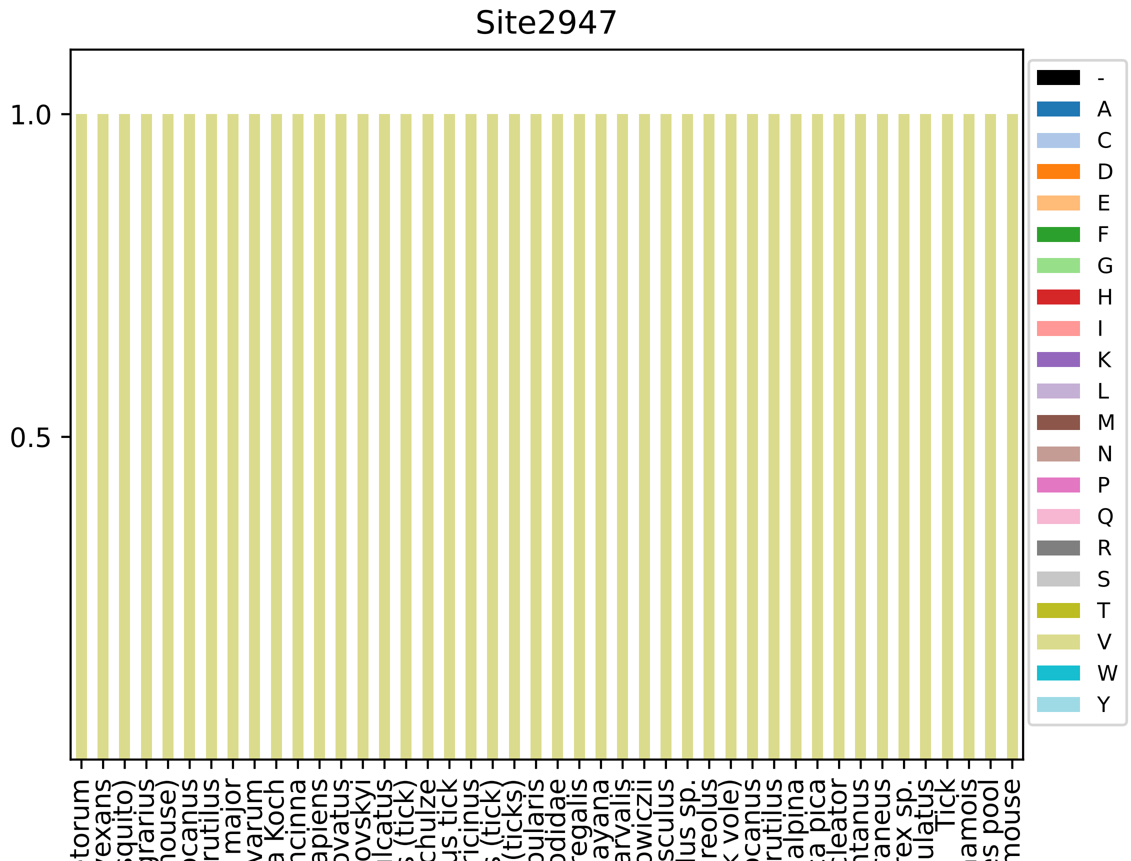
Site2947
1.0
0.5
-
A
C
D
E
F
G
H
I
K
L
M
N
P
Q
R
S
T
V
W
Y

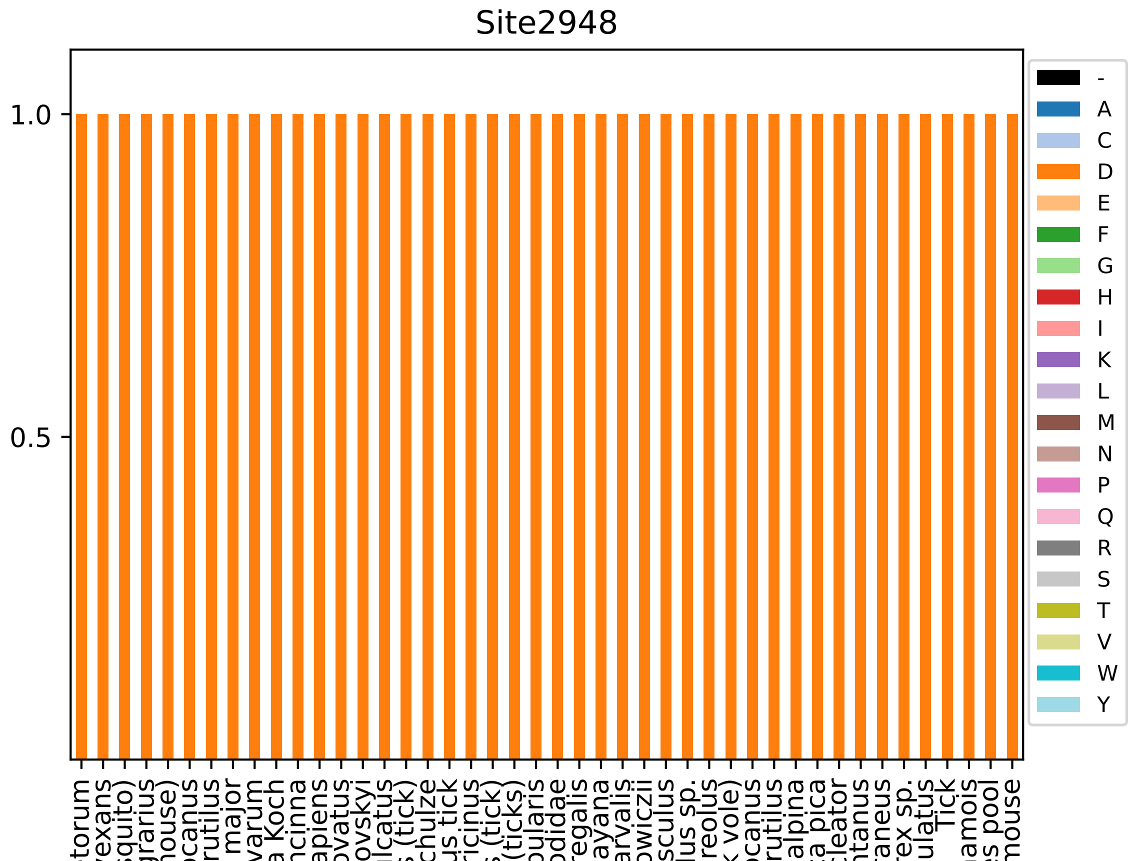
Site2948
1.0
0.5
-
A
C
D
E
F
G
H
I
K
L
M
N
P
Q
R
S
T
V
W
Y

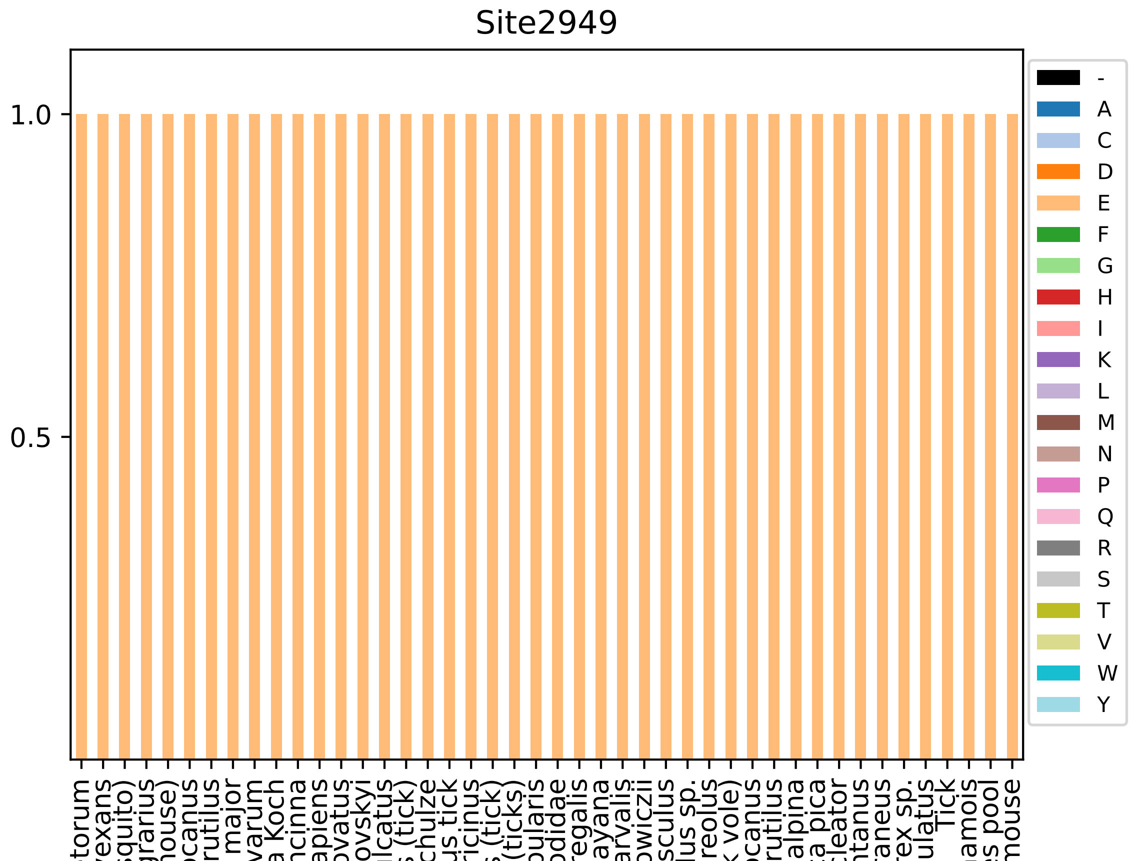
Site2949
1.0
0.5
-
A
C
D
E
F
G
H
I
K
L
M
N
P
Q
R
S
T
V
W
Y
Tick

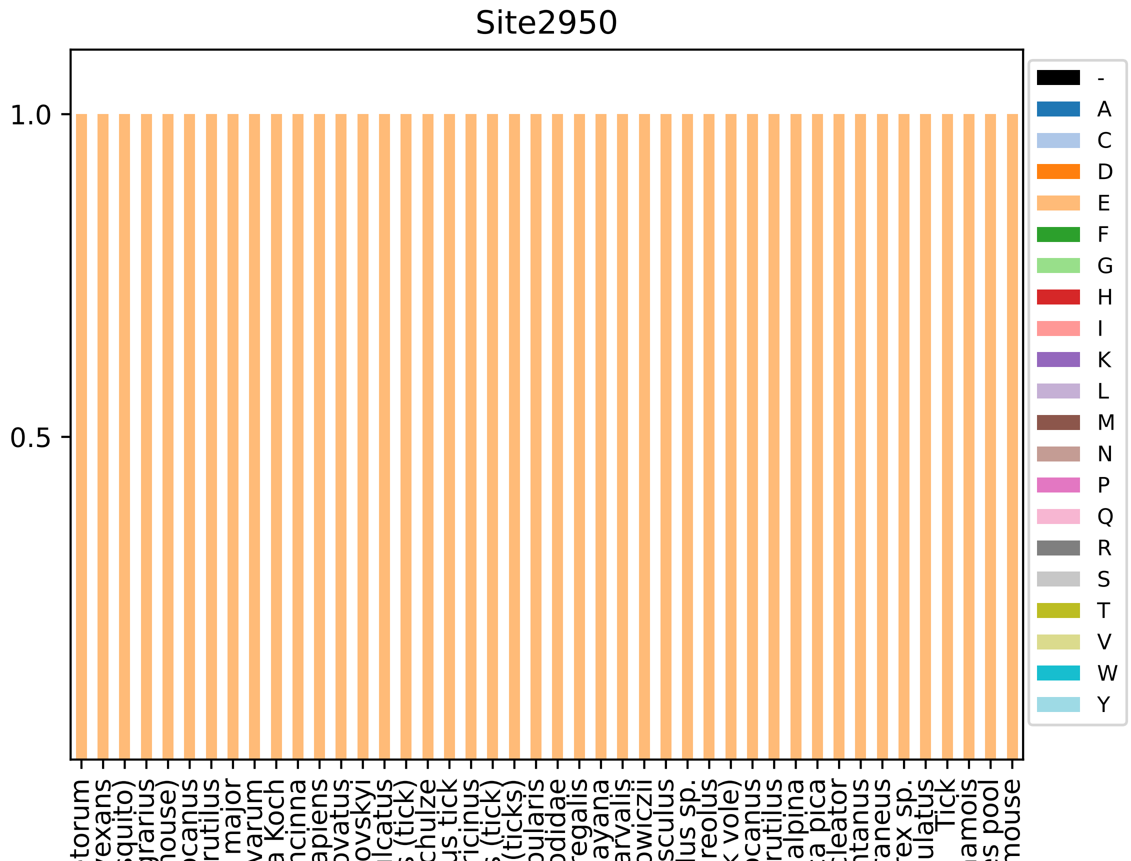
Site2950
1.0
0.5
-
A
C
D
E
F
G
H
I
K
L
M
N
P
Q
R
S
T
V
W
Y

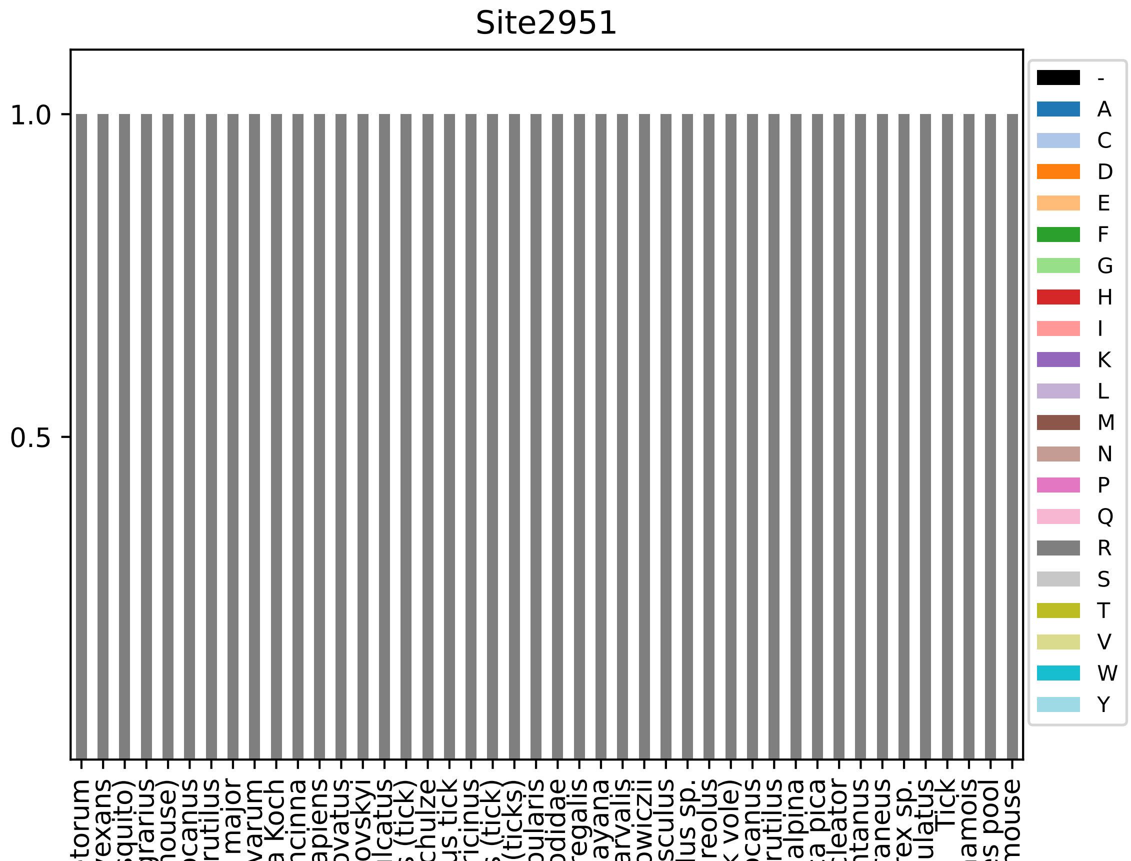
Site2951
1.0
0.5
-
A
C
D
E
F
G
H
I
K
L
M
N
P
Q
R
S
T
V
W
Y

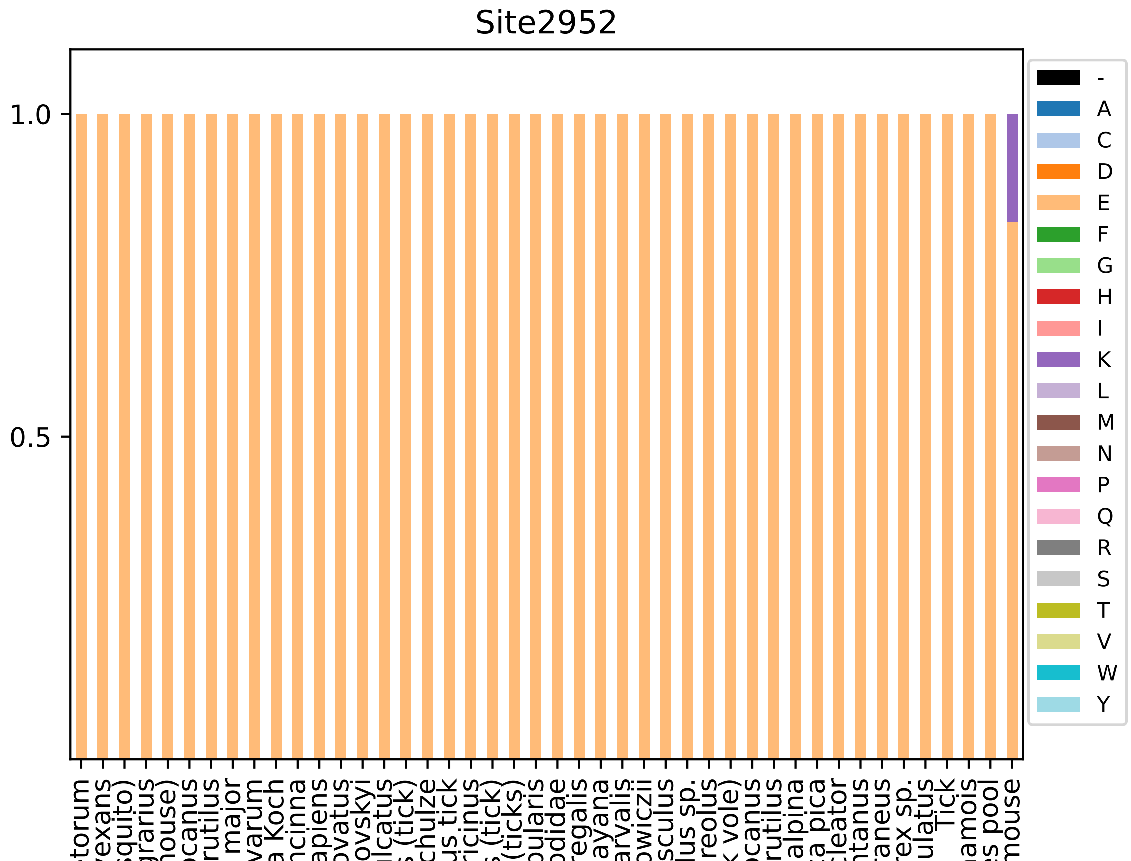
Site2952
1.0
0.5
-
A
C
D
E
F
G
H
I
K
L
M
N
P
Q
R
S
T
V
W
Y

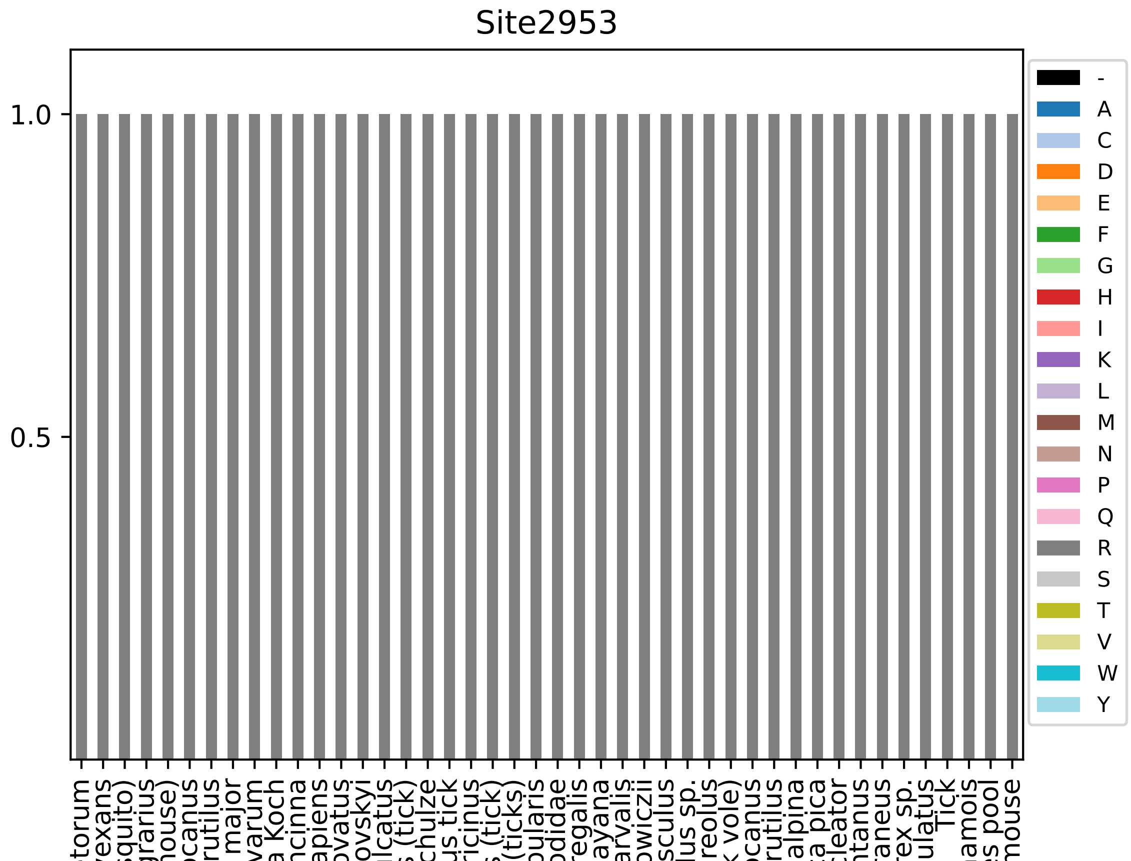

Site2953
1.0
0.5
-
A
C
D
E
F
G
H
I
K
L
M
N
P
Q
R
S
T
V
W
Y

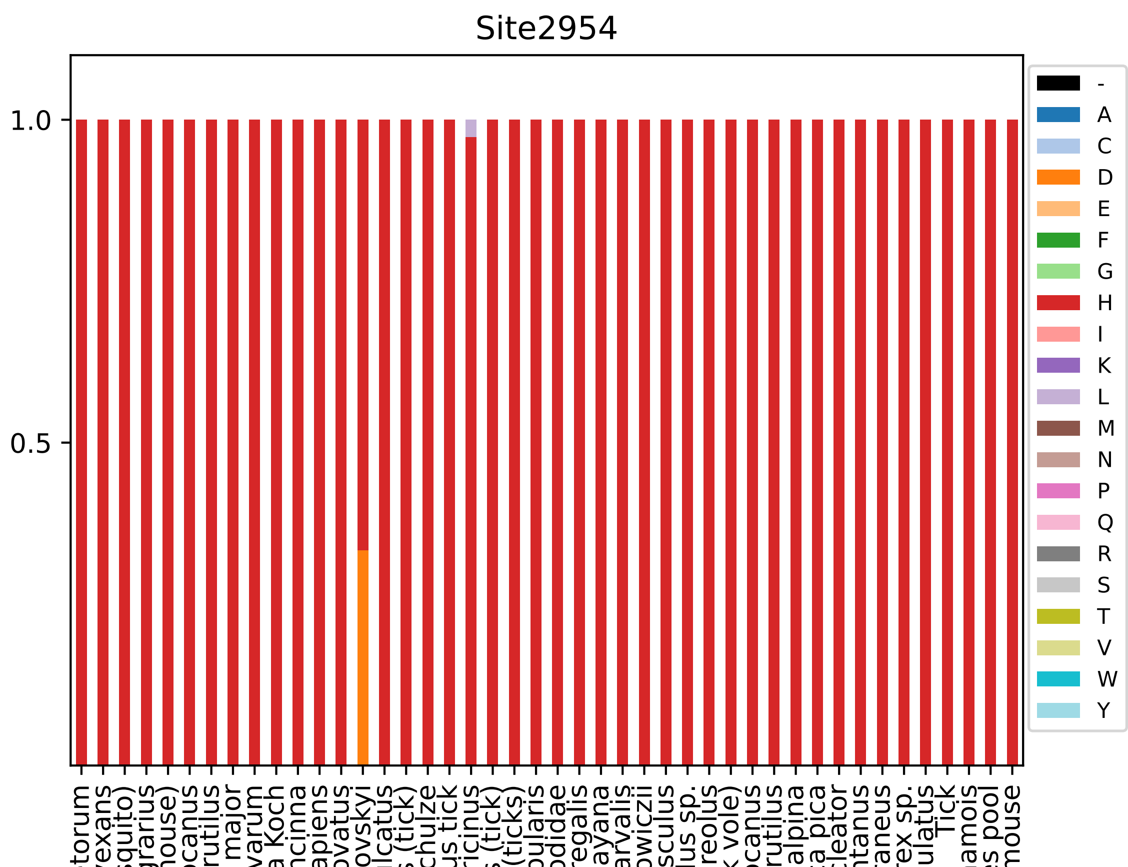
Site2954
1.0
0.5
-
A
C
D
E
F
G
H
I
K
L
M
N
P
Q
R
S
T
V
W
Y

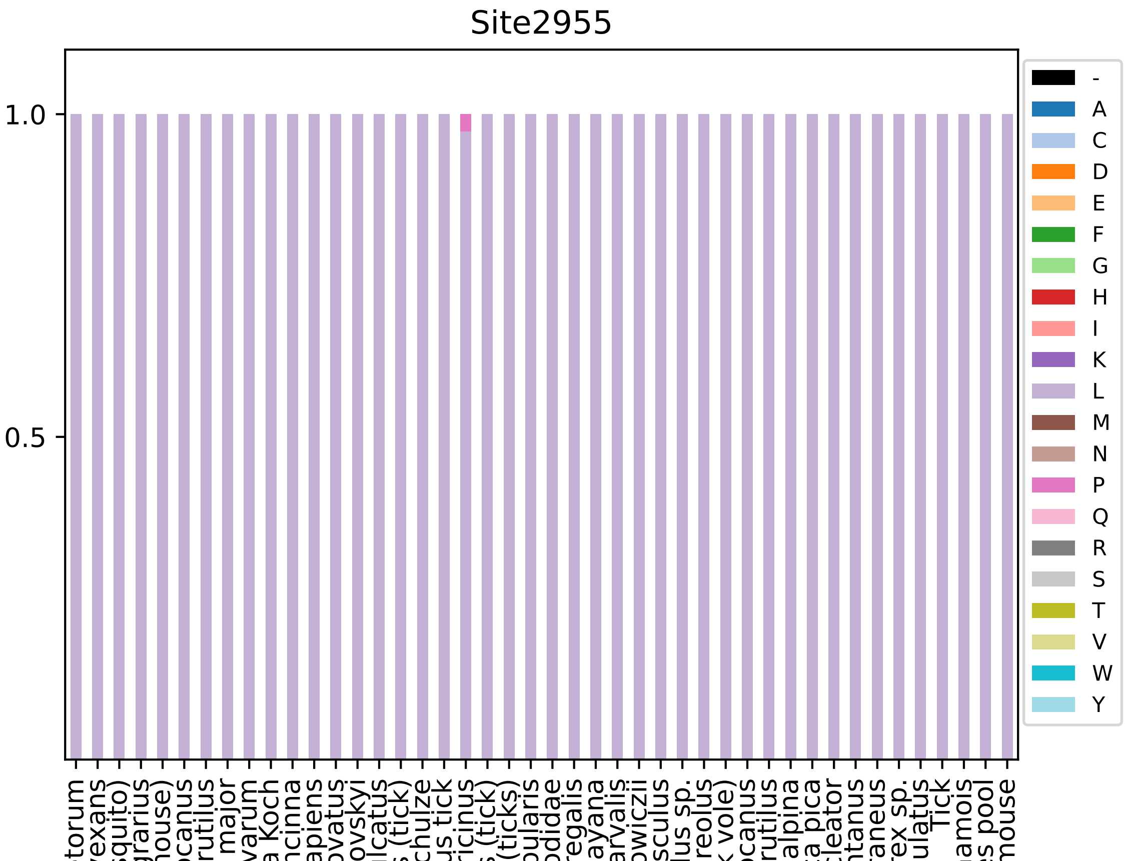
Site2955
1.0
0.5
-
A
C
D
E
F
G
H
I
K
L
M
N
P
Q
R
S
T
V
W
Y

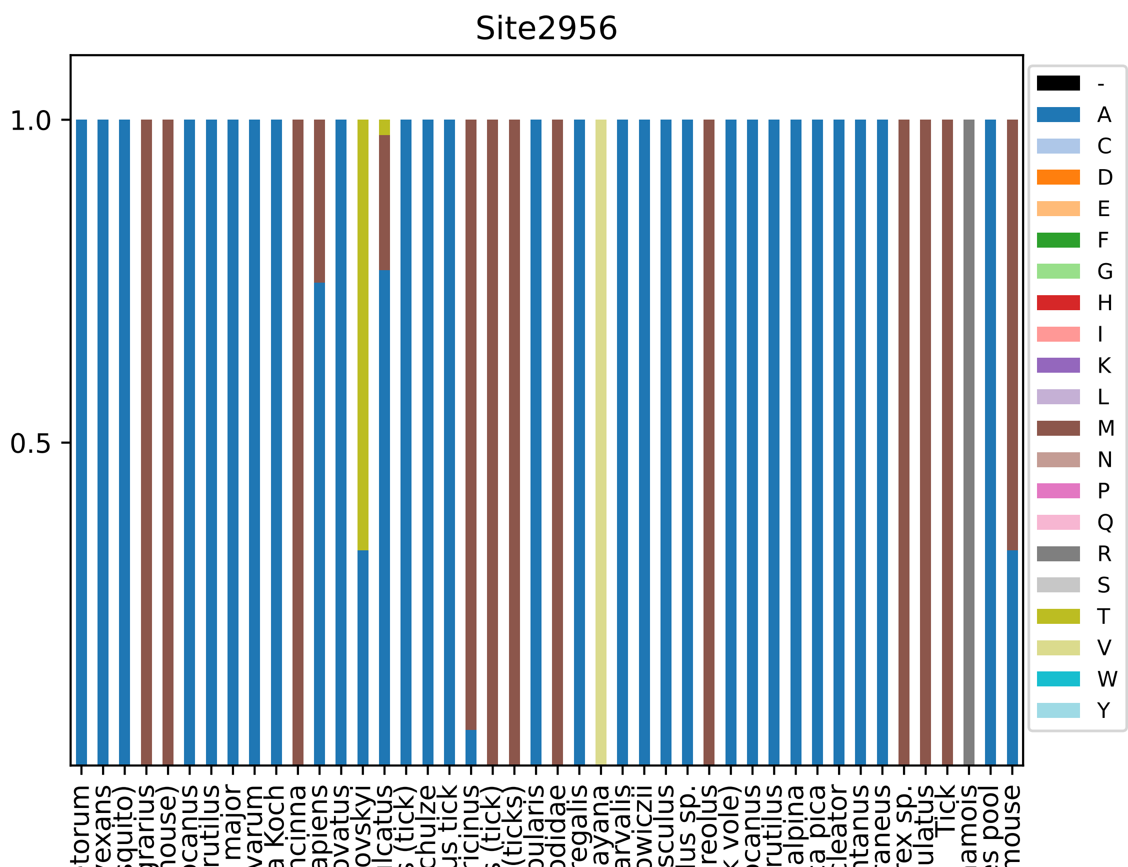
Site2956
1.0
0.5
-
A
C
D
E
F
G
H
I
K
L
M
N
P
Q
R
S
T
V
W
Y

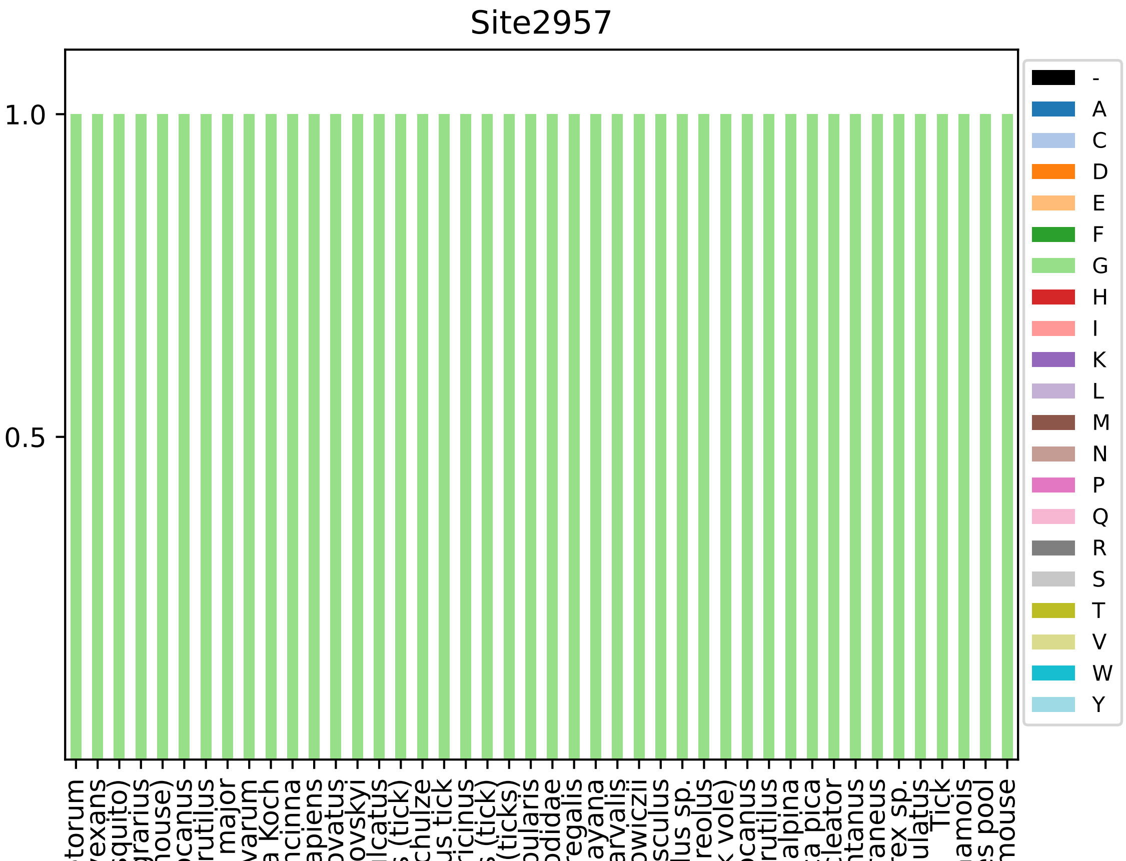
Site2957
1.0
0.5
-
A
C
D
E
F
G
H
I
K
L
M
N
P
Q
R
S
T
V
W
Y

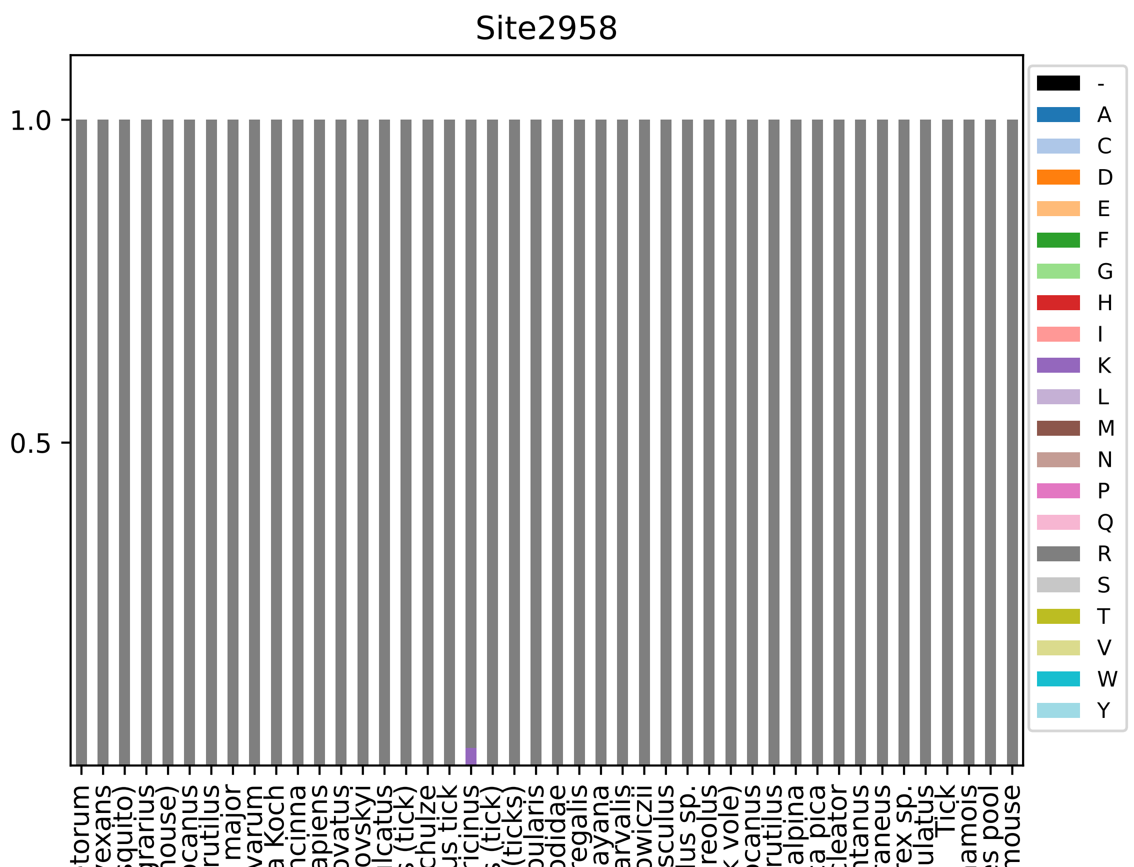
Site2958
1.0
0.5
-
A
C
D
E
F
G
H
I
K
L
M
N
P
Q
R
S
T
V
W
Y

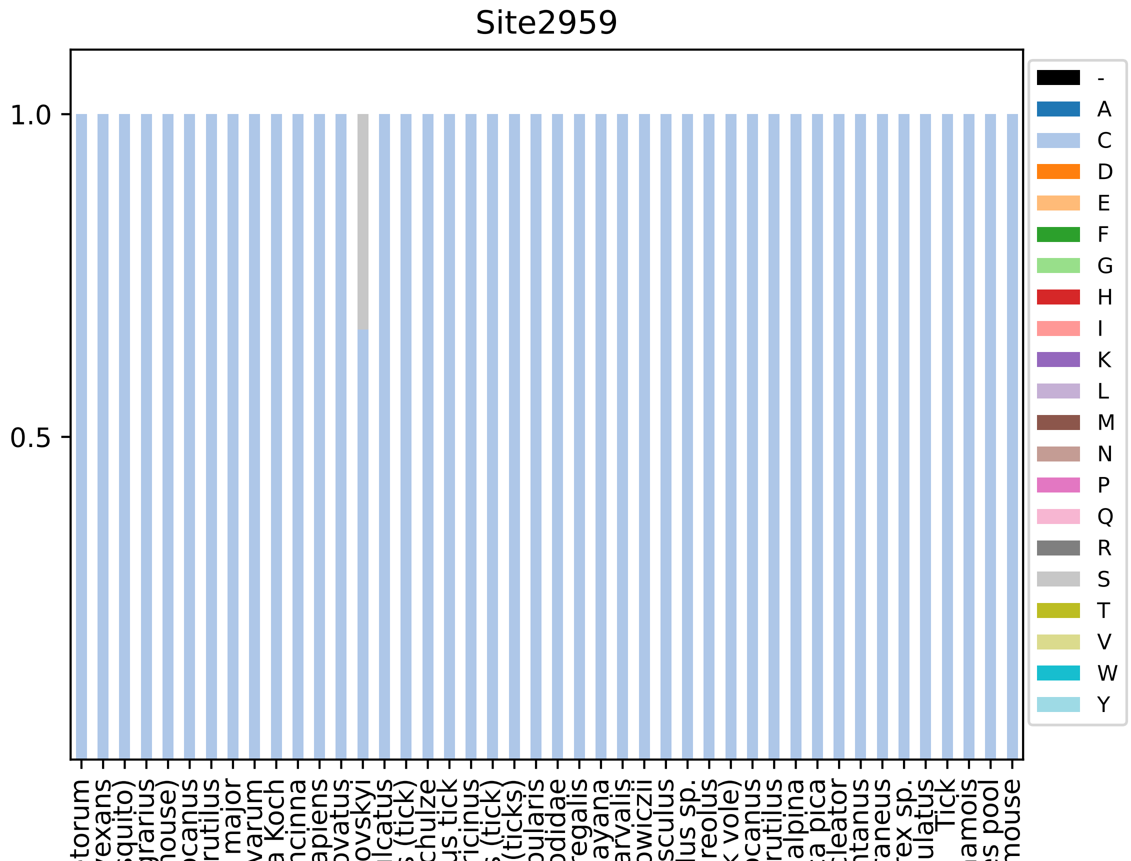

Site2959
1.0
0.5
-
A
C
D
E
F
G
H
I
K
L
M
N
P
Q
R
S
T
V
W
Y

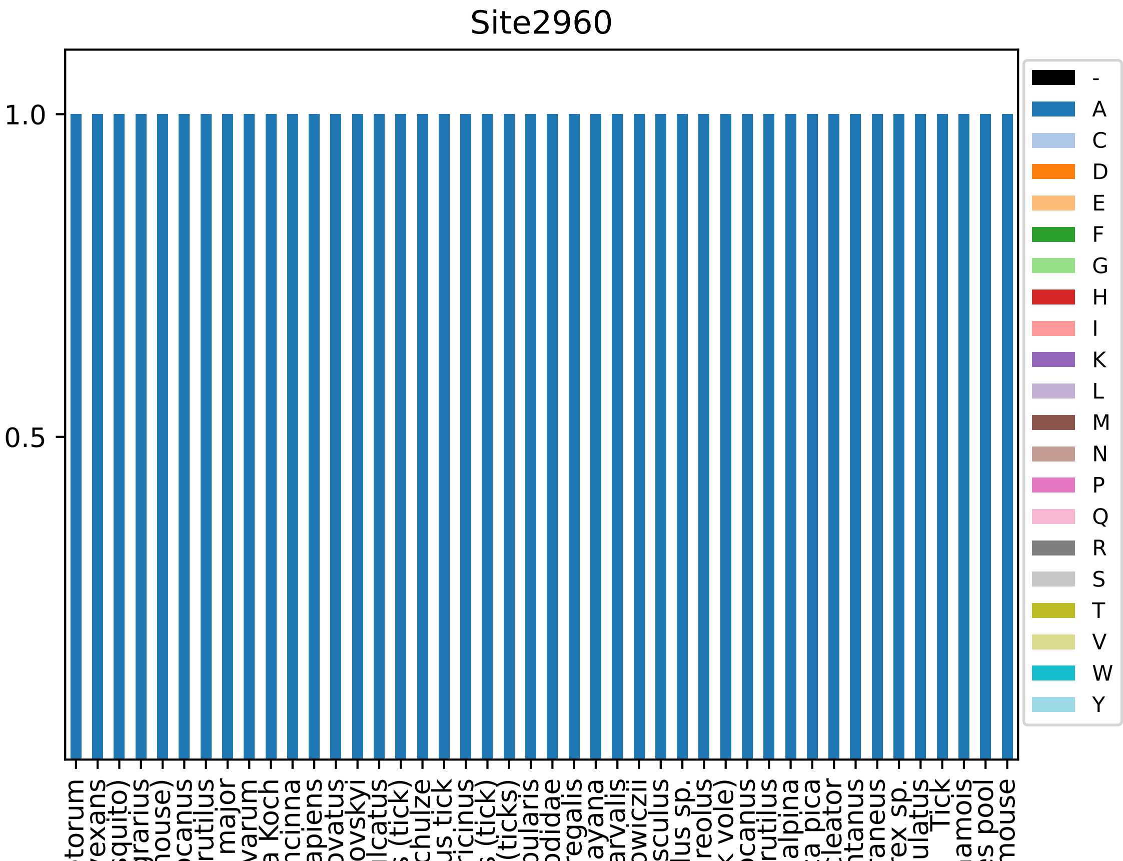
Site2960
1.0
0.5
-
A
C
D
E
F
G
H
I
K
L
M
N
P
Q
R
S
T
V
W
Y

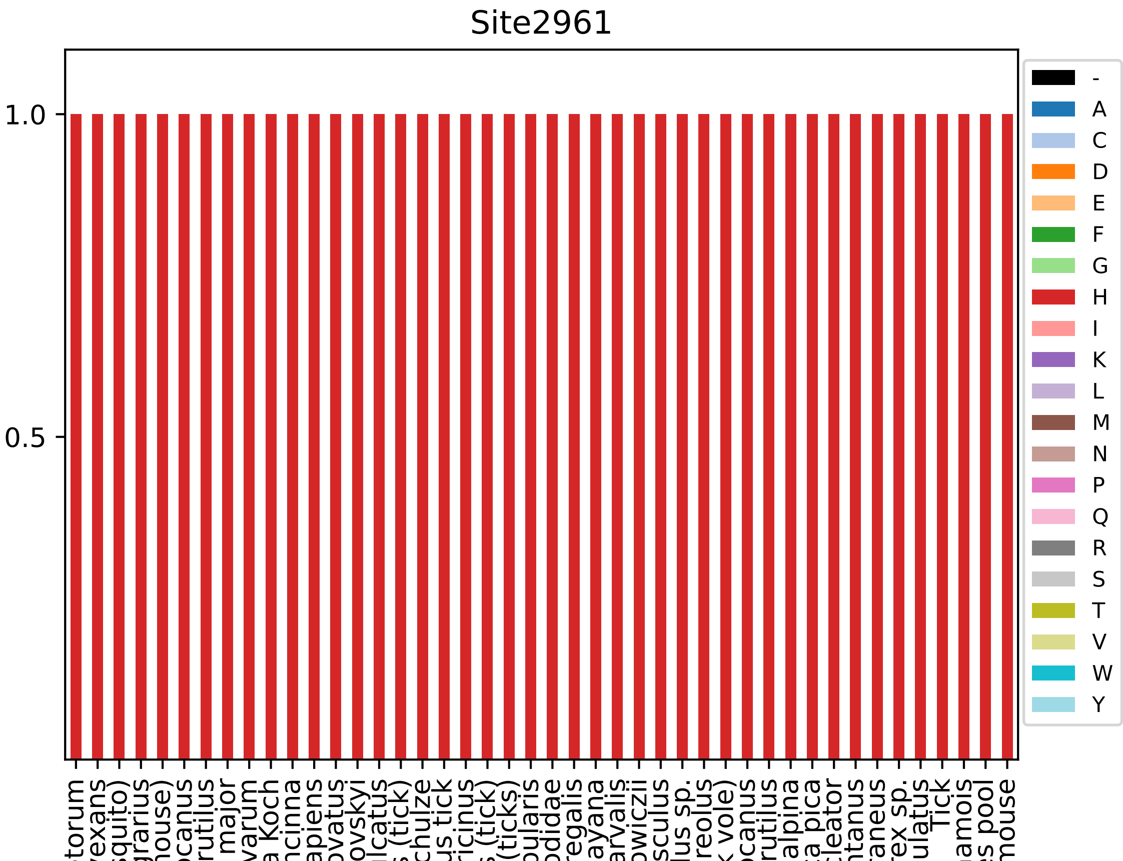
Site2961
1.0
0.5
-
A
C
D
E
F
G
H
I
K
L
M
N
P
Q
R
S
T
V
W
Y

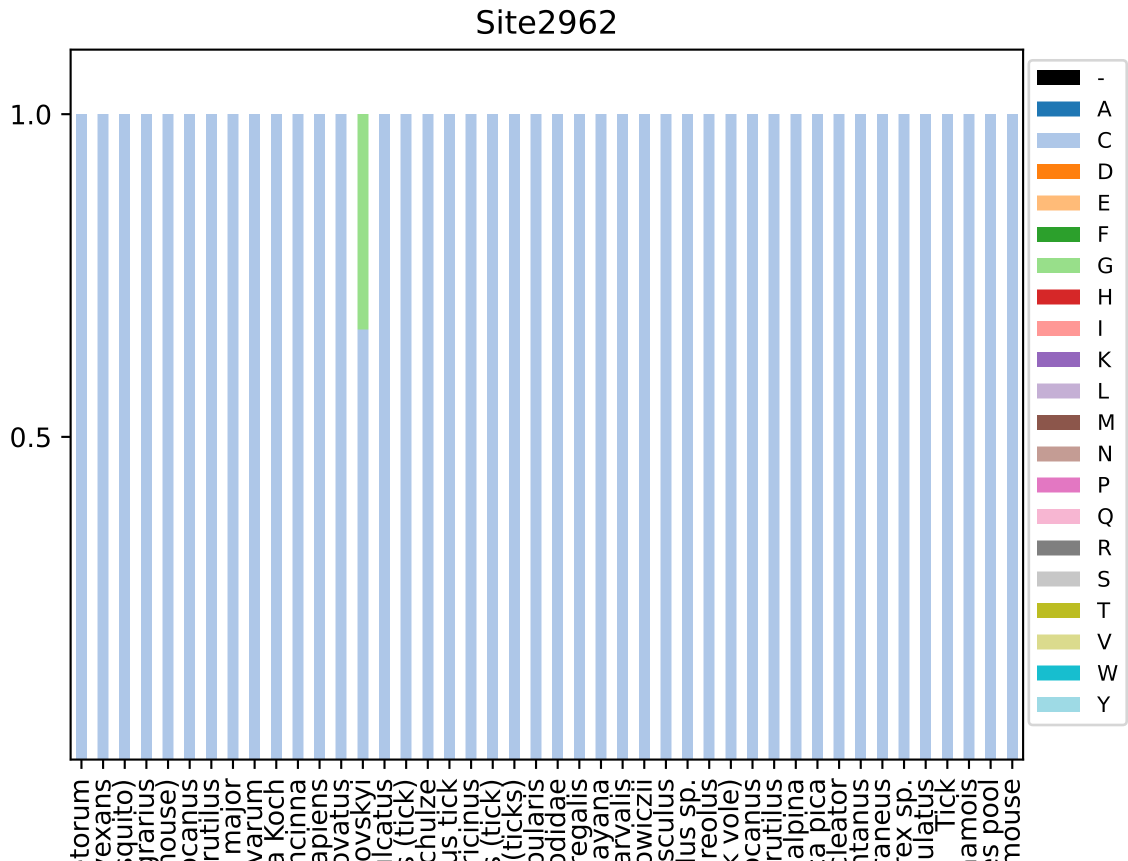
Site2962
1.0
0.5
-
A
C
D
E
F
G
H
I
K
L
M
N
P
Q
R
S
T
V
W
Y

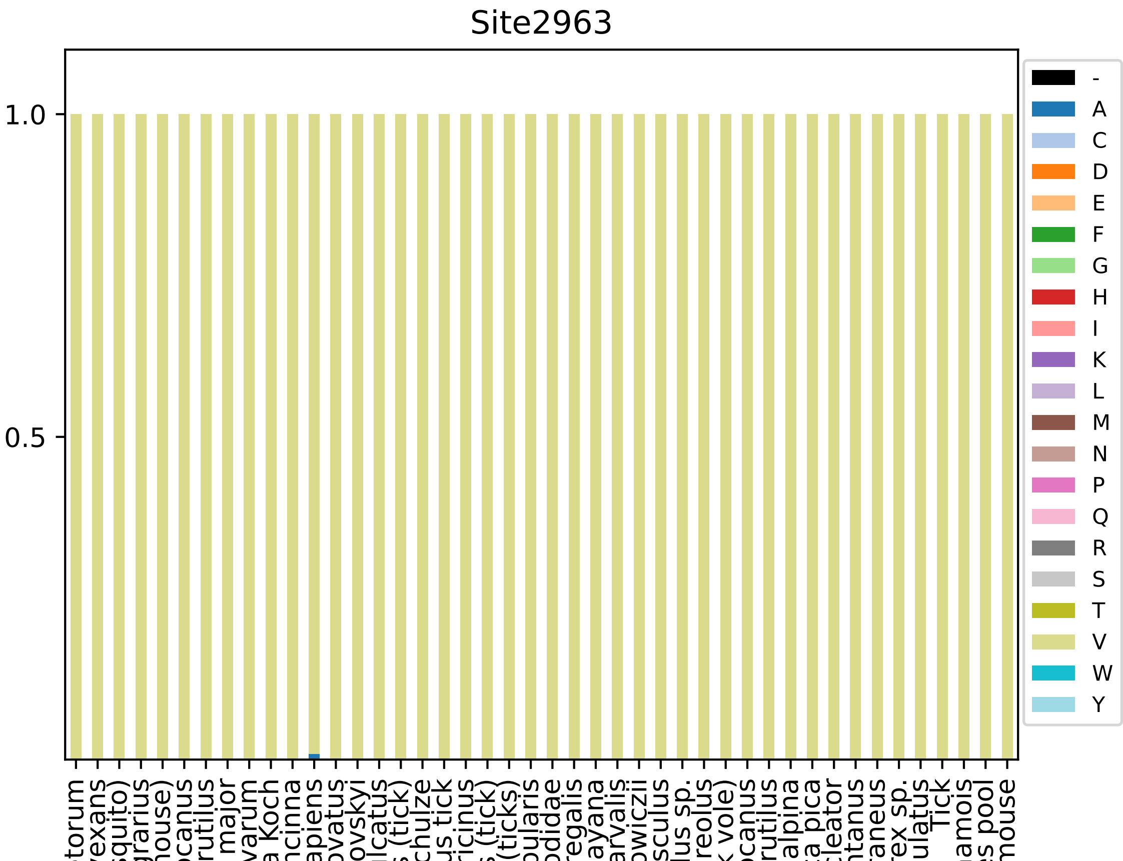
Site2963
1.0
0.5
-
A
C
D
E
F
G
H
I
K
L
M
N
P
Q
R
S
T
V
W
Y

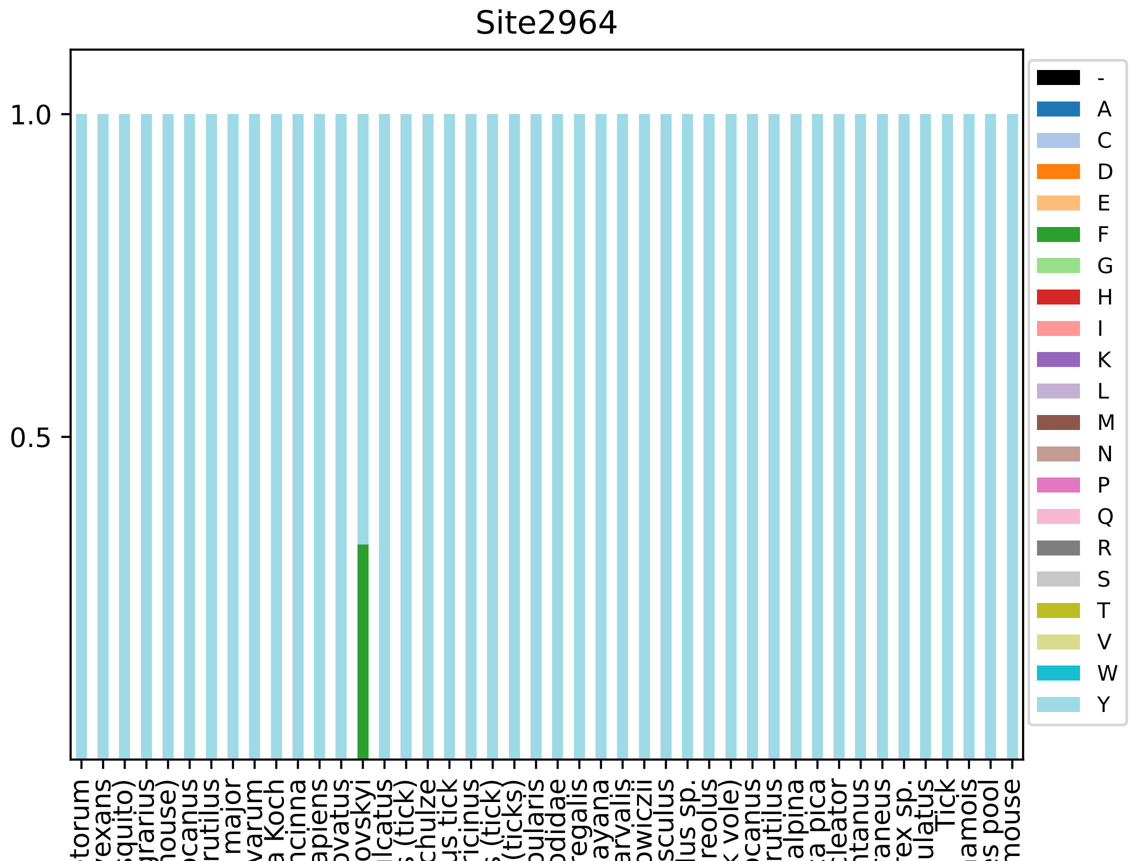

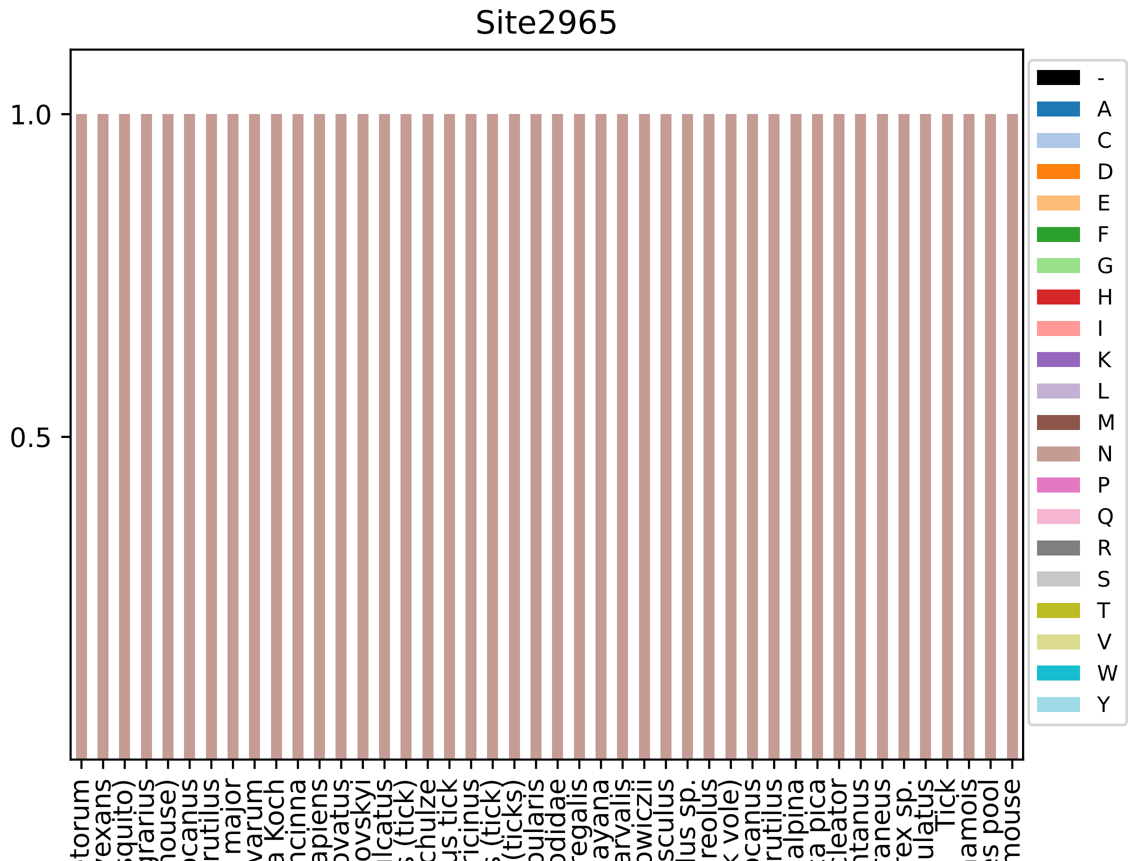
Site2965
1.0
0.5
-
A
C
D
E
F
G
H
I
K
L
M
N
P
Q
R
S
T
V
W
Y

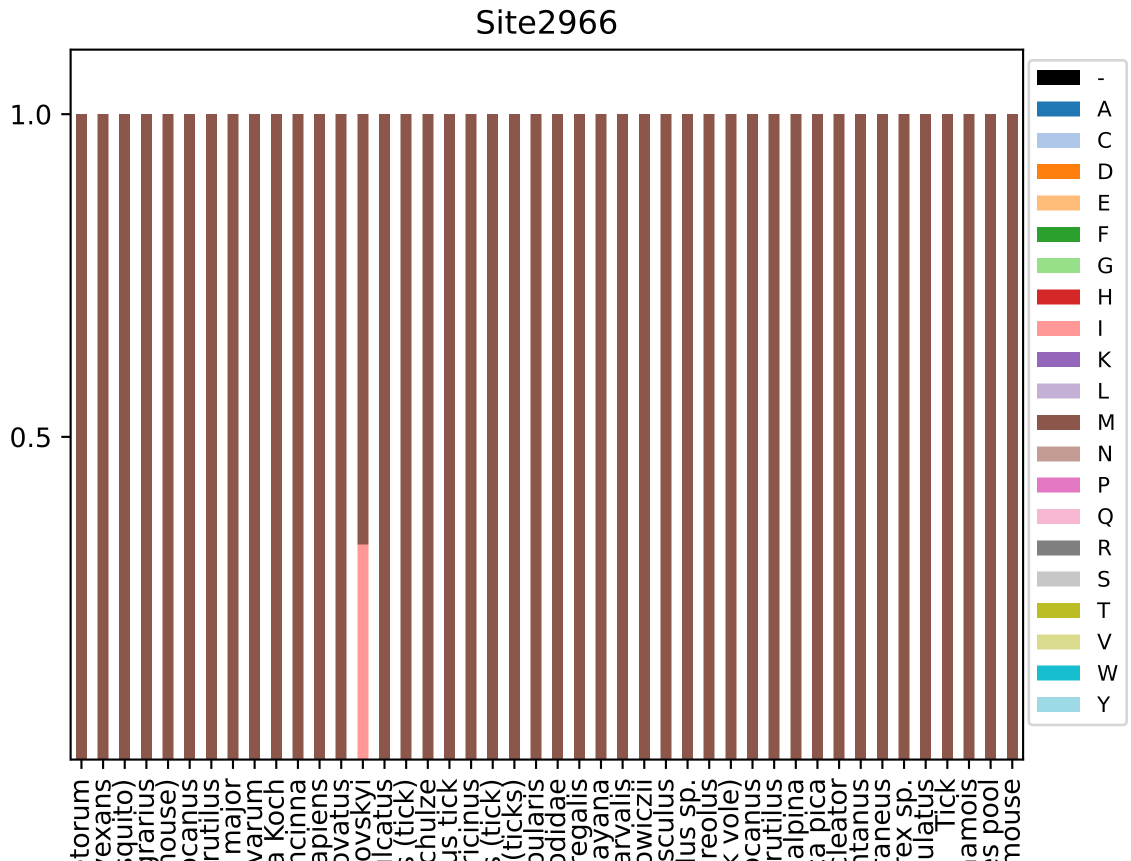
Site2966
1.0
0.5
-
A
C
D
E
F
G
H
I
K
L
M
N
P
Q
R
S
T
V
W
Y

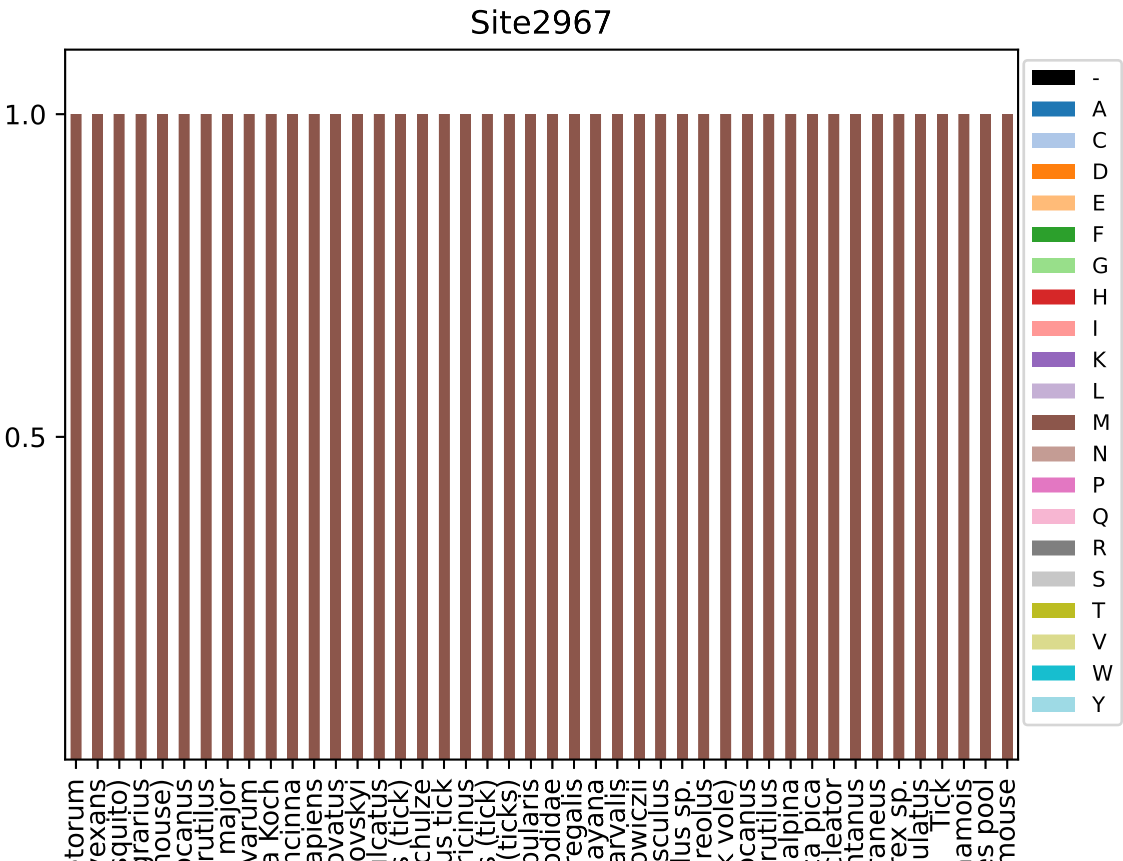
Site2967
1.0
0.5
-
A
C
D
E
F
G
H
I
K
L
M
N
P
Q
R
S
T
V
W
Y

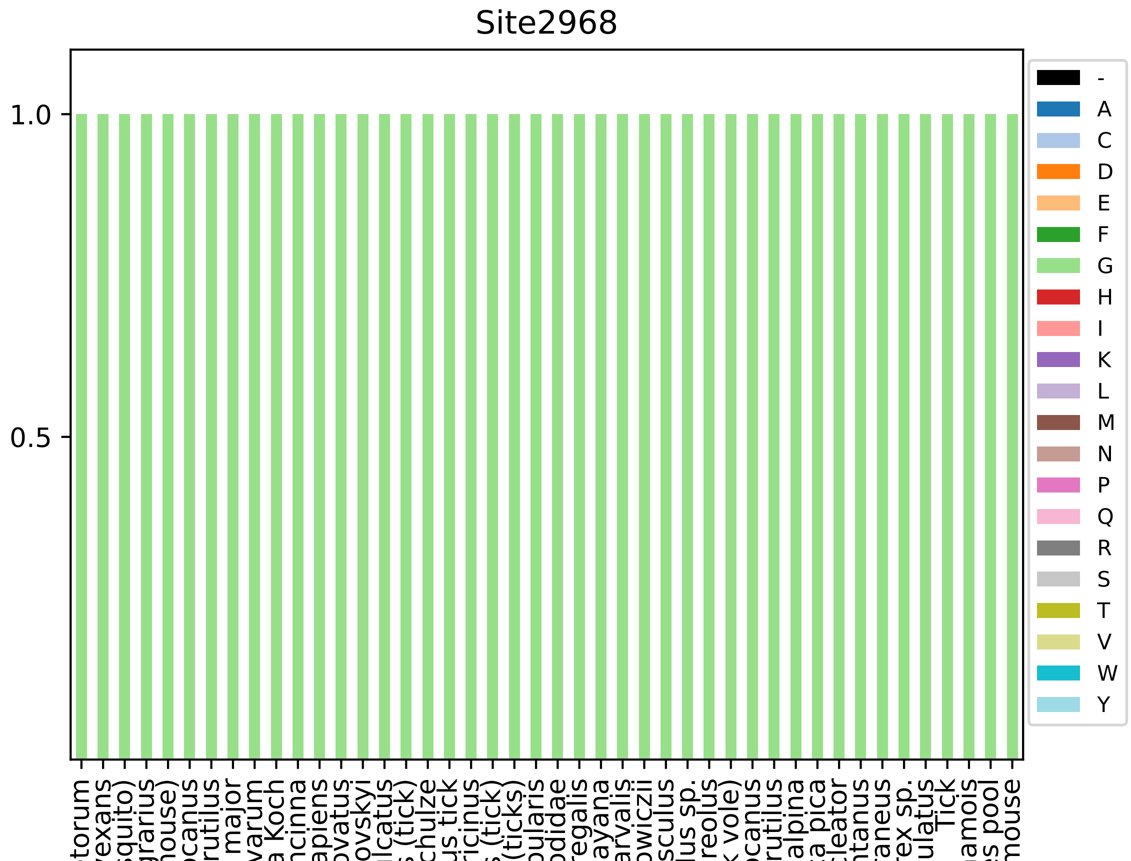
Site2968
1.0
0.5
-
A
C
D
E
F
G
H
I
K
L
M
N
P
Q
R
S
T
V
W
Y

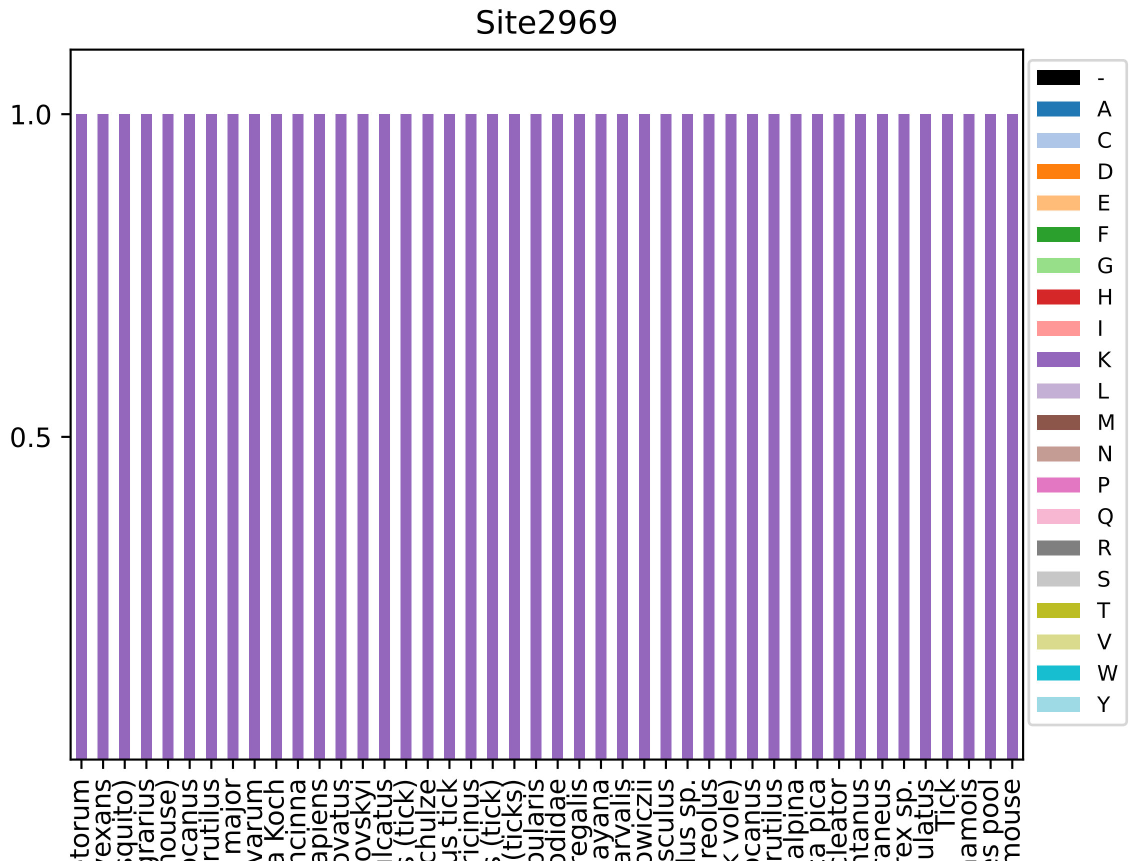
Site2969
1.0
0.5
-
A
C
D
E
F
G
H
I
K
L
M
N
P
Q
R
S
T
V
W
Y
Tick

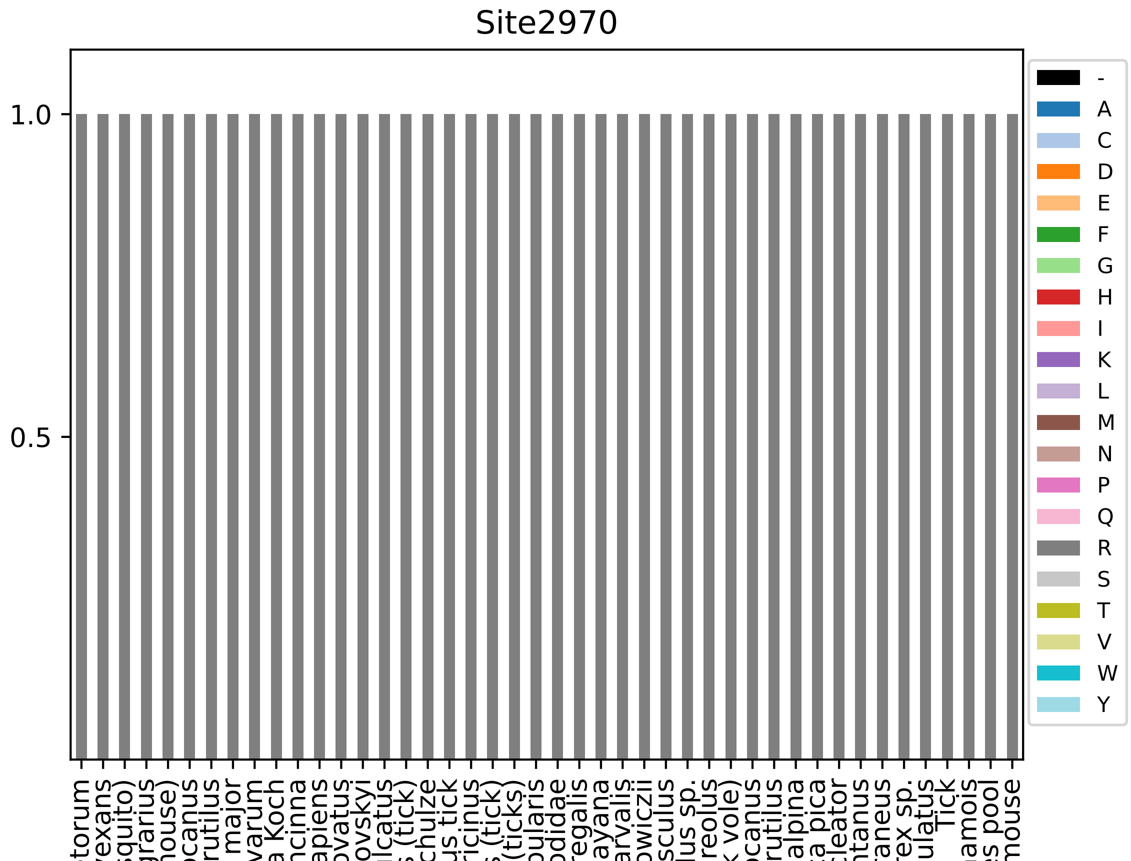
Site2970
1.0
0.5
-
A
C
D
E
F
G
H
I
K
L
M
N
P
Q
R
S
T
V
W
Y

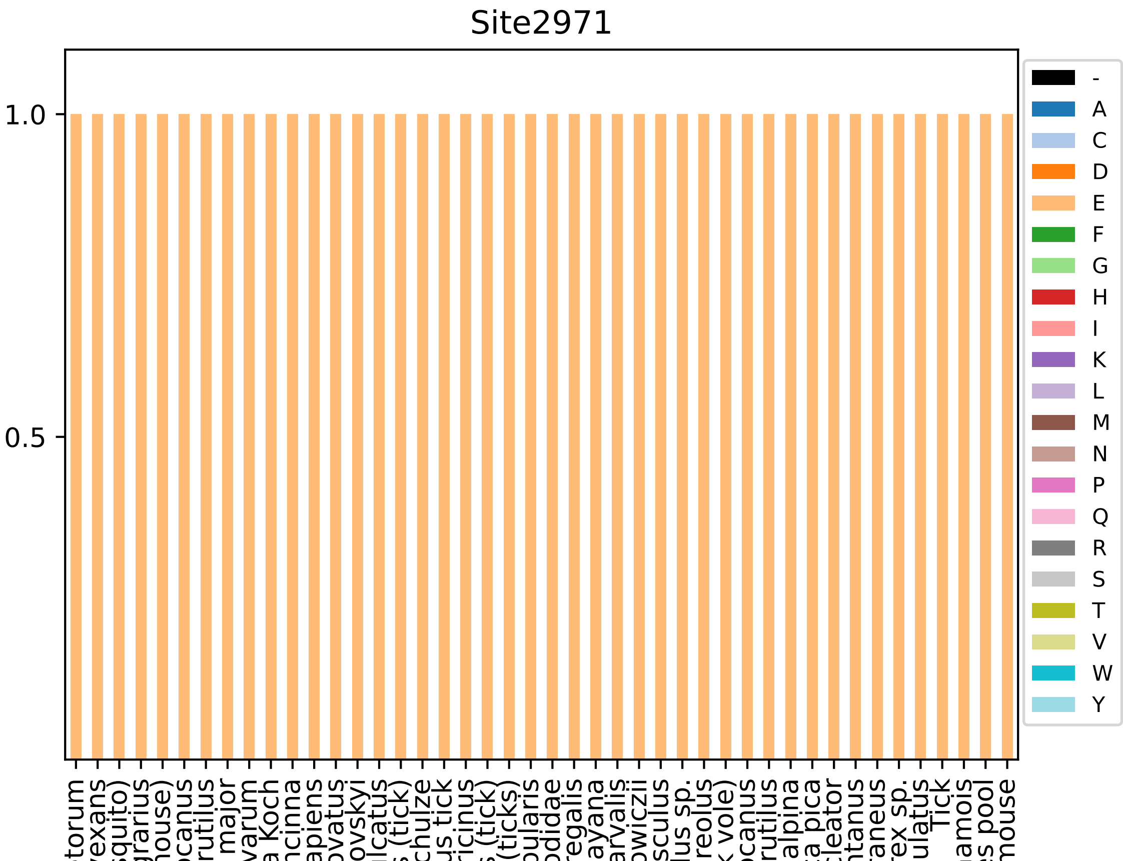

Site2971
1.0
0.5
-
A
C
D
E
F
G
H
I
K
L
M
N
P
Q
R
S
T
V
W
Y
Tick

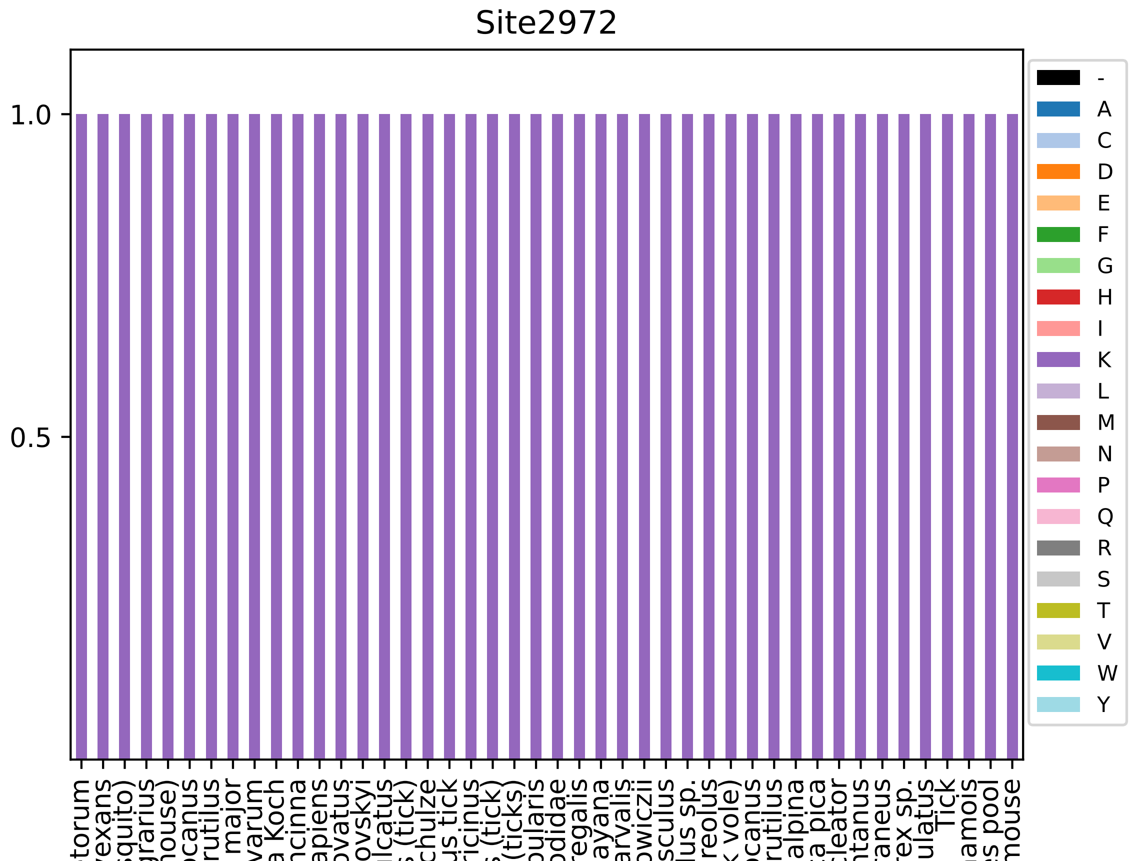
Site2972
1.0
0.5
-
A
C
D
E
F
G
H
I
K
L
M
N
P
Q
R
S
T
V
W
Y

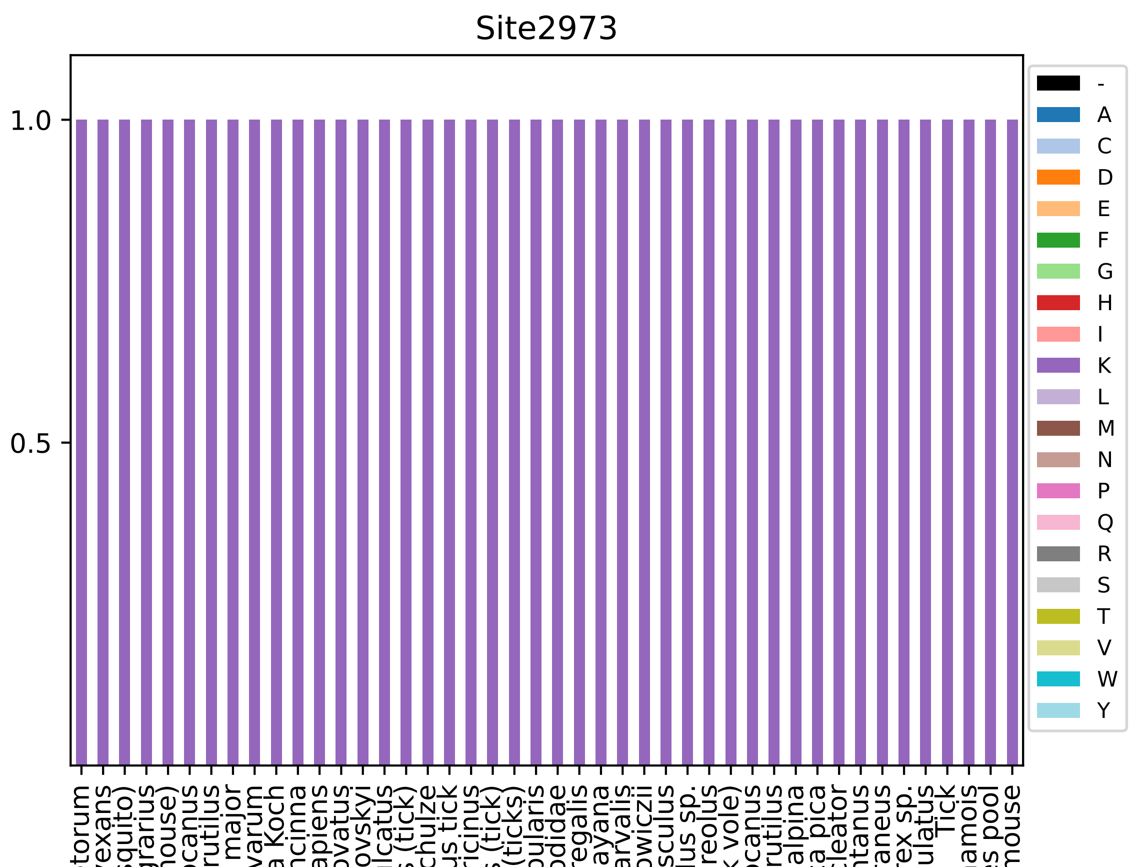
Site2973
1.0
0.5
-
A
C
D
E
F
G
H
I
K
L
M
N
P
Q
R
S
T
V
W
Y

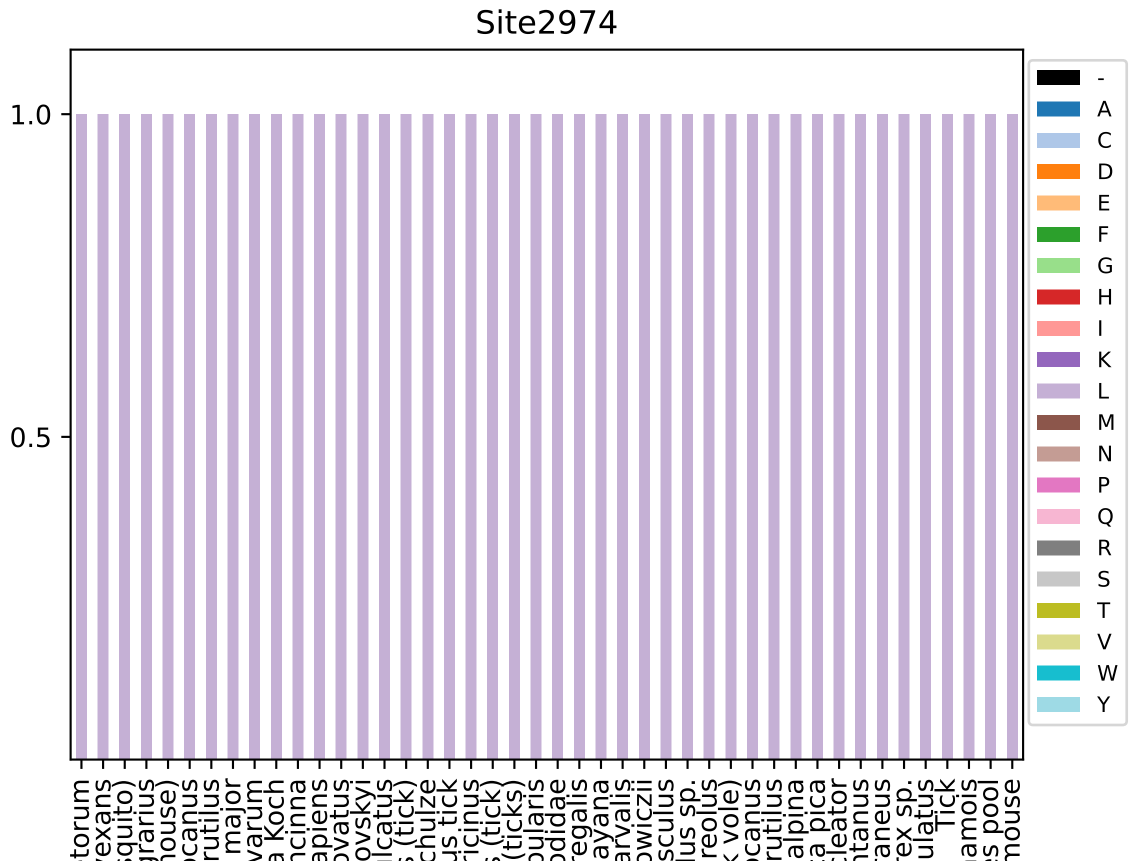
Site2974
1.0
0.5
-
A
C
D
E
F
G
H
I
K
L
M
N
P
Q
R
S
T
V
W
Y

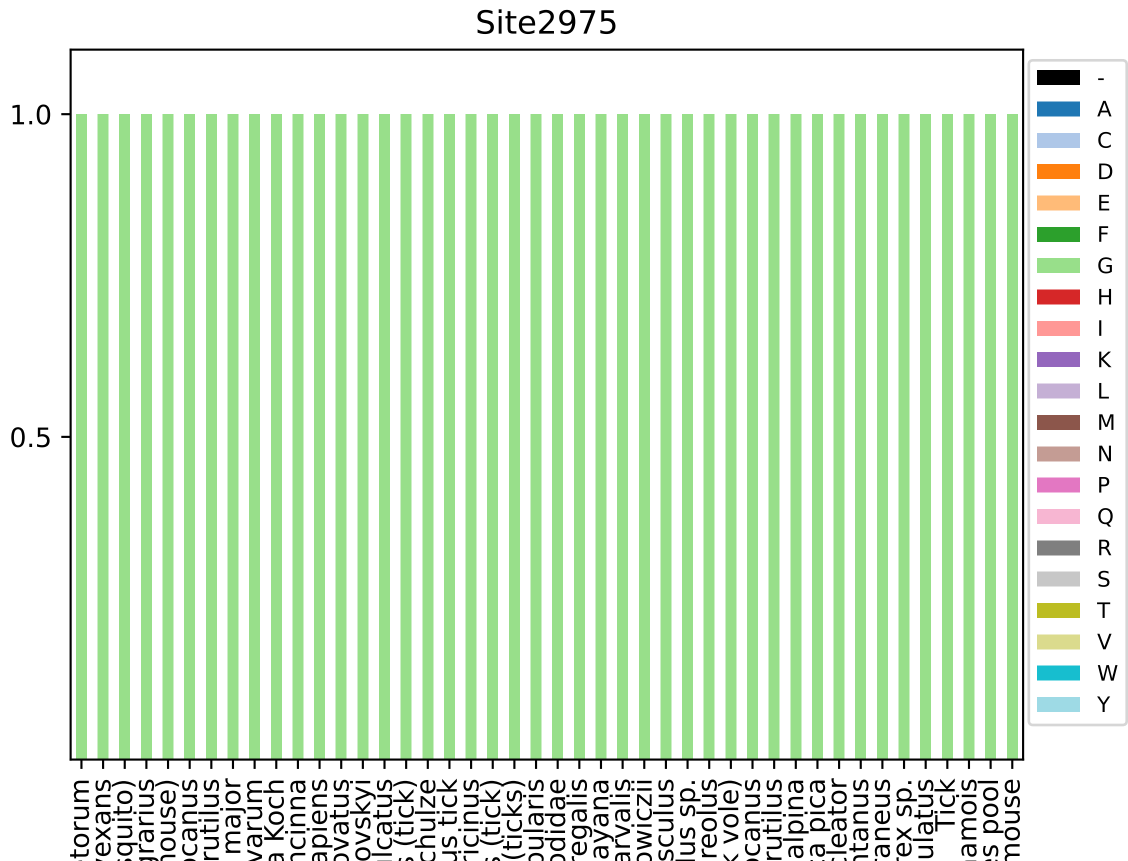
Site2975
1.0
0.5
-
A
C
D
E
F
G
H
I
K
L
M
N
P
Q
R
S
T
V
W
Y

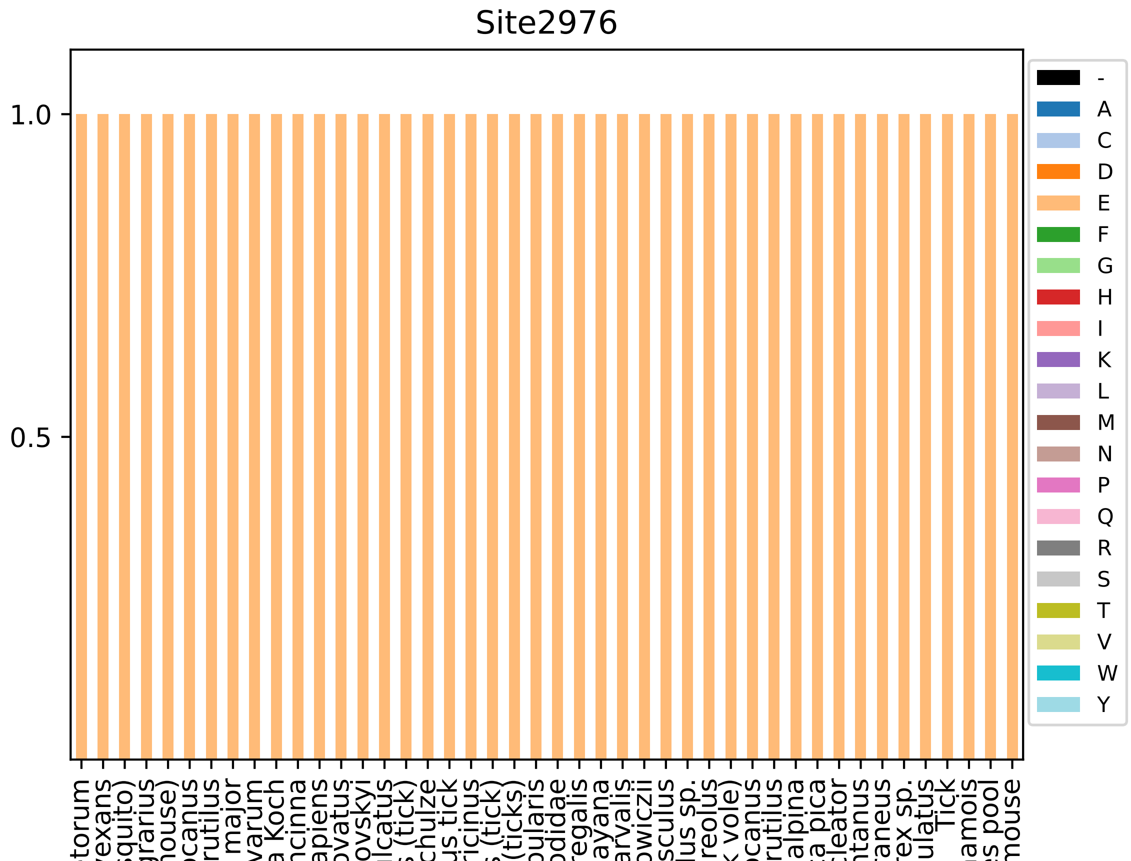
Site2976
1.0
0.5
-
A
C
D
E
F
G
H
I
K
L
M
N
P
Q
R
S
T
V
W
Y

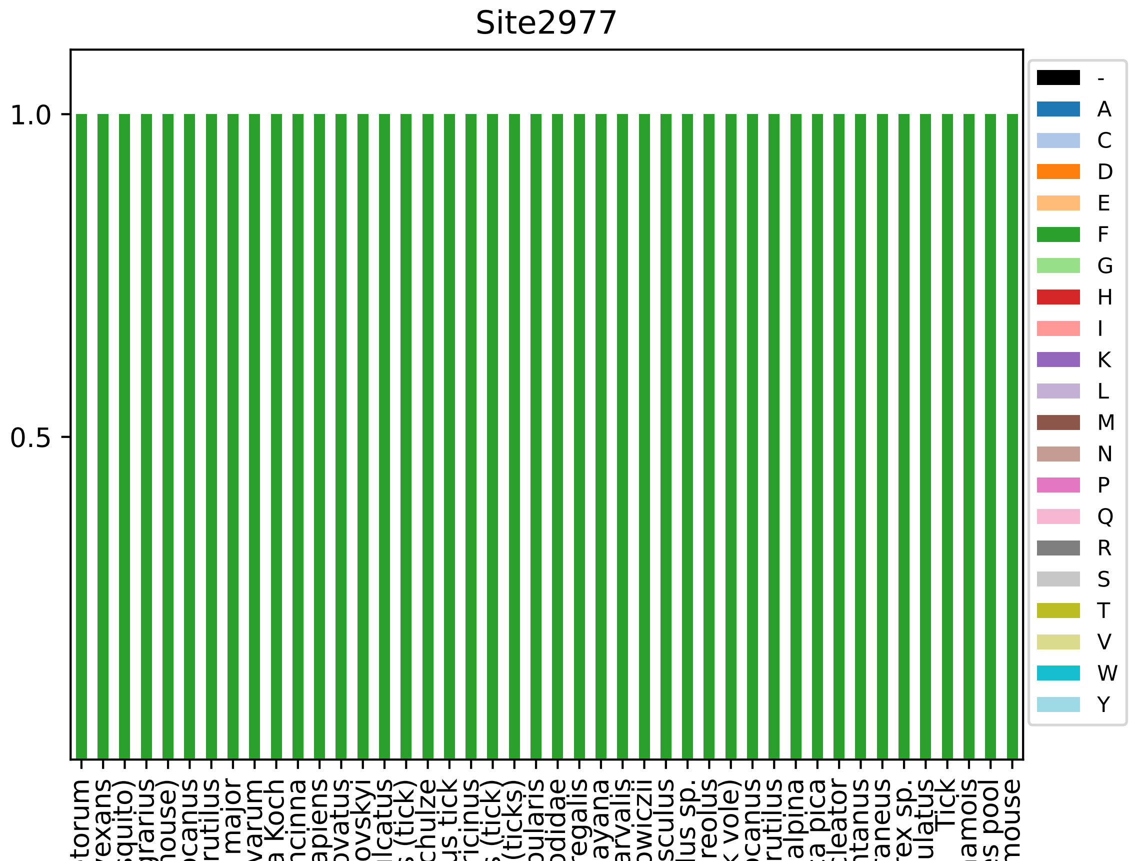
Site2977
1.0
0.5
-
A
C
D
E
F
G
H
I
K
L
M
N
P
Q
R
S
T
V
W
Y

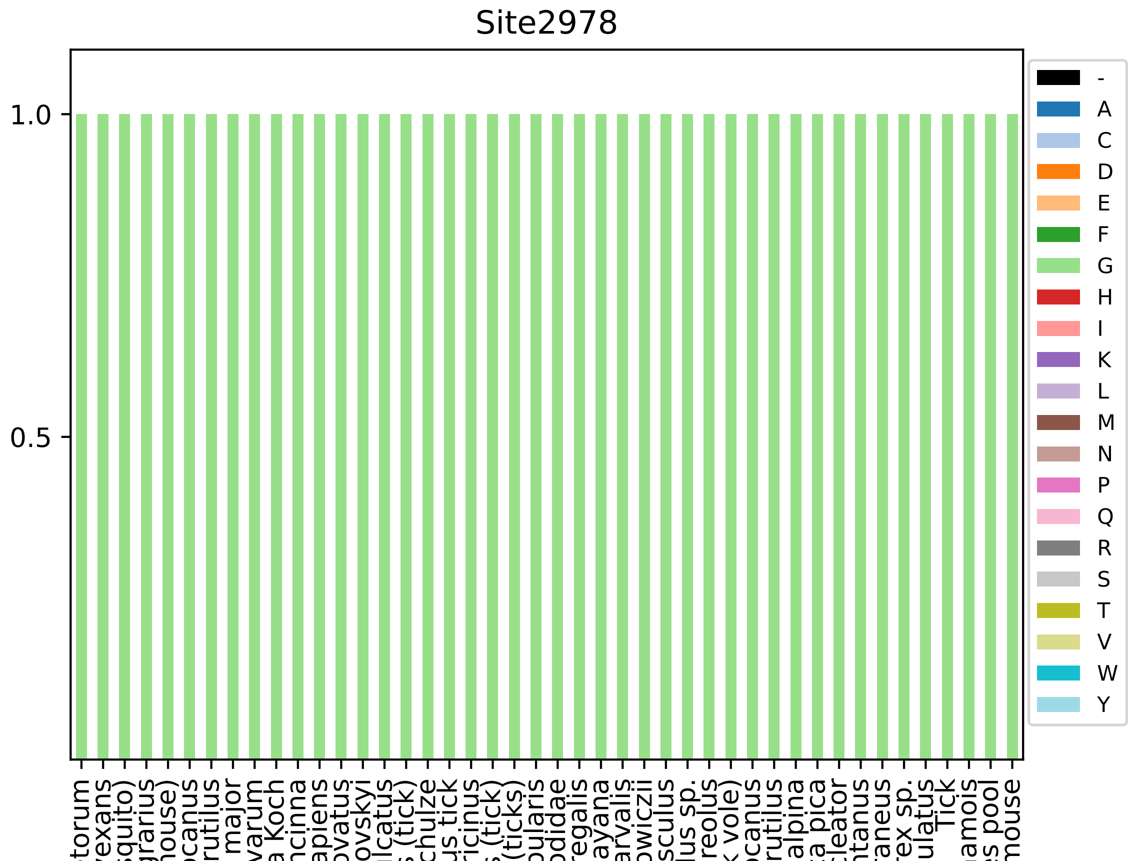

Site2978
1.0
0.5
-
A
C
D
E
F
G
H
I
K
L
M
N
P
Q
R
S
T
V
W
Y

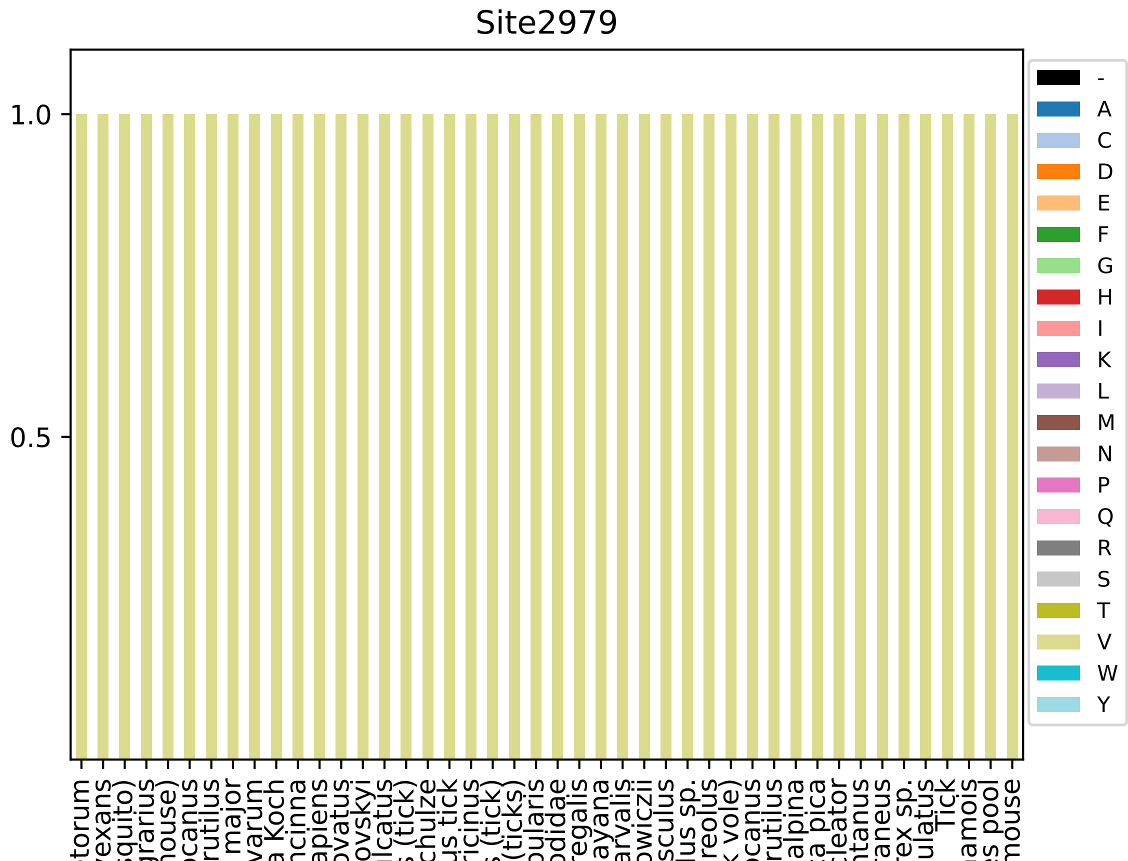

Site2979
1.0
0.5
-
A
C
D
E
F
G
H
I
K
L
M
N
P
Q
R
S
T
V
W
Y

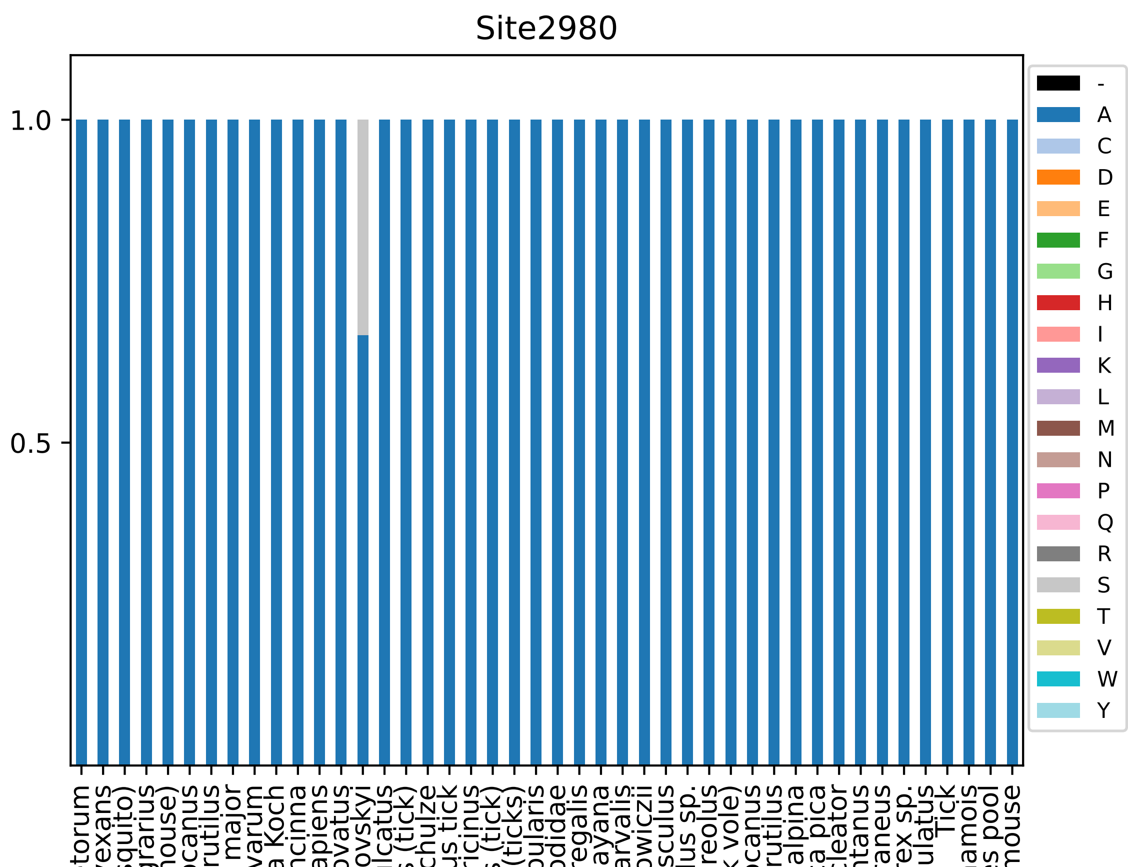

Site2980
1.0
0.5
-
A
C
D
E
F
G
H
I
K
L
M
N
P
Q
R
S
T
V
W
Y

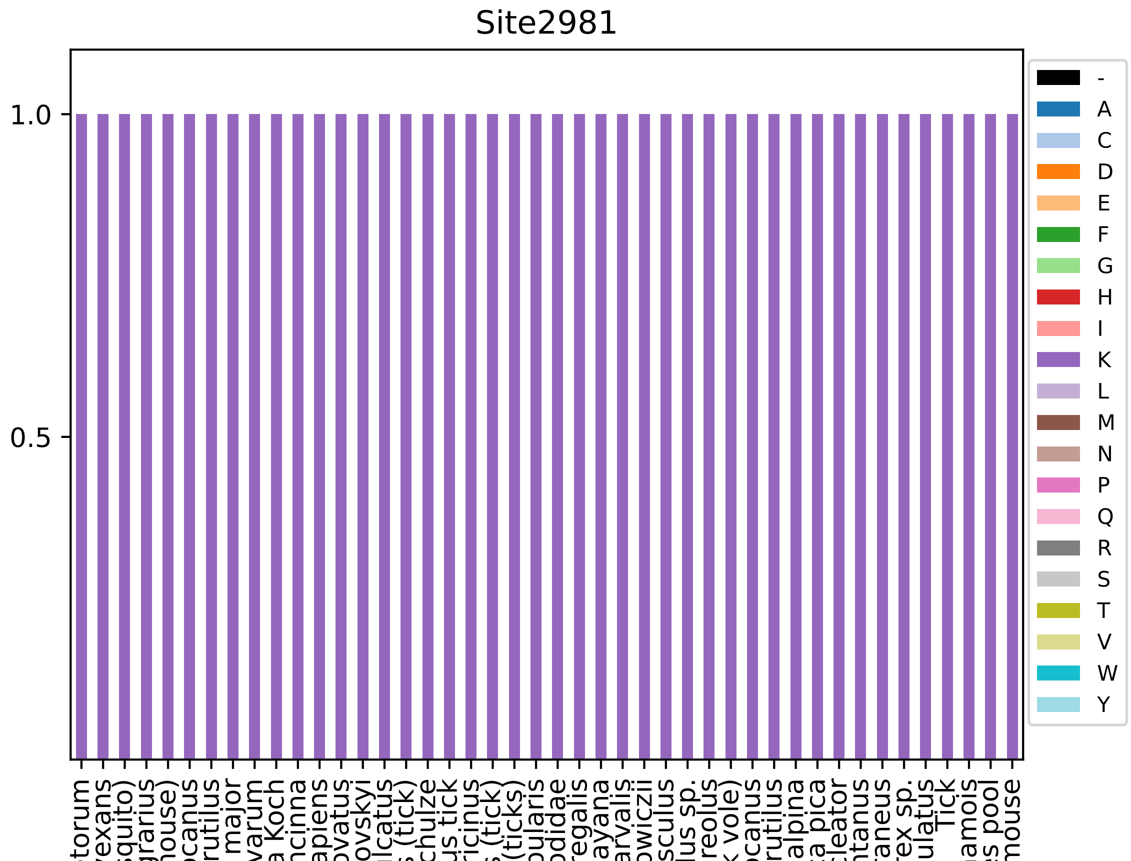

Site2981
1.0
0.5
-
A
C
D
E
F
G
H
I
K
L
M
N
P
Q
R
S
T
V
W
Y

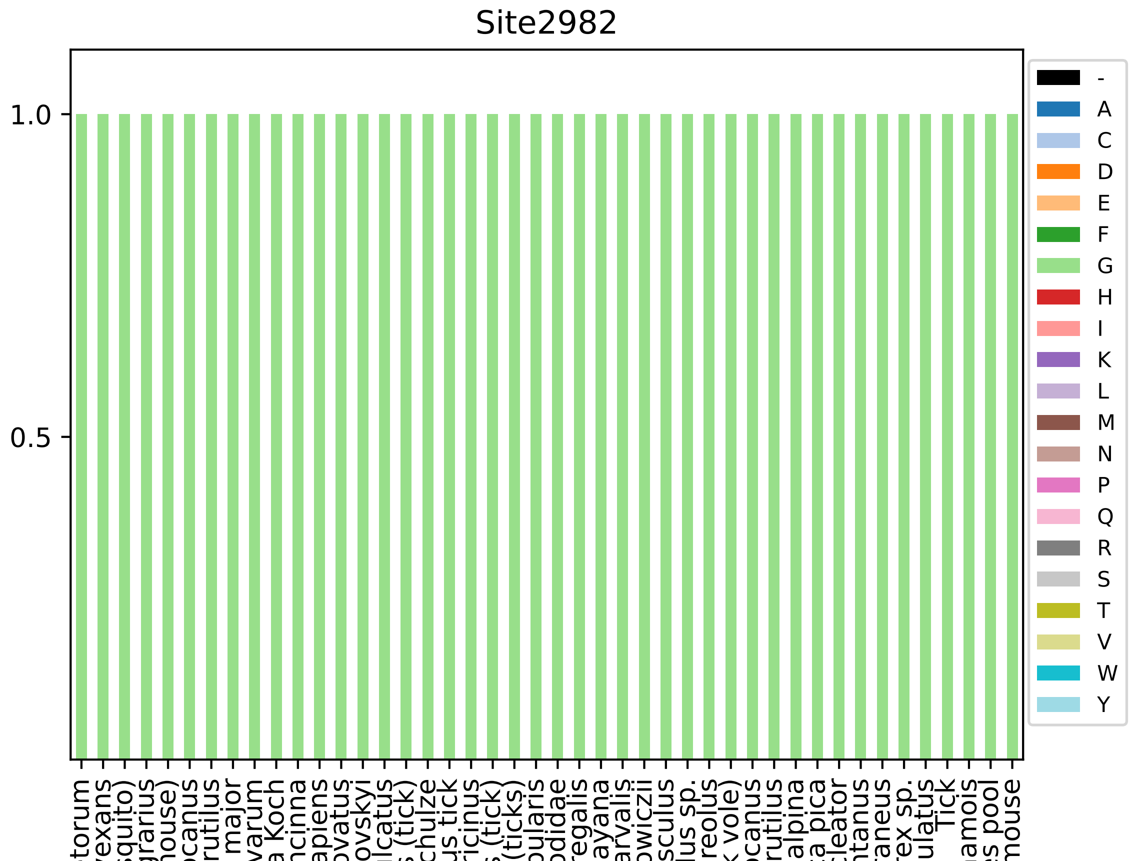
Site2982
1.0
0.5
-
A
C
D
E
F
G
H
I
K
L
M
N
P
Q
R
S
T
V
W
Y

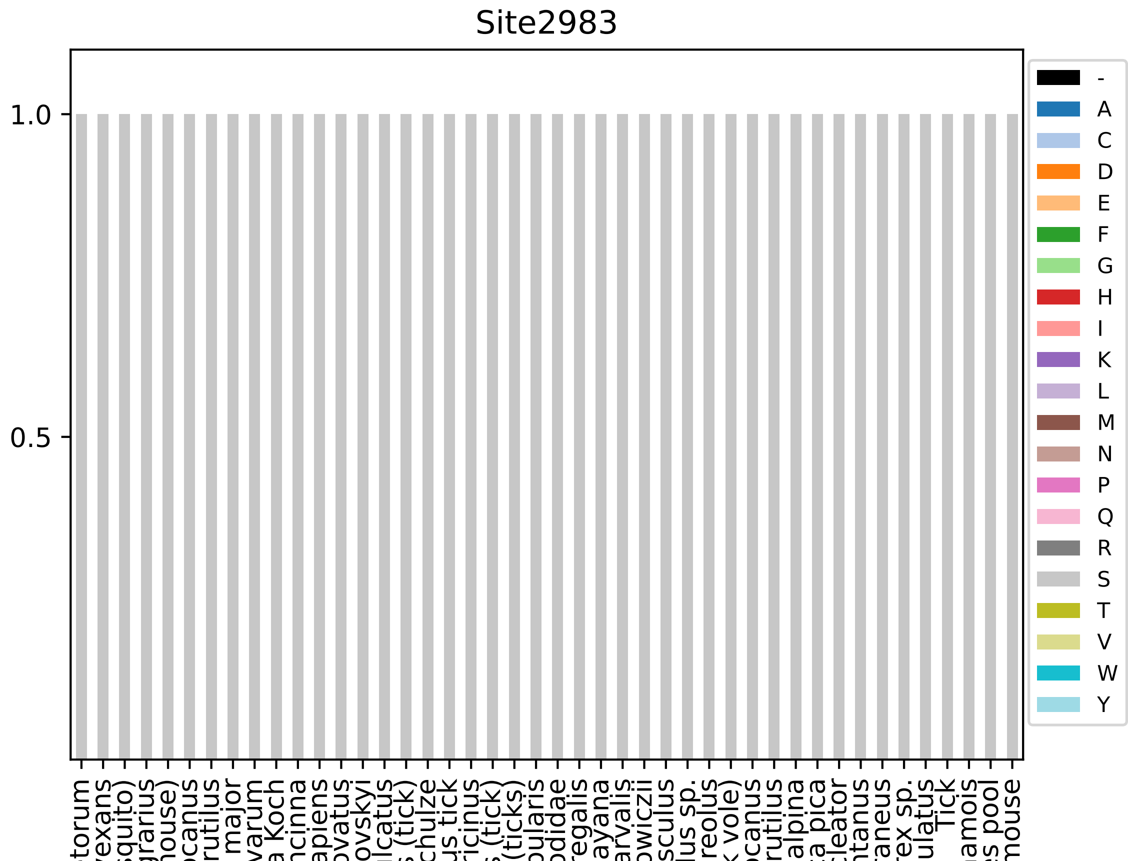
Site2983
1.0
0.5
-
A
C
D
E
F
G
H
I
K
L
M
N
P
Q
R
S
T
V
W
Y

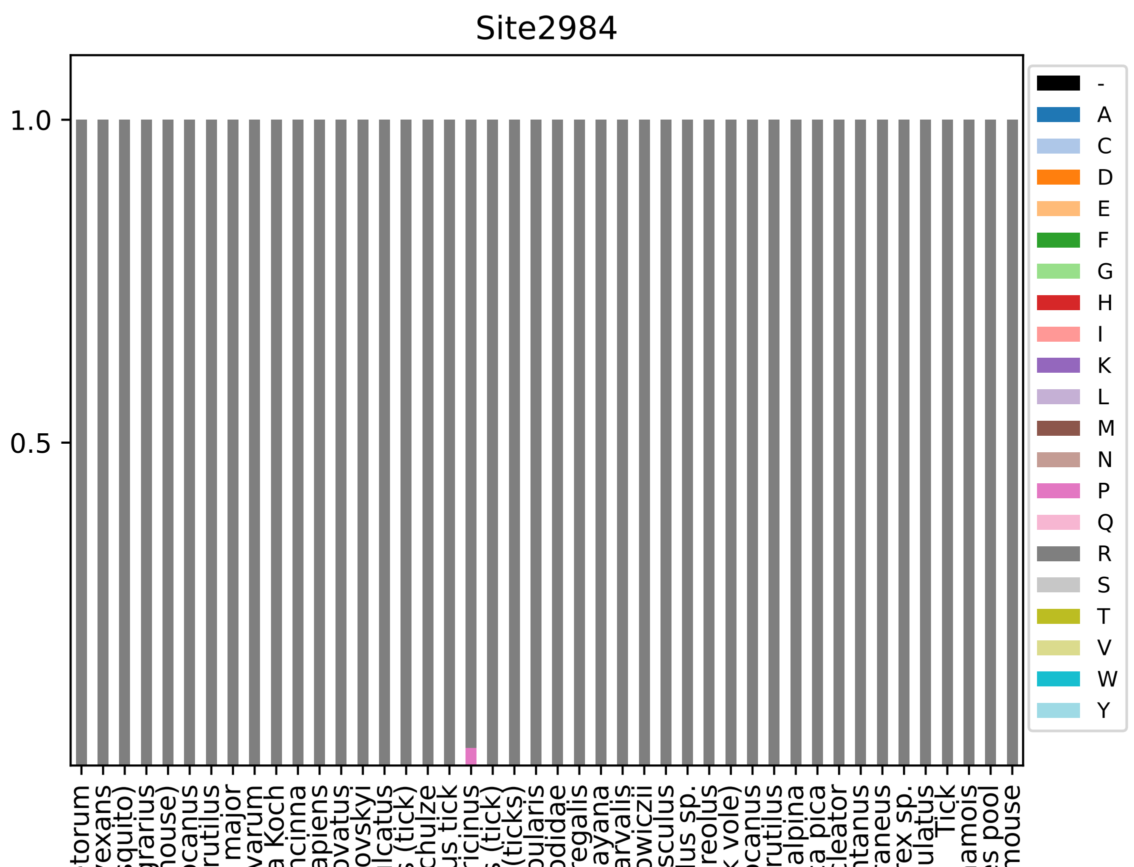
Site2984
1.0
0.5
-
A
C
D
E
F
G
H
I
K
L
M
N
P
Q
R
S
T
V
W
Y

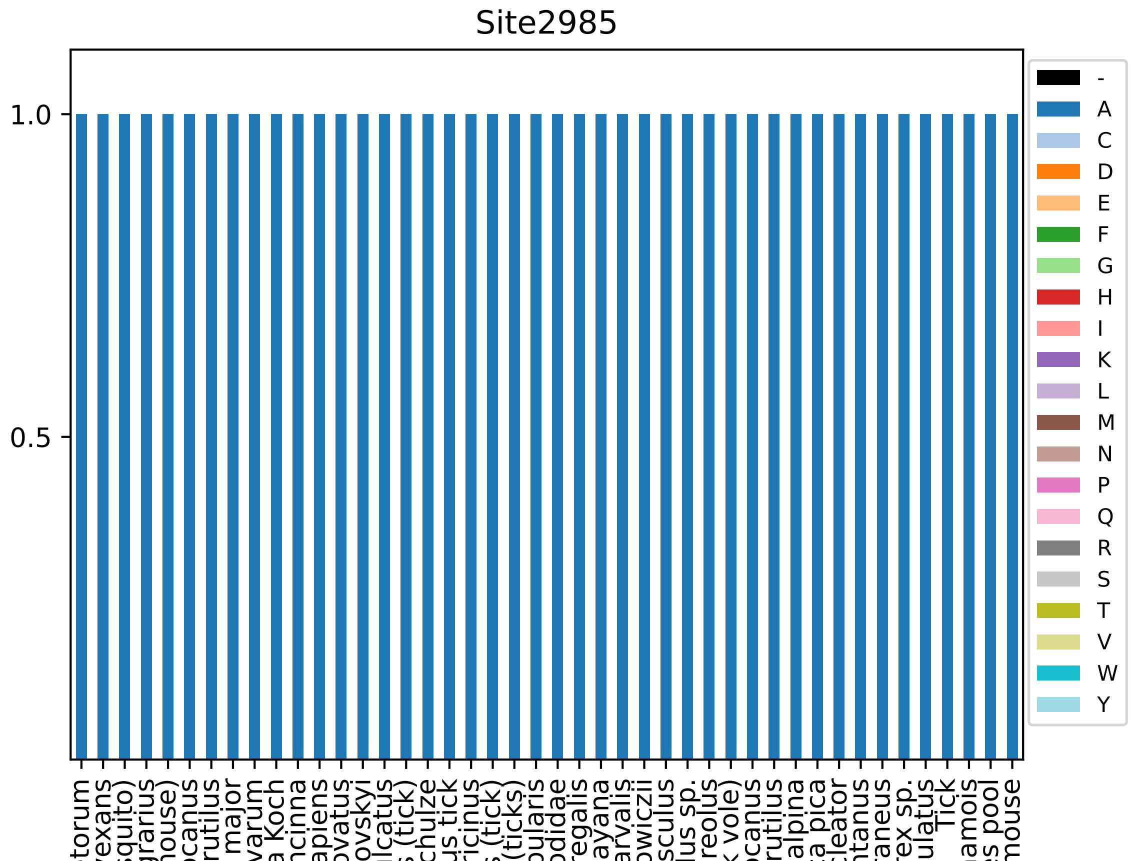

Site2985
1.0
0.5
-
A
C
D
E
F
G
H
I
K
L
M
N
P
Q
R
S
T
V
W
Y

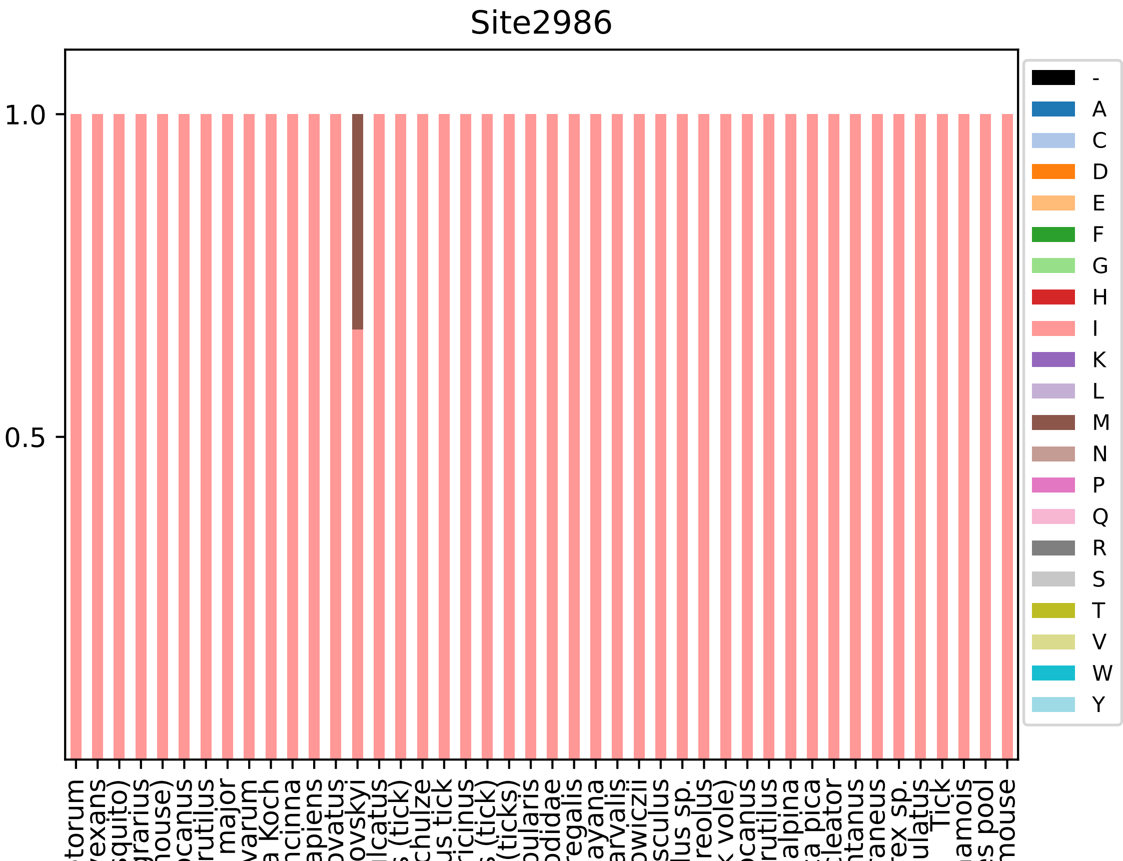

Site2986
1.0
0.5
-
A
C
D
E
F
G
H
I
K
L
M
N
P
Q
R
S
T
V
W
Y
Tick

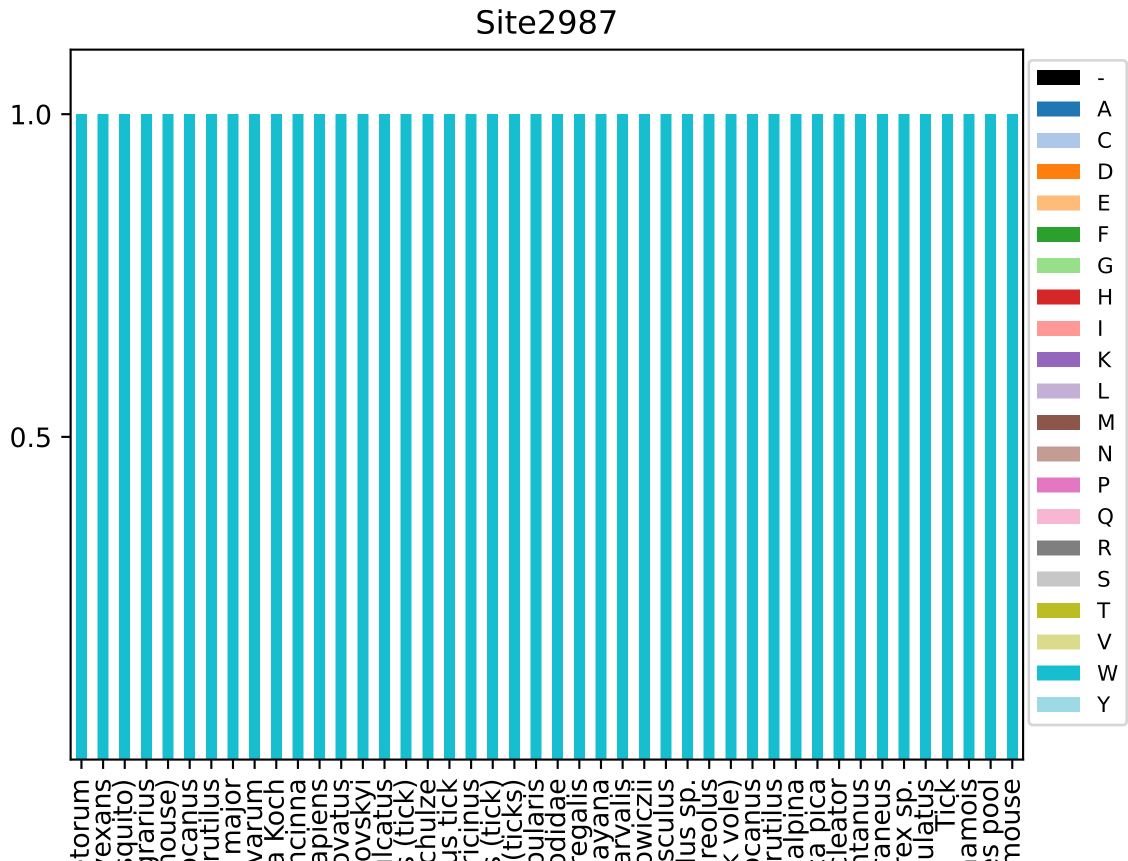
Site2987
1.0
0.5
-
A
C
D
E
F
G
H
I
K
L
M
N
P
Q
R
S
T
V
W
Y

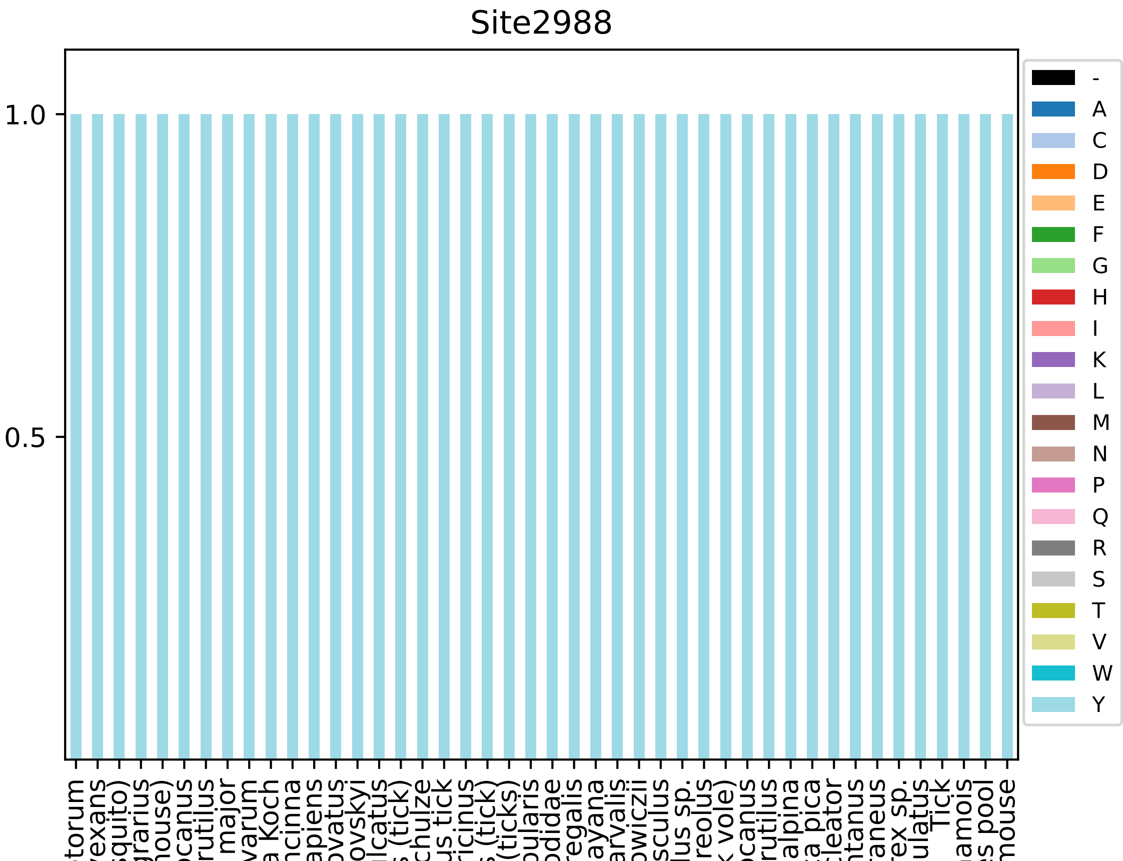
Site2988
1.0
0.5
-
A
C
D
E
F
G
H
I
K
L
M
N
P
Q
R
S
T
V
W
Y

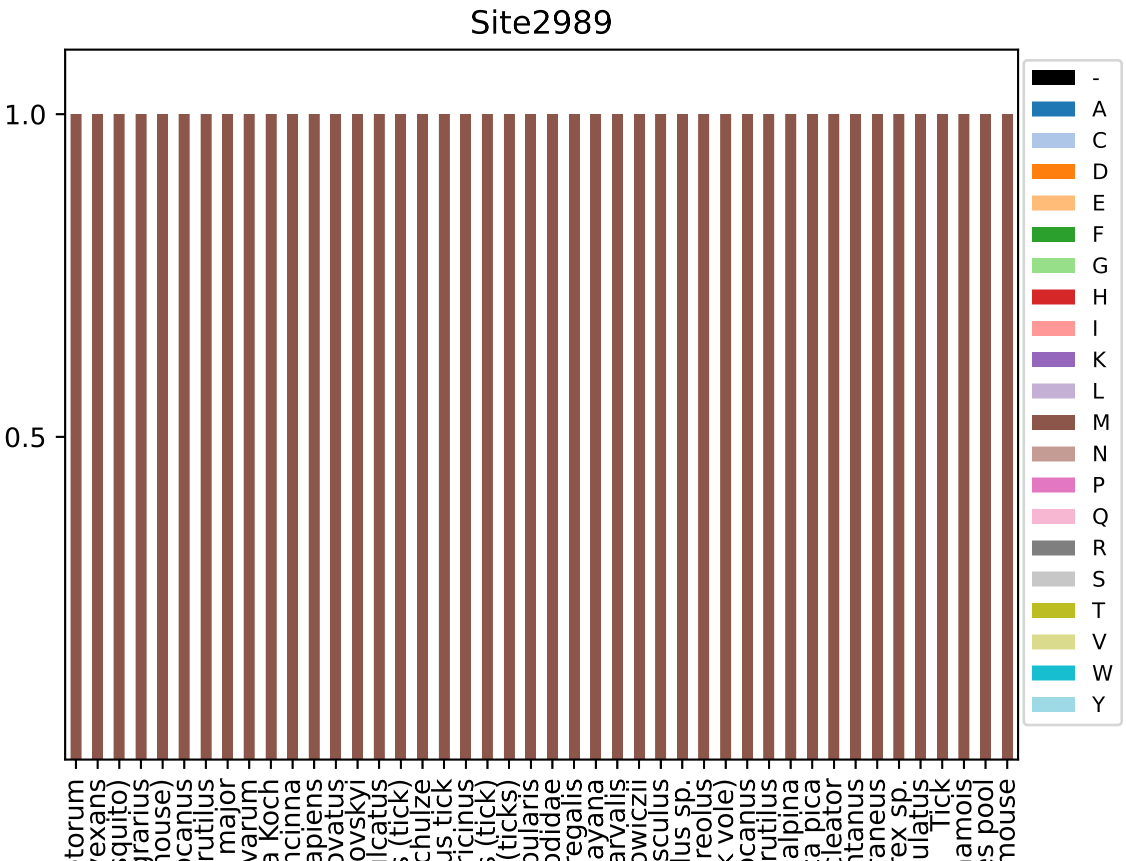
Site2989
1.0
0.5
-
A
C
D
E
F
G
H
I
K
L
M
N
P
Q
R
S
T
V
W
Y

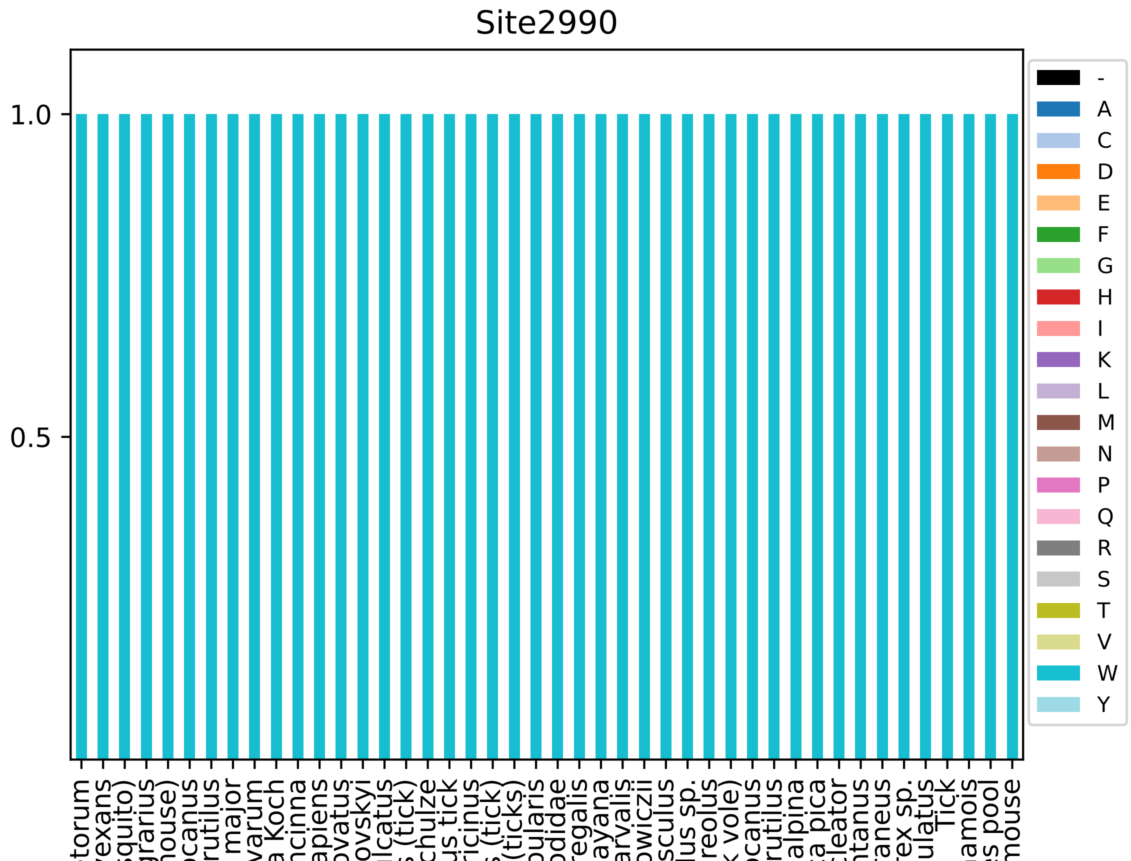

Site2990
1.0
0.5
-
A
C
D
E
F
G
H
I
K
L
M
N
P
Q
R
S
T
V
W
Y

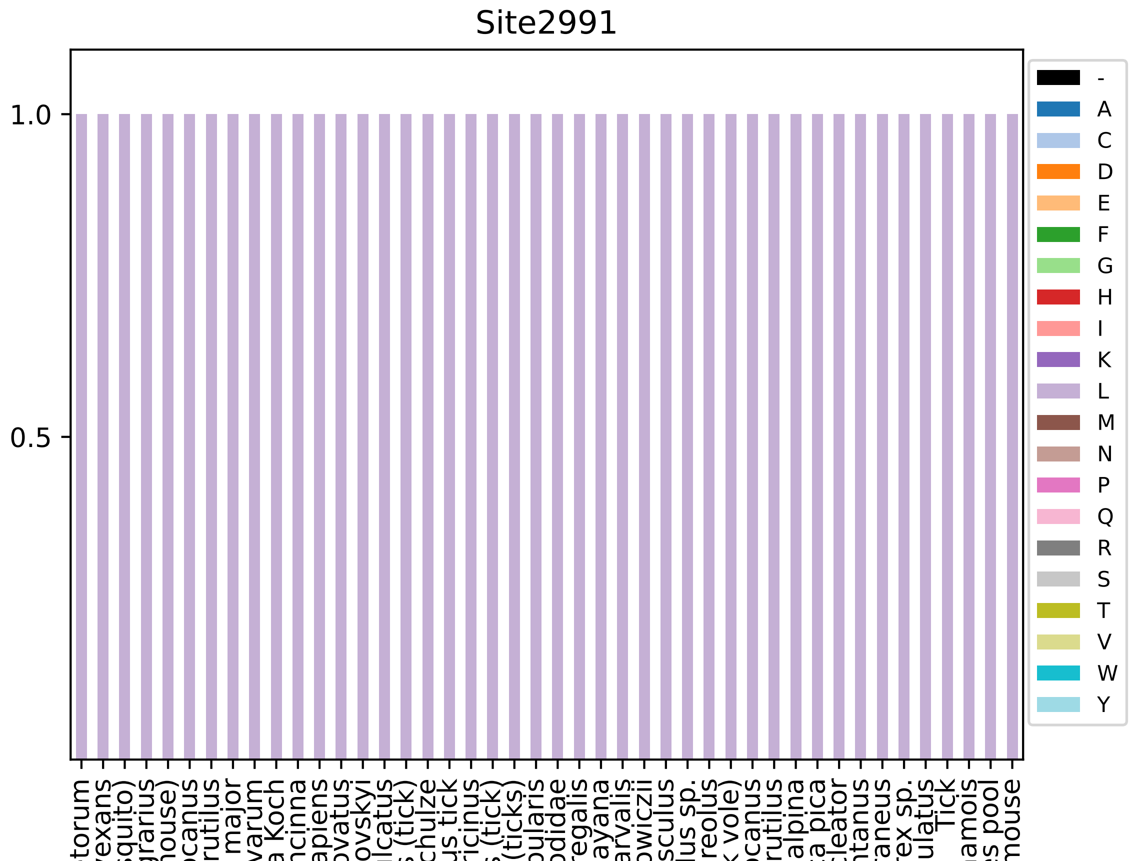

Site2991
1.0
0.5
-
A
C
D
E
F
G
H
I
K
L
M
N
P
Q
R
S
T
V
W
Y

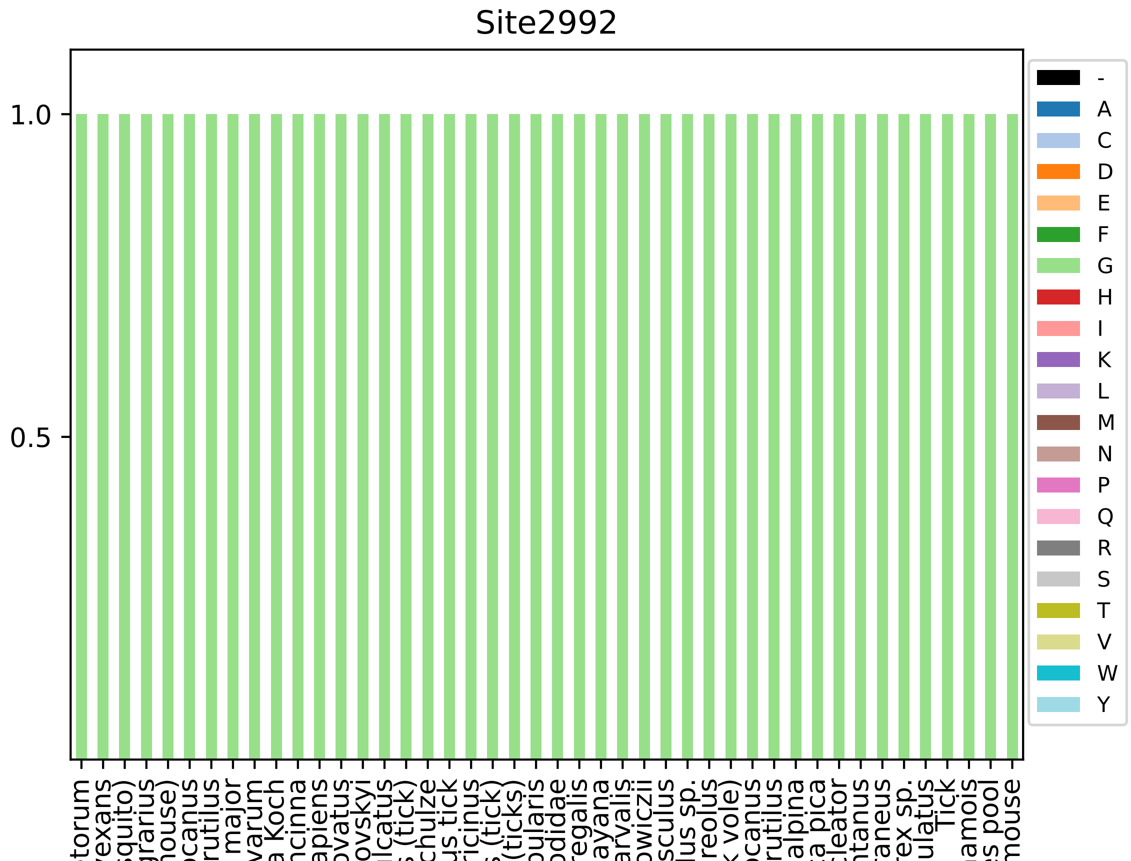
Site2992
1.0
0.5
-
A
C
D
E
F
G
H
I
K
L
M
N
P
Q
R
S
T
V
W
Y

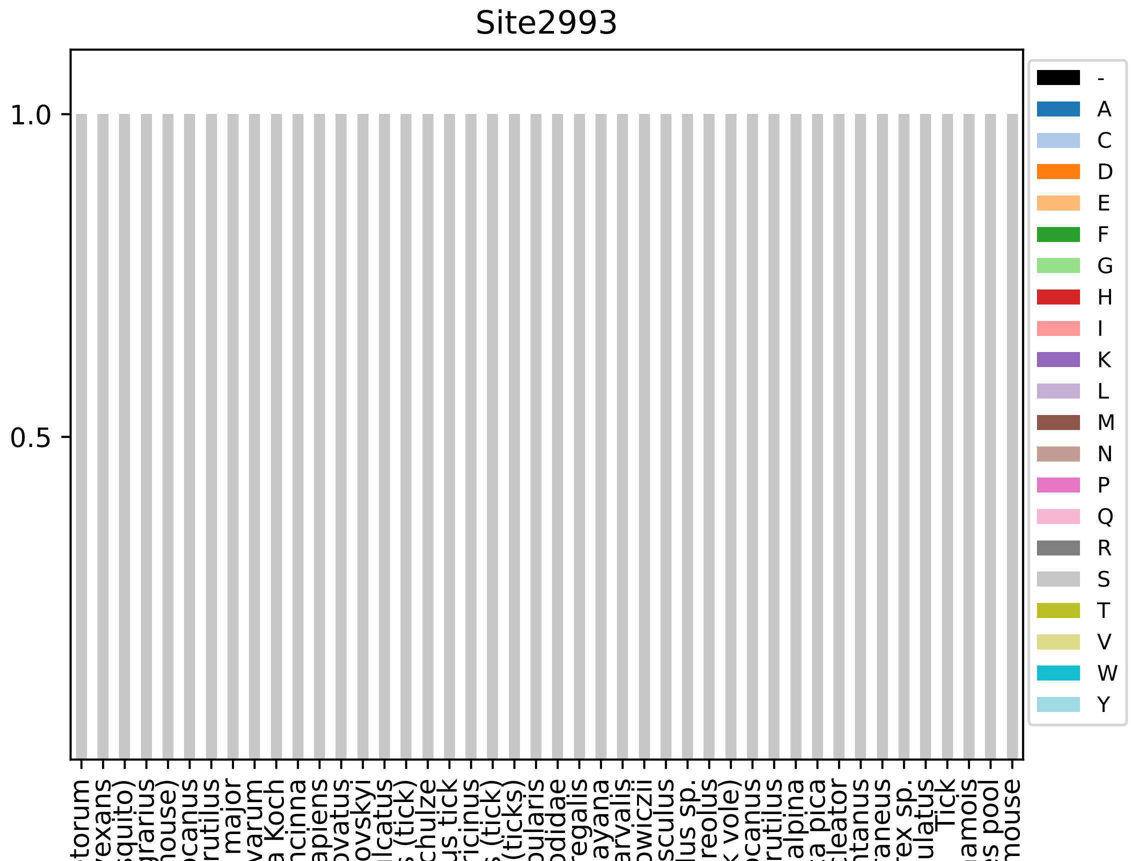
Site2993
1.0
0.5
-
A
C
D
E
F
G
H
I
K
L
M
N
P
Q
R
S
T
V
W
Y
torum
vexans
squito)
grarius
mouse)
ocanus
rutilus
major
varum
a Koch
ncinna
apiens
ovatus
ovskyi
ulcatus
s (tick)
chulze
us tick
ricinus
s (ticks)
pularis
odidae
regalis
ayana
arvalis
owiczii
sculus
lus sp.
reolus
k vole)
ocanus
rutilus
alpina
a pica
cleator
ntanus
raneus
rex sp.
ulatus
Tick
amois
es pool
mouse

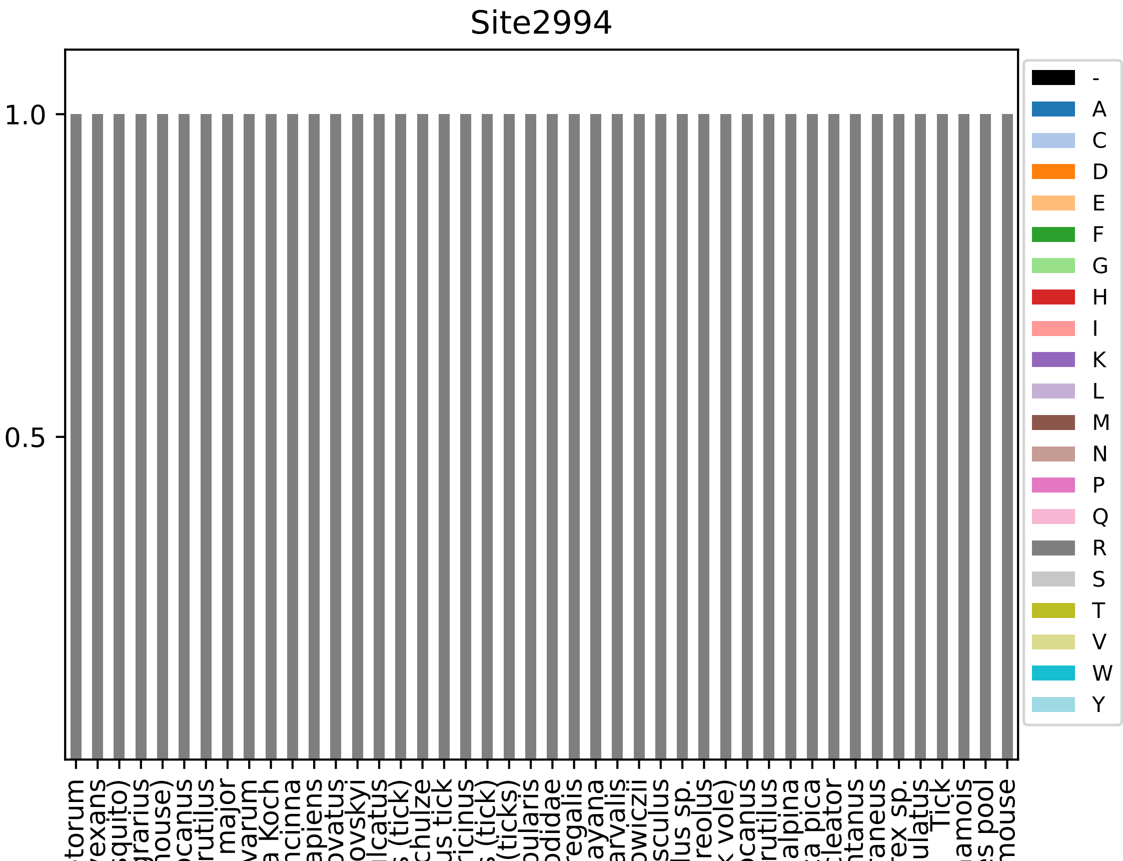

Site2994
1.0
0.5
-
A
C
D
E
F
G
H
I
K
L
M
N
P
Q
R
S
T
V
W
Y

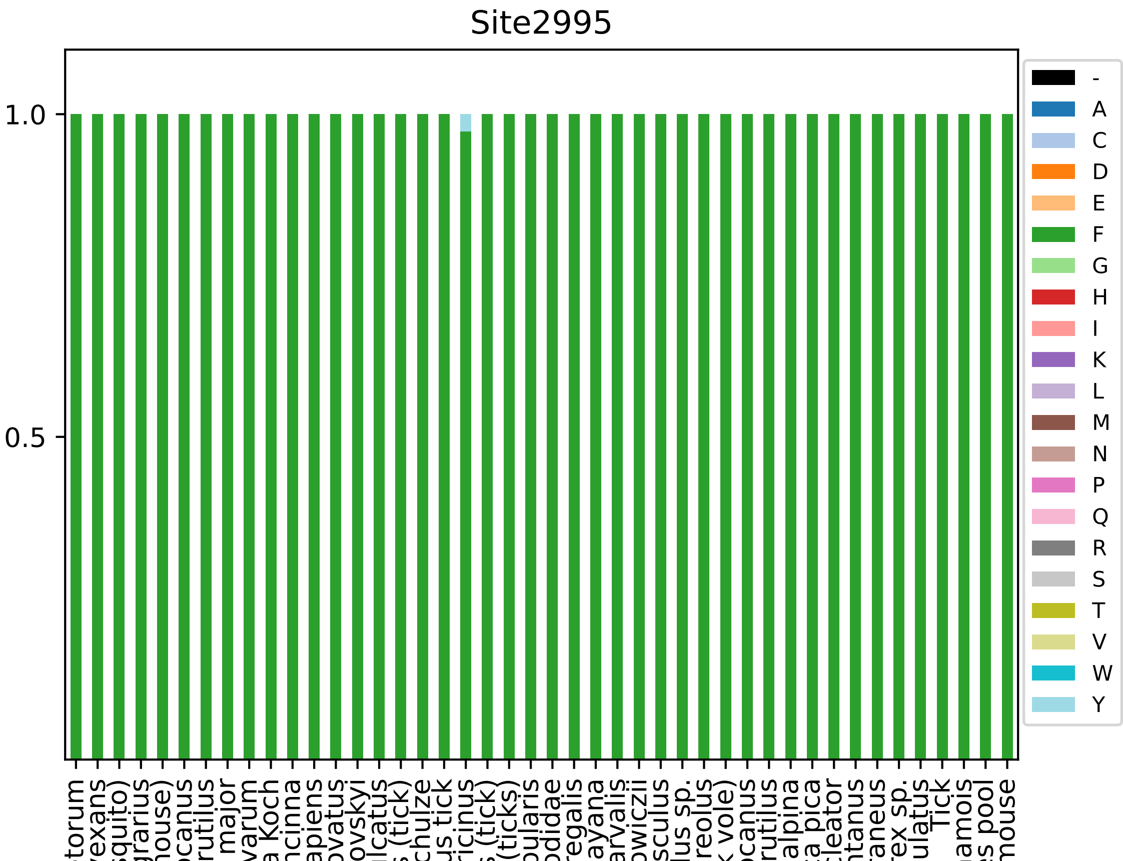
Site2995
1.0
0.5
-
A
C
D
E
F
G
H
I
K
L
M
N
P
Q
R
S
T
V
W
Y

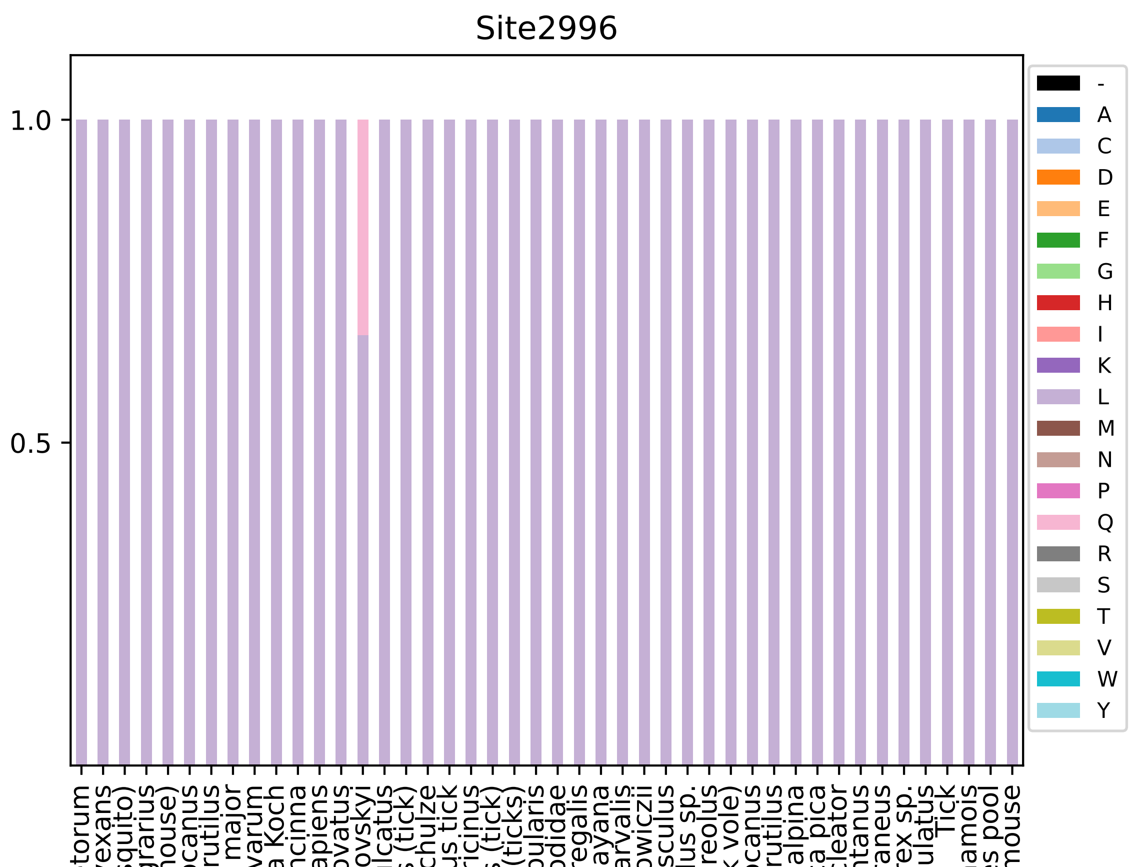

Site2996
1.0
0.5
-
A
C
D
E
F
G
H
I
K
L
M
N
P
Q
R
S
T
V
W
Y

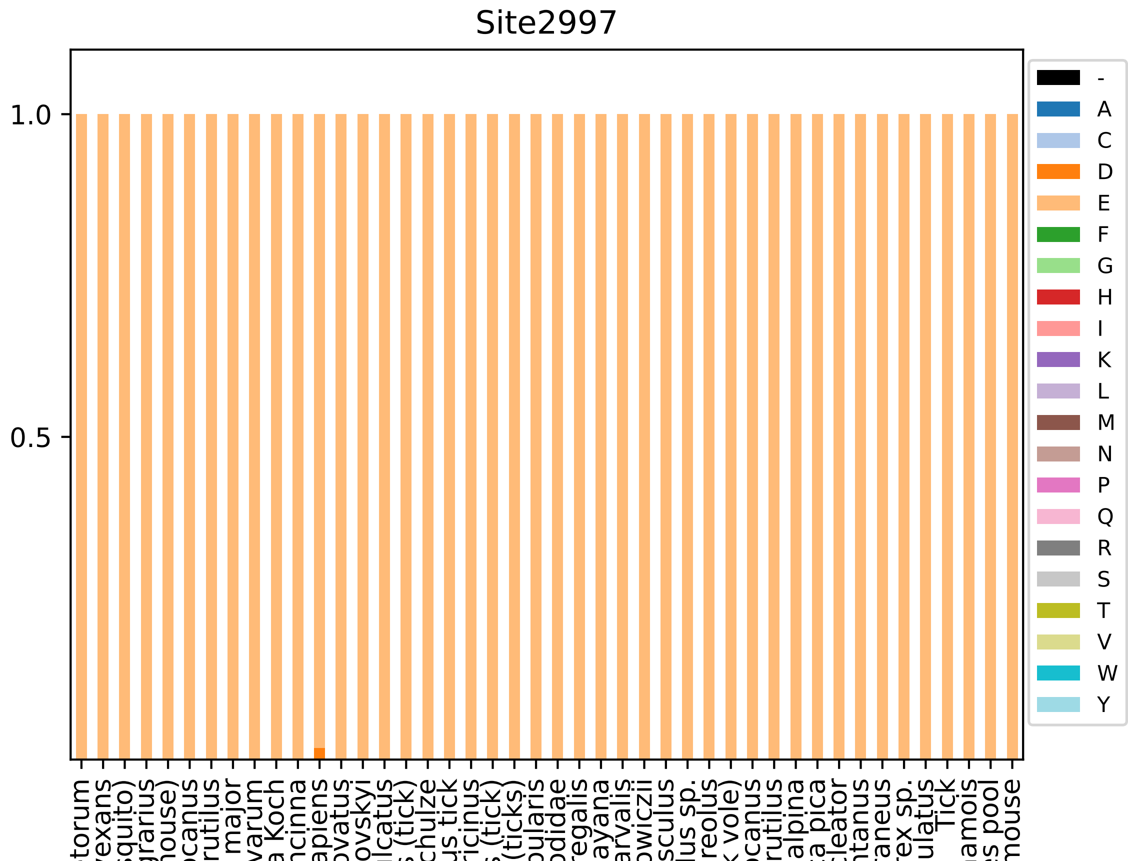
Site2997
1.0
0.5
-
A
C
D
E
F
G
H
I
K
L
M
N
P
Q
R
S
T
V
W
Y

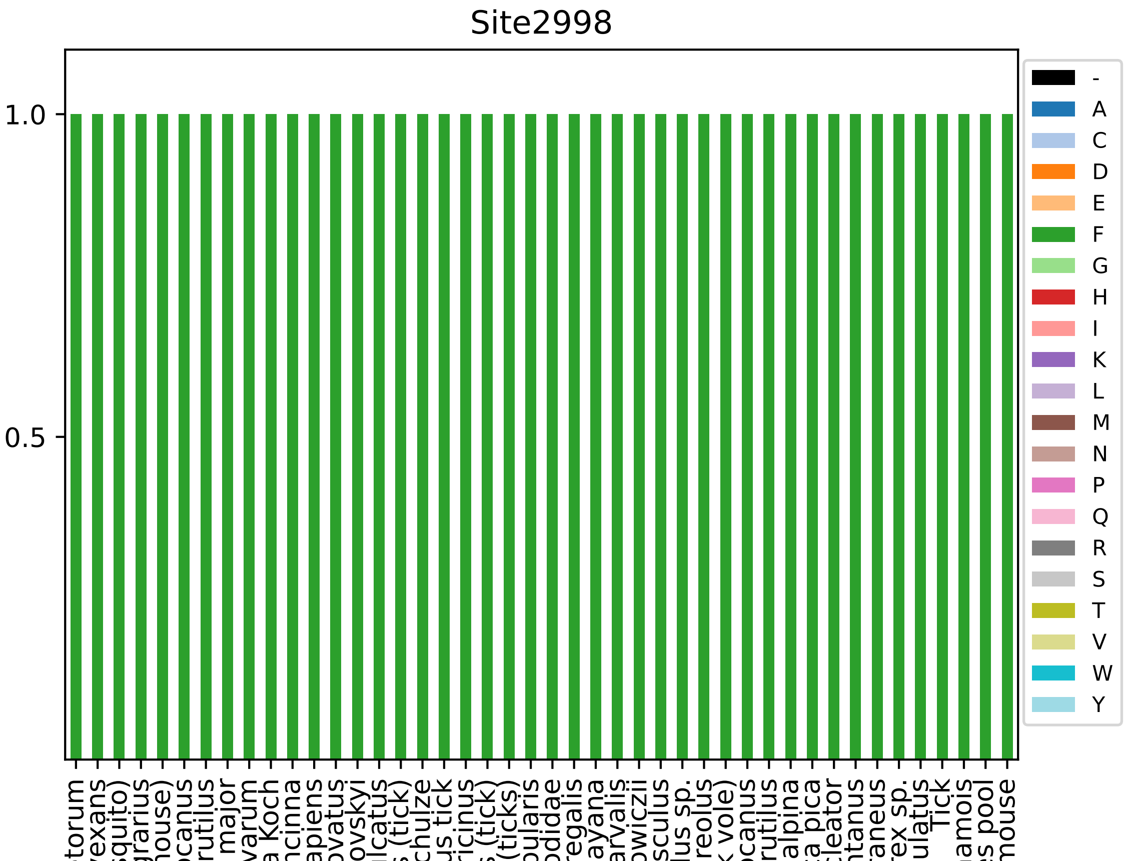

Site2998
1.0
0.5
-
A
C
D
E
F
G
H
I
K
L
M
N
P
Q
R
S
T
V
W
Y

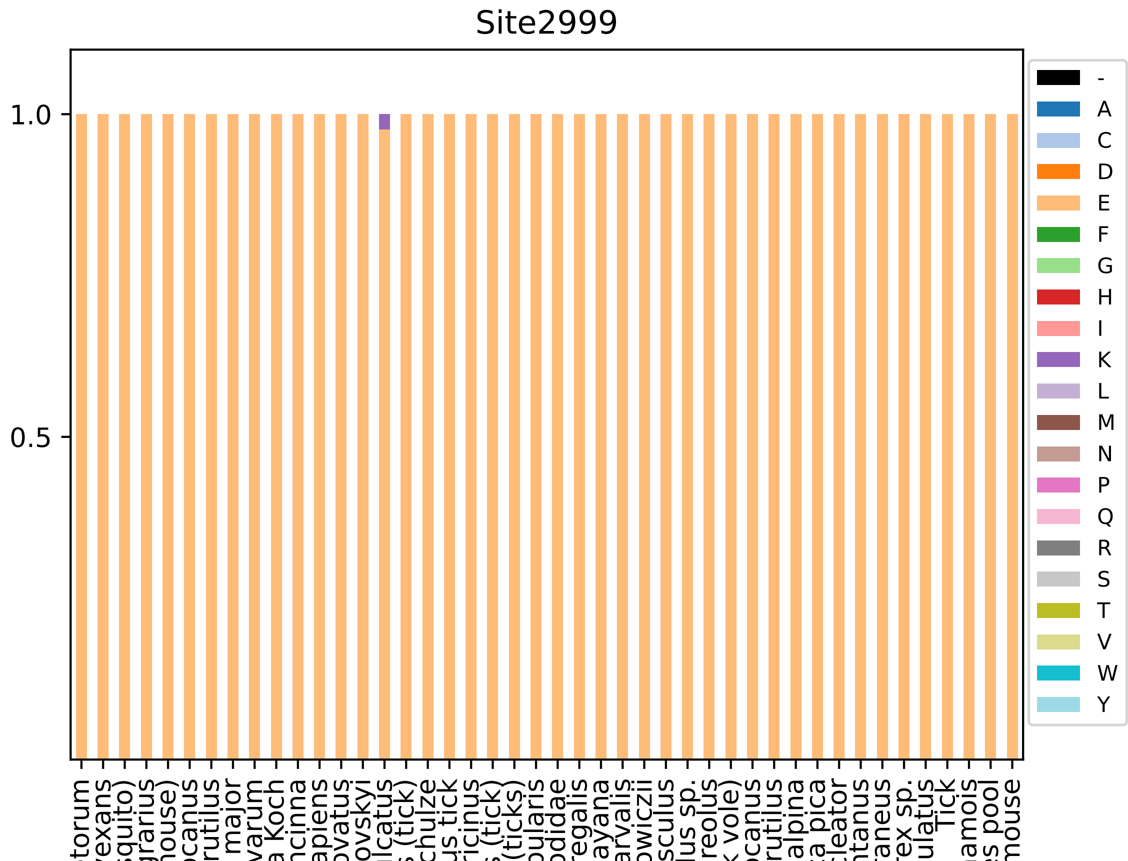
Site2999
1.0
0.5
-
A
C
D
E
F
G
H
I
K
L
M
N
P
Q
R
S
T
V
W
Y

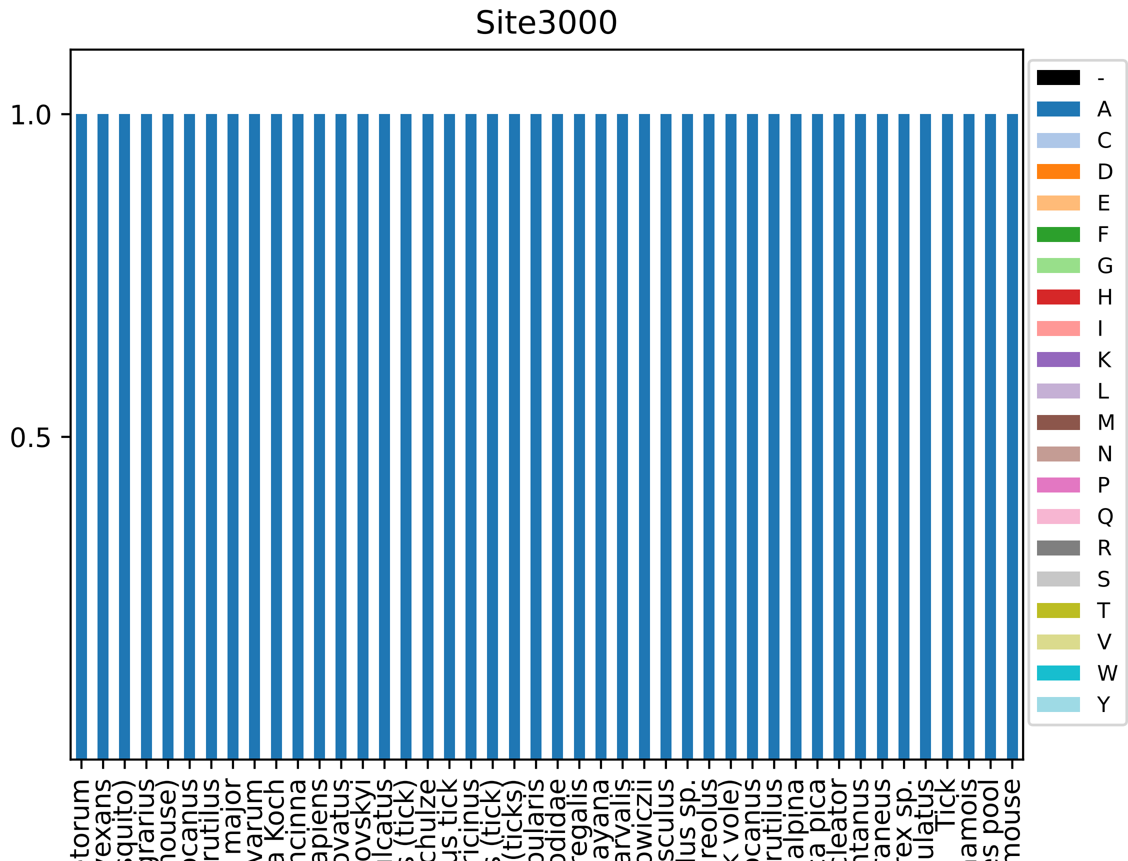
Site3000
1.0
0.5
-
A
C
D
E
F
G
H
I
K
L
M
N
P
Q
R
S
T
V
W
Y

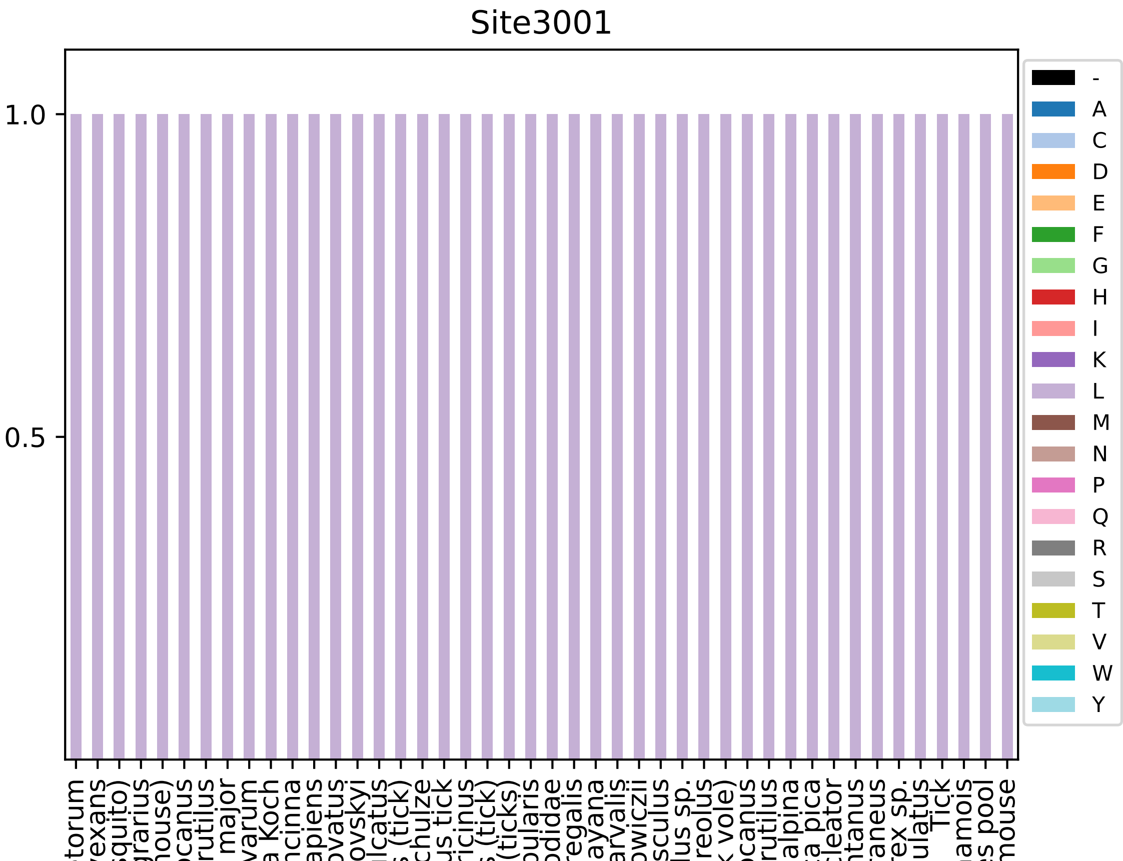
Site3001
1.0
0.5
-
A
C
D
E
F
G
H
I
K
L
M
N
P
Q
R
S
T
V
W
Y

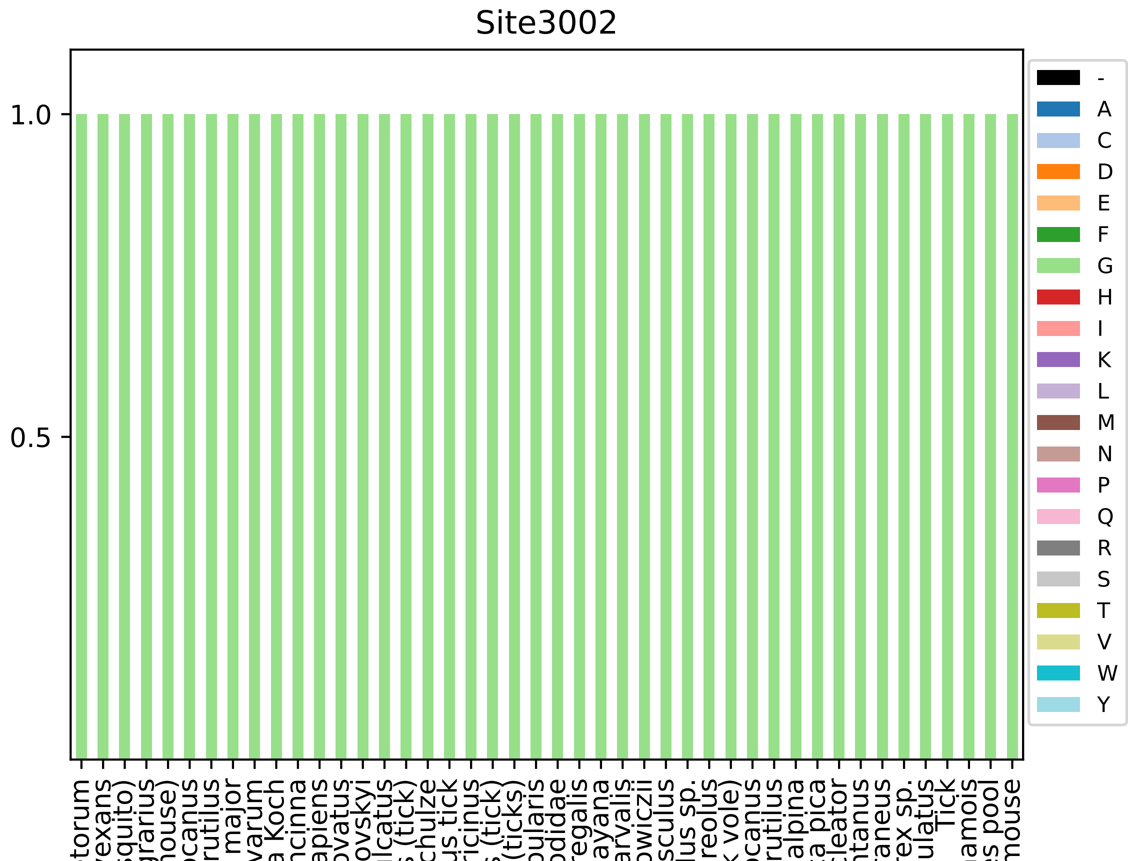
Site3002
1.0
0.5
-
A
C
D
E
F
G
H
I
K
L
M
N
P
Q
R
S
T
V
W
Y

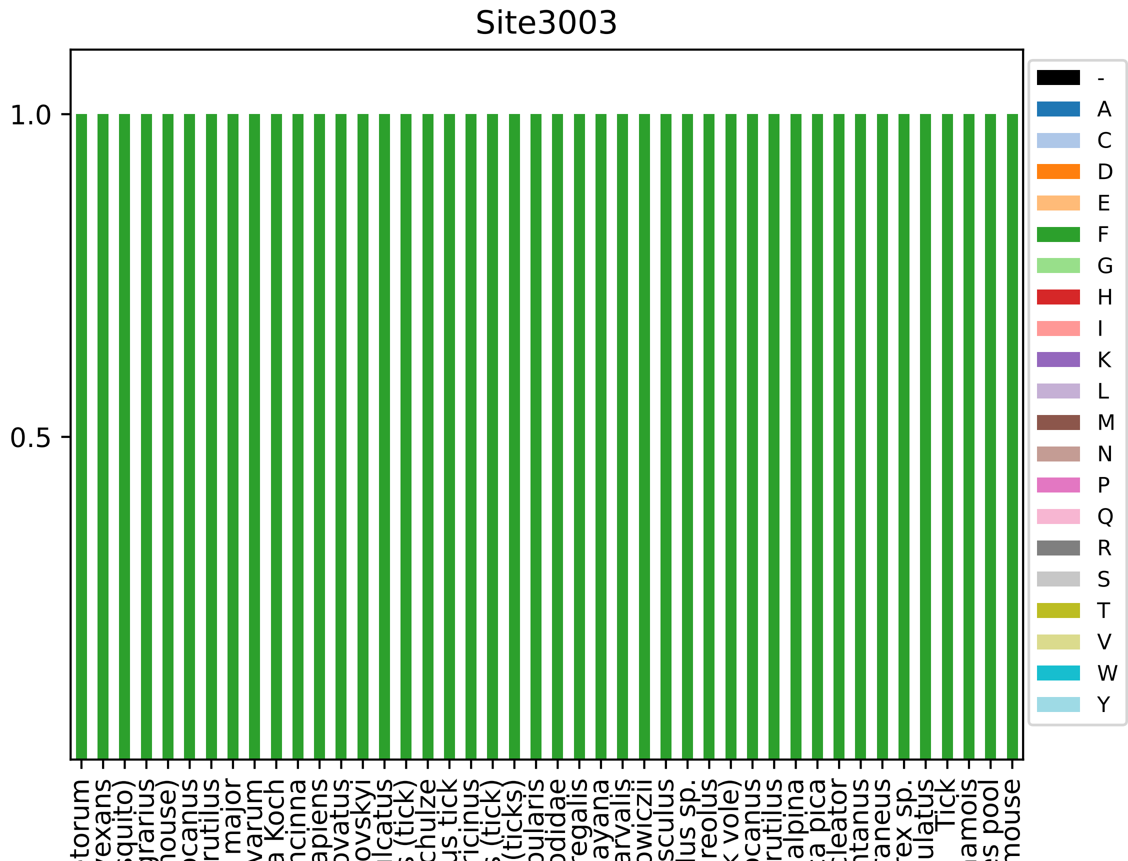
Site3003
1.0
0.5
-
A
C
D
E
F
G
H
I
K
L
M
N
P
Q
R
S
T
V
W
Y

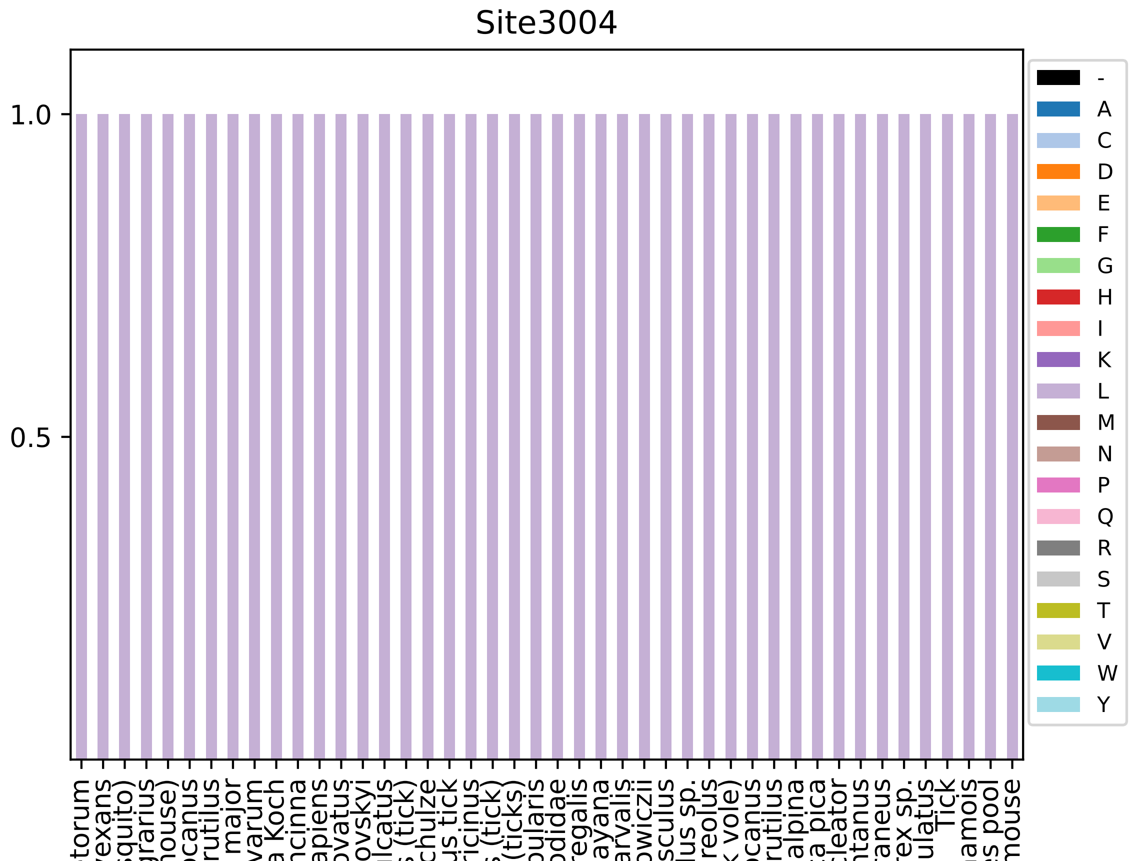

Site3004
1.0
0.5
-
A
C
D
E
F
G
H
I
K
L
M
N
P
Q
R
S
T
V
W
Y

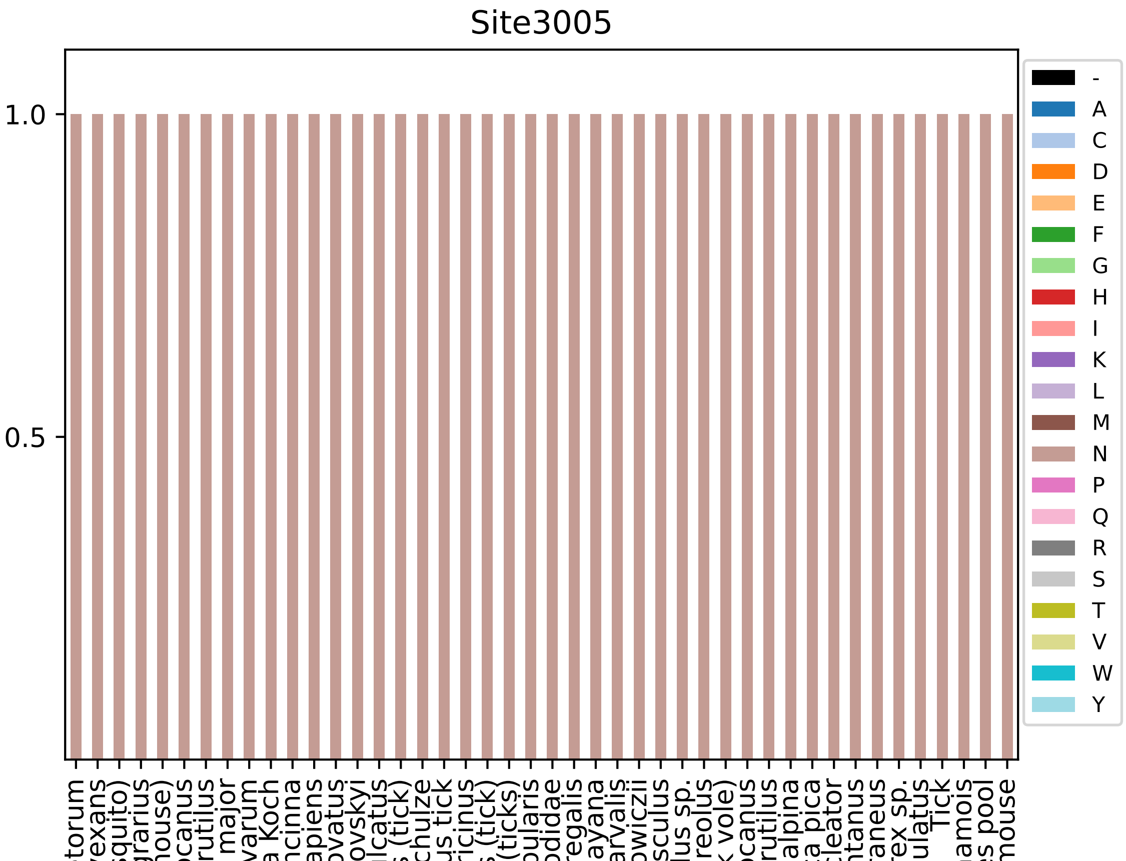
Site3005
1.0
0.5
-
A
C
D
E
F
G
H
I
K
L
M
N
P
Q
R
S
T
V
W
Y

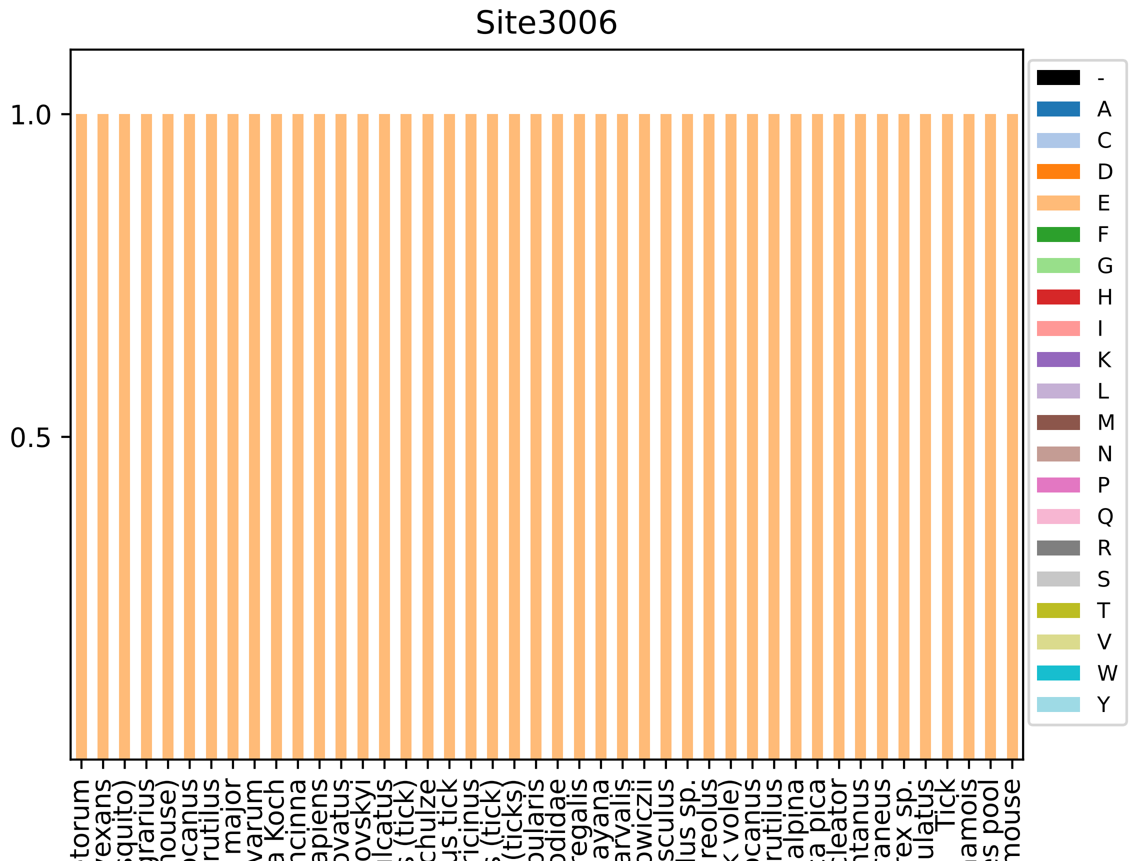

Site3006
1.0
0.5
-
A
C
D
E
F
G
H
I
K
L
M
N
P
Q
R
S
T
V
W
Y

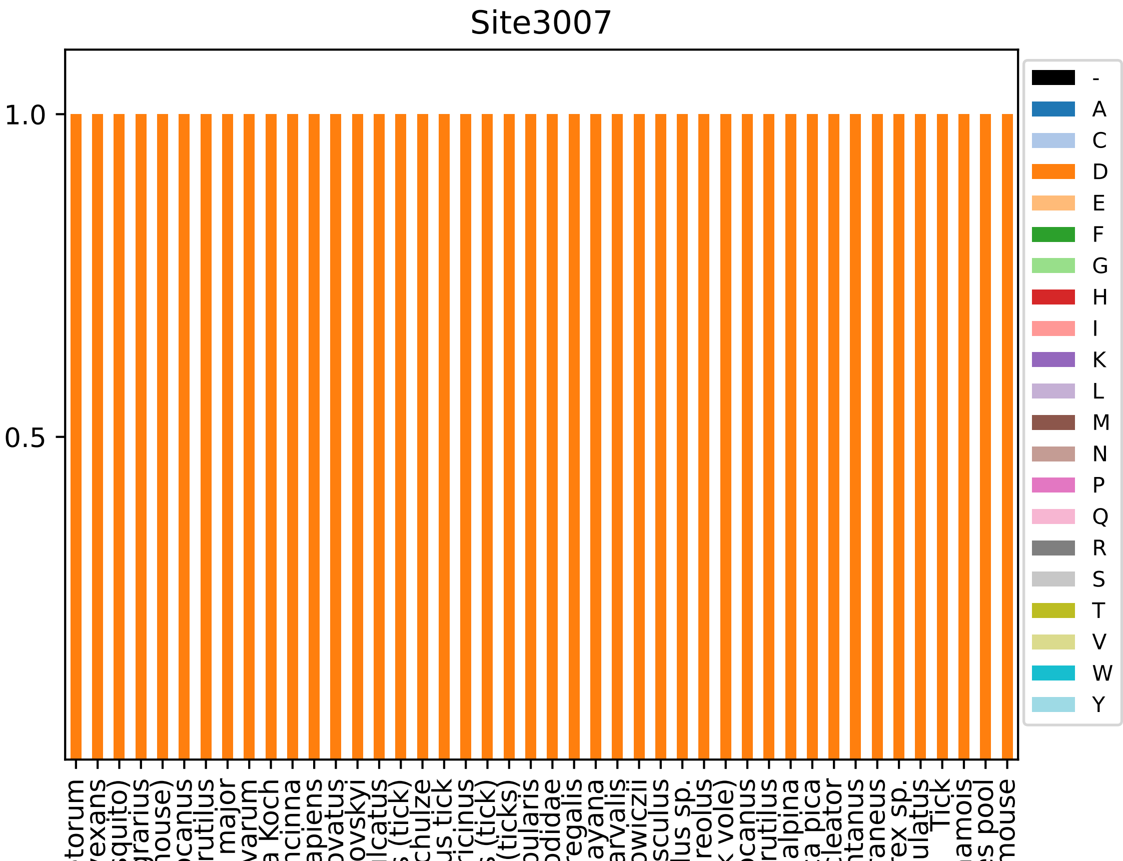
Site3007
1.0
0.5
-
A
C
D
E
F
G
H
I
K
L
M
N
P
Q
R
S
T
V
W
Y

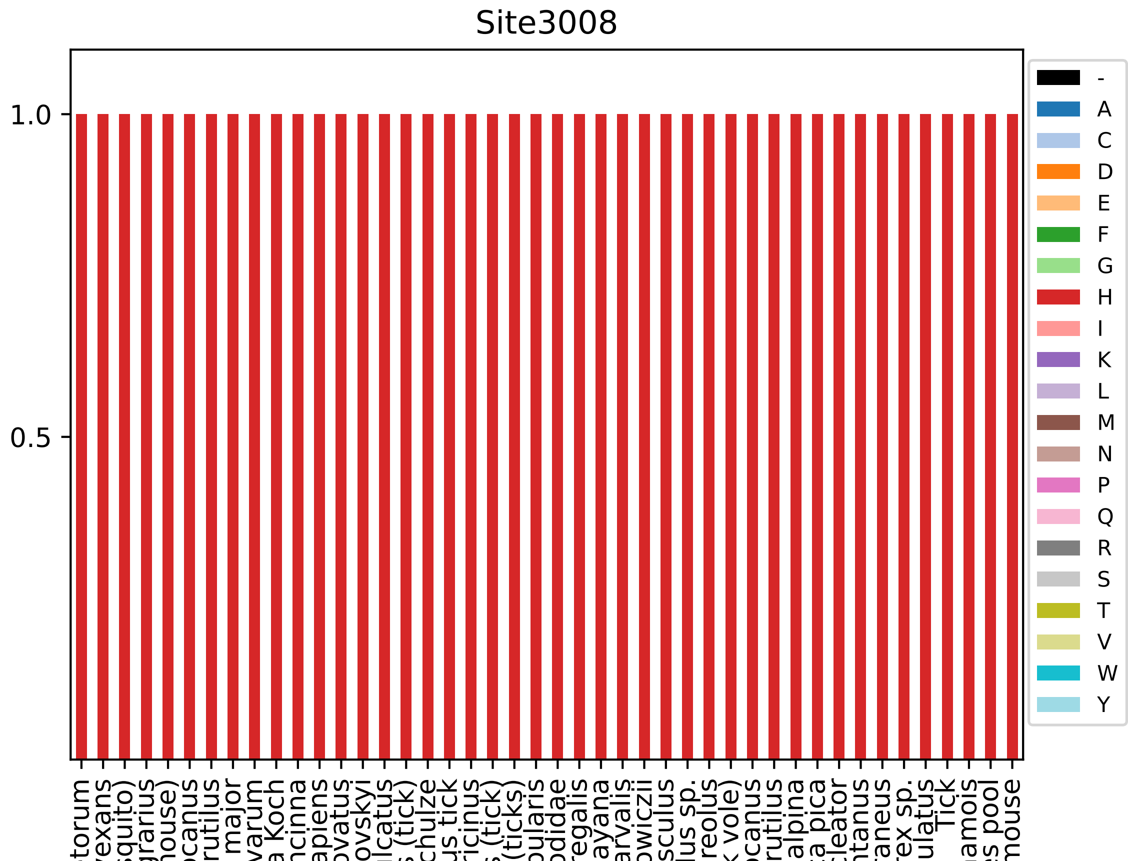
Site3008
1.0
0.5
-
A
C
D
E
F
G
H
I
K
L
M
N
P
Q
R
S
T
V
W
Y

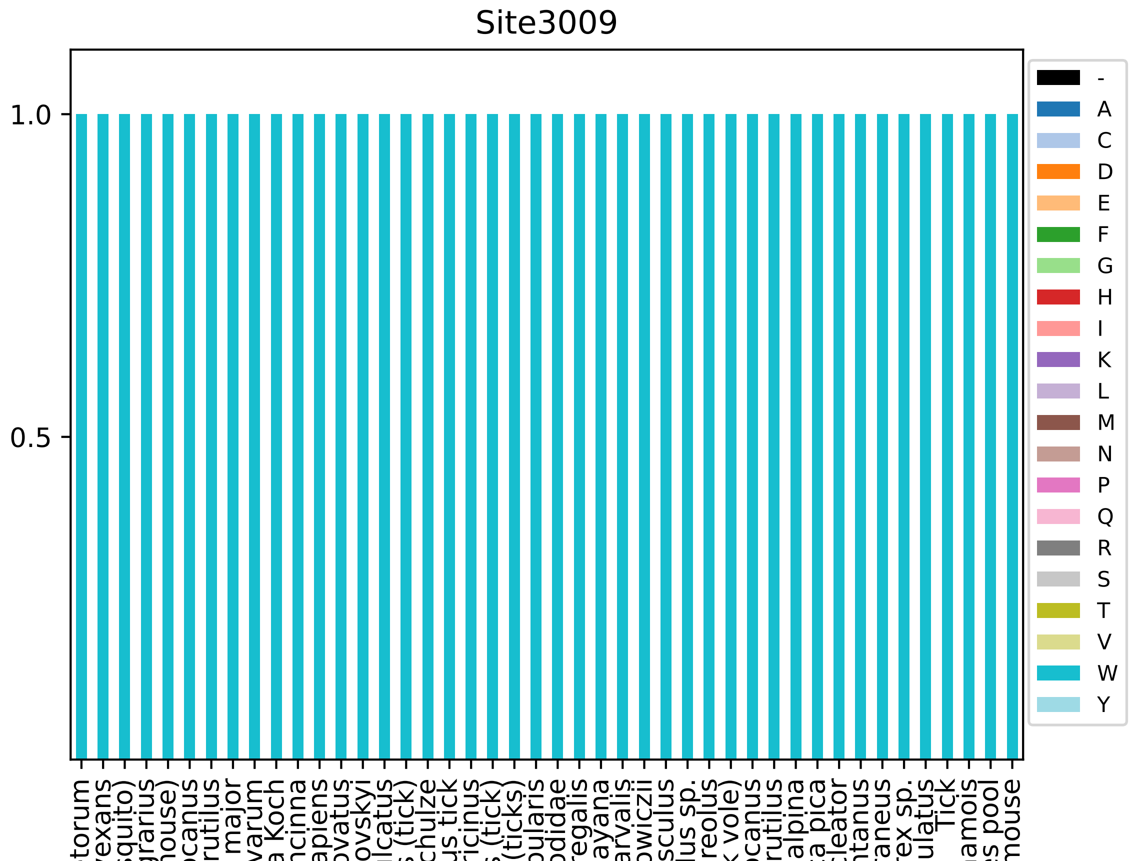
Site3009
1.0
0.5
-
A
C
D
E
F
G
H
I
K
L
M
N
P
Q
R
S
T
V
W
Y

Site3010

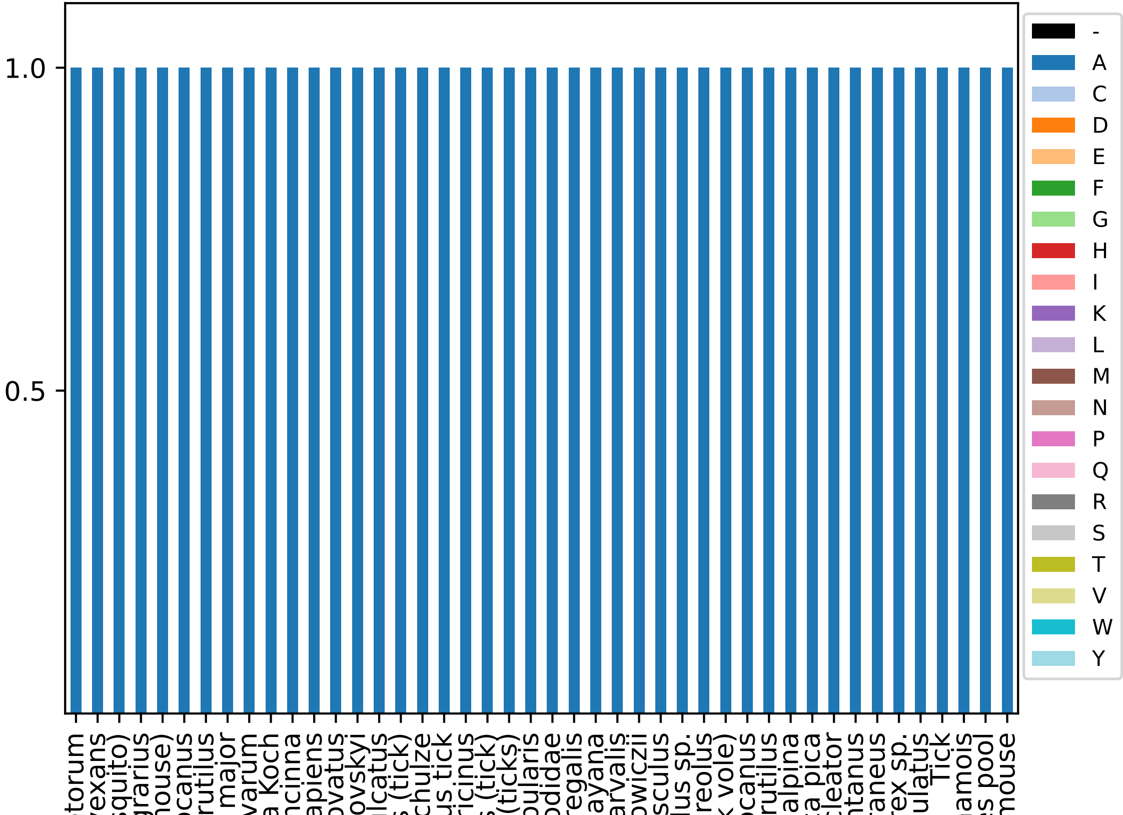

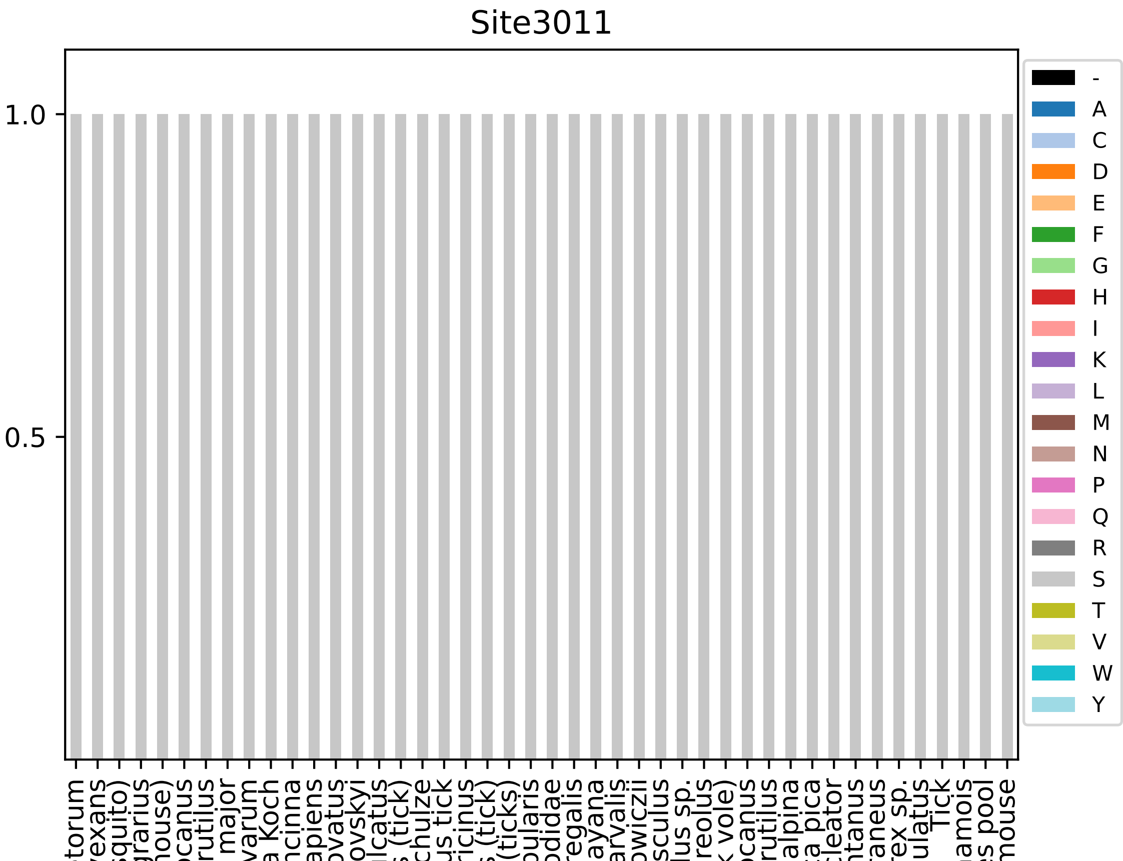
Site3011
1.0
0.5
-
A
C
D
E
F
G
H
I
K
L
M
N
P
Q
R
S
T
V
W
Y

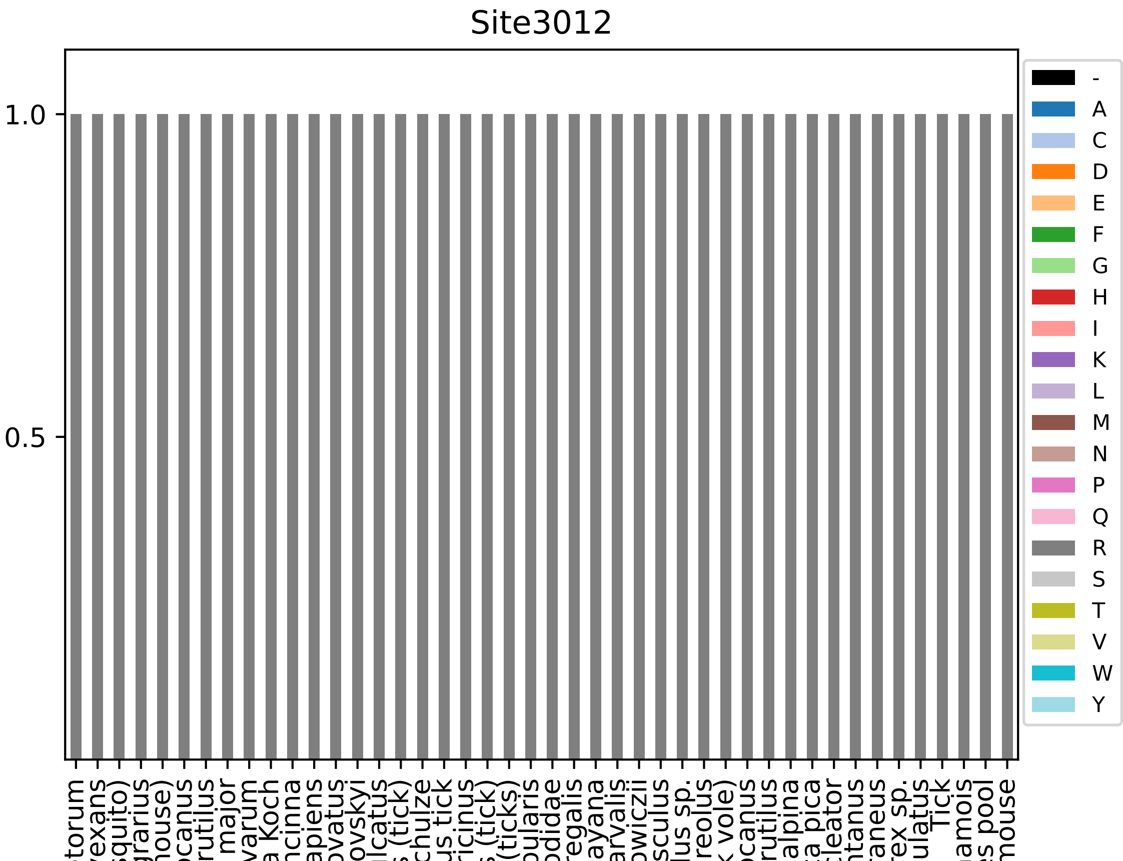
Site3012
1.0
0.5
-
A
C
D
E
F
G
H
I
K
L
M
N
P
Q
R
S
T
V
W
Y

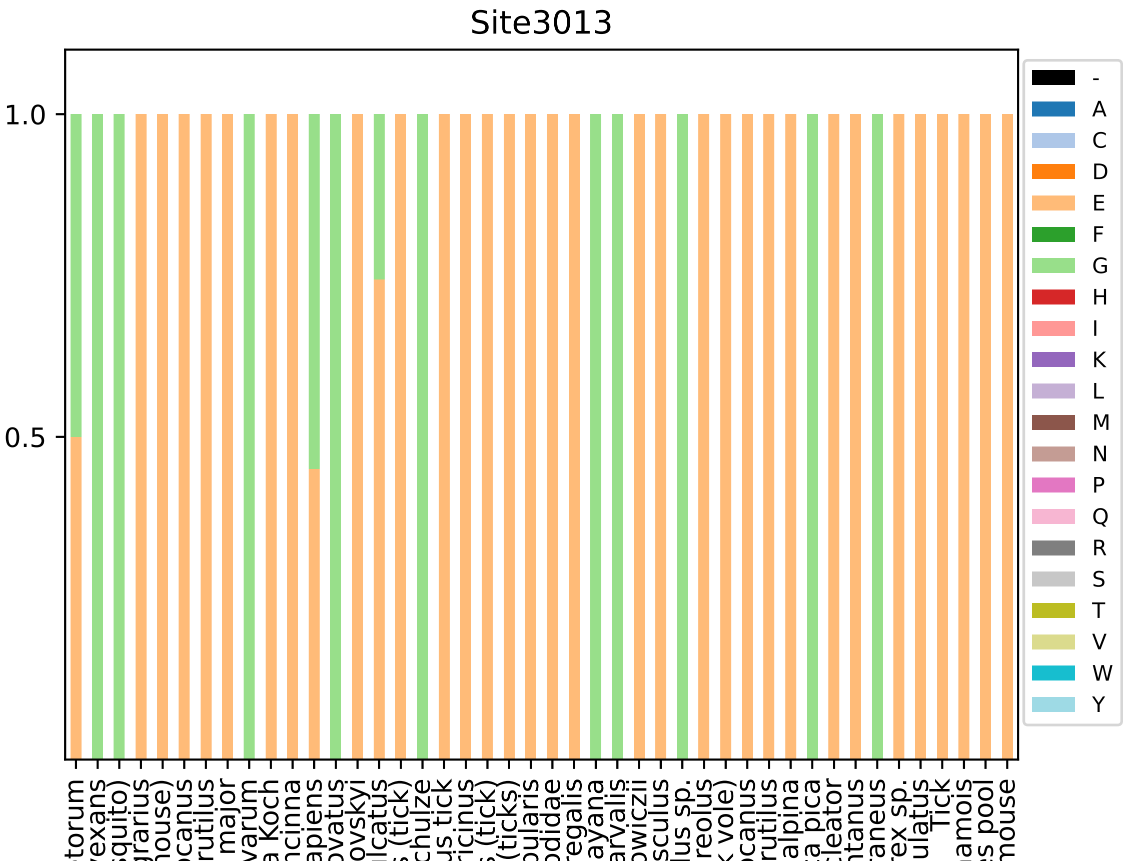
Site3013
1.0
0.5
-
A
C
D
E
F
G
H
I
K
L
M
N
P
Q
R
S
T
V
W
Y

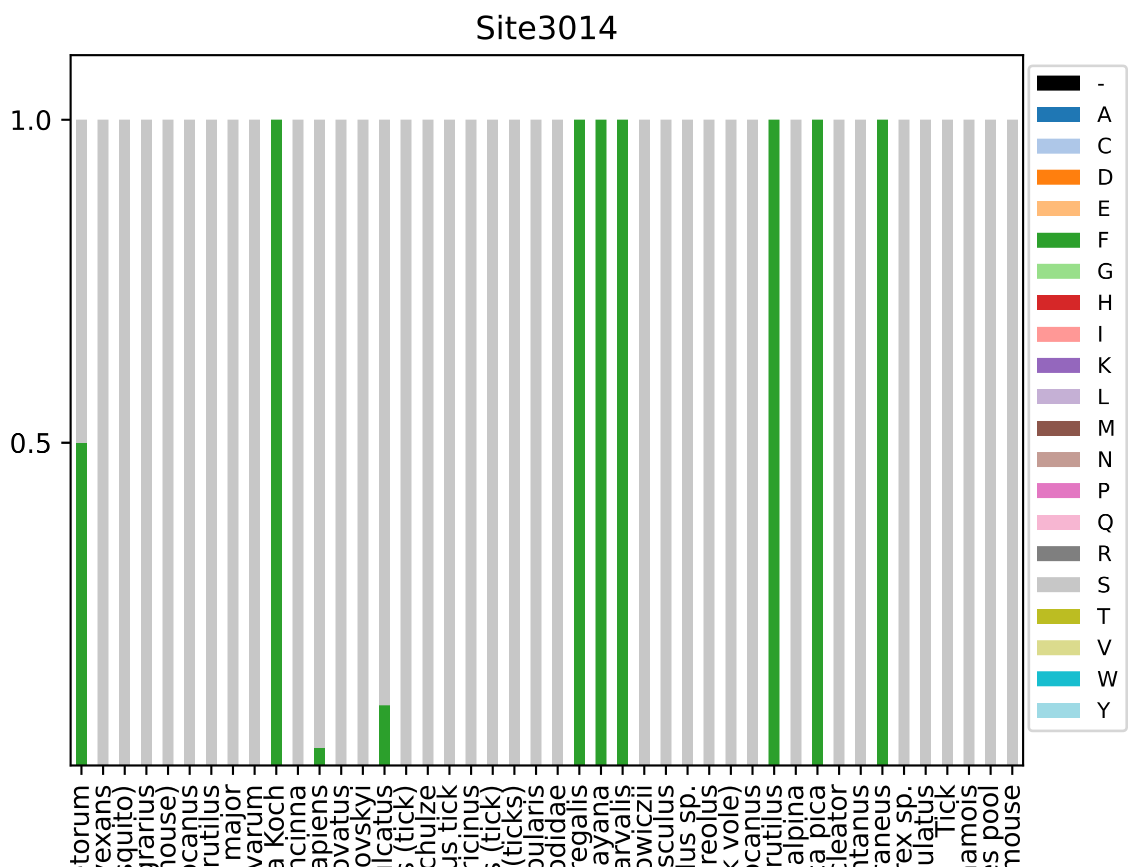

Site3014
1.0
0.5
-
A
C
D
E
F
G
H
I
K
L
M
N
P
Q
R
S
T
V
W
Y

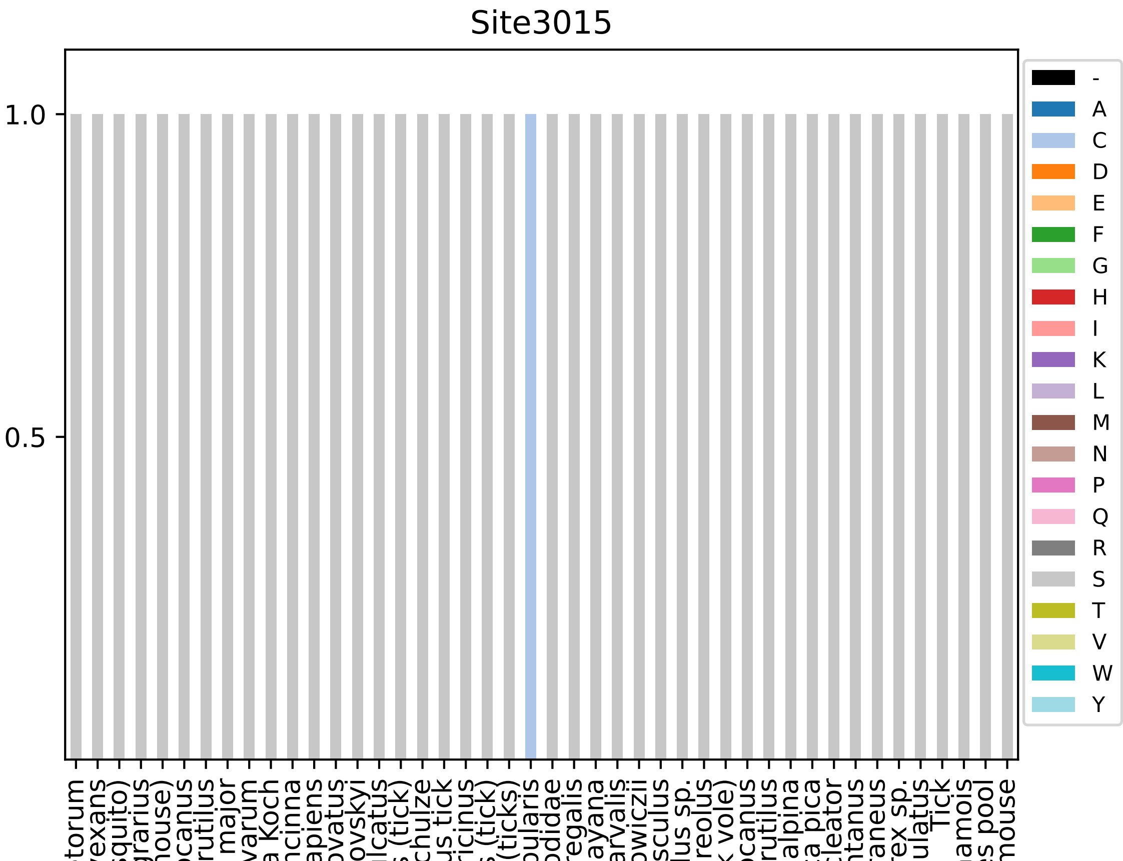

Site3015
1.0
0.5
-
A
C
D
E
F
G
H
I
K
L
M
N
P
Q
R
S
T
V
W
Y

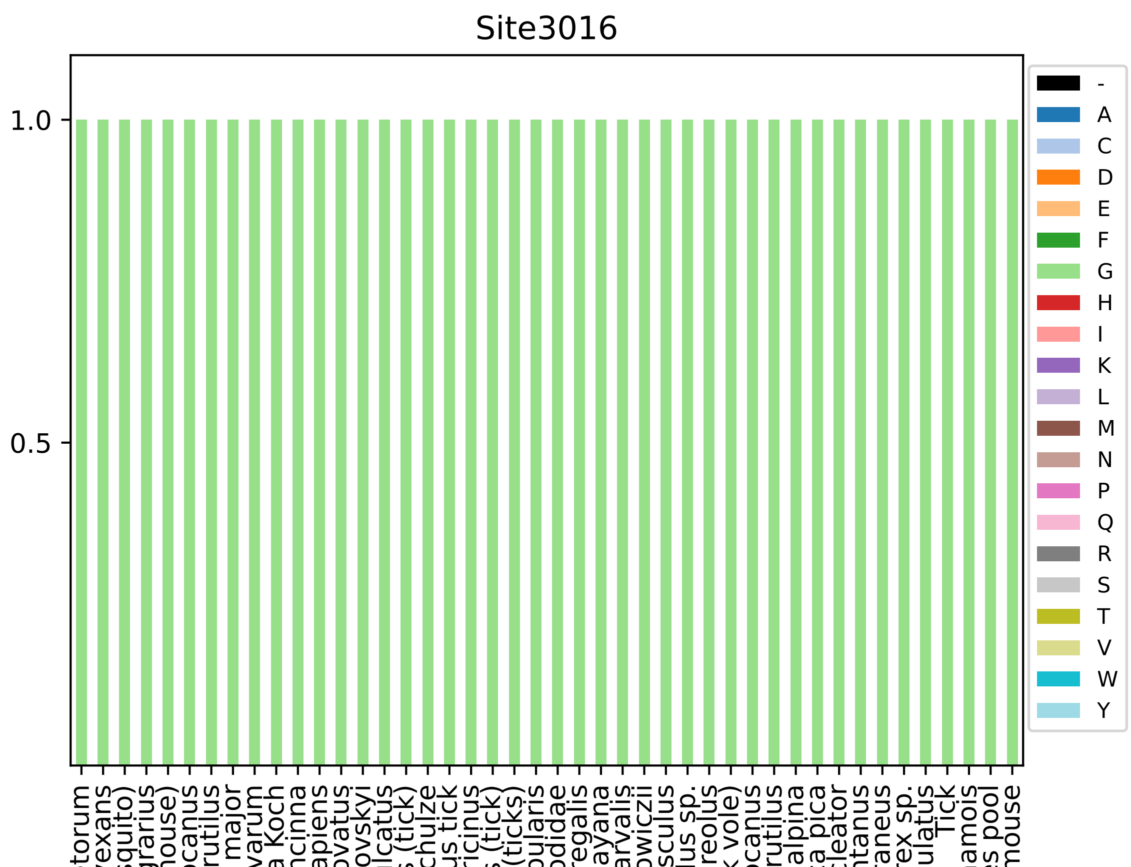
Site3016
1.0
0.5
-
A
C
D
E
F
G
H
I
K
L
M
N
P
Q
R
S
T
V
W
Y

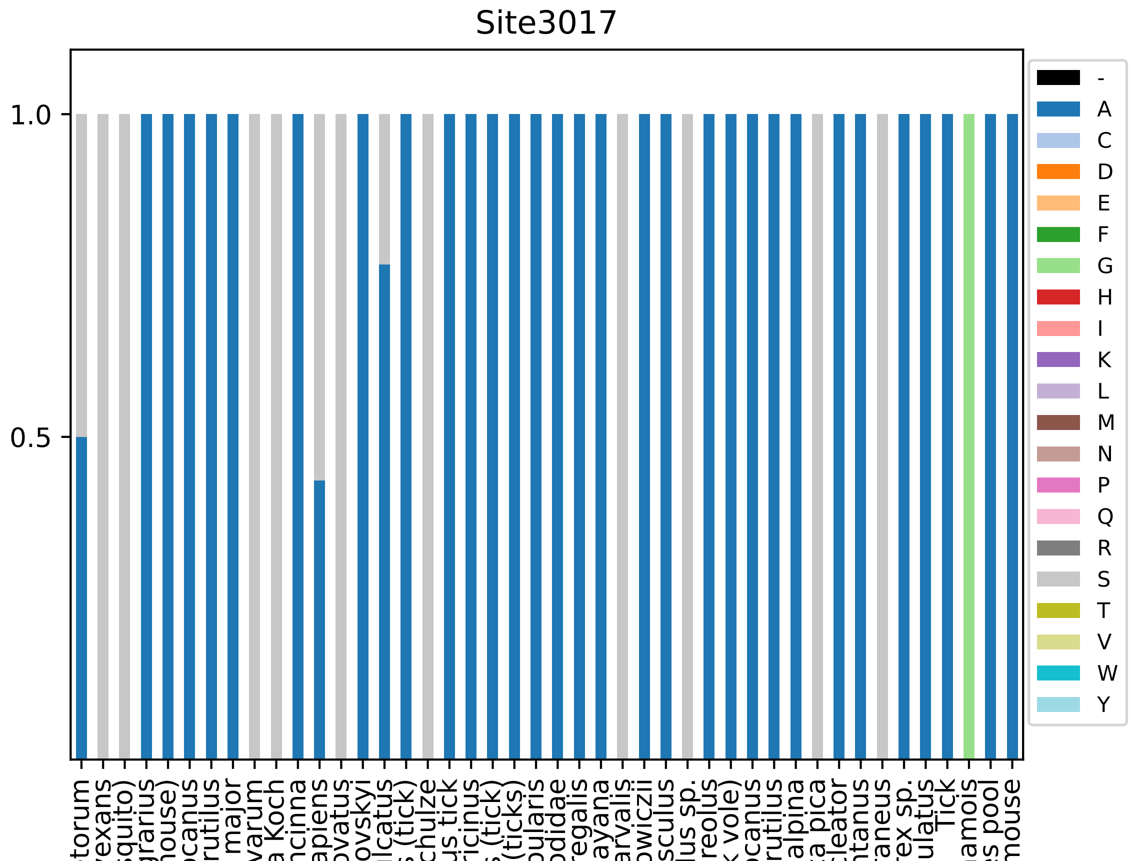

Site3017
1.0
0.5
-
A
C
D
E
F
G
H
I
K
L
M
N
P
Q
R
S
T
V
W
Y

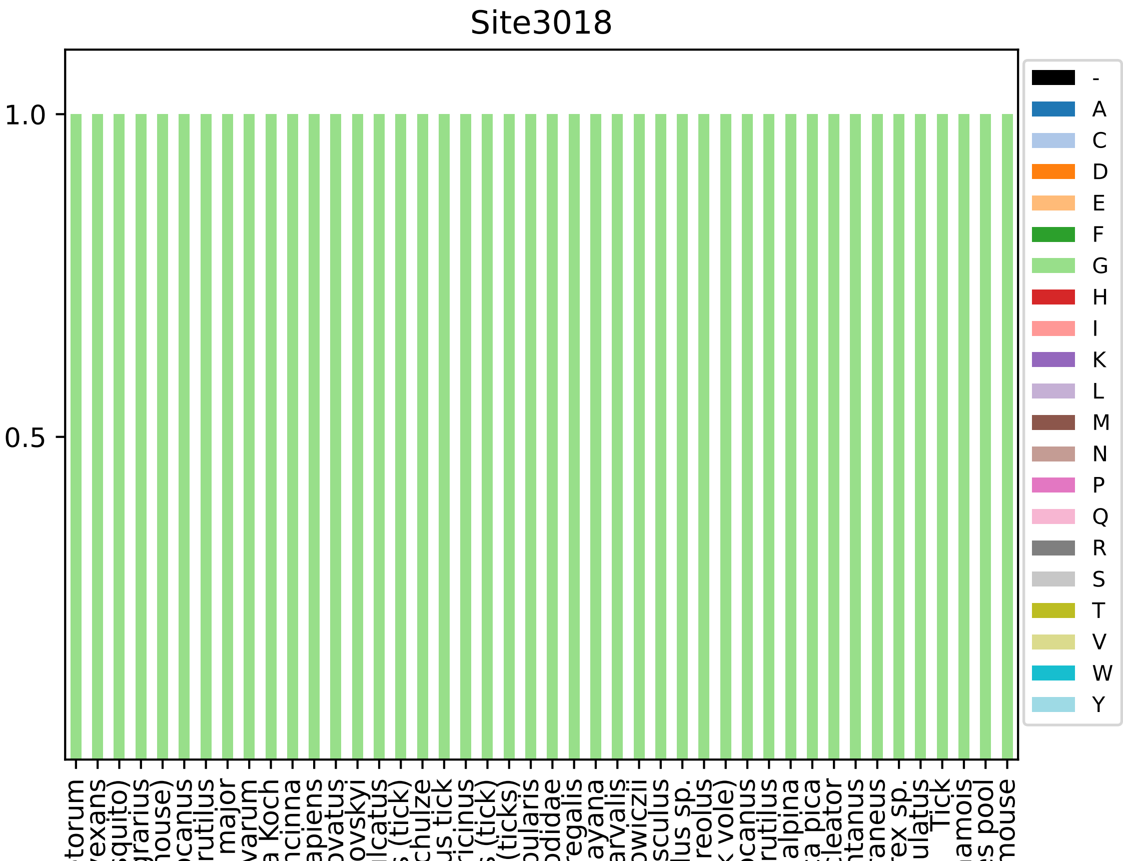
Site3018
1.0
0.5
-
A
C
D
E
F
G
H
I
K
L
M
N
P
Q
R
S
T
V
W
Y

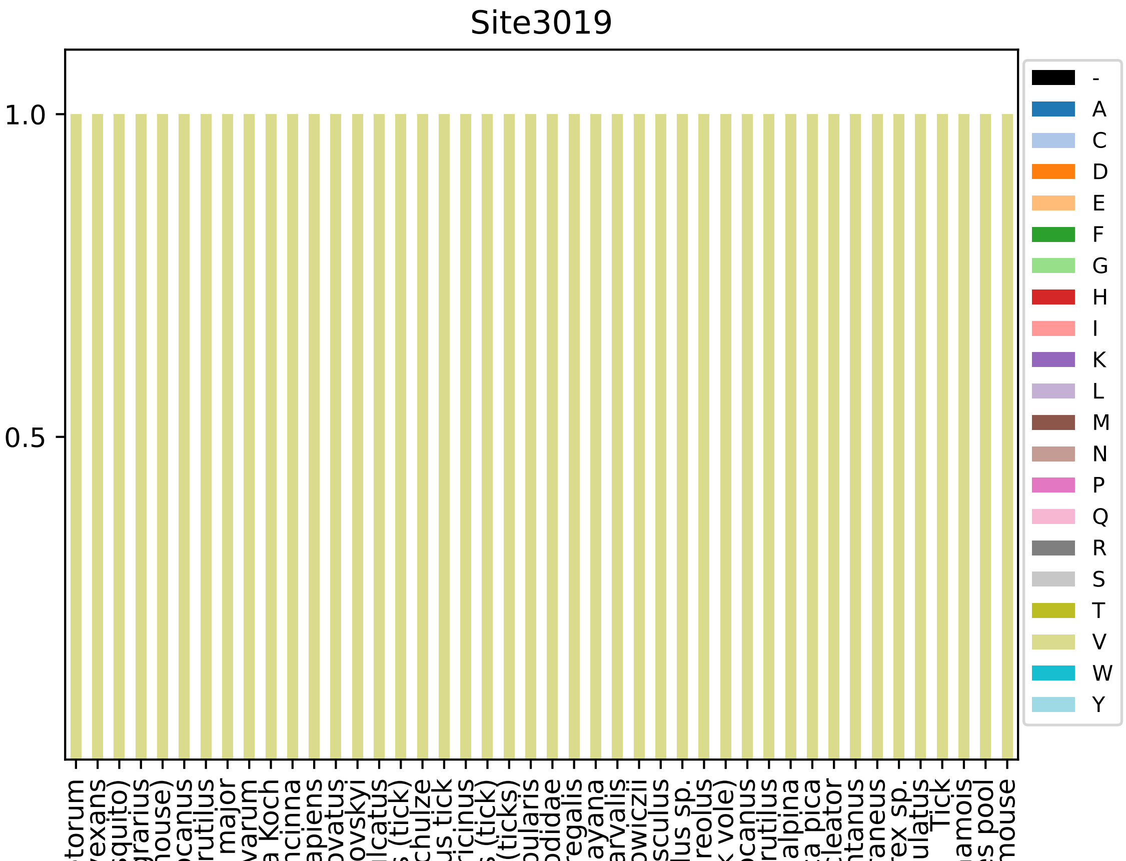
Site3019
1.0
0.5
-
A
C
D
E
F
G
H
I
K
L
M
N
P
Q
R
S
T
V
W
Y

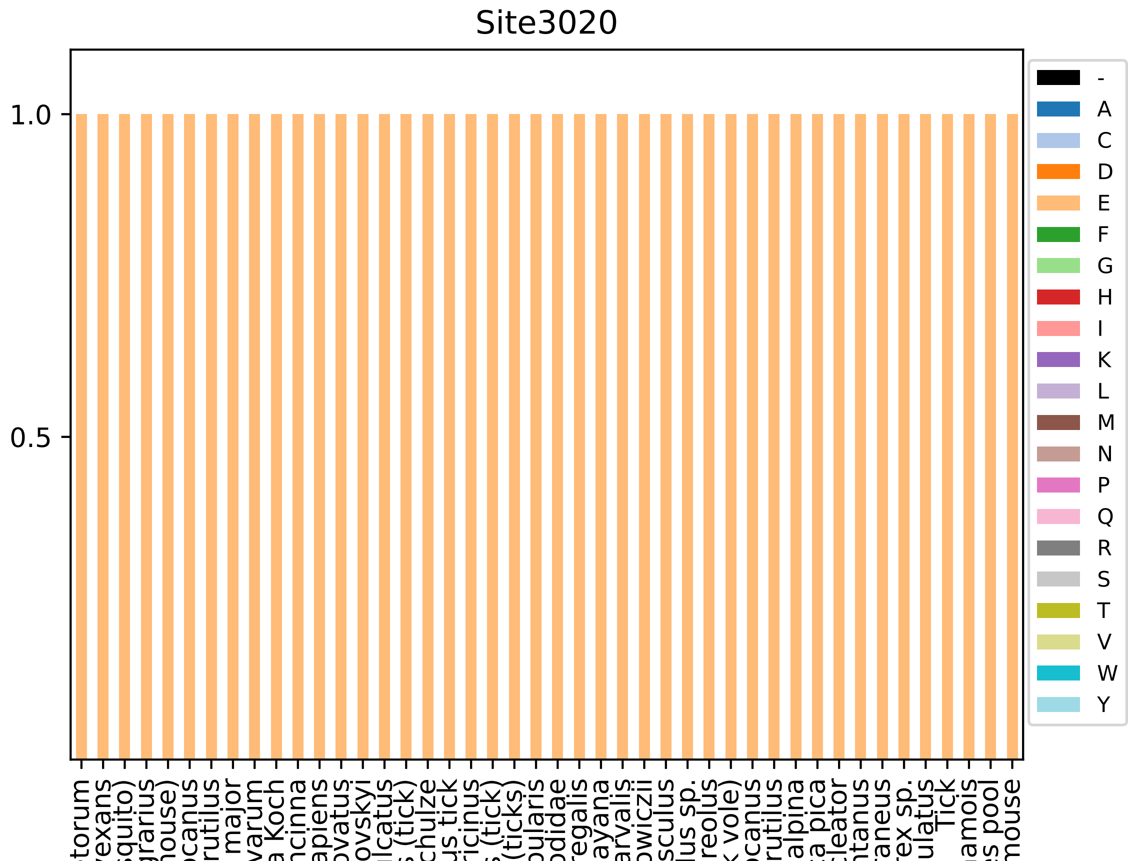

Site3020
1.0
0.5
-
A
C
D
E
F
G
H
I
K
L
M
N
P
Q
R
S
T
V
W
Y

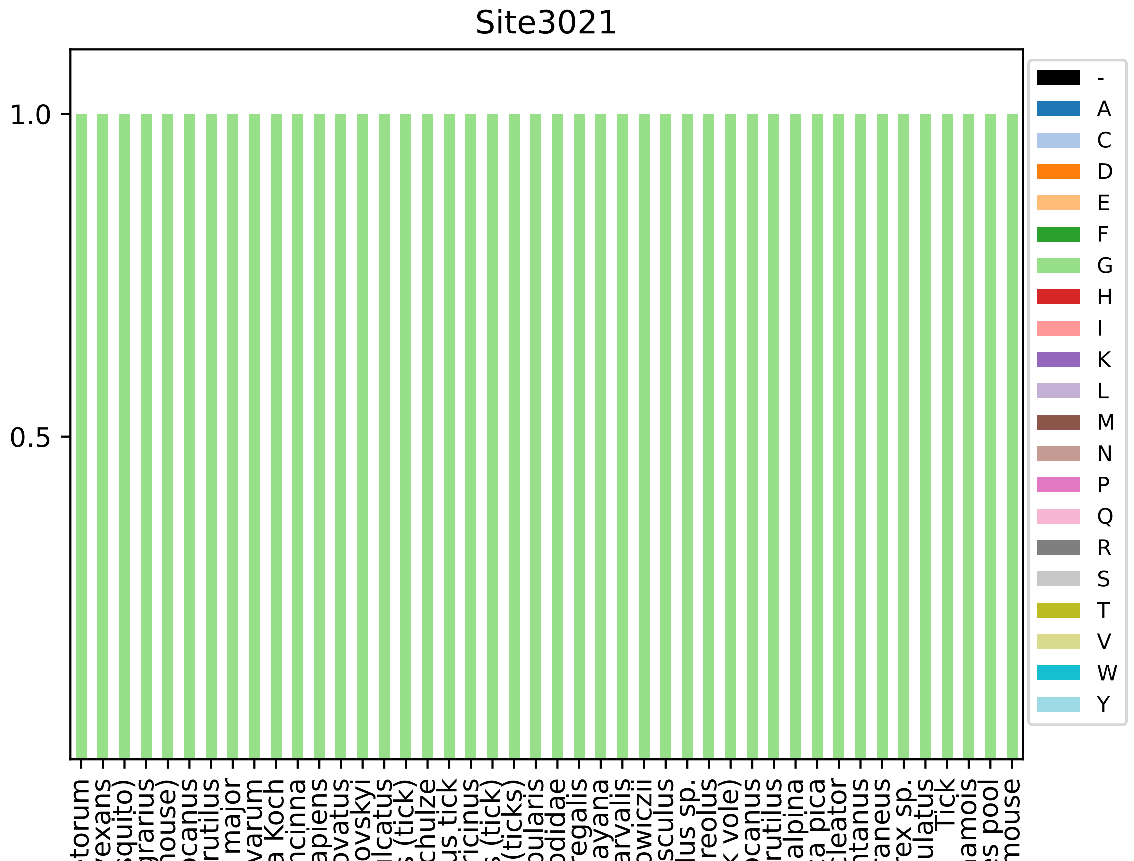

Site3021
1.0
0.5
-
A
C
D
E
F
G
H
I
K
L
M
N
P
Q
R
S
T
V
W
Y

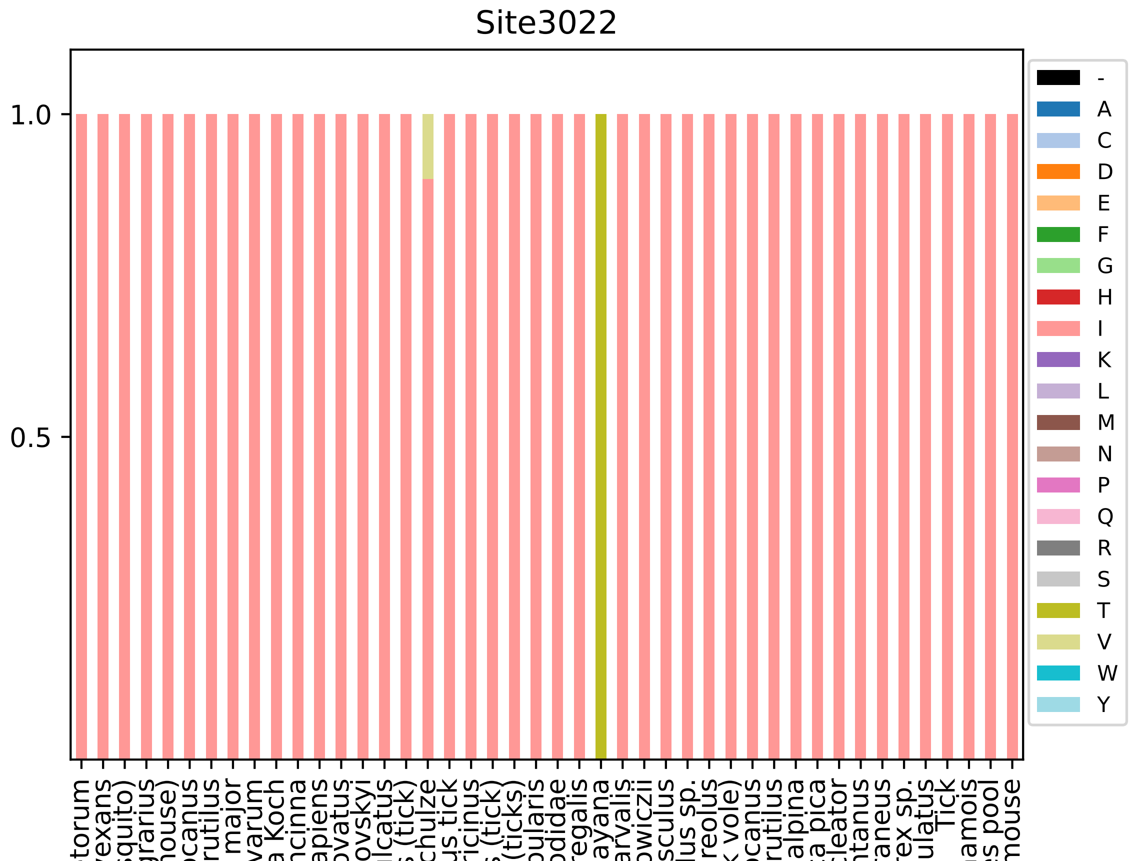
Site3022
1.0
0.5
-
A
C
D
E
F
G
H
I
K
L
M
N
P
Q
R
S
T
V
W
Y

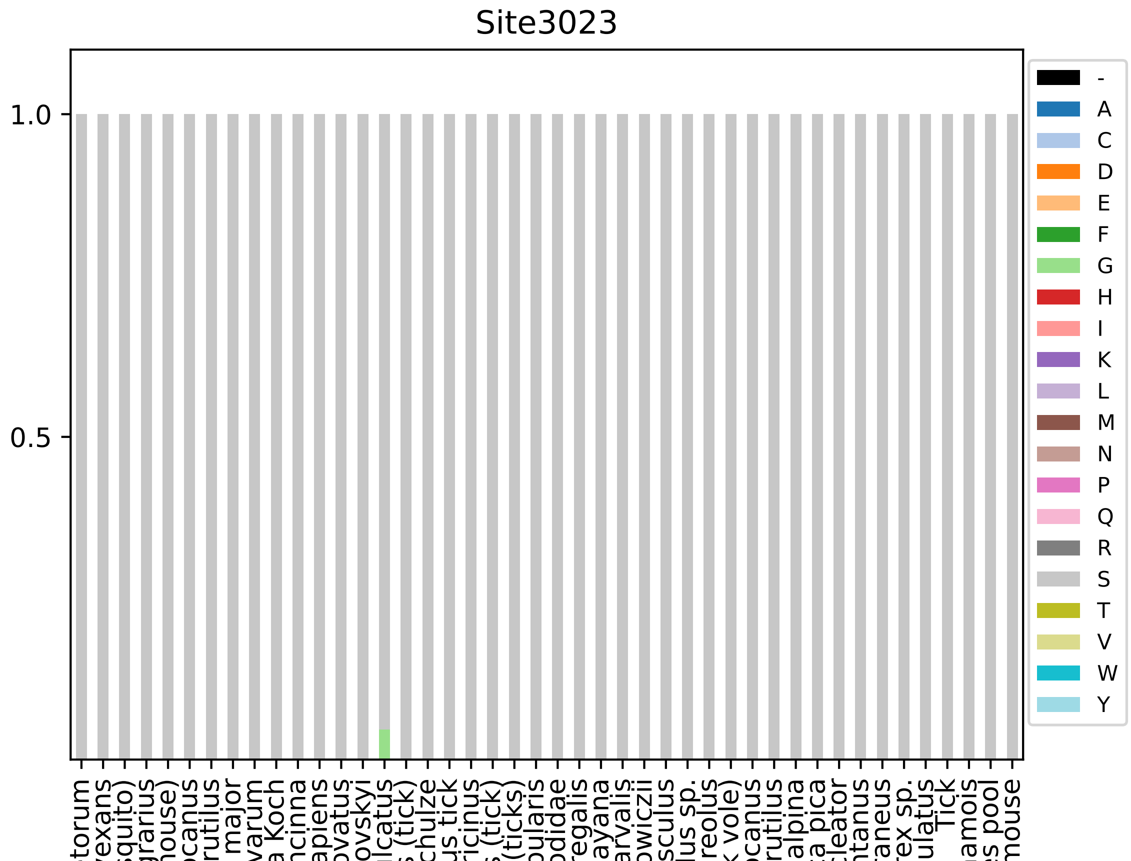

Site3023
1.0
0.5
-
A
C
D
E
F
G
H
I
K
L
M
N
P
Q
R
S
T
V
W
Y

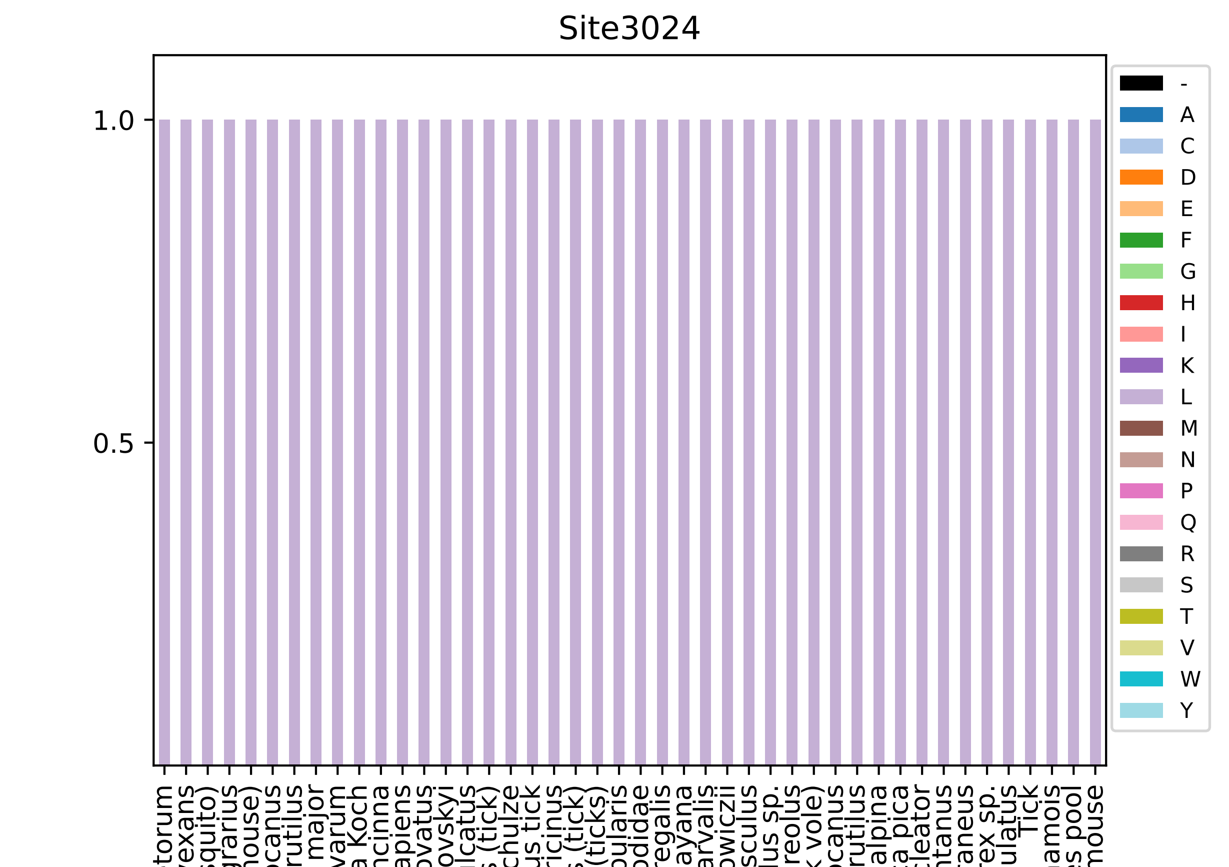

Site3024
1.0
0.5
-
A
C
D
E
F
G
H
I
K
L
M
N
P
Q
R
S
T
V
W
Y

Site3025

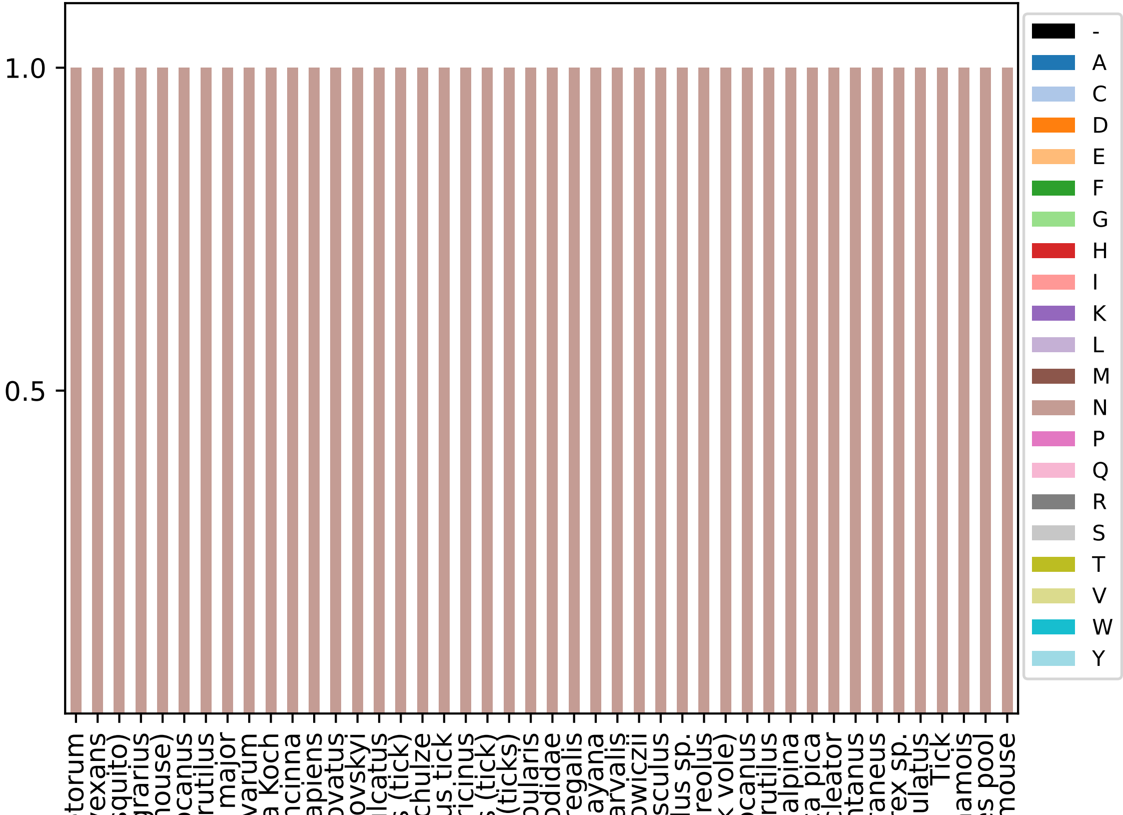

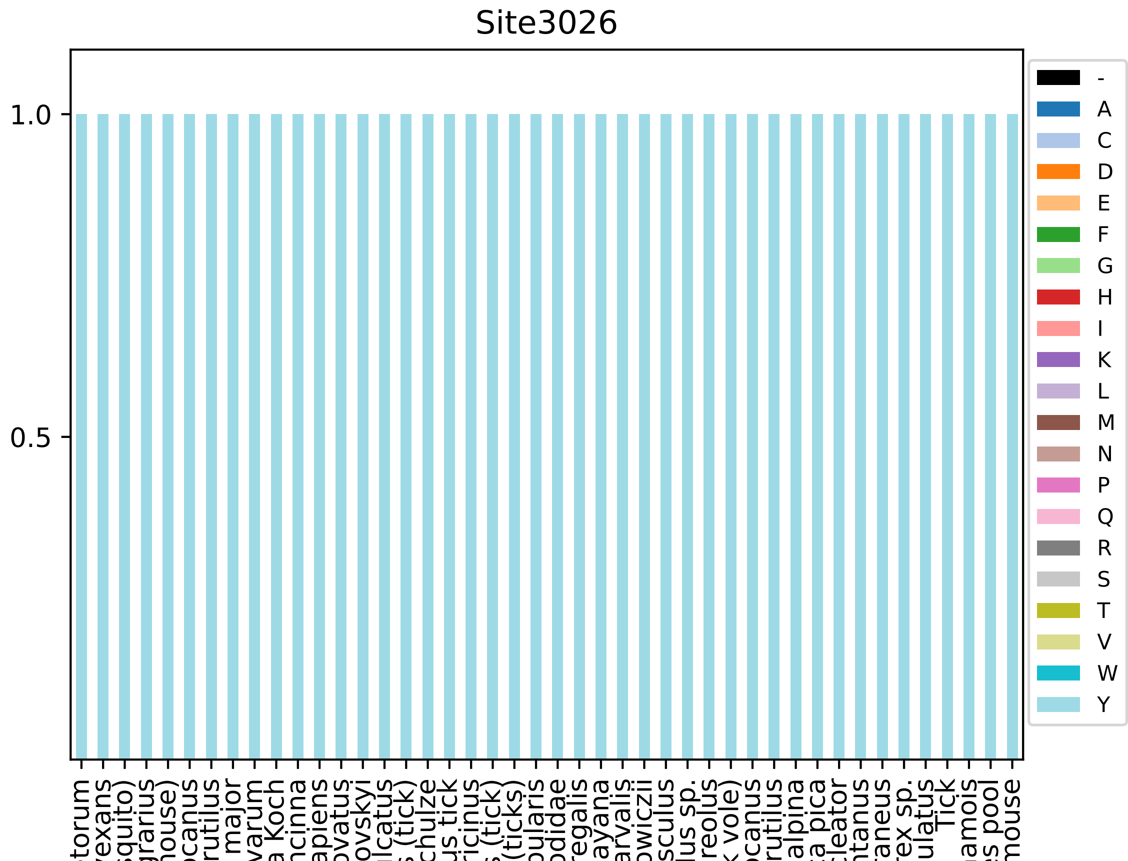
Site3026
1.0
0.5
-
A
C
D
E
F
G
H
I
K
L
M
N
P
Q
R
S
T
V
W
Y

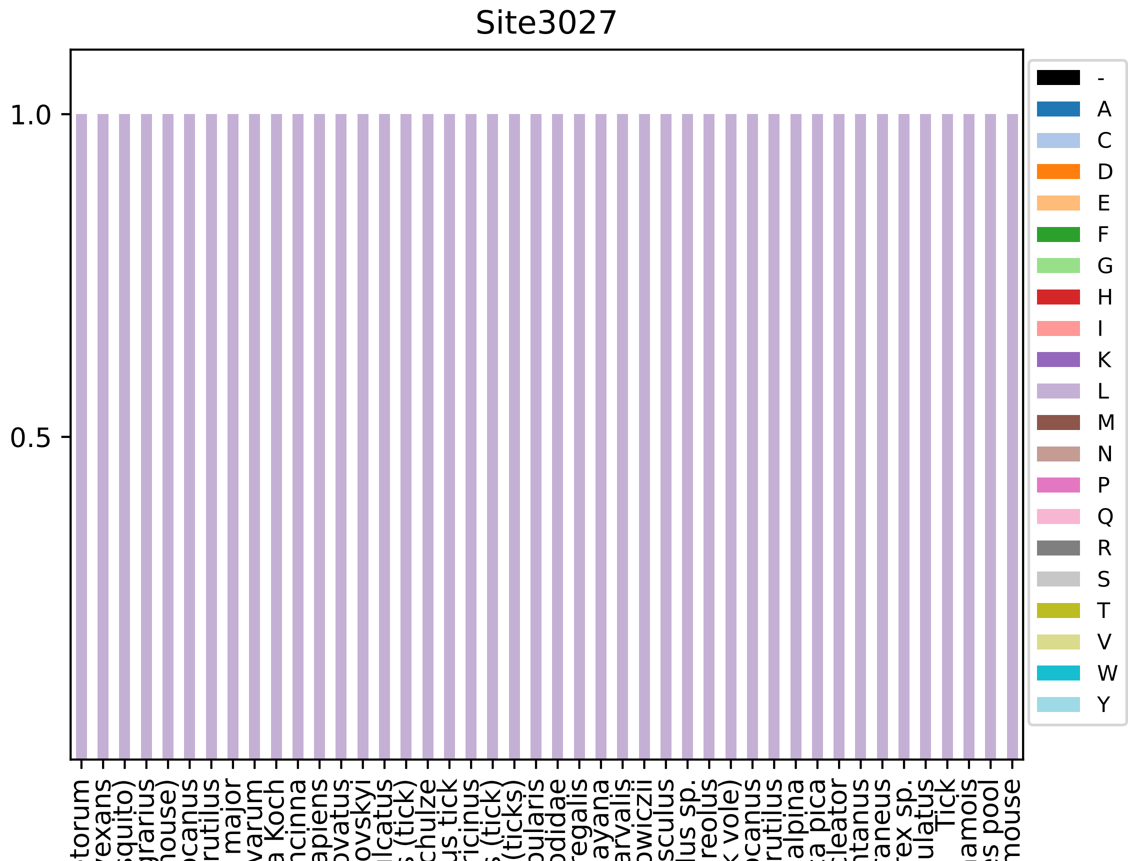
Site3027
1.0
0.5
-
A
C
D
E
F
G
H
I
K
L
M
N
P
Q
R
S
T
V
W
Y

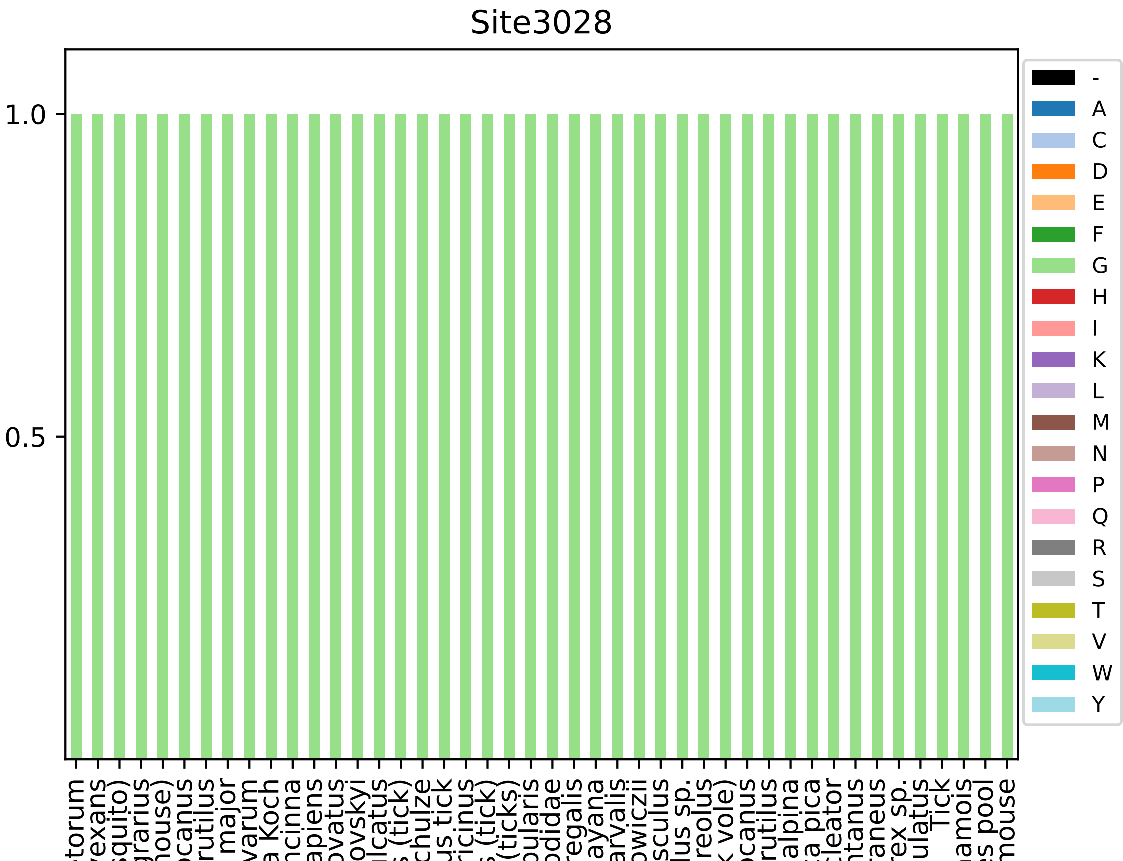

Site3028
1.0
0.5
-
A
C
D
E
F
G
H
I
K
L
M
N
P
Q
R
S
T
V
W
Y

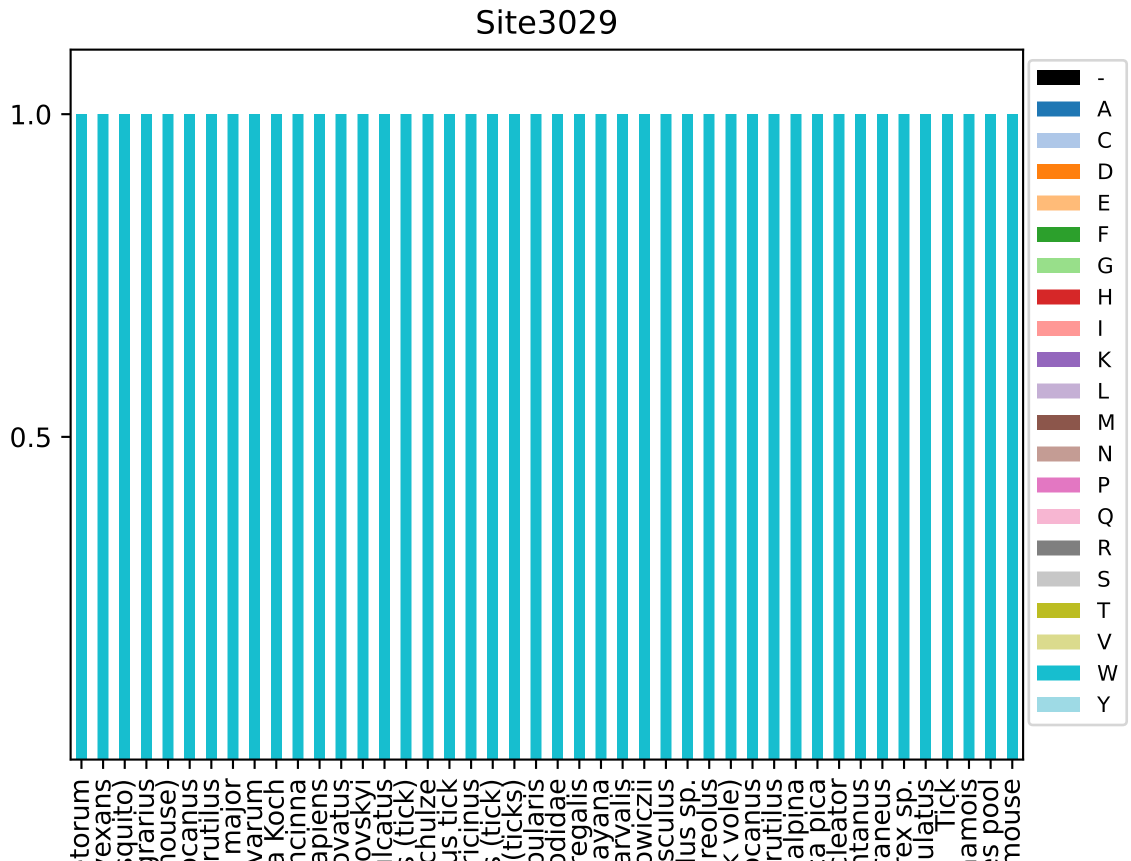
Site3029
1.0
0.5
-
A
C
D
E
F
G
H
I
K
L
M
N
P
Q
R
S
T
V
W
Y

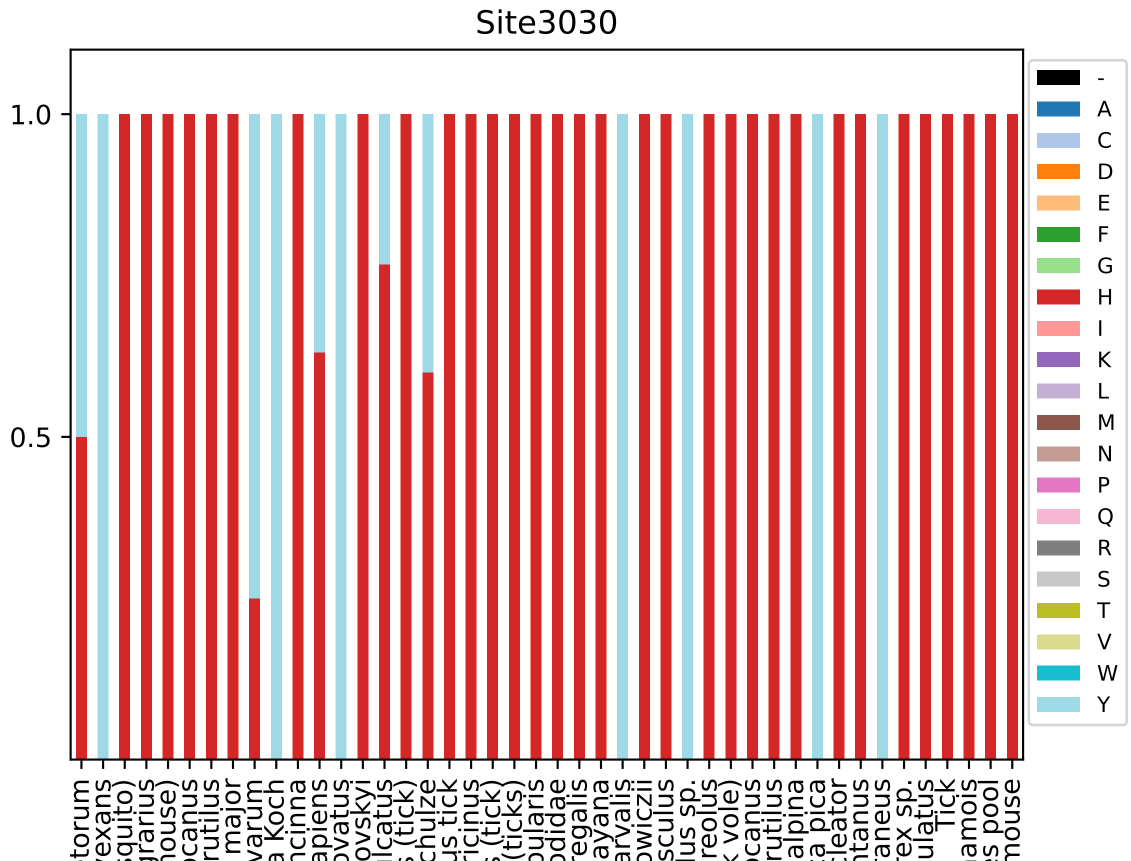
Site3030
1.0
0.5
-
A
C
D
E
F
G
H
I
K
L
M
N
P
Q
R
S
T
V
W
Y

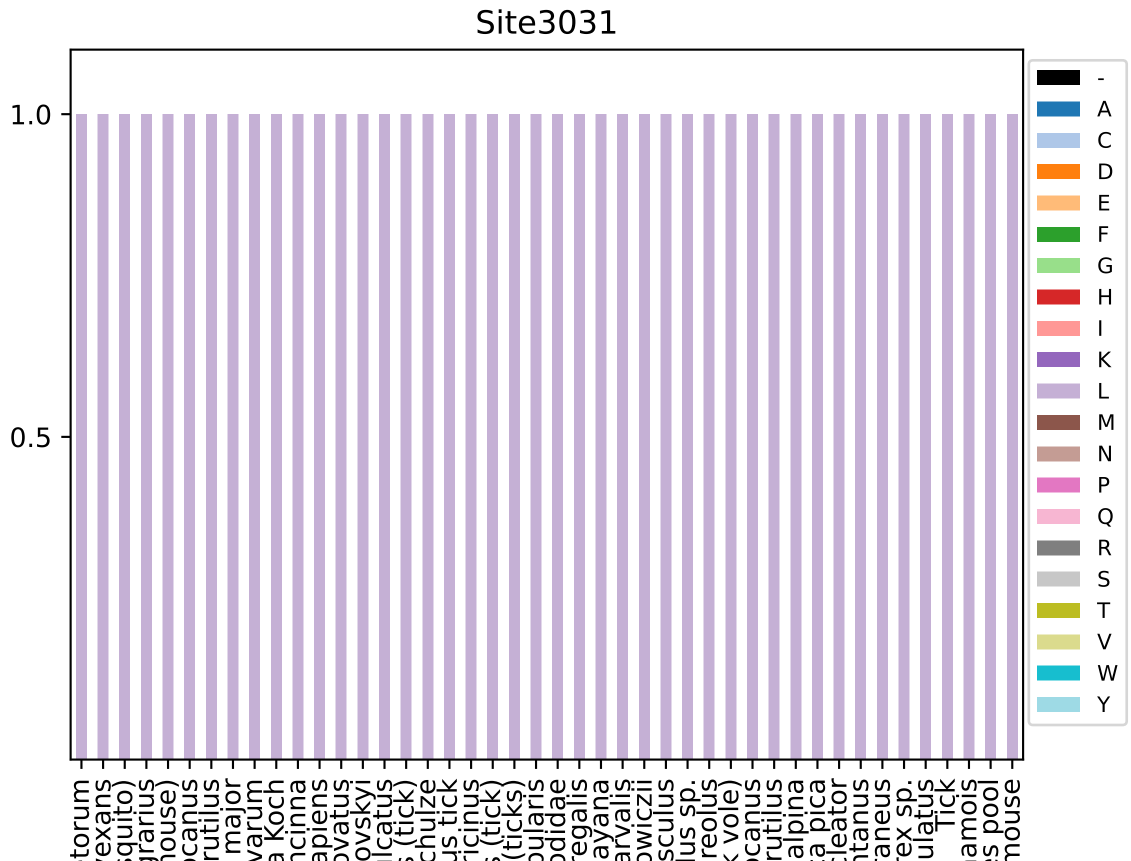

Site3031
1.0
0.5
-
A
C
D
E
F
G
H
I
K
L
M
N
P
Q
R
S
T
V
W
Y

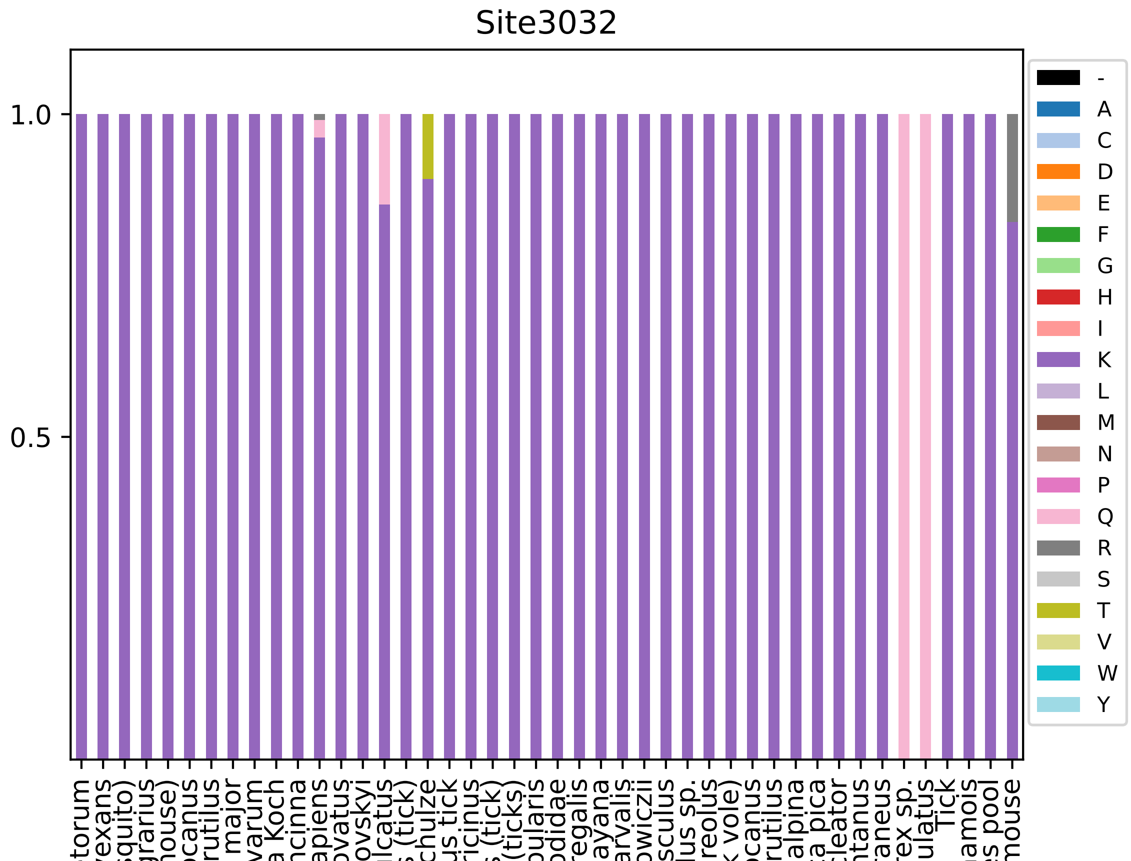

Site3032
1.0
0.5
-
A
C
D
E
F
G
H
I
K
L
M
N
P
Q
R
S
T
V
W
Y

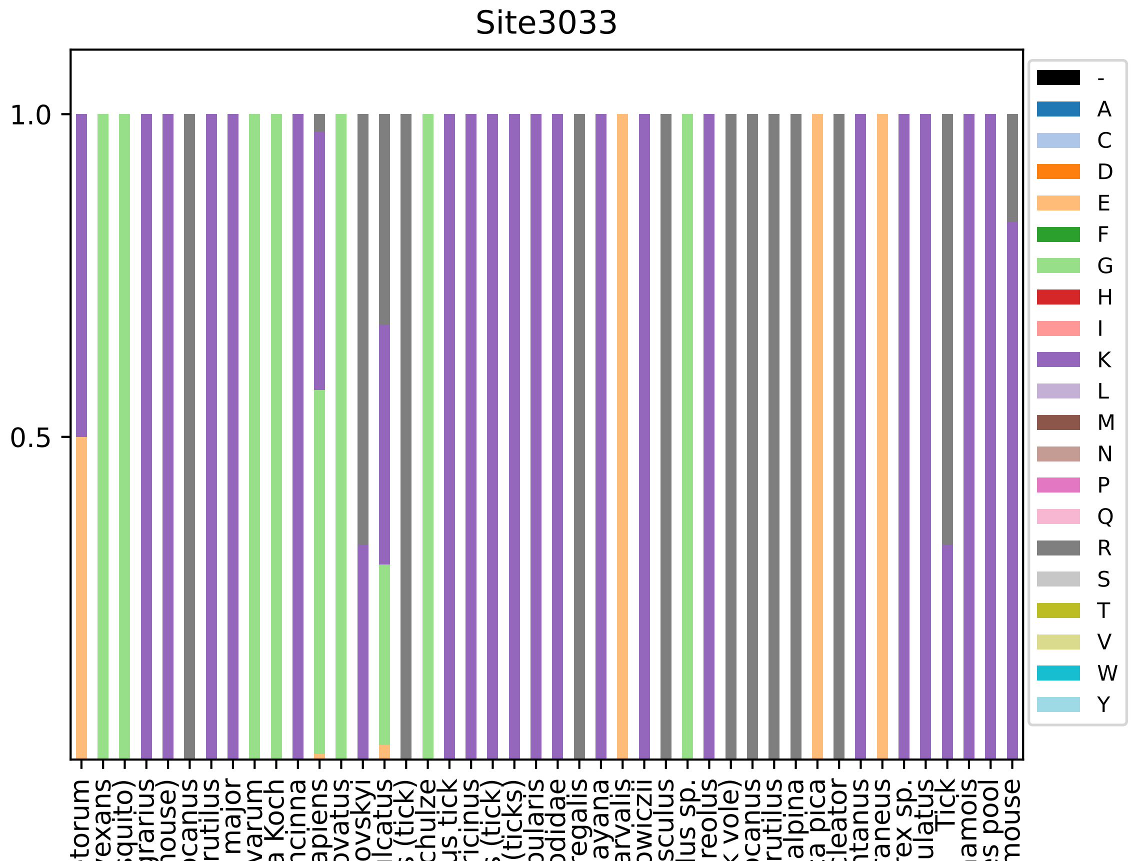

Site3033
1.0
0.5
-
A
C
D
E
F
G
H
I
K
L
M
N
P
Q
R
S
T
V
W
Y

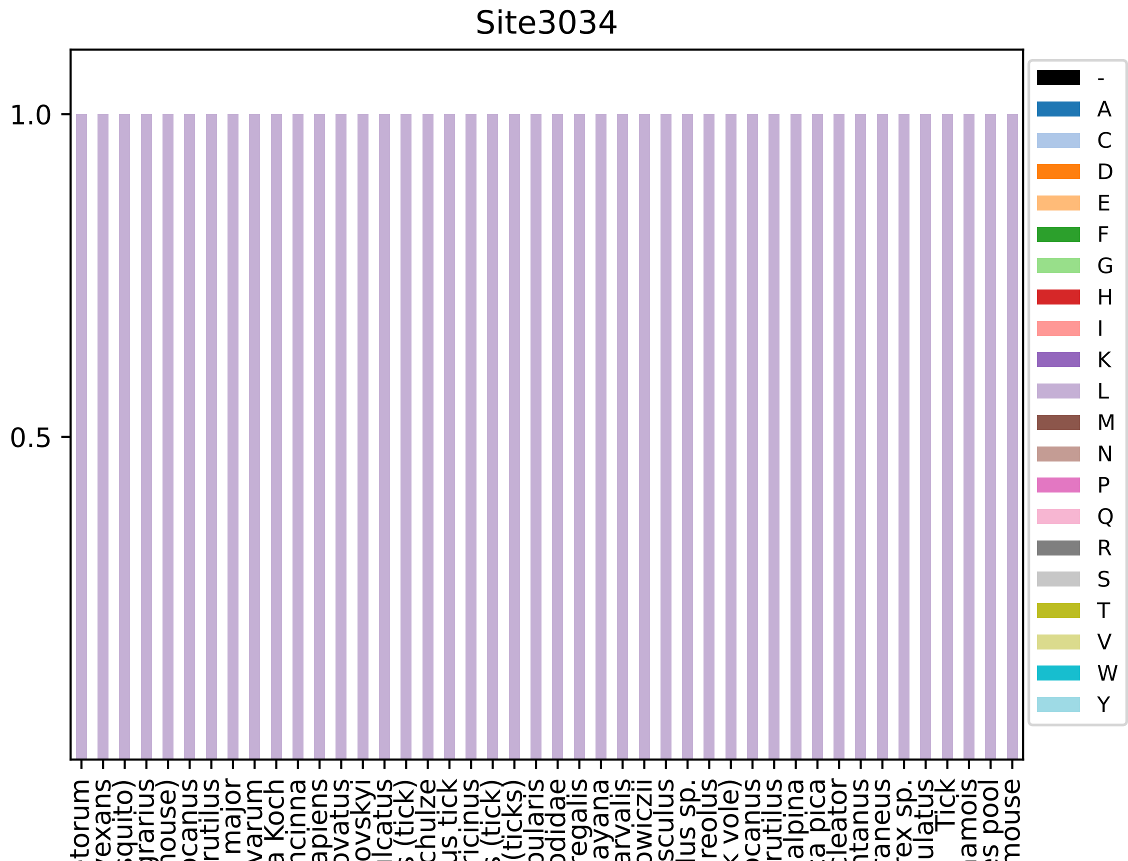

Site3034
1.0
0.5
-
A
C
D
E
F
G
H
I
K
L
M
N
P
Q
R
S
T
V
W
Y

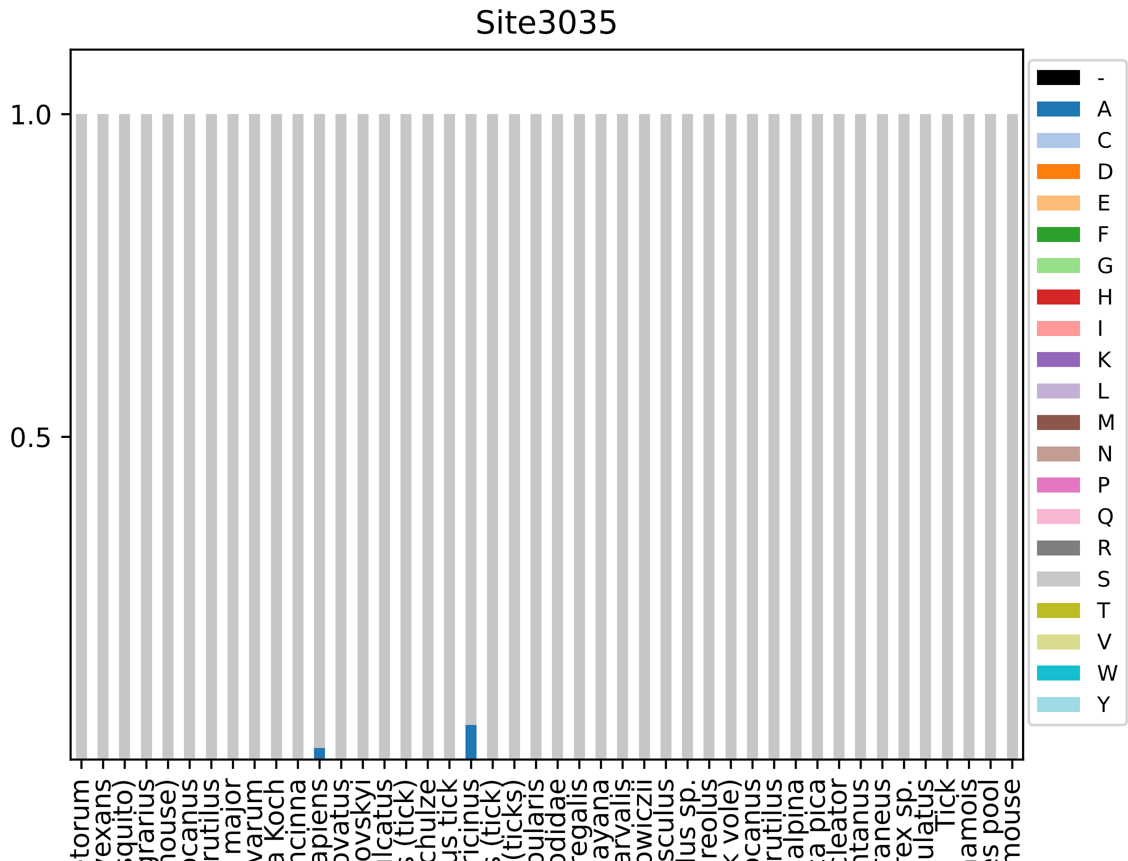
Site3035
1.0
0.5
-
A
C
D
E
F
G
H
I
K
L
M
N
P
Q
R
S
T
V
W
Y
torum
vexans
squito)
garius
house)
canus
rutilus
major
varum
a Koch
ncinna
apiens
ovatus
ovskyi
lcatus
s (tick)
chulze
us tick
ricinus
s (tick)
(ticks)
pularis
podidae
regalis
ayana
arvalis
owiczii
sculus
lus sp.
reolus
k vole)
canus
rutilus
alpina
a pica
cleator
ntanus
raneus
rex sp.
ulatus
Tick
amois
es pool
mouse

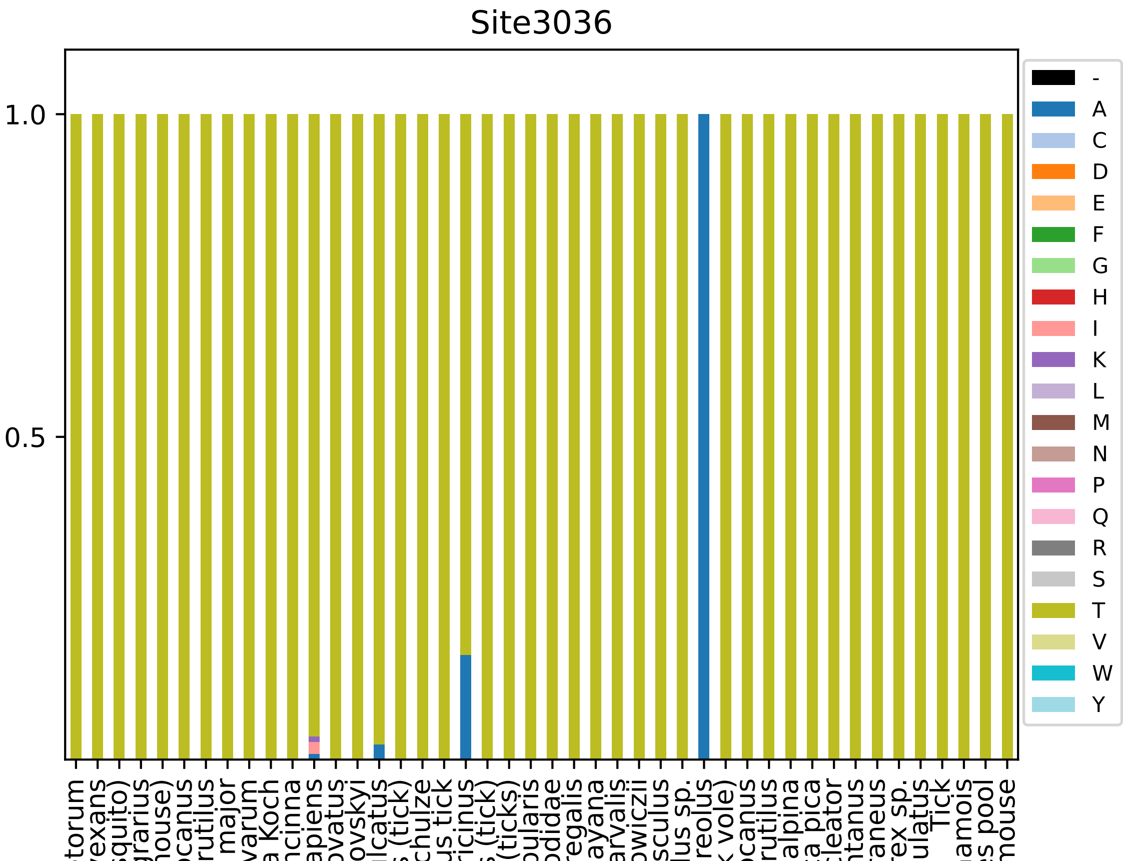
Site3036
1.0
0.5
-
A
C
D
E
F
G
H
I
K
L
M
N
P
Q
R
S
T
V
W
Y

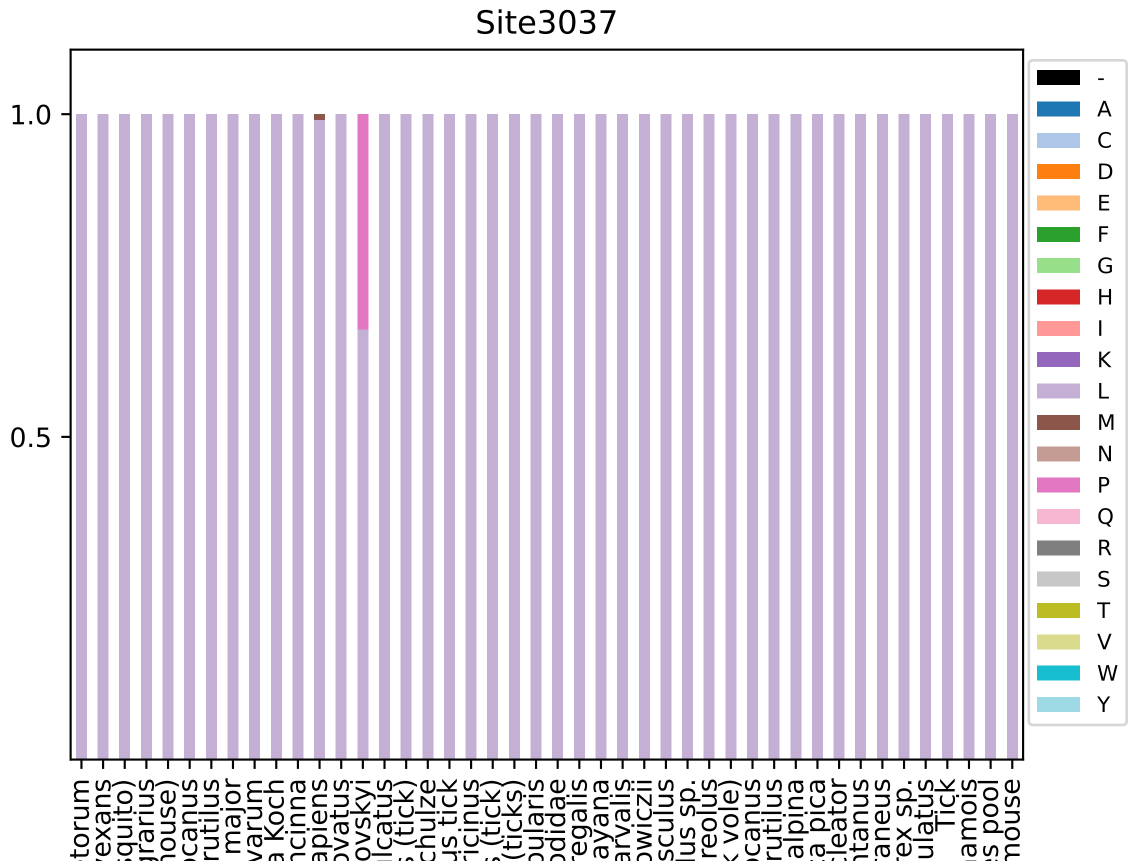

Site3037
1.0
0.5
-
A
C
D
E
F
G
H
I
K
L
M
N
P
Q
R
S
T
V
W
Y

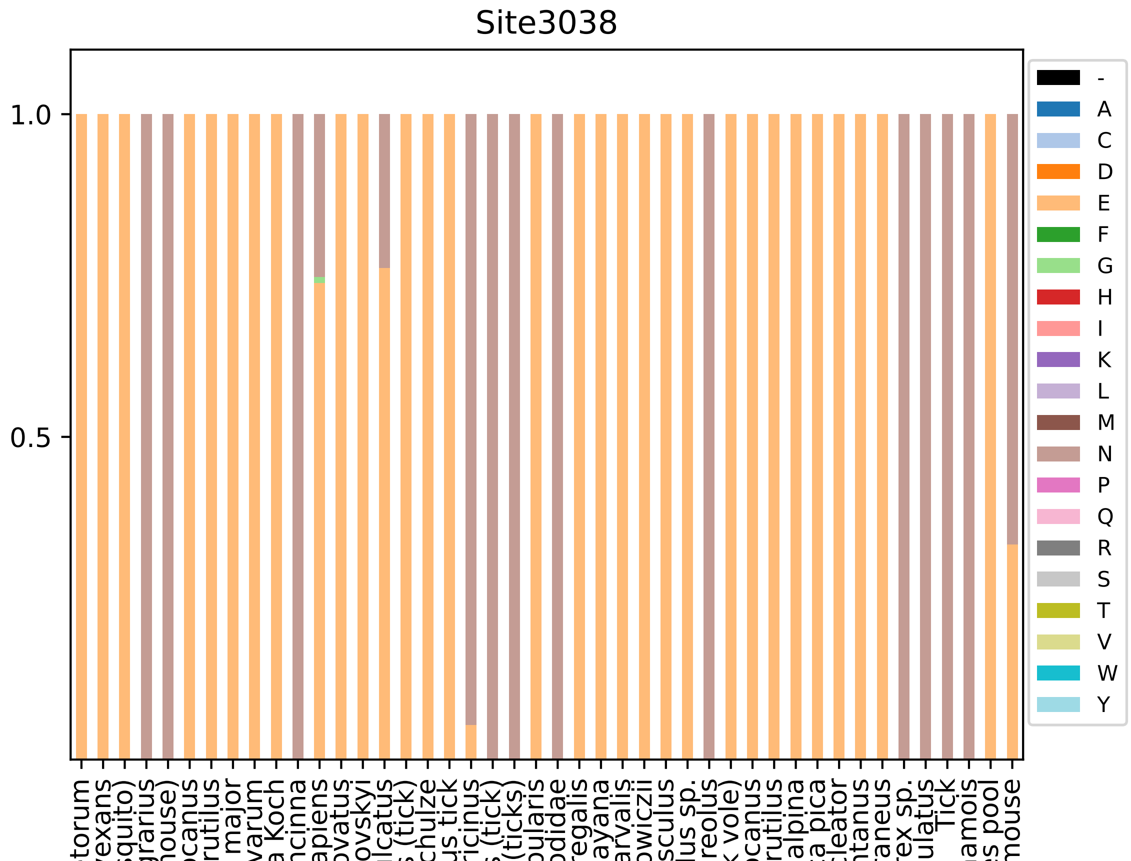
Site3038
1.0
0.5
-
A
C
D
E
F
G
H
I
K
L
M
N
P
Q
R
S
T
V
W
Y

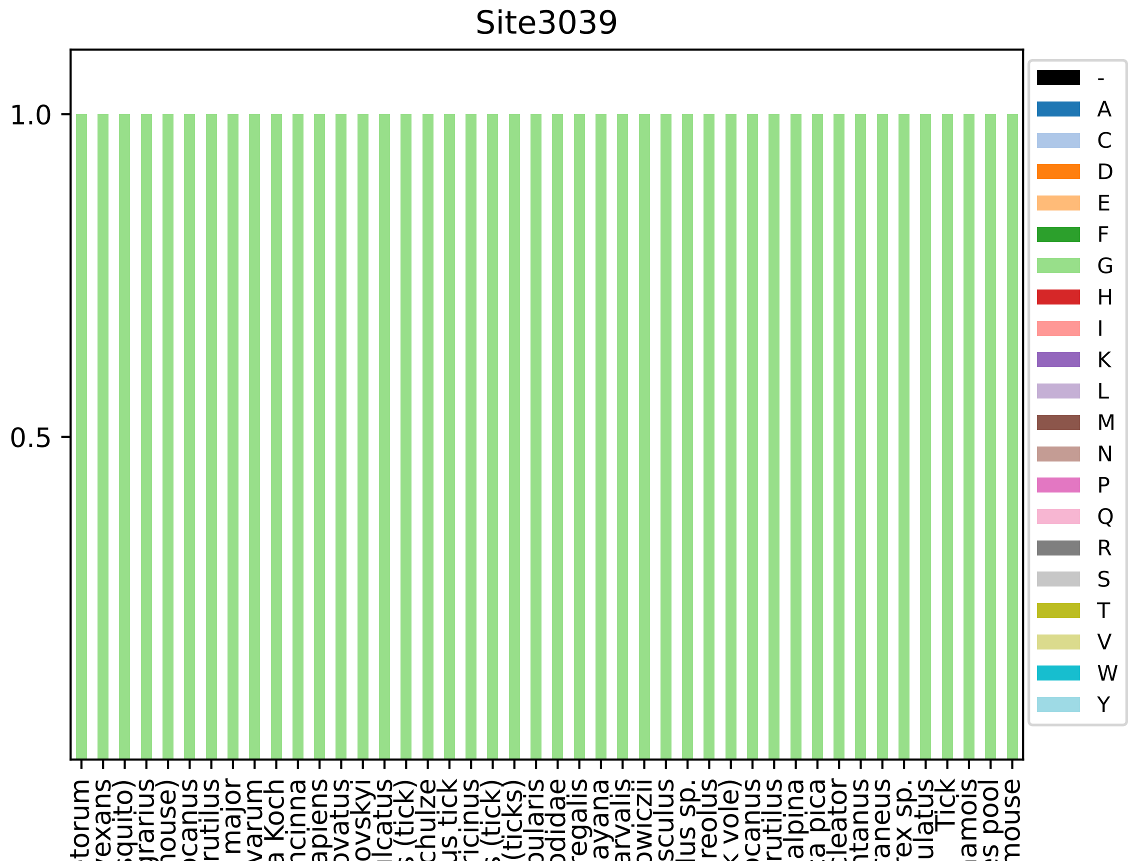
Site3039
1.0
0.5
-
A
C
D
E
F
G
H
I
K
L
M
N
P
Q
R
S
T
V
W
Y

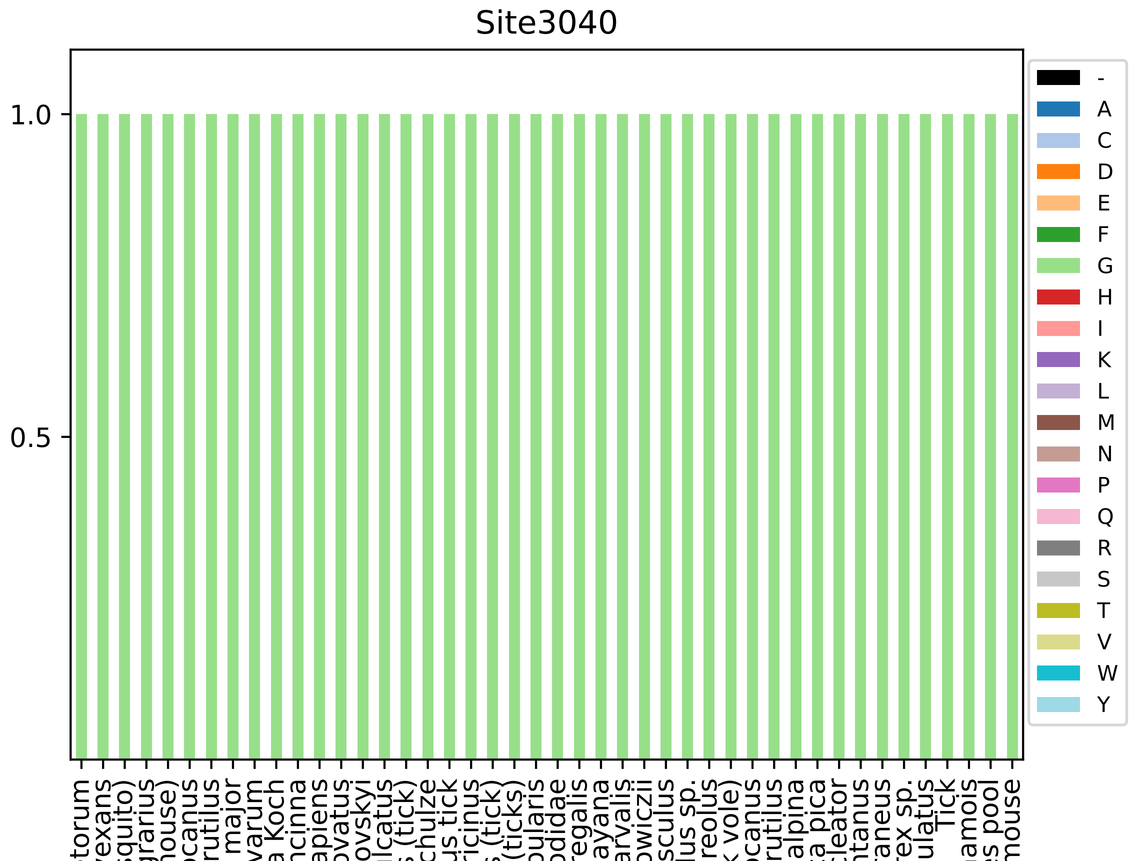

Site3040
1.0
0.5
-
A
C
D
E
F
G
H
I
K
L
M
N
P
Q
R
S
T
V
W
Y

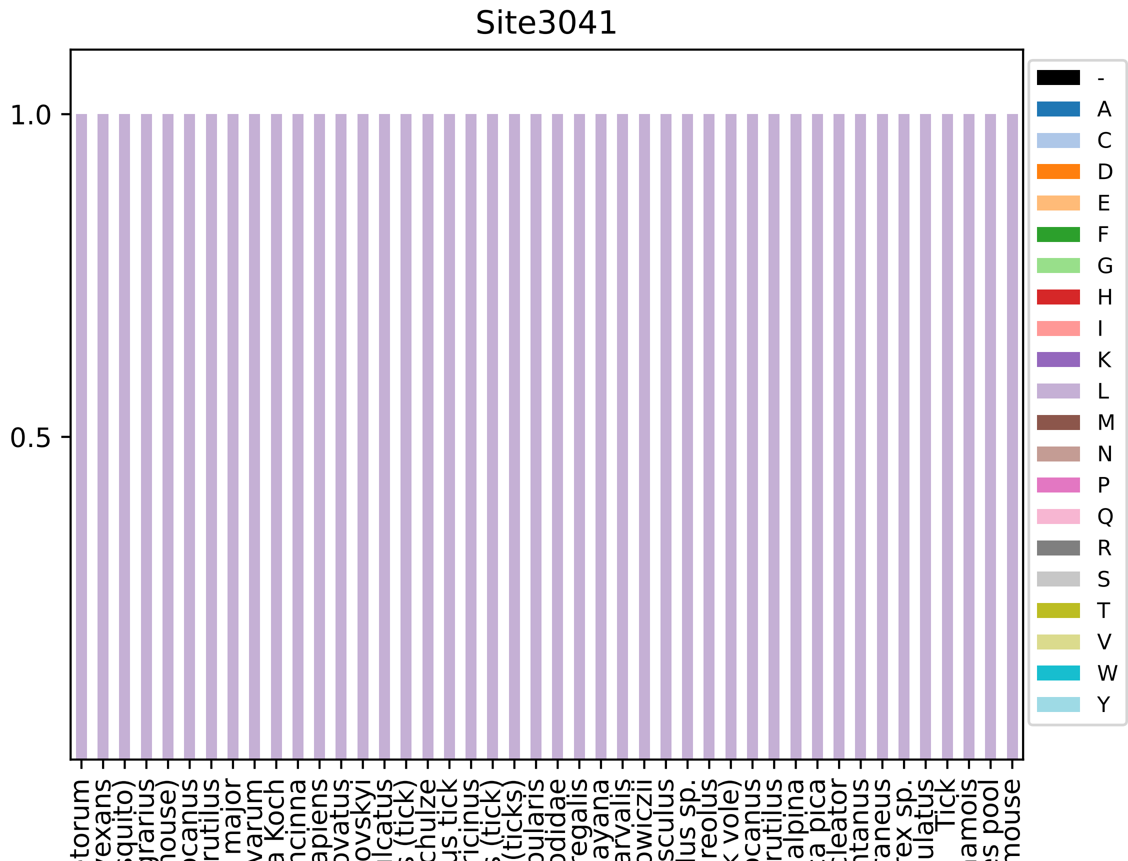

Site3041
1.0
0.5
-
A
C
D
E
F
G
H
I
K
L
M
N
P
Q
R
S
T
V
W
Y

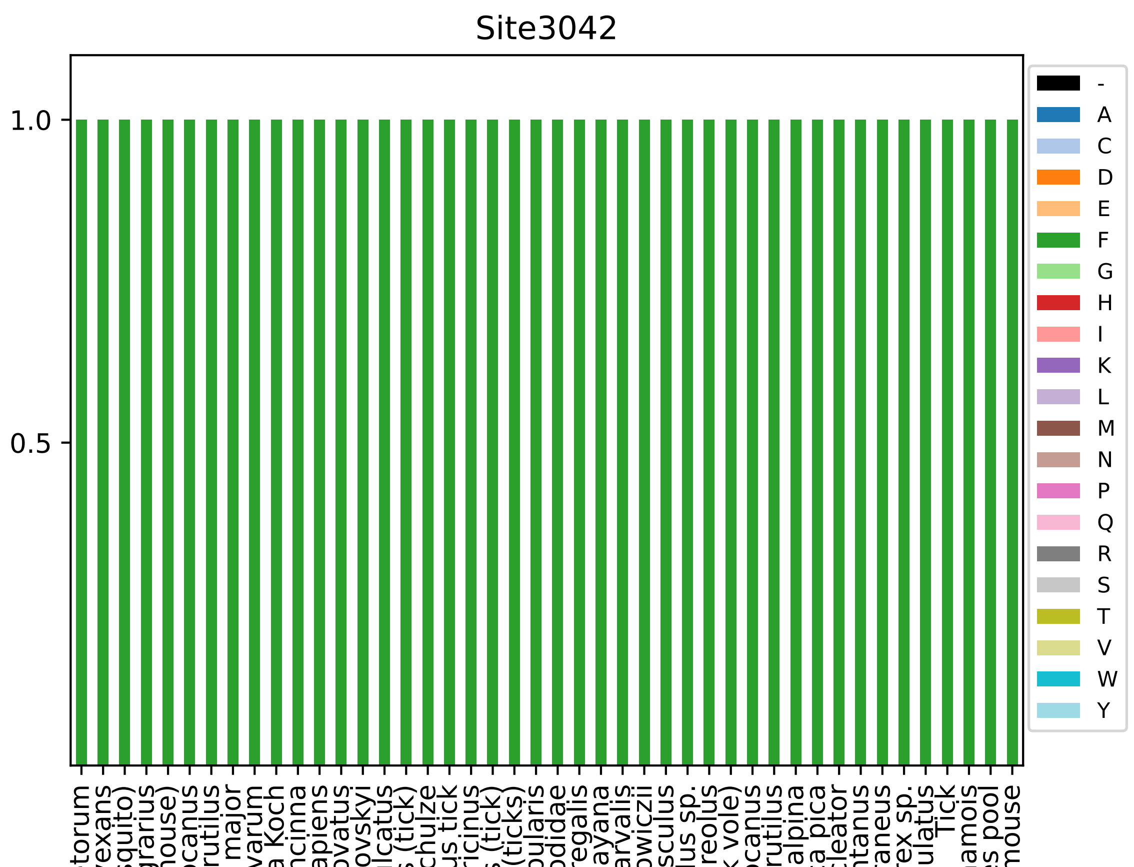
Site3042
1.0
0.5
-
A
C
D
E
F
G
H
I
K
L
M
N
P
Q
R
S
T
V
W
Y

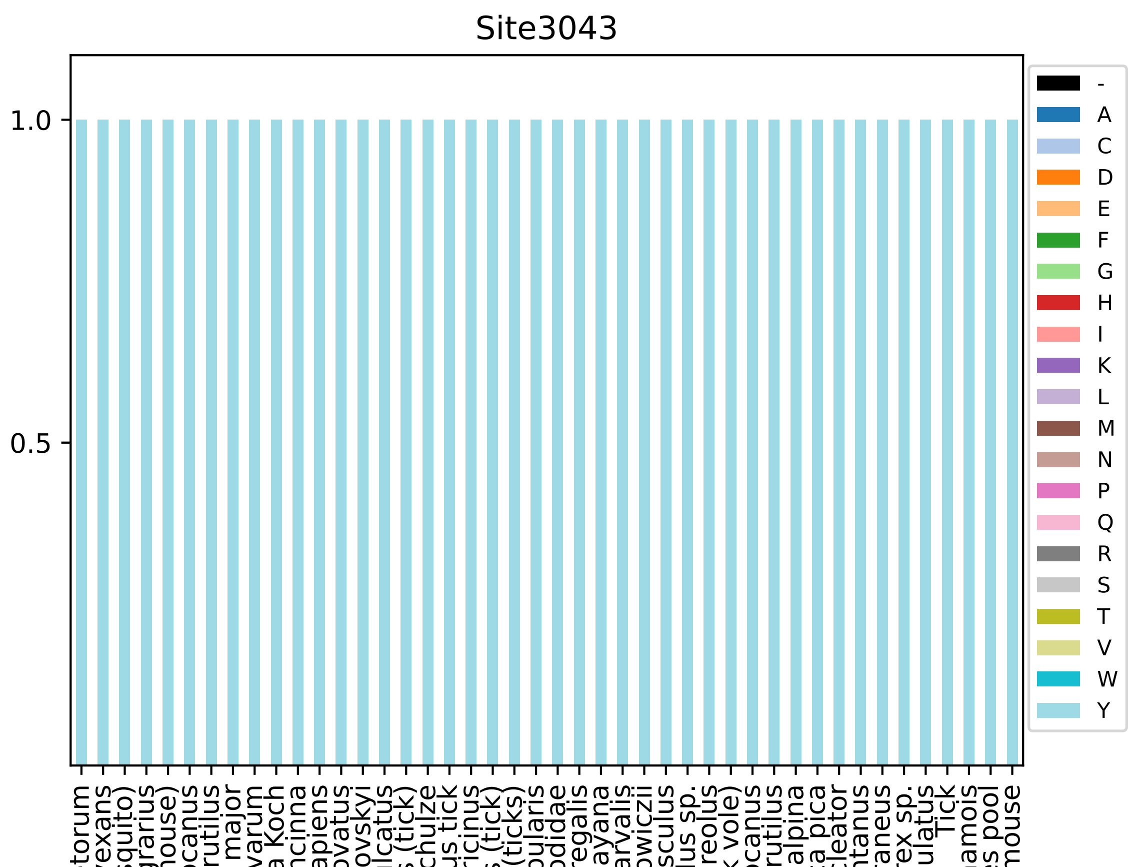

Site3043
1.0
0.5
-
A
C
D
E
F
G
H
I
K
L
M
N
P
Q
R
S
T
V
W
Y

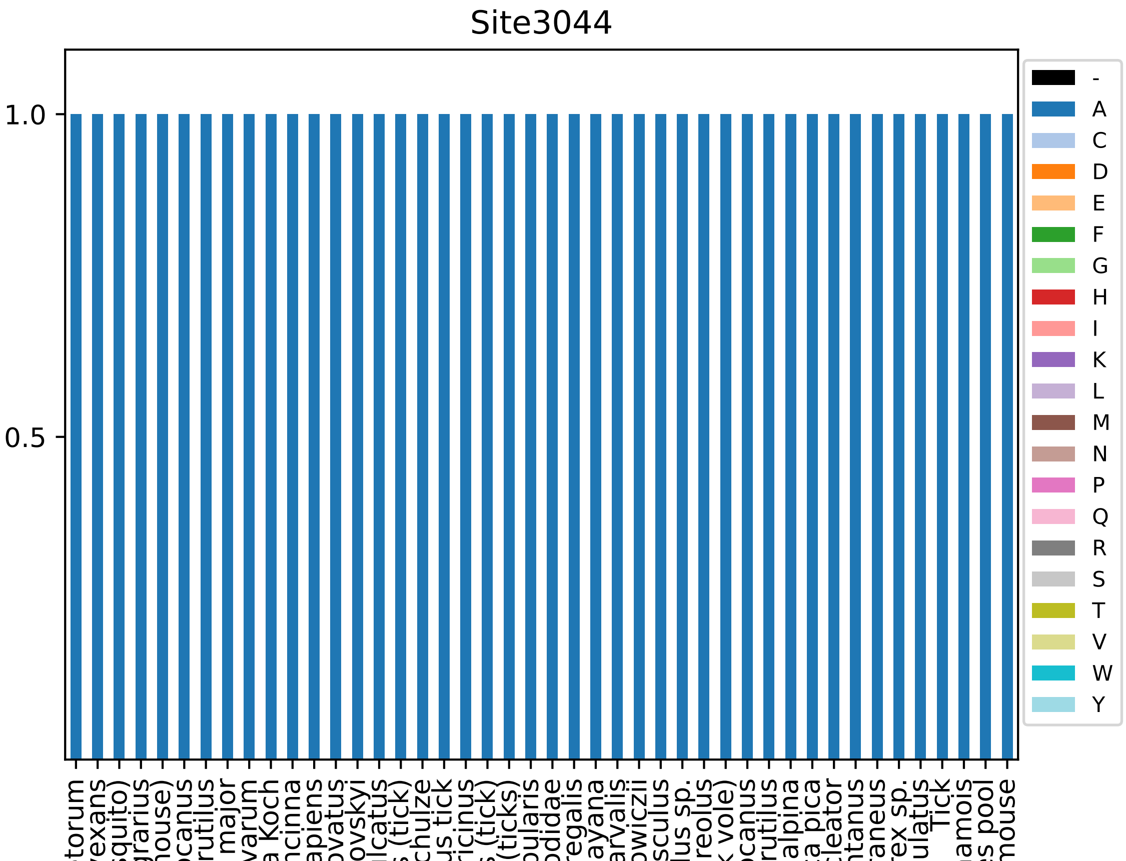
Site3044
1.0
0.5
-
A
C
D
E
F
G
H
I
K
L
M
N
P
Q
R
S
T
V
W
Y

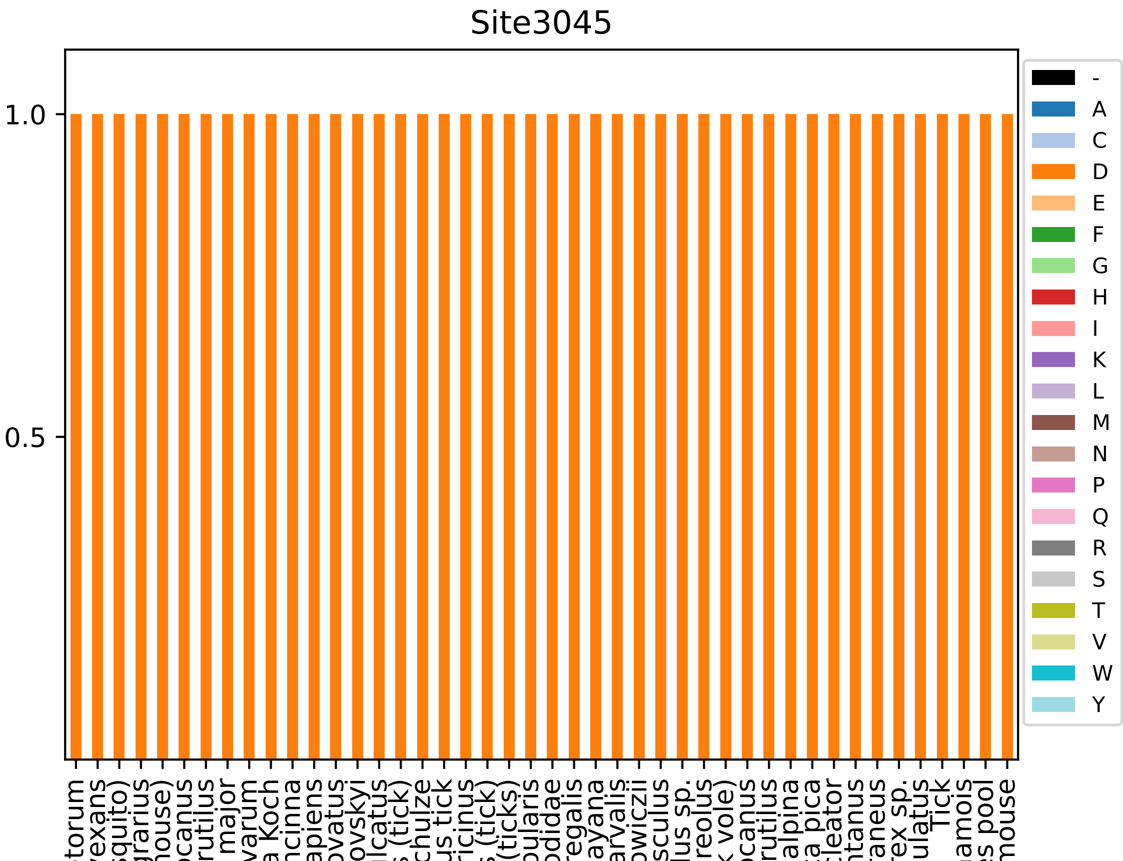
Site3045
1.0
0.5
-
A
C
D
E
F
G
H
I
K
L
M
N
P
Q
R
S
T
V
W
Y

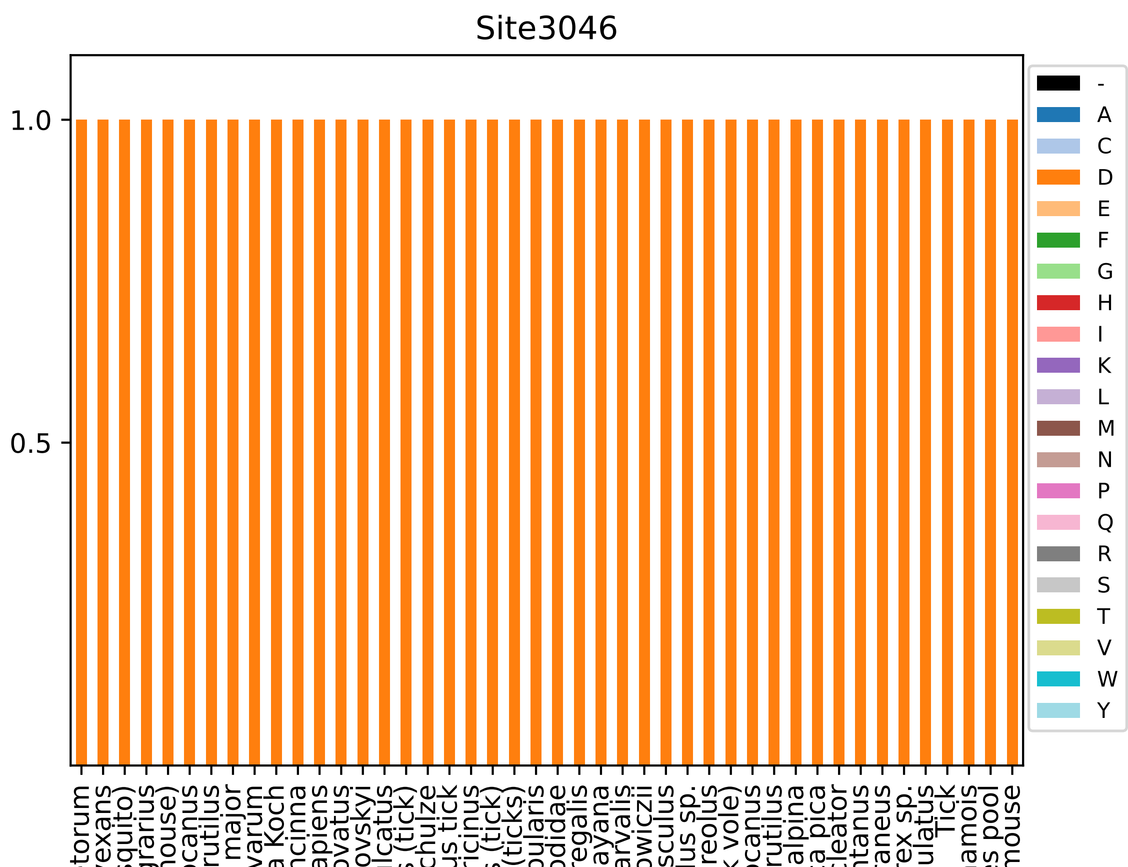
Site3046
1.0
0.5
-
A
C
D
E
F
G
H
I
K
L
M
N
P
Q
R
S
T
V
W
Y

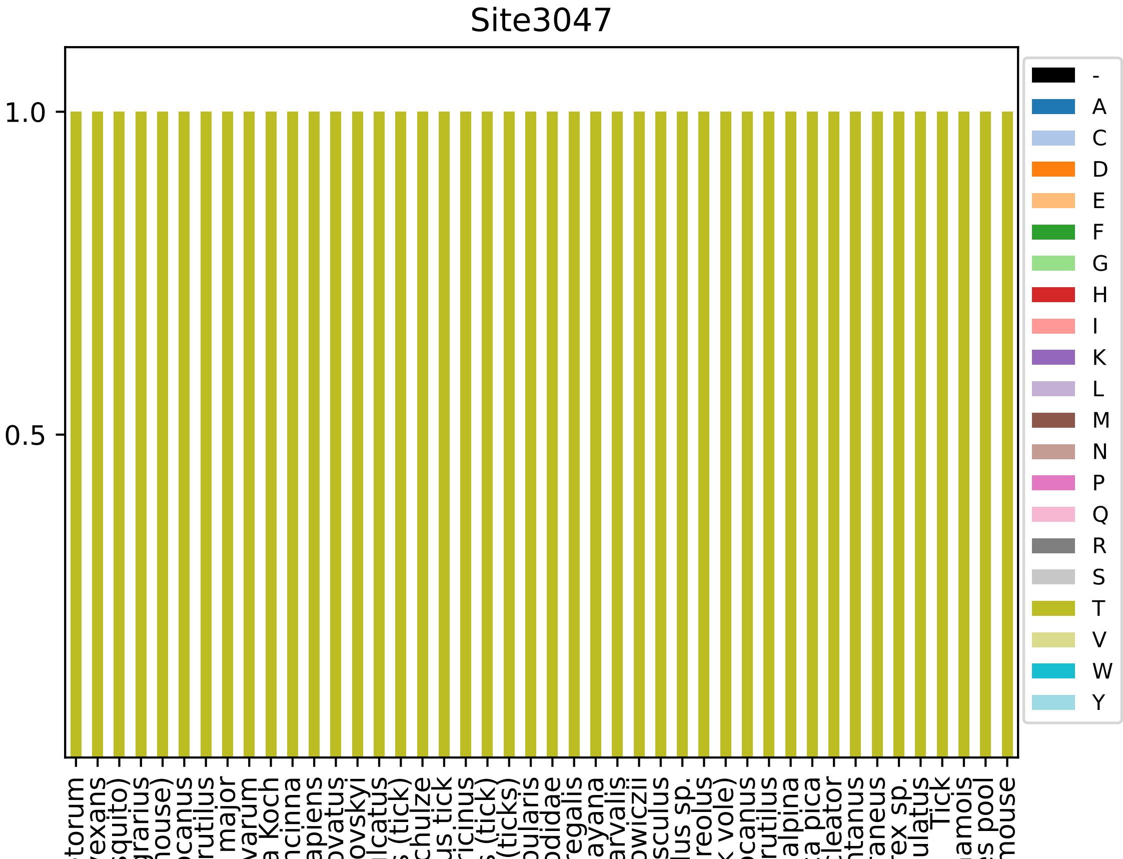
Site3047
1.0
0.5
-
A
C
D
E
F
G
H
I
K
L
M
N
P
Q
R
S
T
V
W
Y

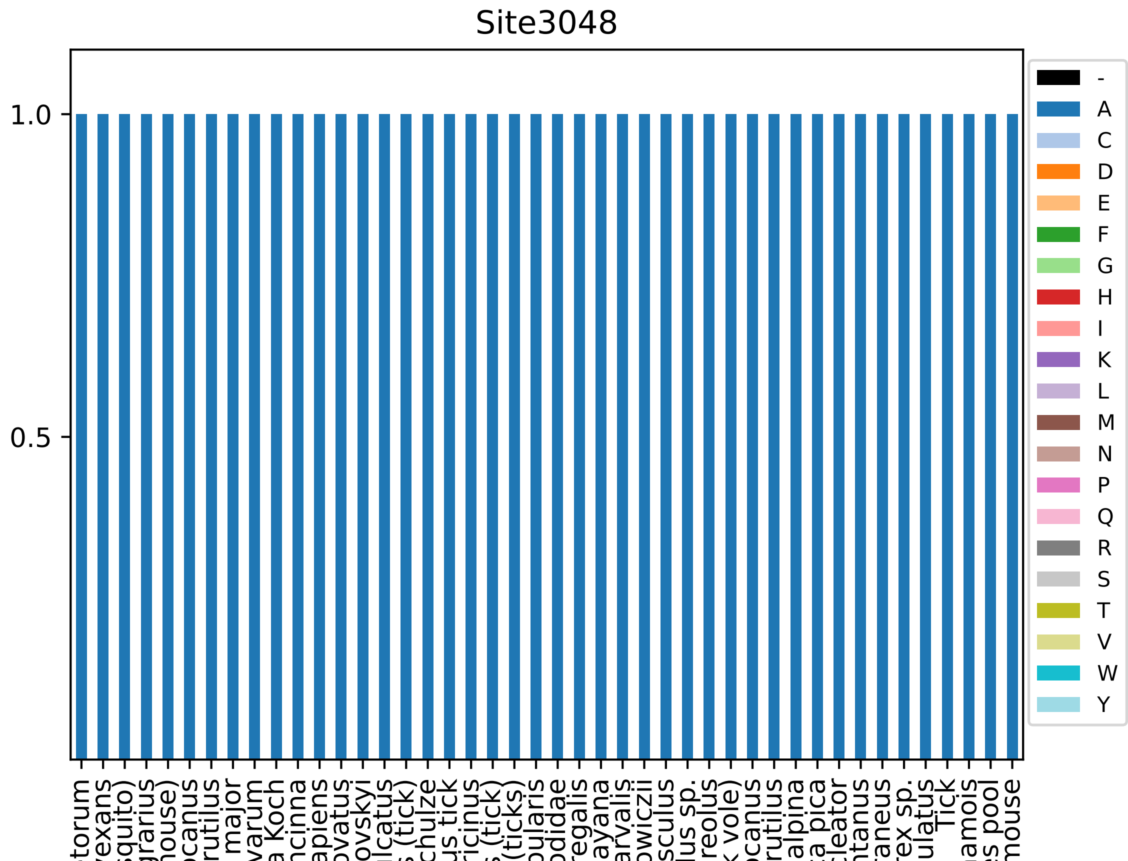

Site3048
1.0
0.5
-
A
C
D
E
F
G
H
I
K
L
M
N
P
Q
R
S
T
V
W
Y

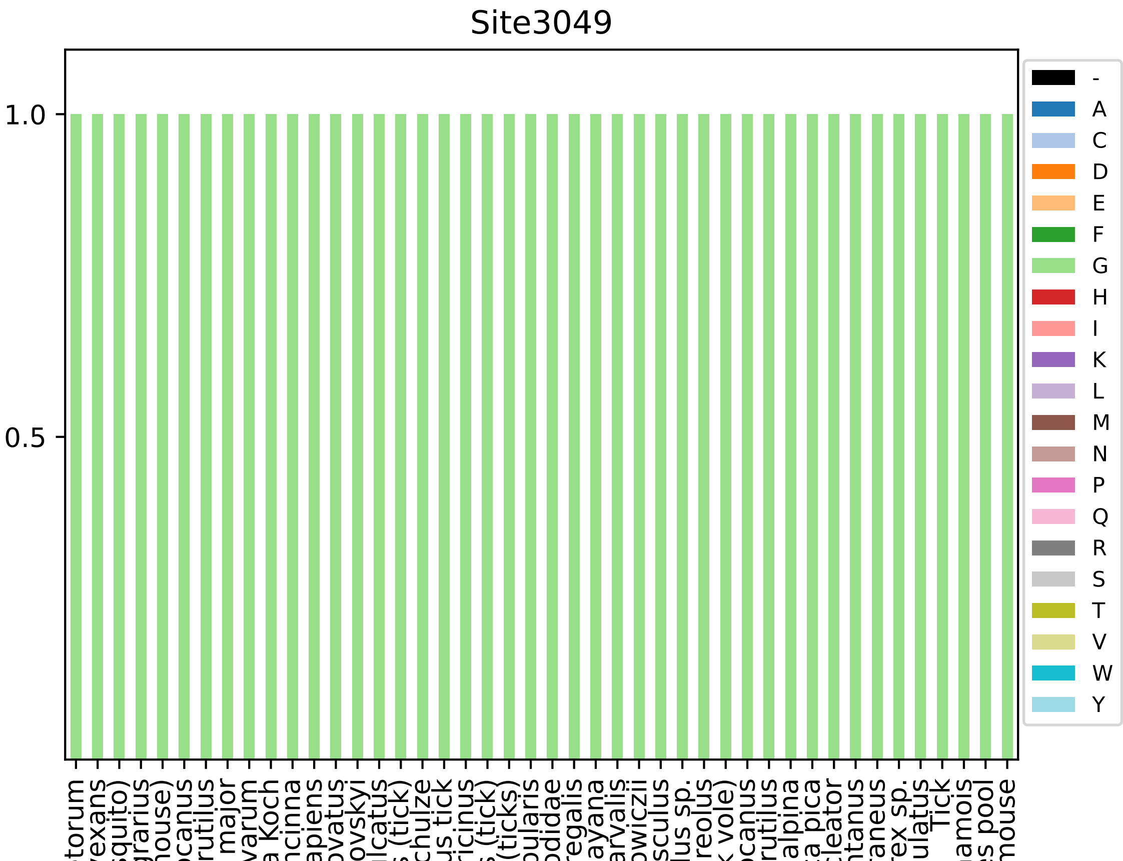

Site3049
1.0
0.5
-
A
C
D
E
F
G
H
I
K
L
M
N
P
Q
R
S
T
V
W
Y

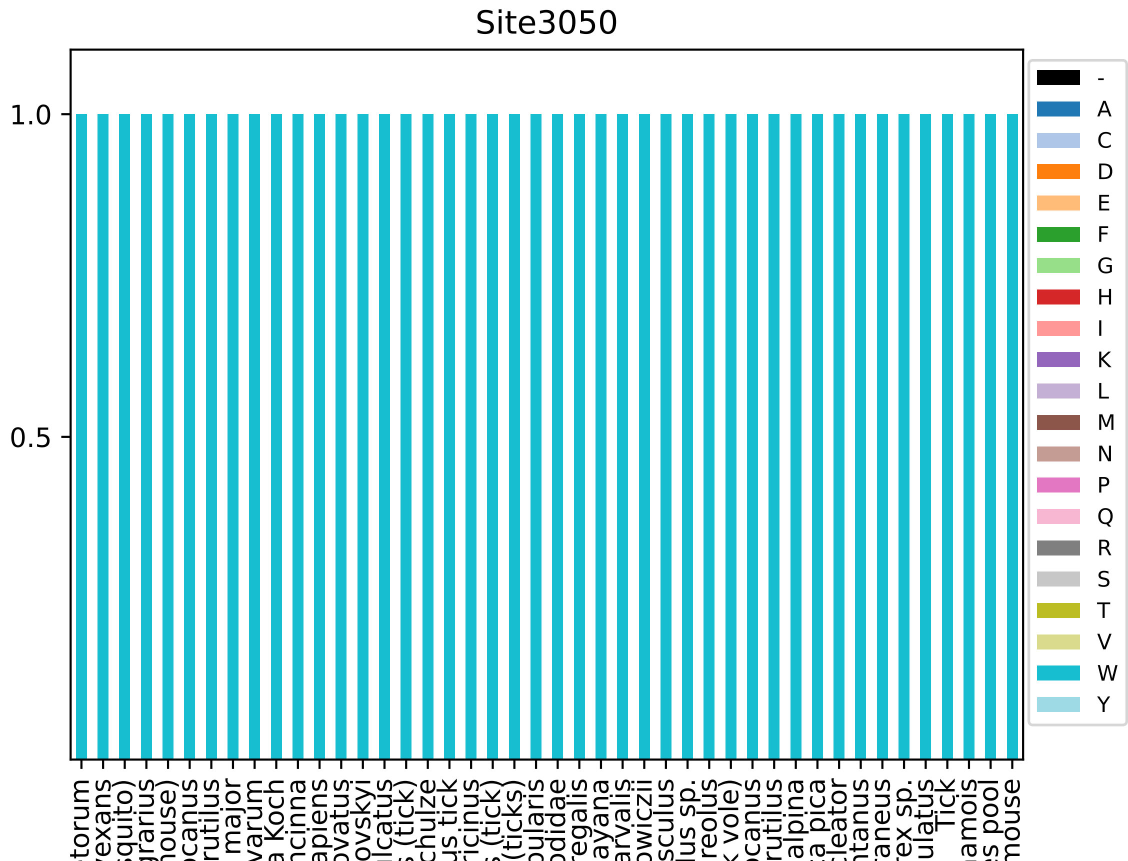
Site3050
1.0
0.5
-
A
C
D
E
F
G
H
I
K
L
M
N
P
Q
R
S
T
V
W
Y

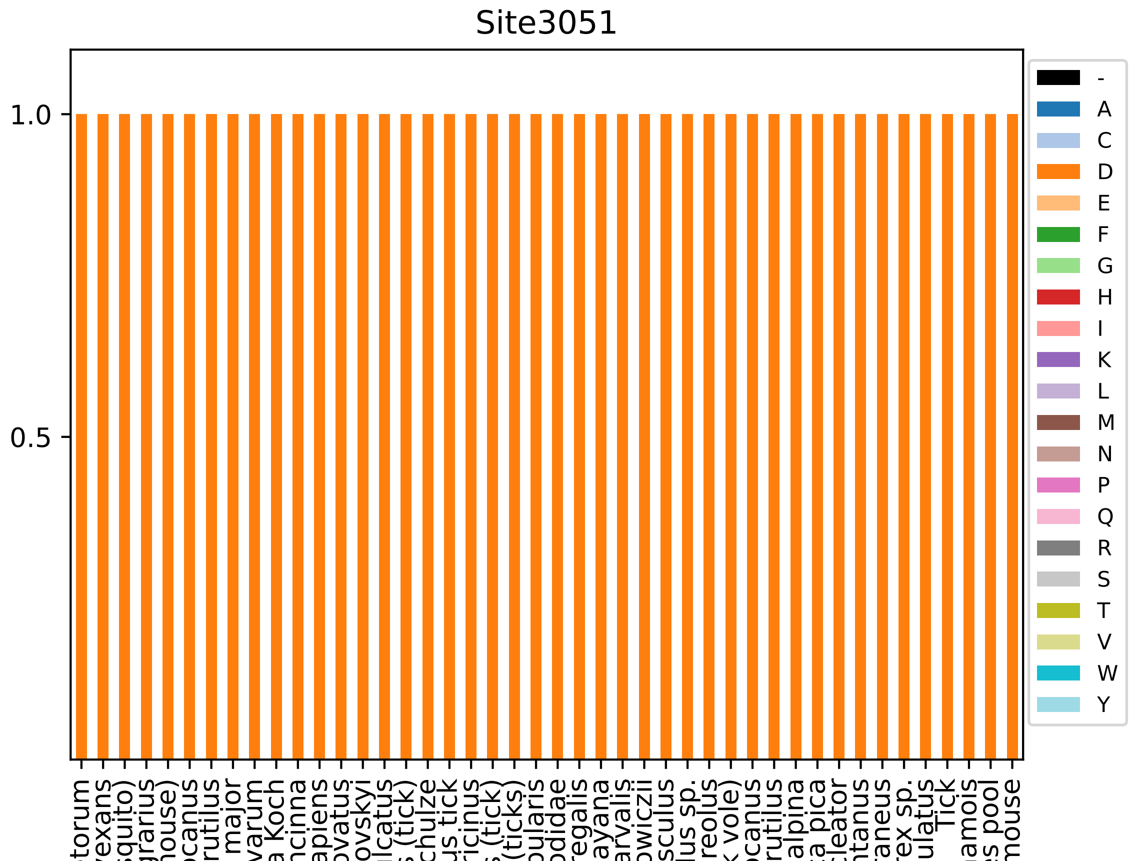
Site3051
1.0
0.5
-
A
C
D
E
F
G
H
I
K
L
M
N
P
Q
R
S
T
V
W
Y
torum
vexans
squito)
garius
nouse)
ocanus
rutilus
major
varum
a Koch
ncinna
apiens
ovatus
ovskyi
ulcatus
(tick)
chulze
us tick
ricinus
(tick)
(ticks)
pularis
odidae
regalis
ayana
arvalis
owiczii
sculus
lus sp.
reolus
vole)
ocanus
rutilus
alpina
a pica
leator
ntanus
raneus
rex sp.
ulatus
Tick
amois
s pool
mouse

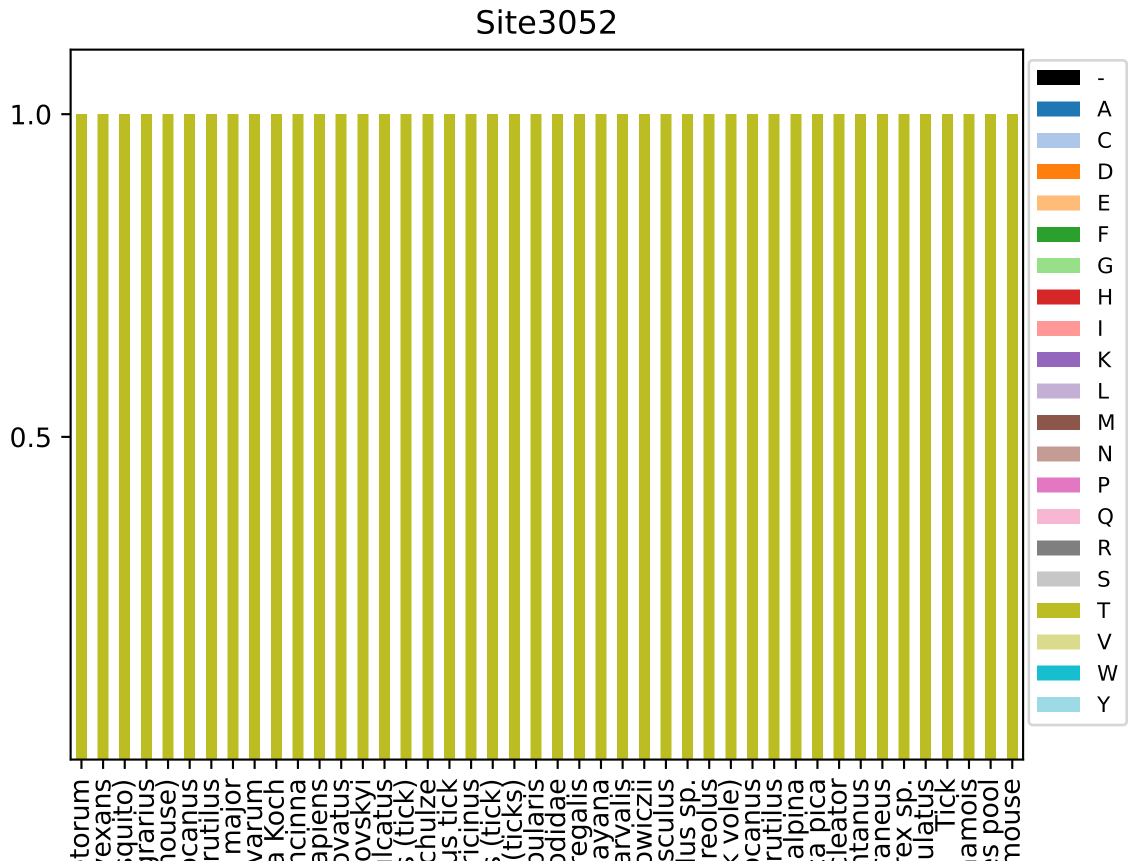

Site3052
1.0
0.5
-
A
C
D
E
F
G
H
I
K
L
M
N
P
Q
R
S
T
V
W
Y

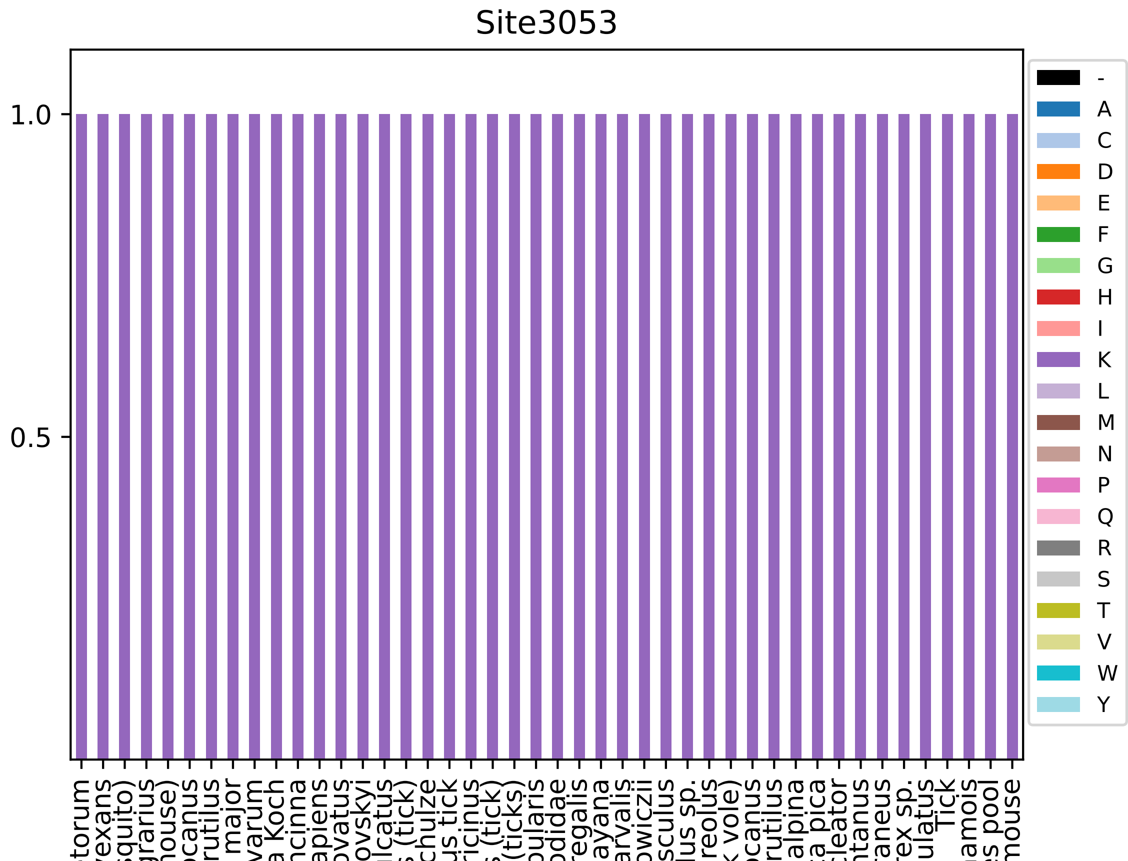

Site3053
1.0
0.5
-
A
C
D
E
F
G
H
I
K
L
M
N
P
Q
R
S
T
V
W
Y

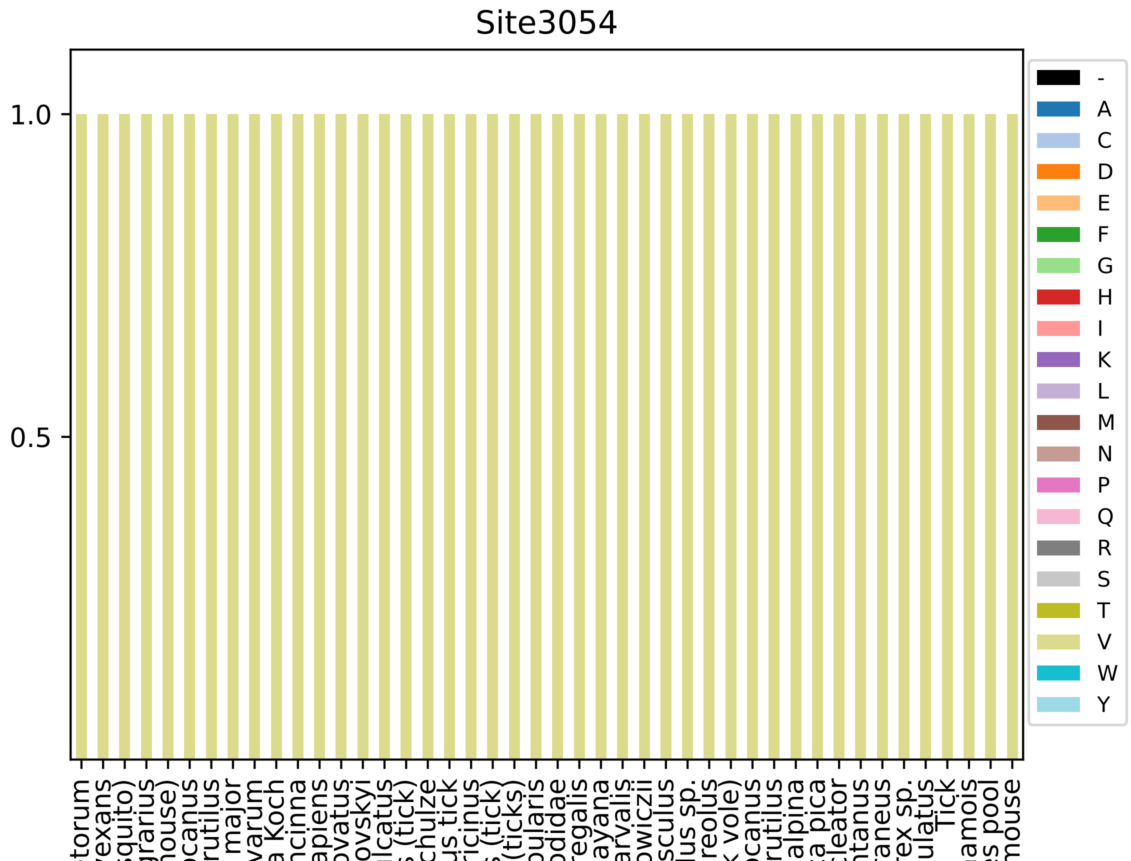

Site3054
1.0
0.5
-
A
C
D
E
F
G
H
I
K
L
M
N
P
Q
R
S
T
V
W
Y

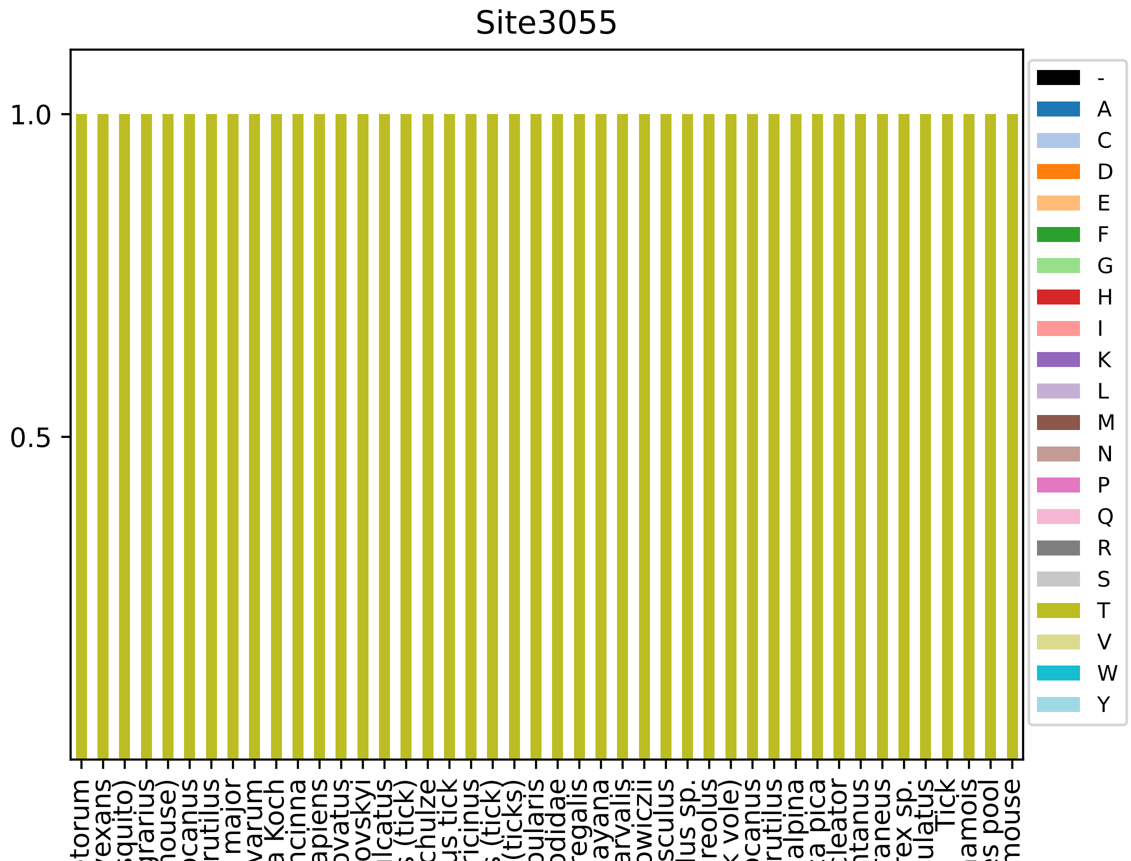
Site3055
1.0
0.5
-
A
C
D
E
F
G
H
I
K
L
M
N
P
Q
R
S
T
V
W
Y

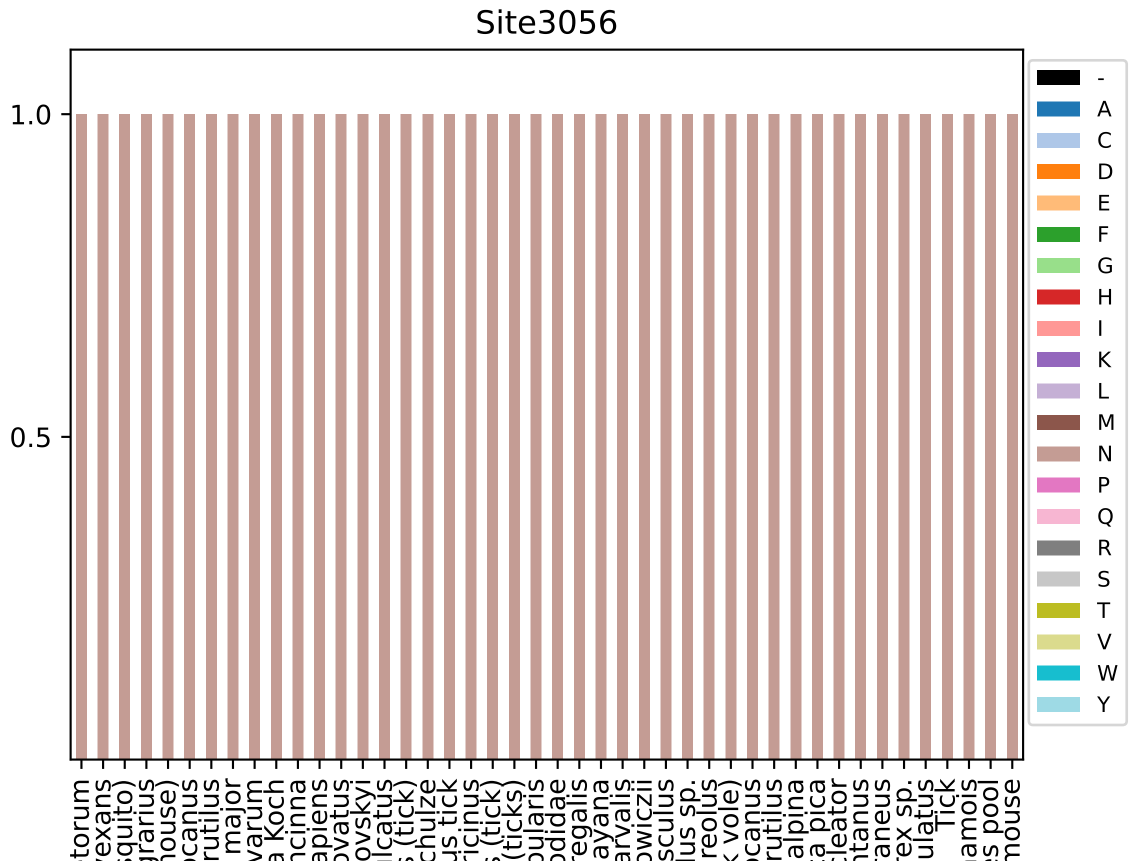

Site3056
1.0
0.5
-
A
C
D
E
F
G
H
I
K
L
M
N
P
Q
R
S
T
V
W
Y

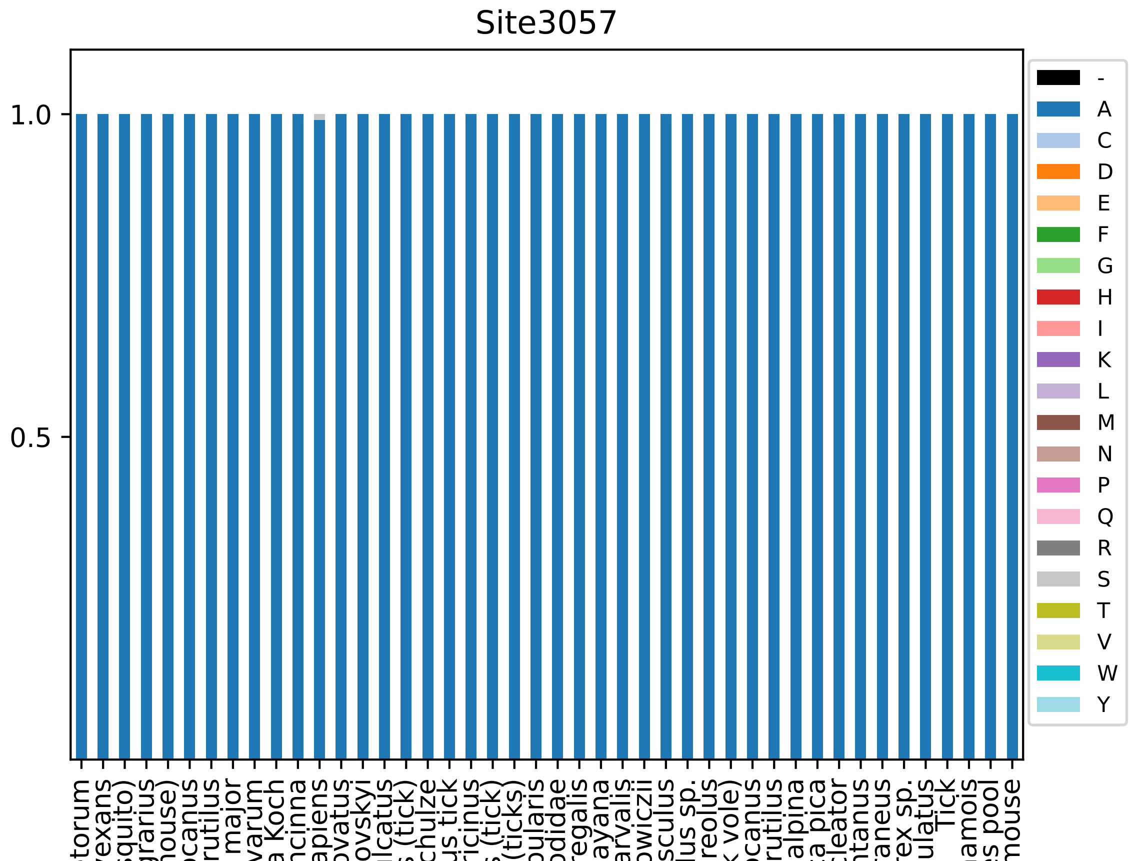
Site3057
1.0
0.5
-
A
C
D
E
F
G
H
I
K
L
M
N
P
Q
R
S
T
V
W
Y

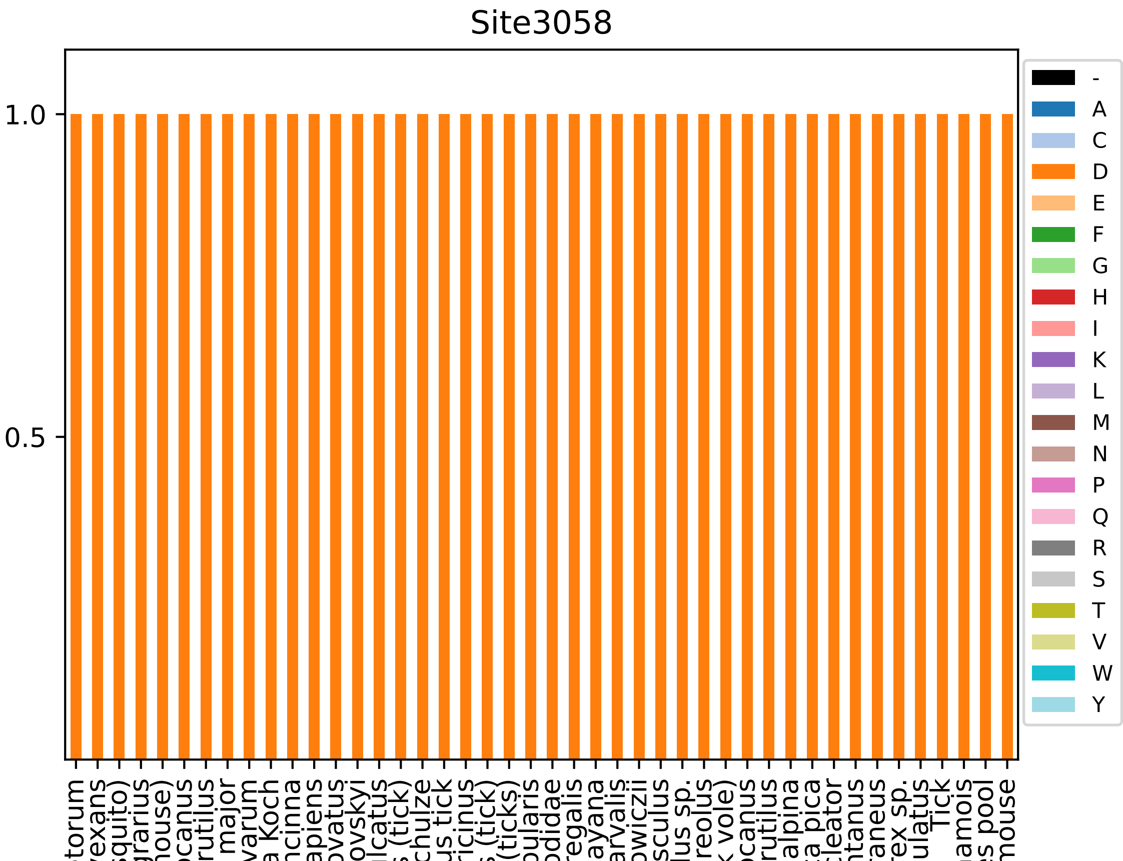
Site3058
1.0
0.5
-
A
C
D
E
F
G
H
I
K
L
M
N
P
Q
R
S
T
V
W
Y

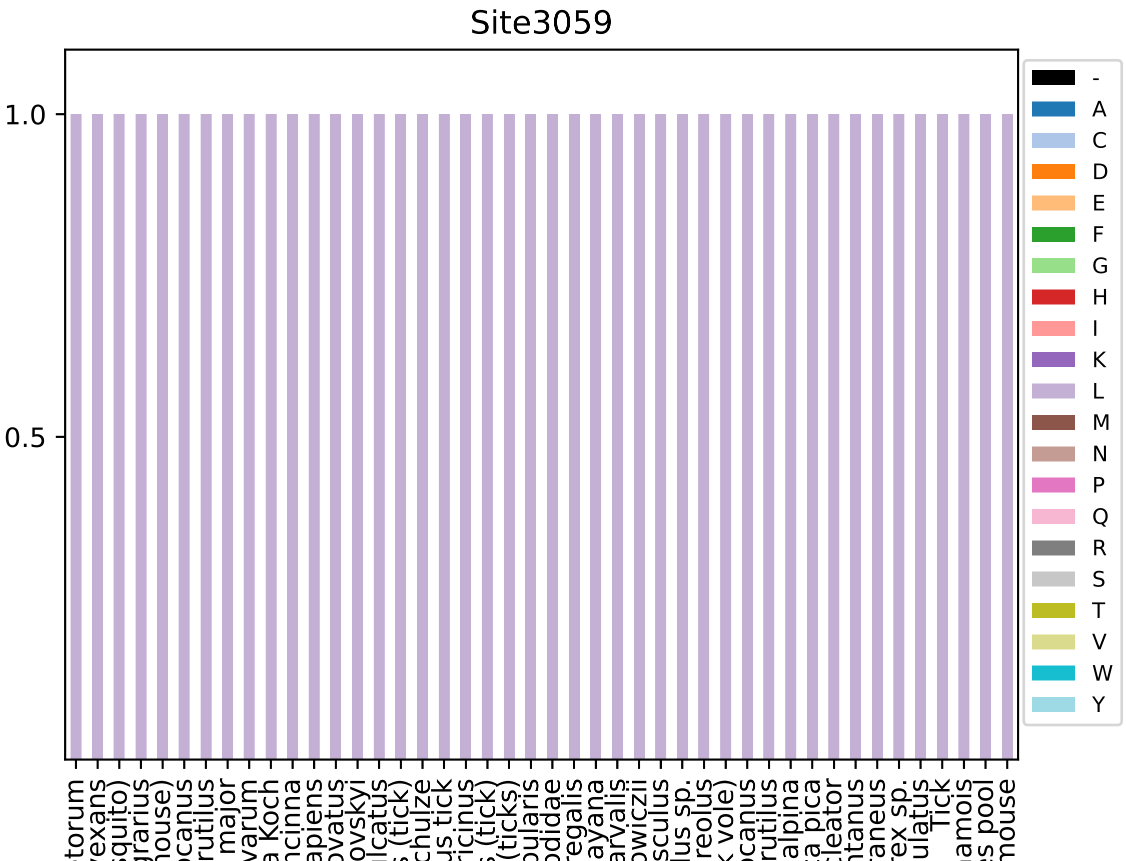

Site3059
1.0
0.5
-
A
C
D
E
F
G
H
I
K
L
M
N
P
Q
R
S
T
V
W
Y

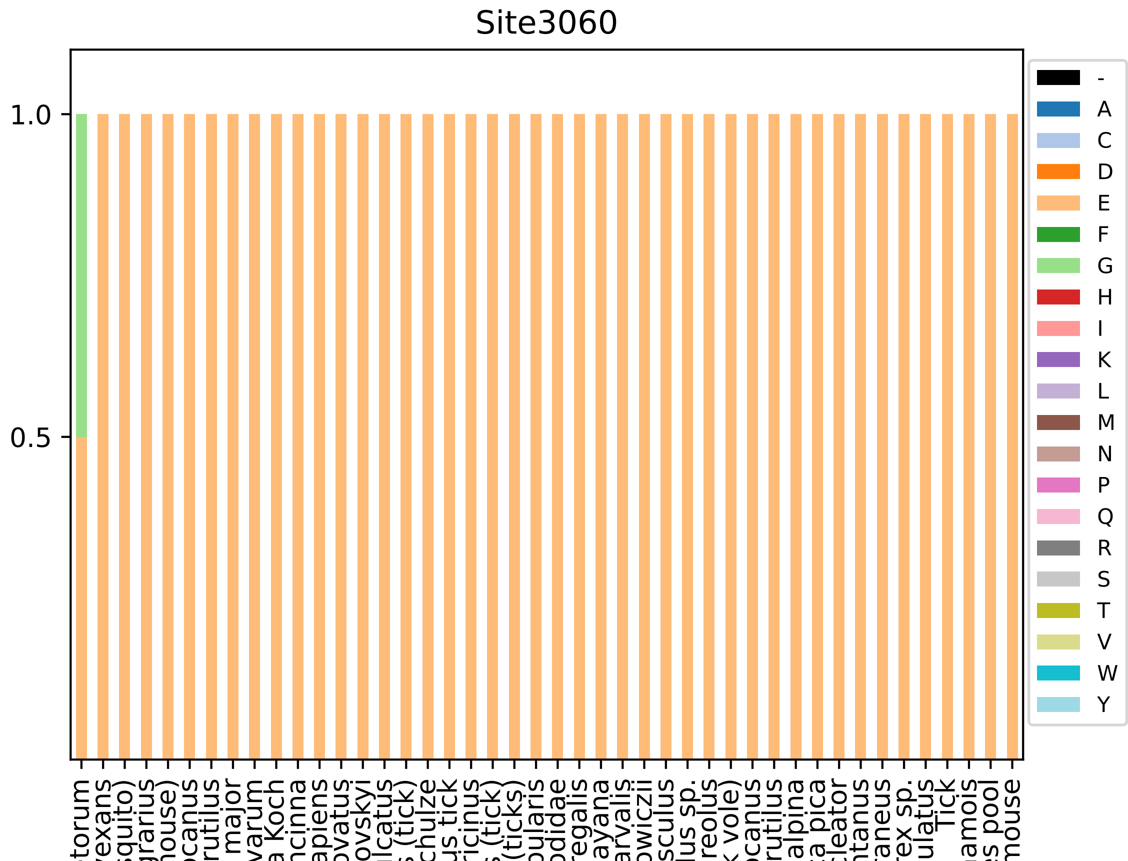
Site3060
1.0
0.5
-
A
C
D
E
F
G
H
I
K
L
M
N
P
Q
R
S
T
V
W
Y

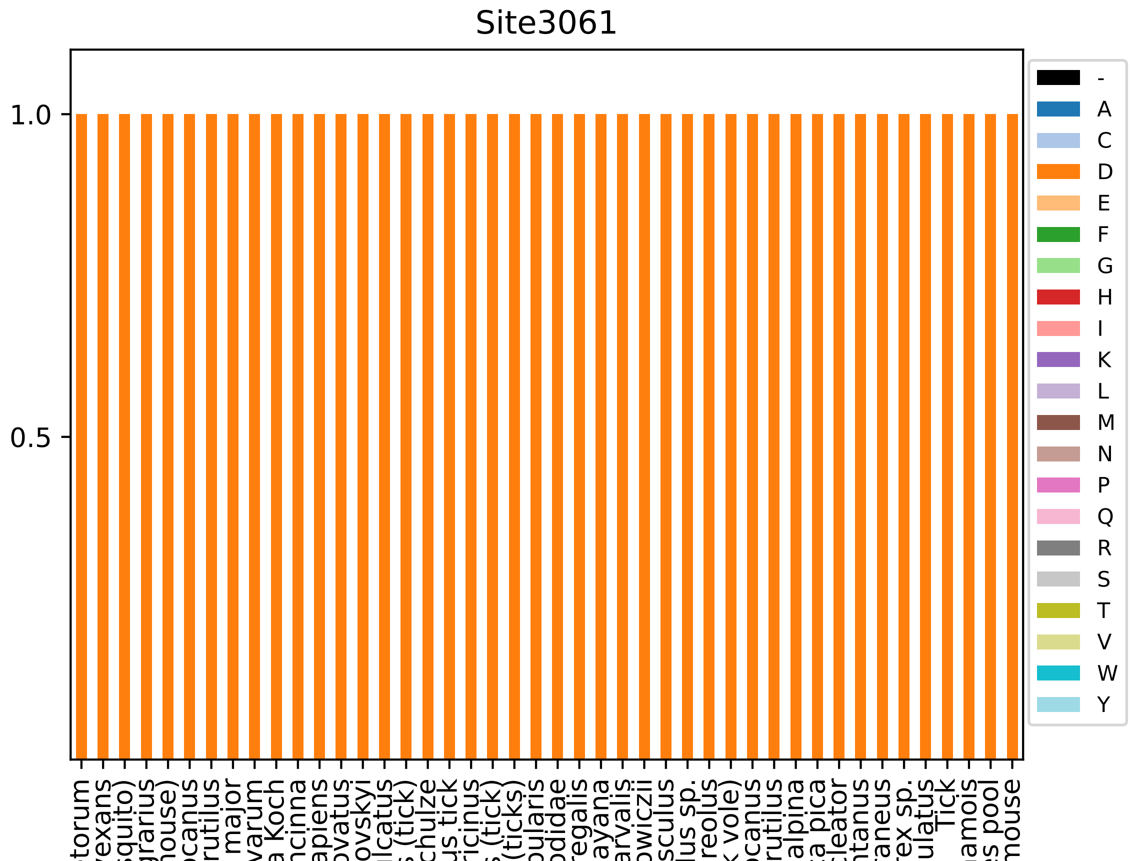

Site3061
1.0
0.5
-
A
C
D
E
F
G
H
I
K
L
M
N
P
Q
R
S
T
V
W
Y

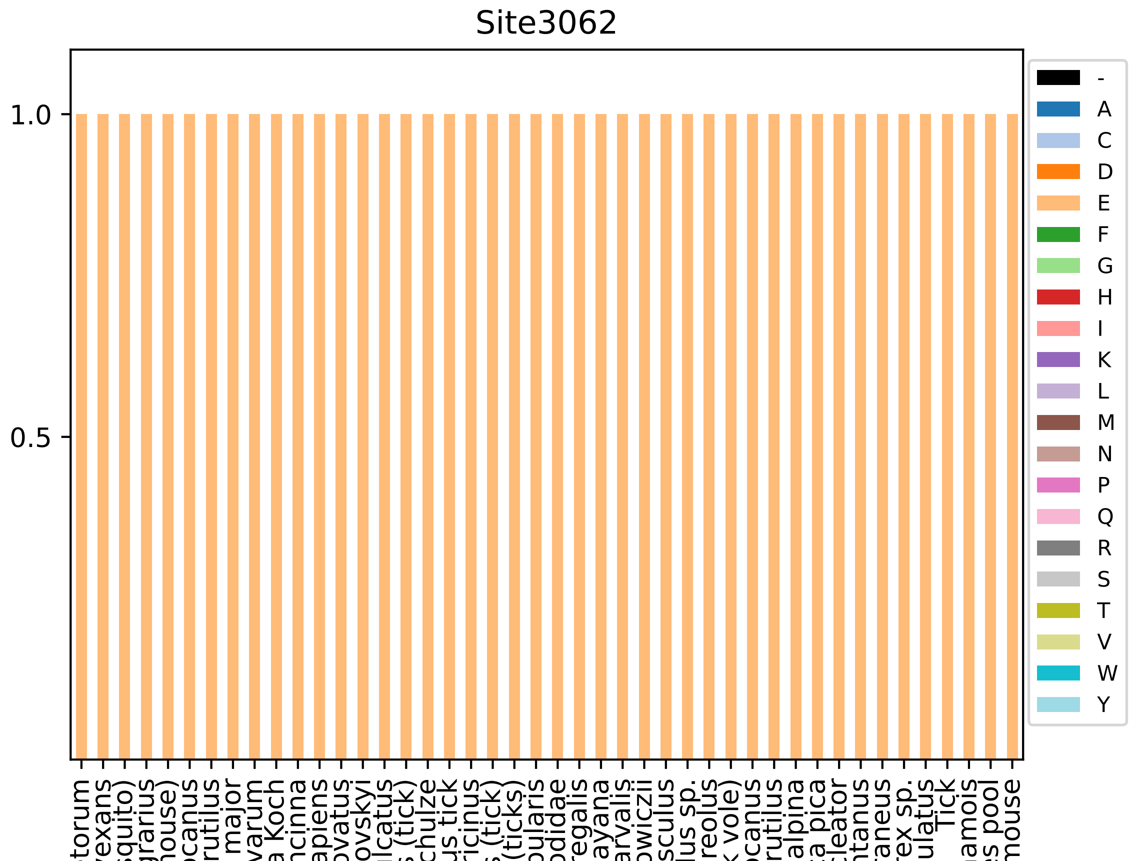

Site3062
1.0
0.5
-
A
C
D
E
F
G
H
I
K
L
M
N
P
Q
R
S
T
V
W
Y

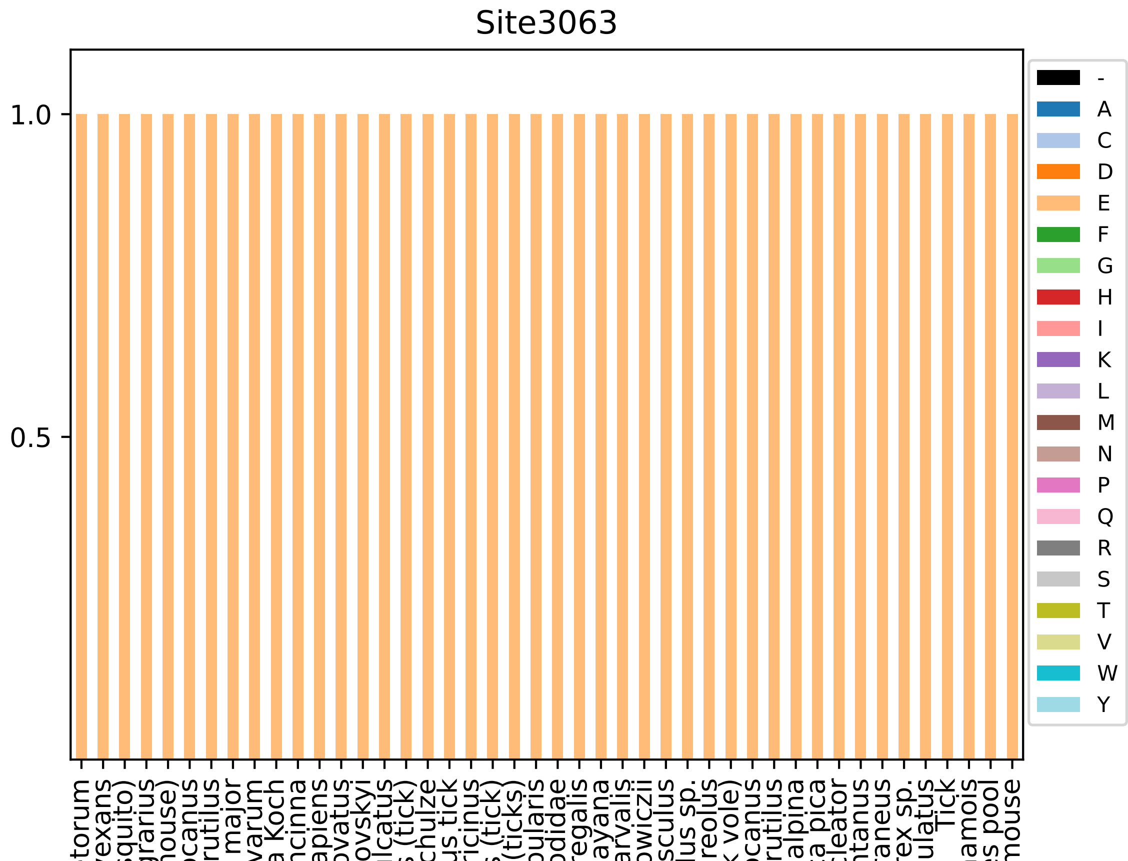
Site3063
1.0
0.5
-
A
C
D
E
F
G
H
I
K
L
M
N
P
Q
R
S
T
V
W
Y

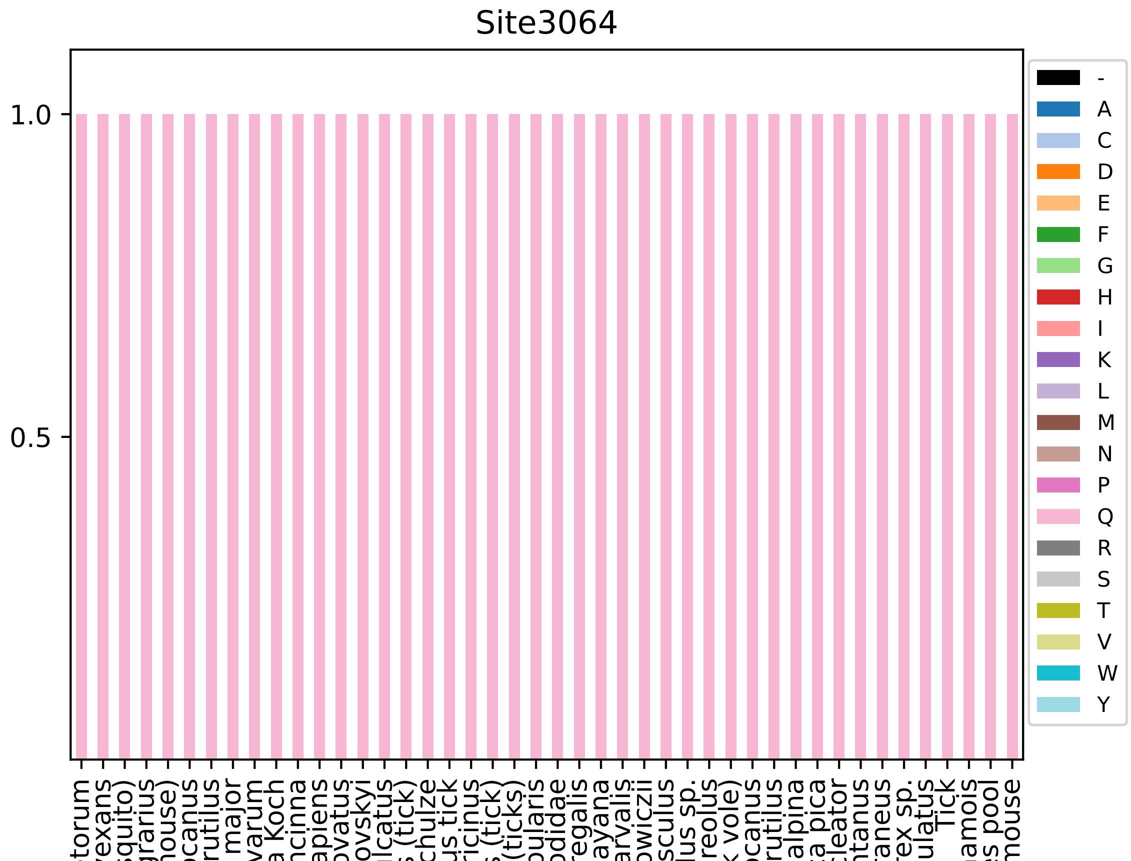

Site3064
1.0
0.5
-
A
C
D
E
F
G
H
I
K
L
M
N
P
Q
R
S
T
V
W
Y

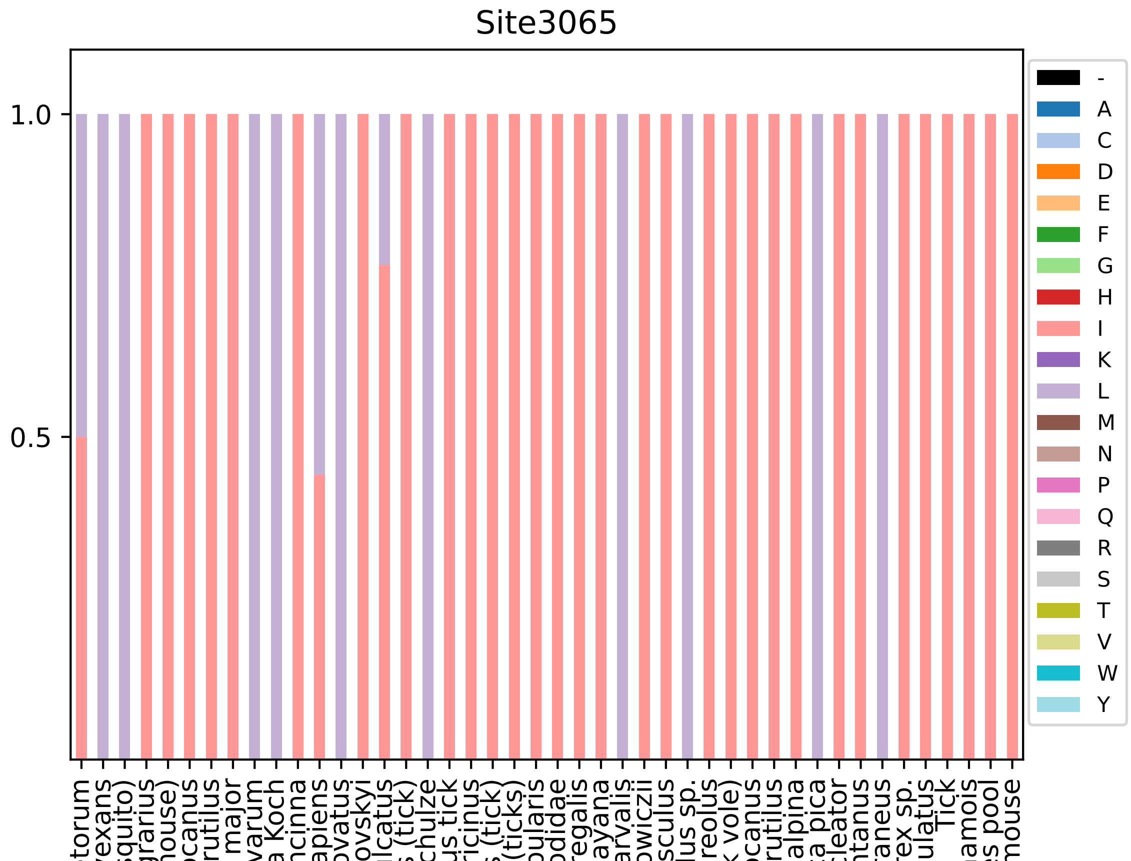

Site3065
1.0
0.5
-
A
C
D
E
F
G
H
I
K
L
M
N
P
Q
R
S
T
V
W
Y

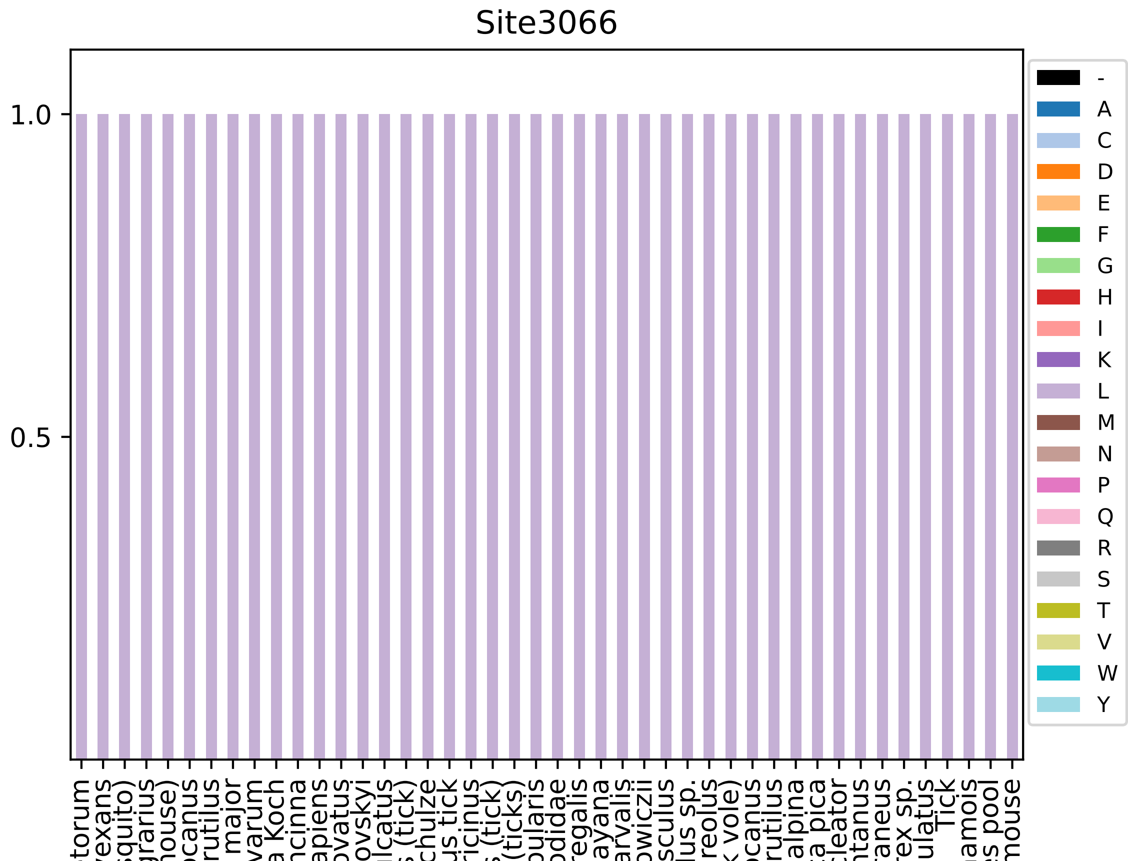
Site3066
1.0
0.5
-
A
C
D
E
F
G
H
I
K
L
M
N
P
Q
R
S
T
V
W
Y

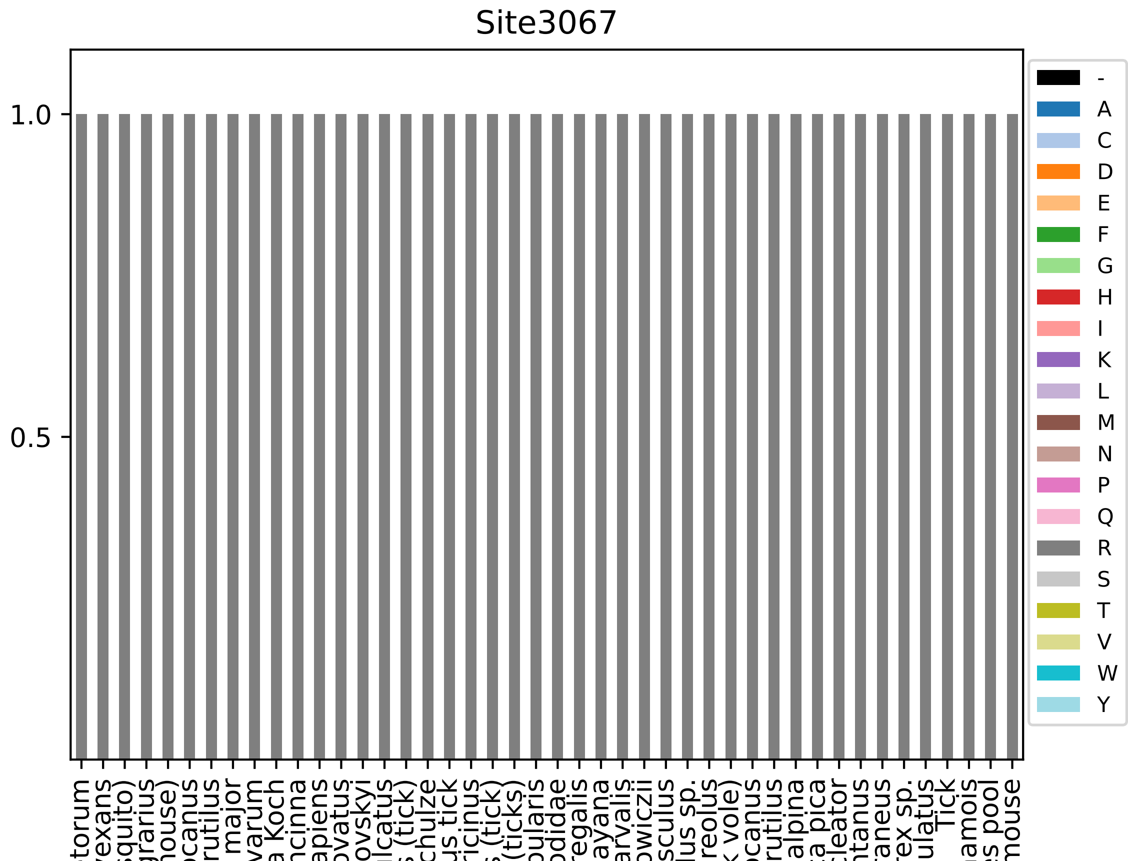

Site3067
1.0
0.5
-
A
C
D
E
F
G
H
I
K
L
M
N
P
Q
R
S
T
V
W
Y

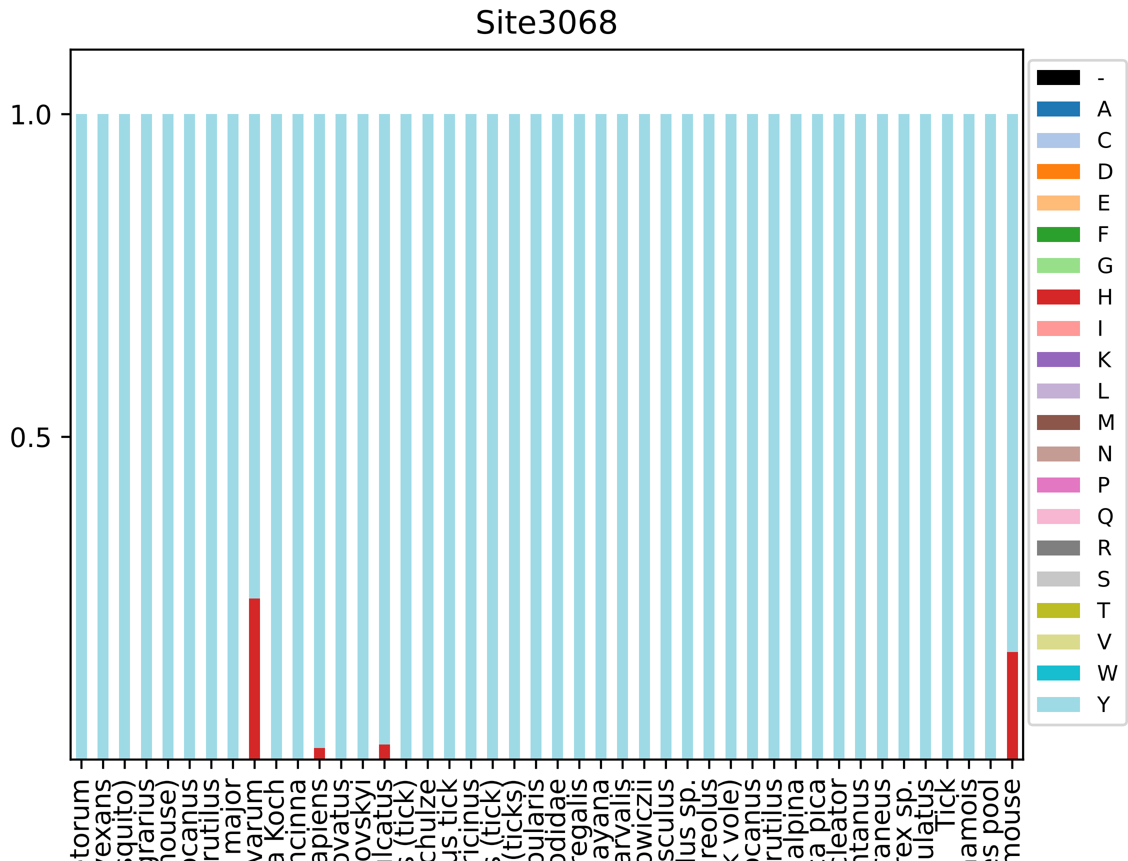
Site3068
1.0
0.5
-
A
C
D
E
F
G
H
I
K
L
M
N
P
Q
R
S
T
V
W
Y

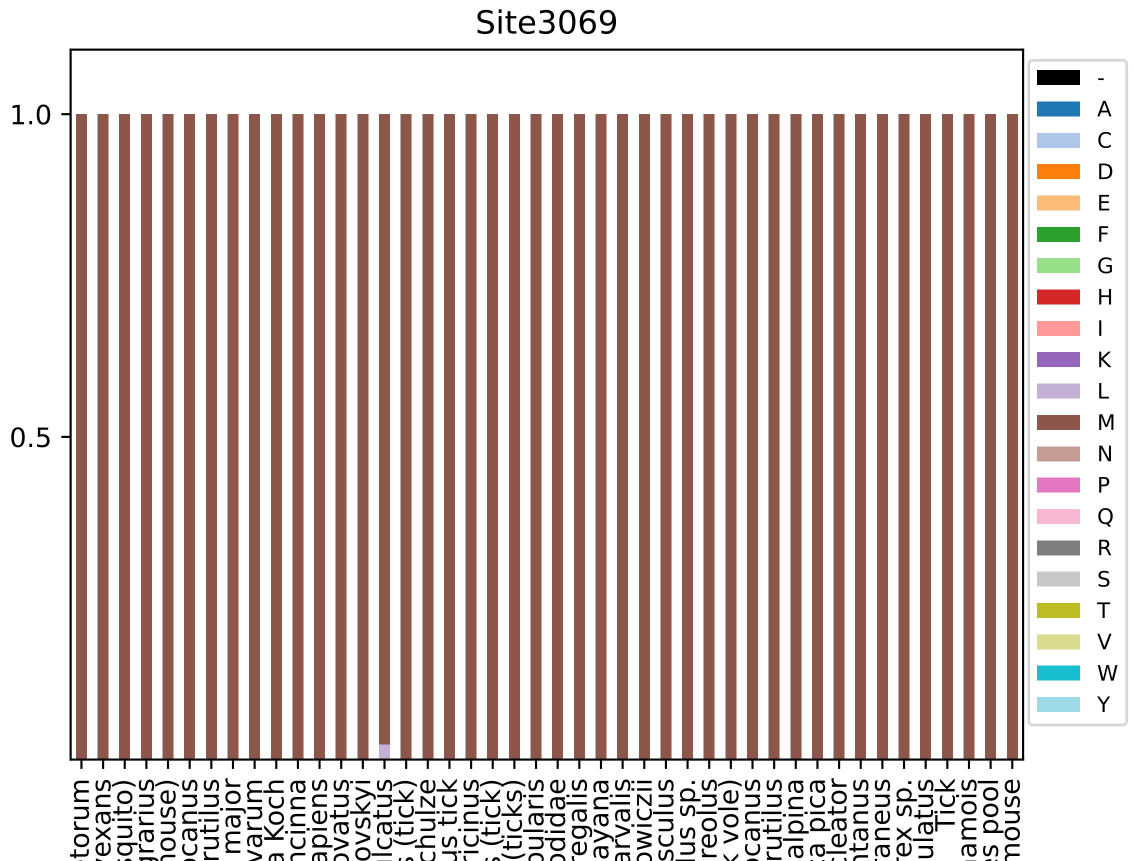

Site3069
1.0
0.5
-
A
C
D
E
F
G
H
I
K
L
M
N
P
Q
R
S
T
V
W
Y

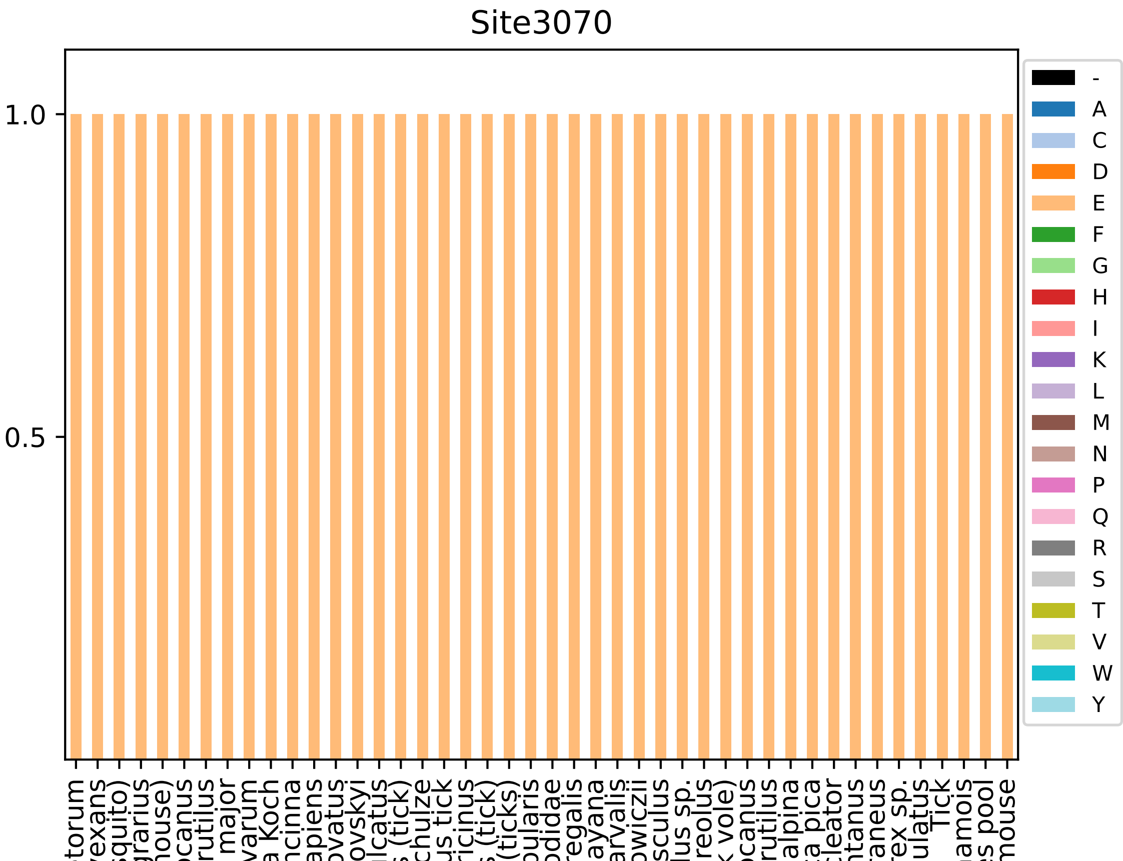
Site3070
1.0
0.5
-
A
C
D
E
F
G
H
I
K
L
M
N
P
Q
R
S
T
V
W
Y

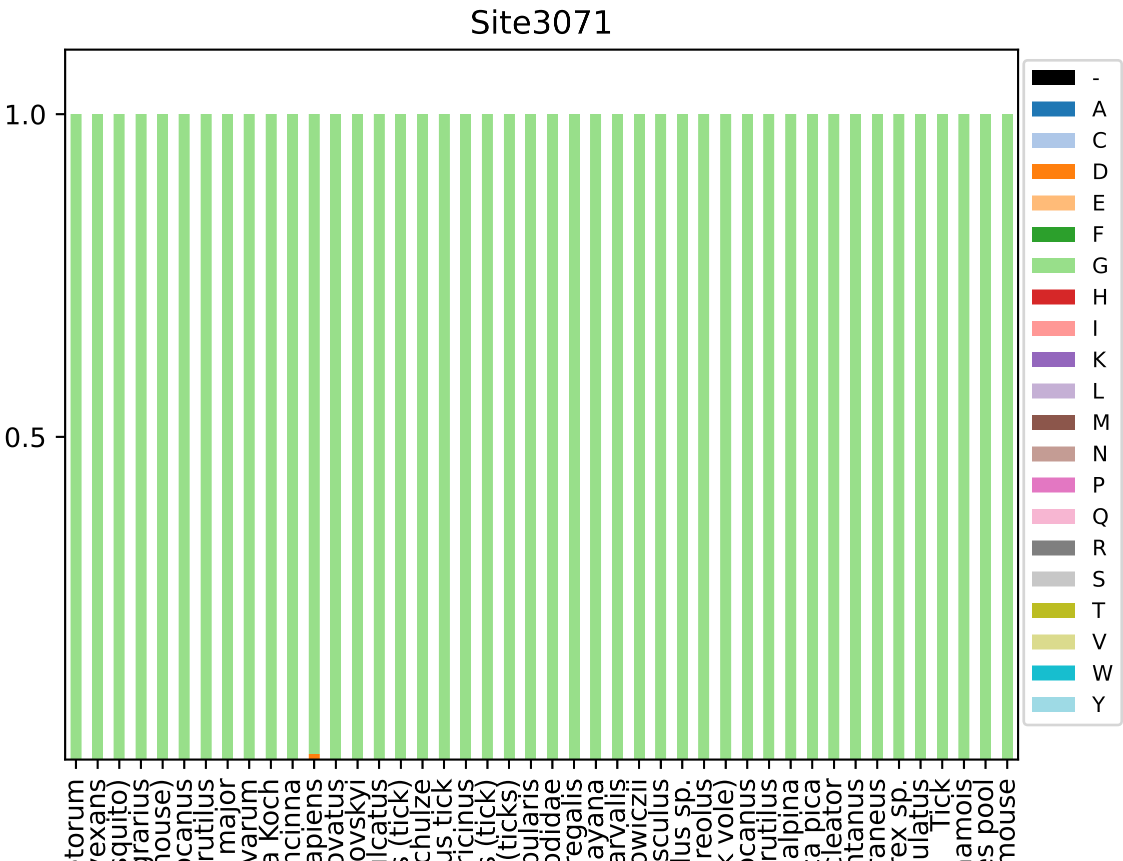
Site3071
1.0
0.5
-
A
C
D
E
F
G
H
I
K
L
M
N
P
Q
R
S
T
V
W
Y

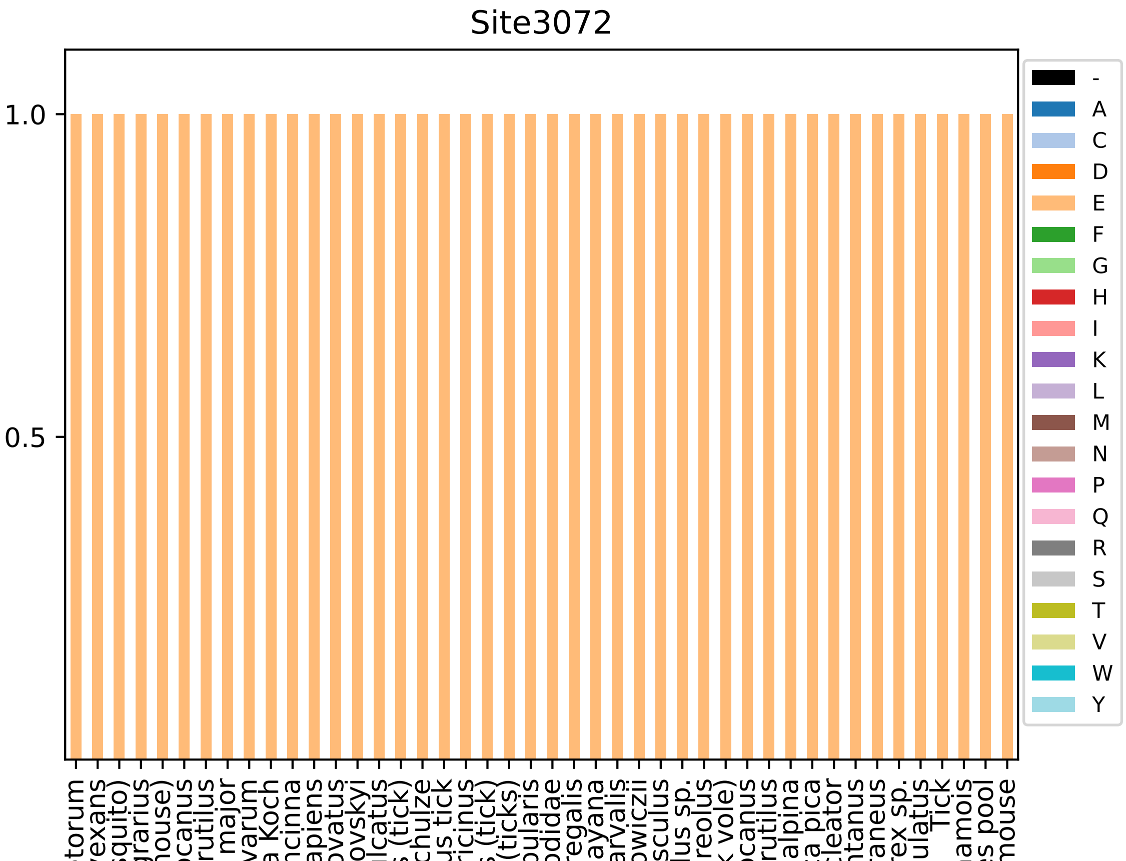

Site3072
1.0
0.5
-
A
C
D
E
F
G
H
I
K
L
M
N
P
Q
R
S
T
V
W
Y

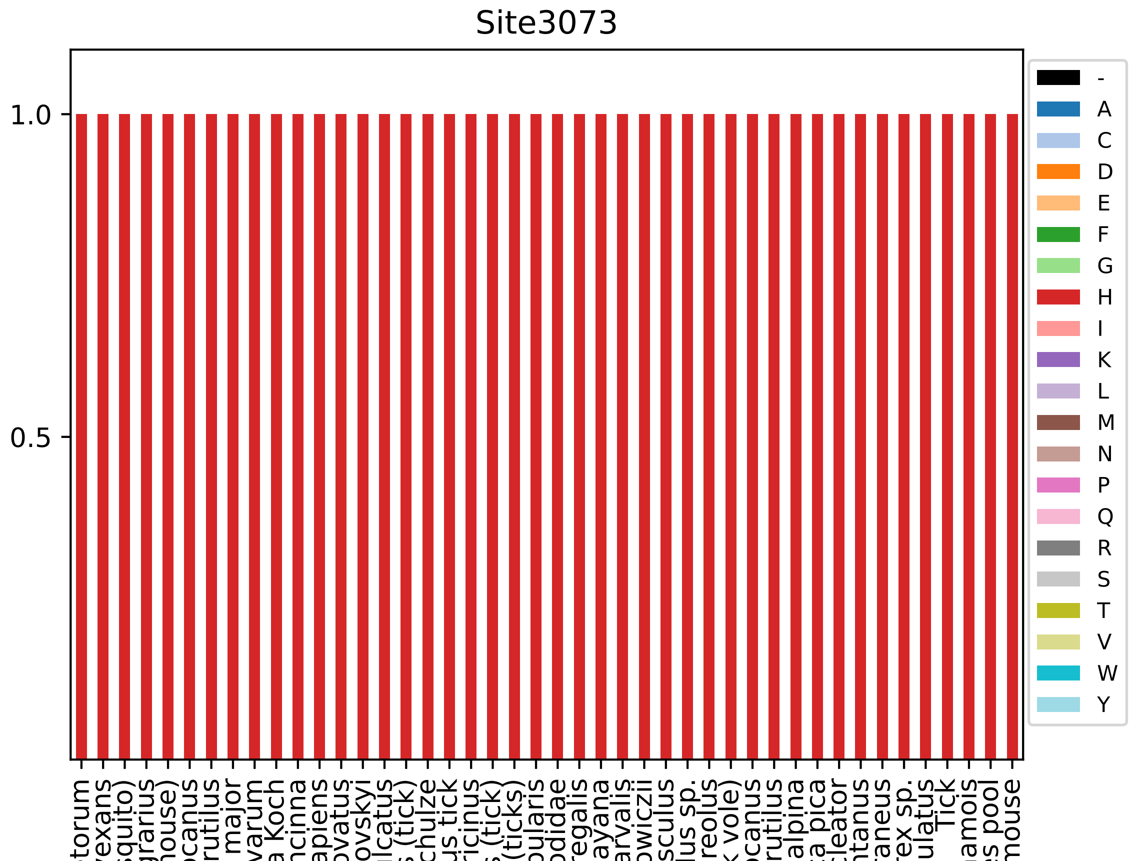
Site3073
1.0
0.5
-
A
C
D
E
F
G
H
I
K
L
M
N
P
Q
R
S
T
V
W
Y

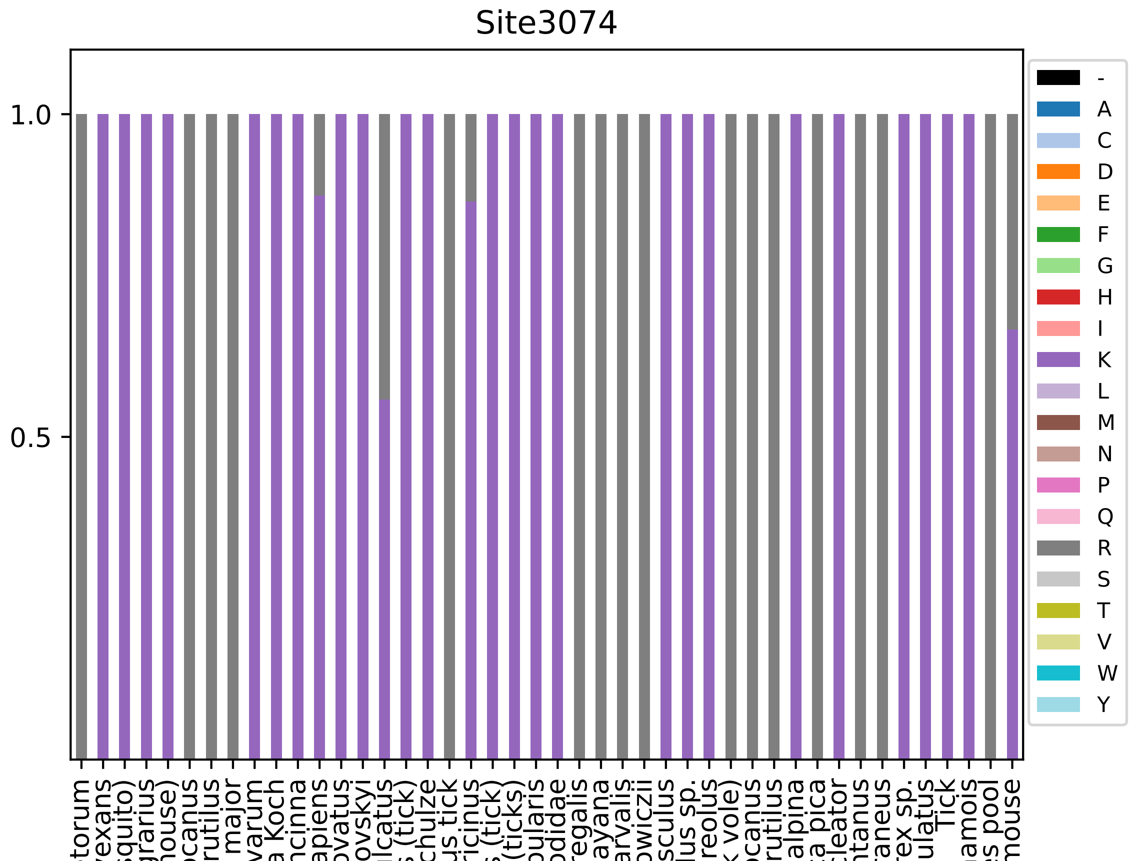

Site3074
1.0
0.5
-
A
C
D
E
F
G
H
I
K
L
M
N
P
Q
R
S
T
V
W
Y

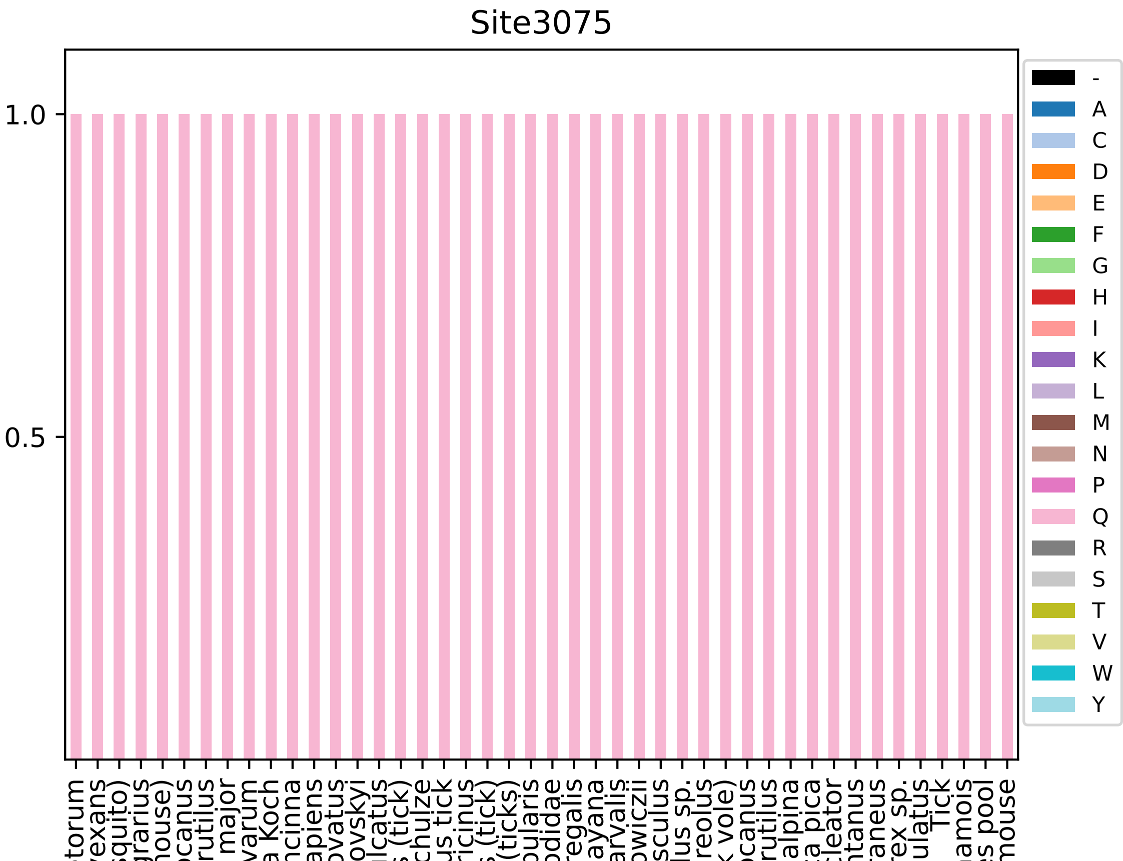
Site3075
1.0
0.5
-
A
C
D
E
F
G
H
I
K
L
M
N
P
Q
R
S
T
V
W
Y

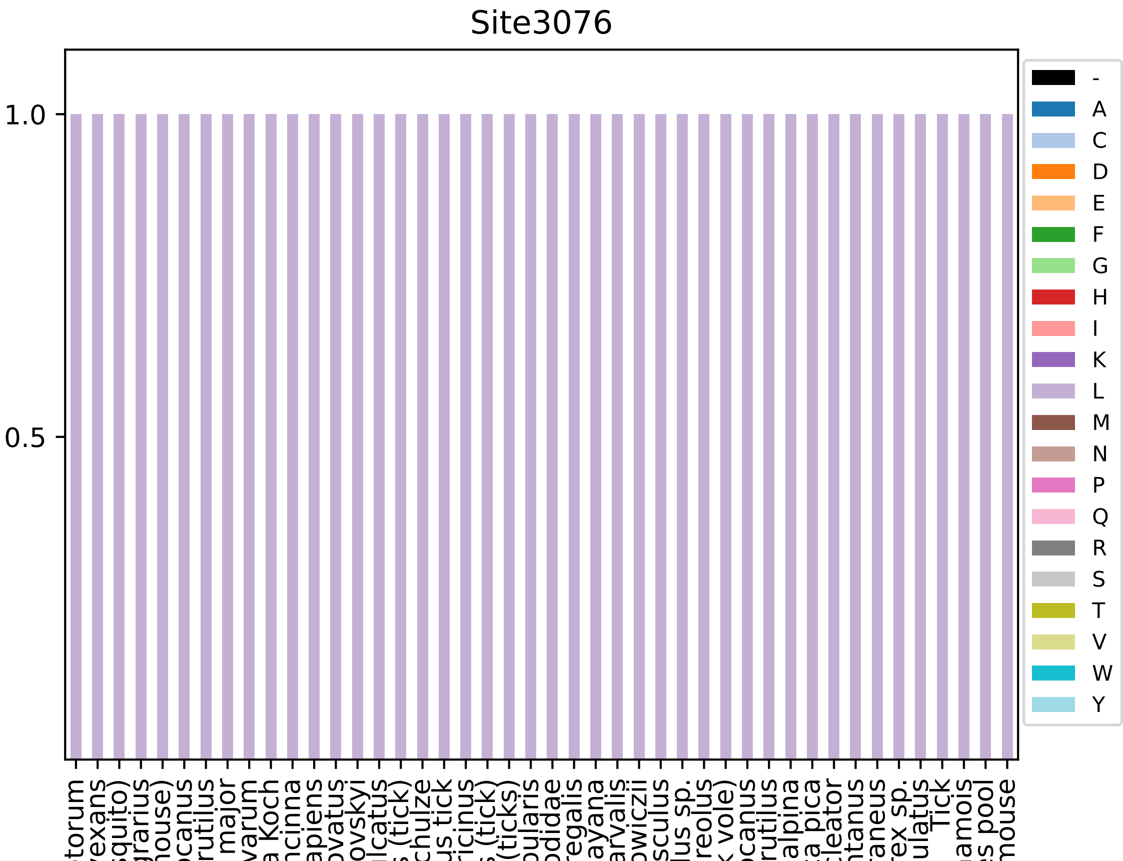
Site3076
1.0
0.5
-
A
C
D
E
F
G
H
I
K
L
M
N
P
Q
R
S
T
V
W
Y

Site3077

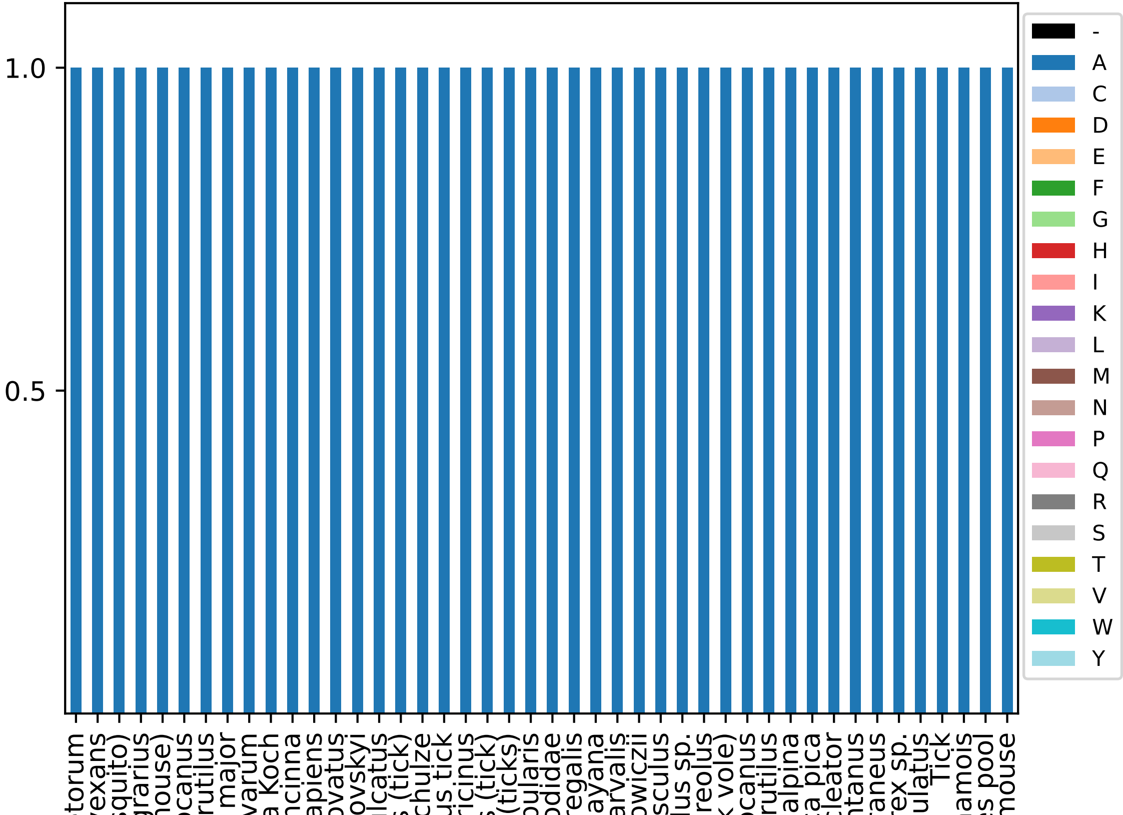

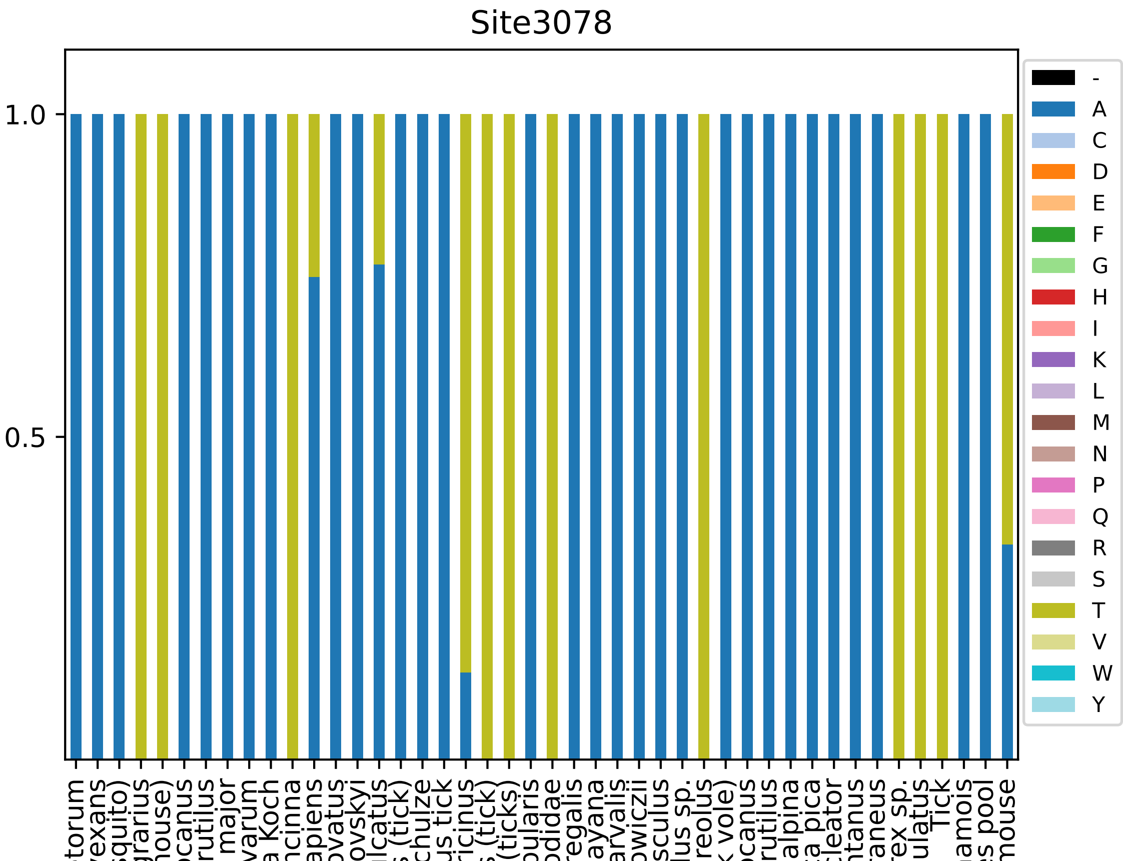

Site3078
1.0
0.5
-
A
C
D
E
F
G
H
I
K
L
M
N
P
Q
R
S
T
V
W
Y

Site3079

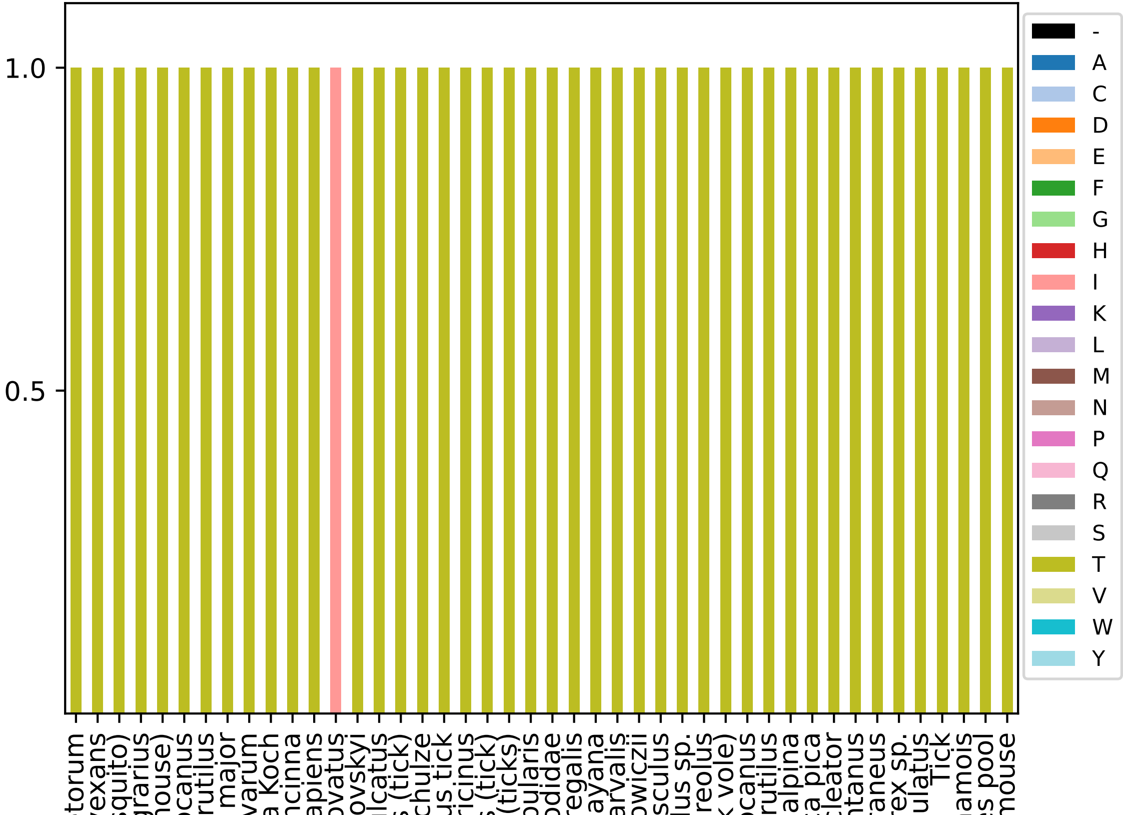

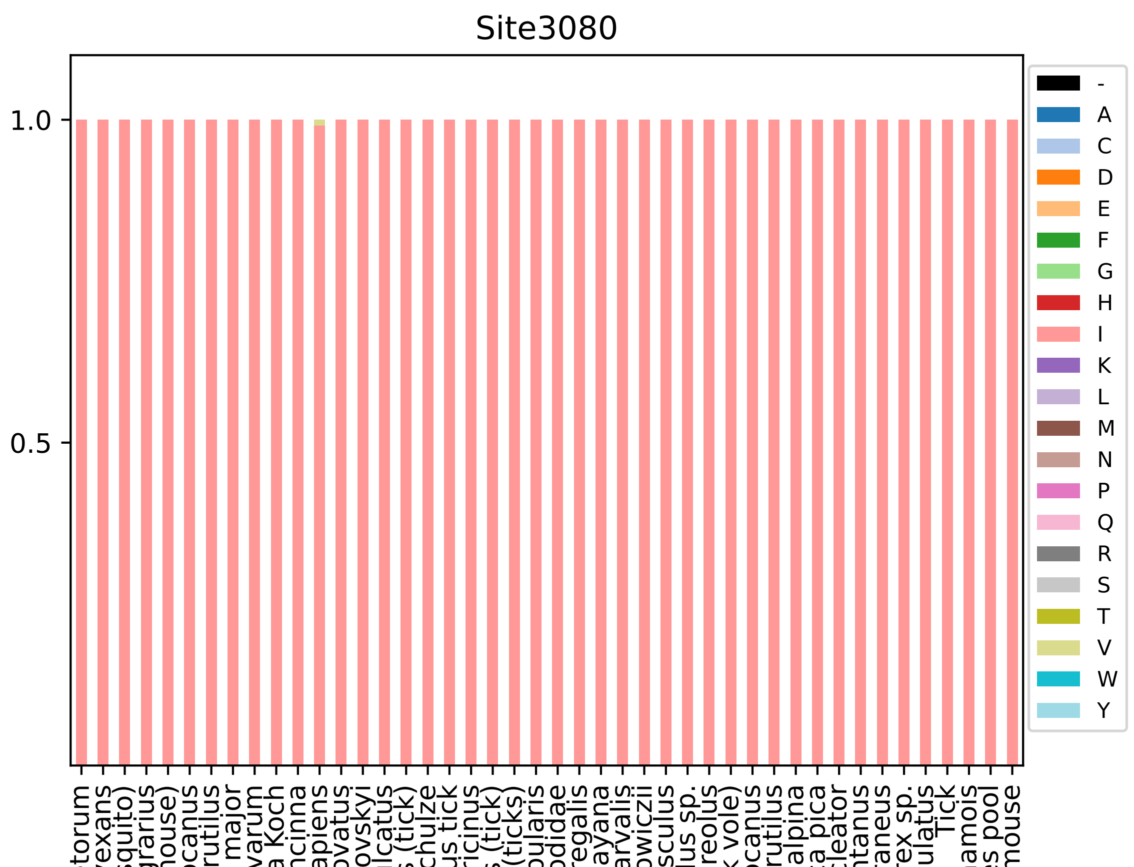
Site3080
1.0
0.5
-
A
C
D
E
F
G
H
I
K
L
M
N
P
Q
R
S
T
V
W
Y

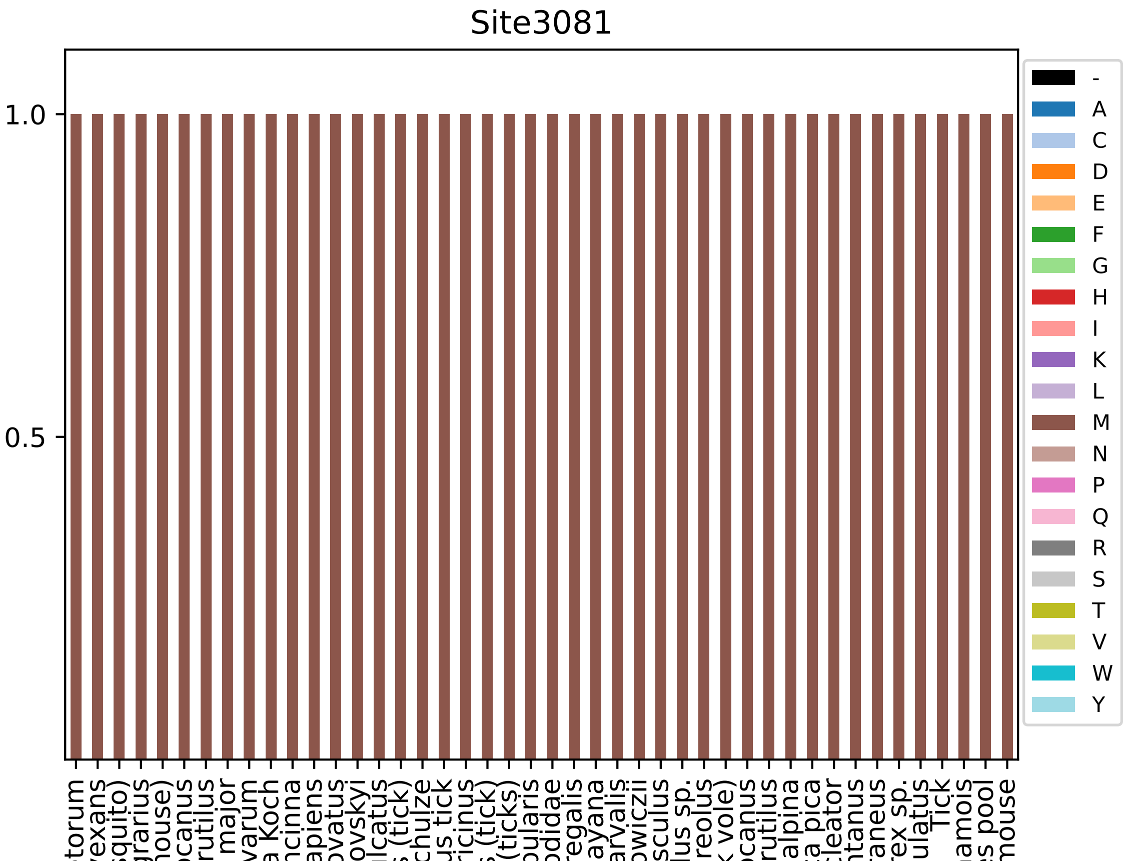
Site3081
1.0
0.5
-
A
C
D
E
F
G
H
I
K
L
M
N
P
Q
R
S
T
V
W
Y

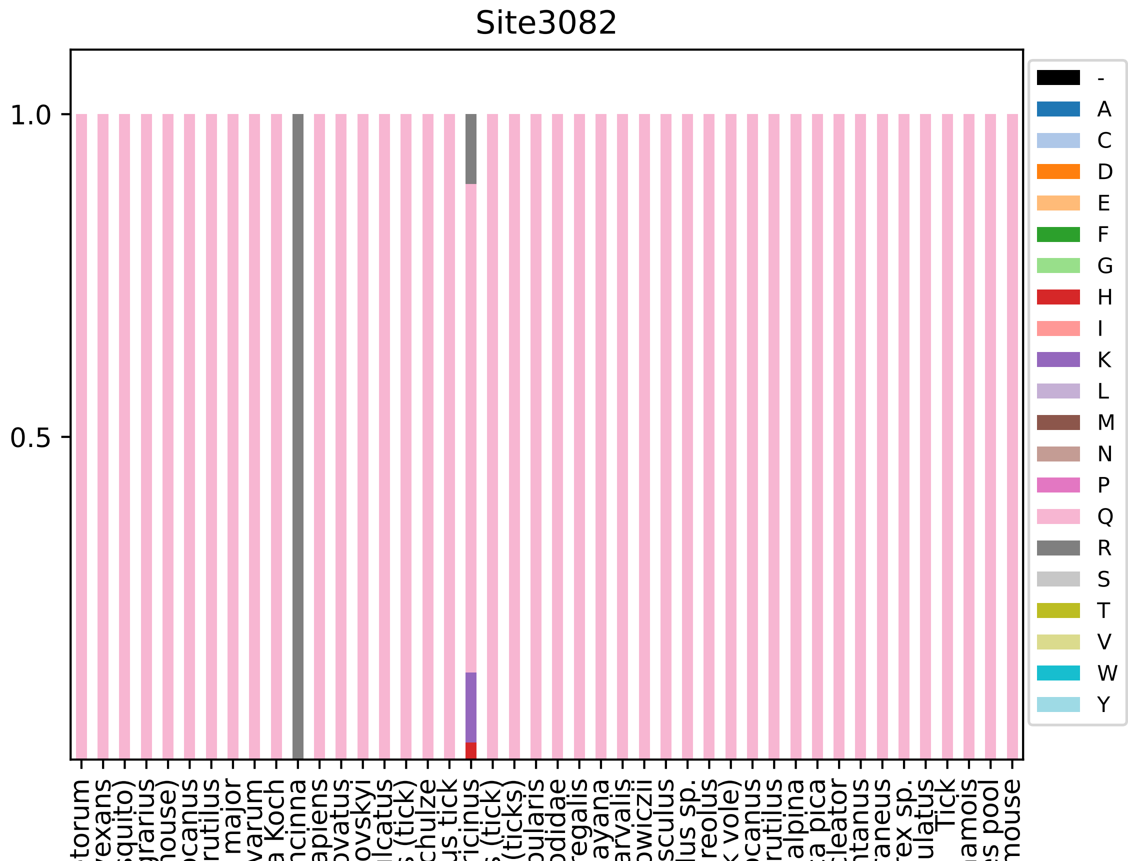
Site3082
1.0
0.5
-
A
C
D
E
F
G
H
I
K
L
M
N
P
Q
R
S
T
V
W
Y

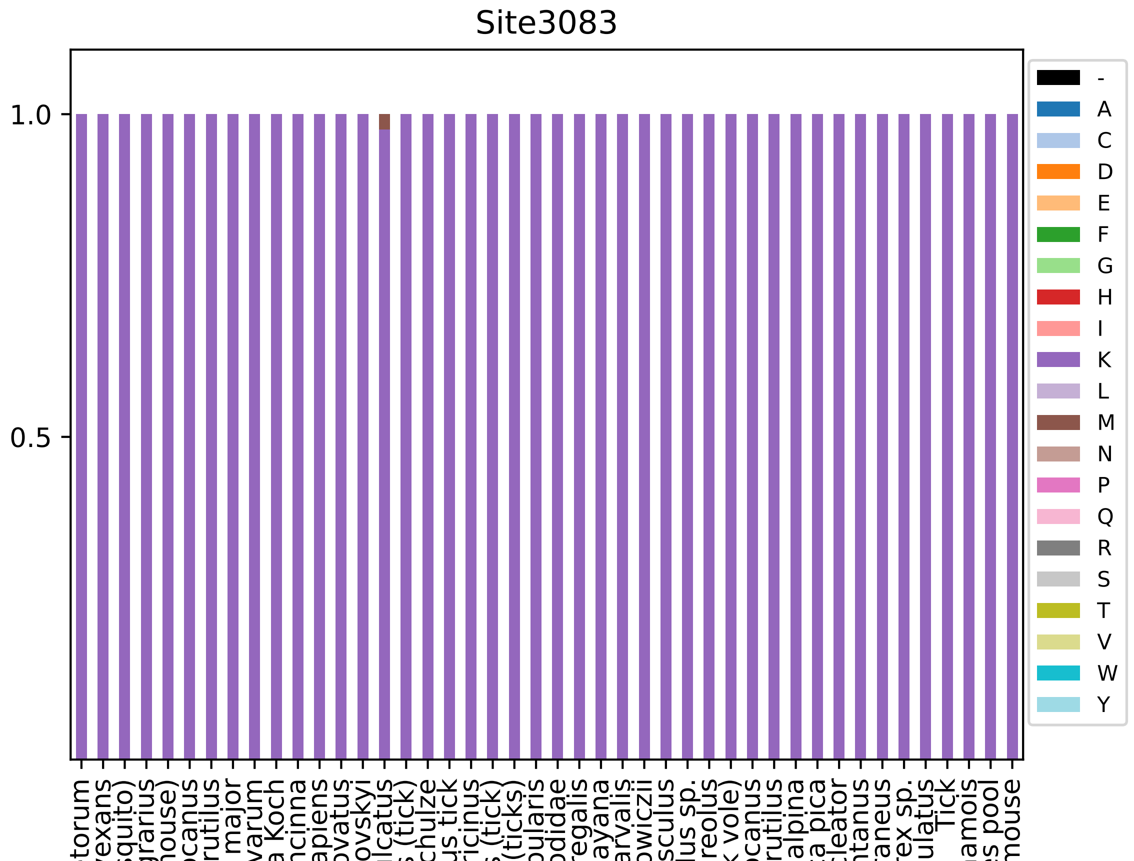
Site3083
1.0
0.5
-
A
C
D
E
F
G
H
I
K
L
M
N
P
Q
R
S
T
V
W
Y
Tick

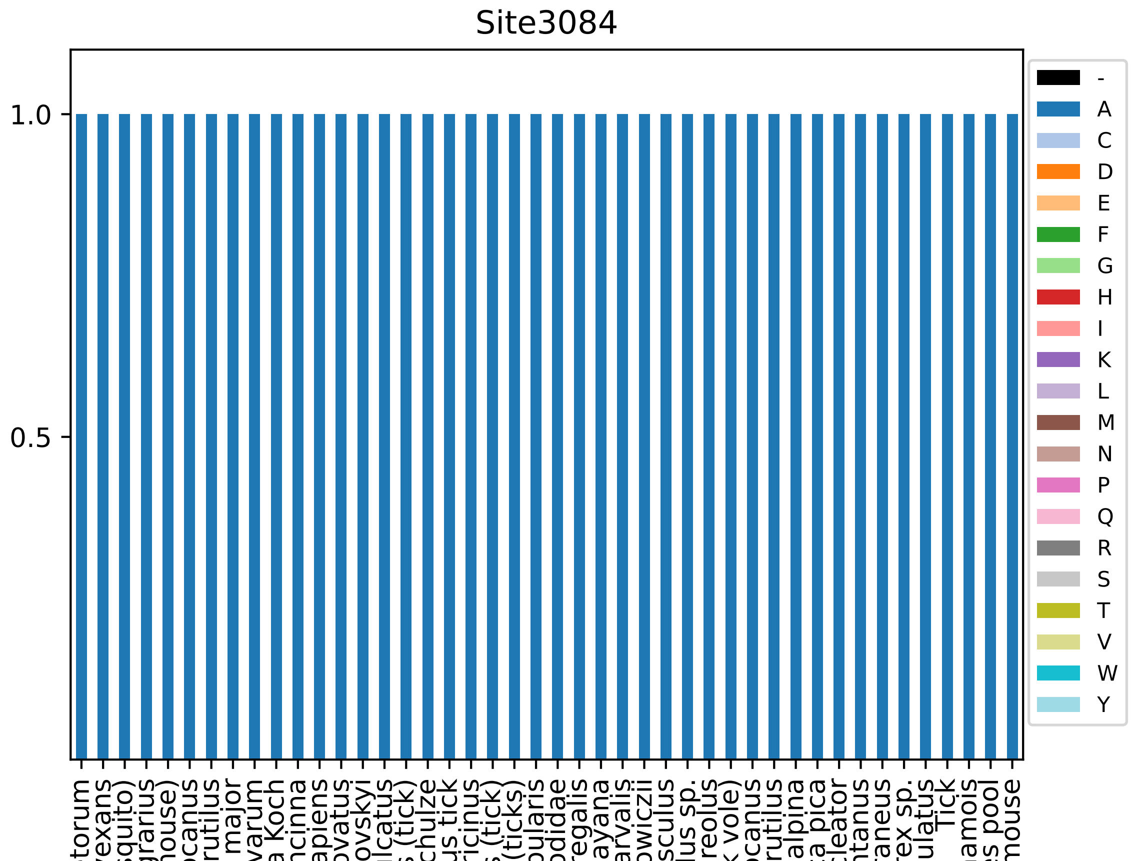

Site3084
1.0
0.5
-
A
C
D
E
F
G
H
I
K
L
M
N
P
Q
R
S
T
V
W
Y

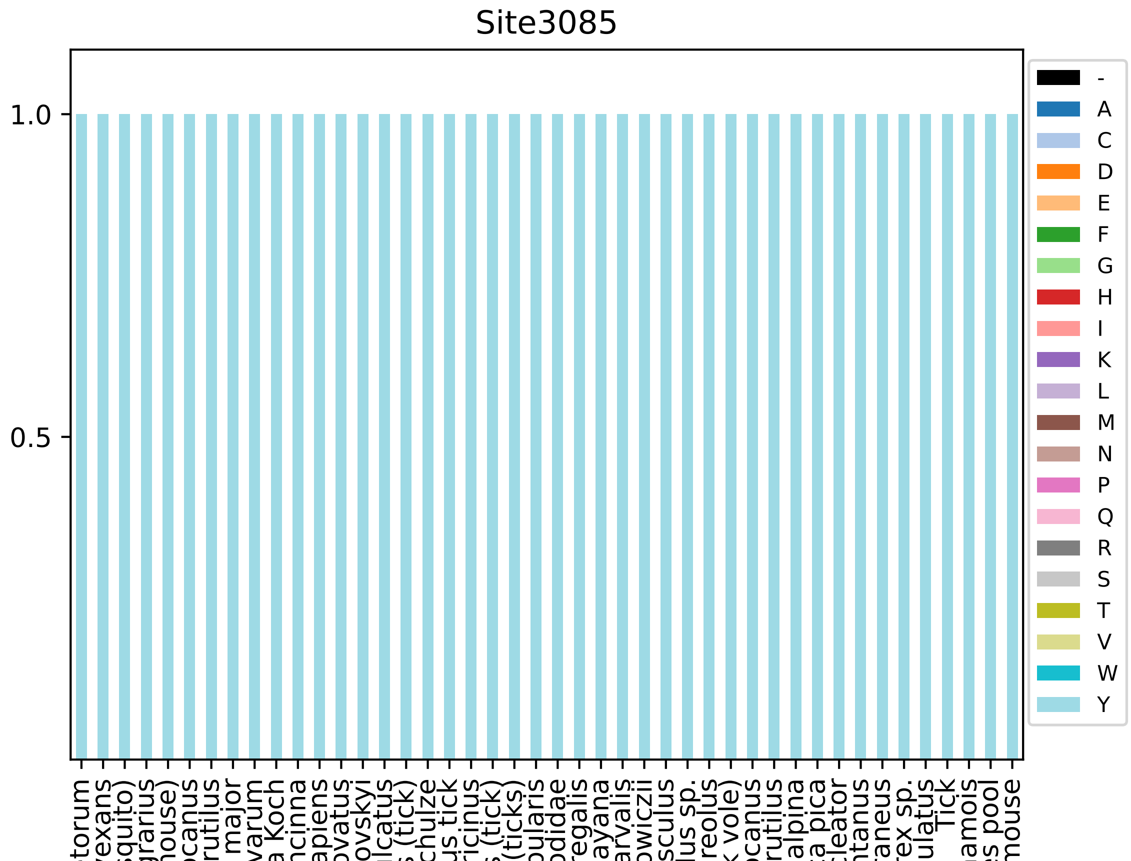

Site3085
1.0
0.5
-
A
C
D
E
F
G
H
I
K
L
M
N
P
Q
R
S
T
V
W
Y

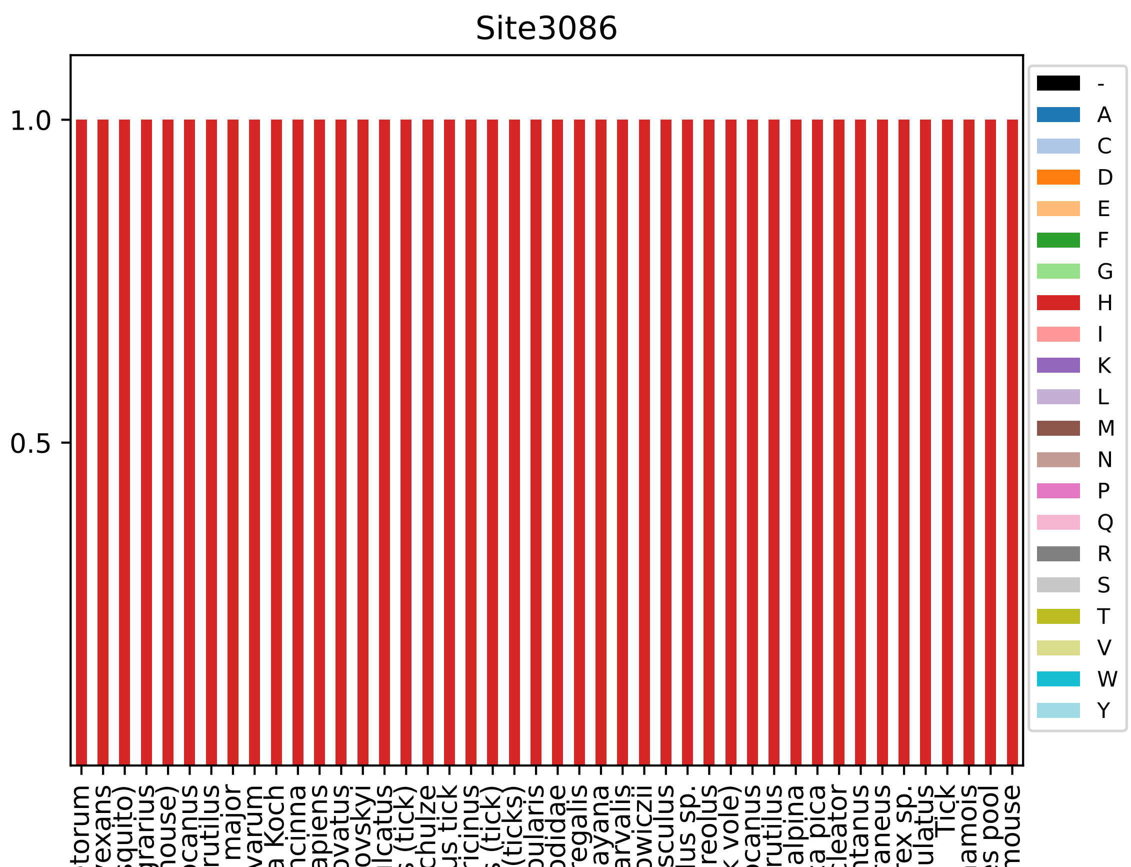

Site3086
1.0
0.5
-
A
C
D
E
F
G
H
I
K
L
M
N
P
Q
R
S
T
V
W
Y

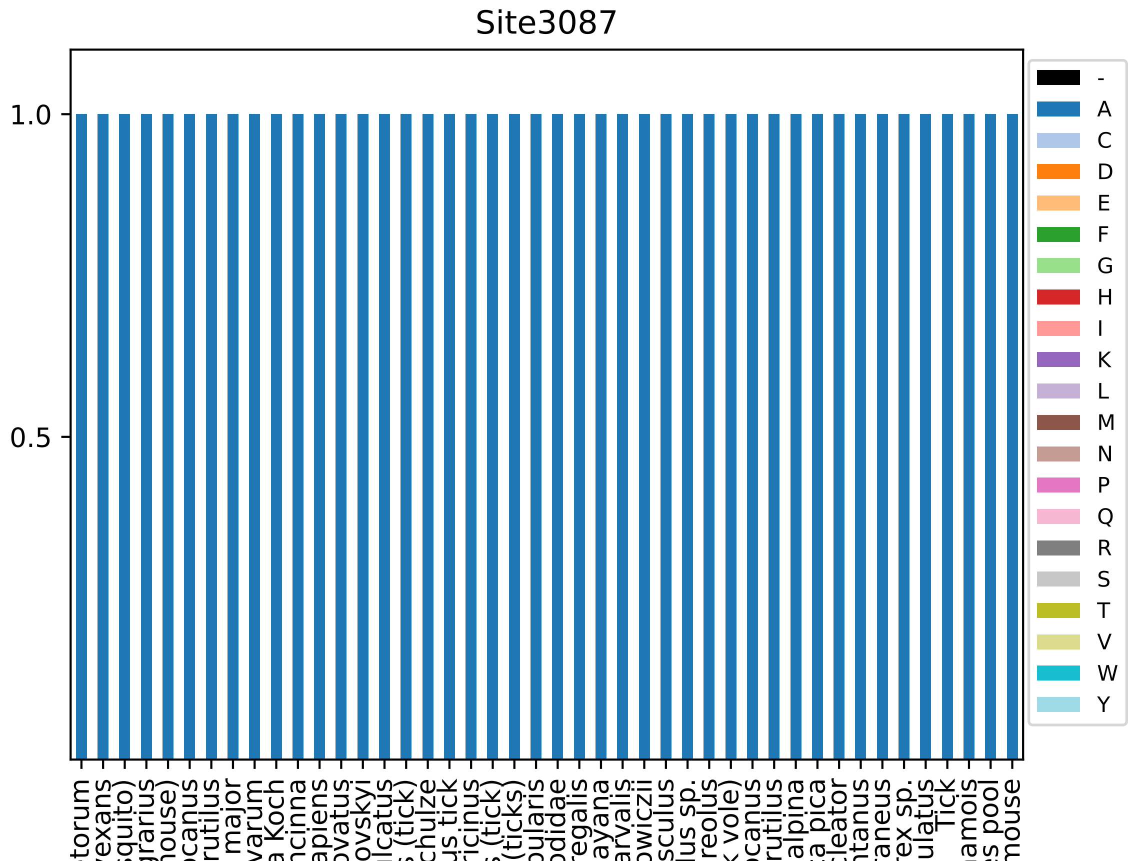
Site3087
1.0
0.5
-
A
C
D
E
F
G
H
I
K
L
M
N
P
Q
R
S
T
V
W
Y

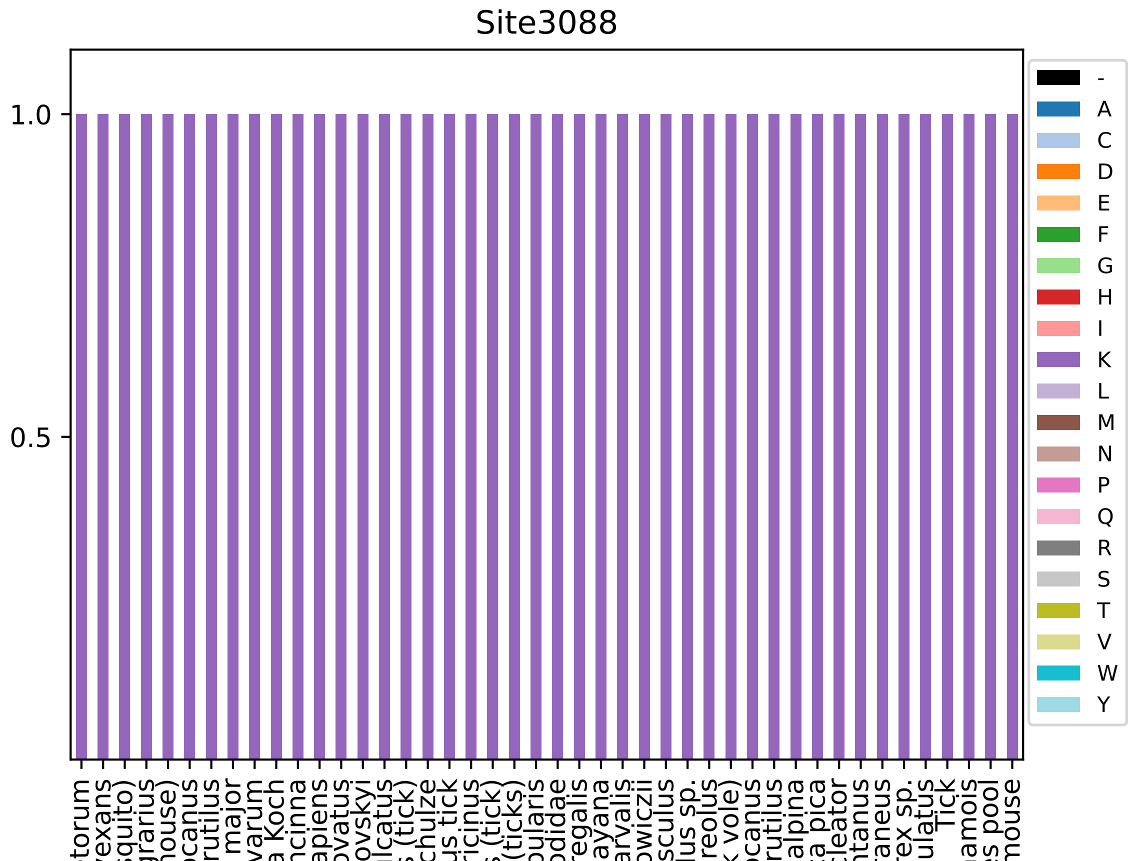
Site3088
1.0
0.5
-
A
C
D
E
F
G
H
I
K
L
M
N
P
Q
R
S
T
V
W
Y

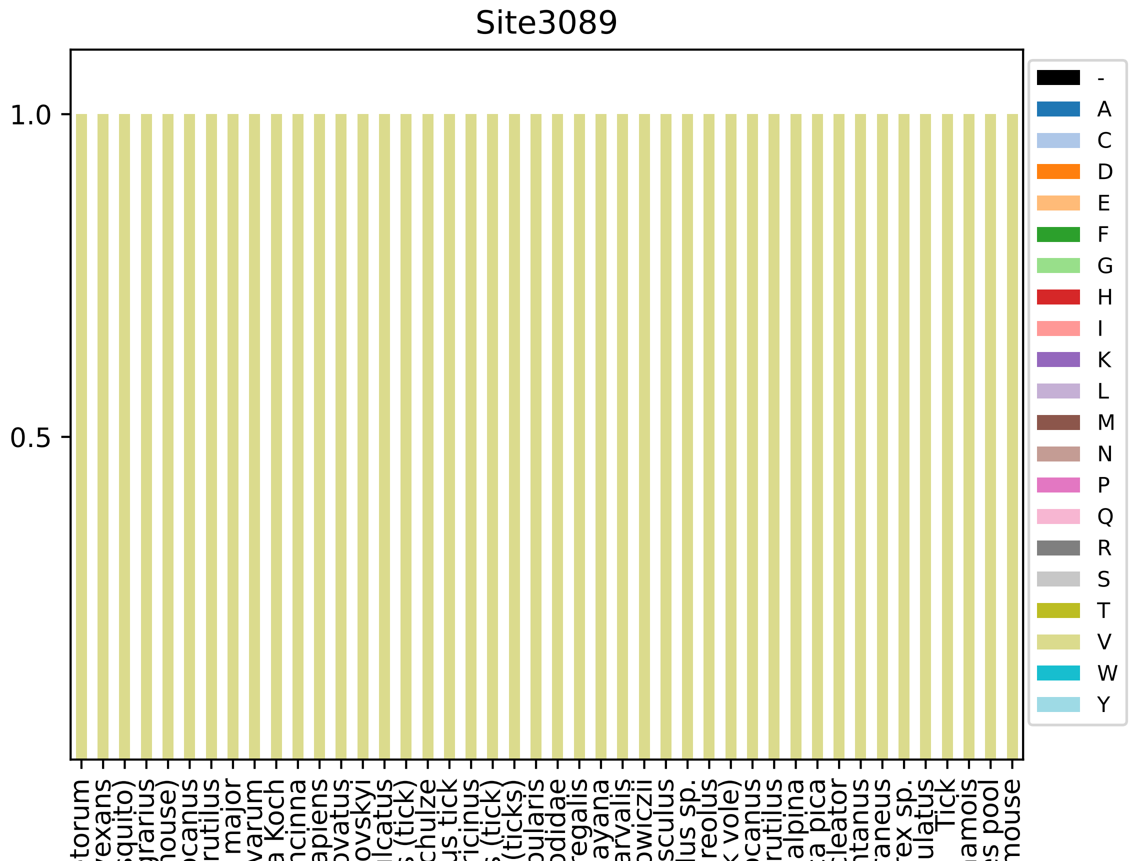
Site3089
1.0
0.5
-
A
C
D
E
F
G
H
I
K
L
M
N
P
Q
R
S
T
V
W
Y

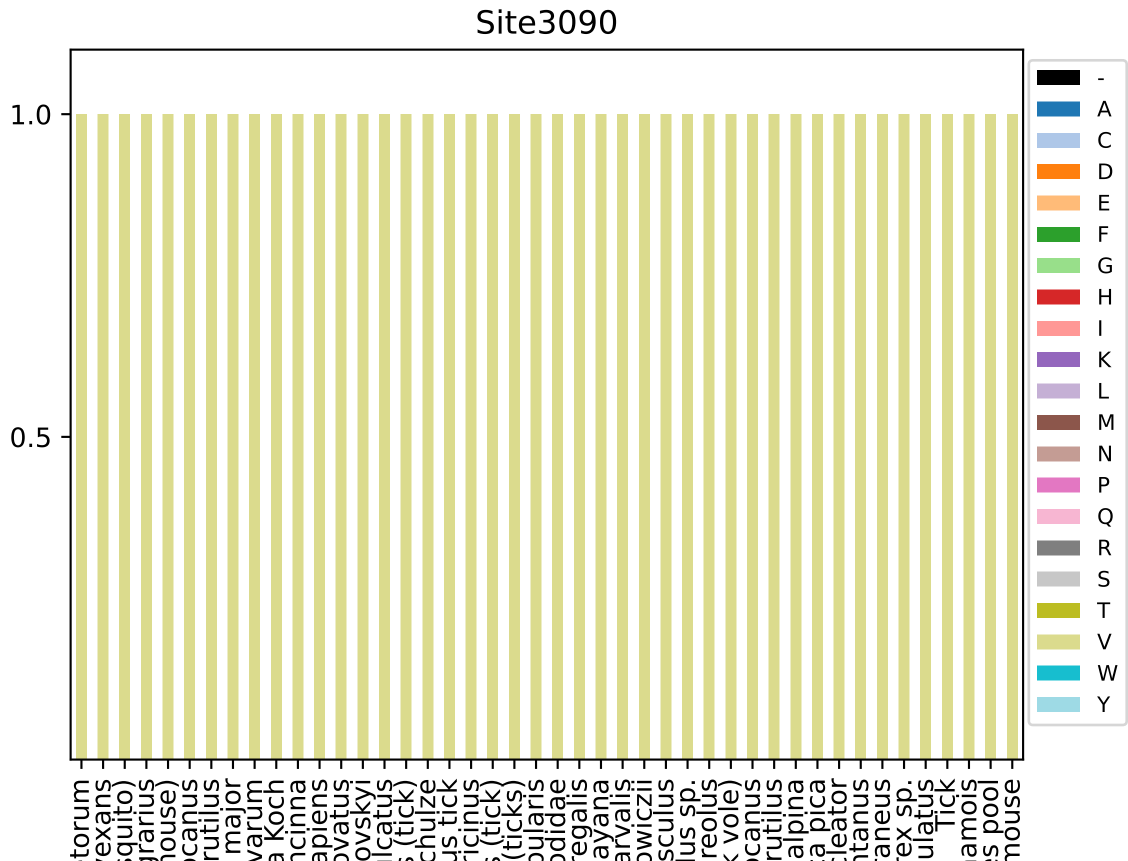
Site3090
1.0
0.5
-
A
C
D
E
F
G
H
I
K
L
M
N
P
Q
R
S
T
V
W
Y

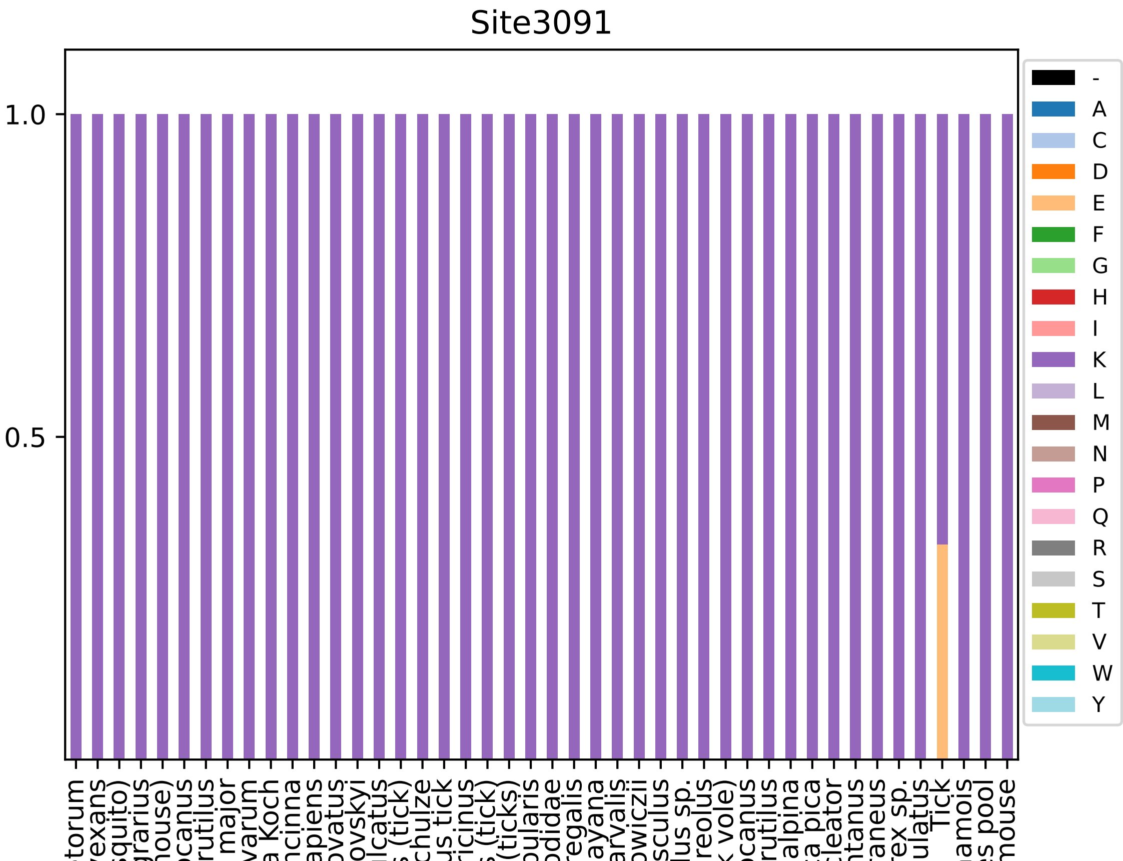

Site3091
1.0
0.5
-
A
C
D
E
F
G
H
I
K
L
M
N
P
Q
R
S
T
V
W
Y
Tick

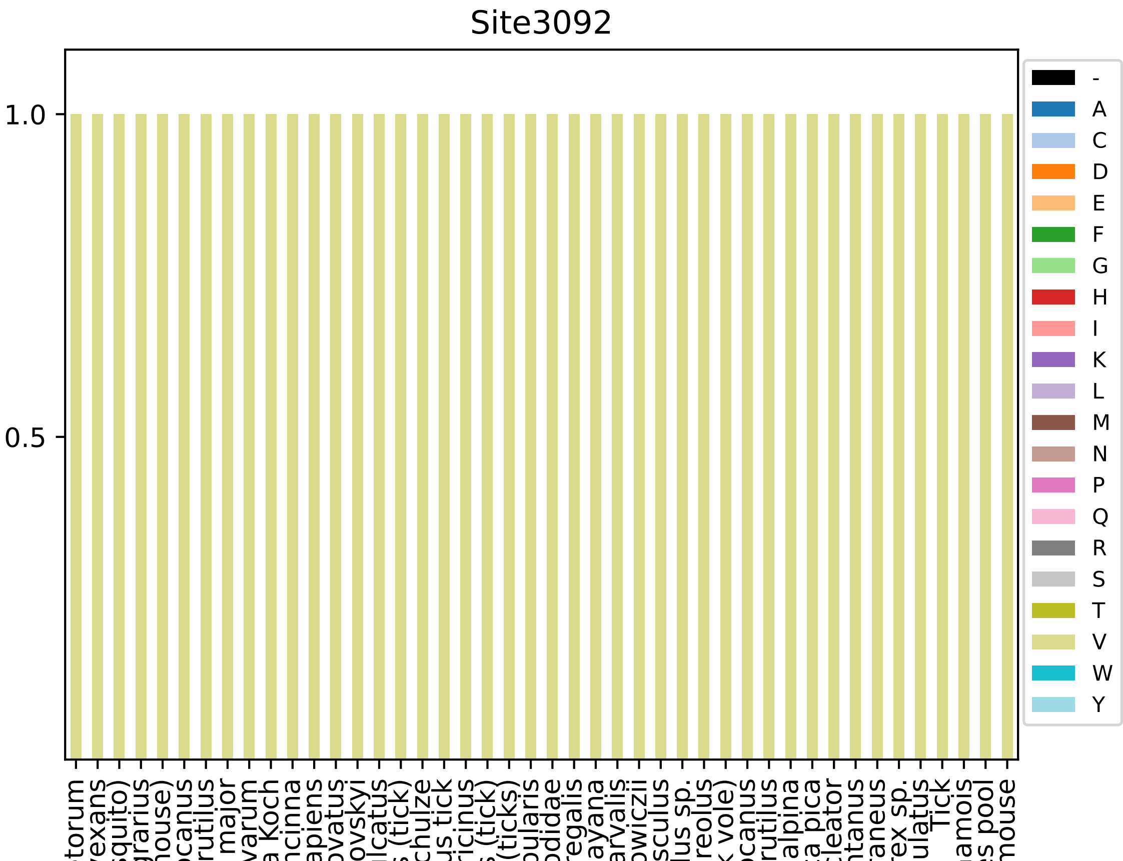
Site3092
1.0
0.5
-
A
C
D
E
F
G
H
I
K
L
M
N
P
Q
R
S
T
V
W
Y

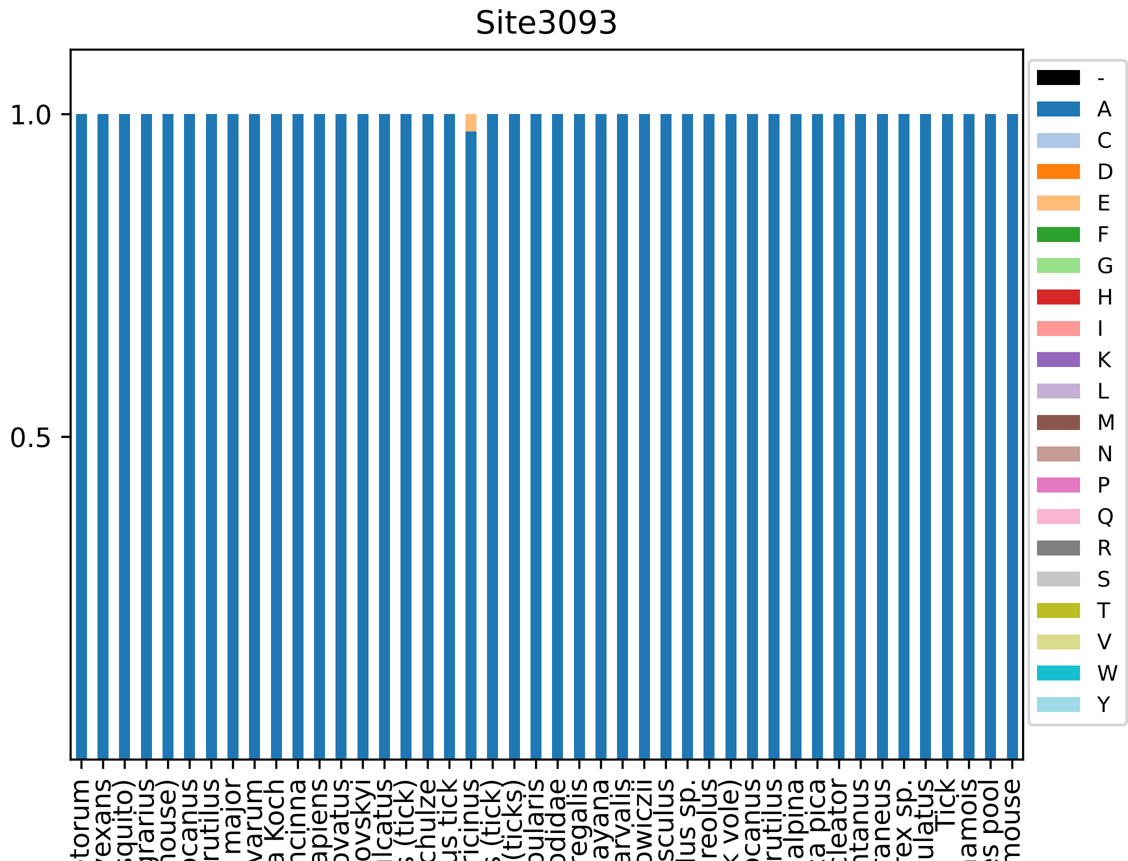
Site3093
1.0
0.5
-
A
C
D
E
F
G
H
I
K
L
M
N
P
Q
R
S
T
V
W
Y

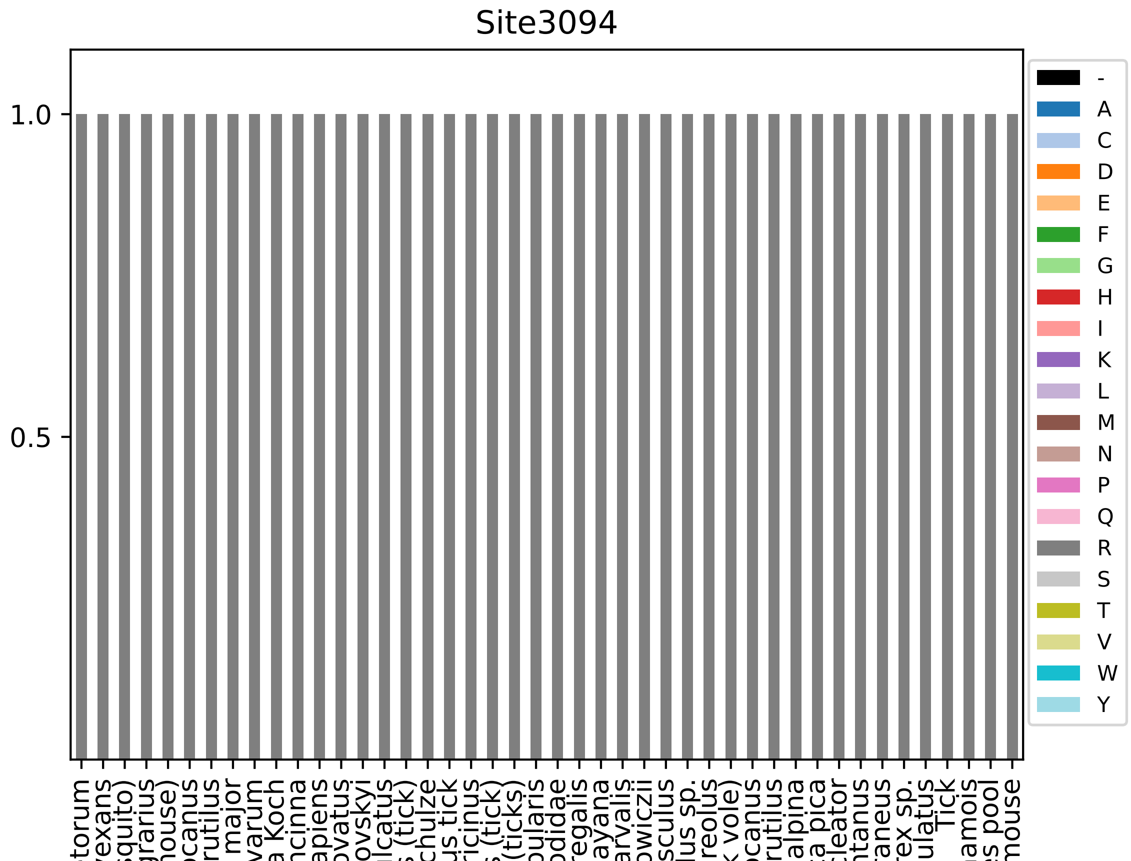
Site3094
1.0
0.5
-
A
C
D
E
F
G
H
I
K
L
M
N
P
Q
R
S
T
V
W
Y

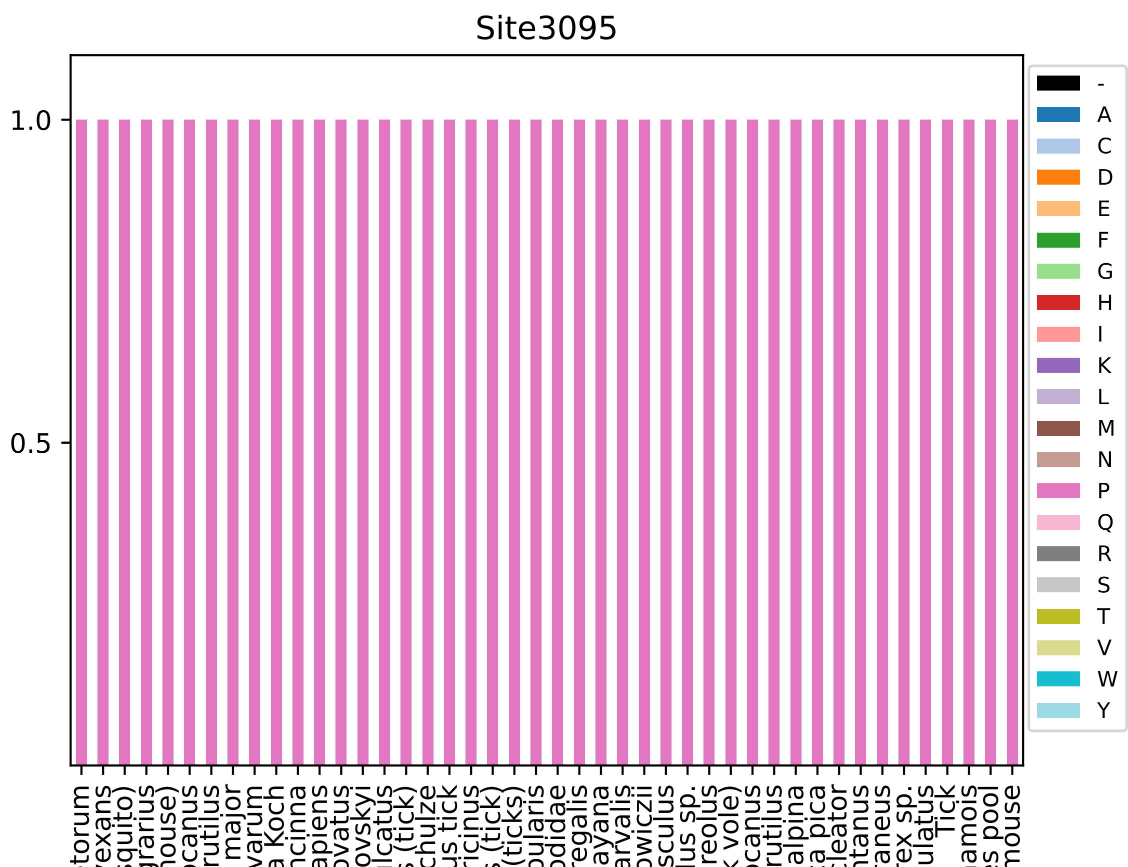
Site3095
1.0
0.5
-
A
C
D
E
F
G
H
I
K
L
M
N
P
Q
R
S
T
V
W
Y
Tick

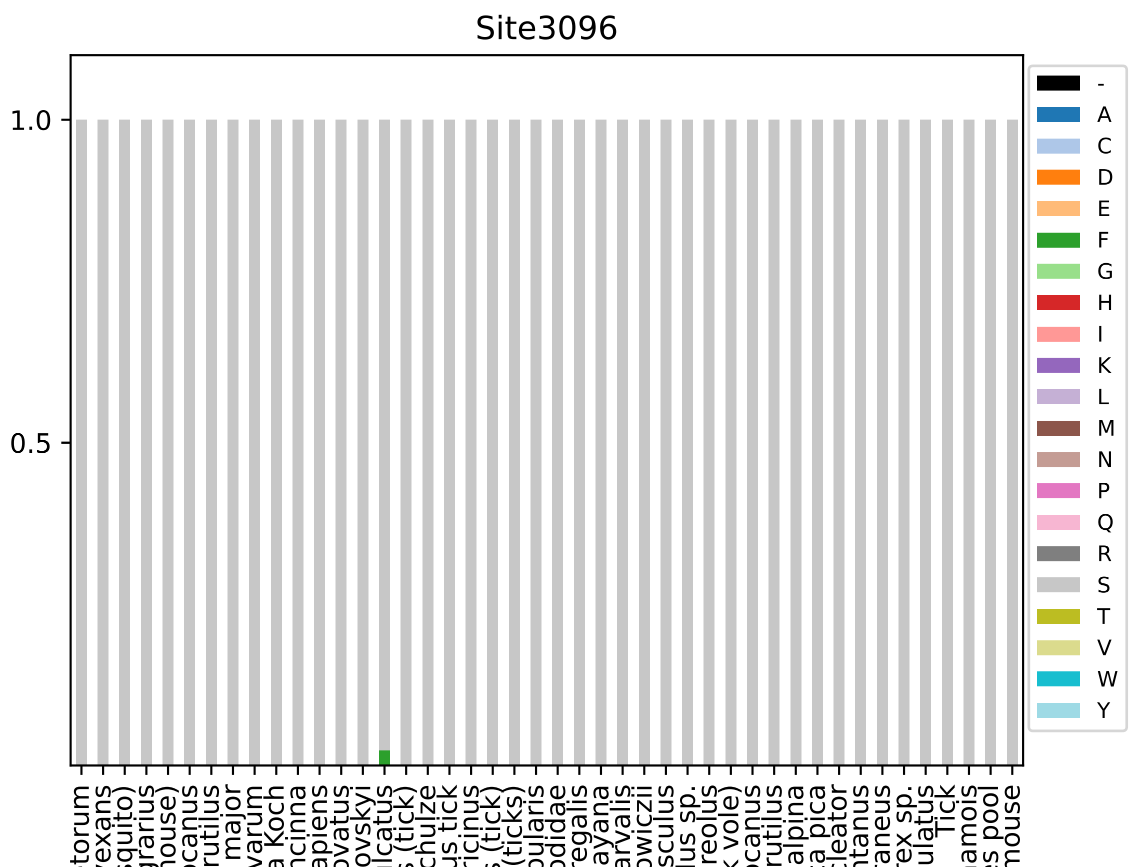
Site3096
1.0
0.5
-
A
C
D
E
F
G
H
I
K
L
M
N
P
Q
R
S
T
V
W
Y

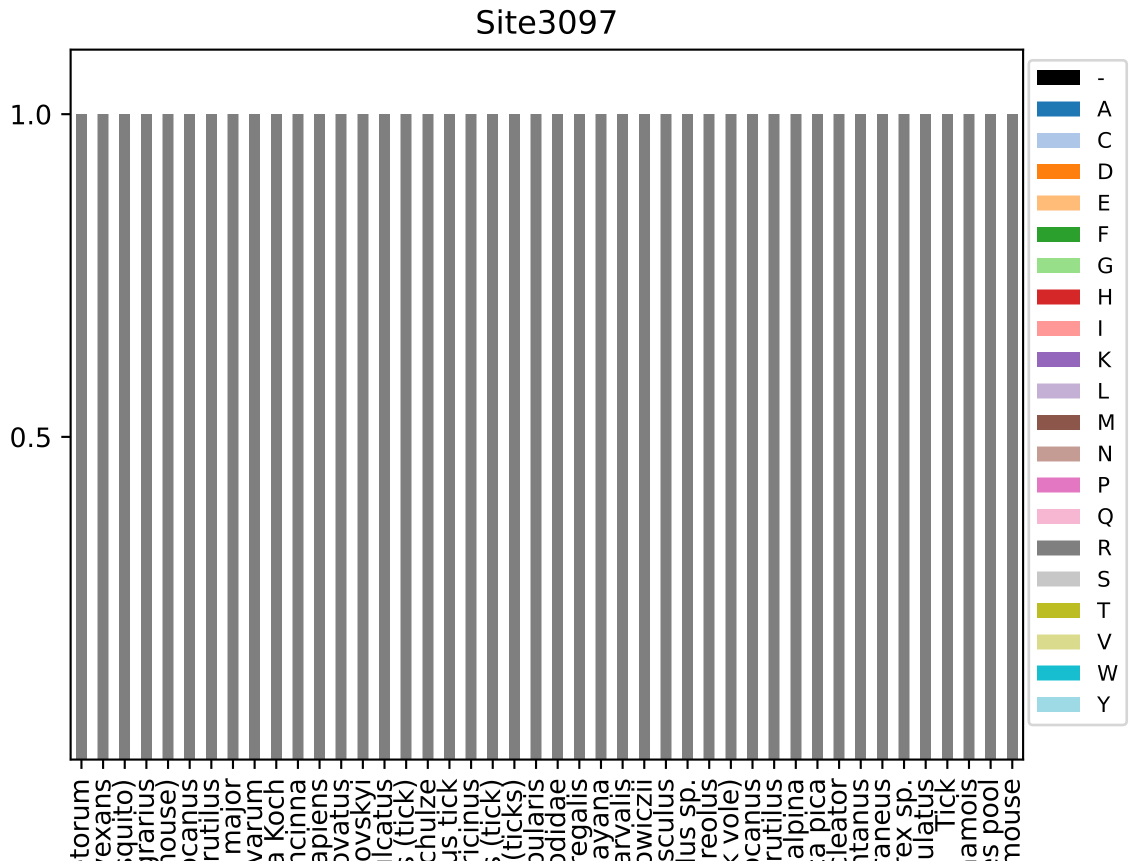
Site3097
1.0
0.5
-
A
C
D
E
F
G
H
I
K
L
M
N
P
Q
R
S
T
V
W
Y

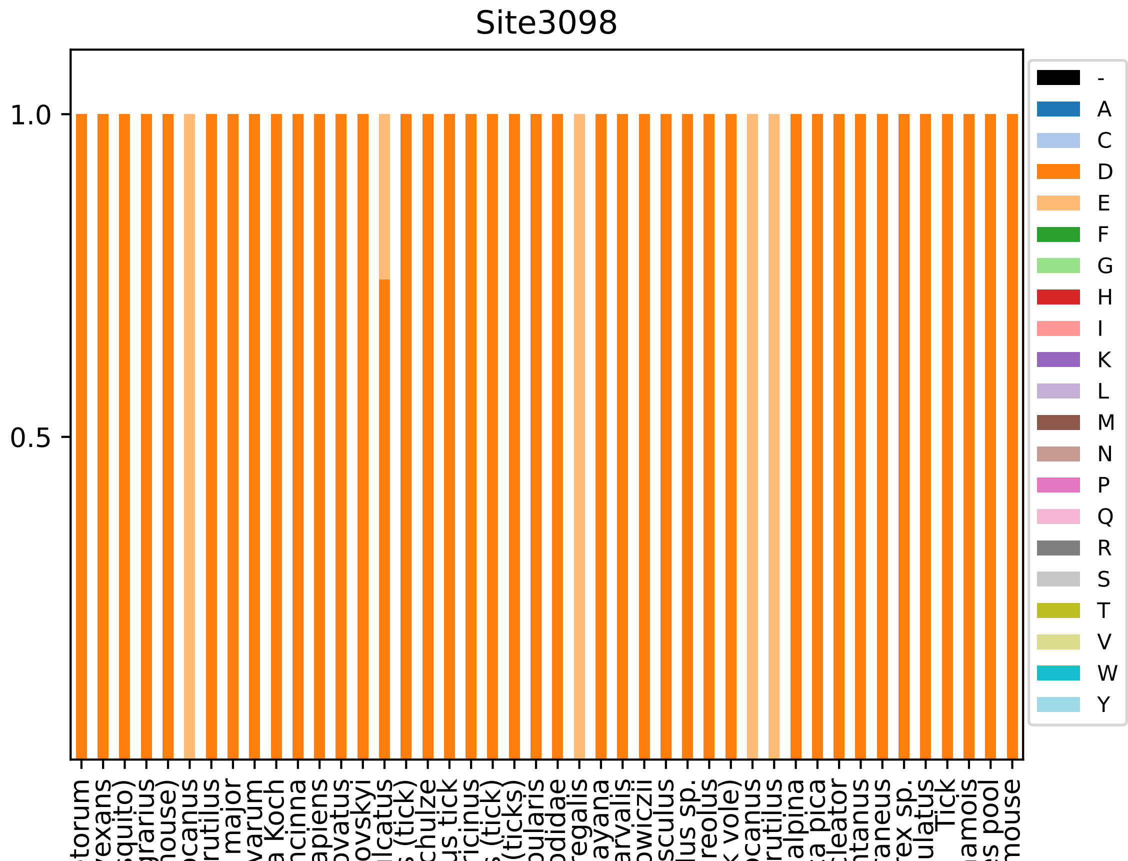
Site3098
1.0
0.5
-
A
C
D
E
F
G
H
I
K
L
M
N
P
Q
R
S
T
V
W
Y

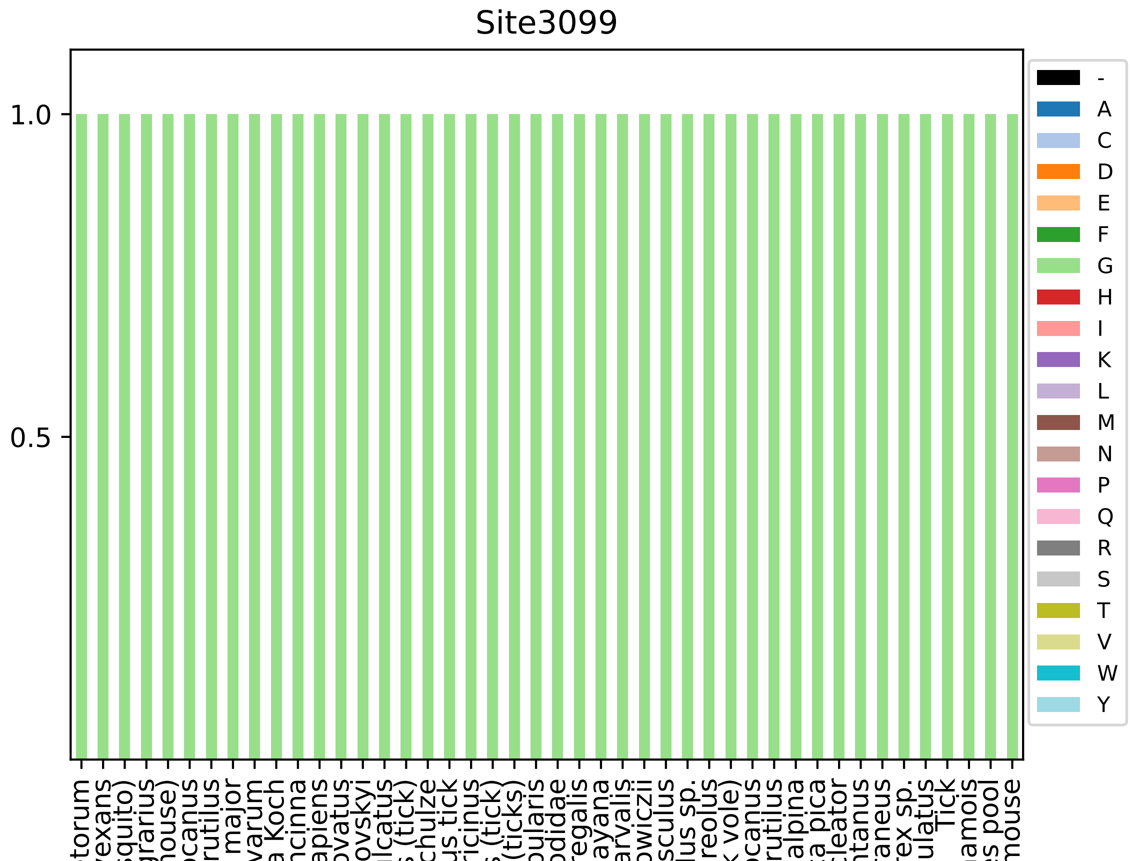
Site3099
1.0
0.5
-
A
C
D
E
F
G
H
I
K
L
M
N
P
Q
R
S
T
V
W
Y

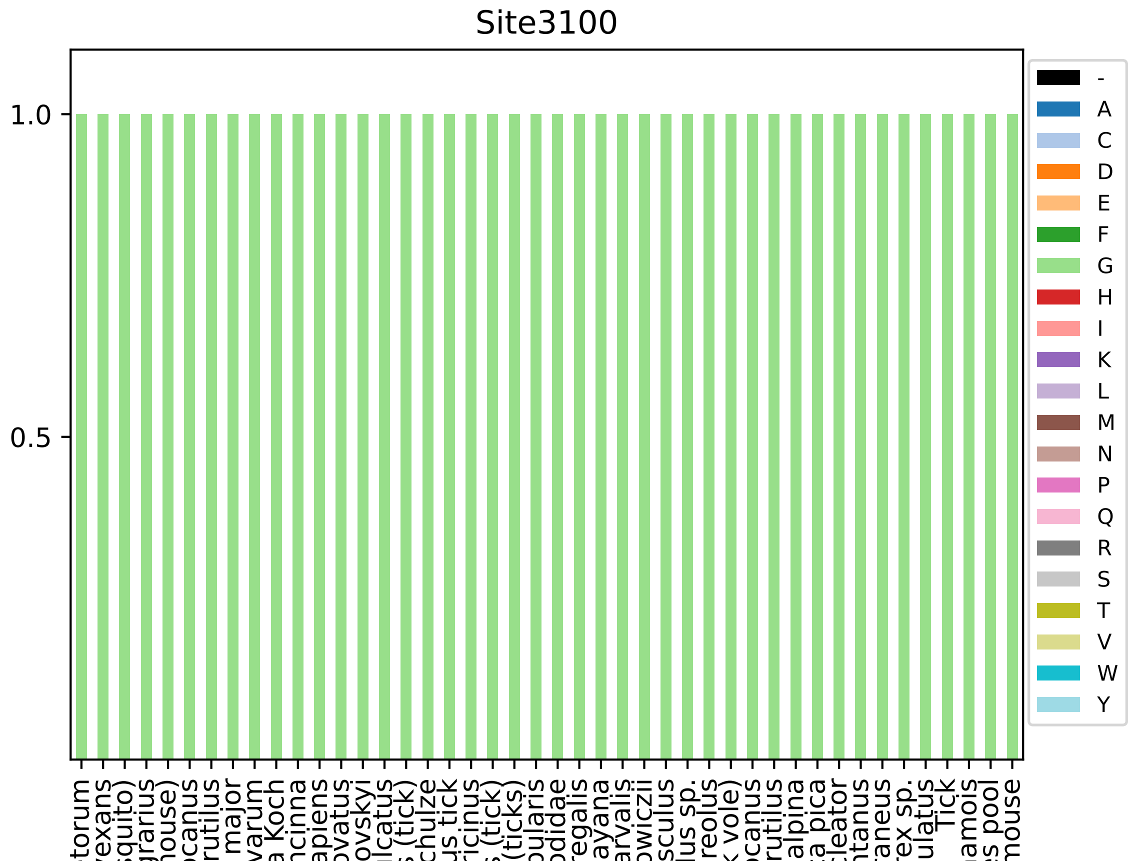
Site3100
1.0
0.5
-
A
C
D
E
F
G
H
I
K
L
M
N
P
Q
R
S
T
V
W
Y

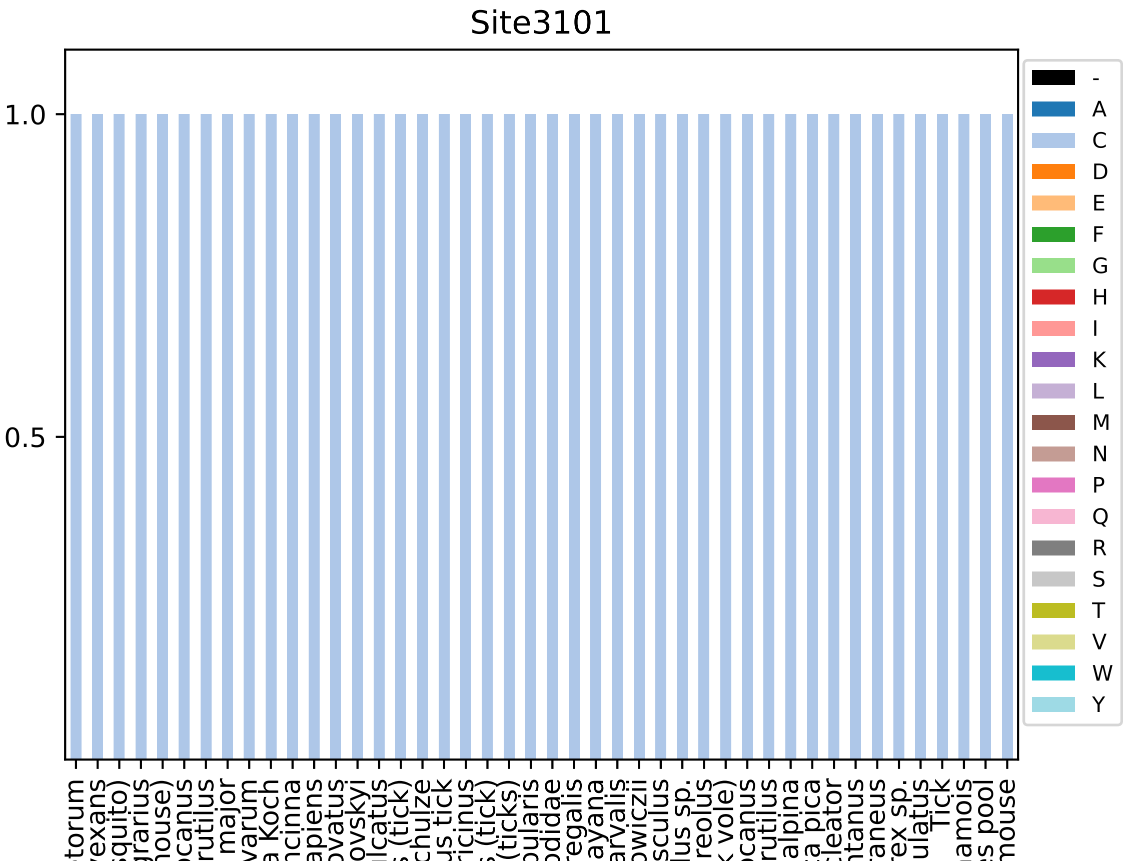

Site3101
1.0
0.5
-
A
C
D
E
F
G
H
I
K
L
M
N
P
Q
R
S
T
V
W
Y

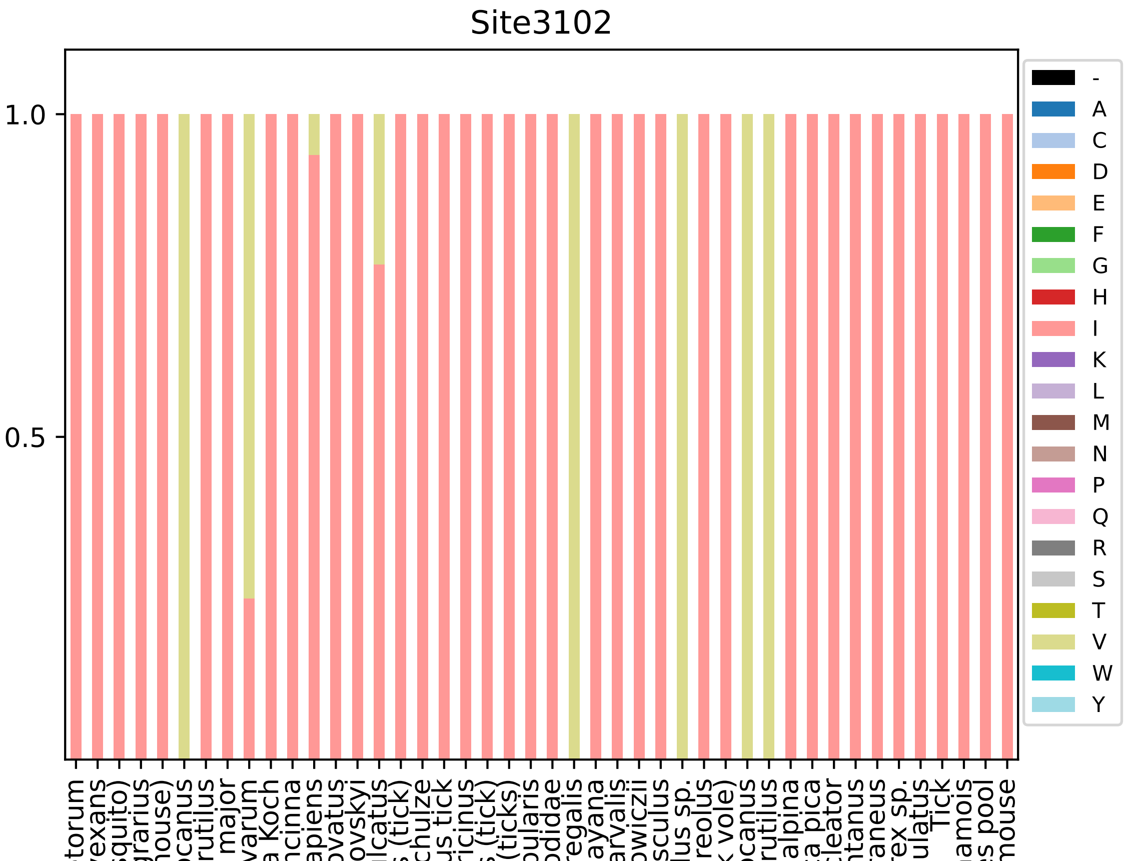

Site3102
1.0
0.5
-
A
C
D
E
F
G
H
I
K
L
M
N
P
Q
R
S
T
V
W
Y

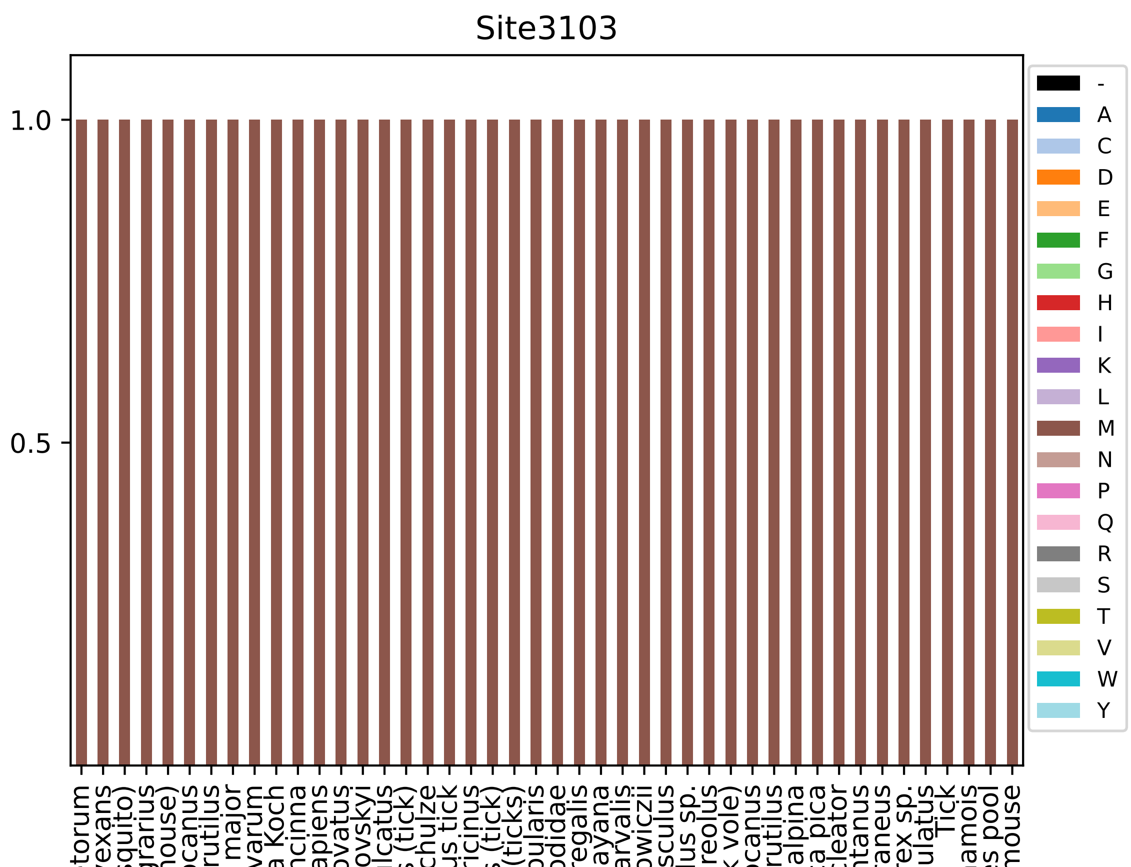
Site3103
1.0
0.5
-
A
C
D
E
F
G
H
I
K
L
M
N
P
Q
R
S
T
V
W
Y

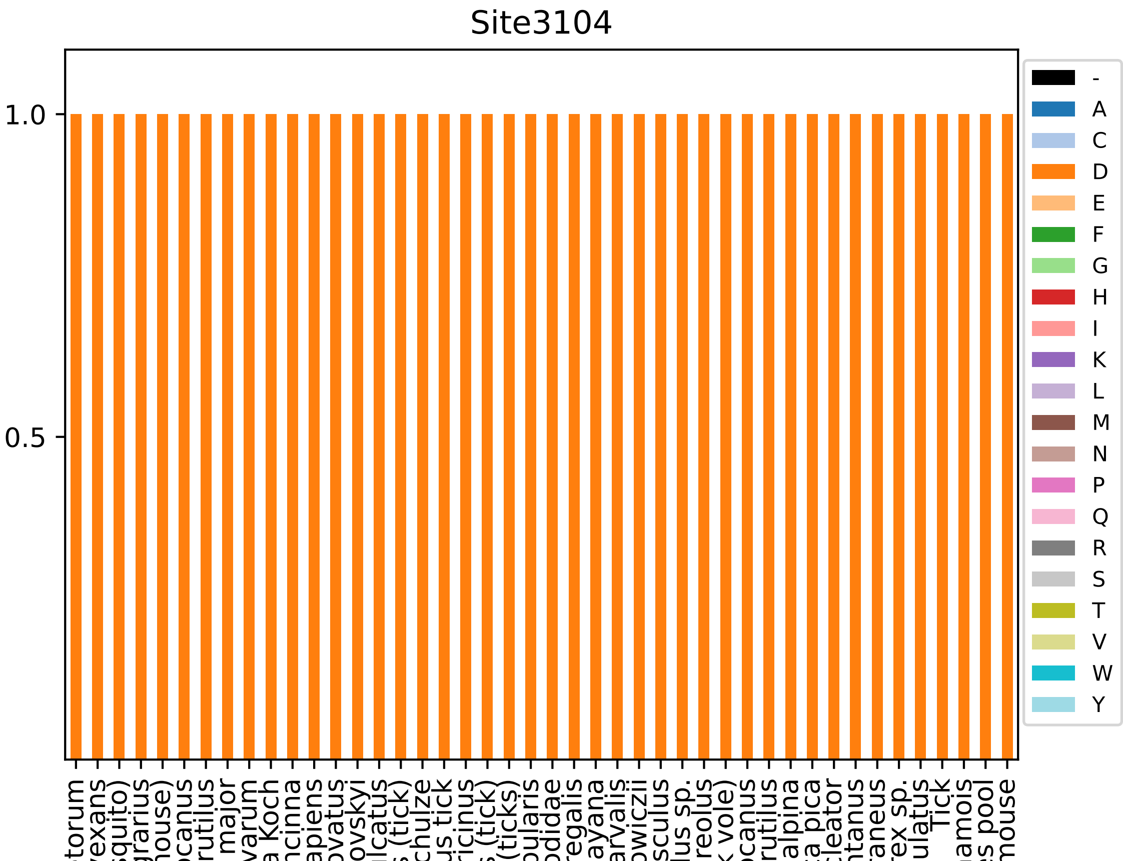
Site3104
1.0
0.5
-
A
C
D
E
F
G
H
I
K
L
M
N
P
Q
R
S
T
V
W
Y

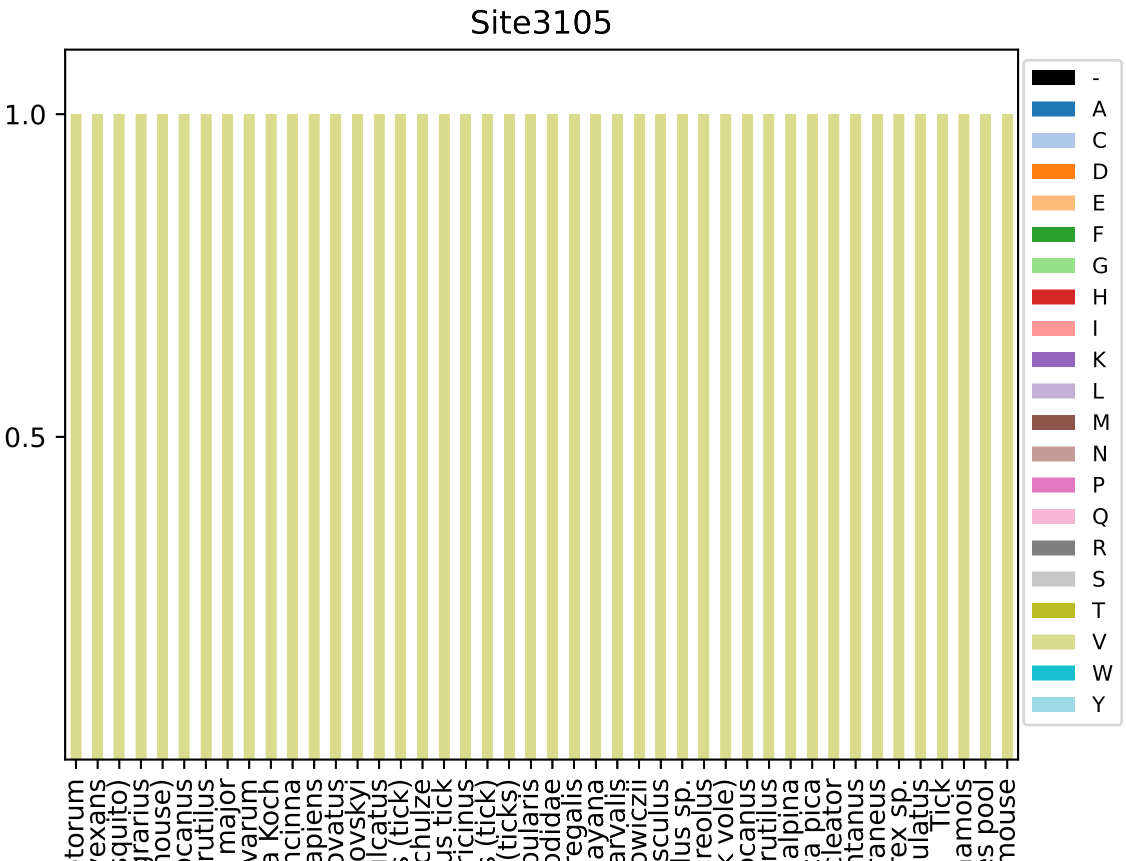
Site3105
1.0
0.5
-
A
C
D
E
F
G
H
I
K
L
M
N
P
Q
R
S
T
V
W
Y

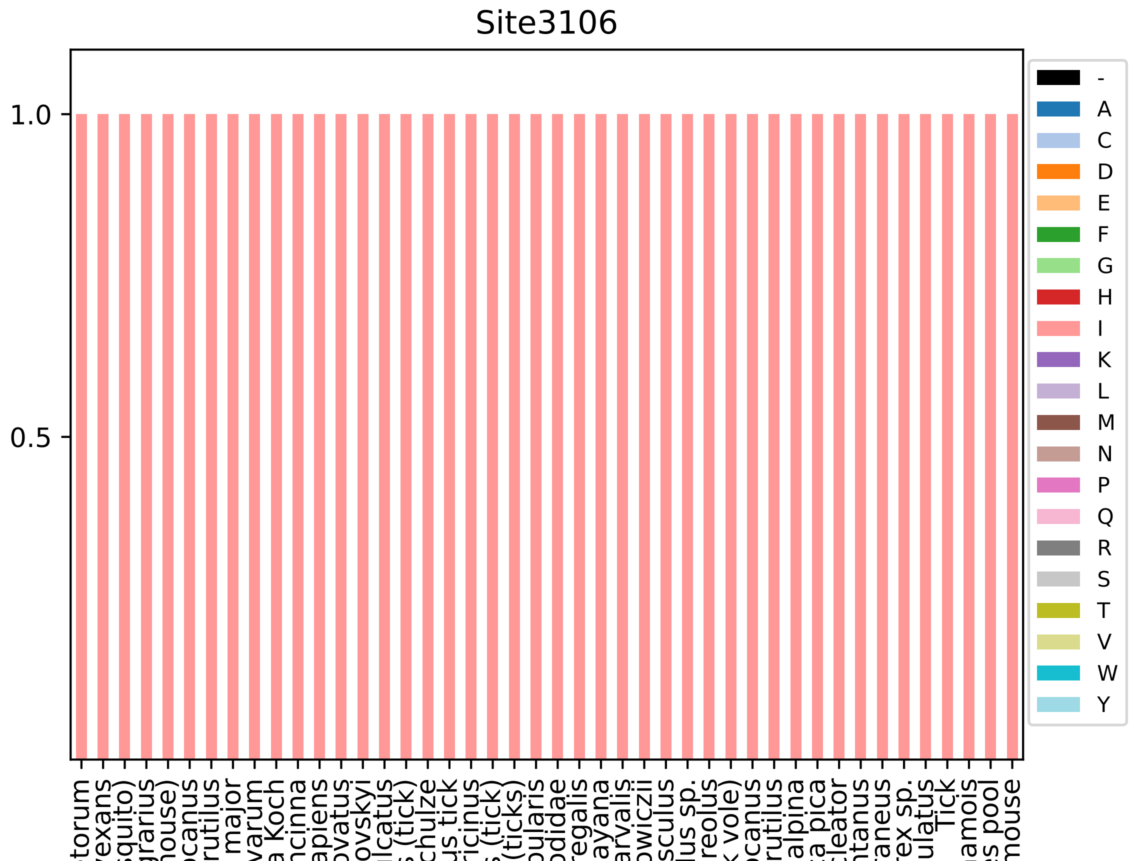
Site3106
1.0
0.5
-
A
C
D
E
F
G
H
I
K
L
M
N
P
Q
R
S
T
V
W
Y
Tick

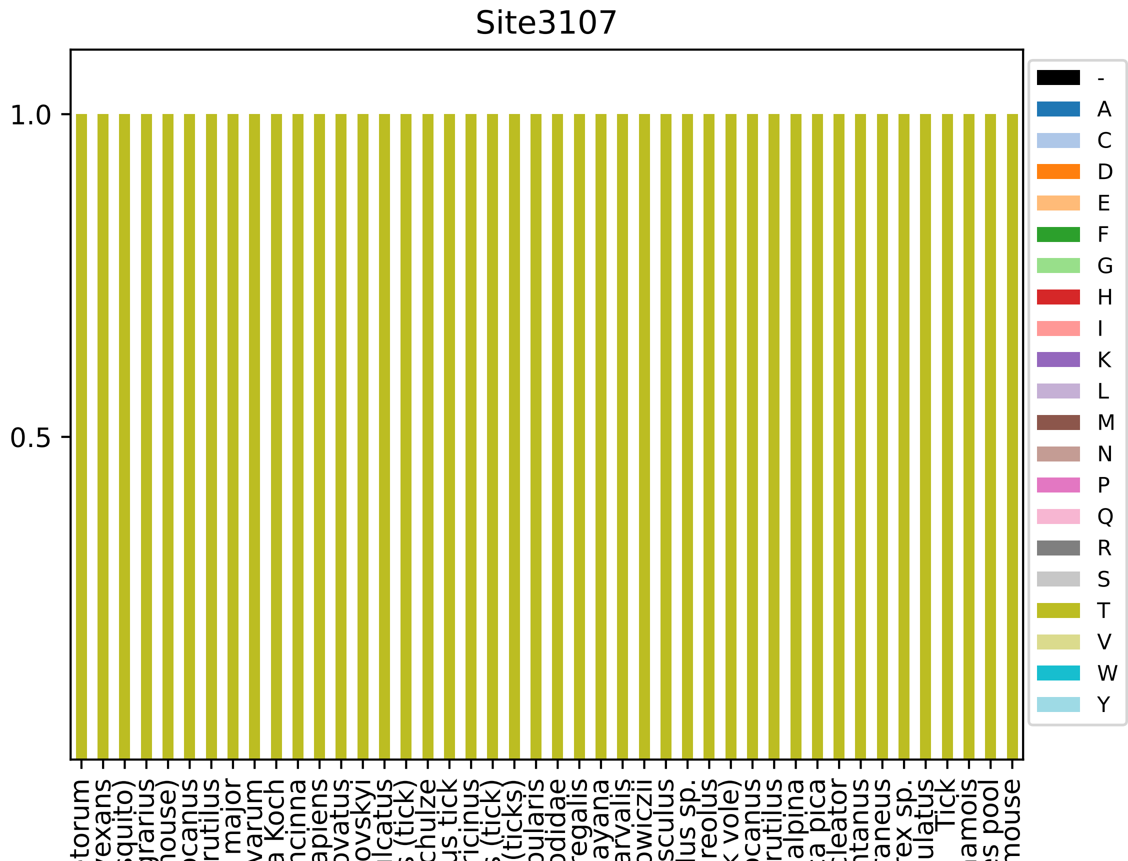

Site3107
1.0
0.5
-
A
C
D
E
F
G
H
I
K
L
M
N
P
Q
R
S
T
V
W
Y

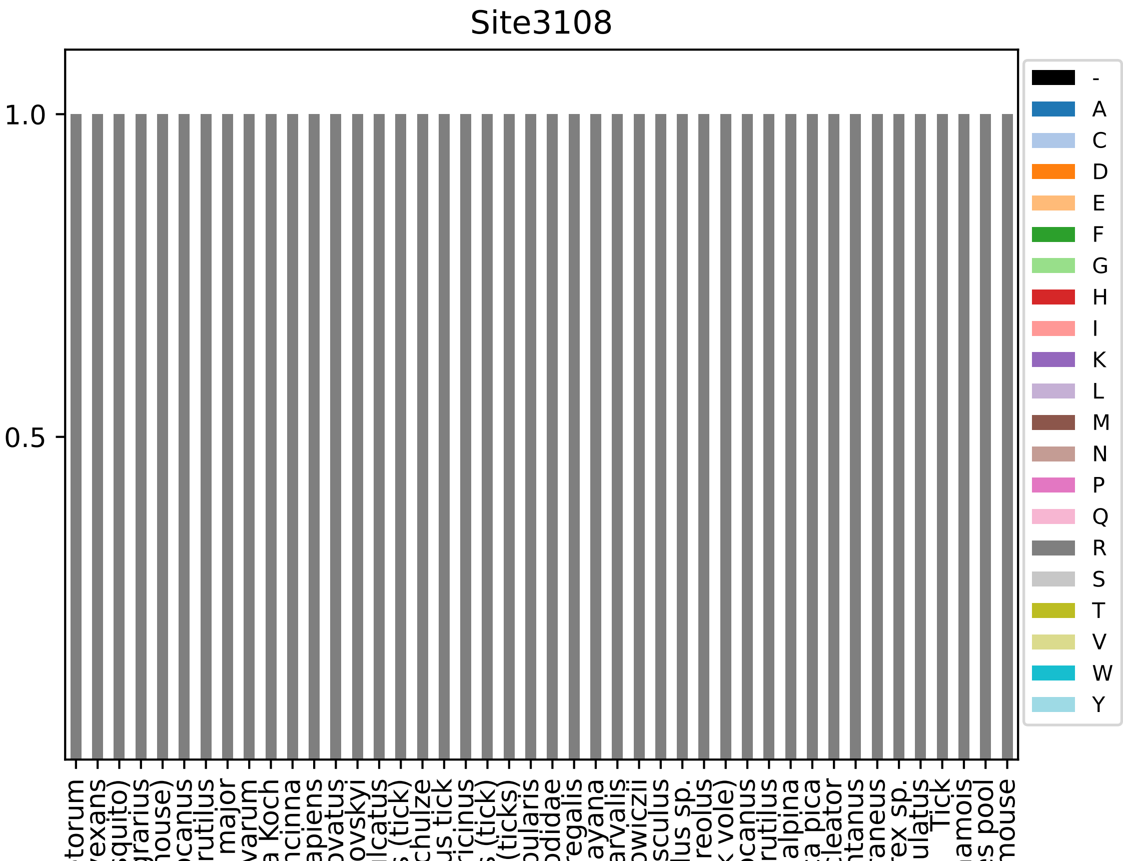
Site3108
1.0
0.5
-
A
C
D
E
F
G
H
I
K
L
M
N
P
Q
R
S
T
V
W
Y

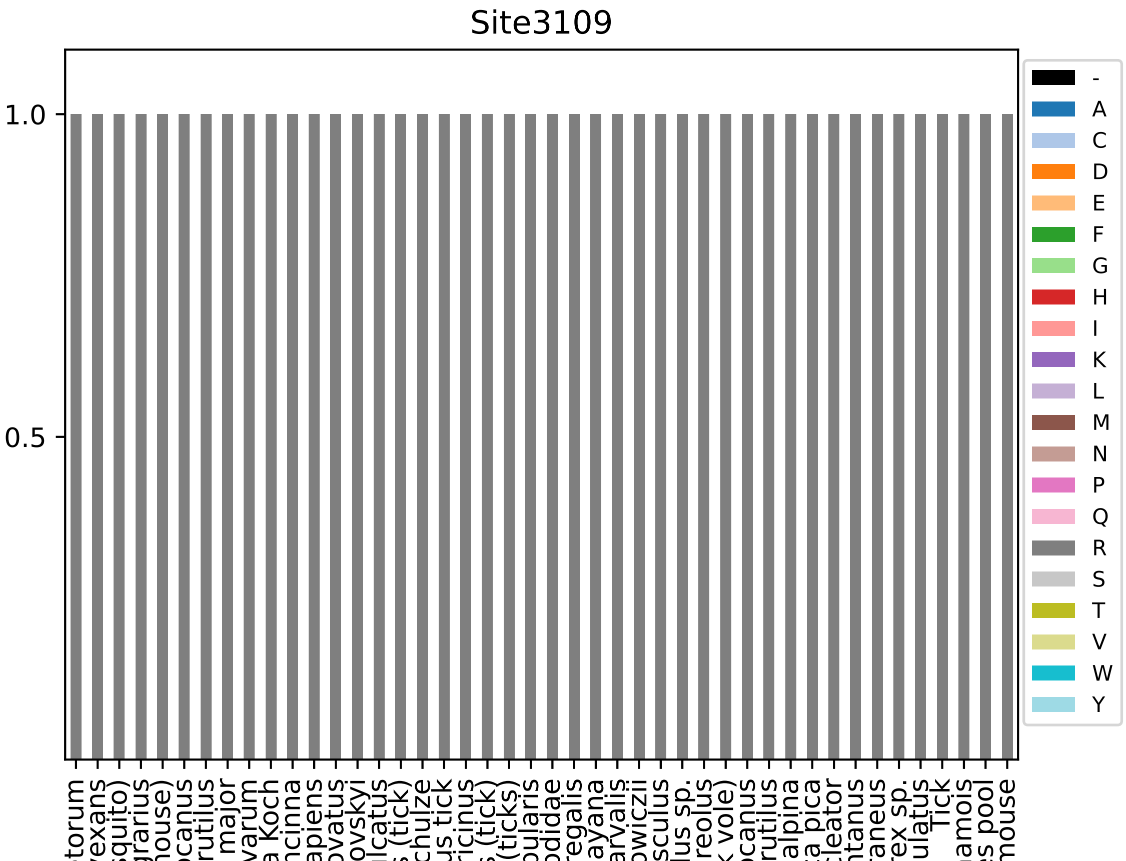
Site3109
1.0
0.5
-
A
C
D
E
F
G
H
I
K
L
M
N
P
Q
R
S
T
V
W
Y

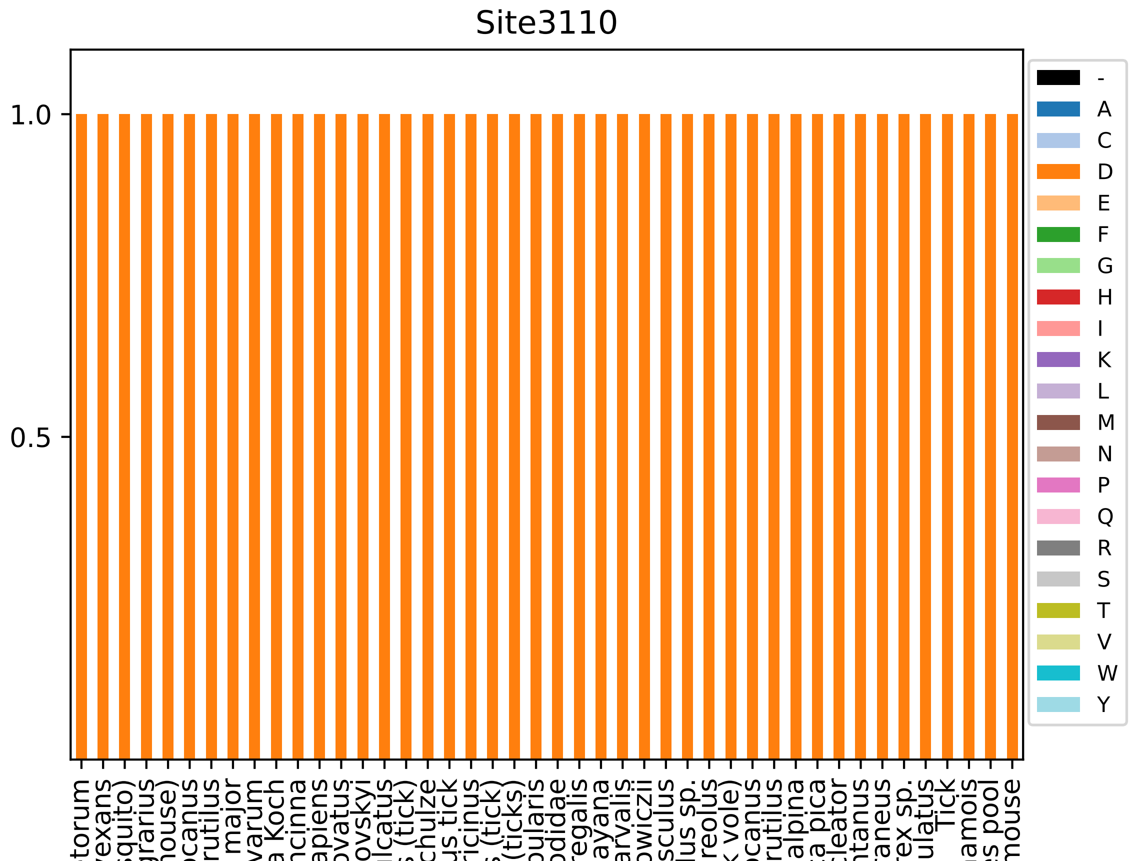

Site3110
1.0
0.5
-
A
C
D
E
F
G
H
I
K
L
M
N
P
Q
R
S
T
V
W
Y

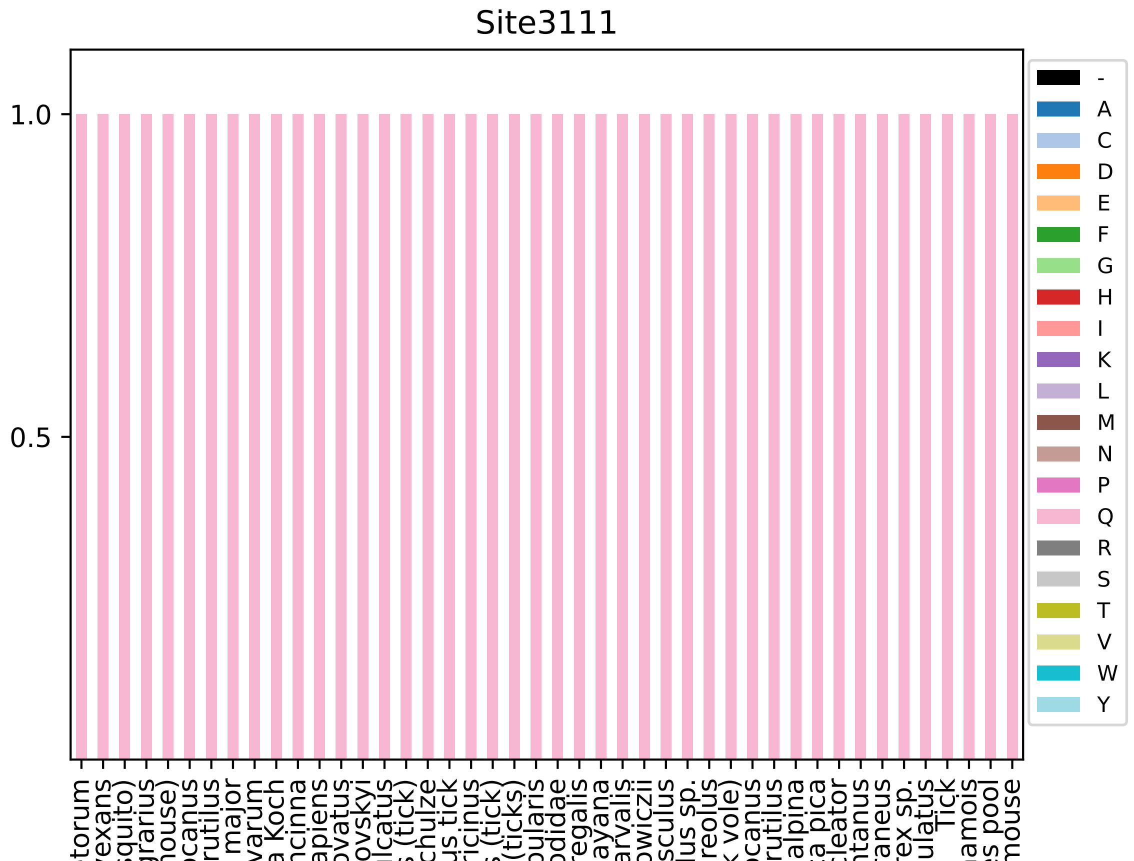

Site3111
1.0
0.5
-
A
C
D
E
F
G
H
I
K
L
M
N
P
Q
R
S
T
V
W
Y

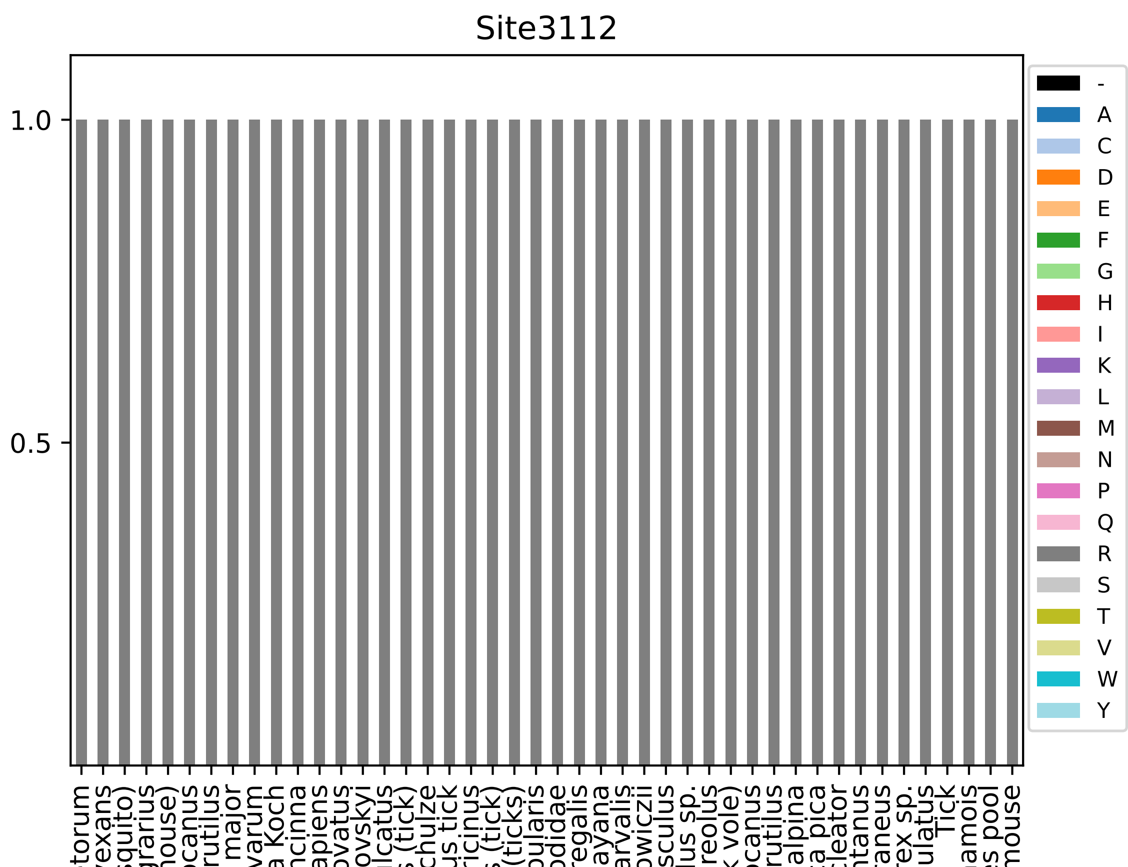
Site3112
1.0
0.5
-
A
C
D
E
F
G
H
I
K
L
M
N
P
Q
R
S
T
V
W
Y

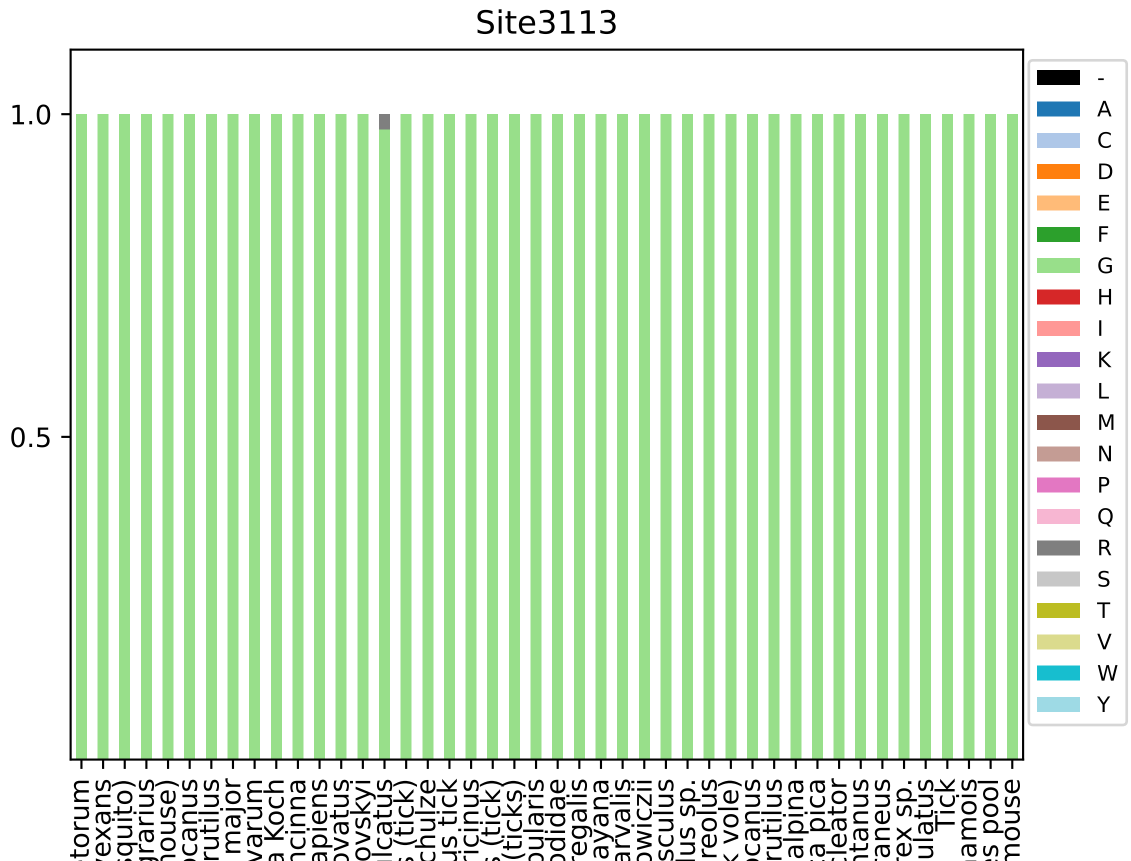
Site3113
1.0
0.5
-
A
C
D
E
F
G
H
I
K
L
M
N
P
Q
R
S
T
V
W
Y

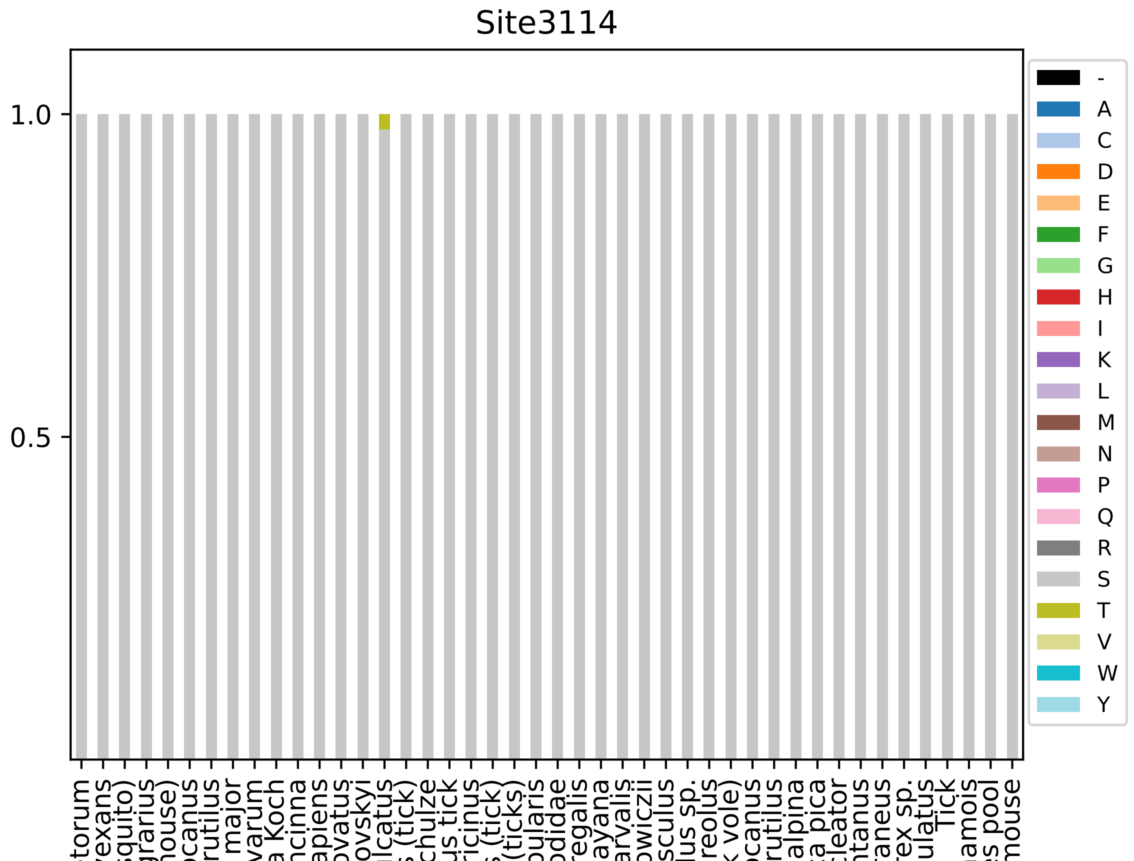

Site3114
1.0
0.5
-
A
C
D
E
F
G
H
I
K
L
M
N
P
Q
R
S
T
V
W
Y

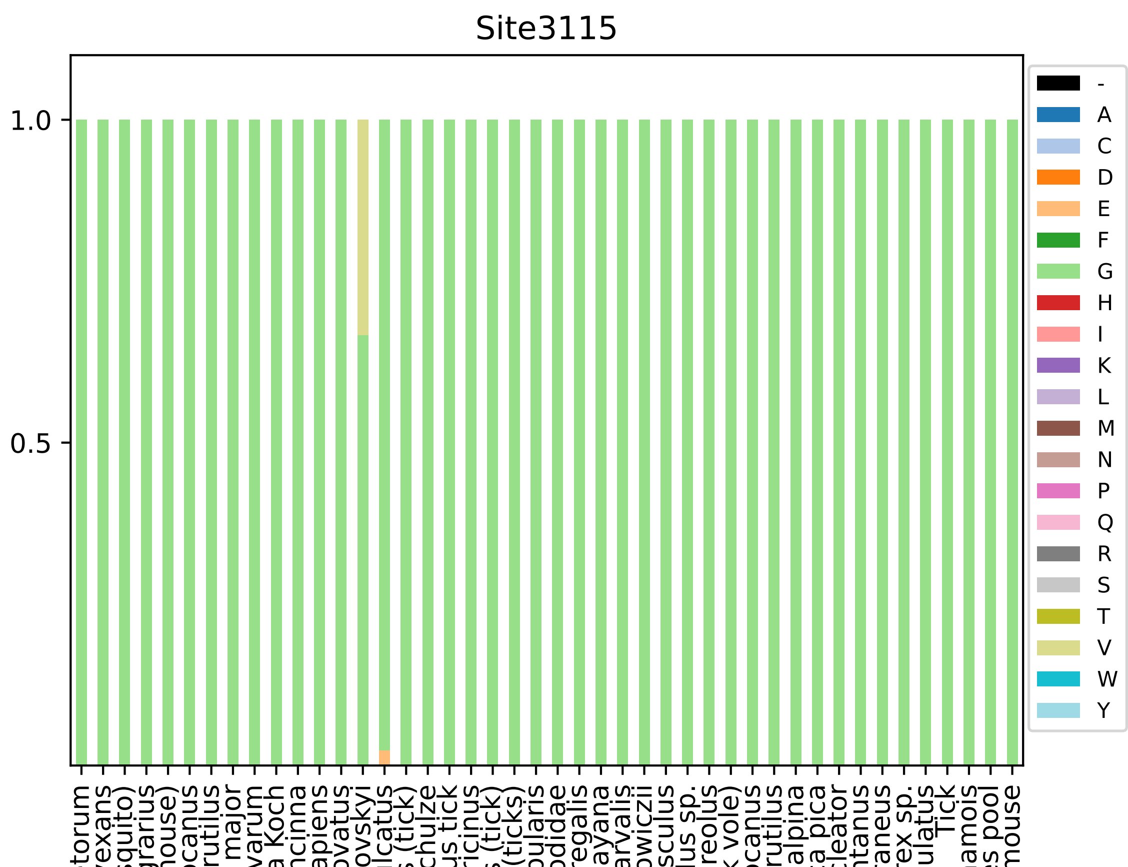

Site3115
1.0
0.5
-
A
C
D
E
F
G
H
I
K
L
M
N
P
Q
R
S
T
V
W
Y
torum
vexans
squito)
grarius
nouse)
ocanus
rutilus
major
varum
a Koch
ncinna
apiens
ovatus
ovskyi
ulcatus
s (tick)
chulze
us tick
ricinus
s (tick)
(ticks)
pularis
podidae
regalis
ayana
arvalis
owiczii
sculus
lus sp.
reolus
k vole)
ocanus
rutilus
alpina
a pica
cleator
ntanus
raneus
rex sp.
ulatus
Tick
amois
s pool
mouse

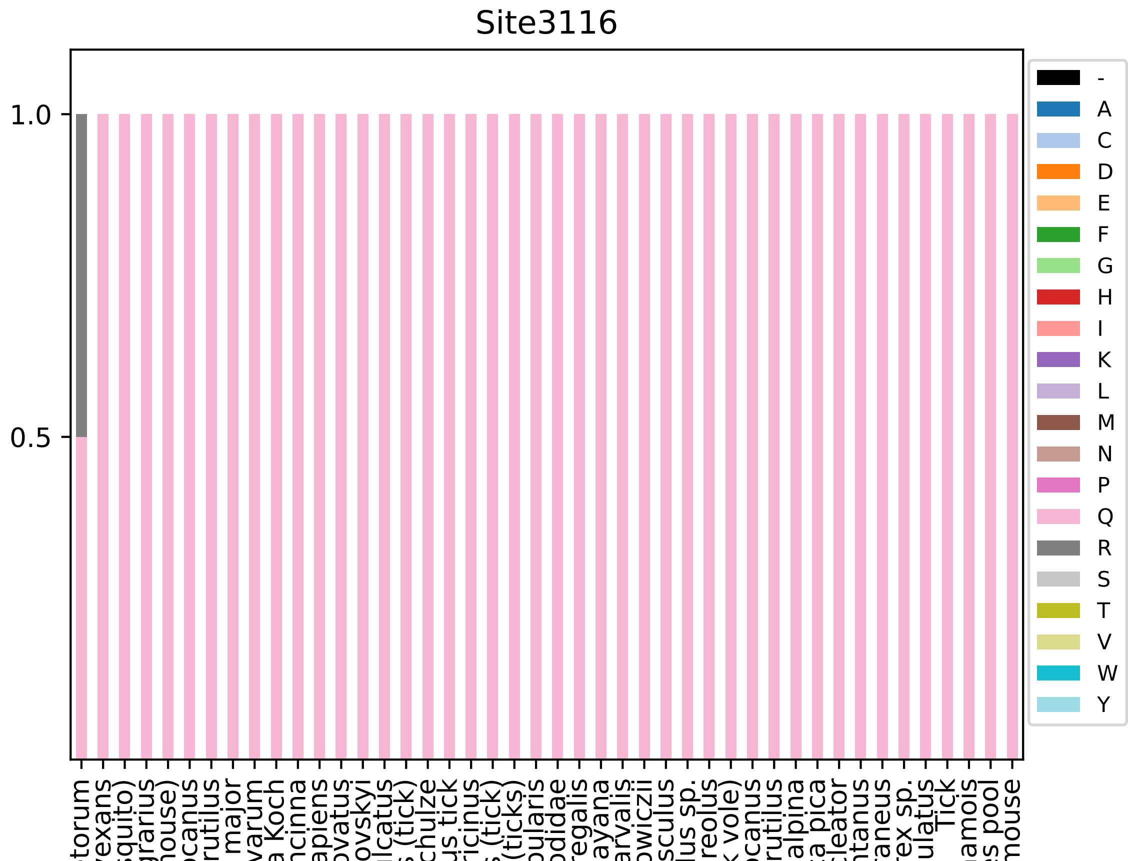
Site3116
1.0
0.5
-
A
C
D
E
F
G
H
I
K
L
M
N
P
Q
R
S
T
V
W
Y

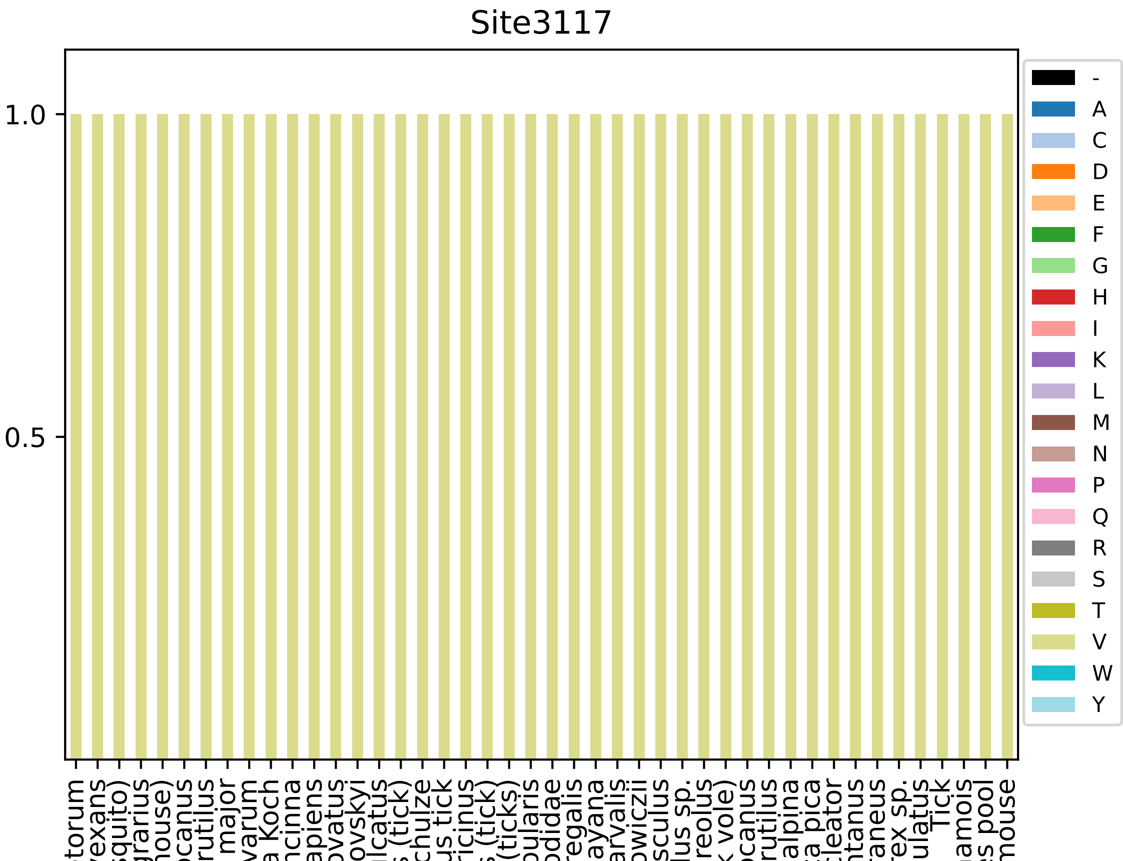
Site3117
1.0
0.5
-
A
C
D
E
F
G
H
I
K
L
M
N
P
Q
R
S
T
V
W
Y

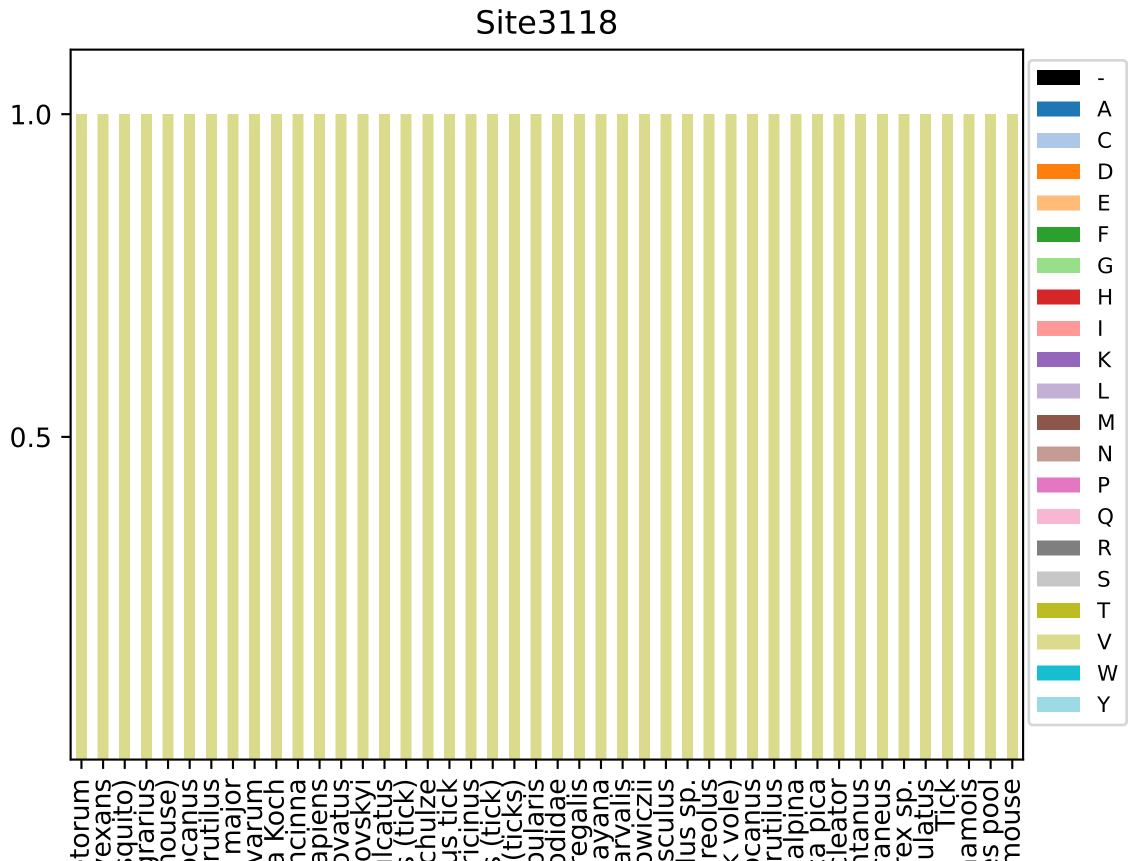
Site3118
1.0
0.5
-
A
C
D
E
F
G
H
I
K
L
M
N
P
Q
R
S
T
V
W
Y

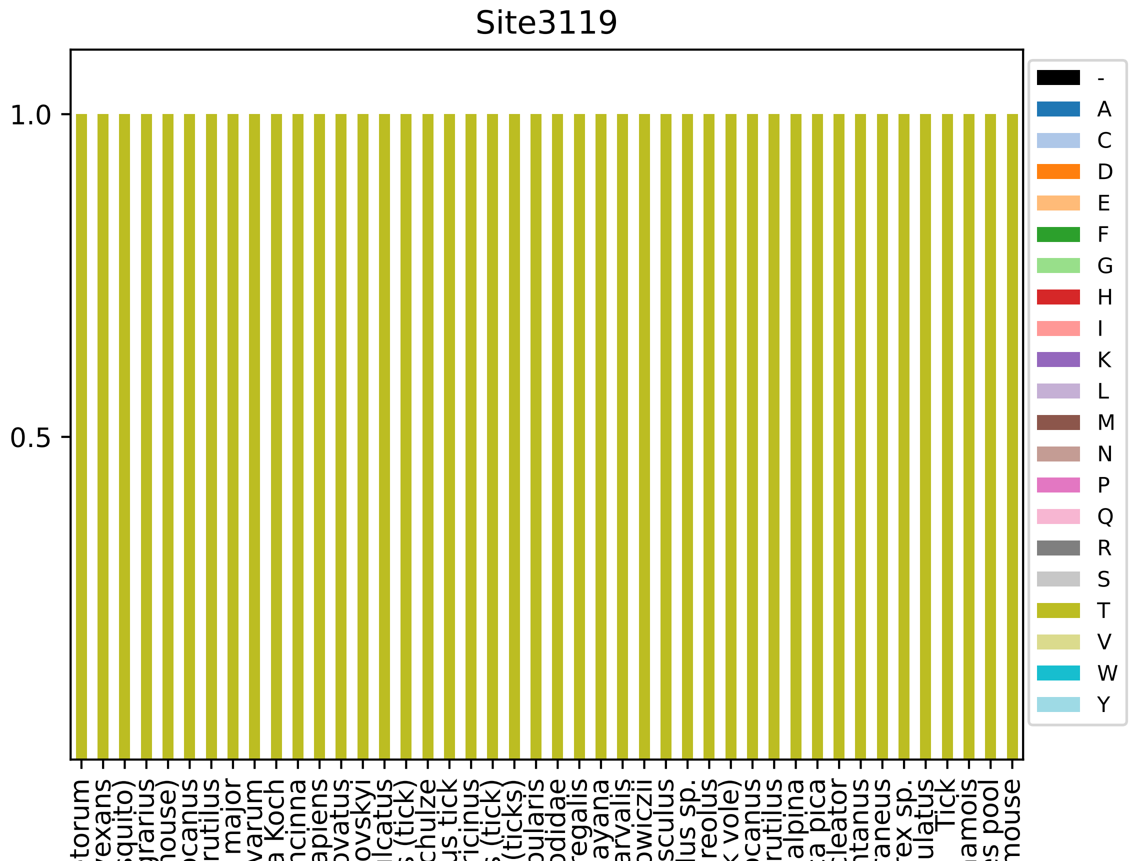
Site3119
1.0
0.5
-
A
C
D
E
F
G
H
I
K
L
M
N
P
Q
R
S
T
V
W
Y

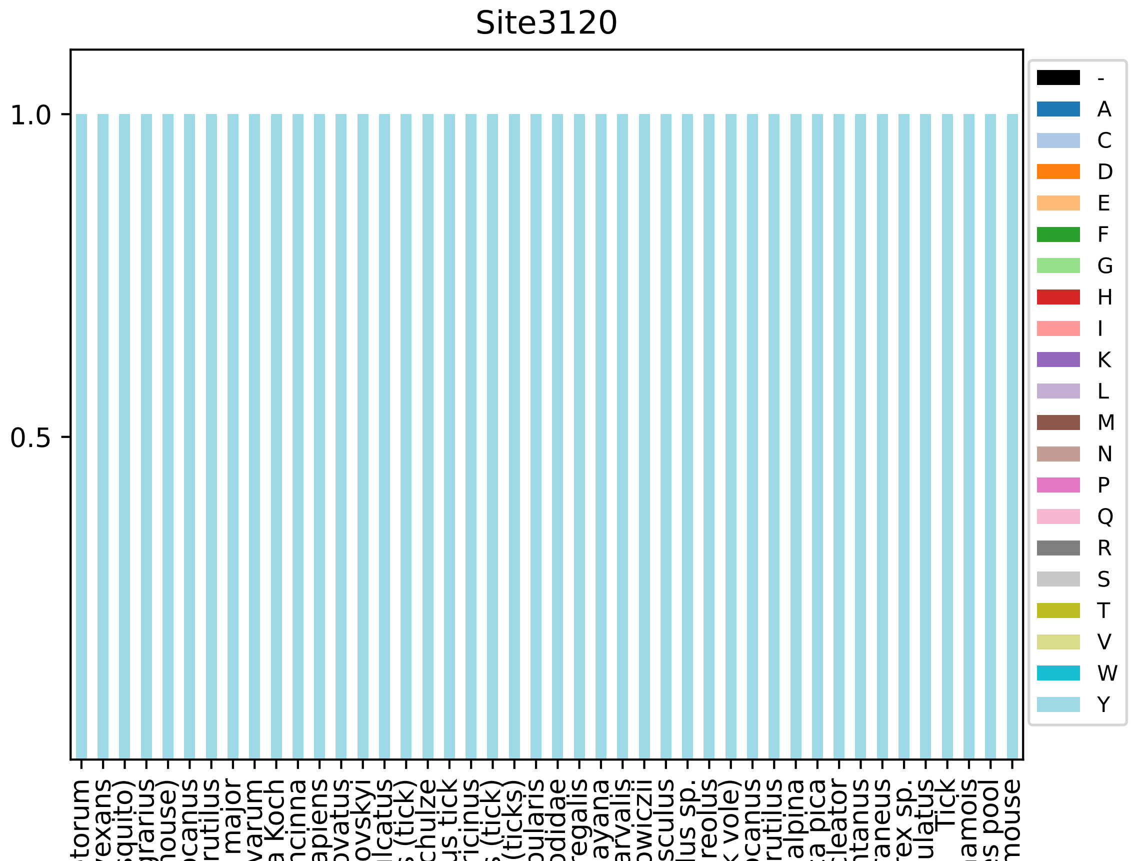
Site3120
1.0
0.5
-
A
C
D
E
F
G
H
I
K
L
M
N
P
Q
R
S
T
V
W
Y

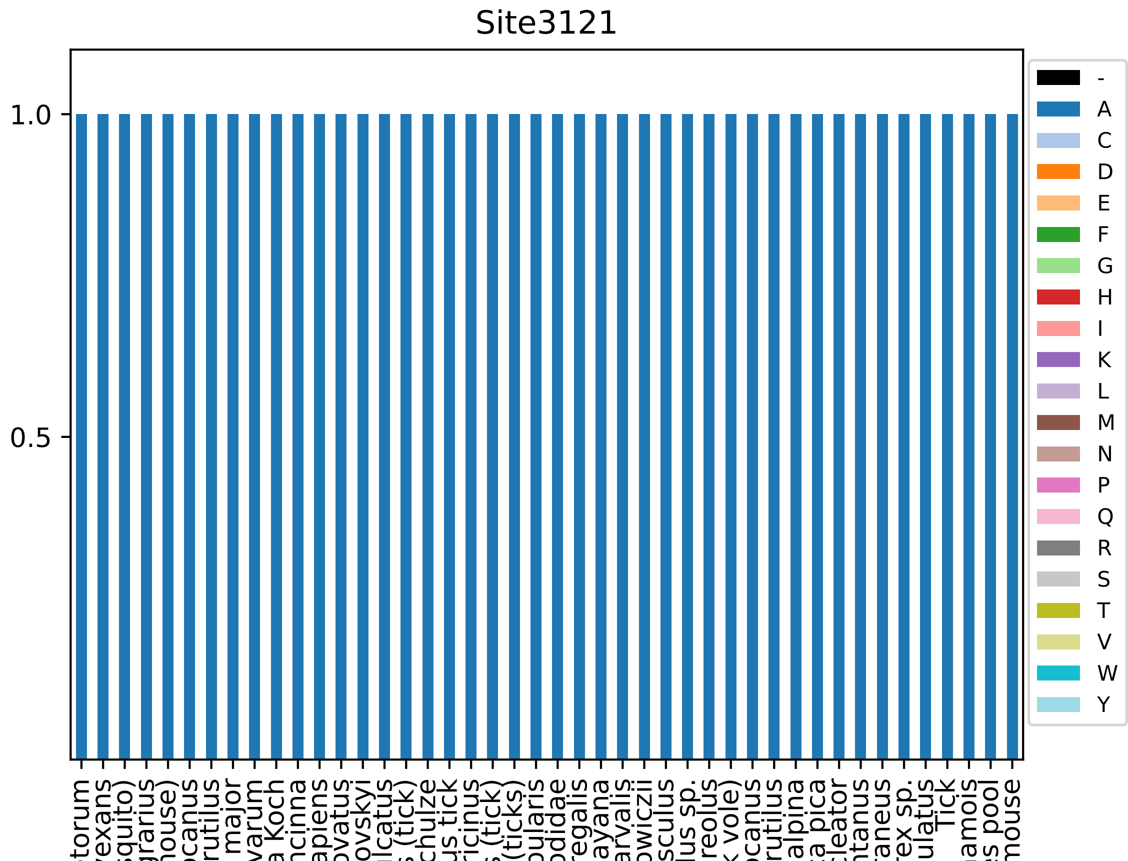
Site3121
1.0
0.5
-
A
C
D
E
F
G
H
I
K
L
M
N
P
Q
R
S
T
V
W
Y

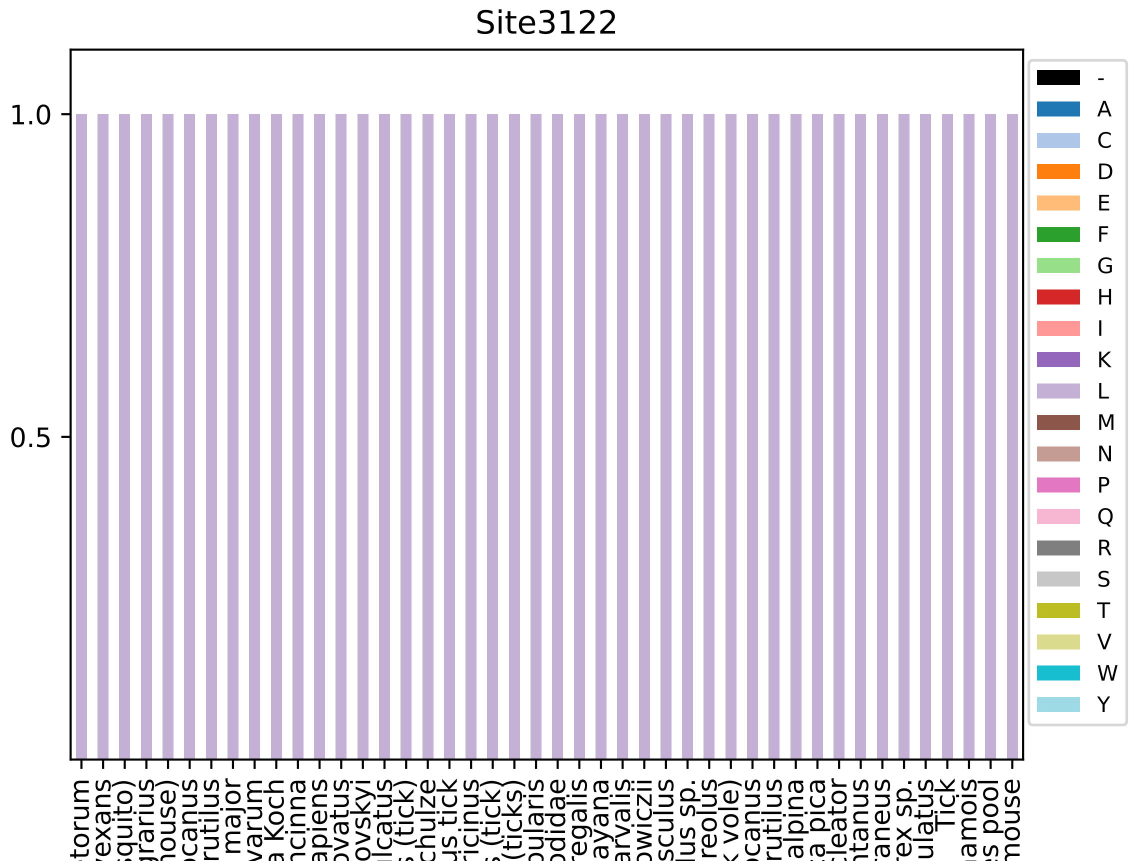
Site3122
1.0
0.5
-
A
C
D
E
F
G
H
I
K
L
M
N
P
Q
R
S
T
V
W
Y

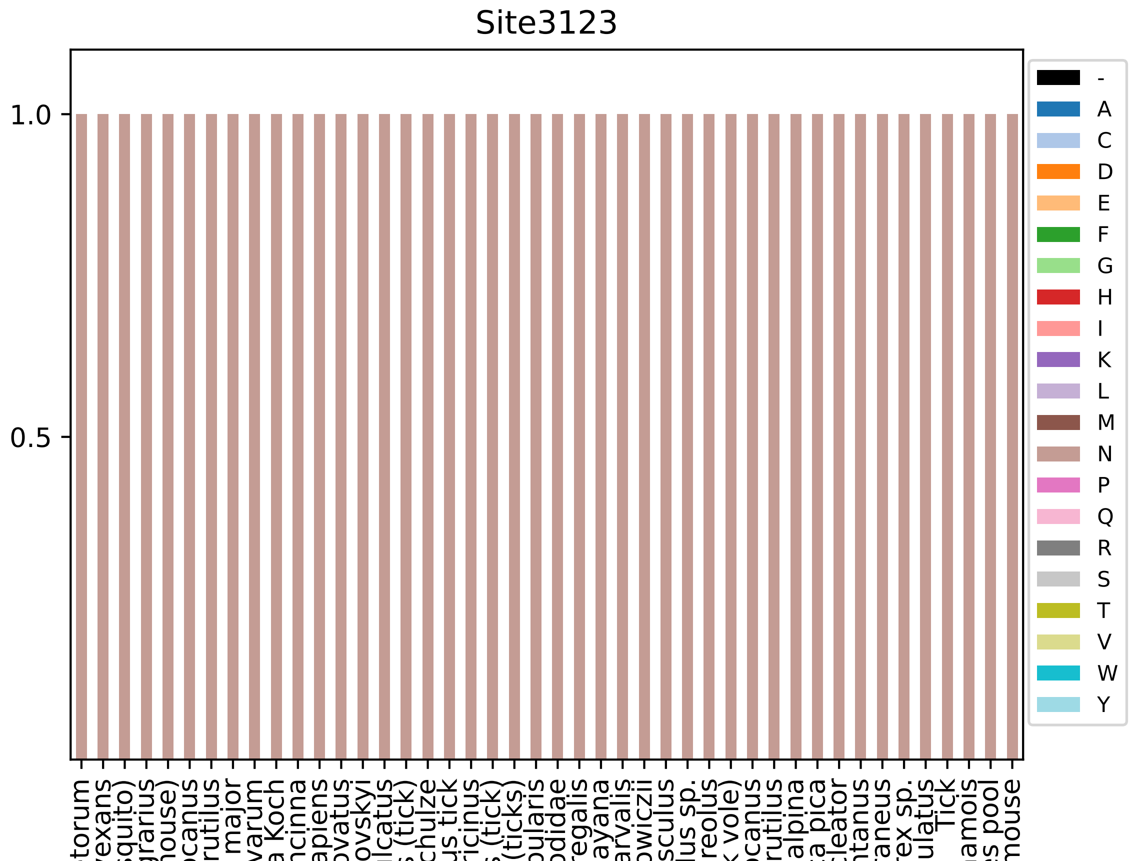

Site3123
1.0
0.5
-
A
C
D
E
F
G
H
I
K
L
M
N
P
Q
R
S
T
V
W
Y

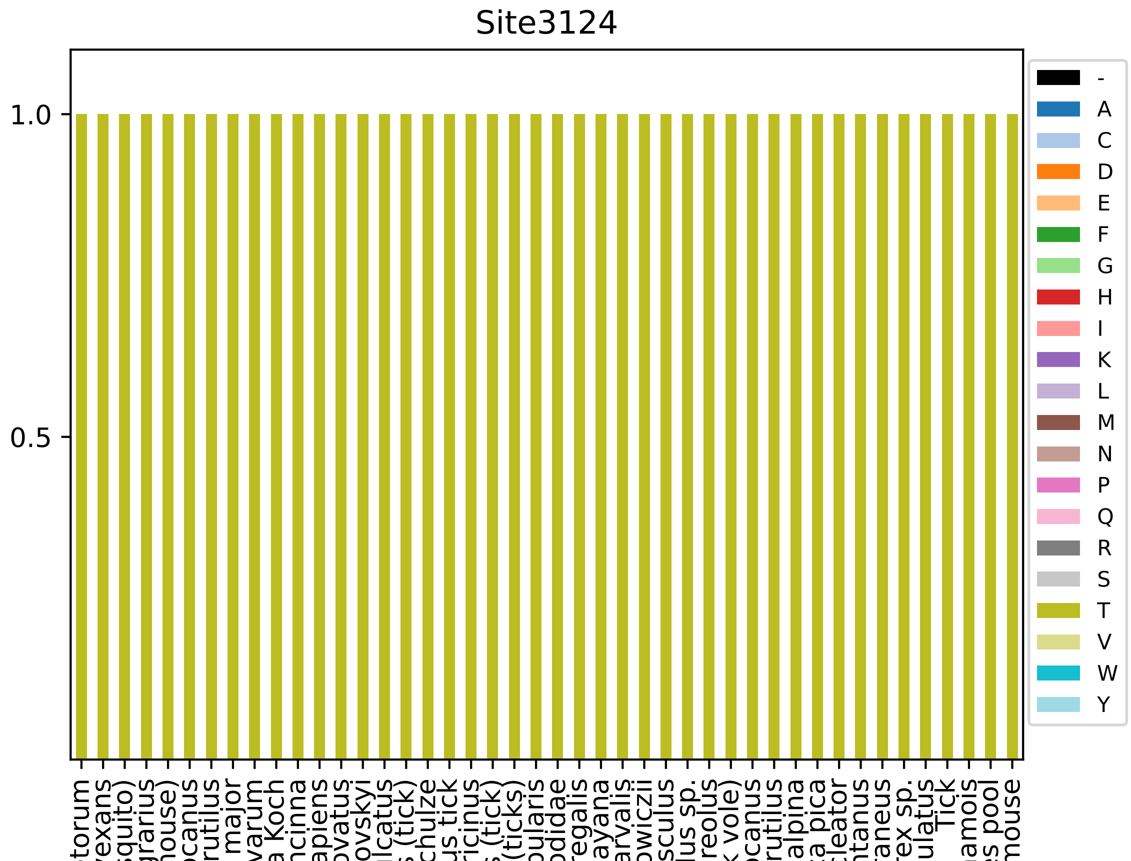

Site3124
1.0
0.5
-
A
C
D
E
F
G
H
I
K
L
M
N
P
Q
R
S
T
V
W
Y

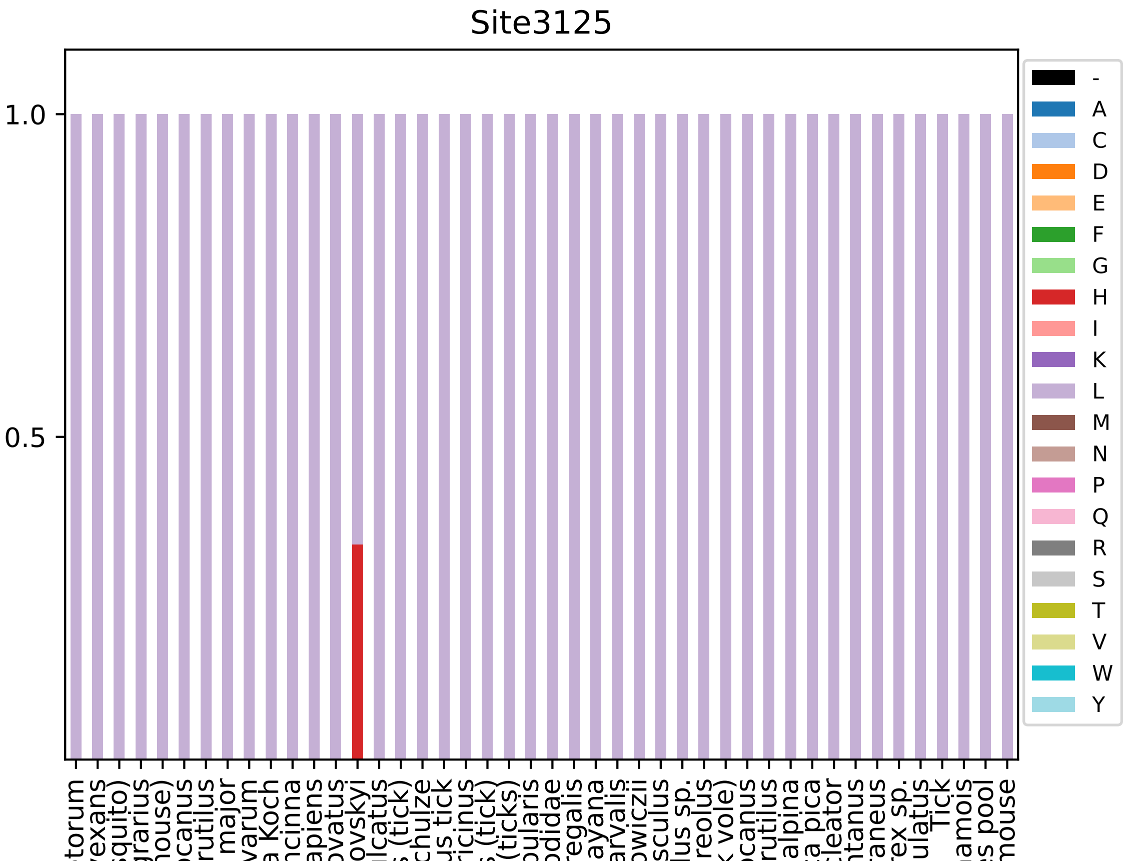

Site3125
1.0
0.5
-
A
C
D
E
F
G
H
I
K
L
M
N
P
Q
R
S
T
V
W
Y

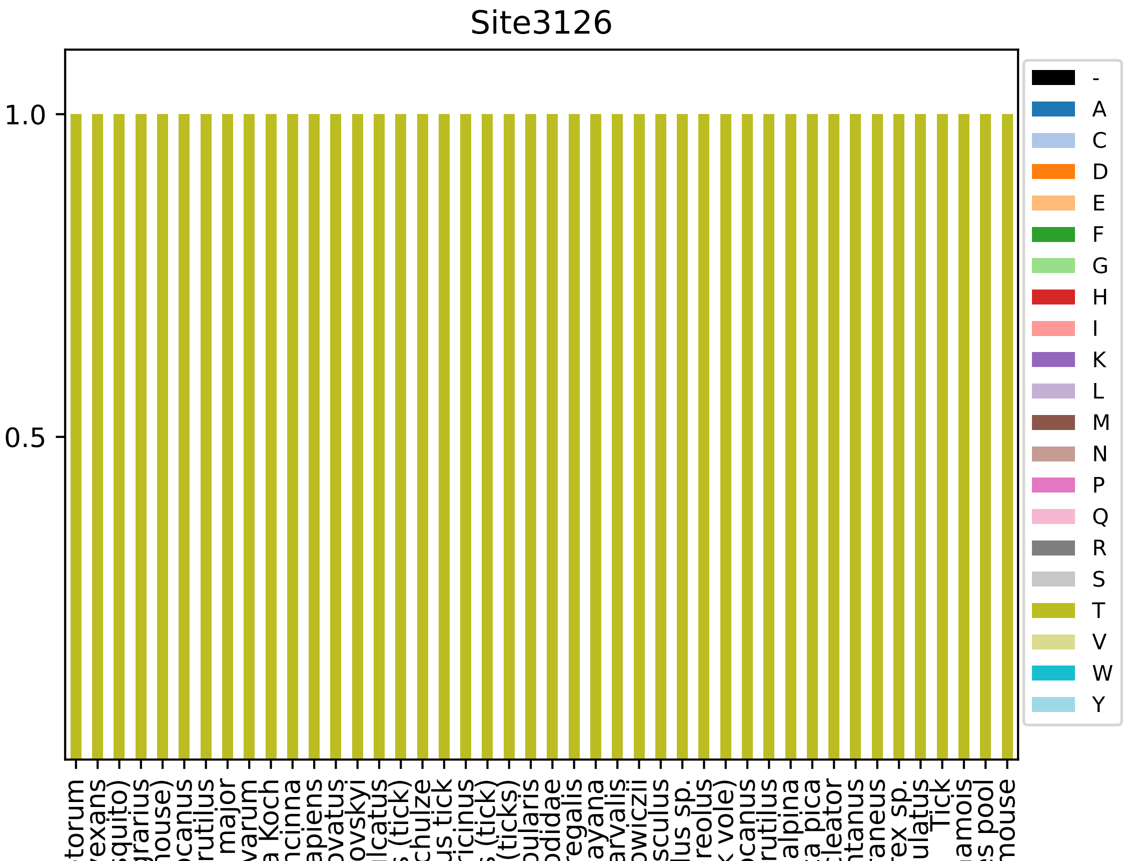
Site3126
1.0
0.5
-
A
C
D
E
F
G
H
I
K
L
M
N
P
Q
R
S
T
V
W
Y

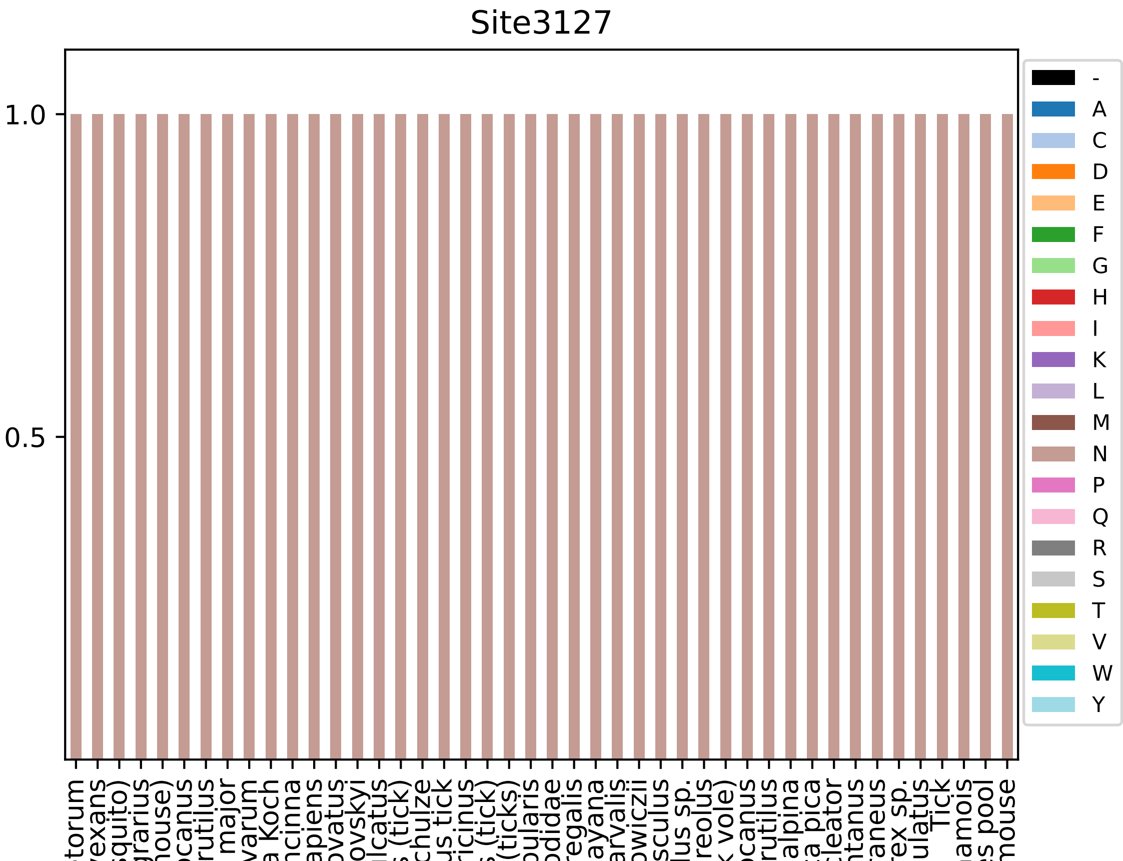
Site3127
1.0
0.5
-
A
C
D
E
F
G
H
I
K
L
M
N
P
Q
R
S
T
V
W
Y

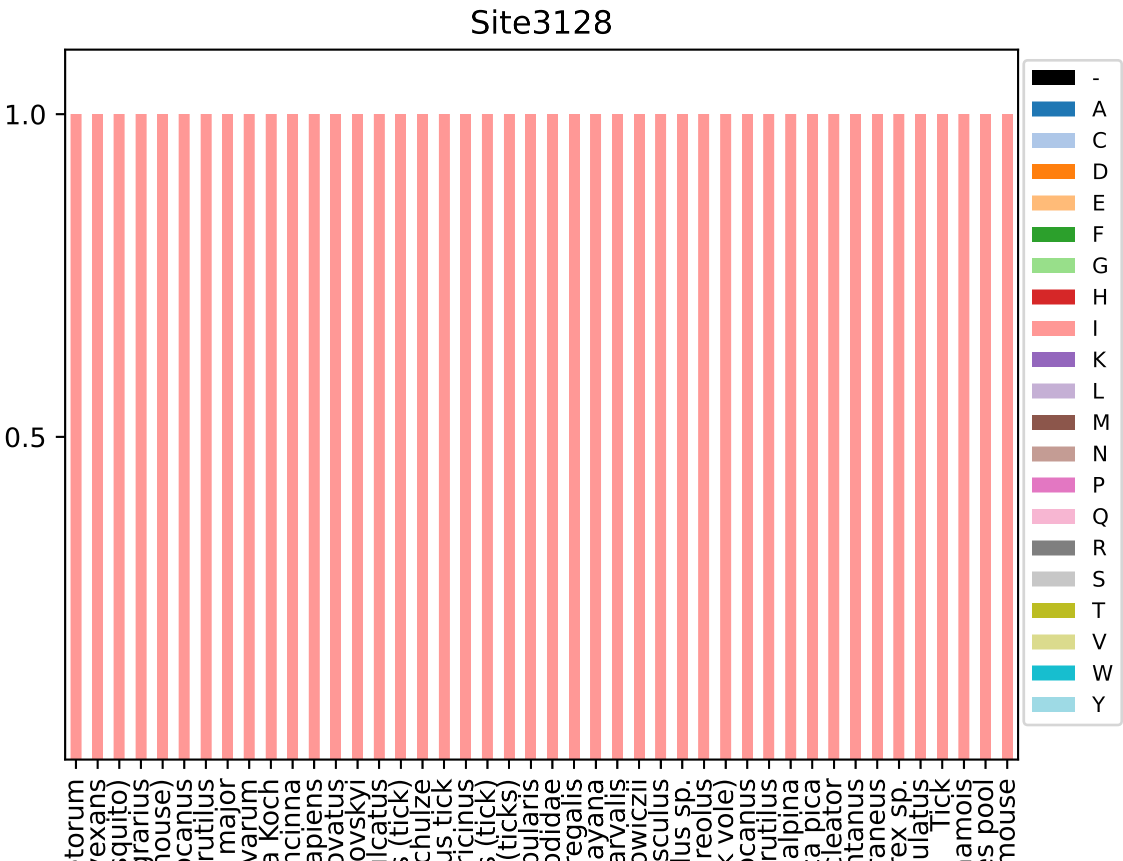
Site3128
1.0
0.5
-
A
C
D
E
F
G
H
I
K
L
M
N
P
Q
R
S
T
V
W
Y

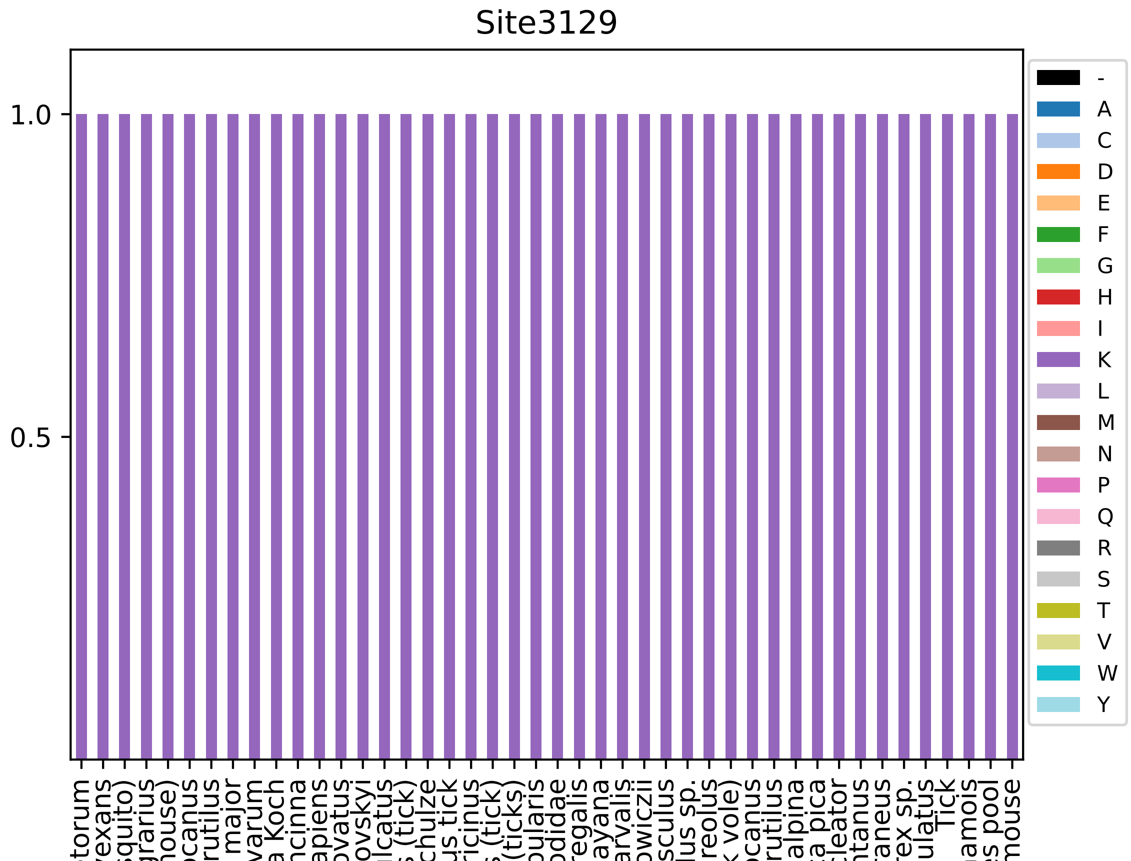
Site3129
1.0
0.5
-
A
C
D
E
F
G
H
I
K
L
M
N
P
Q
R
S
T
V
W
Y

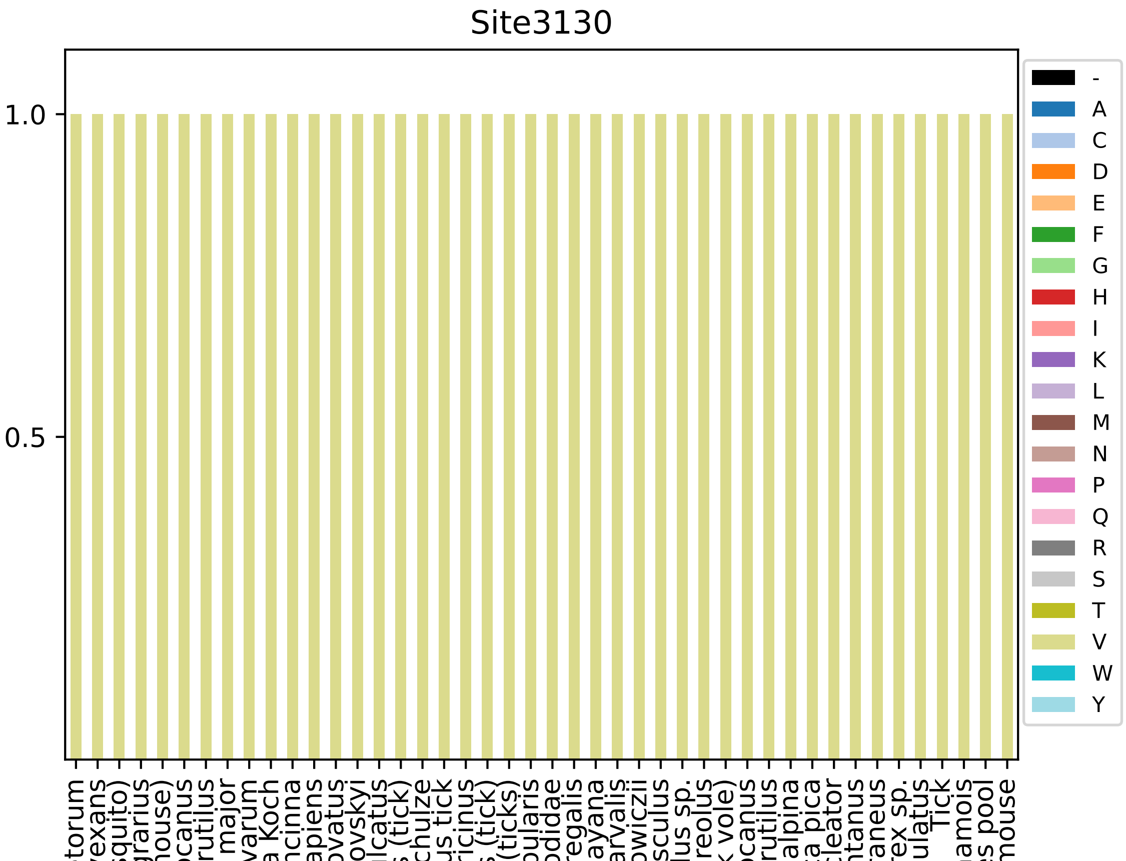
Site3130
1.0
0.5
-
A
C
D
E
F
G
H
I
K
L
M
N
P
Q
R
S
T
V
W
Y

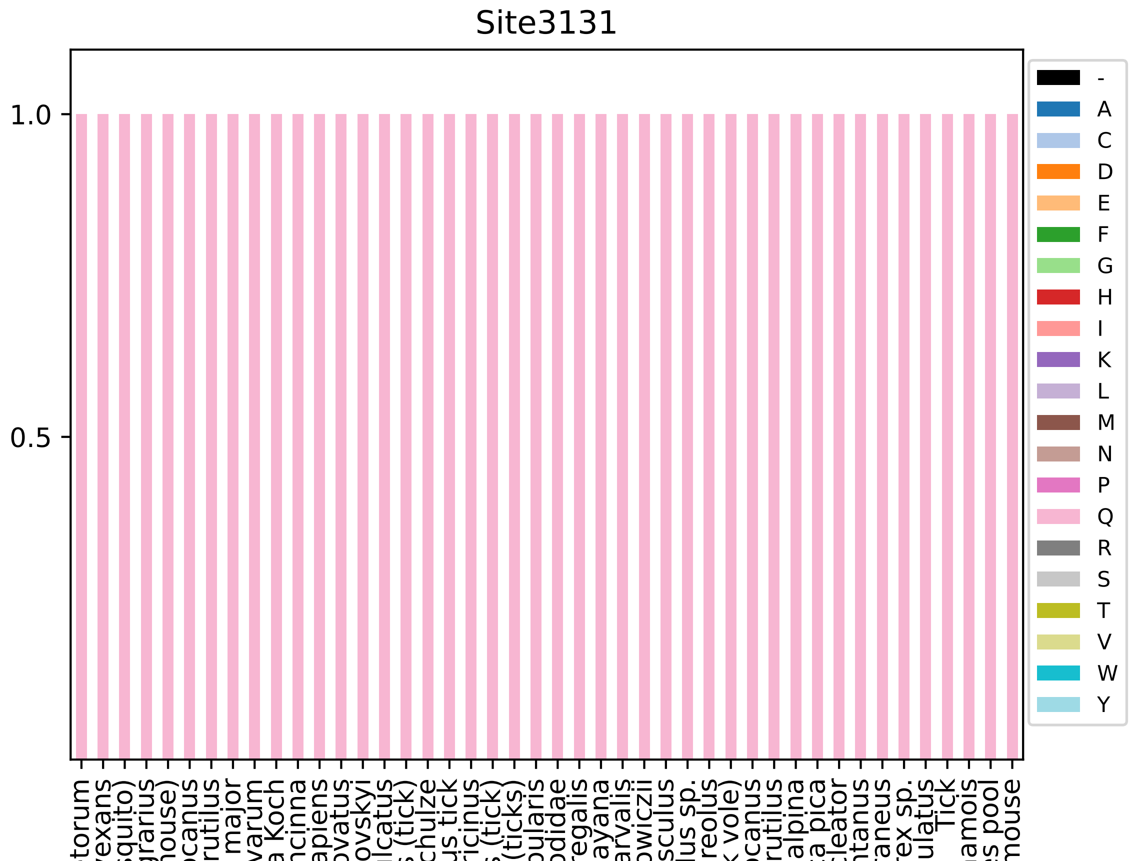
Site3131
1.0
0.5
-
A
C
D
E
F
G
H
I
K
L
M
N
P
Q
R
S
T
V
W
Y

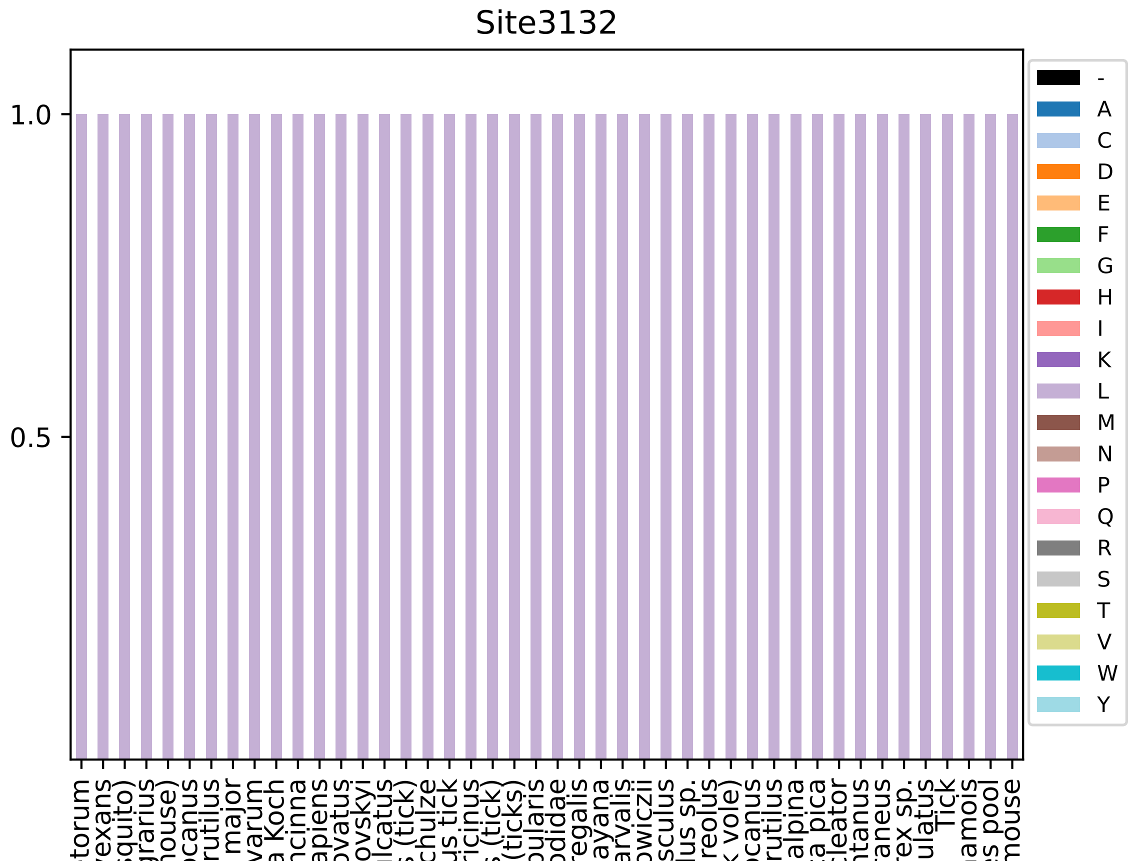
Site3132
1.0
0.5
-
A
C
D
E
F
G
H
I
K
L
M
N
P
Q
R
S
T
V
W
Y

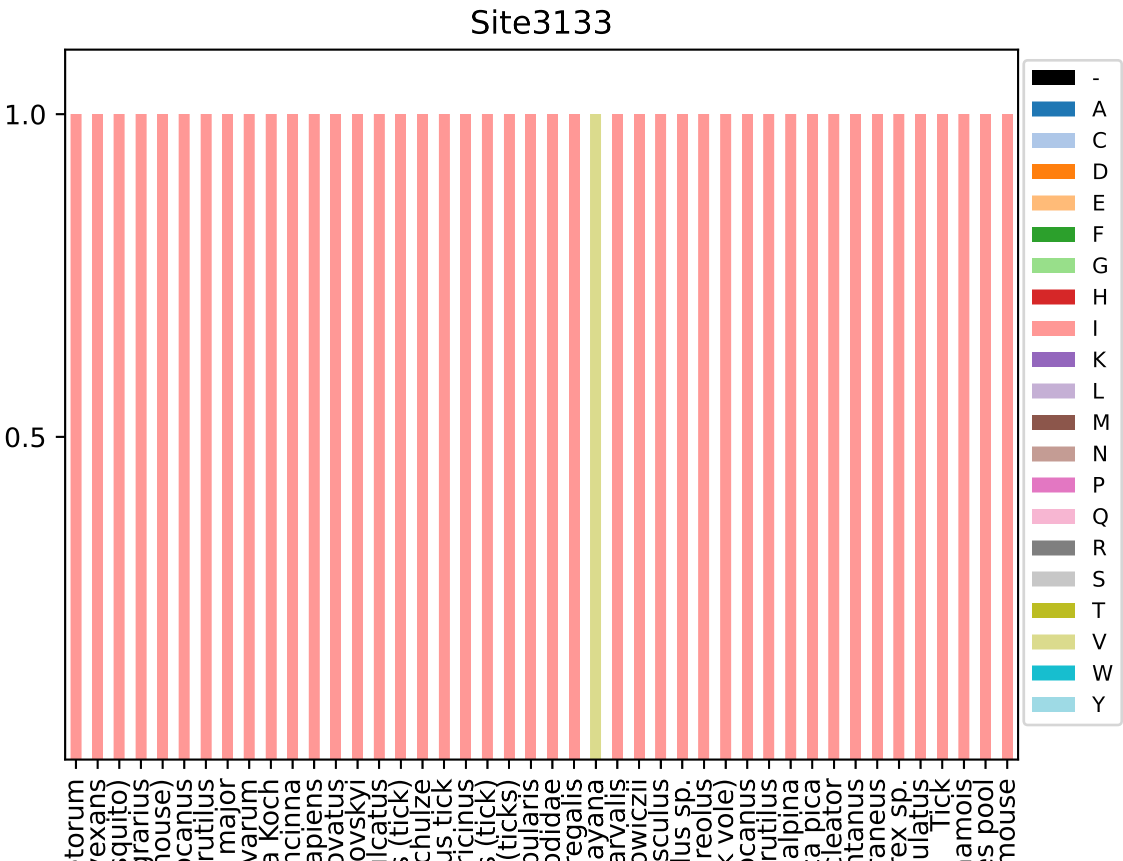
Site3133
1.0
0.5
-
A
C
D
E
F
G
H
I
K
L
M
N
P
Q
R
S
T
V
W
Y
Tick

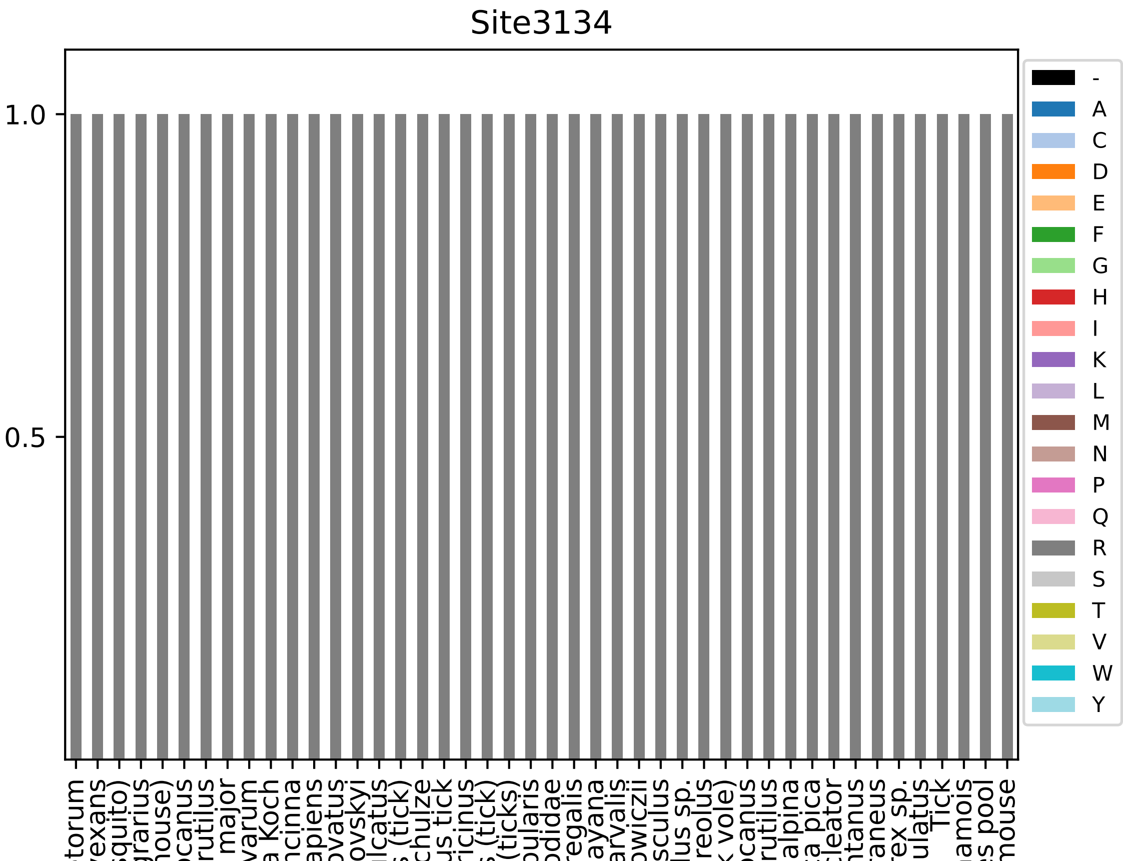
Site3134
1.0
0.5
-
A
C
D
E
F
G
H
I
K
L
M
N
P
Q
R
S
T
V
W
Y

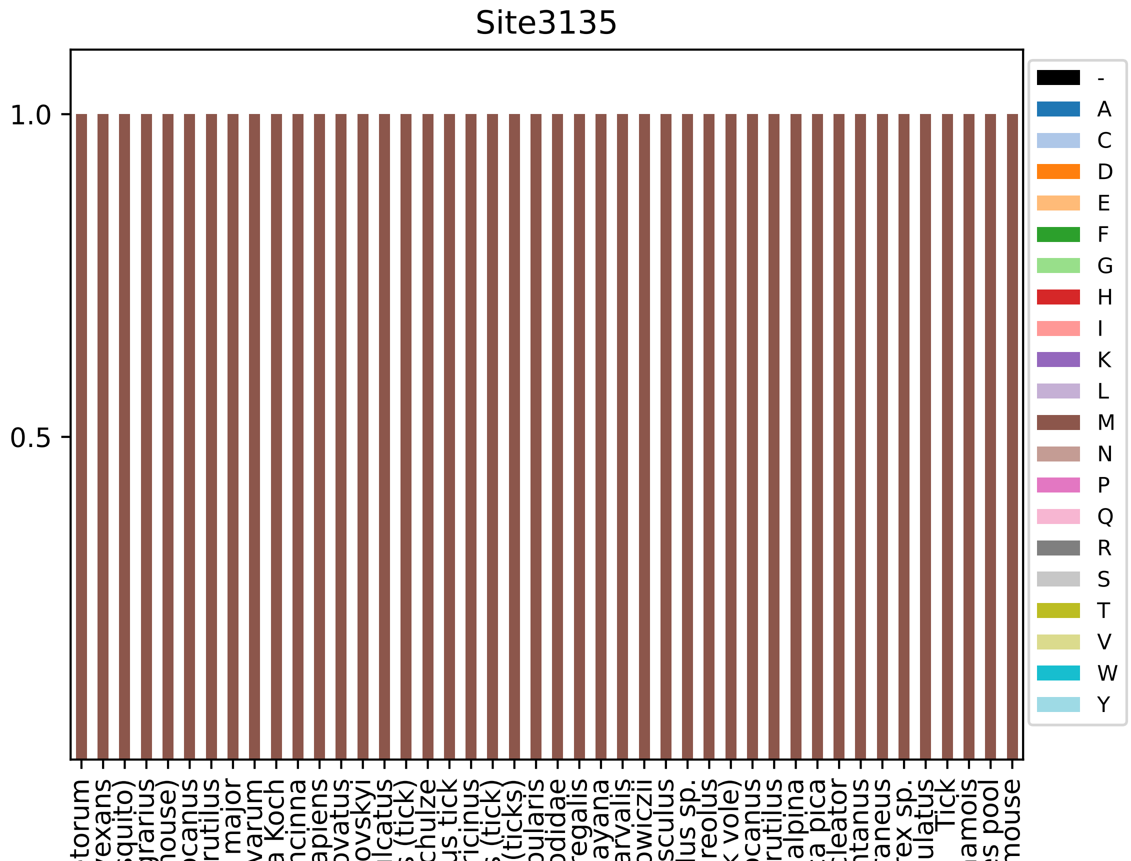
Site3135
1.0
0.5
-
A
C
D
E
F
G
H
I
K
L
M
N
P
Q
R
S
T
V
W
Y

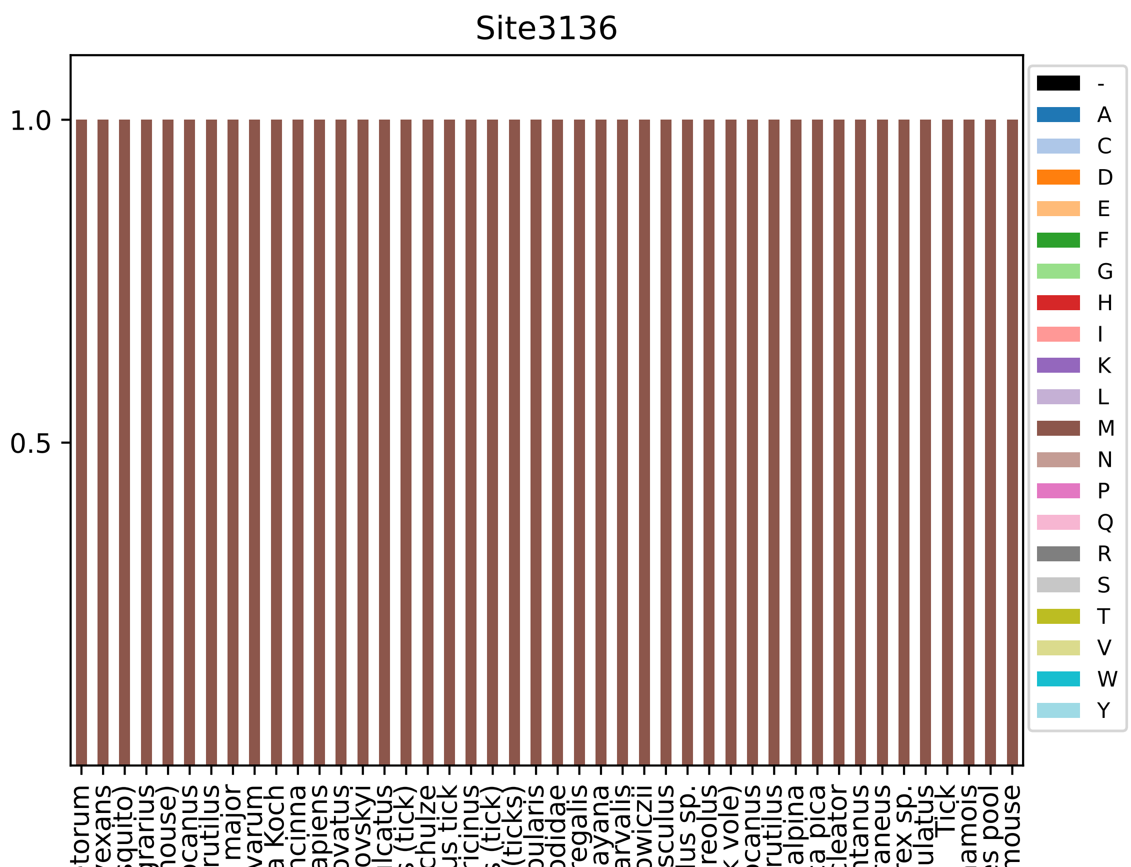

Site3136
1.0
0.5
-
A
C
D
E
F
G
H
I
K
L
M
N
P
Q
R
S
T
V
W
Y

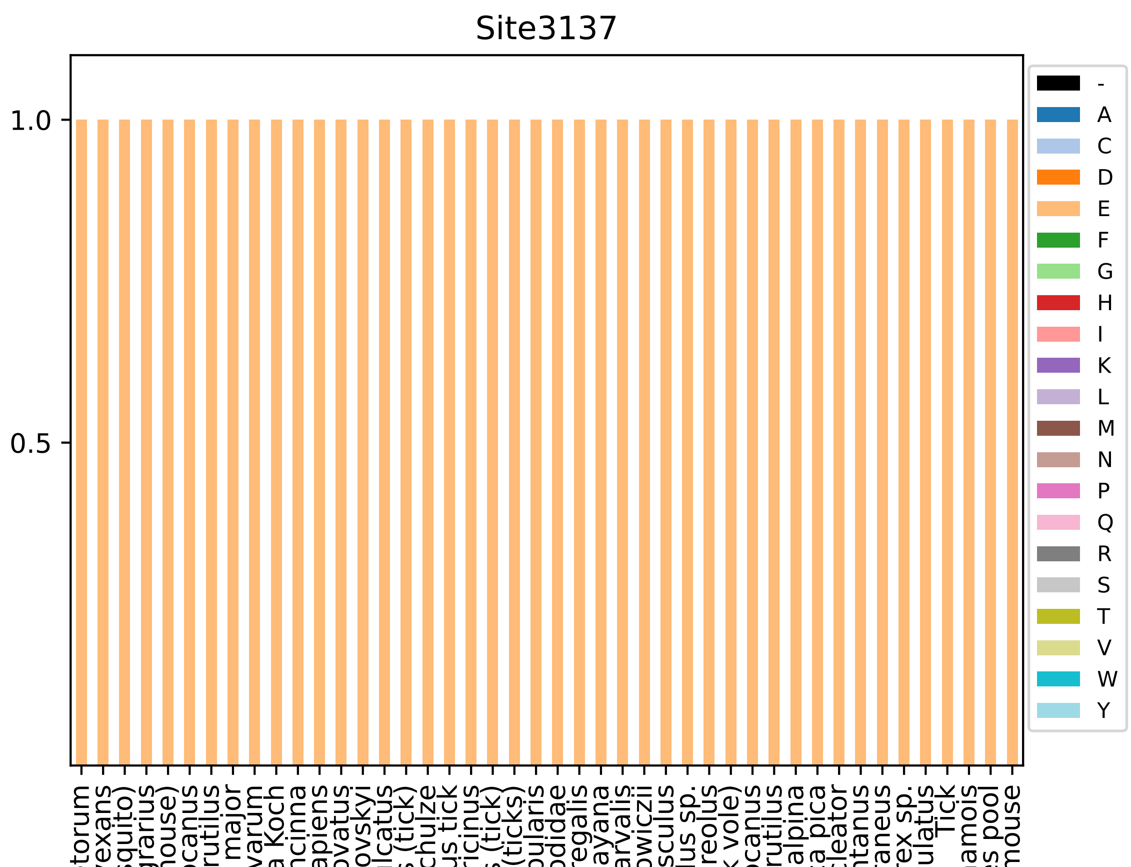
Site3137
1.0
0.5
-
A
C
D
E
F
G
H
I
K
L
M
N
P
Q
R
S
T
V
W
Y

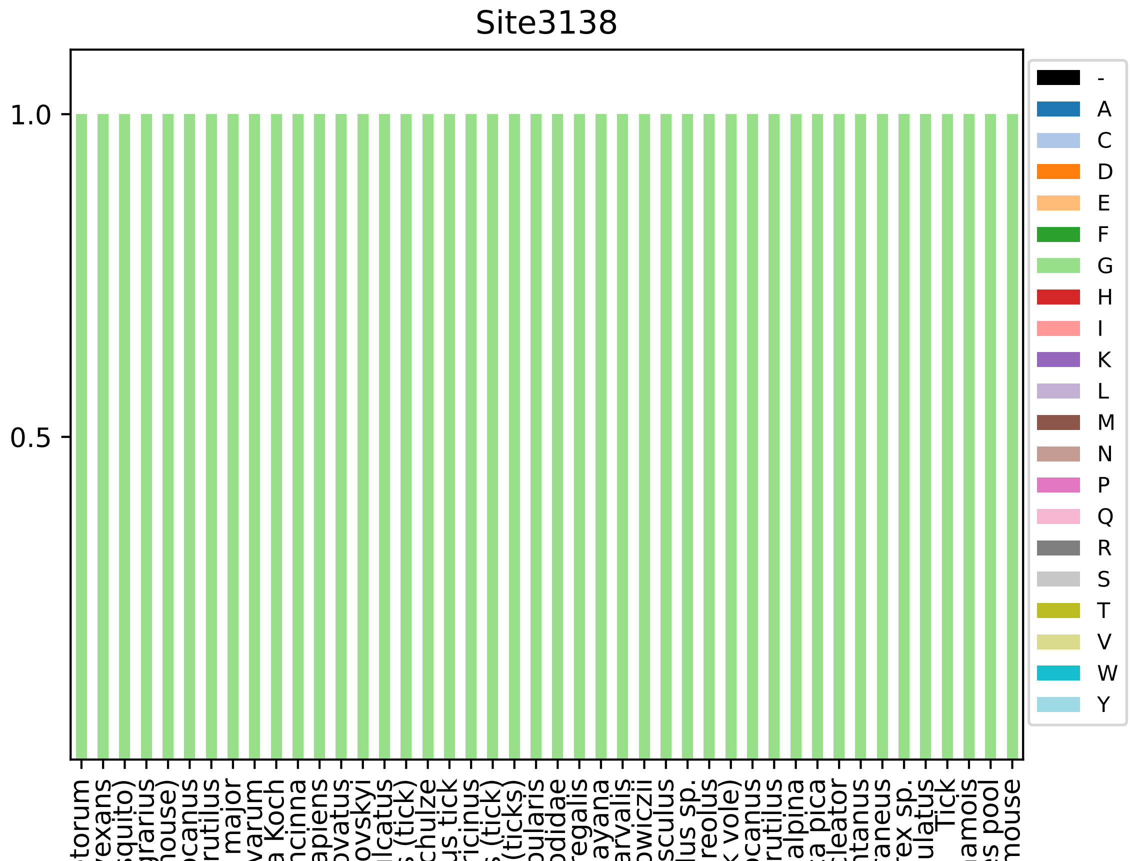
Site3138
1.0
0.5
-
A
C
D
E
F
G
H
I
K
L
M
N
P
Q
R
S
T
V
W
Y

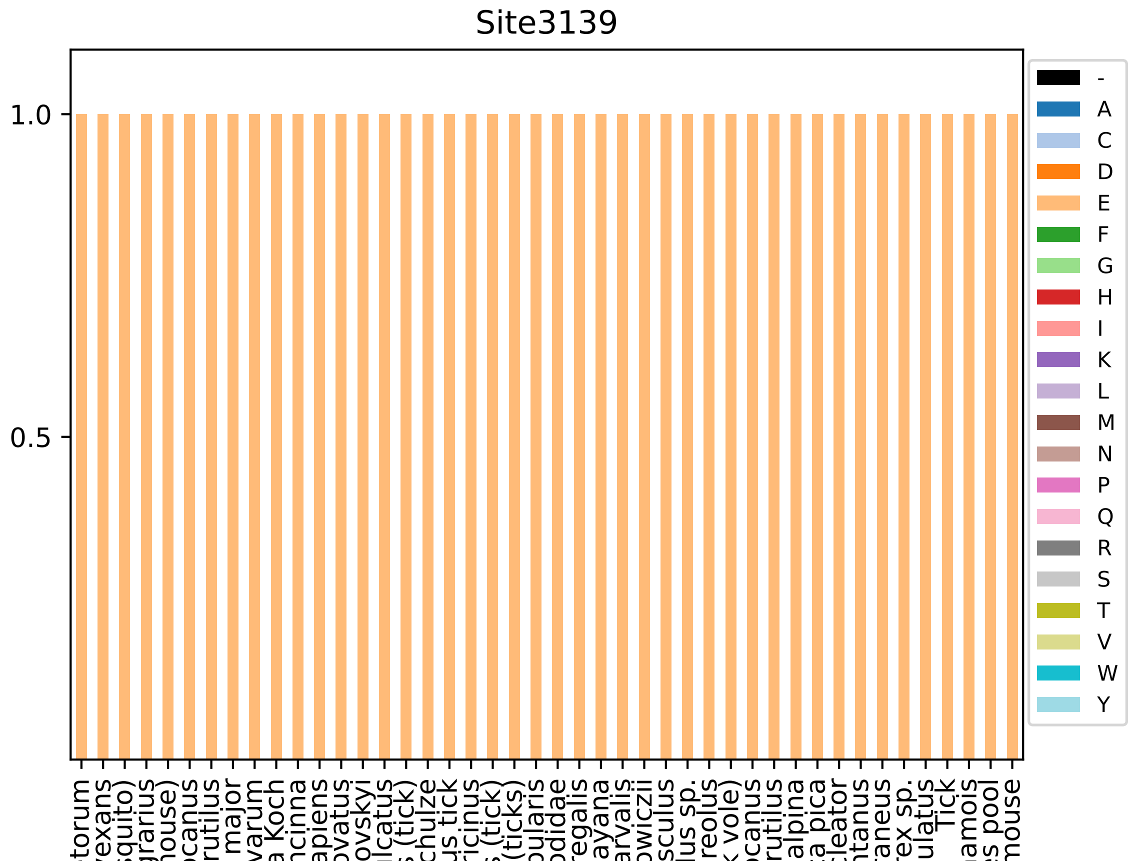
Site3139
1.0
0.5
-
A
C
D
E
F
G
H
I
K
L
M
N
P
Q
R
S
T
V
W
Y

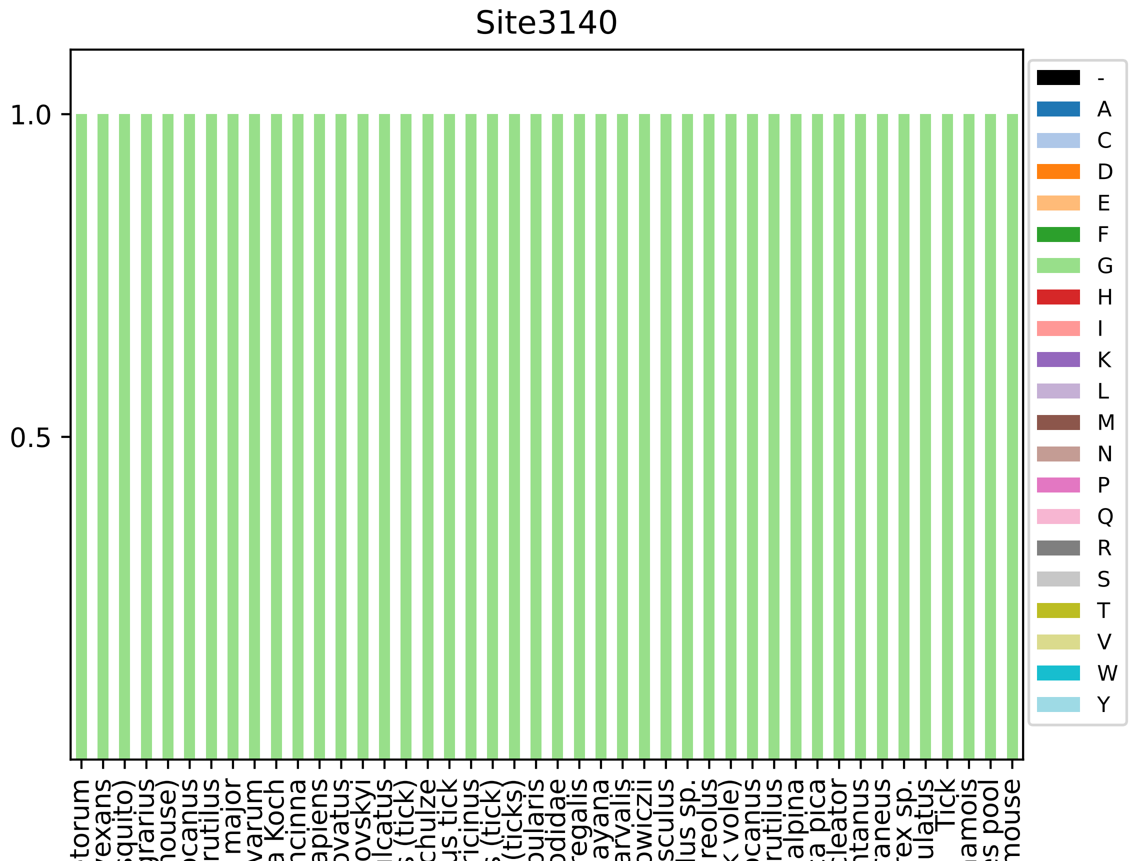
Site3140
1.0
0.5
-
A
C
D
E
F
G
H
I
K
L
M
N
P
Q
R
S
T
V
W
Y

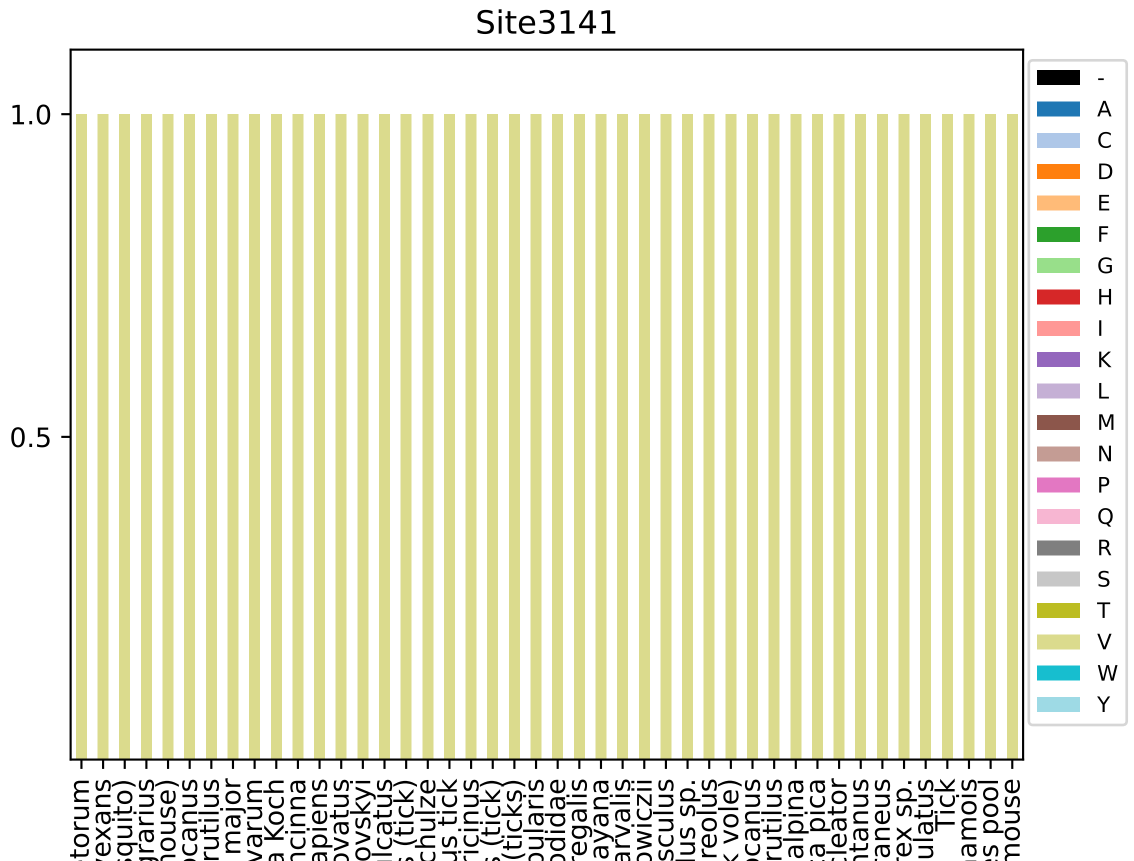
Site3141
1.0
0.5
-
A
C
D
E
F
G
H
I
K
L
M
N
P
Q
R
S
T
V
W
Y

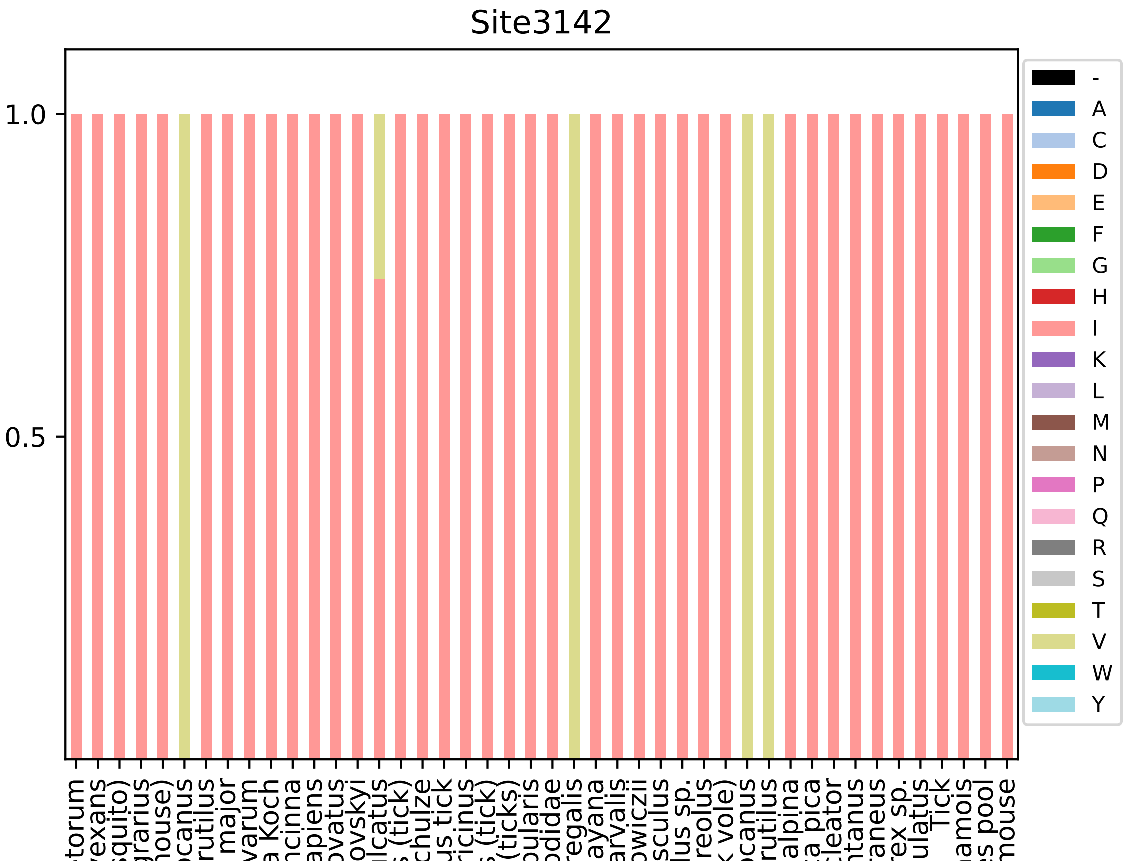
Site3142
1.0
0.5
-
A
C
D
E
F
G
H
I
K
L
M
N
P
Q
R
S
T
V
W
Y
Tick

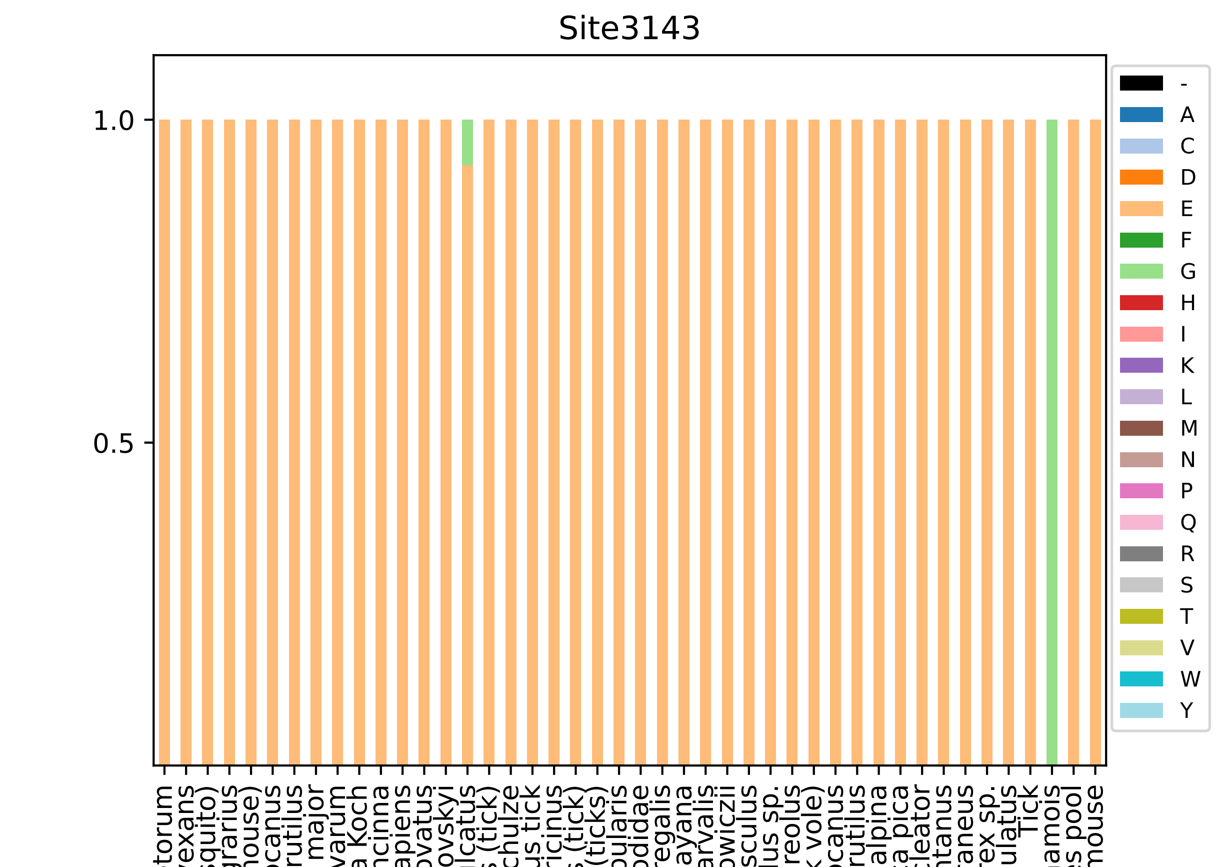

Site3143
1.0
0.5
-
A
C
D
E
F
G
H
I
K
L
M
N
P
Q
R
S
T
V
W
Y

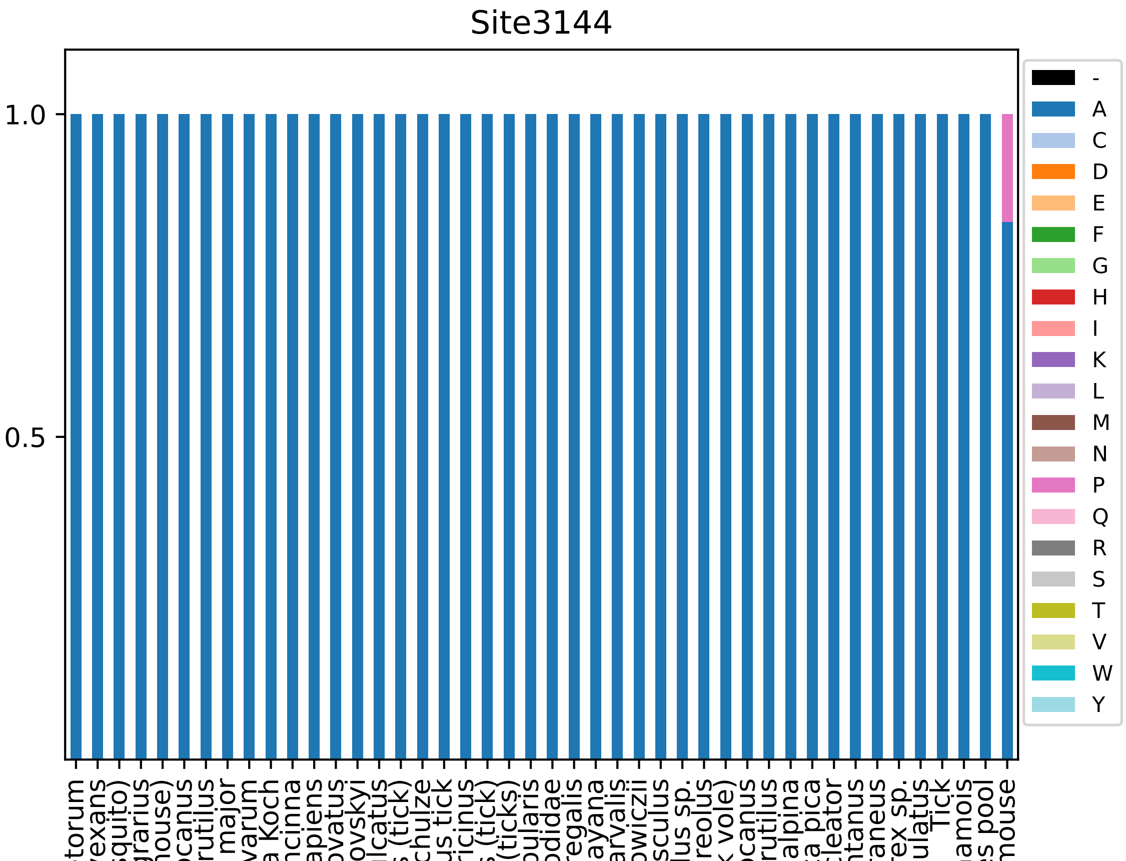

Site3144
1.0
0.5
-
A
C
D
E
F
G
H
I
K
L
M
N
P
Q
R
S
T
V
W
Y

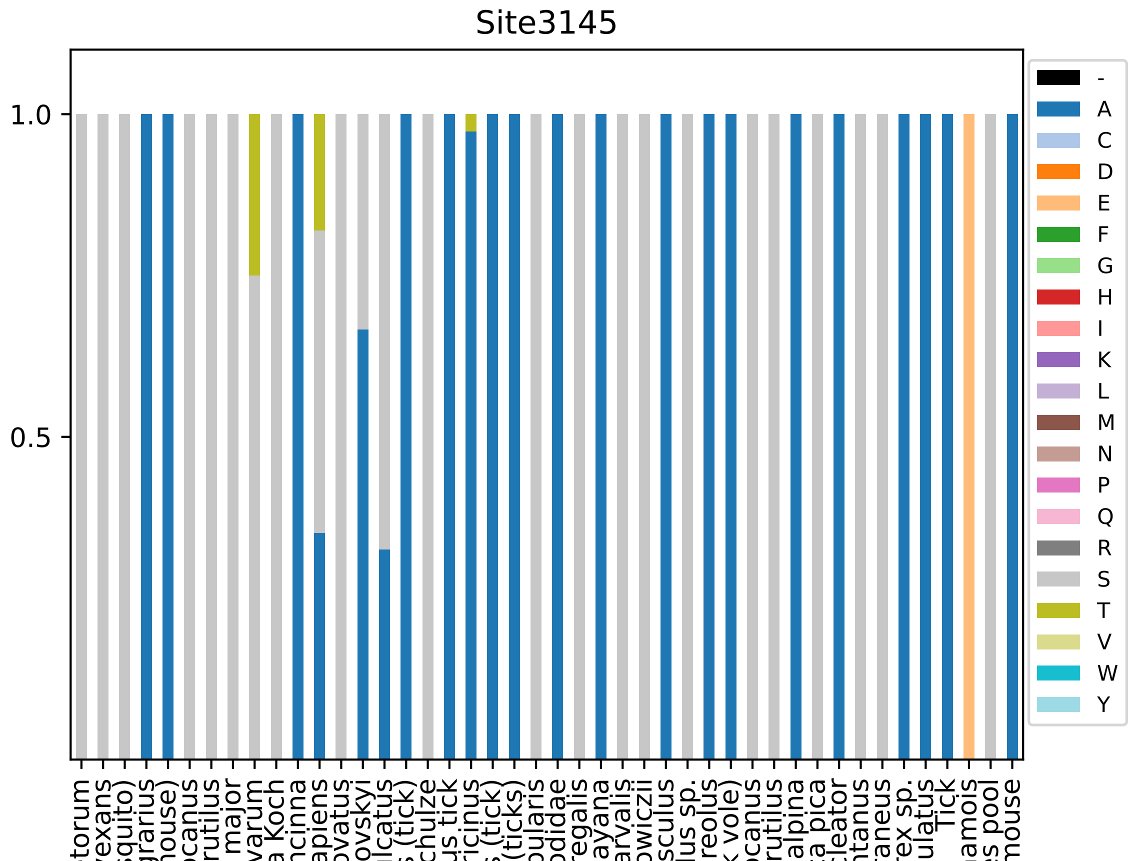
Site3145
1.0
0.5
-
A
C
D
E
F
G
H
I
K
L
M
N
P
Q
R
S
T
V
W
Y

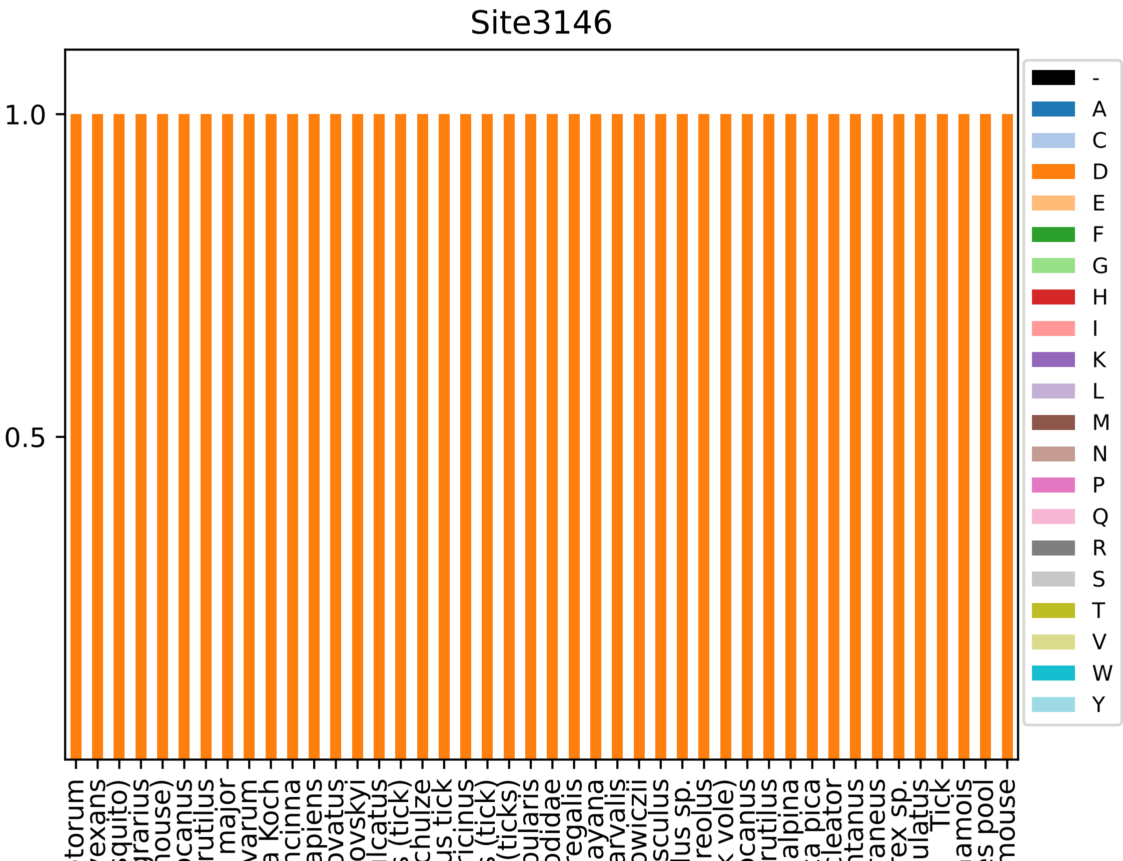
Site3146
1.0
0.5
-
A
C
D
E
F
G
H
I
K
L
M
N
P
Q
R
S
T
V
W
Y

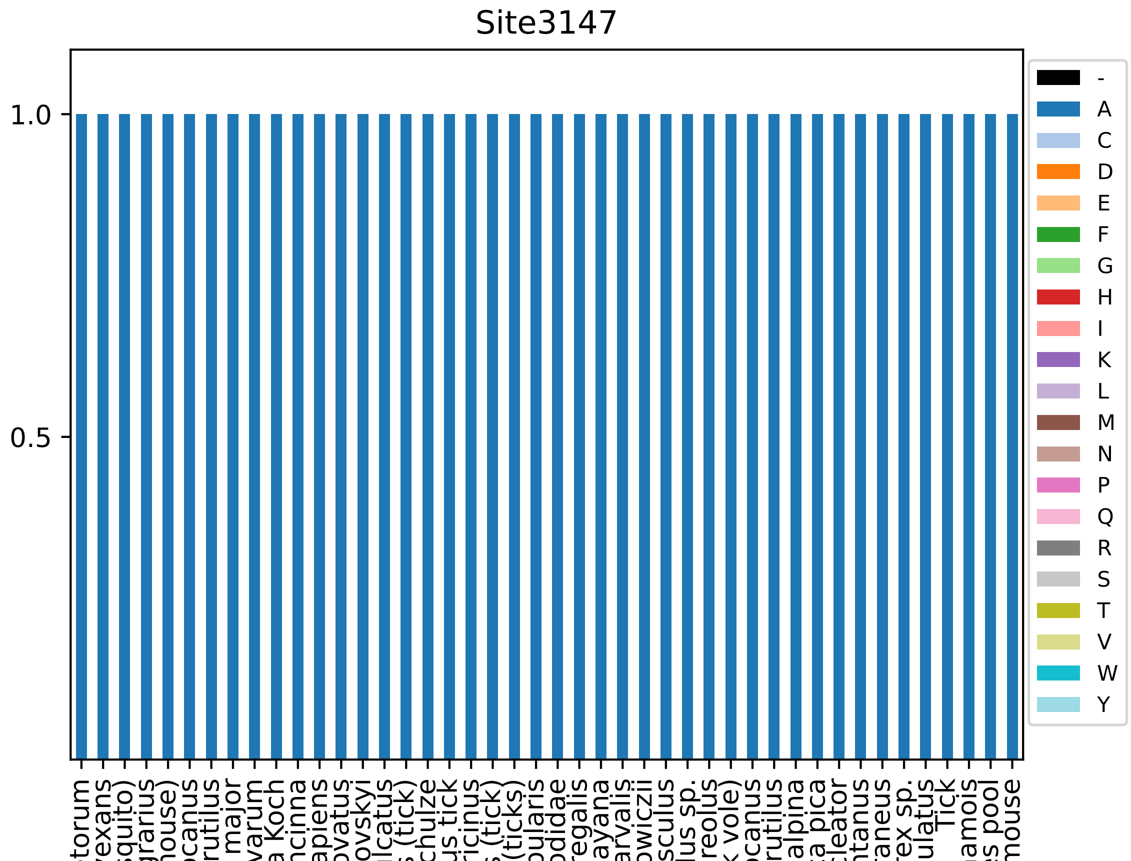
Site3147
1.0
0.5
-
A
C
D
E
F
G
H
I
K
L
M
N
P
Q
R
S
T
V
W
Y

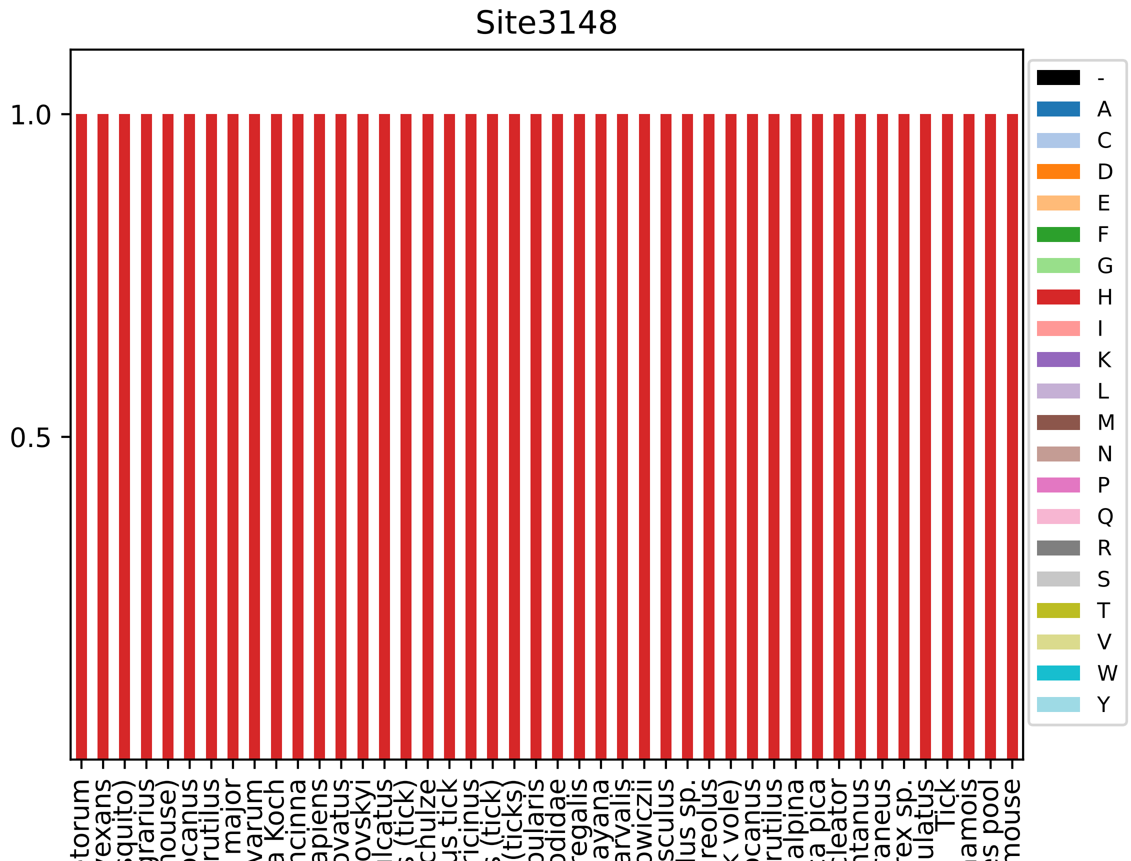
Site3148
1.0
0.5
-
A
C
D
E
F
G
H
I
K
L
M
N
P
Q
R
S
T
V
W
Y

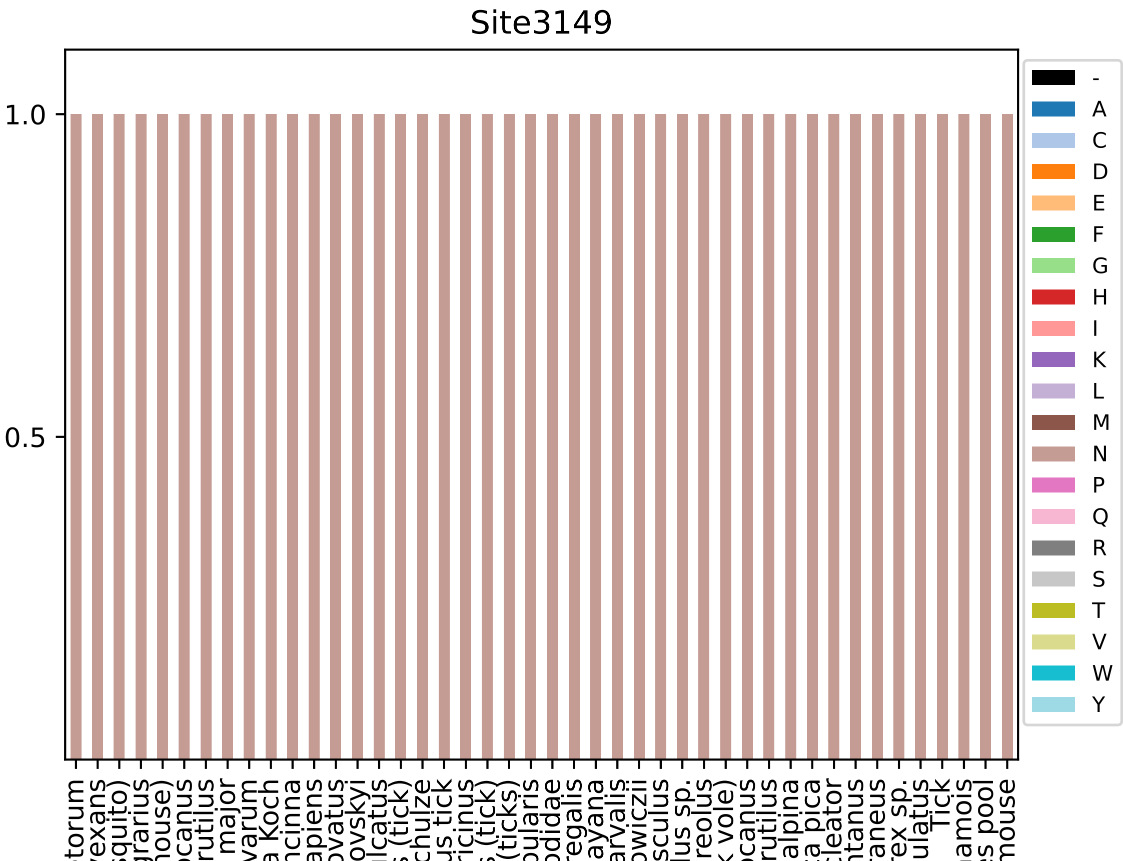
Site3149
1.0
0.5
-
A
C
D
E
F
G
H
I
K
L
M
N
P
Q
R
S
T
V
W
Y

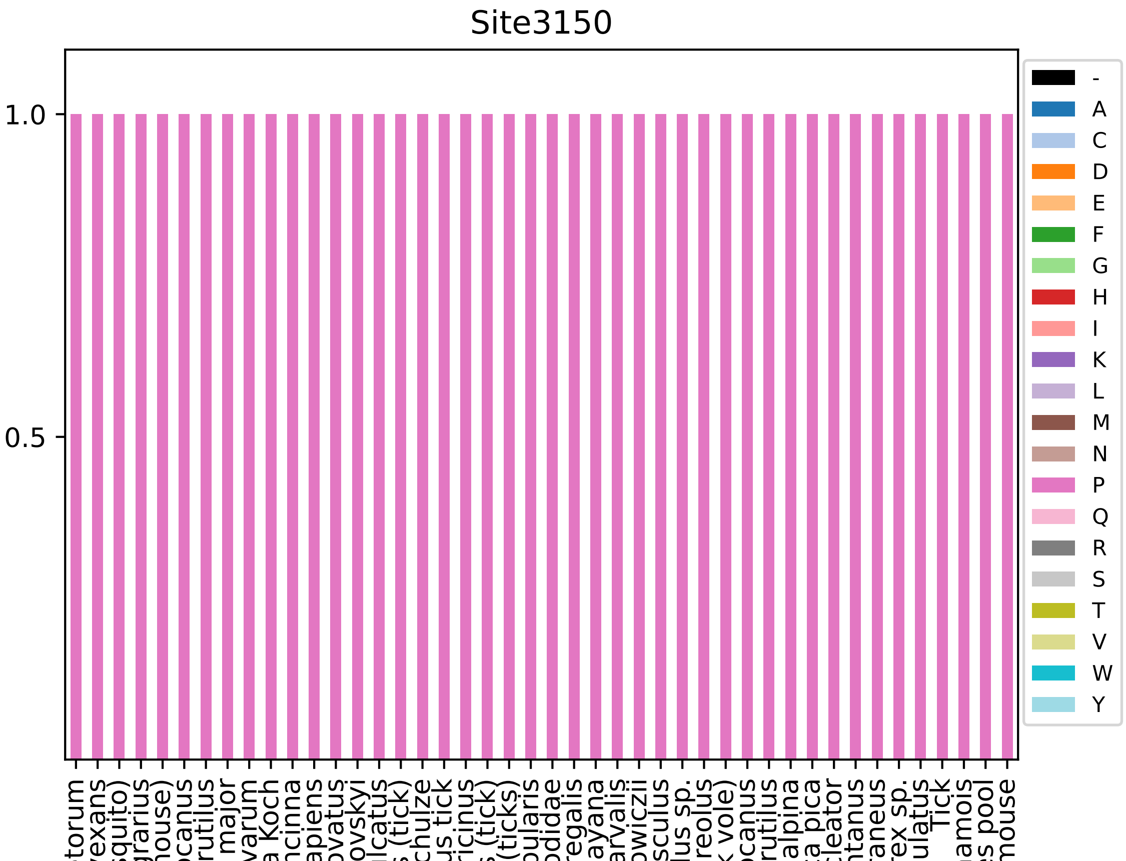

Site3150
1.0
0.5
-
A
C
D
E
F
G
H
I
K
L
M
N
P
Q
R
S
T
V
W
Y

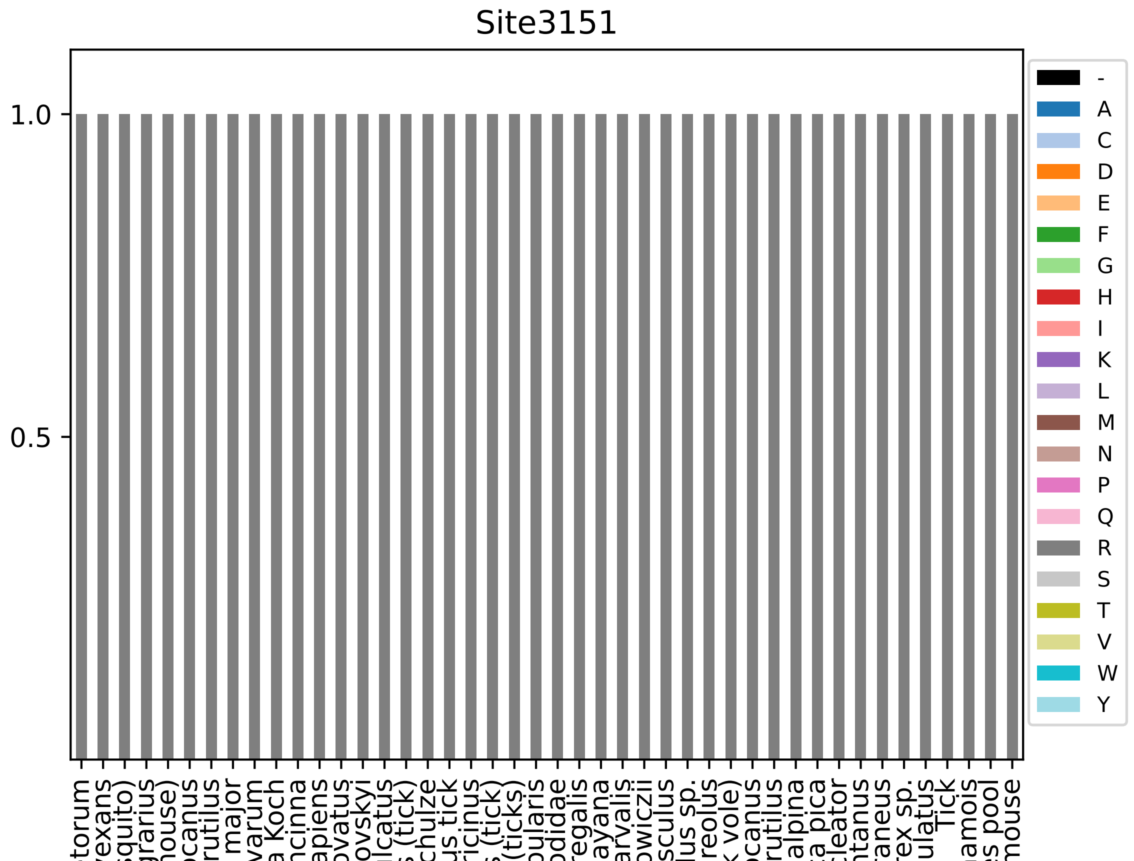

Site3151
1.0
0.5
-
A
C
D
E
F
G
H
I
K
L
M
N
P
Q
R
S
T
V
W
Y

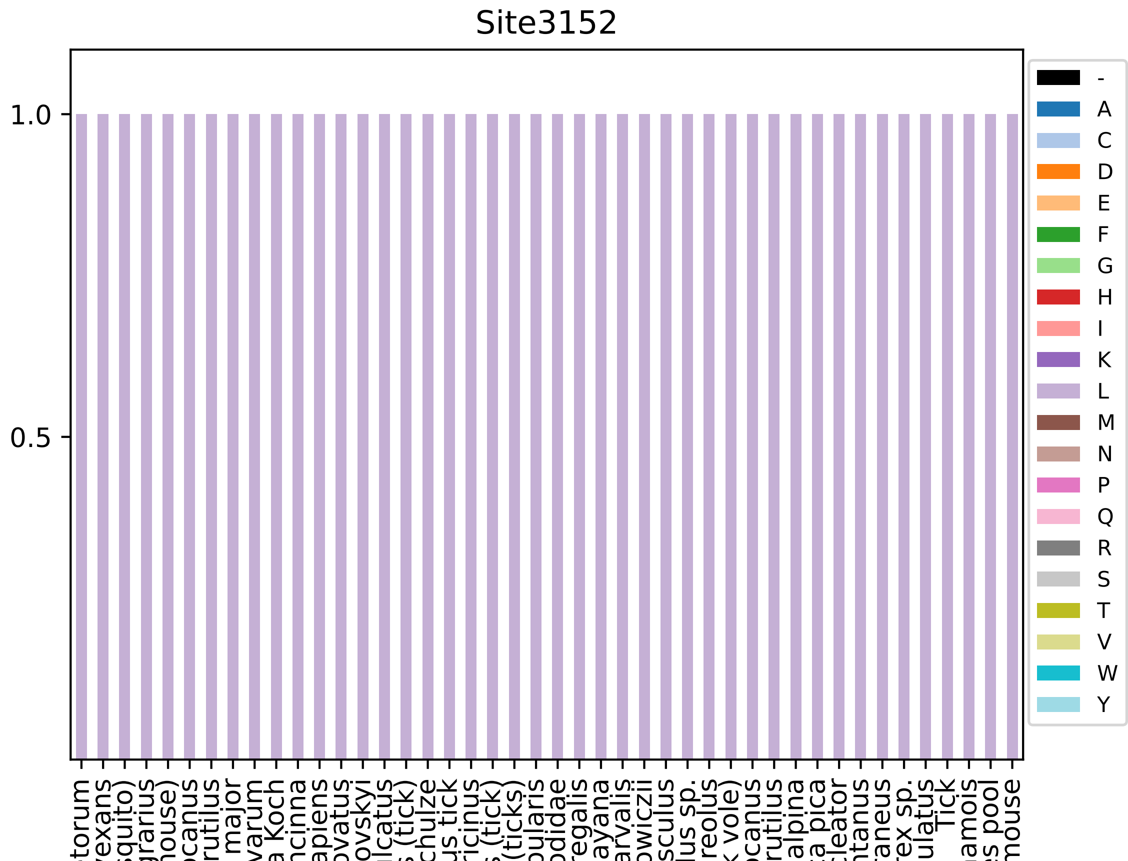
Site3152
1.0
0.5
-
A
C
D
E
F
G
H
I
K
L
M
N
P
Q
R
S
T
V
W
Y

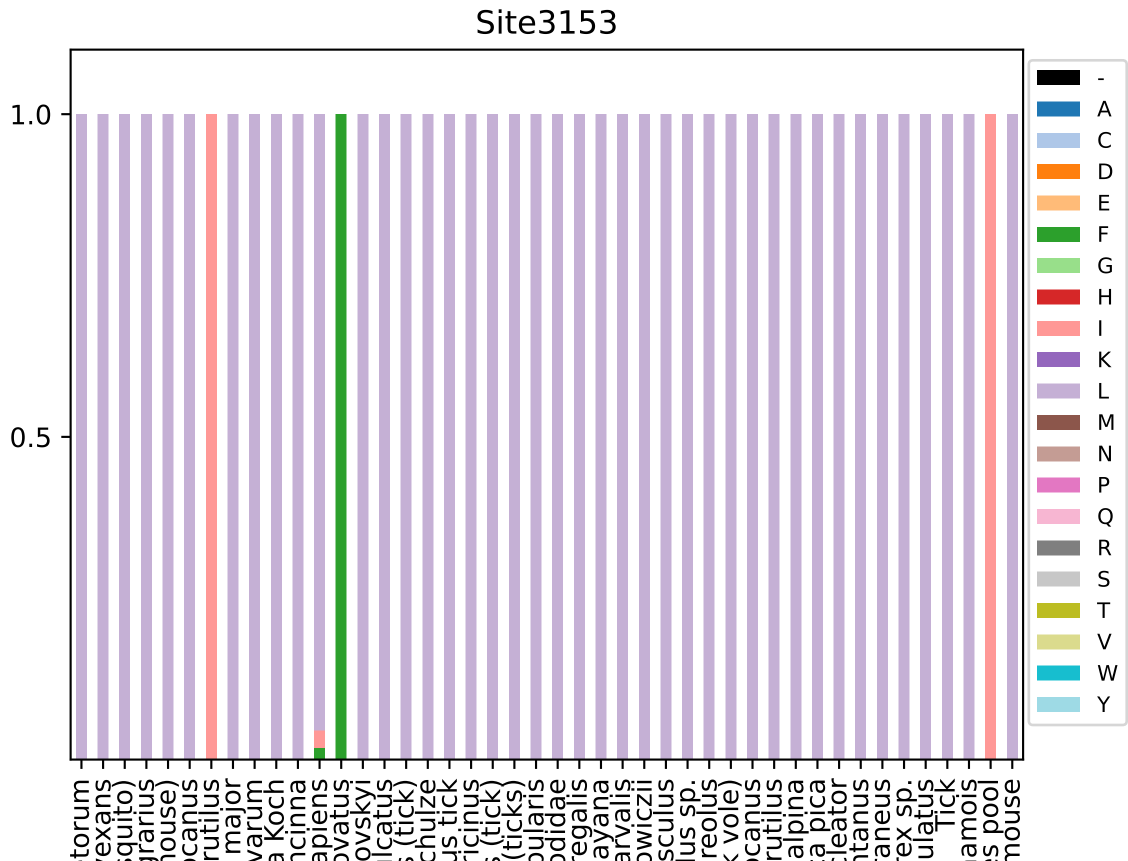

Site3153
1.0
0.5
-
A
C
D
E
F
G
H
I
K
L
M
N
P
Q
R
S
T
V
W
Y

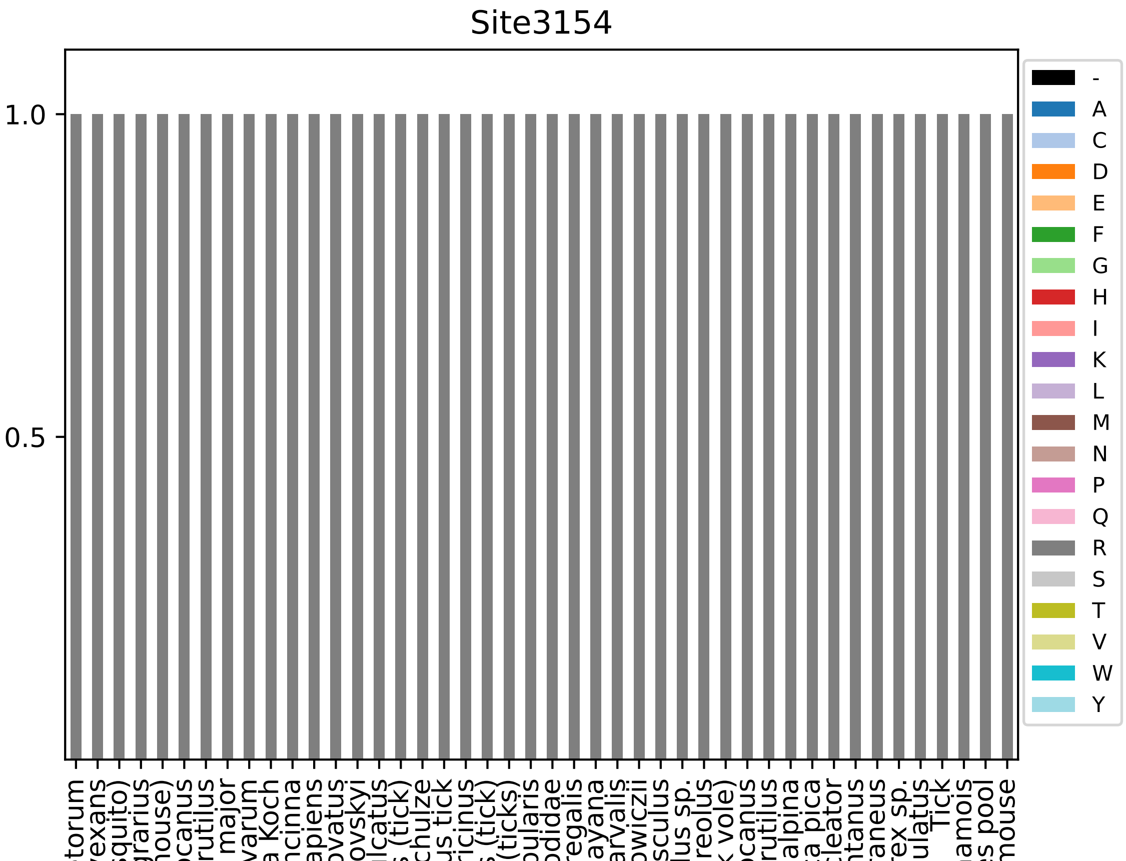
Site3154
1.0
0.5
-
A
C
D
E
F
G
H
I
K
L
M
N
P
Q
R
S
T
V
W
Y
Tick

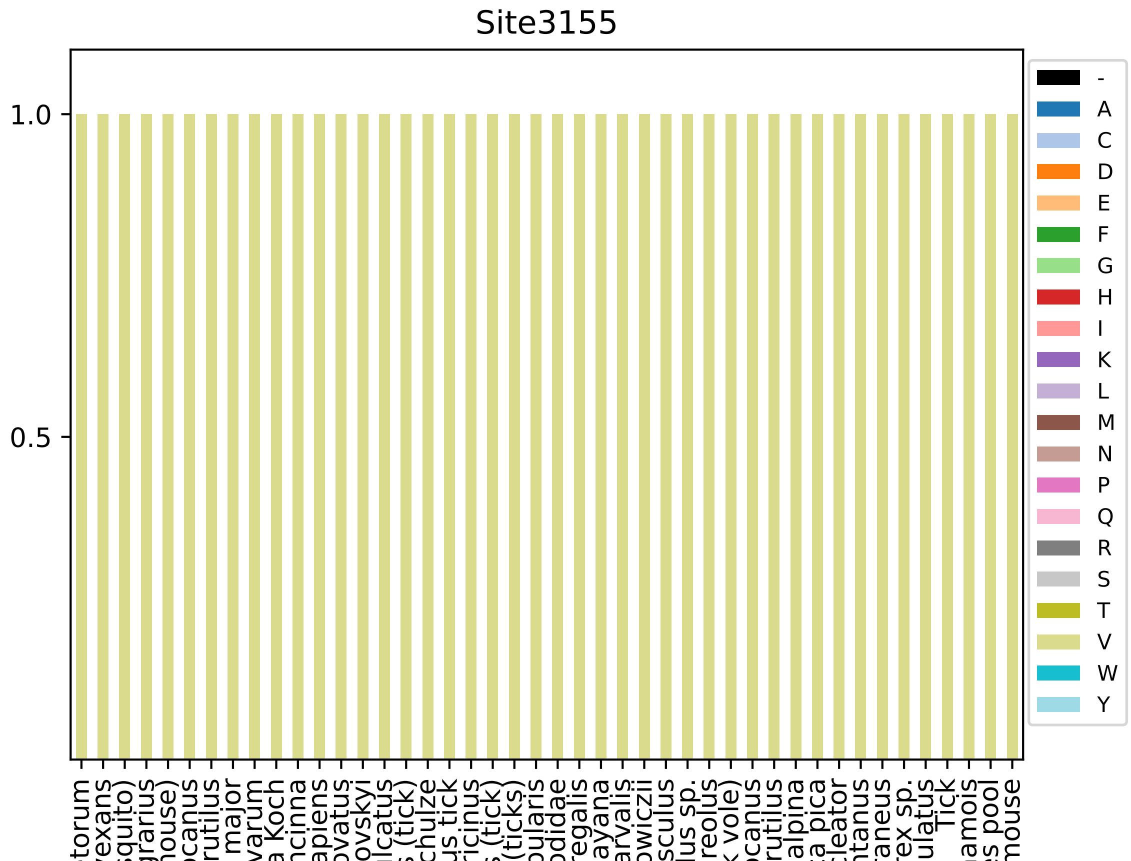
Site3155
1.0
0.5
-
A
C
D
E
F
G
H
I
K
L
M
N
P
Q
R
S
T
V
W
Y

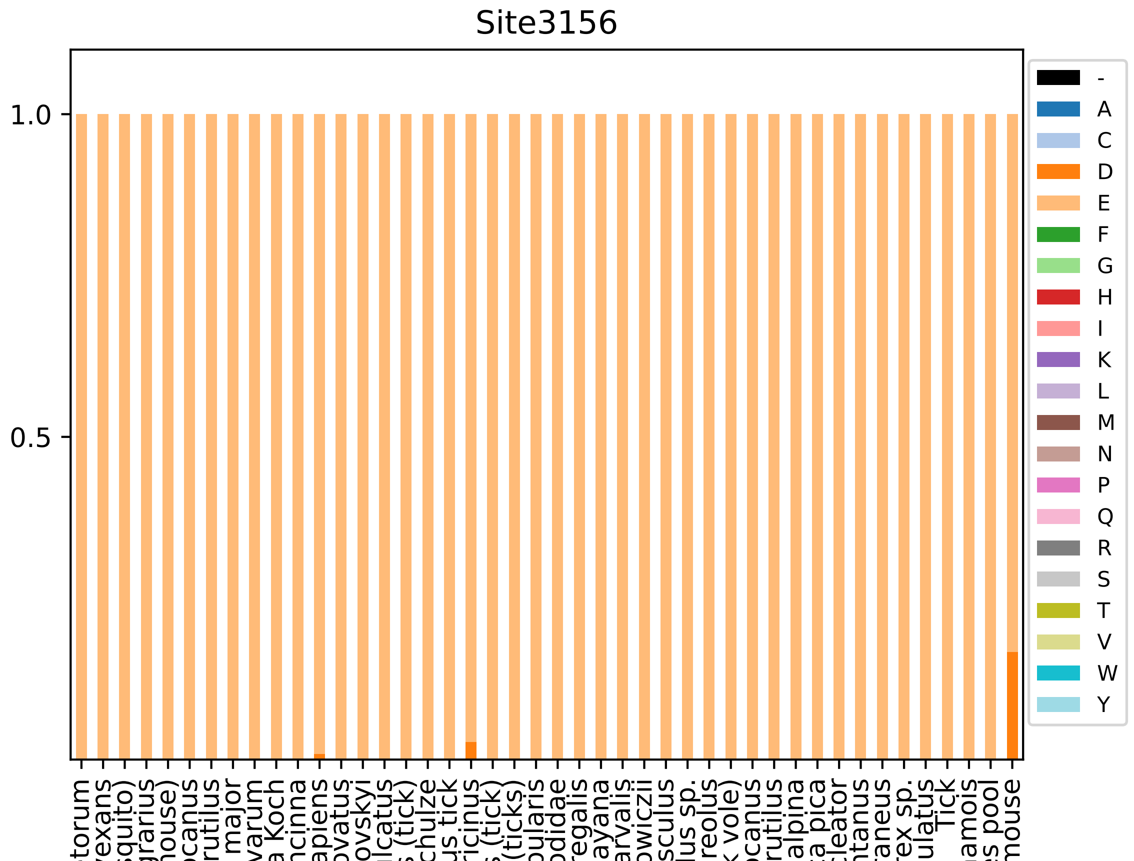

Site3156
1.0
0.5
-
A
C
D
E
F
G
H
I
K
L
M
N
P
Q
R
S
T
V
W
Y

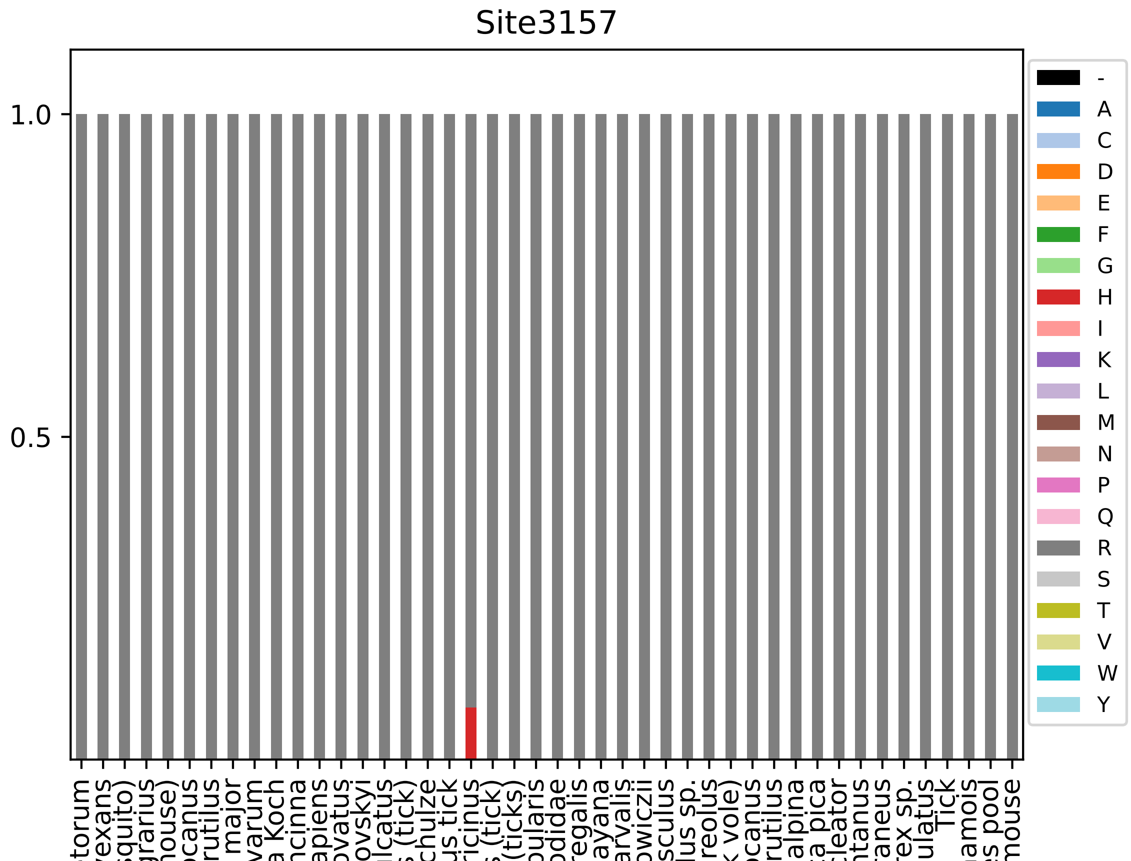

Site3157
1.0
0.5
-
A
C
D
E
F
G
H
I
K
L
M
N
P
Q
R
S
T
V
W
Y

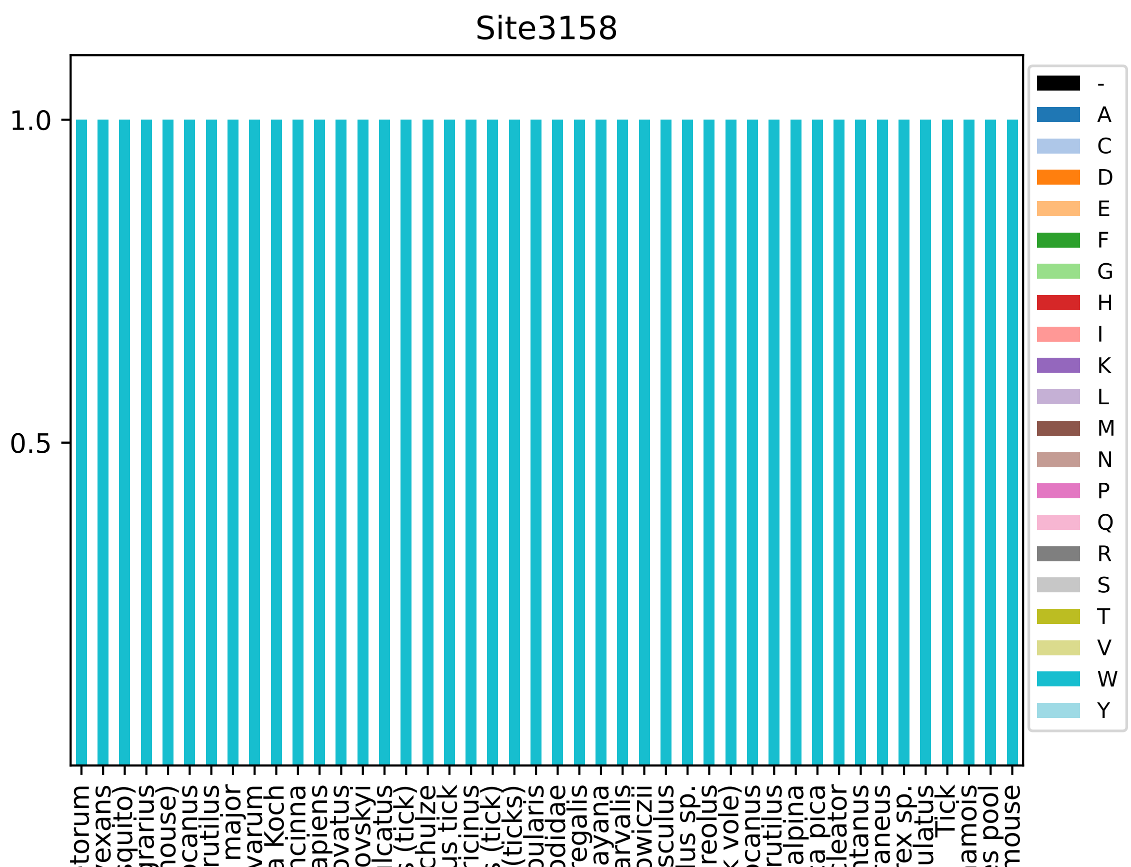

Site3158
1.0
0.5
-
A
C
D
E
F
G
H
I
K
L
M
N
P
Q
R
S
T
V
W
Y

Site3159

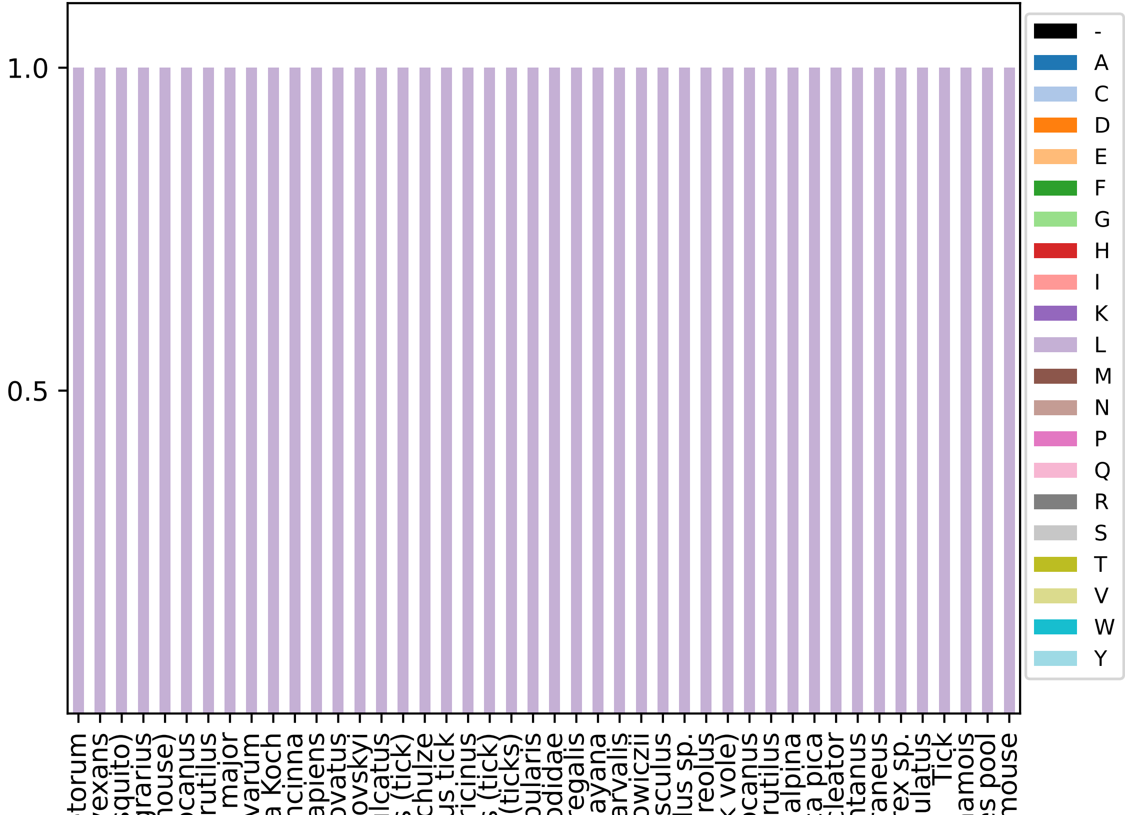

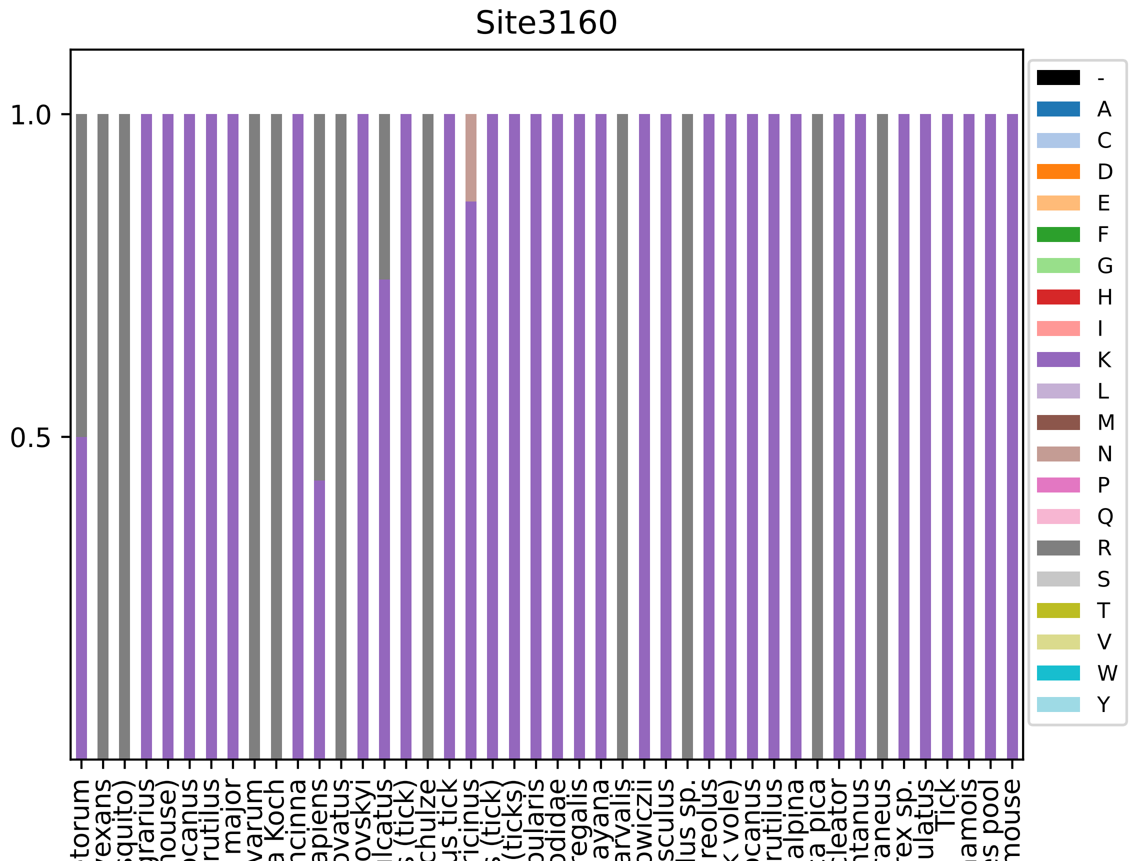

Site3160
1.0
0.5
-
A
C
D
E
F
G
H
I
K
L
M
N
P
Q
R
S
T
V
W
Y

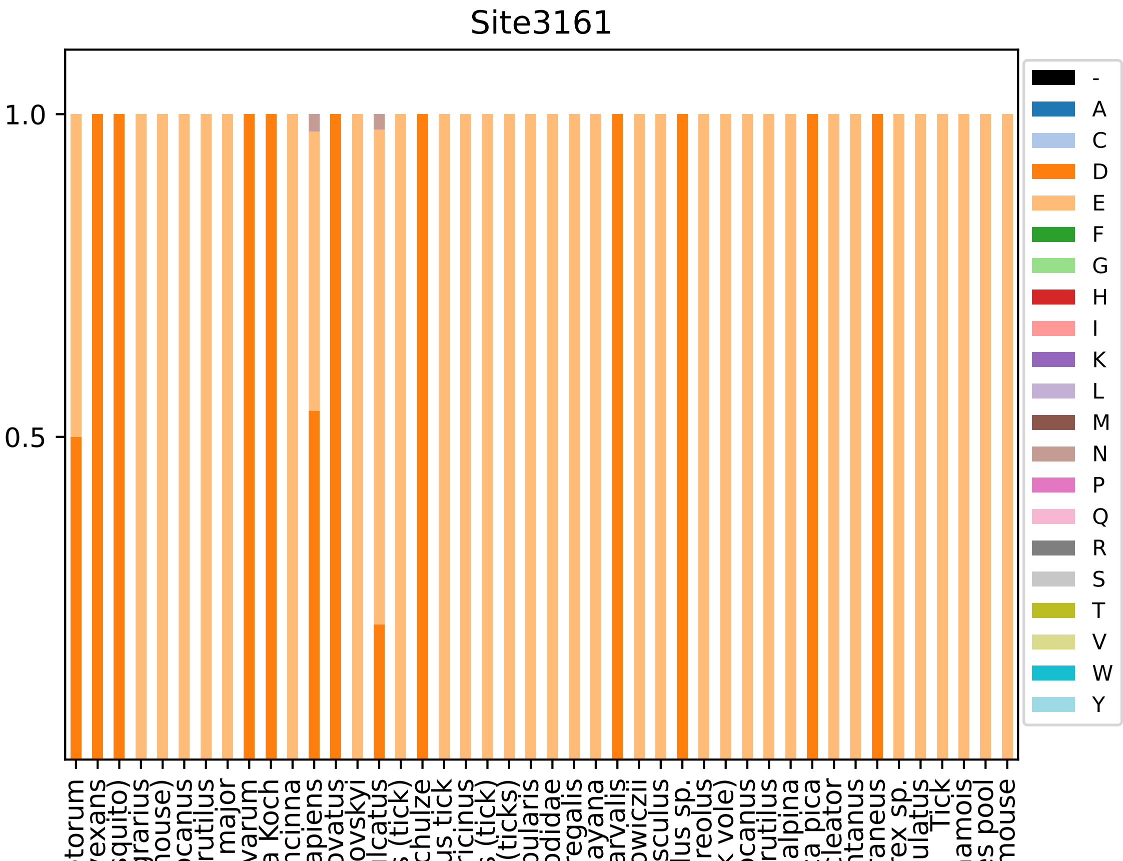
Site3161
1.0
0.5
-
A
C
D
E
F
G
H
I
K
L
M
N
P
Q
R
S
T
V
W
Y
Tick

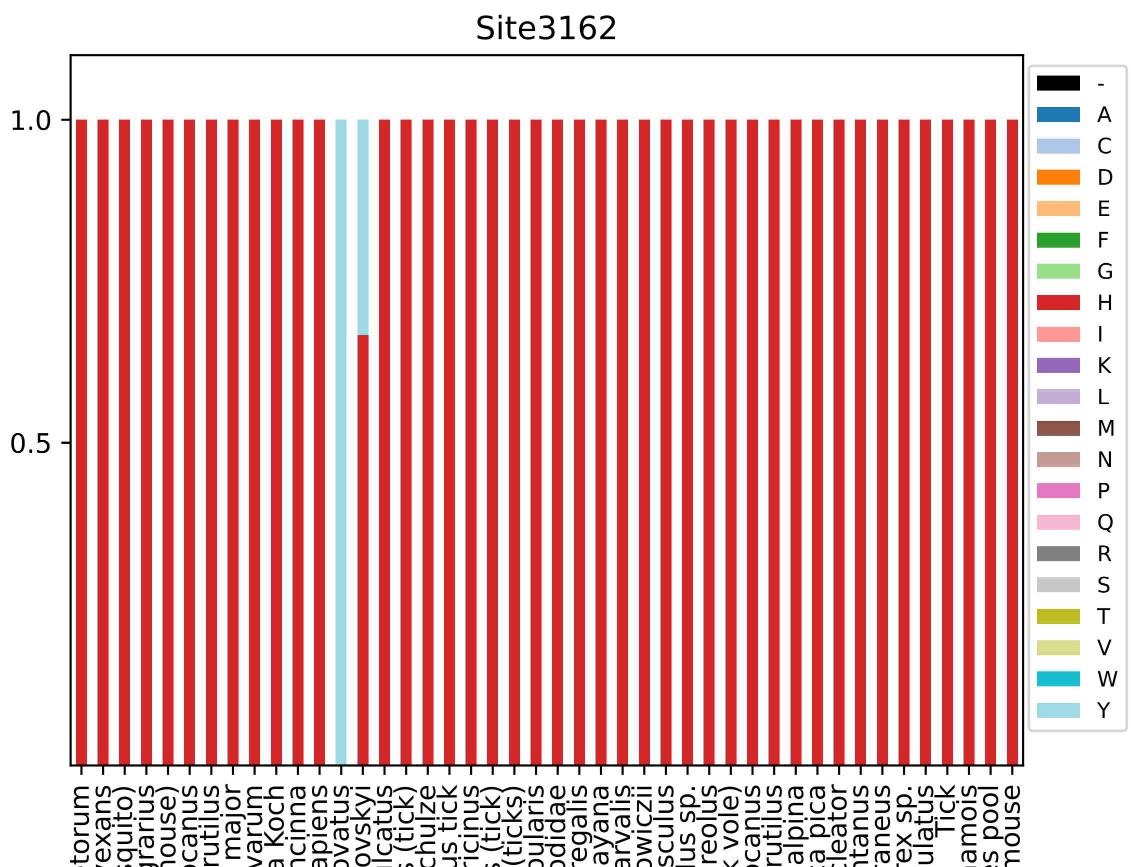
Site3162
1.0
0.5
-
A
C
D
E
F
G
H
I
K
L
M
N
P
Q
R
S
T
V
W
Y
torum
exans
squito)
grarius
ouse)
ocanus
rutilus
major
varum
a Koch
ncinna
apiens
ovatus
ovskyi
lcatus
(tick)
chulze
us tick
icinus
(tick)
(ticks)
pularis
odidae
regalis
ayana
arvalis
owiczii
sculus
lus sp.
reolus
k vole)
ocanus
rutilus
alpina
a pica
leator
ntanus
aneus
rex sp.
ulatus
Tick
amois
es pool
mouse

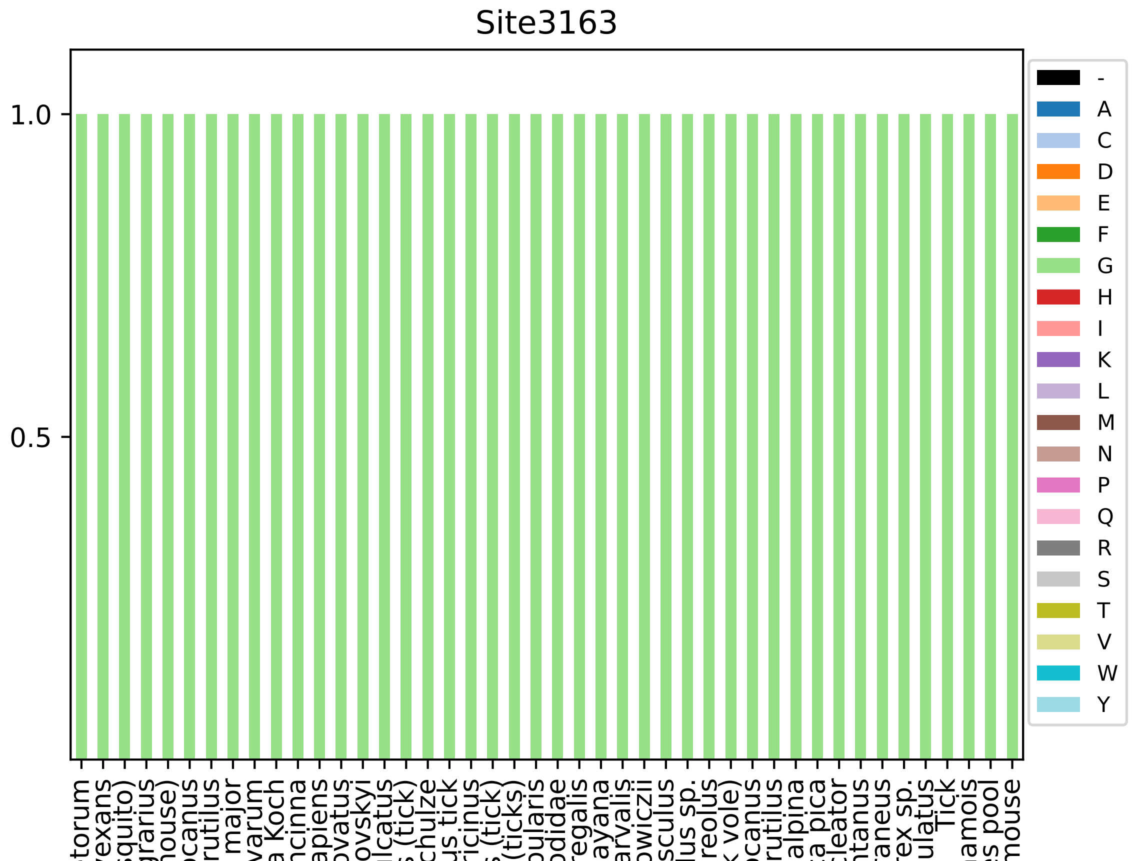
Site3163
1.0
0.5
-
A
C
D
E
F
G
H
I
K
L
M
N
P
Q
R
S
T
V
W
Y
Tick

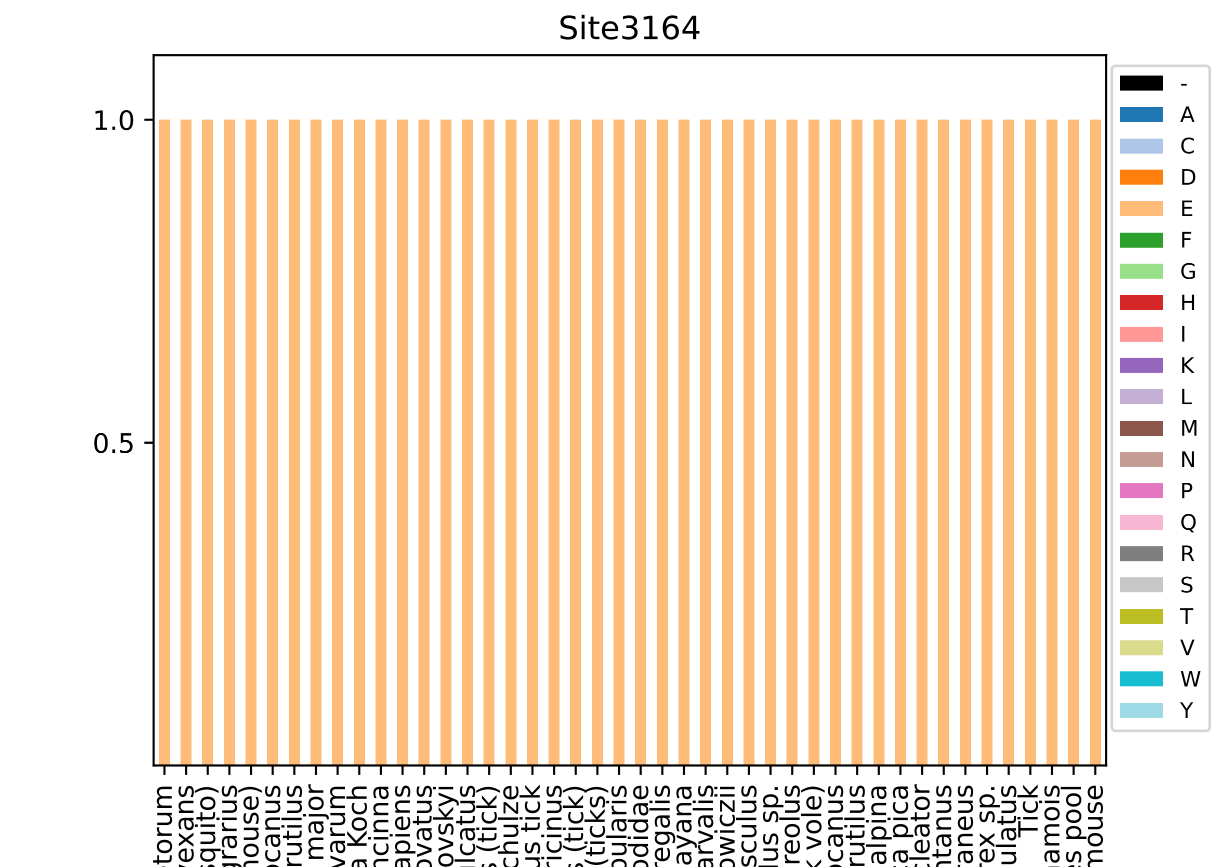
Site3164
1.0
0.5
-
A
C
D
E
F
G
H
I
K
L
M
N
P
Q
R
S
T
V
W
Y

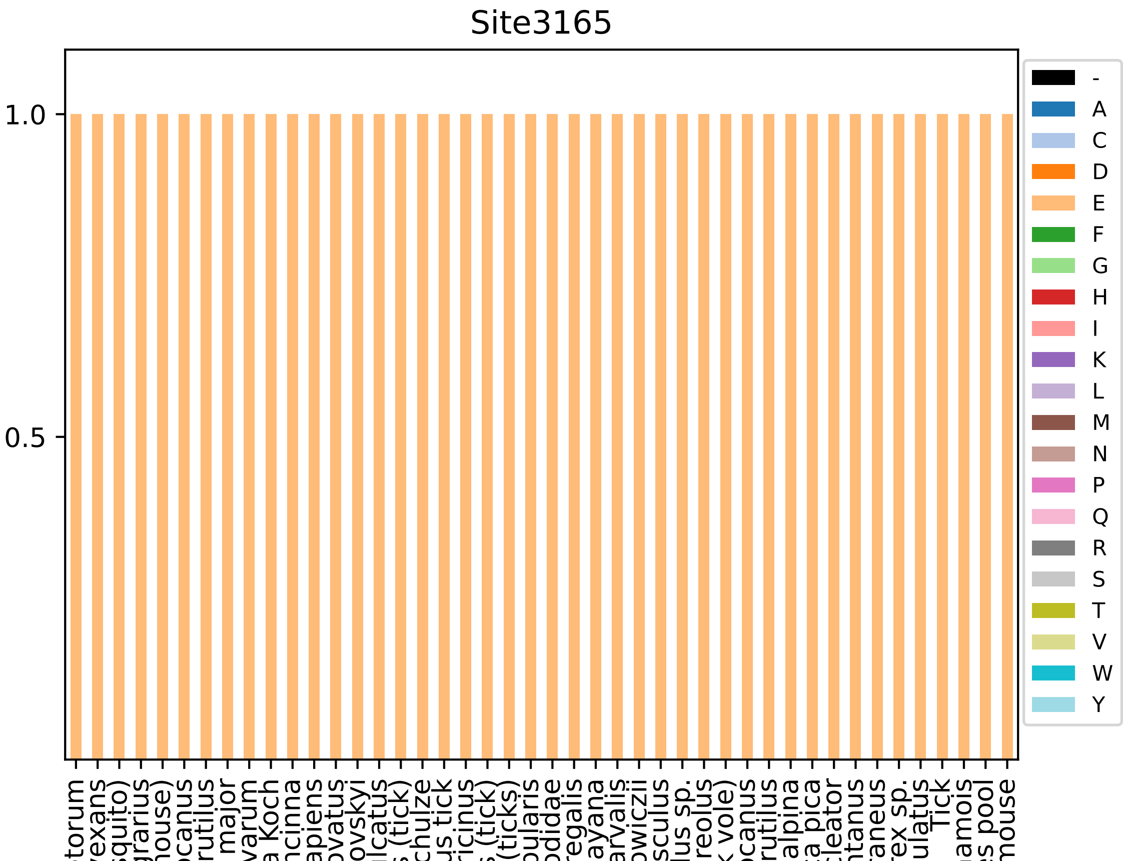
Site3165
1.0
0.5
-
A
C
D
E
F
G
H
I
K
L
M
N
P
Q
R
S
T
V
W
Y

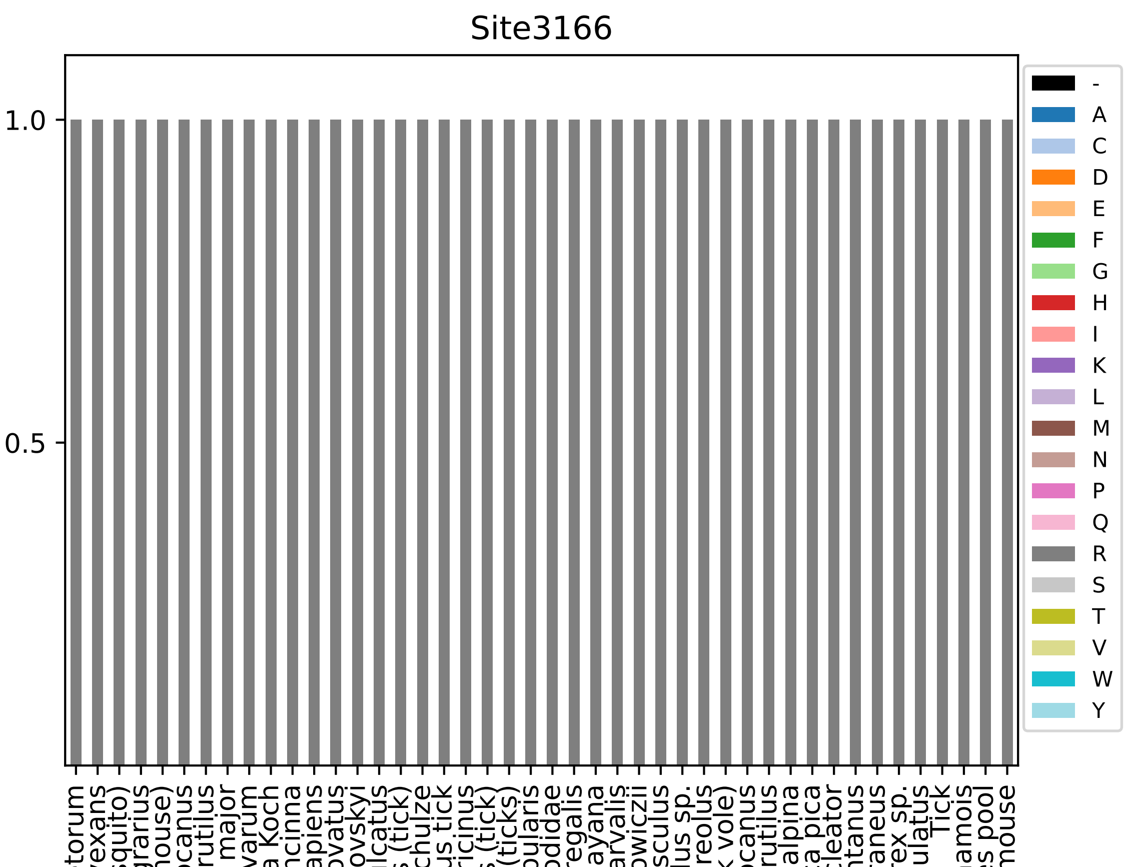

Site3166
1.0
0.5
-
A
C
D
E
F
G
H
I
K
L
M
N
P
Q
R
S
T
V
W
Y

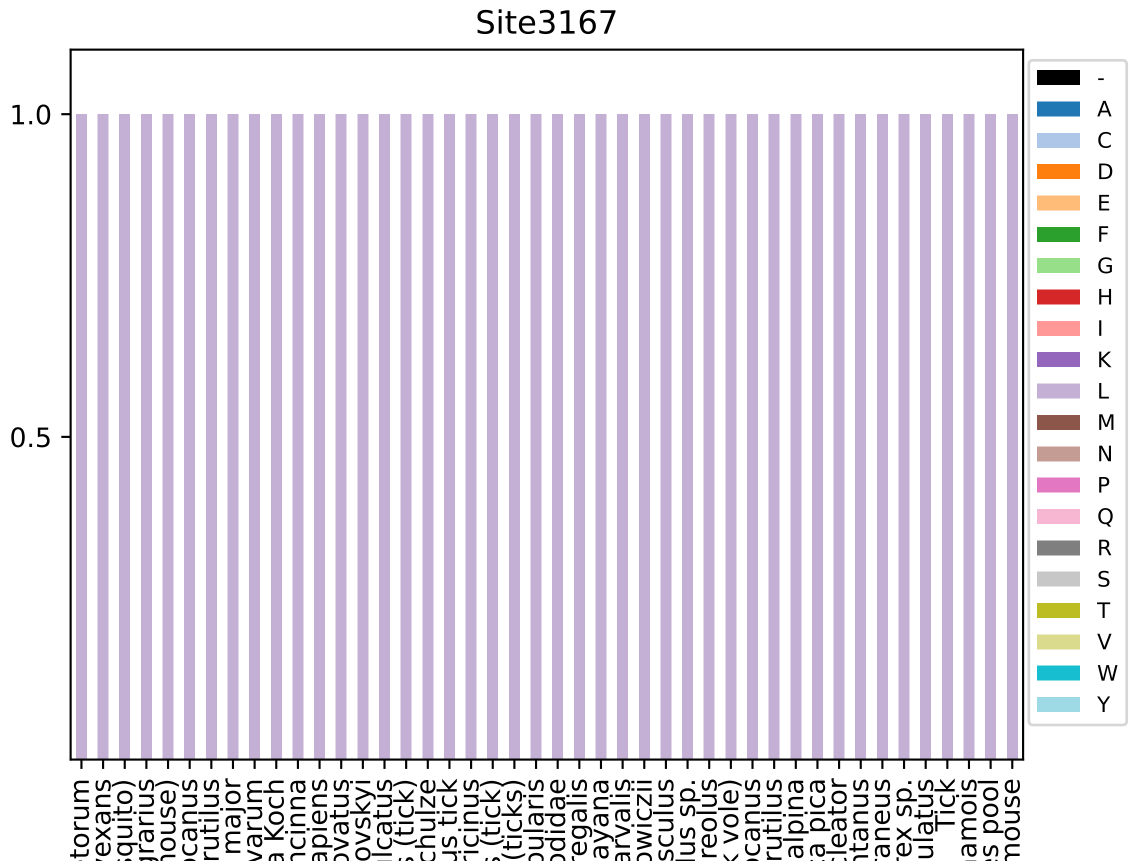

Site3167
1.0
0.5
-
A
C
D
E
F
G
H
I
K
L
M
N
P
Q
R
S
T
V
W
Y

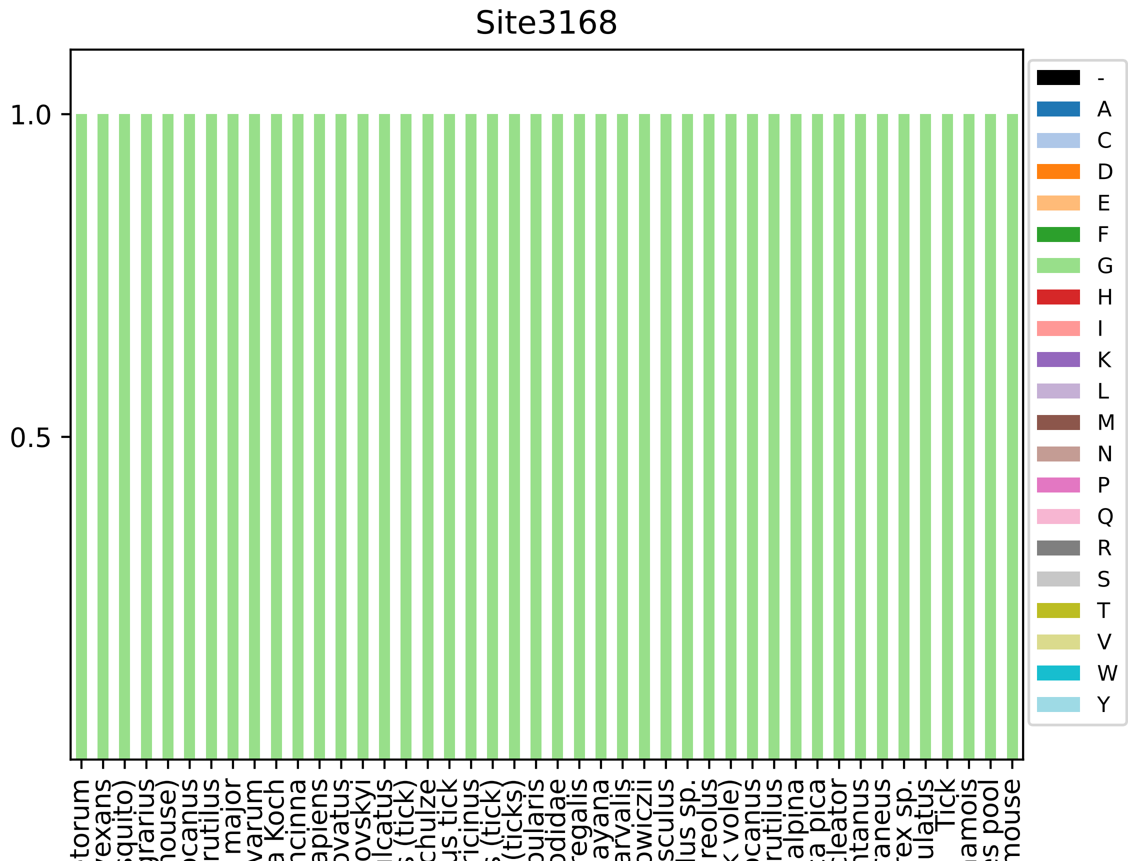
Site3168
1.0
0.5
-
A
C
D
E
F
G
H
I
K
L
M
N
P
Q
R
S
T
V
W
Y

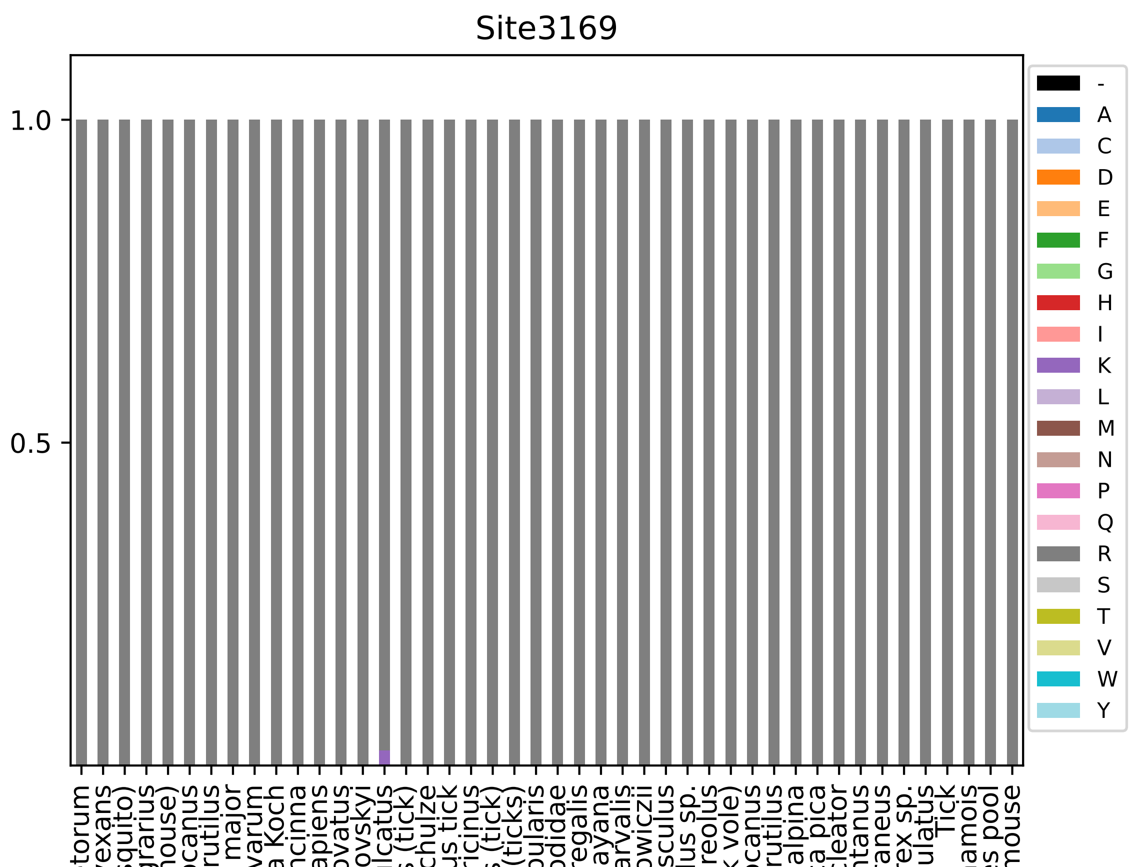
Site3169
1.0
0.5
-
A
C
D
E
F
G
H
I
K
L
M
N
P
Q
R
S
T
V
W
Y
Tick

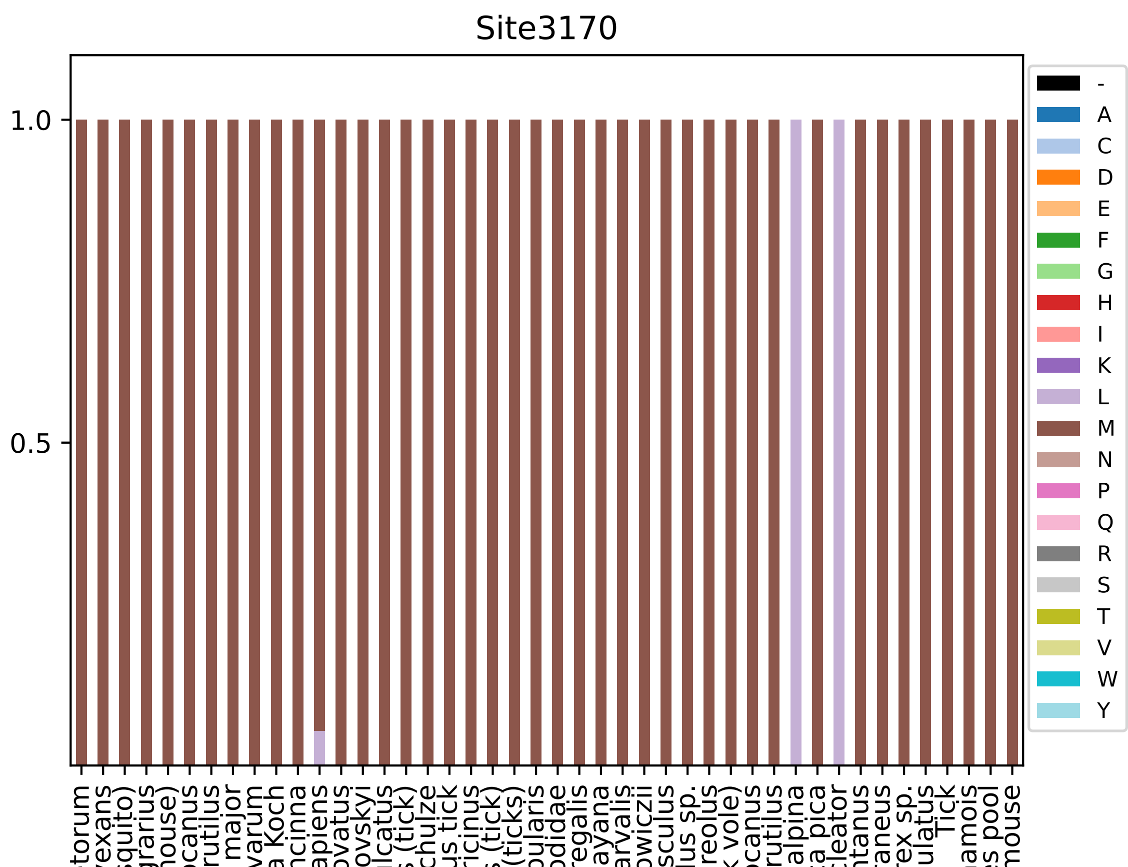

Site3170
1.0
0.5
-
A
C
D
E
F
G
H
I
K
L
M
N
P
Q
R
S
T
V
W
Y

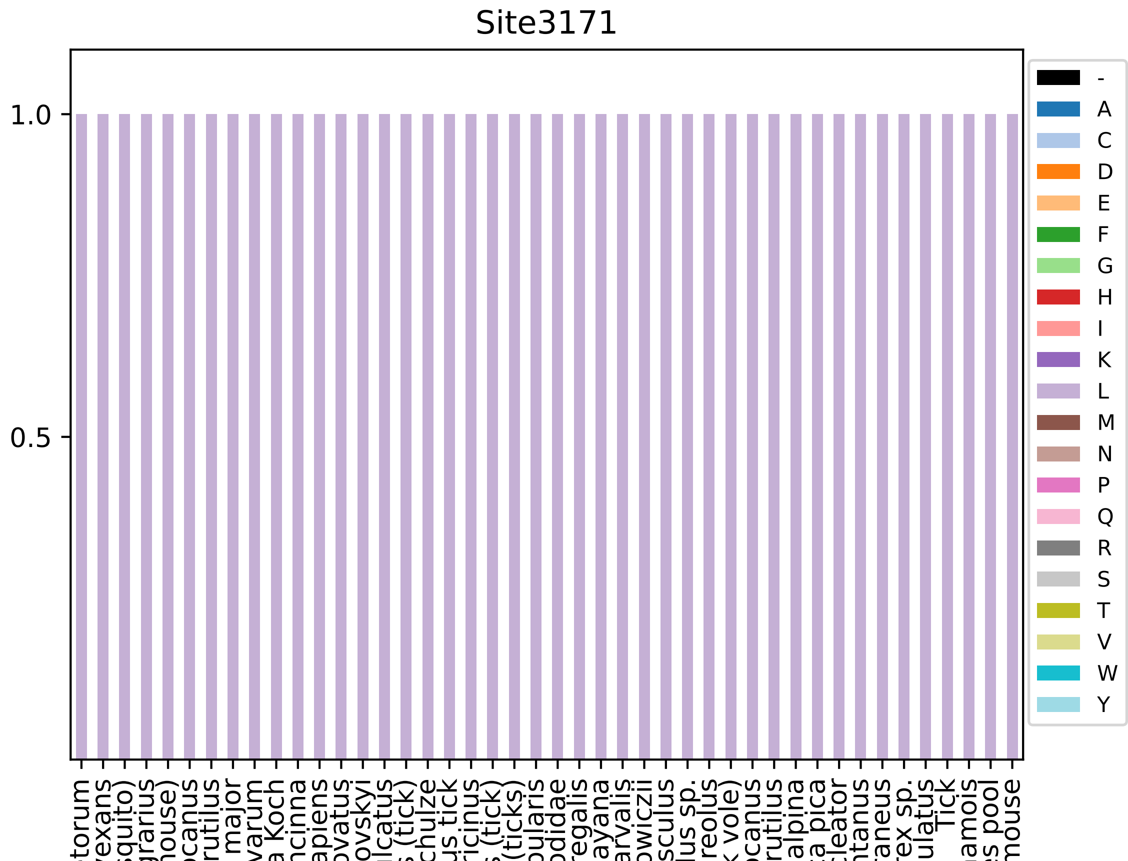
Site3171
1.0
0.5
-
A
C
D
E
F
G
H
I
K
L
M
N
P
Q
R
S
T
V
W
Y
Tick

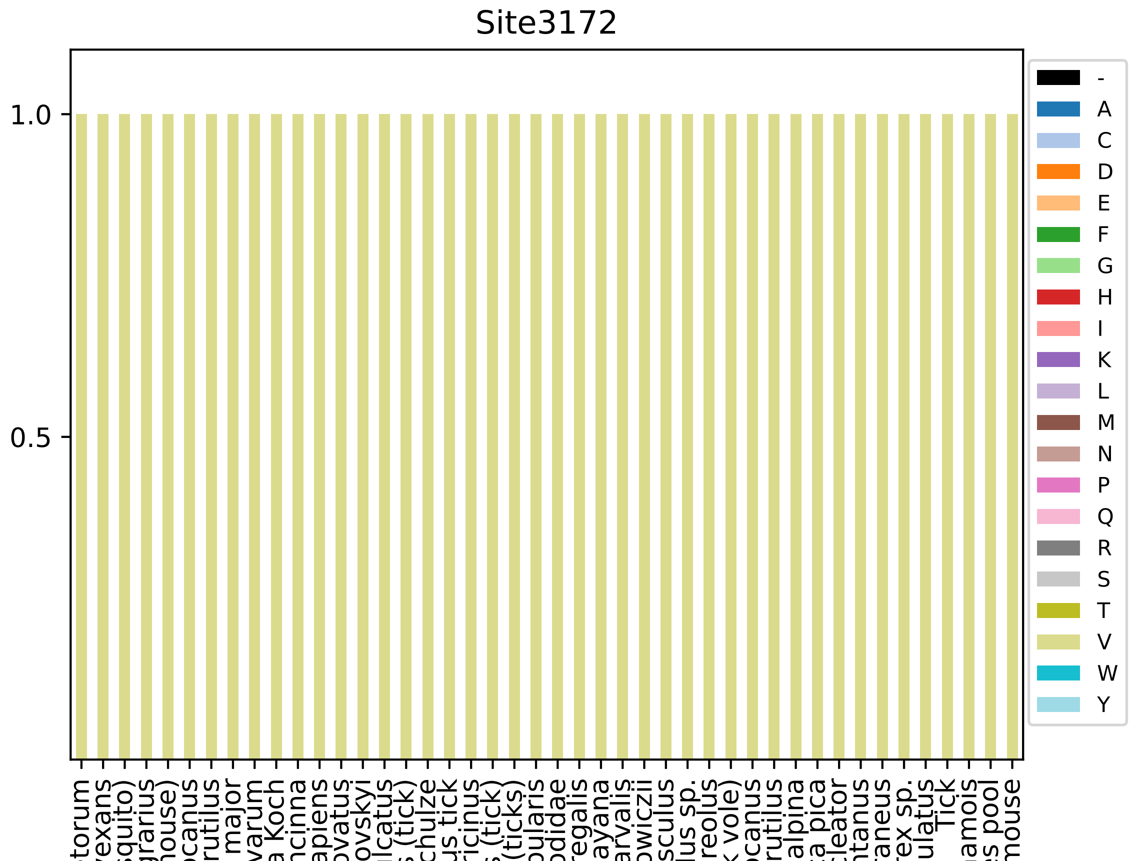
Site3172
1.0
0.5
-
A
C
D
E
F
G
H
I
K
L
M
N
P
Q
R
S
T
V
W
Y

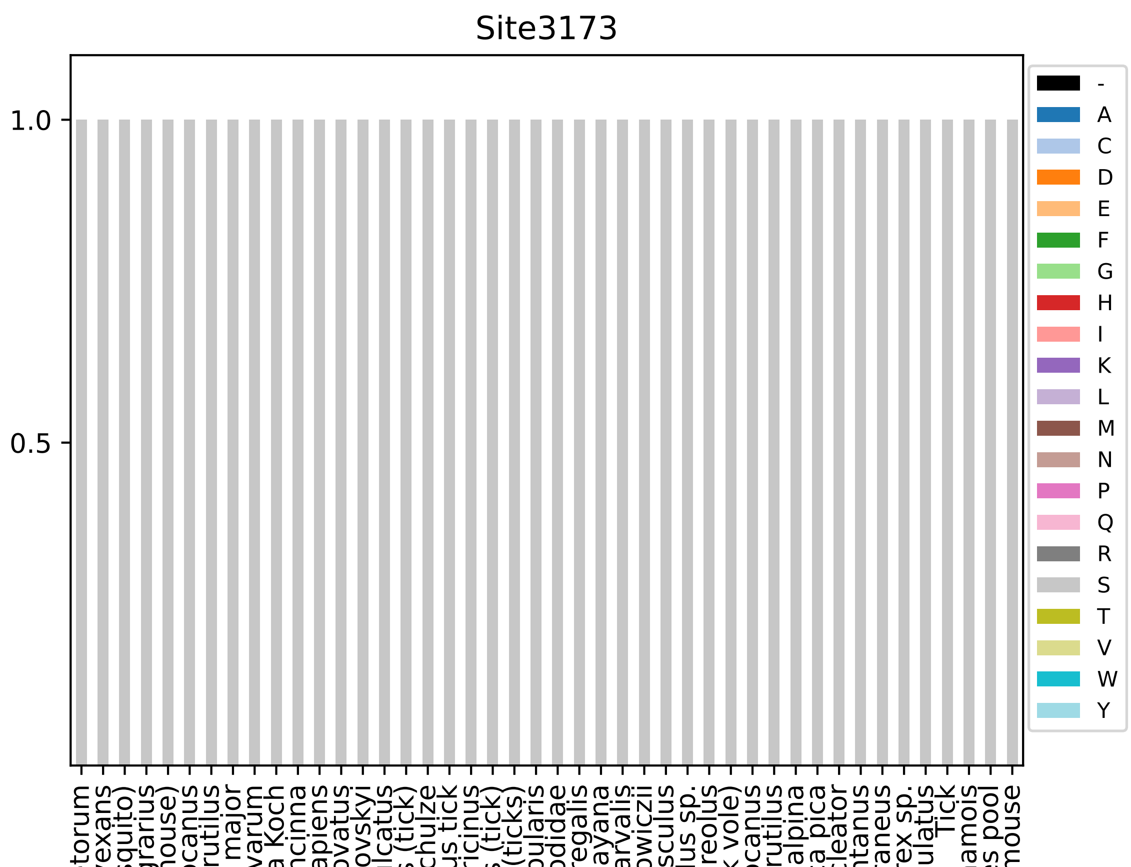
Site3173
1.0
0.5
-
A
C
D
E
F
G
H
I
K
L
M
N
P
Q
R
S
T
V
W
Y
Tick

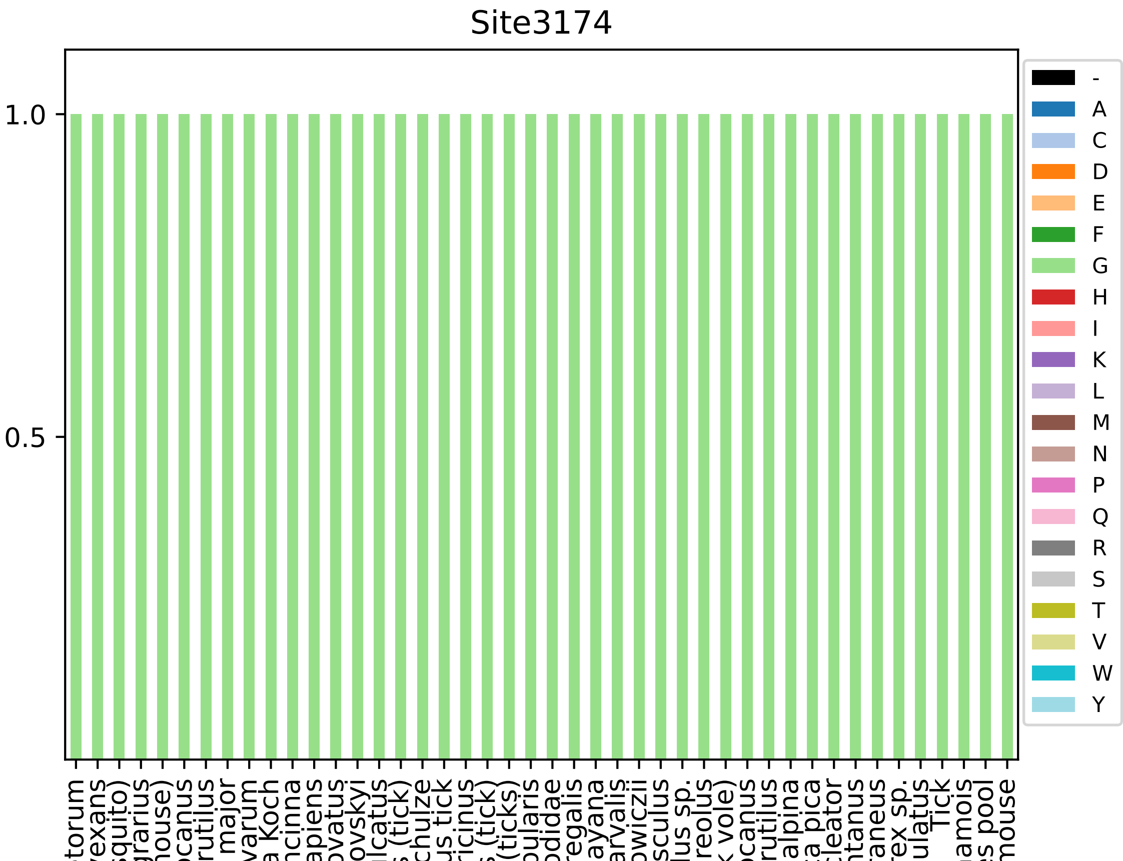
Site3174
1.0
0.5
-
A
C
D
E
F
G
H
I
K
L
M
N
P
Q
R
S
T
V
W
Y

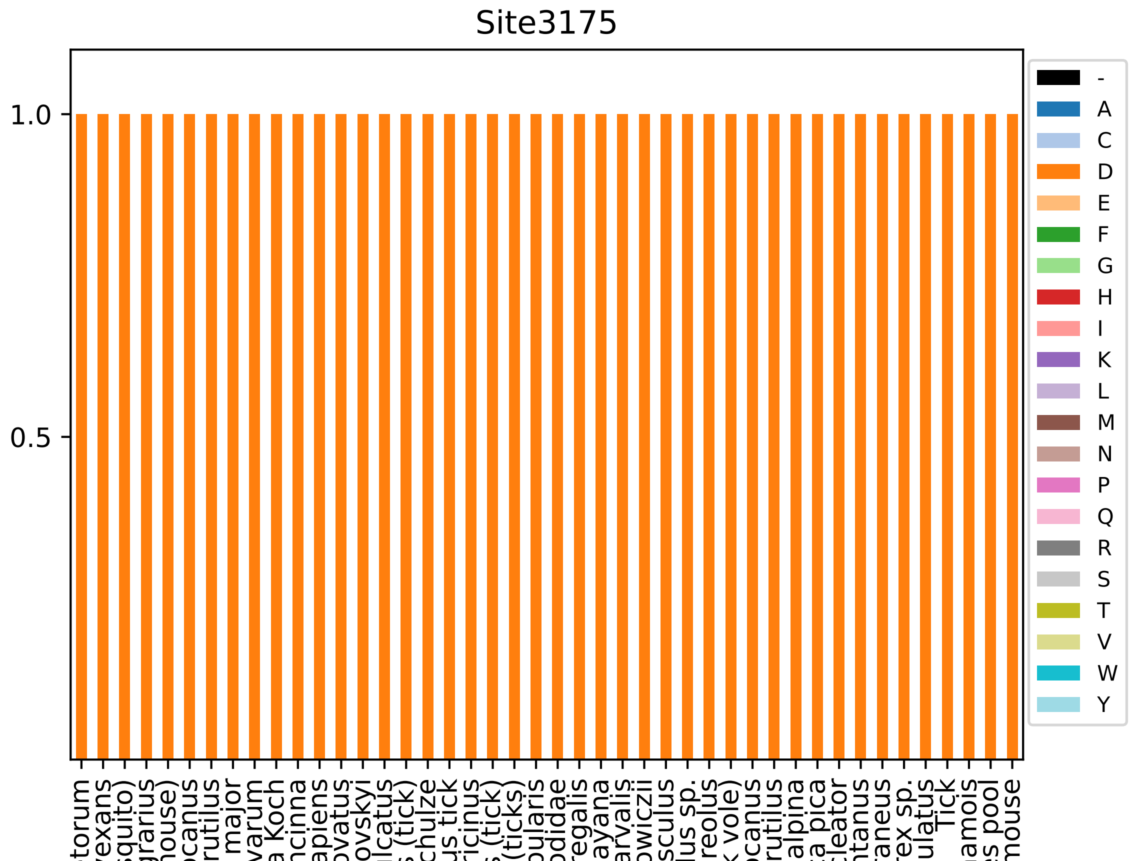

Site3175
1.0
0.5
-
A
C
D
E
F
G
H
I
K
L
M
N
P
Q
R
S
T
V
W
Y

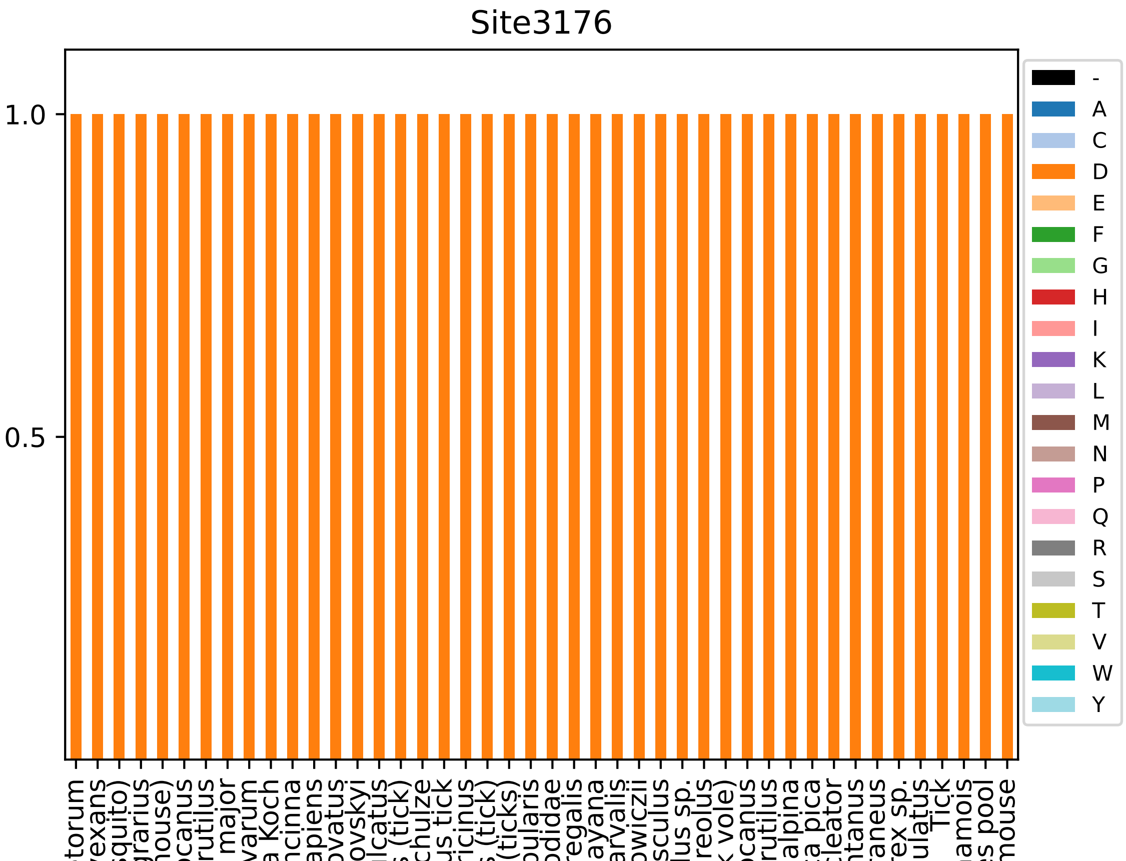
Site3176
1.0
0.5
-
A
C
D
E
F
G
H
I
K
L
M
N
P
Q
R
S
T
V
W
Y

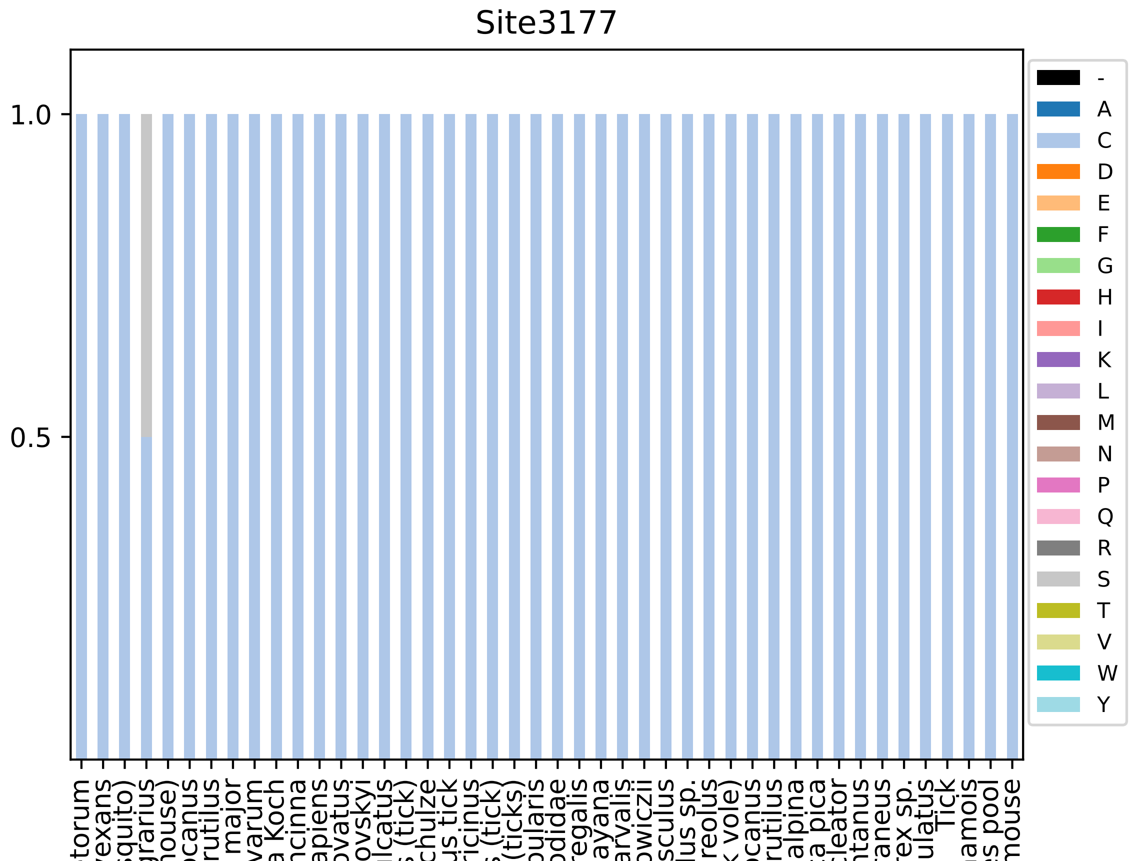
Site3177
1.0
0.5
-
A
C
D
E
F
G
H
I
K
L
M
N
P
Q
R
S
T
V
W
Y

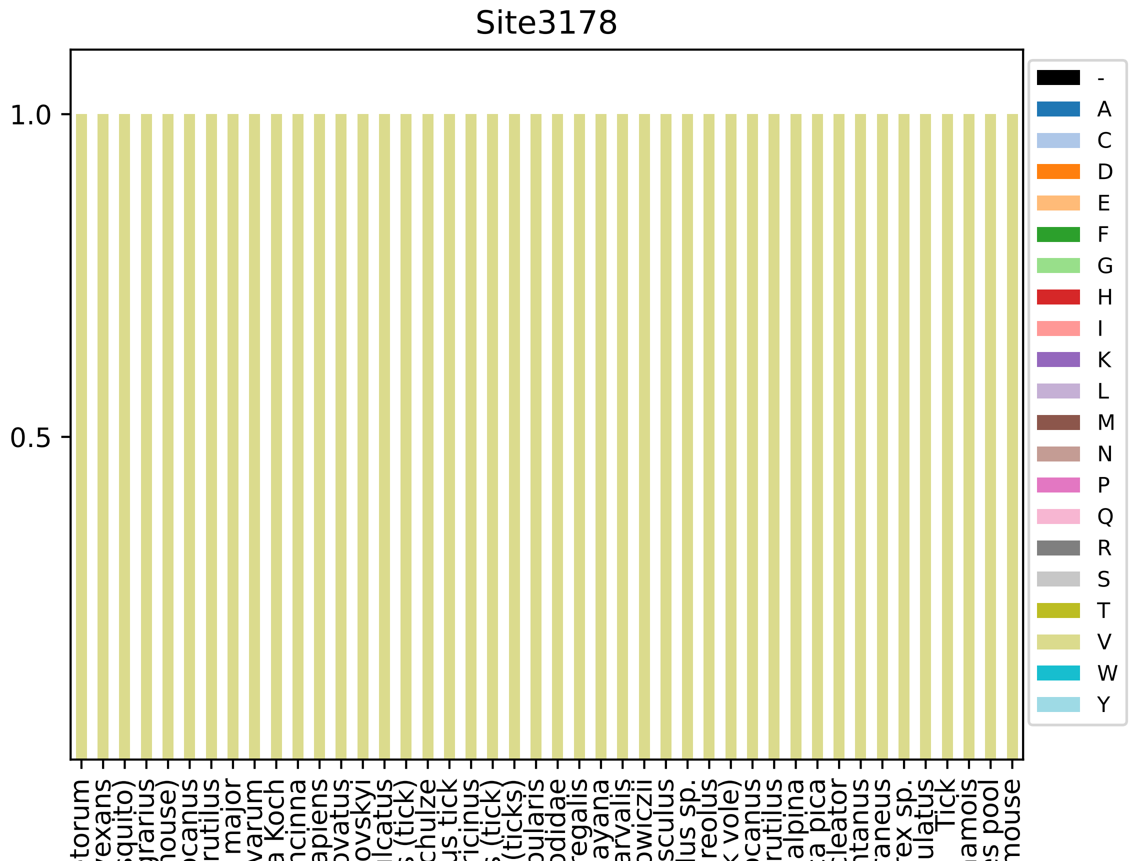
Site3178
1.0
0.5
-
A
C
D
E
F
G
H
I
K
L
M
N
P
Q
R
S
T
V
W
Y

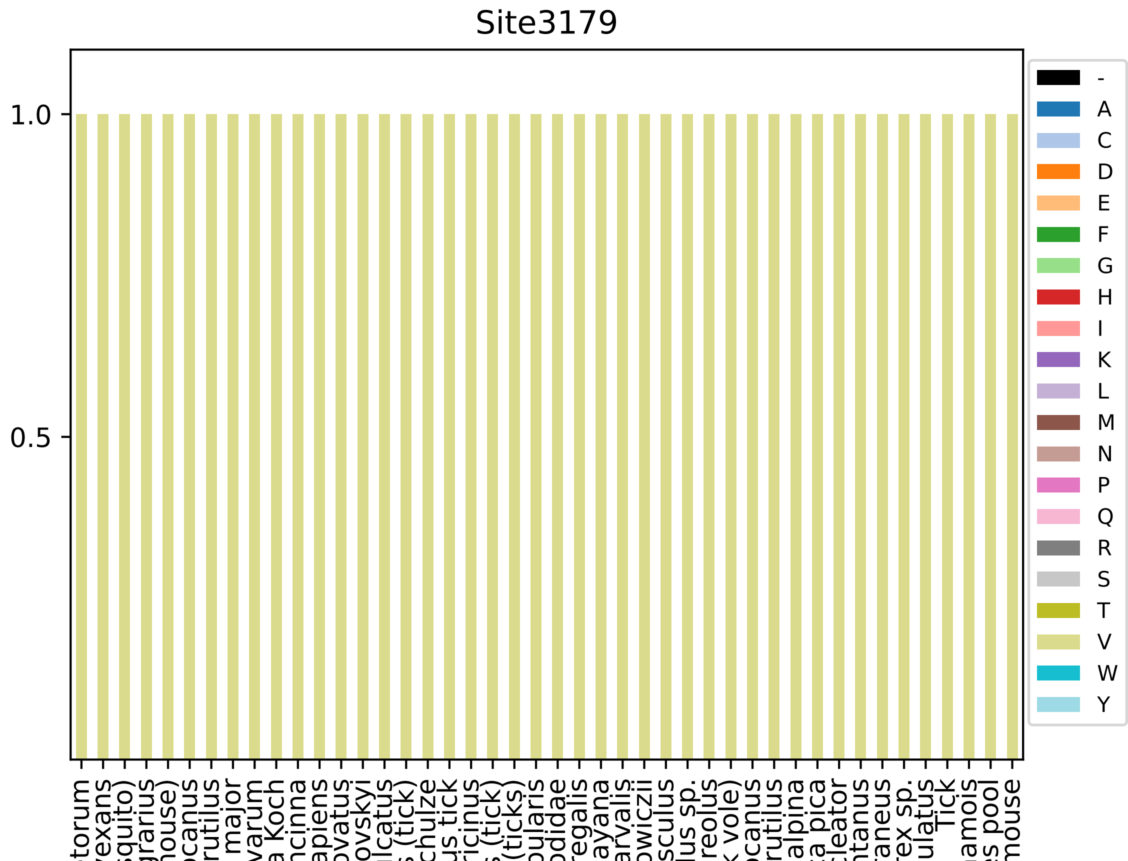
Site3179
1.0
0.5
-
A
C
D
E
F
G
H
I
K
L
M
N
P
Q
R
S
T
V
W
Y

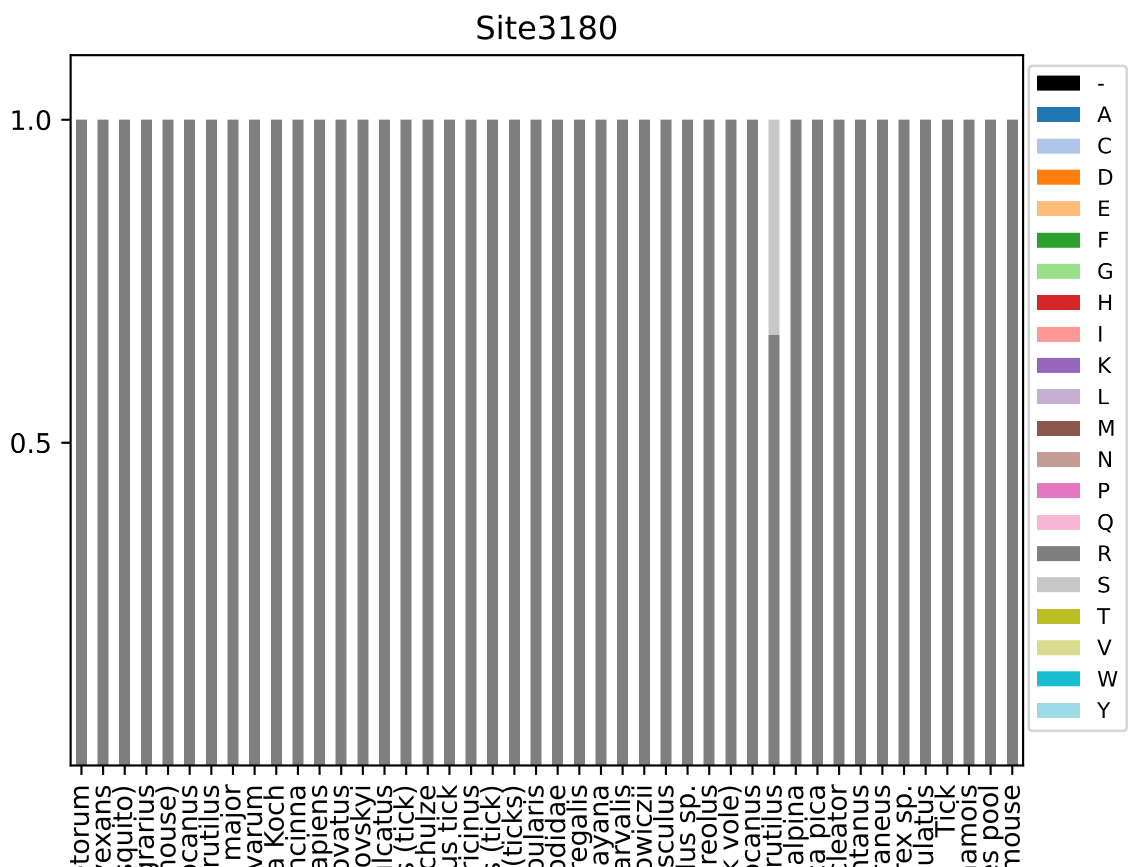
Site3180
1.0
0.5
-
A
C
D
E
F
G
H
I
K
L
M
N
P
Q
R
S
T
V
W
Y

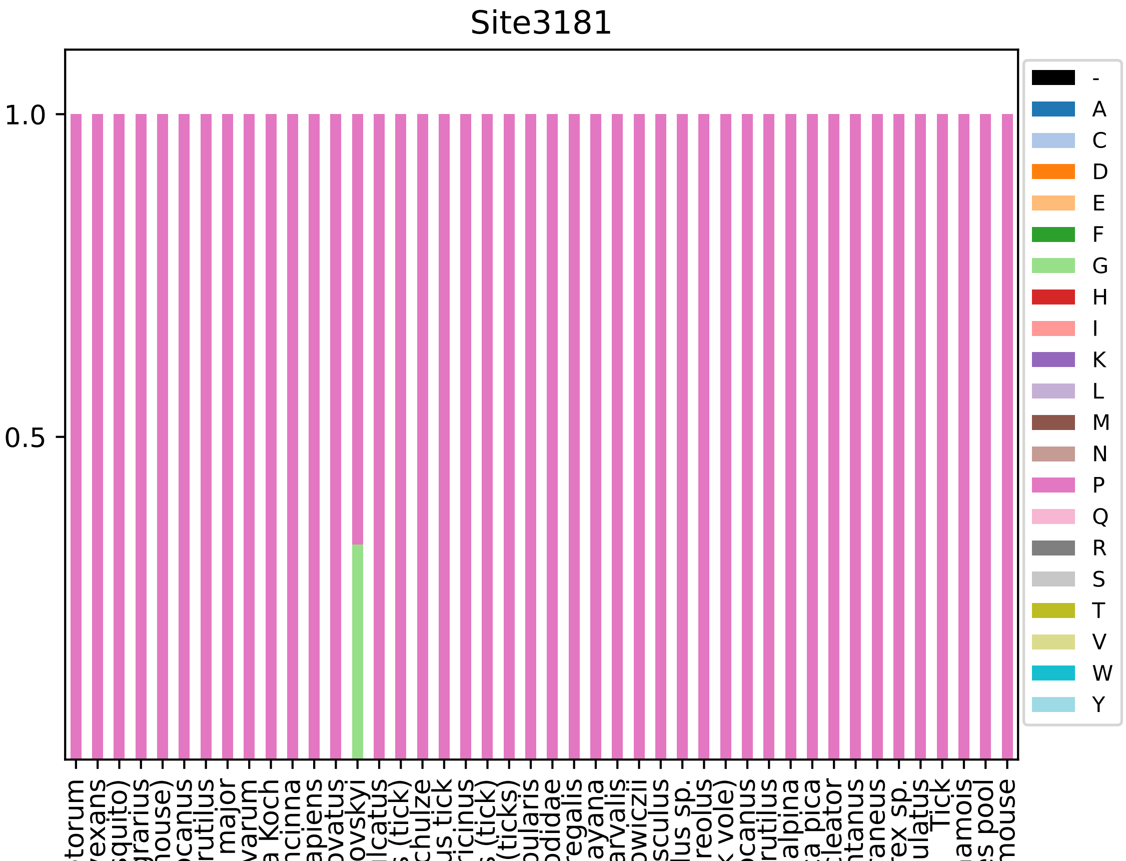

Site3181
1.0
0.5
-
A
C
D
E
F
G
H
I
K
L
M
N
P
Q
R
S
T
V
W
Y

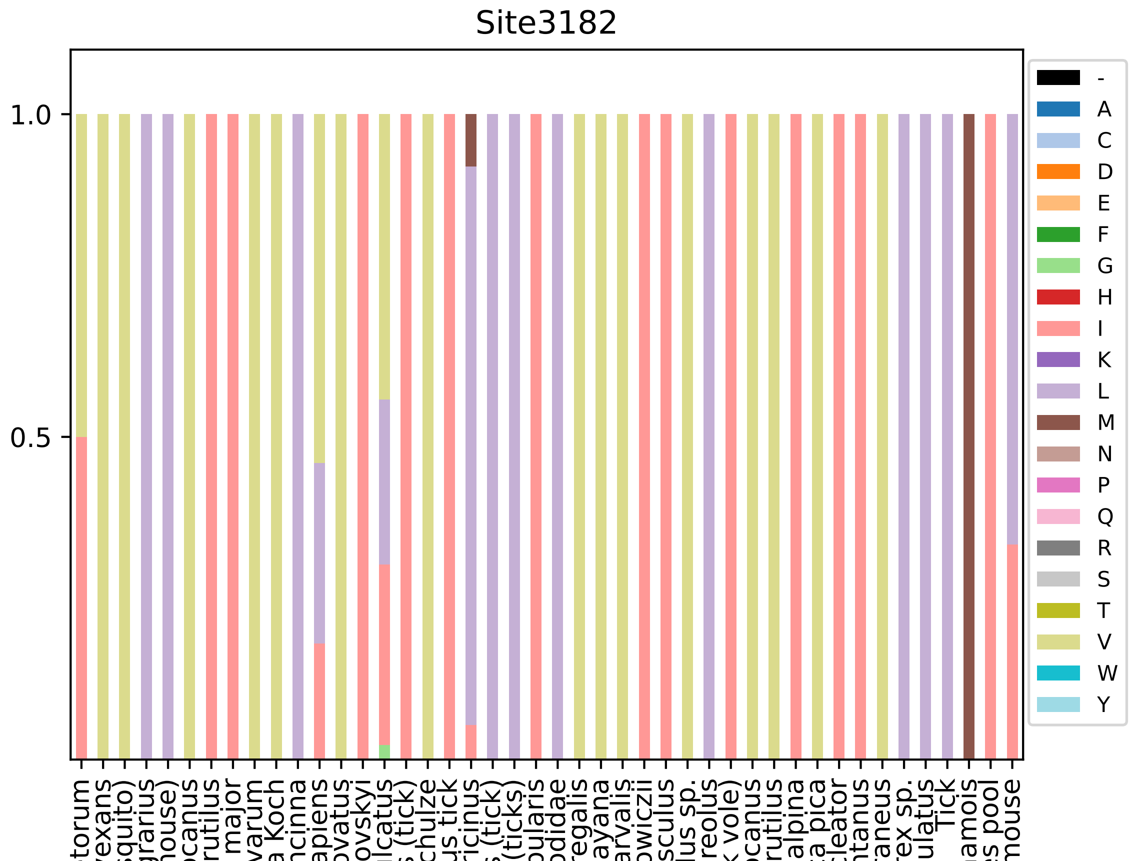
Site3182
1.0
0.5
-
A
C
D
E
F
G
H
I
K
L
M
N
P
Q
R
S
T
V
W
Y

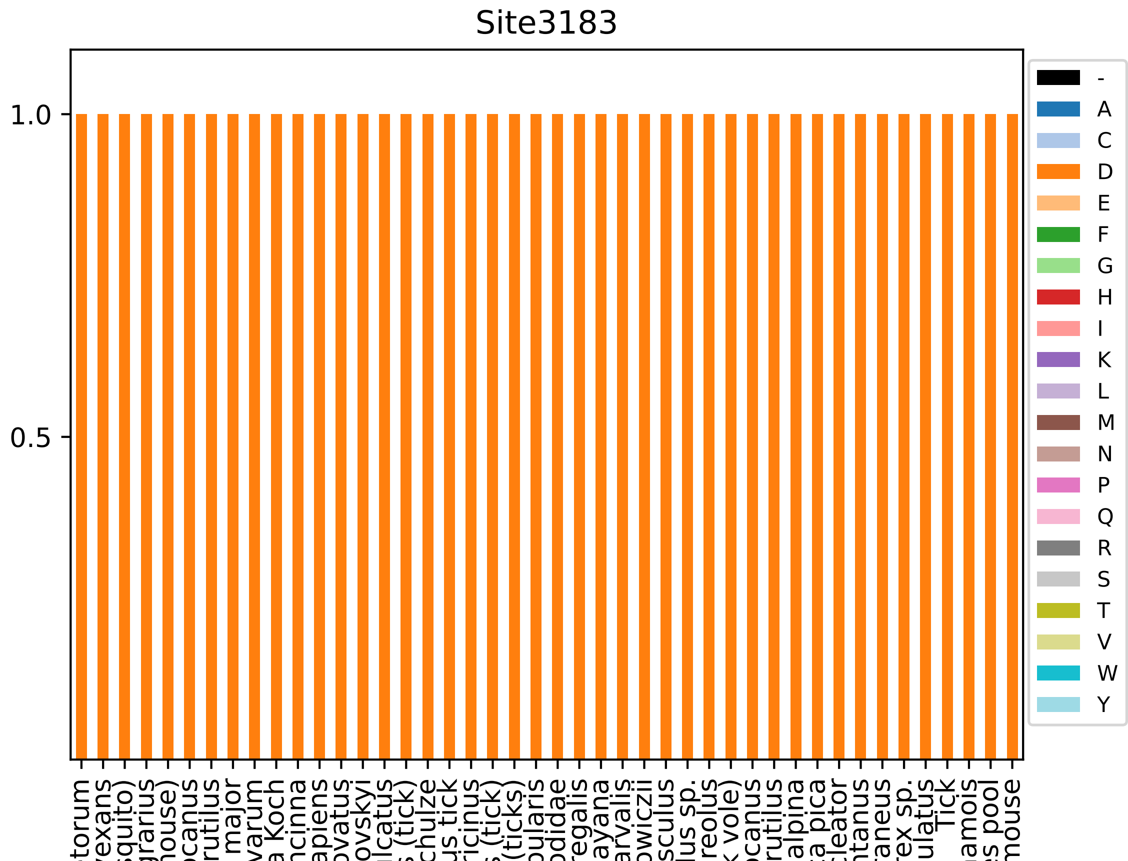
Site3183
1.0
0.5
-
A
C
D
E
F
G
H
I
K
L
M
N
P
Q
R
S
T
V
W
Y

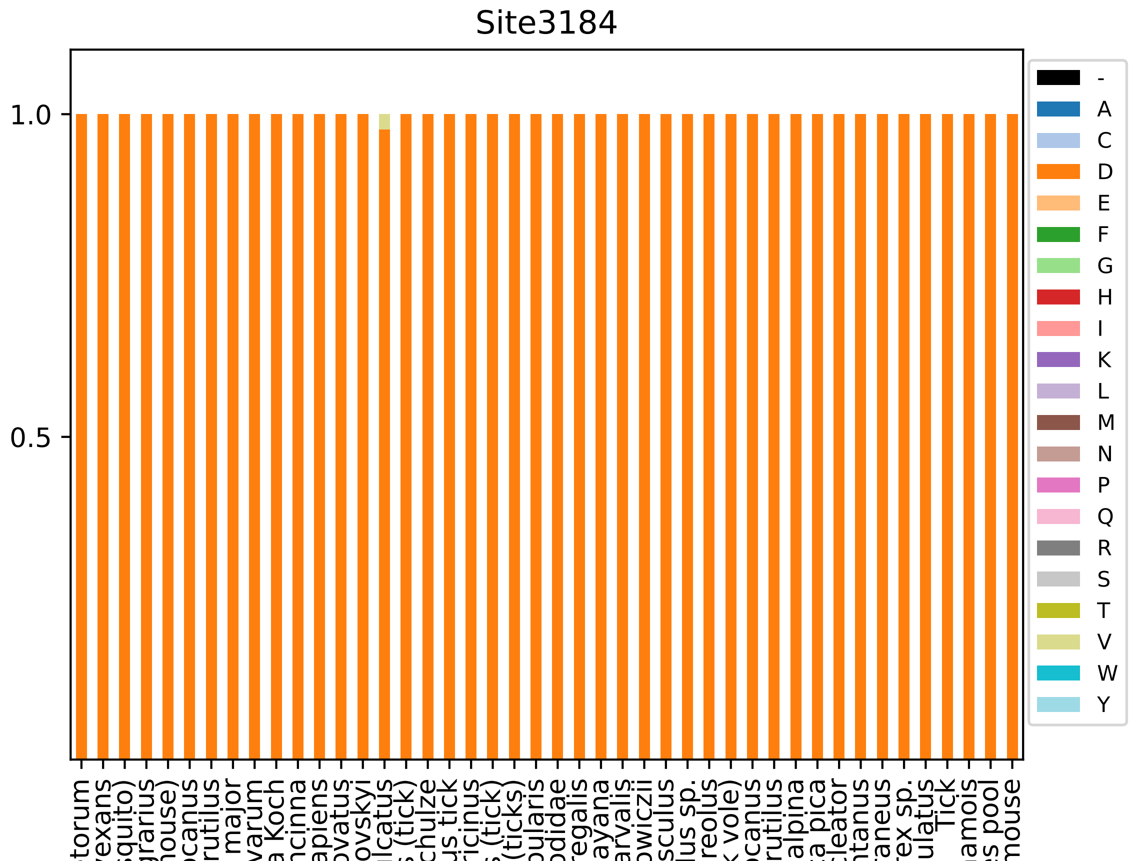
Site3184
1.0
0.5
-
A
C
D
E
F
G
H
I
K
L
M
N
P
Q
R
S
T
V
W
Y

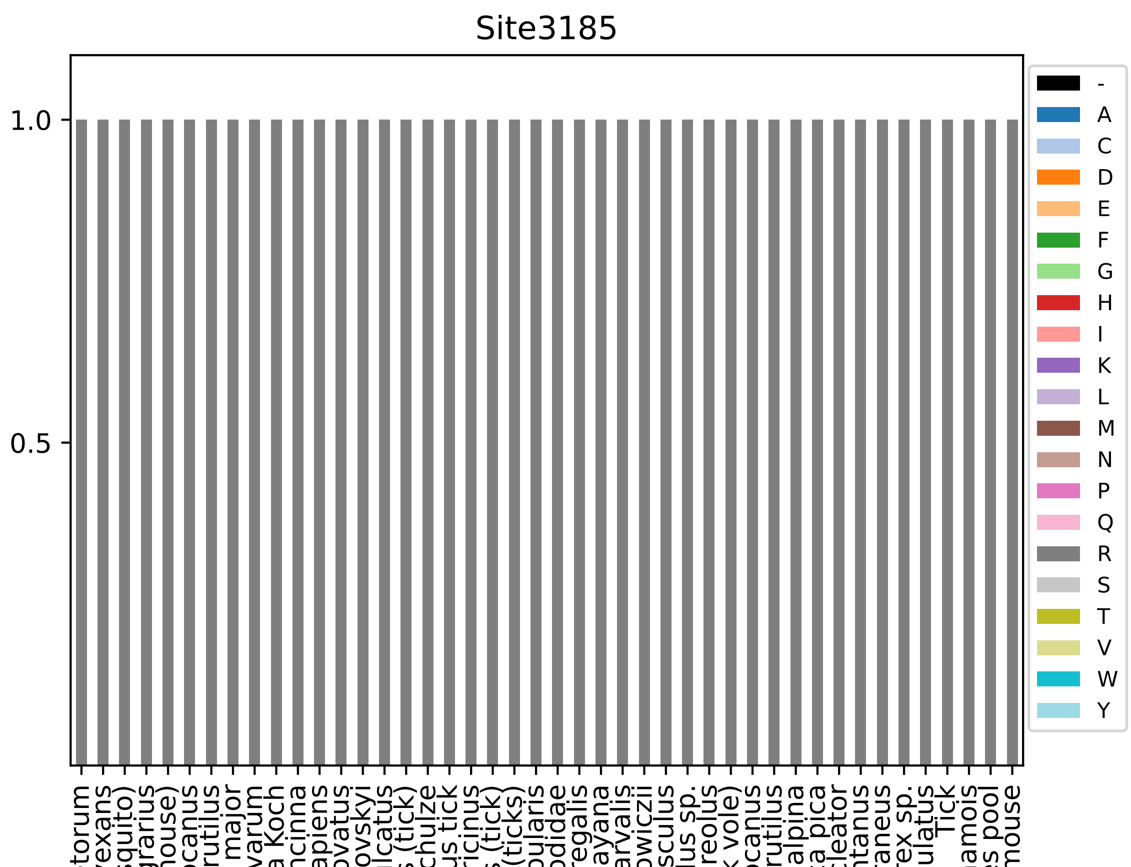
Site3185
1.0
0.5
-
A
C
D
E
F
G
H
I
K
L
M
N
P
Q
R
S
T
V
W
Y

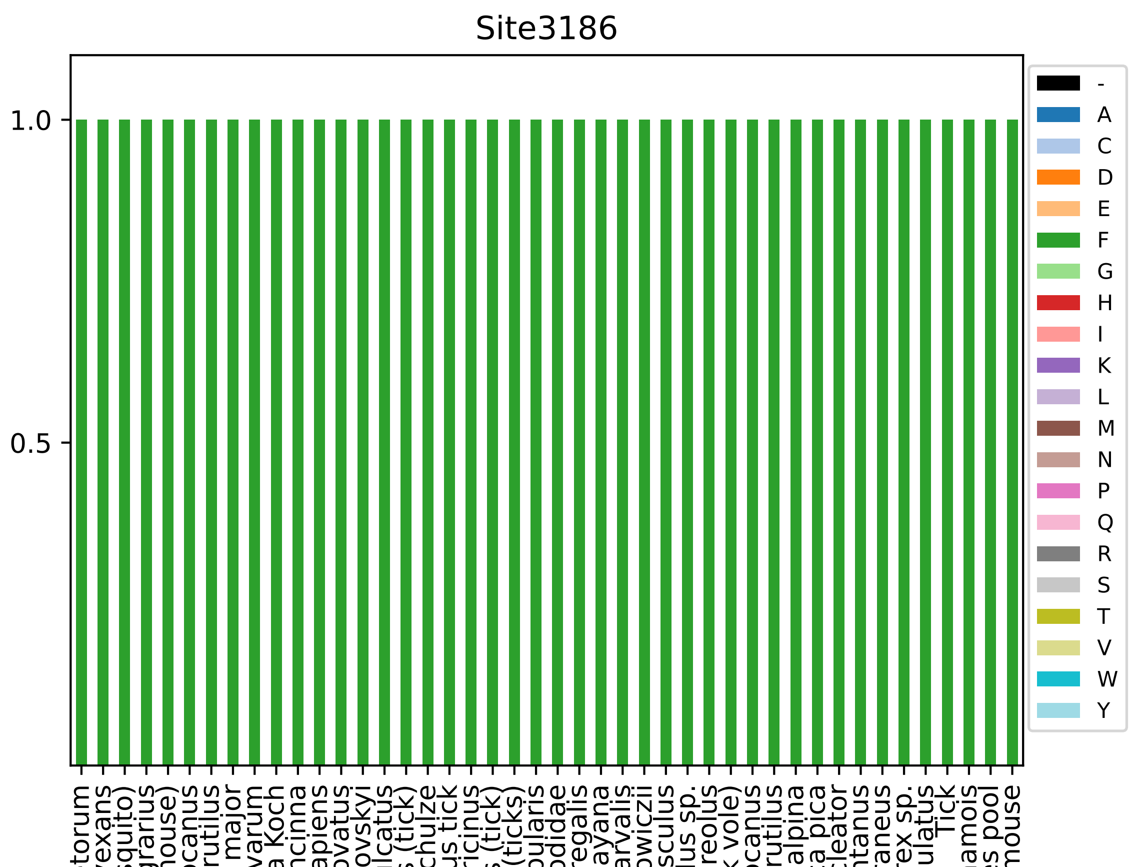

Site3186
1.0
0.5
-
A
C
D
E
F
G
H
I
K
L
M
N
P
Q
R
S
T
V
W
Y

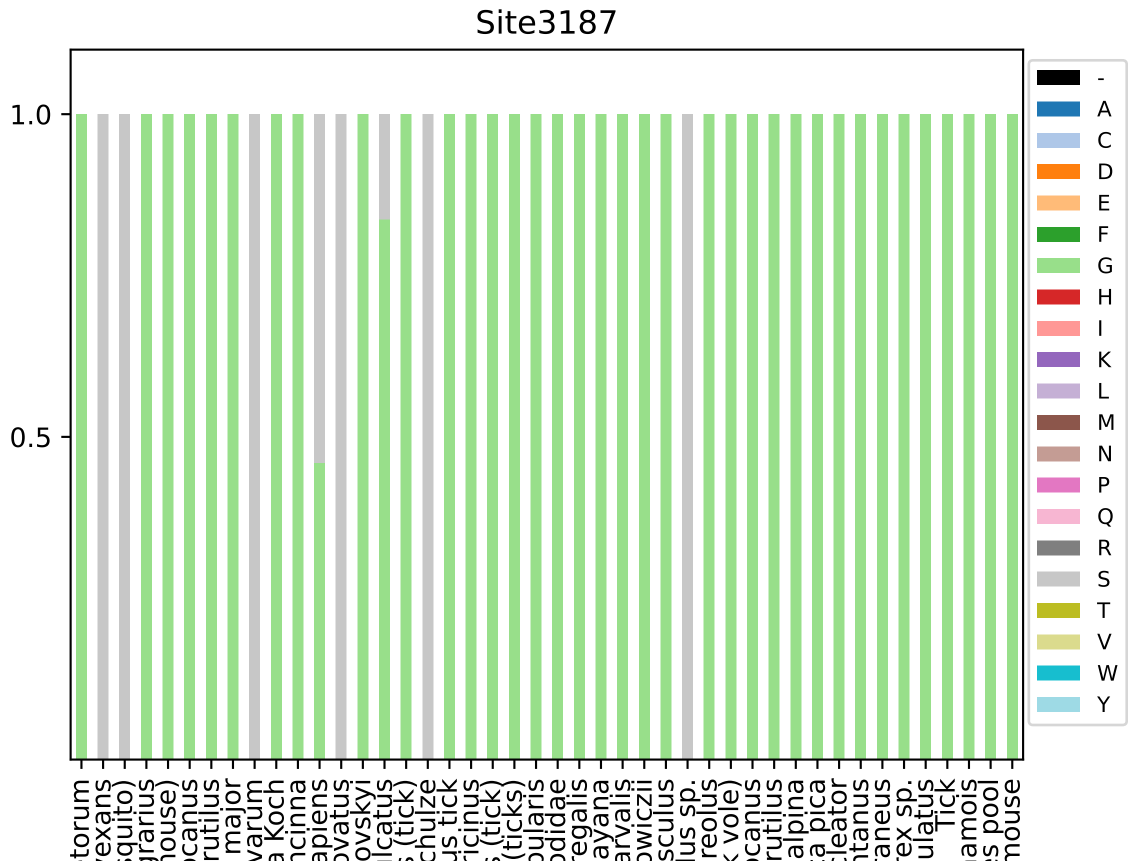

Site3187
1.0
0.5
-
A
C
D
E
F
G
H
I
K
L
M
N
P
Q
R
S
T
V
W
Y

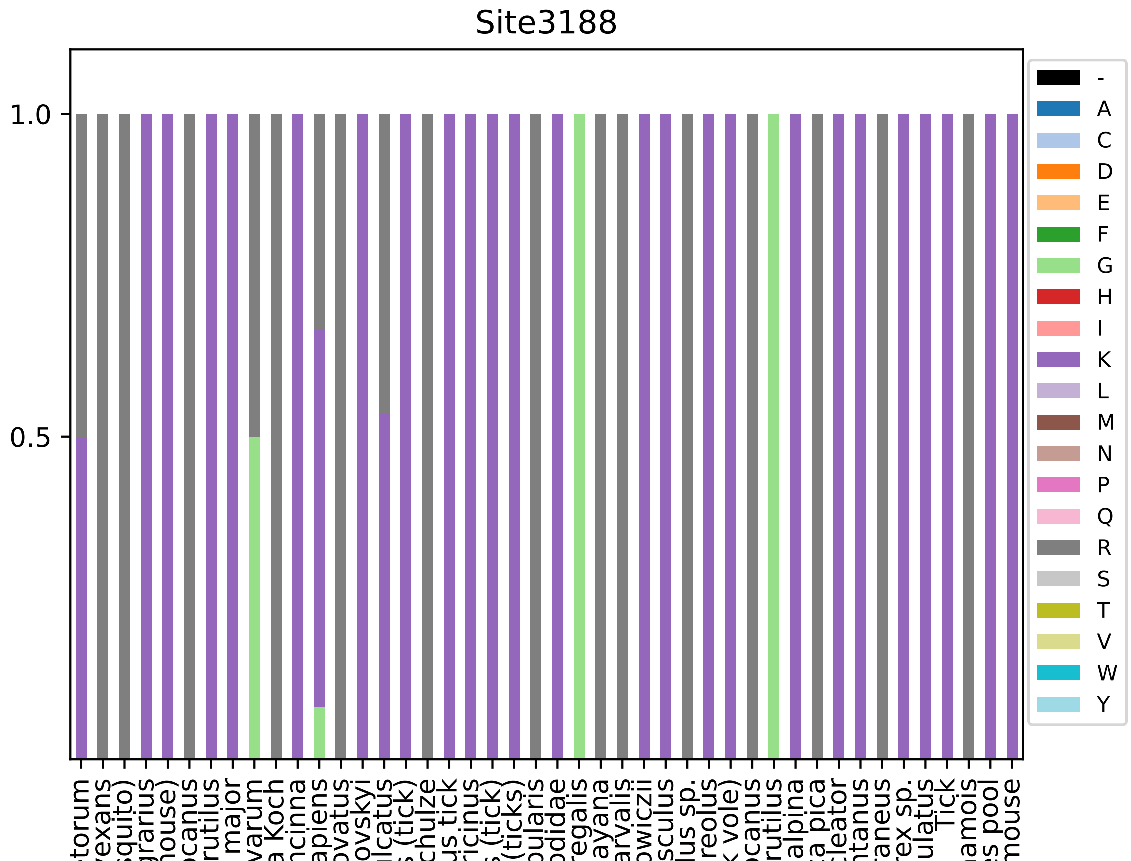
Site3188
1.0
0.5
-
A
C
D
E
F
G
H
I
K
L
M
N
P
Q
R
S
T
V
W
Y

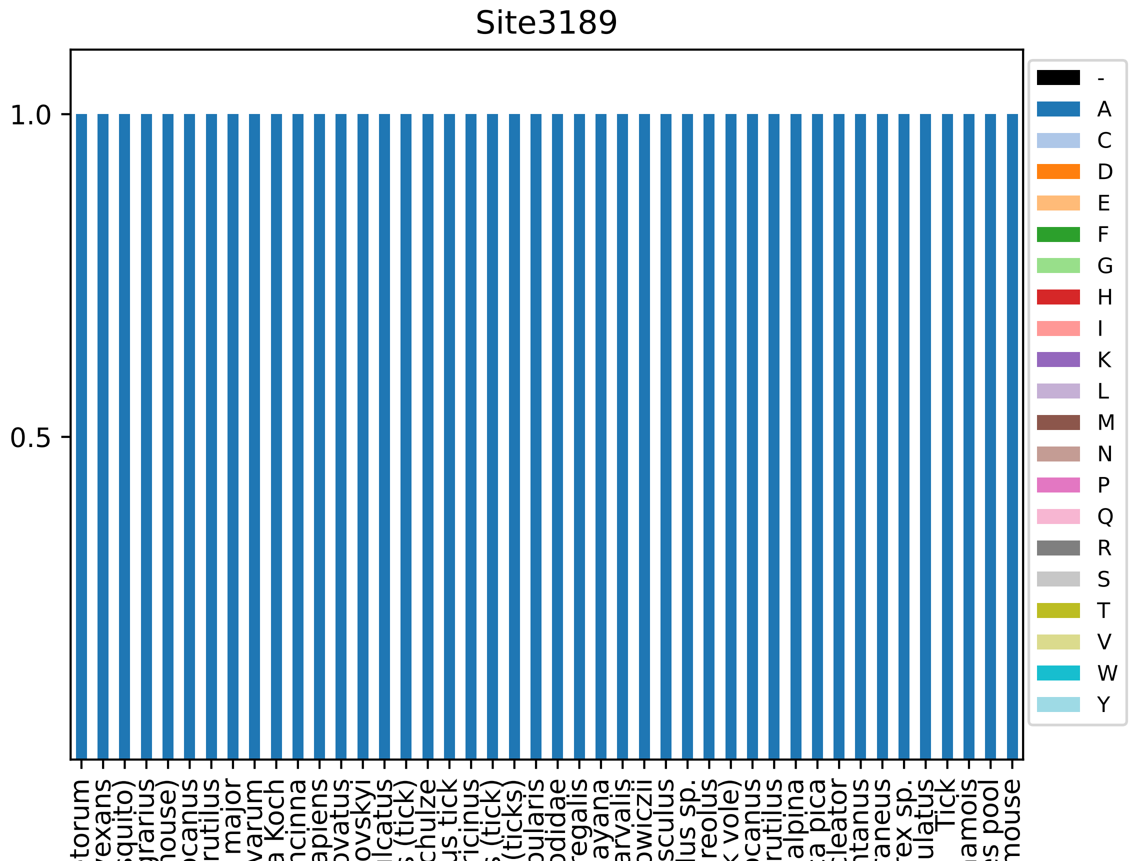
Site3189
1.0
0.5
-
A
C
D
E
F
G
H
I
K
L
M
N
P
Q
R
S
T
V
W
Y

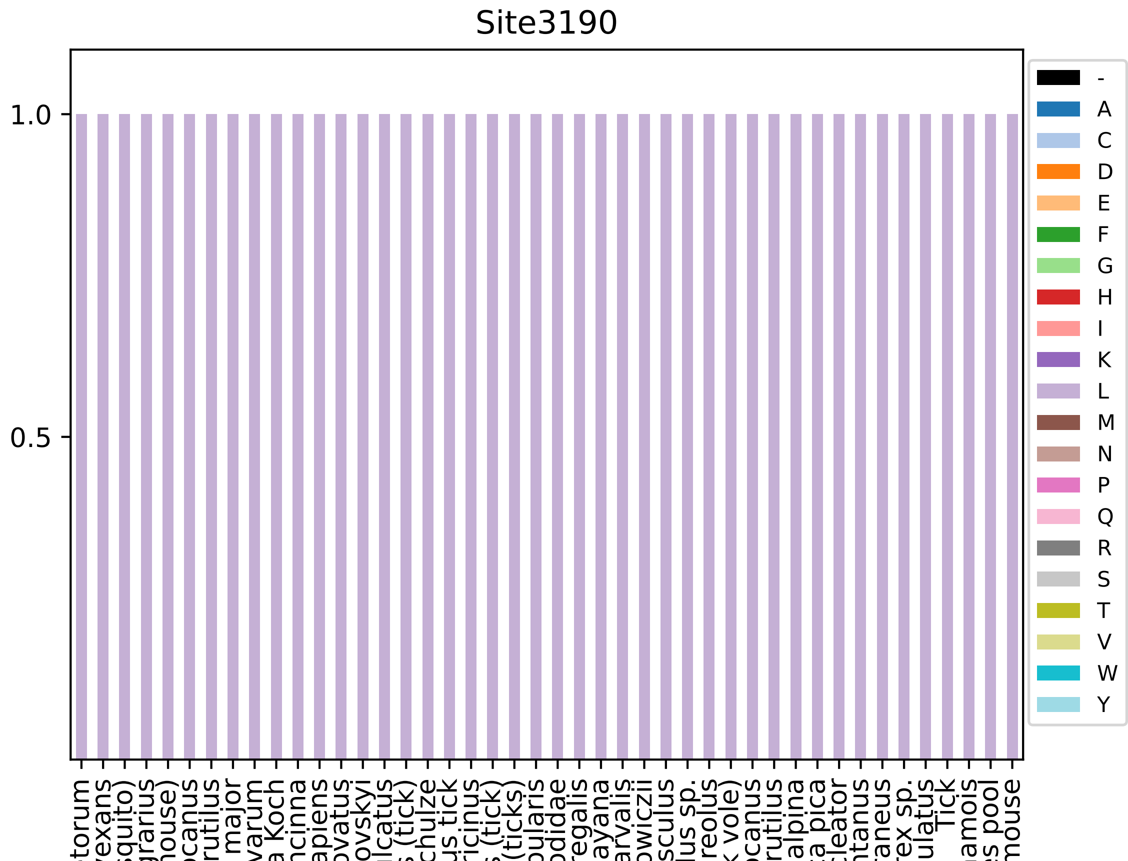

Site3190
1.0
0.5
-
A
C
D
E
F
G
H
I
K
L
M
N
P
Q
R
S
T
V
W
Y

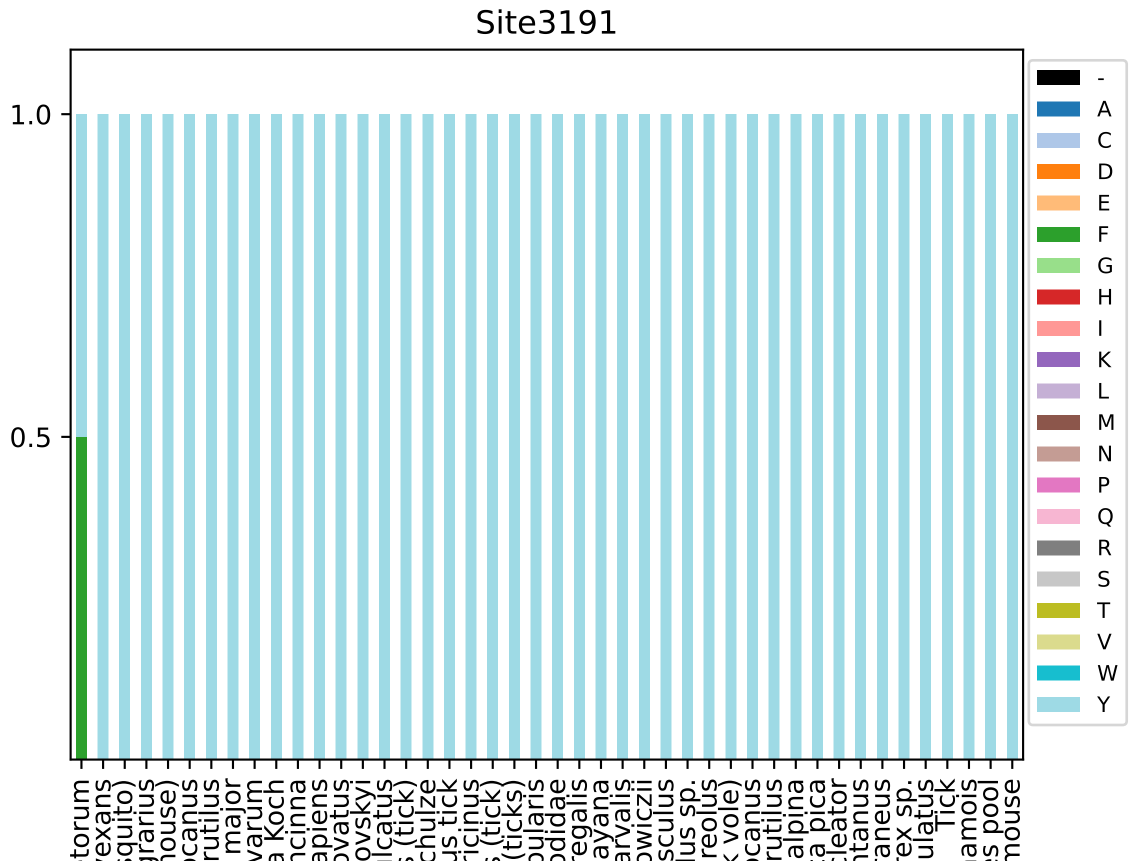
Site3191
1.0
0.5
-
A
C
D
E
F
G
H
I
K
L
M
N
P
Q
R
S
T
V
W
Y
torum
vexans
squito)
grarius
nouse)
ocanus
rutilus
major
varum
a Koch
ncinna
apiens
ovatus
ovskyi
ulcatus
s (tick)
chulze
us tick
ricinus
s (tick)
(ticks)
pularis
podidae
regalis
ayana
arvalis
owiczii
sculus
lus sp.
reolus
k vole)
ocanus
rutilus
alpina
a pica
cleator
ntanus
raneus
rex sp.
ulatus
Tick
amois
es pool
mouse

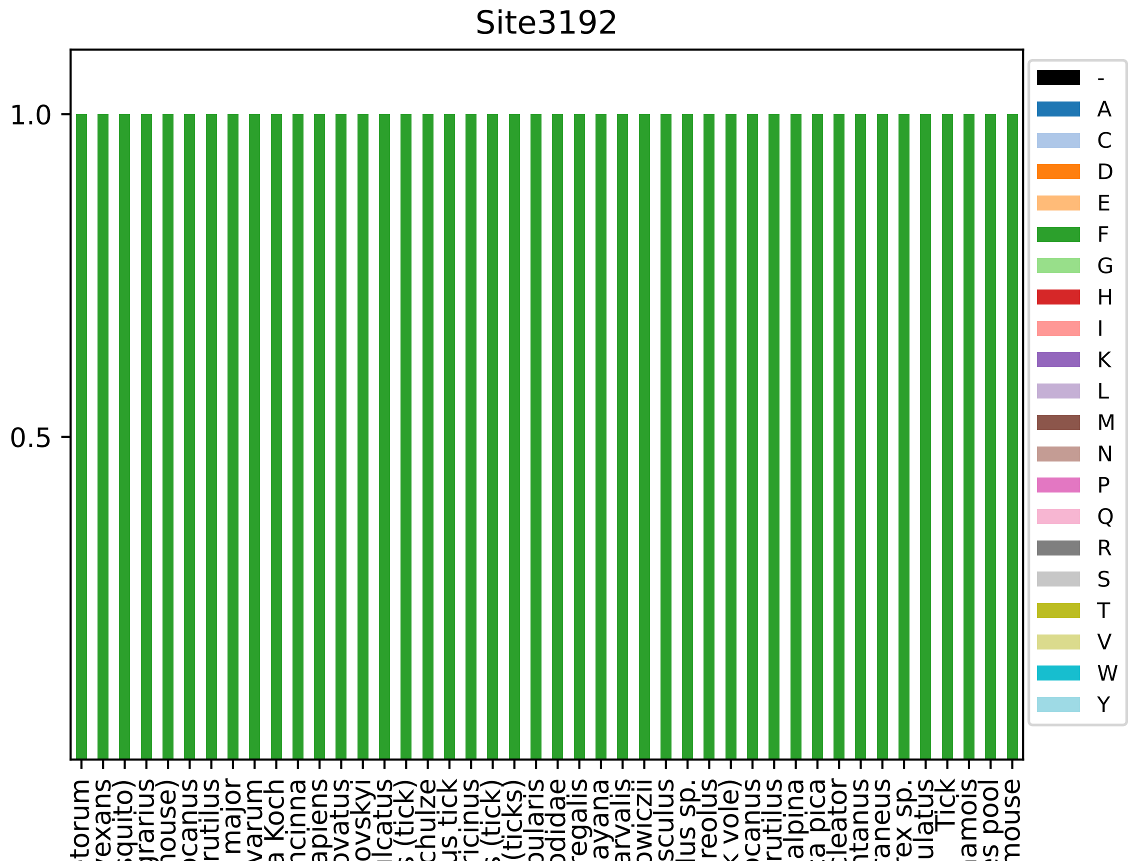
Site3192
1.0
0.5
-
A
C
D
E
F
G
H
I
K
L
M
N
P
Q
R
S
T
V
W
Y

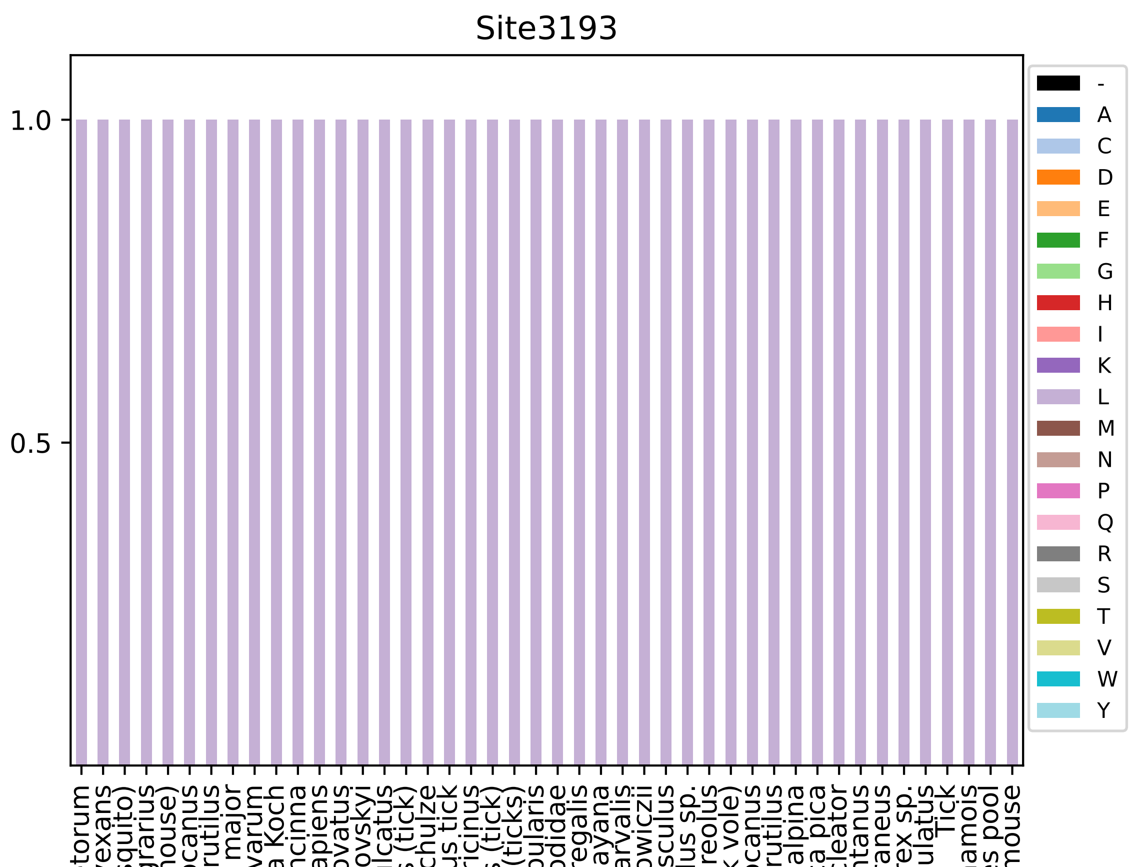
Site3193
1.0
0.5
-
A
C
D
E
F
G
H
I
K
L
M
N
P
Q
R
S
T
V
W
Y

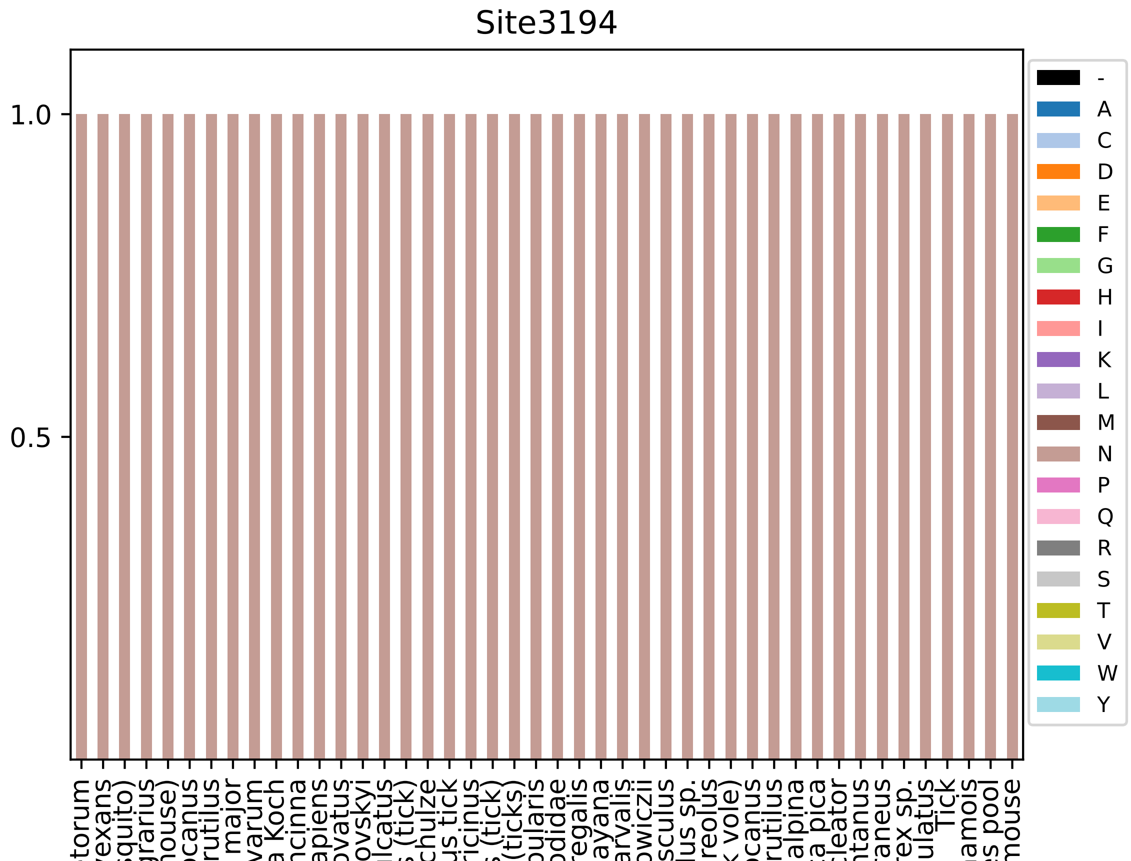

Site3194
1.0
0.5
-
A
C
D
E
F
G
H
I
K
L
M
N
P
Q
R
S
T
V
W
Y

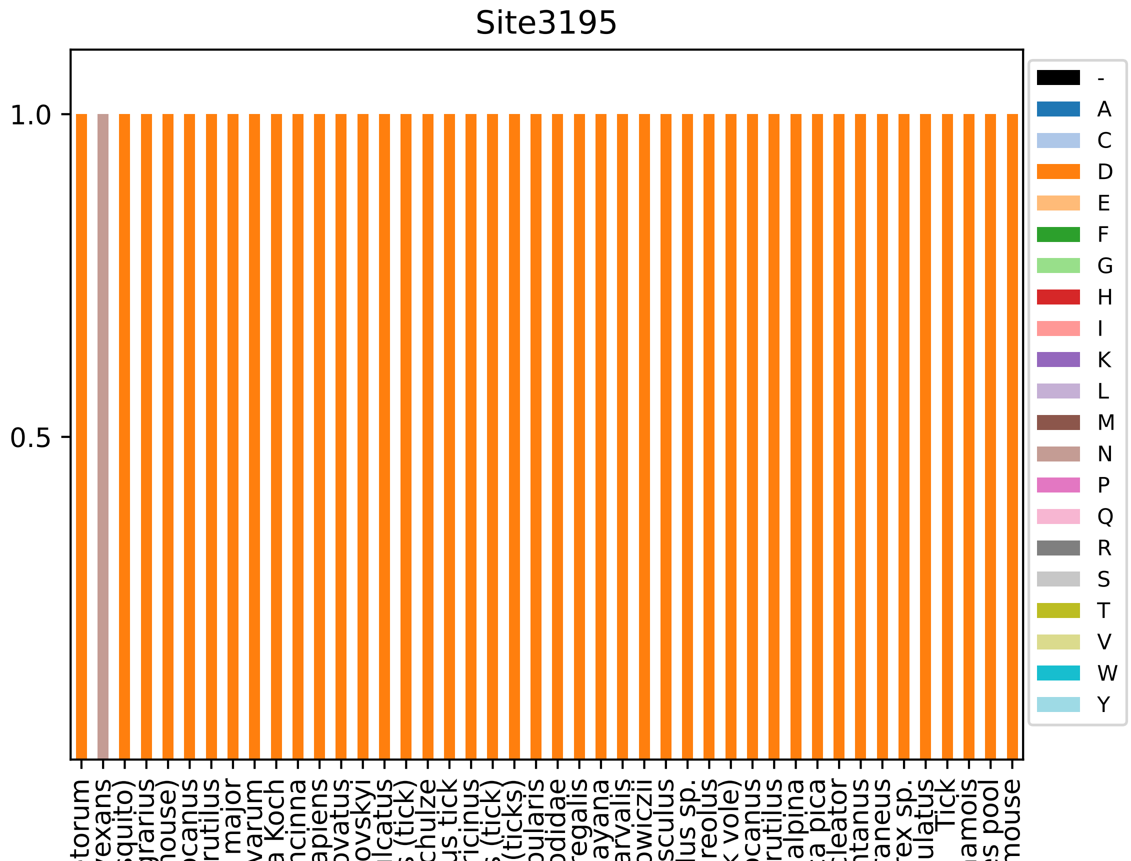
Site3195
1.0
0.5
-
A
C
D
E
F
G
H
I
K
L
M
N
P
Q
R
S
T
V
W
Y

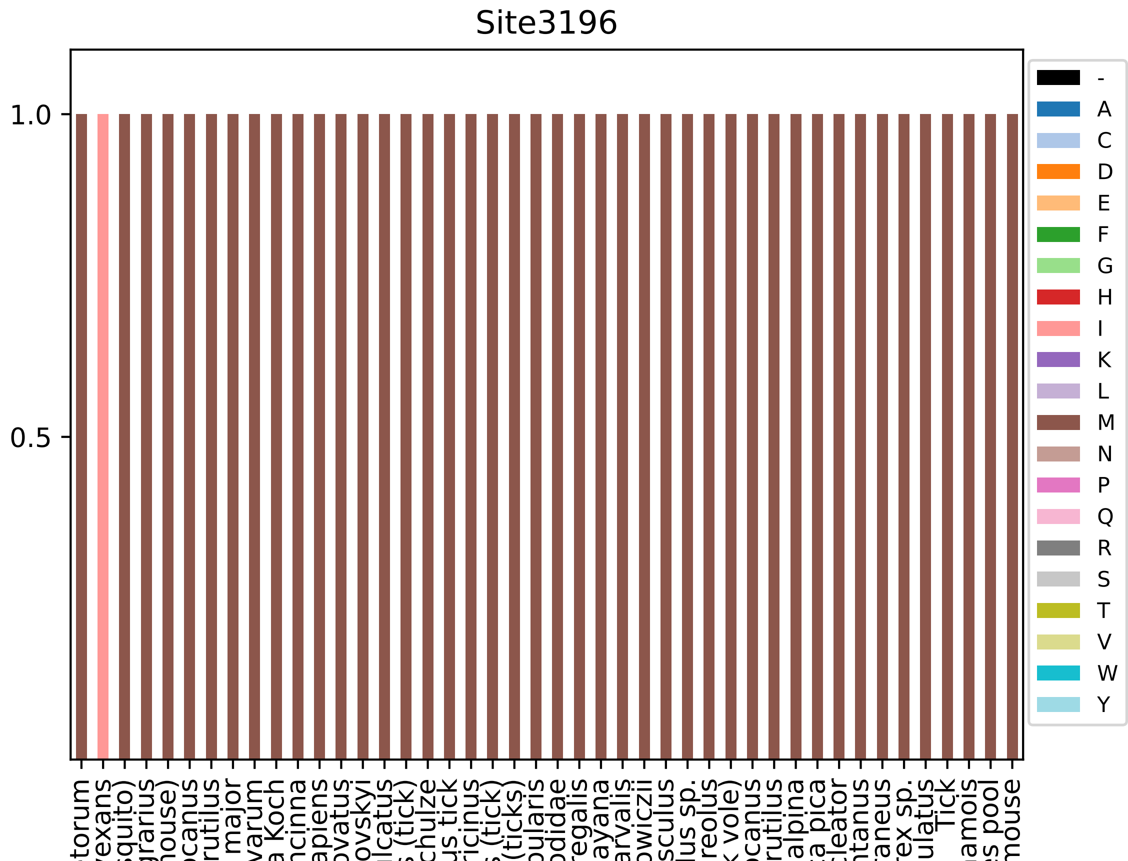
Site3196
1.0
0.5
-
A
C
D
E
F
G
H
I
K
L
M
N
P
Q
R
S
T
V
W
Y

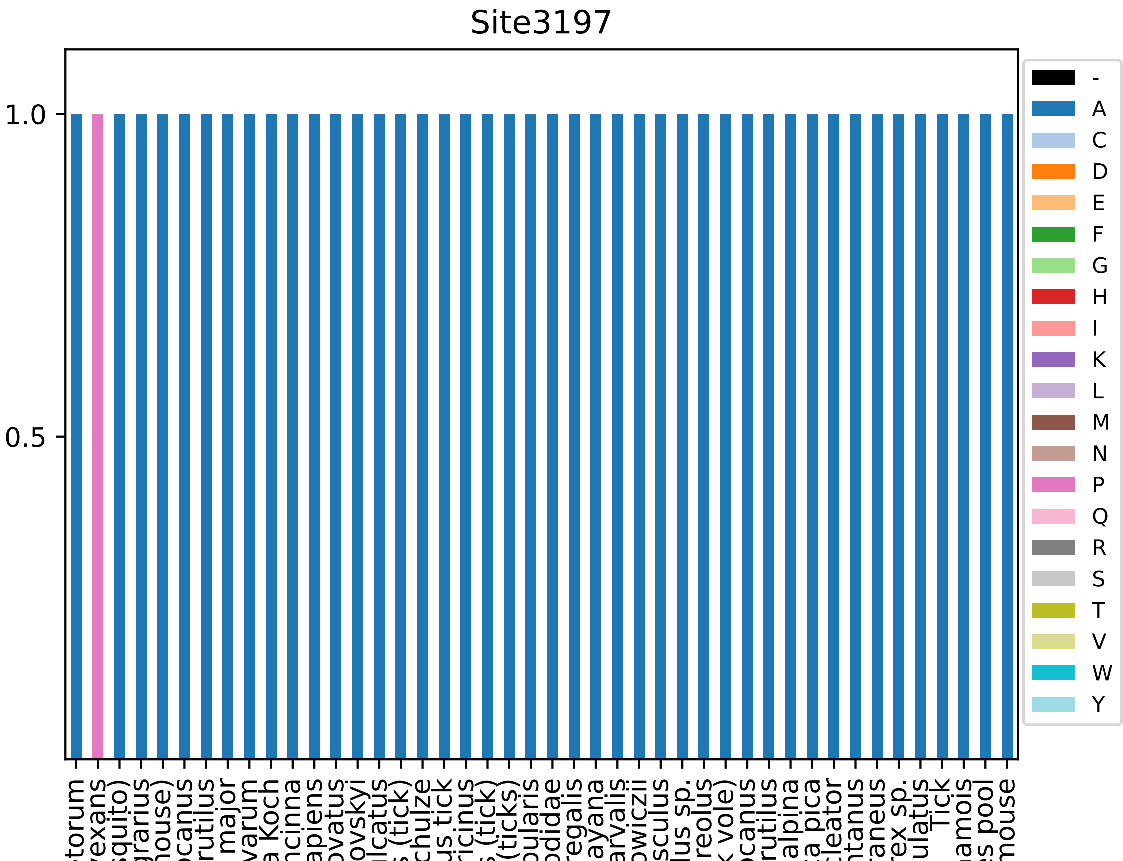
Site3197
1.0
0.5
-
A
C
D
E
F
G
H
I
K
L
M
N
P
Q
R
S
T
V
W
Y

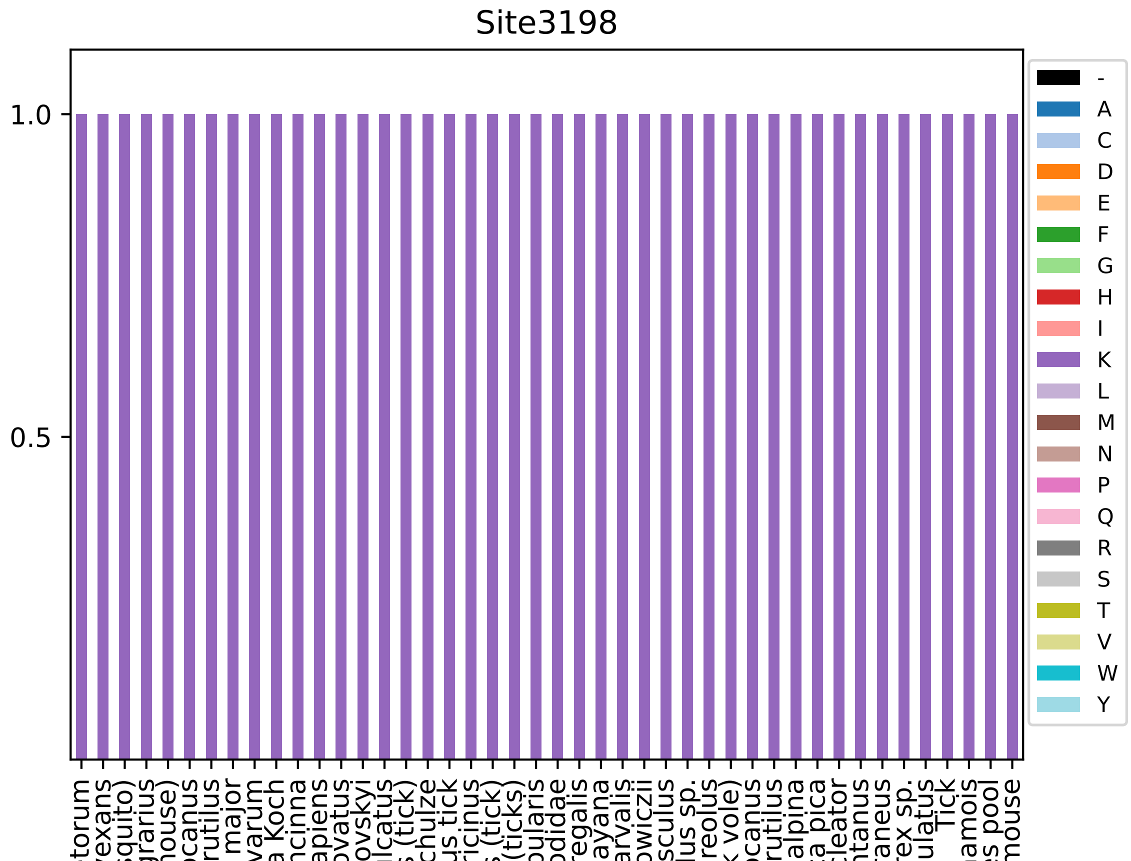
Site3198
1.0
0.5
-
A
C
D
E
F
G
H
I
K
L
M
N
P
Q
R
S
T
V
W
Y

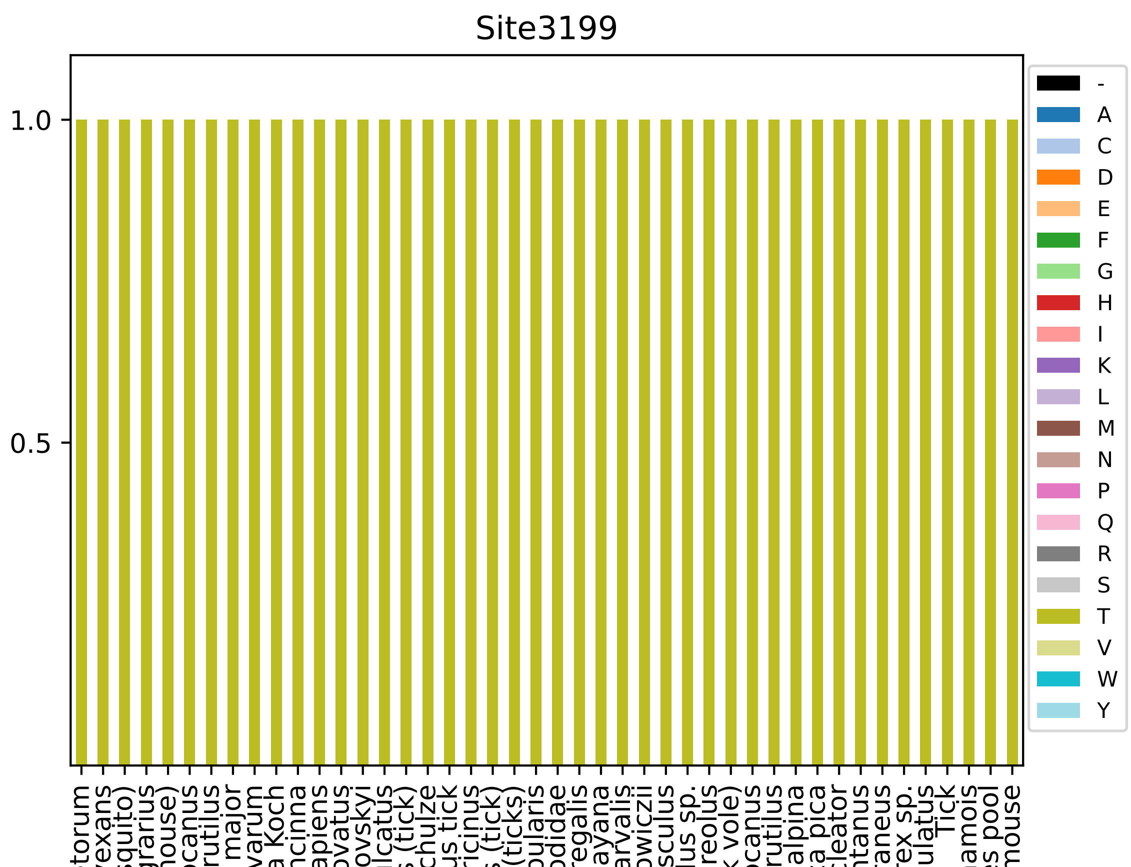
Site3199
1.0
0.5
-
A
C
D
E
F
G
H
I
K
L
M
N
P
Q
R
S
T
V
W
Y
torum
vexans
squito)
grarius
mouse)
ocanus
rutilus
major
varum
a Koch
ncinna
apiens
ovatus
ovskyi
lcatus
s (tick)
chulze
us tick
ricinus
s (tick)
(ticks)
pularis
odidae
regalis
ayana
arvalis
owiczii
sculus
lus sp.
reolus
k vole)
ocanus
rutilus
alpina
a pica
cleator
ntanus
aneus
rex sp.
ulatus
Tick
amois
es pool
mouse

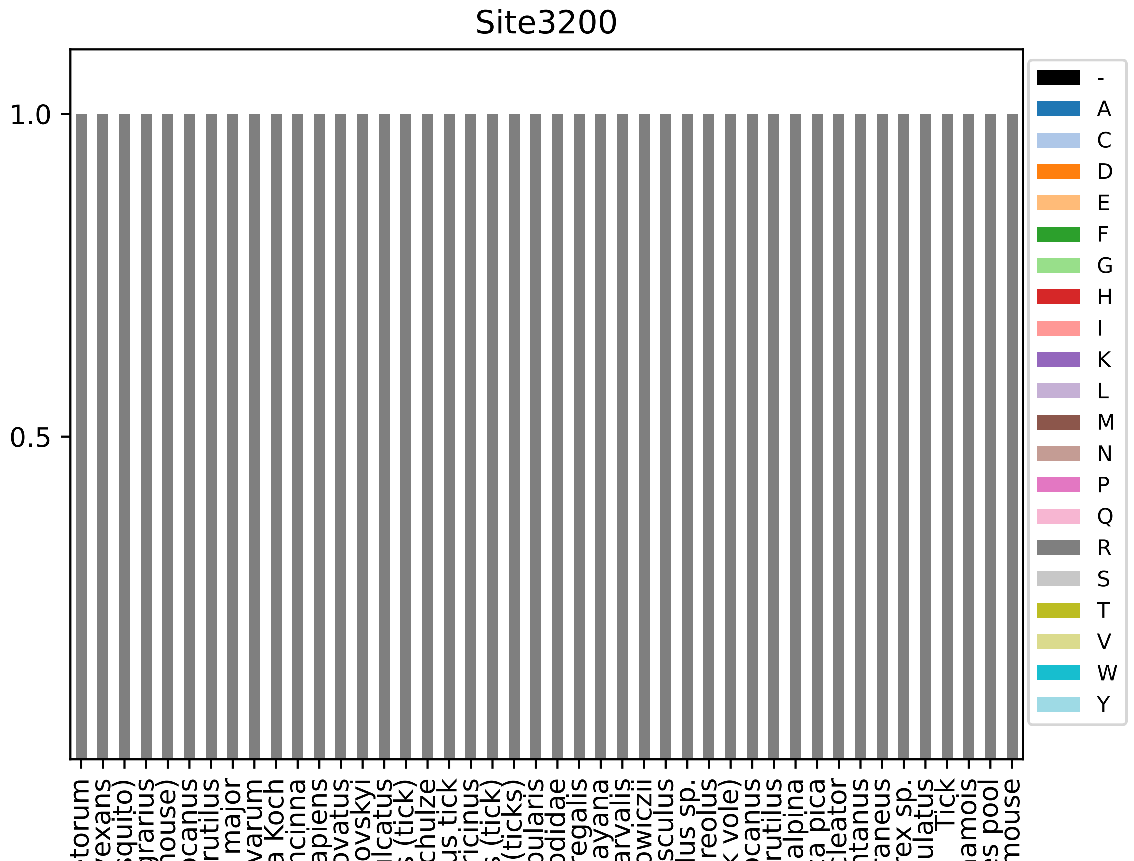
Site3200
1.0
0.5
-
A
C
D
E
F
G
H
I
K
L
M
N
P
Q
R
S
T
V
W
Y

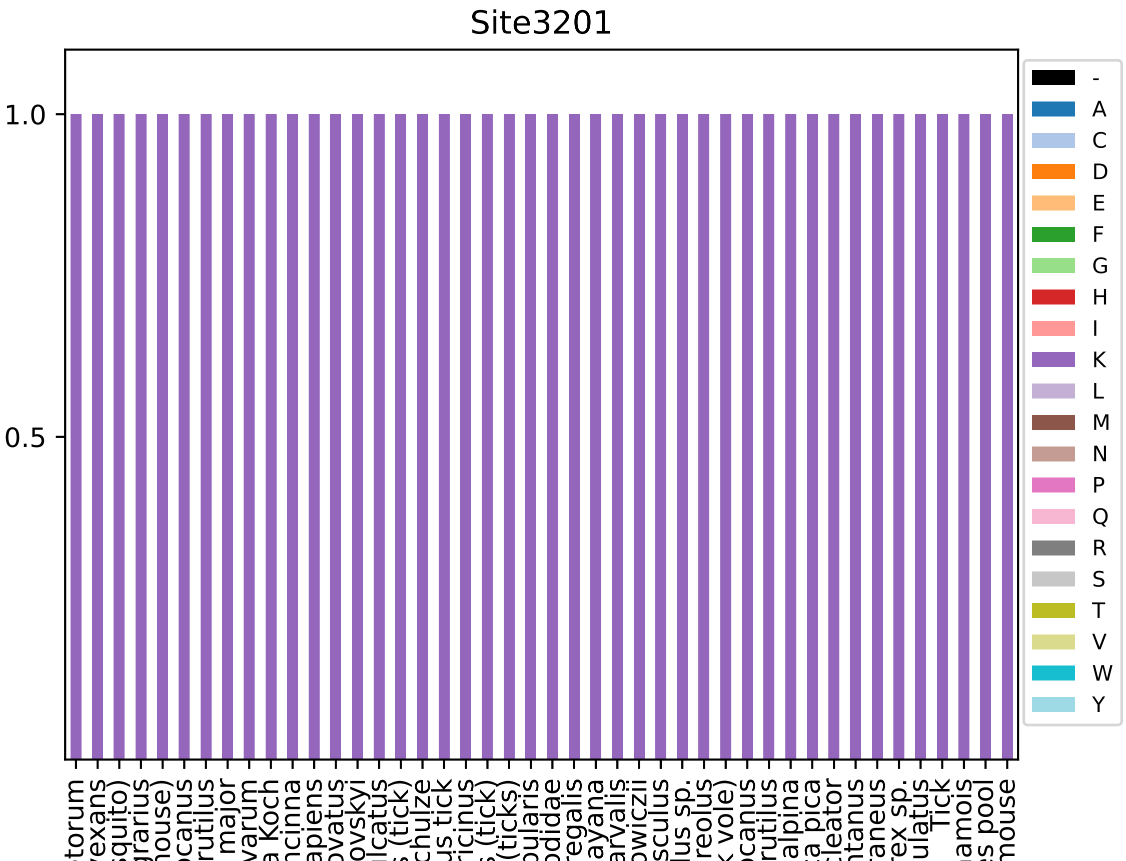
Site3201
1.0
0.5
-
A
C
D
E
F
G
H
I
K
L
M
N
P
Q
R
S
T
V
W
Y

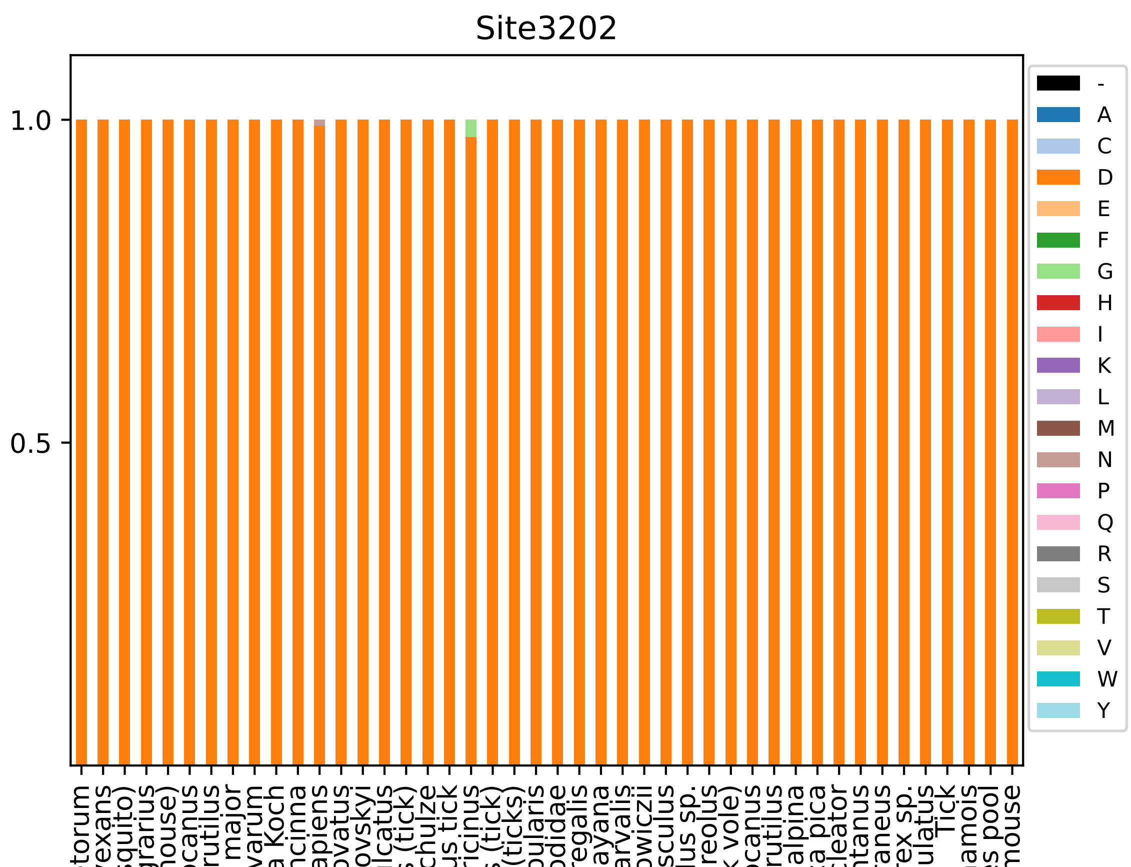

Site3202
1.0
0.5
-
A
C
D
E
F
G
H
I
K
L
M
N
P
Q
R
S
T
V
W
Y

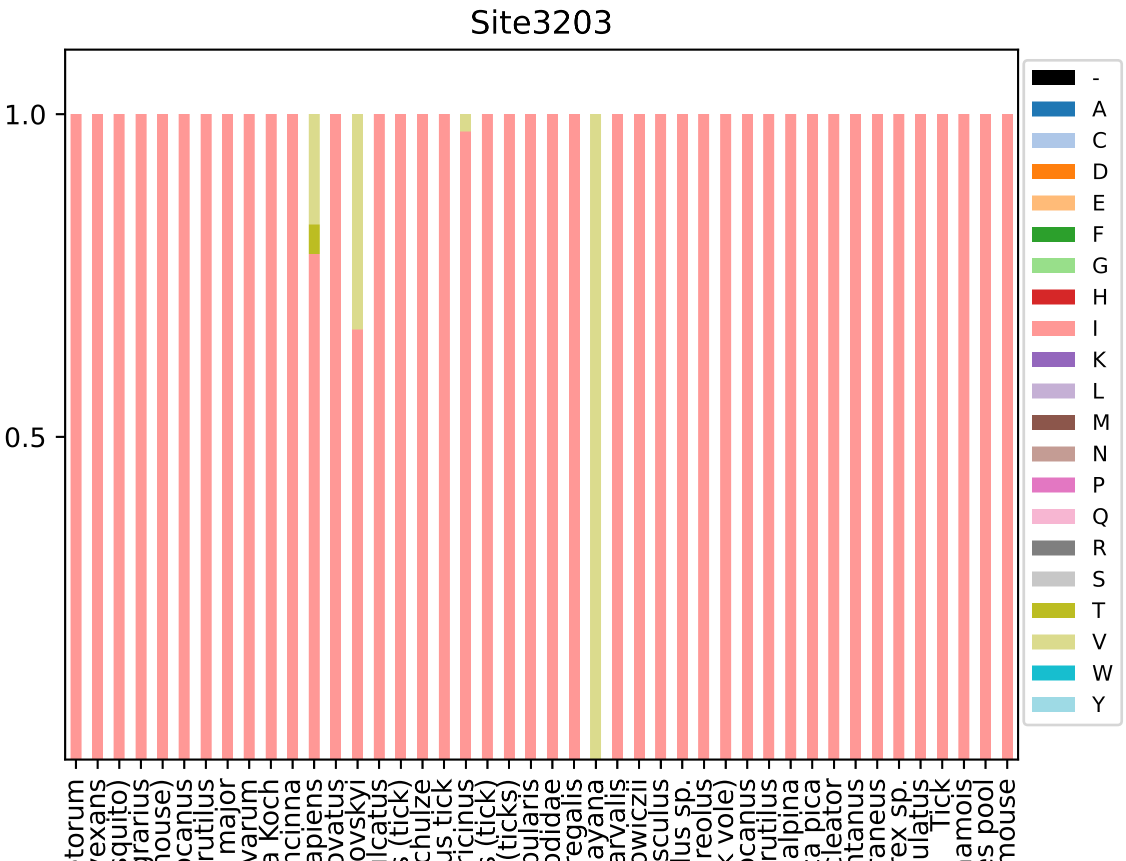

Site3203
1.0
0.5
-
A
C
D
E
F
G
H
I
K
L
M
N
P
Q
R
S
T
V
W
Y

Site3204

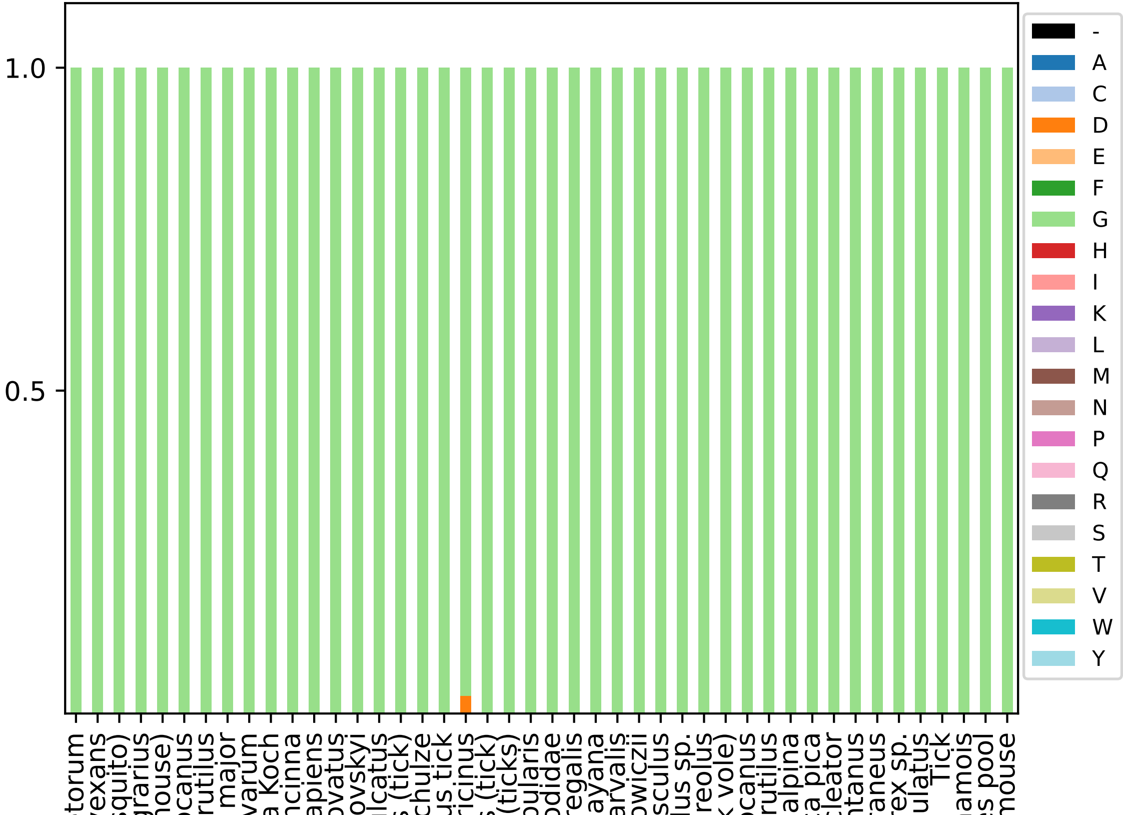

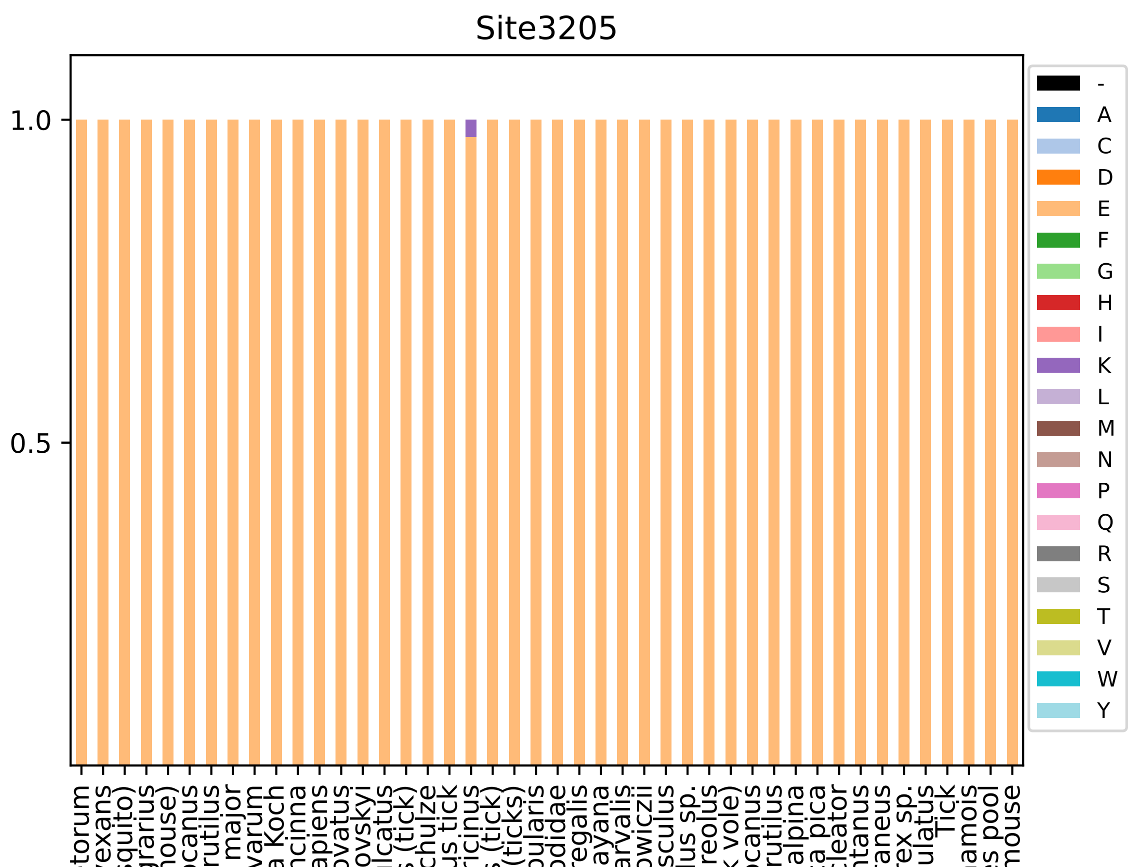
Site3205
1.0
0.5
-
A
C
D
E
F
G
H
I
K
L
M
N
P
Q
R
S
T
V
W
Y

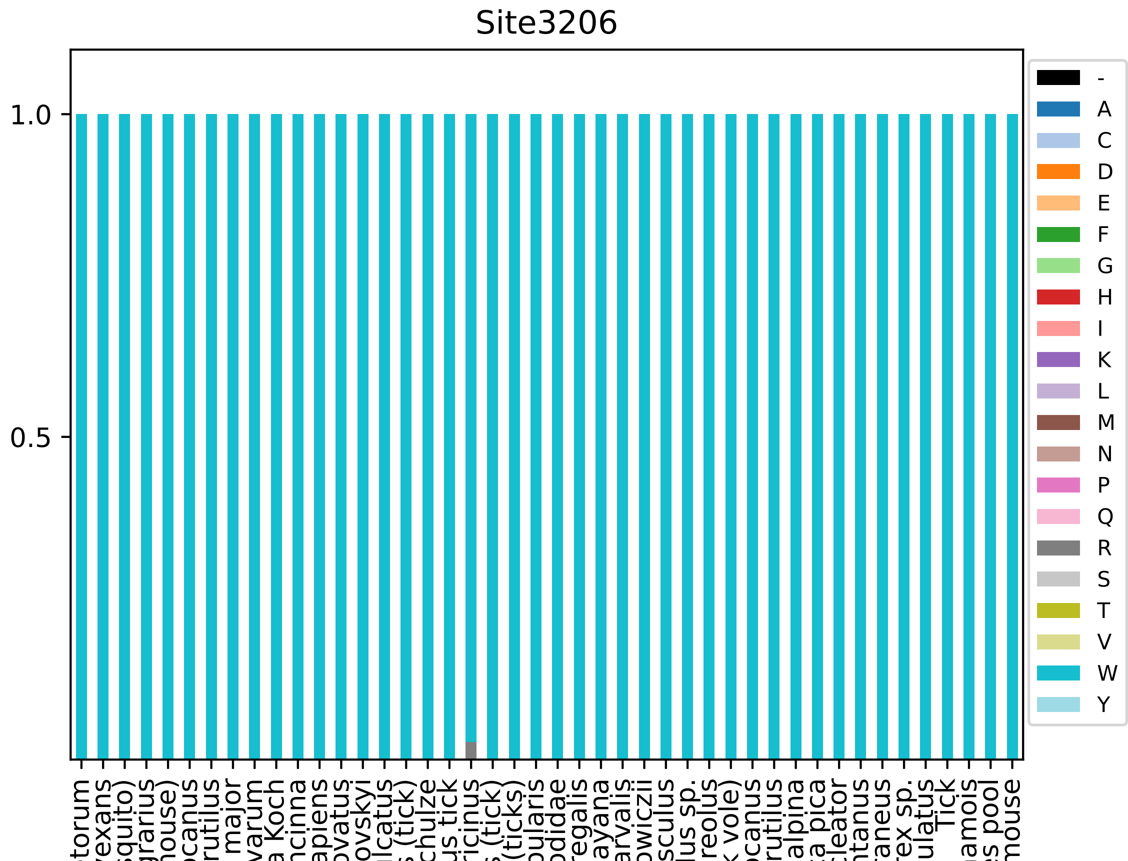

Site3206
1.0
0.5
-
A
C
D
E
F
G
H
I
K
L
M
N
P
Q
R
S
T
V
W
Y

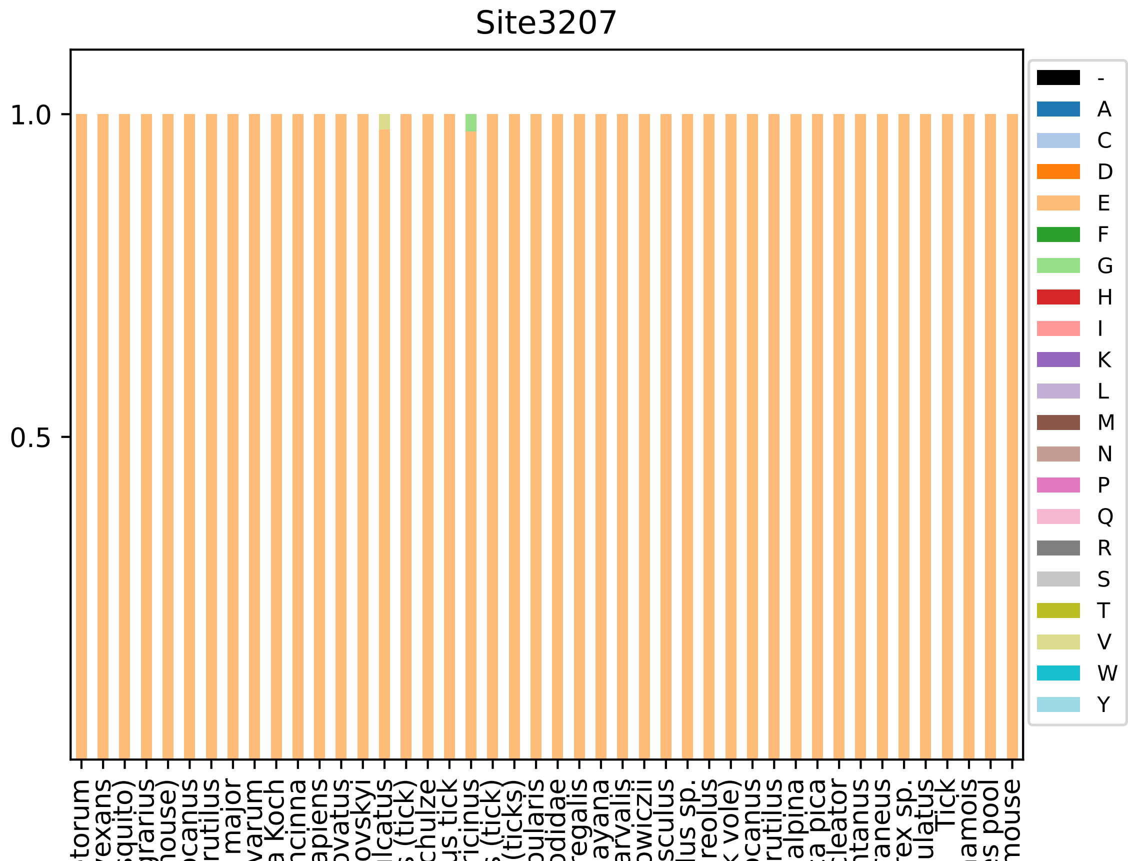

Site3207
1.0
0.5
-
A
C
D
E
F
G
H
I
K
L
M
N
P
Q
R
S
T
V
W
Y

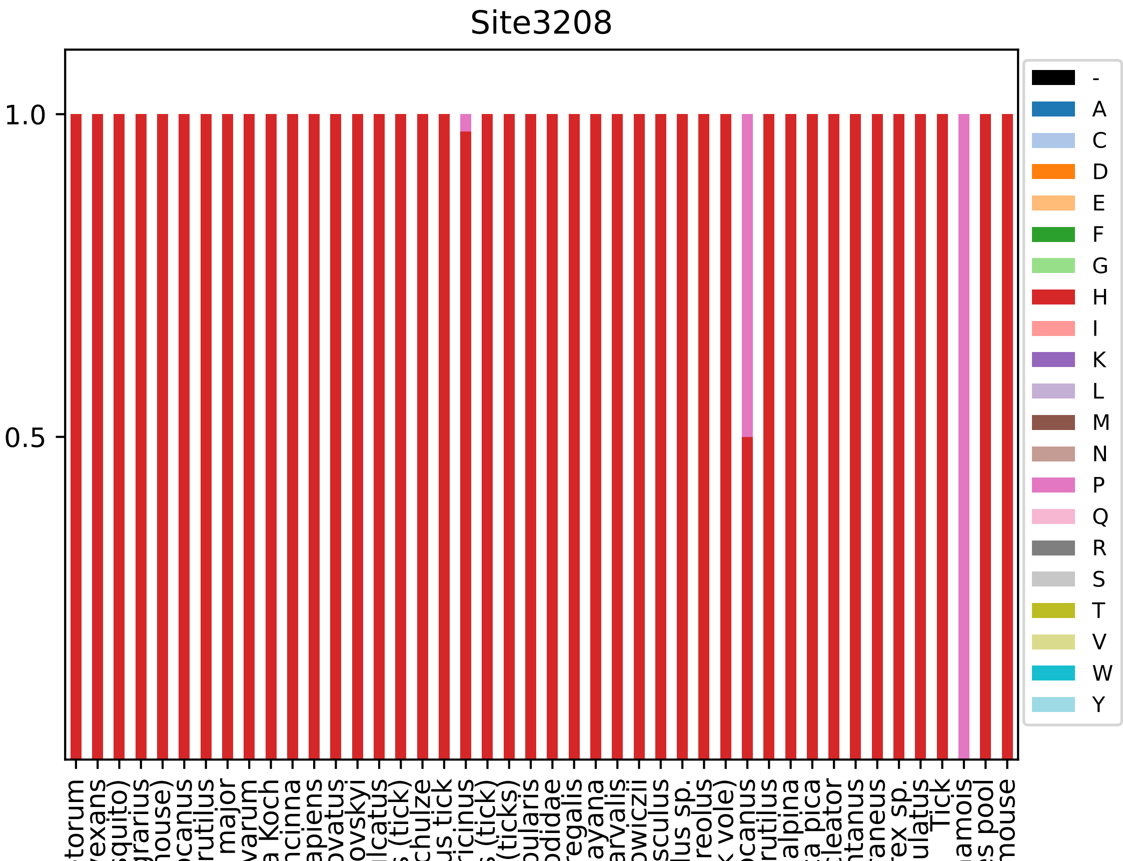

Site3208
1.0
0.5
-
A
C
D
E
F
G
H
I
K
L
M
N
P
Q
R
S
T
V
W
Y

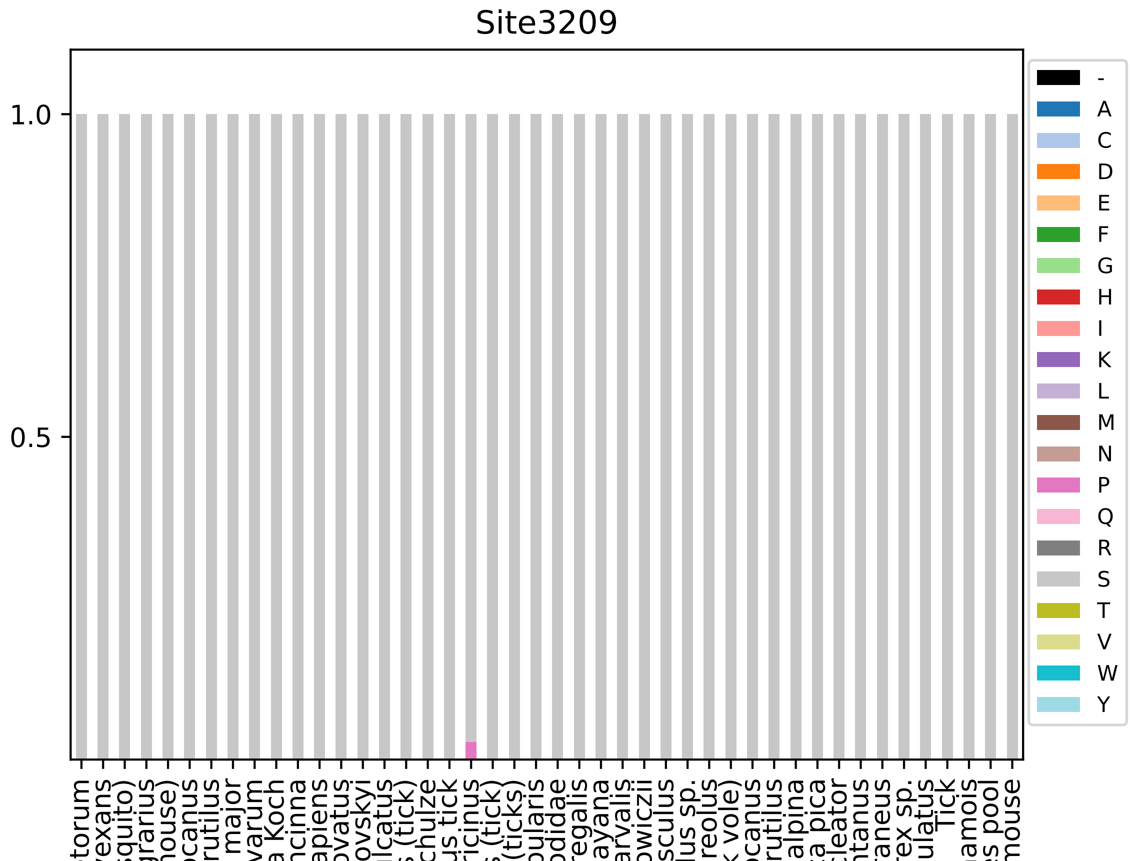

Site3209
1.0
0.5
-
A
C
D
E
F
G
H
I
K
L
M
N
P
Q
R
S
T
V
W
Y

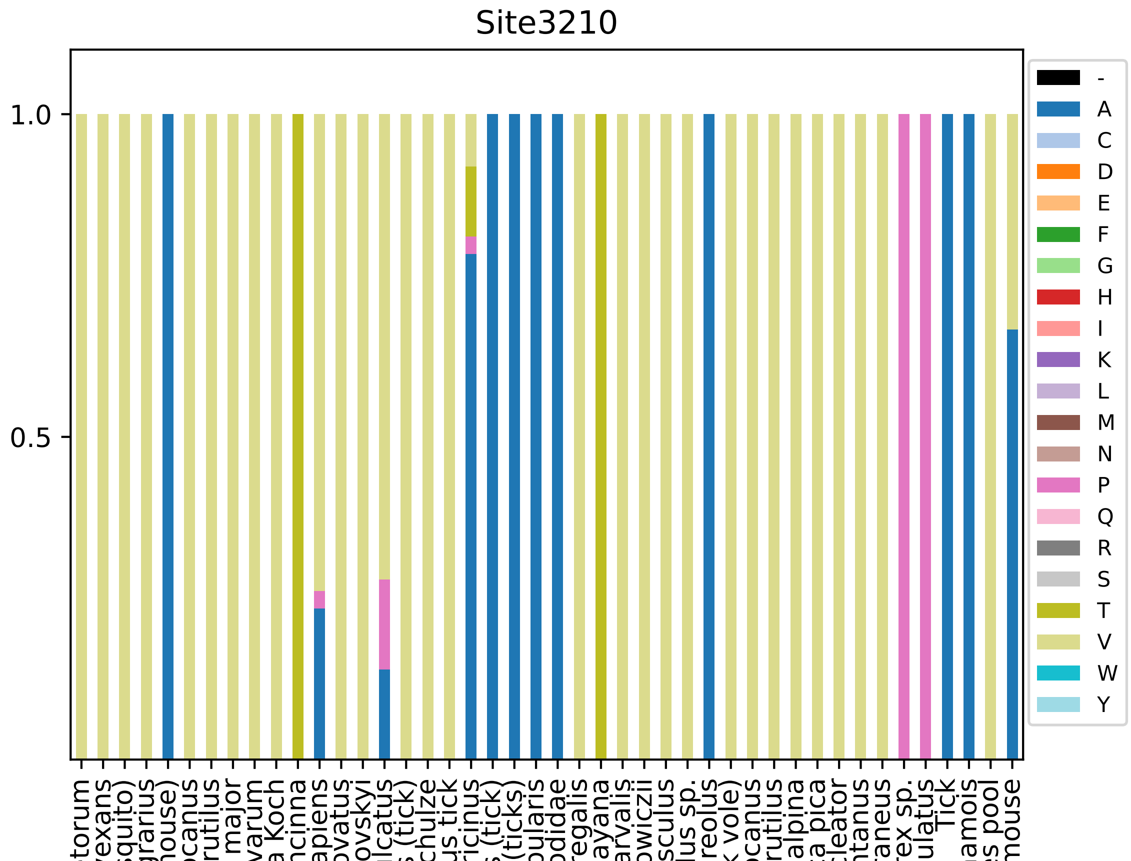

Site3210
1.0
0.5
-
A
C
D
E
F
G
H
I
K
L
M
N
P
Q
R
S
T
V
W
Y
torum
vexans
squito)
grarius
house)
canus
rutilus
major
varum
a Koch
ncinna
apiens
ovatus
ovskyi
lcatus
(tick)
chulze
us tick
ricinus
(tick)
(ticks)
pularis
podidae
regalis
ayana
arvalis
owiczii
sculus
lus sp.
reolus
k vole)
canus
rutilus
alpina
a pica
leator
ntanus
aneus
rex sp.
ulatus
Tick
amois
s pool
mouse

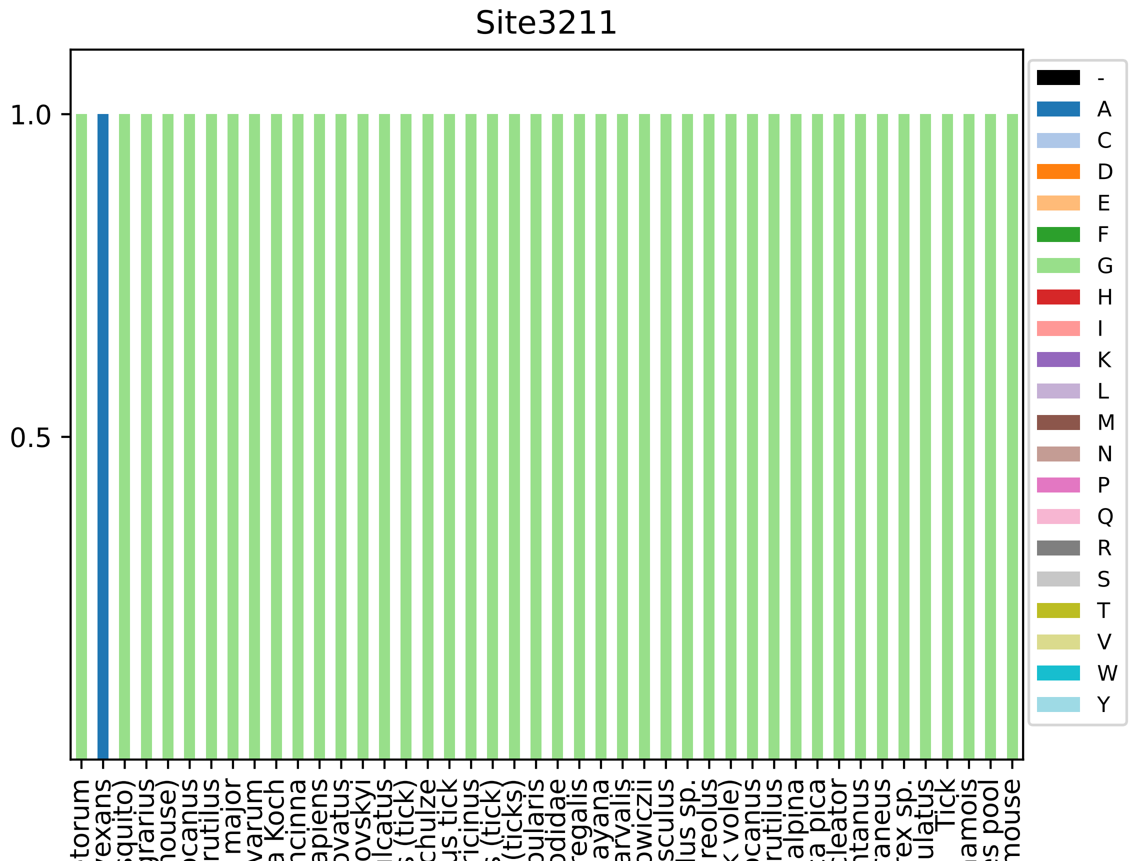
Site3211
1.0
0.5
-
A
C
D
E
F
G
H
I
K
L
M
N
P
Q
R
S
T
V
W
Y

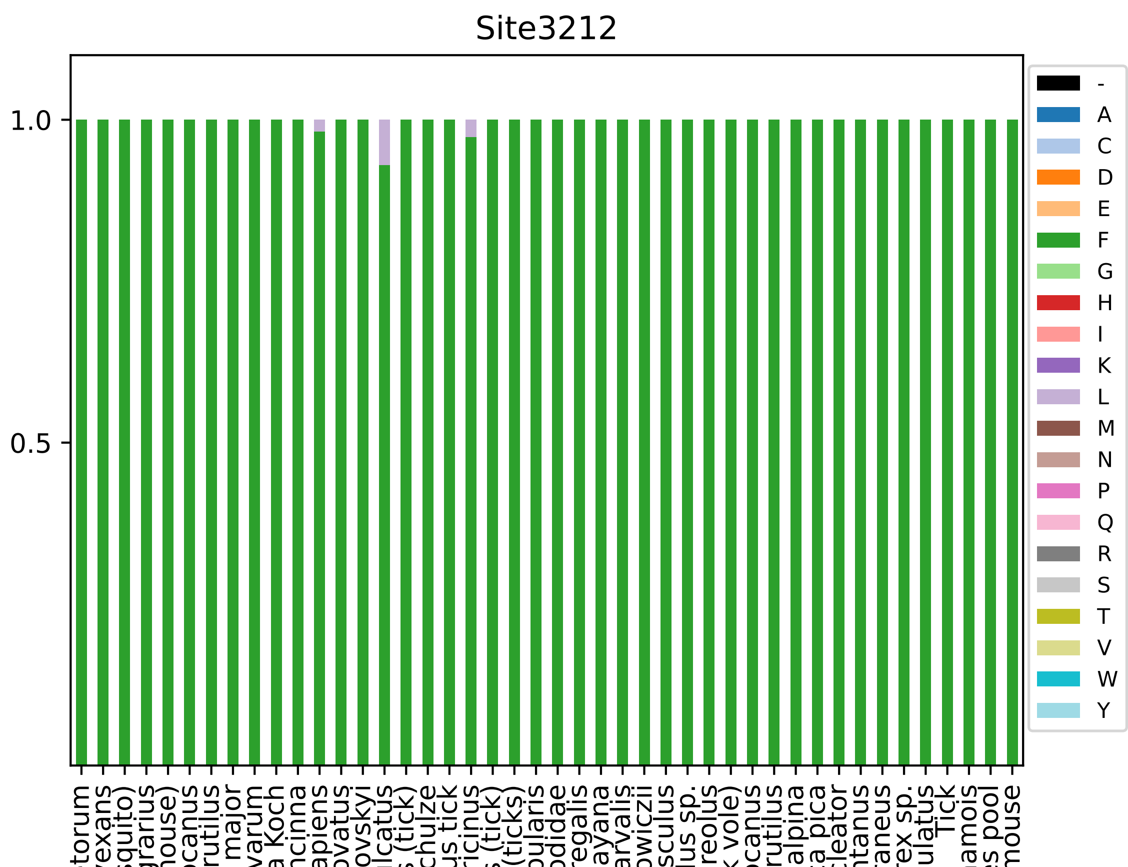
Site3212
1.0
0.5
-
A
C
D
E
F
G
H
I
K
L
M
N
P
Q
R
S
T
V
W
Y

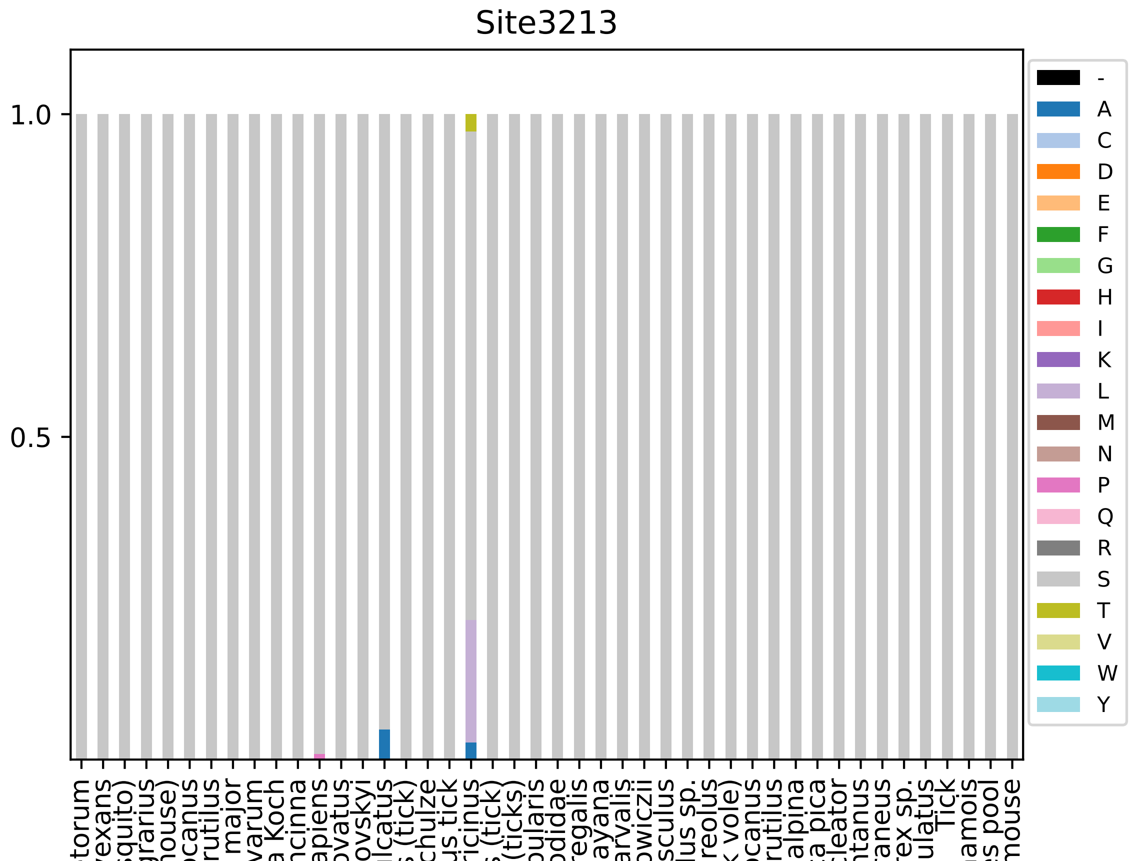
Site3213
1.0
0.5
-
A
C
D
E
F
G
H
I
K
L
M
N
P
Q
R
S
T
V
W
Y

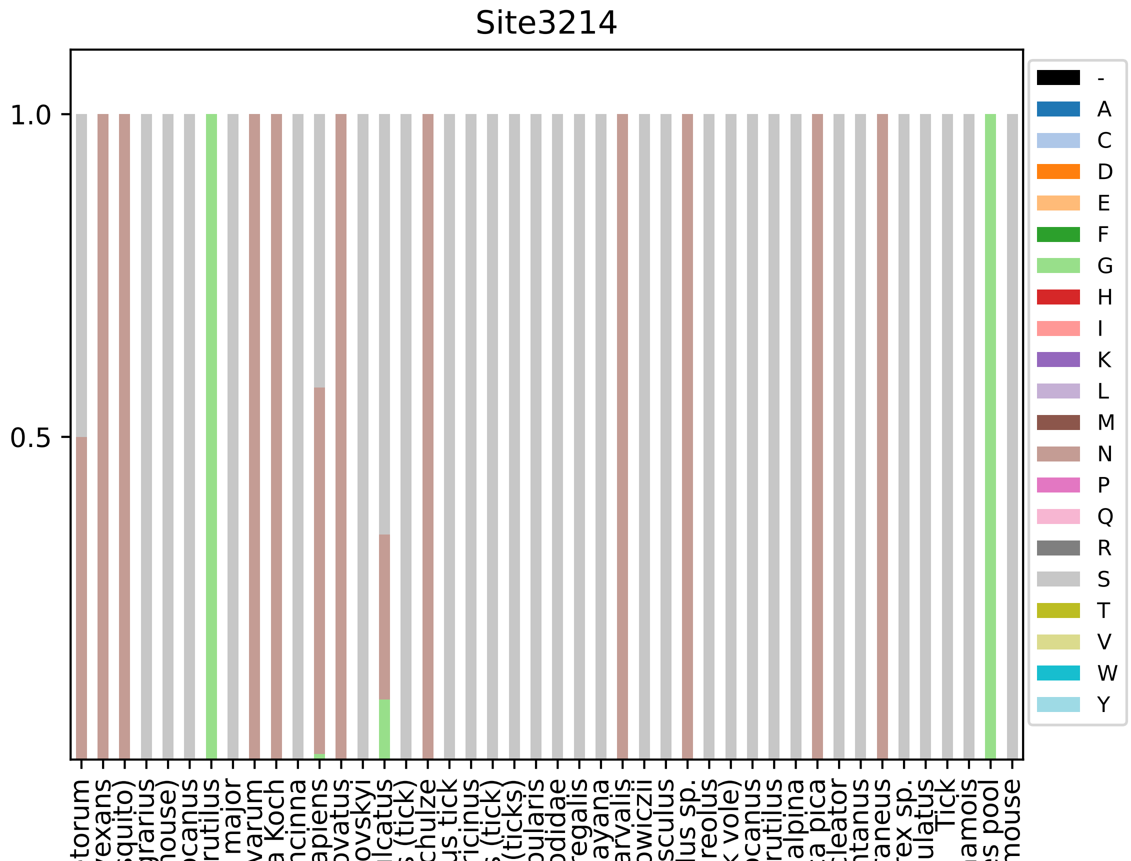
Site3214
1.0
0.5
-
A
C
D
E
F
G
H
I
K
L
M
N
P
Q
R
S
T
V
W
Y

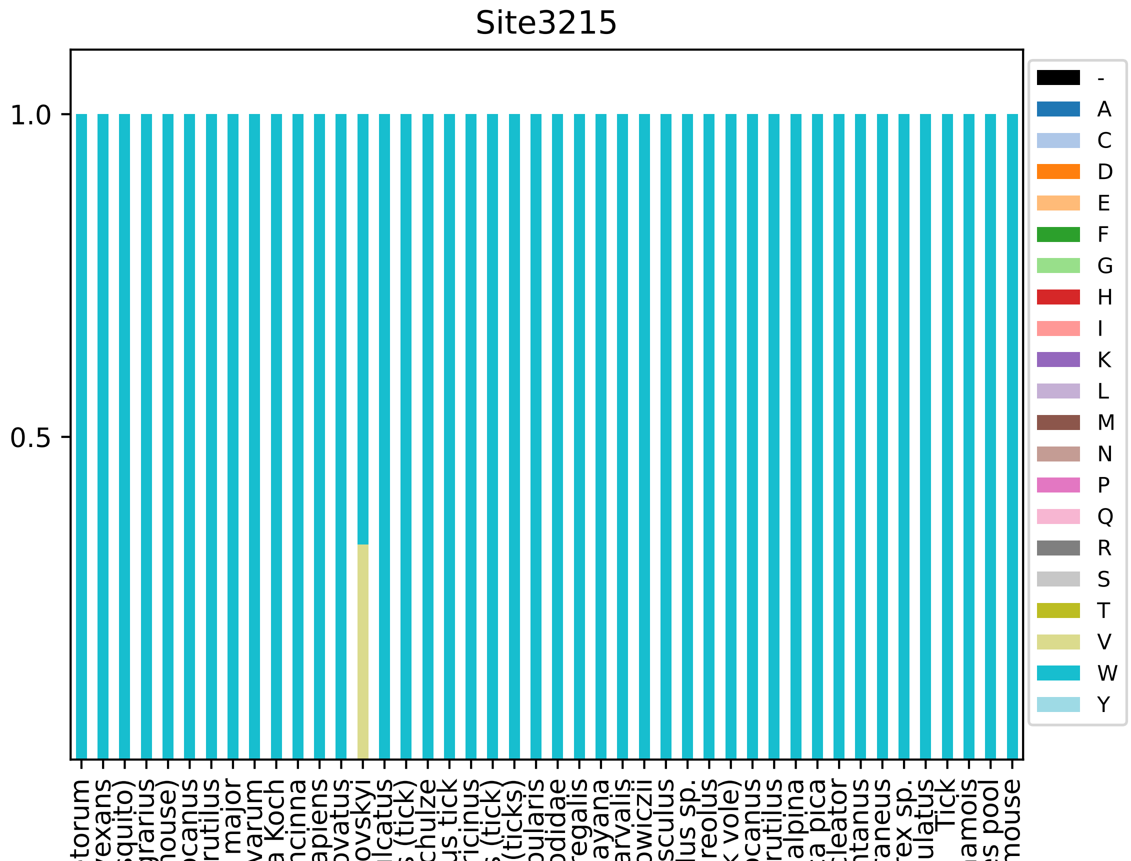
Site3215
1.0
0.5
-
A
C
D
E
F
G
H
I
K
L
M
N
P
Q
R
S
T
V
W
Y

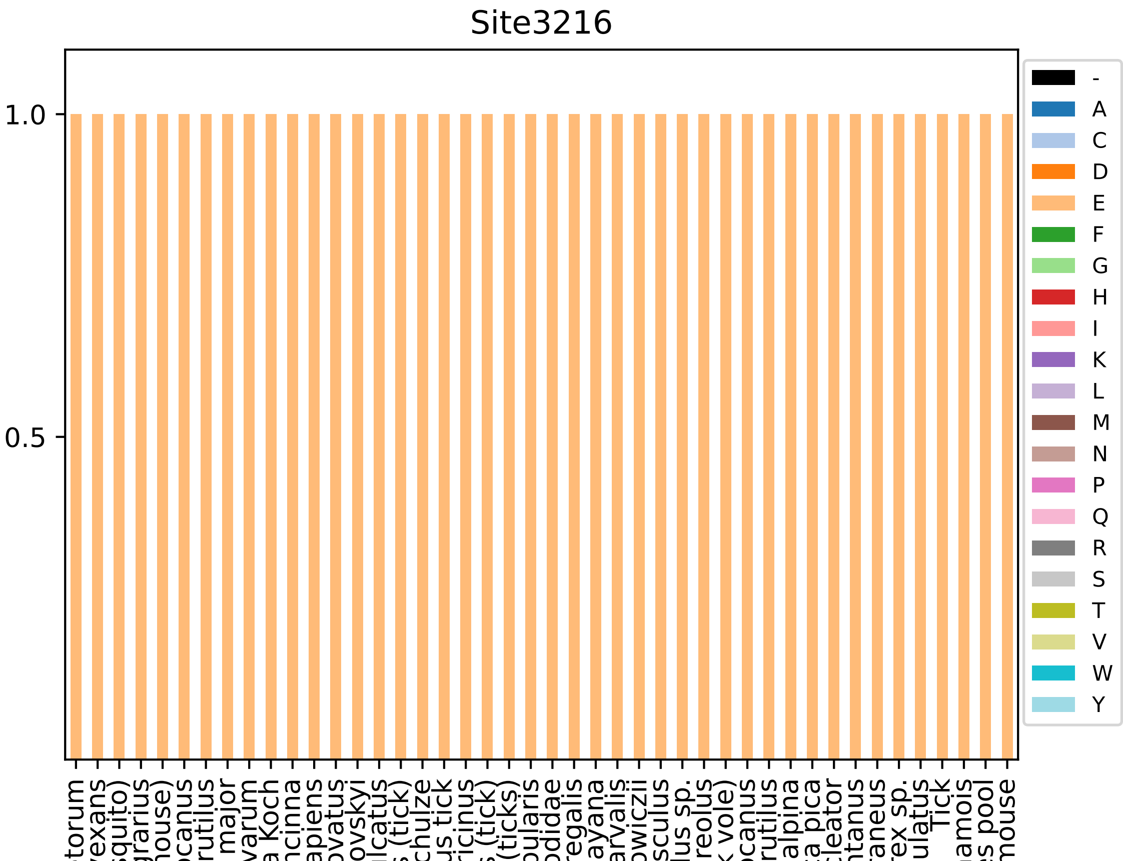
Site3216
1.0
0.5
-
A
C
D
E
F
G
H
I
K
L
M
N
P
Q
R
S
T
V
W
Y

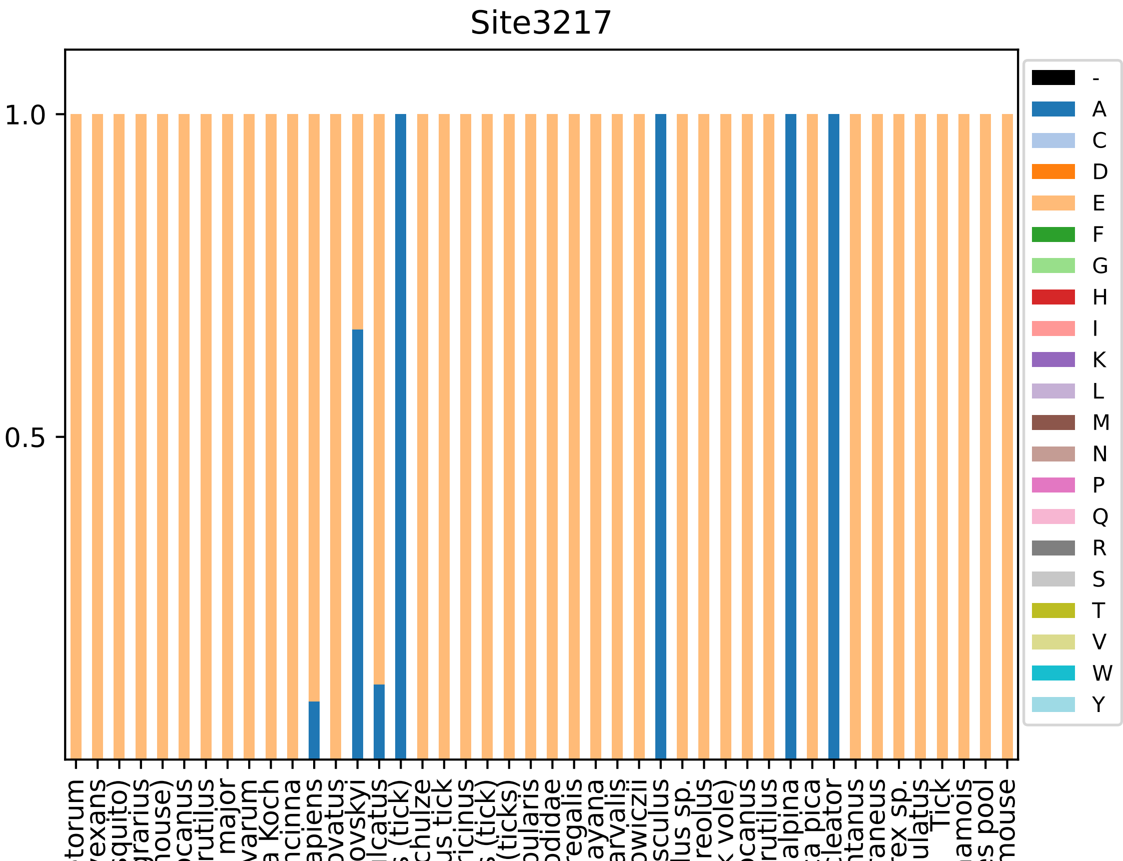

Site3217
1.0
0.5
-
A
C
D
E
F
G
H
I
K
L
M
N
P
Q
R
S
T
V
W
Y

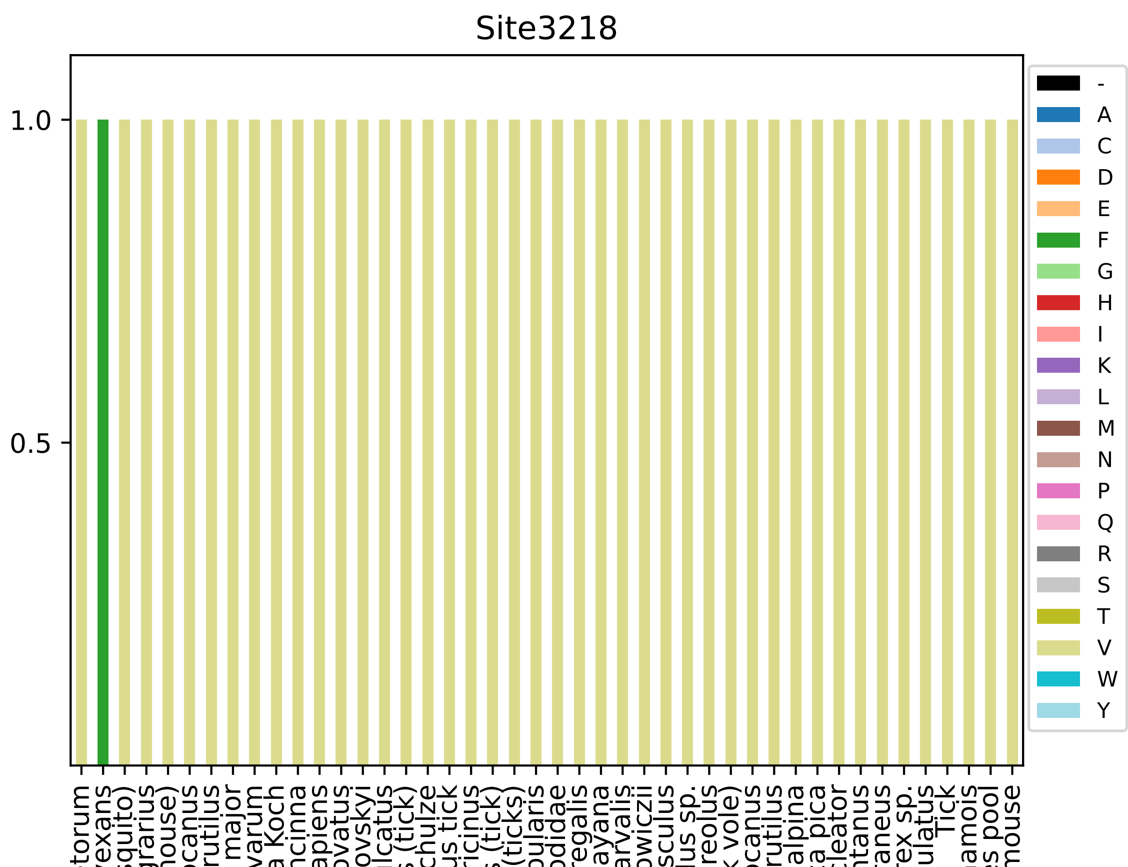
Site3218
1.0
0.5
-
A
C
D
E
F
G
H
I
K
L
M
N
P
Q
R
S
T
V
W
Y

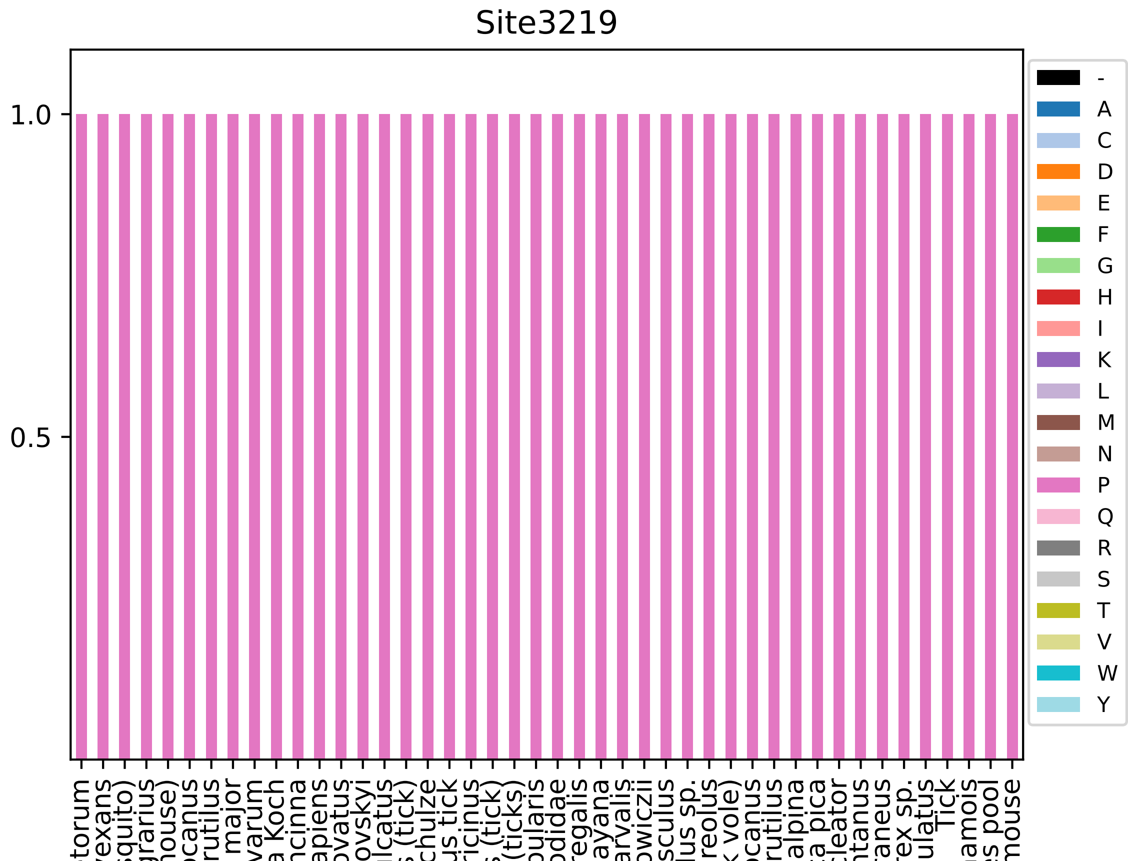
Site3219
1.0
0.5
-
A
C
D
E
F
G
H
I
K
L
M
N
P
Q
R
S
T
V
W
Y

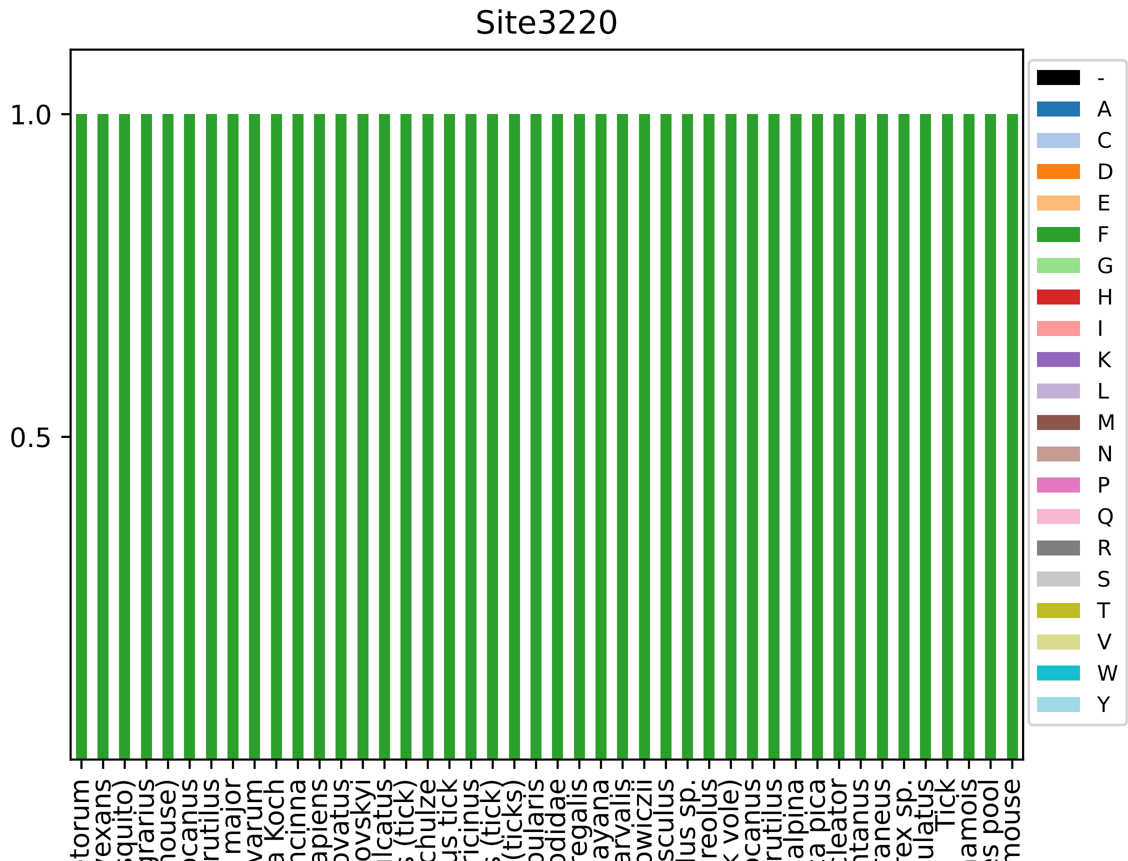
Site3220
1.0
0.5
-
A
C
D
E
F
G
H
I
K
L
M
N
P
Q
R
S
T
V
W
Y

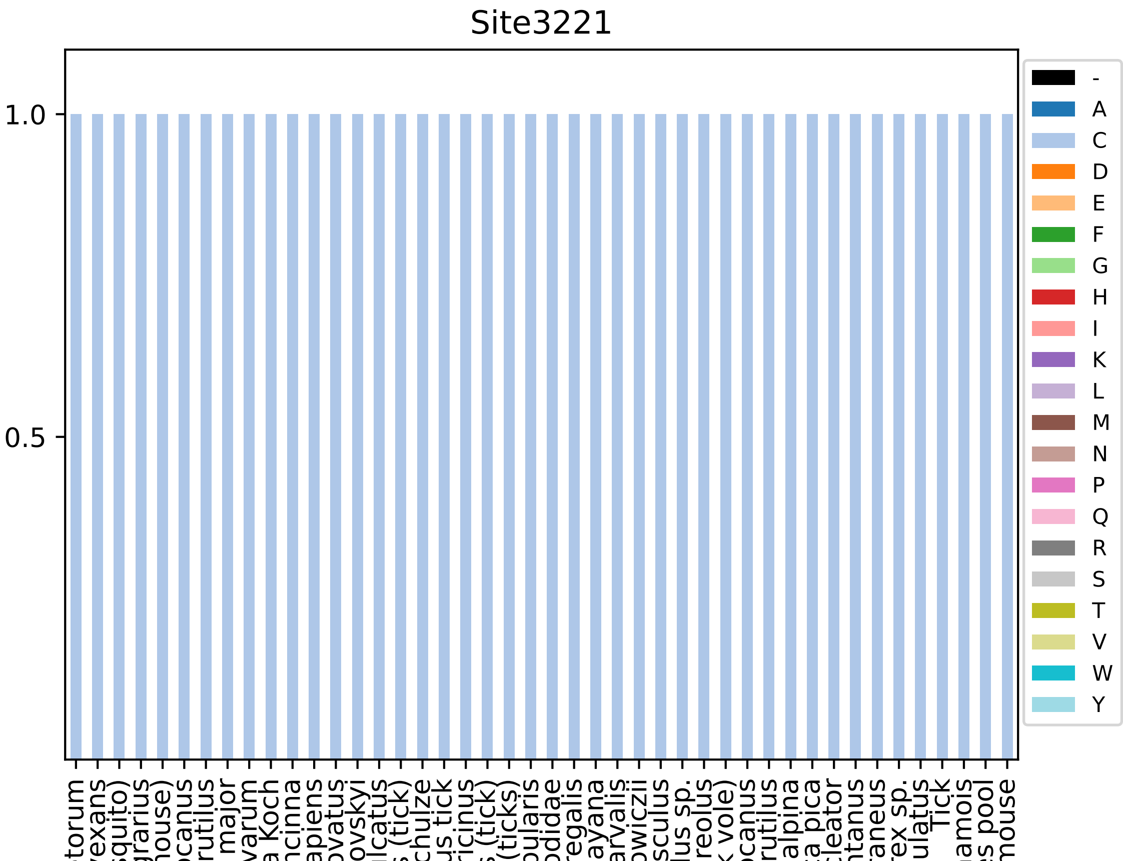
Site3221
1.0
0.5
-
A
C
D
E
F
G
H
I
K
L
M
N
P
Q
R
S
T
V
W
Y

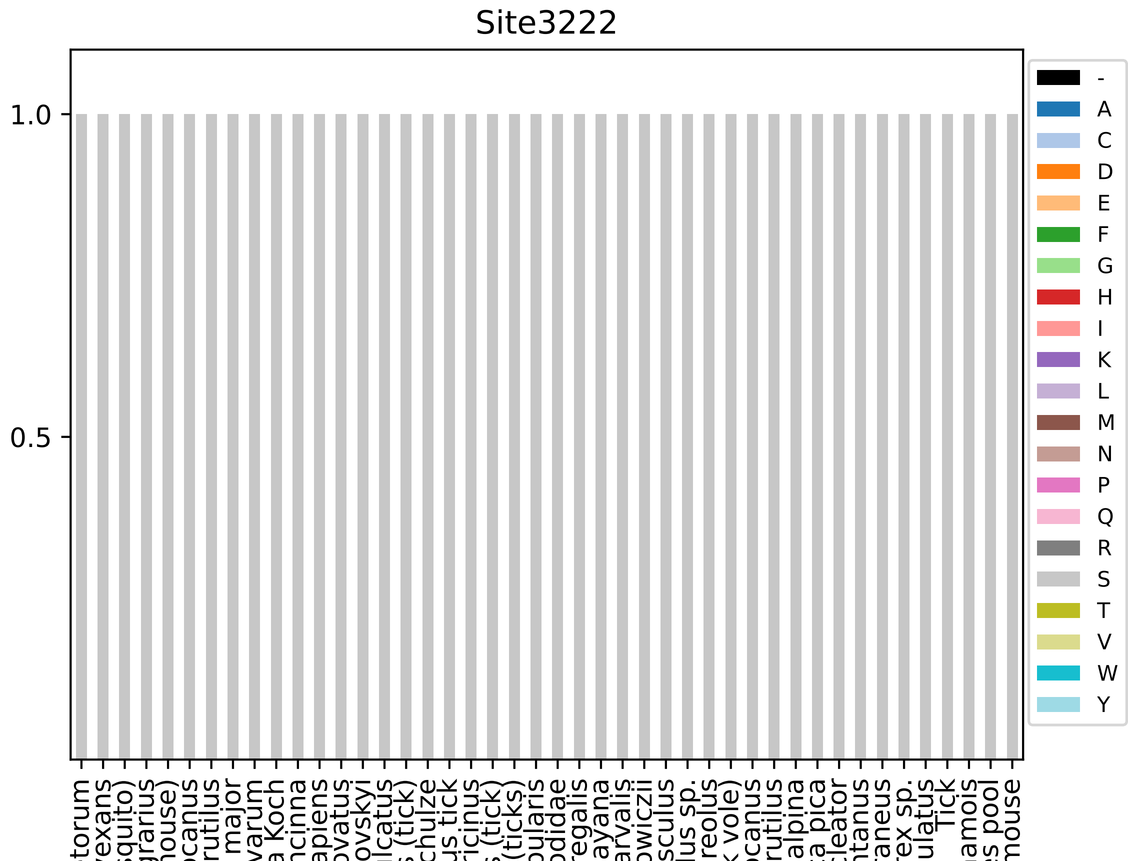
Site3222
1.0
0.5
-
A
C
D
E
F
G
H
I
K
L
M
N
P
Q
R
S
T
V
W
Y

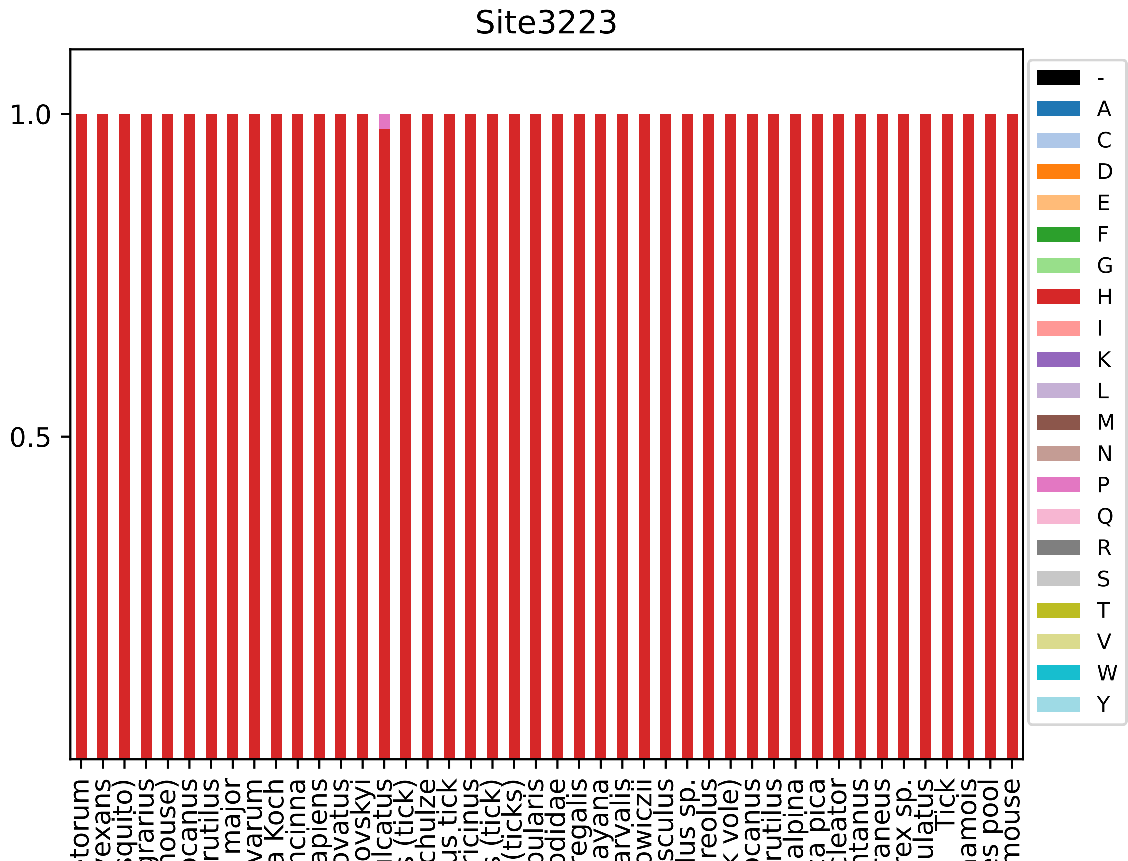

Site3223
1.0
0.5
-
A
C
D
E
F
G
H
I
K
L
M
N
P
Q
R
S
T
V
W
Y

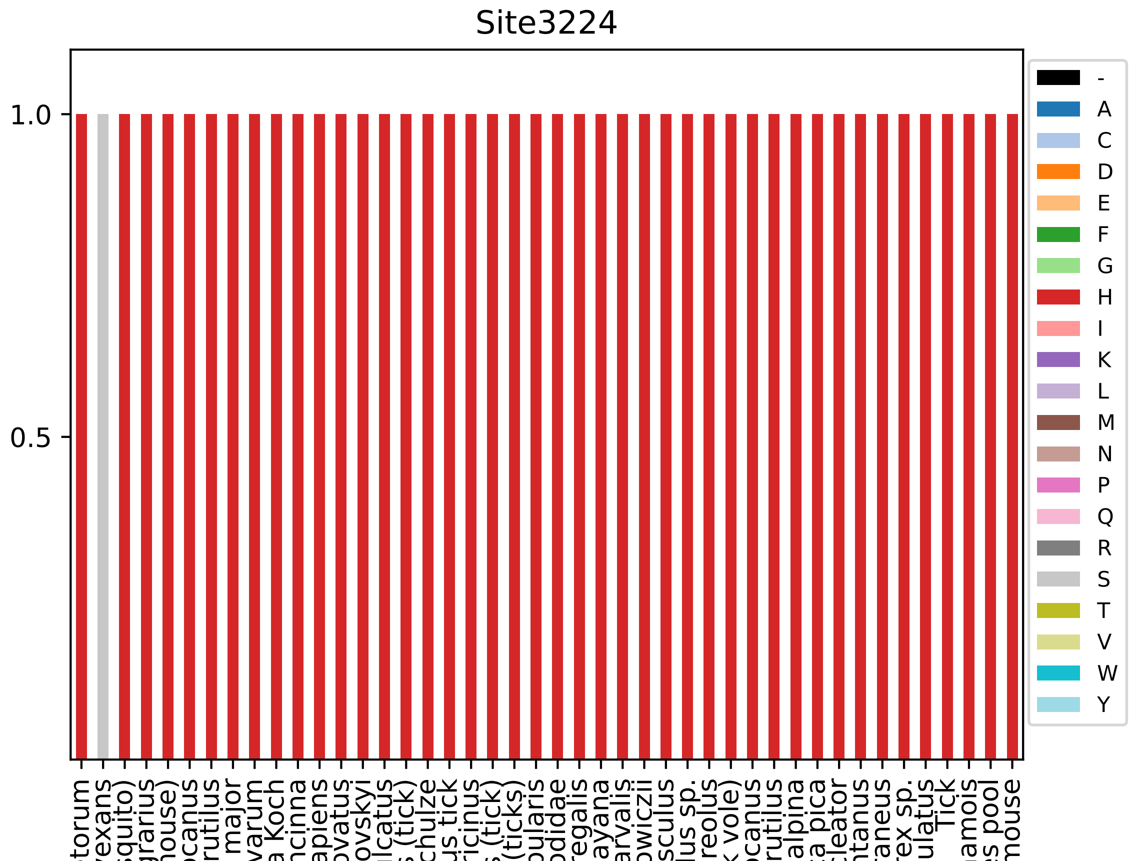
Site3224
1.0
0.5
-
A
C
D
E
F
G
H
I
K
L
M
N
P
Q
R
S
T
V
W
Y
torum
vexans
squito)
garius
nouse)
ocanus
rutilus
major
varum
a Koch
ncinna
apiens
ovatus
ovskyi
ulcatus
s (tick)
chulze
us tick
ricinus
s (tick)
s (ticks)
pularis
odidae
regalis
ayana
arvalis
owiczii
usculus
lus sp.
reolus
x vole)
ocanus
rutilus
alpina
ca pica
cleator
ntanus
raneus
rex sp.
ulatus
Tick
amois
es pool
mouse

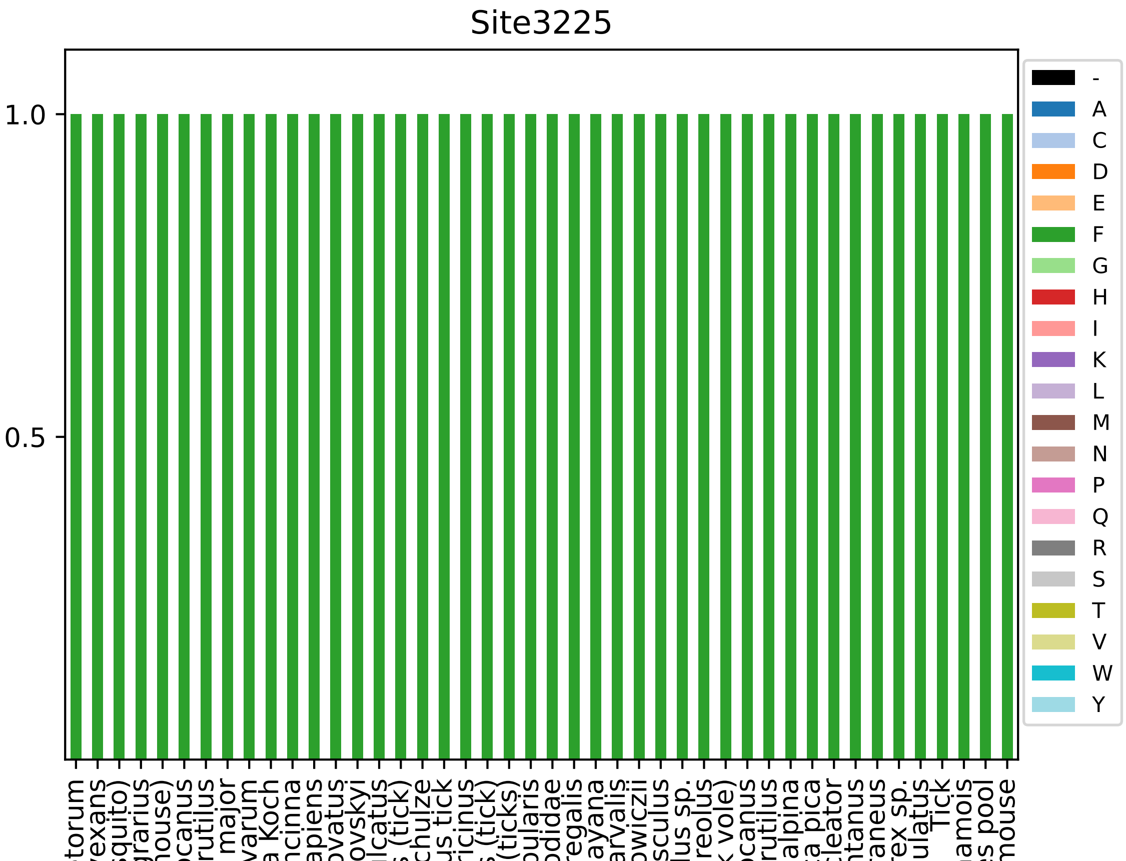
Site3225
1.0
0.5
-
A
C
D
E
F
G
H
I
K
L
M
N
P
Q
R
S
T
V
W
Y
Tick

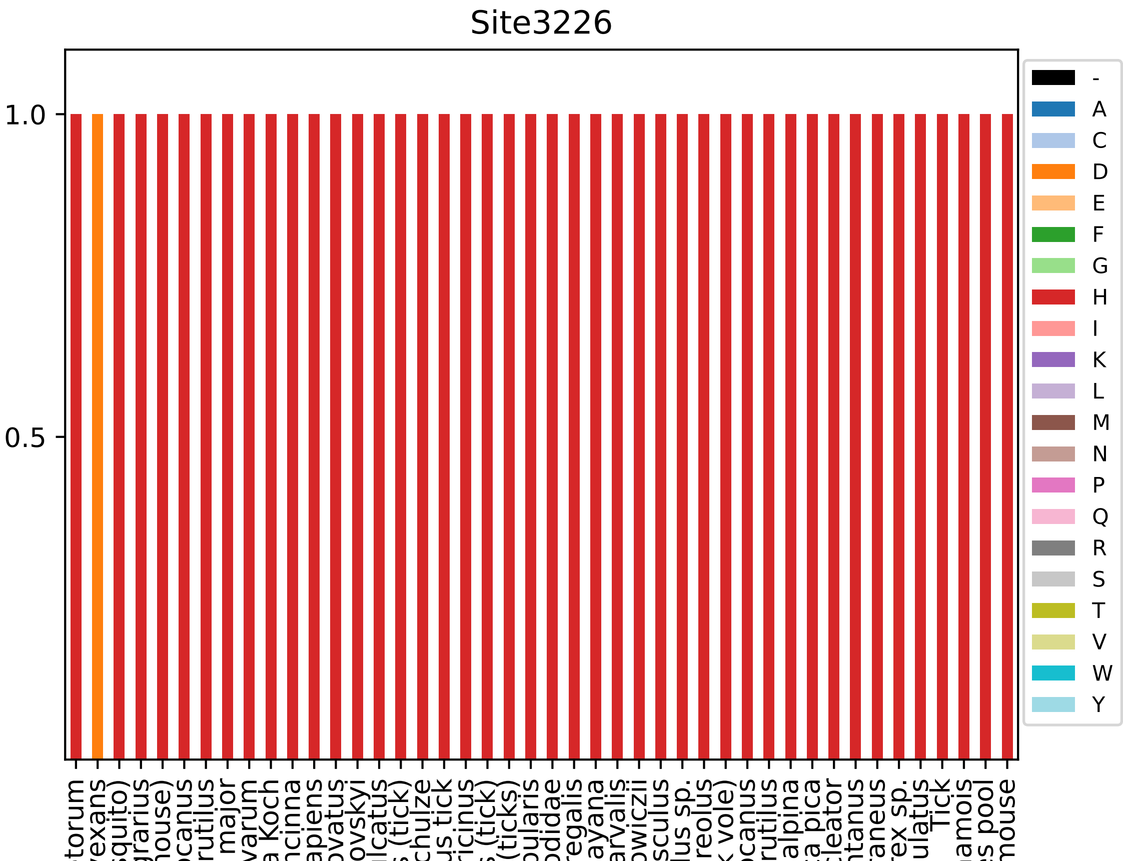
Site3226
1.0
0.5
-
A
C
D
E
F
G
H
I
K
L
M
N
P
Q
R
S
T
V
W
Y

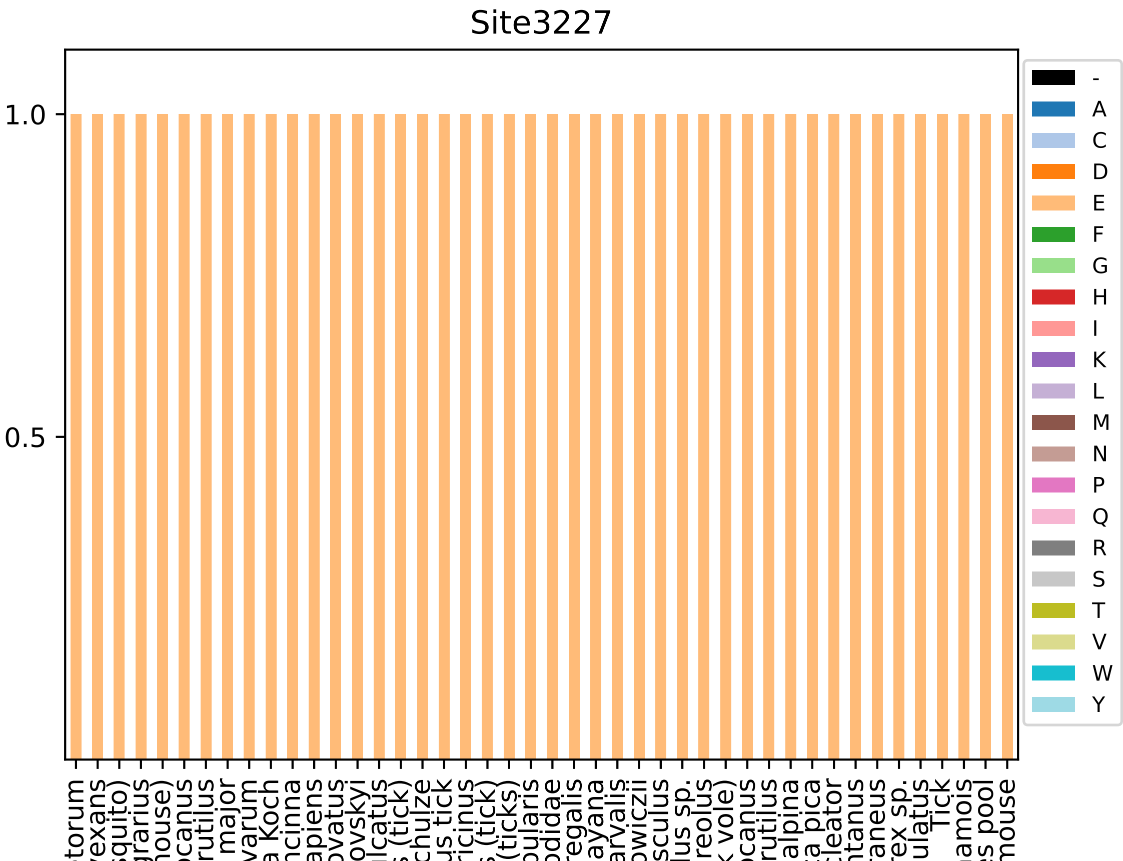

Site3227
1.0
0.5
-
A
C
D
E
F
G
H
I
K
L
M
N
P
Q
R
S
T
V
W
Y

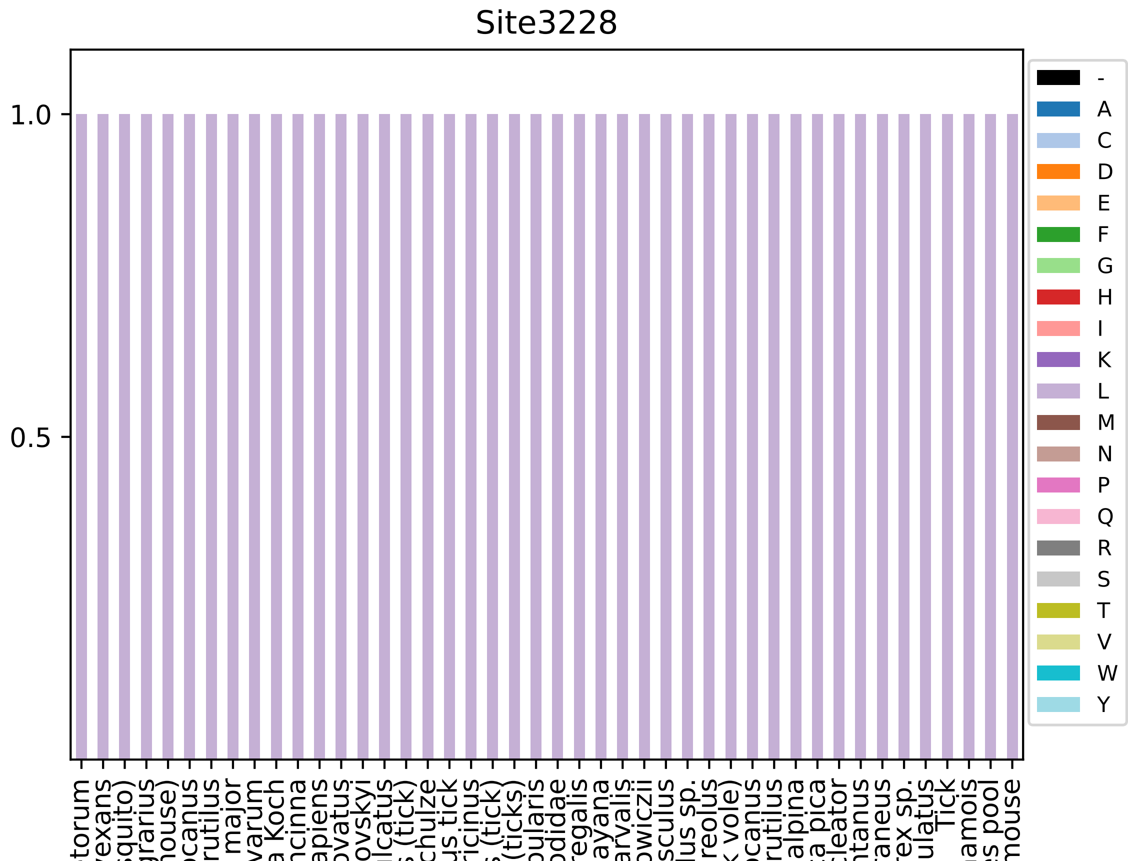
Site3228
1.0
0.5
-
A
C
D
E
F
G
H
I
K
L
M
N
P
Q
R
S
T
V
W
Y

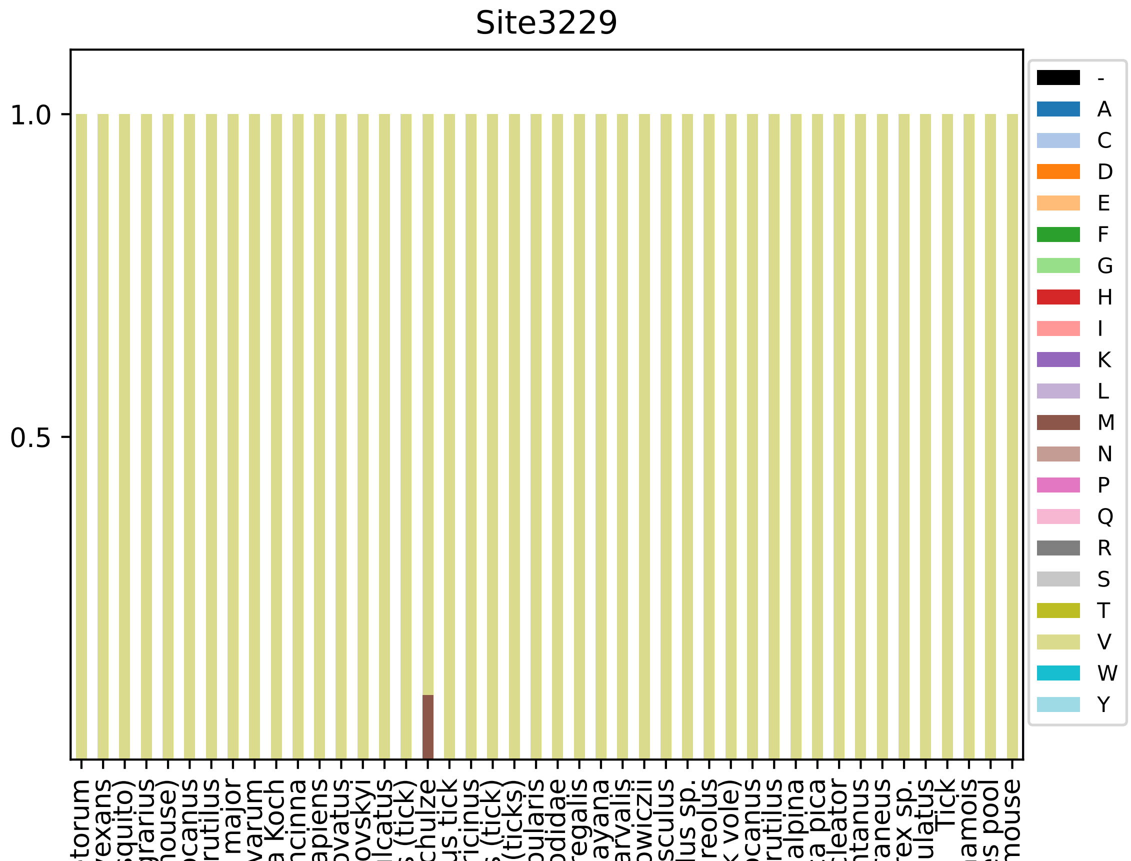

Site3229
1.0
0.5
-
A
C
D
E
F
G
H
I
K
L
M
N
P
Q
R
S
T
V
W
Y

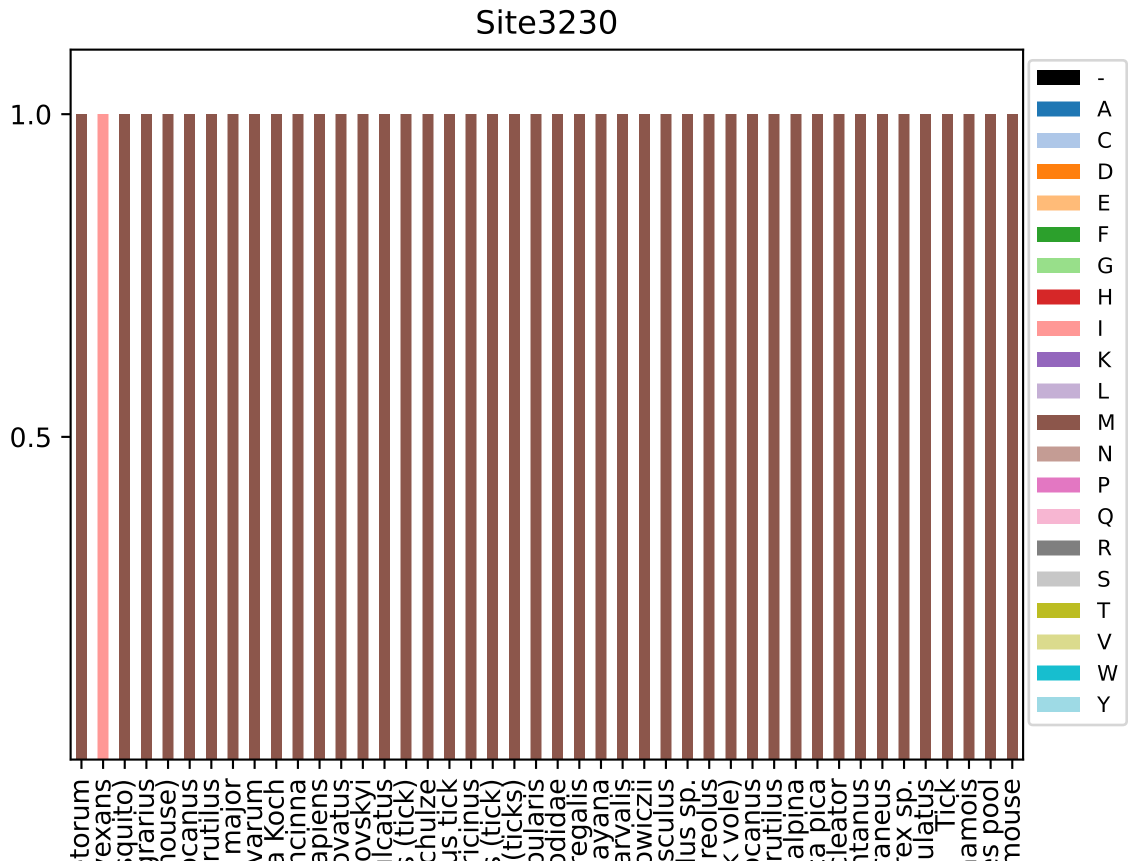

Site3230
1.0
0.5
-
A
C
D
E
F
G
H
I
K
L
M
N
P
Q
R
S
T
V
W
Y
Tick

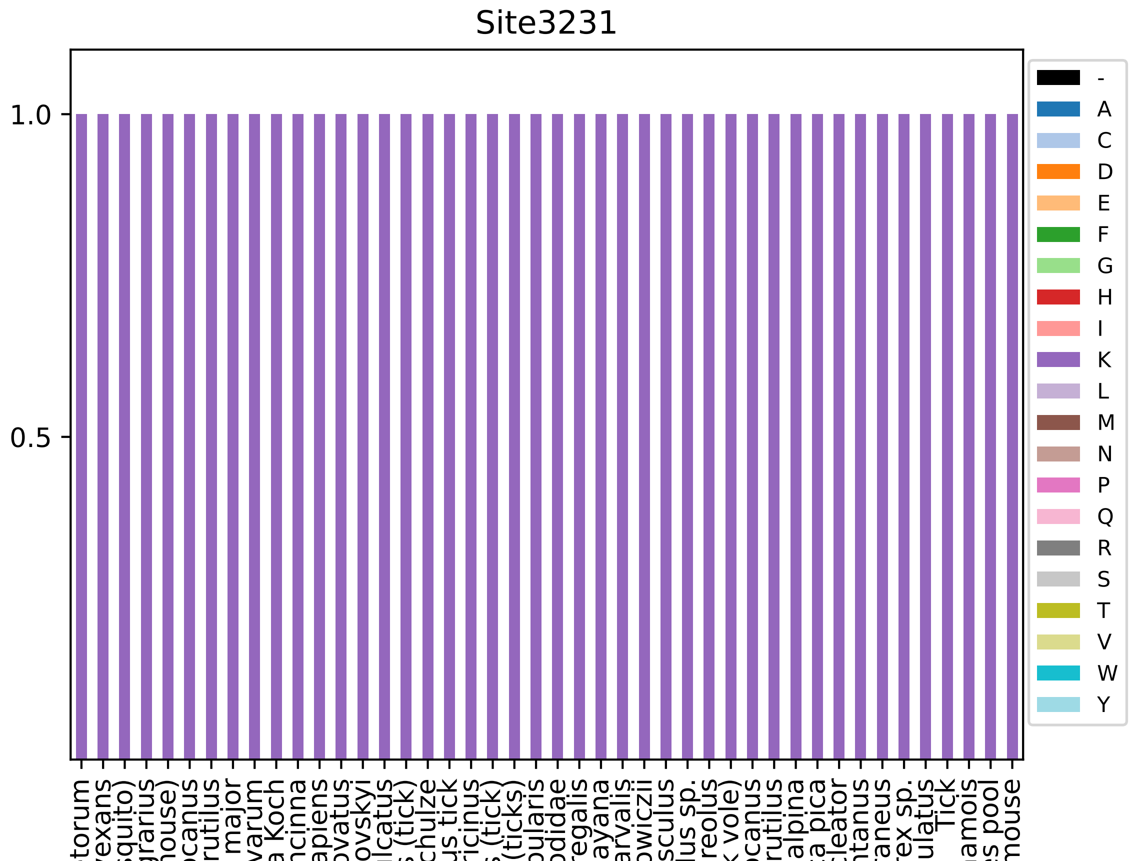
Site3231
1.0
0.5
-
A
C
D
E
F
G
H
I
K
L
M
N
P
Q
R
S
T
V
W
Y

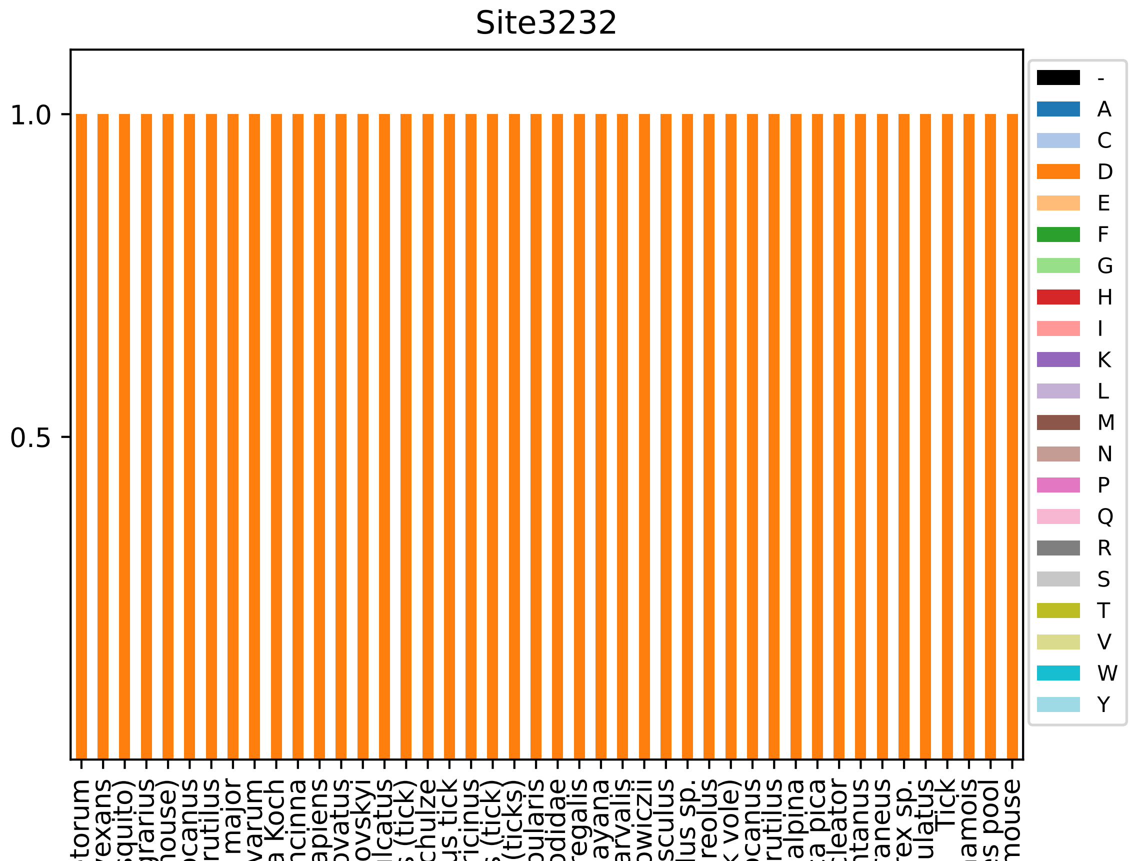
Site3232
1.0
0.5
-
A
C
D
E
F
G
H
I
K
L
M
N
P
Q
R
S
T
V
W
Y

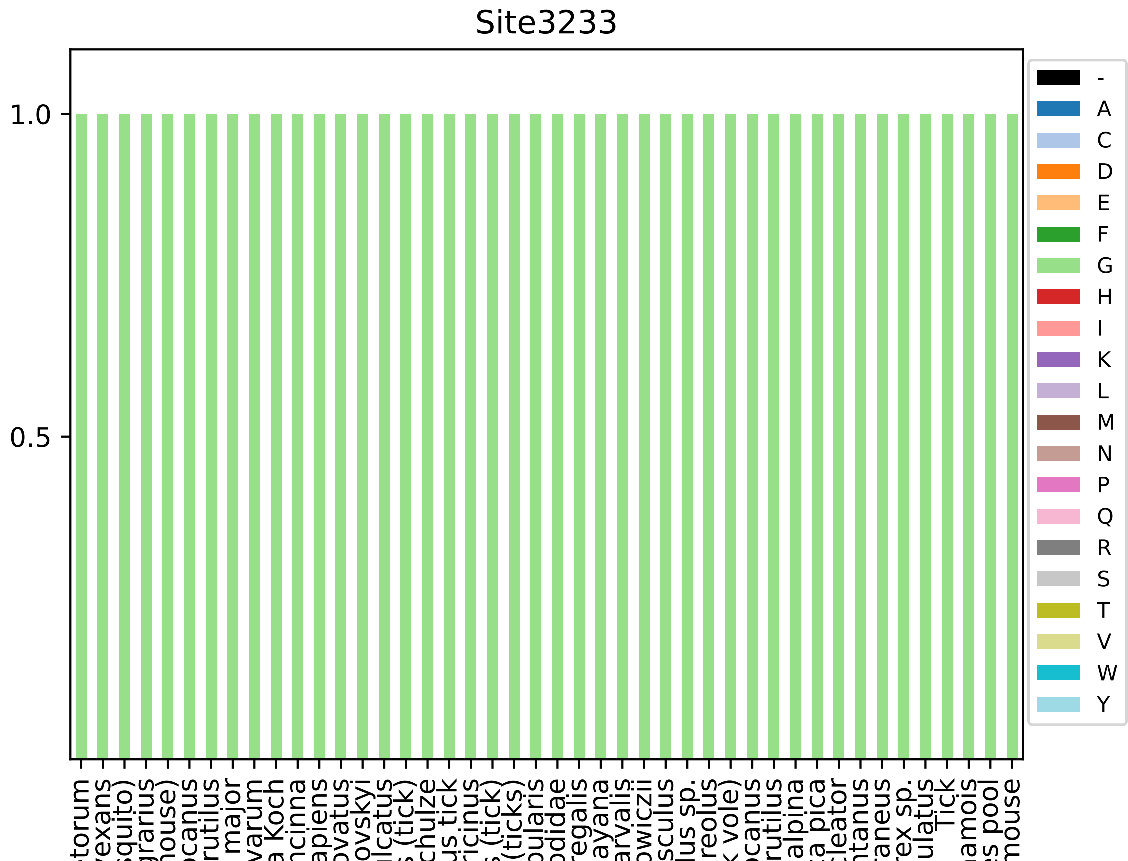

Site3233
1.0
0.5
-
A
C
D
E
F
G
H
I
K
L
M
N
P
Q
R
S
T
V
W
Y

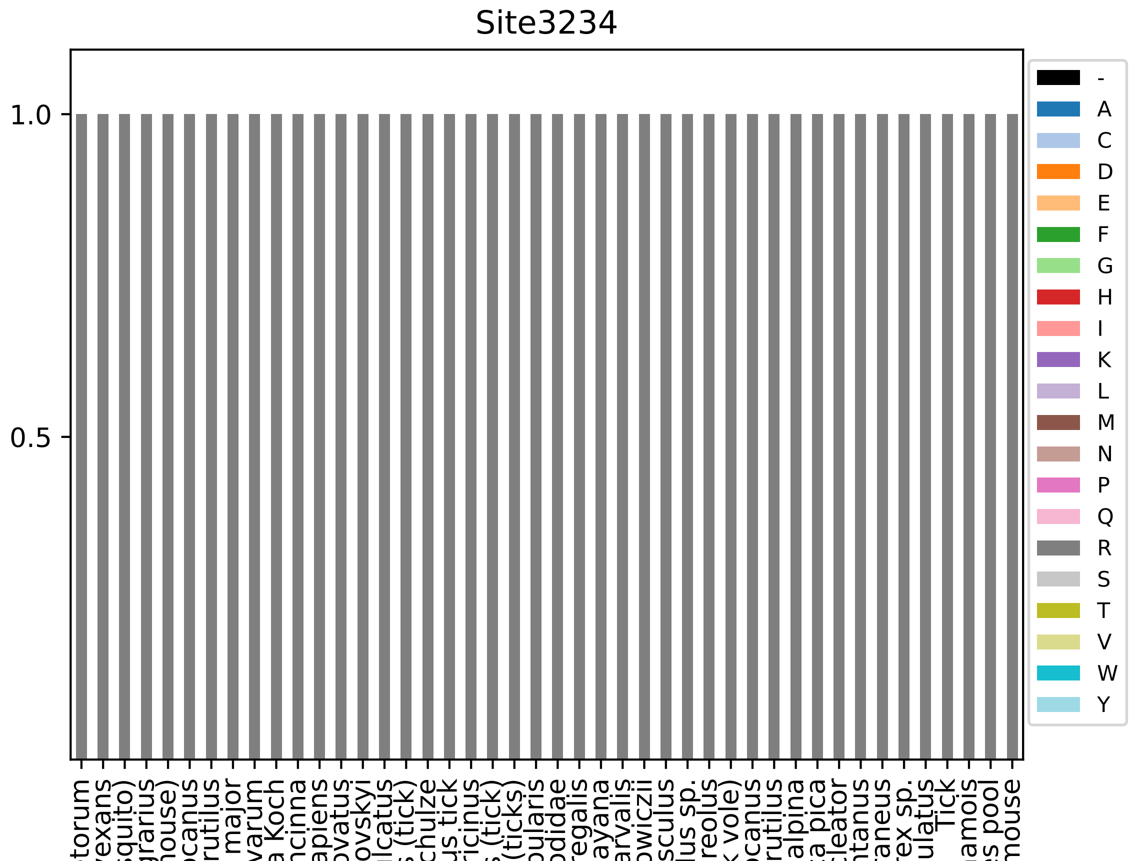

Site3234
1.0
0.5
-
A
C
D
E
F
G
H
I
K
L
M
N
P
Q
R
S
T
V
W
Y

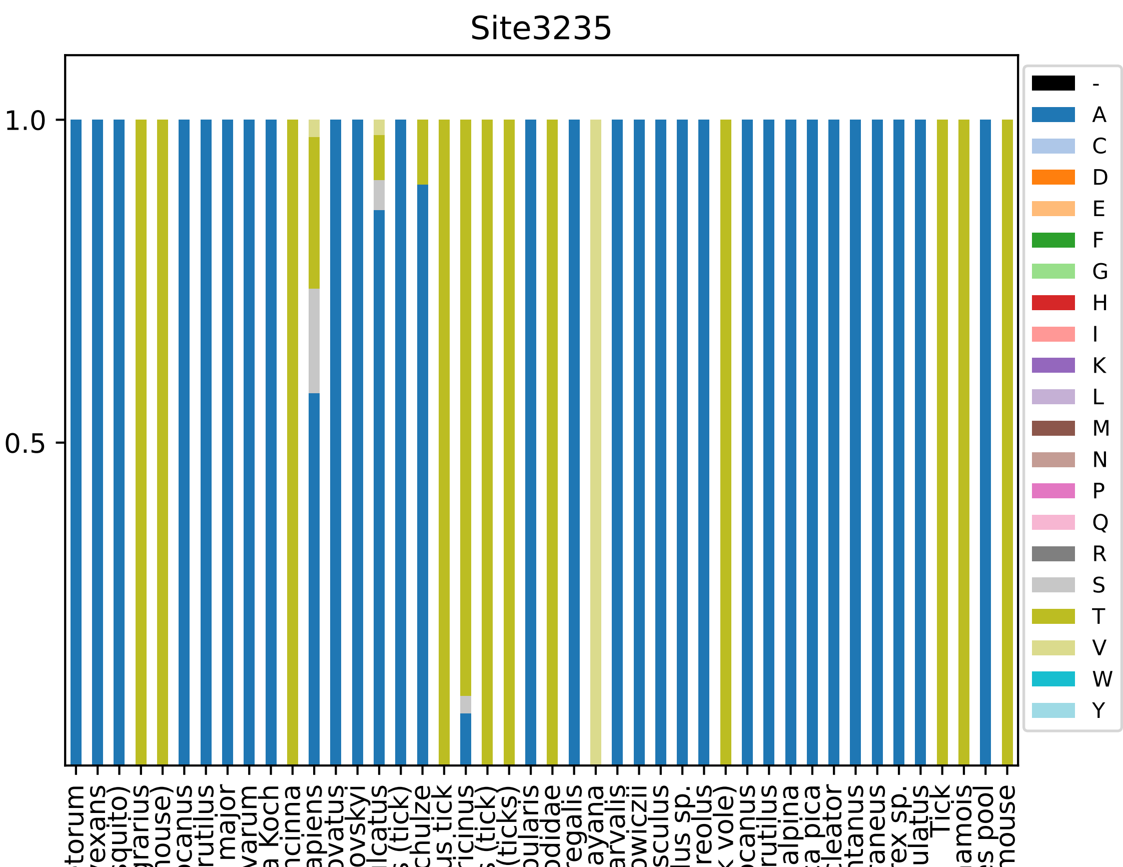
Site3235
1.0
0.5
-
A
C
D
E
F
G
H
I
K
L
M
N
P
Q
R
S
T
V
W
Y
major
Tick

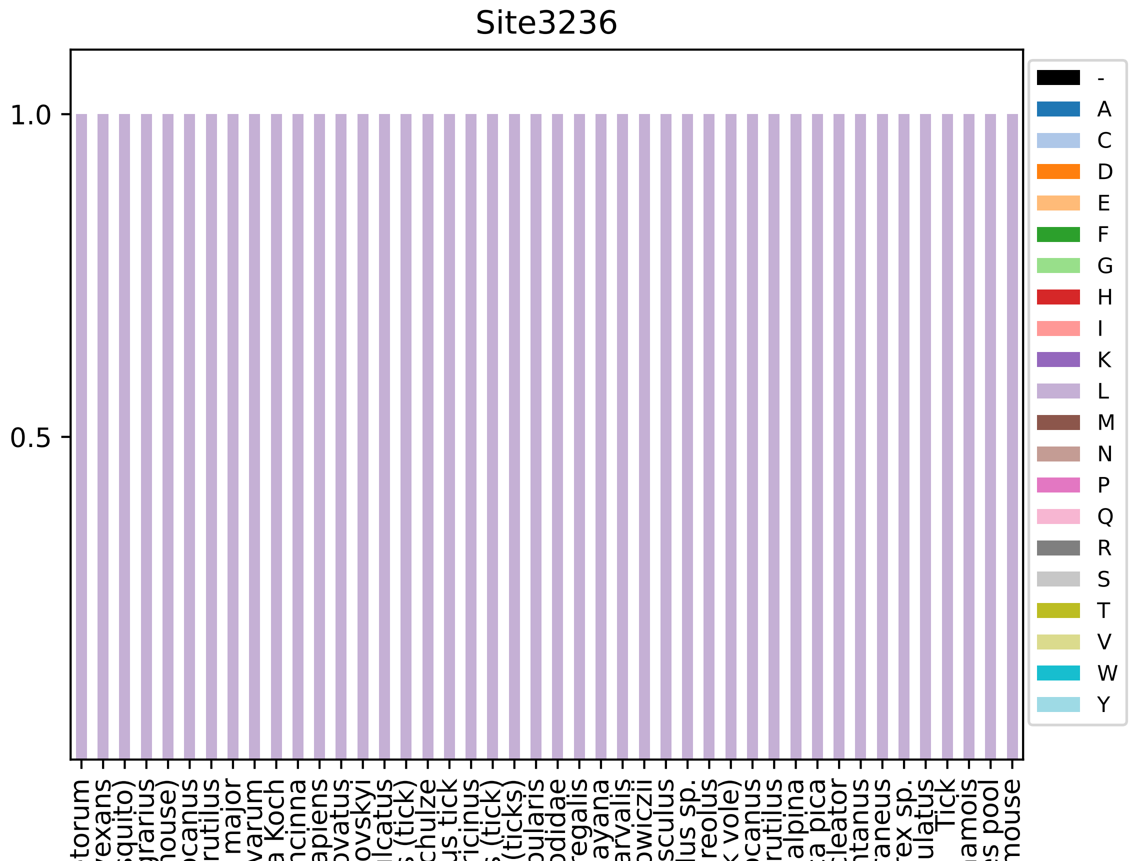

Site3236
1.0
0.5
-
A
C
D
E
F
G
H
I
K
L
M
N
P
Q
R
S
T
V
W
Y

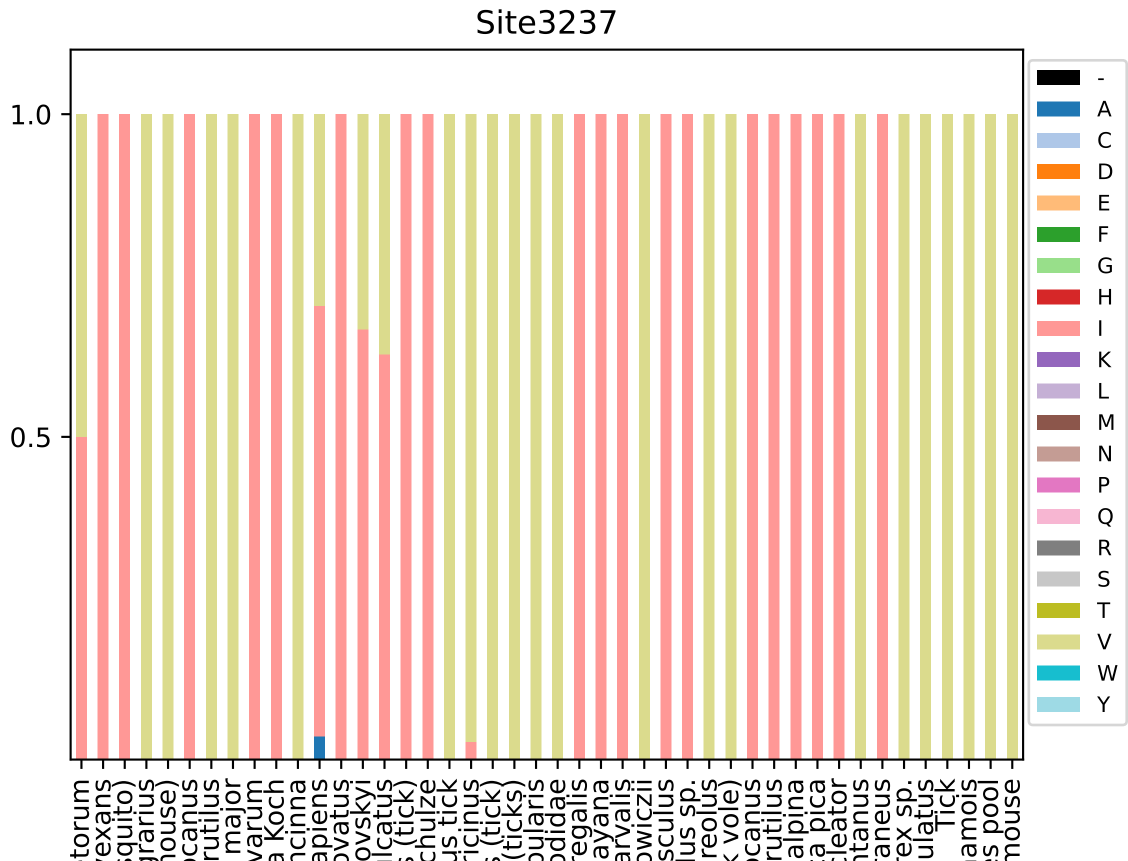

Site3237
1.0
0.5
-
A
C
D
E
F
G
H
I
K
L
M
N
P
Q
R
S
T
V
W
Y
Tick

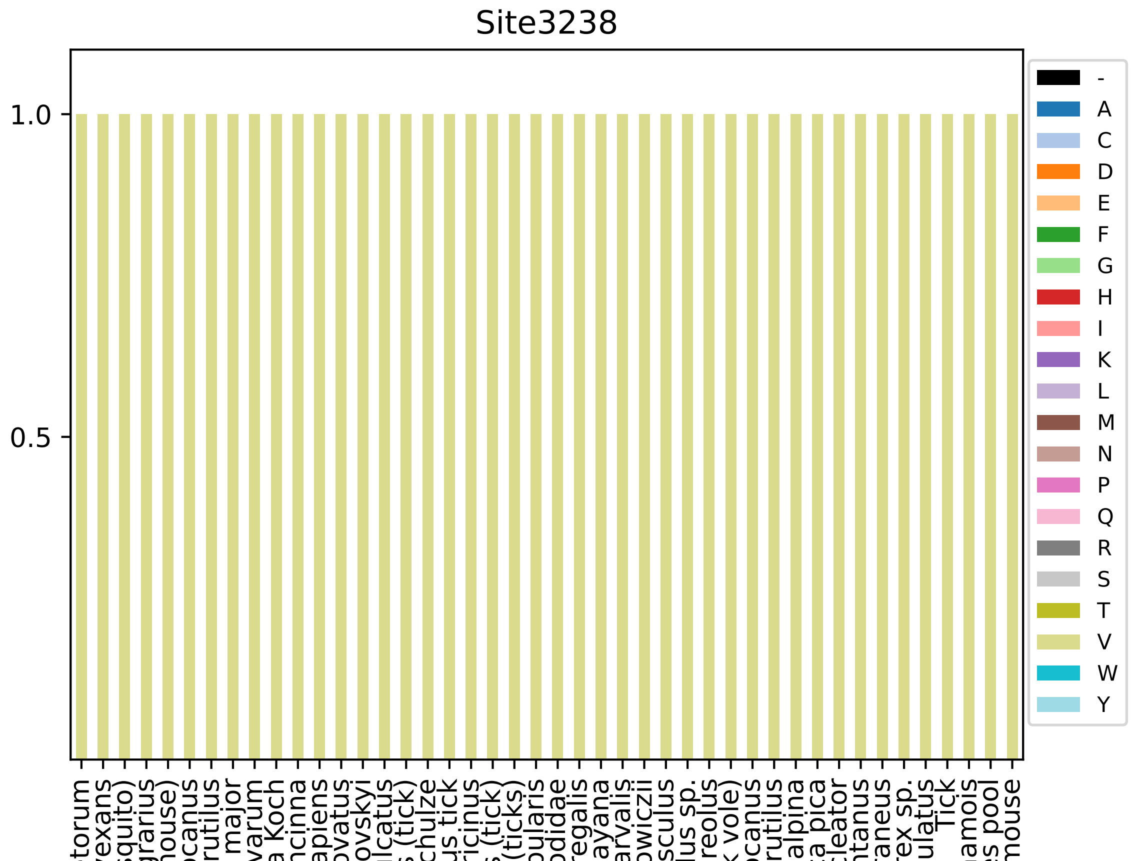

Site3238
1.0
0.5
-
A
C
D
E
F
G
H
I
K
L
M
N
P
Q
R
S
T
V
W
Y

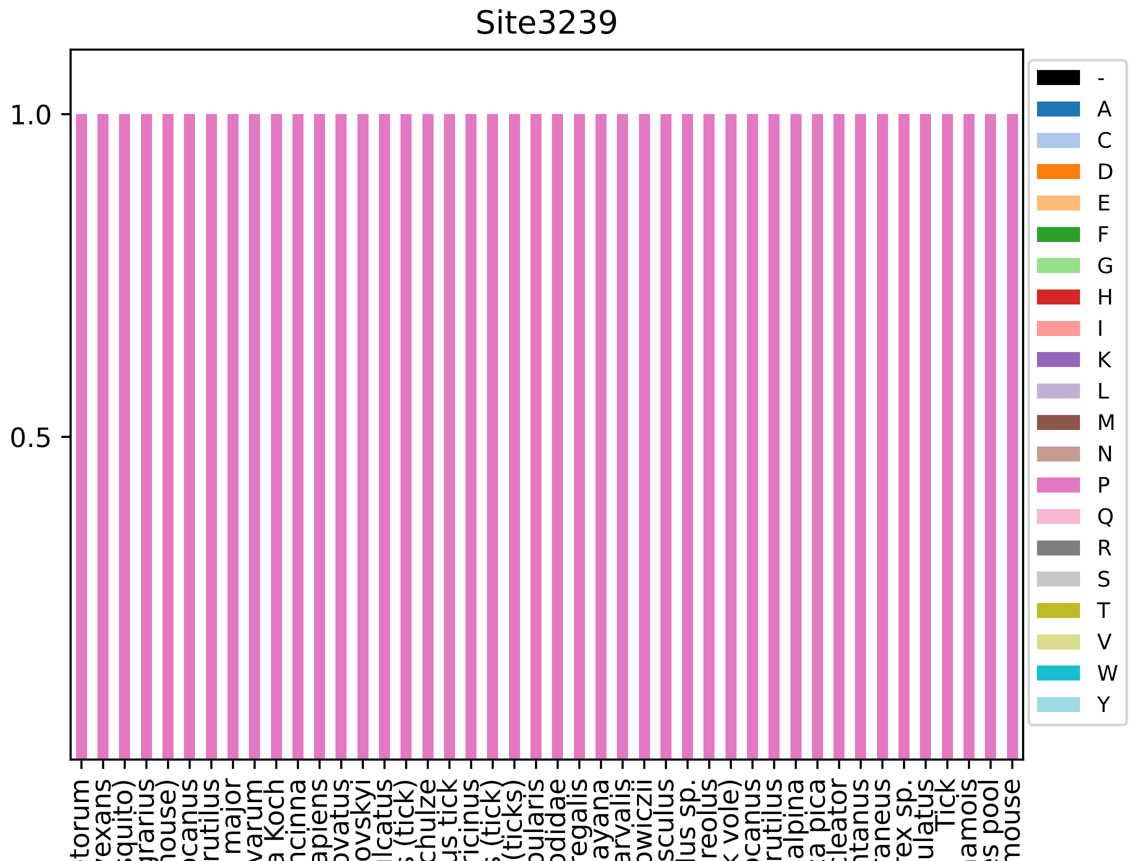
Site3239
1.0
0.5
-
A
C
D
E
F
G
H
I
K
L
M
N
P
Q
R
S
T
V
W
Y

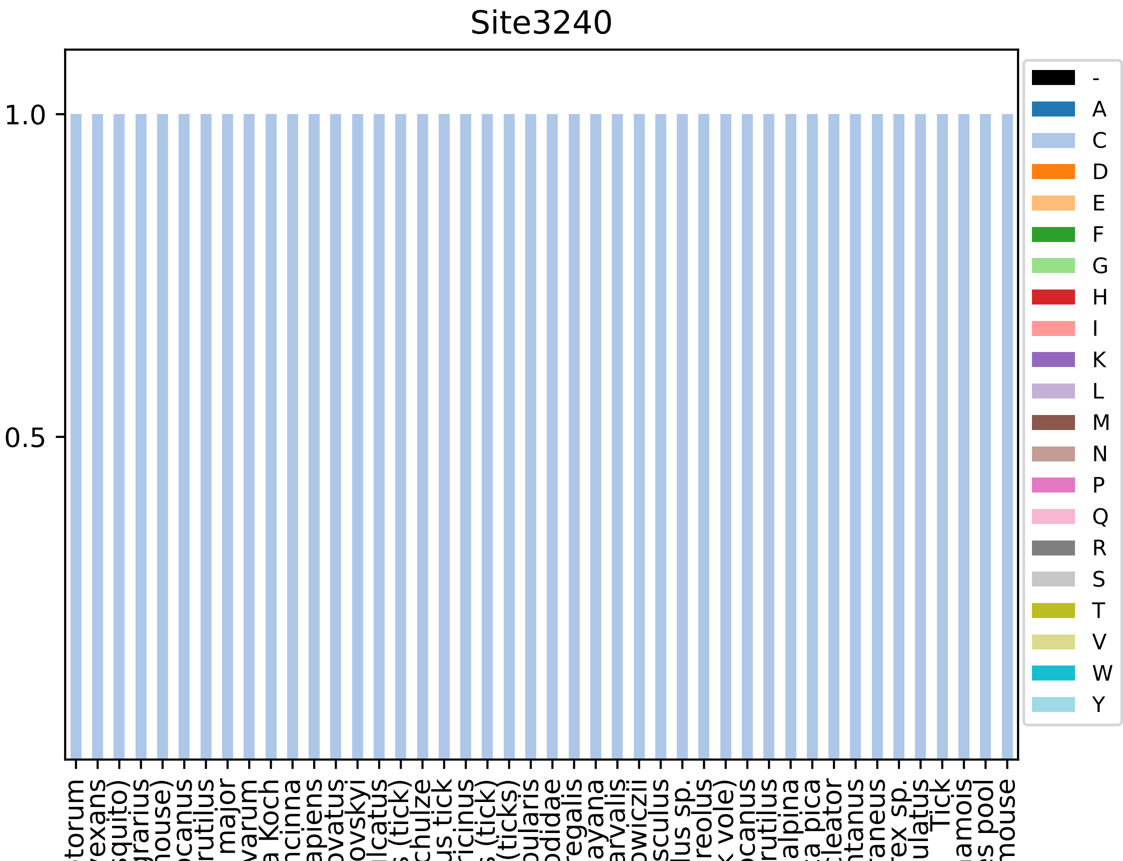
Site3240
1.0
0.5
-
A
C
D
E
F
G
H
I
K
L
M
N
P
Q
R
S
T
V
W
Y

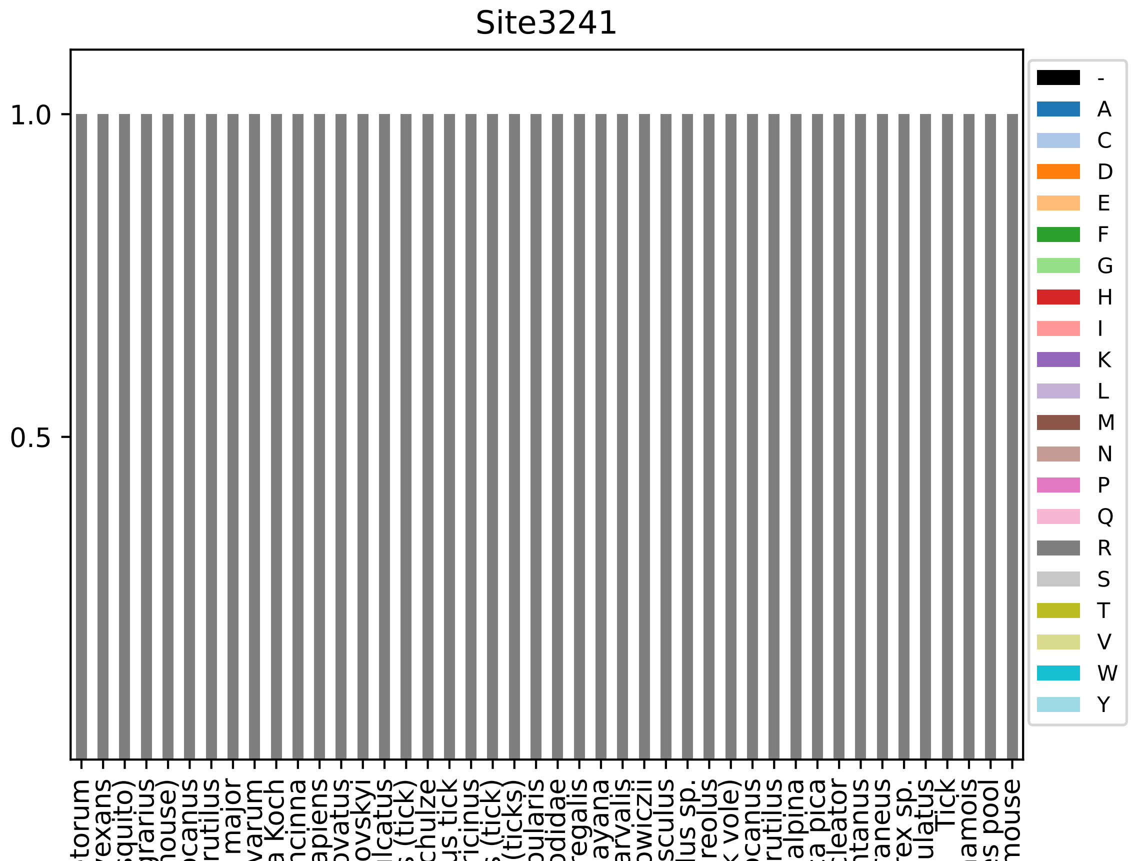
Site3241
1.0
0.5
-
A
C
D
E
F
G
H
I
K
L
M
N
P
Q
R
S
T
V
W
Y

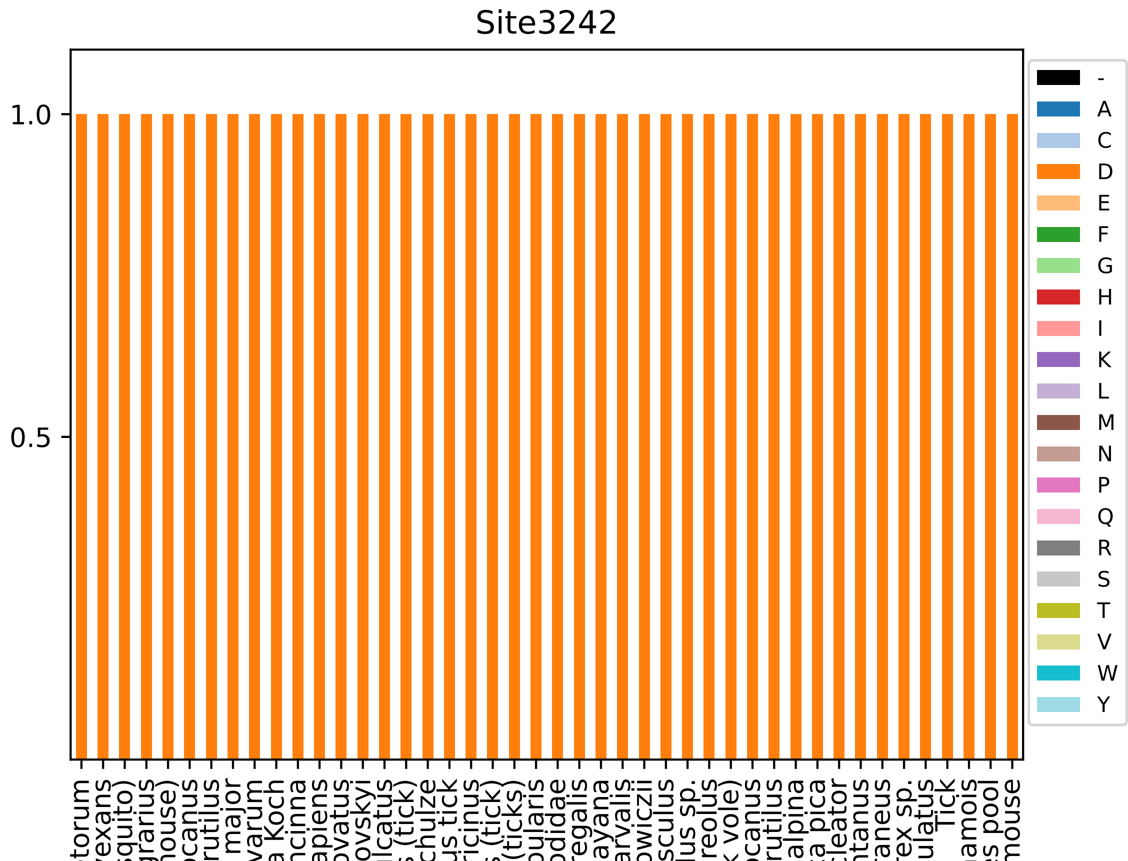

Site3242
1.0
0.5
-
A
C
D
E
F
G
H
I
K
L
M
N
P
Q
R
S
T
V
W
Y

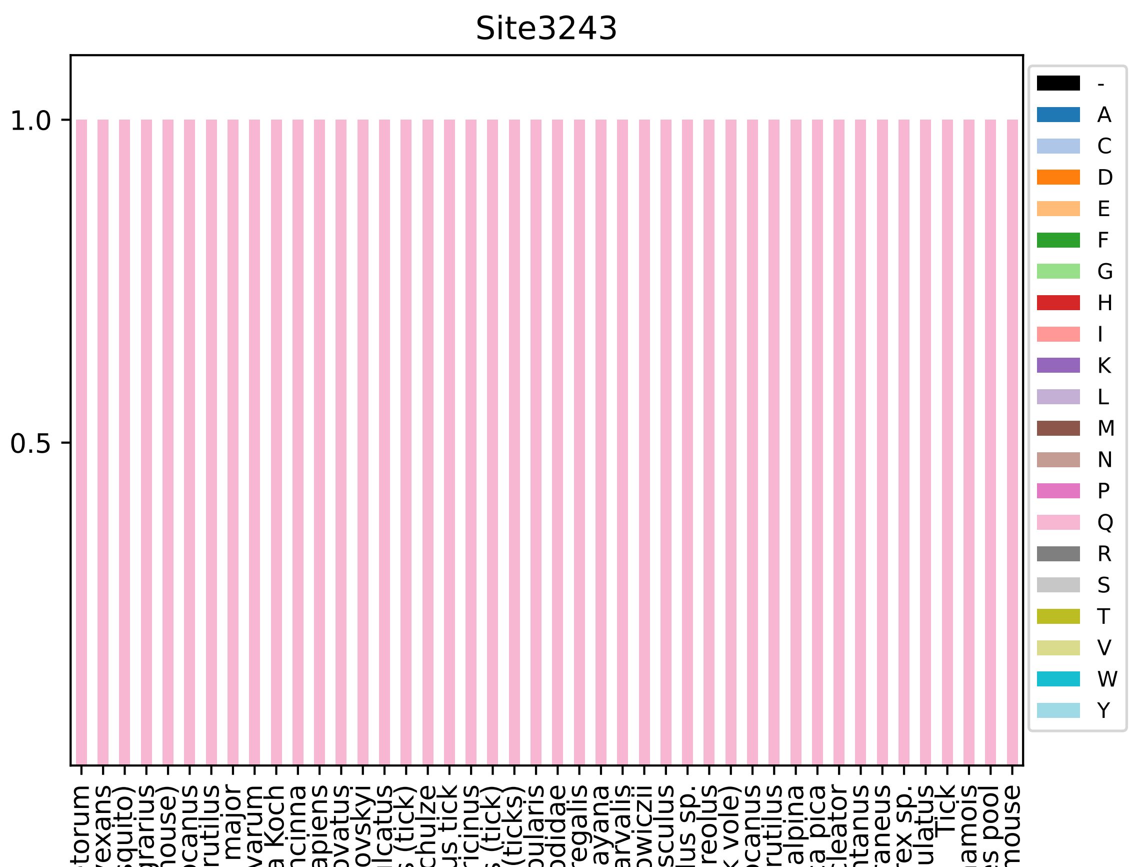
Site3243
1.0
0.5
-
A
C
D
E
F
G
H
I
K
L
M
N
P
Q
R
S
T
V
W
Y

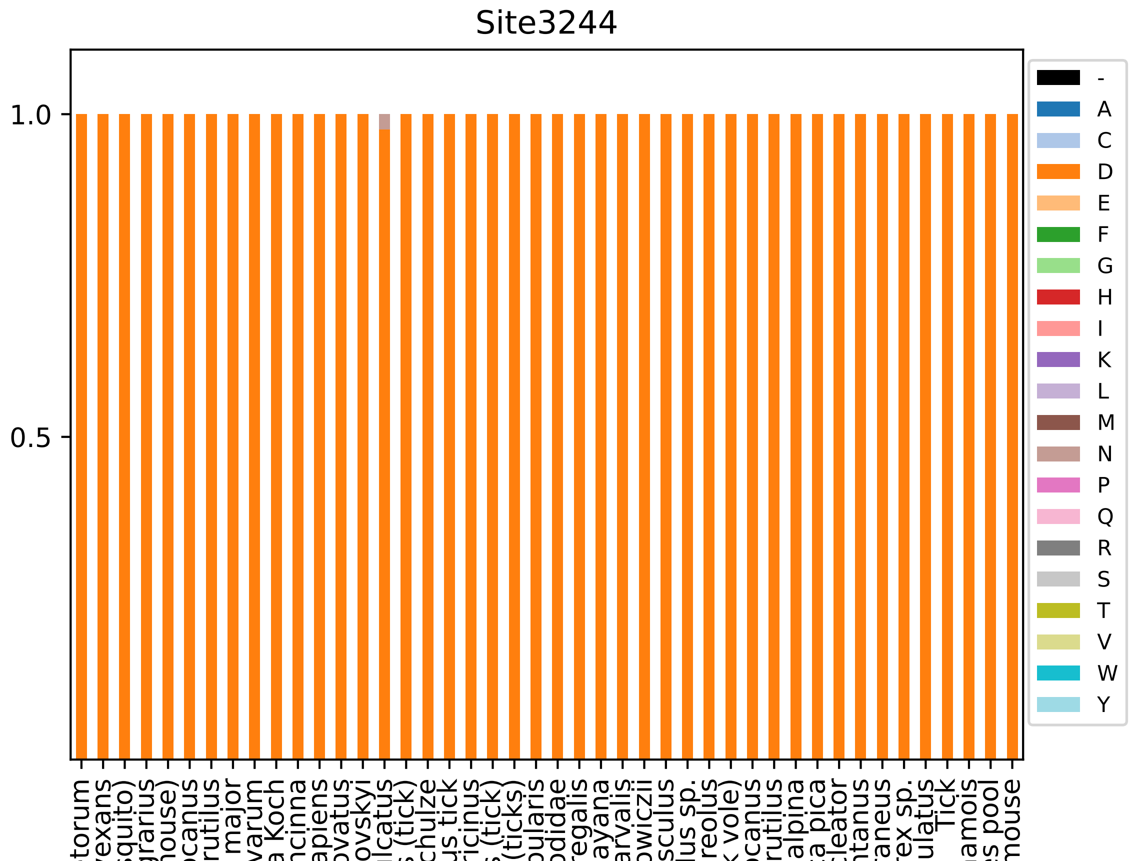
Site3244
1.0
0.5
-
A
C
D
E
F
G
H
I
K
L
M
N
P
Q
R
S
T
V
W
Y

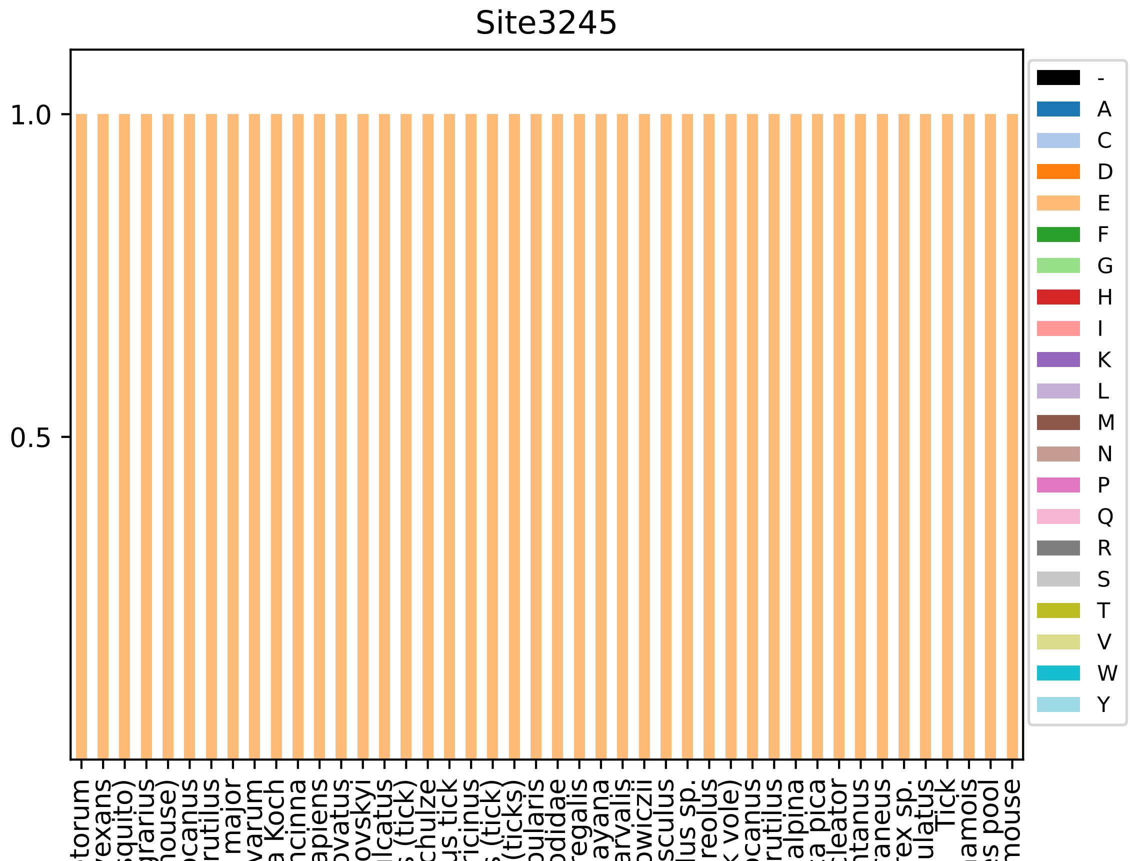
Site3245
1.0
0.5
-
A
C
D
E
F
G
H
I
K
L
M
N
P
Q
R
S
T
V
W
Y
Tick

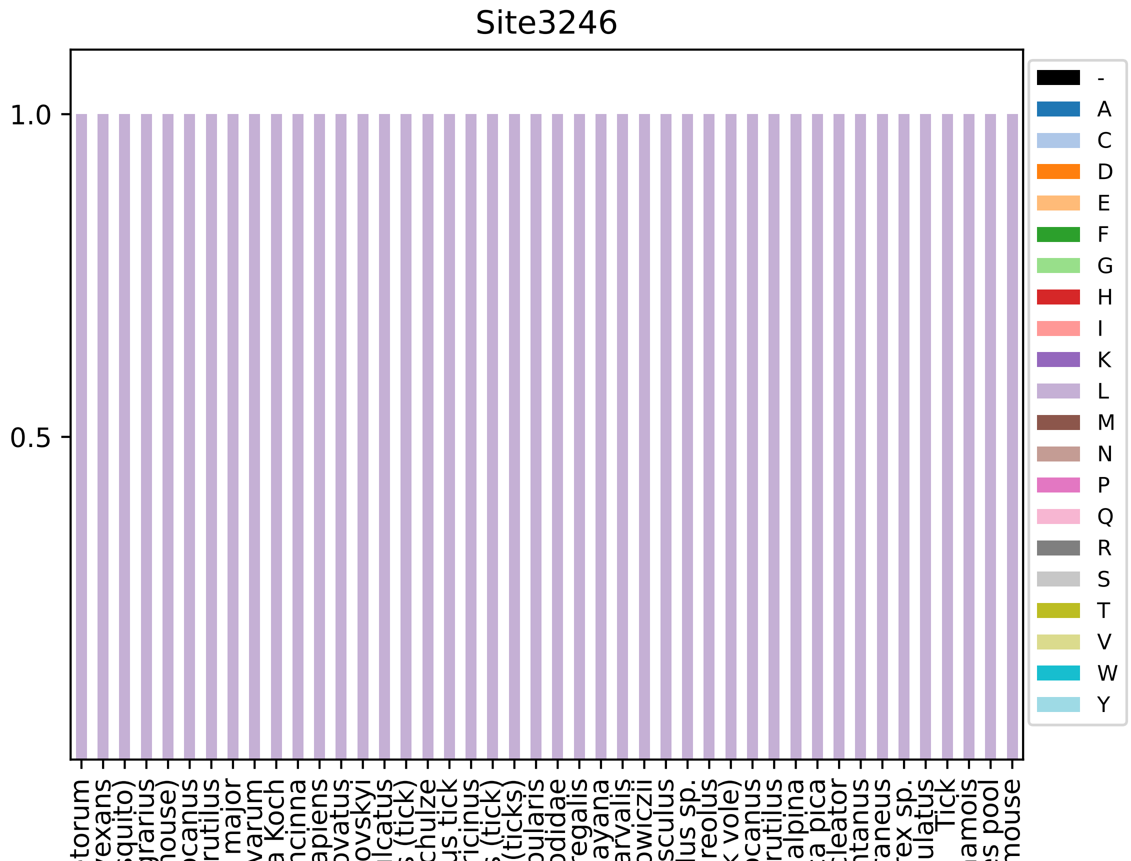
Site3246
1.0
0.5
-
A
C
D
E
F
G
H
I
K
L
M
N
P
Q
R
S
T
V
W
Y

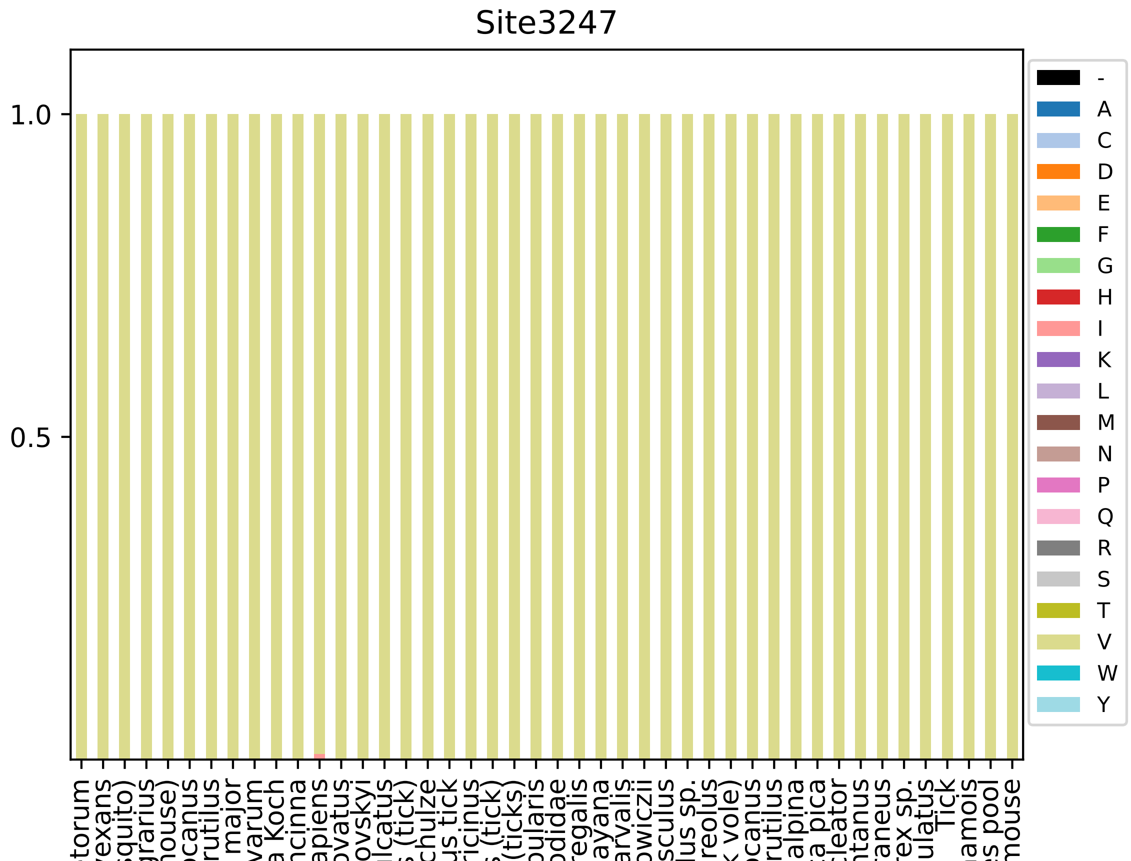
Site3247
1.0
0.5
-
A
C
D
E
F
G
H
I
K
L
M
N
P
Q
R
S
T
V
W
Y

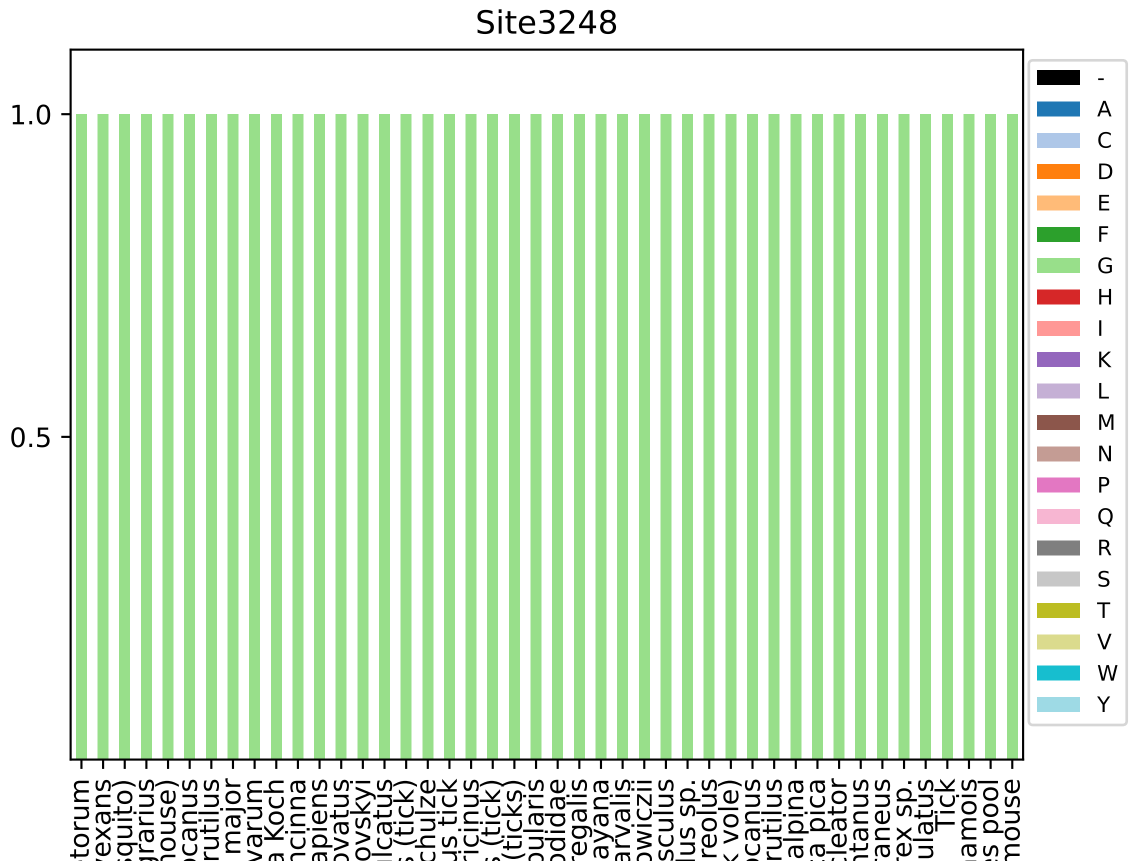

Site3248
1.0
0.5
-
A
C
D
E
F
G
H
I
K
L
M
N
P
Q
R
S
T
V
W
Y

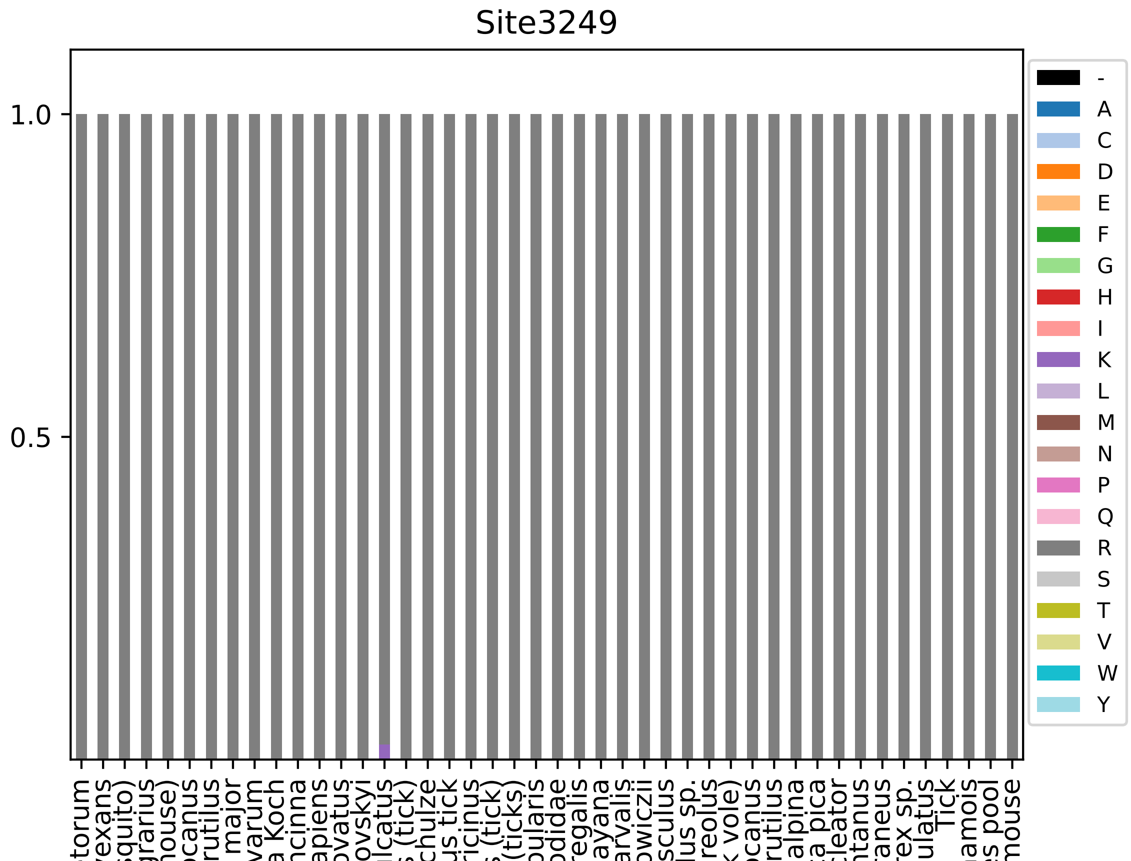
Site3249
1.0
0.5
-
A
C
D
E
F
G
H
I
K
L
M
N
P
Q
R
S
T
V
W
Y

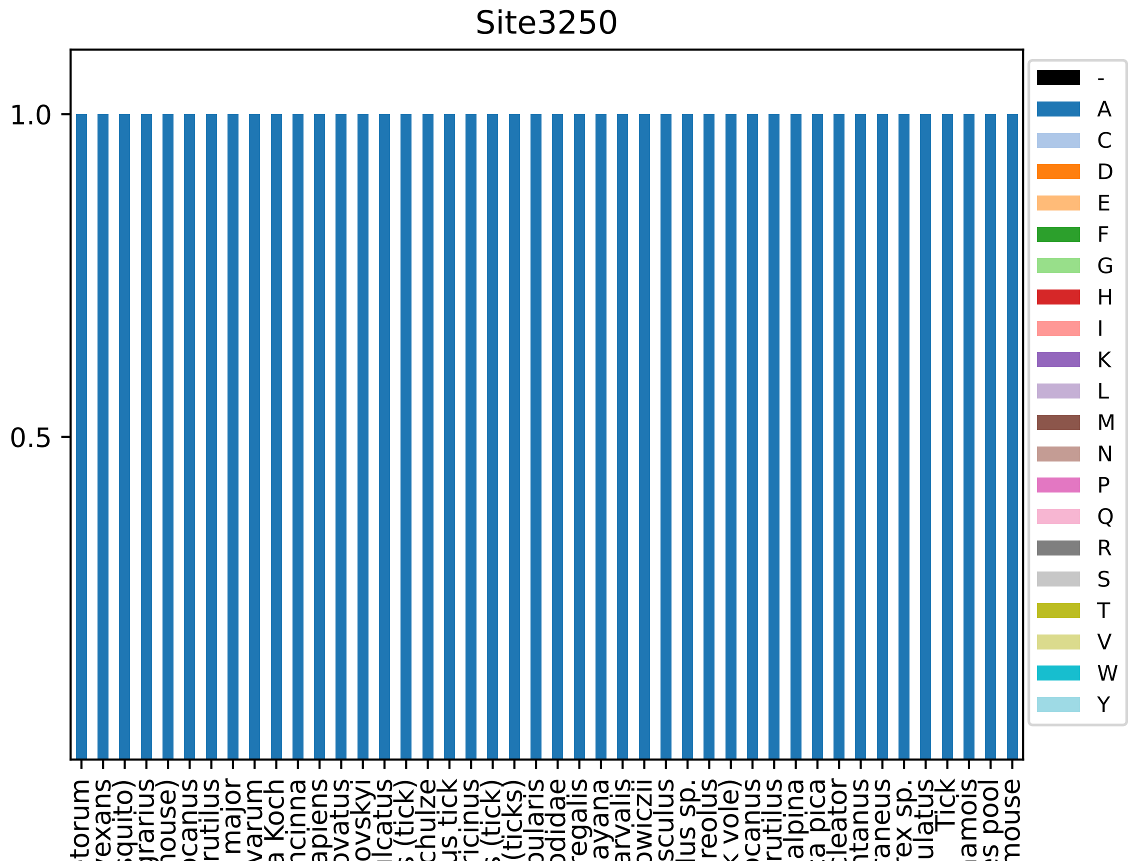

Site3250
1.0
0.5
-
A
C
D
E
F
G
H
I
K
L
M
N
P
Q
R
S
T
V
W
Y

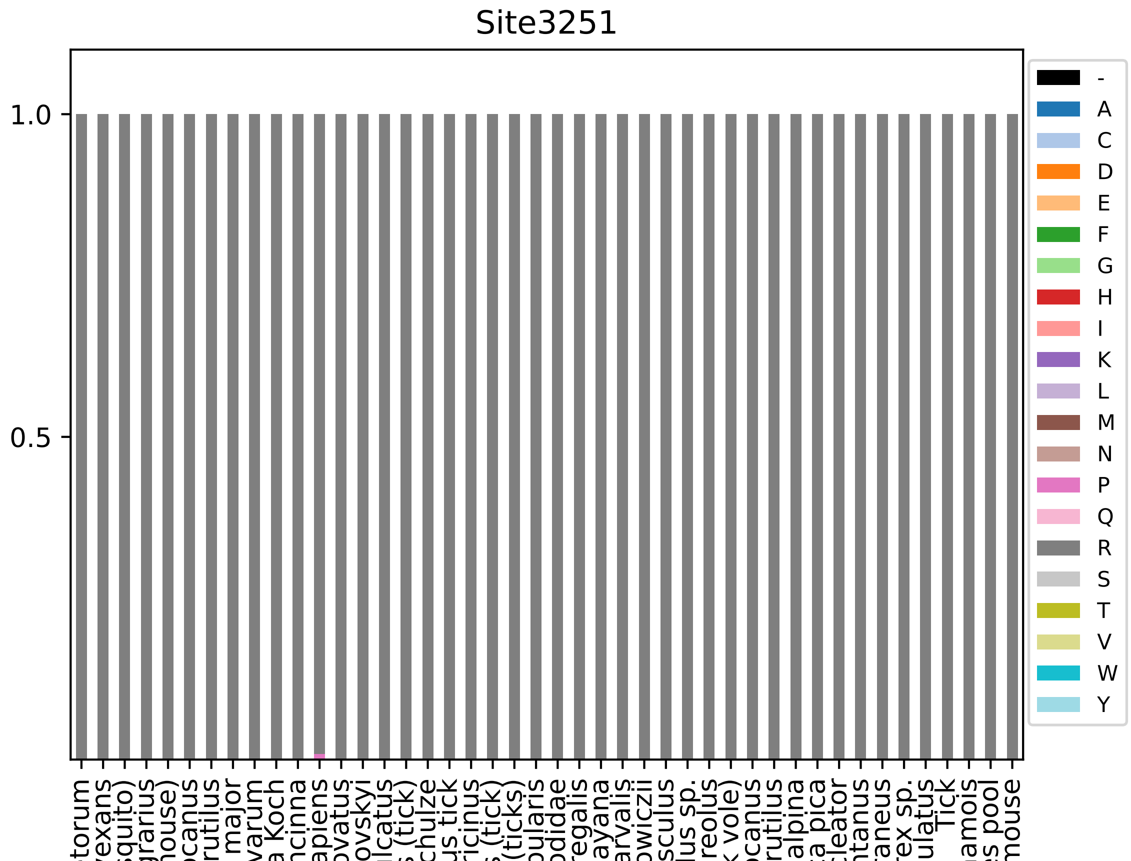
Site3251
1.0
0.5
-
A
C
D
E
F
G
H
I
K
L
M
N
P
Q
R
S
T
V
W
Y
Tick

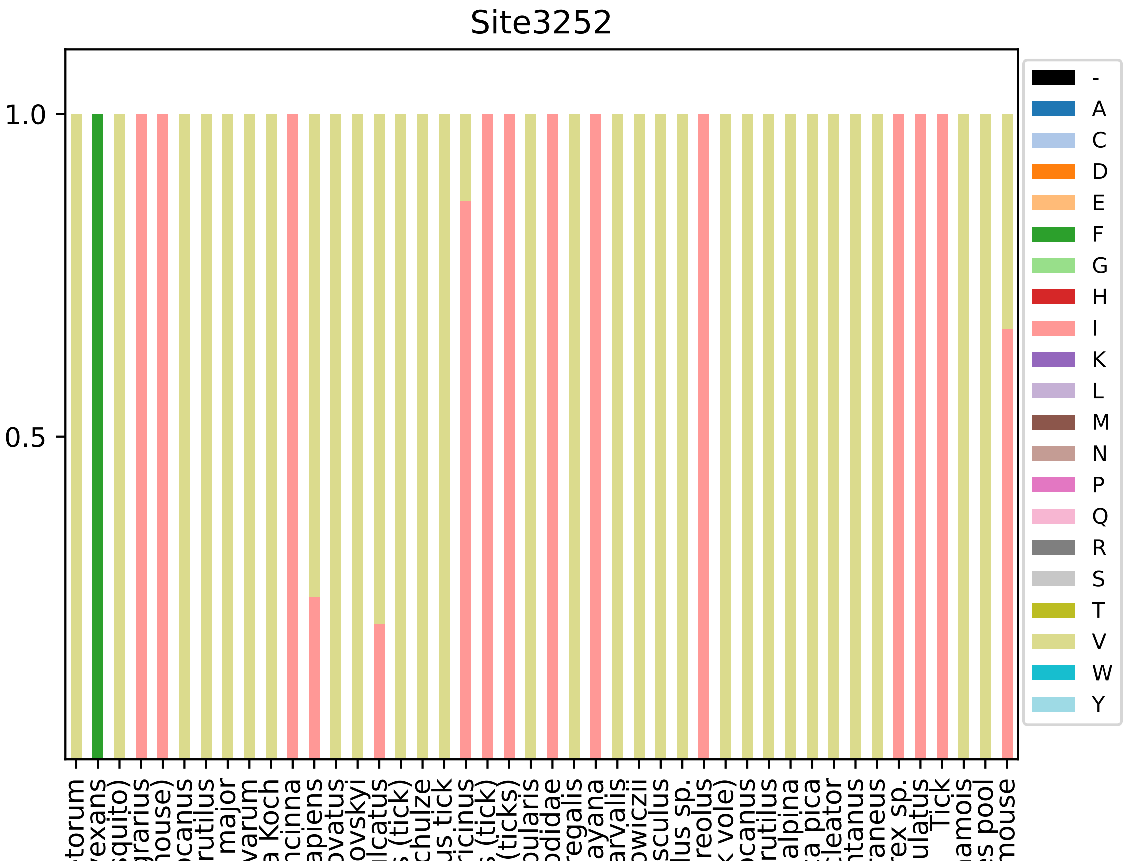
Site3252
1.0
0.5
-
A
C
D
E
F
G
H
I
K
L
M
N
P
Q
R
S
T
V
W
Y
Tick

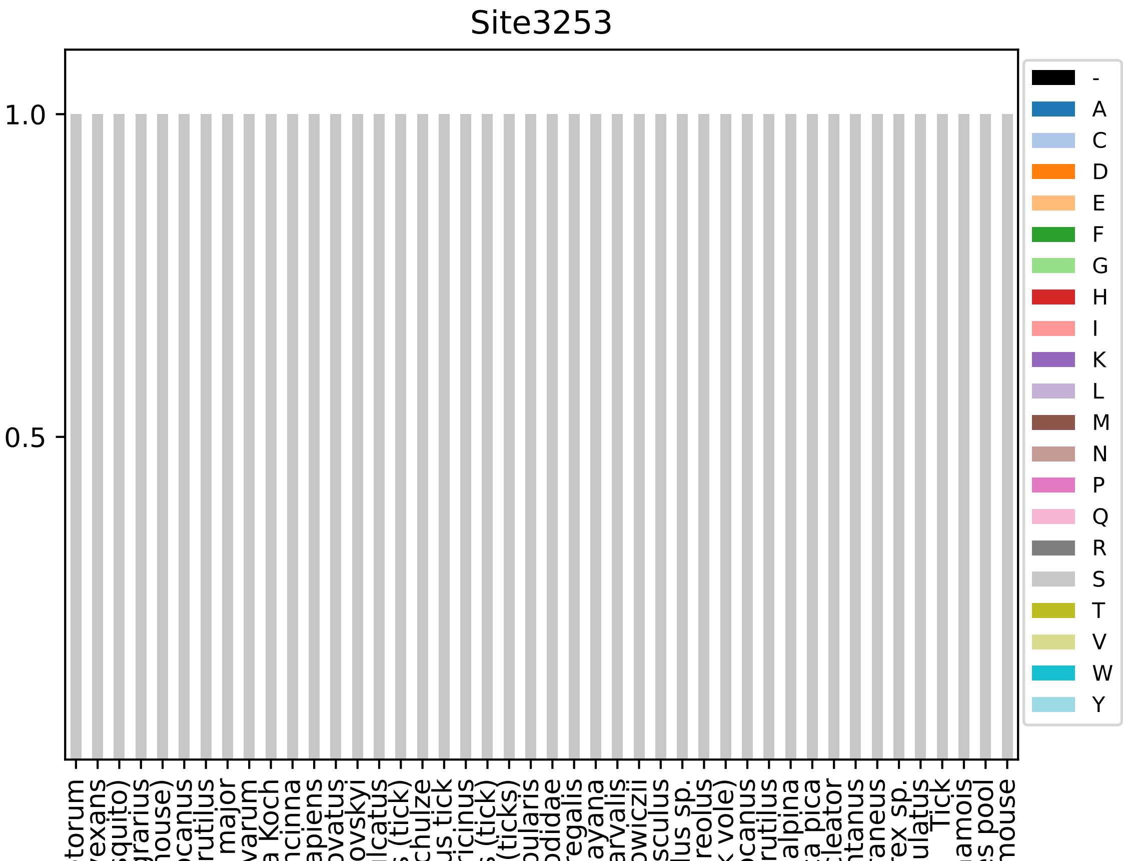

Site3253
1.0
0.5
-
A
C
D
E
F
G
H
I
K
L
M
N
P
Q
R
S
T
V
W
Y

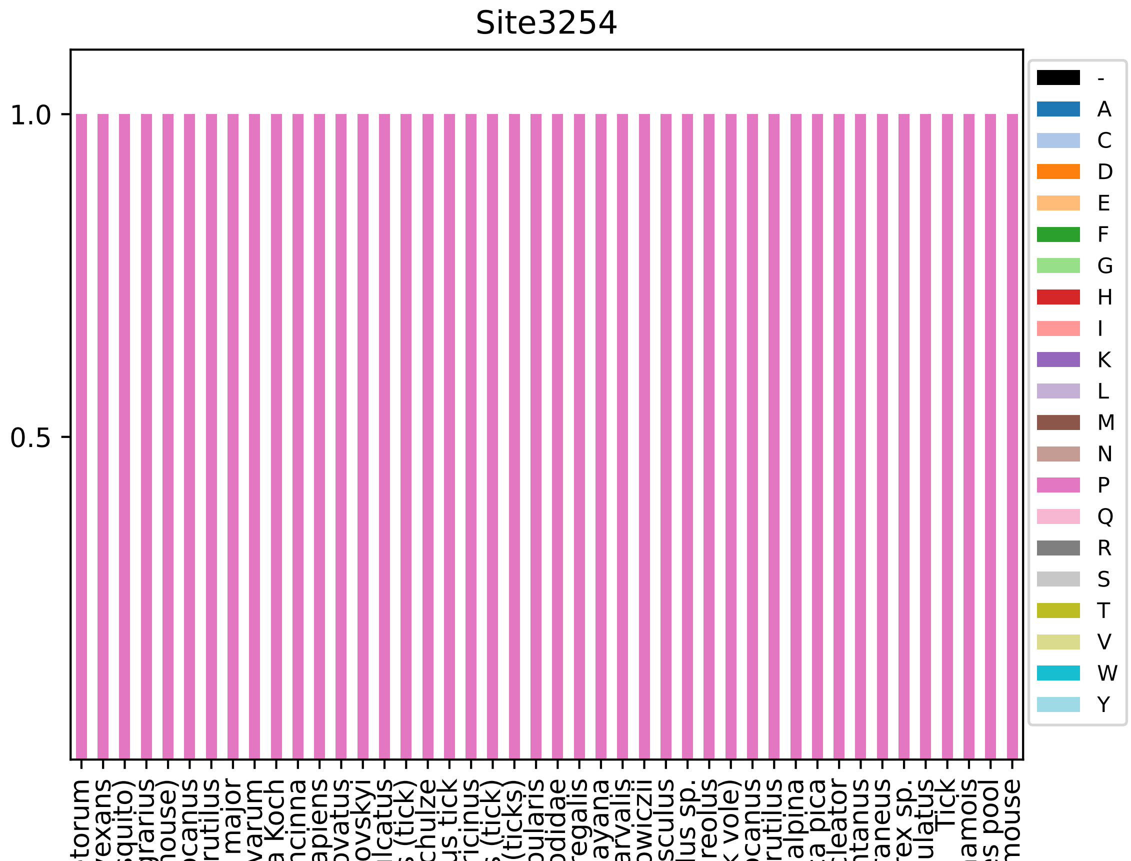
Site3254
1.0
0.5
-
A
C
D
E
F
G
H
I
K
L
M
N
P
Q
R
S
T
V
W
Y

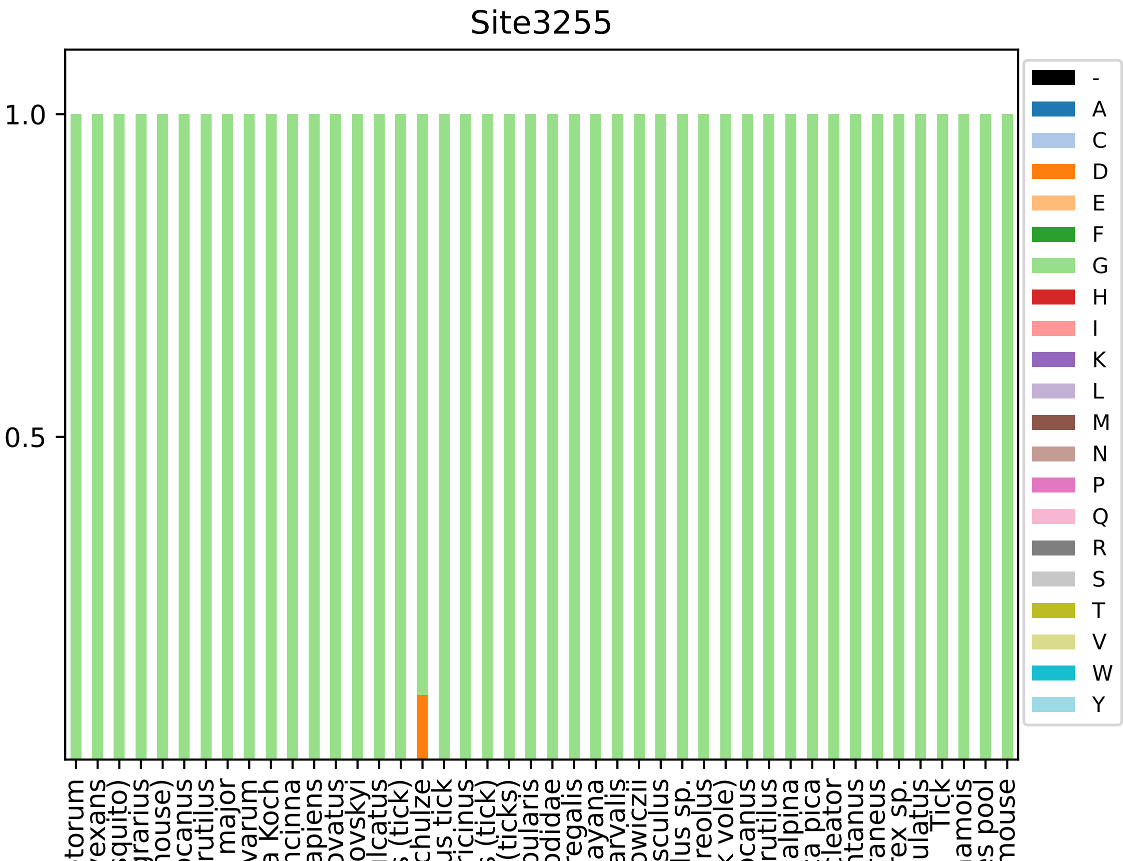
Site3255
1.0
0.5
-
A
C
D
E
F
G
H
I
K
L
M
N
P
Q
R
S
T
V
W
Y

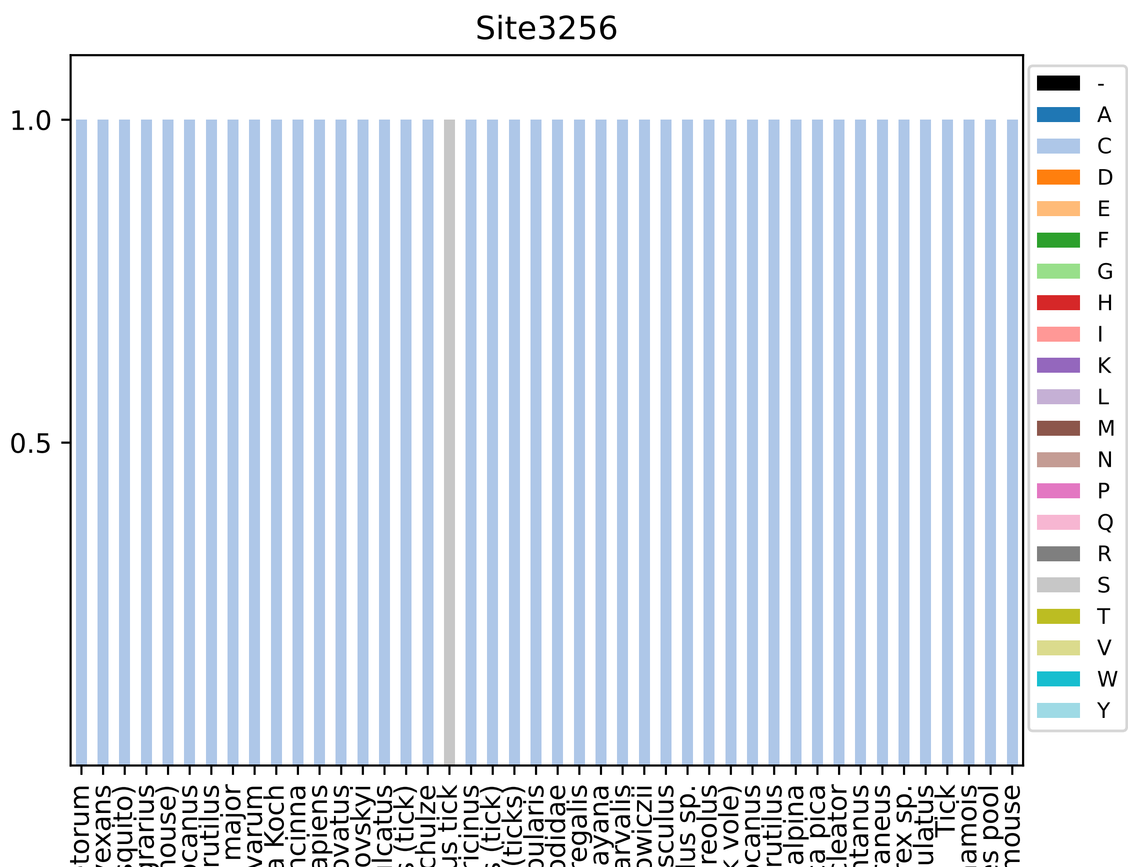
Site3256
1.0
0.5
-
A
C
D
E
F
G
H
I
K
L
M
N
P
Q
R
S
T
V
W
Y

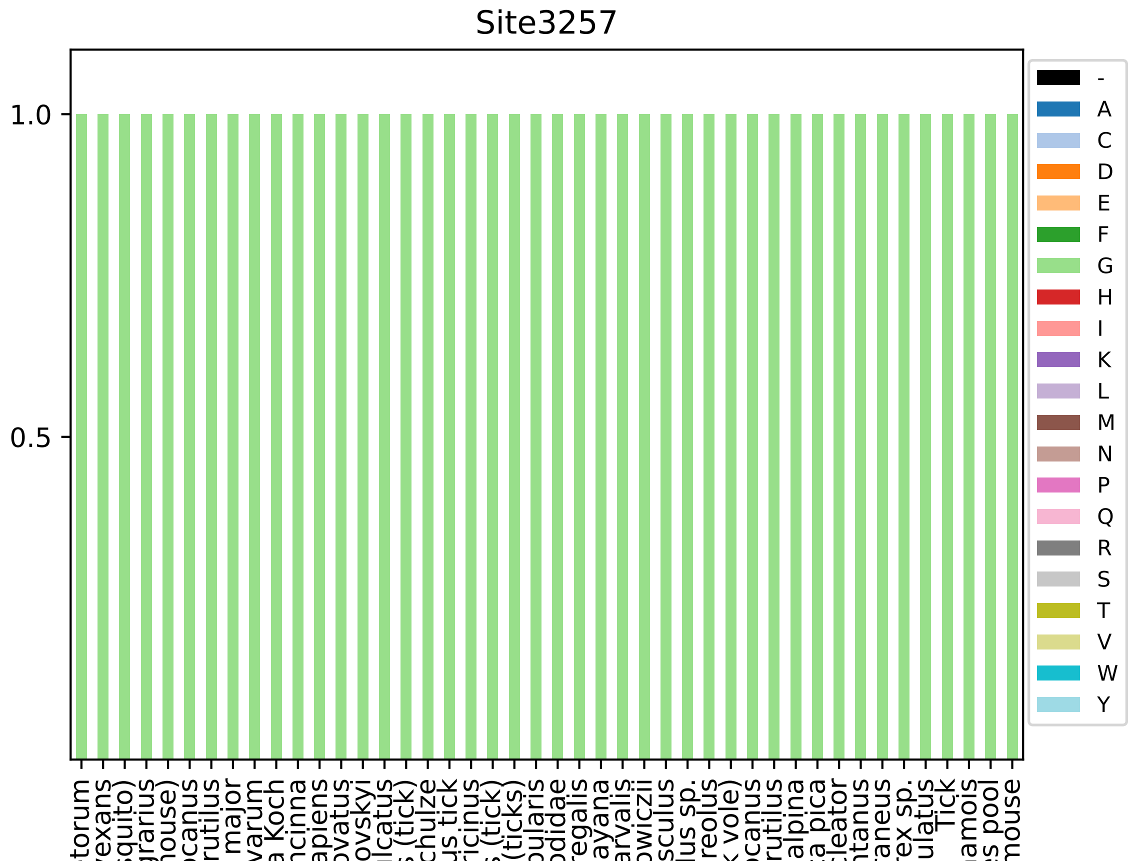

Site3257
1.0
0.5
-
A
C
D
E
F
G
H
I
K
L
M
N
P
Q
R
S
T
V
W
Y

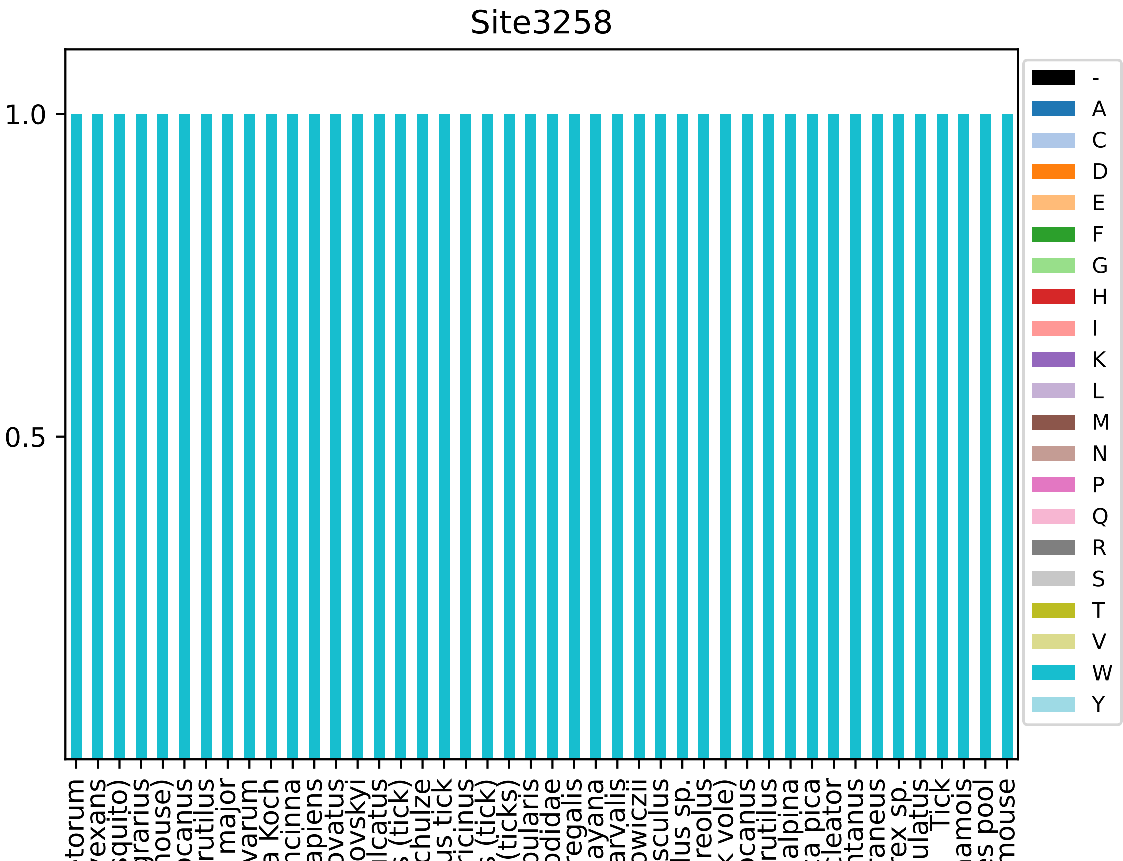
Site3258
1.0
0.5
-
A
C
D
E
F
G
H
I
K
L
M
N
P
Q
R
S
T
V
W
Y

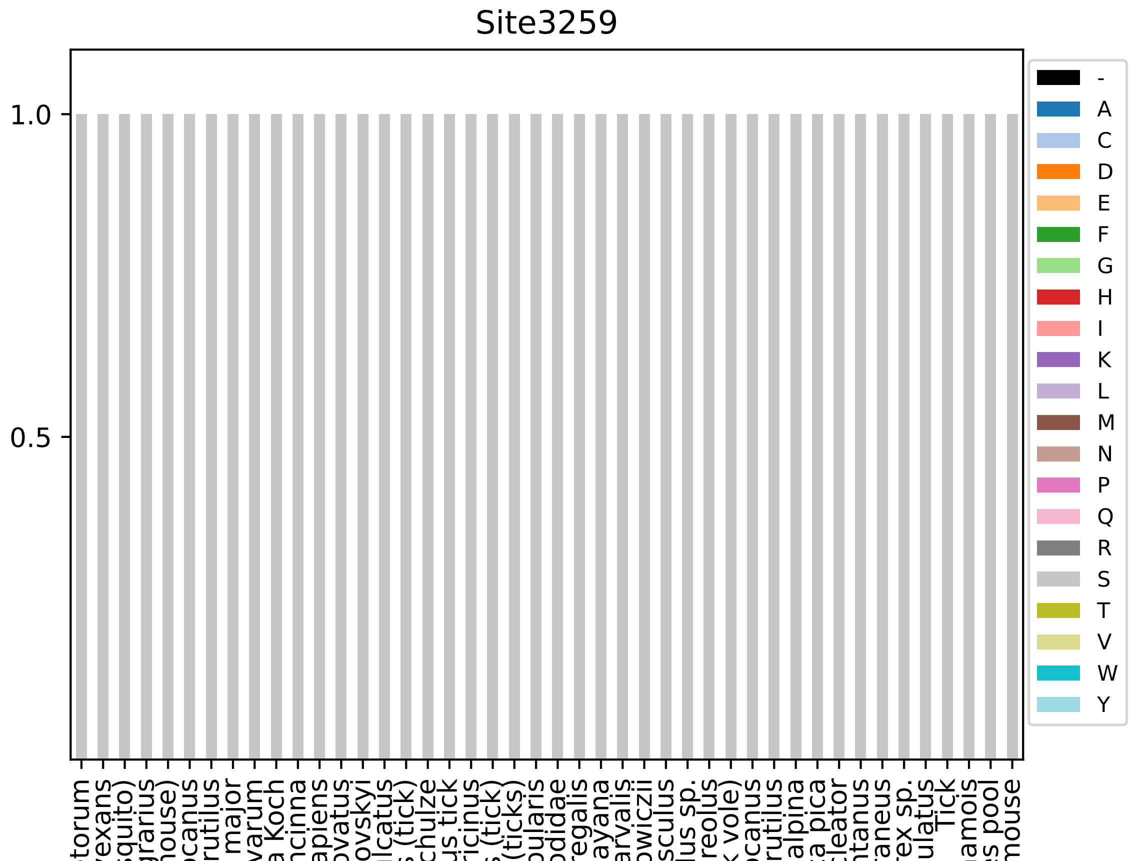
Site3259
1.0
0.5
-
A
C
D
E
F
G
H
I
K
L
M
N
P
Q
R
S
T
V
W
Y

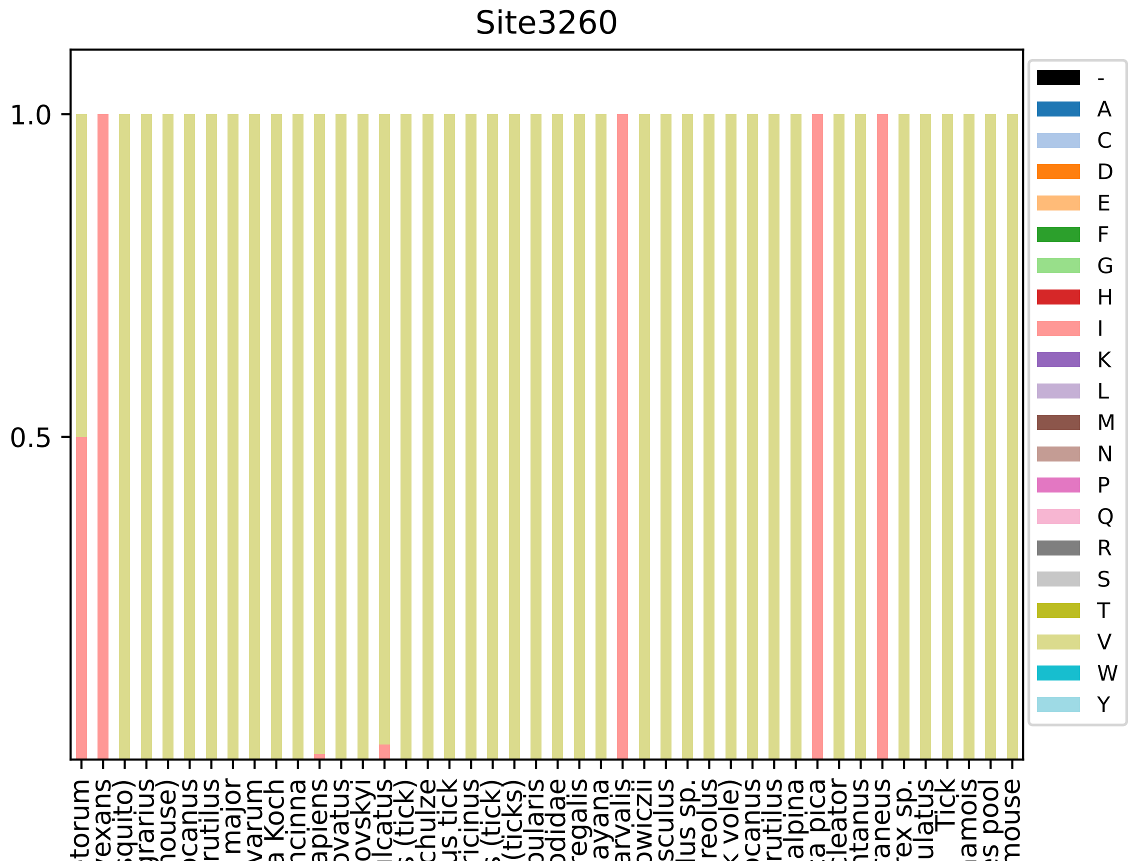

Site3260
1.0
0.5
-
A
C
D
E
F
G
H
I
K
L
M
N
P
Q
R
S
T
V
W
Y

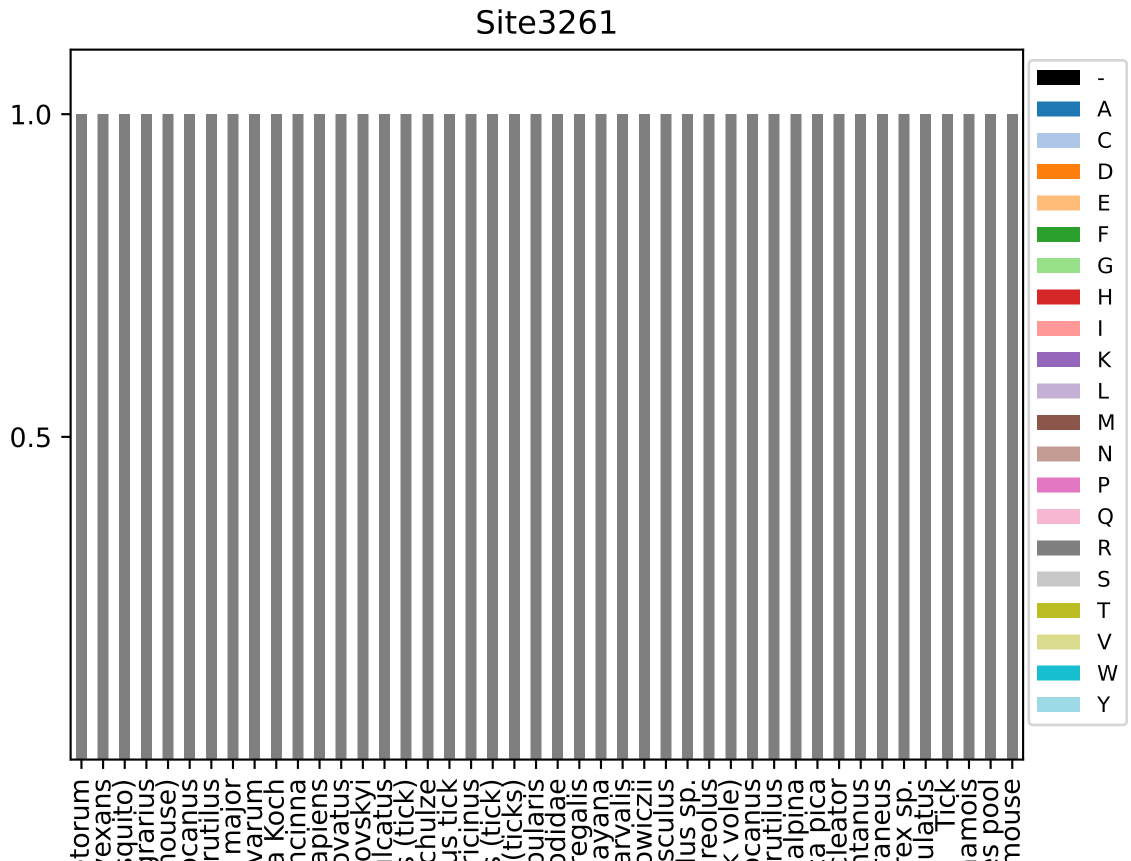
Site3261
1.0
0.5
-
A
C
D
E
F
G
H
I
K
L
M
N
P
Q
R
S
T
V
W
Y

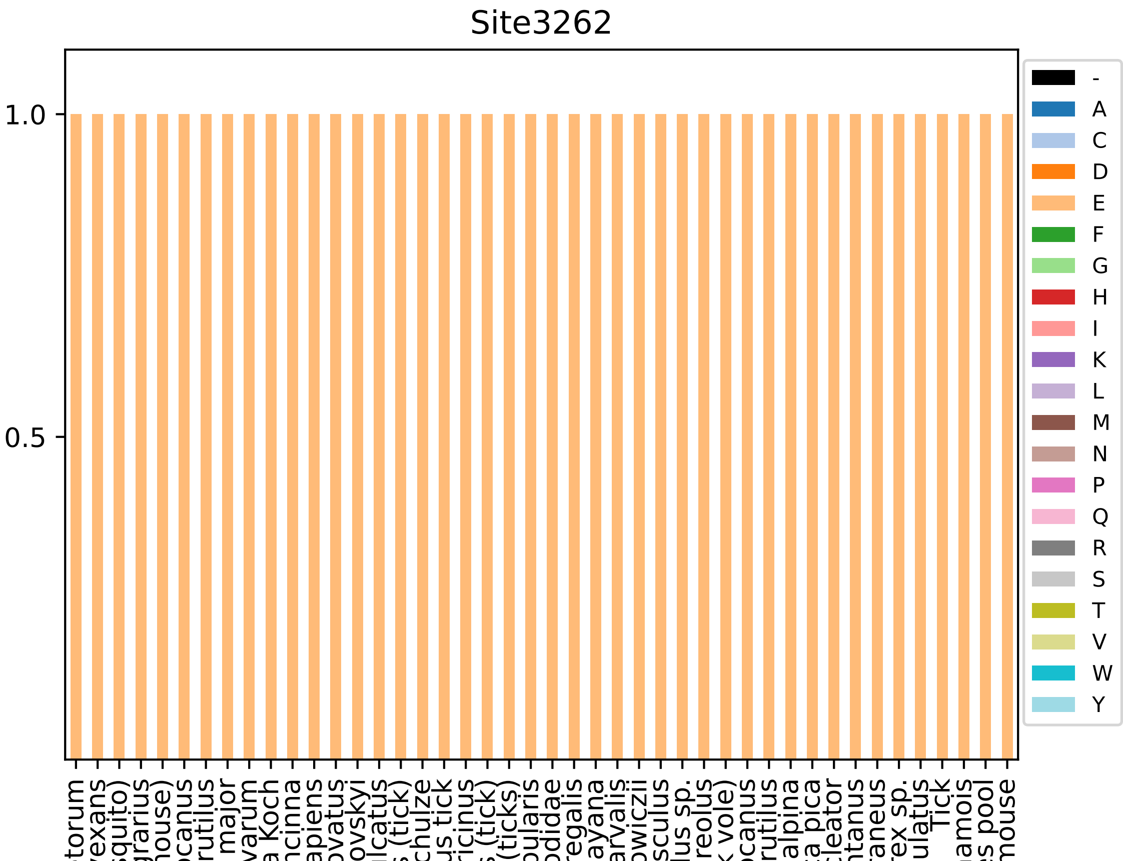
Site3262
1.0
0.5
-
A
C
D
E
F
G
H
I
K
L
M
N
P
Q
R
S
T
V
W
Y

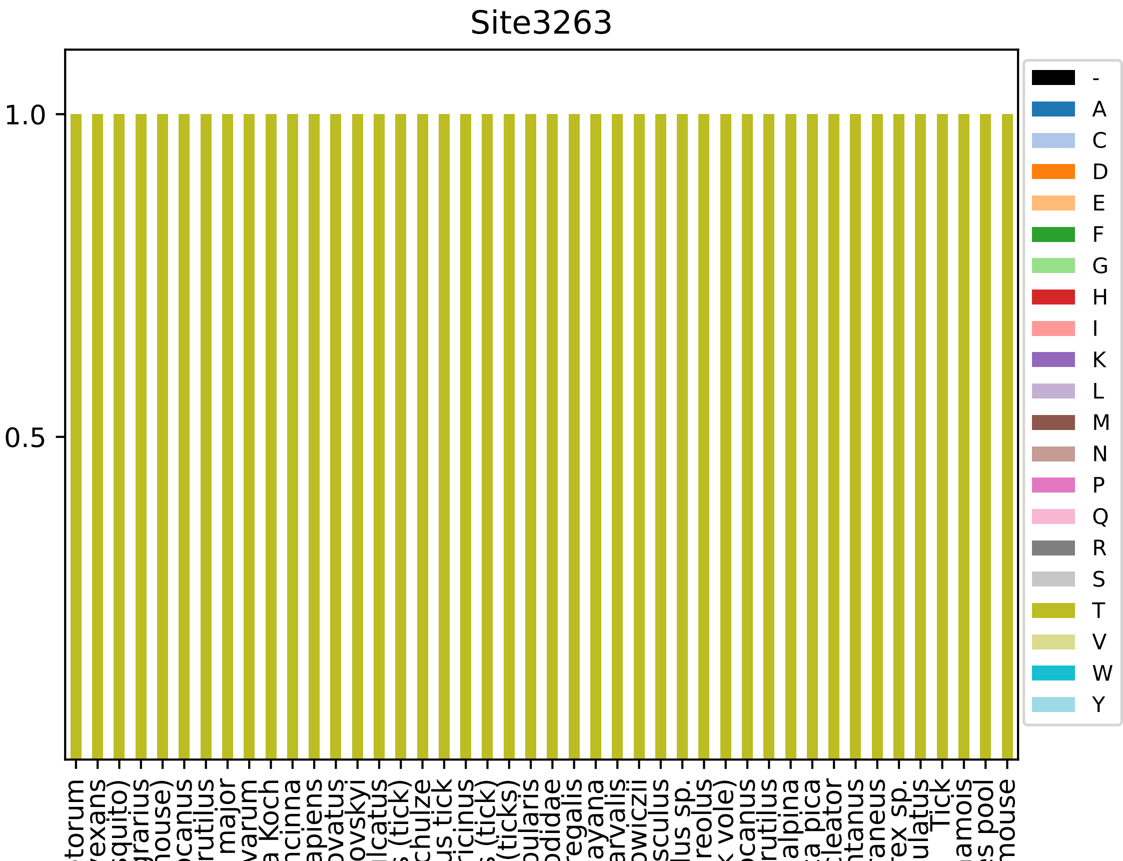
Site3263
1.0
0.5
-
A
C
D
E
F
G
H
I
K
L
M
N
P
Q
R
S
T
V
W
Y

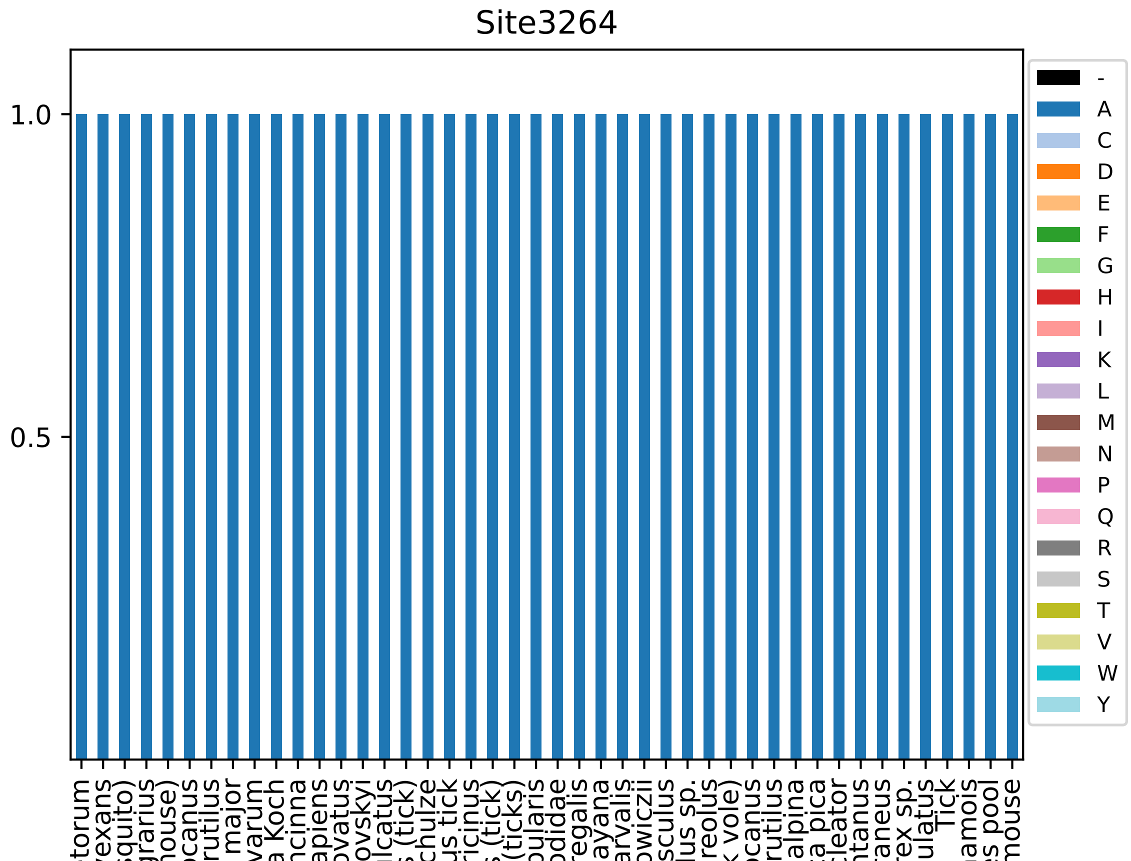

Site3264
1.0
0.5
-
A
C
D
E
F
G
H
I
K
L
M
N
P
Q
R
S
T
V
W
Y

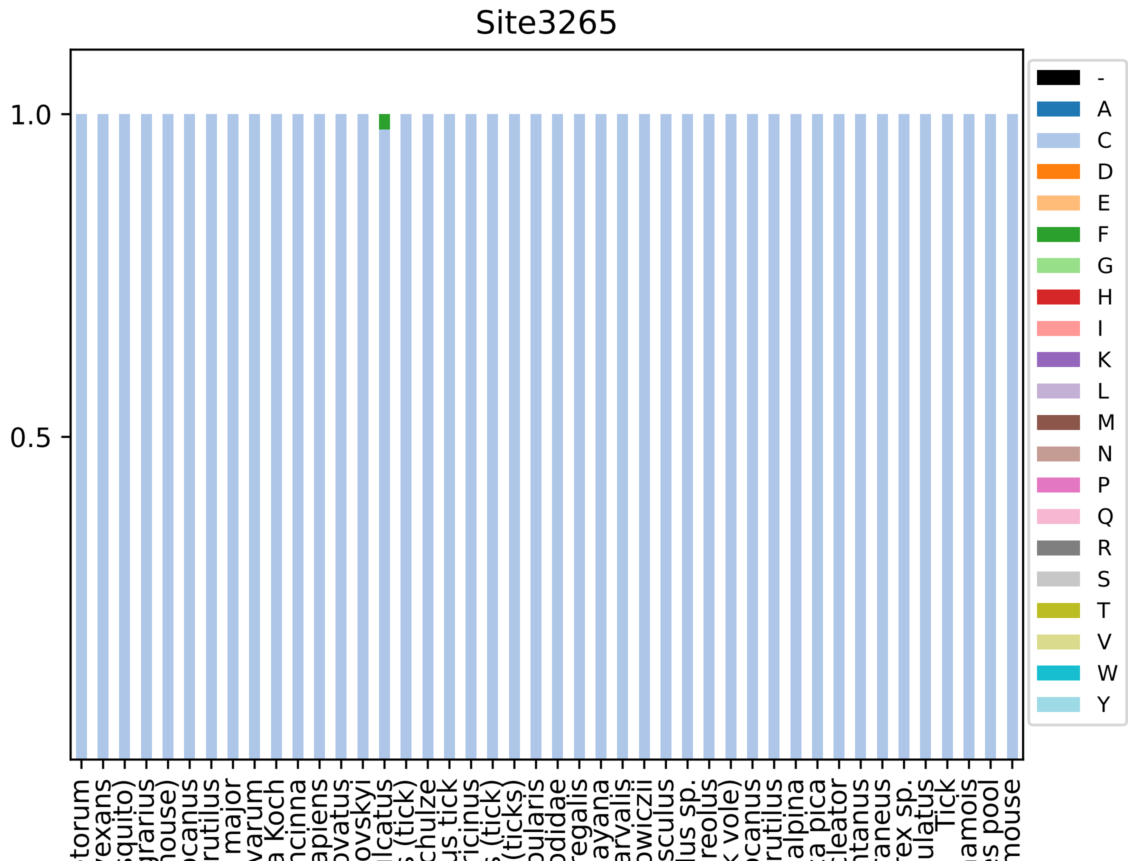

Site3265
1.0
0.5
-
A
C
D
E
F
G
H
I
K
L
M
N
P
Q
R
S
T
V
W
Y

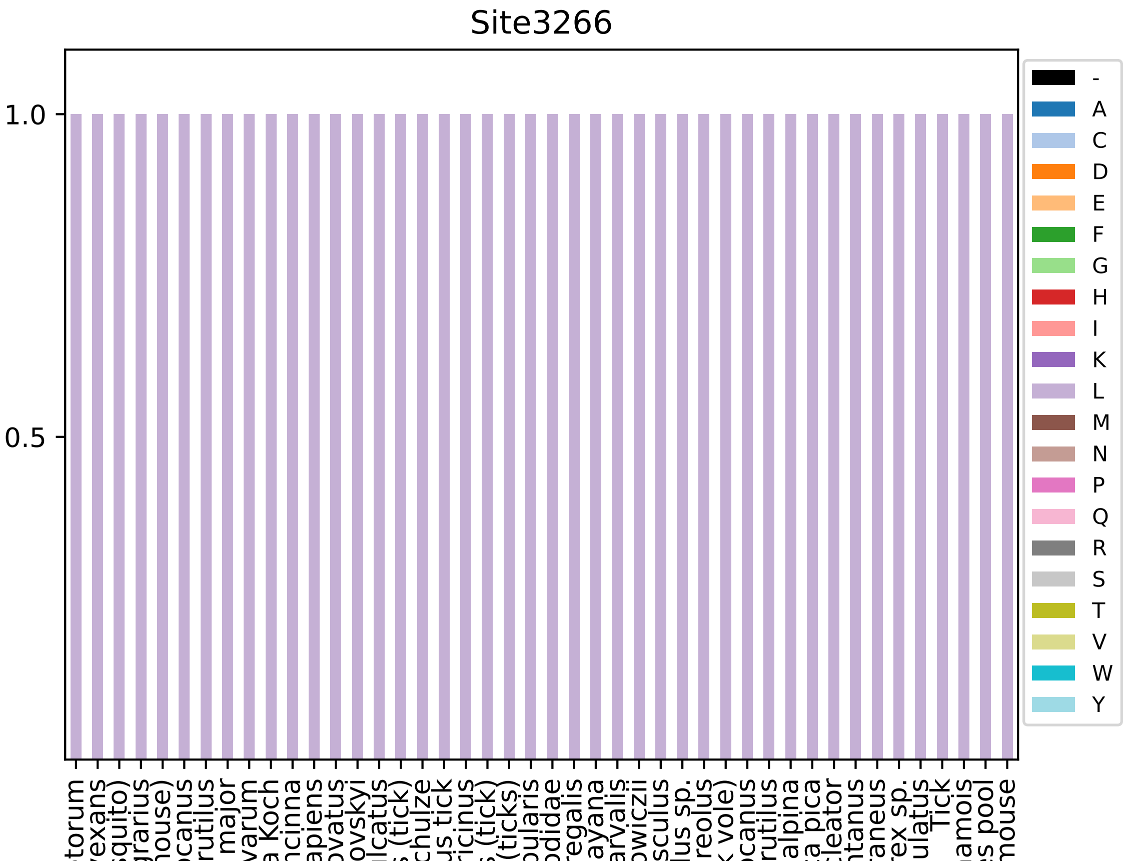
Site3266
1.0
0.5
-
A
C
D
E
F
G
H
I
K
L
M
N
P
Q
R
S
T
V
W
Y

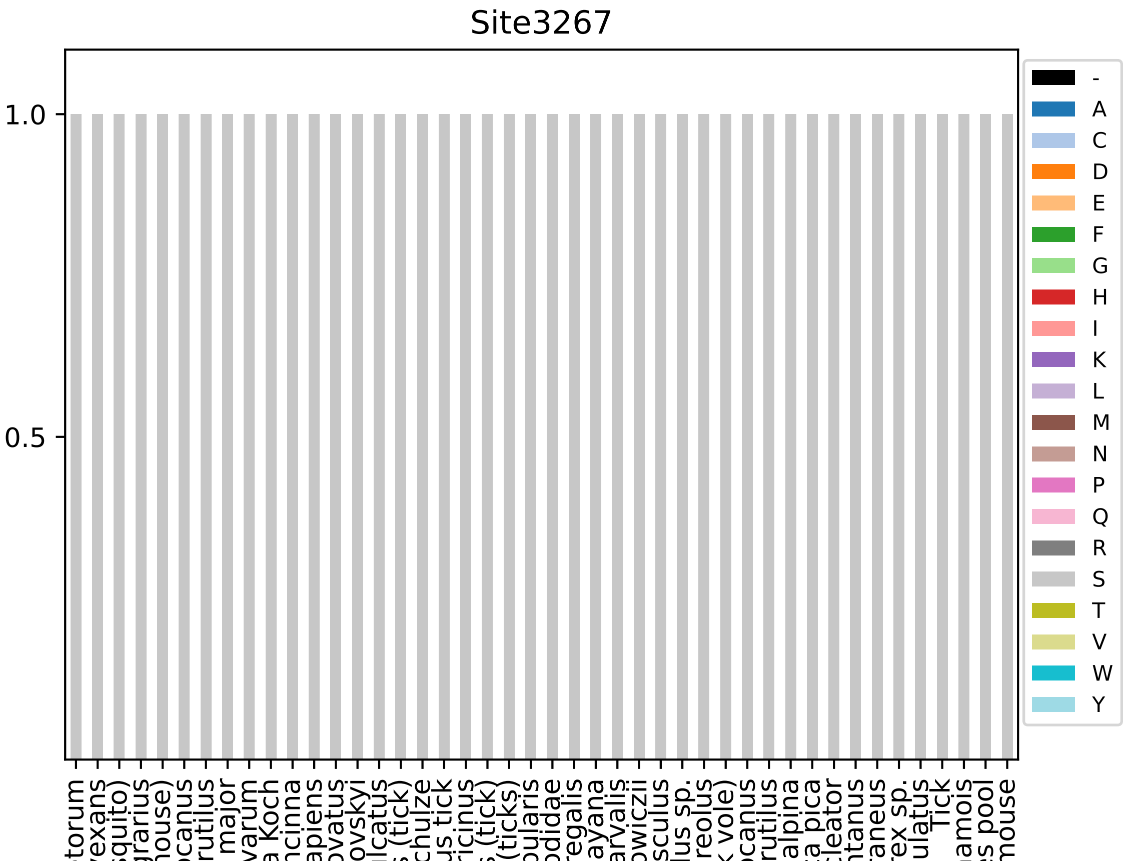
Site3267
1.0
0.5
-
A
C
D
E
F
G
H
I
K
L
M
N
P
Q
R
S
T
V
W
Y

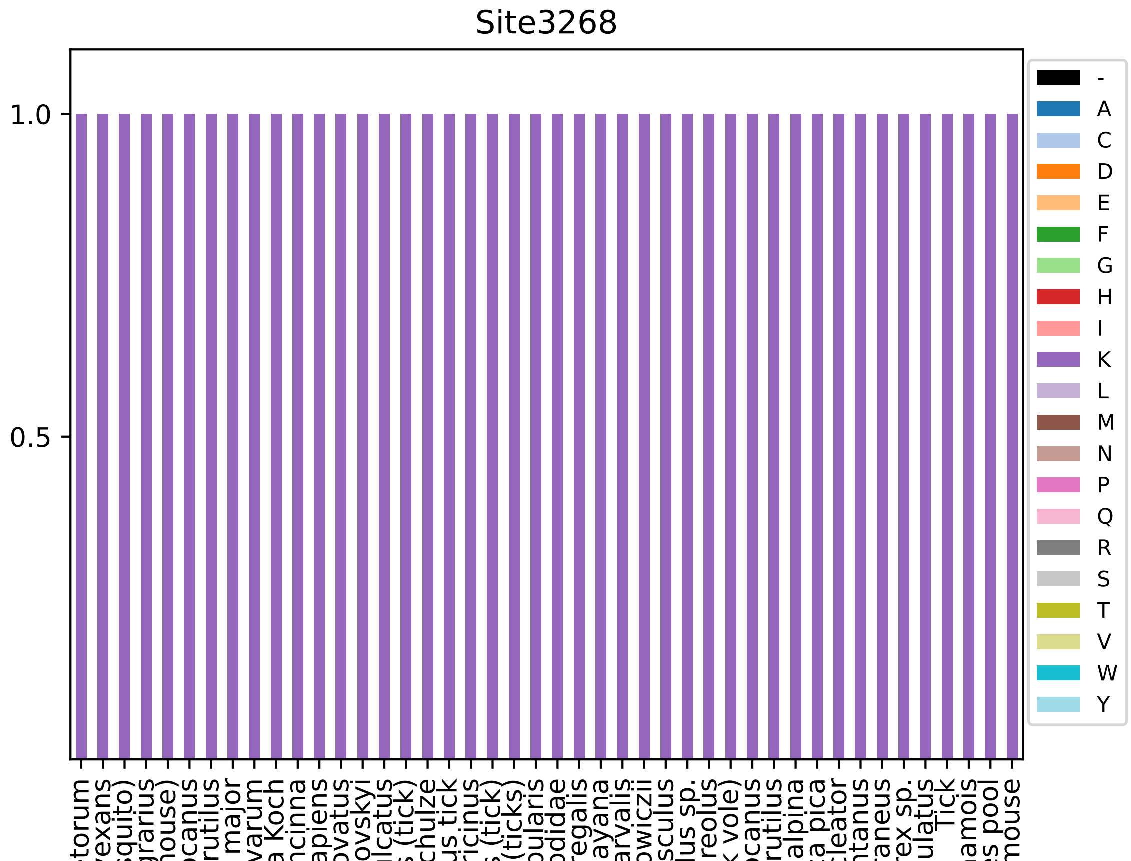
Site3268
1.0
0.5
-
A
C
D
E
F
G
H
I
K
L
M
N
P
Q
R
S
T
V
W
Y

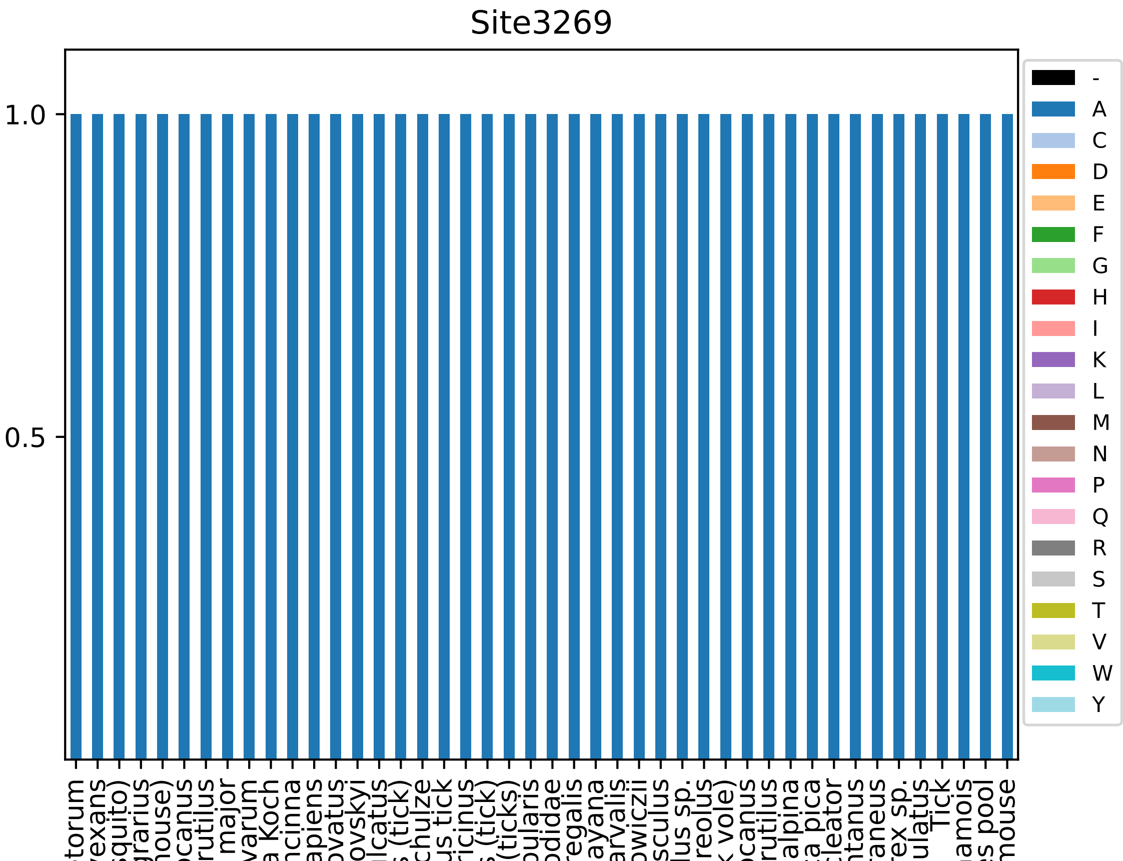

Site3269
1.0
0.5
-
A
C
D
E
F
G
H
I
K
L
M
N
P
Q
R
S
T
V
W
Y

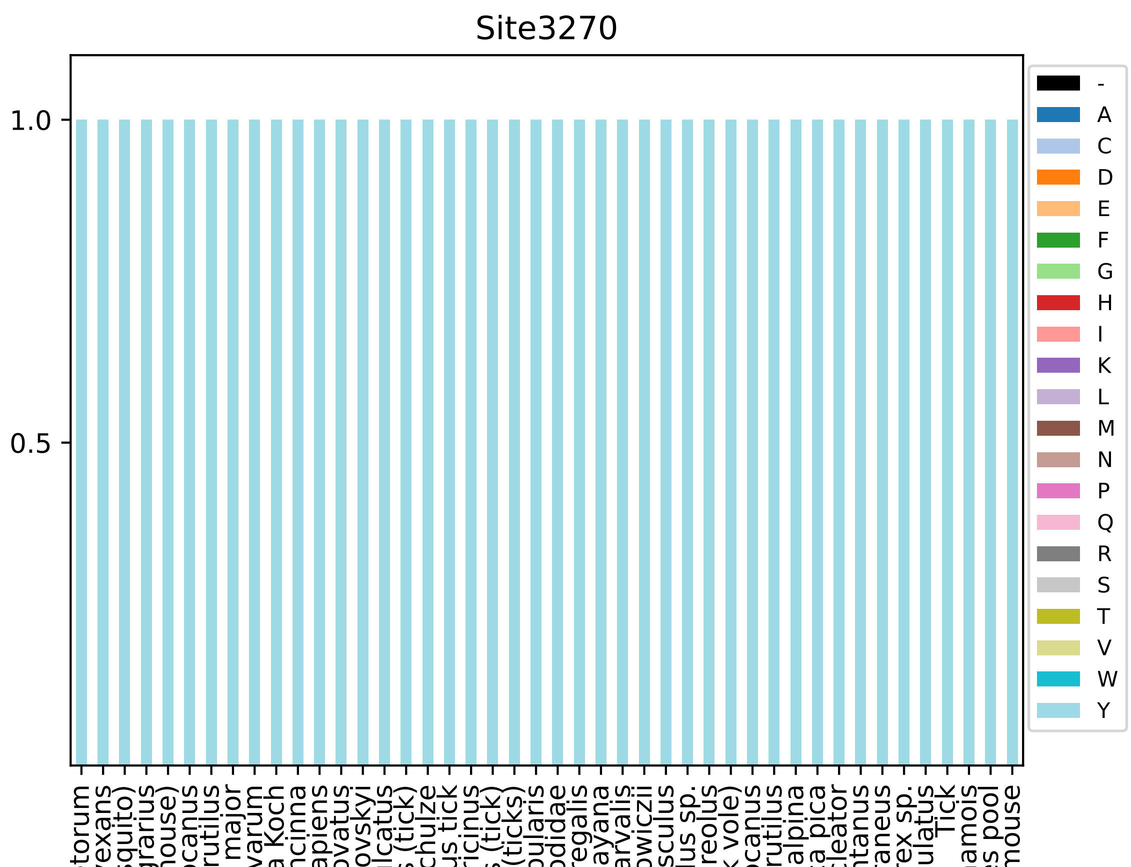

Site3270
1.0
0.5
-
A
C
D
E
F
G
H
I
K
L
M
N
P
Q
R
S
T
V
W
Y

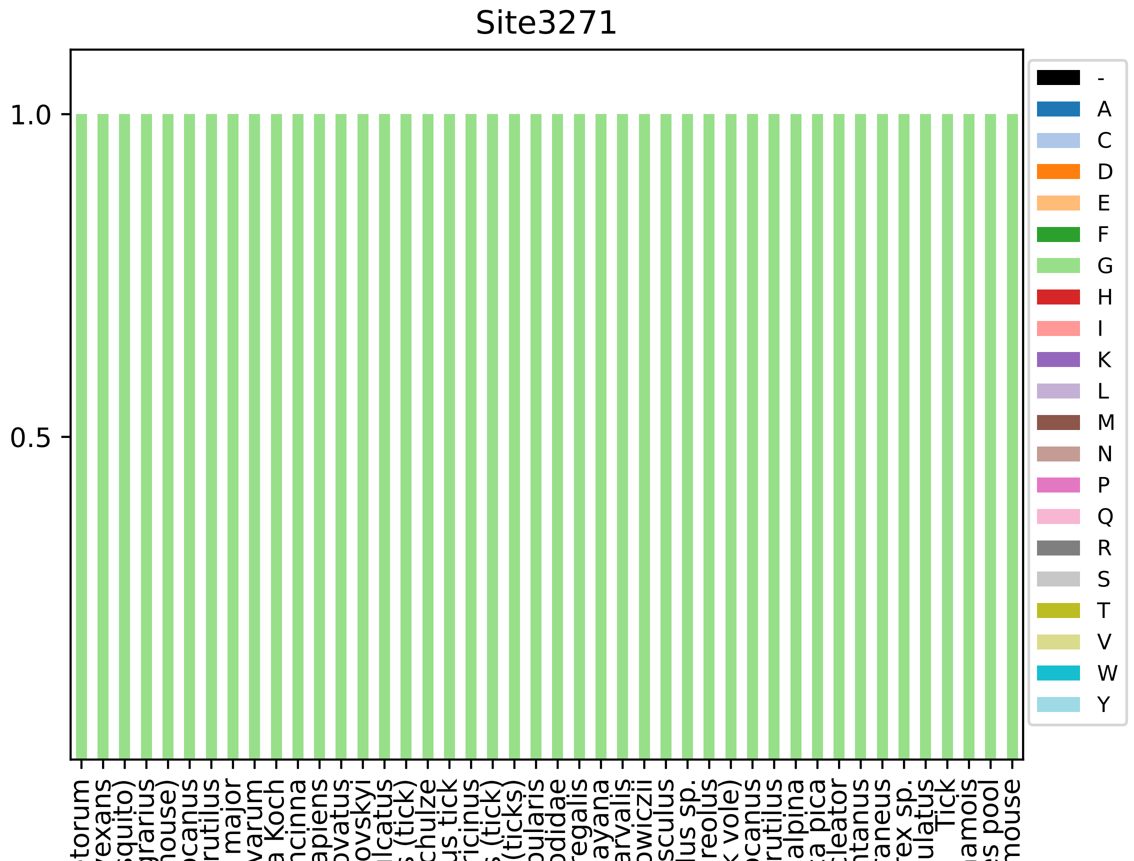
Site3271
1.0
0.5
-
A
C
D
E
F
G
H
I
K
L
M
N
P
Q
R
S
T
V
W
Y

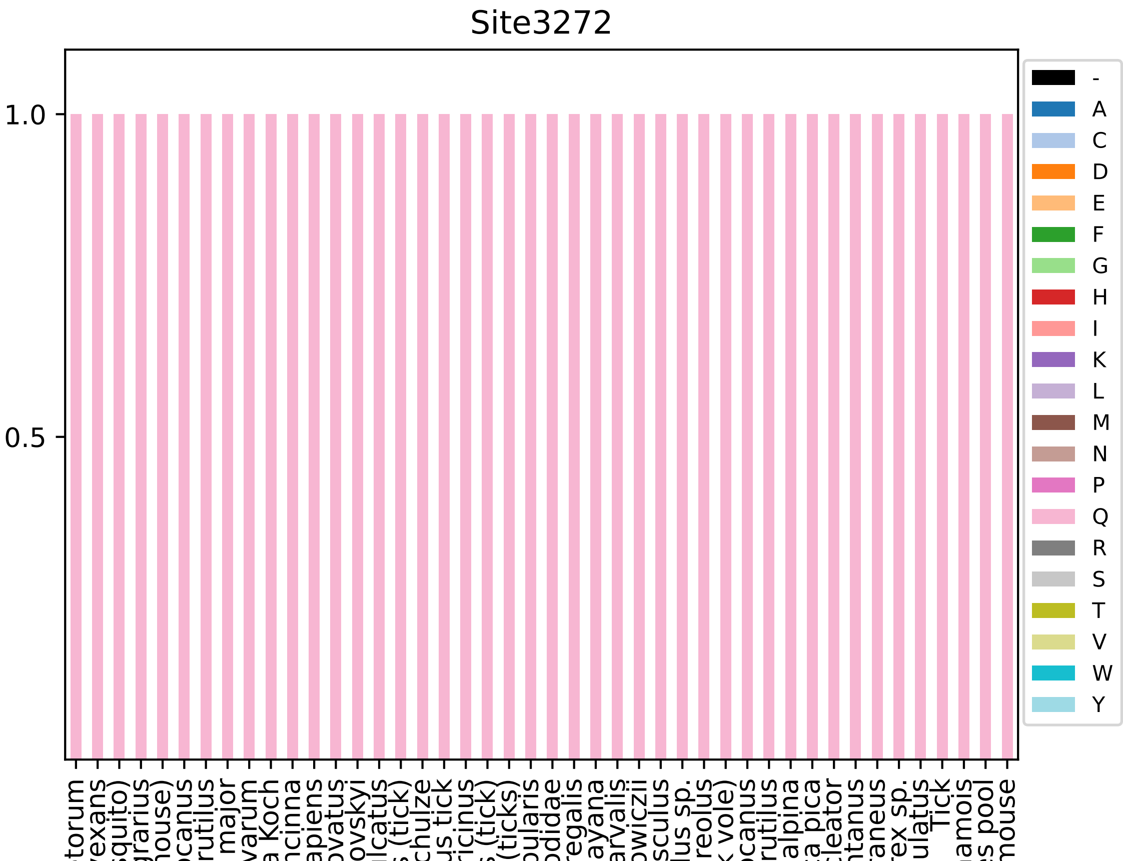

Site3272
1.0
0.5
-
A
C
D
E
F
G
H
I
K
L
M
N
P
Q
R
S
T
V
W
Y

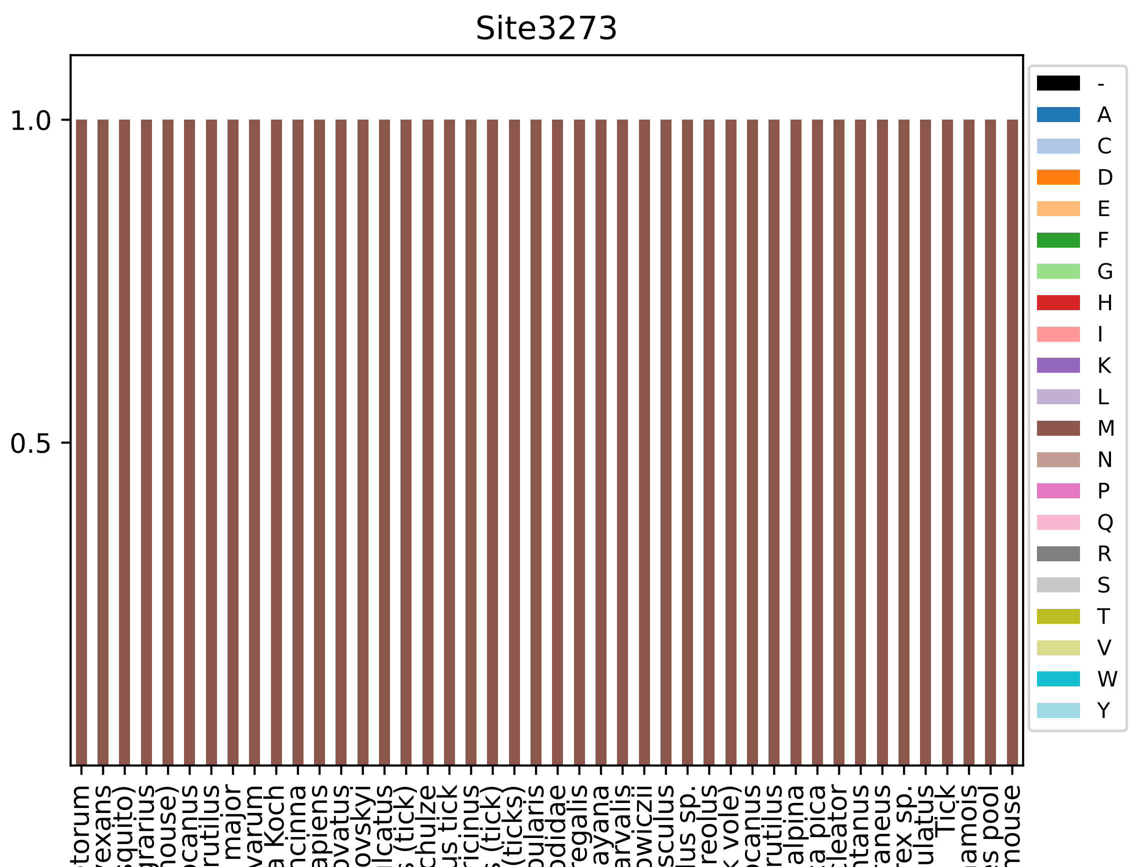
Site3273
1.0
0.5
-
A
C
D
E
F
G
H
I
K
L
M
N
P
Q
R
S
T
V
W
Y

Site3274

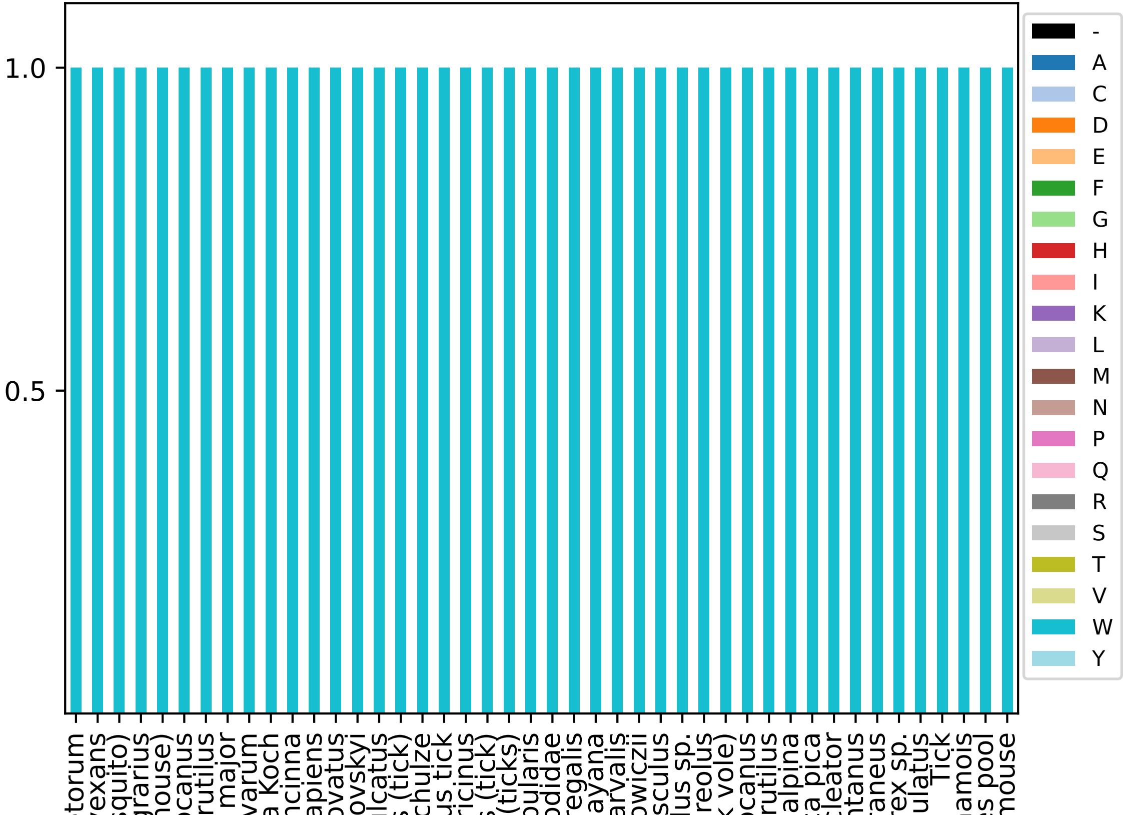

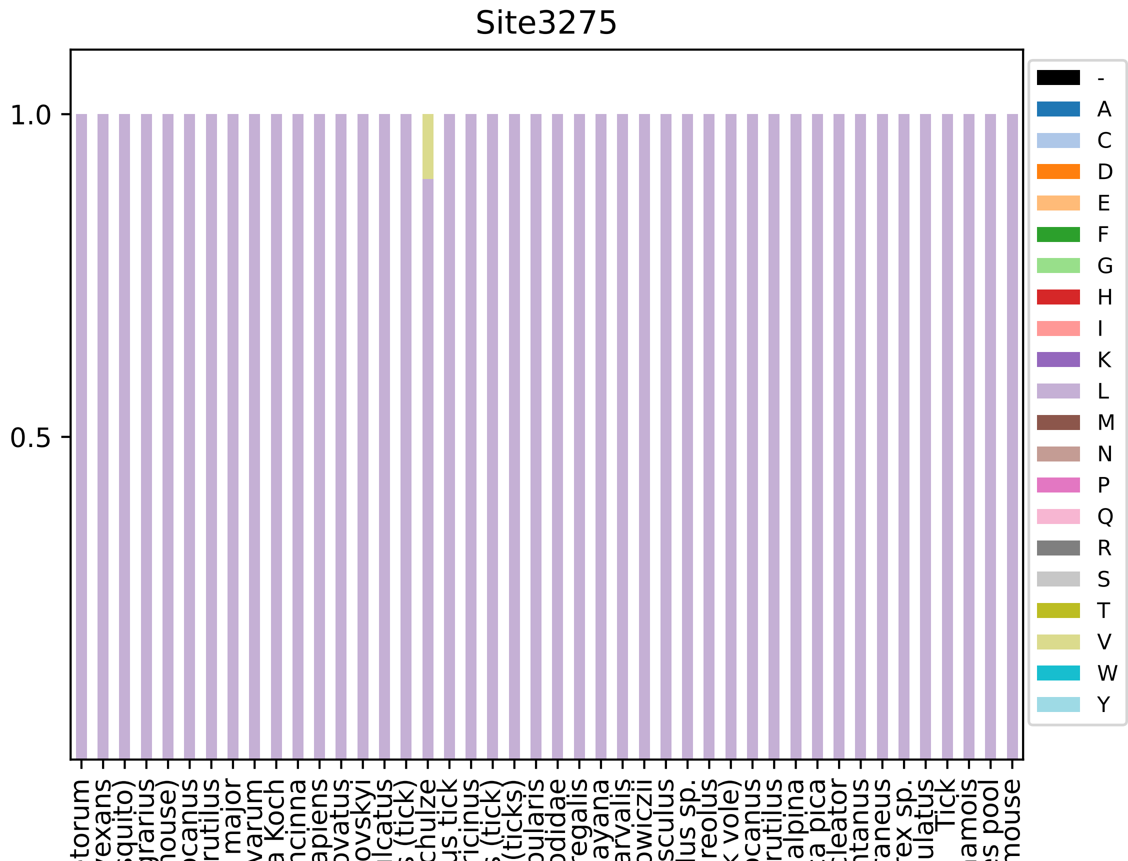
Site3275
1.0
0.5
-
A
C
D
E
F
G
H
I
K
L
M
N
P
Q
R
S
T
V
W
Y

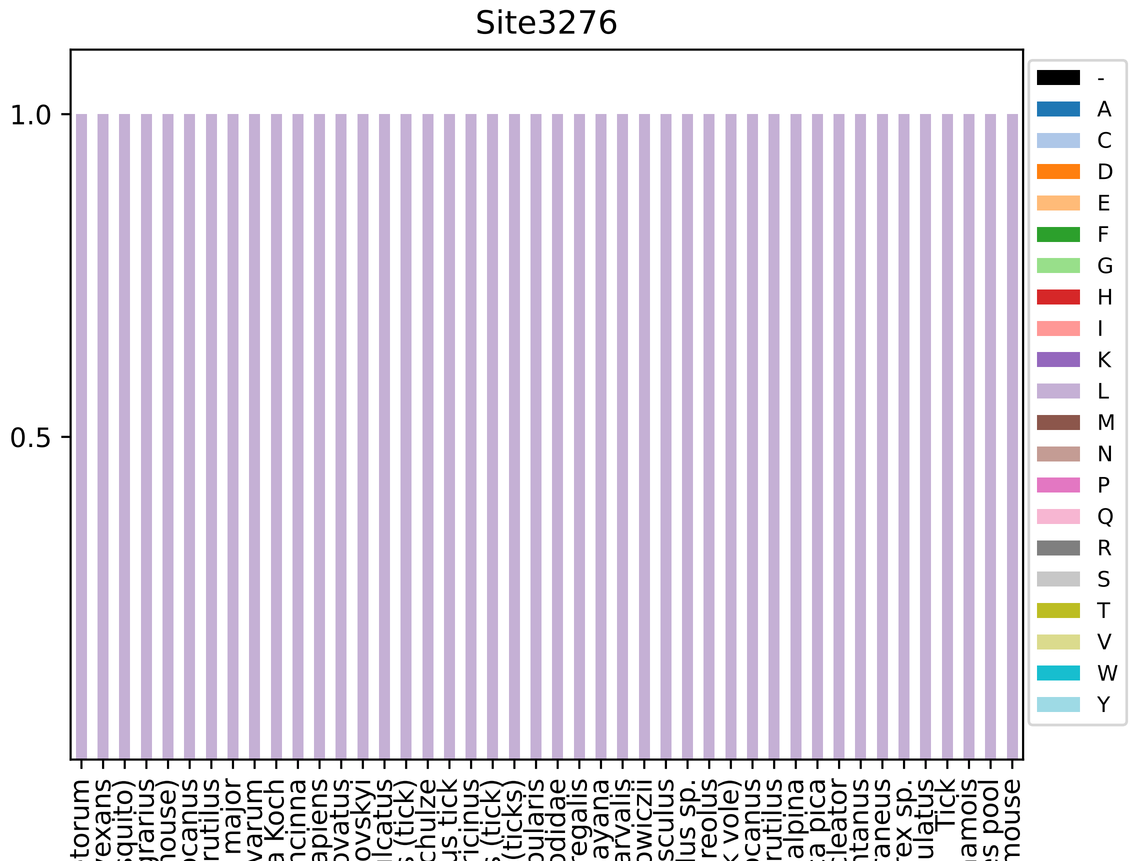
Site3276
1.0
0.5
-
A
C
D
E
F
G
H
I
K
L
M
N
P
Q
R
S
T
V
W
Y

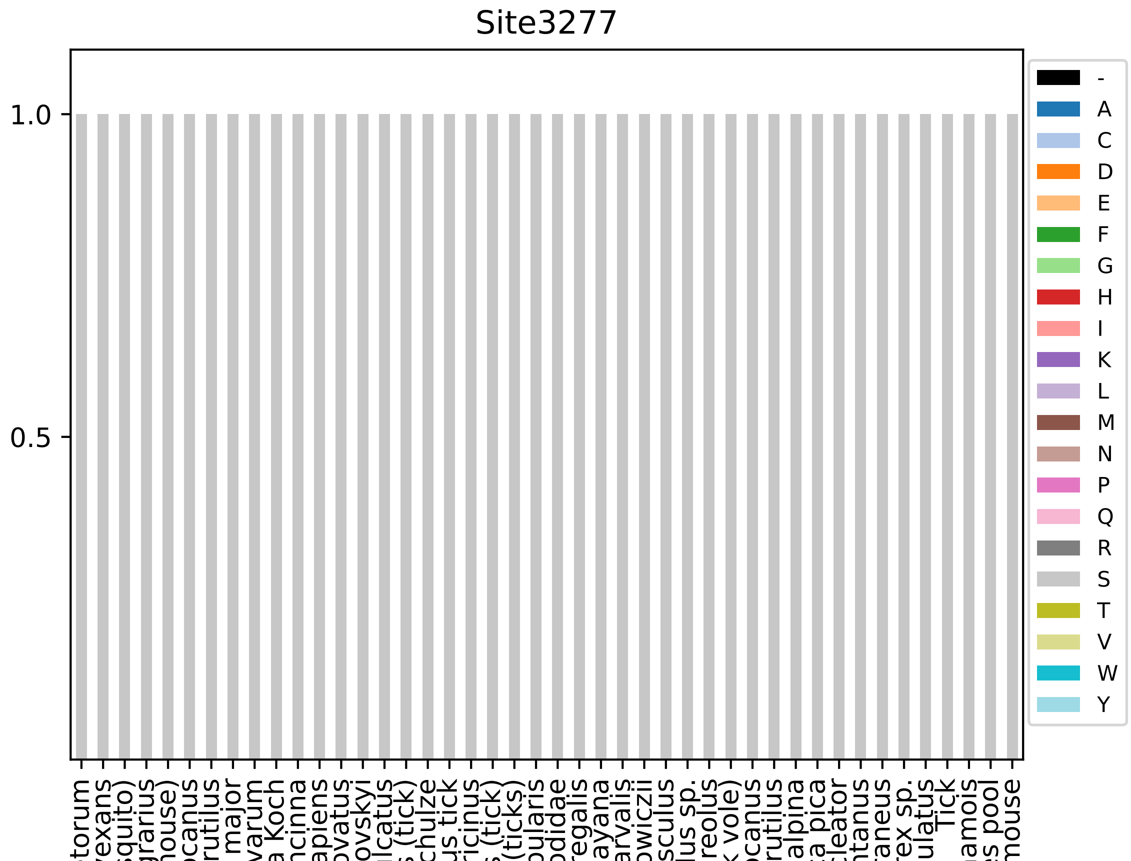

Site3277
1.0
0.5
-
A
C
D
E
F
G
H
I
K
L
M
N
P
Q
R
S
T
V
W
Y

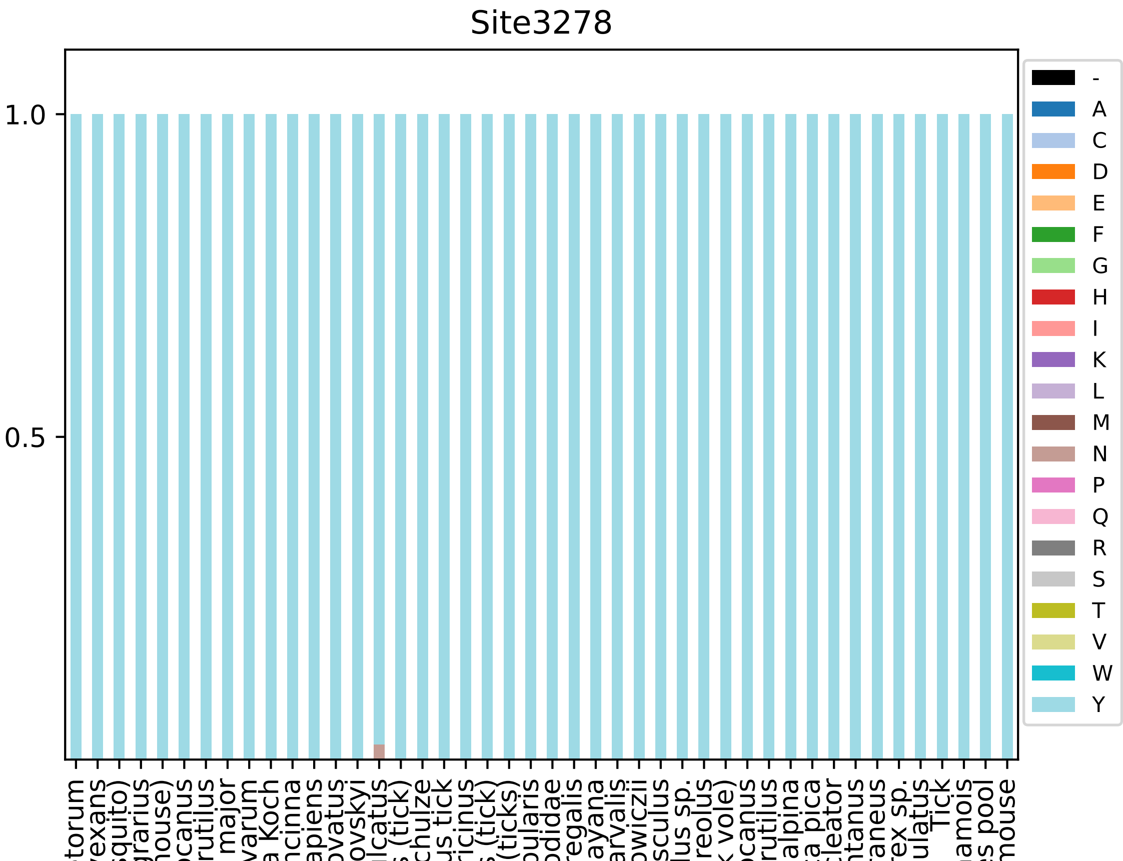

Site3278
1.0
0.5
-
A
C
D
E
F
G
H
I
K
L
M
N
P
Q
R
S
T
V
W
Y

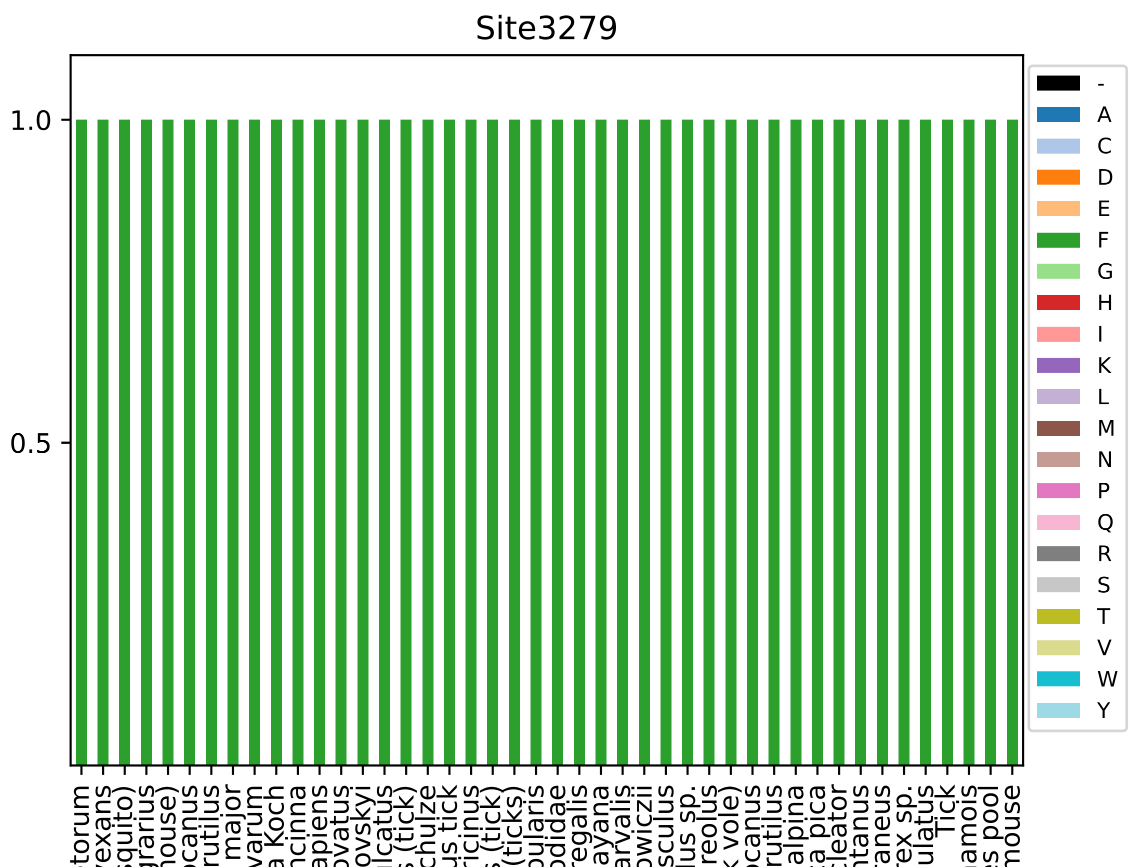
Site3279
1.0
0.5
-
A
C
D
E
F
G
H
I
K
L
M
N
P
Q
R
S
T
V
W
Y

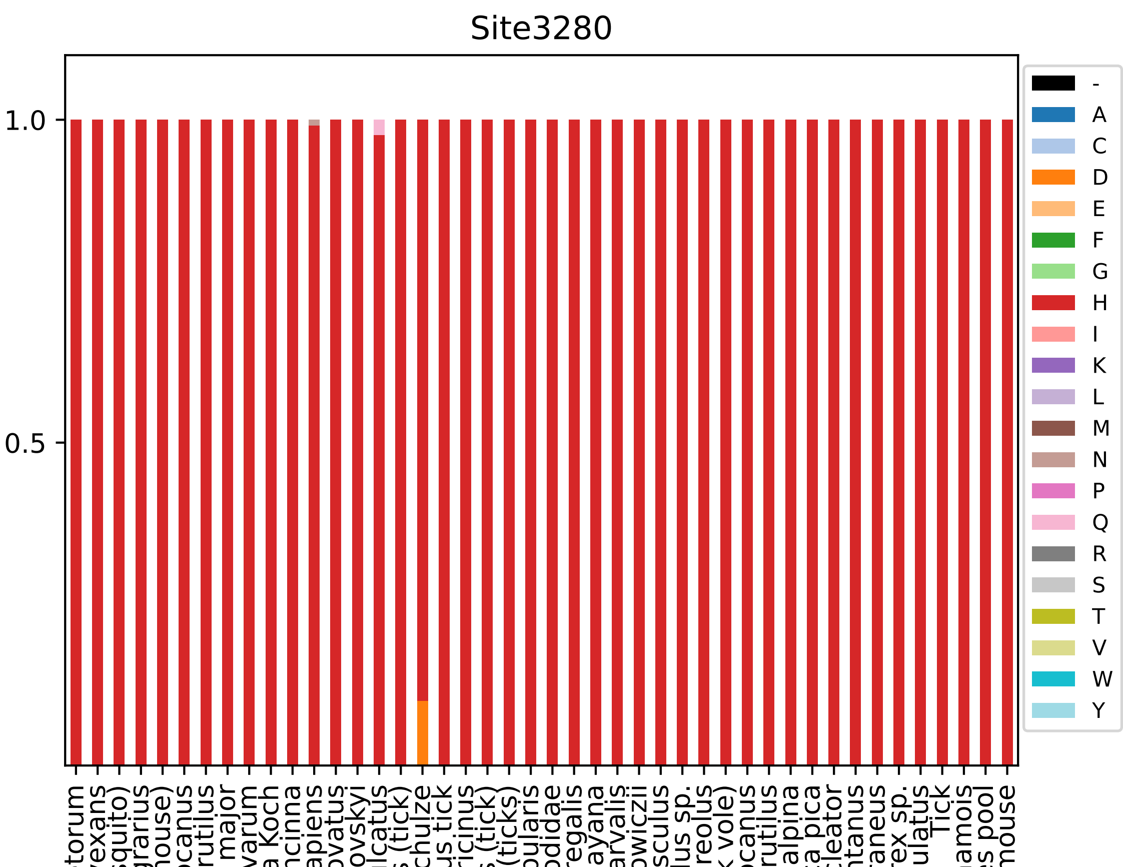
Site3280
1.0
0.5
-
A
C
D
E
F
G
H
I
K
L
M
N
P
Q
R
S
T
V
W
Y

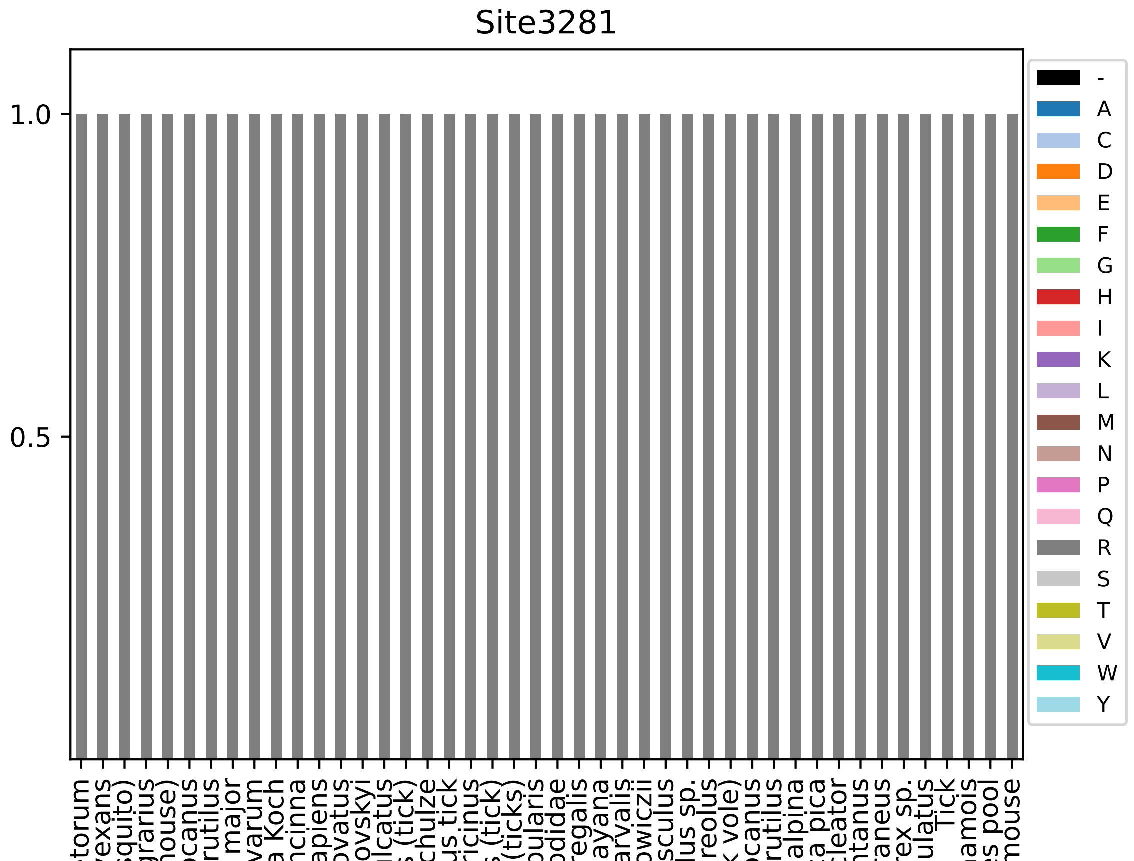
Site3281
1.0
0.5
-
A
C
D
E
F
G
H
I
K
L
M
N
P
Q
R
S
T
V
W
Y

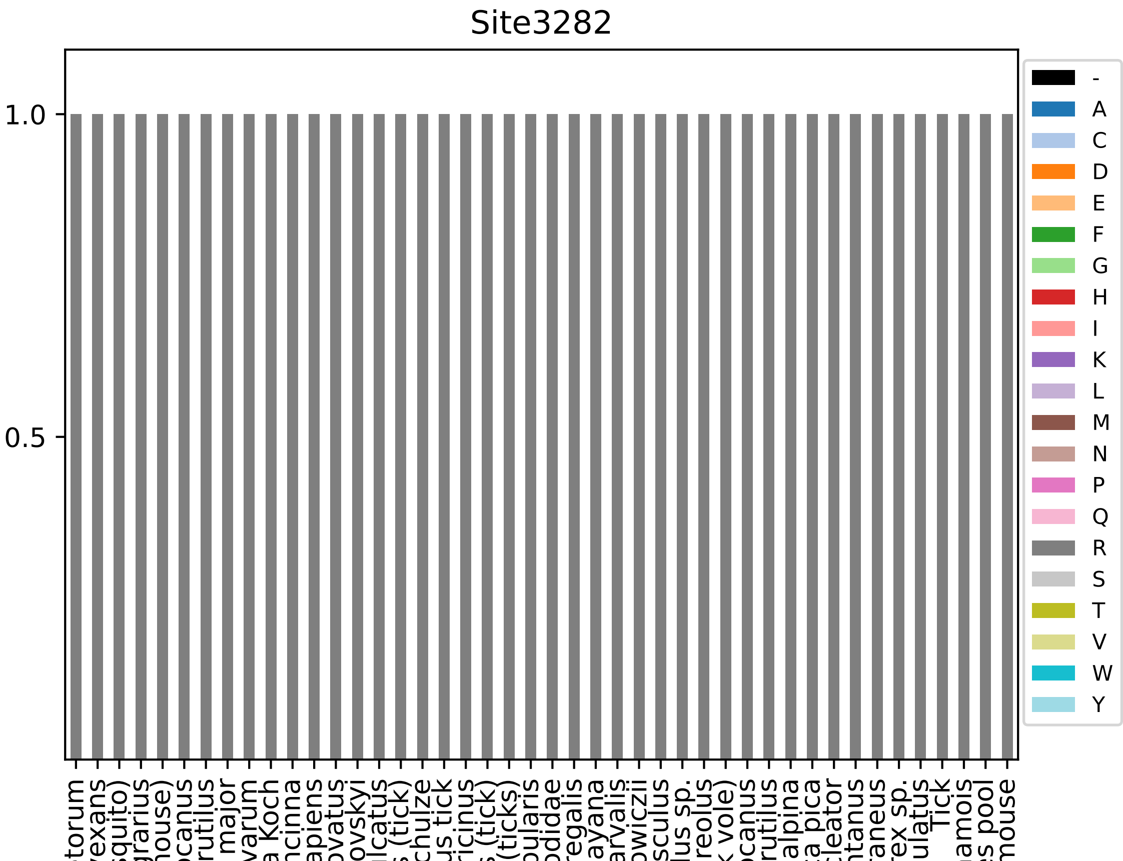
Site3282
1.0
0.5
-
A
C
D
E
F
G
H
I
K
L
M
N
P
Q
R
S
T
V
W
Y
Tick

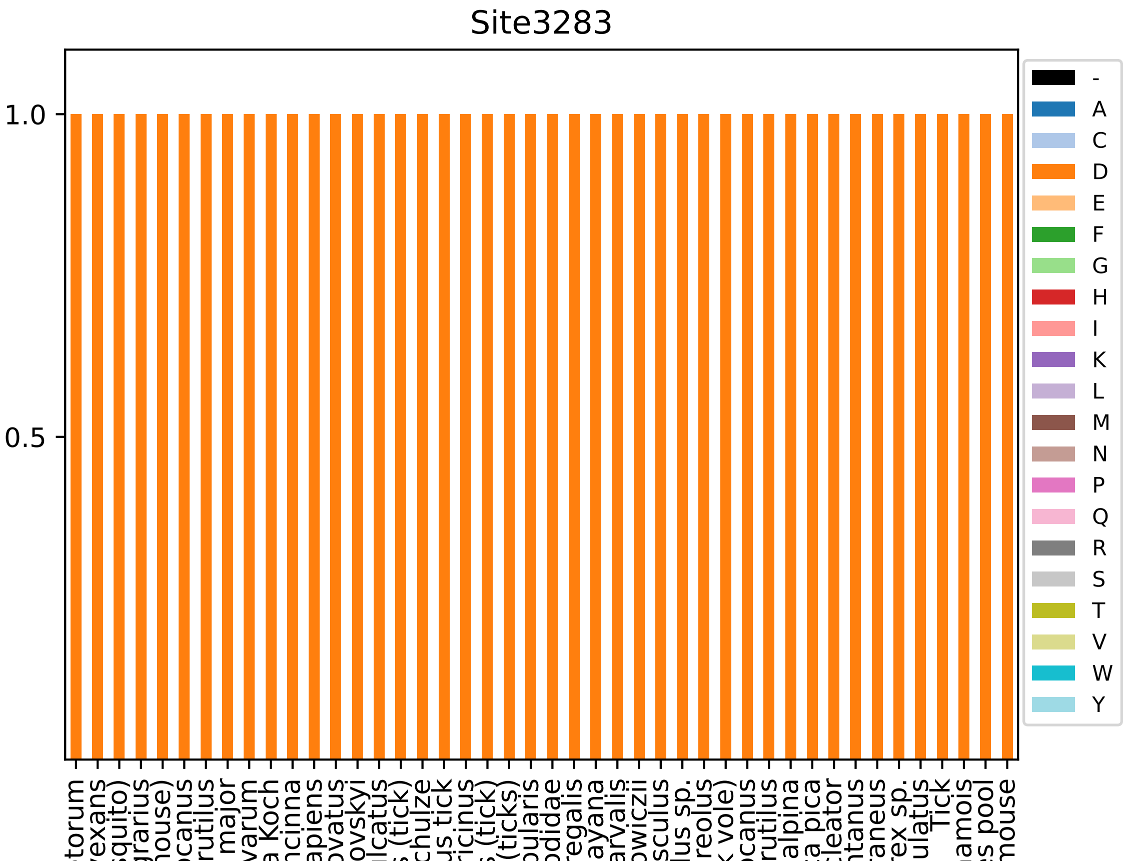
Site3283
1.0
0.5
-
A
C
D
E
F
G
H
I
K
L
M
N
P
Q
R
S
T
V
W
Y
torum
vexans
squito)
grarius
nouse)
ocanus
rutilus
major
varum
a Koch
ncinna
apiens
ovatus
ovskyi
ulcatus
s (tick)
chulze
us tick
ricinus
s (tick)
(ticks)
oularis
odidae
regalis
ayana
arvalis
owiczii
sculus
lus sp.
reolus
x vole)
ocanus
rutilus
alpina
a pica
leator
ntanus
raneus
rex sp.
ulatus
Tick
amois
es pool
mouse

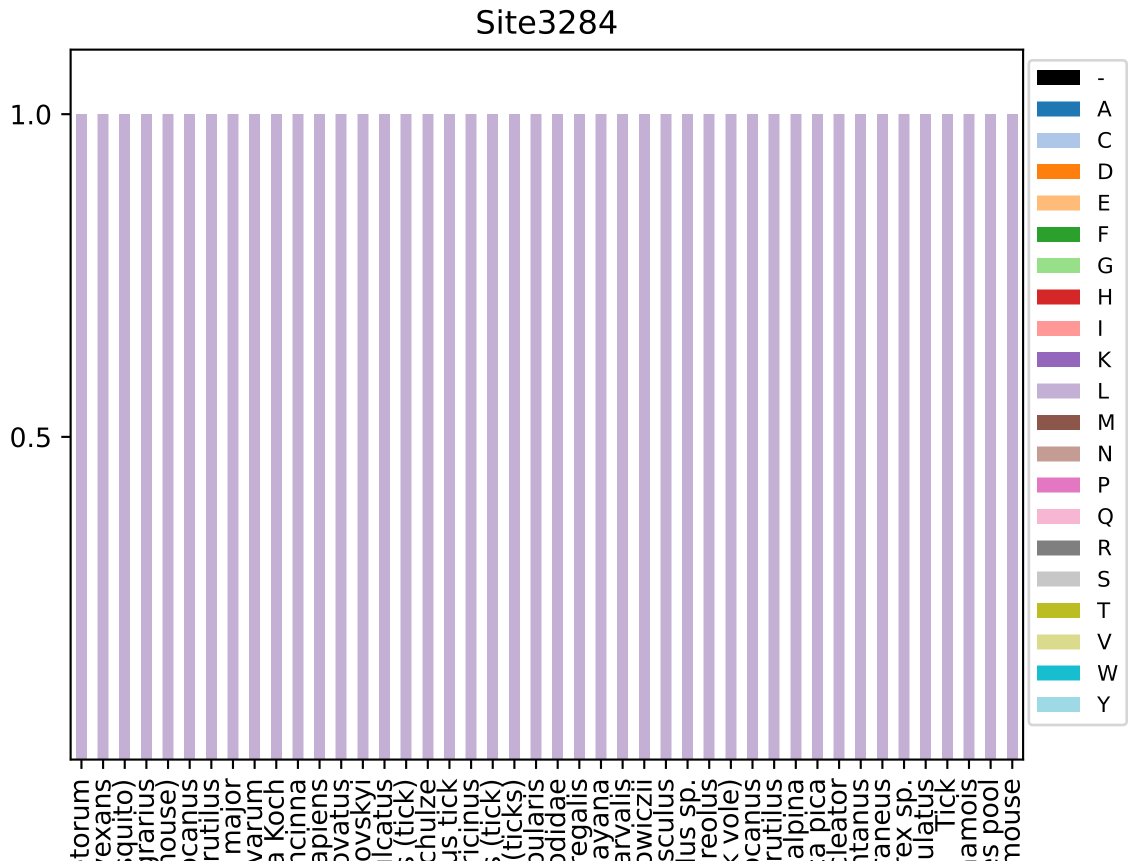
Site3284
1.0
0.5
-
A
C
D
E
F
G
H
I
K
L
M
N
P
Q
R
S
T
V
W
Y

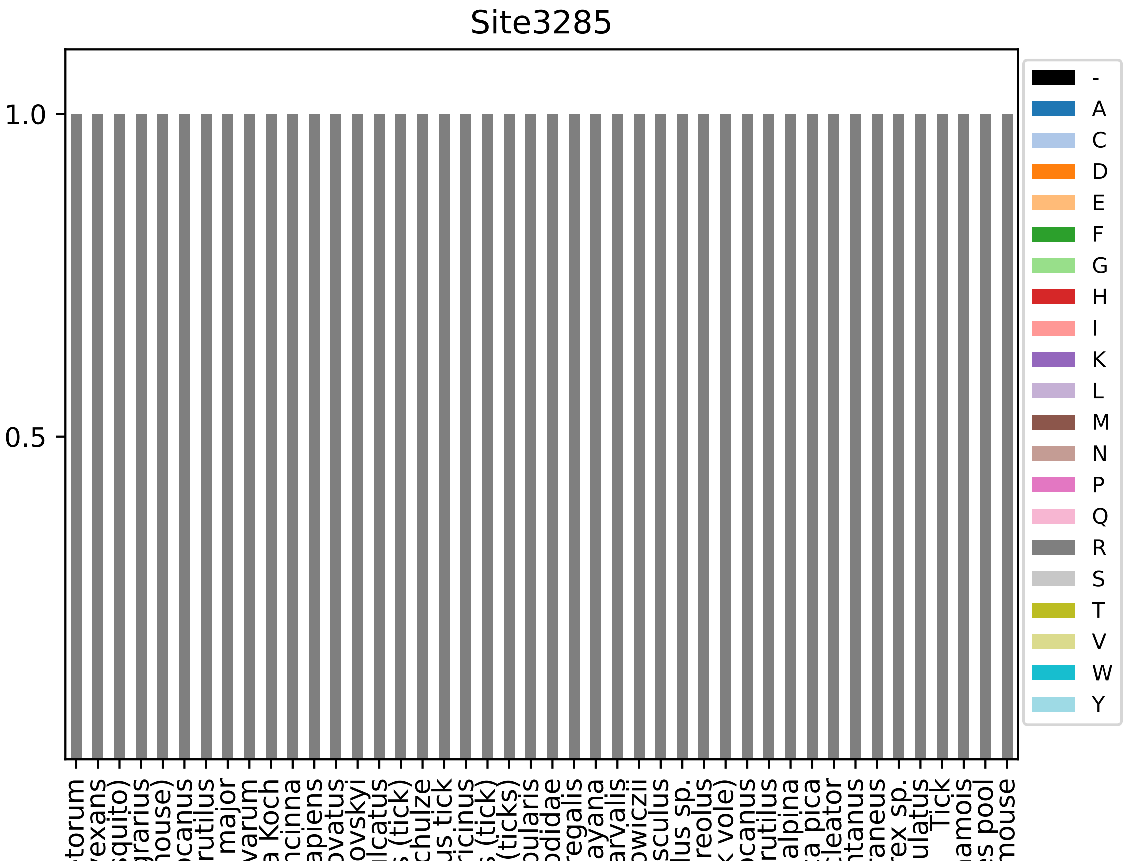

Site3285
1.0
0.5
-
A
C
D
E
F
G
H
I
K
L
M
N
P
Q
R
S
T
V
W
Y

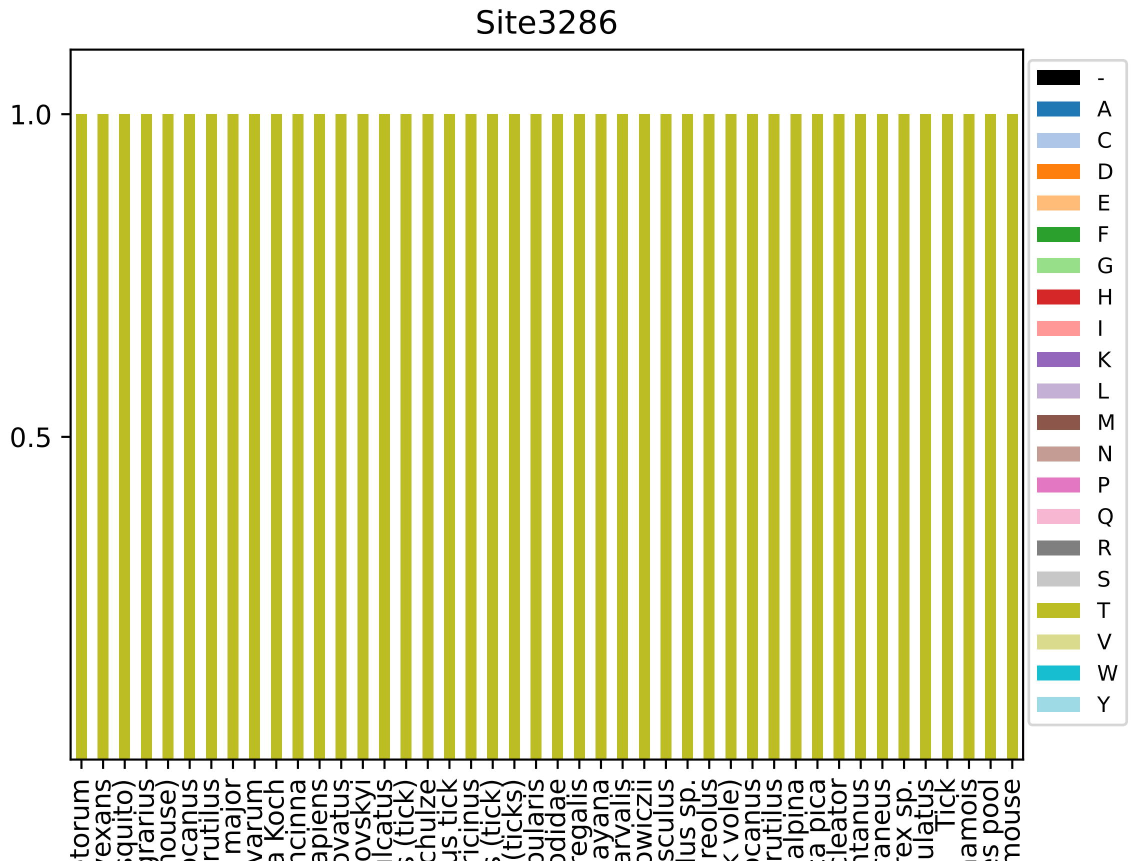
Site3286
1.0
0.5
-
A
C
D
E
F
G
H
I
K
L
M
N
P
Q
R
S
T
V
W
Y

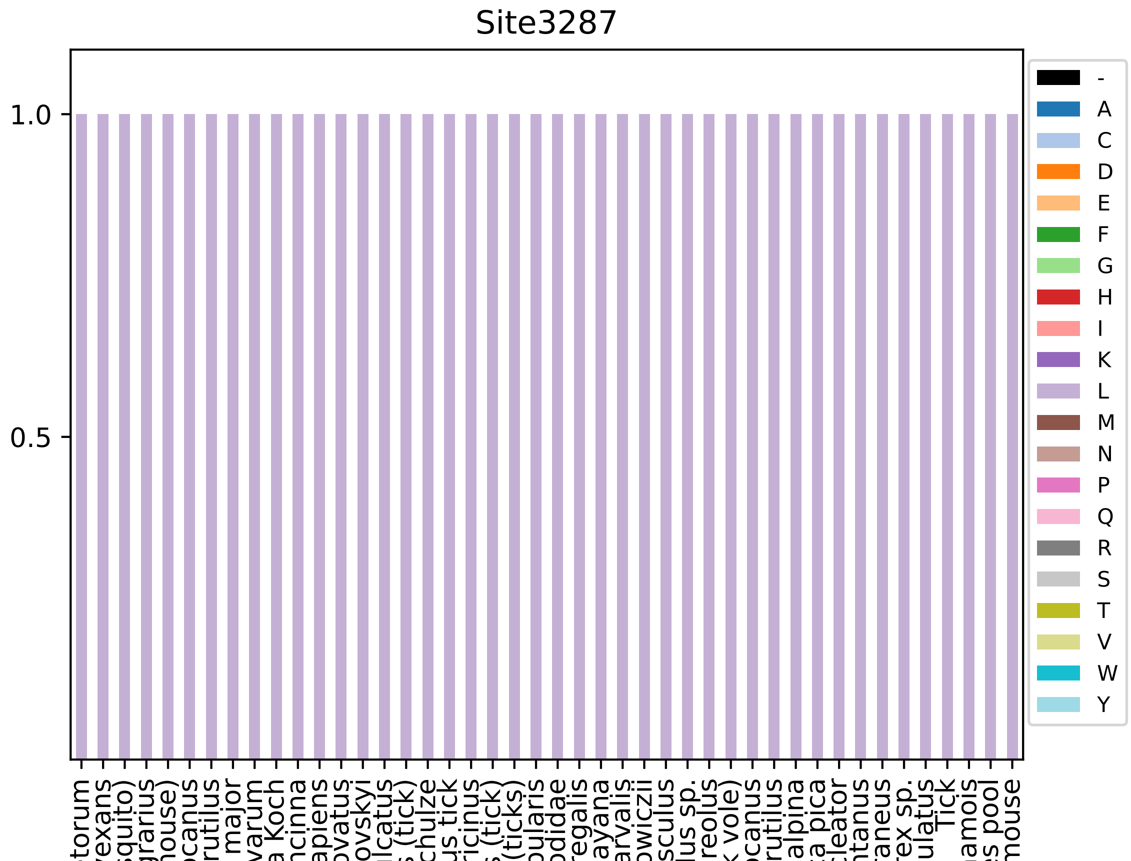
Site3287
1.0
0.5
-
A
C
D
E
F
G
H
I
K
L
M
N
P
Q
R
S
T
V
W
Y

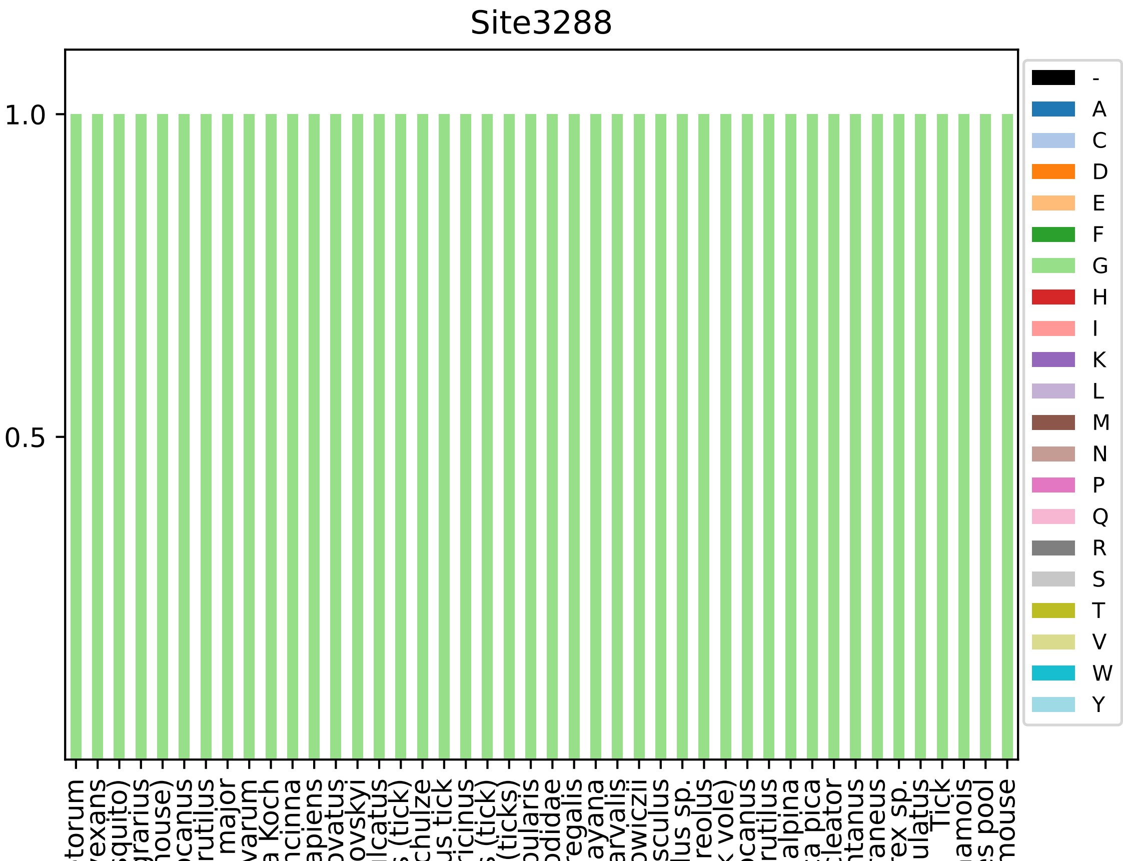

Site3288
1.0
0.5
-
A
C
D
E
F
G
H
I
K
L
M
N
P
Q
R
S
T
V
W
Y

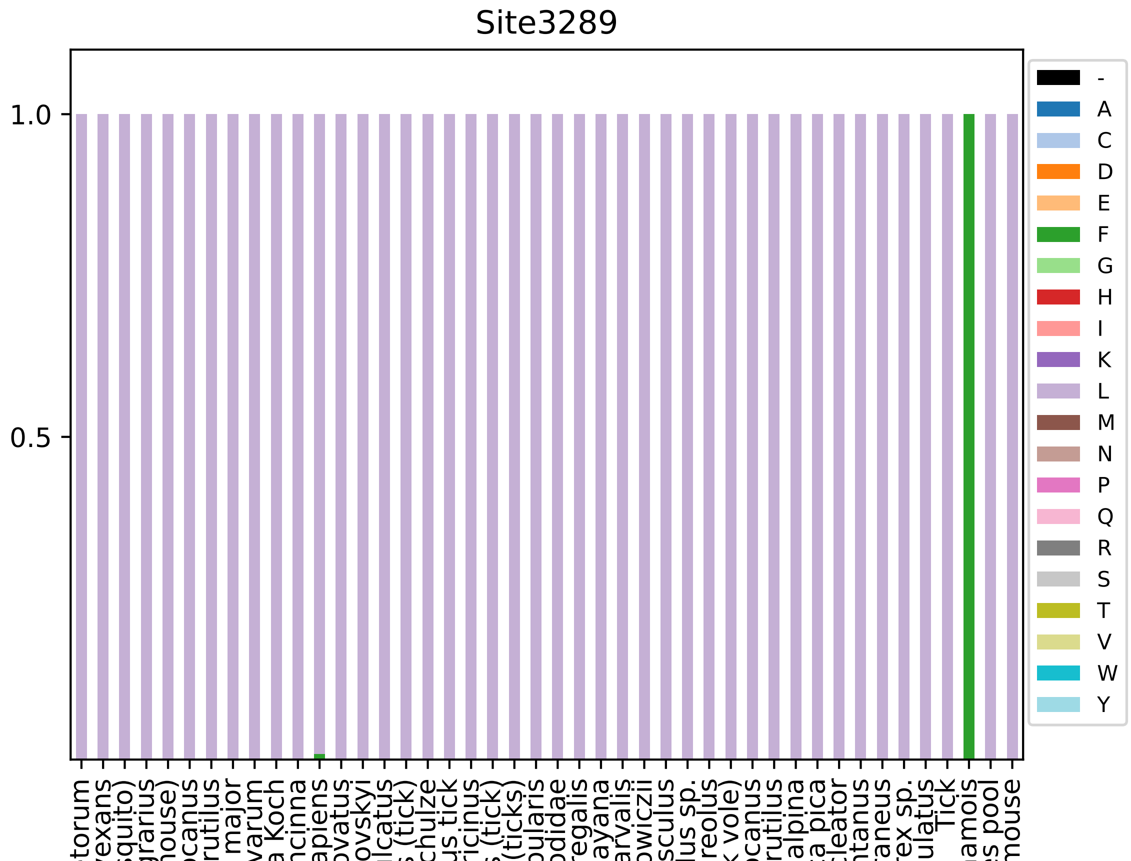
Site3289
1.0
0.5
-
A
C
D
E
F
G
H
I
K
L
M
N
P
Q
R
S
T
V
W
Y

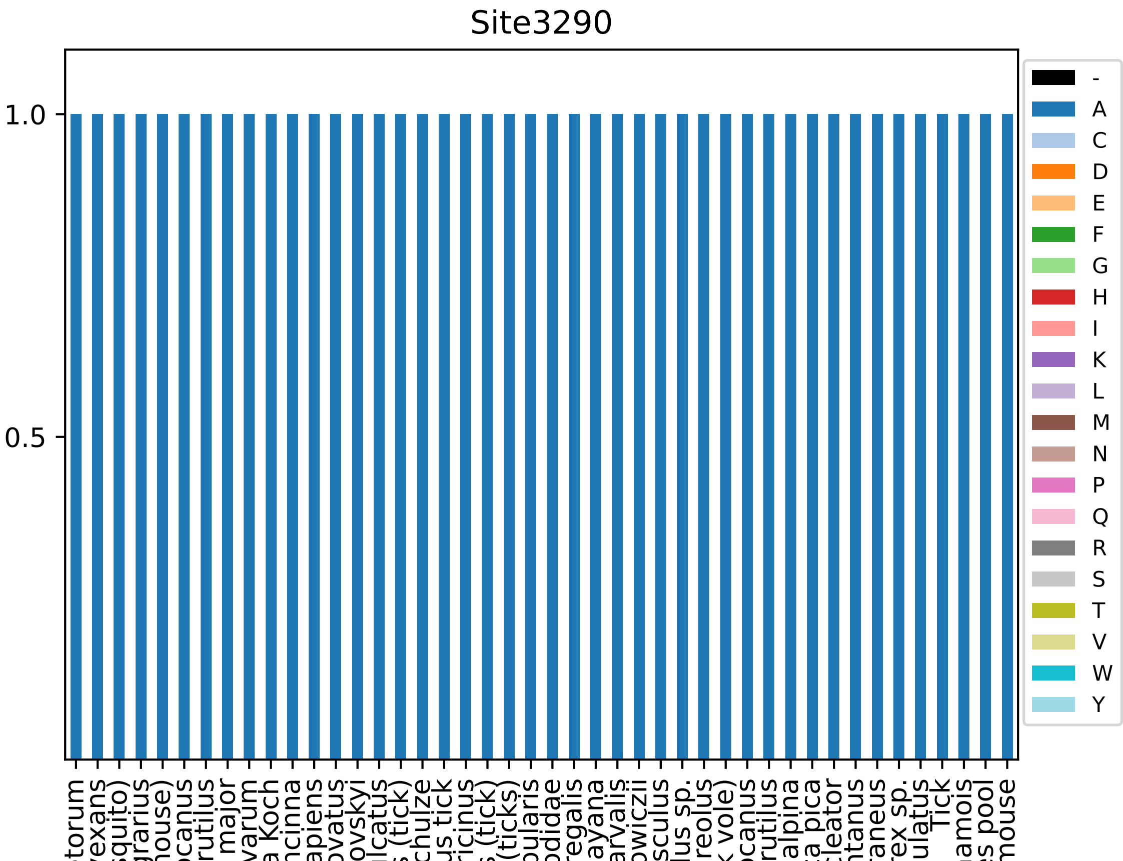
Site3290
1.0
0.5
-
A
C
D
E
F
G
H
I
K
L
M
N
P
Q
R
S
T
V
W
Y

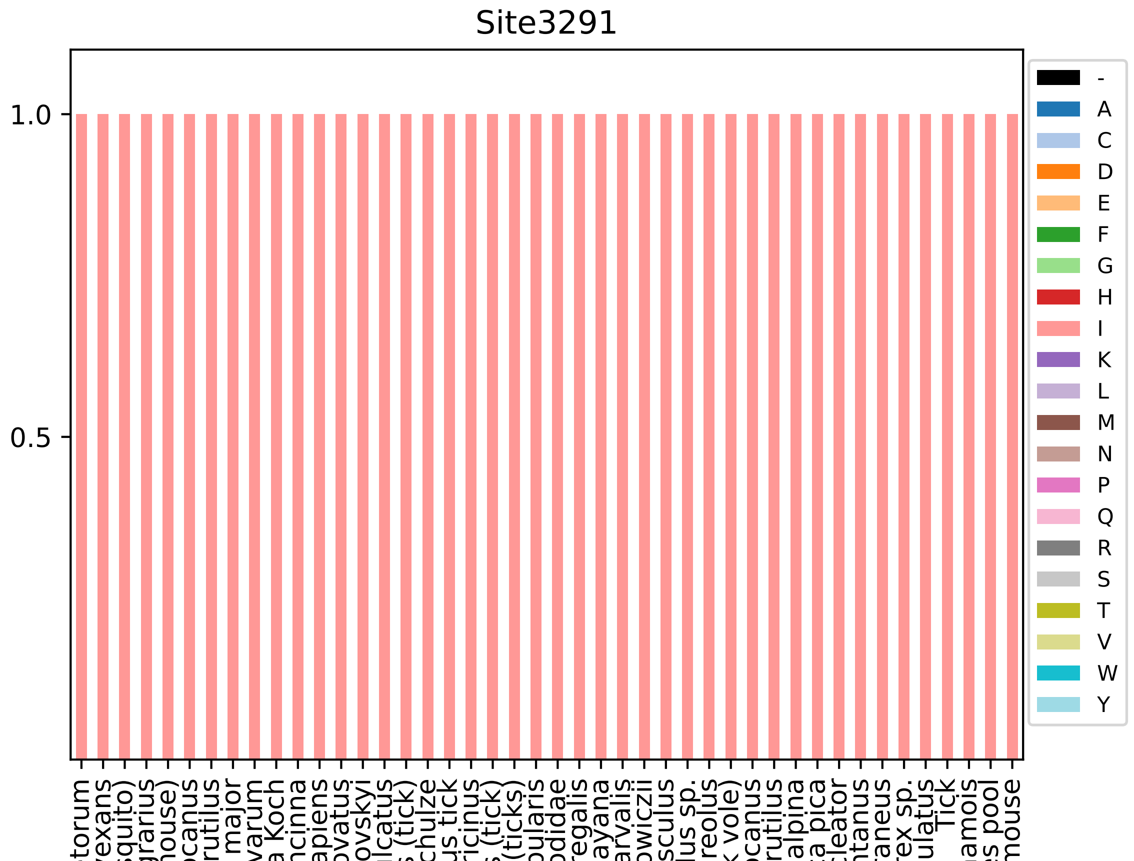
Site3291
1.0
0.5
-
A
C
D
E
F
G
H
I
K
L
M
N
P
Q
R
S
T
V
W
Y
Tick

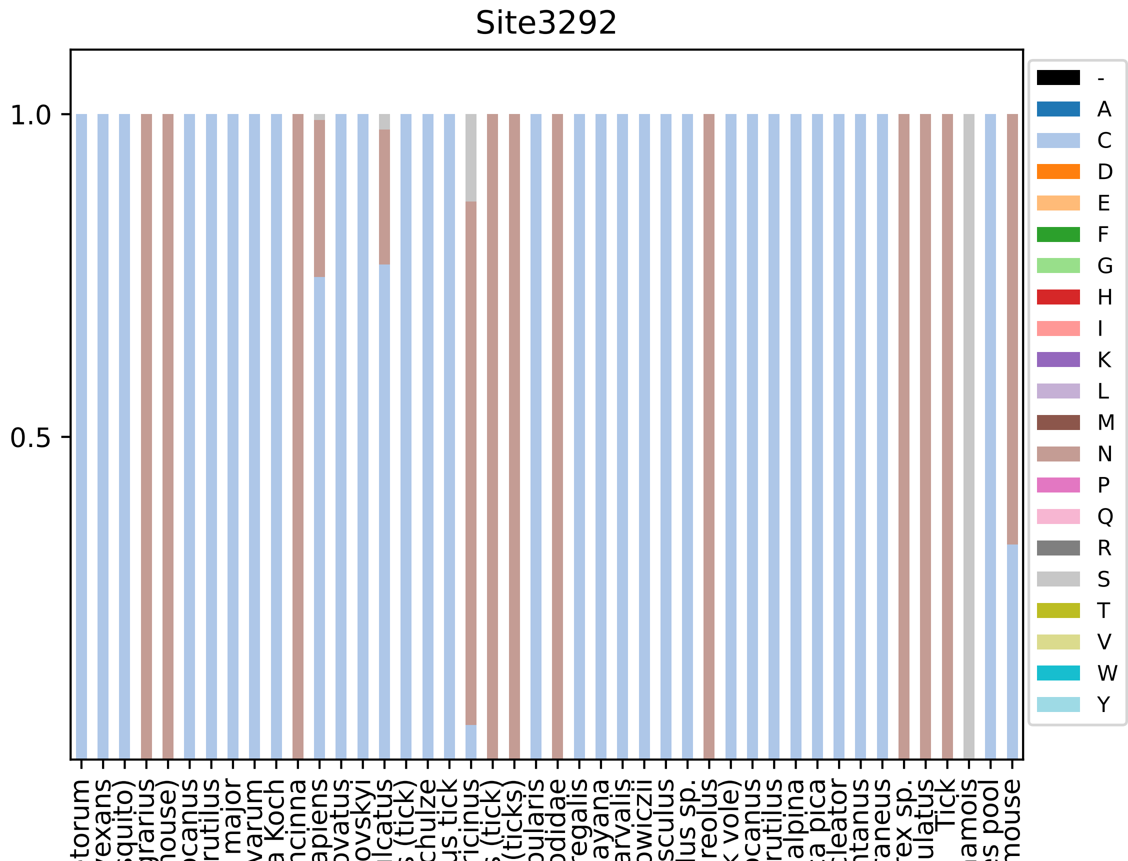

Site3292
1.0
0.5
-
A
C
D
E
F
G
H
I
K
L
M
N
P
Q
R
S
T
V
W
Y

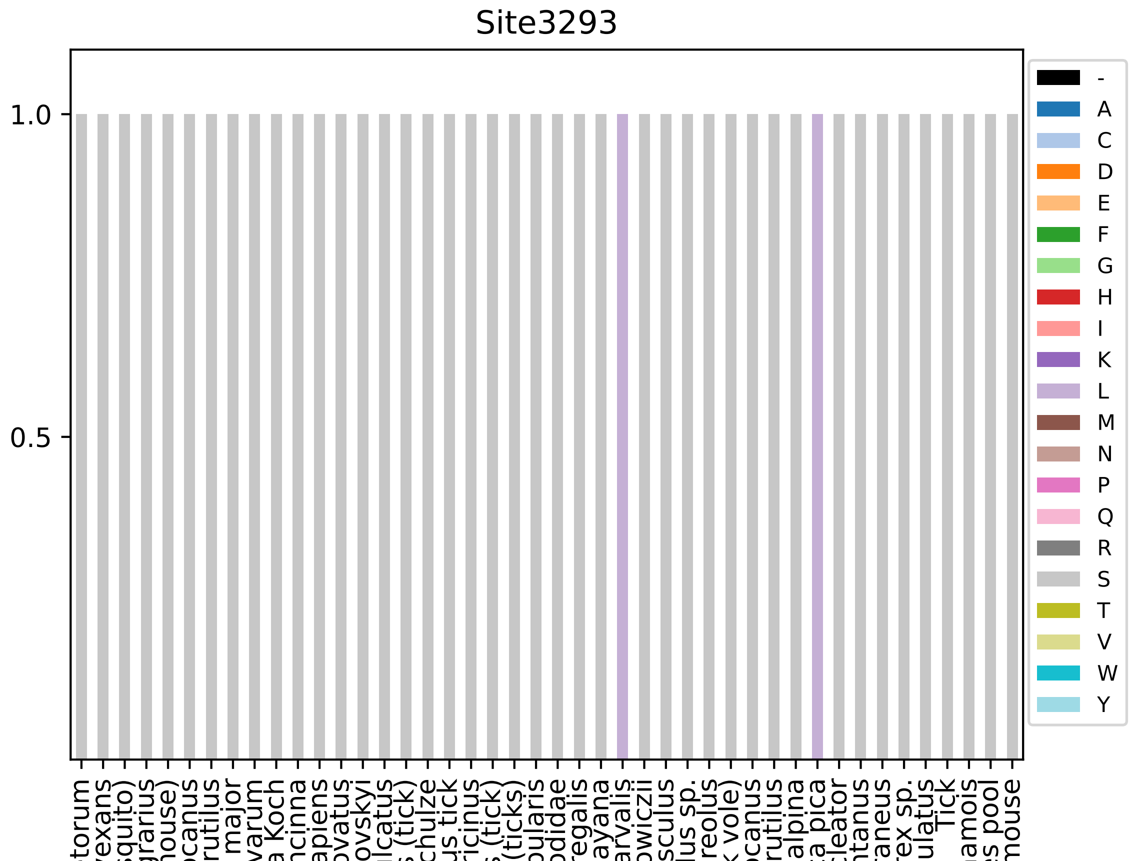
Site3293
1.0
0.5
-
A
C
D
E
F
G
H
I
K
L
M
N
P
Q
R
S
T
V
W
Y
Tick

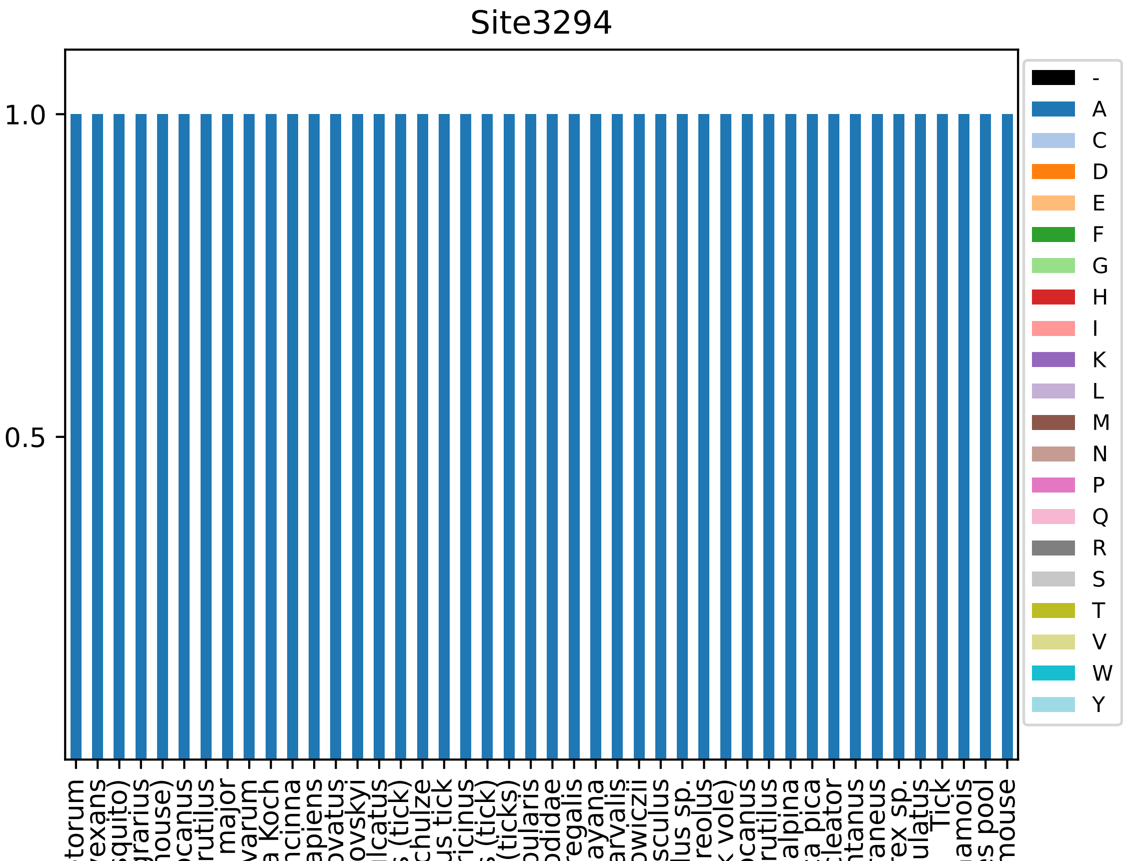
Site3294
1.0
0.5
-
A
C
D
E
F
G
H
I
K
L
M
N
P
Q
R
S
T
V
W
Y

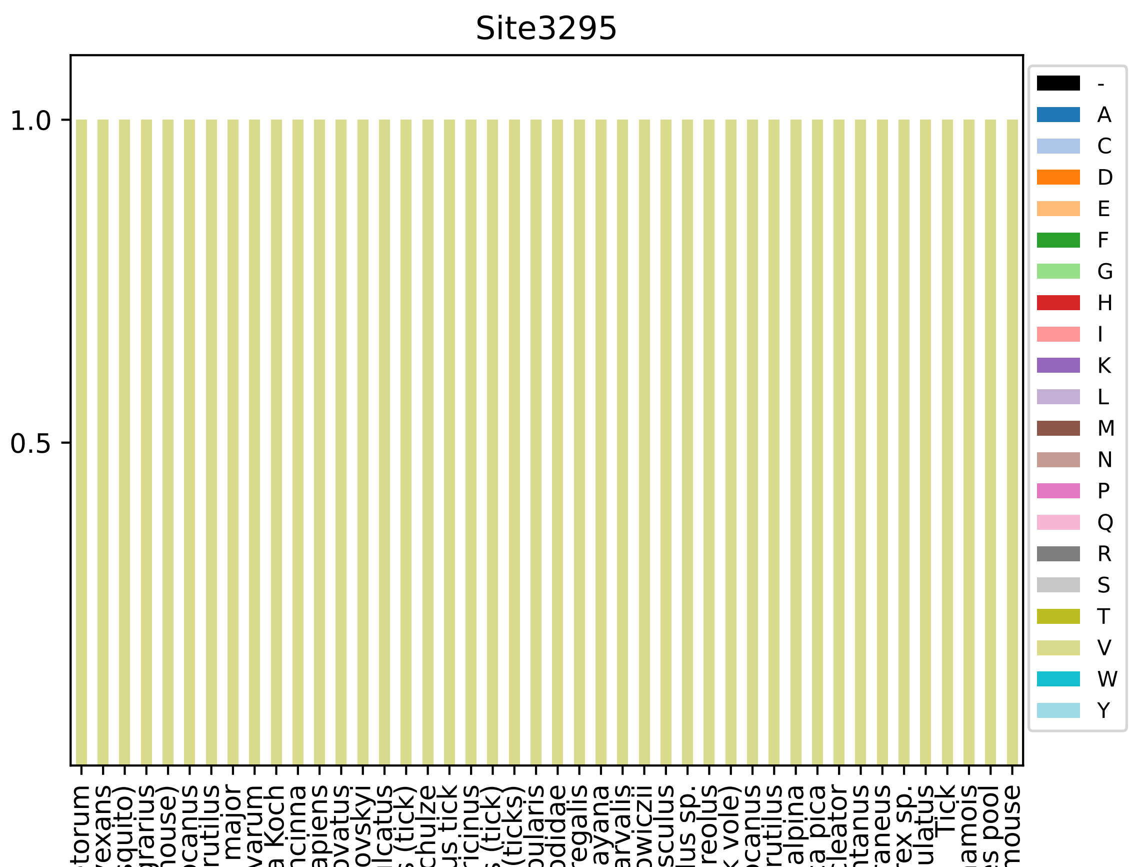
Site3295
1.0
0.5
-
A
C
D
E
F
G
H
I
K
L
M
N
P
Q
R
S
T
V
W
Y

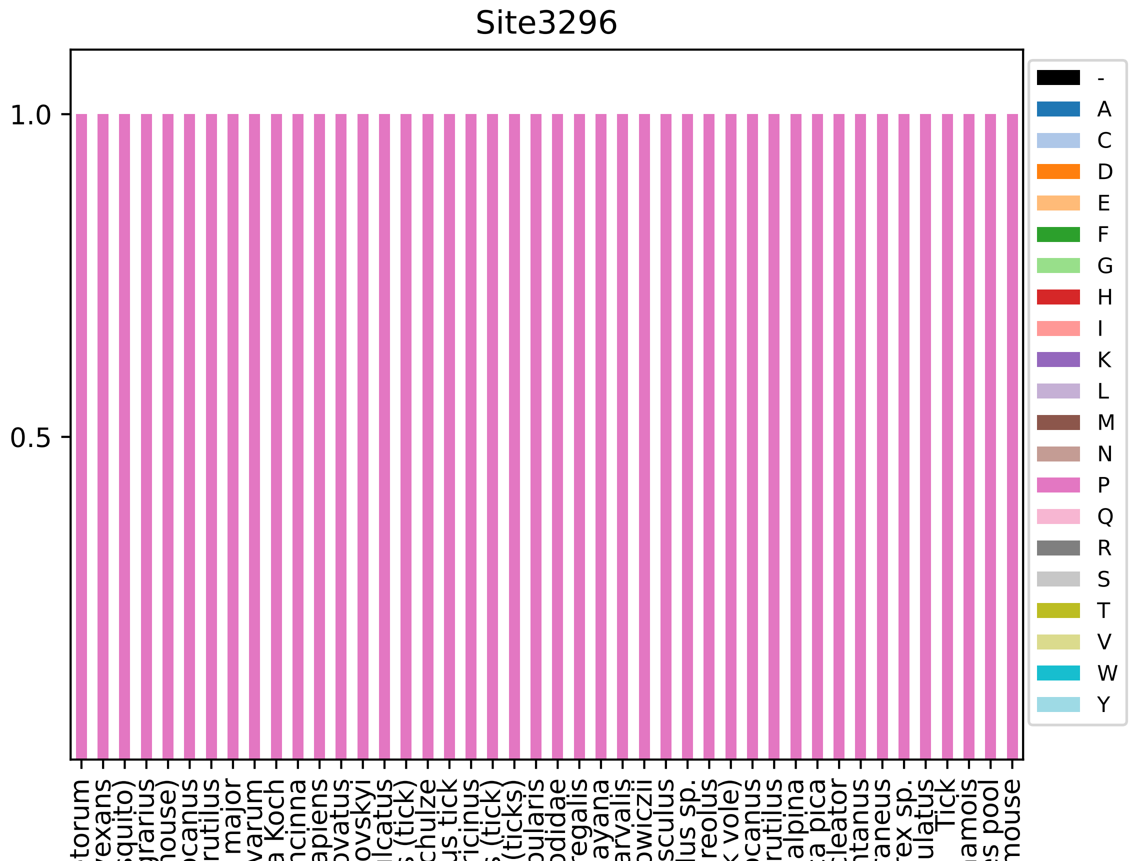
Site3296
1.0
0.5
-
A
C
D
E
F
G
H
I
K
L
M
N
P
Q
R
S
T
V
W
Y

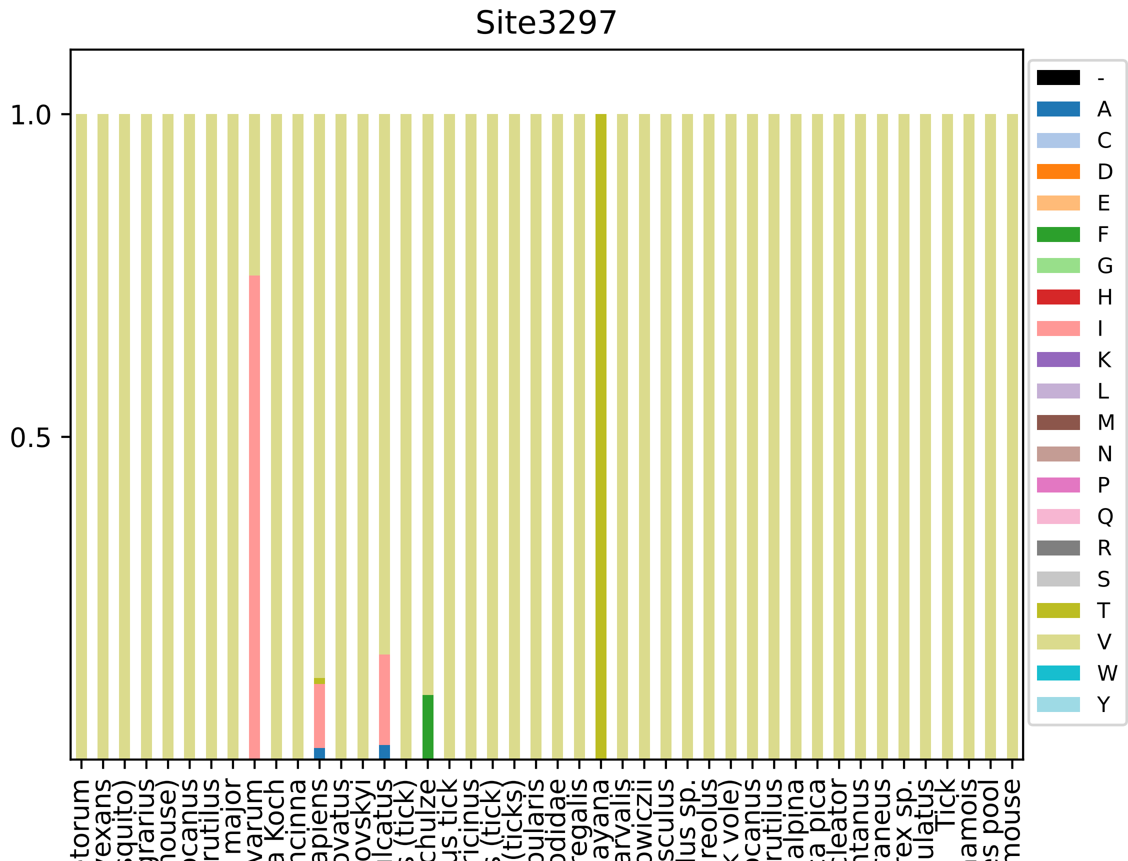

Site3297
1.0
0.5
-
A
C
D
E
F
G
H
I
K
L
M
N
P
Q
R
S
T
V
W
Y

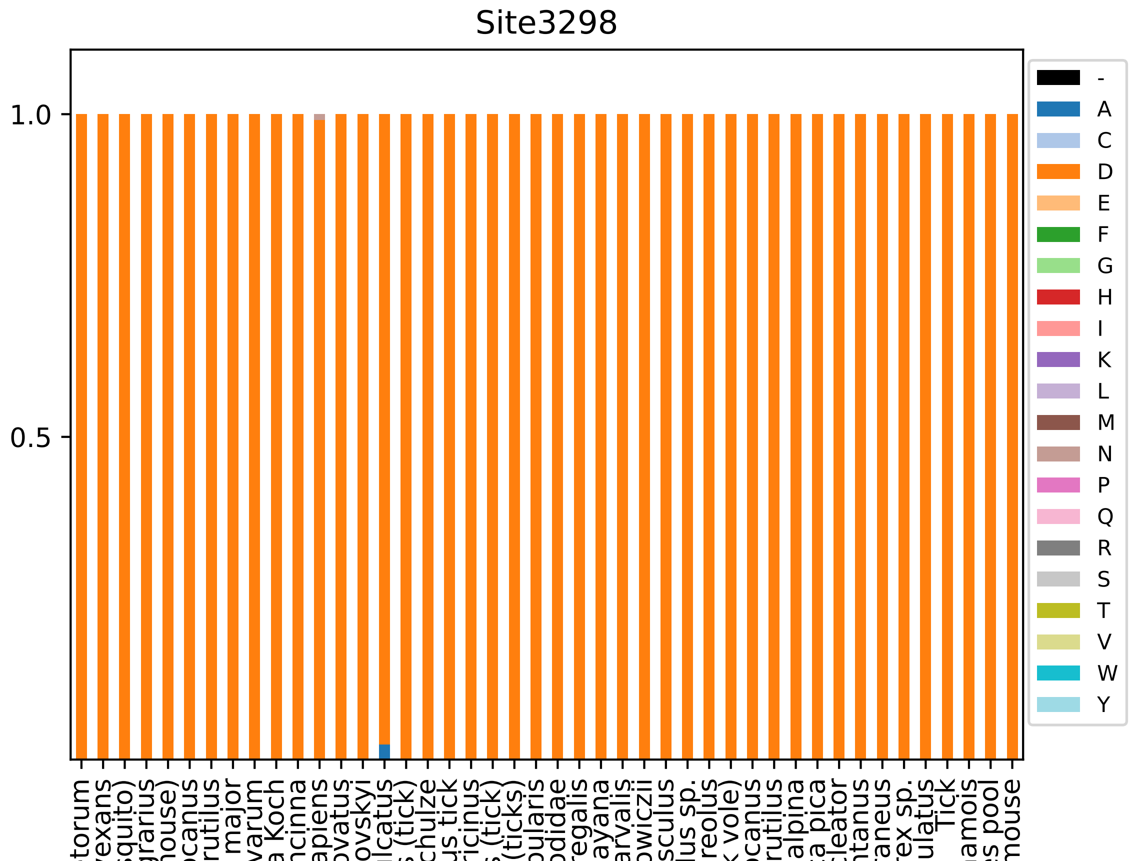
Site3298
1.0
0.5
-
A
C
D
E
F
G
H
I
K
L
M
N
P
Q
R
S
T
V
W
Y

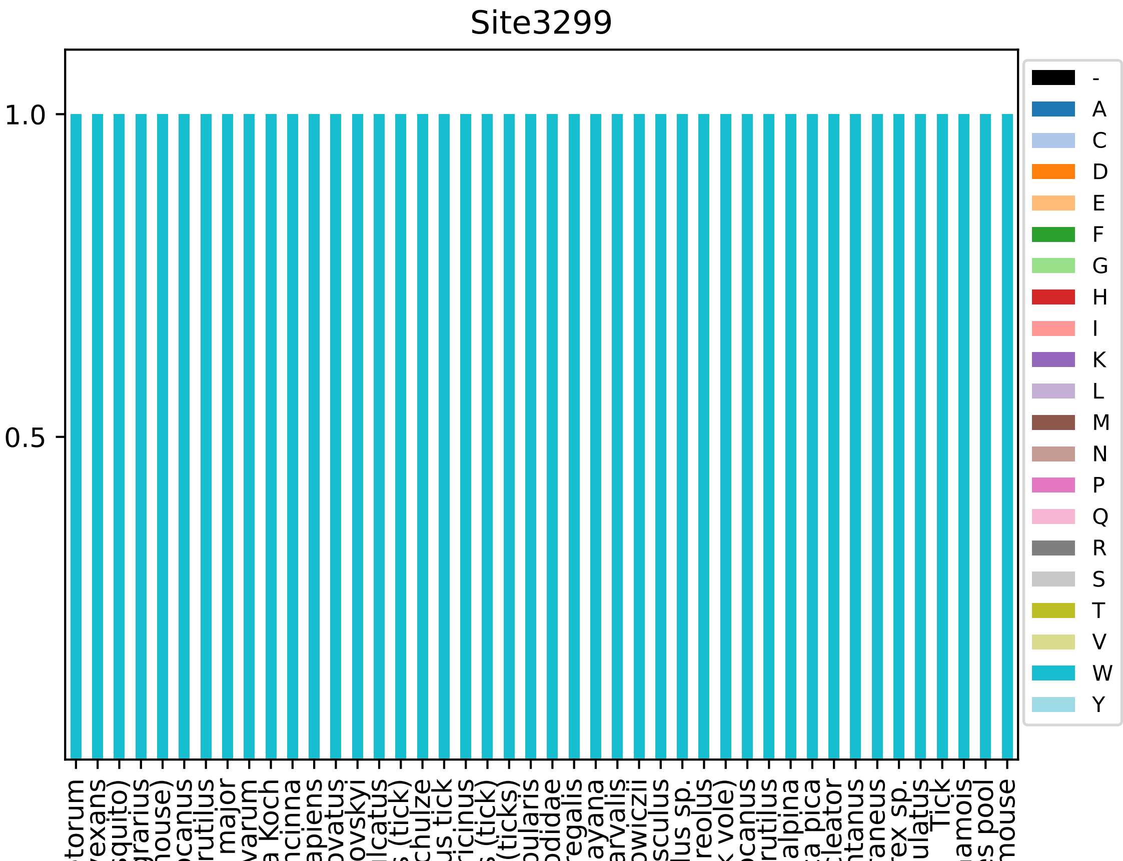

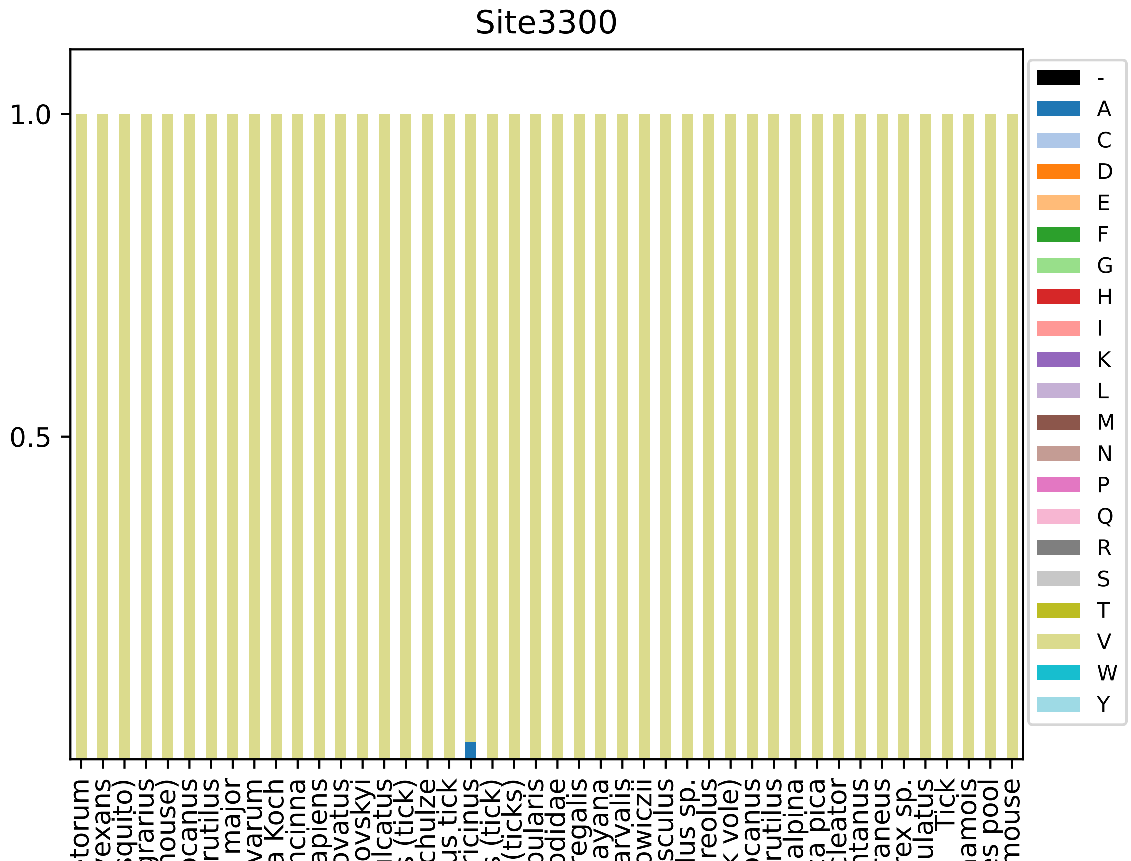
Site3300
1.0
0.5
-
A
C
D
E
F
G
H
I
K
L
M
N
P
Q
R
S
T
V
W
Y

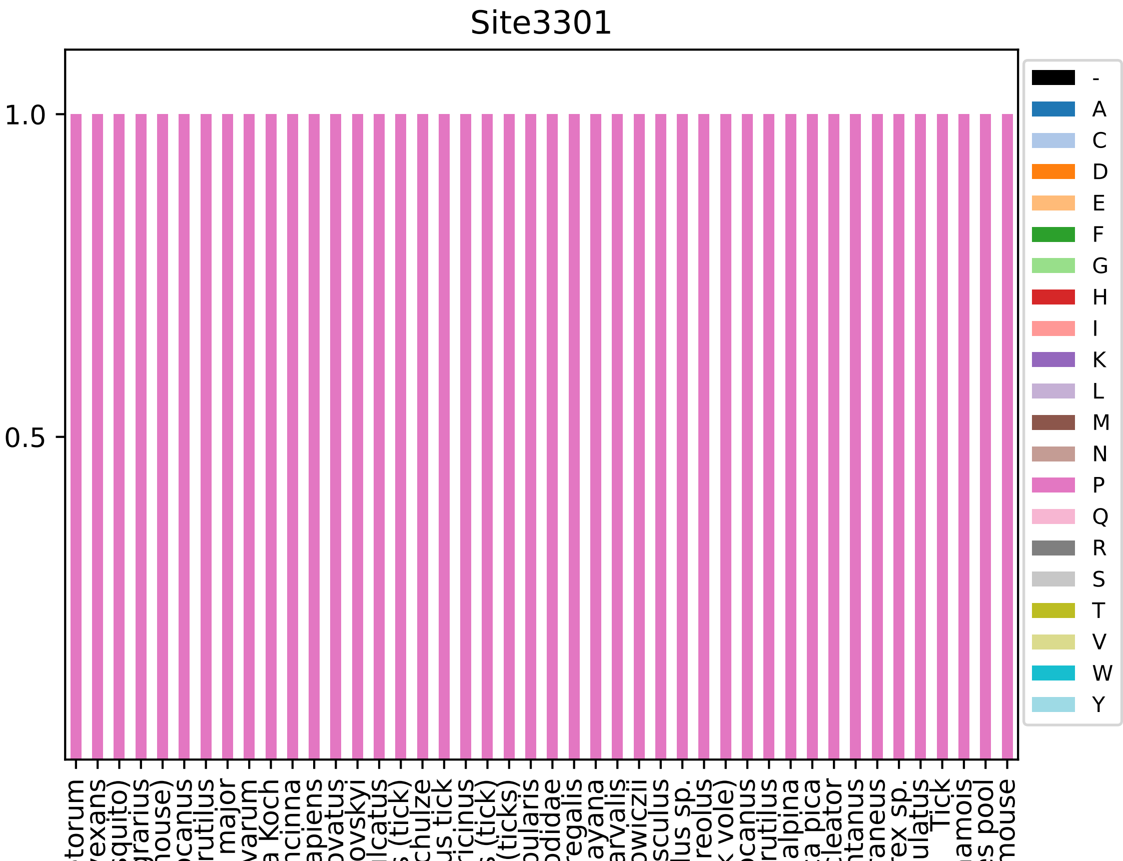
Site3301
1.0
0.5
-
A
C
D
E
F
G
H
I
K
L
M
N
P
Q
R
S
T
V
W
Y

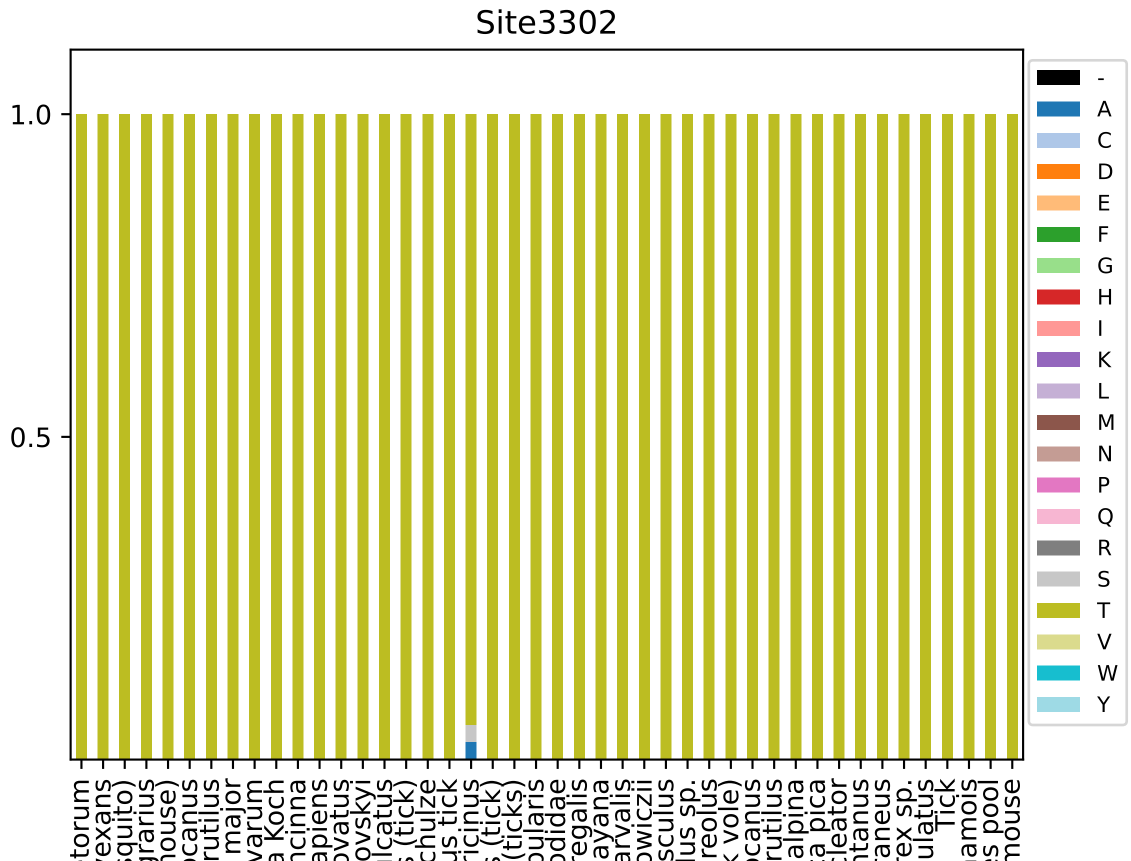
Site3302
1.0
0.5
-
A
C
D
E
F
G
H
I
K
L
M
N
P
Q
R
S
T
V
W
Y

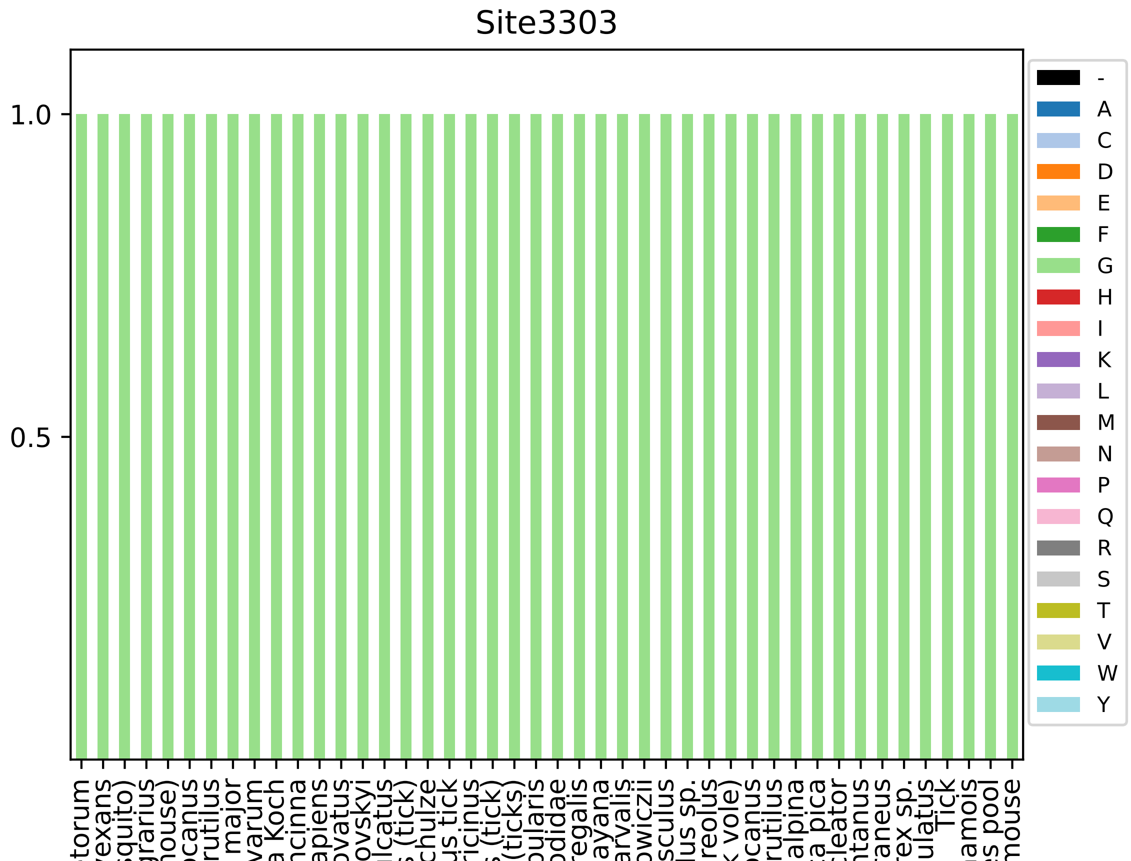
Site3303
1.0
0.5
-
A
C
D
E
F
G
H
I
K
L
M
N
P
Q
R
S
T
V
W
Y

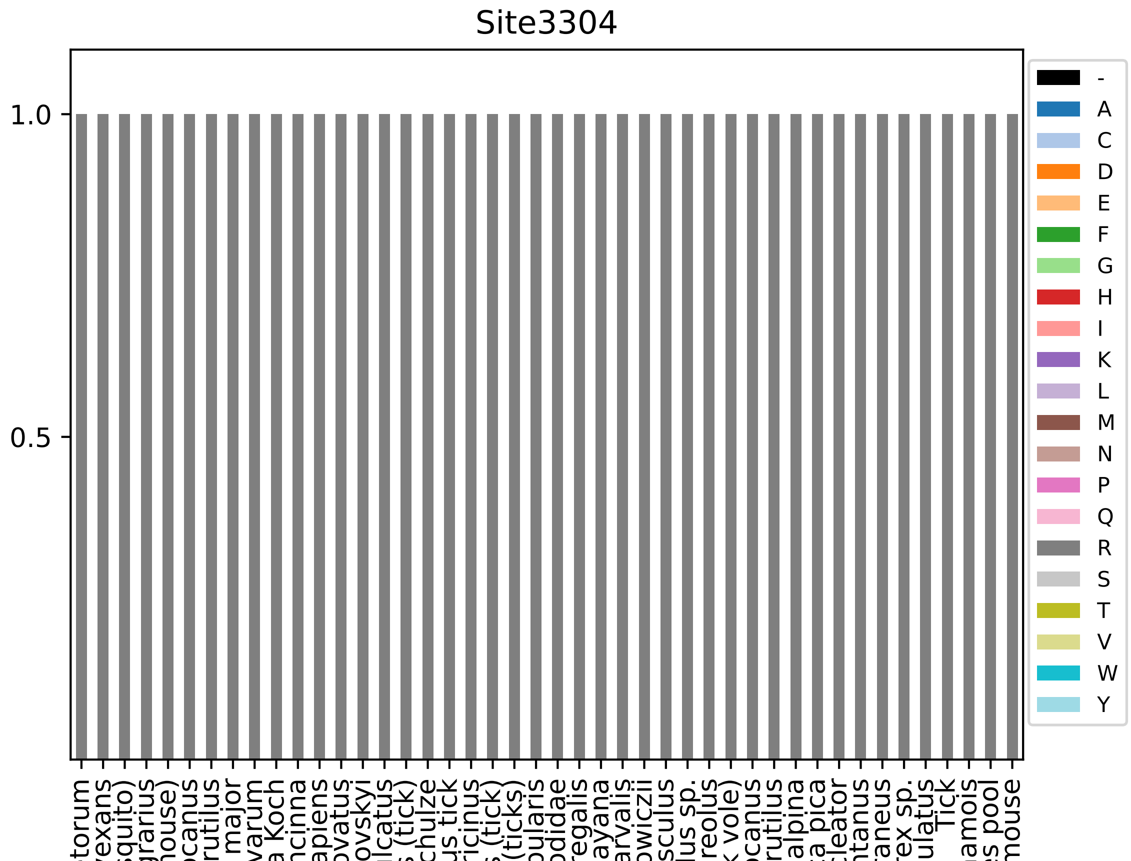
Site3304
1.0
0.5
-
A
C
D
E
F
G
H
I
K
L
M
N
P
Q
R
S
T
V
W
Y

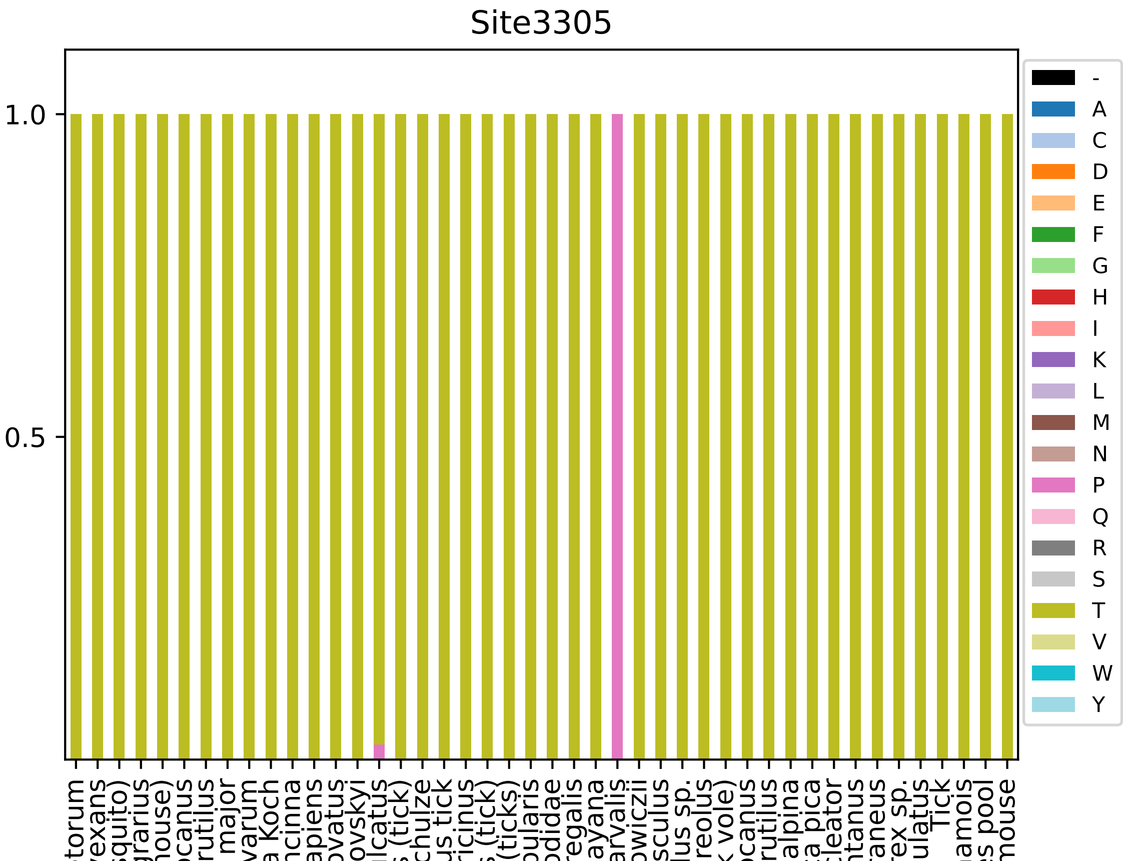
Site3305
1.0
0.5
-
A
C
D
E
F
G
H
I
K
L
M
N
P
Q
R
S
T
V
W
Y

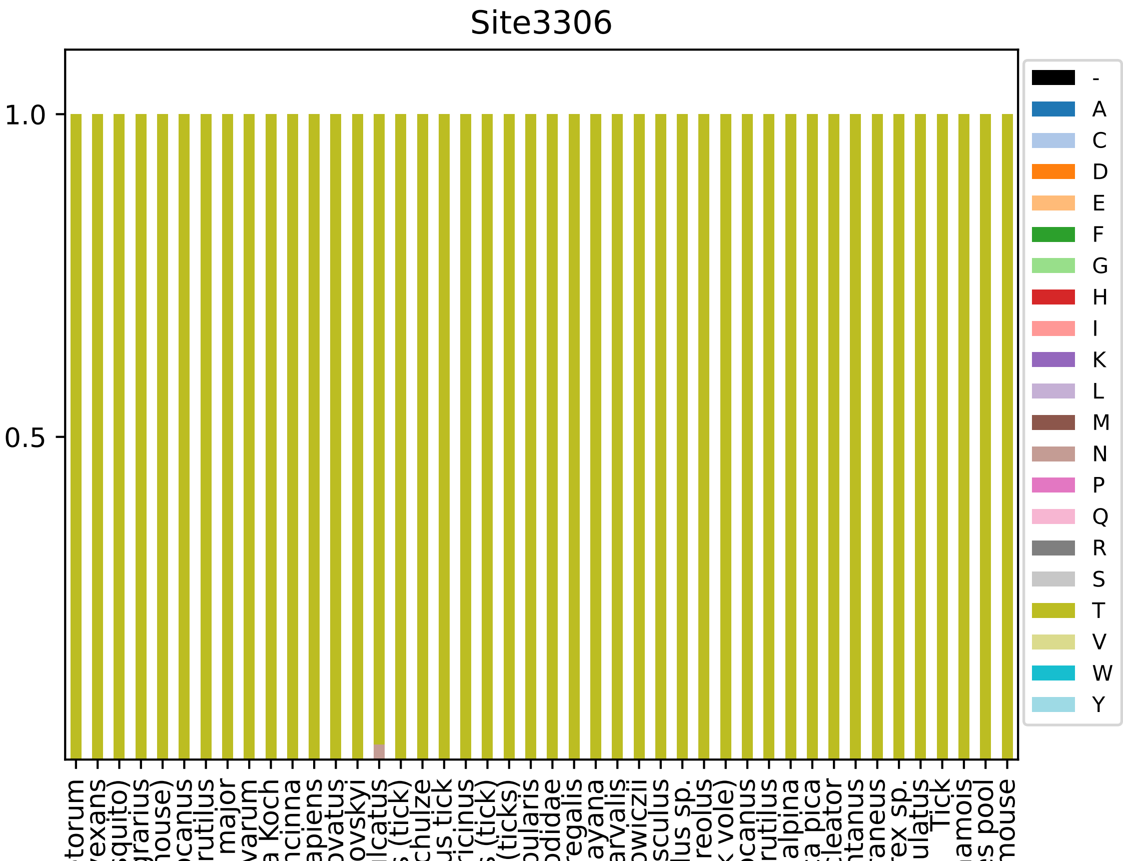
Site3306
1.0
0.5
-
A
C
D
E
F
G
H
I
K
L
M
N
P
Q
R
S
T
V
W
Y

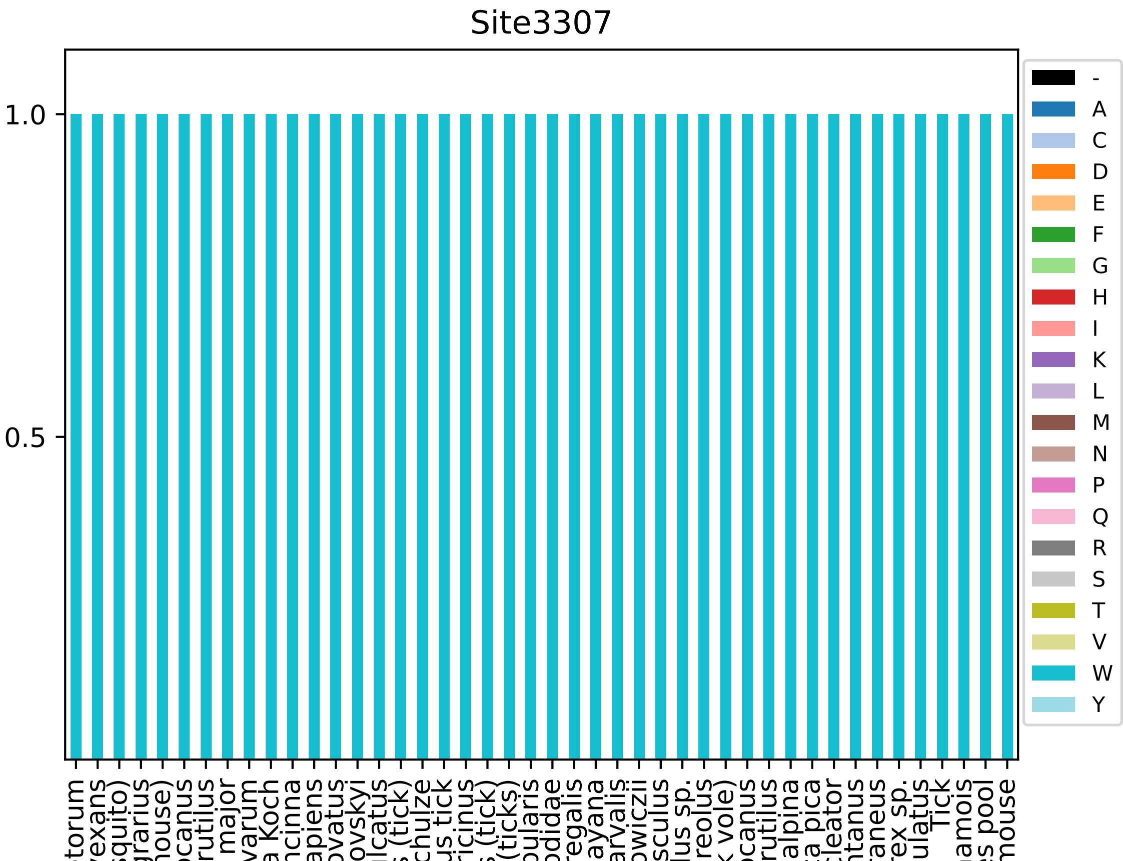
Site3307
1.0
0.5
-
A
C
D
E
F
G
H
I
K
L
M
N
P
Q
R
S
T
V
W
Y

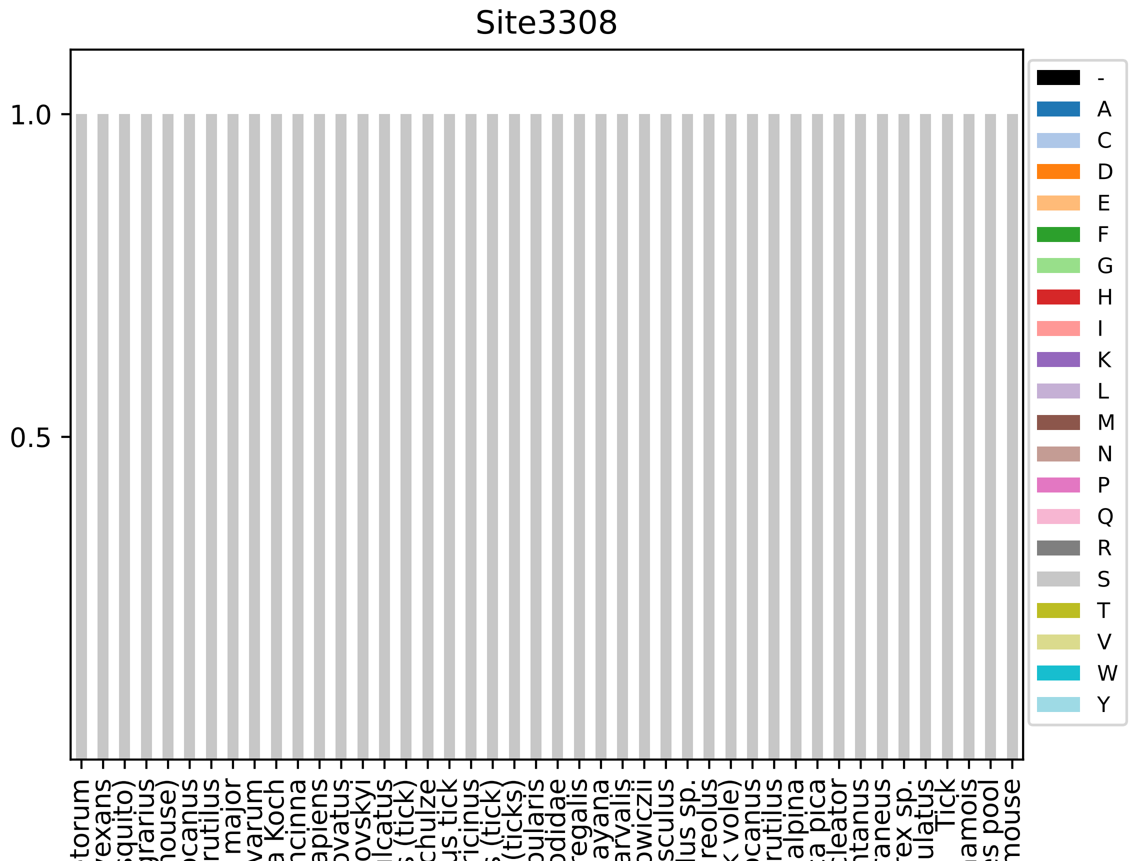
Site3308
1.0
0.5
-
A
C
D
E
F
G
H
I
K
L
M
N
P
Q
R
S
T
V
W
Y

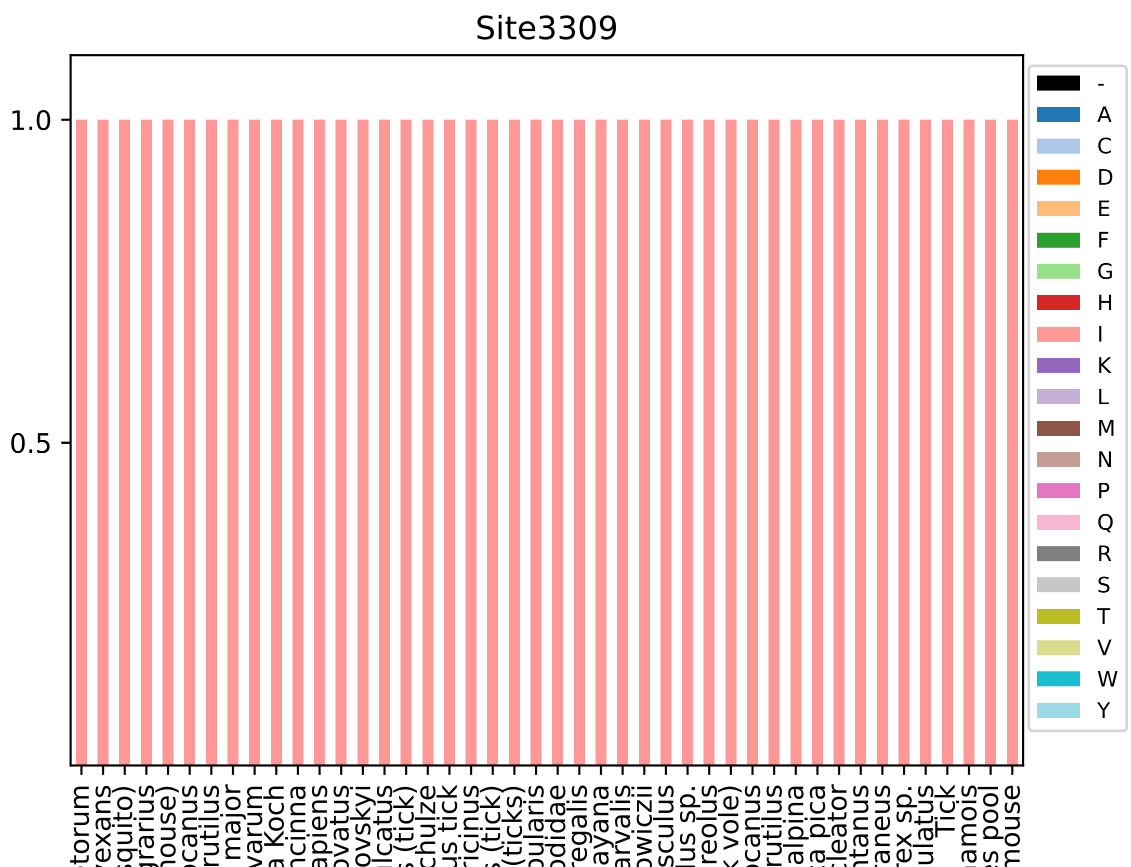

Site3309
1.0
0.5
-
A
C
D
E
F
G
H
I
K
L
M
N
P
Q
R
S
T
V
W
Y

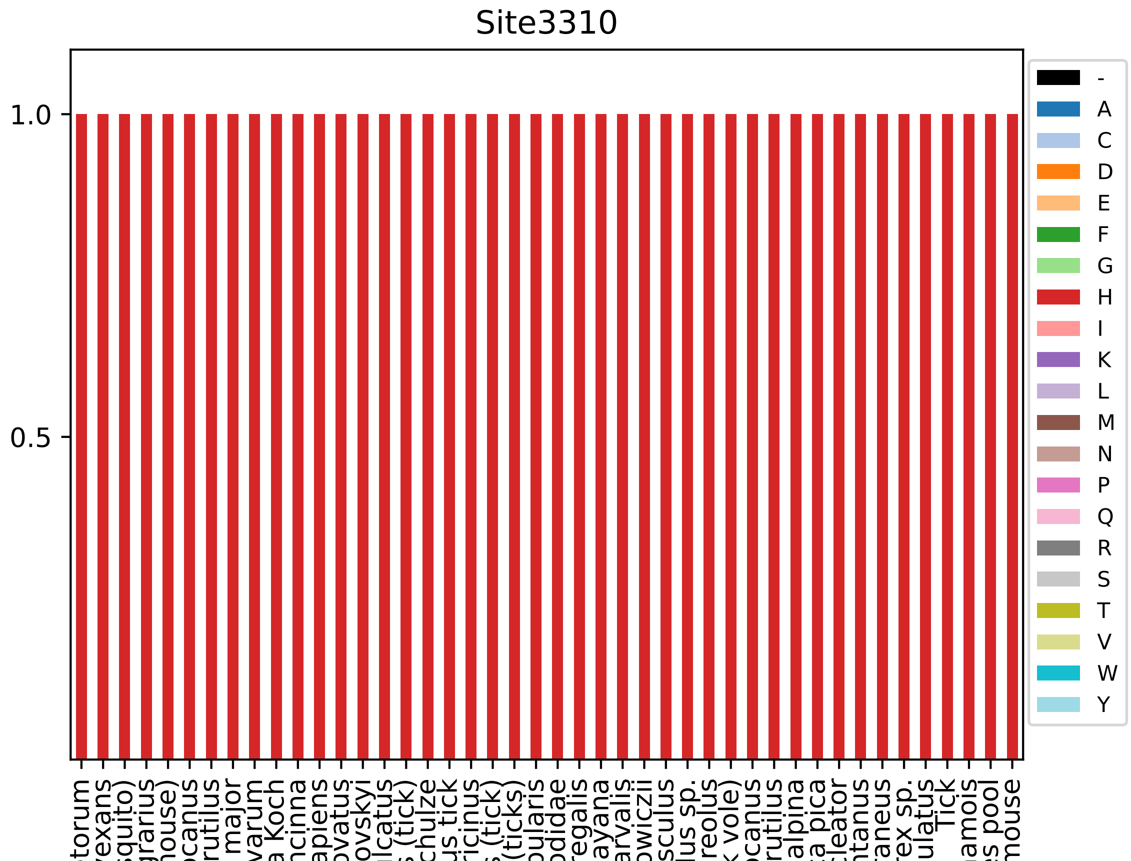

Site3310
1.0
0.5
-
A
C
D
E
F
G
H
I
K
L
M
N
P
Q
R
S
T
V
W
Y
Tick

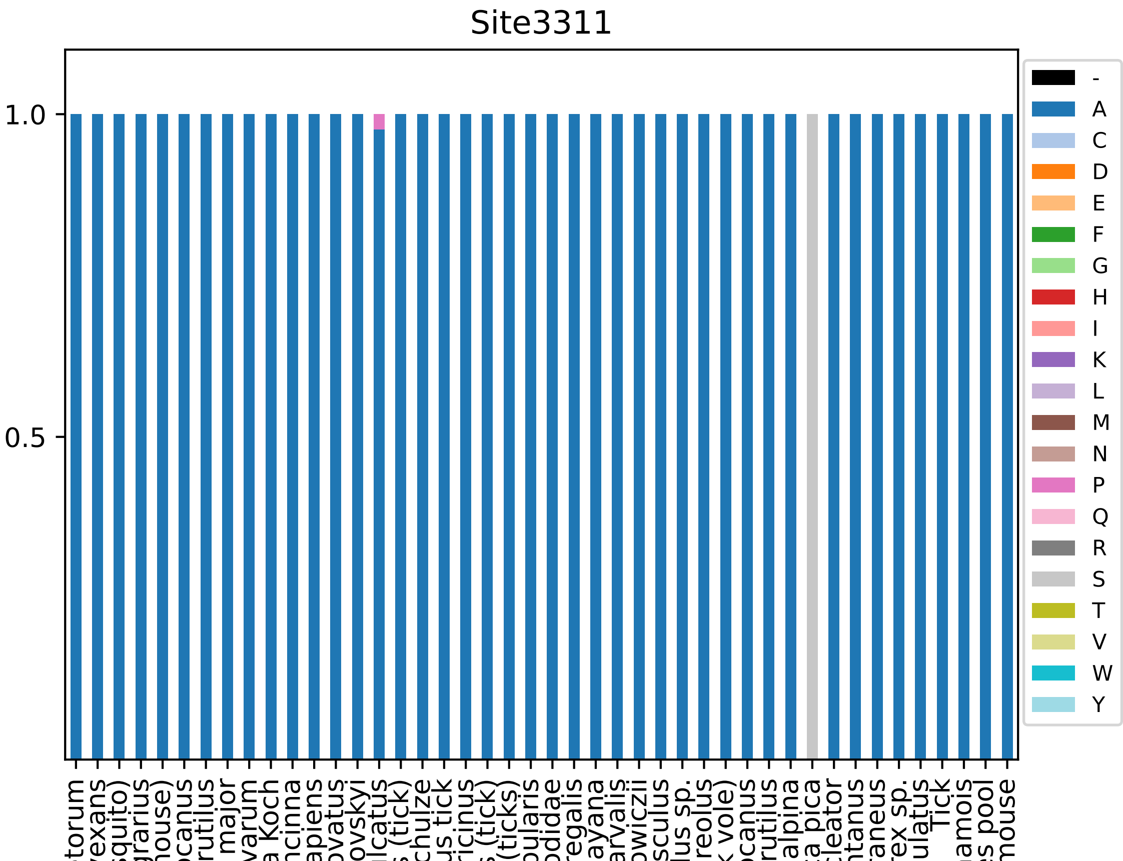
Site3311
1.0
0.5
-
A
C
D
E
F
G
H
I
K
L
M
N
P
Q
R
S
T
V
W
Y

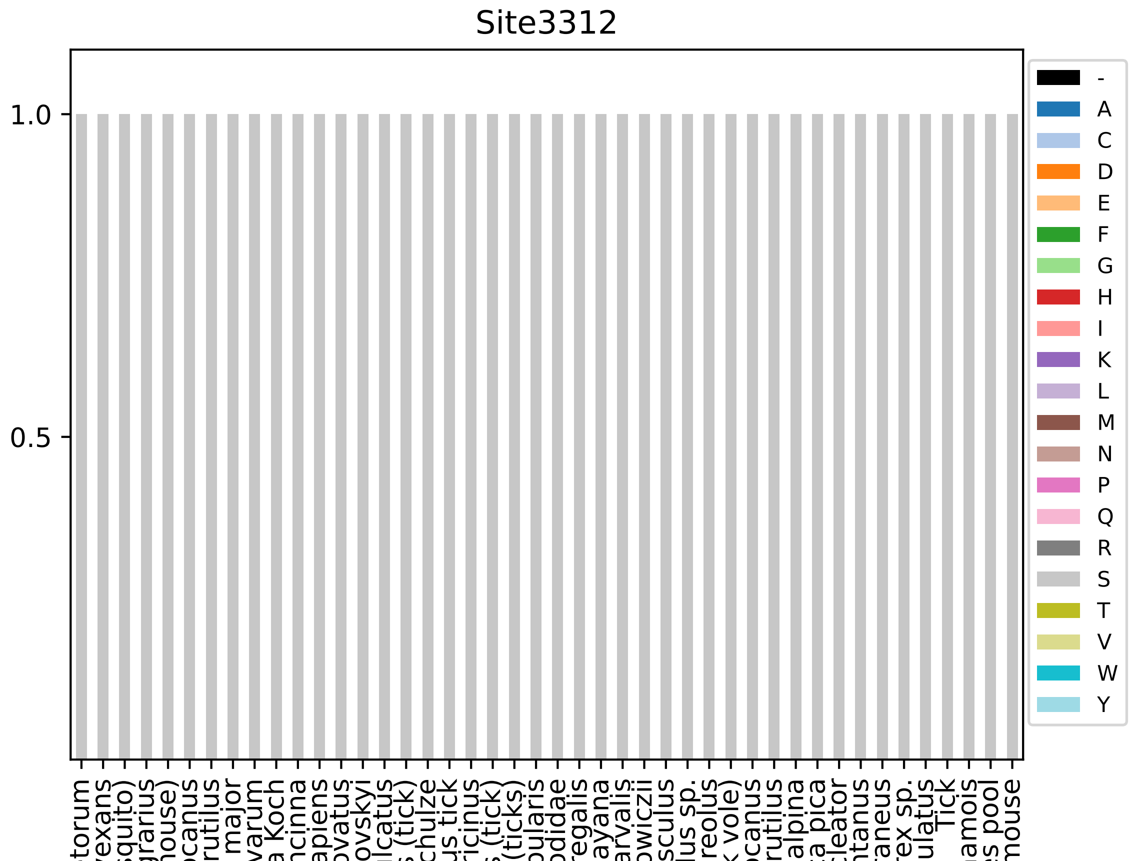

Site3312
1.0
0.5
-
A
C
D
E
F
G
H
I
K
L
M
N
P
Q
R
S
T
V
W
Y

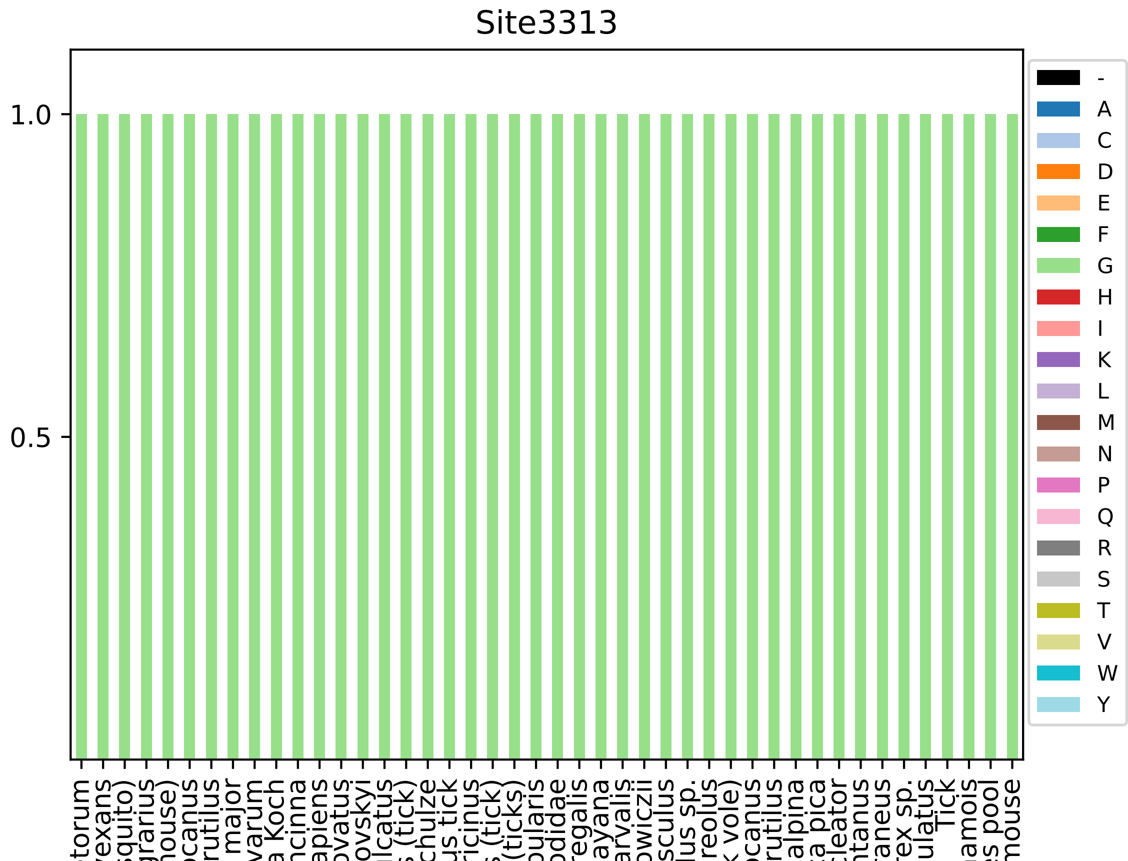
Site3313
1.0
0.5
-
A
C
D
E
F
G
H
I
K
L
M
N
P
Q
R
S
T
V
W
Y

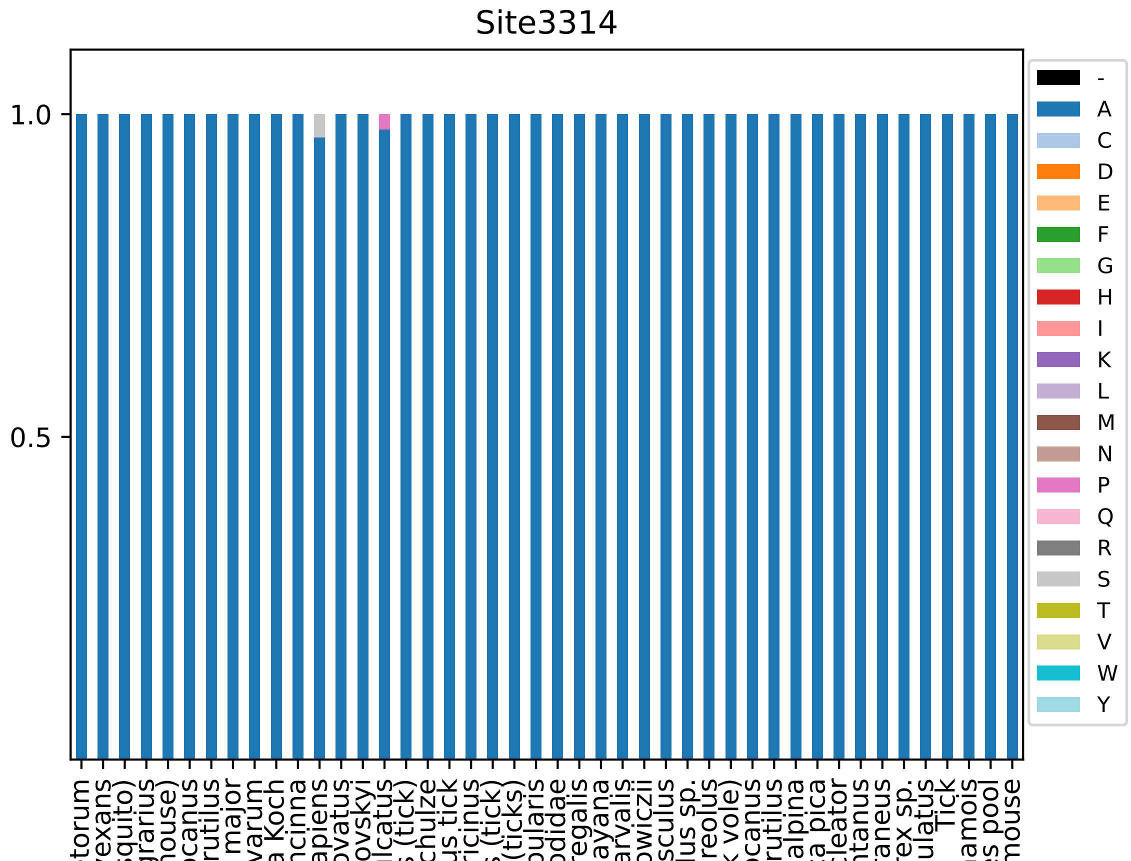
Site3314
1.0
0.5
-
A
C
D
E
F
G
H
I
K
L
M
N
P
Q
R
S
T
V
W
Y

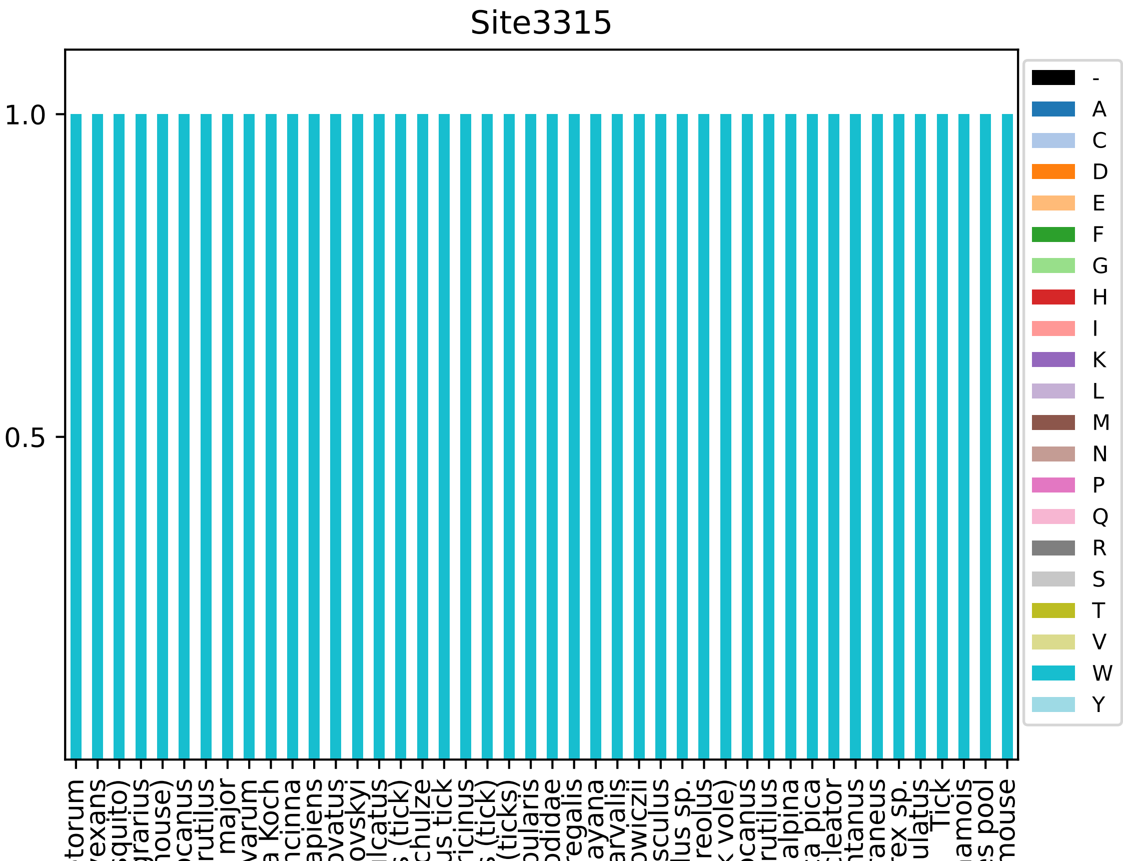
Site3315
1.0
0.5
-
A
C
D
E
F
G
H
I
K
L
M
N
P
Q
R
S
T
V
W
Y

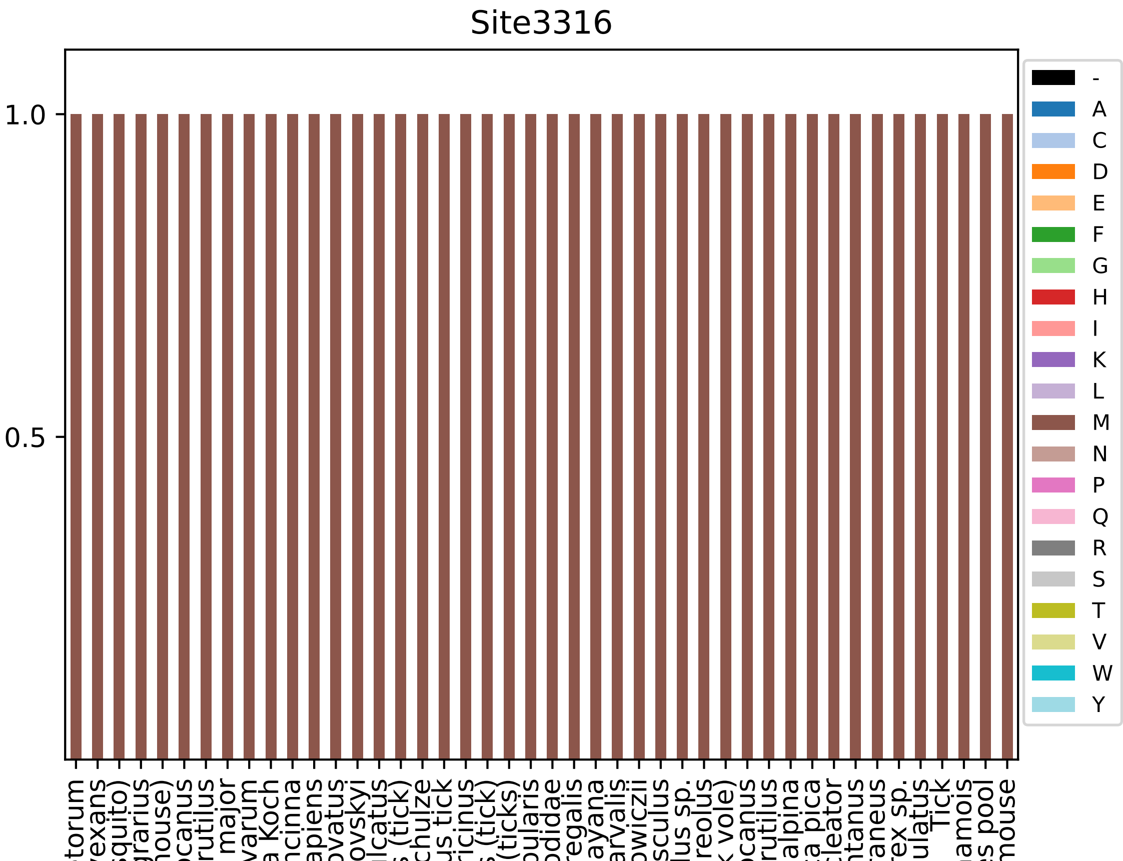
Site3316
1.0
0.5
-
A
C
D
E
F
G
H
I
K
L
M
N
P
Q
R
S
T
V
W
Y

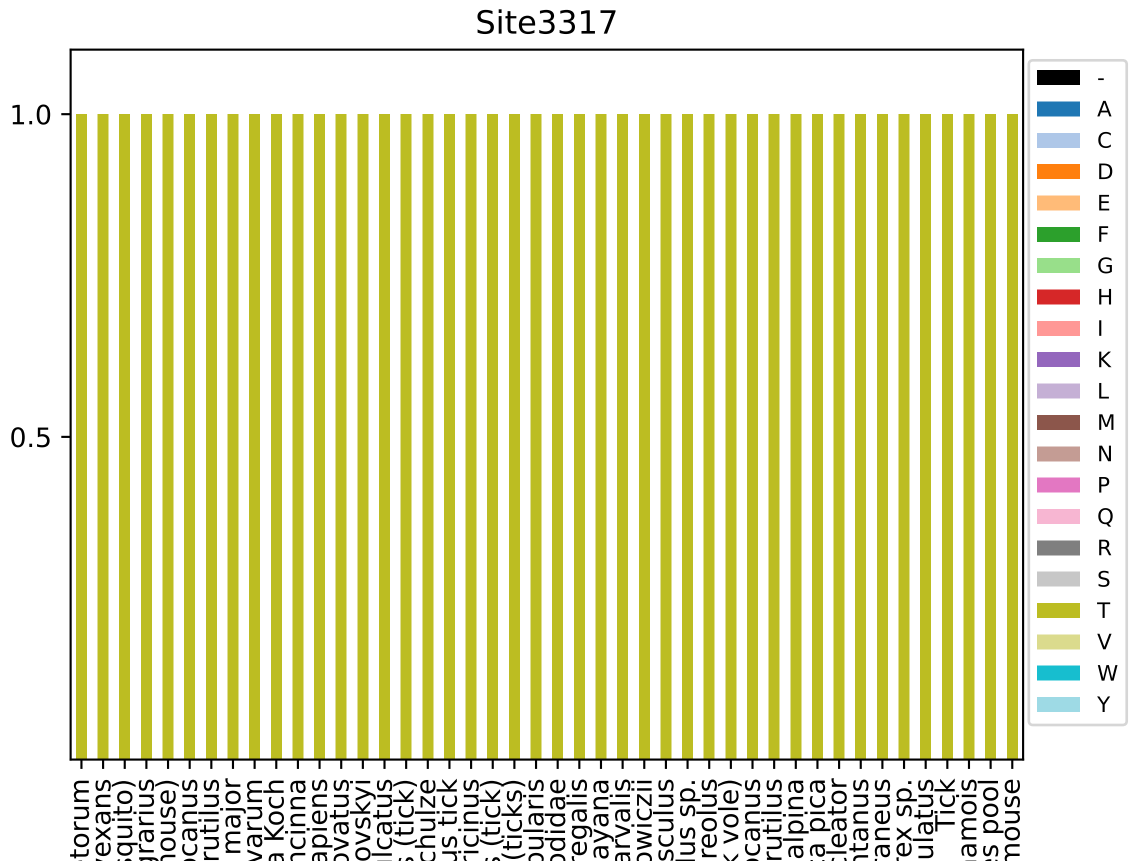
Site3317
1.0
0.5
-
A
C
D
E
F
G
H
I
K
L
M
N
P
Q
R
S
T
V
W
Y
Tick

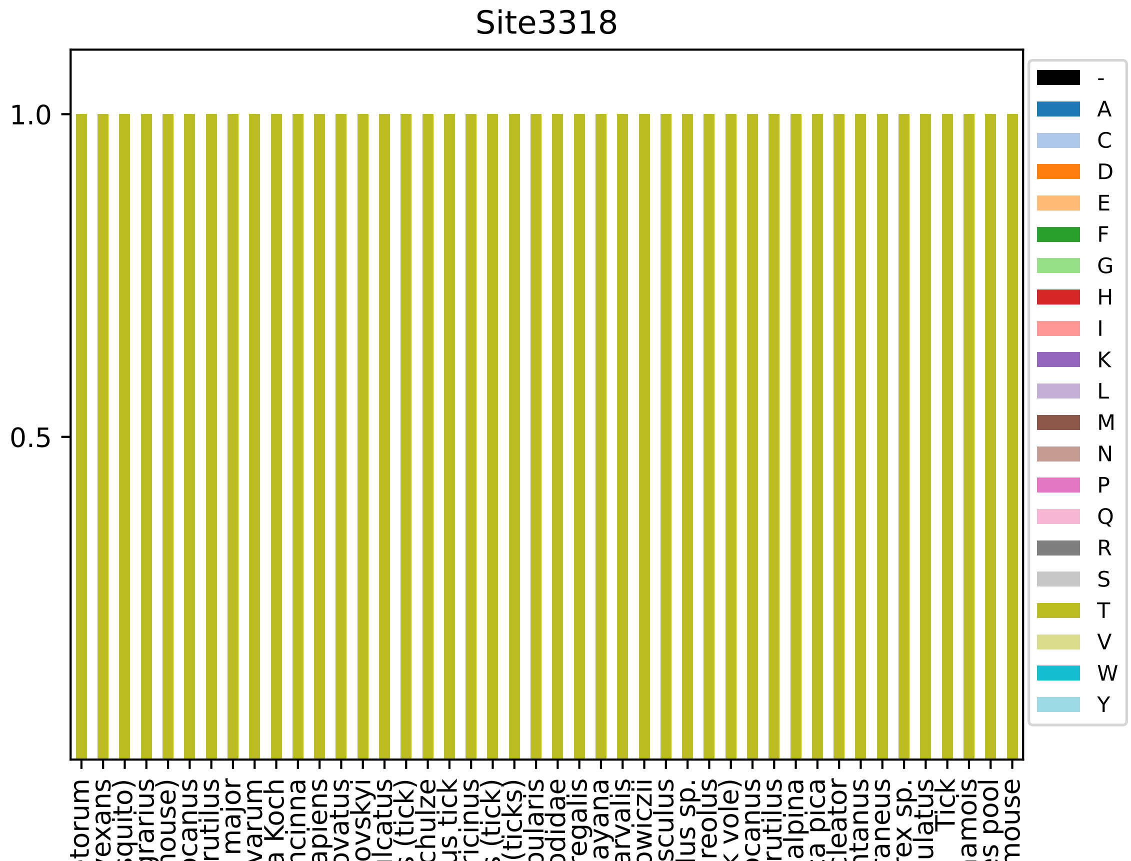
Site3318
1.0
0.5
-
A
C
D
E
F
G
H
I
K
L
M
N
P
Q
R
S
T
V
W
Y

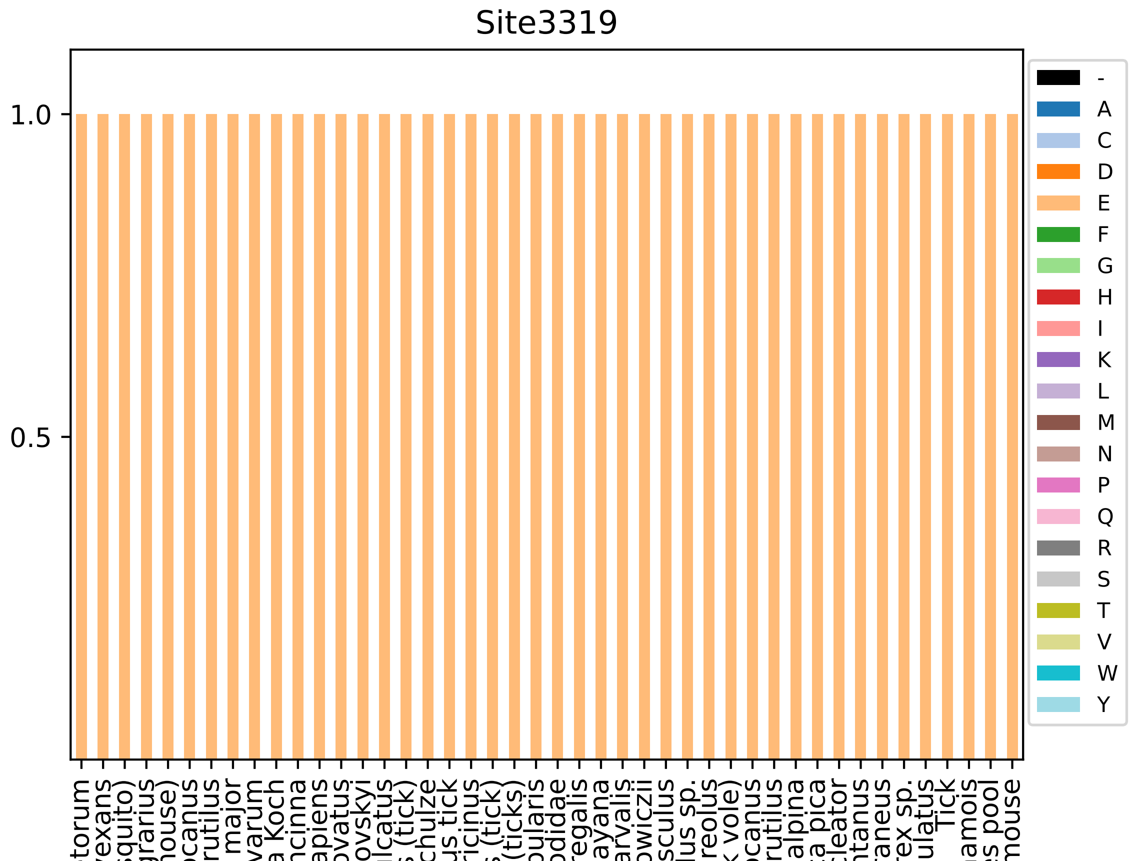

Site3319
1.0
0.5
-
A
C
D
E
F
G
H
I
K
L
M
N
P
Q
R
S
T
V
W
Y

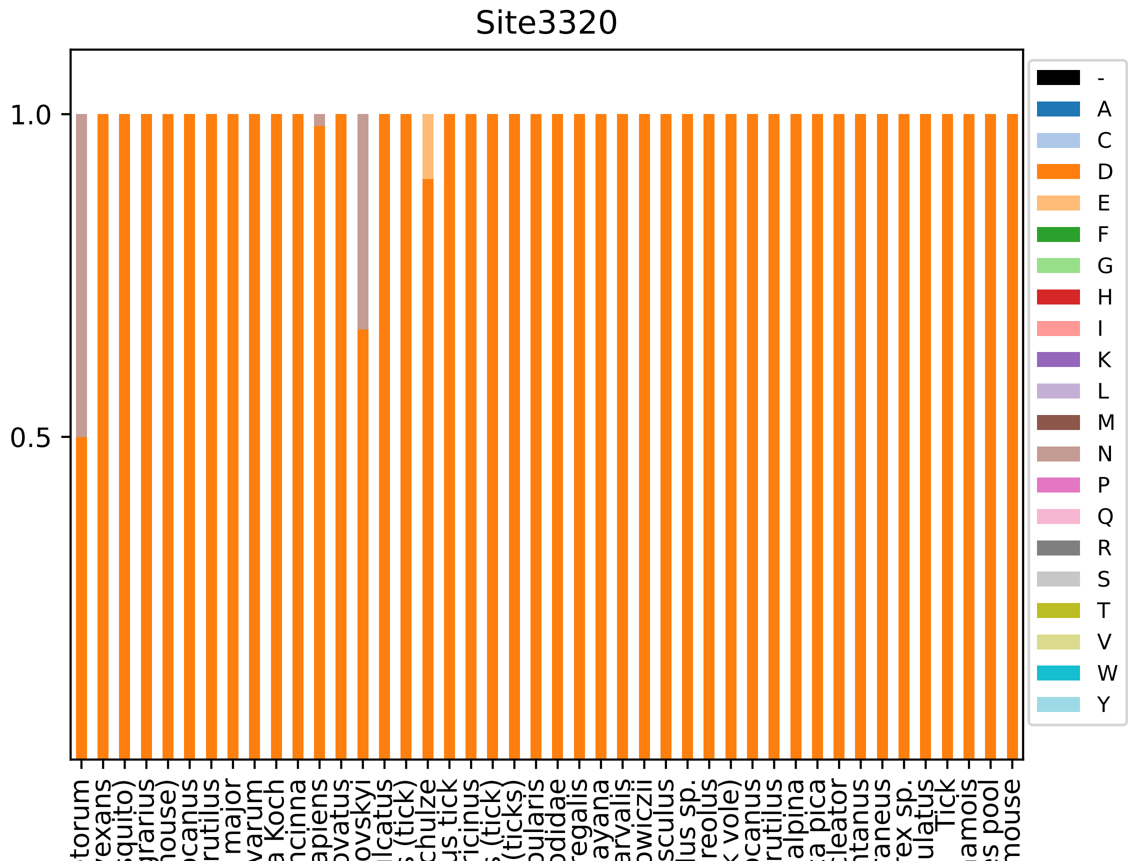
Site3320
1.0
0.5
-
A
C
D
E
F
G
H
I
K
L
M
N
P
Q
R
S
T
V
W
Y

Site3321

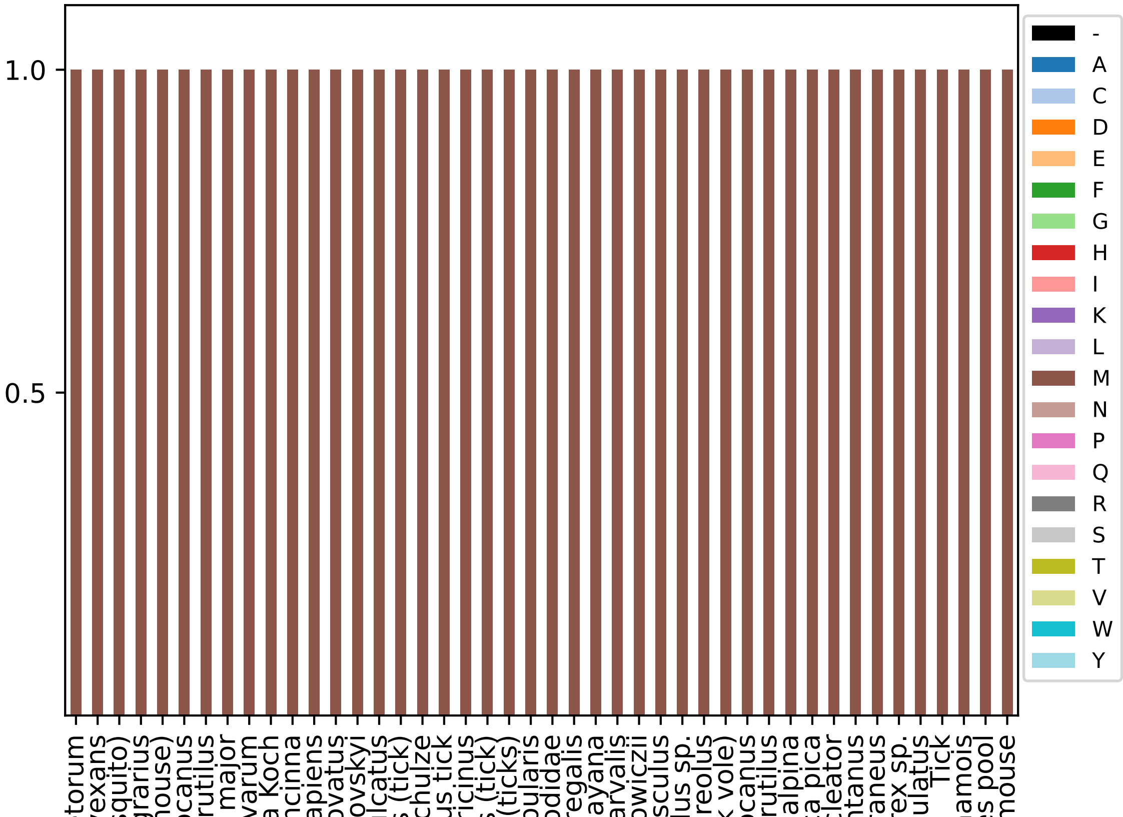

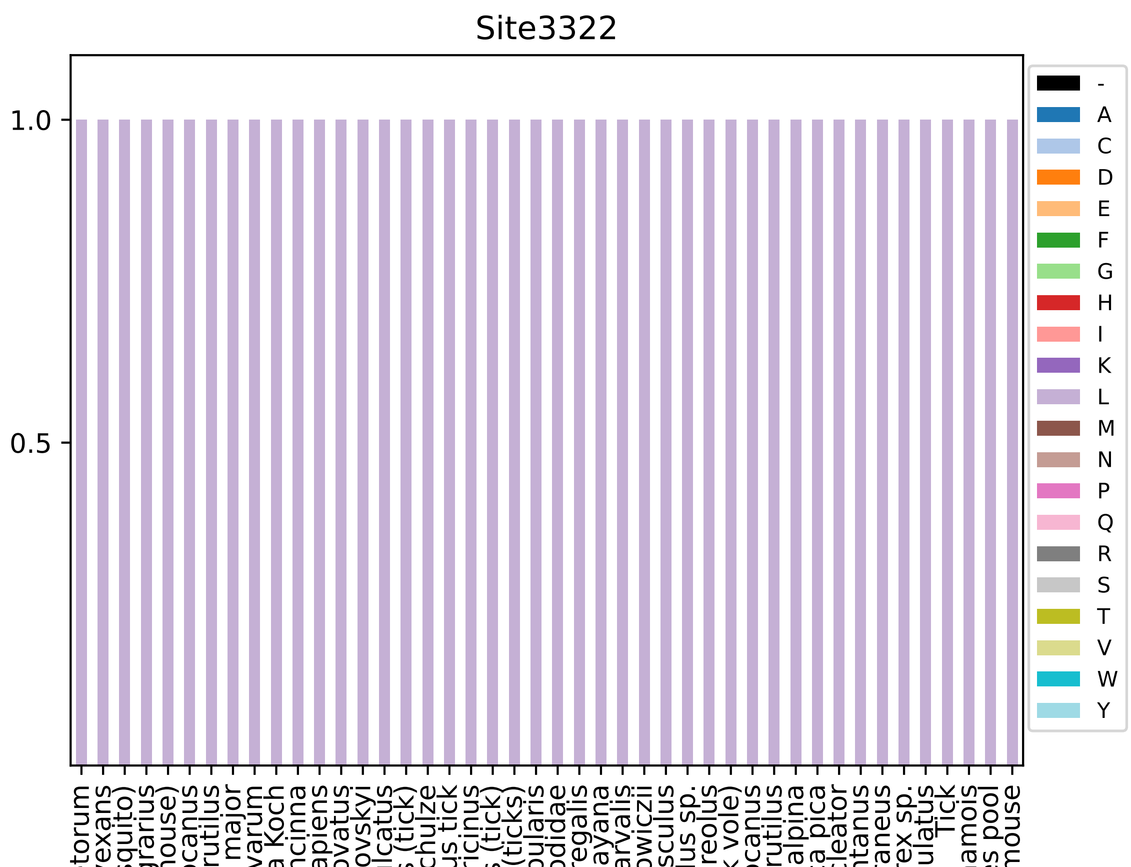
Site3322
1.0
0.5
-
A
C
D
E
F
G
H
I
K
L
M
N
P
Q
R
S
T
V
W
Y

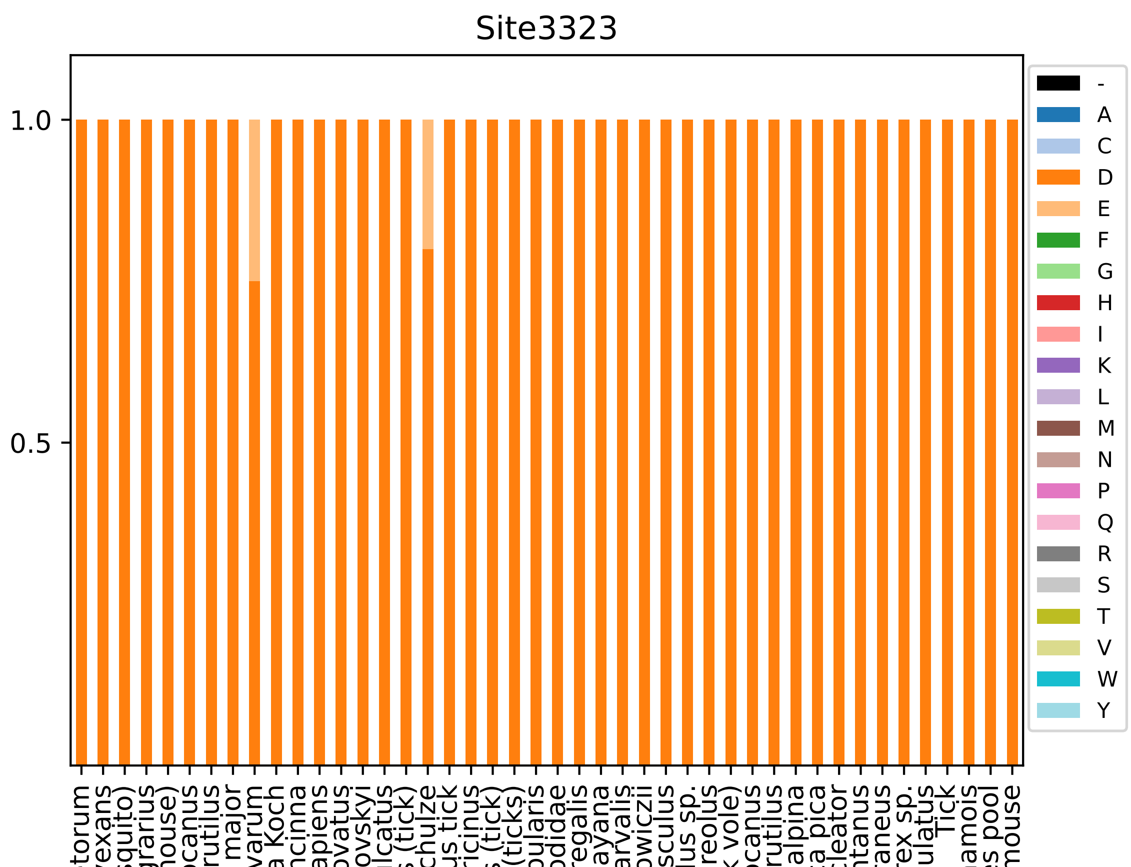
Site3323
1.0
0.5
-
A
C
D
E
F
G
H
I
K
L
M
N
P
Q
R
S
T
V
W
Y

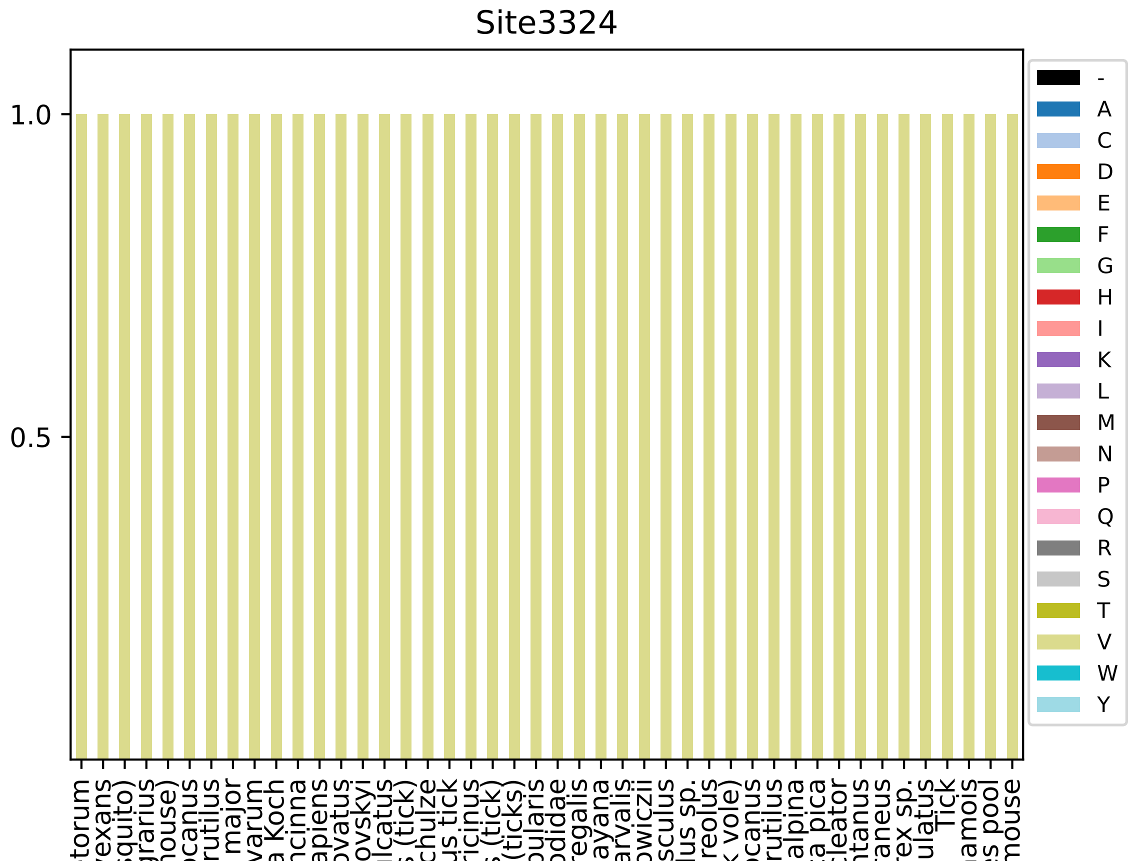
Site3324
1.0
0.5
-
A
C
D
E
F
G
H
I
K
L
M
N
P
Q
R
S
T
V
W
Y

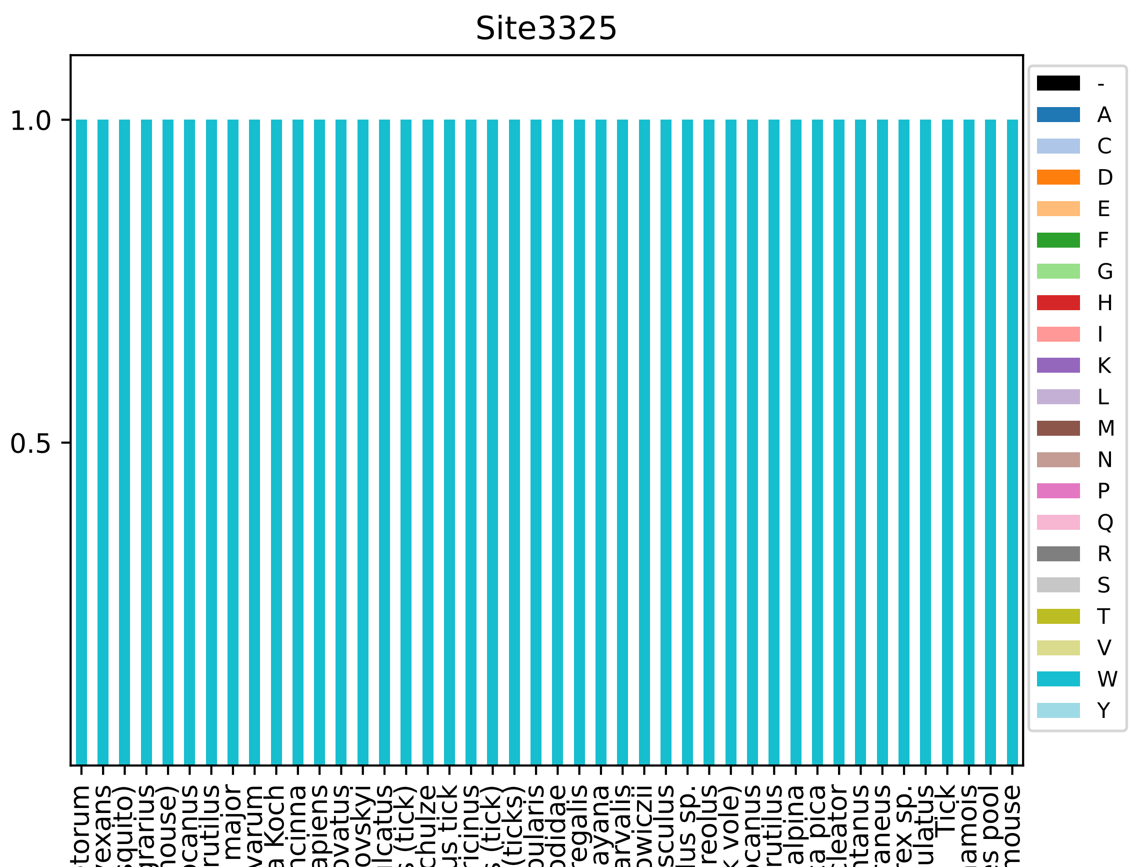
Site3325
1.0
0.5
-
A
C
D
E
F
G
H
I
K
L
M
N
P
Q
R
S
T
V
W
Y

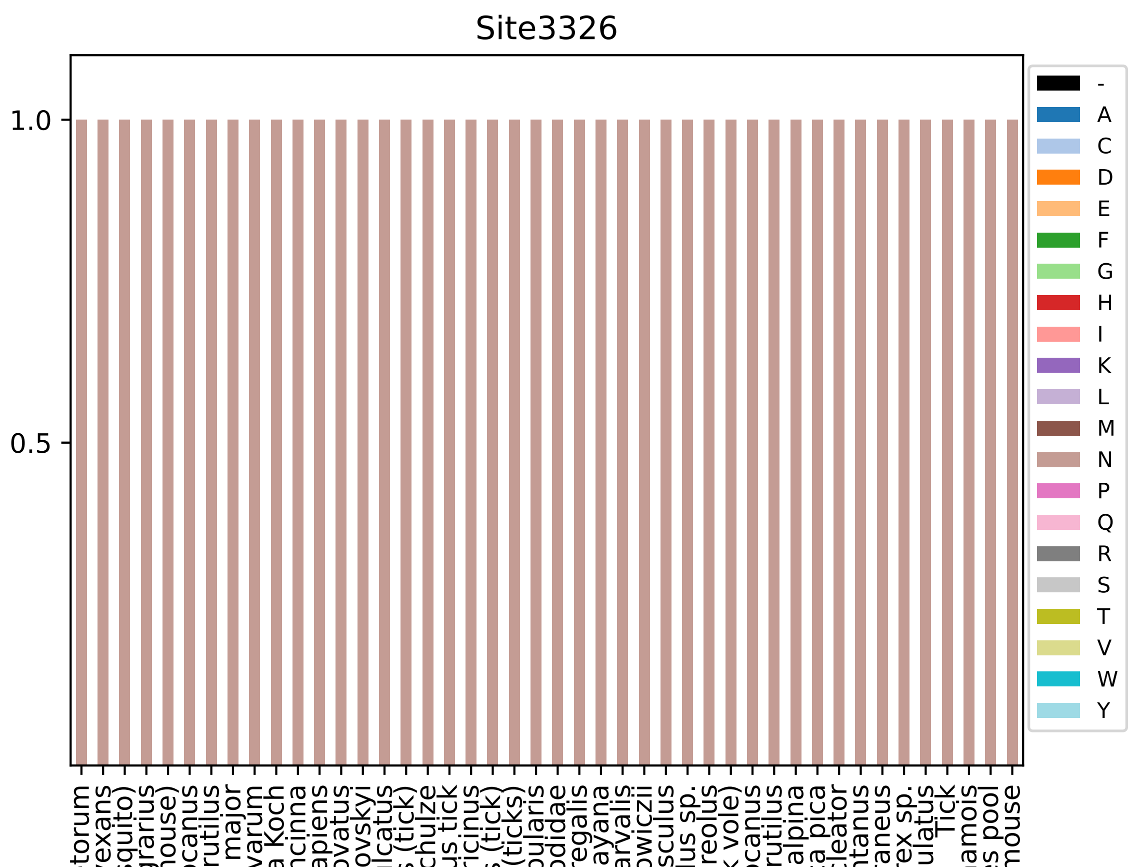
Site3326
1.0
0.5
-
A
C
D
E
F
G
H
I
K
L
M
N
P
Q
R
S
T
V
W
Y
Tick

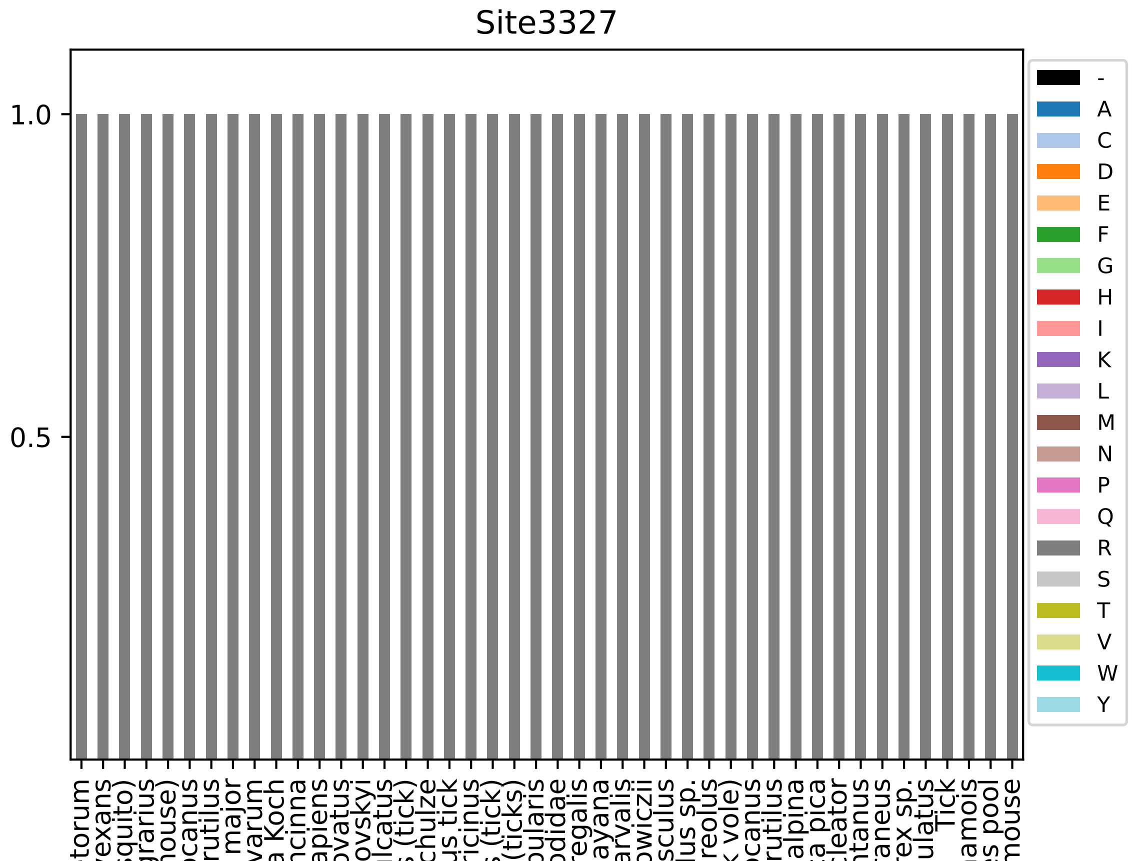

Site3327
1.0
0.5
-
A
C
D
E
F
G
H
I
K
L
M
N
P
Q
R
S
T
V
W
Y

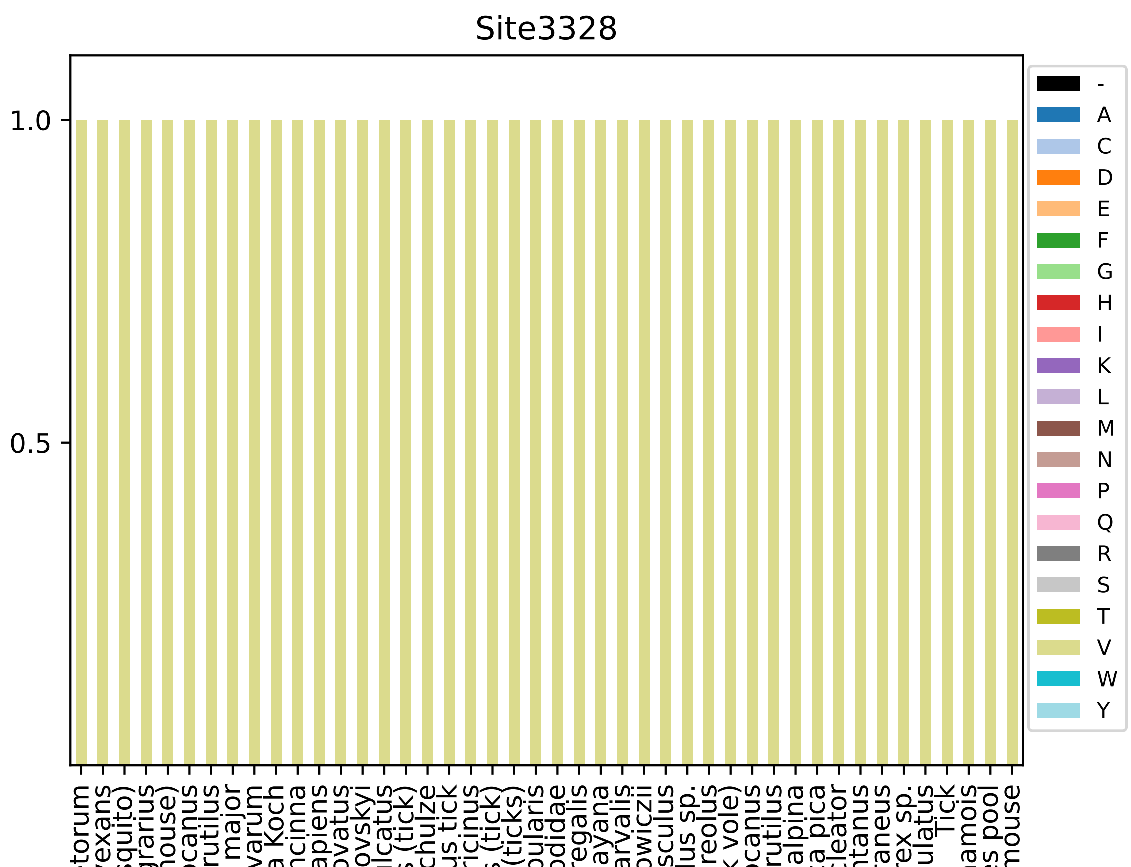

Site3328
1.0
0.5
-
A
C
D
E
F
G
H
I
K
L
M
N
P
Q
R
S
T
V
W
Y

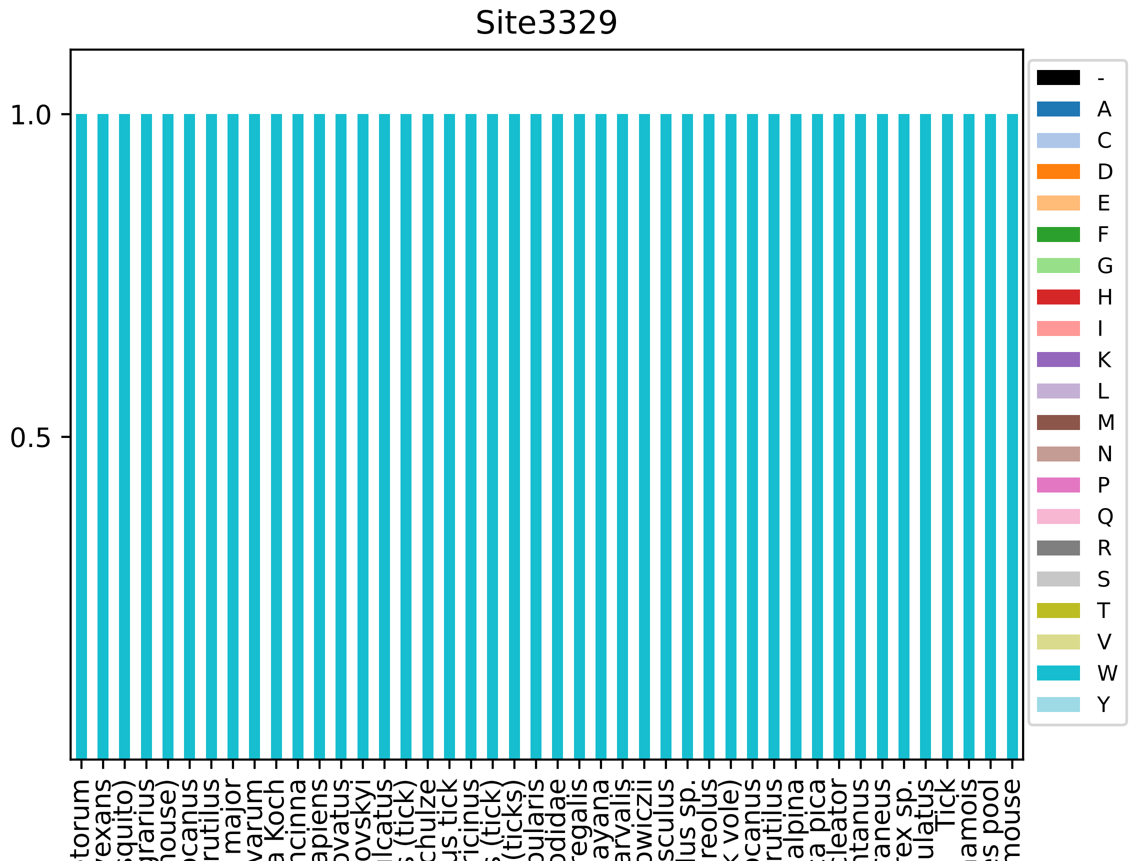
Site3329
1.0
0.5
-
A
C
D
E
F
G
H
I
K
L
M
N
P
Q
R
S
T
V
W
Y

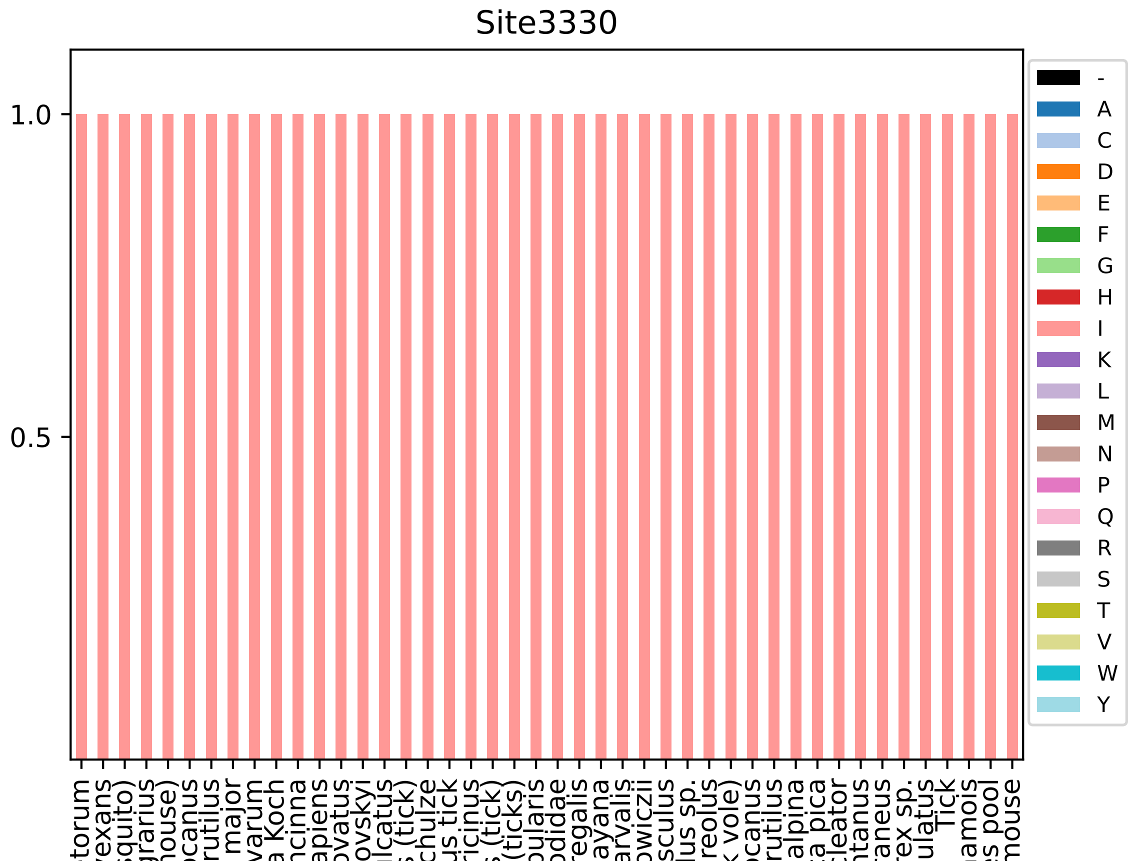
Site3330
1.0
0.5
-
A
C
D
E
F
G
H
I
K
L
M
N
P
Q
R
S
T
V
W
Y

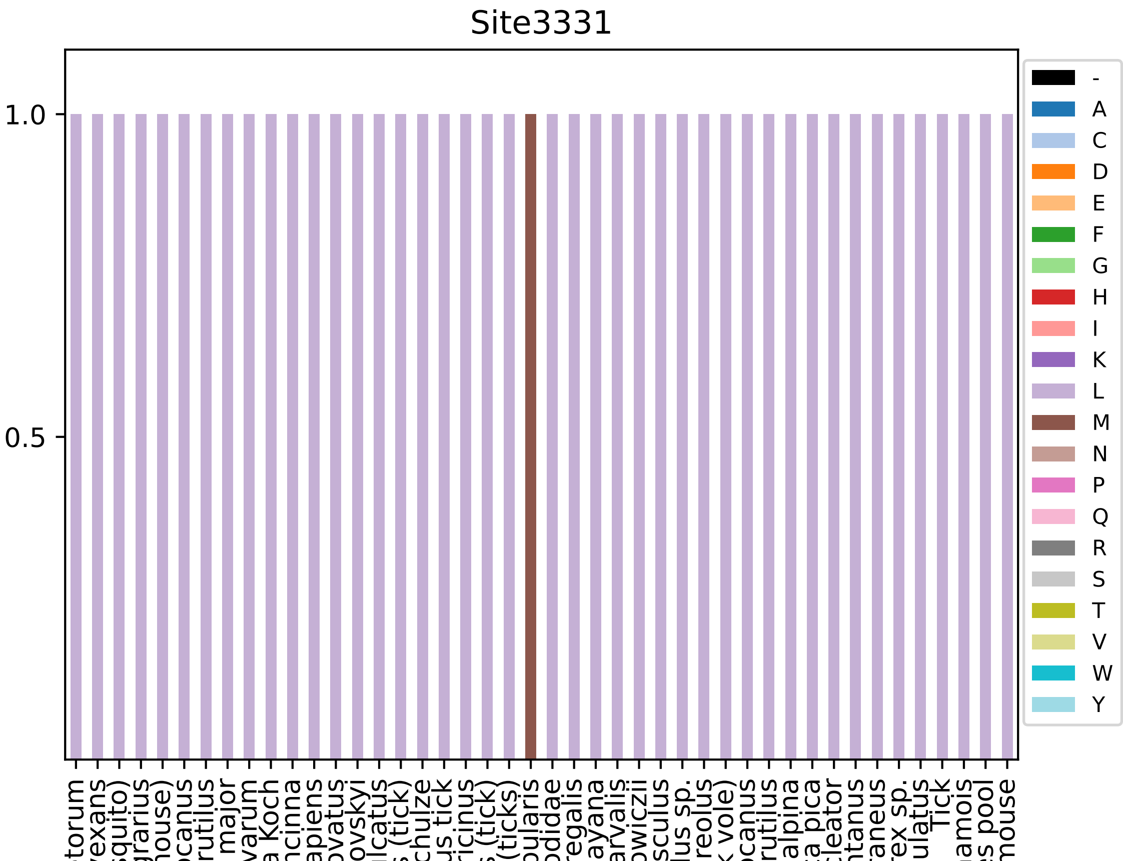
Site3331
1.0
0.5
-
A
C
D
E
F
G
H
I
K
L
M
N
P
Q
R
S
T
V
W
Y

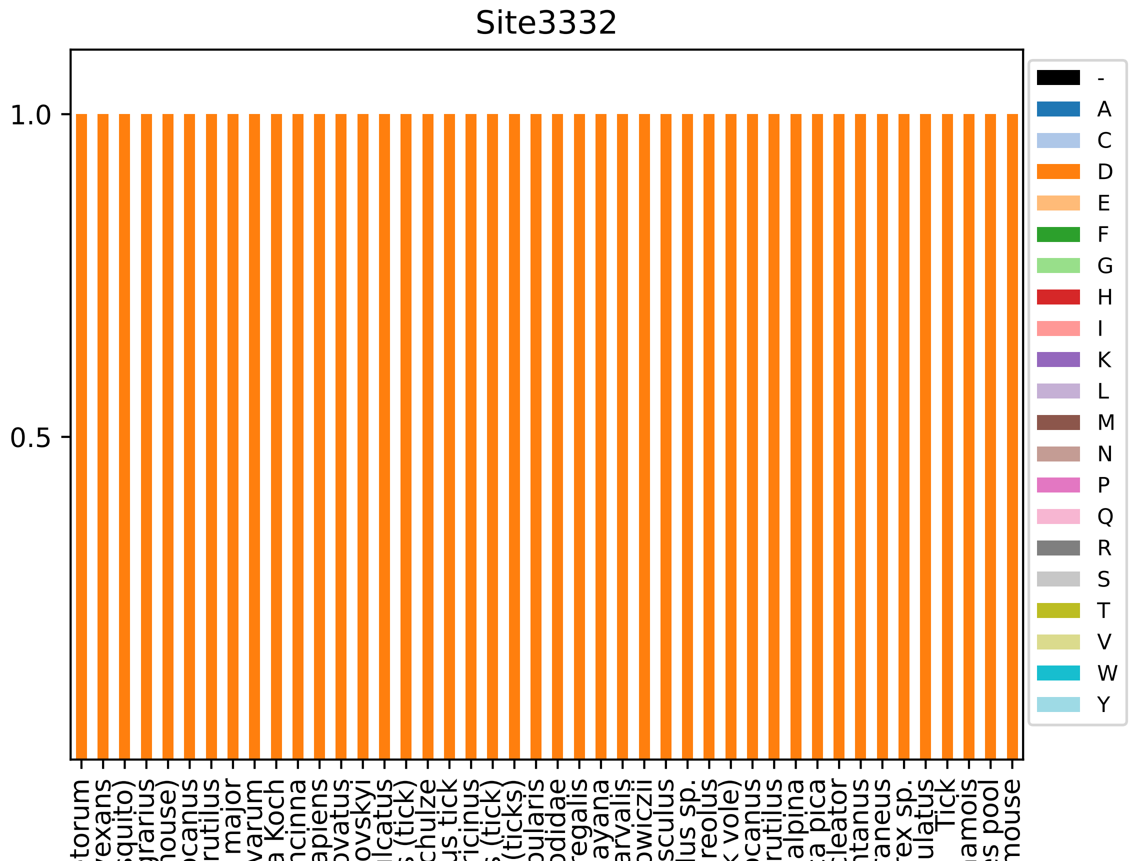
Site3332
1.0
0.5
-
A
C
D
E
F
G
H
I
K
L
M
N
P
Q
R
S
T
V
W
Y

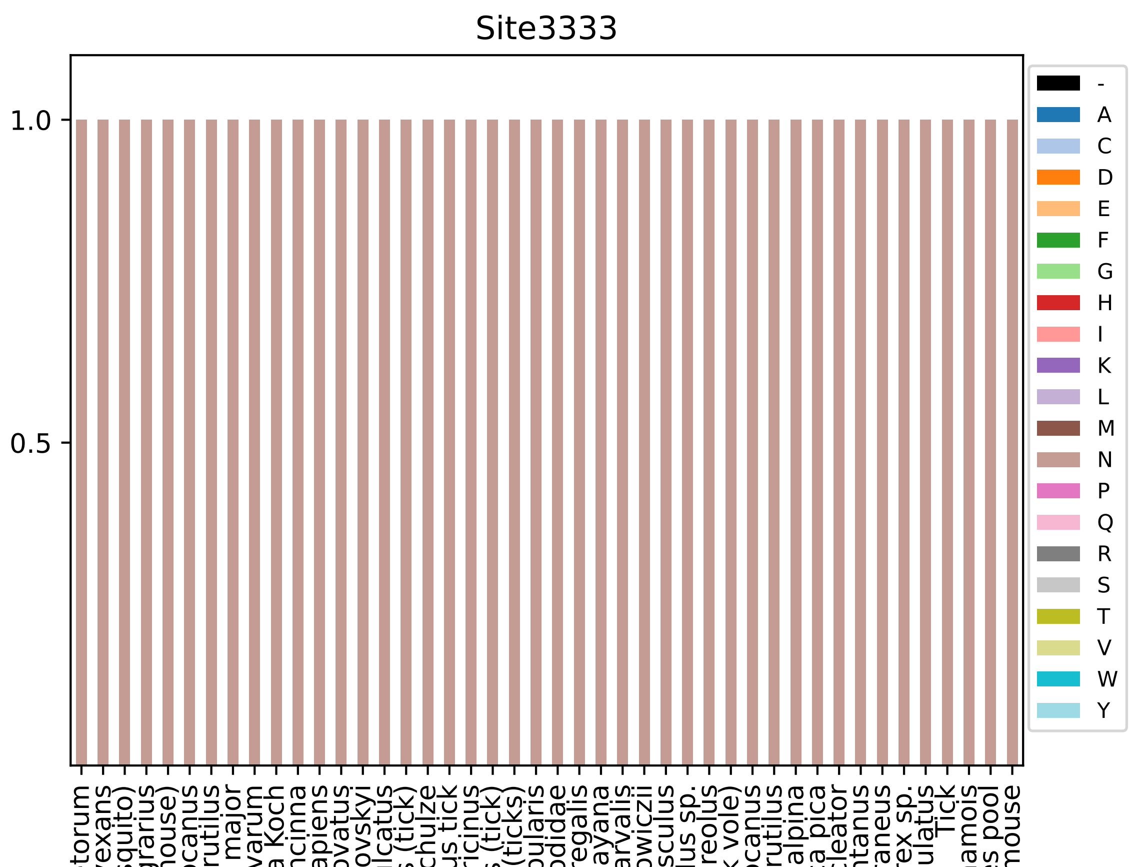
Site3333
1.0
0.5
-
A
C
D
E
F
G
H
I
K
L
M
N
P
Q
R
S
T
V
W
Y

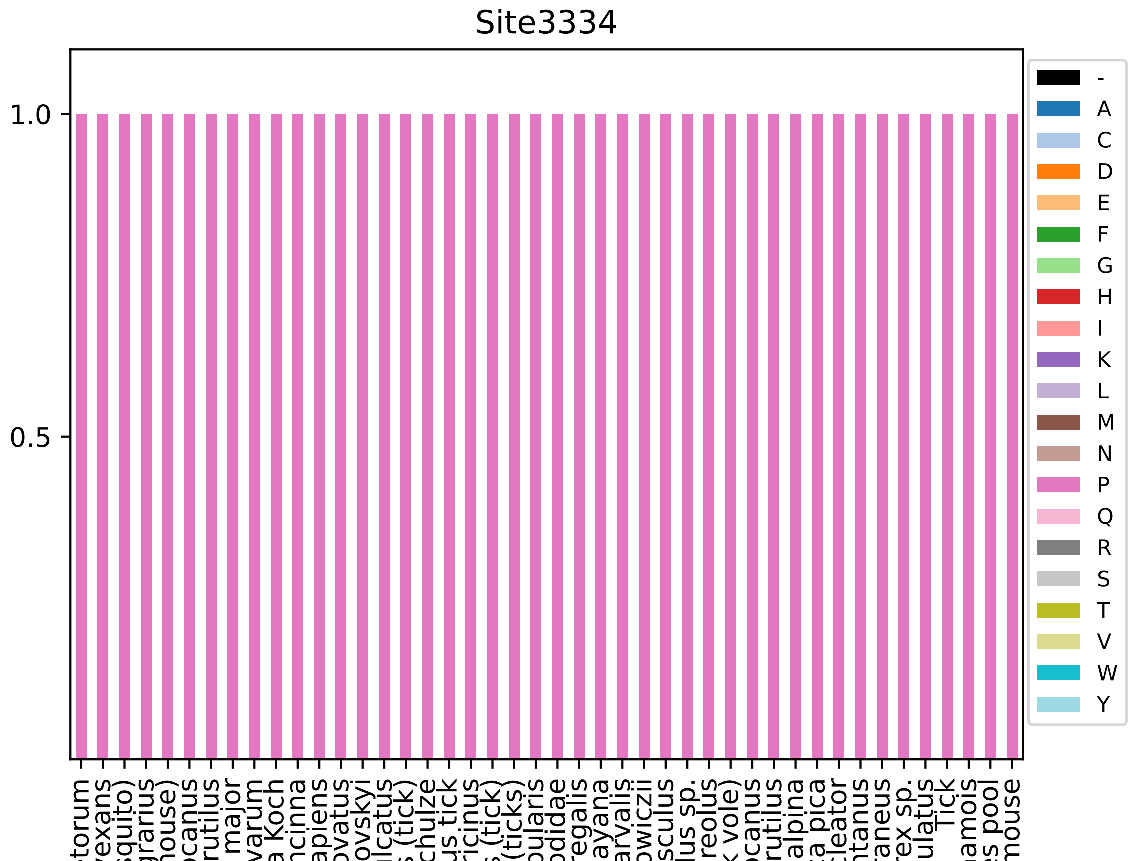
Site3334
1.0
0.5
-
A
C
D
E
F
G
H
I
K
L
M
N
P
Q
R
S
T
V
W
Y
torum
vexans
squito)
grarius
house)
ocanus
rutilus
major
varum
a Koch
ncinna
apiens
ovatus
ovskyi
lcatus
(tick)
chulze
us tick
ricinus
(tick)
(ticks)
pularis
odidae
regalis
ayana
arvalis
owiczii
sculus
lus sp.
reolus
k vole)
ocanus
rutilus
alpina
a pica
leator
ntanus
raneus
rex sp.
ulatus
Tick
amois
es pool
mouse

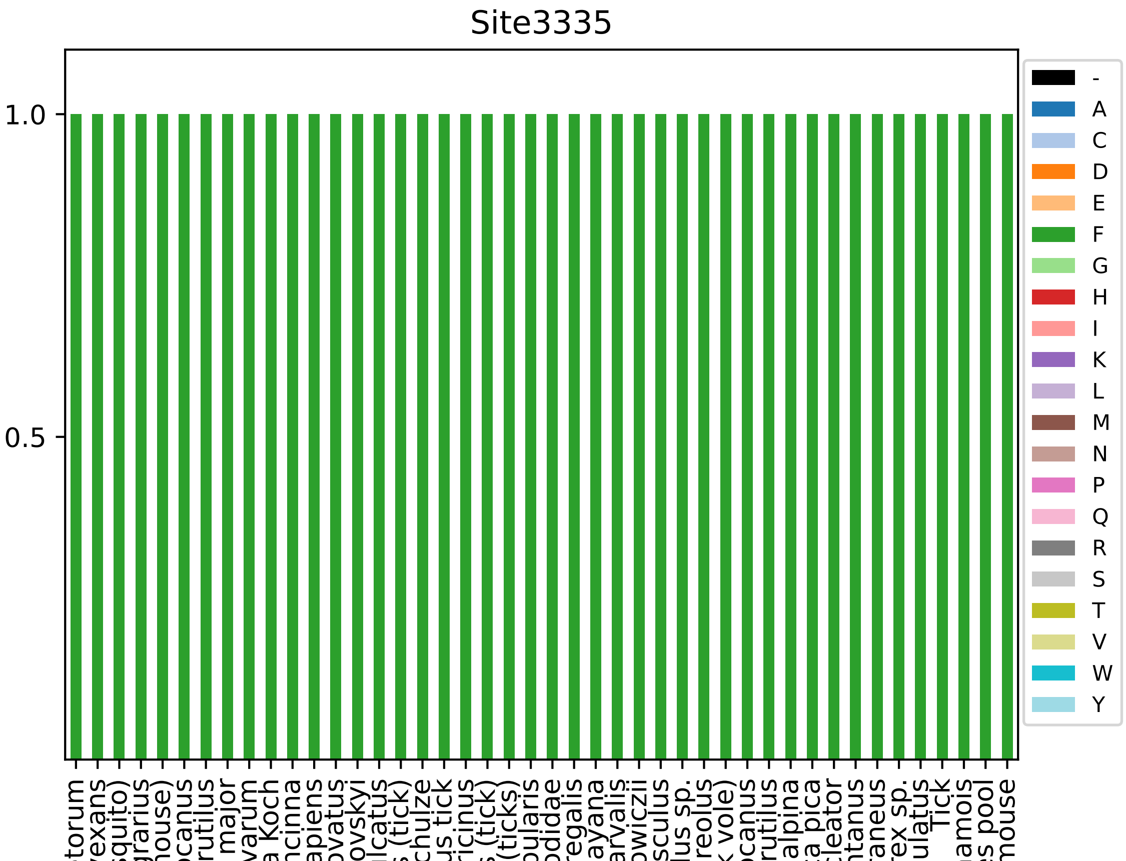

Site3335
1.0
0.5
-
A
C
D
E
F
G
H
I
K
L
M
N
P
Q
R
S
T
V
W
Y

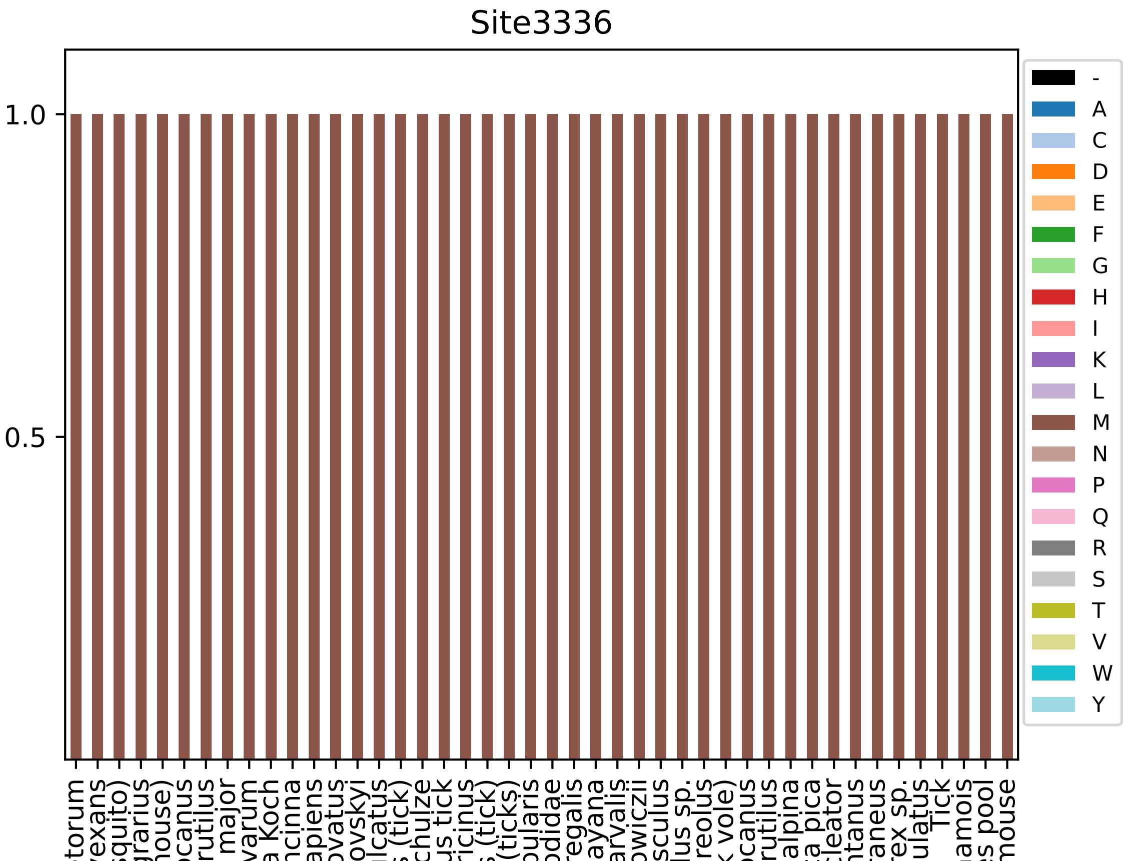
Site3336
1.0
0.5
-
A
C
D
E
F
G
H
I
K
L
M
N
P
Q
R
S
T
V
W
Y

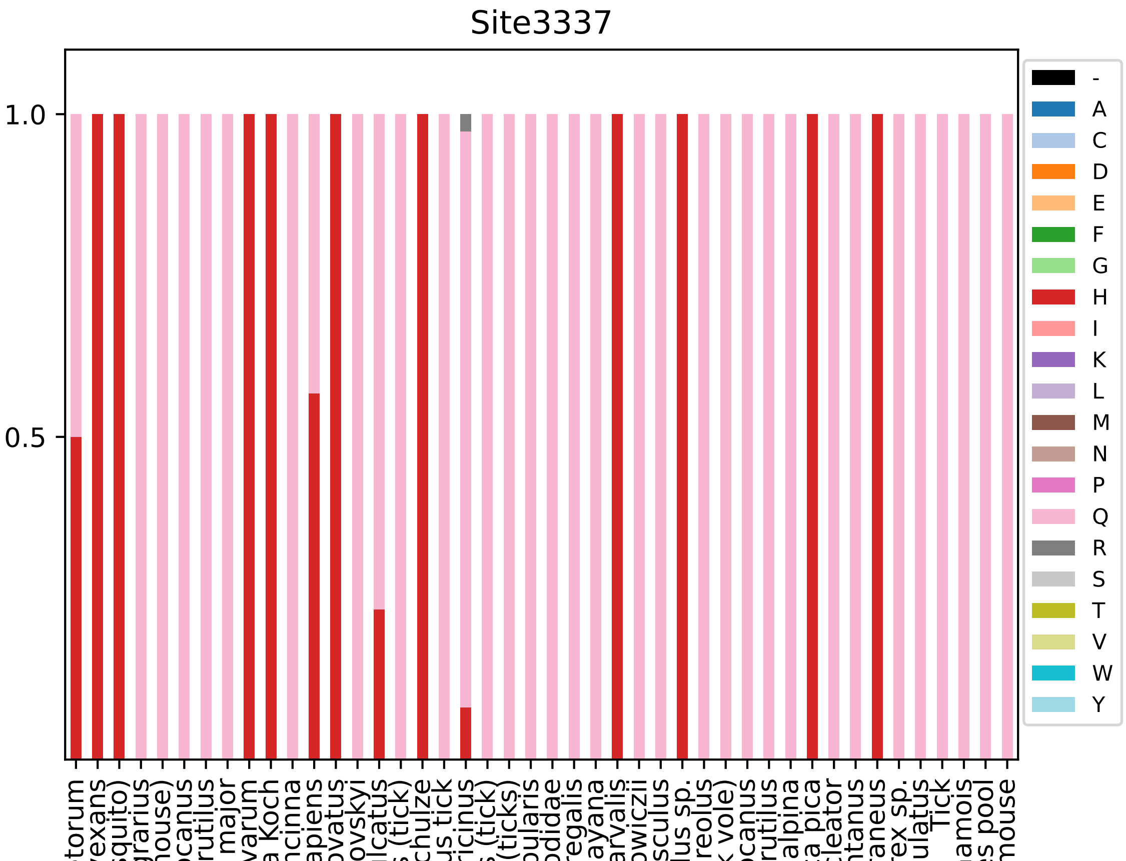
Site3337
1.0
0.5
-
A
C
D
E
F
G
H
I
K
L
M
N
P
Q
R
S
T
V
W
Y

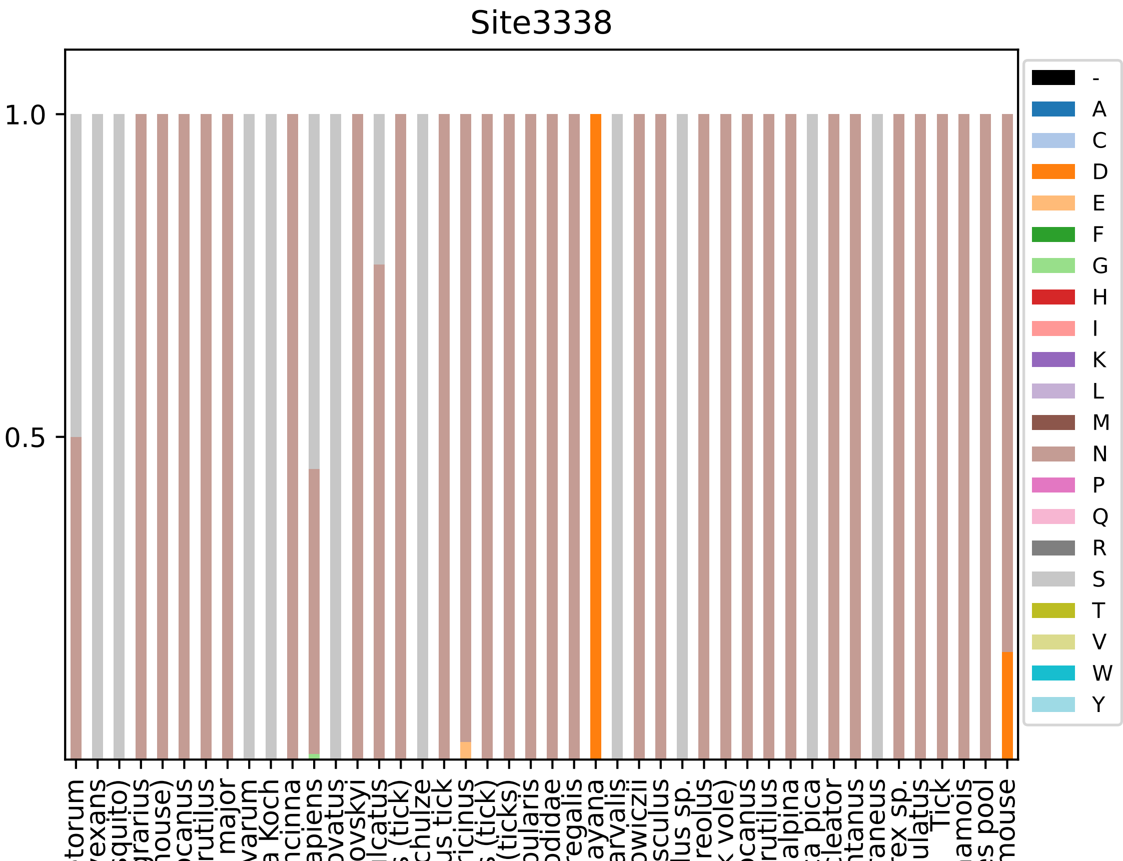

Site3338
1.0
0.5
-
A
C
D
E
F
G
H
I
K
L
M
N
P
Q
R
S
T
V
W
Y

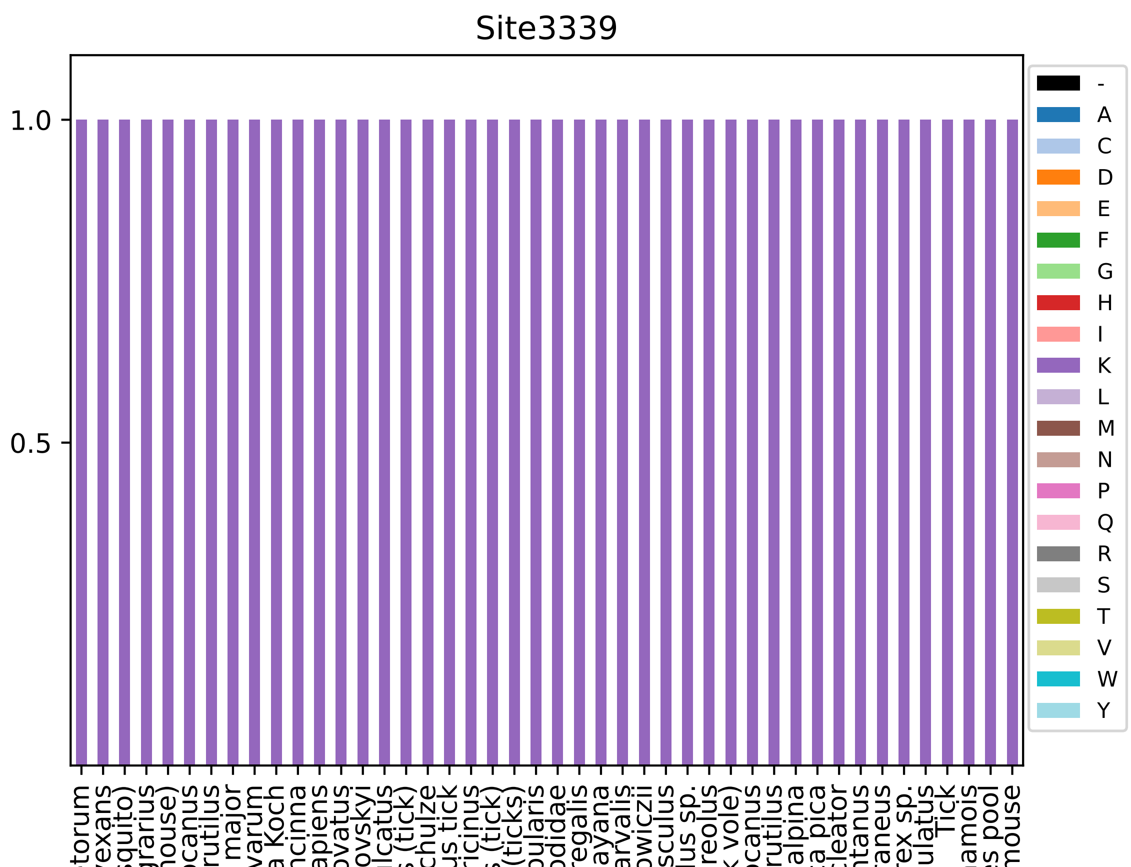
Site3339
1.0
0.5
-
A
C
D
E
F
G
H
I
K
L
M
N
P
Q
R
S
T
V
W
Y
Tick

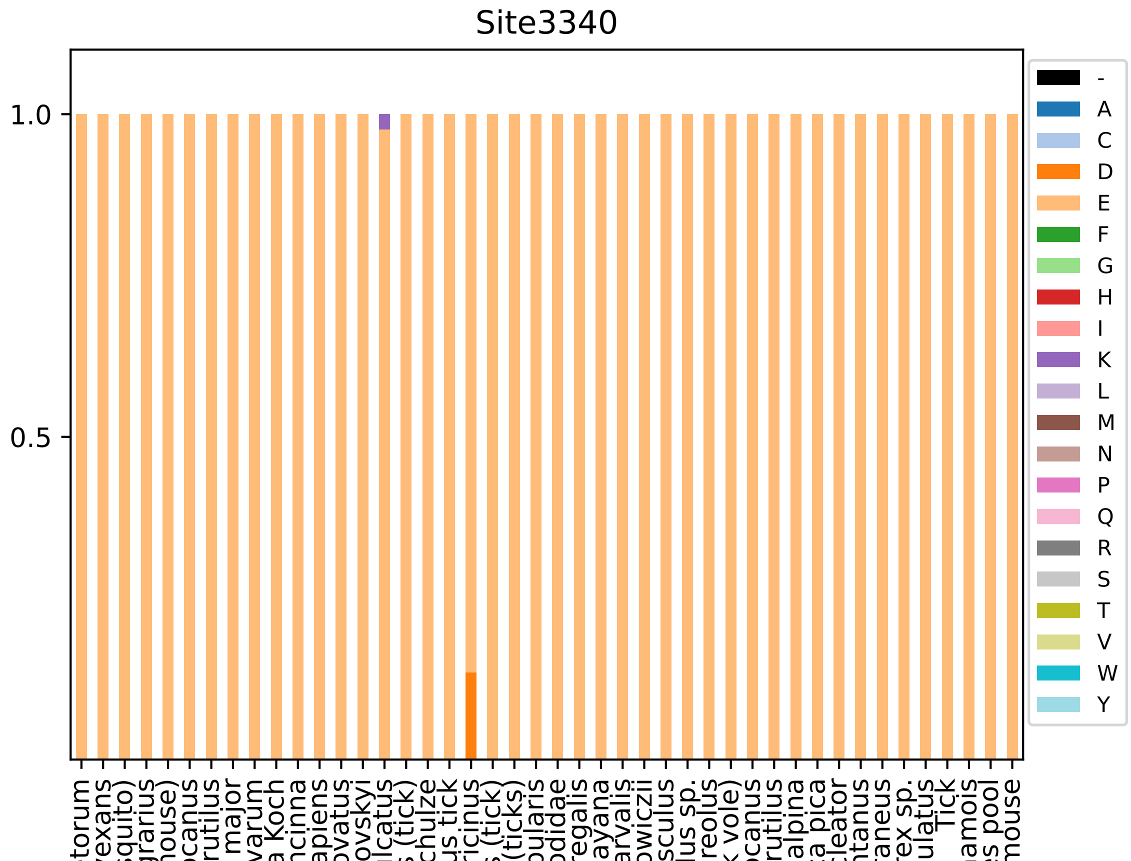
Site3340
1.0
0.5
-
A
C
D
E
F
G
H
I
K
L
M
N
P
Q
R
S
T
V
W
Y

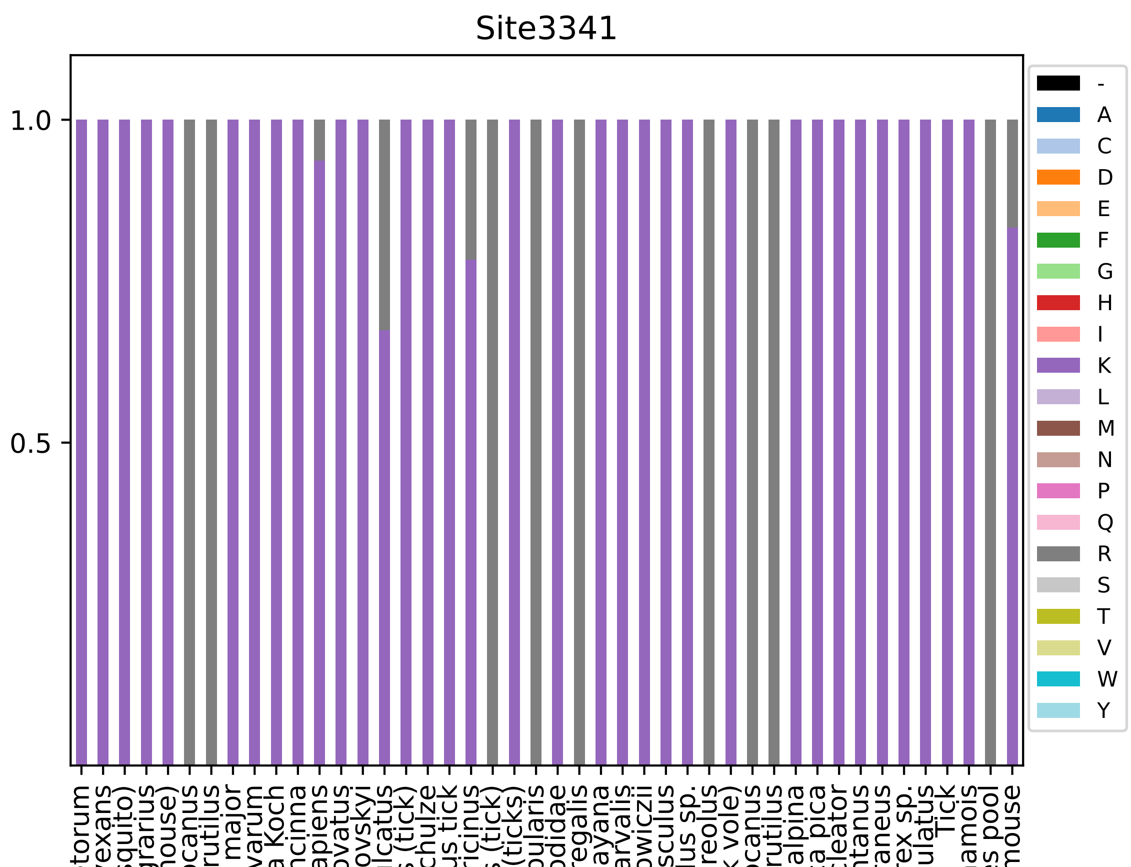
Site3341
1.0
0.5
-
A
C
D
E
F
G
H
I
K
L
M
N
P
Q
R
S
T
V
W
Y

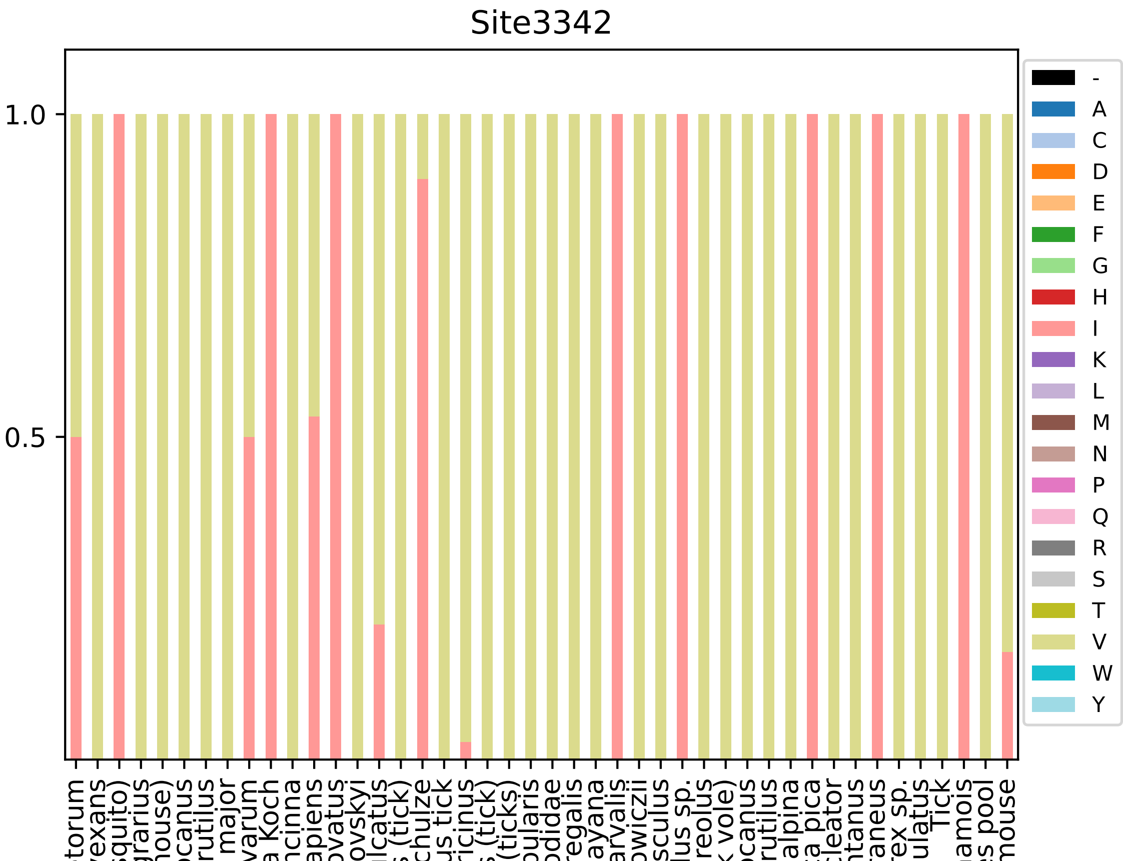

Site3342
1.0
0.5
-
A
C
D
E
F
G
H
I
K
L
M
N
P
Q
R
S
T
V
W
Y

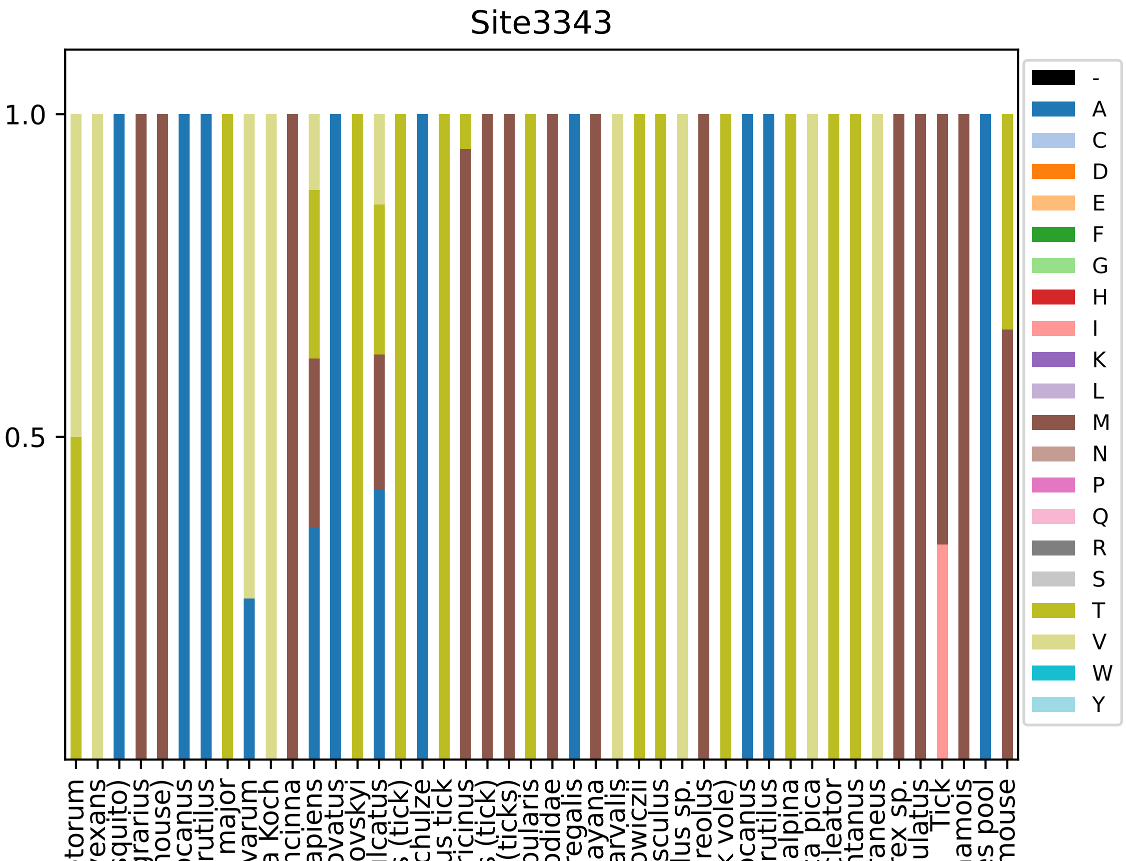
Site3343
1.0
0.5
-
A
C
D
E
F
G
H
I
K
L
M
N
P
Q
R
S
T
V
W
Y

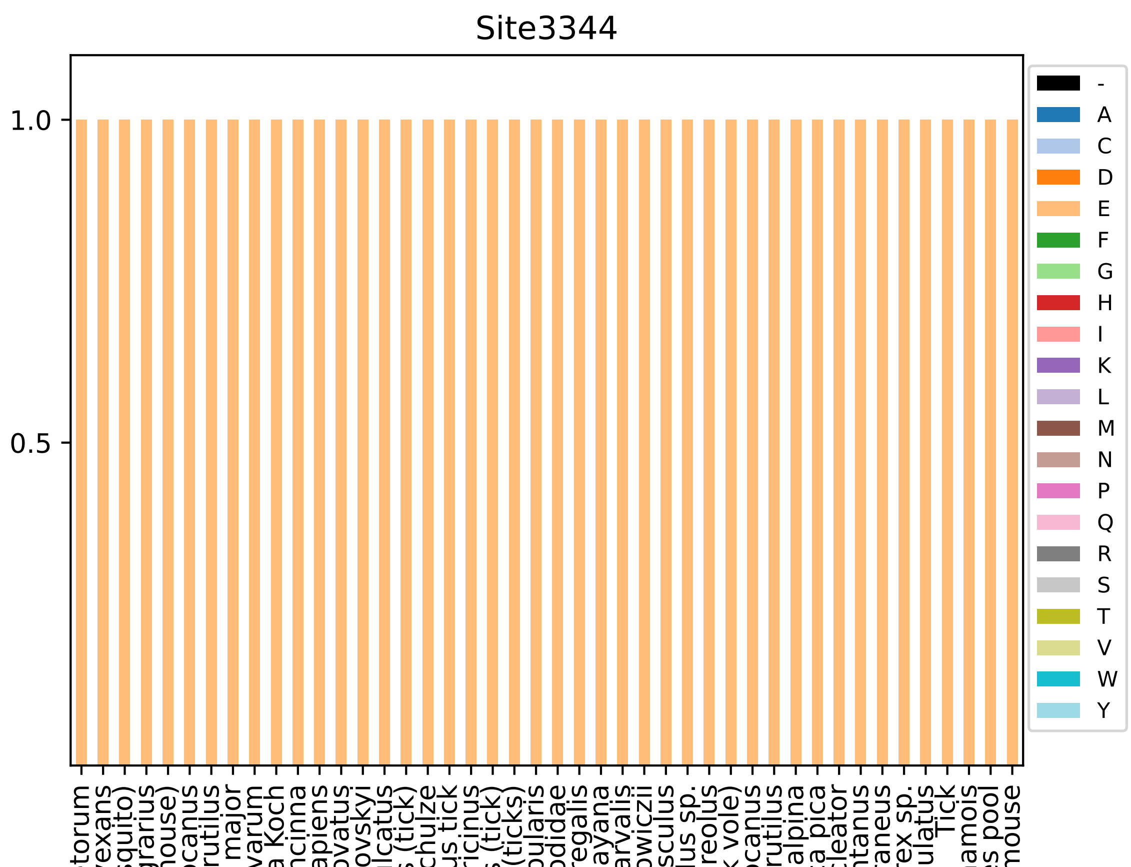
Site3344
1.0
0.5
-
A
C
D
E
F
G
H
I
K
L
M
N
P
Q
R
S
T
V
W
Y

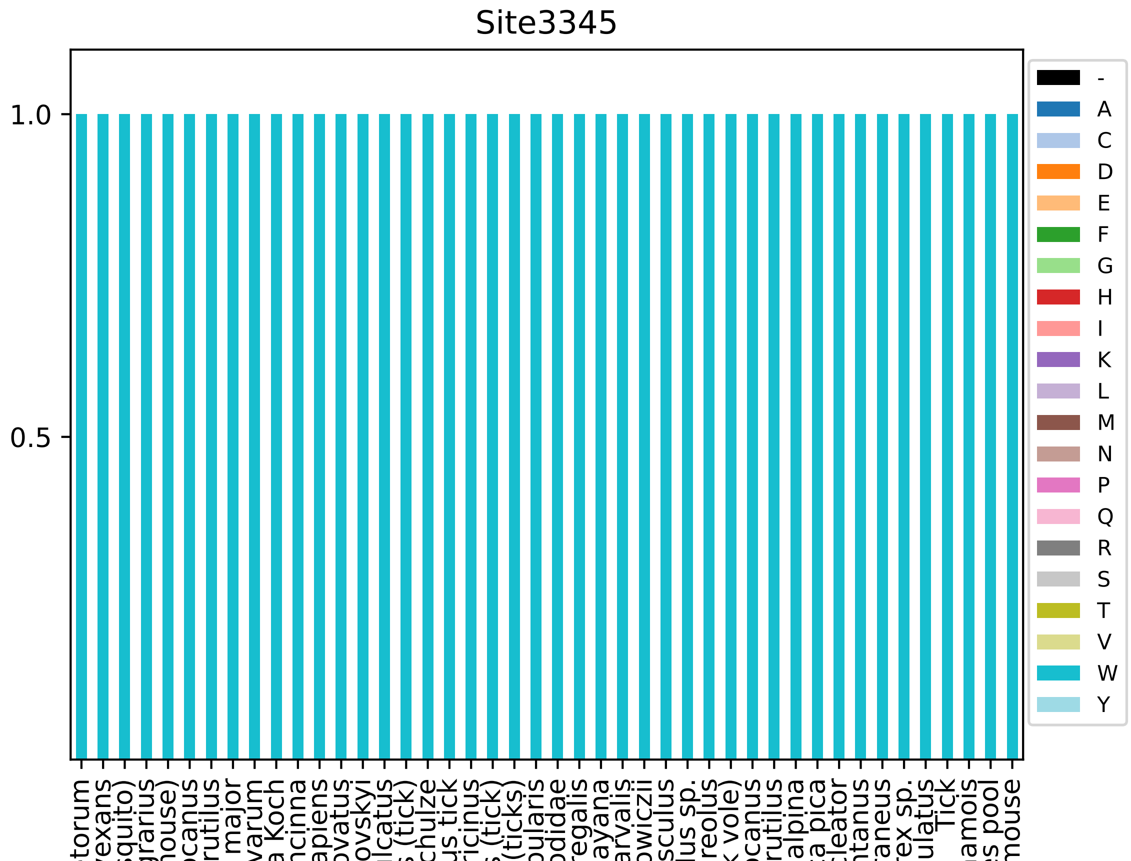
Site3345
1.0
0.5
-
A
C
D
E
F
G
H
I
K
L
M
N
P
Q
R
S
T
V
W
Y

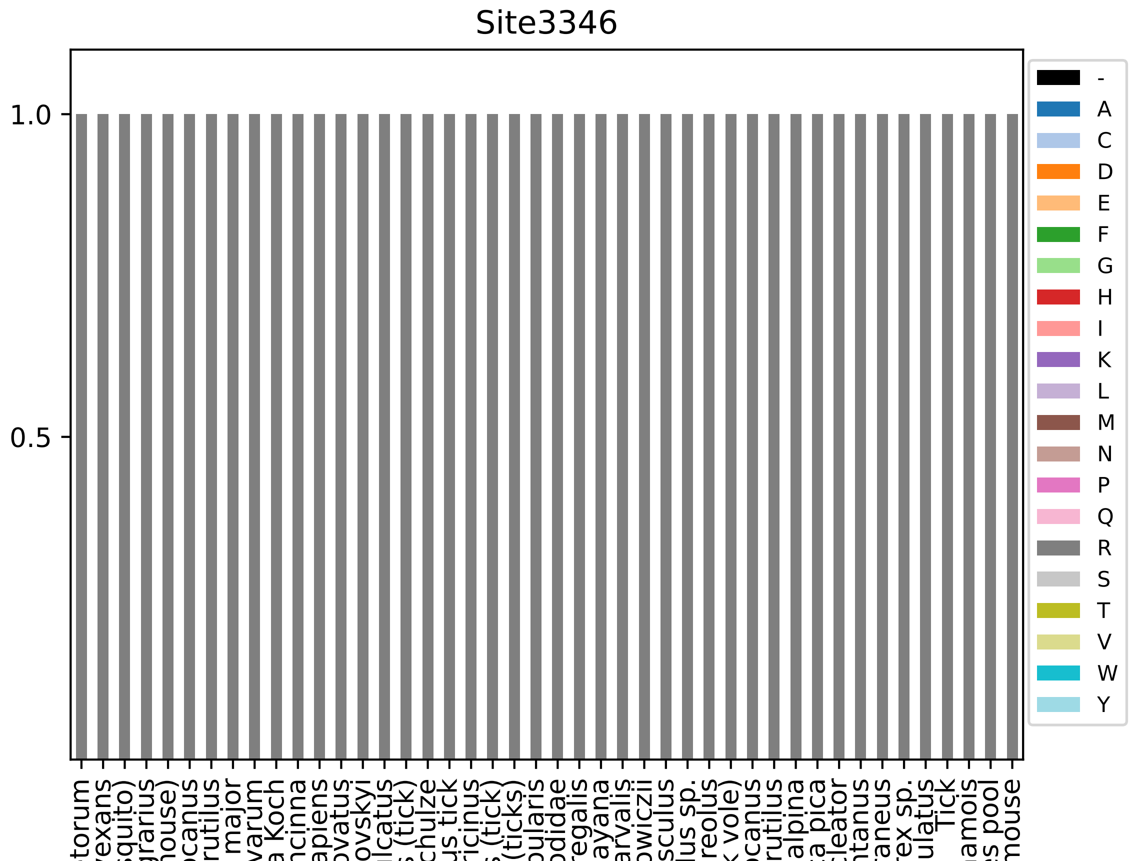
Site3346
1.0
0.5
-
A
C
D
E
F
G
H
I
K
L
M
N
P
Q
R
S
T
V
W
Y

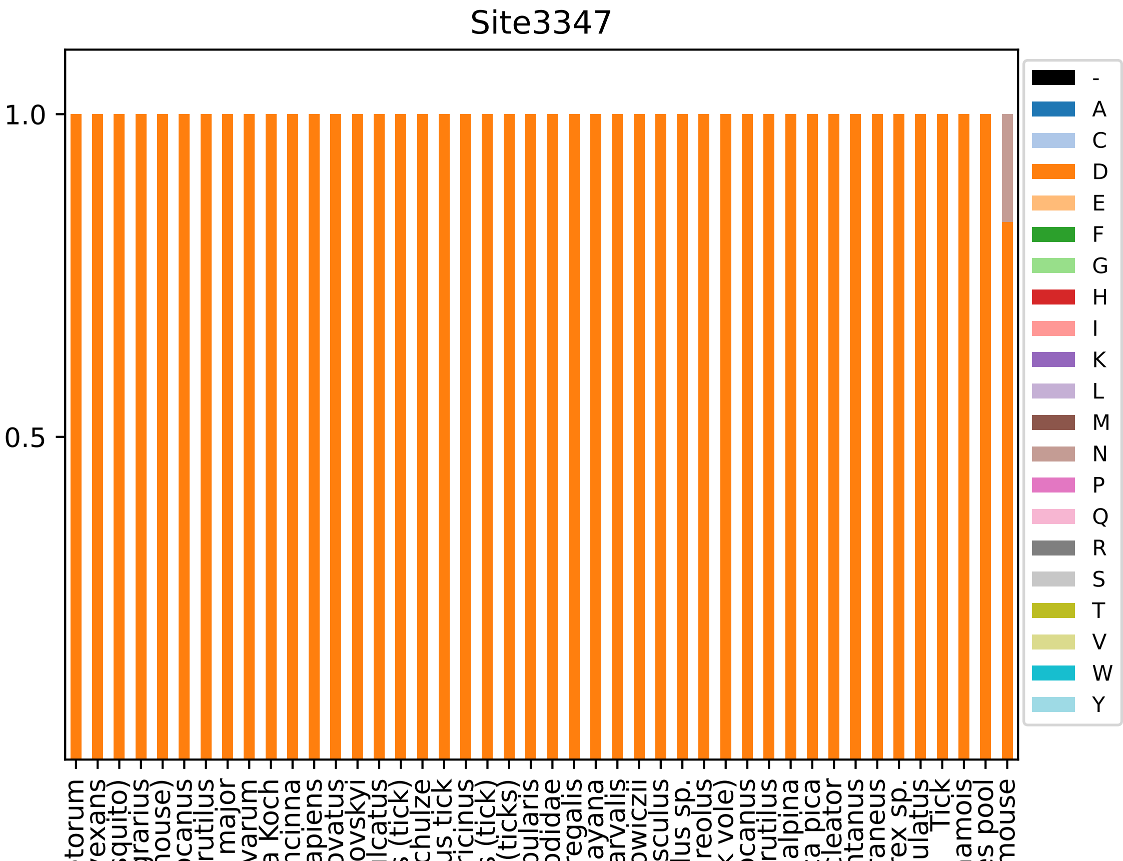

Site3347
1.0
0.5
-
A
C
D
E
F
G
H
I
K
L
M
N
P
Q
R
S
T
V
W
Y

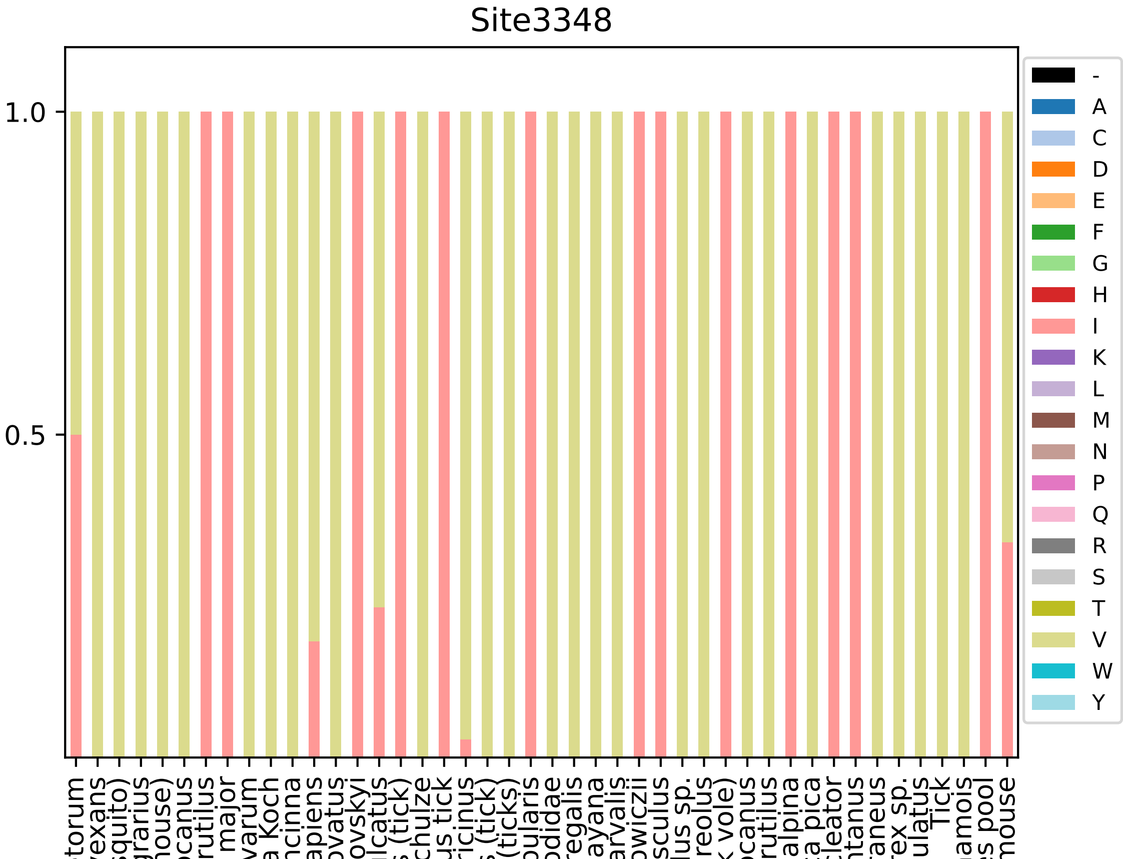
Site3348
1.0
0.5
-
A
C
D
E
F
G
H
I
K
L
M
N
P
Q
R
S
T
V
W
Y

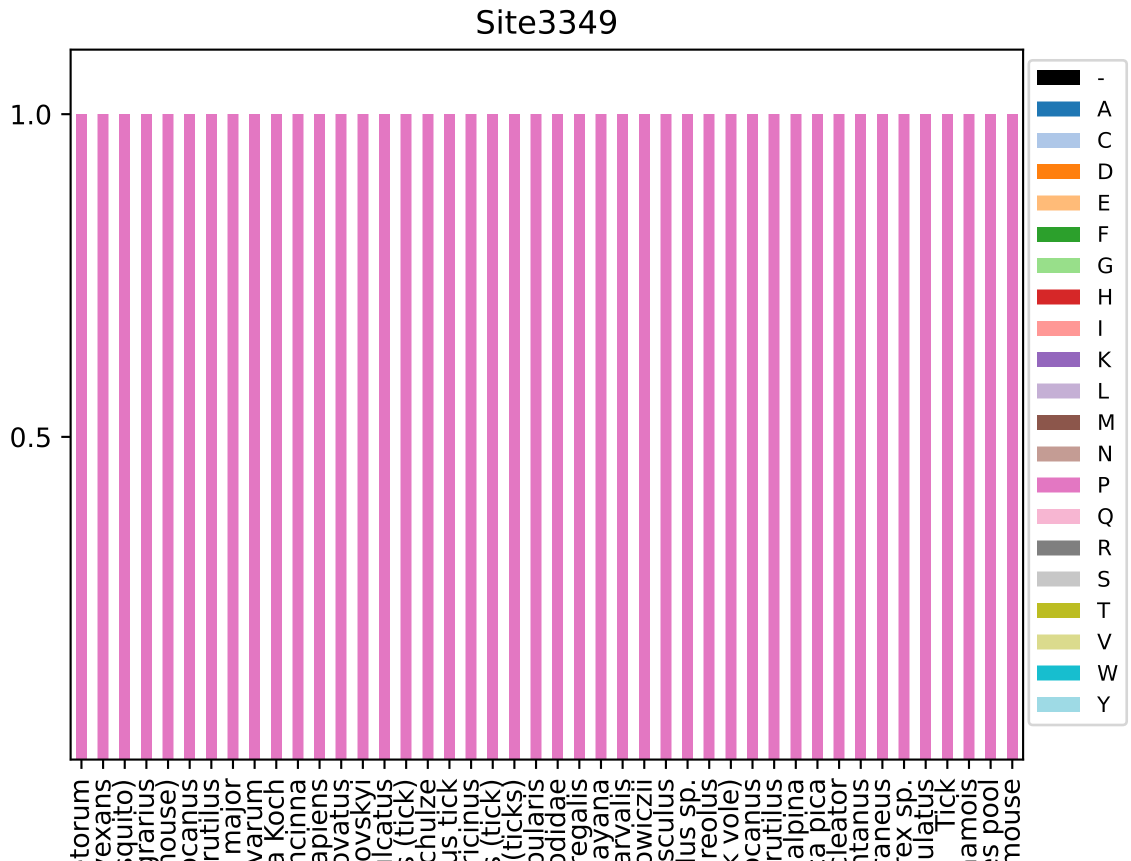
Site3349
1.0
0.5
-
A
C
D
E
F
G
H
I
K
L
M
N
P
Q
R
S
T
V
W
Y

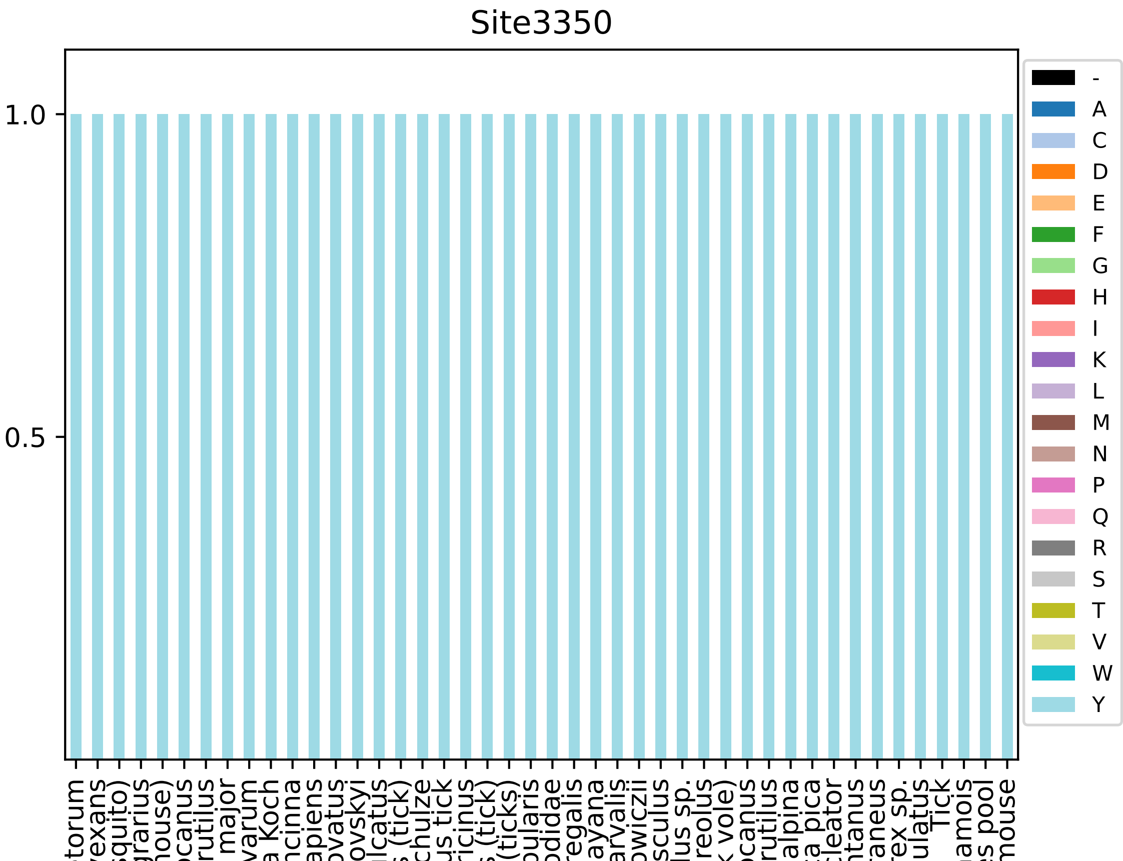
Site3350
1.0
0.5
-
A
C
D
E
F
G
H
I
K
L
M
N
P
Q
R
S
T
V
W
Y

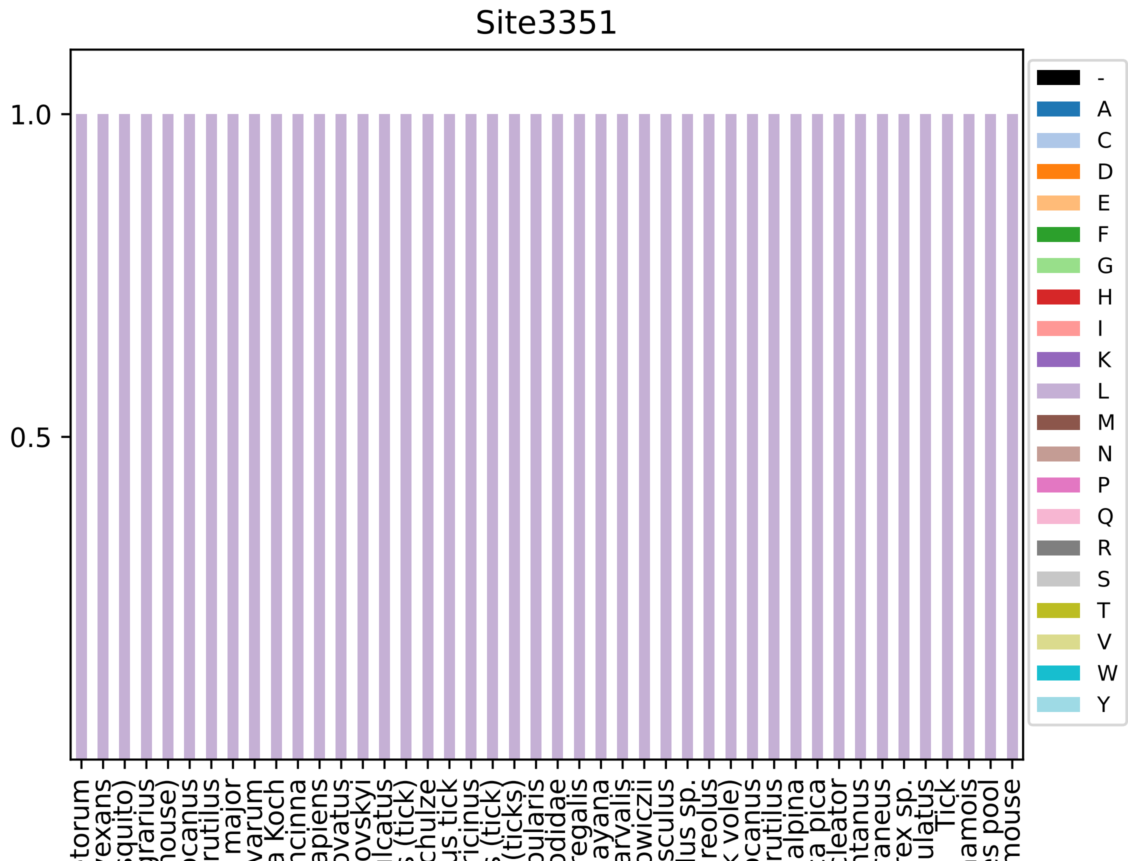
Site3351
1.0
0.5
-
A
C
D
E
F
G
H
I
K
L
M
N
P
Q
R
S
T
V
W
Y

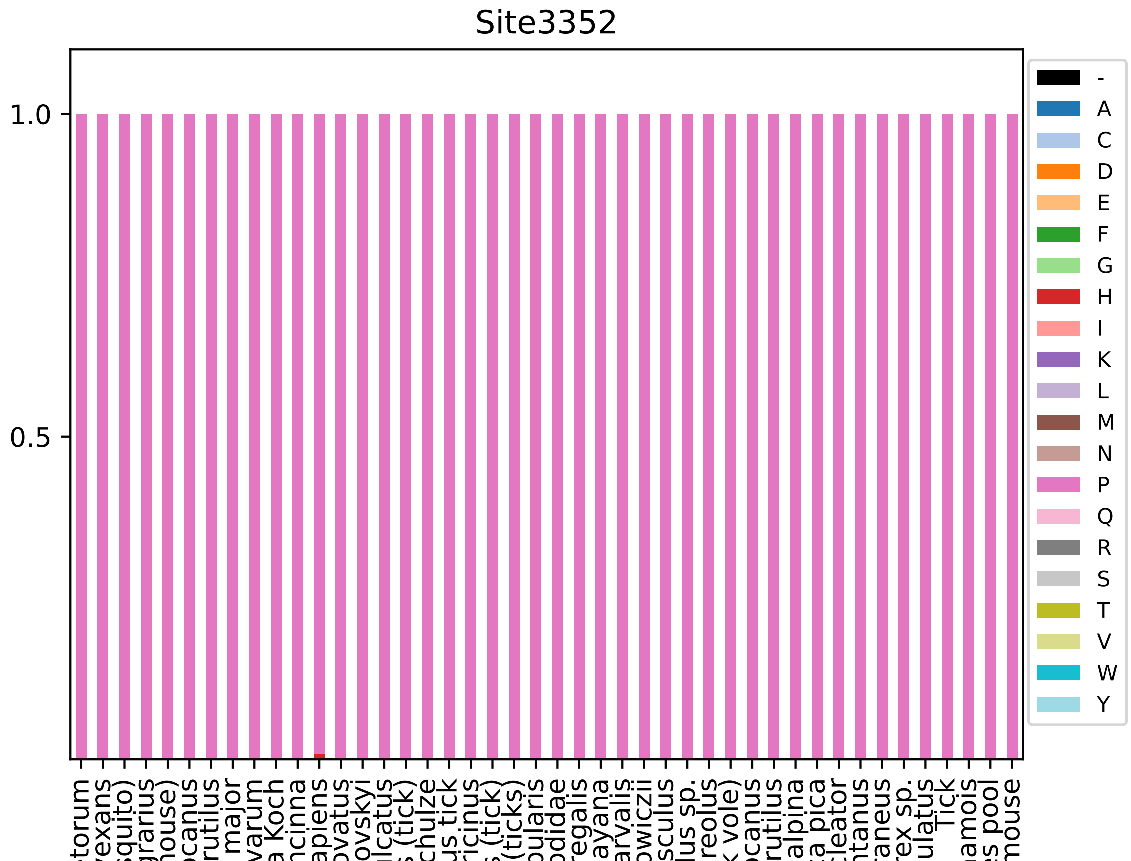
Site3352
1.0
0.5
-
A
C
D
E
F
G
H
I
K
L
M
N
P
Q
R
S
T
V
W
Y

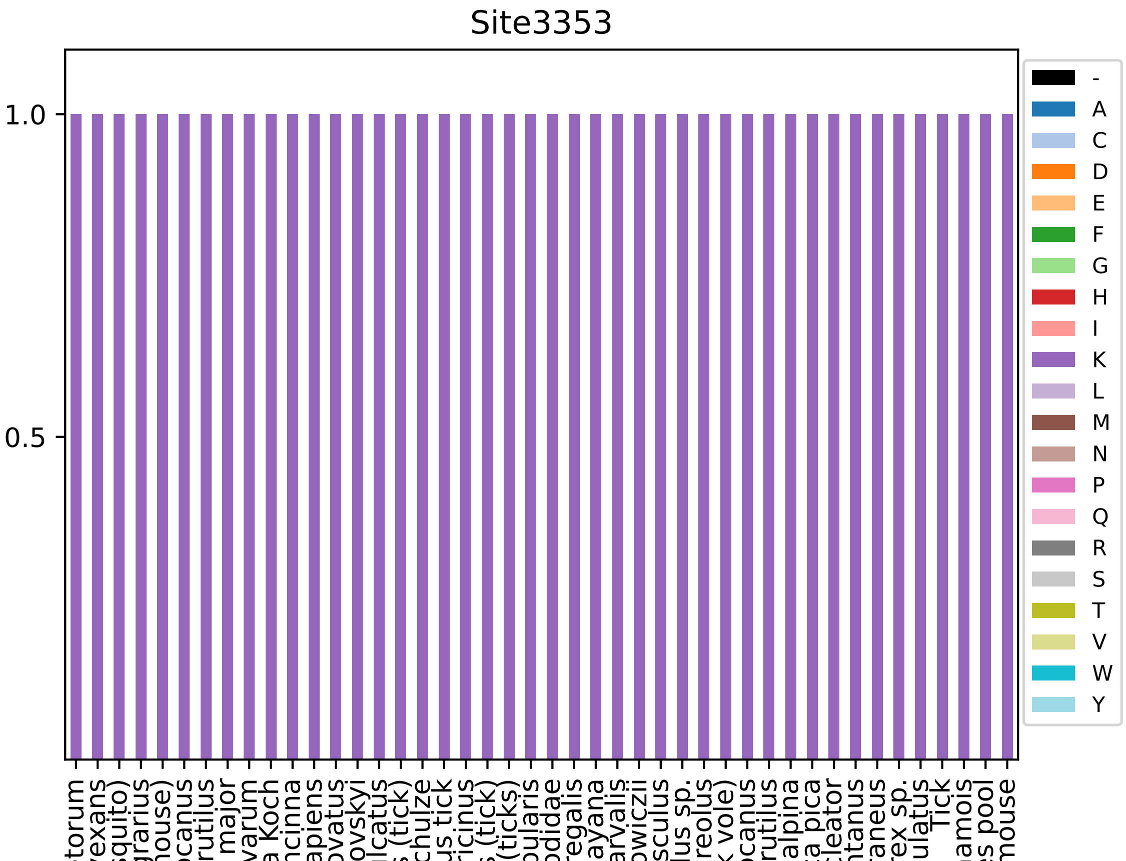
Site3353
1.0
0.5
-
A
C
D
E
F
G
H
I
K
L
M
N
P
Q
R
S
T
V
W
Y

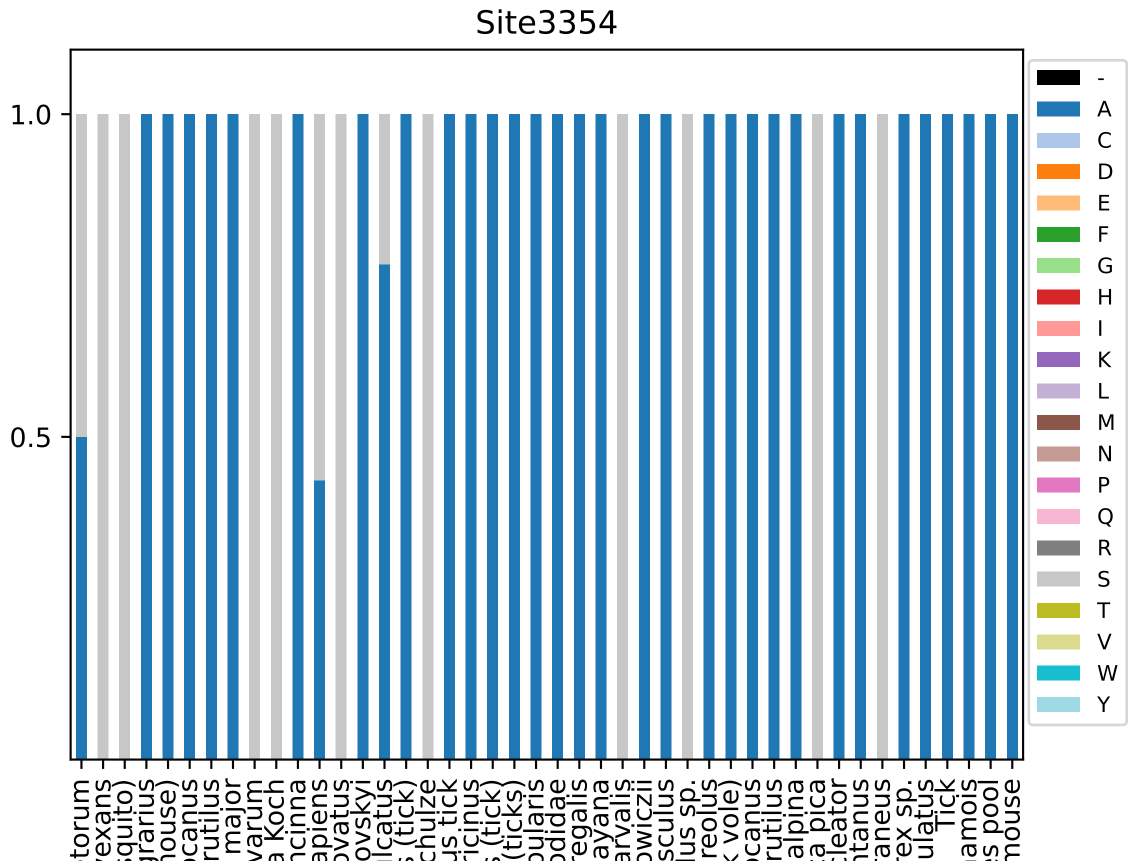
Site3354
1.0
0.5
-
A
C
D
E
F
G
H
I
K
L
M
N
P
Q
R
S
T
V
W
Y

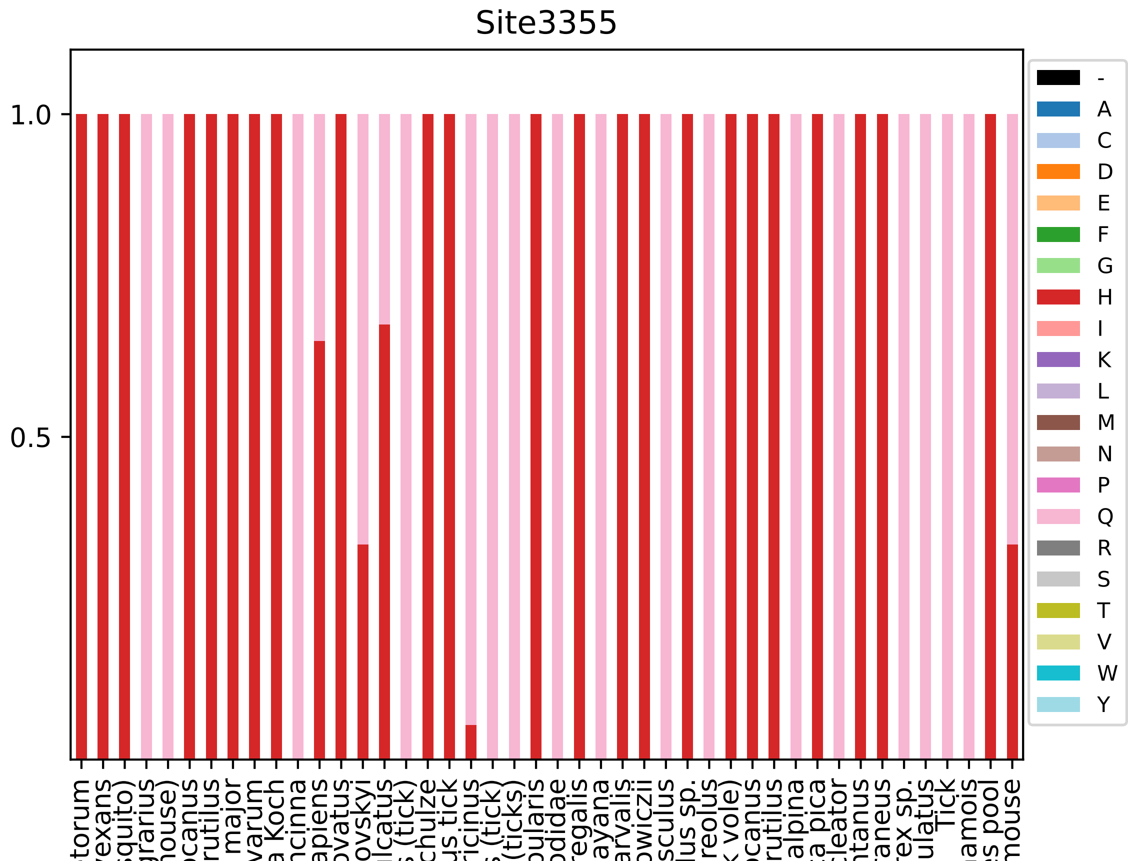
Site3355
1.0
0.5
-
A
C
D
E
F
G
H
I
K
L
M
N
P
Q
R
S
T
V
W
Y
Tick

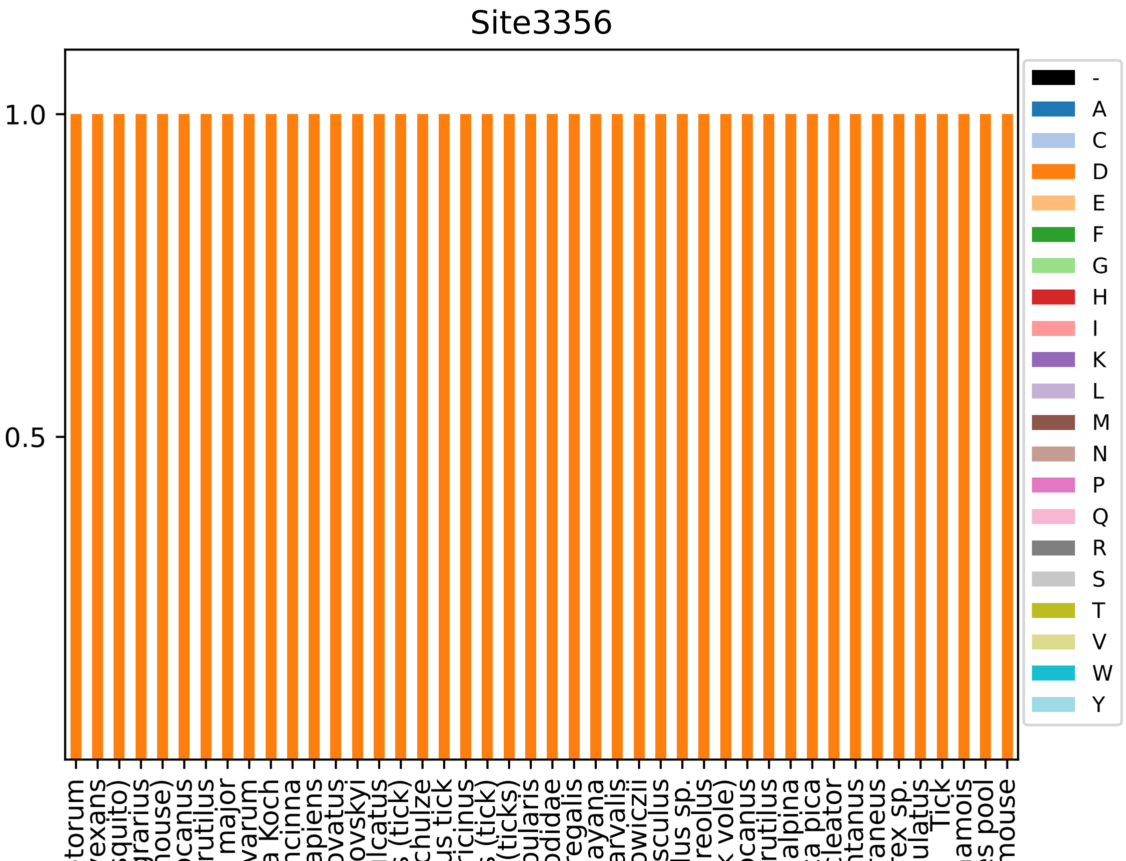
Site3356
1.0
0.5
-
A
C
D
E
F
G
H
I
K
L
M
N
P
Q
R
S
T
V
W
Y

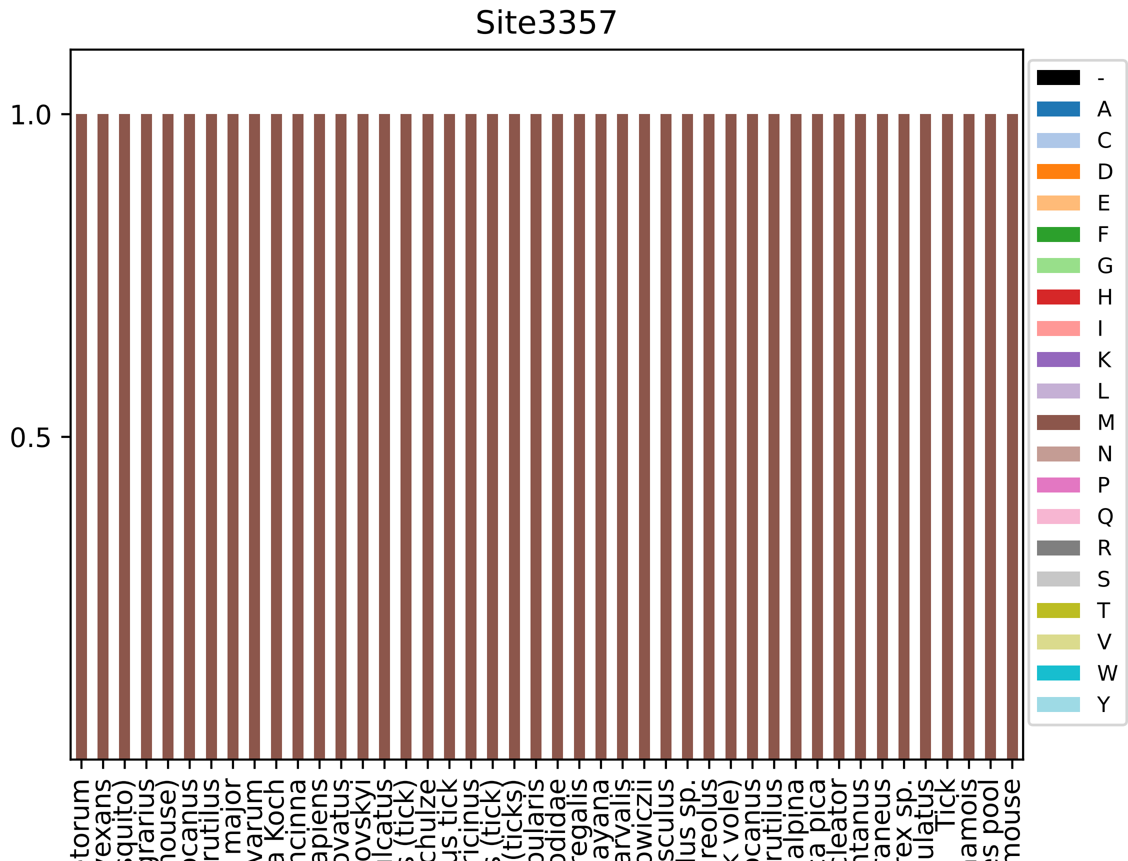
Site3357
1.0
0.5
-
A
C
D
E
F
G
H
I
K
L
M
N
P
Q
R
S
T
V
W
Y

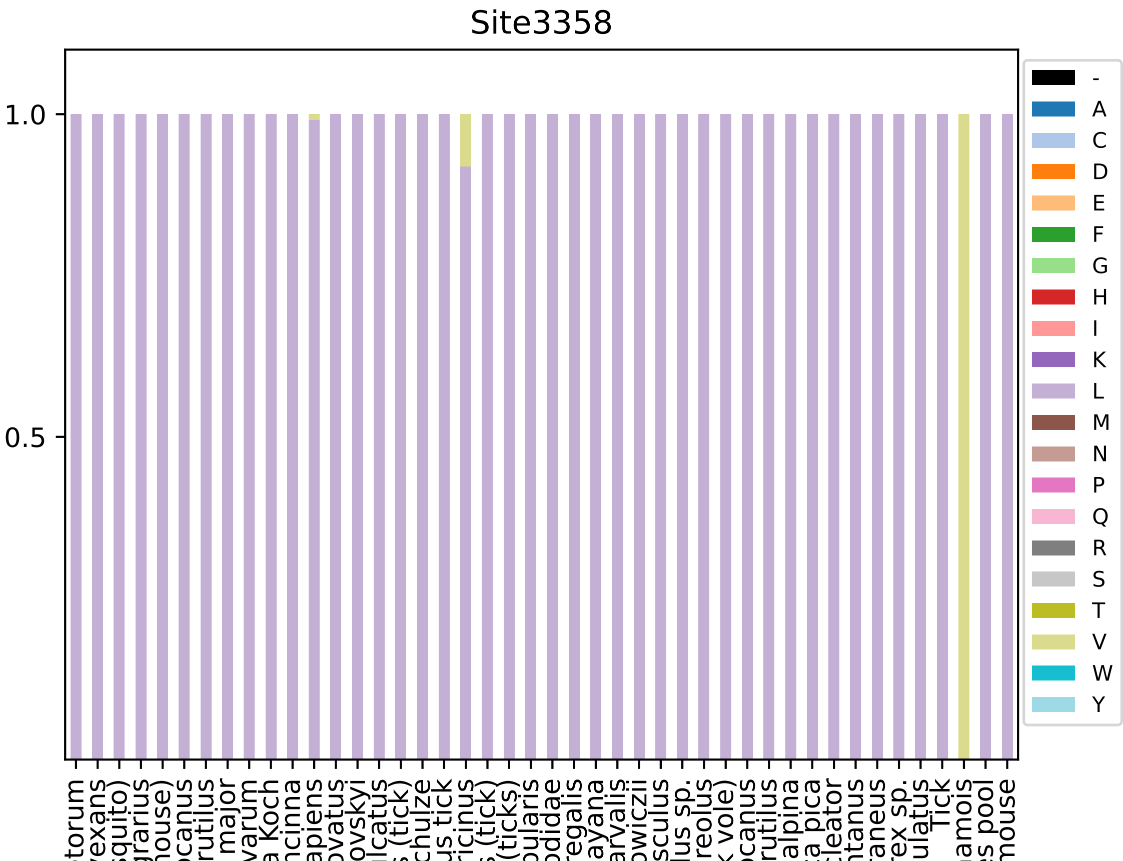
Site3358
1.0
0.5
-
A
C
D
E
F
G
H
I
K
L
M
N
P
Q
R
S
T
V
W
Y

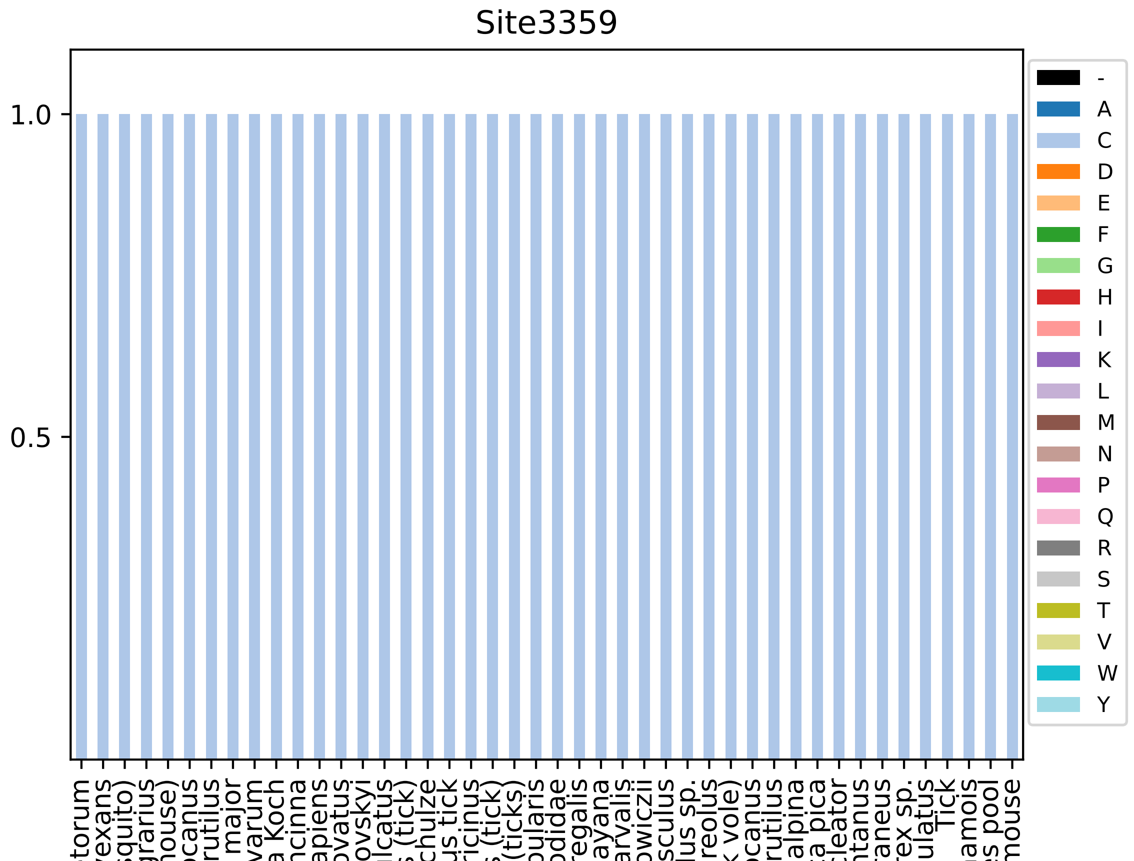
Site3359
1.0
0.5
-
A
C
D
E
F
G
H
I
K
L
M
N
P
Q
R
S
T
V
W
Y

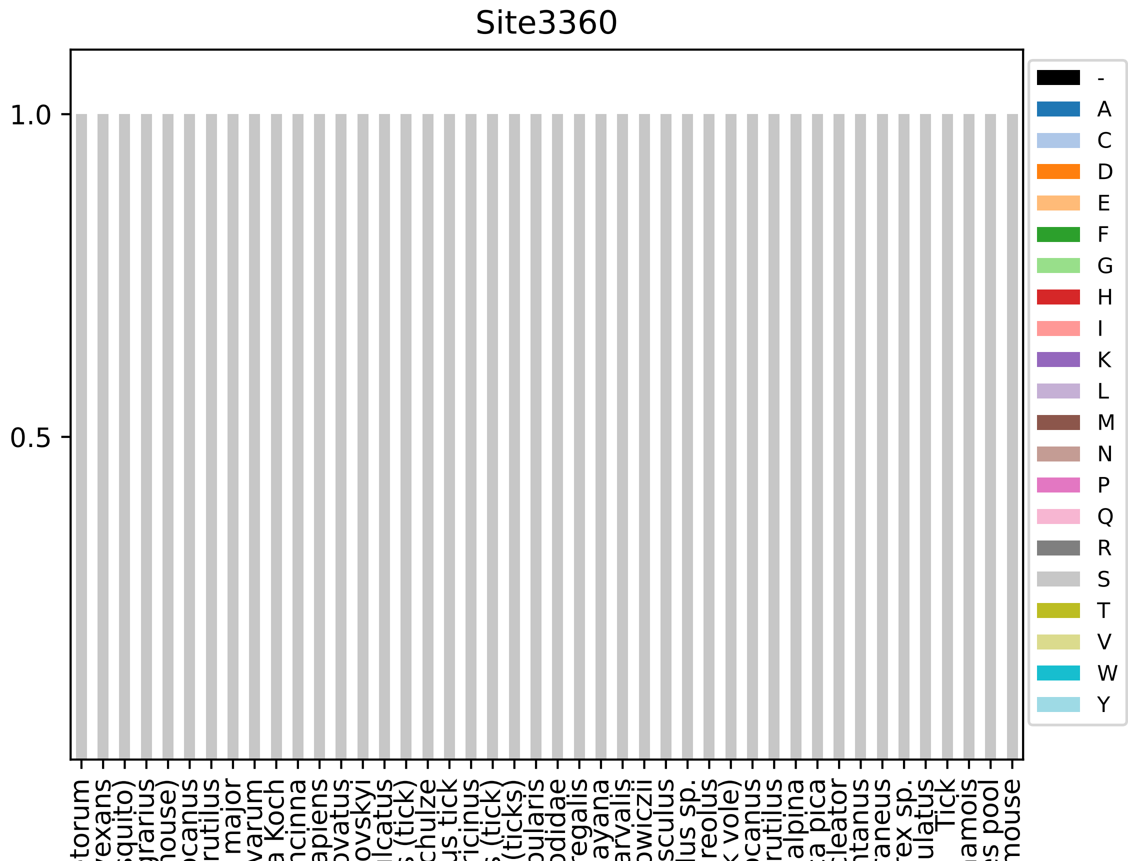
Site3360
1.0
0.5
-
A
C
D
E
F
G
H
I
K
L
M
N
P
Q
R
S
T
V
W
Y

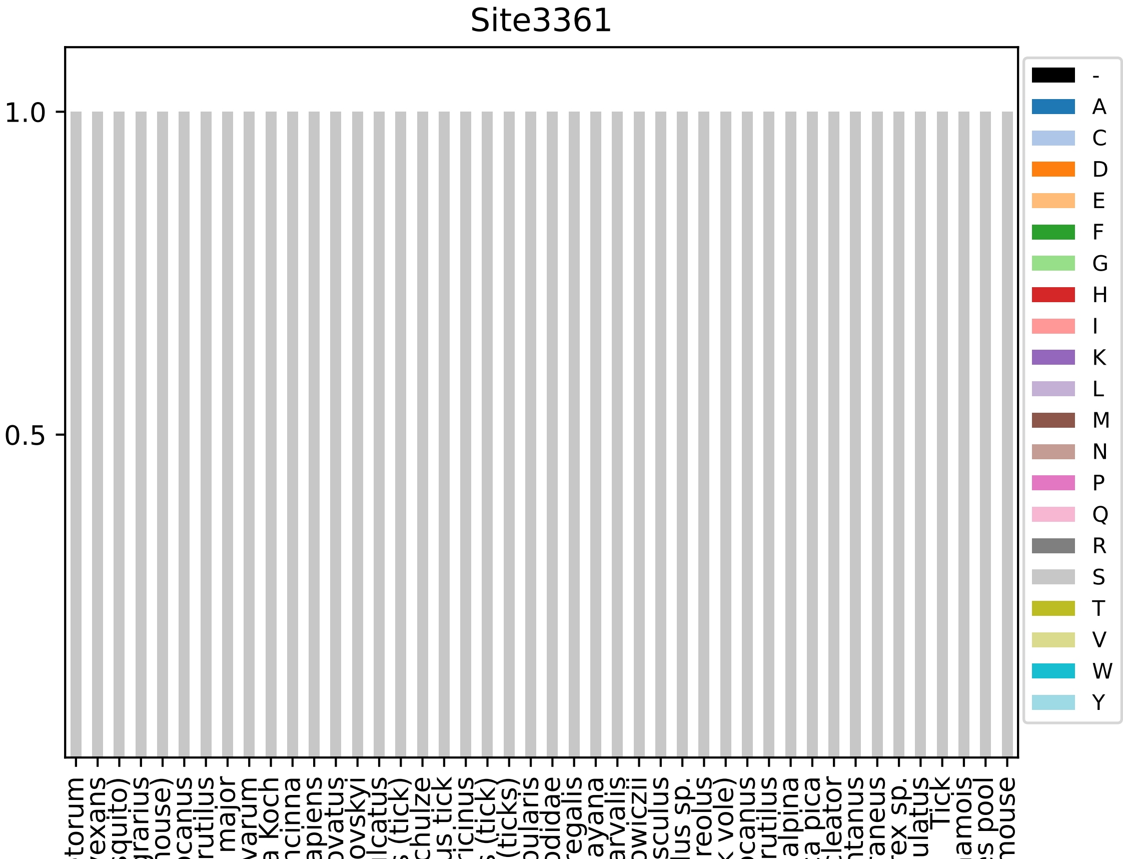
Site3361
1.0
0.5
-
A
C
D
E
F
G
H
I
K
L
M
N
P
Q
R
S
T
V
W
Y

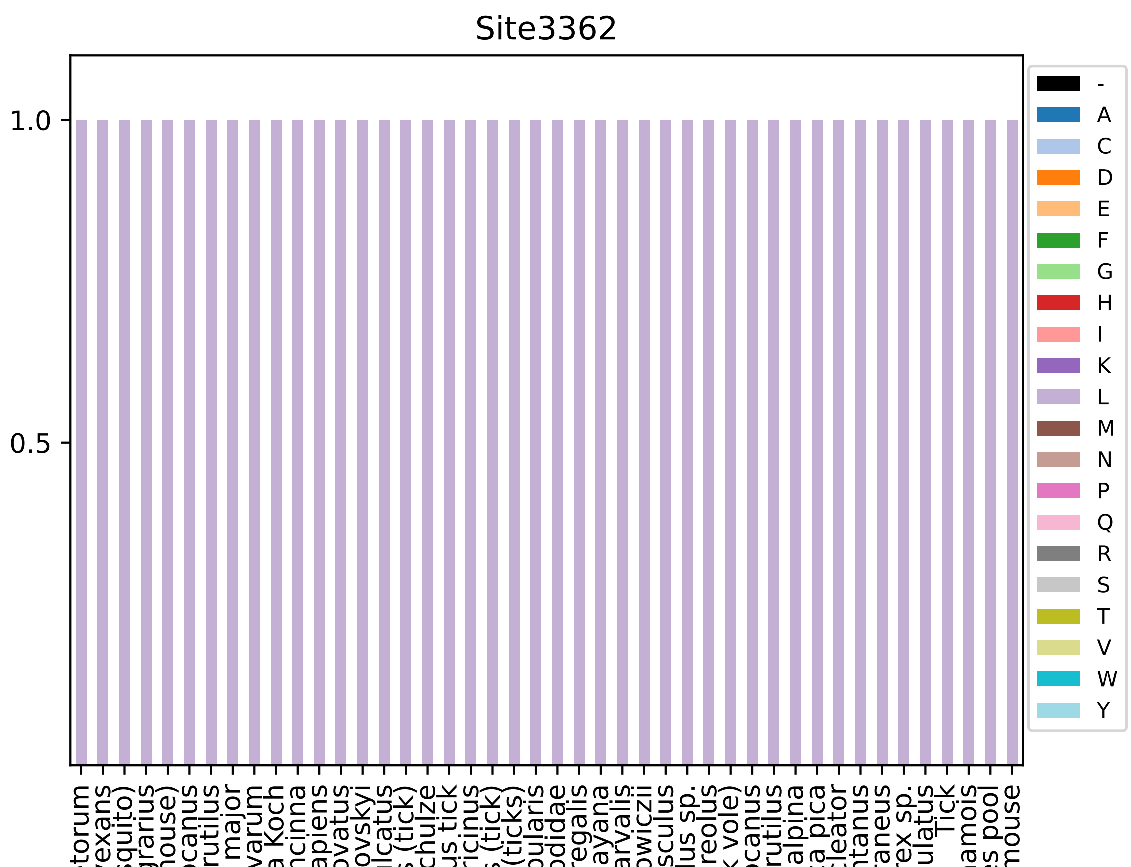
Site3362
1.0
0.5
-
A
C
D
E
F
G
H
I
K
L
M
N
P
Q
R
S
T
V
W
Y

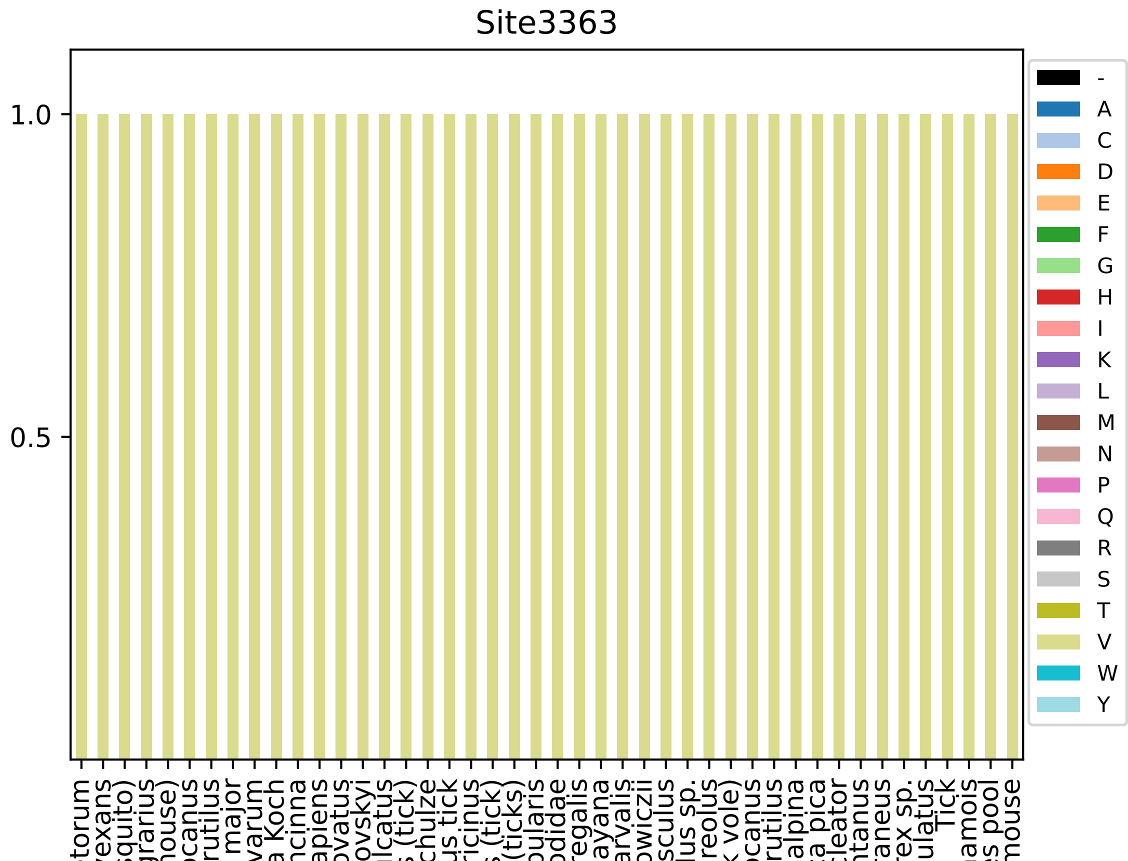

Site3363
1.0
0.5
-
A
C
D
E
F
G
H
I
K
L
M
N
P
Q
R
S
T
V
W
Y

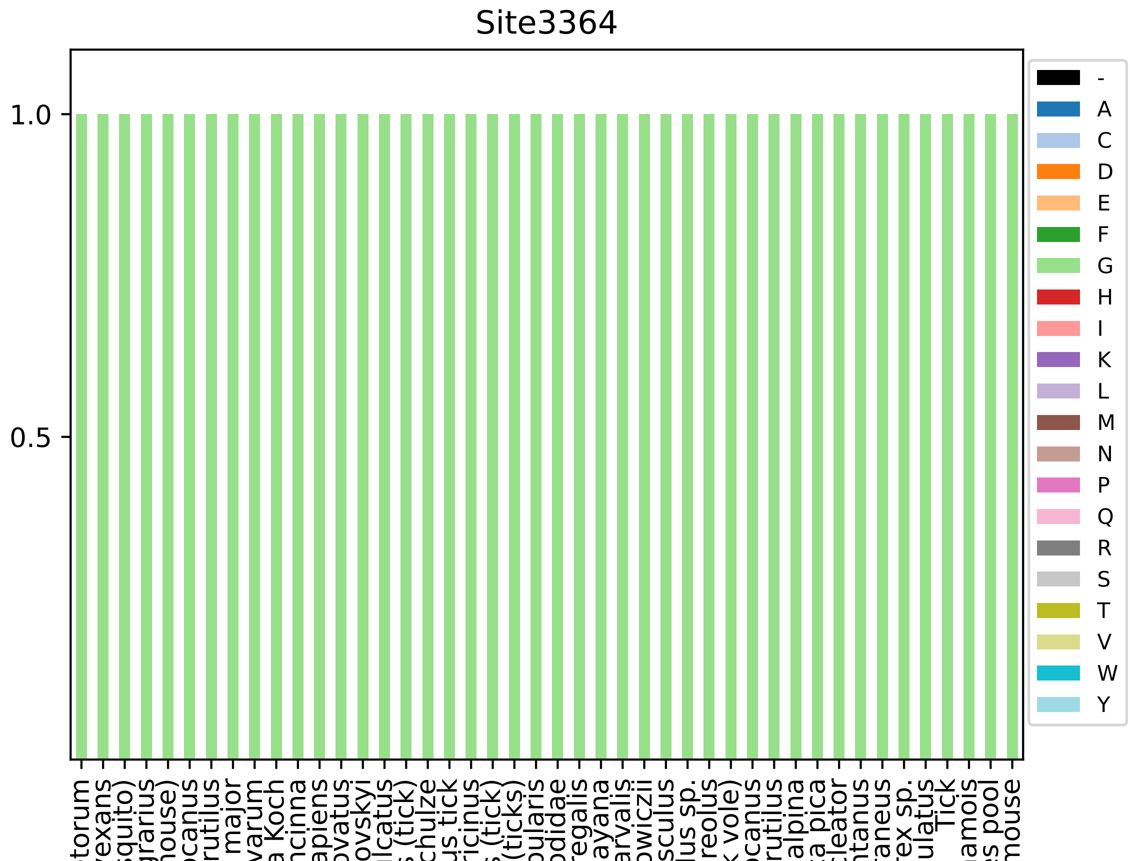
Site3364
1.0
0.5
-
A
C
D
E
F
G
H
I
K
L
M
N
P
Q
R
S
T
V
W
Y

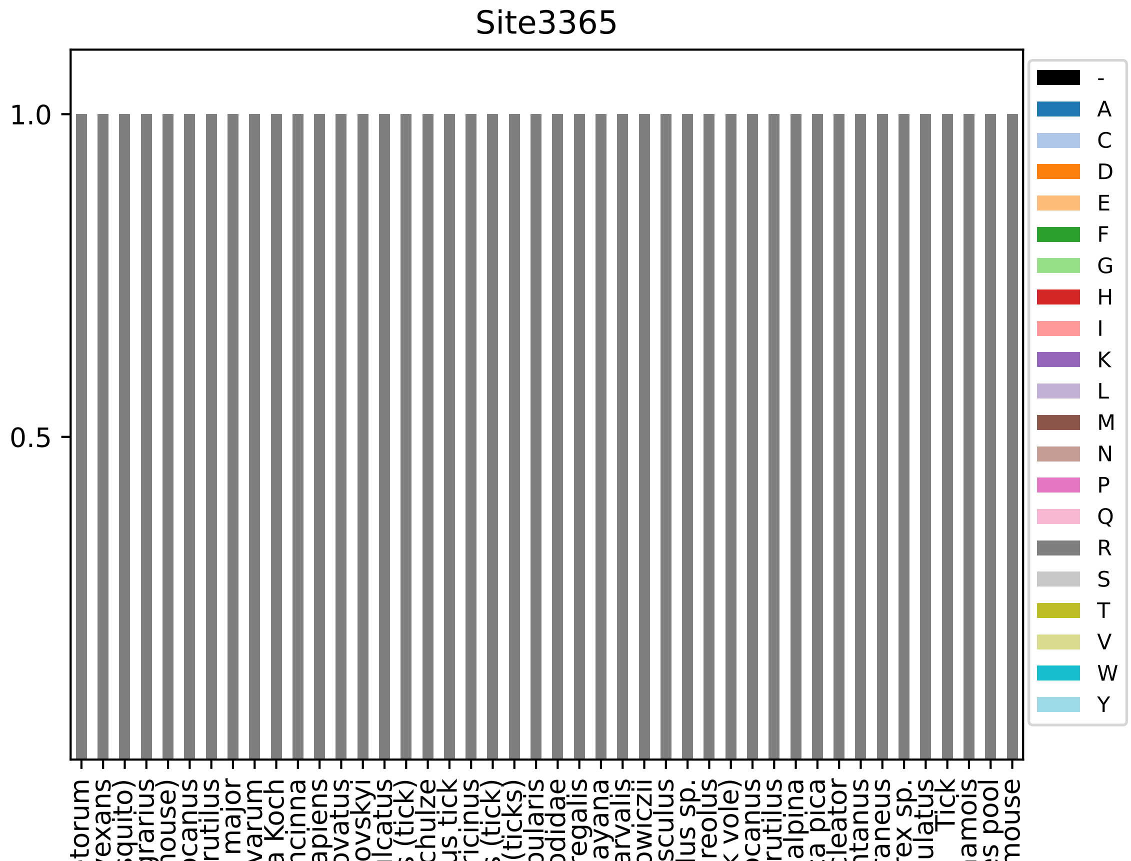
Site3365
1.0
0.5
-
A
C
D
E
F
G
H
I
K
L
M
N
P
Q
R
S
T
V
W
Y

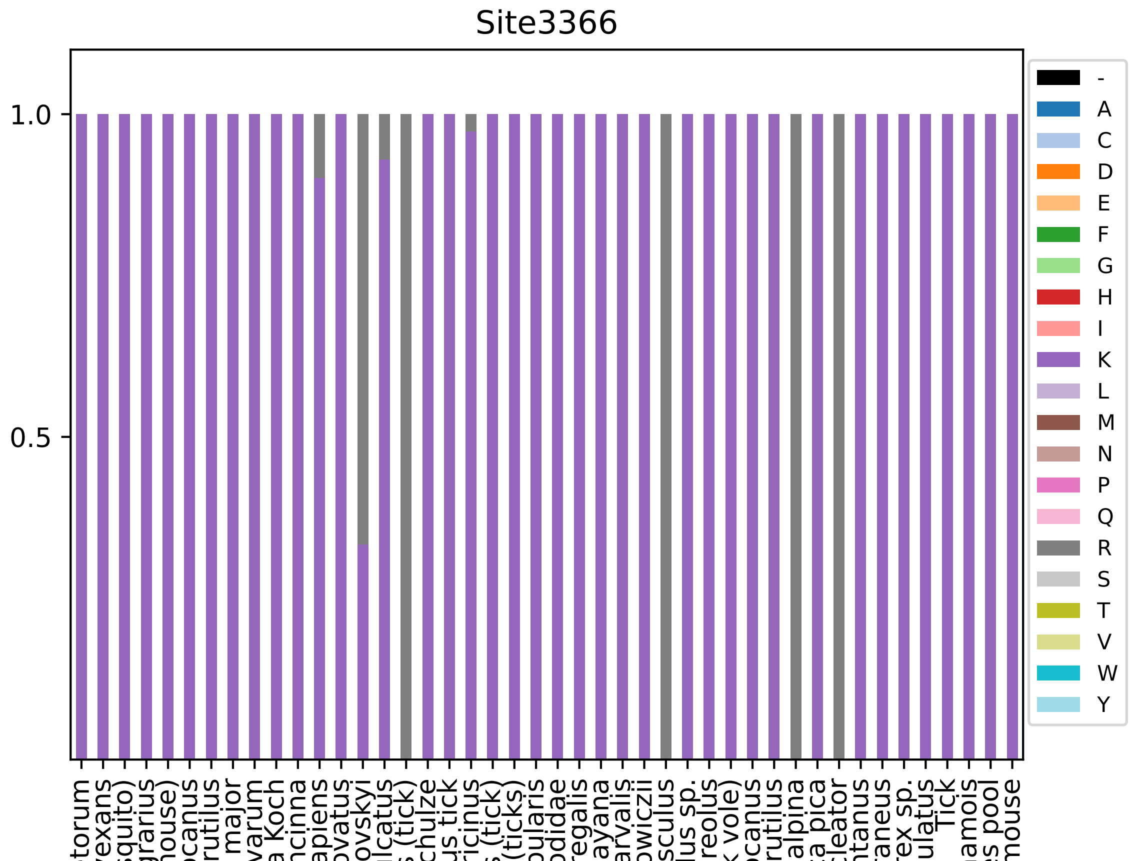

Site3366
1.0
0.5
-
A
C
D
E
F
G
H
I
K
L
M
N
P
Q
R
S
T
V
W
Y

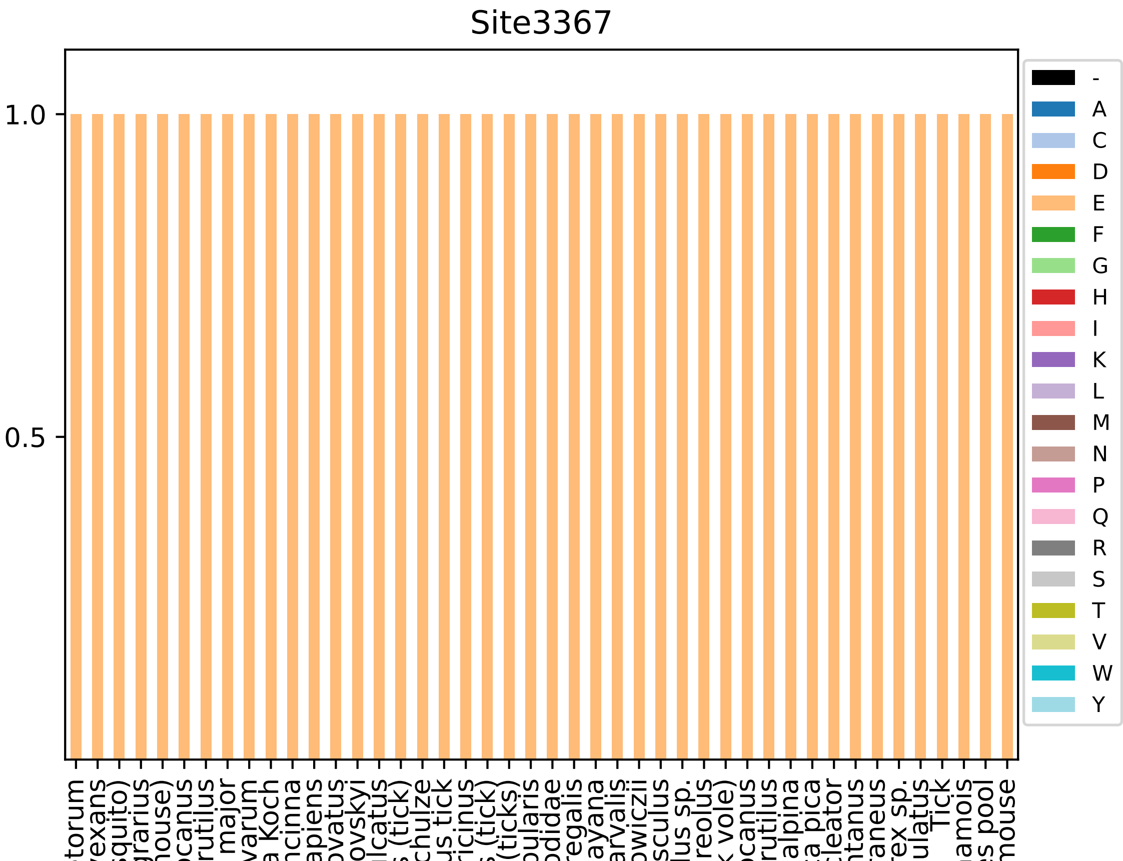
Site3367
1.0
0.5
-
A
C
D
E
F
G
H
I
K
L
M
N
P
Q
R
S
T
V
W
Y

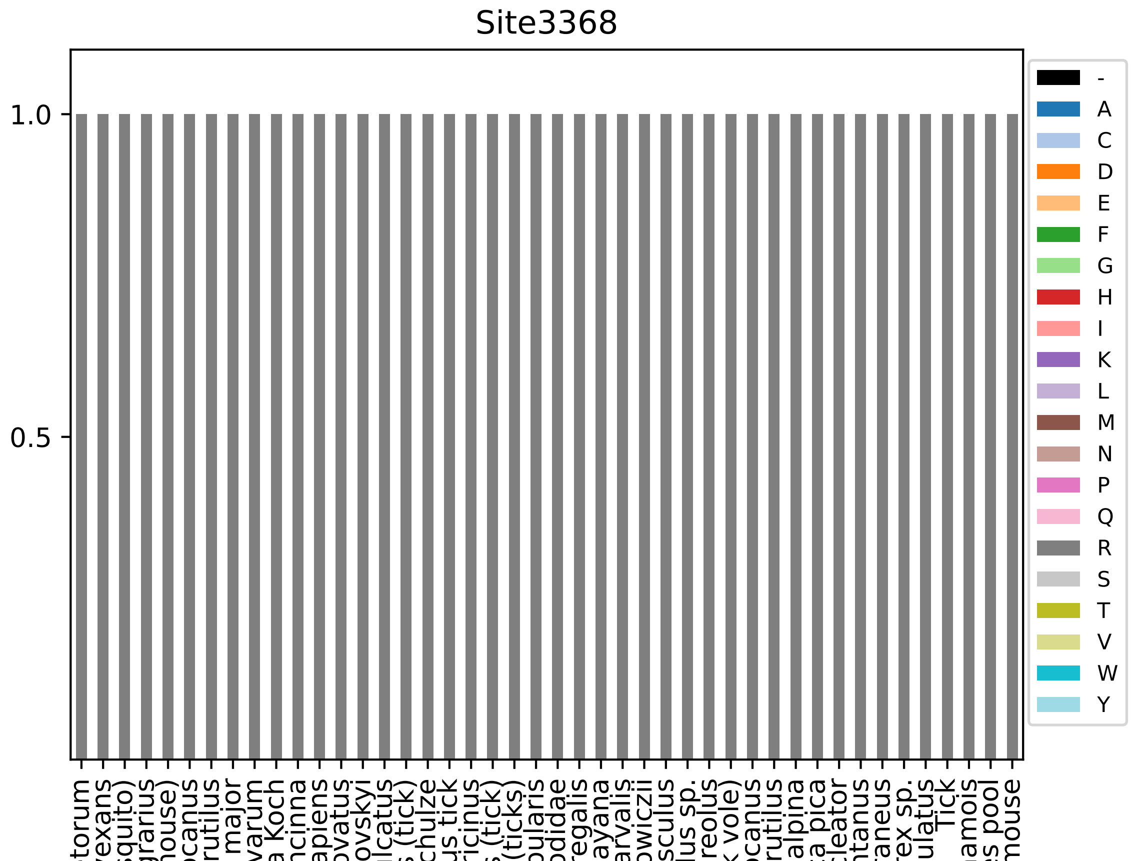
Site3368
1.0
0.5
-
A
C
D
E
F
G
H
I
K
L
M
N
P
Q
R
S
T
V
W
Y

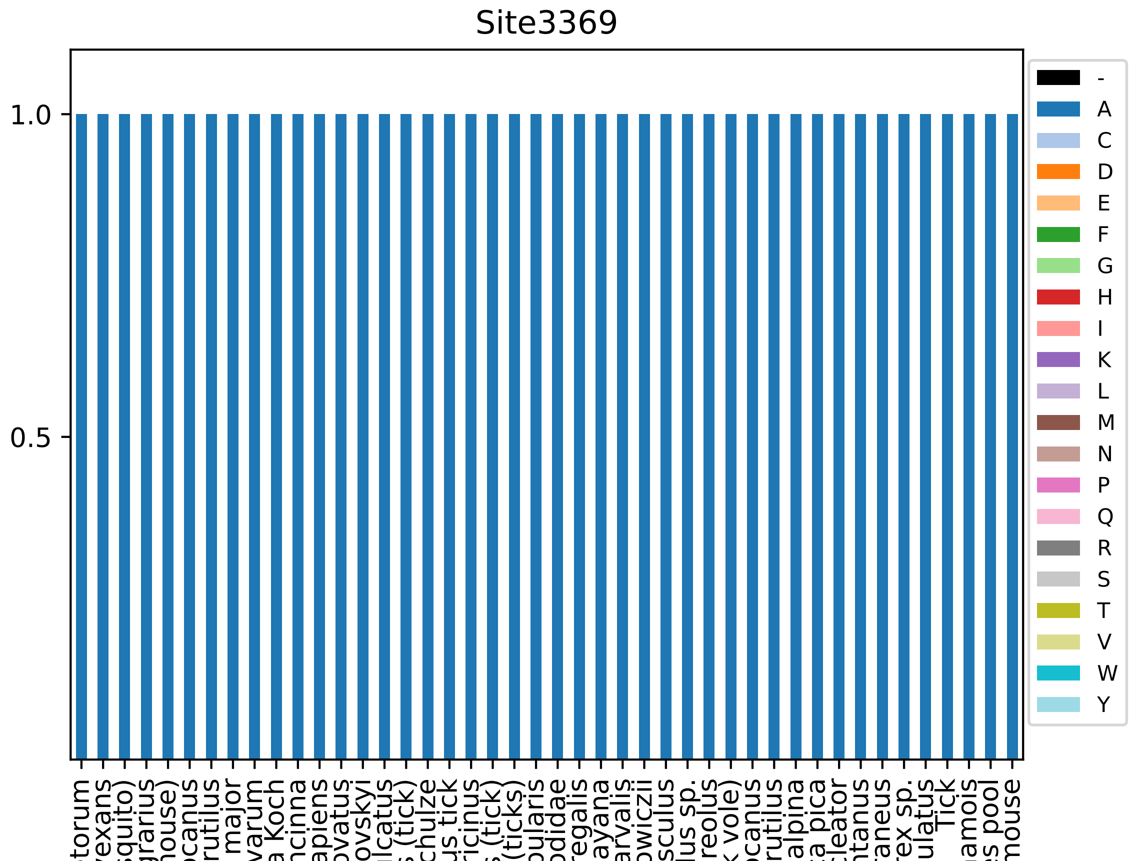
Site3369
1.0
0.5
-
A
C
D
E
F
G
H
I
K
L
M
N
P
Q
R
S
T
V
W
Y

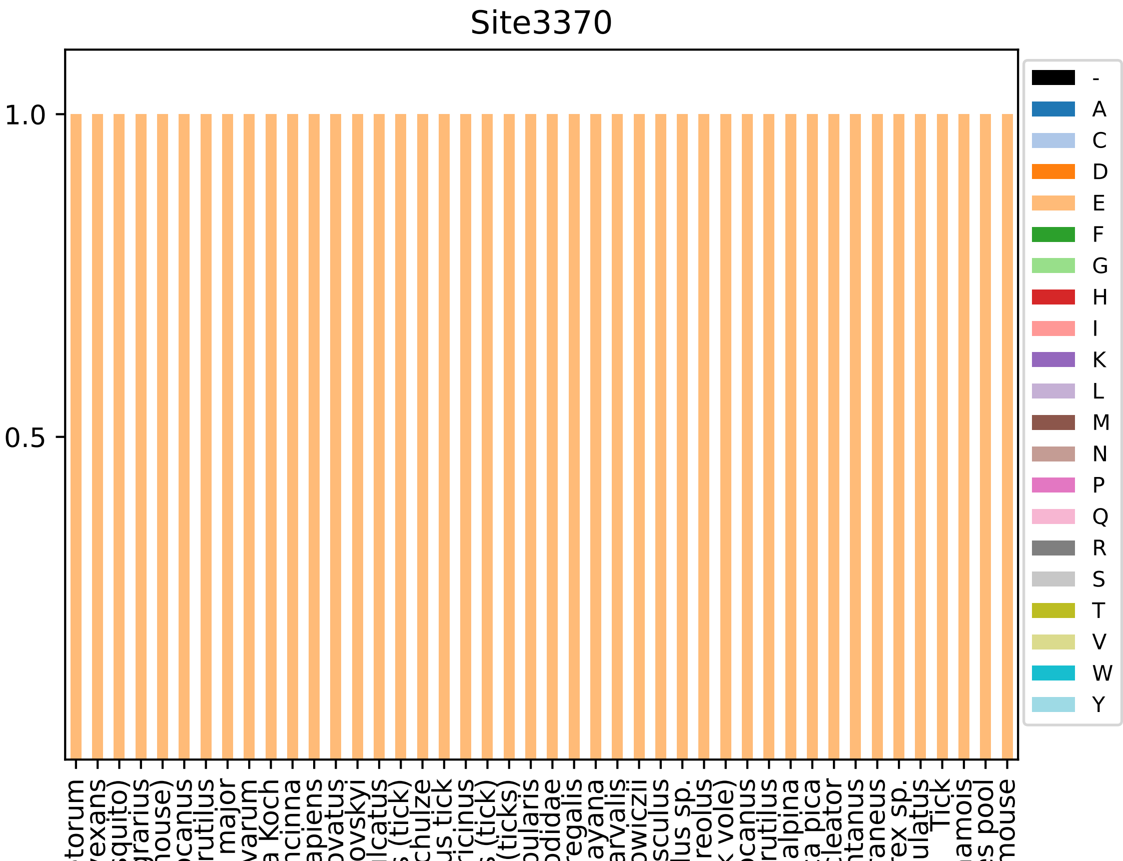
Site3370
1.0
0.5
-
A
C
D
E
F
G
H
I
K
L
M
N
P
Q
R
S
T
V
W
Y

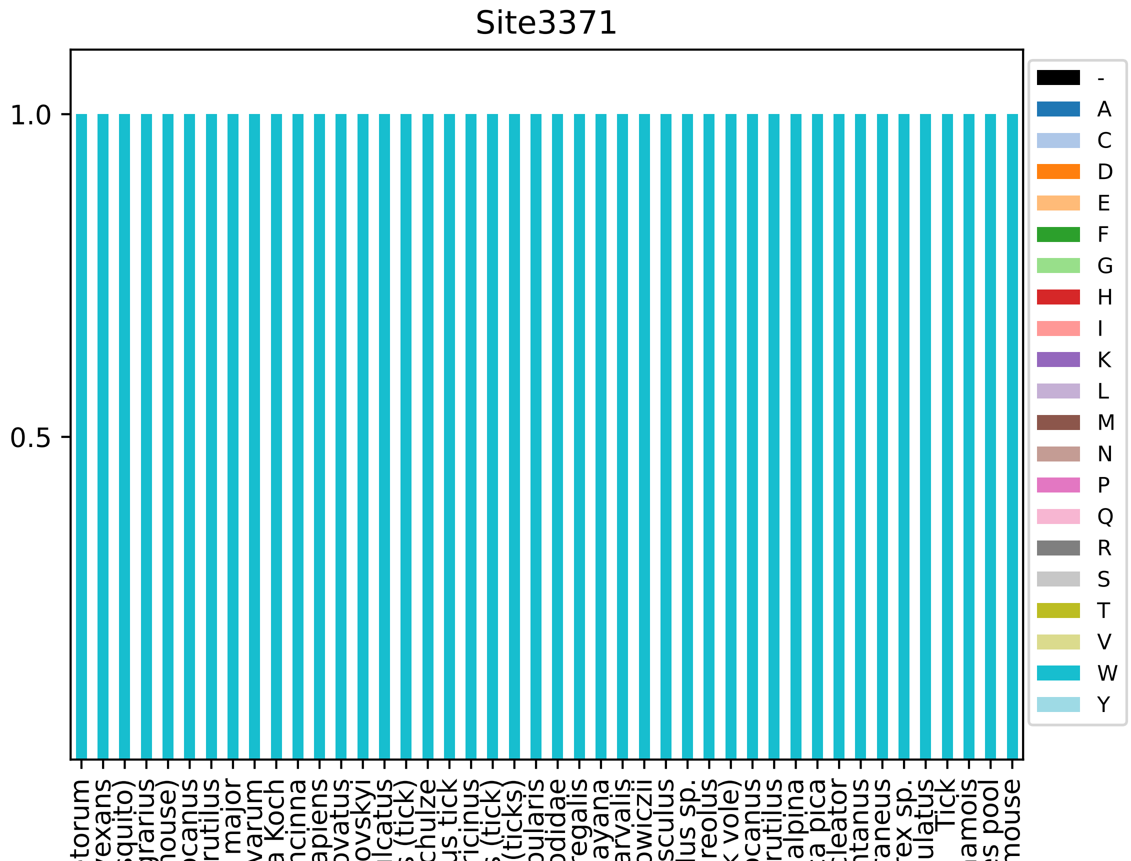
Site3371
1.0
0.5
-
A
C
D
E
F
G
H
I
K
L
M
N
P
Q
R
S
T
V
W
Y

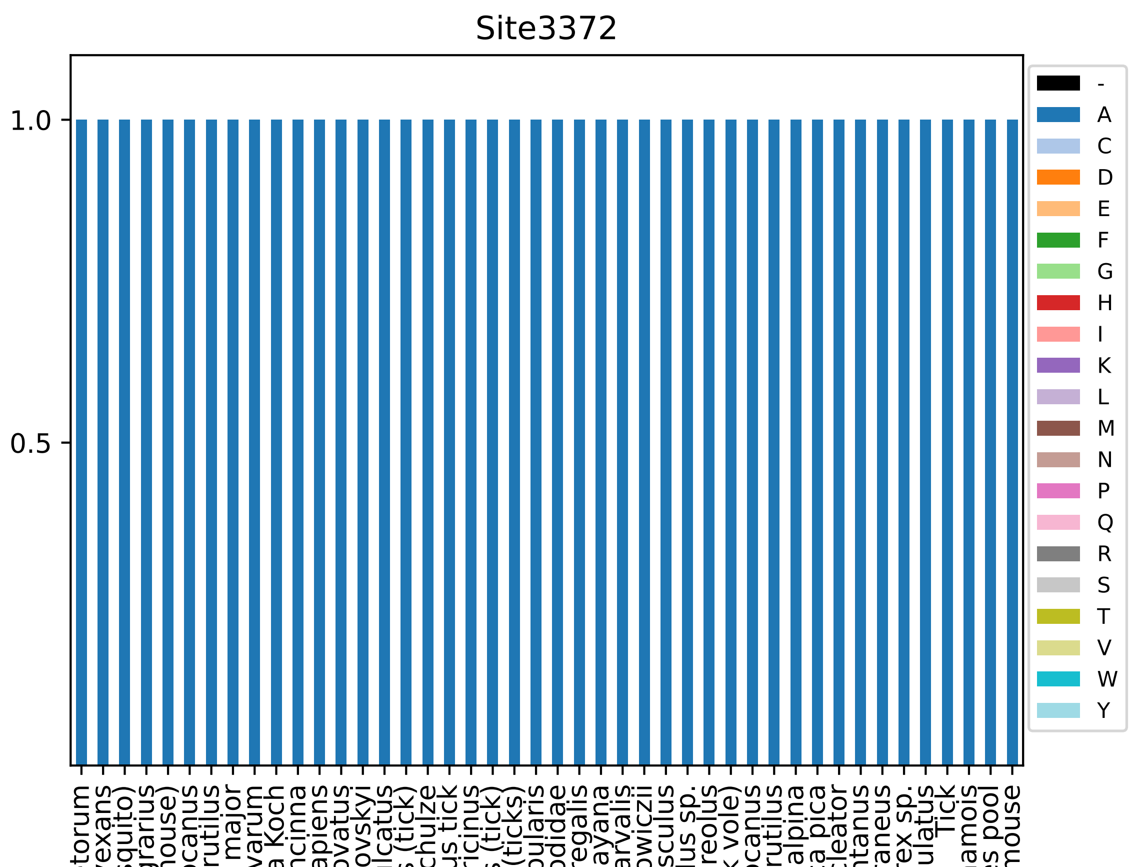

Site3372
1.0
0.5
-
A
C
D
E
F
G
H
I
K
L
M
N
P
Q
R
S
T
V
W
Y

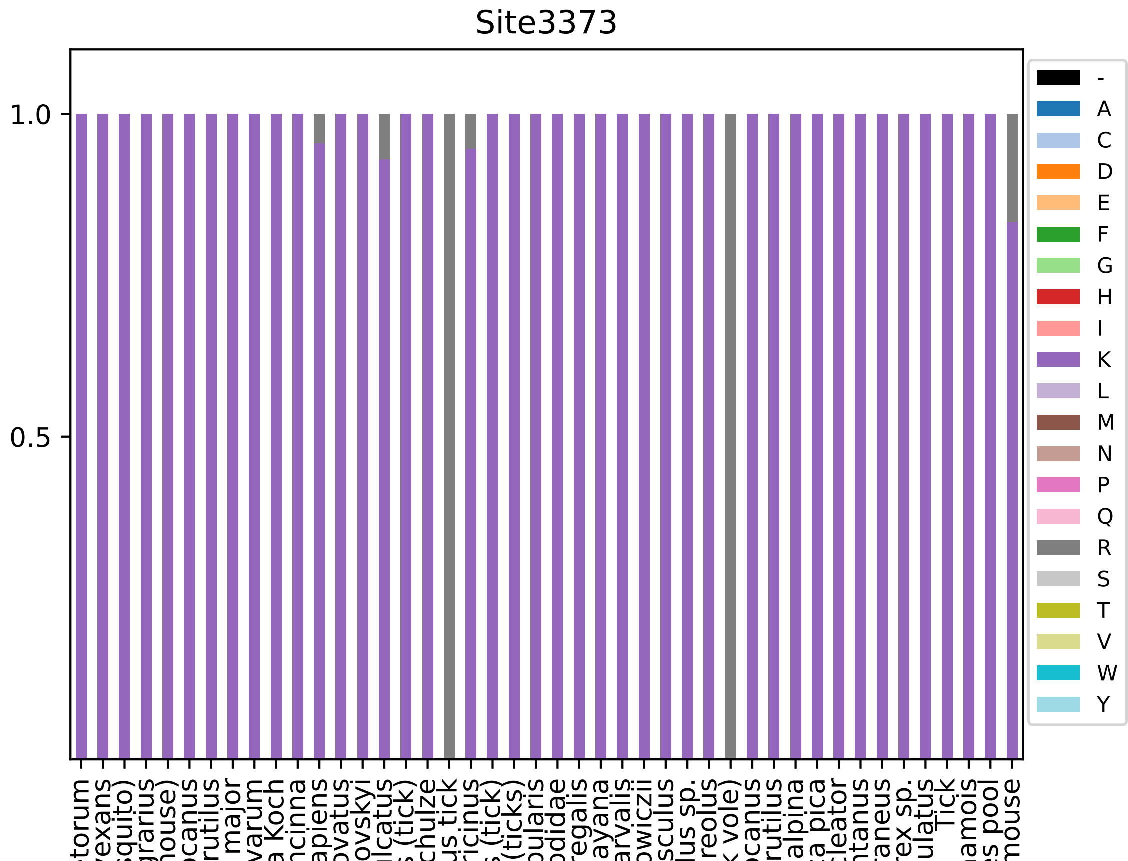

Site3373
1.0
0.5
-
A
C
D
E
F
G
H
I
K
L
M
N
P
Q
R
S
T
V
W
Y

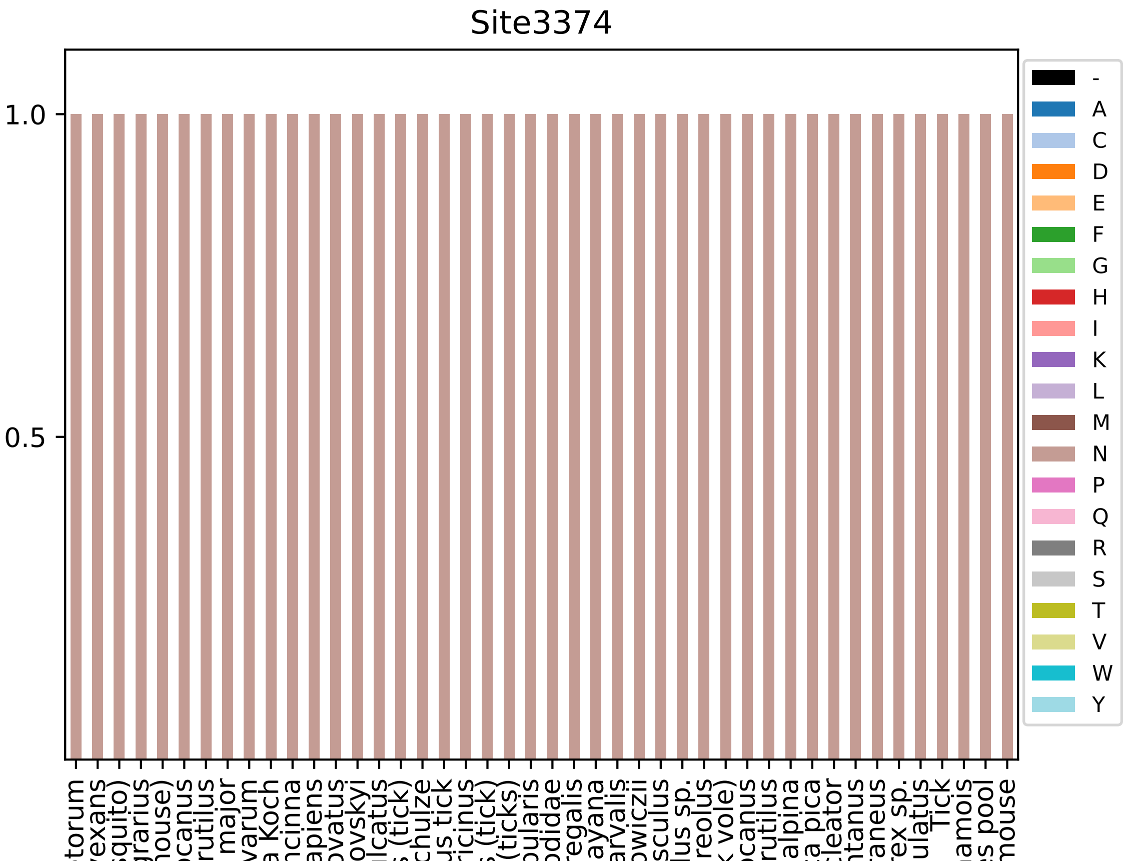
Site3374
1.0
0.5
-
A
C
D
E
F
G
H
I
K
L
M
N
P
Q
R
S
T
V
W
Y

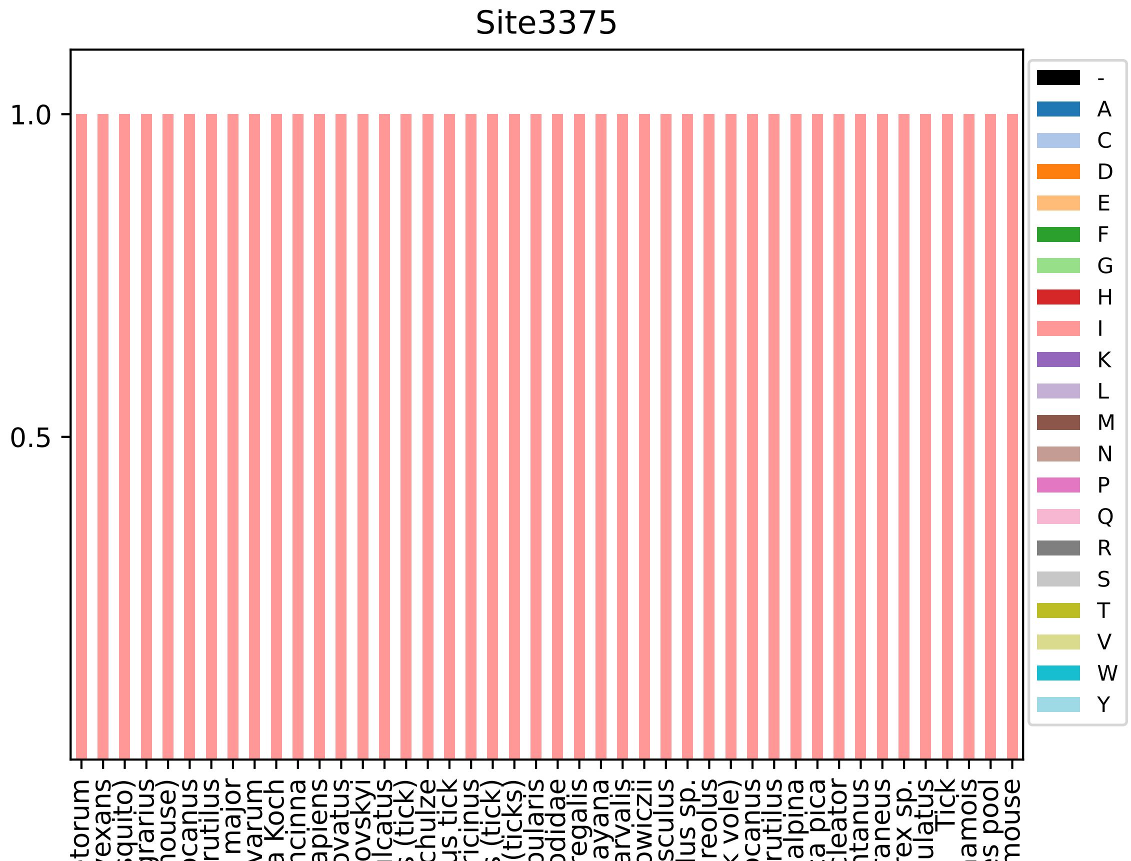
Site3375
1.0
0.5
-
A
C
D
E
F
G
H
I
K
L
M
N
P
Q
R
S
T
V
W
Y

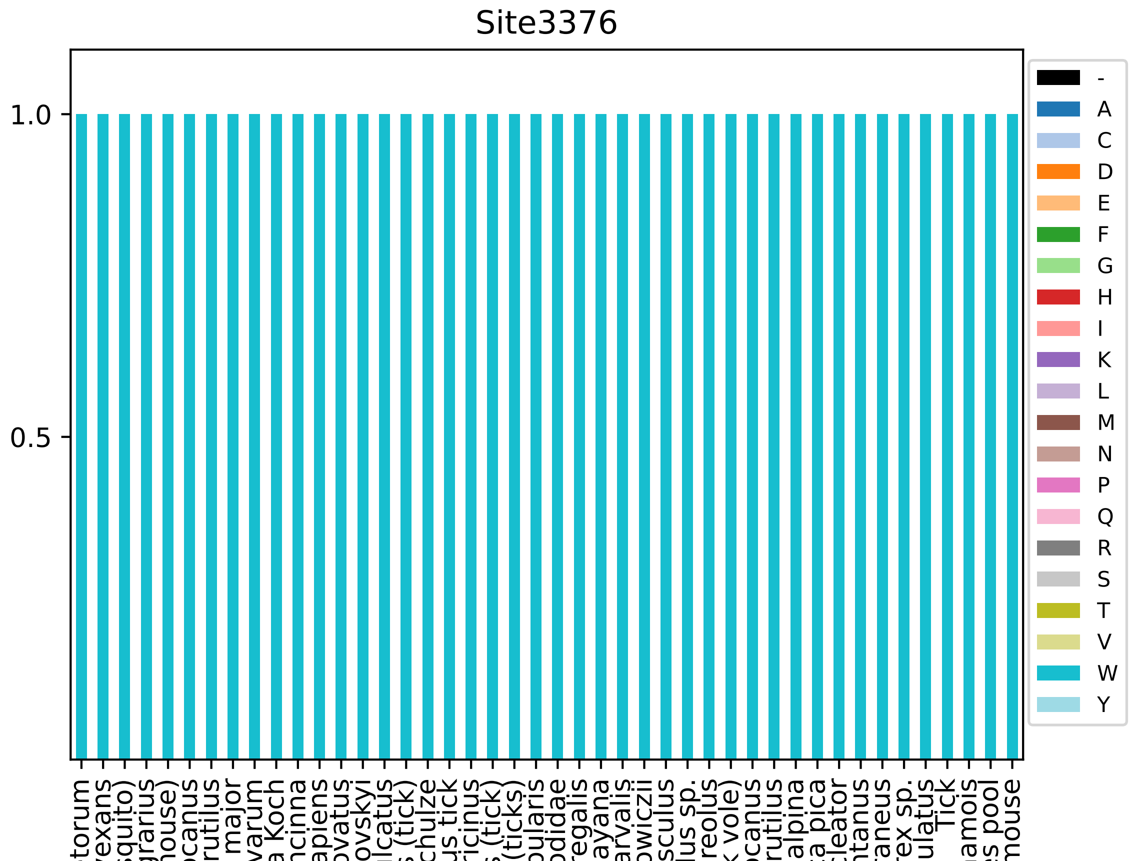

Site3376
1.0
0.5
-
A
C
D
E
F
G
H
I
K
L
M
N
P
Q
R
S
T
V
W
Y

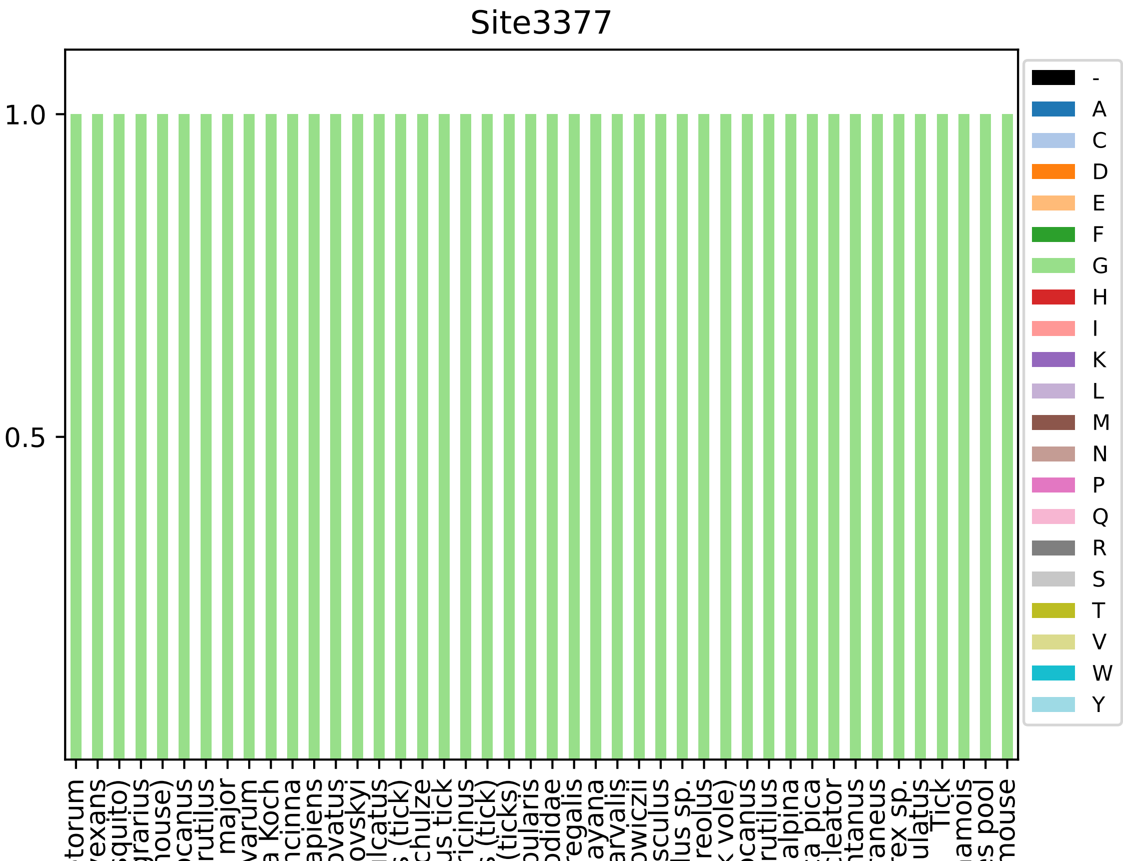

Site3377
1.0
0.5
-
A
C
D
E
F
G
H
I
K
L
M
N
P
Q
R
S
T
V
W
Y

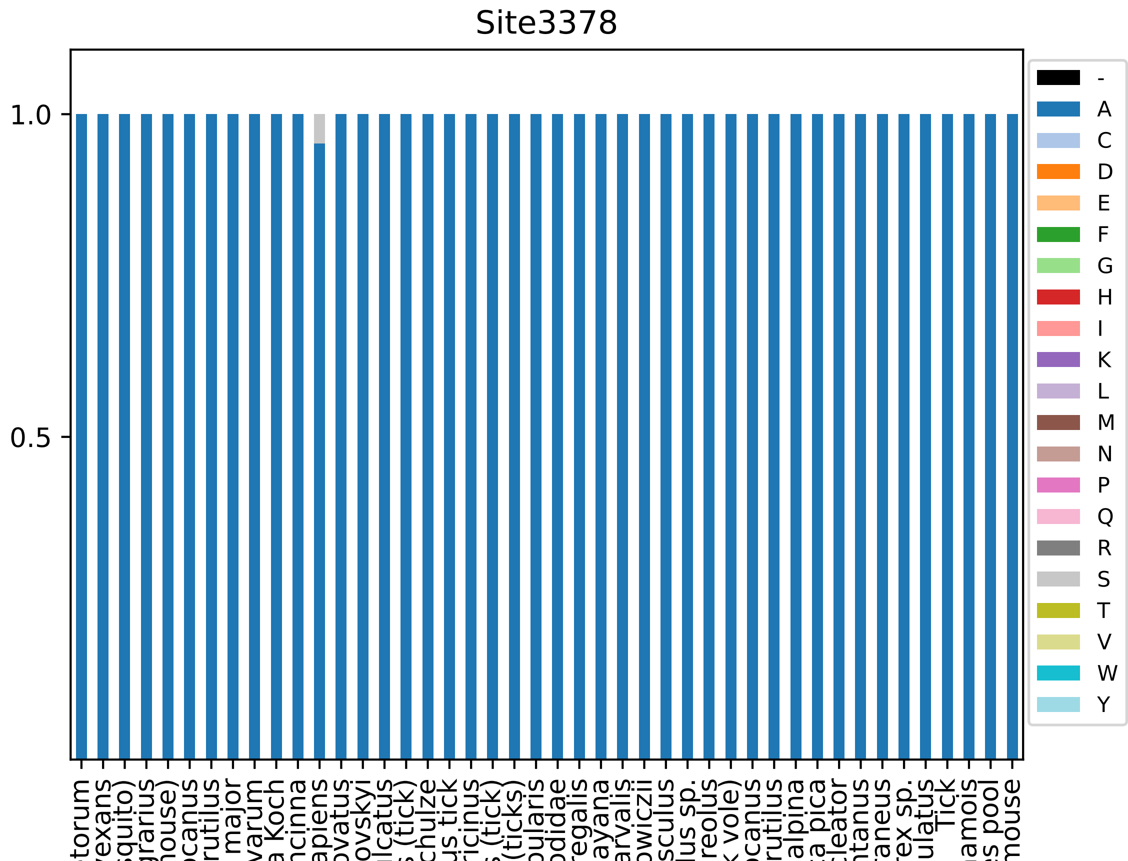
Site3378
1.0
0.5
-
A
C
D
E
F
G
H
I
K
L
M
N
P
Q
R
S
T
V
W
Y

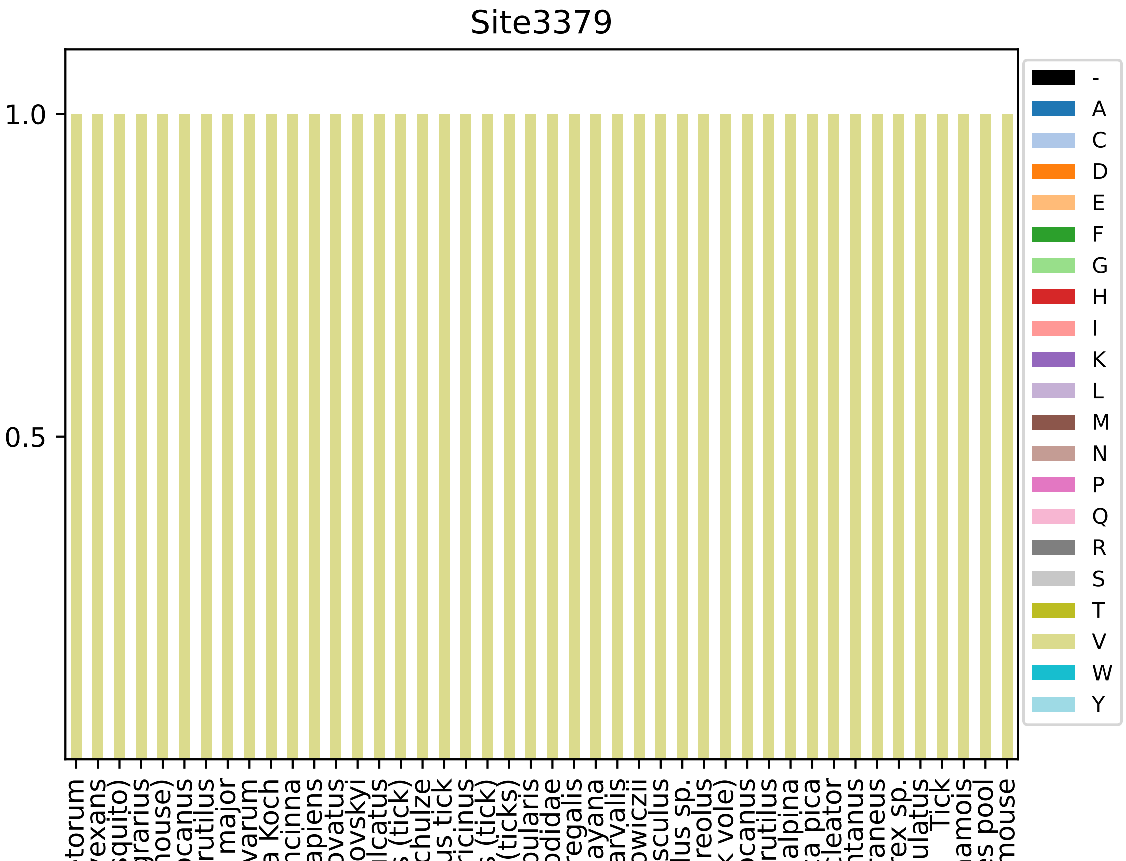
Site3379
1.0
0.5
-
A
C
D
E
F
G
H
I
K
L
M
N
P
Q
R
S
T
V
W
Y

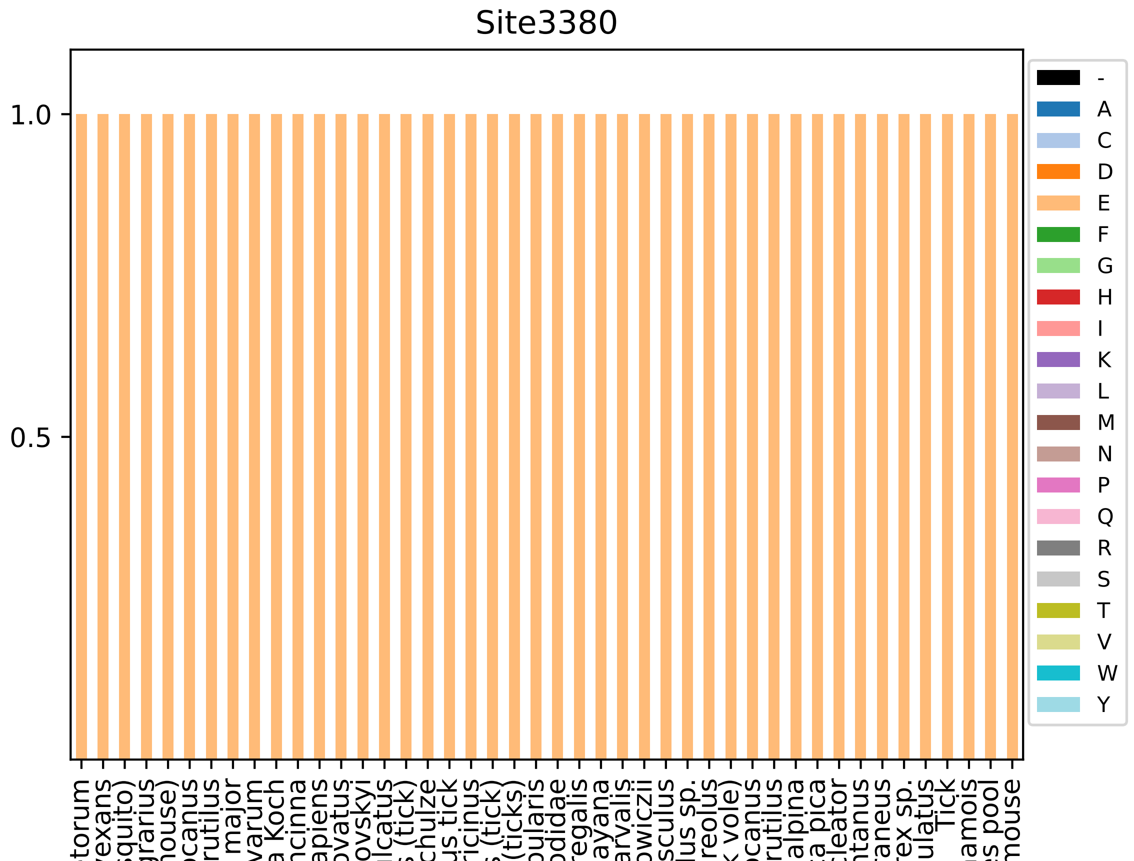
Site3380
1.0
0.5
-
A
C
D
E
F
G
H
I
K
L
M
N
P
Q
R
S
T
V
W
Y

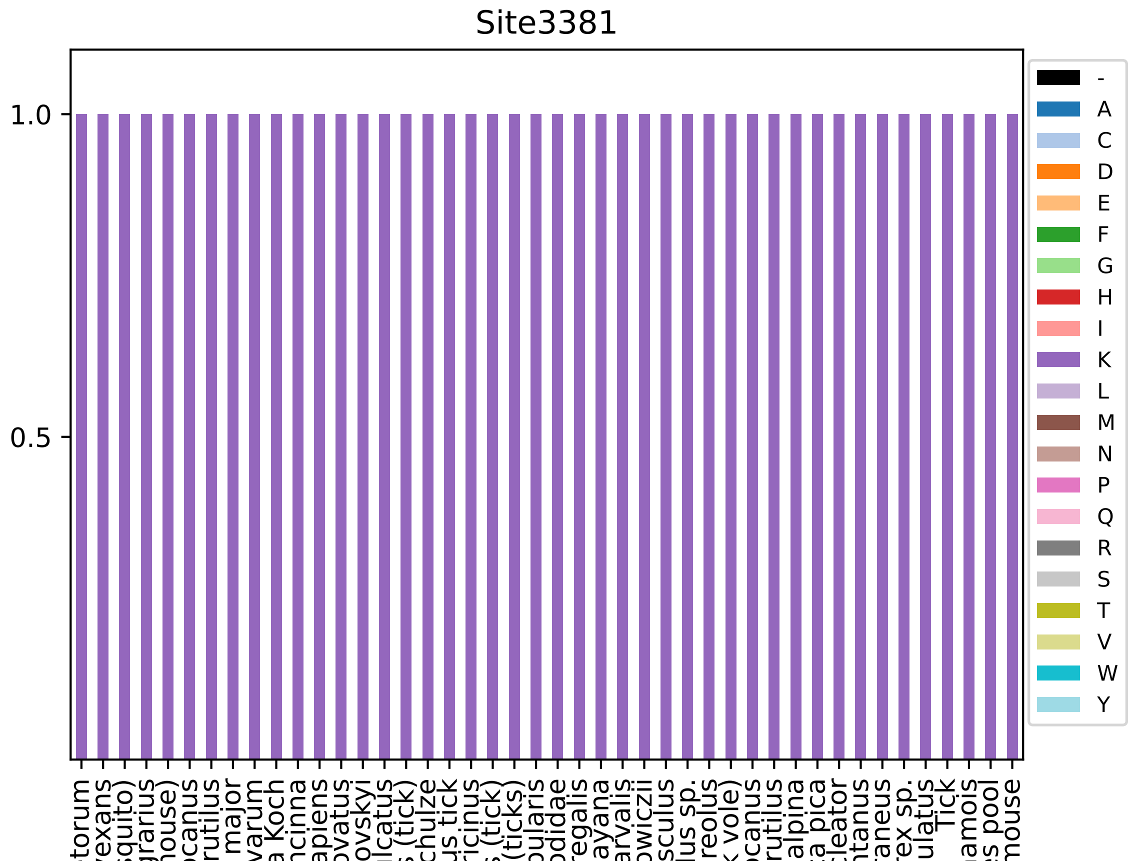
Site3381
1.0
0.5
-
A
C
D
E
F
G
H
I
K
L
M
N
P
Q
R
S
T
V
W
Y
torum
vexans
squito)
grarius
nouse)
ocanus
rutilus
major
varum
a Koch
ncinna
apiens
ovatus
ovskyi
ulcatus
s (tick)
chulze
us tick
ricinus
s (tick)
(ticks)
pularis
podidae
regalis
ayana
arvalis
owiczii
sculus
lus sp.
reolus
k vole)
ocanus
rutilus
alpina
a pica
cleator
ntanus
raneus
rex sp.
ulatus
Tick
amois
es pool
mouse

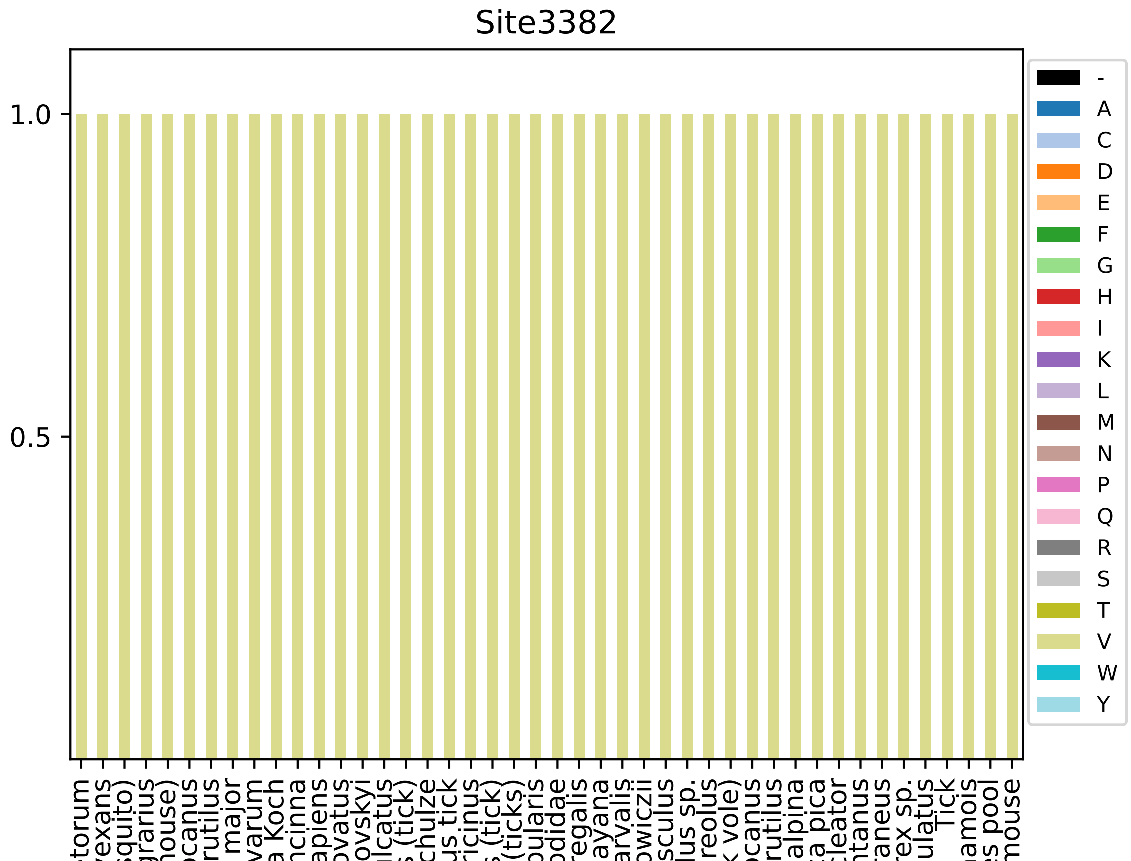

Site3382
1.0
0.5
-
A
C
D
E
F
G
H
I
K
L
M
N
P
Q
R
S
T
V
W
Y

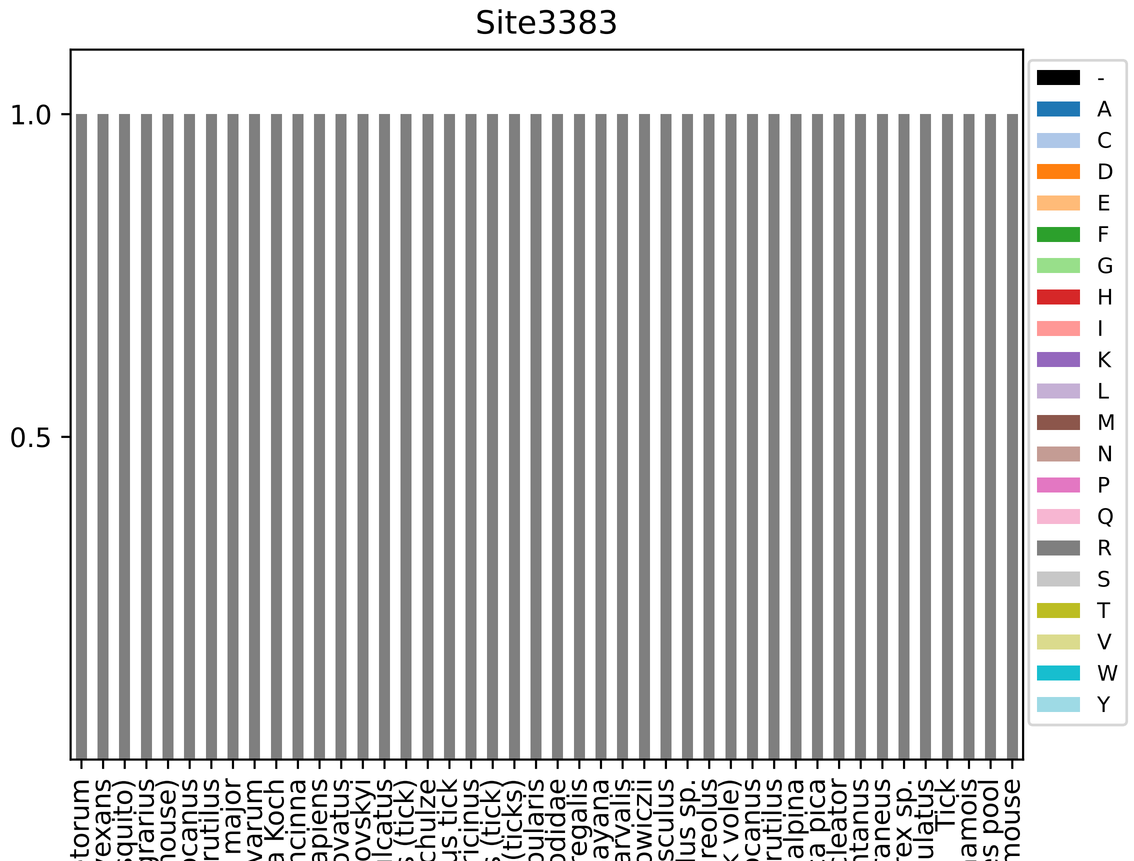

Site3383
1.0
0.5
-
A
C
D
E
F
G
H
I
K
L
M
N
P
Q
R
S
T
V
W
Y

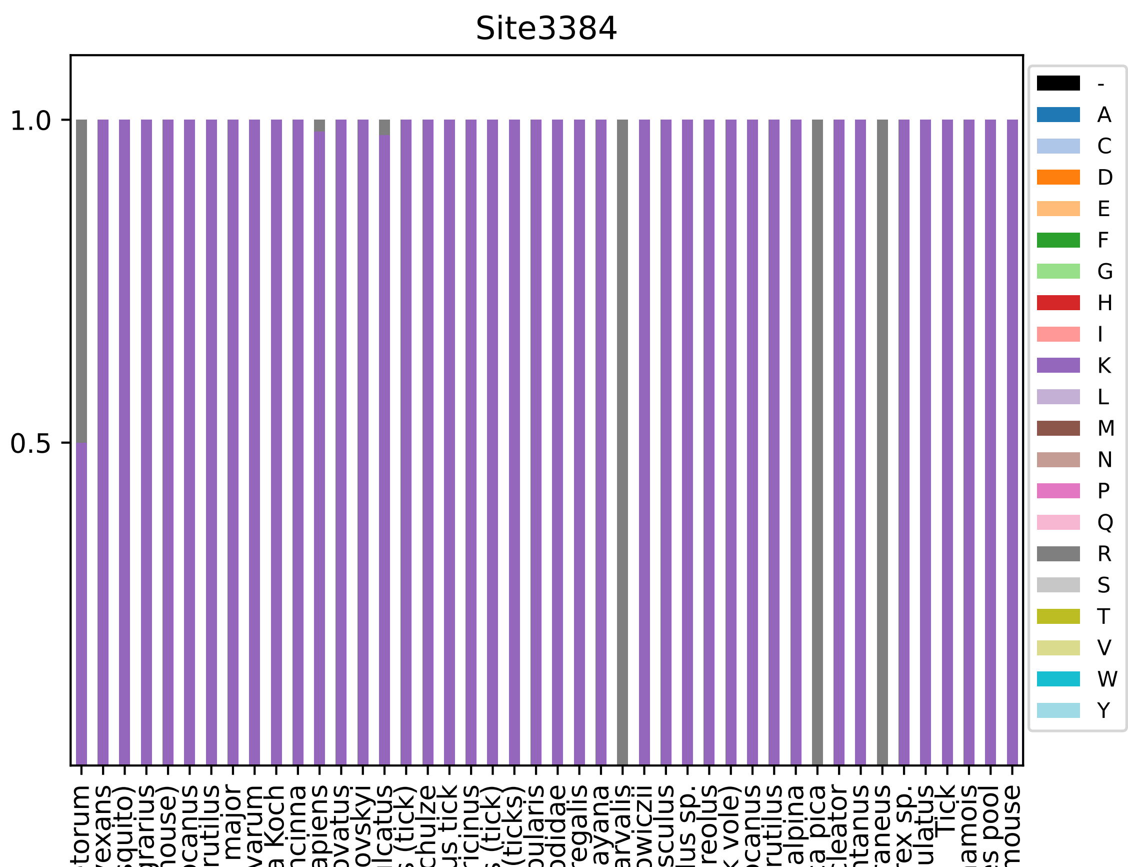
Site3384
1.0
0.5
-
A
C
D
E
F
G
H
I
K
L
M
N
P
Q
R
S
T
V
W
Y

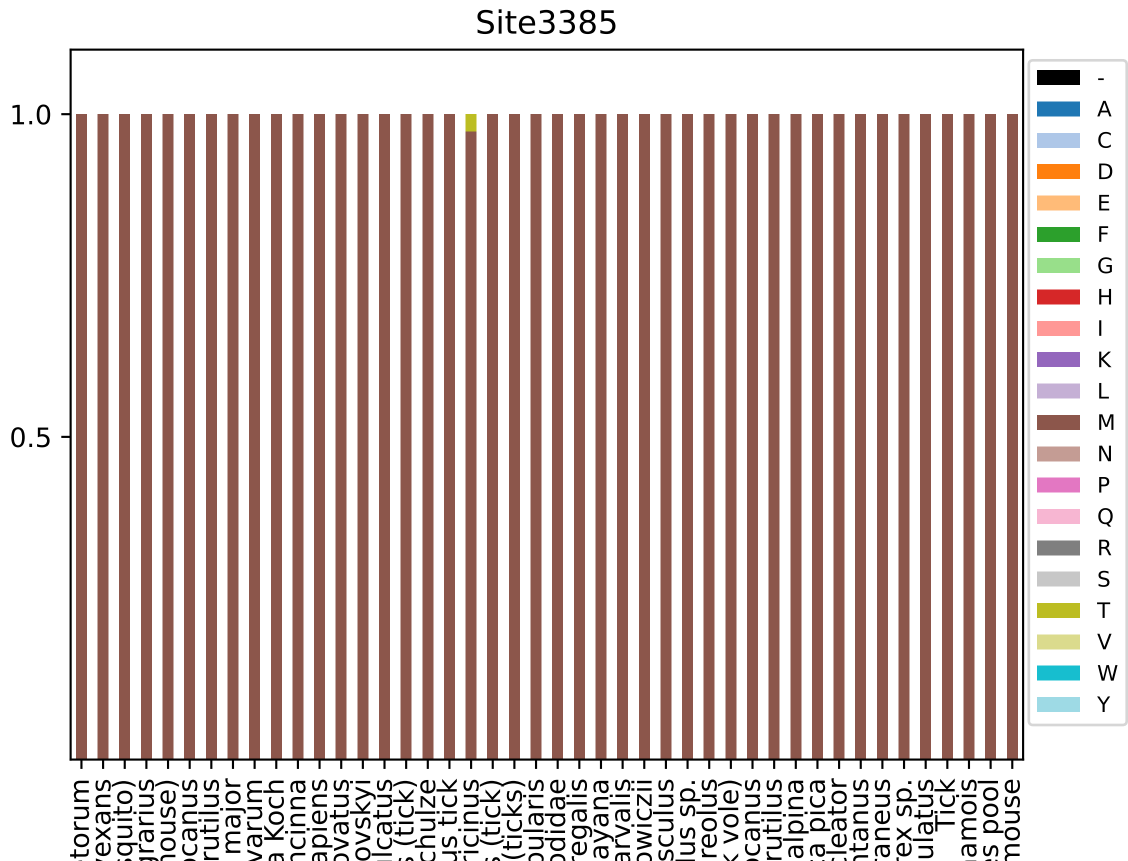
Site3385
1.0
0.5
-
A
C
D
E
F
G
H
I
K
L
M
N
P
Q
R
S
T
V
W
Y
Tick

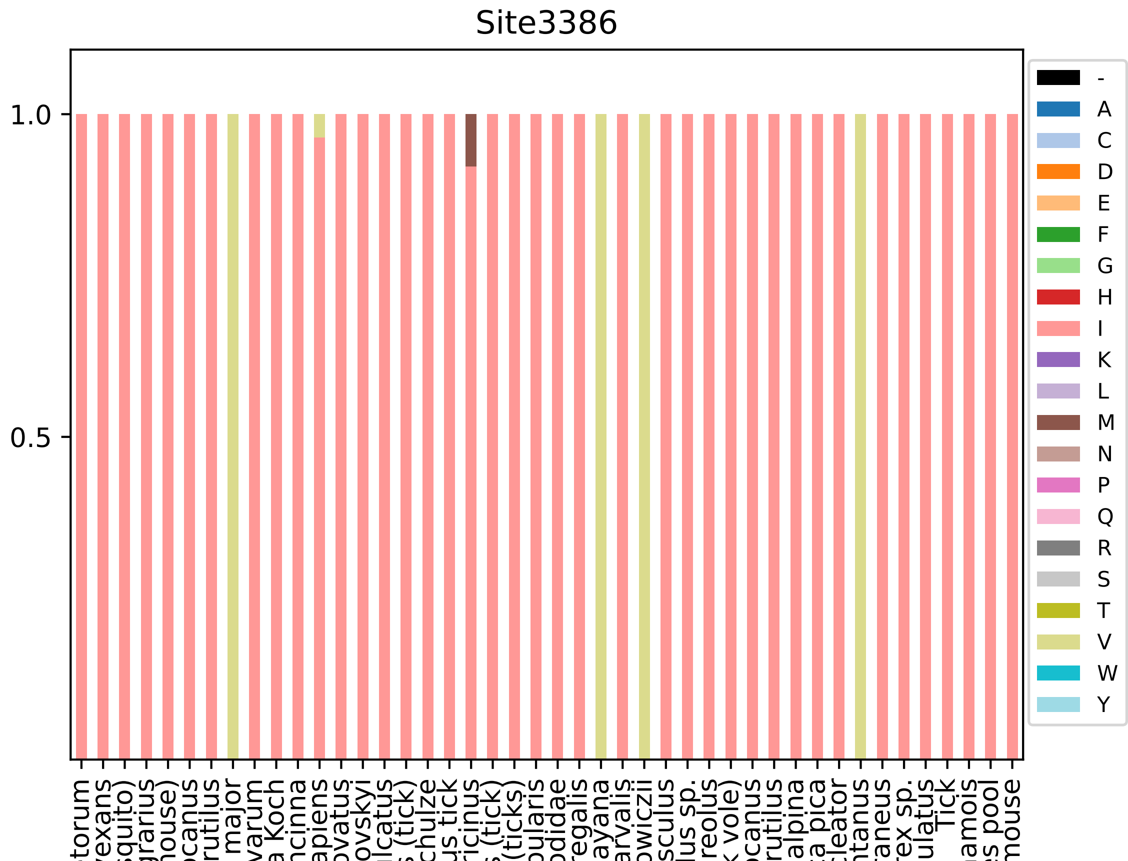
Site3386
1.0
0.5
-
A
C
D
E
F
G
H
I
K
L
M
N
P
Q
R
S
T
V
W
Y

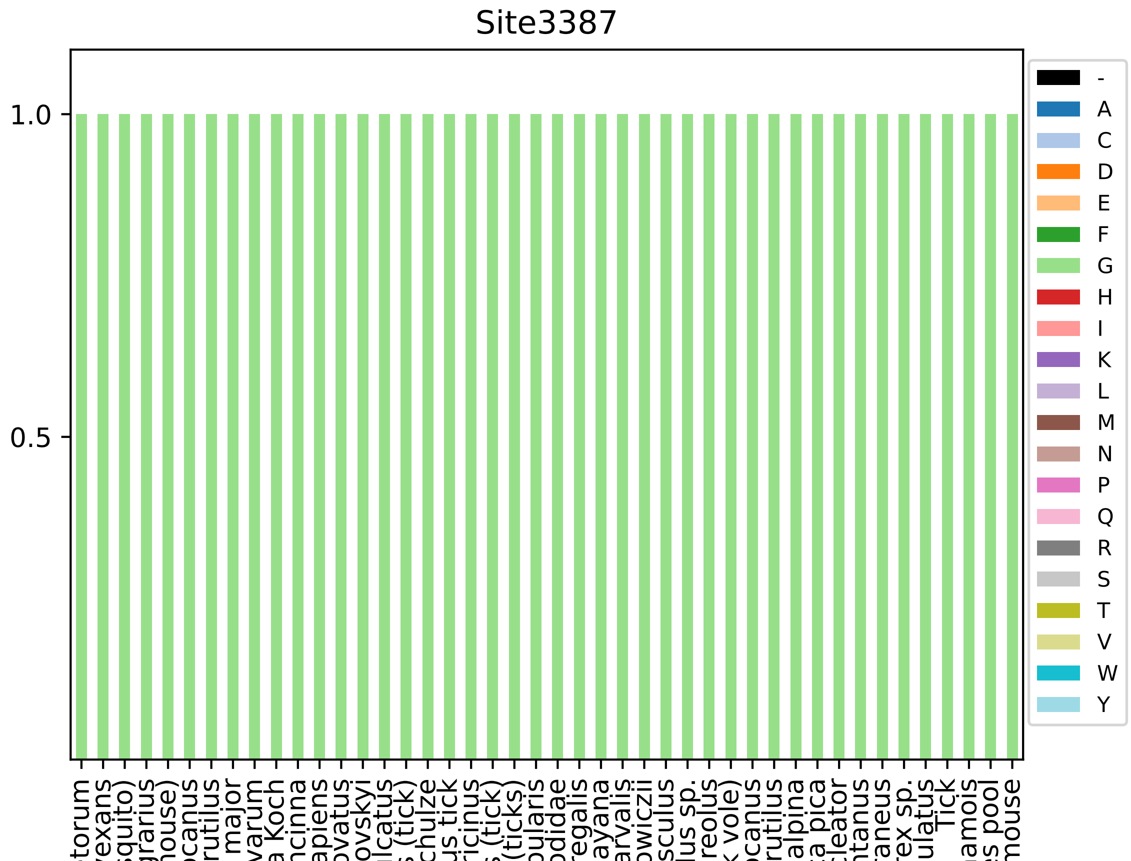
Site3387
1.0
0.5
-
A
C
D
E
F
G
H
I
K
L
M
N
P
Q
R
S
T
V
W
Y

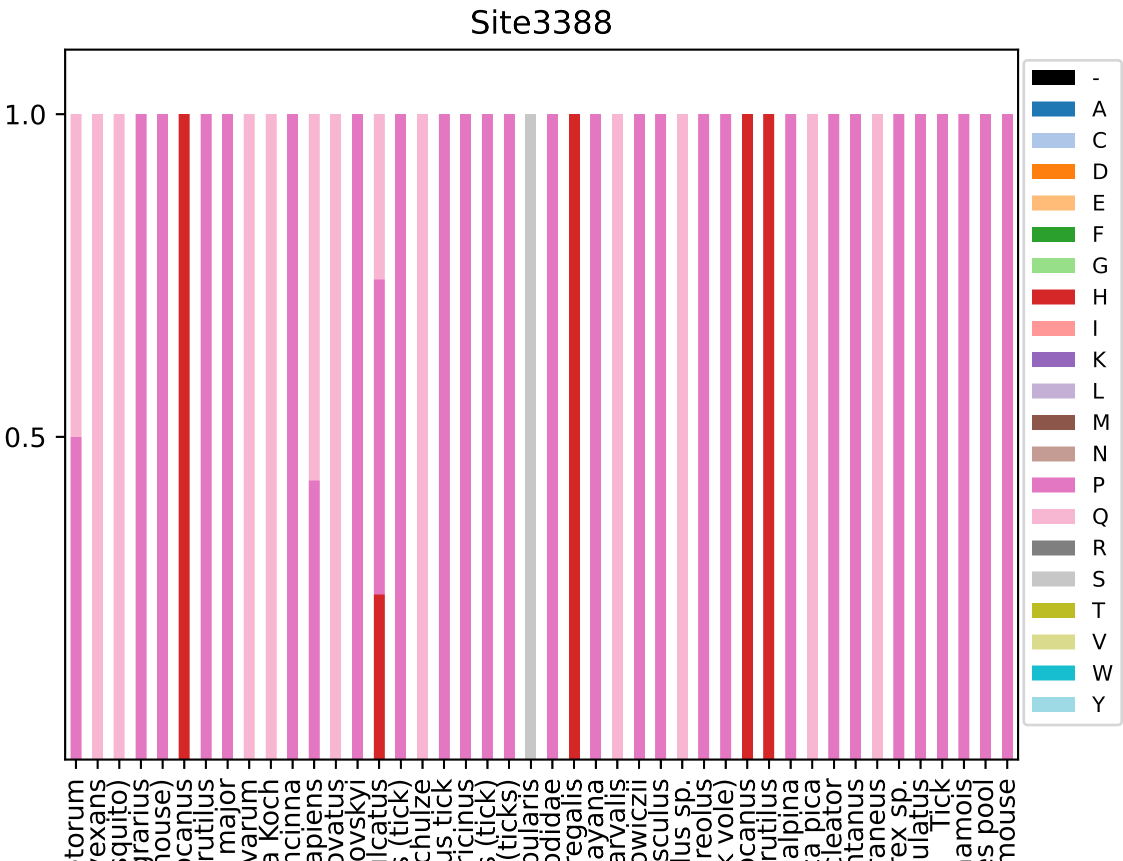

Site3388
1.0
0.5
-
A
C
D
E
F
G
H
I
K
L
M
N
P
Q
R
S
T
V
W
Y
torum
exans
squito)
garius
nouse)
ocanus
rutilus
major
varum
a Koch
ncinna
apiens
ovatus
ovskyi
ilcatus
chulze
us tick
ricinus
(ticks)
pularis
podidae
regalis
ayana
arvalis
owiczii
sculus
lus sp.
reolus
k vole)
ocanus
rutilus
alpina
a pica
cleator
ntanus
raneus
rex sp.
ulatus
Tick
amois
es pool
mouse

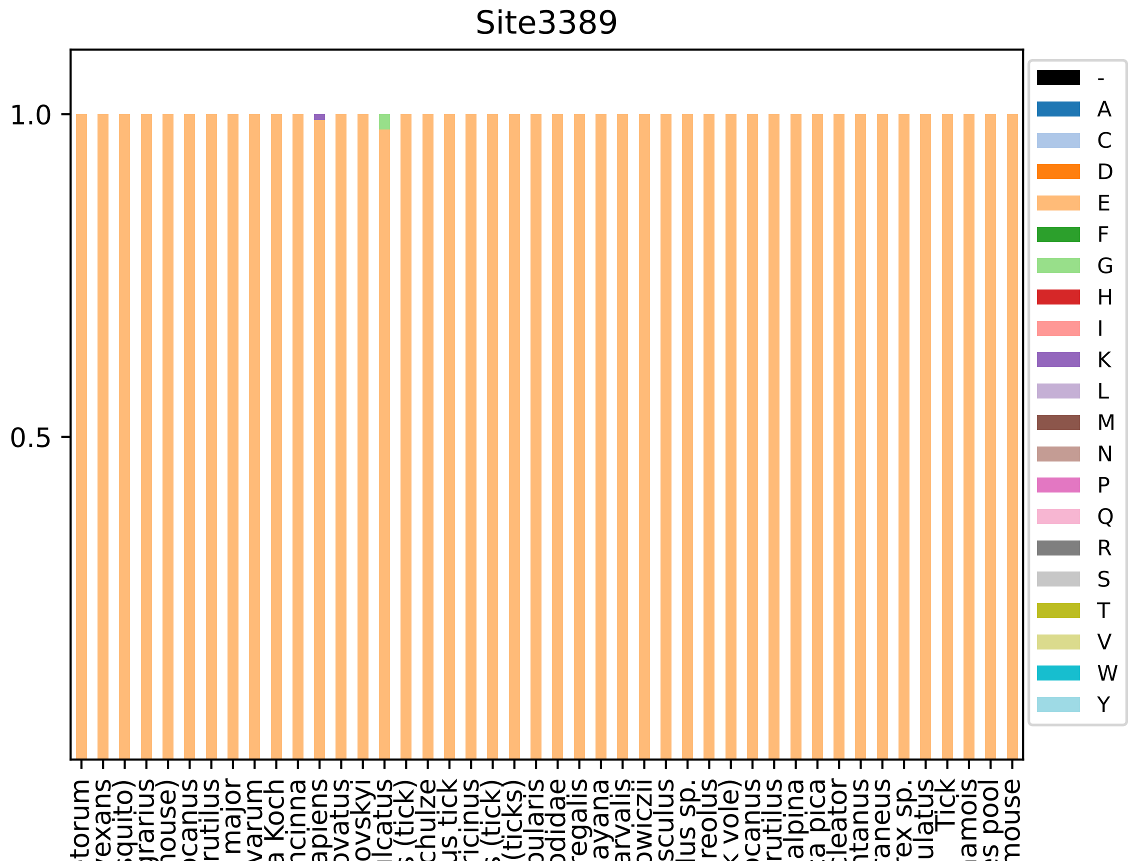
Site3389
1.0
0.5
-
A
C
D
E
F
G
H
I
K
L
M
N
P
Q
R
S
T
V
W
Y
torum
vexans
squito)
grarius
nouse)
ocanus
rutilus
major
varum
a Koch
ncinna
apiens
ovatus
ovskyi
ulcatus
s (tick)
chulze
us tick
ricinus
s (tick)
(ticks)
pularis
odidae
regalis
ayana
arvalis
owiczii
sculus
lus sp.
reolus
k vole)
ocanus
rutilus
alpina
a pica
cleator
ntanus
raneus
rex sp.
ulatus
Tick
amois
es pool
mouse

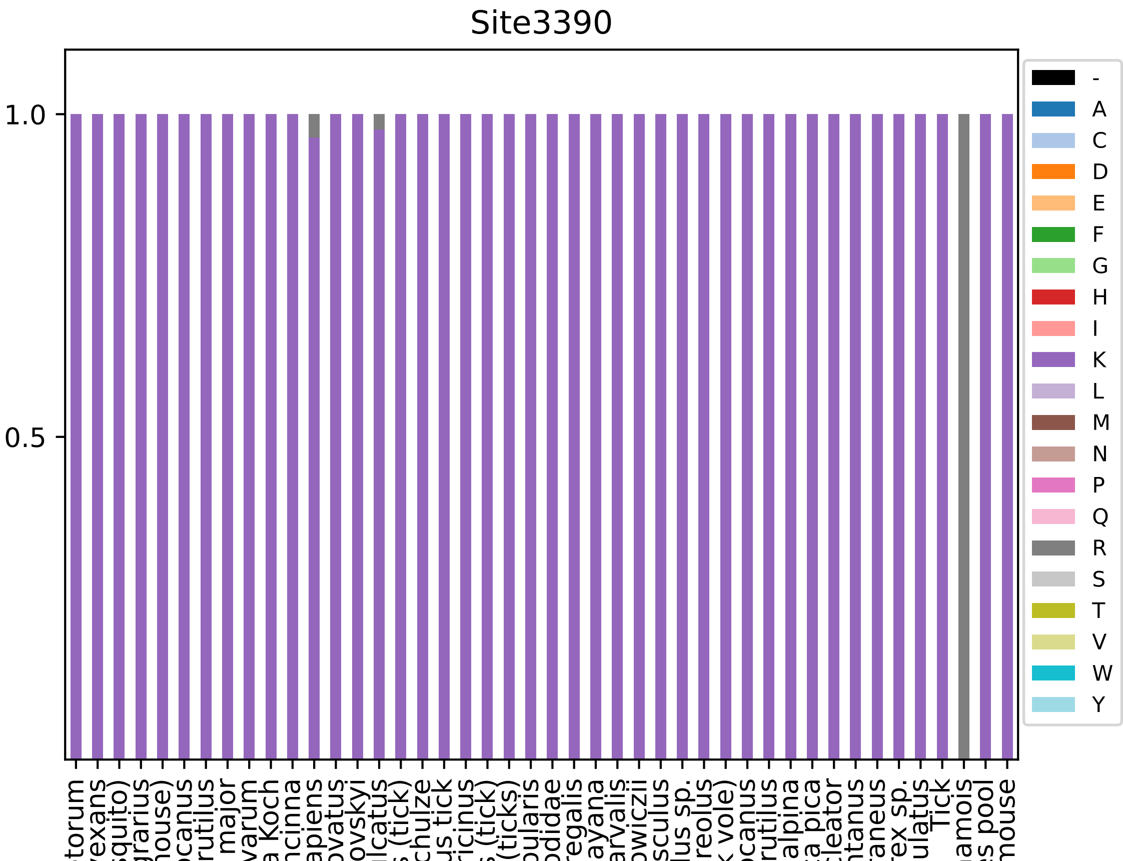
Site3390
1.0
0.5
-
A
C
D
E
F
G
H
I
K
L
M
N
P
Q
R
S
T
V
W
Y

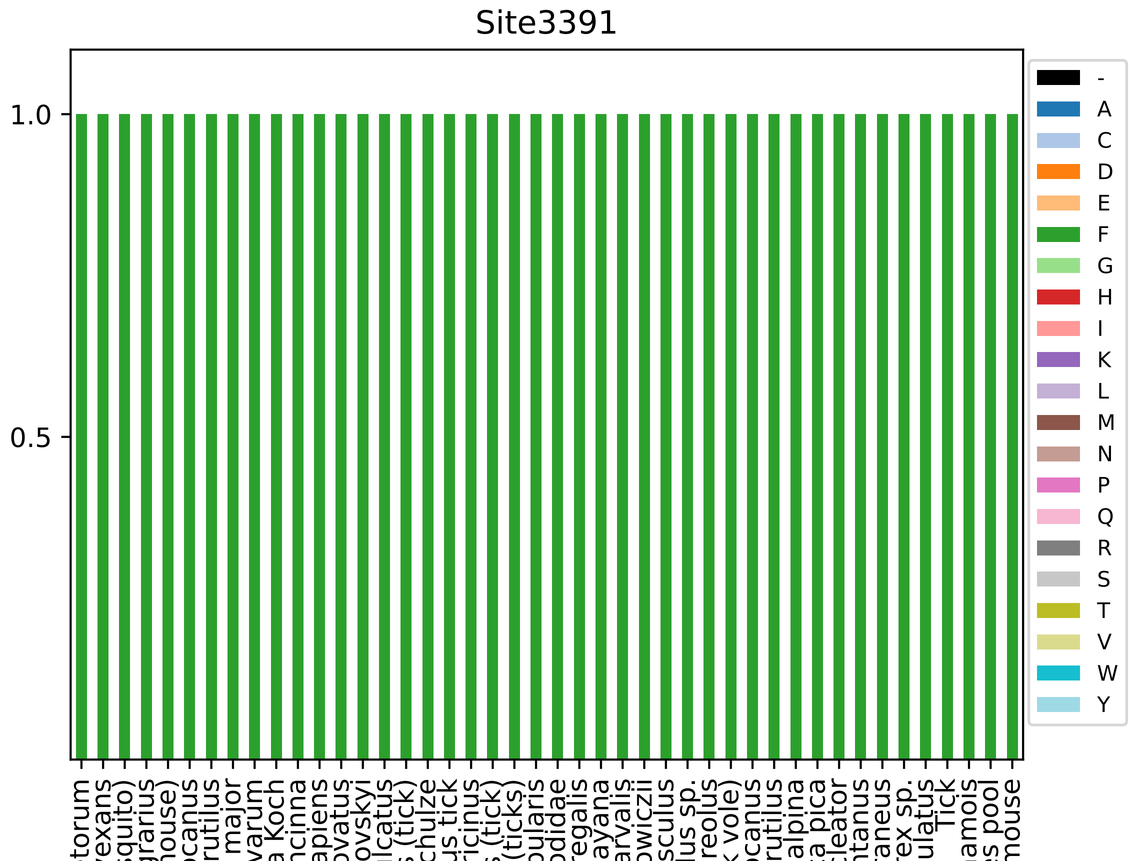
Site3391
1.0
0.5
-
A
C
D
E
F
G
H
I
K
L
M
N
P
Q
R
S
T
V
W
Y

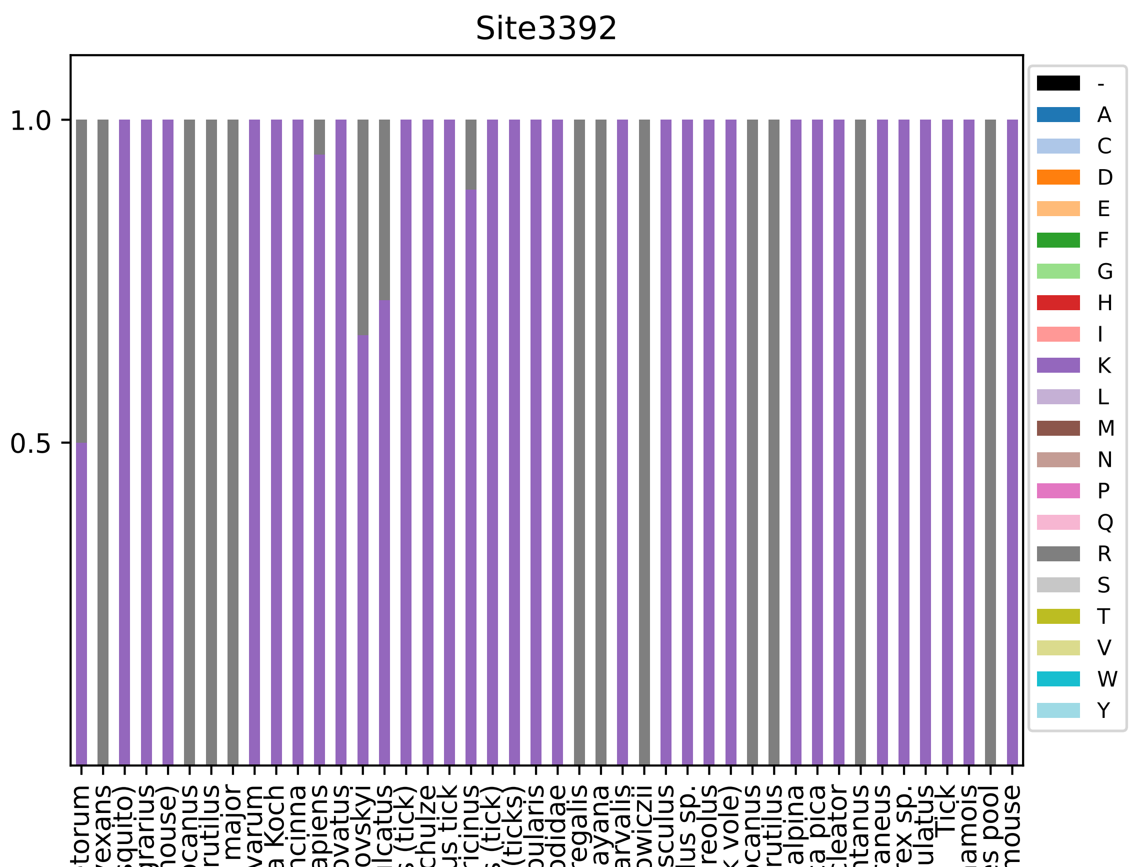
Site3392
1.0
0.5
-
A
C
D
E
F
G
H
I
K
L
M
N
P
Q
R
S
T
V
W
Y

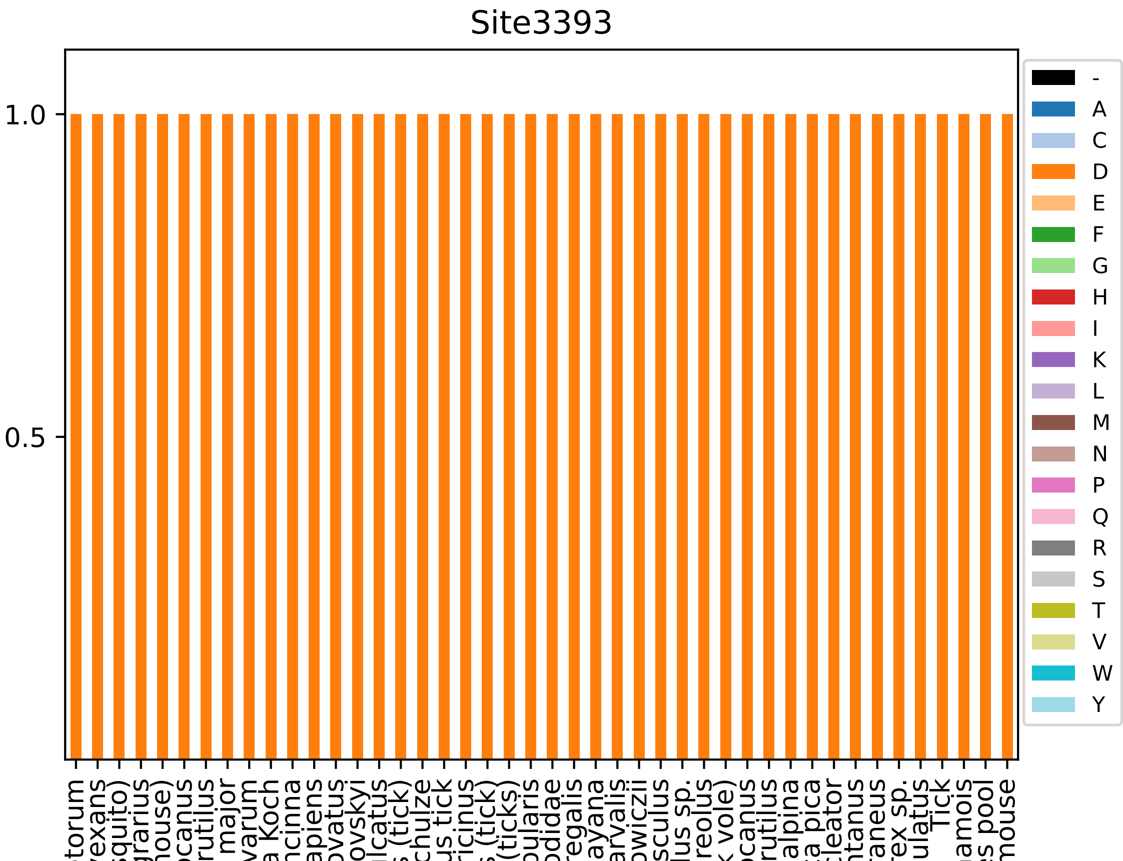
Site3393
1.0
0.5
-
A
C
D
E
F
G
H
I
K
L
M
N
P
Q
R
S
T
V
W
Y

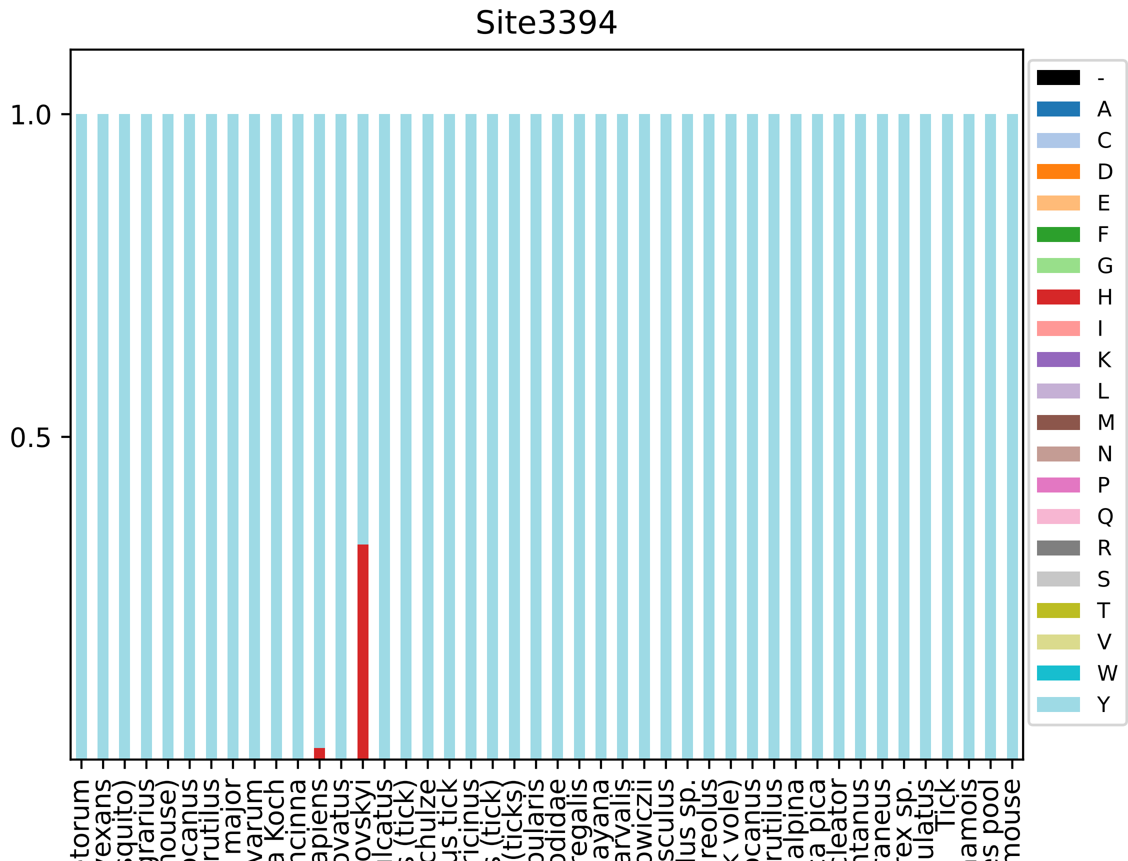
Site3394
1.0
0.5
-
A
C
D
E
F
G
H
I
K
L
M
N
P
Q
R
S
T
V
W
Y

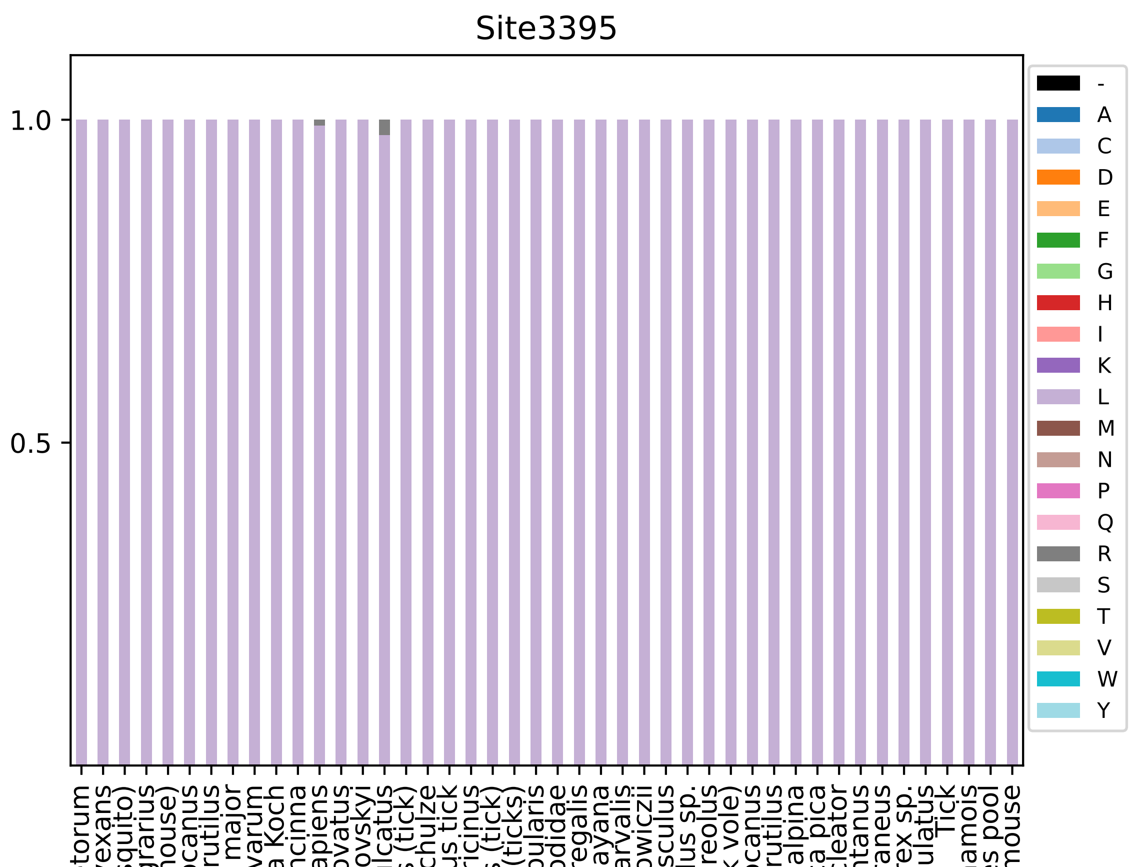
Site3395
1.0
0.5
-
A
C
D
E
F
G
H
I
K
L
M
N
P
Q
R
S
T
V
W
Y

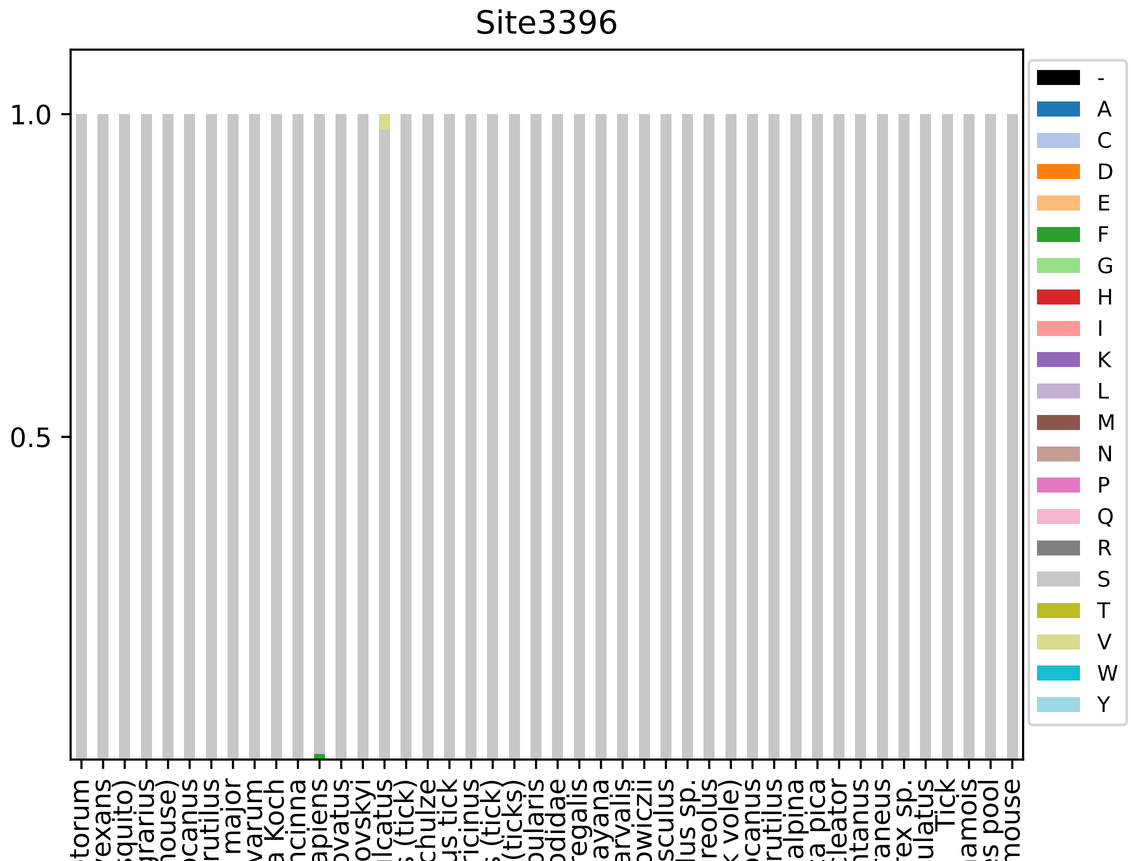
Site3396
1.0
0.5
-
A
C
D
E
F
G
H
I
K
L
M
N
P
Q
R
S
T
V
W
Y

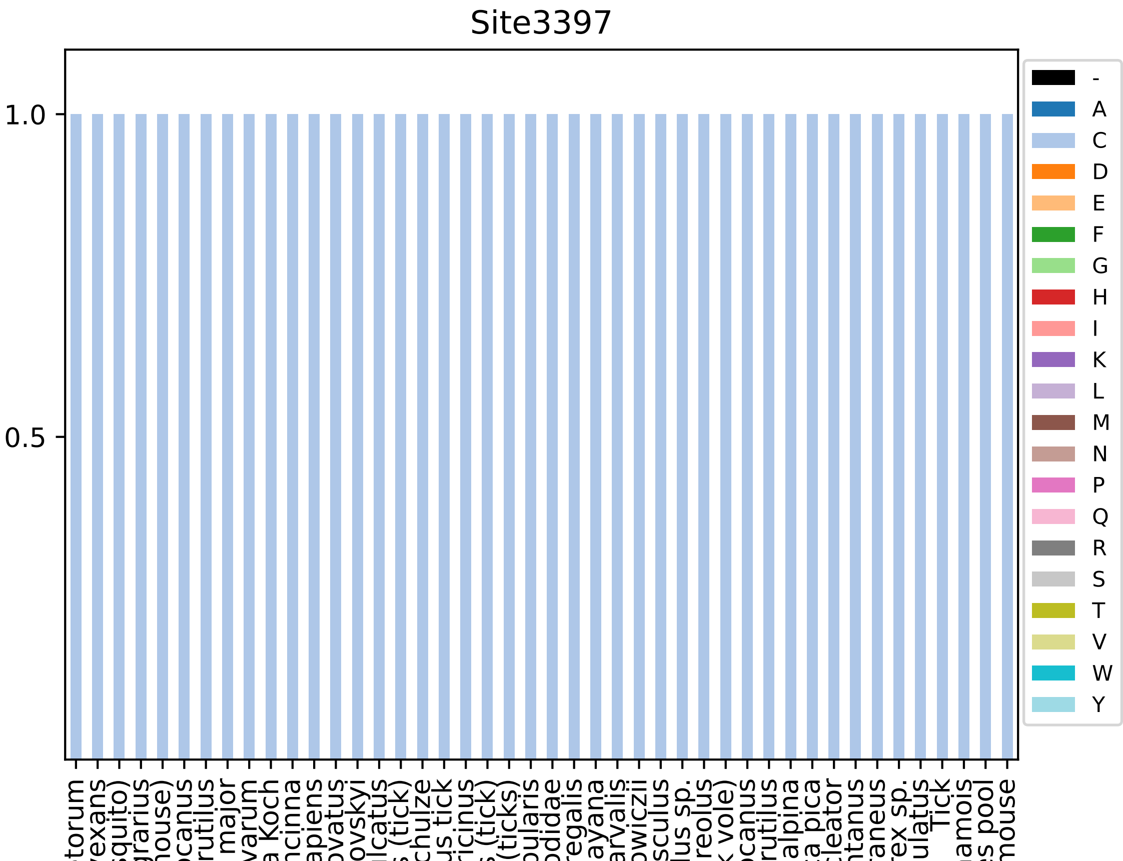
Site3397
1.0
0.5
-
A
C
D
E
F
G
H
I
K
L
M
N
P
Q
R
S
T
V
W
Y

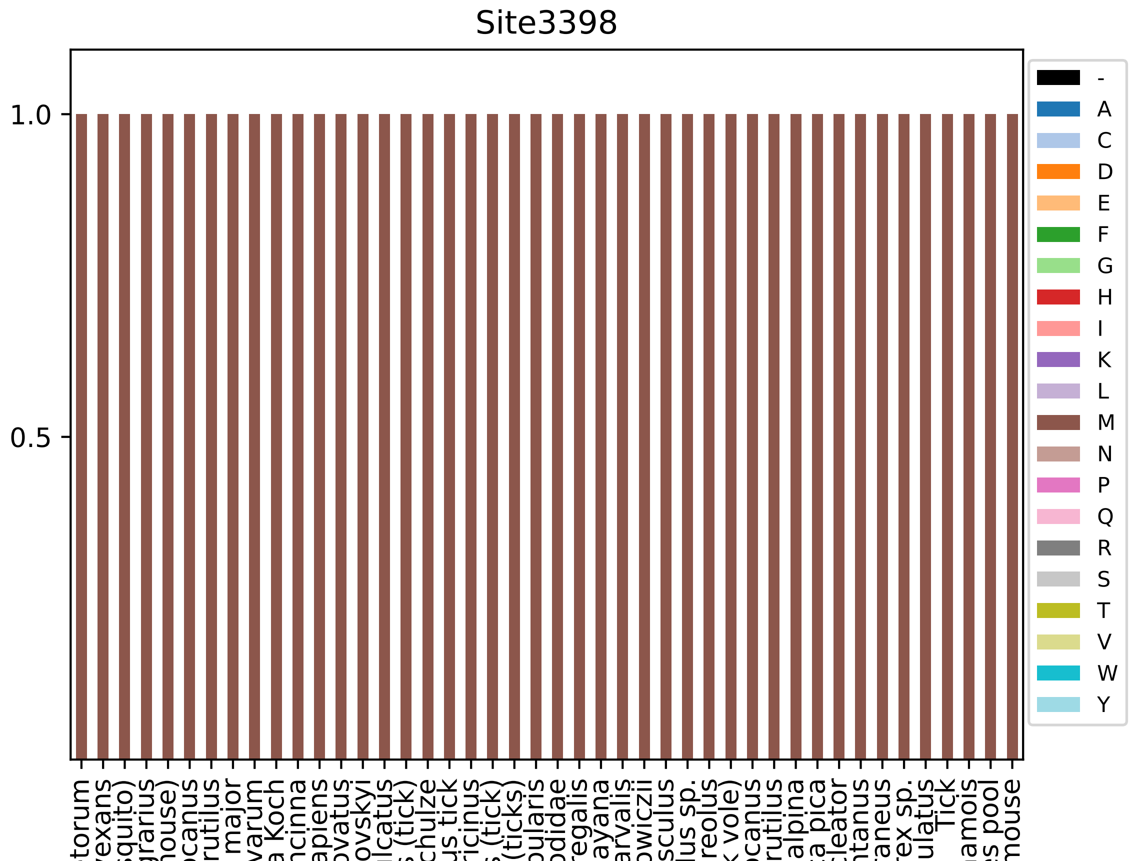
Site3398
1.0
0.5
-
A
C
D
E
F
G
H
I
K
L
M
N
P
Q
R
S
T
V
W
Y

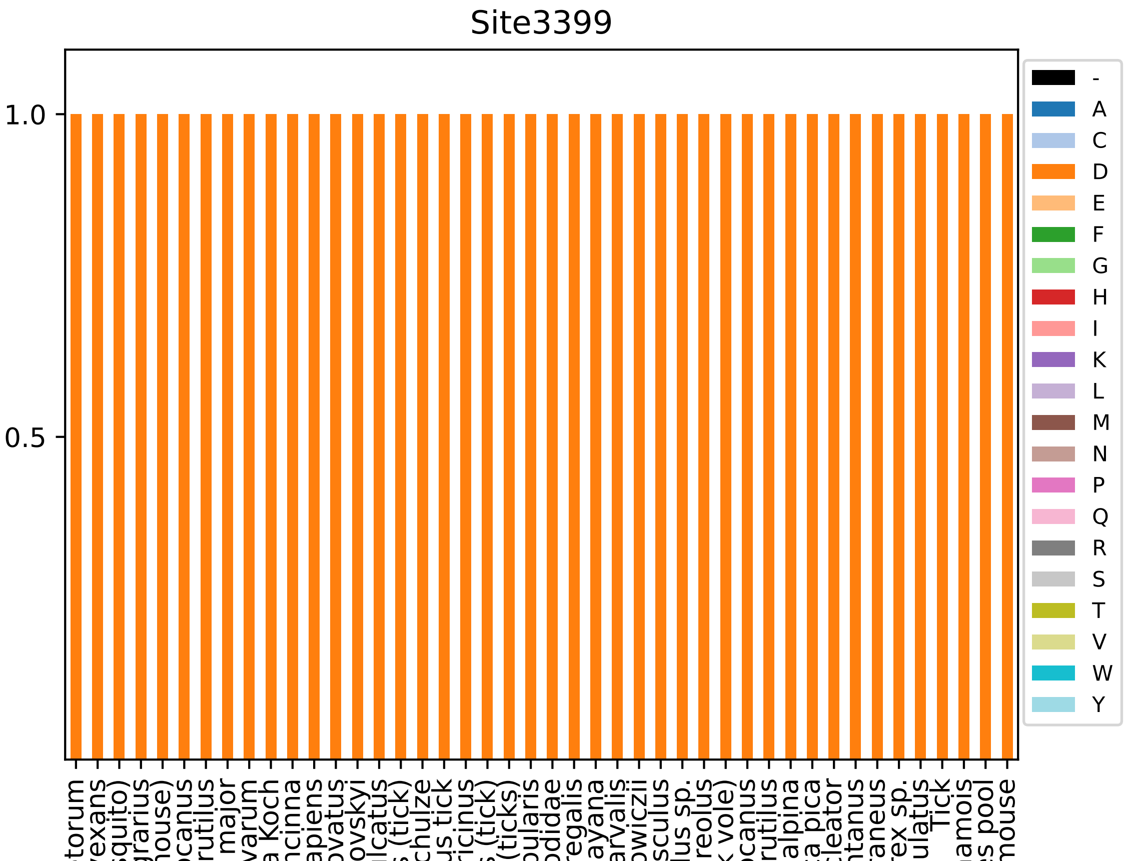

Site3399
1.0
0.5
-
A
C
D
E
F
G
H
I
K
L
M
N
P
Q
R
S
T
V
W
Y

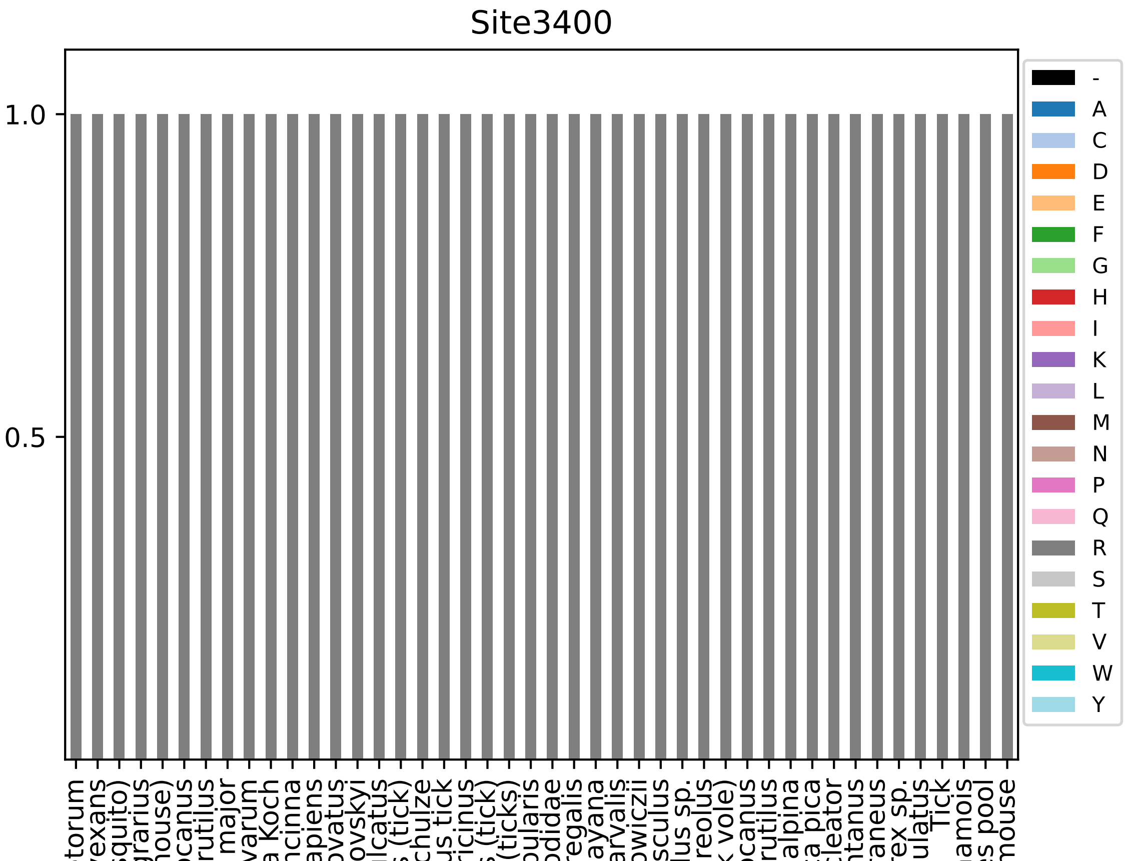

Site3400
1.0
0.5
-
A
C
D
E
F
G
H
I
K
L
M
N
P
Q
R
S
T
V
W
Y

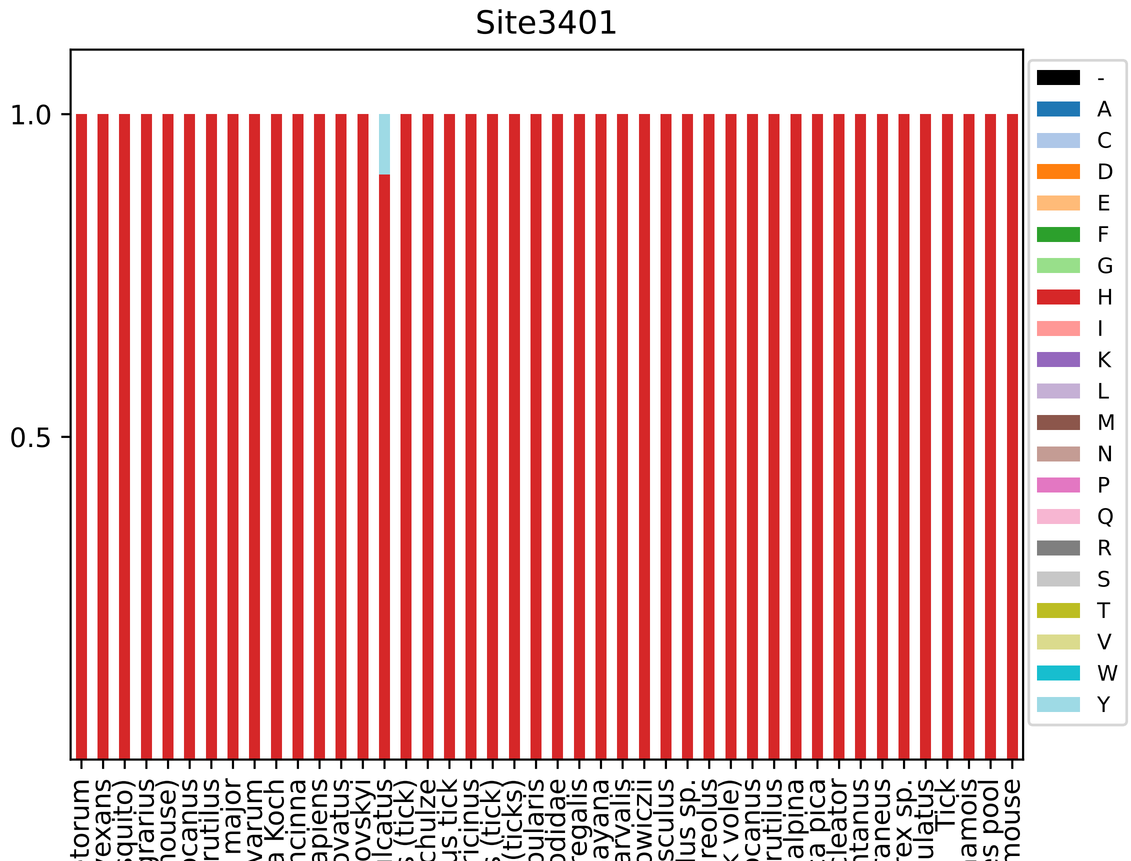
Site3401
1.0
0.5
-
A
C
D
E
F
G
H
I
K
L
M
N
P
Q
R
S
T
V
W
Y

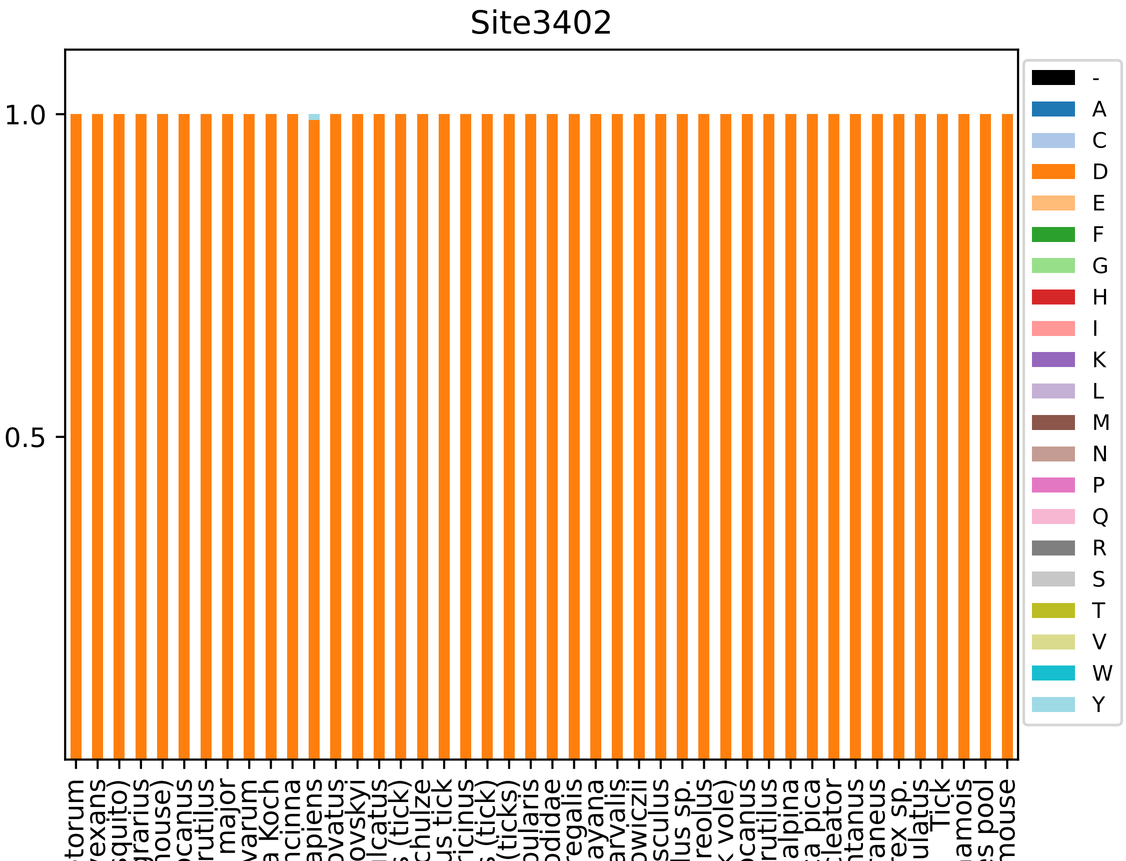
Site3402
1.0
0.5
-
A
C
D
E
F
G
H
I
K
L
M
N
P
Q
R
S
T
V
W
Y

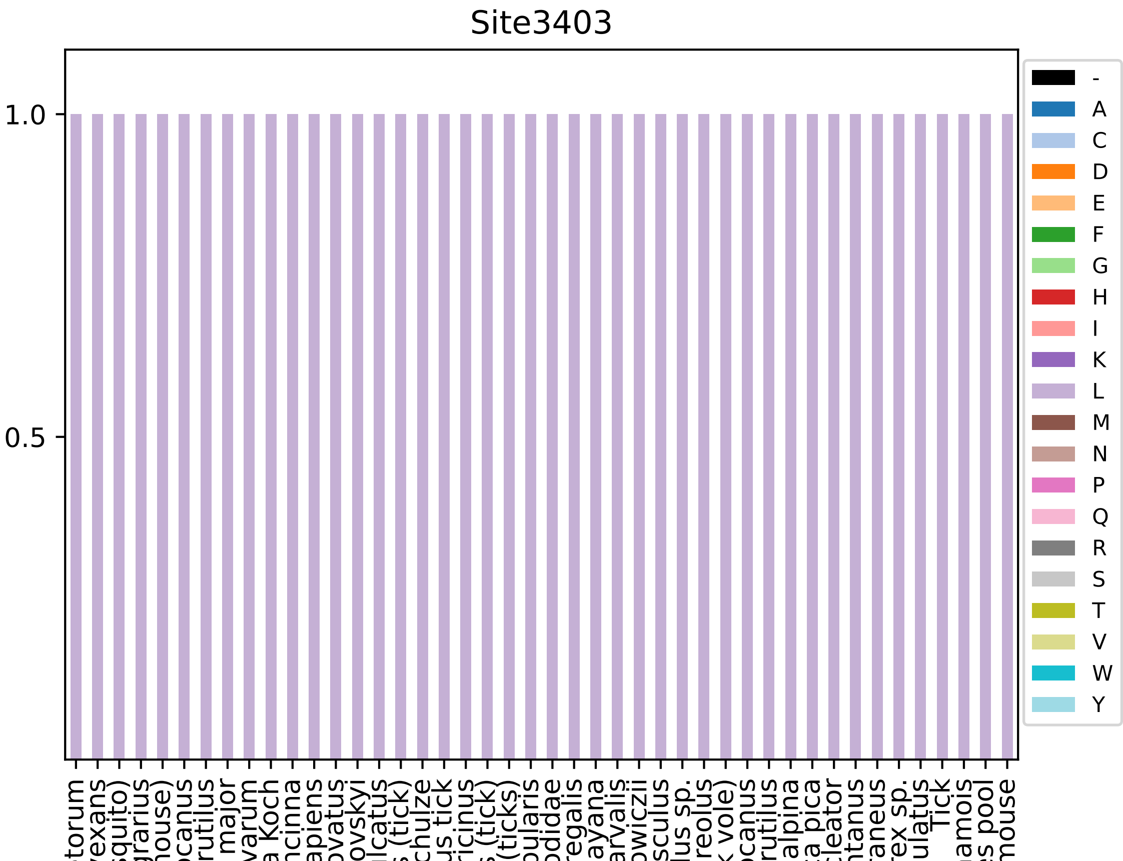

Site3403
1.0
0.5
-
A
C
D
E
F
G
H
I
K
L
M
N
P
Q
R
S
T
V
W
Y

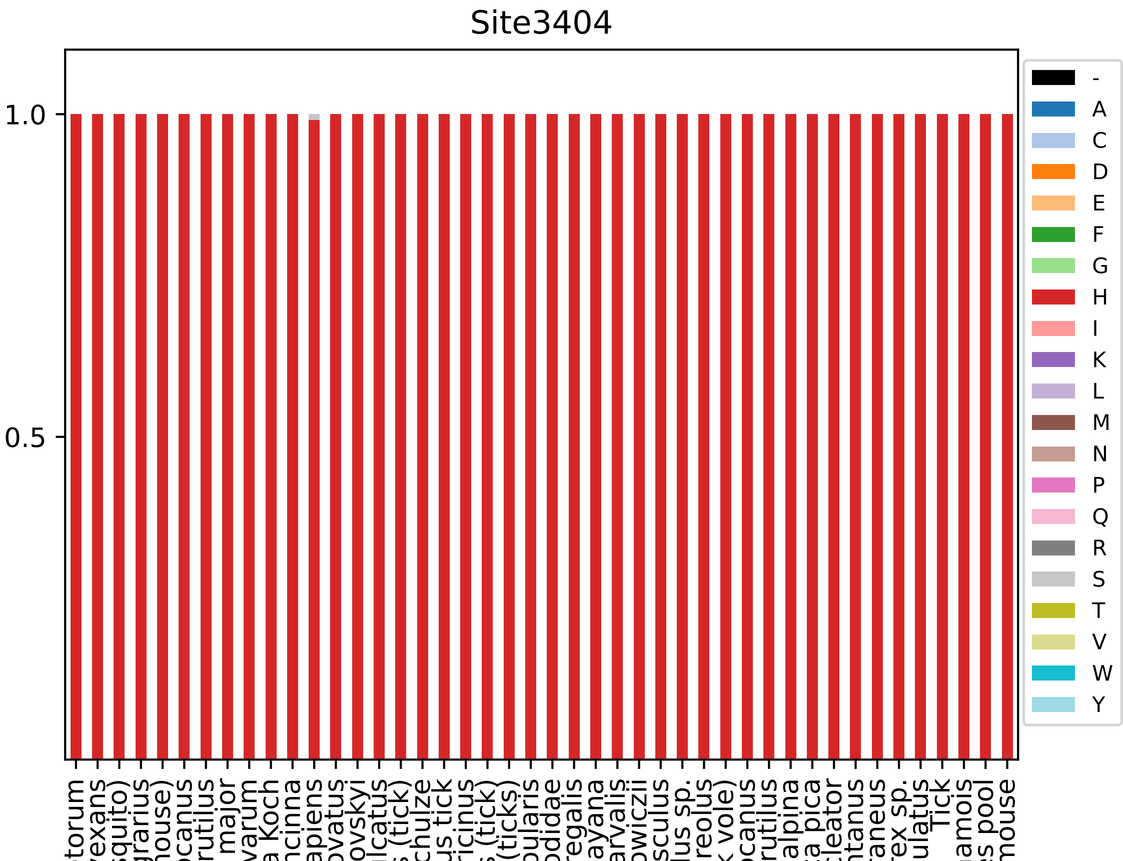

Site3404
1.0
0.5
-
A
C
D
E
F
G
H
I
K
L
M
N
P
Q
R
S
T
V
W
Y

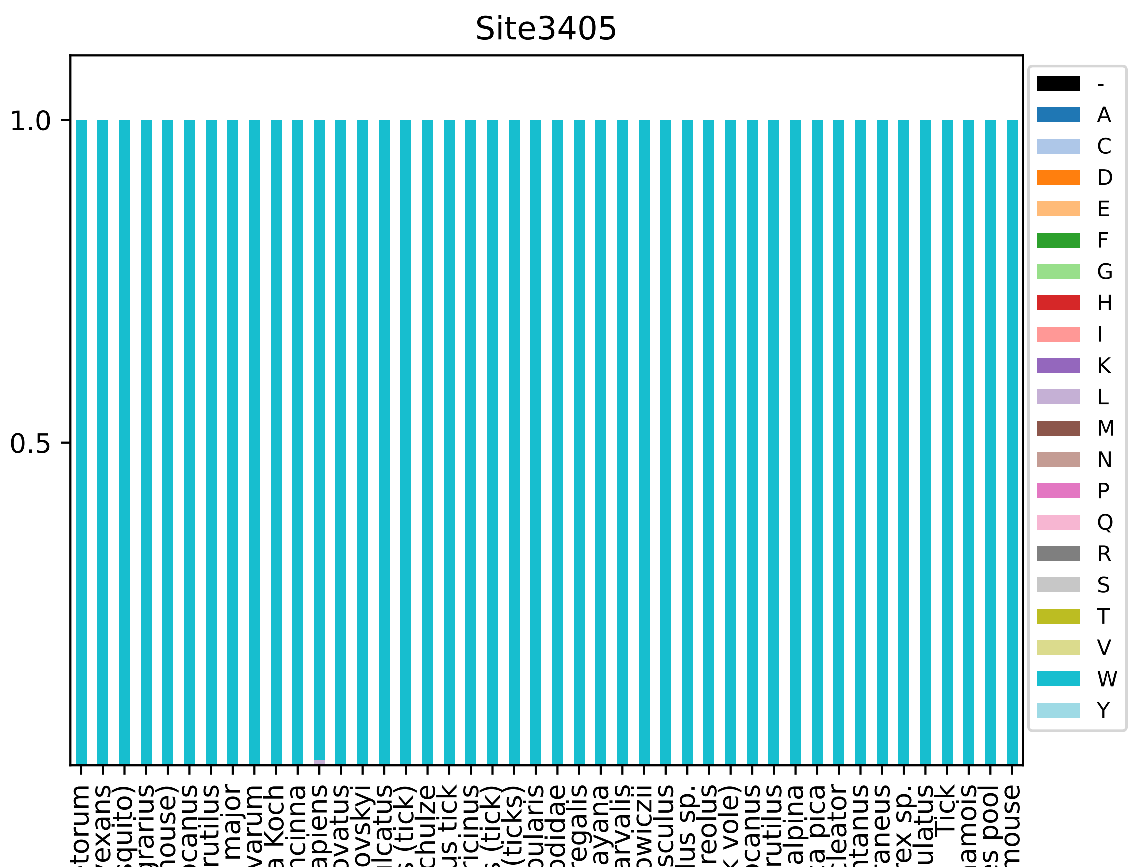
Site3405
1.0
0.5
-
A
C
D
E
F
G
H
I
K
L
M
N
P
Q
R
S
T
V
W
Y

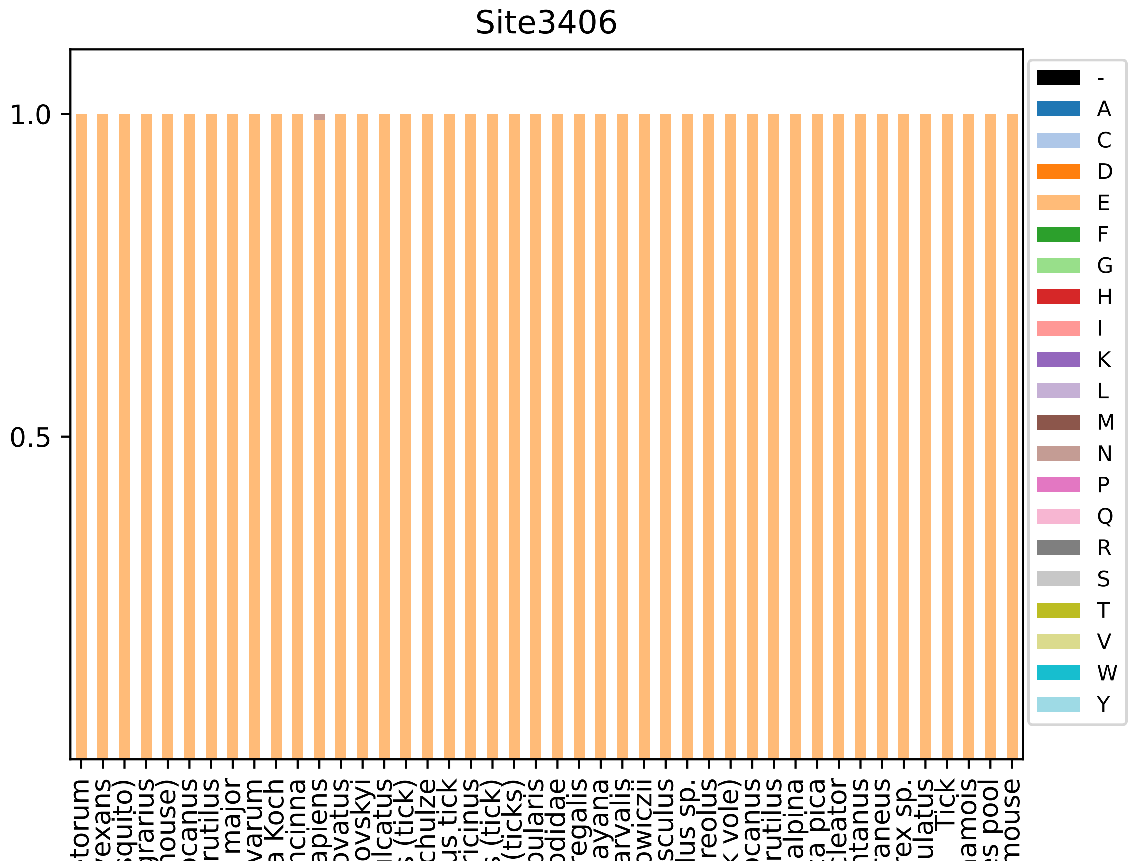
Site3406
1.0
0.5
-
A
C
D
E
F
G
H
I
K
L
M
N
P
Q
R
S
T
V
W
Y

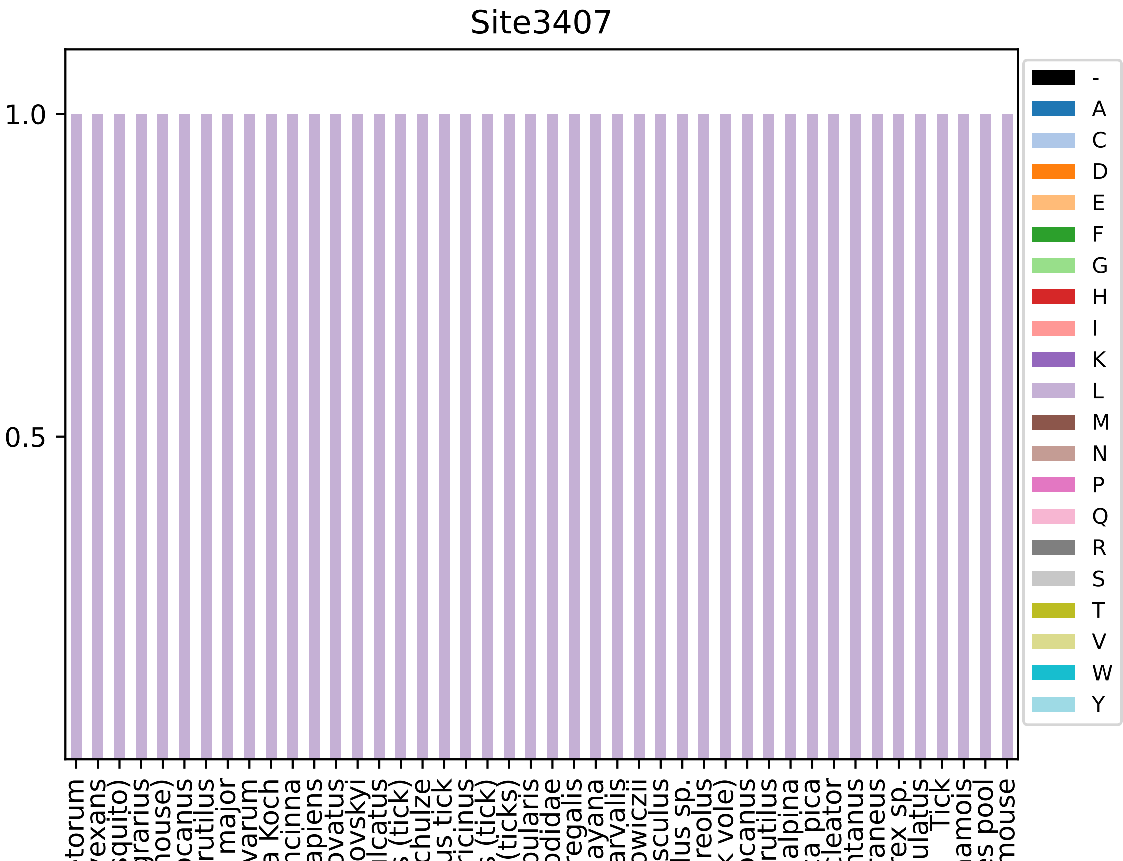
Site3407
1.0
0.5
-
A
C
D
E
F
G
H
I
K
L
M
N
P
Q
R
S
T
V
W
Y

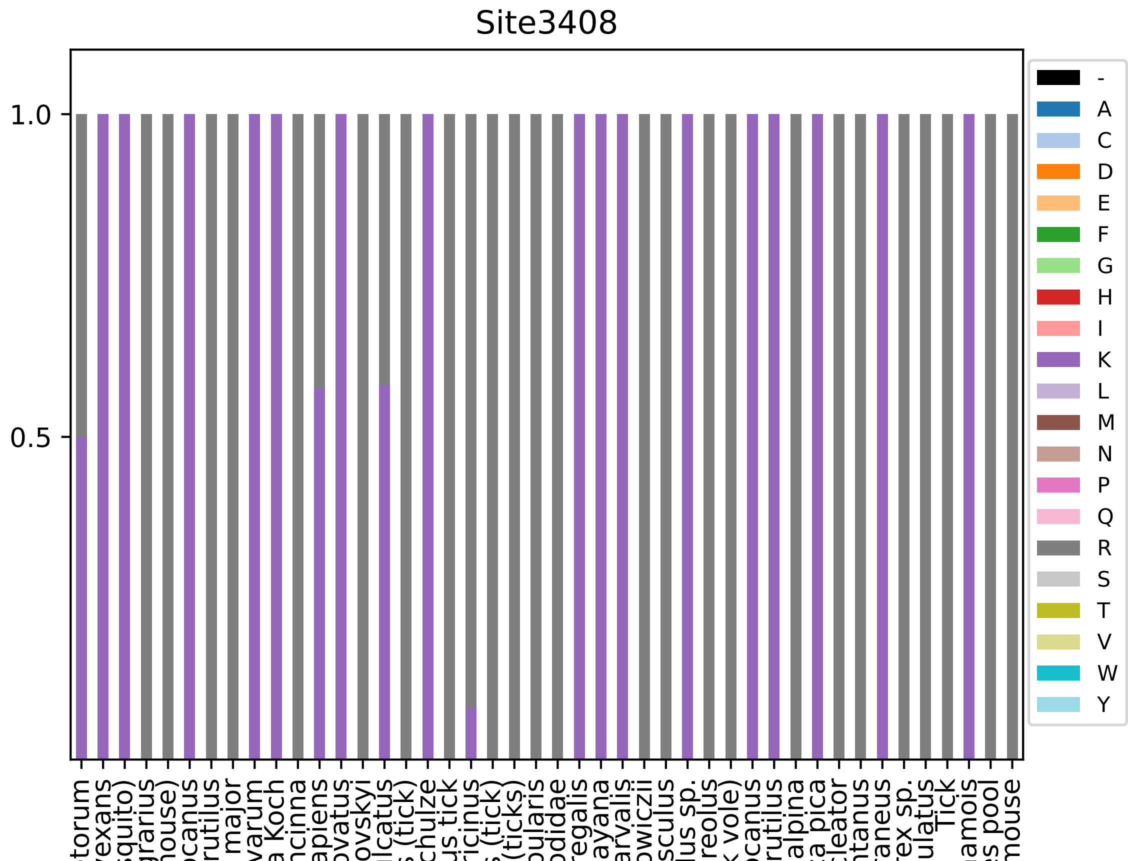
Site3408
1.0
0.5
-
A
C
D
E
F
G
H
I
K
L
M
N
P
Q
R
S
T
V
W
Y
torum
vexans
squito)
grarius
house)
ocanus
rutilus
major
varum
a Koch
ncinna
apiens
ovatus
ovskyi
ulcatus
s (tick)
chulze
us tick
ricinus
s (tick)
(ticks)
pularis
odidae
regalis
ayana
arvalis
owiczii
sculus
lus sp.
reolus
x vole)
ocanus
rutilus
alpina
a pica
tleator
ntanus
raneus
rex sp.
ulatus
Tick
amois
es pool
mouse

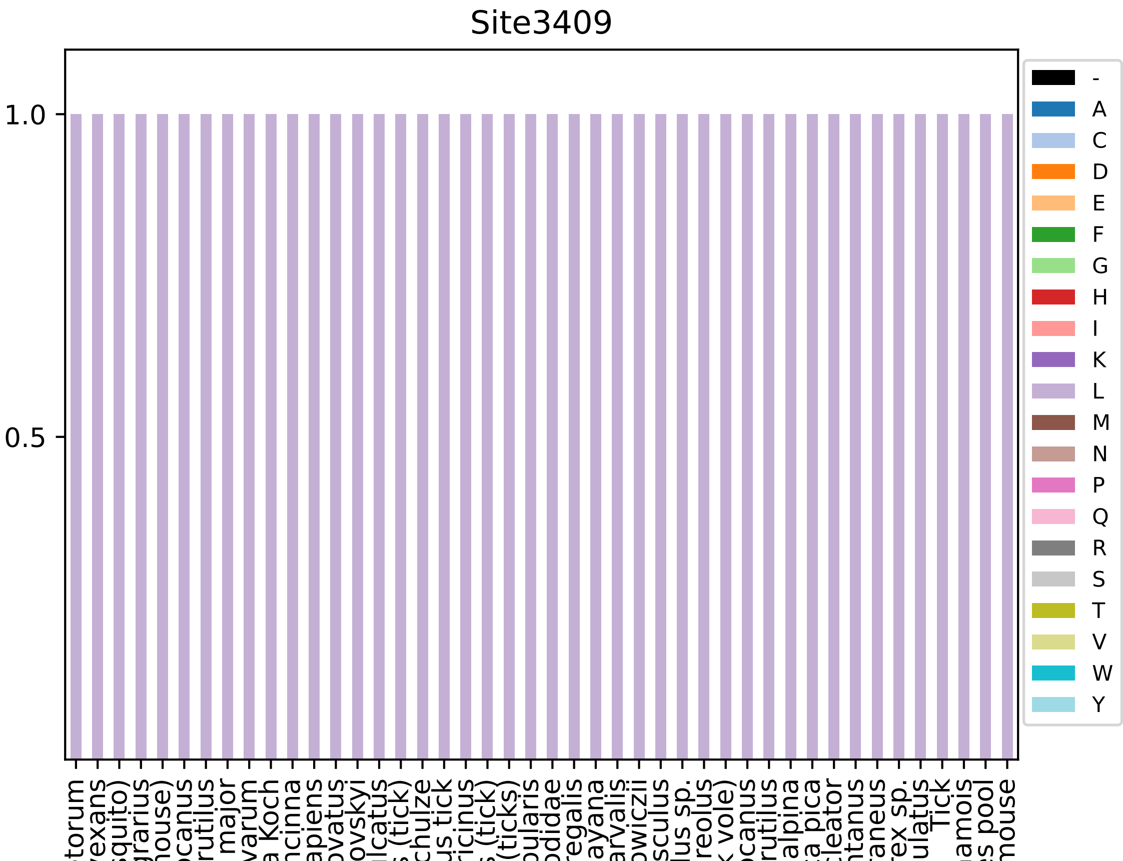
Site3409
1.0
0.5
-
A
C
D
E
F
G
H
I
K
L
M
N
P
Q
R
S
T
V
W
Y
Tick

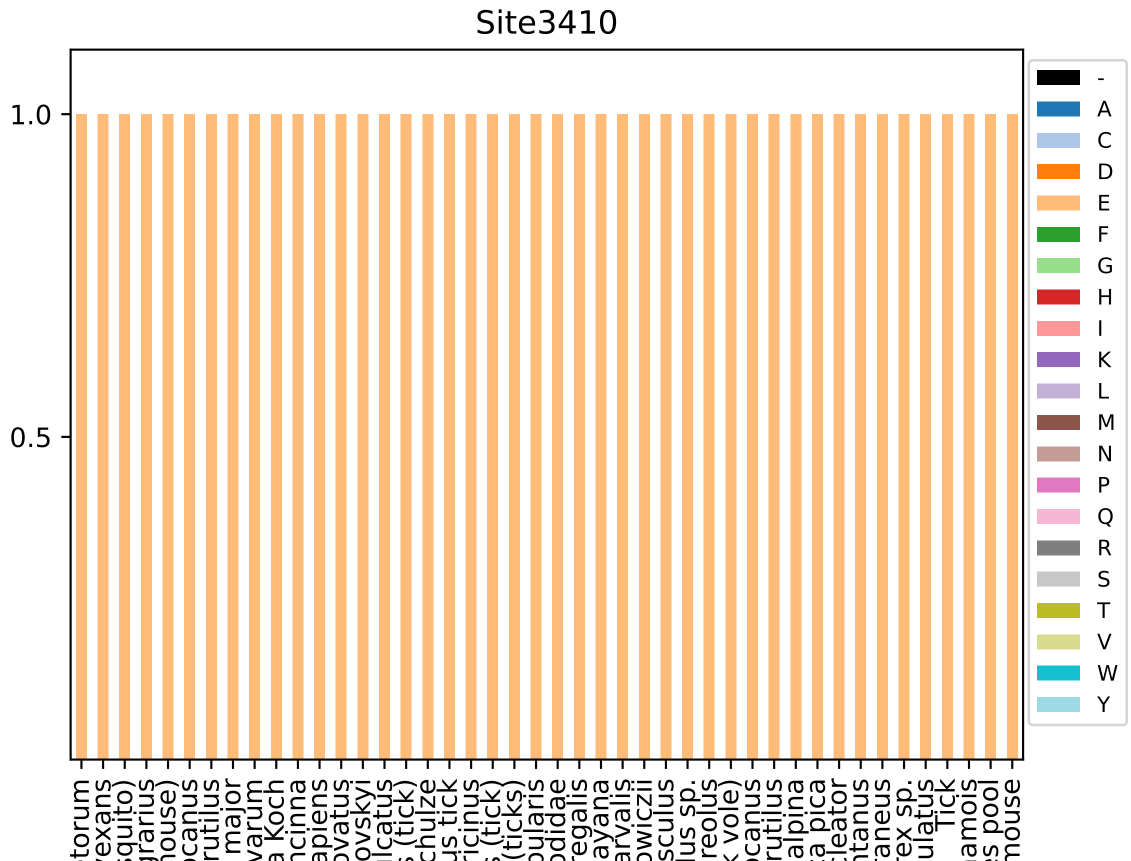
Site3410
1.0
0.5
-
A
C
D
E
F
G
H
I
K
L
M
N
P
Q
R
S
T
V
W
Y

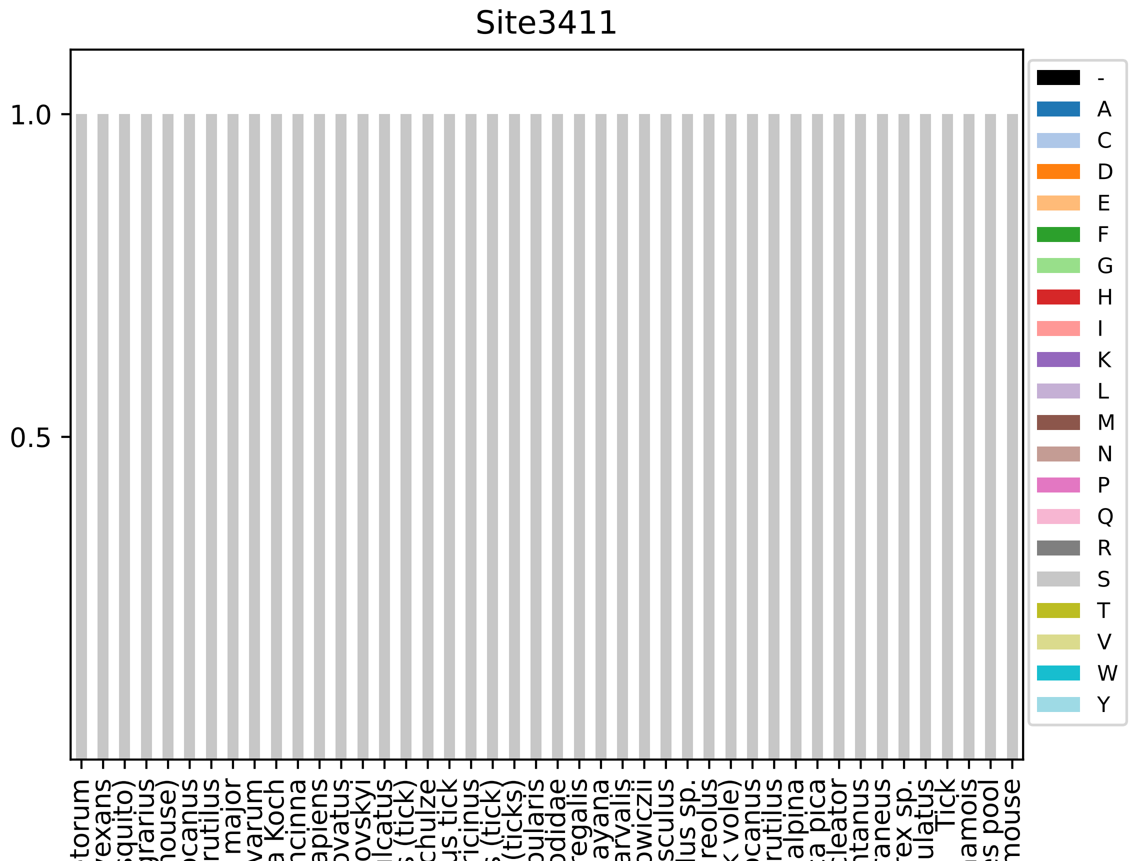

Site3411
1.0
0.5
-
A
C
D
E
F
G
H
I
K
L
M
N
P
Q
R
S
T
V
W
Y

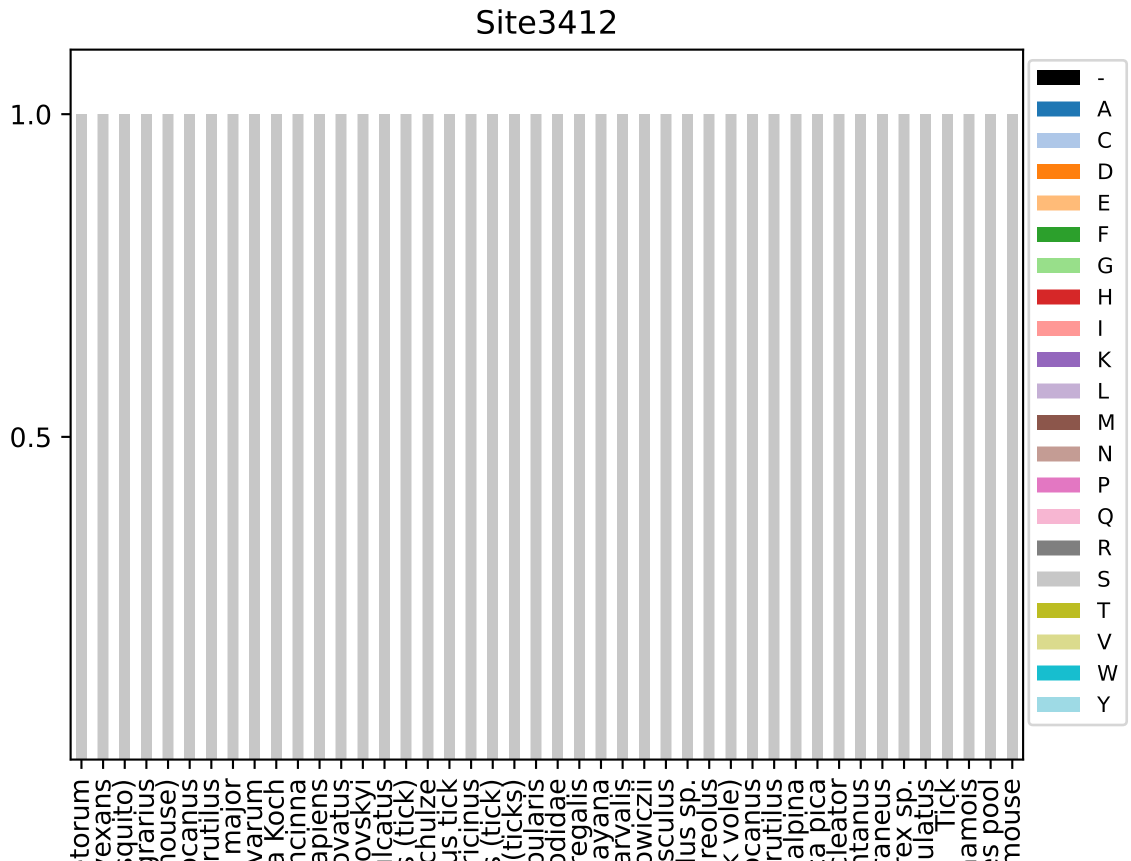
Site3412
1.0
0.5
-
A
C
D
E
F
G
H
I
K
L
M
N
P
Q
R
S
T
V
W
Y

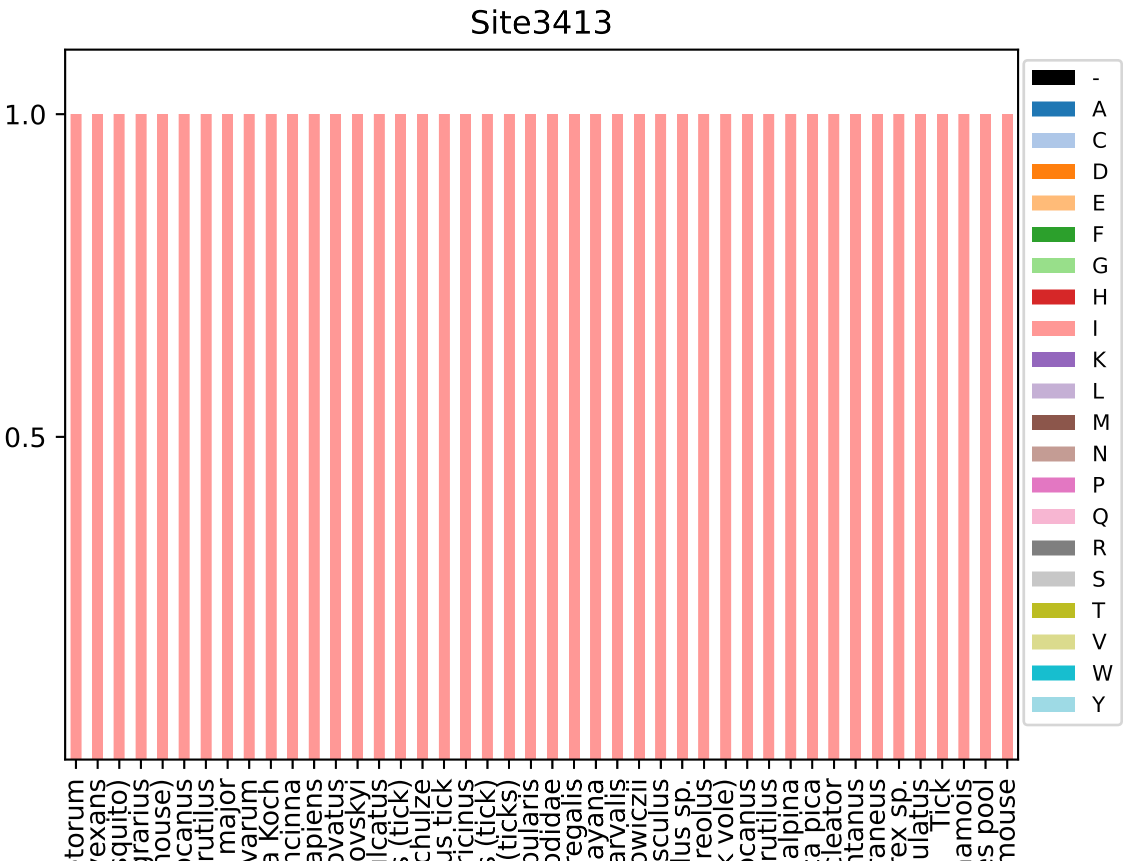
Site3413
1.0
0.5
-
A
C
D
E
F
G
H
I
K
L
M
N
P
Q
R
S
T
V
W
Y

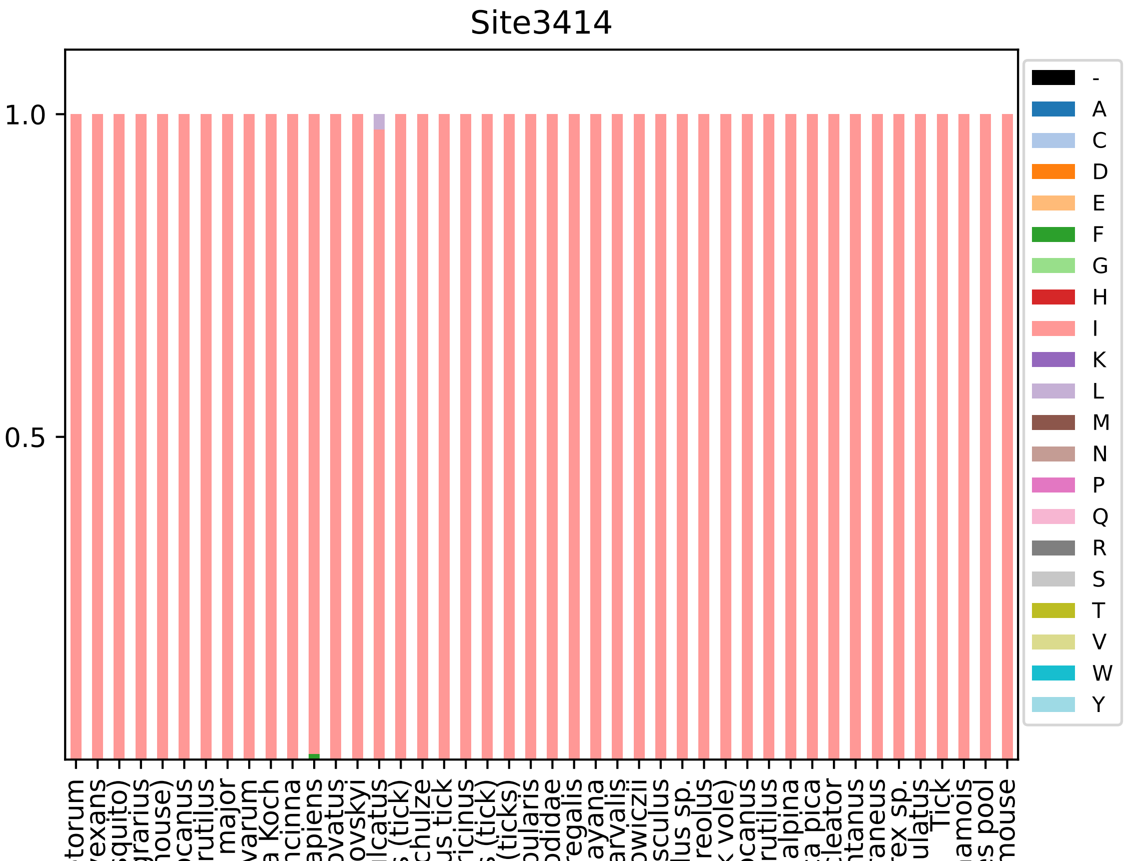
Site3414
1.0
0.5
-
A
C
D
E
F
G
H
I
K
L
M
N
P
Q
R
S
T
V
W
Y

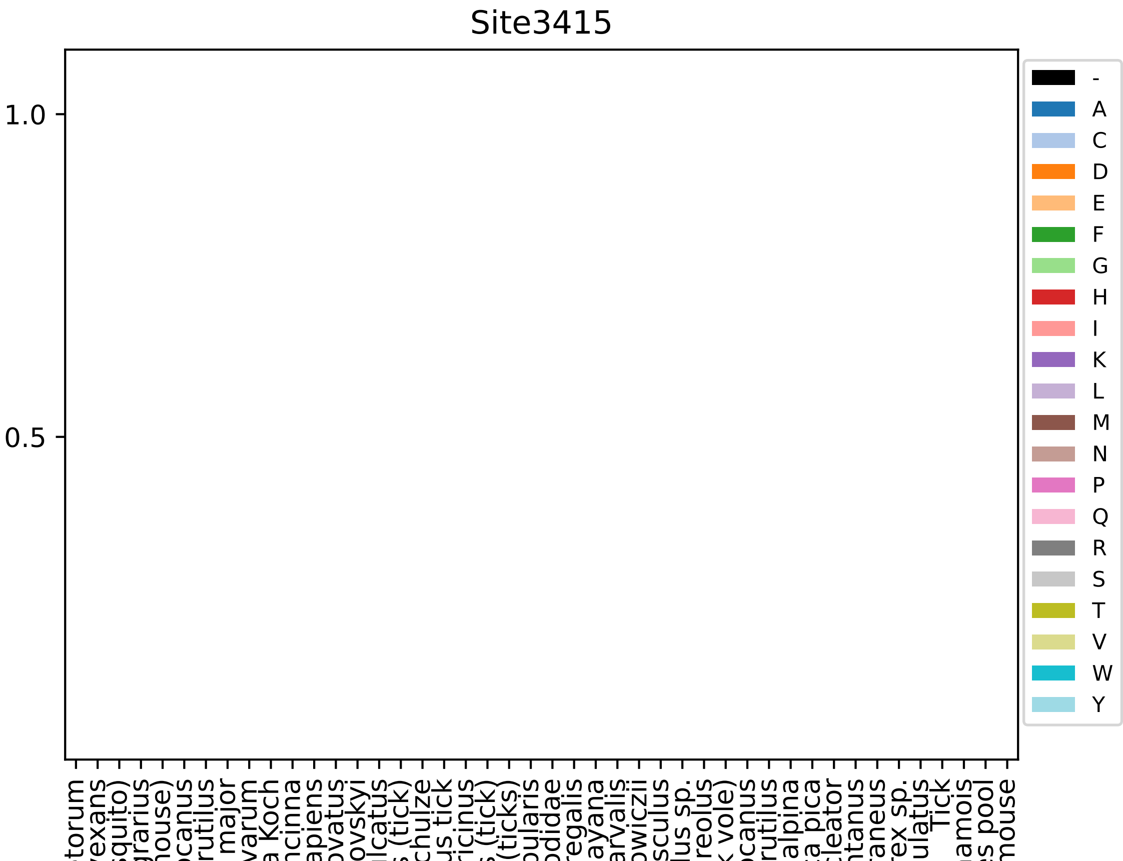
Site3415
1.0
0.5
-
A
C
D
E
F
G
H
I
K
L
M
N
P
Q
R
S
T
V
W
Y
