# Supplementary material for: Evolutionary Patterns and Genotype-Specific Amino Acid Mutations of Tick-Borne Encephalitis Virus
Source: Int J Mol Sci. 2025 Jan 23;26(3):954. doi: 10.3390/ijms26030954 (PMC11817229; doi:10.3390/ijms26030954)
Supplement: Supplementary file 1 [file ijms-26-00954-s001.zip › ijms-3423653-supplementary/Supplementary Data S2.zip!/year/year_aa_mutation.pdf]

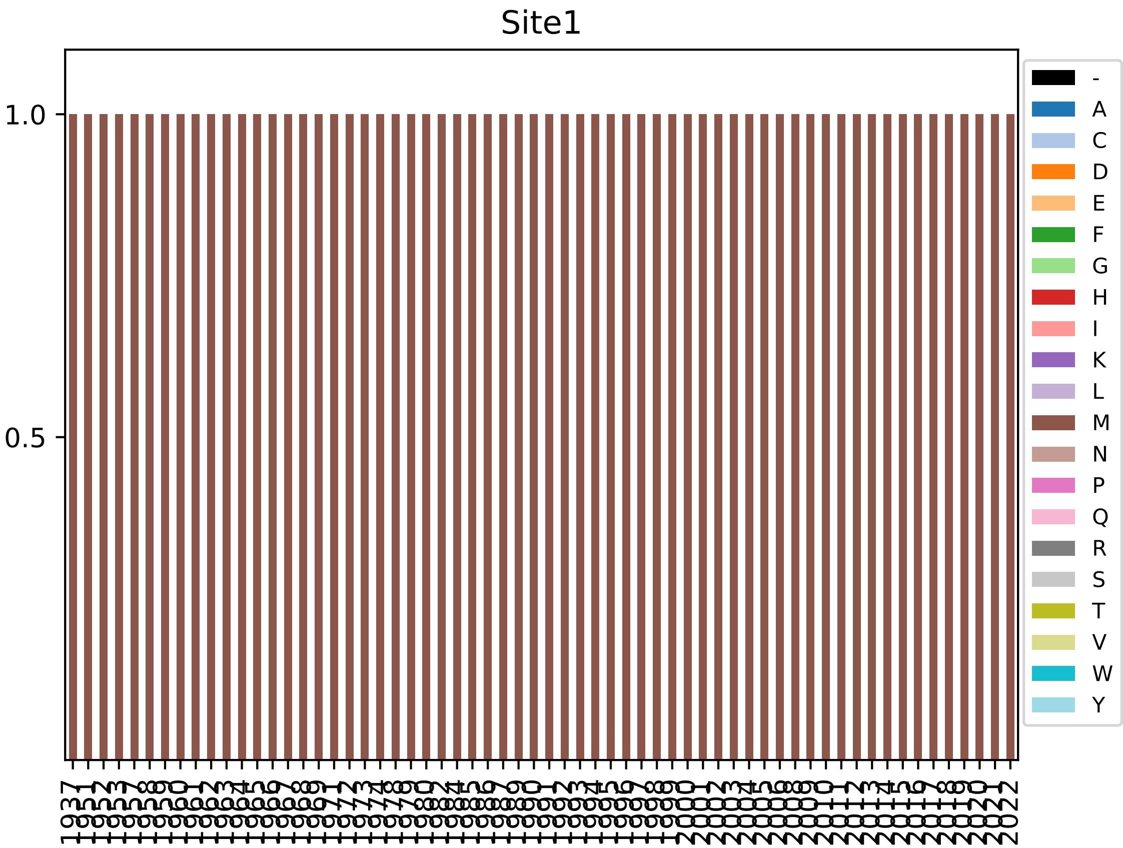
Site1
1.0
0.5
-
A
C
D
E
F
G
H
I
K
L
M
N
P
Q
R
S
T
V
W
Y

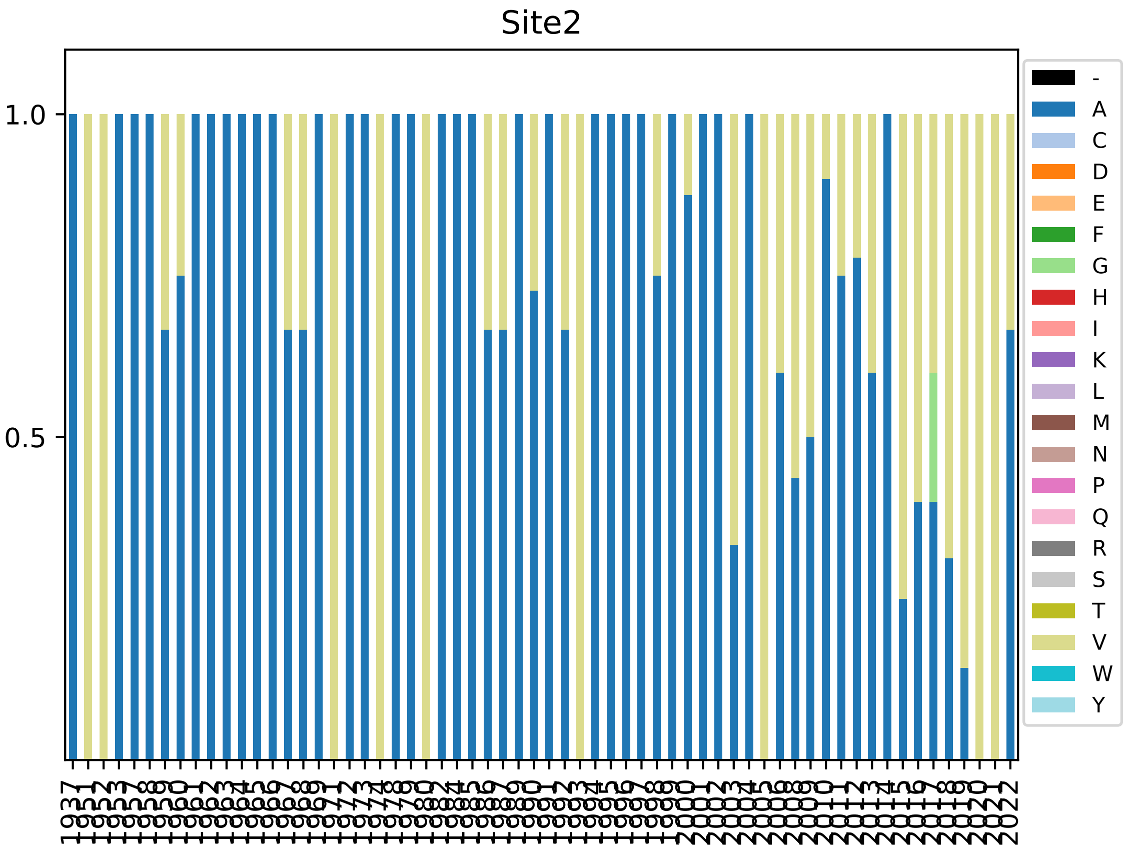
Site2
1.0
0.5
-
A
C
D
E
F
G
H
I
K
L
M
N
P
Q
R
S
T
V
W
Y

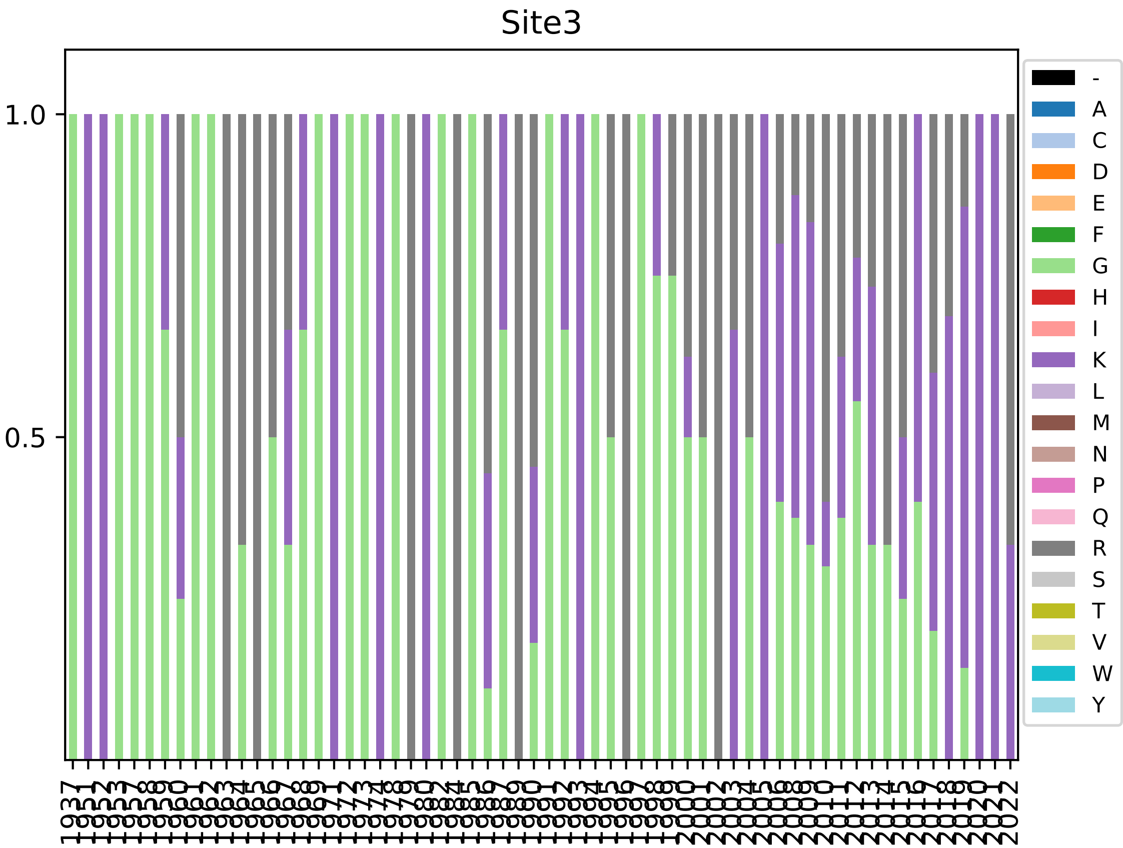
Site3
1.0
0.5
-
A
C
D
E
F
G
H
I
K
L
M
N
P
Q
R
S
T
V
W
Y

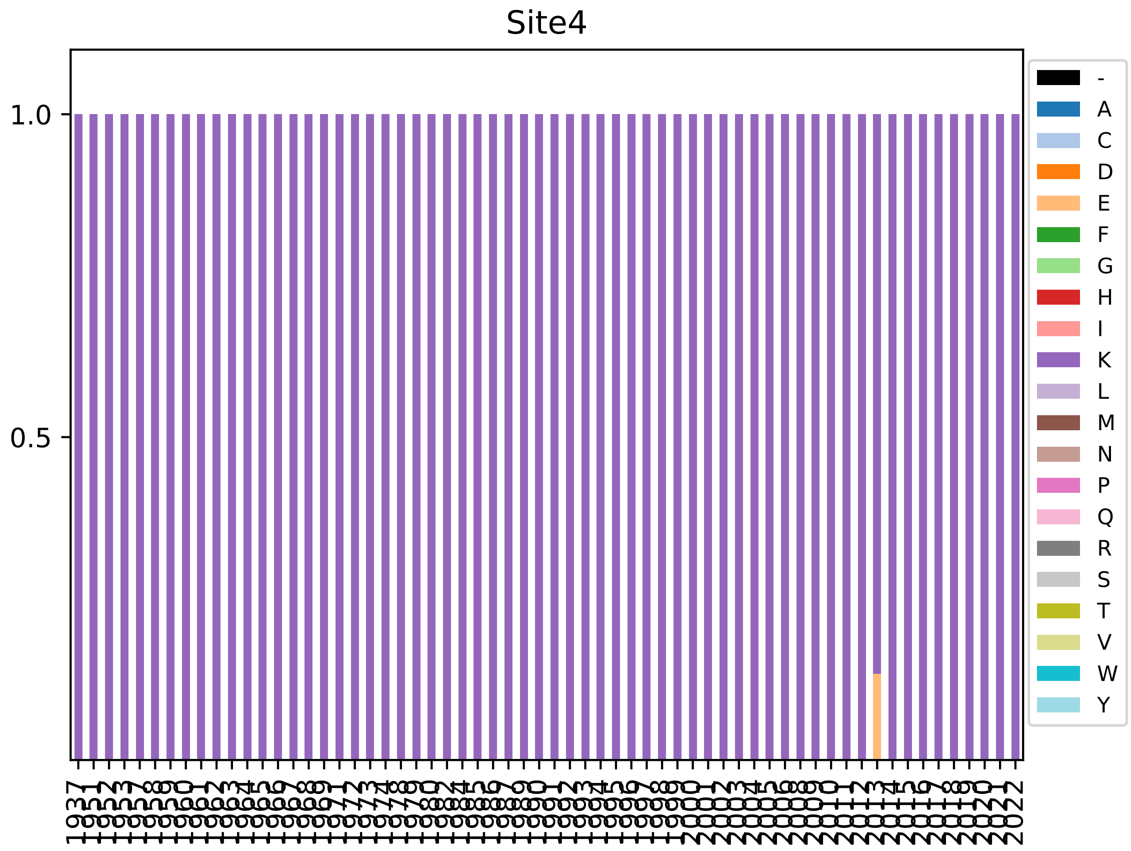
Site4
1.0
0.5
-
A
C
D
E
F
G
H
I
K
L
M
N
P
Q
R
S
T
V
W
Y

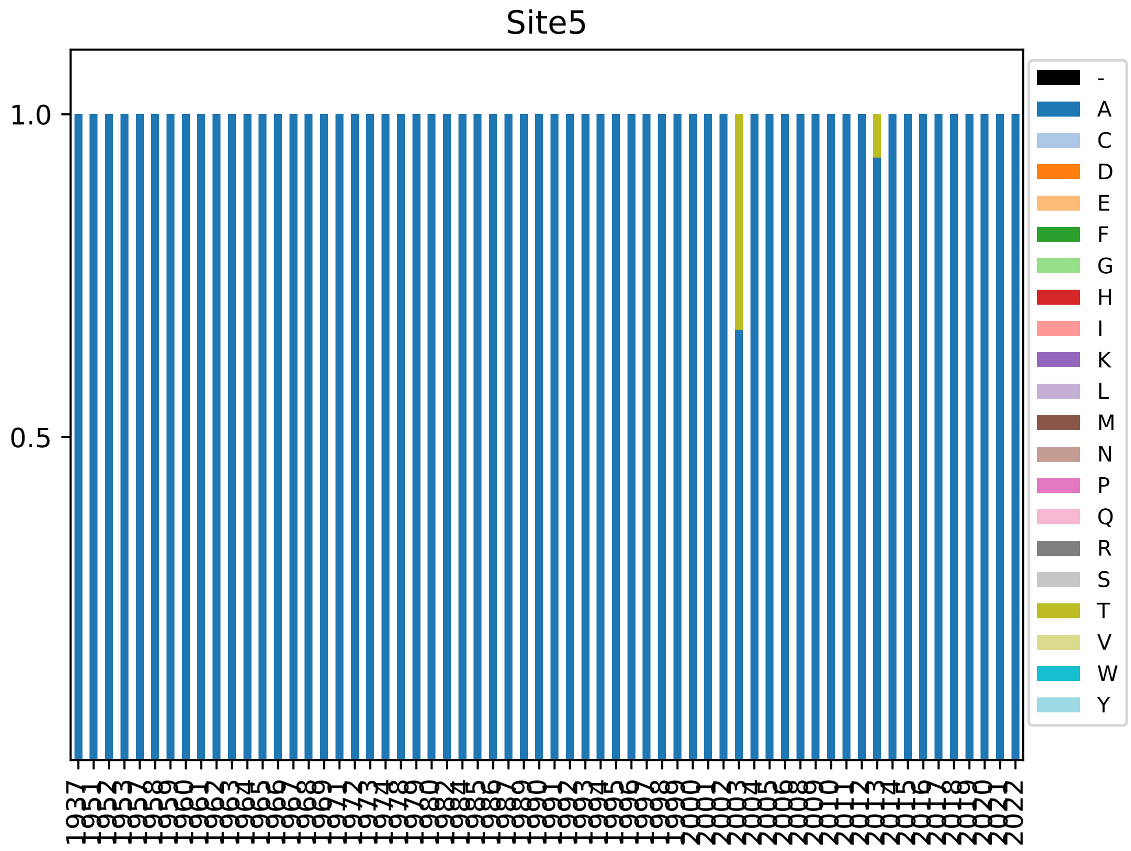
Site5
1.0
0.5
-
A
C
D
E
F
G
H
I
K
L
M
N
P
Q
R
S
T
V
W
Y

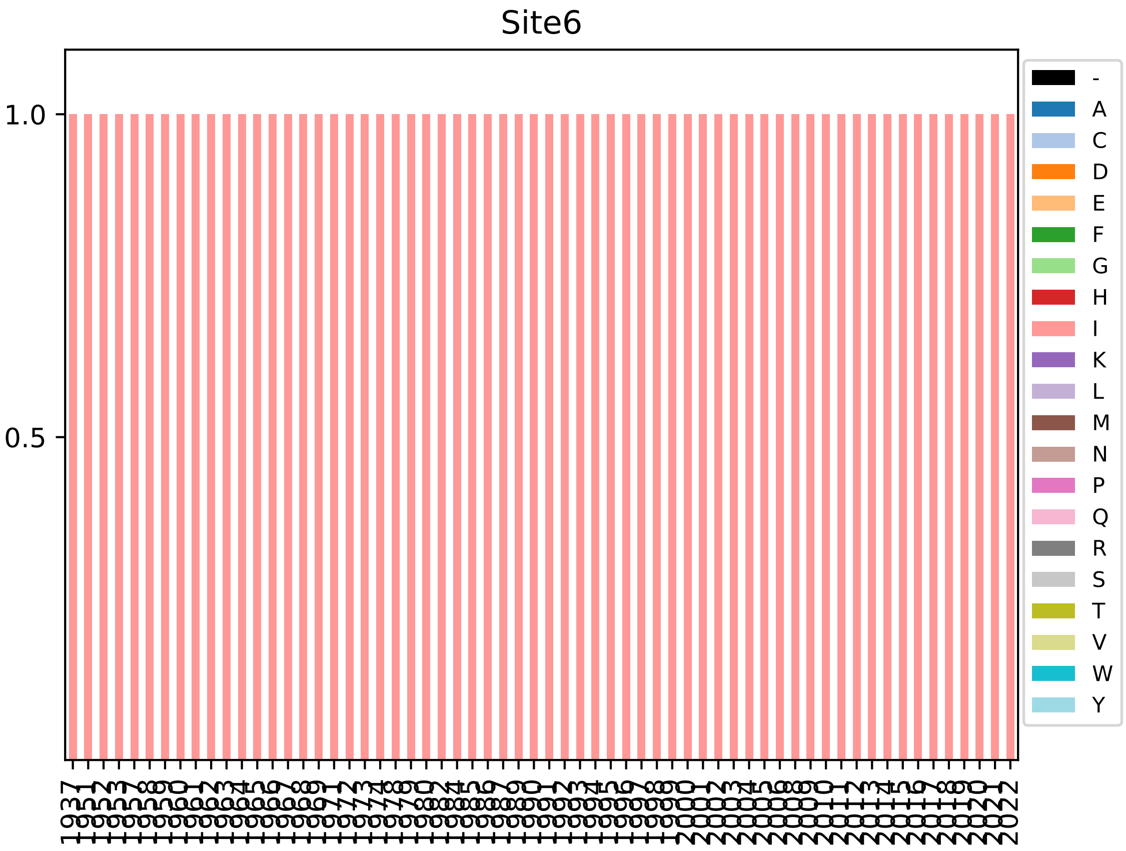
Site6
1.0
0.5
-
A
C
D
E
F
G
H
I
K
L
M
N
P
Q
R
S
T
V
W
Y

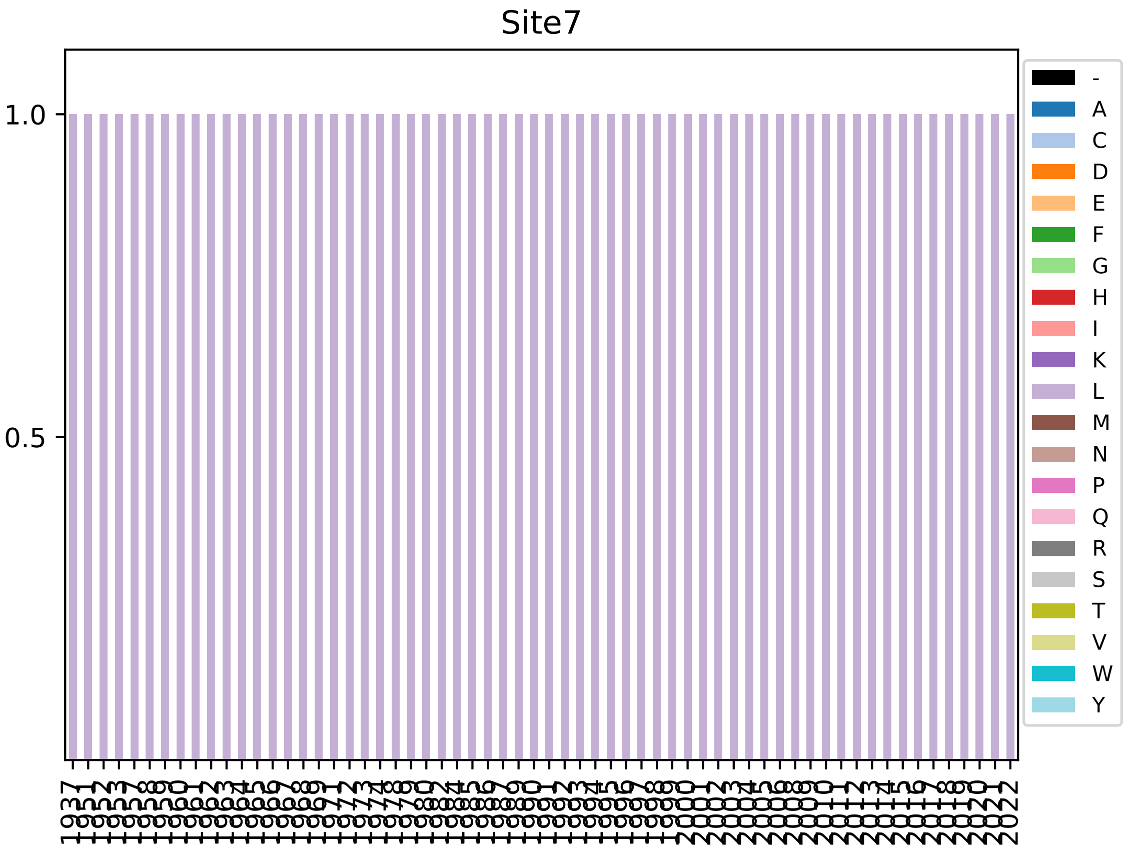
Site7
1.0
0.5
-
A
C
D
E
F
G
H
I
K
L
M
N
P
Q
R
S
T
V
W
Y

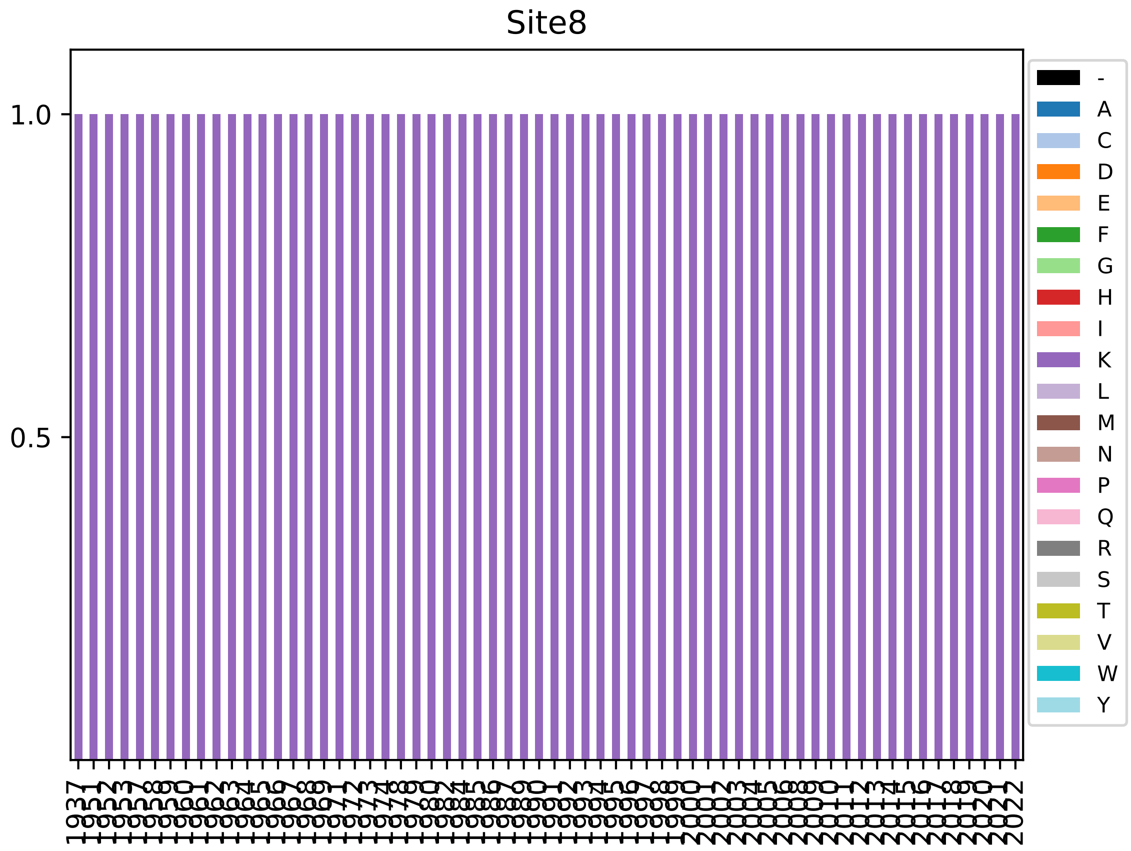
Site8
1.0
0.5
-
A
C
D
E
F
G
H
I
K
L
M
N
P
Q
R
S
T
V
W
Y

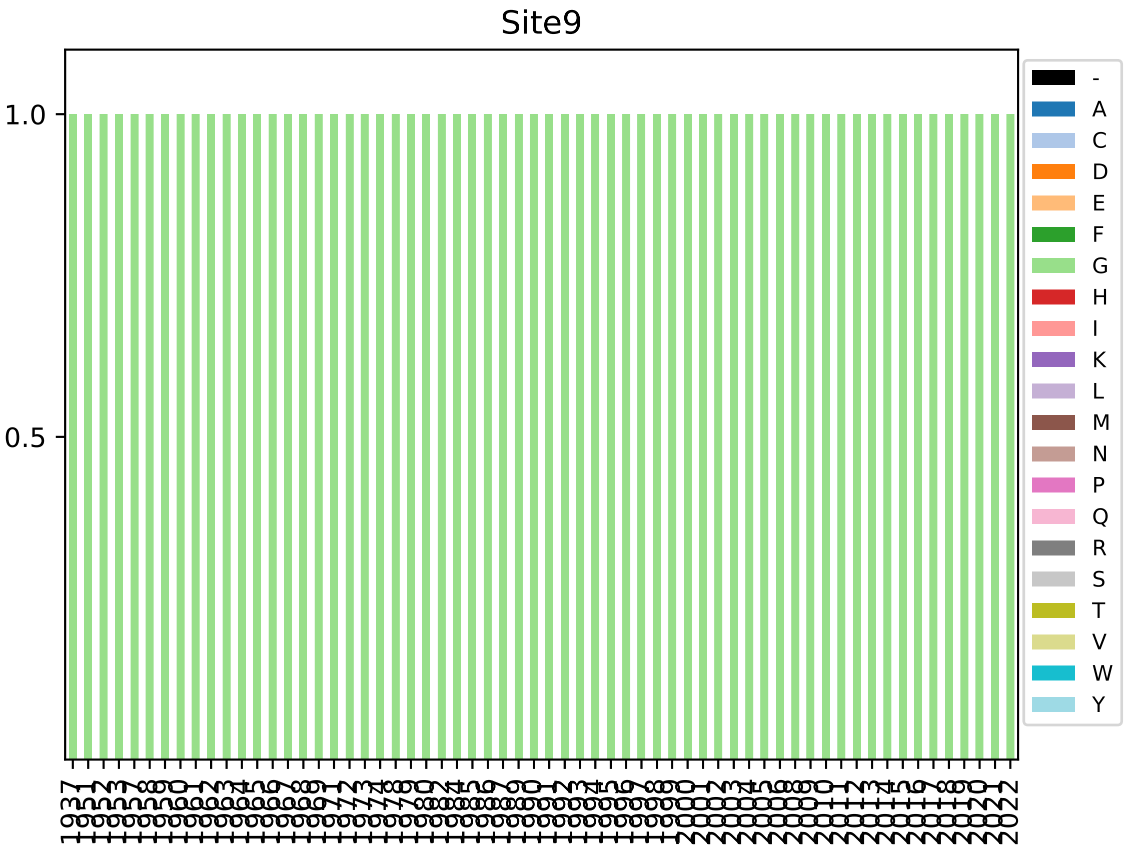
Site9
1.0
0.5
-
A
C
D
E
F
G
H
I
K
L
M
N
P
Q
R
S
T
V
W
Y

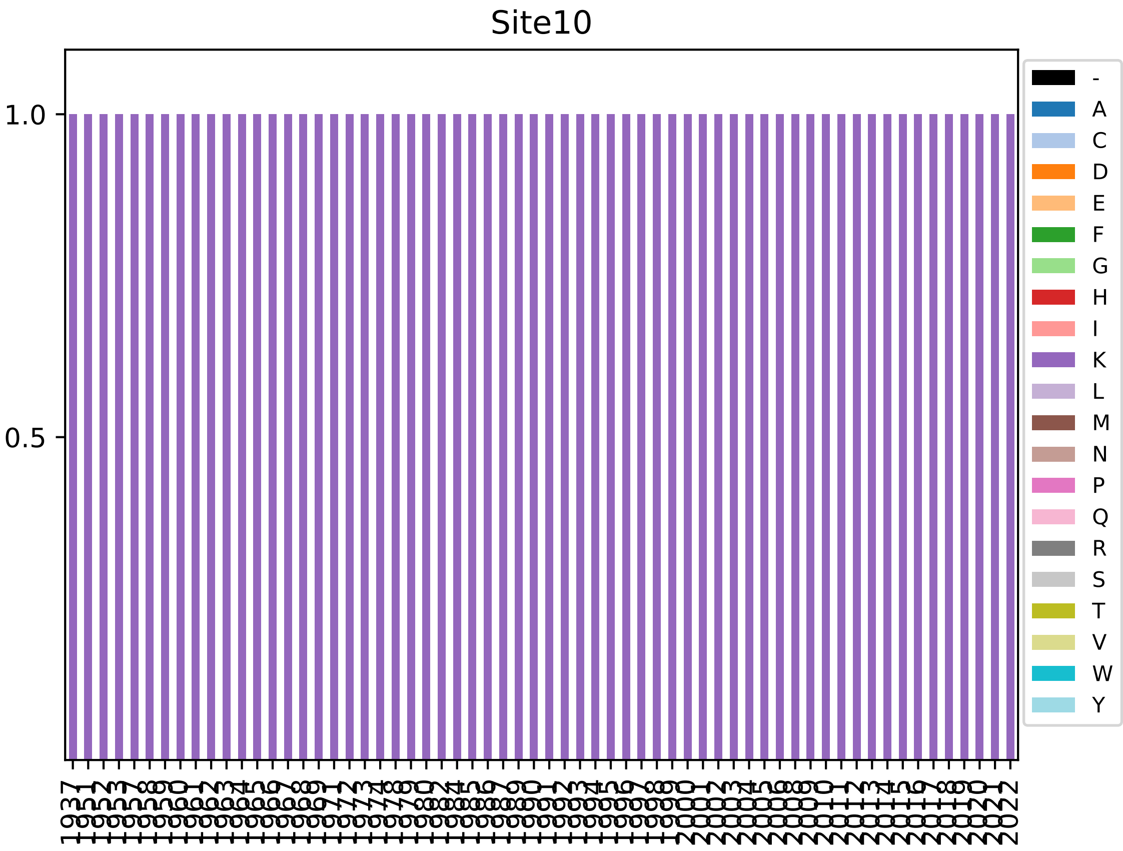
Site10
1.0
0.5
-
A
C
D
E
F
G
H
I
K
L
M
N
P
Q
R
S
T
V
W
Y

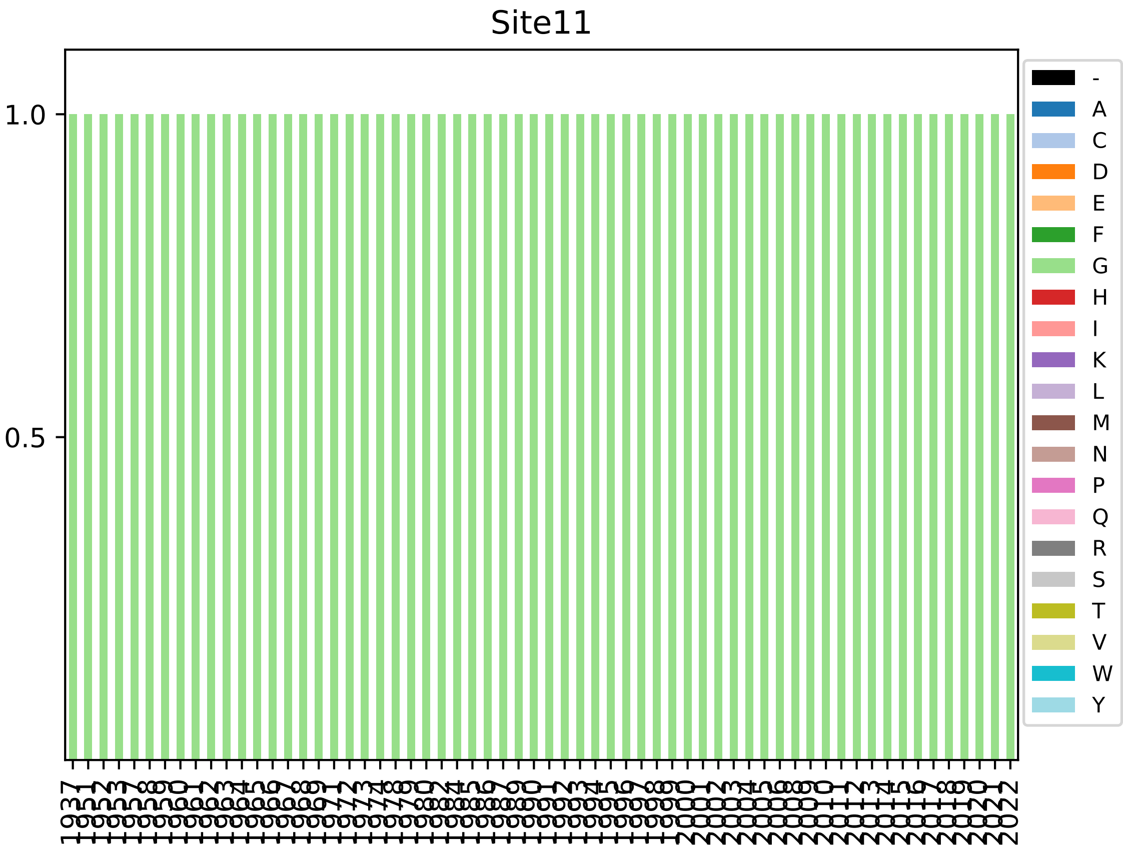
Site11
1.0
0.5
-
A
C
D
E
F
G
H
I
K
L
M
N
P
Q
R
S
T
V
W
Y

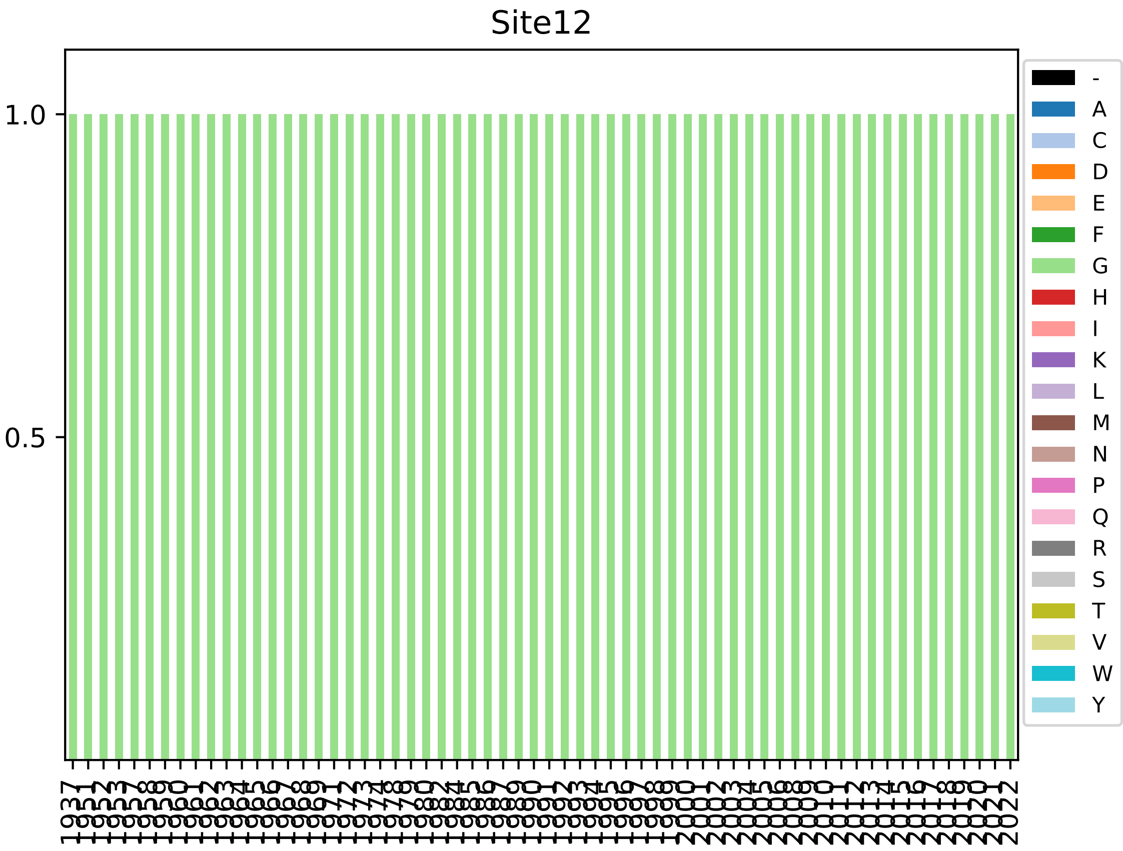
Site12
1.0
0.5
-
A
C
D
E
F
G
H
I
K
L
M
N
P
Q
R
S
T
V
W
Y

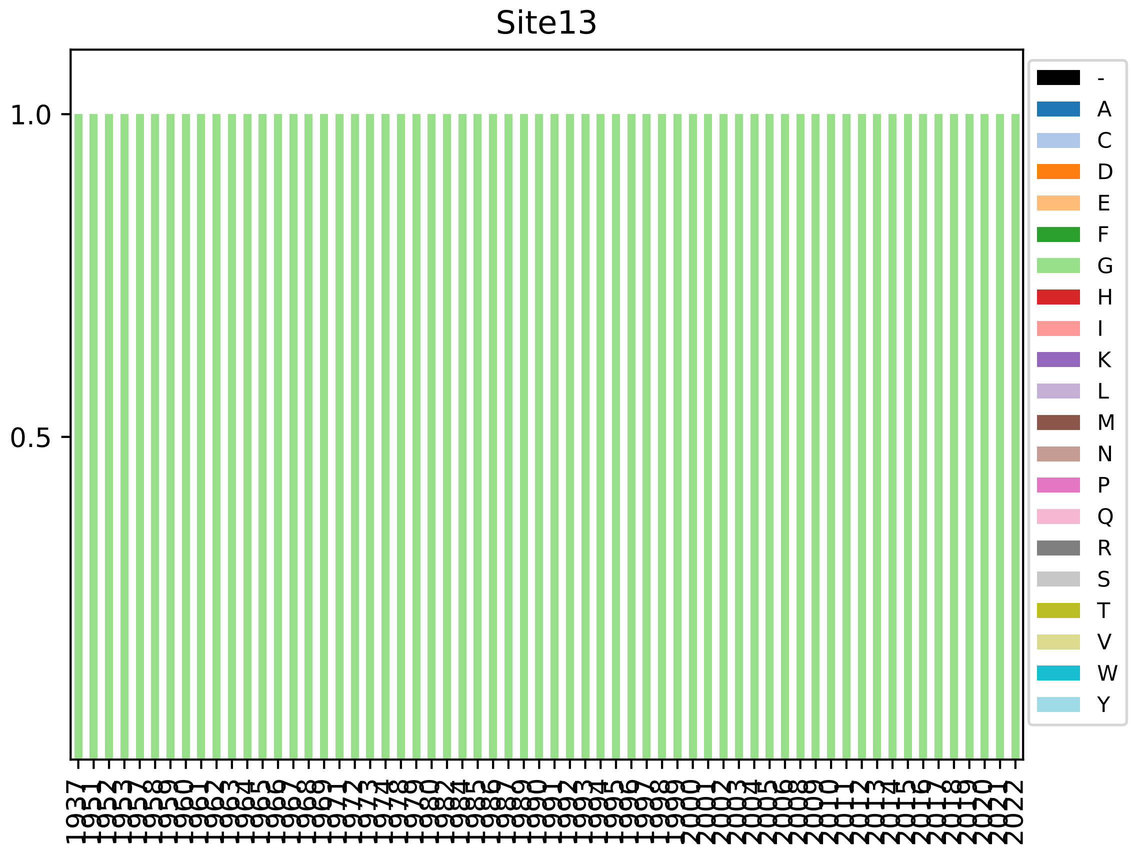
Site13
1.0
0.5
-
A
C
D
E
F
G
H
I
K
L
M
N
P
Q
R
S
T
V
W
Y

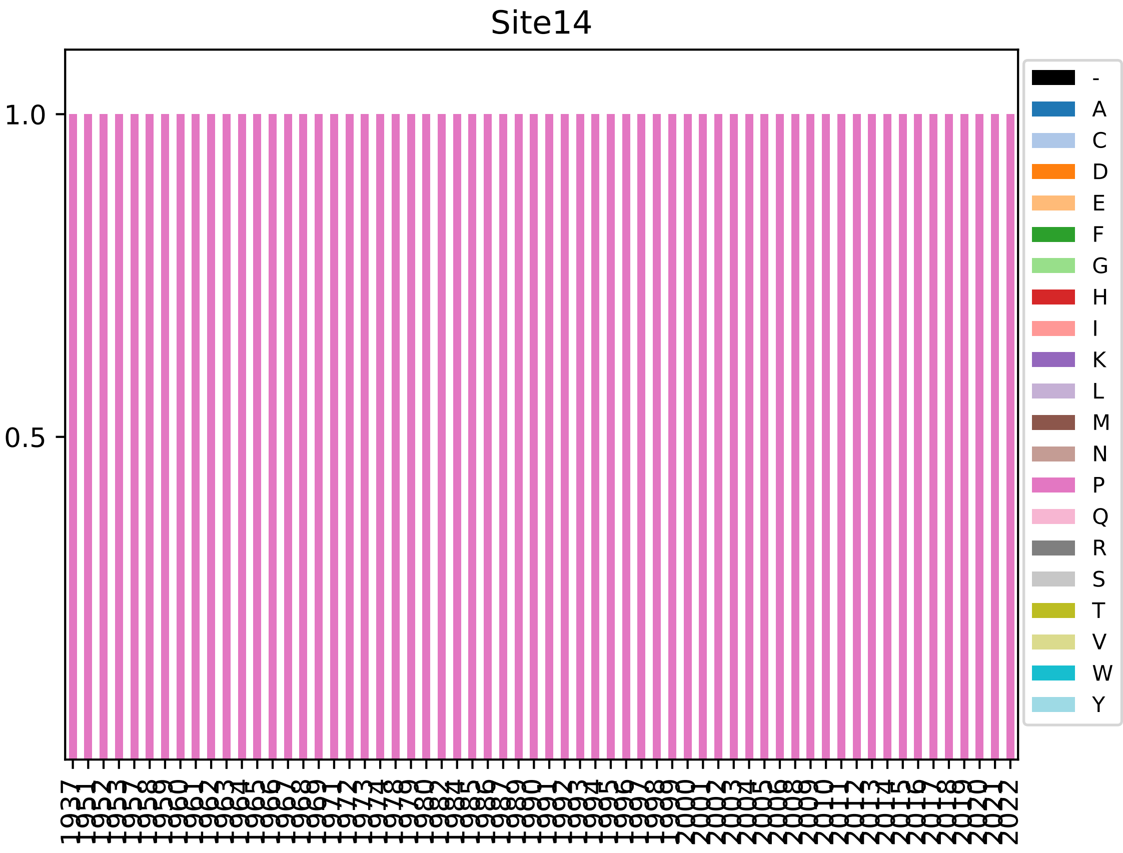
Site14
1.0
0.5
-
A
C
D
E
F
G
H
I
K
L
M
N
P
Q
R
S
T
V
W
Y

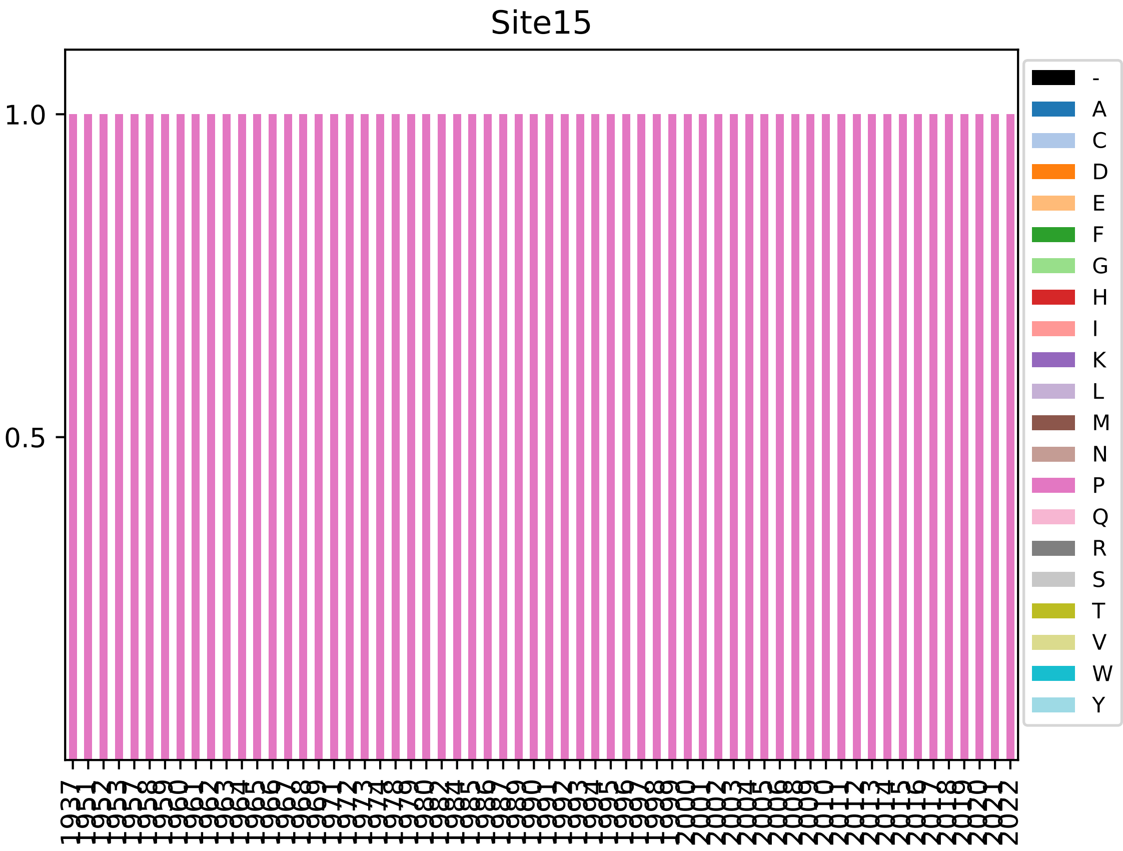
Site15
1.0
0.5
-
A
C
D
E
F
G
H
I
K
L
M
N
P
Q
R
S
T
V
W
Y

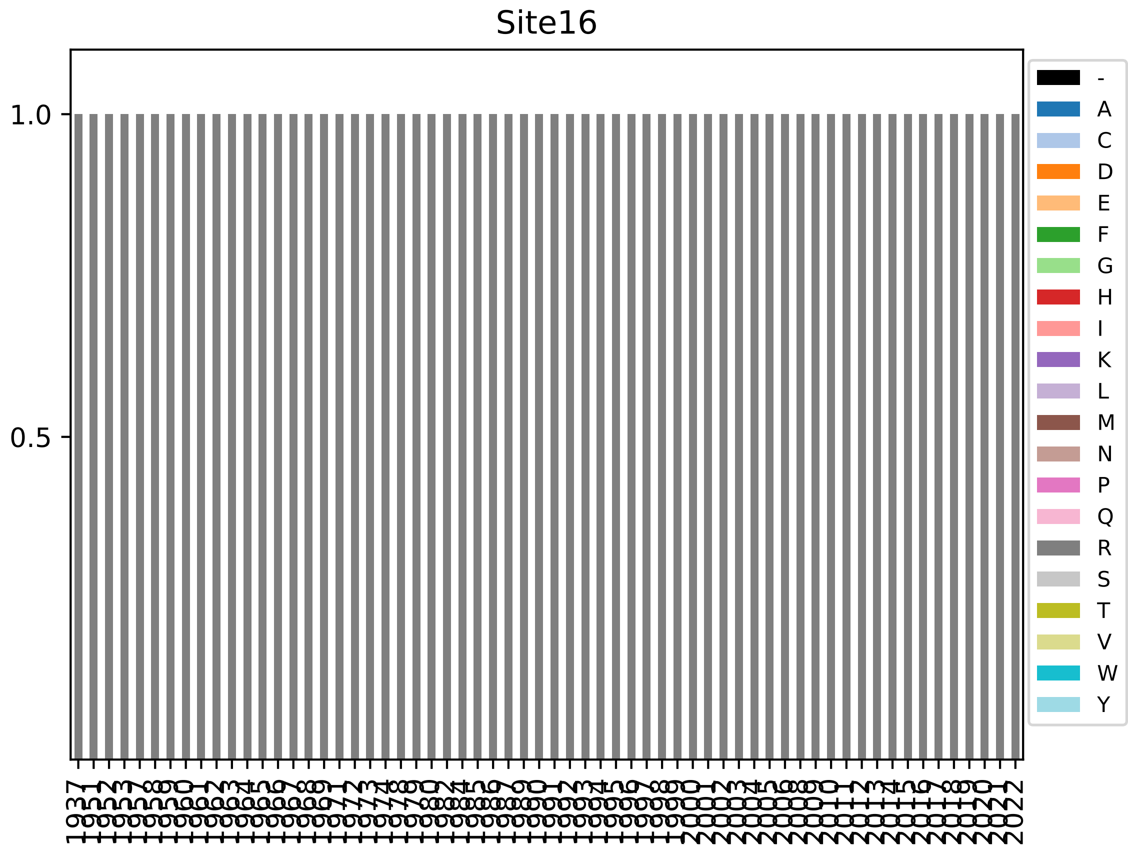
Site16
1.0
0.5
-
A
C
D
E
F
G
H
I
K
L
M
N
P
Q
R
S
T
V
W
Y

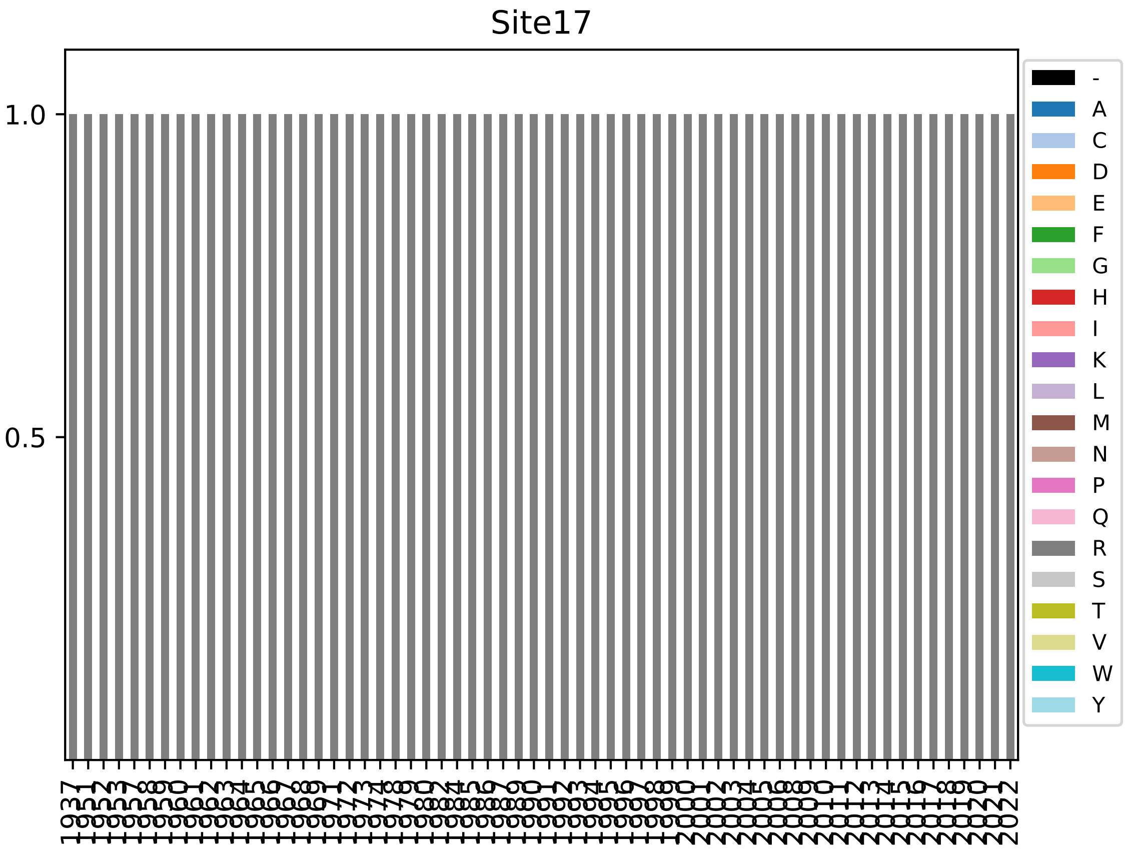
Site17
1.0
0.5
-
A
C
D
E
F
G
H
I
K
L
M
N
P
Q
R
S
T
V
W
Y

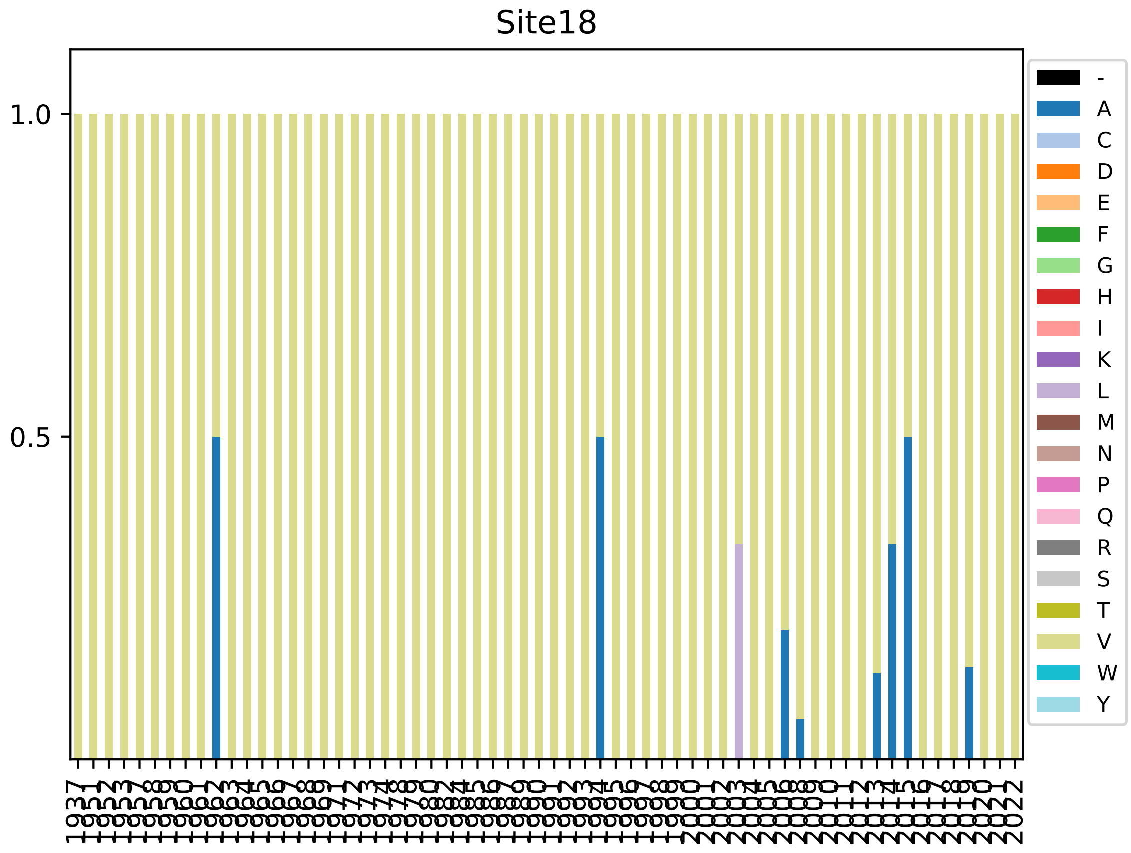
Site18
1.0
0.5
-
A
C
D
E
F
G
H
I
K
L
M
N
P
Q
R
S
T
V
W
Y

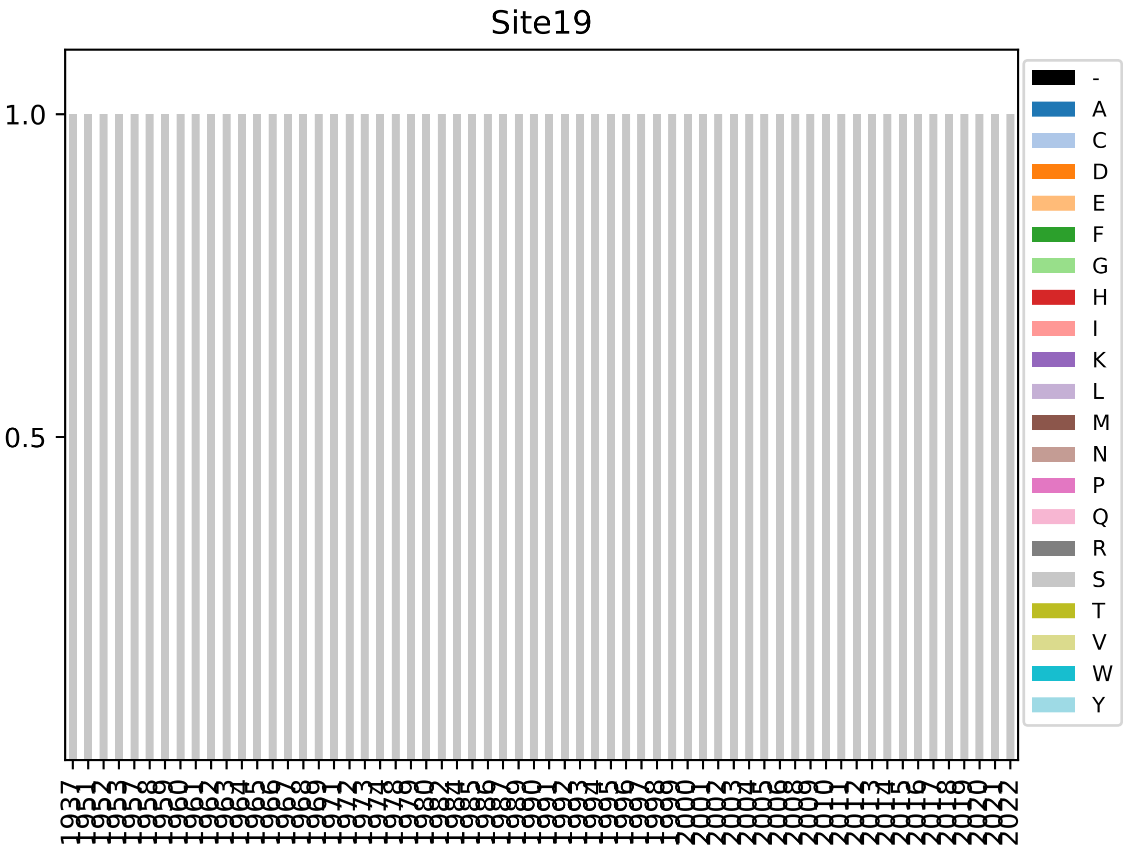
Site19
1.0
0.5
-
A
C
D
E
F
G
H
I
K
L
M
N
P
Q
R
S
T
V
W
Y

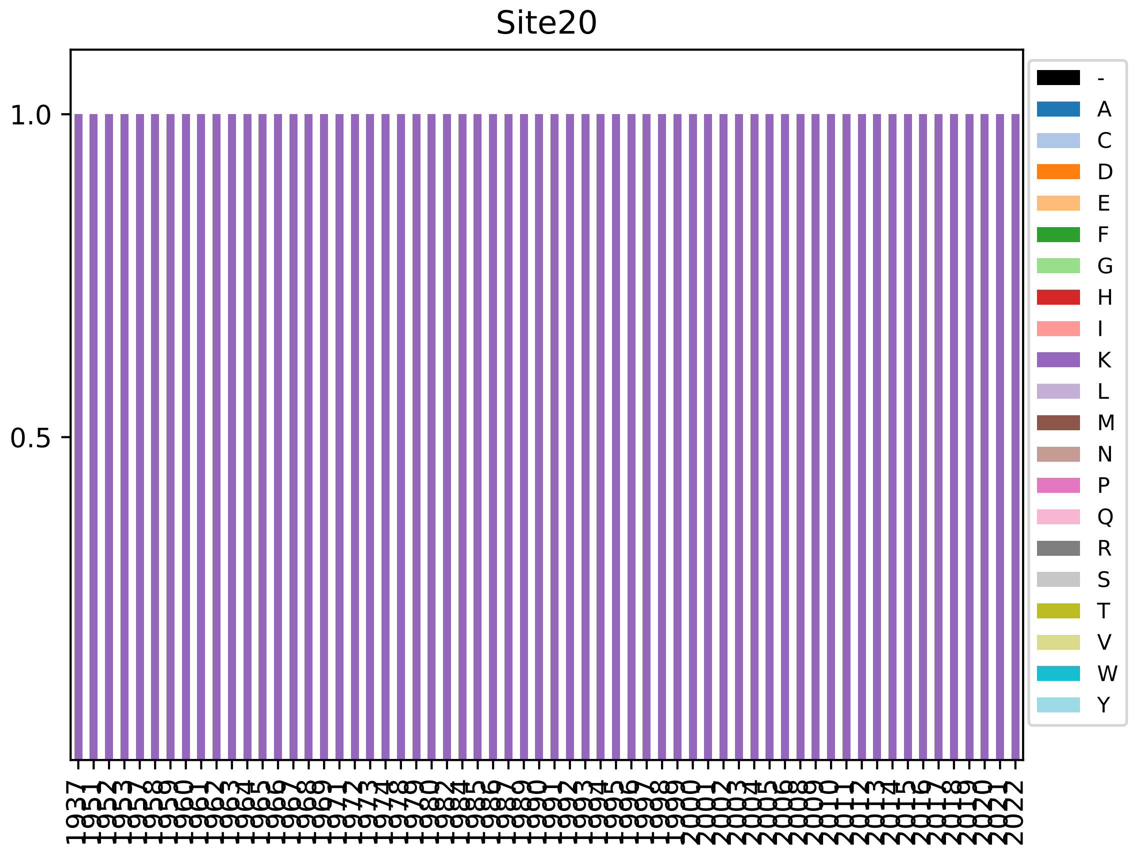
Site20
1.0
0.5
-
A
C
D
E
F
G
H
I
K
L
M
N
P
Q
R
S
T
V
W
Y

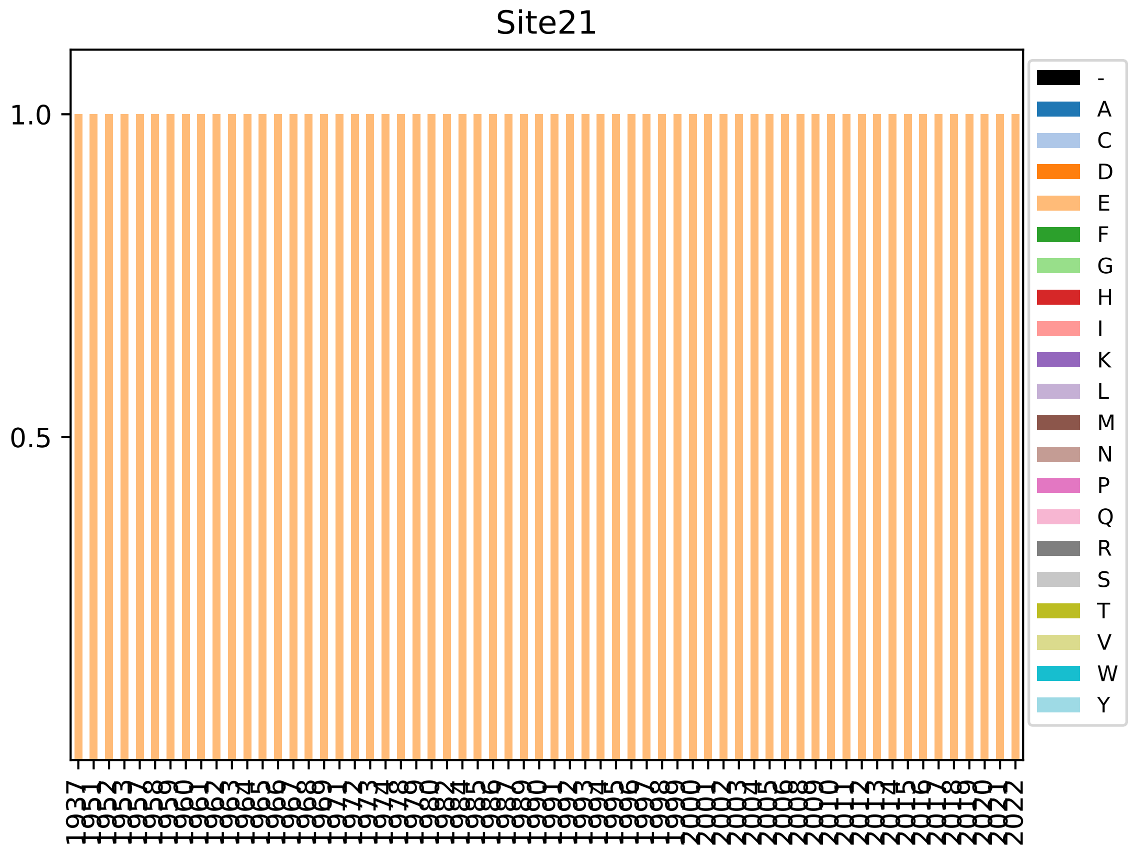
Site21
1.0
0.5
-
A
C
D
E
F
G
H
I
K
L
M
N
P
Q
R
S
T
V
W
Y

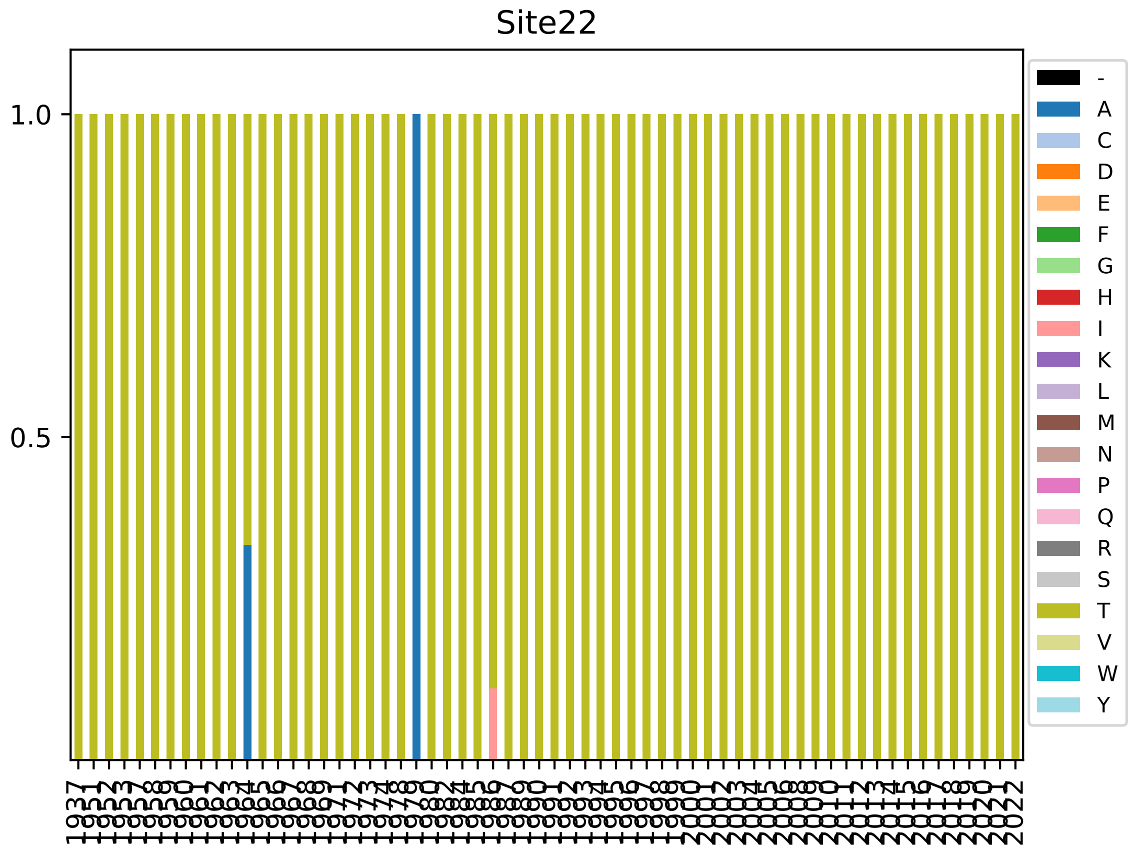
Site22
1.0
0.5
-
A
C
D
E
F
G
H
I
K
L
M
N
P
Q
R
S
T
V
W
Y

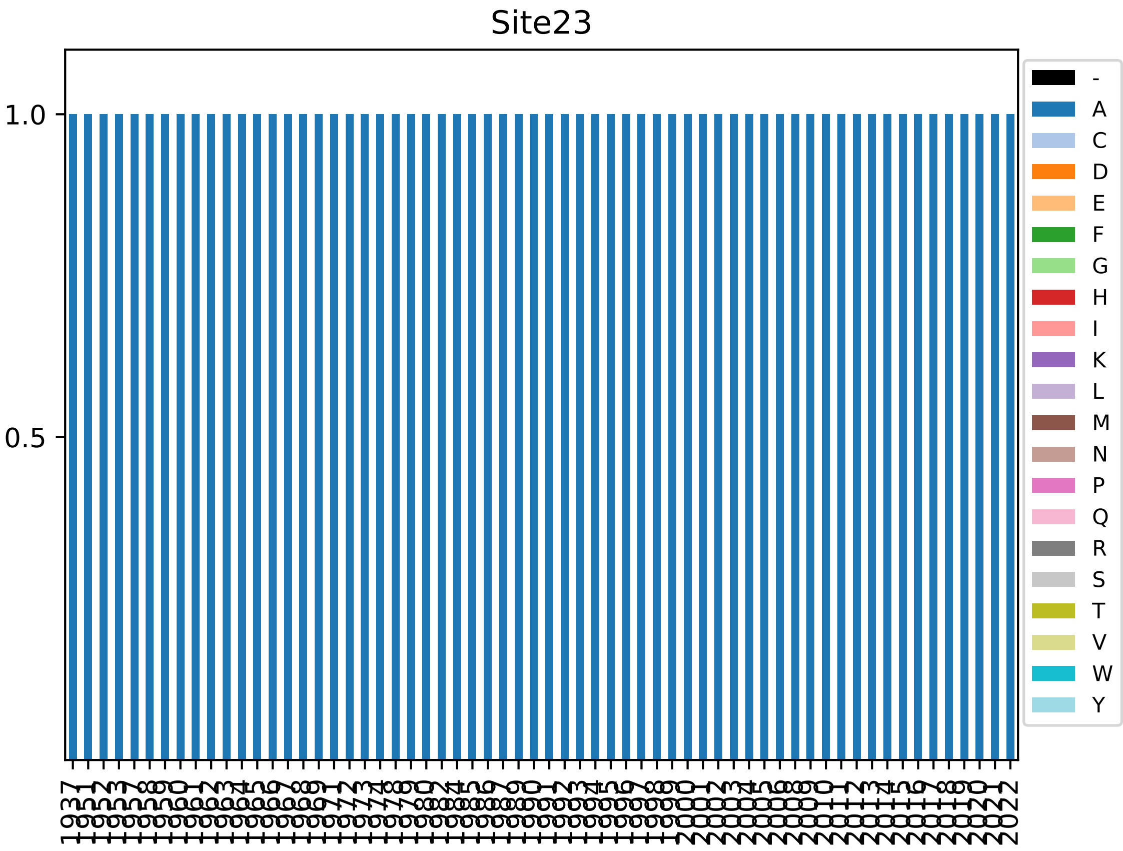
Site23
1.0
0.5
-
A
C
D
E
F
G
H
I
K
L
M
N
P
Q
R
S
T
V
W
Y

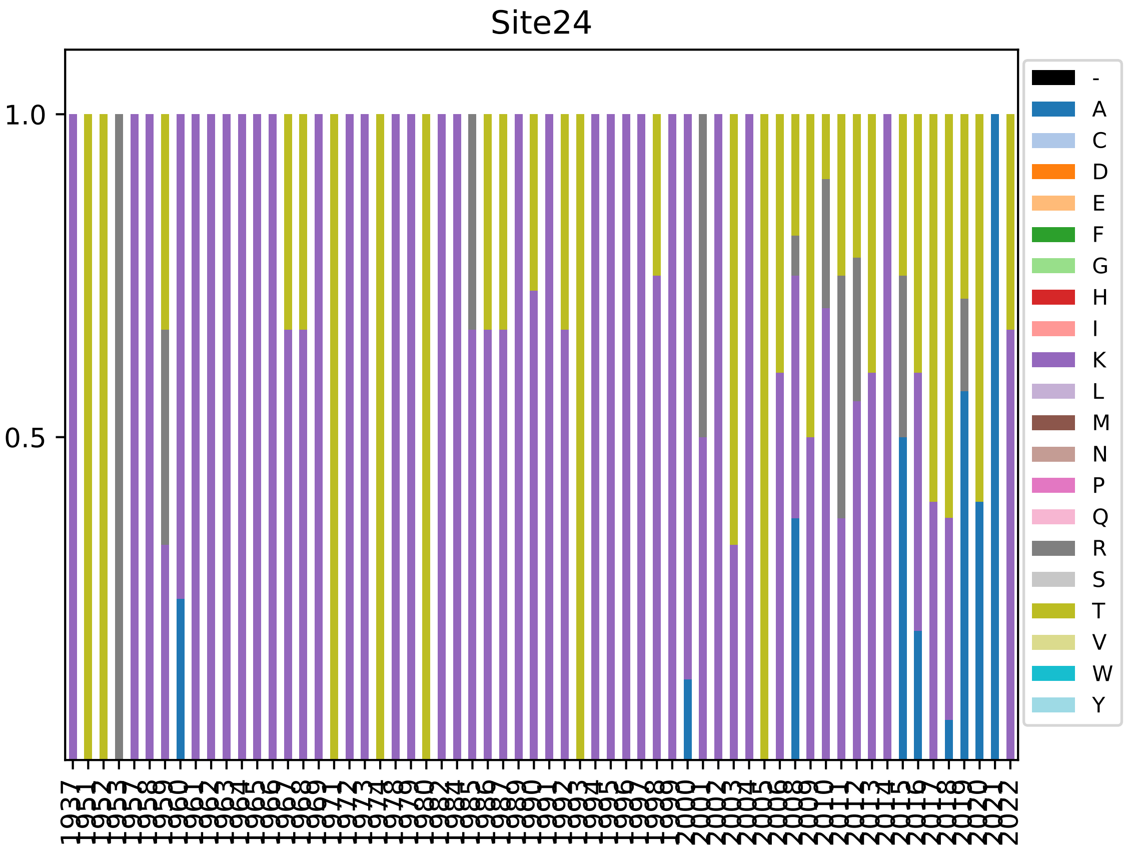
Site24
1.0
0.5
-
A
C
D
E
F
G
H
I
K
L
M
N
P
Q
R
S
T
V
W
Y

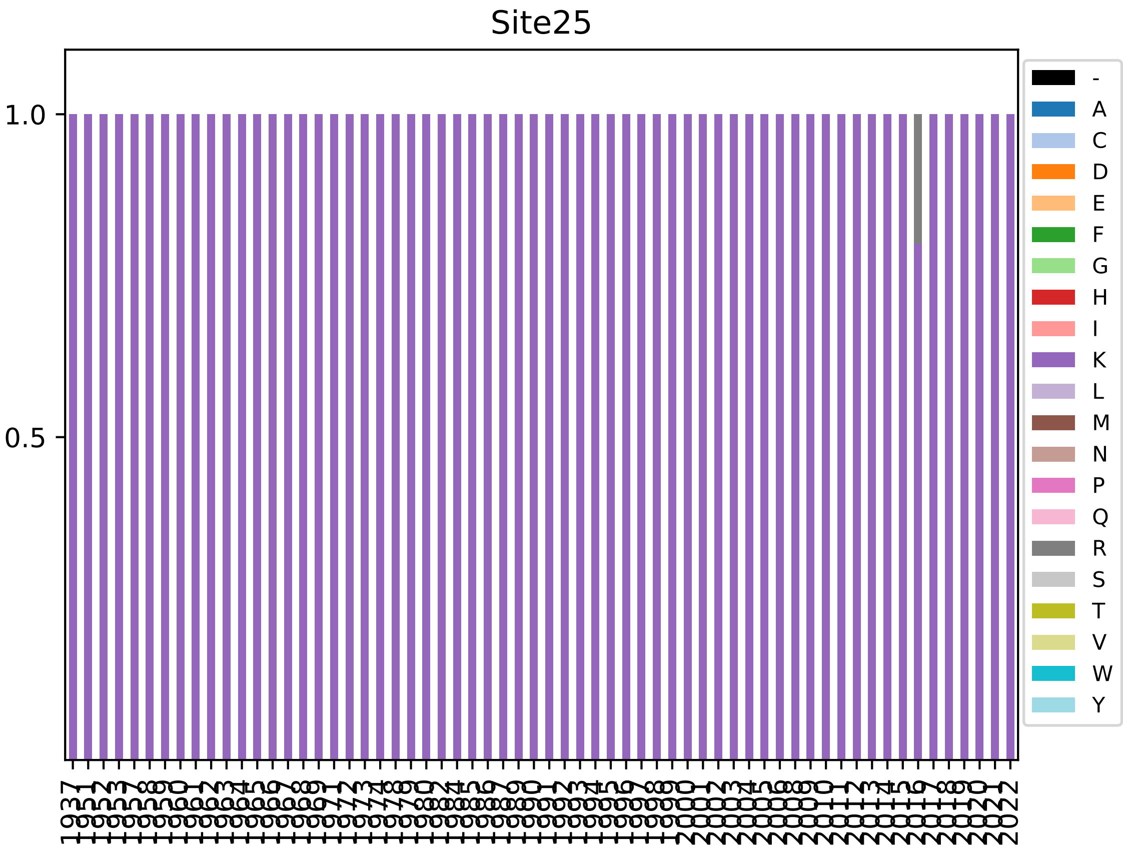
Site25
1.0
0.5
-
A
C
D
E
F
G
H
I
K
L
M
N
P
Q
R
S
T
V
W
Y

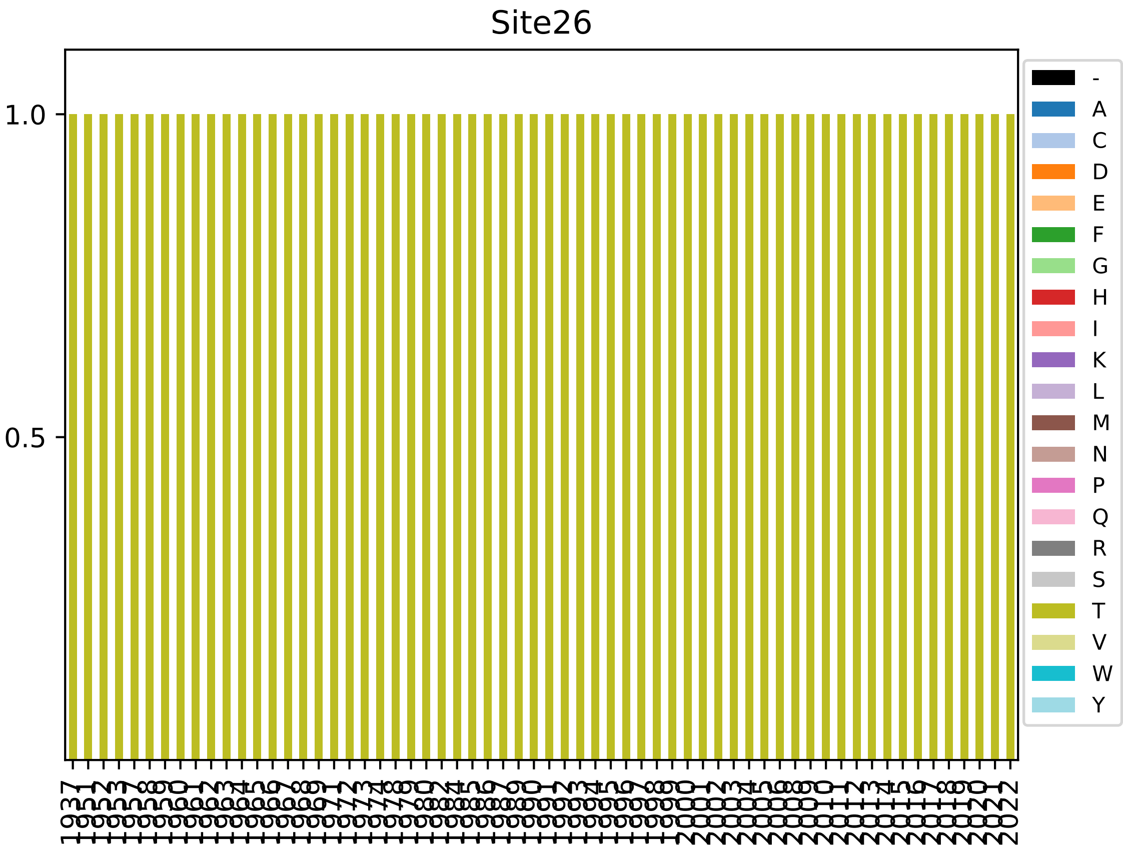
Site26
1.0
0.5
-
A
C
D
E
F
G
H
I
K
L
M
N
P
Q
R
S
T
V
W
Y

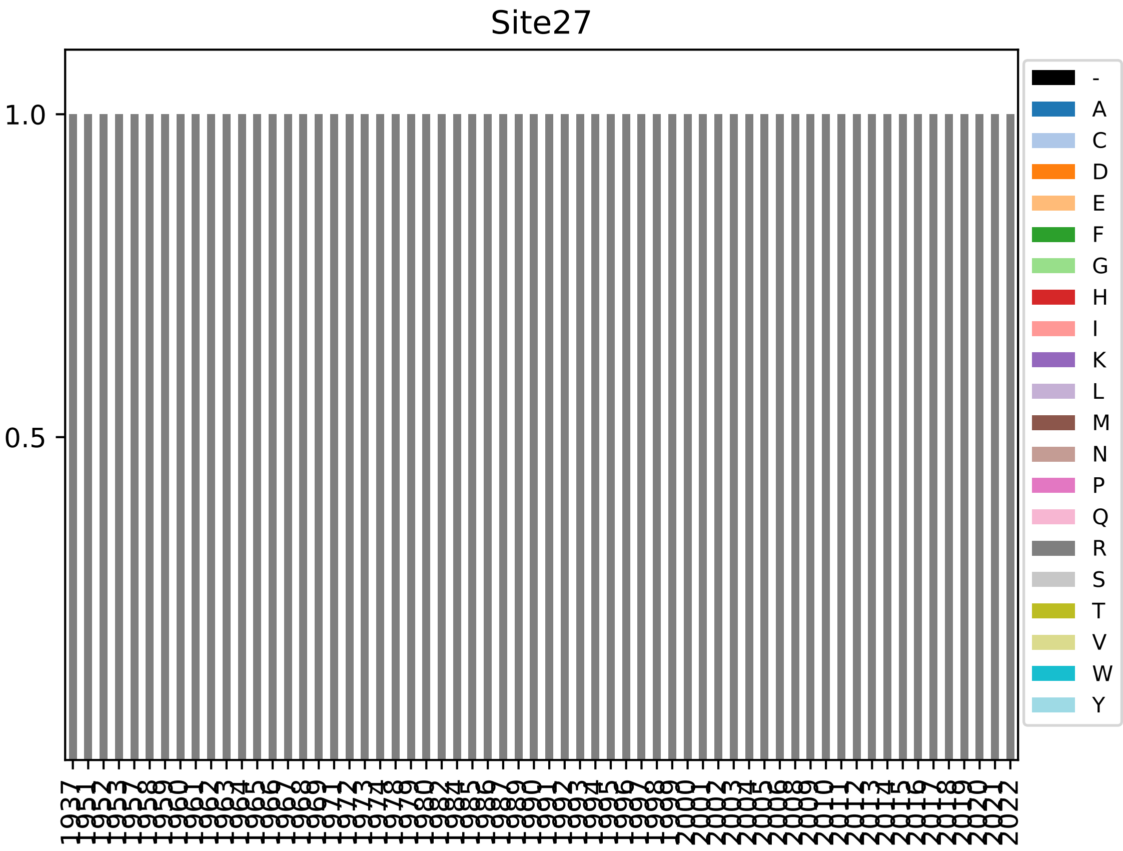
Site27
1.0
0.5
-
A
C
D
E
F
G
H
I
K
L
M
N
P
Q
R
S
T
V
W
Y

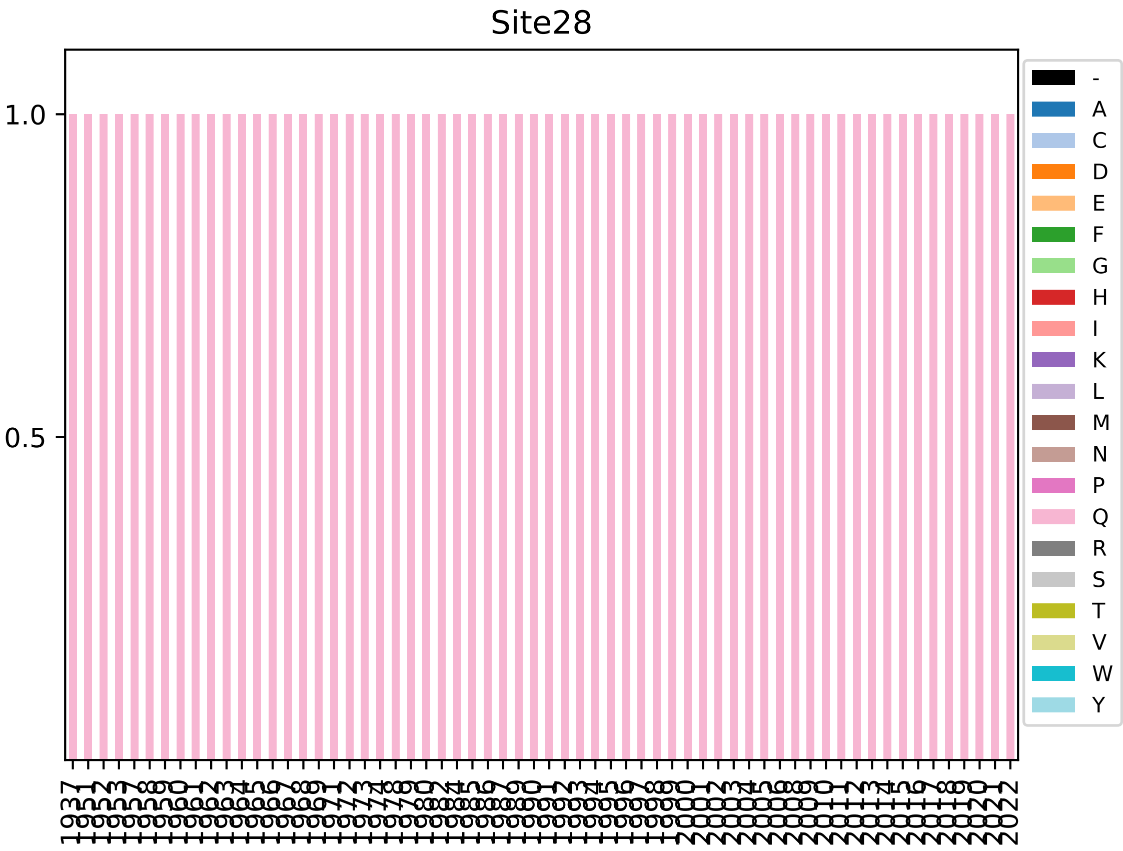
Site28
1.0
0.5
-
A
C
D
E
F
G
H
I
K
L
M
N
P
Q
R
S
T
V
W
Y

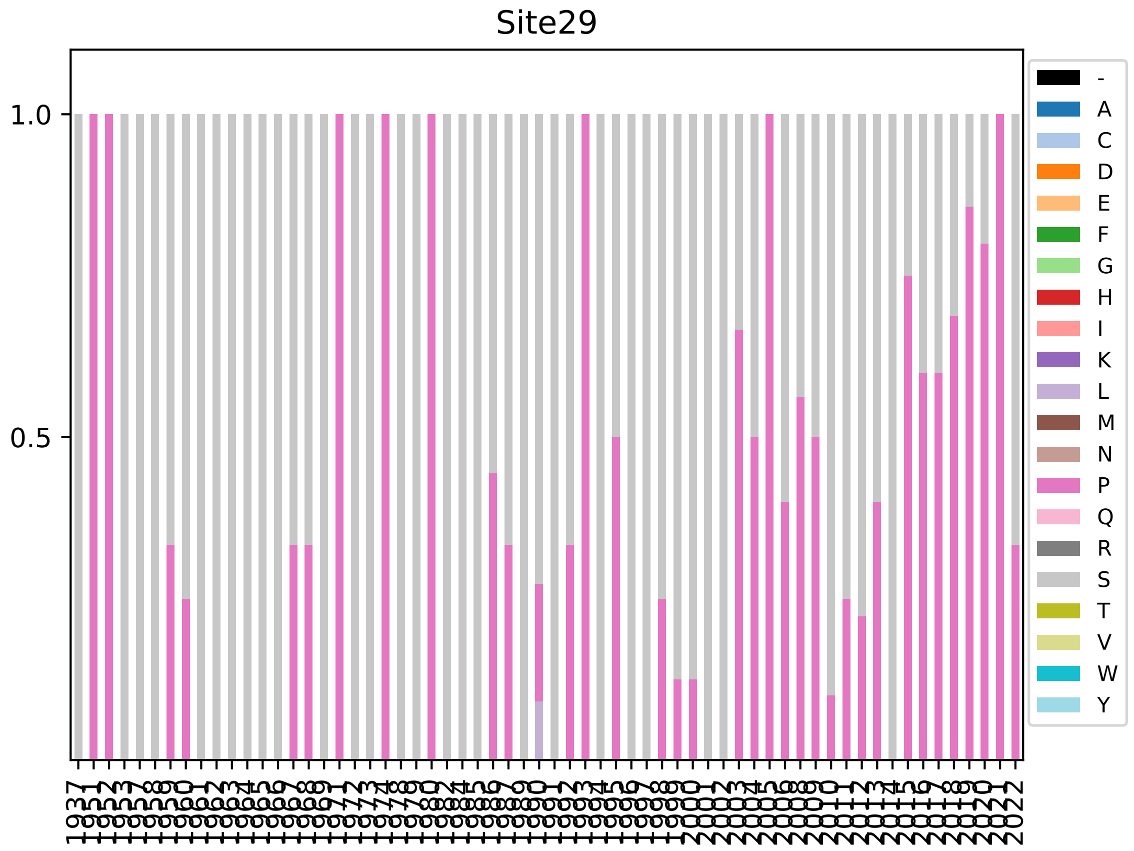
Site29
1.0
0.5
-
A
C
D
E
F
G
H
I
K
L
M
N
P
Q
R
S
T
V
W
Y

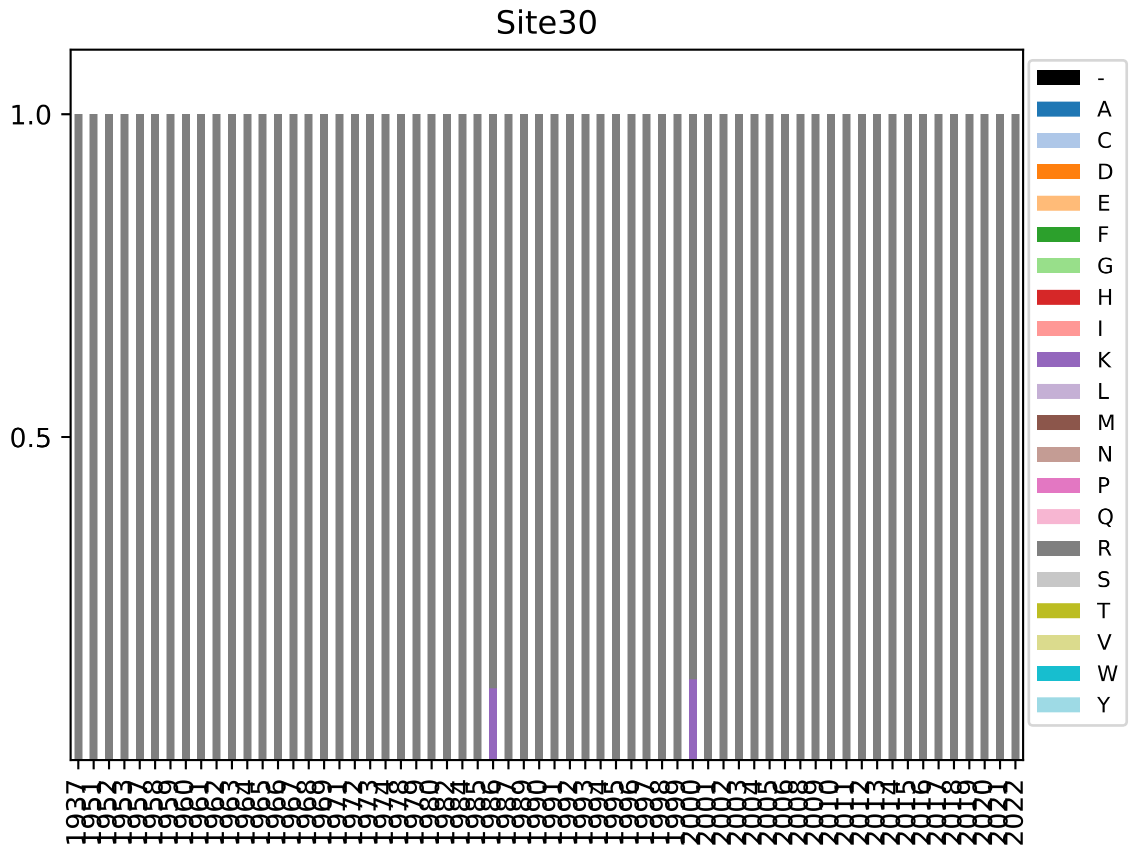
Site30
1.0
0.5
-
A
C
D
E
F
G
H
I
K
L
M
N
P
Q
R
S
T
V
W
Y

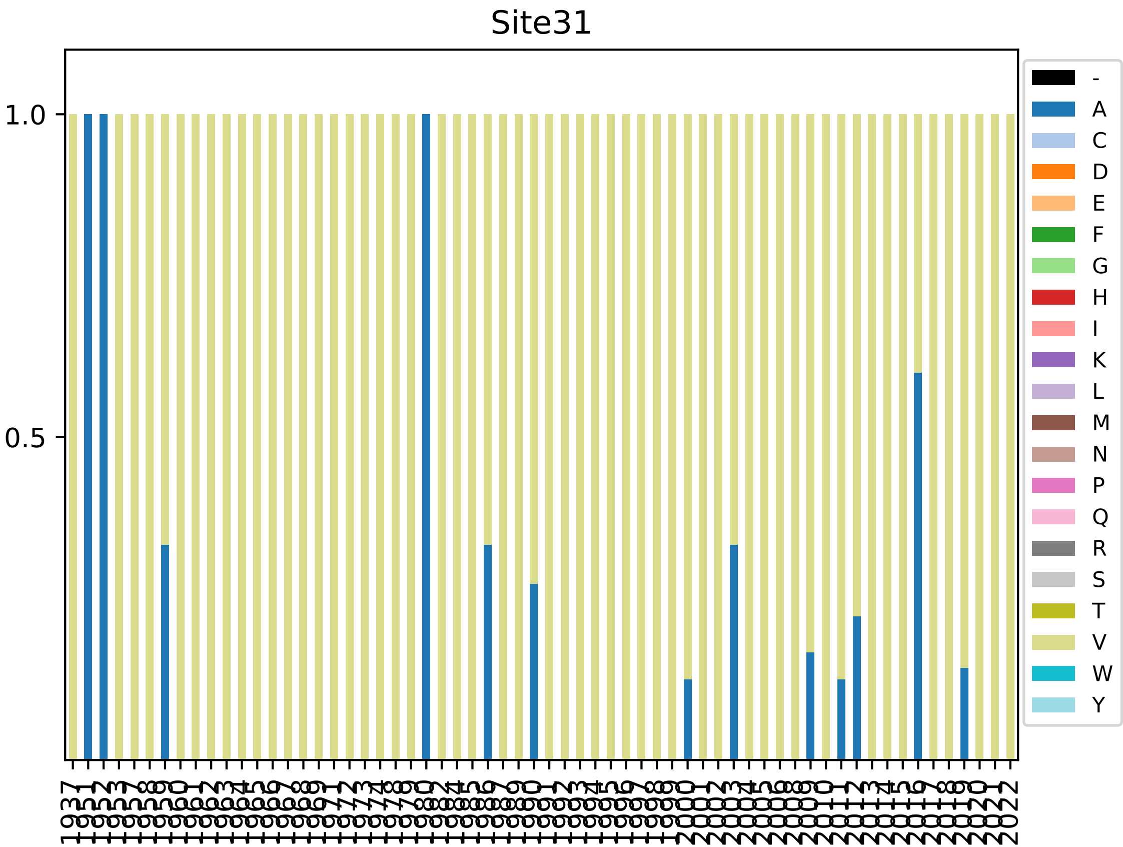

Site31
1.0
0.5
-
A
C
D
E
F
G
H
I
K
L
M
N
P
Q
R
S
T
V
W
Y

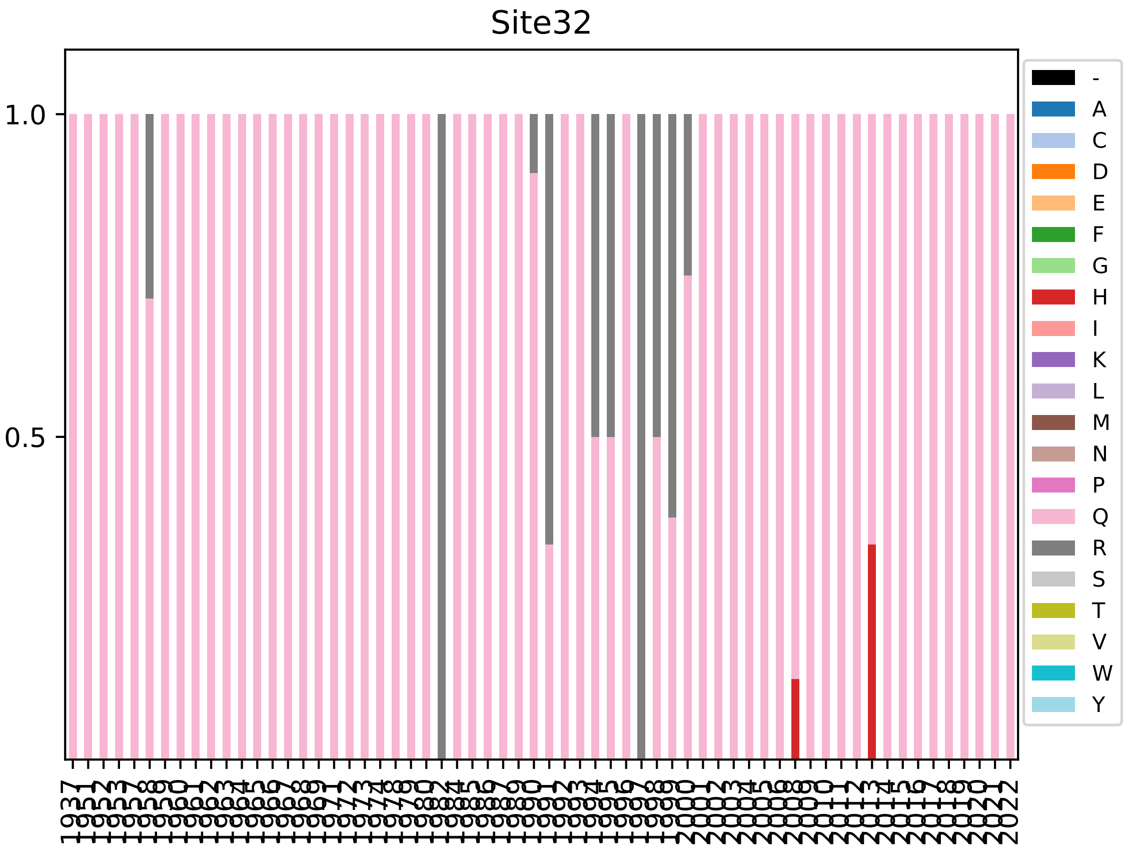
Site32
1.0
0.5
-
A
C
D
E
F
G
H
I
K
L
M
N
P
Q
R
S
T
V
W
Y

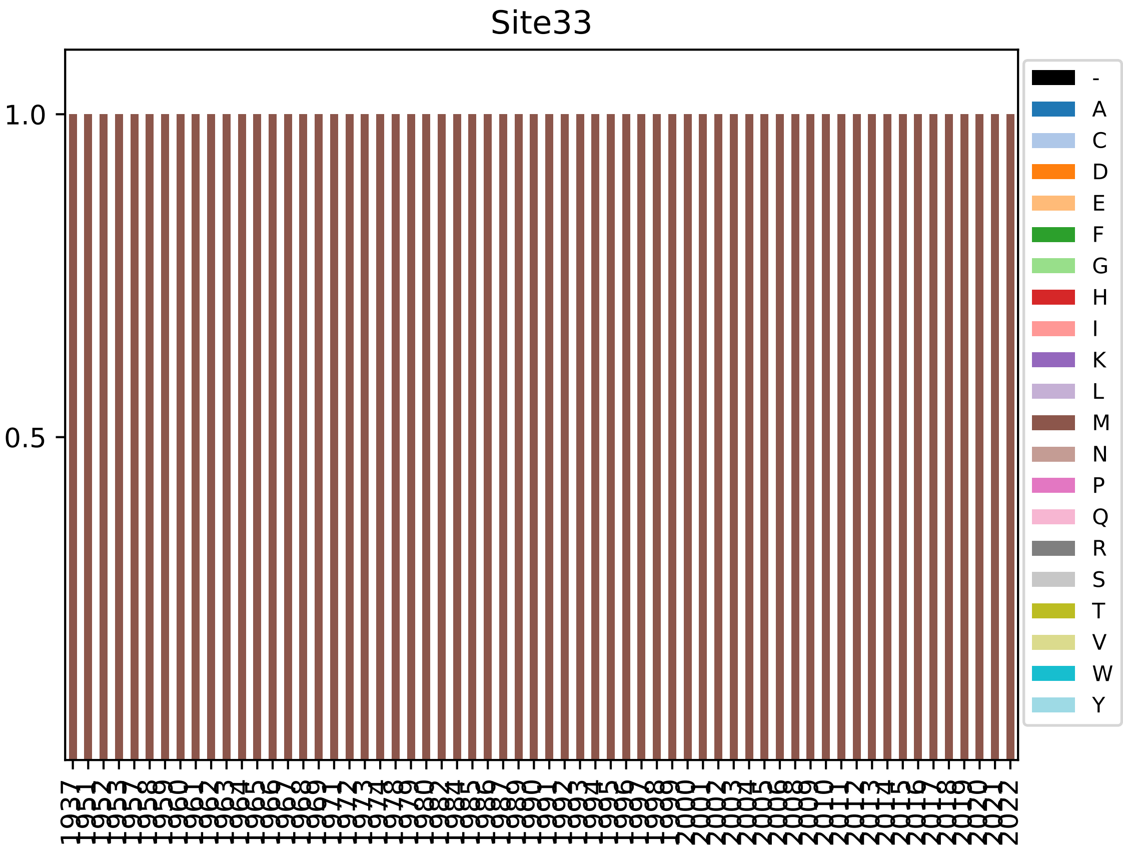
Site33
1.0
0.5
-
A
C
D
E
F
G
H
I
K
L
M
N
P
Q
R
S
T
V
W
Y

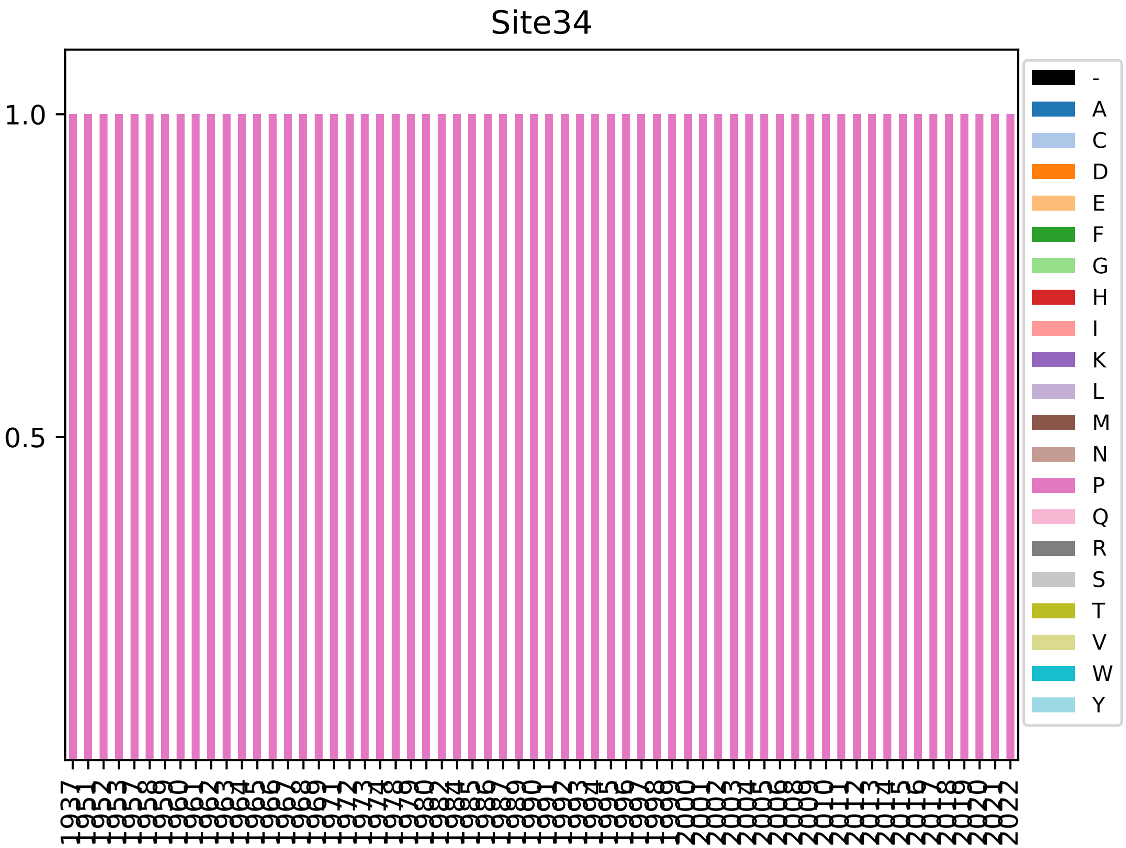
Site34
1.0
0.5
-
A
C
D
E
F
G
H
I
K
L
M
N
P
Q
R
S
T
V
W
Y

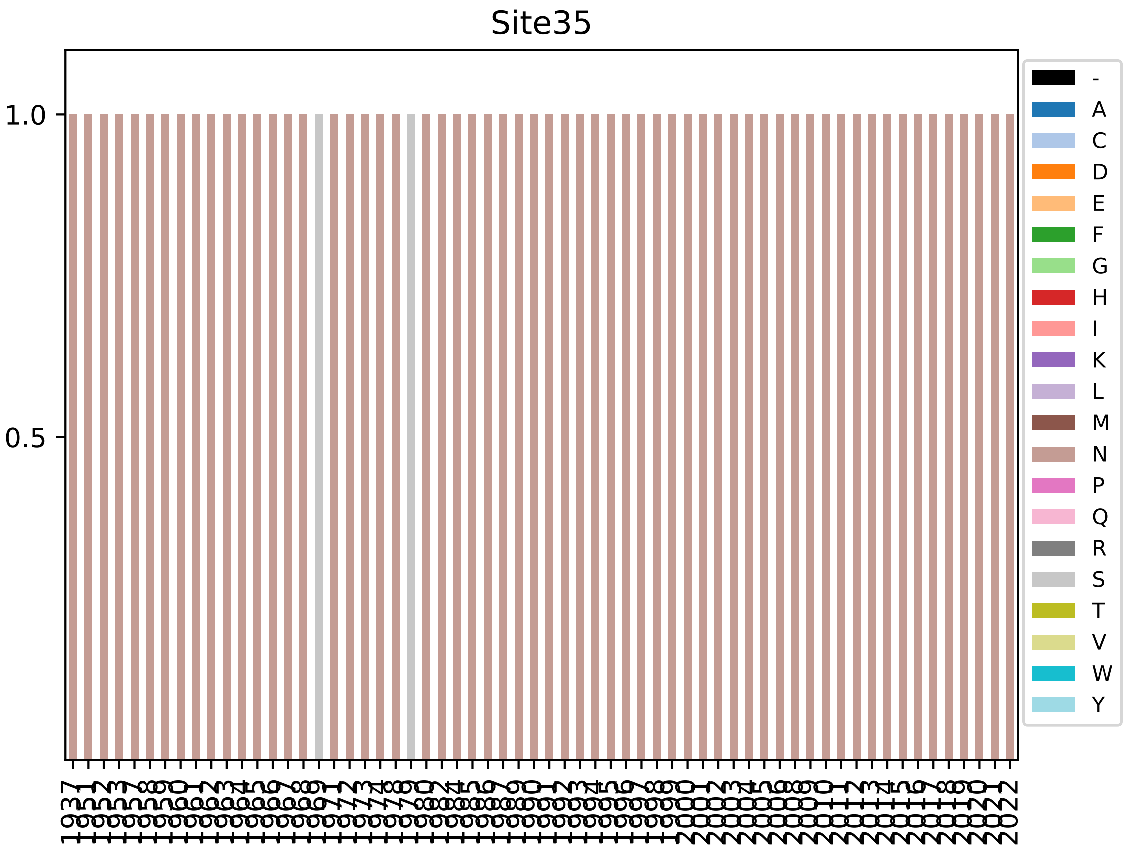
Site35
1.0
0.5
-
A
C
D
E
F
G
H
I
K
L
M
N
P
Q
R
S
T
V
W
Y

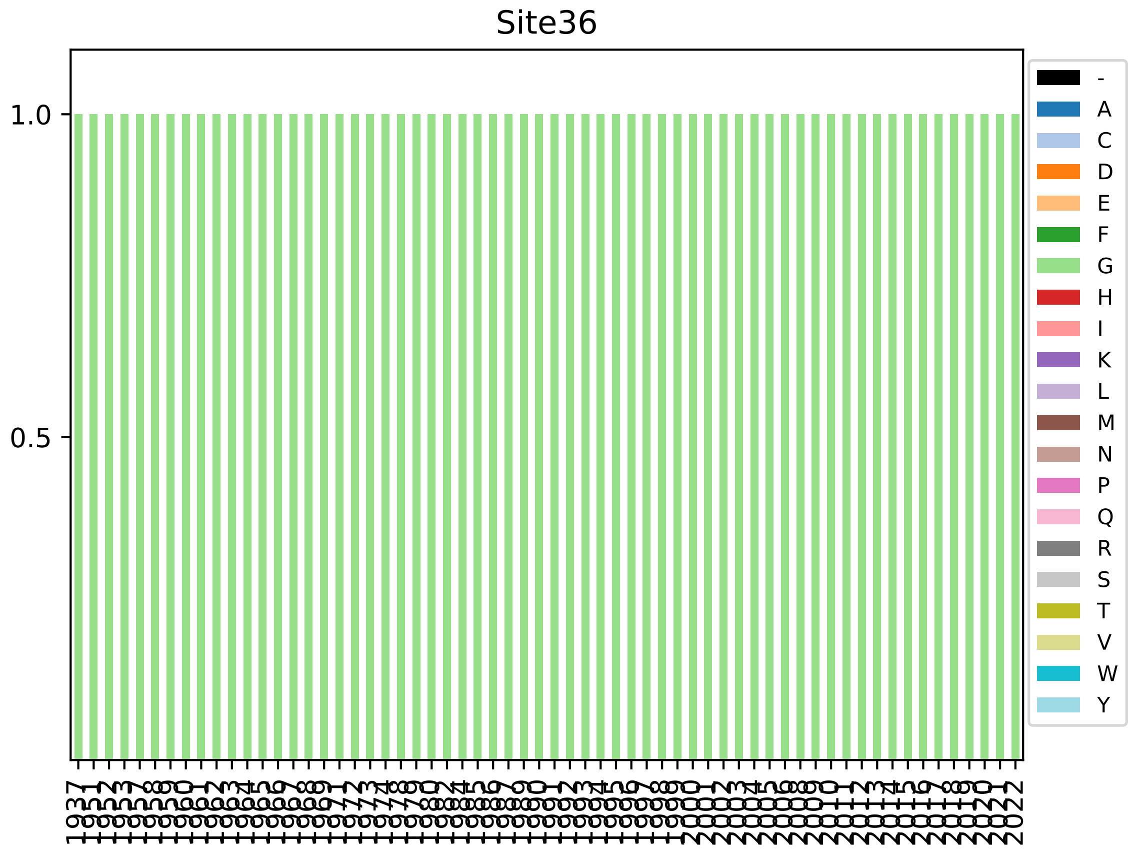
Site36
1.0
0.5
-
A
C
D
E
F
G
H
I
K
L
M
N
P
Q
R
S
T
V
W
Y

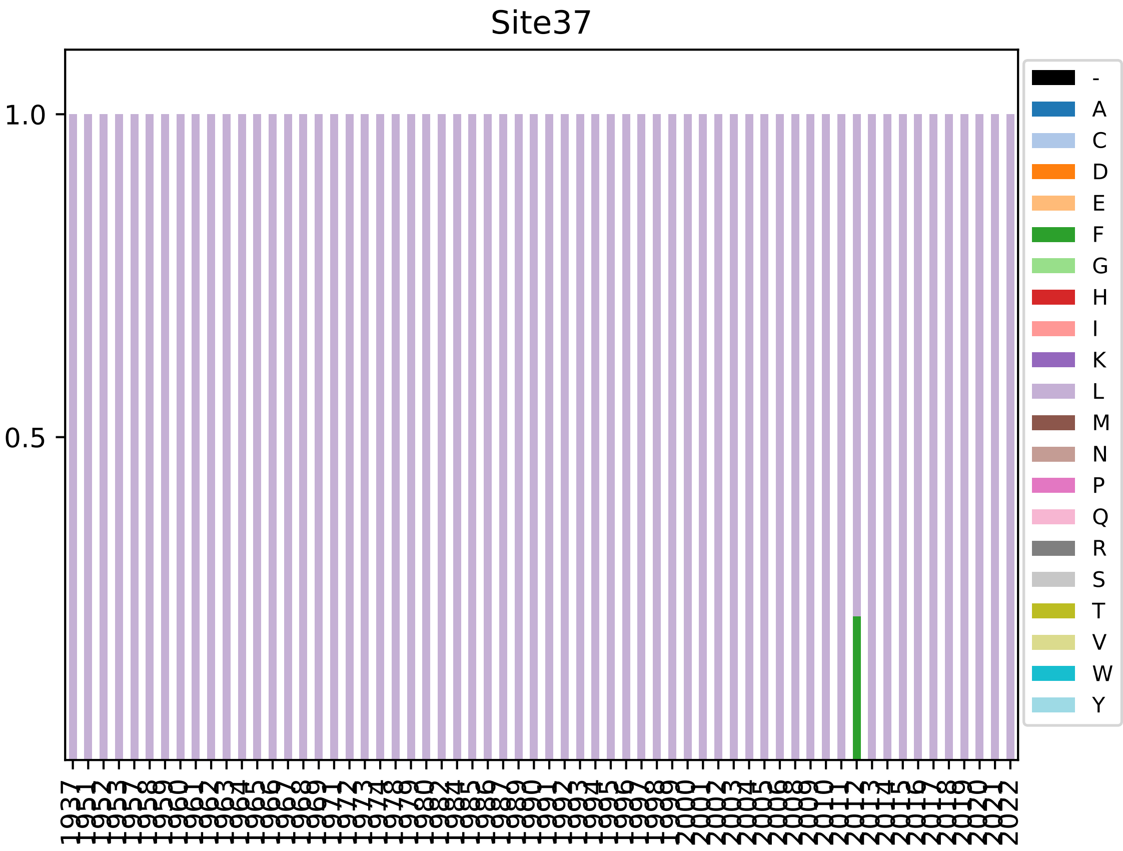

Site37
1.0
0.5
-
A
C
D
E
F
G
H
I
K
L
M
N
P
Q
R
S
T
V
W
Y

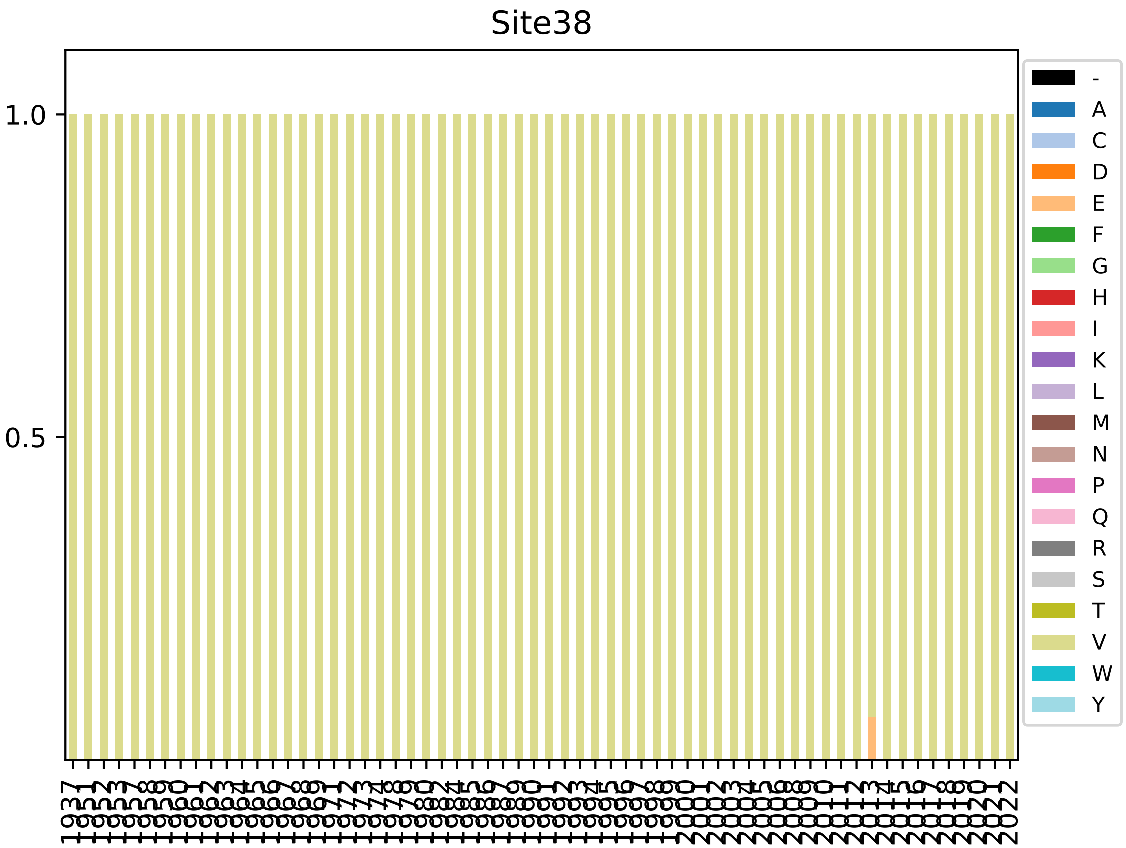
Site38
1.0
0.5
-
A
C
D
E
F
G
H
I
K
L
M
N
P
Q
R
S
T
V
W
Y

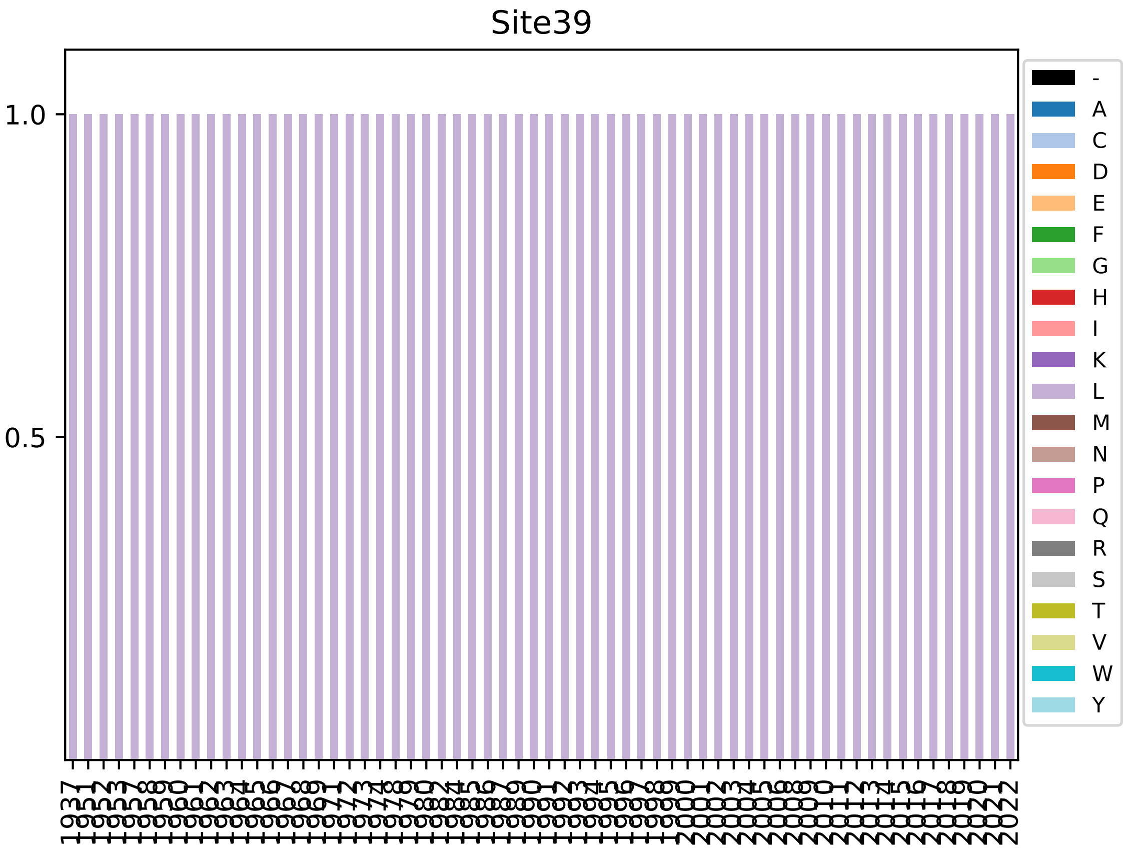
Site39
1.0
0.5
-
A
C
D
E
F
G
H
I
K
L
M
N
P
Q
R
S
T
V
W
Y

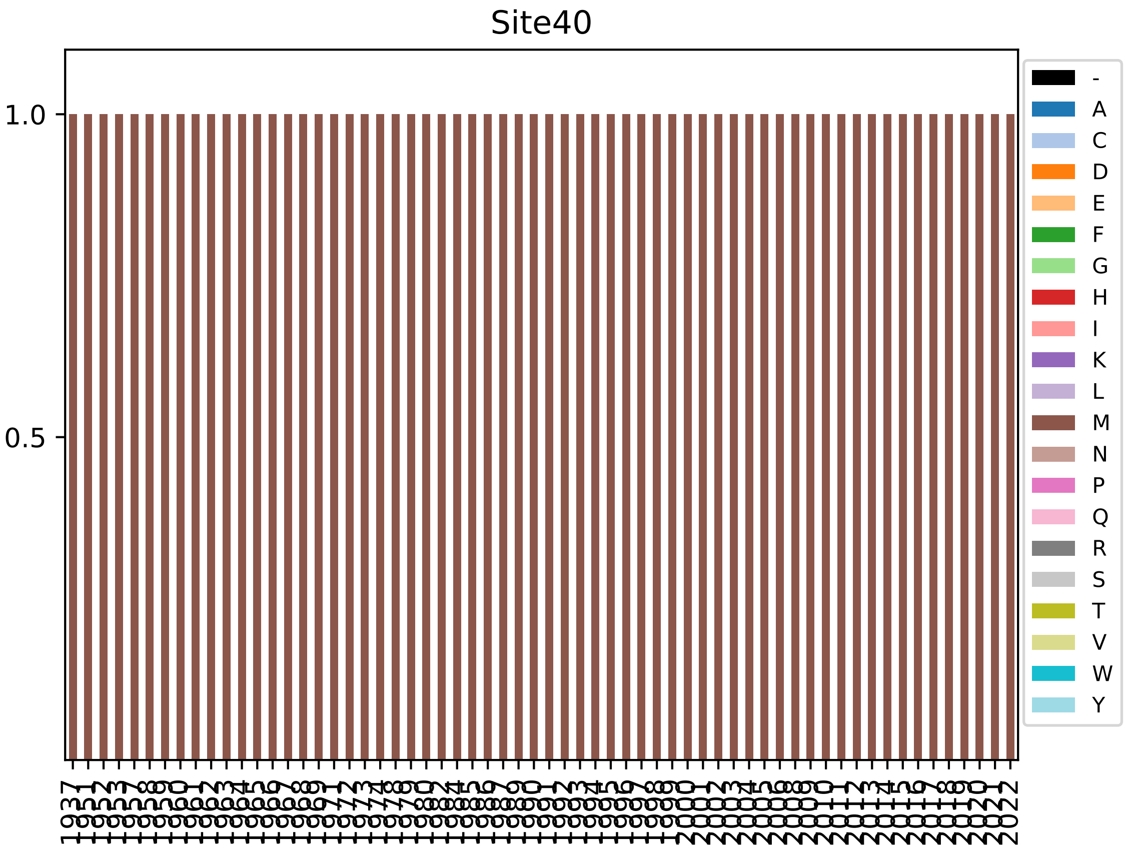
Site40
1.0
0.5
-
A
C
D
E
F
G
H
I
K
L
M
N
P
Q
R
S
T
V
W
Y

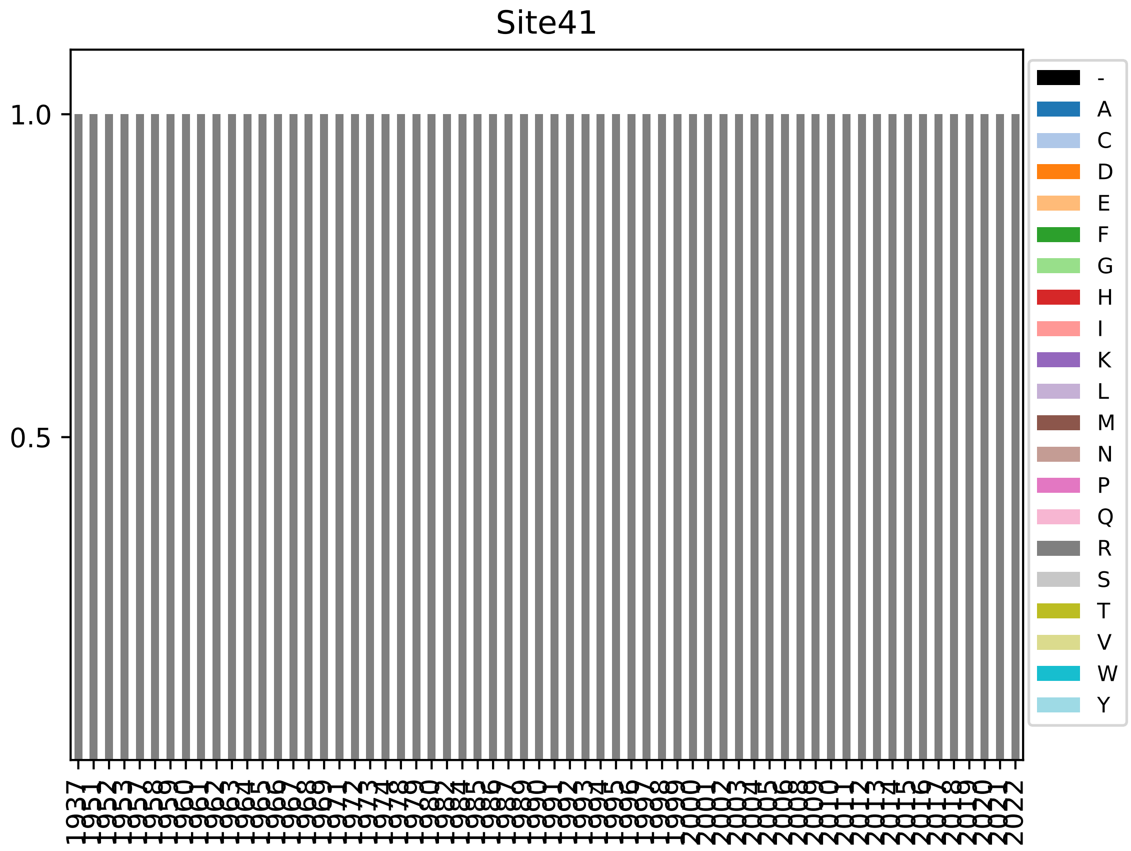
Site41
1.0
0.5
-
A
C
D
E
F
G
H
I
K
L
M
N
P
Q
R
S
T
V
W
Y

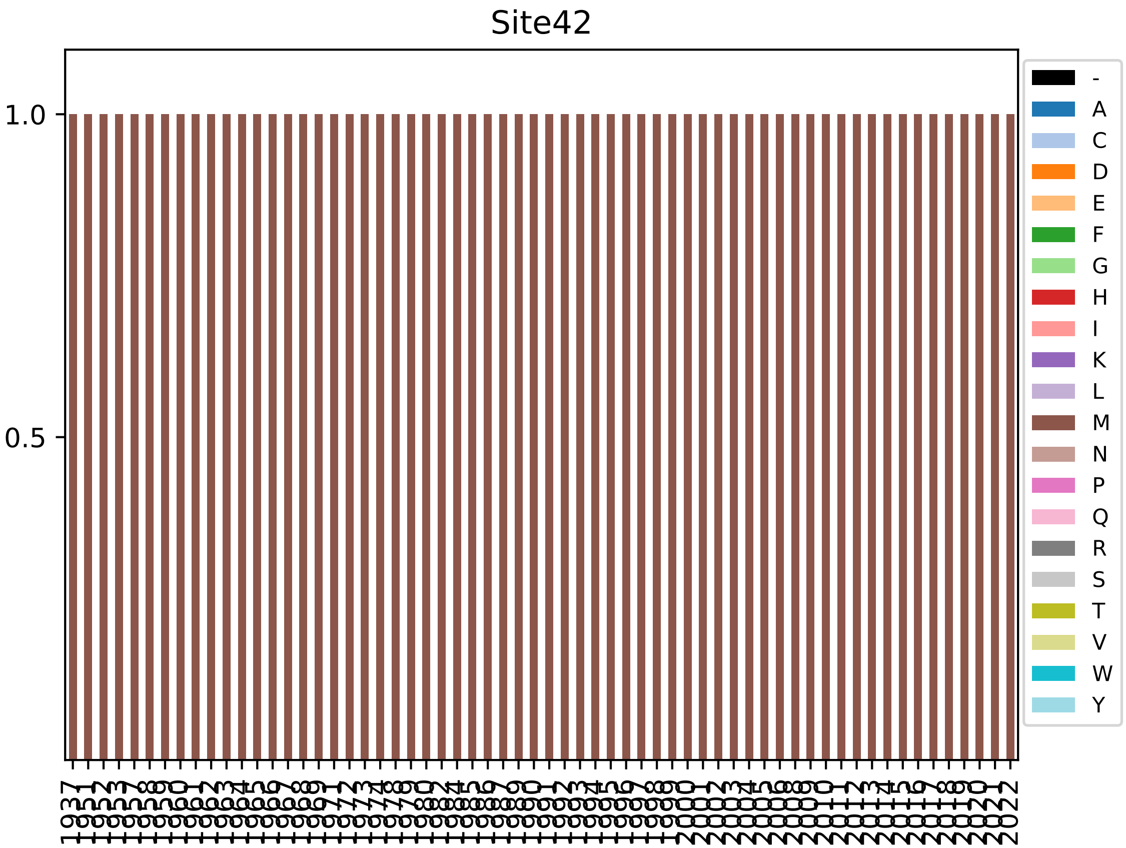
Site42
1.0
0.5
-
A
C
D
E
F
G
H
I
K
L
M
N
P
Q
R
S
T
V
W
Y

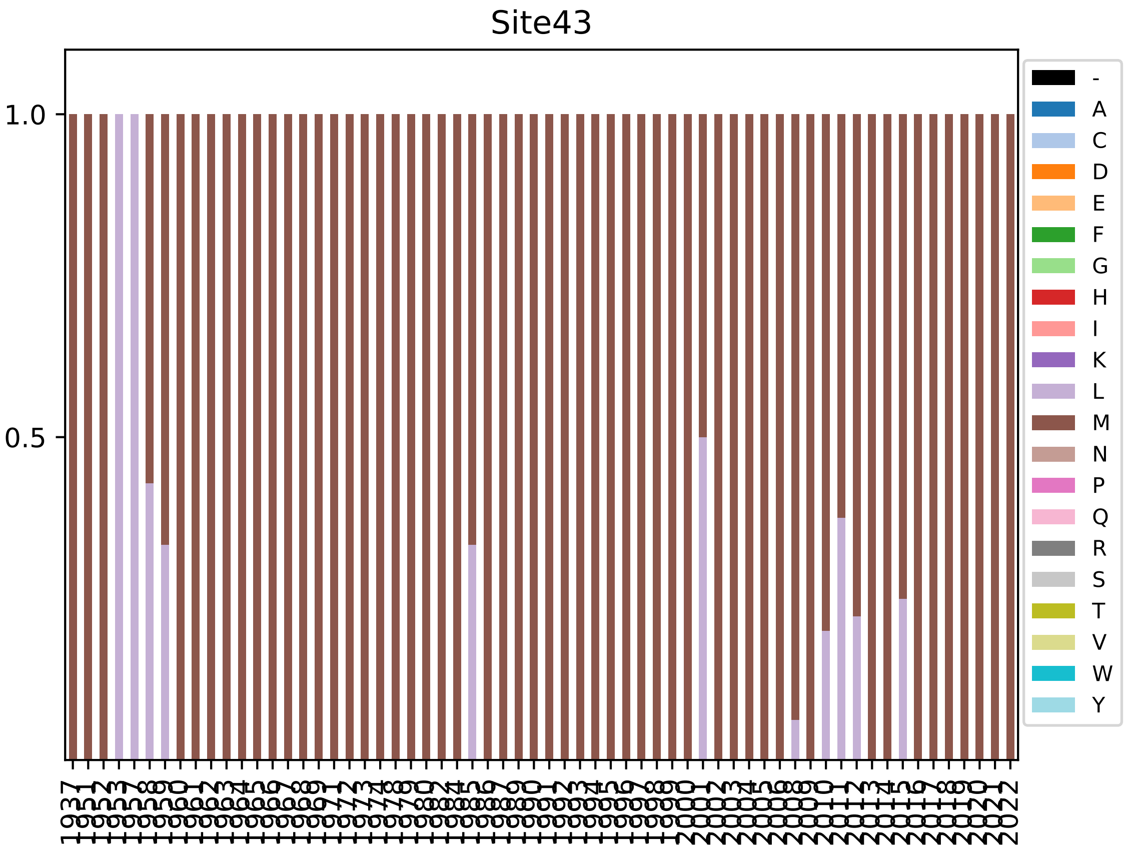

Site43
1.0
0.5
-
A
C
D
E
F
G
H
I
K
L
M
N
P
Q
R
S
T
V
W
Y

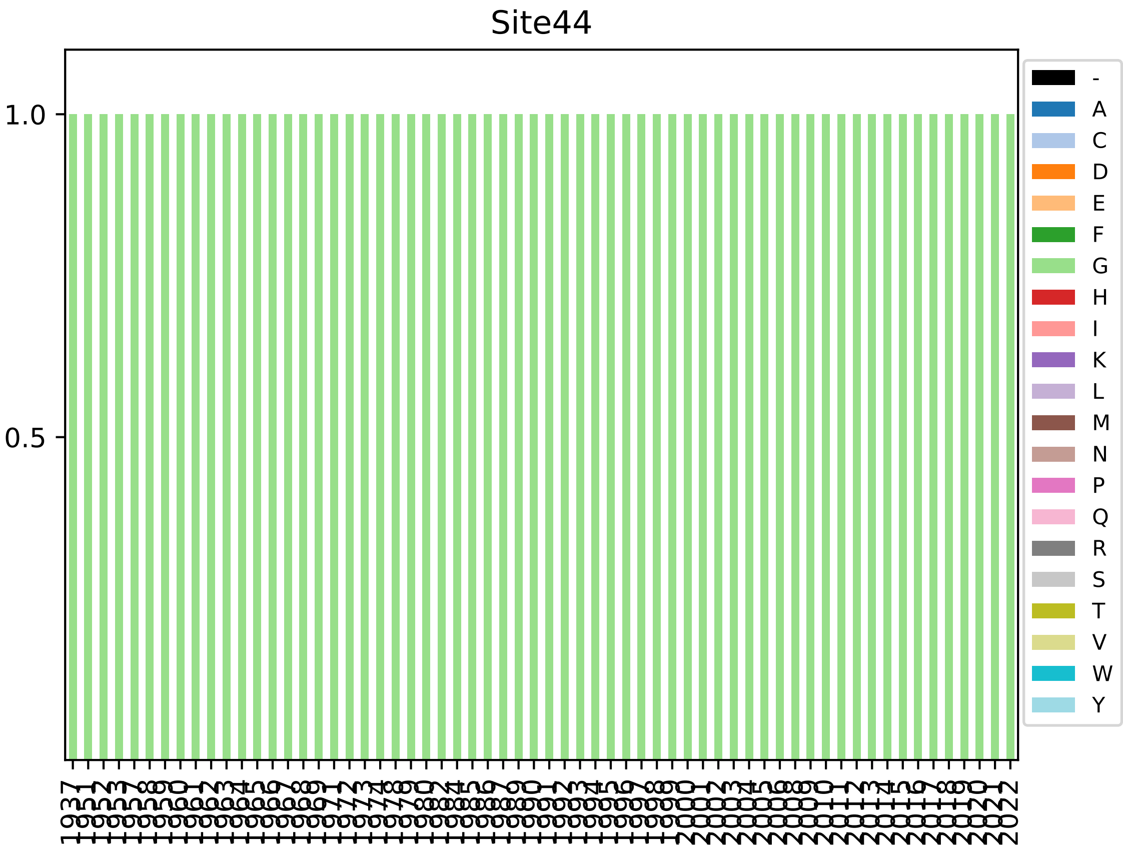
Site44
1.0
0.5
-
A
C
D
E
F
G
H
I
K
L
M
N
P
Q
R
S
T
V
W
Y

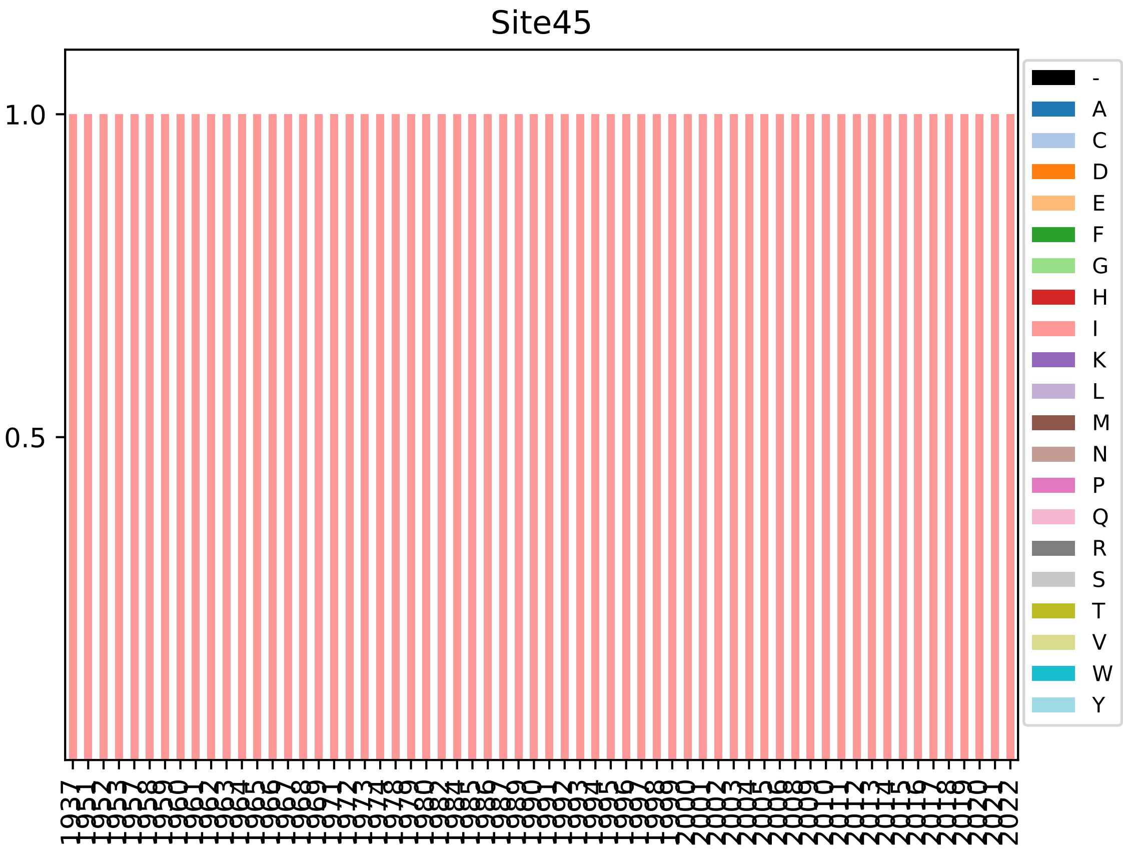
Site45
1.0
0.5
-
A
C
D
E
F
G
H
I
K
L
M
N
P
Q
R
S
T
V
W
Y

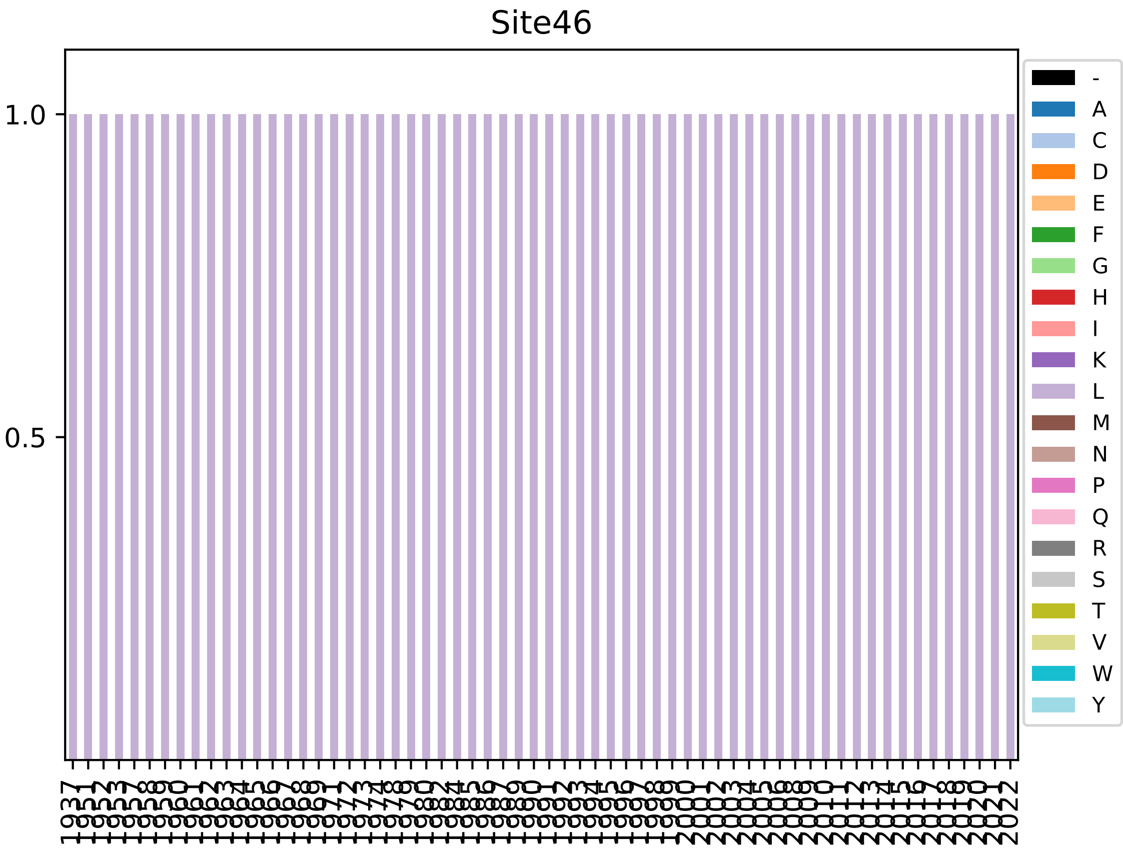
Site46
1.0
0.5
-
A
C
D
E
F
G
H
I
K
L
M
N
P
Q
R
S
T
V
W
Y

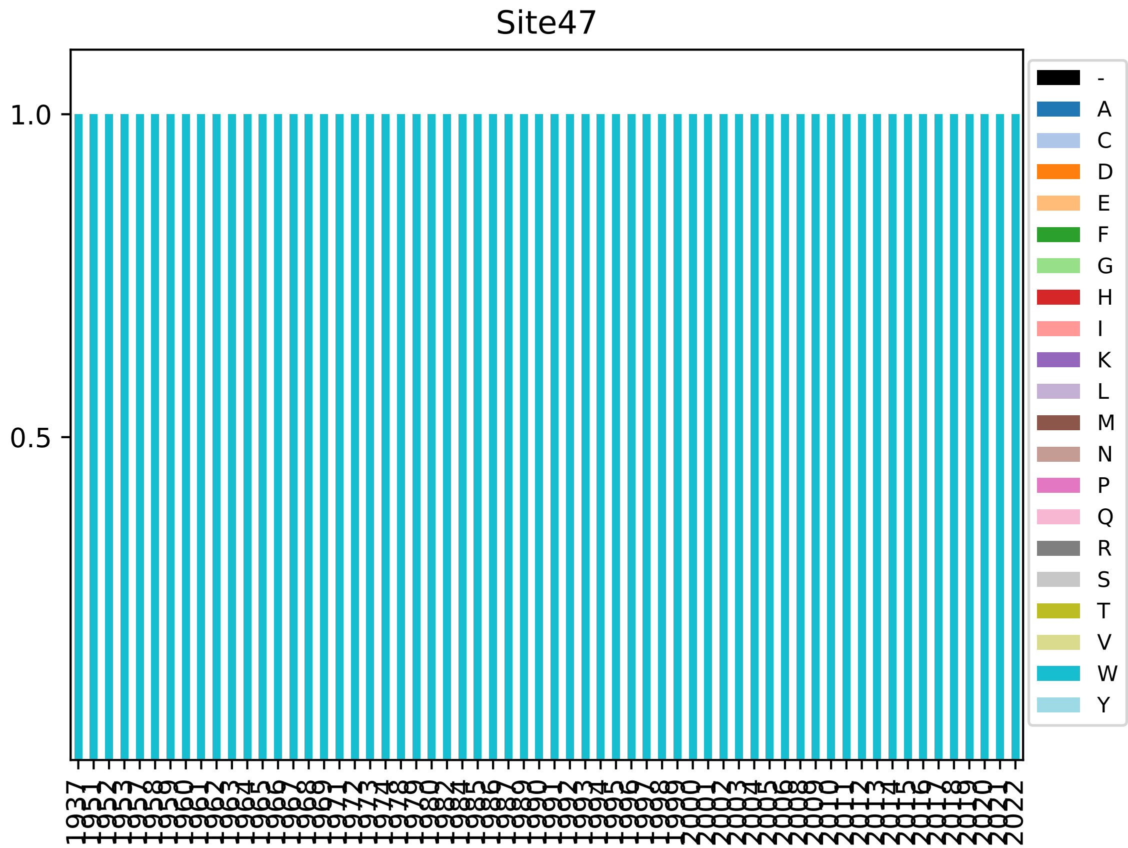
Site47
1.0
0.5
-
A
C
D
E
F
G
H
I
K
L
M
N
P
Q
R
S
T
V
W
Y

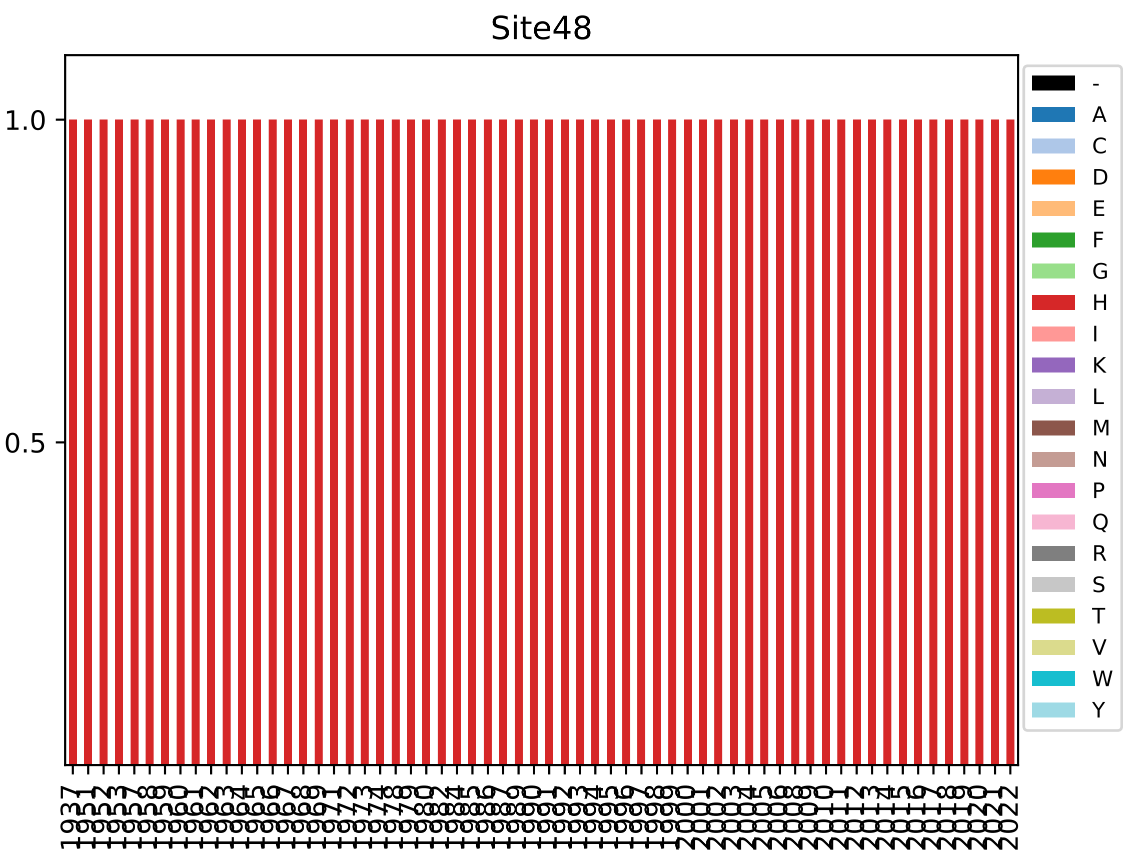
Site48
1.0
0.5
-
A
C
D
E
F
G
H
I
K
L
M
N
P
Q
R
S
T
V
W
Y

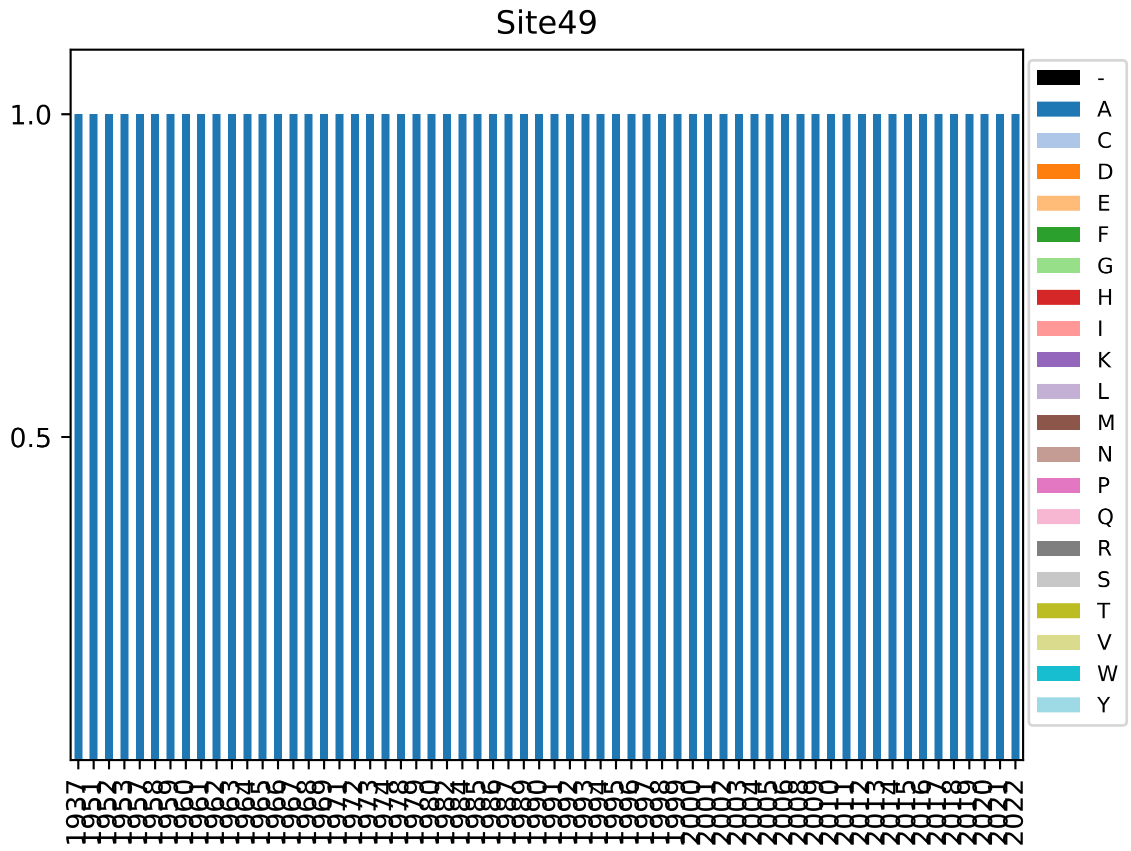
Site49
1.0
0.5
-
A
C
D
E
F
G
H
I
K
L
M
N
P
Q
R
S
T
V
W
Y

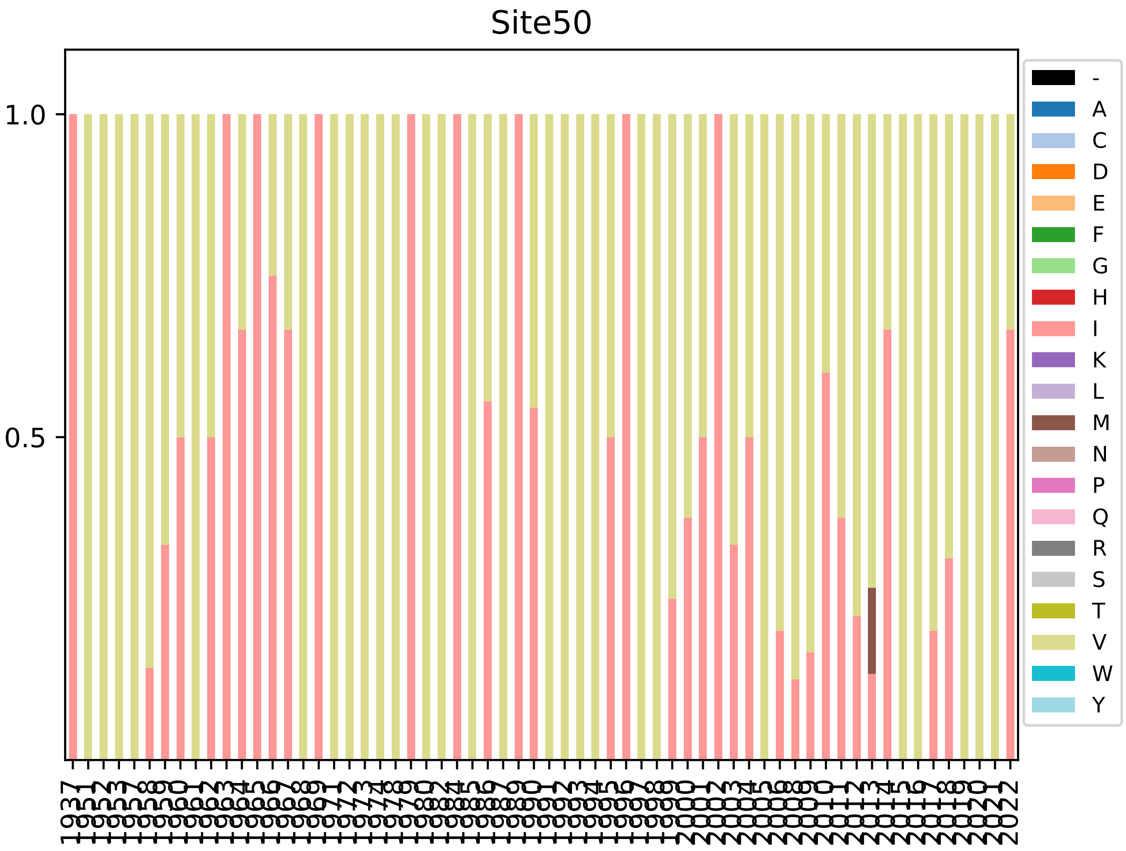

Site50
1.0
0.5
-
A
C
D
E
F
G
H
I
K
L
M
N
P
Q
R
S
T
V
W
Y

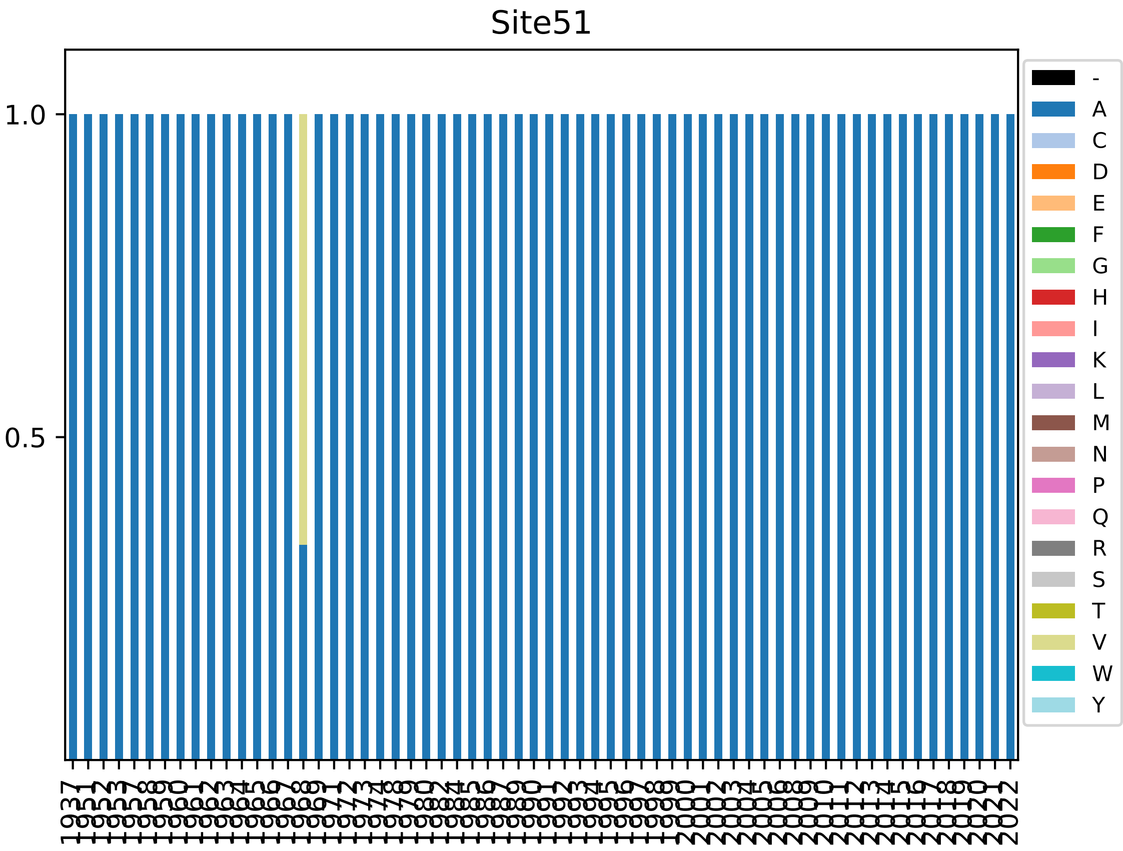
Site51
1.0
0.5
-
A
C
D
E
F
G
H
I
K
L
M
N
P
Q
R
S
T
V
W
Y

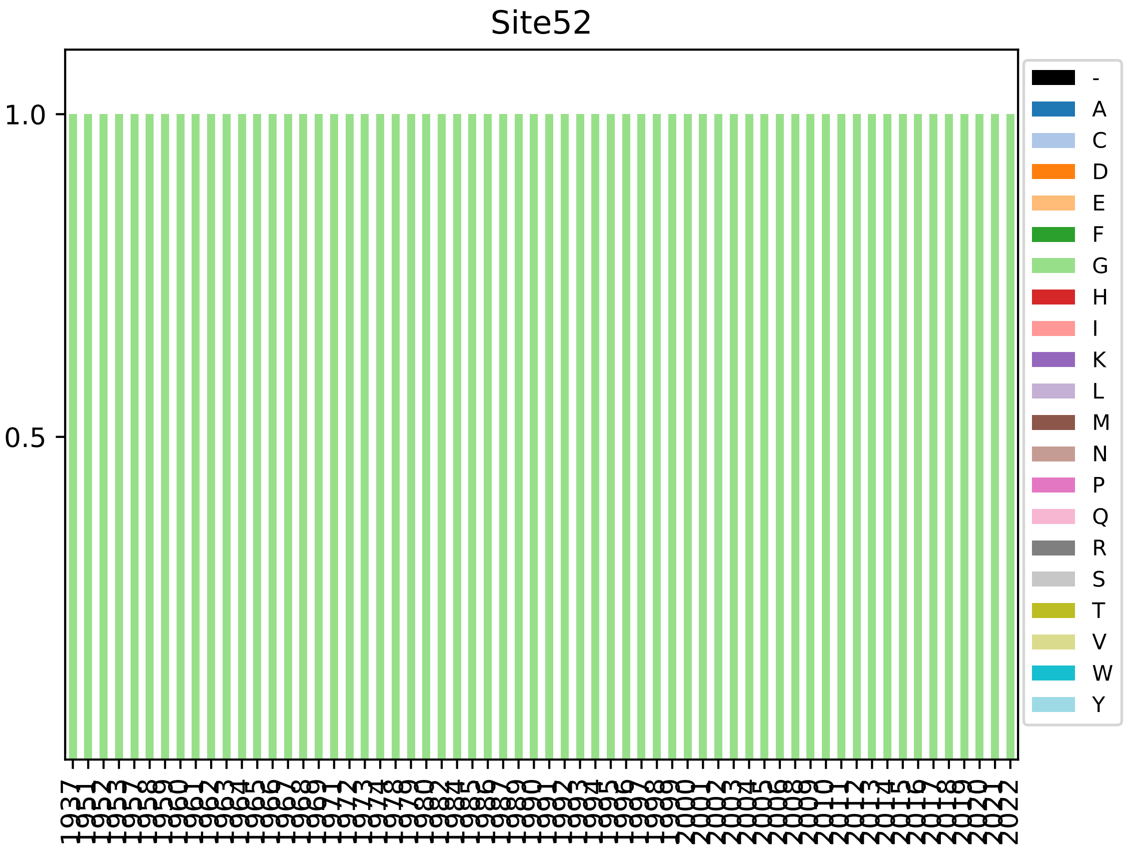
Site52
1.0
0.5
-
A
C
D
E
F
G
H
I
K
L
M
N
P
Q
R
S
T
V
W
Y

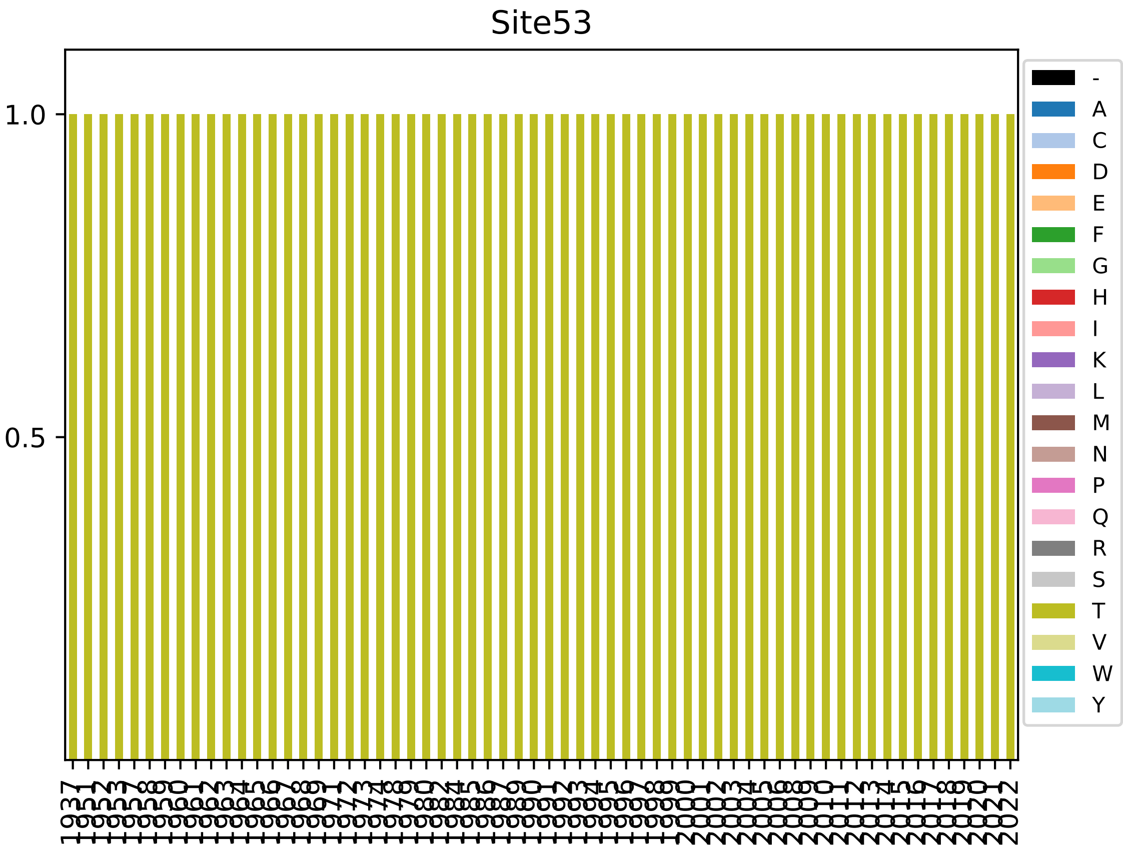
Site53
1.0
0.5
-
A
C
D
E
F
G
H
I
K
L
M
N
P
Q
R
S
T
V
W
Y

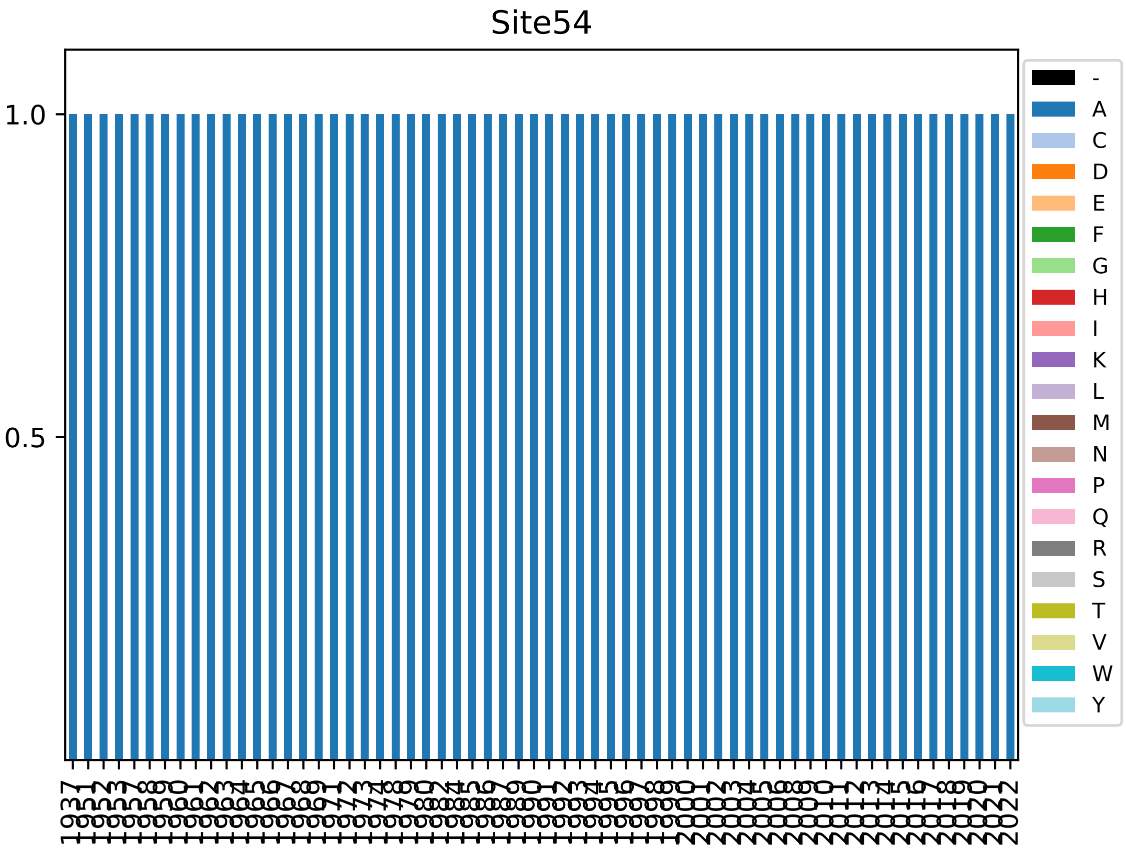
Site54
1.0
0.5
-
A
C
D
E
F
G
H
I
K
L
M
N
P
Q
R
S
T
V
W
Y

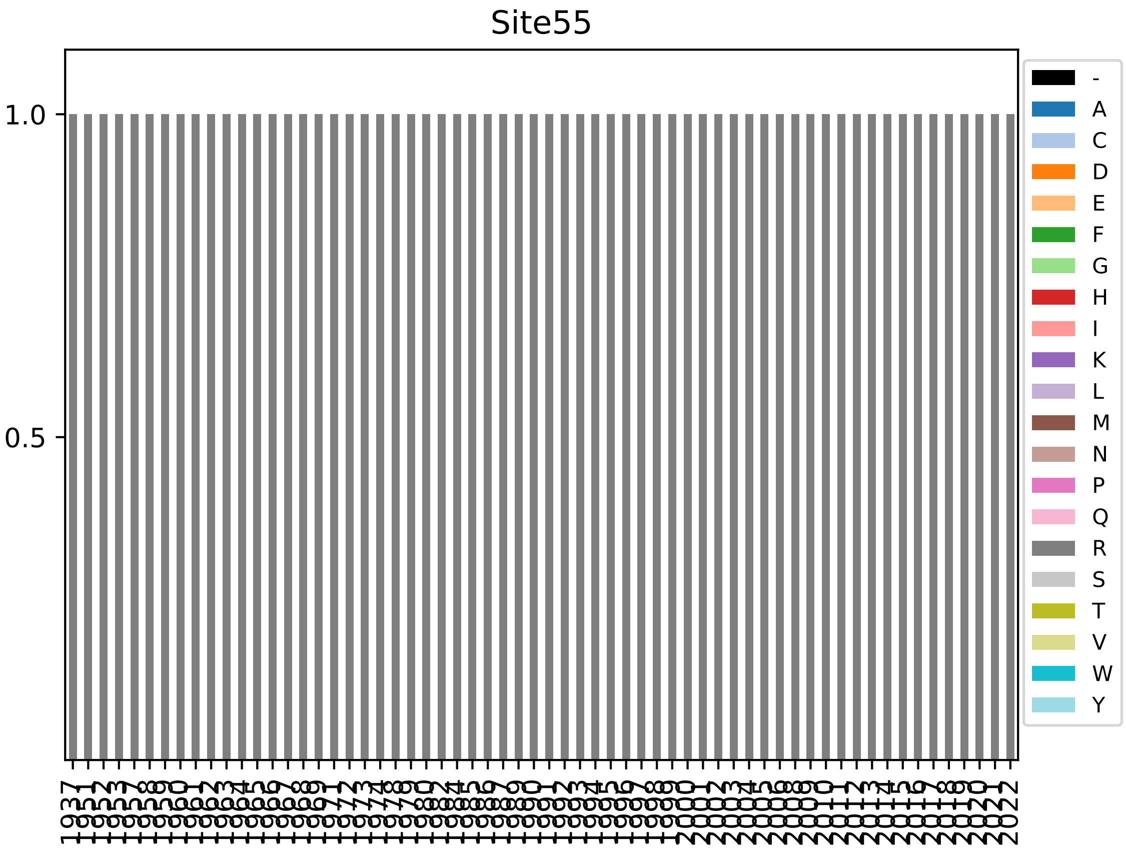

Site55
1.0
0.5
-
A
C
D
E
F
G
H
I
K
L
M
N
P
Q
R
S
T
V
W
Y

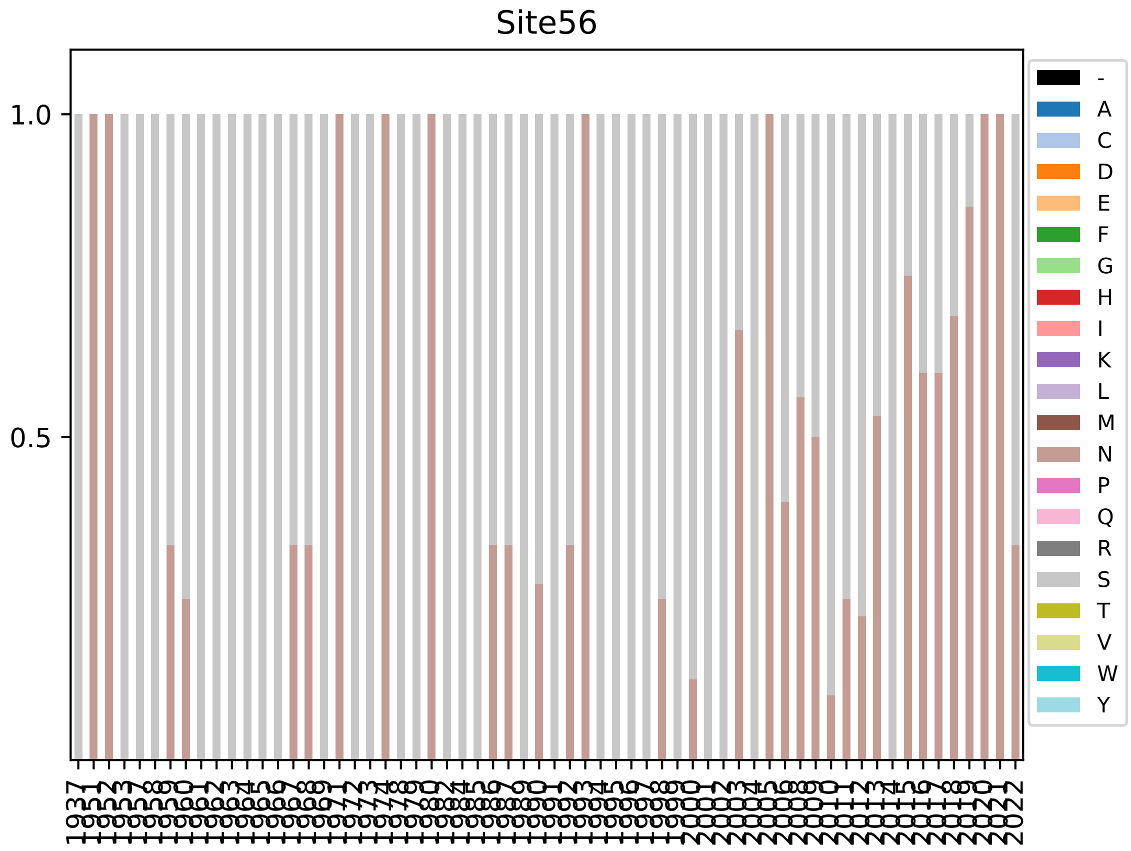
Site56
1.0
0.5
-
A
C
D
E
F
G
H
I
K
L
M
N
P
Q
R
S
T
V
W
Y

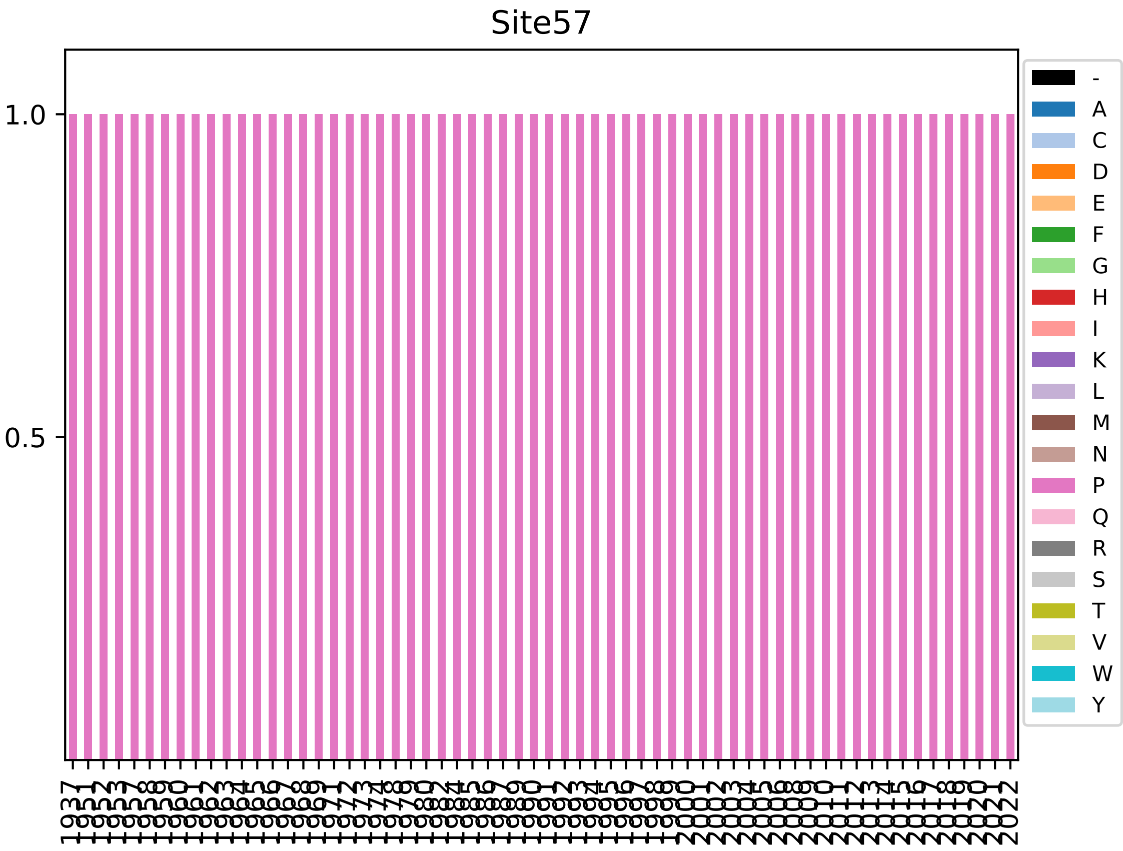
Site57
1.0
0.5
-
A
C
D
E
F
G
H
I
K
L
M
N
P
Q
R
S
T
V
W
Y

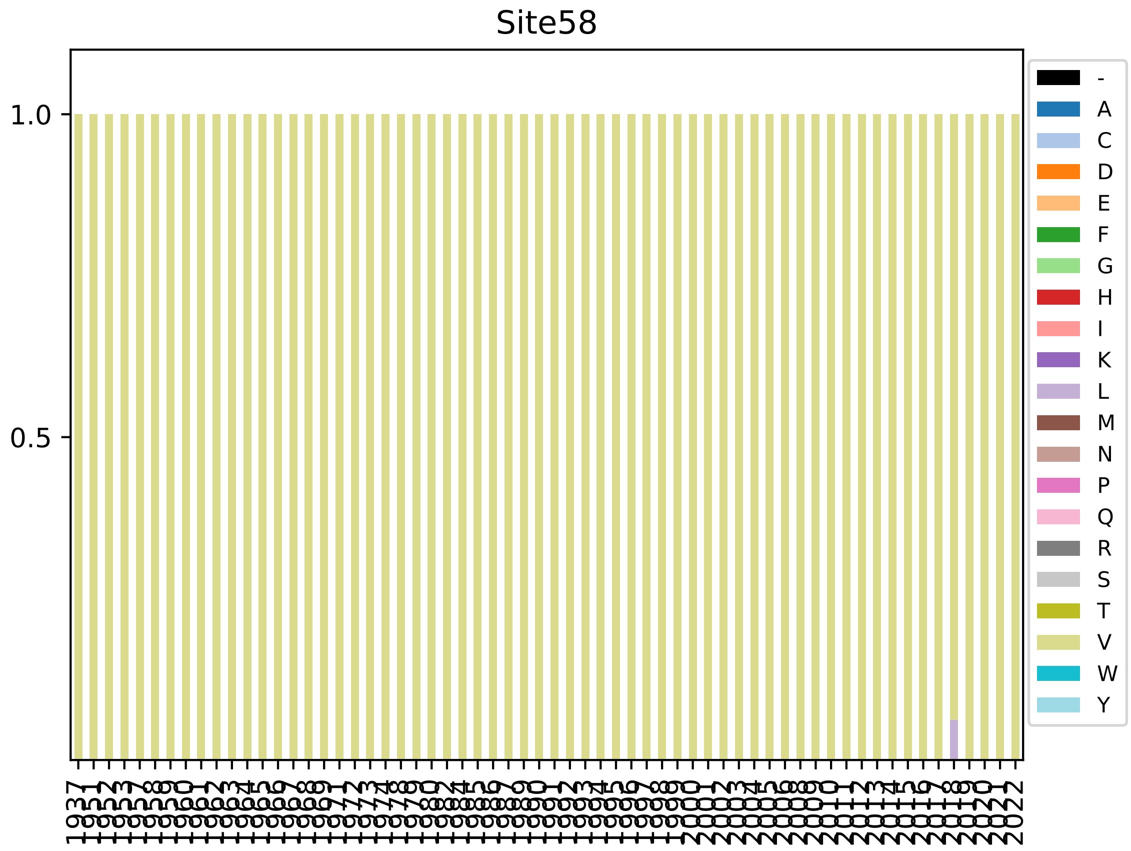
Site58
1.0
0.5
-
A
C
D
E
F
G
H
I
K
L
M
N
P
Q
R
S
T
V
W
Y

Site59

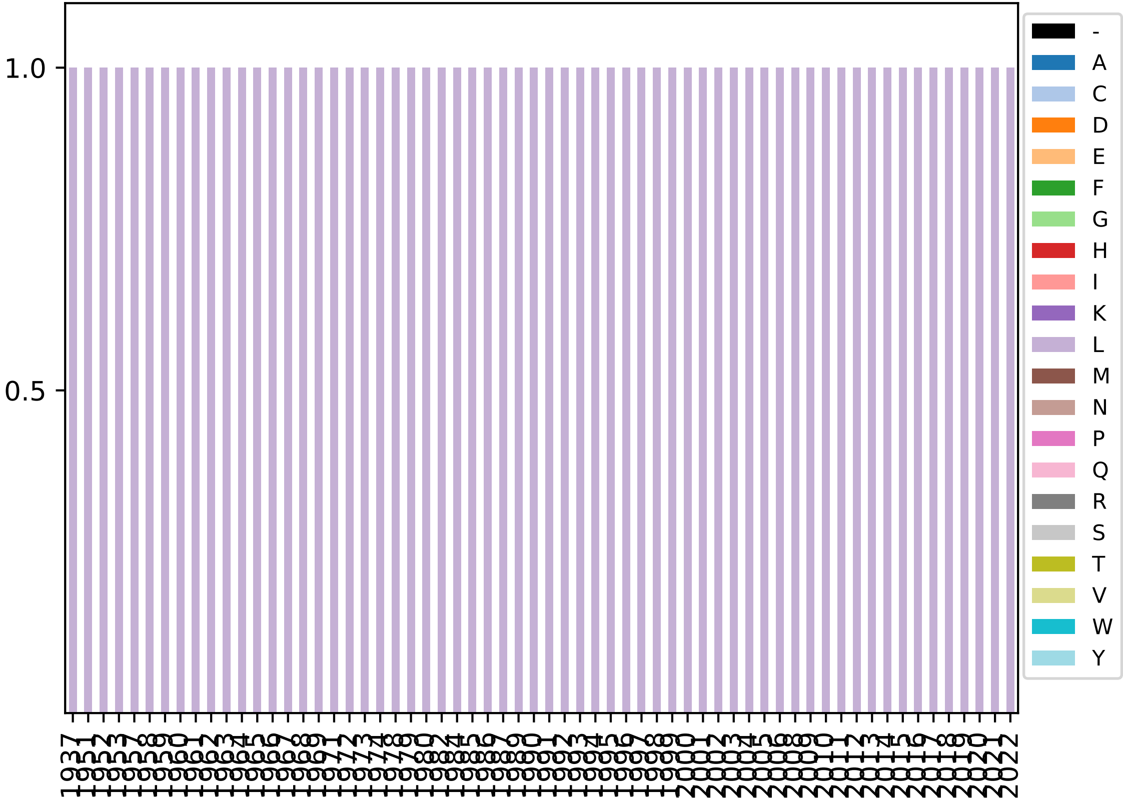

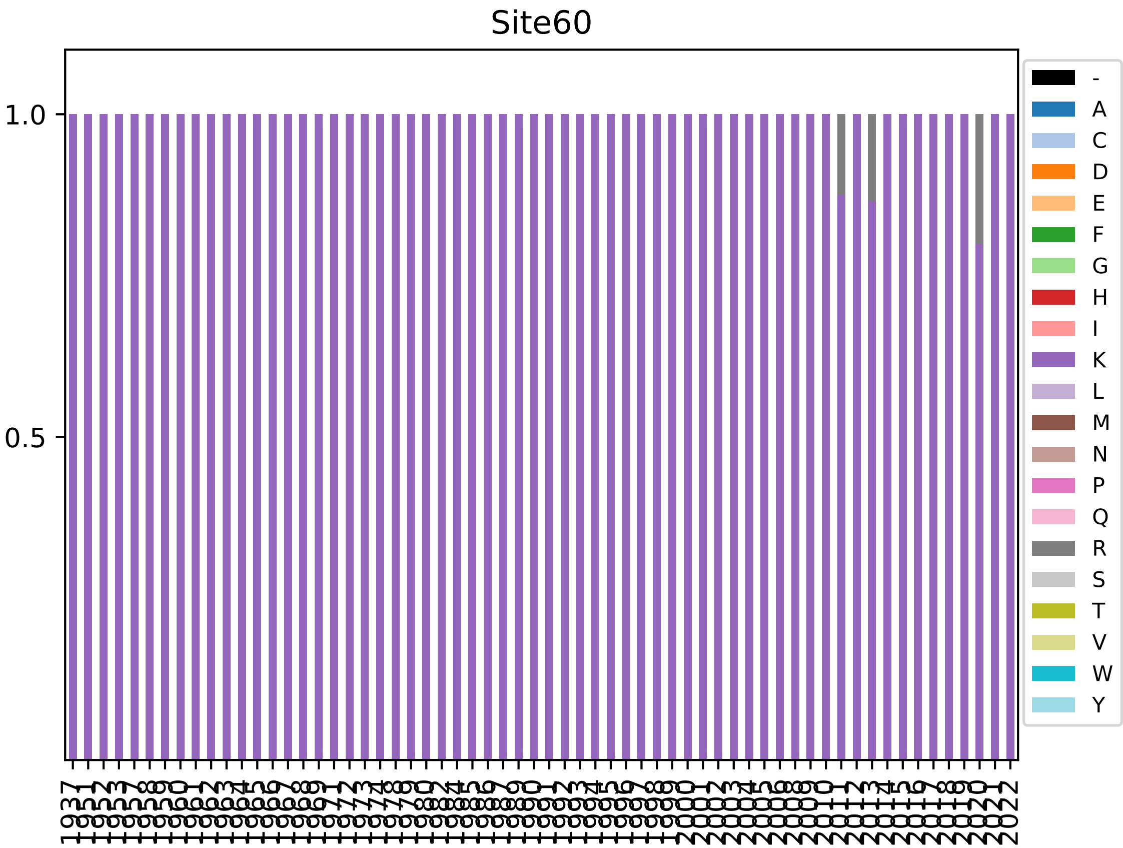
Site60
1.0
0.5
-
A
C
D
E
F
G
H
I
K
L
M
N
P
Q
R
S
T
V
W
Y

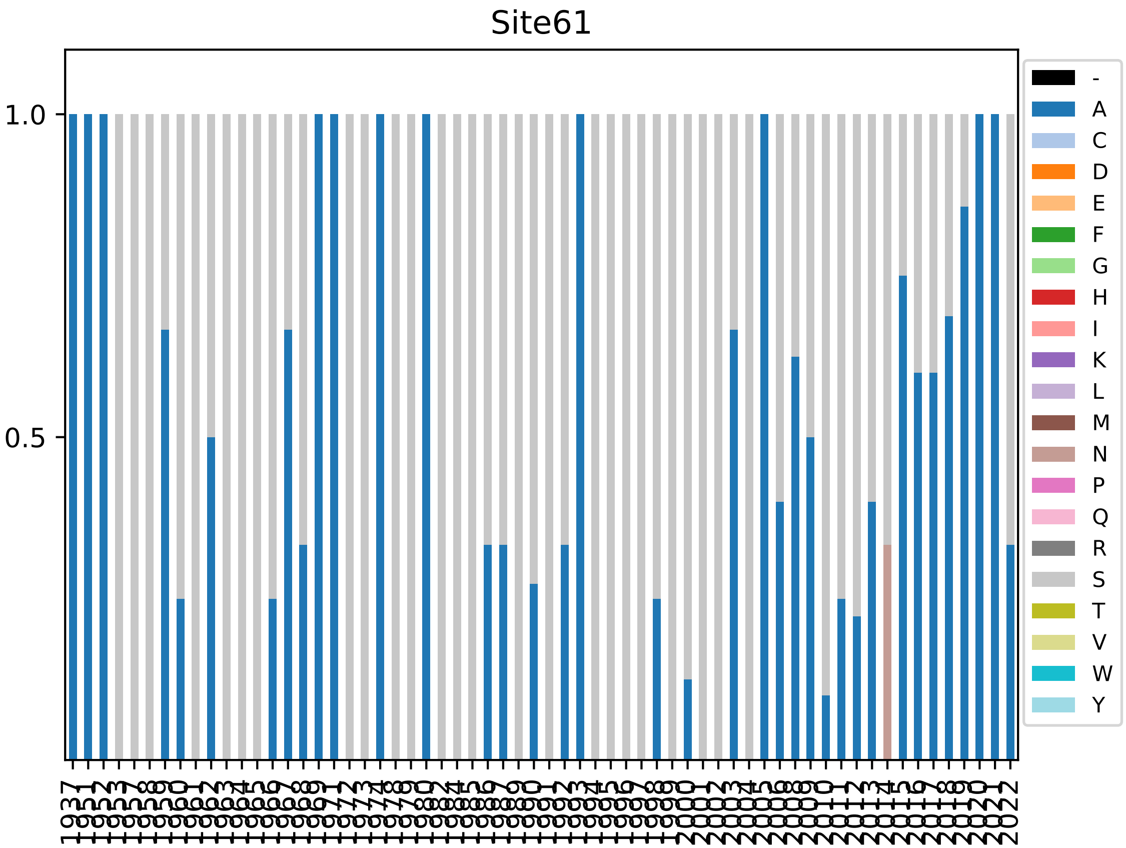
Site61
1.0
0.5
-
A
C
D
E
F
G
H
I
K
L
M
N
P
Q
R
S
T
V
W
Y

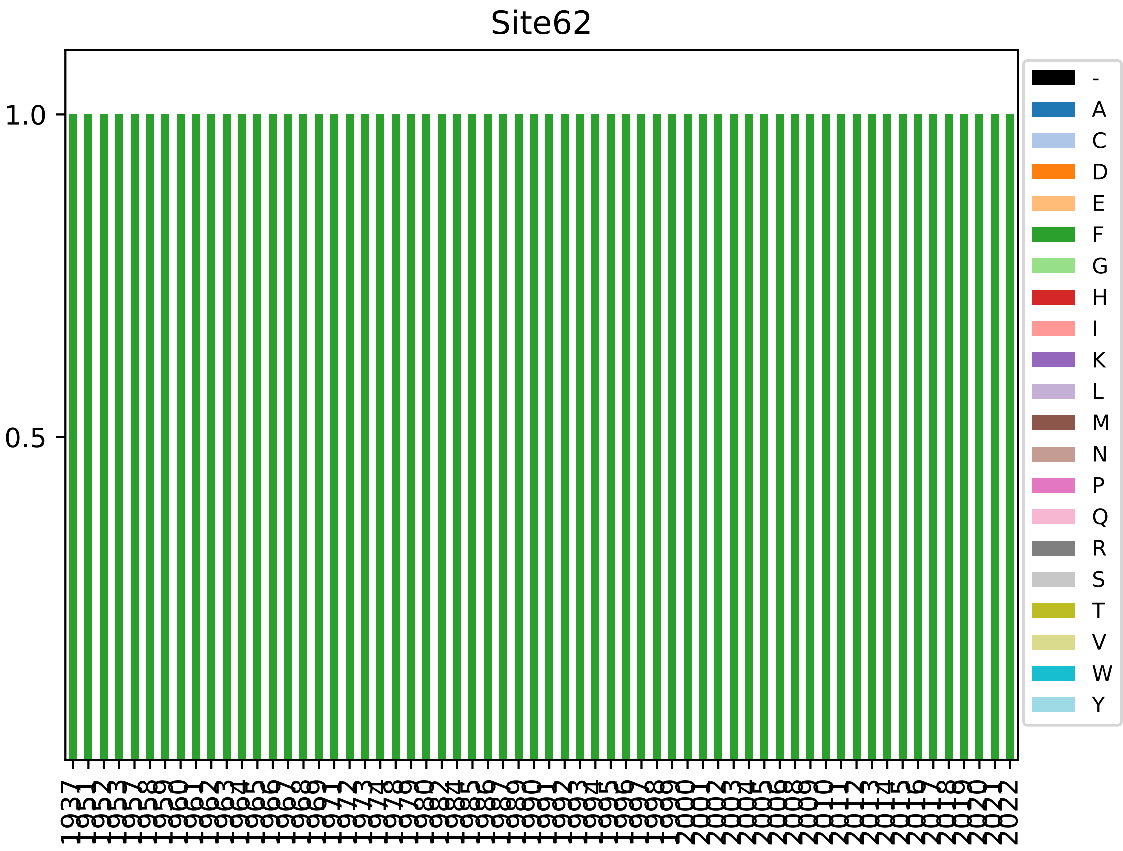
Site62
1.0
0.5
-
A
C
D
E
F
G
H
I
K
L
M
N
P
Q
R
S
T
V
W
Y

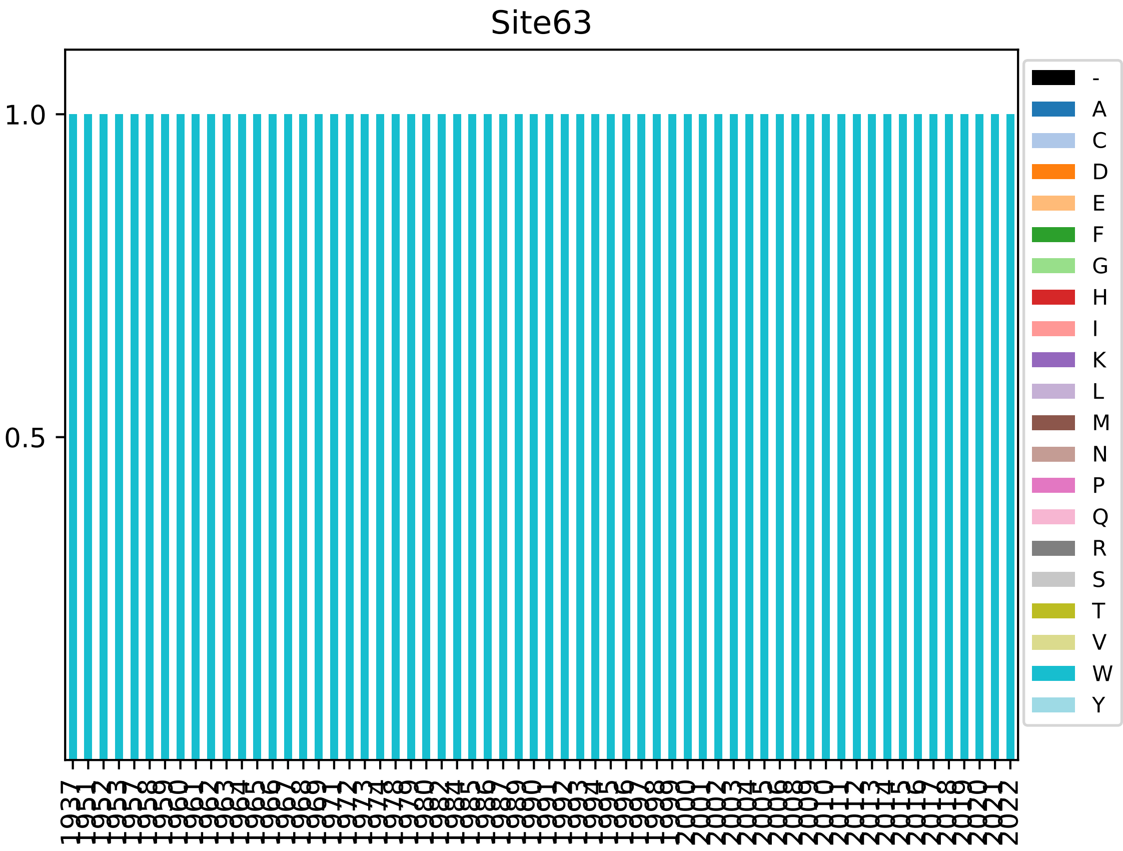
Site63
1.0
0.5
-
A
C
D
E
F
G
H
I
K
L
M
N
P
Q
R
S
T
V
W
Y

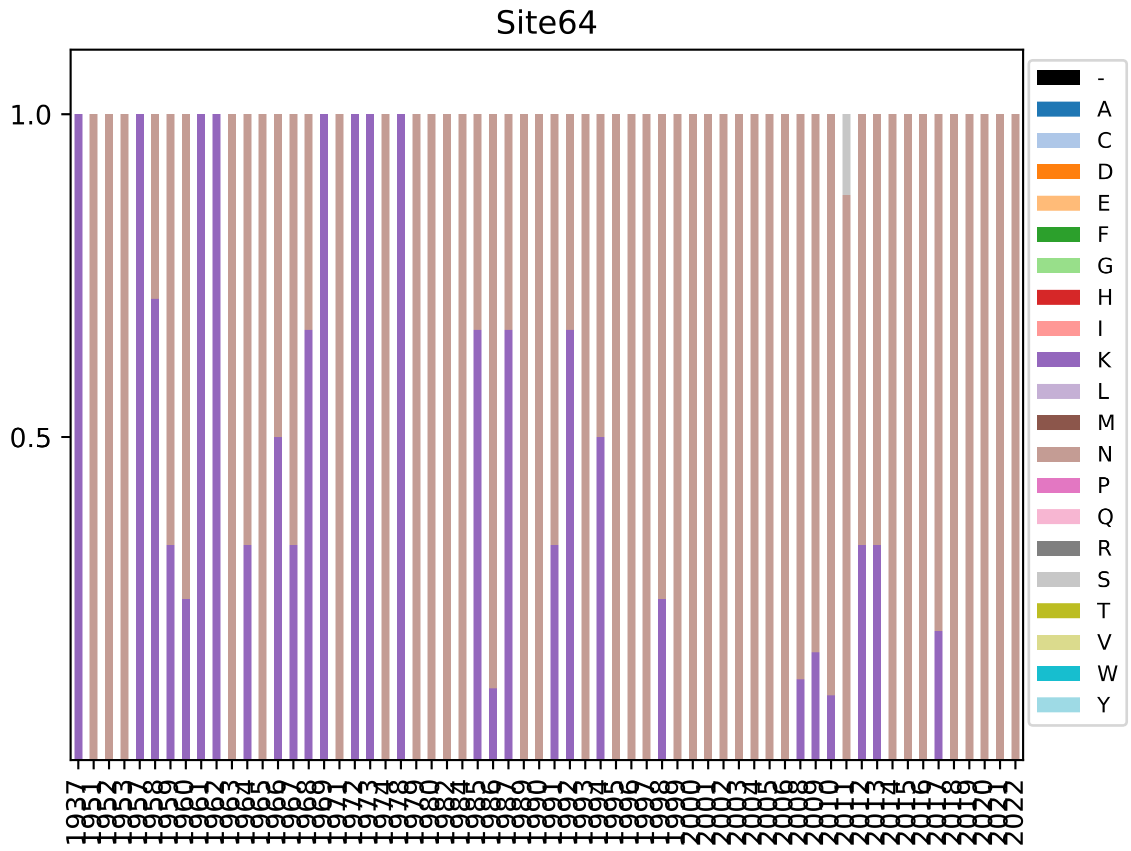
Site64
1.0
0.5
-
A
C
D
E
F
G
H
I
K
L
M
N
P
Q
R
S
T
V
W
Y

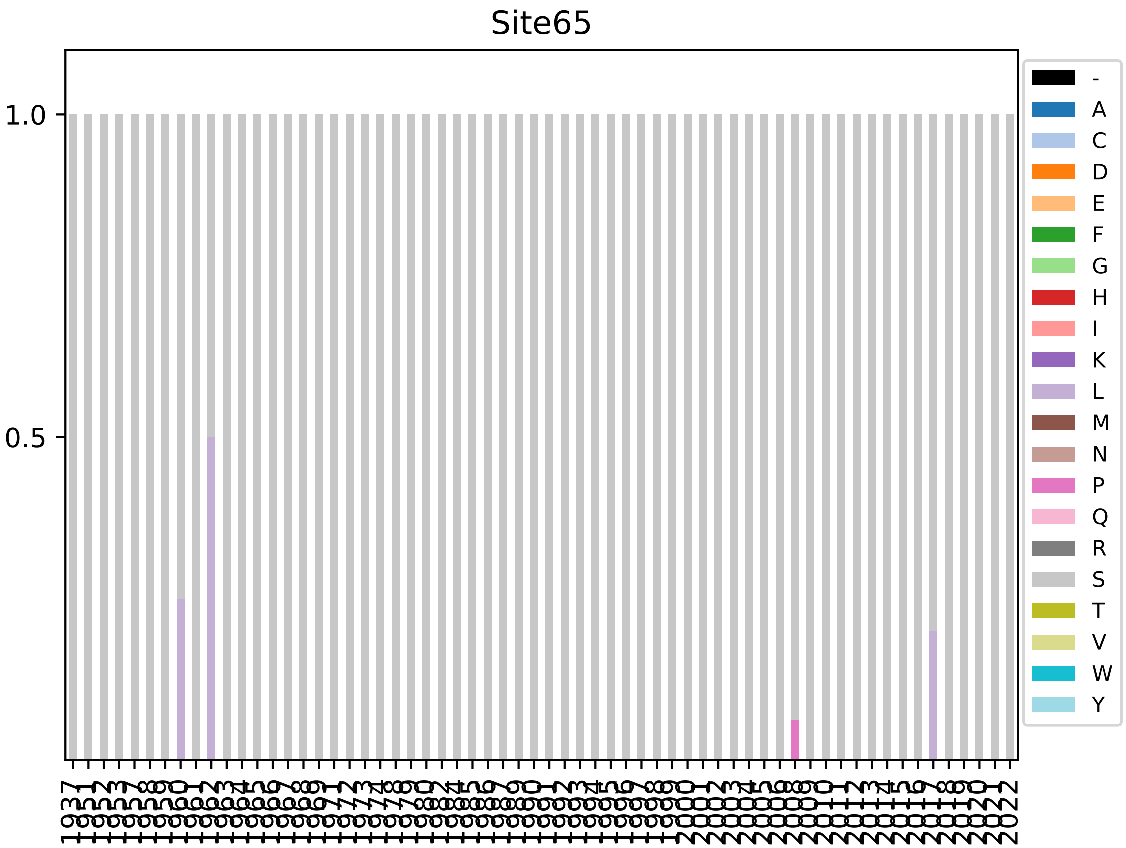

Site65
1.0
0.5
-
A
C
D
E
F
G
H
I
K
L
M
N
P
Q
R
S
T
V
W
Y

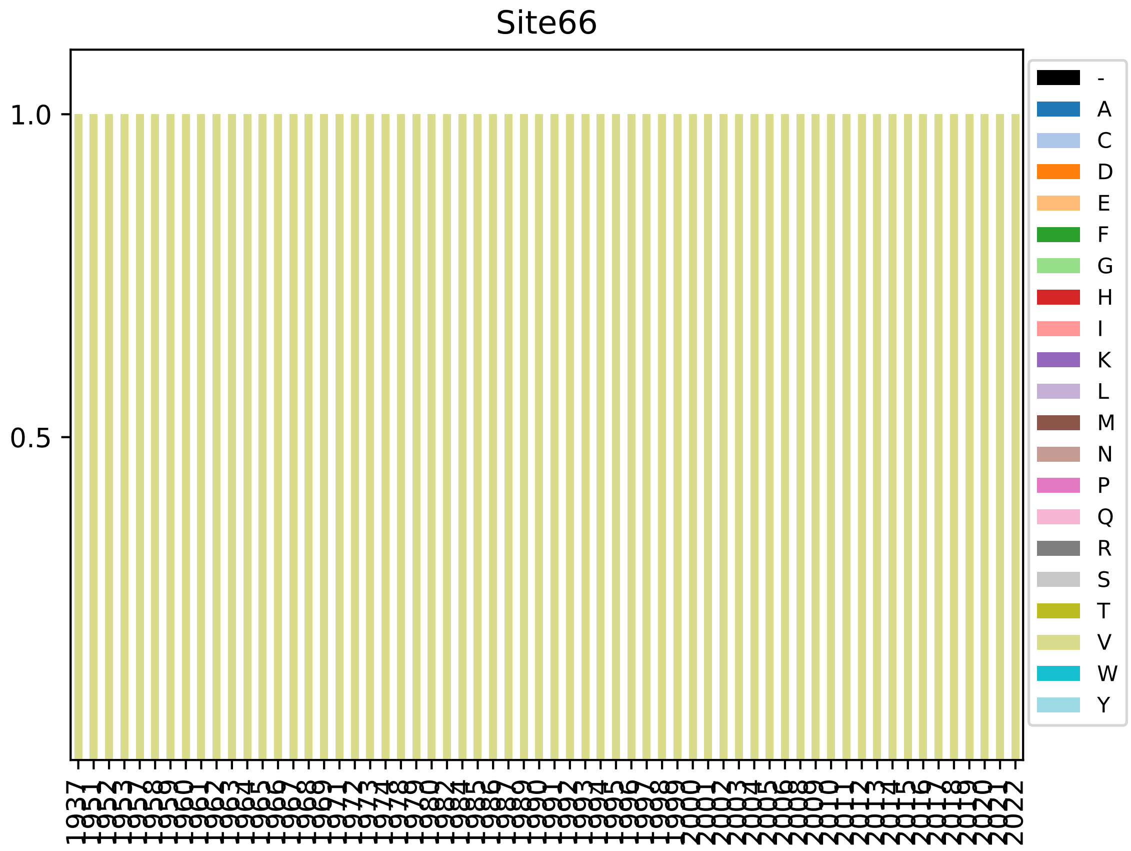
Site66
1.0
0.5
-
A
C
D
E
F
G
H
I
K
L
M
N
P
Q
R
S
T
V
W
Y

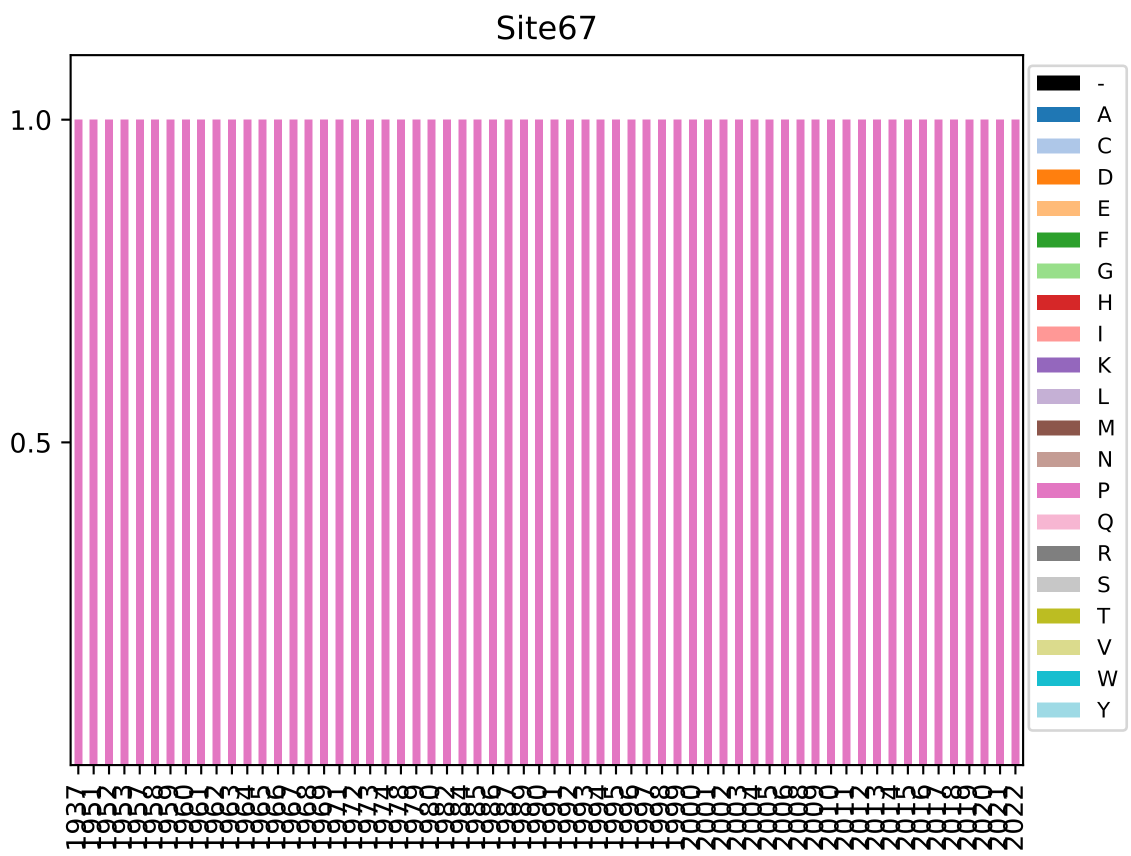
Site67
1.0
0.5
-
A
C
D
E
F
G
H
I
K
L
M
N
P
Q
R
S
T
V
W
Y

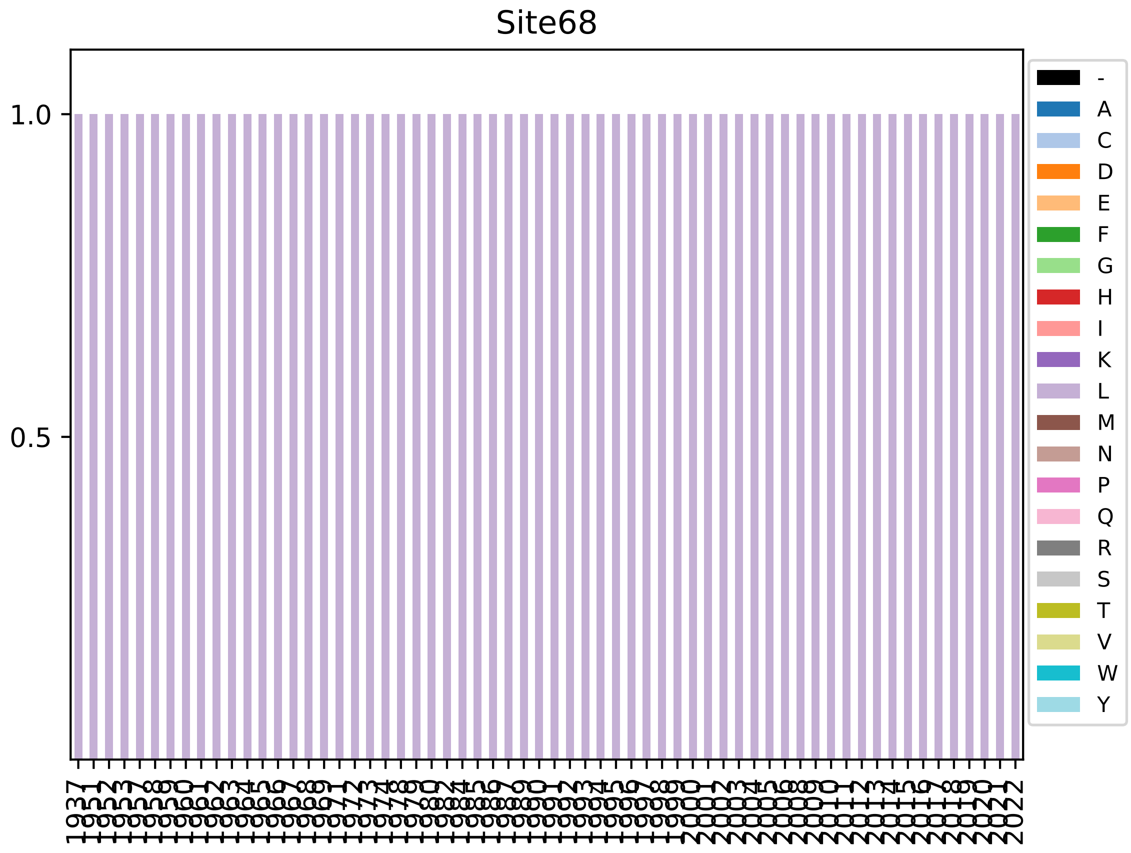
Site68
1.0
0.5
-
A
C
D
E
F
G
H
I
K
L
M
N
P
Q
R
S
T
V
W
Y

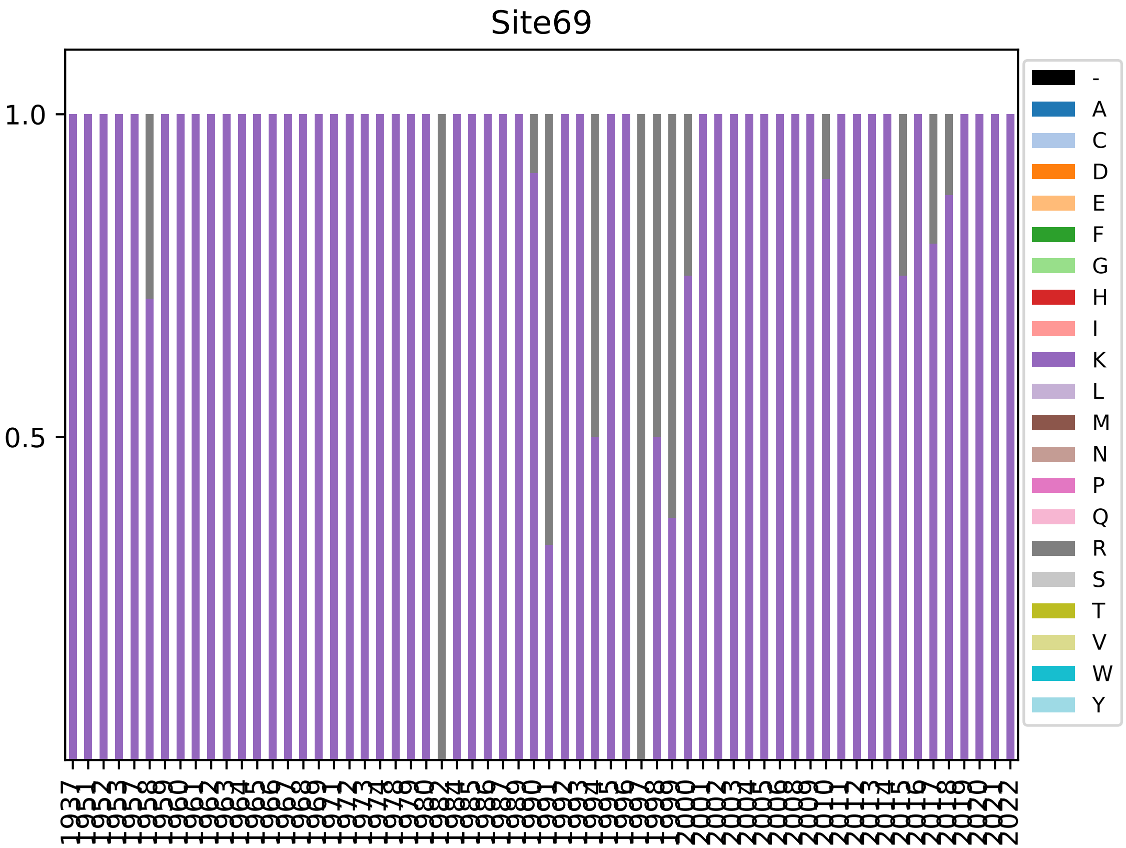
Site69
1.0
0.5
-
A
C
D
E
F
G
H
I
K
L
M
N
P
Q
R
S
T
V
W
Y

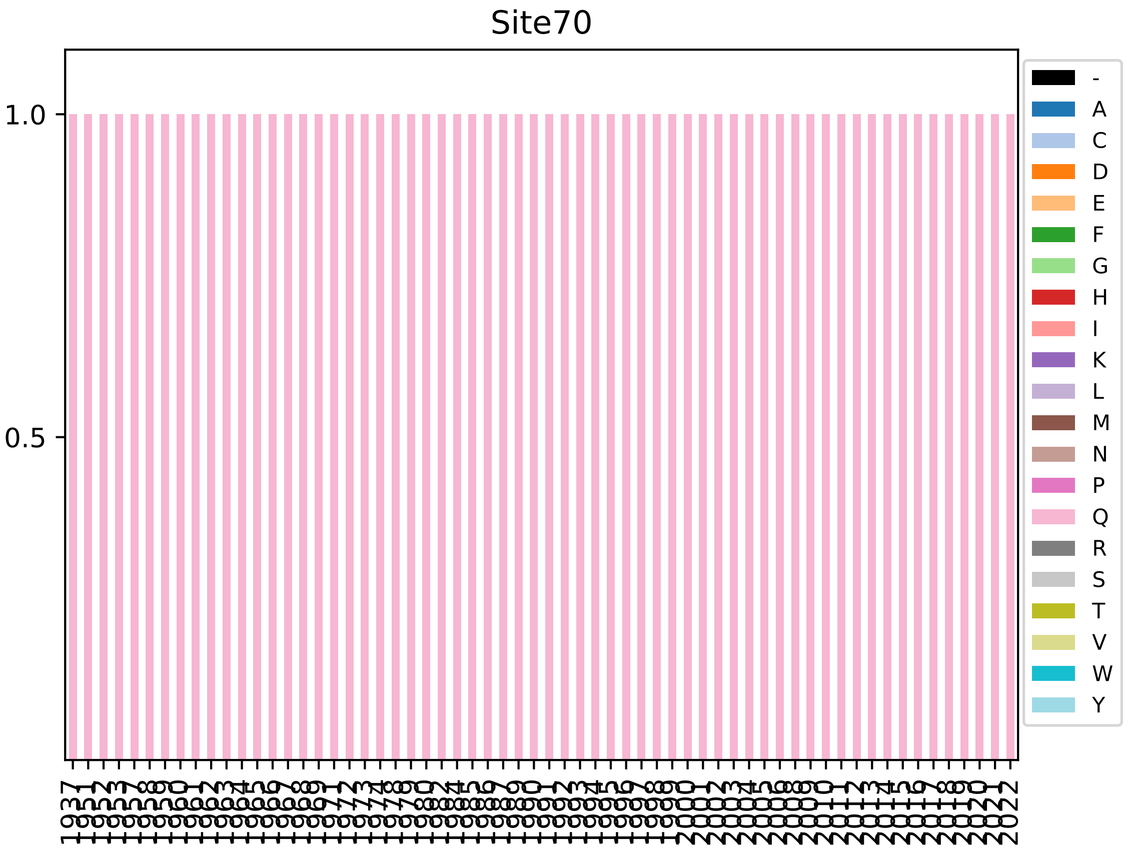
Site70
1.0
0.5
-
A
C
D
E
F
G
H
I
K
L
M
N
P
Q
R
S
T
V
W
Y

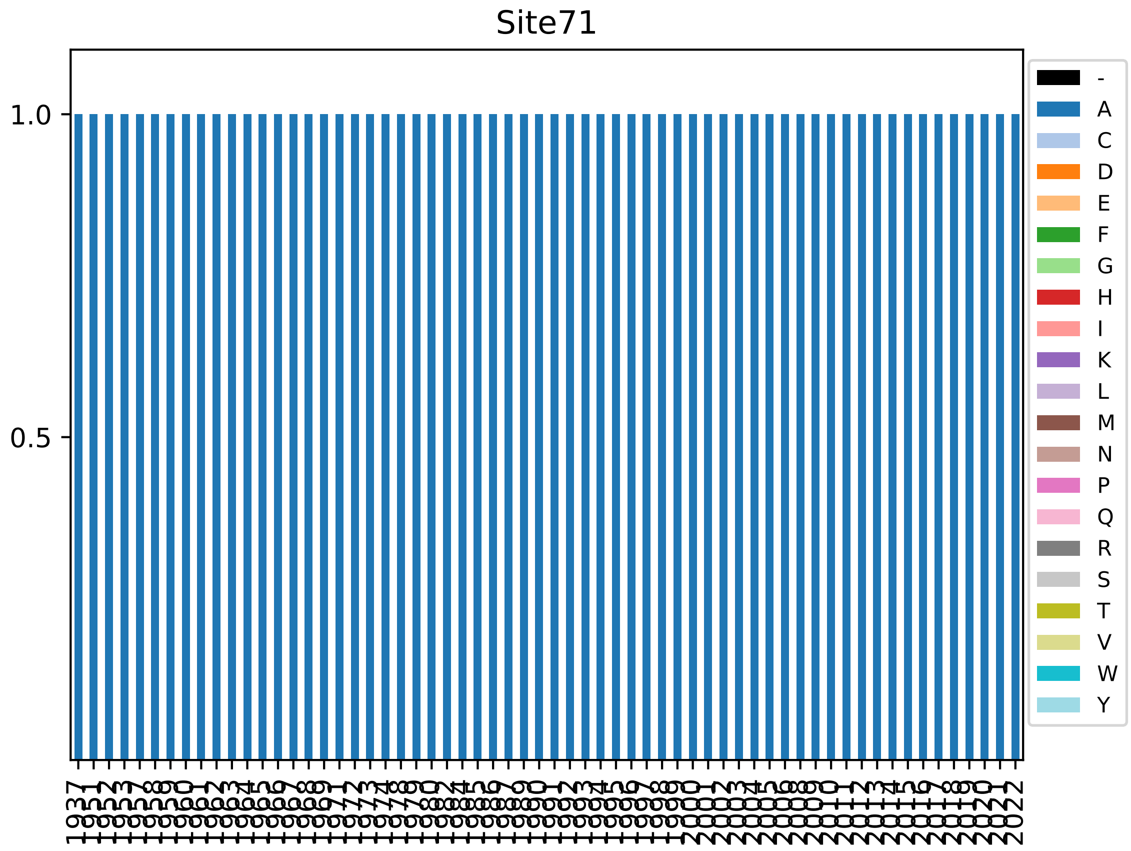
Site71
1.0
0.5
-
A
C
D
E
F
G
H
I
K
L
M
N
P
Q
R
S
T
V
W
Y

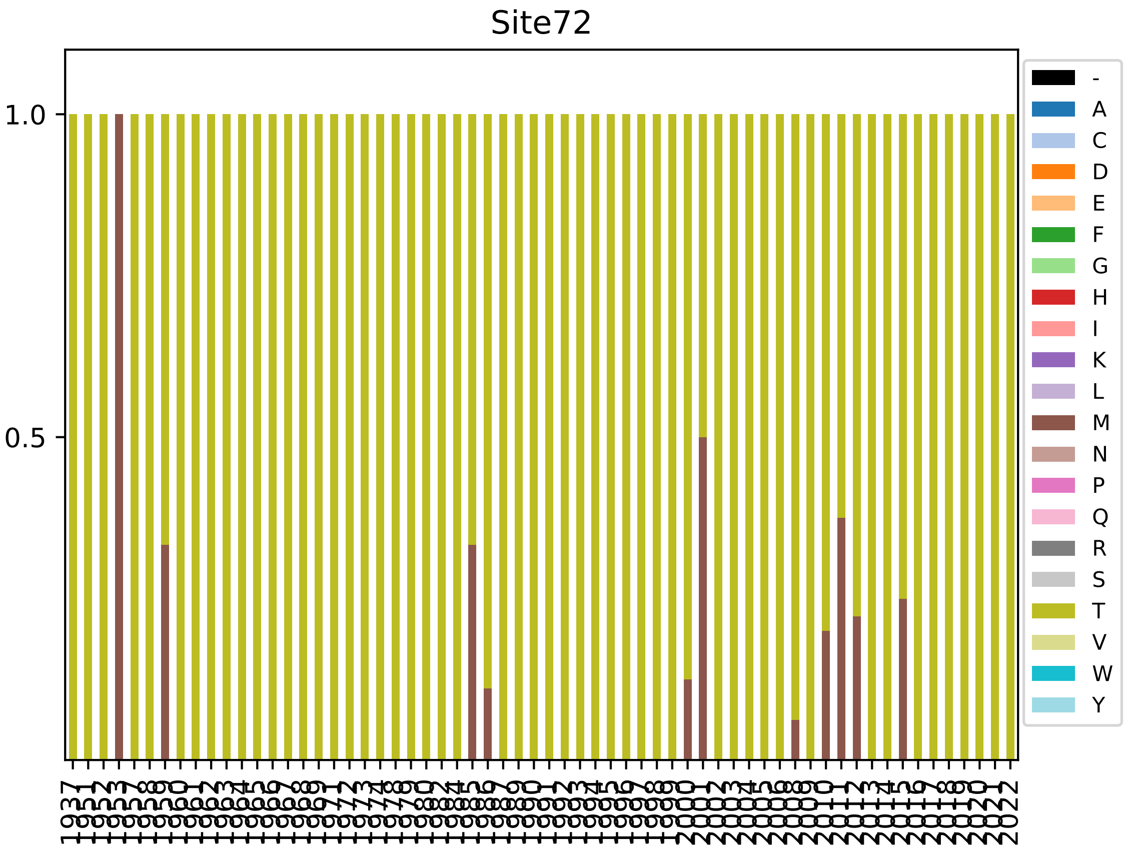
Site72
1.0
0.5
-
A
C
D
E
F
G
H
I
K
L
M
N
P
Q
R
S
T
V
W
Y

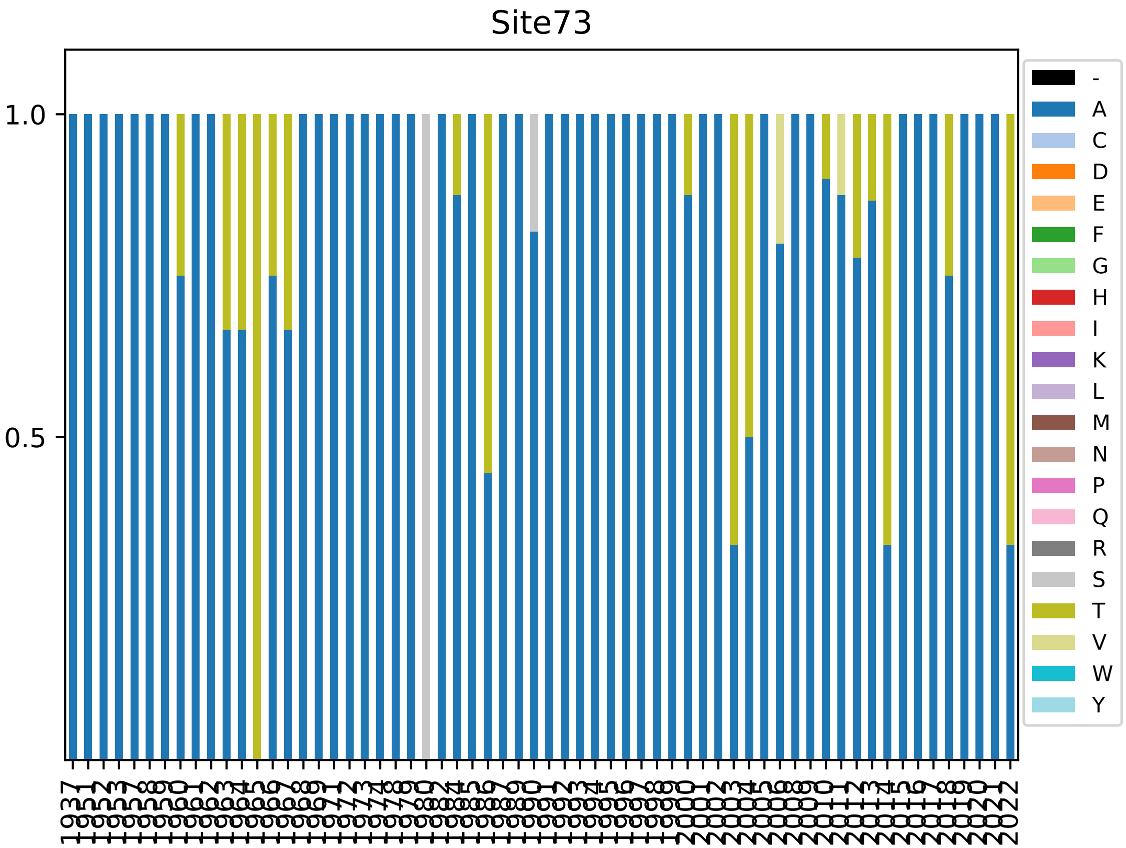
Site73
1.0
0.5
-
A
C
D
E
F
G
H
I
K
L
M
N
P
Q
R
S
T
V
W
Y

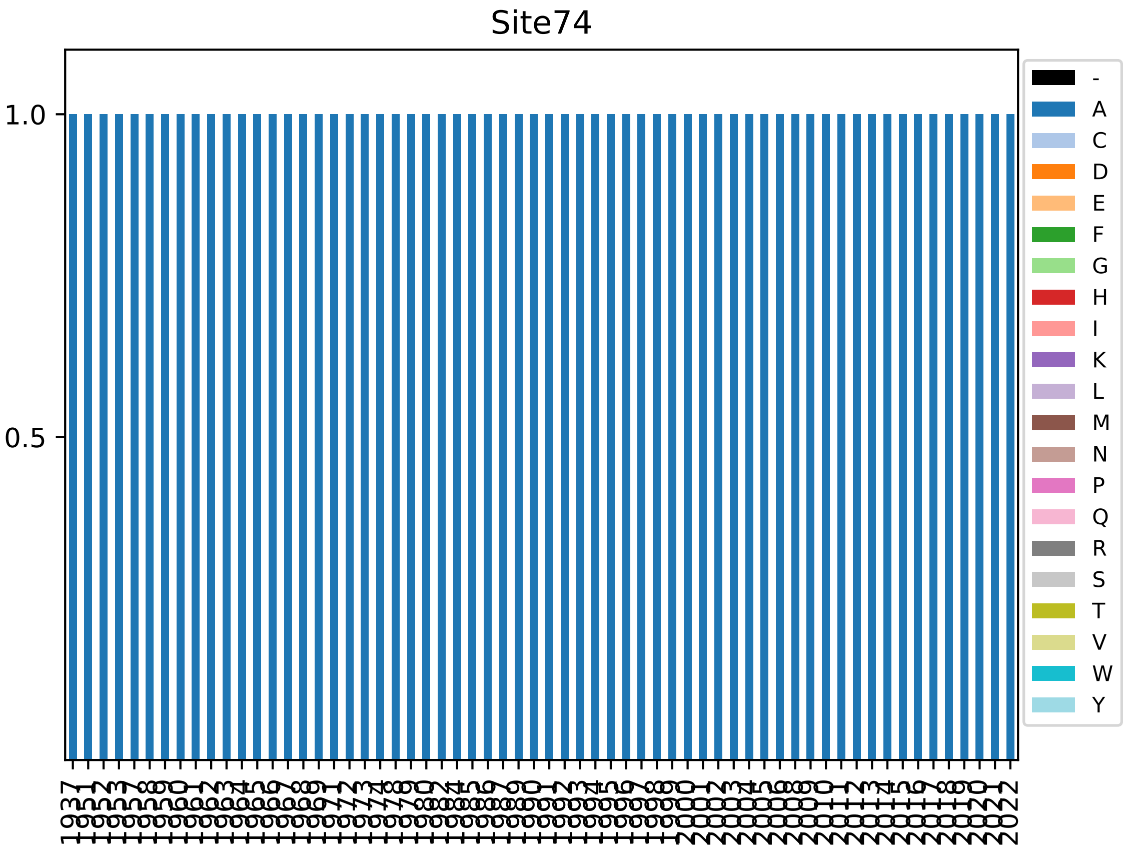
Site74
1.0
0.5
-
A
C
D
E
F
G
H
I
K
L
M
N
P
Q
R
S
T
V
W
Y

Site75

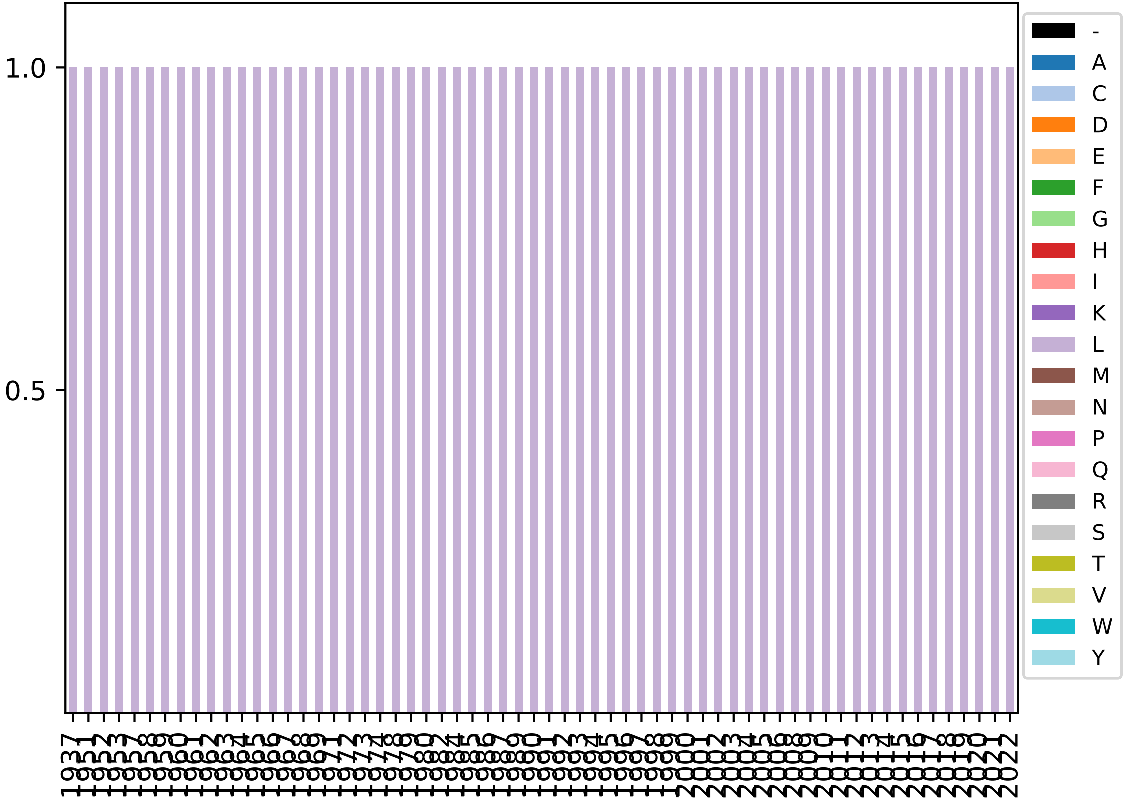

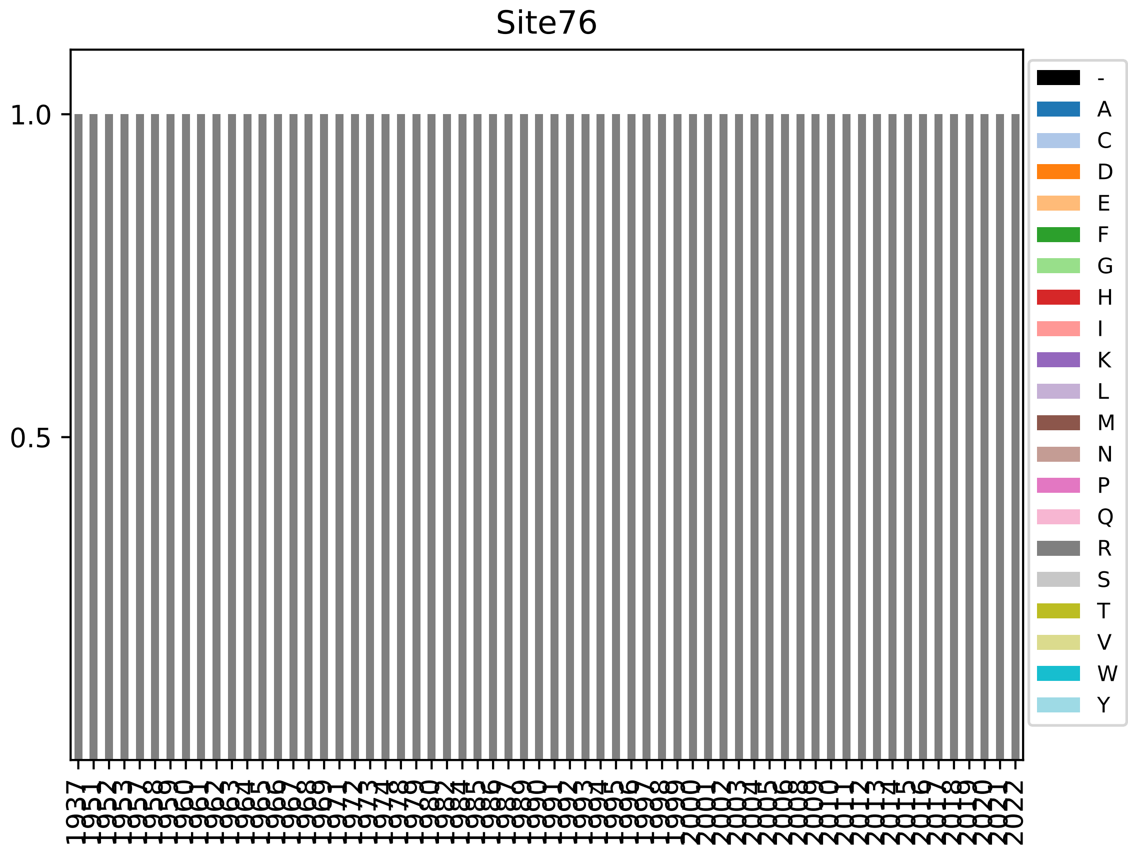

Site76
1.0
0.5
-
A
C
D
E
F
G
H
I
K
L
M
N
P
Q
R
S
T
V
W
Y

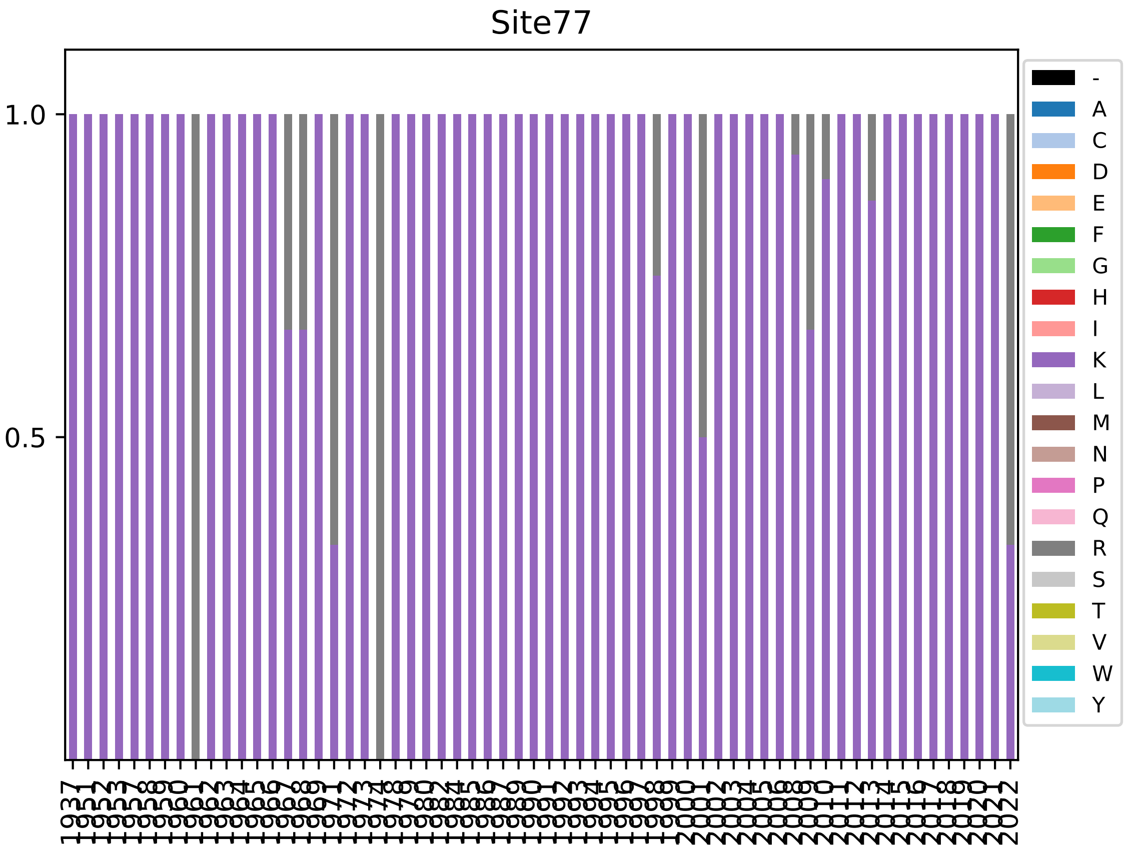
Site77
1.0
0.5
-
A
C
D
E
F
G
H
I
K
L
M
N
P
Q
R
S
T
V
W
Y

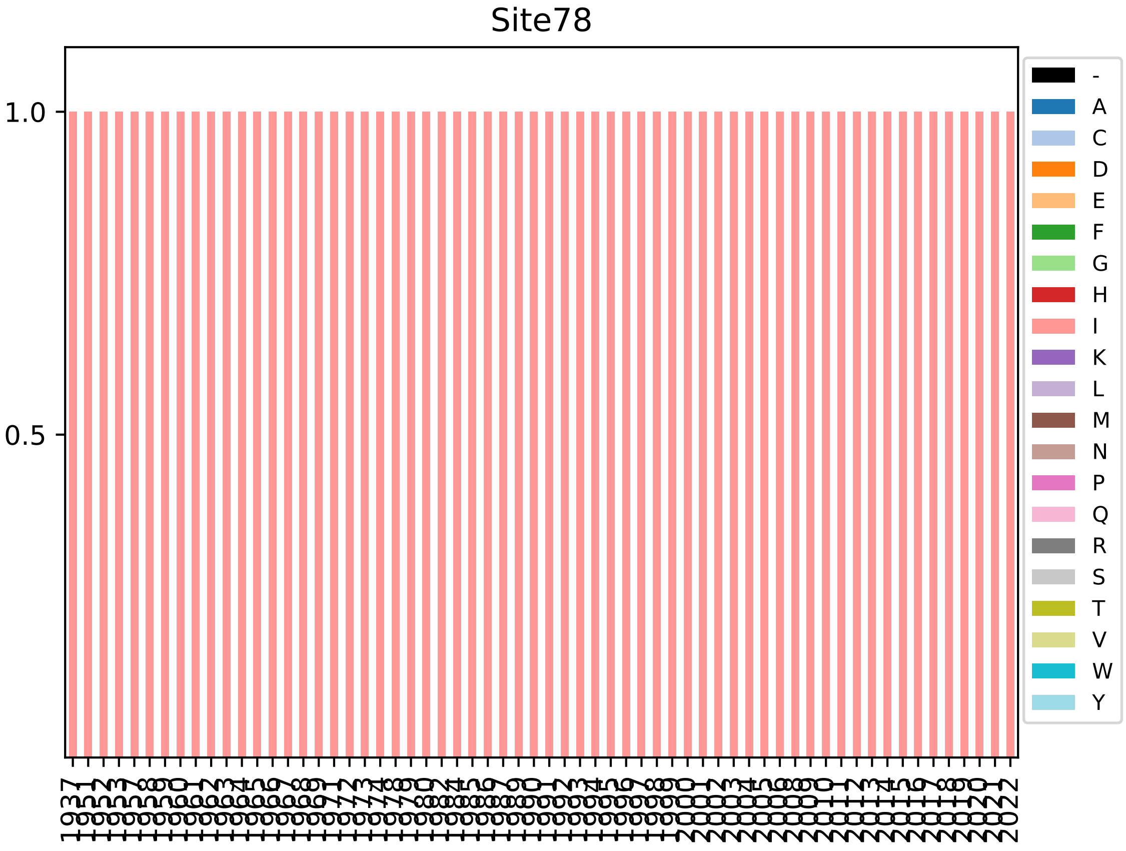

Site78
1.0
0.5
-
A
C
D
E
F
G
H
I
K
L
M
N
P
Q
R
S
T
V
W
Y

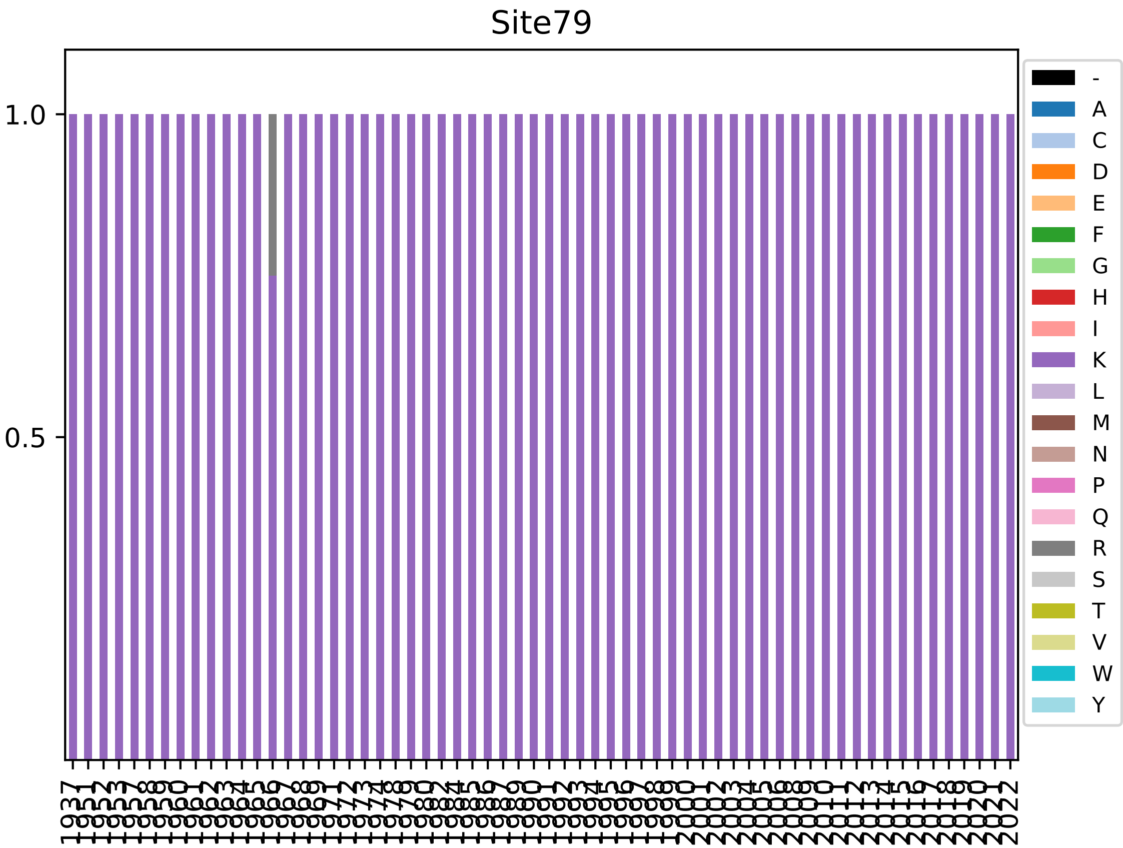
Site79
1.0
0.5
-
A
C
D
E
F
G
H
I
K
L
M
N
P
Q
R
S
T
V
W
Y

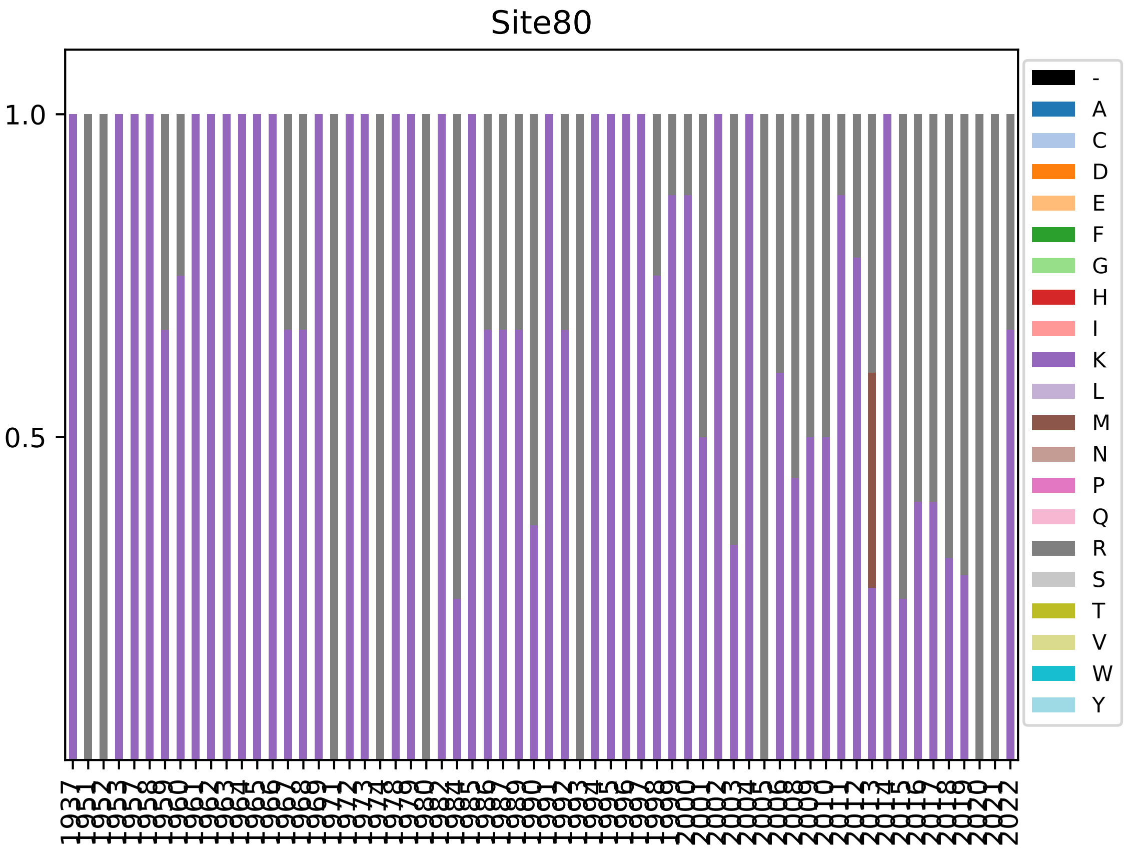
Site80
1.0
0.5
-
A
C
D
E
F
G
H
I
K
L
M
N
P
Q
R
S
T
V
W
Y

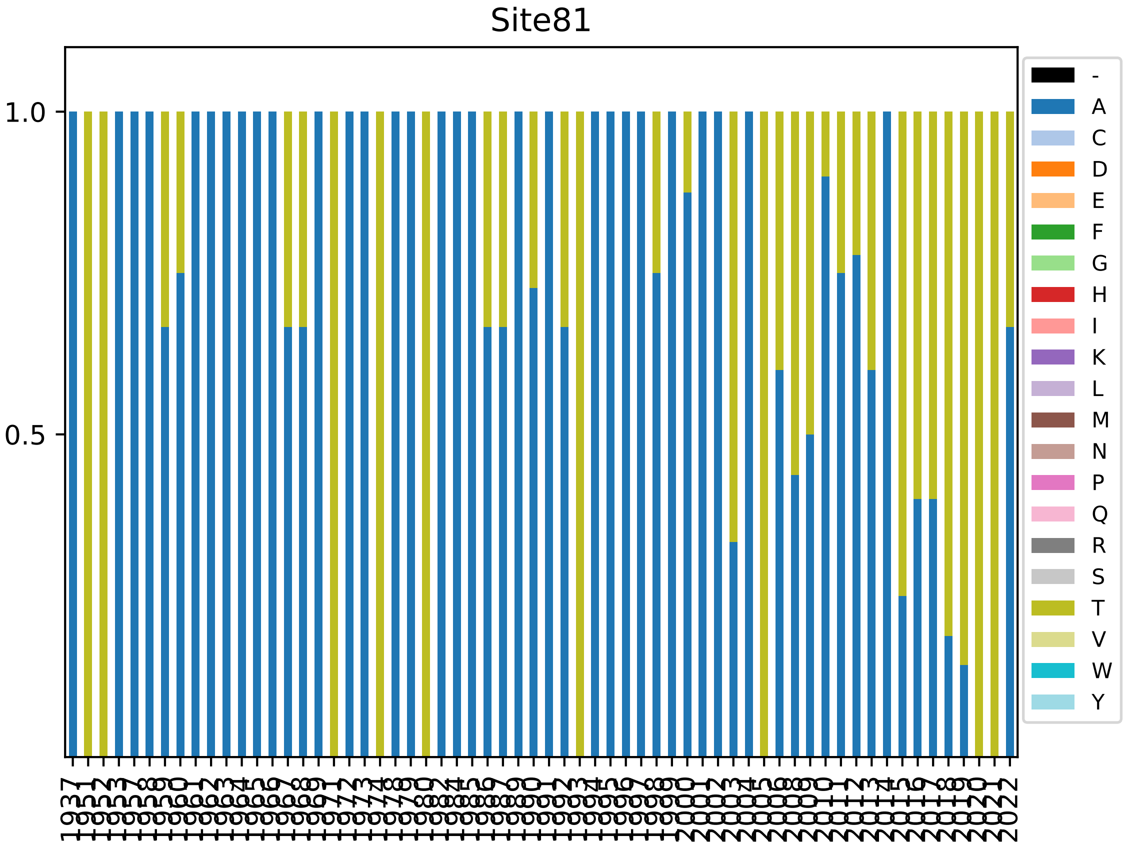
Site81
1.0
0.5
-
A
C
D
E
F
G
H
I
K
L
M
N
P
Q
R
S
T
V
W
Y

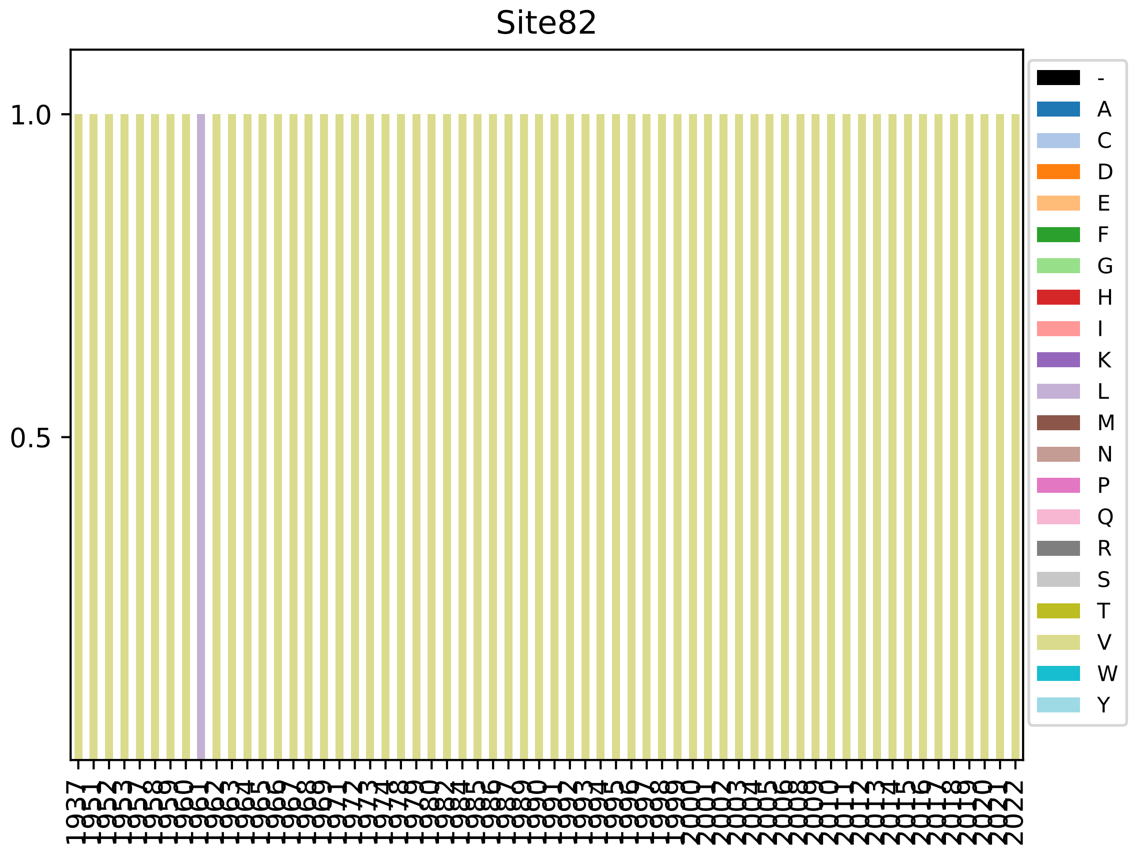
Site82
1.0
0.5
-
A
C
D
E
F
G
H
I
K
L
M
N
P
Q
R
S
T
V
W
Y

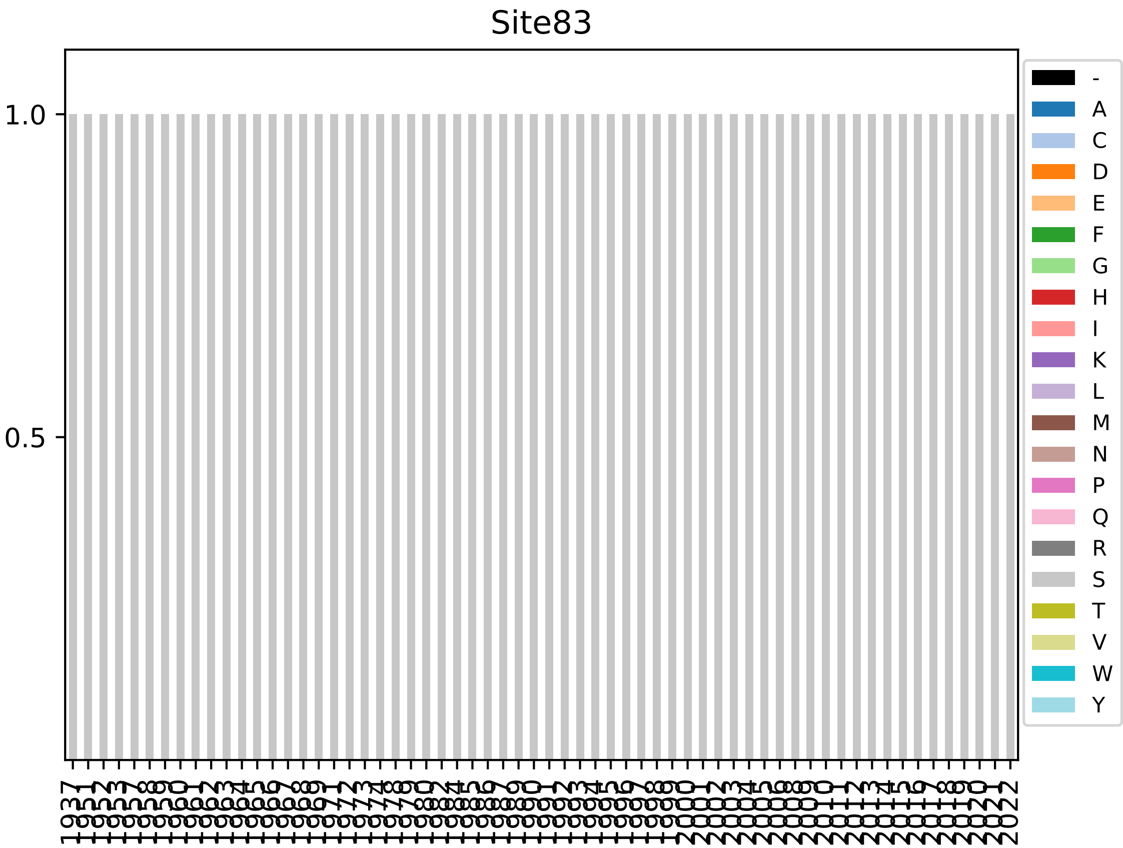
Site83
1.0
0.5
-
A
C
D
E
F
G
H
I
K
L
M
N
P
Q
R
S
T
V
W
Y

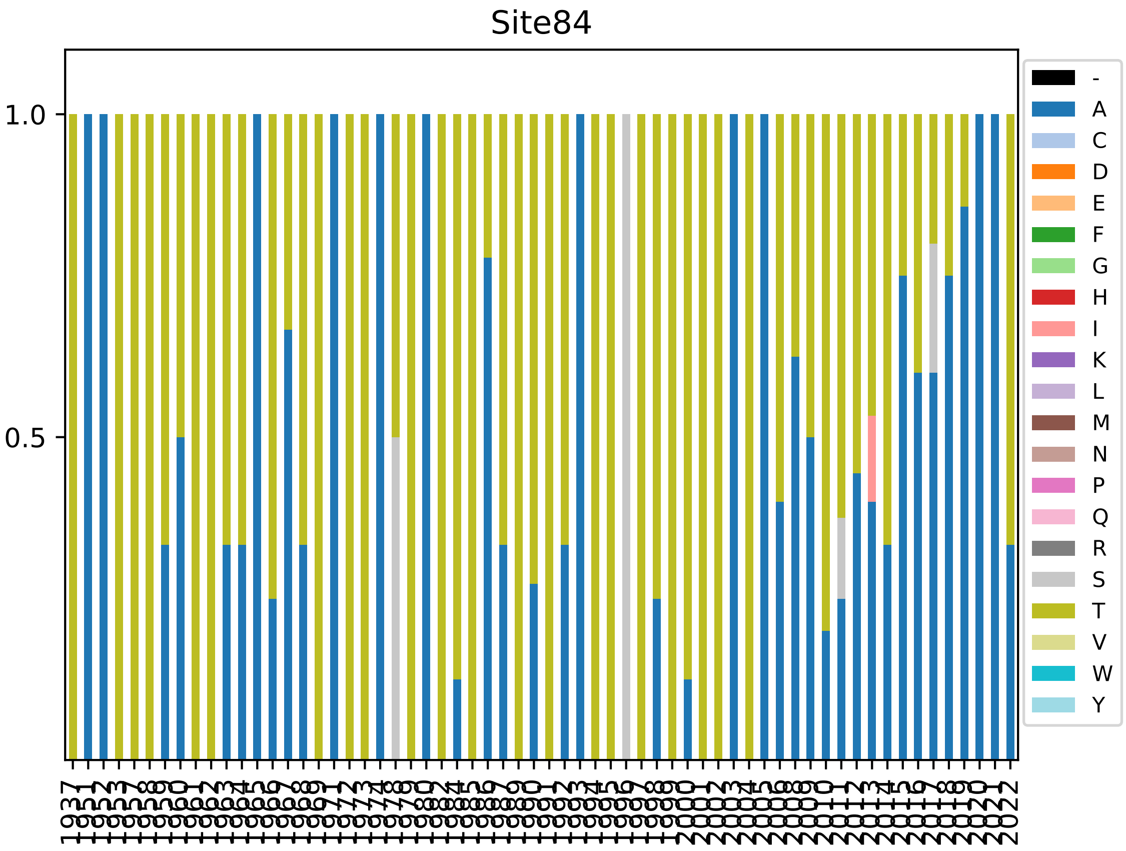
Site84
1.0
0.5
-
A
C
D
E
F
G
H
I
K
L
M
N
P
Q
R
S
T
V
W
Y

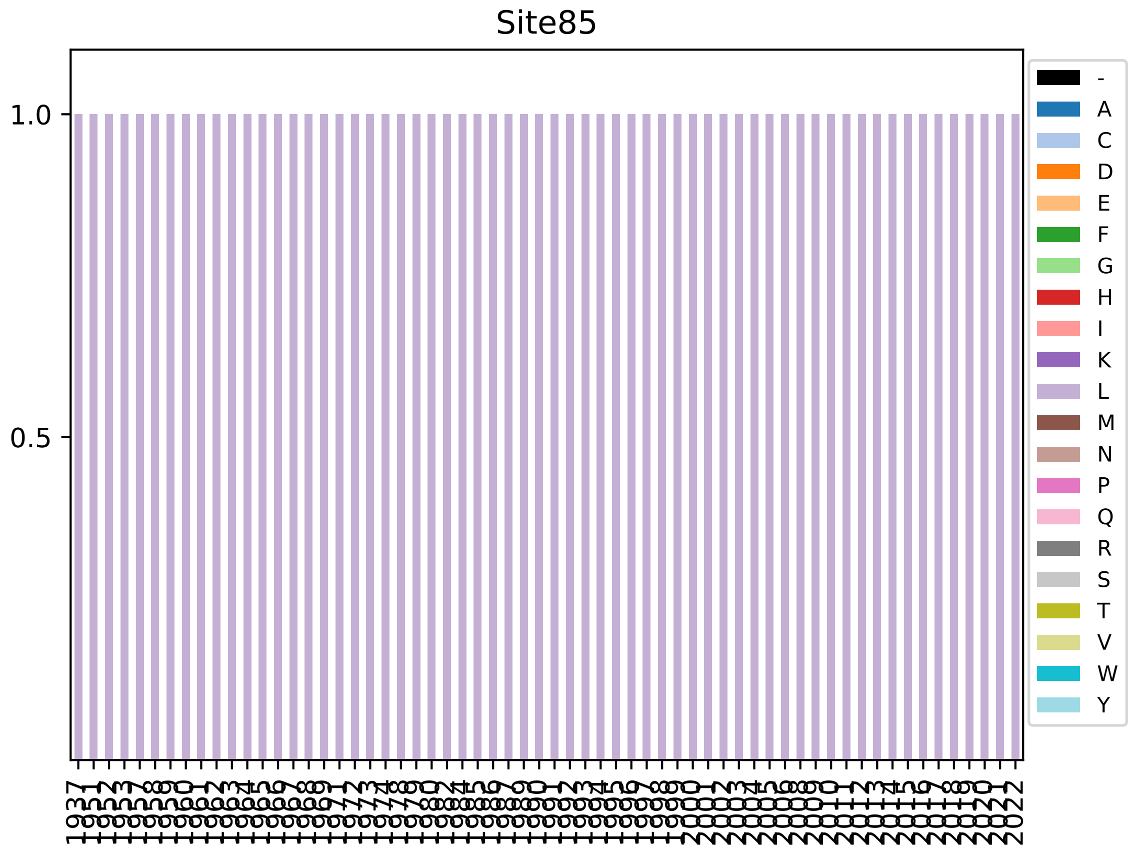
Site85
1.0
0.5
-
A
C
D
E
F
G
H
I
K
L
M
N
P
Q
R
S
T
V
W
Y

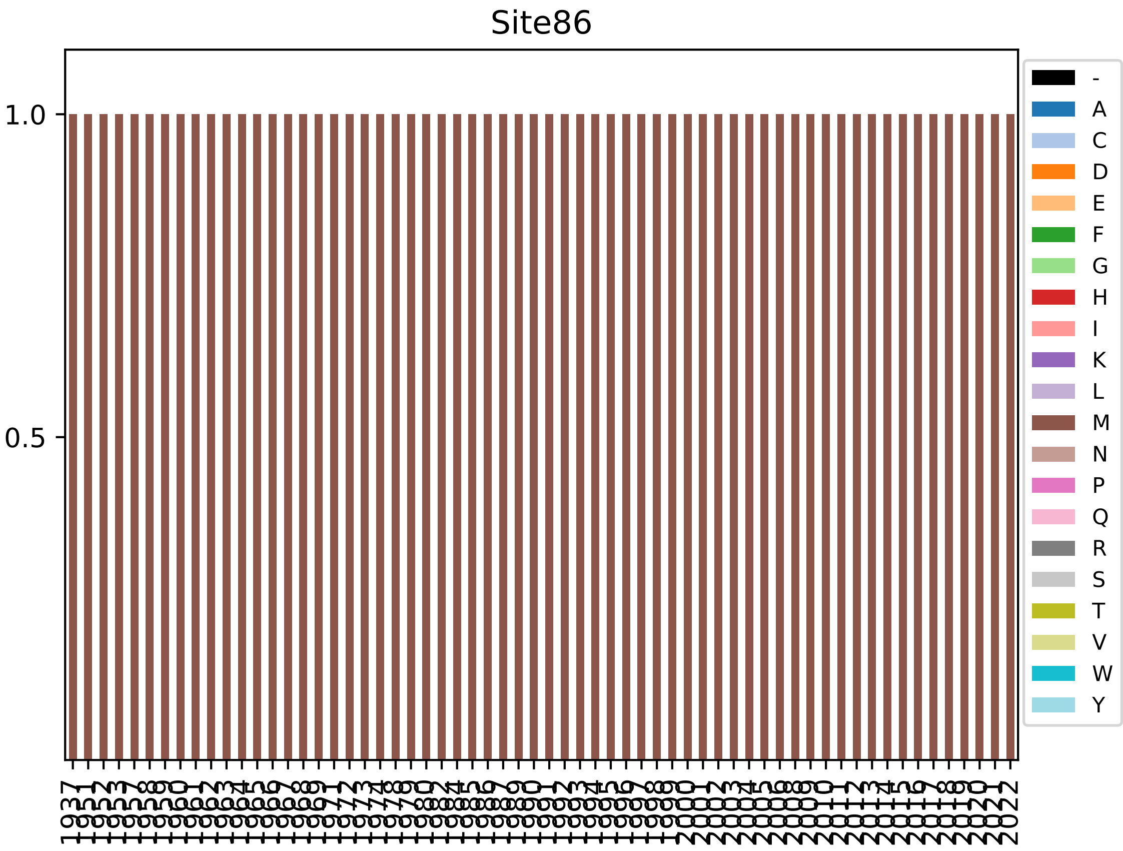
Site86
1.0
0.5
-
A
C
D
E
F
G
H
I
K
L
M
N
P
Q
R
S
T
V
W
Y

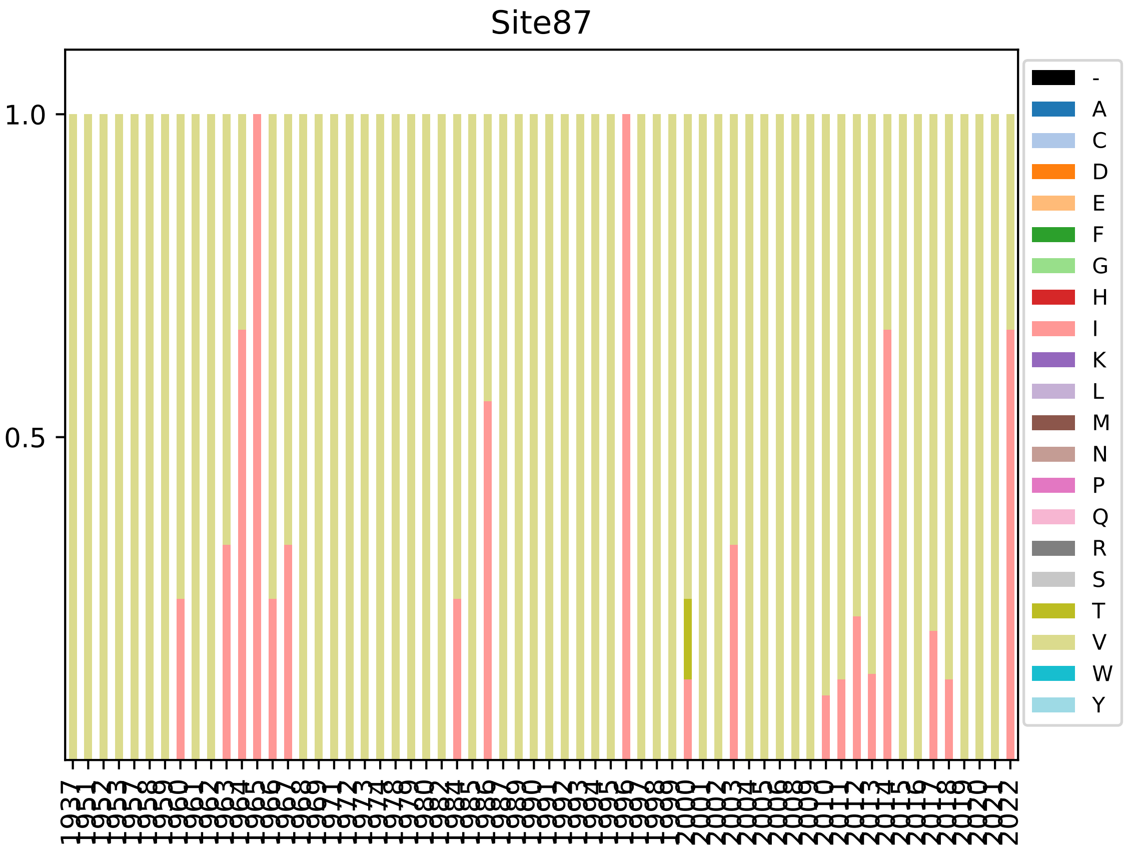
Site87
1.0
0.5
-
A
C
D
E
F
G
H
I
K
L
M
N
P
Q
R
S
T
V
W
Y

Site88

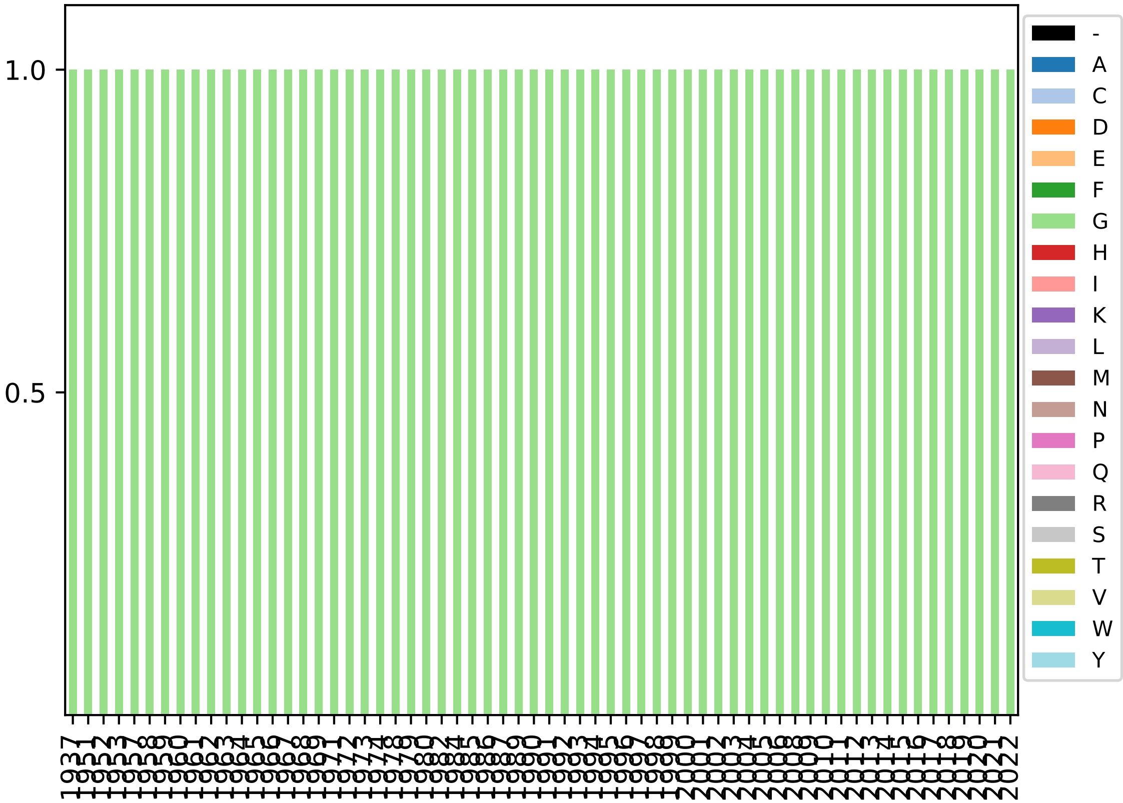

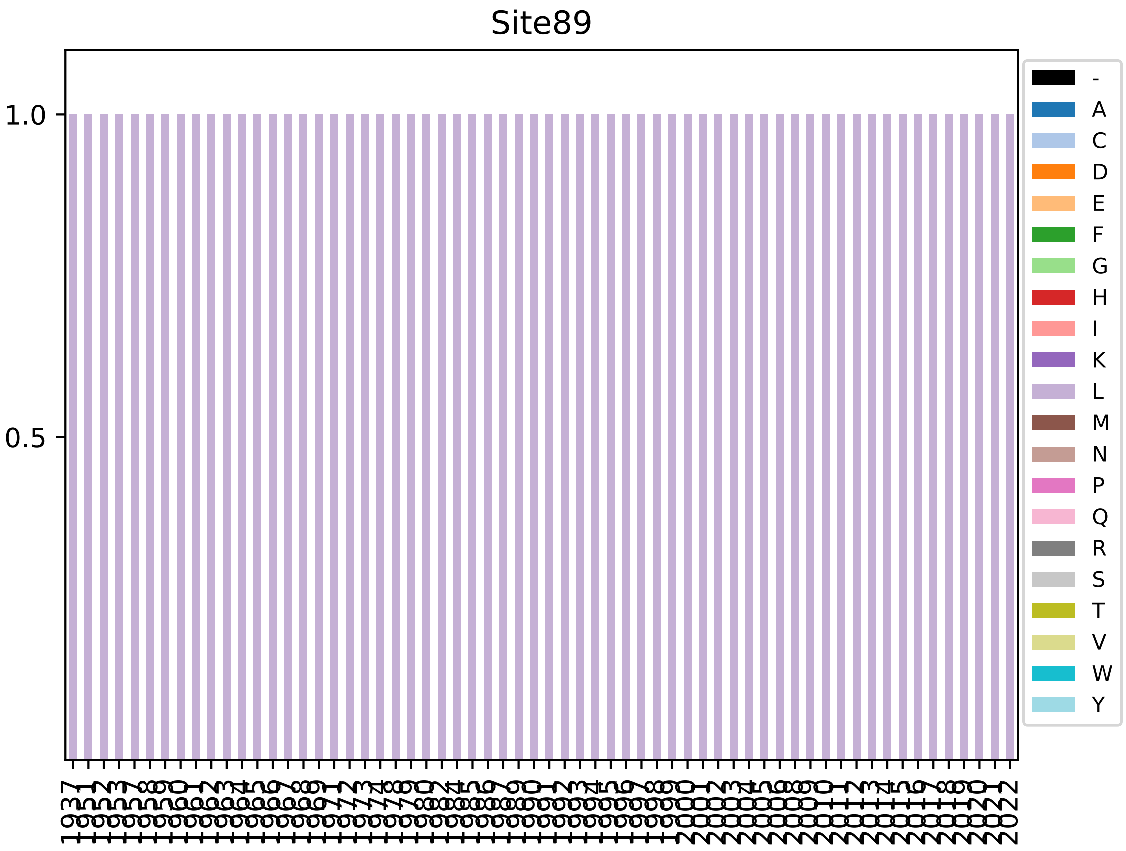
Site89
1.0
0.5
-
A
C
D
E
F
G
H
I
K
L
M
N
P
Q
R
S
T
V
W
Y

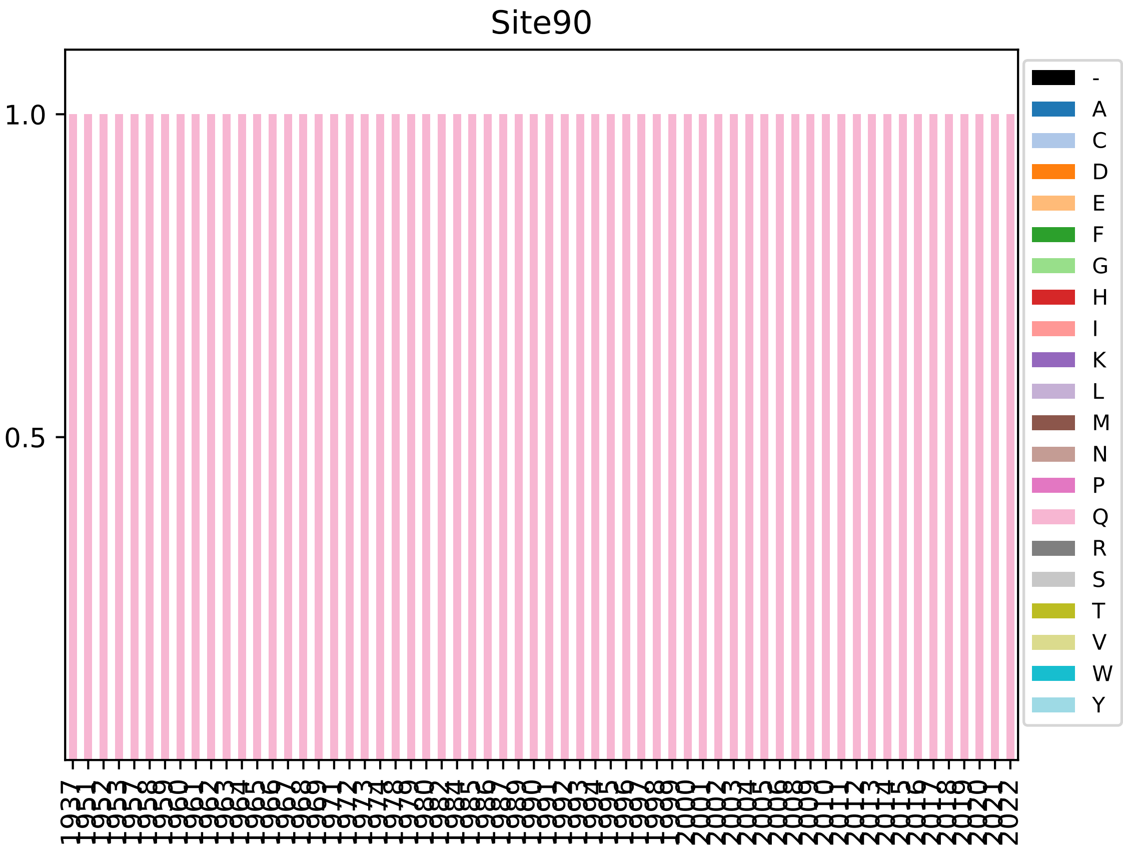
Site90
1.0
0.5
-
A
C
D
E
F
G
H
I
K
L
M
N
P
Q
R
S
T
V
W
Y

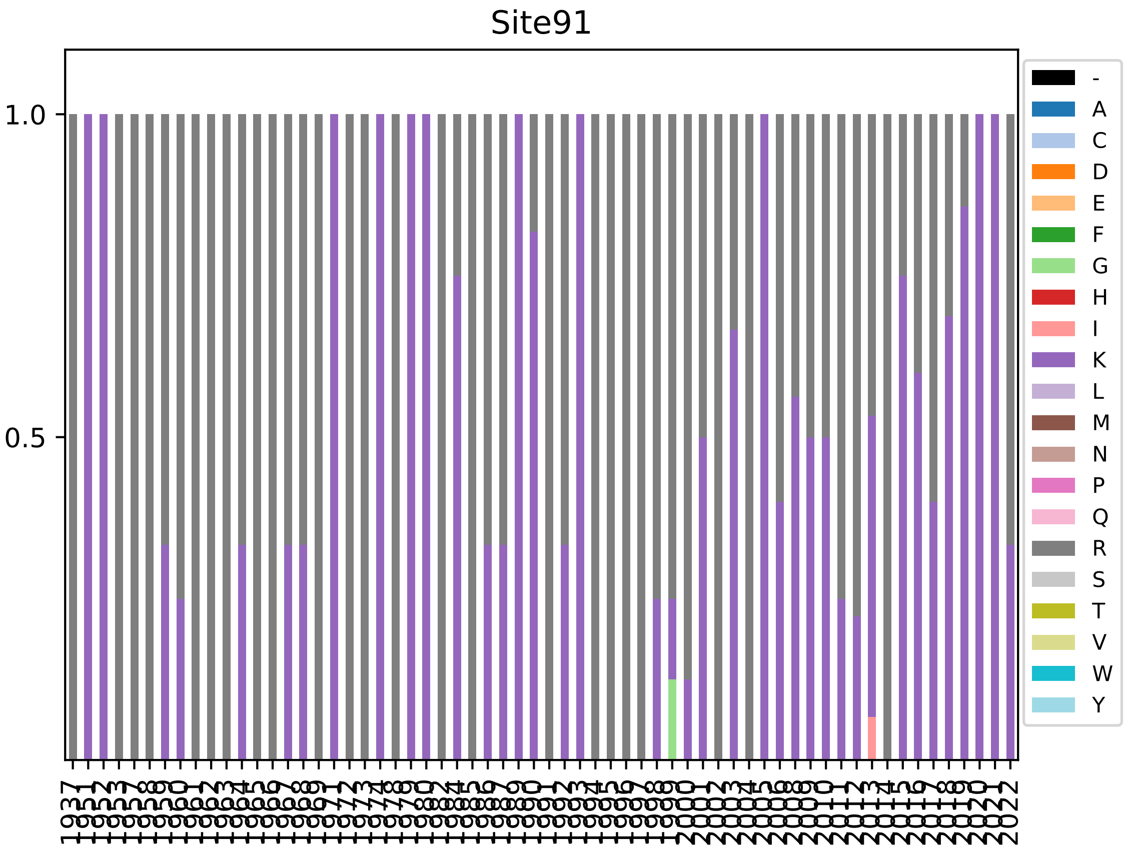
Site91
1.0
0.5
-
A
C
D
E
F
G
H
I
K
L
M
N
P
Q
R
S
T
V
W
Y

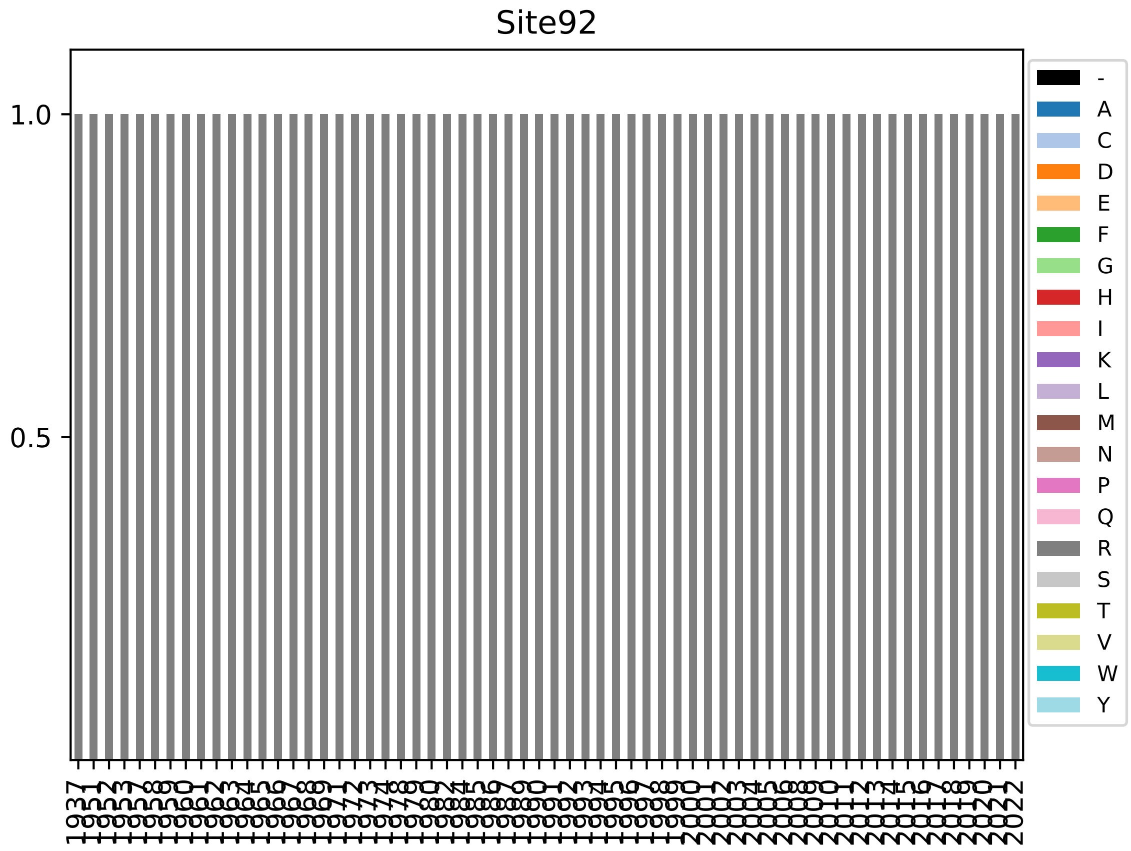
Site92
1.0
0.5
-
A
C
D
E
F
G
H
I
K
L
M
N
P
Q
R
S
T
V
W
Y

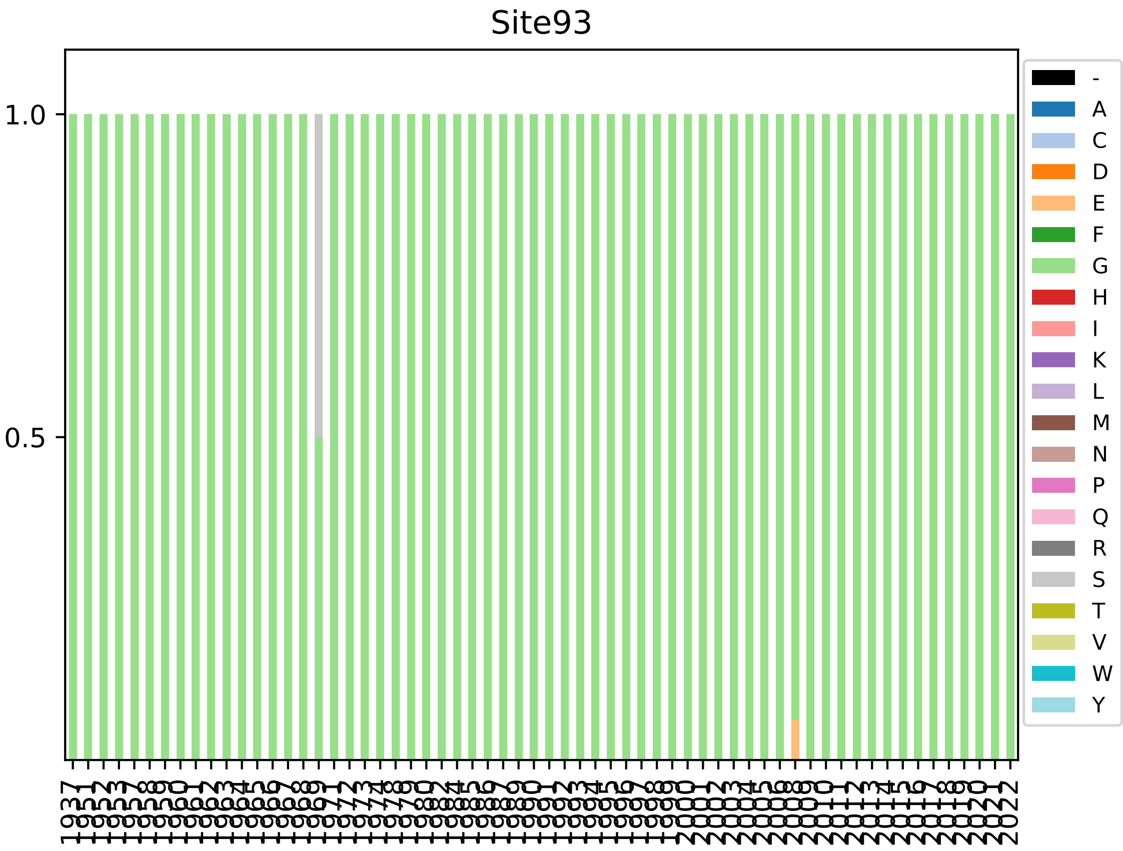

Site93
1.0
0.5
-
A
C
D
E
F
G
H
I
K
L
M
N
P
Q
R
S
T
V
W
Y

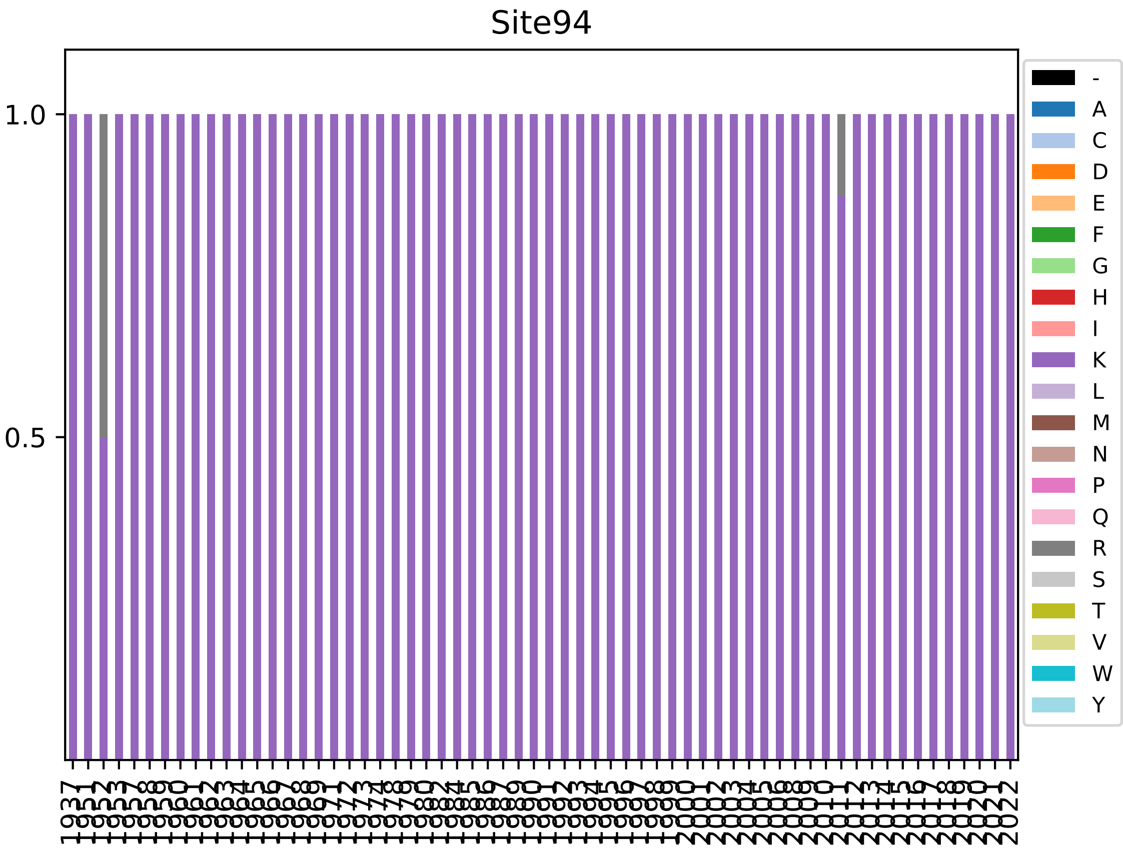
Site94
1.0
0.5
-
A
C
D
E
F
G
H
I
K
L
M
N
P
Q
R
S
T
V
W
Y

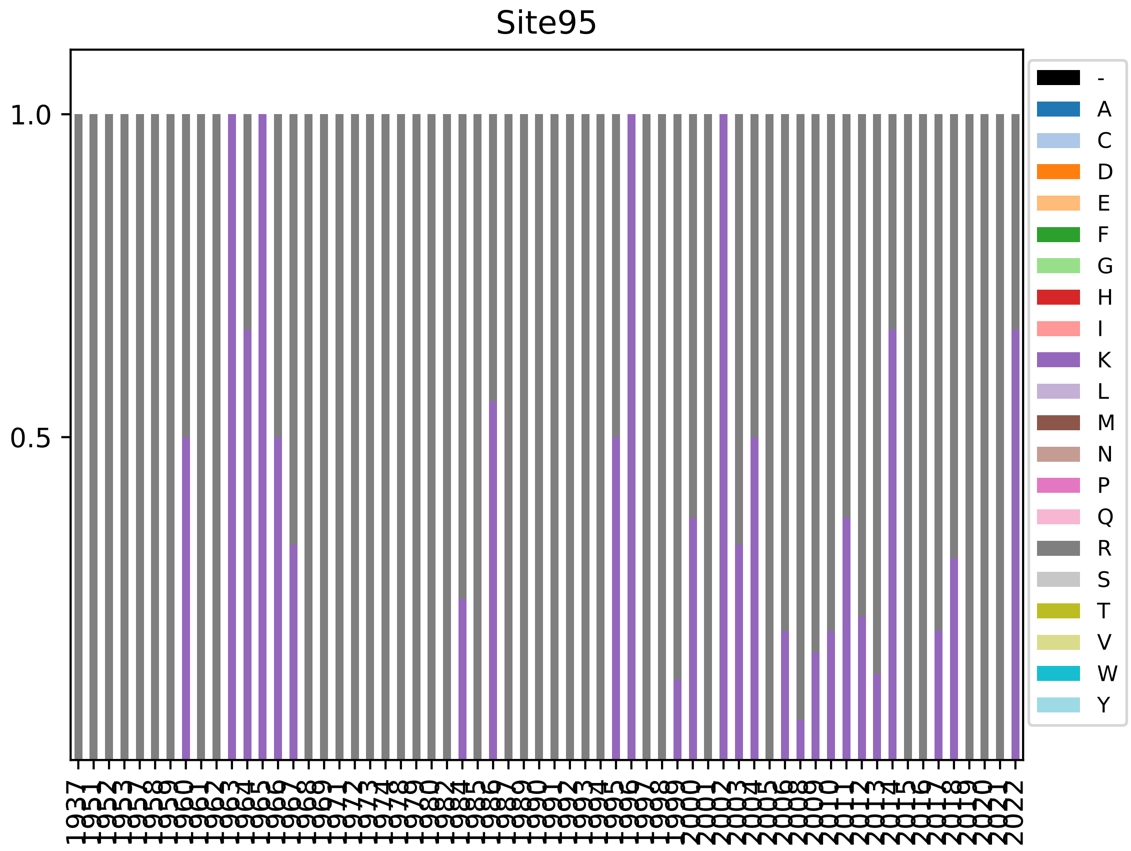
Site95
1.0
0.5
-
A
C
D
E
F
G
H
I
K
L
M
N
P
Q
R
S
T
V
W
Y

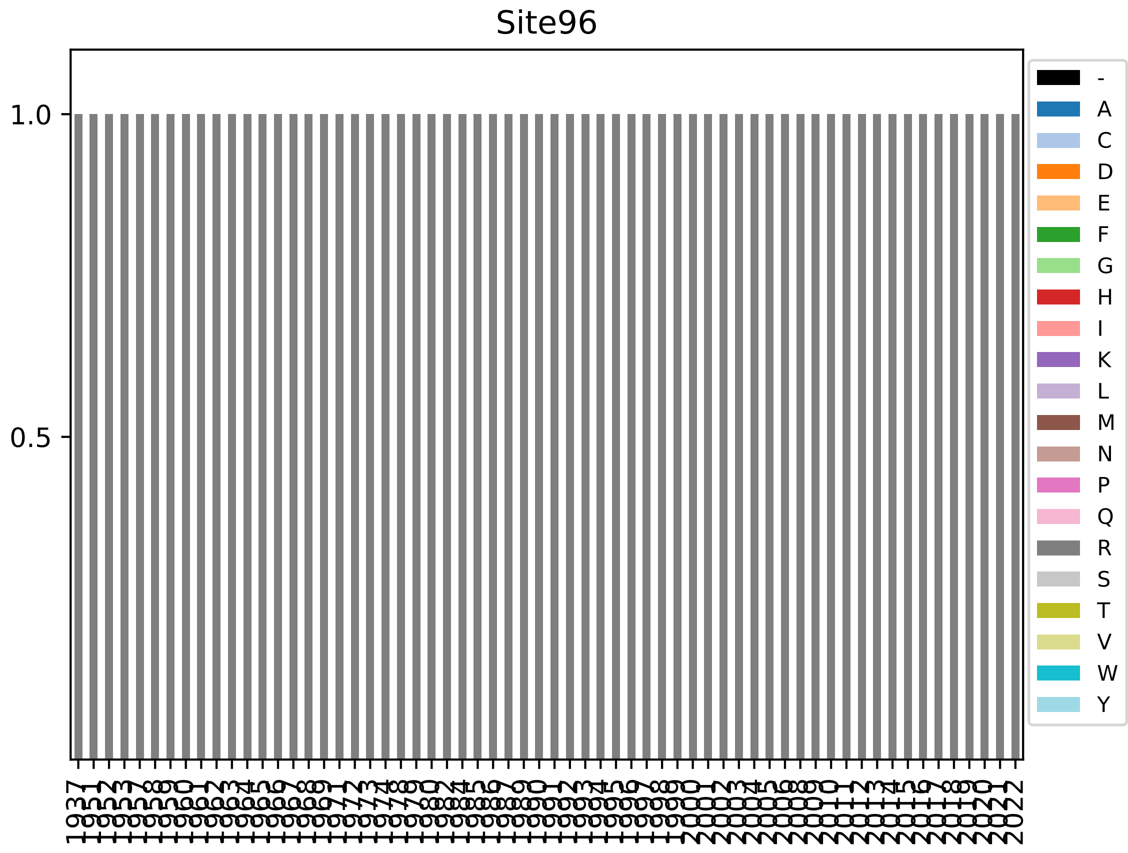
Site96
1.0
0.5
-
A
C
D
E
F
G
H
I
K
L
M
N
P
Q
R
S
T
V
W
Y

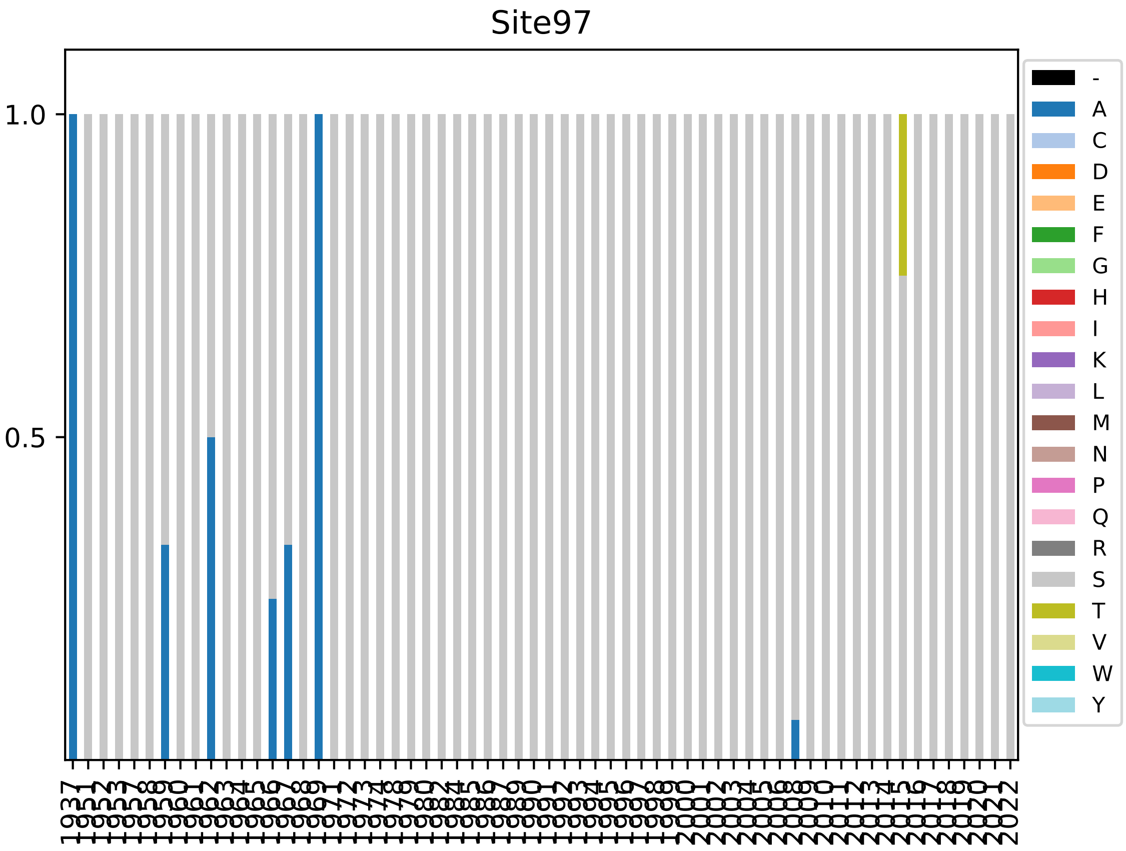
Site97
1.0
0.5
-
A
C
D
E
F
G
H
I
K
L
M
N
P
Q
R
S
T
V
W
Y

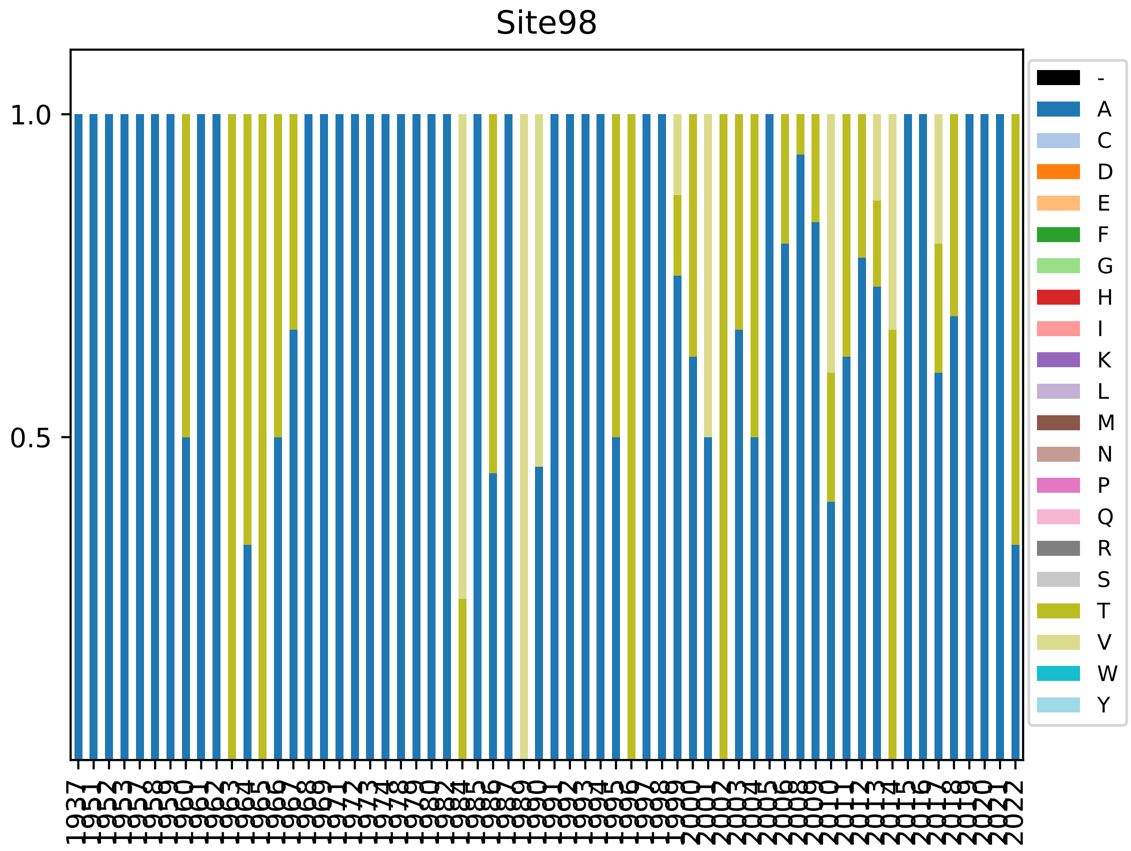
Site98
1.0
0.5
-
A
C
D
E
F
G
H
I
K
L
M
N
P
Q
R
S
T
V
W
Y

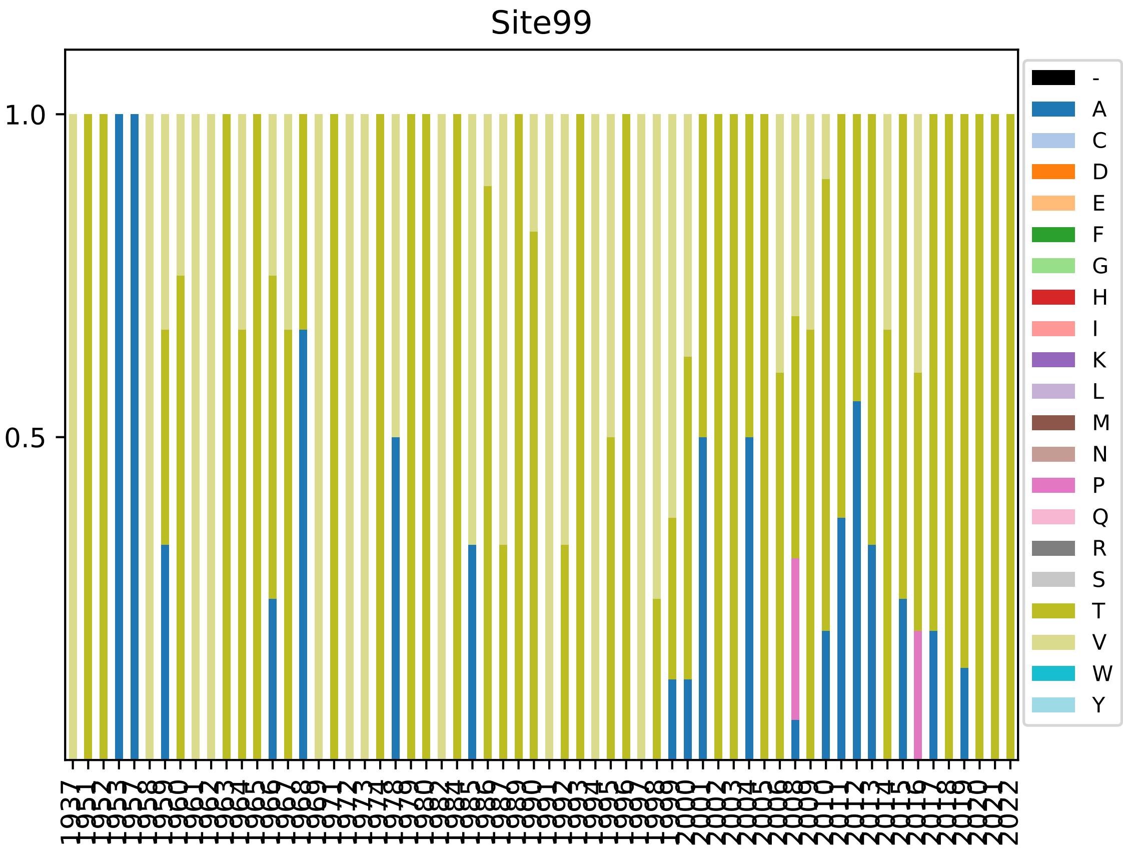
Site99
1.0
0.5
-
A
C
D
E
F
G
H
I
K
L
M
N
P
Q
R
S
T
V
W
Y

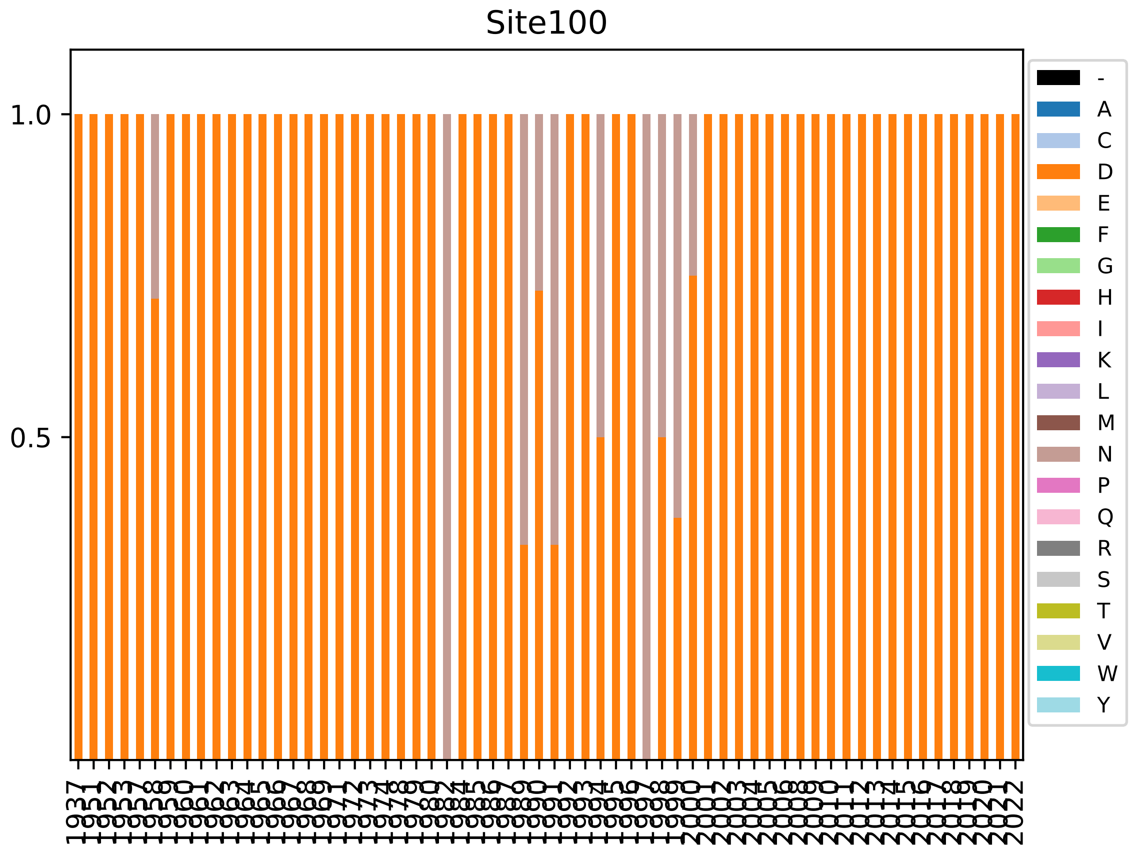
Site100
1.0
0.5
-
A
C
D
E
F
G
H
I
K
L
M
N
P
Q
R
S
T
V
W
Y

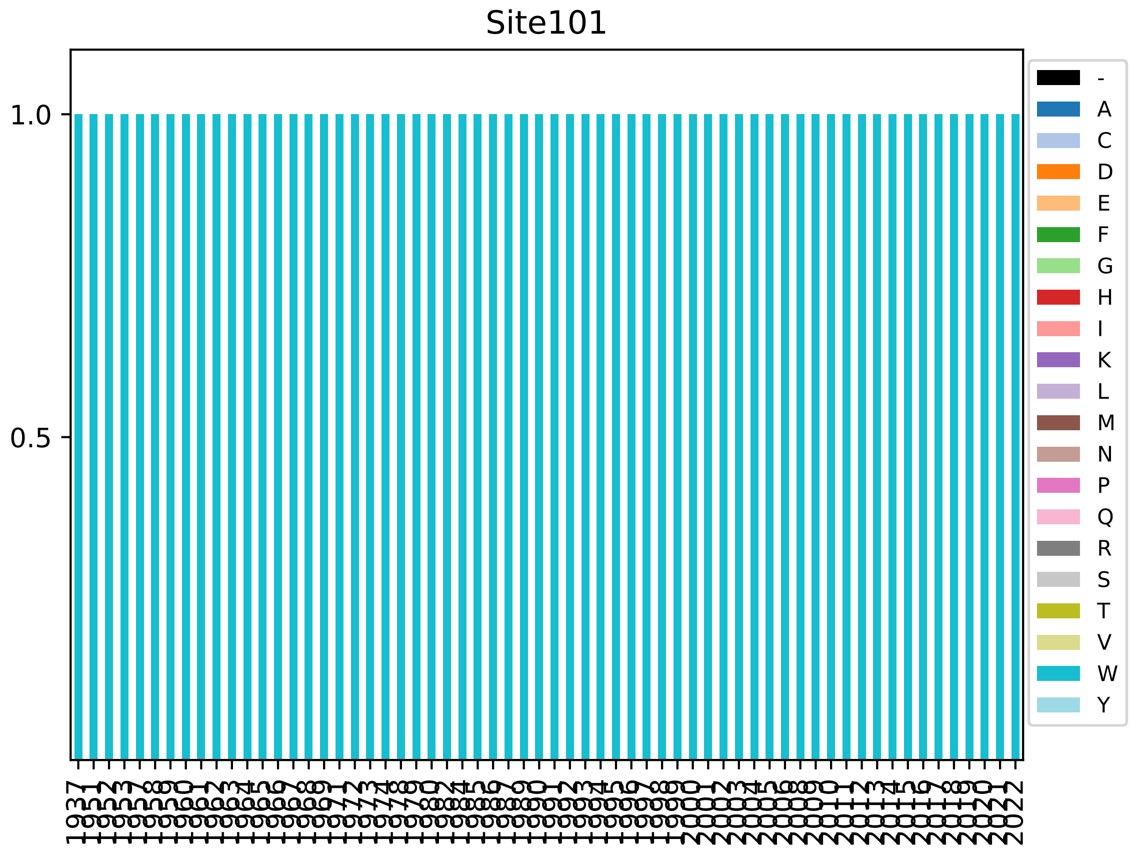
Site101
1.0
0.5
-
A
C
D
E
F
G
H
I
K
L
M
N
P
Q
R
S
T
V
W
Y

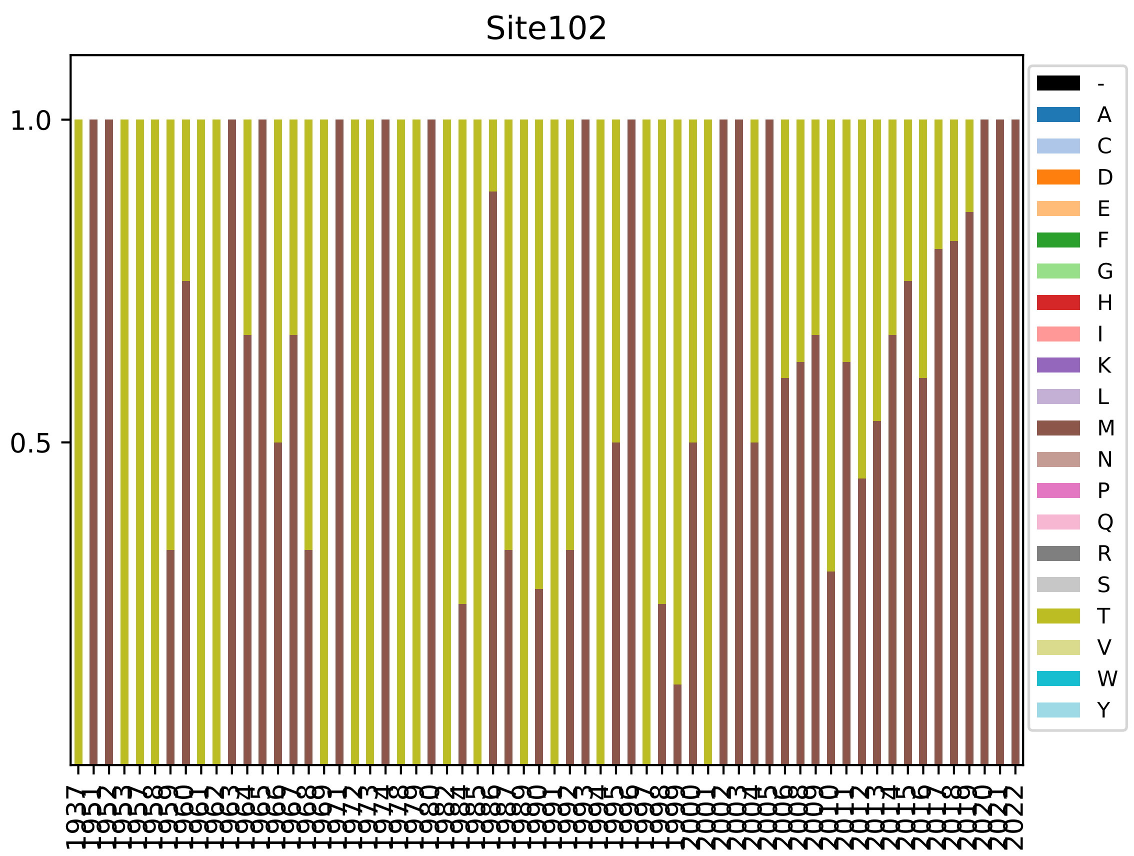

Site102
1.0
0.5
-
A
C
D
E
F
G
H
I
K
L
M
N
P
Q
R
S
T
V
W
Y

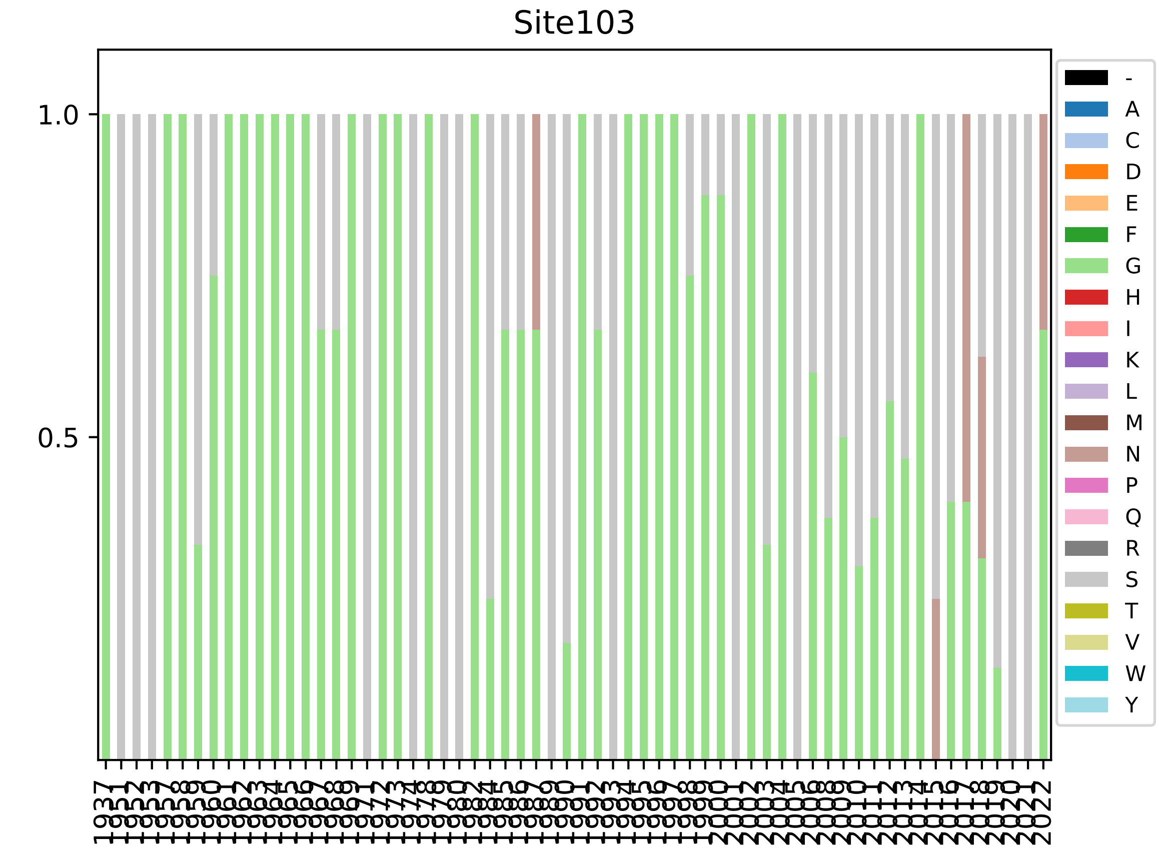
Site103
1.0
0.5
-
A
C
D
E
F
G
H
I
K
L
M
N
P
Q
R
S
T
V
W
Y

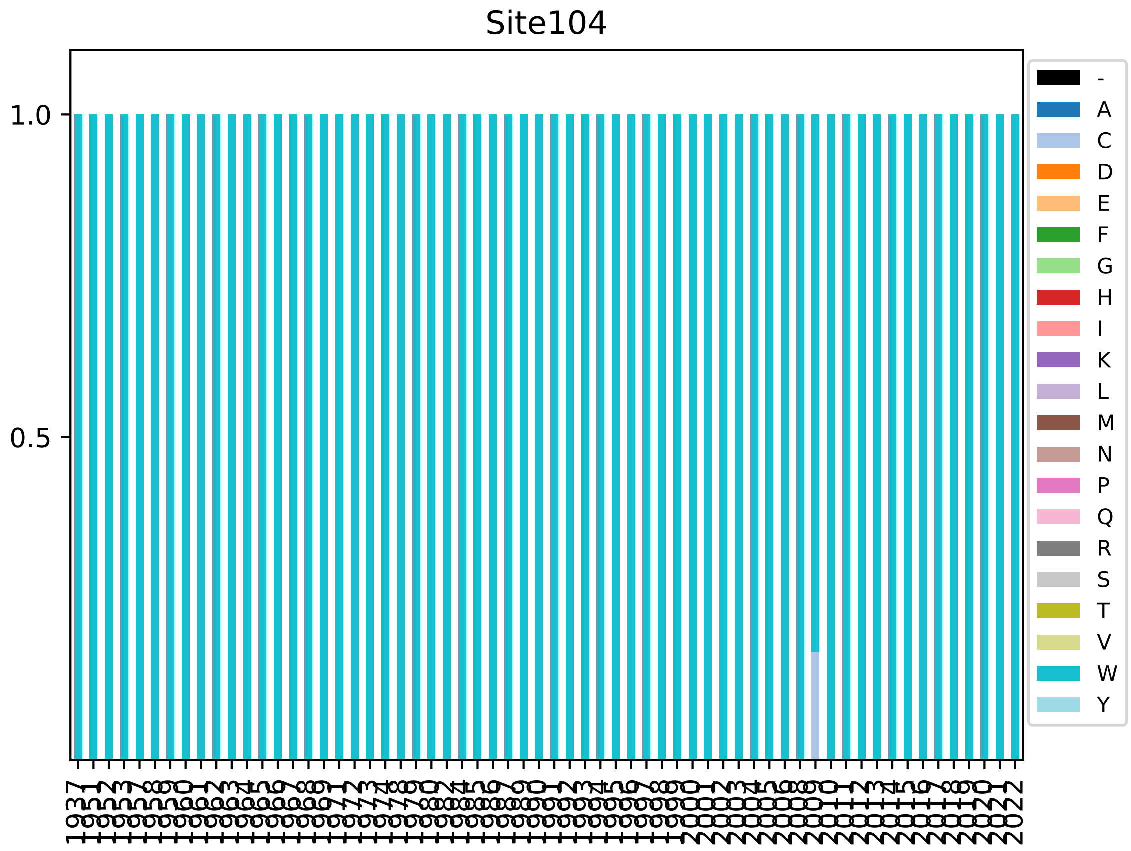
Site104
1.0
0.5
-
A
C
D
E
F
G
H
I
K
L
M
N
P
Q
R
S
T
V
W
Y

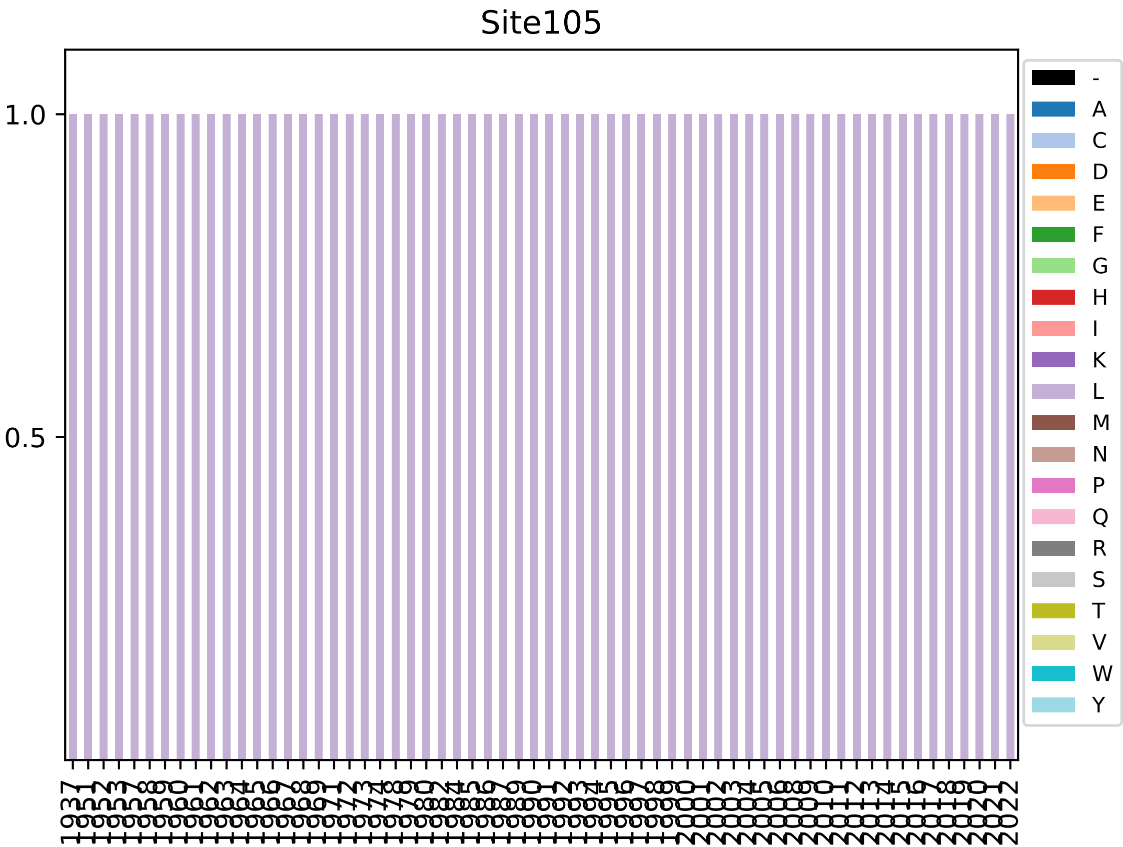
Site105
1.0
0.5
-
A
C
D
E
F
G
H
I
K
L
M
N
P
Q
R
S
T
V
W
Y

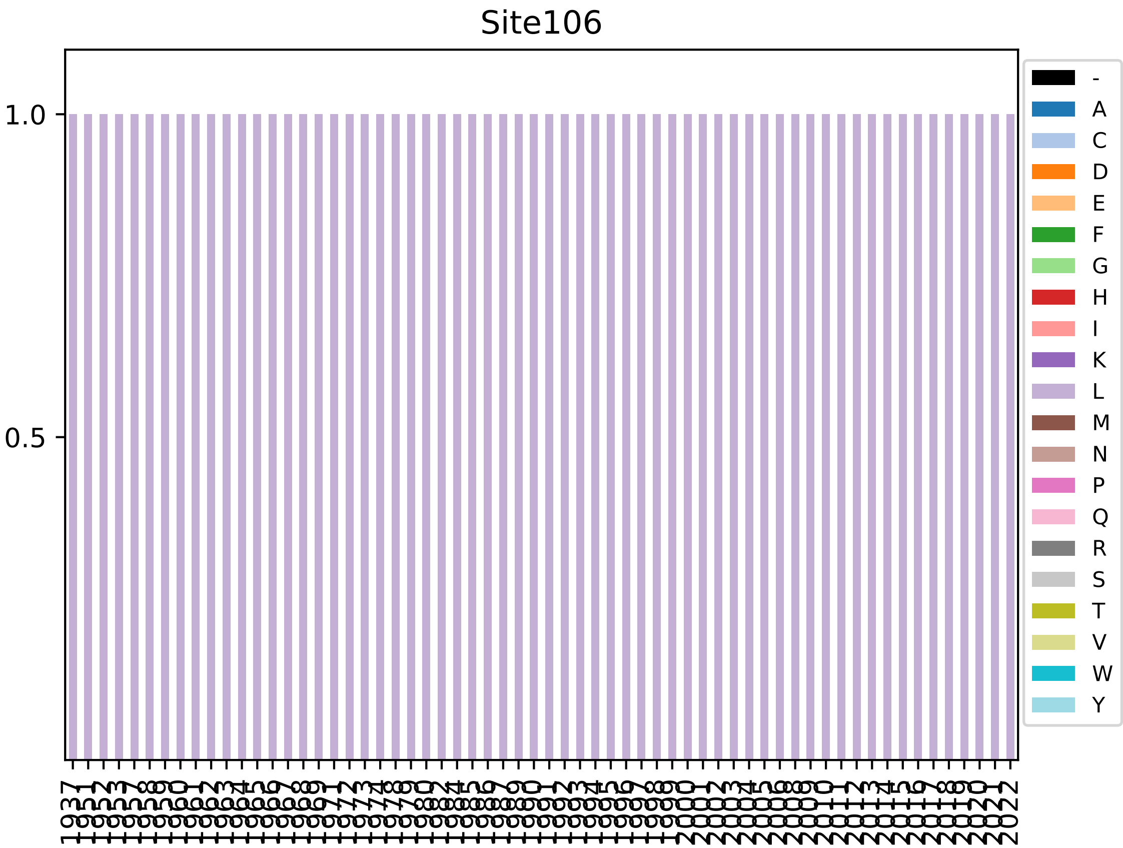
Site106
1.0
0.5
-
A
C
D
E
F
G
H
I
K
L
M
N
P
Q
R
S
T
V
W
Y

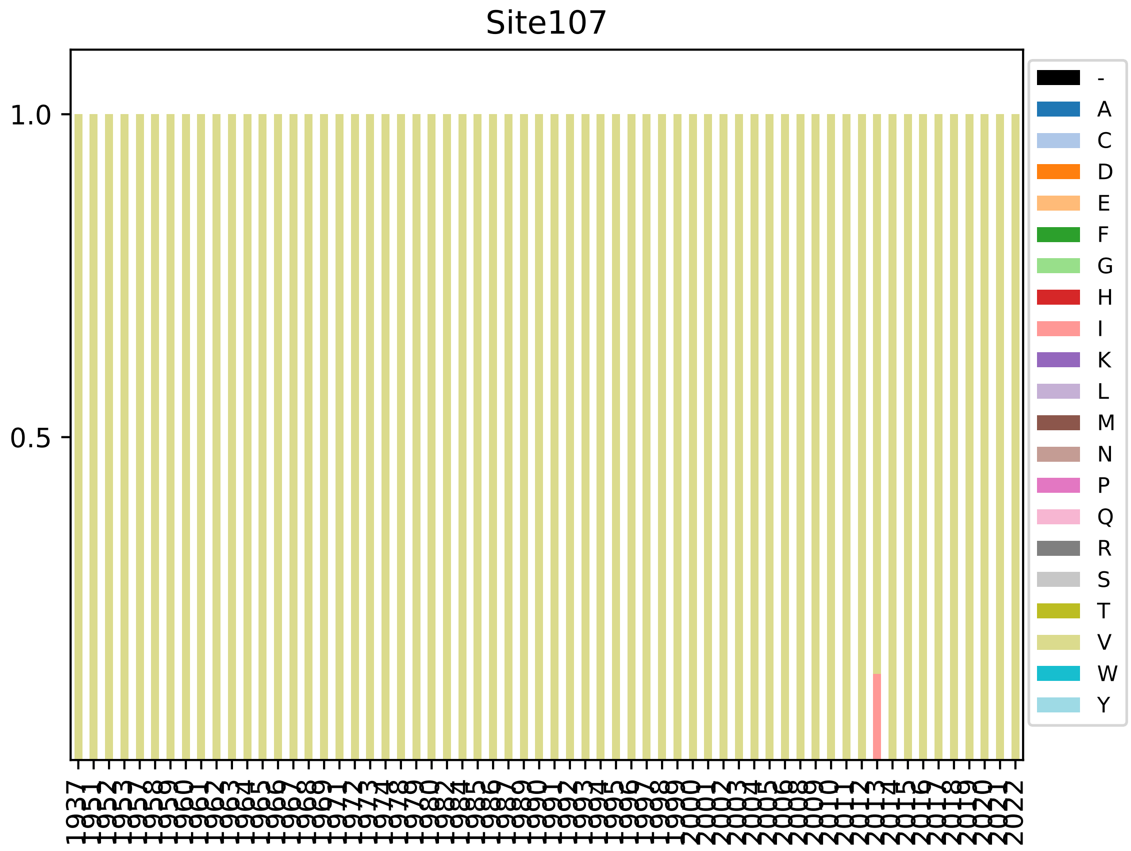

Site107
1.0
0.5
-
A
C
D
E
F
G
H
I
K
L
M
N
P
Q
R
S
T
V
W
Y

Site108

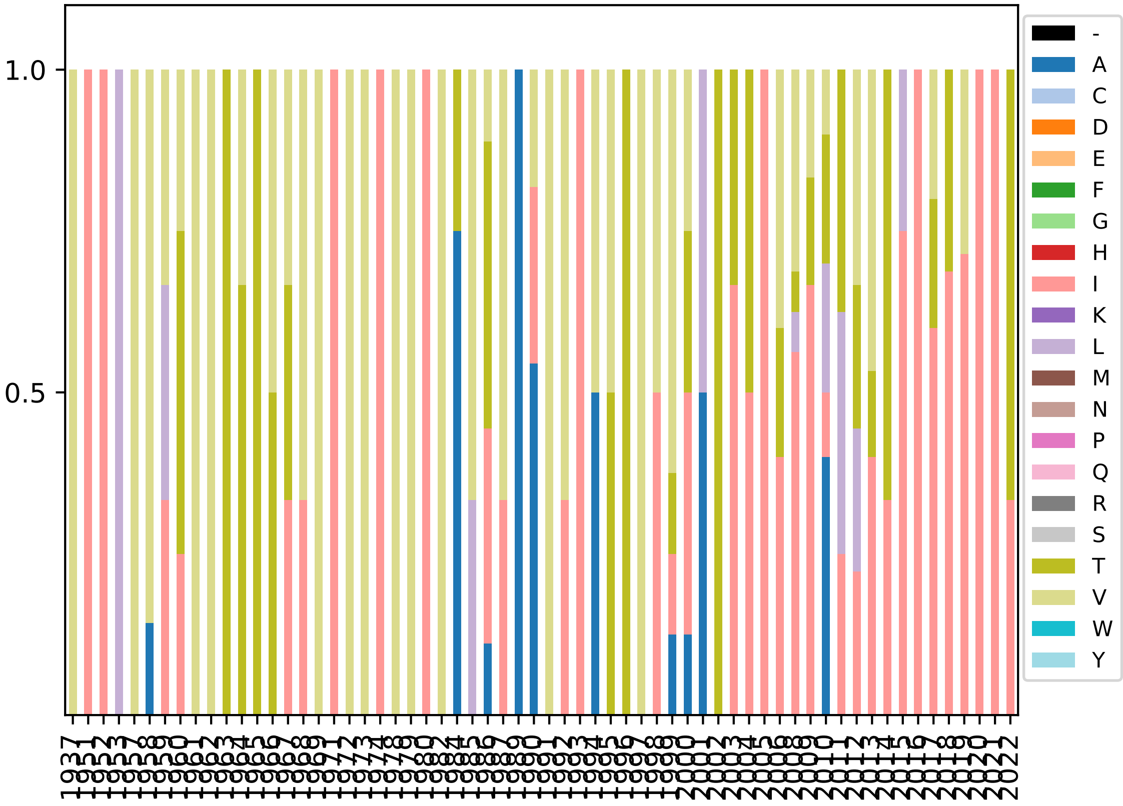

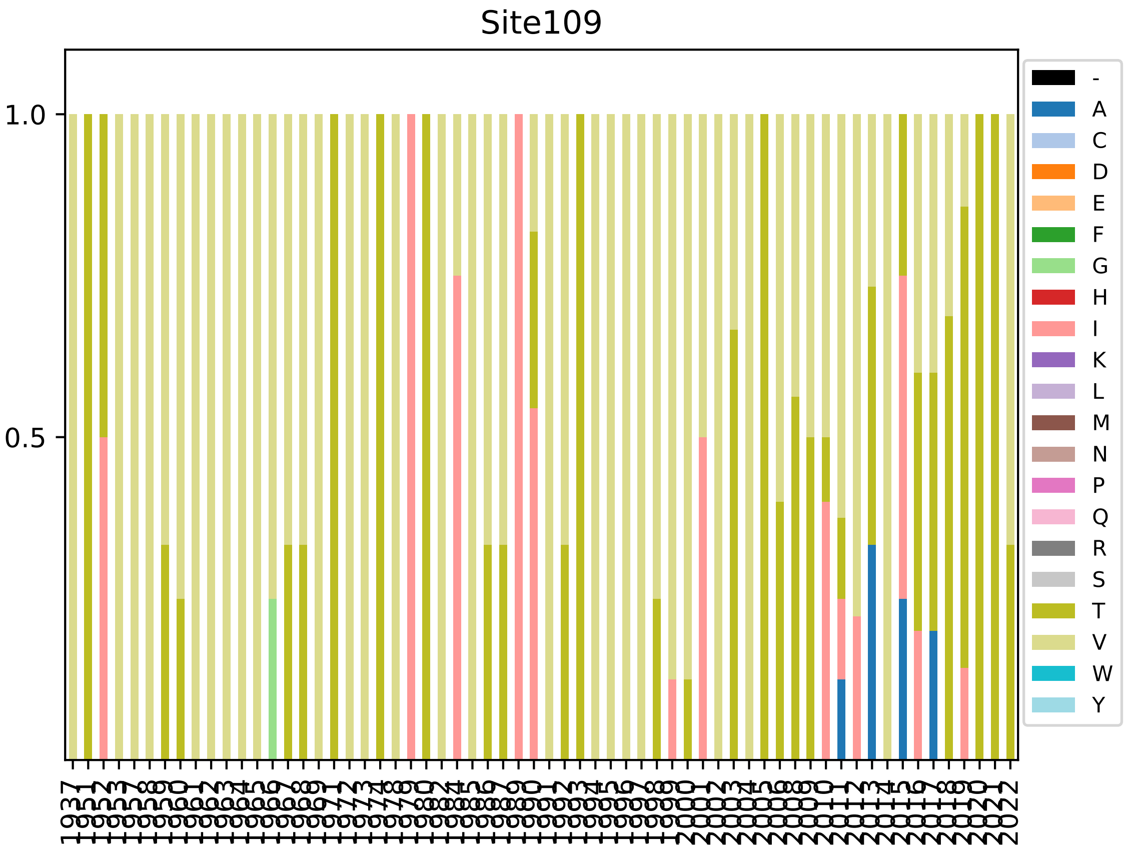
Site109
1.0
0.5
-
A
C
D
E
F
G
H
I
K
L
M
N
P
Q
R
S
T
V
W
Y

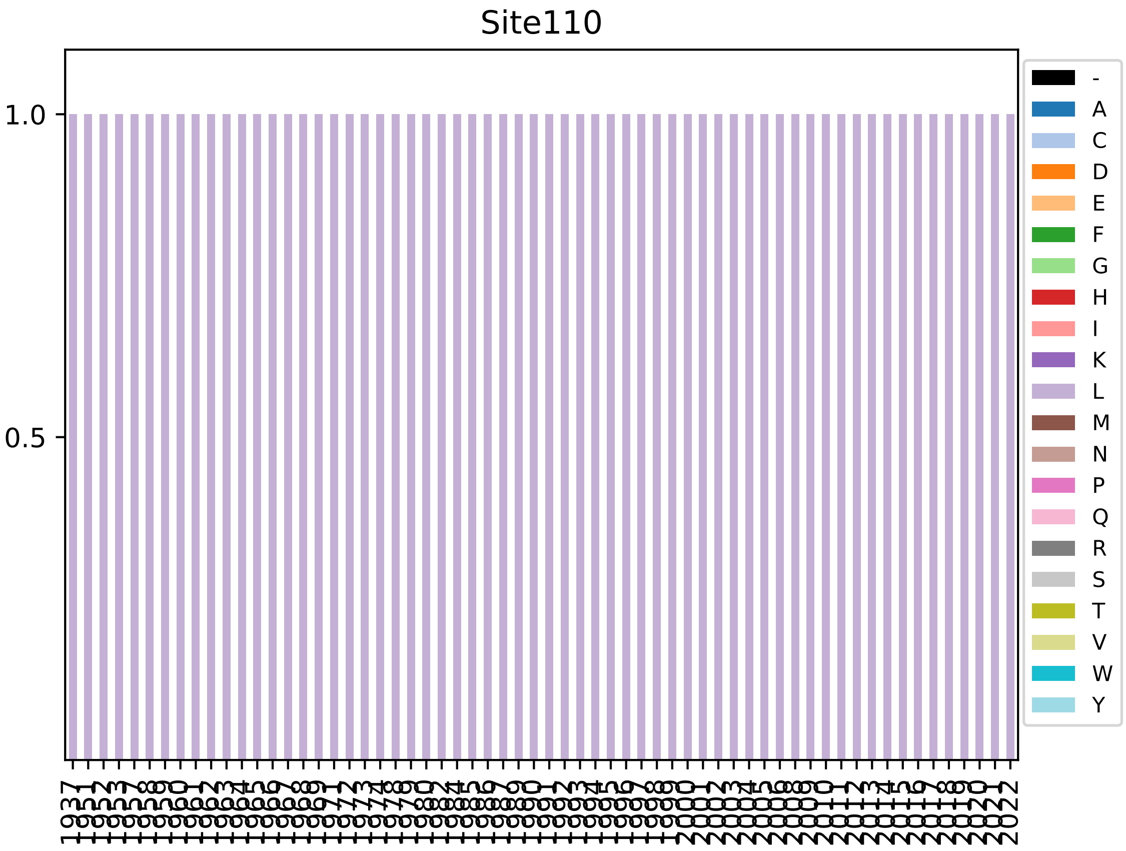
Site110
1.0
0.5
-
A
C
D
E
F
G
H
I
K
L
M
N
P
Q
R
S
T
V
W
Y

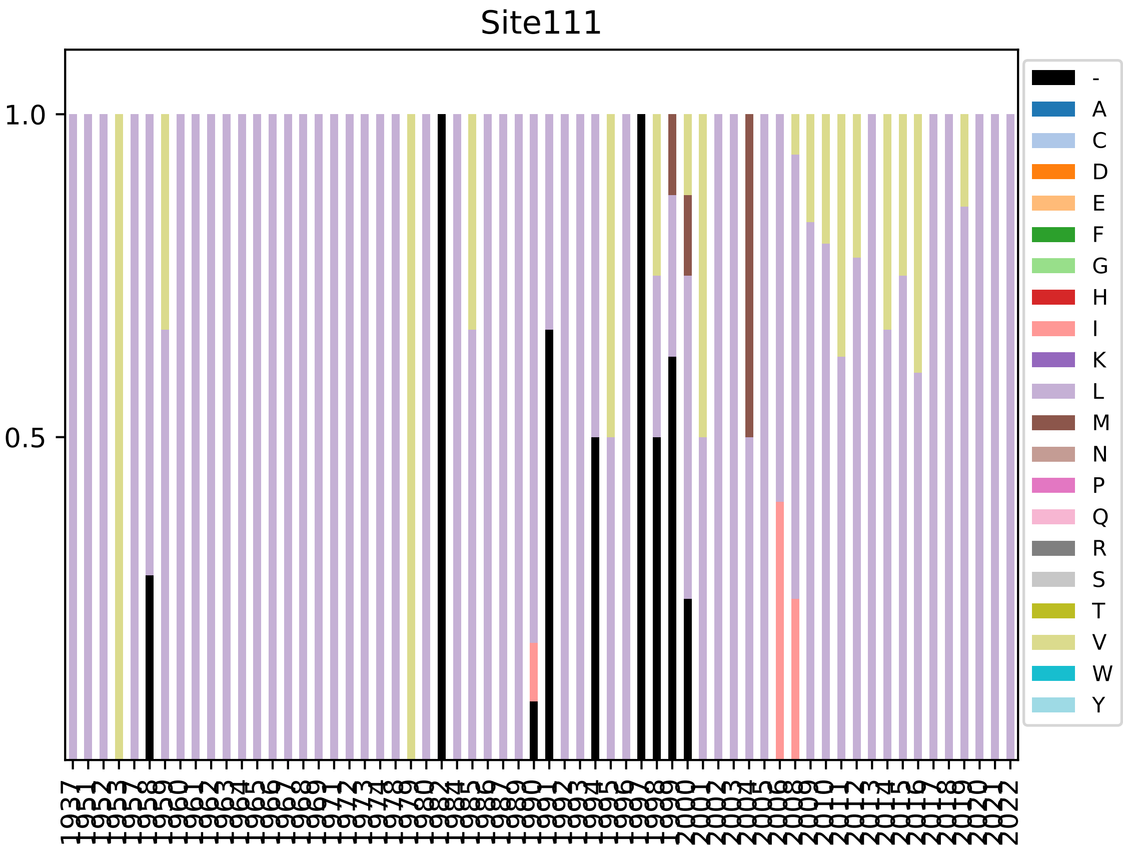
Site111
1.0
0.5
-
A
C
D
E
F
G
H
I
K
L
M
N
P
Q
R
S
T
V
W
Y

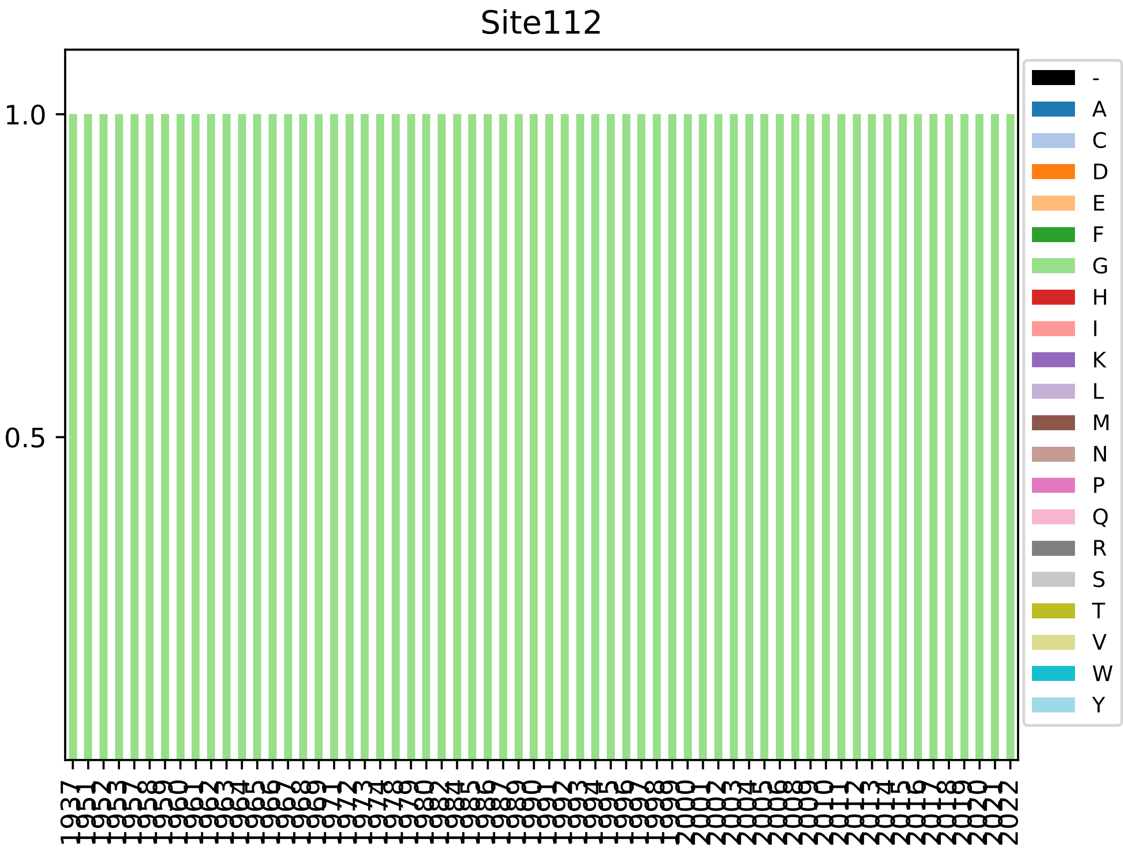
Site112
1.0
0.5
-
A
C
D
E
F
G
H
I
K
L
M
N
P
Q
R
S
T
V
W
Y

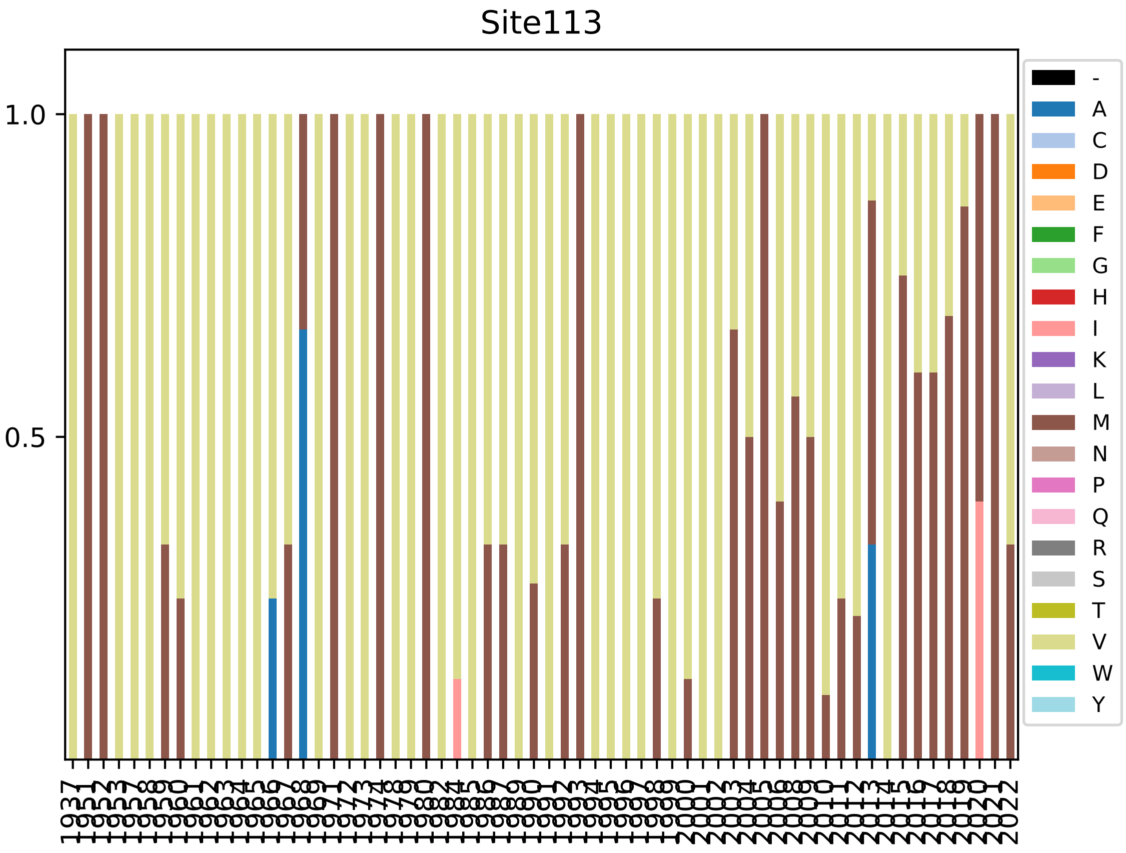
Site113
1.0
0.5
-
A
C
D
E
F
G
H
I
K
L
M
N
P
Q
R
S
T
V
W
Y

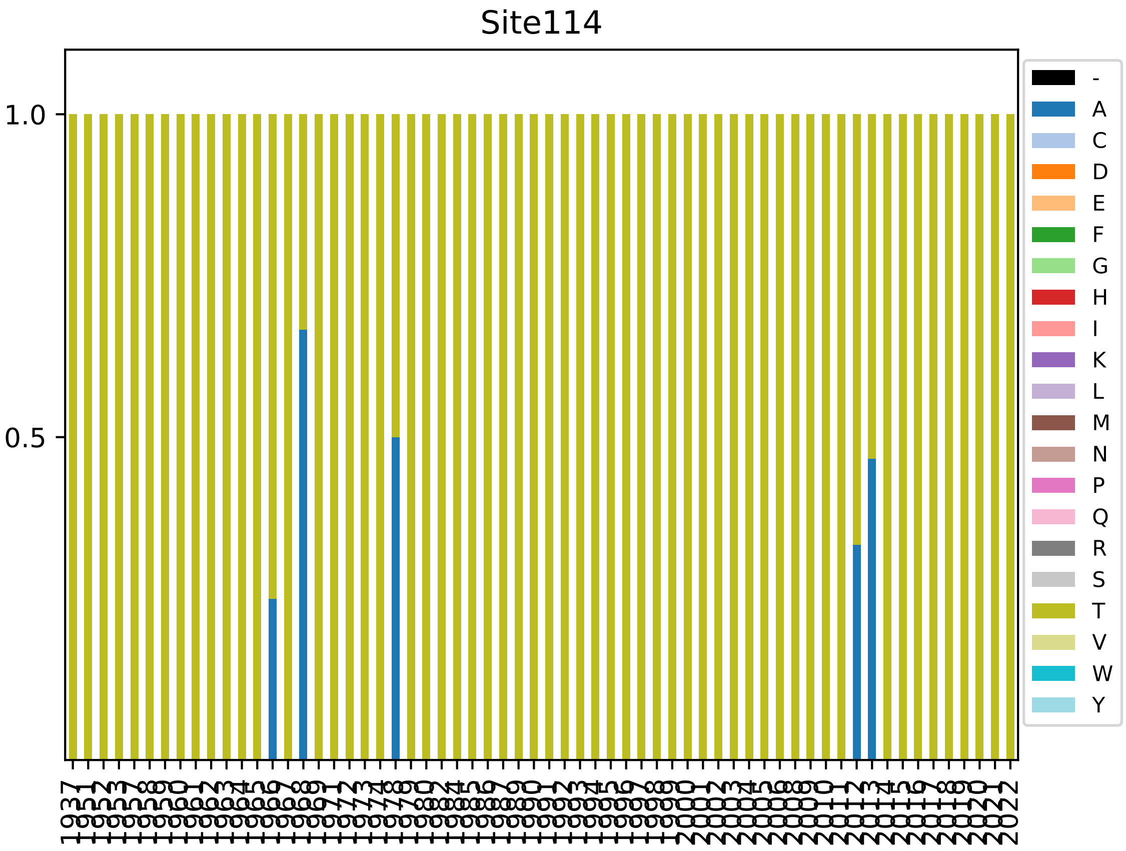
Site114
1.0
0.5
-
A
C
D
E
F
G
H
I
K
L
M
N
P
Q
R
S
T
V
W
Y

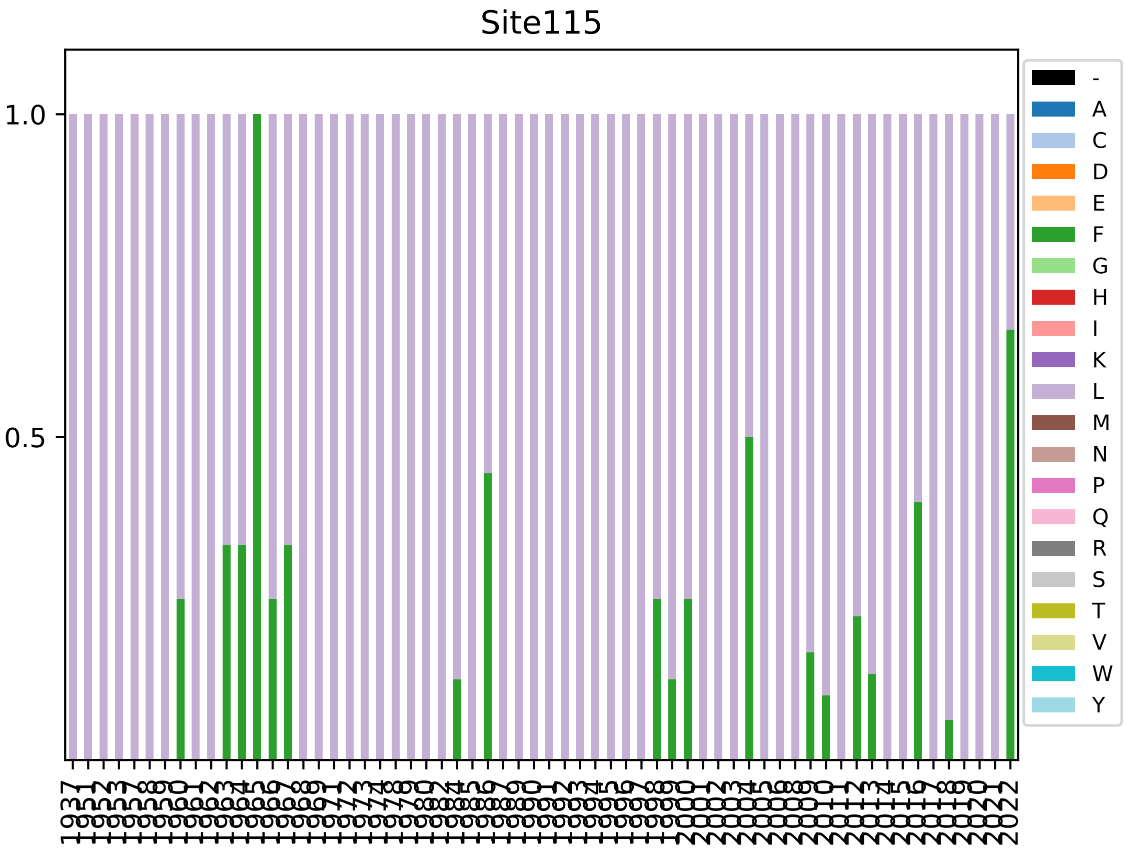
Site115
1.0
0.5
-
A
C
D
E
F
G
H
I
K
L
M
N
P
Q
R
S
T
V
W
Y

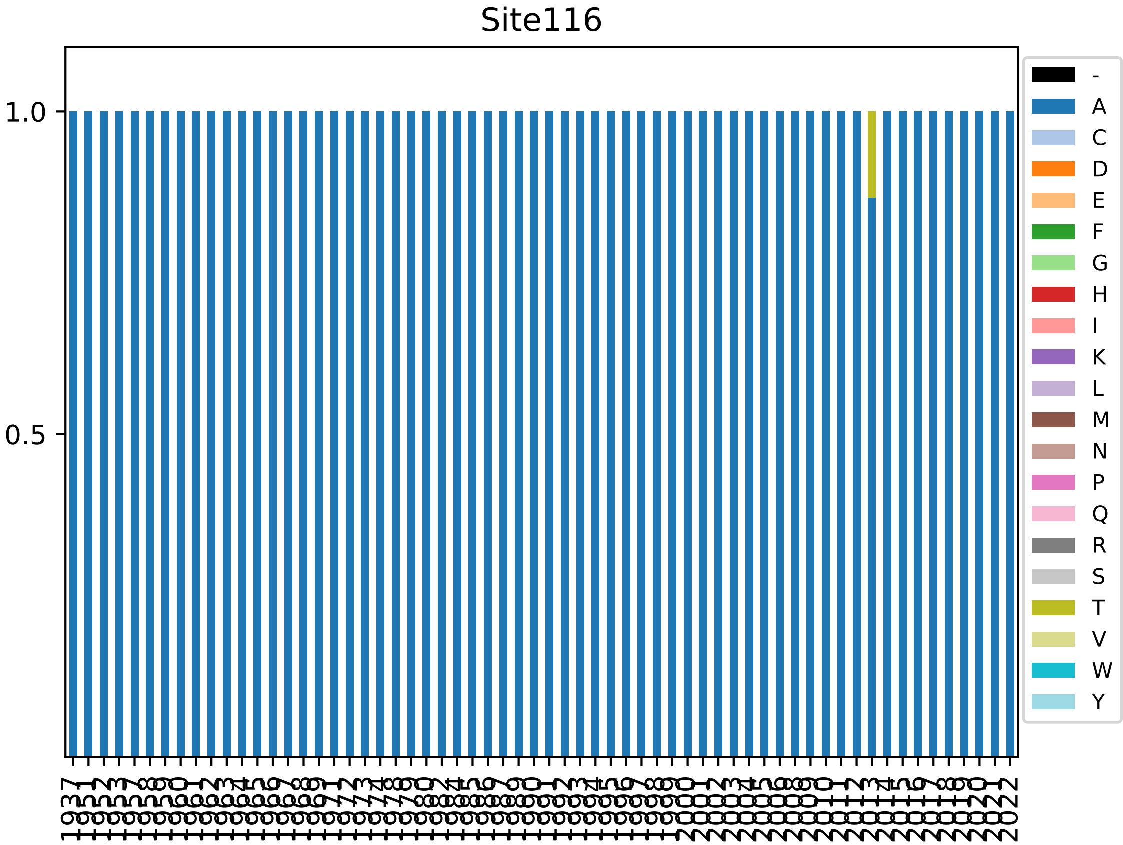
Site116
1.0
0.5
-
A
C
D
E
F
G
H
I
K
L
M
N
P
Q
R
S
T
V
W
Y

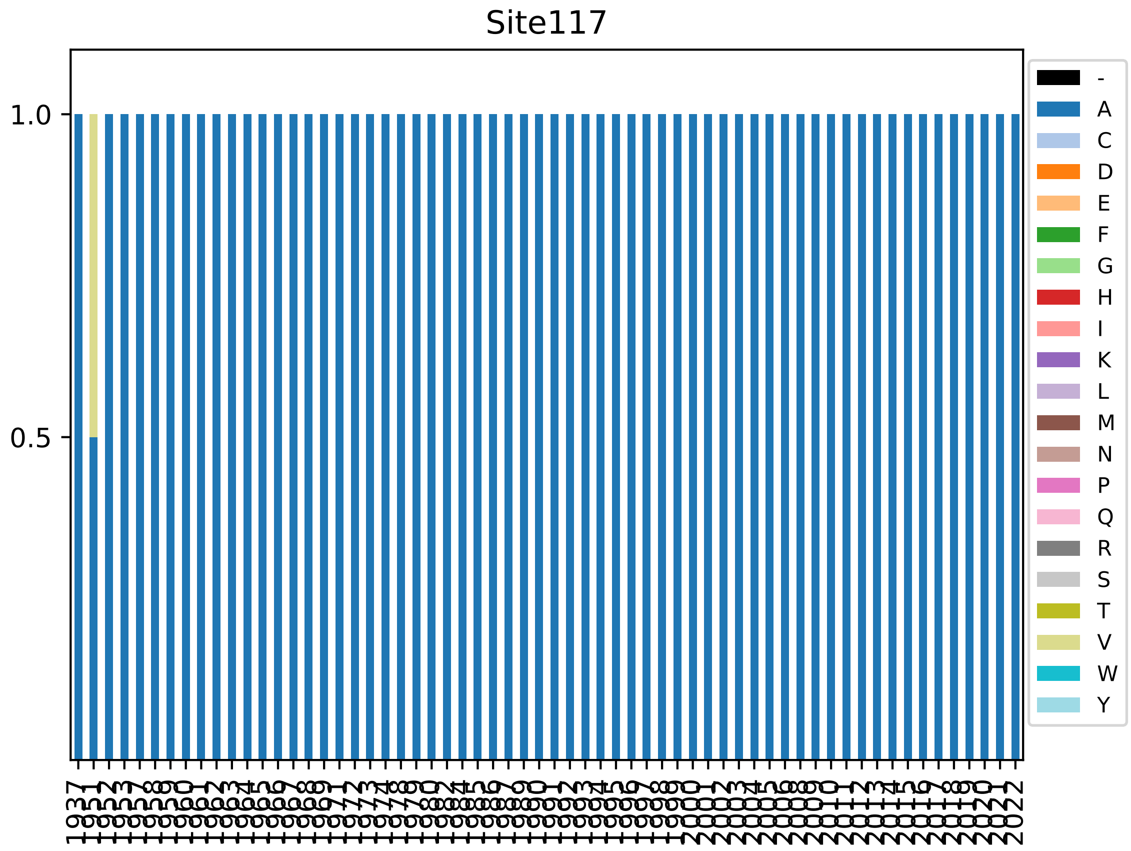
Site117
1.0
0.5
-
A
C
D
E
F
G
H
I
K
L
M
N
P
Q
R
S
T
V
W
Y

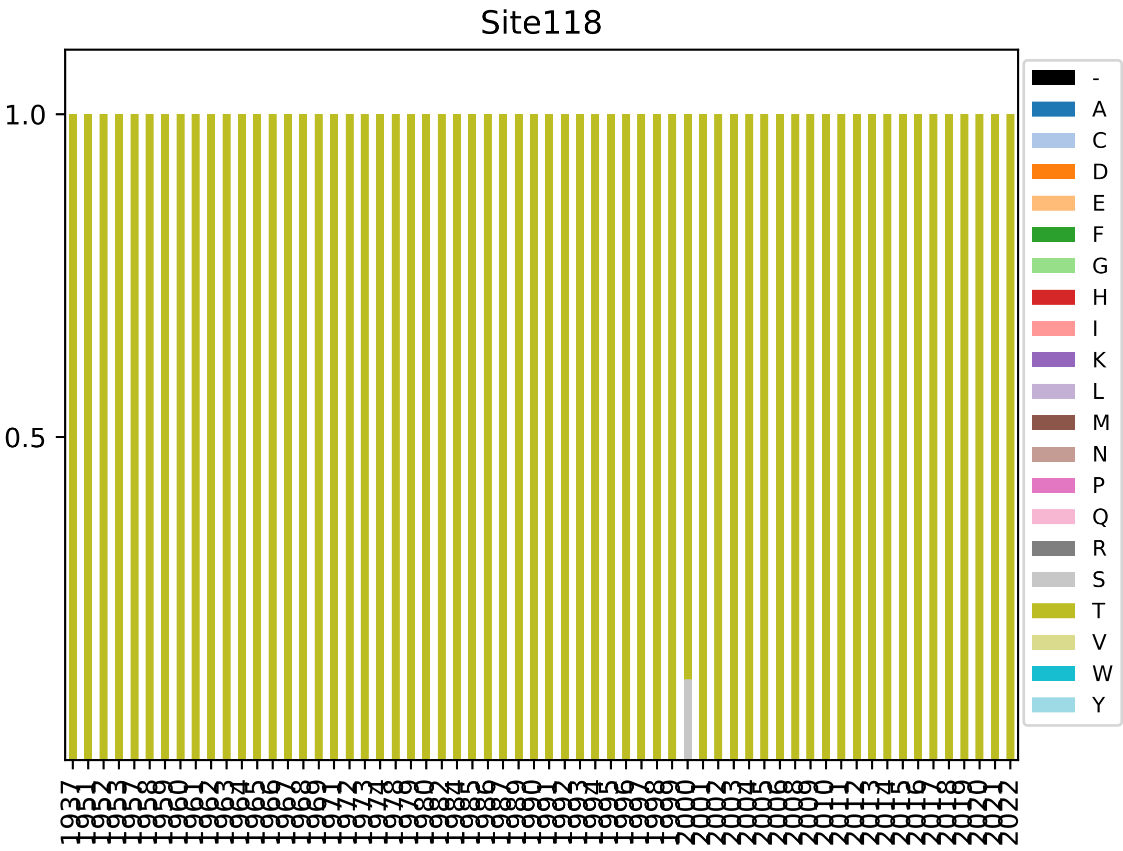
Site118
1.0
0.5
-
A
C
D
E
F
G
H
I
K
L
M
N
P
Q
R
S
T
V
W
Y

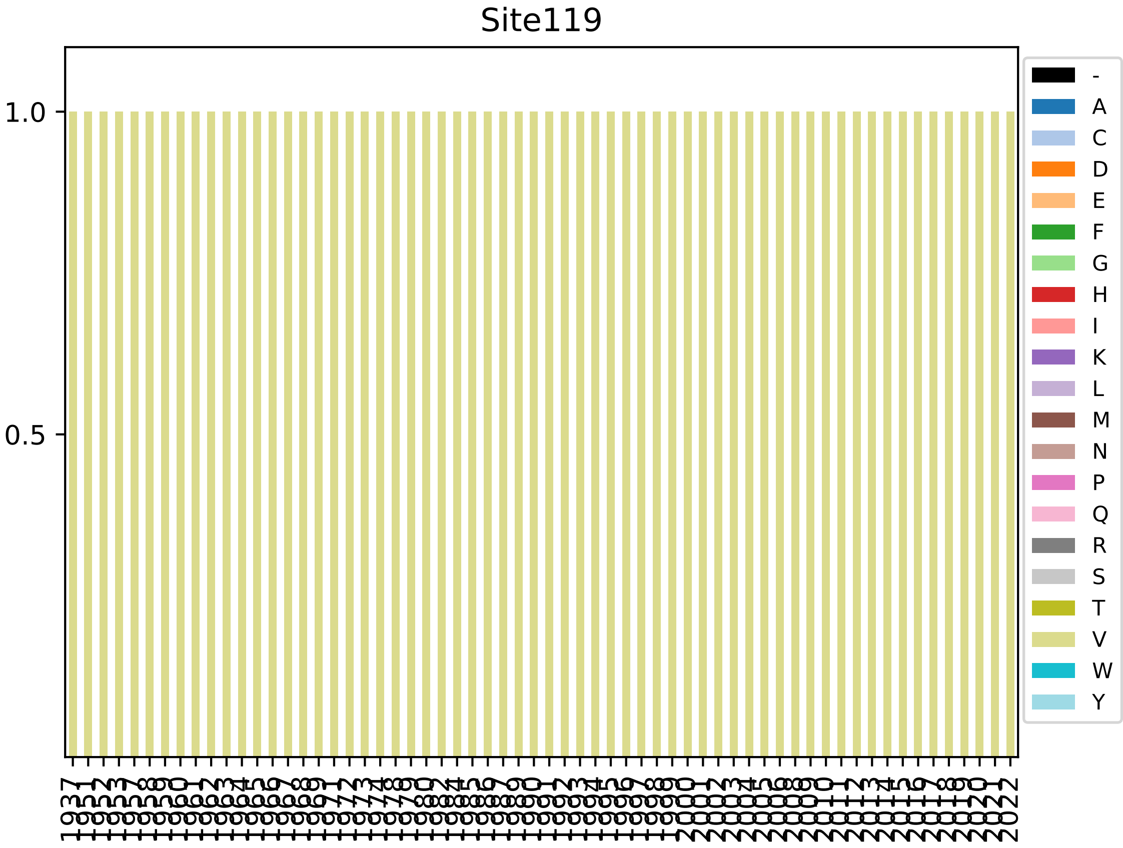
Site119
1.0
0.5
-
A
C
D
E
F
G
H
I
K
L
M
N
P
Q
R
S
T
V
W
Y

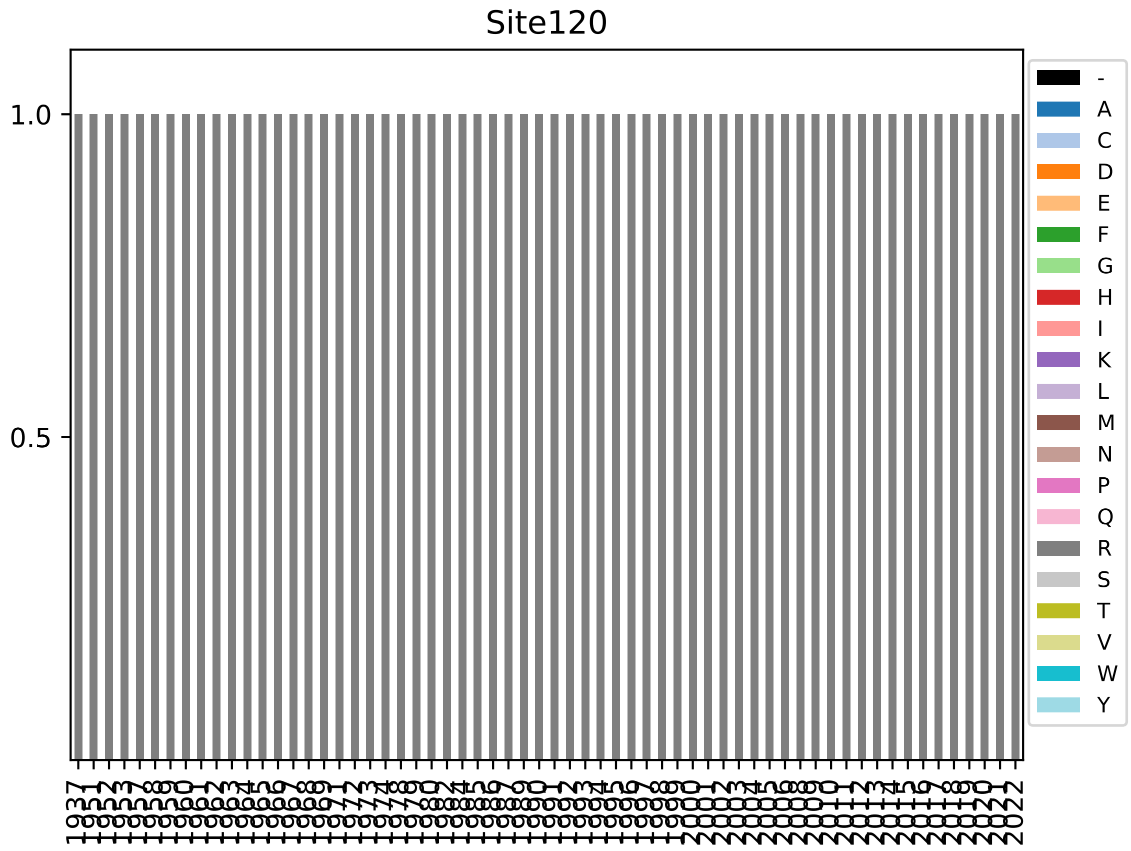
Site120
1.0
0.5
-
A
C
D
E
F
G
H
I
K
L
M
N
P
Q
R
S
T
V
W
Y

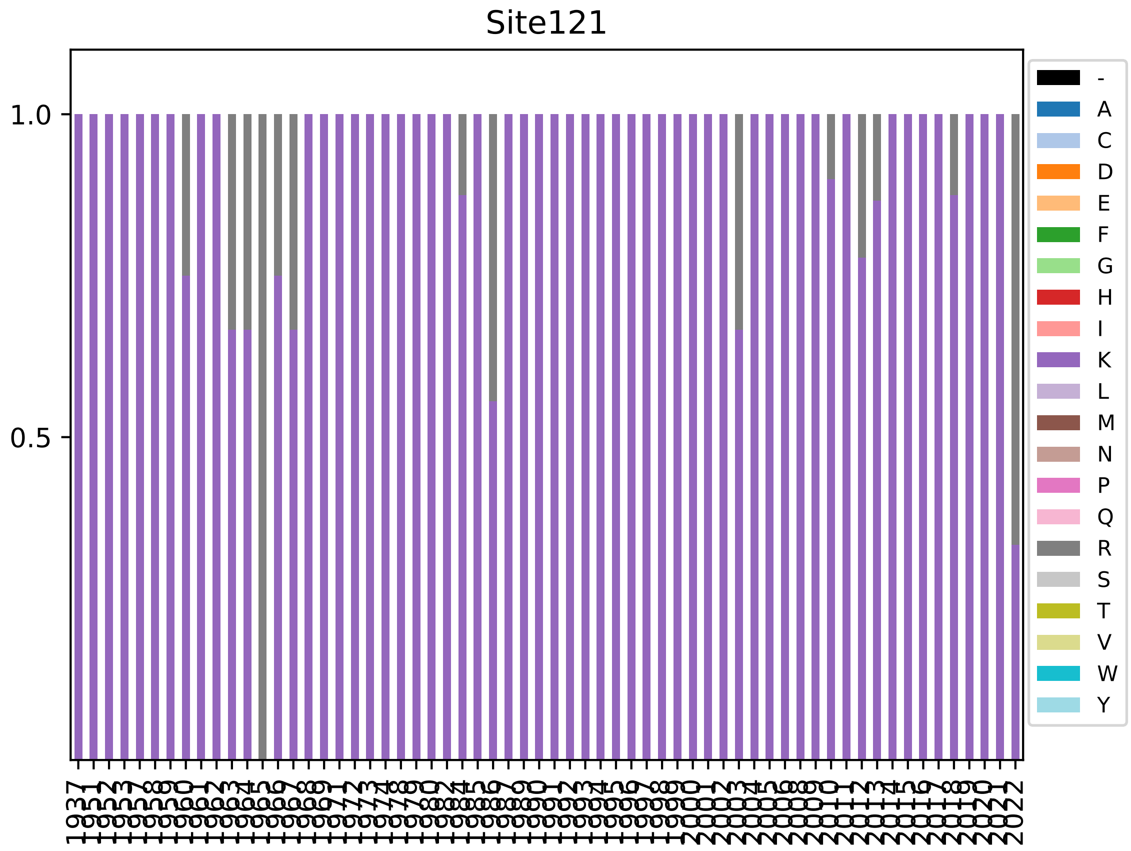
Site121
1.0
0.5
-
A
C
D
E
F
G
H
I
K
L
M
N
P
Q
R
S
T
V
W
Y

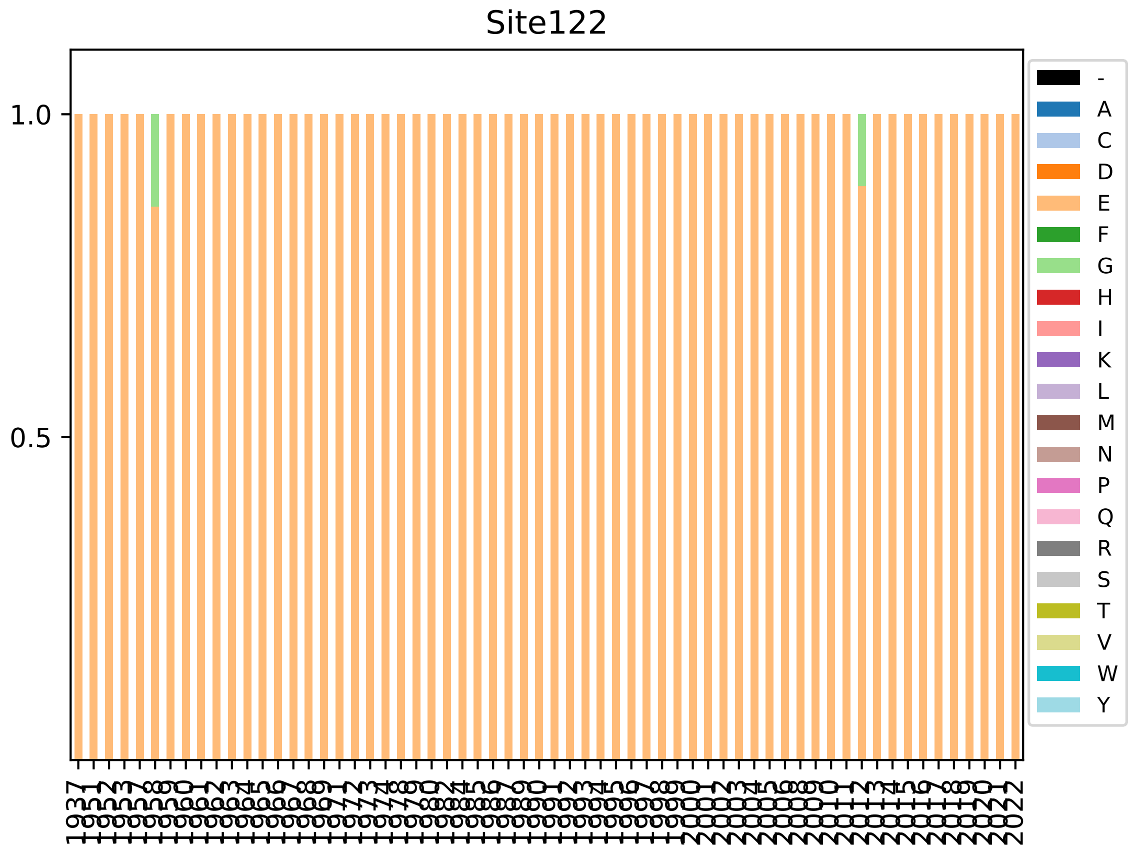
Site122
1.0
0.5
-
A
C
D
E
F
G
H
I
K
L
M
N
P
Q
R
S
T
V
W
Y

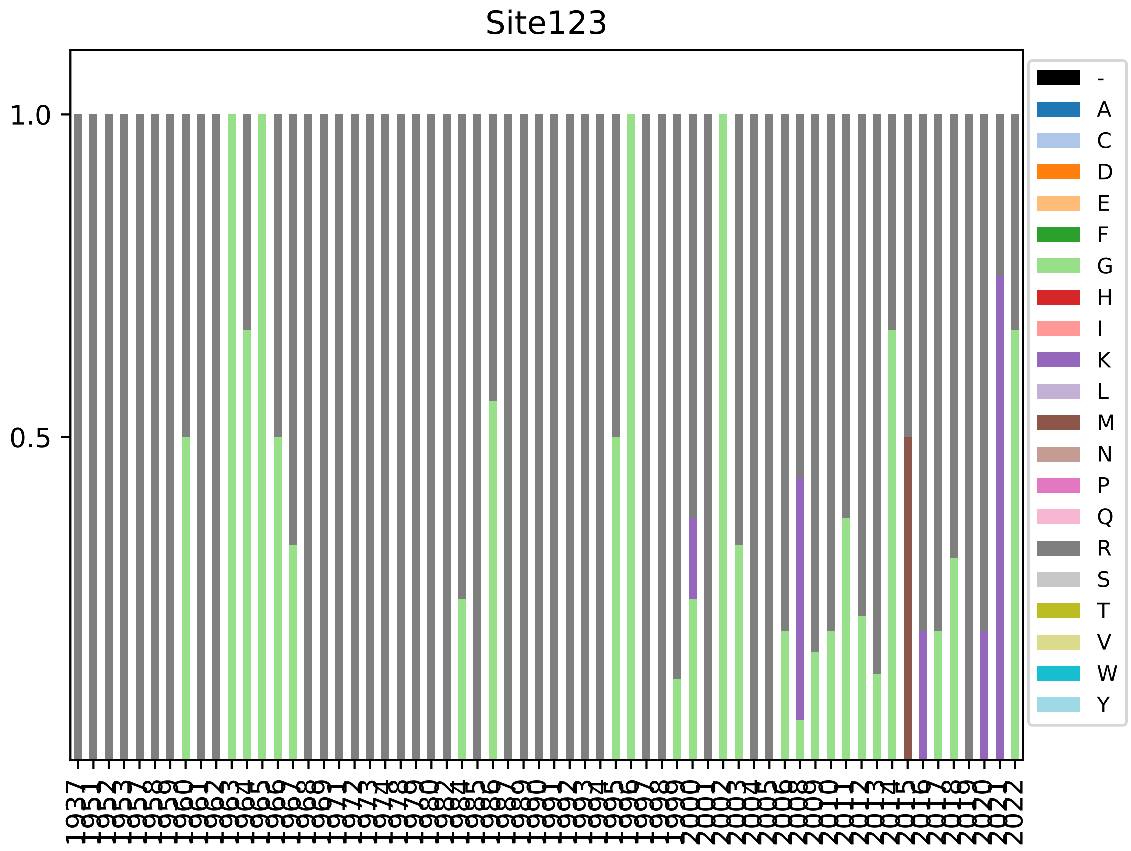
Site123
1.0
0.5
-
A
C
D
E
F
G
H
I
K
L
M
N
P
Q
R
S
T
V
W
Y

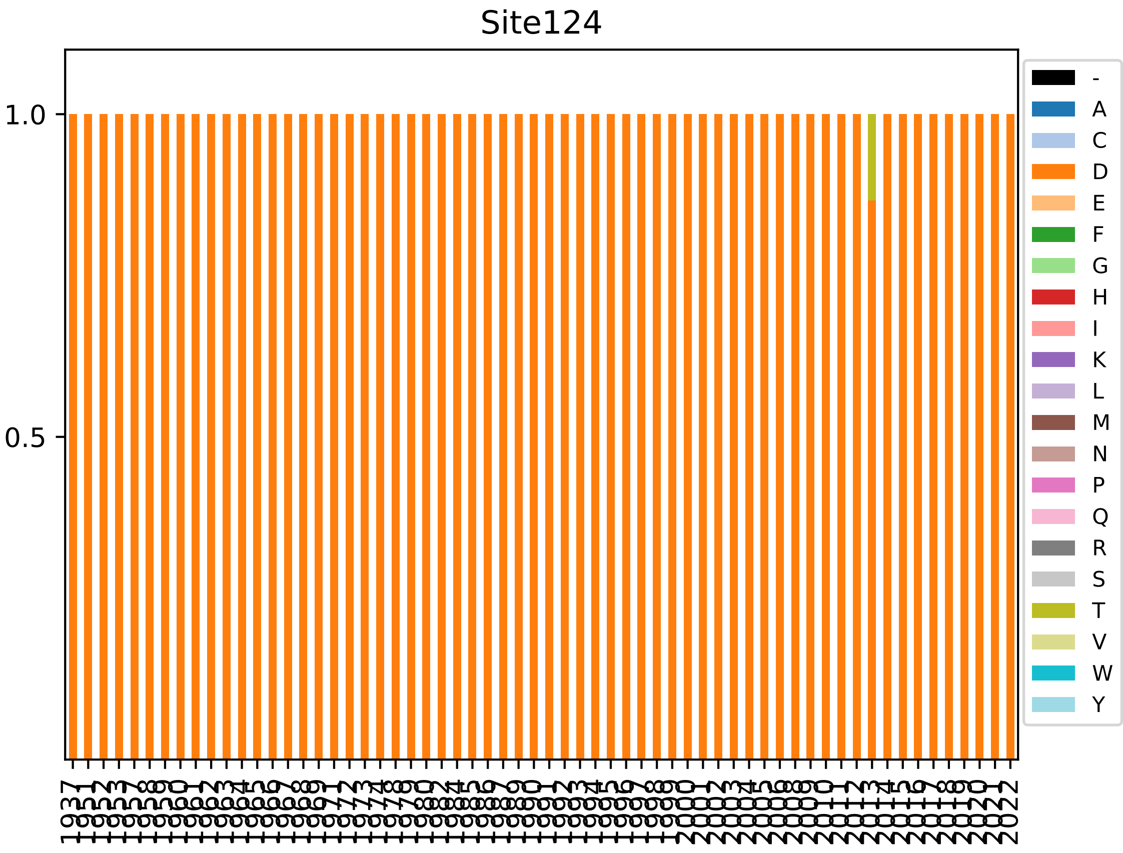
Site124
1.0
0.5
-
A
C
D
E
F
G
H
I
K
L
M
N
P
Q
R
S
T
V
W
Y

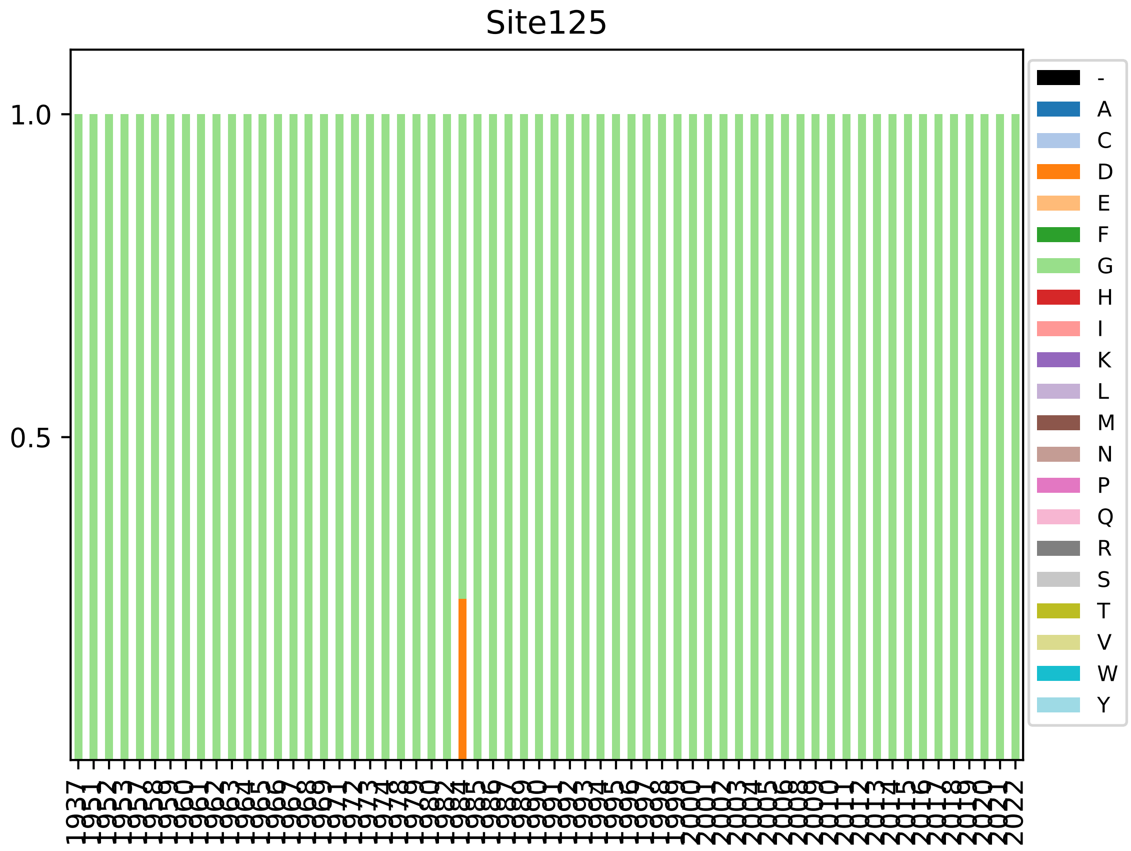
Site125
1.0
0.5
-
A
C
D
E
F
G
H
I
K
L
M
N
P
Q
R
S
T
V
W
Y

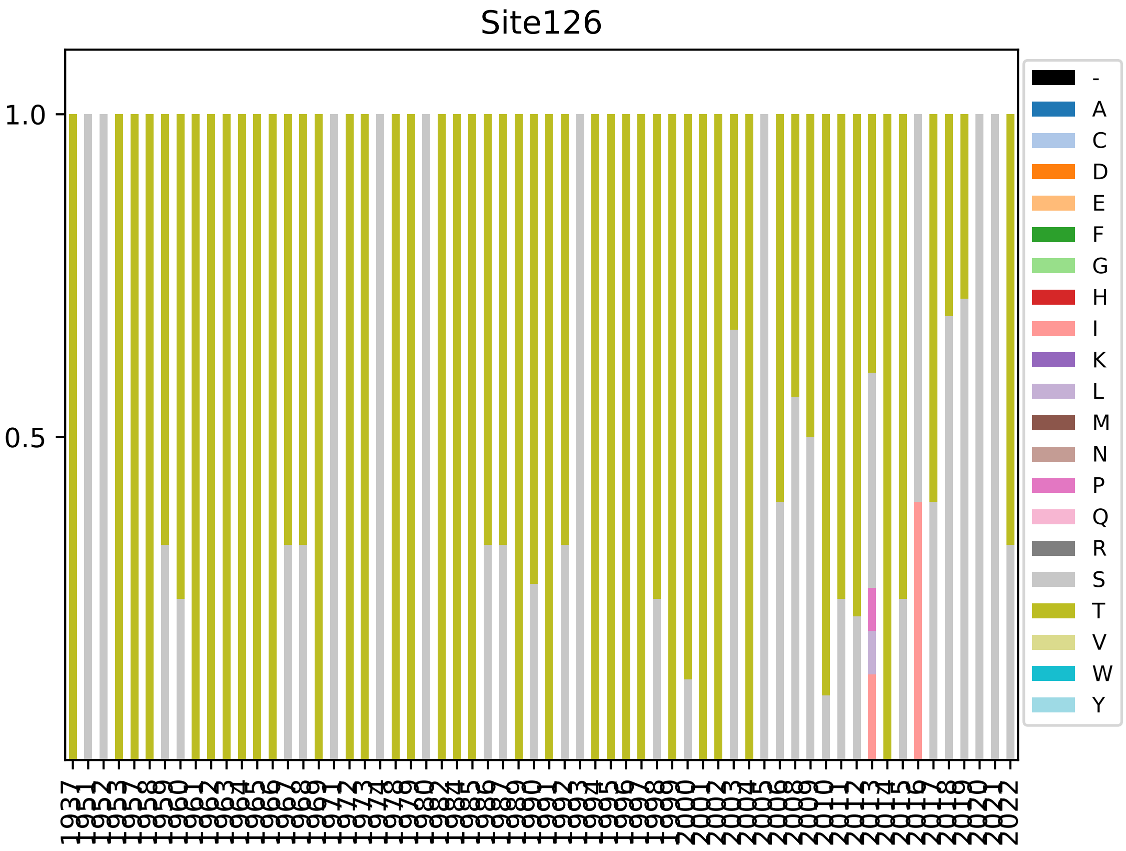
Site126
1.0
0.5
-
A
C
D
E
F
G
H
I
K
L
M
N
P
Q
R
S
T
V
W
Y

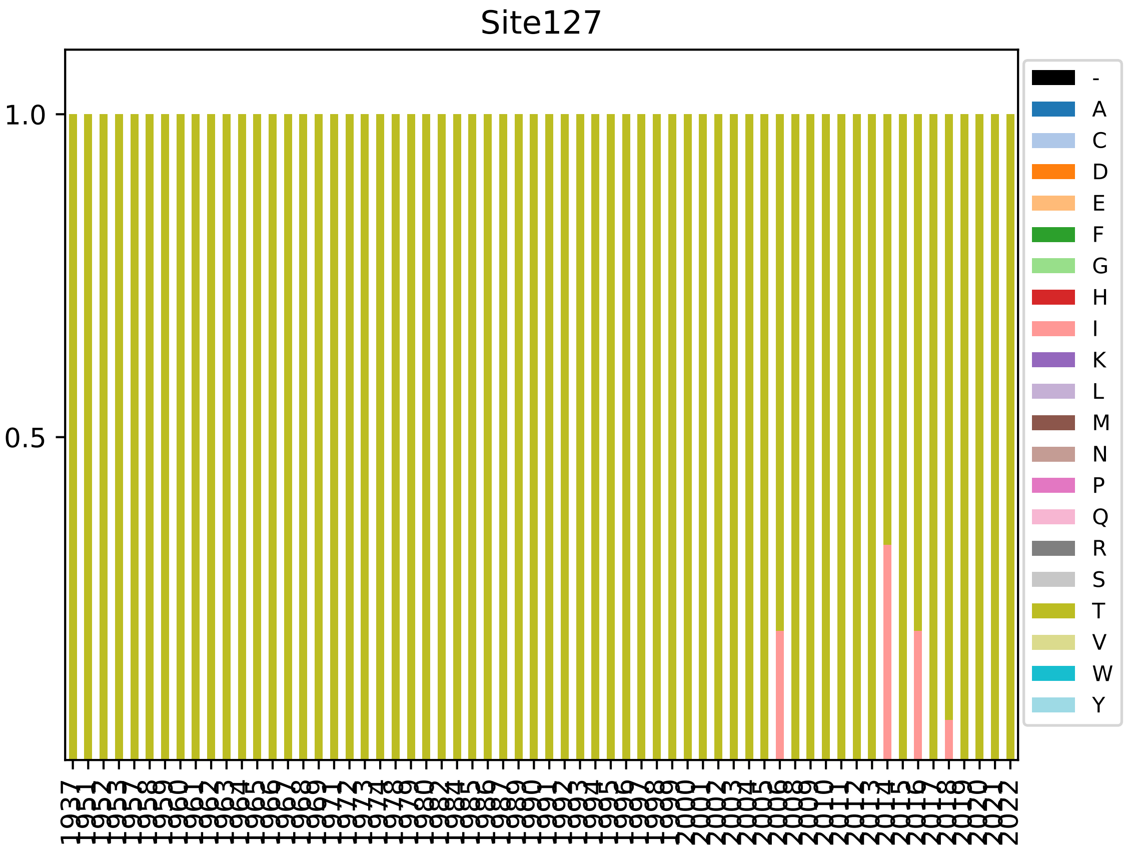

Site127
1.0
0.5
-
A
C
D
E
F
G
H
I
K
L
M
N
P
Q
R
S
T
V
W
Y

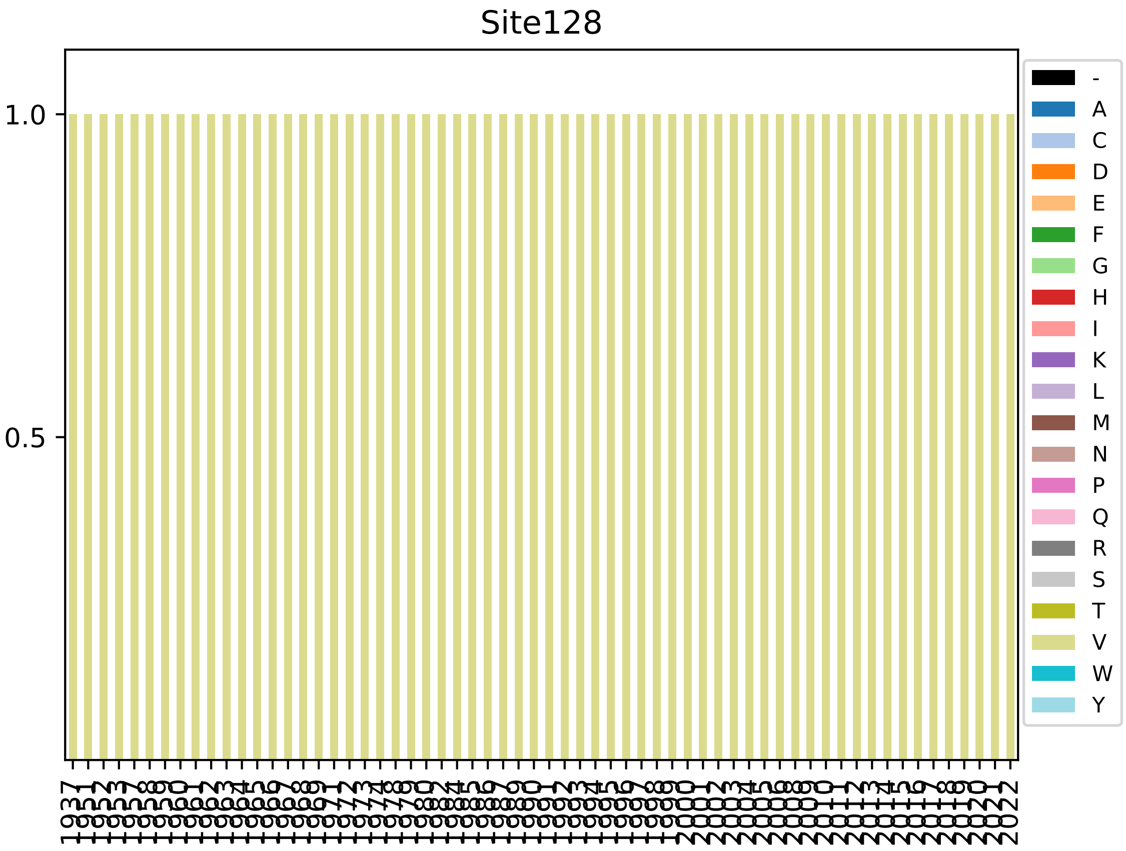
Site128
1.0
0.5
-
A
C
D
E
F
G
H
I
K
L
M
N
P
Q
R
S
T
V
W
Y

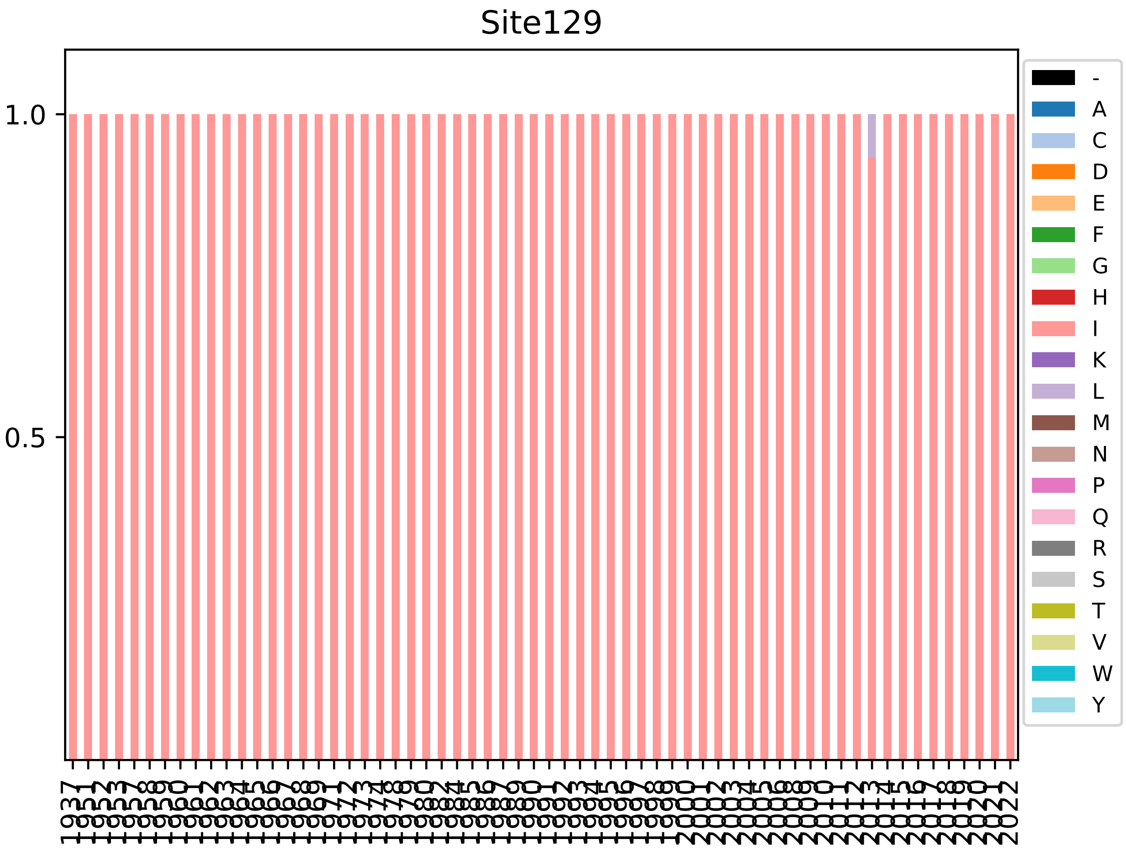
Site129
1.0
0.5
-
A
C
D
E
F
G
H
I
K
L
M
N
P
Q
R
S
T
V
W
Y

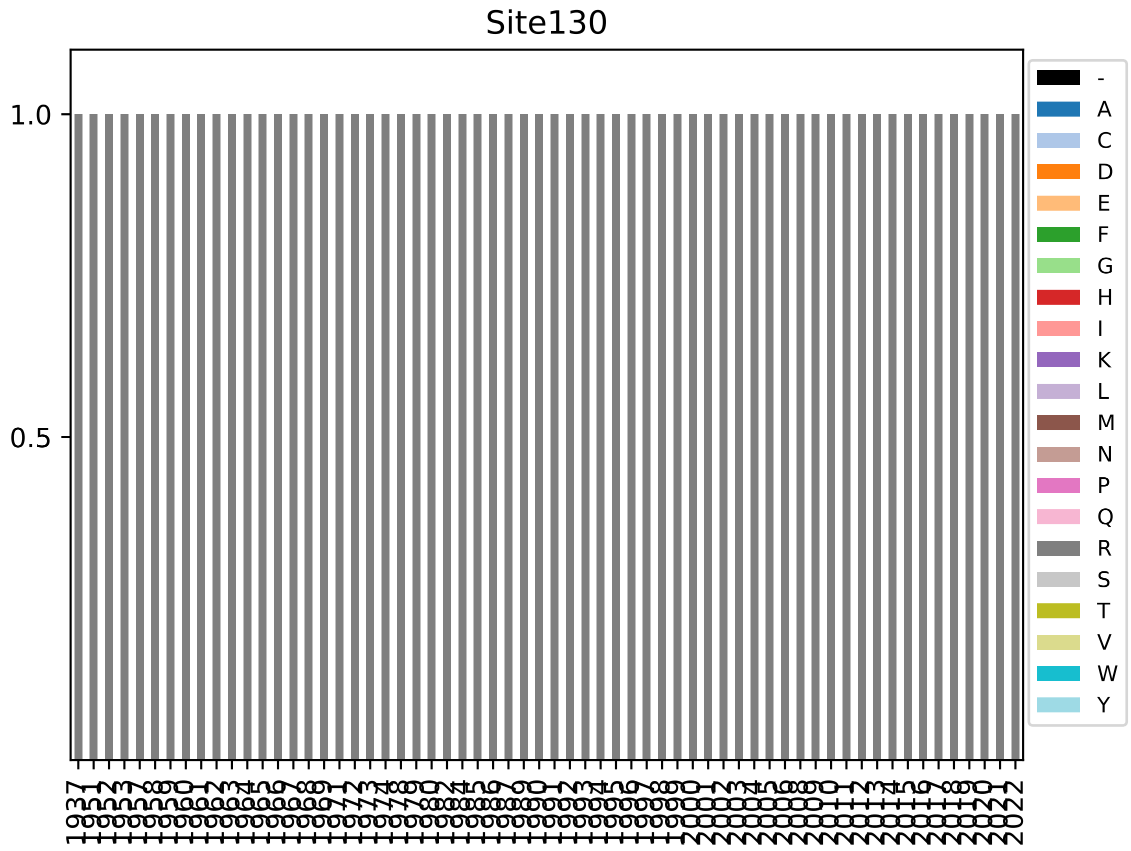
Site130
1.0
0.5
-
A
C
D
E
F
G
H
I
K
L
M
N
P
Q
R
S
T
V
W
Y

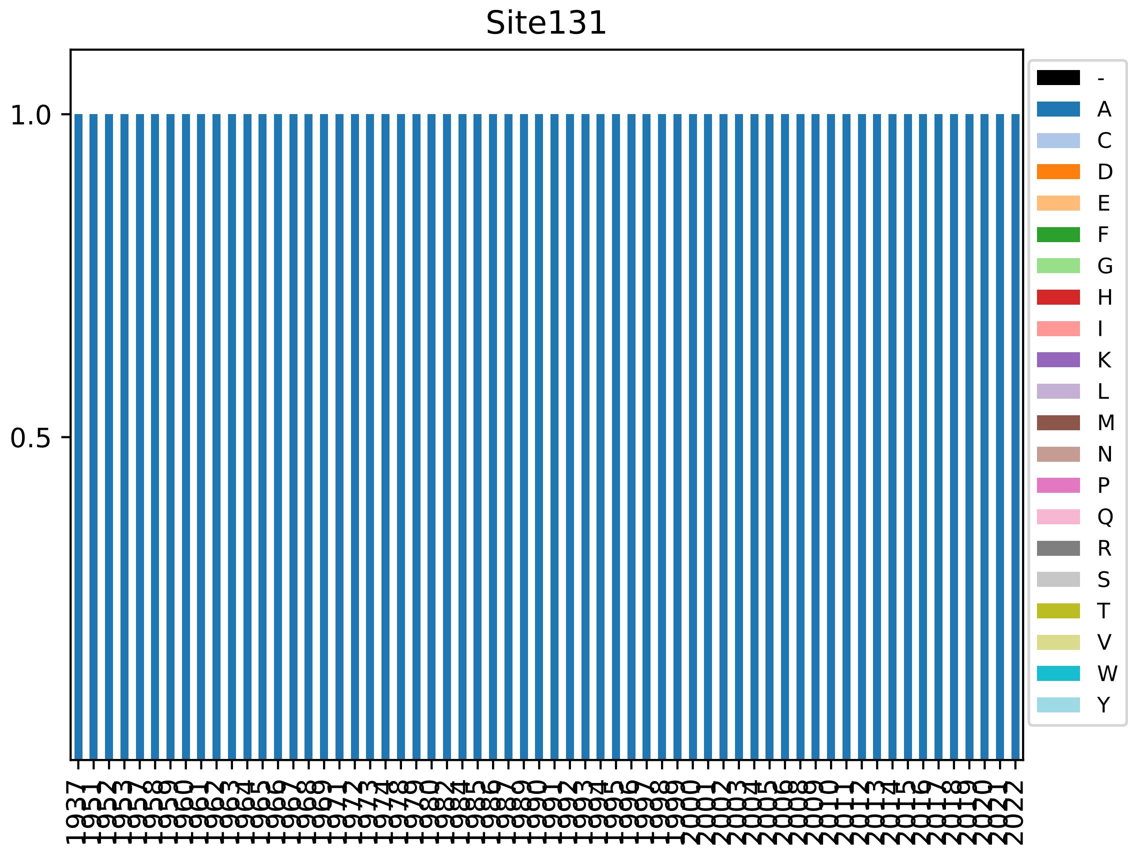
Site131
1.0
0.5
-
A
C
D
E
F
G
H
I
K
L
M
N
P
Q
R
S
T
V
W
Y

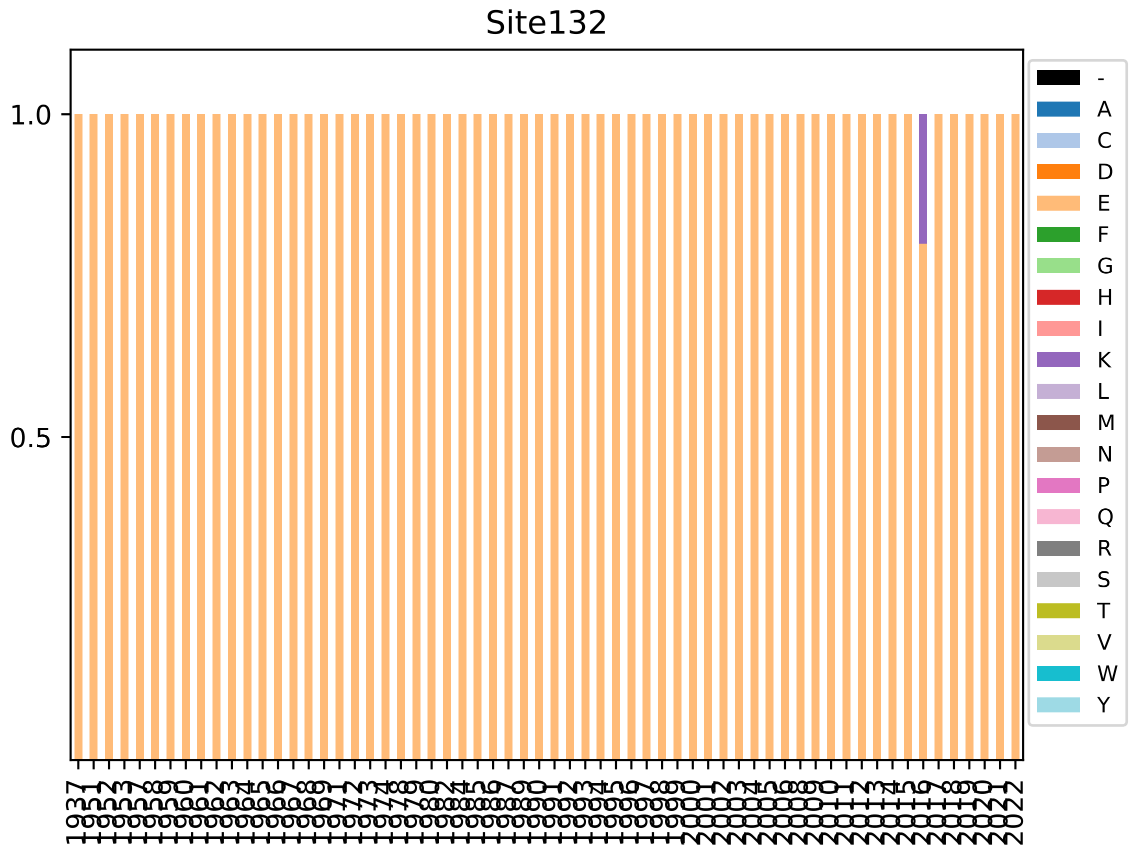

Site132
1.0
0.5
-
A
C
D
E
F
G
H
I
K
L
M
N
P
Q
R
S
T
V
W
Y

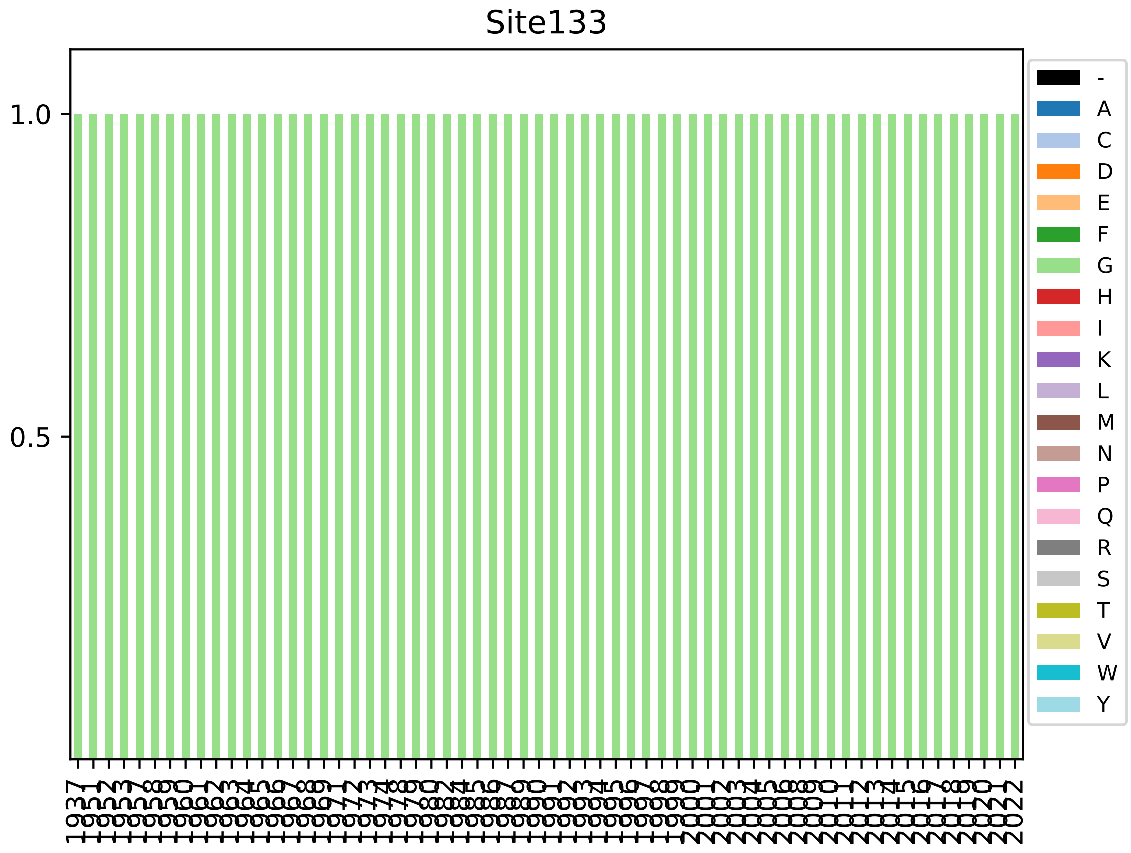

Site133
1.0
0.5
-
A
C
D
E
F
G
H
I
K
L
M
N
P
Q
R
S
T
V
W
Y

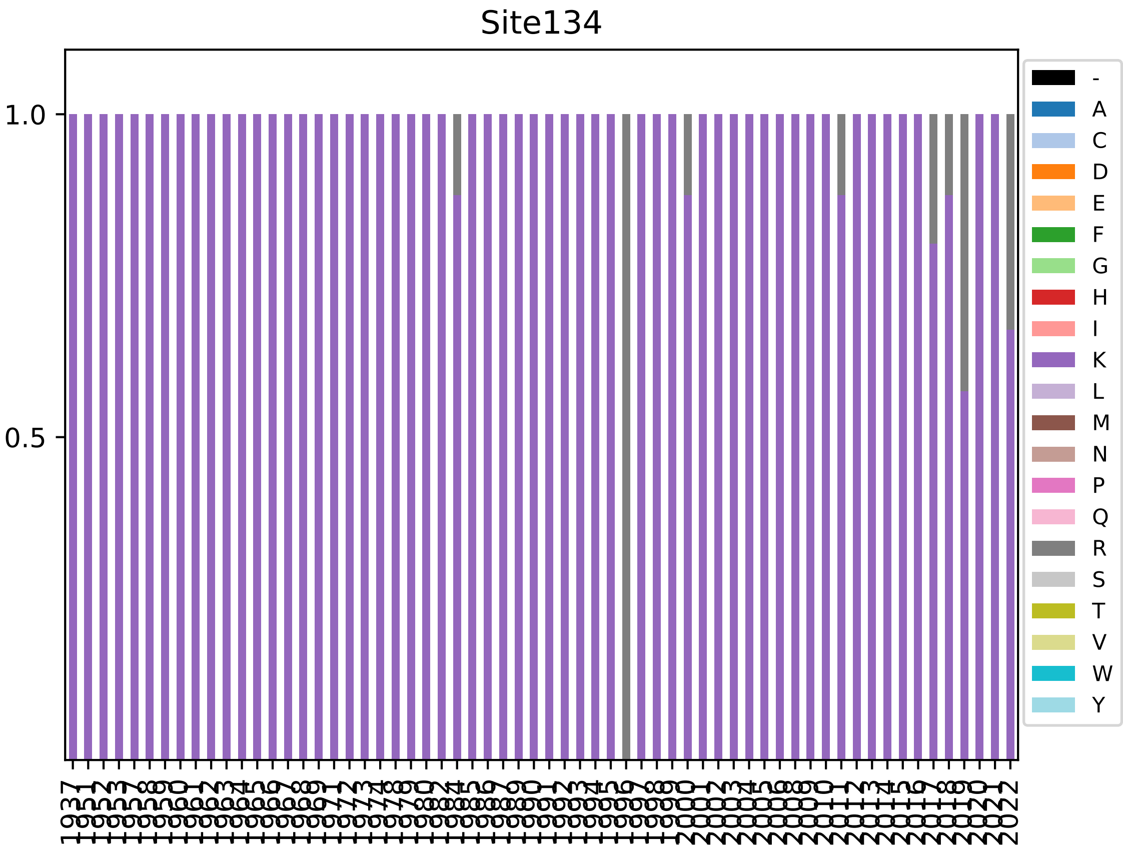
Site134
1.0
0.5
-
A
C
D
E
F
G
H
I
K
L
M
N
P
Q
R
S
T
V
W
Y

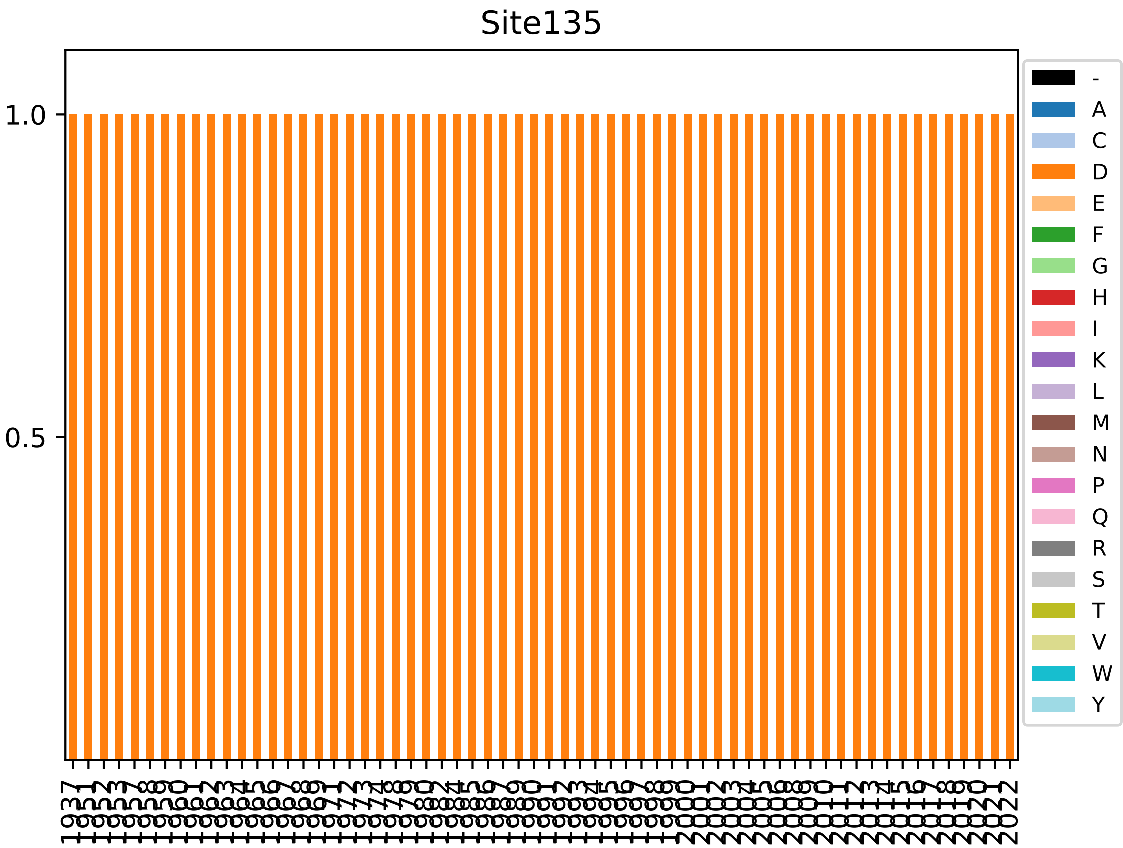
Site135
1.0
0.5
-
A
C
D
E
F
G
H
I
K
L
M
N
P
Q
R
S
T
V
W
Y

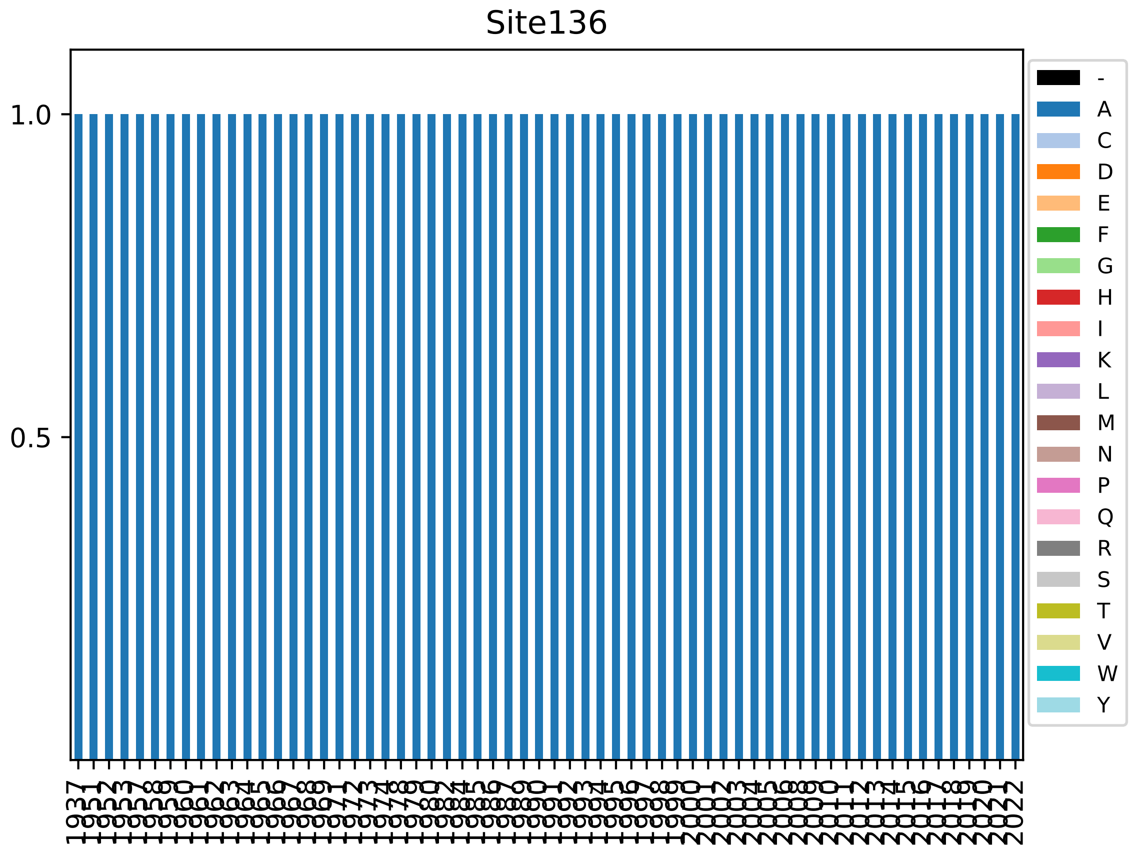
Site136
1.0
0.5
-
A
C
D
E
F
G
H
I
K
L
M
N
P
Q
R
S
T
V
W
Y

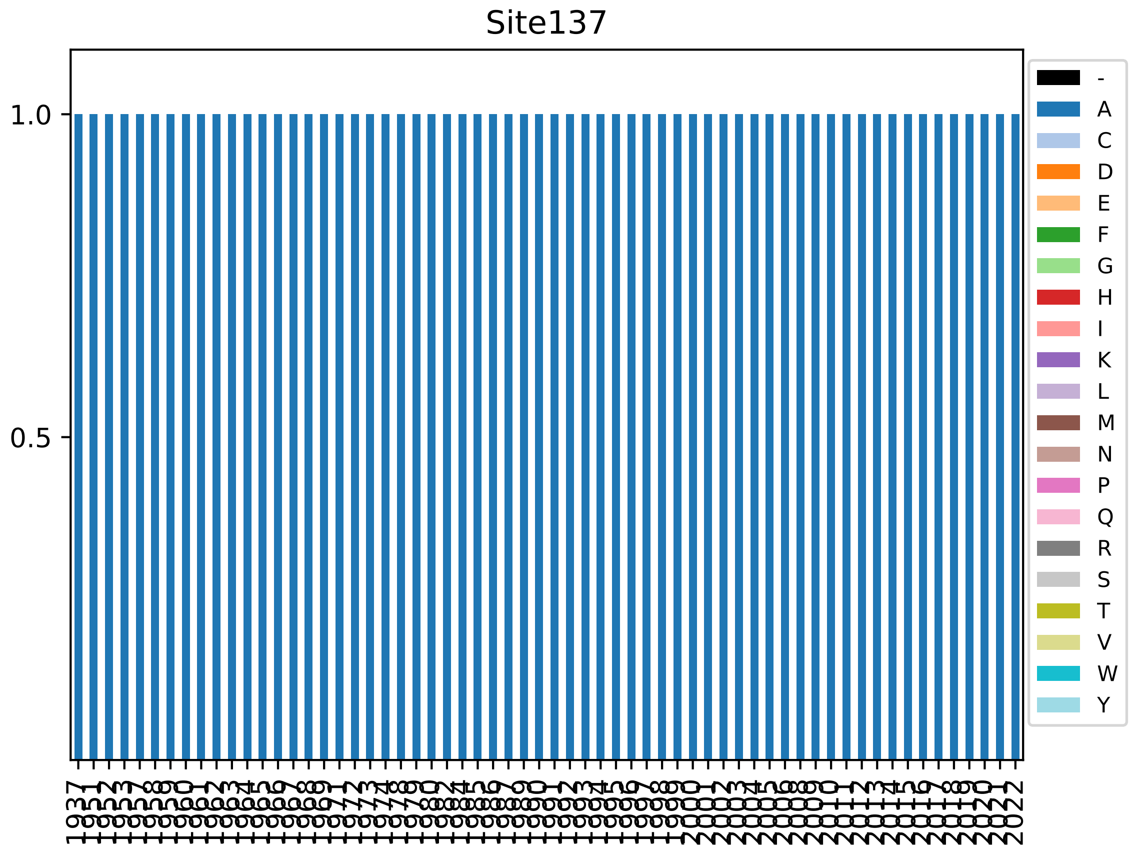
Site137
1.0
0.5
-
A
C
D
E
F
G
H
I
K
L
M
N
P
Q
R
S
T
V
W
Y

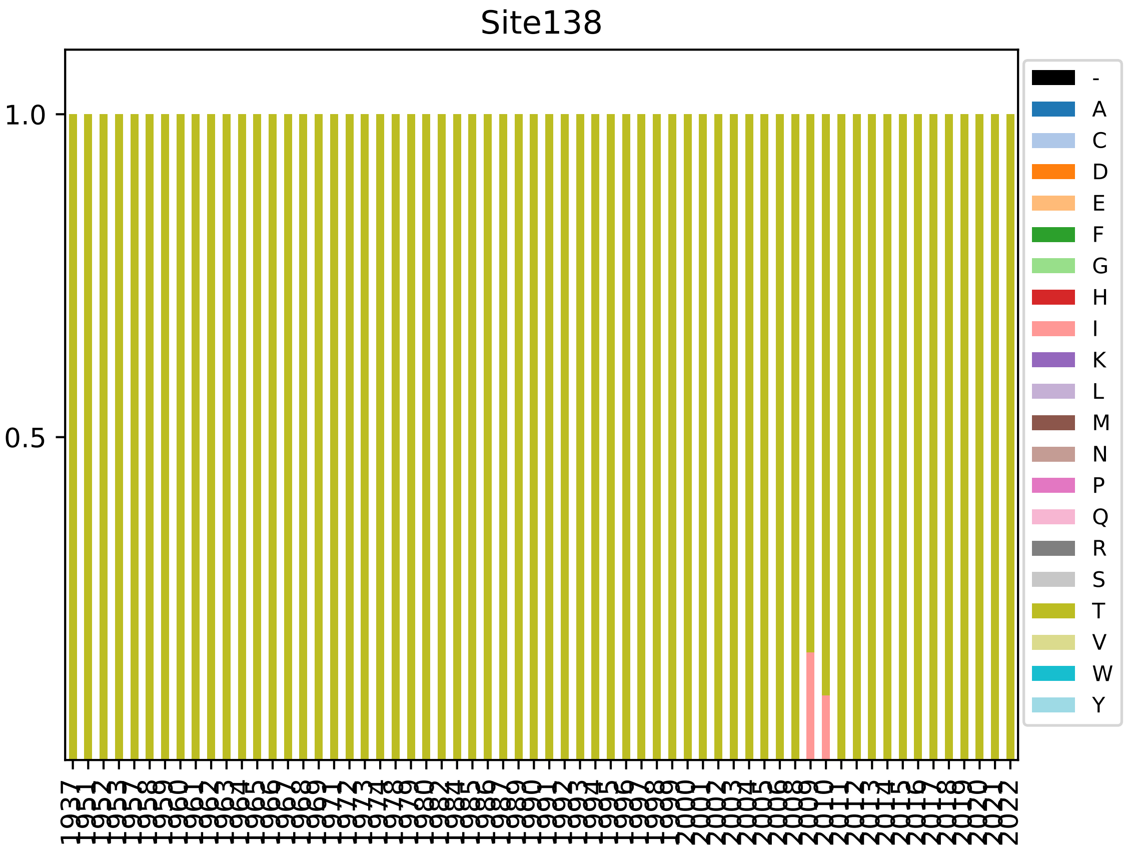
Site138
1.0
0.5
-
A
C
D
E
F
G
H
I
K
L
M
N
P
Q
R
S
T
V
W
Y

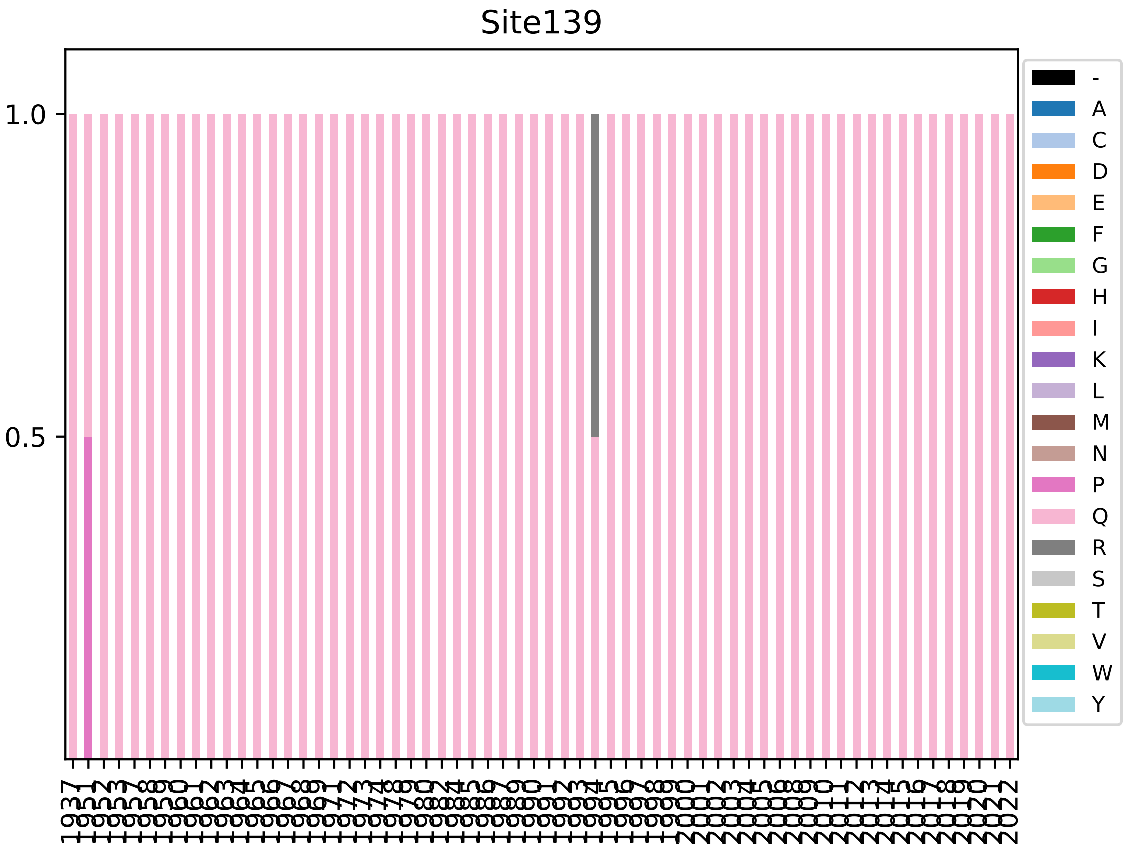
Site139
1.0
0.5
-
A
C
D
E
F
G
H
I
K
L
M
N
P
Q
R
S
T
V
W
Y

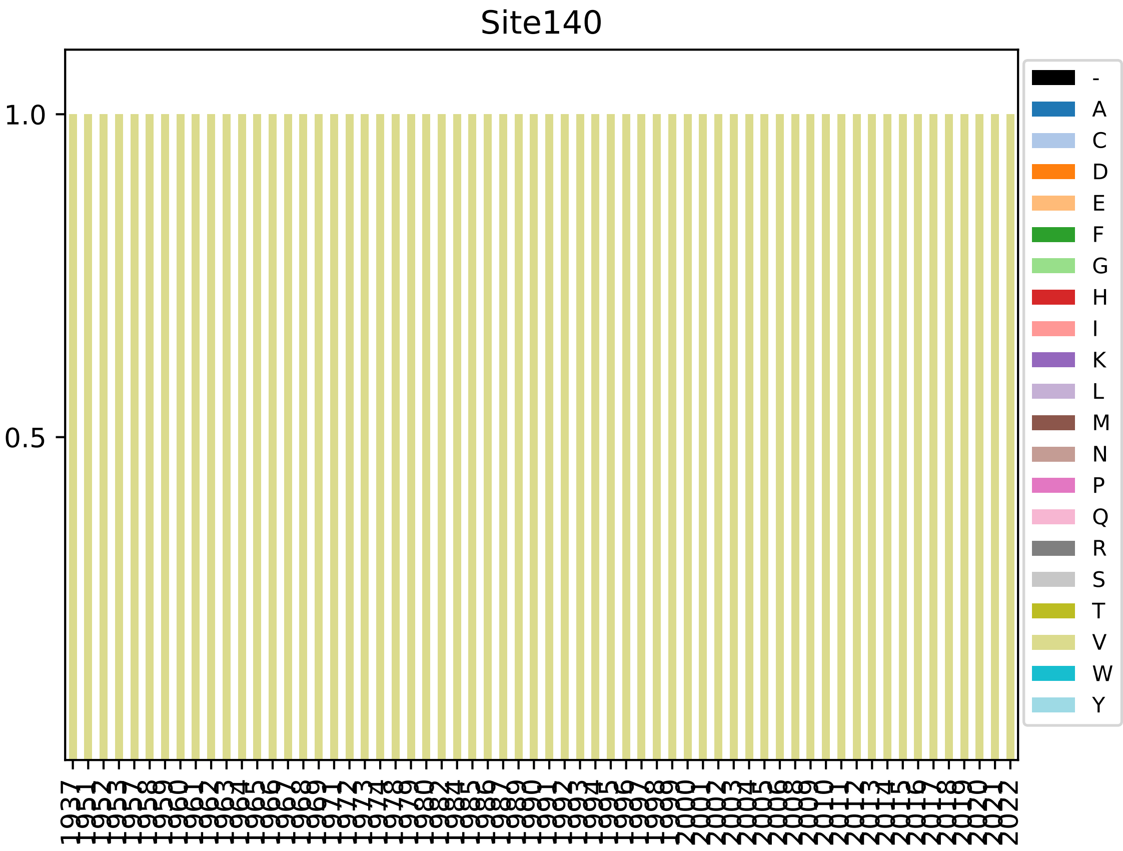
Site140
1.0
0.5
-
A
C
D
E
F
G
H
I
K
L
M
N
P
Q
R
S
T
V
W
Y

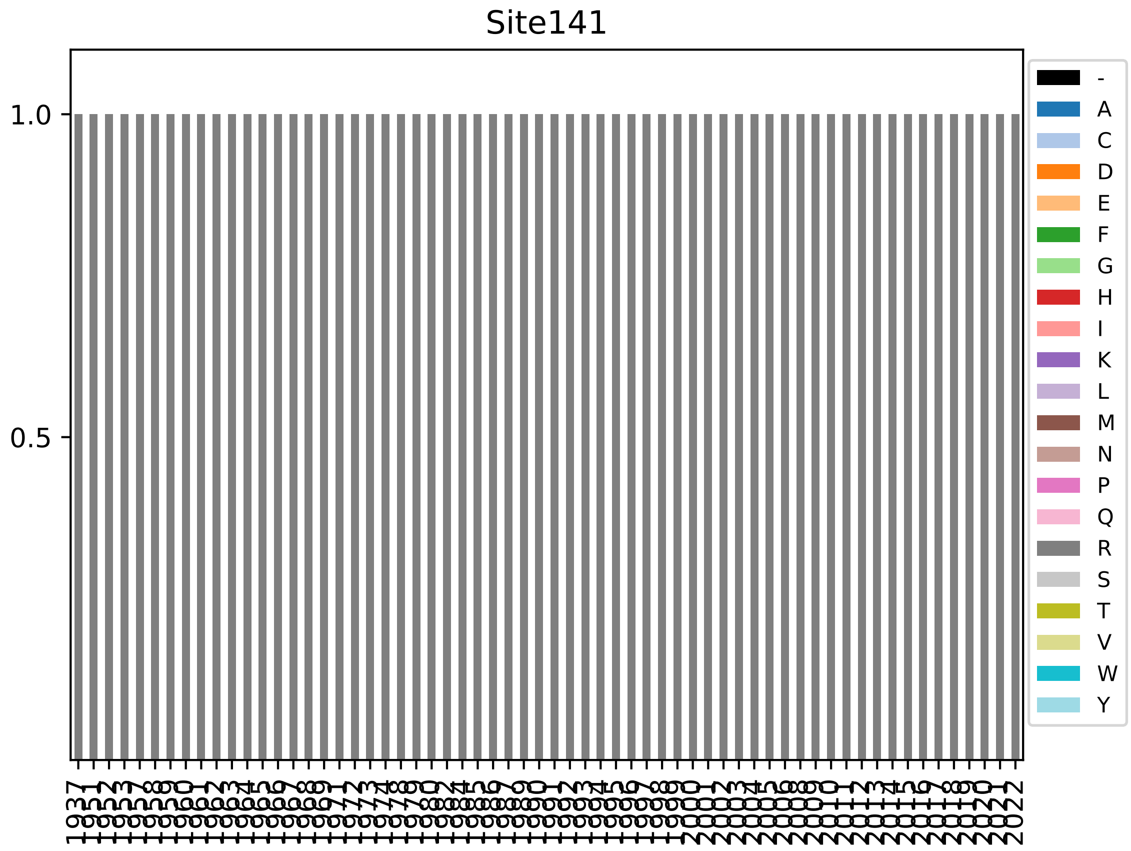
Site141
1.0
0.5
-
A
C
D
E
F
G
H
I
K
L
M
N
P
Q
R
S
T
V
W
Y

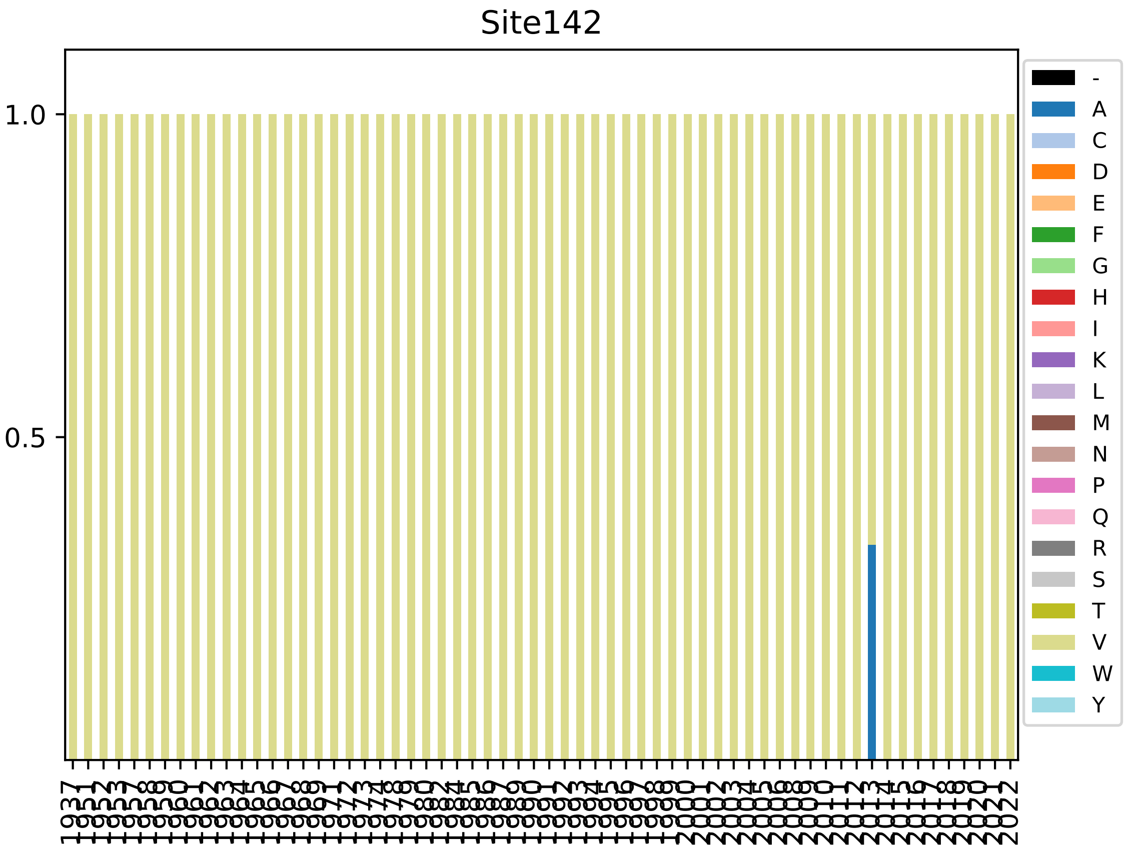

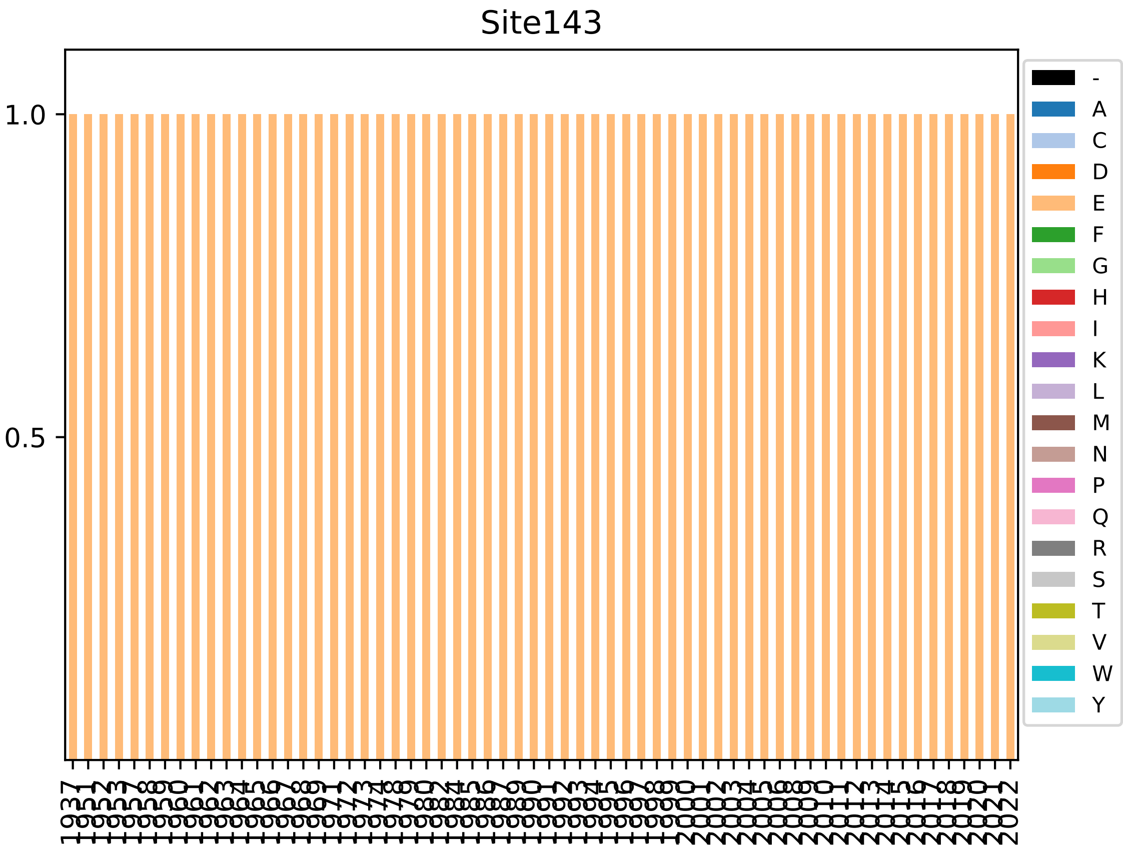
Site143
1.0
0.5
-
A
C
D
E
F
G
H
I
K
L
M
N
P
Q
R
S
T
V
W
Y

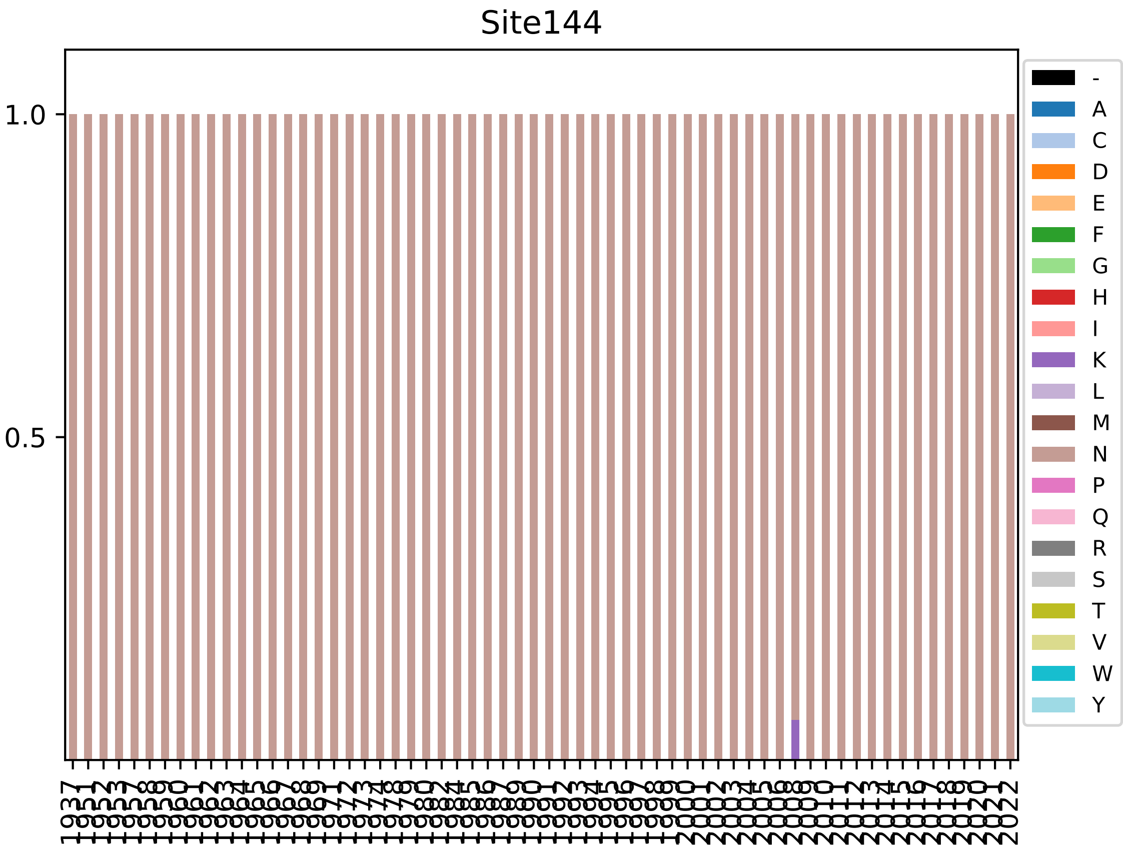
Site144
1.0
0.5
-
A
C
D
E
F
G
H
I
K
L
M
N
P
Q
R
S
T
V
W
Y

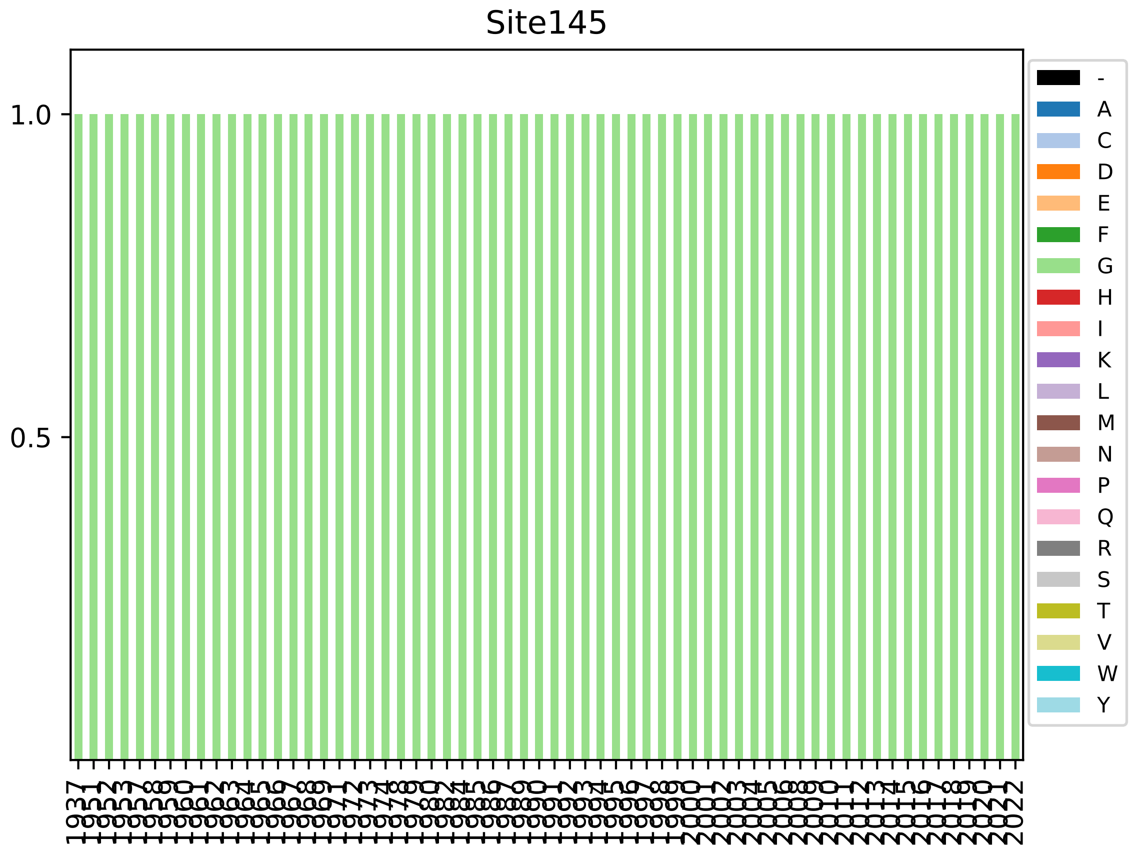
Site145
1.0
0.5
-
A
C
D
E
F
G
H
I
K
L
M
N
P
Q
R
S
T
V
W
Y

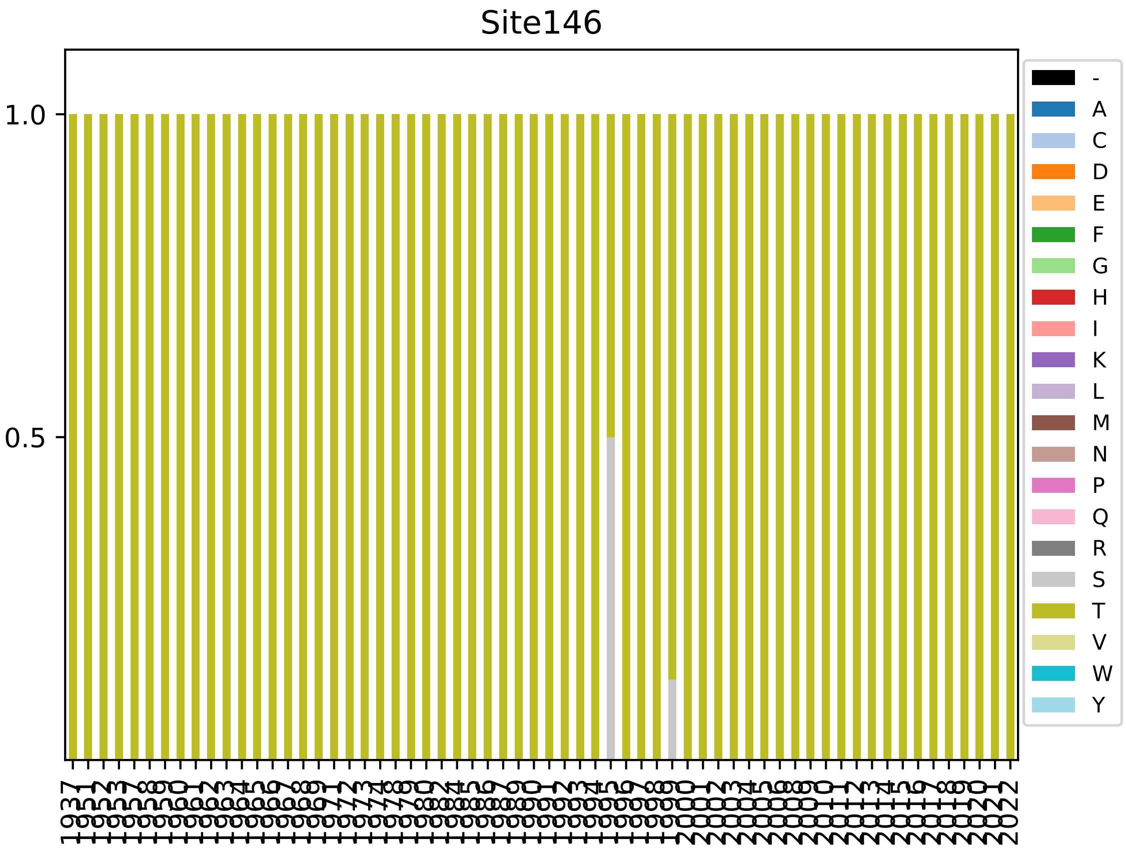

Site146
1.0
0.5
-
A
C
D
E
F
G
H
I
K
L
M
N
P
Q
R
S
T
V
W
Y

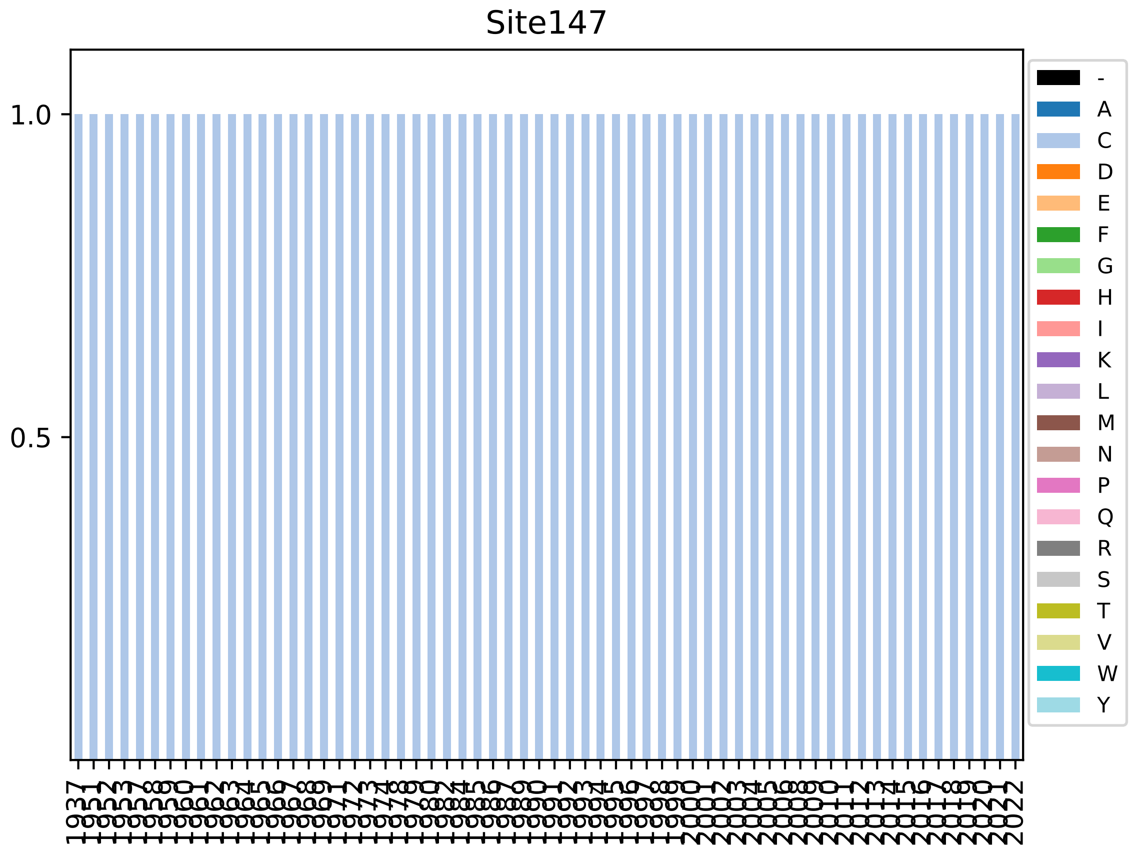
Site147
1.0
0.5
-
A
C
D
E
F
G
H
I
K
L
M
N
P
Q
R
S
T
V
W
Y

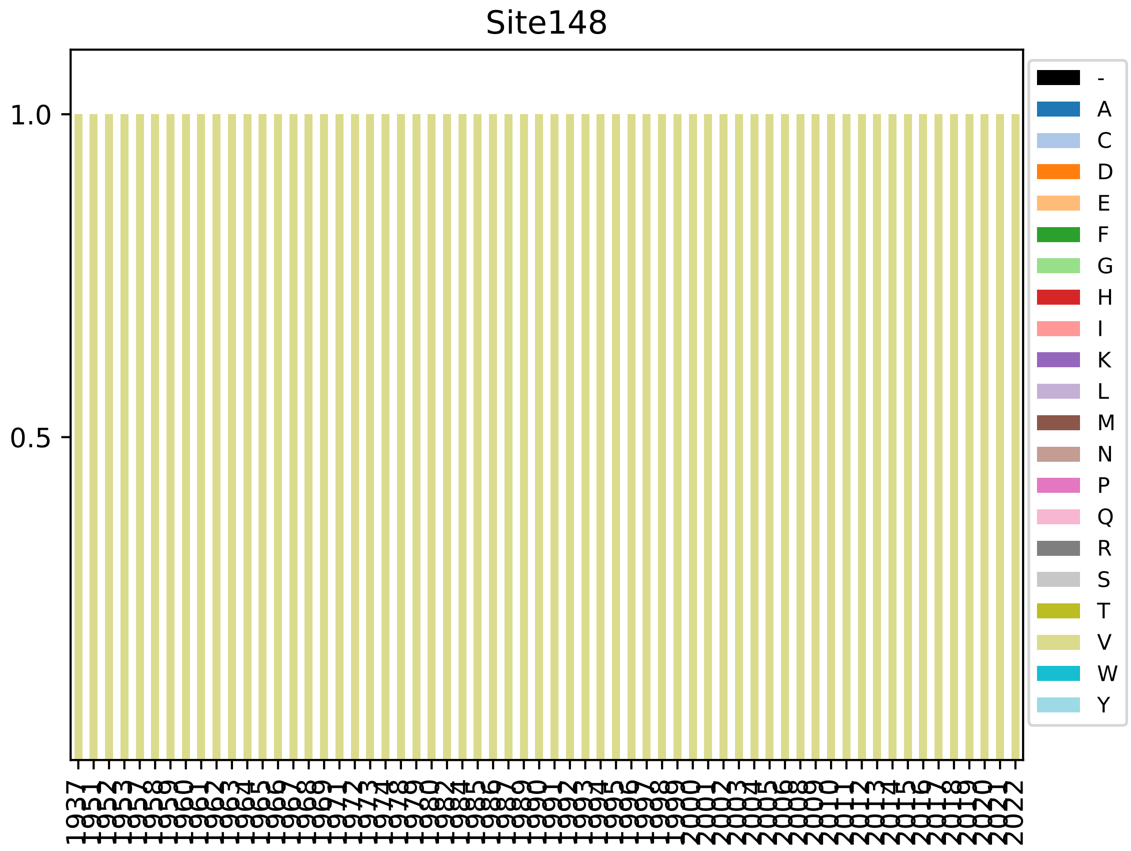
Site148
1.0
0.5
-
A
C
D
E
F
G
H
I
K
L
M
N
P
Q
R
S
T
V
W
Y

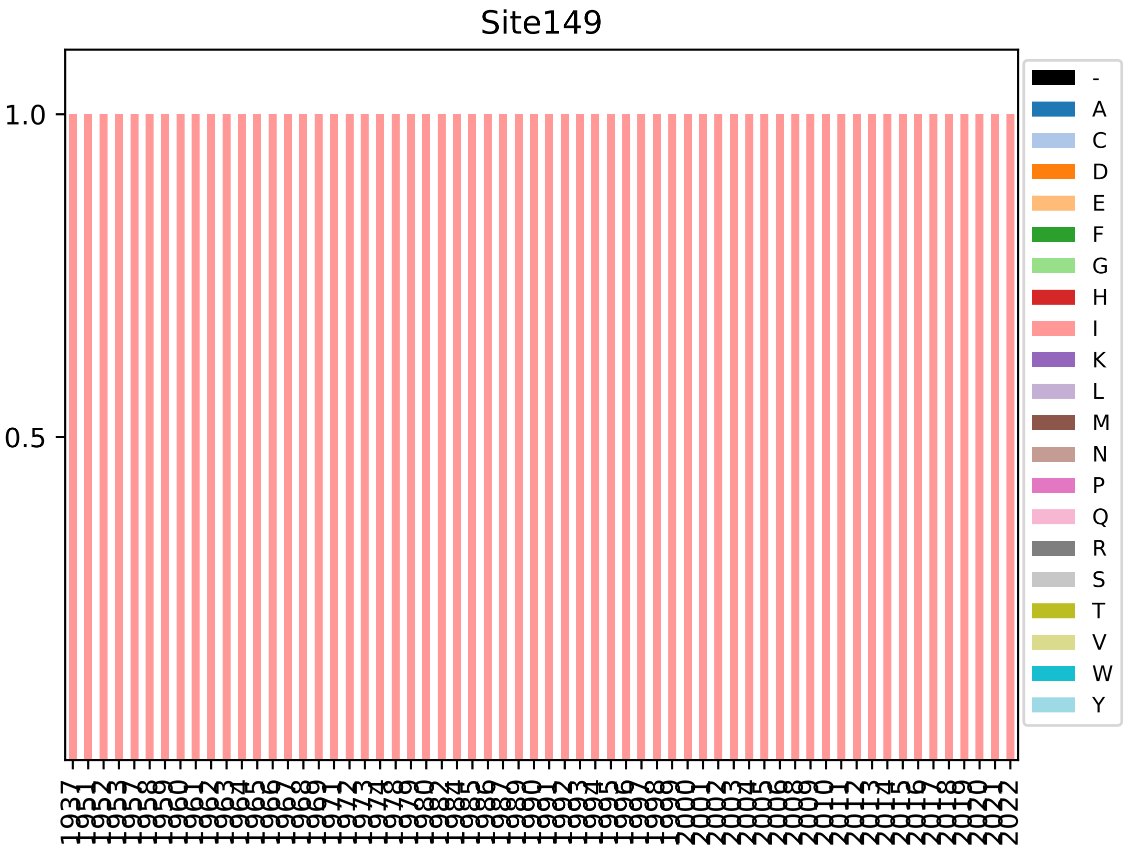
Site149
1.0
0.5
-
A
C
D
E
F
G
H
I
K
L
M
N
P
Q
R
S
T
V
W
Y

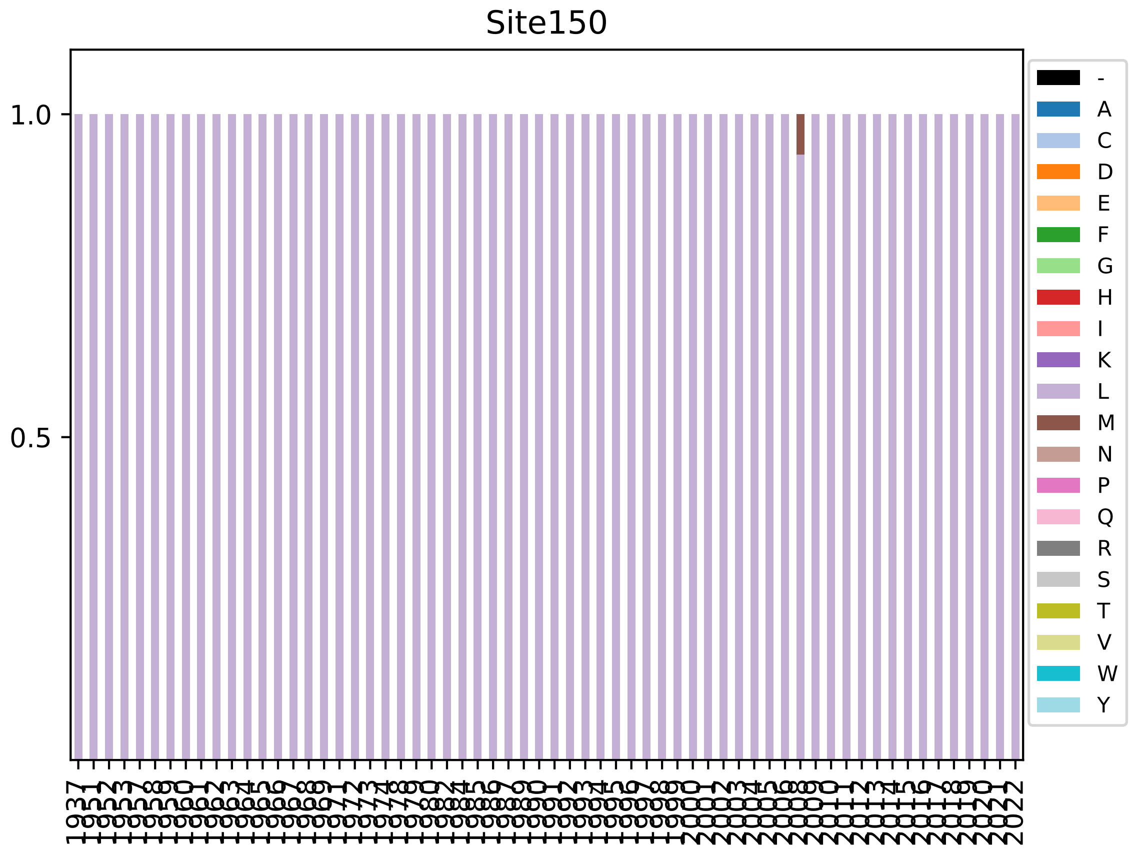
Site150
1.0
0.5
-
A
C
D
E
F
G
H
I
K
L
M
N
P
Q
R
S
T
V
W
Y

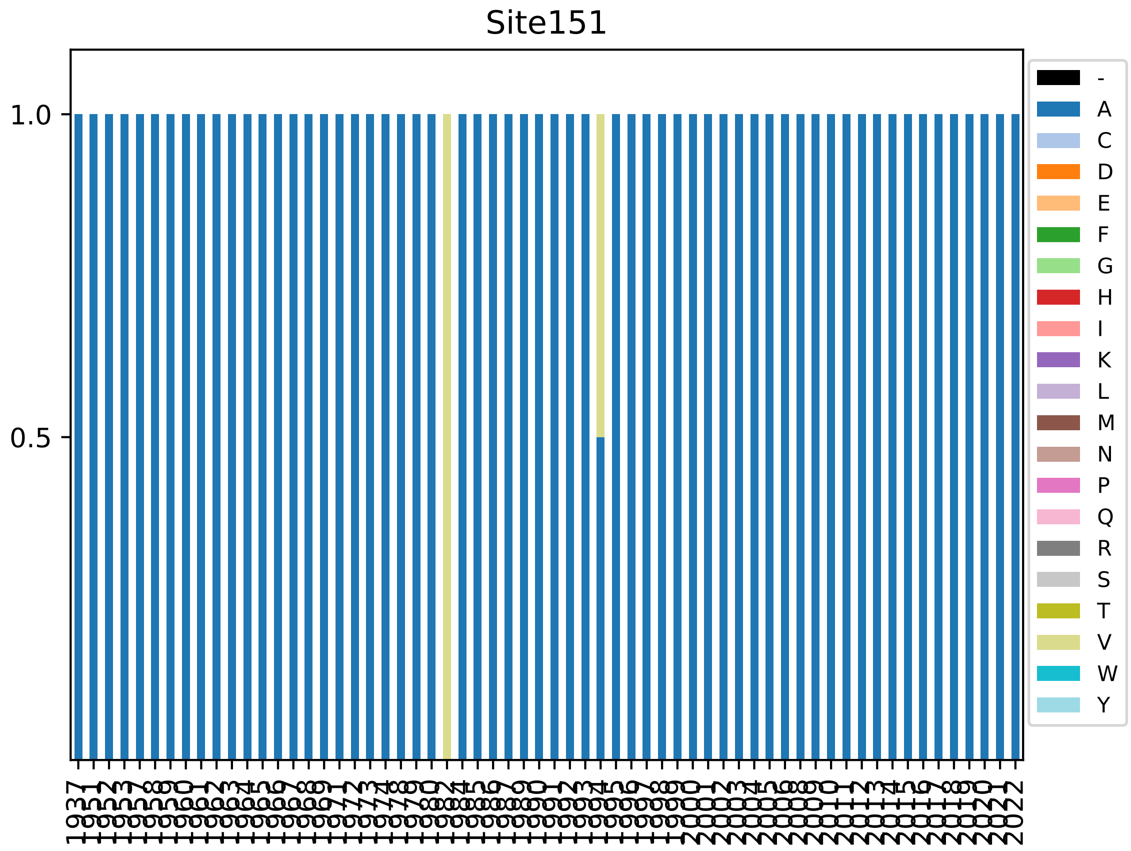
Site151
1.0
0.5
-
A
C
D
E
F
G
H
I
K
L
M
N
P
Q
R
S
T
V
W
Y

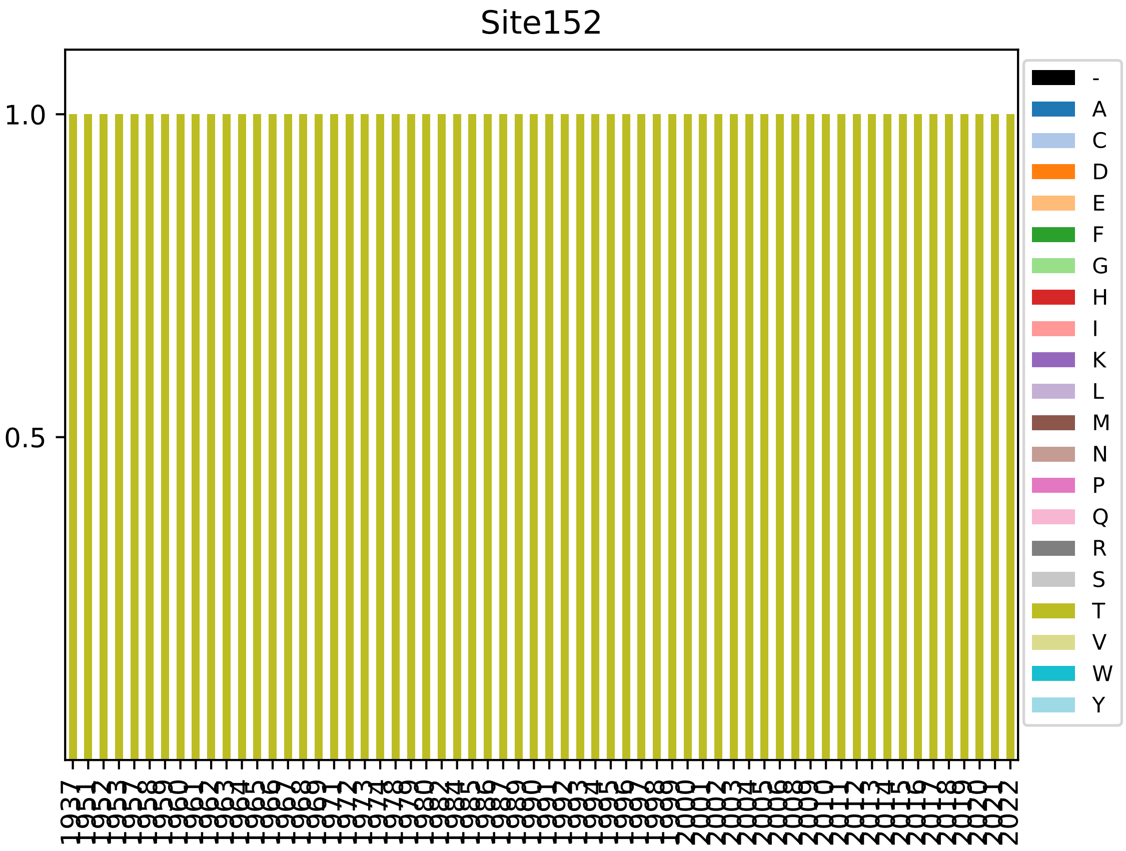

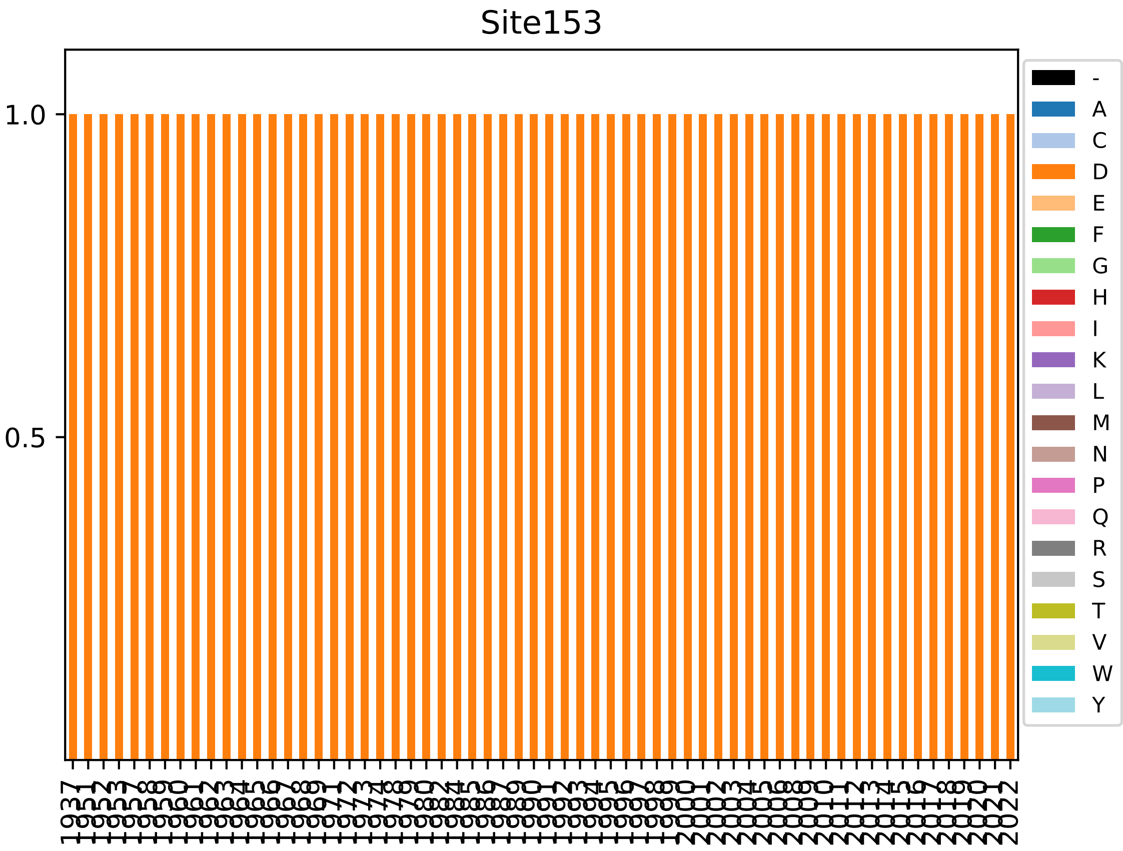
Site153
1.0
0.5
-
A
C
D
E
F
G
H
I
K
L
M
N
P
Q
R
S
T
V
W
Y

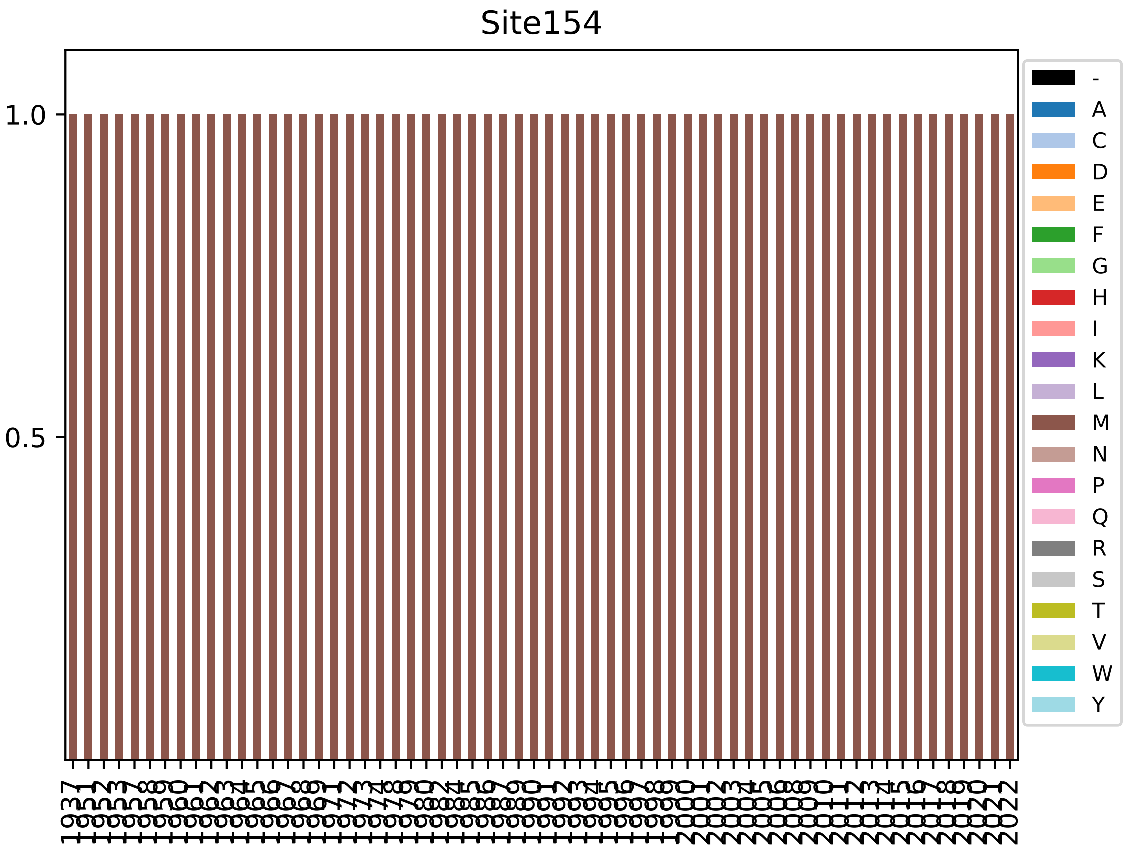
Site154
1.0
0.5
-
A
C
D
E
F
G
H
I
K
L
M
N
P
Q
R
S
T
V
W
Y

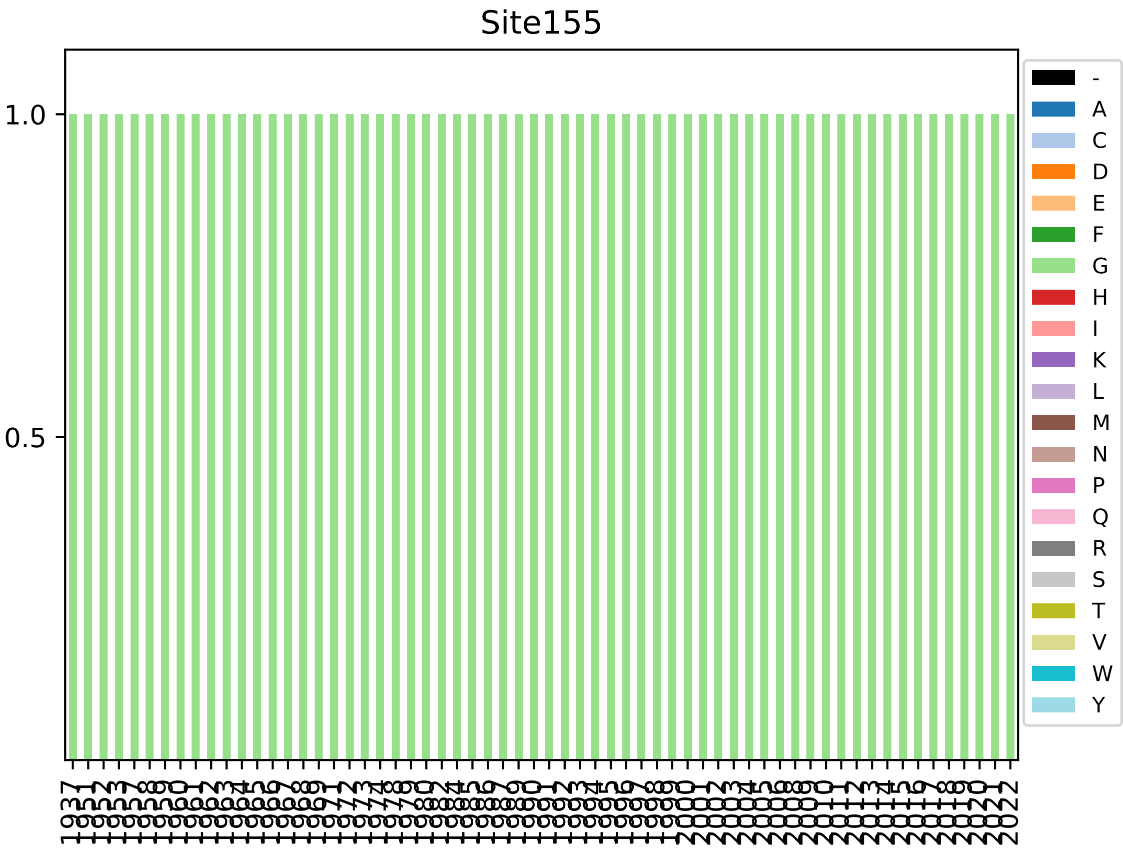
Site155
1.0
0.5
-
A
C
D
E
F
G
H
I
K
L
M
N
P
Q
R
S
T
V
W
Y

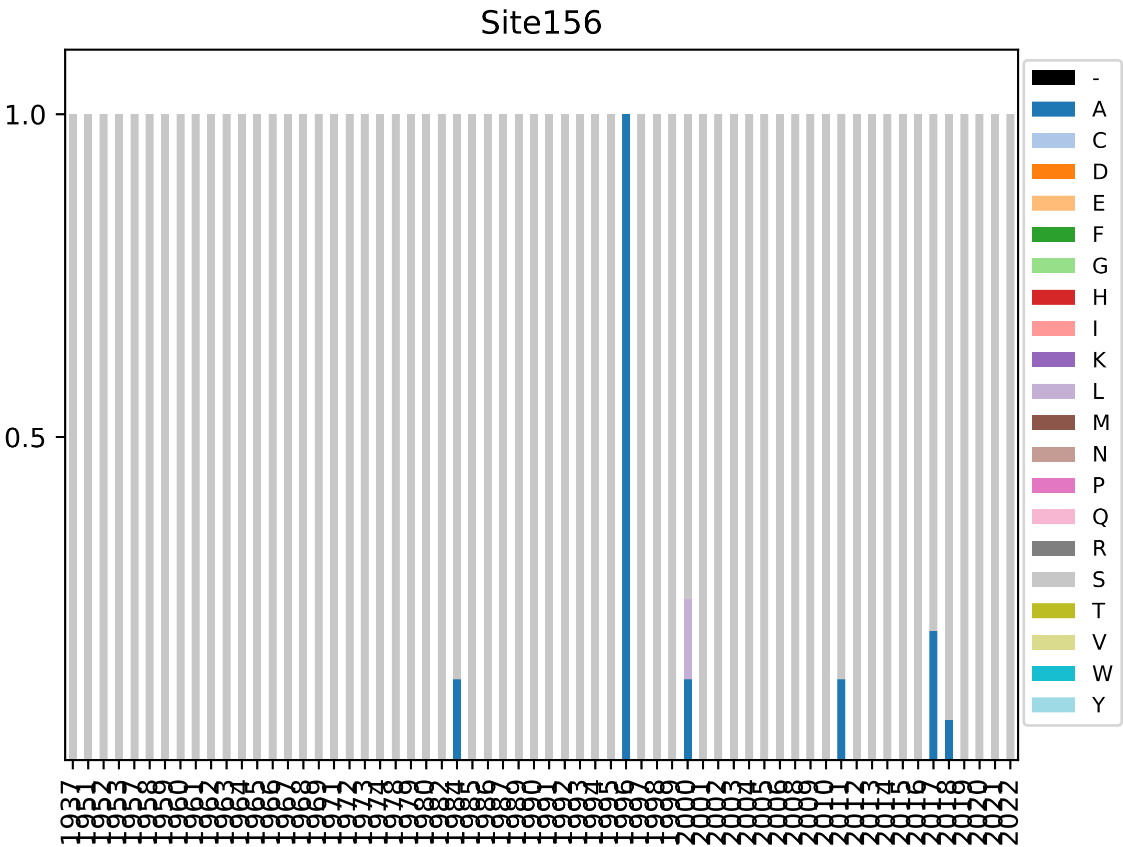
Site156
1.0
0.5
-
A
C
D
E
F
G
H
I
K
L
M
N
P
Q
R
S
T
V
W
Y

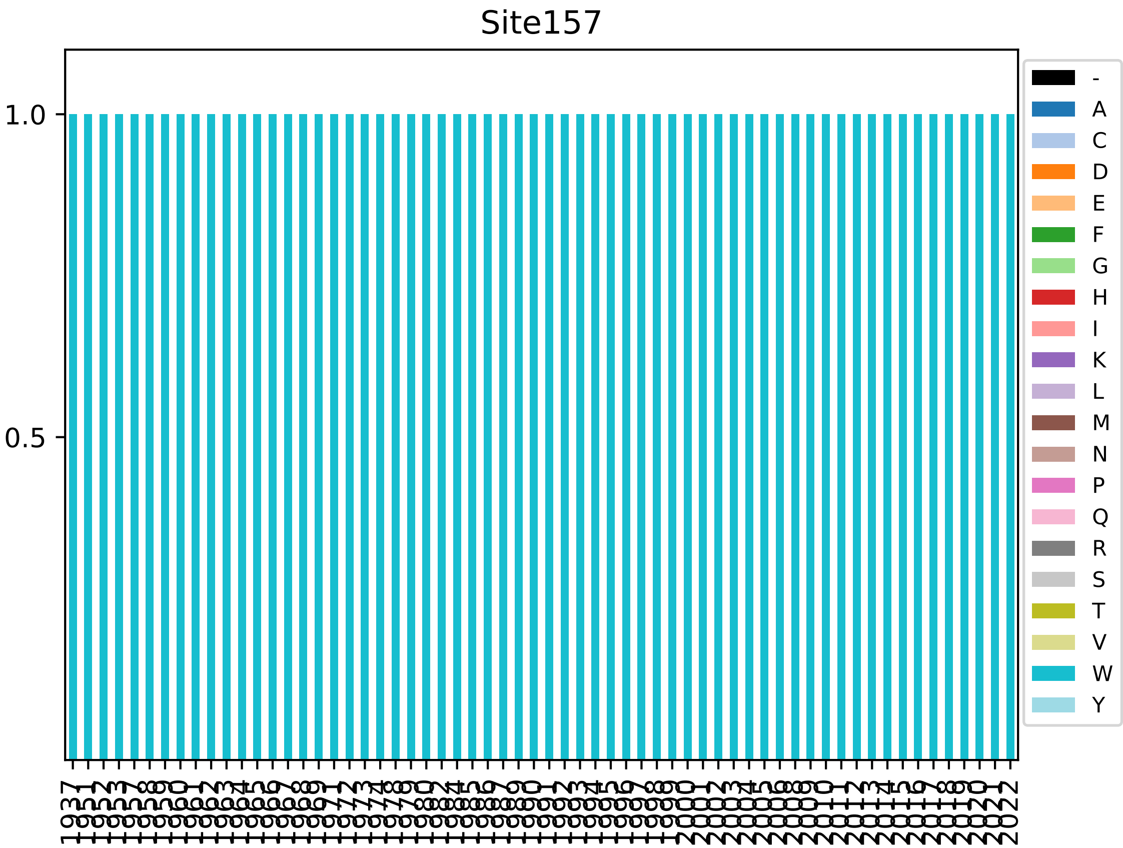
Site157
1.0
0.5
-
A
C
D
E
F
G
H
I
K
L
M
N
P
Q
R
S
T
V
W
Y

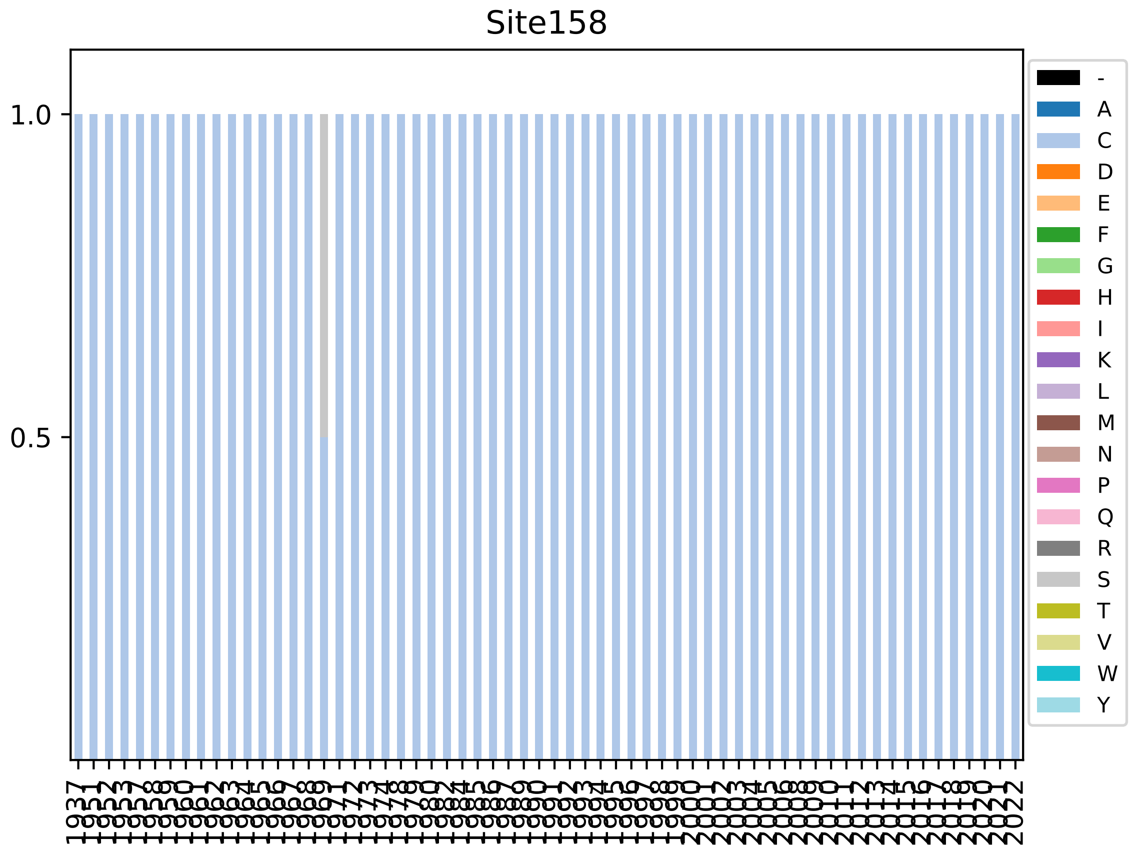
Site158
1.0
0.5
-
A
C
D
E
F
G
H
I
K
L
M
N
P
Q
R
S
T
V
W
Y

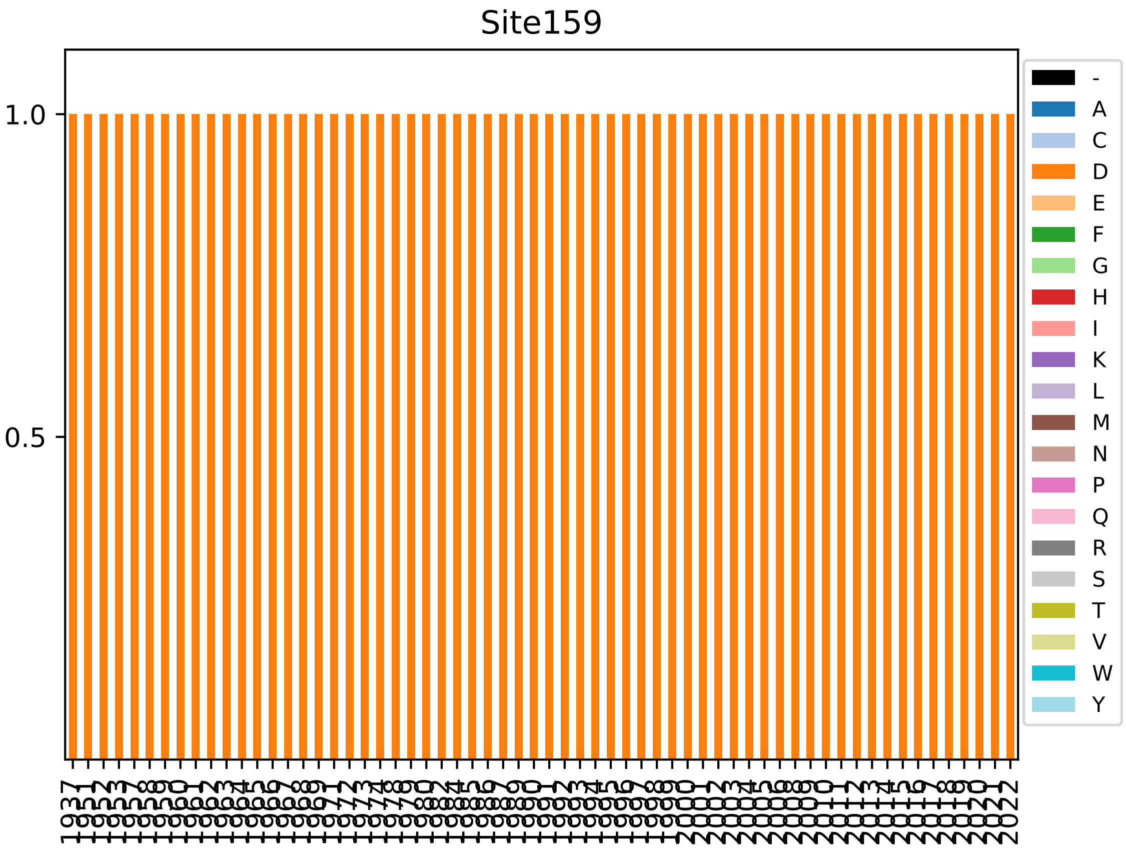
Site159
1.0
0.5
-
A
C
D
E
F
G
H
I
K
L
M
N
P
Q
R
S
T
V
W
Y

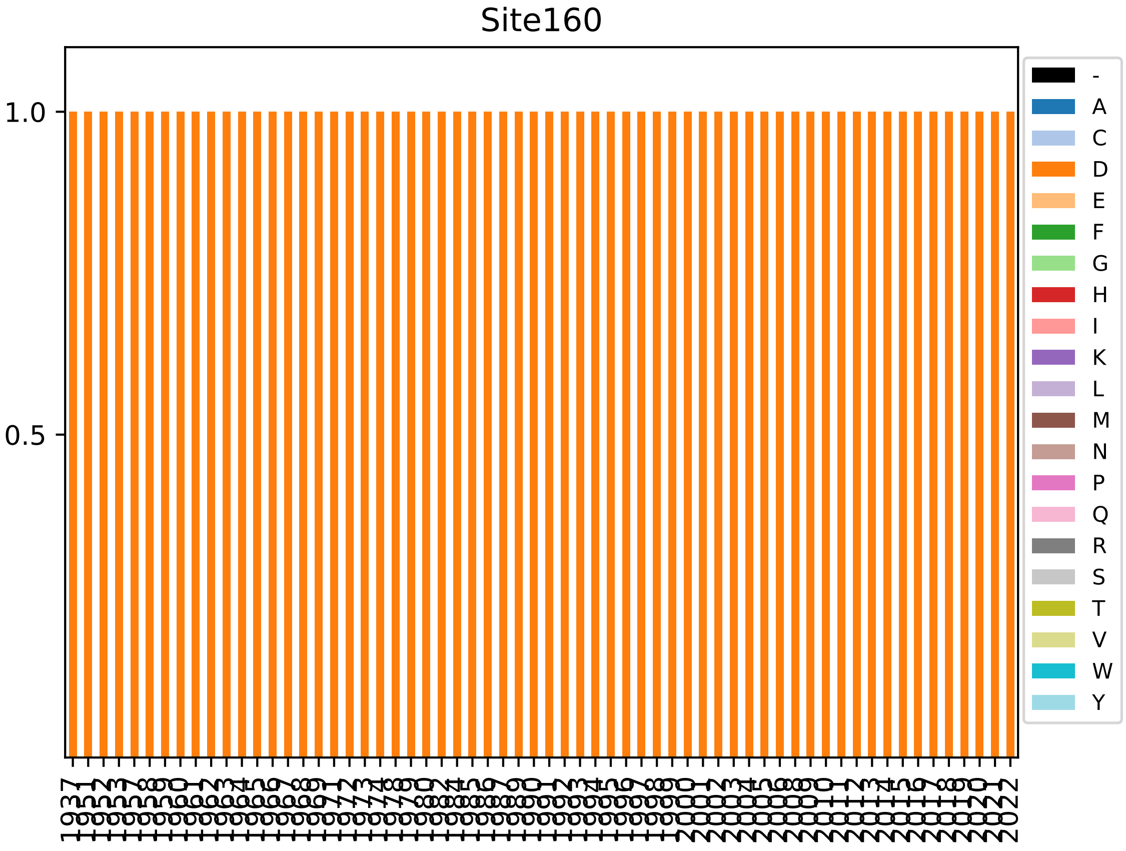
Site160
1.0
0.5
-
A
C
D
E
F
G
H
I
K
L
M
N
P
Q
R
S
T
V
W
Y

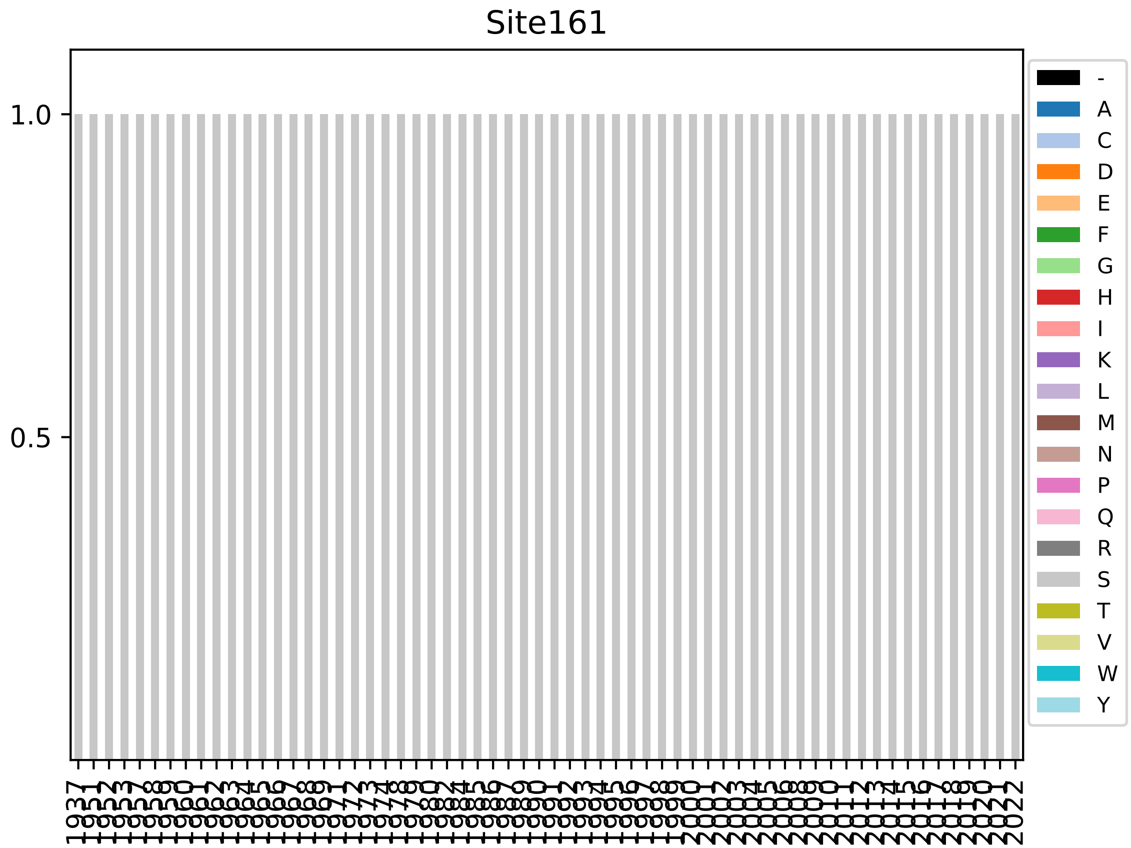
Site161
1.0
0.5
-
A
C
D
E
F
G
H
I
K
L
M
N
P
Q
R
S
T
V
W
Y

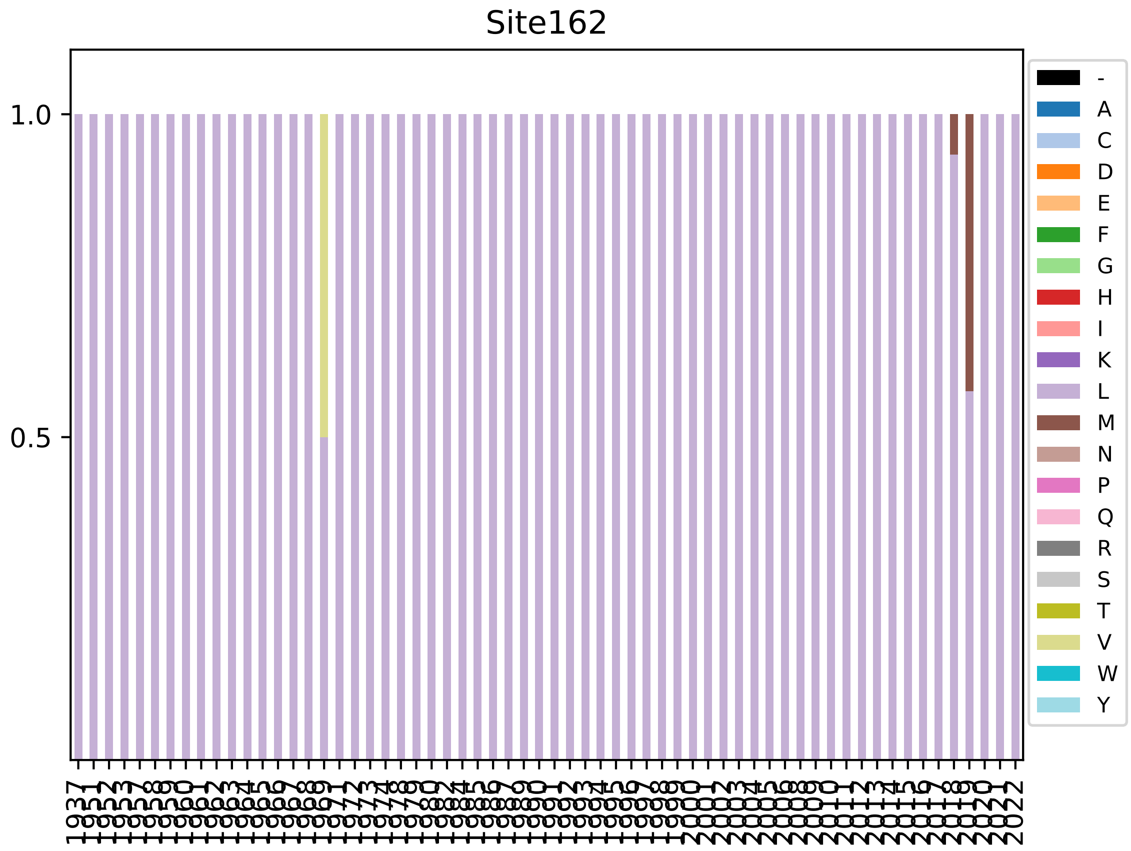
Site162
1.0
0.5
-
A
C
D
E
F
G
H
I
K
L
M
N
P
Q
R
S
T
V
W
Y

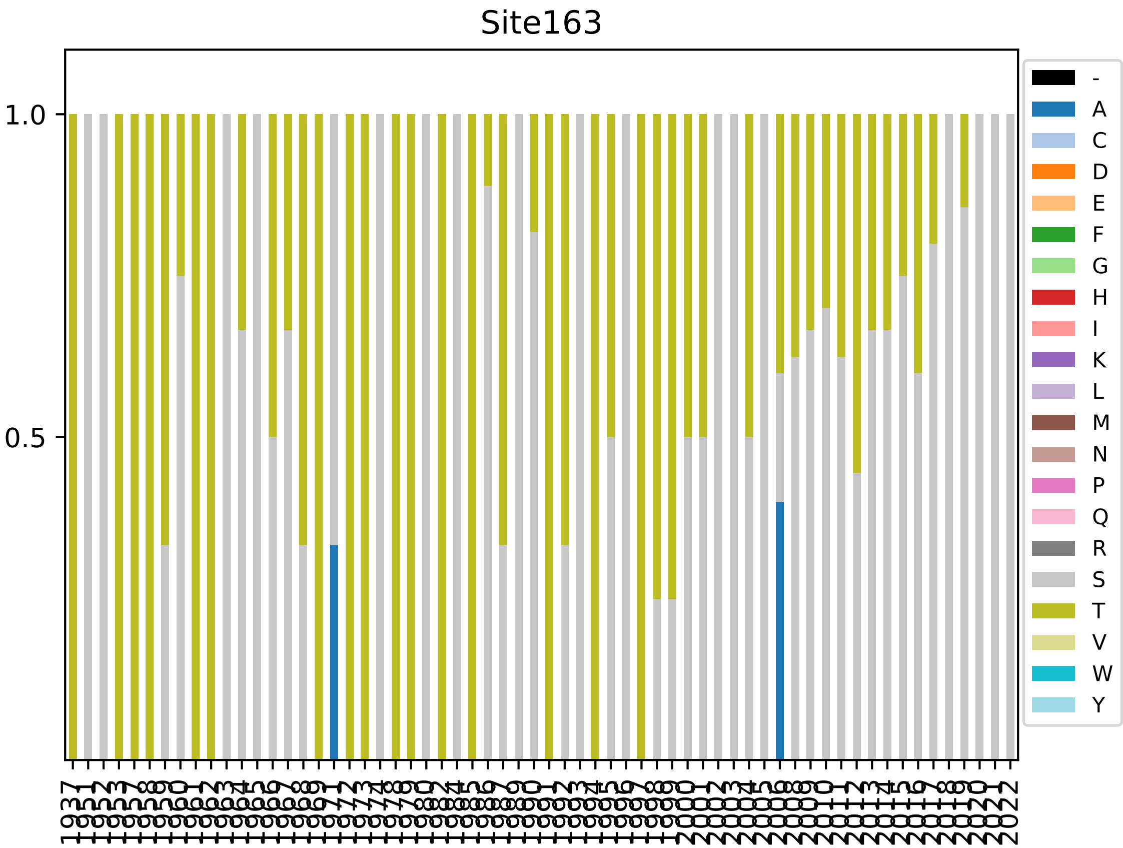
Site163
1.0
0.5
-
A
C
D
E
F
G
H
I
K
L
M
N
P
Q
R
S
T
V
W
Y

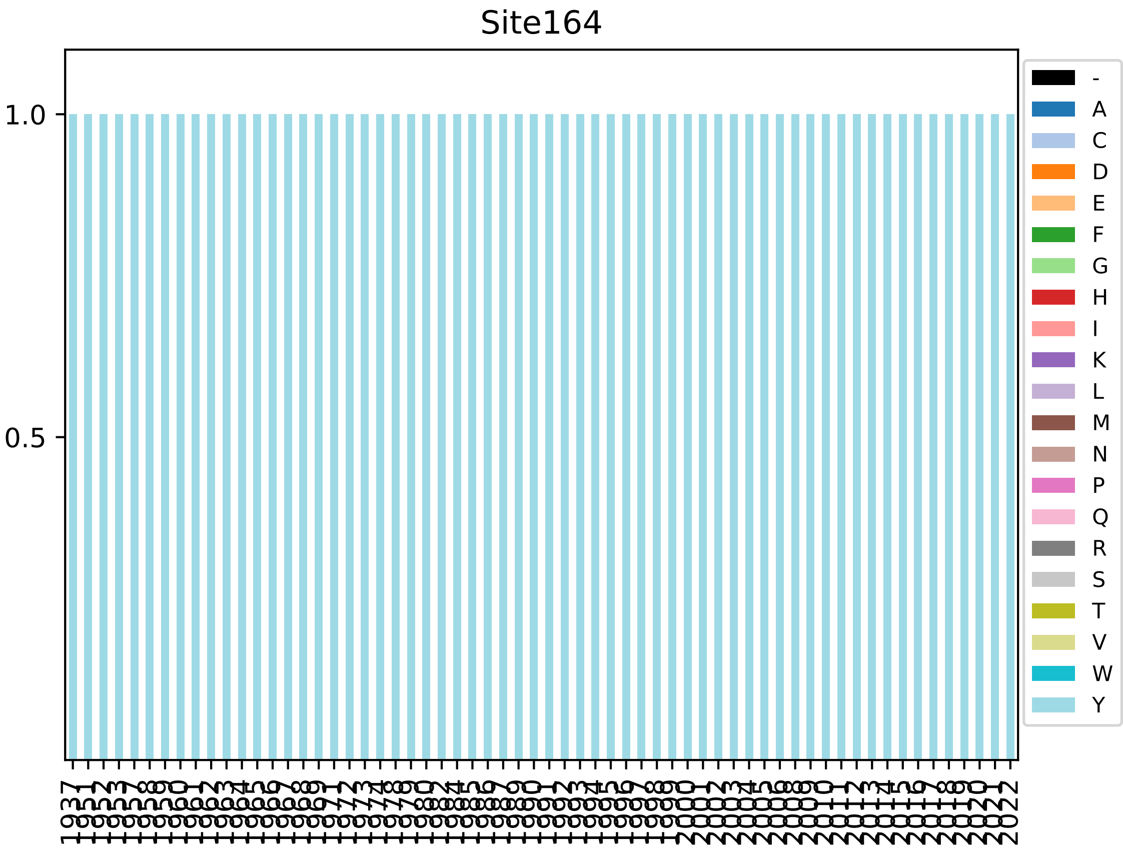
Site164
1.0
0.5
-
A
C
D
E
F
G
H
I
K
L
M
N
P
Q
R
S
T
V
W
Y

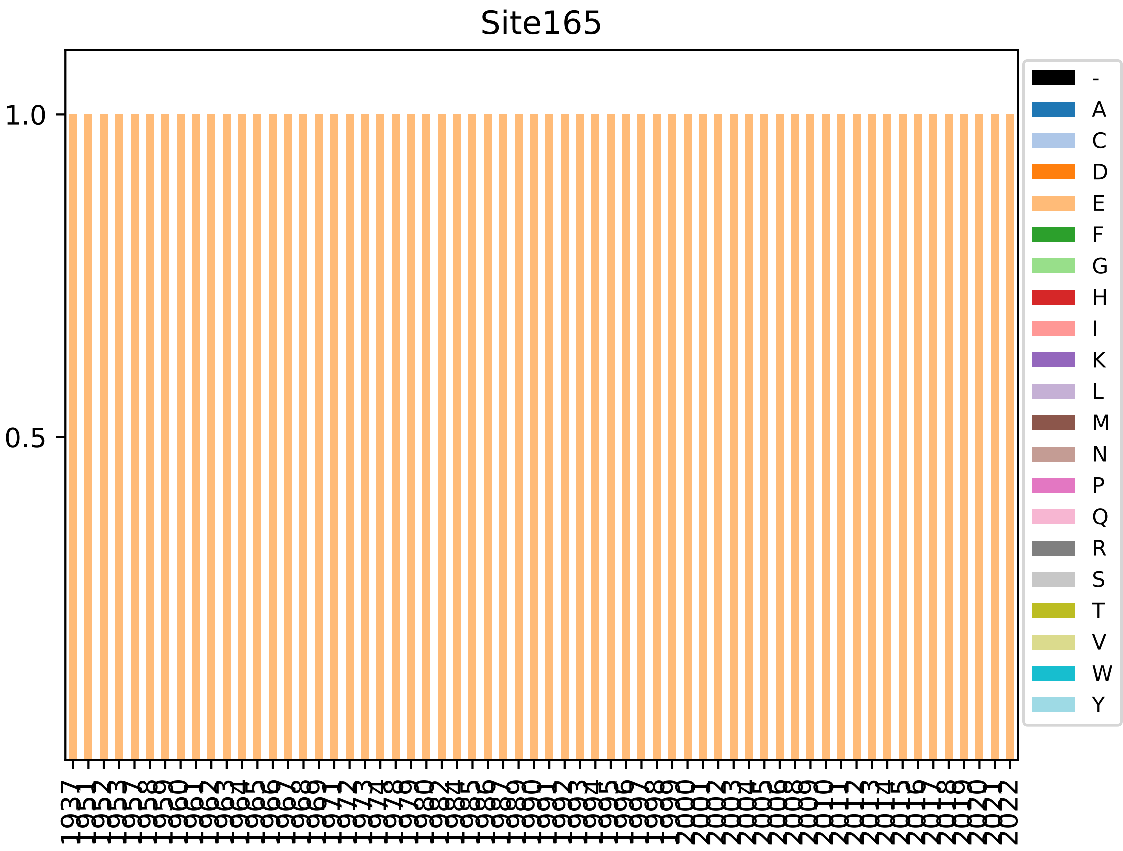
Site165
1.0
0.5
-
A
C
D
E
F
G
H
I
K
L
M
N
P
Q
R
S
T
V
W
Y

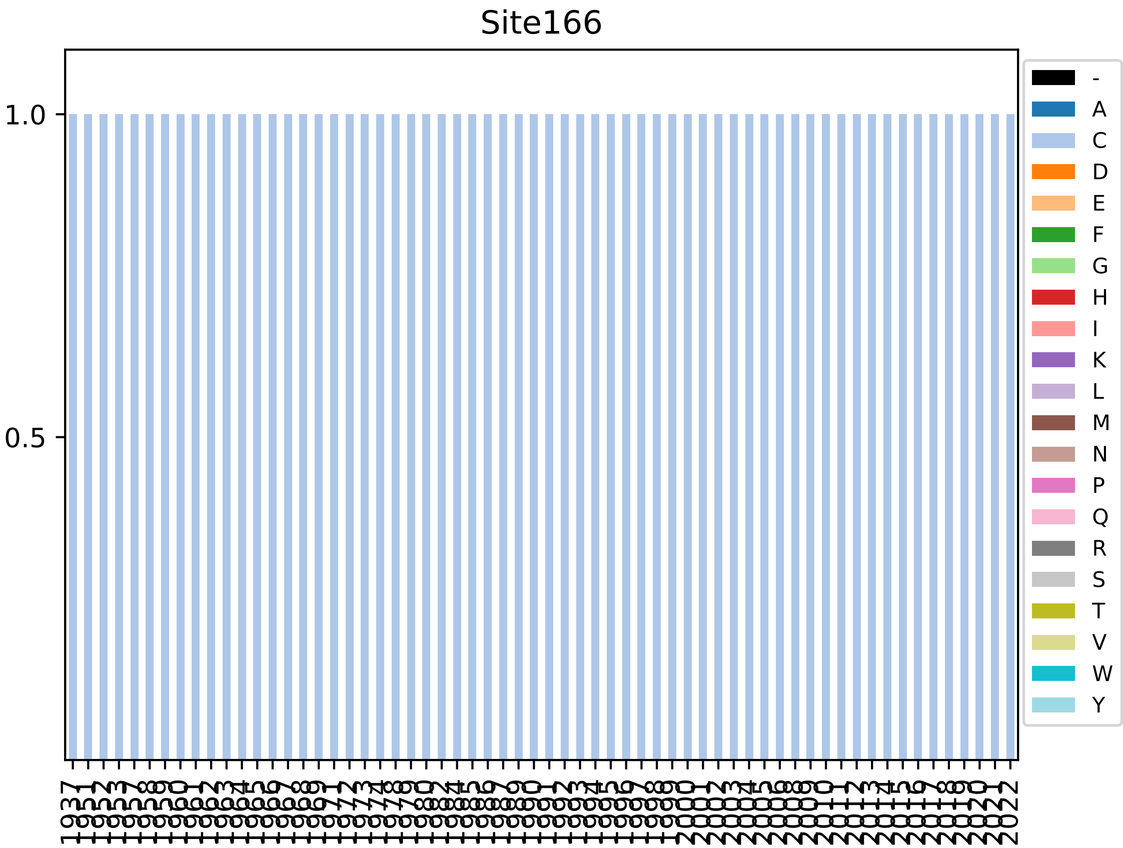
Site166
1.0
0.5
-
A
C
D
E
F
G
H
I
K
L
M
N
P
Q
R
S
T
V
W
Y

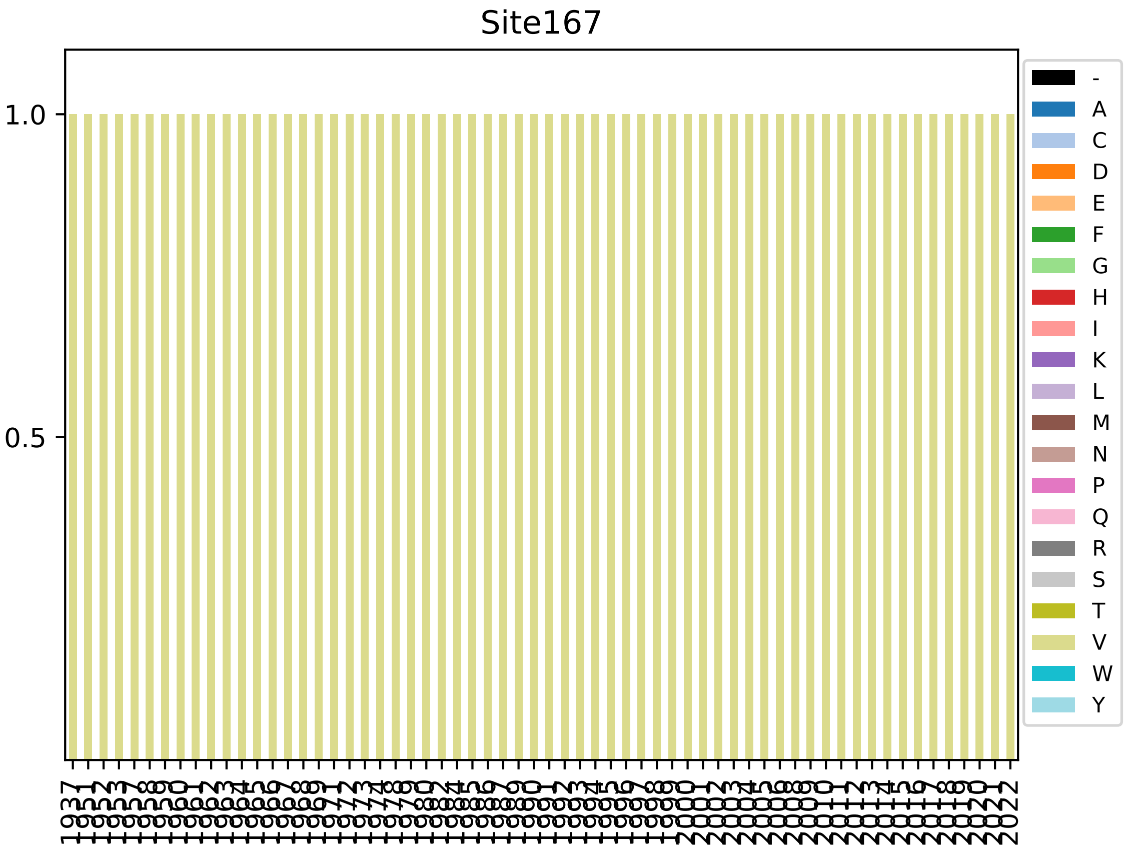
Site167
1.0
0.5
-
A
C
D
E
F
G
H
I
K
L
M
N
P
Q
R
S
T
V
W
Y

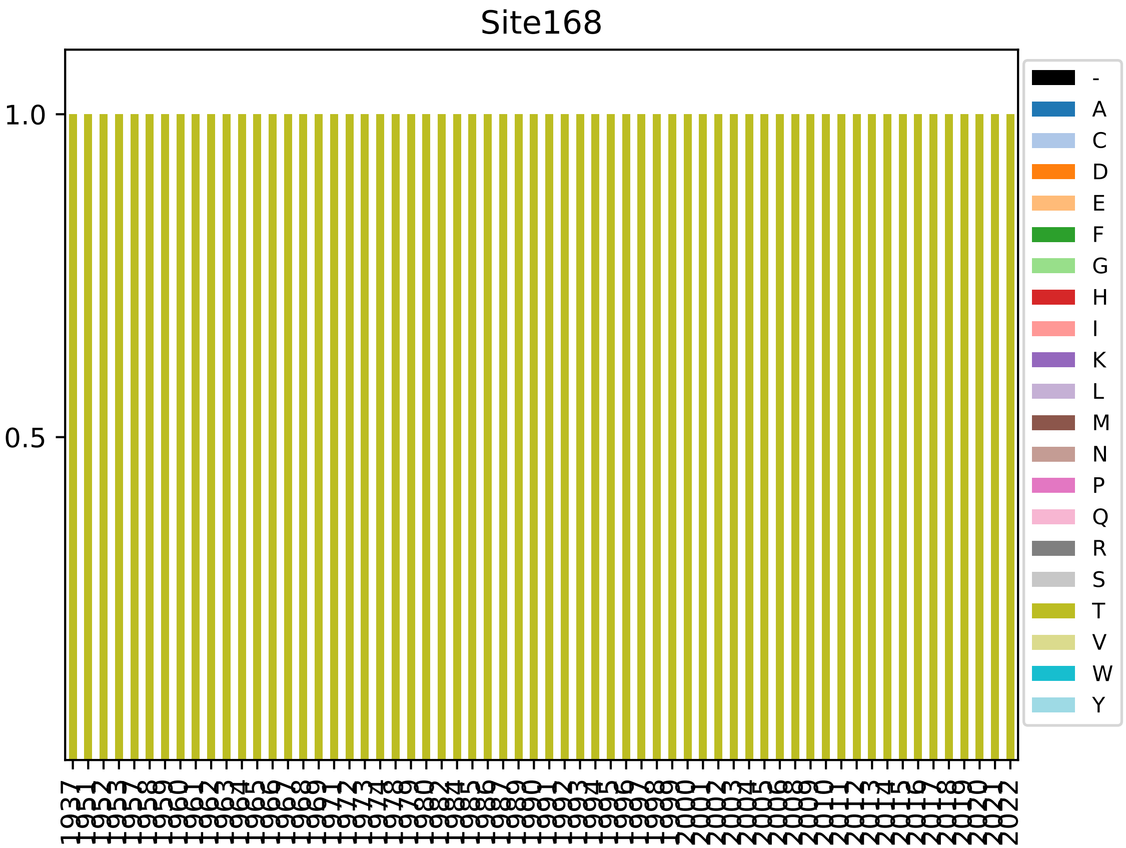
Site168
1.0
0.5
-
A
C
D
E
F
G
H
I
K
L
M
N
P
Q
R
S
T
V
W
Y

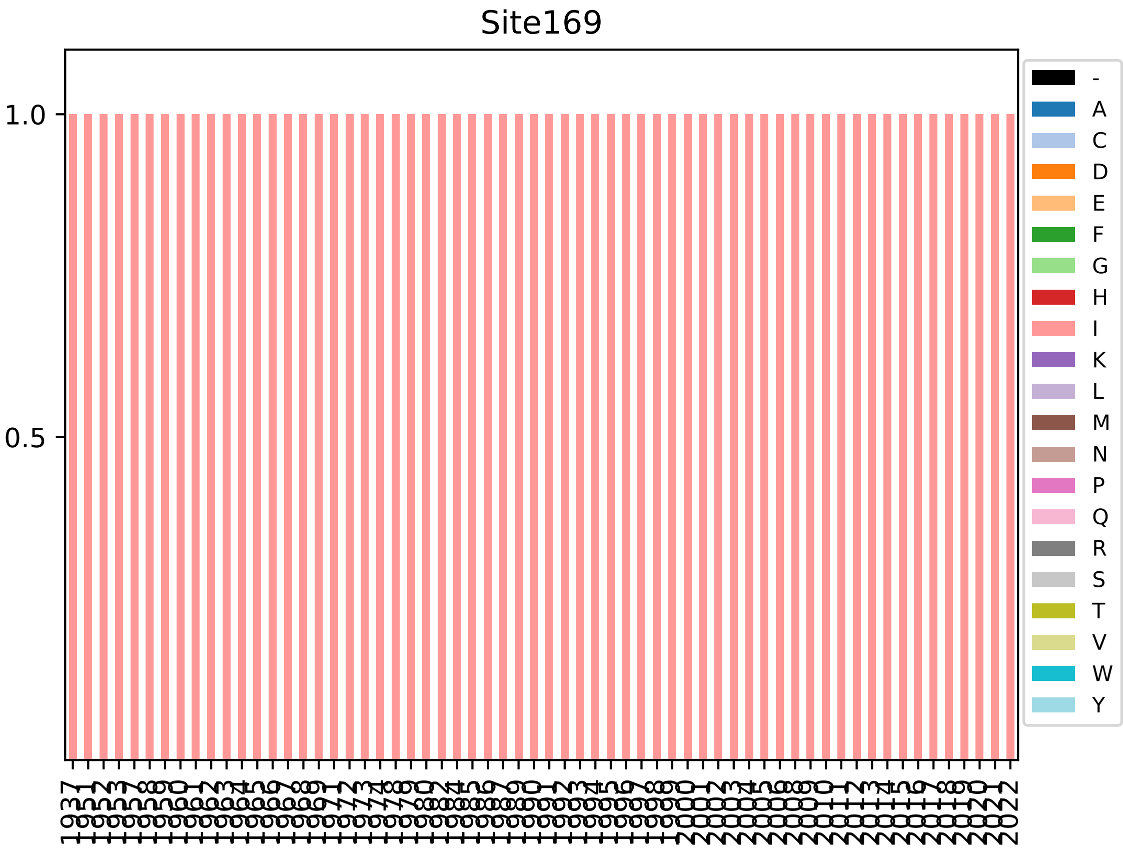
Site169
1.0
0.5
-
A
C
D
E
F
G
H
I
K
L
M
N
P
Q
R
S
T
V
W
Y

Site170

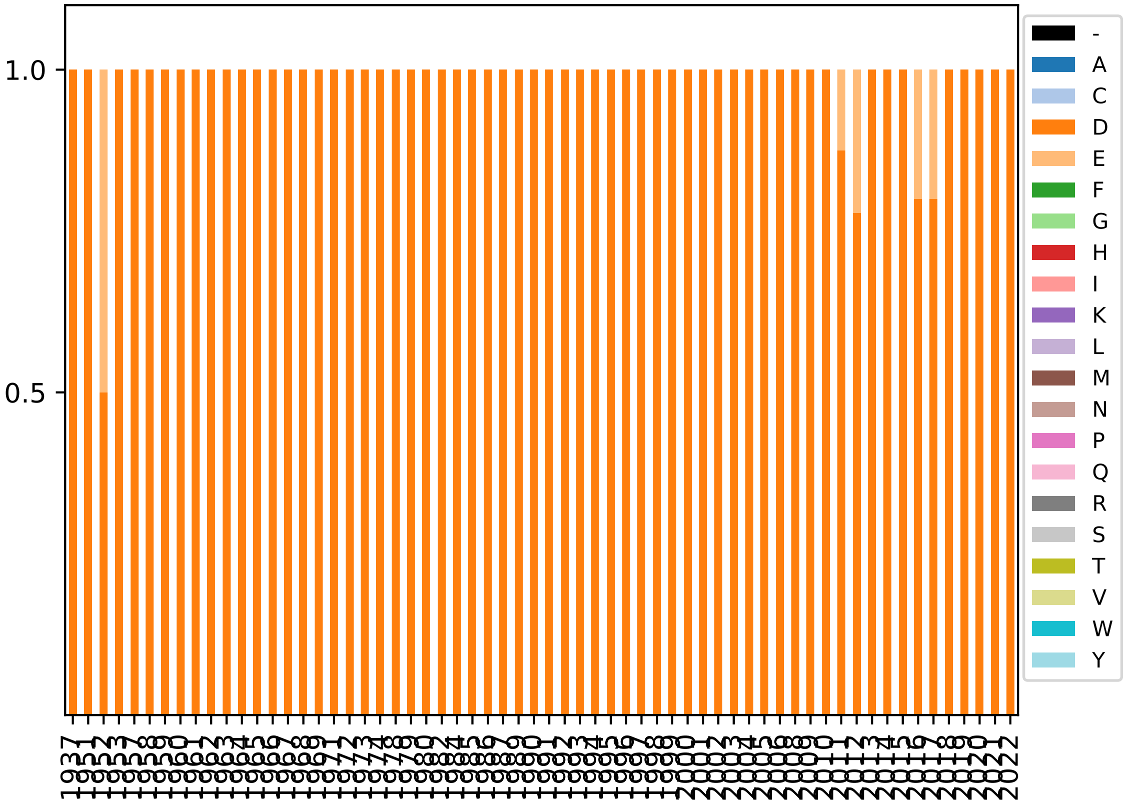

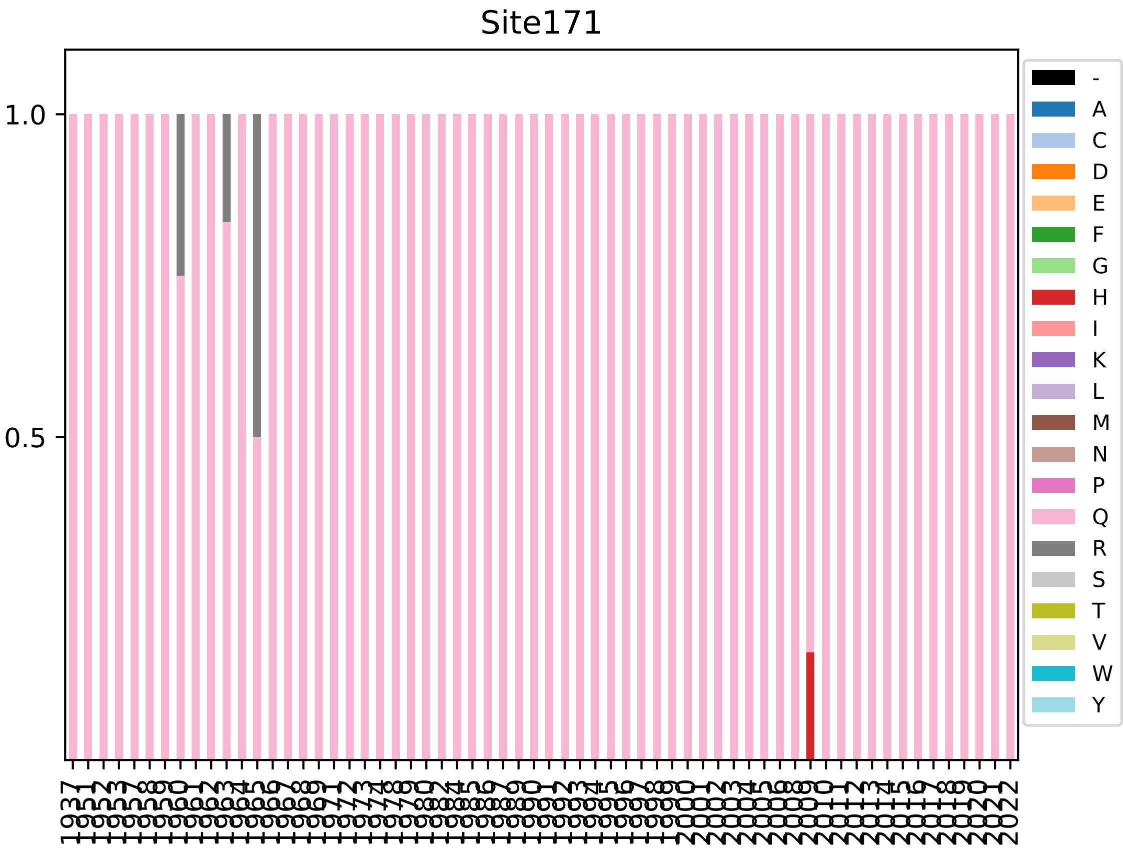
Site171
1.0
0.5
-
A
C
D
E
F
G
H
I
K
L
M
N
P
Q
R
S
T
V
W
Y

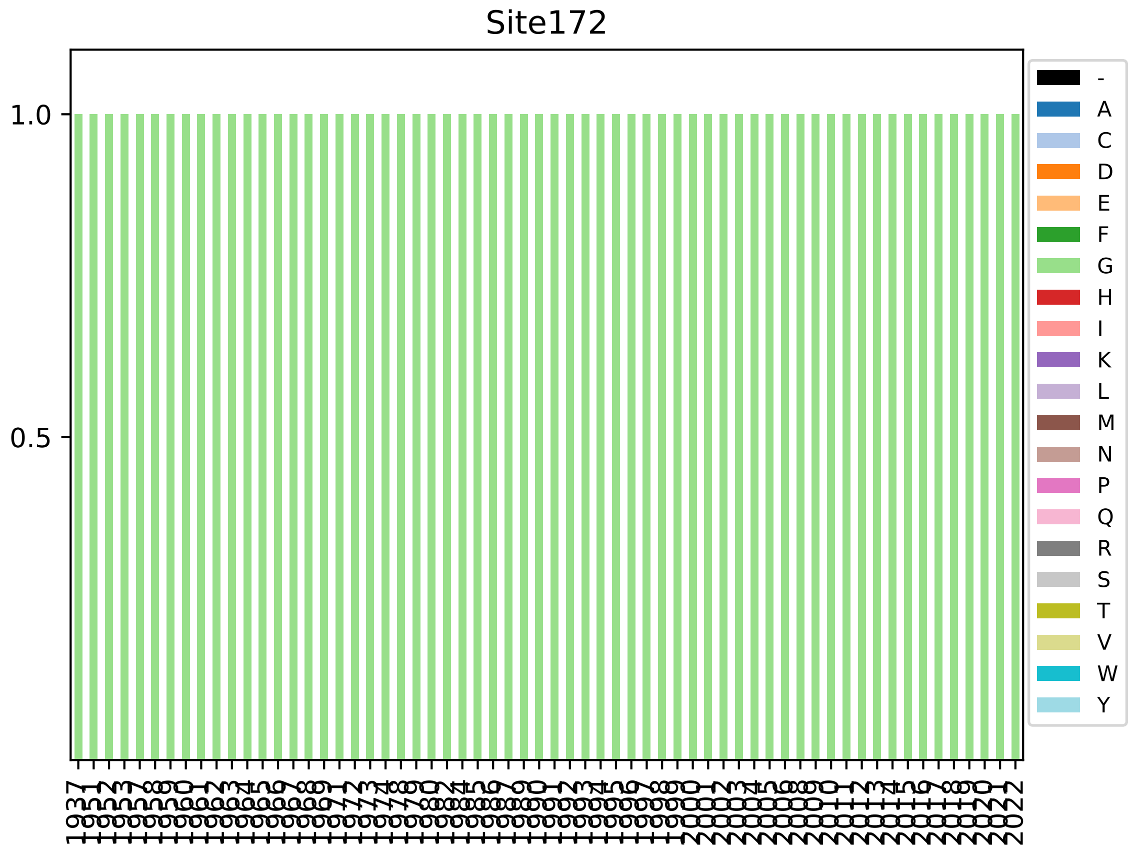
Site172
1.0
0.5
-
A
C
D
E
F
G
H
I
K
L
M
N
P
Q
R
S
T
V
W
Y

Site173

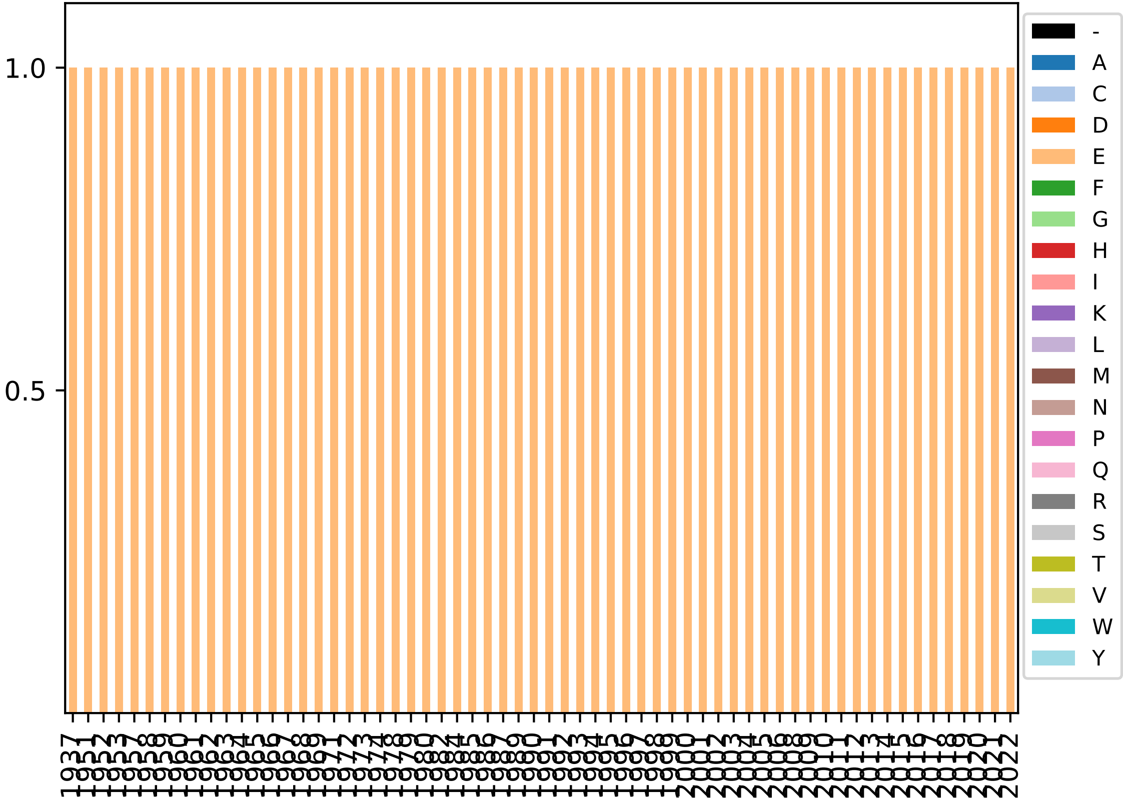

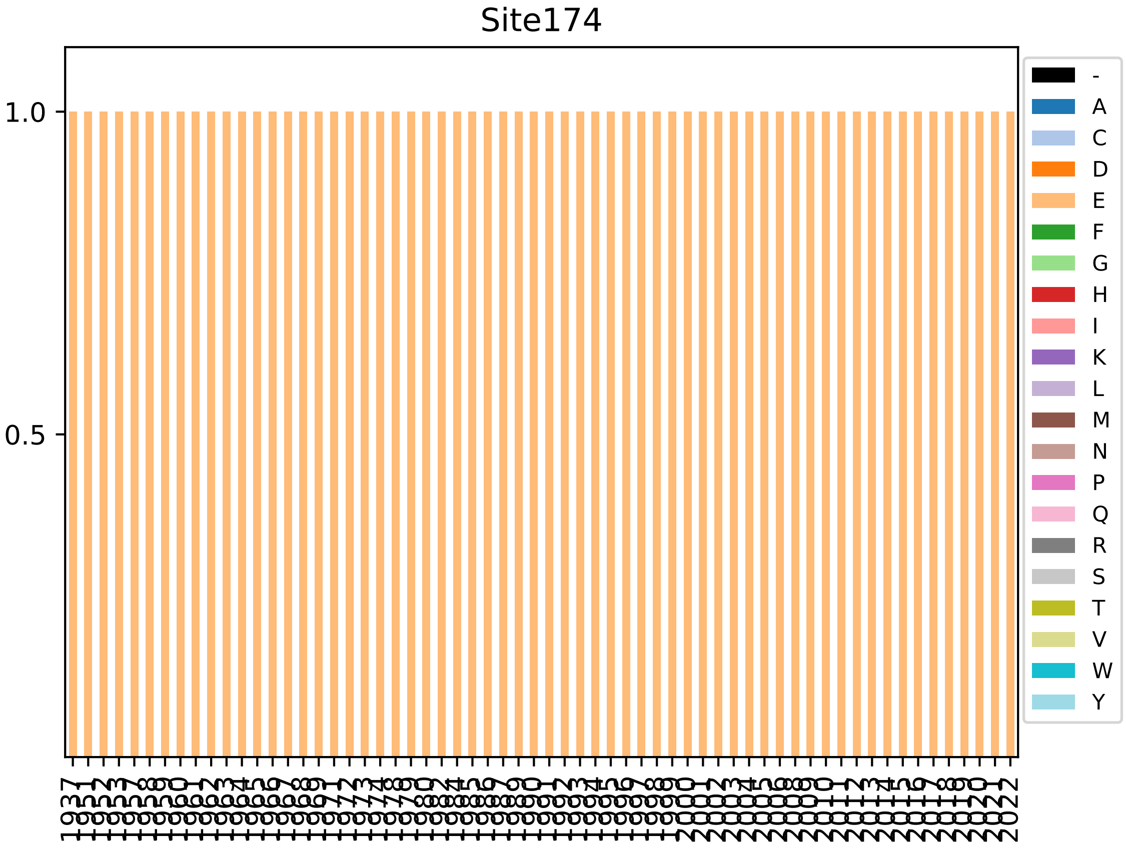
Site174
1.0
0.5
-
A
C
D
E
F
G
H
I
K
L
M
N
P
Q
R
S
T
V
W
Y

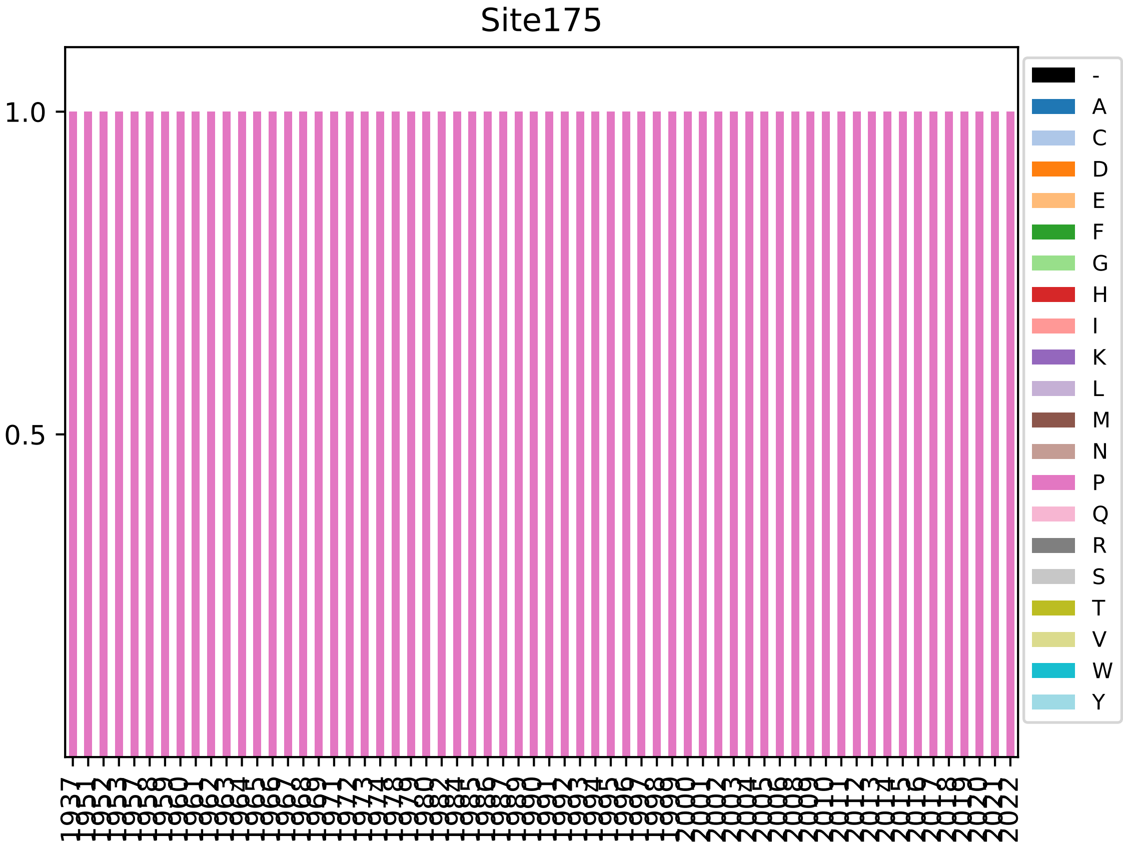

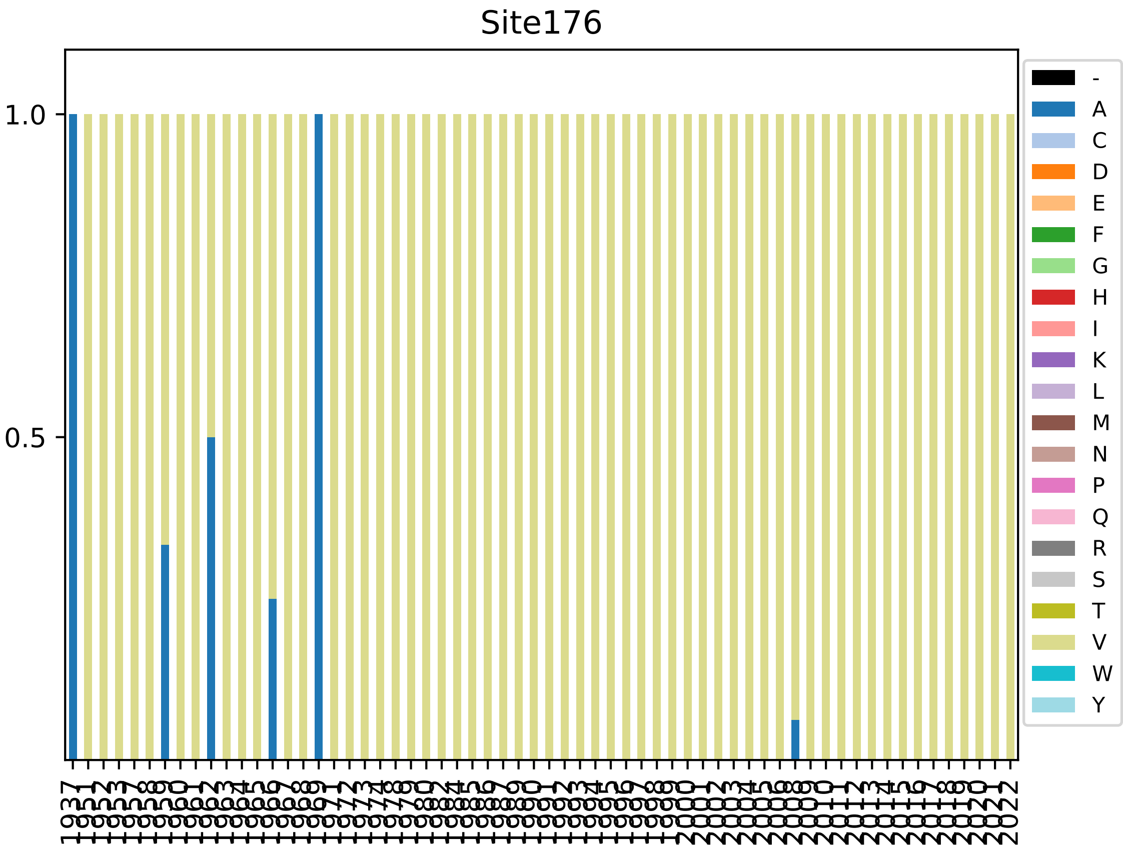
Site176
1.0
0.5
-
A
C
D
E
F
G
H
I
K
L
M
N
P
Q
R
S
T
V
W
Y

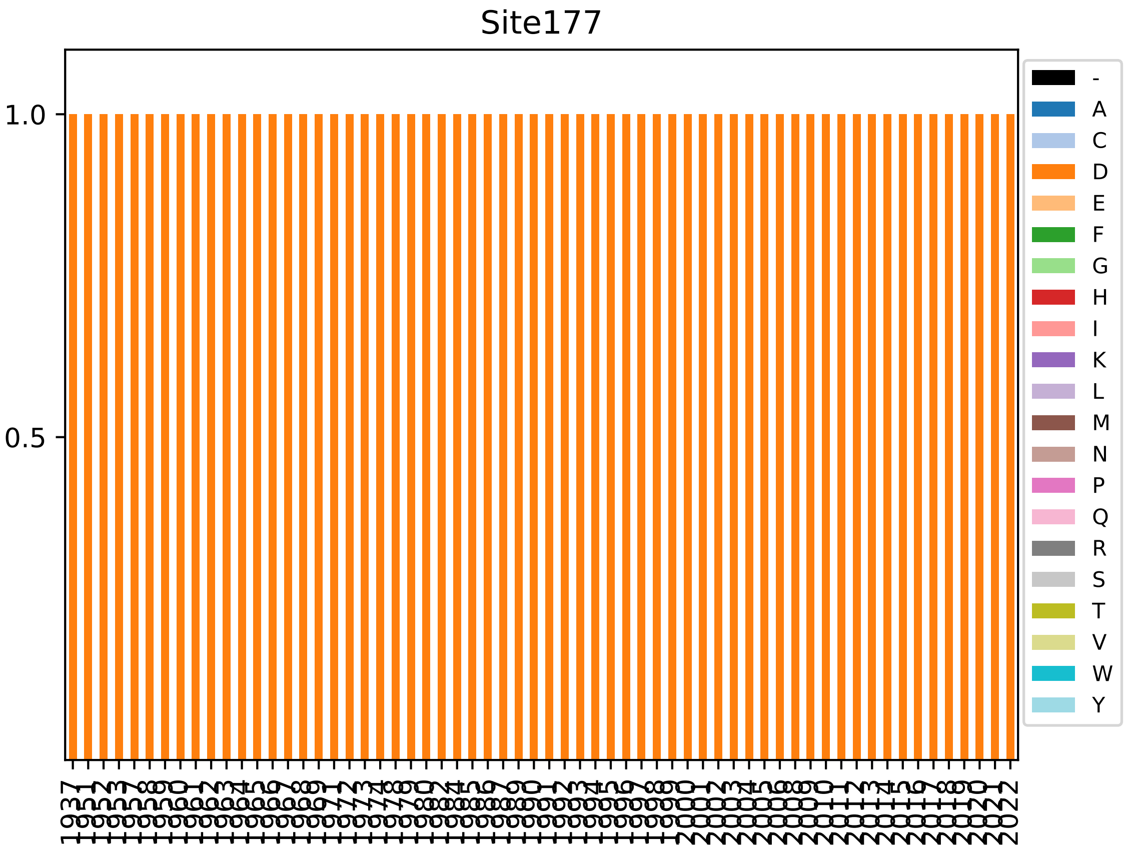
Site177
1.0
0.5
-
A
C
D
E
F
G
H
I
K
L
M
N
P
Q
R
S
T
V
W
Y

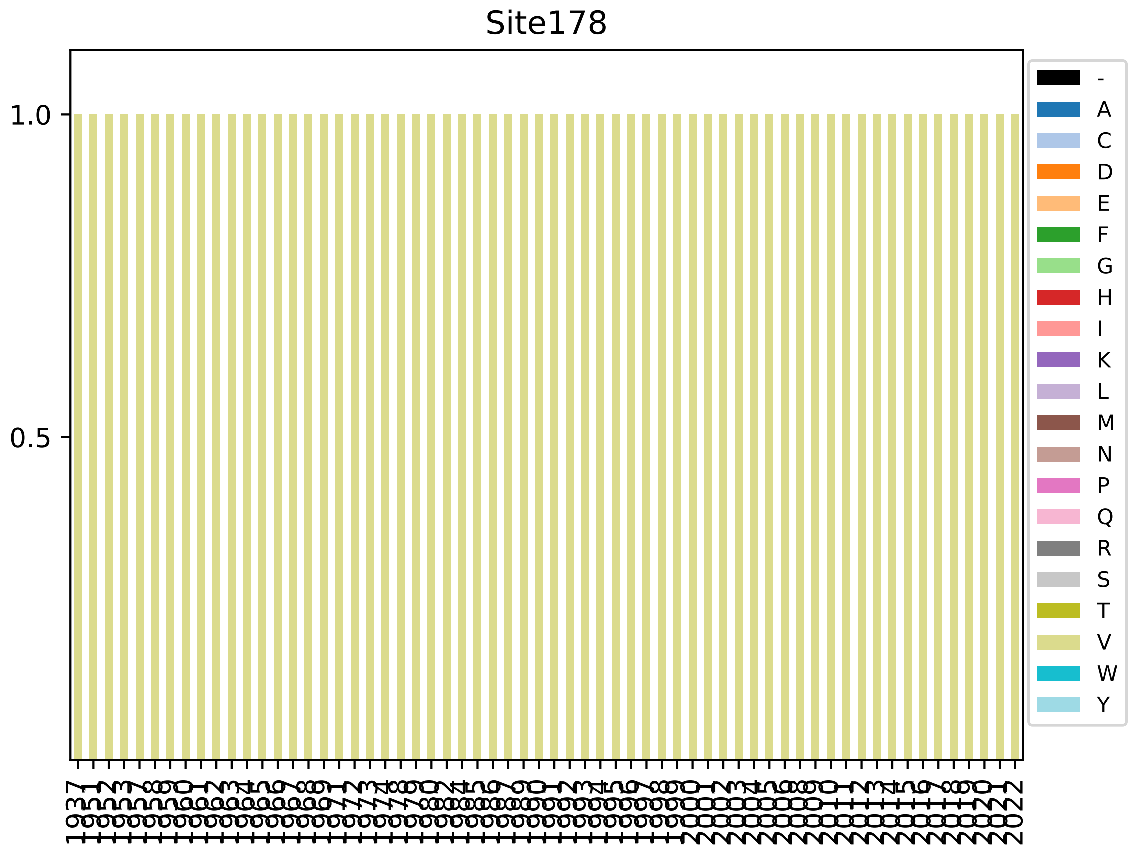
Site178
1.0
0.5
-
A
C
D
E
F
G
H
I
K
L
M
N
P
Q
R
S
T
V
W
Y

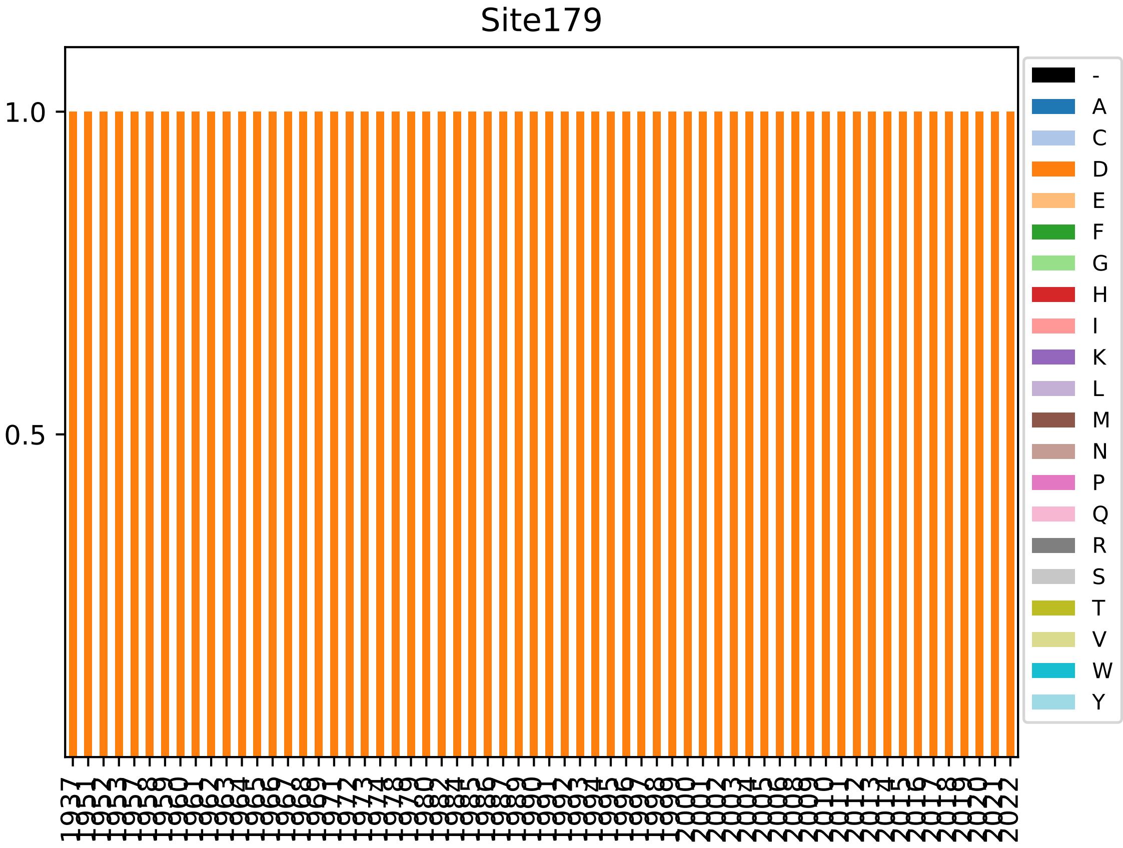
Site179
1.0
0.5
-
A
C
D
E
F
G
H
I
K
L
M
N
P
Q
R
S
T
V
W
Y

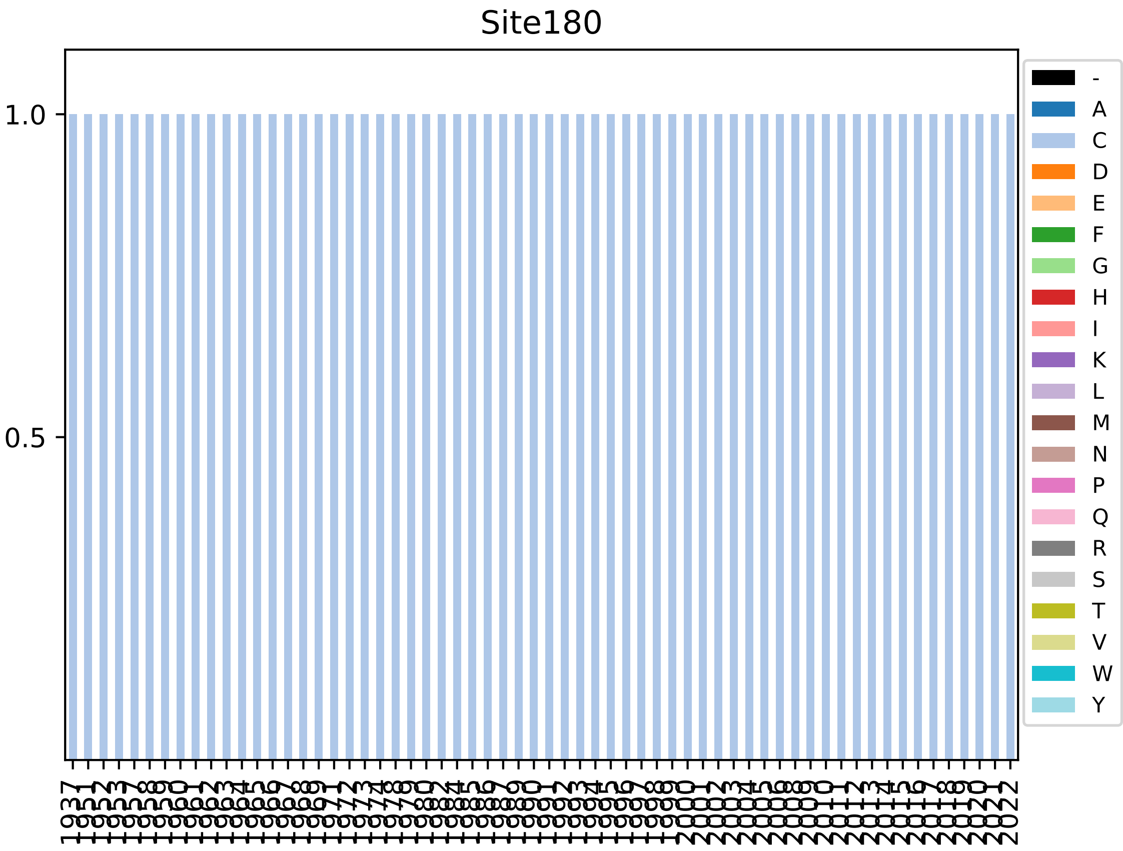
Site180
1.0
0.5
-
A
C
D
E
F
G
H
I
K
L
M
N
P
Q
R
S
T
V
W
Y

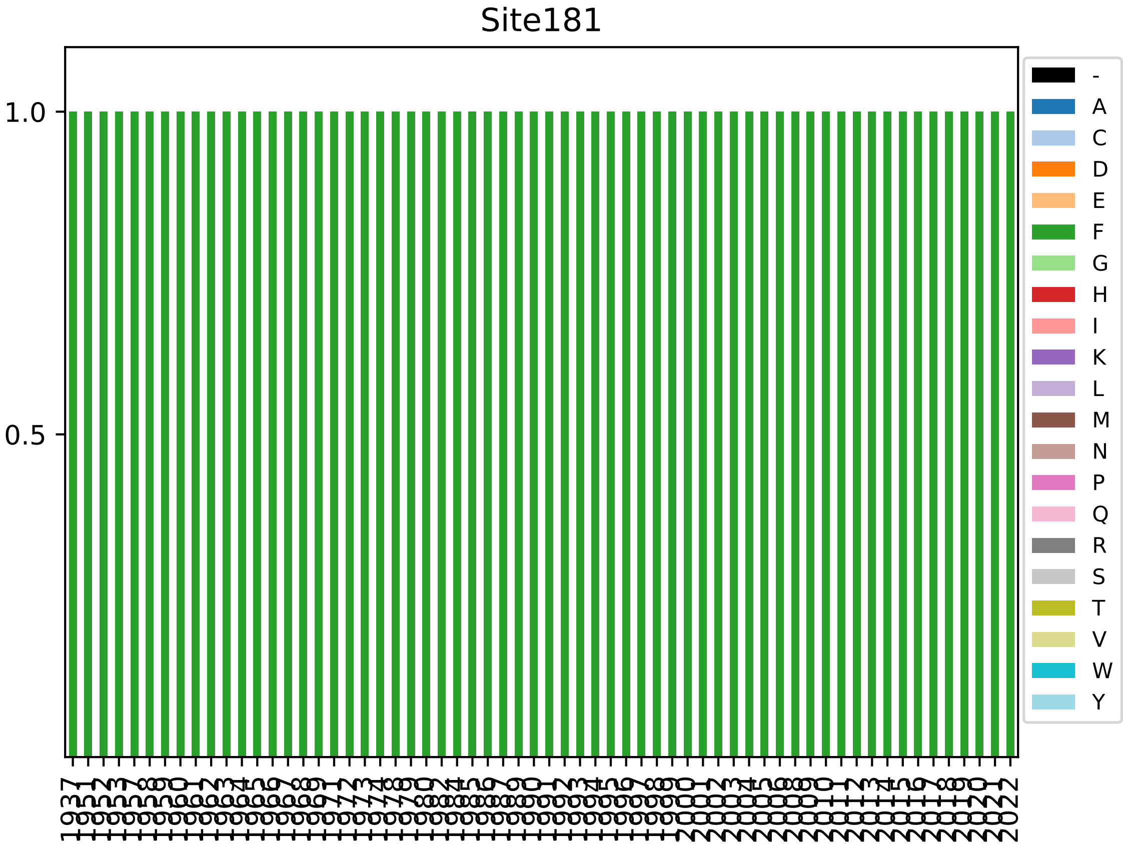
Site181
1.0
0.5
-
A
C
D
E
F
G
H
I
K
L
M
N
P
Q
R
S
T
V
W
Y

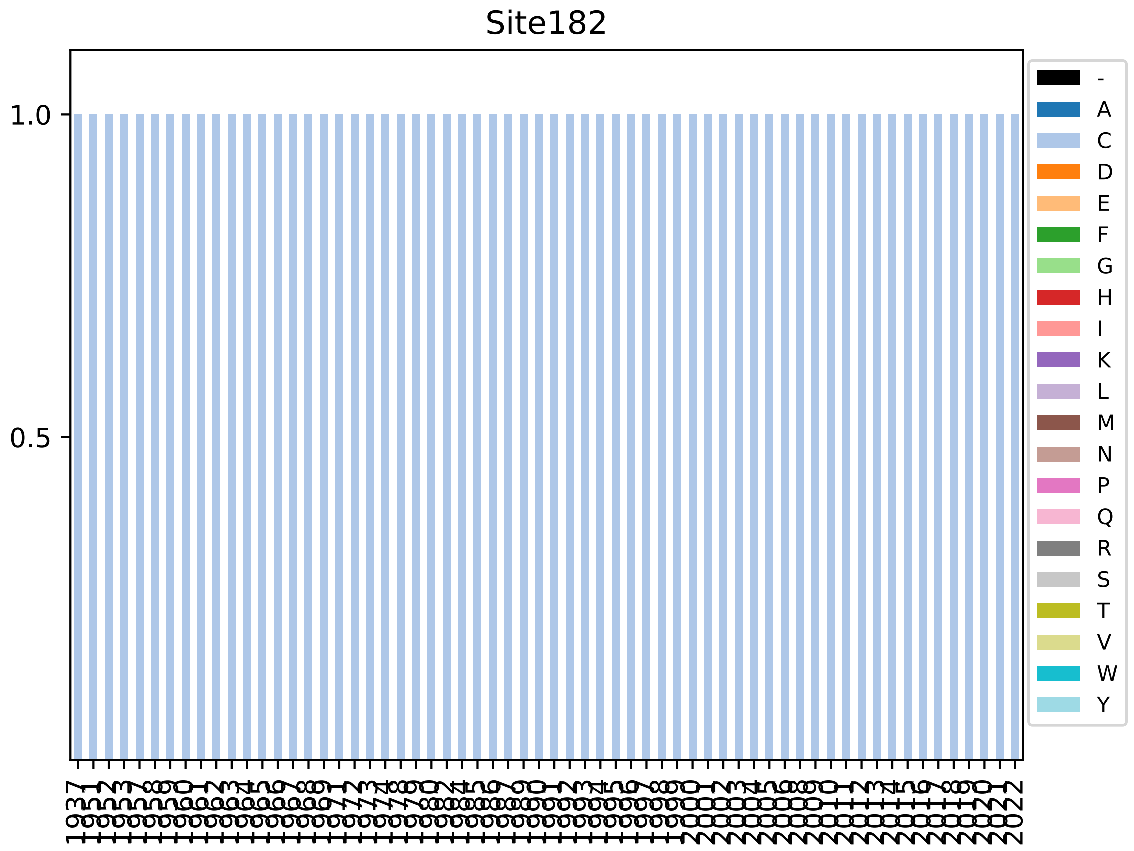
Site182
1.0
0.5
-
A
C
D
E
F
G
H
I
K
L
M
N
P
Q
R
S
T
V
W
Y

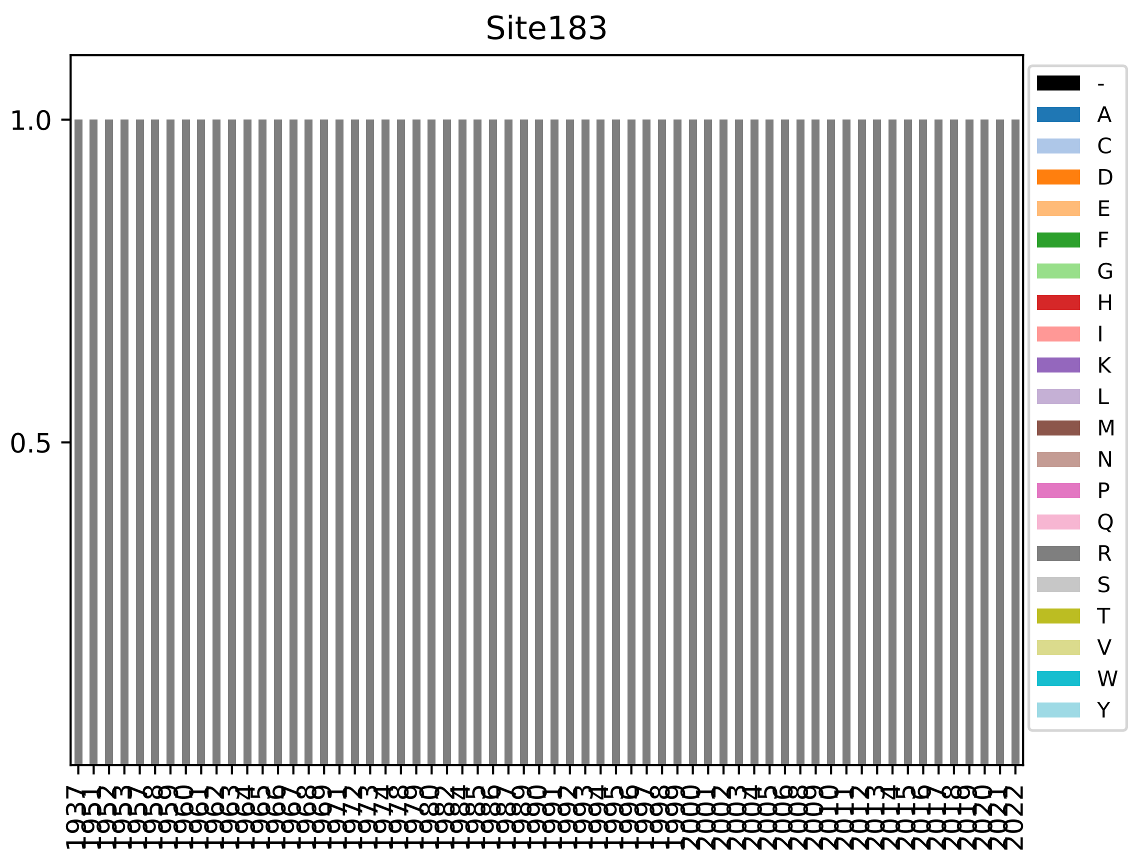
Site183
1.0
0.5
-
A
C
D
E
F
G
H
I
K
L
M
N
P
Q
R
S
T
V
W
Y

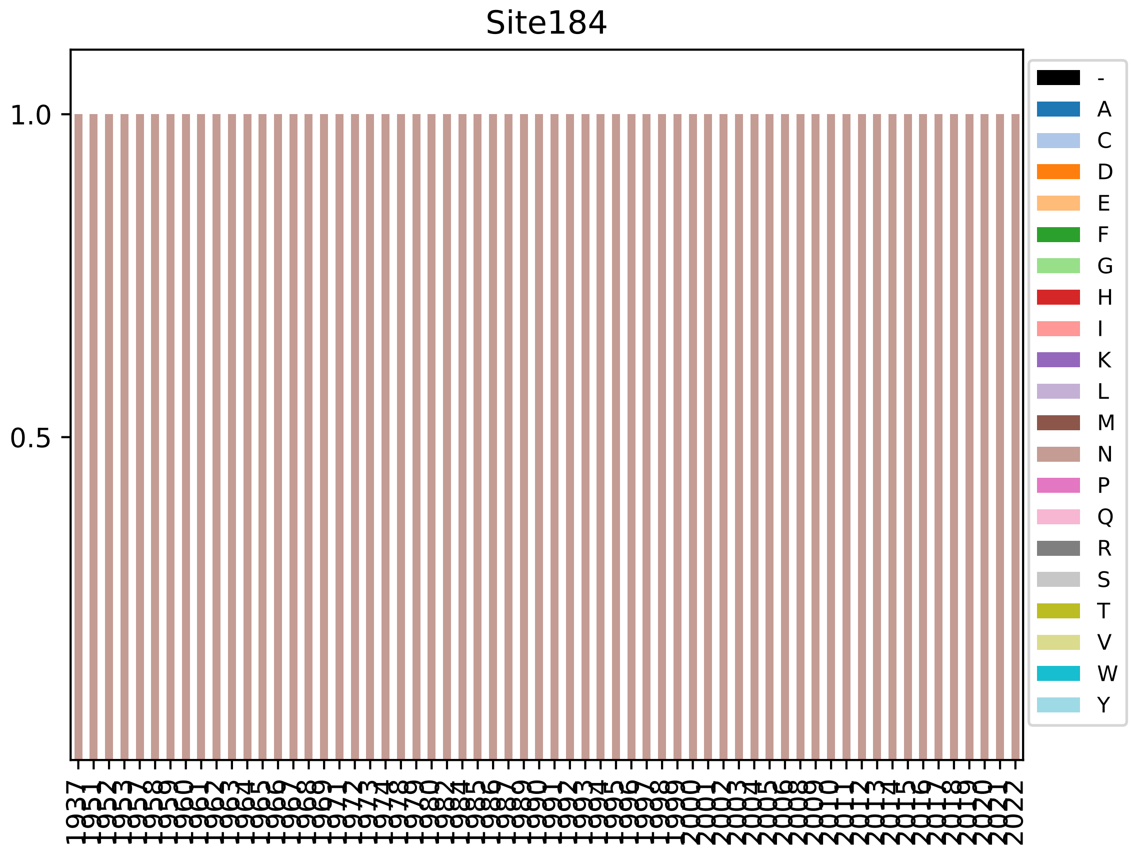
Site184
1.0
0.5
-
A
C
D
E
F
G
H
I
K
L
M
N
P
Q
R
S
T
V
W
Y

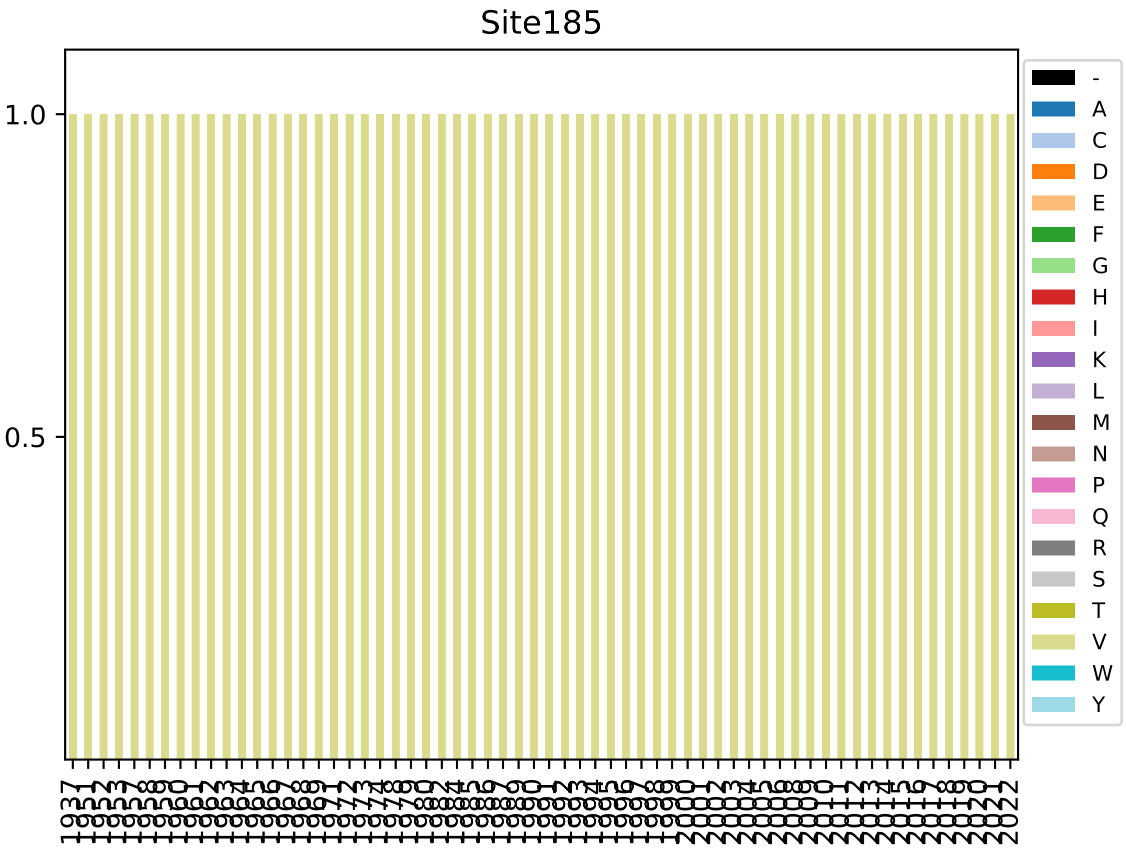
Site185
1.0
0.5
-
A
C
D
E
F
G
H
I
K
L
M
N
P
Q
R
S
T
V
W
Y

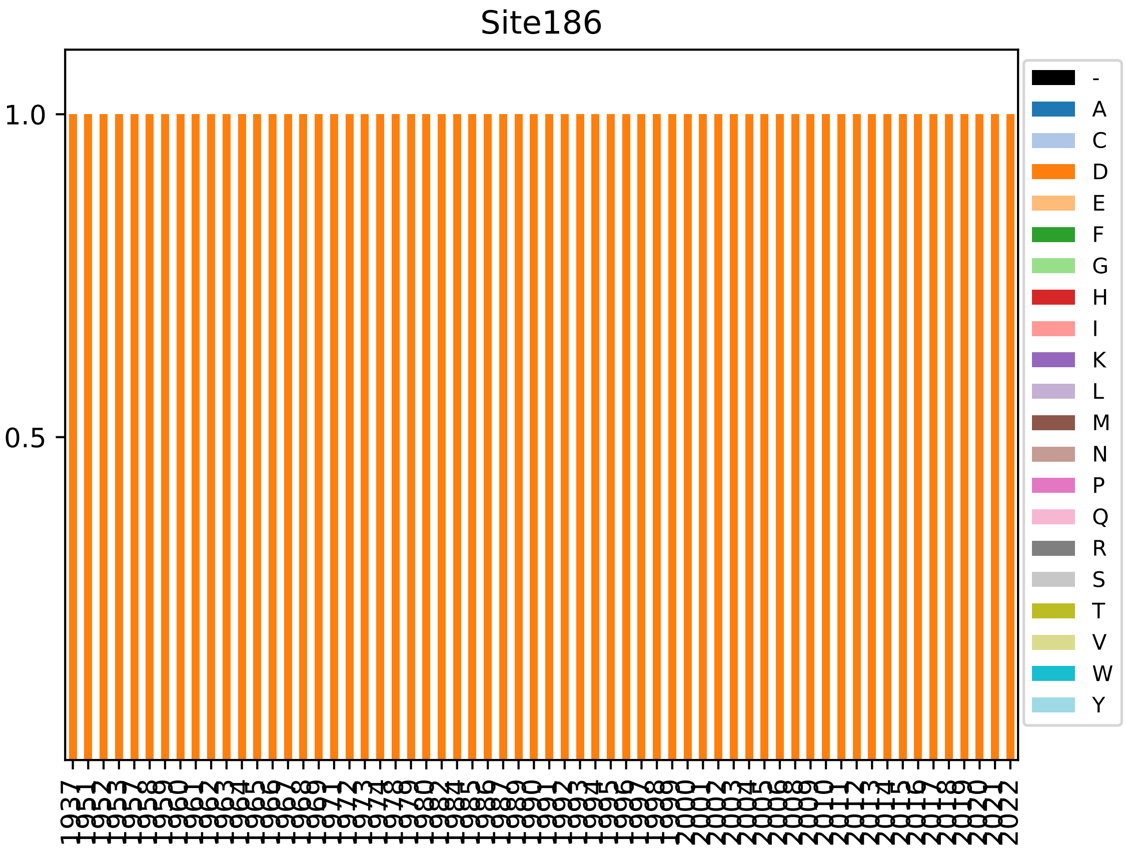
Site186
1.0
0.5
-
A
C
D
E
F
G
H
I
K
L
M
N
P
Q
R
S
T
V
W
Y

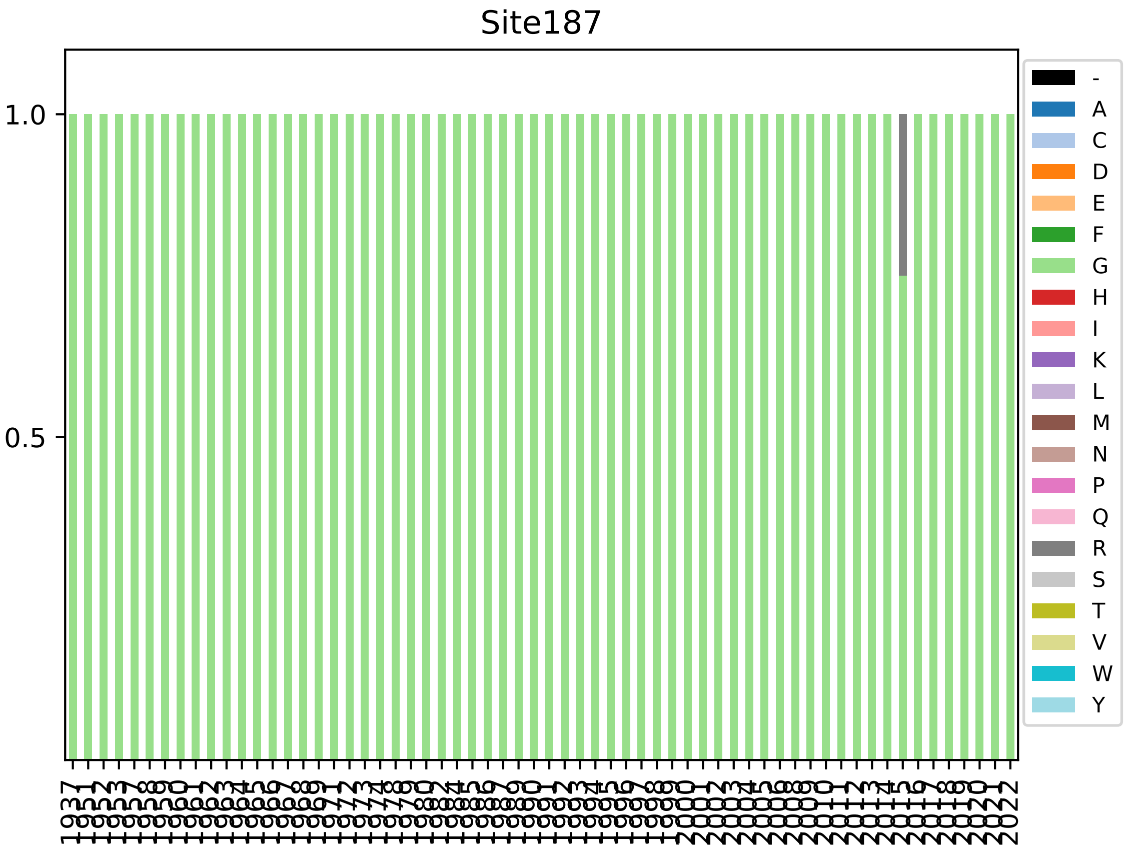
Site187
1.0
0.5
-
A
C
D
E
F
G
H
I
K
L
M
N
P
Q
R
S
T
V
W
Y

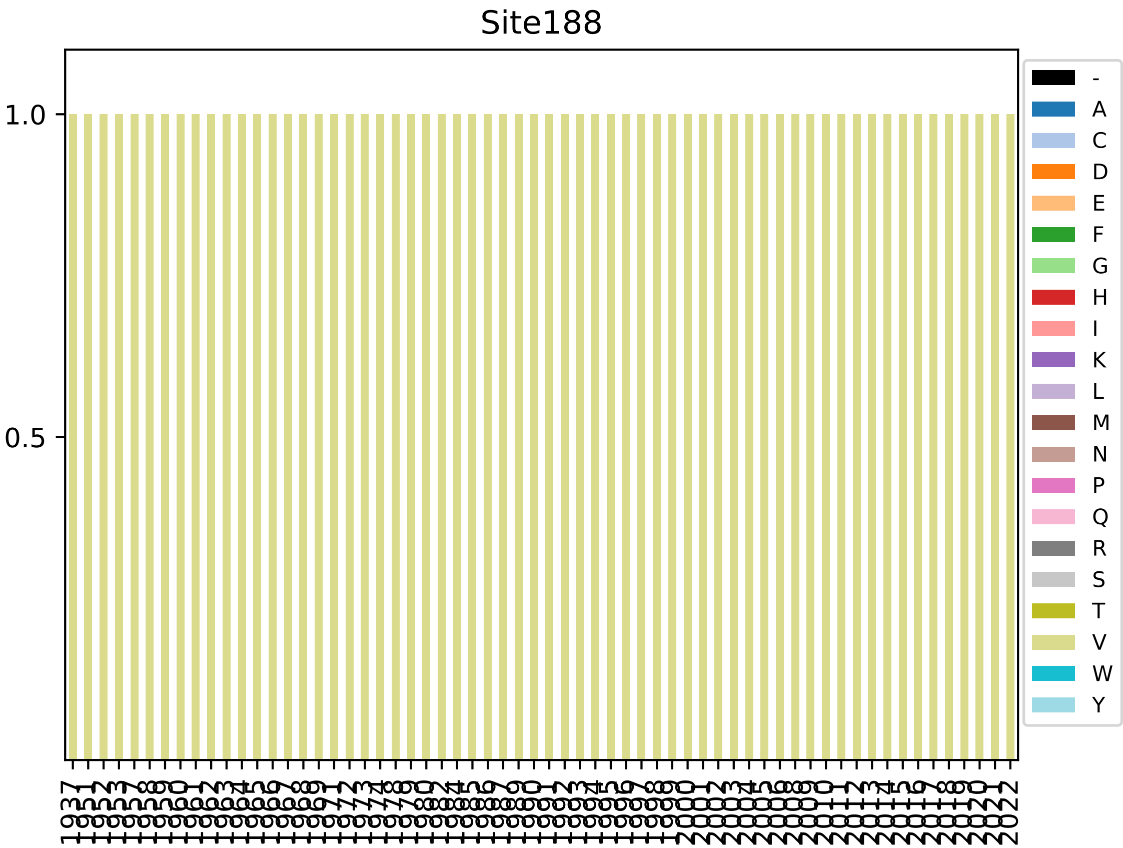
Site188
1.0
0.5
-
A
C
D
E
F
G
H
I
K
L
M
N
P
Q
R
S
T
V
W
Y

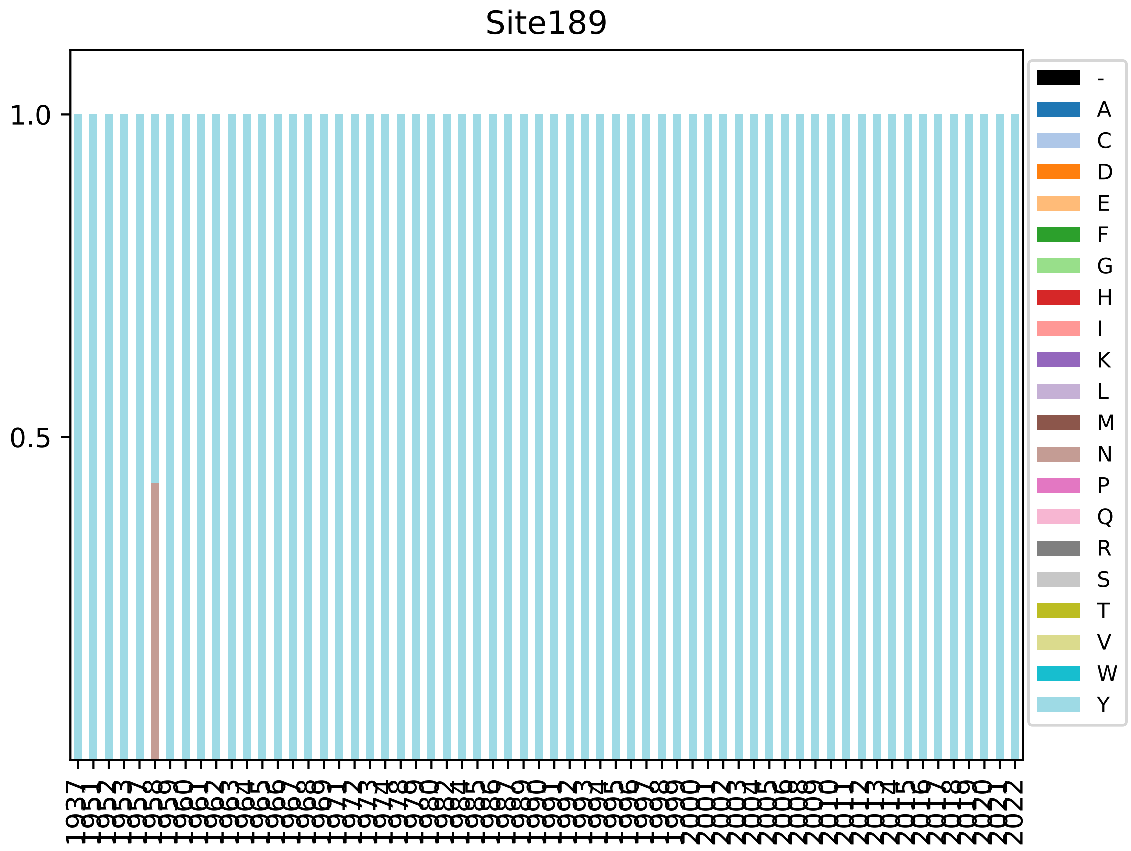
Site189
1.0
0.5
-
A
C
D
E
F
G
H
I
K
L
M
N
P
Q
R
S
T
V
W
Y

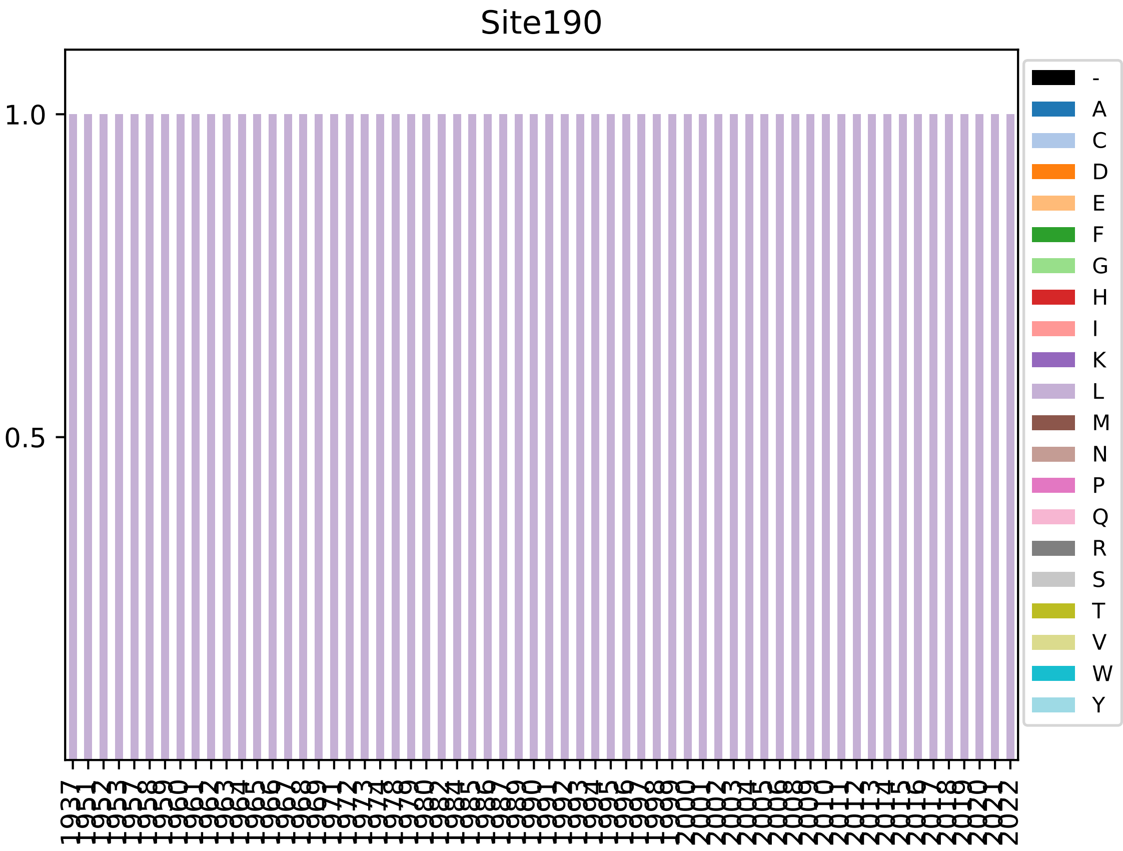
Site190
1.0
0.5
-
A
C
D
E
F
G
H
I
K
L
M
N
P
Q
R
S
T
V
W
Y

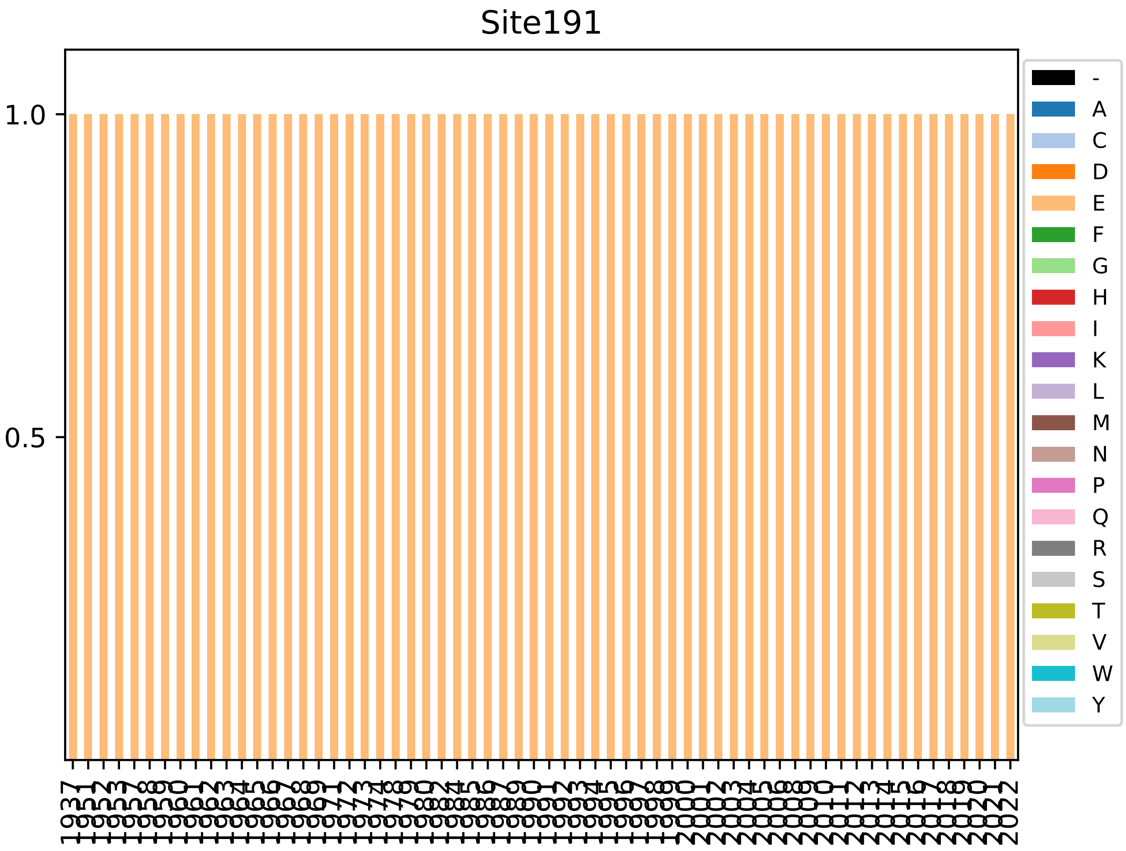
Site191
1.0
0.5
-
A
C
D
E
F
G
H
I
K
L
M
N
P
Q
R
S
T
V
W
Y

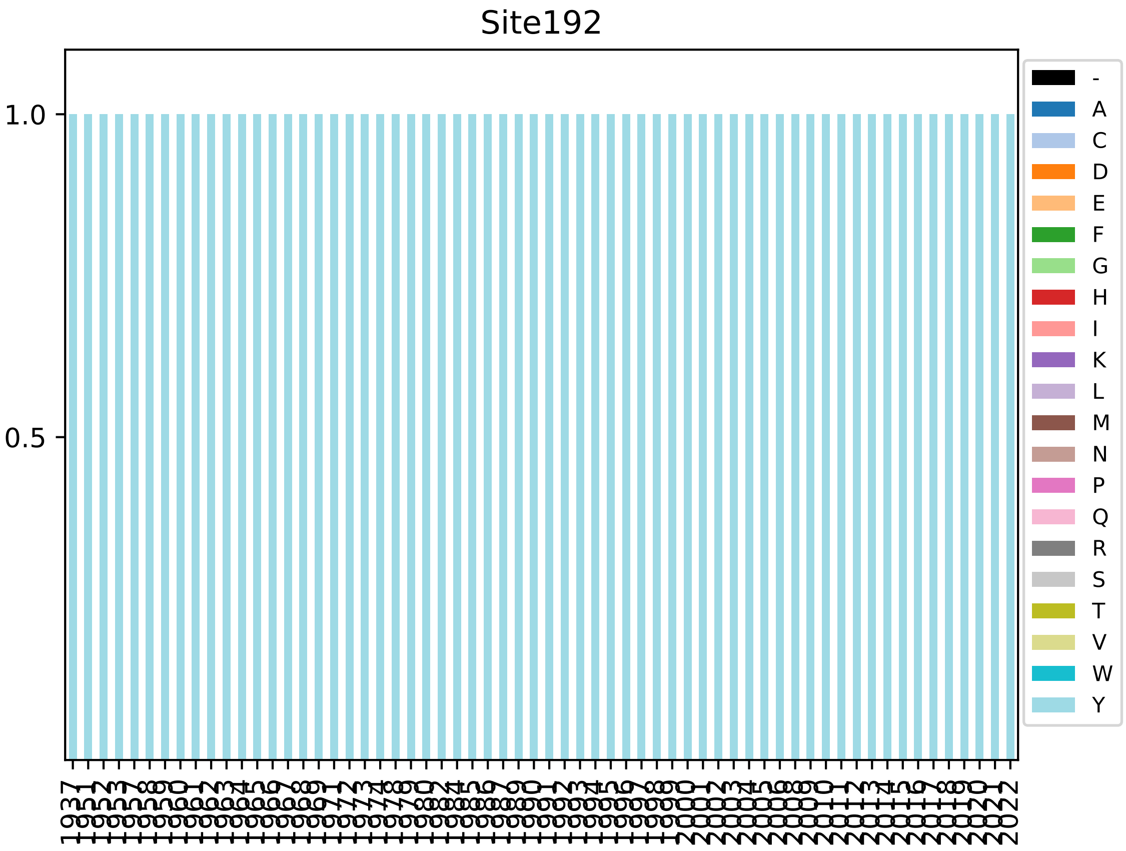
Site192
1.0
0.5
-
A
C
D
E
F
G
H
I
K
L
M
N
P
Q
R
S
T
V
W
Y

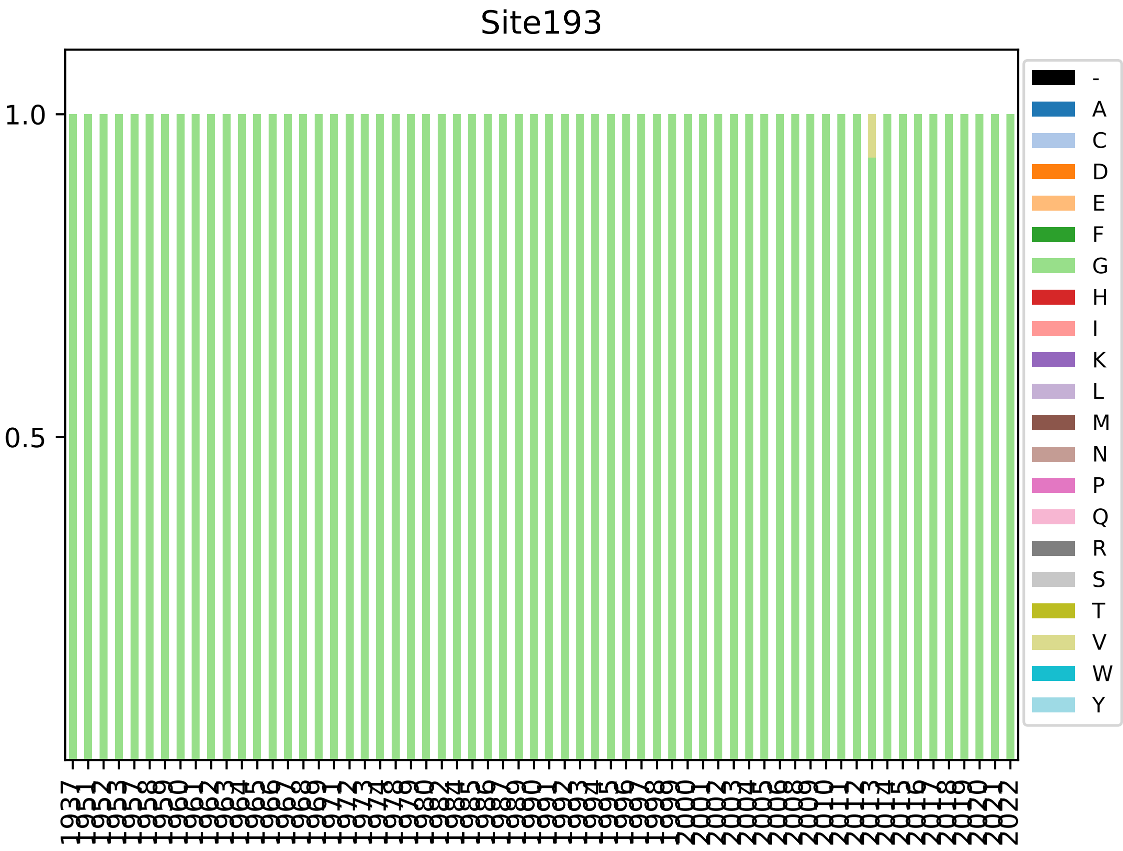

Site193
1.0
0.5
-
A
C
D
E
F
G
H
I
K
L
M
N
P
Q
R
S
T
V
W
Y

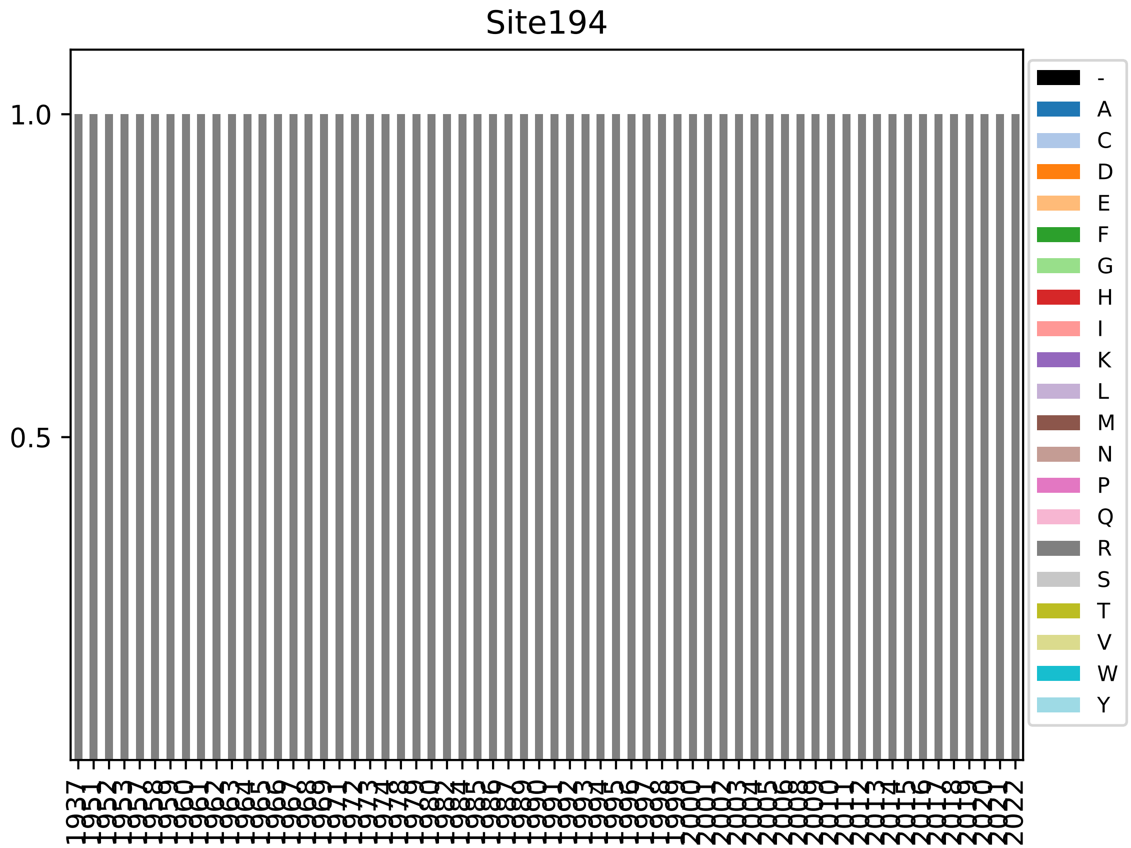
Site194
1.0
0.5
-
A
C
D
E
F
G
H
I
K
L
M
N
P
Q
R
S
T
V
W
Y

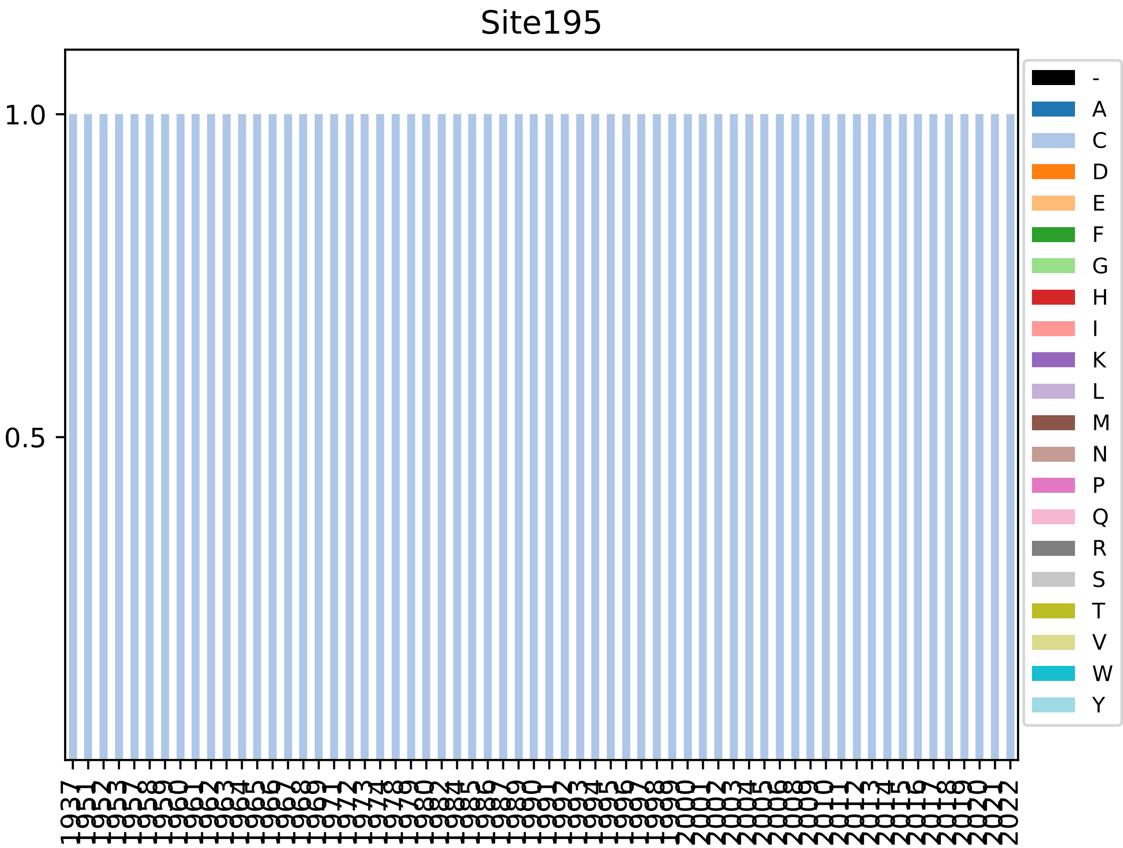
Site195
1.0
0.5
-
A
C
D
E
F
G
H
I
K
L
M
N
P
Q
R
S
T
V
W
Y

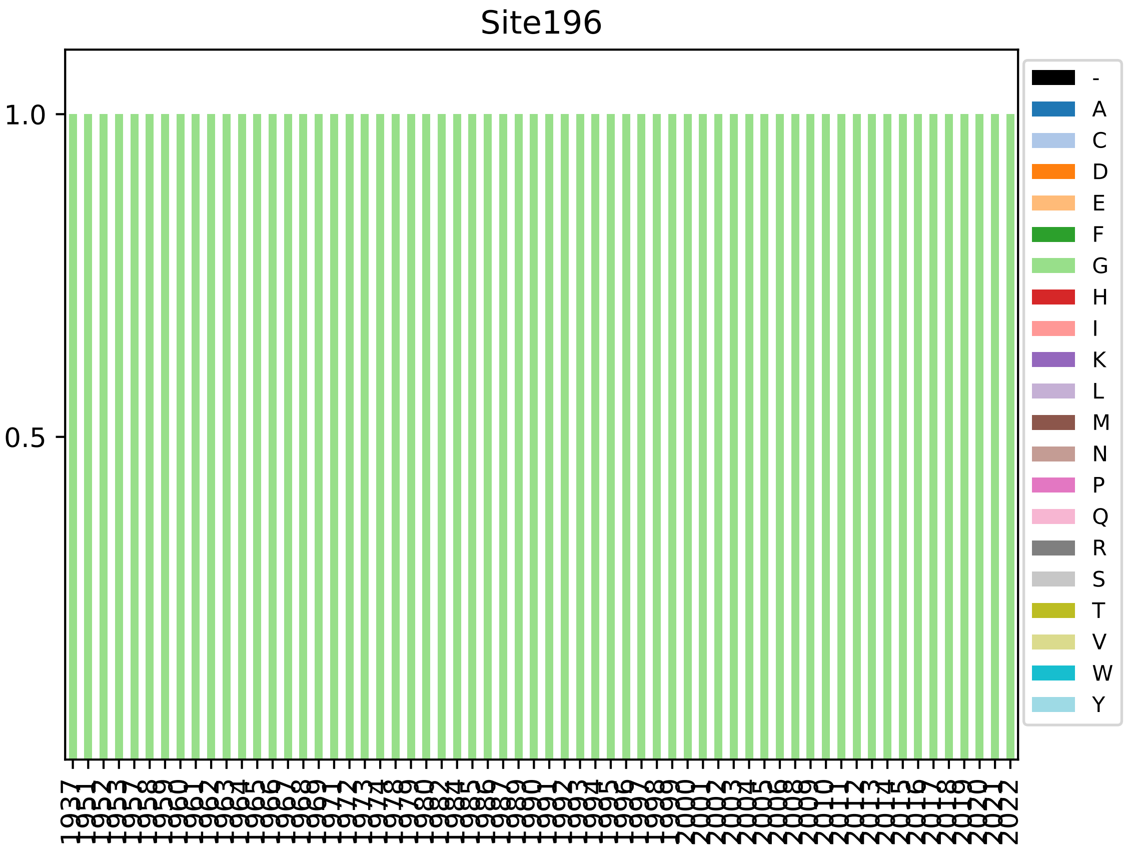
Site196
1.0
0.5
-
A
C
D
E
F
G
H
I
K
L
M
N
P
Q
R
S
T
V
W
Y

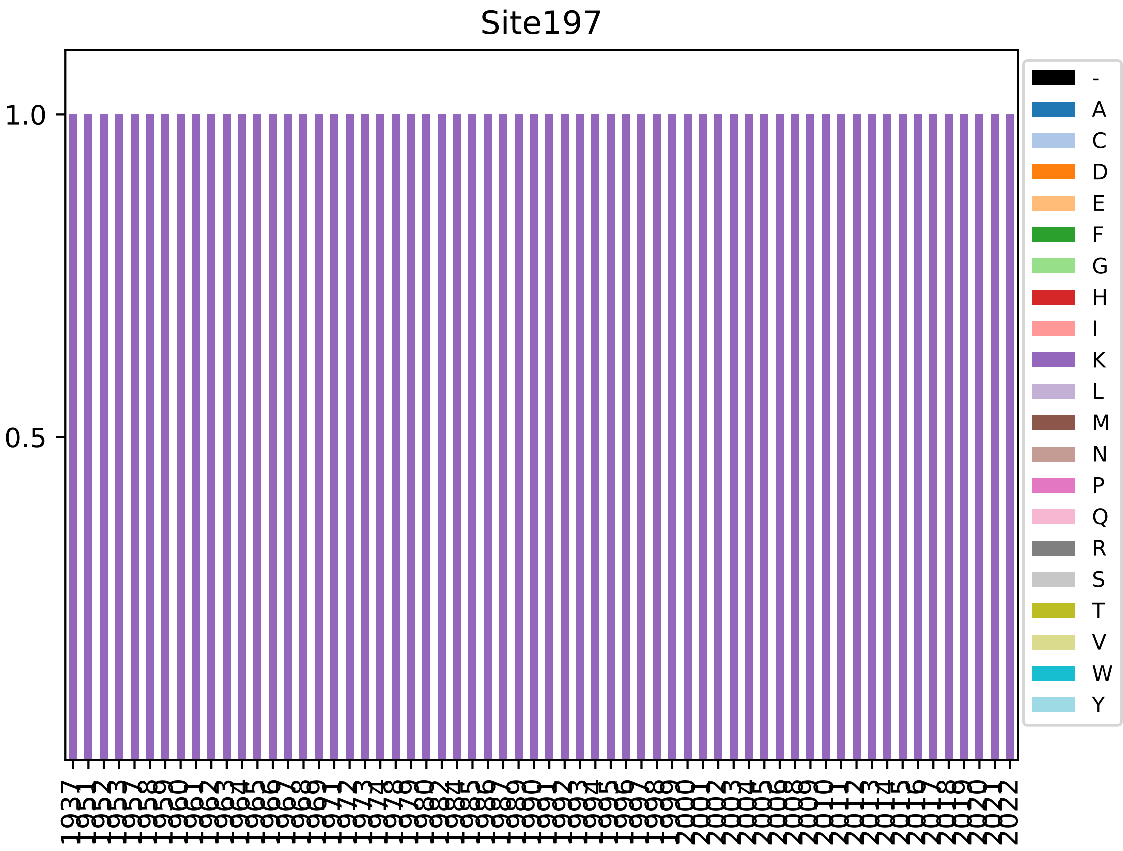
Site197
1.0
0.5
-
A
C
D
E
F
G
H
I
K
L
M
N
P
Q
R
S
T
V
W
Y

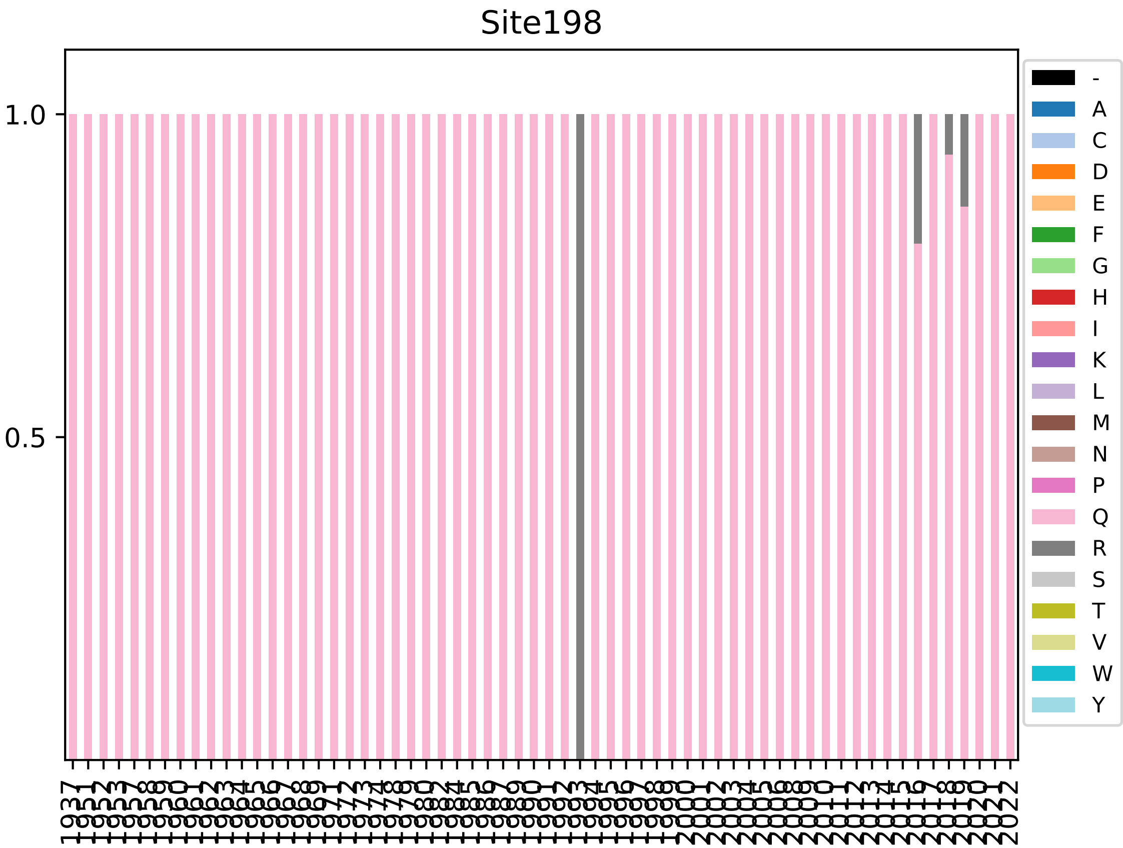
Site198
1.0
0.5
-
A
C
D
E
F
G
H
I
K
L
M
N
P
Q
R
S
T
V
W
Y

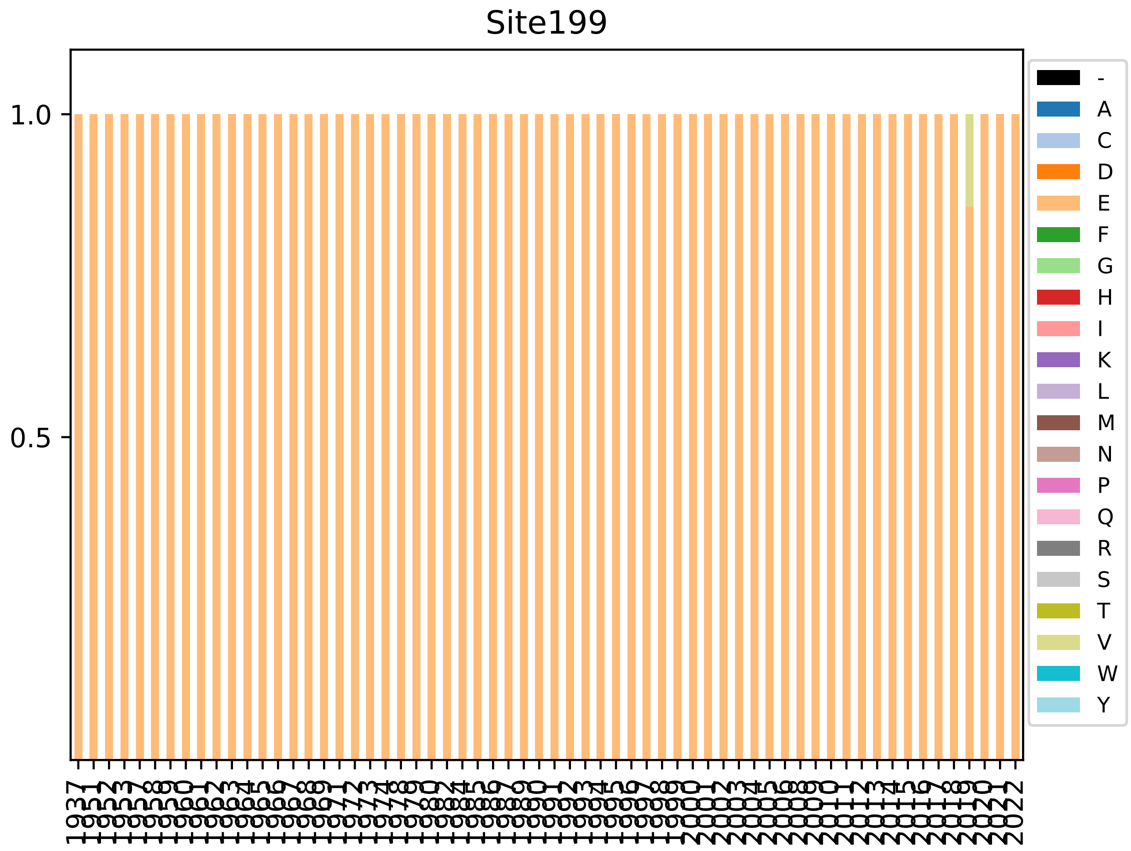
Site199
1.0
0.5
-
A
C
D
E
F
G
H
I
K
L
M
N
P
Q
R
S
T
V
W
Y

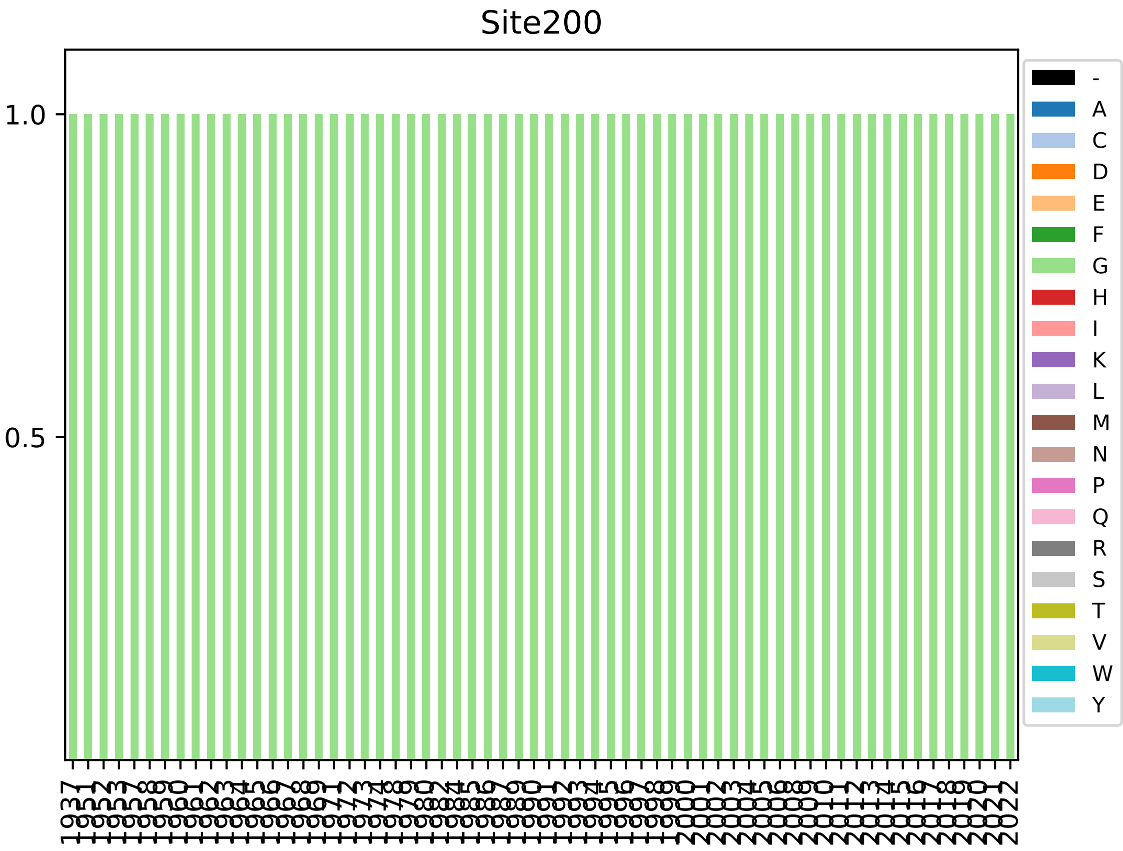
Site200
1.0
0.5
-
A
C
D
E
F
G
H
I
K
L
M
N
P
Q
R
S
T
V
W
Y

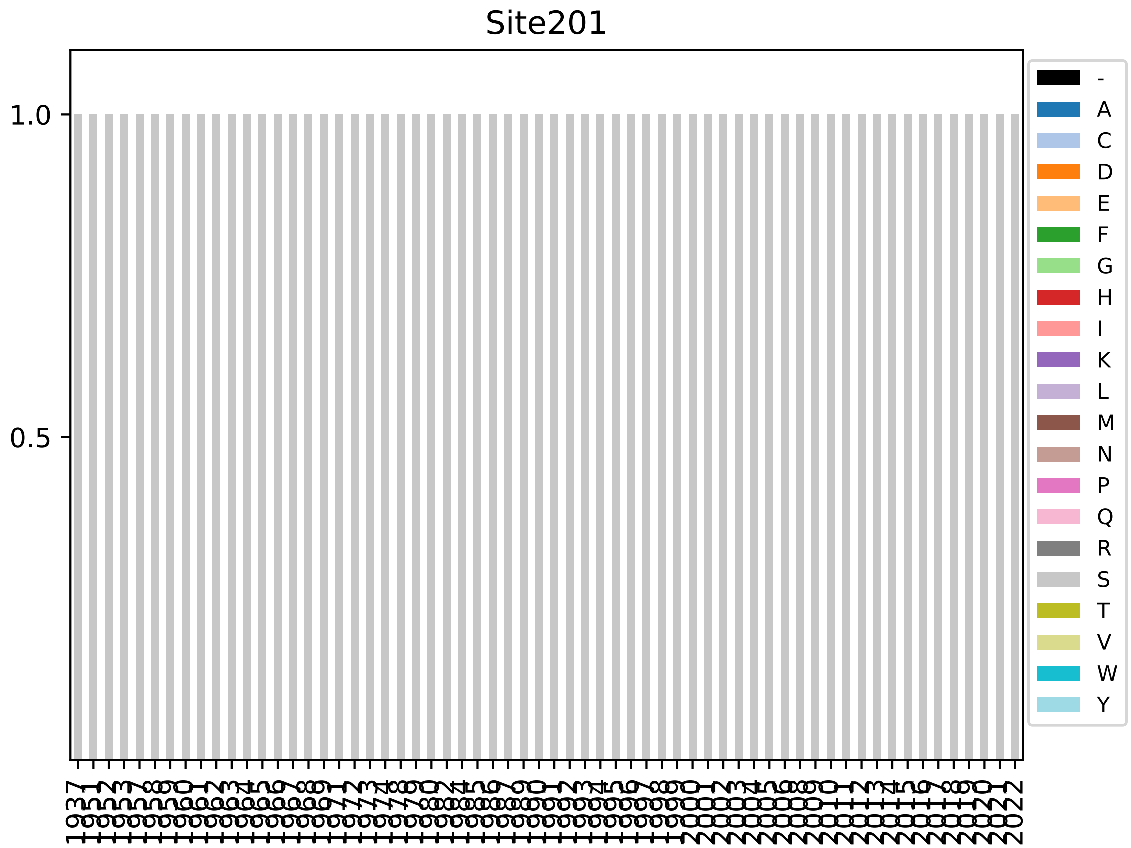
Site201
1.0
0.5
-
A
C
D
E
F
G
H
I
K
L
M
N
P
Q
R
S
T
V
W
Y

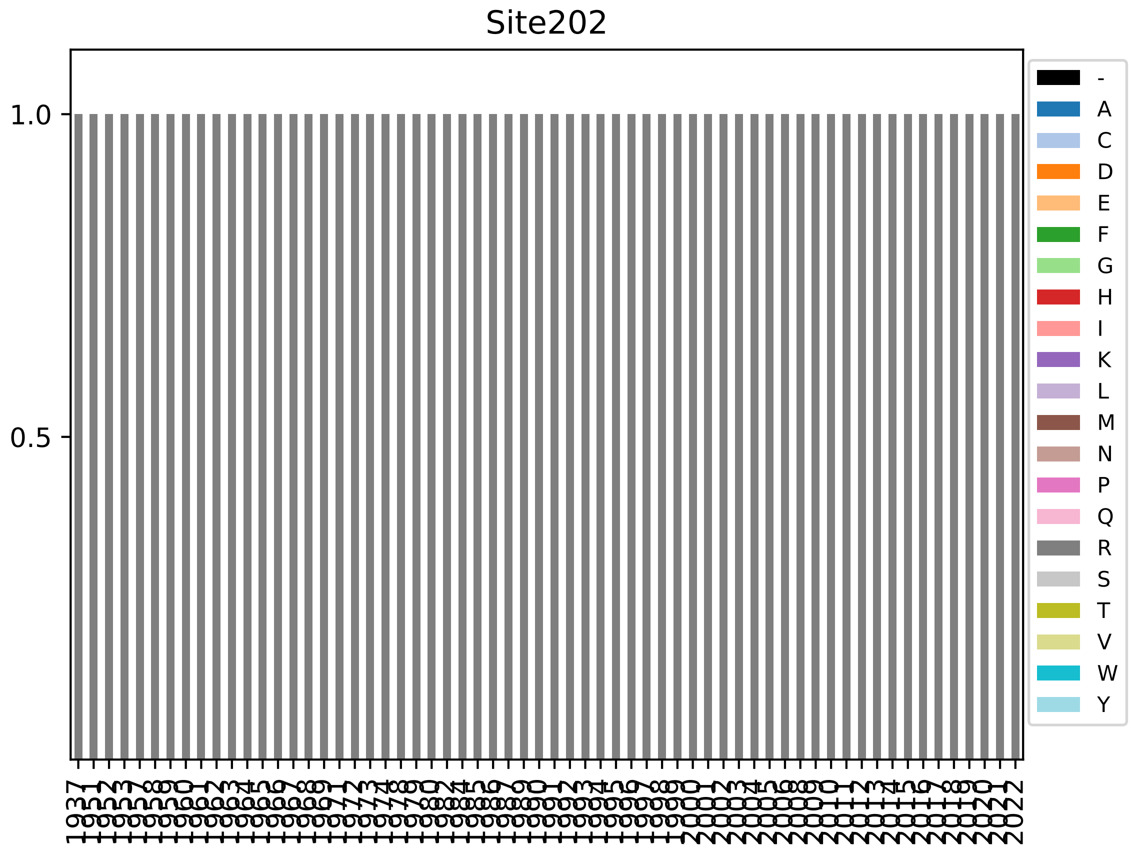
Site202
1.0
0.5
-
A
C
D
E
F
G
H
I
K
L
M
N
P
Q
R
S
T
V
W
Y

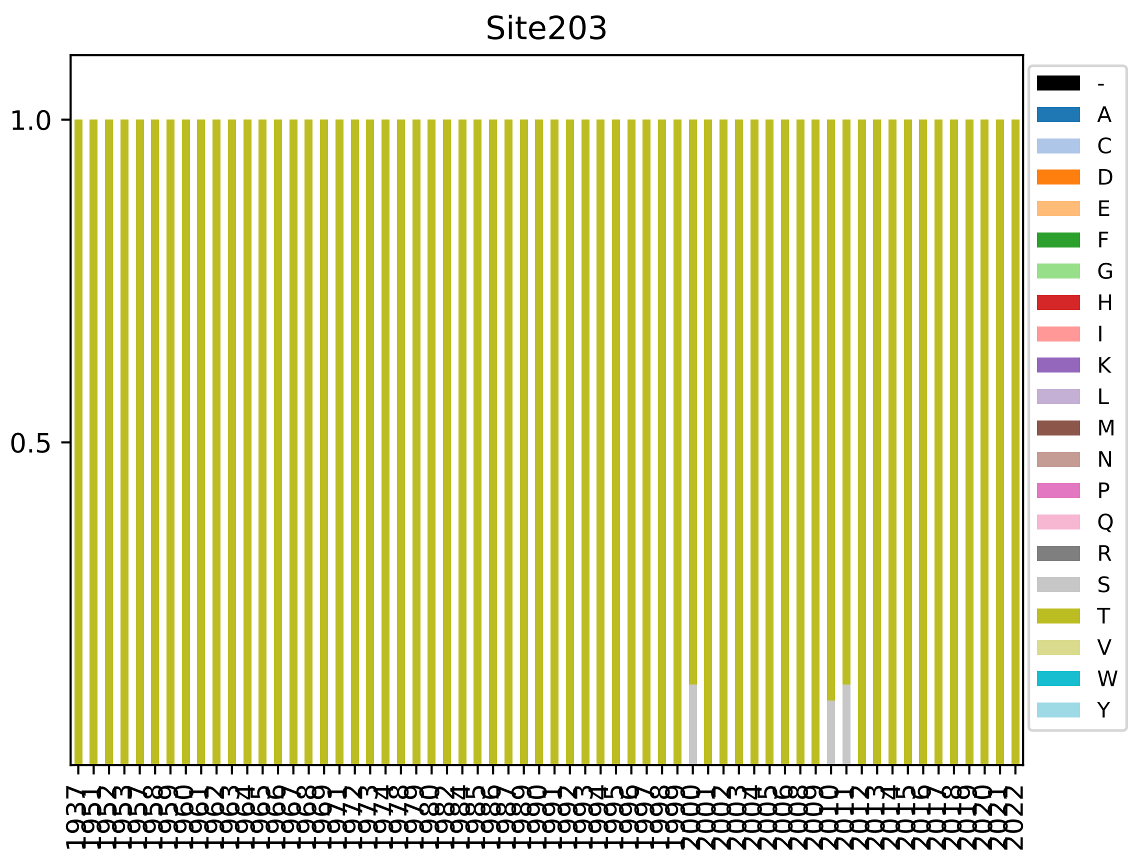
Site203
1.0
0.5
-
A
C
D
E
F
G
H
I
K
L
M
N
P
Q
R
S
T
V
W
Y

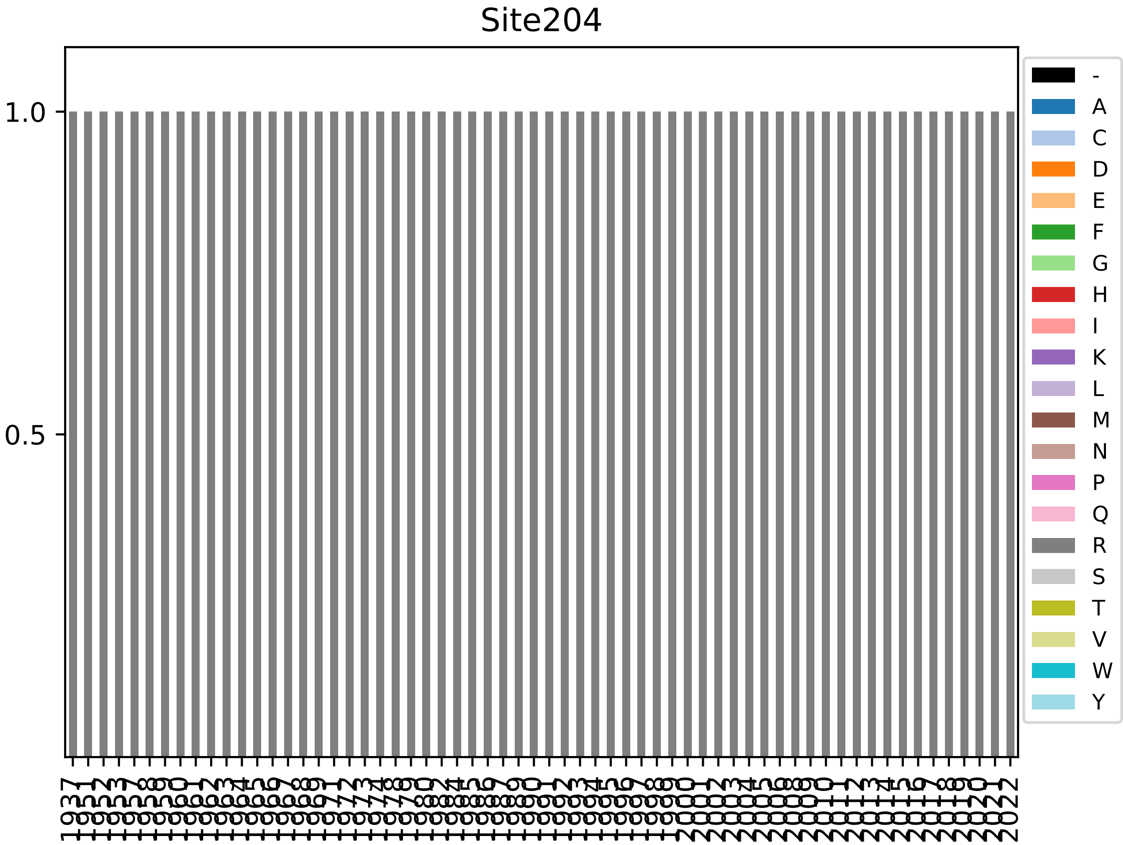
Site204
1.0
0.5
-
A
C
D
E
F
G
H
I
K
L
M
N
P
Q
R
S
T
V
W
Y

Site205

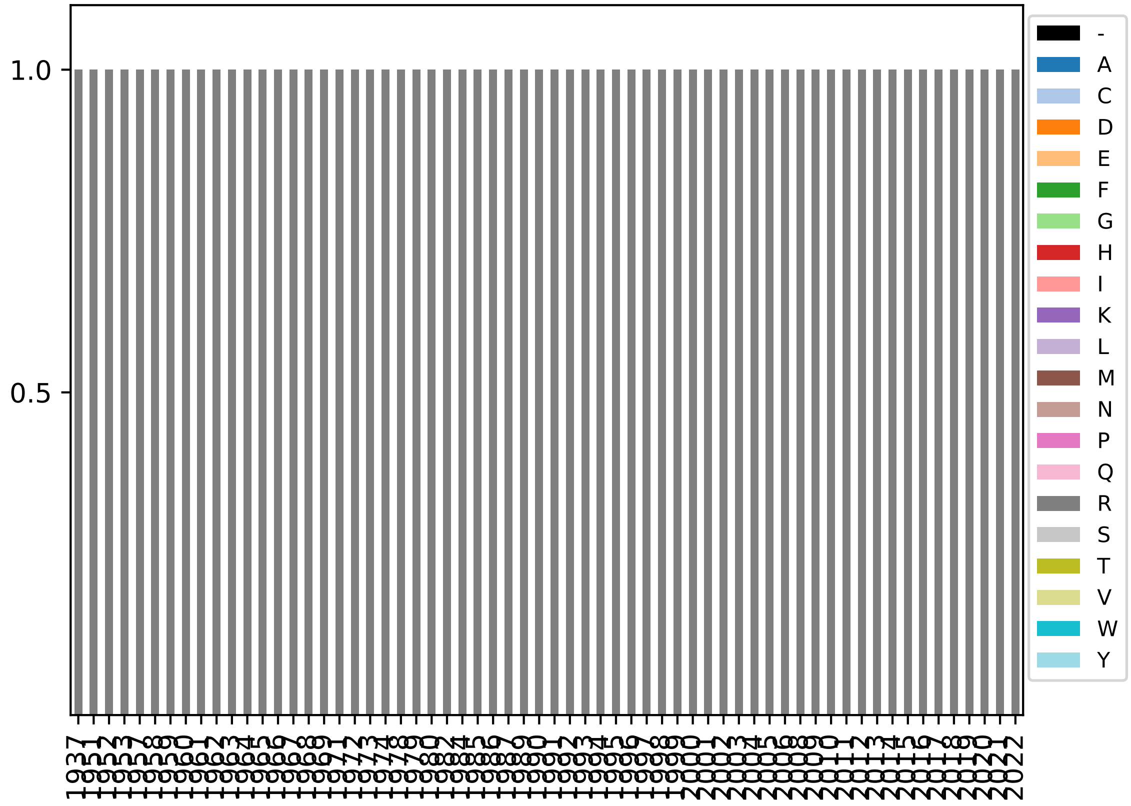

Site206

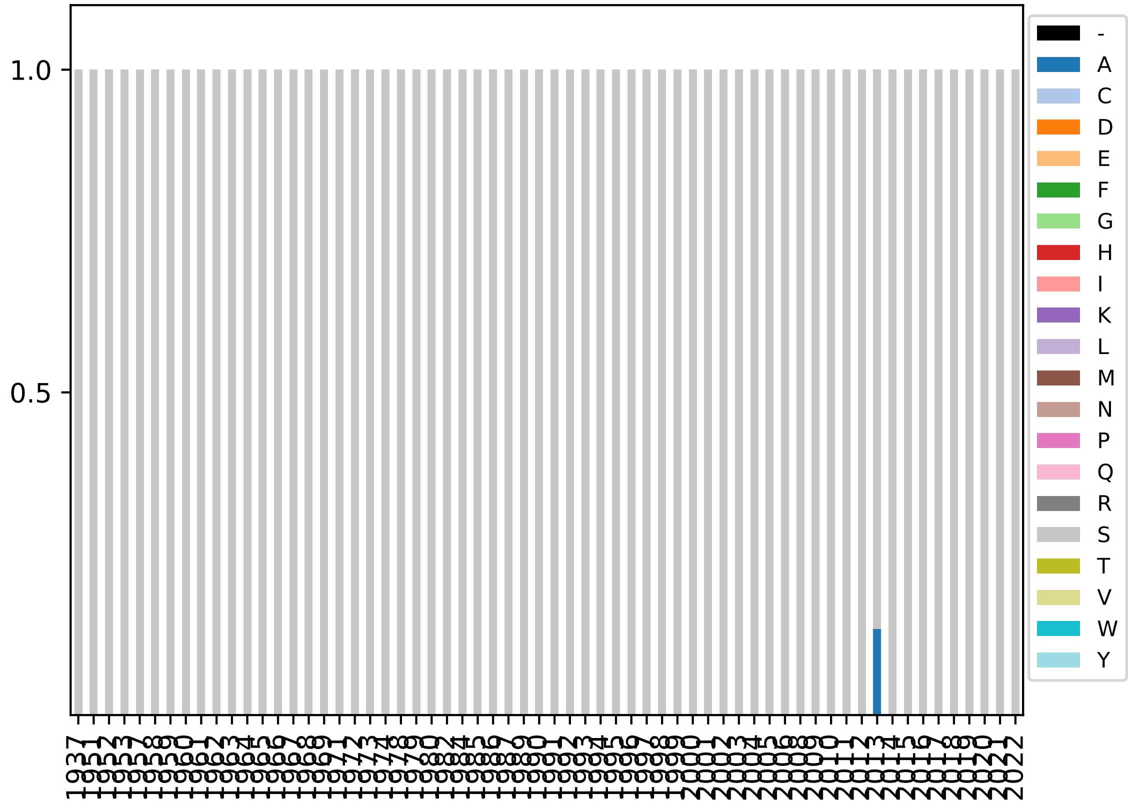

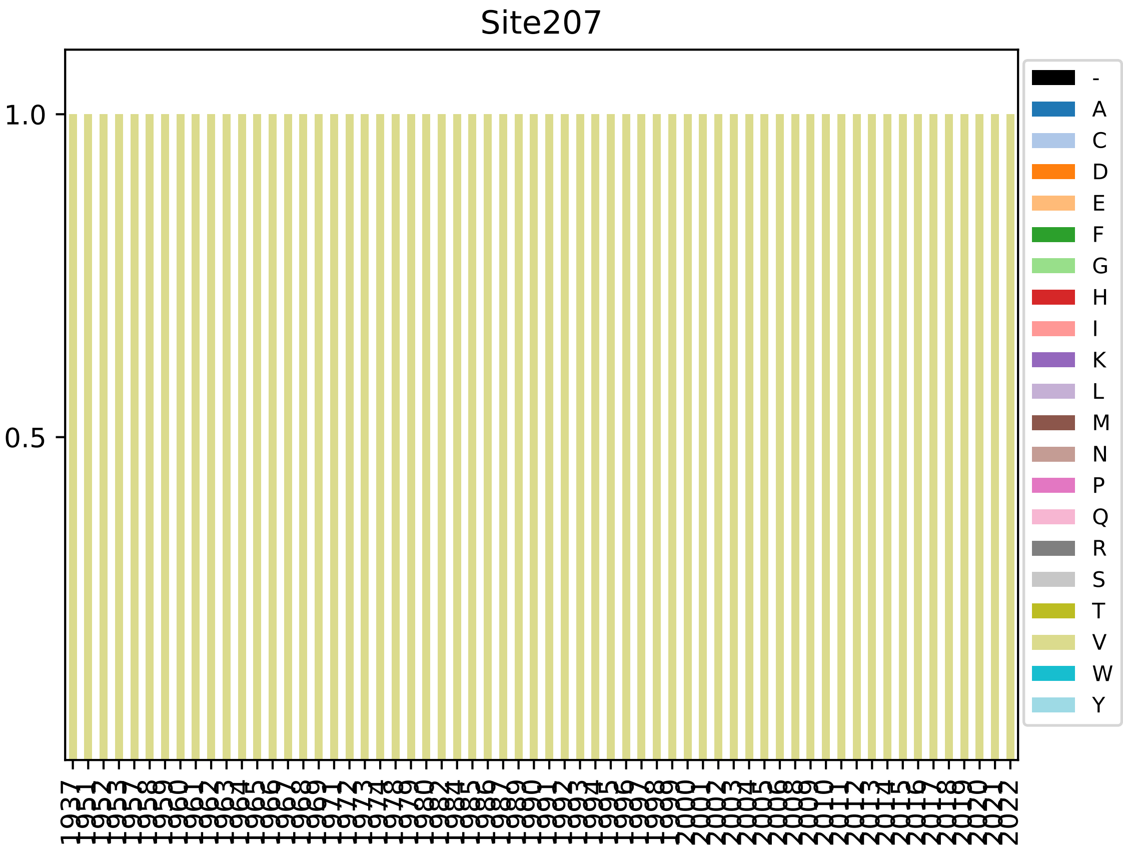
Site207
1.0
0.5
-
A
C
D
E
F
G
H
I
K
L
M
N
P
Q
R
S
T
V
W
Y

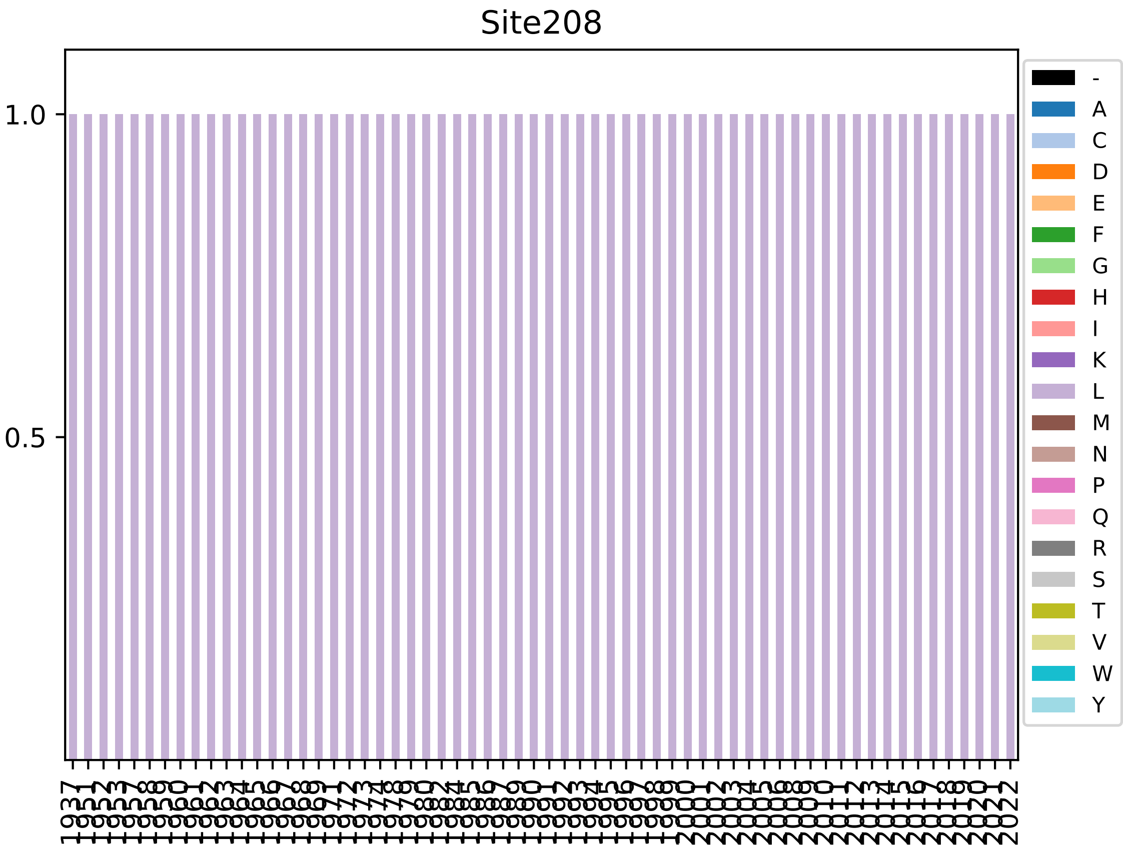

Site208
1.0
0.5
-
A
C
D
E
F
G
H
I
K
L
M
N
P
Q
R
S
T
V
W
Y

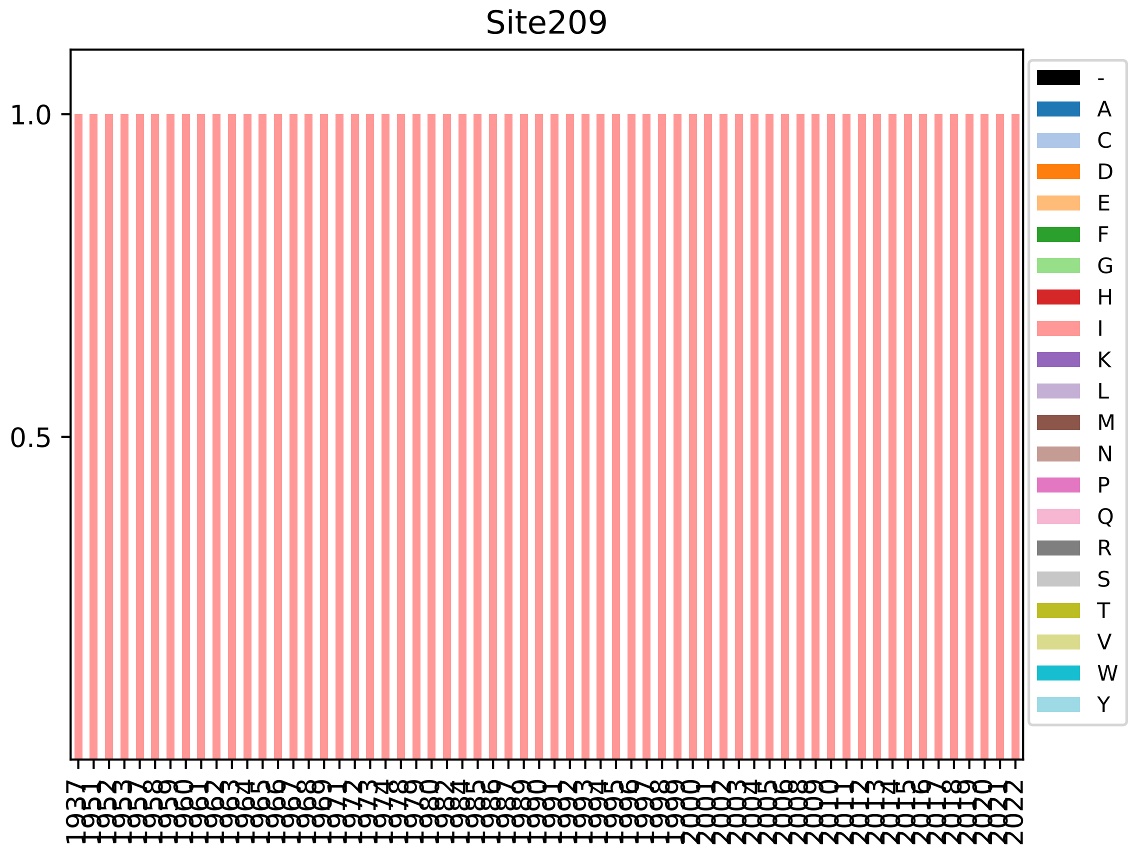
Site209
1.0
0.5
-
A
C
D
E
F
G
H
I
K
L
M
N
P
Q
R
S
T
V
W
Y

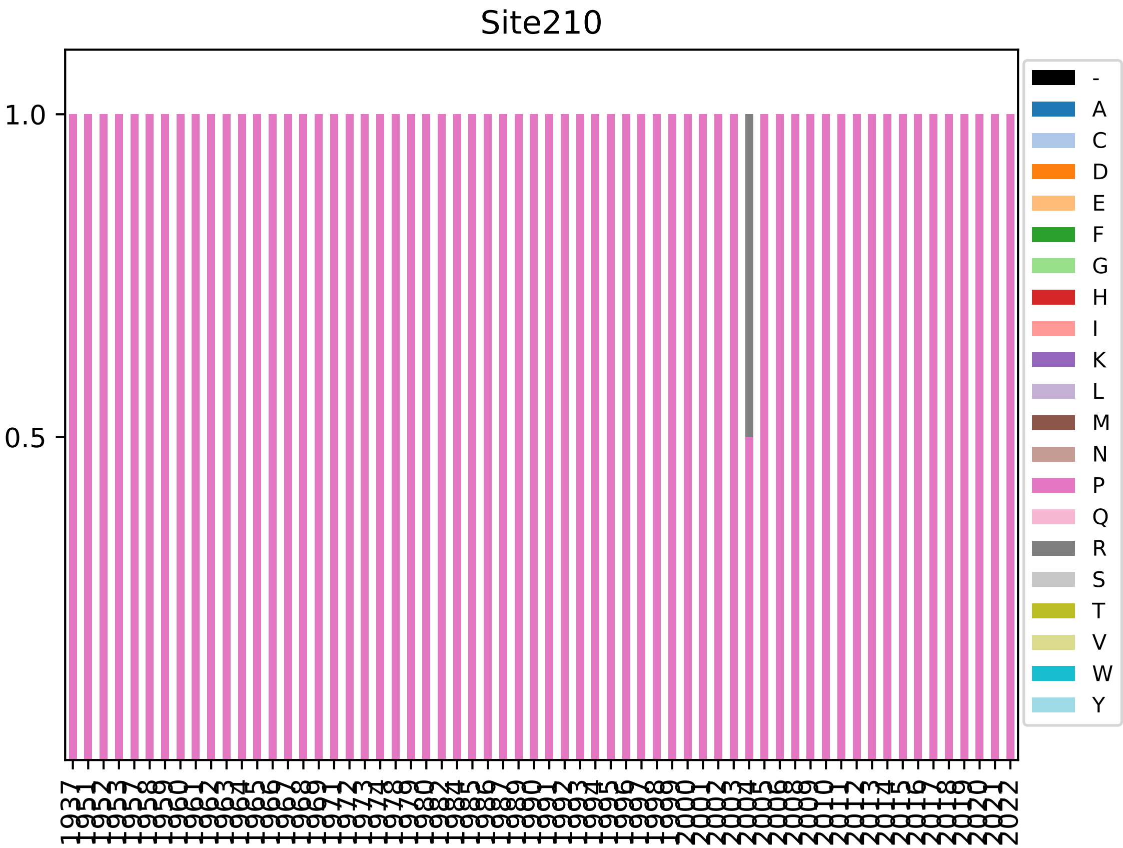
Site210
1.0
0.5
-
A
C
D
E
F
G
H
I
K
L
M
N
P
Q
R
S
T
V
W
Y

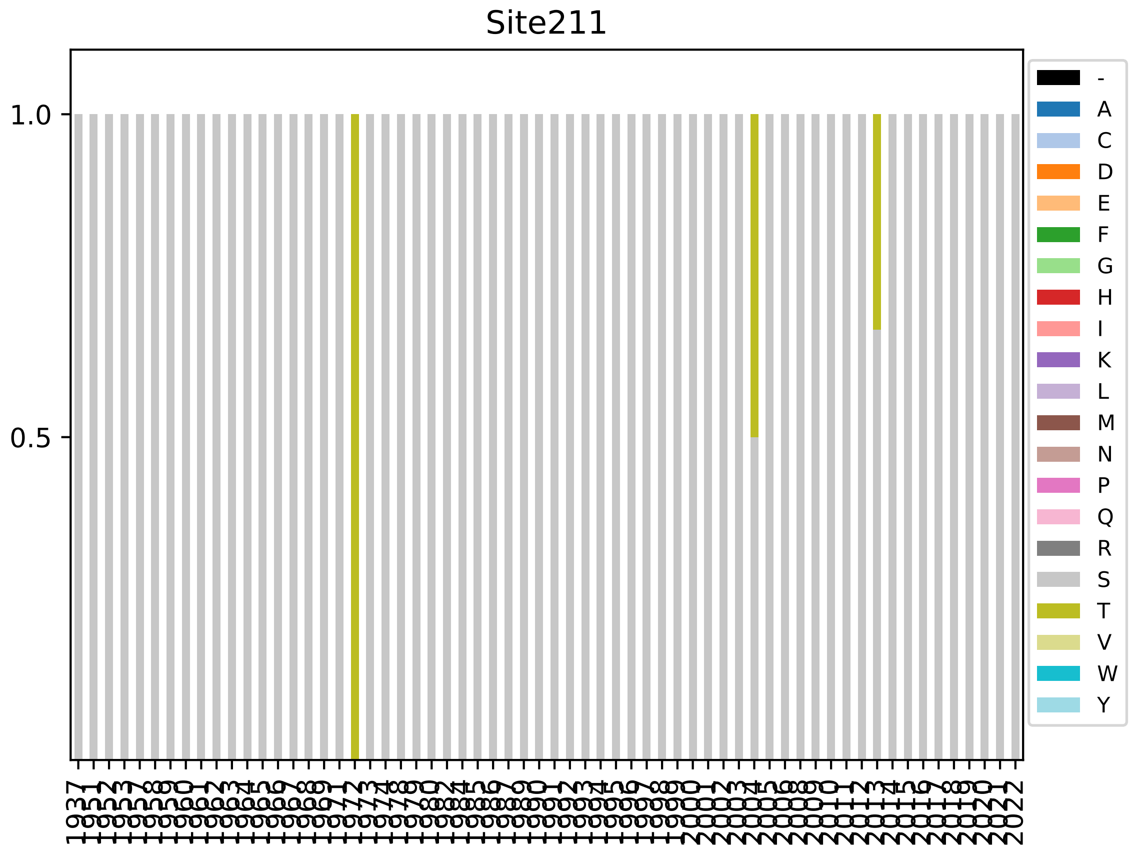
Site211
1.0
0.5
-
A
C
D
E
F
G
H
I
K
L
M
N
P
Q
R
S
T
V
W
Y

Site212

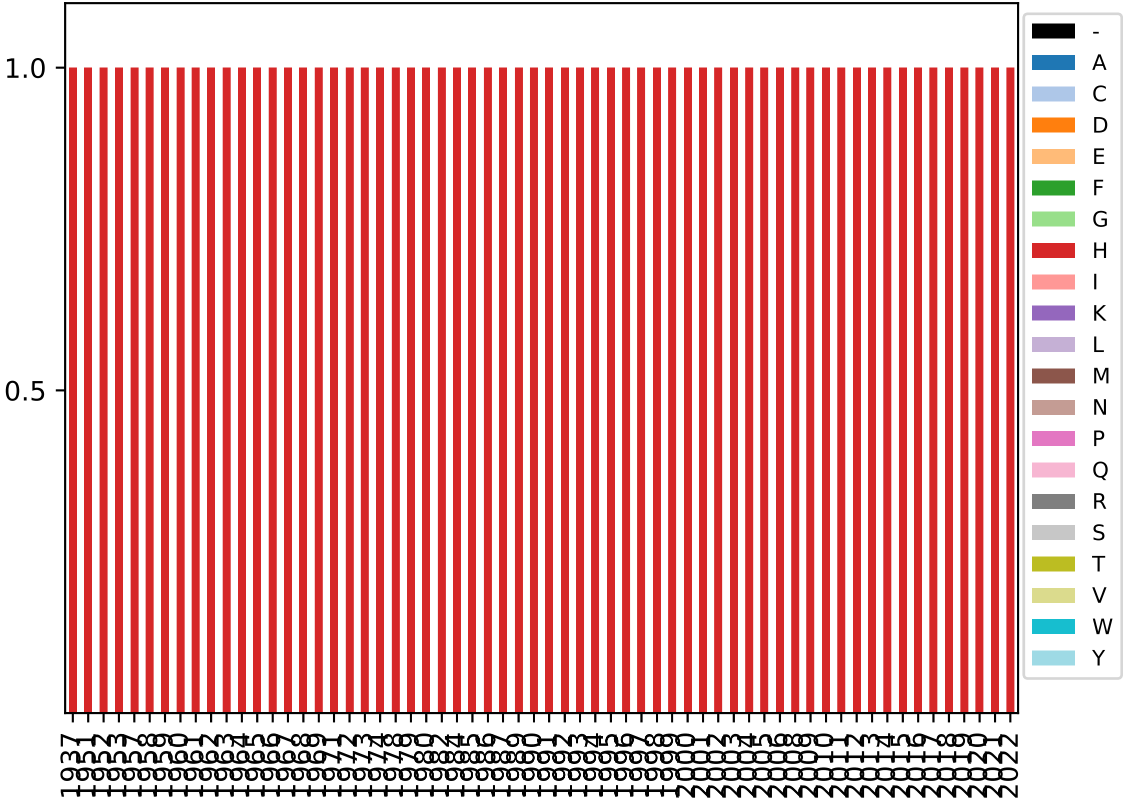

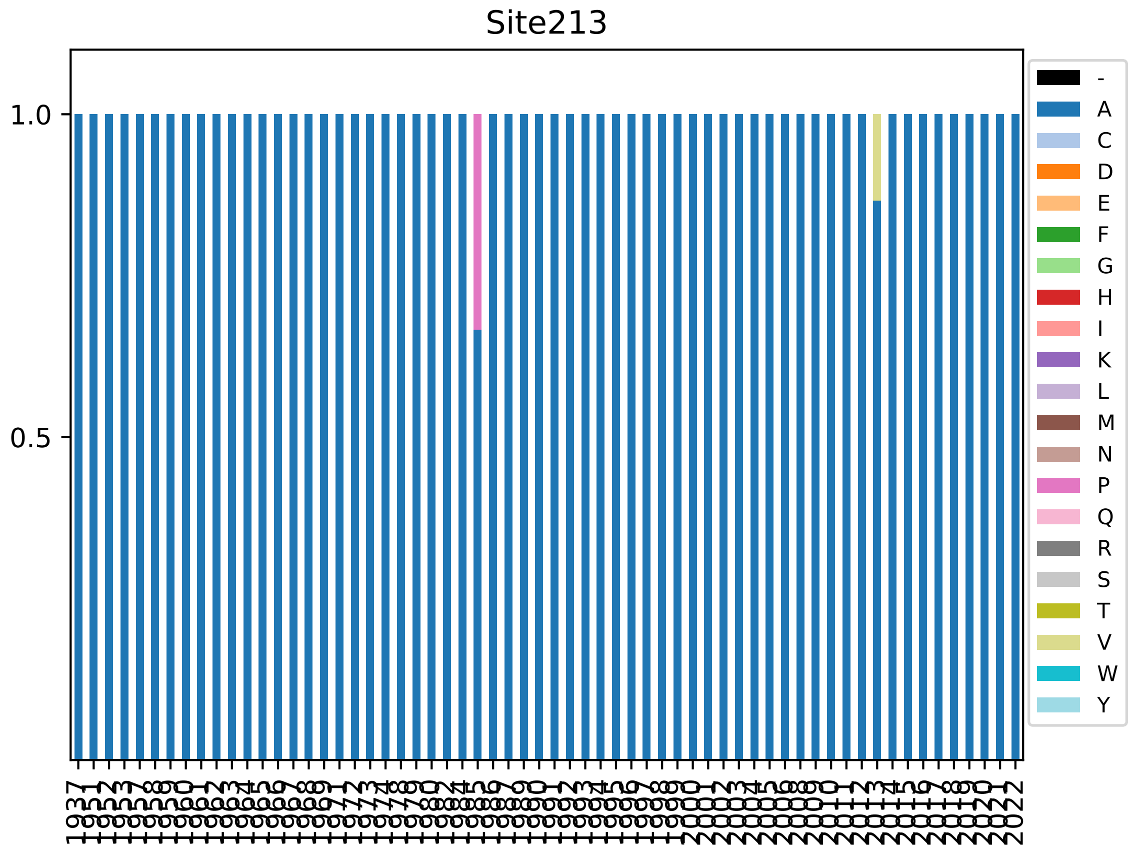
Site213
1.0
0.5
-
A
C
D
E
F
G
H
I
K
L
M
N
P
Q
R
S
T
V
W
Y

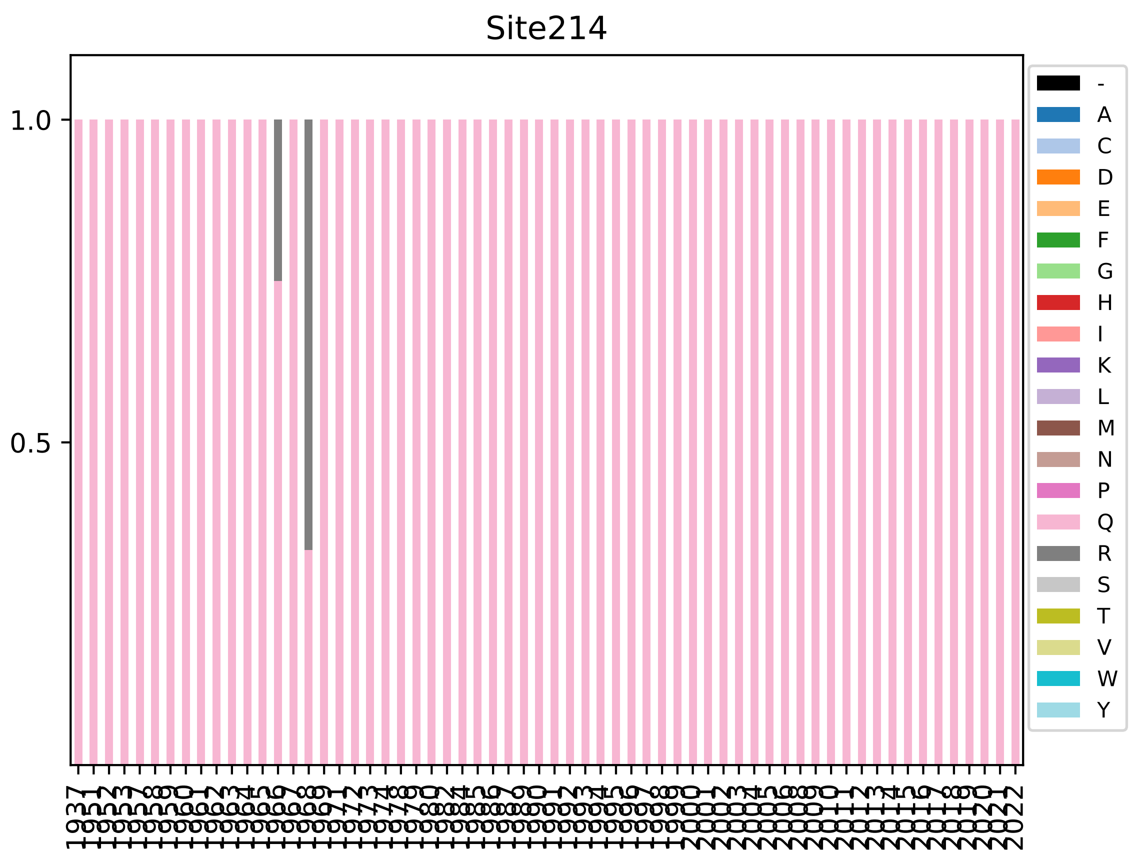
Site214
1.0
0.5
-
A
C
D
E
F
G
H
I
K
L
M
N
P
Q
R
S
T
V
W
Y

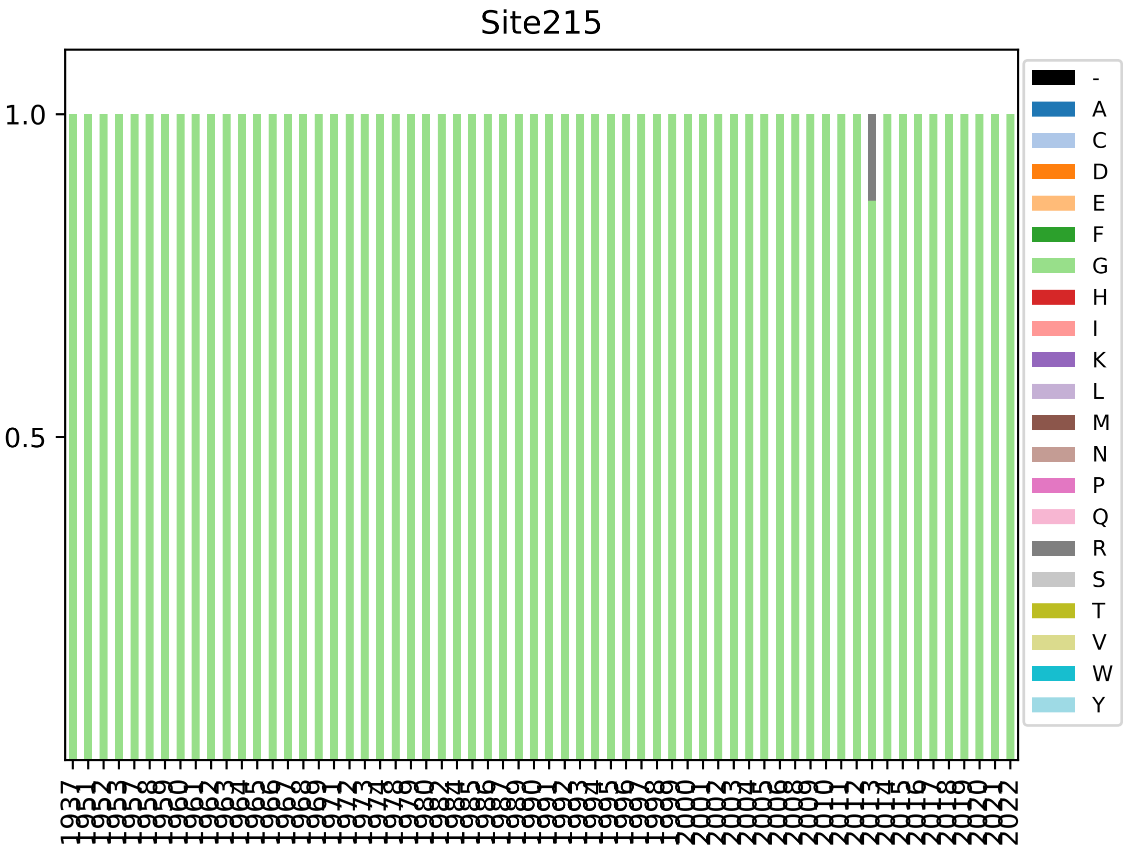
Site215
1.0
0.5
-
A
C
D
E
F
G
H
I
K
L
M
N
P
Q
R
S
T
V
W
Y

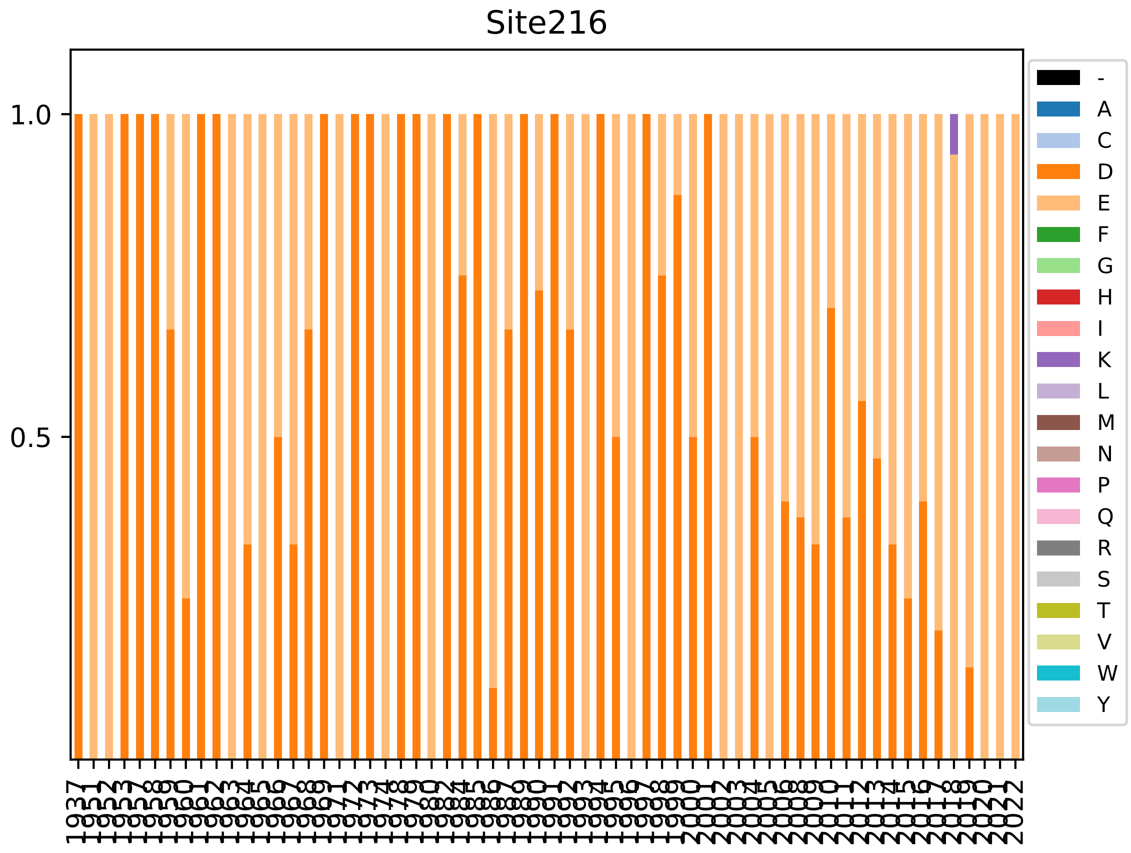
Site216
1.0
0.5
-
A
C
D
E
F
G
H
I
K
L
M
N
P
Q
R
S
T
V
W
Y

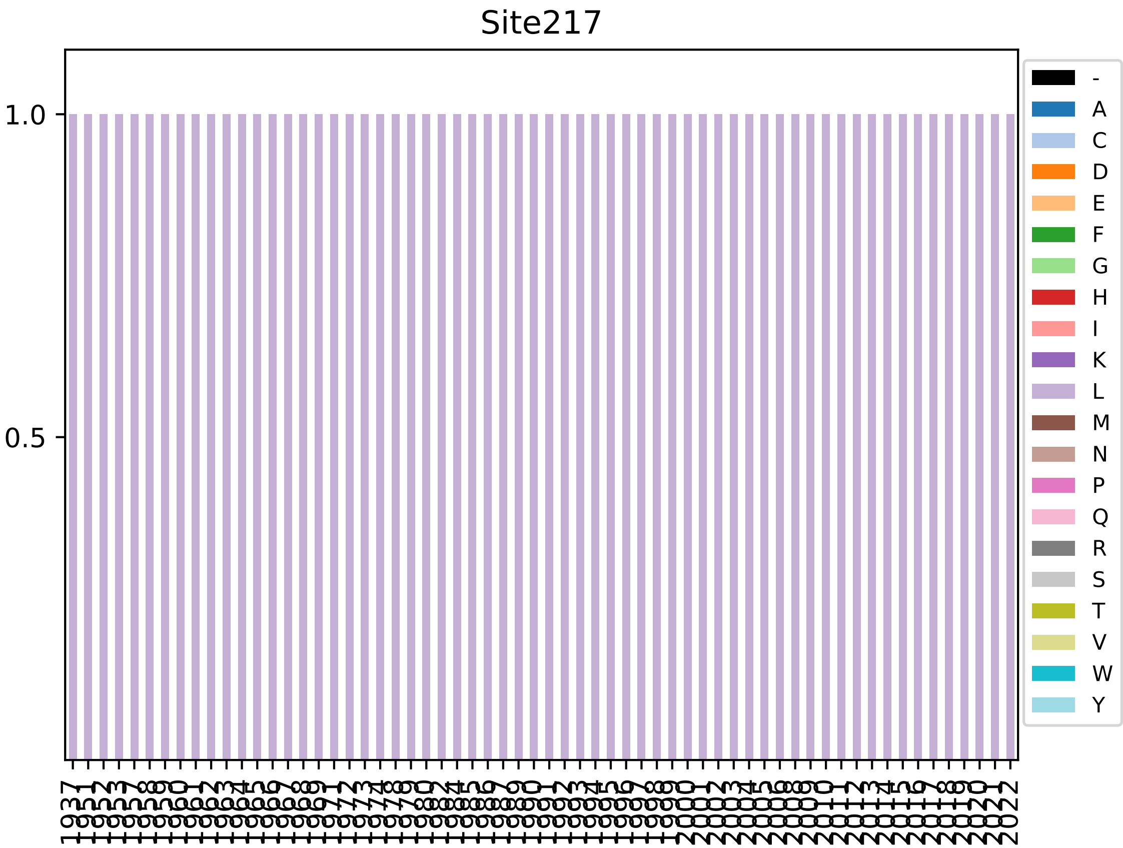

Site217
1.0
0.5
-
A
C
D
E
F
G
H
I
K
L
M
N
P
Q
R
S
T
V
W
Y

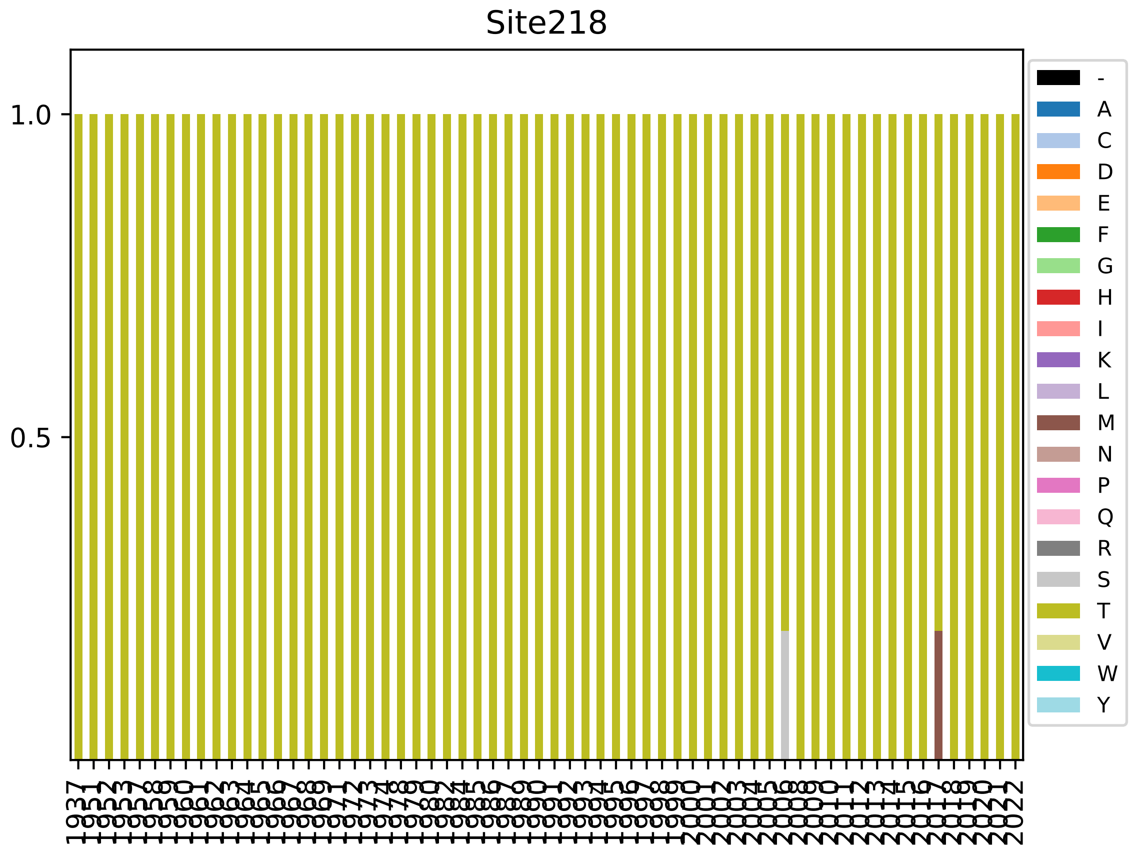
Site218
1.0
0.5
-
A
C
D
E
F
G
H
I
K
L
M
N
P
Q
R
S
T
V
W
Y

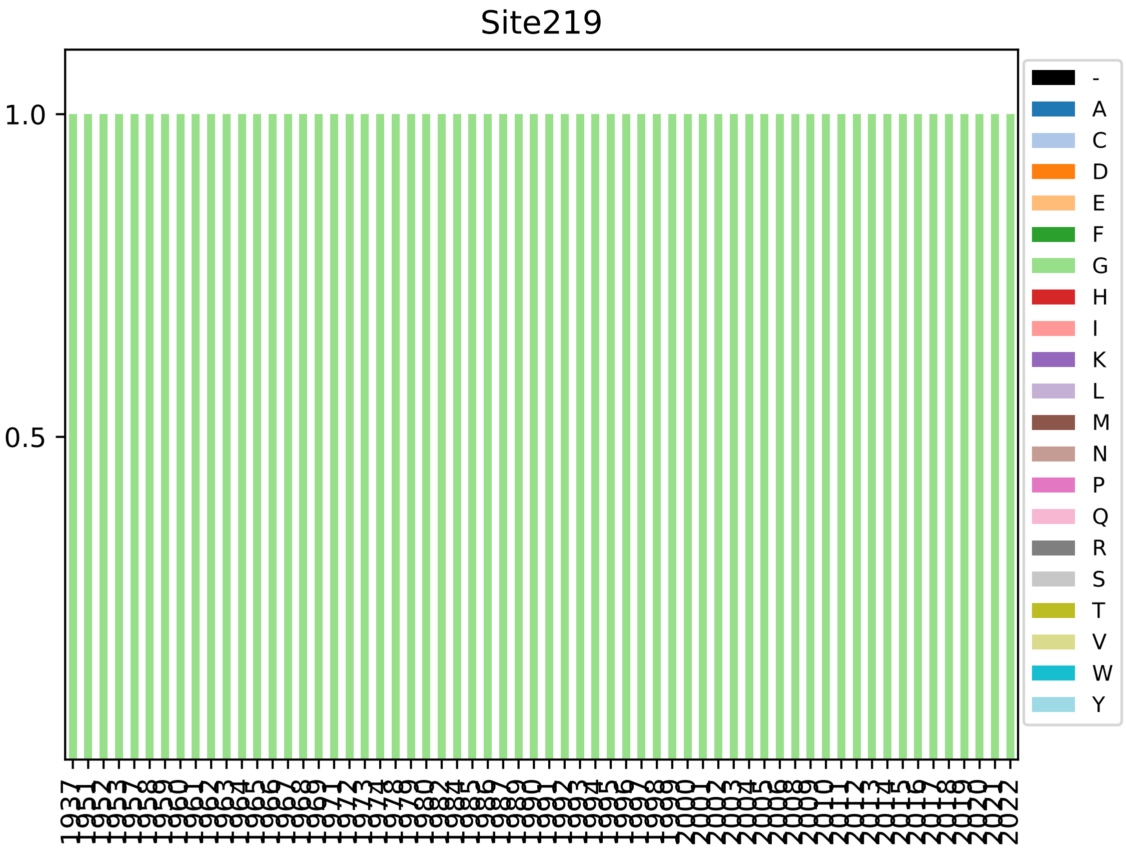
Site219
1.0
0.5
-
A
C
D
E
F
G
H
I
K
L
M
N
P
Q
R
S
T
V
W
Y

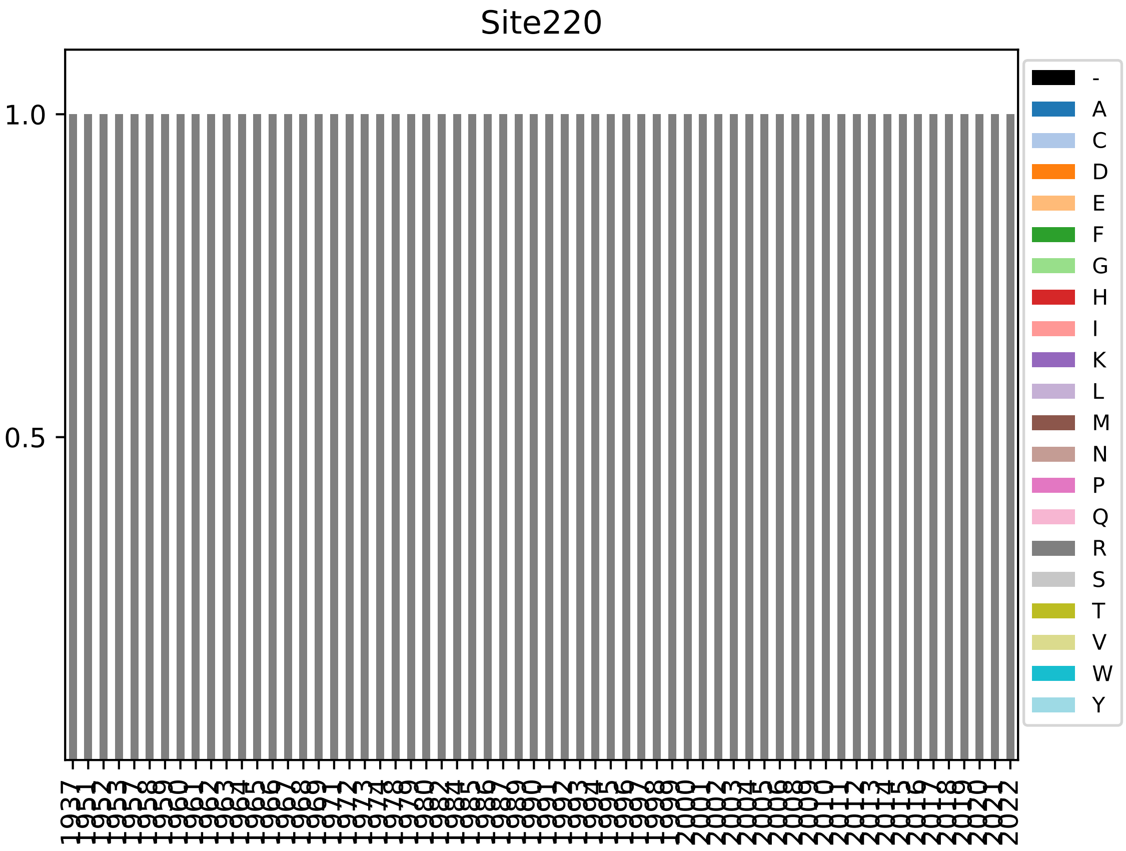
Site220
1.0
0.5
-
A
C
D
E
F
G
H
I
K
L
M
N
P
Q
R
S
T
V
W
Y

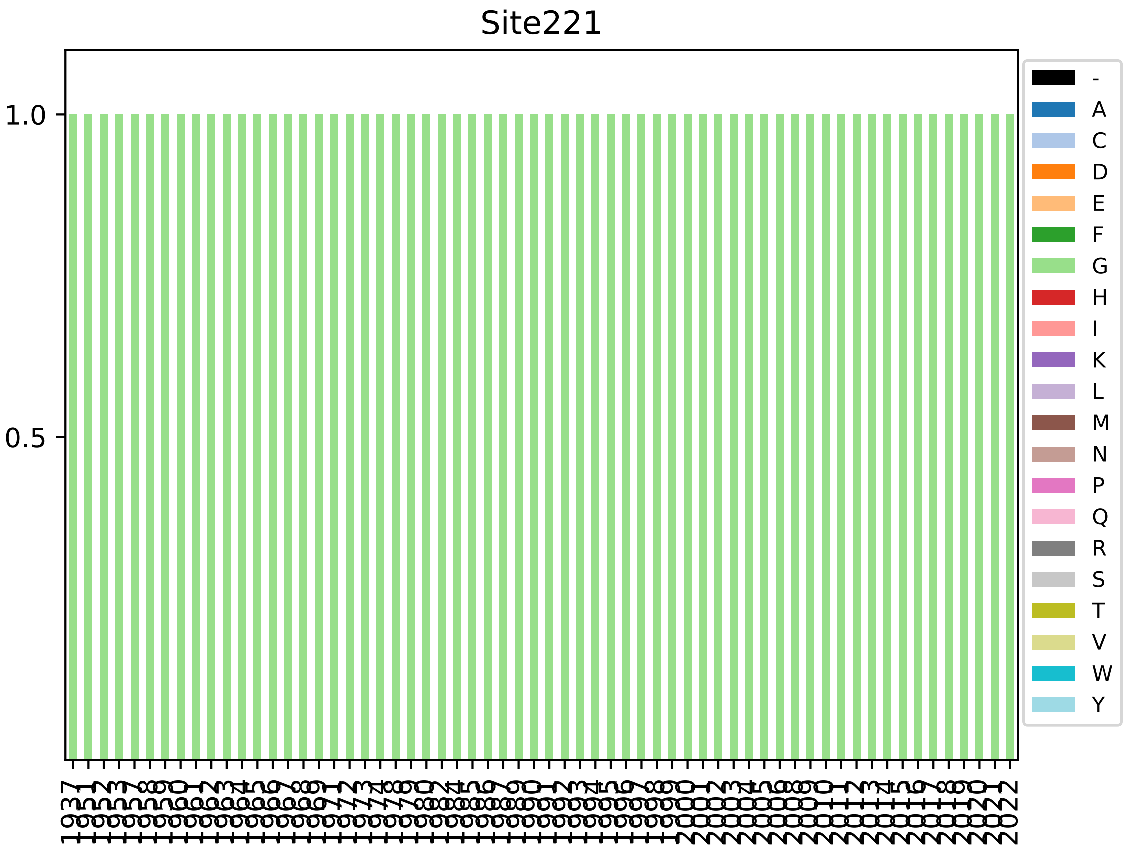
Site221
1.0
0.5
-
A
C
D
E
F
G
H
I
K
L
M
N
P
Q
R
S
T
V
W
Y

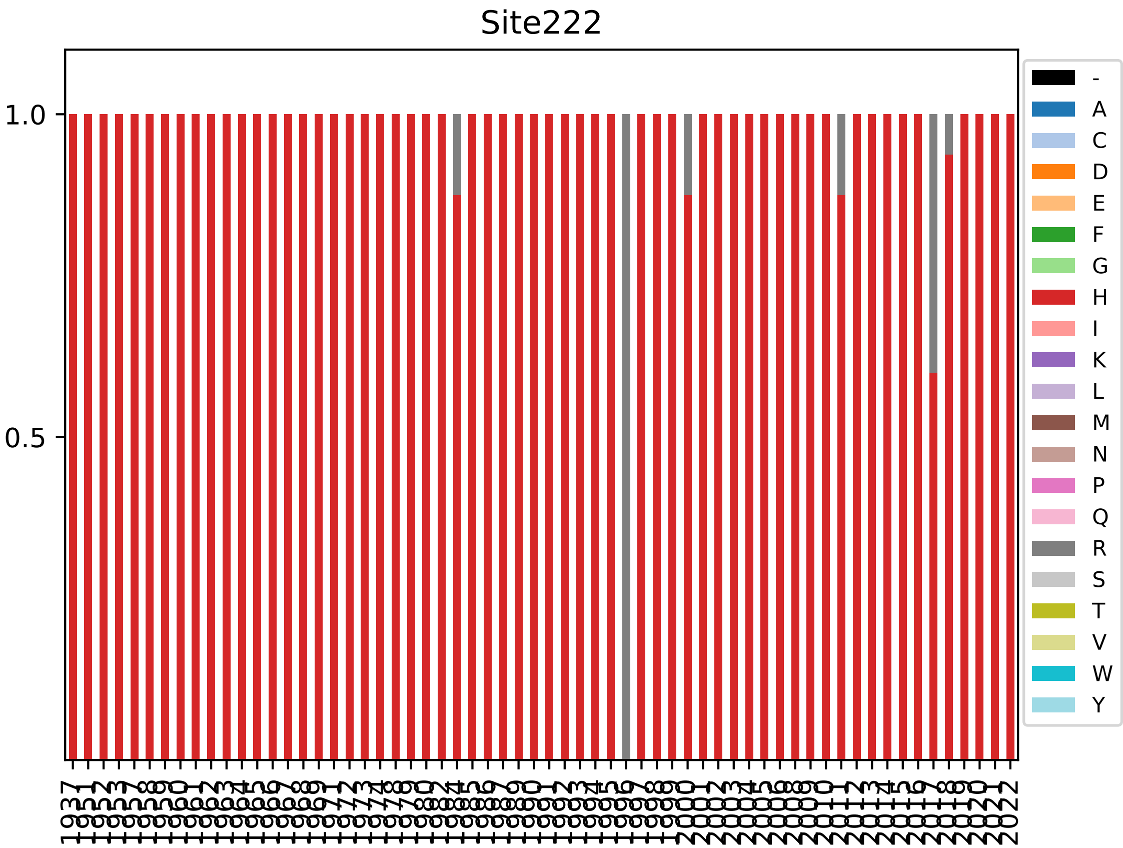
Site222
1.0
0.5
-
A
C
D
E
F
G
H
I
K
L
M
N
P
Q
R
S
T
V
W
Y

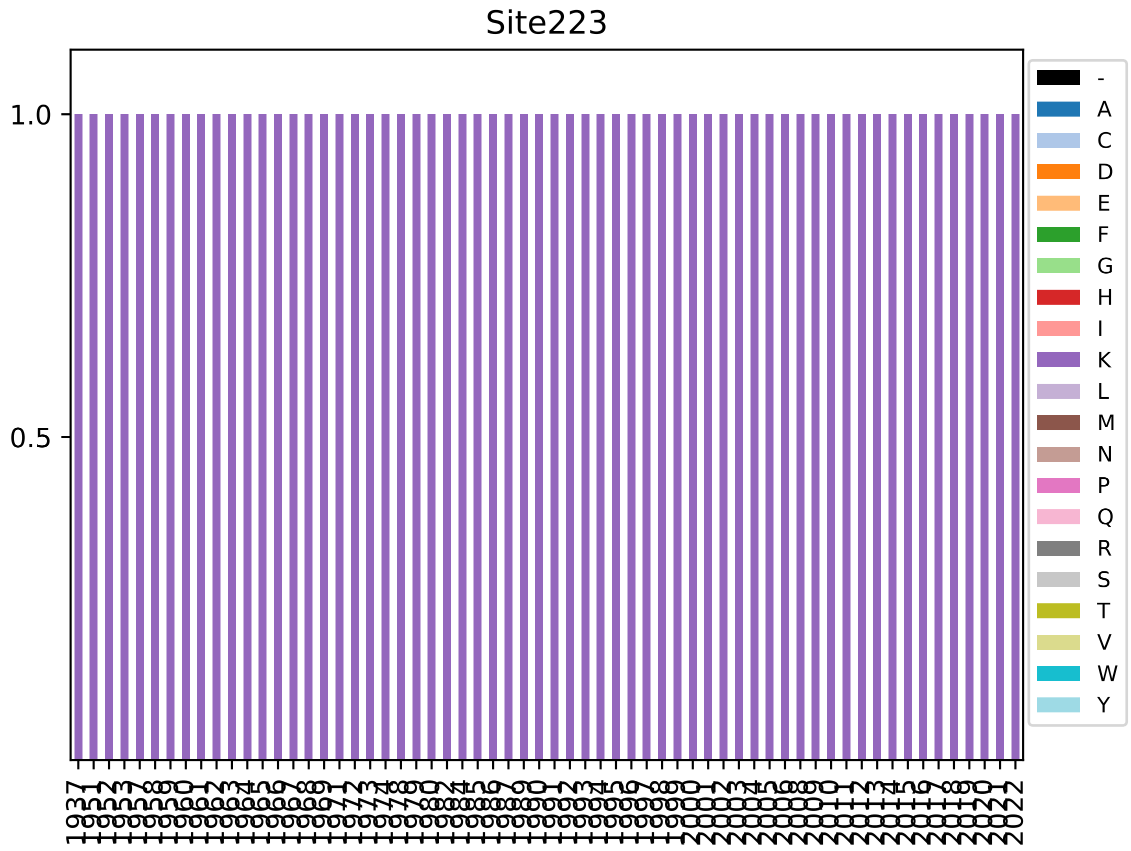
Site223
1.0
0.5
-
A
C
D
E
F
G
H
I
K
L
M
N
P
Q
R
S
T
V
W
Y

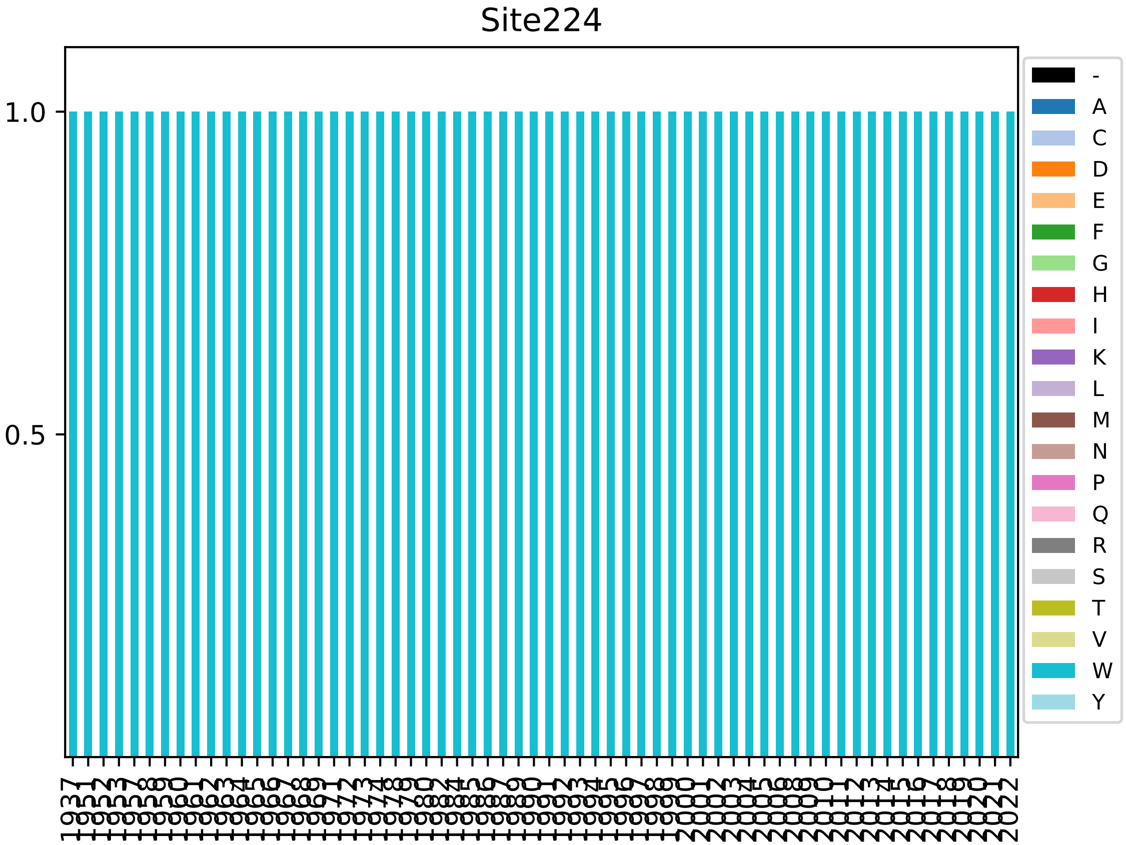
Site224
1.0
0.5
-
A
C
D
E
F
G
H
I
K
L
M
N
P
Q
R
S
T
V
W
Y

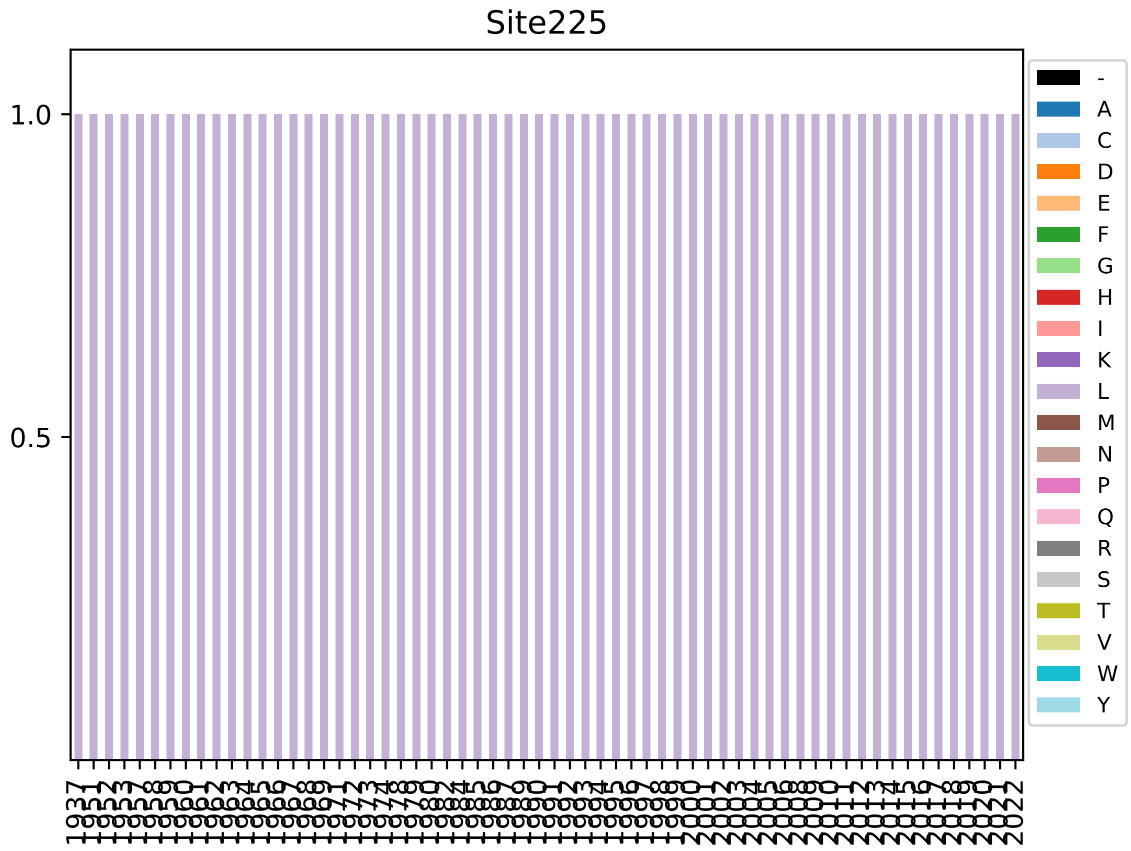
Site225
1.0
0.5
-
A
C
D
E
F
G
H
I
K
L
M
N
P
Q
R
S
T
V
W
Y

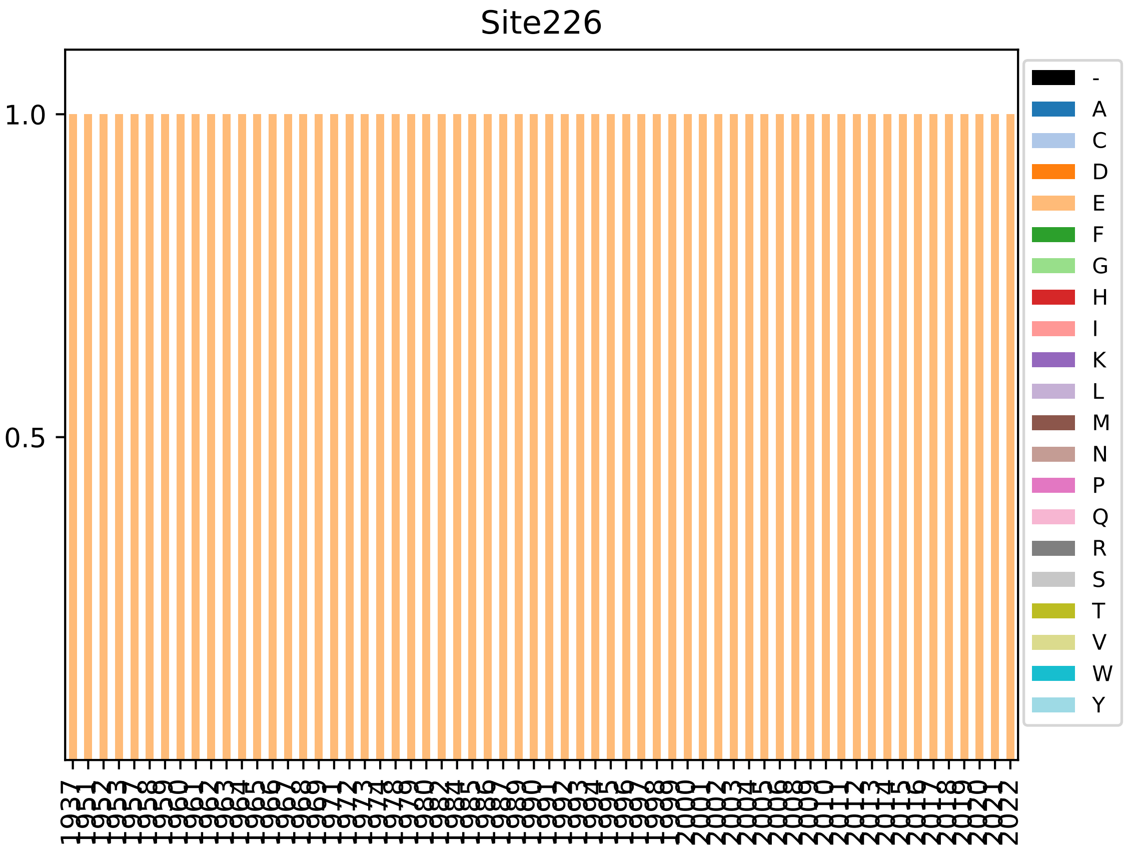
Site226
1.0
0.5
-
A
C
D
E
F
G
H
I
K
L
M
N
P
Q
R
S
T
V
W
Y

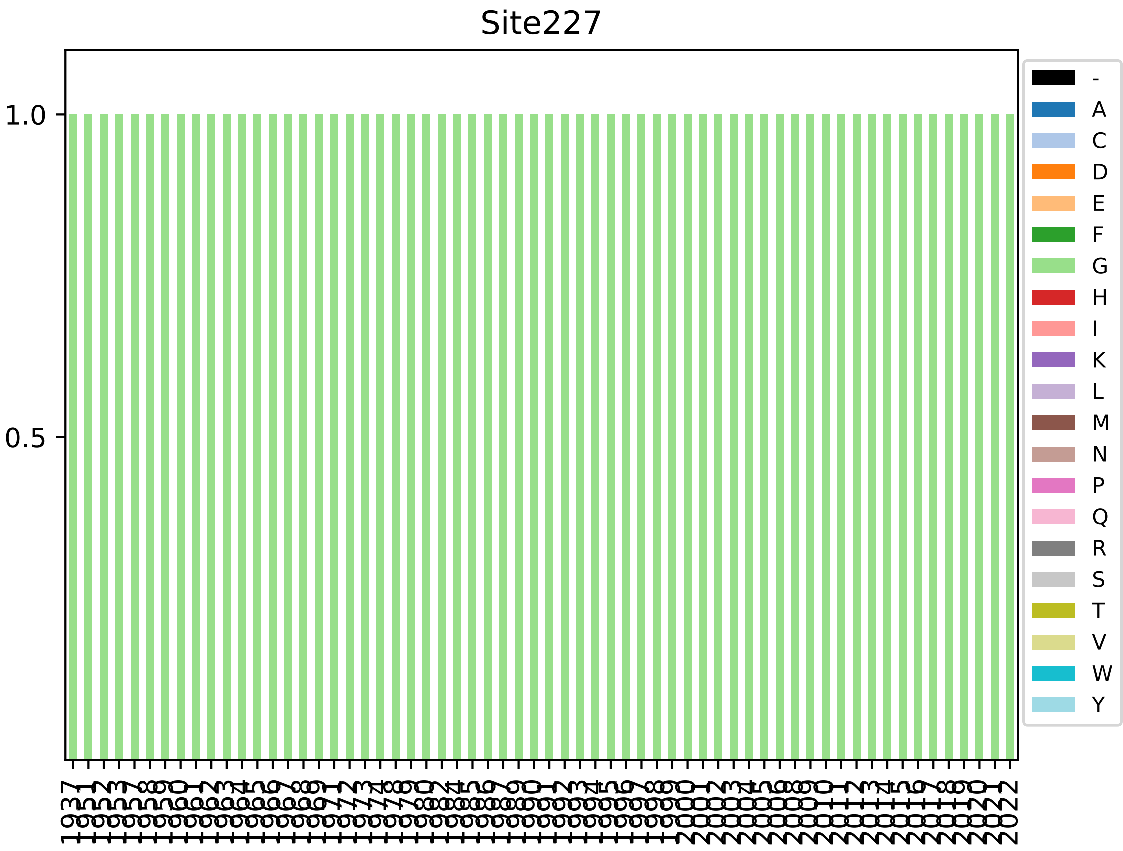
Site227
1.0
0.5
-
A
C
D
E
F
G
H
I
K
L
M
N
P
Q
R
S
T
V
W
Y

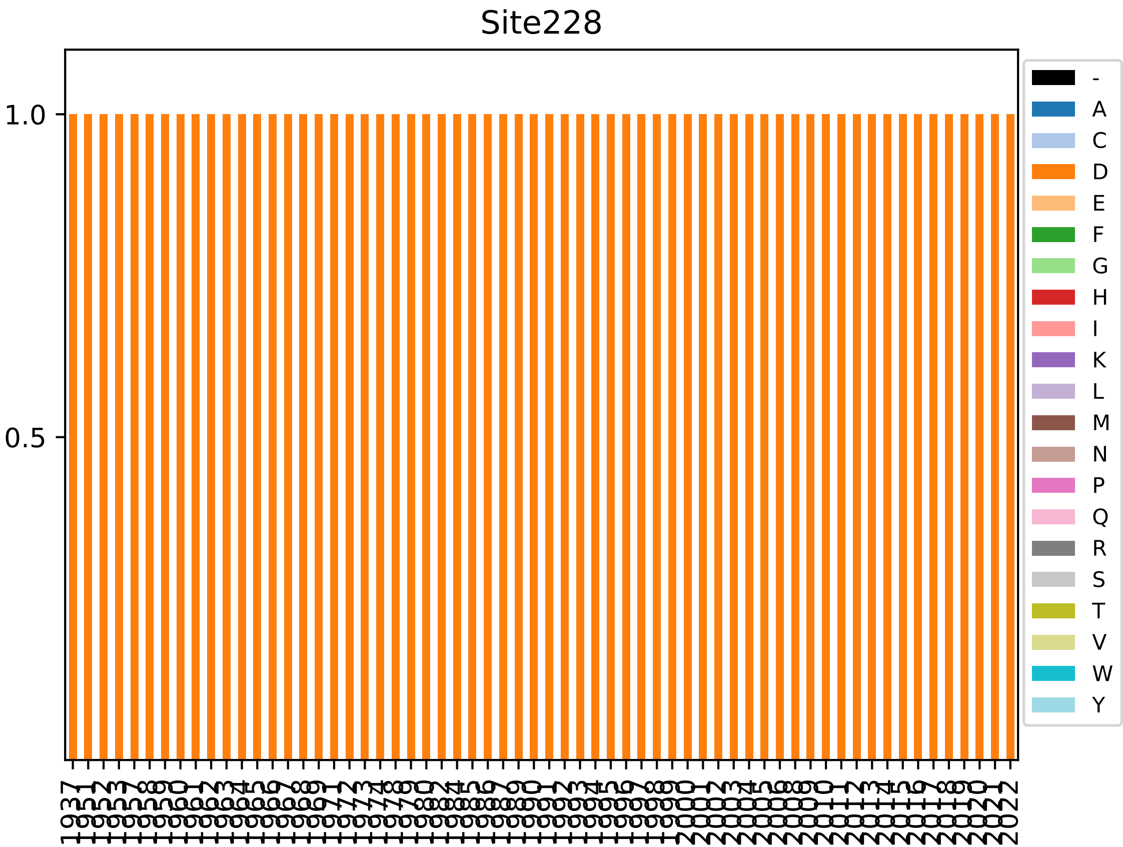
Site228
1.0
0.5
-
A
C
D
E
F
G
H
I
K
L
M
N
P
Q
R
S
T
V
W
Y

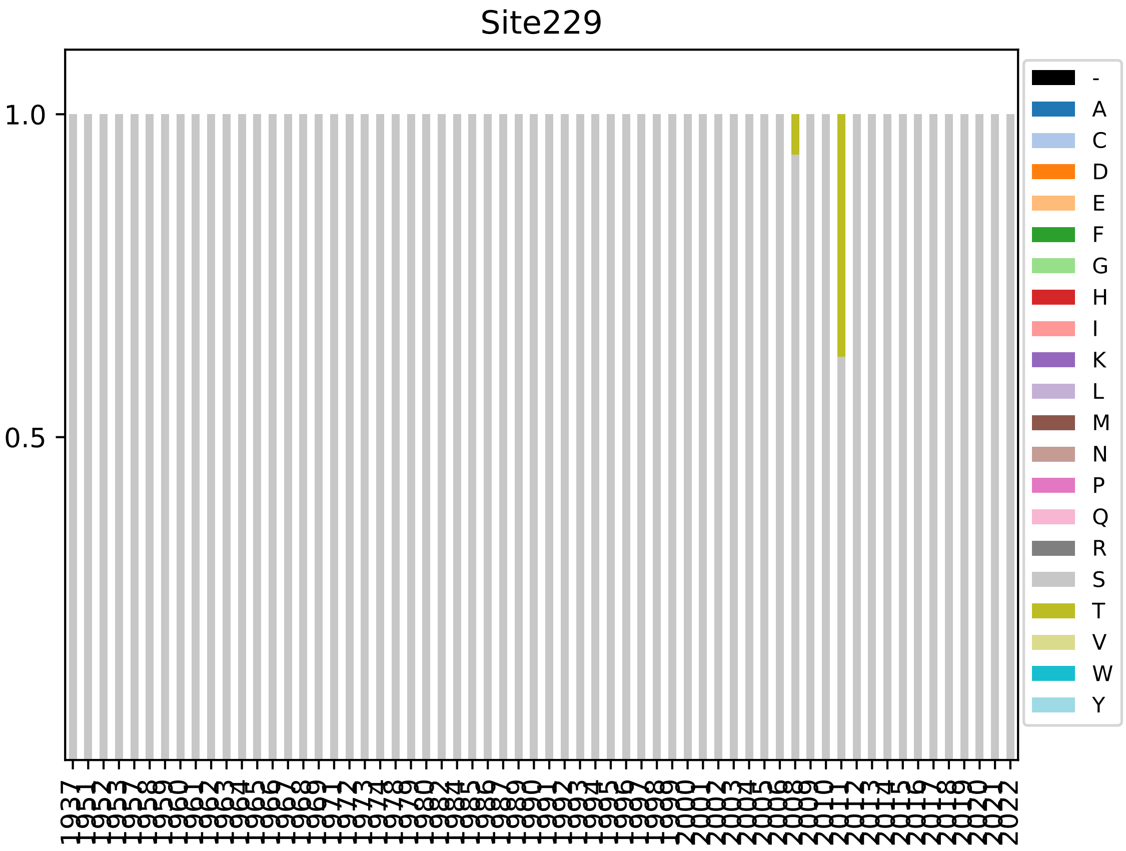
Site229
1.0
0.5
-
A
C
D
E
F
G
H
I
K
L
M
N
P
Q
R
S
T
V
W
Y

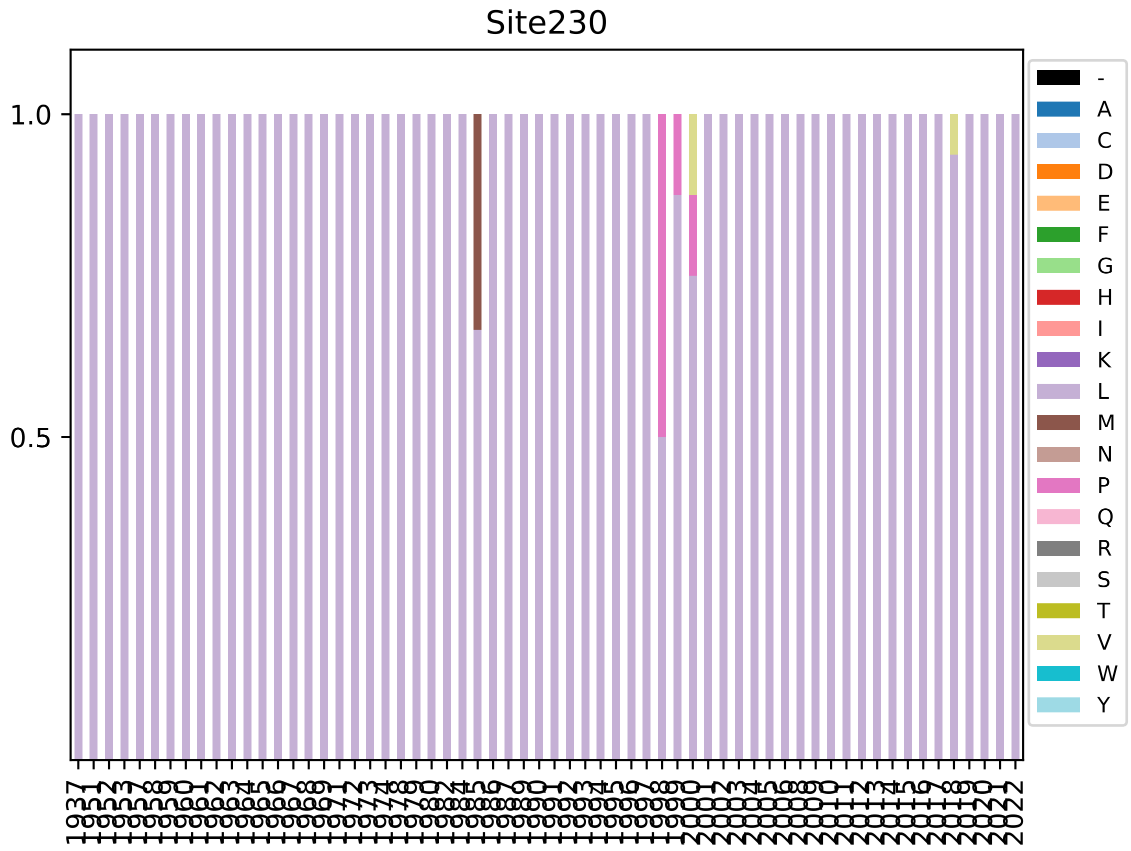

Site230
1.0
0.5
-
A
C
D
E
F
G
H
I
K
L
M
N
P
Q
R
S
T
V
W
Y

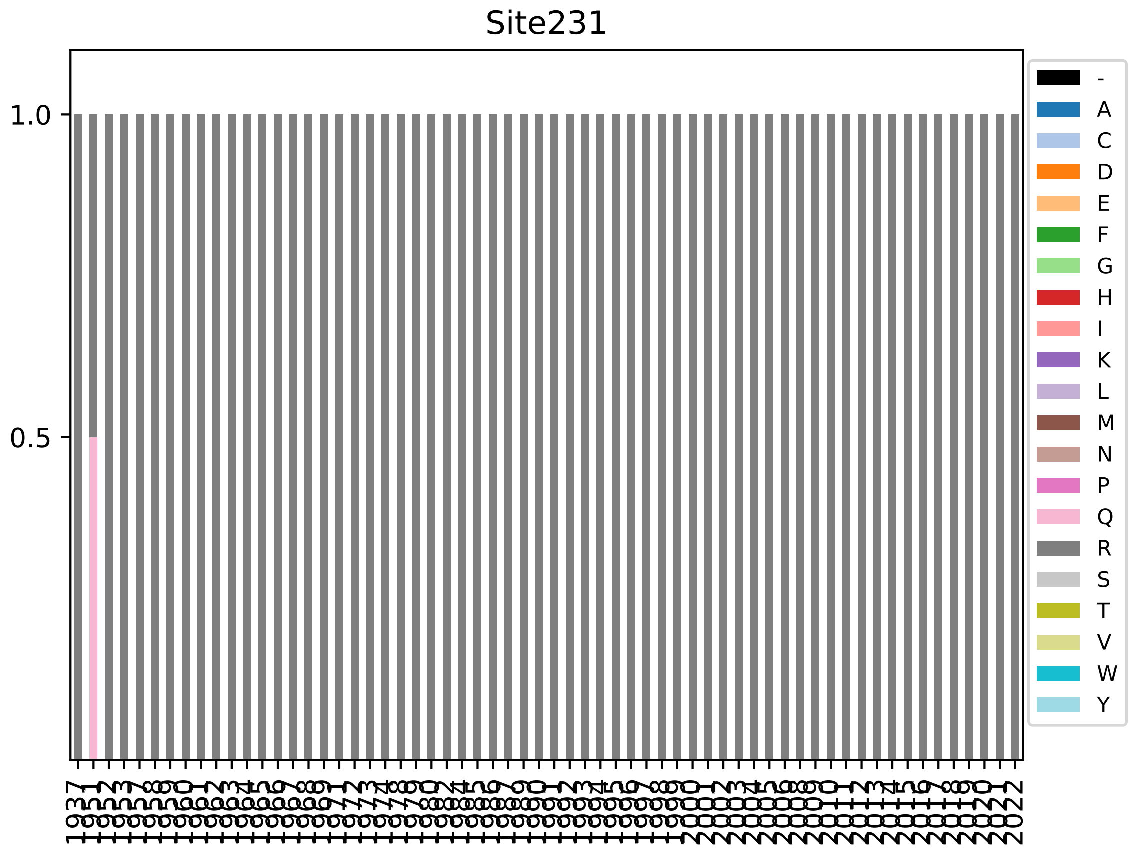
Site231
1.0
0.5
-
A
C
D
E
F
G
H
I
K
L
M
N
P
Q
R
S
T
V
W
Y

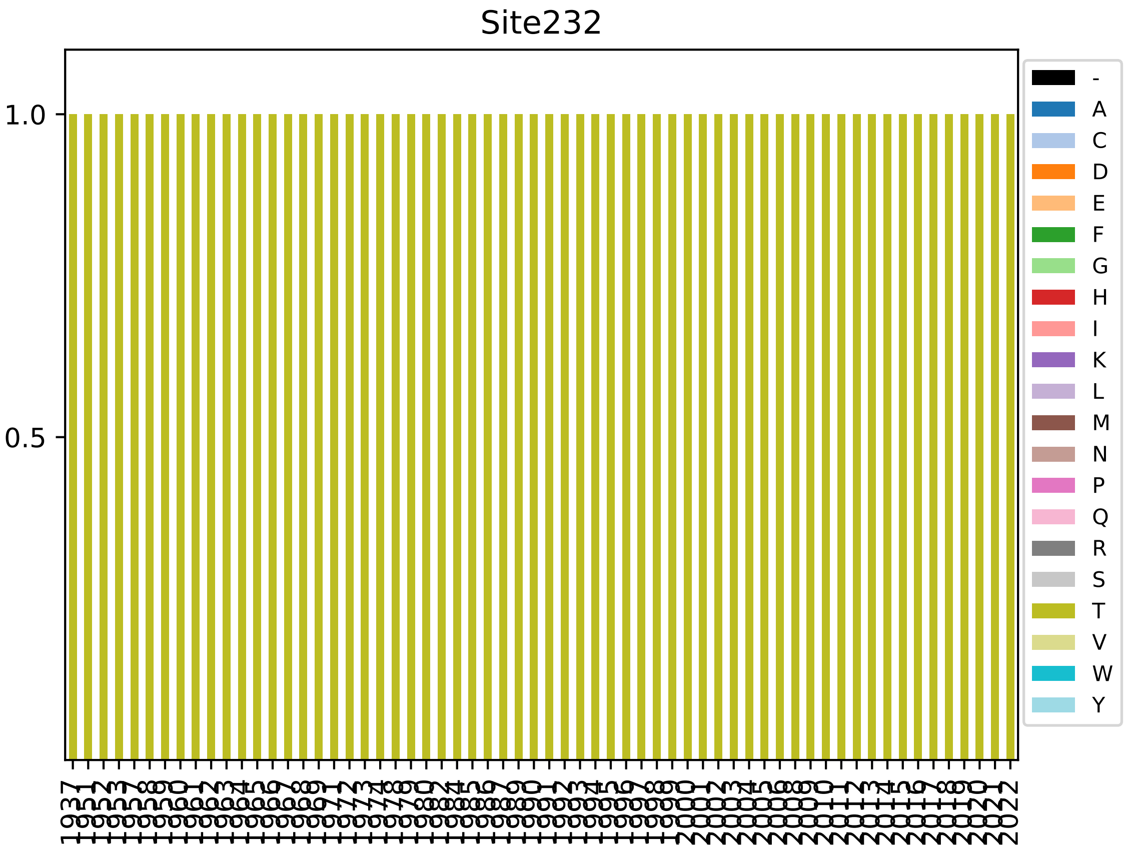
Site232
1.0
0.5
-
A
C
D
E
F
G
H
I
K
L
M
N
P
Q
R
S
T
V
W
Y

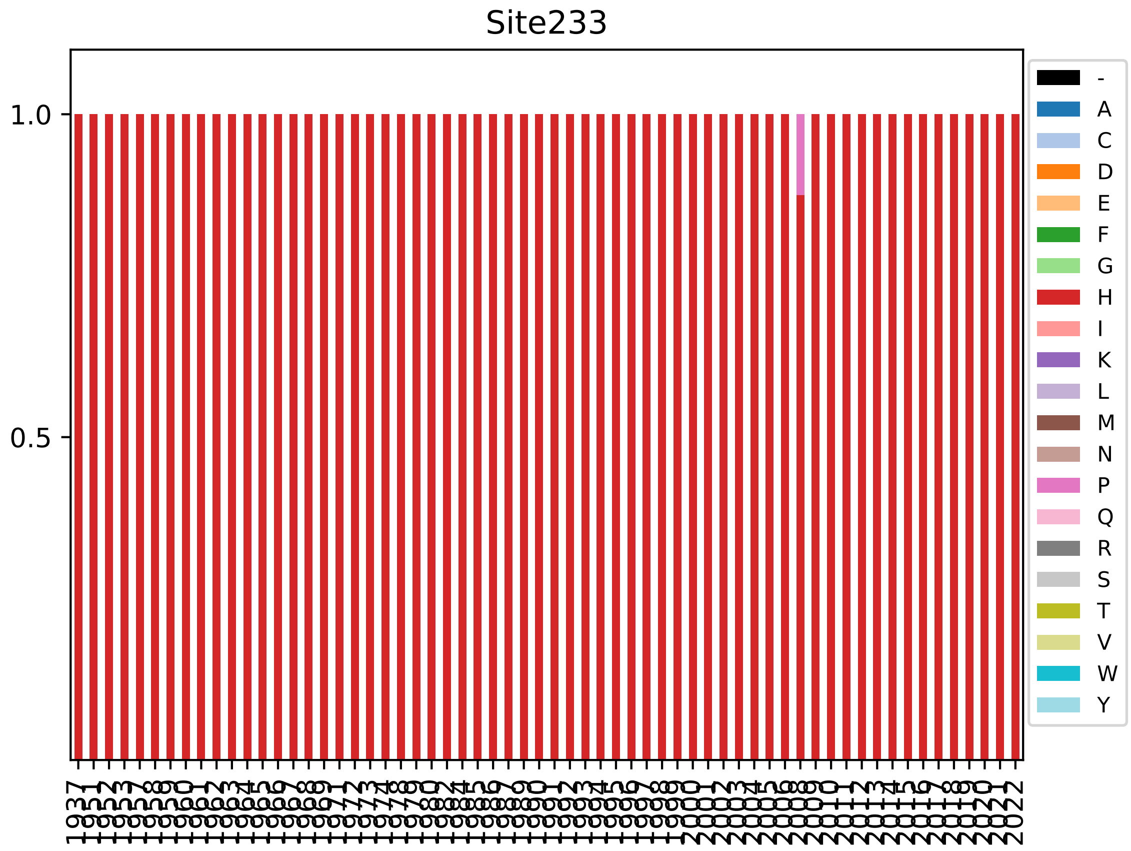
Site233
1.0
0.5
-
A
C
D
E
F
G
H
I
K
L
M
N
P
Q
R
S
T
V
W
Y

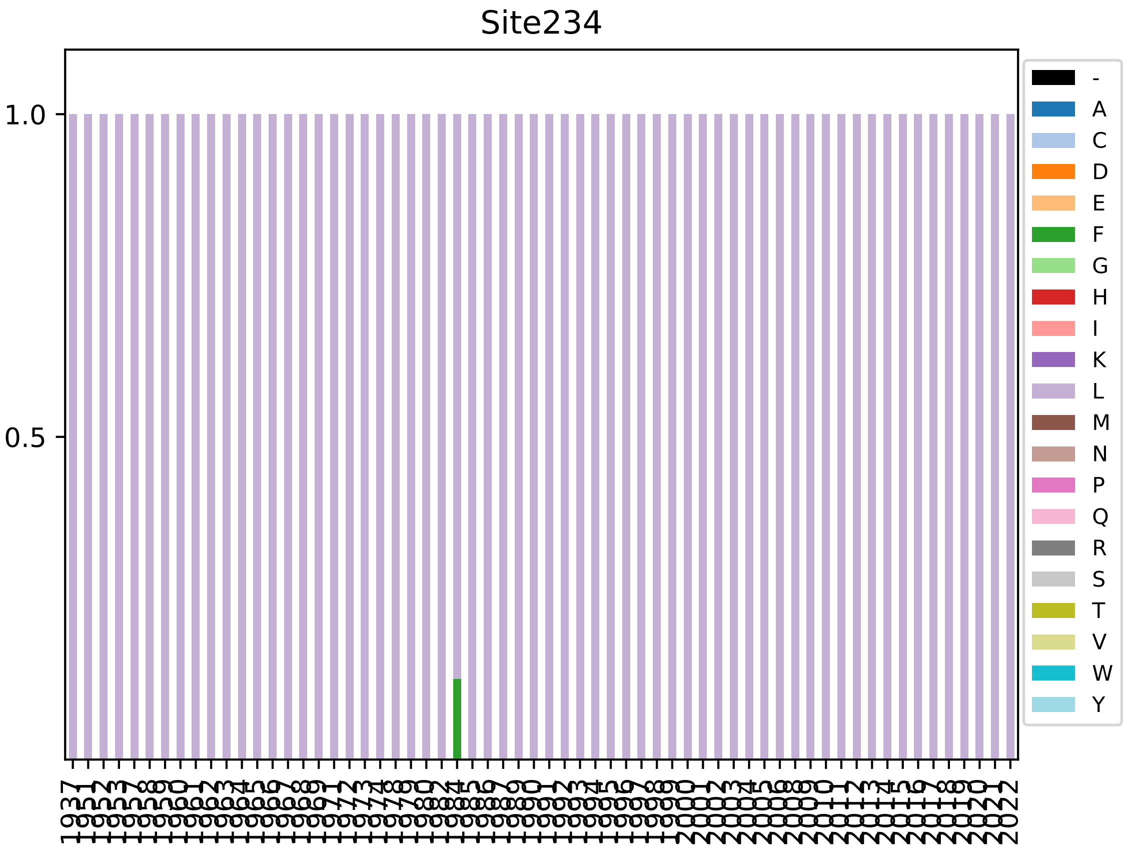
Site234
1.0
0.5
-
A
C
D
E
F
G
H
I
K
L
M
N
P
Q
R
S
T
V
W
Y

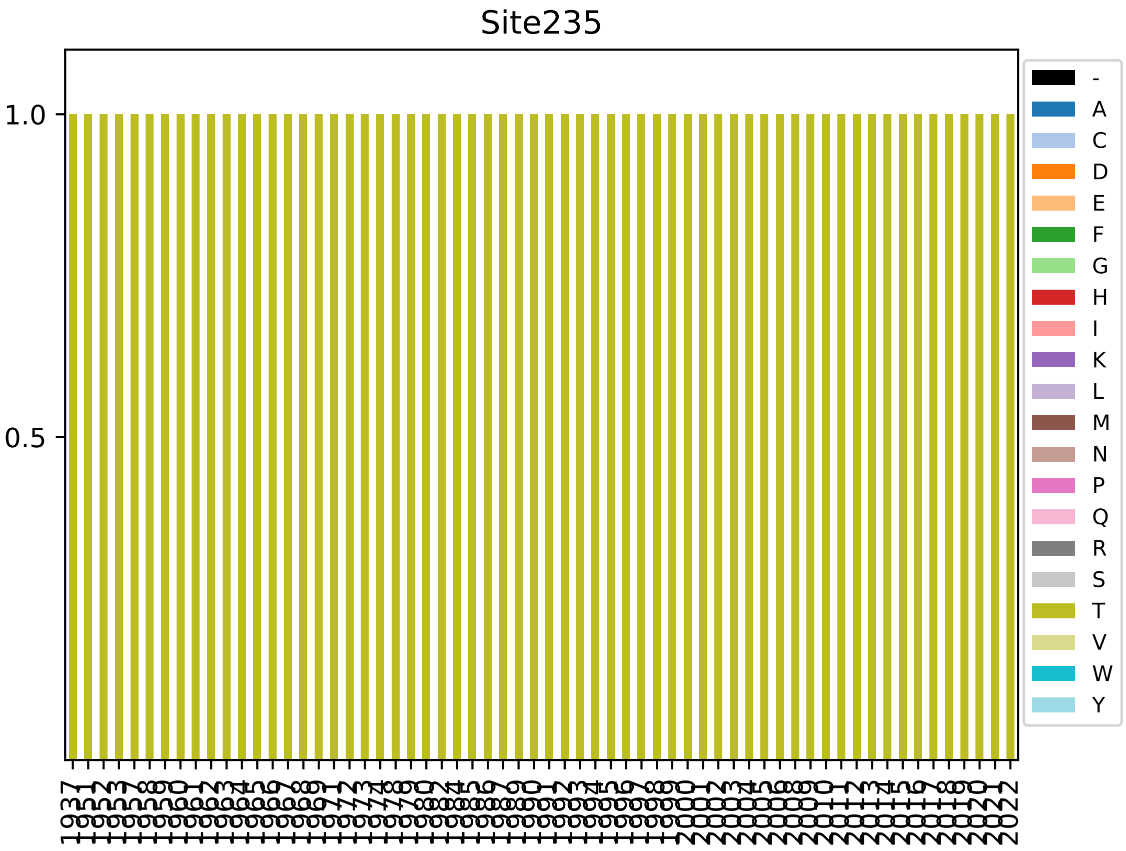
Site235
1.0
0.5
-
A
C
D
E
F
G
H
I
K
L
M
N
P
Q
R
S
T
V
W
Y

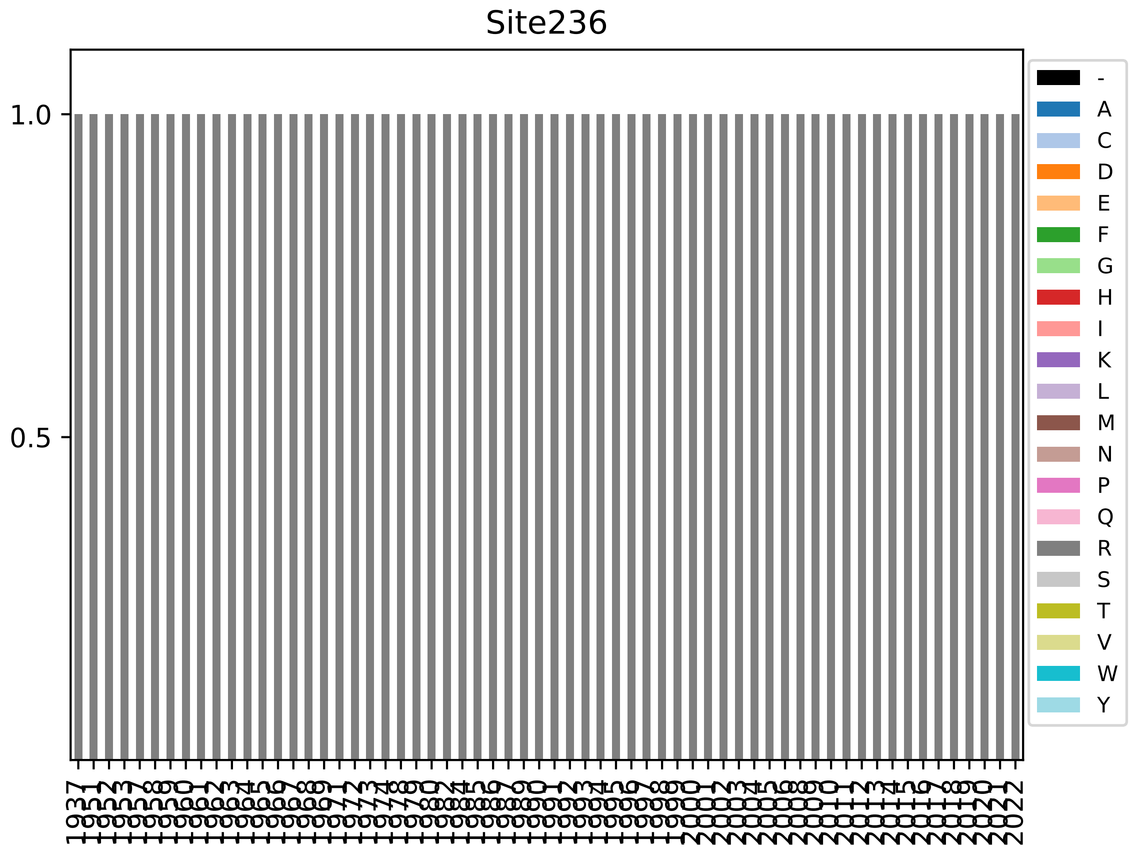
Site236
1.0
0.5
-
A
C
D
E
F
G
H
I
K
L
M
N
P
Q
R
S
T
V
W
Y

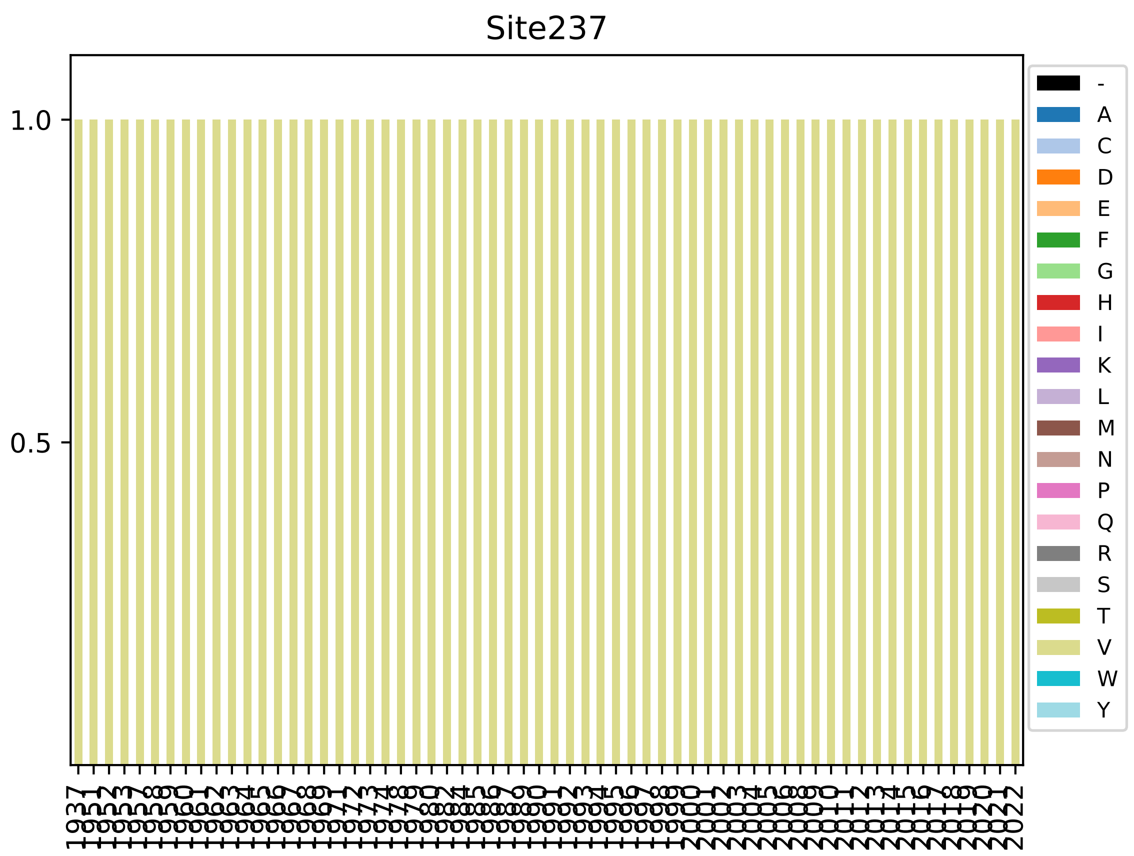
Site237
1.0
0.5
-
A
C
D
E
F
G
H
I
K
L
M
N
P
Q
R
S
T
V
W
Y

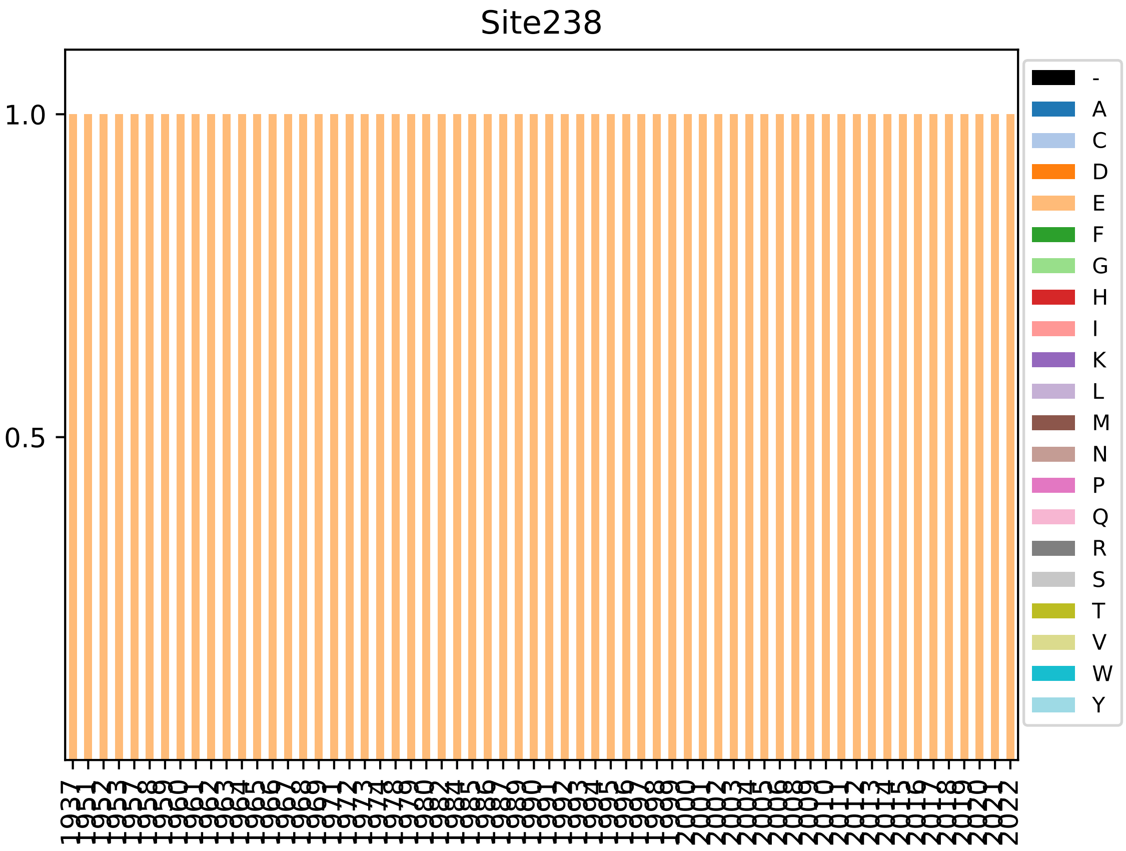

Site238
1.0
0.5
-
A
C
D
E
F
G
H
I
K
L
M
N
P
Q
R
S
T
V
W
Y

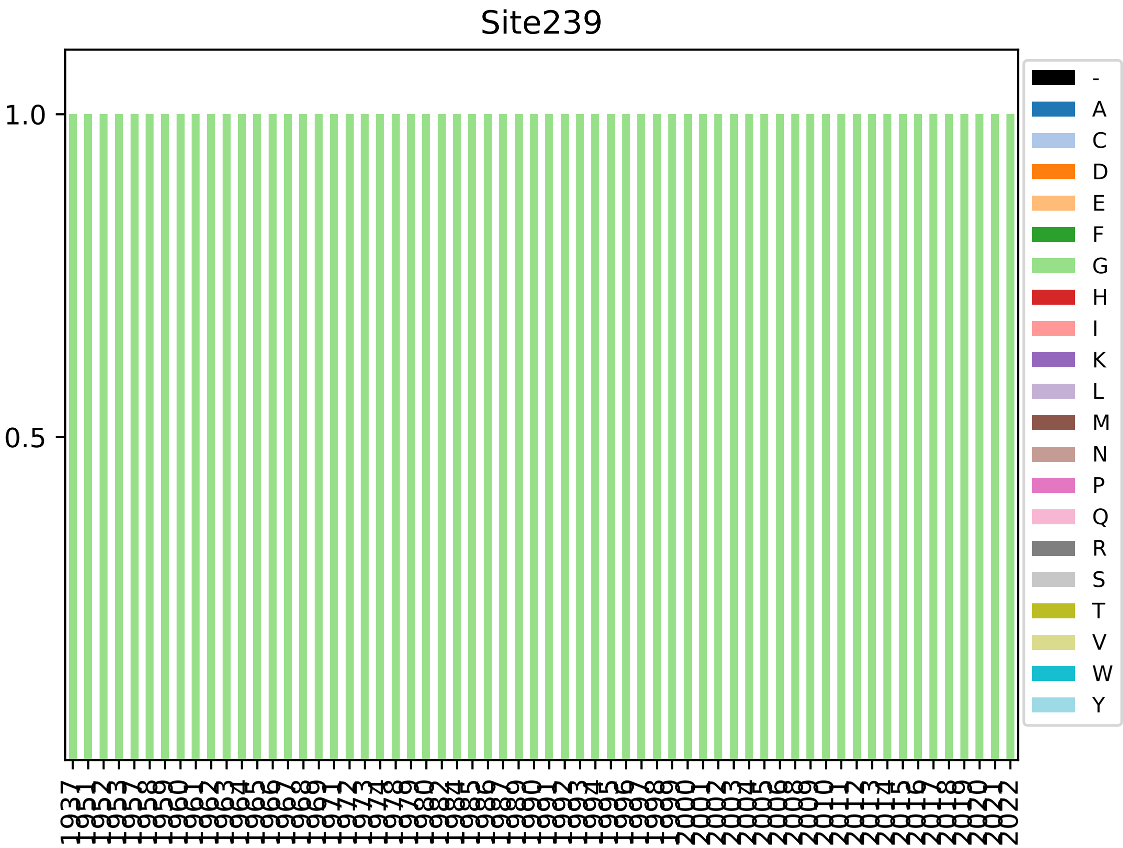
Site239
1.0
0.5
-
A
C
D
E
F
G
H
I
K
L
M
N
P
Q
R
S
T
V
W
Y

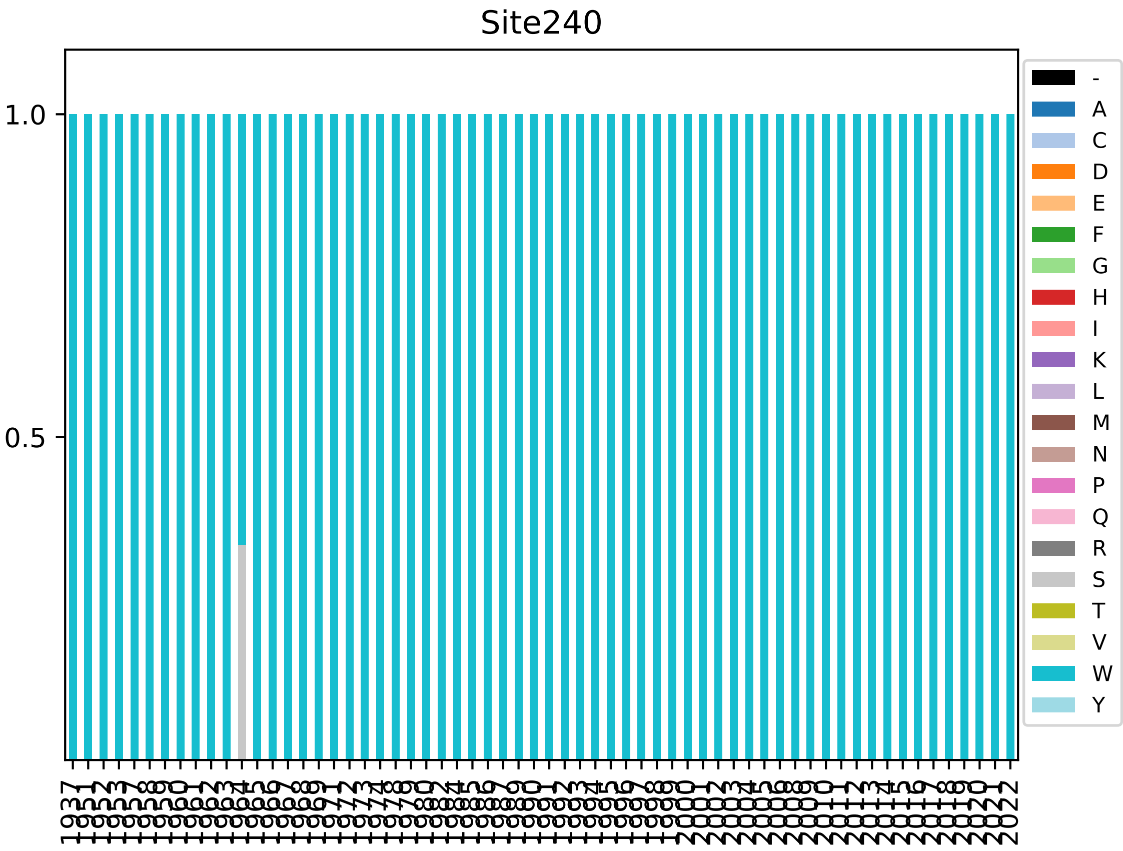
Site240
1.0
0.5
-
A
C
D
E
F
G
H
I
K
L
M
N
P
Q
R
S
T
V
W
Y

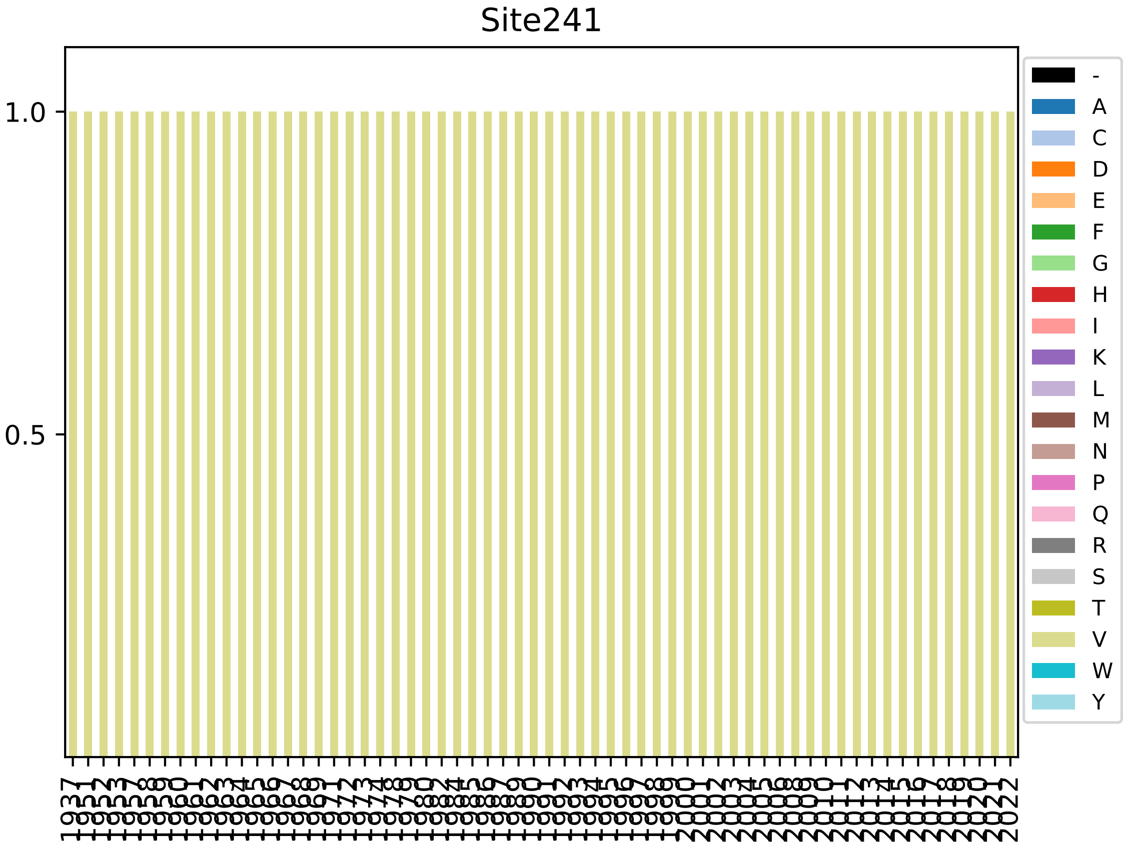
Site241
1.0
0.5
-
A
C
D
E
F
G
H
I
K
L
M
N
P
Q
R
S
T
V
W
Y

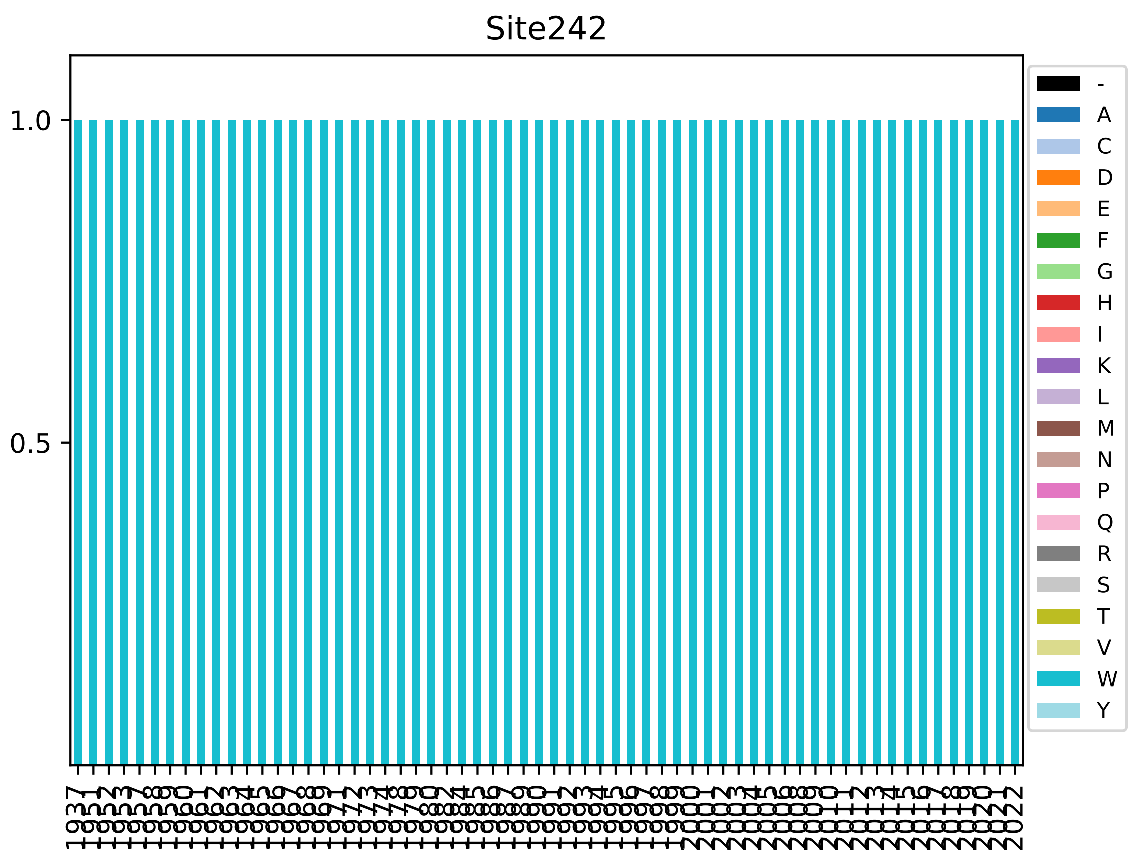
Site242
1.0
0.5
-
A
C
D
E
F
G
H
I
K
L
M
N
P
Q
R
S
T
V
W
Y

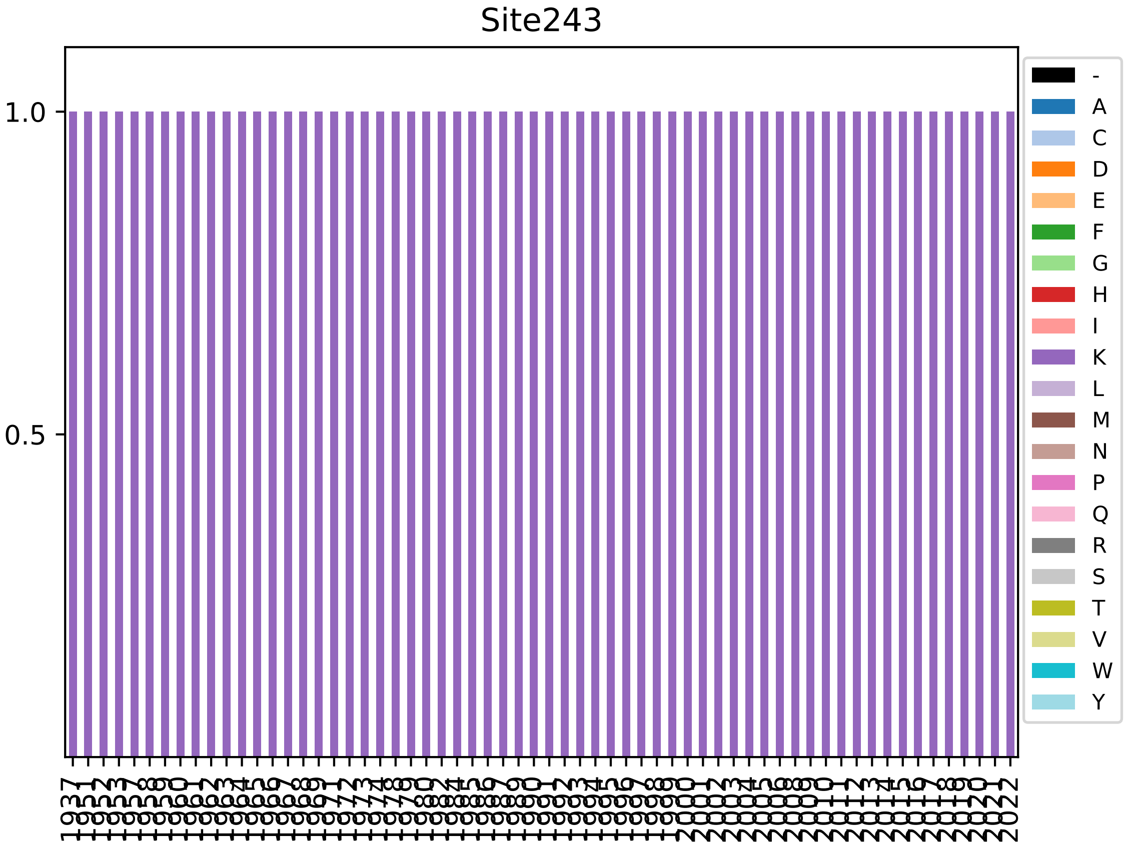
Site243
1.0
0.5
-
A
C
D
E
F
G
H
I
K
L
M
N
P
Q
R
S
T
V
W
Y

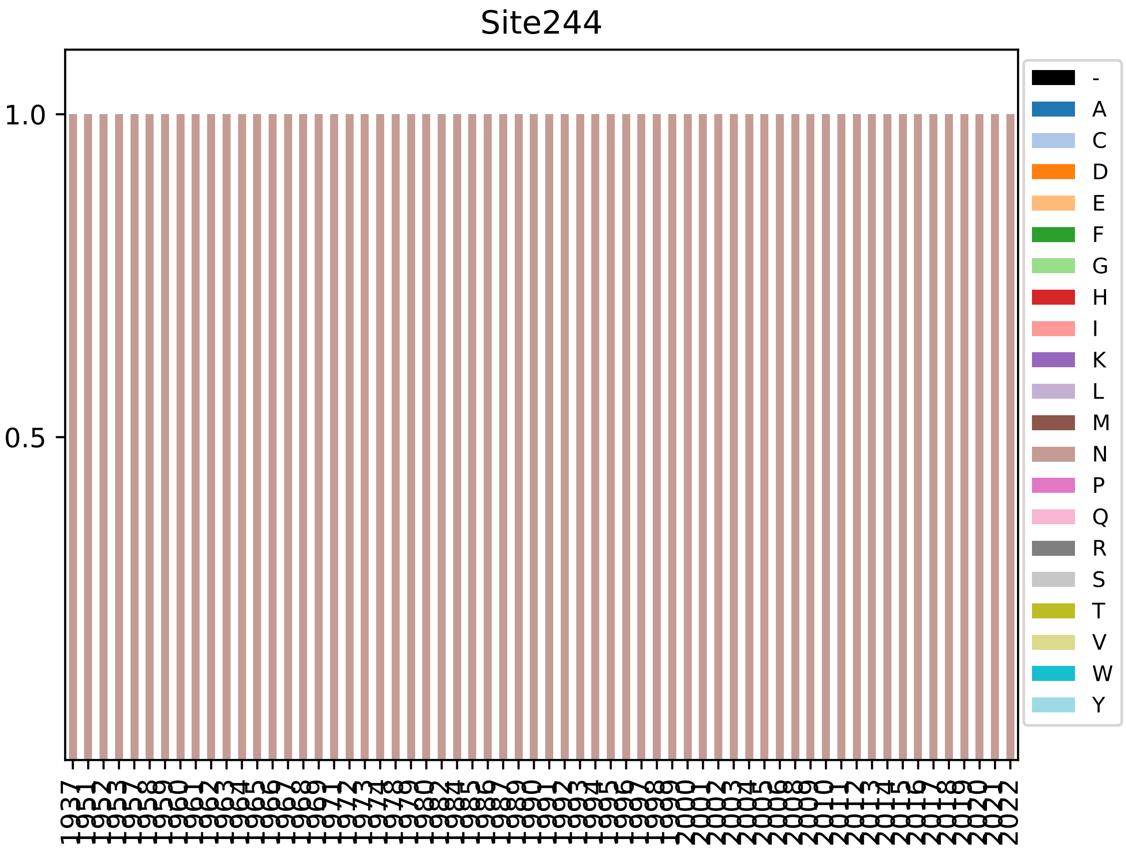
Site244
1.0
0.5
-
A
C
D
E
F
G
H
I
K
L
M
N
P
Q
R
S
T
V
W
Y

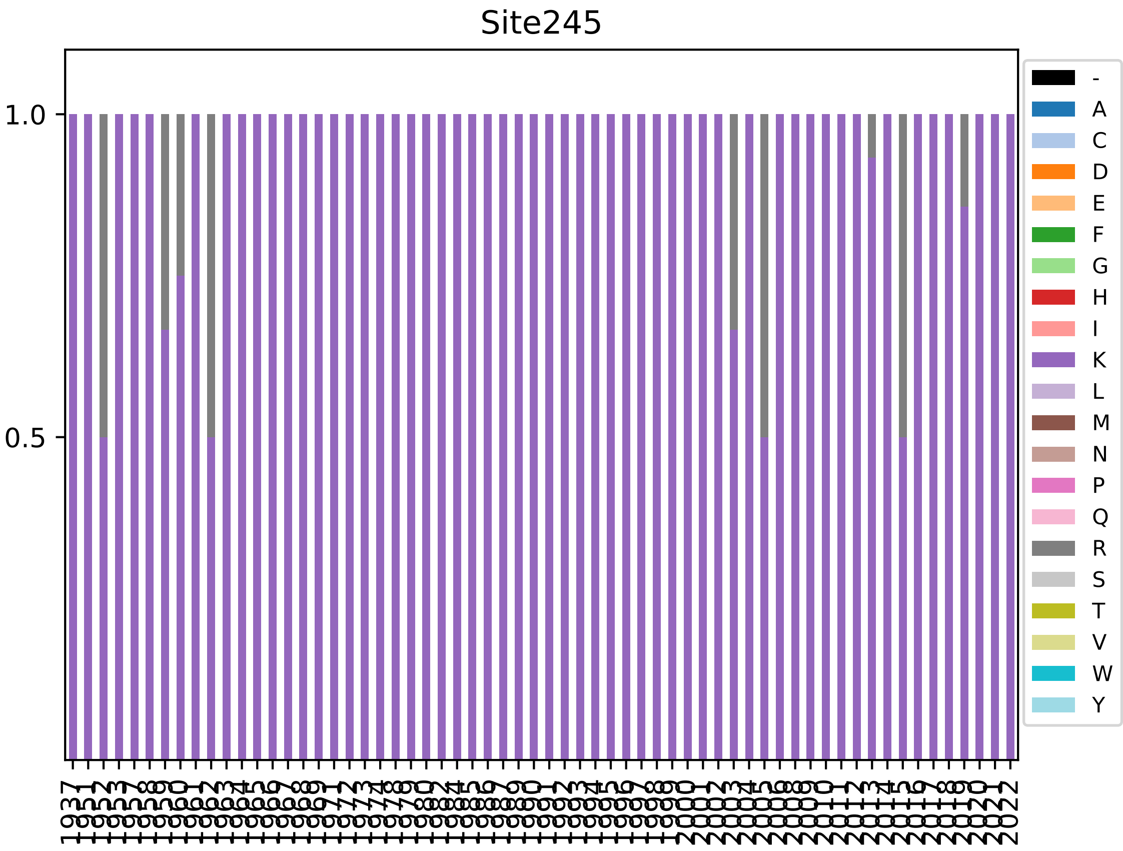

Site245
1.0
0.5
-
A
C
D
E
F
G
H
I
K
L
M
N
P
Q
R
S
T
V
W
Y

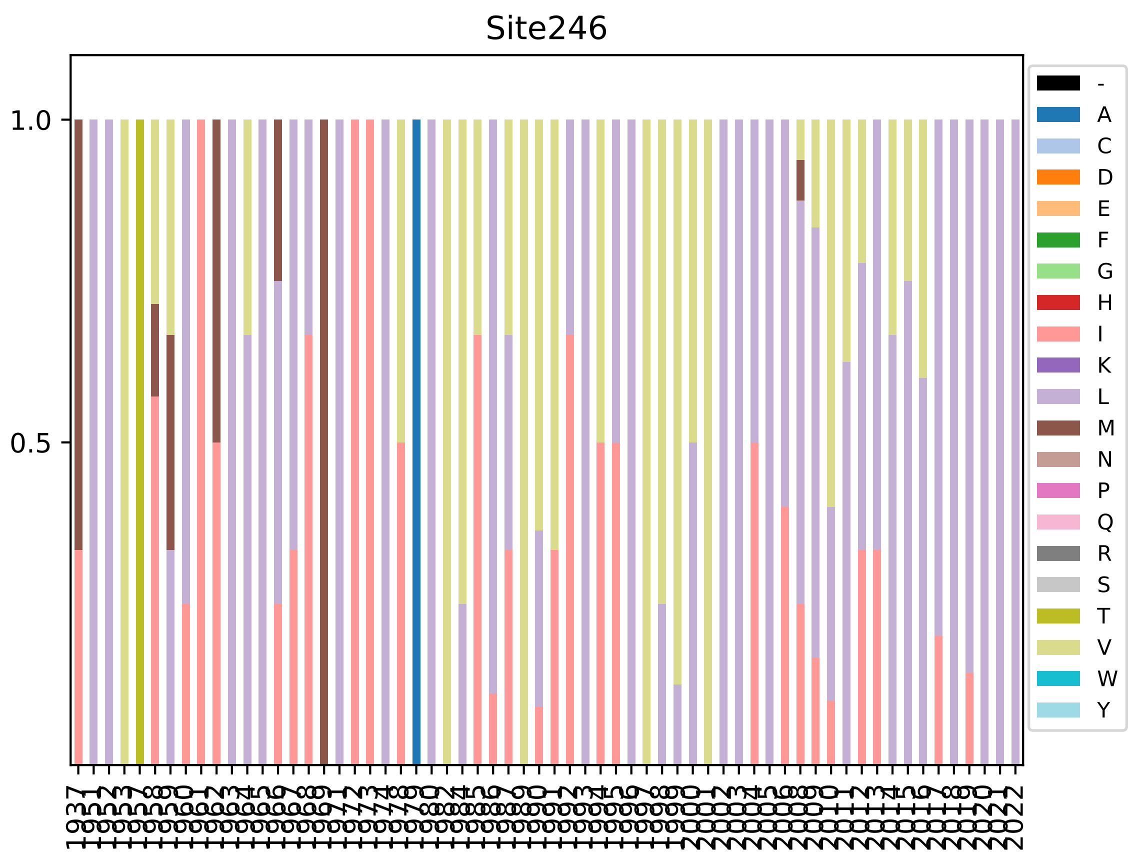
Site246
1.0
0.5
-
A
C
D
E
F
G
H
I
K
L
M
N
P
Q
R
S
T
V
W
Y

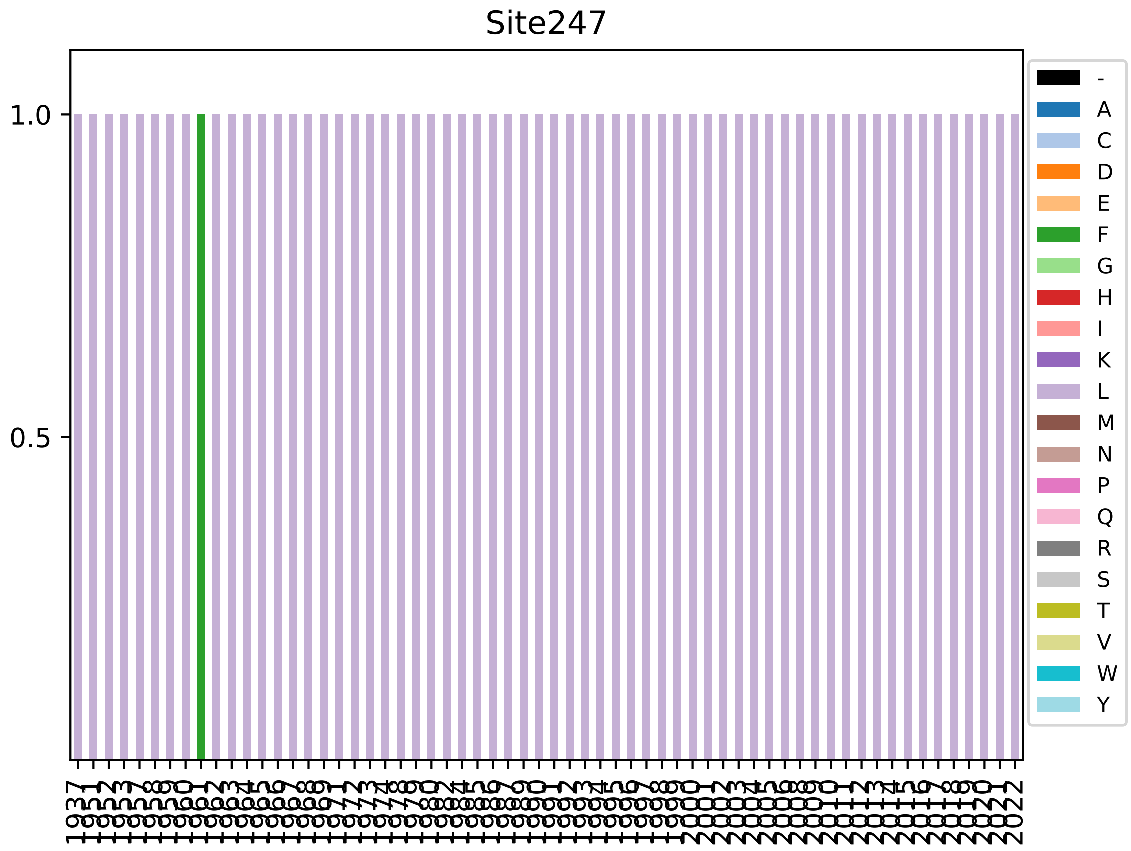
Site247
1.0
0.5
-
A
C
D
E
F
G
H
I
K
L
M
N
P
Q
R
S
T
V
W
Y

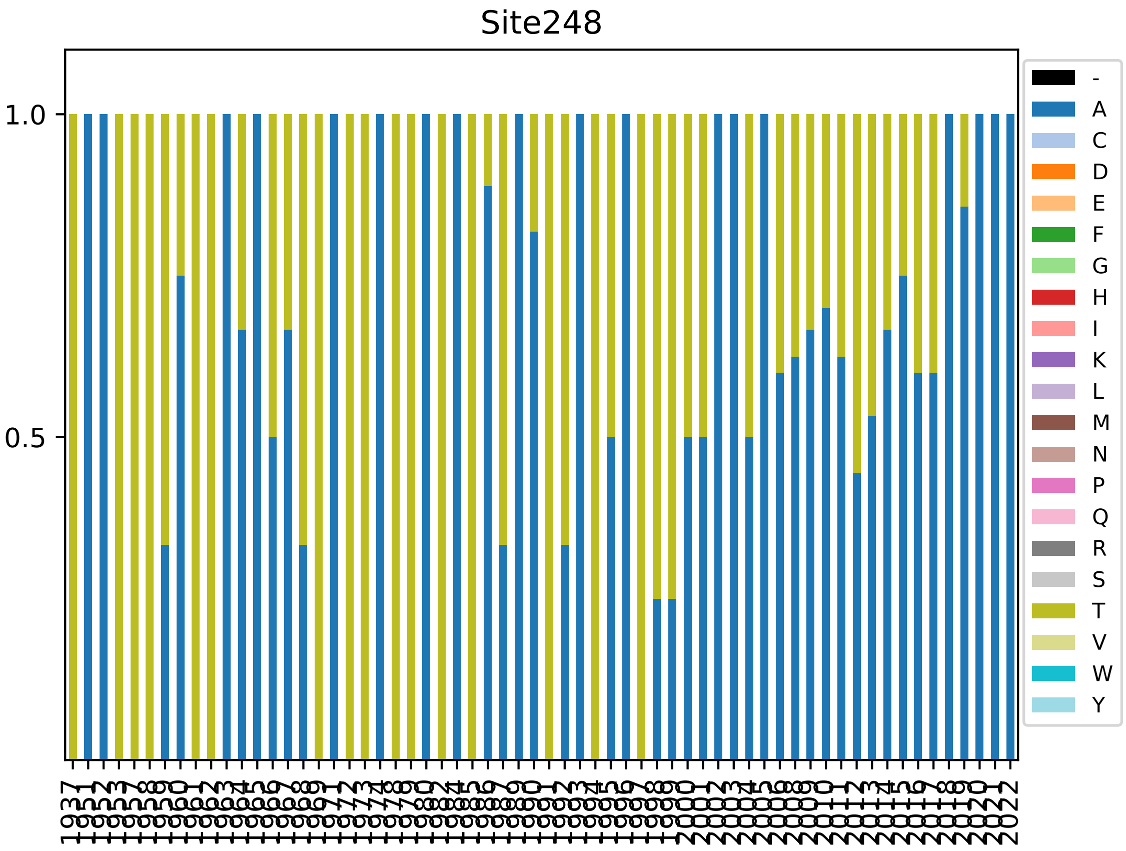
Site248
1.0
0.5
-
A
C
D
E
F
G
H
I
K
L
M
N
P
Q
R
S
T
V
W
Y

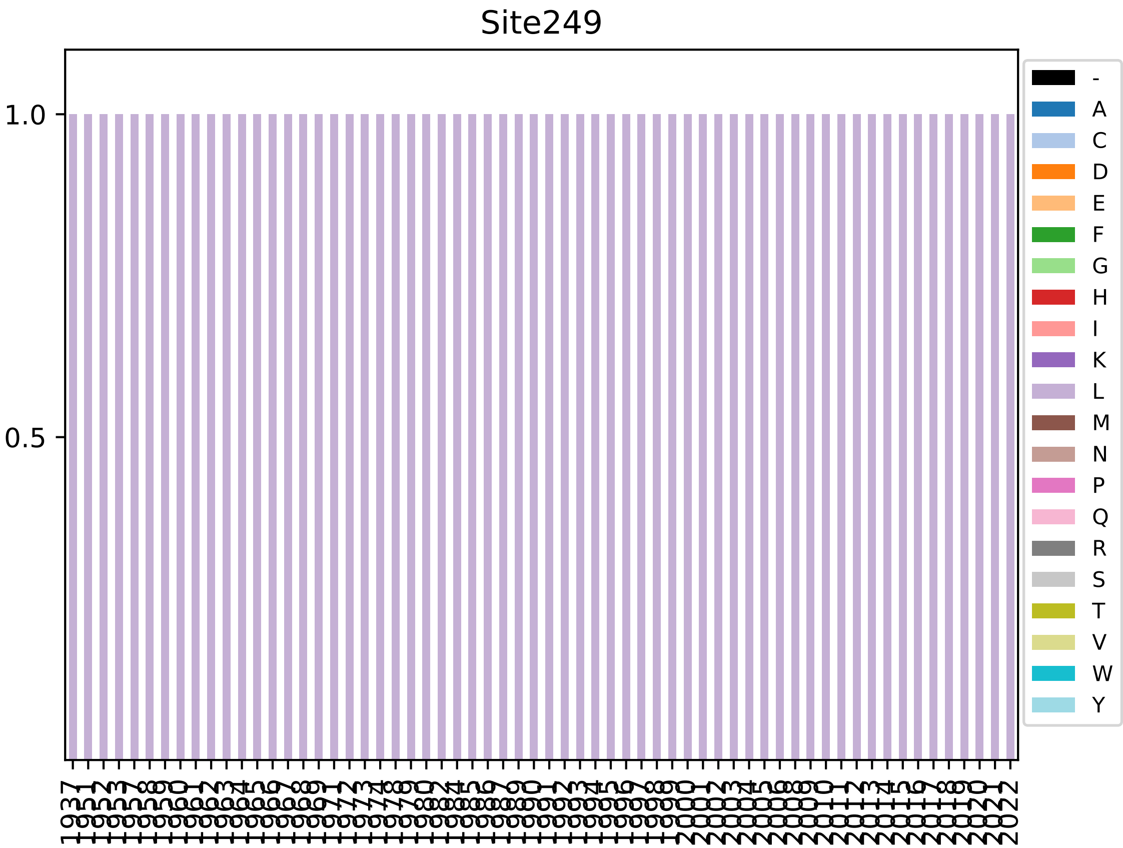
Site249
1.0
0.5
-
A
C
D
E
F
G
H
I
K
L
M
N
P
Q
R
S
T
V
W
Y

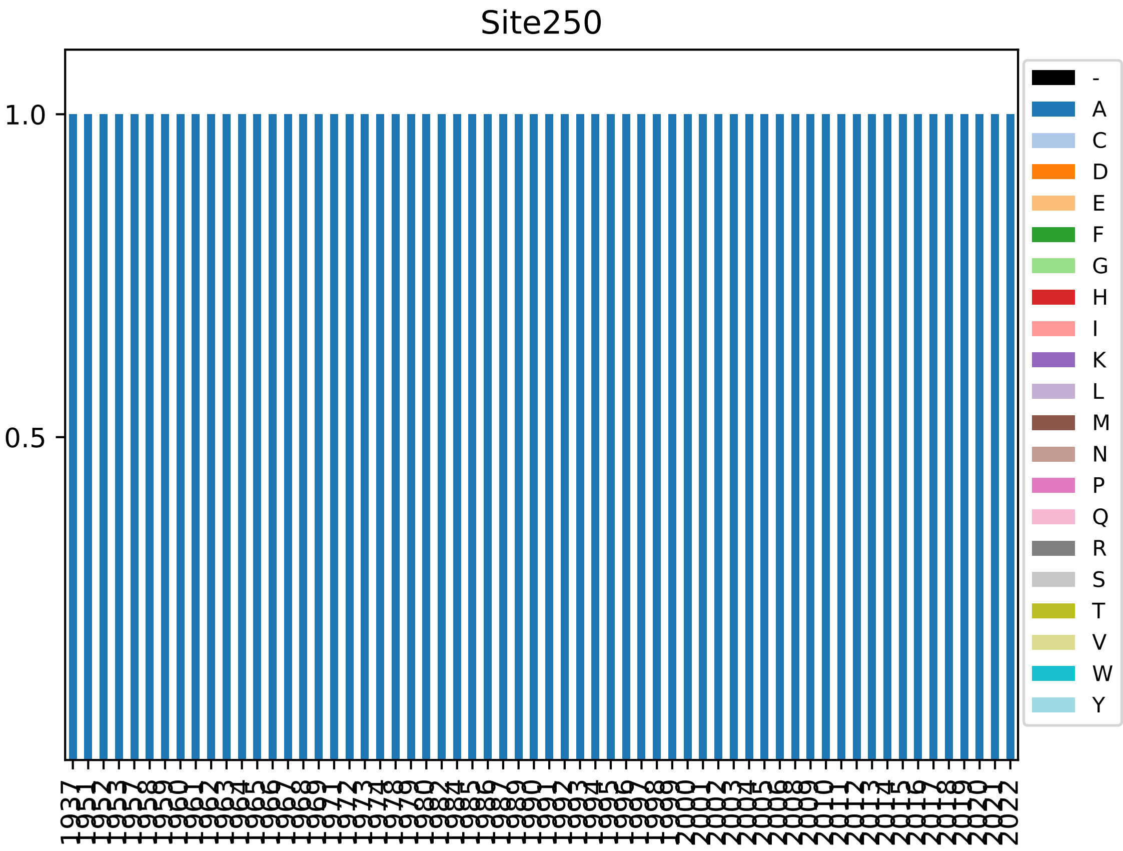
Site250
1.0
0.5
-
A
C
D
E
F
G
H
I
K
L
M
N
P
Q
R
S
T
V
W
Y

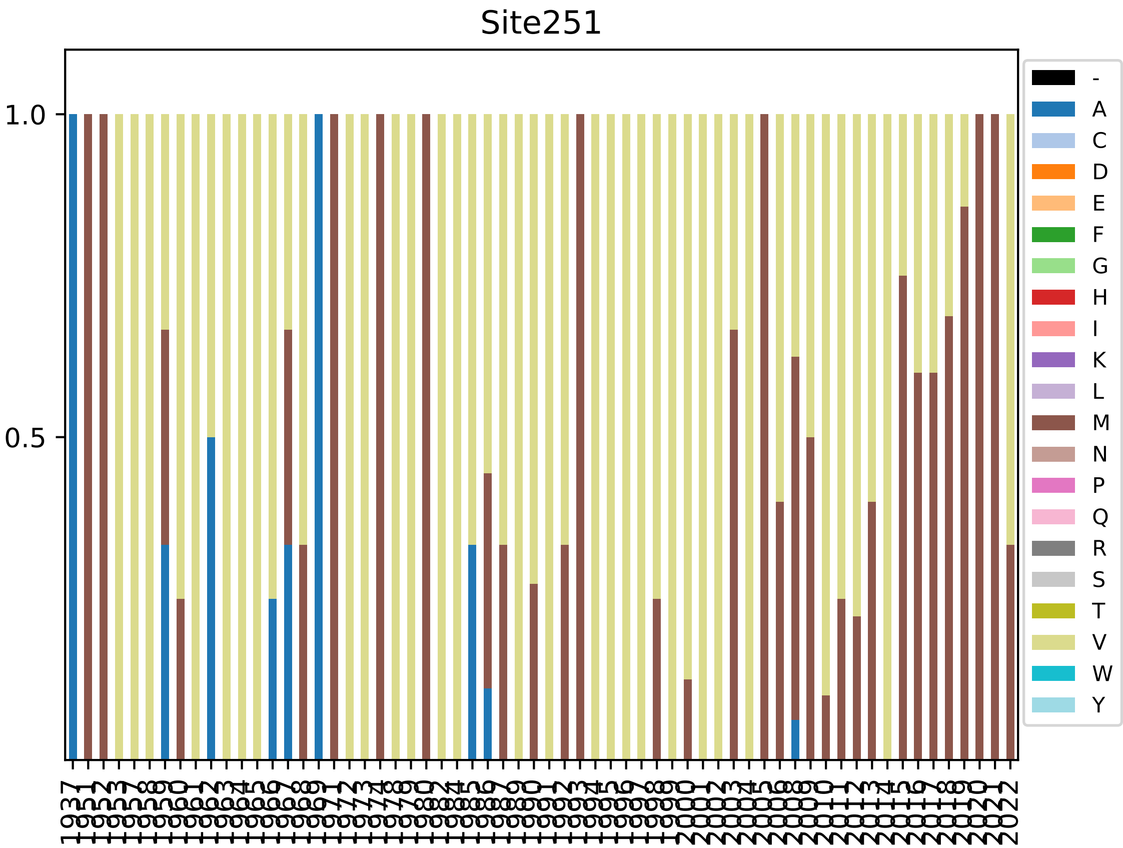
Site251
1.0
0.5
-
A
C
D
E
F
G
H
I
K
L
M
N
P
Q
R
S
T
V
W
Y

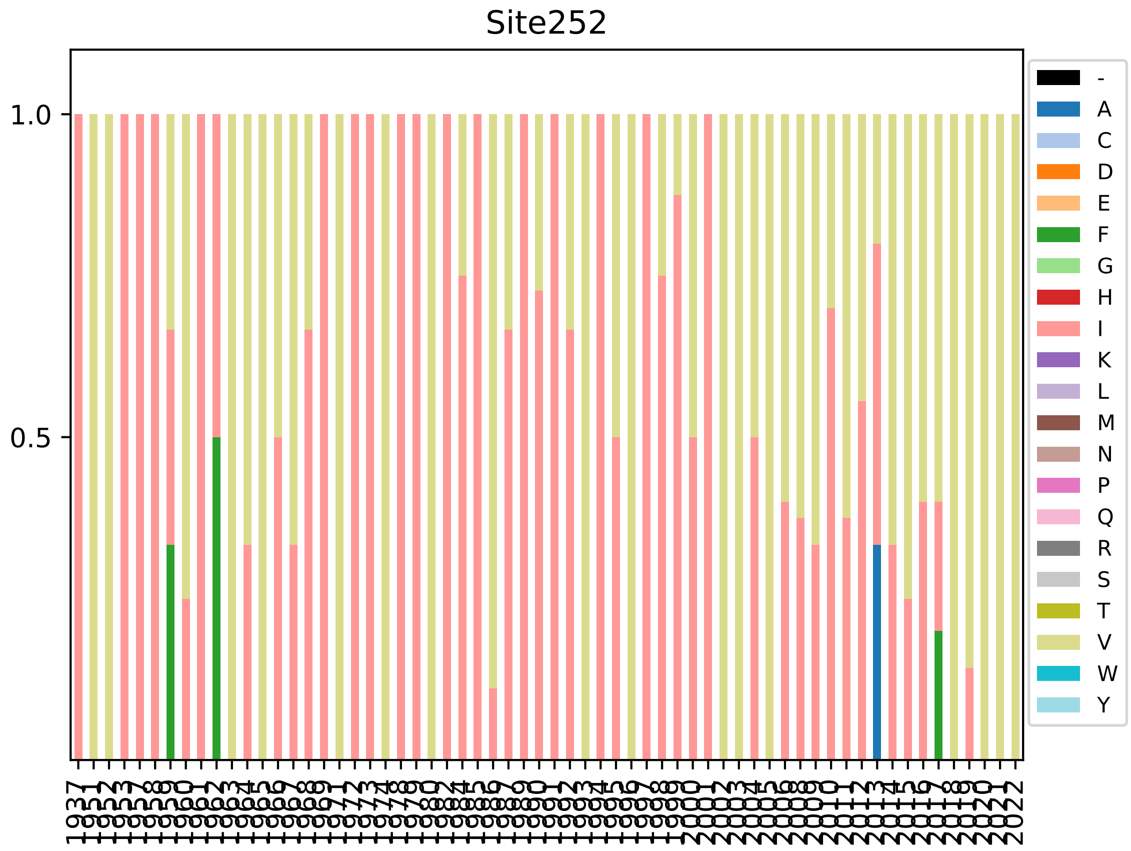
Site252
1.0
0.5
-
A
C
D
E
F
G
H
I
K
L
M
N
P
Q
R
S
T
V
W
Y

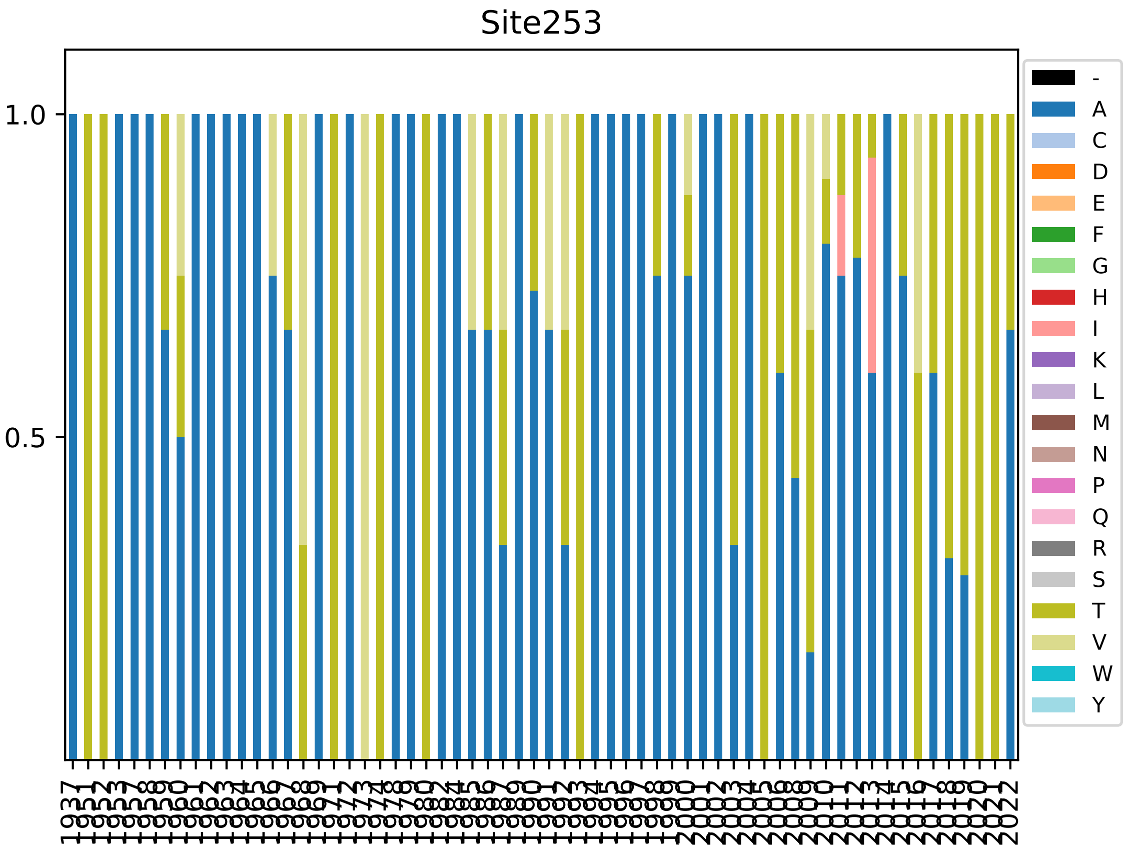
Site253
1.0
0.5
-
A
C
D
E
F
G
H
I
K
L
M
N
P
Q
R
S
T
V
W
Y

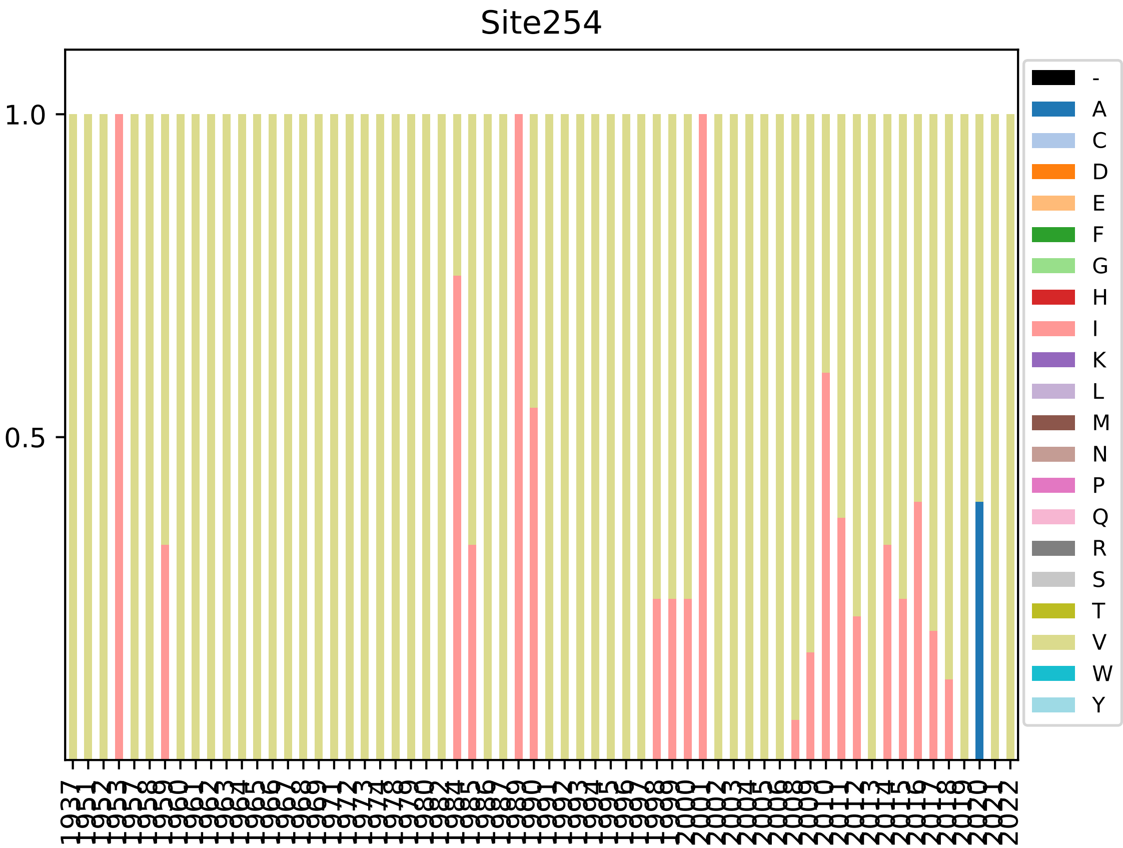
Site254
1.0
0.5
-
A
C
D
E
F
G
H
I
K
L
M
N
P
Q
R
S
T
V
W
Y

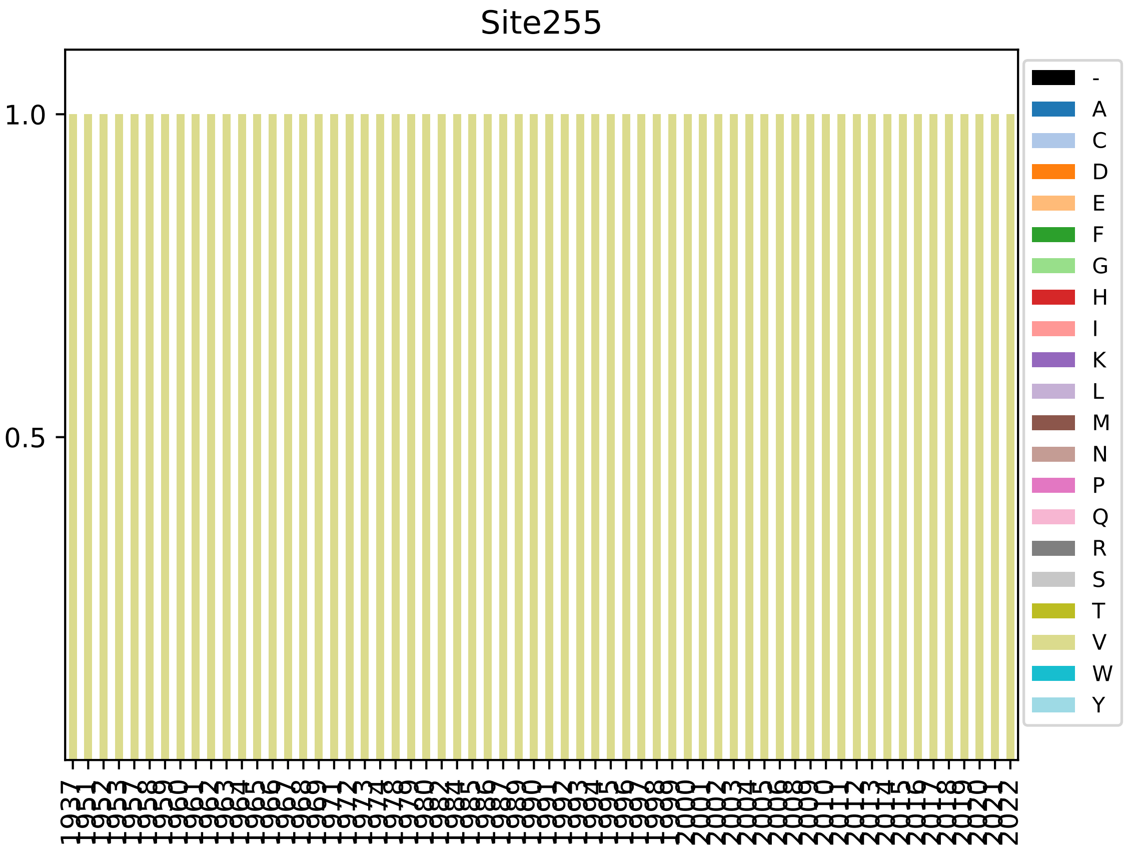
Site255
1.0
0.5
-
A
C
D
E
F
G
H
I
K
L
M
N
P
Q
R
S
T
V
W
Y

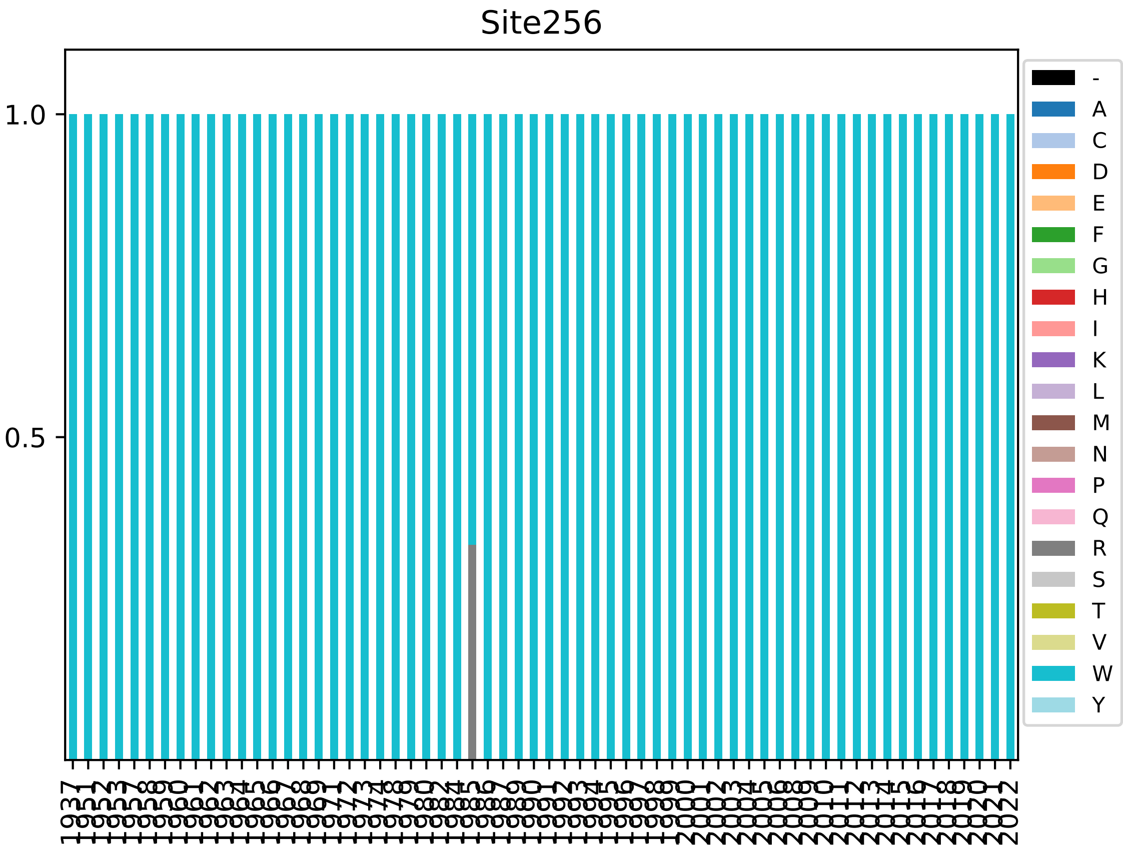
Site256
1.0
0.5
-
A
C
D
E
F
G
H
I
K
L
M
N
P
Q
R
S
T
V
W
Y

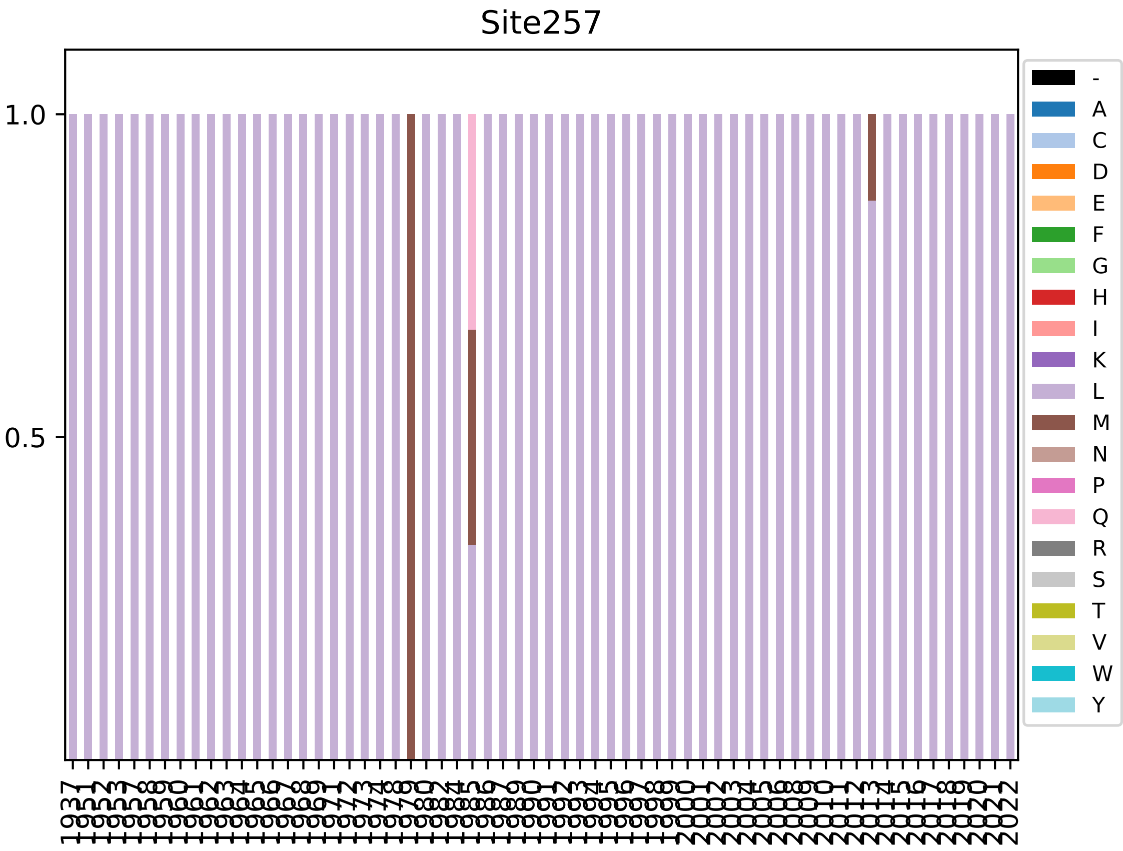
Site257
1.0
0.5
-
A
C
D
E
F
G
H
I
K
L
M
N
P
Q
R
S
T
V
W
Y

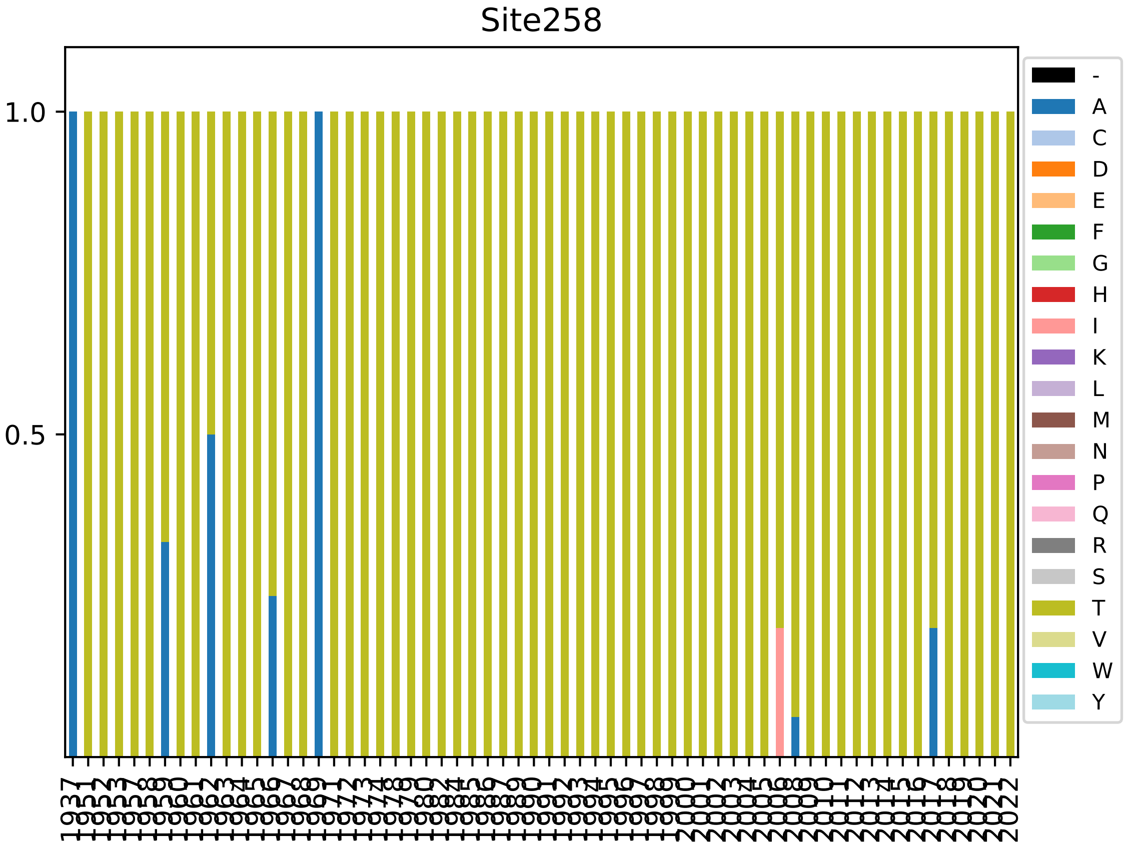
Site258
1.0
0.5
-
A
C
D
E
F
G
H
I
K
L
M
N
P
Q
R
S
T
V
W
Y

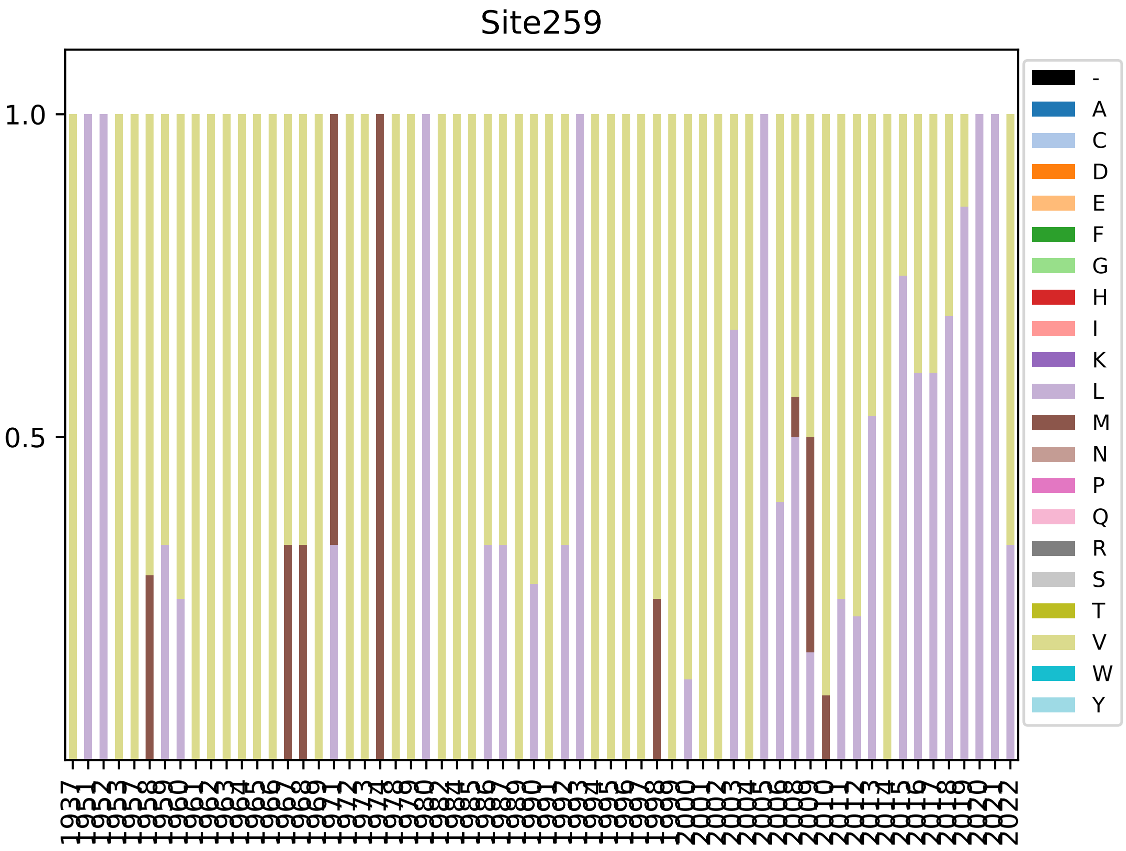
Site259
1.0
0.5
-
A
C
D
E
F
G
H
I
K
L
M
N
P
Q
R
S
T
V
W
Y

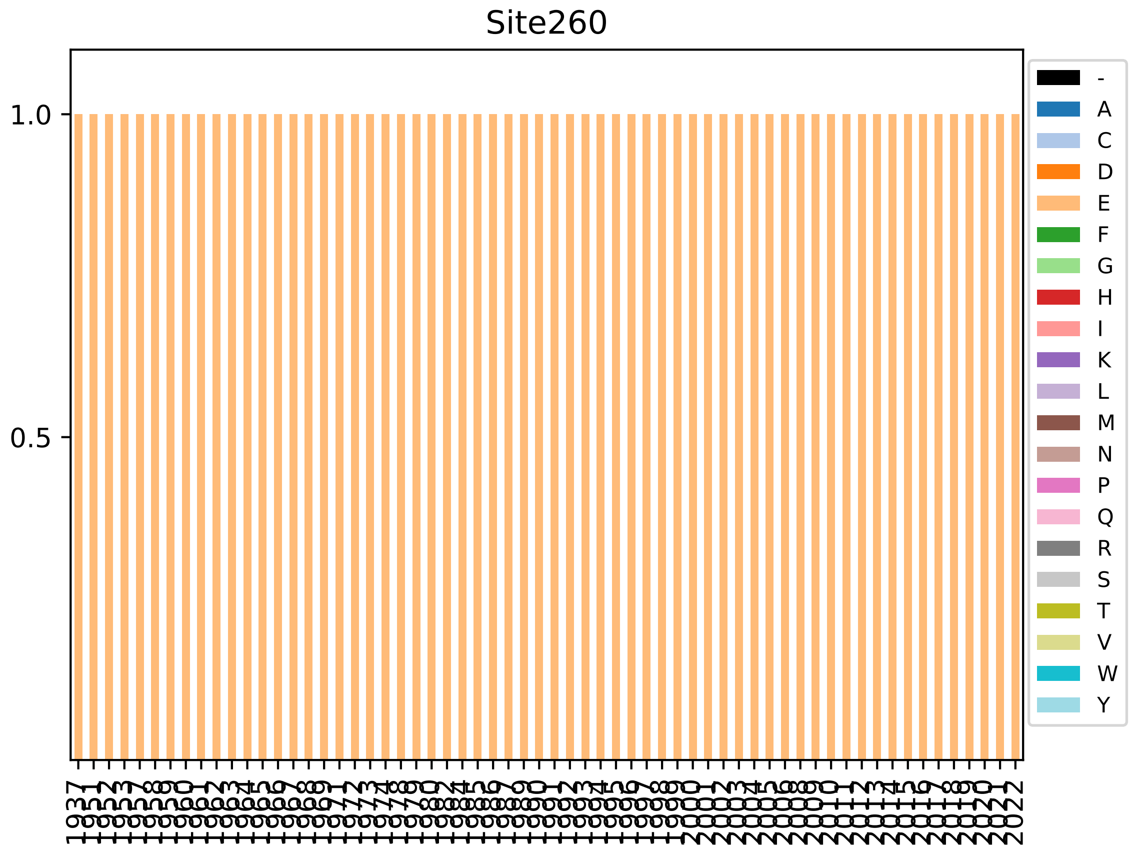
Site260
1.0
0.5
-
A
C
D
E
F
G
H
I
K
L
M
N
P
Q
R
S
T
V
W
Y

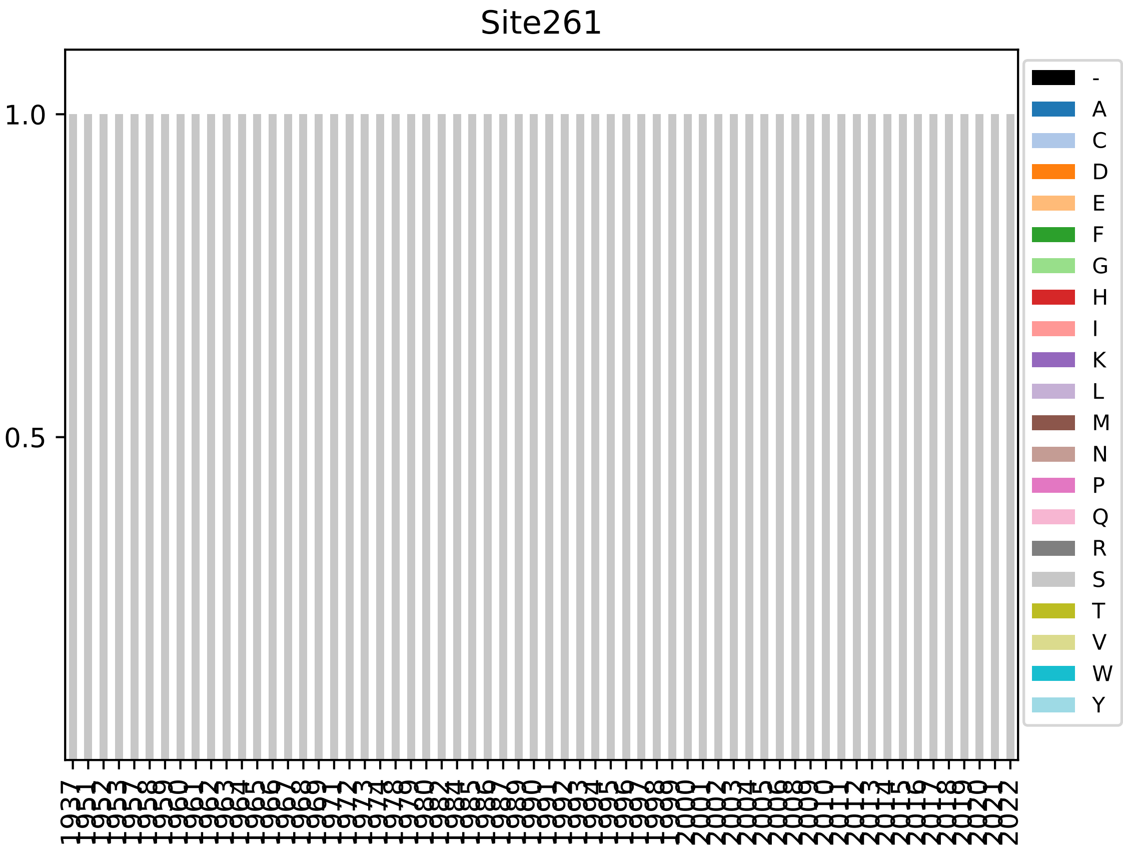
Site261
1.0
0.5
-
A
C
D
E
F
G
H
I
K
L
M
N
P
Q
R
S
T
V
W
Y

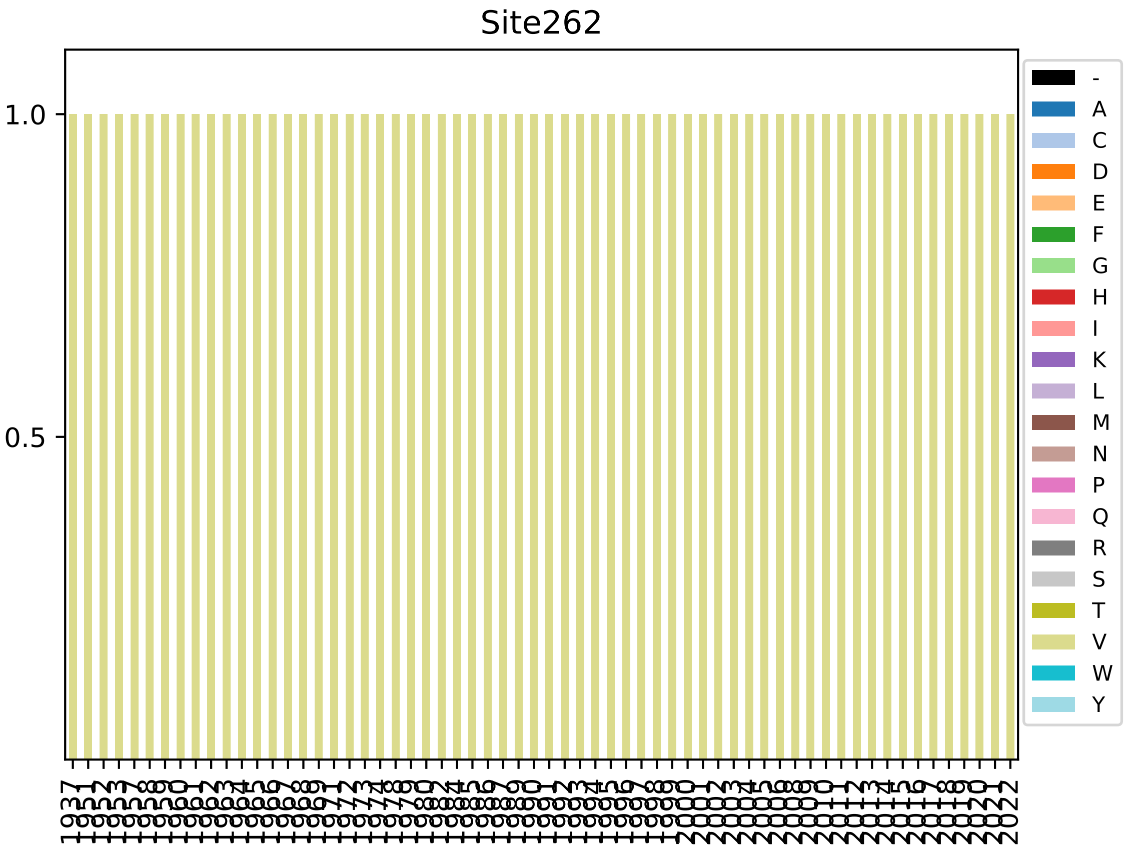
Site262
1.0
0.5
-
A
C
D
E
F
G
H
I
K
L
M
N
P
Q
R
S
T
V
W
Y

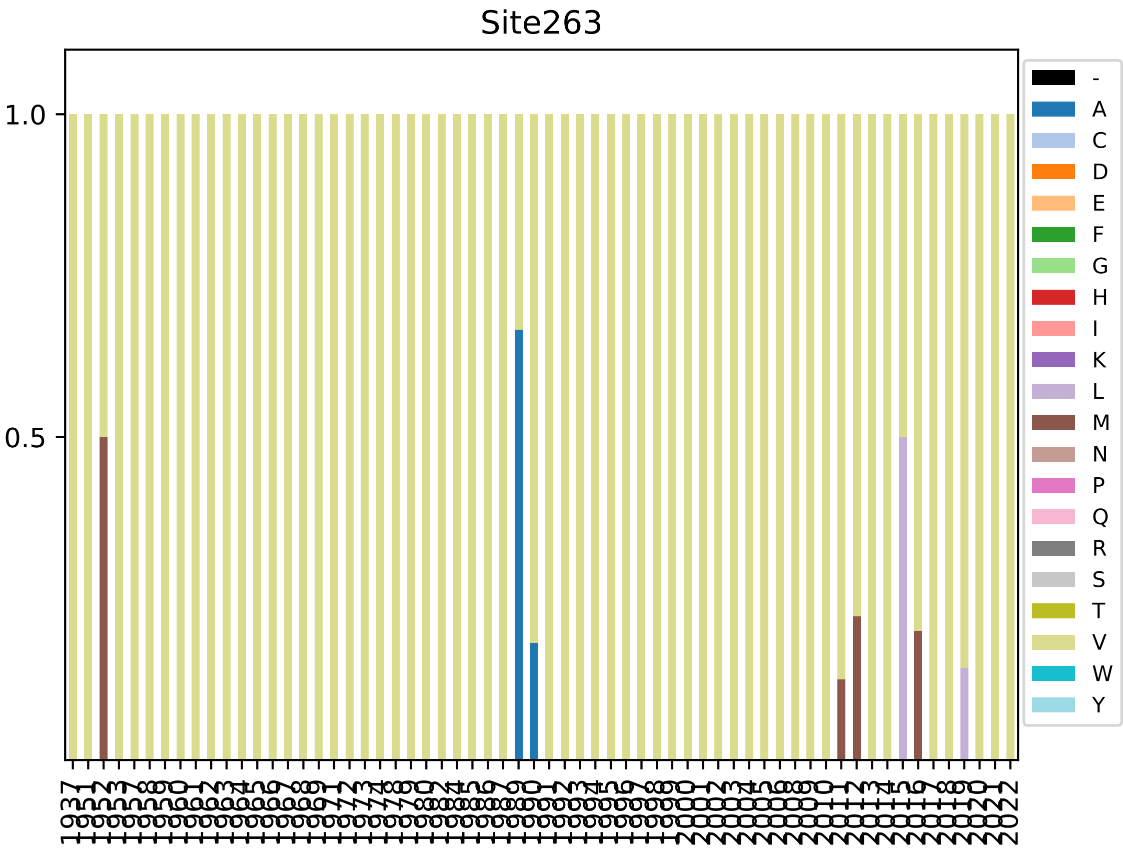
Site263
1.0
0.5
-
A
C
D
E
F
G
H
I
K
L
M
N
P
Q
R
S
T
V
W
Y

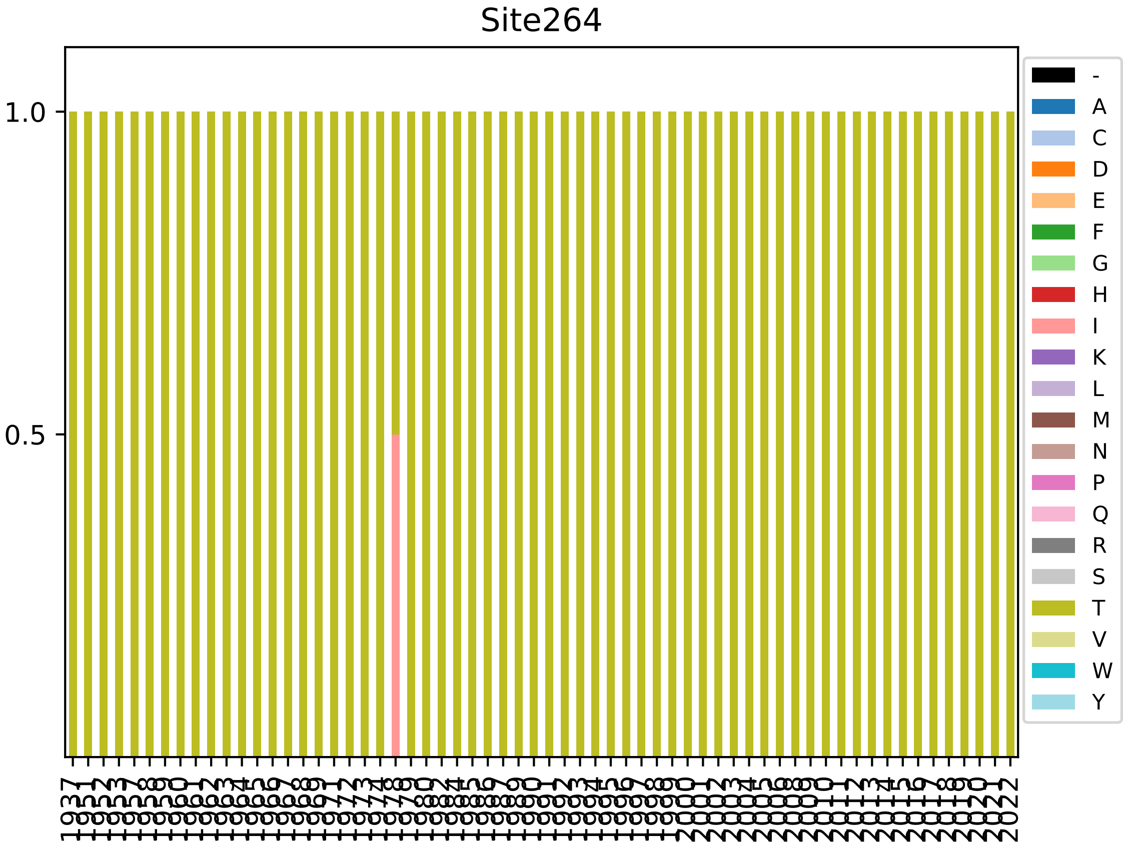
Site264
1.0
0.5
-
A
C
D
E
F
G
H
I
K
L
M
N
P
Q
R
S
T
V
W
Y

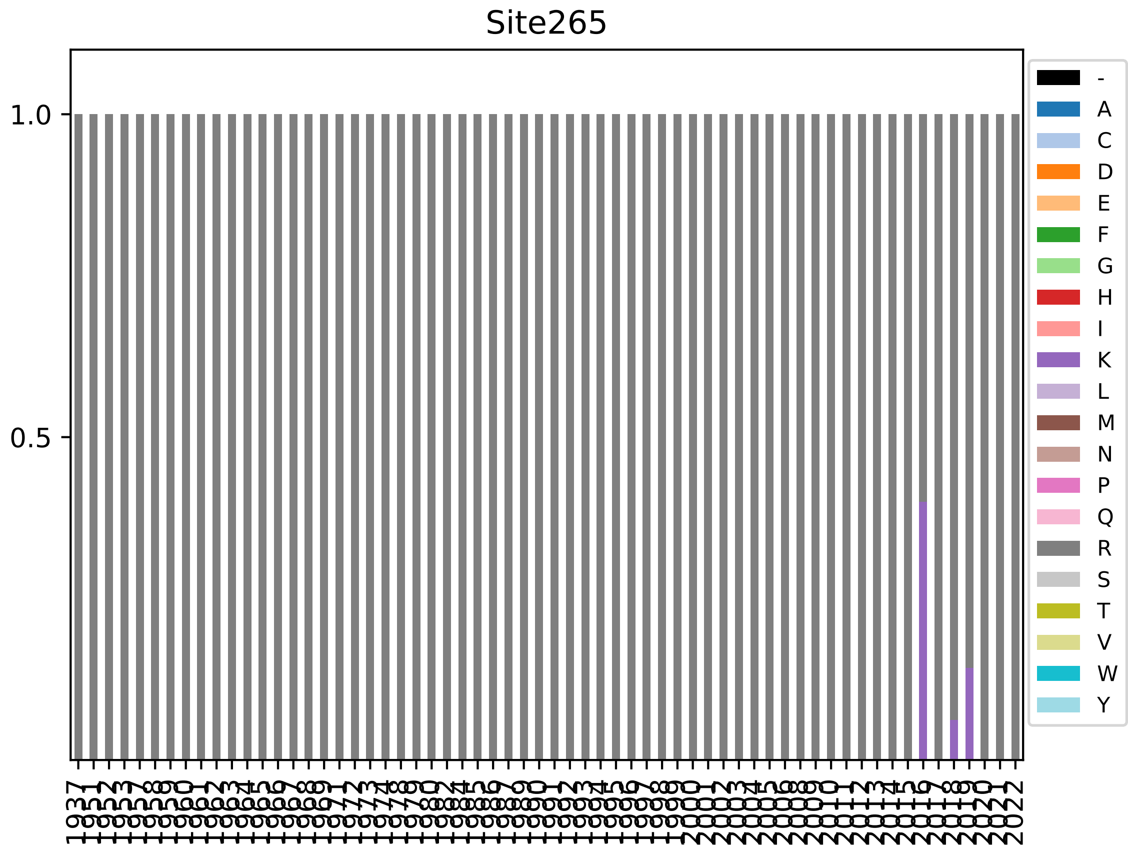
Site265
1.0
0.5
-
A
C
D
E
F
G
H
I
K
L
M
N
P
Q
R
S
T
V
W
Y

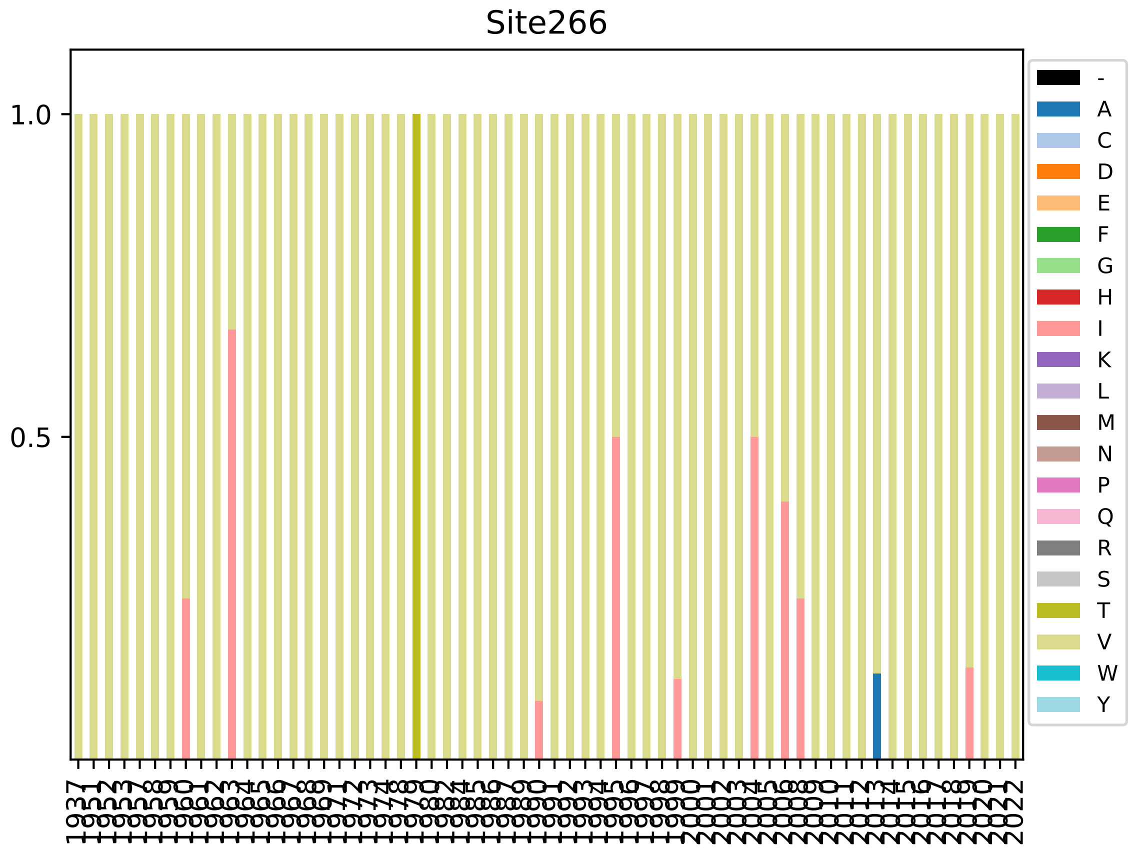
Site266
1.0
0.5
-
A
C
D
E
F
G
H
I
K
L
M
N
P
Q
R
S
T
V
W
Y

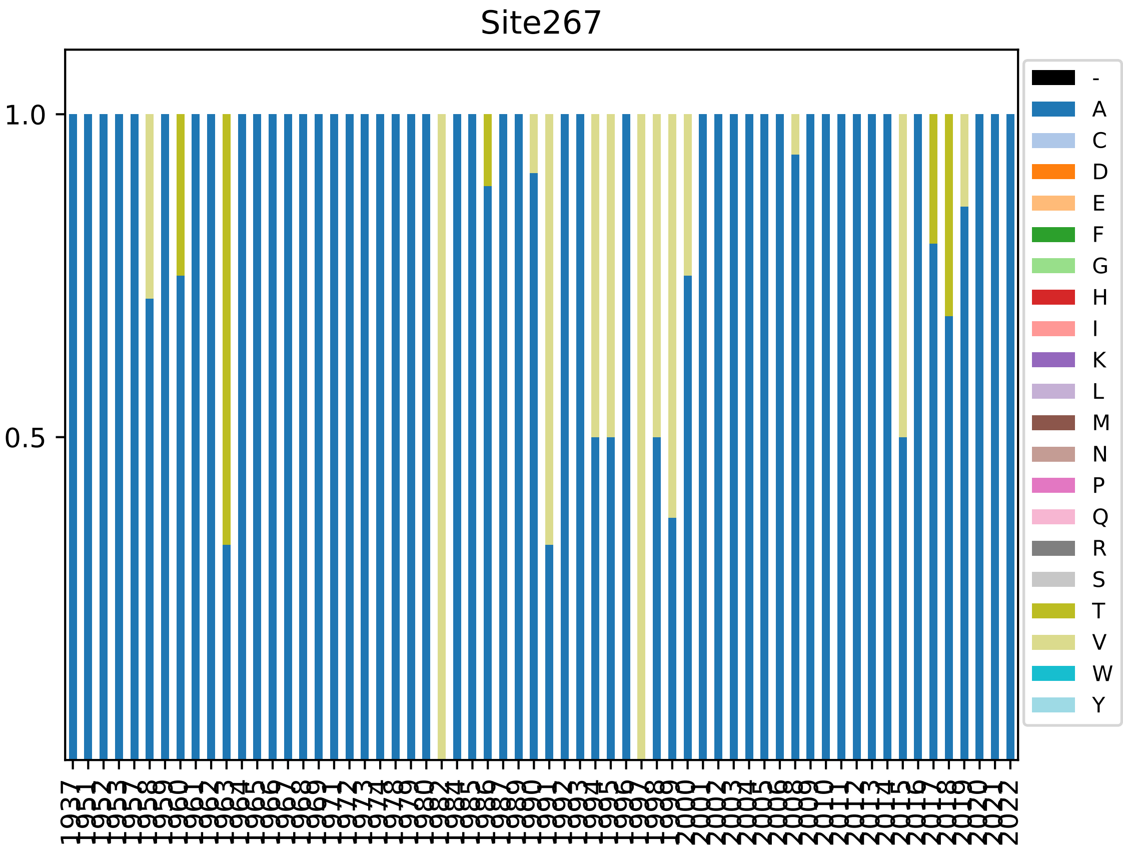
Site267
1.0
0.5
-
A
C
D
E
F
G
H
I
K
L
M
N
P
Q
R
S
T
V
W
Y

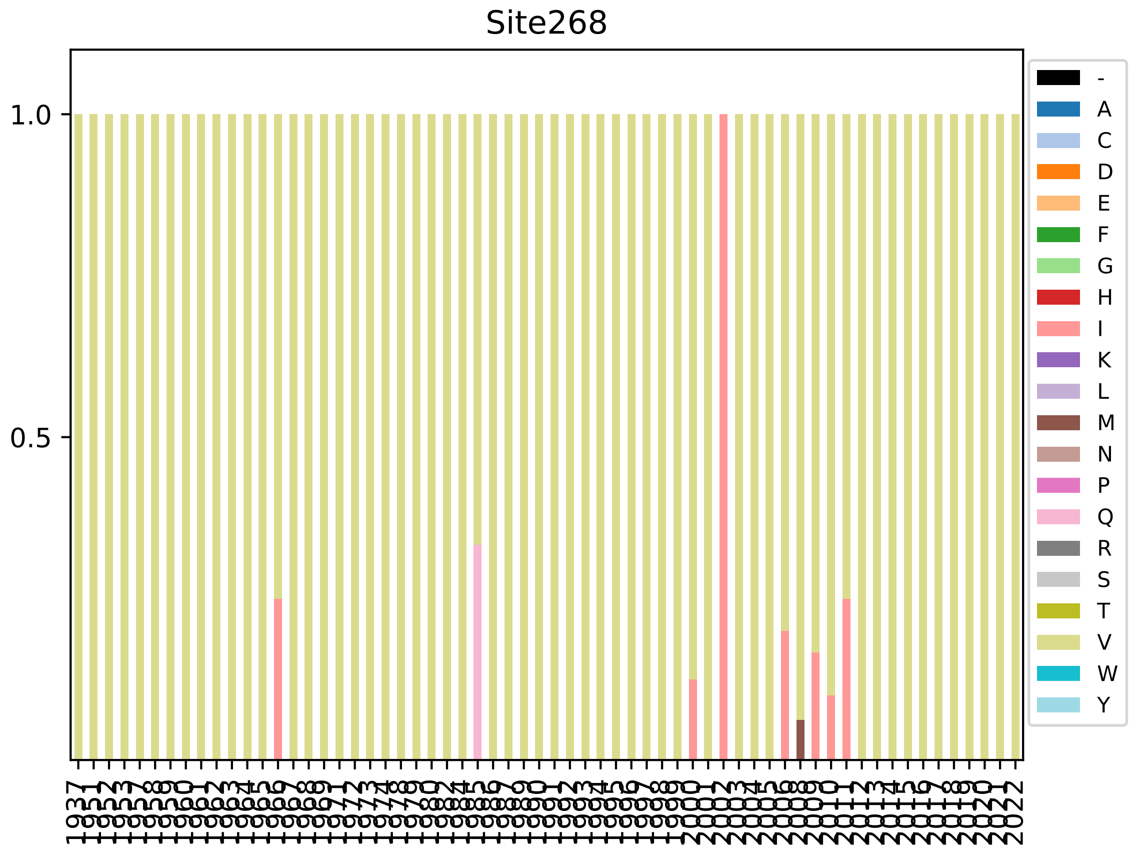
Site268
1.0
0.5
-
A
C
D
E
F
G
H
I
K
L
M
N
P
Q
R
S
T
V
W
Y

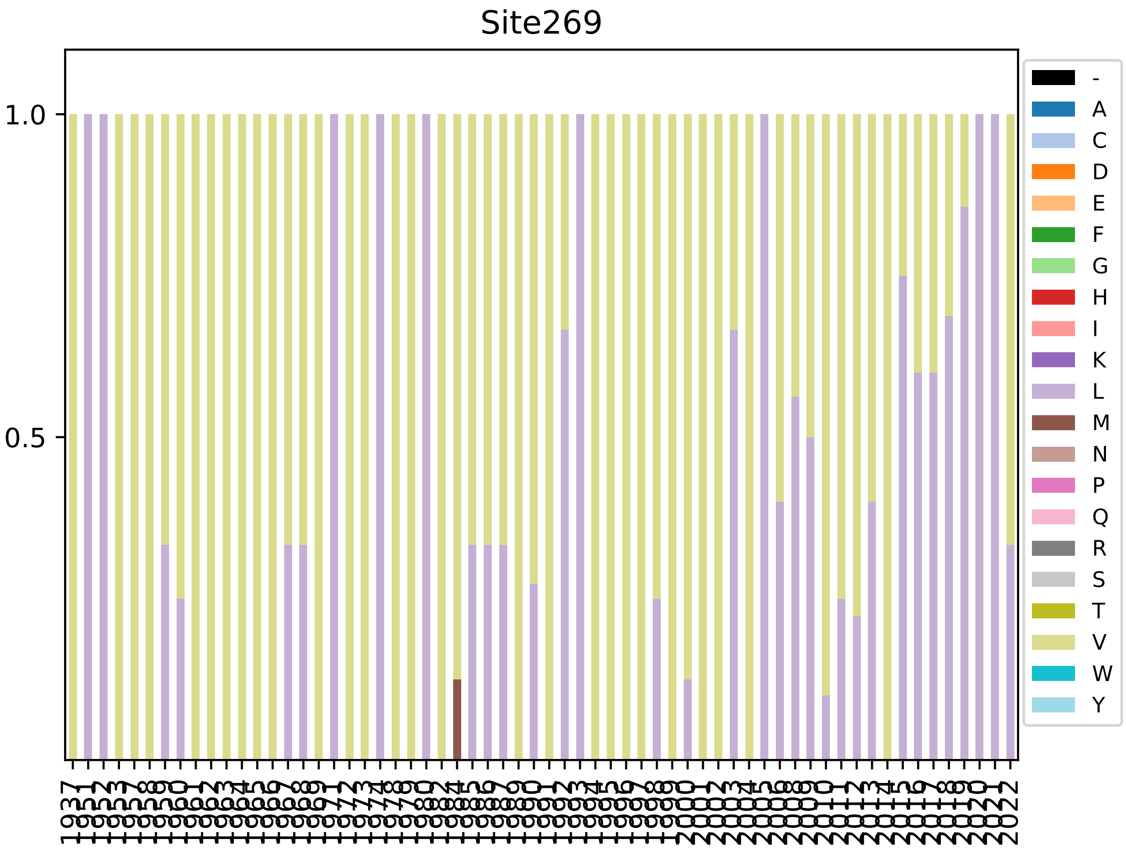
Site269
1.0
0.5
-
A
C
D
E
F
G
H
I
K
L
M
N
P
Q
R
S
T
V
W
Y

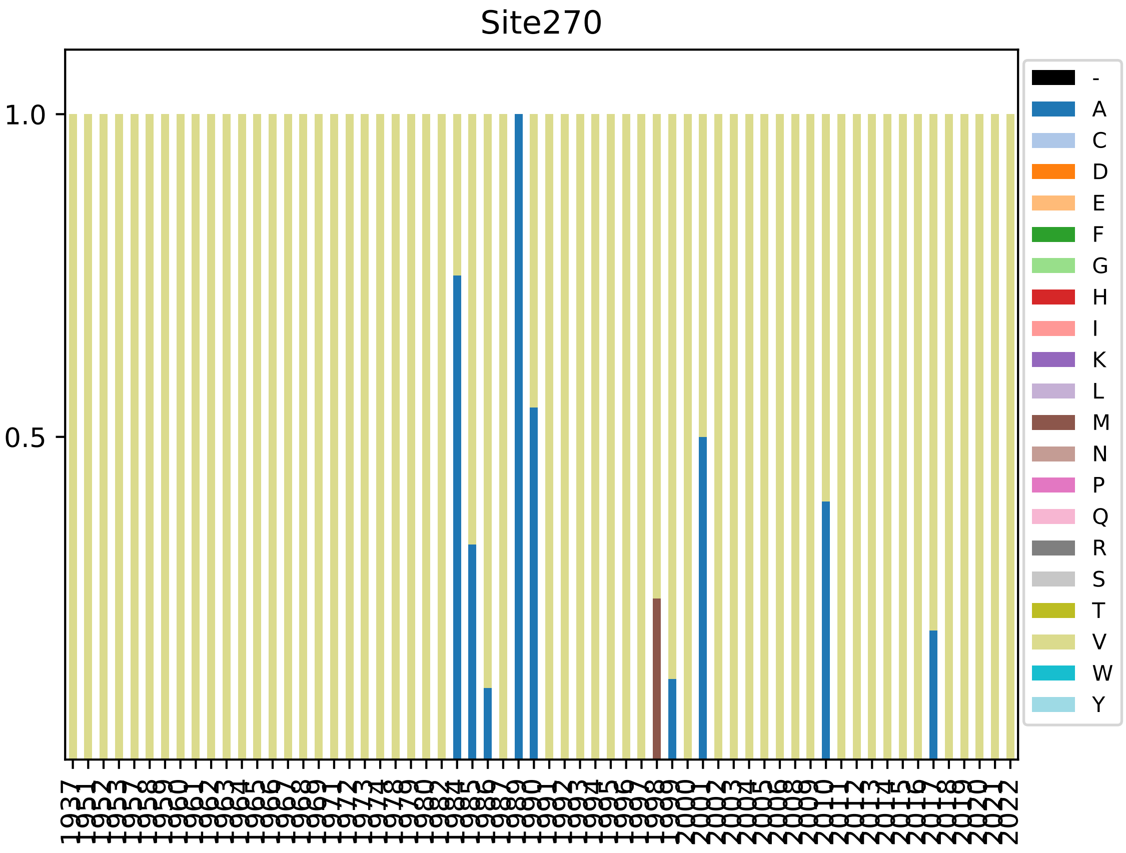
Site270
1.0
0.5
-
A
C
D
E
F
G
H
I
K
L
M
N
P
Q
R
S
T
V
W
Y

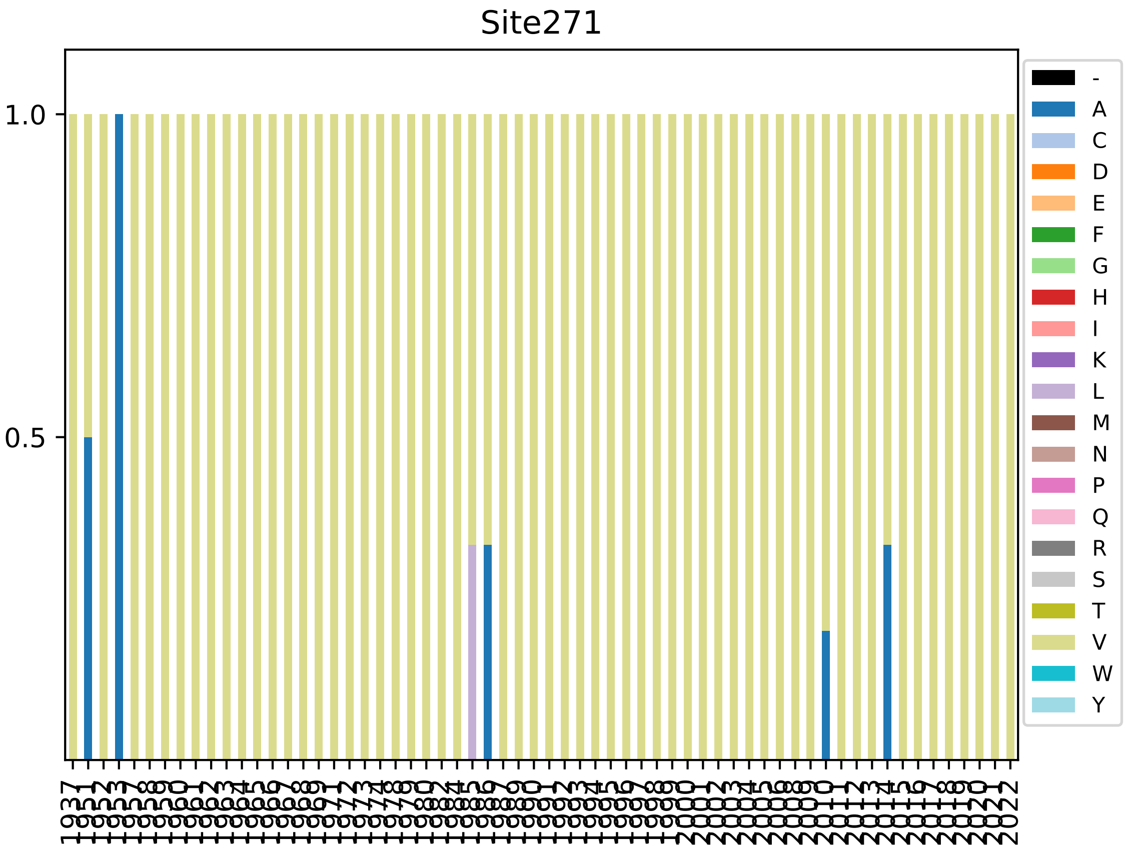
Site271
1.0
0.5
-
A
C
D
E
F
G
H
I
K
L
M
N
P
Q
R
S
T
V
W
Y

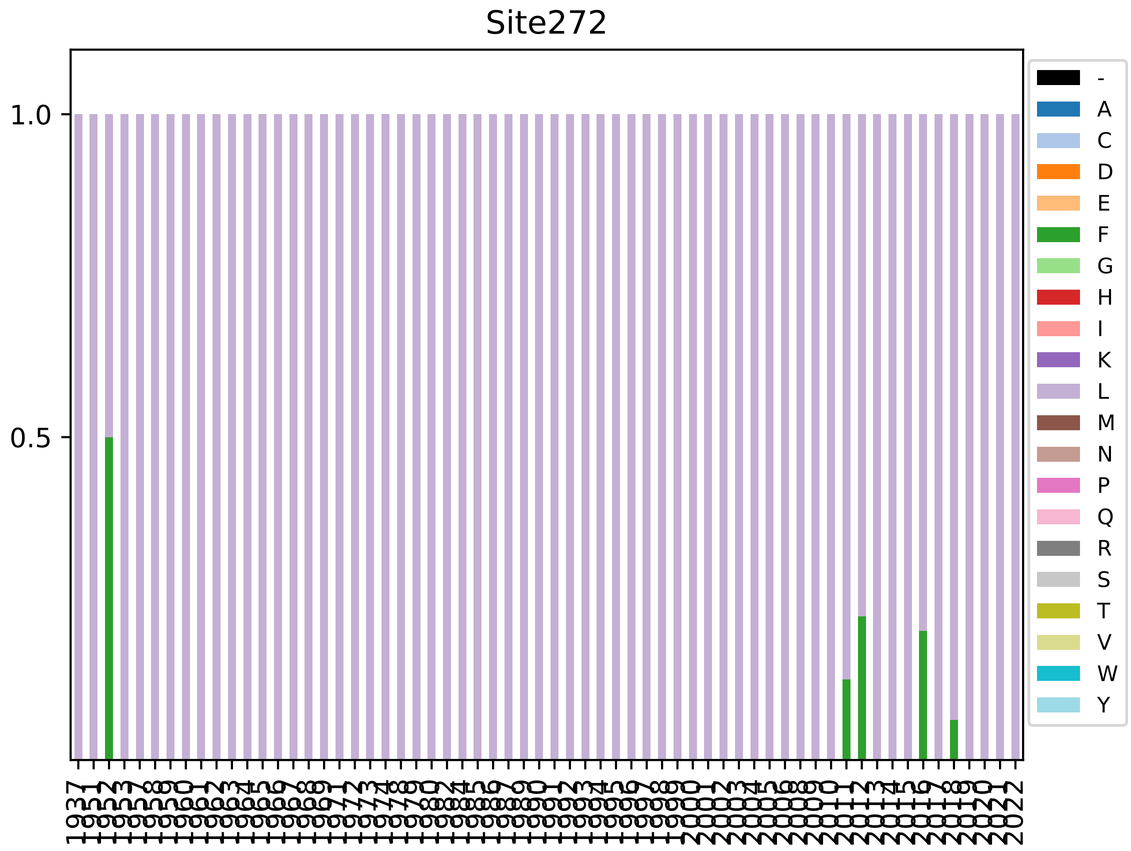
Site272
1.0
0.5
-
A
C
D
E
F
G
H
I
K
L
M
N
P
Q
R
S
T
V
W
Y

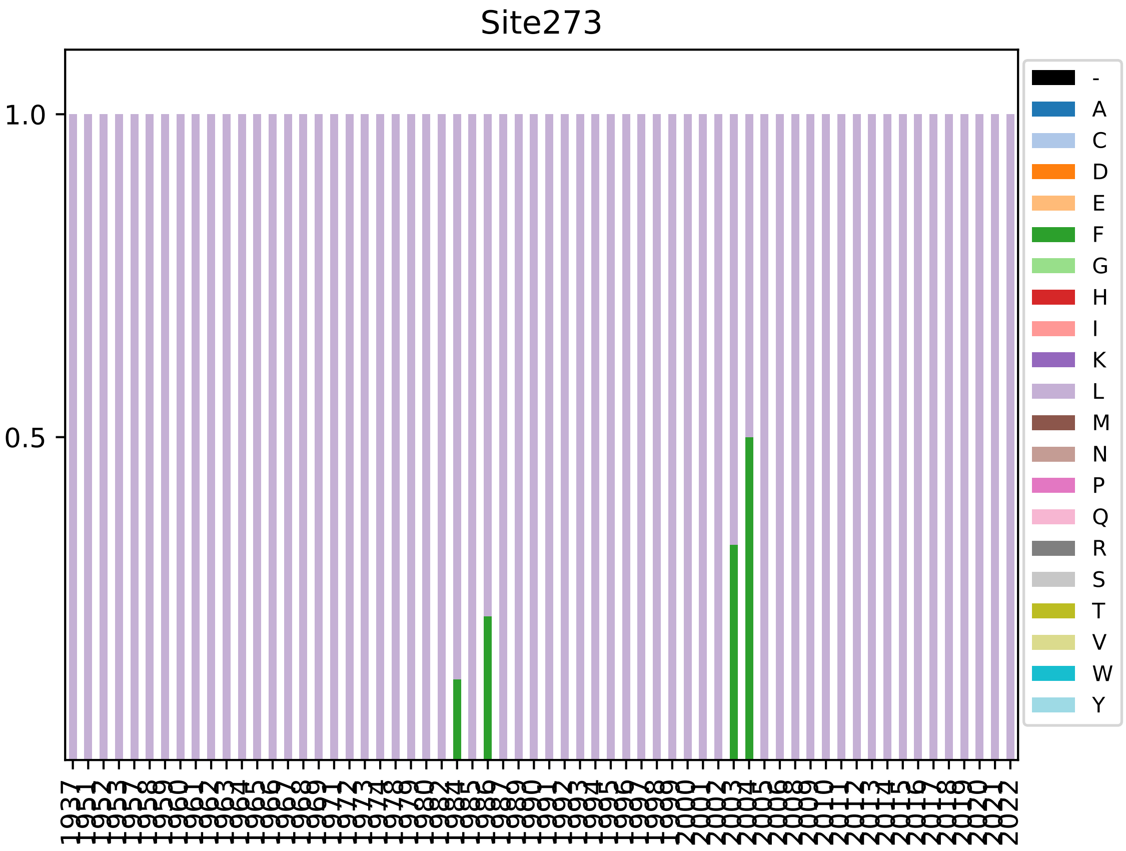
Site273
1.0
0.5
-
A
C
D
E
F
G
H
I
K
L
M
N
P
Q
R
S
T
V
W
Y

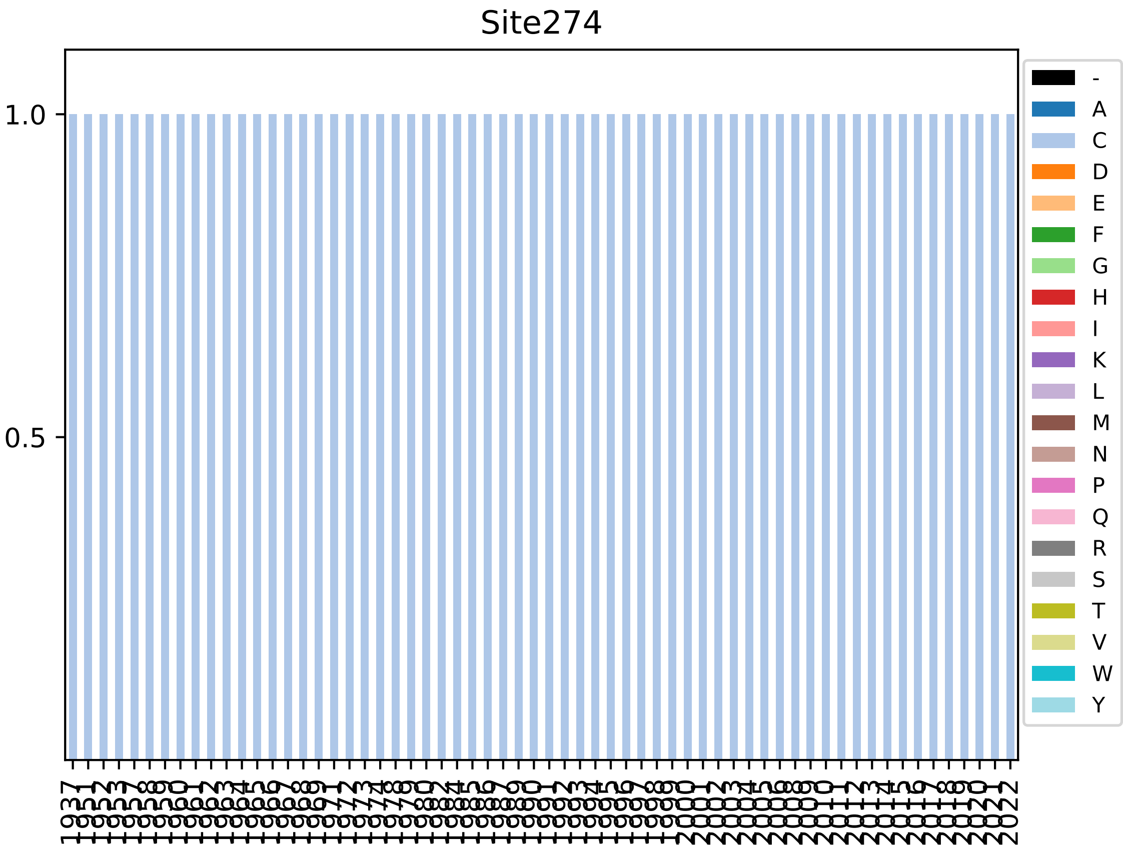

Site274
1.0
0.5
-
A
C
D
E
F
G
H
I
K
L
M
N
P
Q
R
S
T
V
W
Y

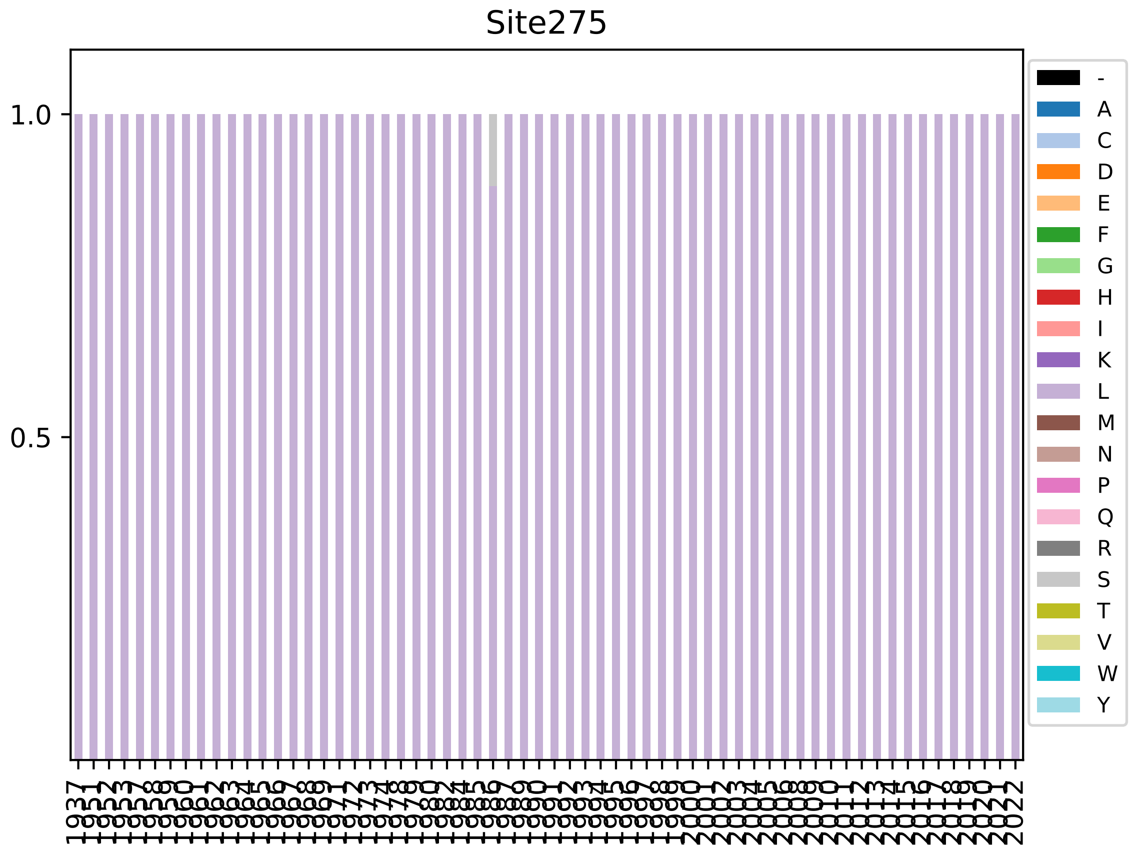
Site275
1.0
0.5
-
A
C
D
E
F
G
H
I
K
L
M
N
P
Q
R
S
T
V
W
Y

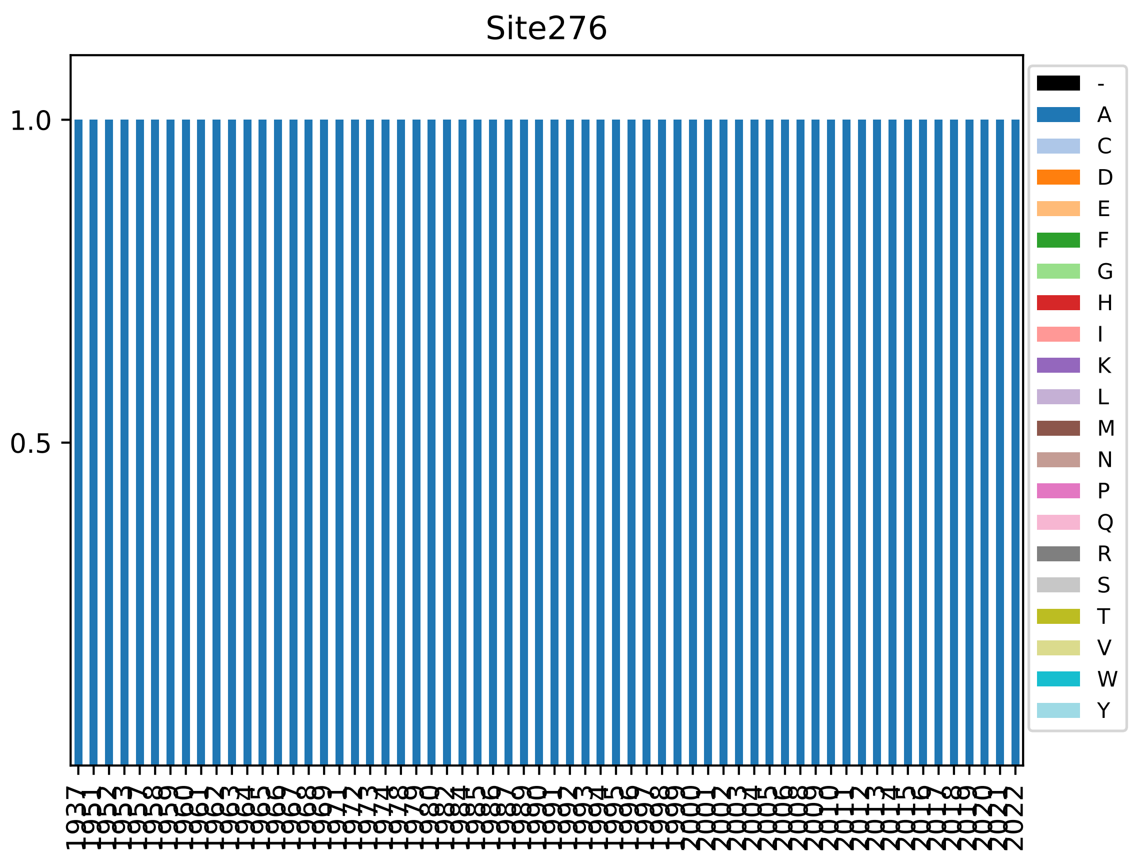
Site276
1.0
0.5
-
A
C
D
E
F
G
H
I
K
L
M
N
P
Q
R
S
T
V
W
Y

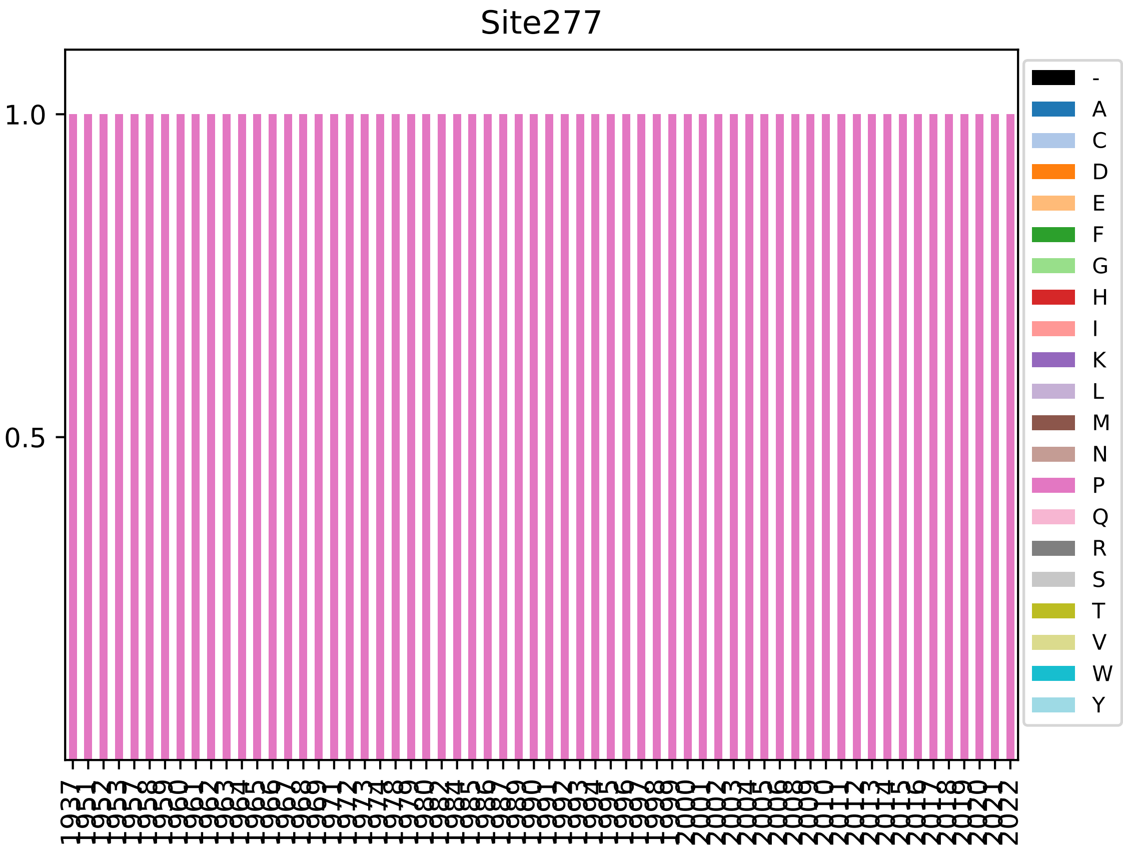
Site277
1.0
0.5
-
A
C
D
E
F
G
H
I
K
L
M
N
P
Q
R
S
T
V
W
Y

Site278

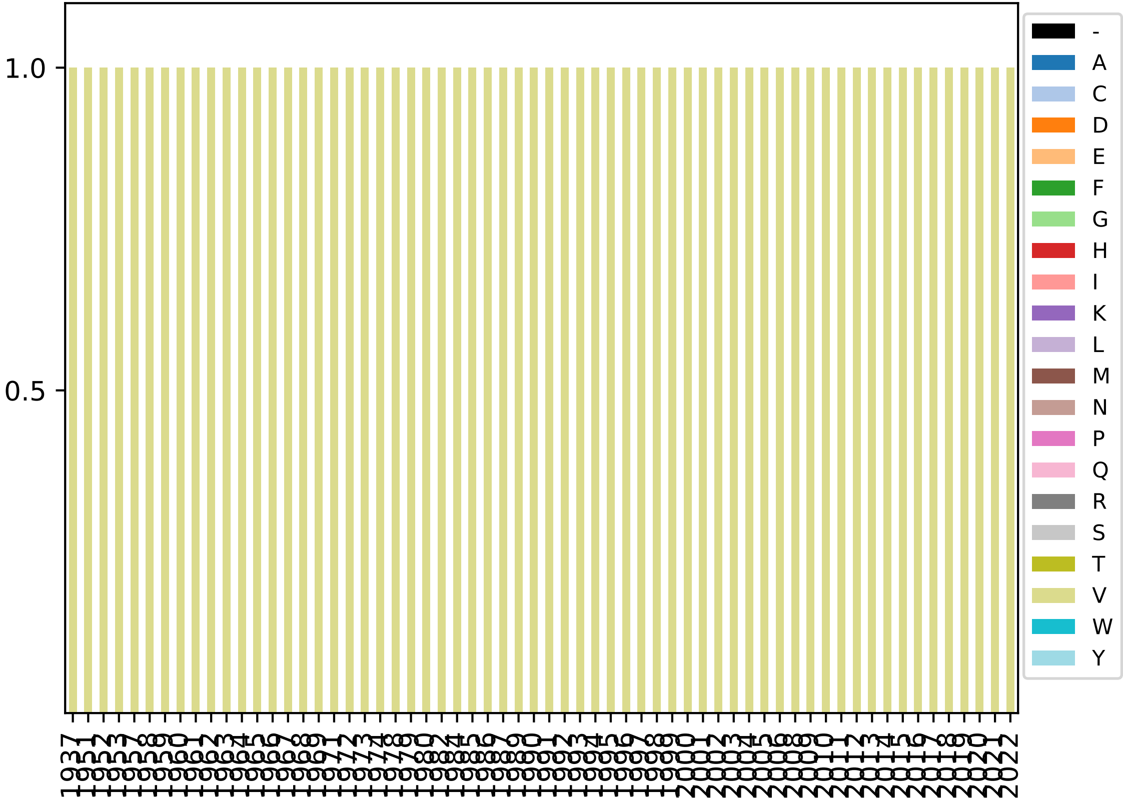

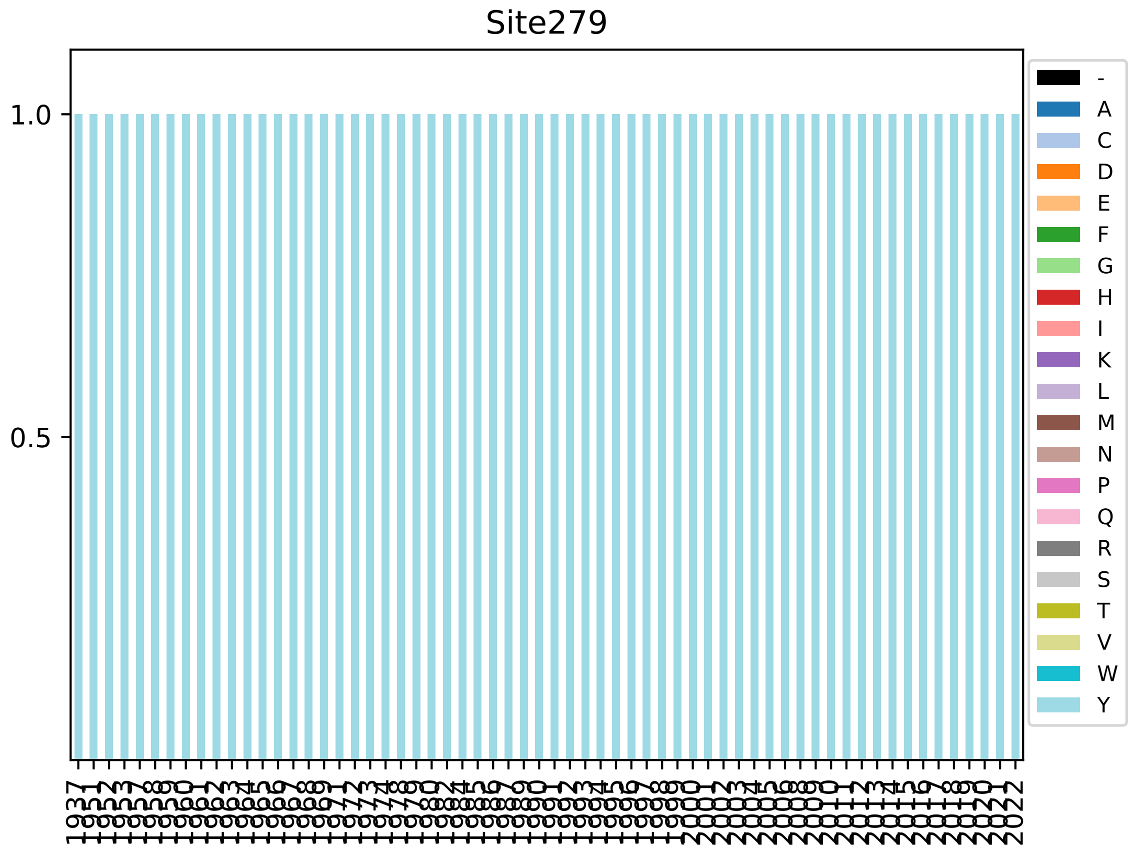

Site279
1.0
0.5
-
A
C
D
E
F
G
H
I
K
L
M
N
P
Q
R
S
T
V
W
Y

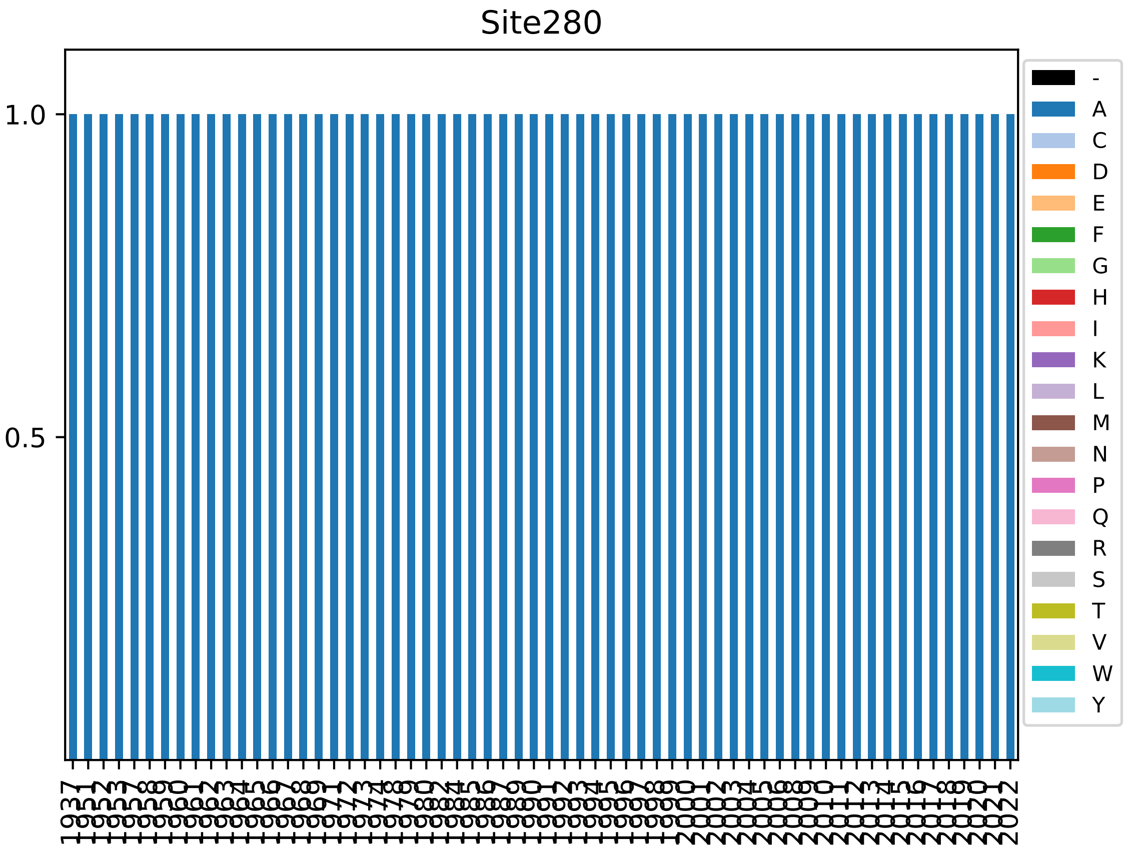
Site280
1.0
0.5
-
A
C
D
E
F
G
H
I
K
L
M
N
P
Q
R
S
T
V
W
Y

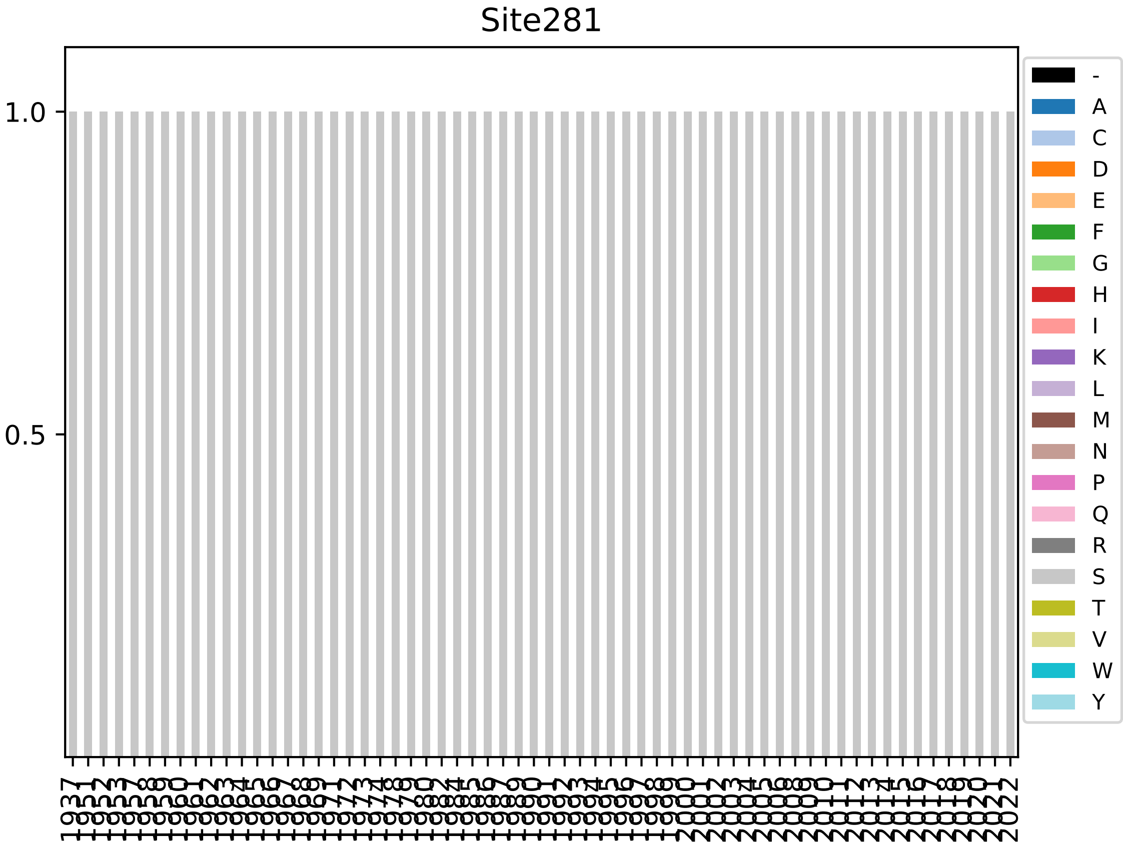
Site281
1.0
0.5
-
A
C
D
E
F
G
H
I
K
L
M
N
P
Q
R
S
T
V
W
Y

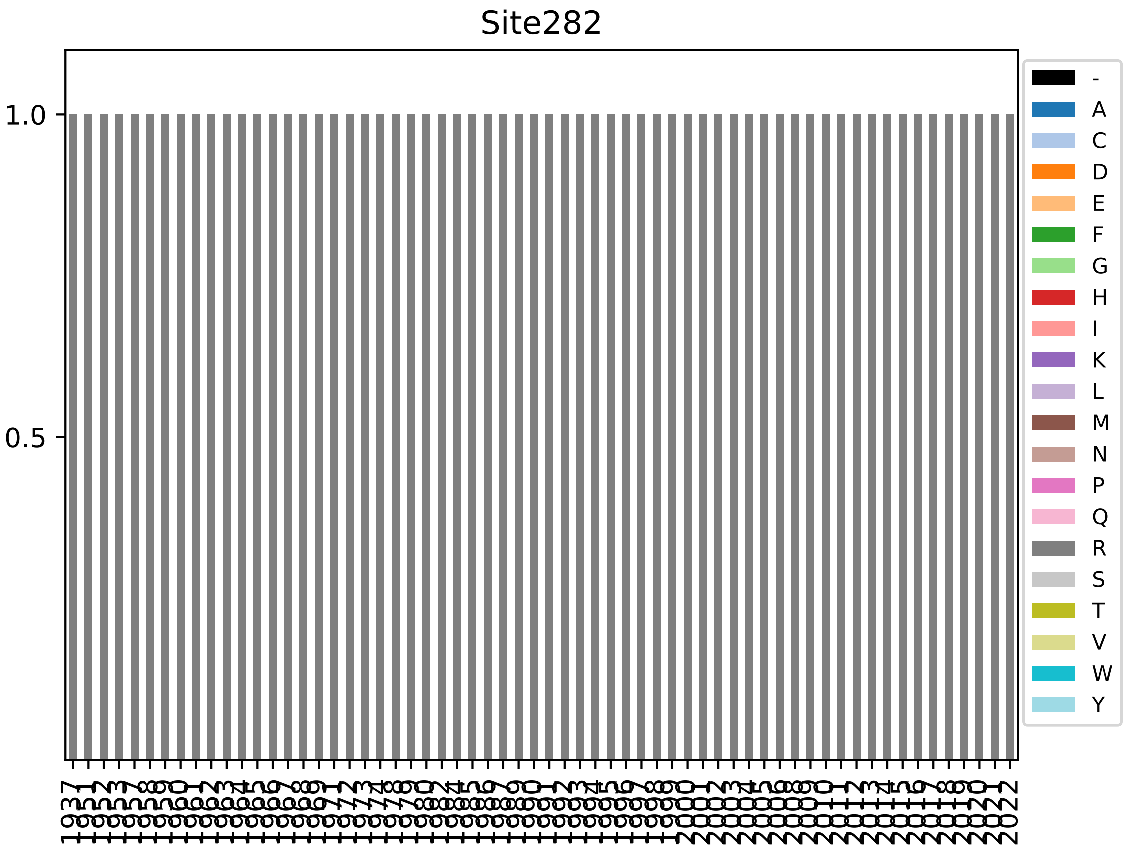
Site282
1.0
0.5
-
A
C
D
E
F
G
H
I
K
L
M
N
P
Q
R
S
T
V
W
Y

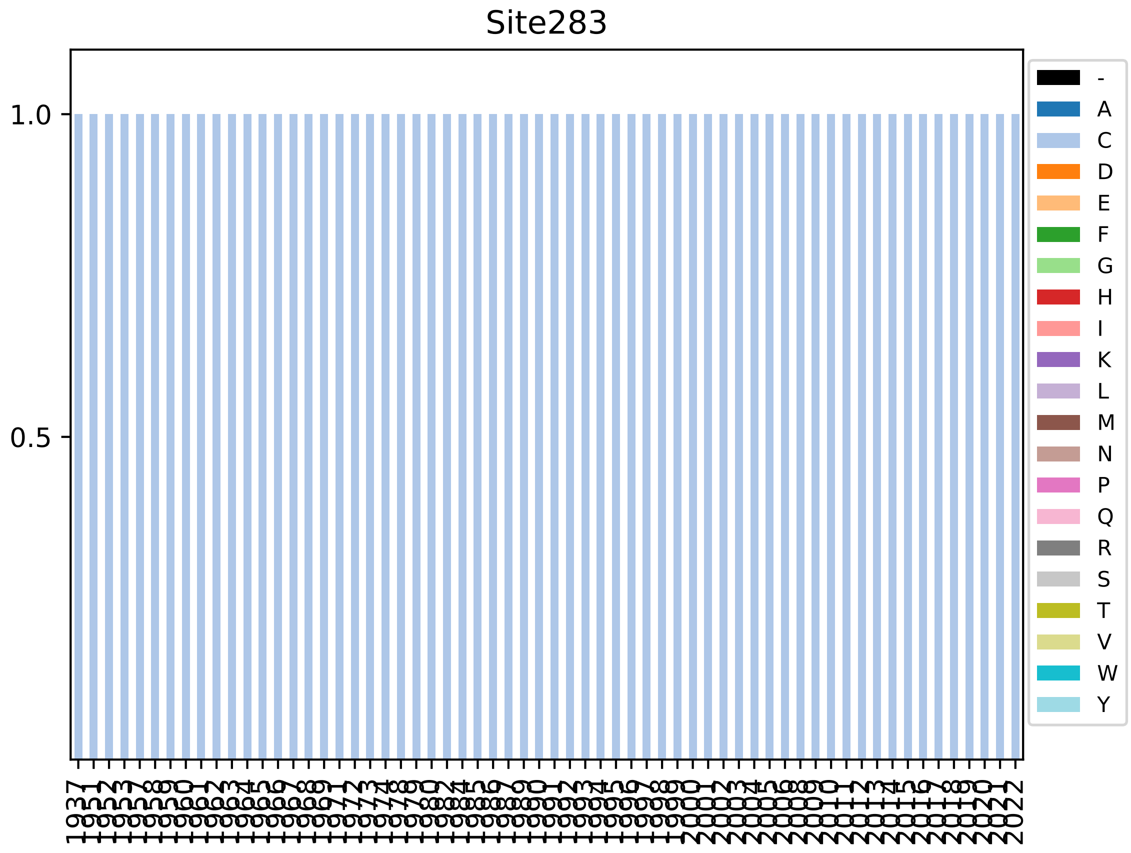
Site283
1.0
0.5
-
A
C
D
E
F
G
H
I
K
L
M
N
P
Q
R
S
T
V
W
Y

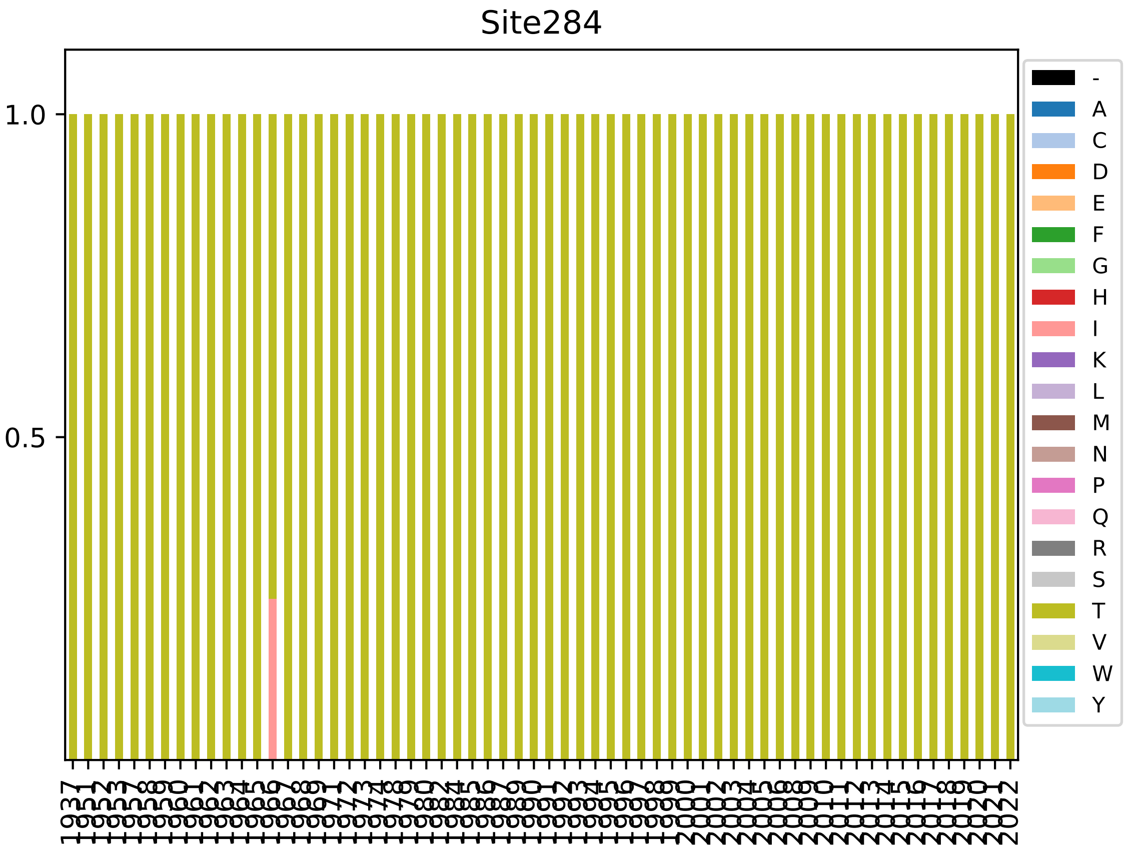
Site284
1.0
0.5
-
A
C
D
E
F
G
H
I
K
L
M
N
P
Q
R
S
T
V
W
Y

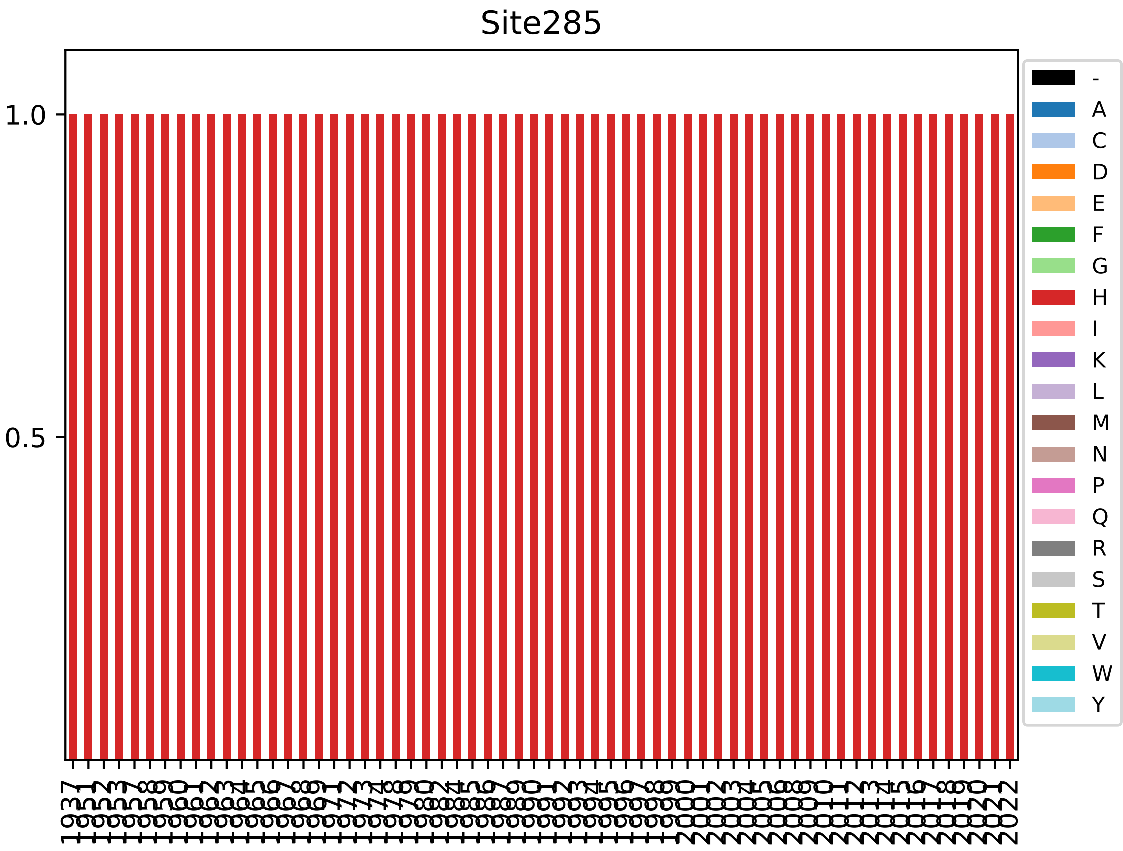

Site285
1.0
0.5
-
A
C
D
E
F
G
H
I
K
L
M
N
P
Q
R
S
T
V
W
Y

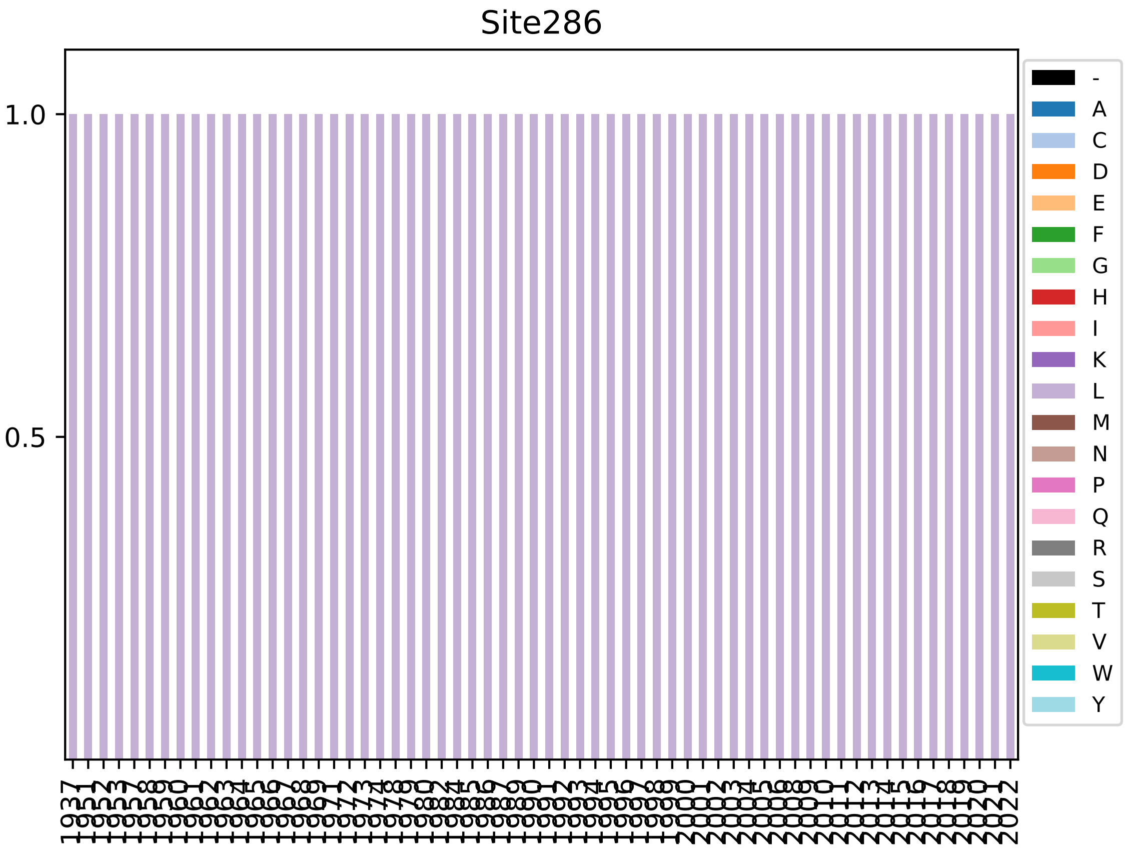

Site286
1.0
0.5
-
A
C
D
E
F
G
H
I
K
L
M
N
P
Q
R
S
T
V
W
Y

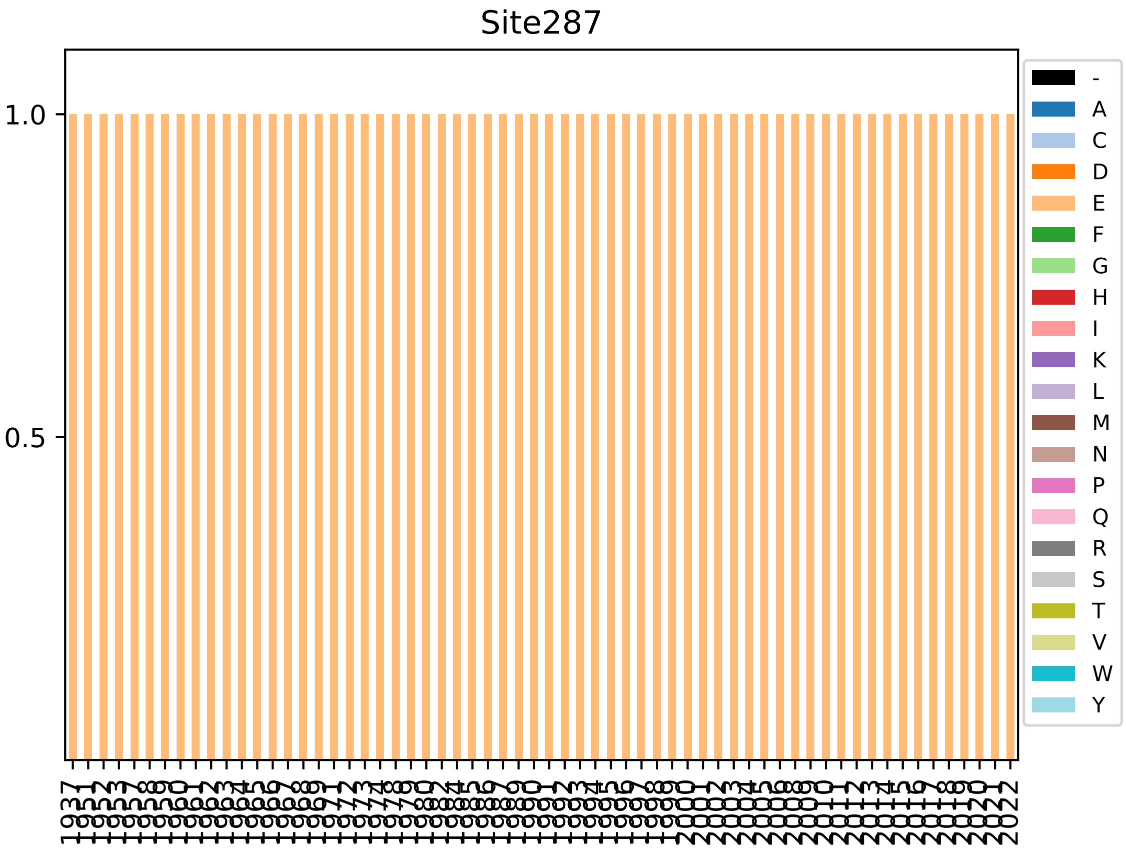
Site287
1.0
0.5
-
A
C
D
E
F
G
H
I
K
L
M
N
P
Q
R
S
T
V
W
Y

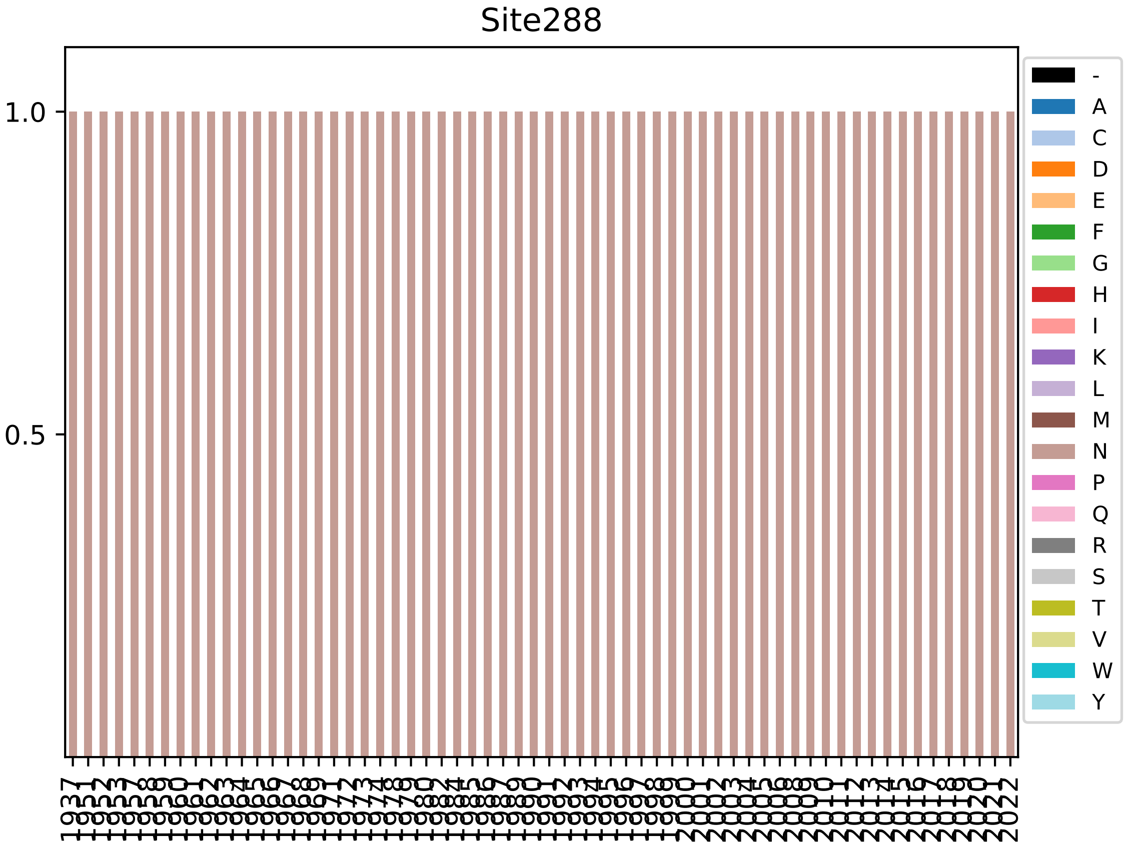
Site288
1.0
0.5
-
A
C
D
E
F
G
H
I
K
L
M
N
P
Q
R
S
T
V
W
Y

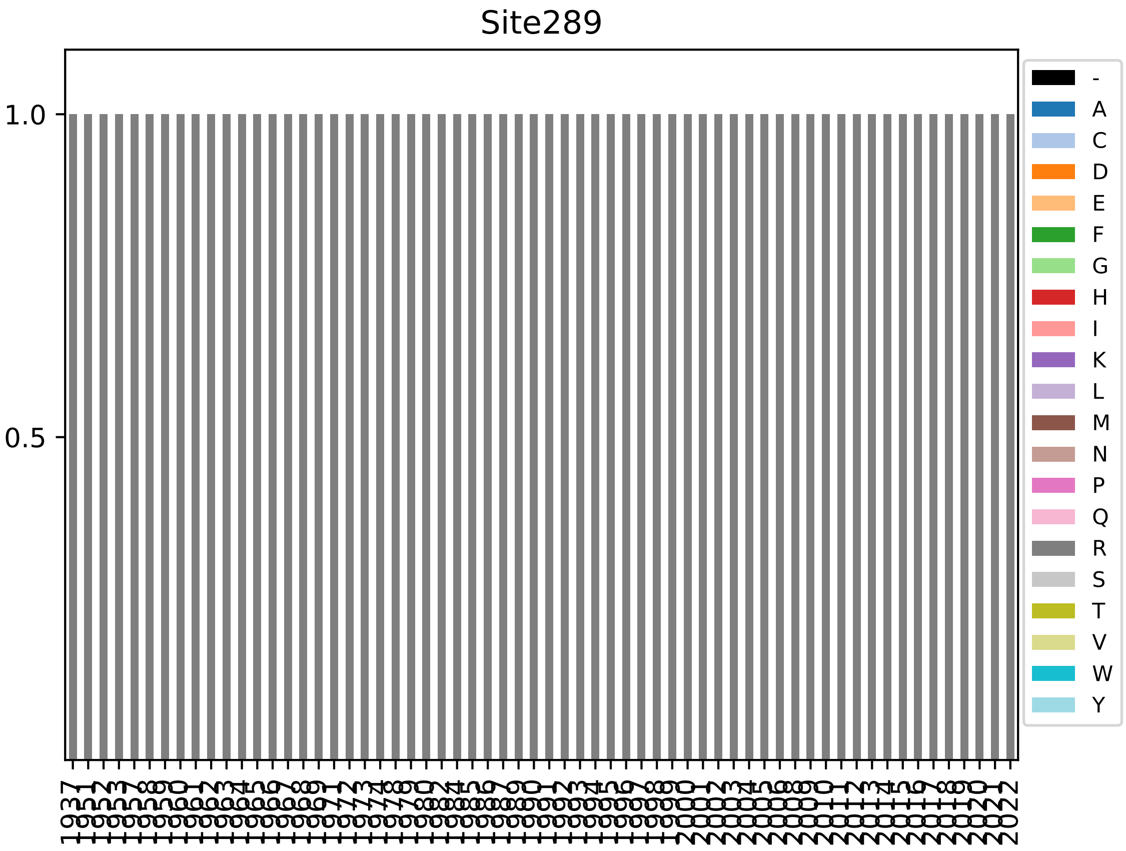
Site289
1.0
0.5
-
A
C
D
E
F
G
H
I
K
L
M
N
P
Q
R
S
T
V
W
Y

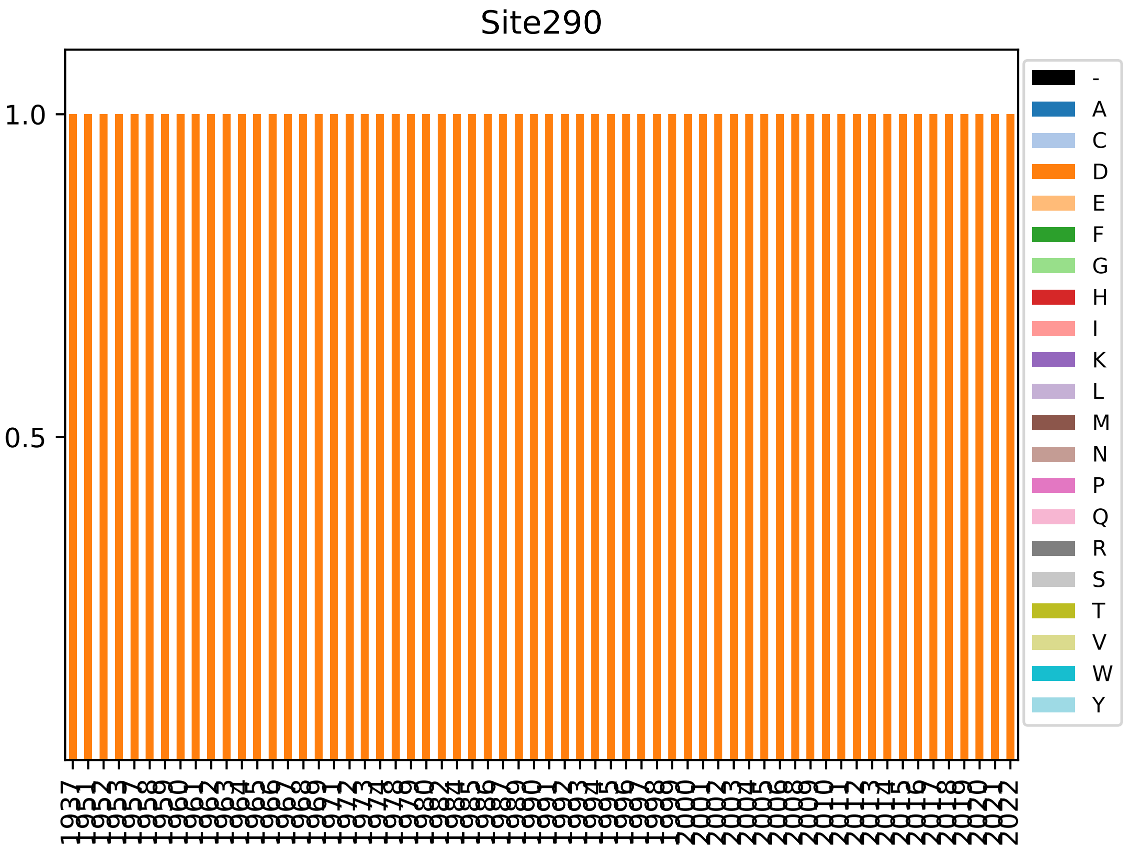
Site290
1.0
0.5
-
A
C
D
E
F
G
H
I
K
L
M
N
P
Q
R
S
T
V
W
Y

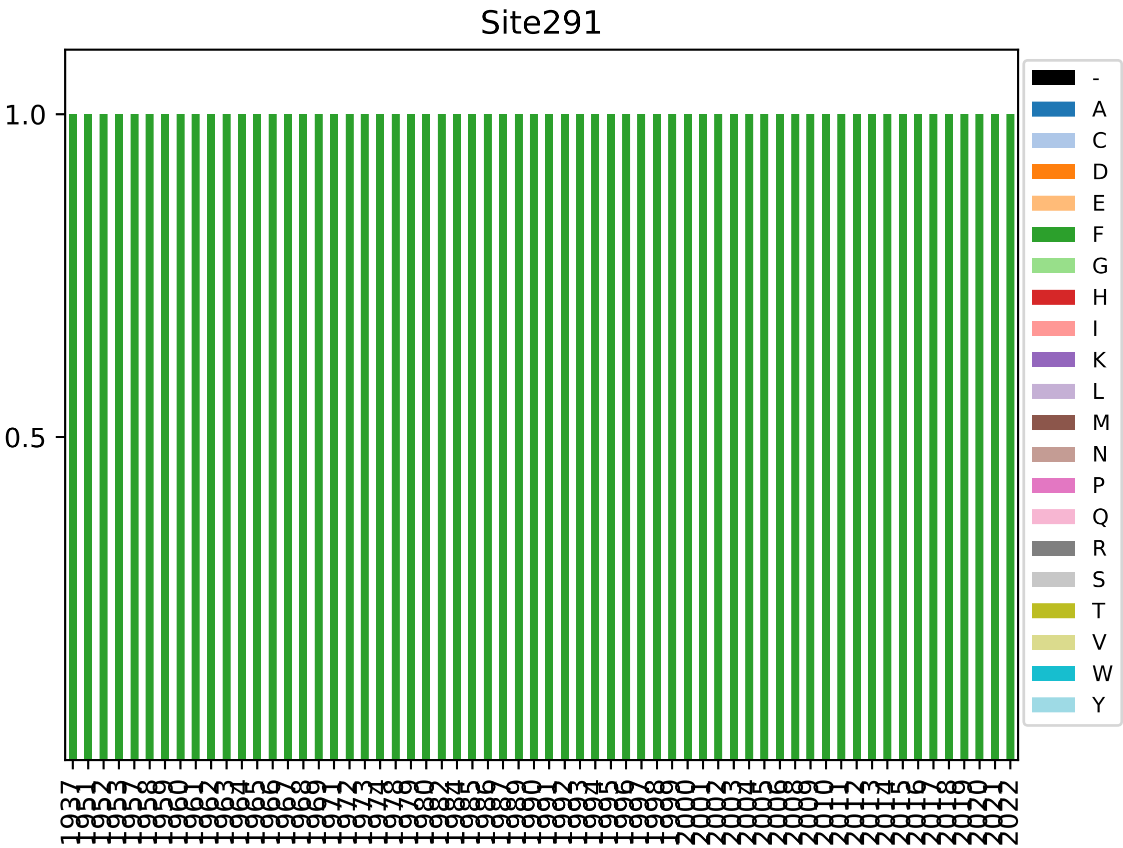
Site291
1.0
0.5
-
A
C
D
E
F
G
H
I
K
L
M
N
P
Q
R
S
T
V
W
Y

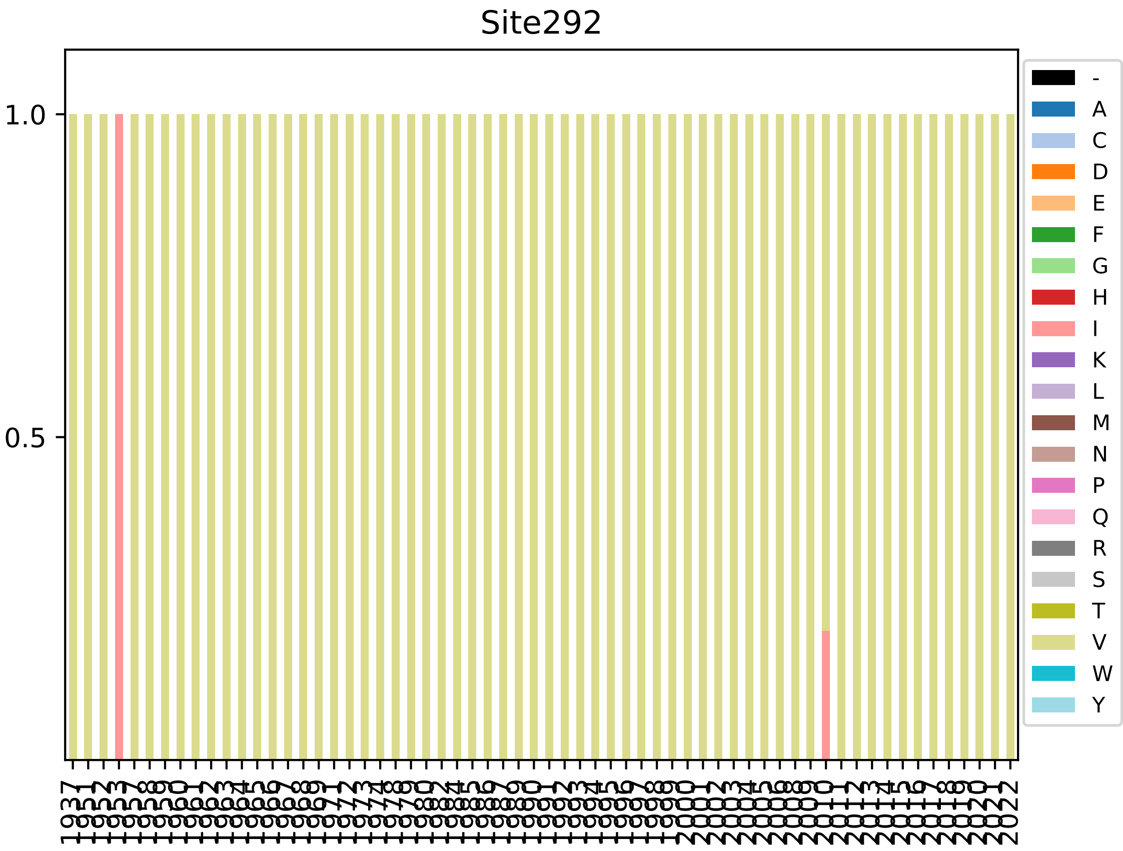
Site292
1.0
0.5
-
A
C
D
E
F
G
H
I
K
L
M
N
P
Q
R
S
T
V
W
Y

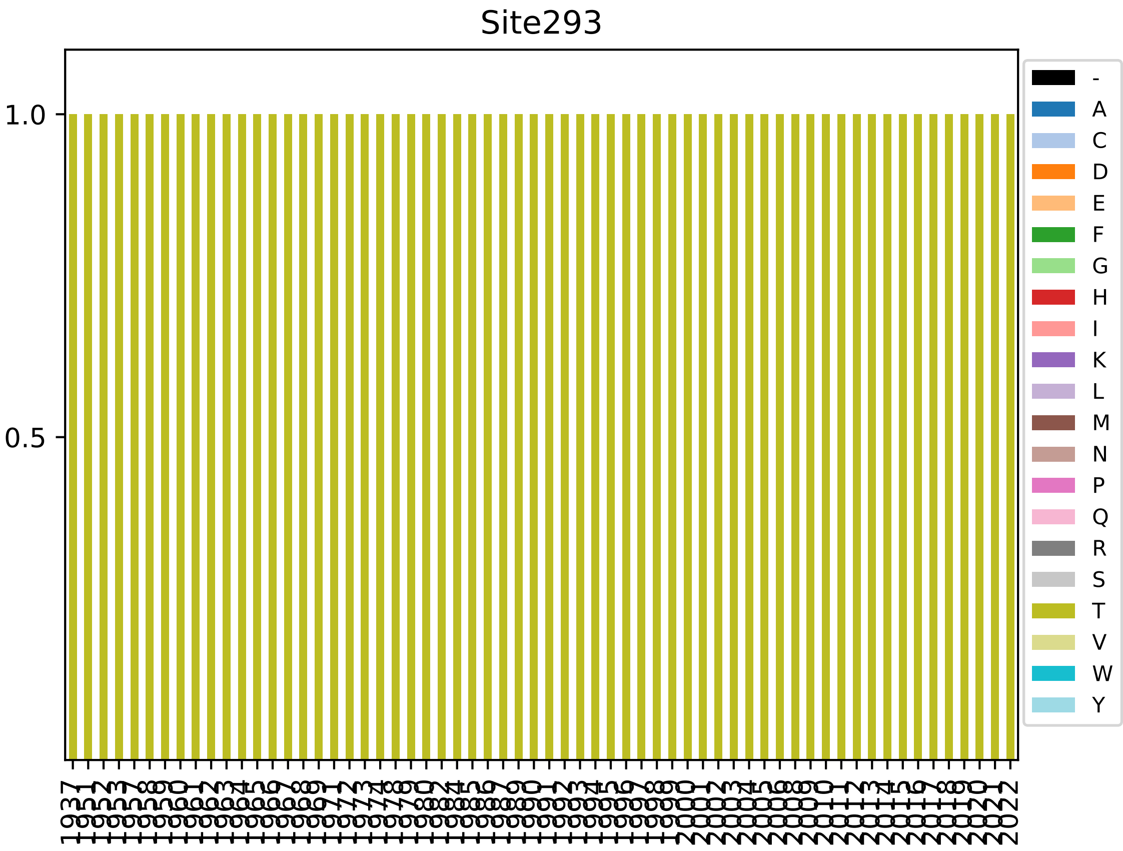
Site293
1.0
0.5
-
A
C
D
E
F
G
H
I
K
L
M
N
P
Q
R
S
T
V
W
Y

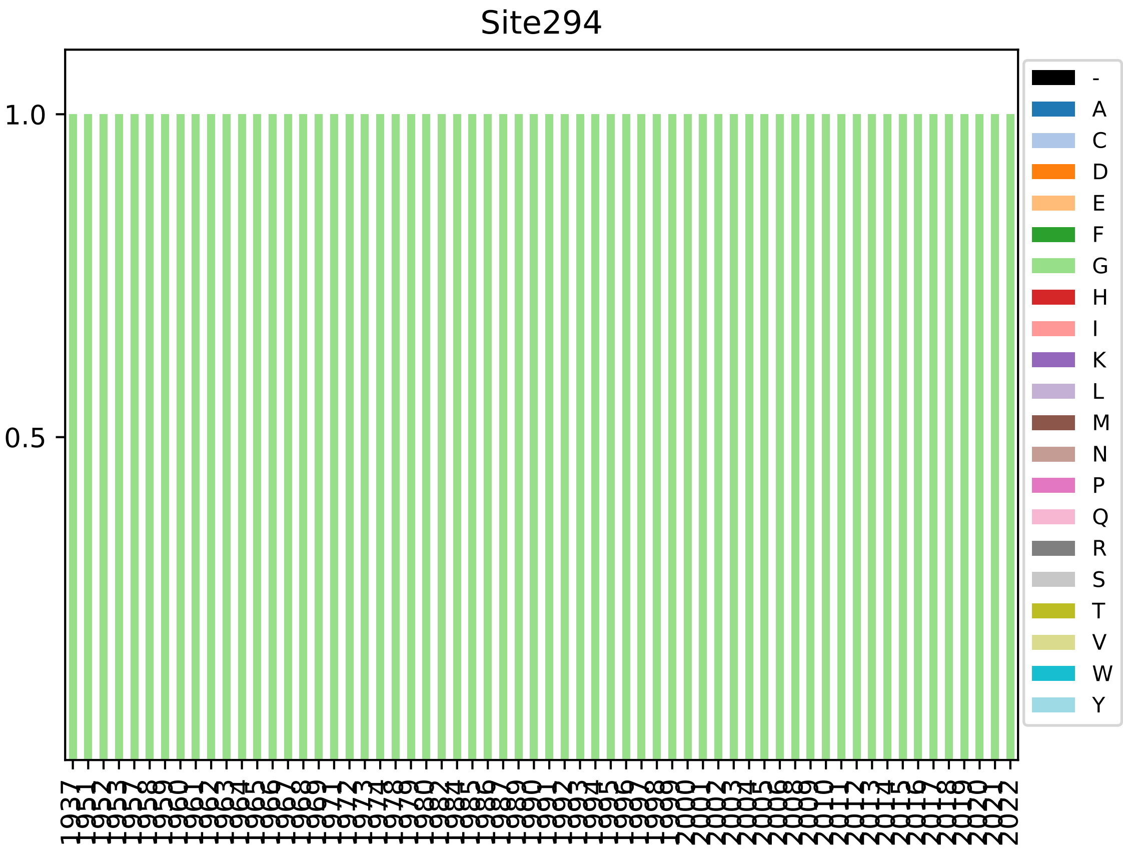
Site294
1.0
0.5
-
A
C
D
E
F
G
H
I
K
L
M
N
P
Q
R
S
T
V
W
Y

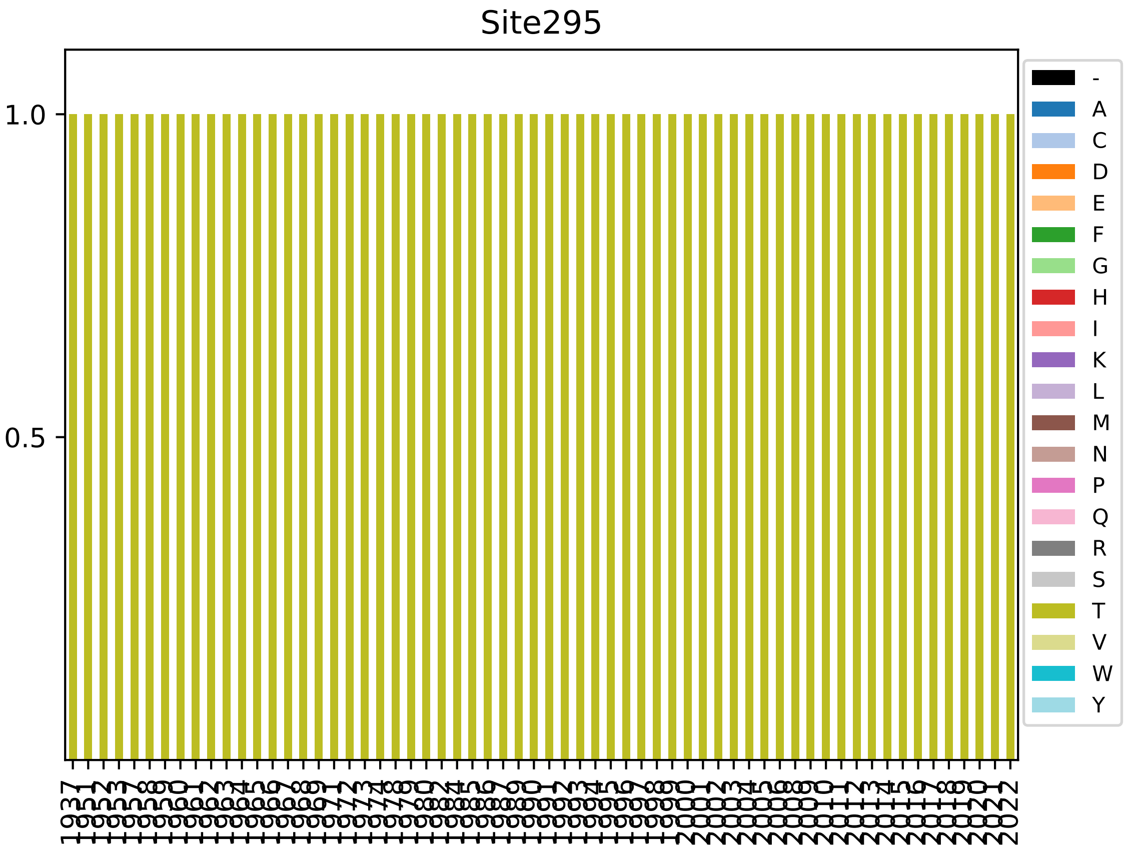
Site295
1.0
0.5
-
A
C
D
E
F
G
H
I
K
L
M
N
P
Q
R
S
T
V
W
Y

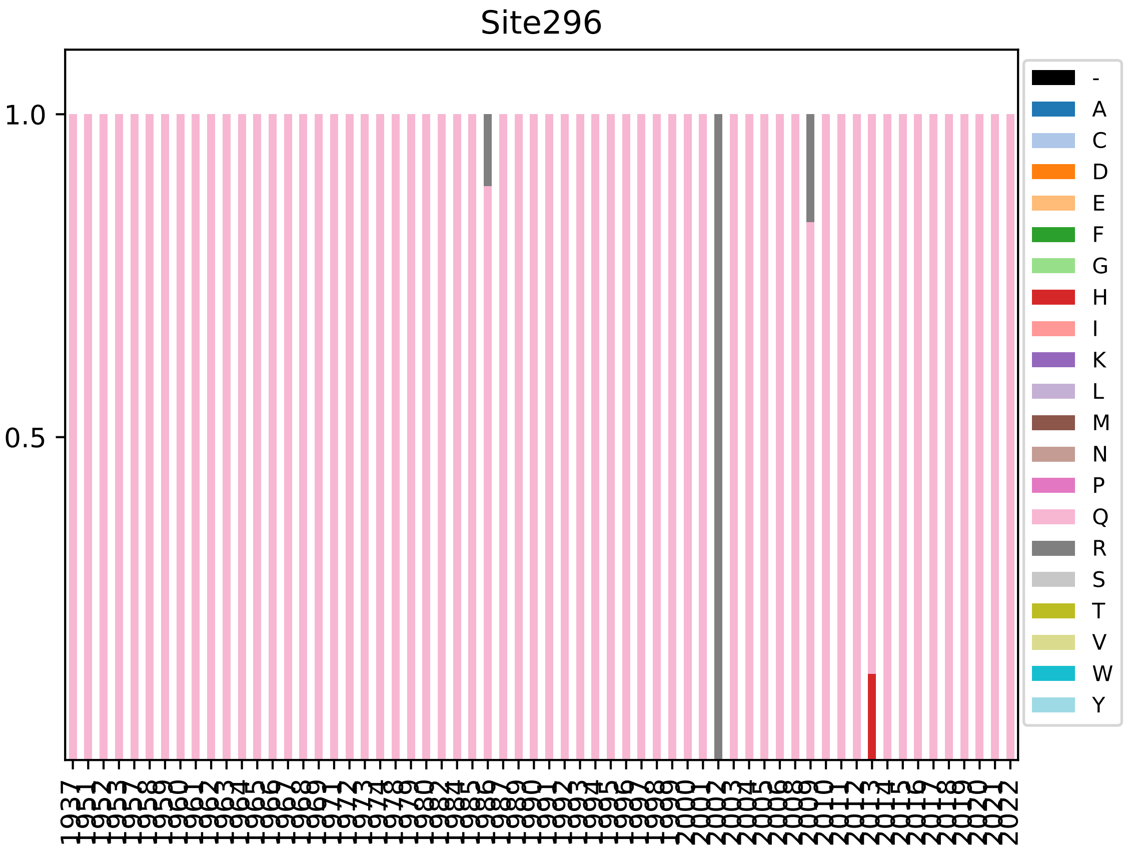

Site296
1.0
0.5
-
A
C
D
E
F
G
H
I
K
L
M
N
P
Q
R
S
T
V
W
Y

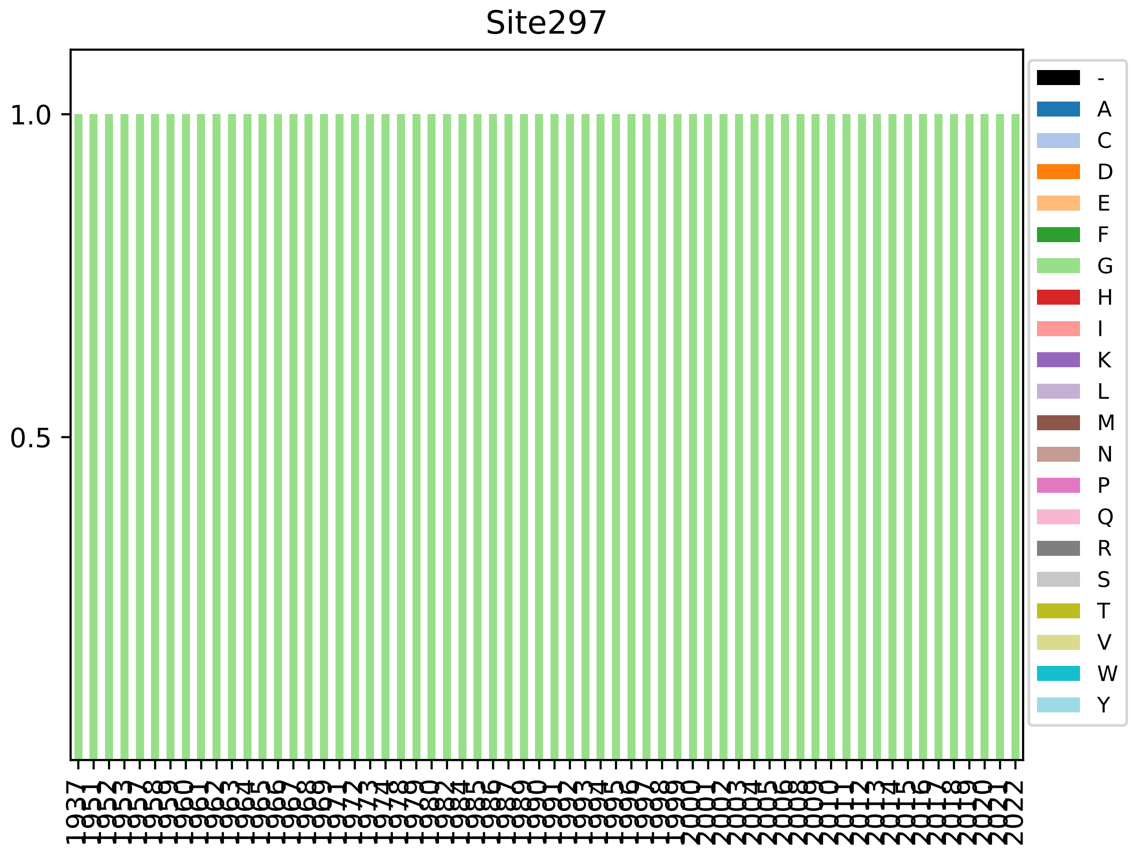
Site297
1.0
0.5
-
A
C
D
E
F
G
H
I
K
L
M
N
P
Q
R
S
T
V
W
Y

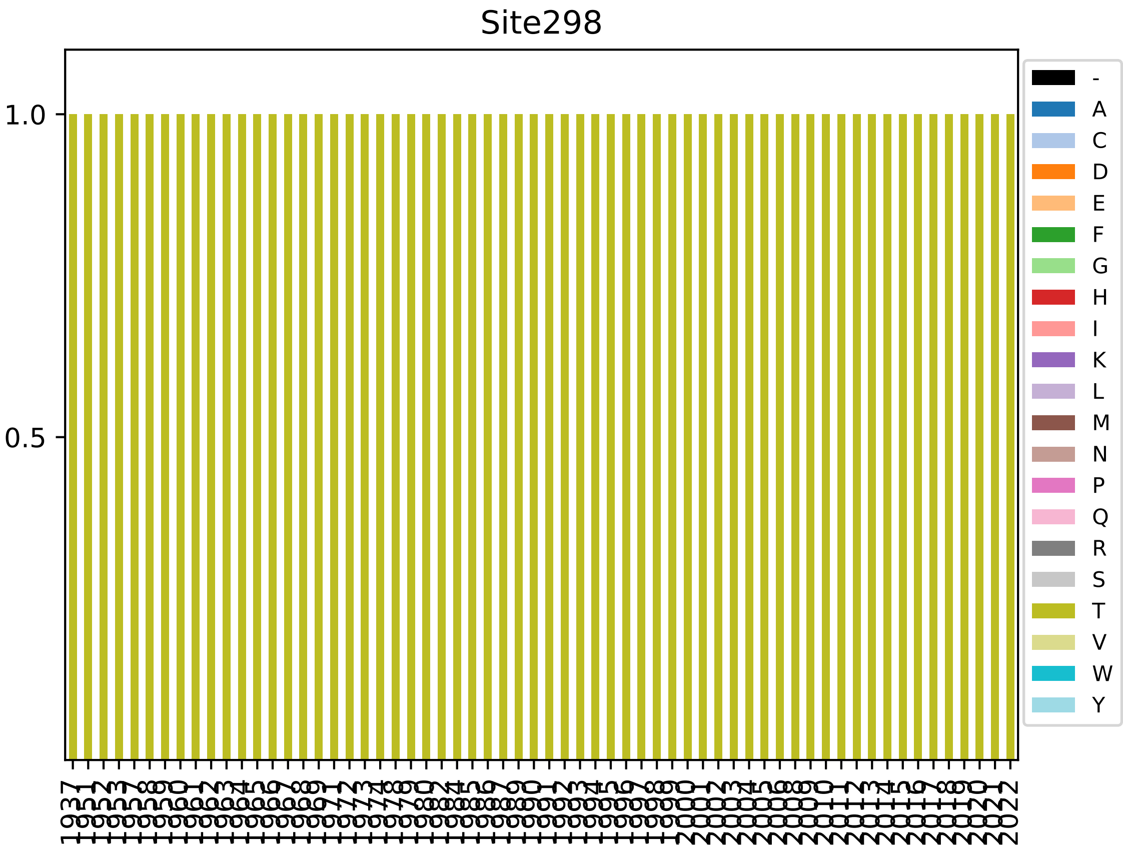
Site298
1.0
0.5
-
A
C
D
E
F
G
H
I
K
L
M
N
P
Q
R
S
T
V
W
Y

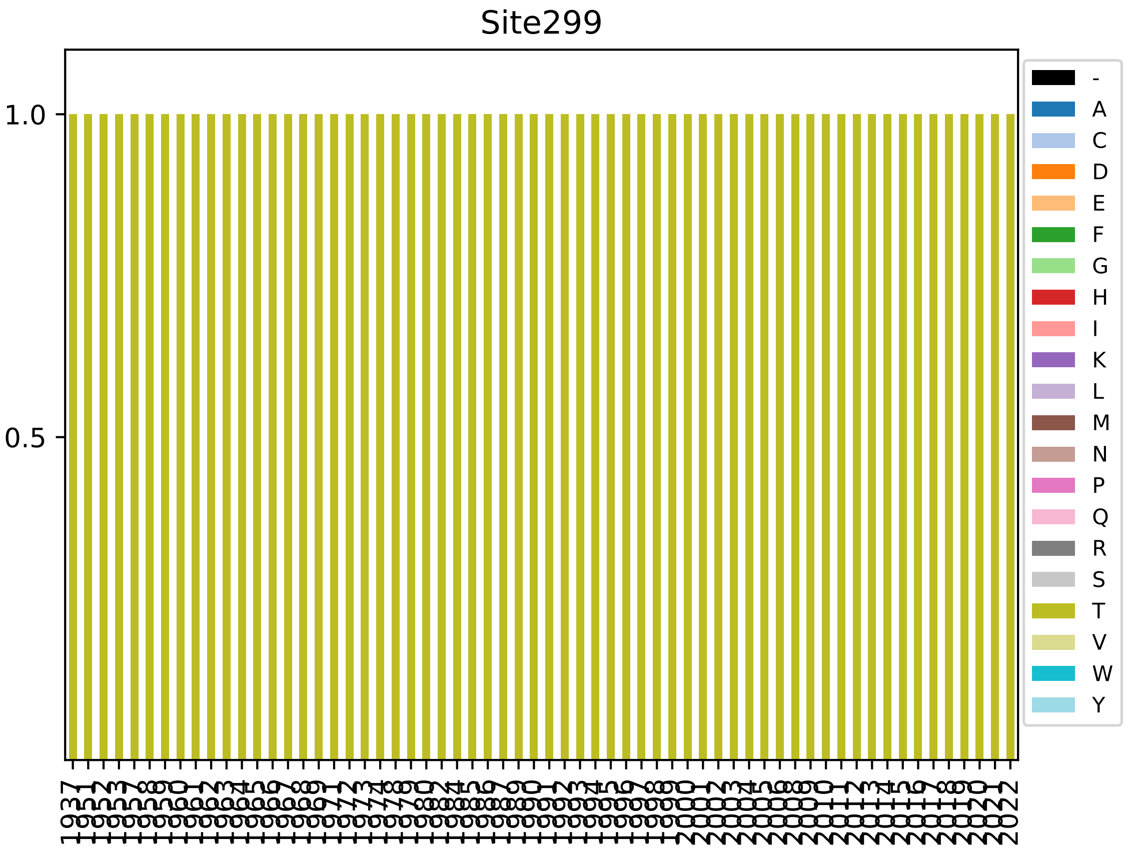
Site299
1.0
0.5
-
A
C
D
E
F
G
H
I
K
L
M
N
P
Q
R
S
T
V
W
Y

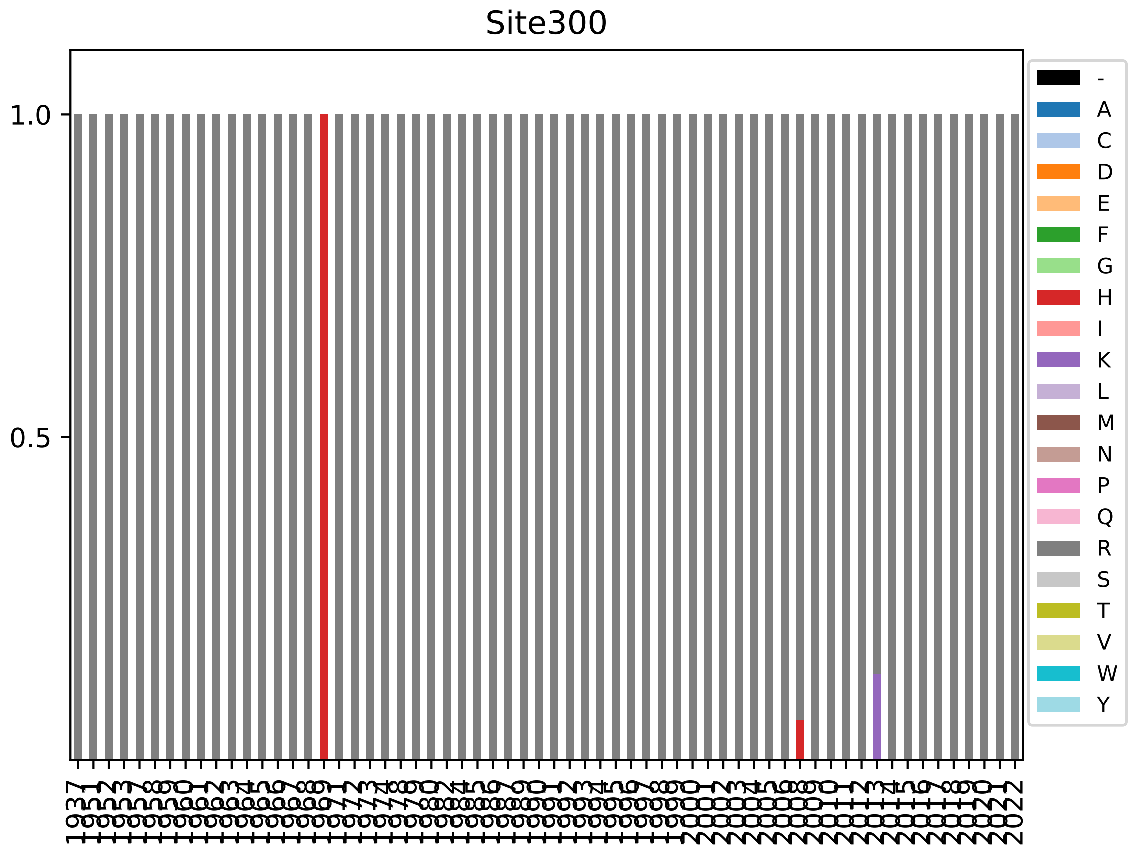

Site300
1.0
0.5
-
A
C
D
E
F
G
H
I
K
L
M
N
P
Q
R
S
T
V
W
Y

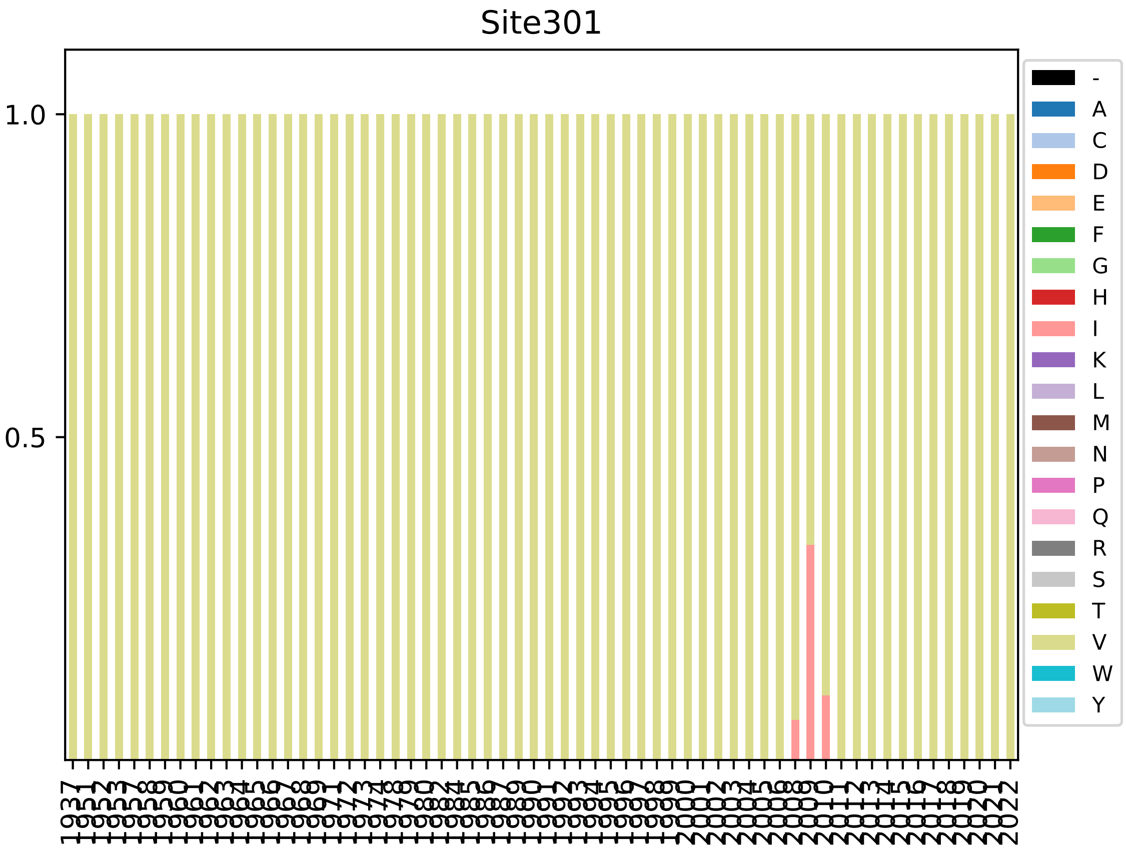
Site301
1.0
0.5
-
A
C
D
E
F
G
H
I
K
L
M
N
P
Q
R
S
T
V
W
Y

Site302

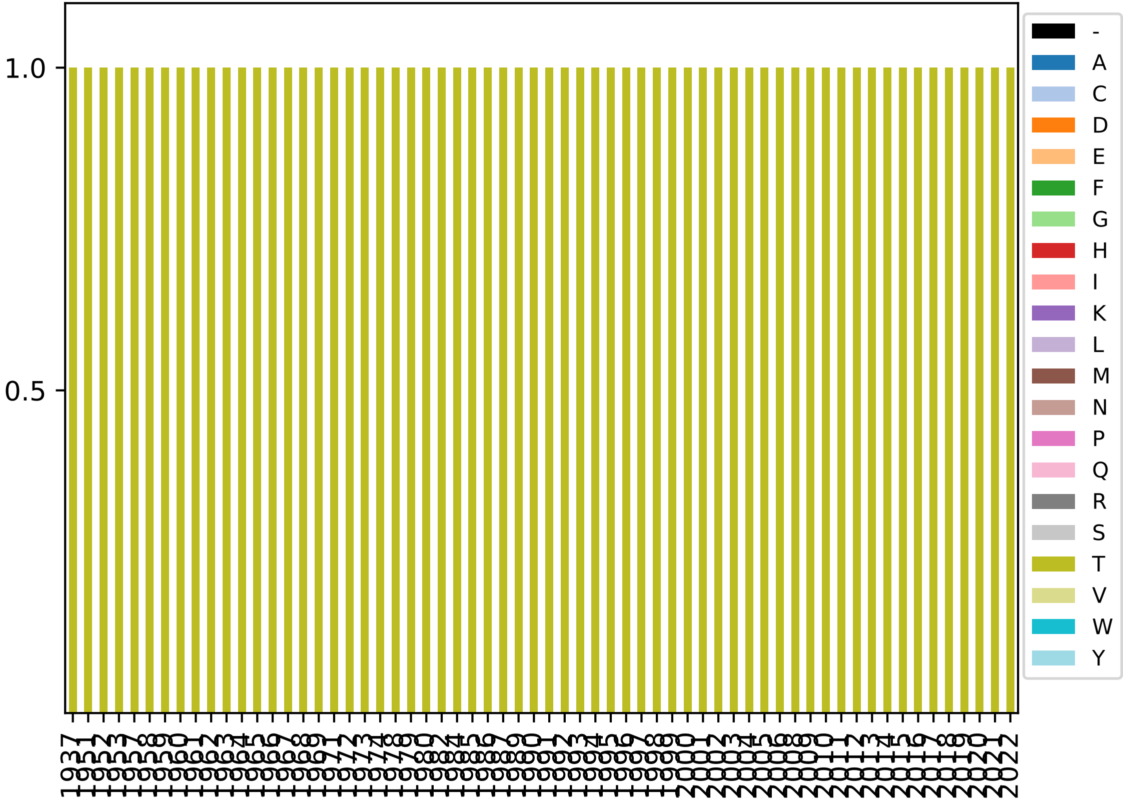

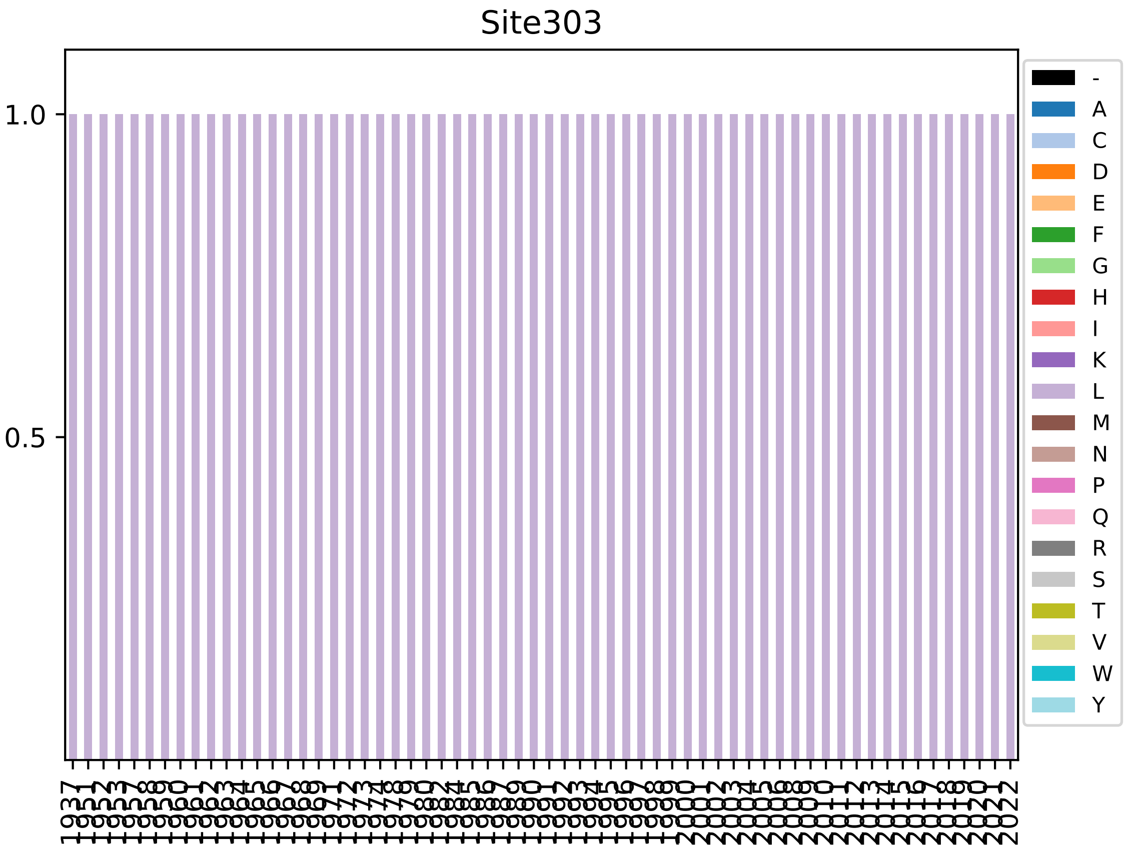
Site303
1.0
0.5
-
A
C
D
E
F
G
H
I
K
L
M
N
P
Q
R
S
T
V
W
Y

Site304

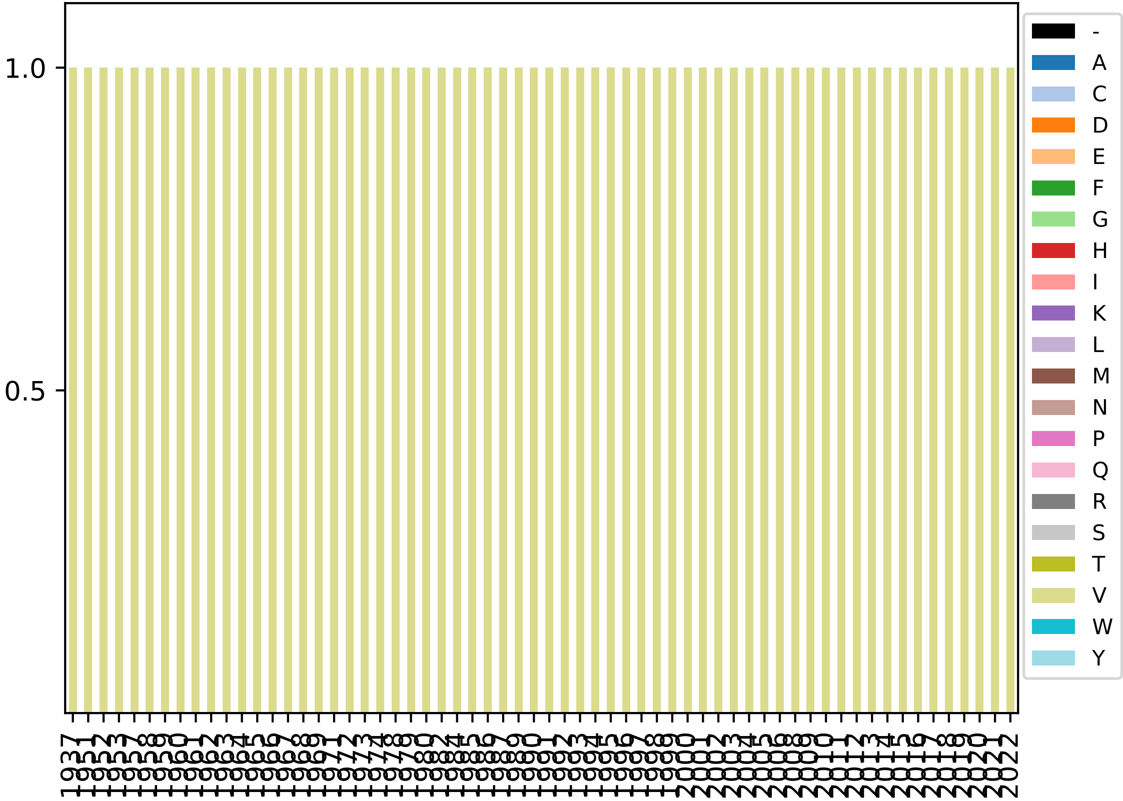

Site305

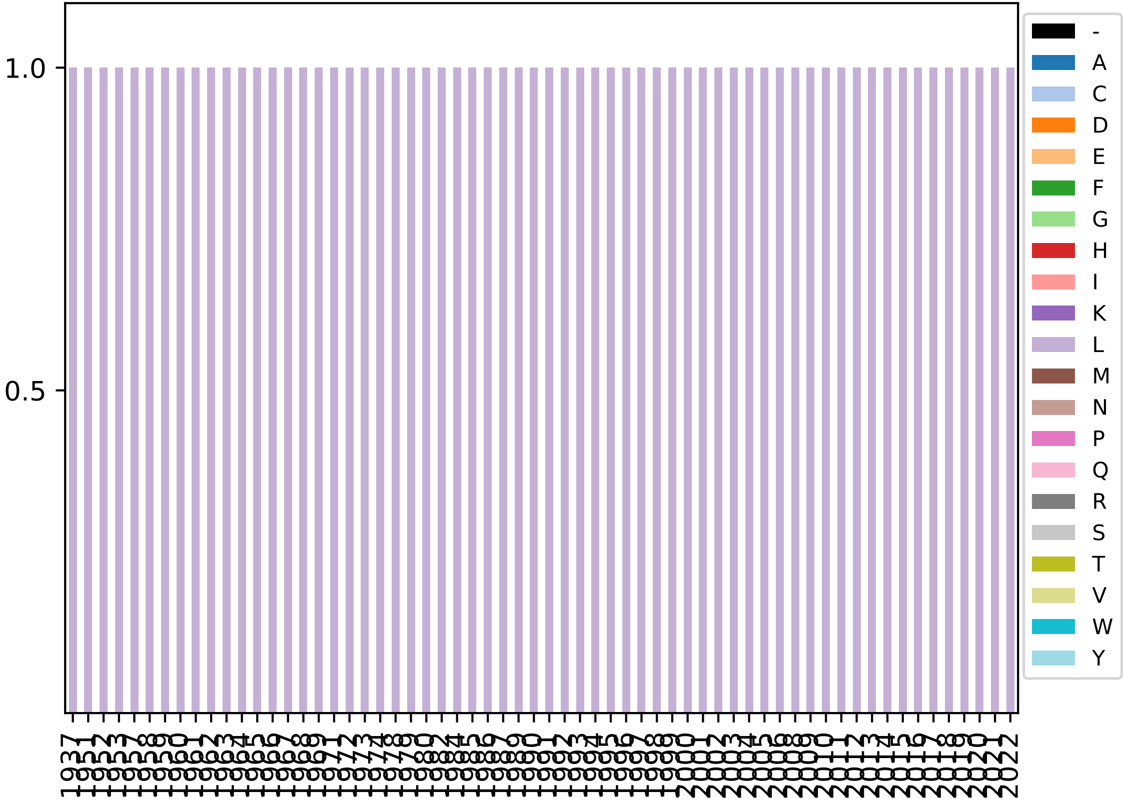

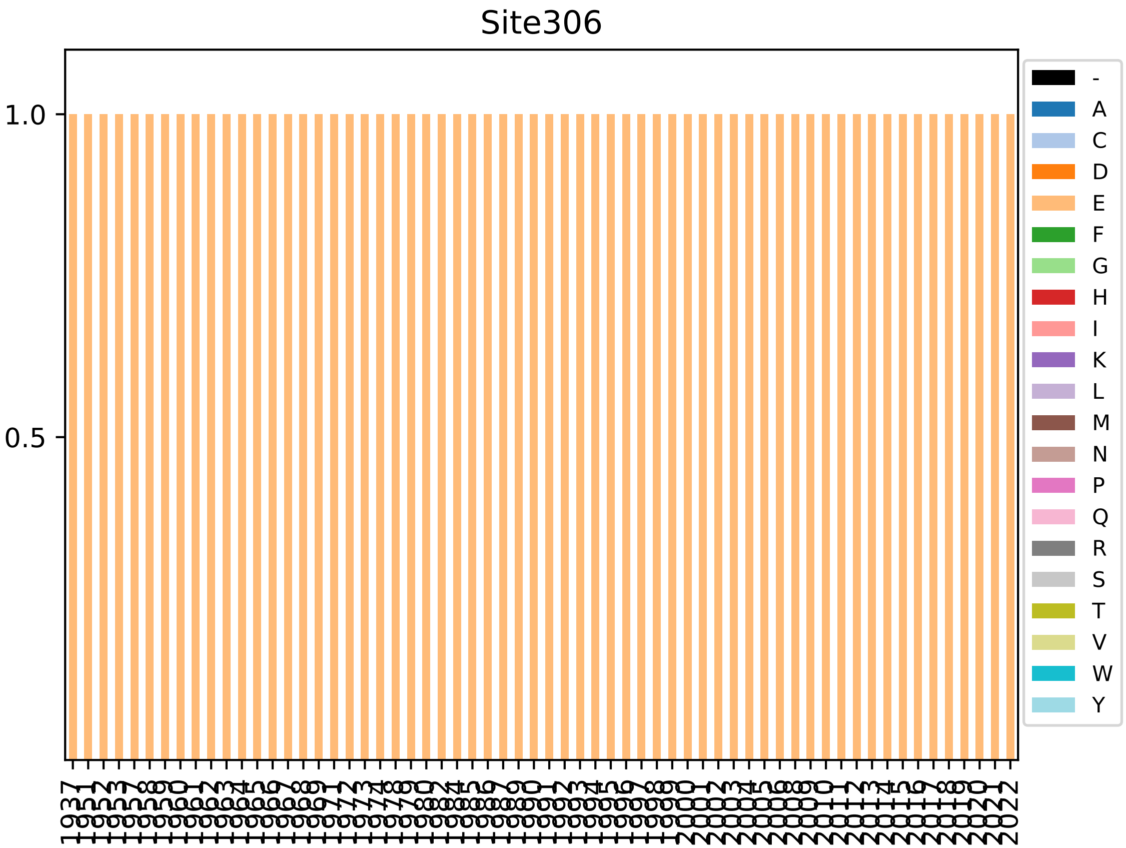
Site306
1.0
0.5
-
A
C
D
E
F
G
H
I
K
L
M
N
P
Q
R
S
T
V
W
Y

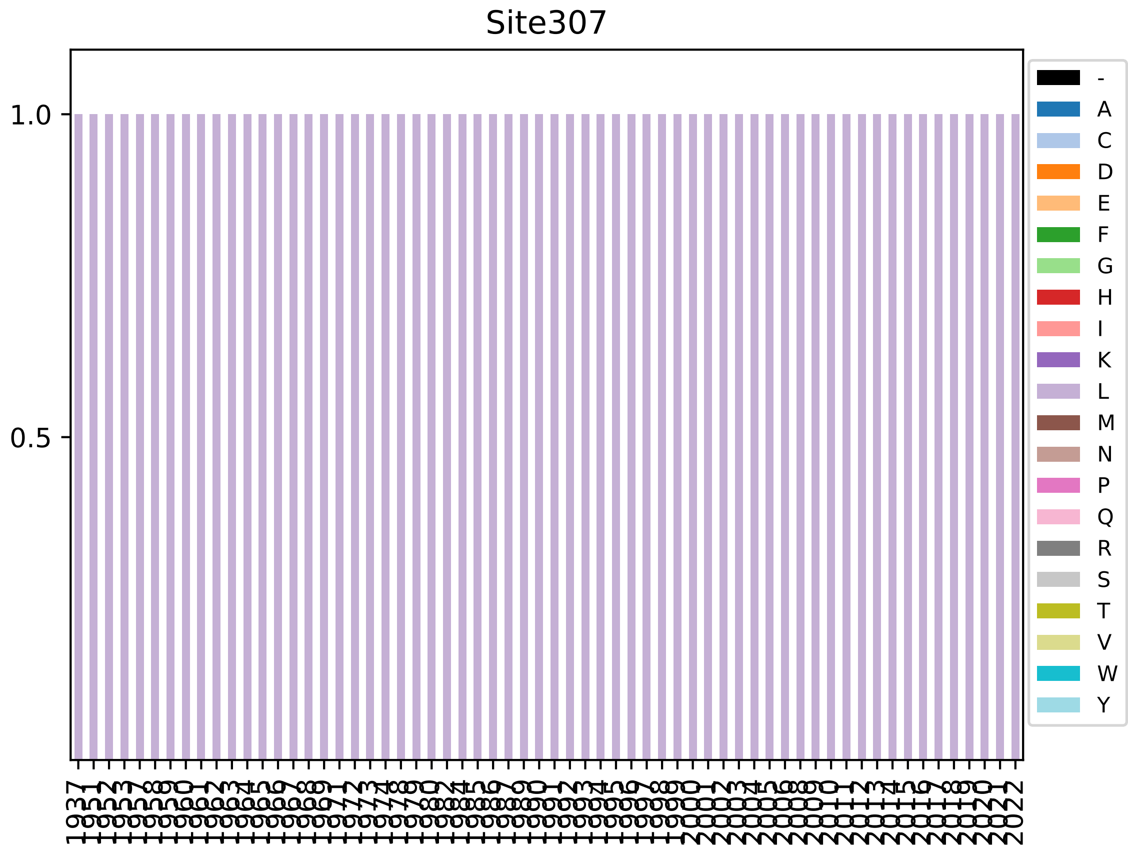
Site307
1.0
0.5
-
A
C
D
E
F
G
H
I
K
L
M
N
P
Q
R
S
T
V
W
Y

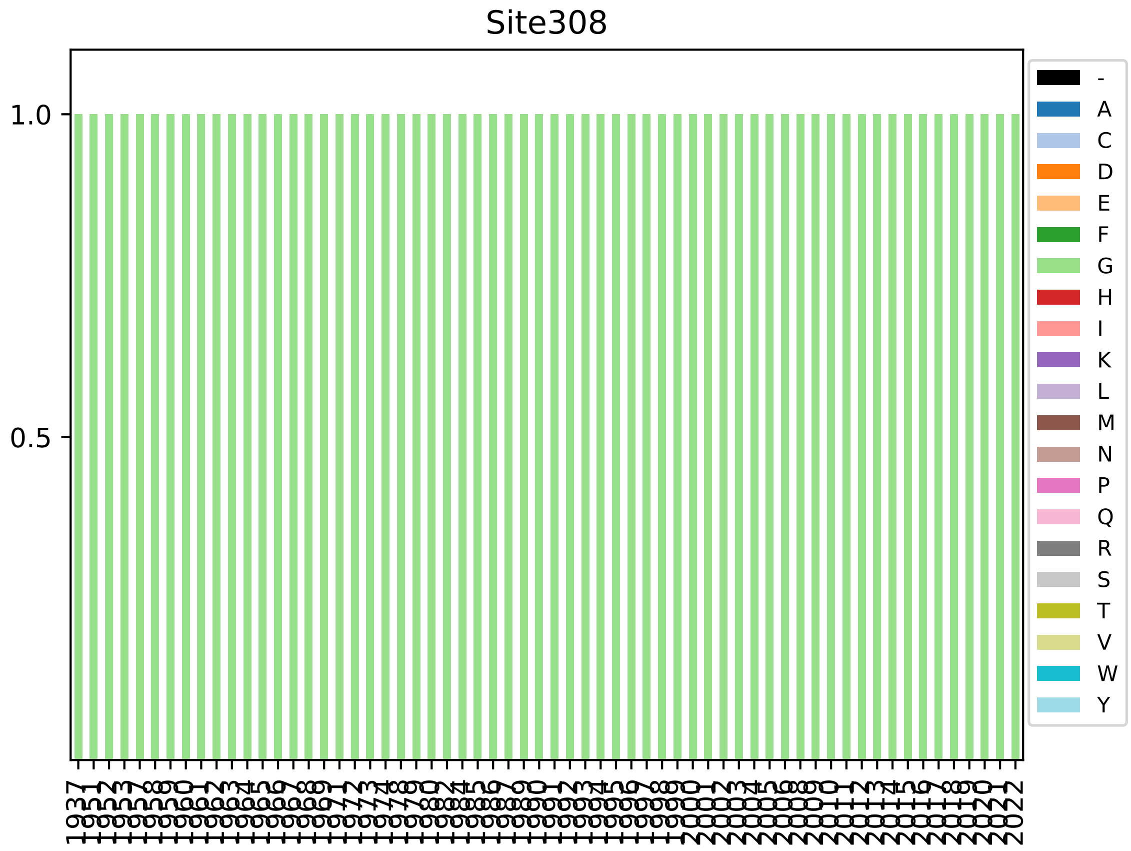
Site308
1.0
0.5
-
A
C
D
E
F
G
H
I
K
L
M
N
P
Q
R
S
T
V
W
Y

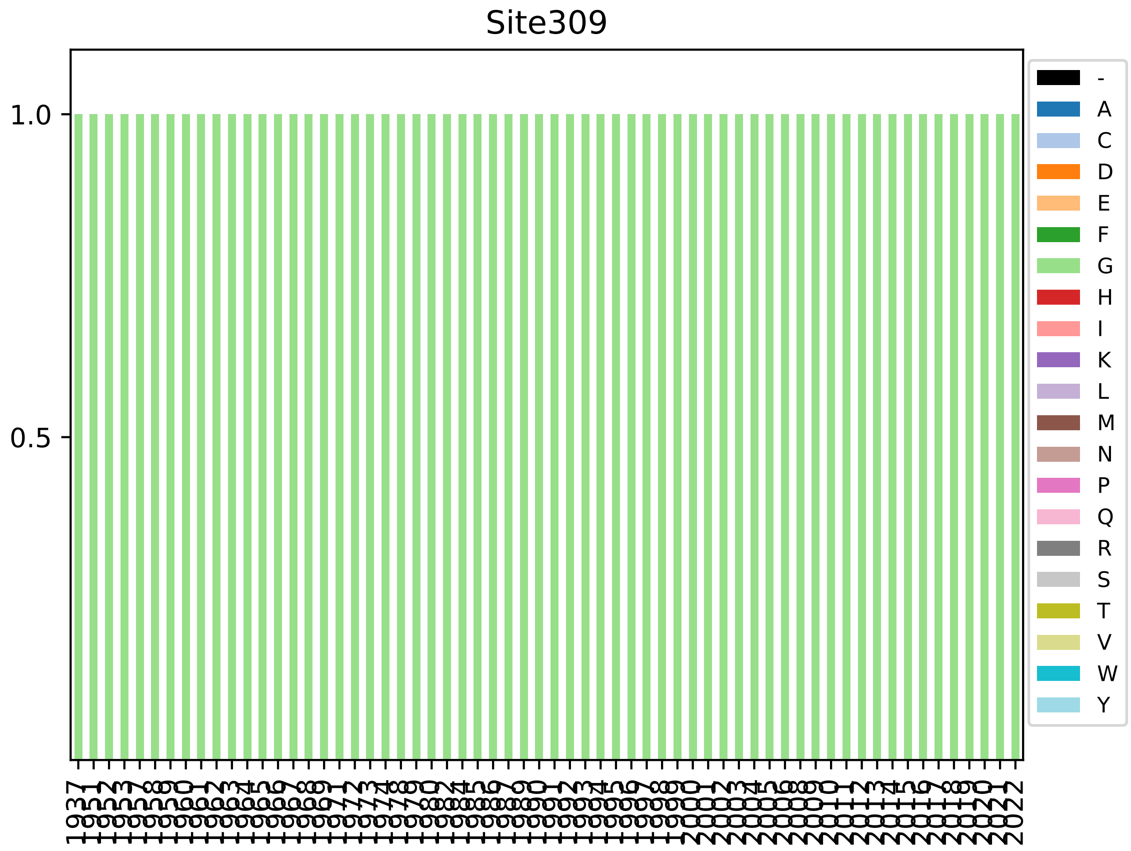
Site309
1.0
0.5
-
A
C
D
E
F
G
H
I
K
L
M
N
P
Q
R
S
T
V
W
Y

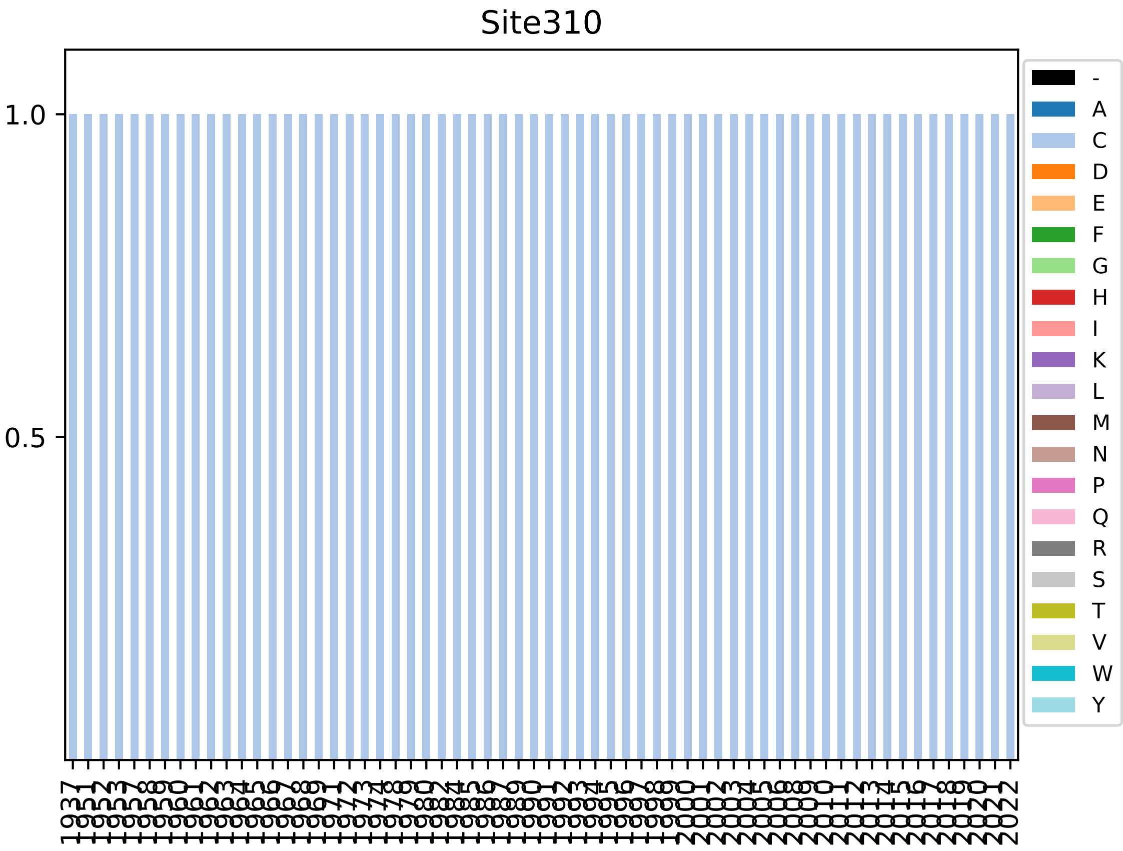
Site310
1.0
0.5
-
A
C
D
E
F
G
H
I
K
L
M
N
P
Q
R
S
T
V
W
Y

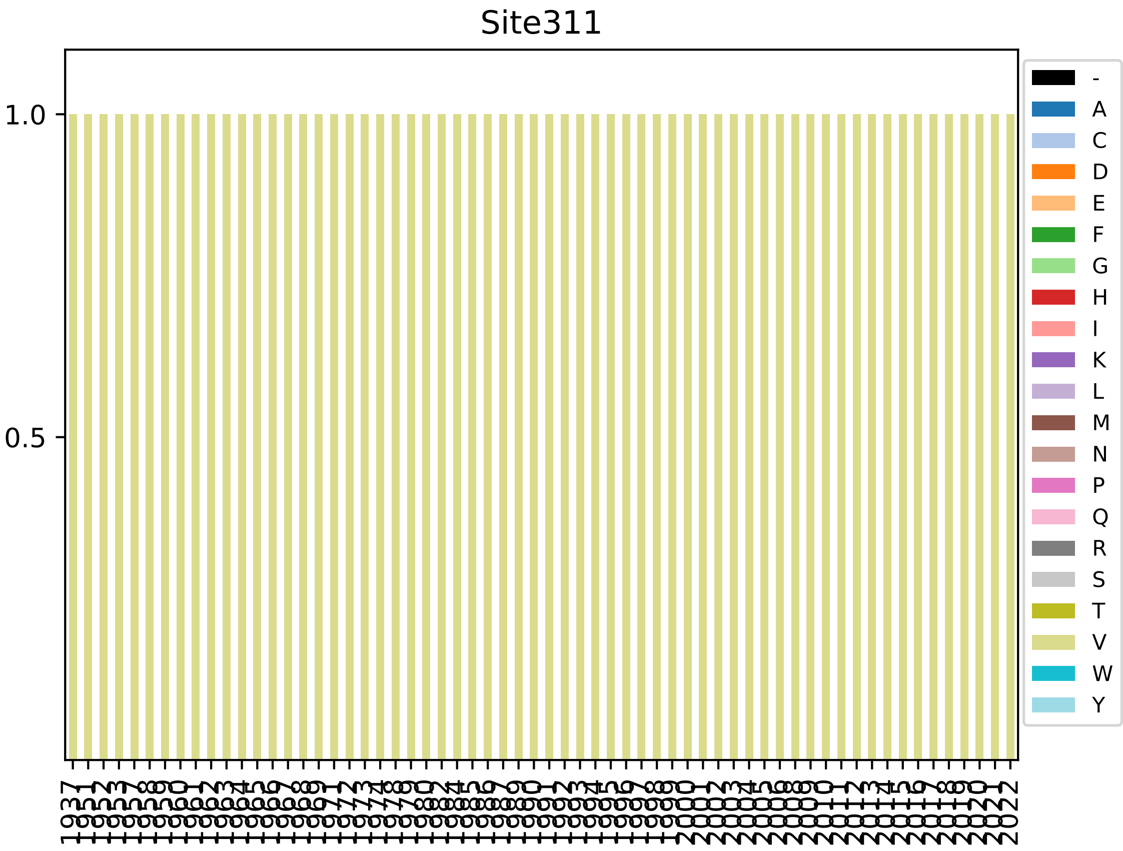
Site311
1.0
0.5
-
A
C
D
E
F
G
H
I
K
L
M
N
P
Q
R
S
T
V
W
Y

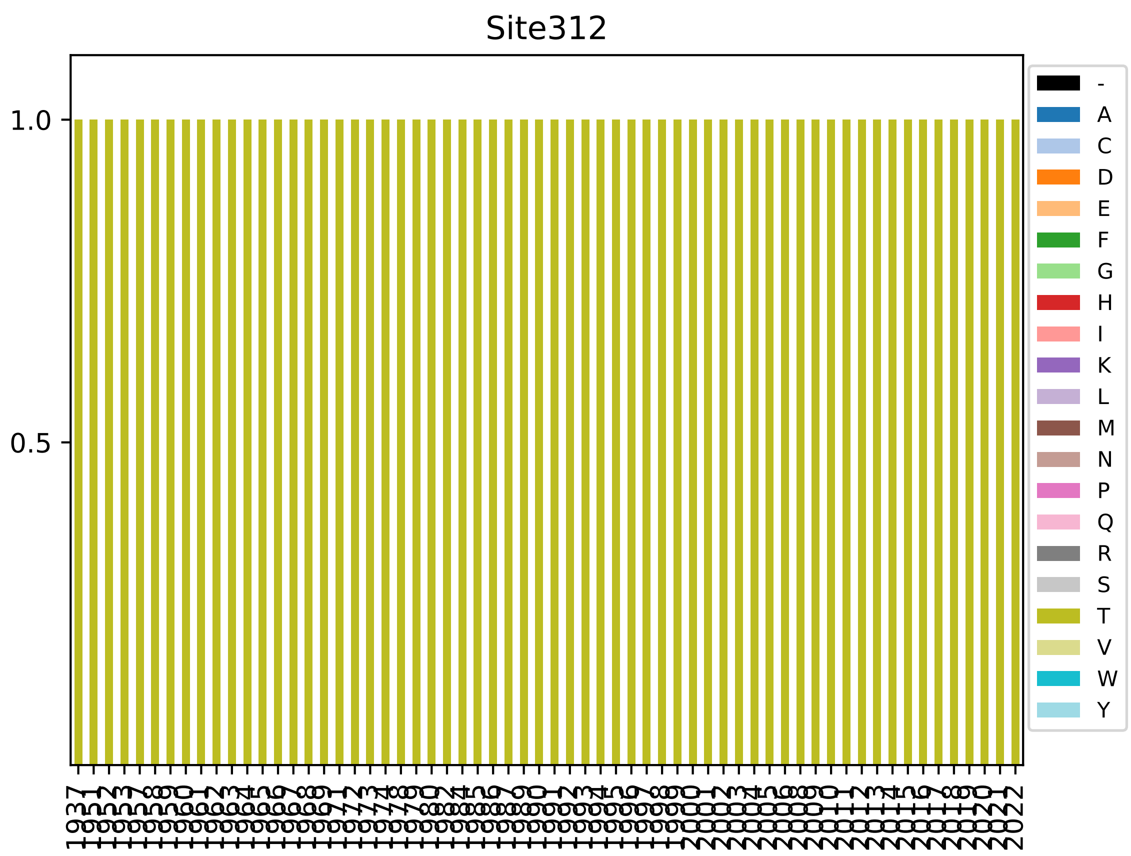

Site312
1.0
0.5
-
A
C
D
E
F
G
H
I
K
L
M
N
P
Q
R
S
T
V
W
Y

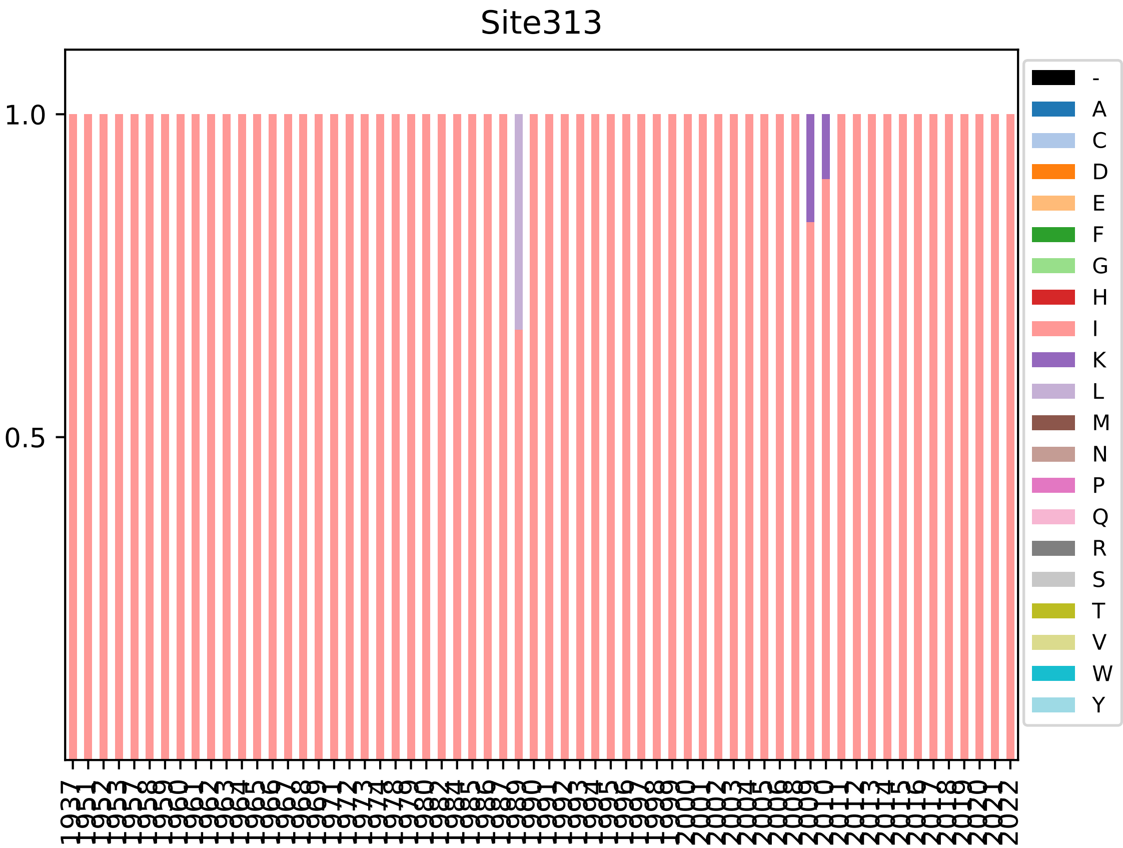
Site313
1.0
0.5
-
A
C
D
E
F
G
H
I
K
L
M
N
P
Q
R
S
T
V
W
Y

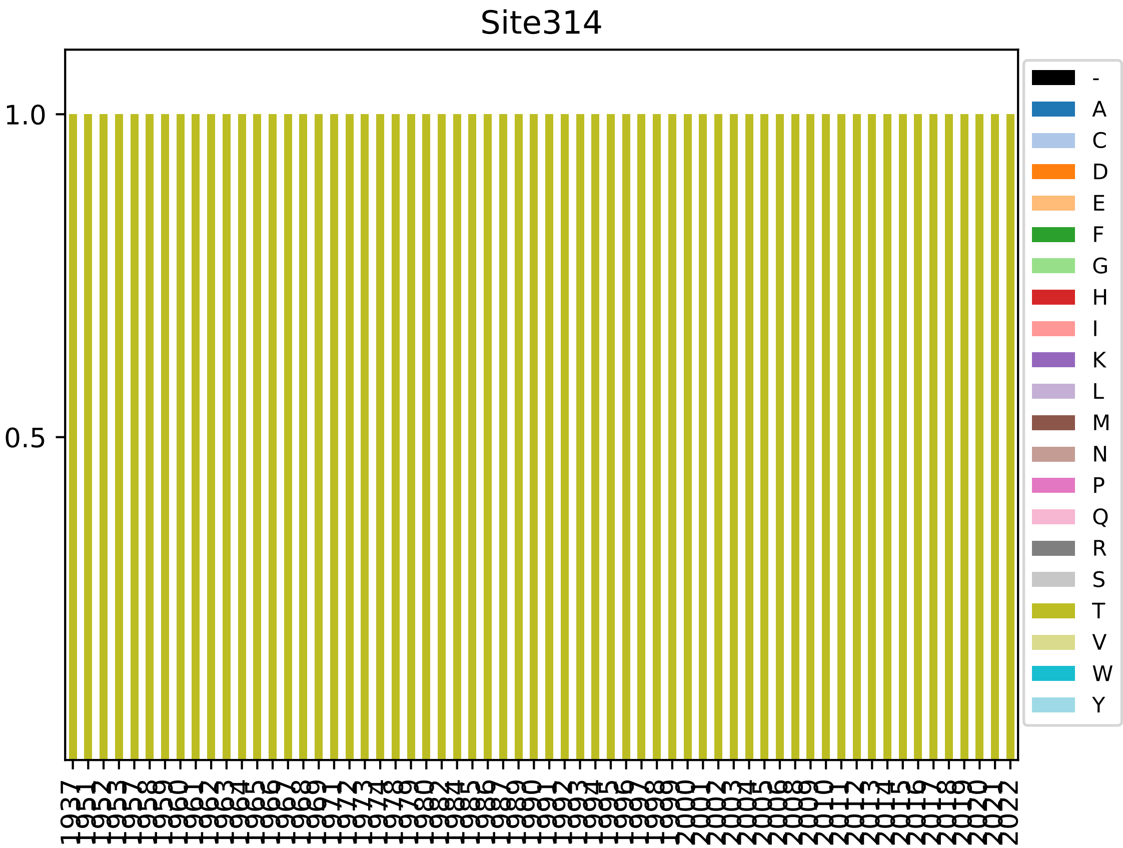
Site314
1.0
0.5
-
A
C
D
E
F
G
H
I
K
L
M
N
P
Q
R
S
T
V
W
Y

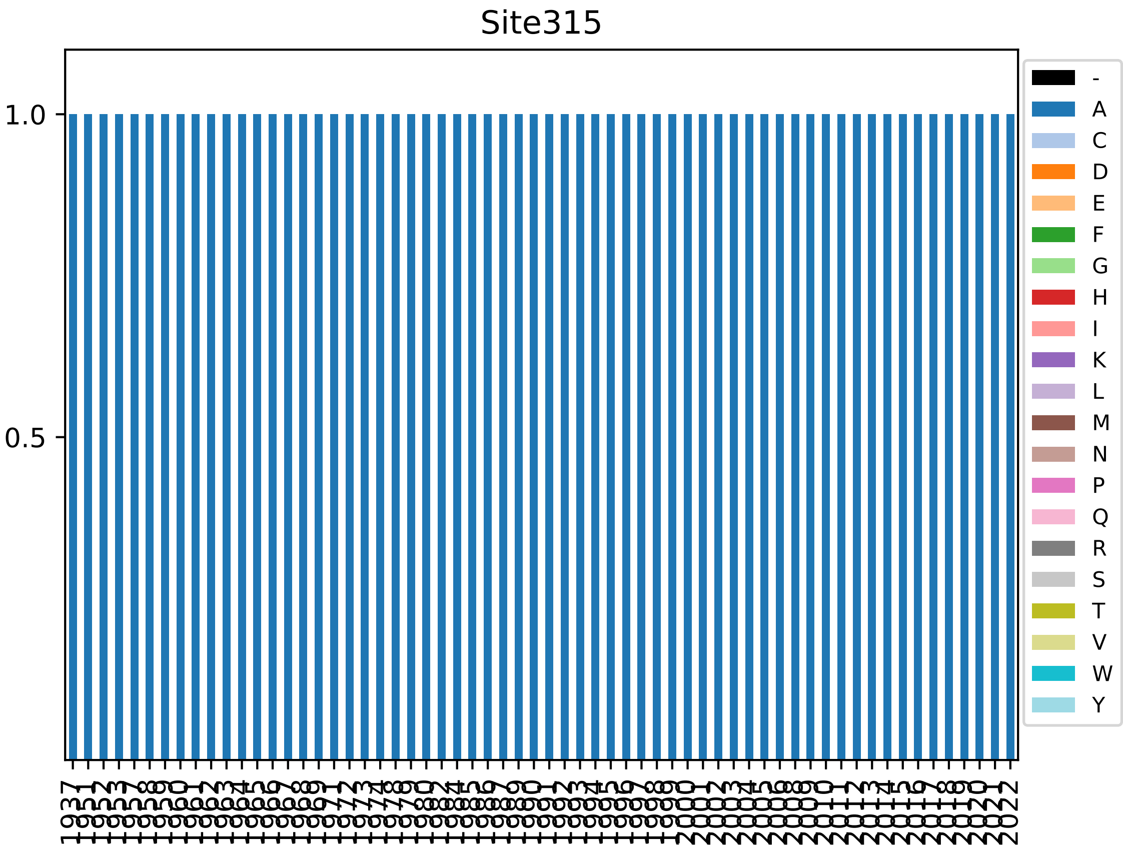
Site315
1.0
0.5
-
A
C
D
E
F
G
H
I
K
L
M
N
P
Q
R
S
T
V
W
Y

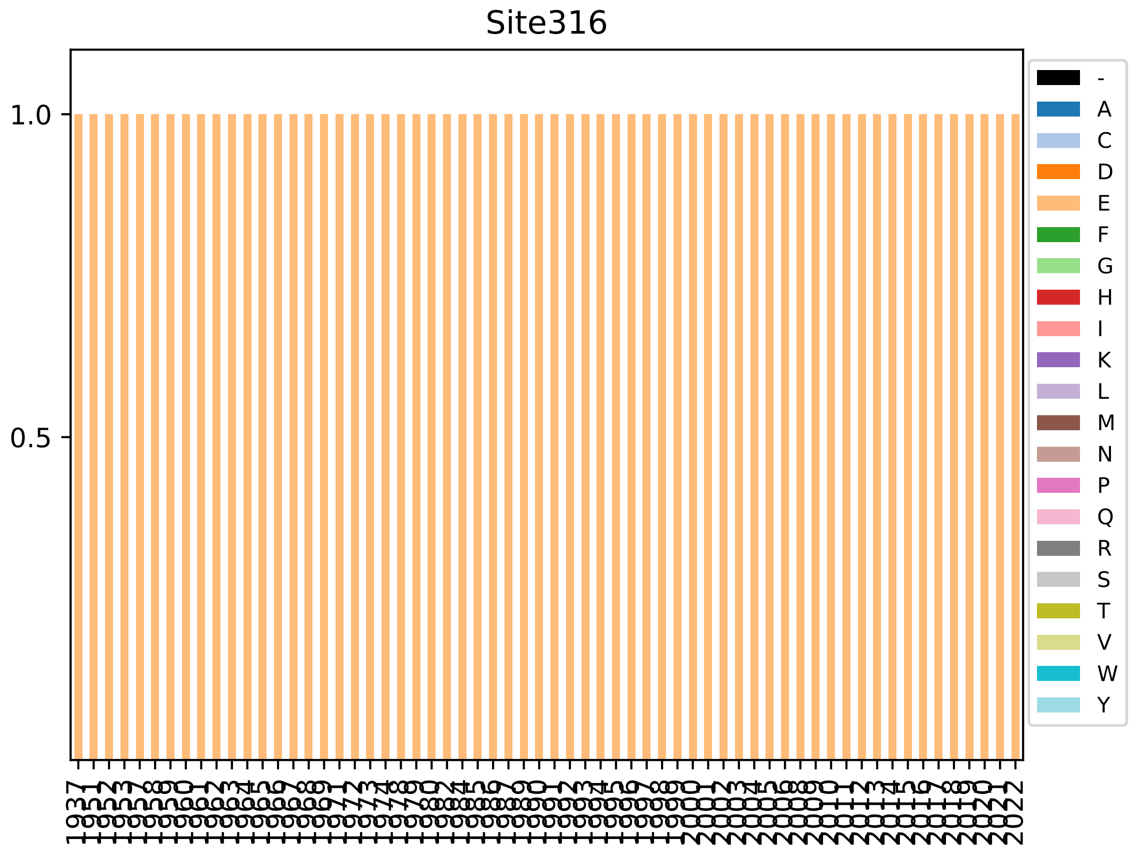
Site316
1.0
0.5
-
A
C
D
E
F
G
H
I
K
L
M
N
P
Q
R
S
T
V
W
Y

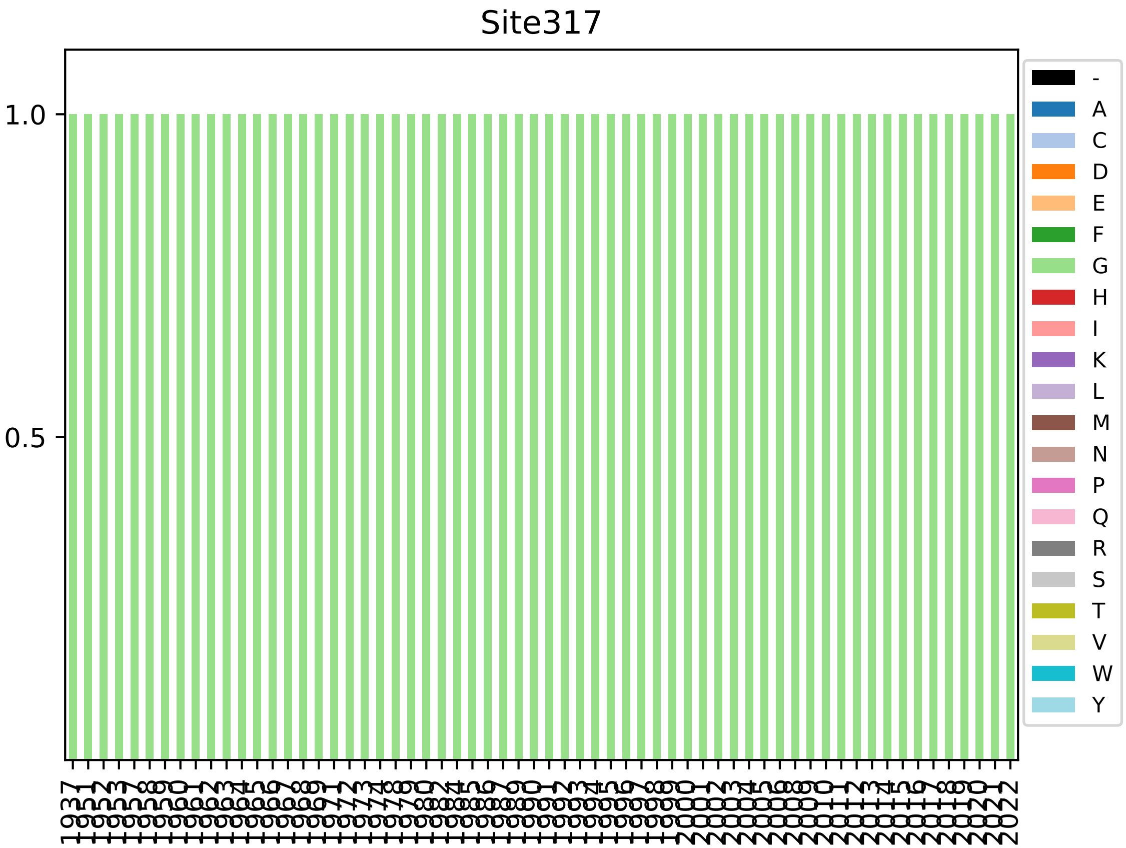

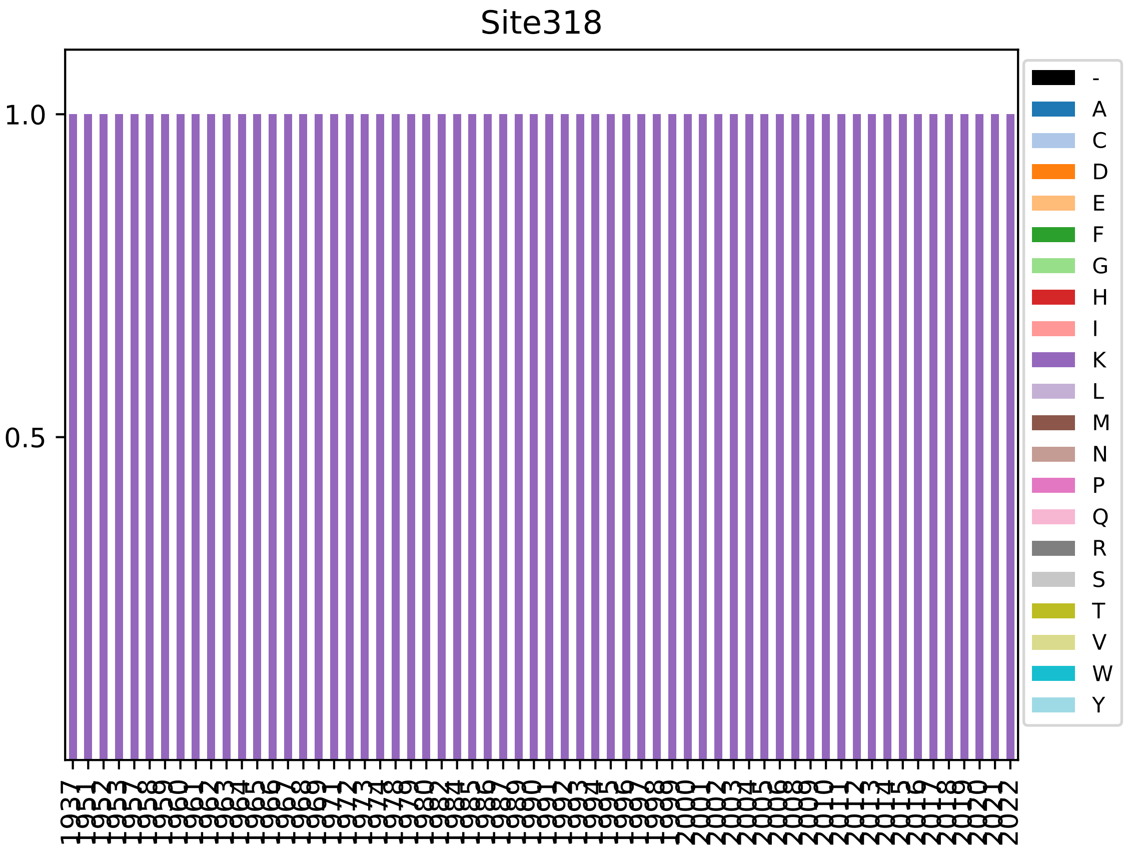
Site318
1.0
0.5
-
A
C
D
E
F
G
H
I
K
L
M
N
P
Q
R
S
T
V
W
Y

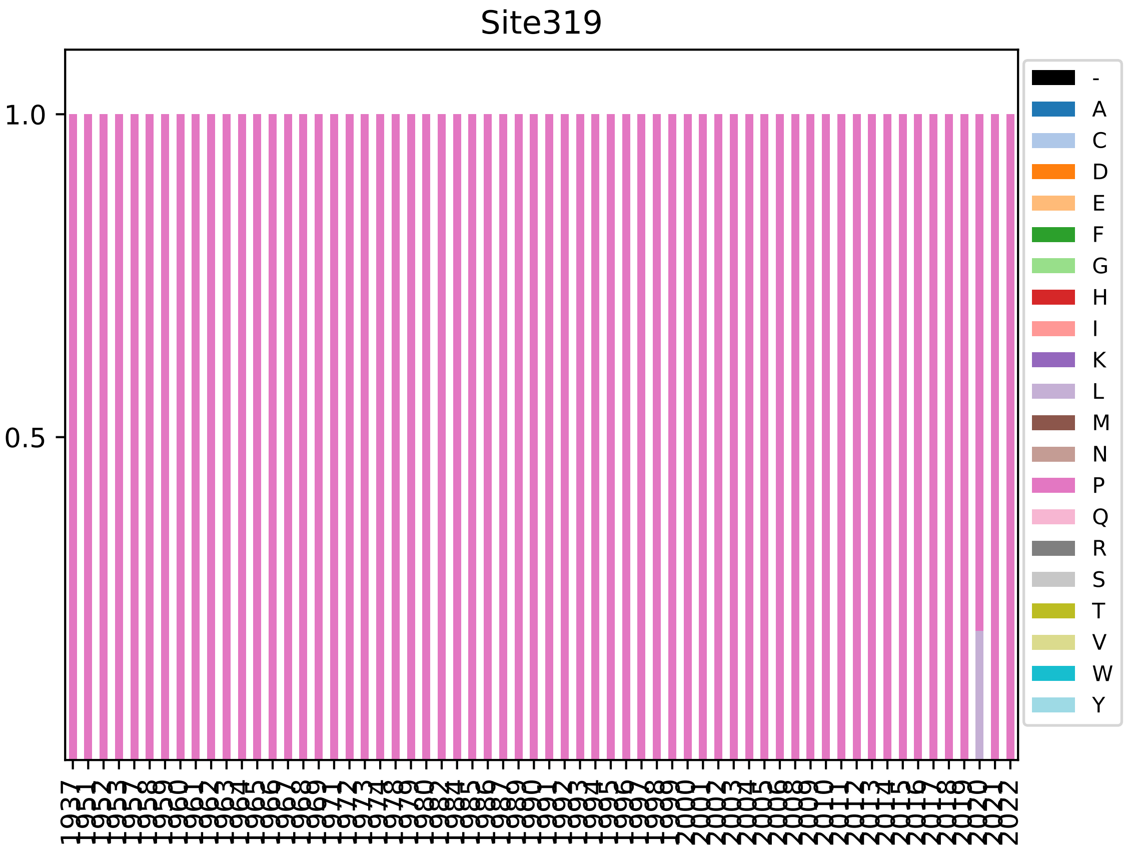
Site319
1.0
0.5
-
A
C
D
E
F
G
H
I
K
L
M
N
P
Q
R
S
T
V
W
Y

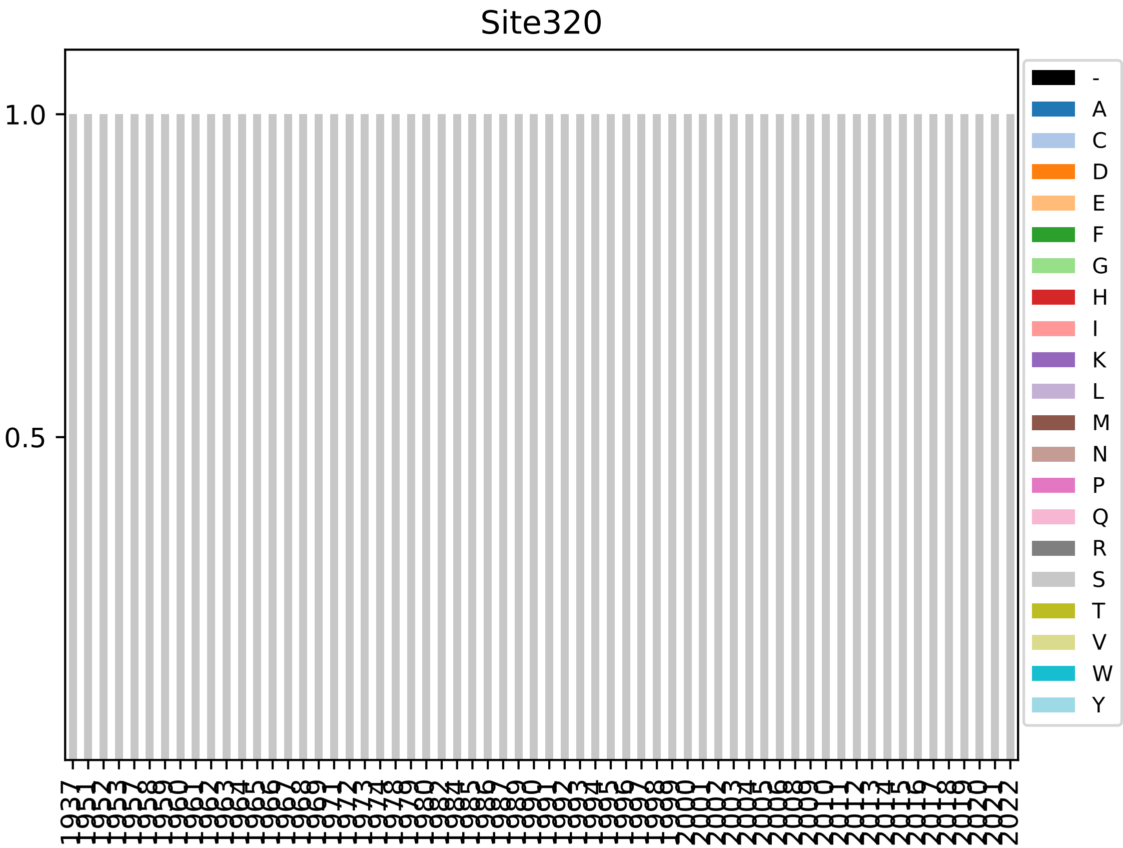

Site320
1.0
0.5
-
A
C
D
E
F
G
H
I
K
L
M
N
P
Q
R
S
T
V
W
Y

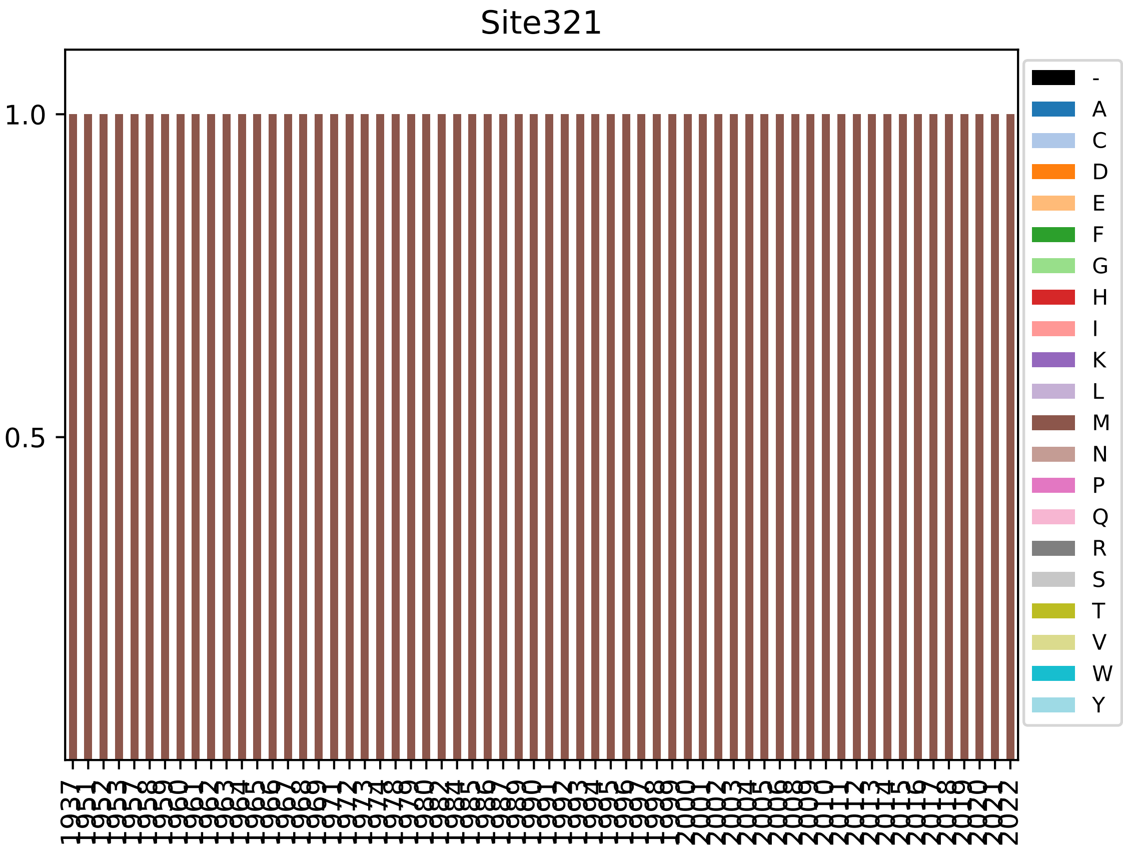
Site321
1.0
0.5
-
A
C
D
E
F
G
H
I
K
L
M
N
P
Q
R
S
T
V
W
Y

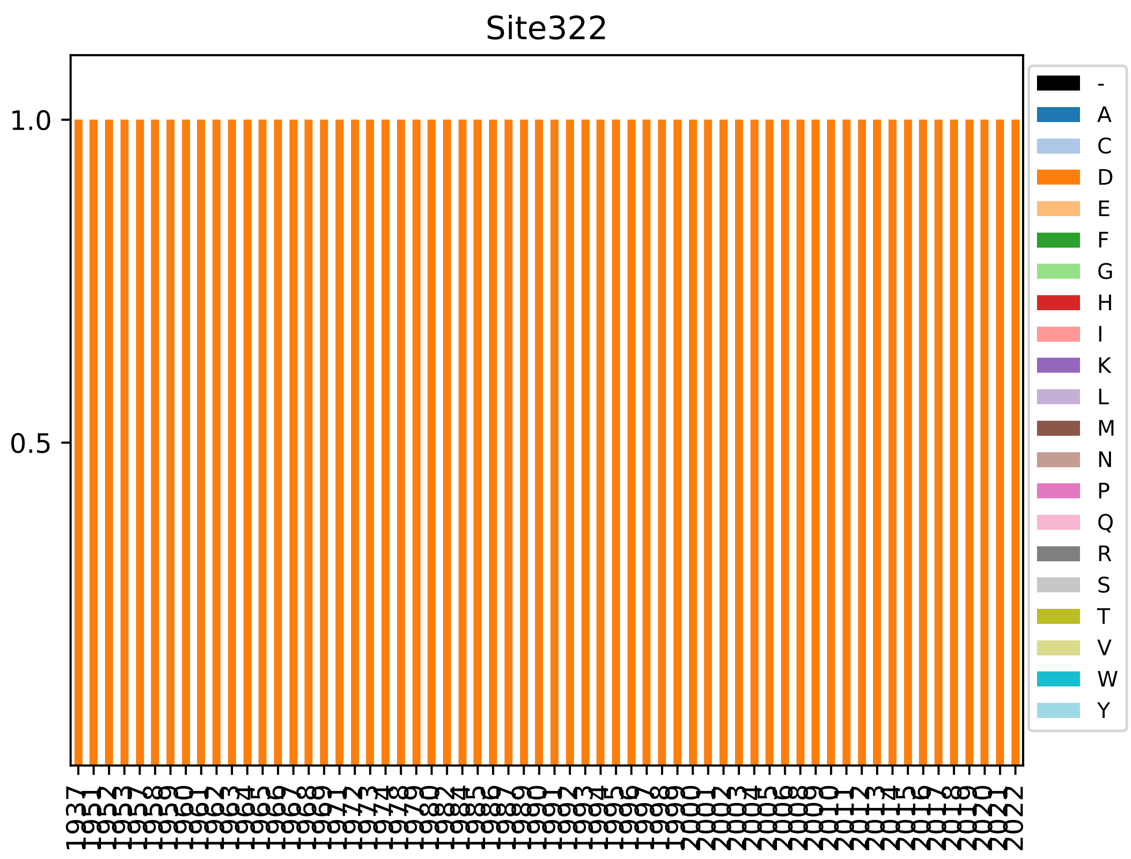

Site322
1.0
0.5
-
A
C
D
E
F
G
H
I
K
L
M
N
P
Q
R
S
T
V
W
Y

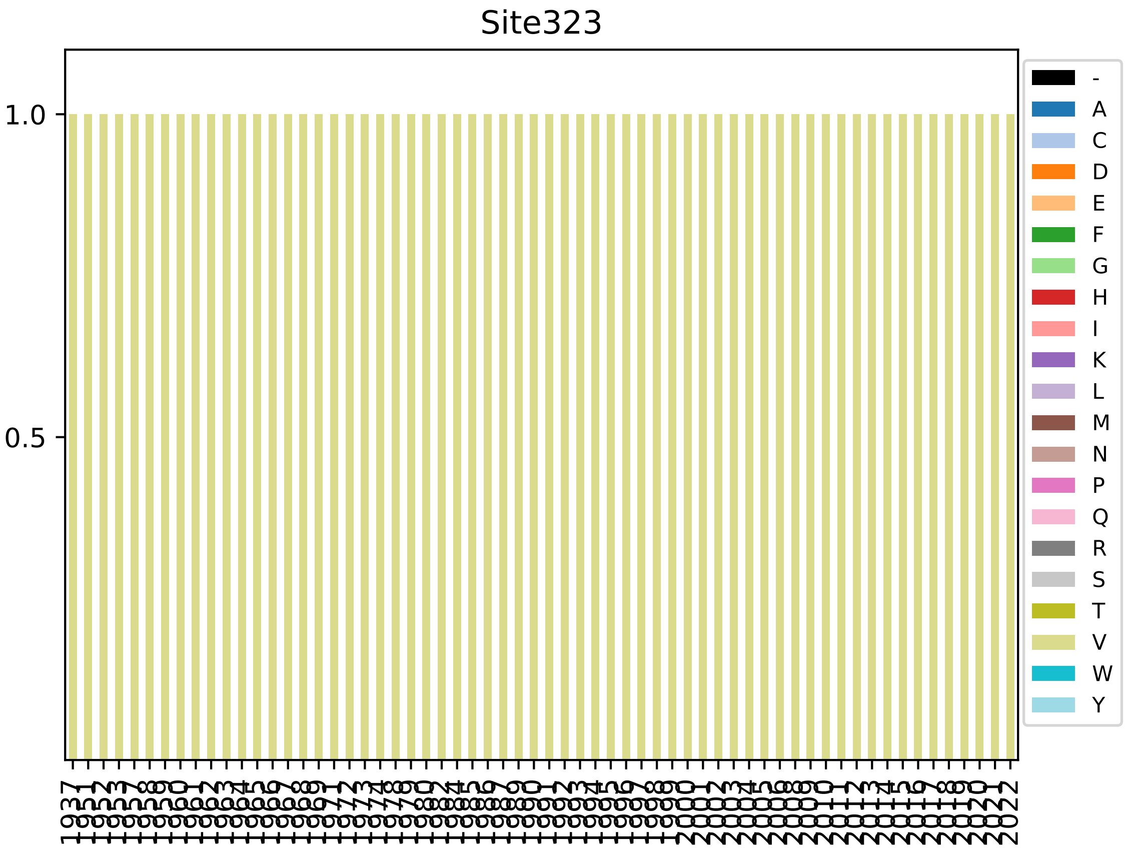
Site323
1.0
0.5
-
A
C
D
E
F
G
H
I
K
L
M
N
P
Q
R
S
T
V
W
Y

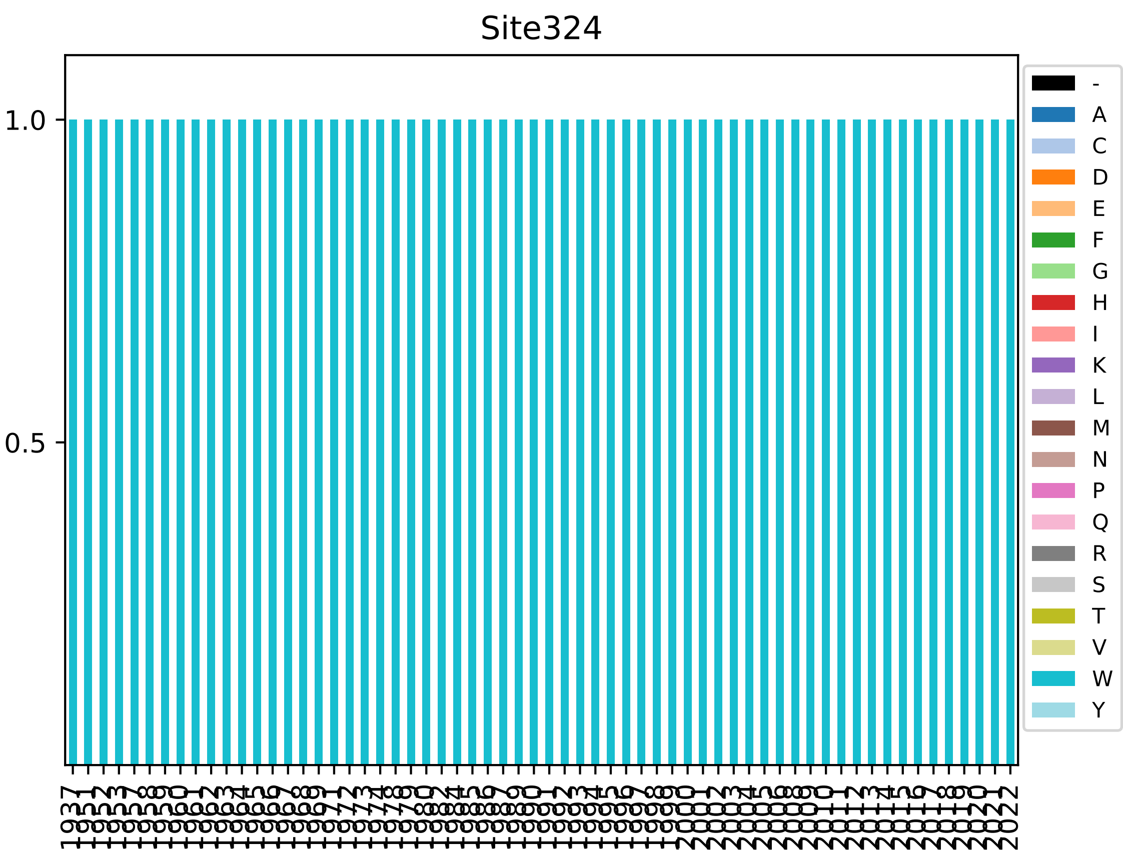
Site324
1.0
0.5
-
A
C
D
E
F
G
H
I
K
L
M
N
P
Q
R
S
T
V
W
Y

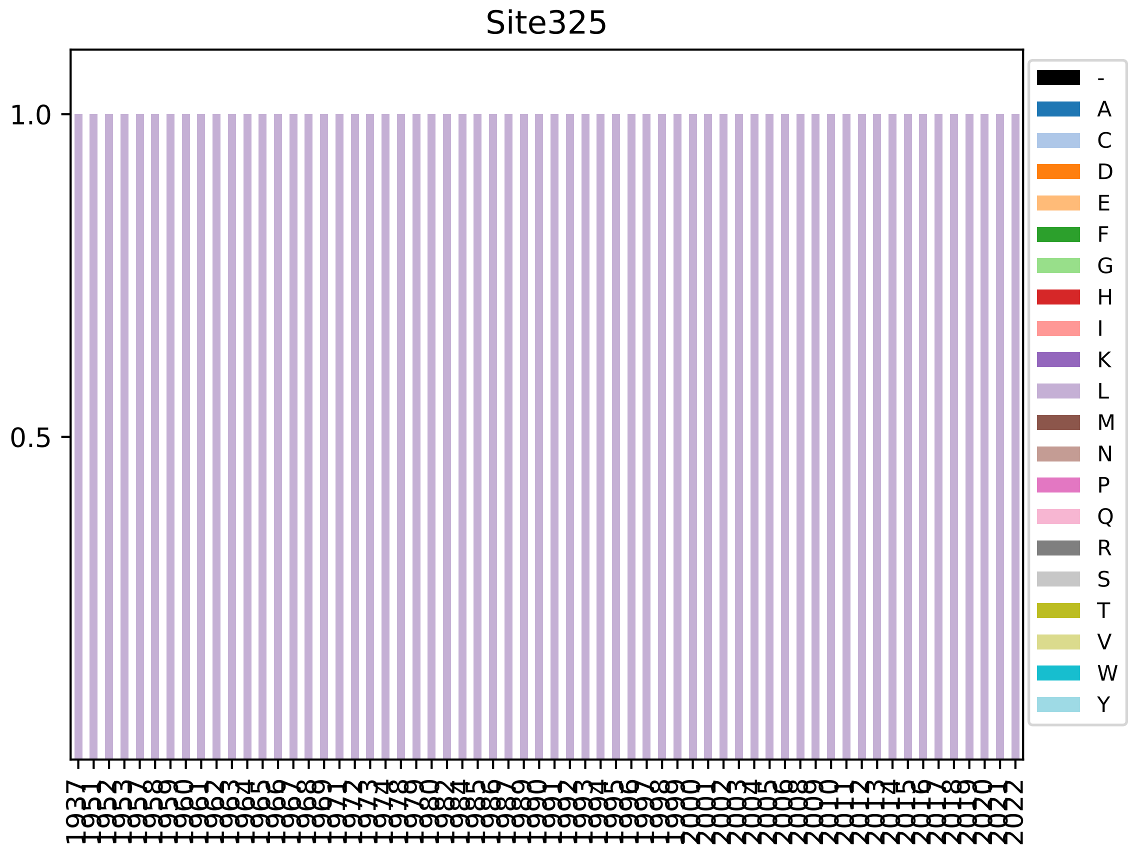
Site325
1.0
0.5
-
A
C
D
E
F
G
H
I
K
L
M
N
P
Q
R
S
T
V
W
Y

Site326

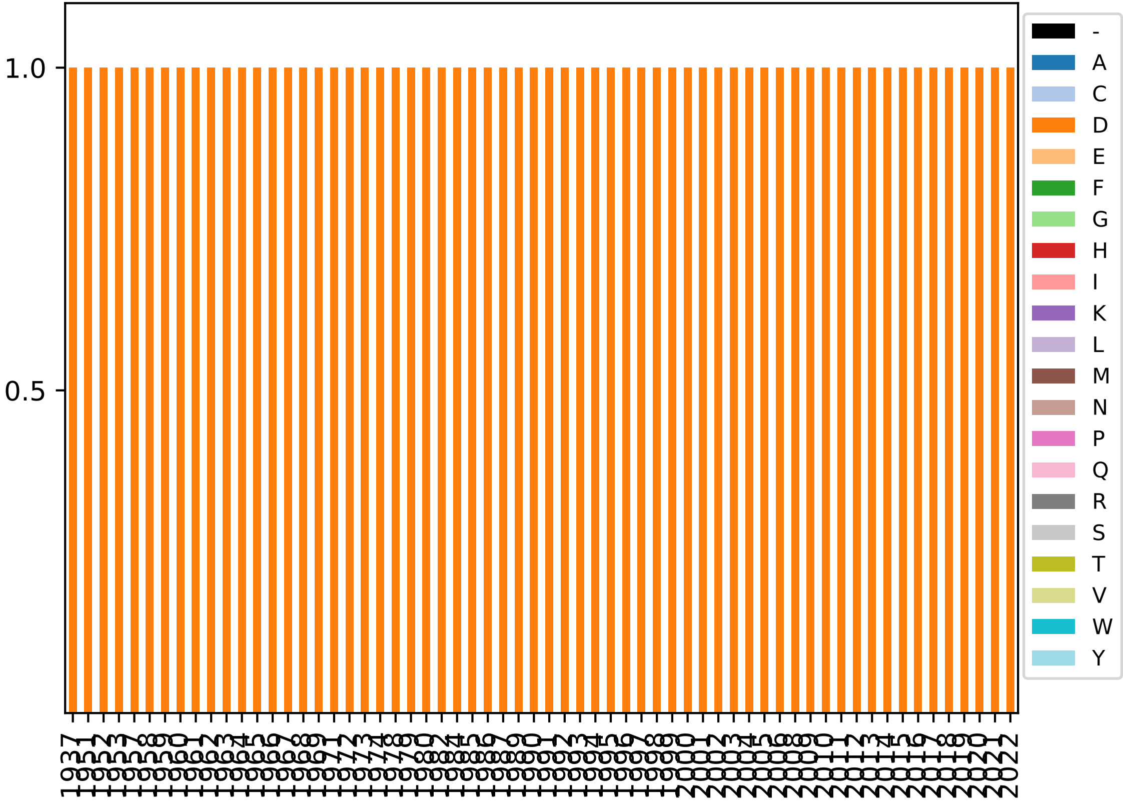

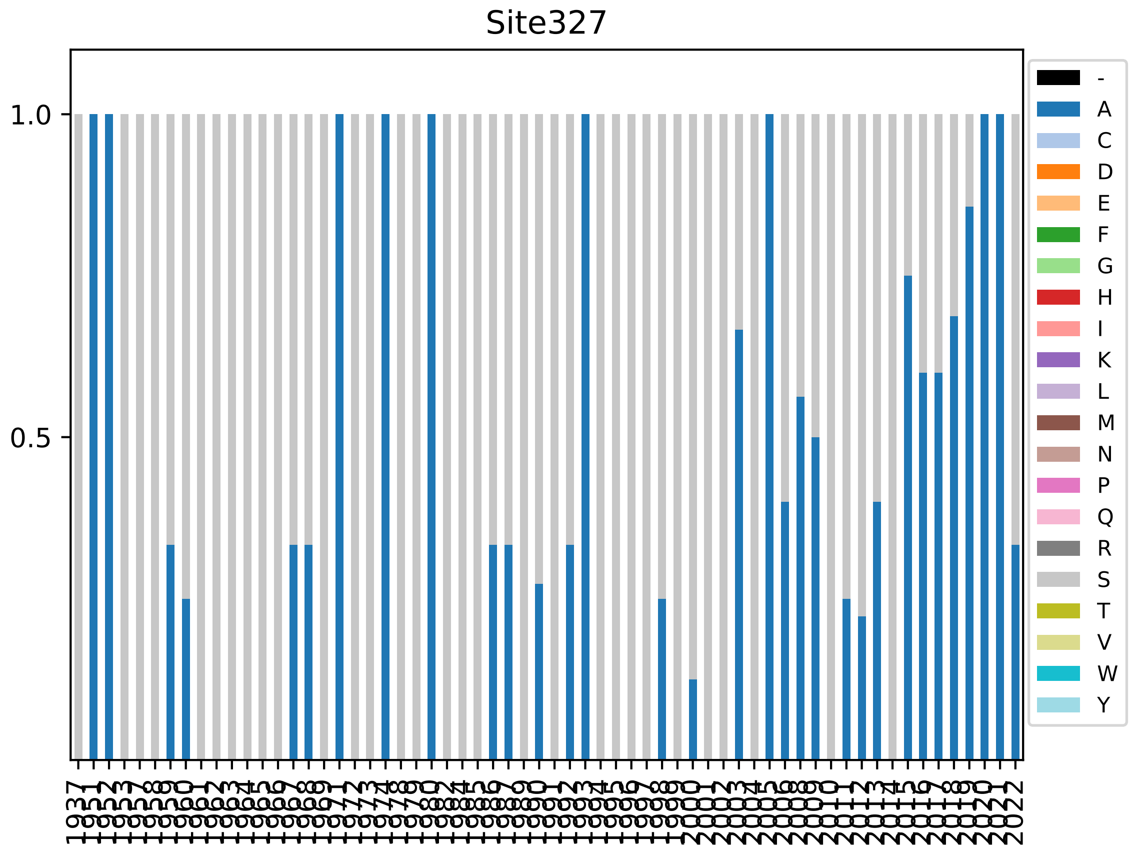

Site327
1.0
0.5
-
A
C
D
E
F
G
H
I
K
L
M
N
P
Q
R
S
T
V
W
Y

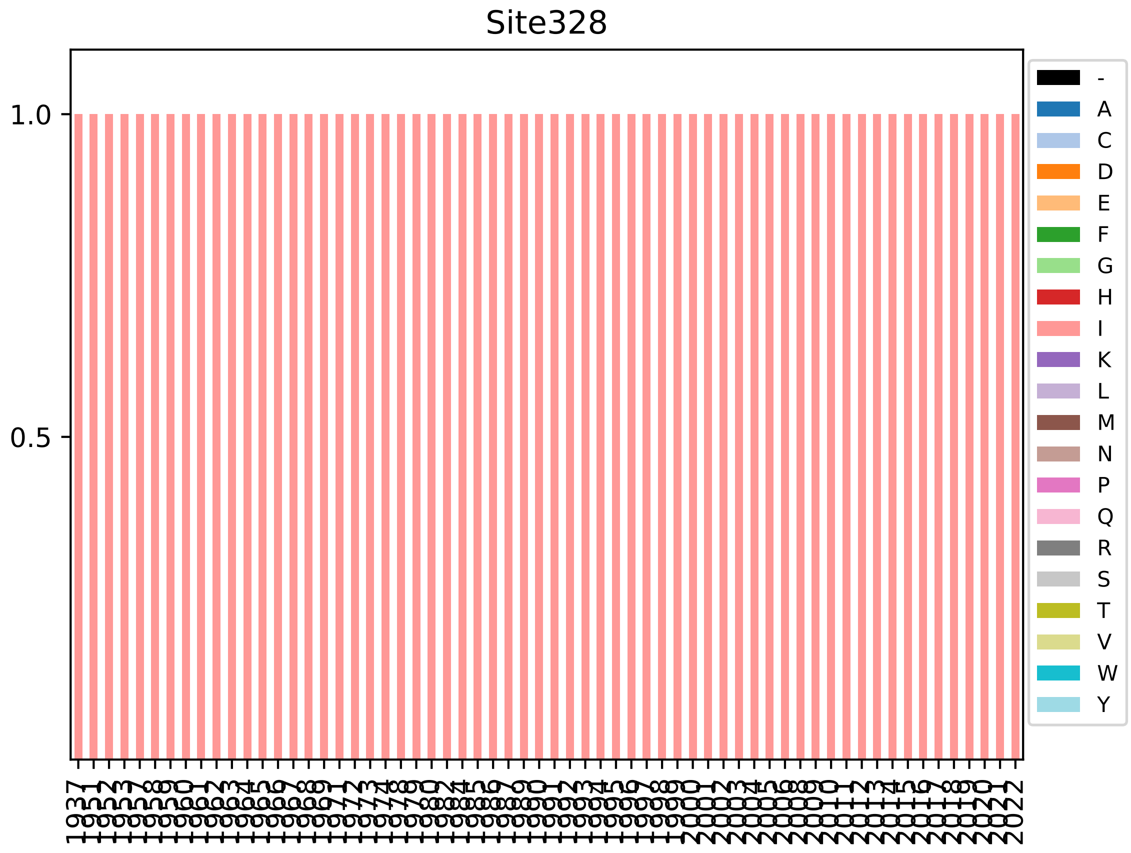
Site328
1.0
0.5
-
A
C
D
E
F
G
H
I
K
L
M
N
P
Q
R
S
T
V
W
Y

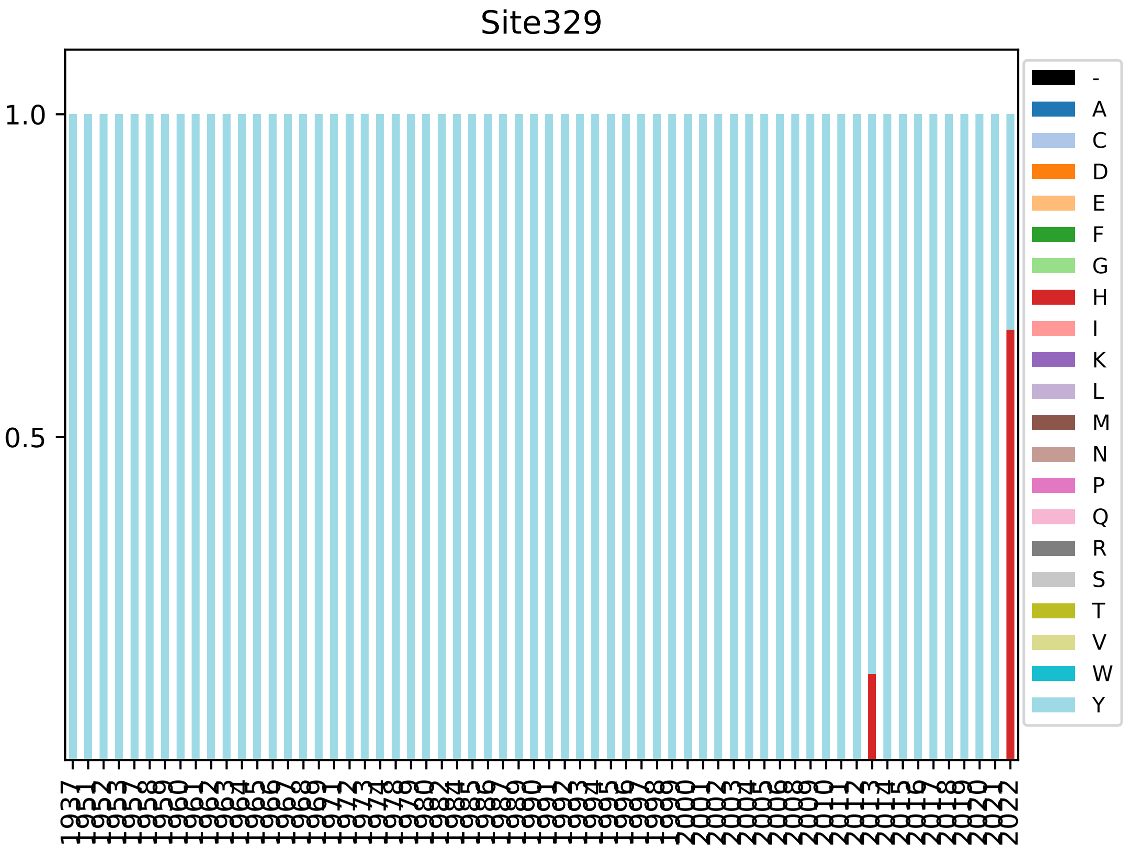
Site329
1.0
0.5
-
A
C
D
E
F
G
H
I
K
L
M
N
P
Q
R
S
T
V
W
Y

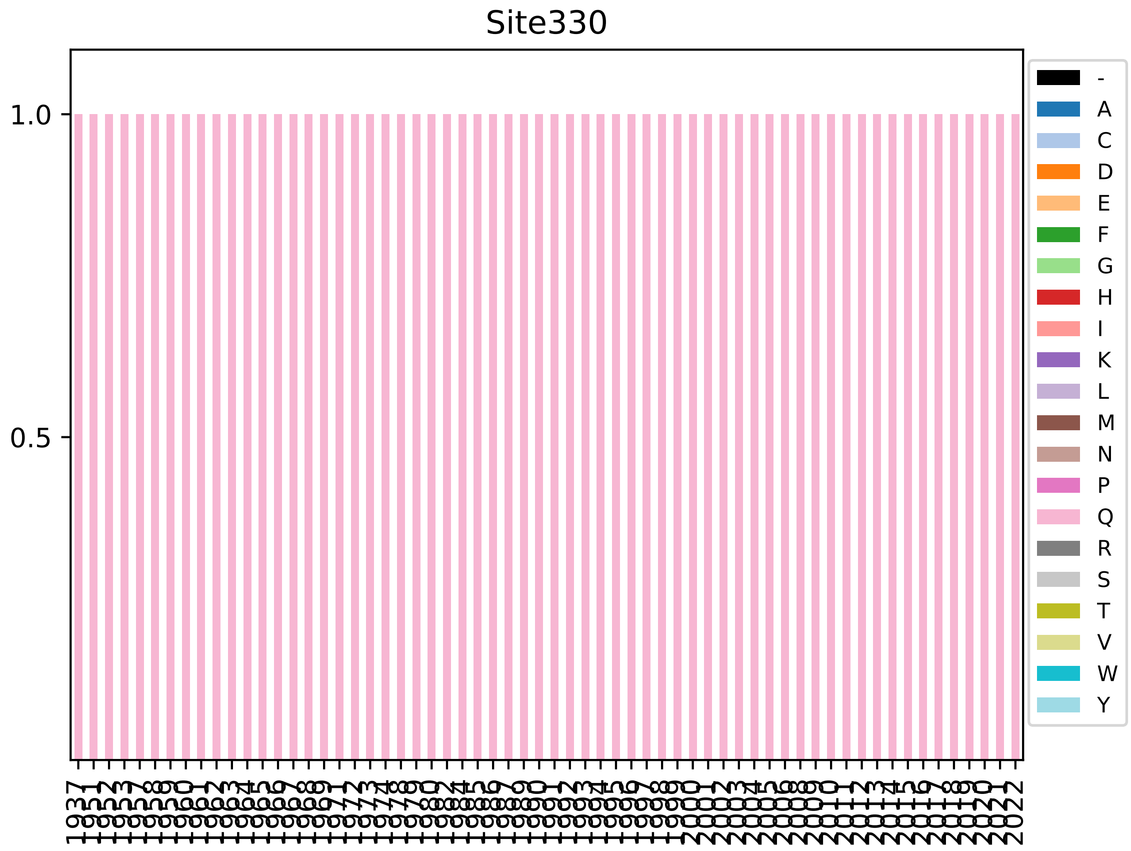

Site330
1.0
0.5
-
A
C
D
E
F
G
H
I
K
L
M
N
P
Q
R
S
T
V
W
Y

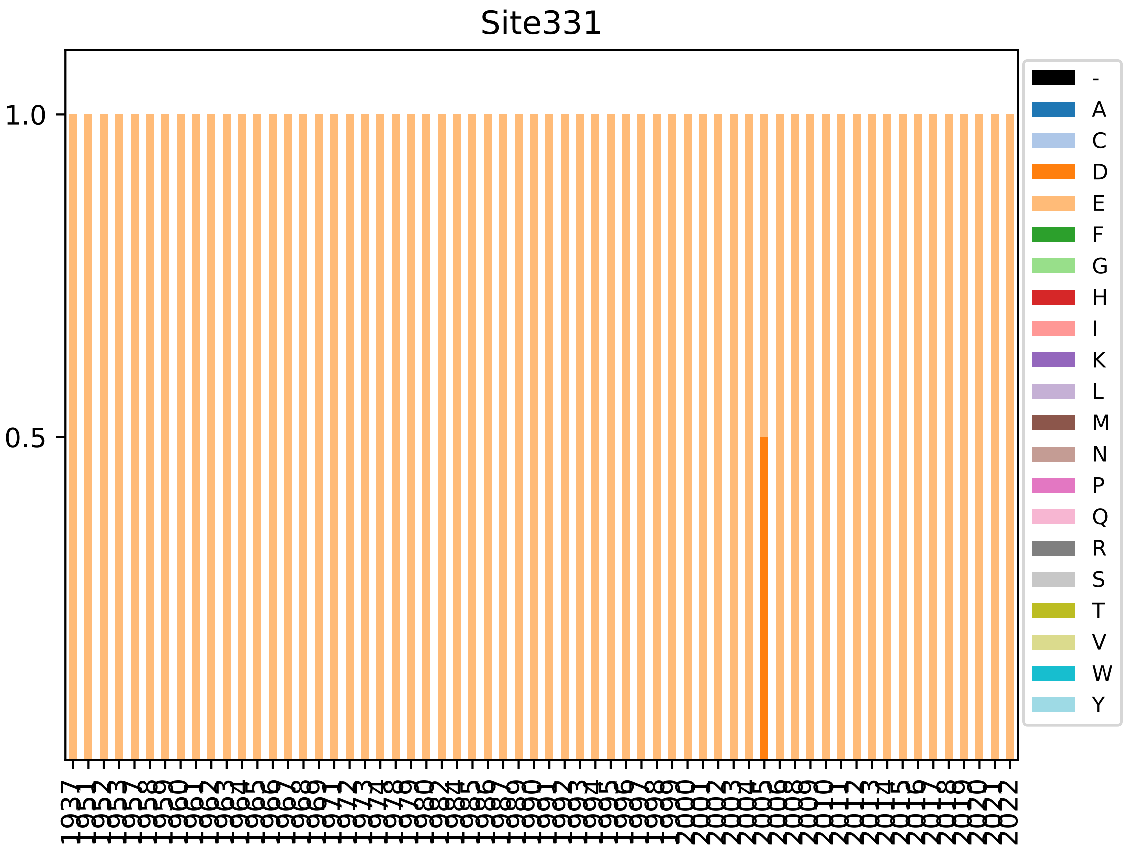
Site331
1.0
0.5
-
A
C
D
E
F
G
H
I
K
L
M
N
P
Q
R
S
T
V
W
Y

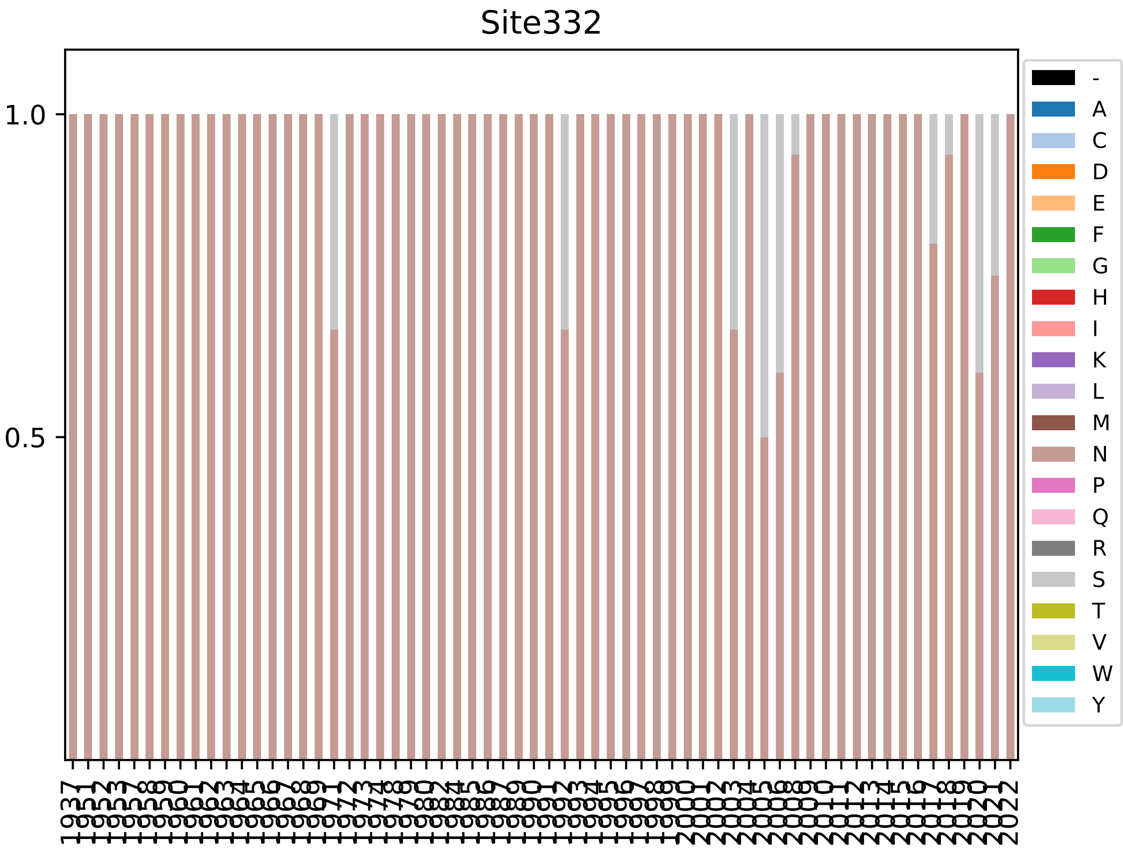
Site332
1.0
0.5
-
A
C
D
E
F
G
H
I
K
L
M
N
P
Q
R
S
T
V
W
Y

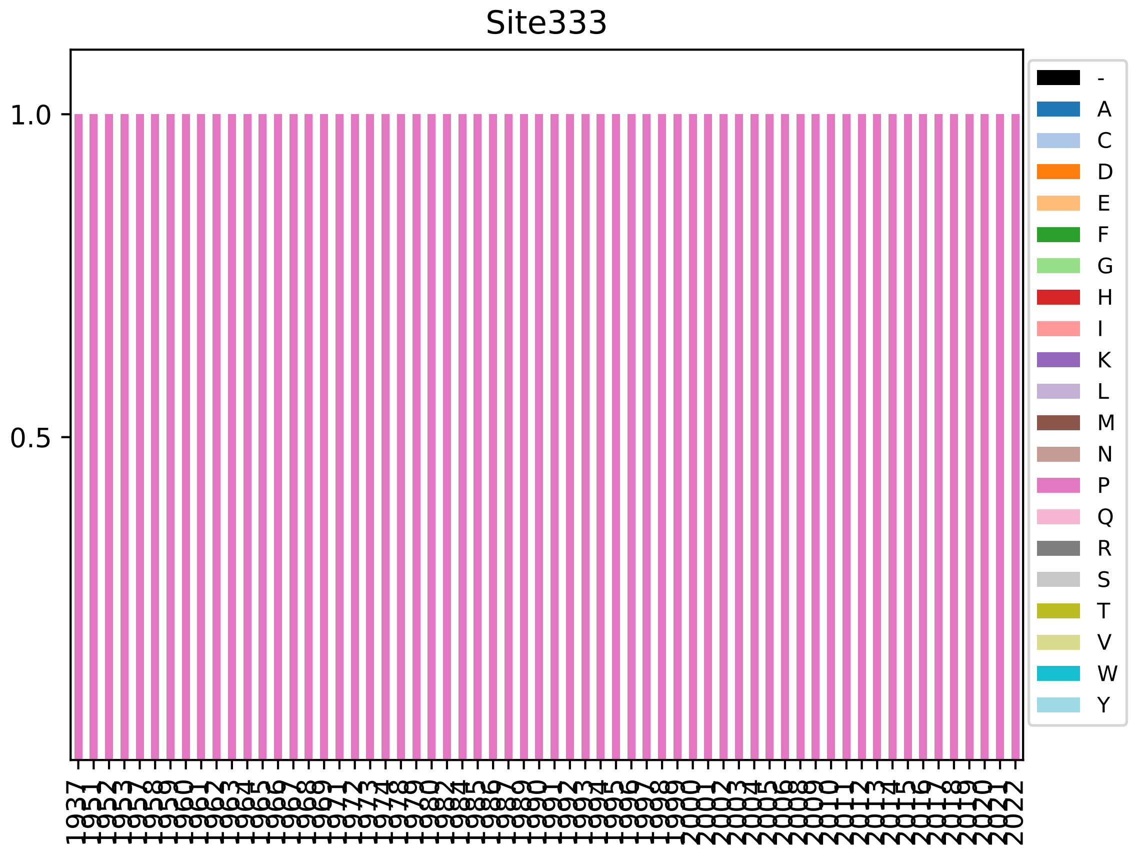
Site333
1.0
0.5
-
A
C
D
E
F
G
H
I
K
L
M
N
P
Q
R
S
T
V
W
Y

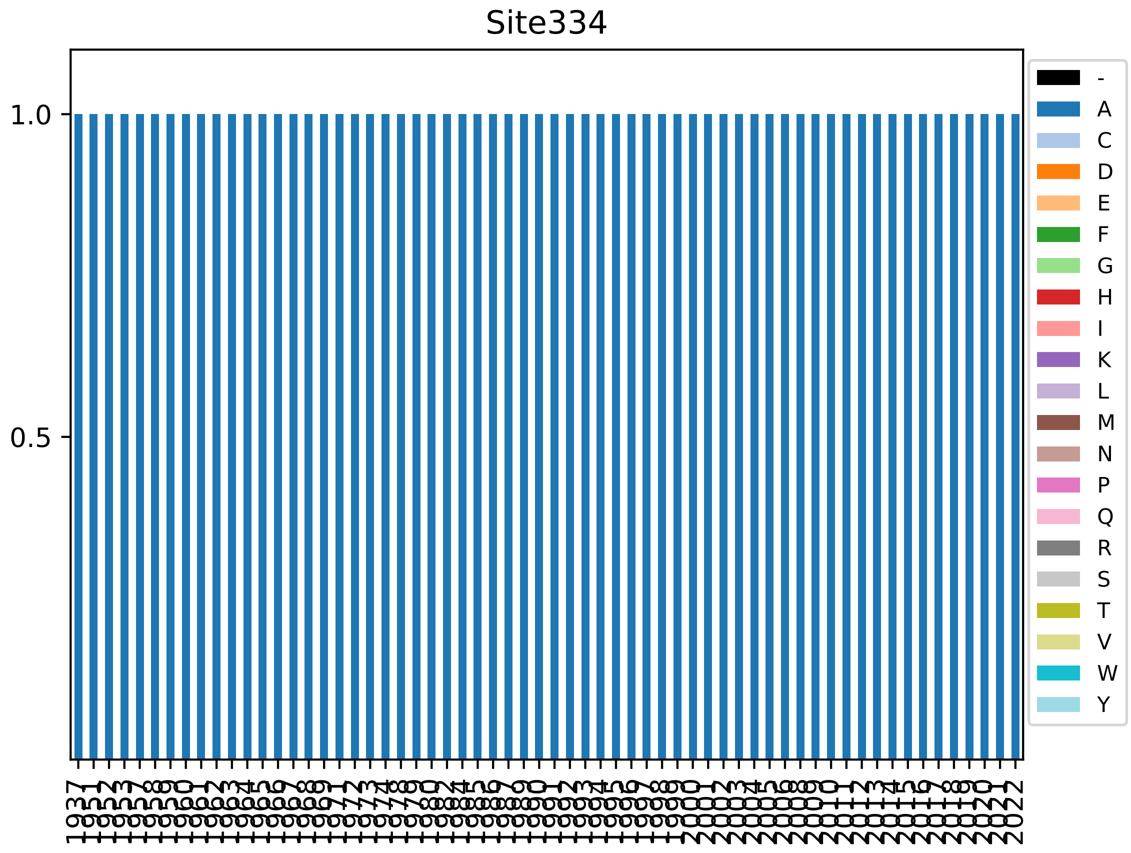
Site334
1.0
0.5
-
A
C
D
E
F
G
H
I
K
L
M
N
P
Q
R
S
T
V
W
Y

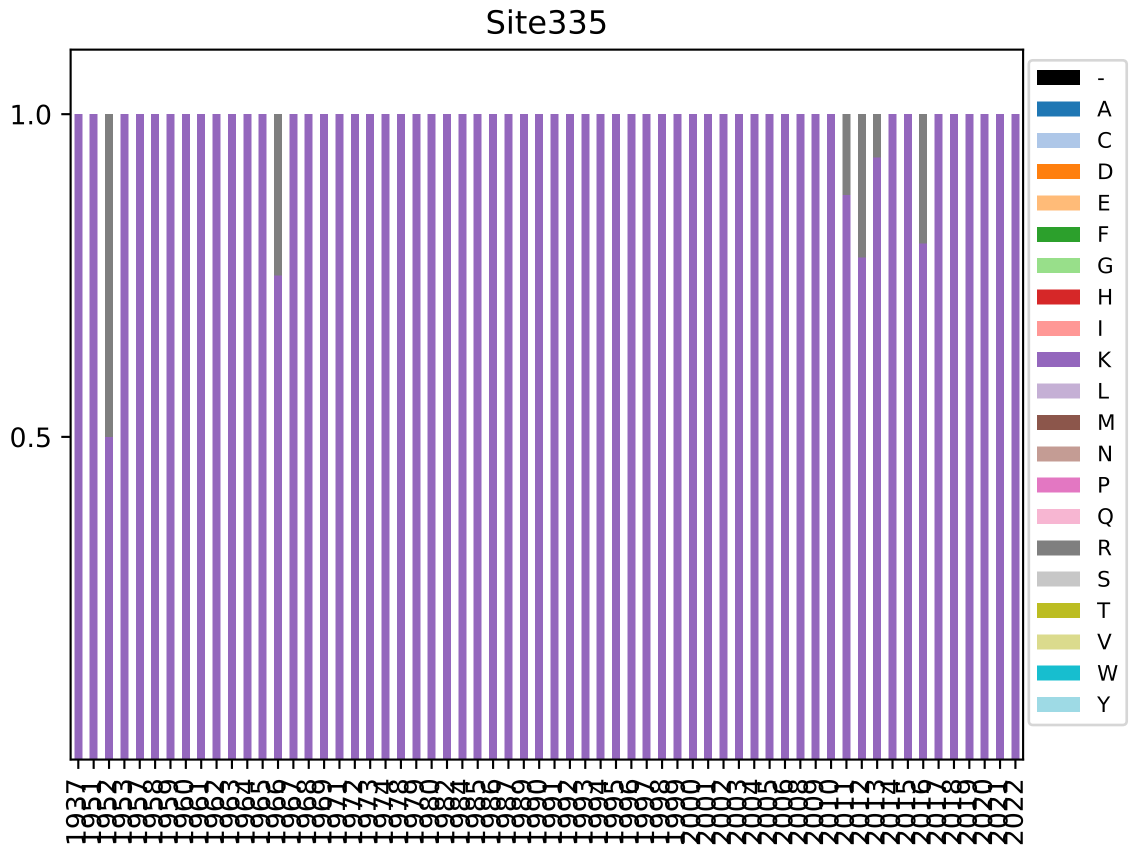
Site335
1.0
0.5
-
A
C
D
E
F
G
H
I
K
L
M
N
P
Q
R
S
T
V
W
Y

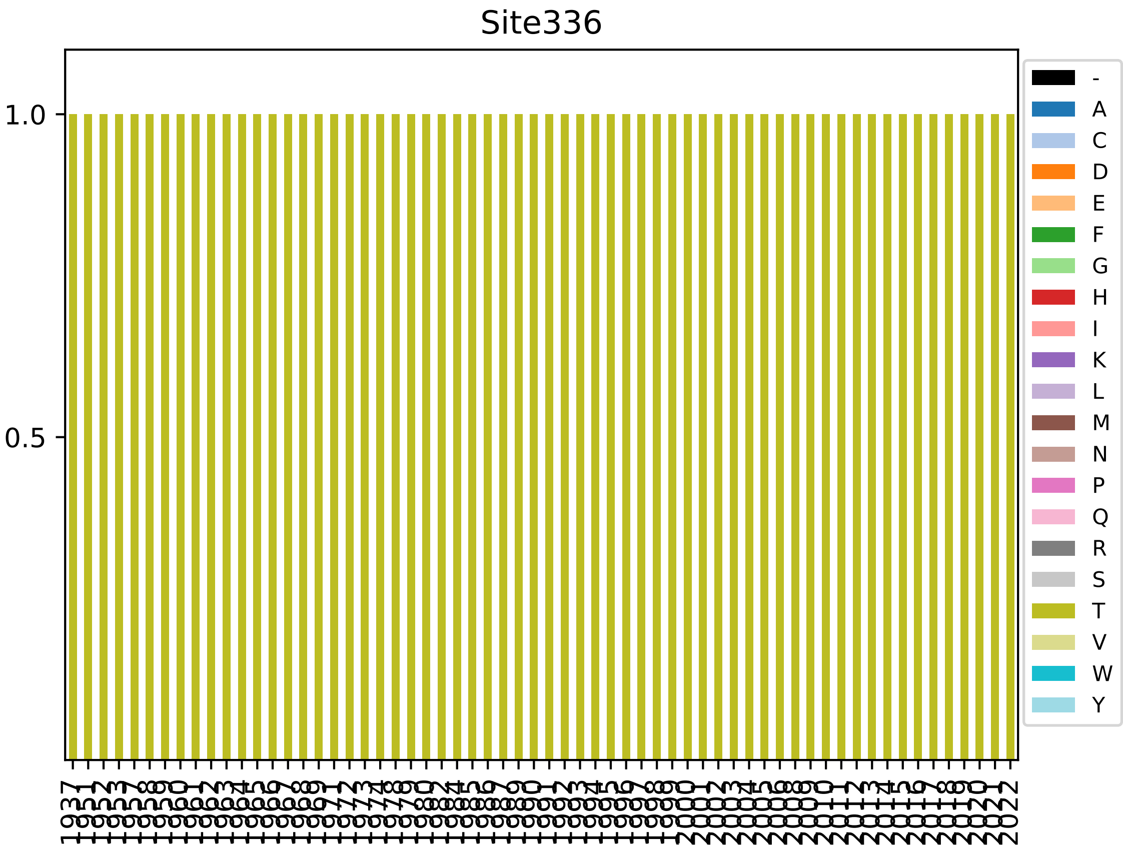
Site336
1.0
0.5
-
A
C
D
E
F
G
H
I
K
L
M
N
P
Q
R
S
T
V
W
Y

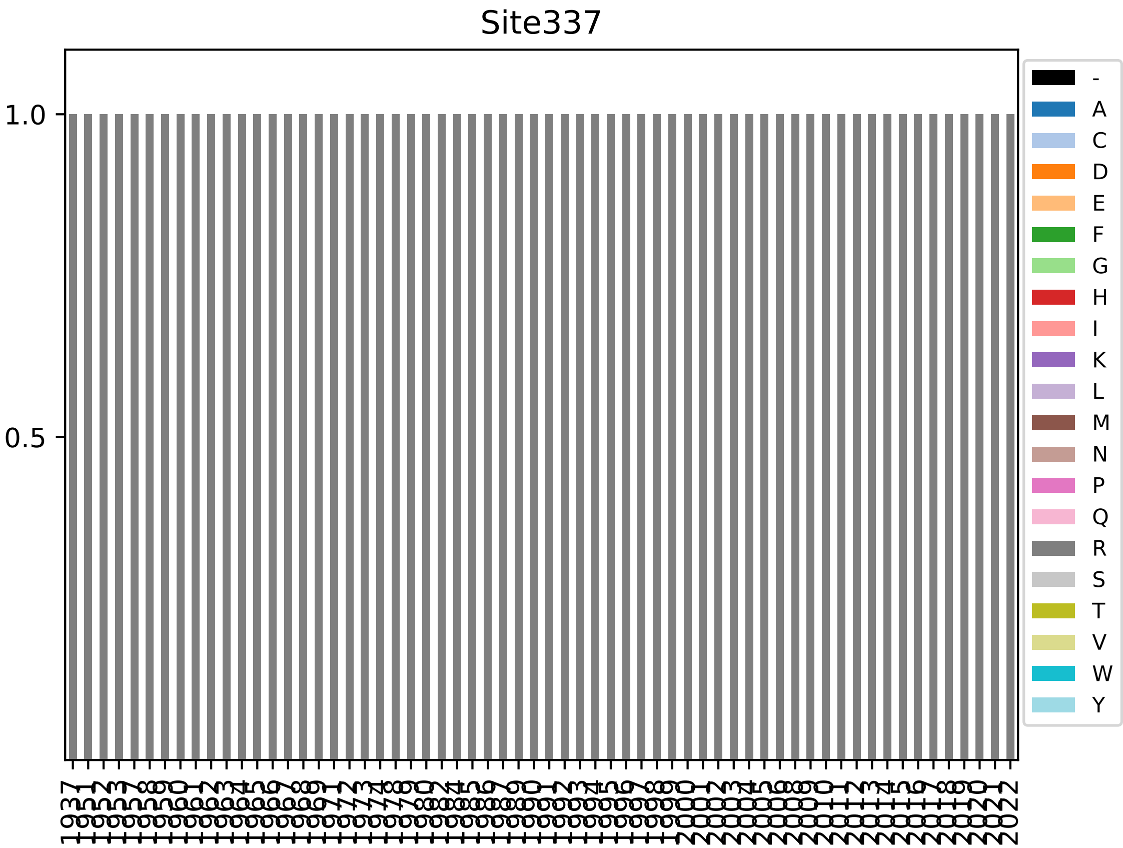
Site337
1.0
0.5
-
A
C
D
E
F
G
H
I
K
L
M
N
P
Q
R
S
T
V
W
Y

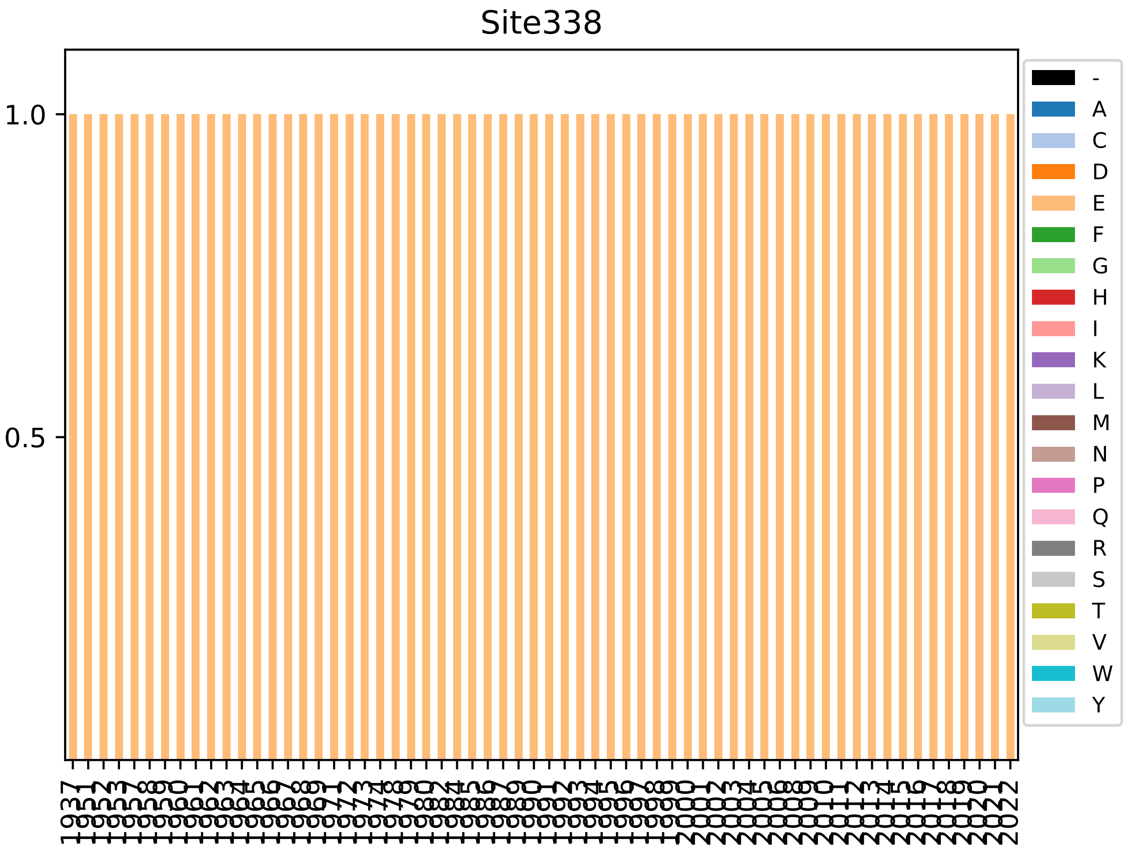
Site338
1.0
0.5
-
A
C
D
E
F
G
H
I
K
L
M
N
P
Q
R
S
T
V
W
Y

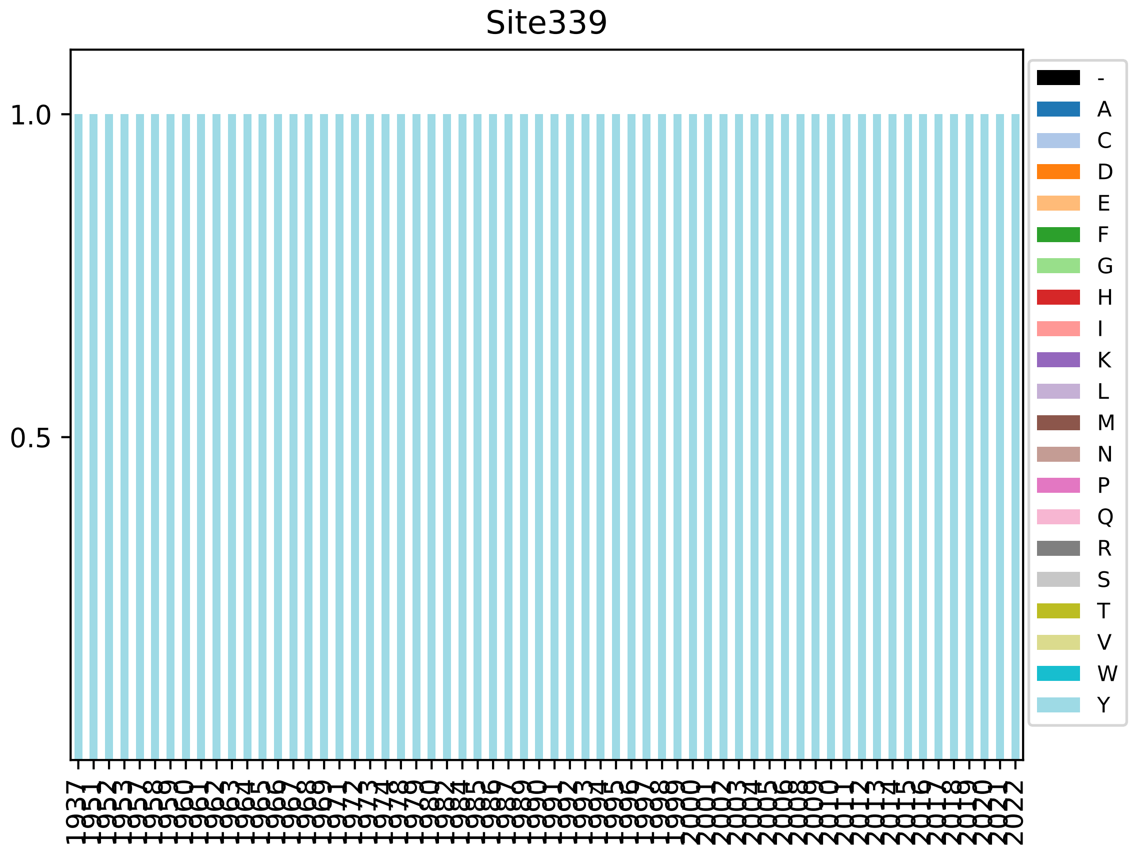
Site339
1.0
0.5
-
A
C
D
E
F
G
H
I
K
L
M
N
P
Q
R
S
T
V
W
Y

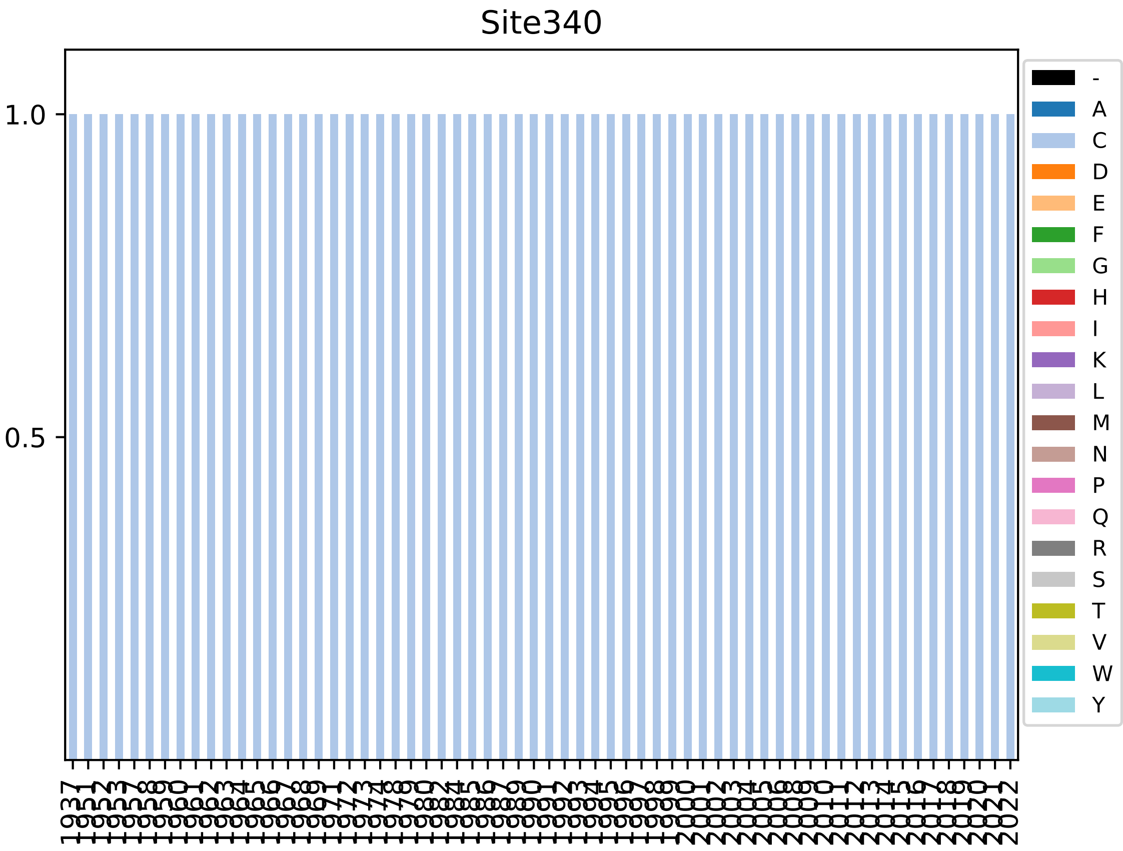
Site340
1.0
0.5
-
A
C
D
E
F
G
H
I
K
L
M
N
P
Q
R
S
T
V
W
Y

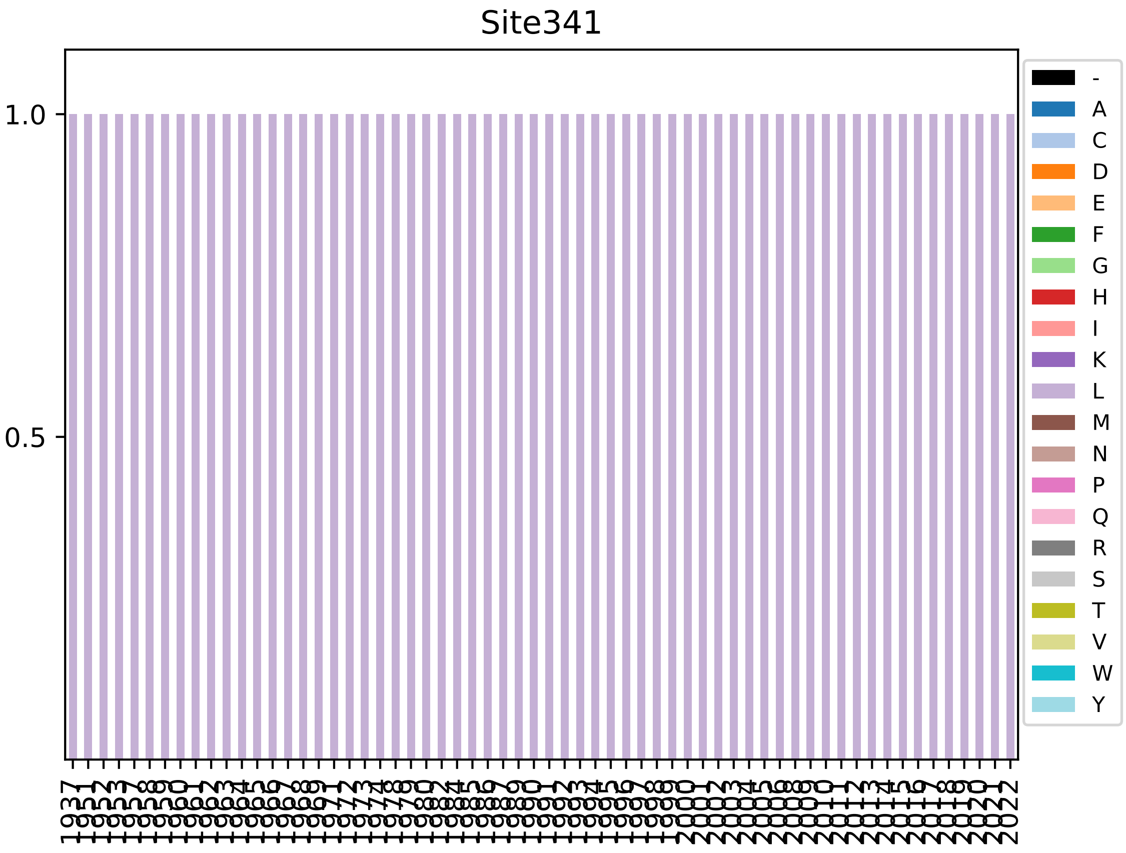
Site341
1.0
0.5
-
A
C
D
E
F
G
H
I
K
L
M
N
P
Q
R
S
T
V
W
Y

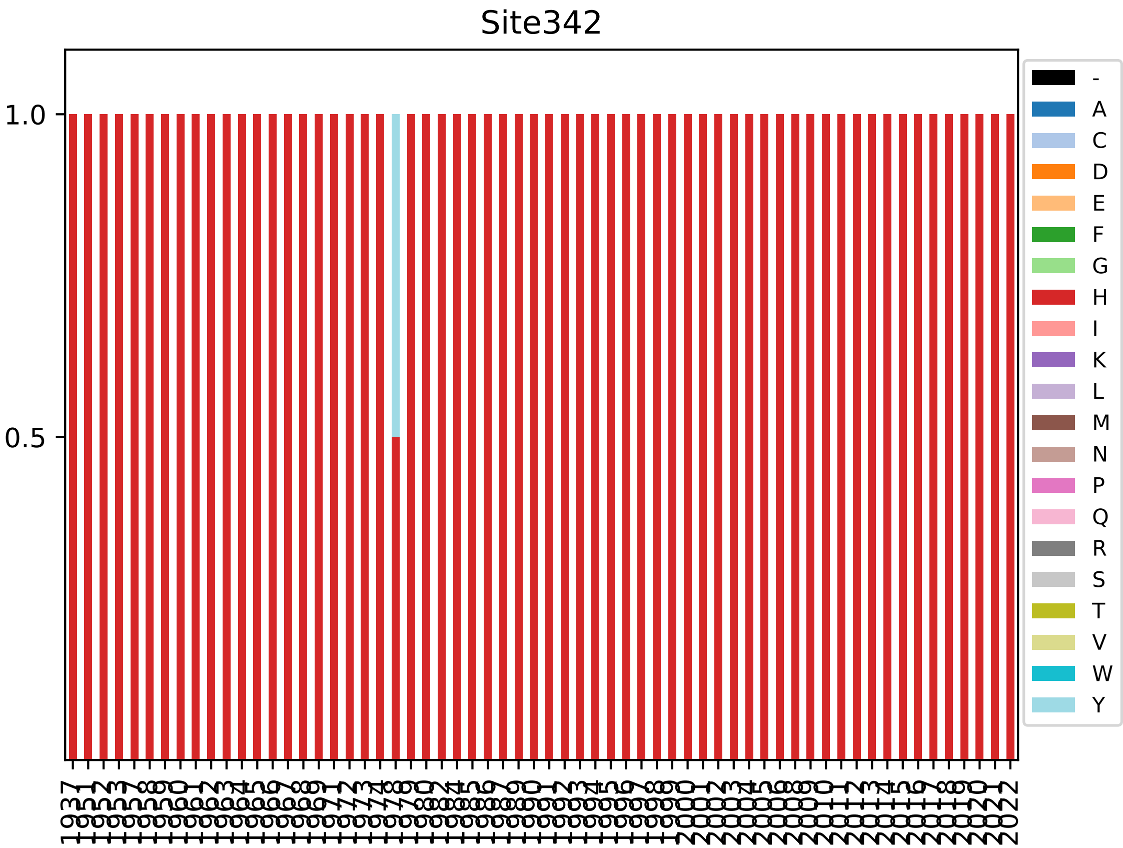
Site342
1.0
0.5
-
A
C
D
E
F
G
H
I
K
L
M
N
P
Q
R
S
T
V
W
Y

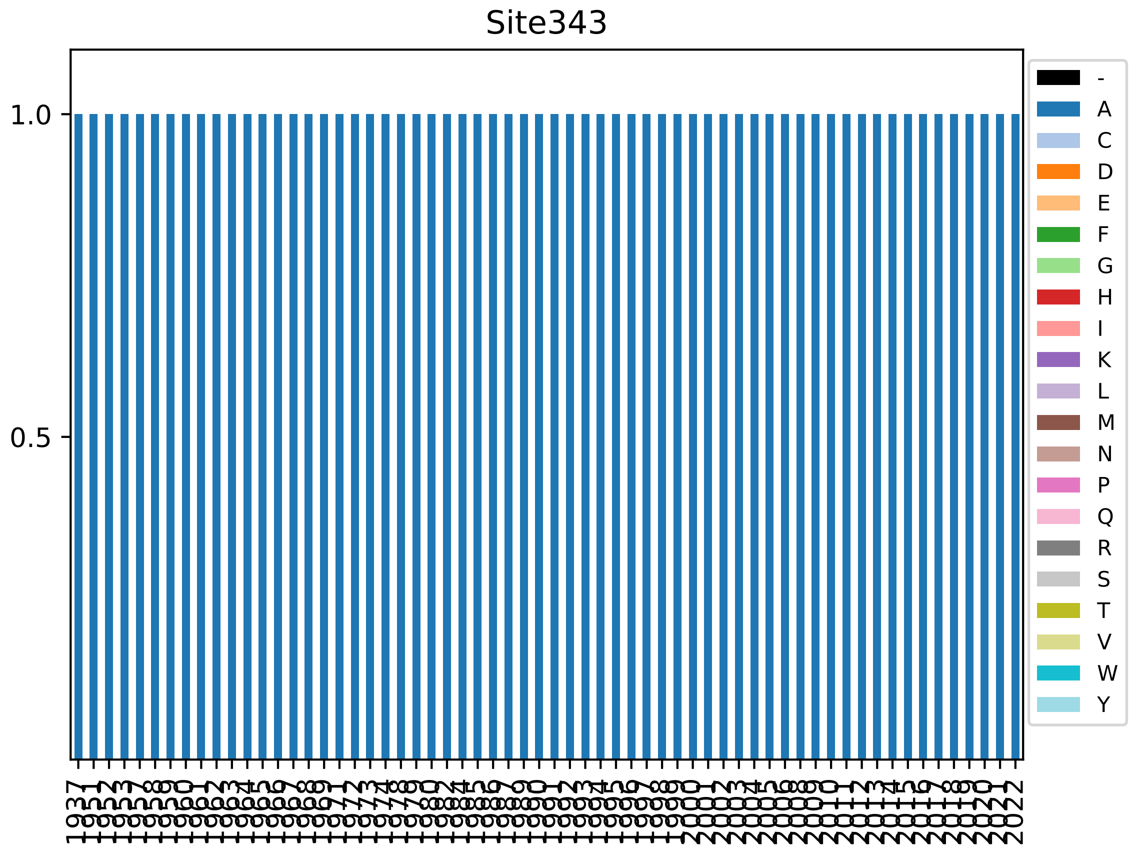
Site343
1.0
0.5
-
A
C
D
E
F
G
H
I
K
L
M
N
P
Q
R
S
T
V
W
Y

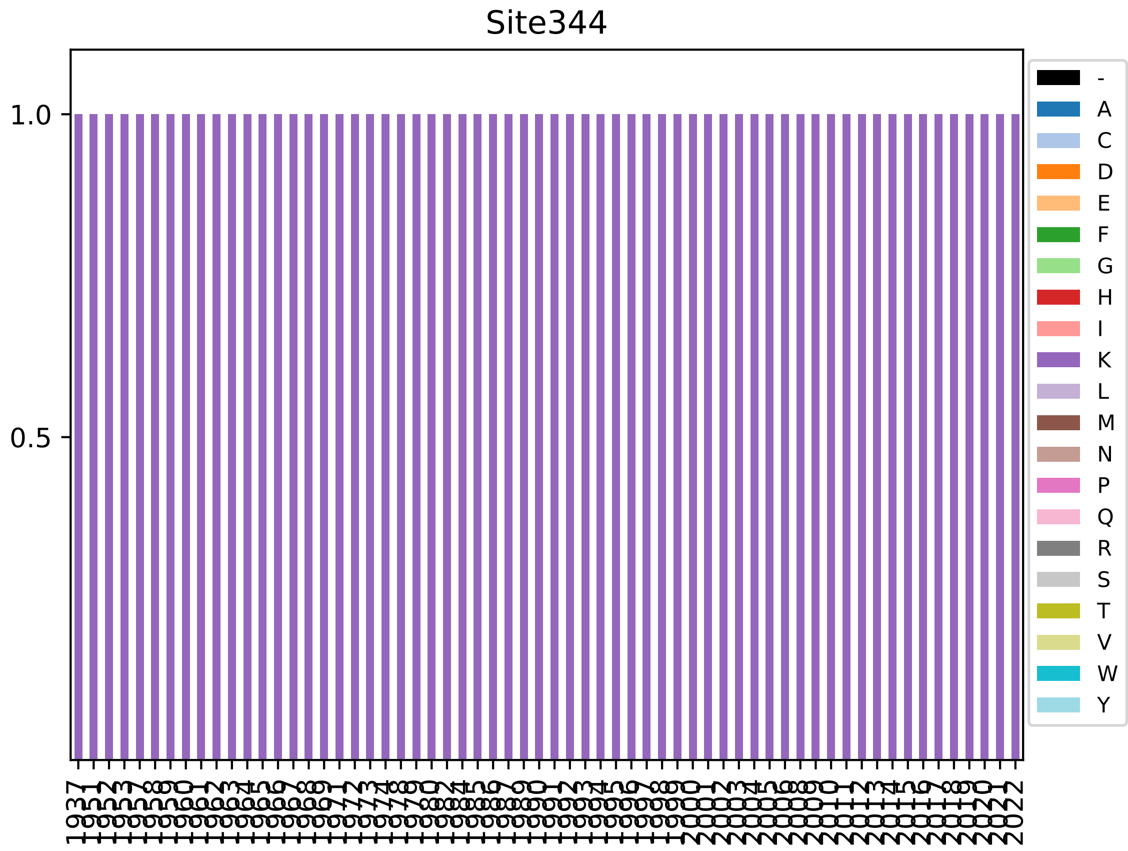
Site344
1.0
0.5
-
A
C
D
E
F
G
H
I
K
L
M
N
P
Q
R
S
T
V
W
Y

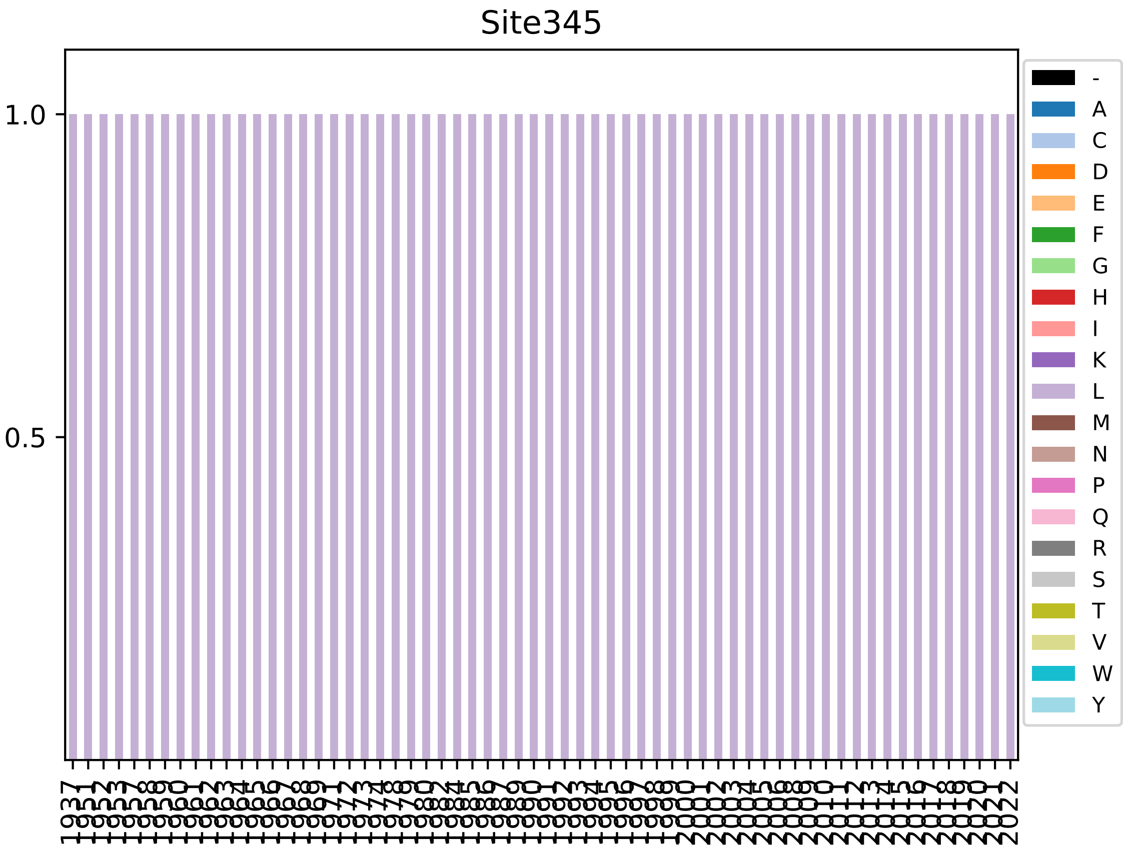
Site345
1.0
0.5
-
A
C
D
E
F
G
H
I
K
L
M
N
P
Q
R
S
T
V
W
Y

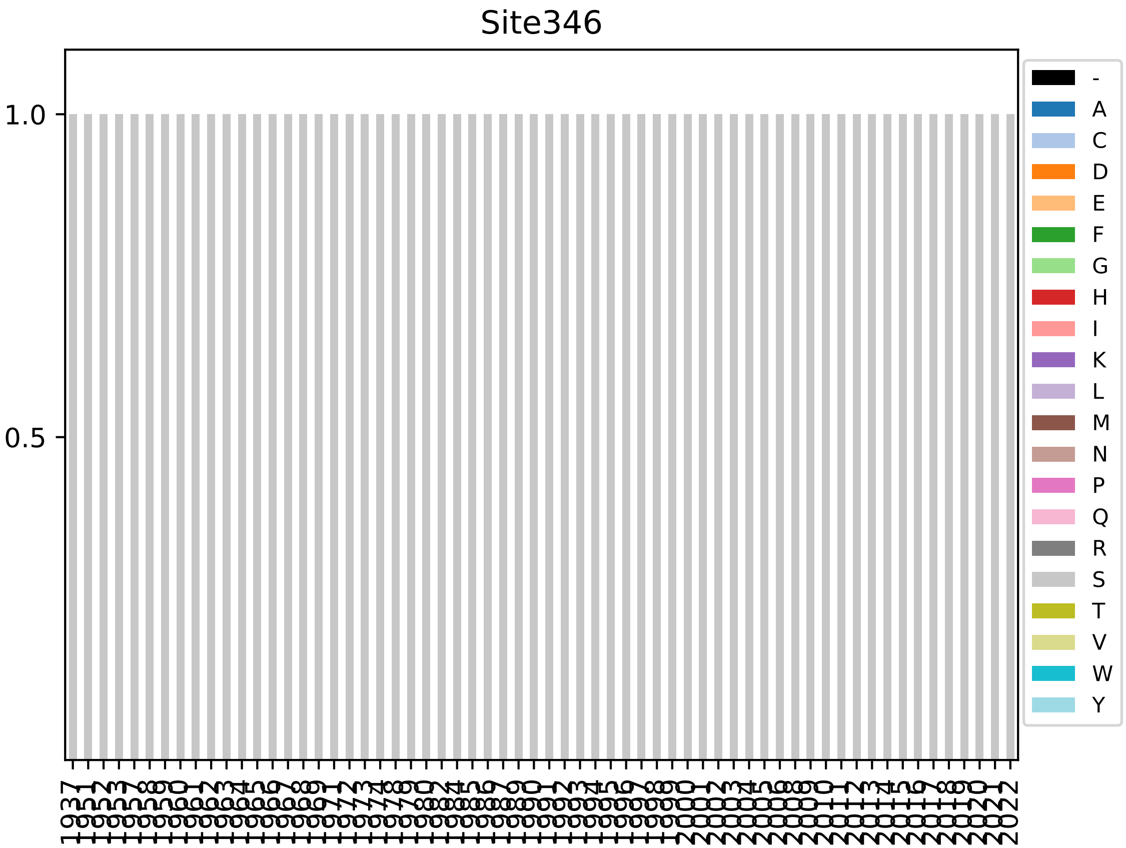
Site346
1.0
0.5
-
A
C
D
E
F
G
H
I
K
L
M
N
P
Q
R
S
T
V
W
Y

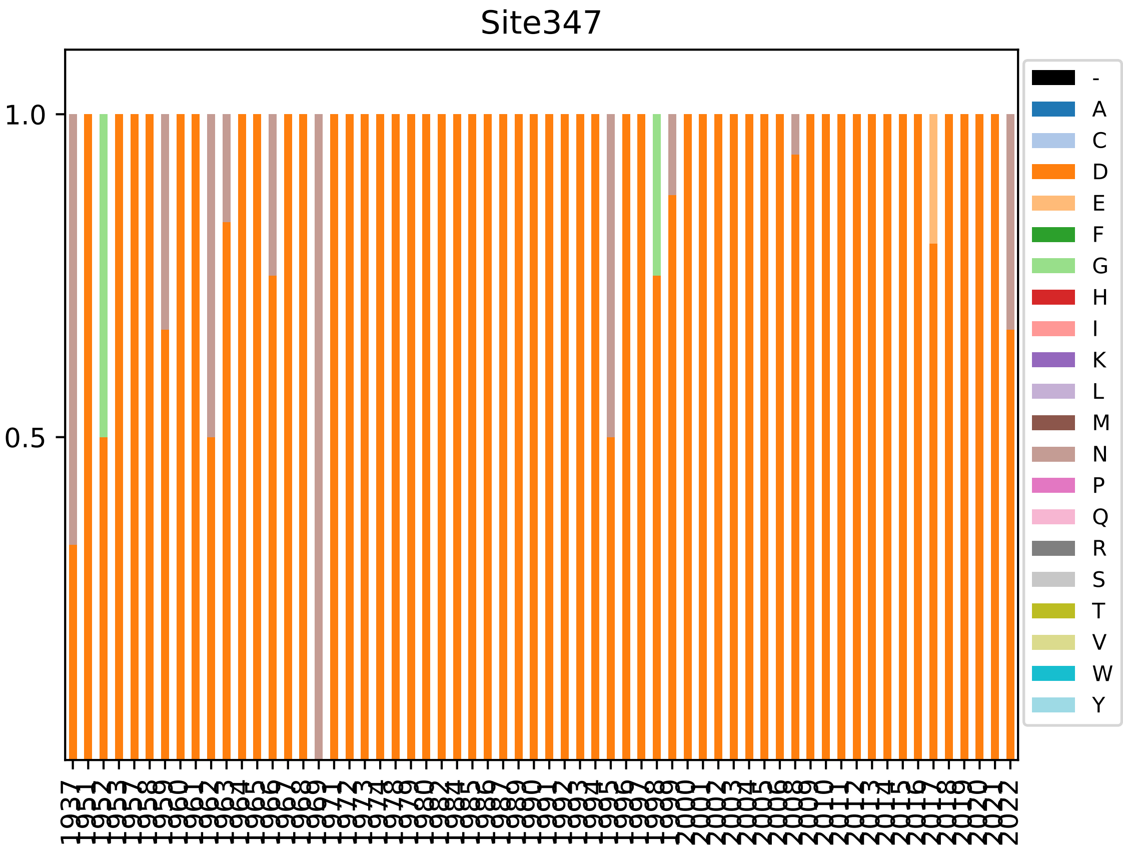

Site347
1.0
0.5
-
A
C
D
E
F
G
H
I
K
L
M
N
P
Q
R
S
T
V
W
Y

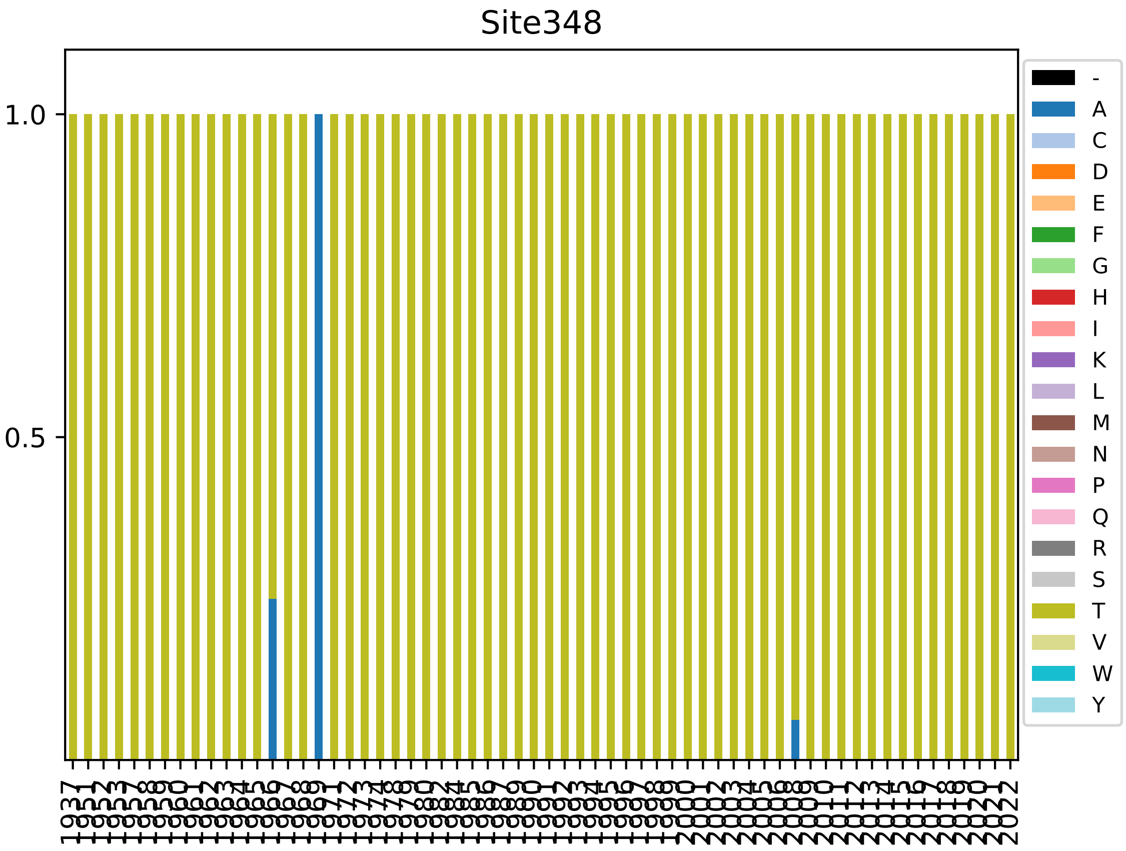
Site348
1.0
0.5
-
A
C
D
E
F
G
H
I
K
L
M
N
P
Q
R
S
T
V
W
Y

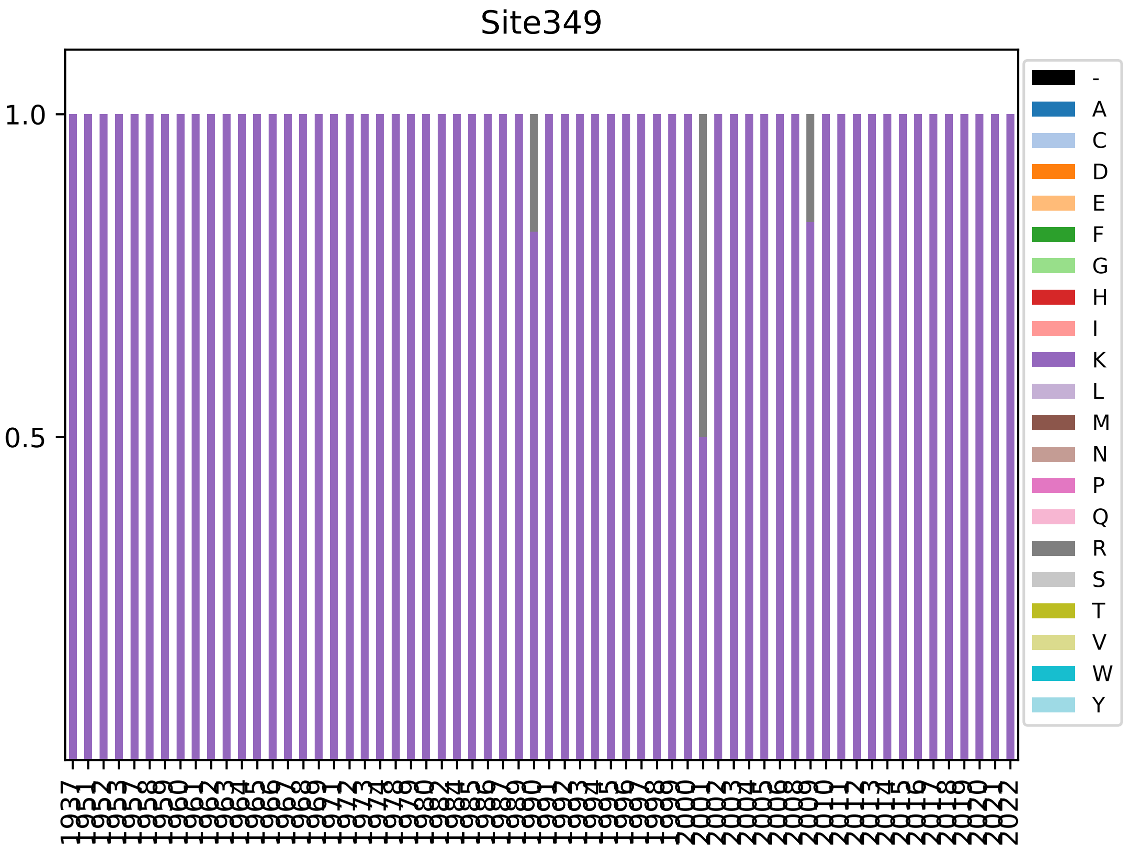
Site349
1.0
0.5
-
A
C
D
E
F
G
H
I
K
L
M
N
P
Q
R
S
T
V
W
Y

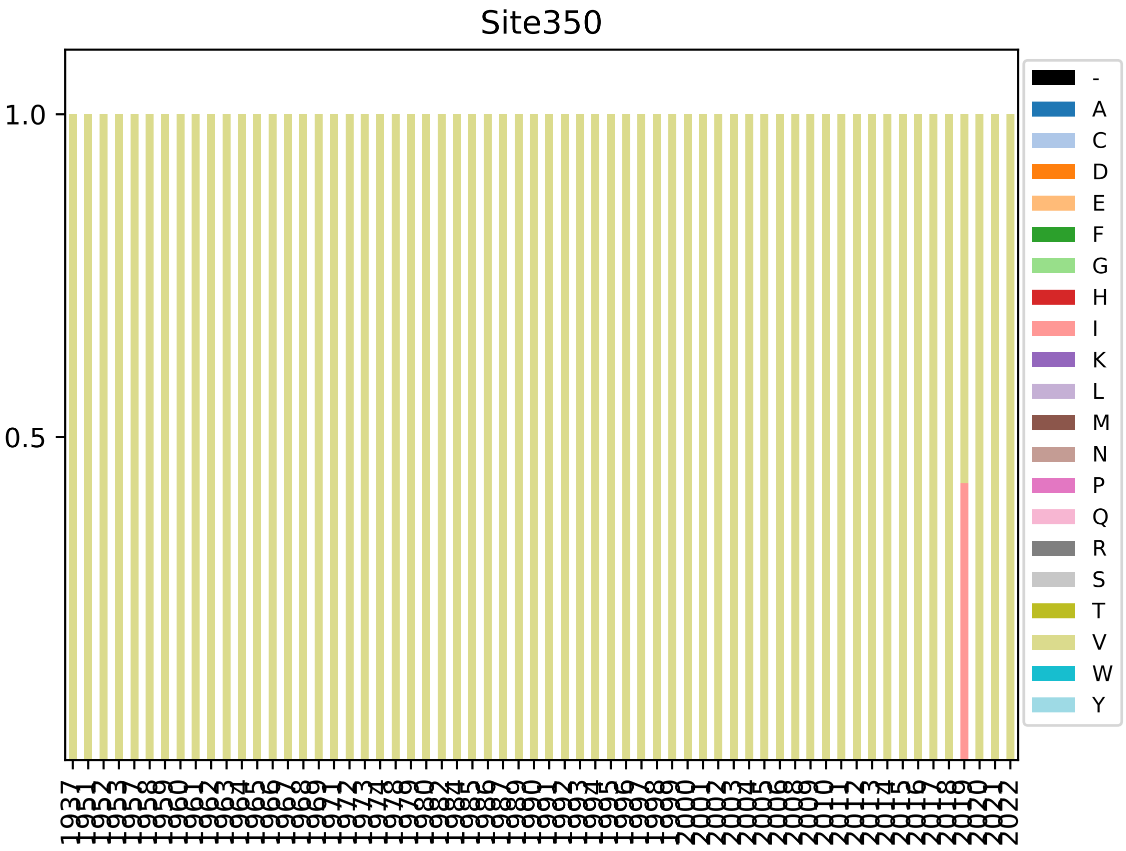

Site350
1.0
0.5
-
A
C
D
E
F
G
H
I
K
L
M
N
P
Q
R
S
T
V
W
Y

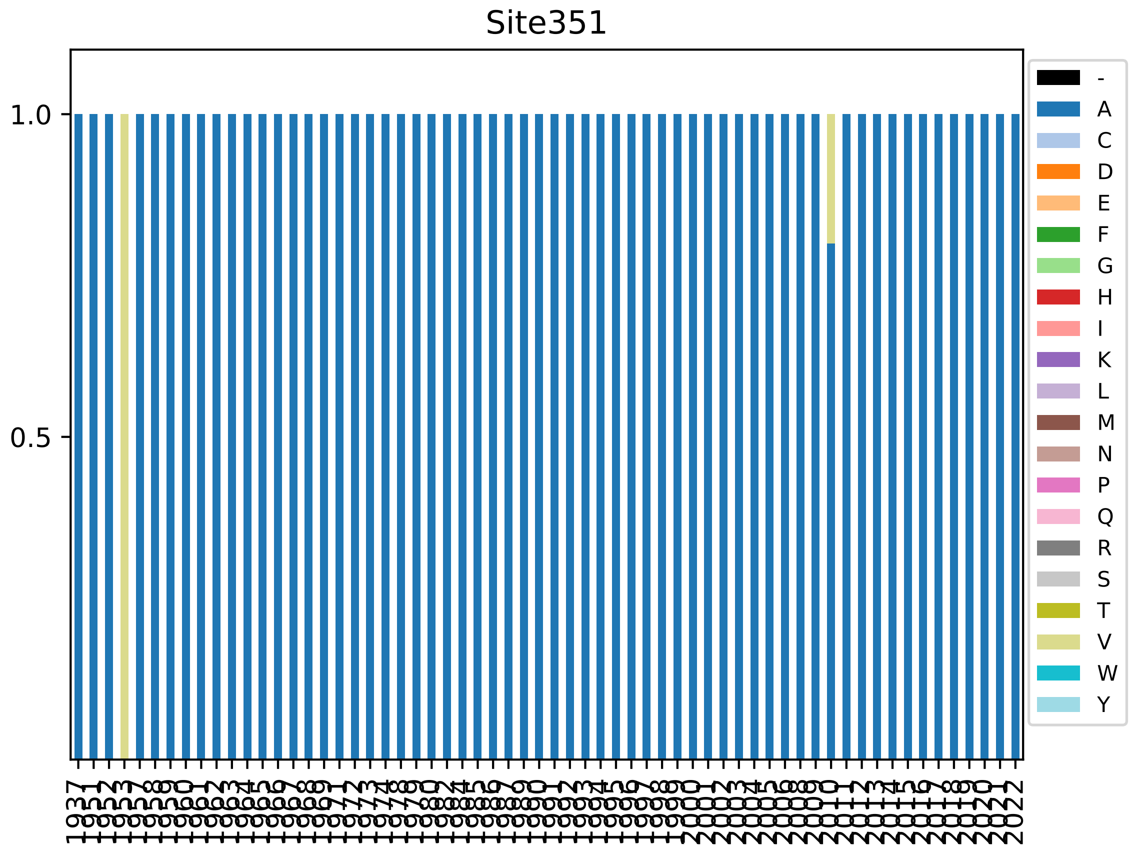
Site351
1.0
0.5
-
A
C
D
E
F
G
H
I
K
L
M
N
P
Q
R
S
T
V
W
Y

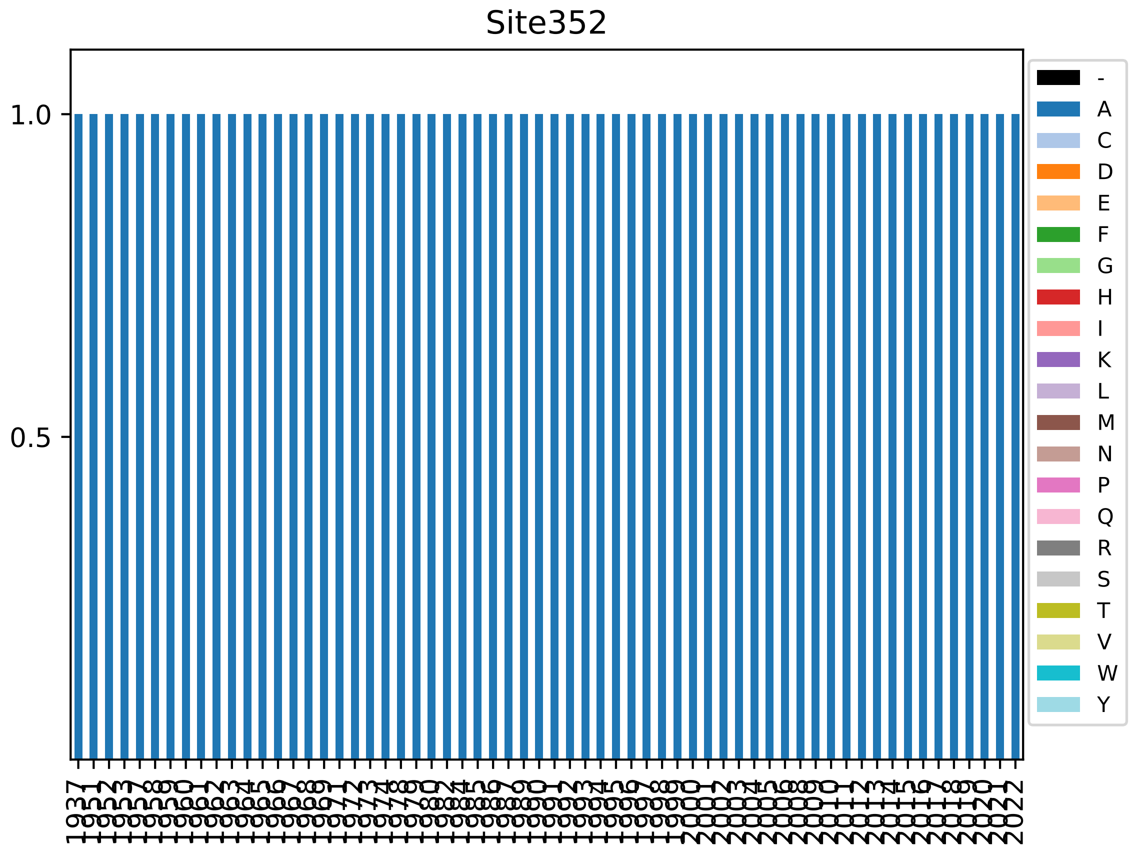
Site352
1.0
0.5
-
A
C
D
E
F
G
H
I
K
L
M
N
P
Q
R
S
T
V
W
Y

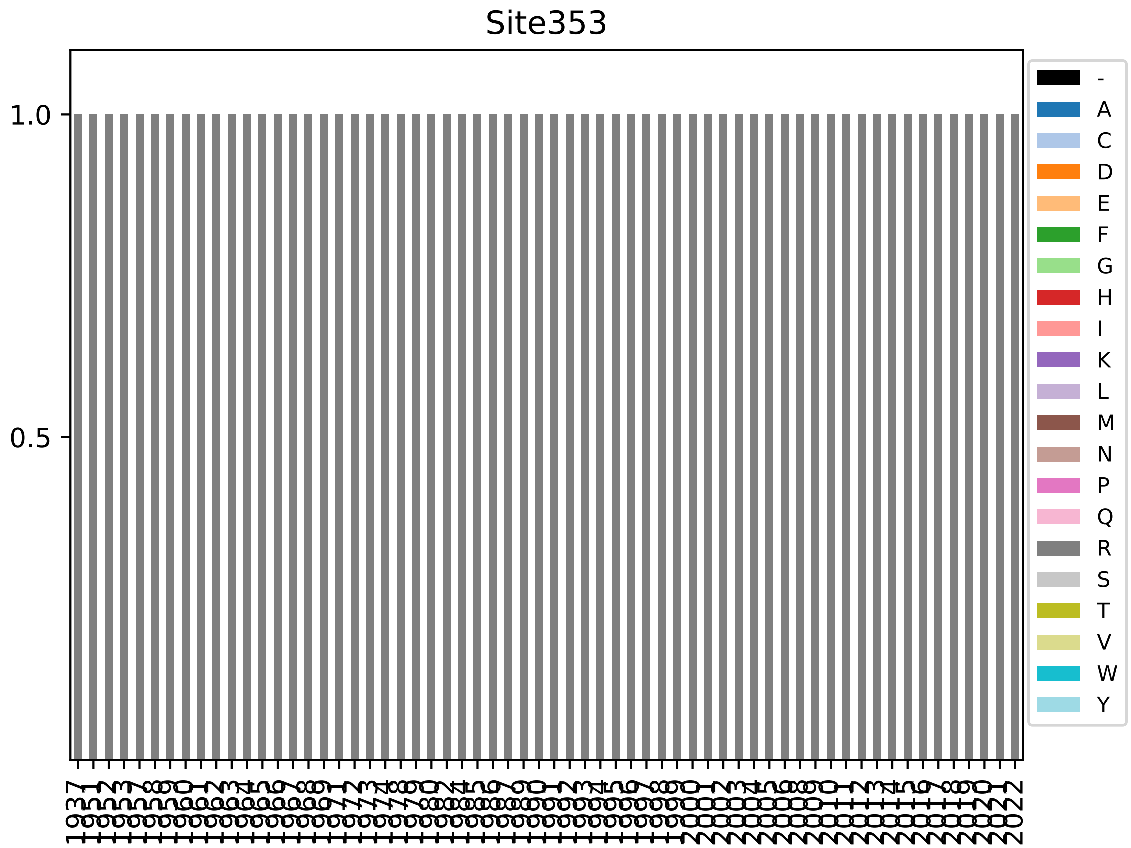
Site353
1.0
0.5
-
A
C
D
E
F
G
H
I
K
L
M
N
P
Q
R
S
T
V
W
Y

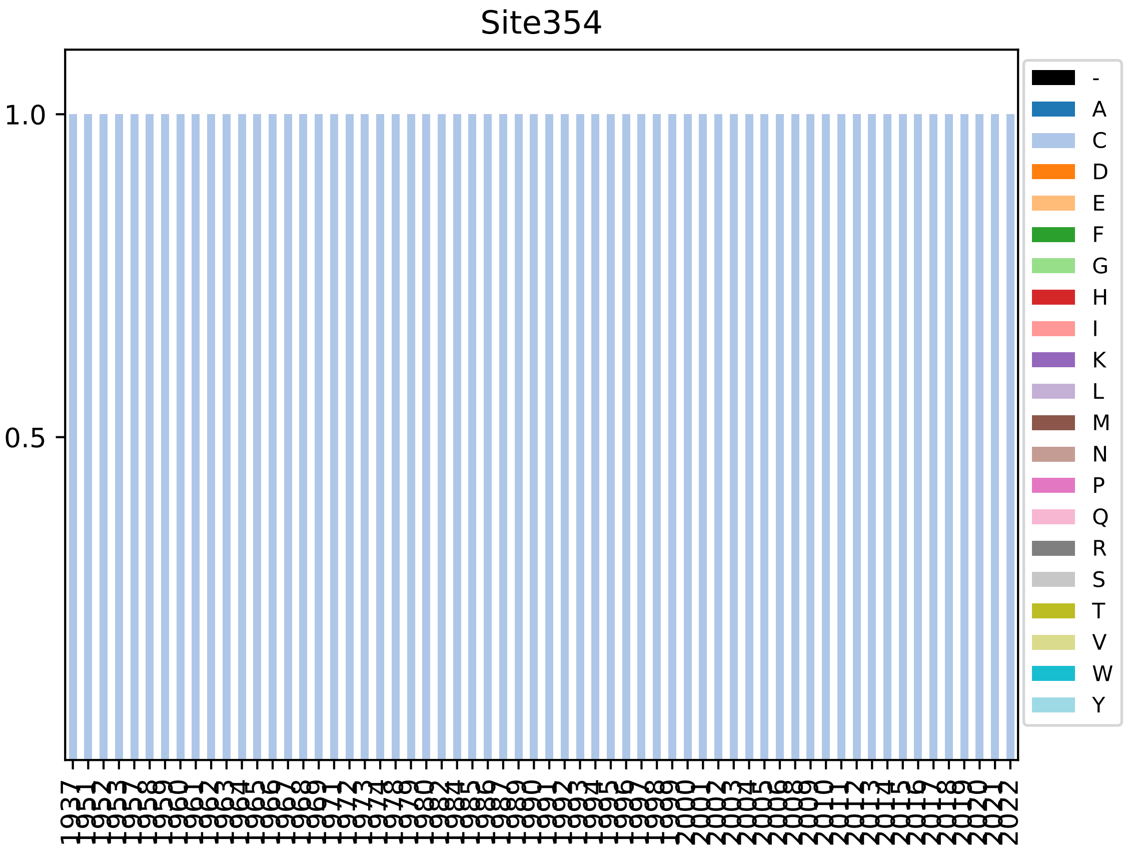
Site354
1.0
0.5
-
A
C
D
E
F
G
H
I
K
L
M
N
P
Q
R
S
T
V
W
Y

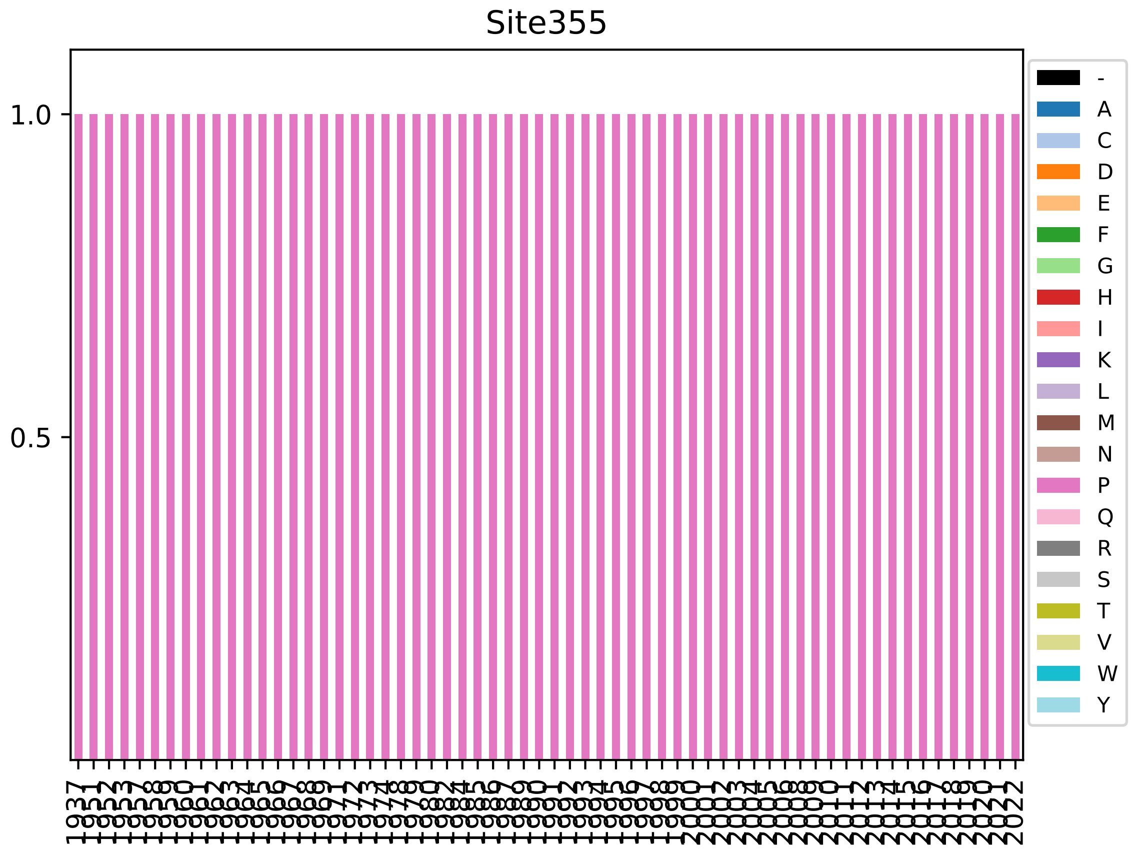
Site355
1.0
0.5
-
A
C
D
E
F
G
H
I
K
L
M
N
P
Q
R
S
T
V
W
Y

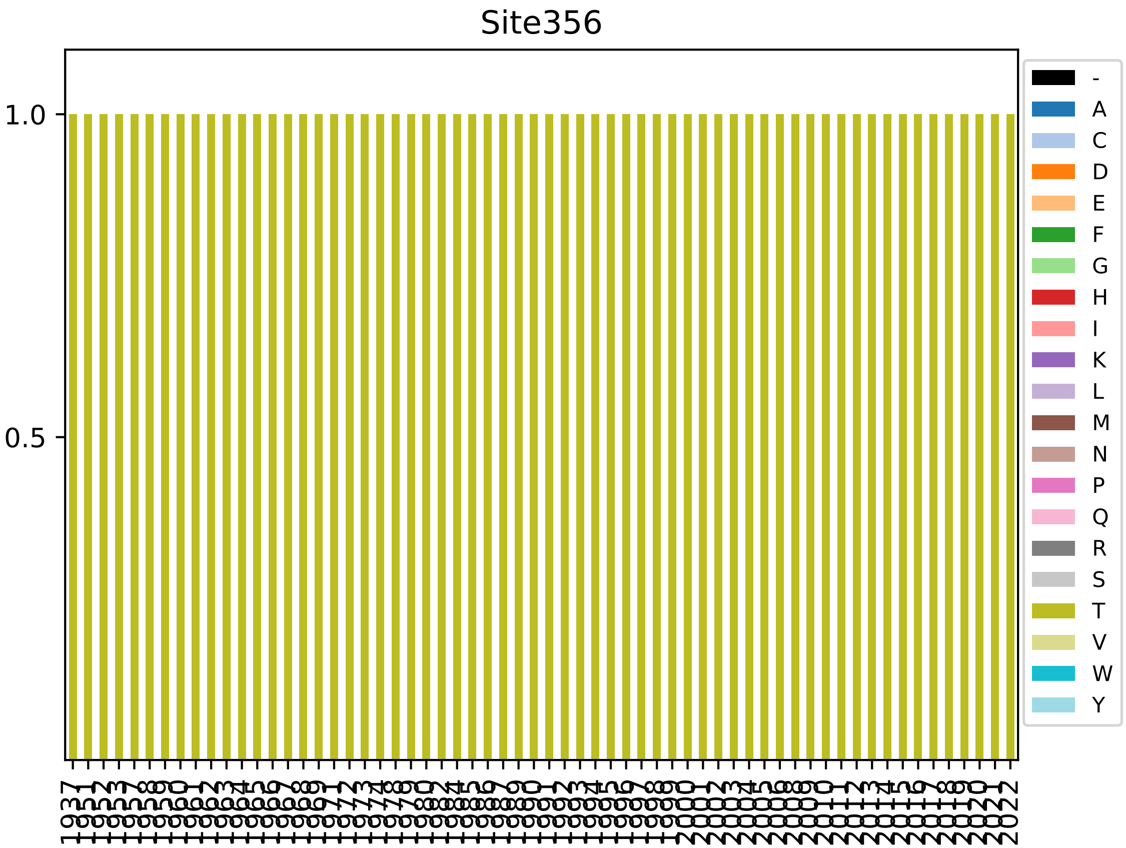
Site356
1.0
0.5
-
A
C
D
E
F
G
H
I
K
L
M
N
P
Q
R
S
T
V
W
Y

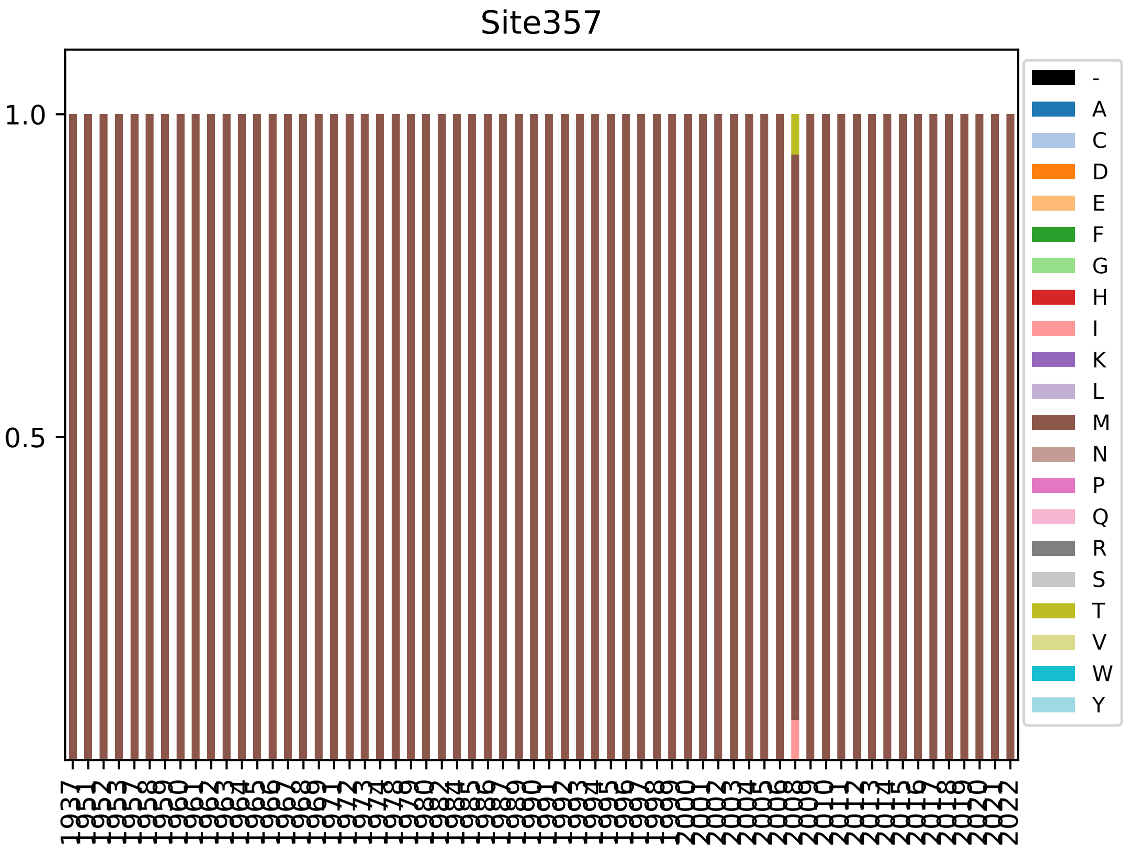

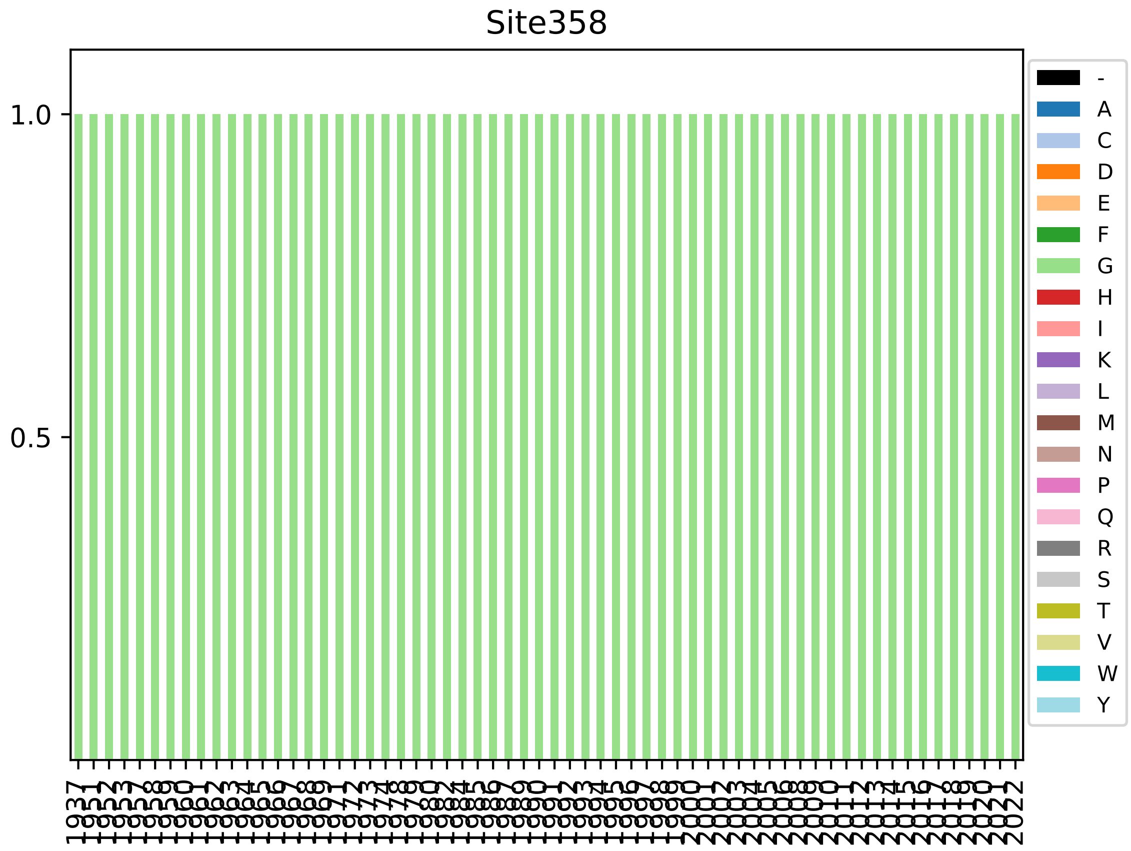
Site358
1.0
0.5
-
A
C
D
E
F
G
H
I
K
L
M
N
P
Q
R
S
T
V
W
Y

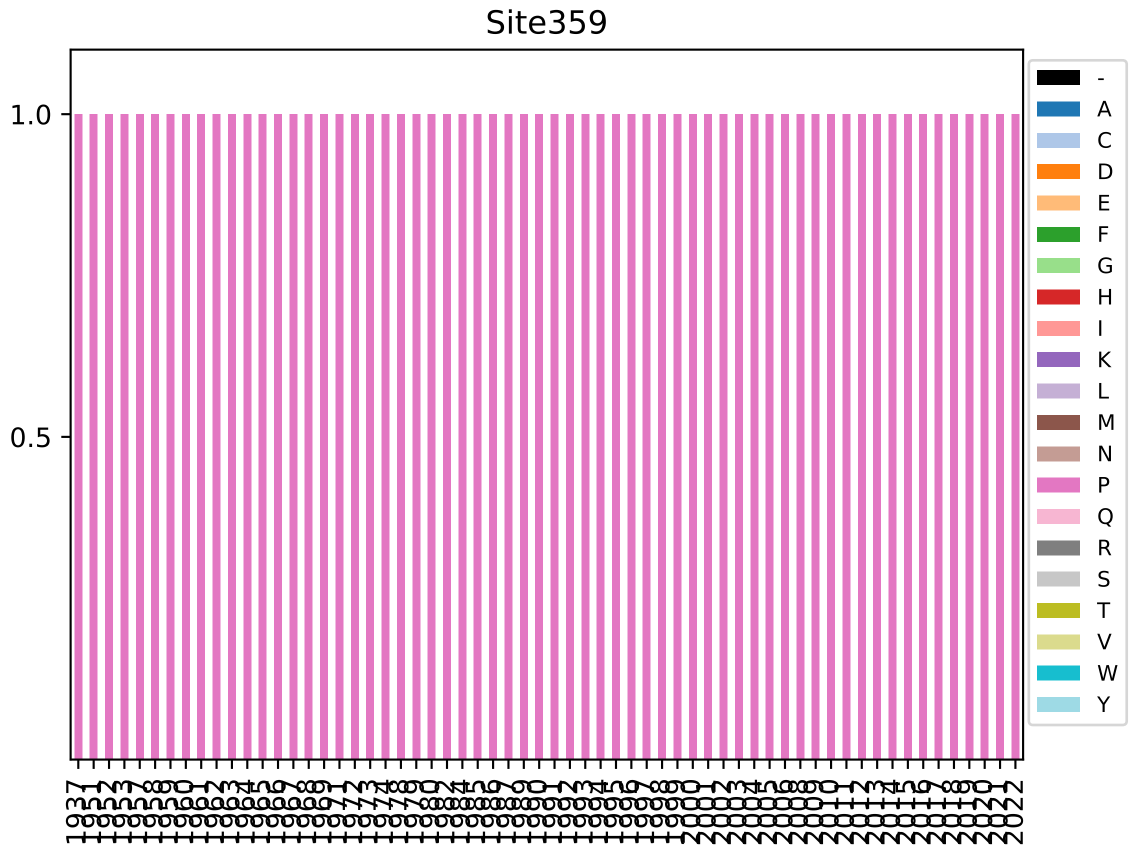
Site359
1.0
0.5
-
A
C
D
E
F
G
H
I
K
L
M
N
P
Q
R
S
T
V
W
Y

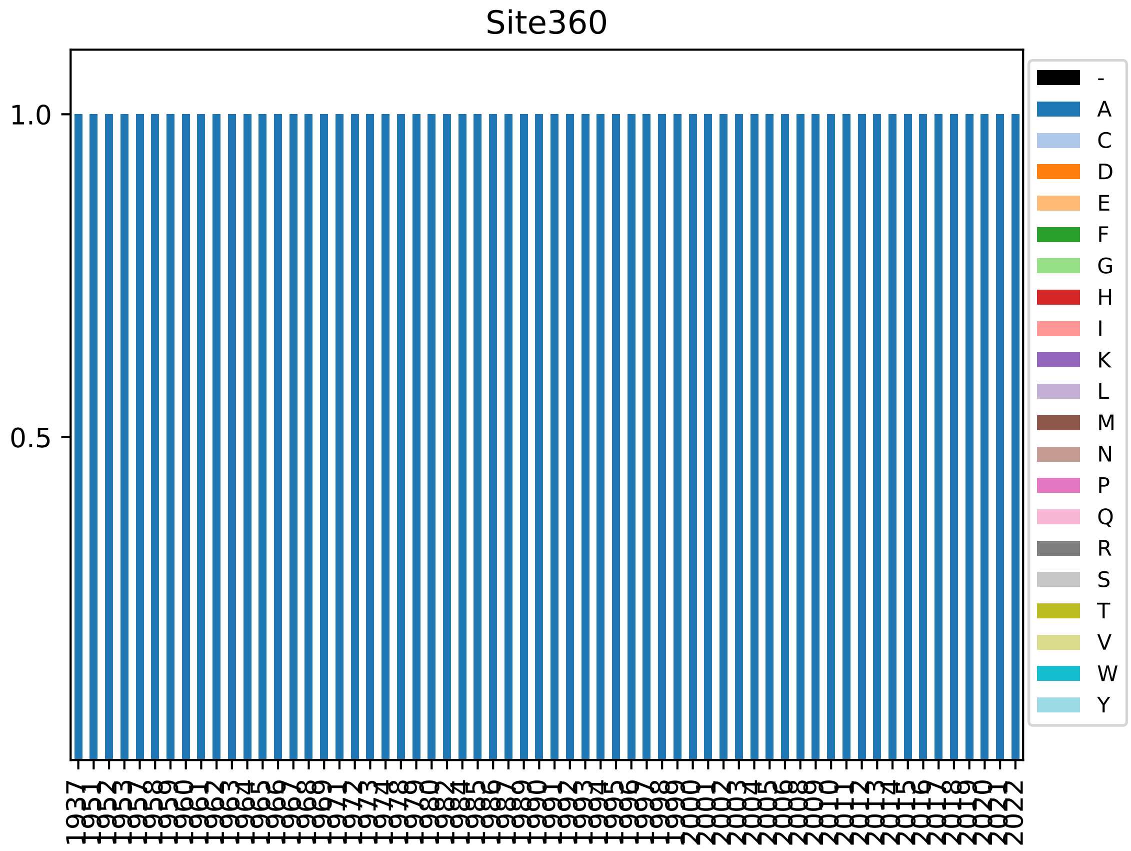
Site360
1.0
0.5
-
A
C
D
E
F
G
H
I
K
L
M
N
P
Q
R
S
T
V
W
Y

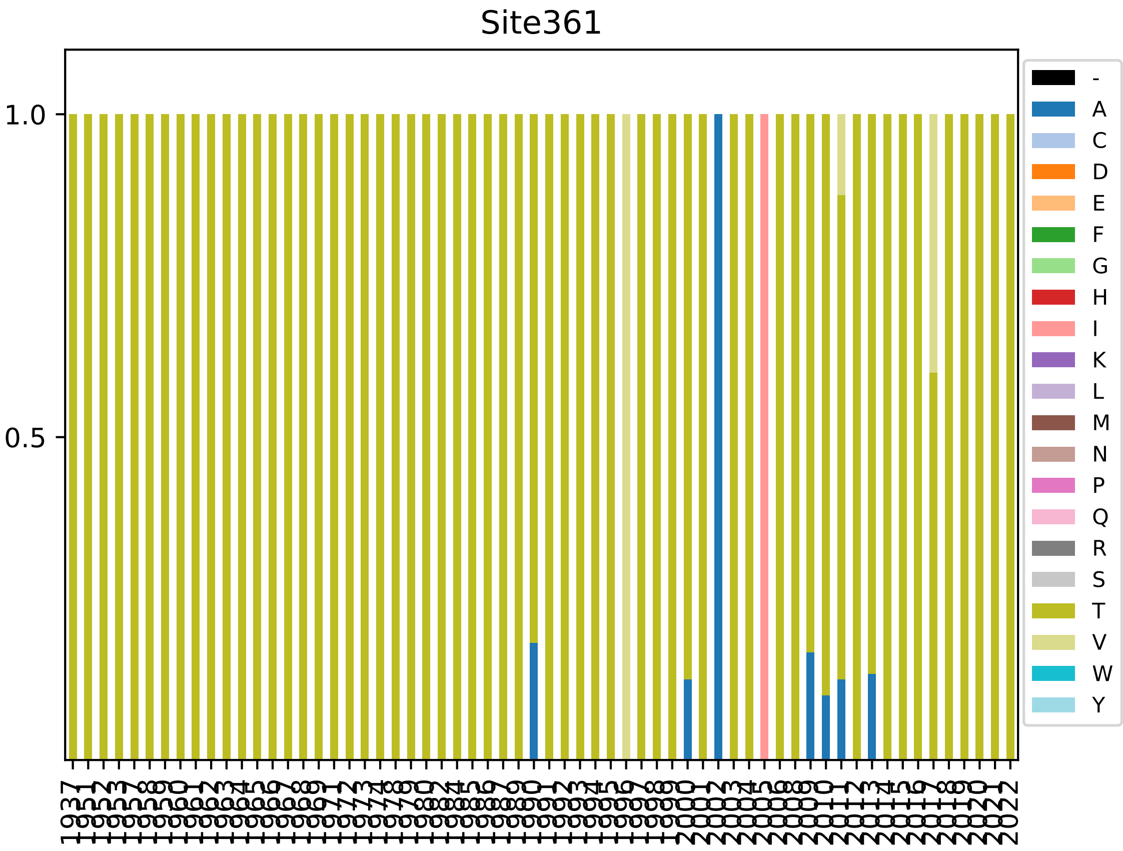
Site361
1.0
0.5
-
A
C
D
E
F
G
H
I
K
L
M
N
P
Q
R
S
T
V
W
Y

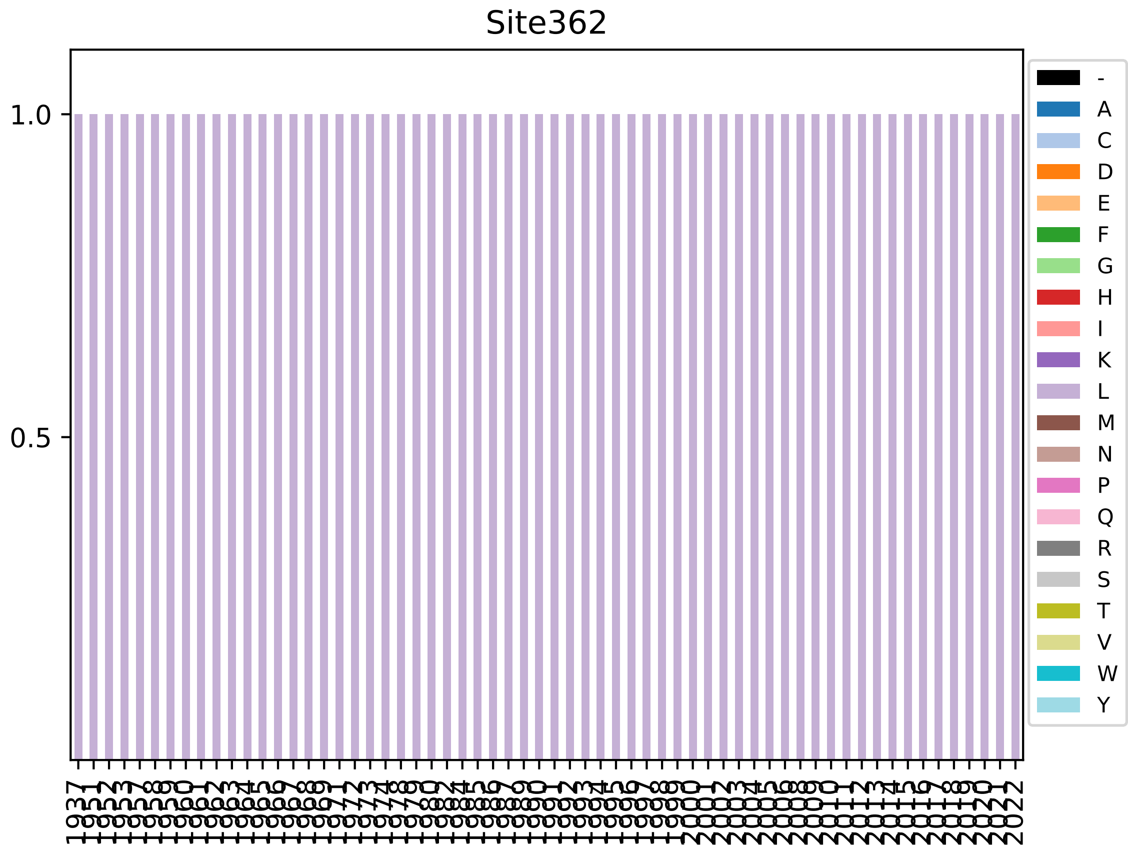
Site362
1.0
0.5
-
A
C
D
E
F
G
H
I
K
L
M
N
P
Q
R
S
T
V
W
Y

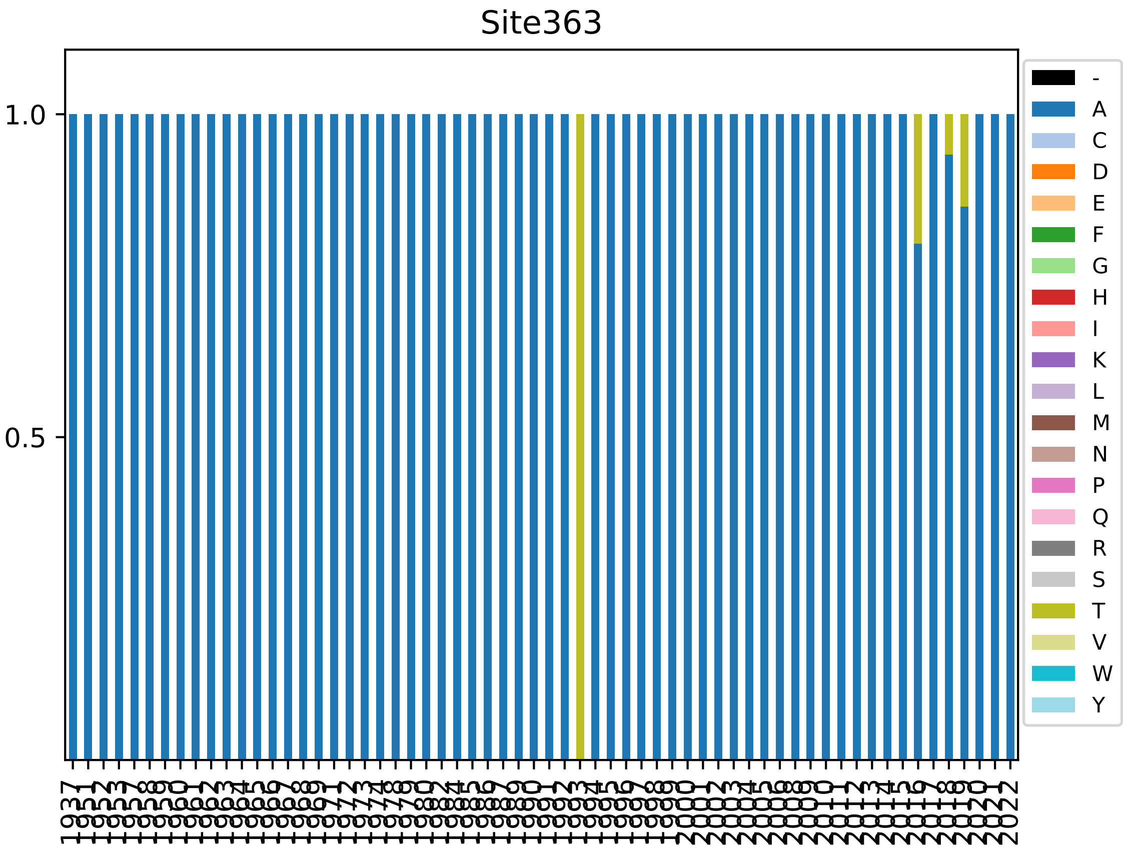
Site363
1.0
0.5
-
A
C
D
E
F
G
H
I
K
L
M
N
P
Q
R
S
T
V
W
Y

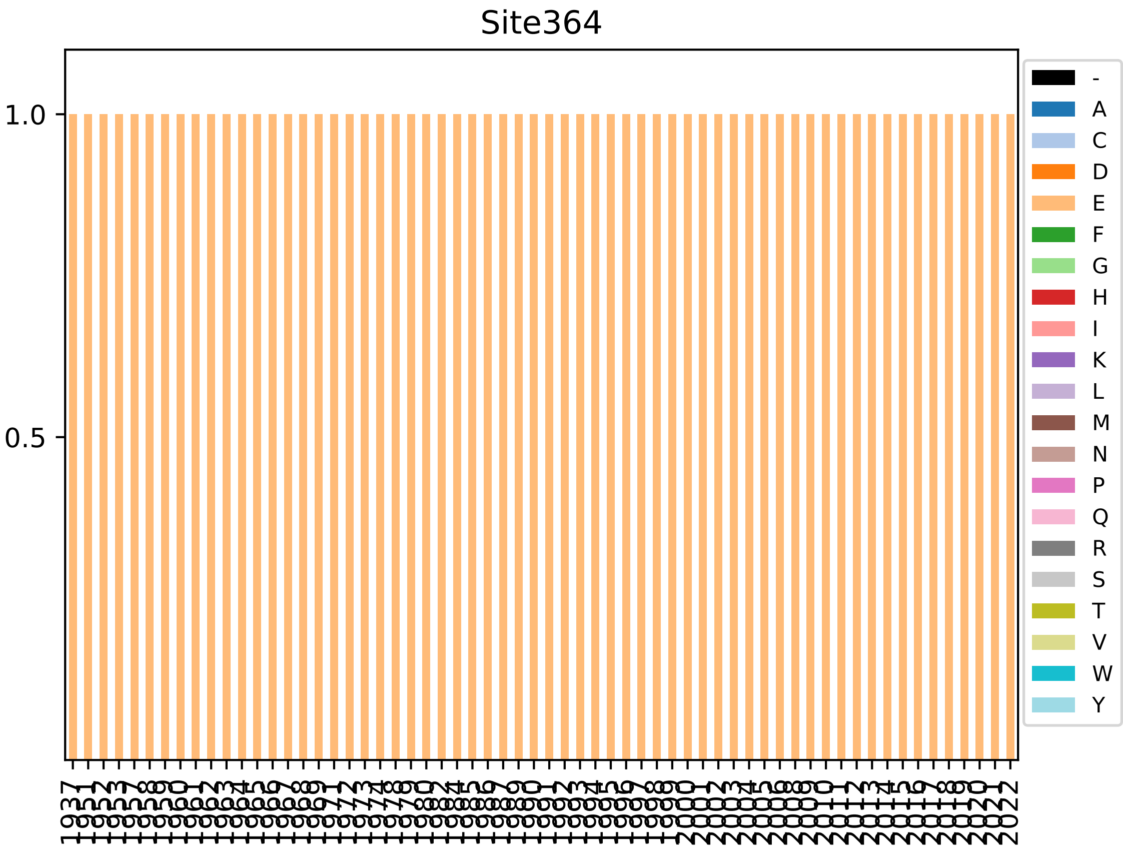

Site364
1.0
0.5
-
A
C
D
E
F
G
H
I
K
L
M
N
P
Q
R
S
T
V
W
Y

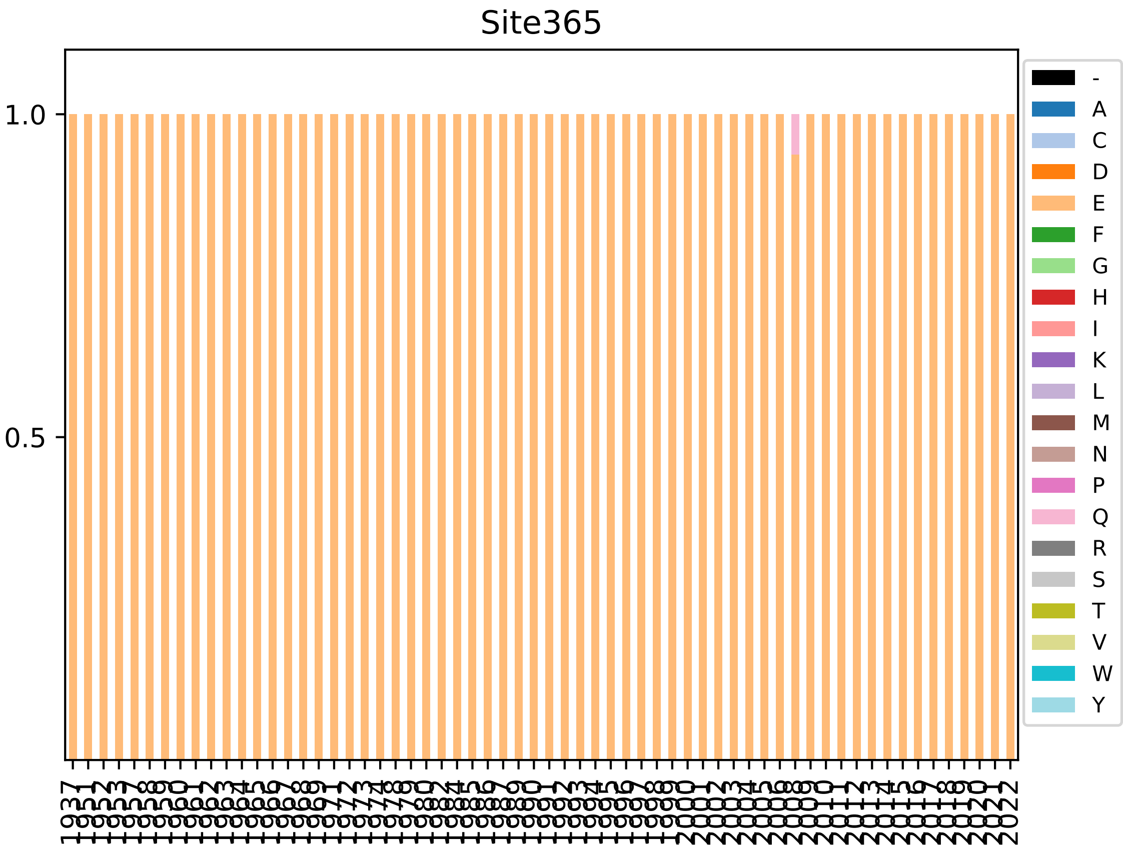
Site365
1.0
0.5
-
A
C
D
E
F
G
H
I
K
L
M
N
P
Q
R
S
T
V
W
Y

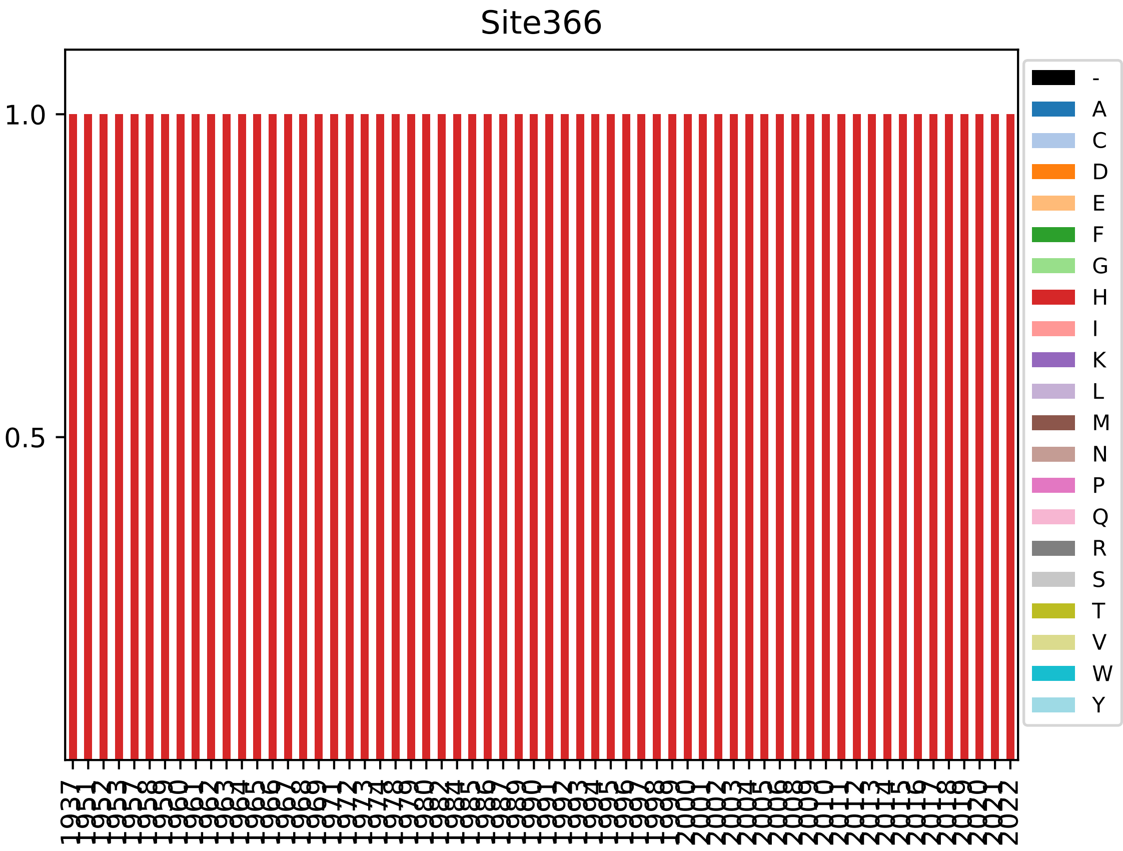
Site366
1.0
0.5
-
A
C
D
E
F
G
H
I
K
L
M
N
P
Q
R
S
T
V
W
Y

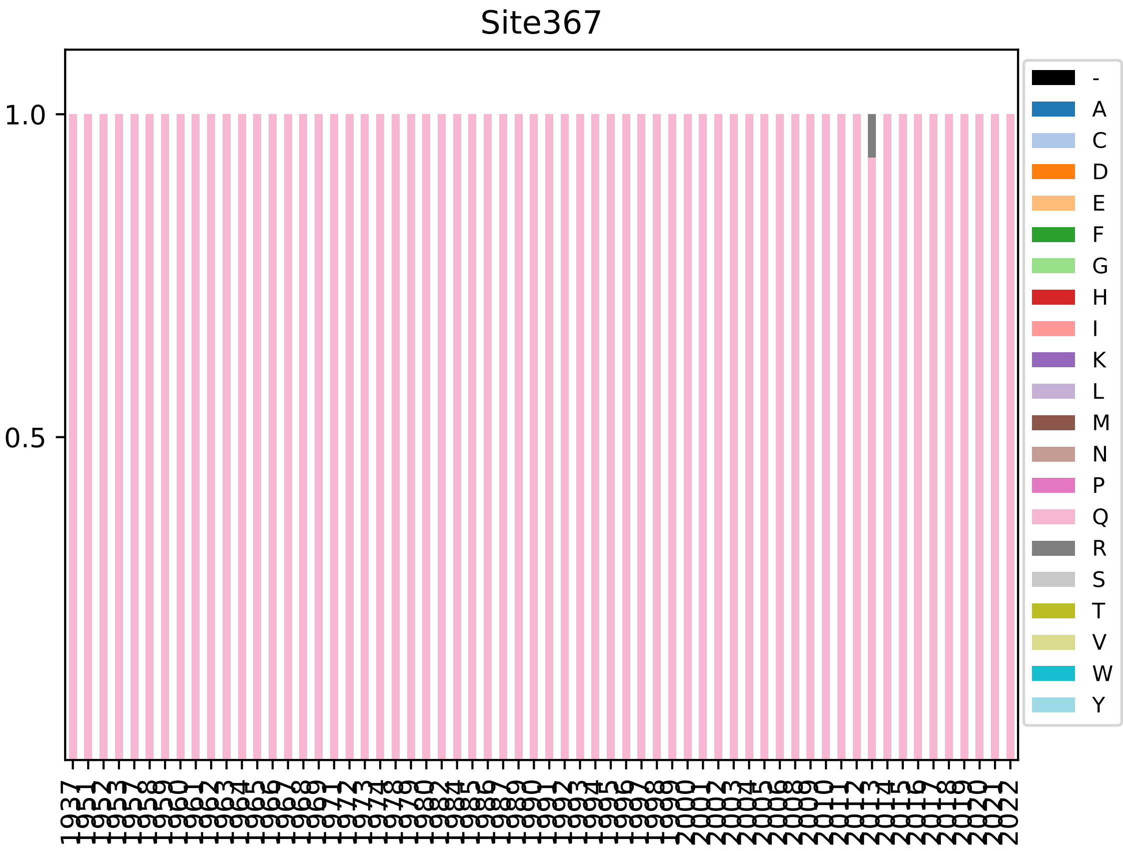
Site367
1.0
0.5
-
A
C
D
E
F
G
H
I
K
L
M
N
P
Q
R
S
T
V
W
Y

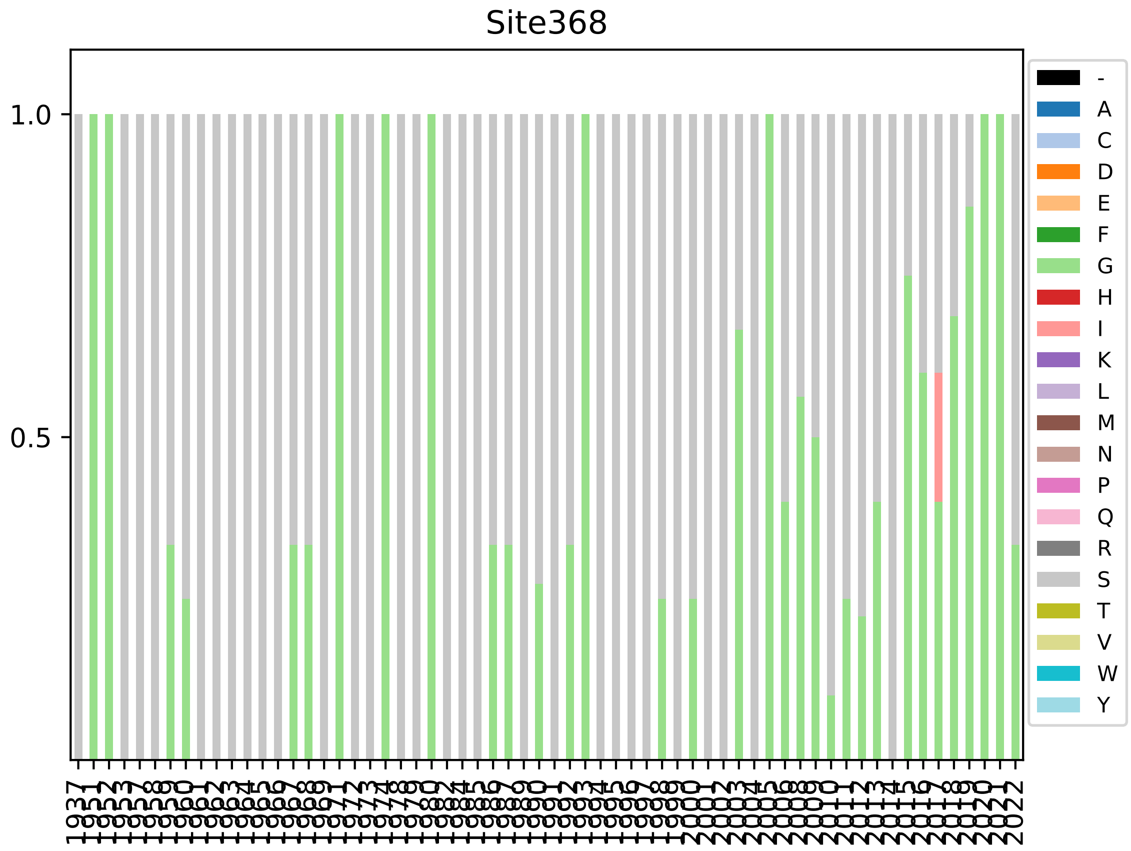
Site368
1.0
0.5
-
A
C
D
E
F
G
H
I
K
L
M
N
P
Q
R
S
T
V
W
Y

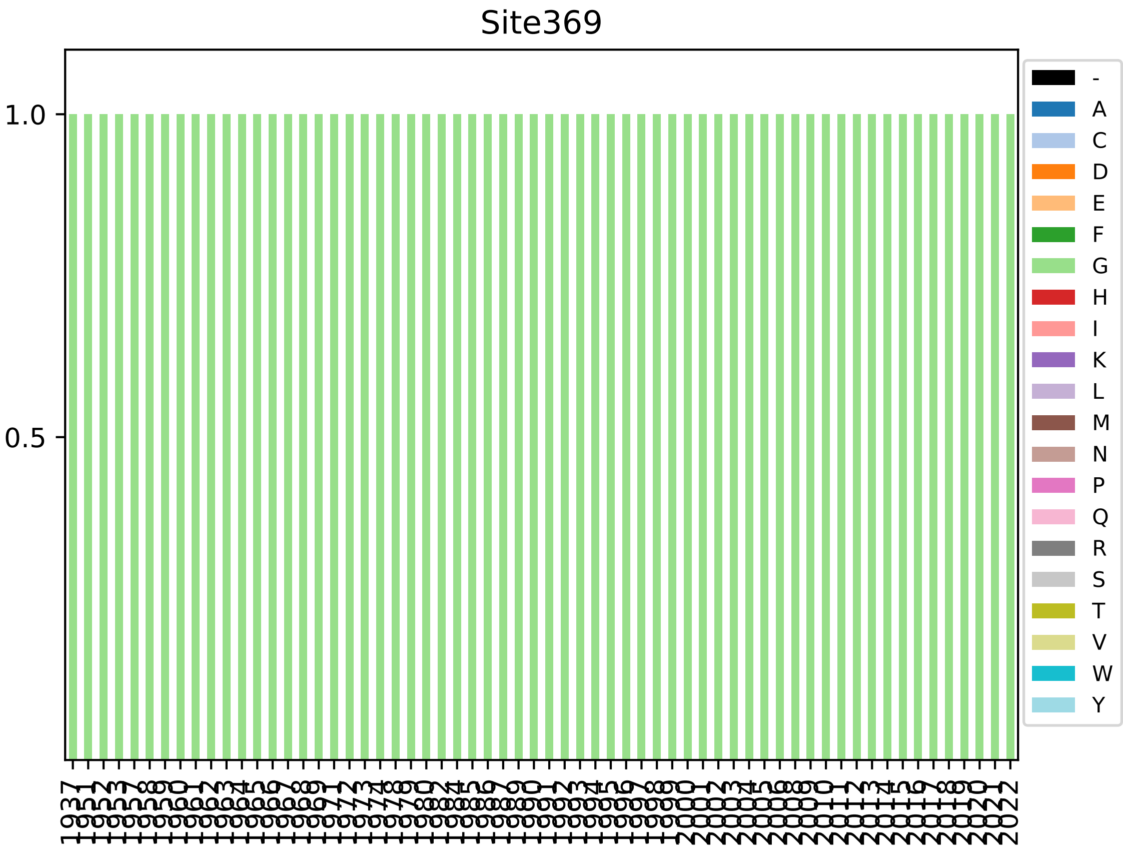
Site369
1.0
0.5
-
A
C
D
E
F
G
H
I
K
L
M
N
P
Q
R
S
T
V
W
Y

Site370

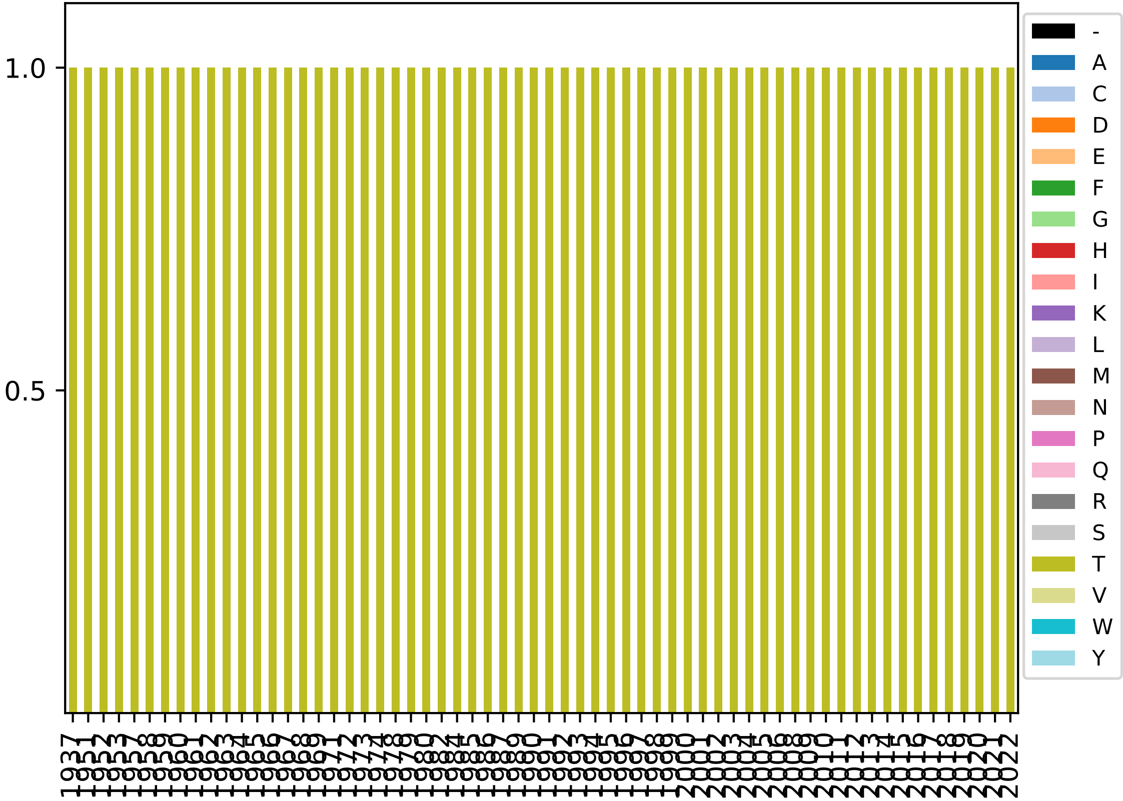

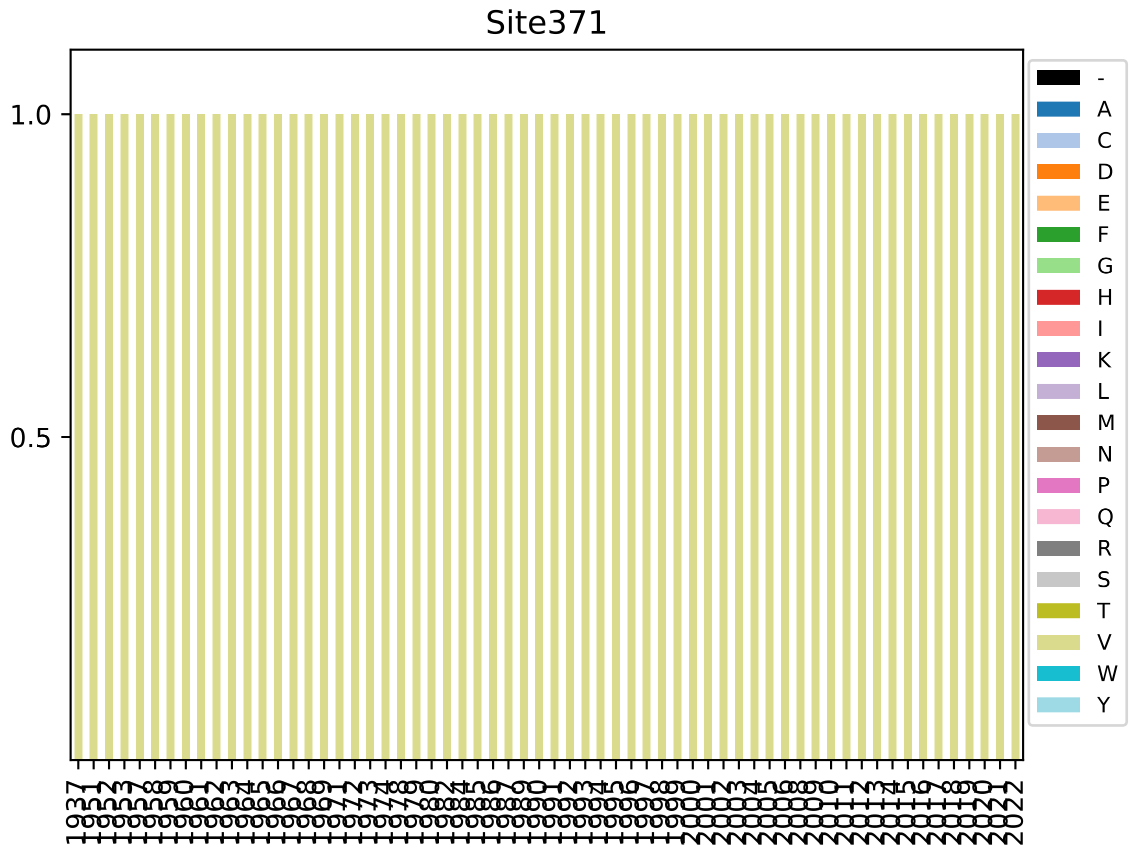
Site371
1.0
0.5
-
A
C
D
E
F
G
H
I
K
L
M
N
P
Q
R
S
T
V
W
Y

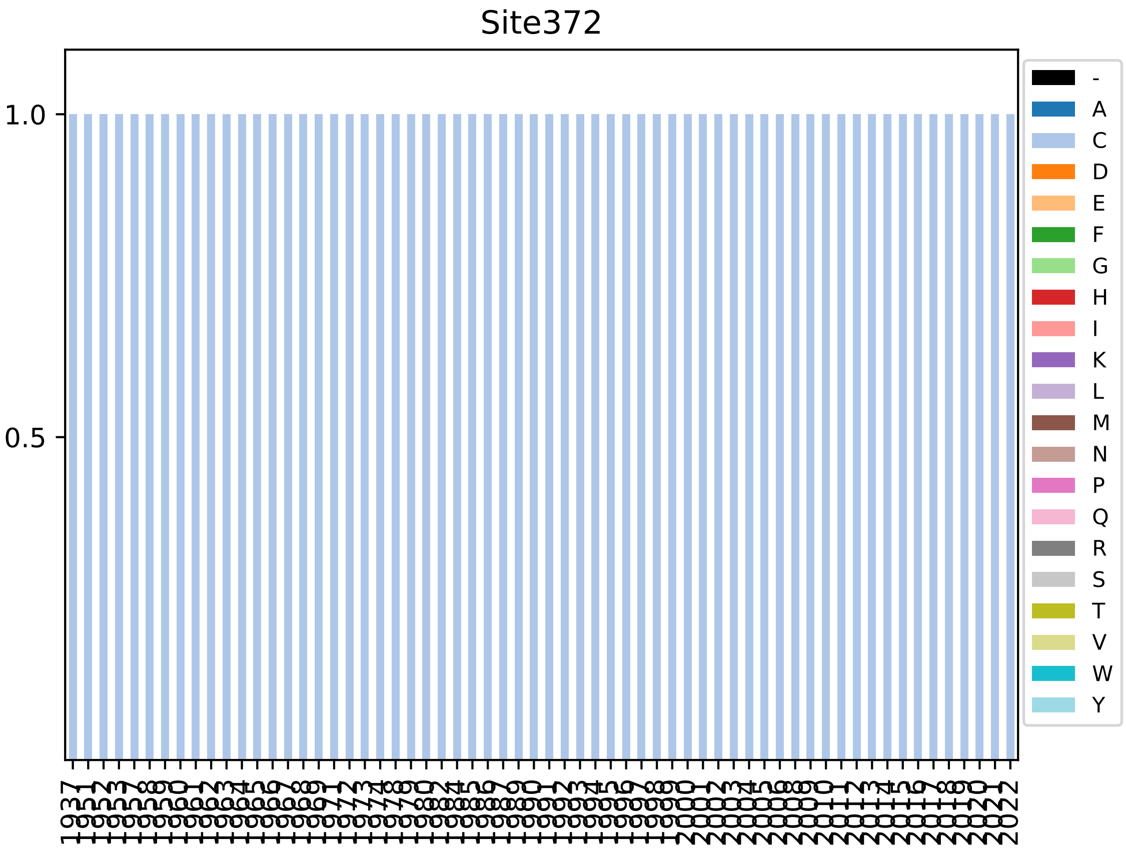
Site372
1.0
0.5
-
A
C
D
E
F
G
H
I
K
L
M
N
P
Q
R
S
T
V
W
Y

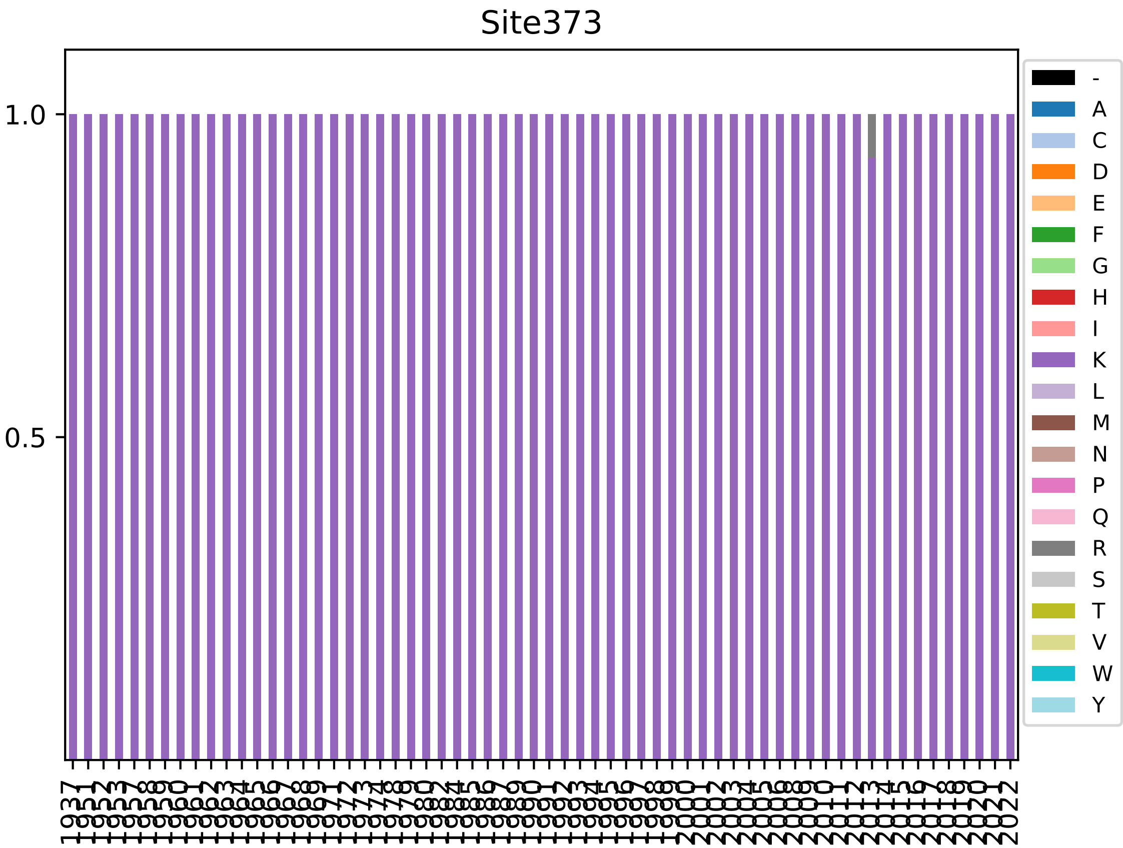
Site373
1.0
0.5
-
A
C
D
E
F
G
H
I
K
L
M
N
P
Q
R
S
T
V
W
Y

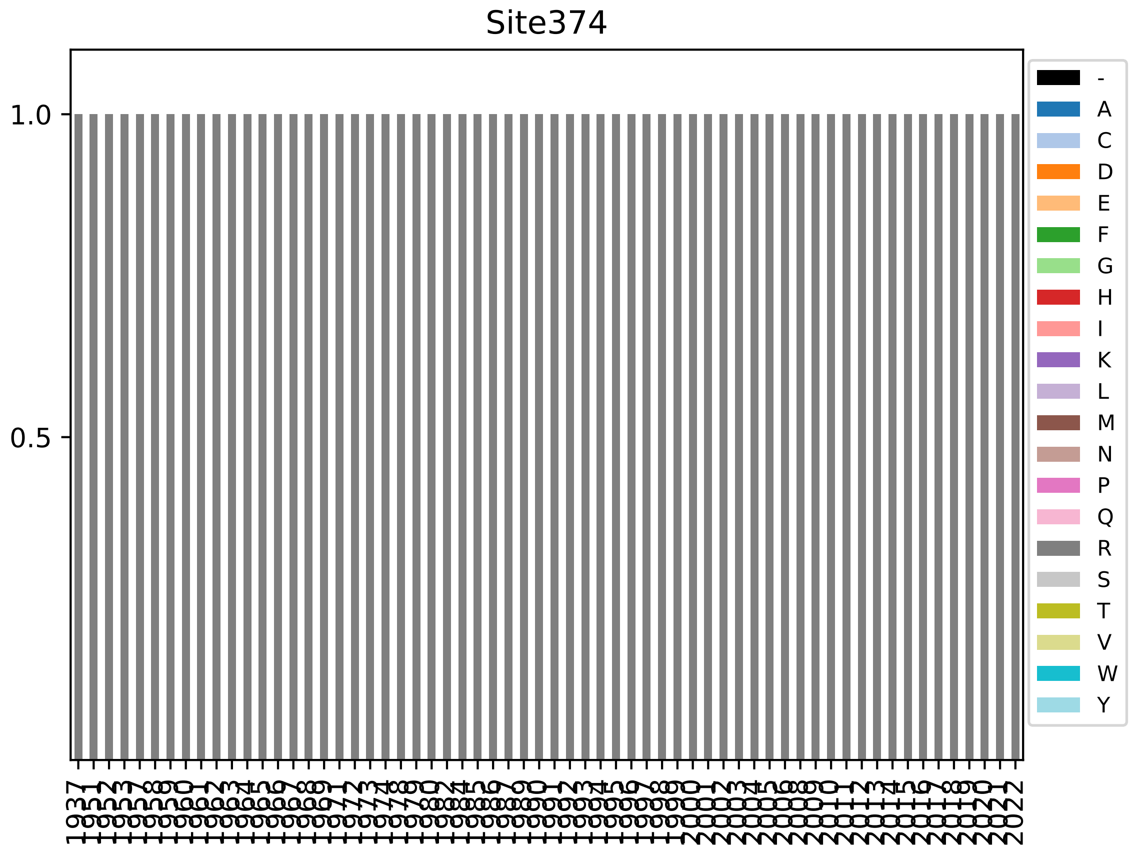
Site374
1.0
0.5
-
A
C
D
E
F
G
H
I
K
L
M
N
P
Q
R
S
T
V
W
Y

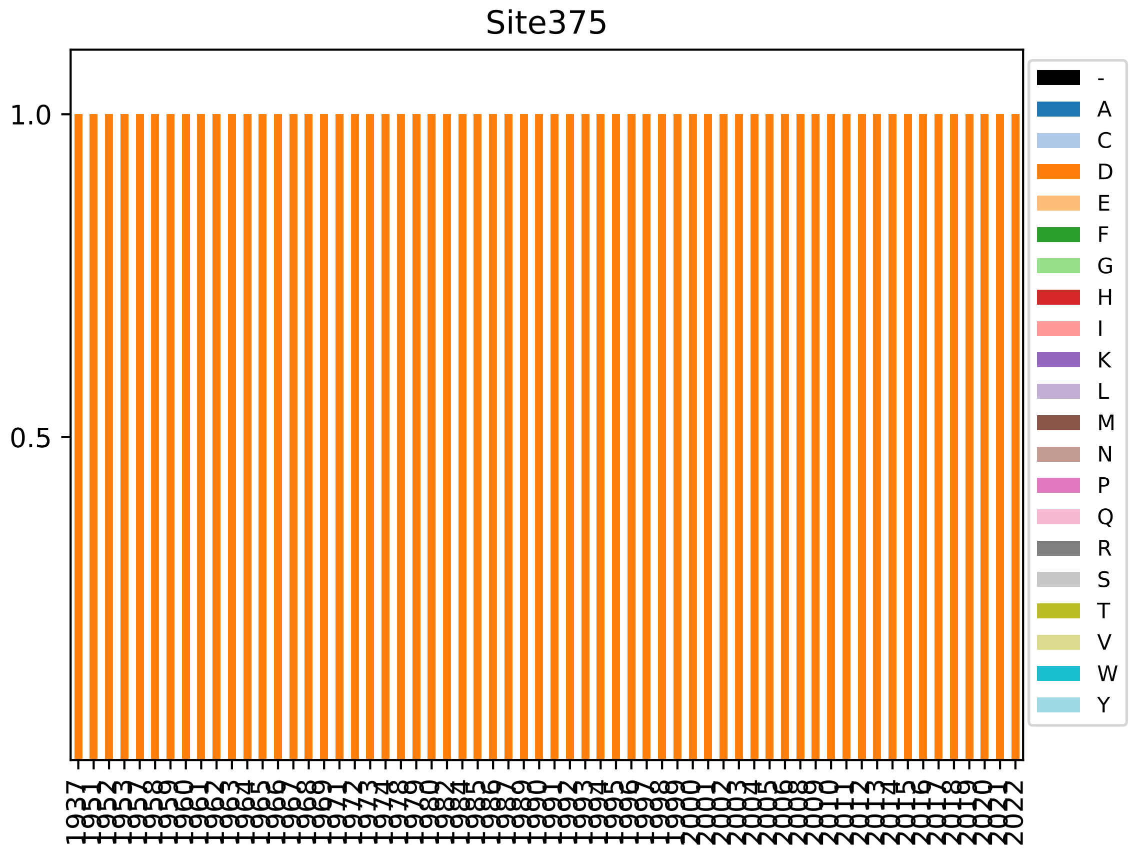
Site375
1.0
0.5
-
A
C
D
E
F
G
H
I
K
L
M
N
P
Q
R
S
T
V
W
Y

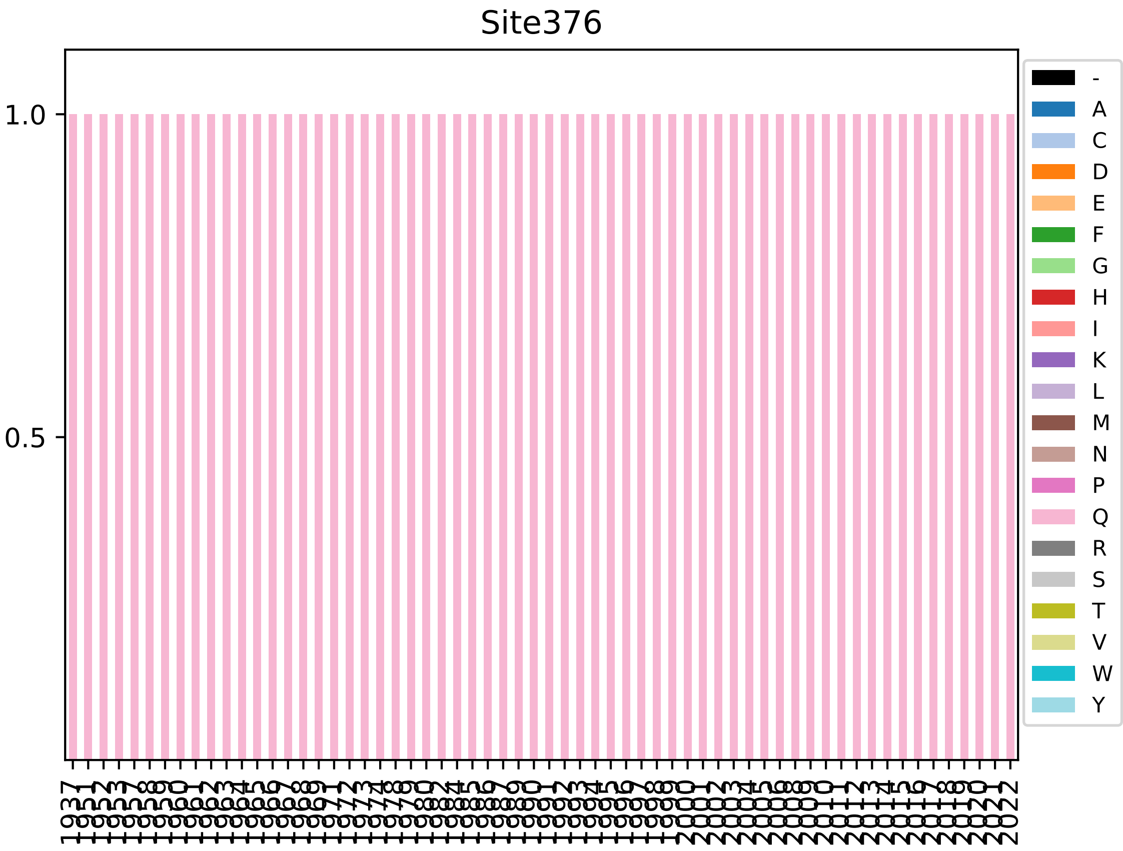
Site376
1.0
0.5
-
A
C
D
E
F
G
H
I
K
L
M
N
P
Q
R
S
T
V
W
Y

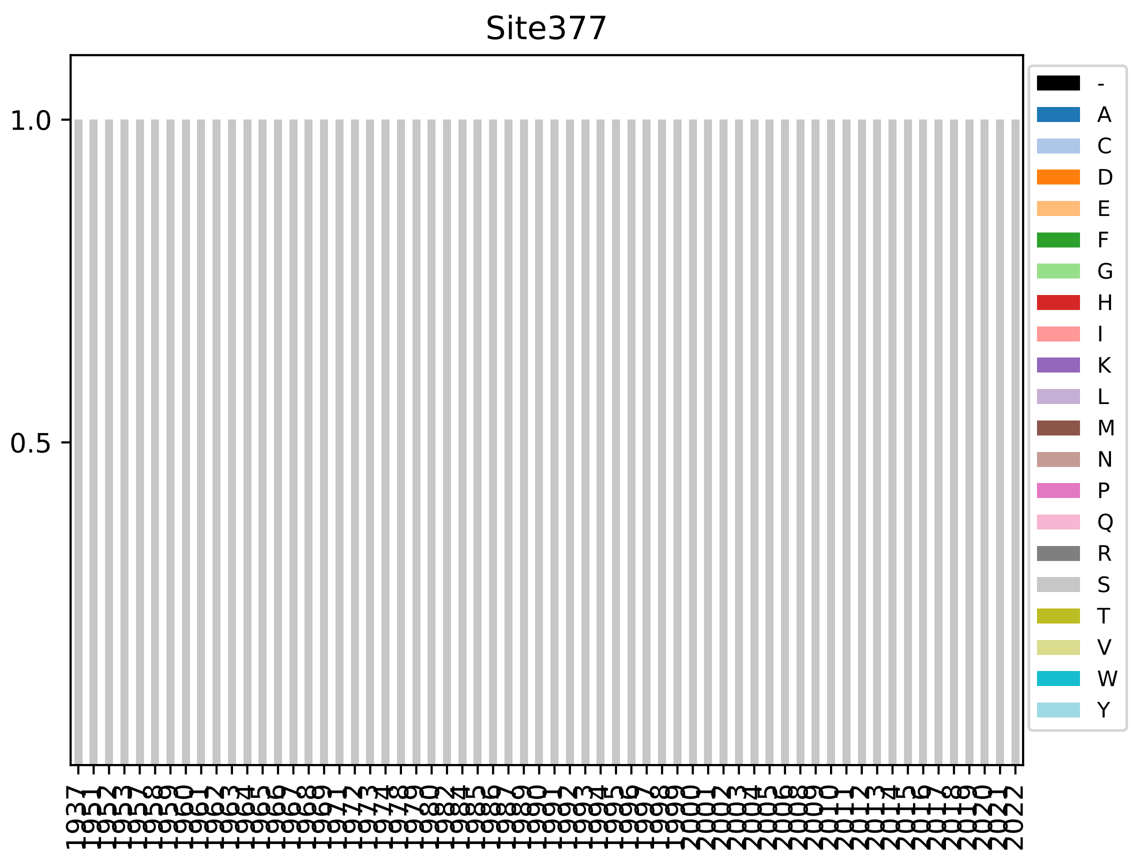
Site377
1.0
0.5
-
A
C
D
E
F
G
H
I
K
L
M
N
P
Q
R
S
T
V
W
Y

Site378

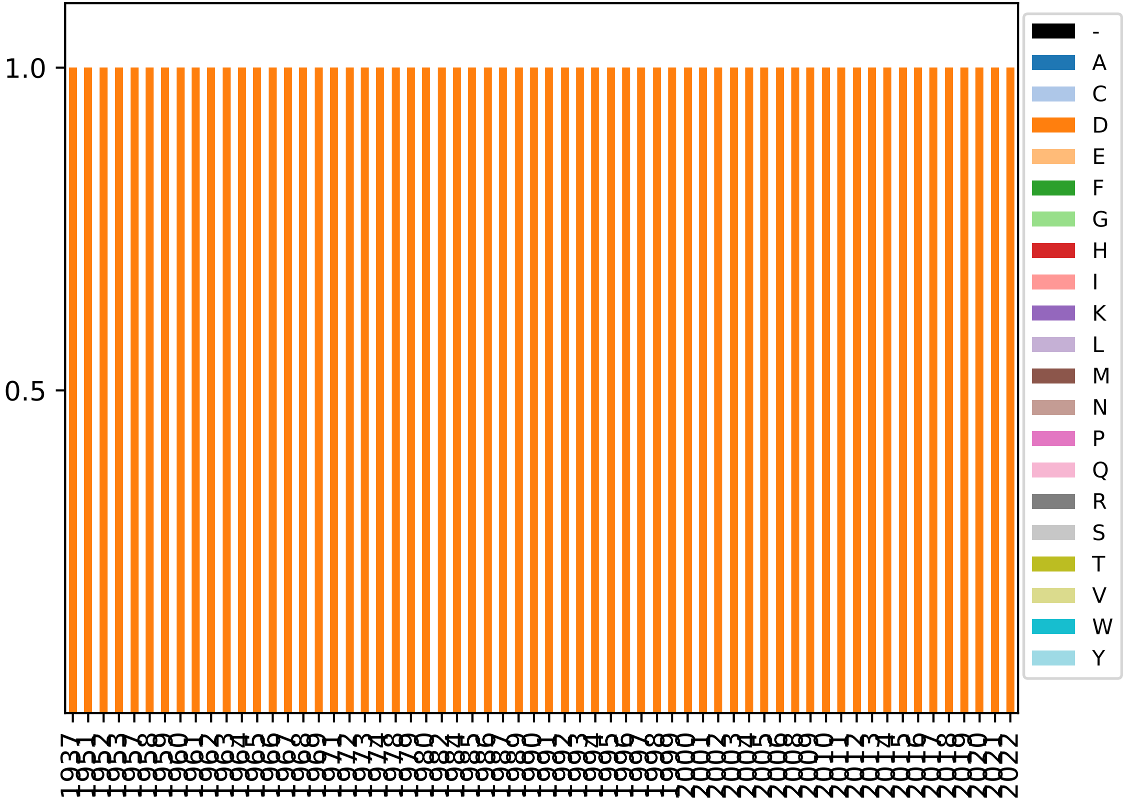

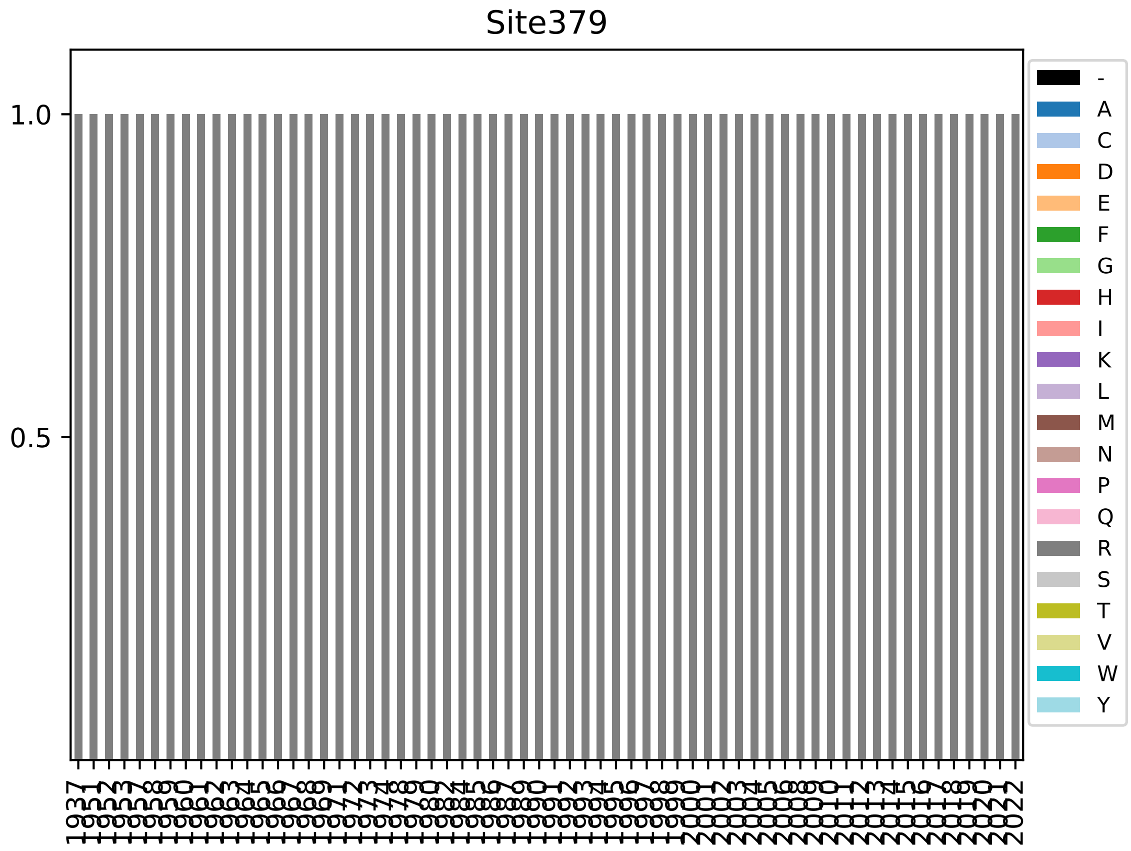
Site379
1.0
0.5
-
A
C
D
E
F
G
H
I
K
L
M
N
P
Q
R
S
T
V
W
Y

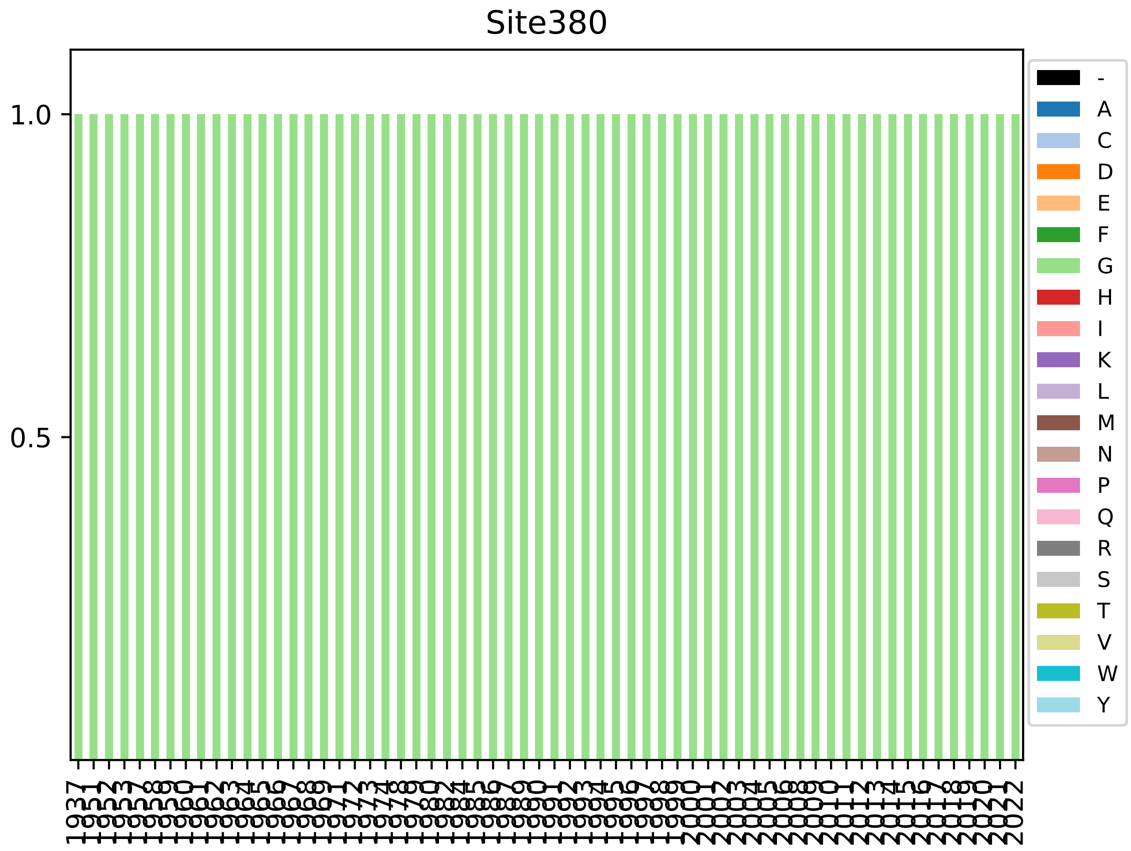

Site380
1.0
0.5
-
A
C
D
E
F
G
H
I
K
L
M
N
P
Q
R
S
T
V
W
Y

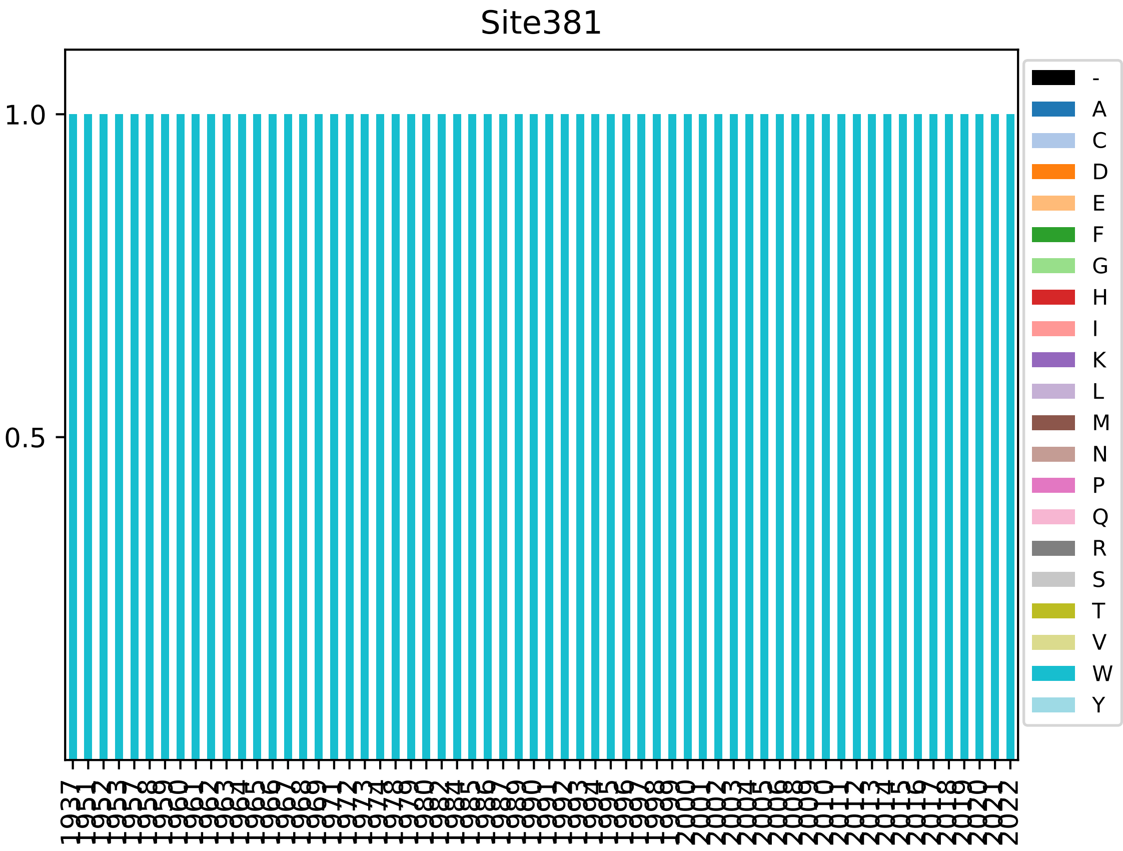
Site381
1.0
0.5
-
A
C
D
E
F
G
H
I
K
L
M
N
P
Q
R
S
T
V
W
Y

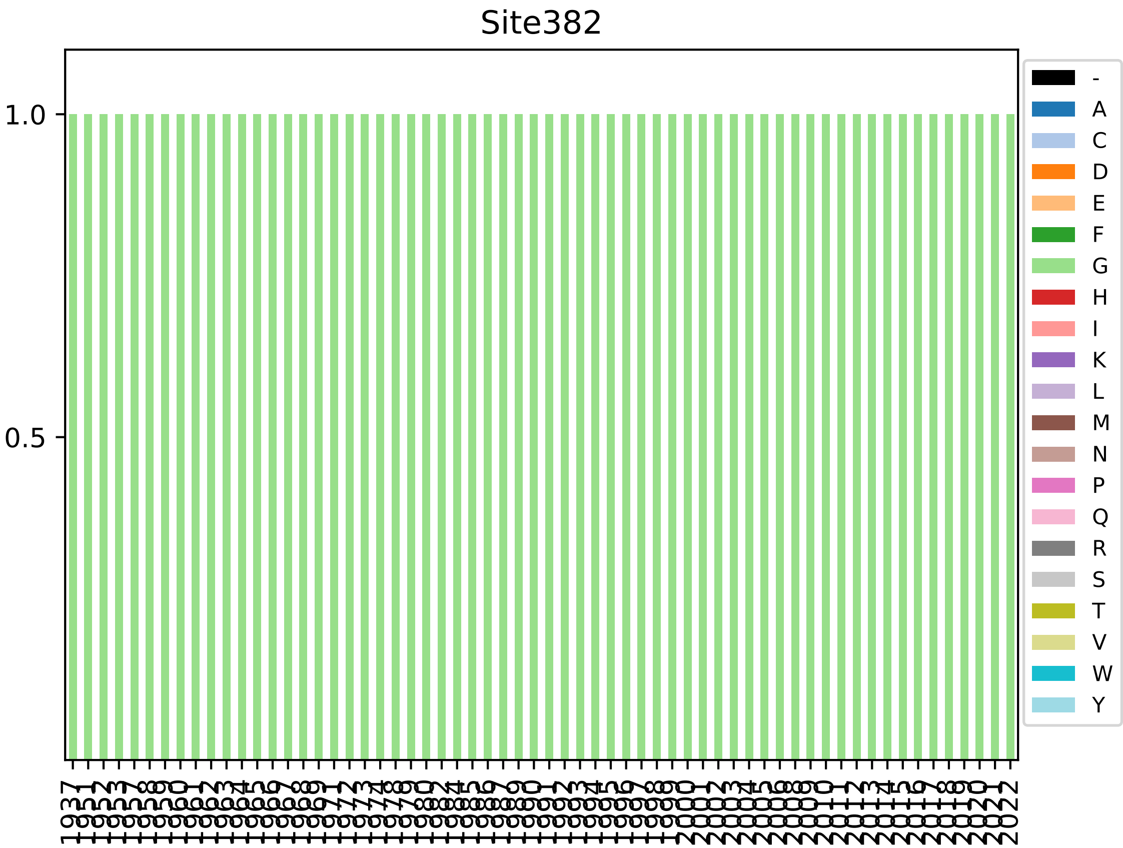
Site382
1.0
0.5
-
A
C
D
E
F
G
H
I
K
L
M
N
P
Q
R
S
T
V
W
Y

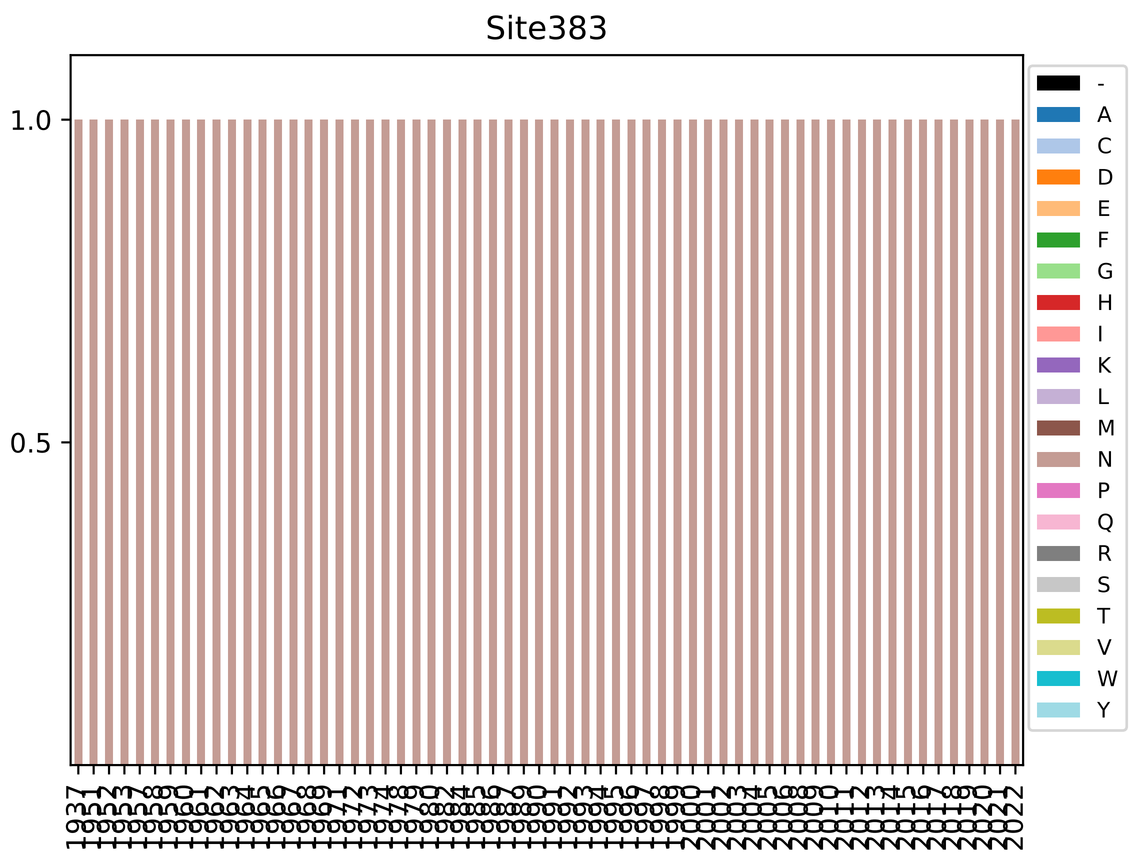
Site383
1.0
0.5
-
A
C
D
E
F
G
H
I
K
L
M
N
P
Q
R
S
T
V
W
Y

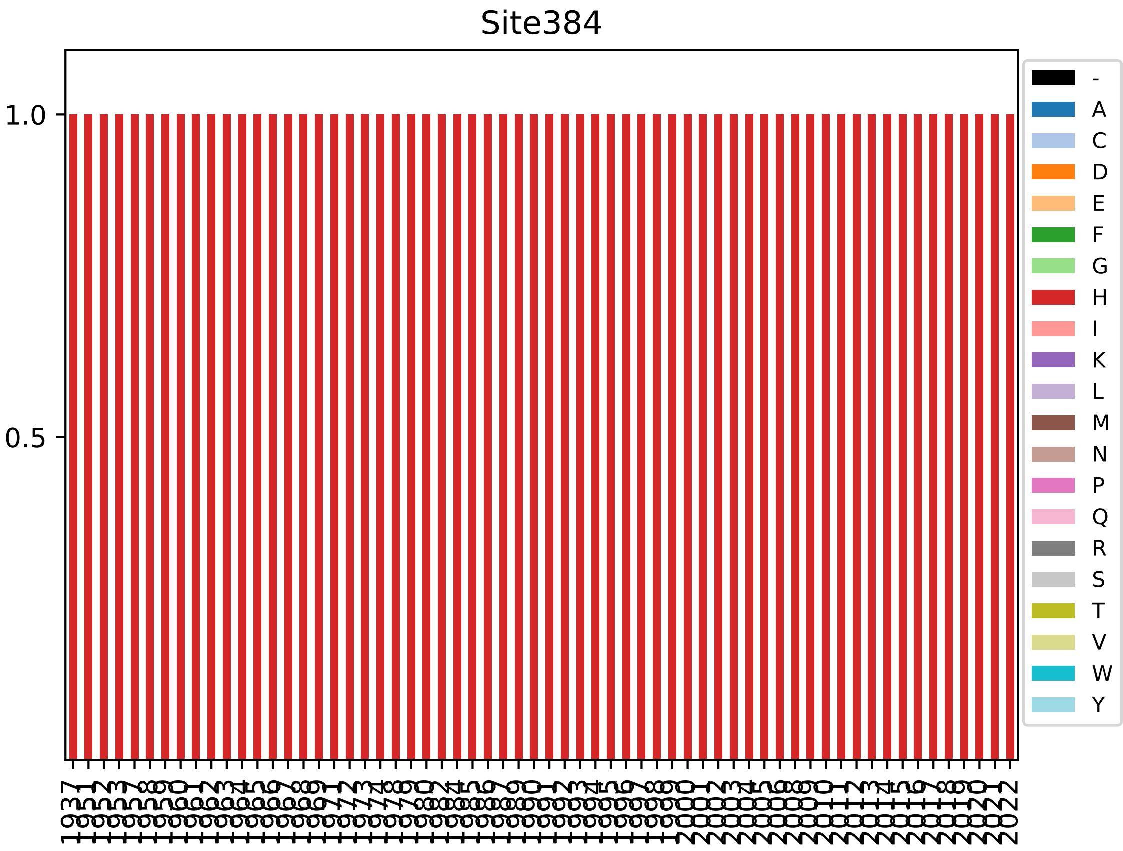
Site384
1.0
0.5
-
A
C
D
E
F
G
H
I
K
L
M
N
P
Q
R
S
T
V
W
Y

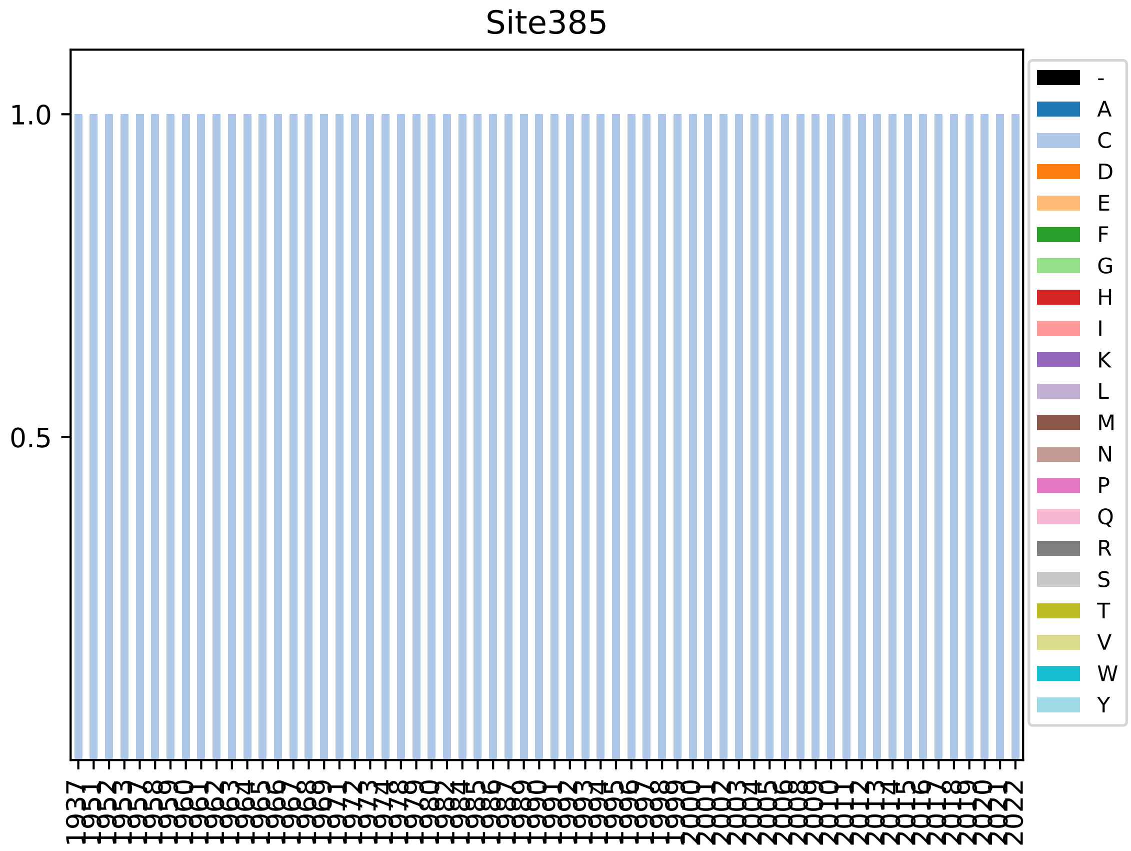
Site385
1.0
0.5
-
A
C
D
E
F
G
H
I
K
L
M
N
P
Q
R
S
T
V
W
Y

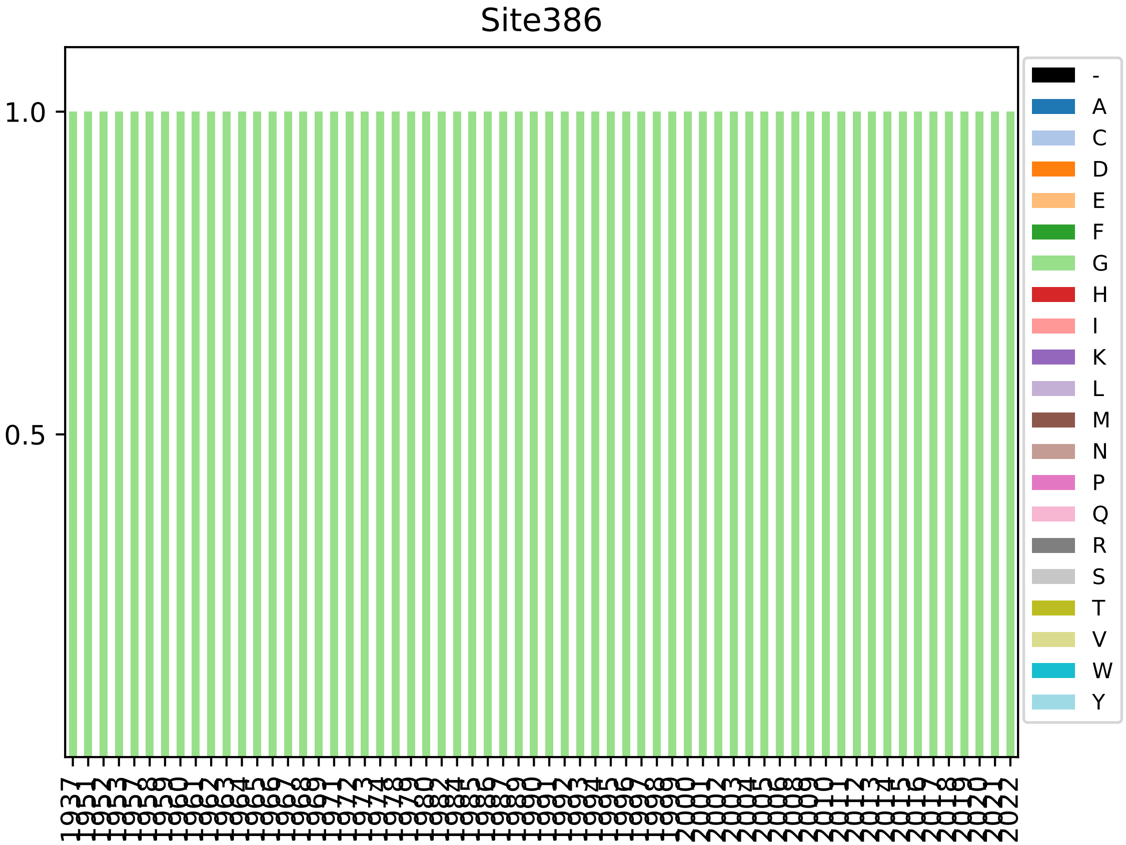

Site386
1.0
0.5
-
A
C
D
E
F
G
H
I
K
L
M
N
P
Q
R
S
T
V
W
Y

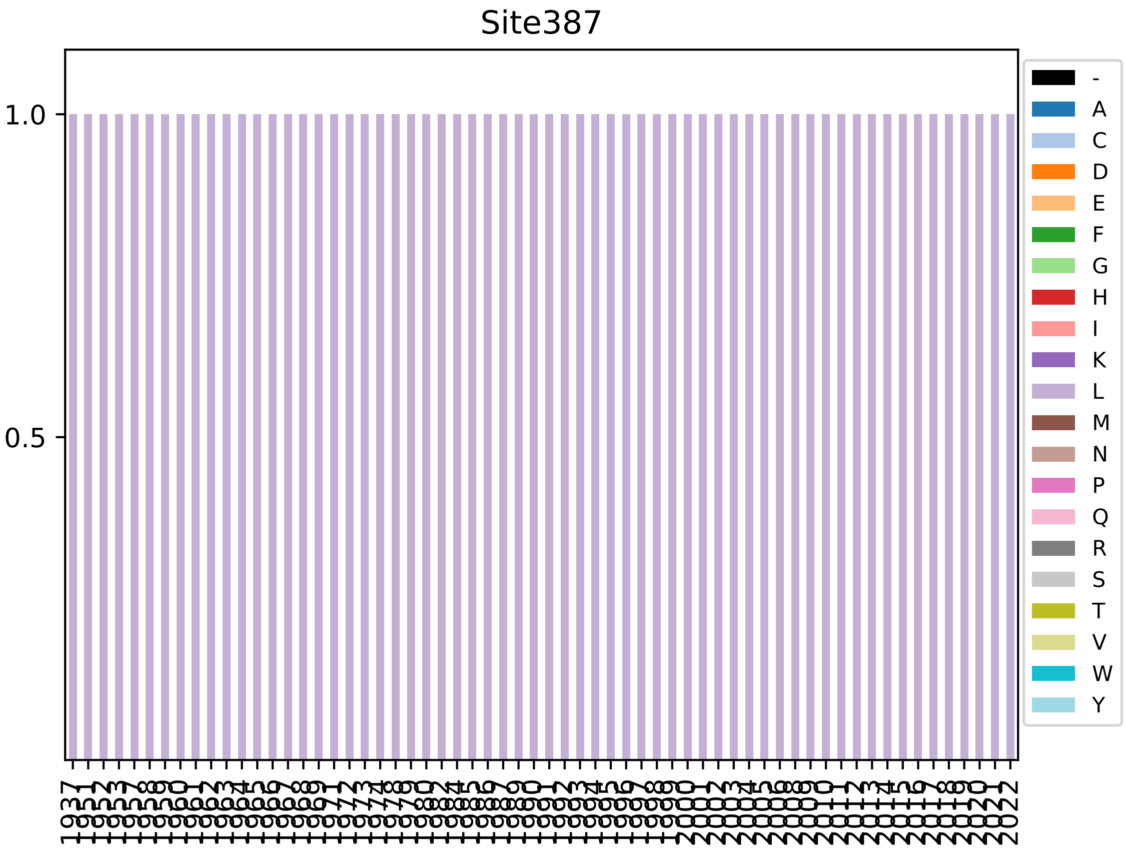

Site387
1.0
0.5
-
A
C
D
E
F
G
H
I
K
L
M
N
P
Q
R
S
T
V
W
Y

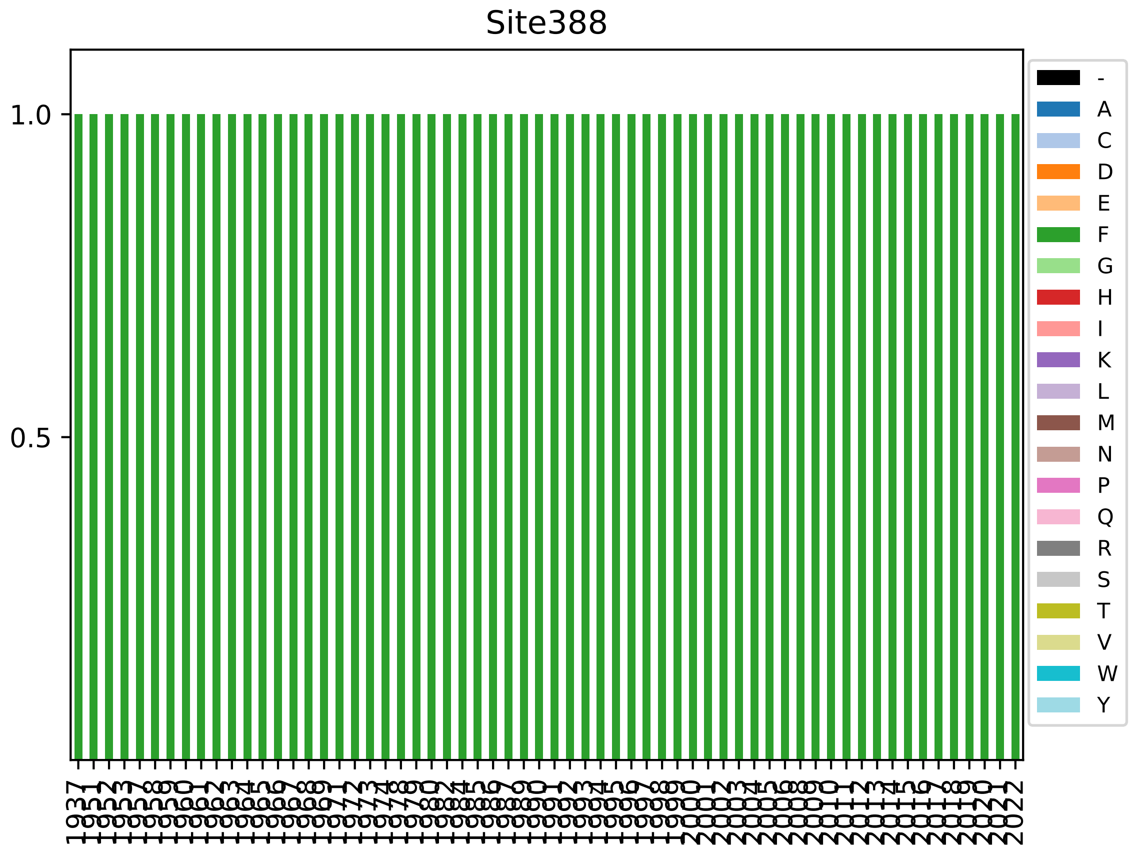
Site388
1.0
0.5
-
A
C
D
E
F
G
H
I
K
L
M
N
P
Q
R
S
T
V
W
Y

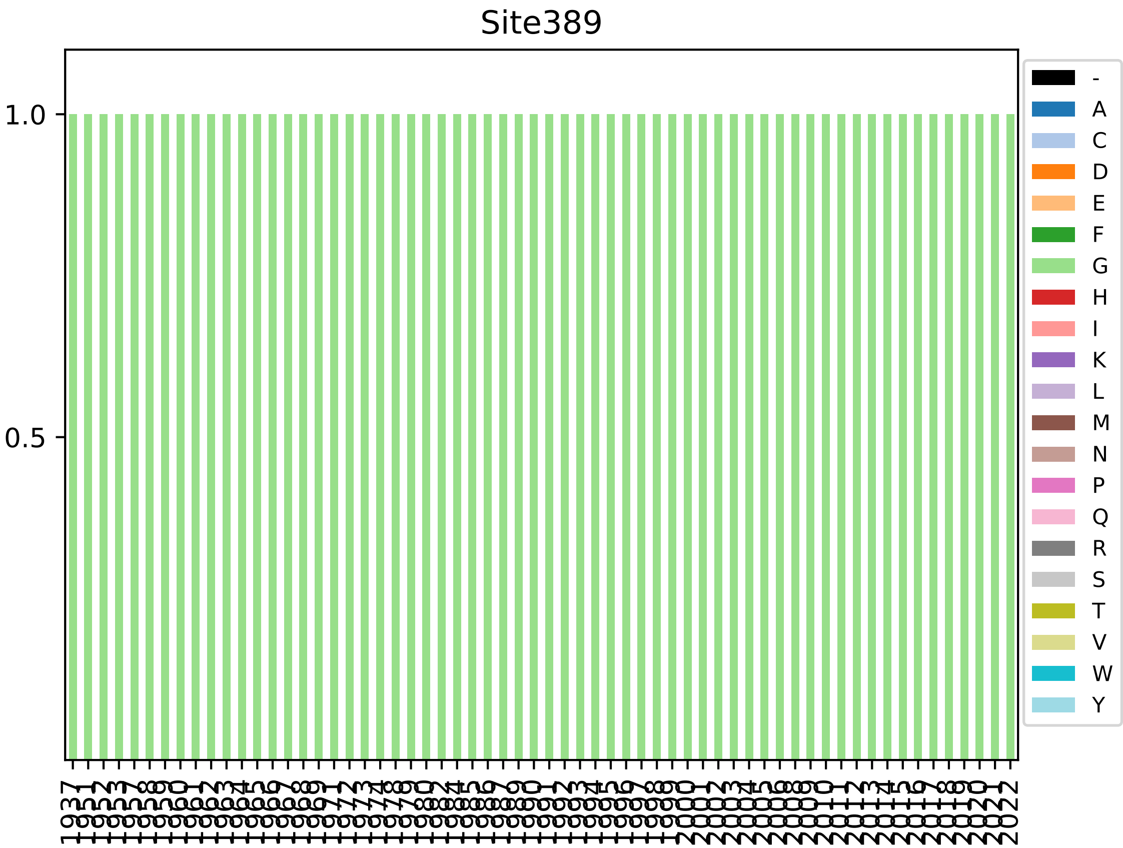
Site389
1.0
0.5
-
A
C
D
E
F
G
H
I
K
L
M
N
P
Q
R
S
T
V
W
Y

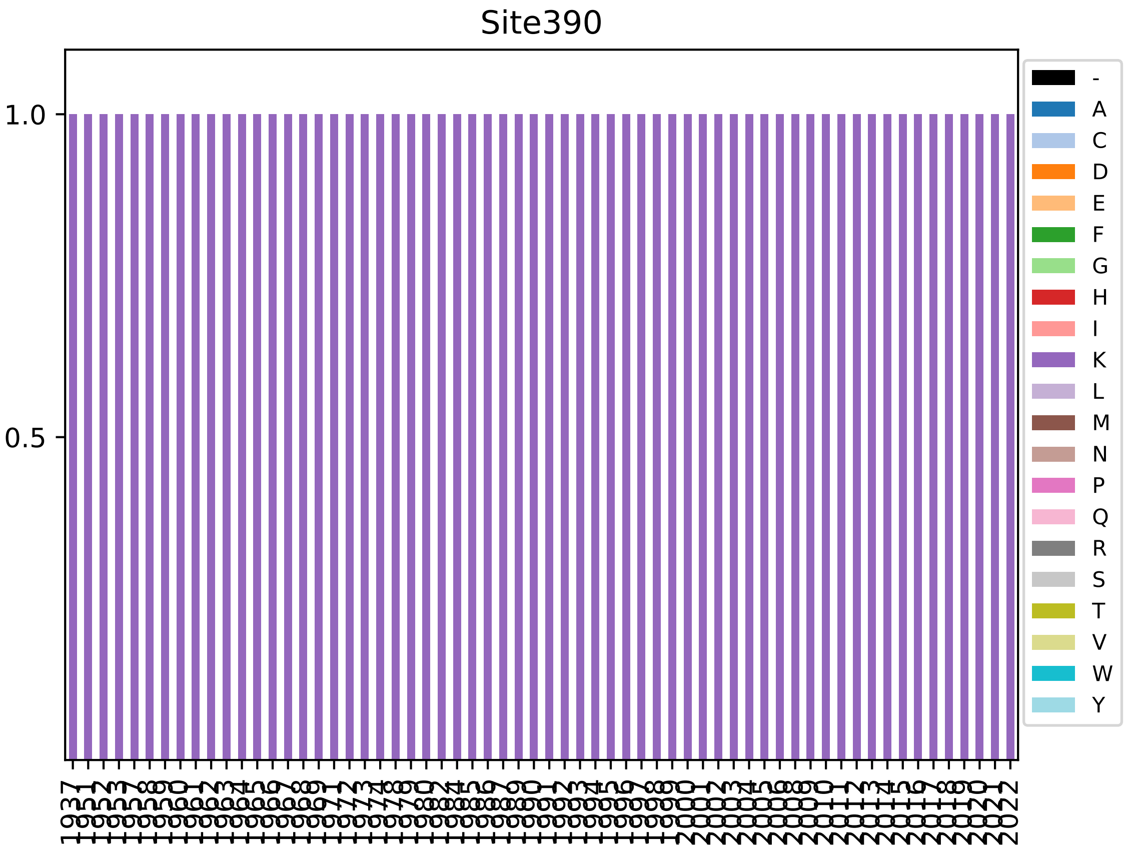
Site390
1.0
0.5
-
A
C
D
E
F
G
H
I
K
L
M
N
P
Q
R
S
T
V
W
Y

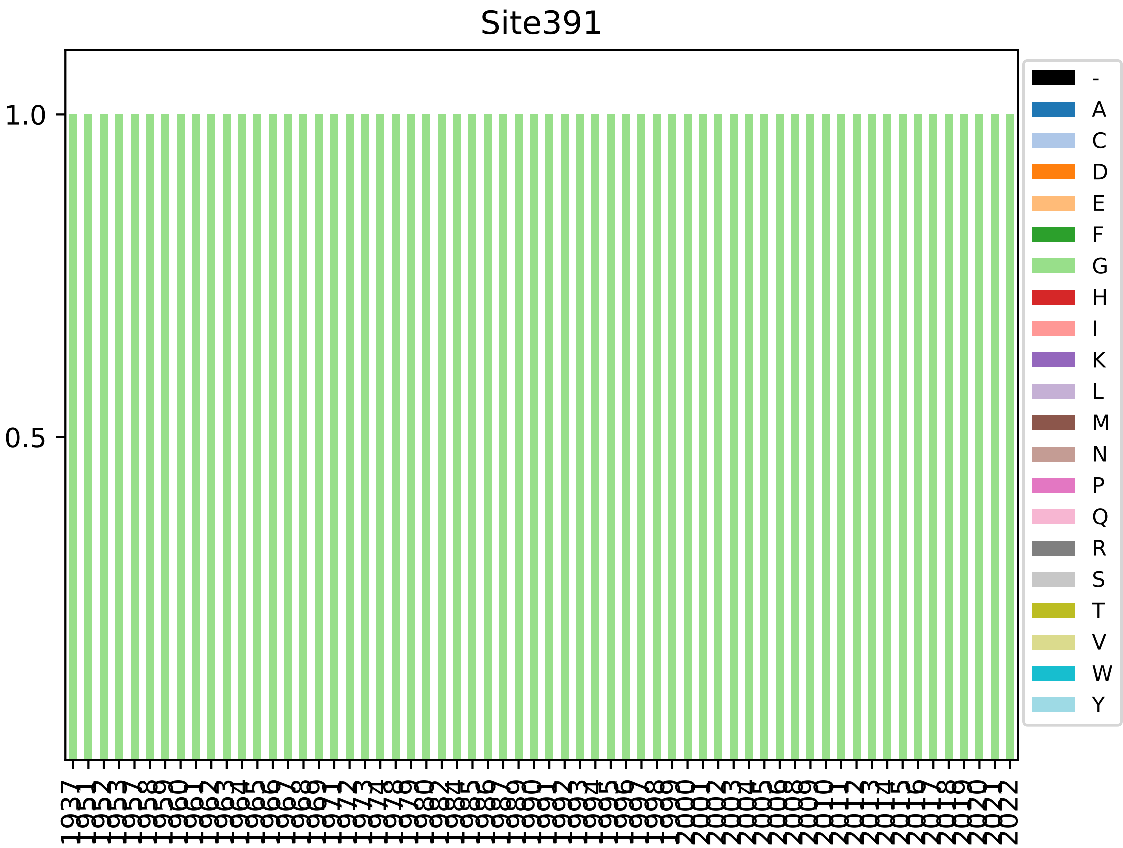
Site391
1.0
0.5
-
A
C
D
E
F
G
H
I
K
L
M
N
P
Q
R
S
T
V
W
Y

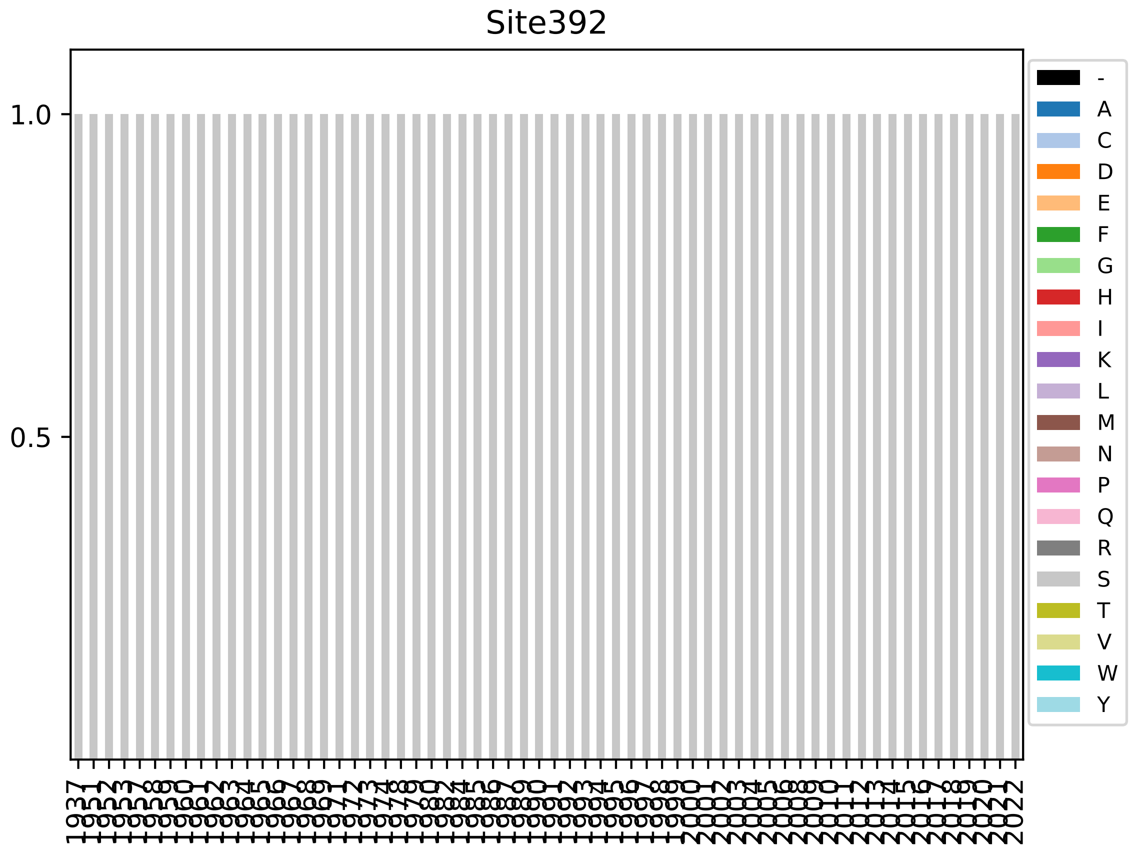
Site392
1.0
0.5
-
A
C
D
E
F
G
H
I
K
L
M
N
P
Q
R
S
T
V
W
Y

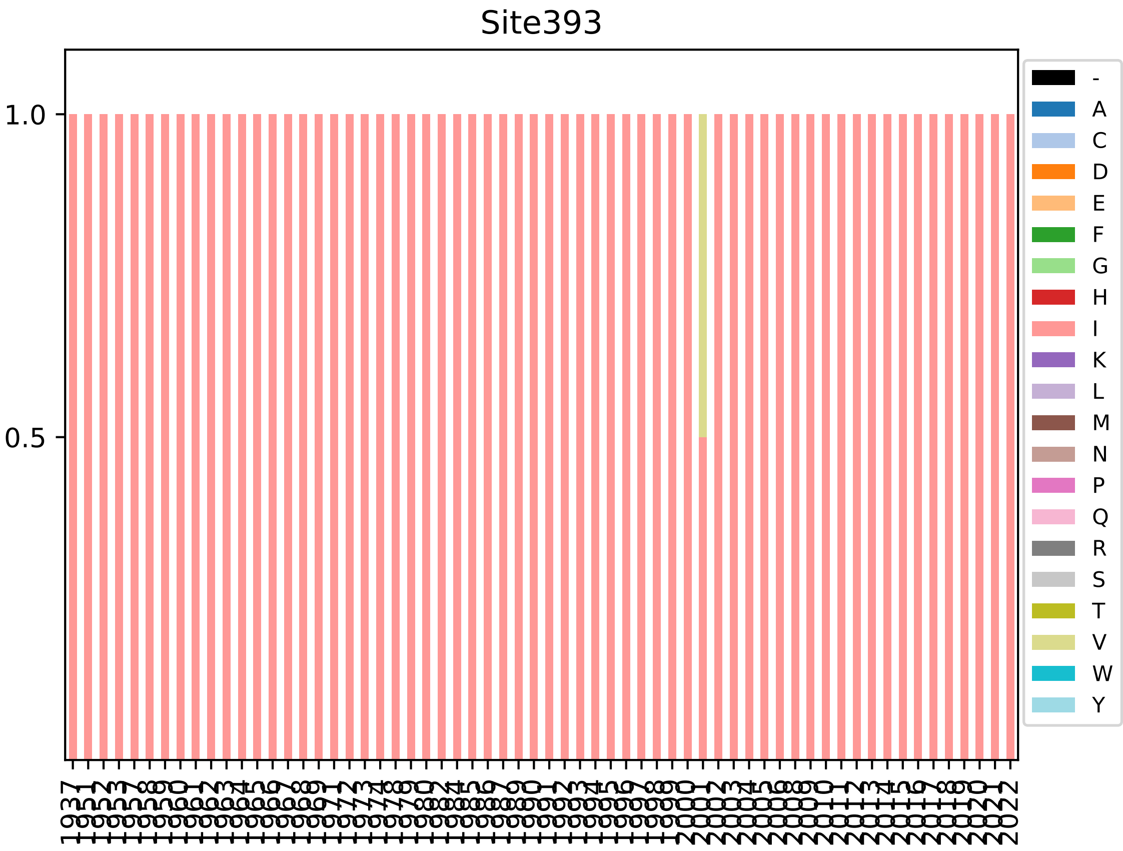
Site393
1.0
0.5
-
A
C
D
E
F
G
H
I
K
L
M
N
P
Q
R
S
T
V
W
Y

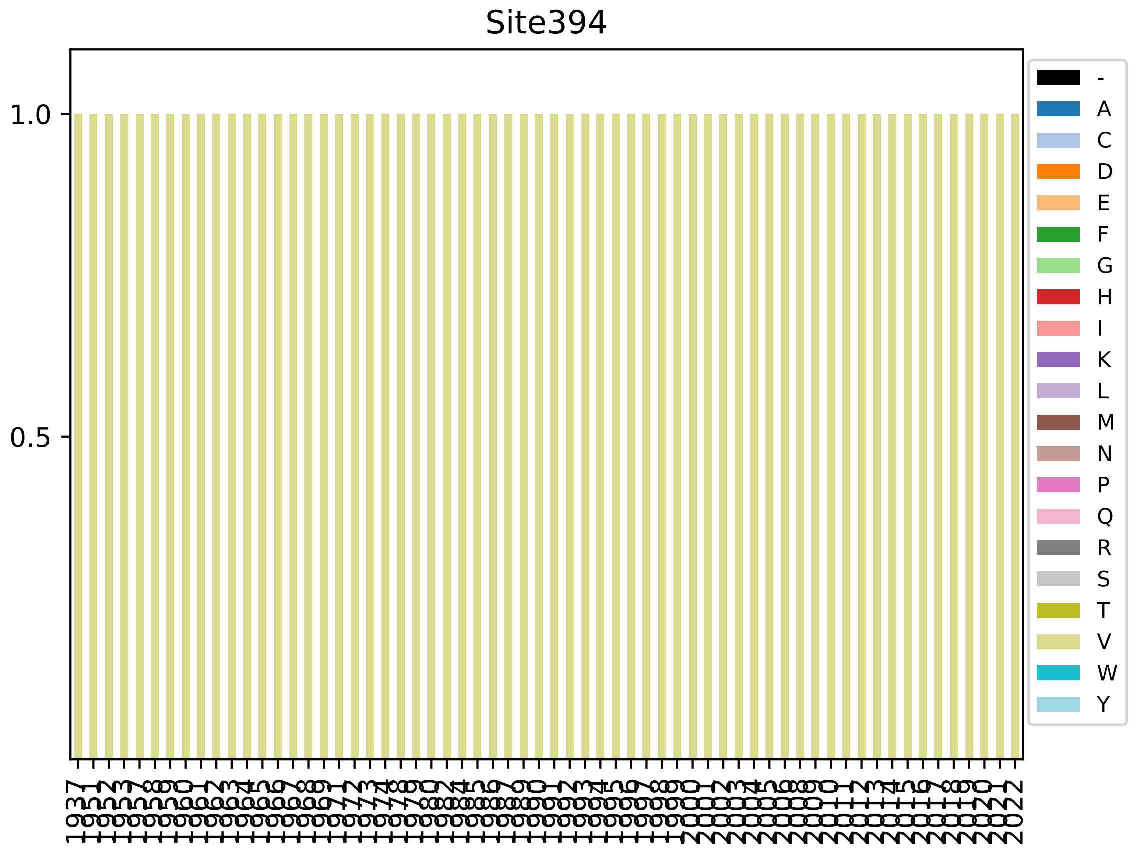
Site394
1.0
0.5
-
A
C
D
E
F
G
H
I
K
L
M
N
P
Q
R
S
T
V
W
Y

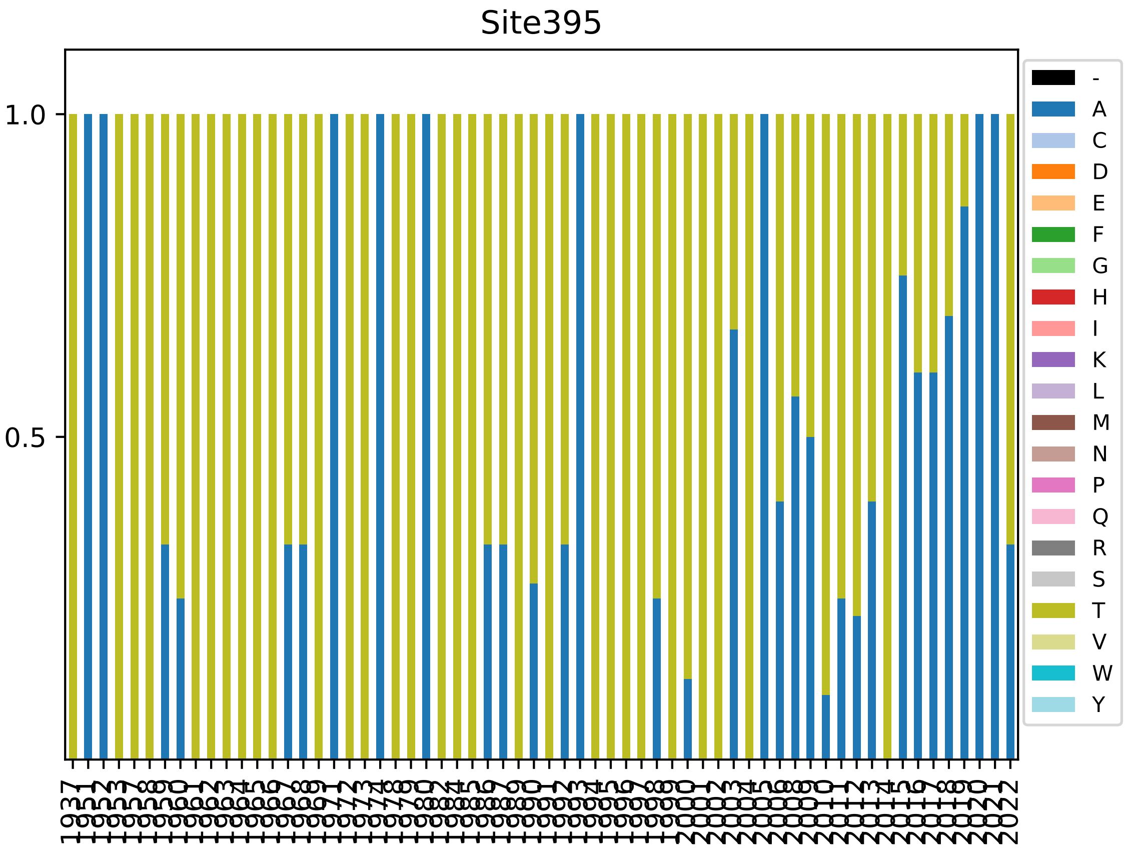
Site395
1.0
0.5
-
A
C
D
E
F
G
H
I
K
L
M
N
P
Q
R
S
T
V
W
Y

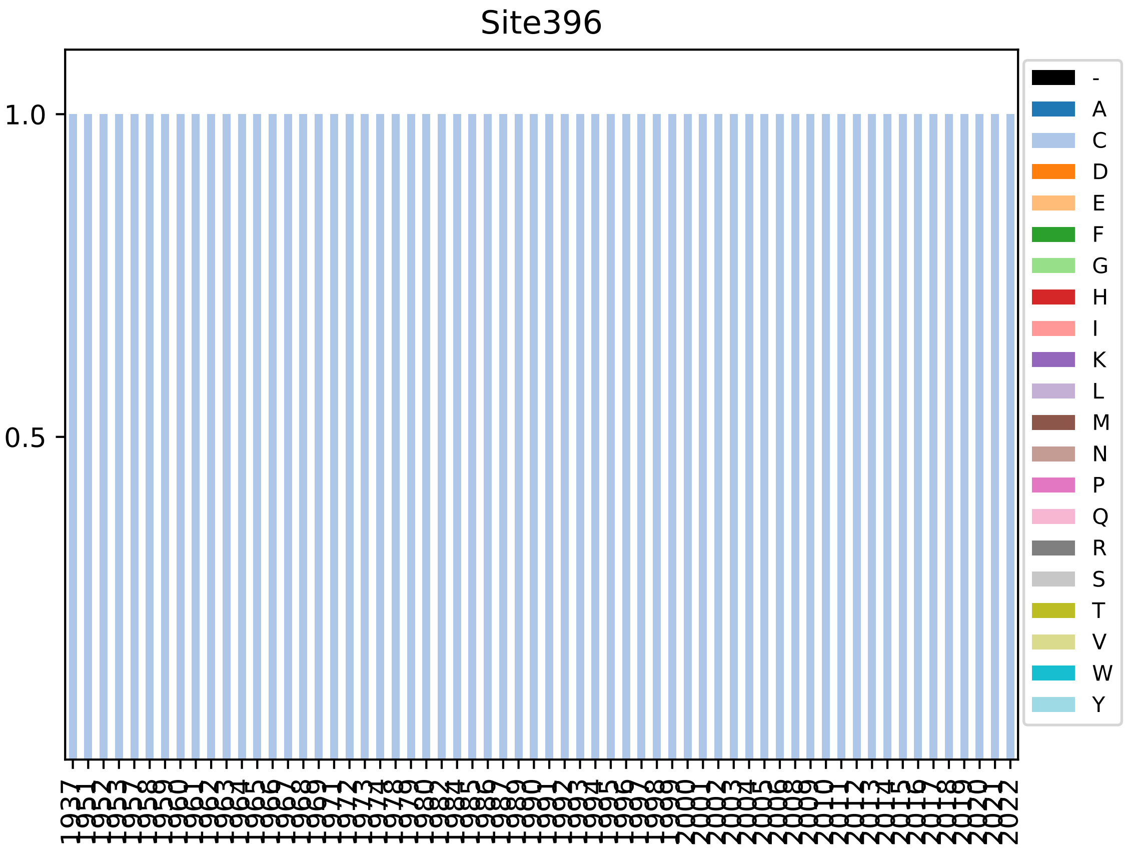
Site396
1.0
0.5
-
A
C
D
E
F
G
H
I
K
L
M
N
P
Q
R
S
T
V
W
Y

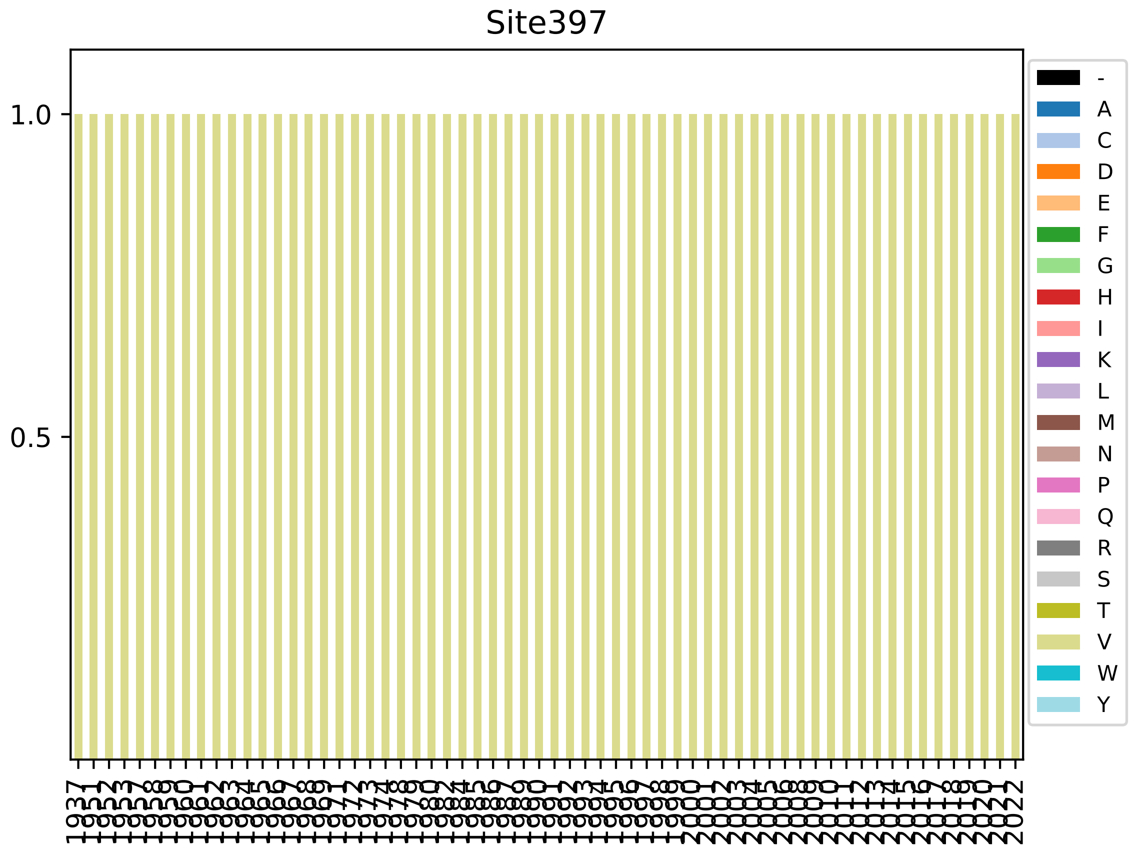
Site397
1.0
0.5
-
A
C
D
E
F
G
H
I
K
L
M
N
P
Q
R
S
T
V
W
Y

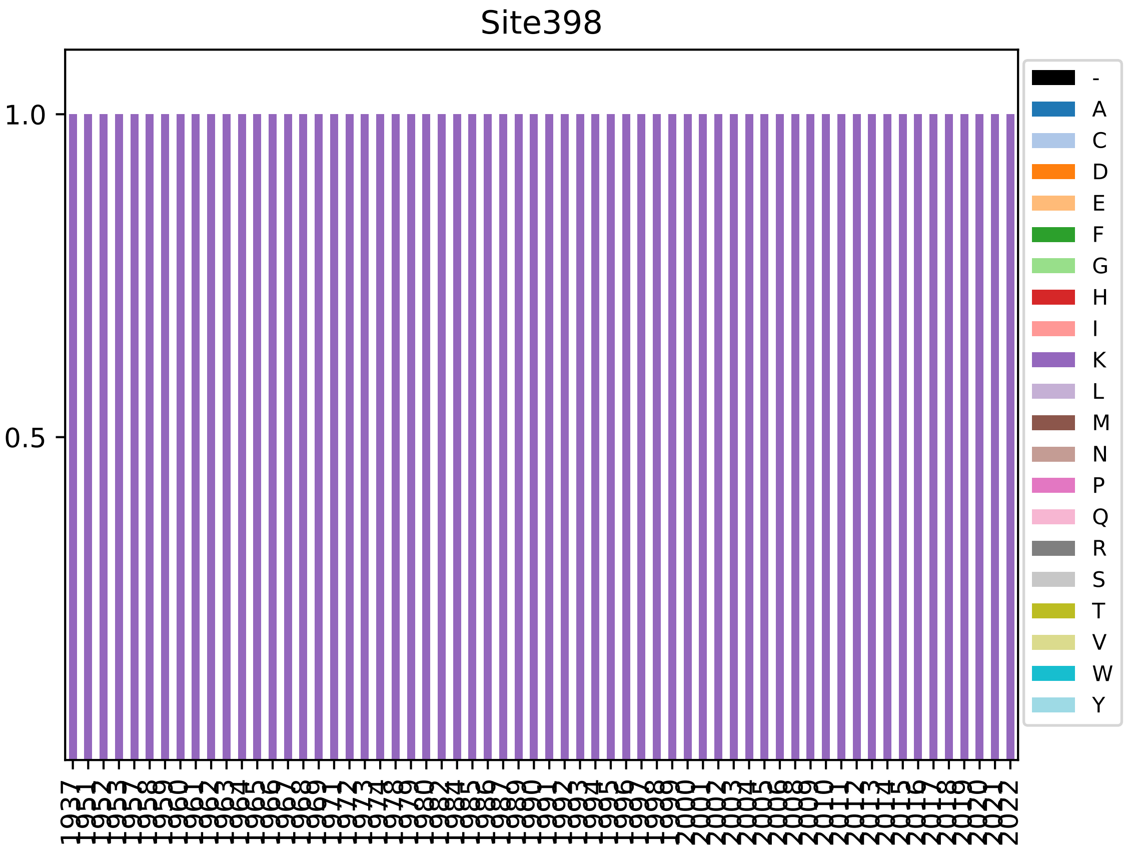
Site398
1.0
0.5
-
A
C
D
E
F
G
H
I
K
L
M
N
P
Q
R
S
T
V
W
Y

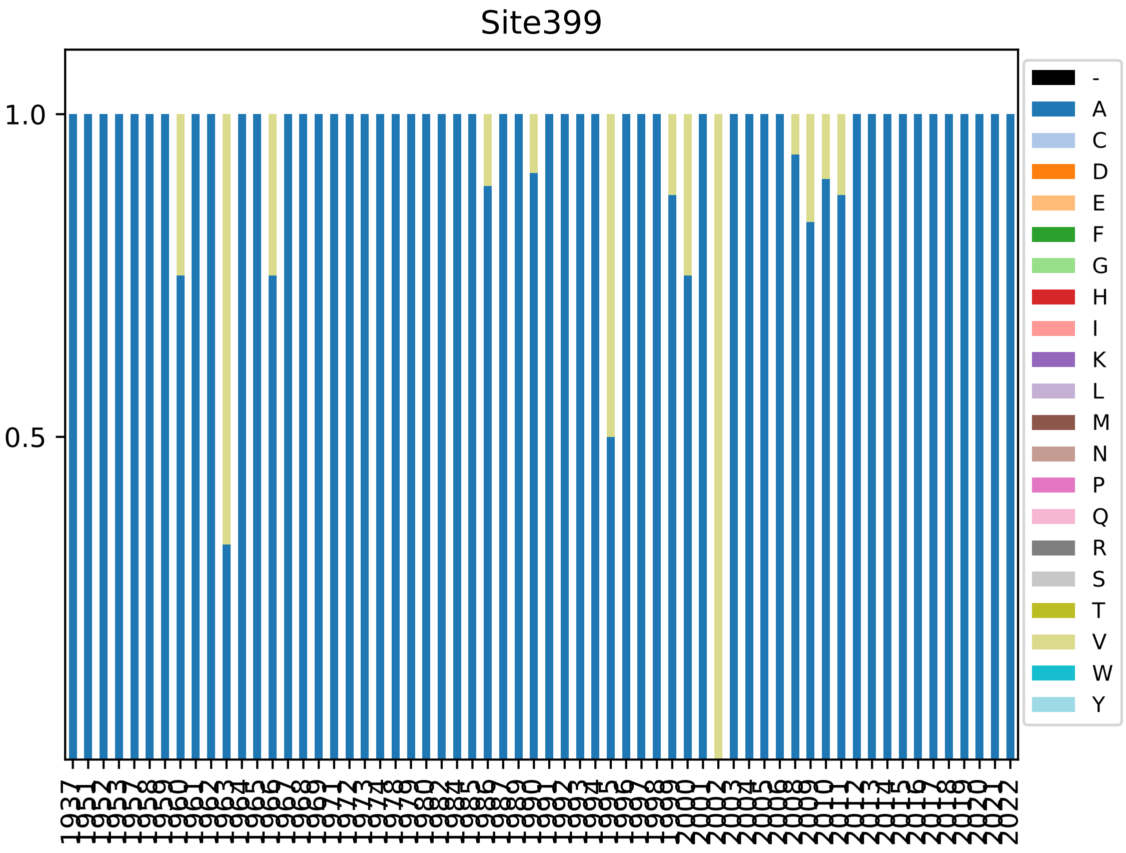
Site399
1.0
0.5
-
A
C
D
E
F
G
H
I
K
L
M
N
P
Q
R
S
T
V
W
Y

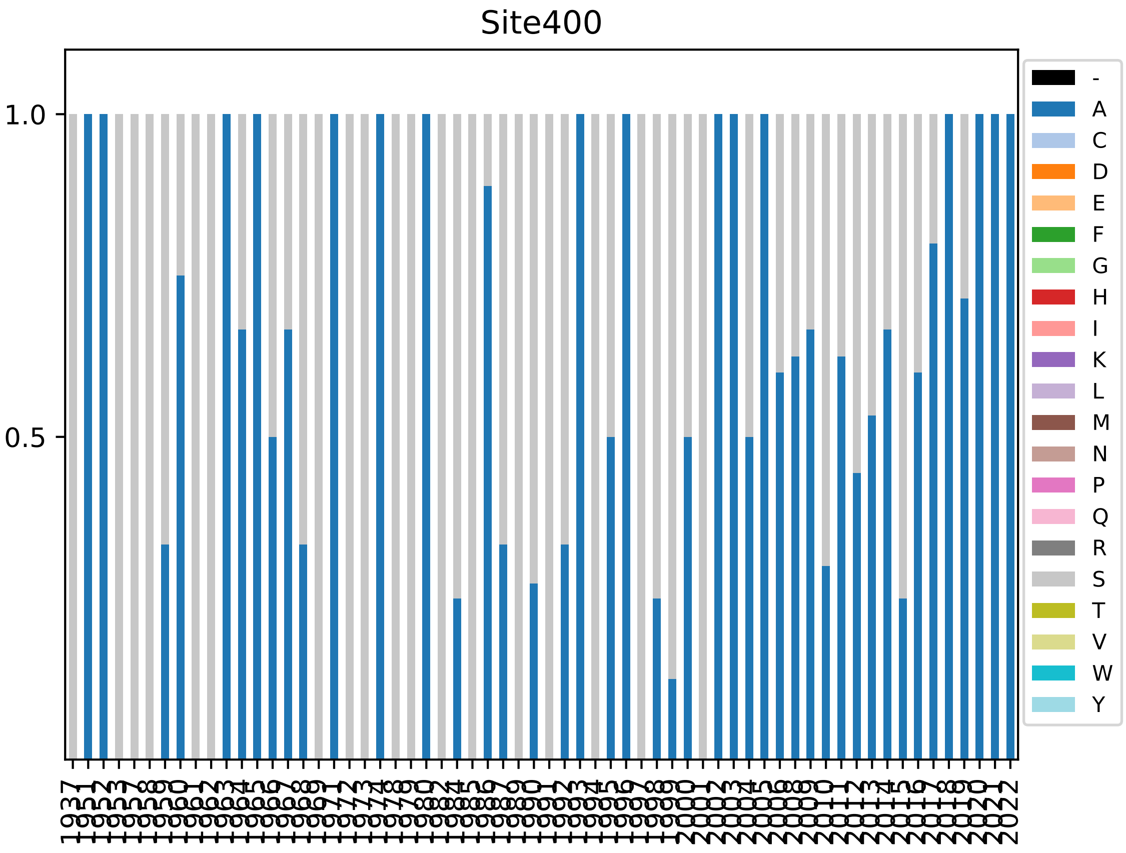
Site400
1.0
0.5
-
A
C
D
E
F
G
H
I
K
L
M
N
P
Q
R
S
T
V
W
Y

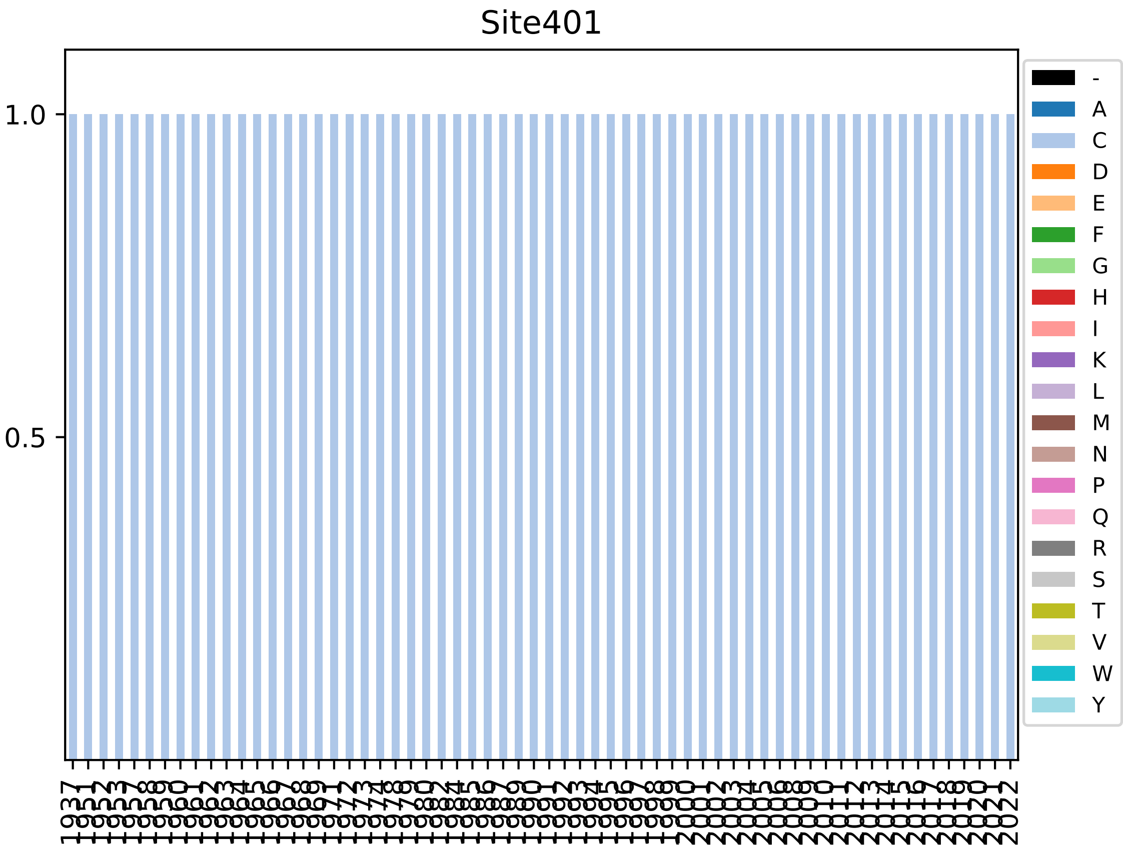
Site401
1.0
0.5
-
A
C
D
E
F
G
H
I
K
L
M
N
P
Q
R
S
T
V
W
Y

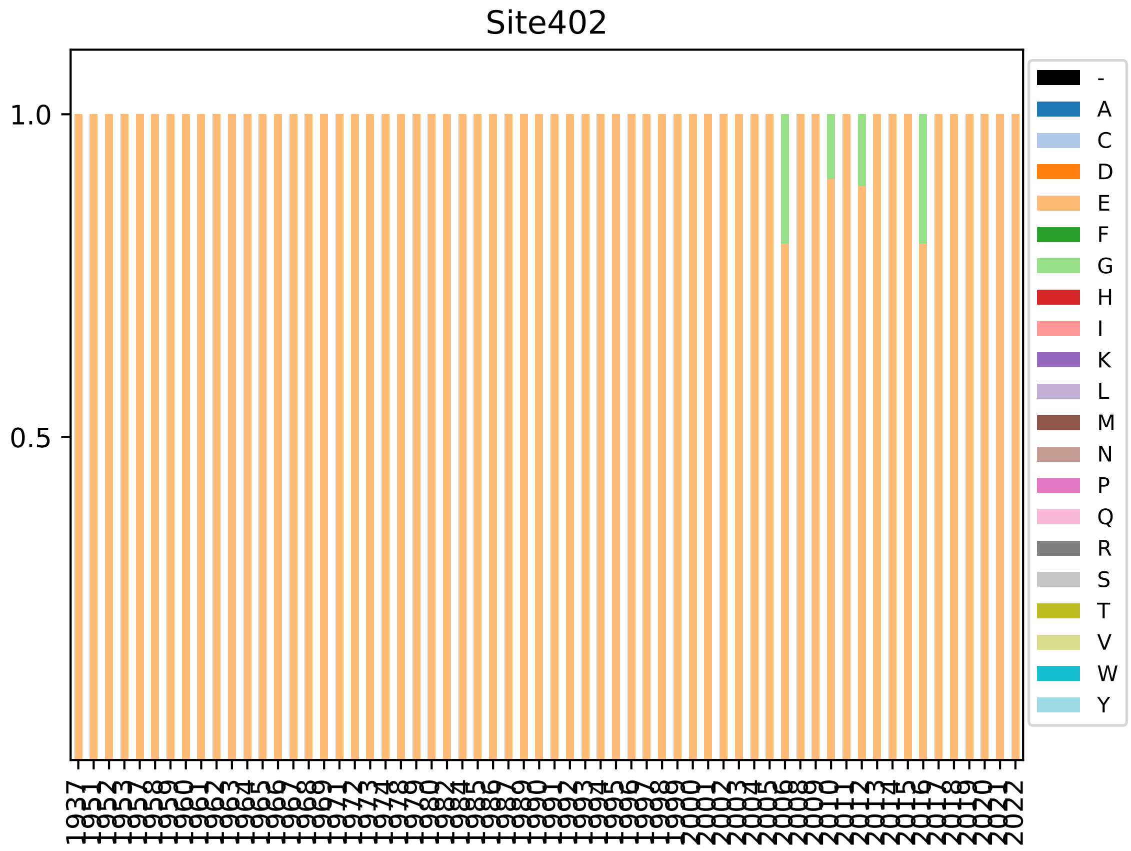
Site402
1.0
0.5
-
A
C
D
E
F
G
H
I
K
L
M
N
P
Q
R
S
T
V
W
Y

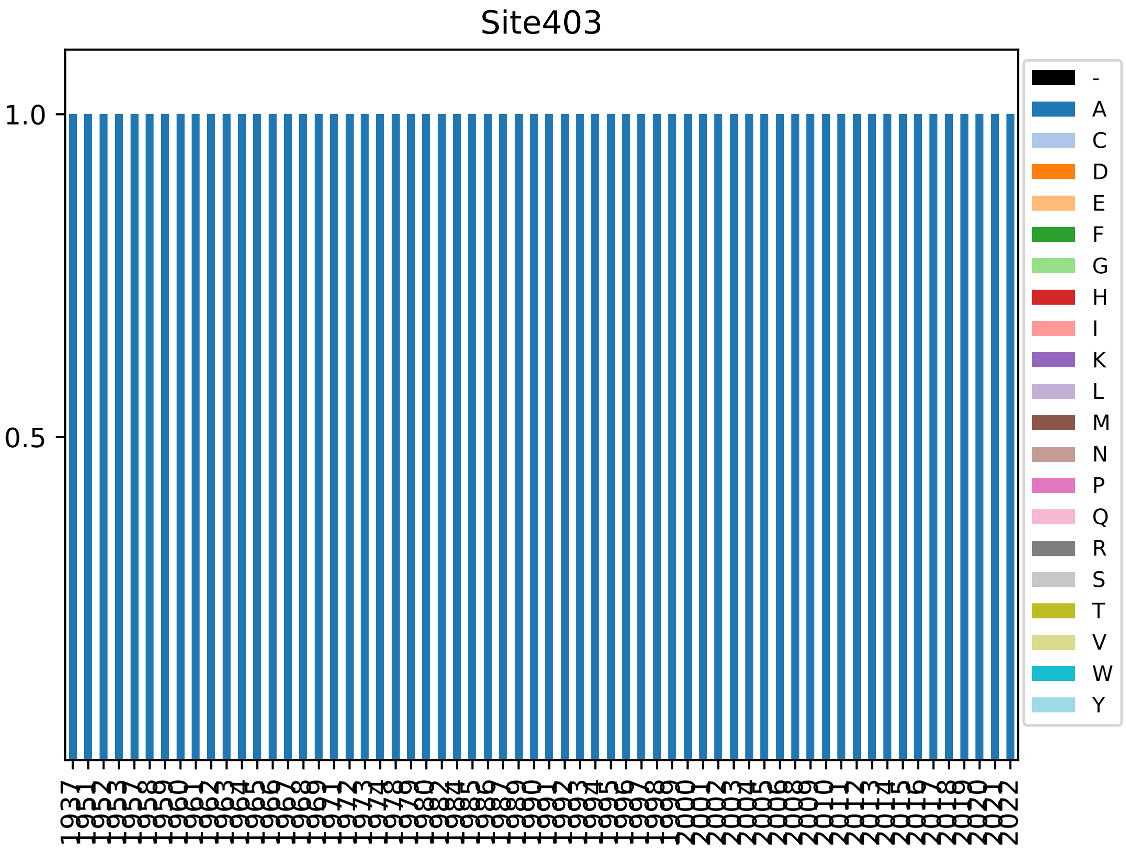
Site403
1.0
0.5
-
A
C
D
E
F
G
H
I
K
L
M
N
P
Q
R
S
T
V
W
Y

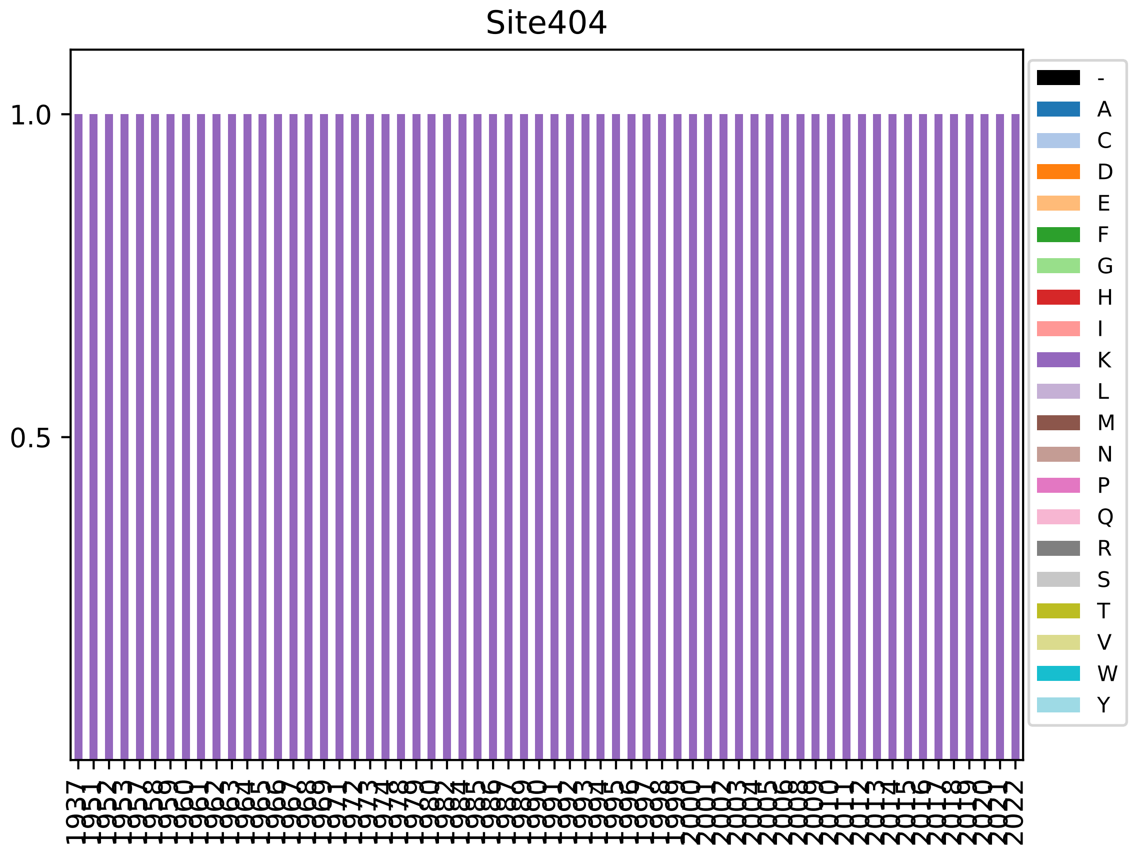
Site404
1.0
0.5
-
A
C
D
E
F
G
H
I
K
L
M
N
P
Q
R
S
T
V
W
Y

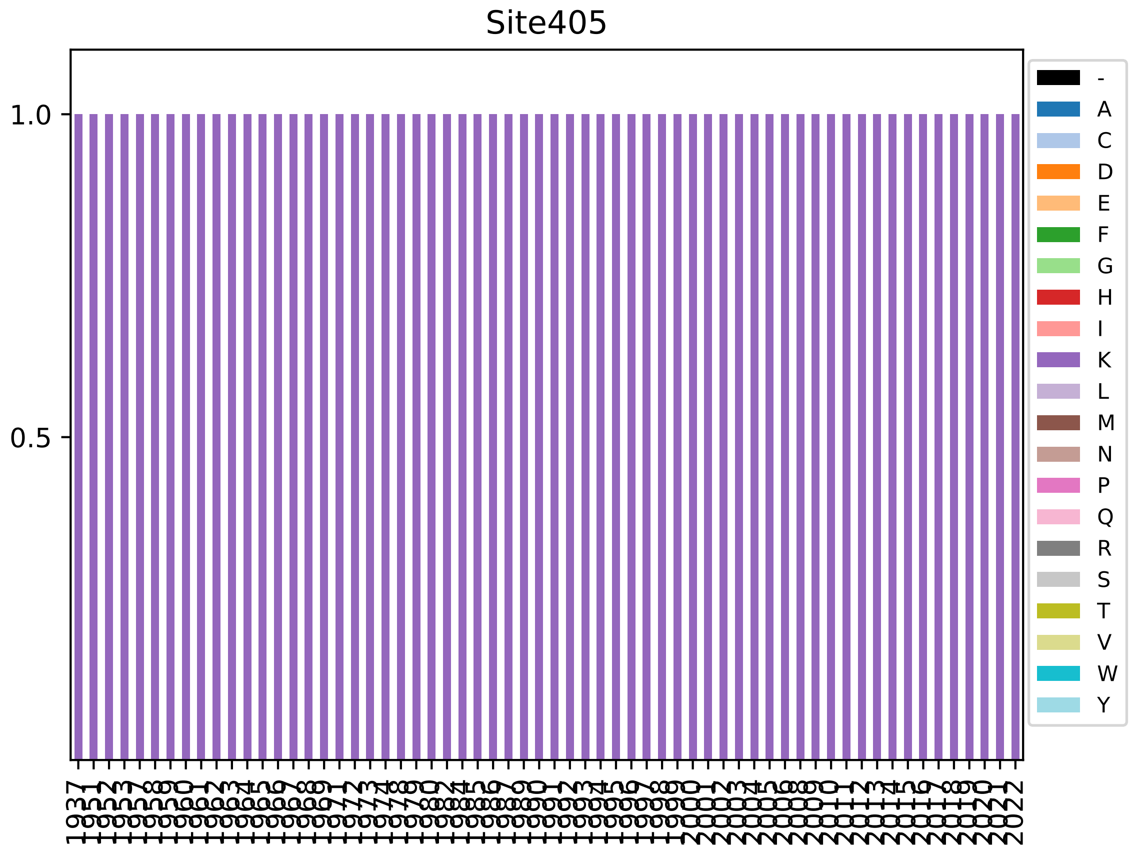
Site405
1.0
0.5
-
A
C
D
E
F
G
H
I
K
L
M
N
P
Q
R
S
T
V
W
Y

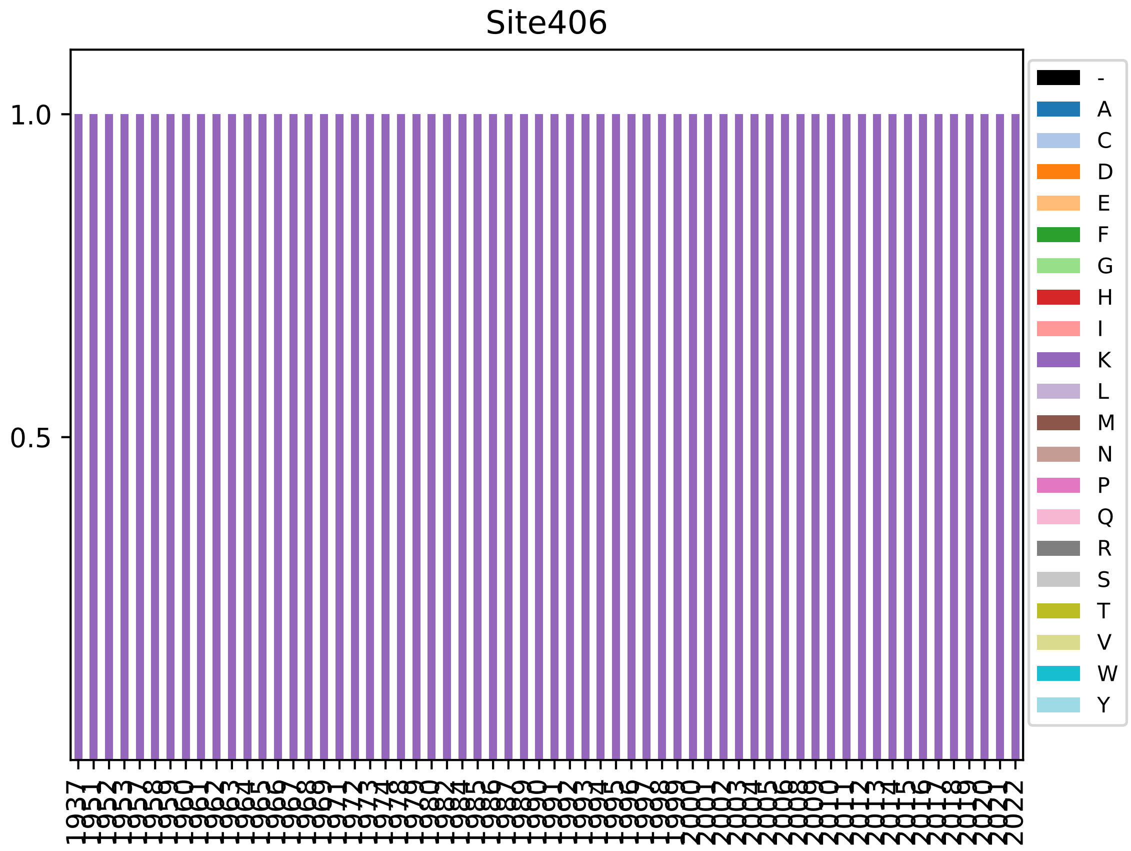
Site406
1.0
0.5
-
A
C
D
E
F
G
H
I
K
L
M
N
P
Q
R
S
T
V
W
Y

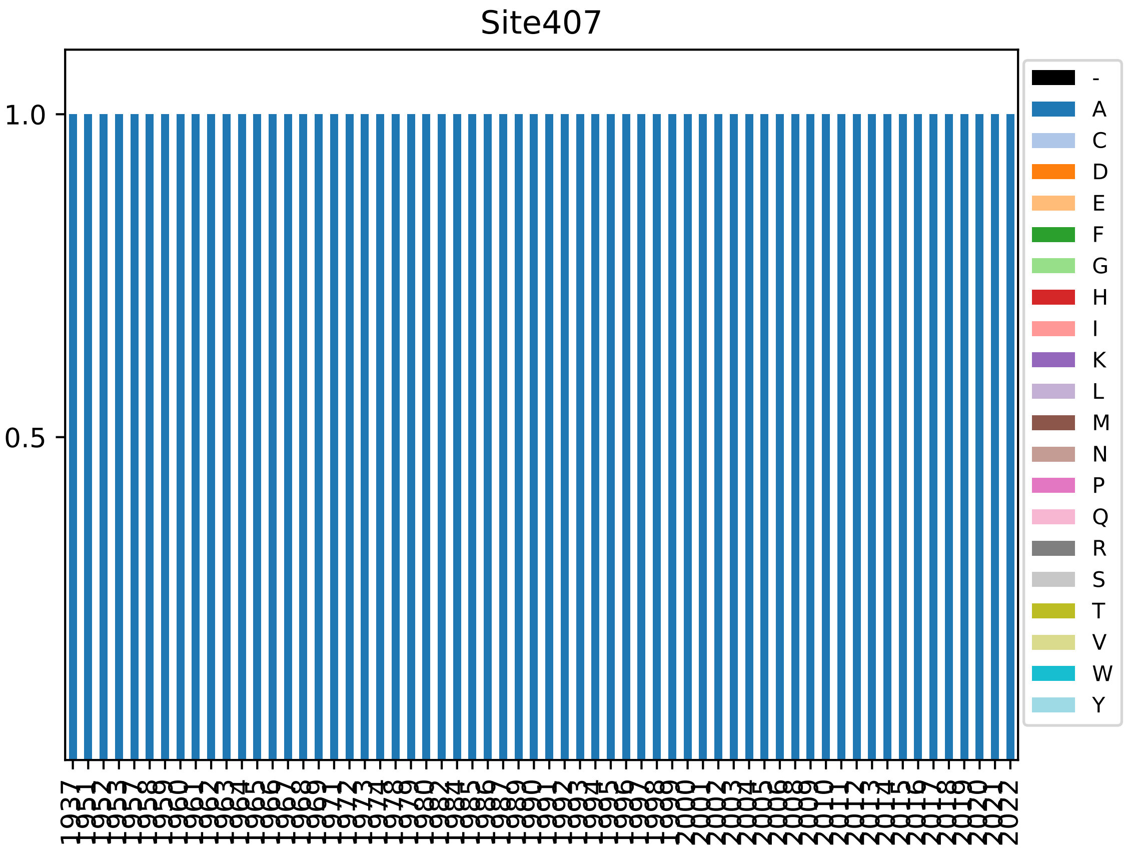
Site407
1.0
0.5
-
A
C
D
E
F
G
H
I
K
L
M
N
P
Q
R
S
T
V
W
Y

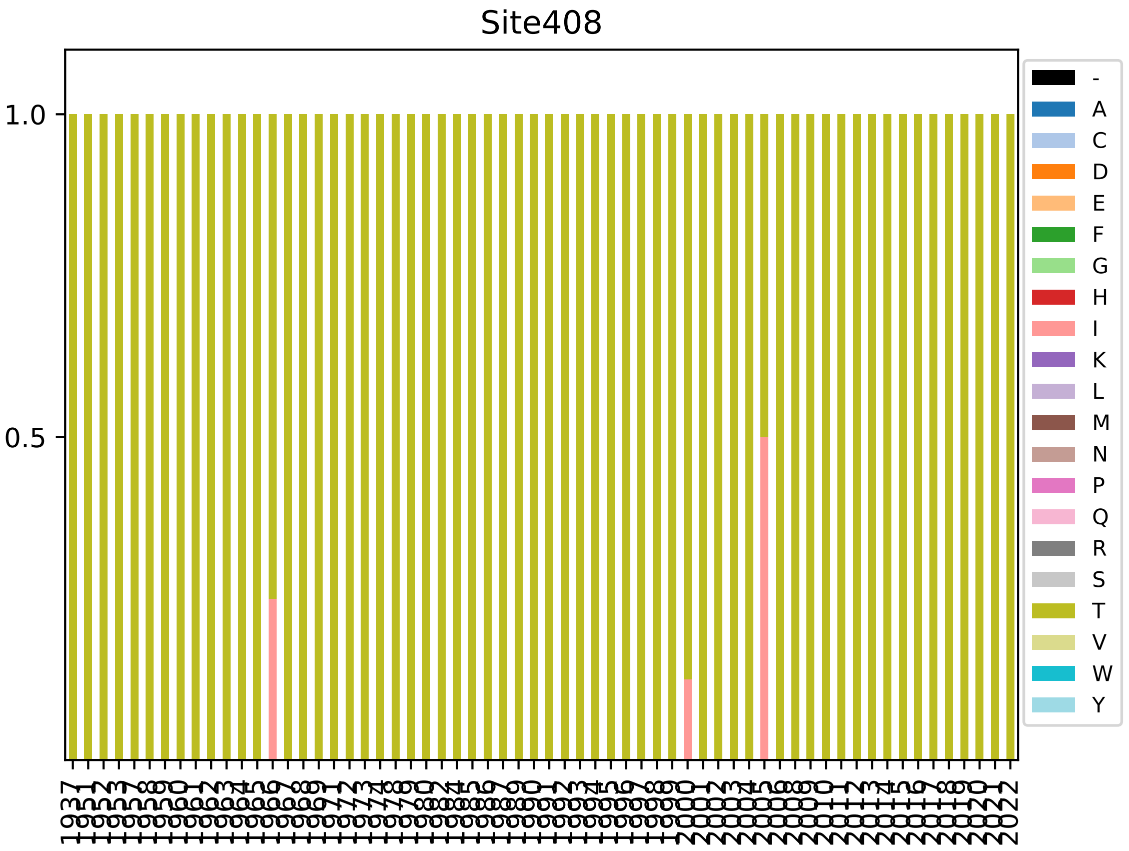

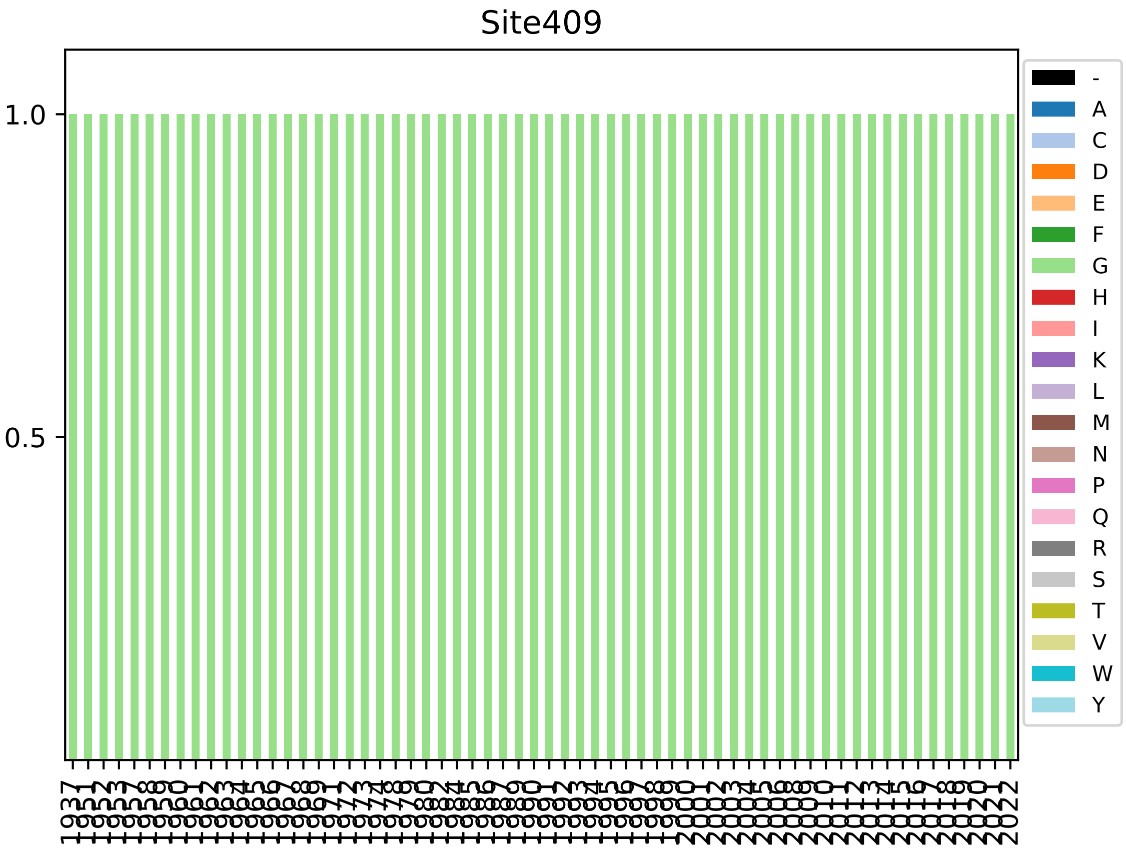
Site409
1.0
0.5
-
A
C
D
E
F
G
H
I
K
L
M
N
P
Q
R
S
T
V
W
Y

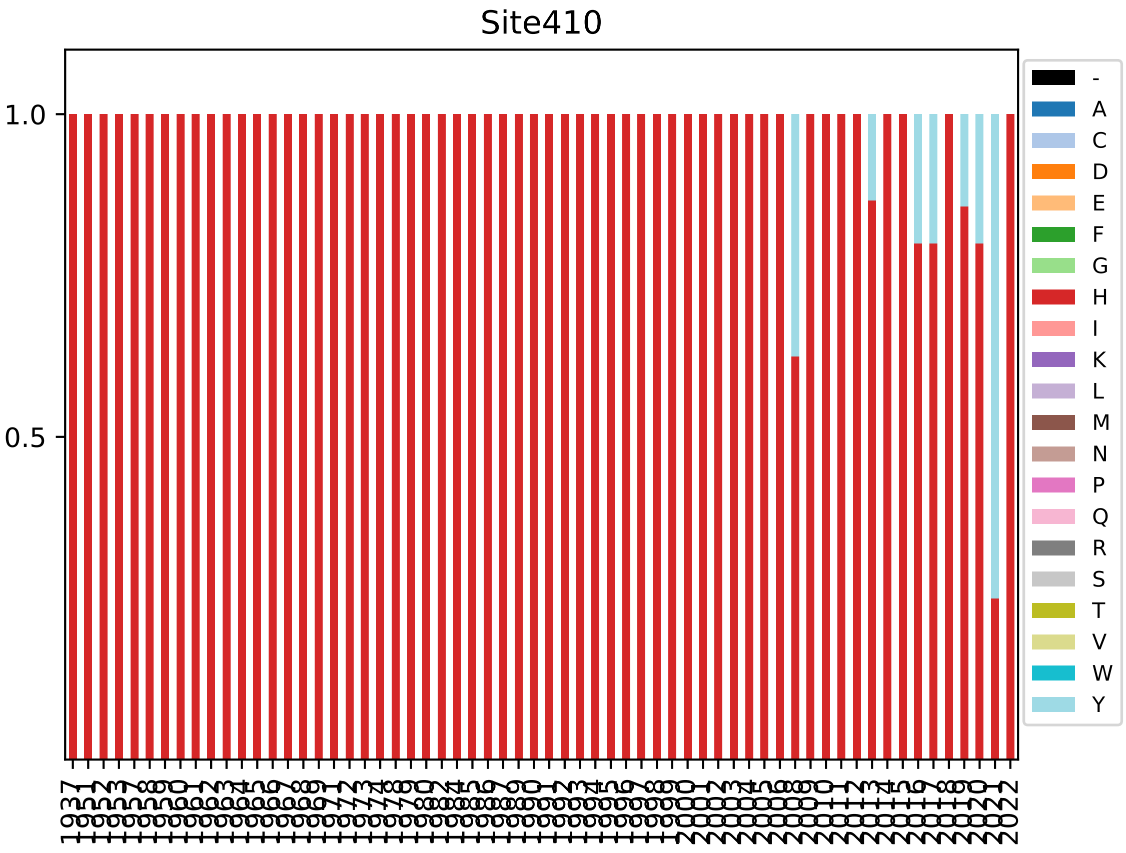
Site410
1.0
0.5
-
A
C
D
E
F
G
H
I
K
L
M
N
P
Q
R
S
T
V
W
Y

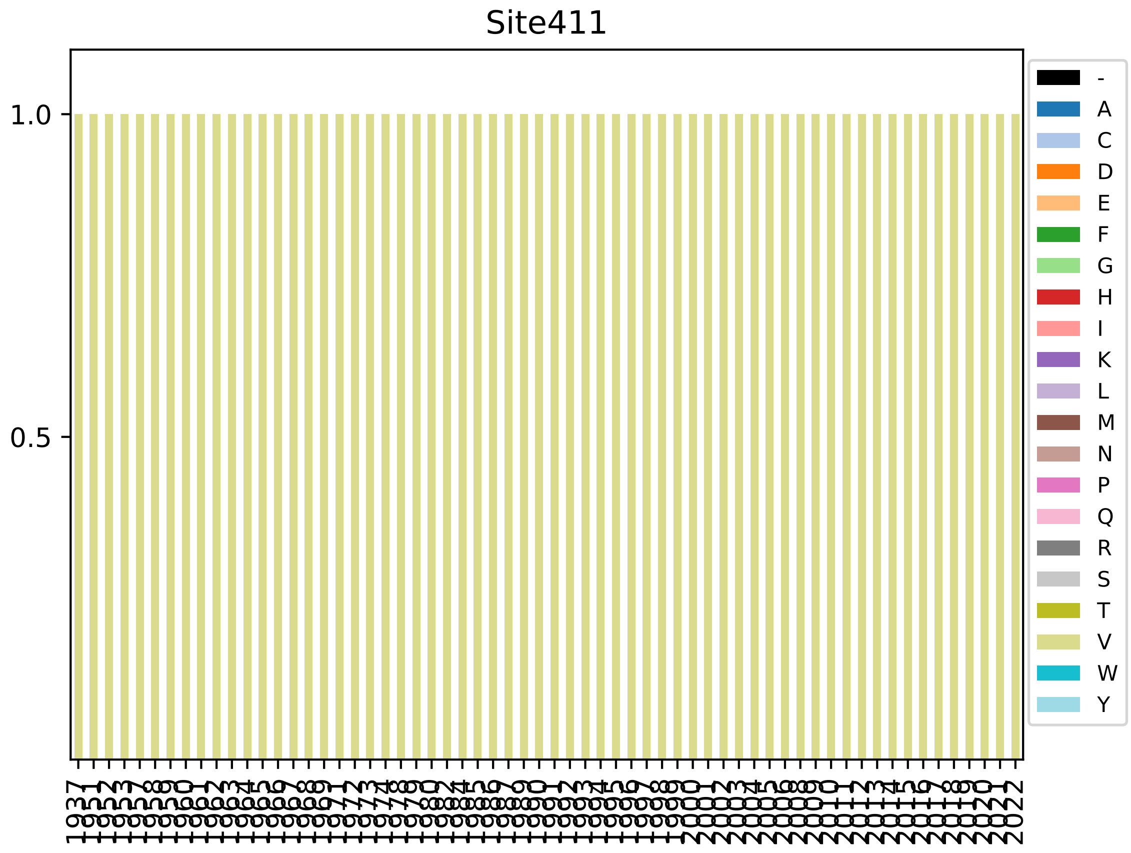
Site411
1.0
0.5
-
A
C
D
E
F
G
H
I
K
L
M
N
P
Q
R
S
T
V
W
Y

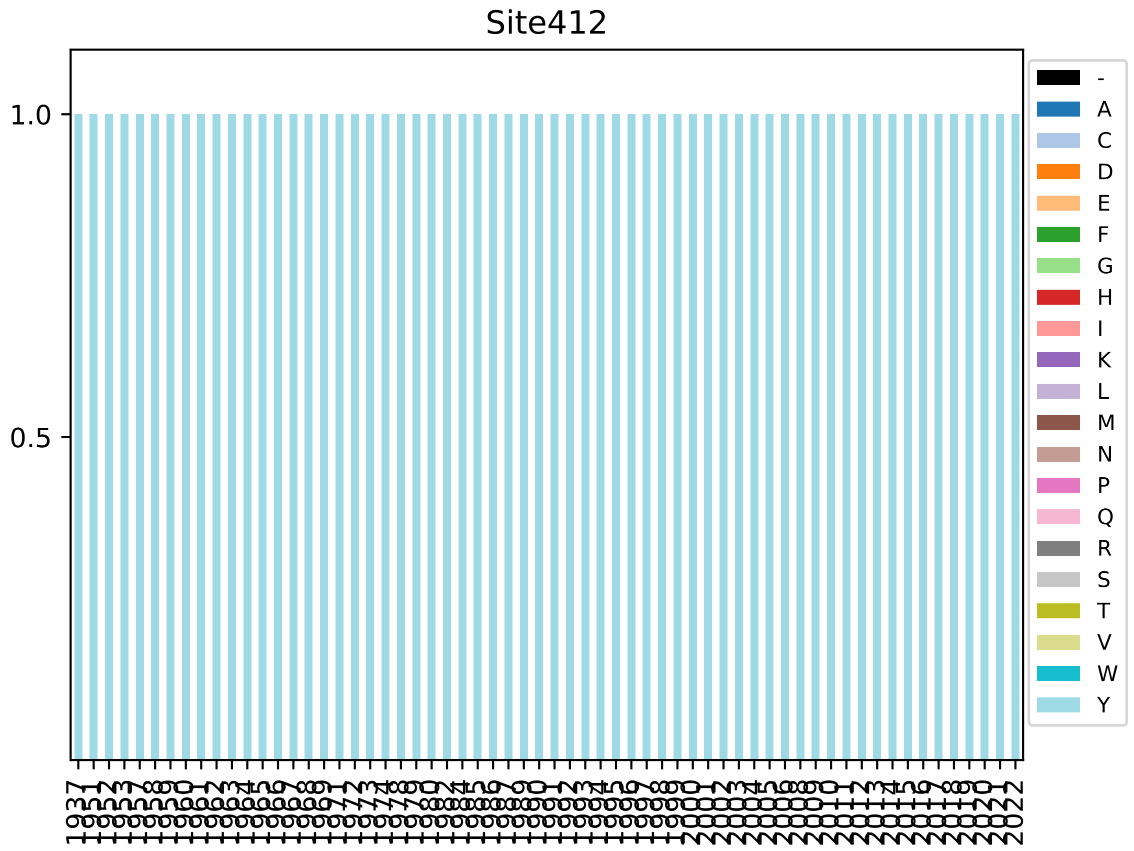
Site412
1.0
0.5
-
A
C
D
E
F
G
H
I
K
L
M
N
P
Q
R
S
T
V
W
Y

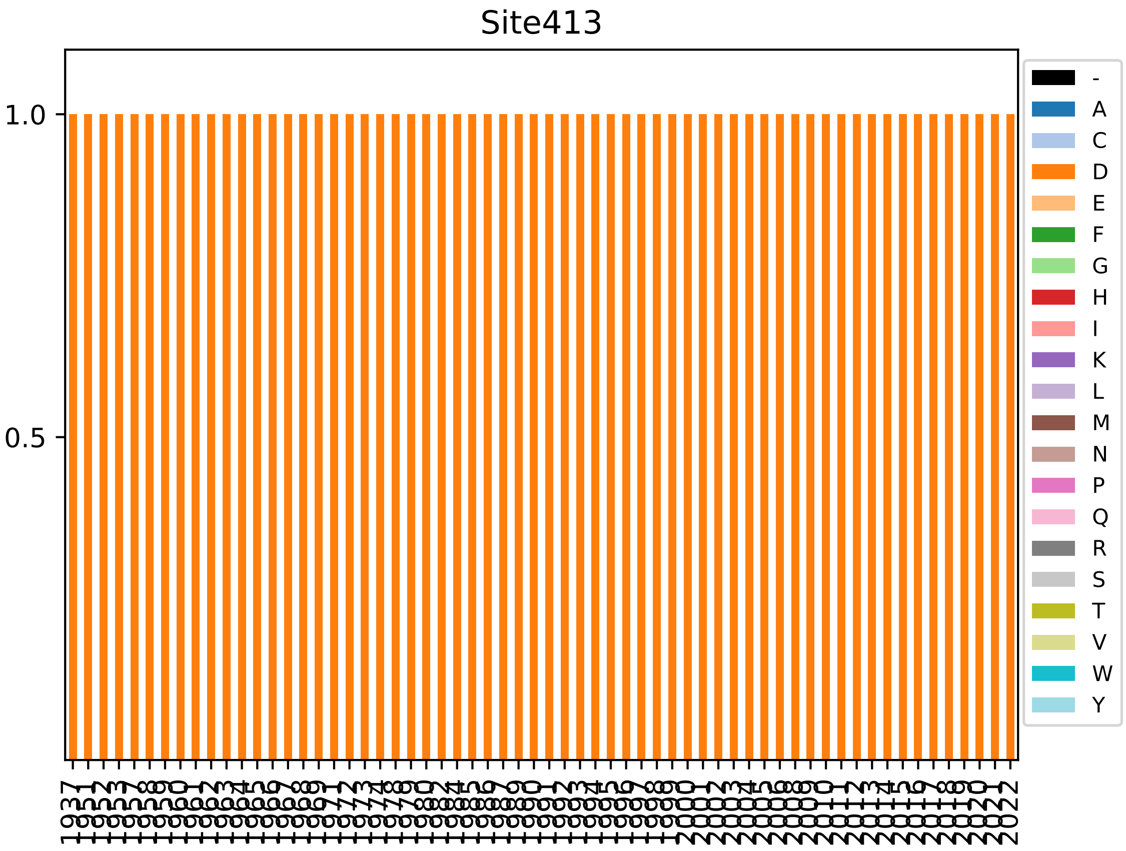
Site413
1.0
0.5
-
A
C
D
E
F
G
H
I
K
L
M
N
P
Q
R
S
T
V
W
Y

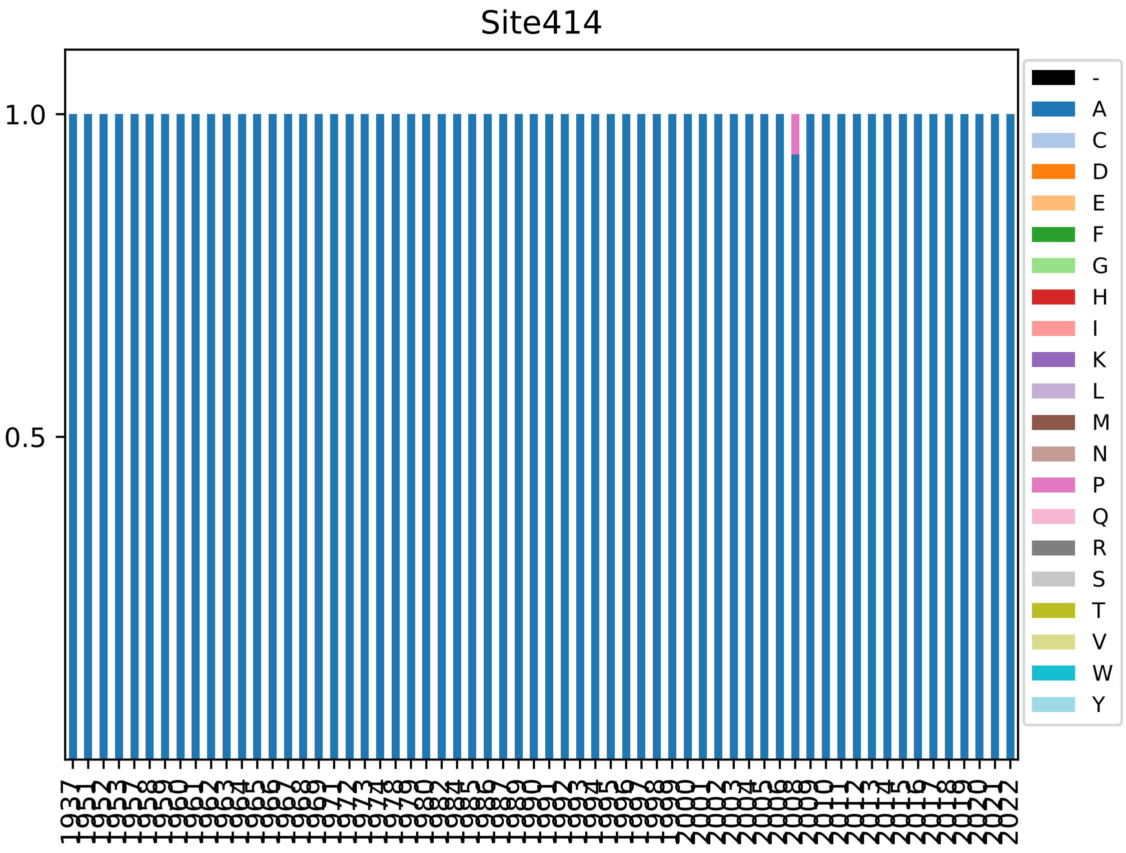
Site414
1.0
0.5
-
A
C
D
E
F
G
H
I
K
L
M
N
P
Q
R
S
T
V
W
Y

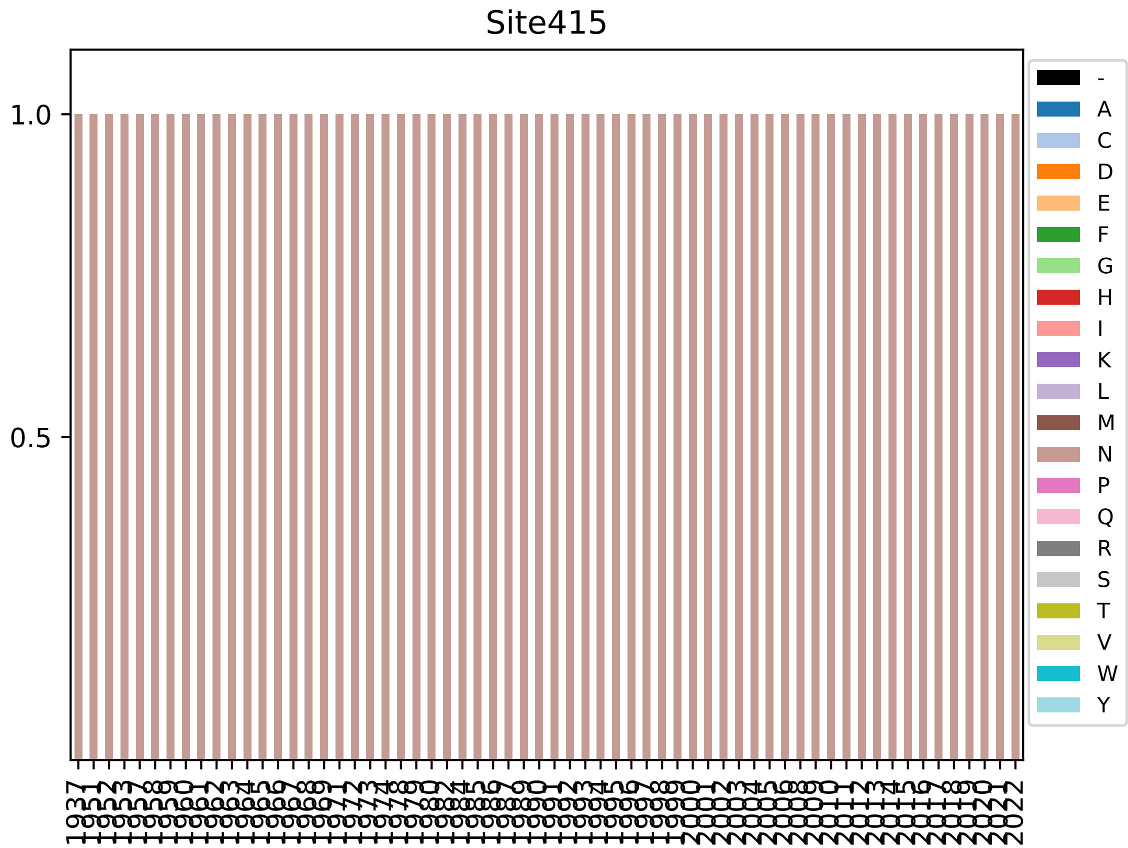
Site415
1.0
0.5
-
A
C
D
E
F
G
H
I
K
L
M
N
P
Q
R
S
T
V
W
Y

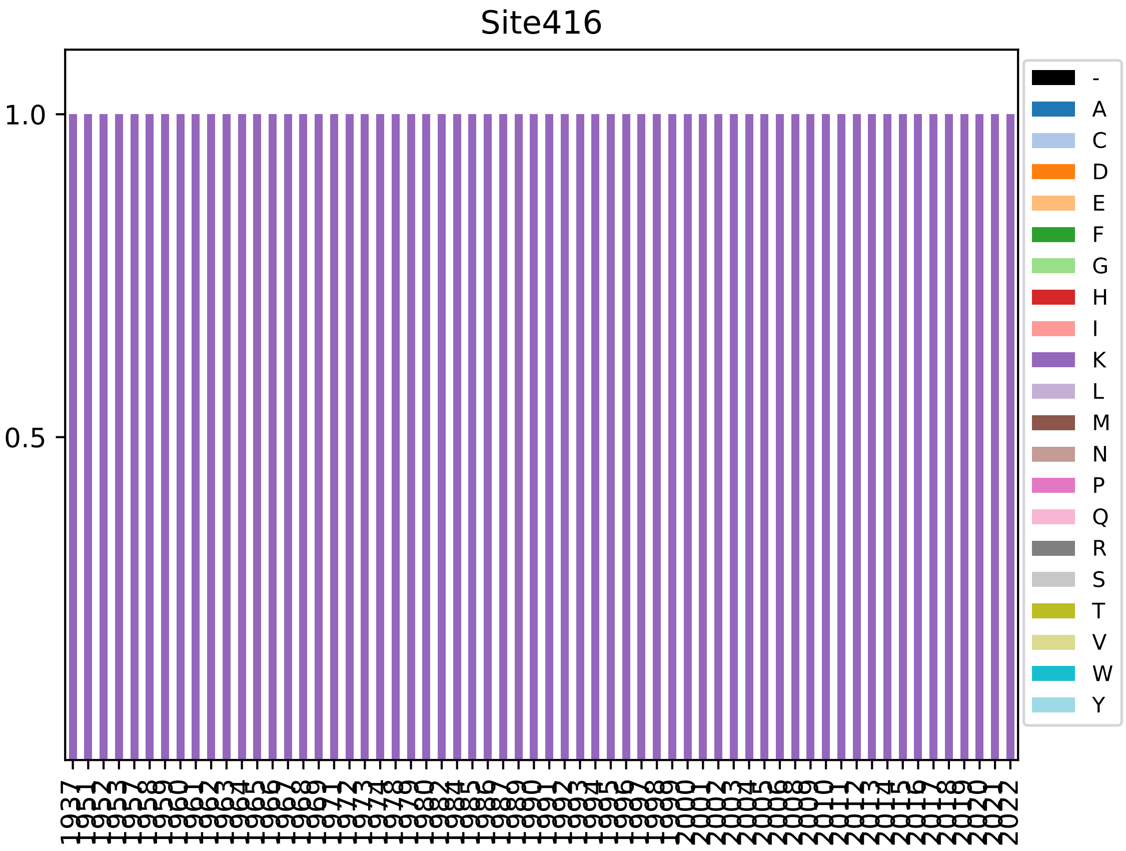
Site416
1.0
0.5
-
A
C
D
E
F
G
H
I
K
L
M
N
P
Q
R
S
T
V
W
Y

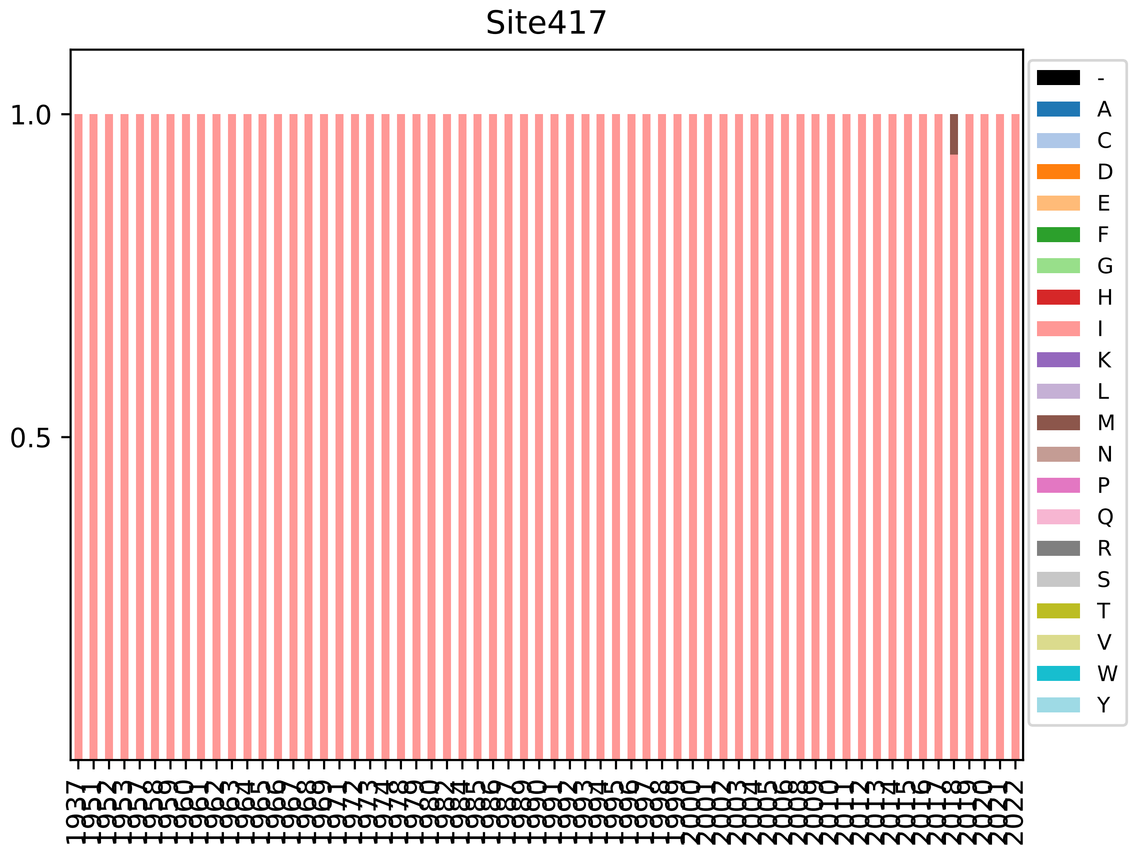
Site417
1.0
0.5
-
A
C
D
E
F
G
H
I
K
L
M
N
P
Q
R
S
T
V
W
Y

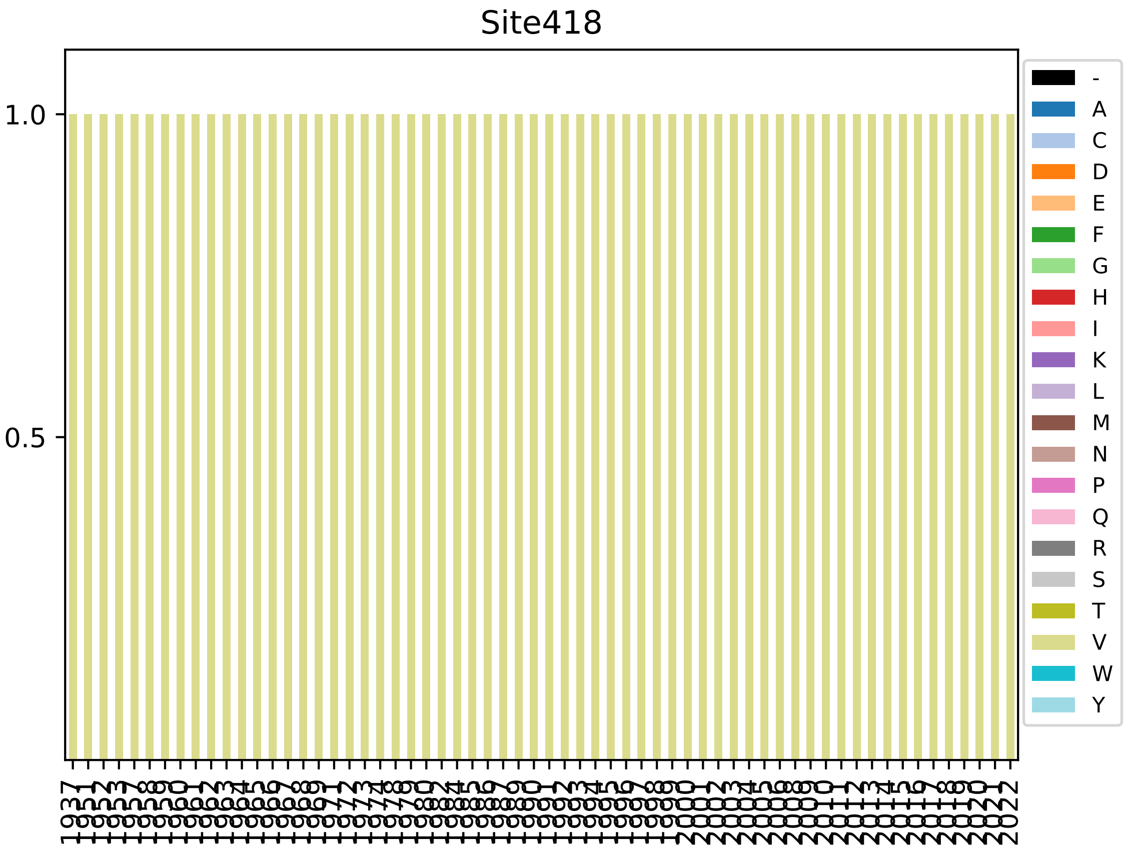
Site418
1.0
0.5
-
A
C
D
E
F
G
H
I
K
L
M
N
P
Q
R
S
T
V
W
Y

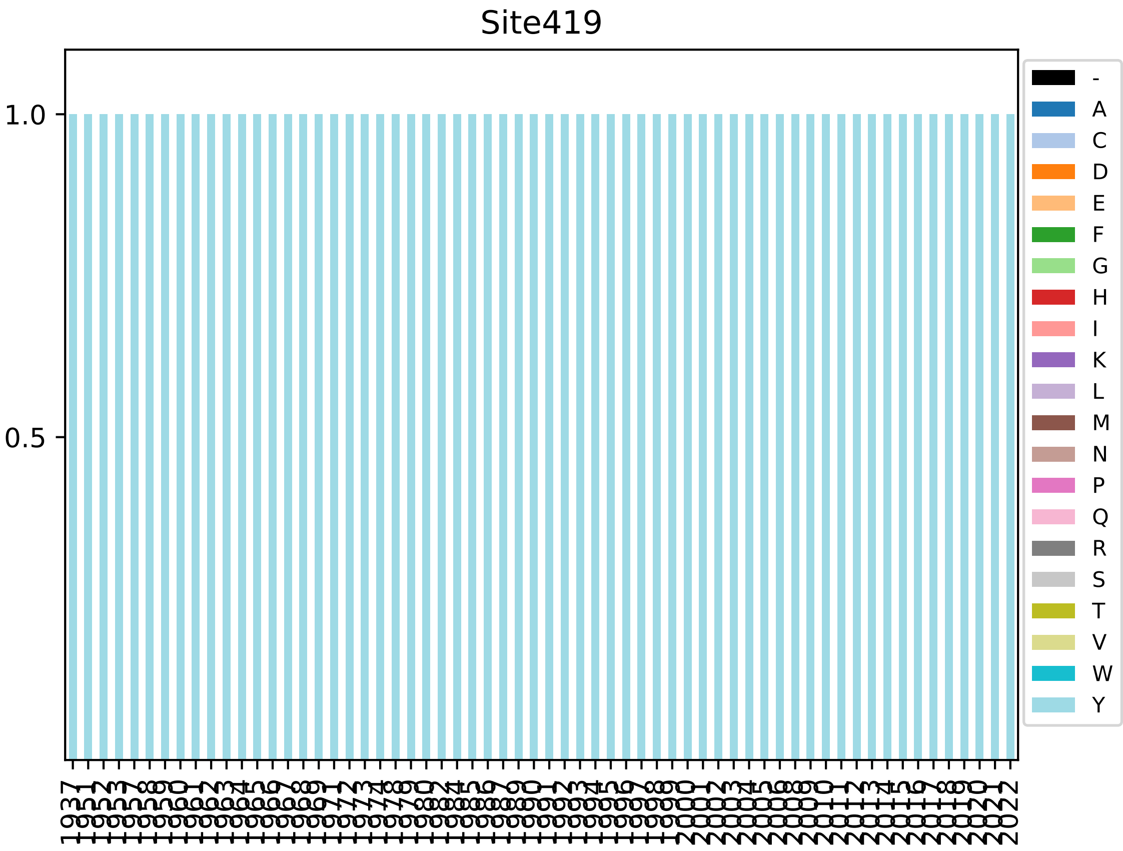
Site419
1.0
0.5
-
A
C
D
E
F
G
H
I
K
L
M
N
P
Q
R
S
T
V
W
Y

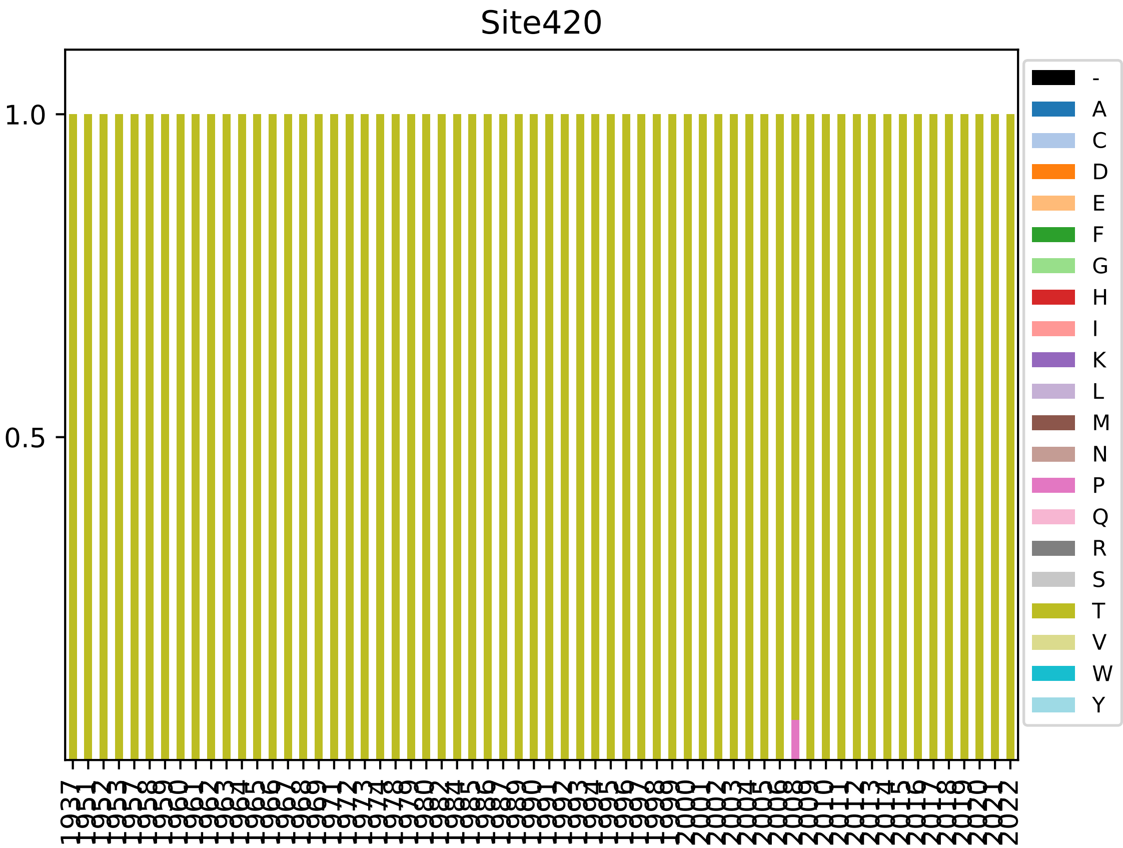
Site420
1.0
0.5
-
A
C
D
E
F
G
H
I
K
L
M
N
P
Q
R
S
T
V
W
Y

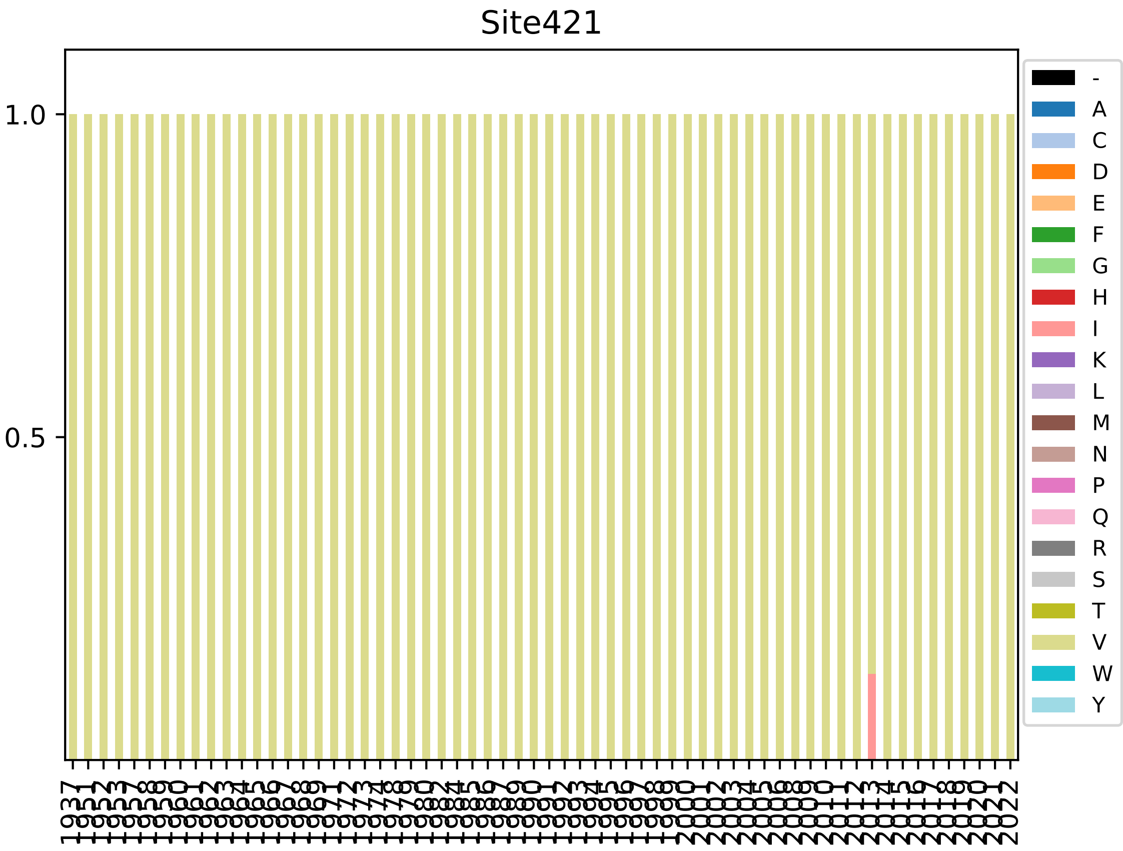
Site421
1.0
0.5
-
A
C
D
E
F
G
H
I
K
L
M
N
P
Q
R
S
T
V
W
Y

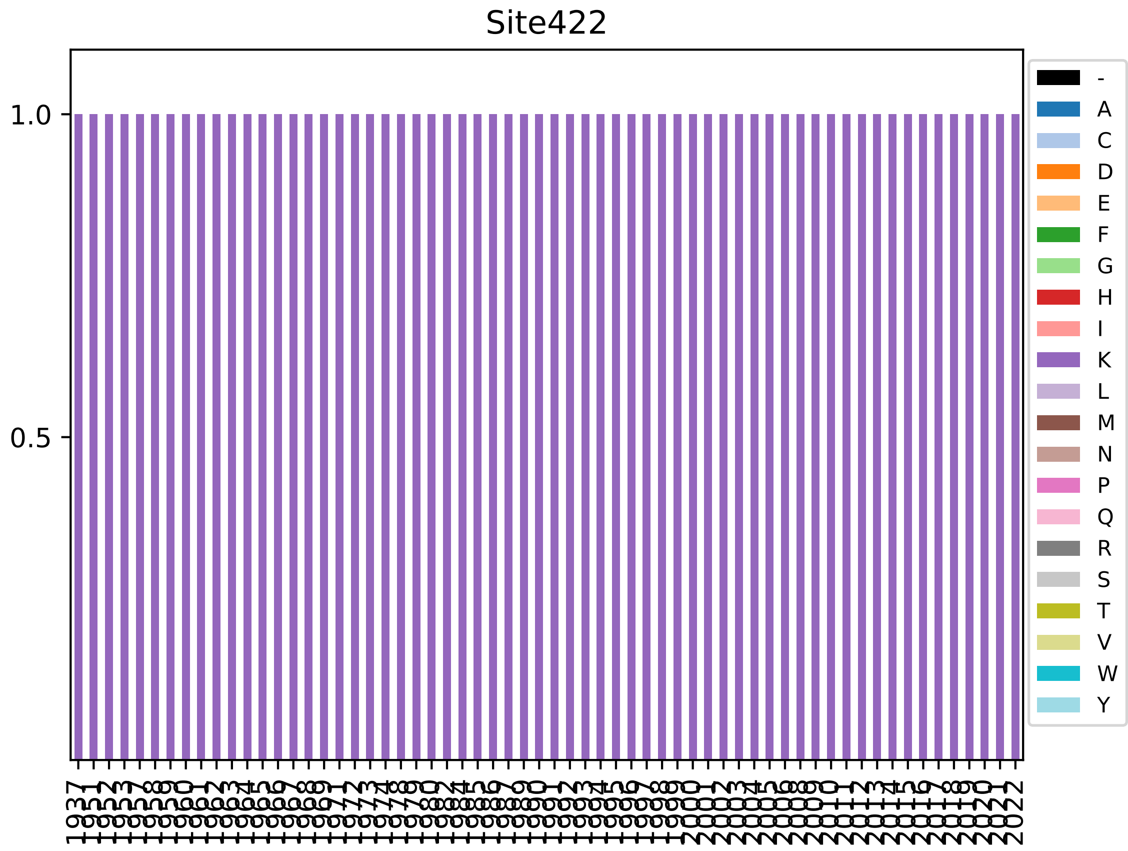
Site422
1.0
0.5
-
A
C
D
E
F
G
H
I
K
L
M
N
P
Q
R
S
T
V
W
Y

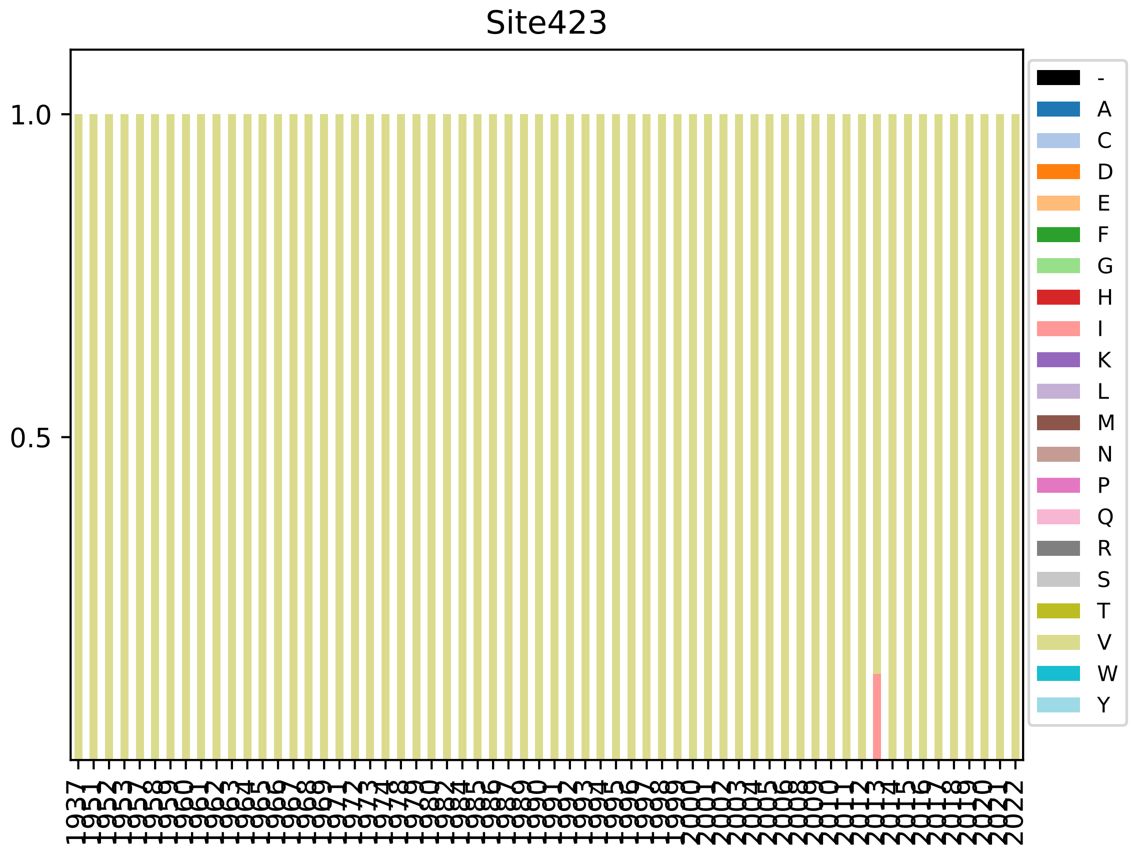
Site423
1.0
0.5
-
A
C
D
E
F
G
H
I
K
L
M
N
P
Q
R
S
T
V
W
Y

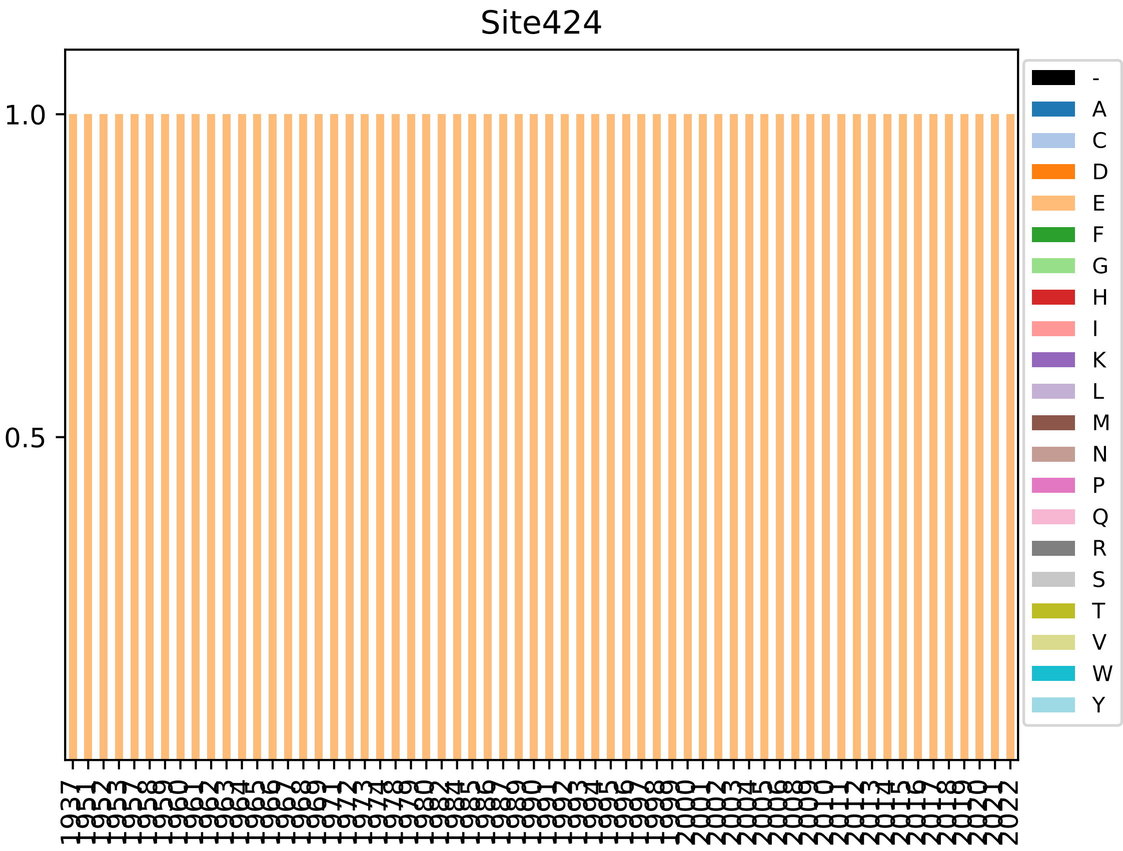
Site424
1.0
0.5
-
A
C
D
E
F
G
H
I
K
L
M
N
P
Q
R
S
T
V
W
Y

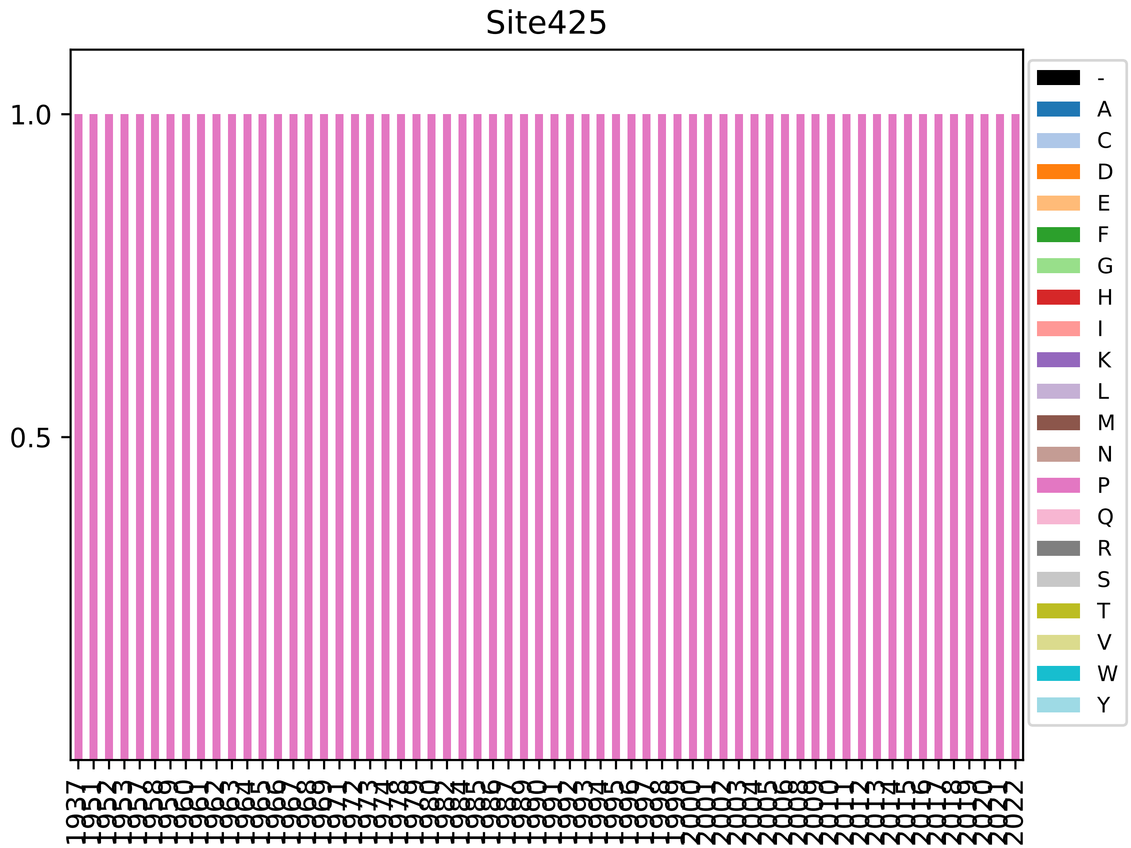
Site425
1.0
0.5
-
A
C
D
E
F
G
H
I
K
L
M
N
P
Q
R
S
T
V
W
Y

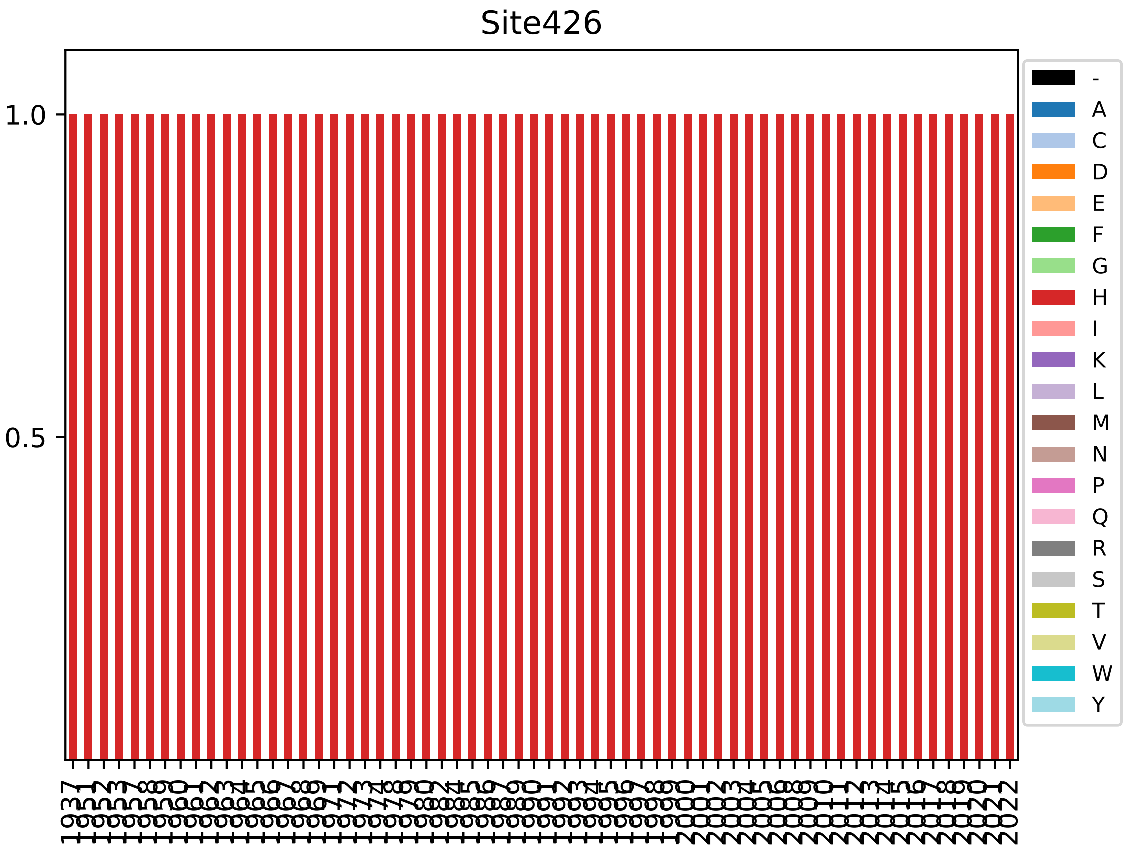

Site426
1.0
0.5
-
A
C
D
E
F
G
H
I
K
L
M
N
P
Q
R
S
T
V
W
Y

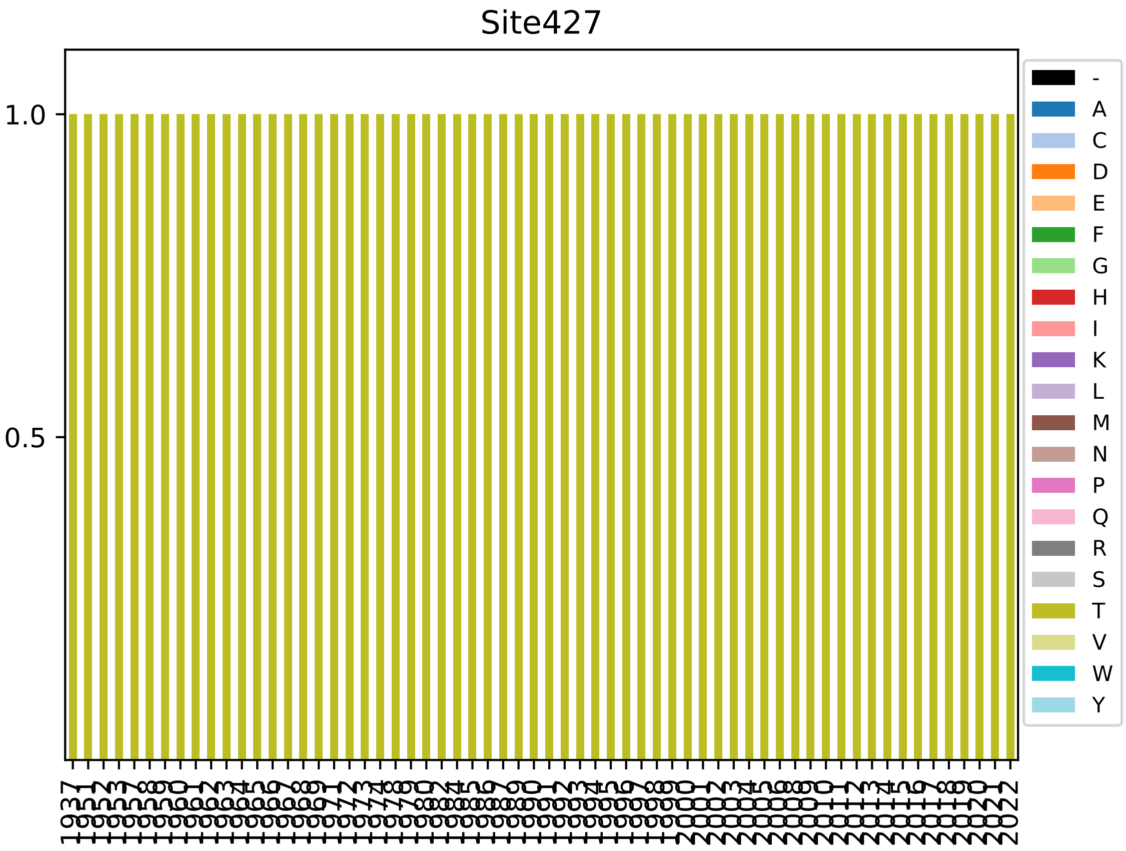
Site427
1.0
0.5
-
A
C
D
E
F
G
H
I
K
L
M
N
P
Q
R
S
T
V
W
Y

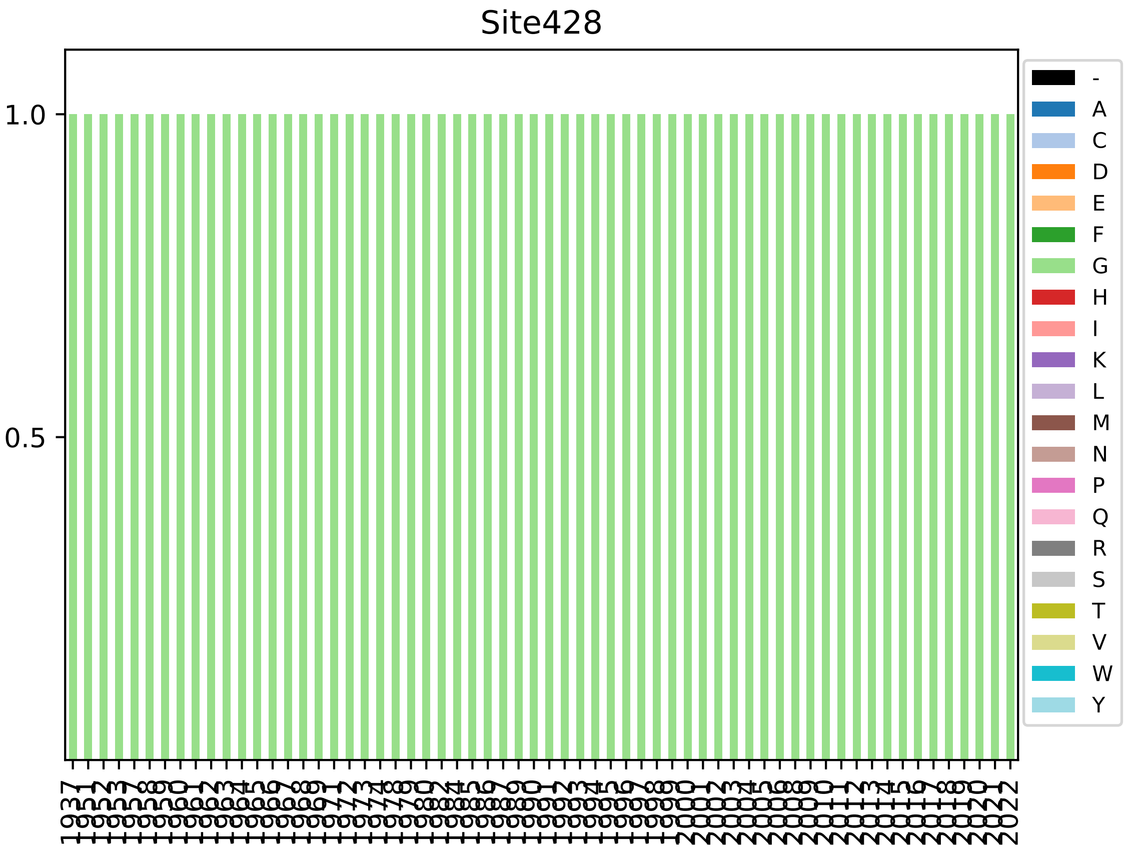
Site428
1.0
0.5
-
A
C
D
E
F
G
H
I
K
L
M
N
P
Q
R
S
T
V
W
Y

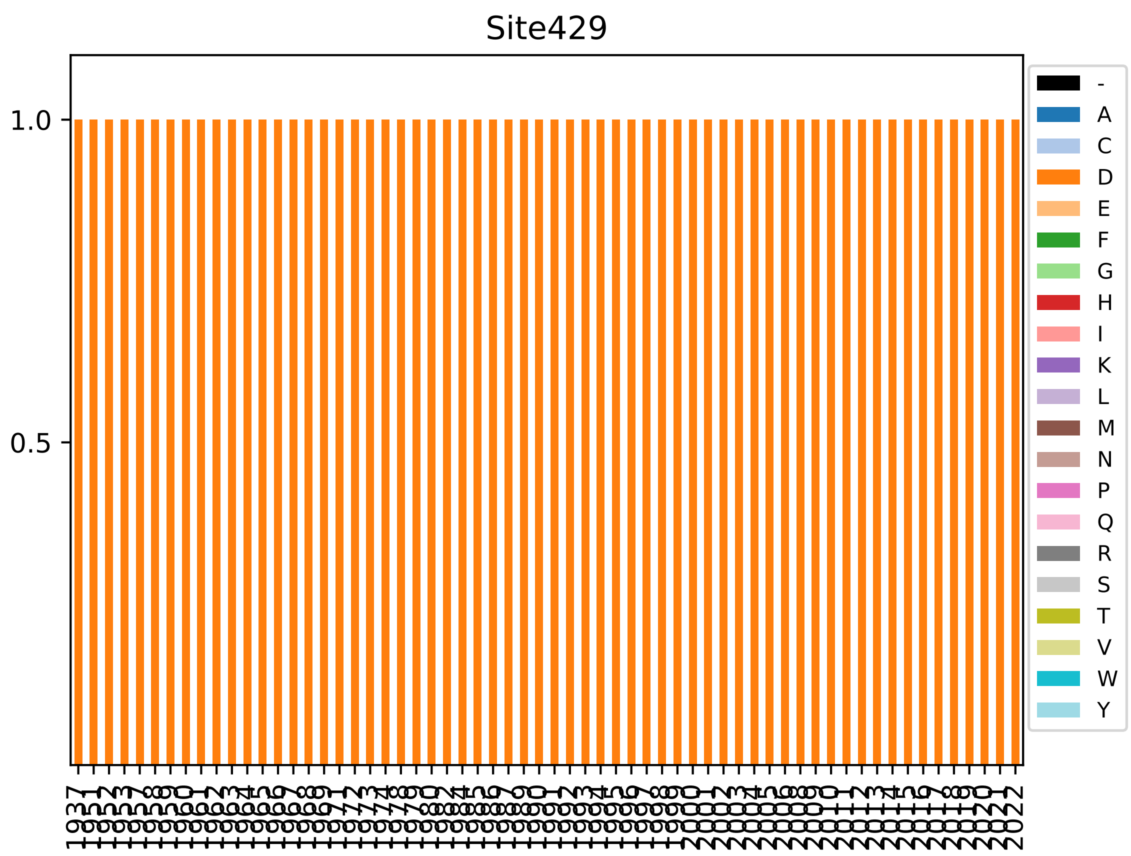

Site429
1.0
0.5
-
A
C
D
E
F
G
H
I
K
L
M
N
P
Q
R
S
T
V
W
Y

Site430

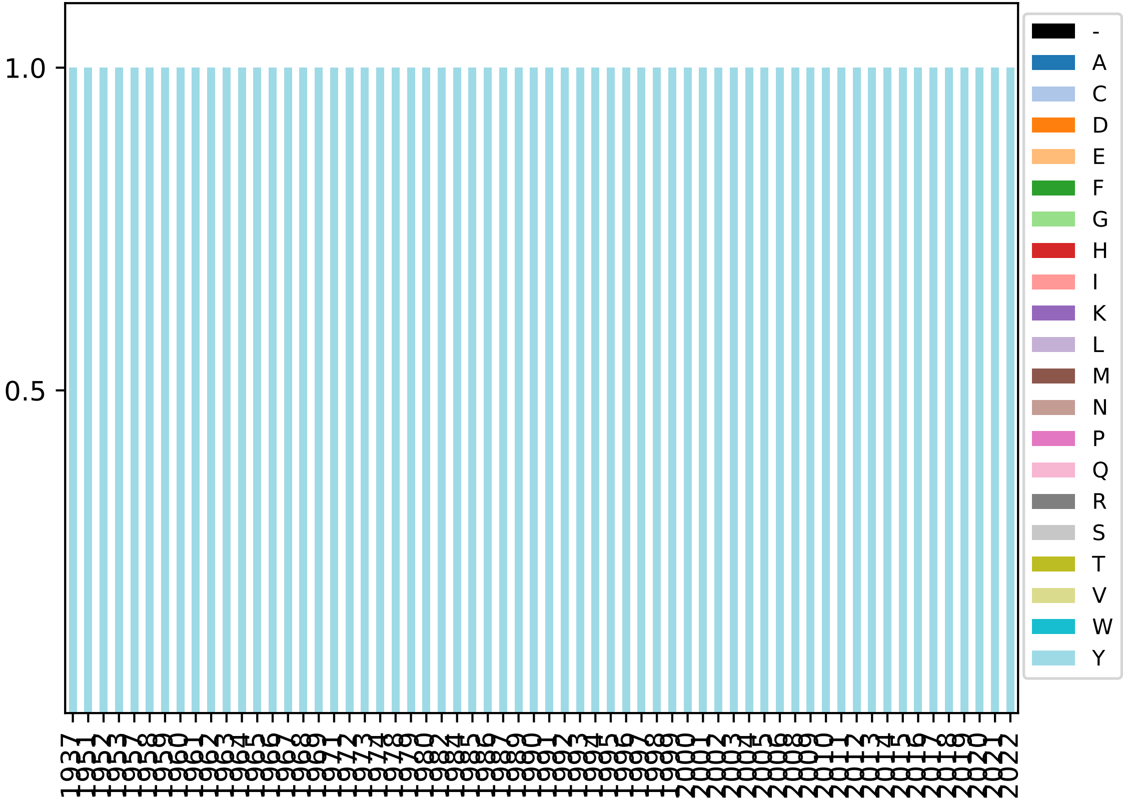

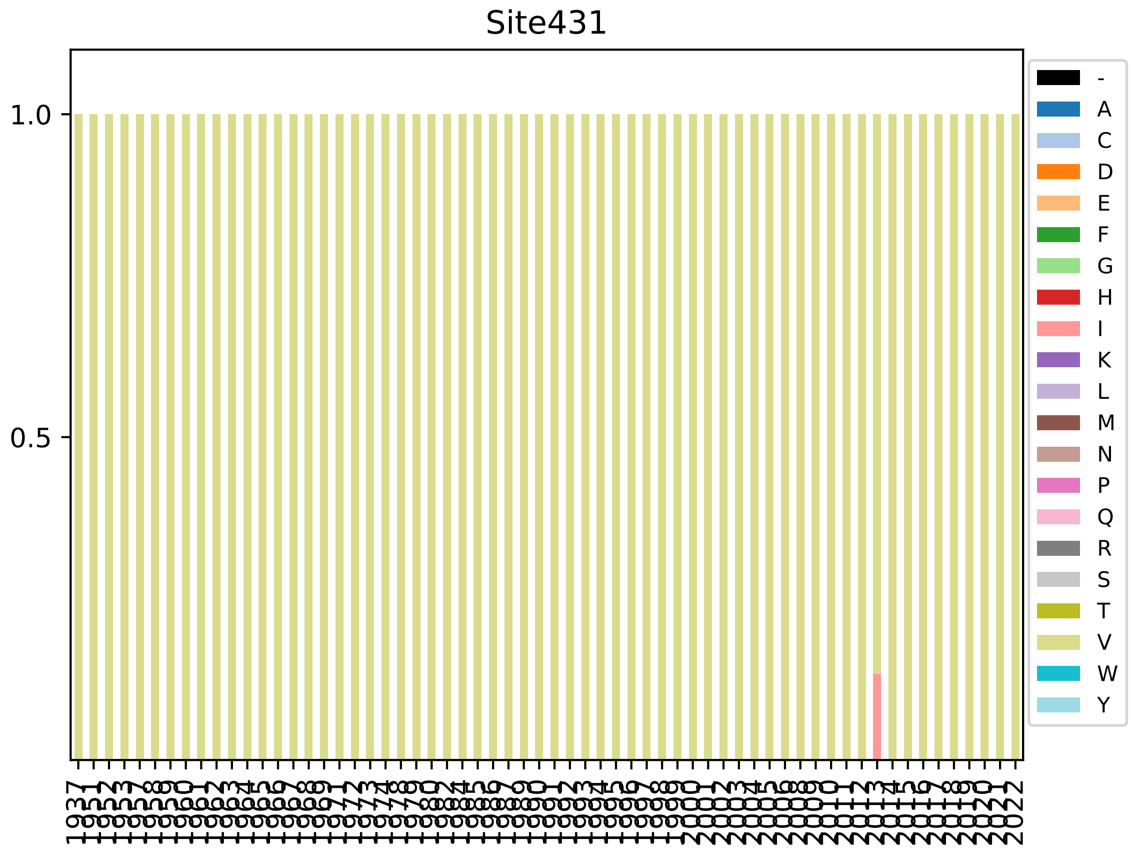
Site431
1.0
0.5
-
A
C
D
E
F
G
H
I
K
L
M
N
P
Q
R
S
T
V
W
Y

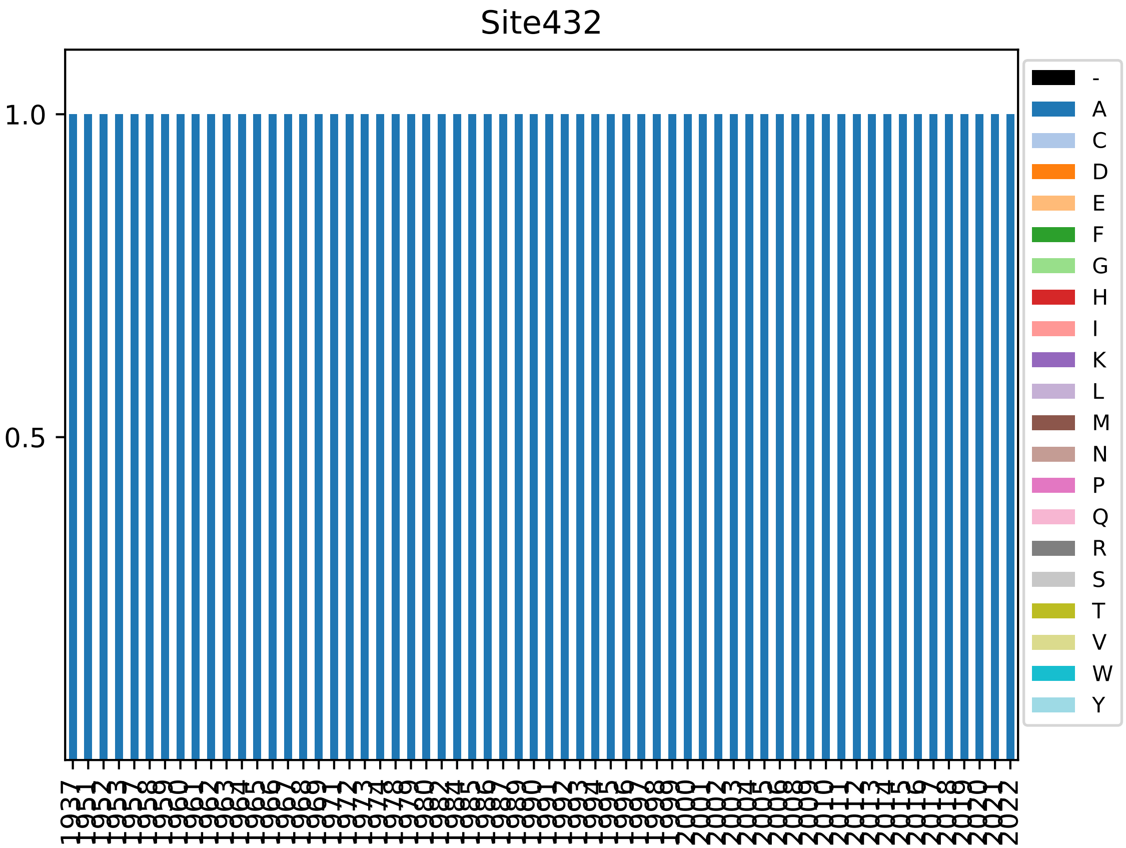
Site432
1.0
0.5
-
A
C
D
E
F
G
H
I
K
L
M
N
P
Q
R
S
T
V
W
Y

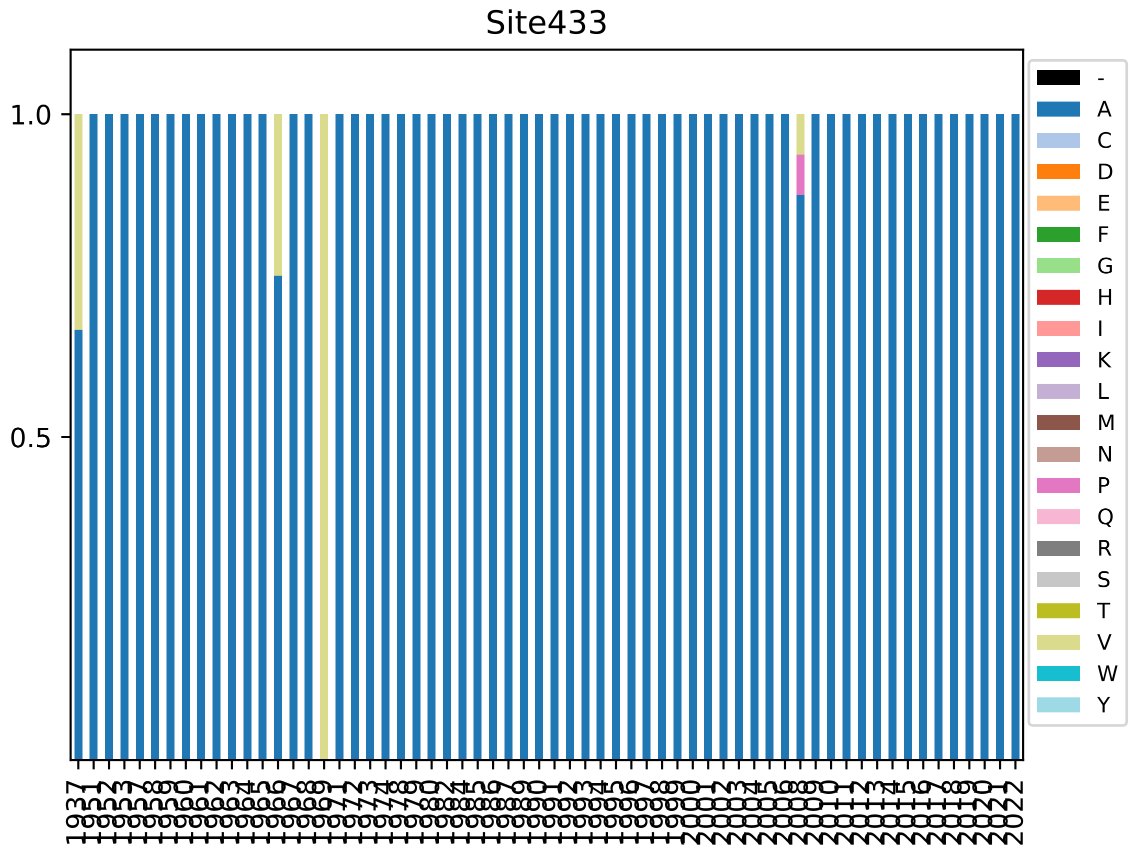

Site433
1.0
0.5
-
A
C
D
E
F
G
H
I
K
L
M
N
P
Q
R
S
T
V
W
Y

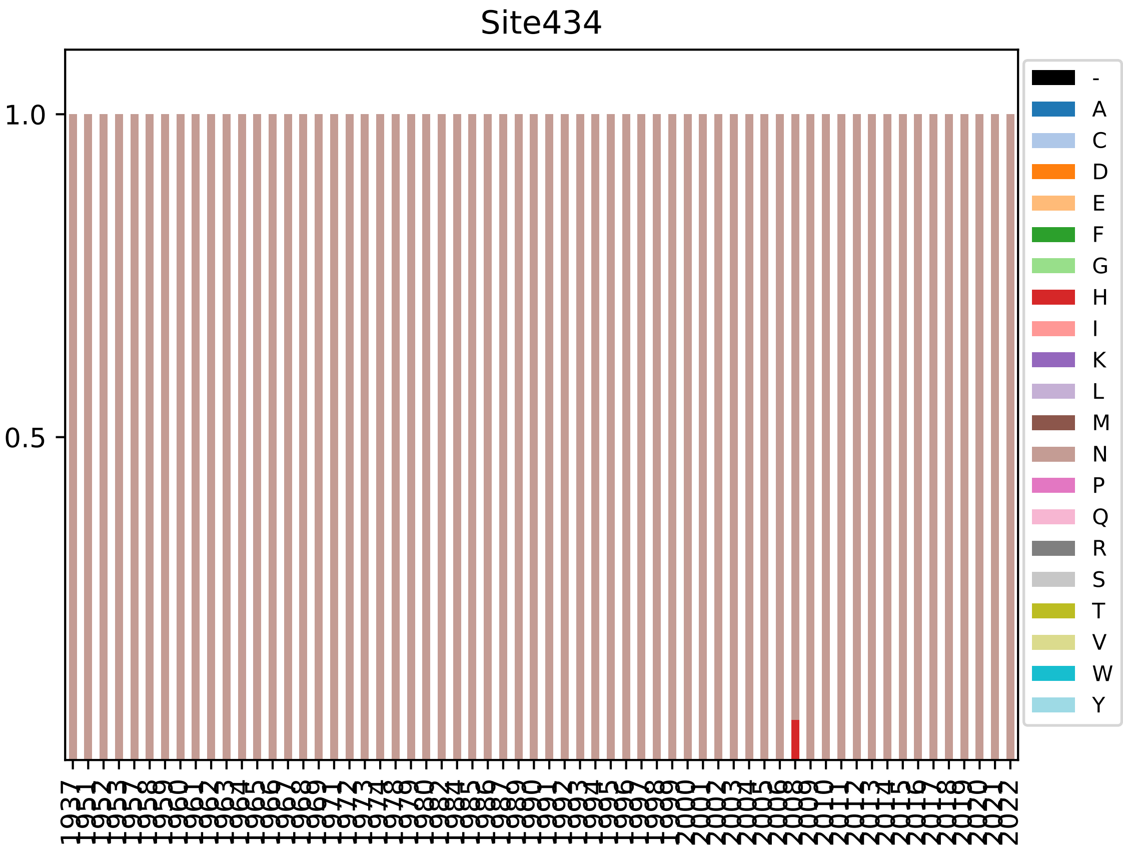
Site434
1.0
0.5
-
A
C
D
E
F
G
H
I
K
L
M
N
P
Q
R
S
T
V
W
Y

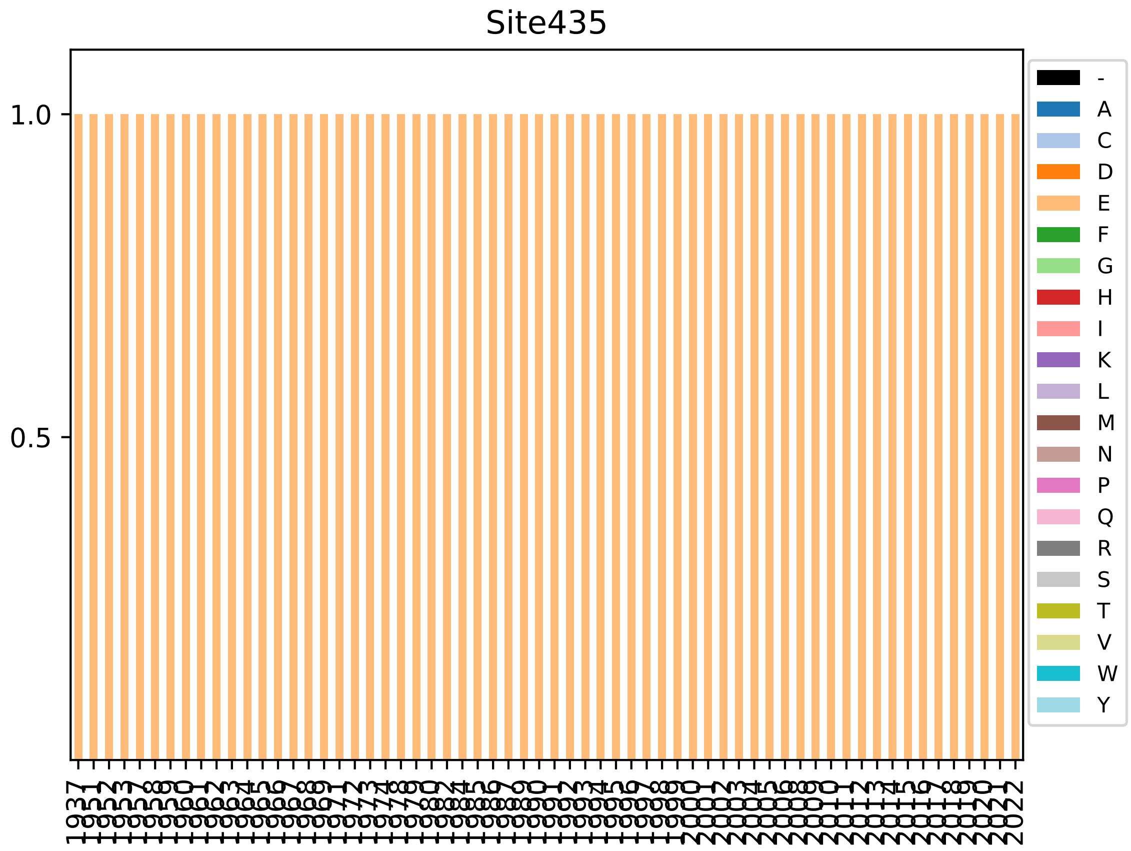
Site435
1.0
0.5
-
A
C
D
E
F
G
H
I
K
L
M
N
P
Q
R
S
T
V
W
Y

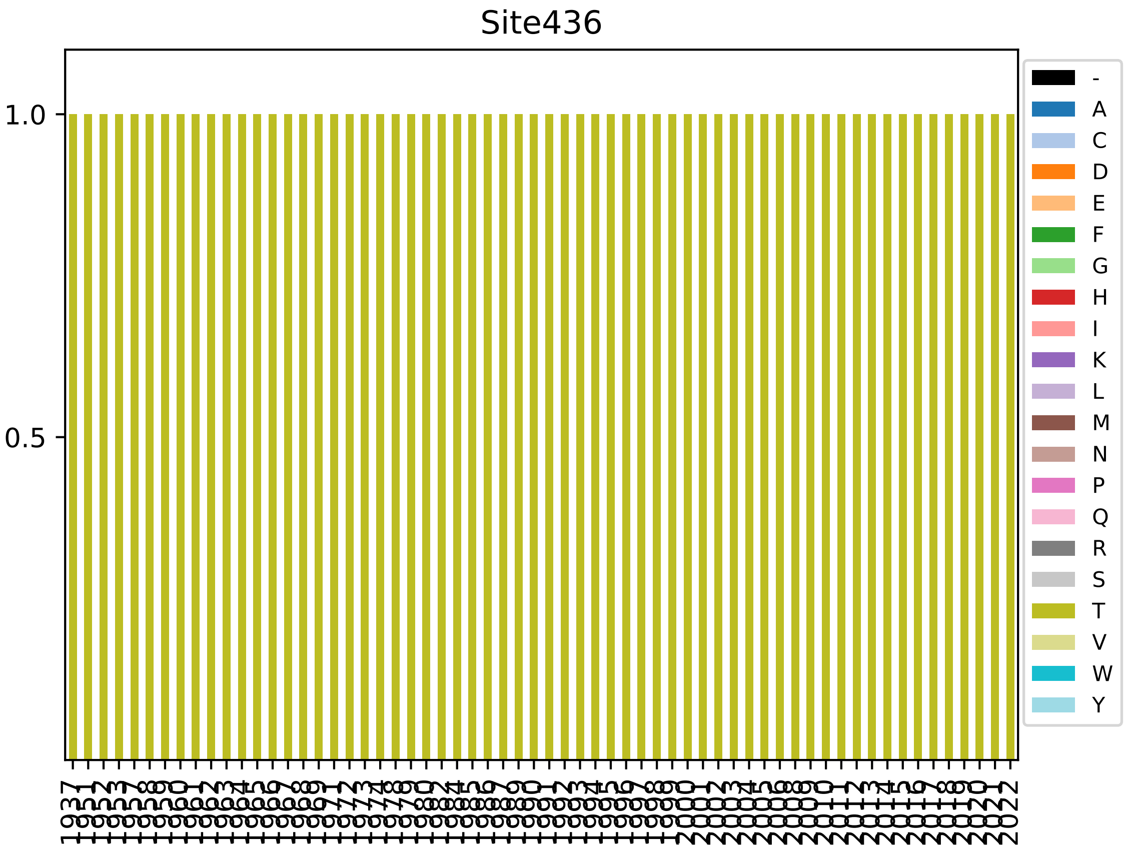

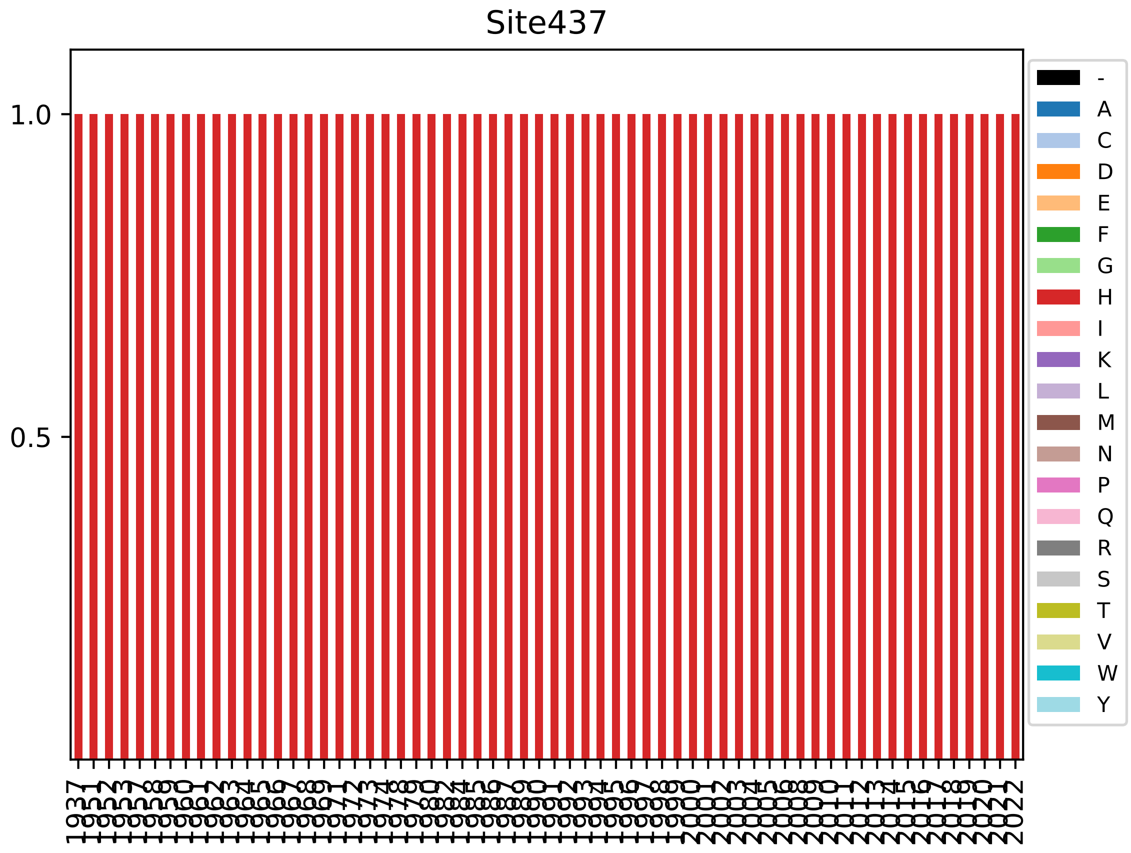
Site437
1.0
0.5
-
A
C
D
E
F
G
H
I
K
L
M
N
P
Q
R
S
T
V
W
Y

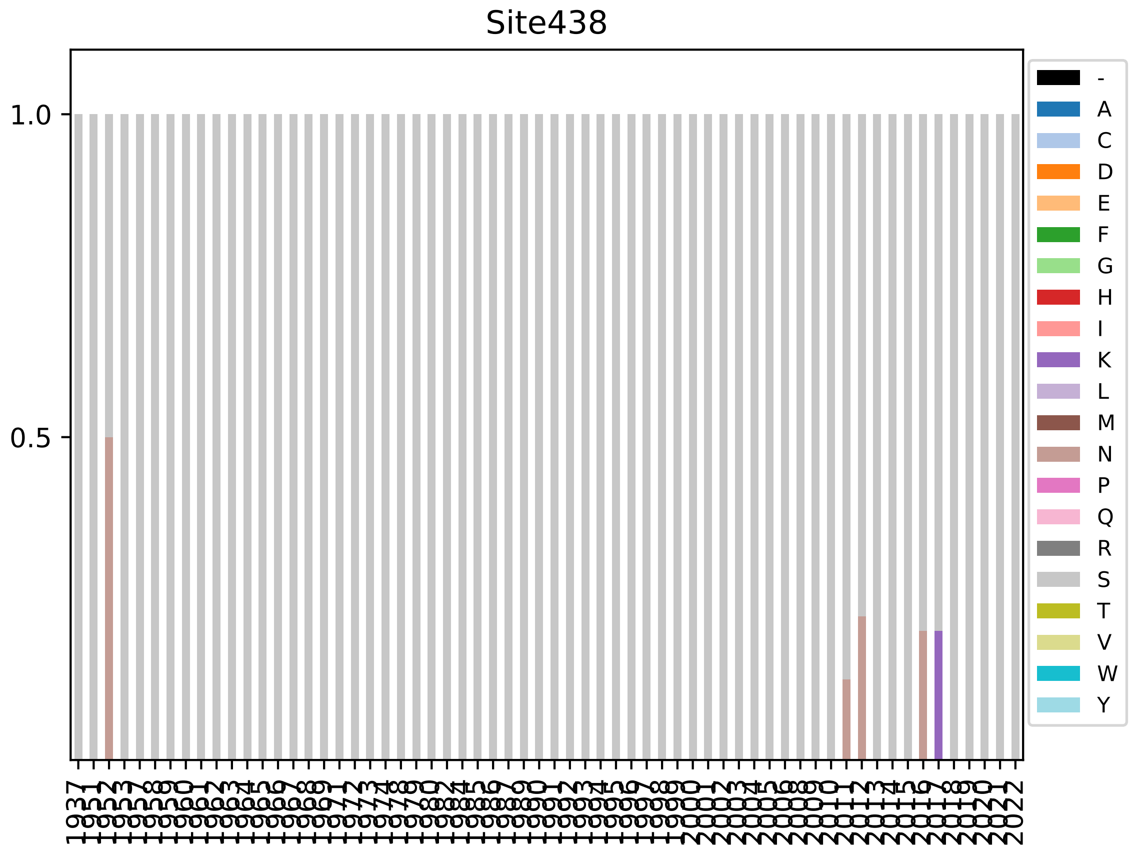
Site438
1.0
0.5
-
A
C
D
E
F
G
H
I
K
L
M
N
P
Q
R
S
T
V
W
Y

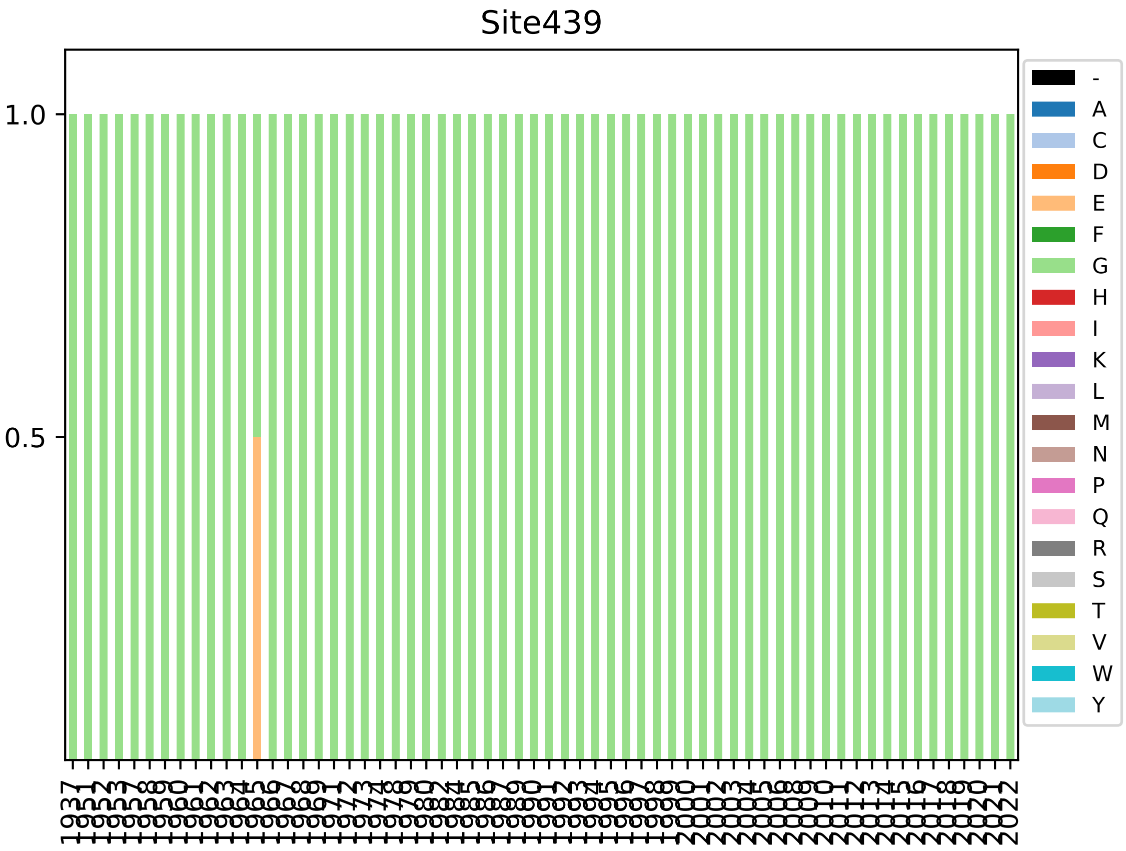
Site439
1.0
0.5
-
A
C
D
E
F
G
H
I
K
L
M
N
P
Q
R
S
T
V
W
Y

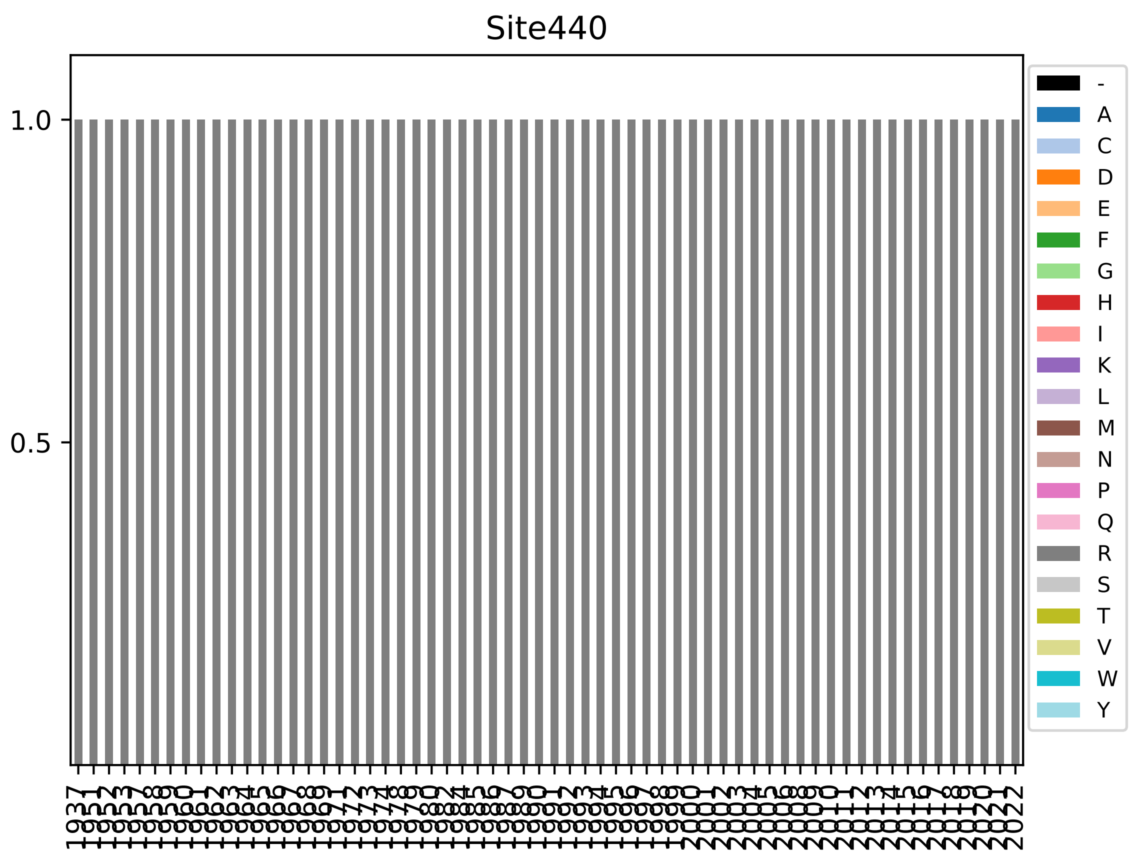
Site440
1.0
0.5
-
A
C
D
E
F
G
H
I
K
L
M
N
P
Q
R
S
T
V
W
Y

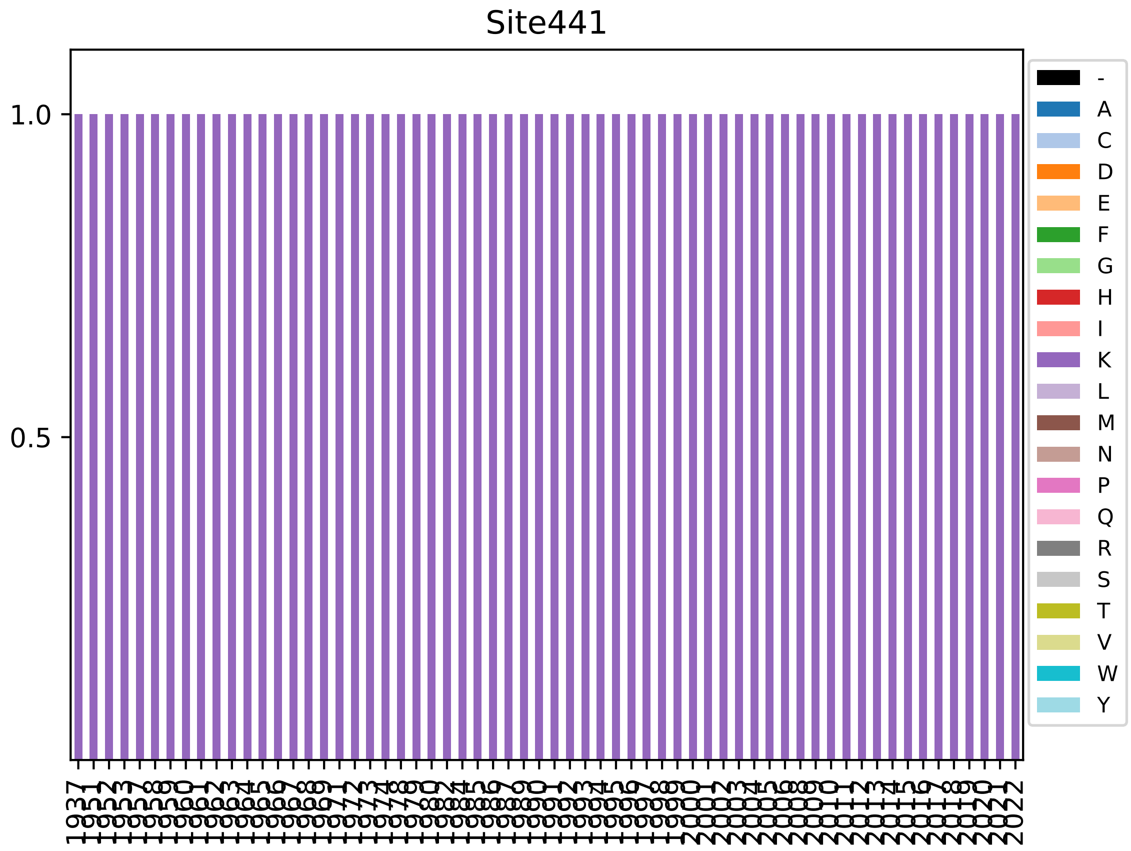
Site441
1.0
0.5
-
A
C
D
E
F
G
H
I
K
L
M
N
P
Q
R
S
T
V
W
Y

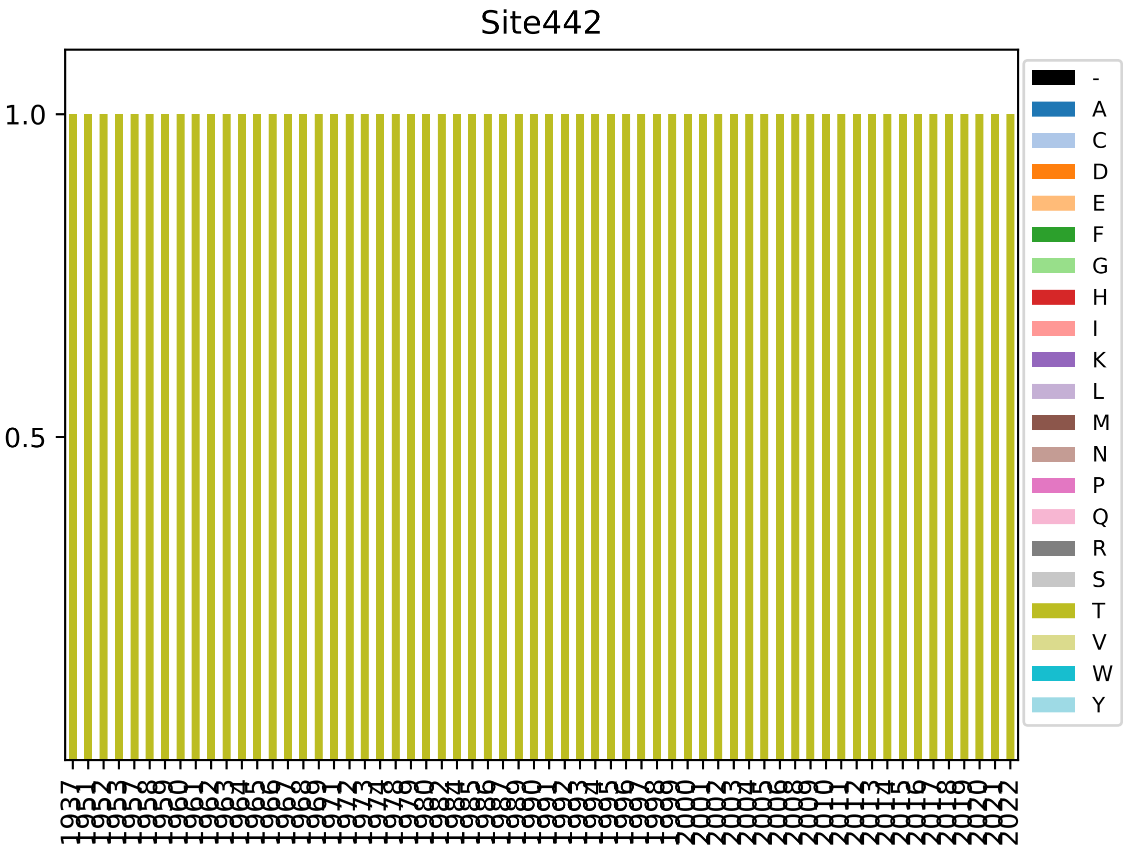
Site442
1.0
0.5
-
A
C
D
E
F
G
H
I
K
L
M
N
P
Q
R
S
T
V
W
Y

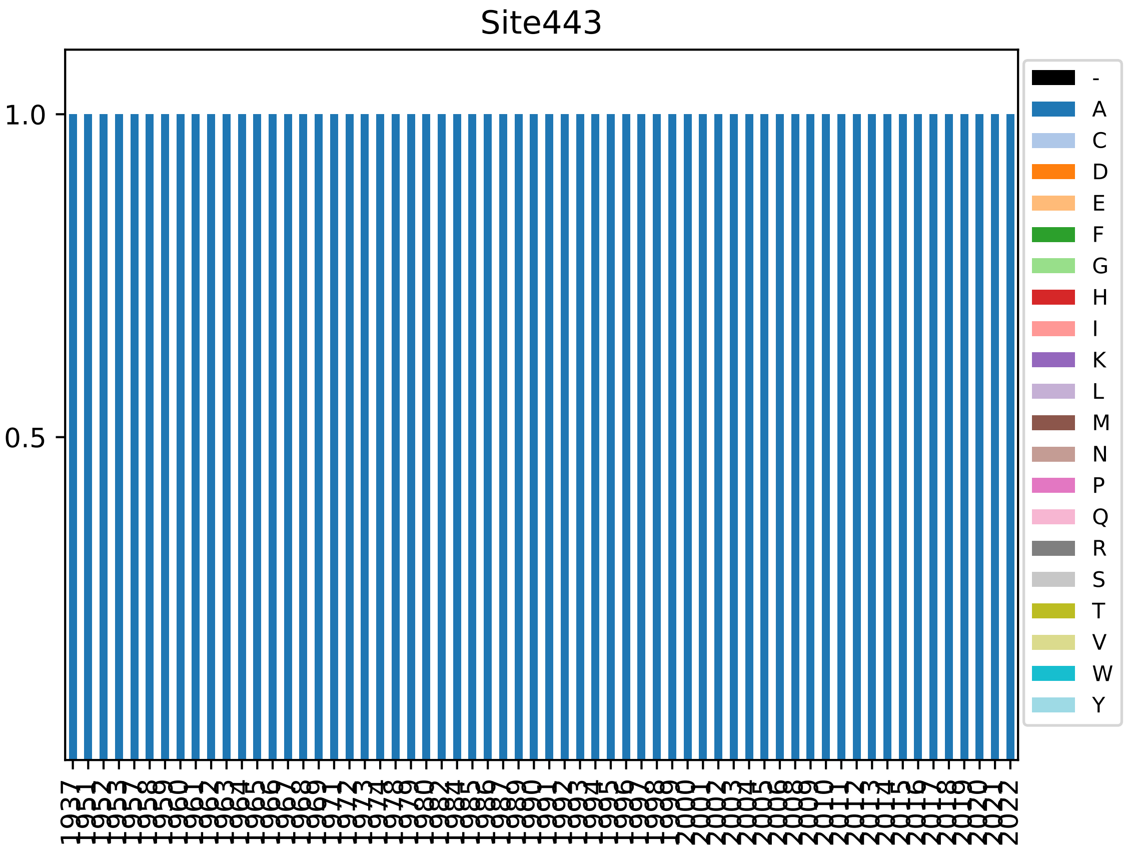
Site443
1.0
0.5
-
A
C
D
E
F
G
H
I
K
L
M
N
P
Q
R
S
T
V
W
Y

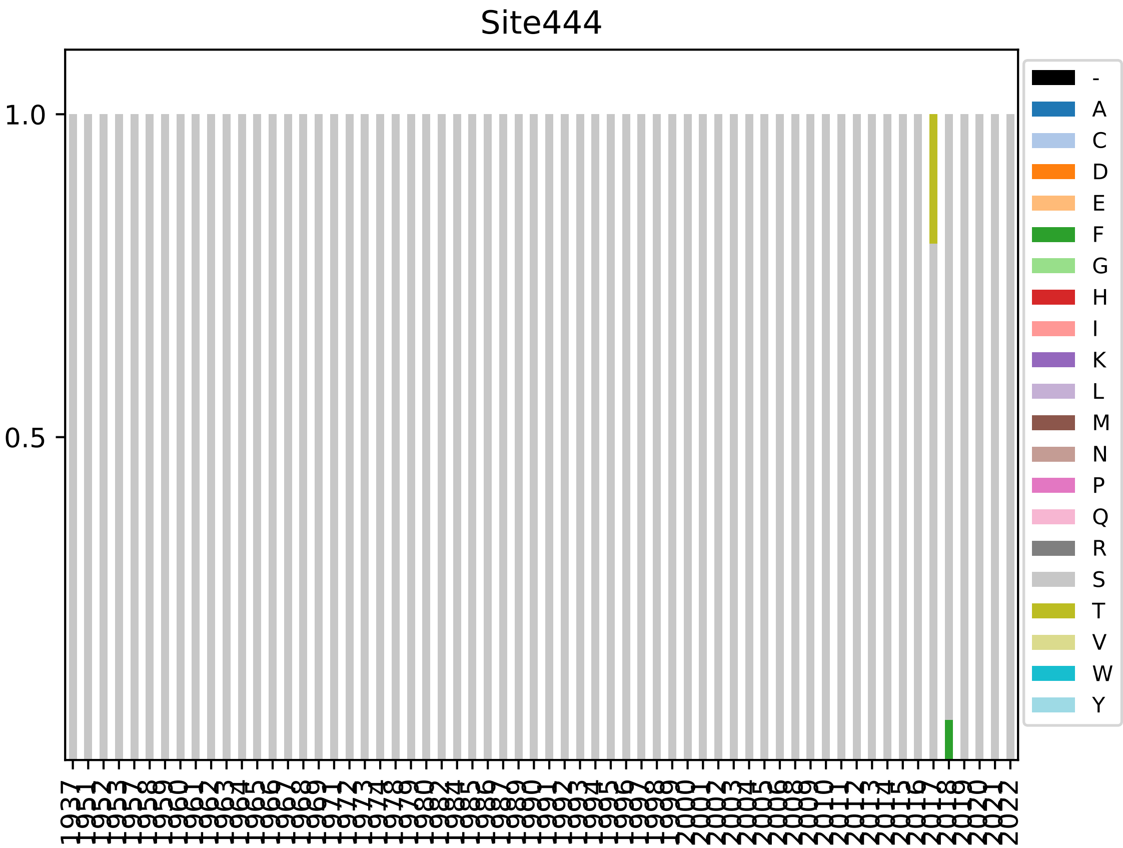
Site444
1.0
0.5
-
A
C
D
E
F
G
H
I
K
L
M
N
P
Q
R
S
T
V
W
Y

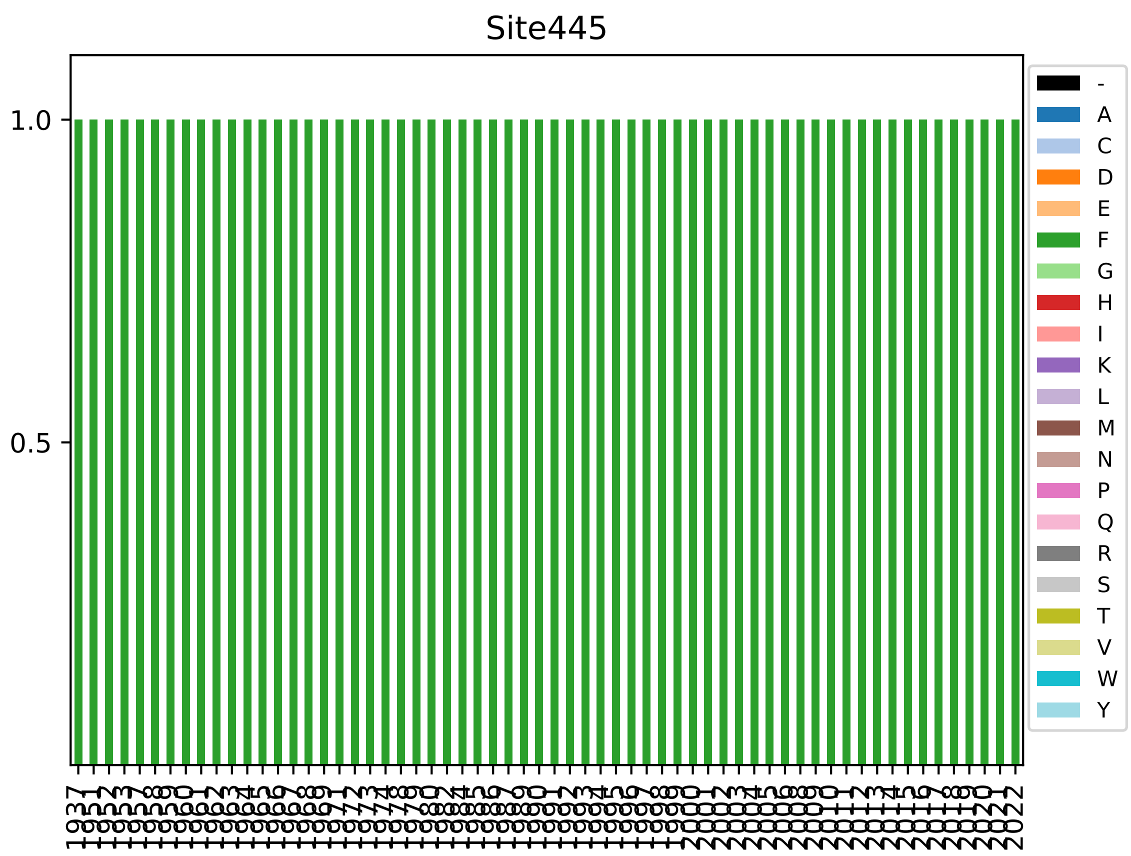
Site445
1.0
0.5
-
A
C
D
E
F
G
H
I
K
L
M
N
P
Q
R
S
T
V
W
Y

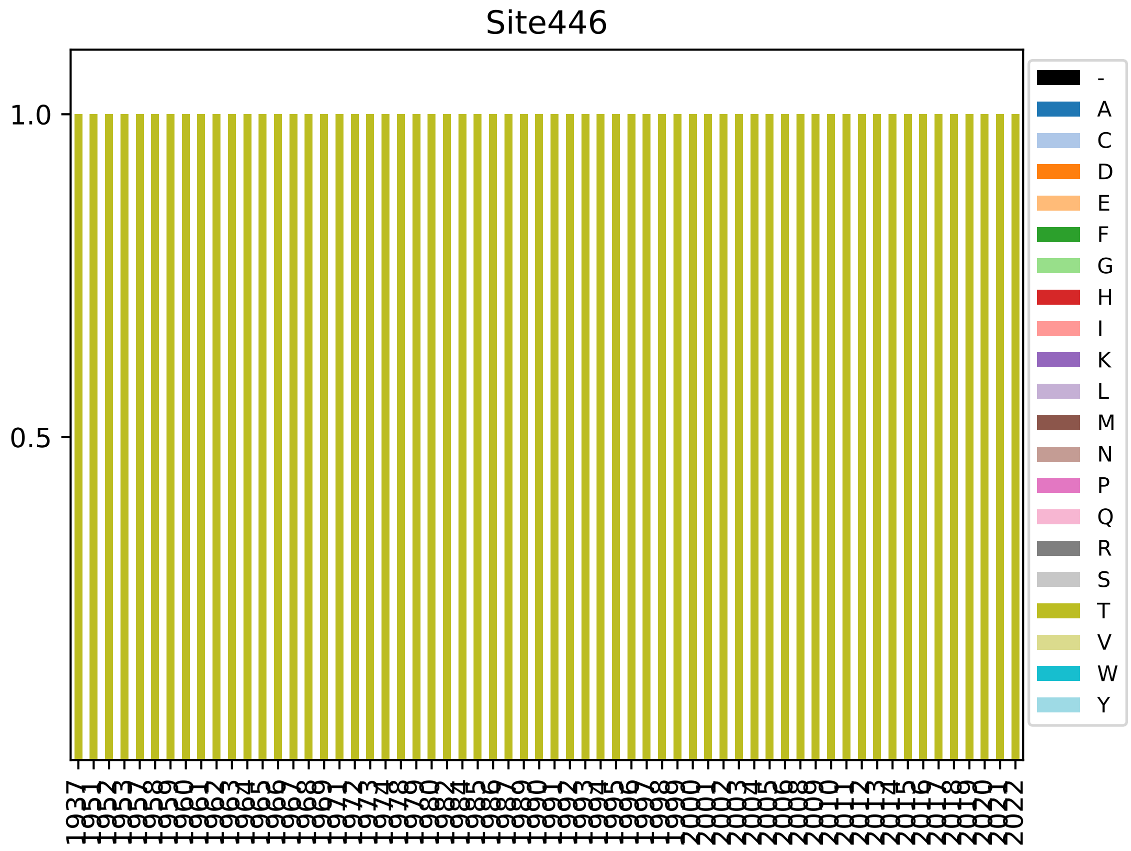
Site446
1.0
0.5
-
A
C
D
E
F
G
H
I
K
L
M
N
P
Q
R
S
T
V
W
Y

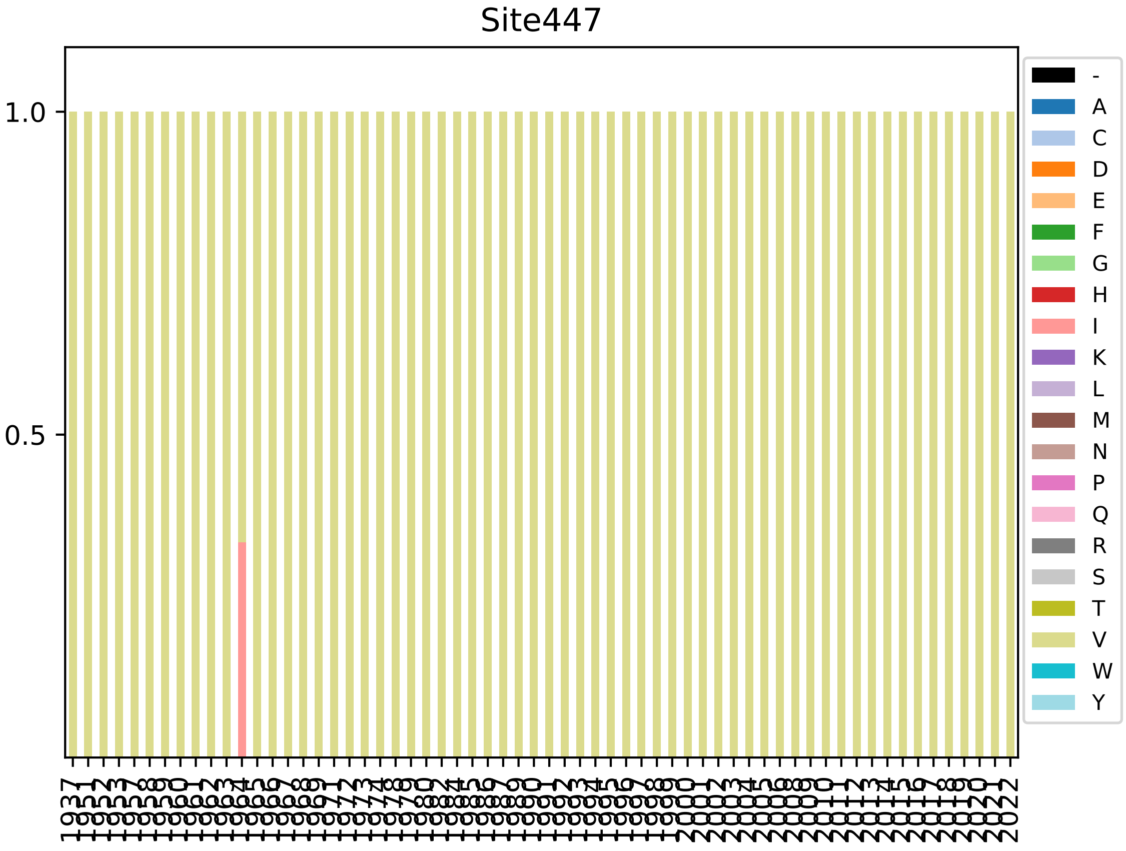
Site447
1.0
0.5
-
A
C
D
E
F
G
H
I
K
L
M
N
P
Q
R
S
T
V
W
Y

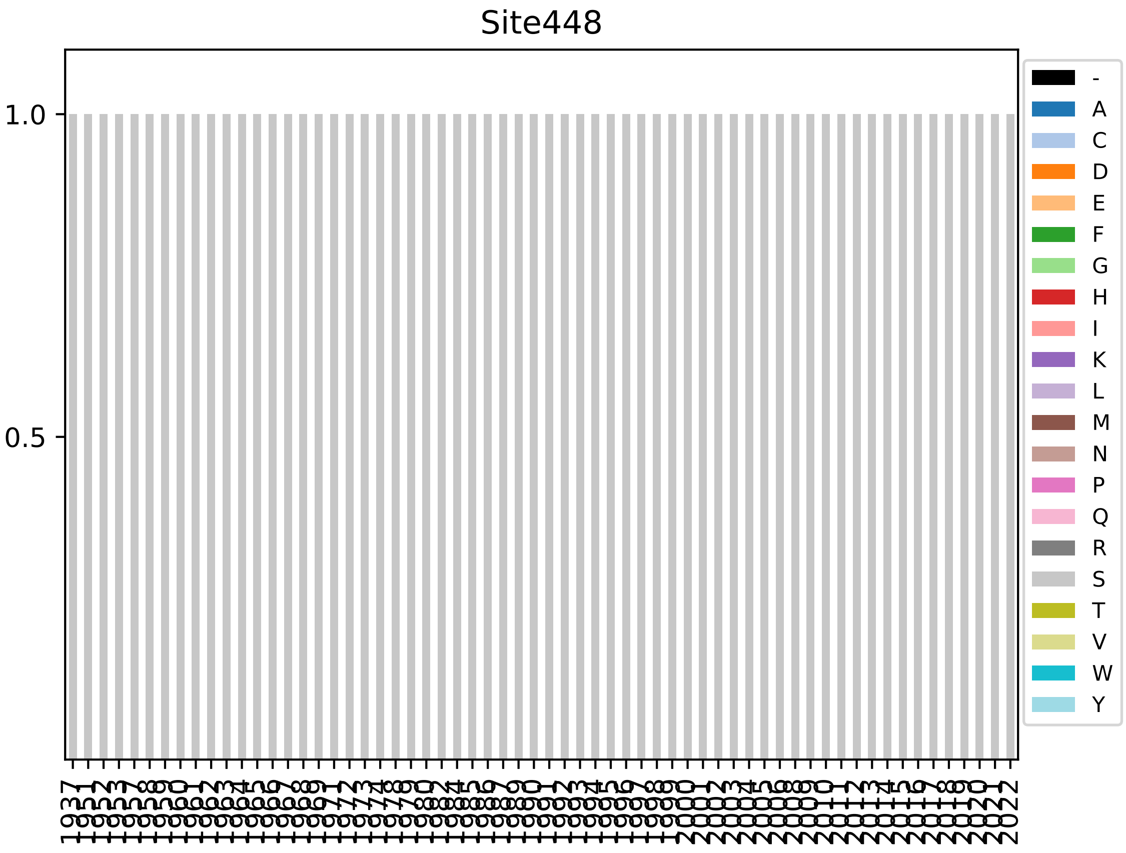
Site448
1.0
0.5
-
A
C
D
E
F
G
H
I
K
L
M
N
P
Q
R
S
T
V
W
Y

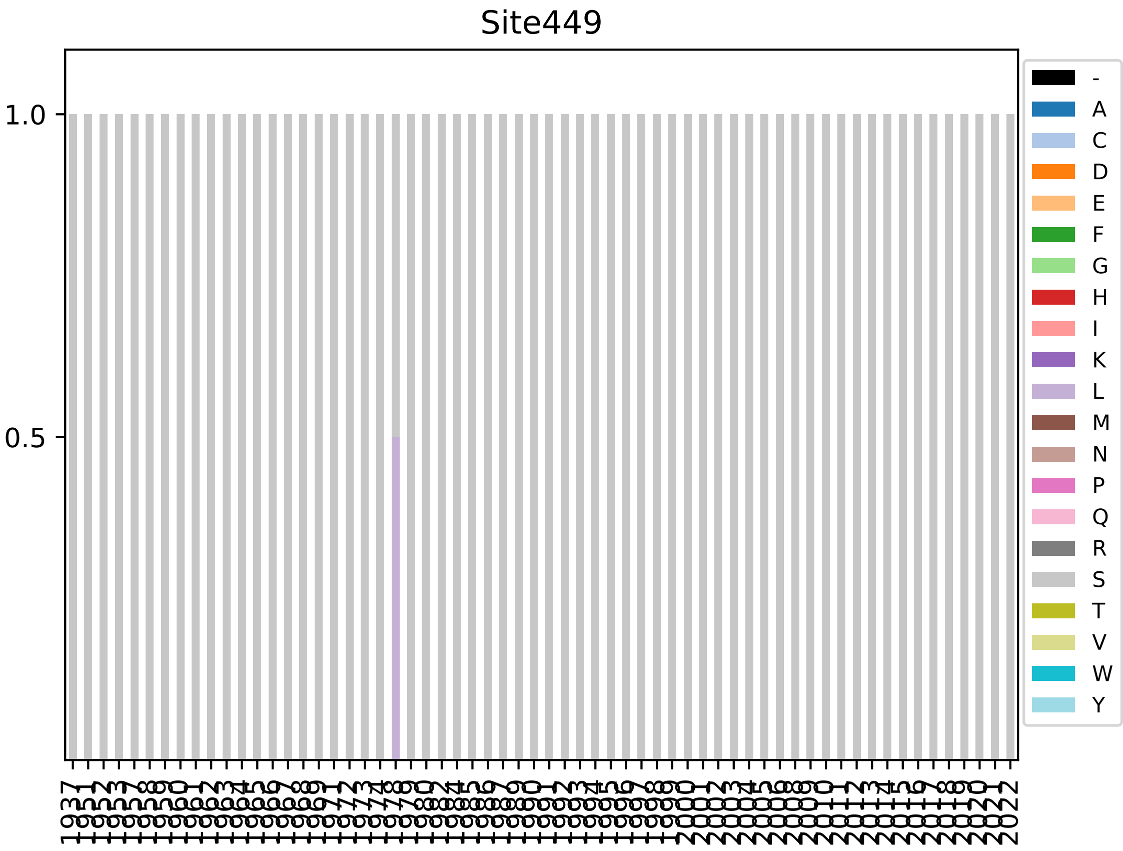
Site449
1.0
0.5
-
A
C
D
E
F
G
H
I
K
L
M
N
P
Q
R
S
T
V
W
Y

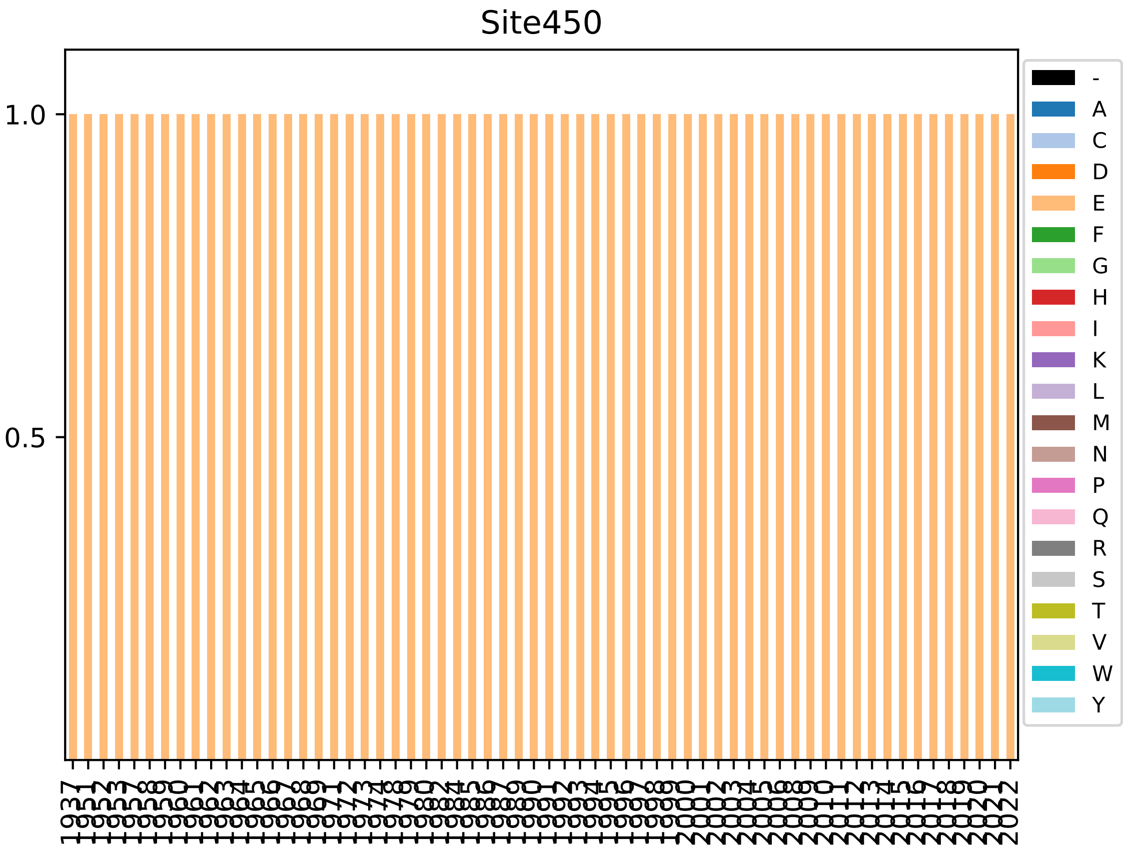
Site450
1.0
0.5
-
A
C
D
E
F
G
H
I
K
L
M
N
P
Q
R
S
T
V
W
Y

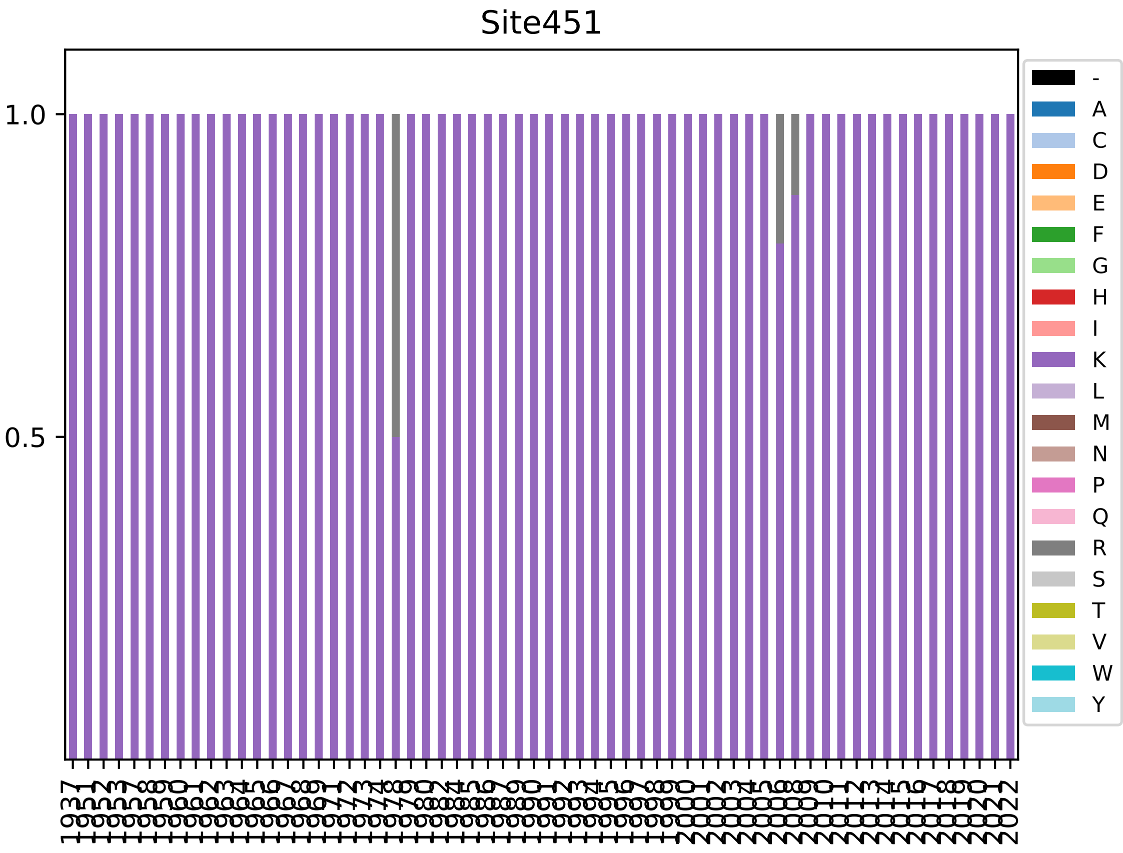
Site451
1.0
0.5
-
A
C
D
E
F
G
H
I
K
L
M
N
P
Q
R
S
T
V
W
Y

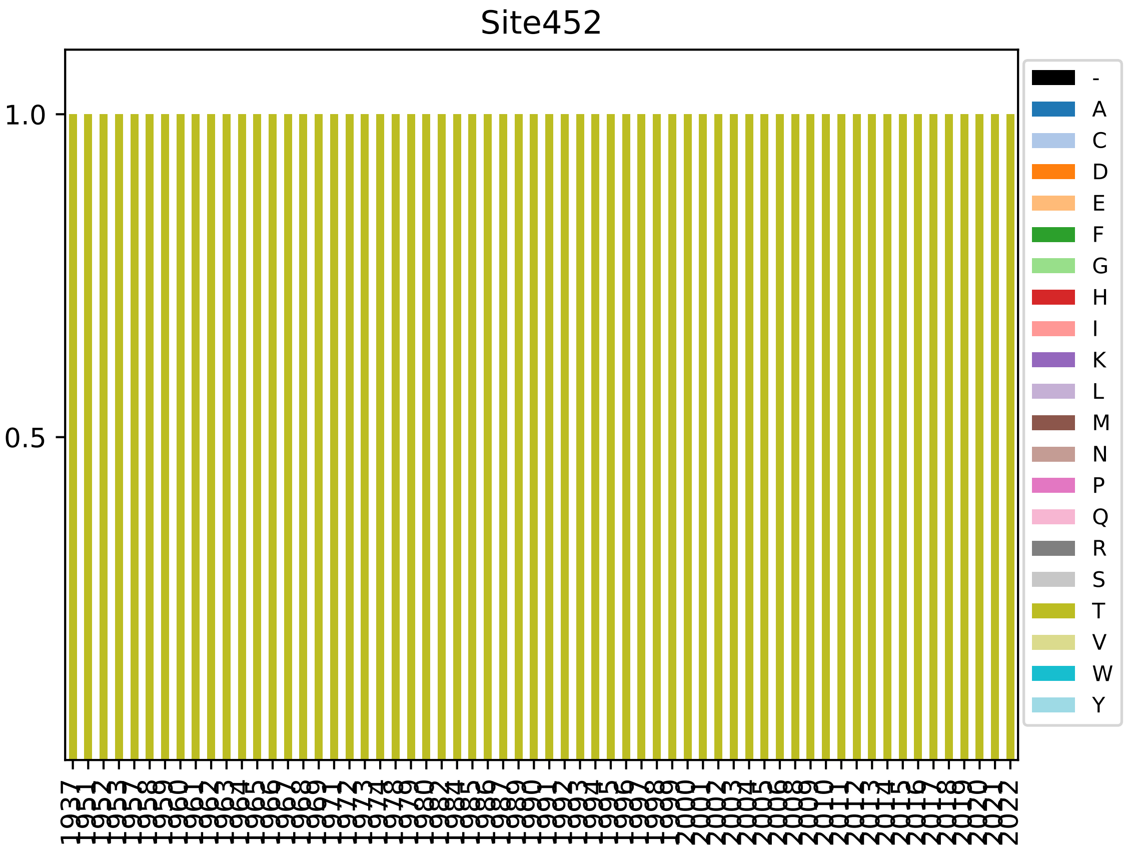
Site452
1.0
0.5
-
A
C
D
E
F
G
H
I
K
L
M
N
P
Q
R
S
T
V
W
Y

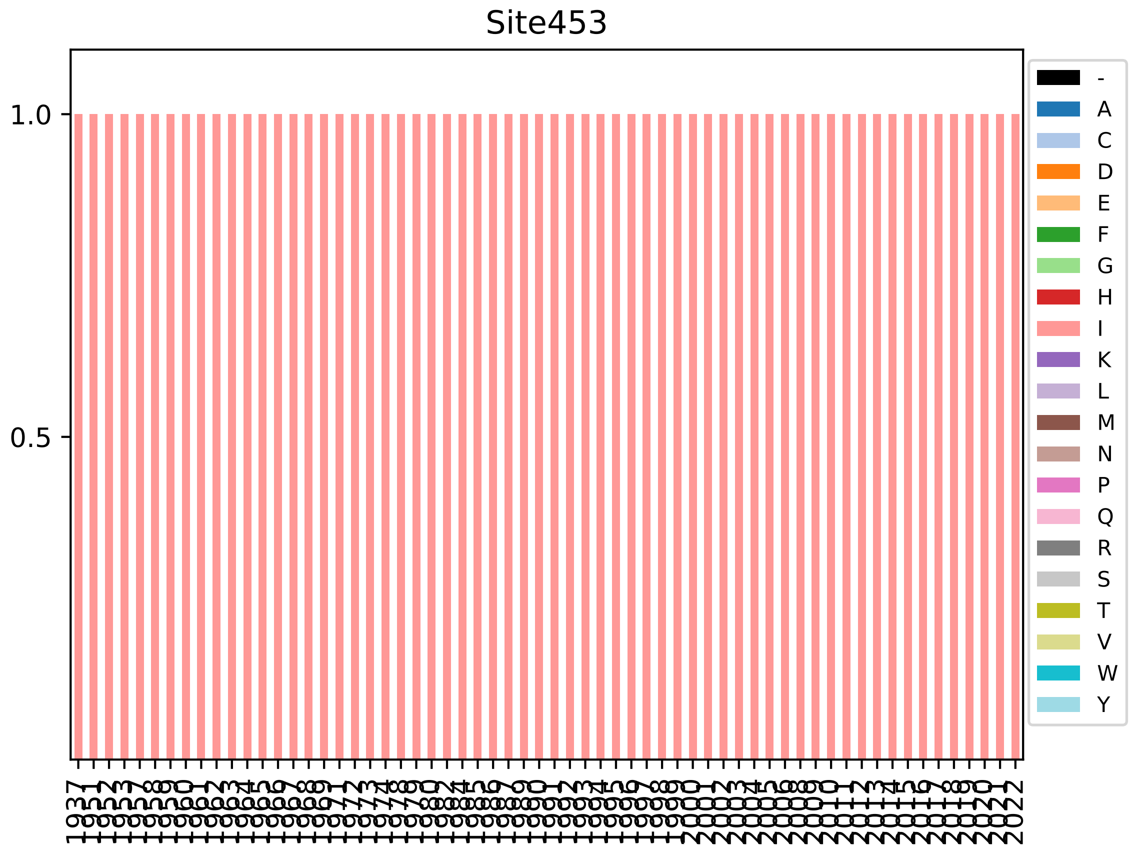
Site453
1.0
0.5
-
A
C
D
E
F
G
H
I
K
L
M
N
P
Q
R
S
T
V
W
Y

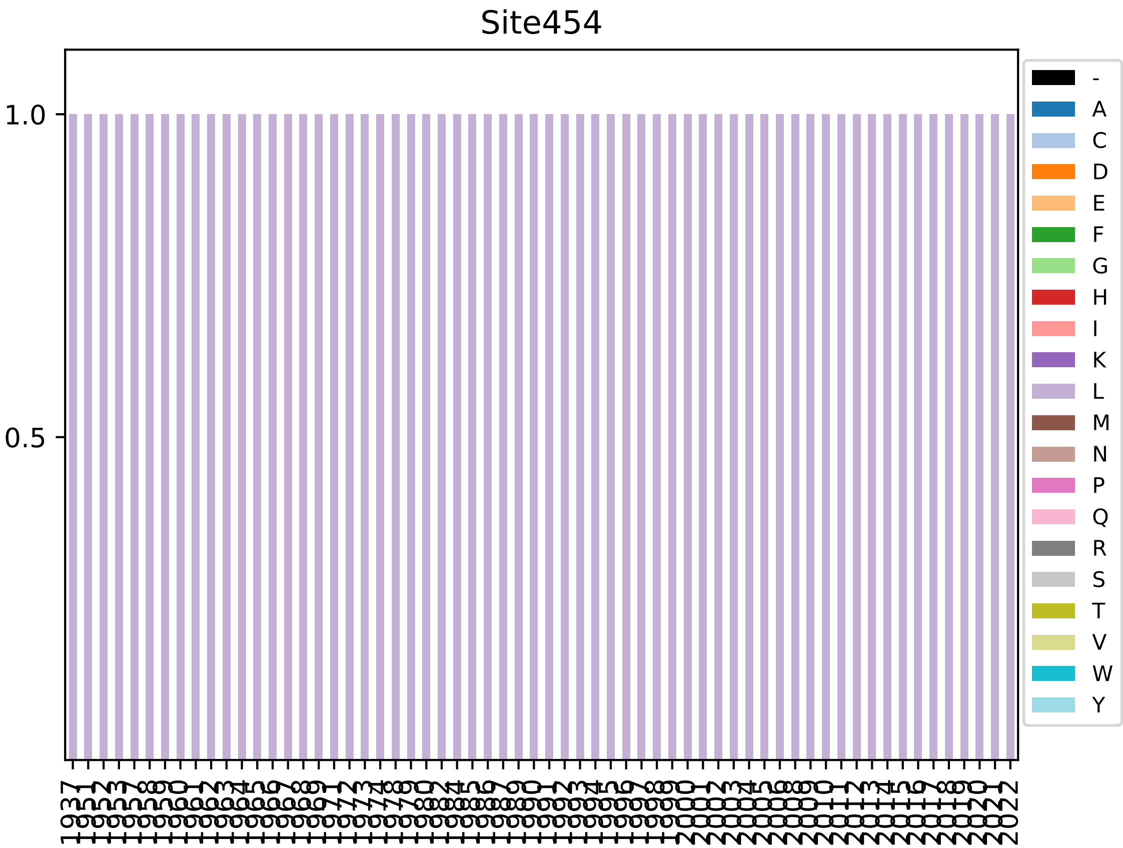
Site454
1.0
0.5
-
A
C
D
E
F
G
H
I
K
L
M
N
P
Q
R
S
T
V
W
Y

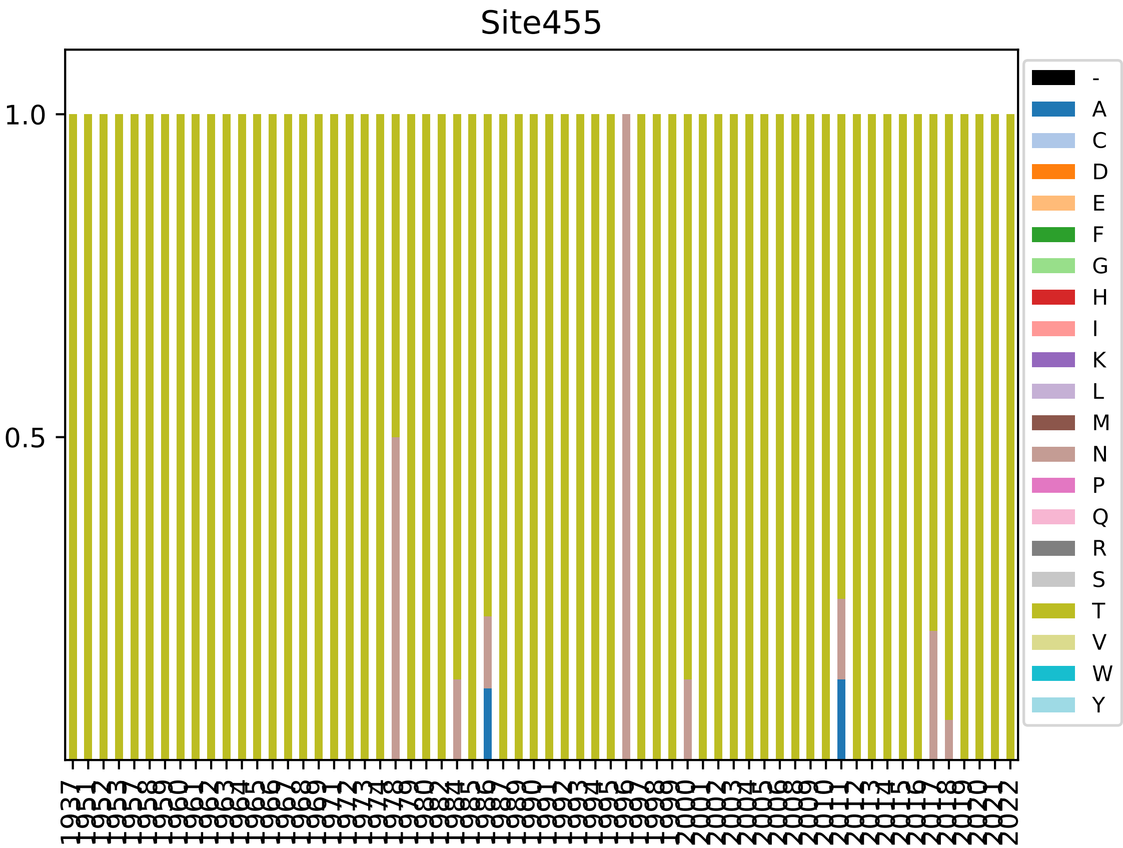
Site455
1.0
0.5
-
A
C
D
E
F
G
H
I
K
L
M
N
P
Q
R
S
T
V
W
Y

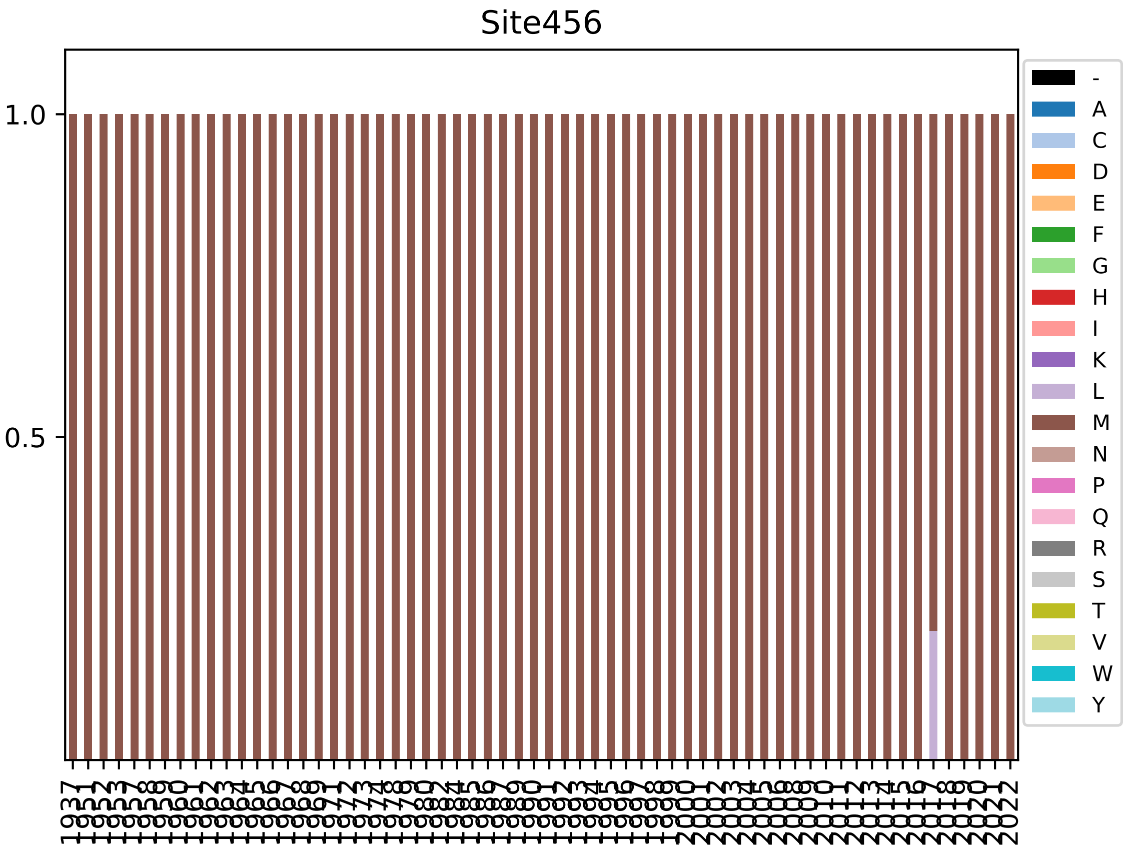
Site456
1.0
0.5
-
A
C
D
E
F
G
H
I
K
L
M
N
P
Q
R
S
T
V
W
Y

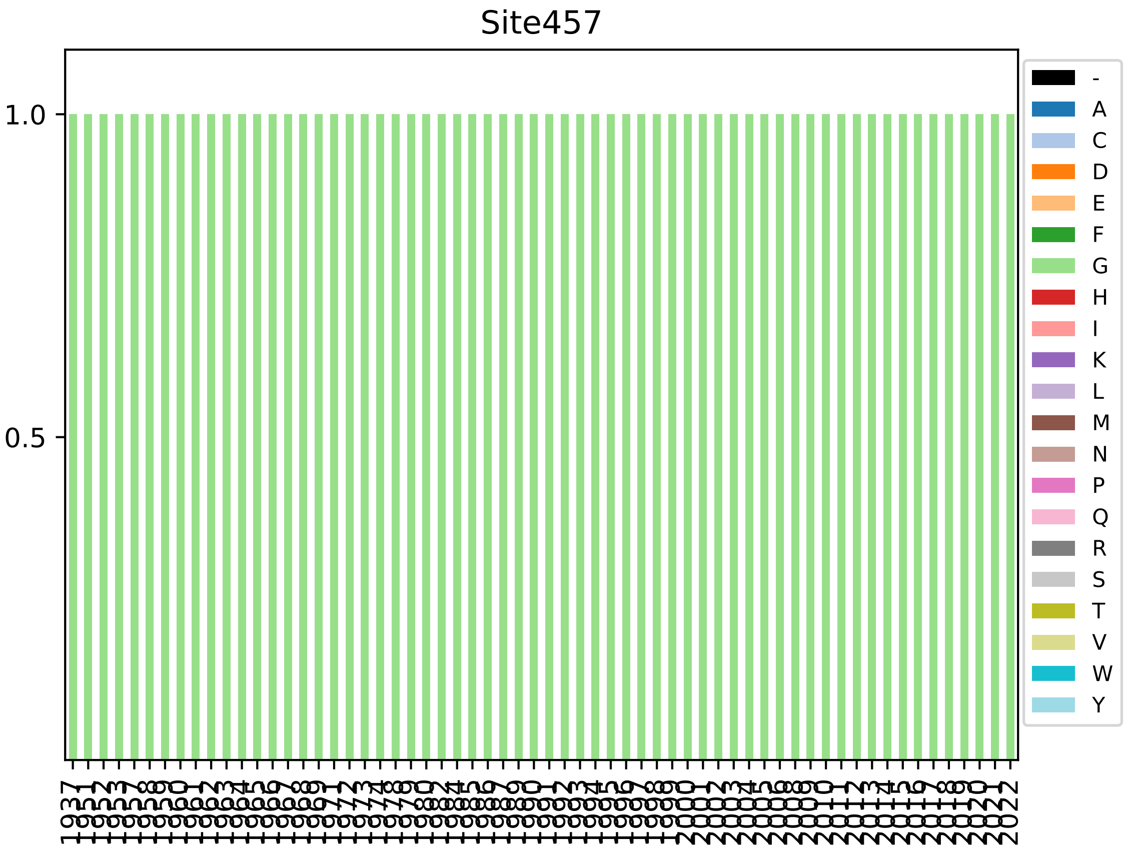
Site457
1.0
0.5
-
A
C
D
E
F
G
H
I
K
L
M
N
P
Q
R
S
T
V
W
Y

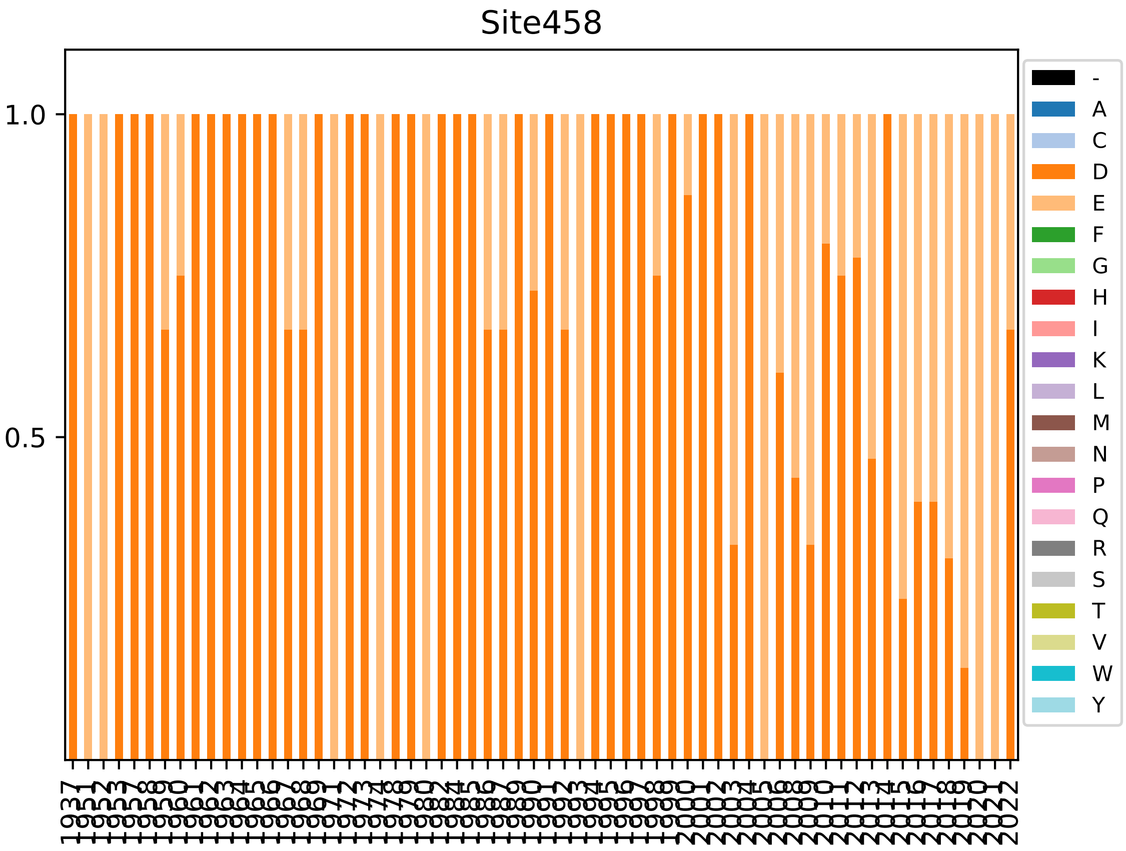
Site458
1.0
0.5
-
A
C
D
E
F
G
H
I
K
L
M
N
P
Q
R
S
T
V
W
Y

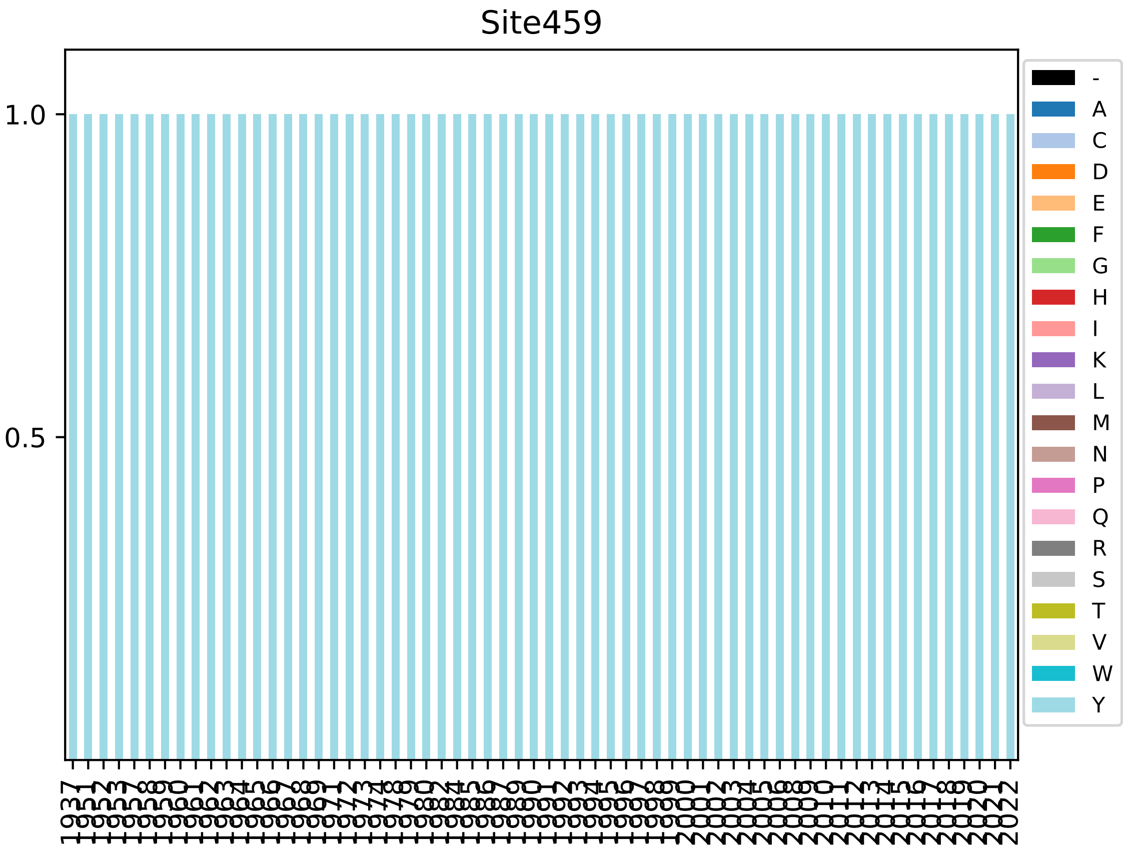
Site459
1.0
0.5
-
A
C
D
E
F
G
H
I
K
L
M
N
P
Q
R
S
T
V
W
Y

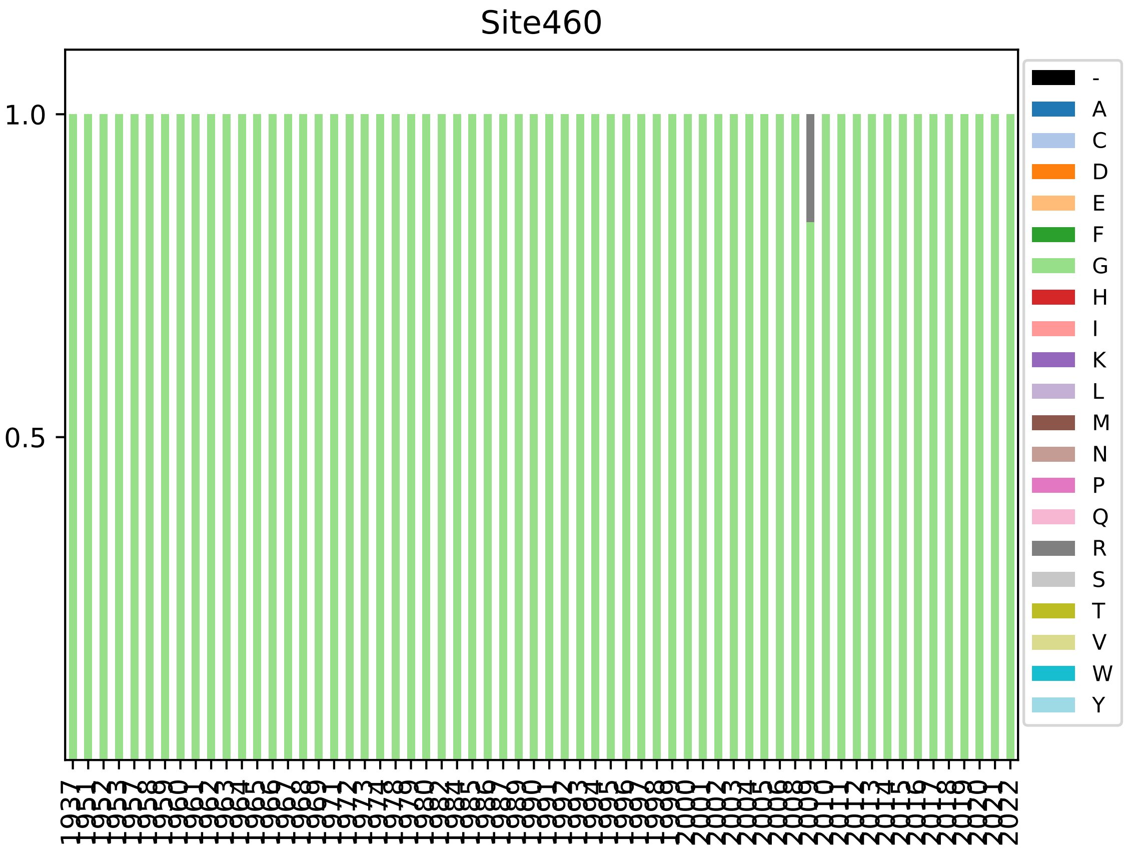

Site460
1.0
0.5
-
A
C
D
E
F
G
H
I
K
L
M
N
P
Q
R
S
T
V
W
Y

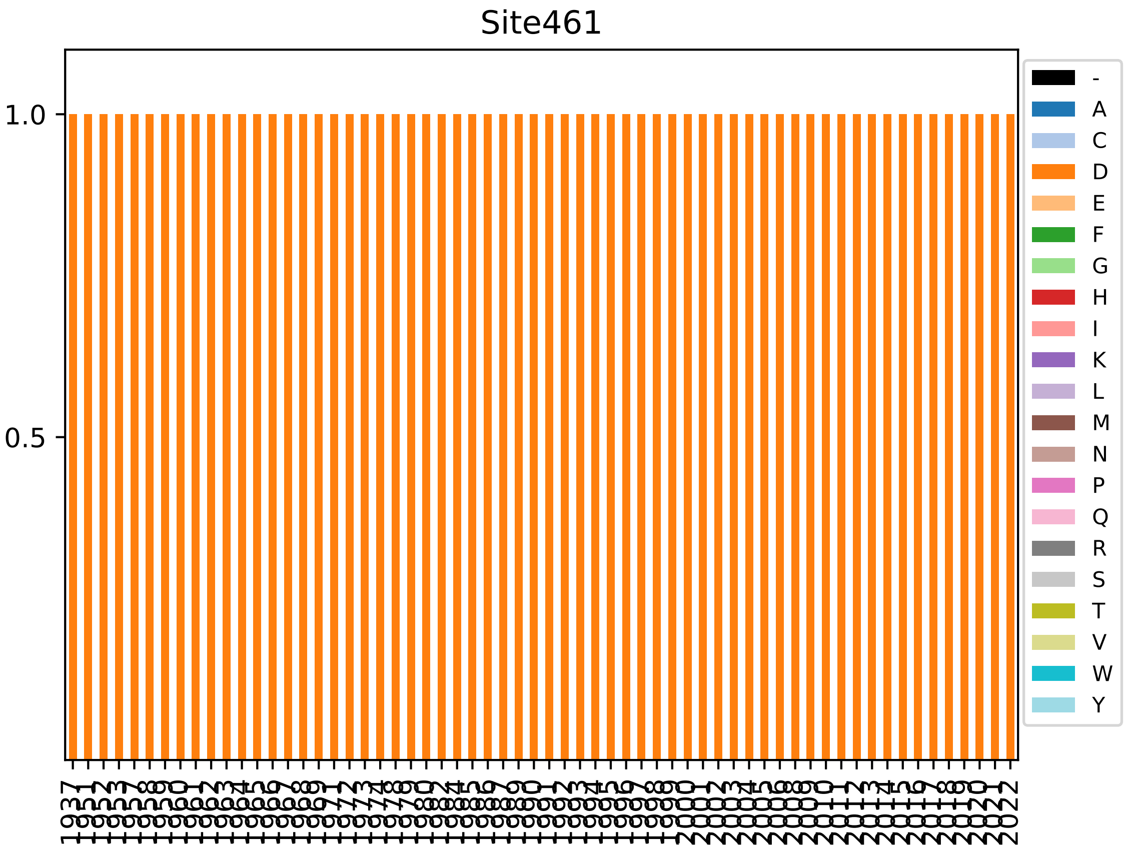
Site461
1.0
0.5
-
A
C
D
E
F
G
H
I
K
L
M
N
P
Q
R
S
T
V
W
Y

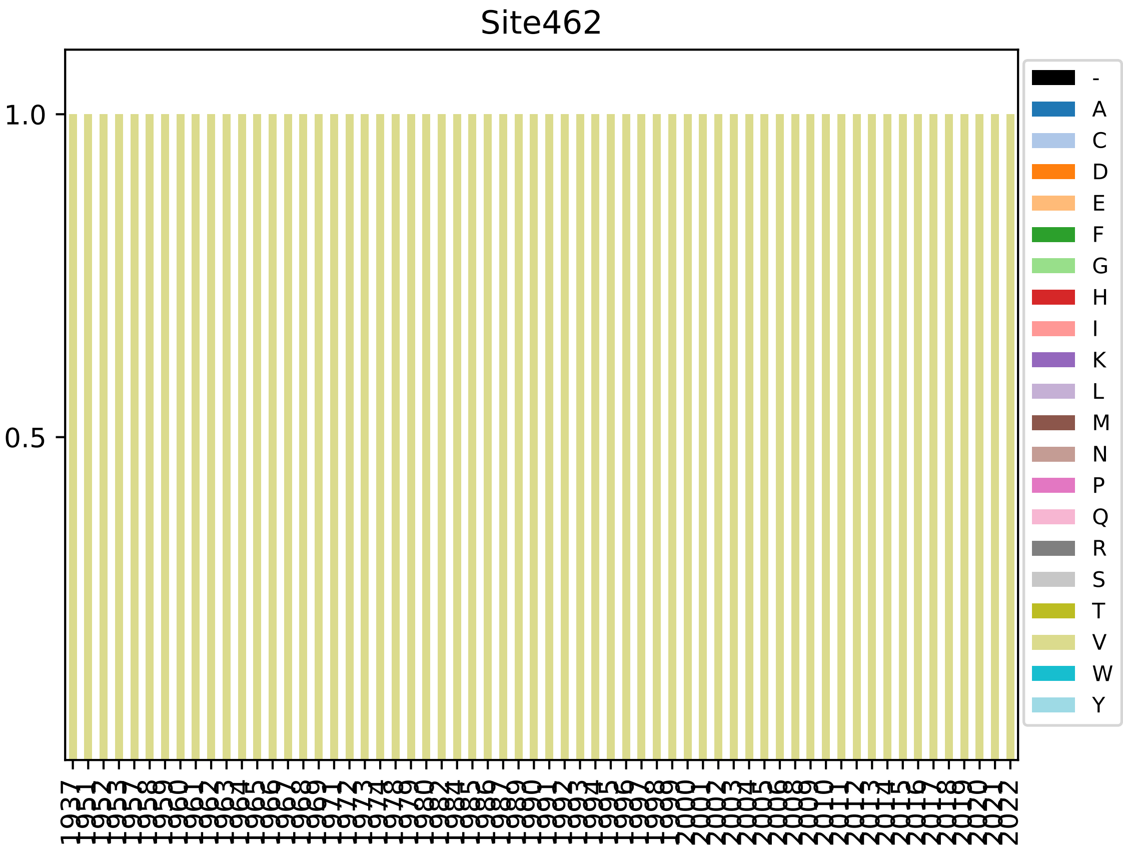
Site462
1.0
0.5
-
A
C
D
E
F
G
H
I
K
L
M
N
P
Q
R
S
T
V
W
Y

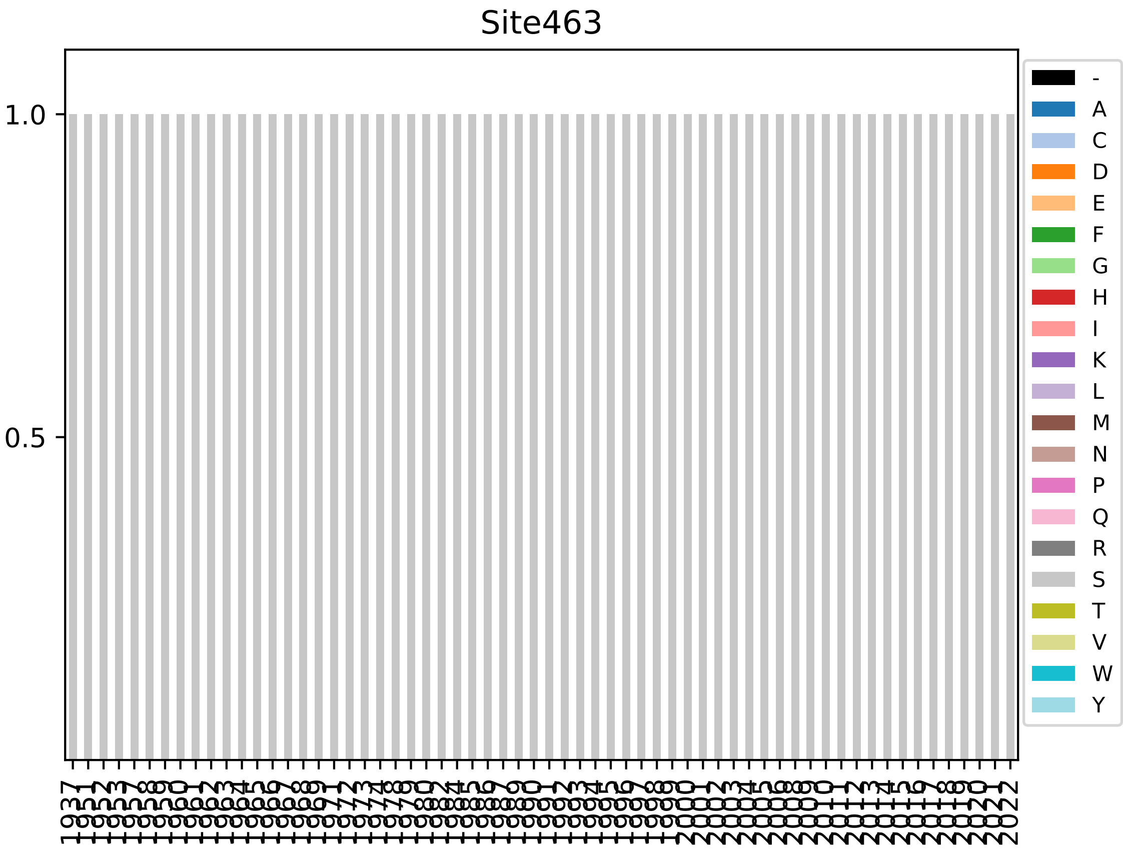
Site463
1.0
0.5
-
A
C
D
E
F
G
H
I
K
L
M
N
P
Q
R
S
T
V
W
Y

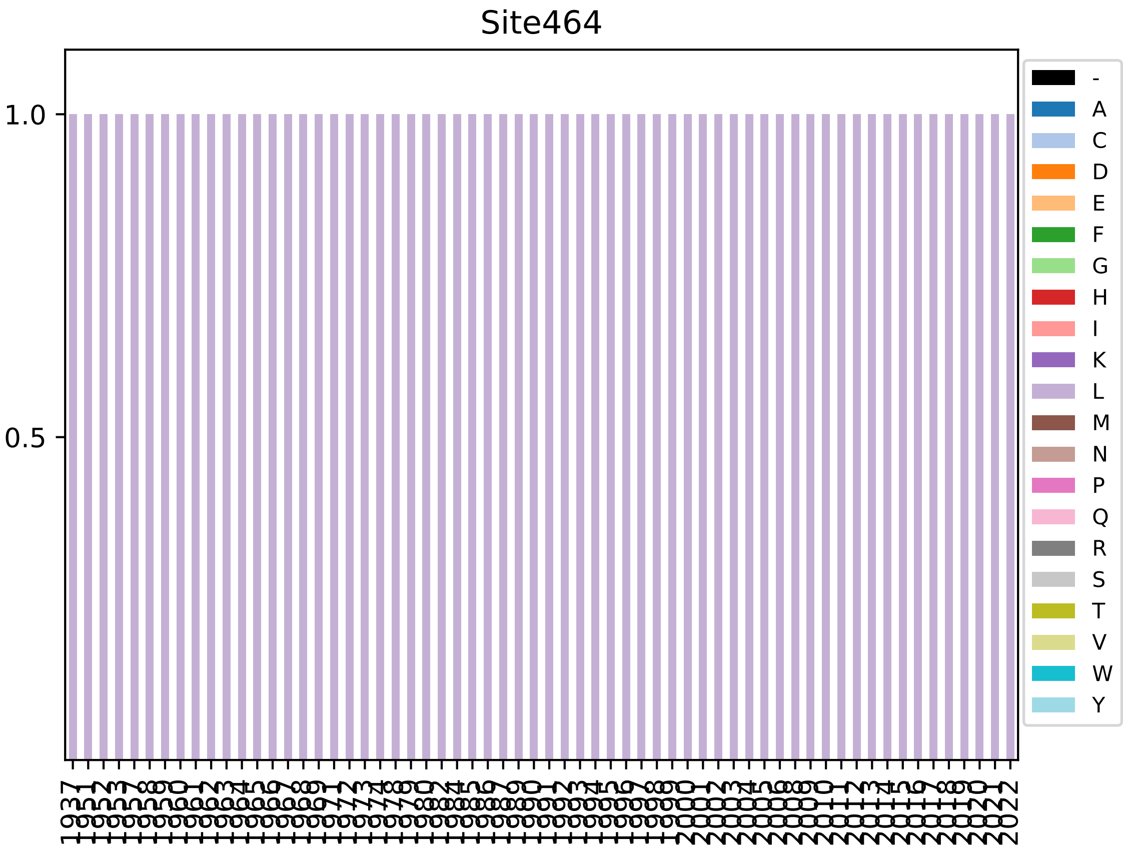
Site464
1.0
0.5
-
A
C
D
E
F
G
H
I
K
L
M
N
P
Q
R
S
T
V
W
Y

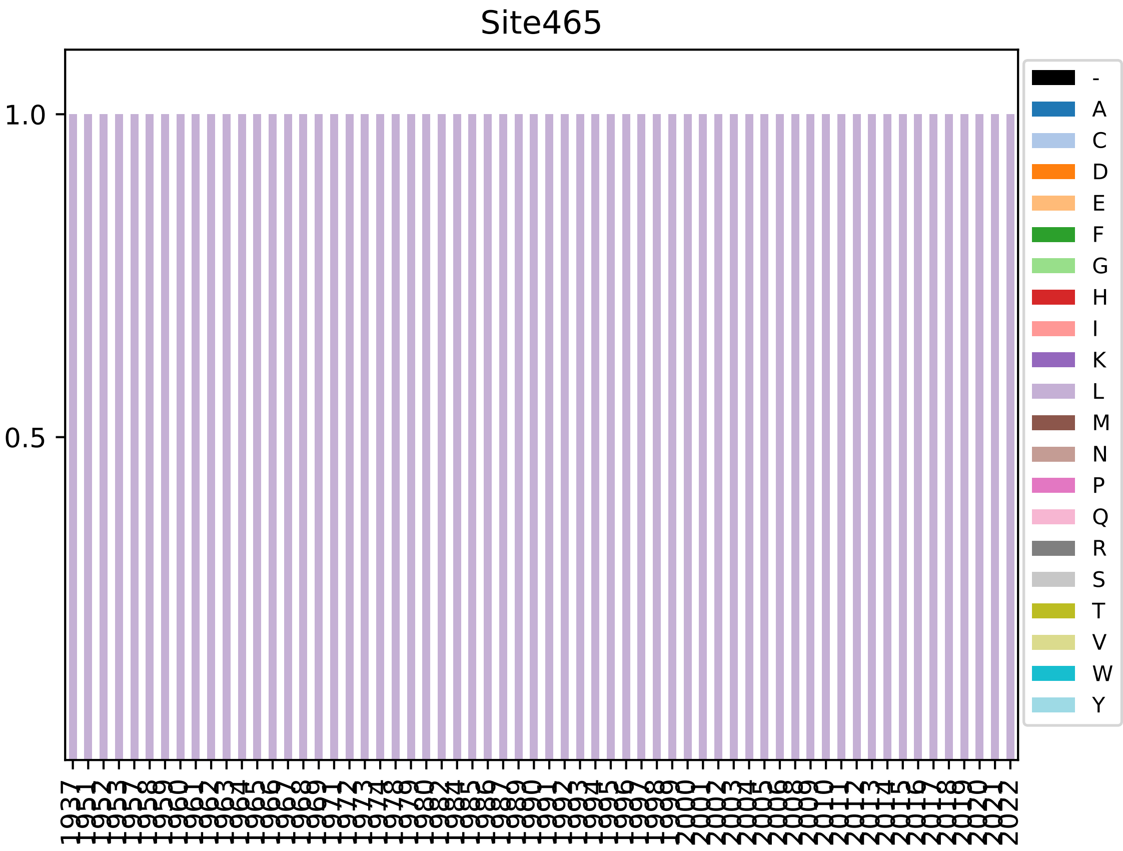
Site465
1.0
0.5
-
A
C
D
E
F
G
H
I
K
L
M
N
P
Q
R
S
T
V
W
Y

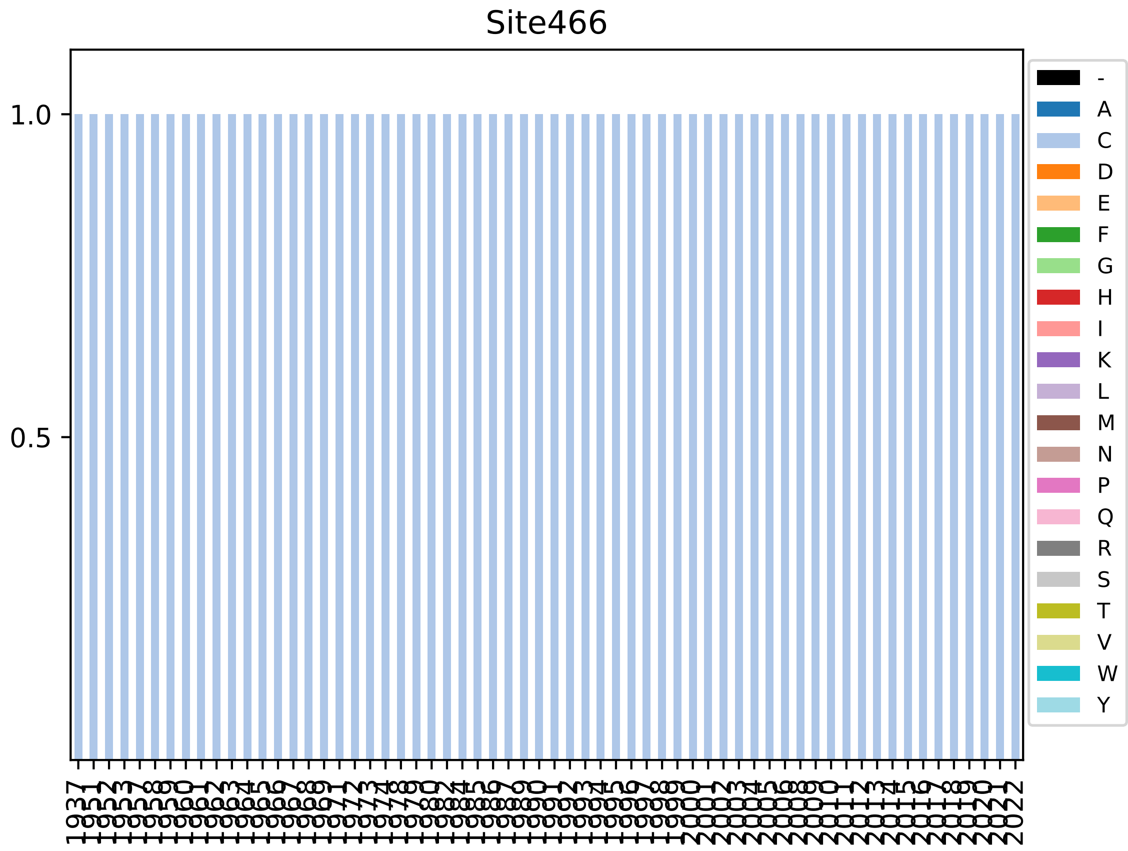
Site466
1.0
0.5
-
A
C
D
E
F
G
H
I
K
L
M
N
P
Q
R
S
T
V
W
Y

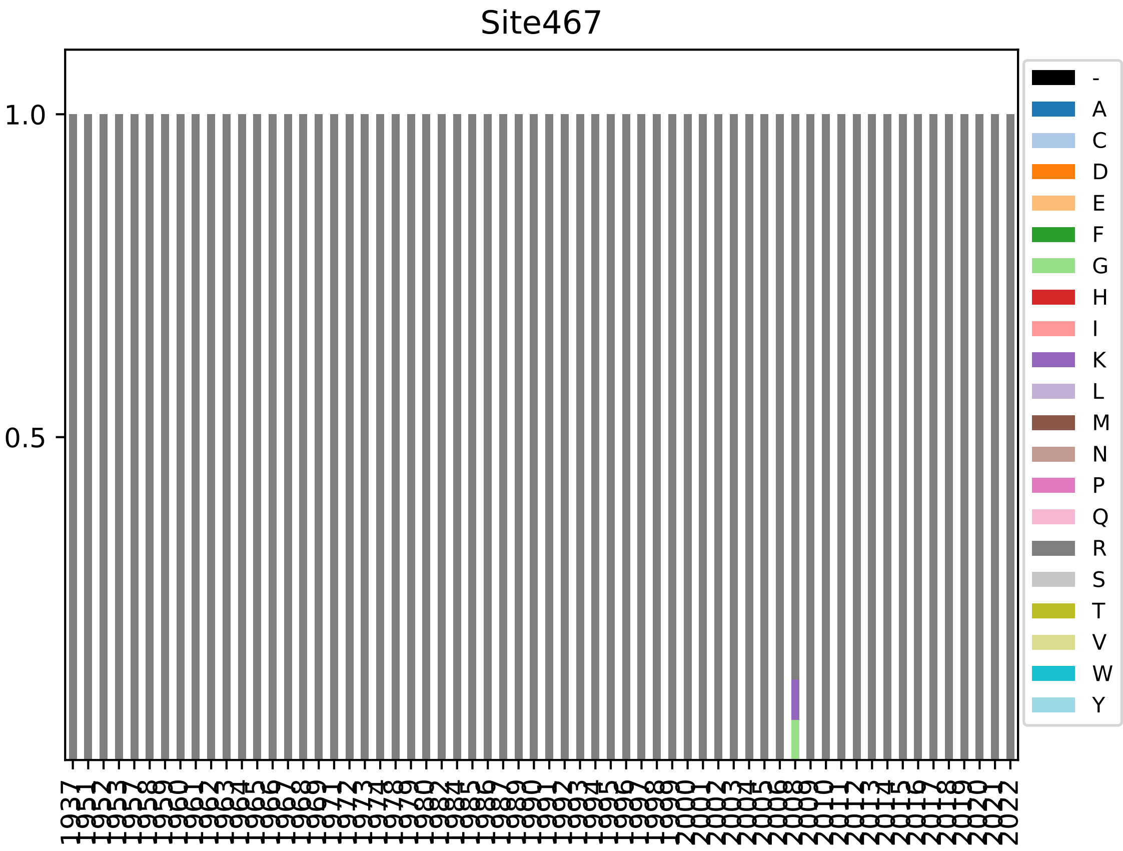
Site467
1.0
0.5
-
A
C
D
E
F
G
H
I
K
L
M
N
P
Q
R
S
T
V
W
Y

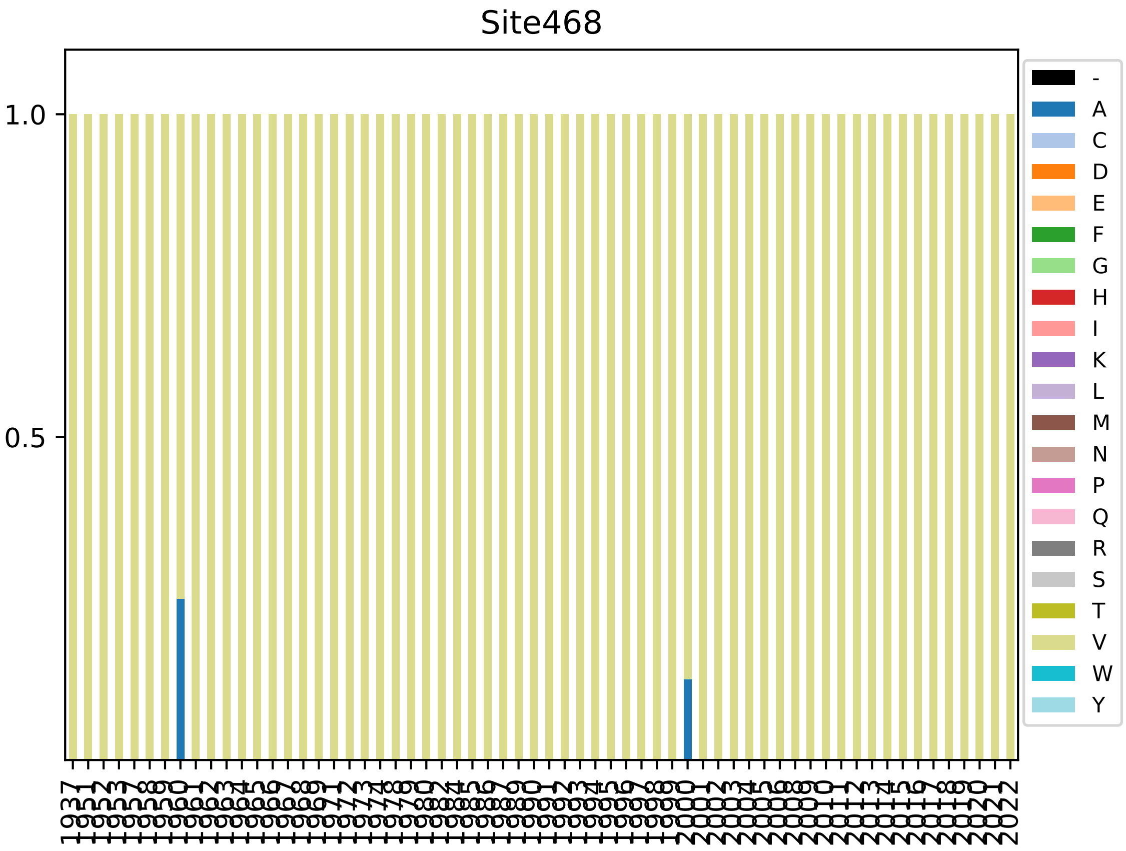
Site468
1.0
0.5
-
A
C
D
E
F
G
H
I
K
L
M
N
P
Q
R
S
T
V
W
Y

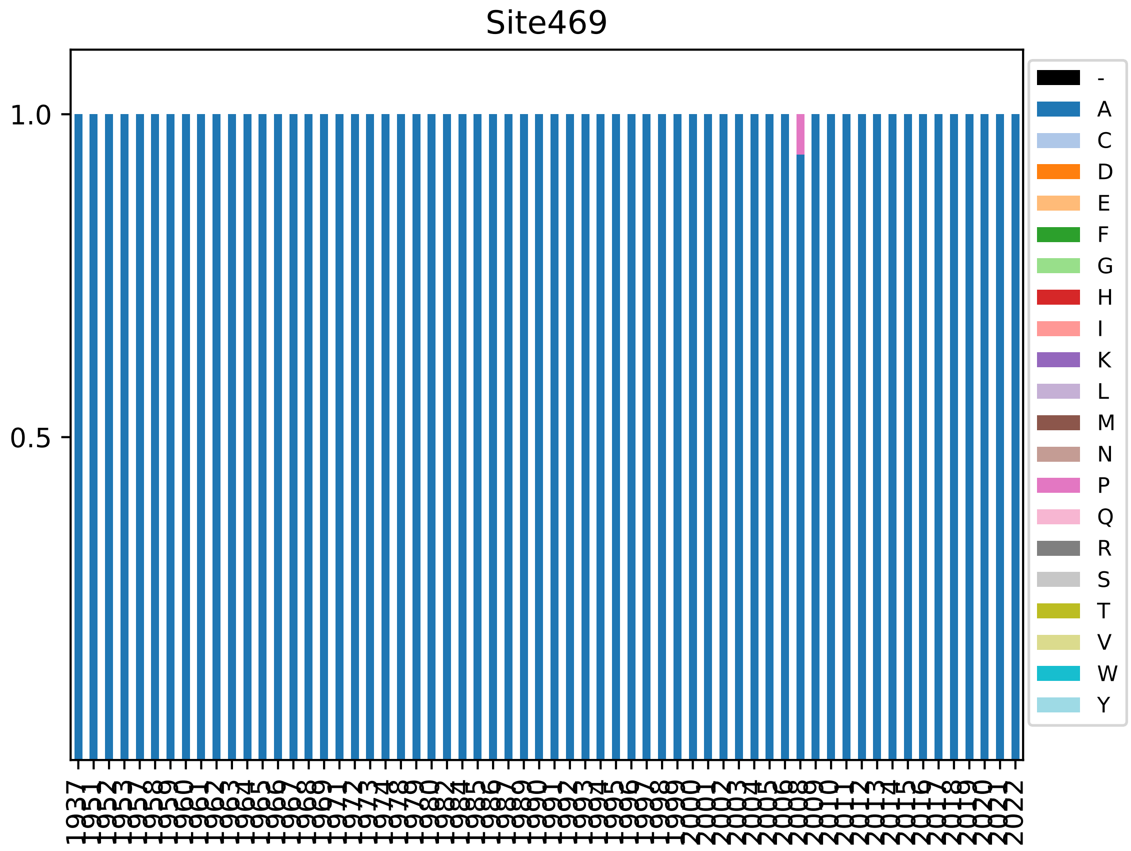
Site469
1.0
0.5
-
A
C
D
E
F
G
H
I
K
L
M
N
P
Q
R
S
T
V
W
Y

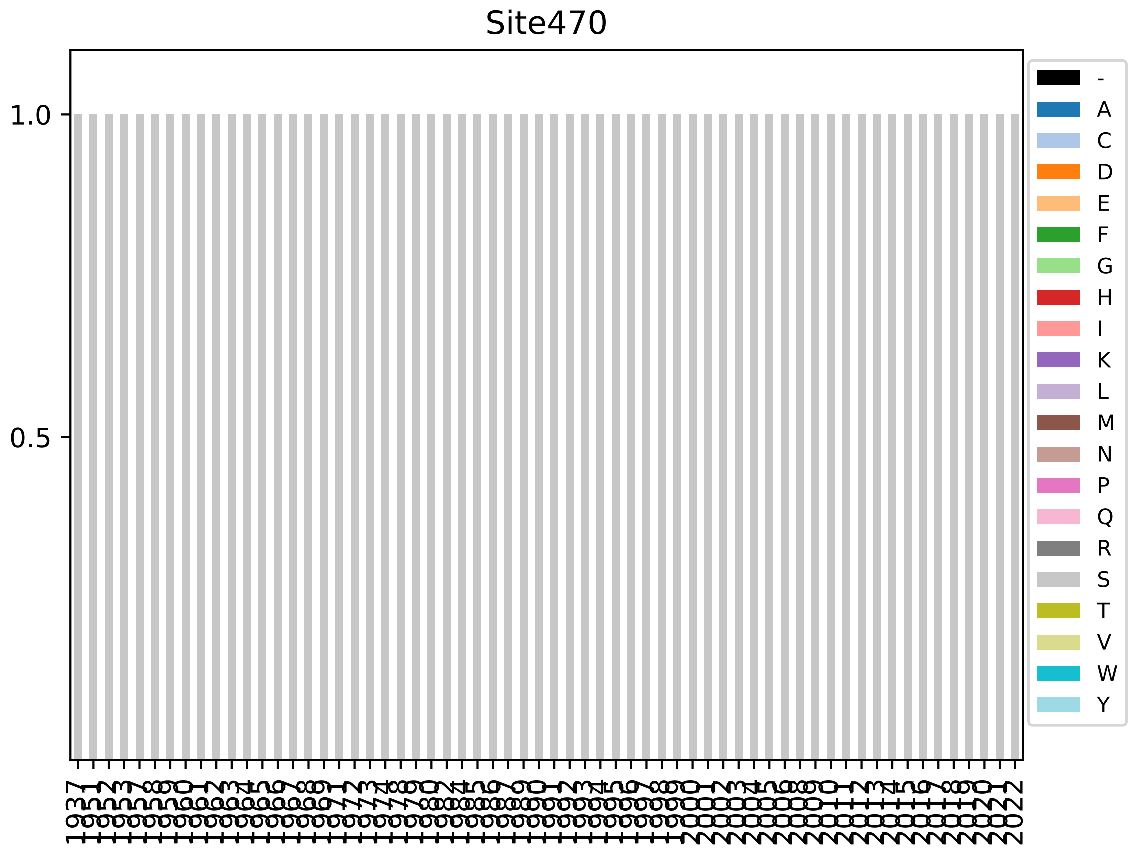
Site470
1.0
0.5
-
A
C
D
E
F
G
H
I
K
L
M
N
P
Q
R
S
T
V
W
Y

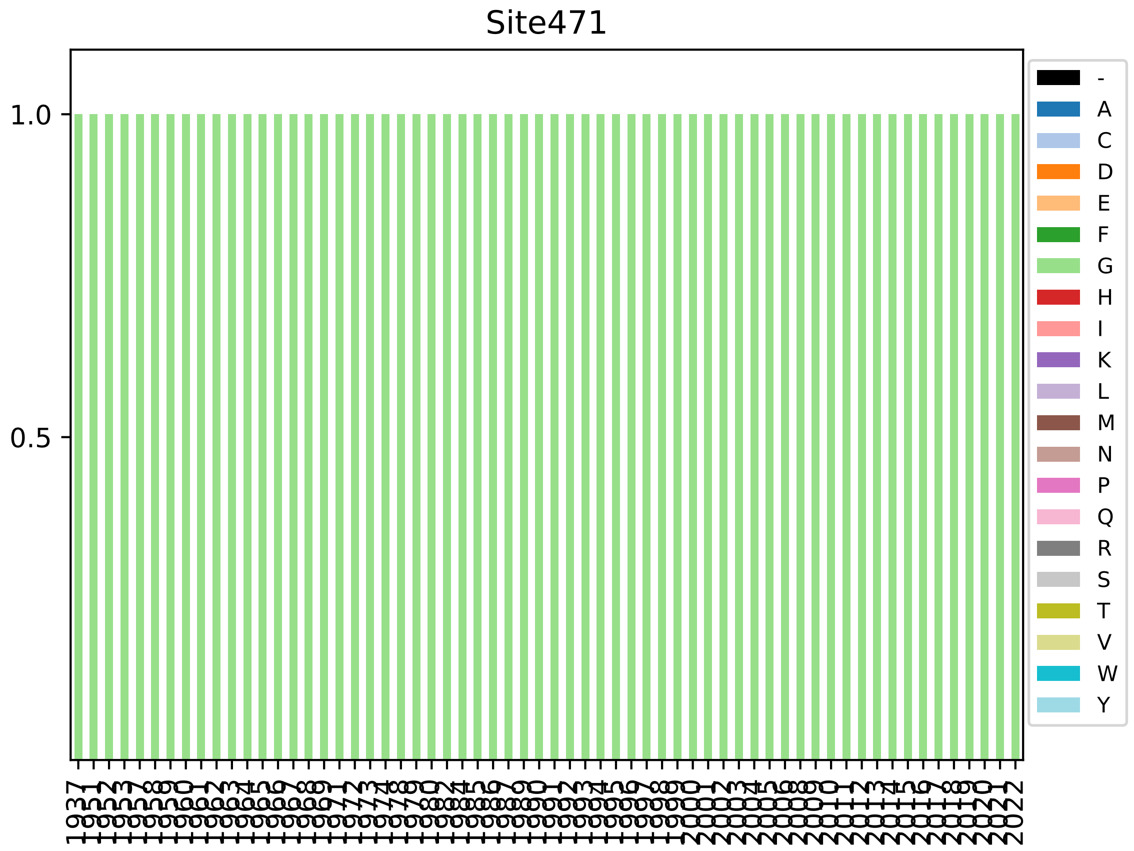
Site471
1.0
0.5
-
A
C
D
E
F
G
H
I
K
L
M
N
P
Q
R
S
T
V
W
Y

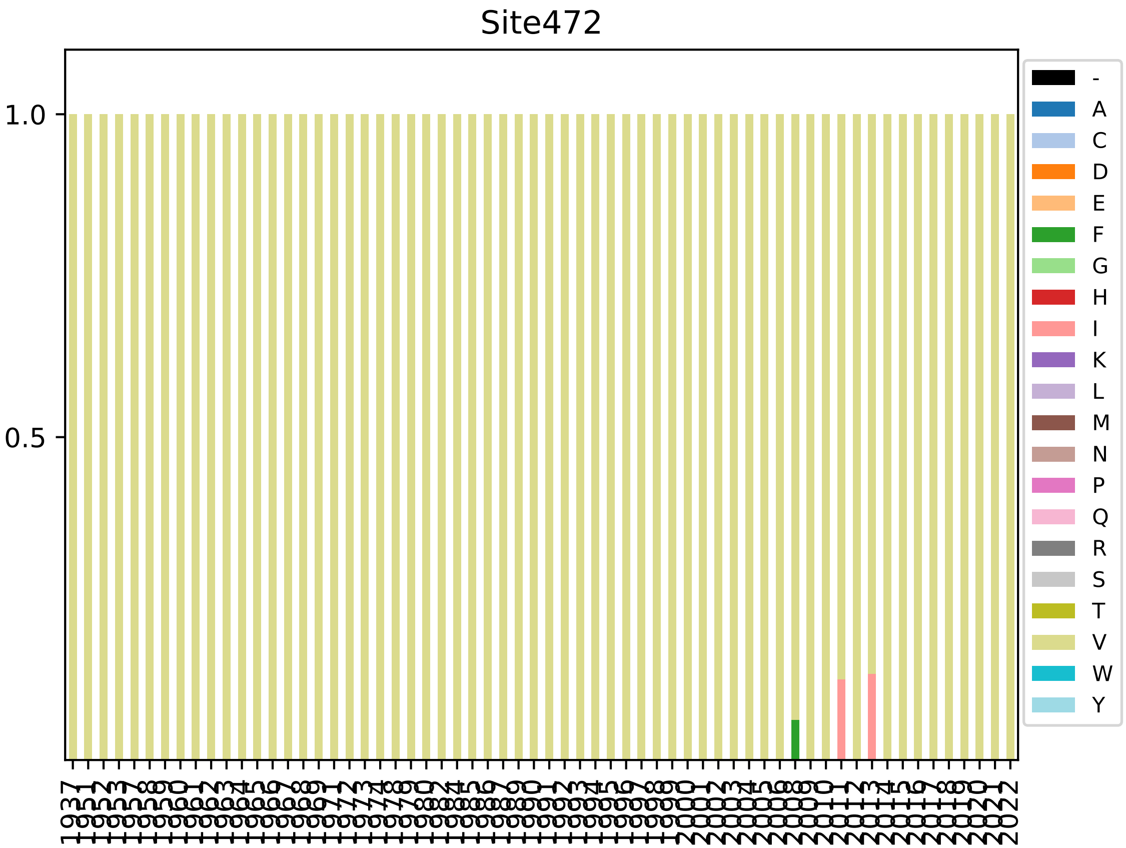
Site472
1.0
0.5
-
A
C
D
E
F
G
H
I
K
L
M
N
P
Q
R
S
T
V
W
Y

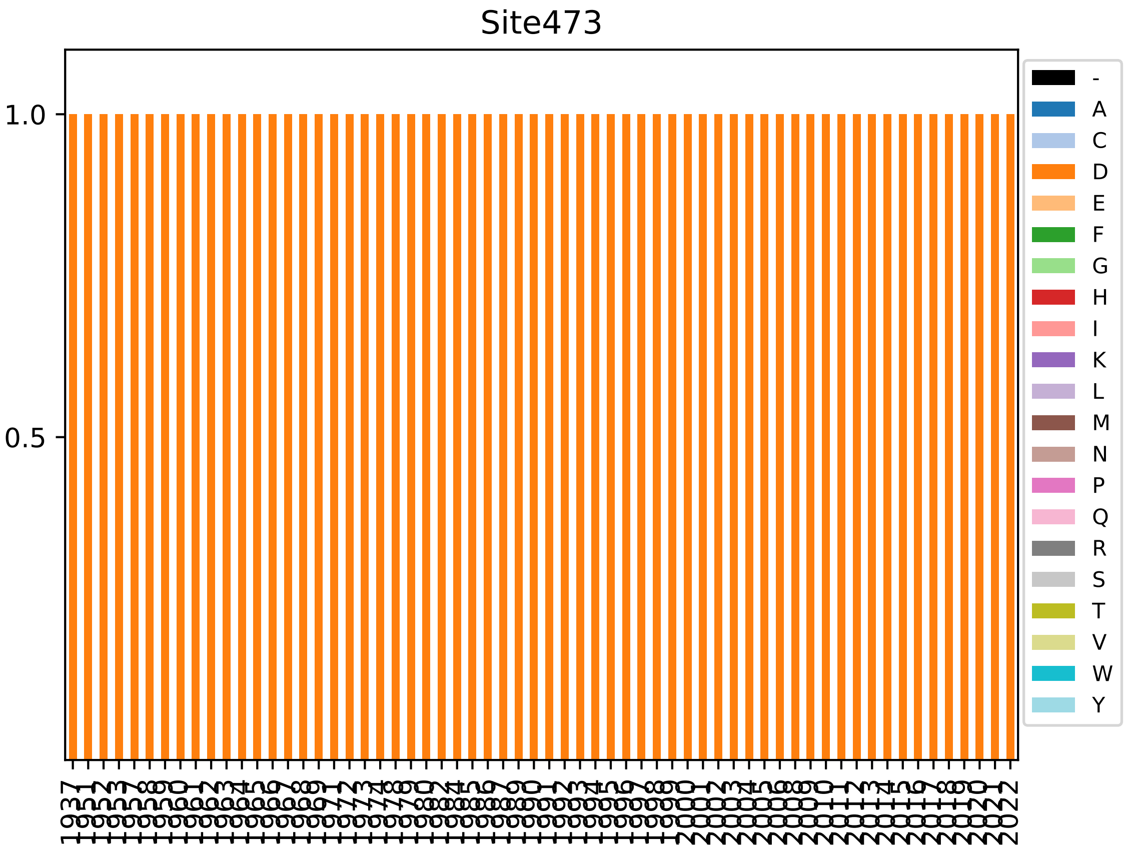
Site473
1.0
0.5
-
A
C
D
E
F
G
H
I
K
L
M
N
P
Q
R
S
T
V
W
Y

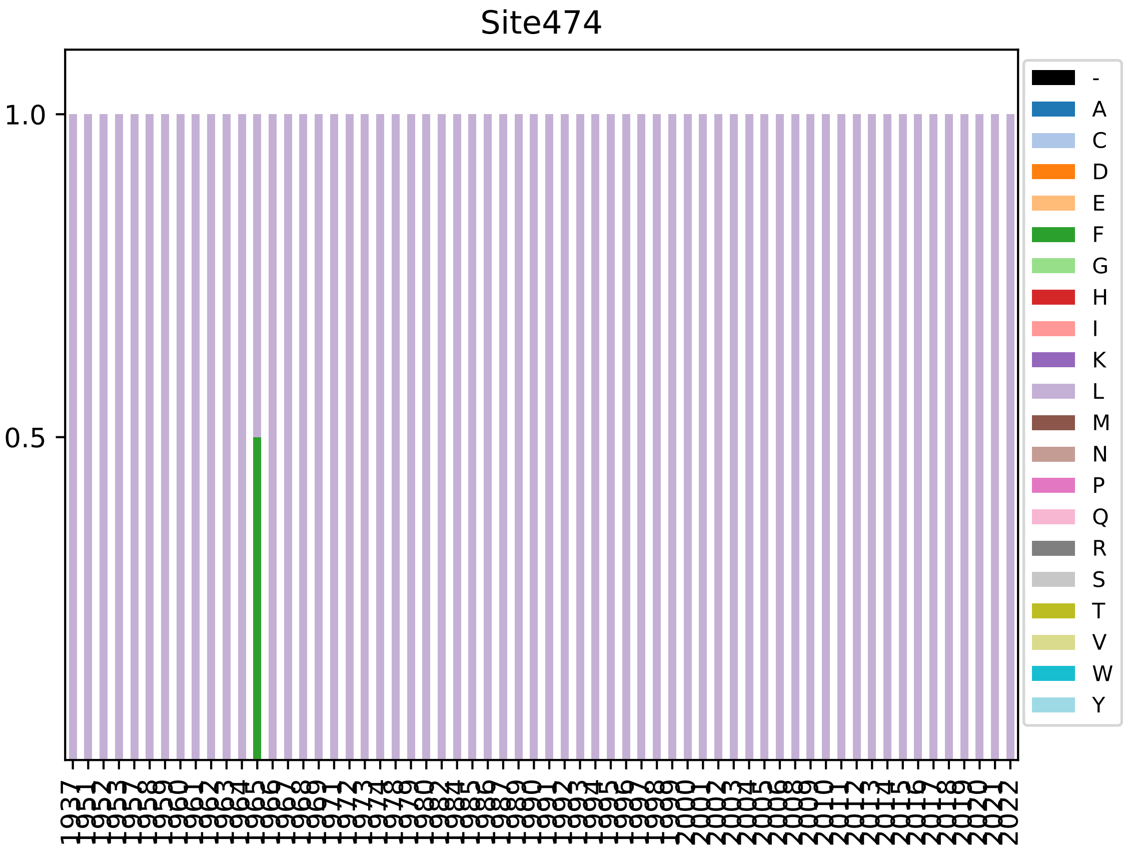
Site474
1.0
0.5
-
A
C
D
E
F
G
H
I
K
L
M
N
P
Q
R
S
T
V
W
Y

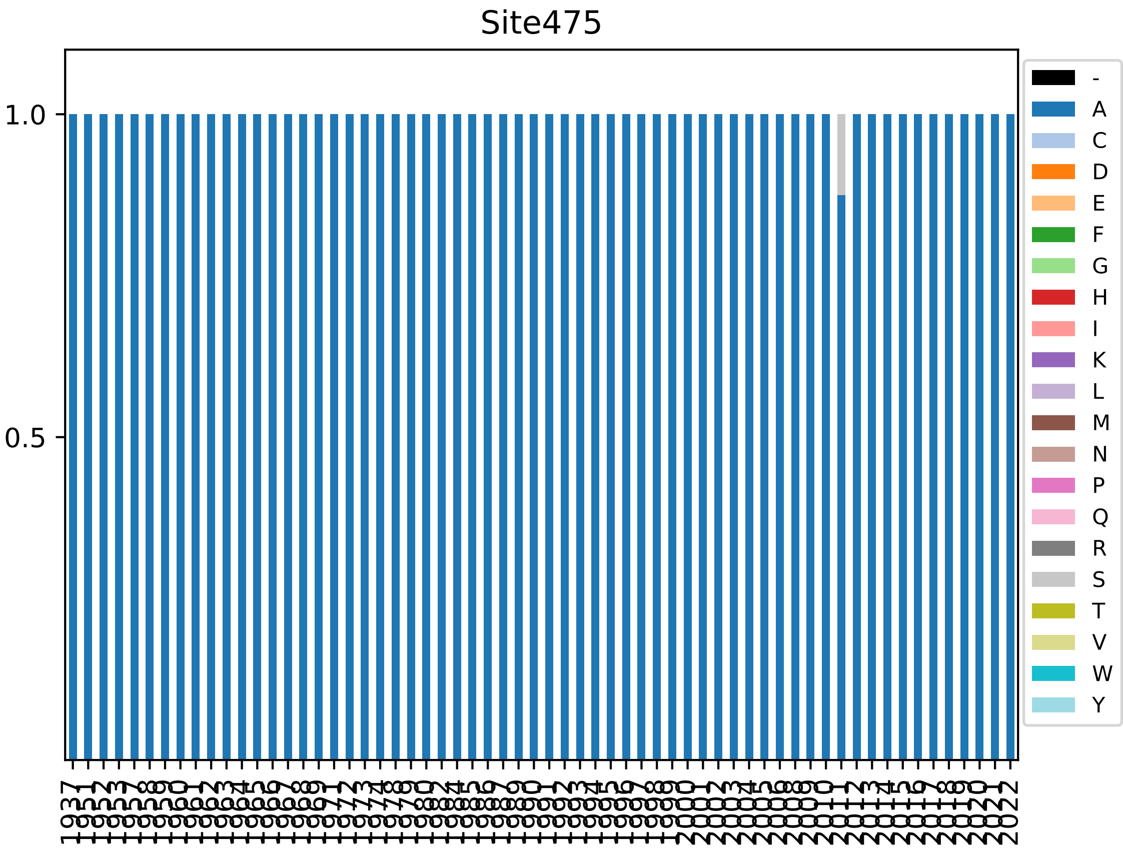
Site475
1.0
0.5
-
A
C
D
E
F
G
H
I
K
L
M
N
P
Q
R
S
T
V
W
Y

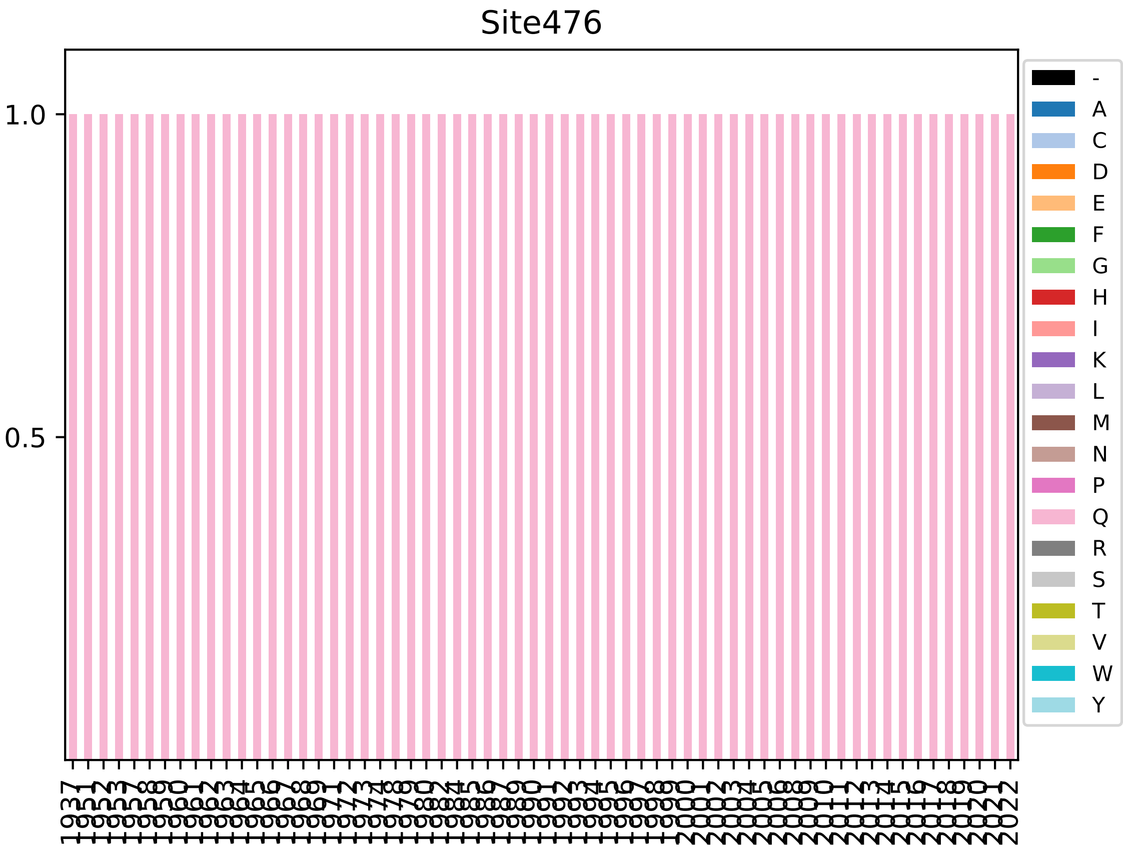
Site476
1.0
0.5
-
A
C
D
E
F
G
H
I
K
L
M
N
P
Q
R
S
T
V
W
Y

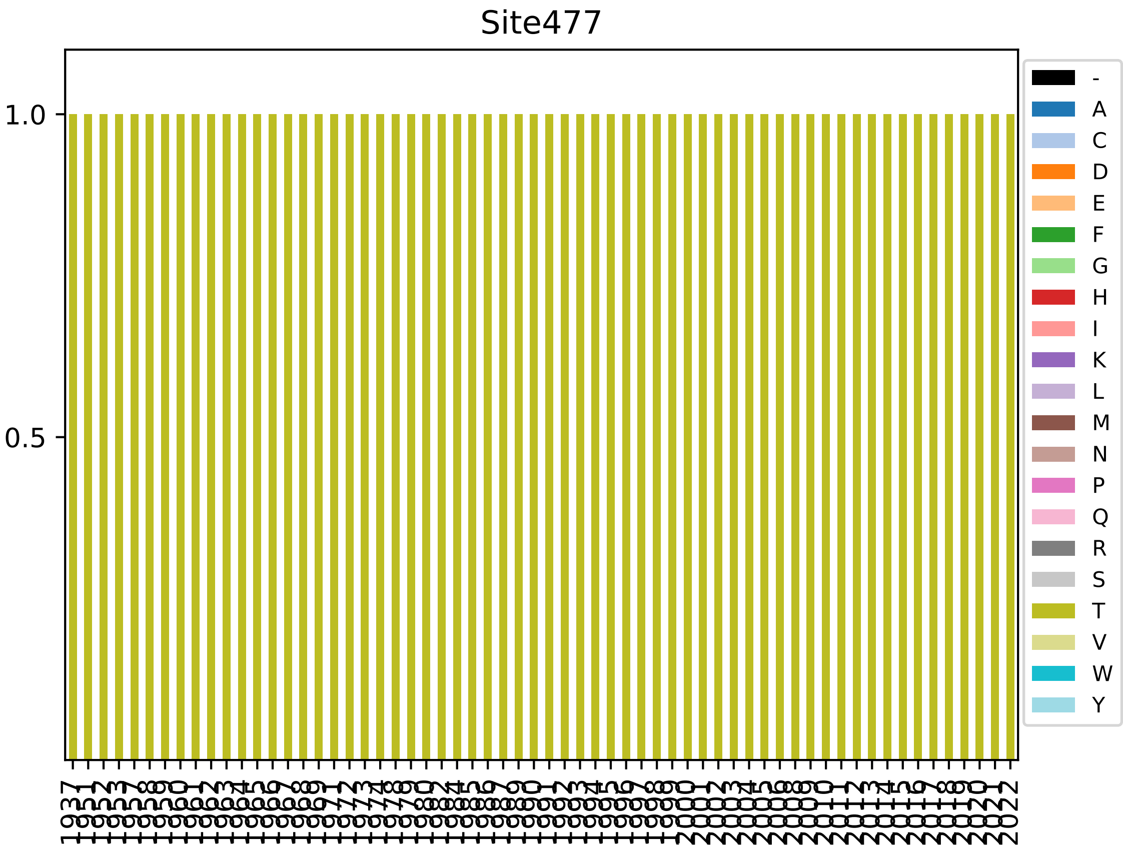
Site477
1.0
0.5
-
A
C
D
E
F
G
H
I
K
L
M
N
P
Q
R
S
T
V
W
Y

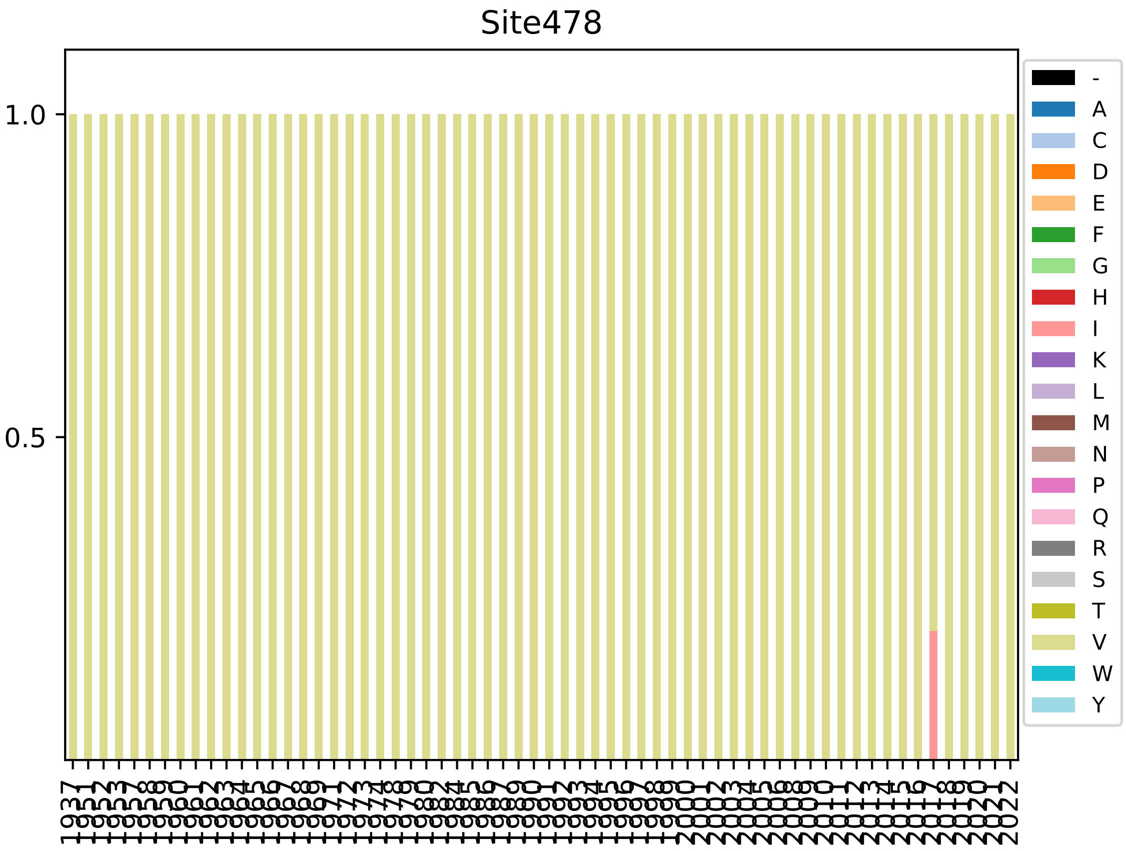

Site478
1.0
0.5
-
A
C
D
E
F
G
H
I
K
L
M
N
P
Q
R
S
T
V
W
Y

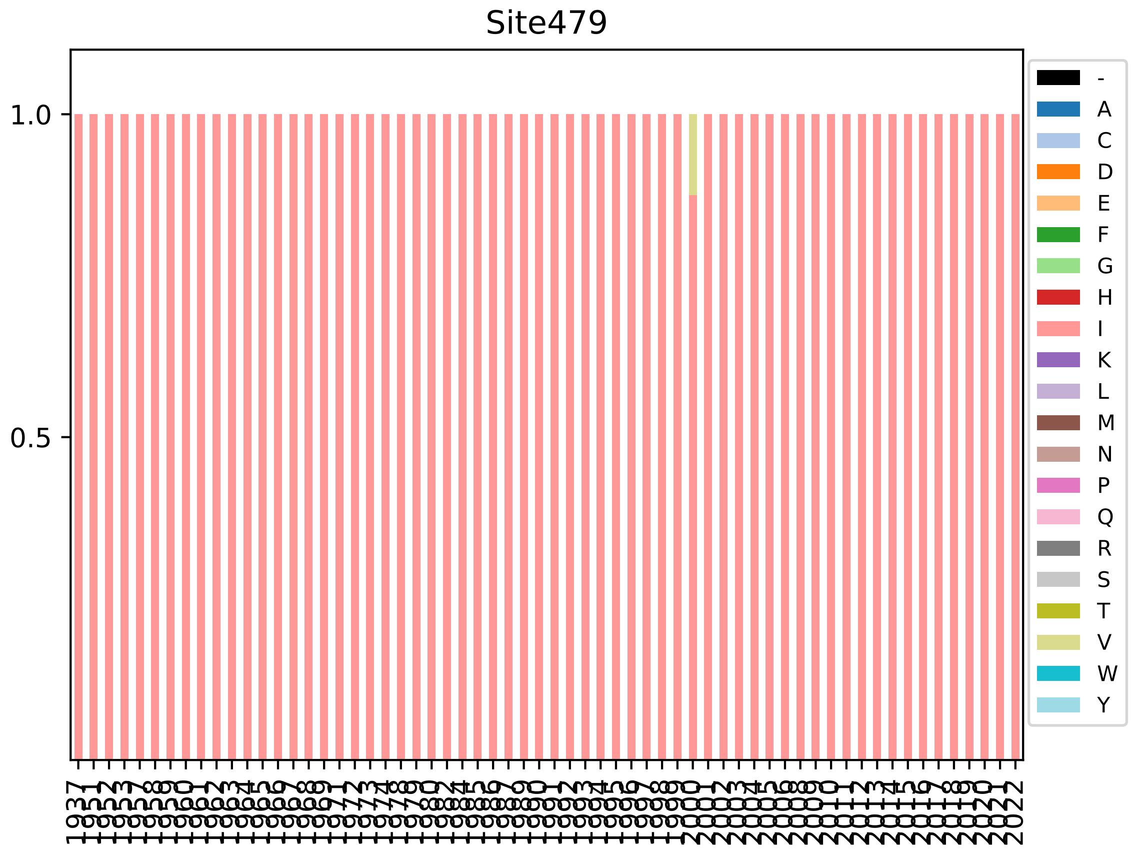
Site479
1.0
0.5
-
A
C
D
E
F
G
H
I
K
L
M
N
P
Q
R
S
T
V
W
Y

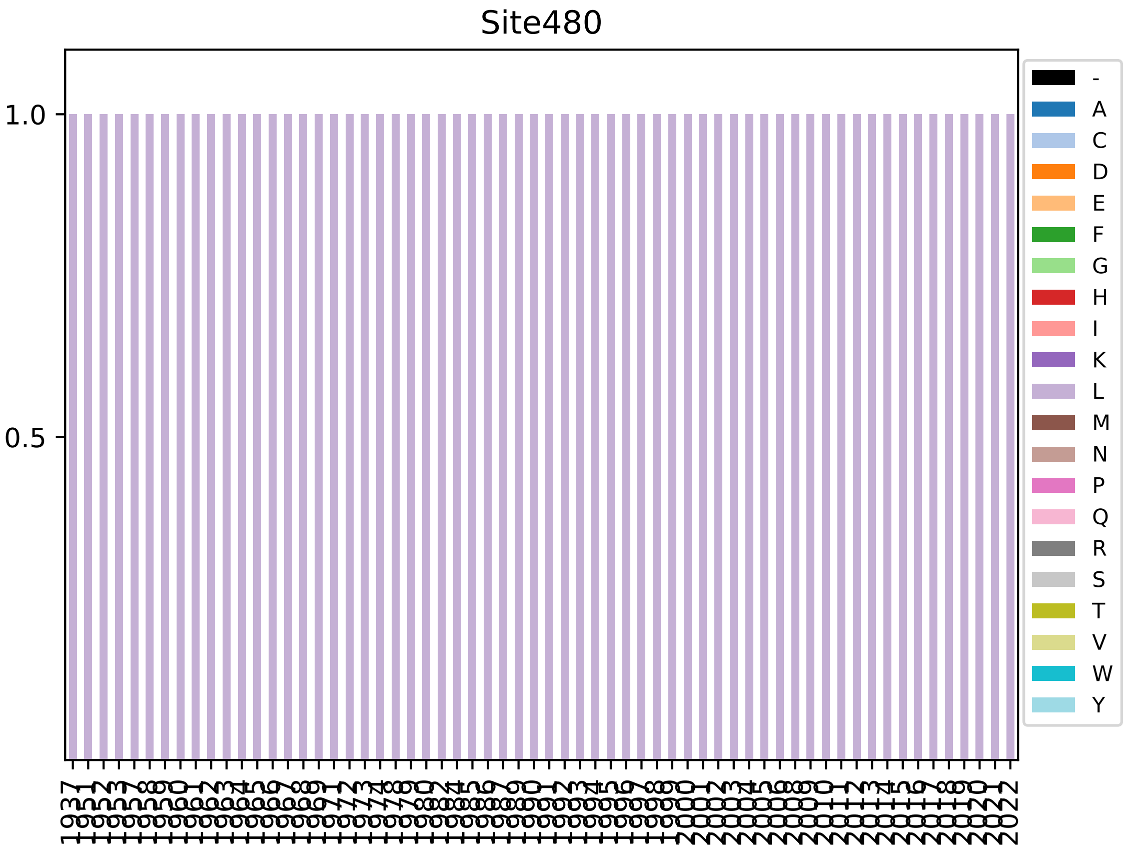
Site480
1.0
0.5
-
A
C
D
E
F
G
H
I
K
L
M
N
P
Q
R
S
T
V
W
Y

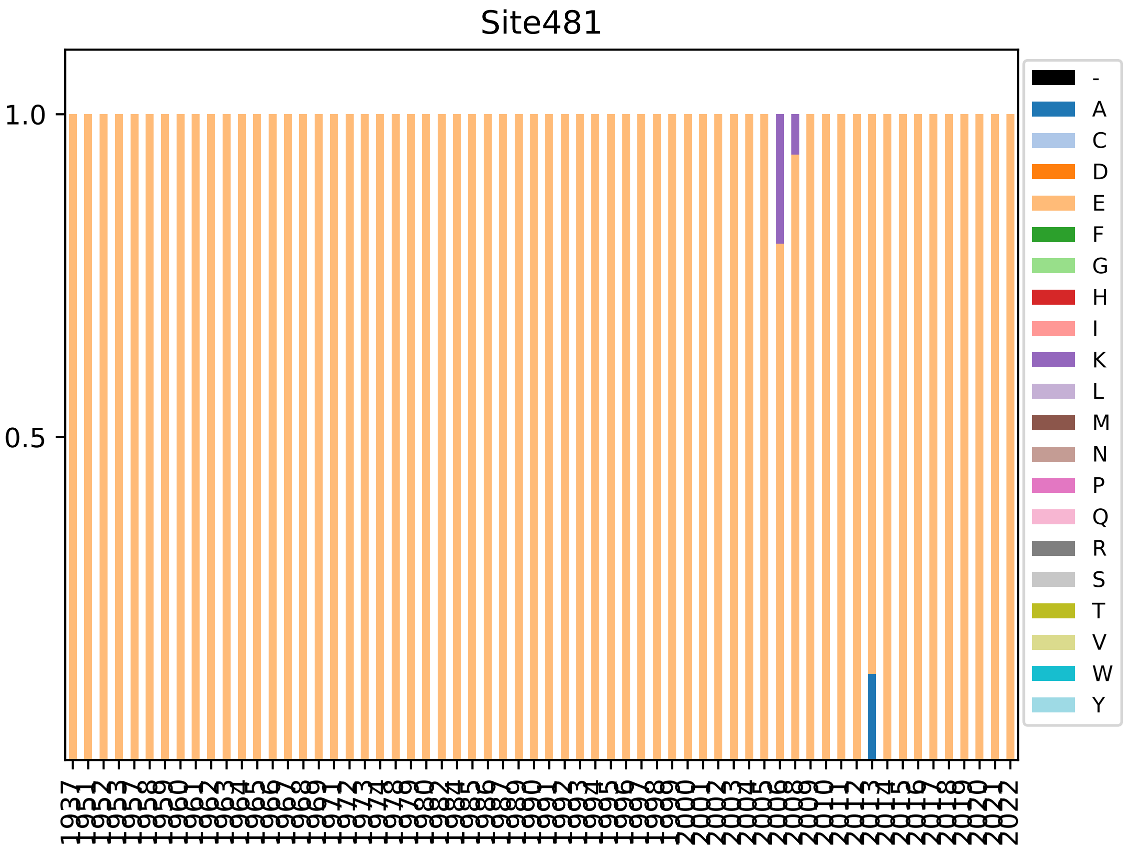
Site481
1.0
0.5
-
A
C
D
E
F
G
H
I
K
L
M
N
P
Q
R
S
T
V
W
Y

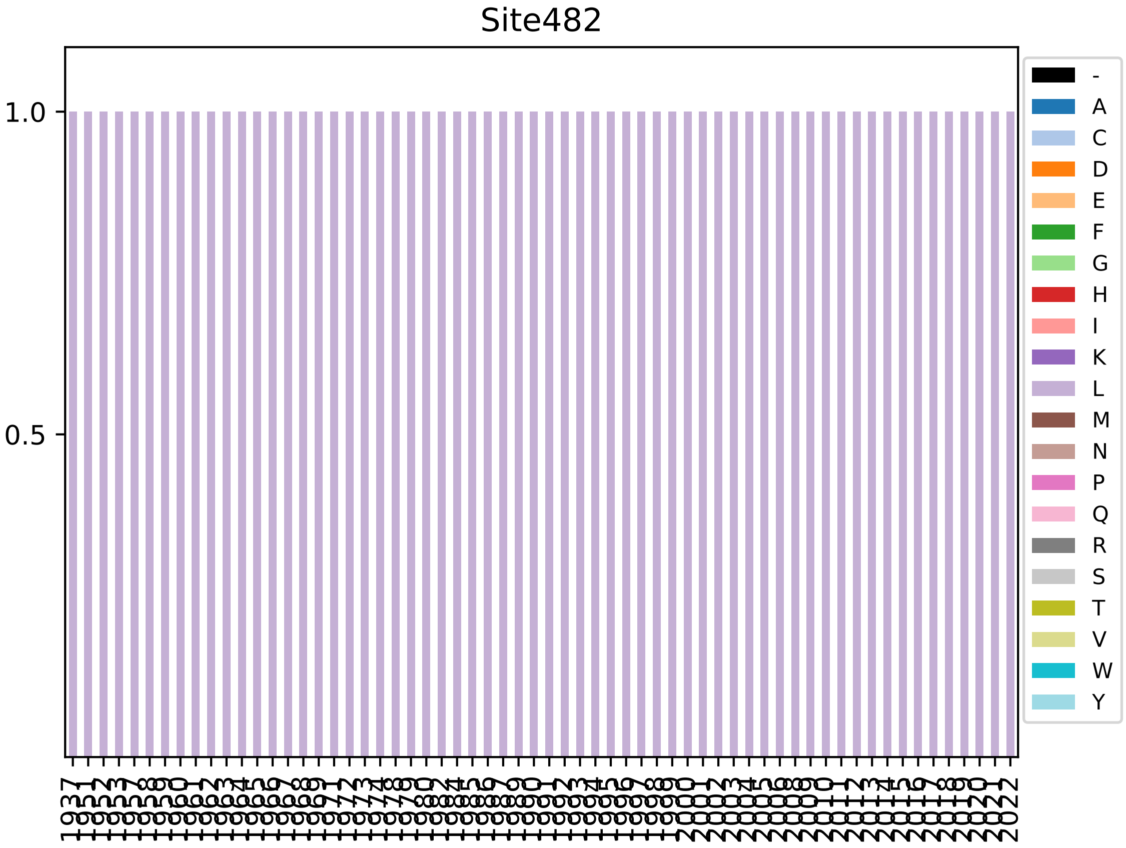
Site482
1.0
0.5
-
A
C
D
E
F
G
H
I
K
L
M
N
P
Q
R
S
T
V
W
Y

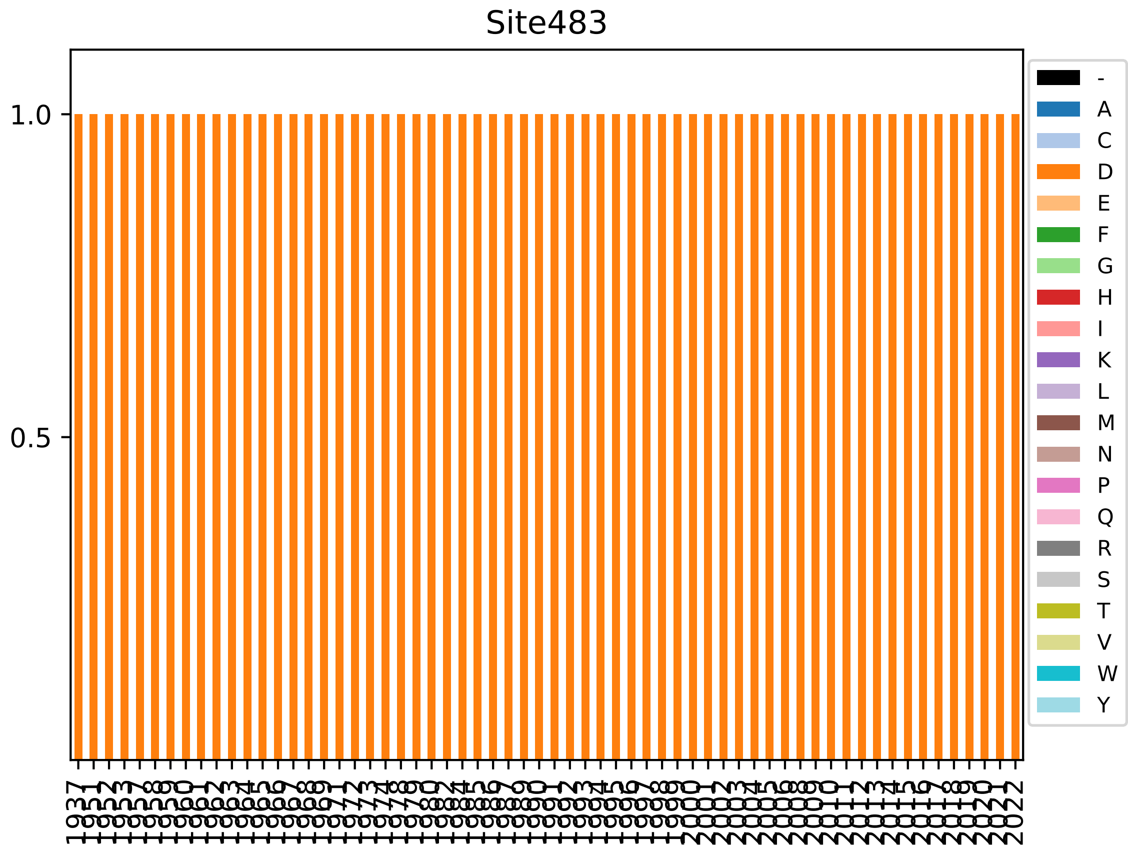
Site483
1.0
0.5
-
A
C
D
E
F
G
H
I
K
L
M
N
P
Q
R
S
T
V
W
Y

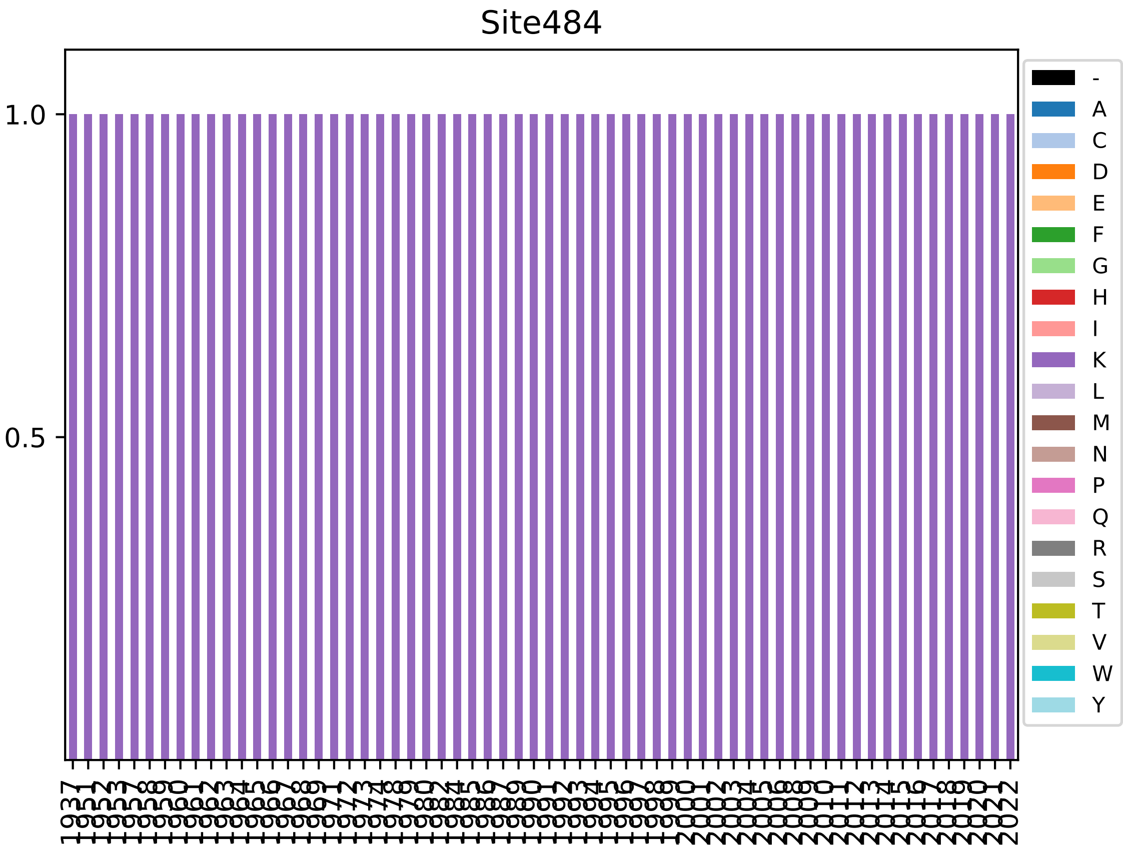
Site484
1.0
0.5
-
A
C
D
E
F
G
H
I
K
L
M
N
P
Q
R
S
T
V
W
Y

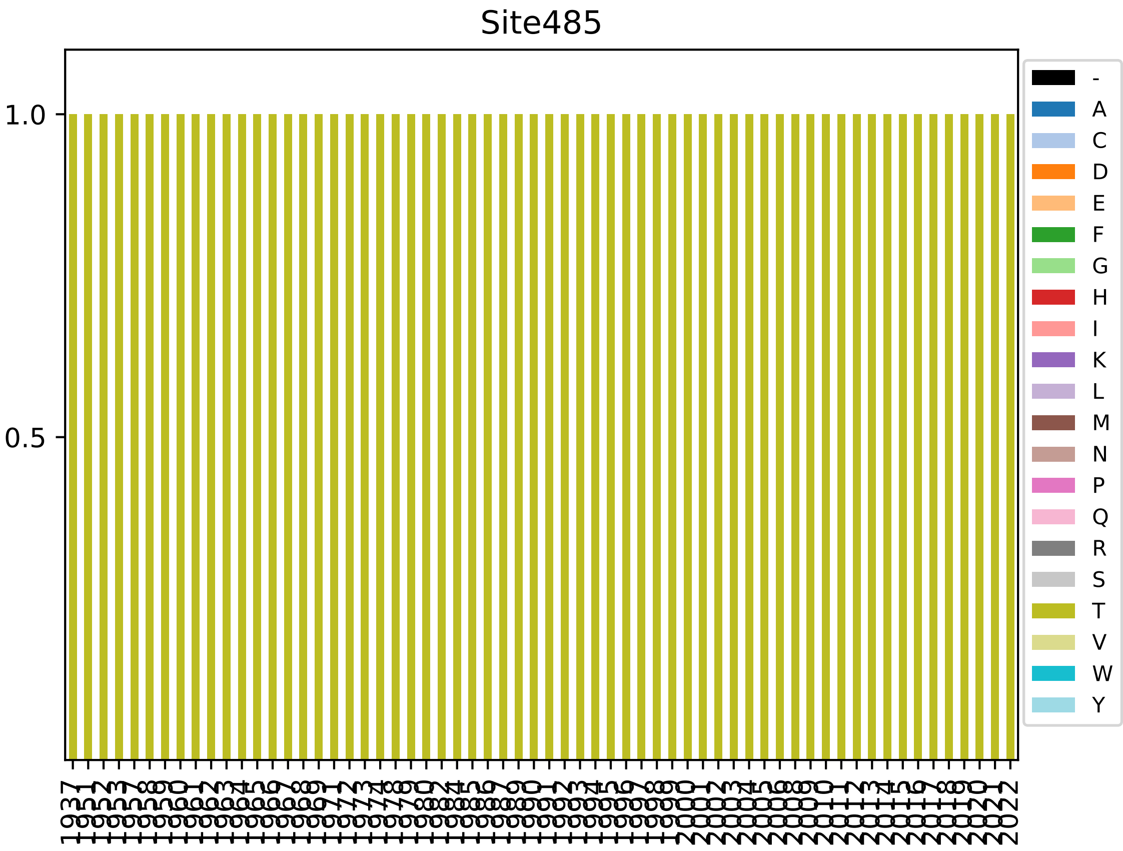
Site485
1.0
0.5
-
A
C
D
E
F
G
H
I
K
L
M
N
P
Q
R
S
T
V
W
Y

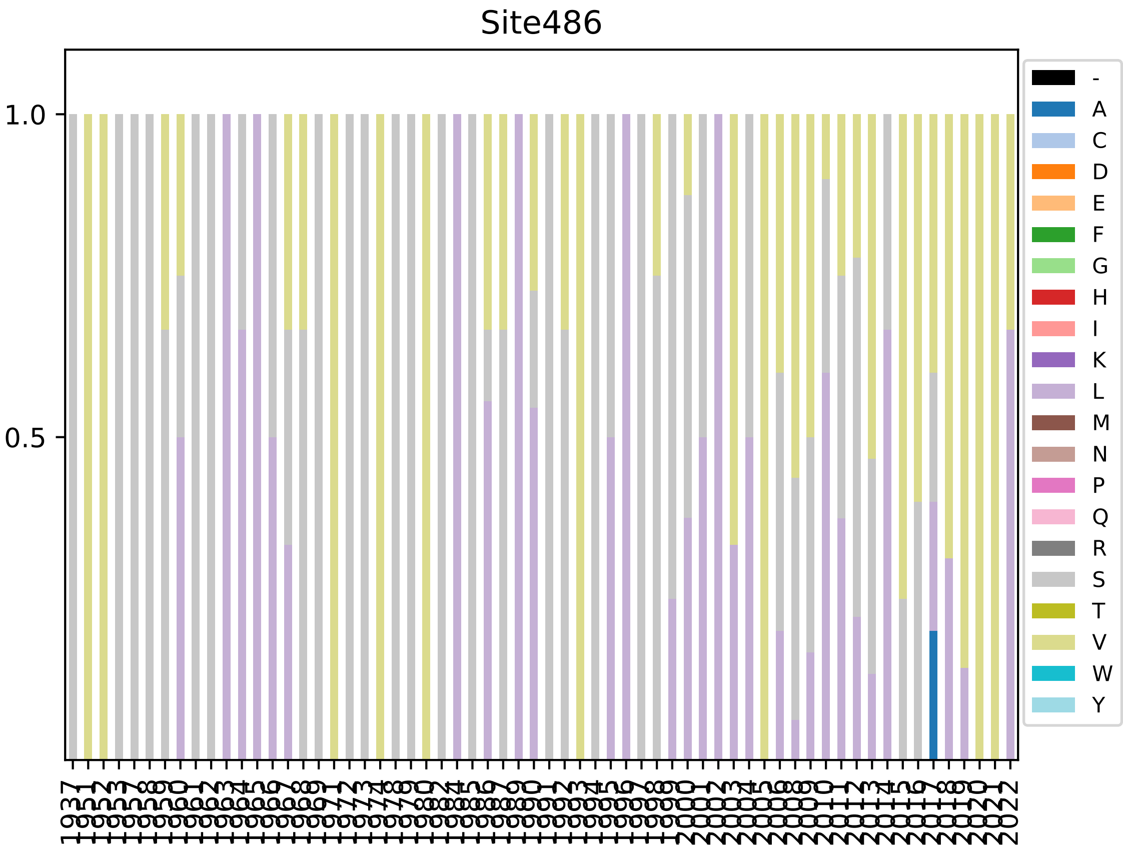
Site486
1.0
0.5
-
A
C
D
E
F
G
H
I
K
L
M
N
P
Q
R
S
T
V
W
Y

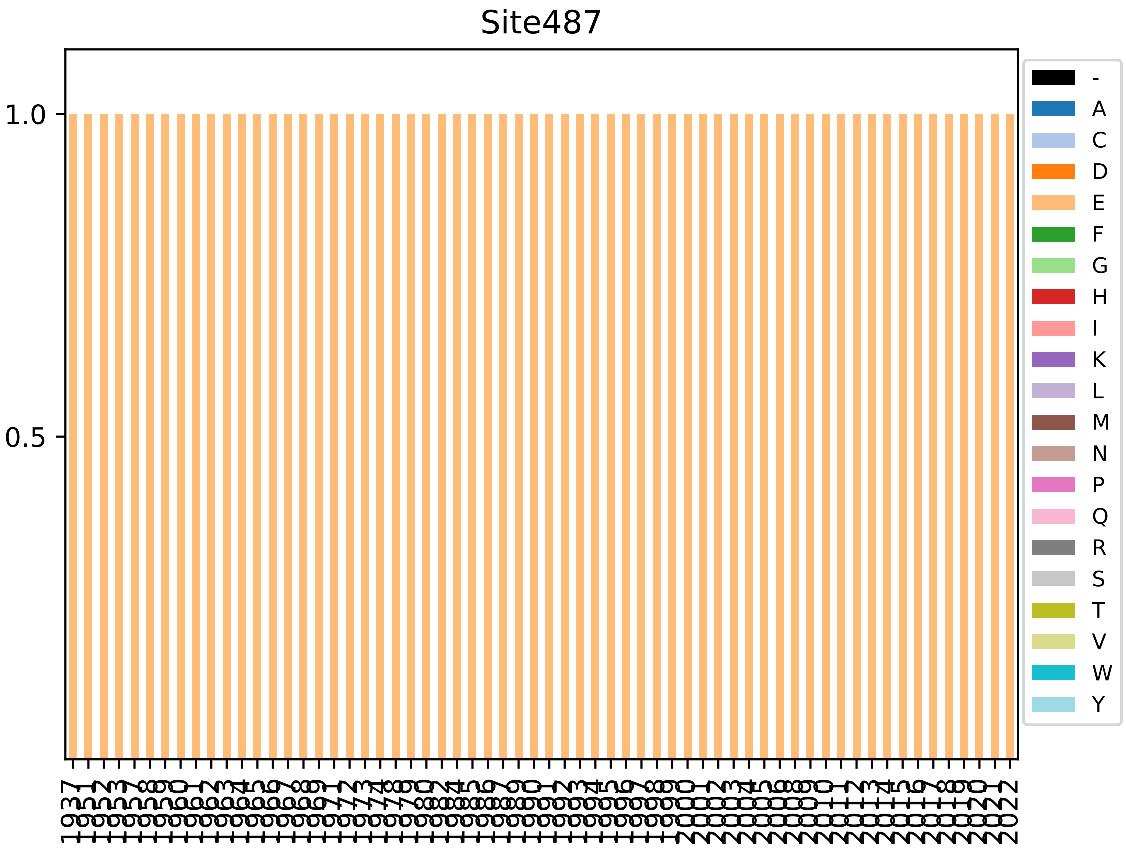
Site487
1.0
0.5
-
A
C
D
E
F
G
H
I
K
L
M
N
P
Q
R
S
T
V
W
Y

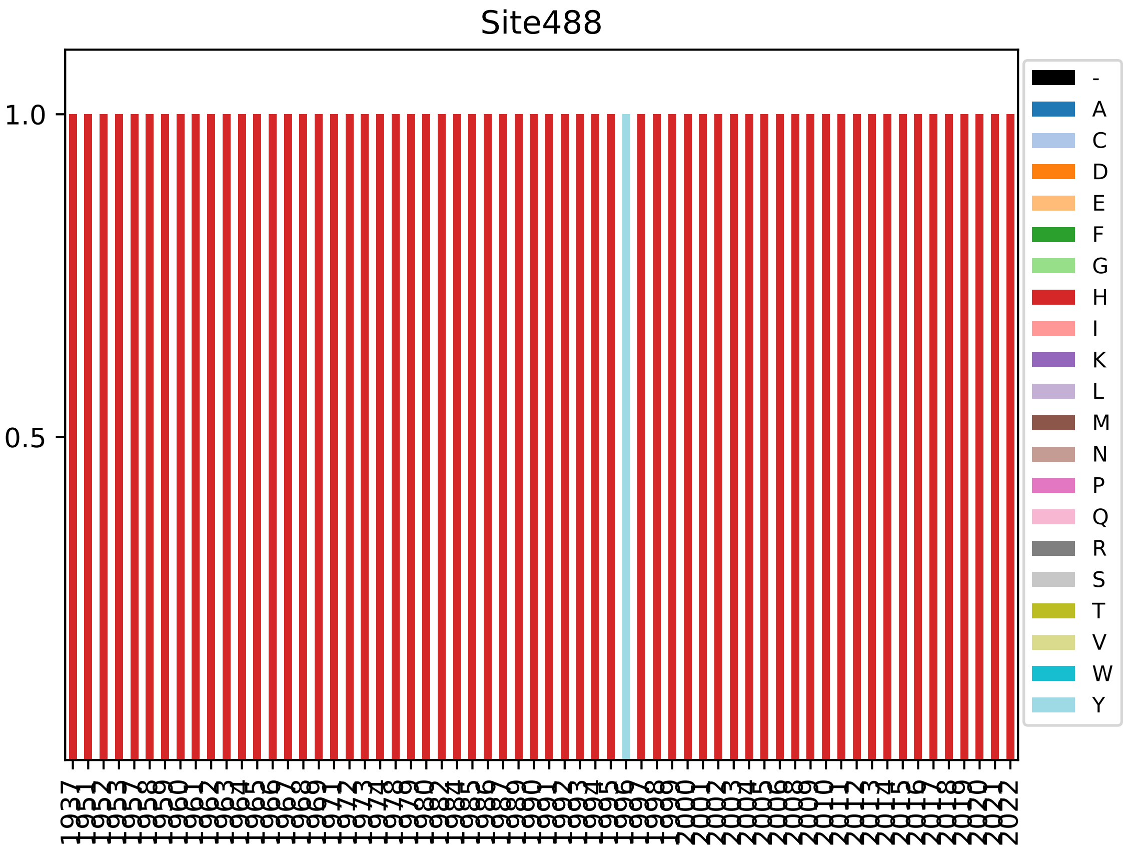
Site488
1.0
0.5
-
A
C
D
E
F
G
H
I
K
L
M
N
P
Q
R
S
T
V
W
Y

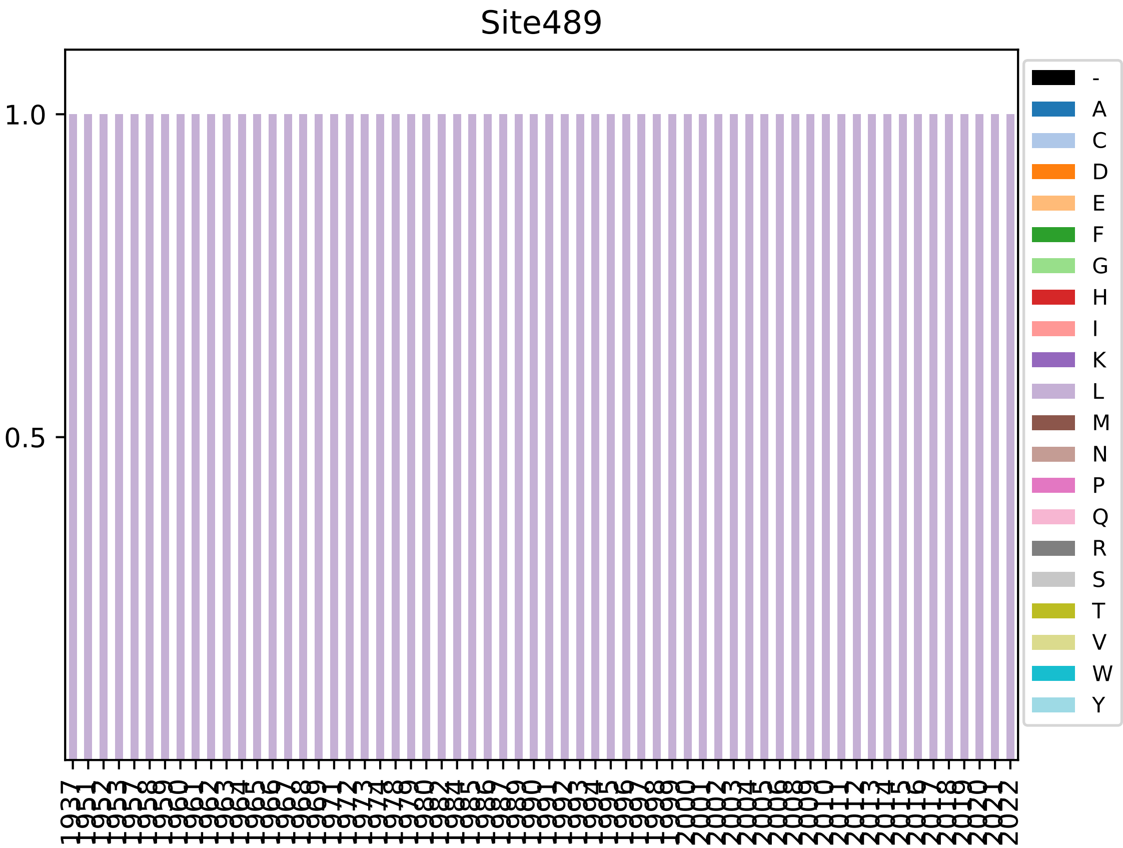
Site489
1.0
0.5
-
A
C
D
E
F
G
H
I
K
L
M
N
P
Q
R
S
T
V
W
Y

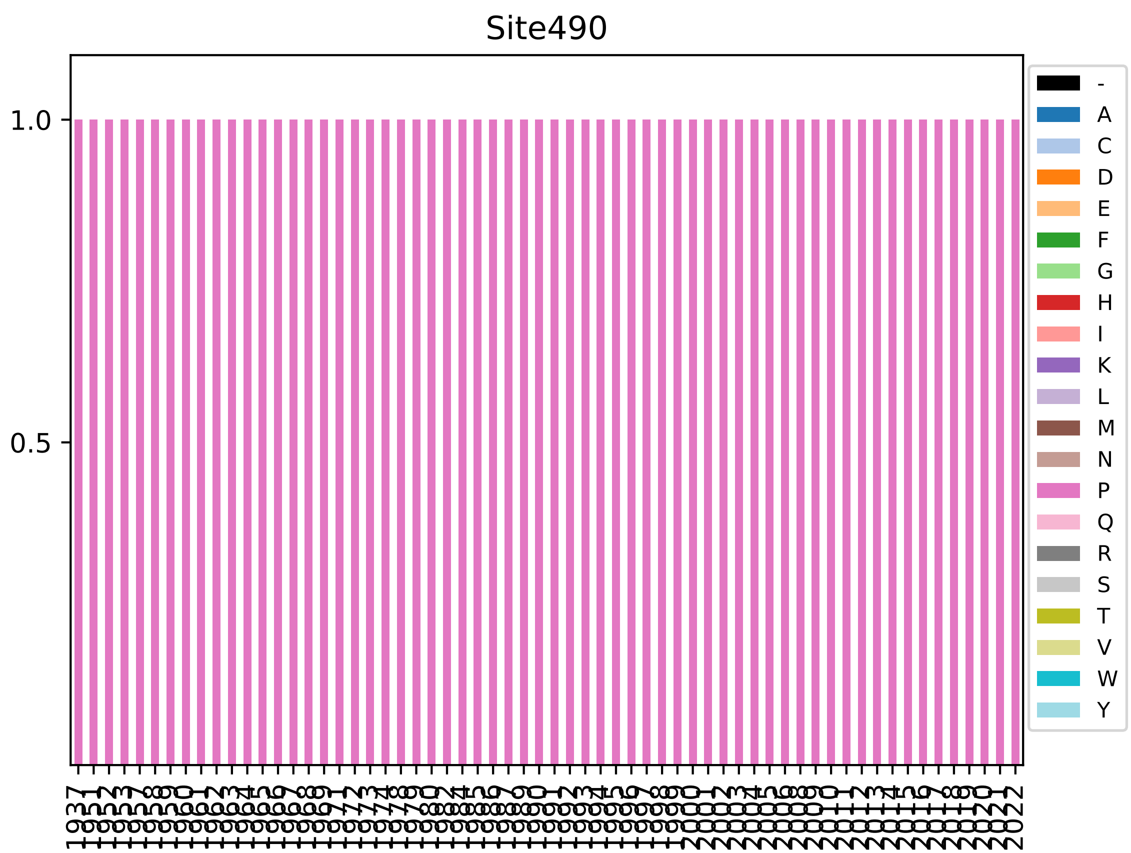
Site490
1.0
0.5
-
A
C
D
E
F
G
H
I
K
L
M
N
P
Q
R
S
T
V
W
Y

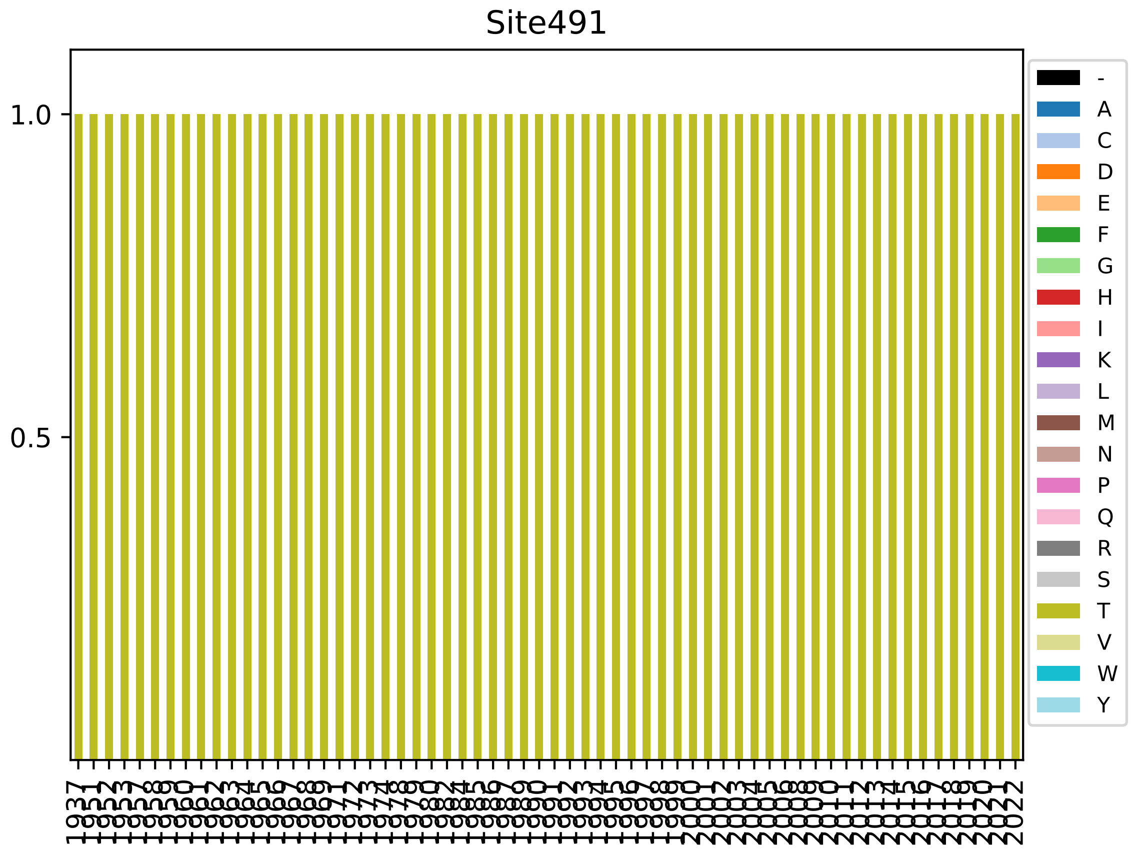
Site491
1.0
0.5
-
A
C
D
E
F
G
H
I
K
L
M
N
P
Q
R
S
T
V
W
Y

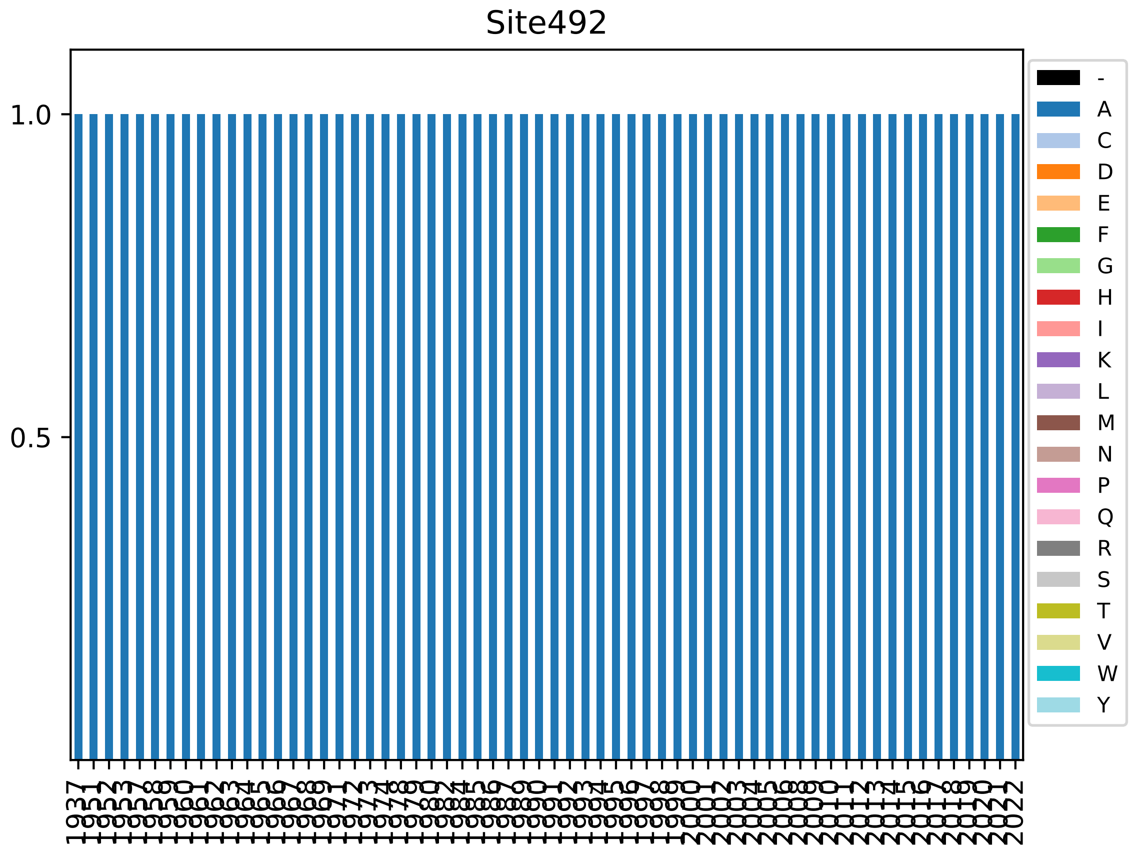
Site492
1.0
0.5
-
A
C
D
E
F
G
H
I
K
L
M
N
P
Q
R
S
T
V
W
Y

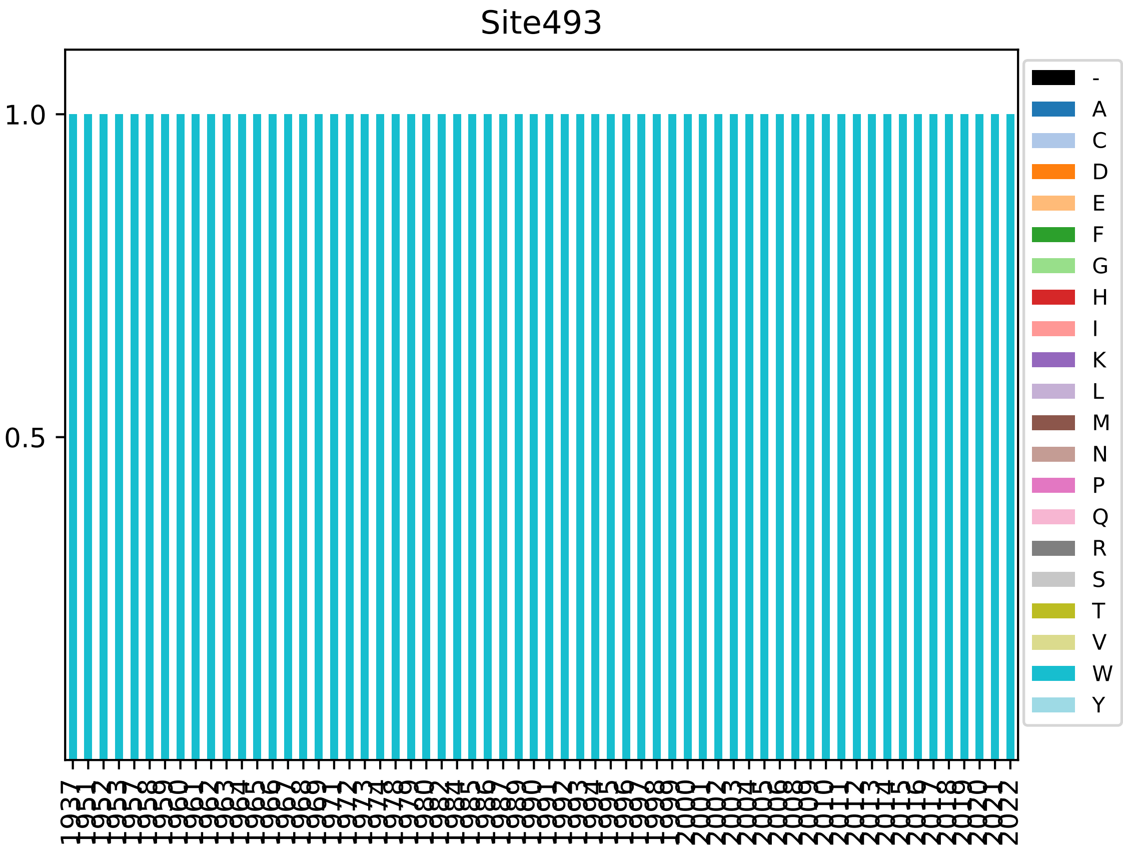
Site493
1.0
0.5
-
A
C
D
E
F
G
H
I
K
L
M
N
P
Q
R
S
T
V
W
Y

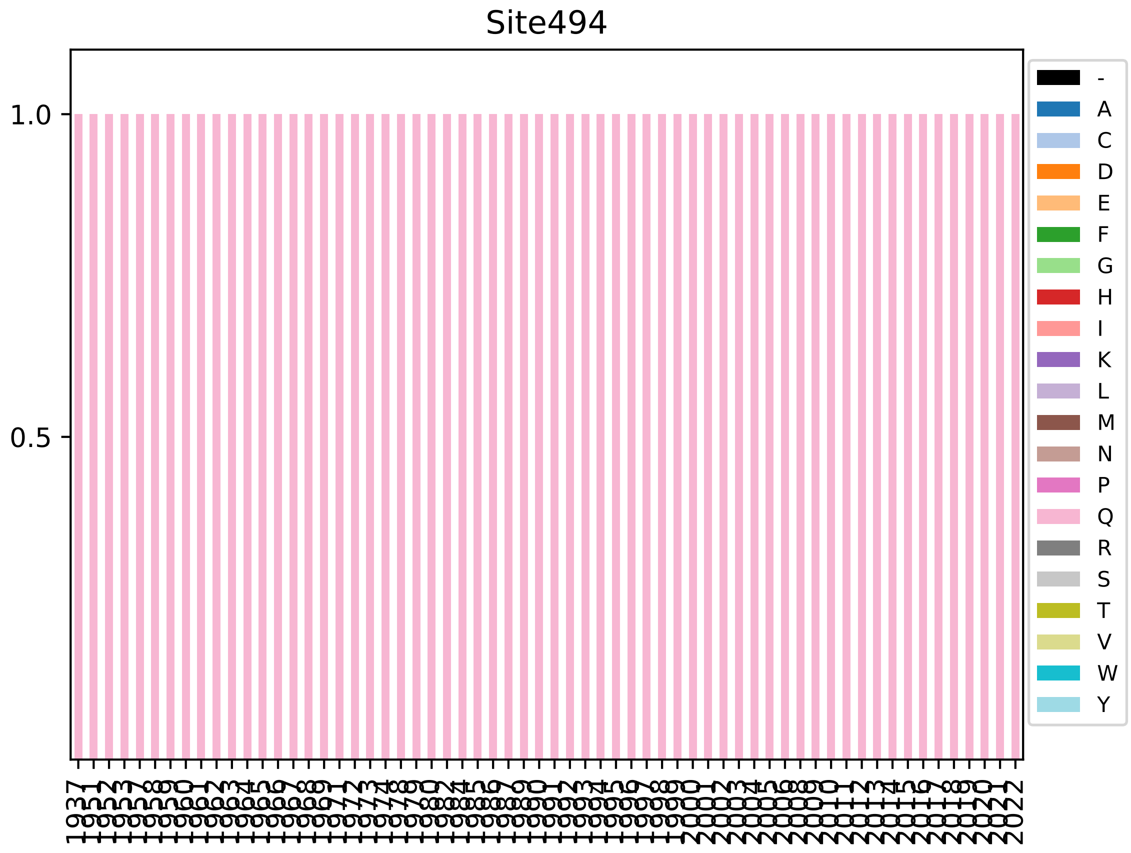
Site494
1.0
0.5
-
A
C
D
E
F
G
H
I
K
L
M
N
P
Q
R
S
T
V
W
Y

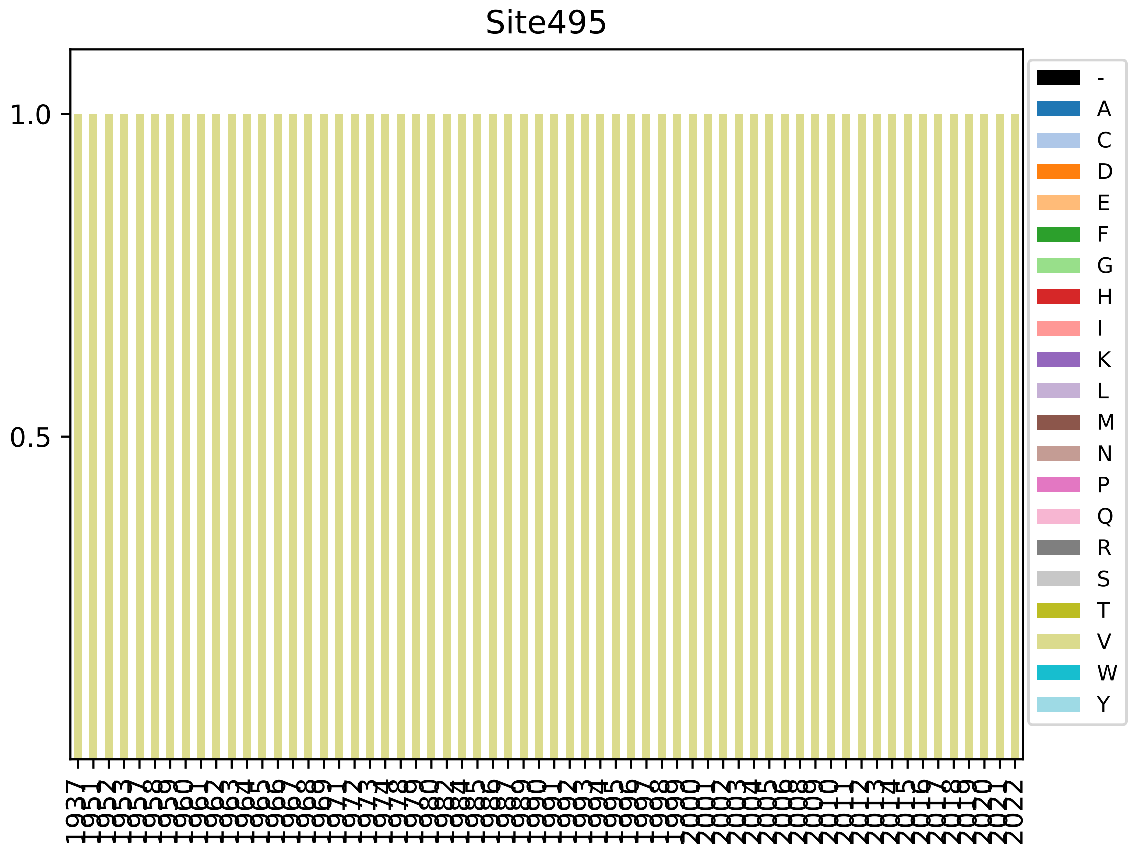

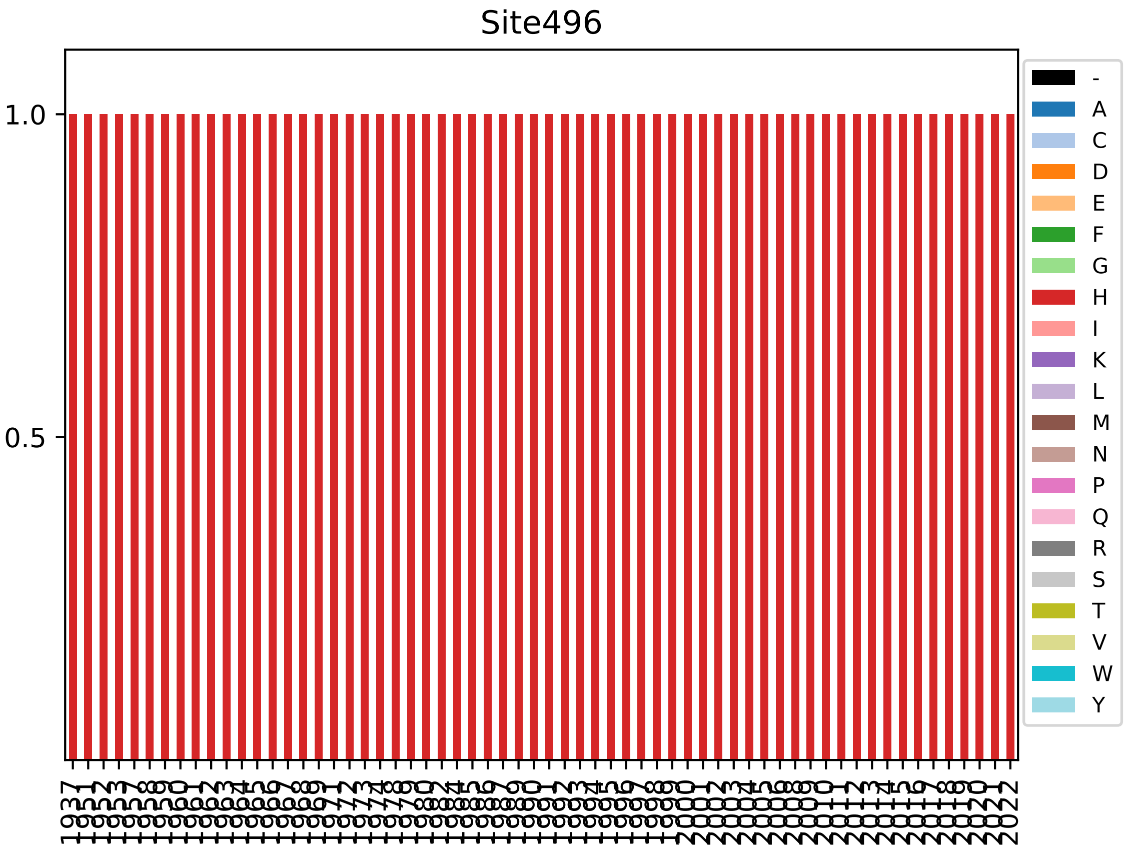
Site496
1.0
0.5
-
A
C
D
E
F
G
H
I
K
L
M
N
P
Q
R
S
T
V
W
Y

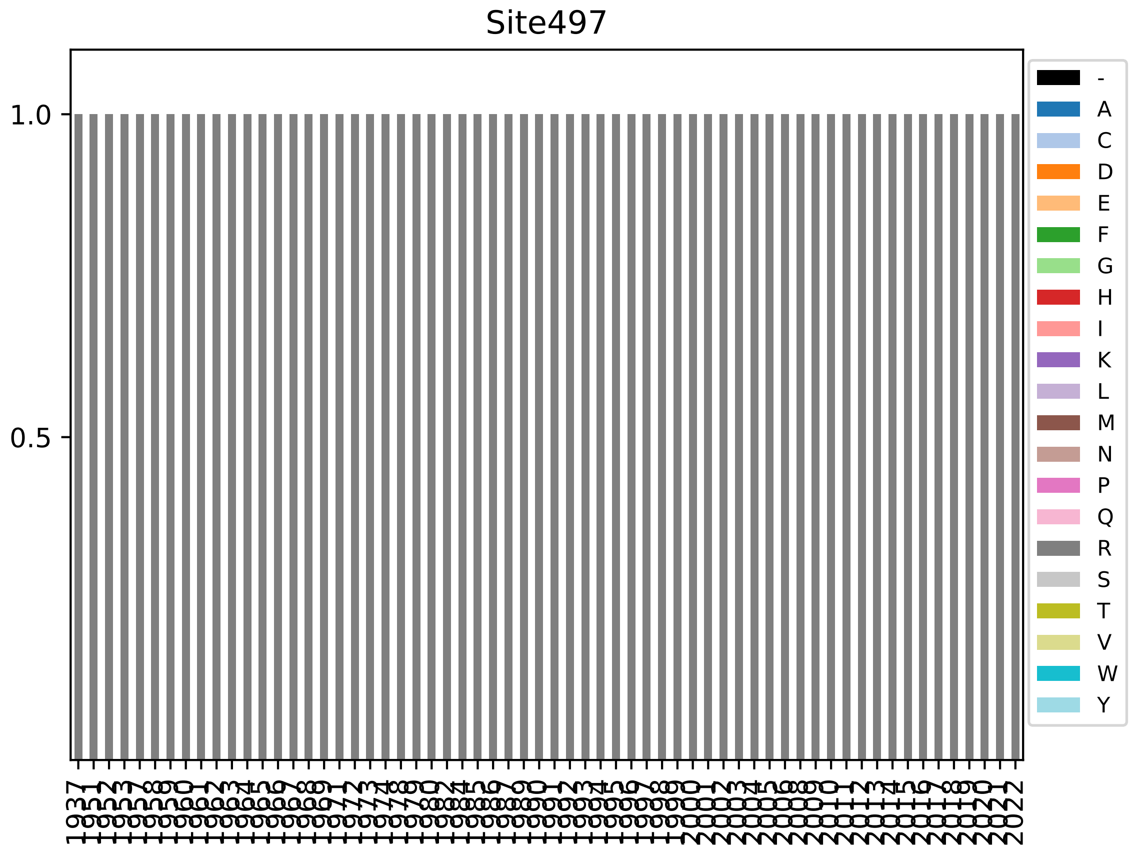
Site497
1.0
0.5
-
A
C
D
E
F
G
H
I
K
L
M
N
P
Q
R
S
T
V
W
Y

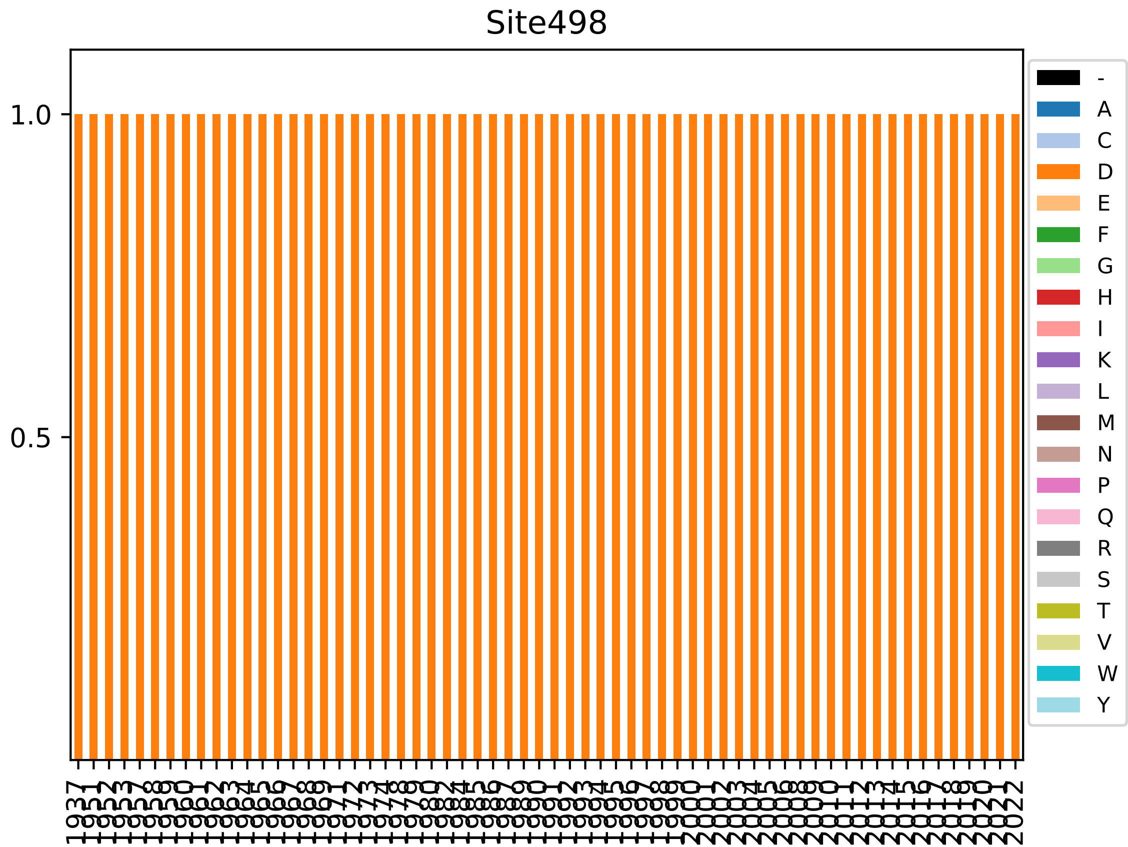
Site498
1.0
0.5
-
A
C
D
E
F
G
H
I
K
L
M
N
P
Q
R
S
T
V
W
Y

Site499

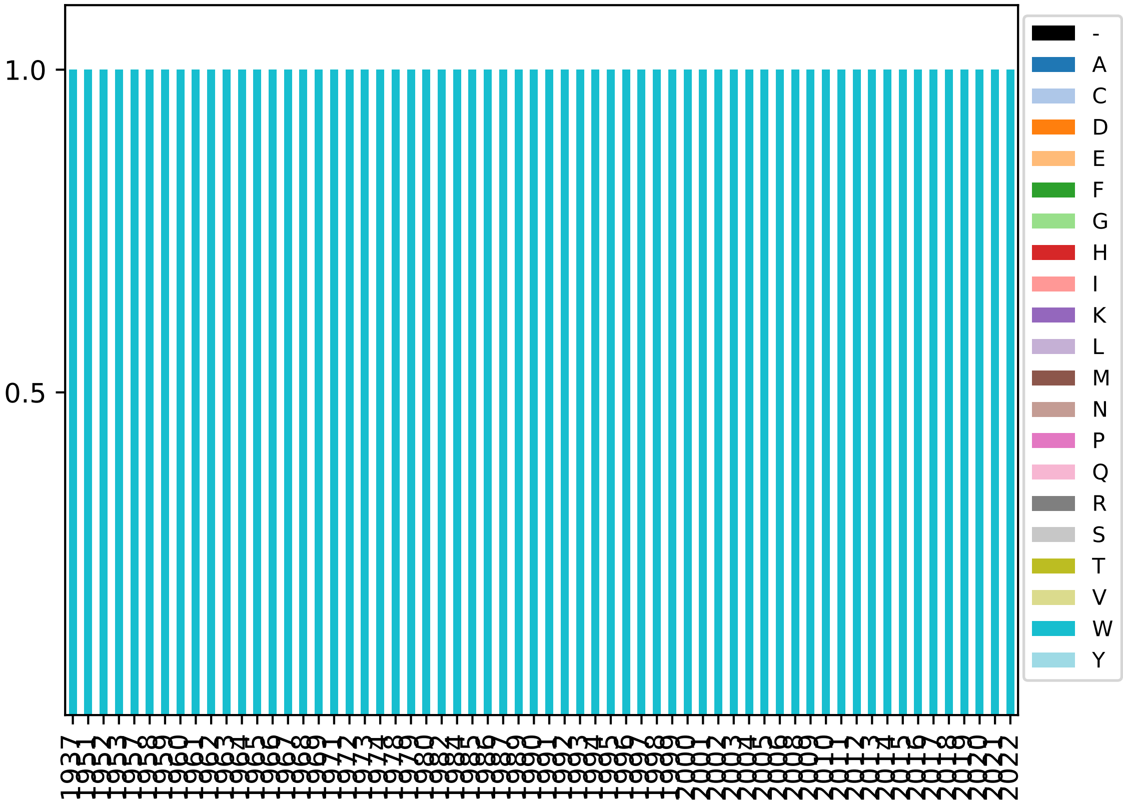

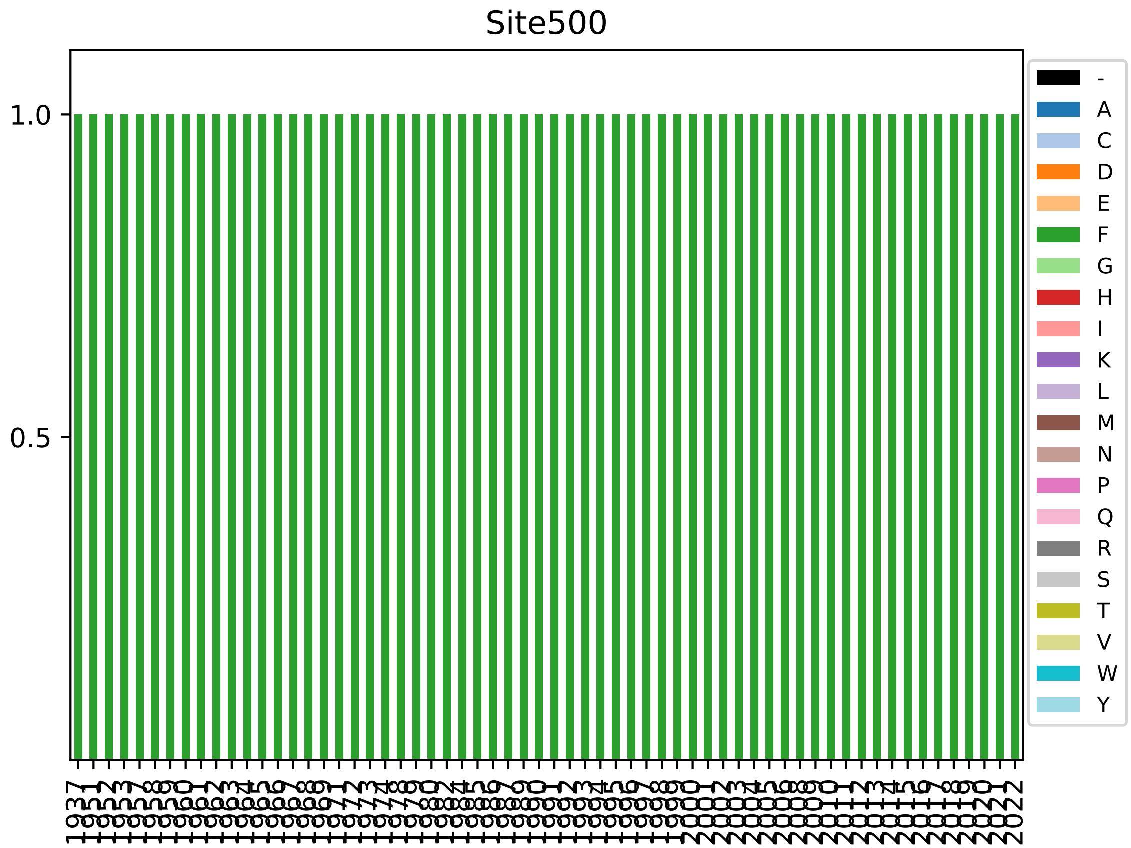
Site500
1.0
0.5
-
A
C
D
E
F
G
H
I
K
L
M
N
P
Q
R
S
T
V
W
Y

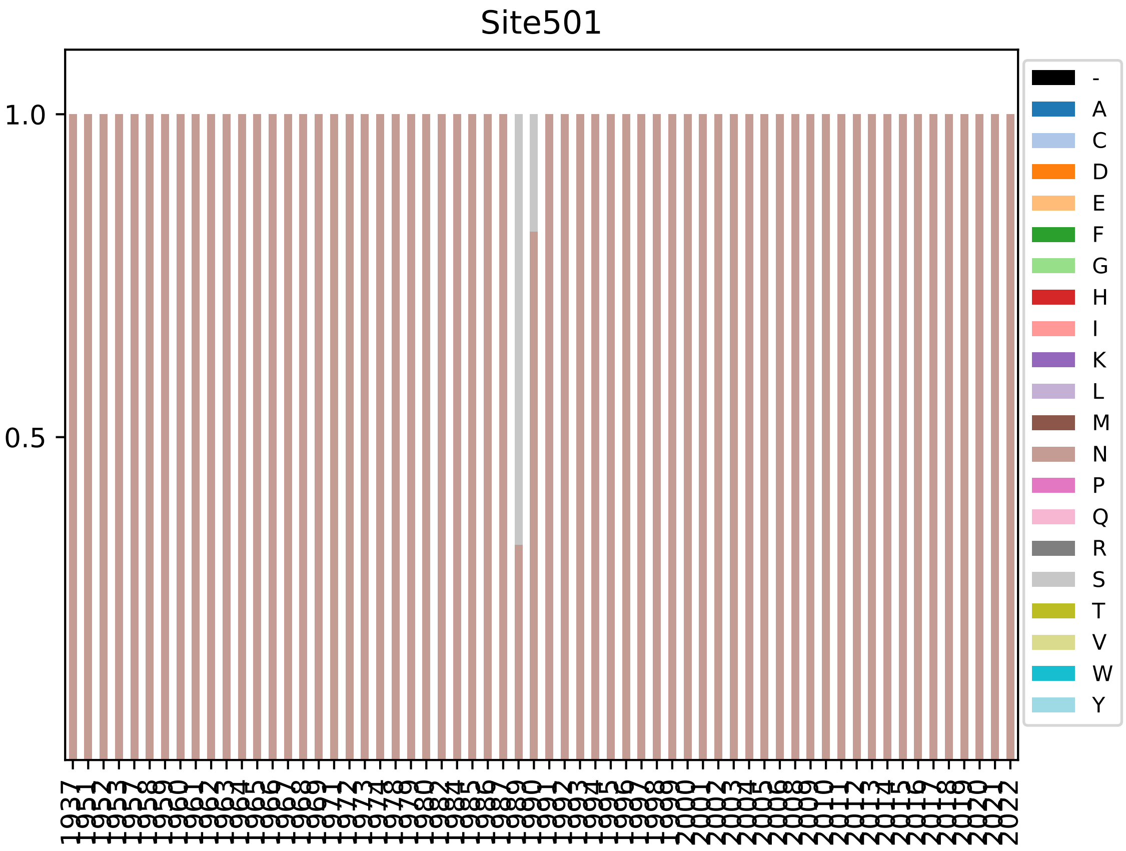
Site501
1.0
0.5
-
A
C
D
E
F
G
H
I
K
L
M
N
P
Q
R
S
T
V
W
Y

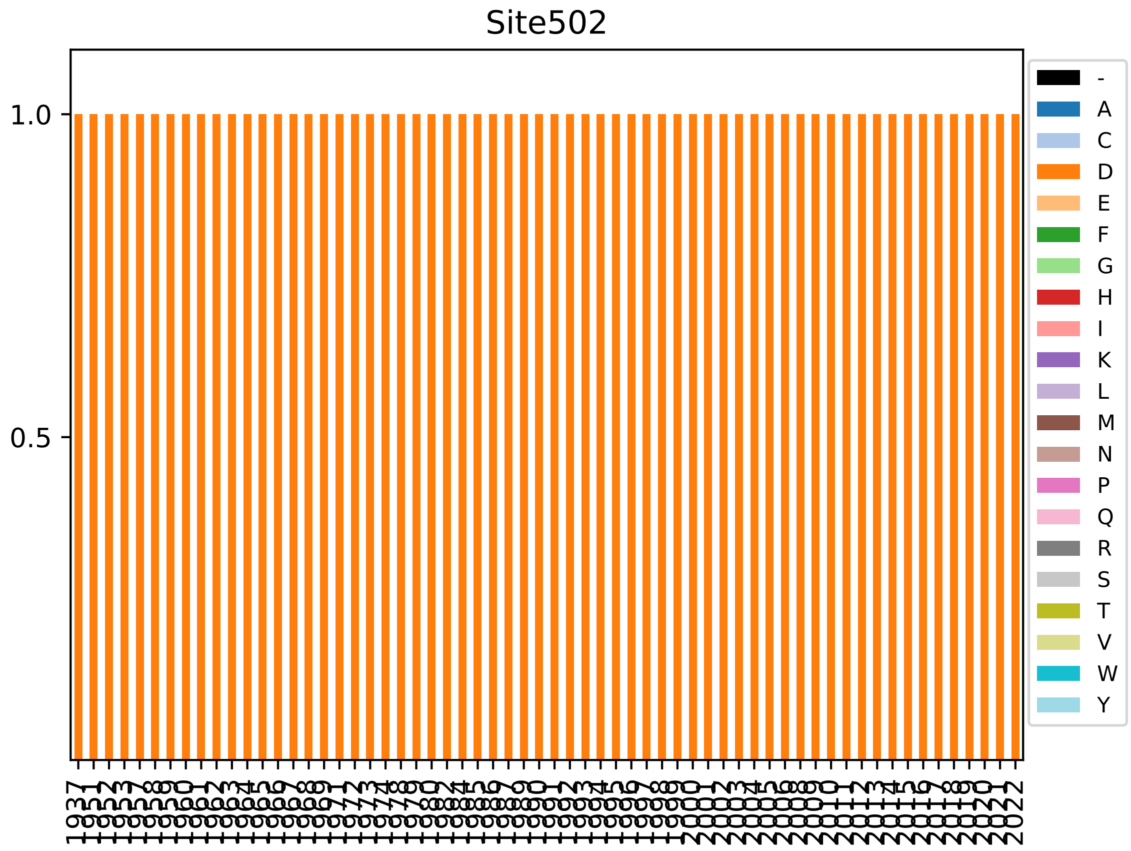
Site502
1.0
0.5
-
A
C
D
E
F
G
H
I
K
L
M
N
P
Q
R
S
T
V
W
Y

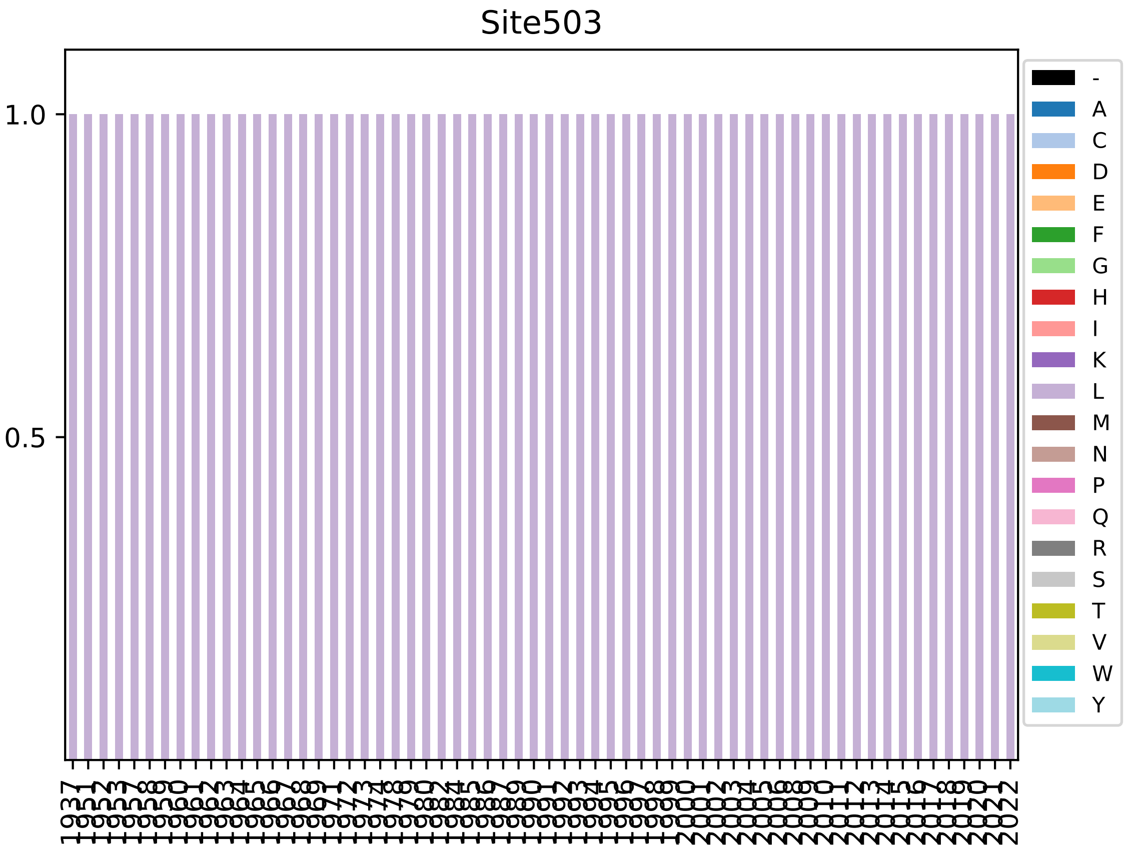
Site503
1.0
0.5
-
A
C
D
E
F
G
H
I
K
L
M
N
P
Q
R
S
T
V
W
Y

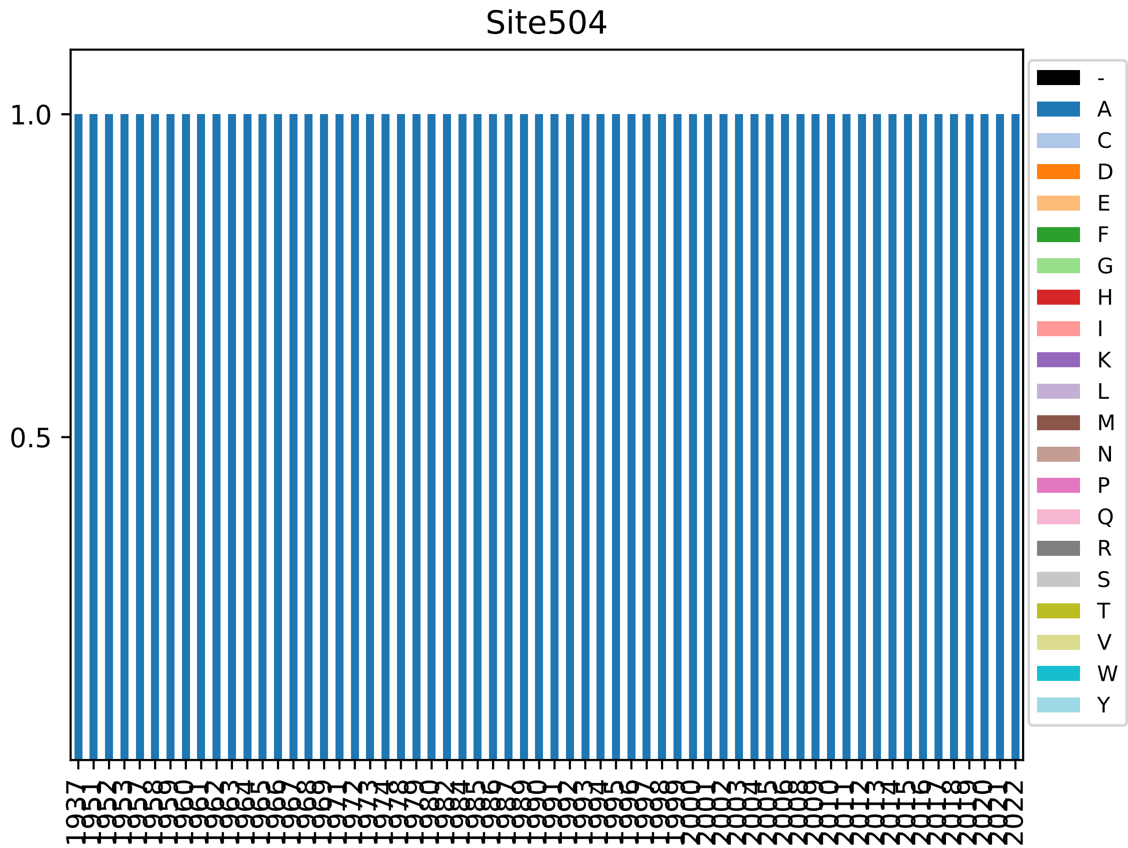
Site504
1.0
0.5
-
A
C
D
E
F
G
H
I
K
L
M
N
P
Q
R
S
T
V
W
Y

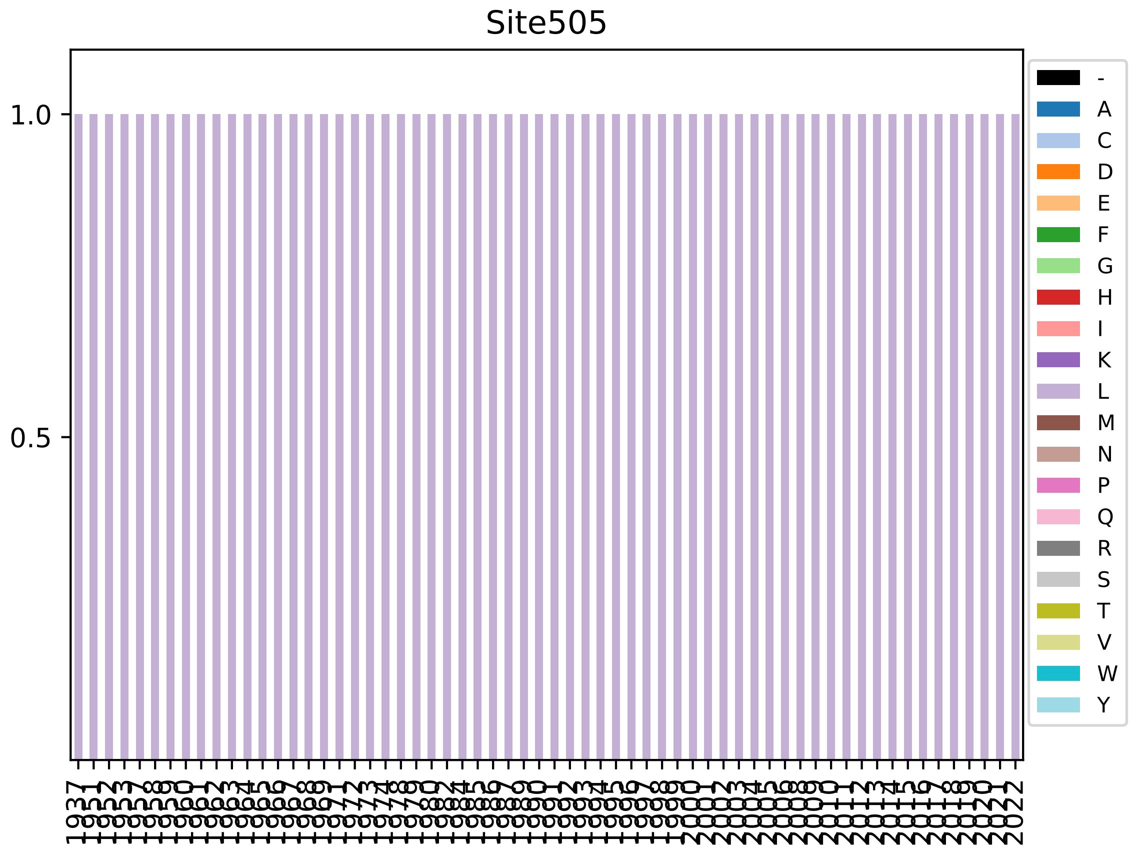

Site505
1.0
0.5
-
A
C
D
E
F
G
H
I
K
L
M
N
P
Q
R
S
T
V
W
Y

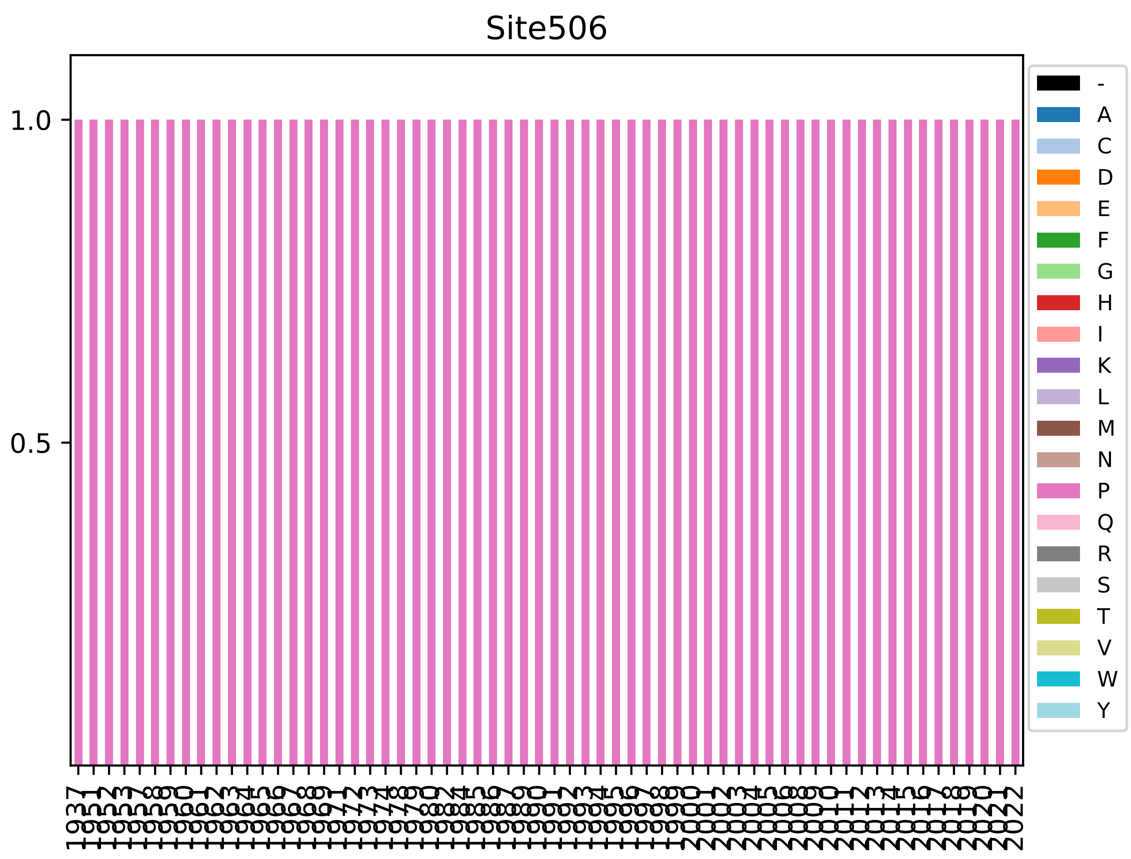
Site506
1.0
0.5
-
A
C
D
E
F
G
H
I
K
L
M
N
P
Q
R
S
T
V
W
Y

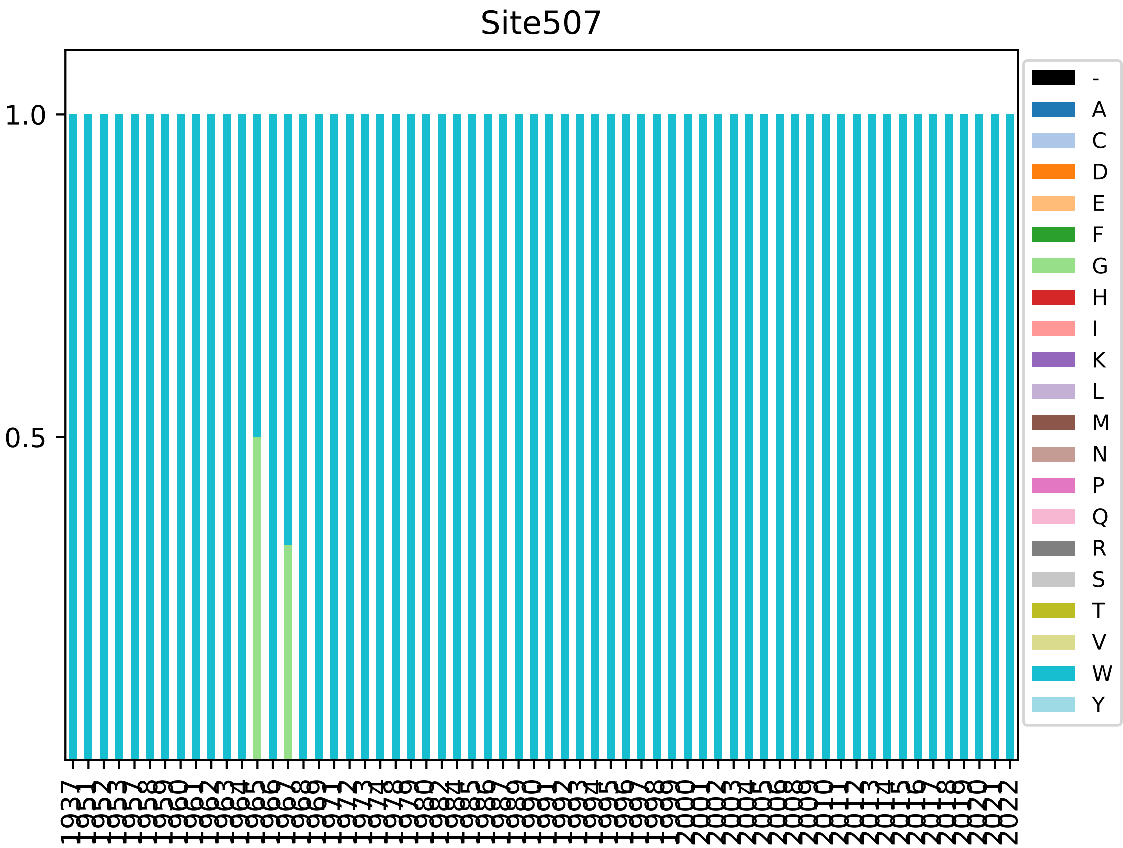
Site507
1.0
0.5
-
A
C
D
E
F
G
H
I
K
L
M
N
P
Q
R
S
T
V
W
Y

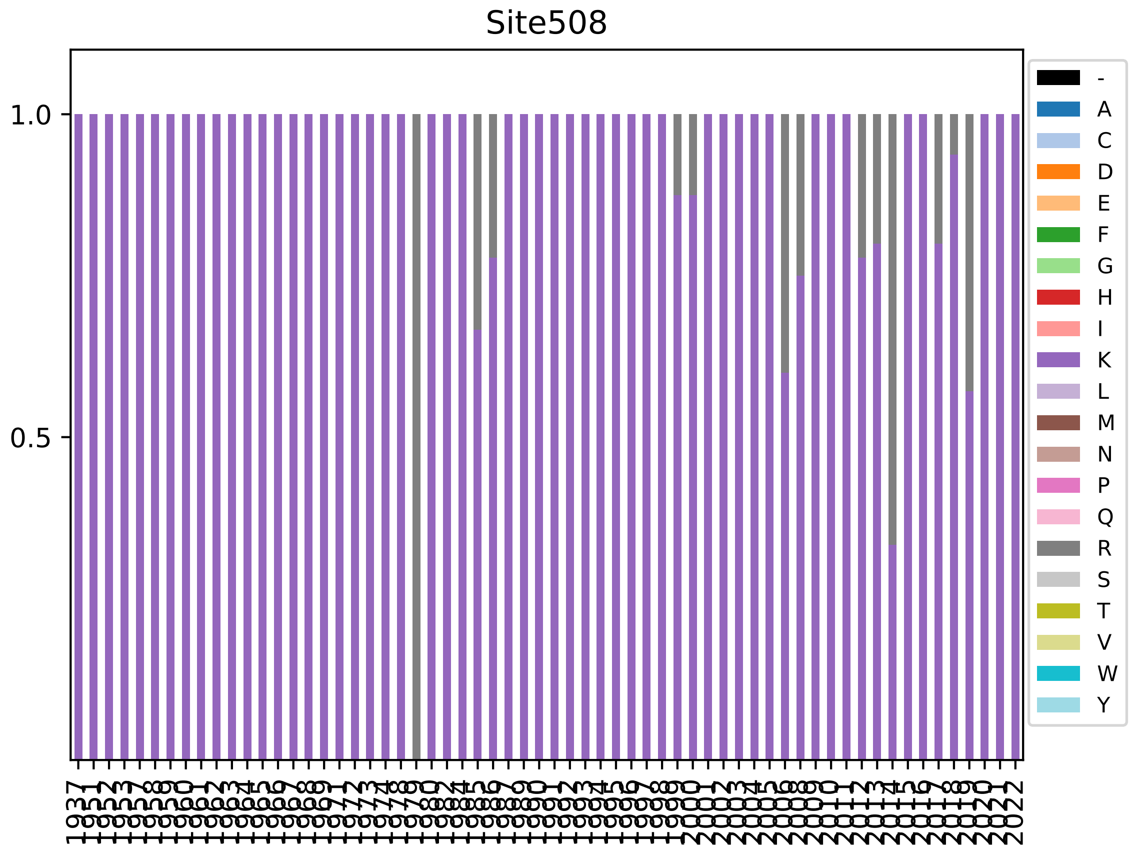
Site508
1.0
0.5
-
A
C
D
E
F
G
H
I
K
L
M
N
P
Q
R
S
T
V
W
Y

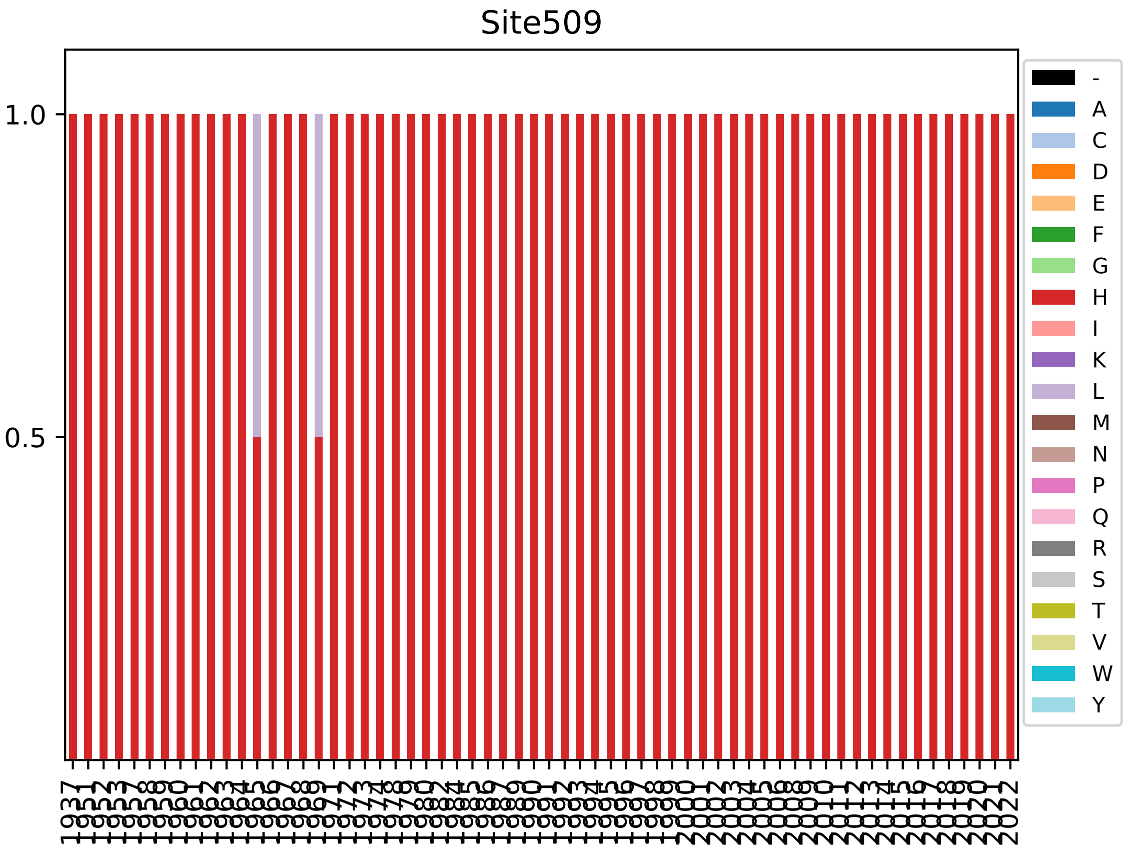
Site509
1.0
0.5
-
A
C
D
E
F
G
H
I
K
L
M
N
P
Q
R
S
T
V
W
Y

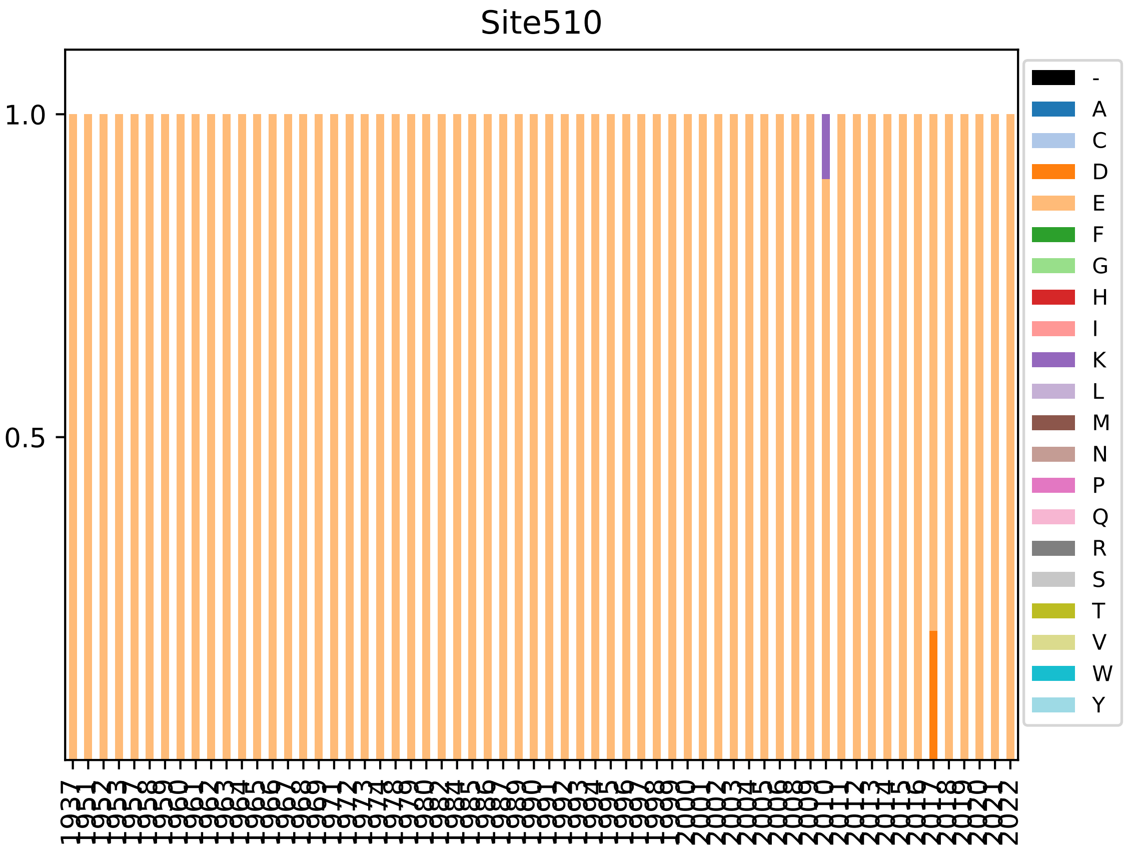

Site510
1.0
0.5
-
A
C
D
E
F
G
H
I
K
L
M
N
P
Q
R
S
T
V
W
Y

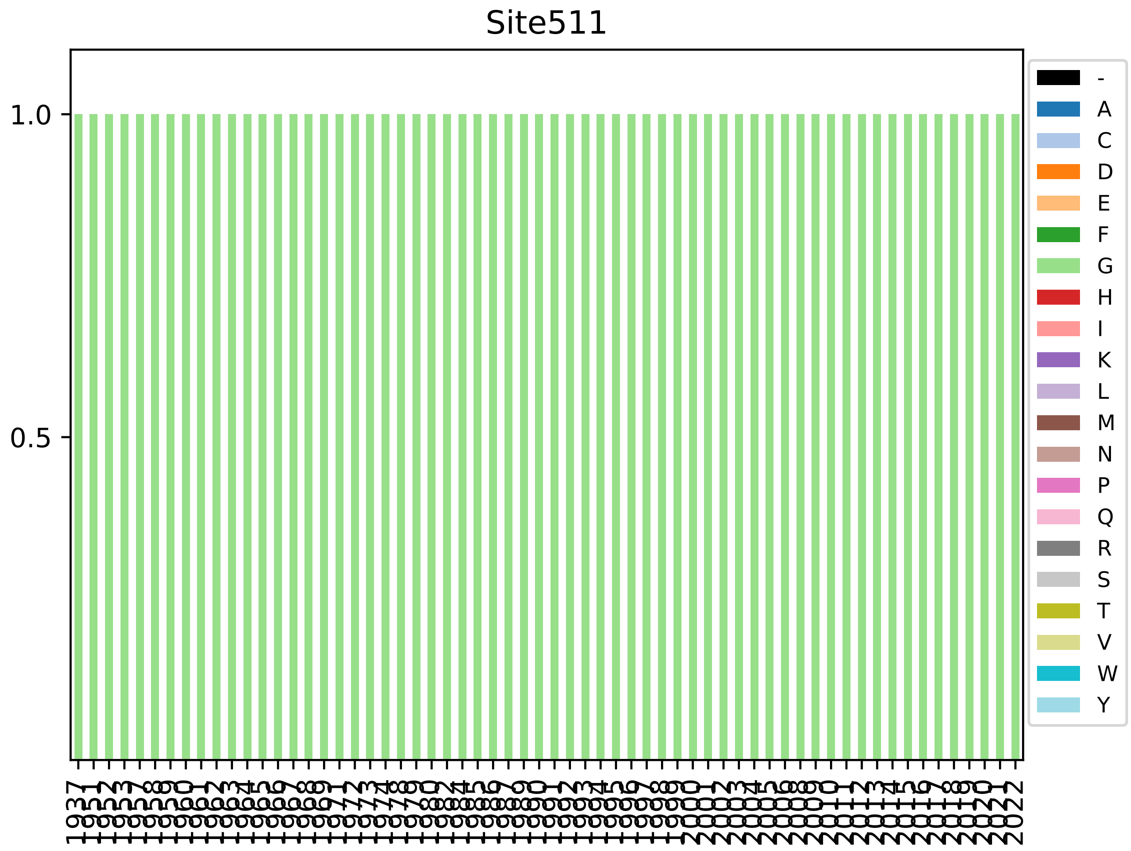
Site511
1.0
0.5
-
A
C
D
E
F
G
H
I
K
L
M
N
P
Q
R
S
T
V
W
Y

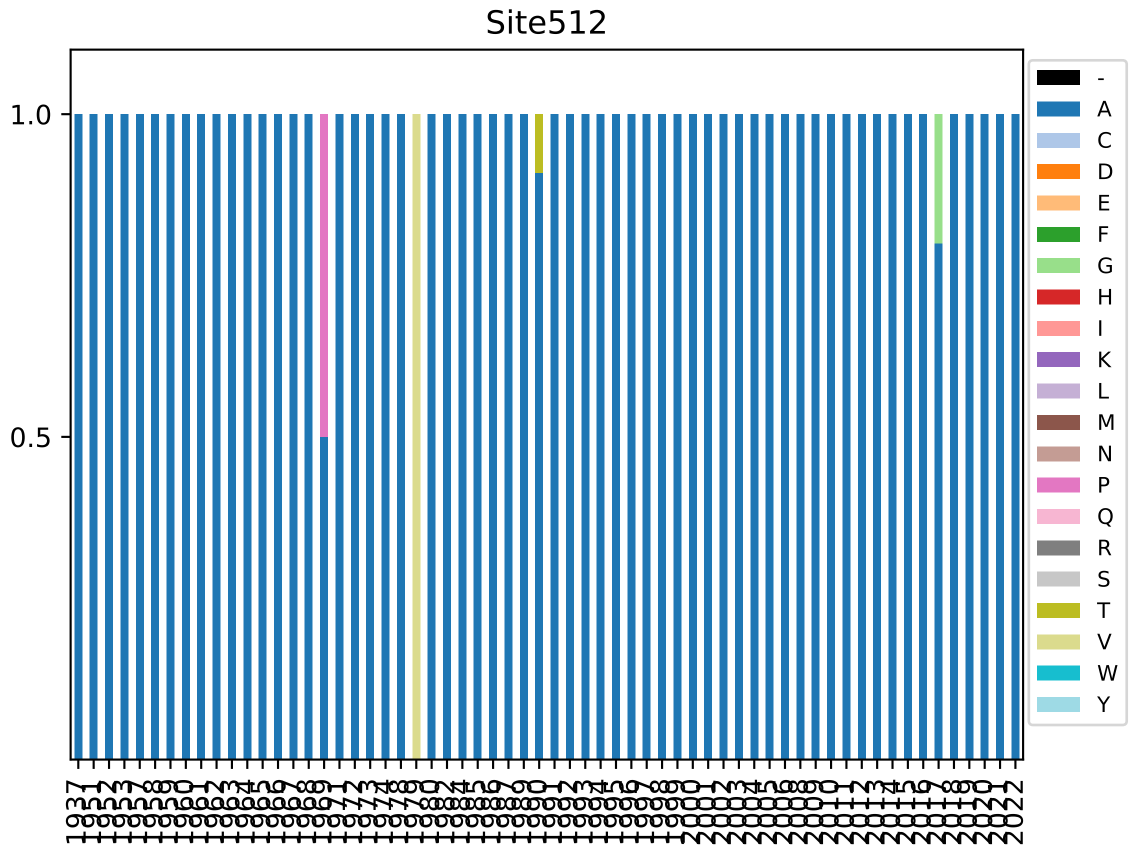
Site512
1.0
0.5
-
A
C
D
E
F
G
H
I
K
L
M
N
P
Q
R
S
T
V
W
Y

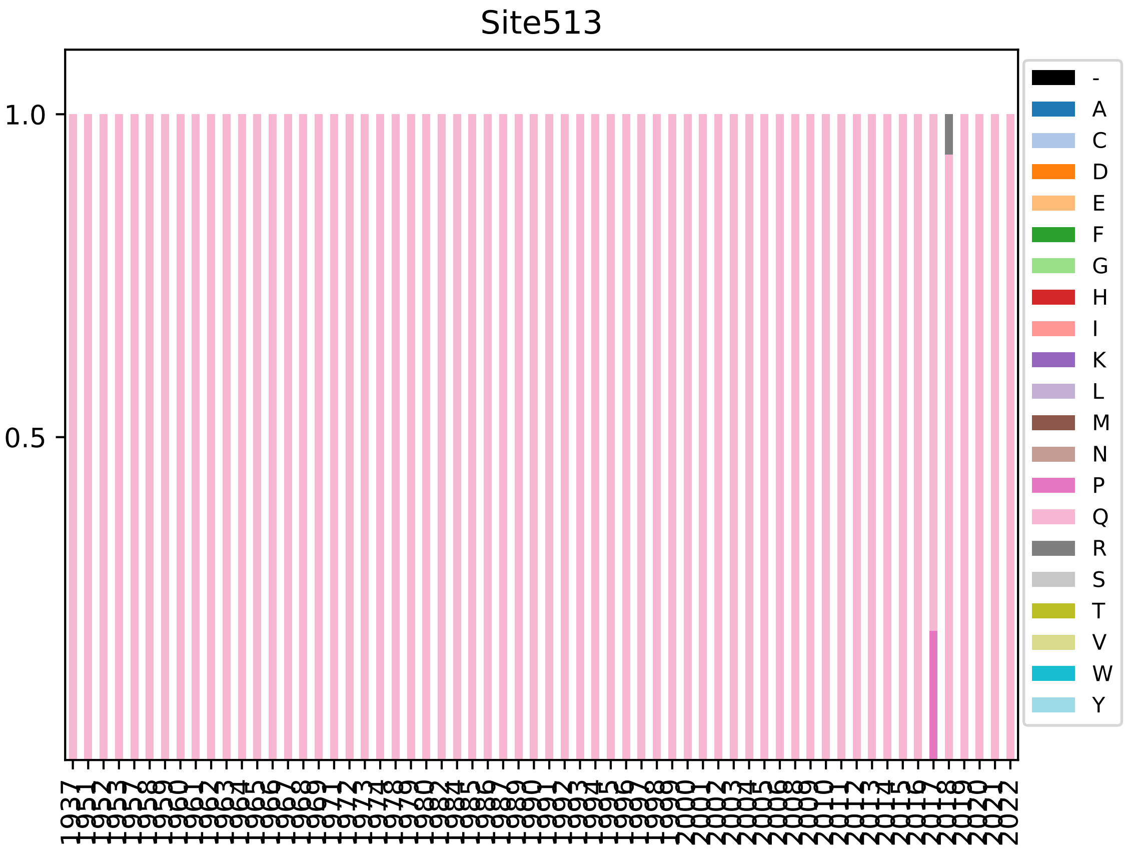
Site513
1.0
0.5
-
A
C
D
E
F
G
H
I
K
L
M
N
P
Q
R
S
T
V
W
Y

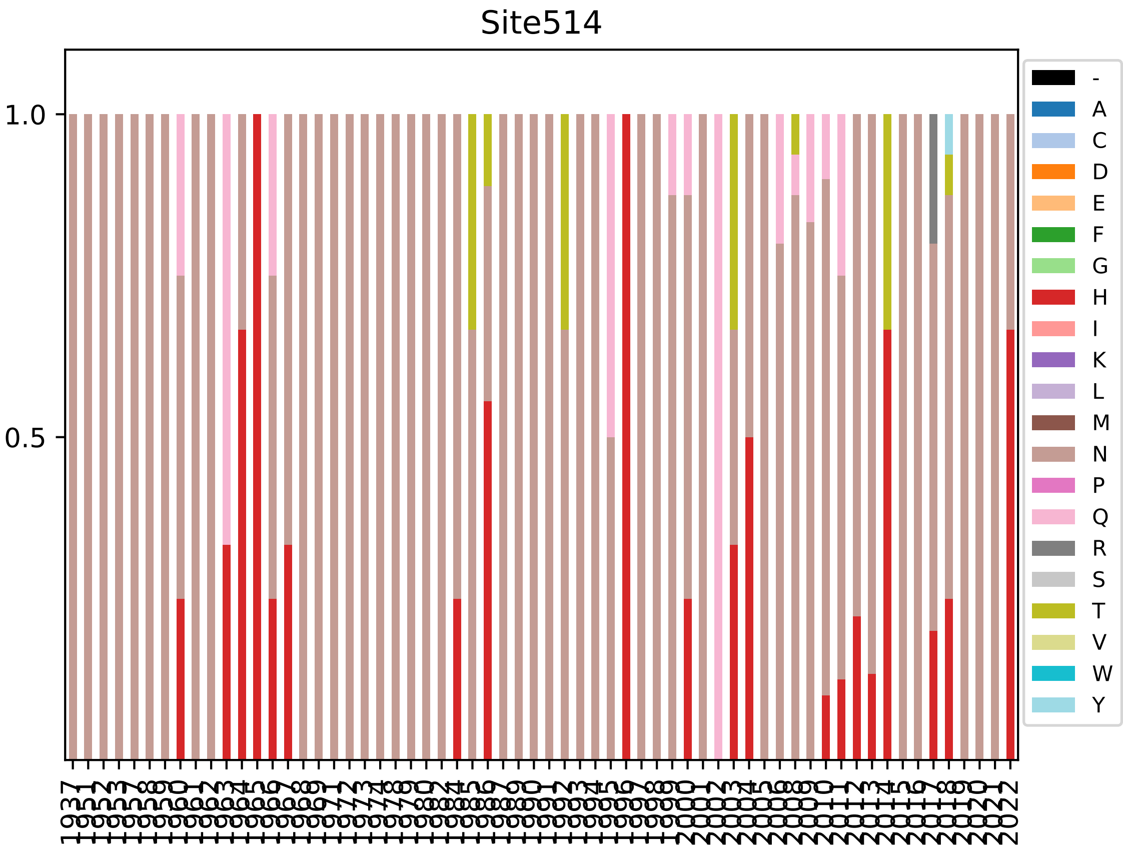

Site514
1.0
0.5
-
A
C
D
E
F
G
H
I
K
L
M
N
P
Q
R
S
T
V
W
Y

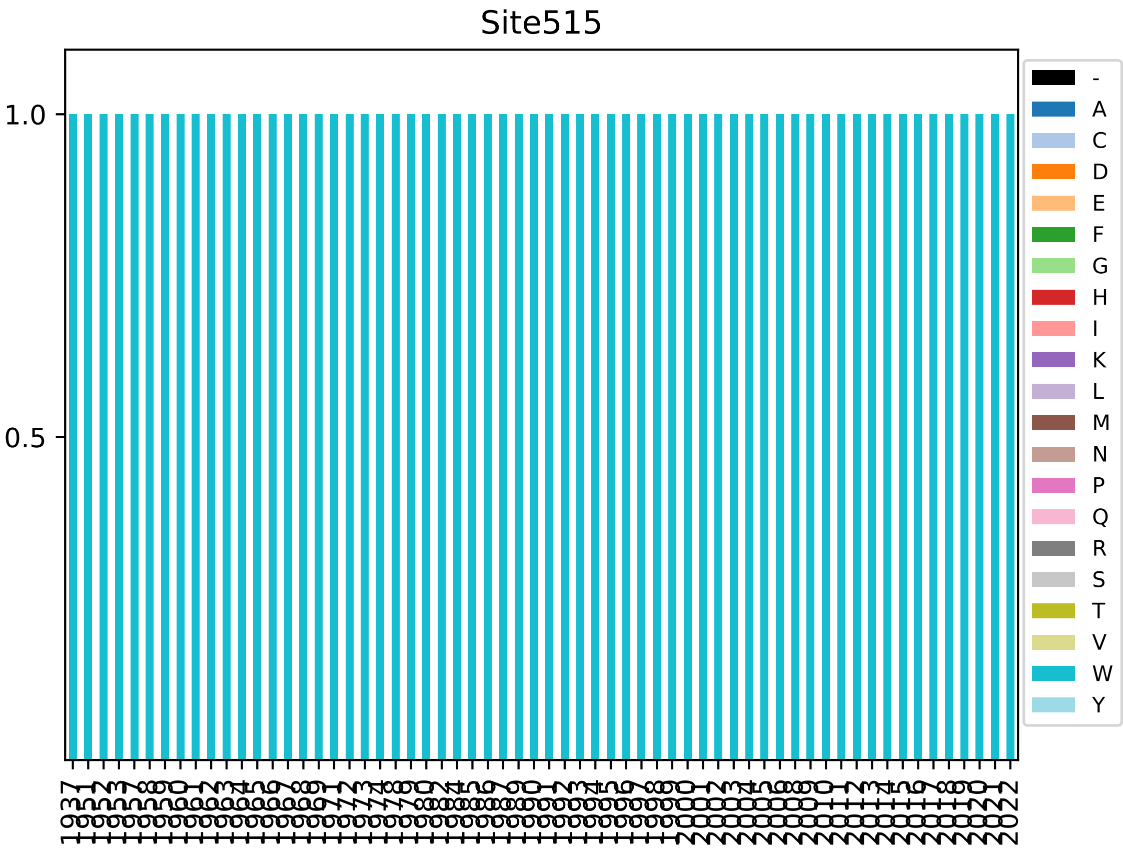
Site515
1.0
0.5
-
A
C
D
E
F
G
H
I
K
L
M
N
P
Q
R
S
T
V
W
Y

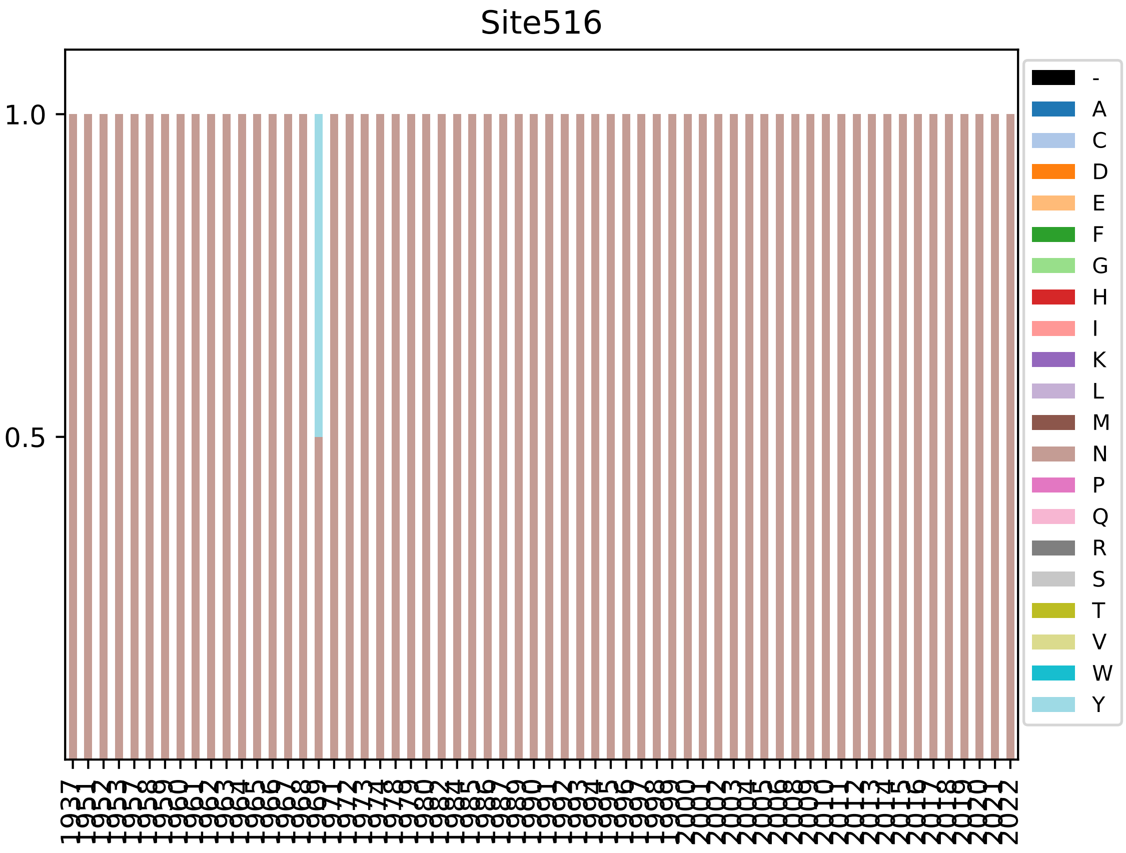
Site516
1.0
0.5
-
A
C
D
E
F
G
H
I
K
L
M
N
P
Q
R
S
T
V
W
Y

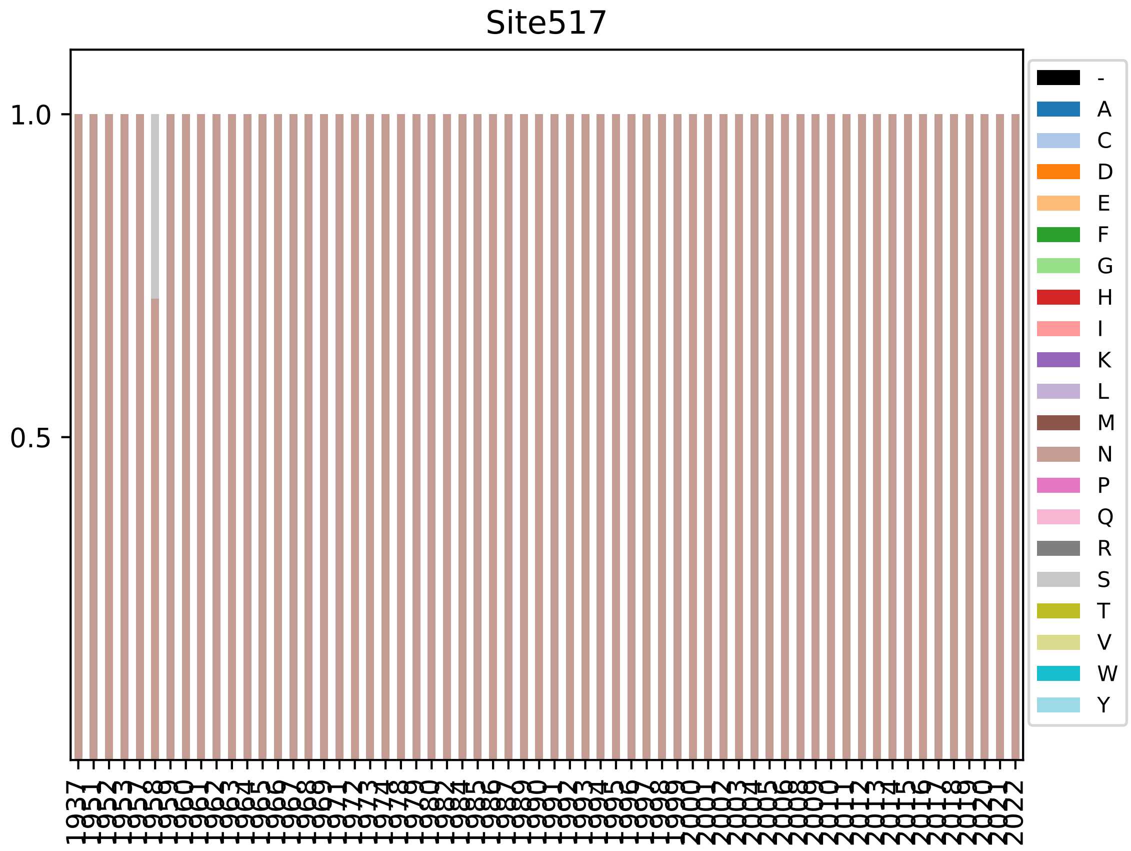
Site517
1.0
0.5
-
A
C
D
E
F
G
H
I
K
L
M
N
P
Q
R
S
T
V
W
Y

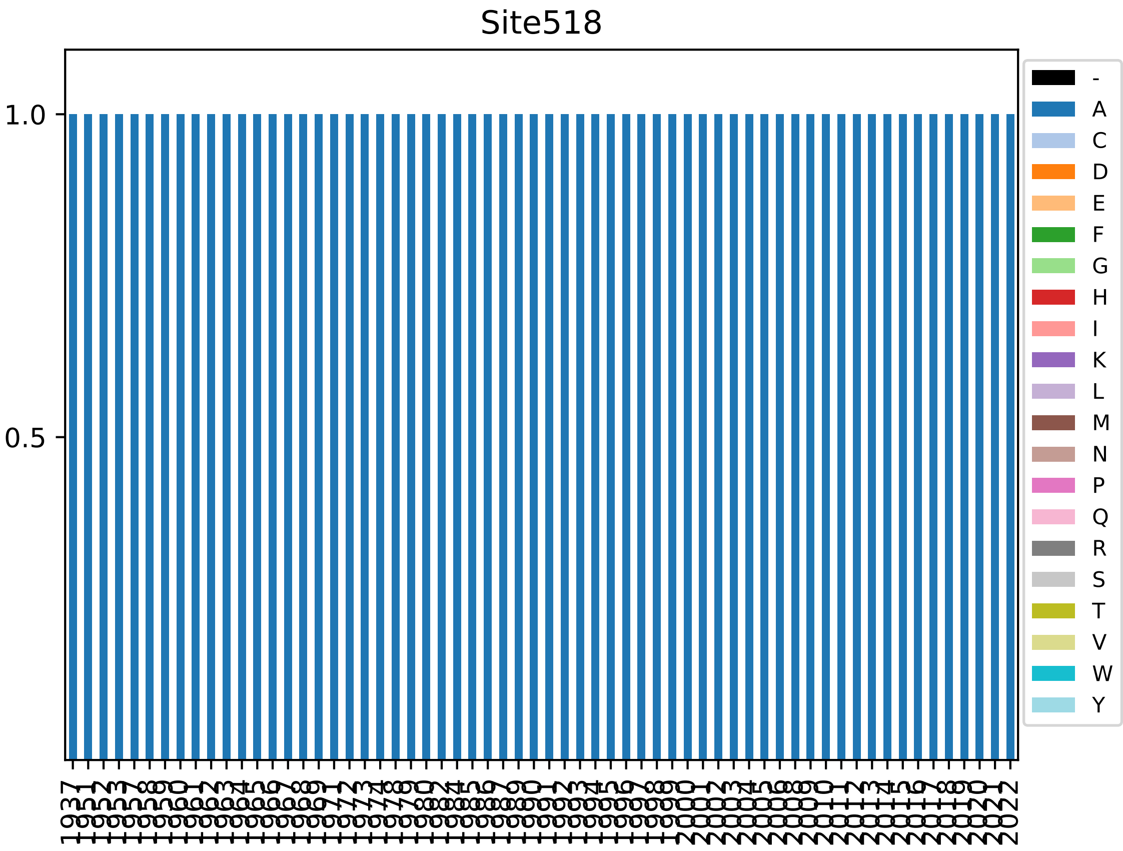
Site518
1.0
0.5
-
A
C
D
E
F
G
H
I
K
L
M
N
P
Q
R
S
T
V
W
Y

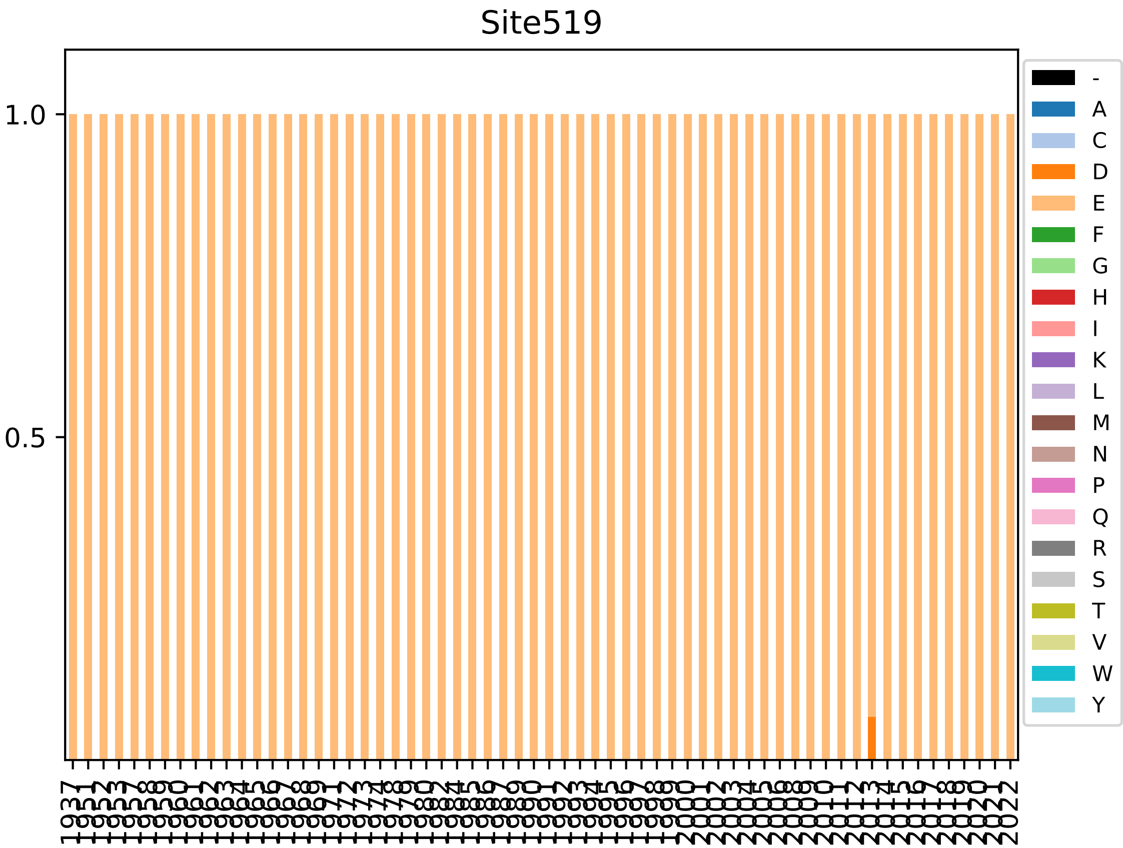
Site519
1.0
0.5
-
A
C
D
E
F
G
H
I
K
L
M
N
P
Q
R
S
T
V
W
Y

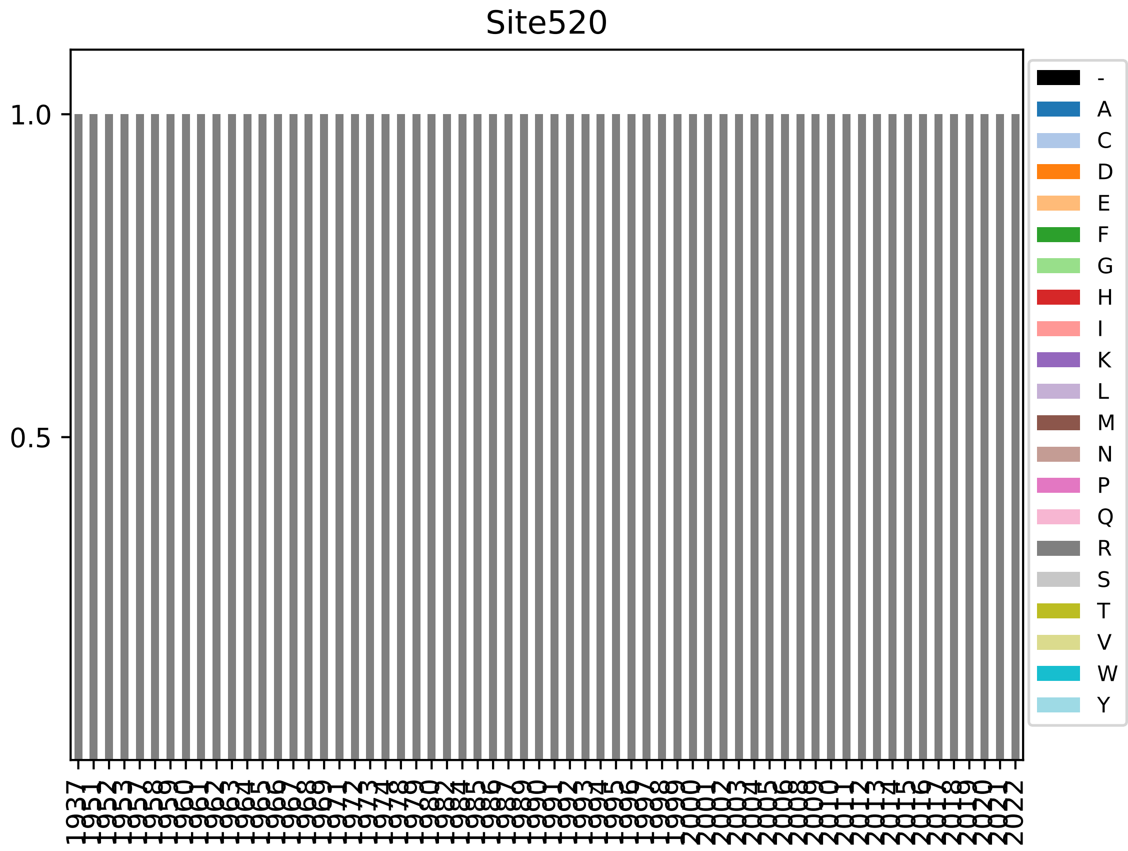
Site520
1.0
0.5
-
A
C
D
E
F
G
H
I
K
L
M
N
P
Q
R
S
T
V
W
Y

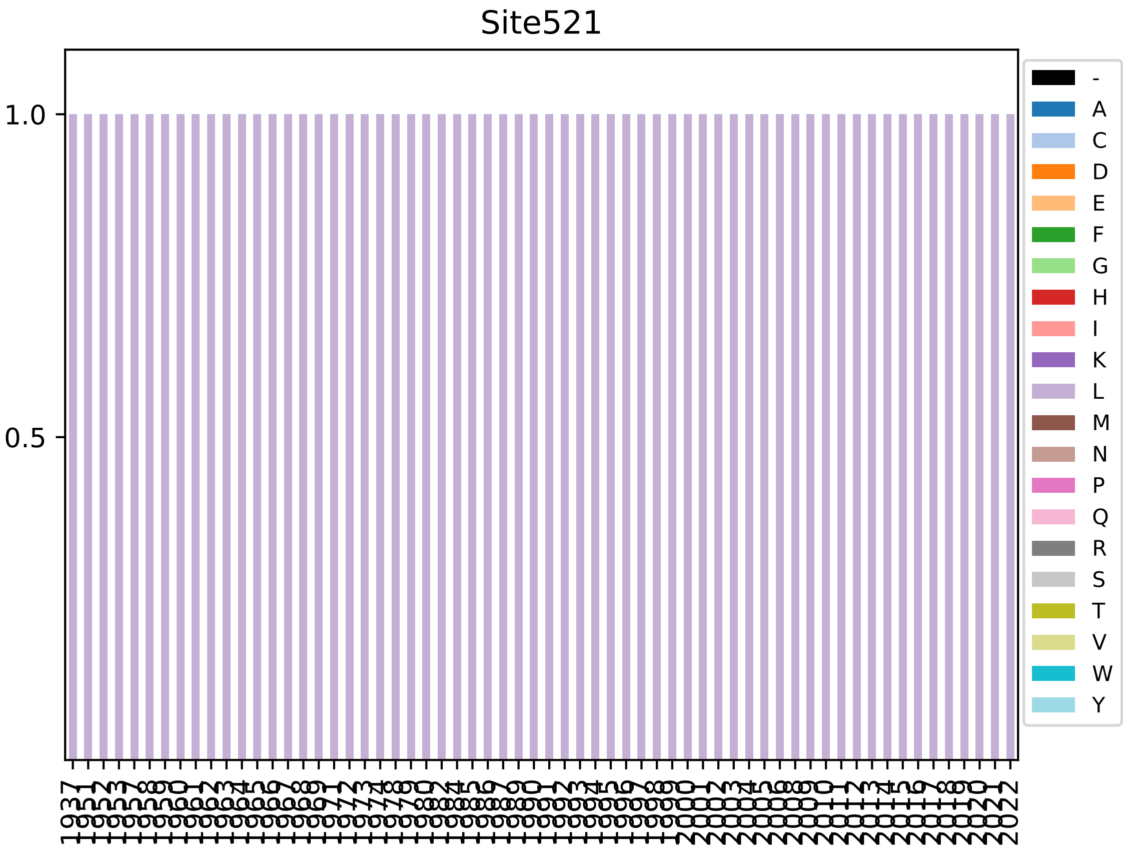
Site521
1.0
0.5
-
A
C
D
E
F
G
H
I
K
L
M
N
P
Q
R
S
T
V
W
Y

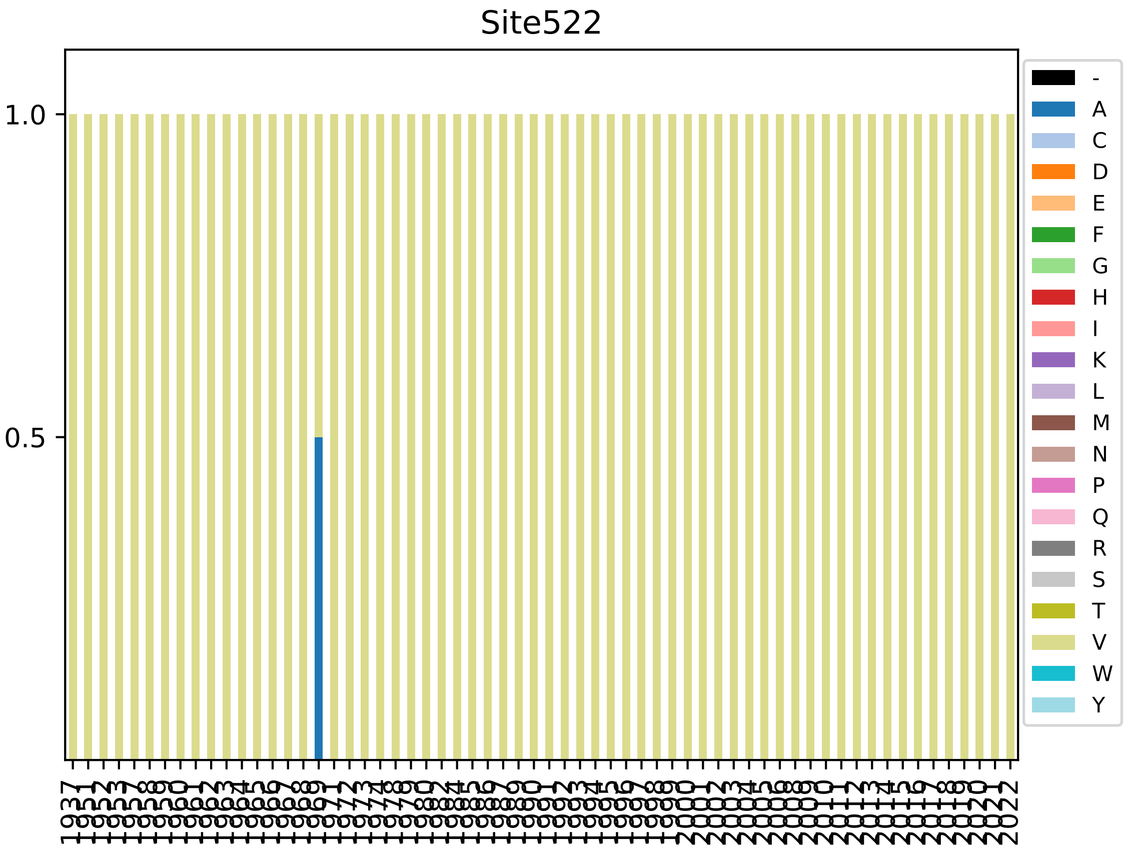
Site522
1.0
0.5
-
A
C
D
E
F
G
H
I
K
L
M
N
P
Q
R
S
T
V
W
Y

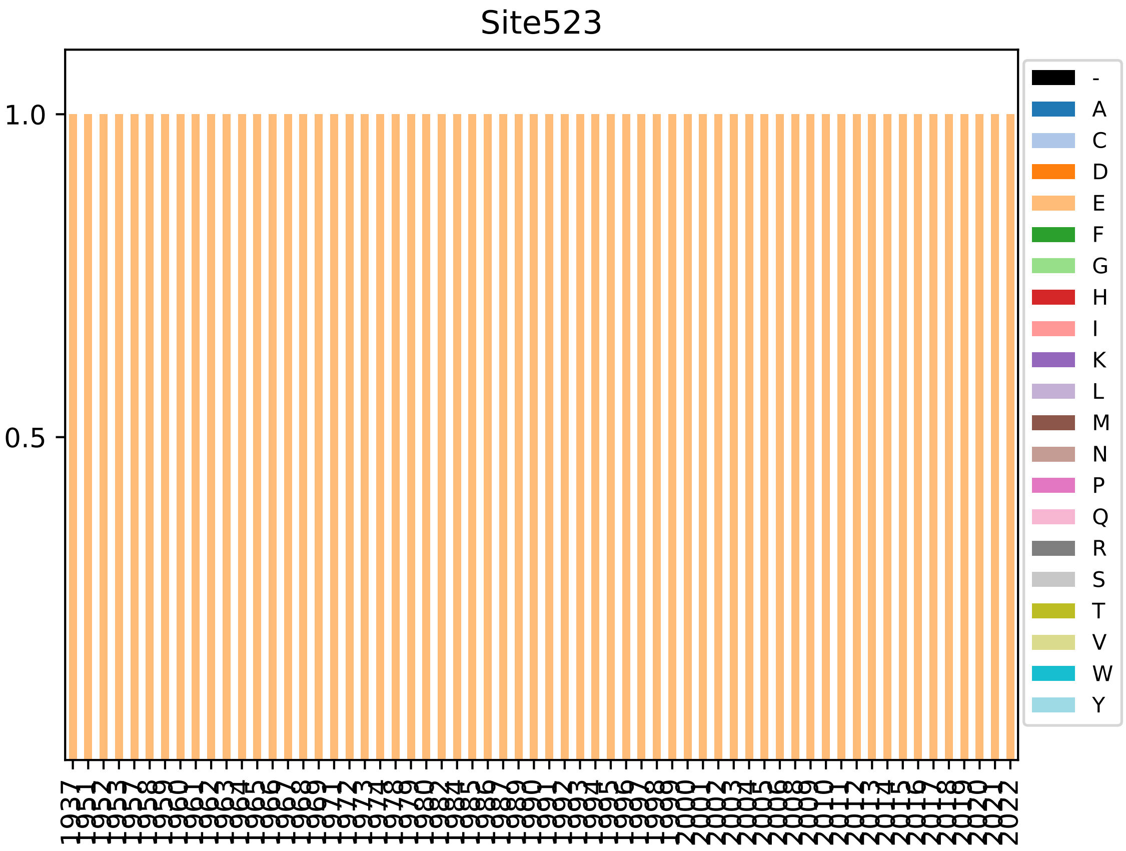
Site523
1.0
0.5
-
A
C
D
E
F
G
H
I
K
L
M
N
P
Q
R
S
T
V
W
Y

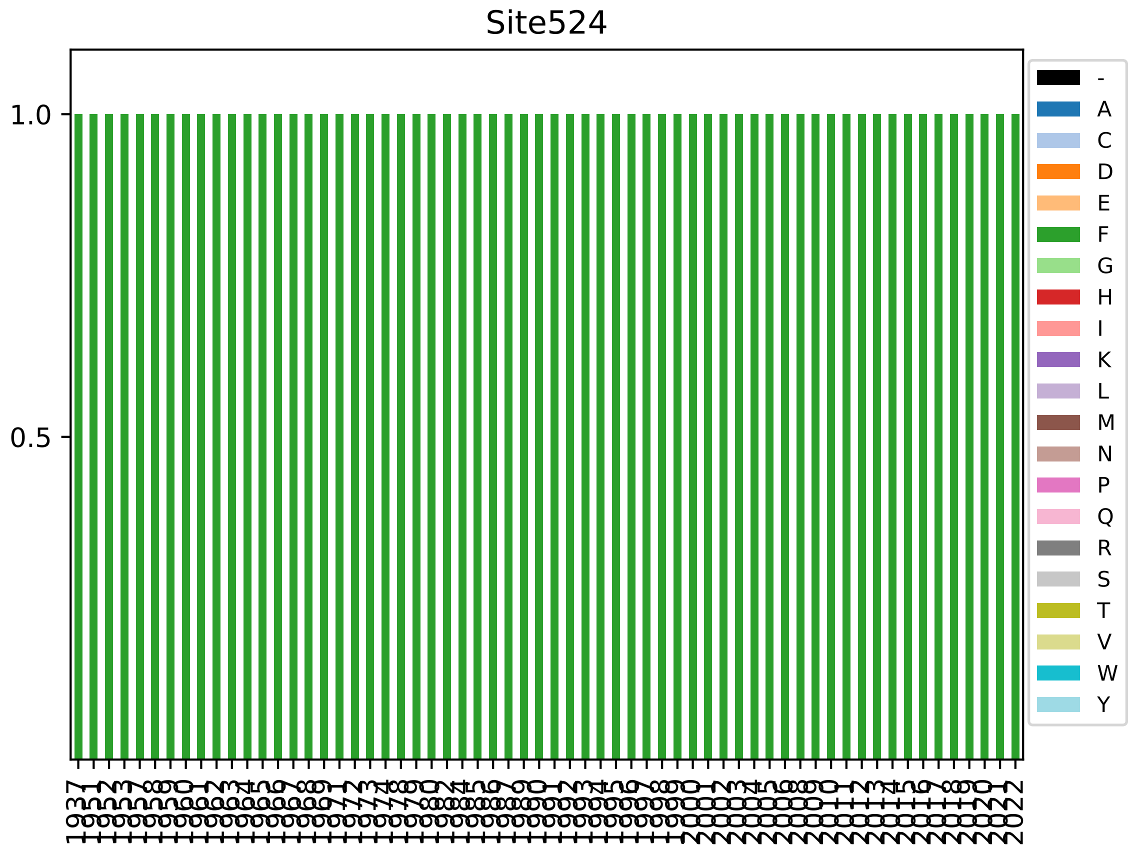
Site524
1.0
0.5
-
A
C
D
E
F
G
H
I
K
L
M
N
P
Q
R
S
T
V
W
Y

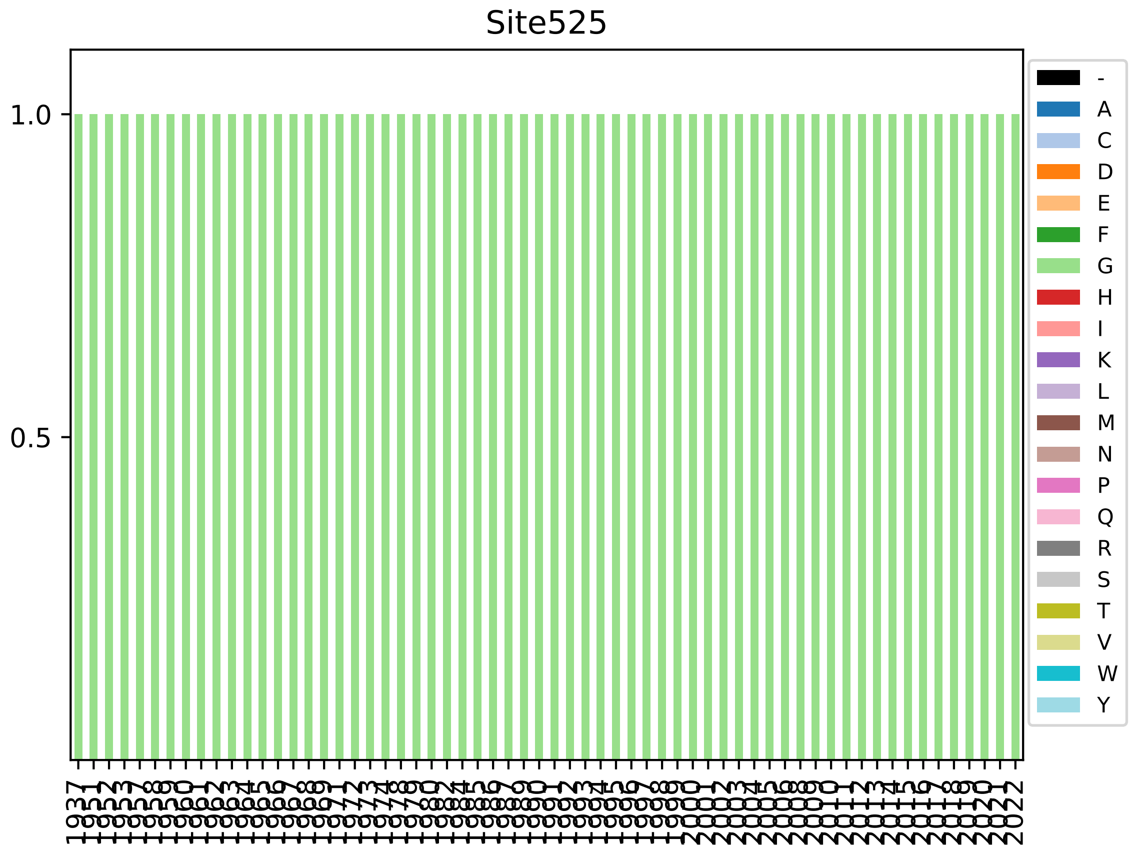
Site525
1.0
0.5
-
A
C
D
E
F
G
H
I
K
L
M
N
P
Q
R
S
T
V
W
Y

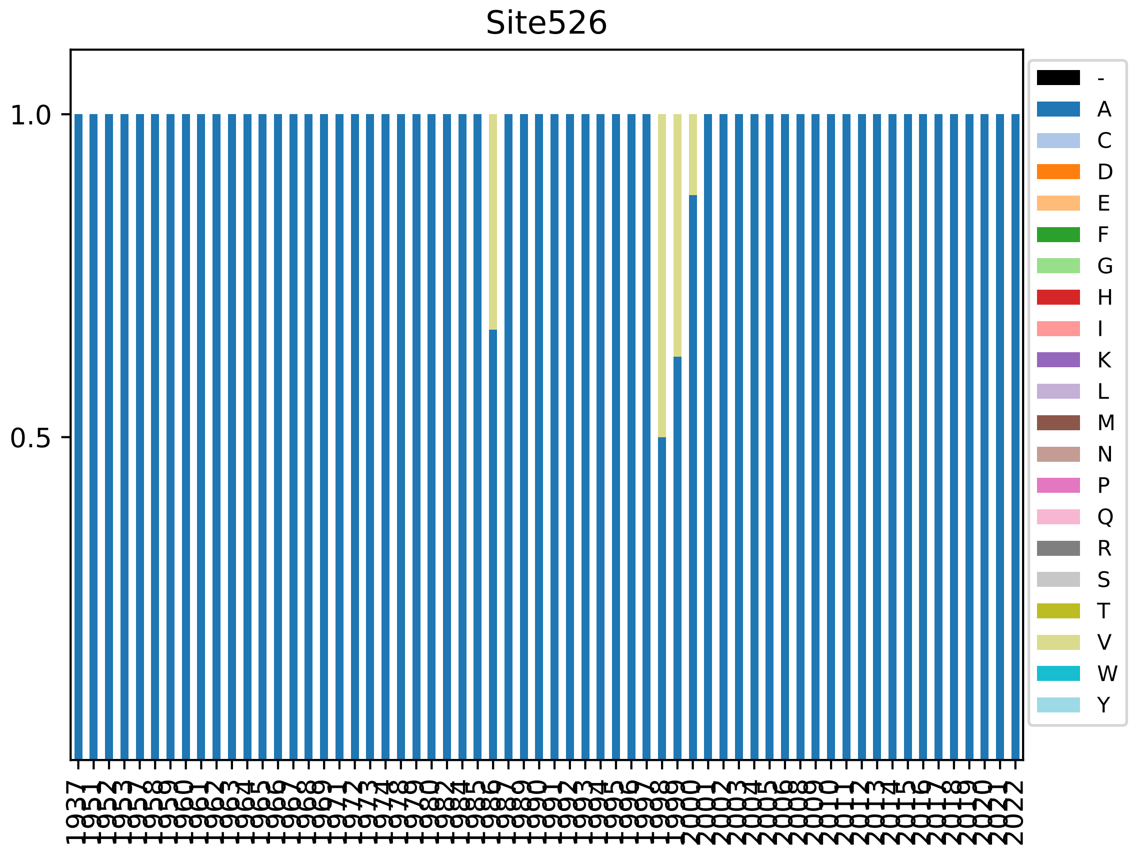

Site526
1.0
0.5
-
A
C
D
E
F
G
H
I
K
L
M
N
P
Q
R
S
T
V
W
Y

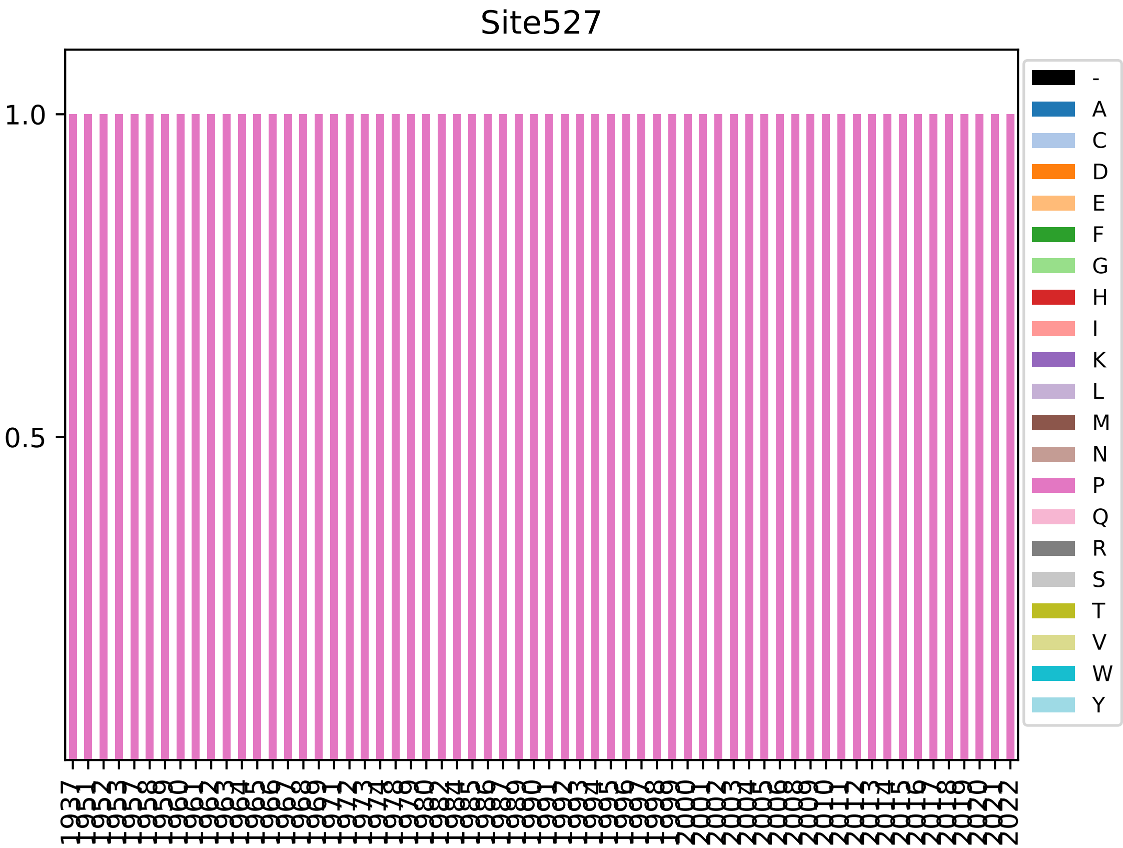
Site527
1.0
0.5
-
A
C
D
E
F
G
H
I
K
L
M
N
P
Q
R
S
T
V
W
Y

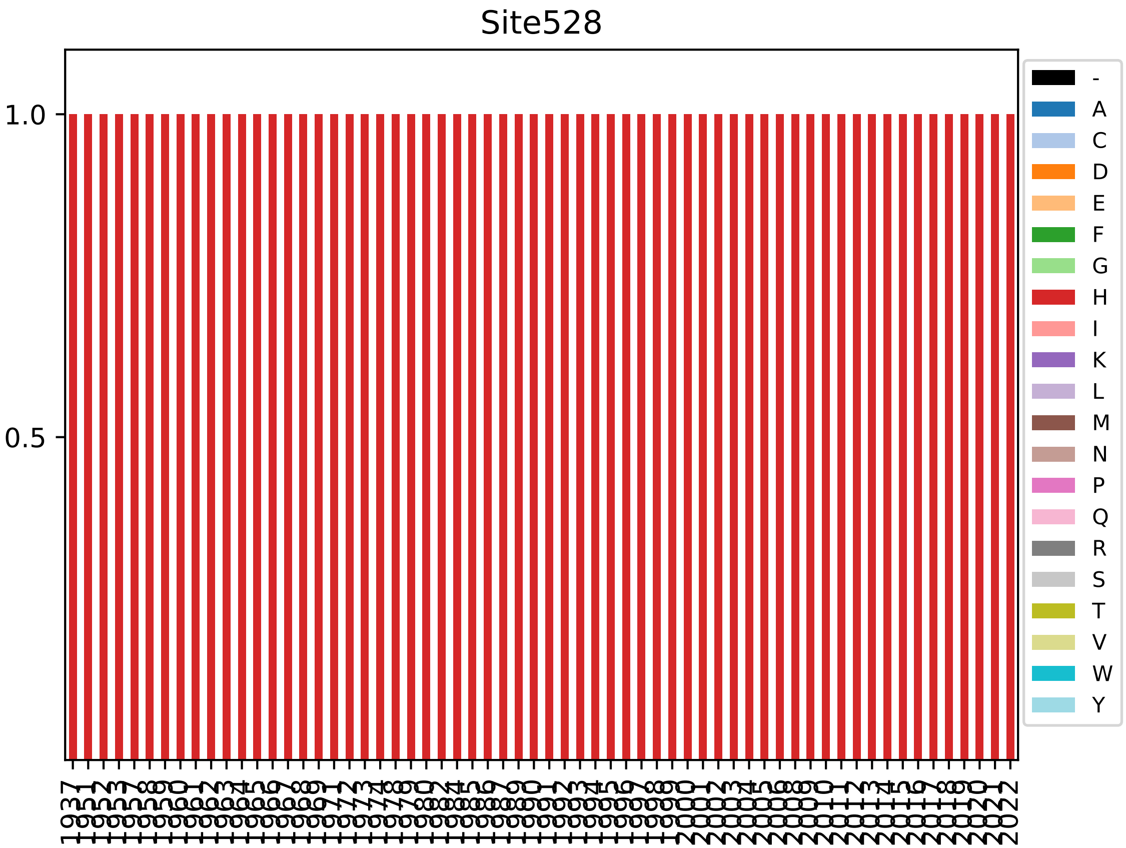
Site528
1.0
0.5
-
A
C
D
E
F
G
H
I
K
L
M
N
P
Q
R
S
T
V
W
Y

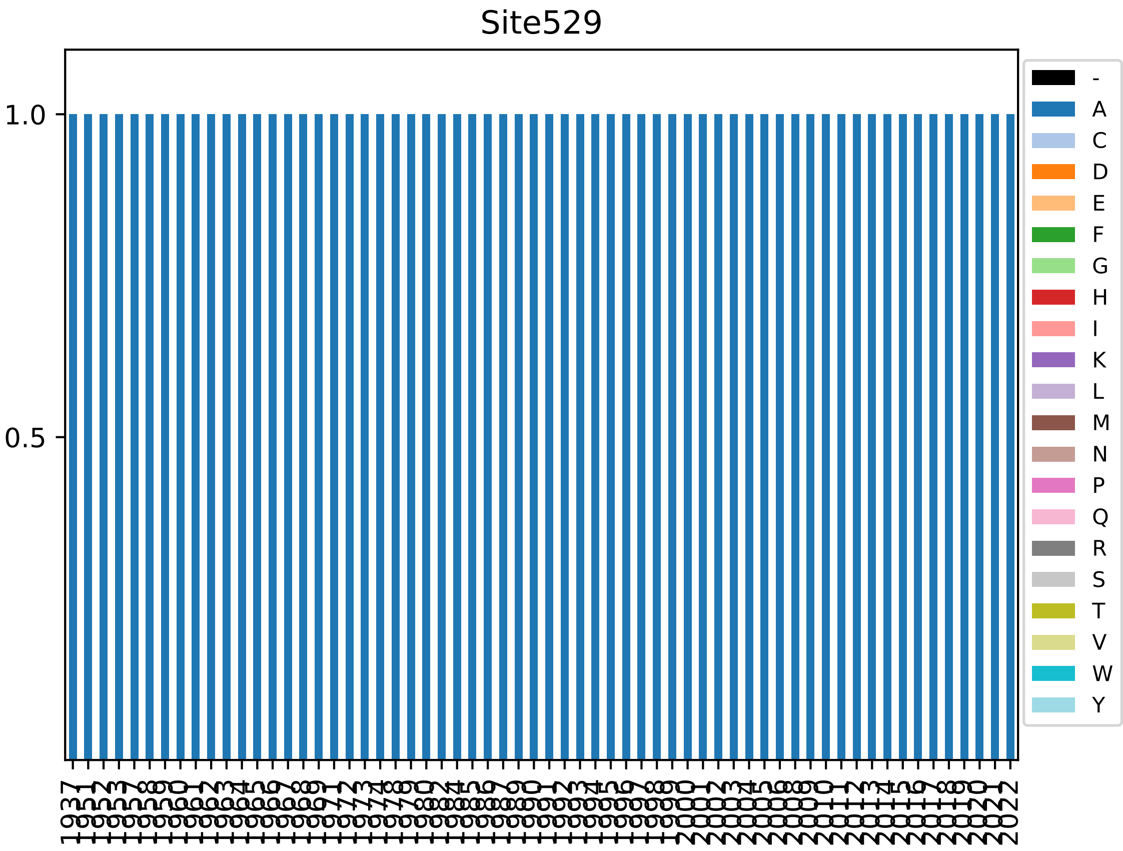
Site529
1.0
0.5
-
A
C
D
E
F
G
H
I
K
L
M
N
P
Q
R
S
T
V
W
Y

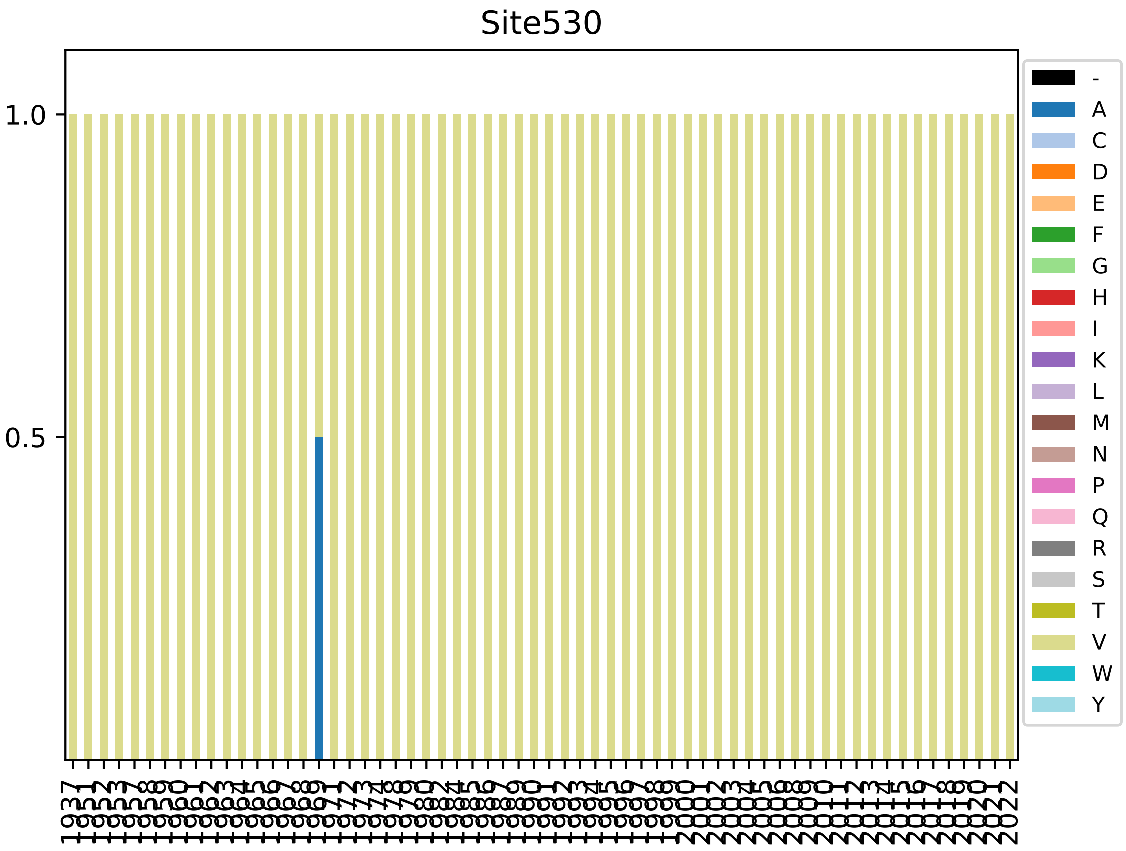
Site530
1.0
0.5
-
A
C
D
E
F
G
H
I
K
L
M
N
P
Q
R
S
T
V
W
Y

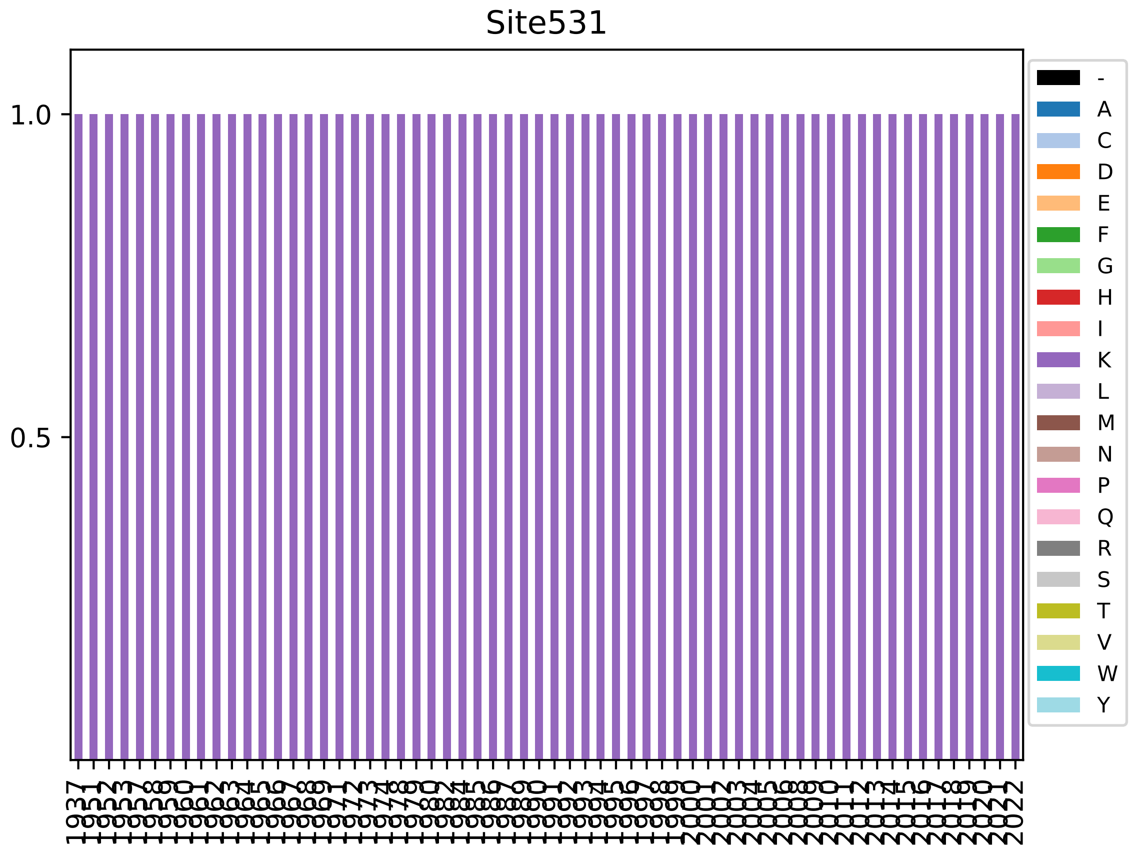
Site531
1.0
0.5
-
A
C
D
E
F
G
H
I
K
L
M
N
P
Q
R
S
T
V
W
Y

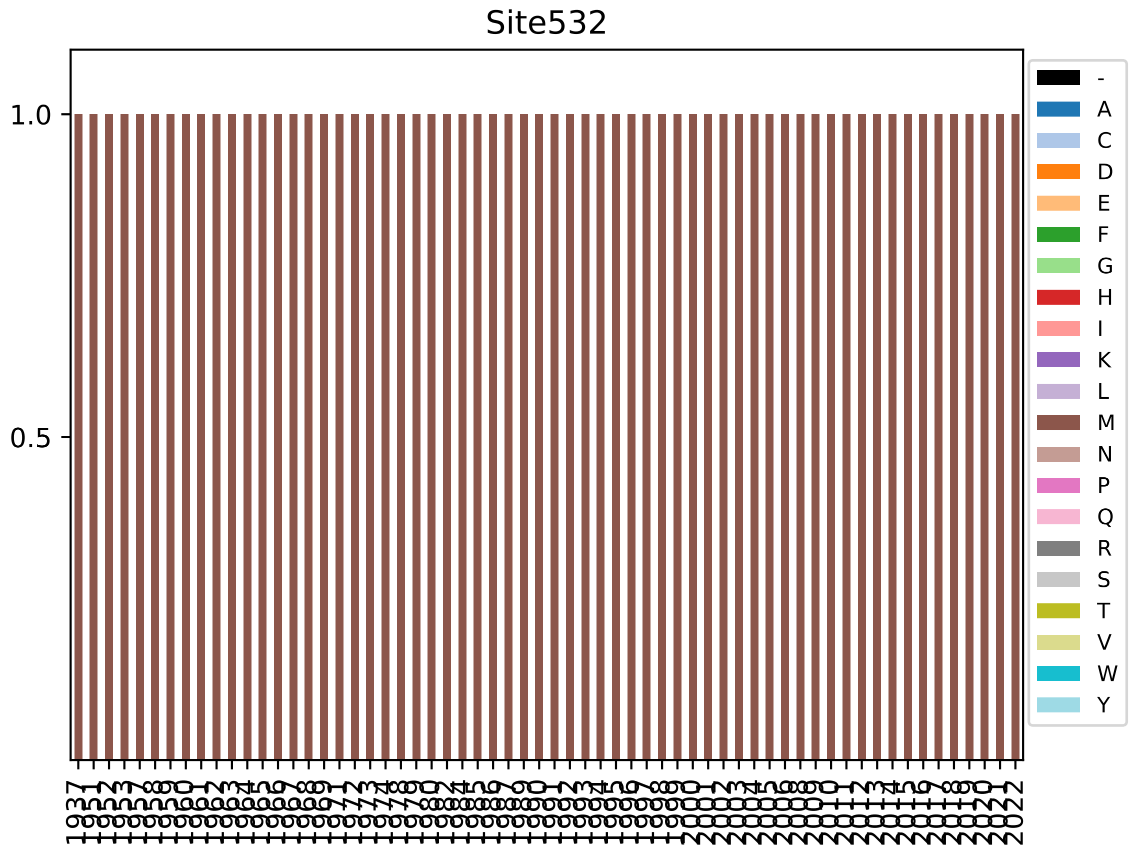
Site532
1.0
0.5
-
A
C
D
E
F
G
H
I
K
L
M
N
P
Q
R
S
T
V
W
Y

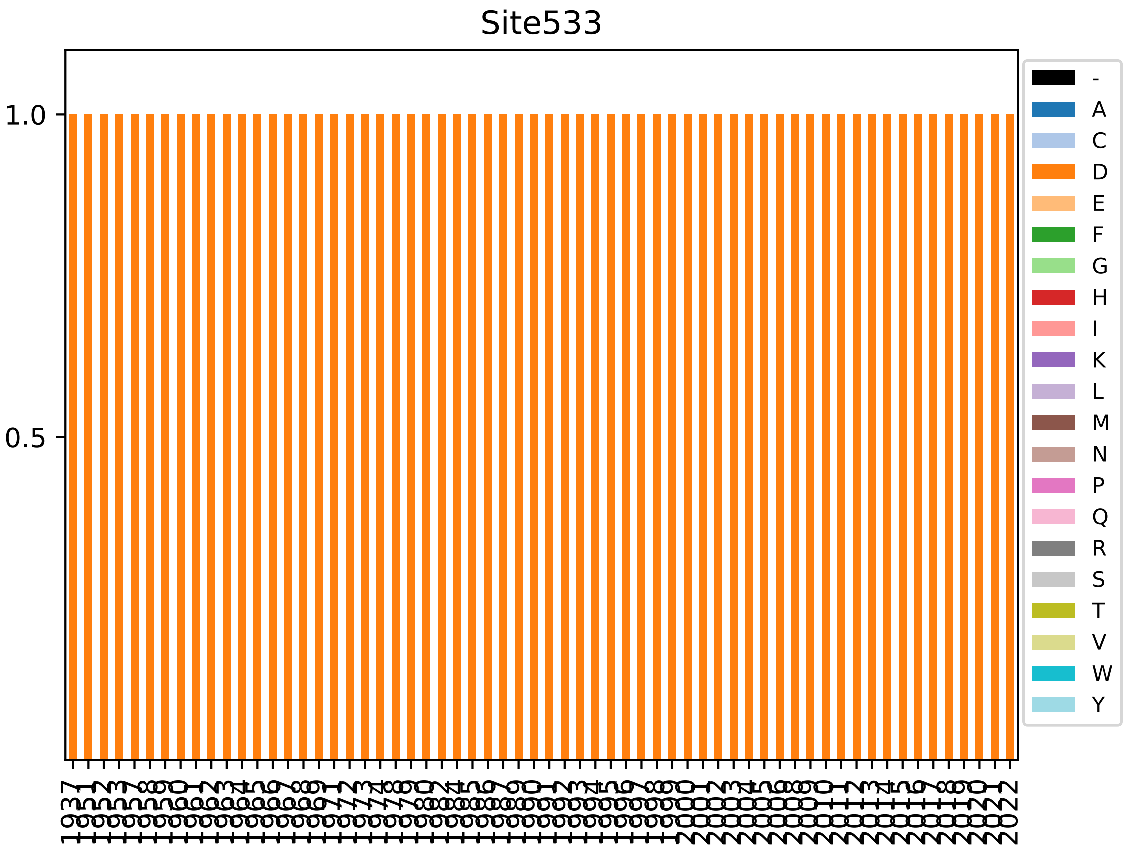
Site533
1.0
0.5
-
A
C
D
E
F
G
H
I
K
L
M
N
P
Q
R
S
T
V
W
Y

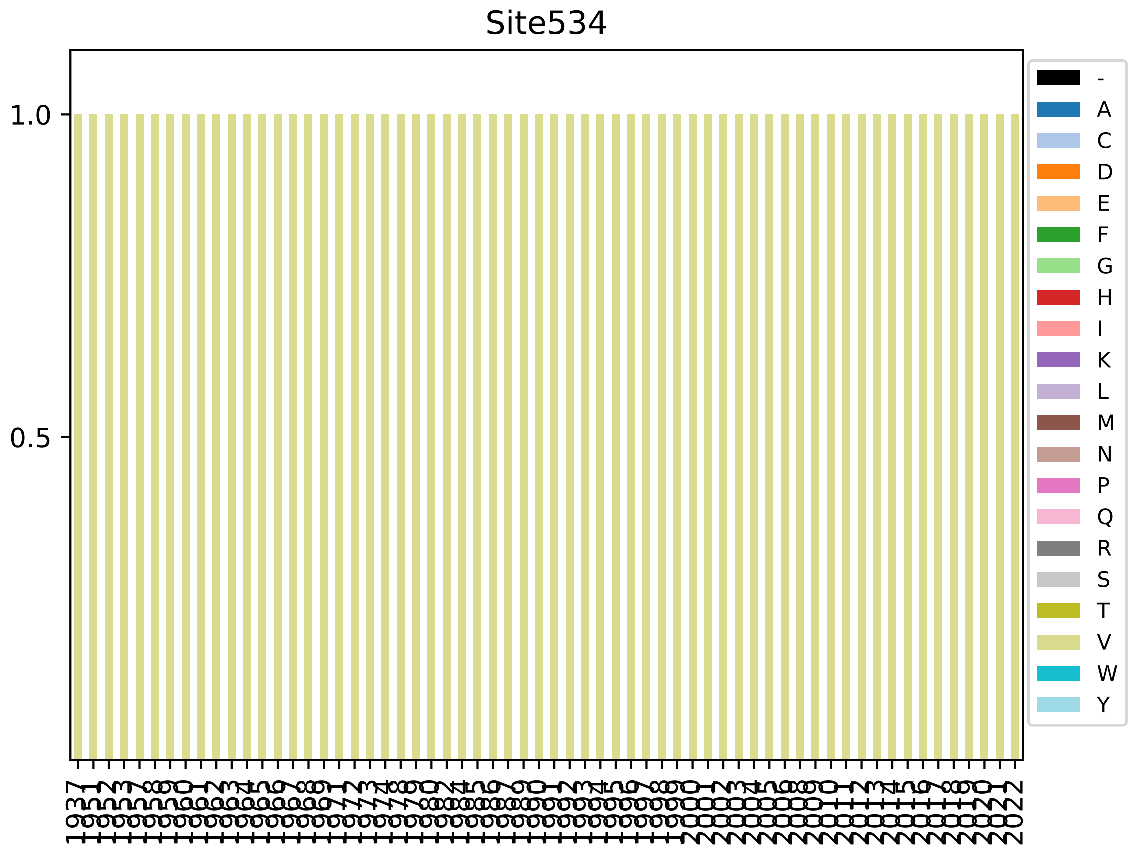
Site534
1.0
0.5
-
A
C
D
E
F
G
H
I
K
L
M
N
P
Q
R
S
T
V
W
Y

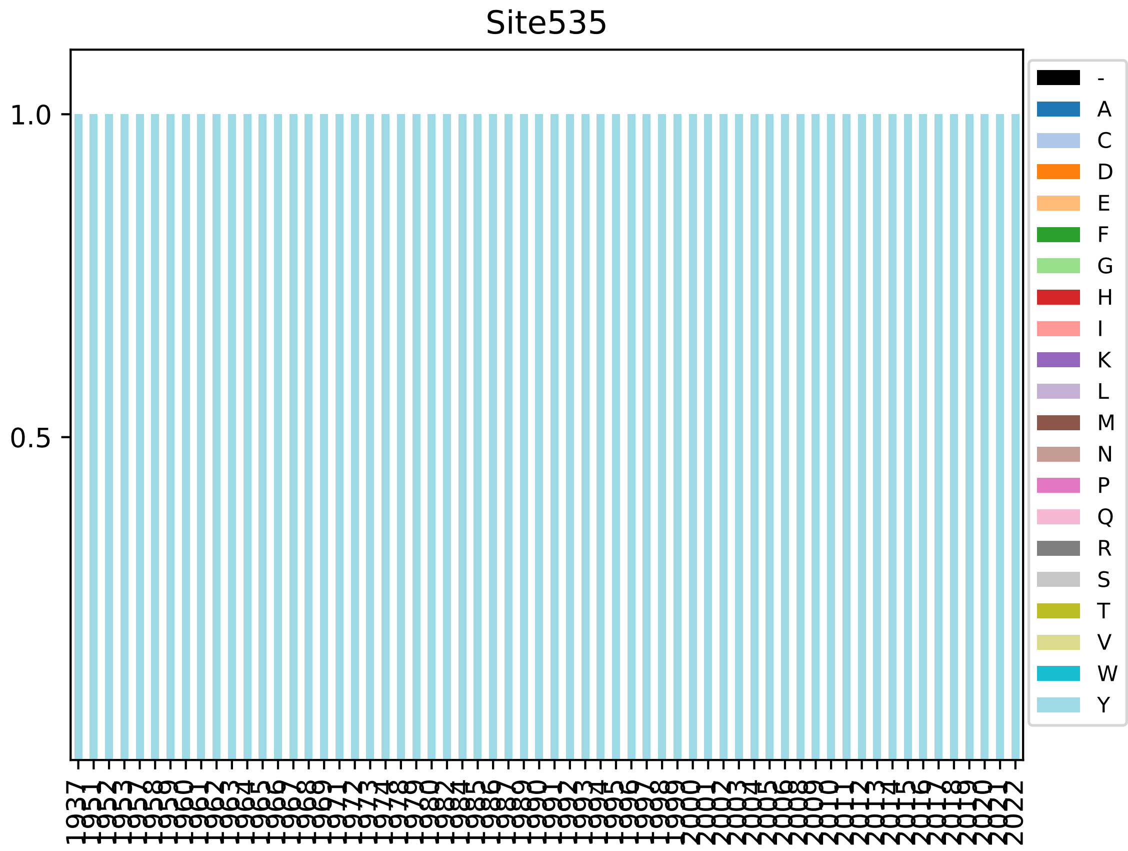
Site535
1.0
0.5
-
A
C
D
E
F
G
H
I
K
L
M
N
P
Q
R
S
T
V
W
Y

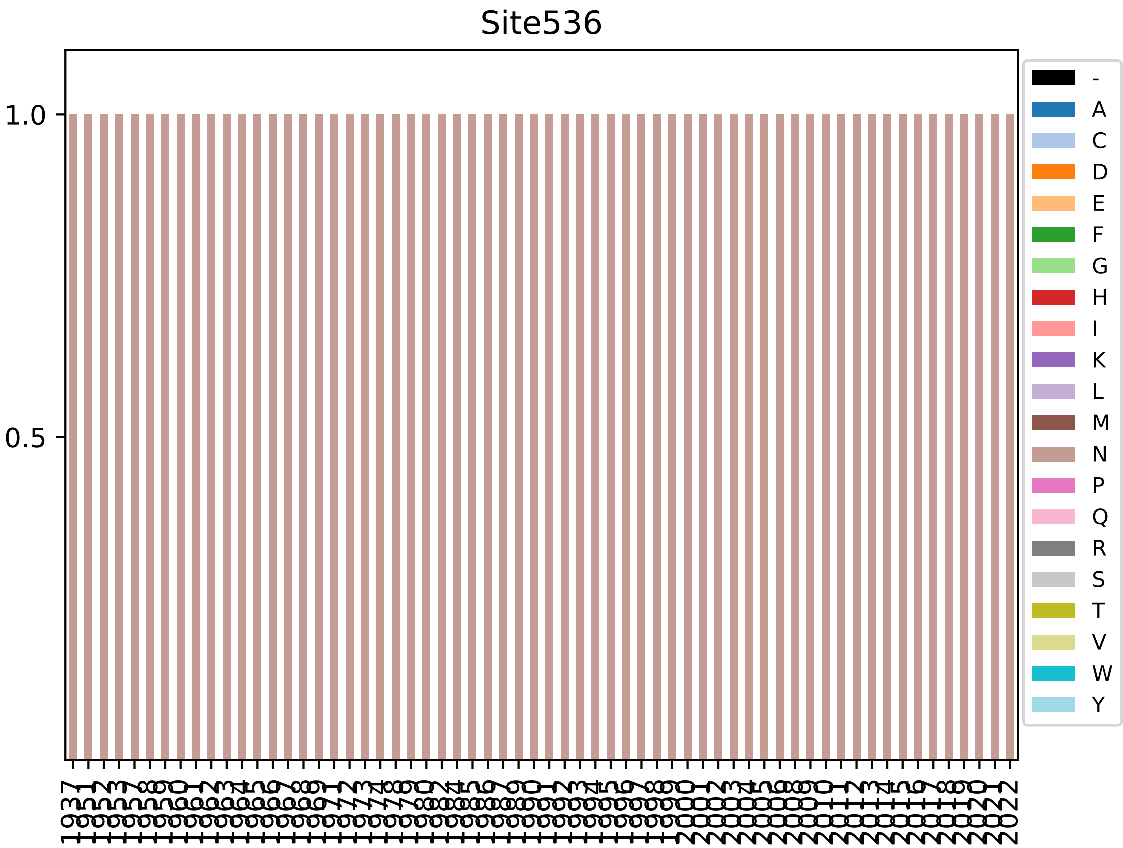
Site536
1.0
0.5
-
A
C
D
E
F
G
H
I
K
L
M
N
P
Q
R
S
T
V
W
Y

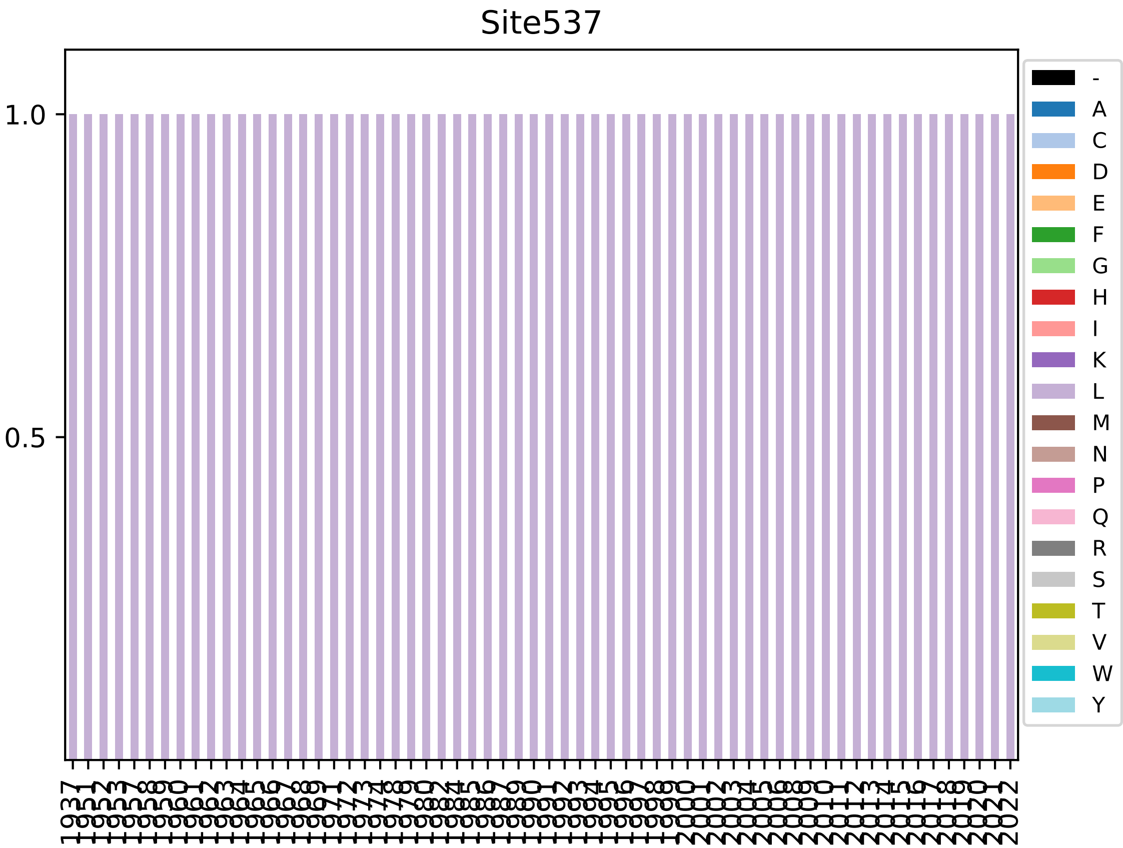
Site537
1.0
0.5
-
A
C
D
E
F
G
H
I
K
L
M
N
P
Q
R
S
T
V
W
Y

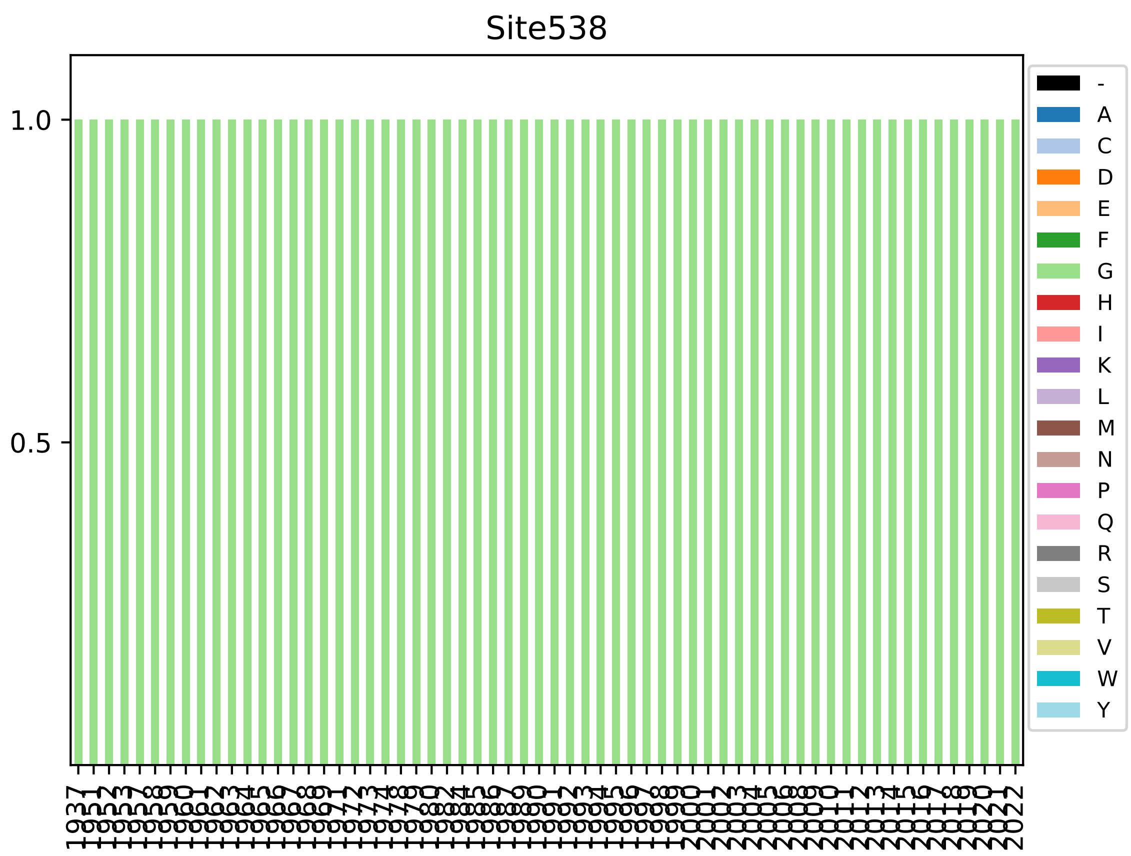
Site538
1.0
0.5
-
A
C
D
E
F
G
H
I
K
L
M
N
P
Q
R
S
T
V
W
Y

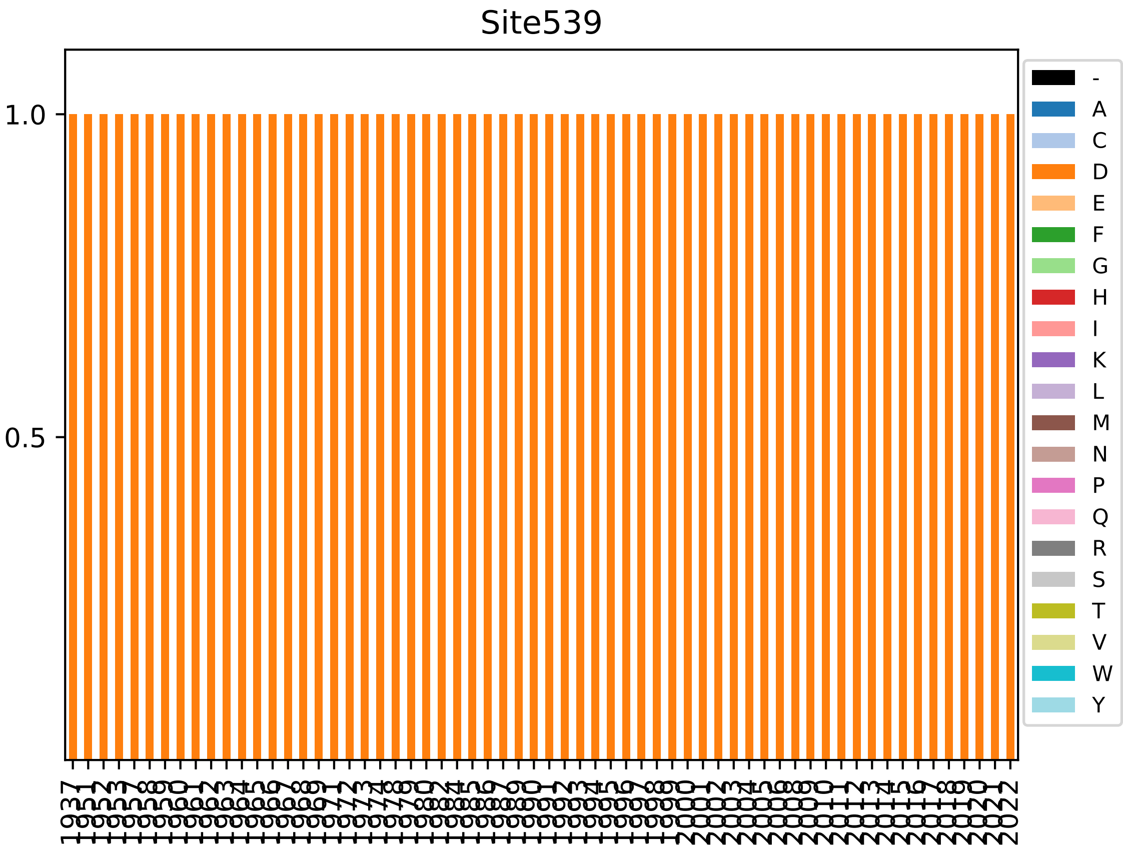
Site539
1.0
0.5
-
A
C
D
E
F
G
H
I
K
L
M
N
P
Q
R
S
T
V
W
Y

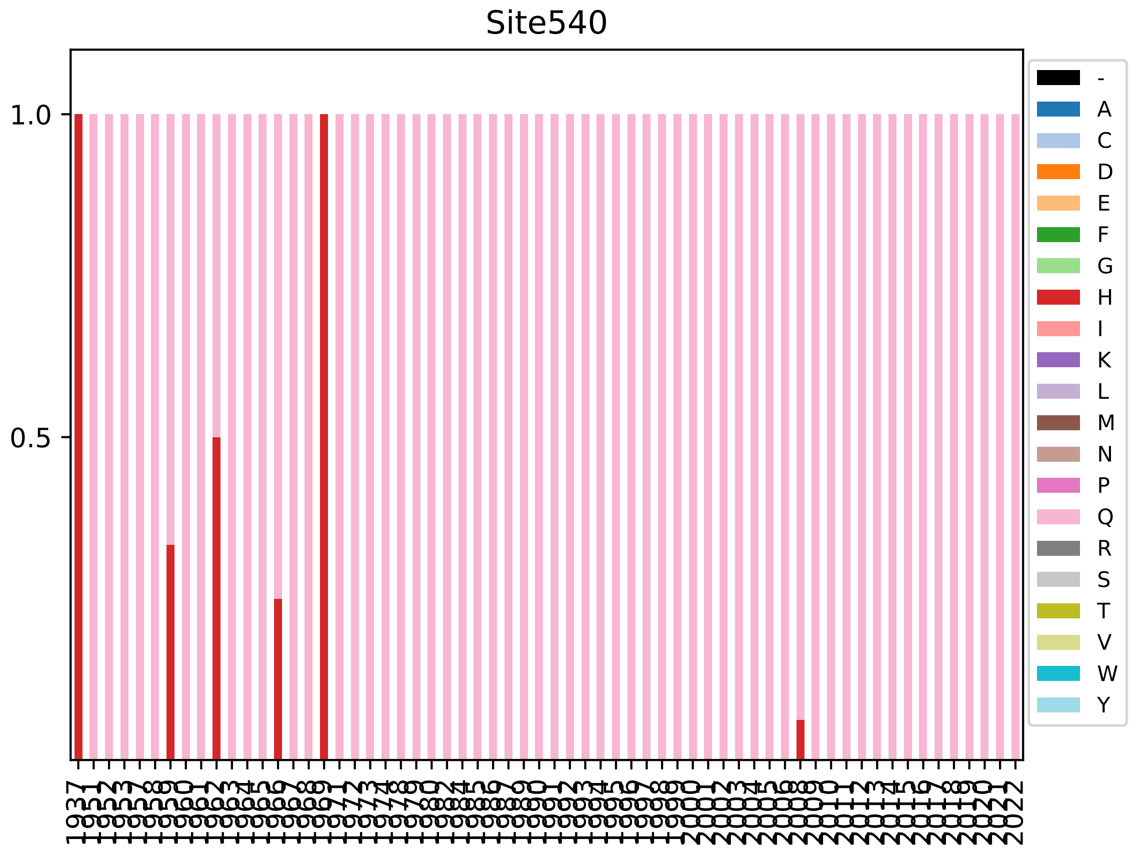
Site540
1.0
0.5
-
A
C
D
E
F
G
H
I
K
L
M
N
P
Q
R
S
T
V
W
Y

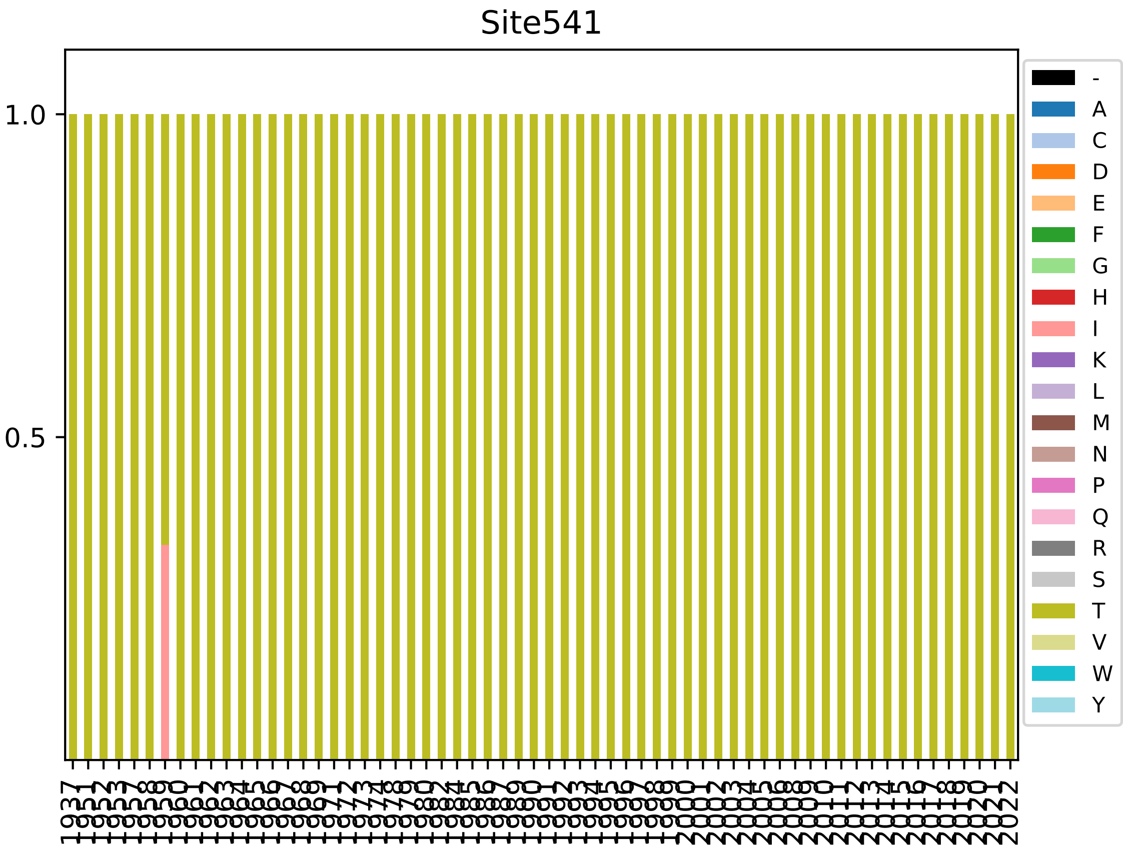
Site541
1.0
0.5
-
A
C
D
E
F
G
H
I
K
L
M
N
P
Q
R
S
T
V
W
Y

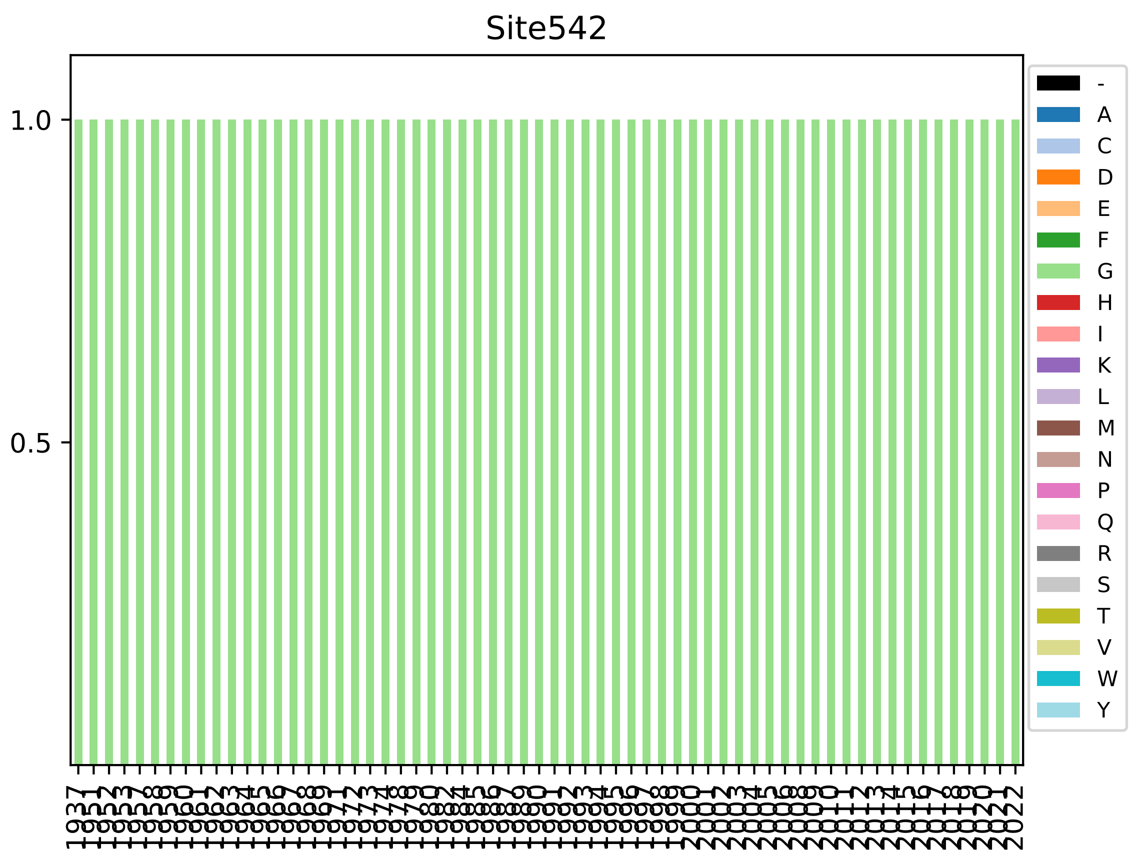
Site542
1.0
0.5
-
A
C
D
E
F
G
H
I
K
L
M
N
P
Q
R
S
T
V
W
Y

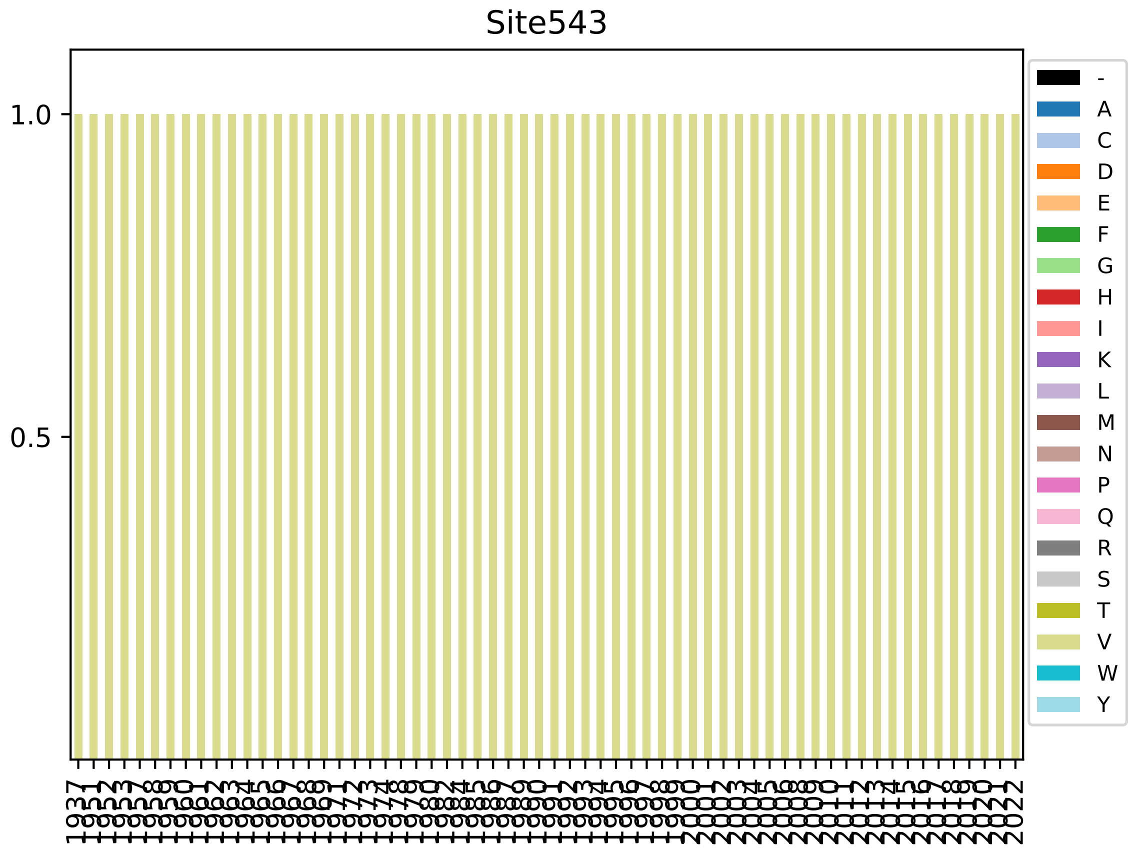
Site543
1.0
0.5
-
A
C
D
E
F
G
H
I
K
L
M
N
P
Q
R
S
T
V
W
Y

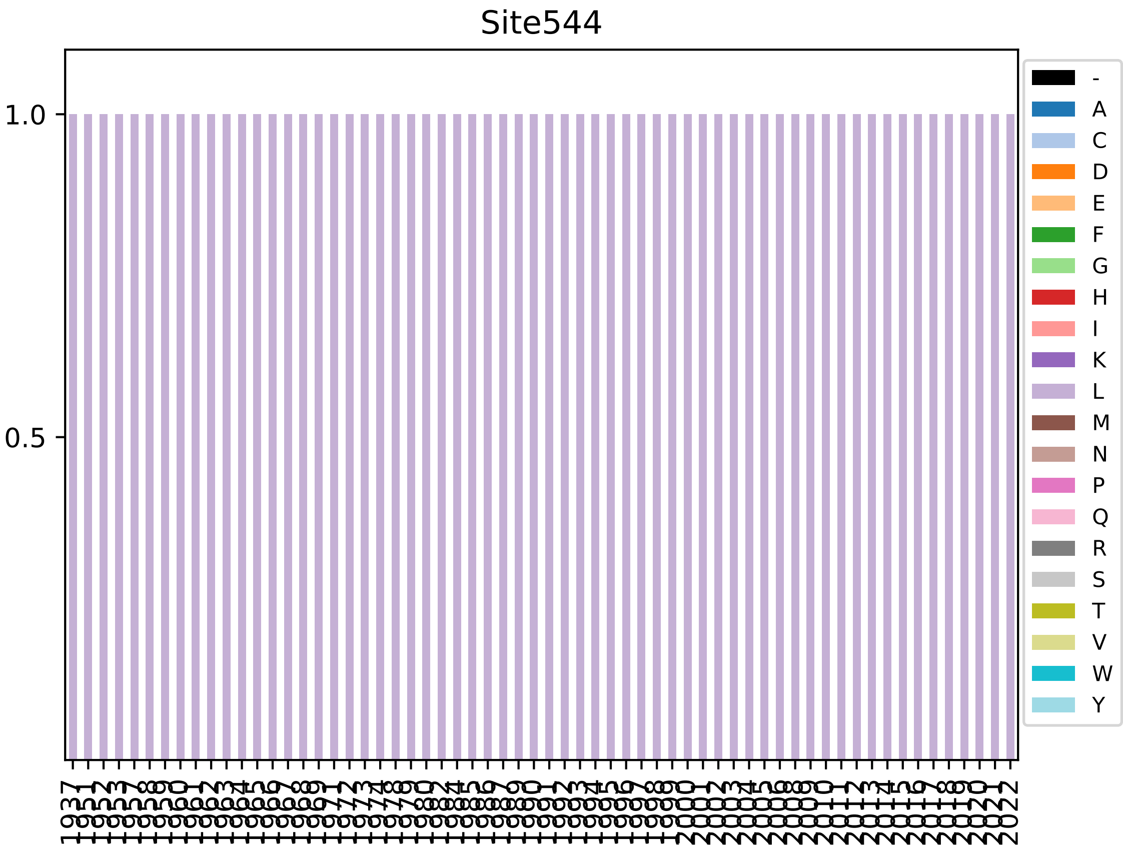
Site544
1.0
0.5
-
A
C
D
E
F
G
H
I
K
L
M
N
P
Q
R
S
T
V
W
Y

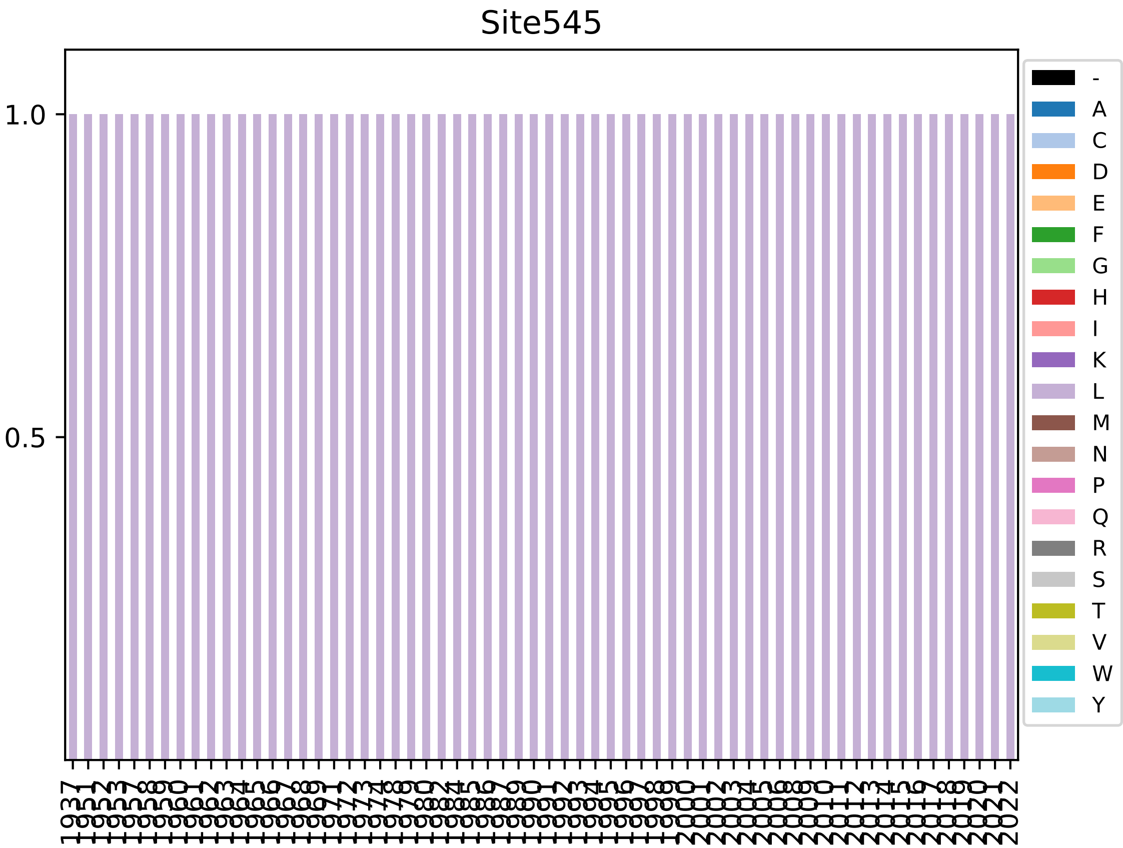
Site545
1.0
0.5
-
A
C
D
E
F
G
H
I
K
L
M
N
P
Q
R
S
T
V
W
Y

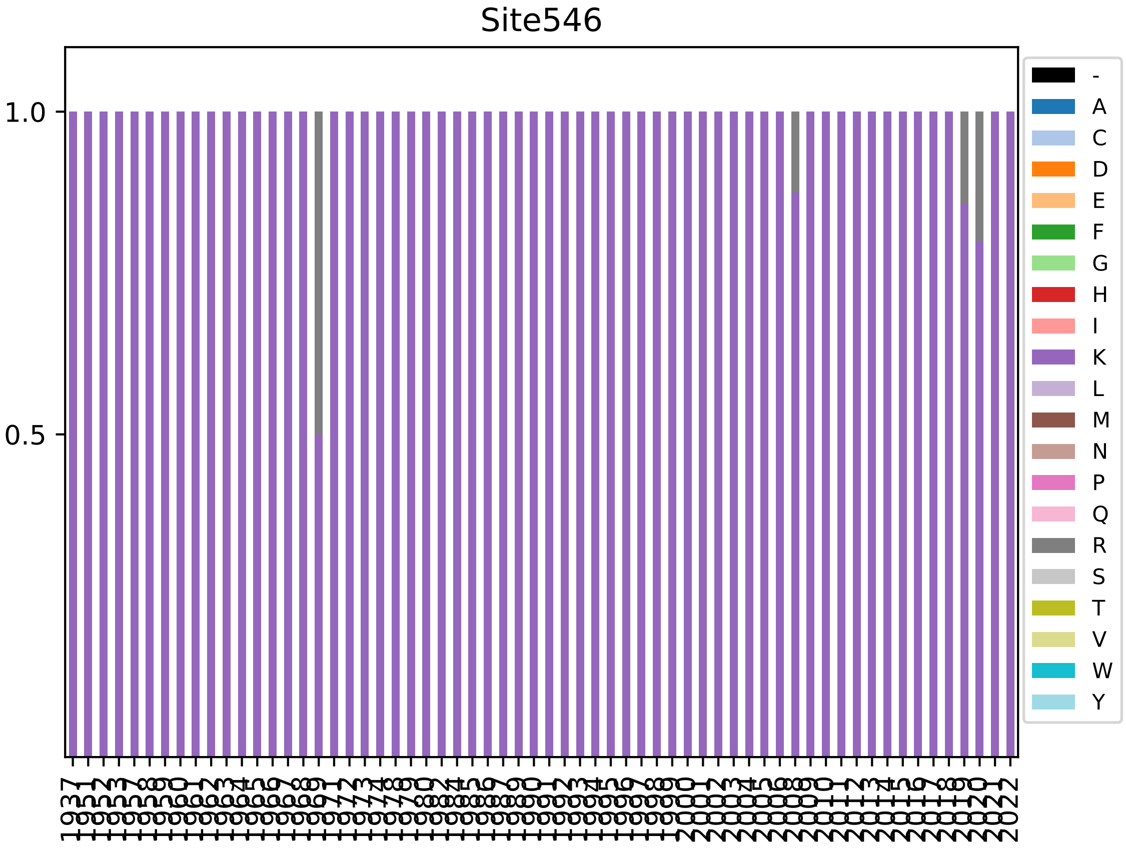
Site546
1.0
0.5
-
A
C
D
E
F
G
H
I
K
L
M
N
P
Q
R
S
T
V
W
Y

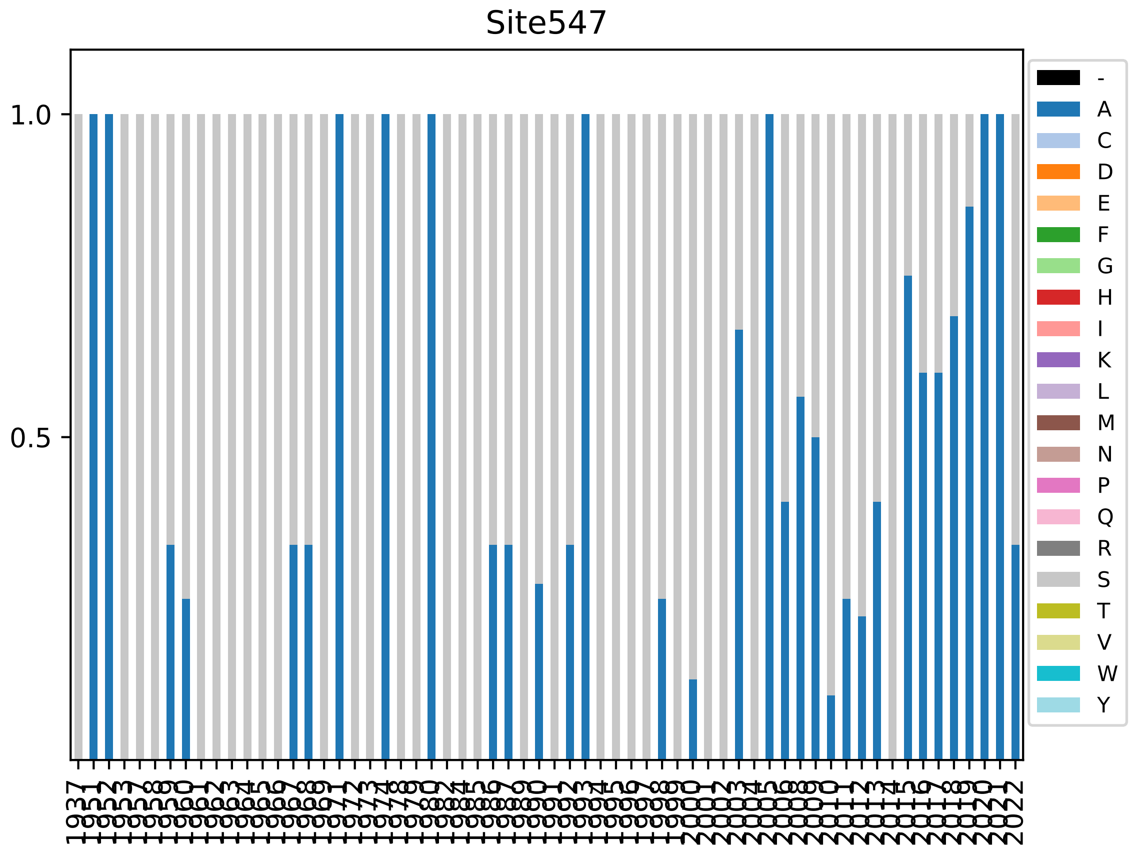
Site547
1.0
0.5
-
A
C
D
E
F
G
H
I
K
L
M
N
P
Q
R
S
T
V
W
Y

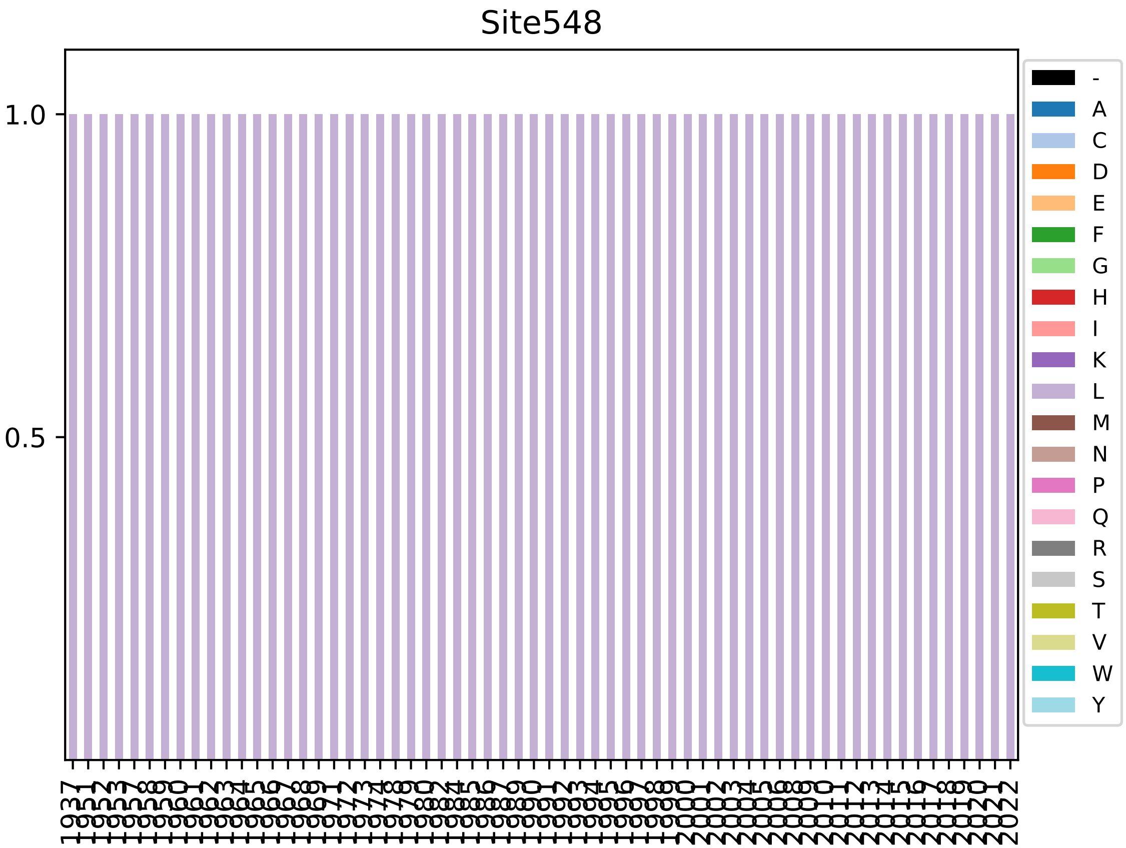
Site548
1.0
0.5
-
A
C
D
E
F
G
H
I
K
L
M
N
P
Q
R
S
T
V
W
Y

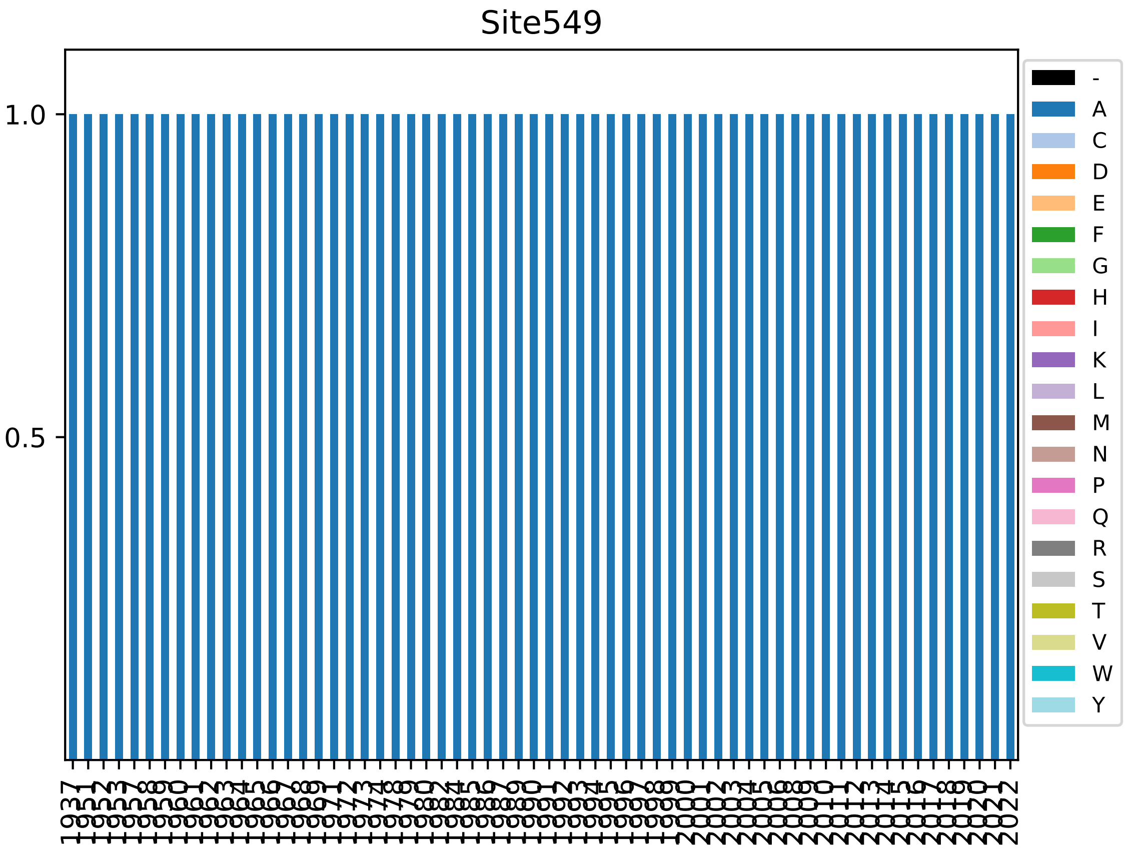
Site549
1.0
0.5
-
A
C
D
E
F
G
H
I
K
L
M
N
P
Q
R
S
T
V
W
Y

Site550

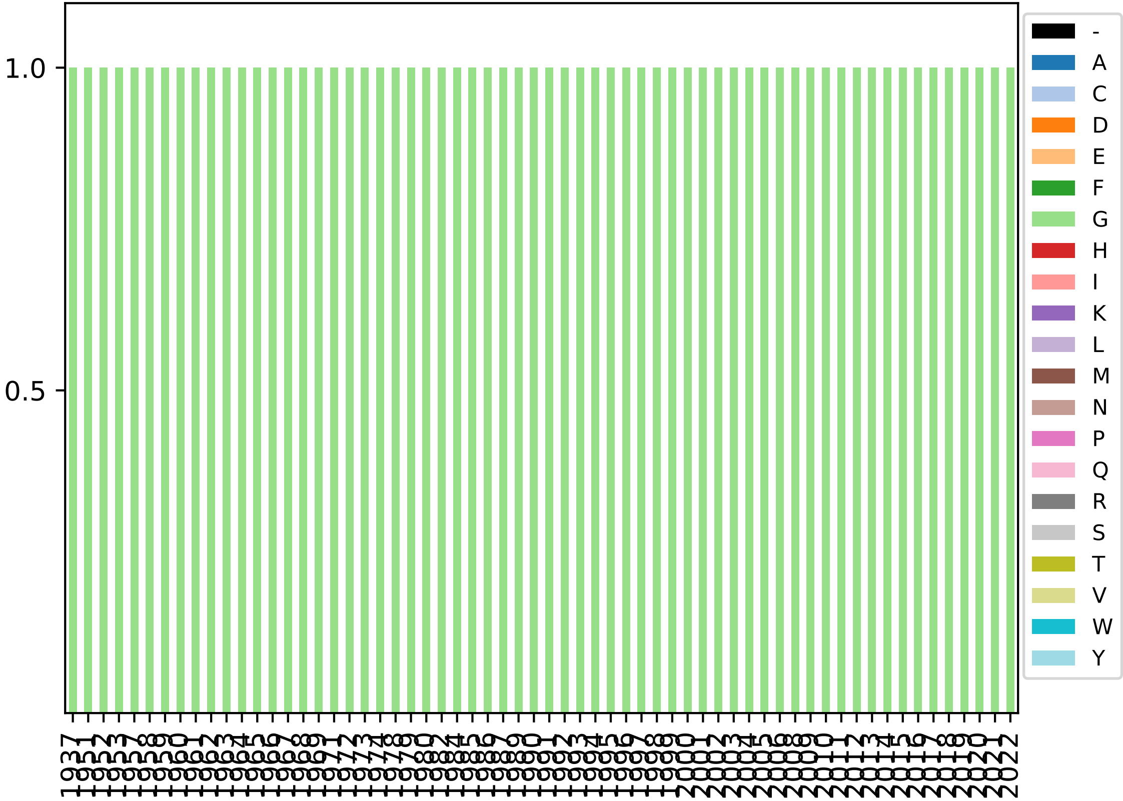

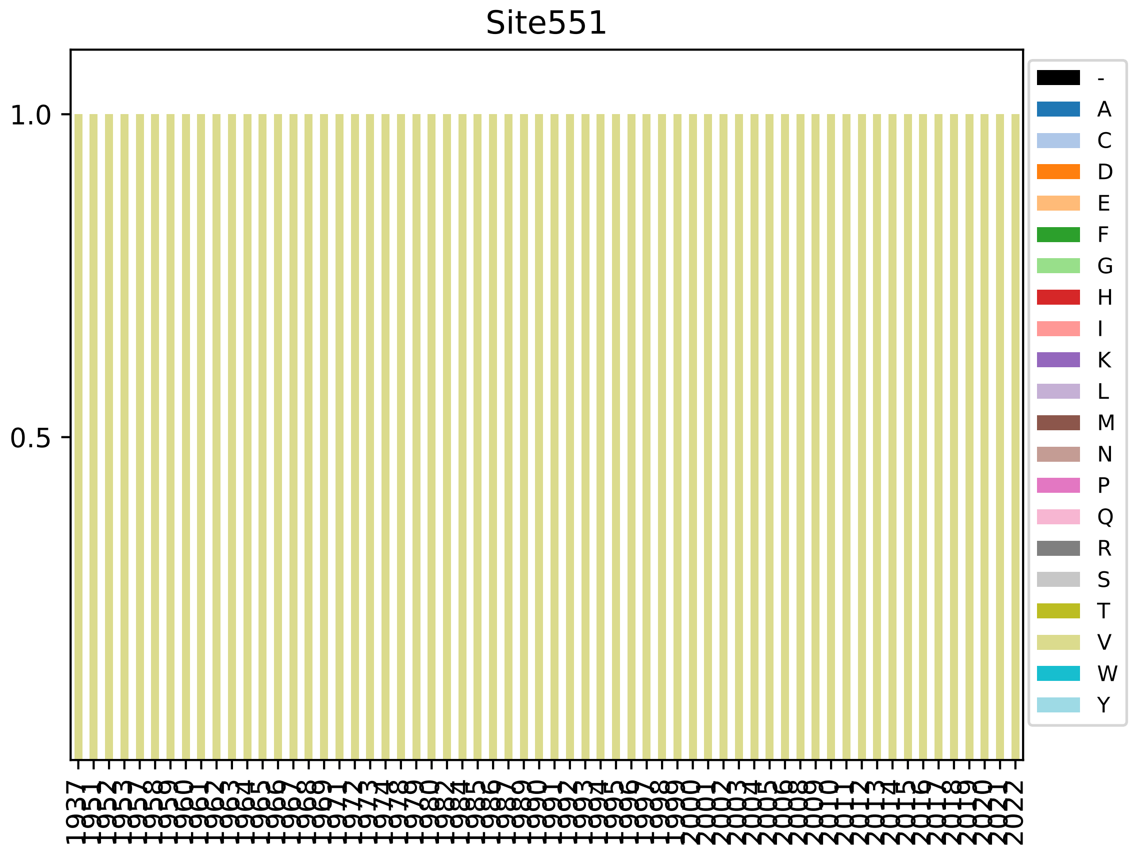
Site551
1.0
0.5
-
A
C
D
E
F
G
H
I
K
L
M
N
P
Q
R
S
T
V
W
Y

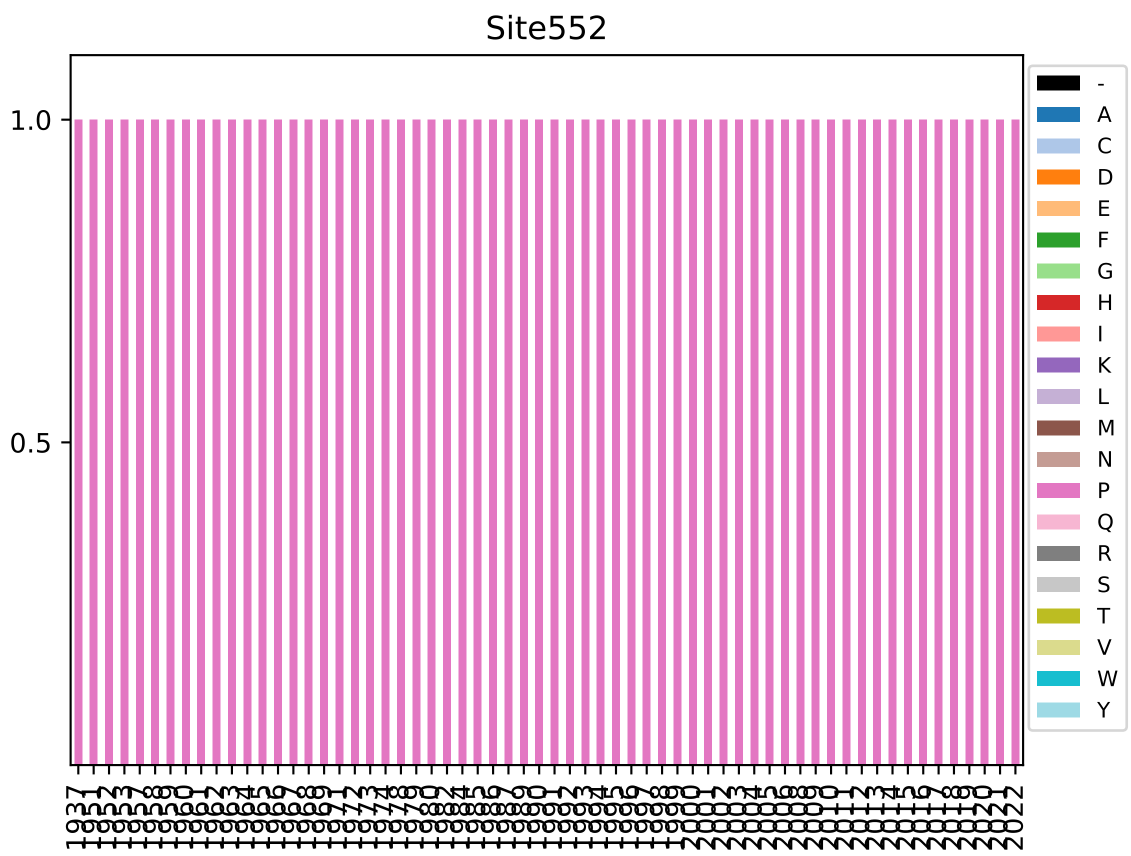
Site552
1.0
0.5
-
A
C
D
E
F
G
H
I
K
L
M
N
P
Q
R
S
T
V
W
Y

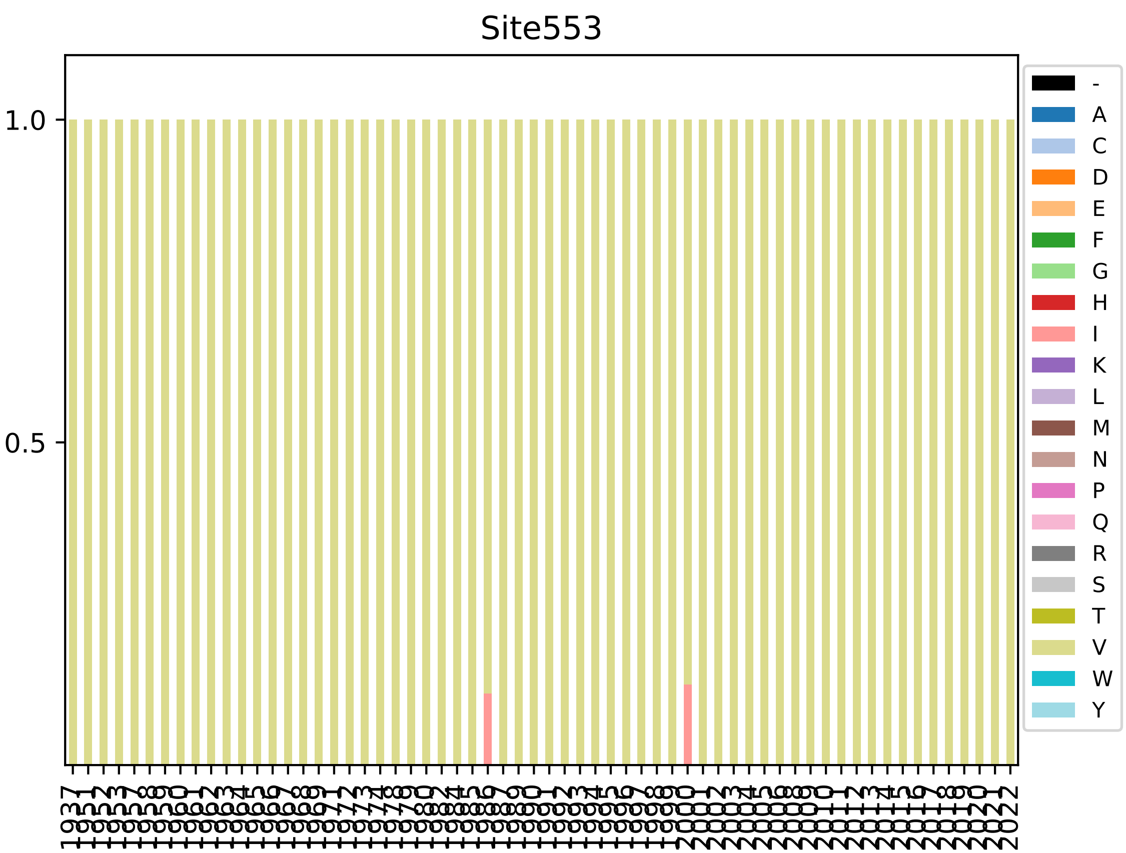
Site553
1.0
0.5
-
A
C
D
E
F
G
H
I
K
L
M
N
P
Q
R
S
T
V
W
Y

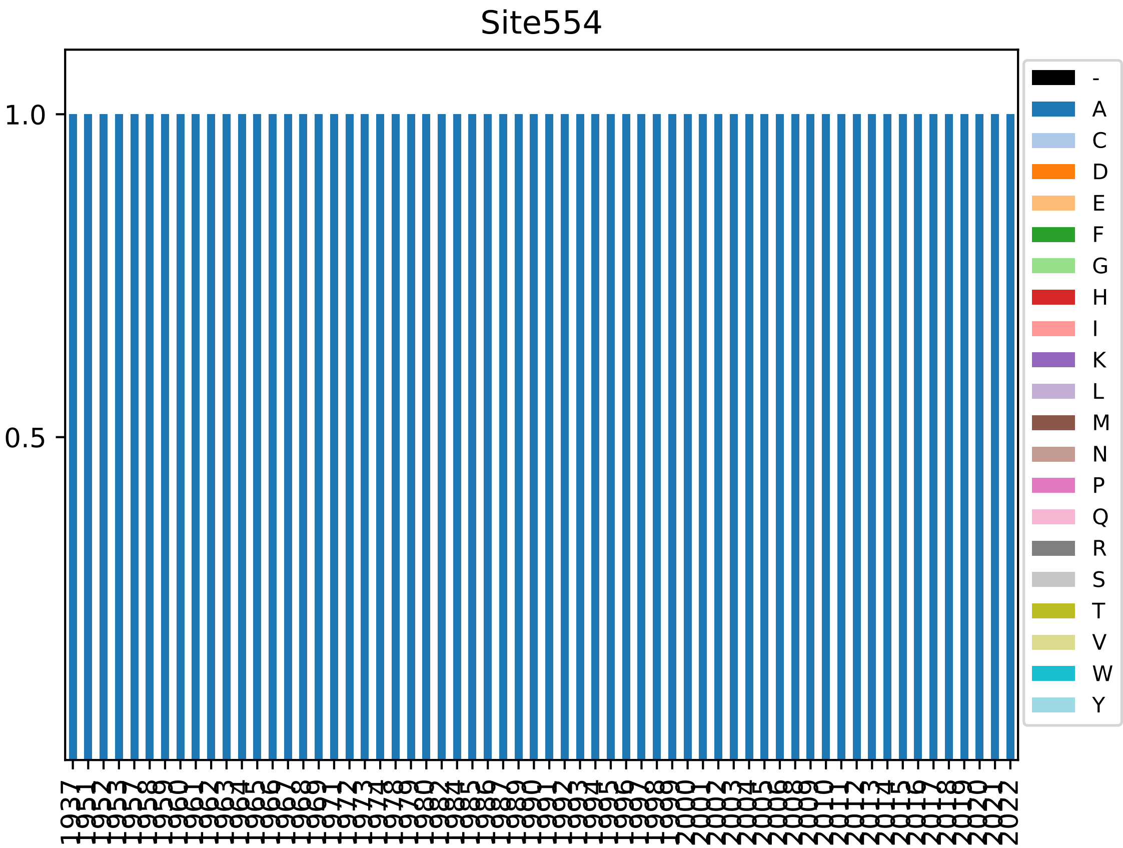
Site554
1.0
0.5
-
A
C
D
E
F
G
H
I
K
L
M
N
P
Q
R
S
T
V
W
Y

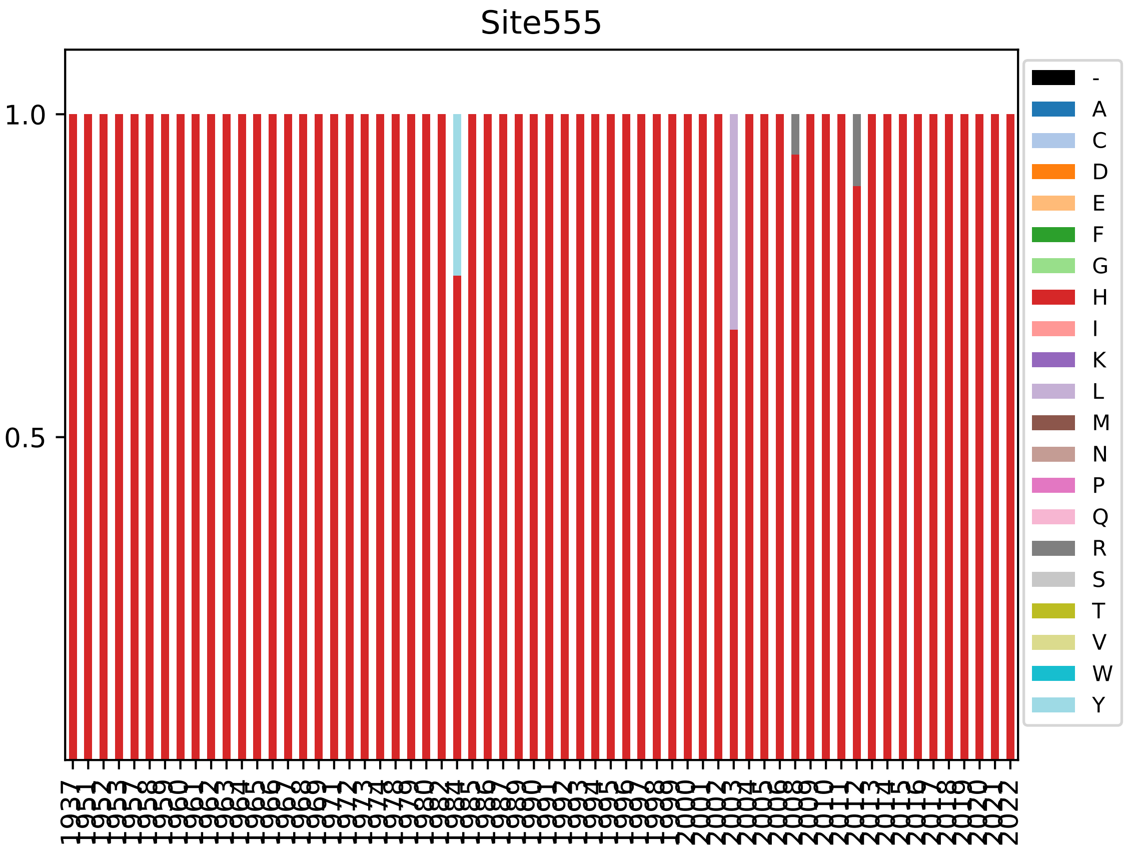
Site555
1.0
0.5
-
A
C
D
E
F
G
H
I
K
L
M
N
P
Q
R
S
T
V
W
Y

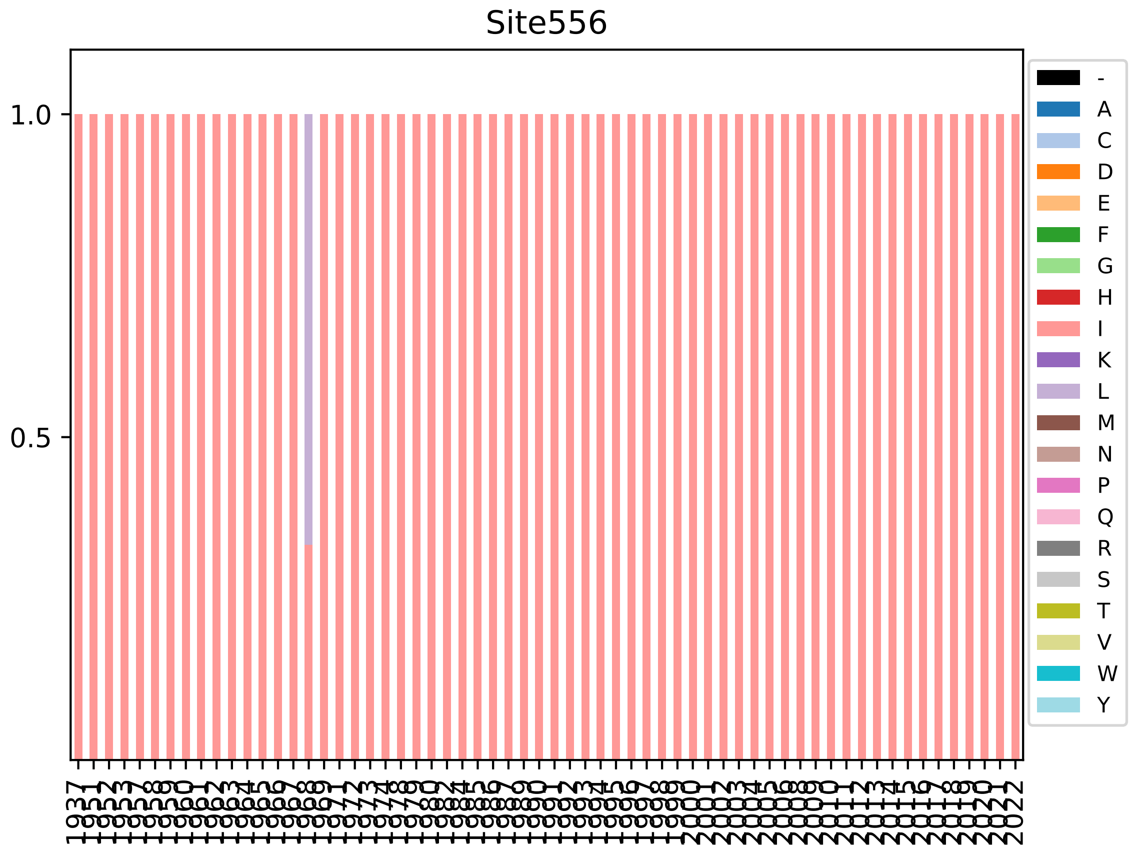
Site556
1.0
0.5
-
A
C
D
E
F
G
H
I
K
L
M
N
P
Q
R
S
T
V
W
Y

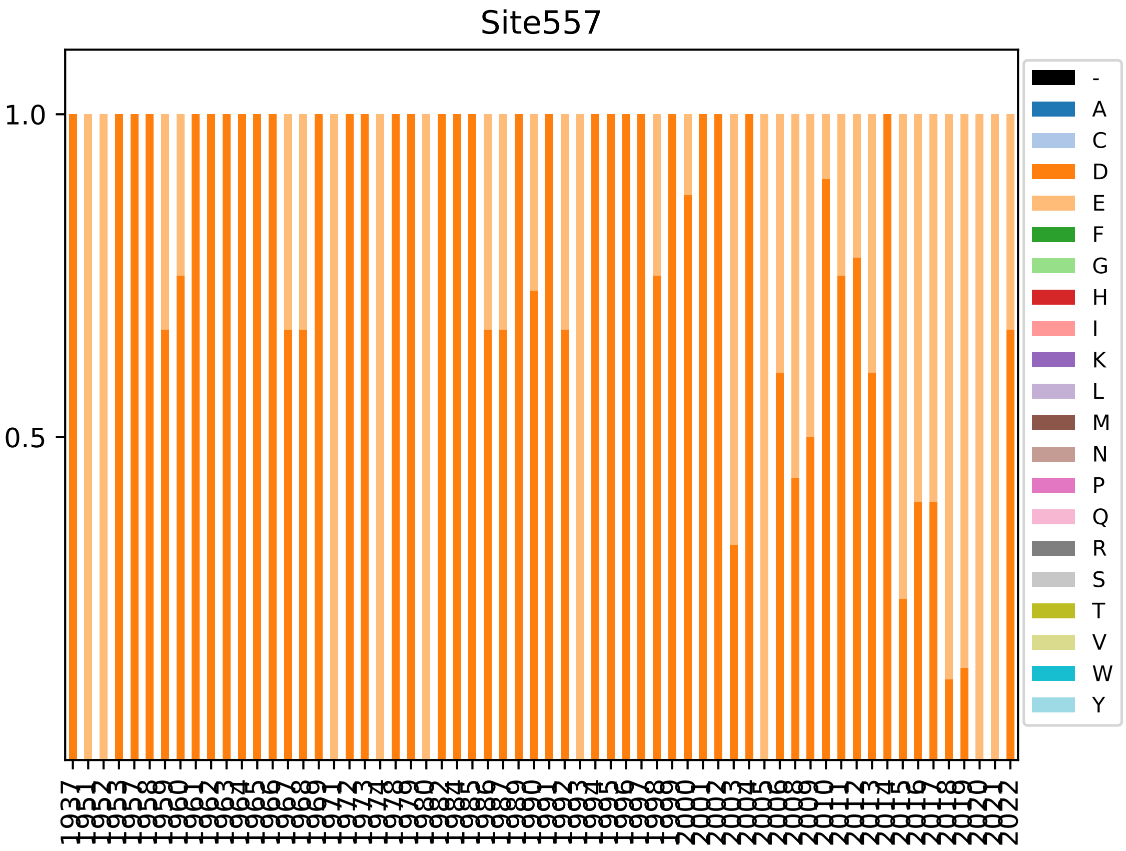
Site557
1.0
0.5
-
A
C
D
E
F
G
H
I
K
L
M
N
P
Q
R
S
T
V
W
Y

Site558

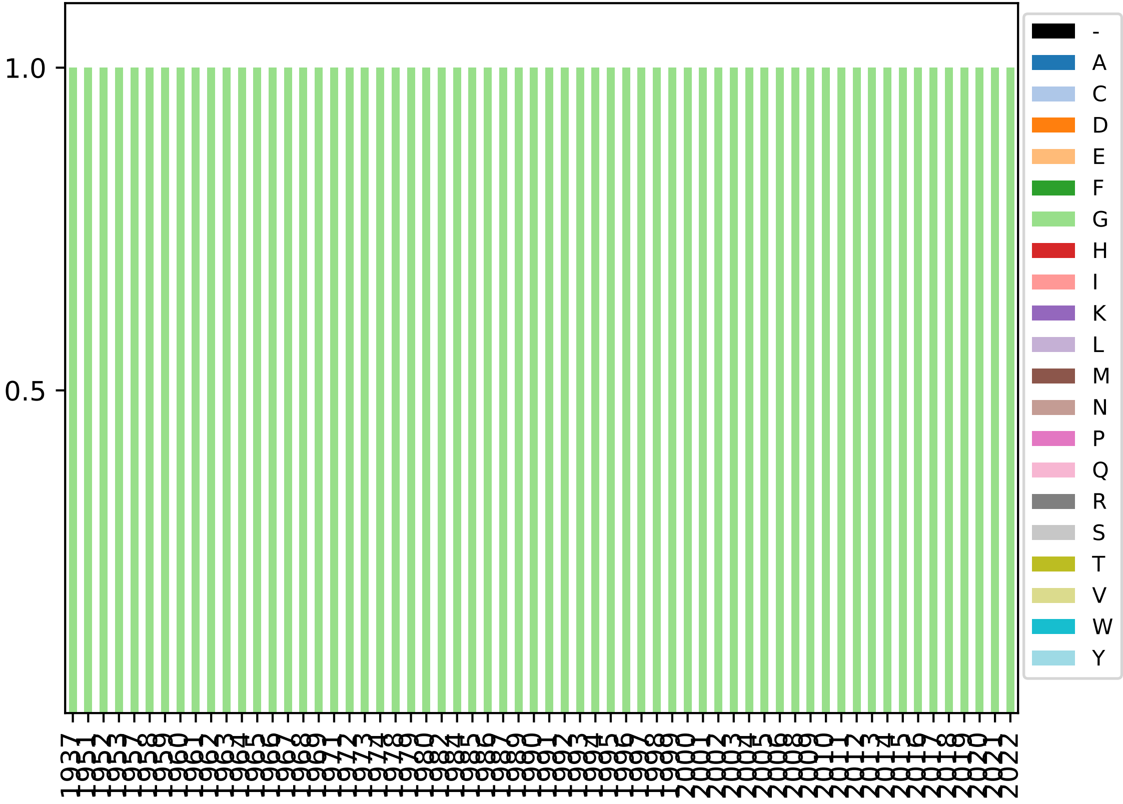

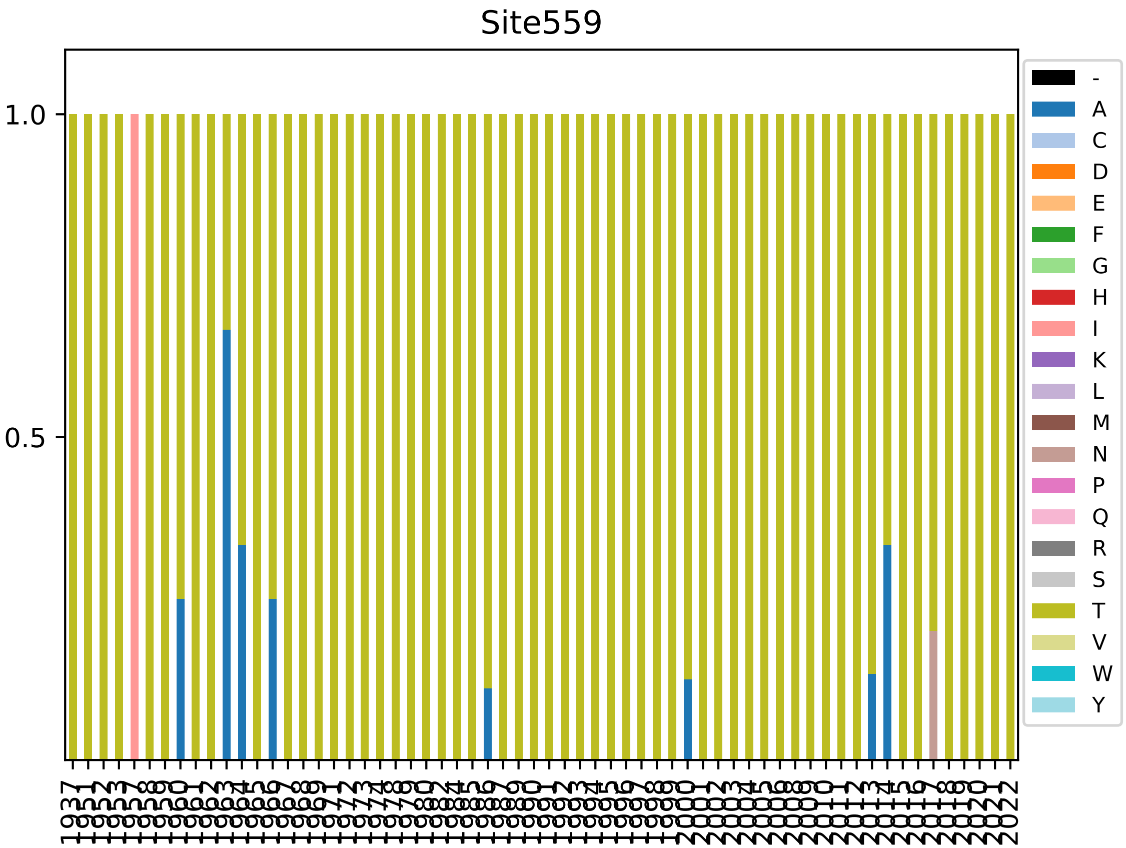
Site559
1.0
0.5
-
A
C
D
E
F
G
H
I
K
L
M
N
P
Q
R
S
T
V
W
Y

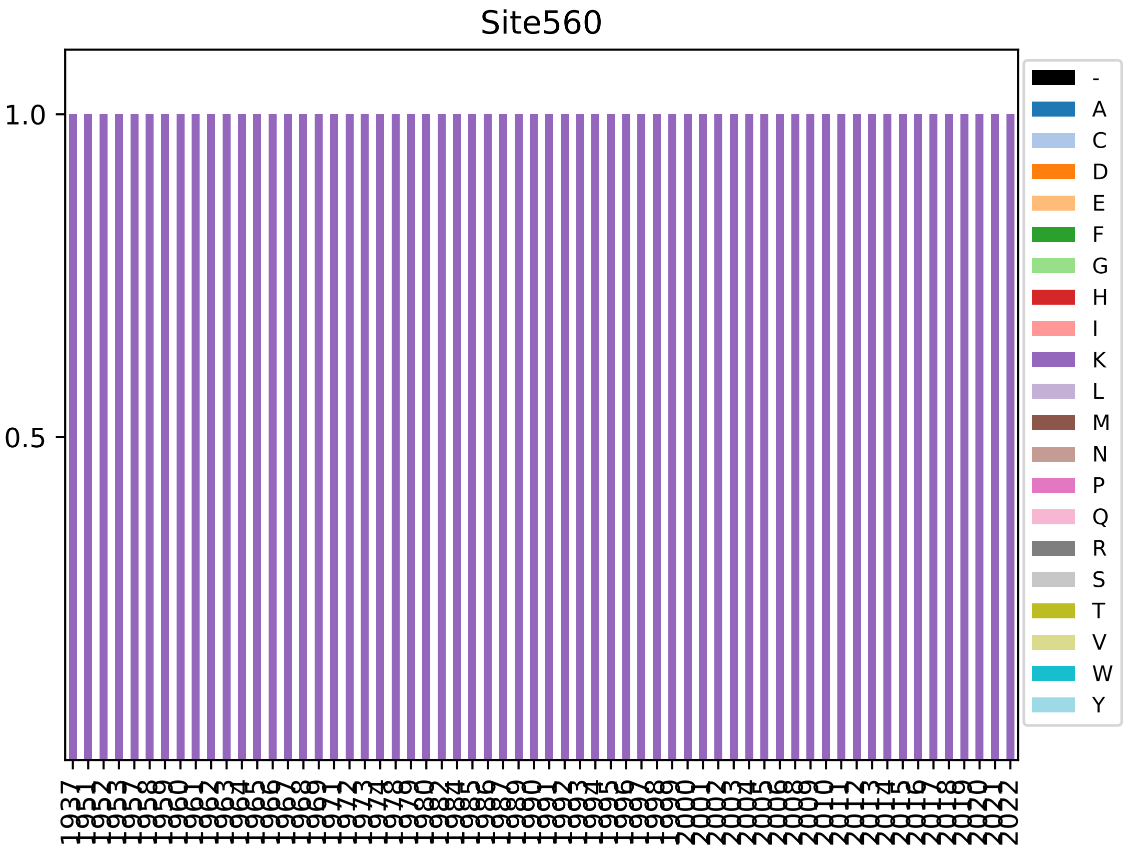
Site560
1.0
0.5
-
A
C
D
E
F
G
H
I
K
L
M
N
P
Q
R
S
T
V
W
Y

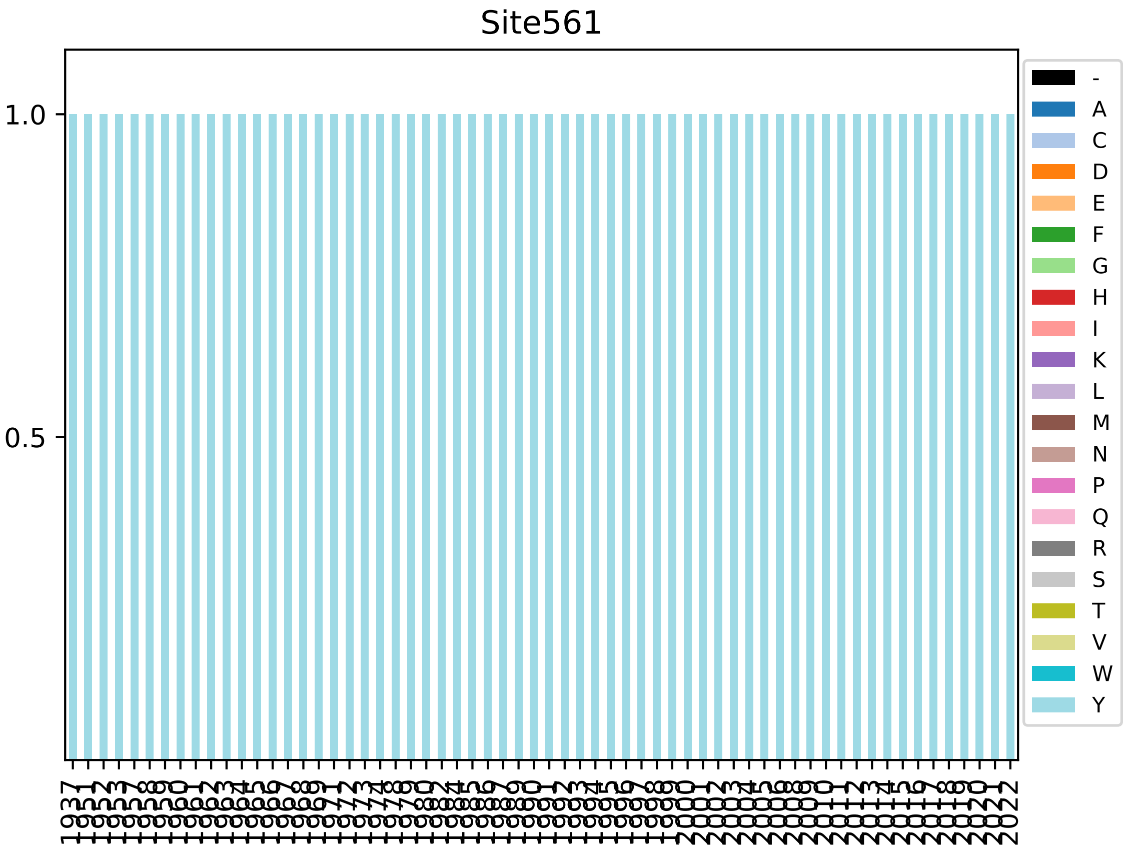

Site561
1.0
0.5
-
A
C
D
E
F
G
H
I
K
L
M
N
P
Q
R
S
T
V
W
Y

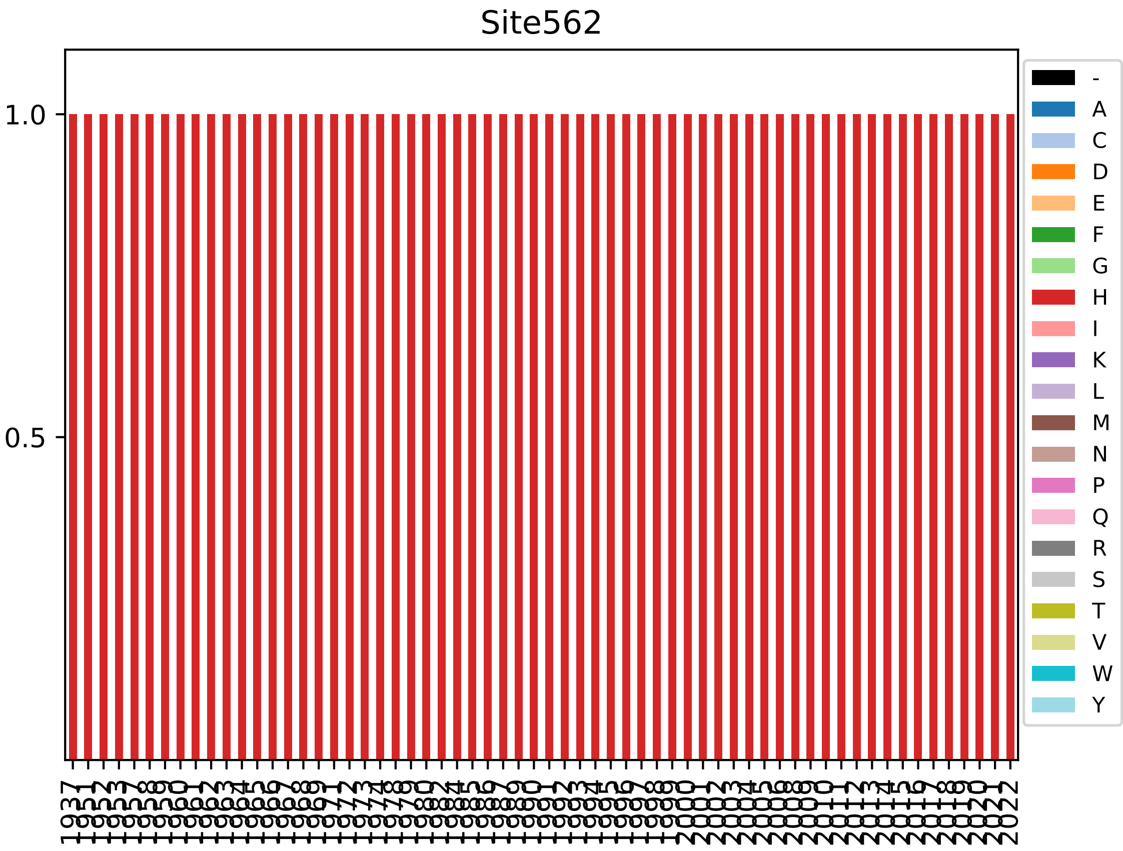
Site562
1.0
0.5
-
A
C
D
E
F
G
H
I
K
L
M
N
P
Q
R
S
T
V
W
Y

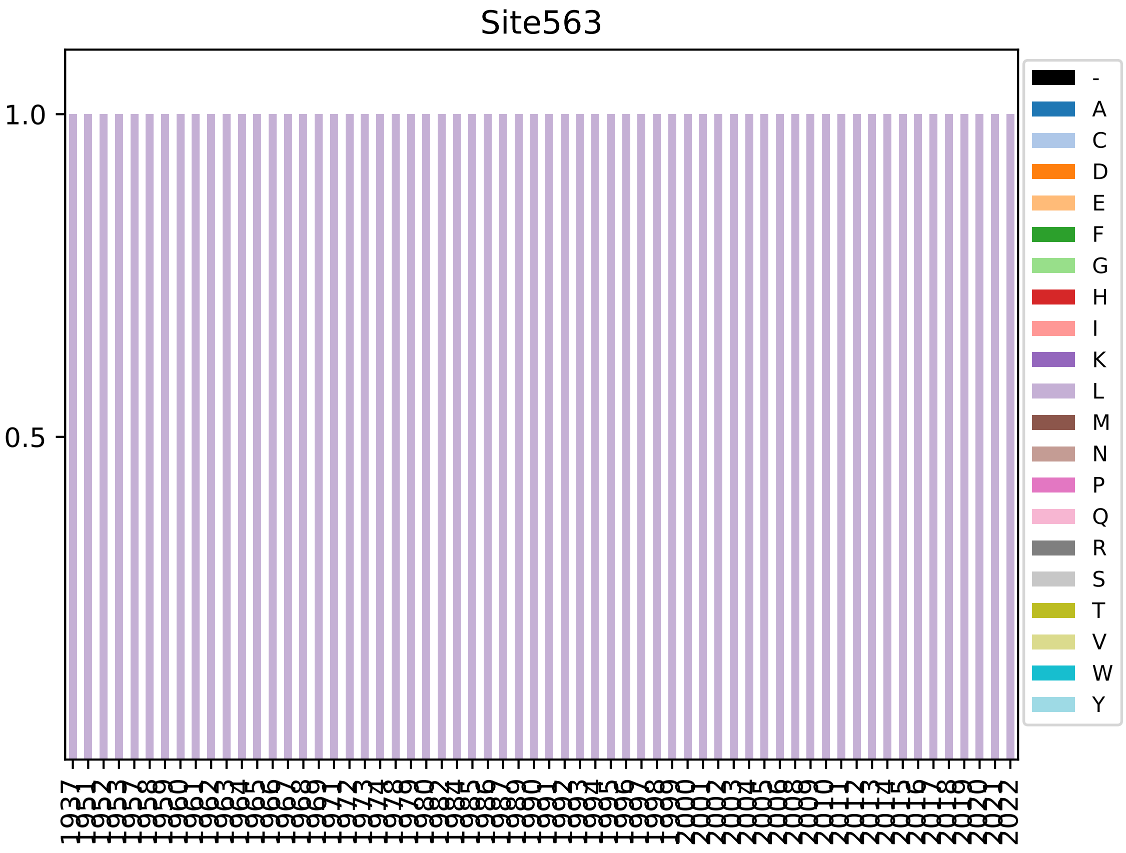
Site563
1.0
0.5
-
A
C
D
E
F
G
H
I
K
L
M
N
P
Q
R
S
T
V
W
Y

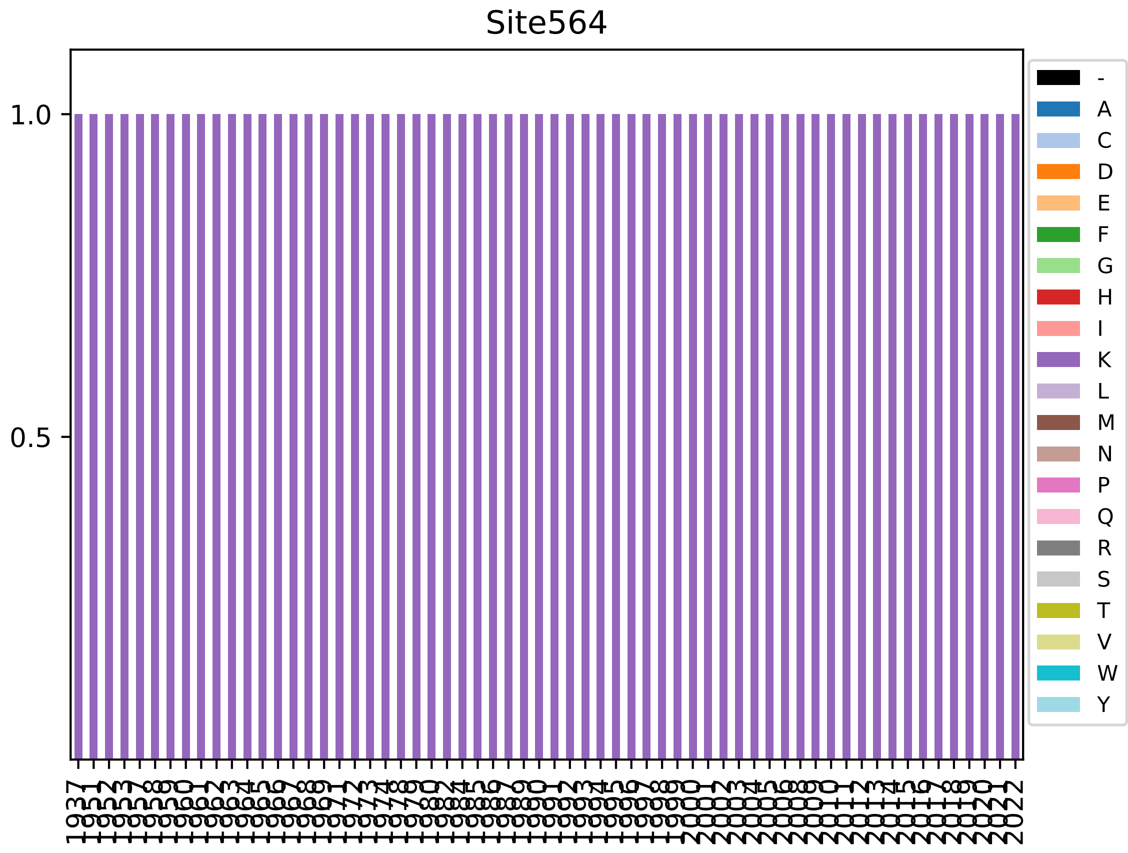
Site564
1.0
0.5
-
A
C
D
E
F
G
H
I
K
L
M
N
P
Q
R
S
T
V
W
Y

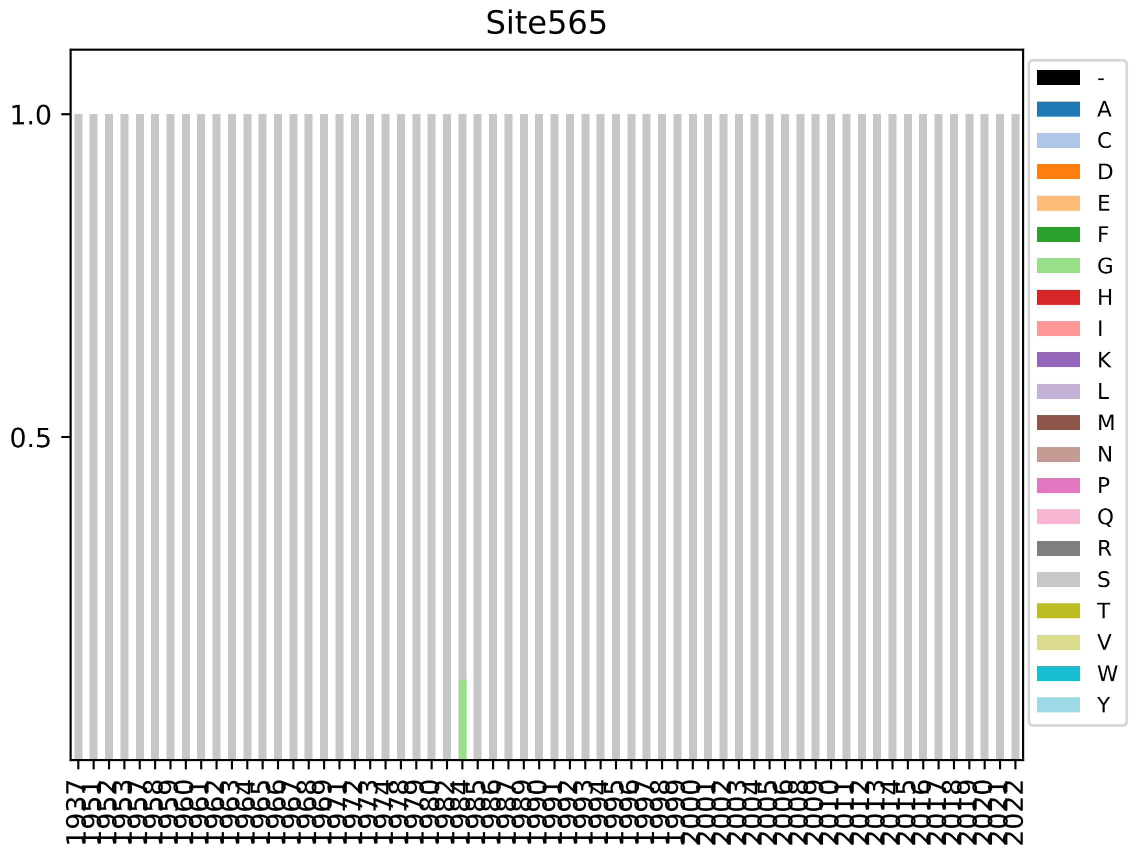
Site565
1.0
0.5
-
A
C
D
E
F
G
H
I
K
L
M
N
P
Q
R
S
T
V
W
Y

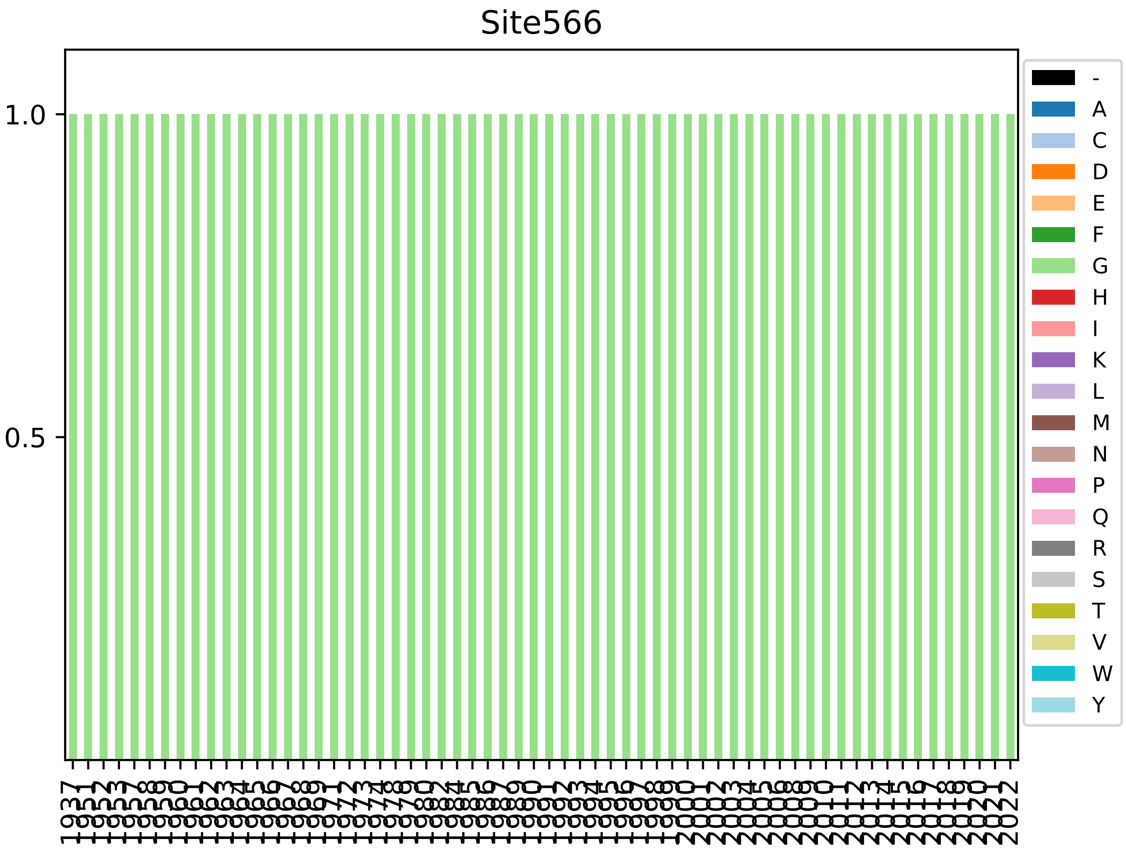
Site566
1.0
0.5
-
A
C
D
E
F
G
H
I
K
L
M
N
P
Q
R
S
T
V
W
Y

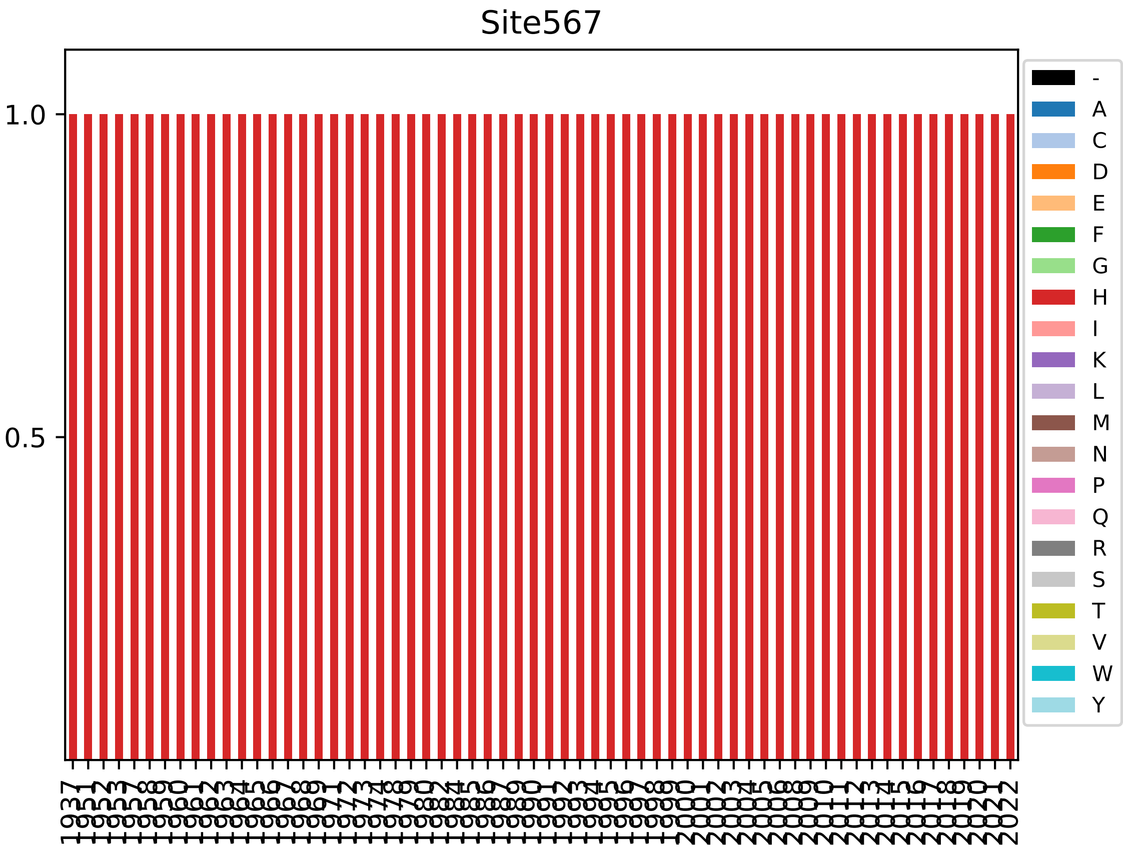
Site567
1.0
0.5
-
A
C
D
E
F
G
H
I
K
L
M
N
P
Q
R
S
T
V
W
Y

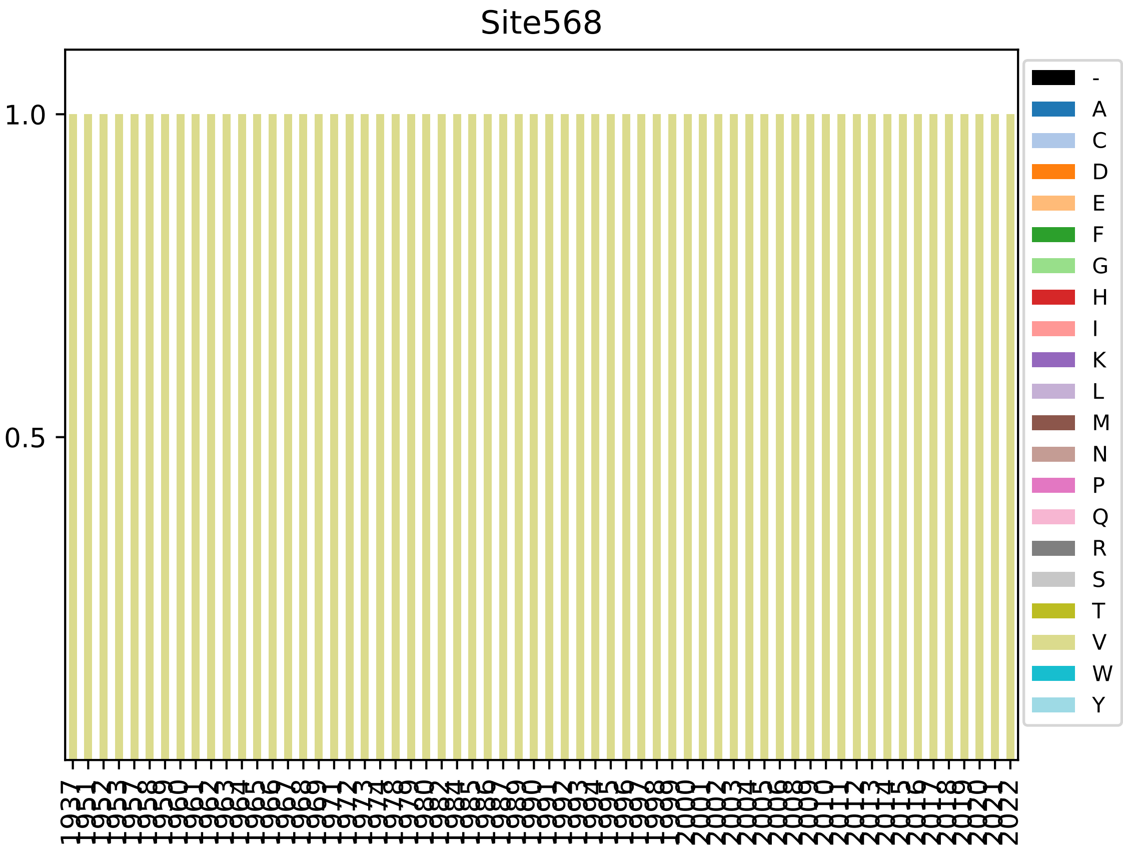
Site568
1.0
0.5
-
A
C
D
E
F
G
H
I
K
L
M
N
P
Q
R
S
T
V
W
Y

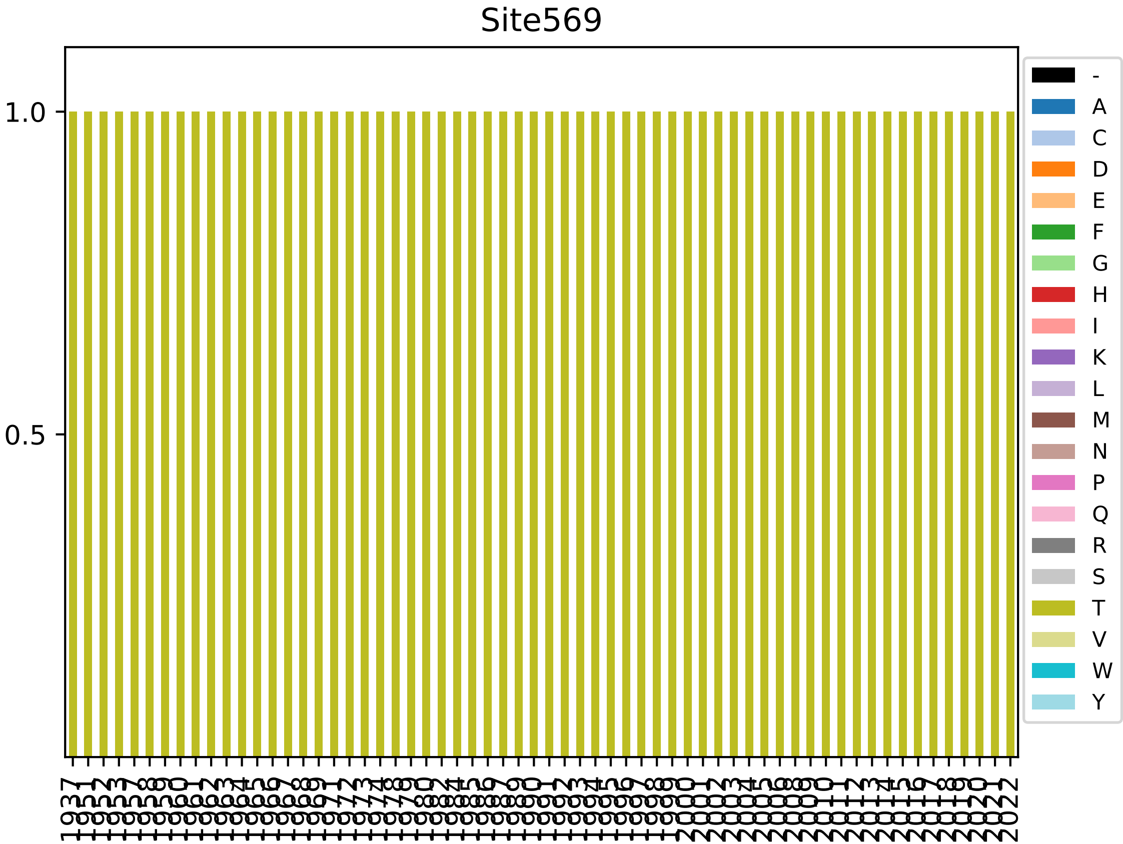
Site569
1.0
0.5
-
A
C
D
E
F
G
H
I
K
L
M
N
P
Q
R
S
T
V
W
Y

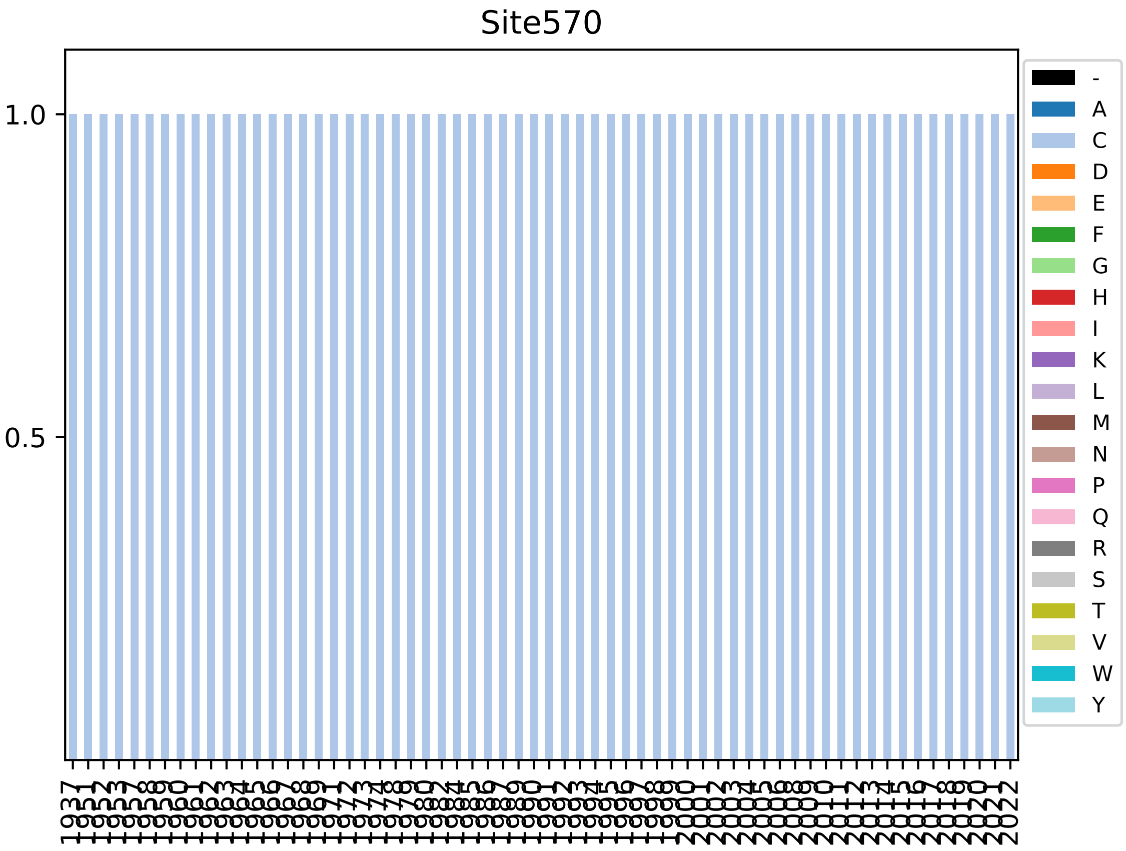
Site570
1.0
0.5
-
A
C
D
E
F
G
H
I
K
L
M
N
P
Q
R
S
T
V
W
Y

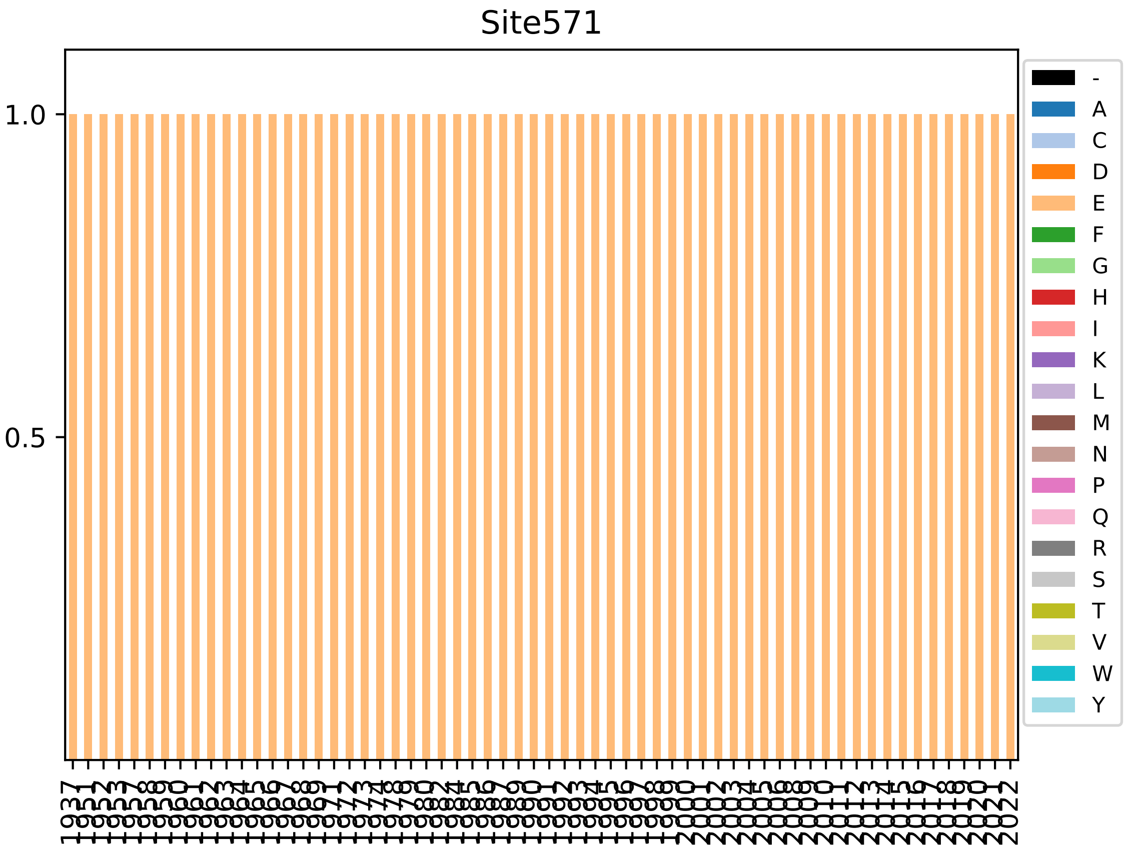
Site571
1.0
0.5
-
A
C
D
E
F
G
H
I
K
L
M
N
P
Q
R
S
T
V
W
Y

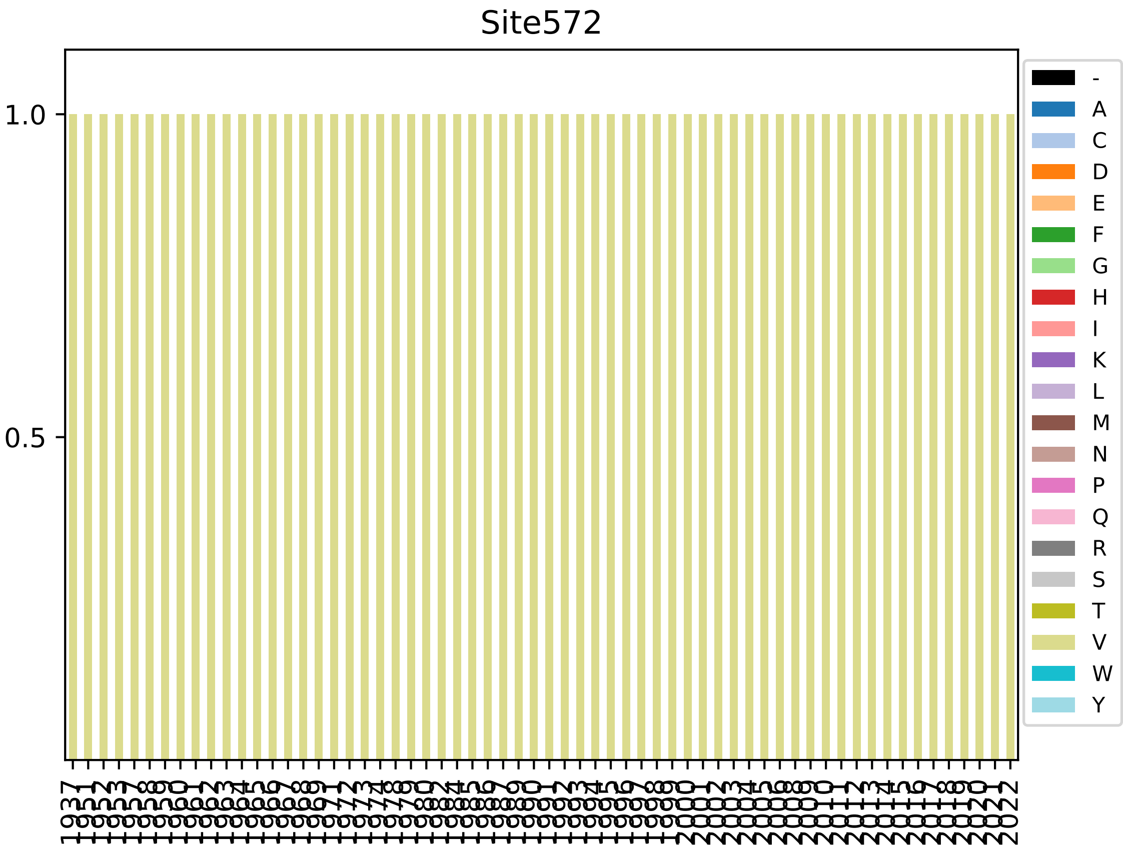
Site572
1.0
0.5
-
A
C
D
E
F
G
H
I
K
L
M
N
P
Q
R
S
T
V
W
Y

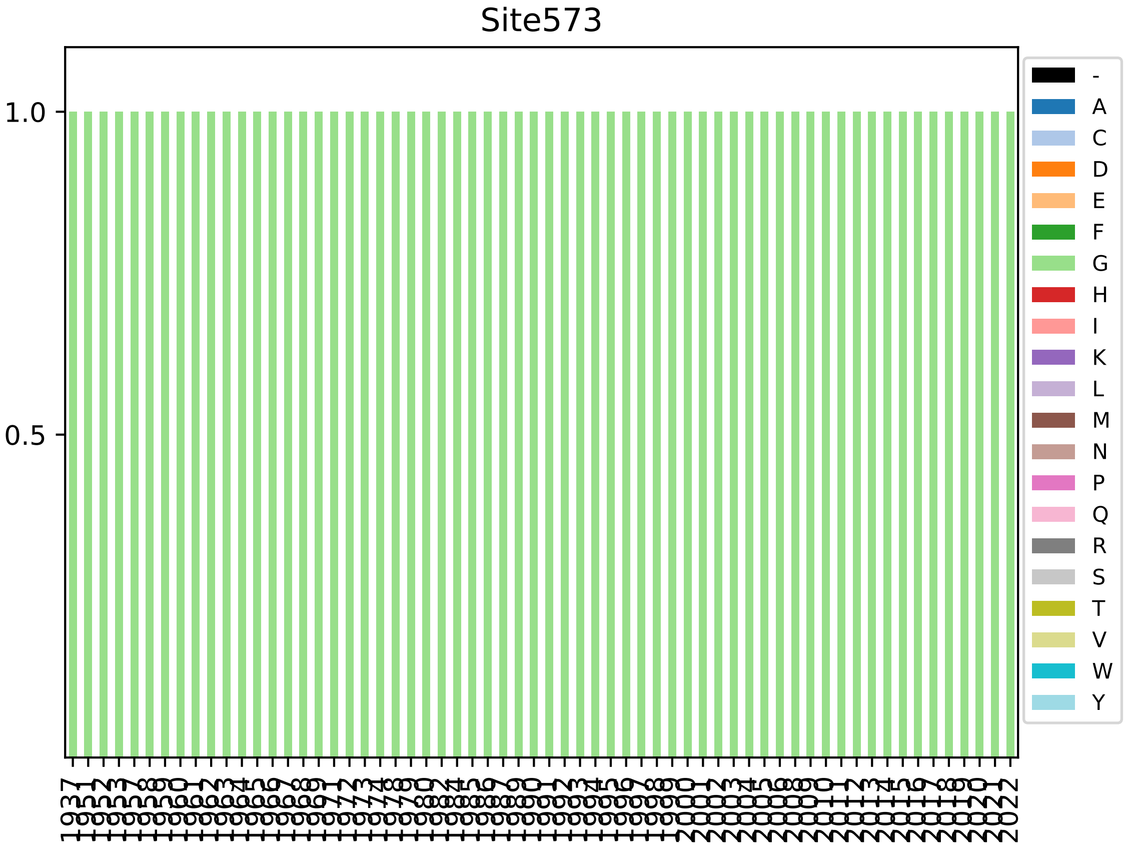
Site573
1.0
0.5
-
A
C
D
E
F
G
H
I
K
L
M
N
P
Q
R
S
T
V
W
Y

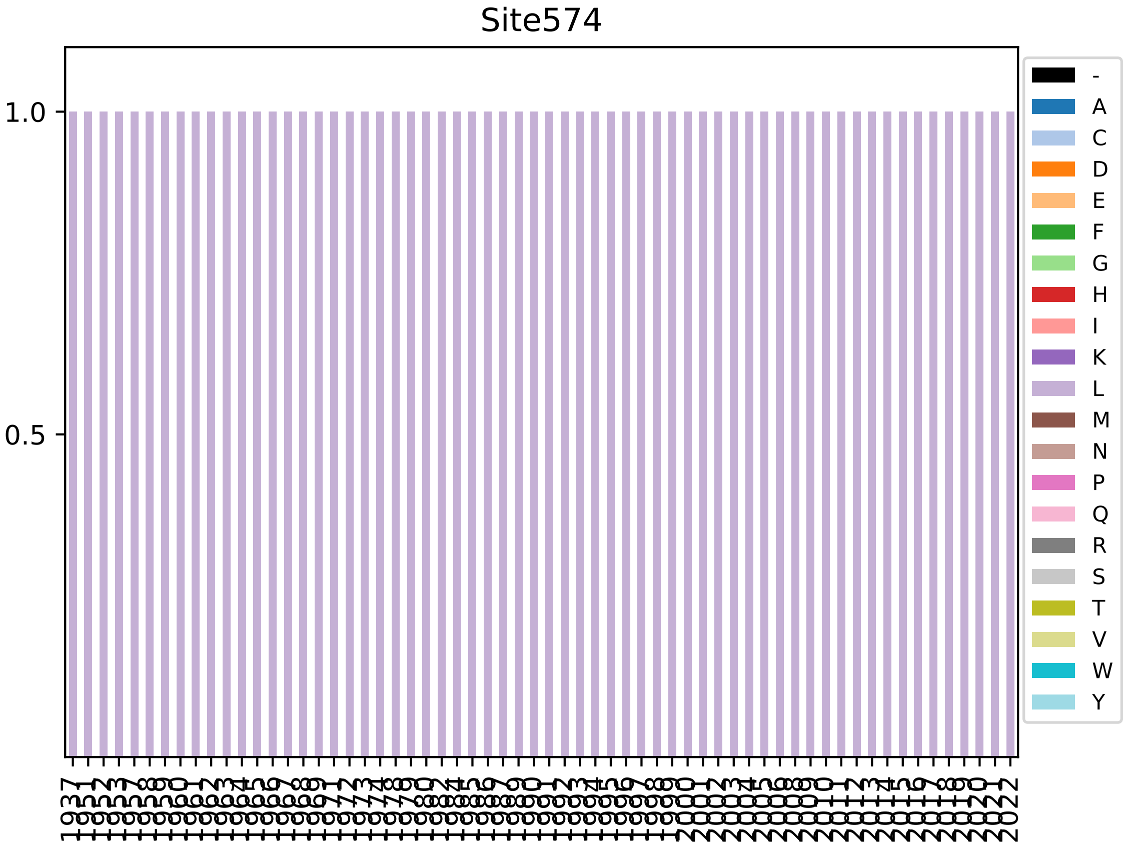
Site574
1.0
0.5
-
A
C
D
E
F
G
H
I
K
L
M
N
P
Q
R
S
T
V
W
Y

Site575

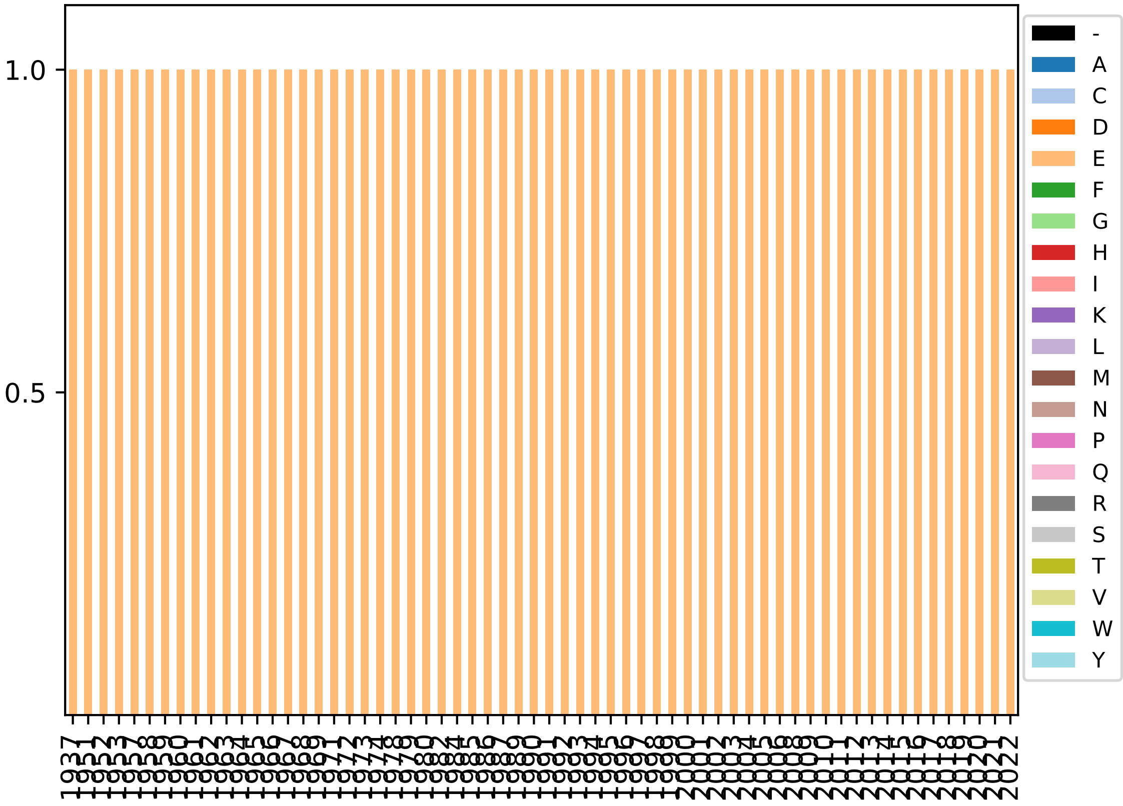

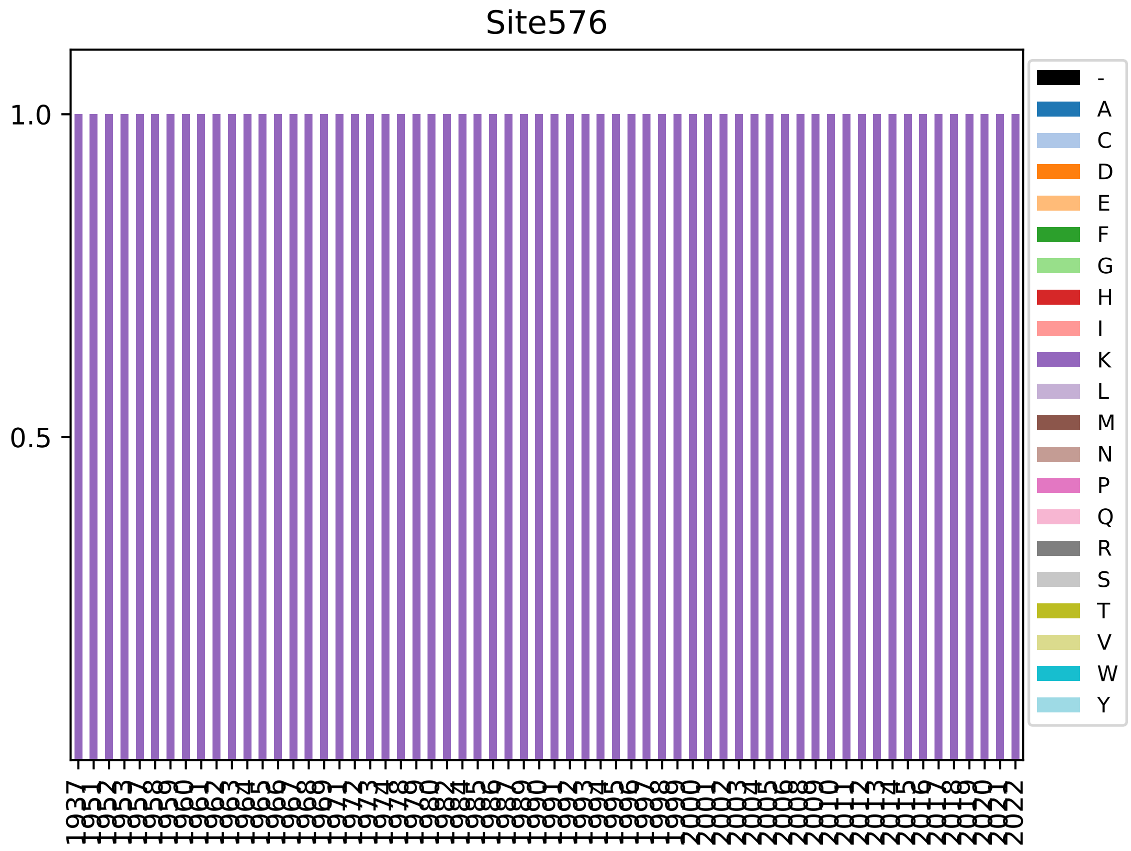
Site576
1.0
0.5
-
A
C
D
E
F
G
H
I
K
L
M
N
P
Q
R
S
T
V
W
Y

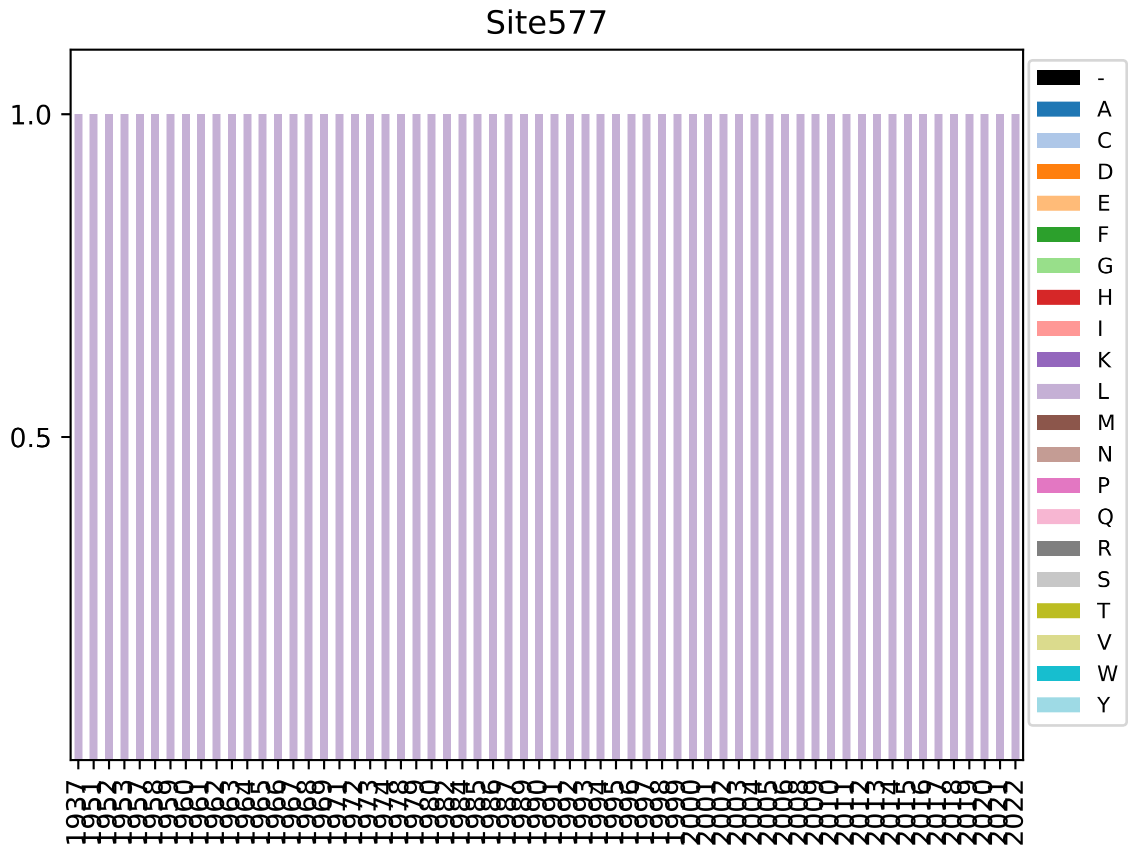
Site577
1.0
0.5
-
A
C
D
E
F
G
H
I
K
L
M
N
P
Q
R
S
T
V
W
Y

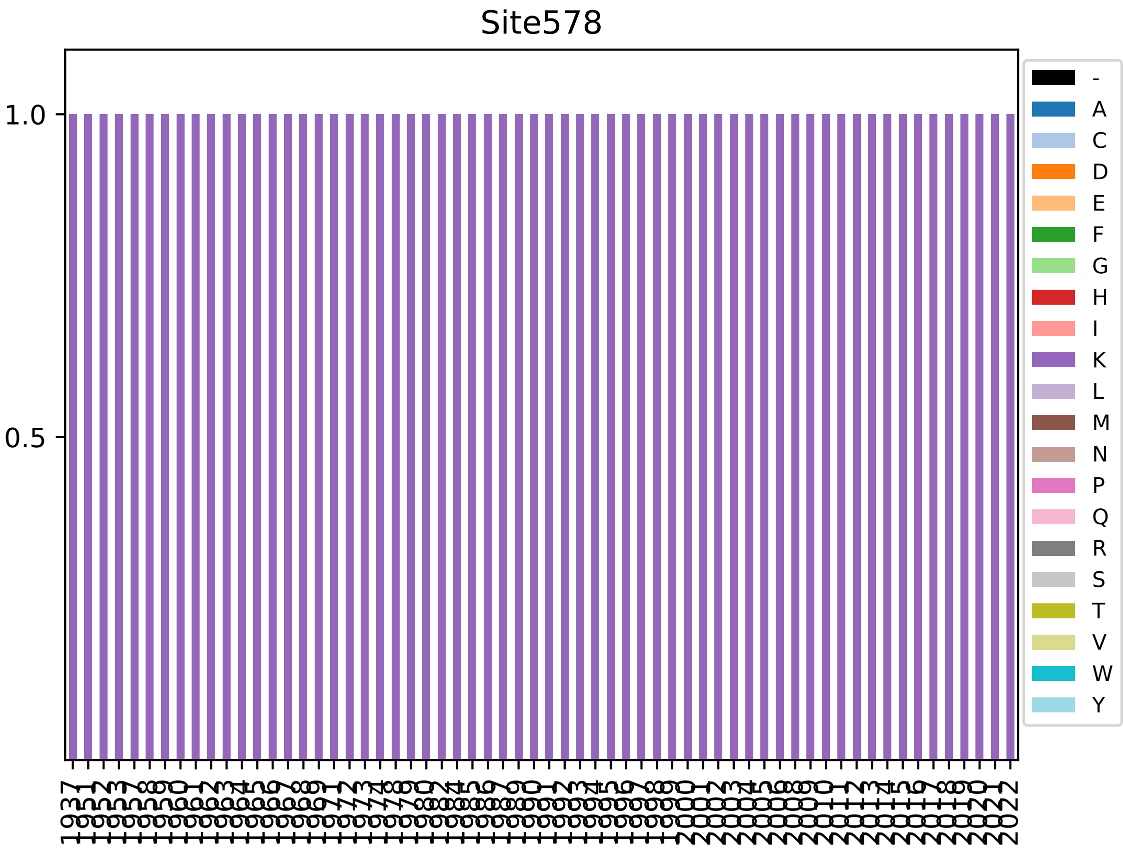
Site578
1.0
0.5
-
A
C
D
E
F
G
H
I
K
L
M
N
P
Q
R
S
T
V
W
Y

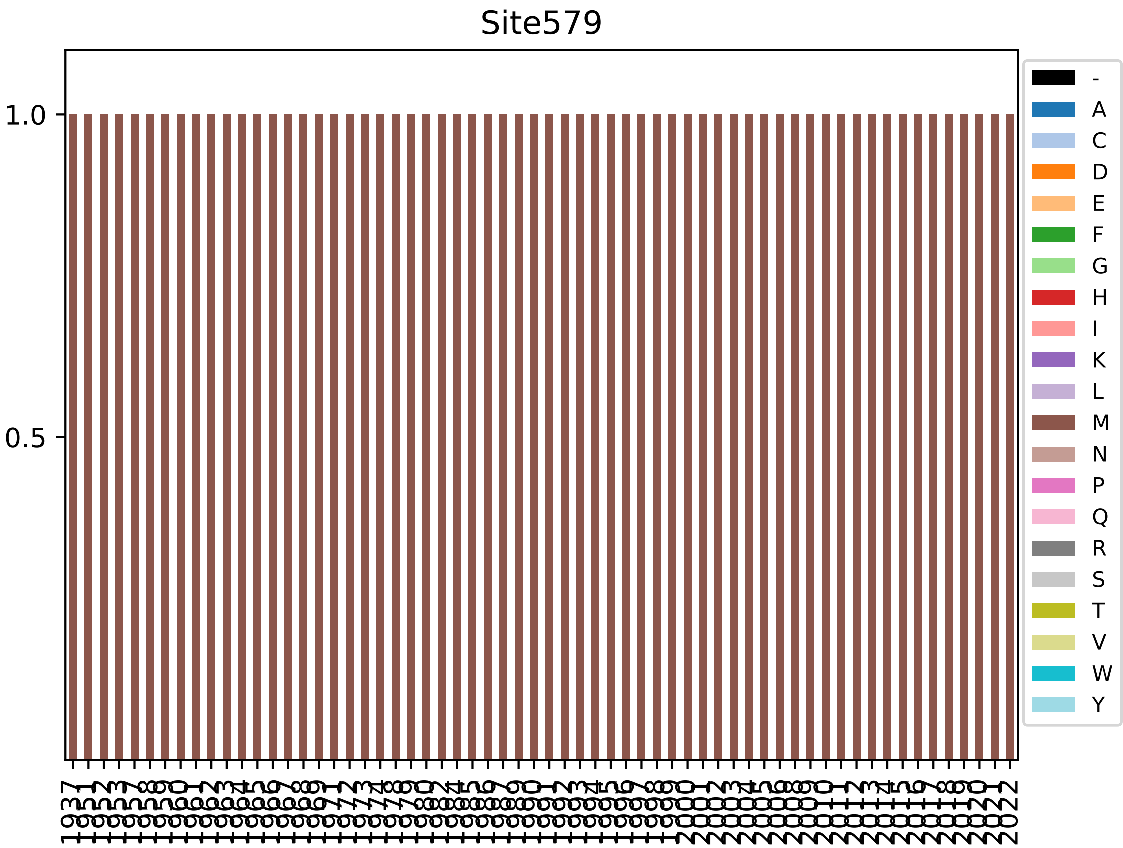
Site579
1.0
0.5
-
A
C
D
E
F
G
H
I
K
L
M
N
P
Q
R
S
T
V
W
Y

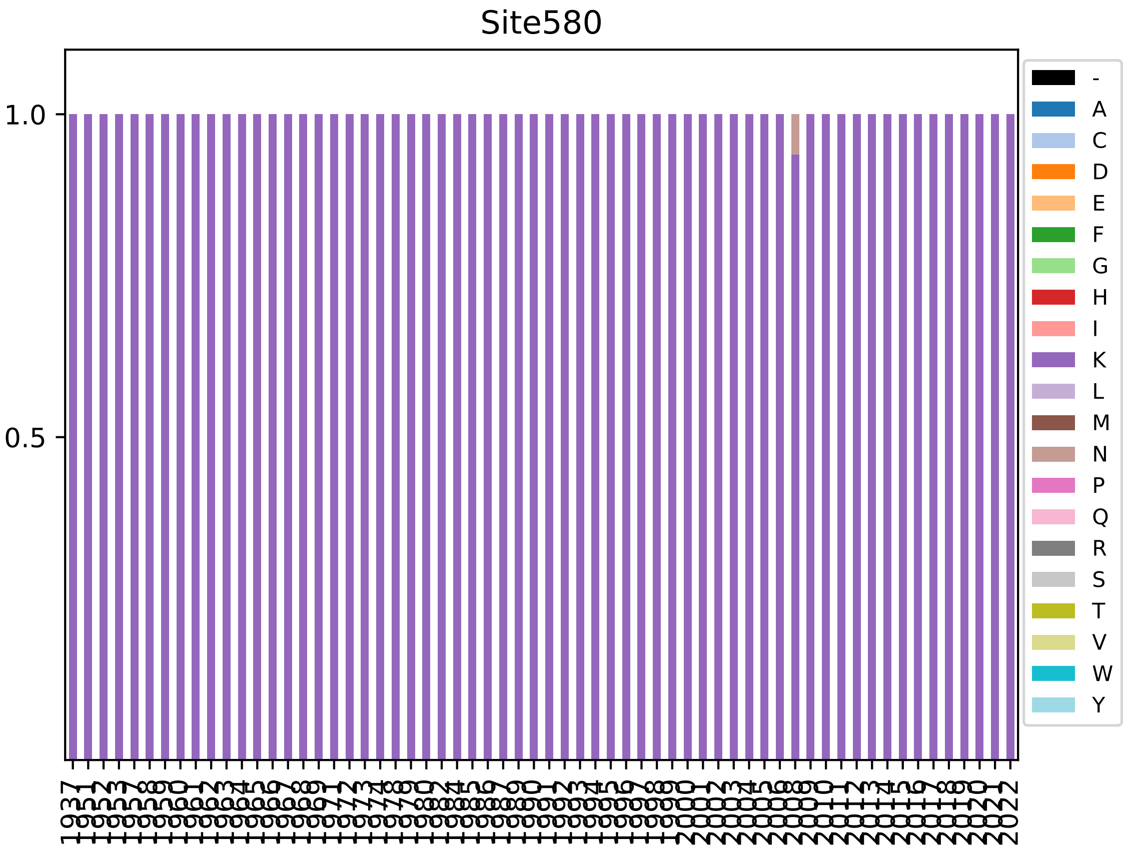
Site580
1.0
0.5
-
A
C
D
E
F
G
H
I
K
L
M
N
P
Q
R
S
T
V
W
Y

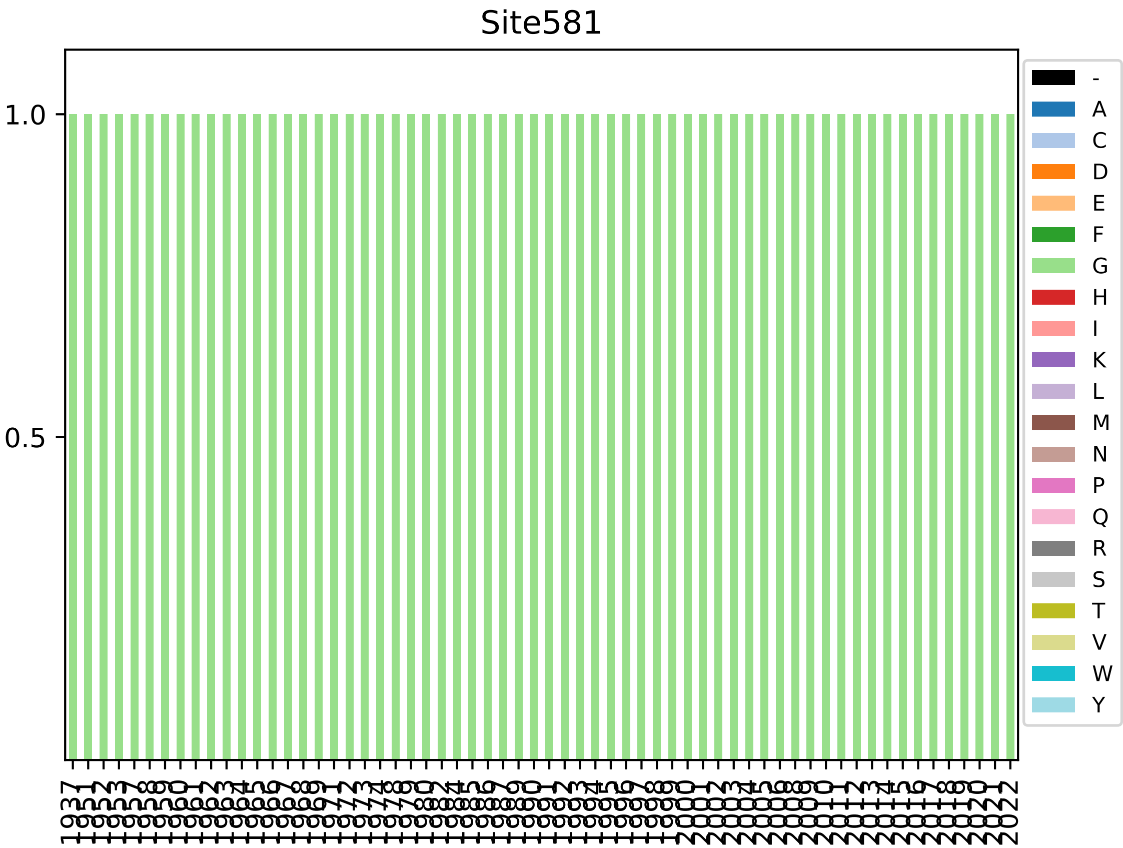
Site581
1.0
0.5
-
A
C
D
E
F
G
H
I
K
L
M
N
P
Q
R
S
T
V
W
Y

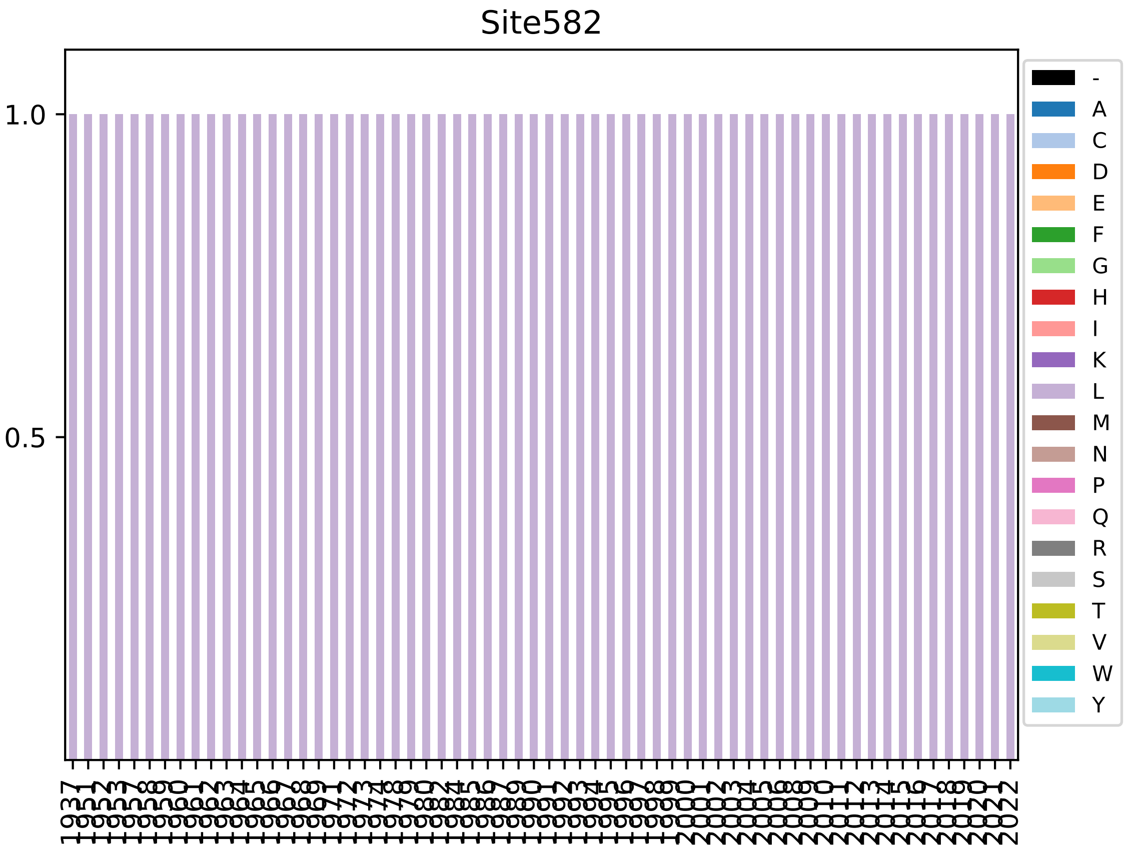
Site582
1.0
0.5
-
A
C
D
E
F
G
H
I
K
L
M
N
P
Q
R
S
T
V
W
Y

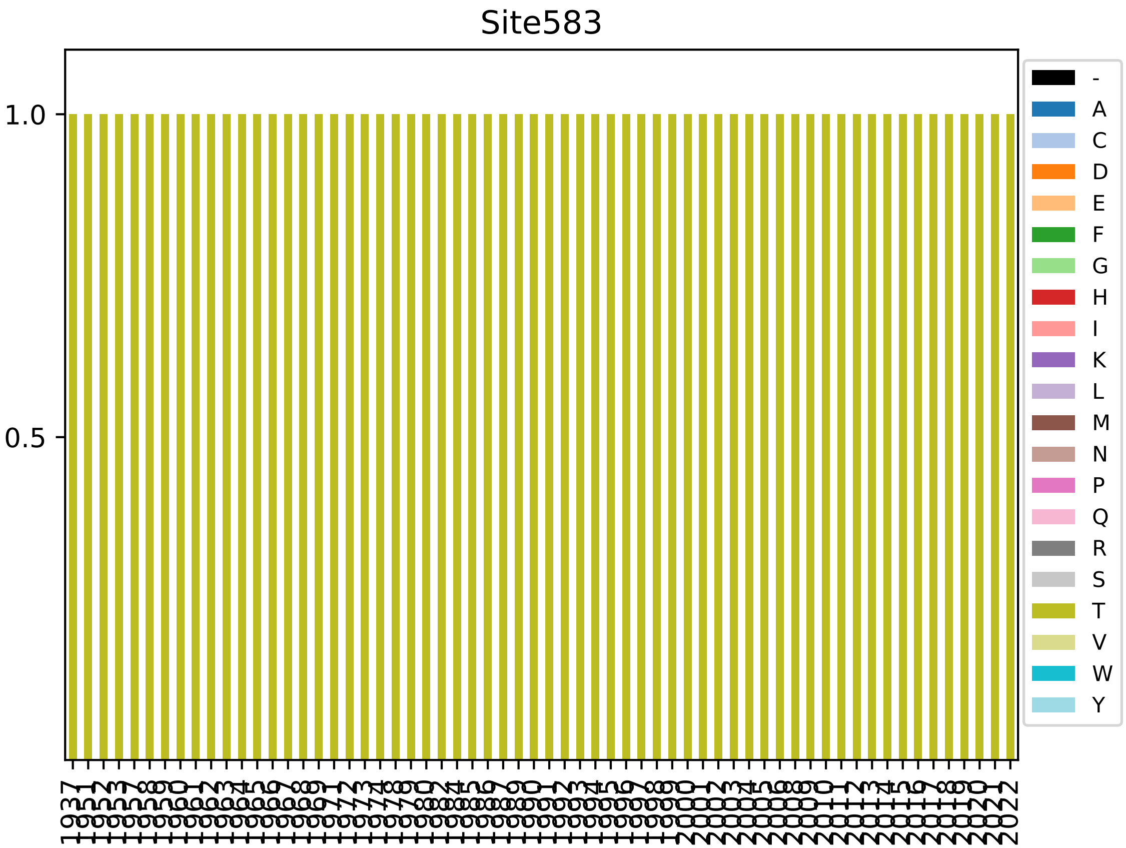
Site583
1.0
0.5
-
A
C
D
E
F
G
H
I
K
L
M
N
P
Q
R
S
T
V
W
Y

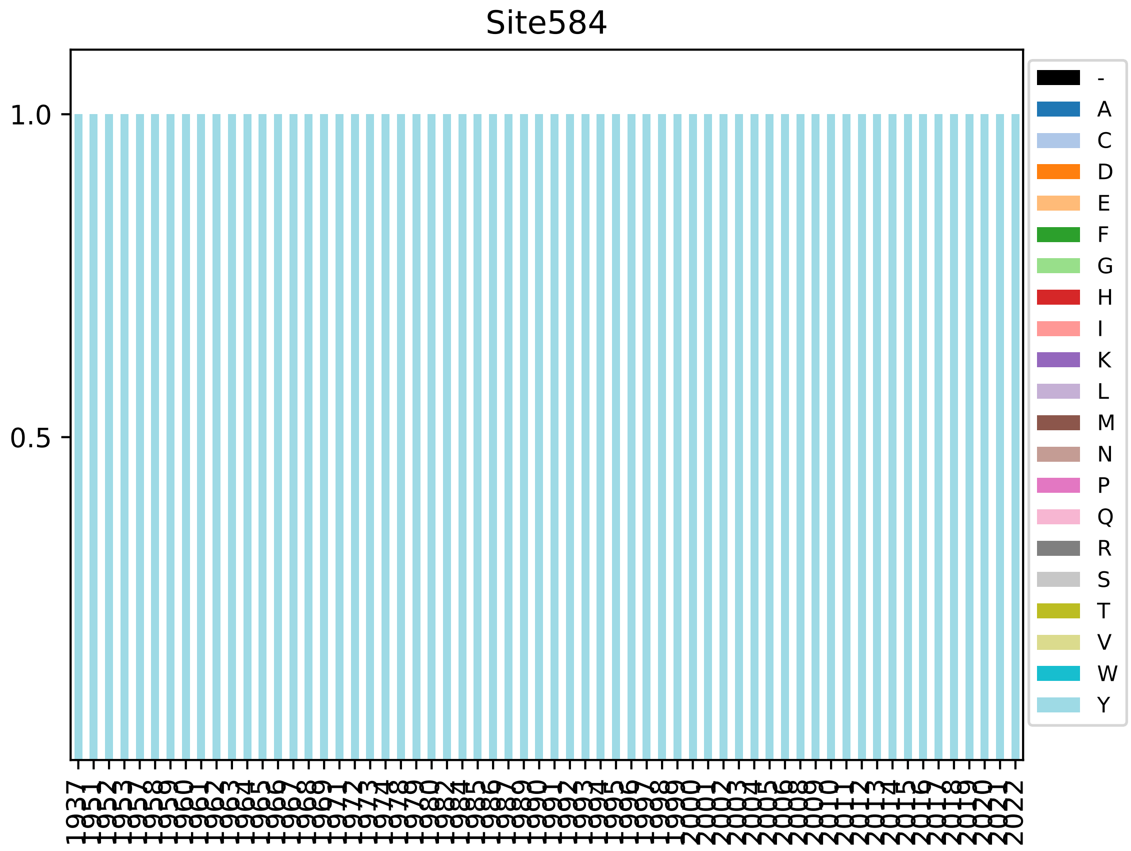
Site584
1.0
0.5
-
A
C
D
E
F
G
H
I
K
L
M
N
P
Q
R
S
T
V
W
Y

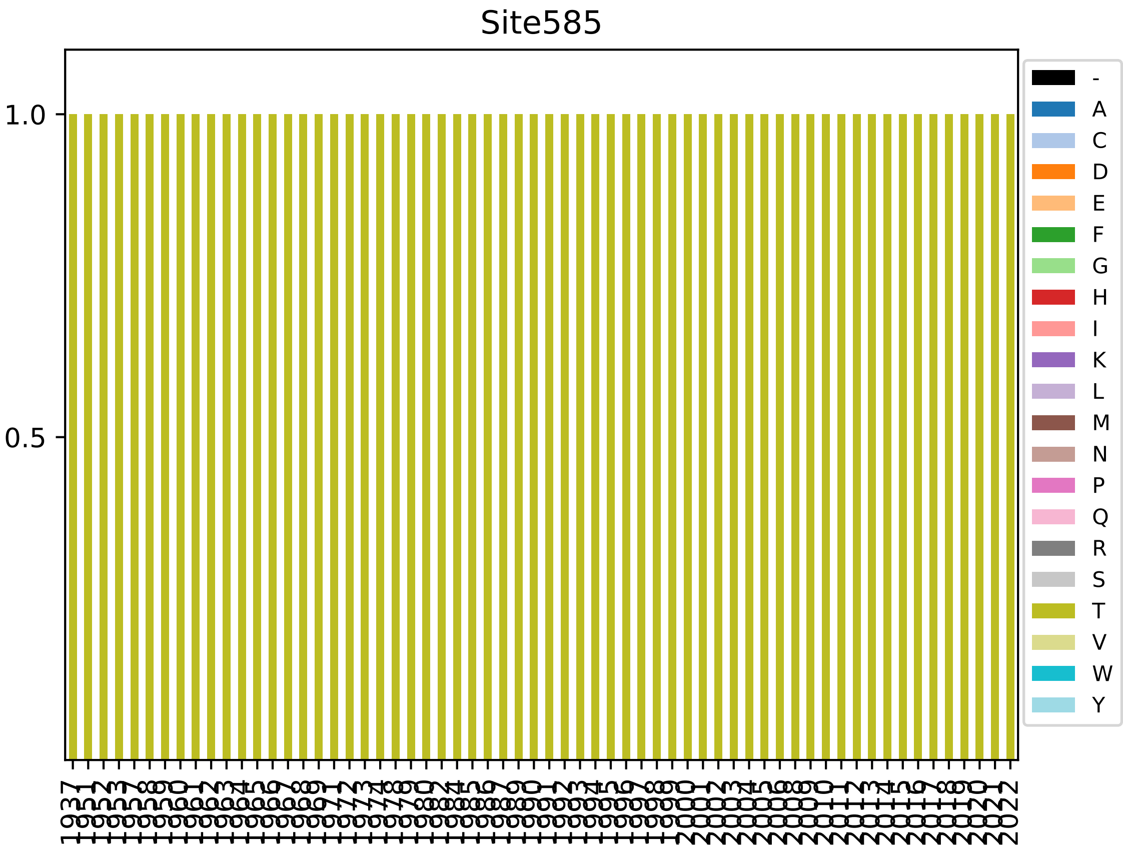

Site585
1.0
0.5
-
A
C
D
E
F
G
H
I
K
L
M
N
P
Q
R
S
T
V
W
Y

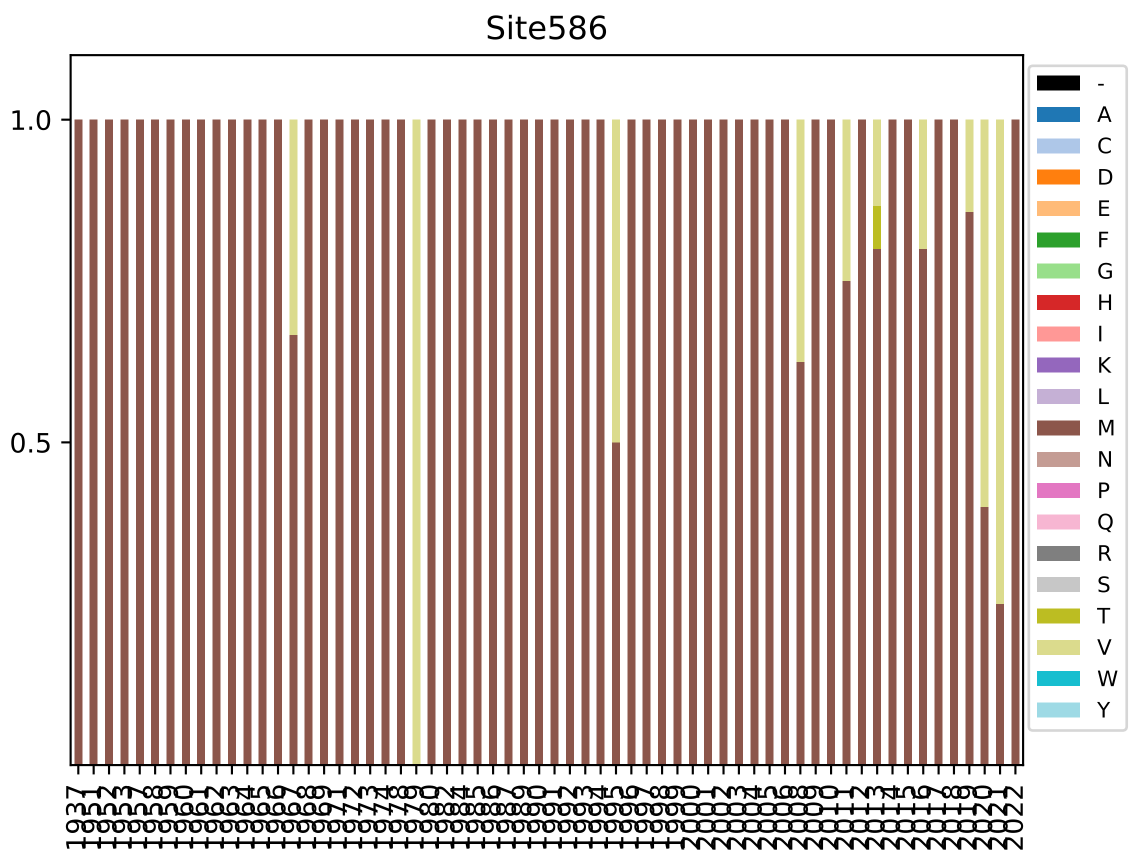

Site586
1.0
0.5
-
A
C
D
E
F
G
H
I
K
L
M
N
P
Q
R
S
T
V
W
Y

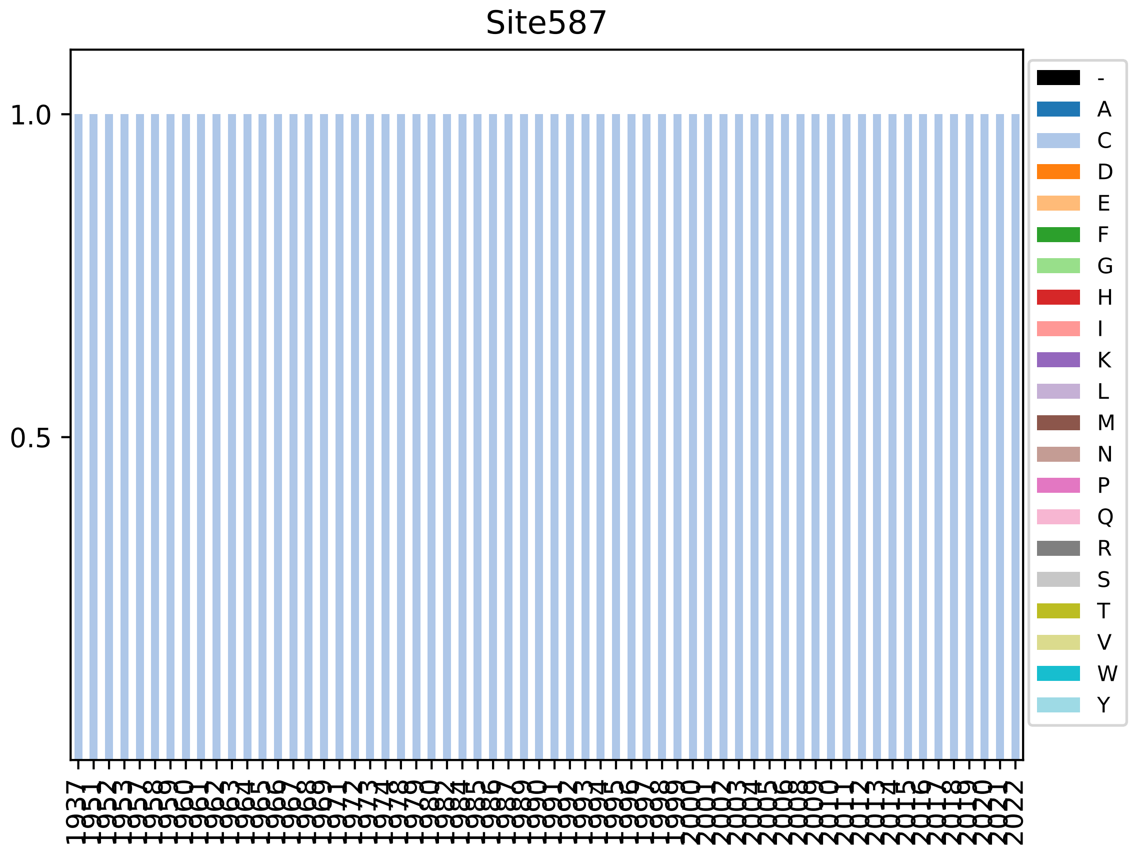
Site587
1.0
0.5
-
A
C
D
E
F
G
H
I
K
L
M
N
P
Q
R
S
T
V
W
Y

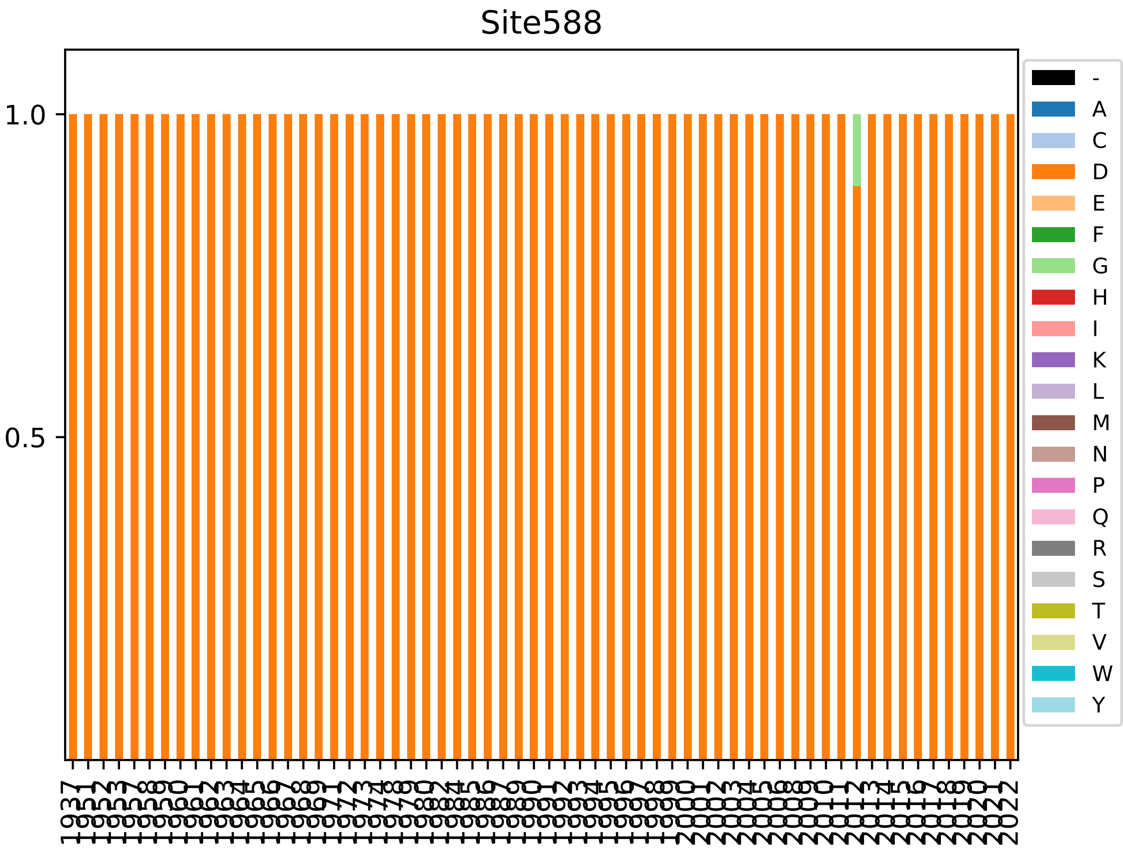
Site588
1.0
0.5
-
A
C
D
E
F
G
H
I
K
L
M
N
P
Q
R
S
T
V
W
Y

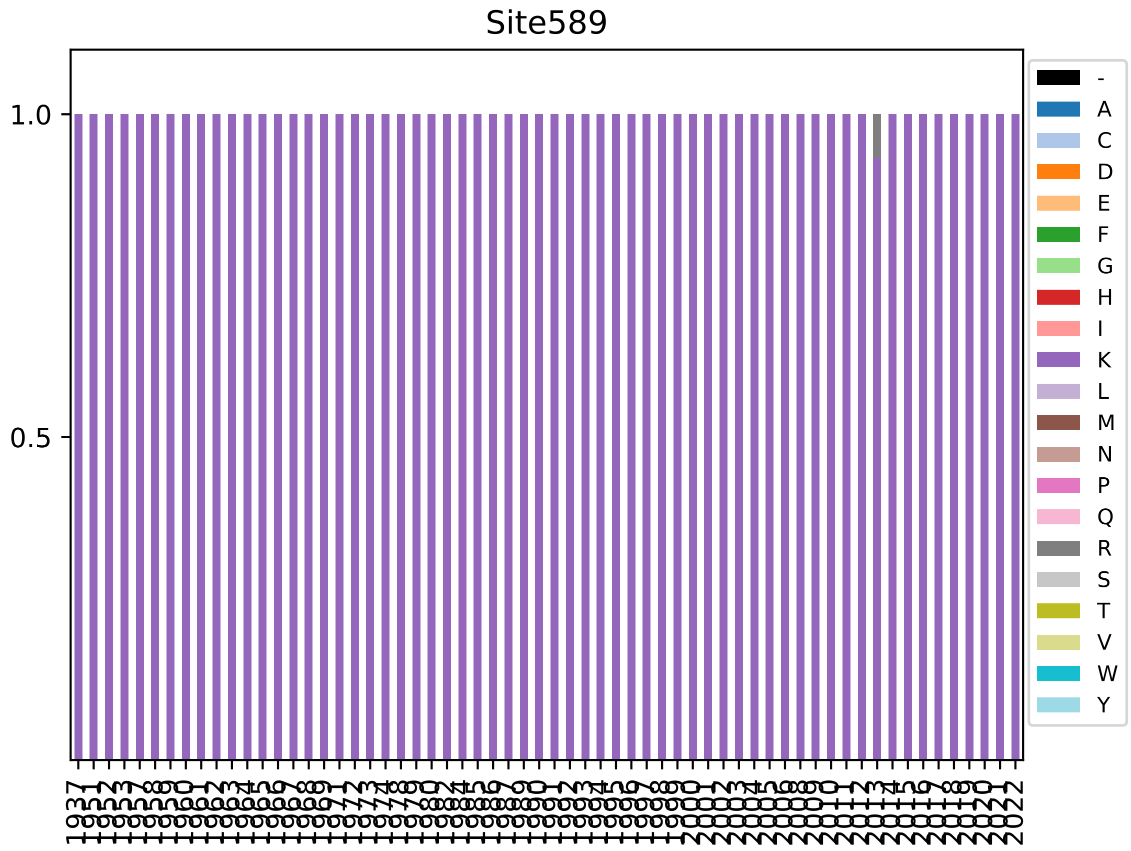
Site589
1.0
0.5
-
A
C
D
E
F
G
H
I
K
L
M
N
P
Q
R
S
T
V
W
Y

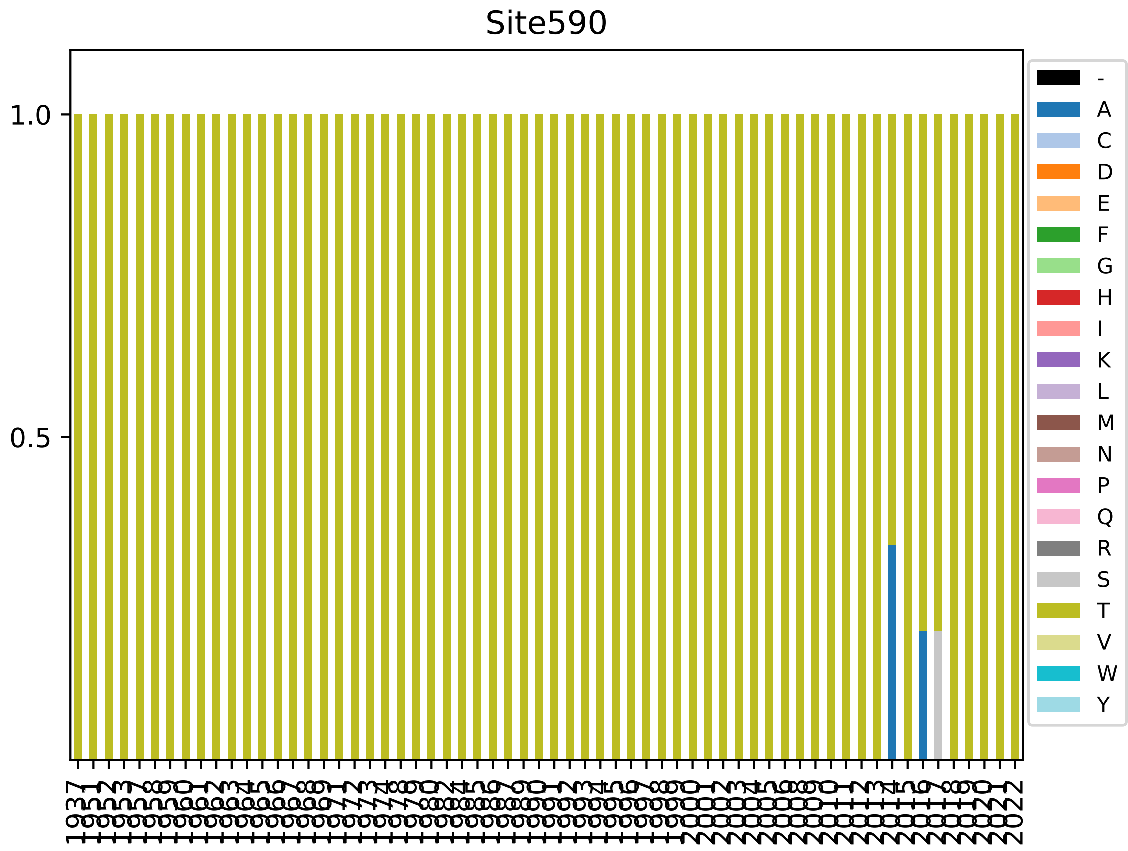
Site590
1.0
0.5
-
A
C
D
E
F
G
H
I
K
L
M
N
P
Q
R
S
T
V
W
Y

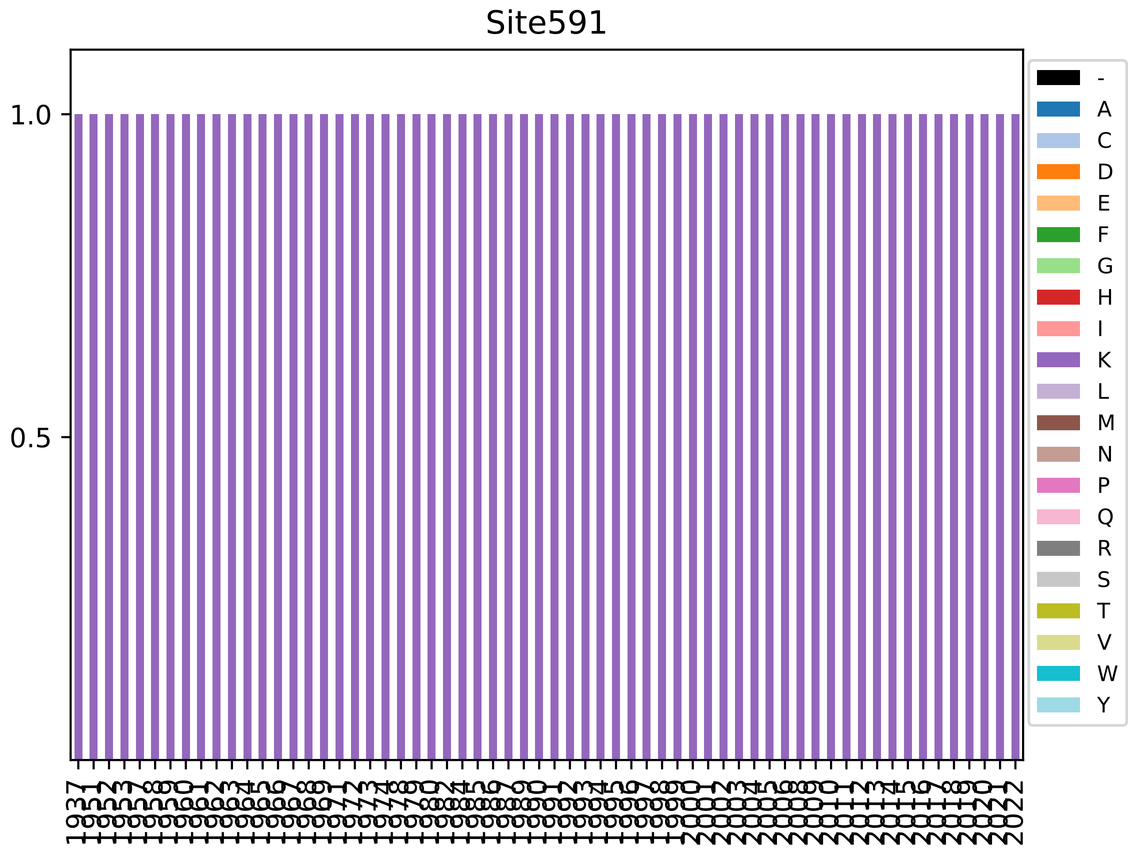
Site591
1.0
0.5
-
A
C
D
E
F
G
H
I
K
L
M
N
P
Q
R
S
T
V
W
Y

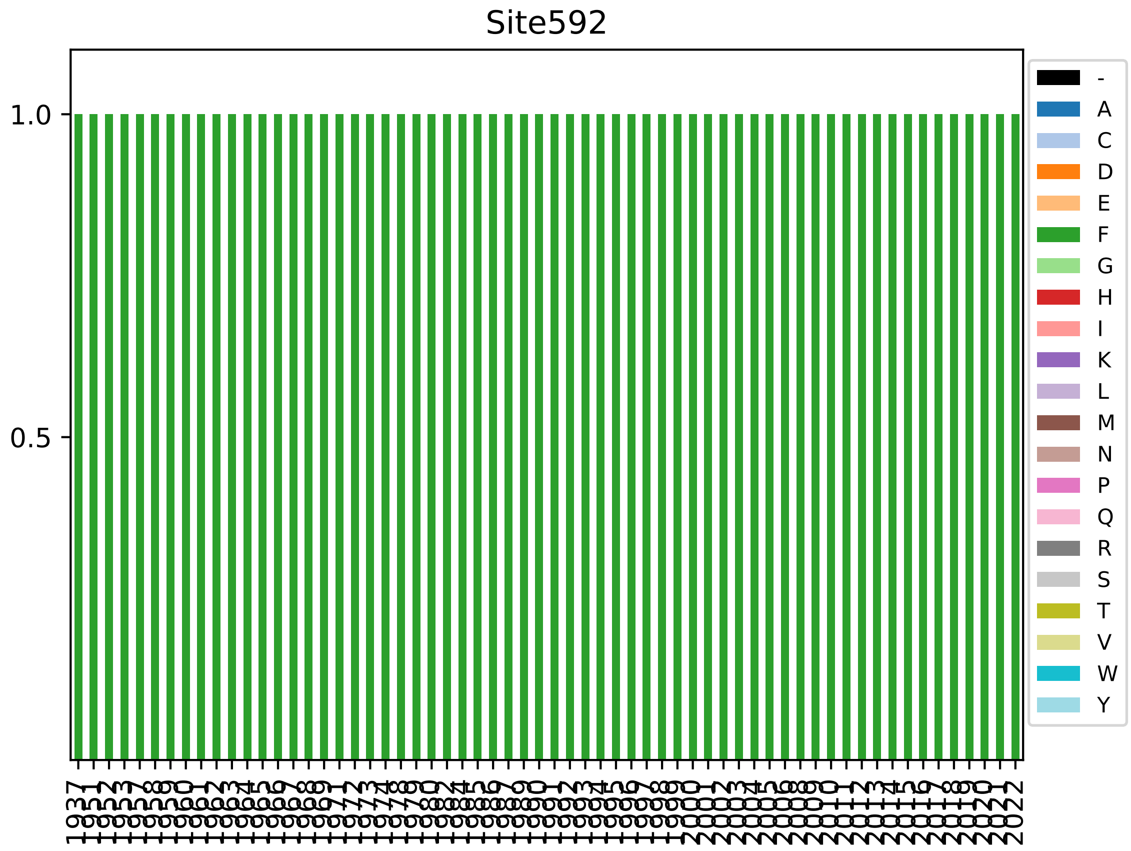
Site592
1.0
0.5
-
A
C
D
E
F
G
H
I
K
L
M
N
P
Q
R
S
T
V
W
Y

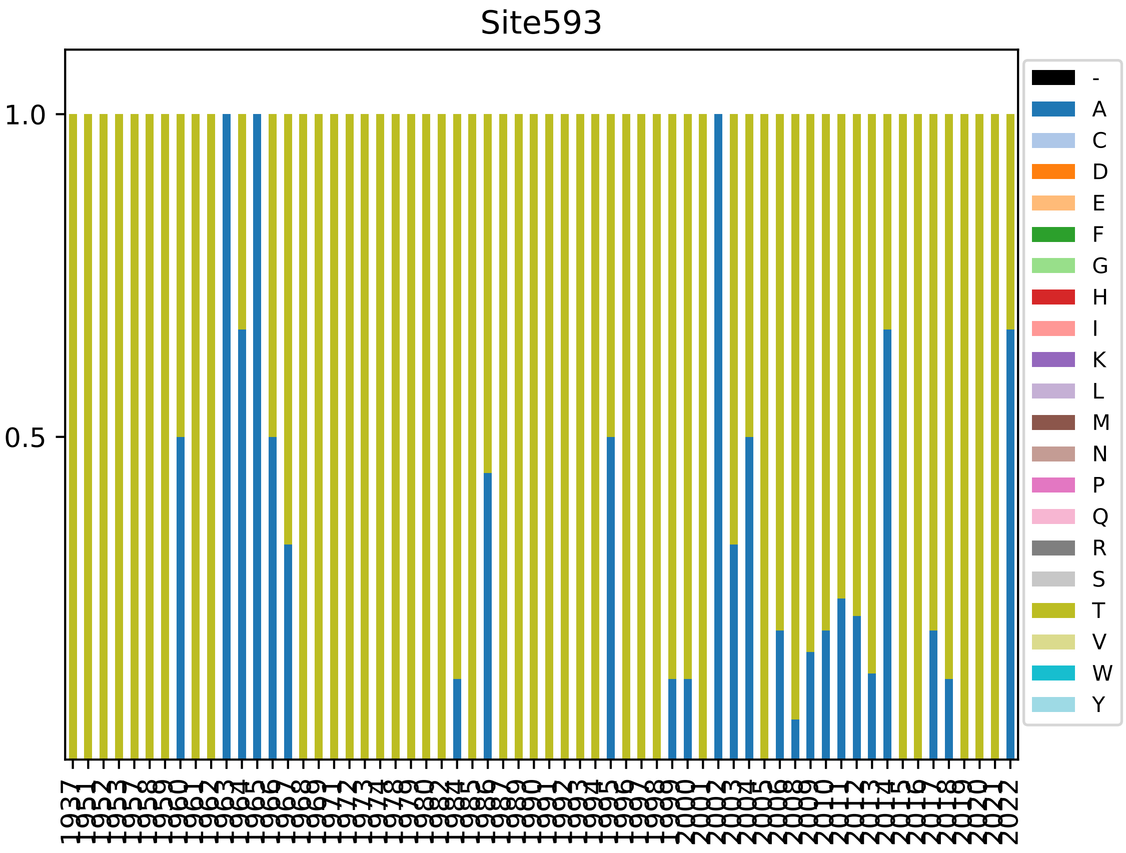
Site593
1.0
0.5
-
A
C
D
E
F
G
H
I
K
L
M
N
P
Q
R
S
T
V
W
Y

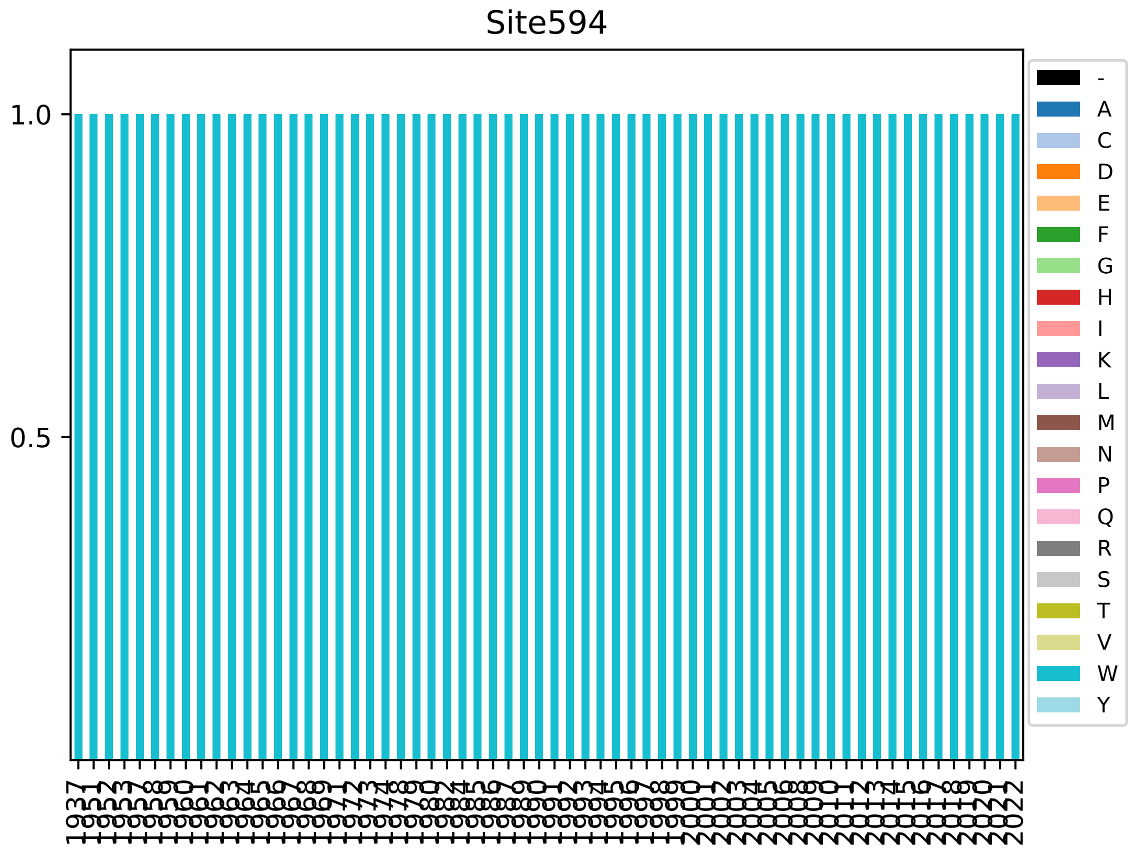
Site594
1.0
0.5
-
A
C
D
E
F
G
H
I
K
L
M
N
P
Q
R
S
T
V
W
Y

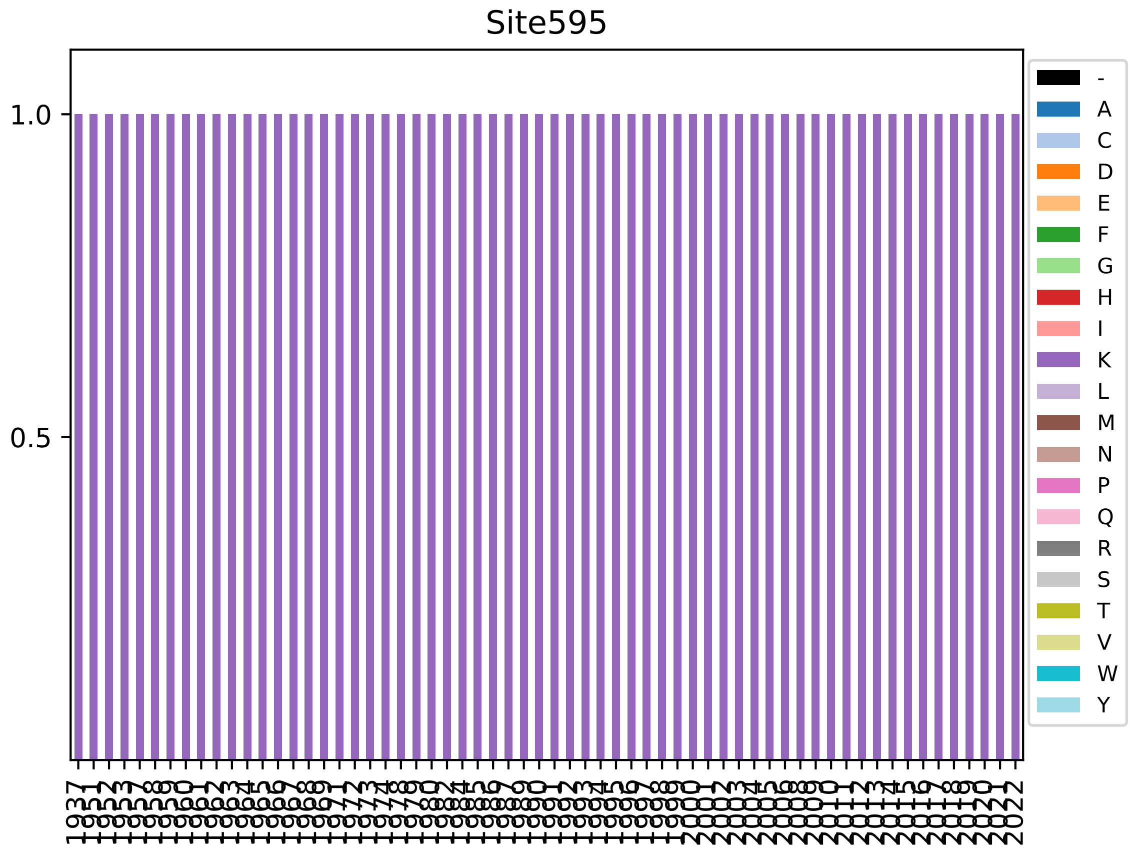
Site595
1.0
0.5
-
A
C
D
E
F
G
H
I
K
L
M
N
P
Q
R
S
T
V
W
Y

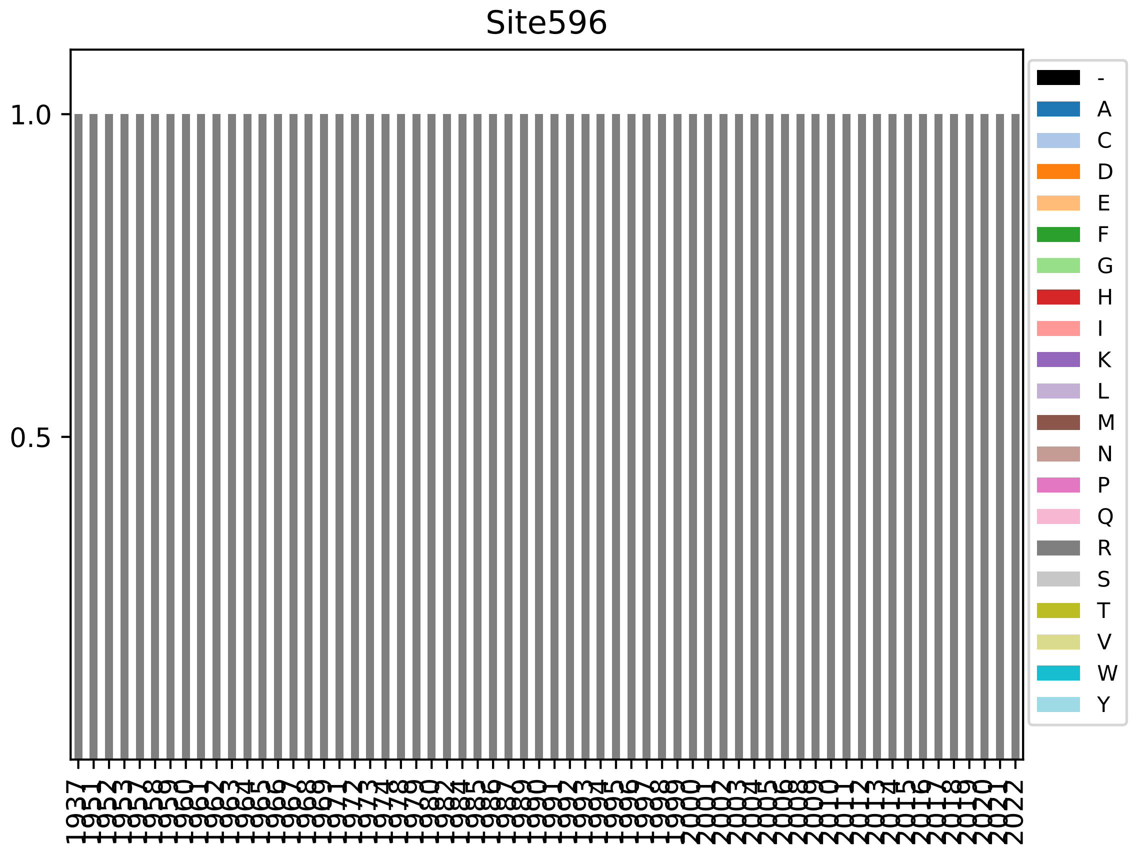
Site596
1.0
0.5
-
A
C
D
E
F
G
H
I
K
L
M
N
P
Q
R
S
T
V
W
Y

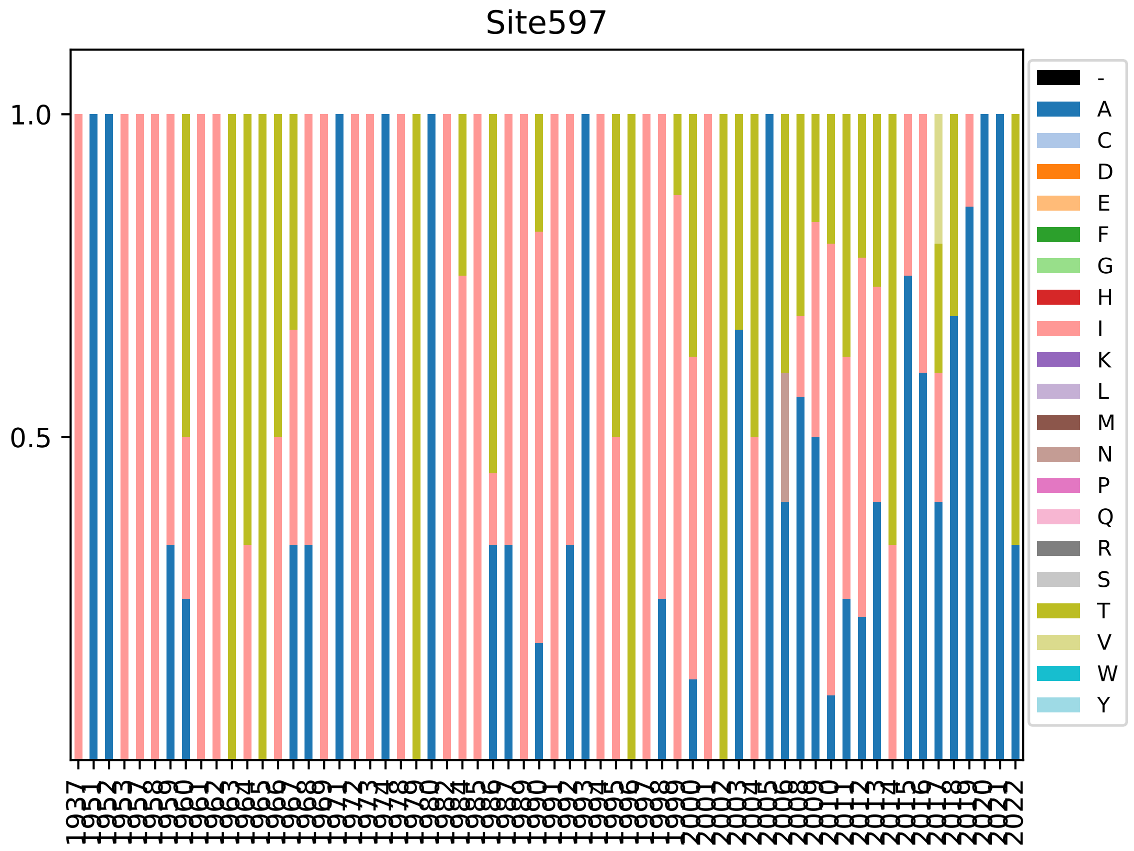

Site597
1.0
0.5
-
A
C
D
E
F
G
H
I
K
L
M
N
P
Q
R
S
T
V
W
Y

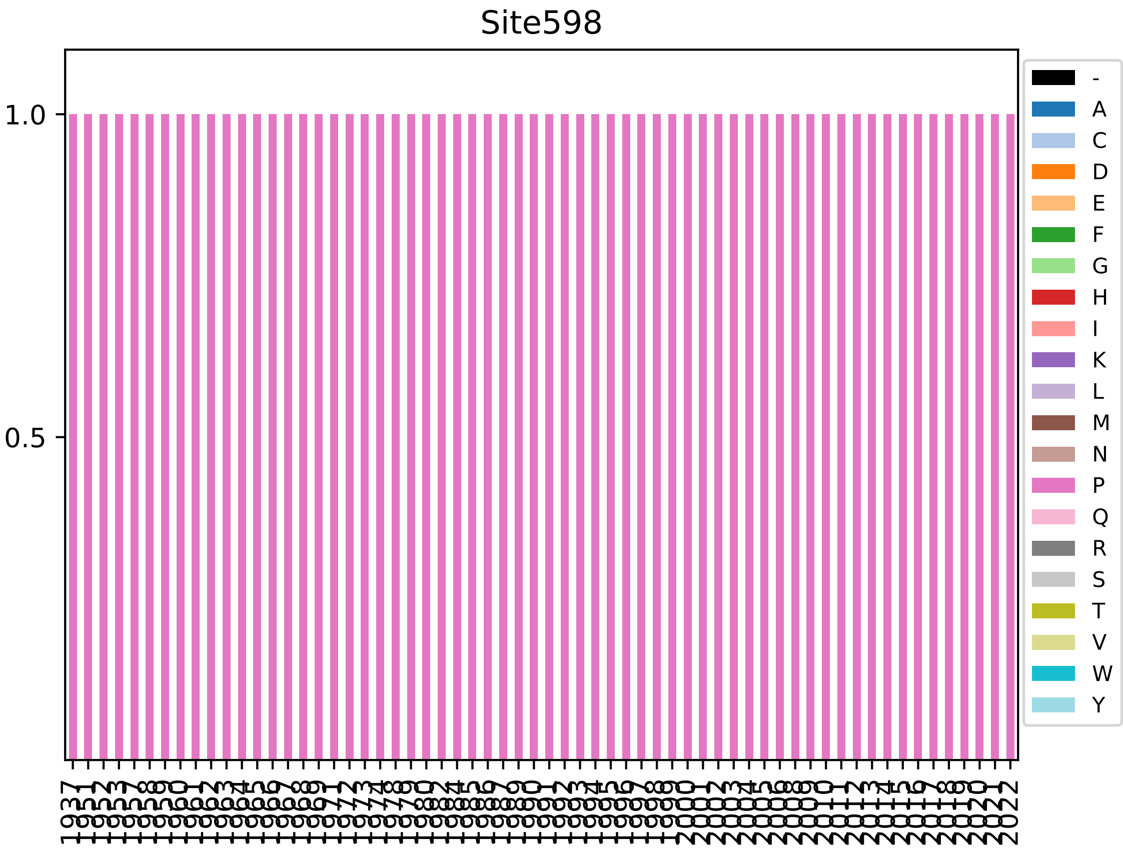
Site598
1.0
0.5
-
A
C
D
E
F
G
H
I
K
L
M
N
P
Q
R
S
T
V
W
Y

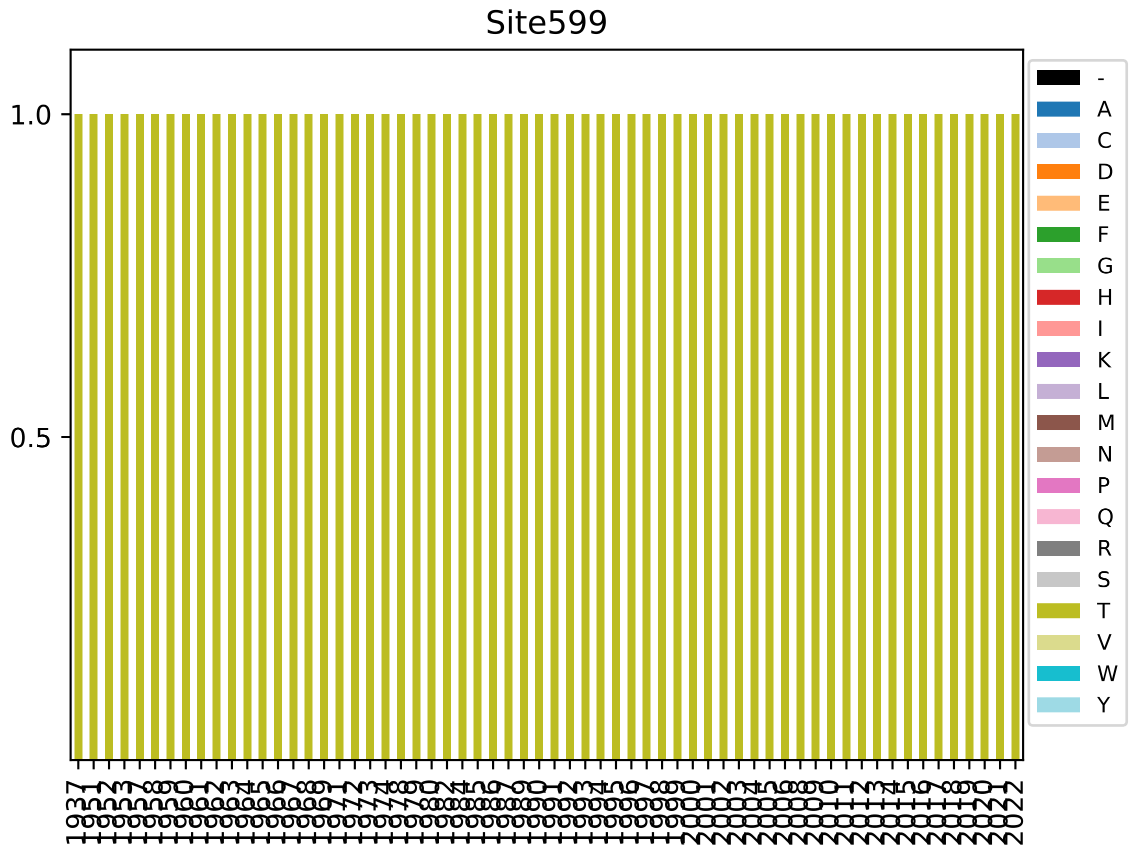
Site599
1.0
0.5
-
A
C
D
E
F
G
H
I
K
L
M
N
P
Q
R
S
T
V
W
Y

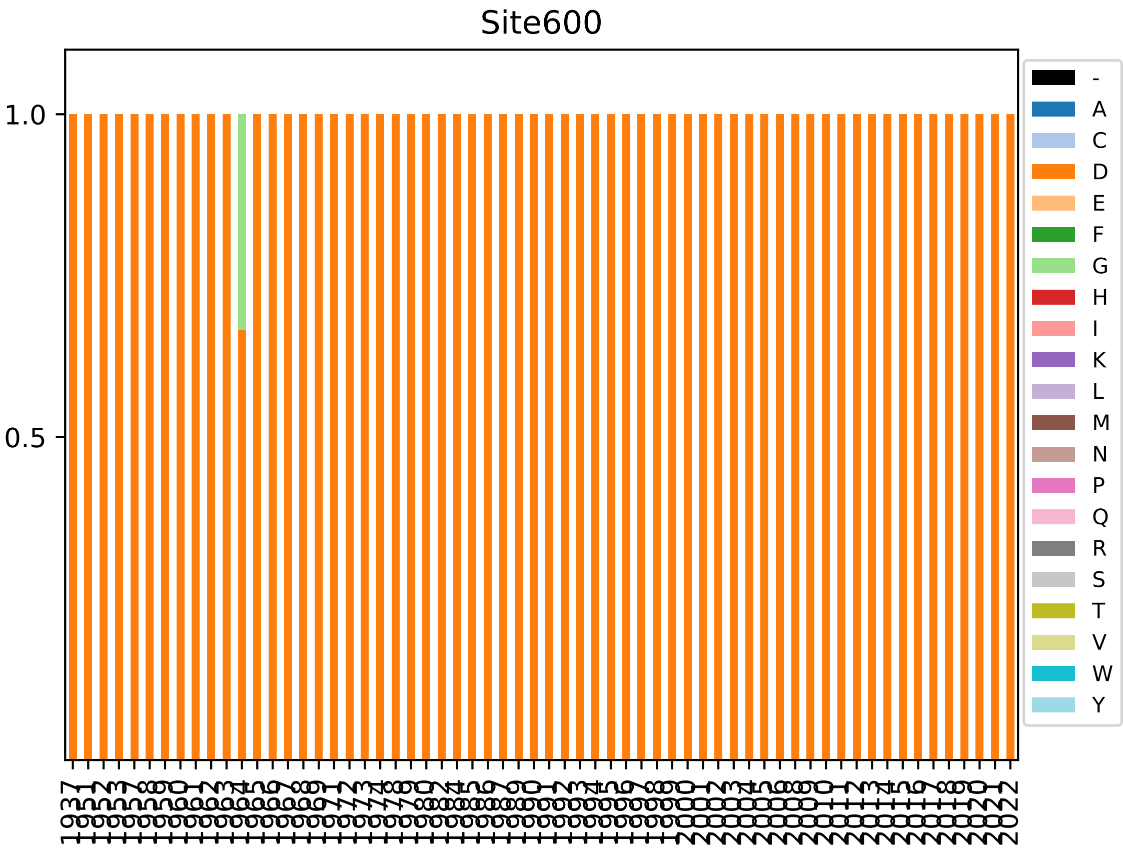
Site600
1.0
0.5
-
A
C
D
E
F
G
H
I
K
L
M
N
P
Q
R
S
T
V
W
Y

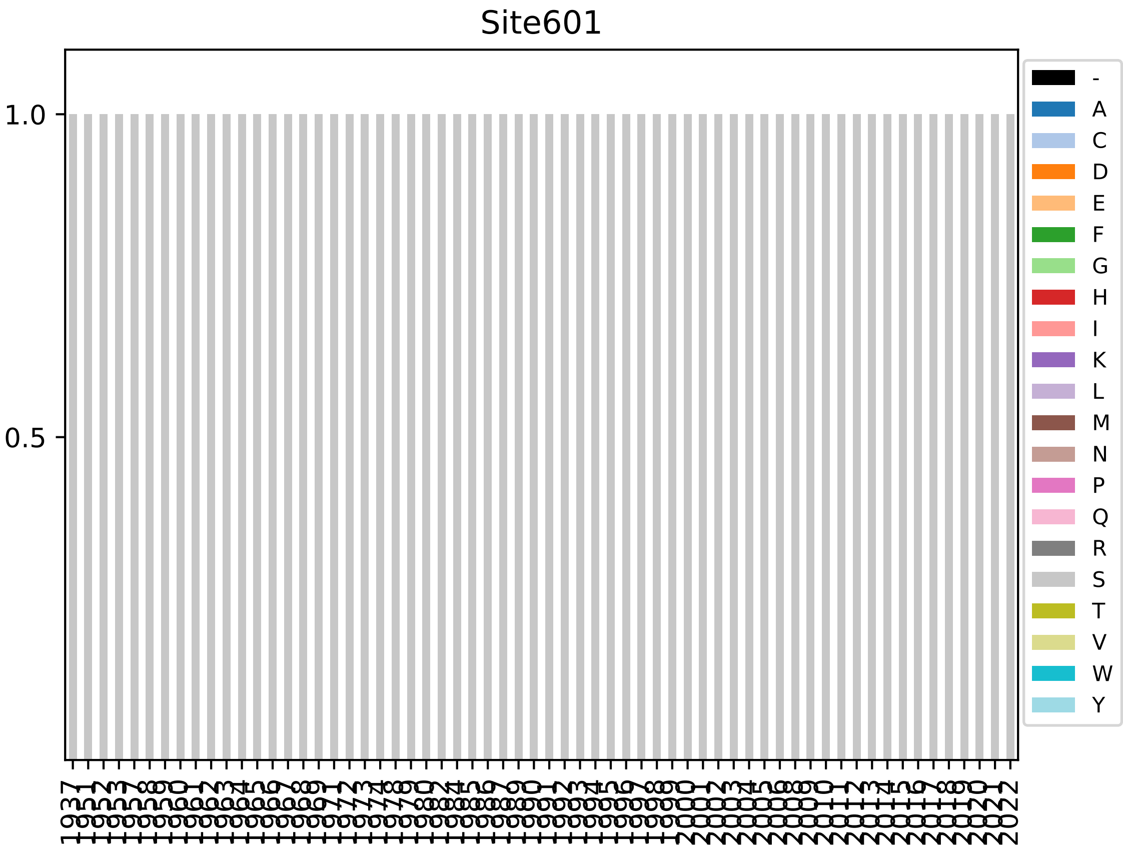
Site601
1.0
0.5
-
A
C
D
E
F
G
H
I
K
L
M
N
P
Q
R
S
T
V
W
Y

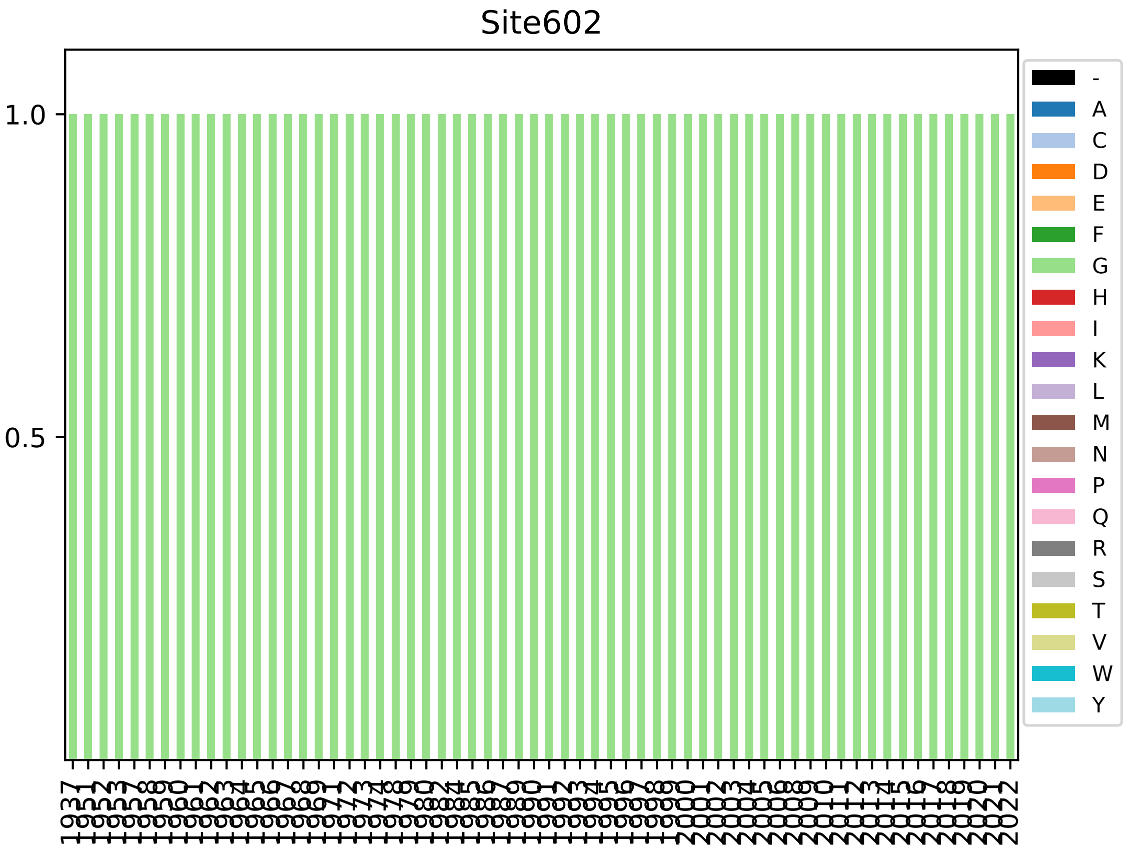
Site602
1.0
0.5
-
A
C
D
E
F
G
H
I
K
L
M
N
P
Q
R
S
T
V
W
Y

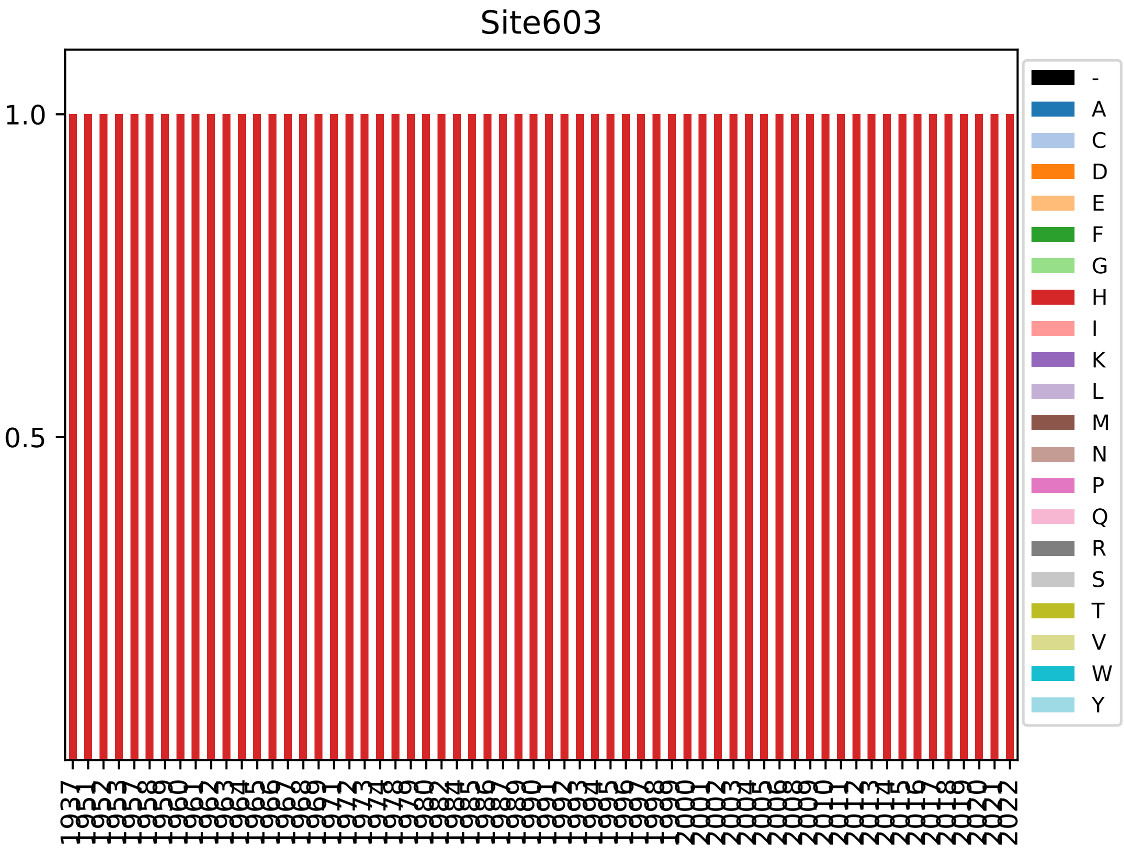
Site603
1.0
0.5
-
A
C
D
E
F
G
H
I
K
L
M
N
P
Q
R
S
T
V
W
Y

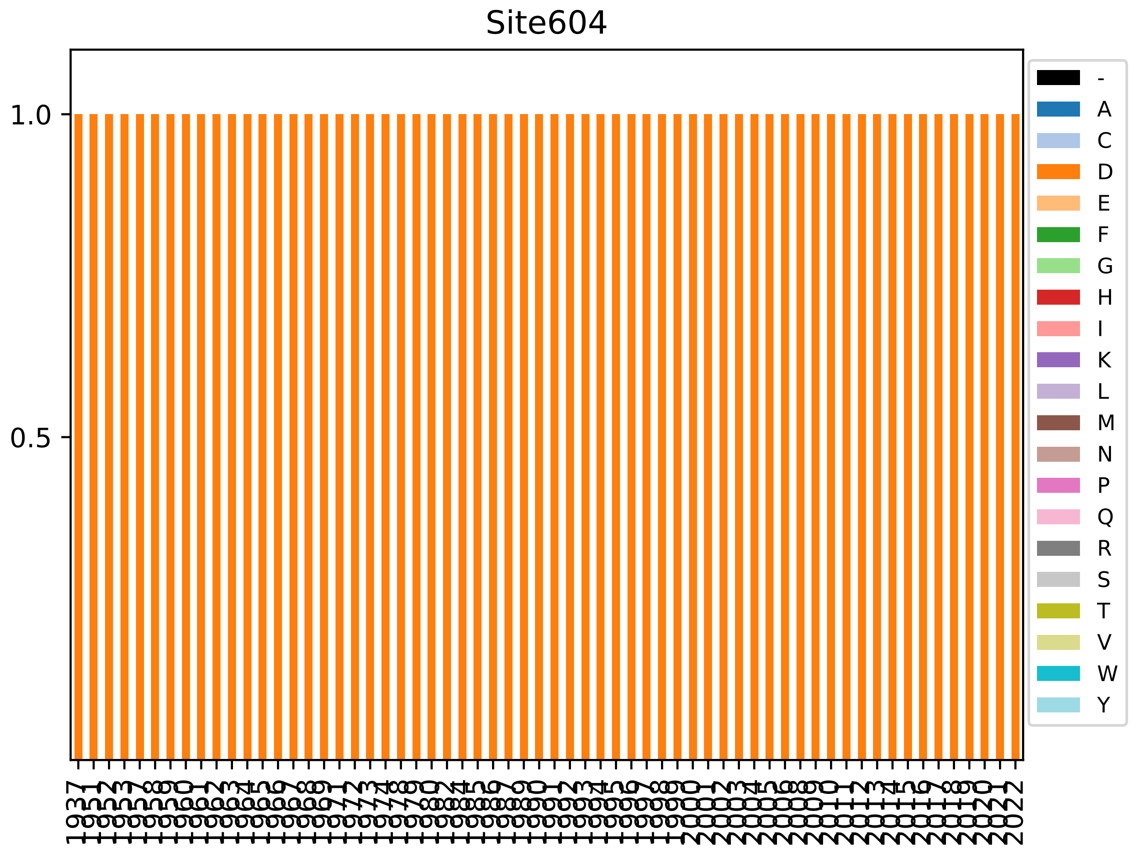
Site604
1.0
0.5
-
A
C
D
E
F
G
H
I
K
L
M
N
P
Q
R
S
T
V
W
Y

Site605

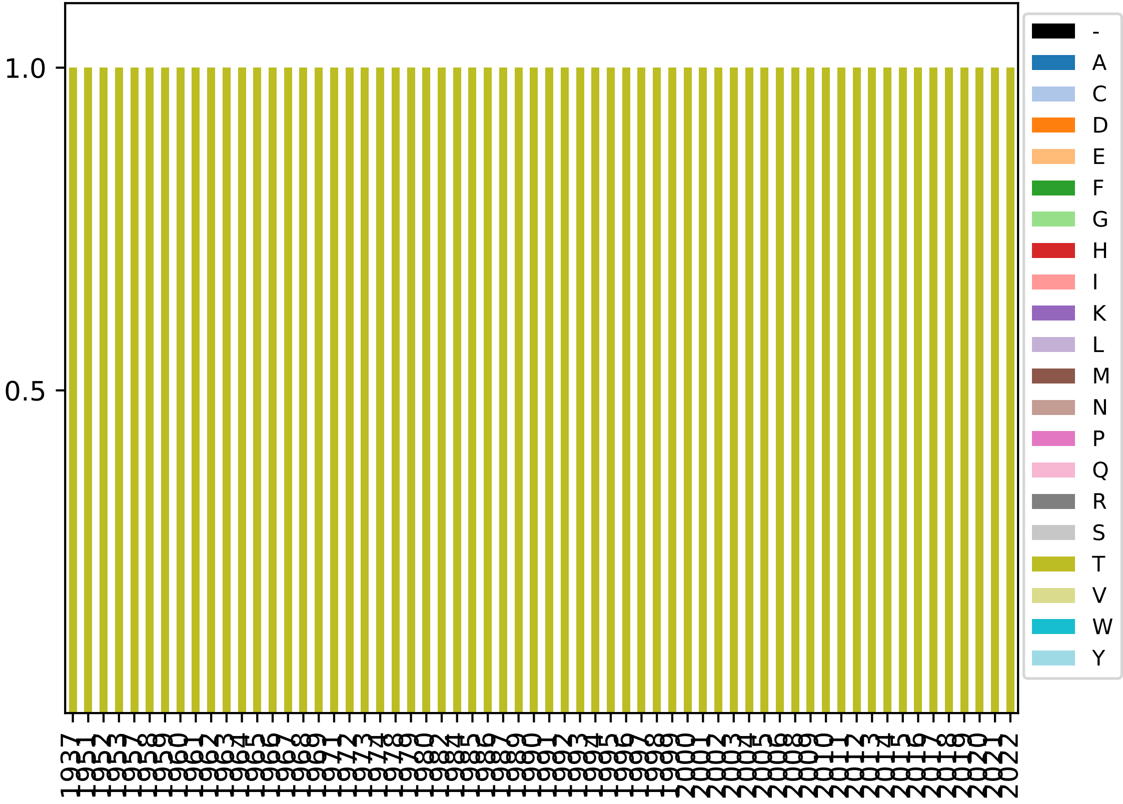

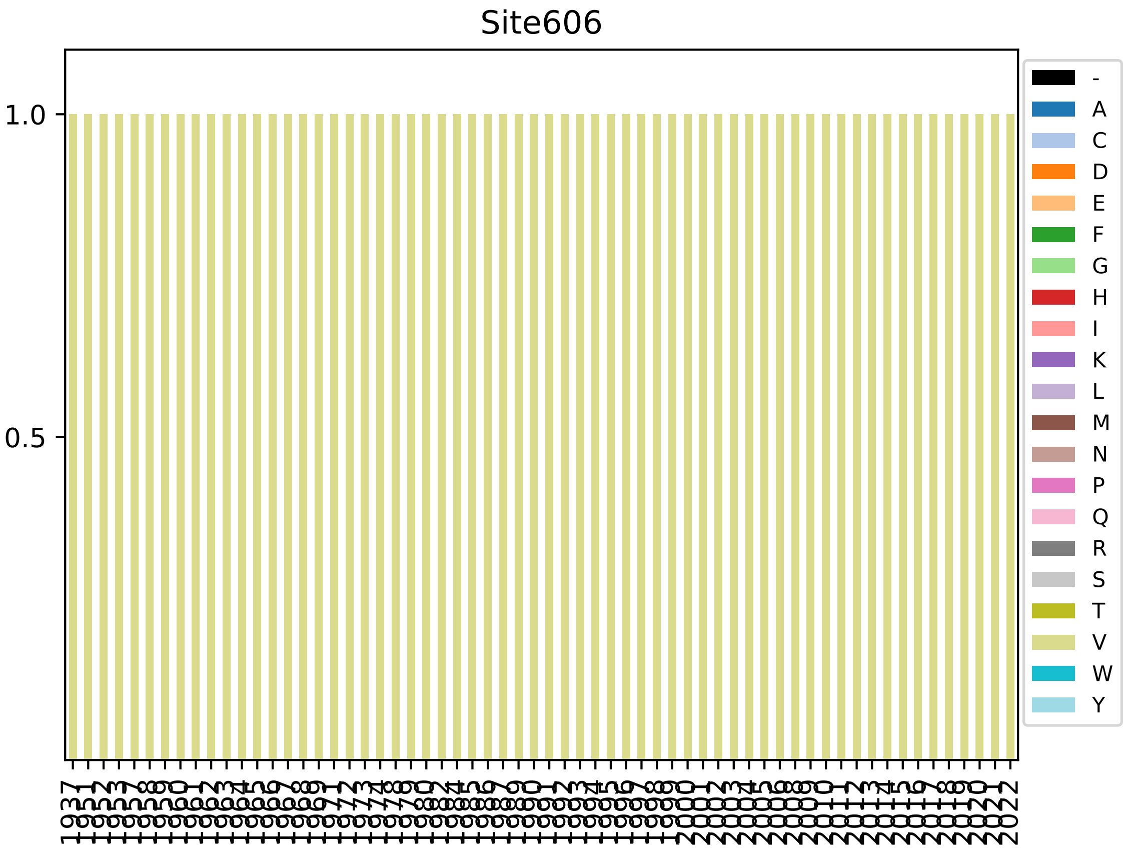
Site606
1.0
0.5
-
A
C
D
E
F
G
H
I
K
L
M
N
P
Q
R
S
T
V
W
Y

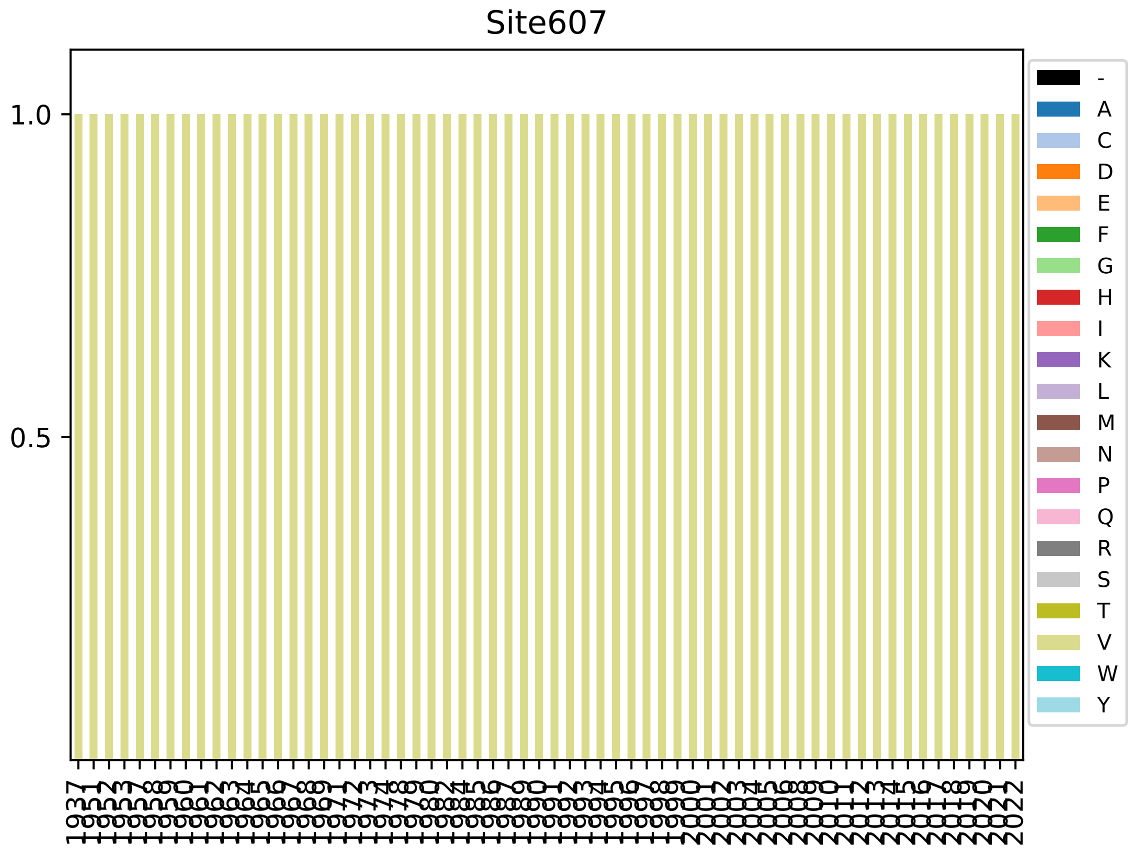
Site607
1.0
0.5
-
A
C
D
E
F
G
H
I
K
L
M
N
P
Q
R
S
T
V
W
Y

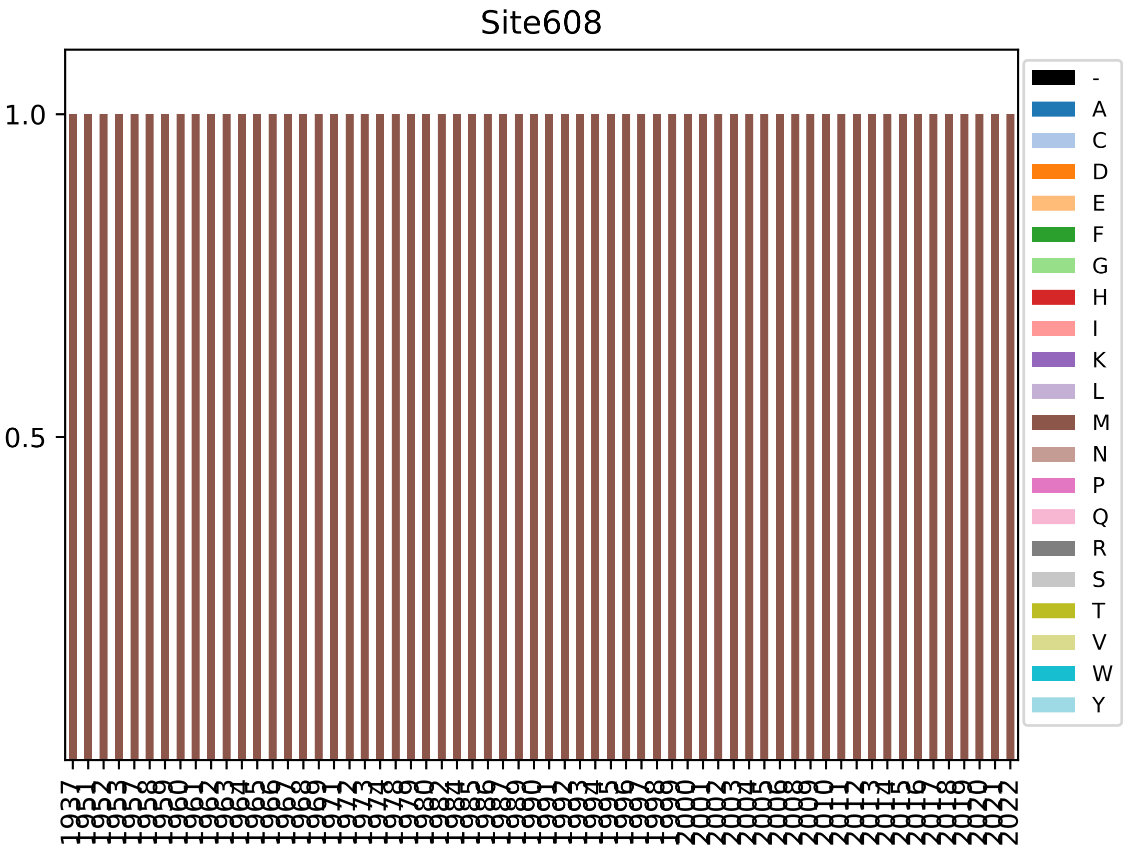
Site608
1.0
0.5
-
A
C
D
E
F
G
H
I
K
L
M
N
P
Q
R
S
T
V
W
Y

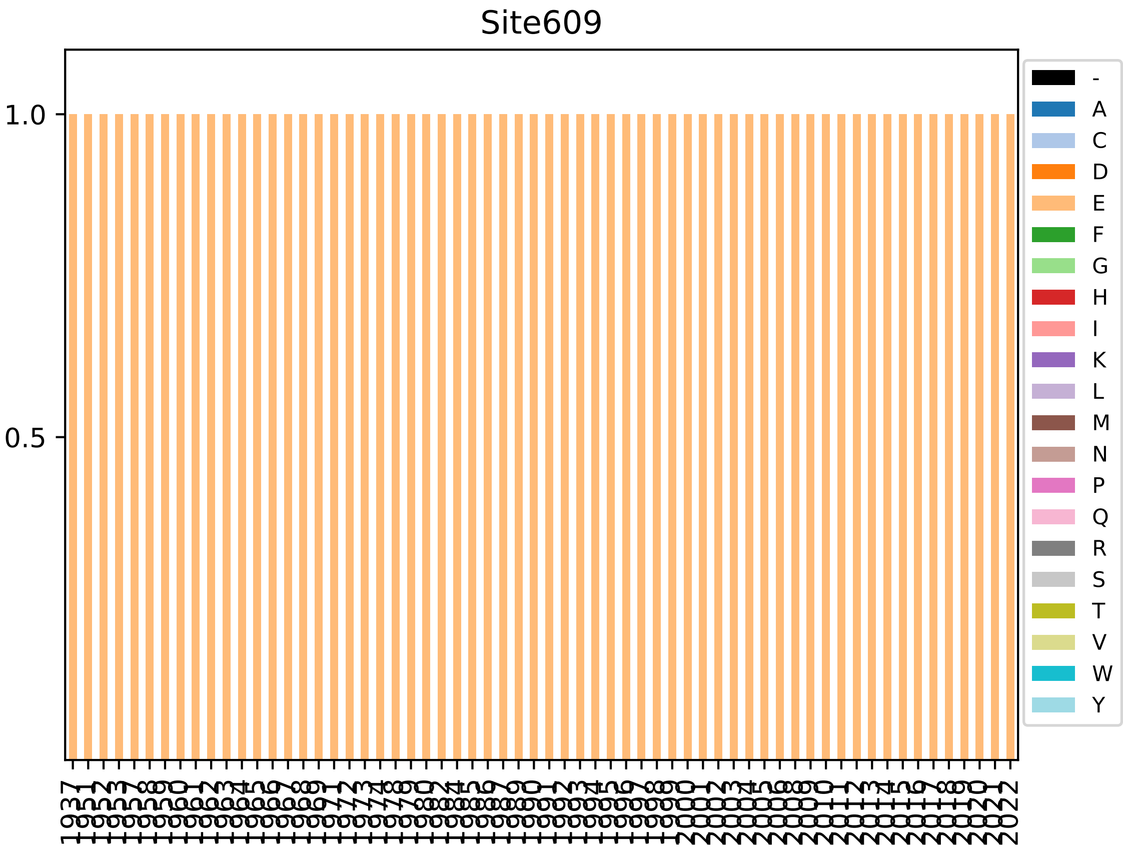
Site609
1.0
0.5
-
A
C
D
E
F
G
H
I
K
L
M
N
P
Q
R
S
T
V
W
Y

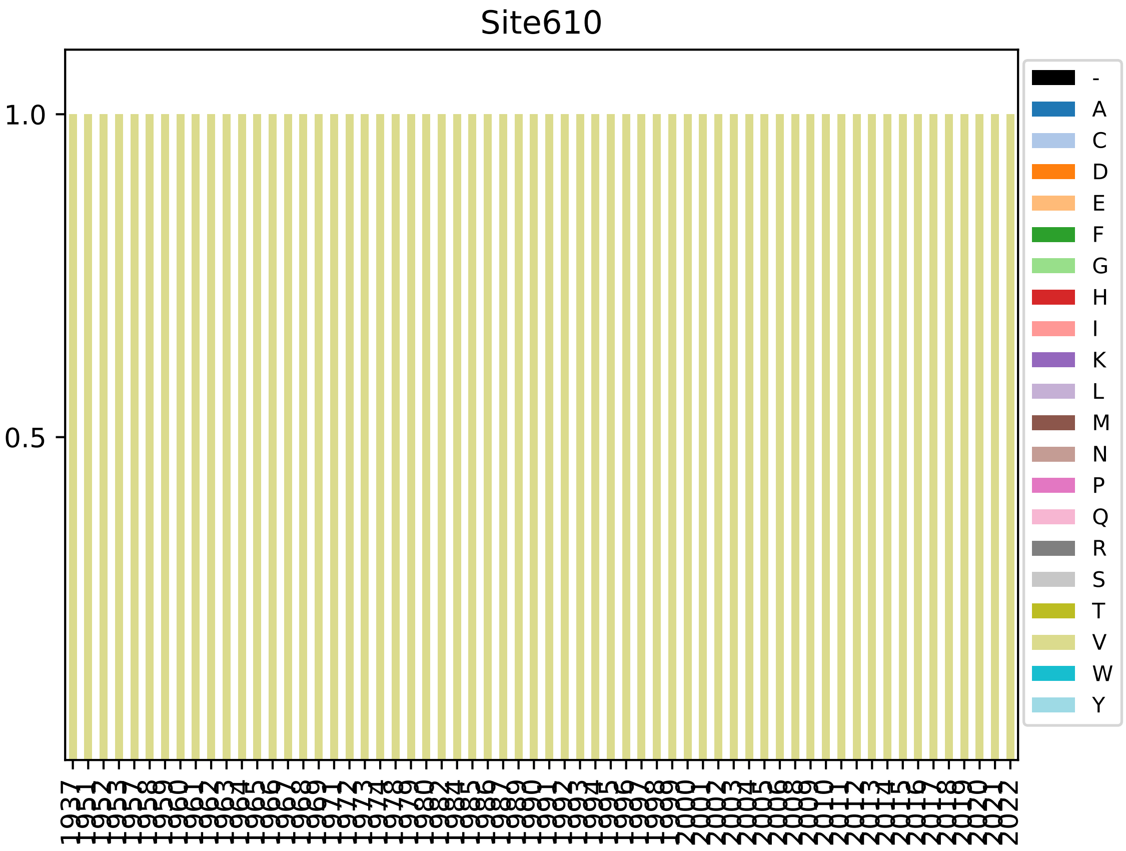
Site610
1.0
0.5
-
A
C
D
E
F
G
H
I
K
L
M
N
P
Q
R
S
T
V
W
Y

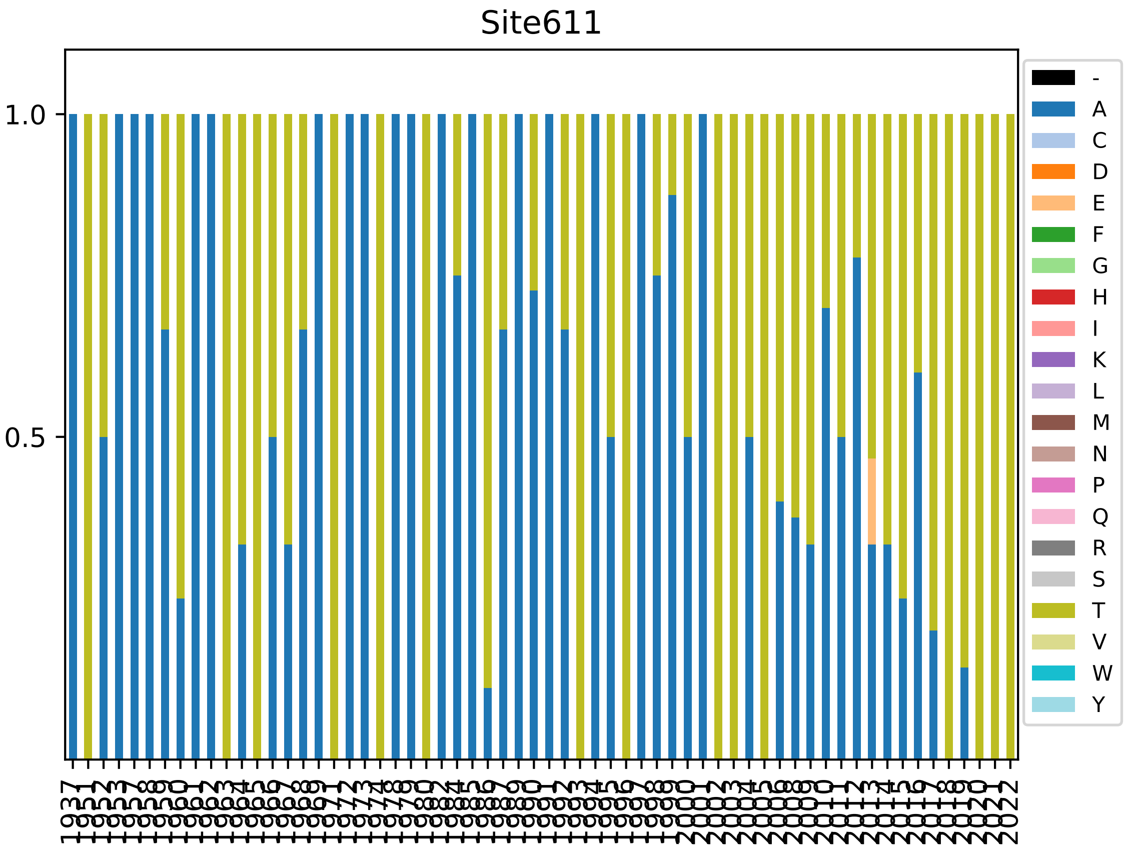
Site611
1.0
0.5
-
A
C
D
E
F
G
H
I
K
L
M
N
P
Q
R
S
T
V
W
Y

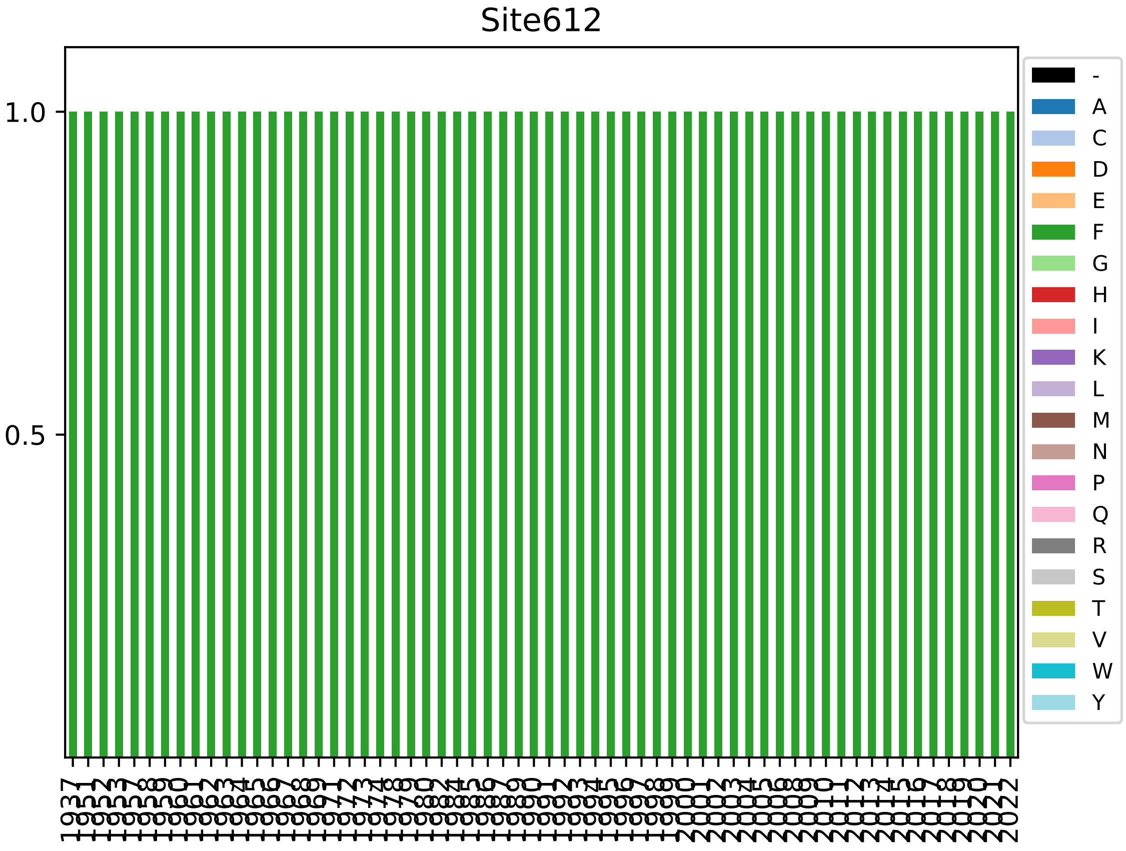
Site612
1.0
0.5
-
A
C
D
E
F
G
H
I
K
L
M
N
P
Q
R
S
T
V
W
Y

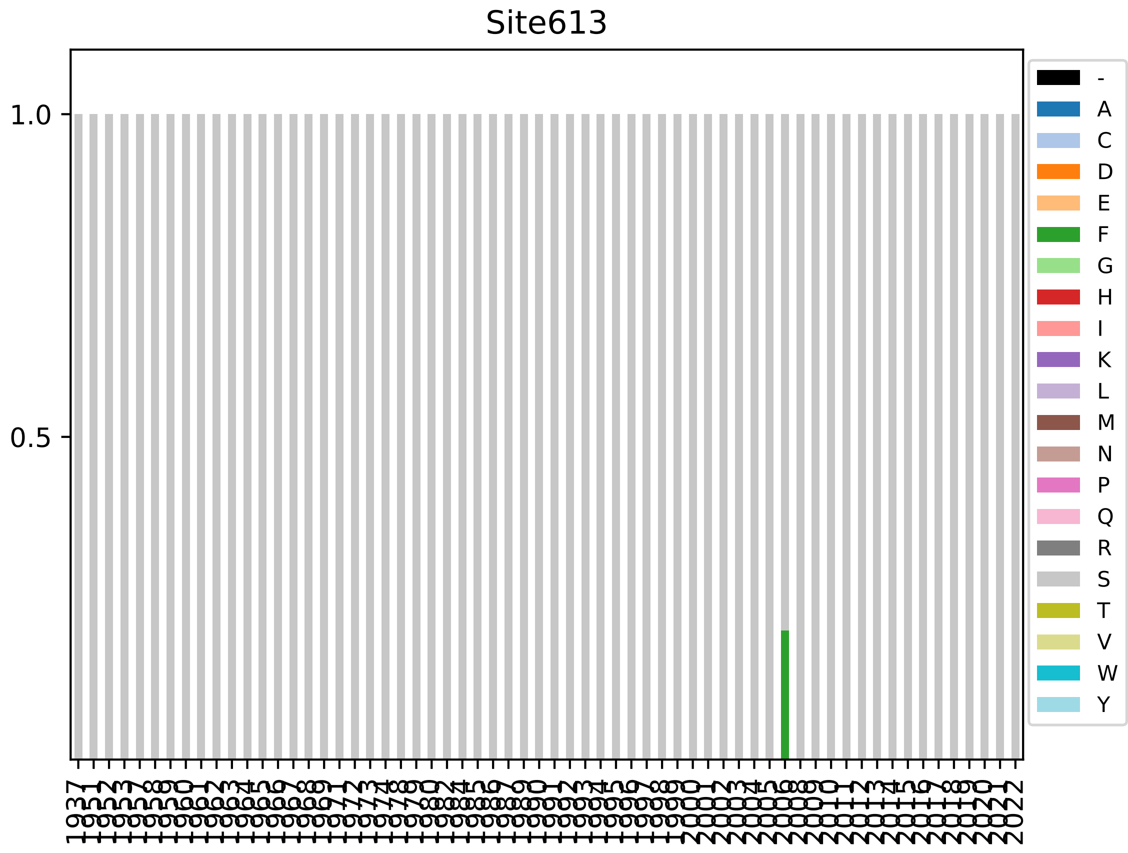
Site613
1.0
0.5
-
A
C
D
E
F
G
H
I
K
L
M
N
P
Q
R
S
T
V
W
Y

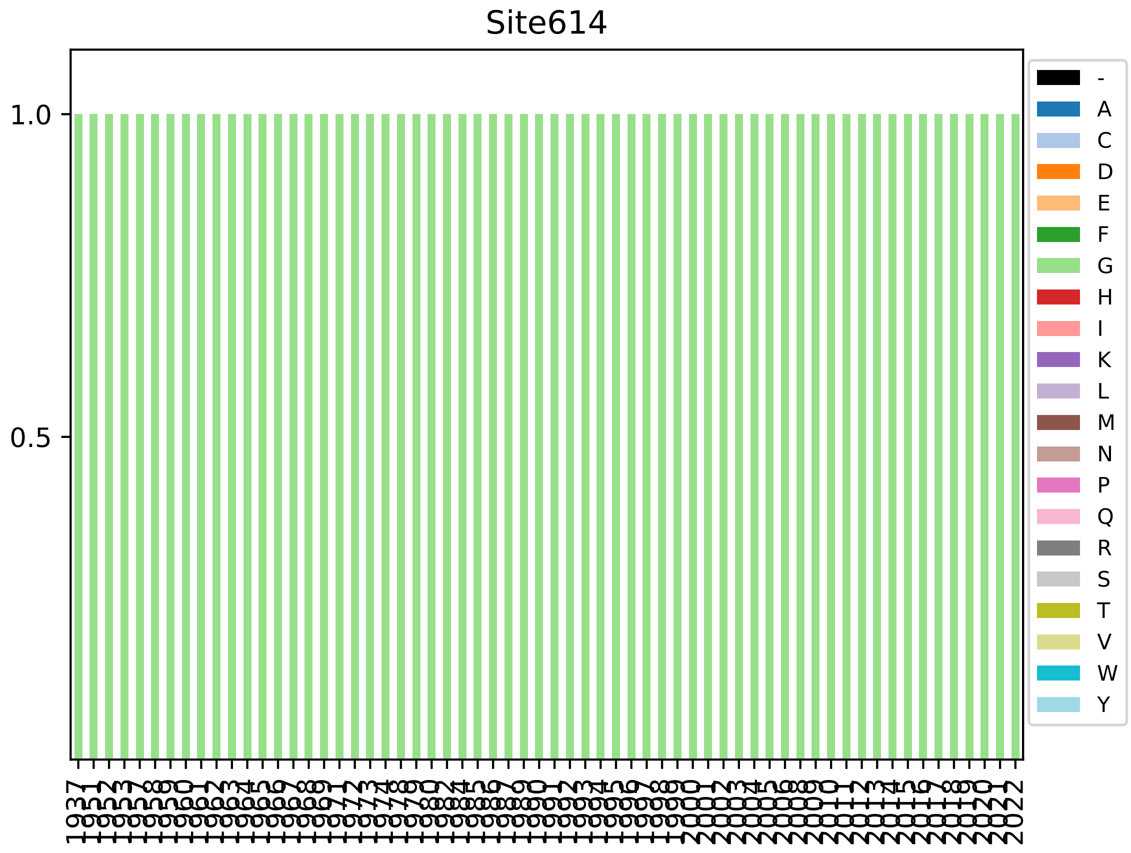
Site614
1.0
0.5
-
A
C
D
E
F
G
H
I
K
L
M
N
P
Q
R
S
T
V
W
Y

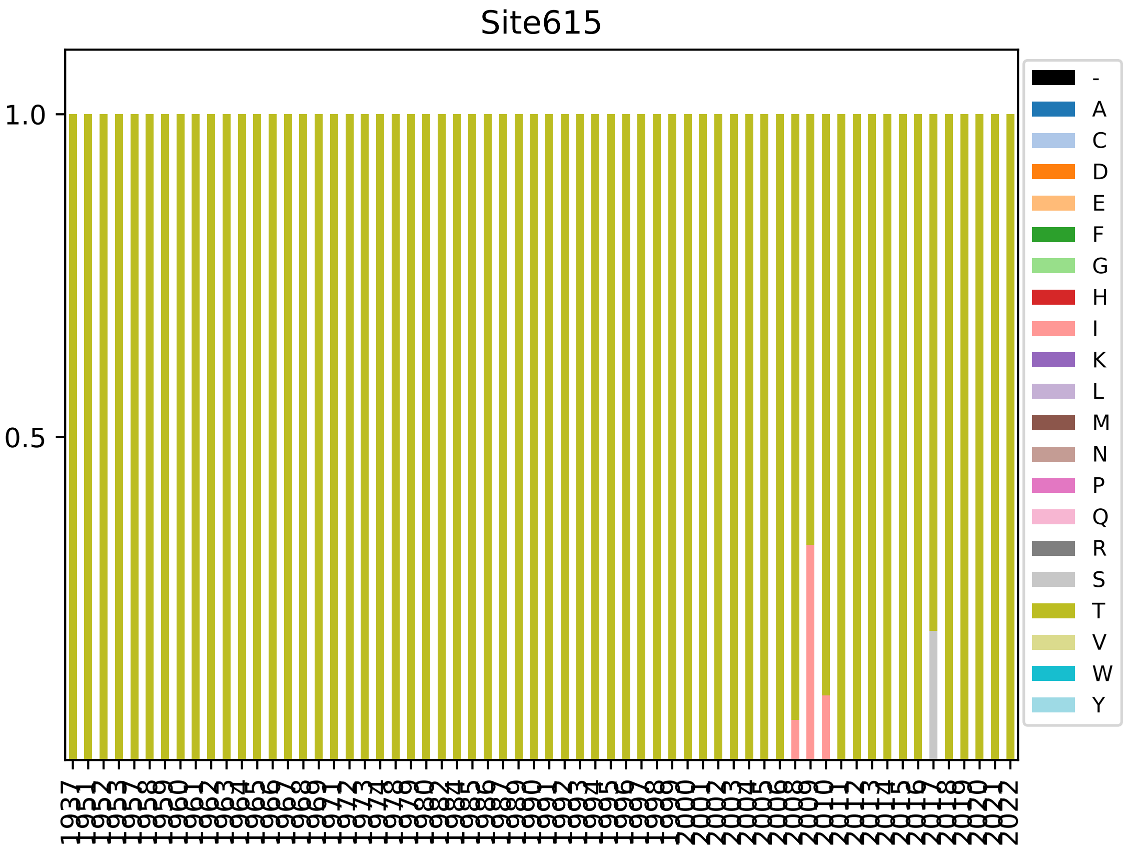
Site615
1.0
0.5
-
A
C
D
E
F
G
H
I
K
L
M
N
P
Q
R
S
T
V
W
Y

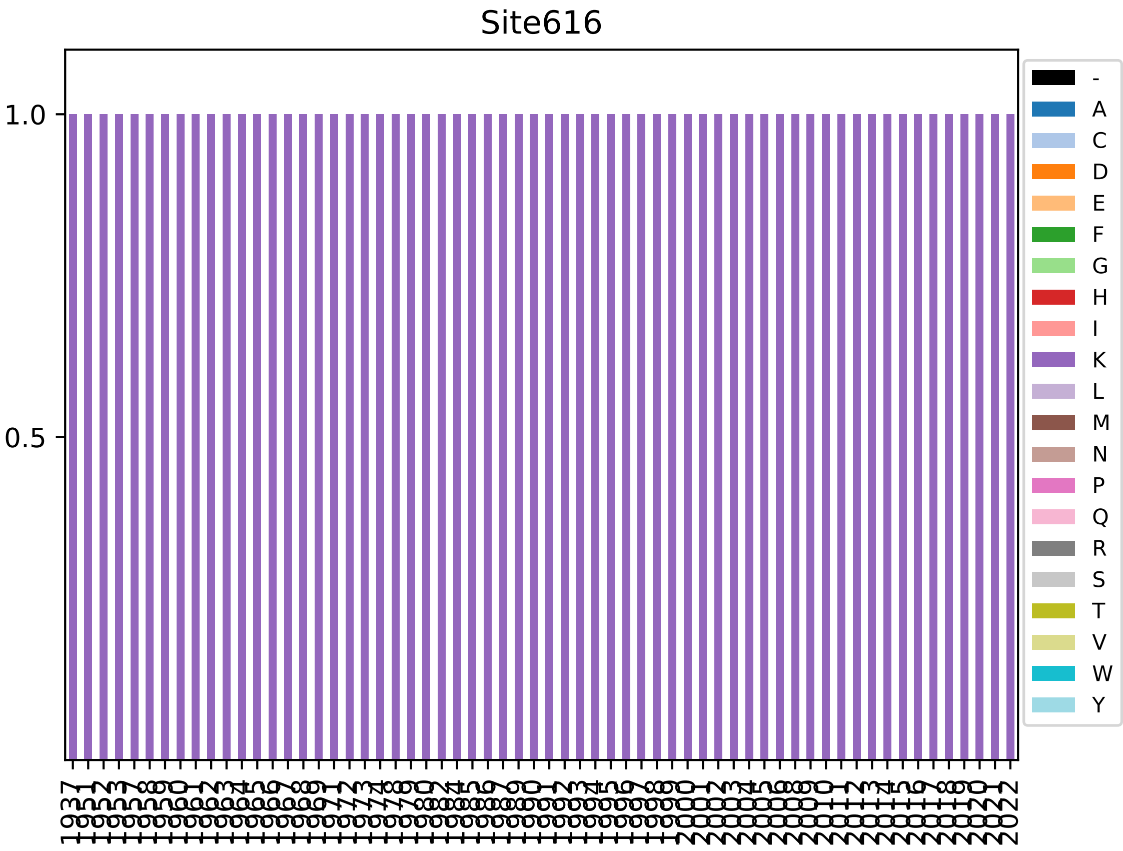
Site616
1.0
0.5
-
A
C
D
E
F
G
H
I
K
L
M
N
P
Q
R
S
T
V
W
Y

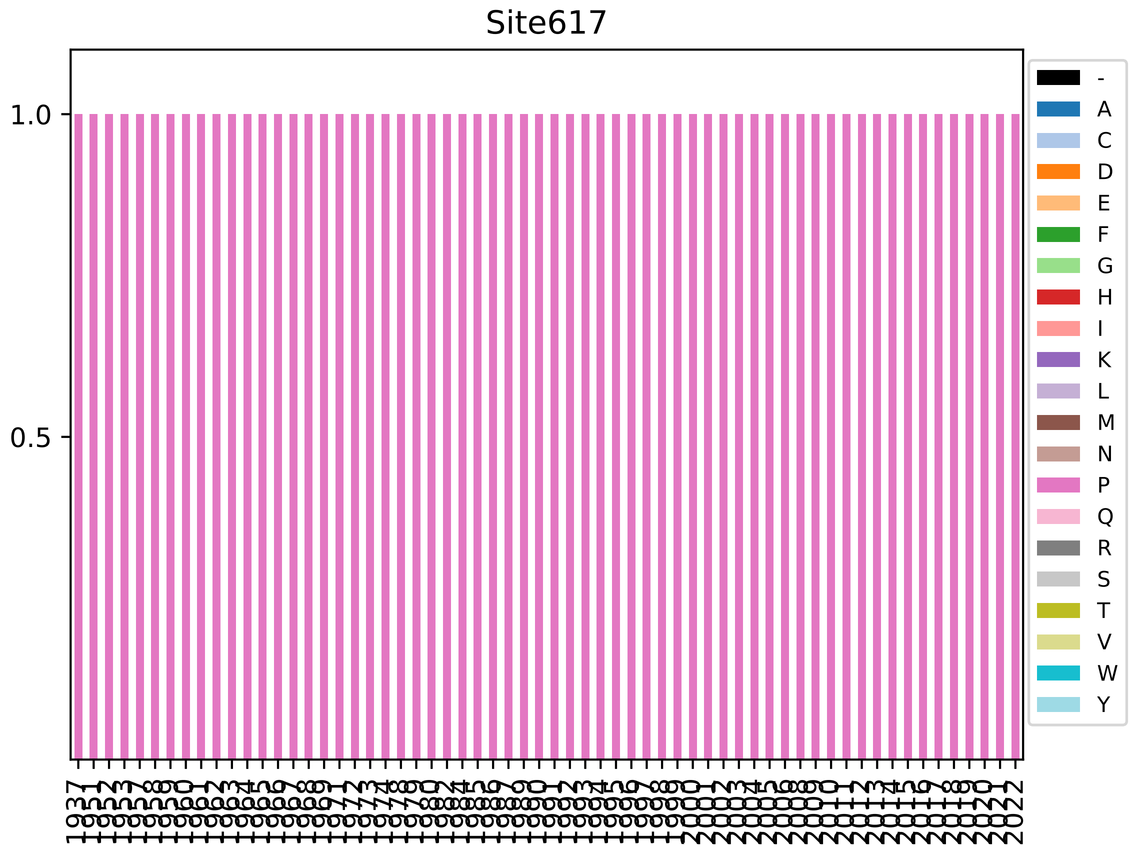
Site617
1.0
0.5
-
A
C
D
E
F
G
H
I
K
L
M
N
P
Q
R
S
T
V
W
Y

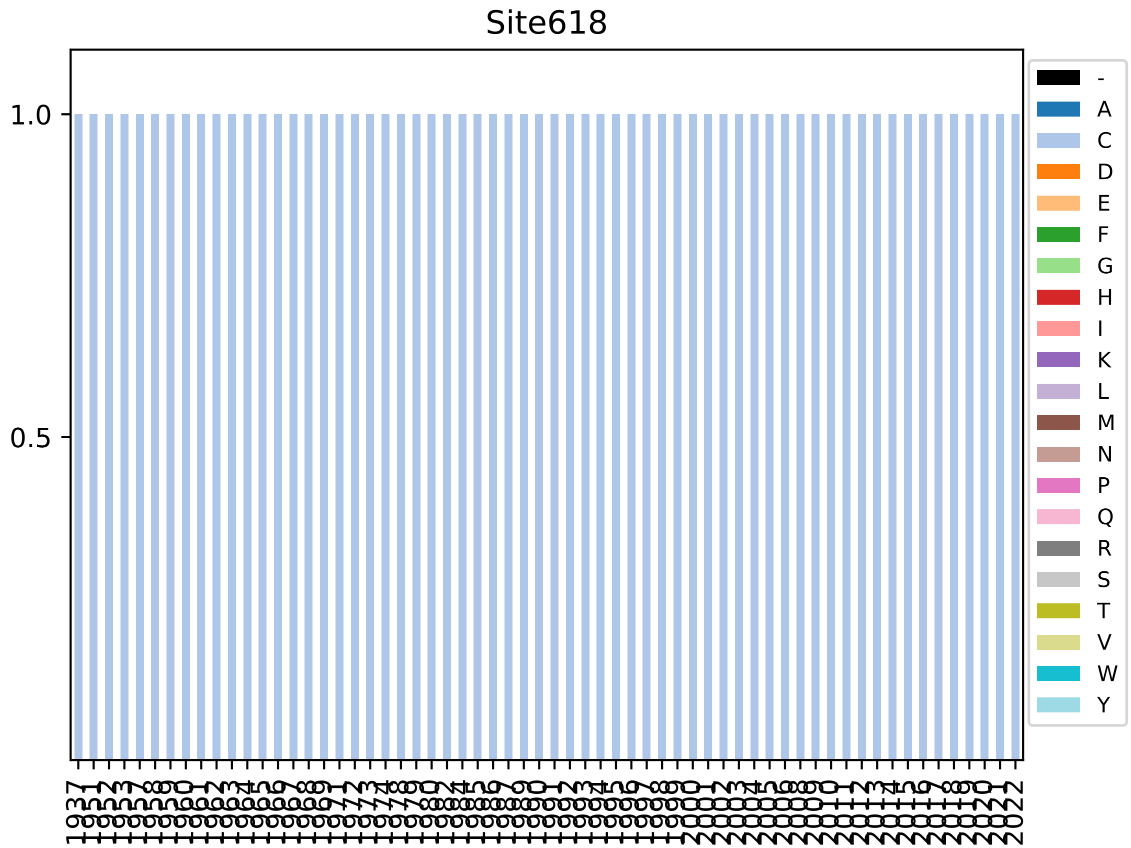
Site618
1.0
0.5
-
A
C
D
E
F
G
H
I
K
L
M
N
P
Q
R
S
T
V
W
Y

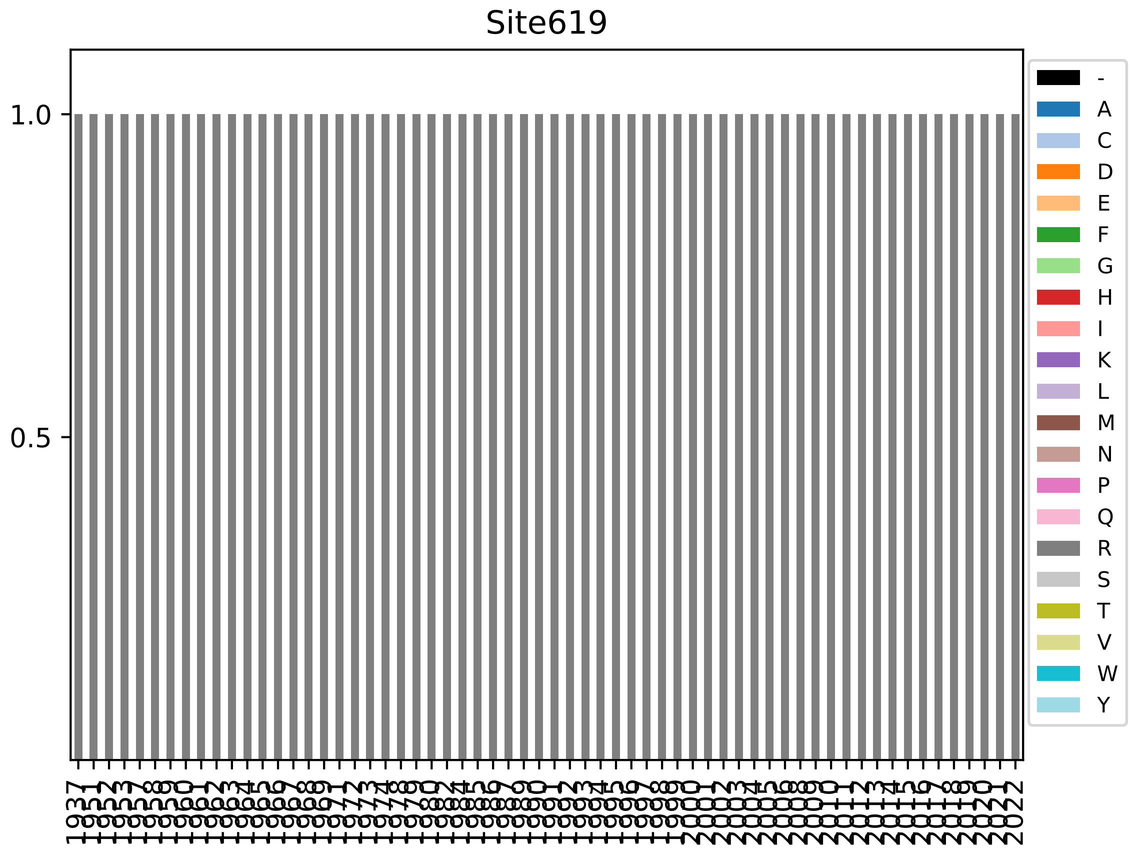
Site619
1.0
0.5
-
A
C
D
E
F
G
H
I
K
L
M
N
P
Q
R
S
T
V
W
Y

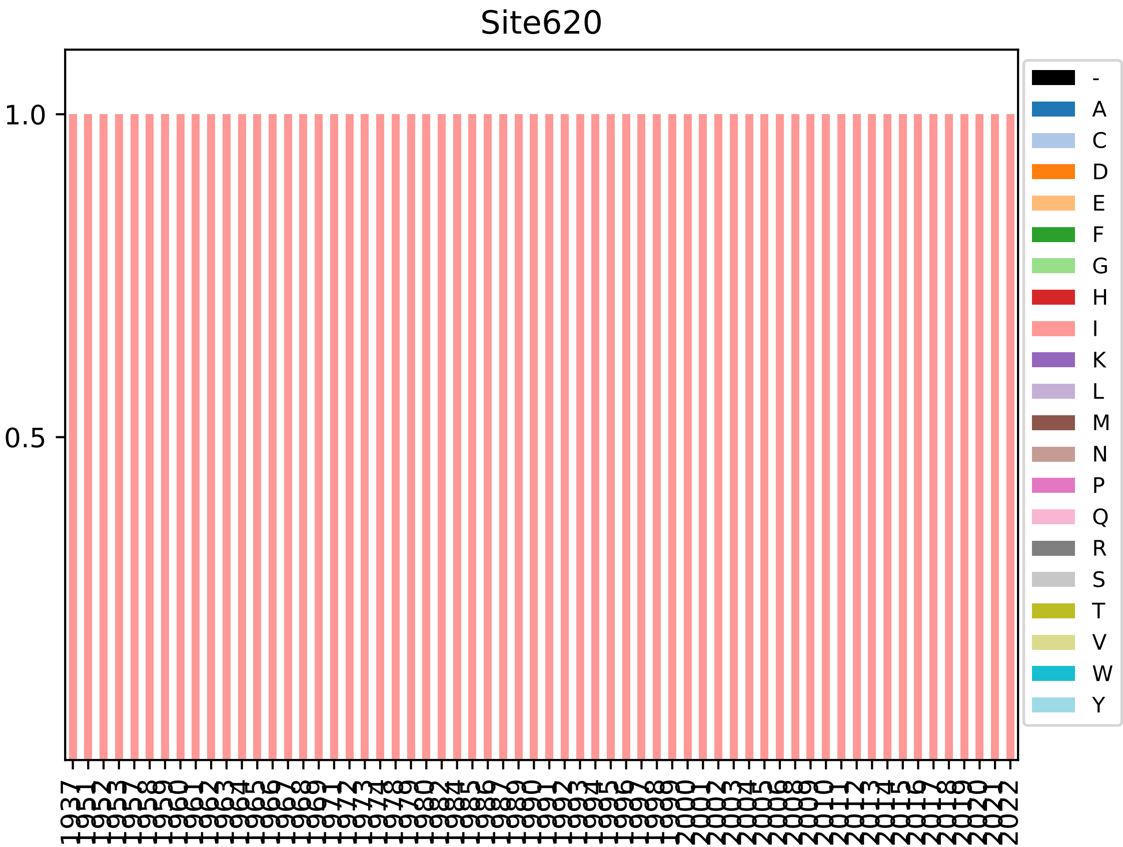
Site620
1.0
0.5
-
A
C
D
E
F
G
H
I
K
L
M
N
P
Q
R
S
T
V
W
Y

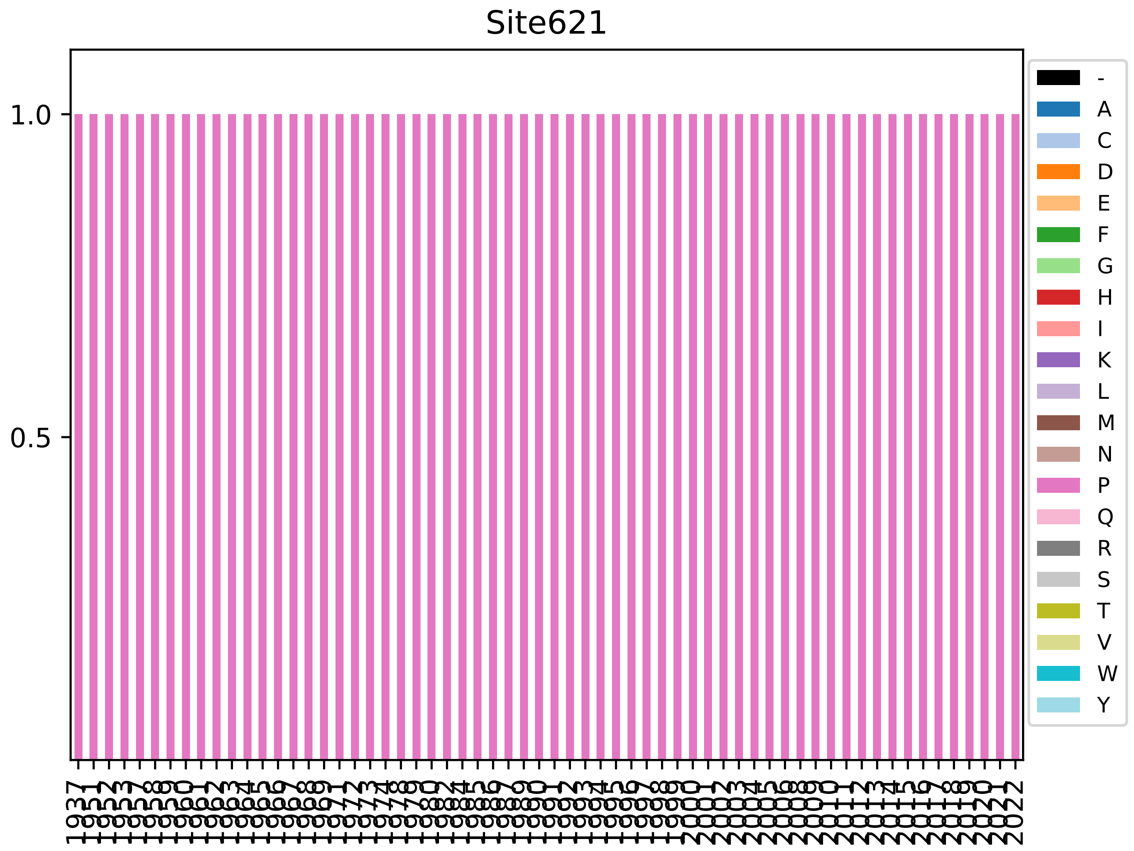
Site621
1.0
0.5
-
A
C
D
E
F
G
H
I
K
L
M
N
P
Q
R
S
T
V
W
Y

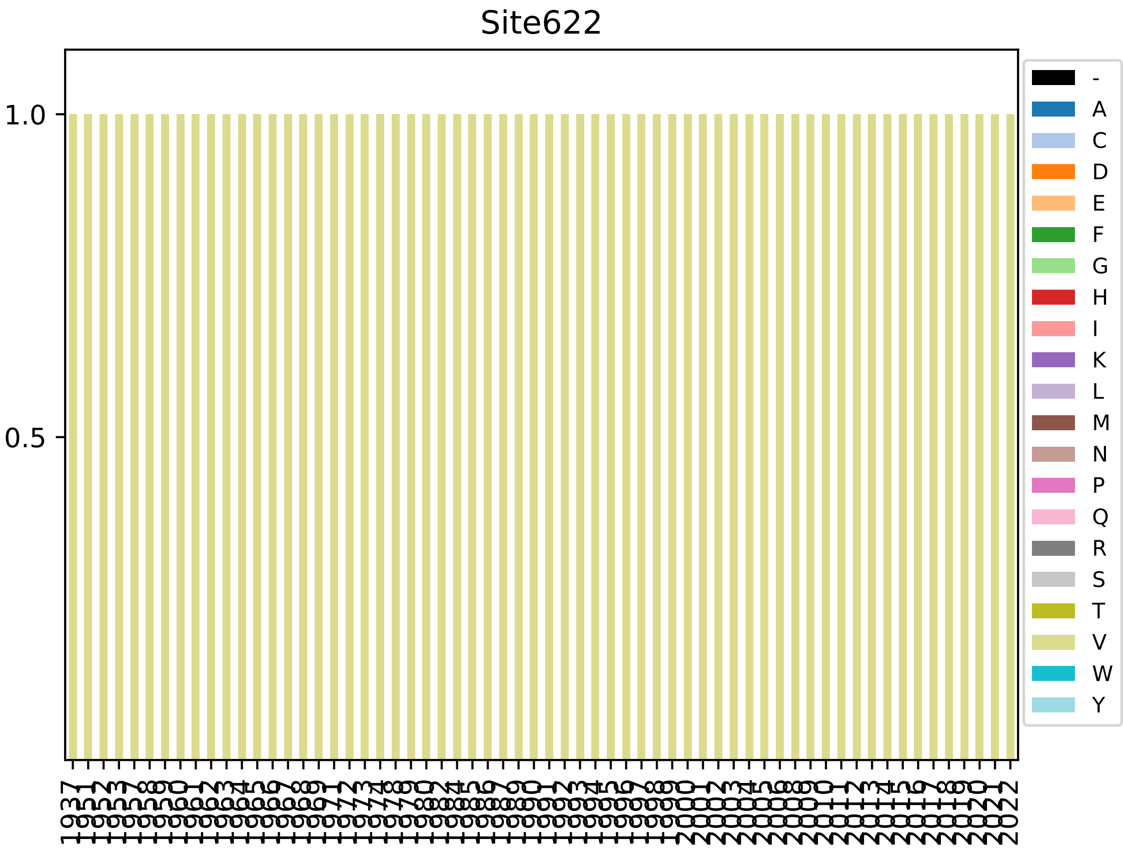

Site622
1.0
0.5
-
A
C
D
E
F
G
H
I
K
L
M
N
P
Q
R
S
T
V
W
Y

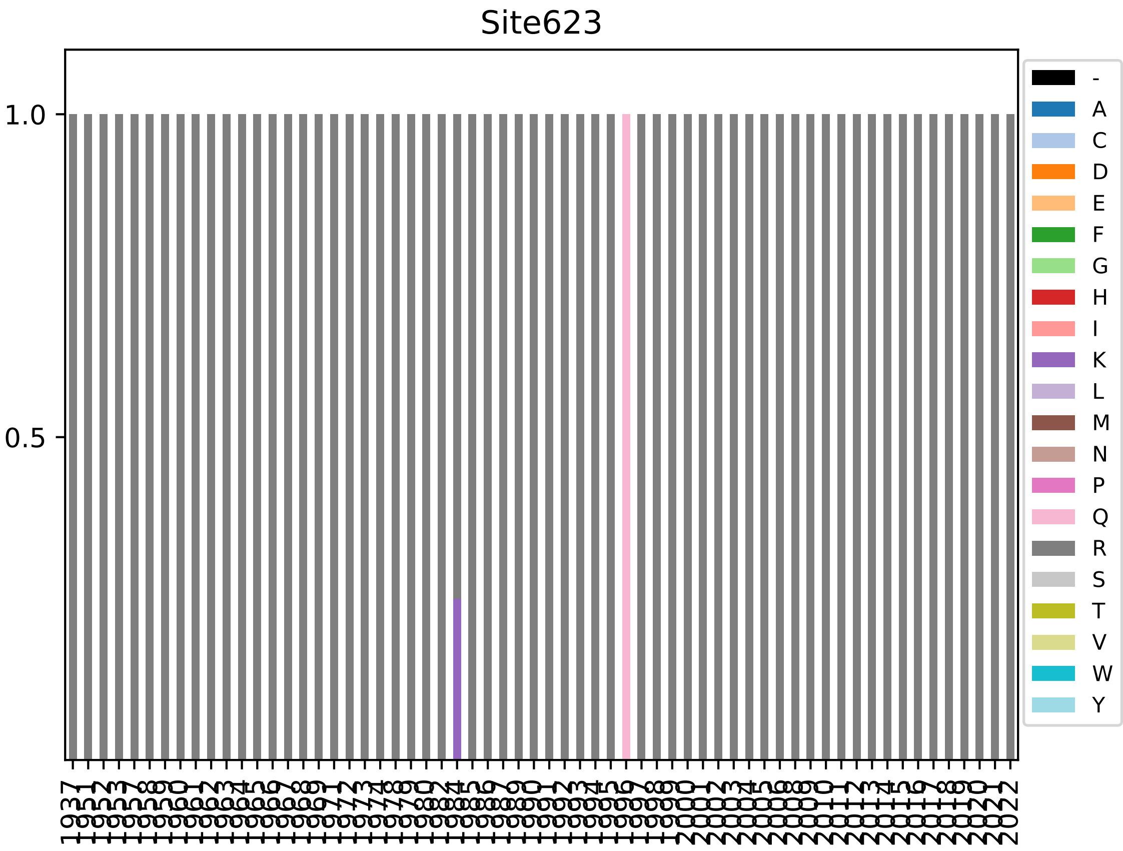
Site623
1.0
0.5
-
A
C
D
E
F
G
H
I
K
L
M
N
P
Q
R
S
T
V
W
Y

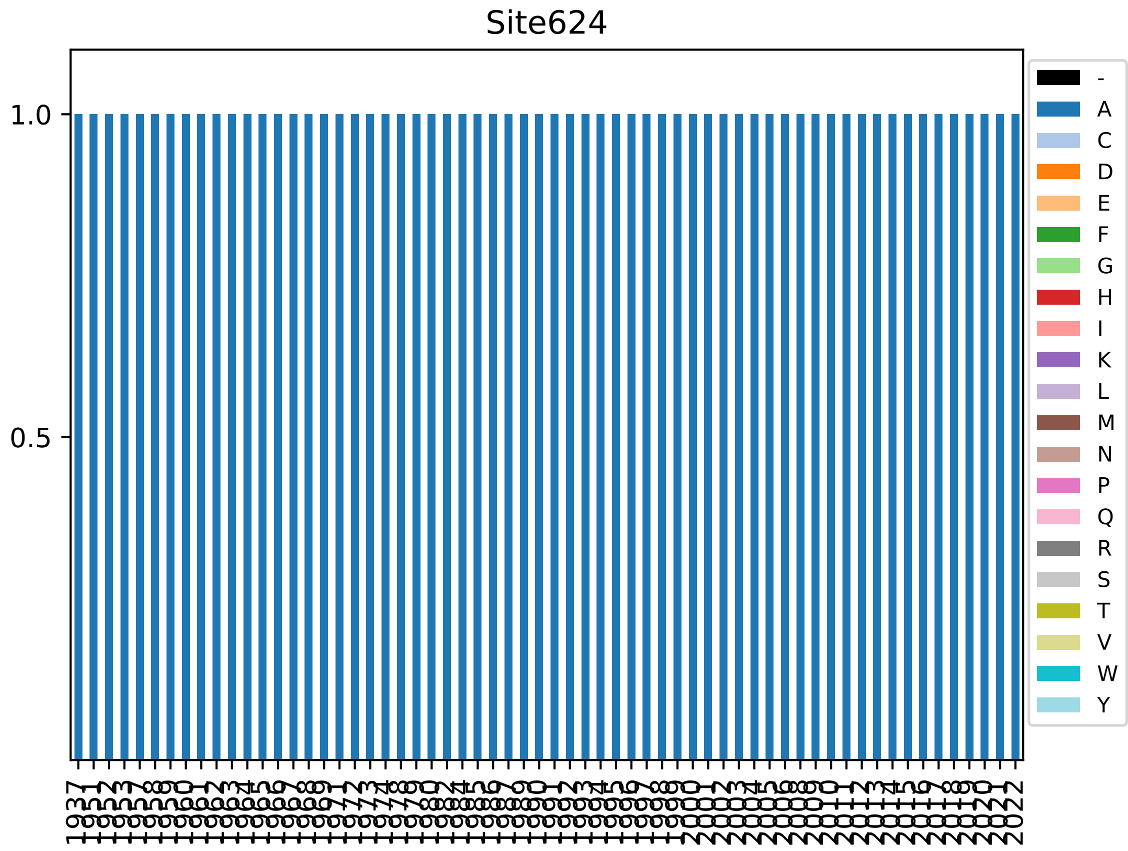
Site624
1.0
0.5
-
A
C
D
E
F
G
H
I
K
L
M
N
P
Q
R
S
T
V
W
Y

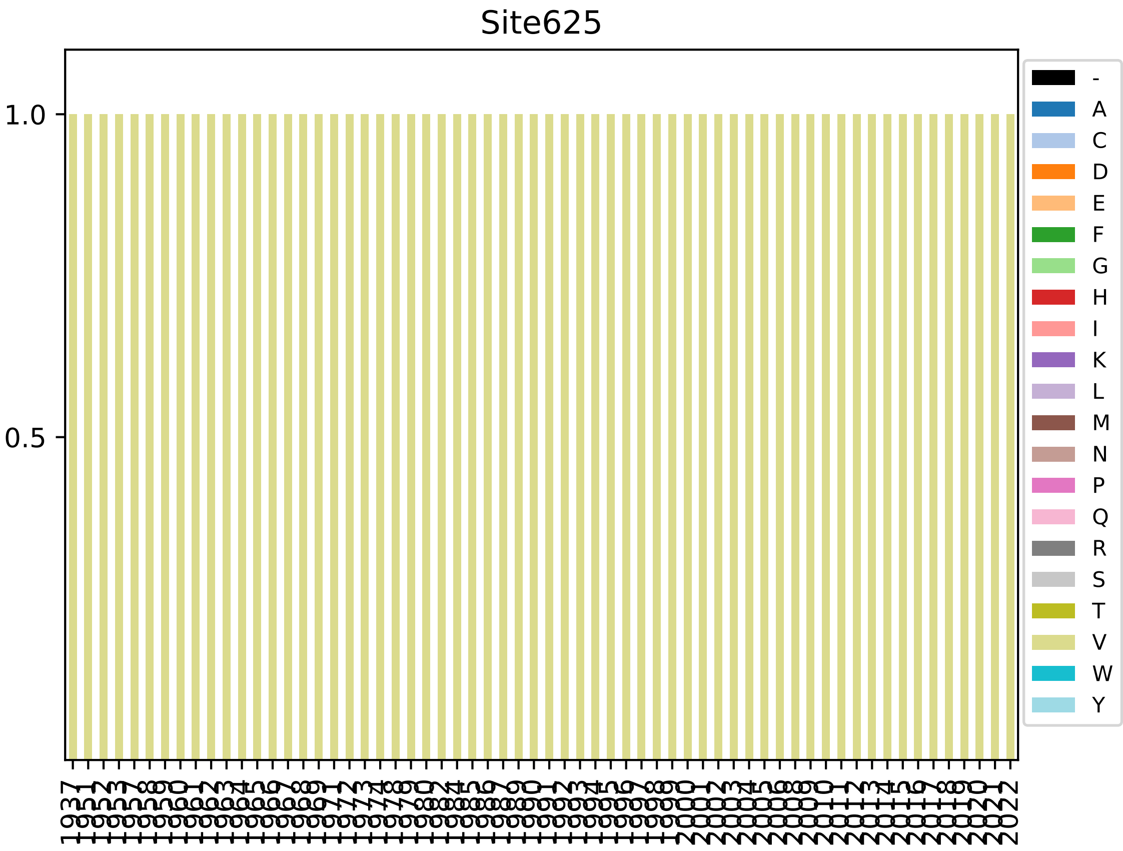
Site625
1.0
0.5
-
A
C
D
E
F
G
H
I
K
L
M
N
P
Q
R
S
T
V
W
Y

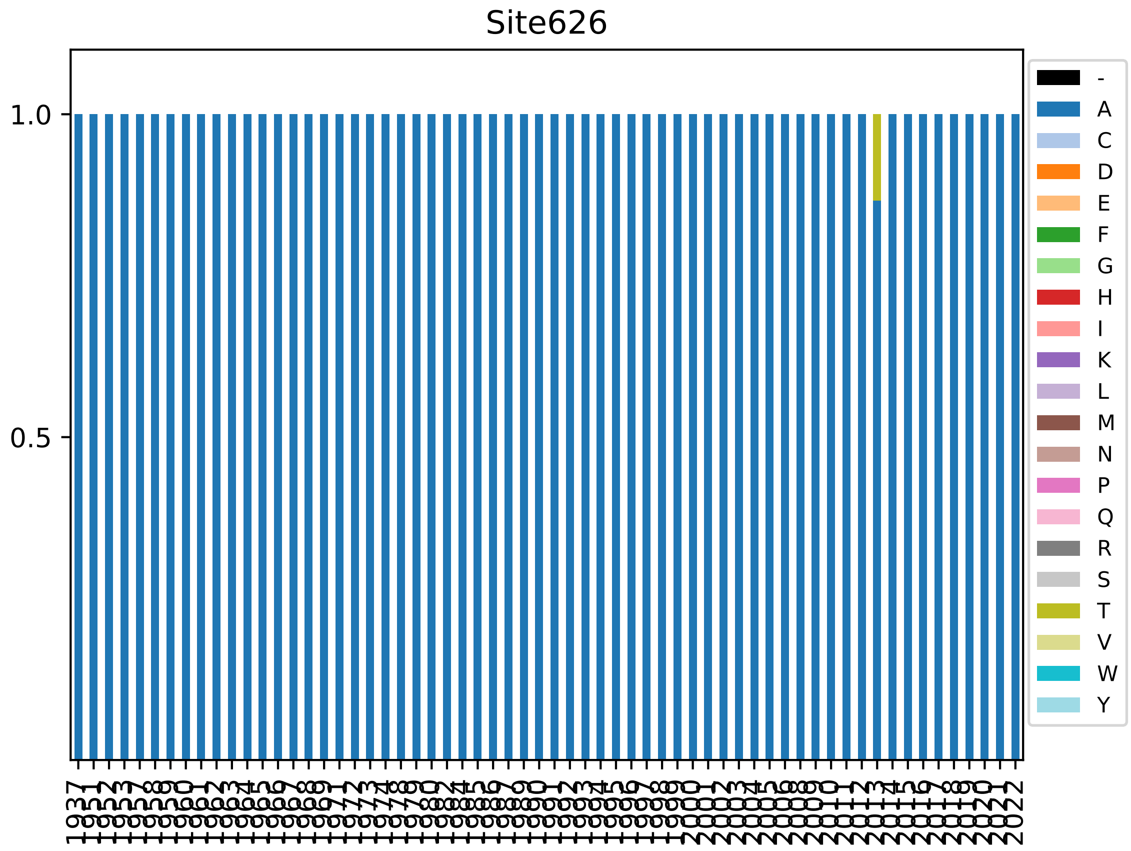
Site626
1.0
0.5
-
A
C
D
E
F
G
H
I
K
L
M
N
P
Q
R
S
T
V
W
Y

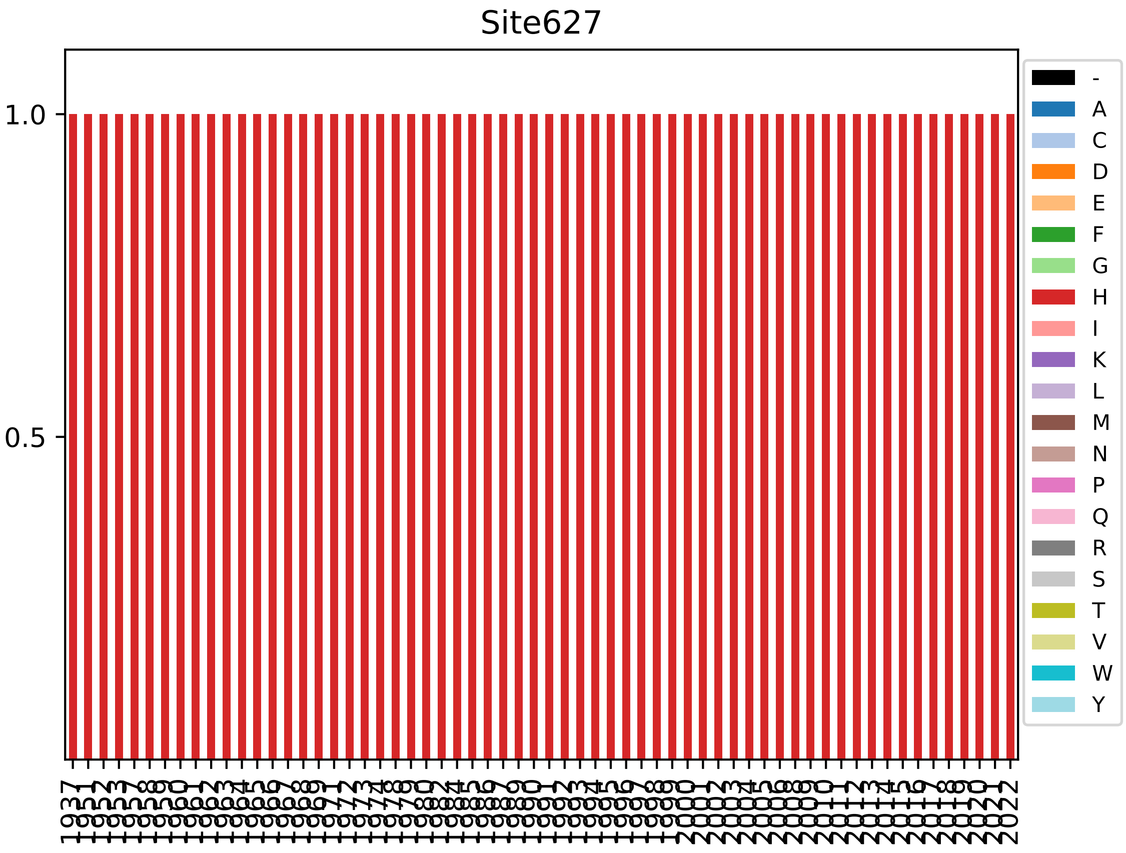
Site627
1.0
0.5
-
A
C
D
E
F
G
H
I
K
L
M
N
P
Q
R
S
T
V
W
Y

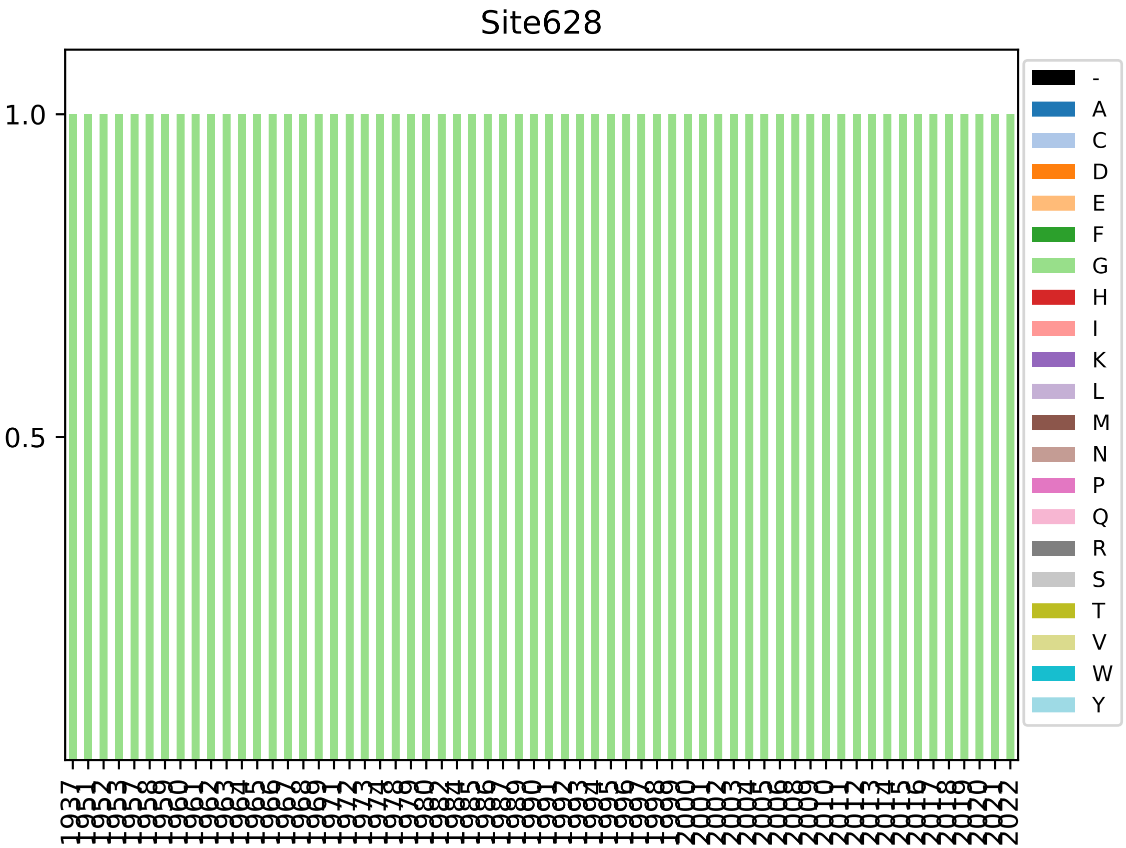
Site628
1.0
0.5
-
A
C
D
E
F
G
H
I
K
L
M
N
P
Q
R
S
T
V
W
Y

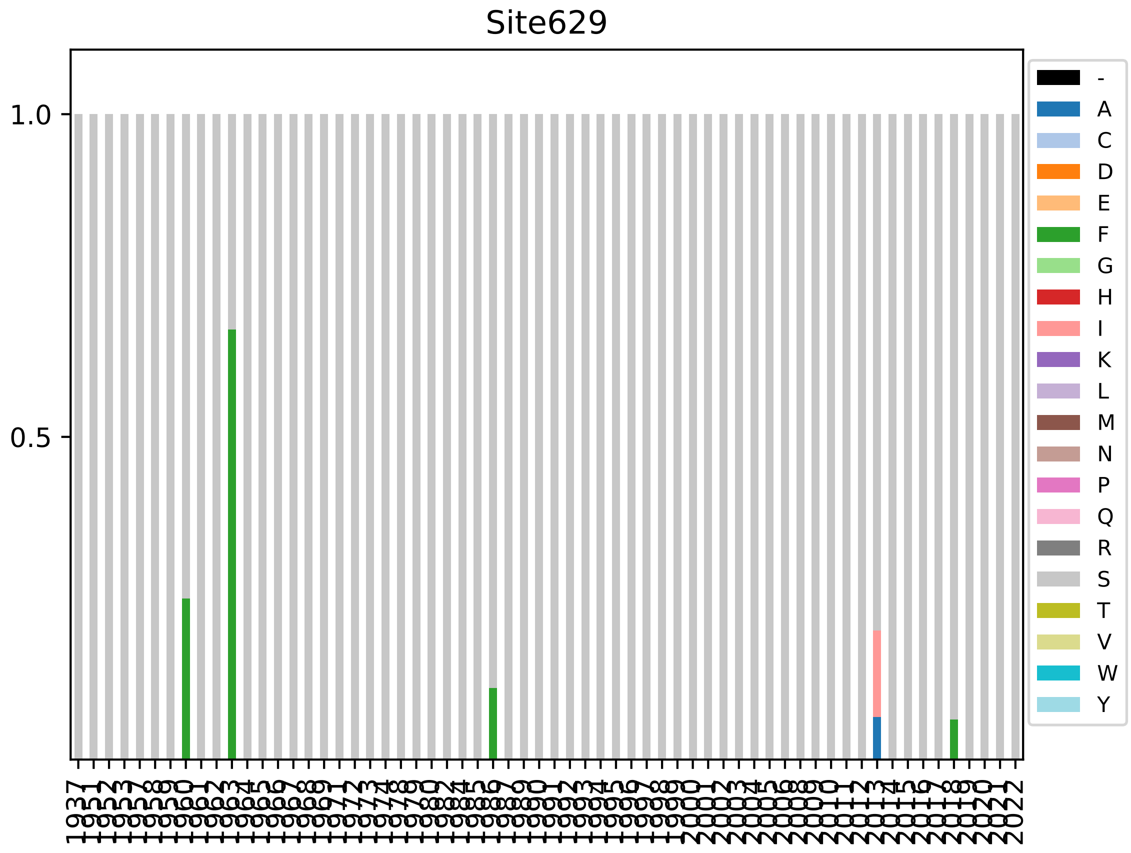
Site629
1.0
0.5
-
A
C
D
E
F
G
H
I
K
L
M
N
P
Q
R
S
T
V
W
Y

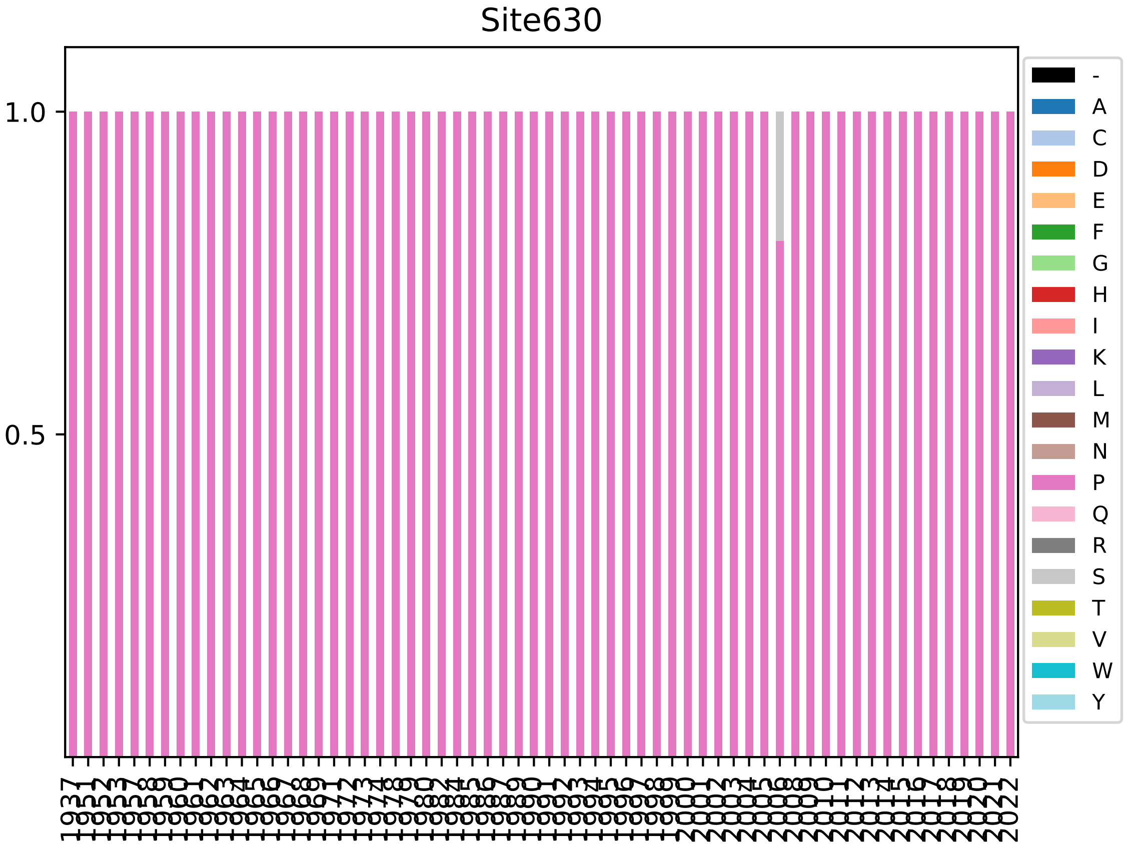

Site630
1.0
0.5
-
A
C
D
E
F
G
H
I
K
L
M
N
P
Q
R
S
T
V
W
Y

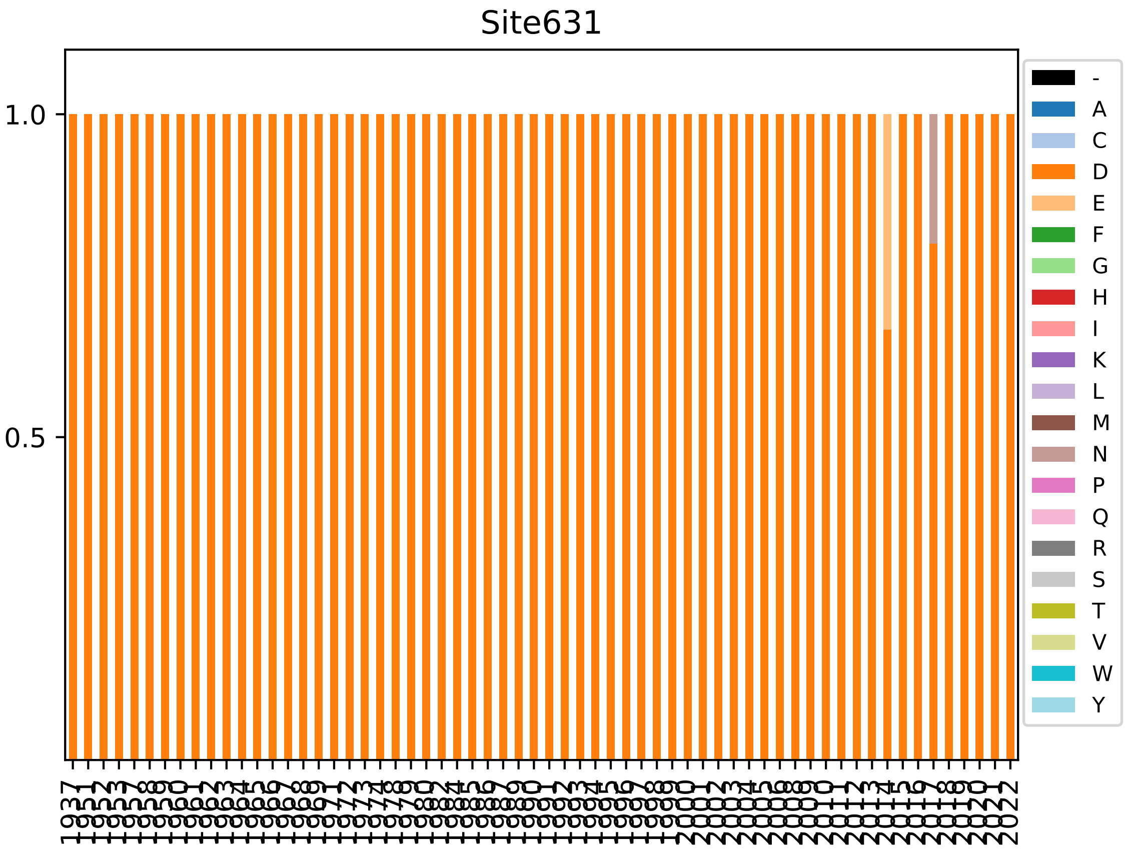
Site631
1.0
0.5
-
A
C
D
E
F
G
H
I
K
L
M
N
P
Q
R
S
T
V
W
Y

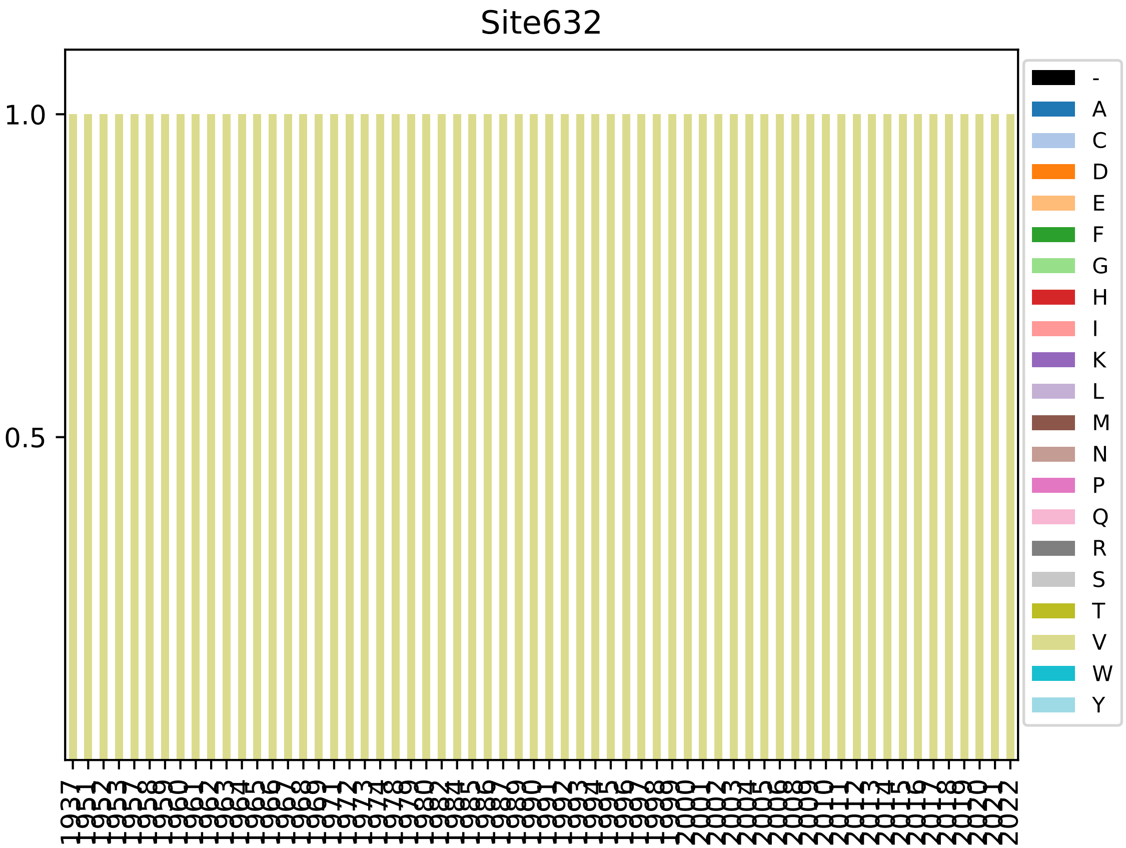

Site632
1.0
0.5
-
A
C
D
E
F
G
H
I
K
L
M
N
P
Q
R
S
T
V
W
Y

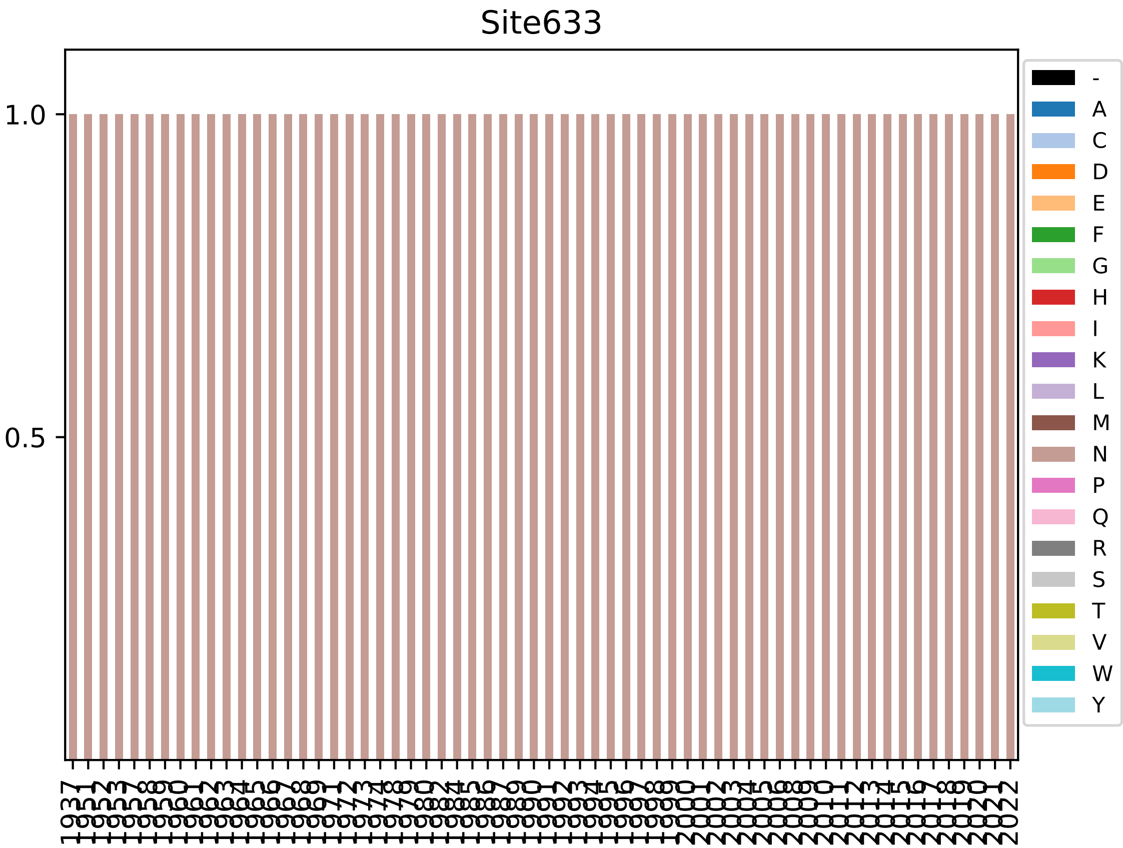
Site633
1.0
0.5
-
A
C
D
E
F
G
H
I
K
L
M
N
P
Q
R
S
T
V
W
Y

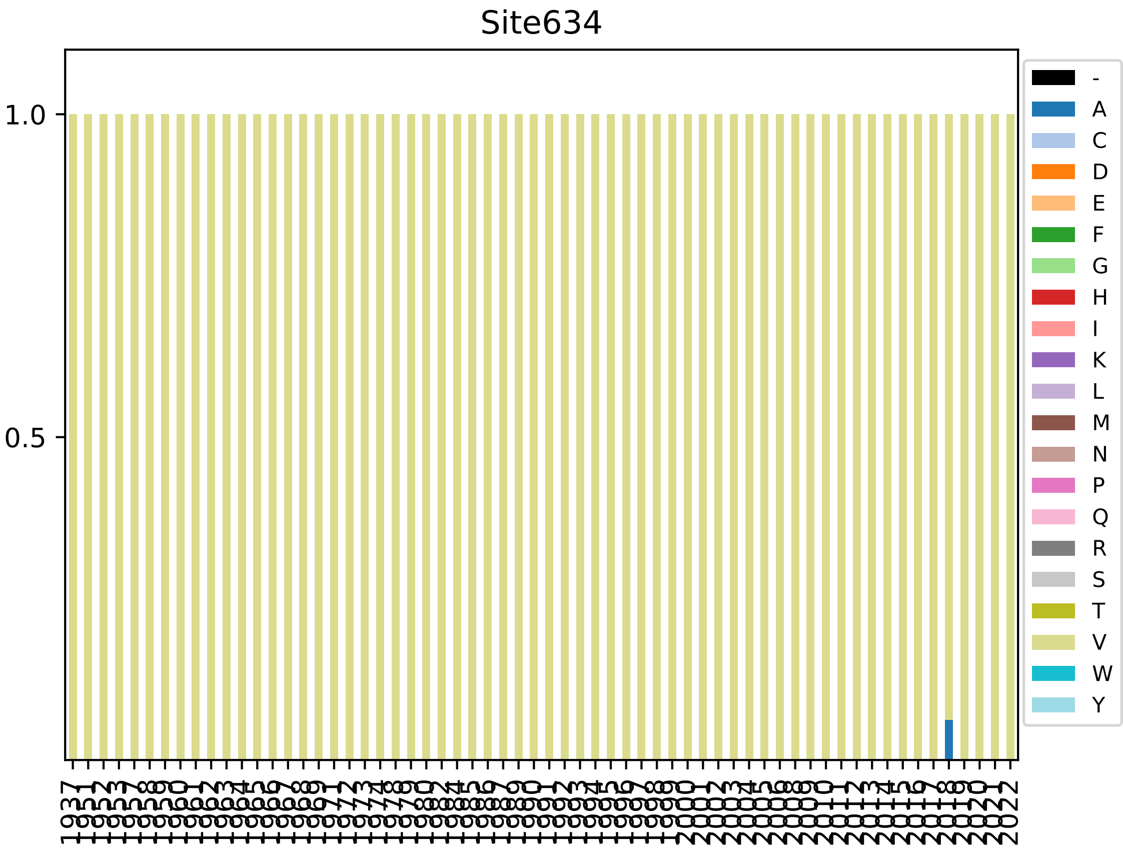
Site634
1.0
0.5
-
A
C
D
E
F
G
H
I
K
L
M
N
P
Q
R
S
T
V
W
Y

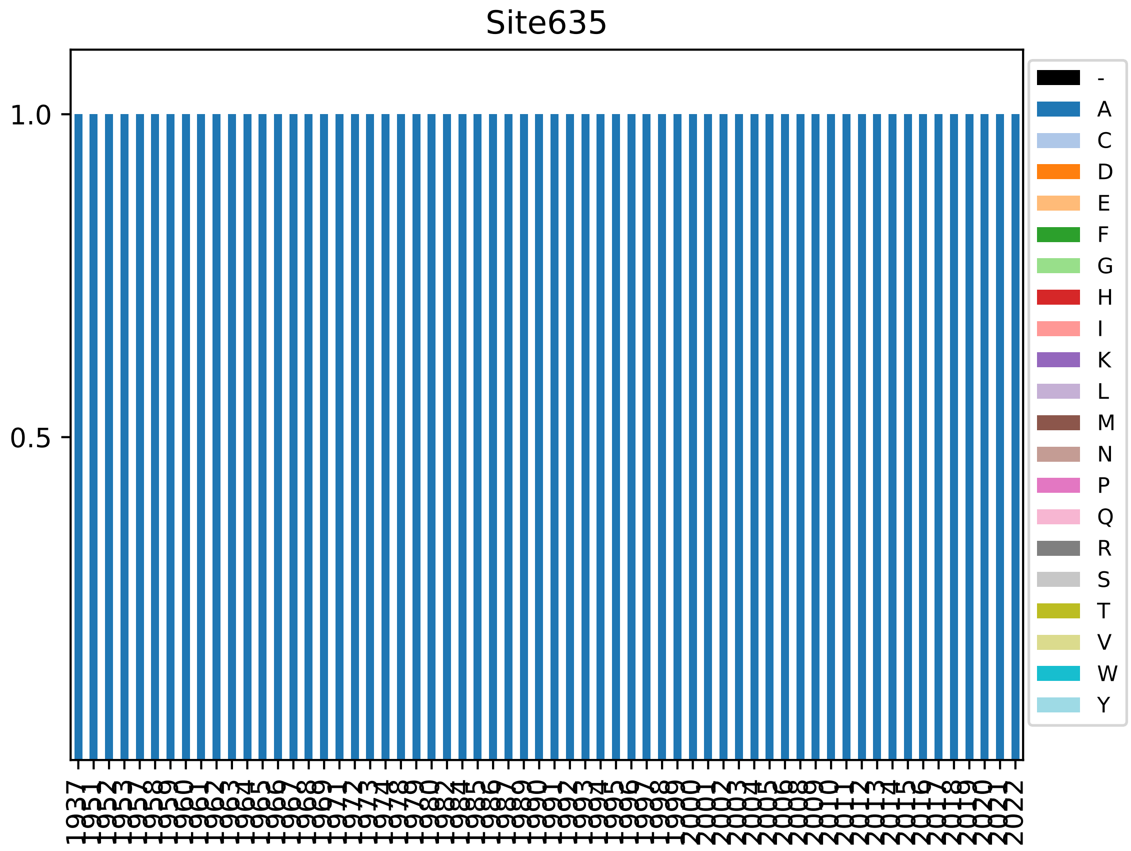
Site635
1.0
0.5
-
A
C
D
E
F
G
H
I
K
L
M
N
P
Q
R
S
T
V
W
Y

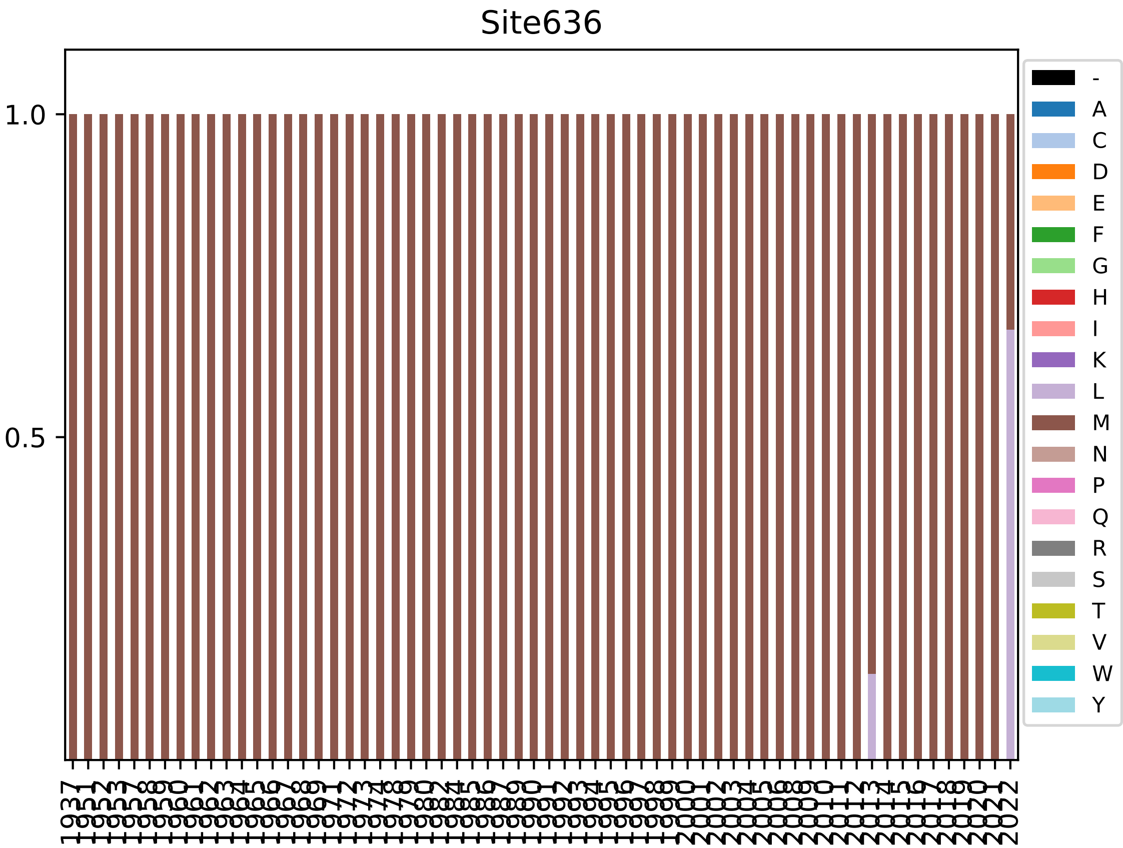
Site636
1.0
0.5
-
A
C
D
E
F
G
H
I
K
L
M
N
P
Q
R
S
T
V
W
Y

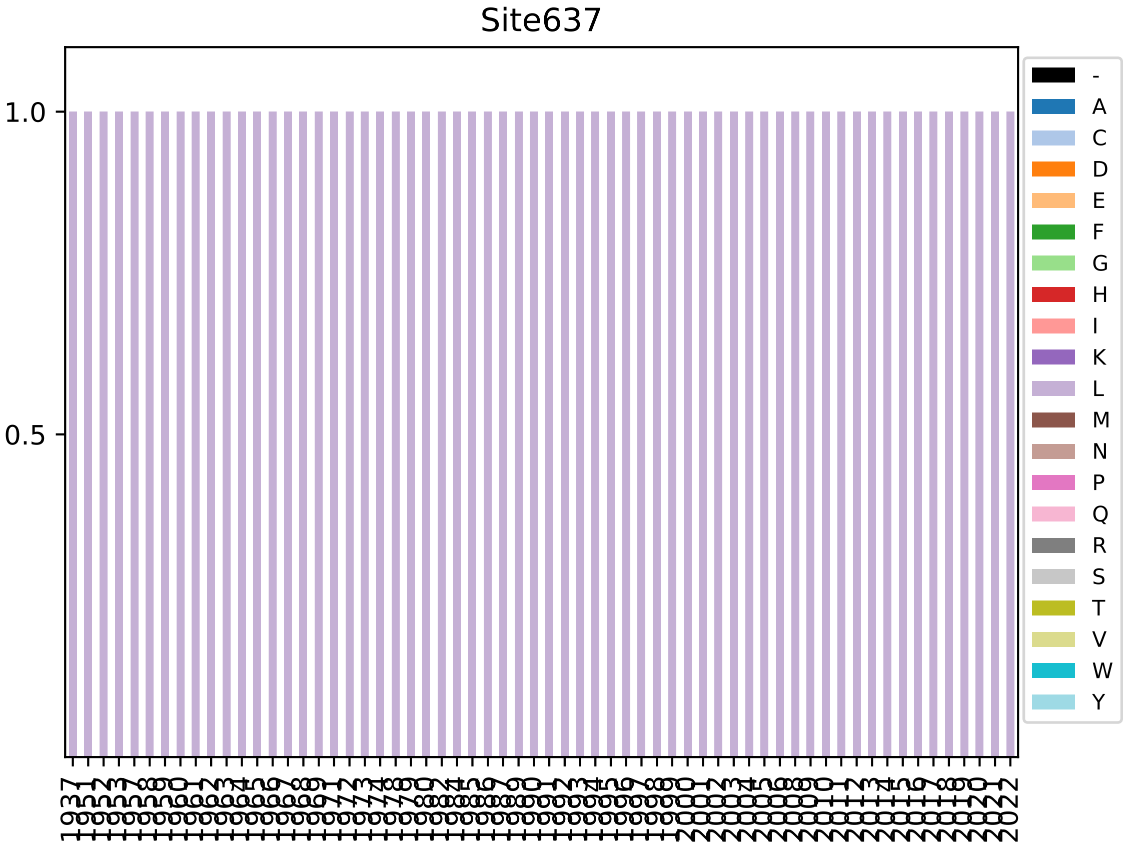
Site637
1.0
0.5
-
A
C
D
E
F
G
H
I
K
L
M
N
P
Q
R
S
T
V
W
Y

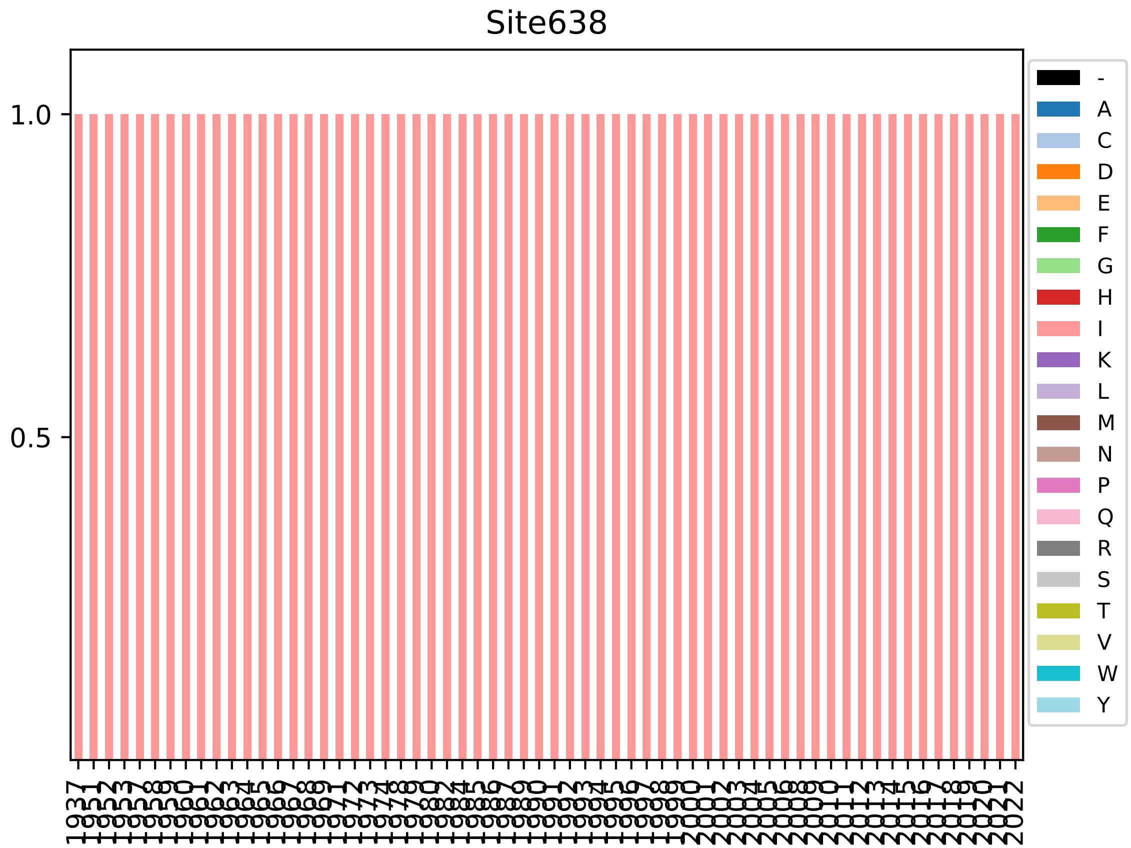
Site638
1.0
0.5
-
A
C
D
E
F
G
H
I
K
L
M
N
P
Q
R
S
T
V
W
Y

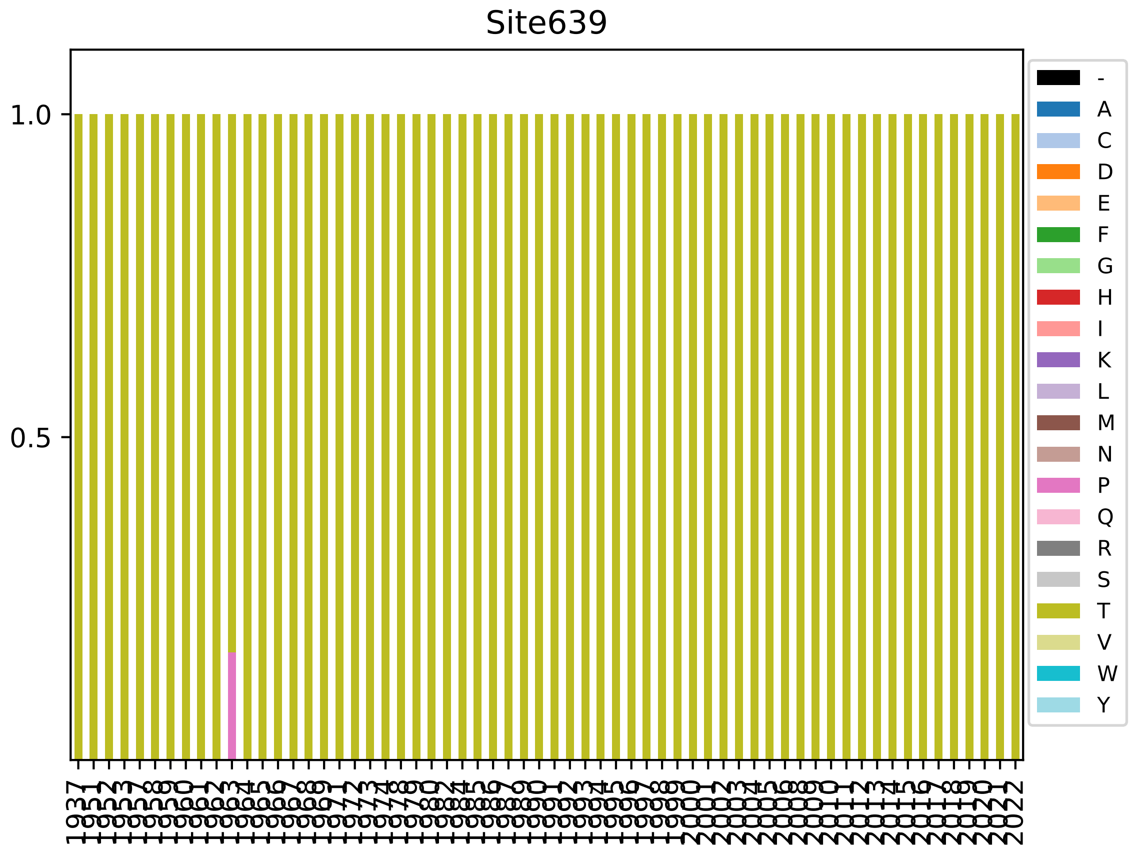
Site639
1.0
0.5
-
A
C
D
E
F
G
H
I
K
L
M
N
P
Q
R
S
T
V
W
Y

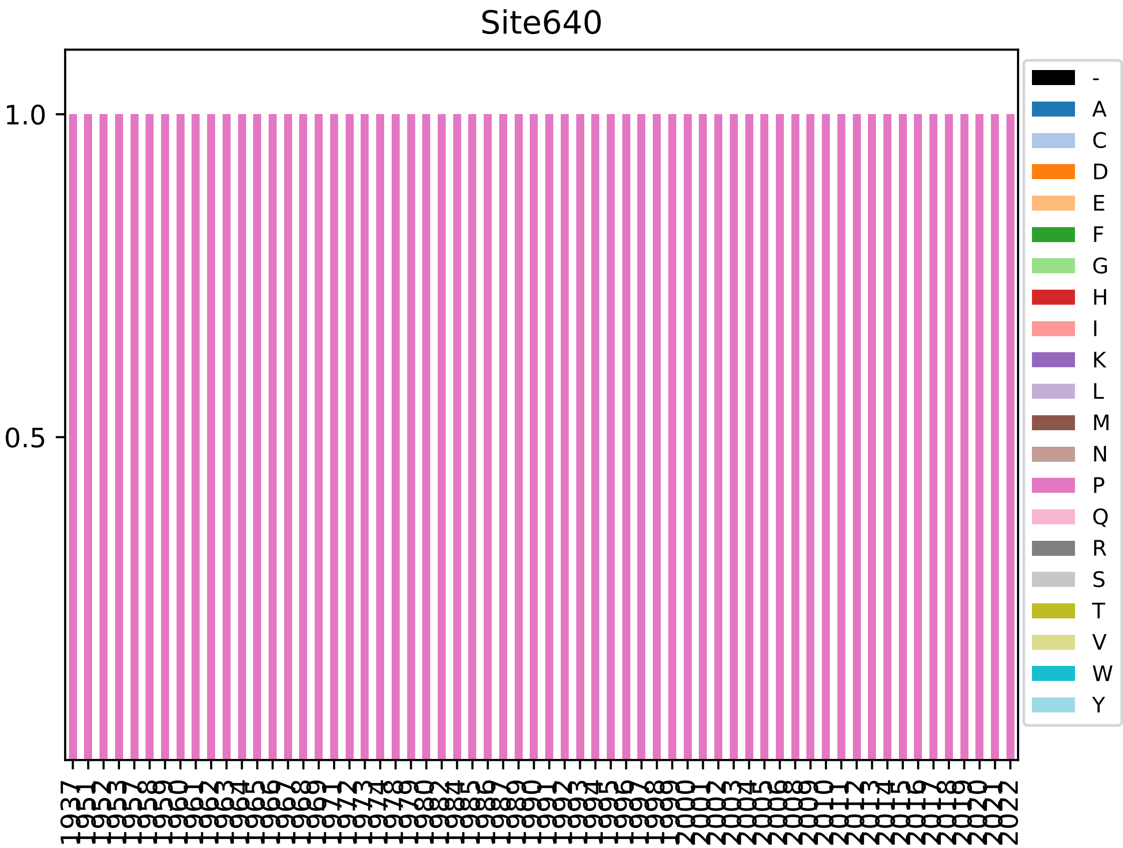
Site640
1.0
0.5
-
A
C
D
E
F
G
H
I
K
L
M
N
P
Q
R
S
T
V
W
Y

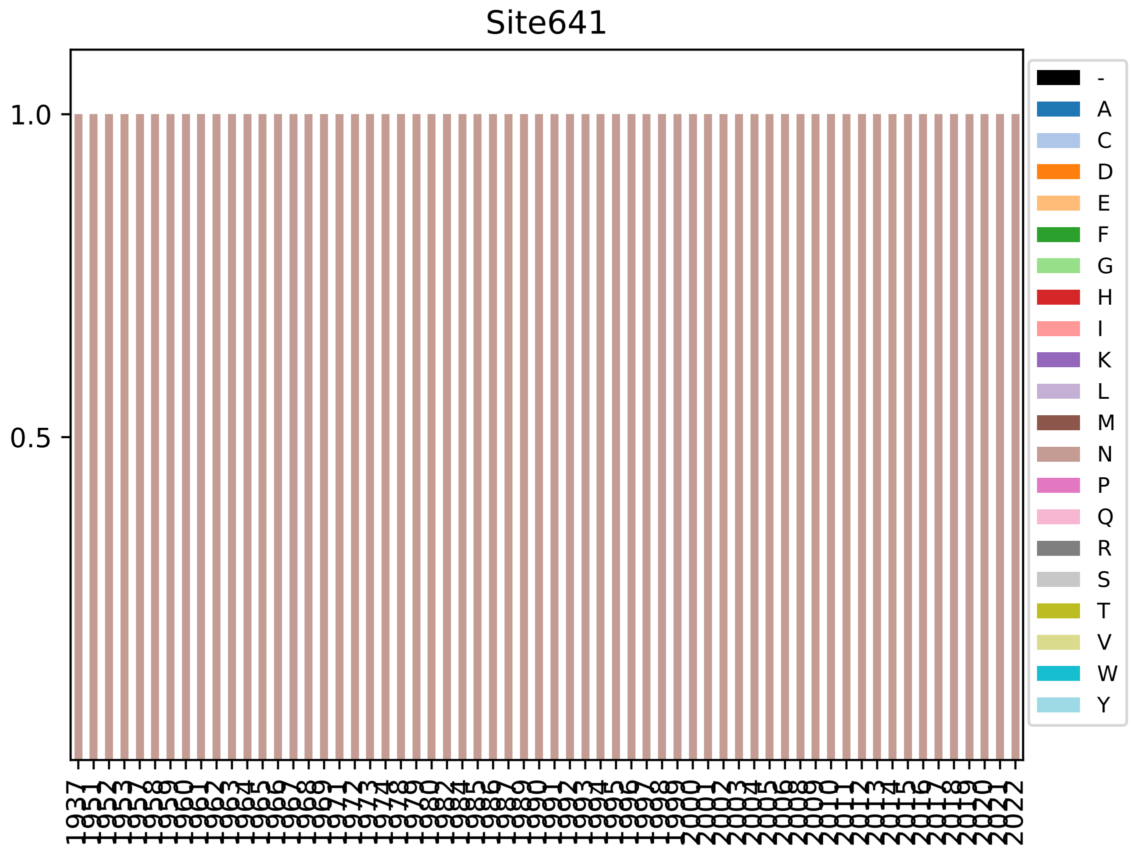
Site641
1.0
0.5
-
A
C
D
E
F
G
H
I
K
L
M
N
P
Q
R
S
T
V
W
Y

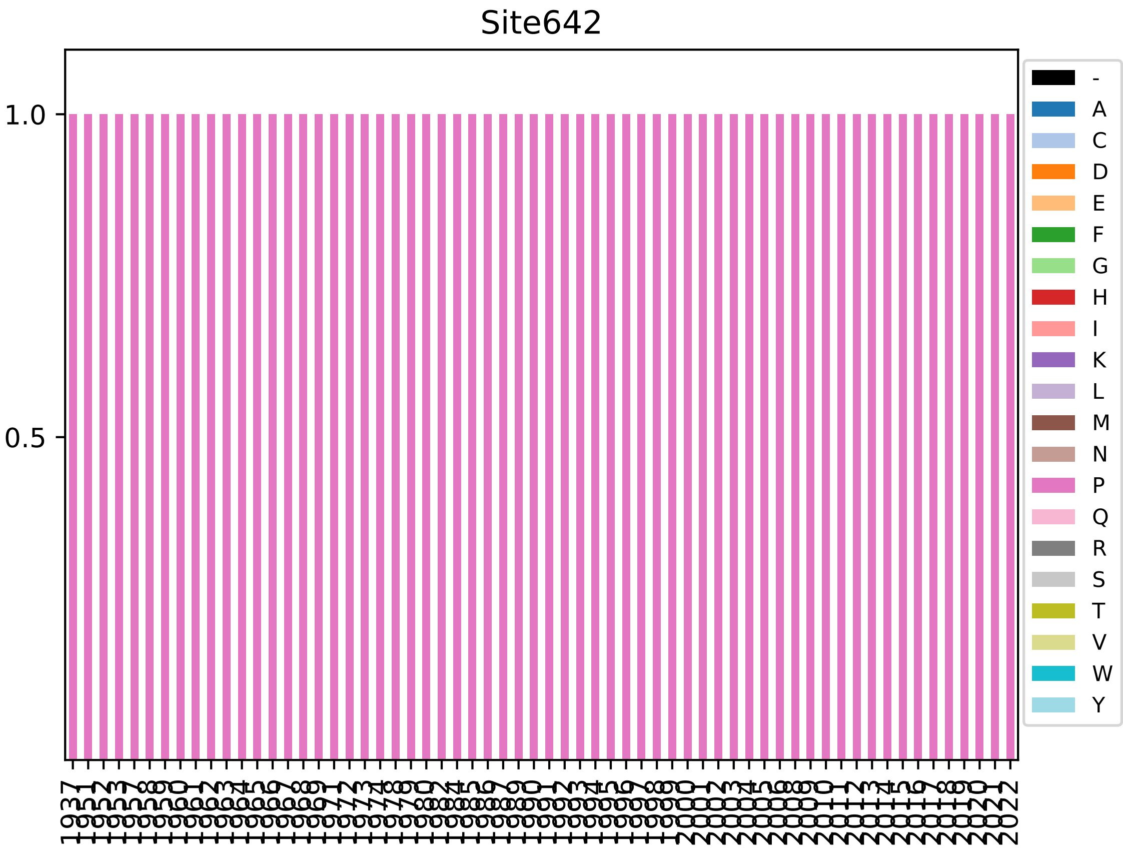
Site642
1.0
0.5
-
A
C
D
E
F
G
H
I
K
L
M
N
P
Q
R
S
T
V
W
Y

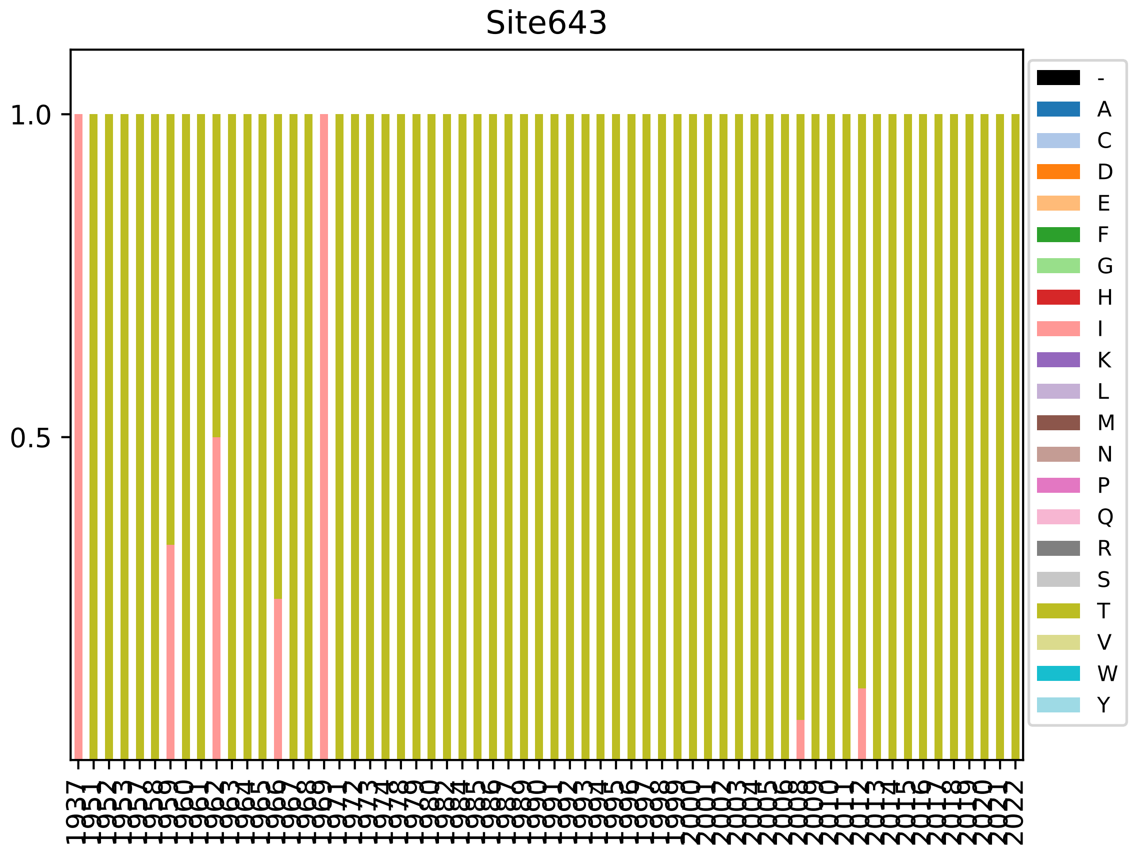
Site643
1.0
0.5
-
A
C
D
E
F
G
H
I
K
L
M
N
P
Q
R
S
T
V
W
Y

Site644

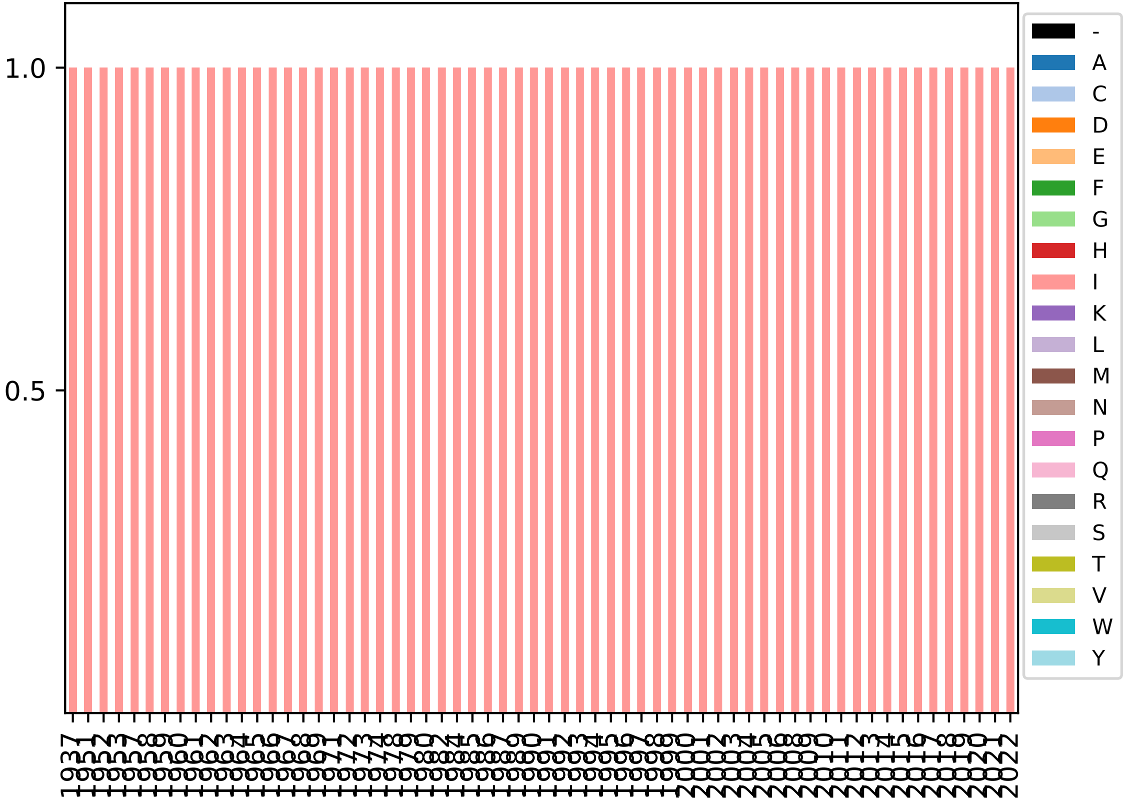

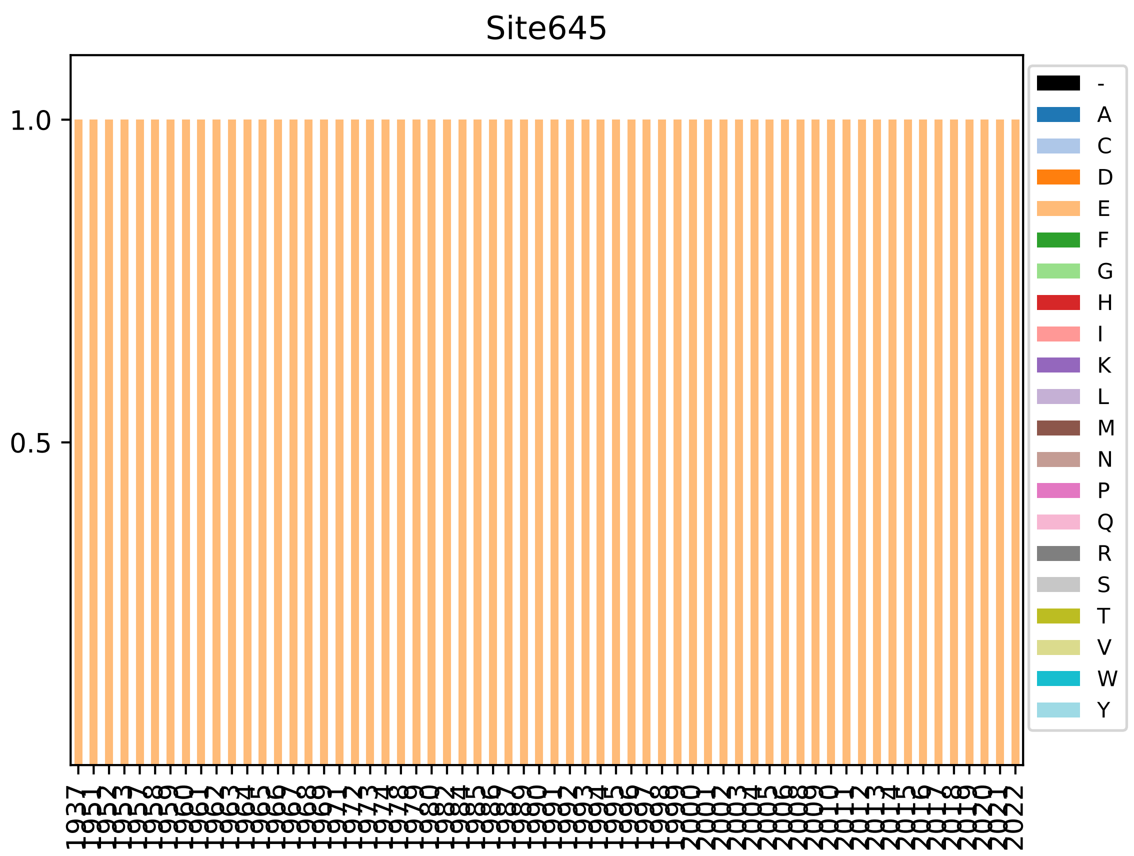

Site645
1.0
0.5
-
A
C
D
E
F
G
H
I
K
L
M
N
P
Q
R
S
T
V
W
Y

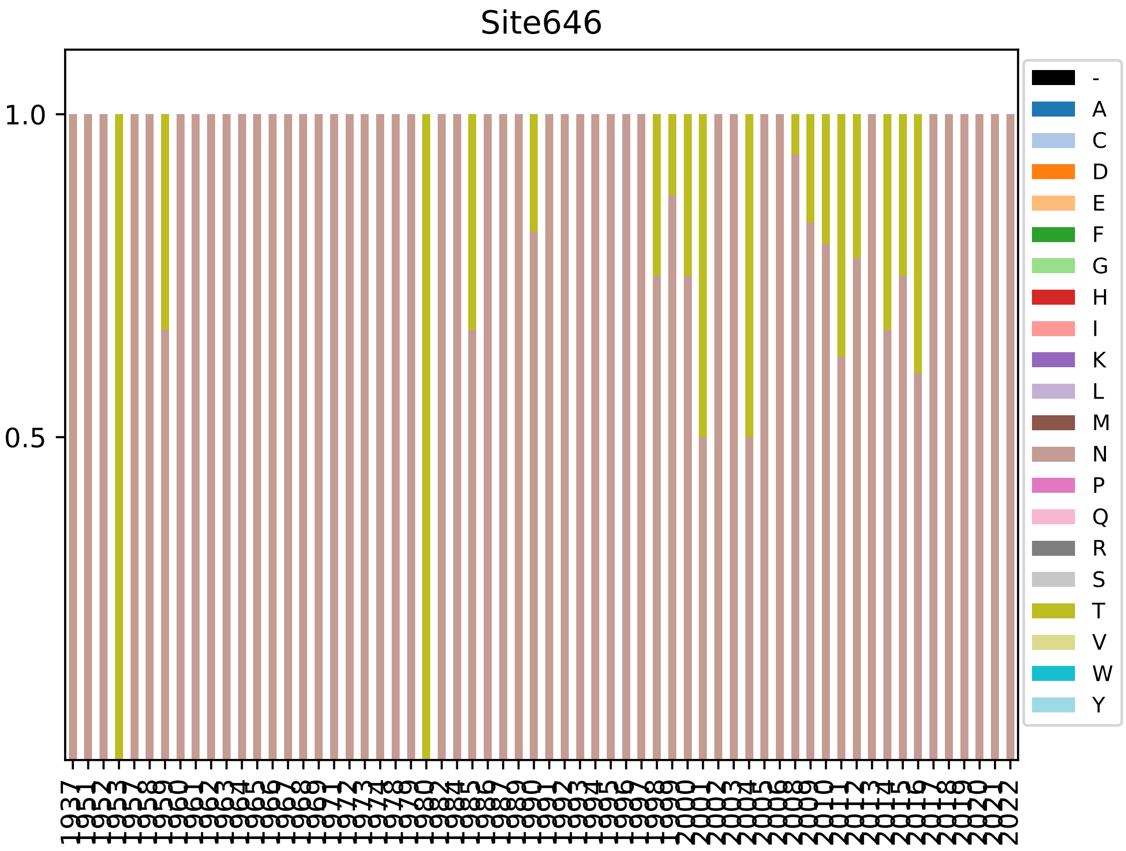
Site646
1.0
0.5
-
A
C
D
E
F
G
H
I
K
L
M
N
P
Q
R
S
T
V
W
Y

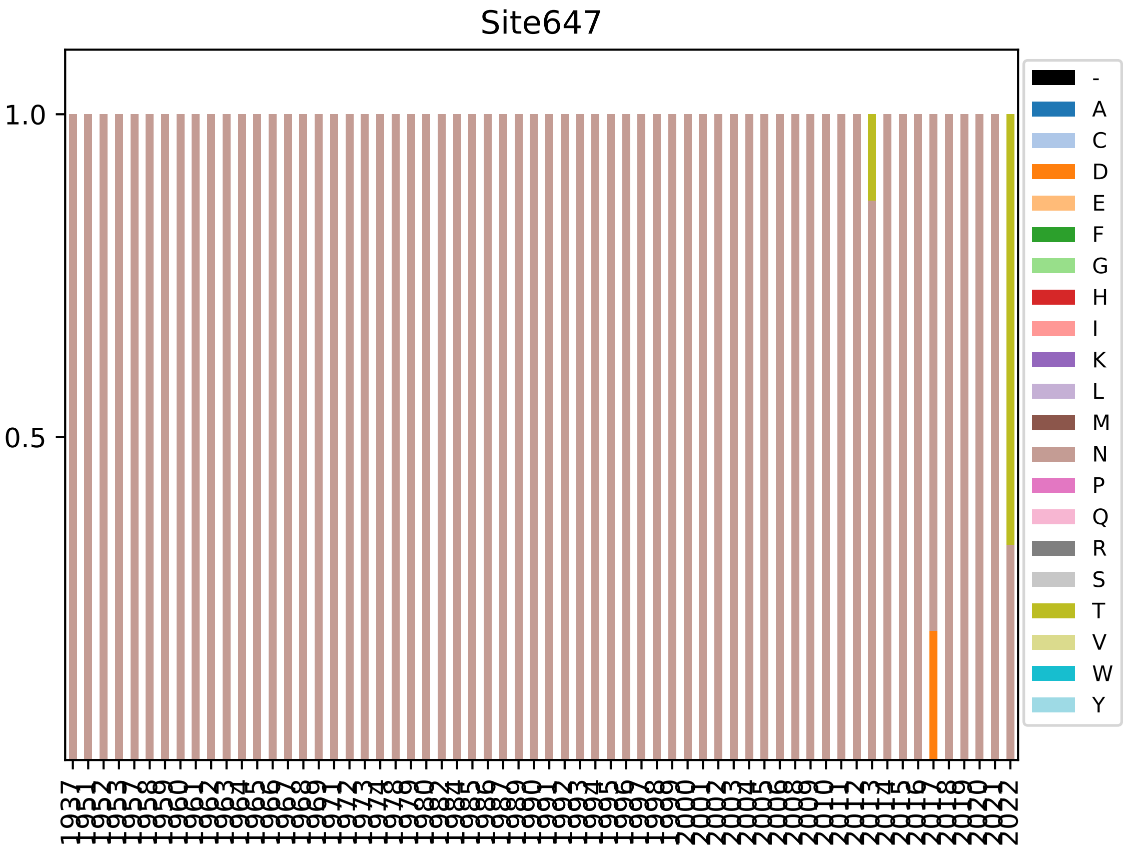
Site647
1.0
0.5
-
A
C
D
E
F
G
H
I
K
L
M
N
P
Q
R
S
T
V
W
Y

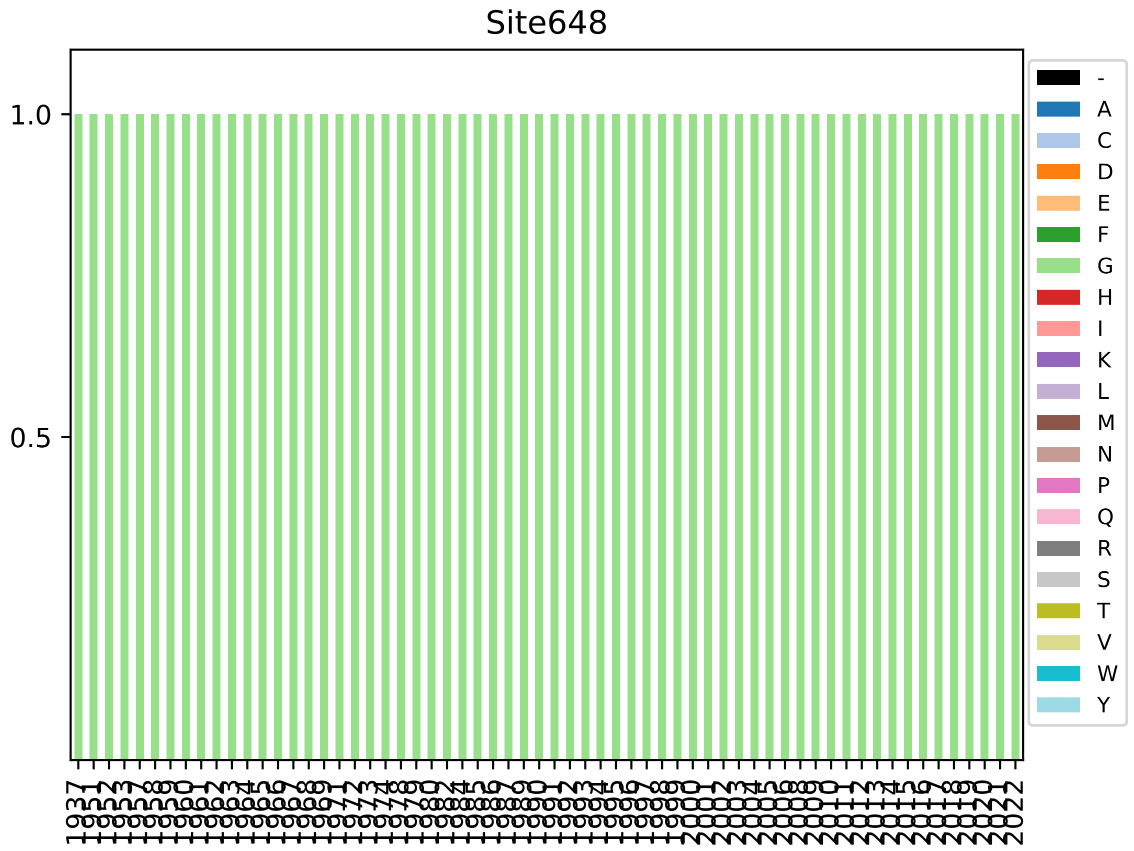
Site648
1.0
0.5
-
A
C
D
E
F
G
H
I
K
L
M
N
P
Q
R
S
T
V
W
Y

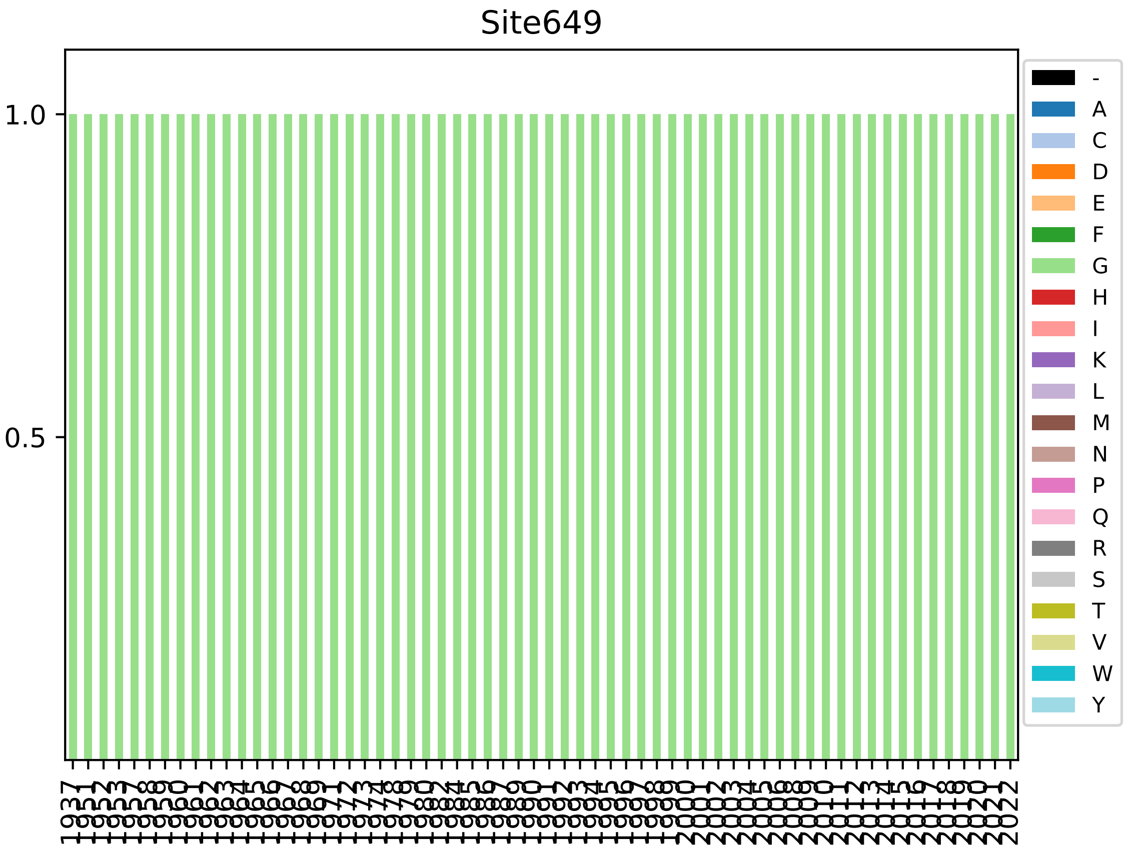
Site649
1.0
0.5
-
A
C
D
E
F
G
H
I
K
L
M
N
P
Q
R
S
T
V
W
Y

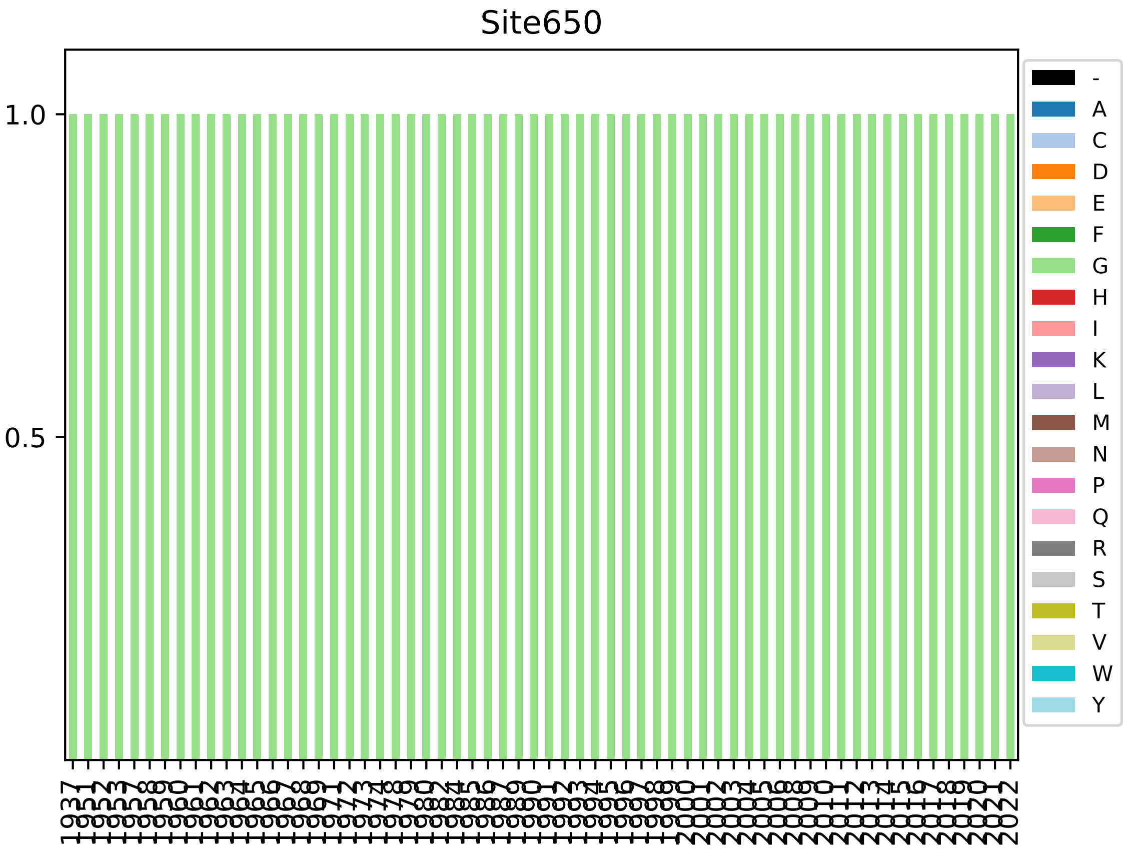
Site650
1.0
0.5
-
A
C
D
E
F
G
H
I
K
L
M
N
P
Q
R
S
T
V
W
Y

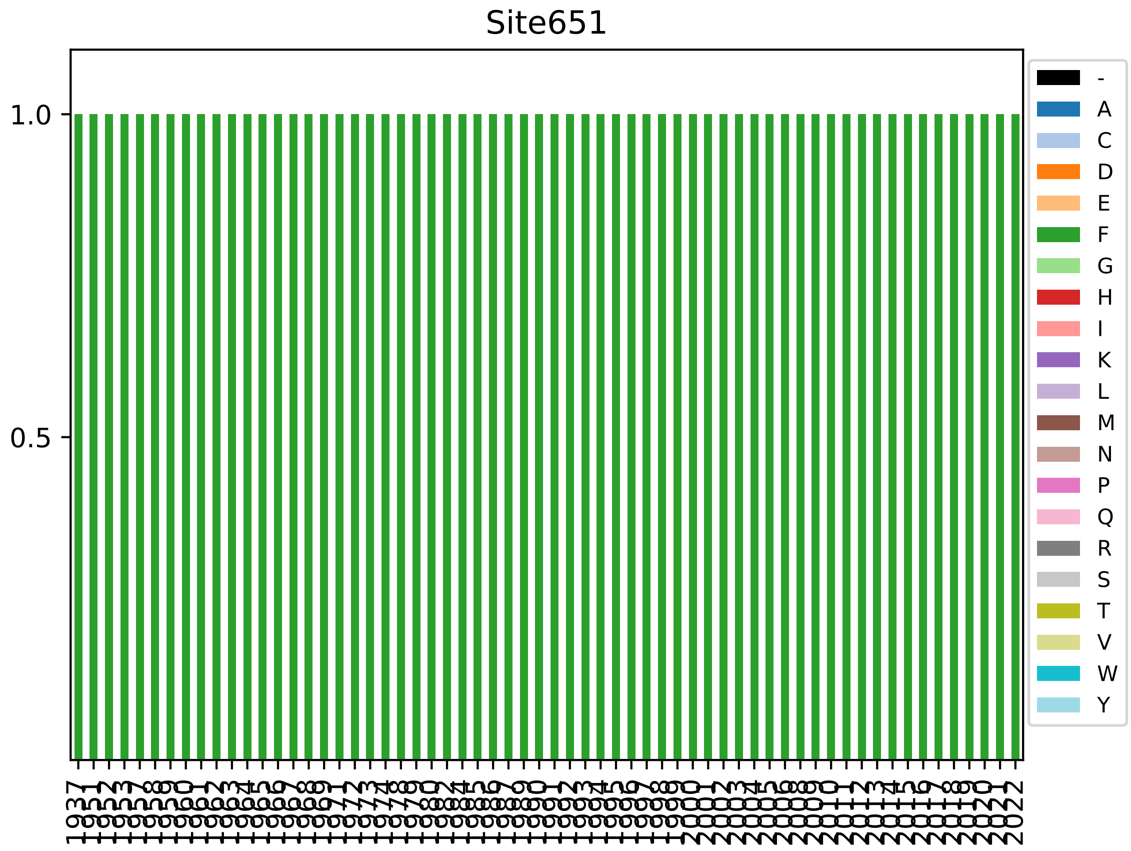
Site651
1.0
0.5
-
A
C
D
E
F
G
H
I
K
L
M
N
P
Q
R
S
T
V
W
Y

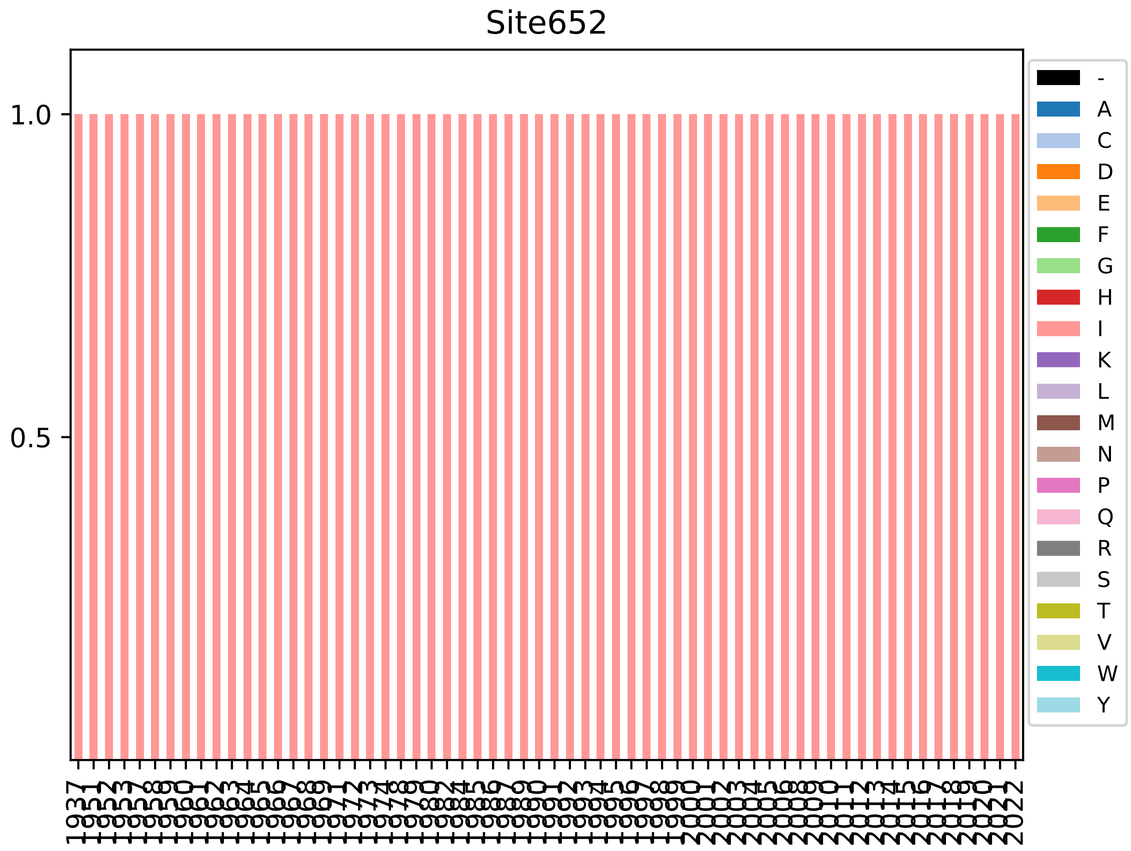
Site652
1.0
0.5
-
A
C
D
E
F
G
H
I
K
L
M
N
P
Q
R
S
T
V
W
Y

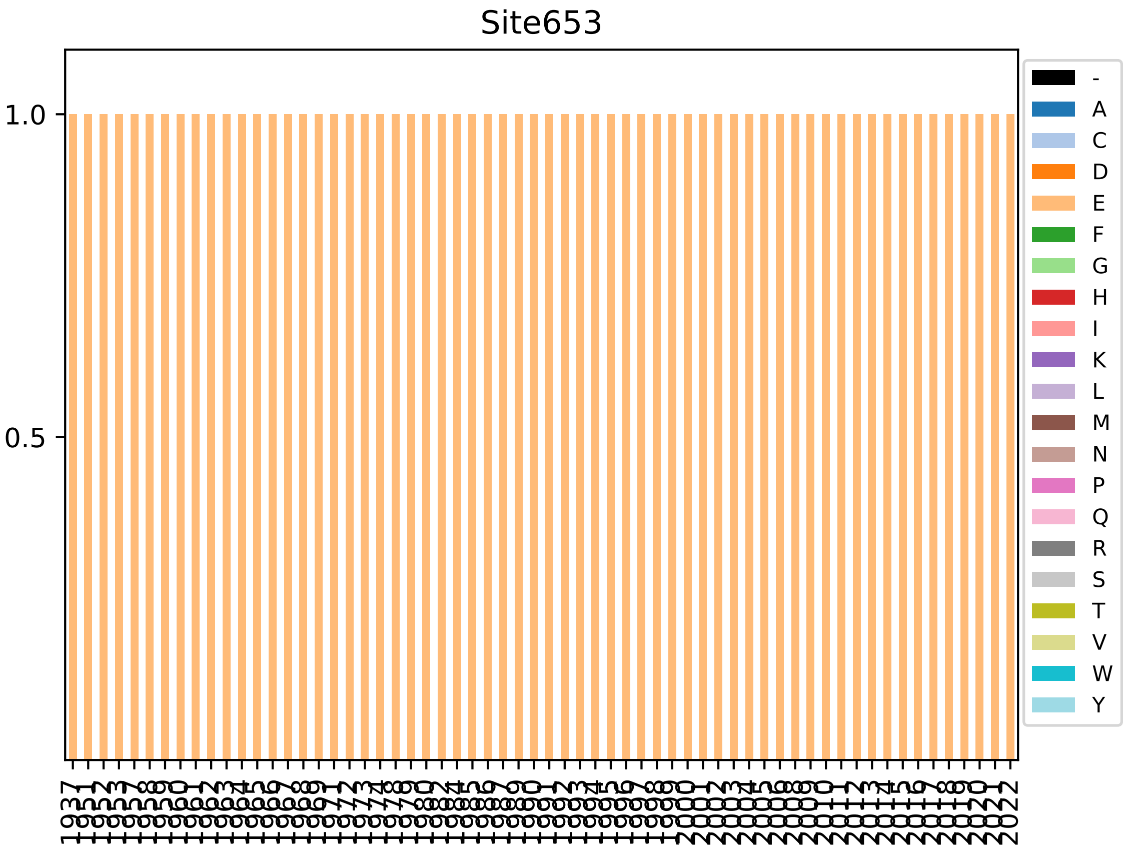

Site653
1.0
0.5
-
A
C
D
E
F
G
H
I
K
L
M
N
P
Q
R
S
T
V
W
Y

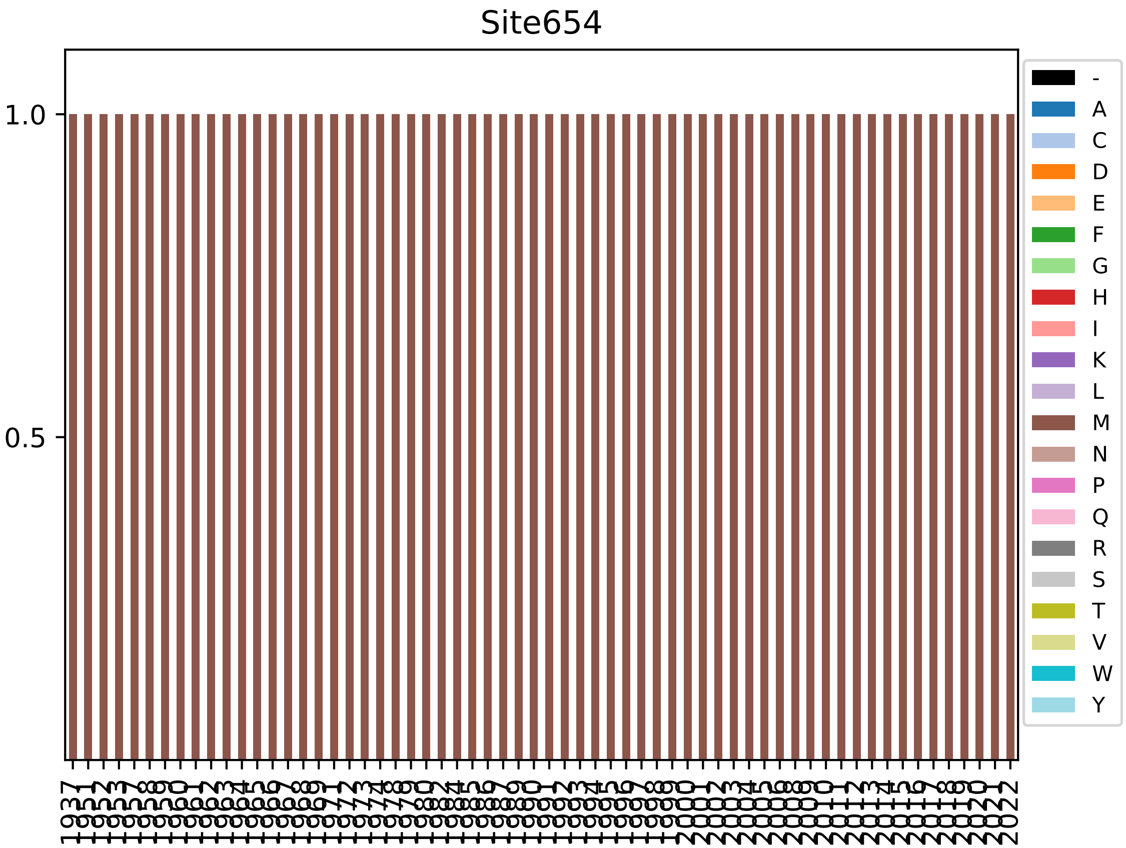
Site654
1.0
0.5
-
A
C
D
E
F
G
H
I
K
L
M
N
P
Q
R
S
T
V
W
Y

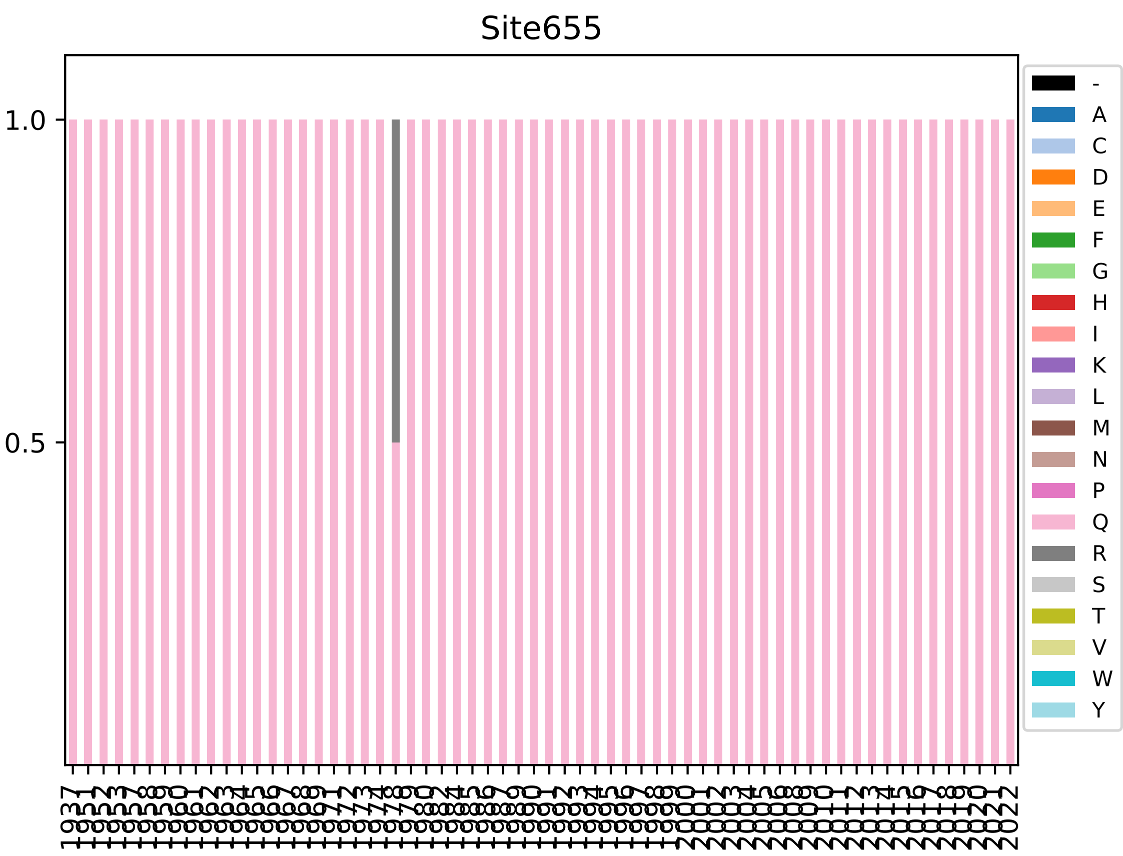
Site655
1.0
0.5
-
A
C
D
E
F
G
H
I
K
L
M
N
P
Q
R
S
T
V
W
Y

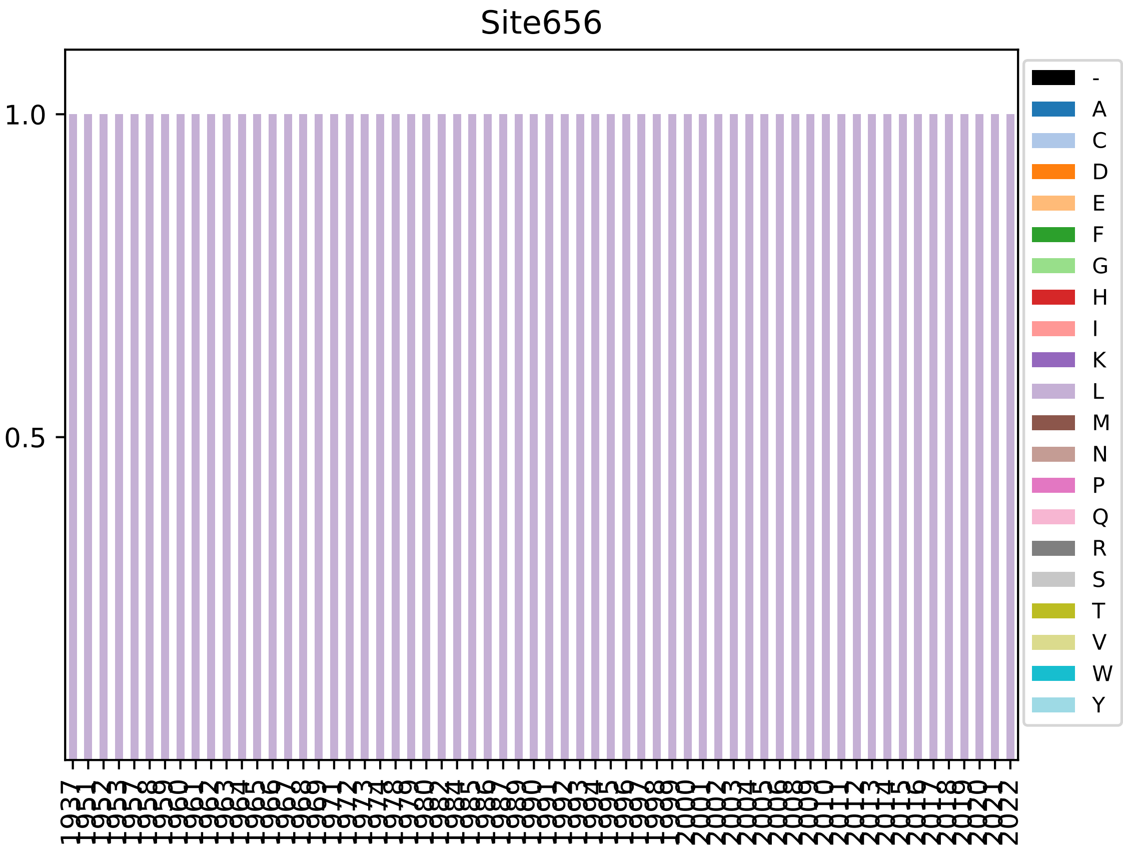
Site656
1.0
0.5
-
A
C
D
E
F
G
H
I
K
L
M
N
P
Q
R
S
T
V
W
Y

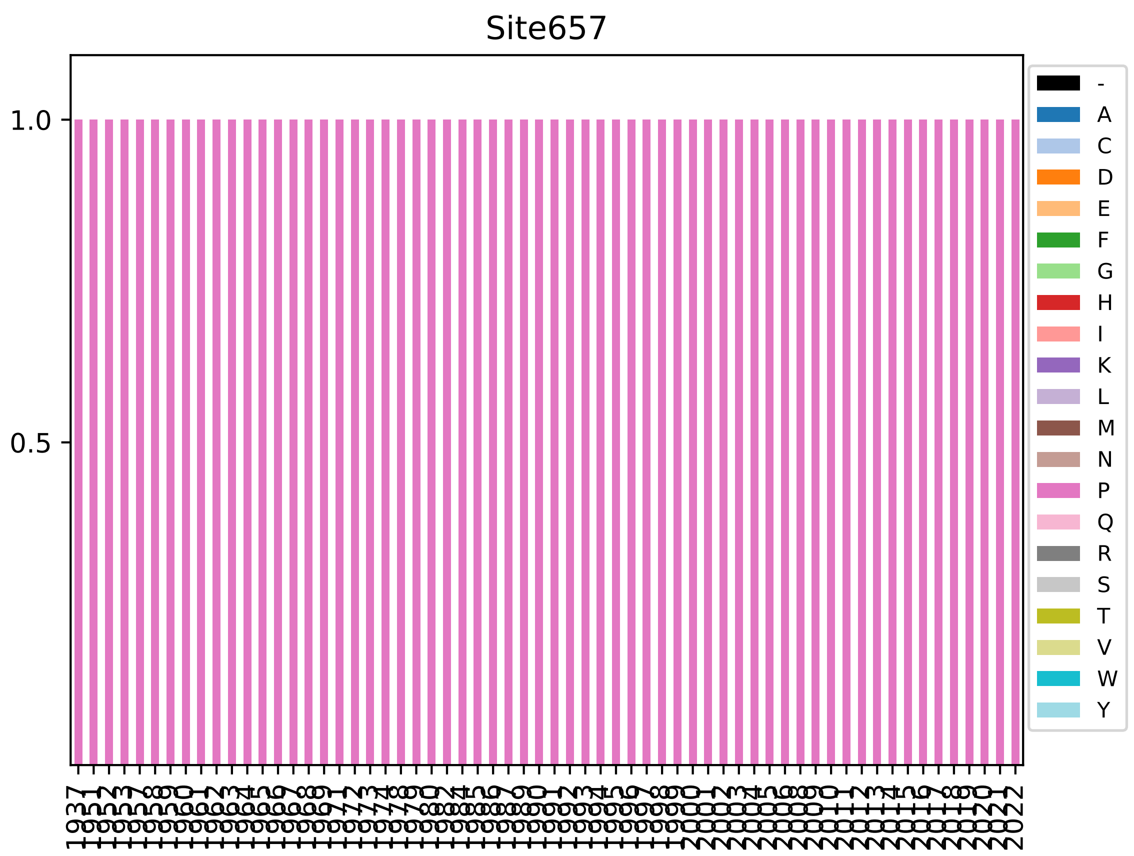

Site657
1.0
0.5
-
A
C
D
E
F
G
H
I
K
L
M
N
P
Q
R
S
T
V
W
Y

Site658

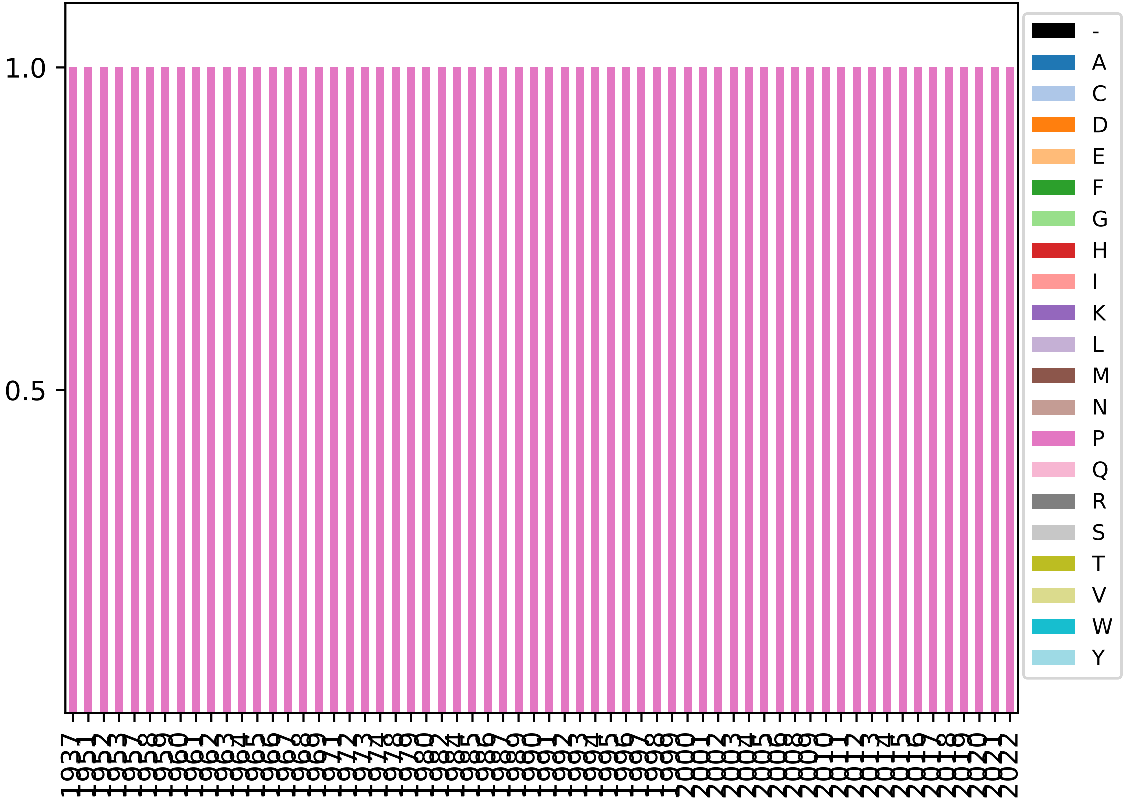

Site659

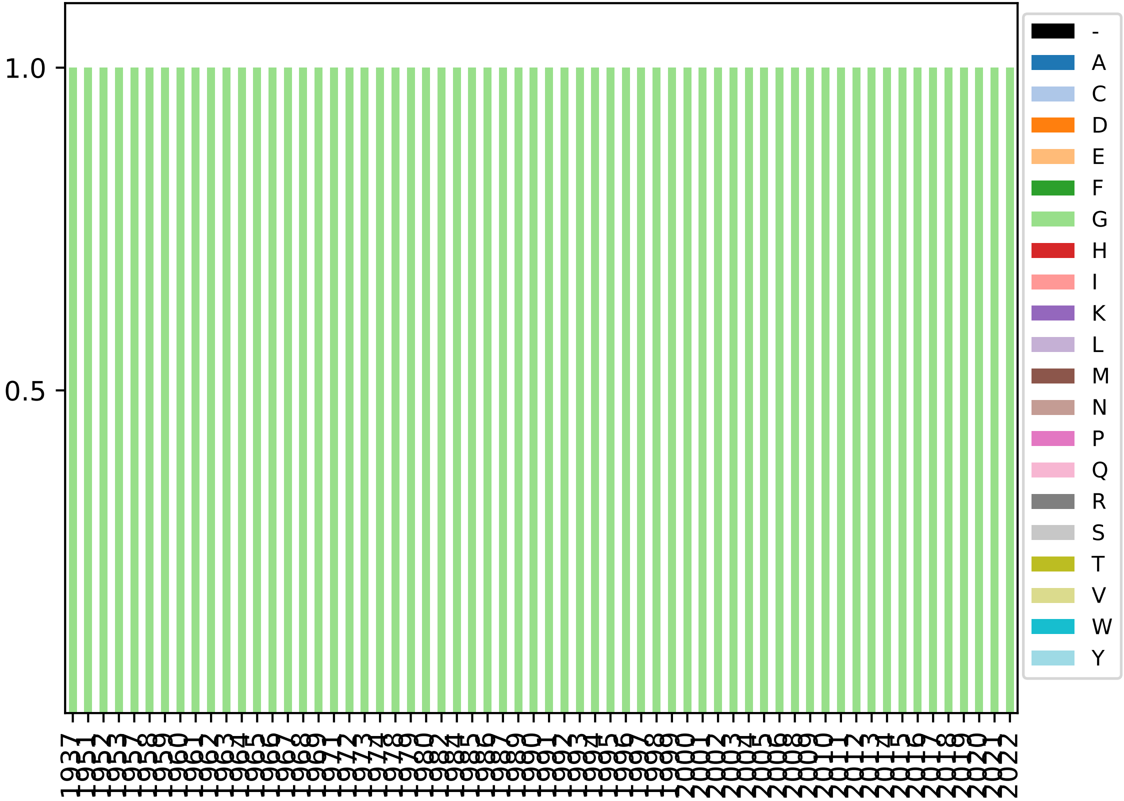

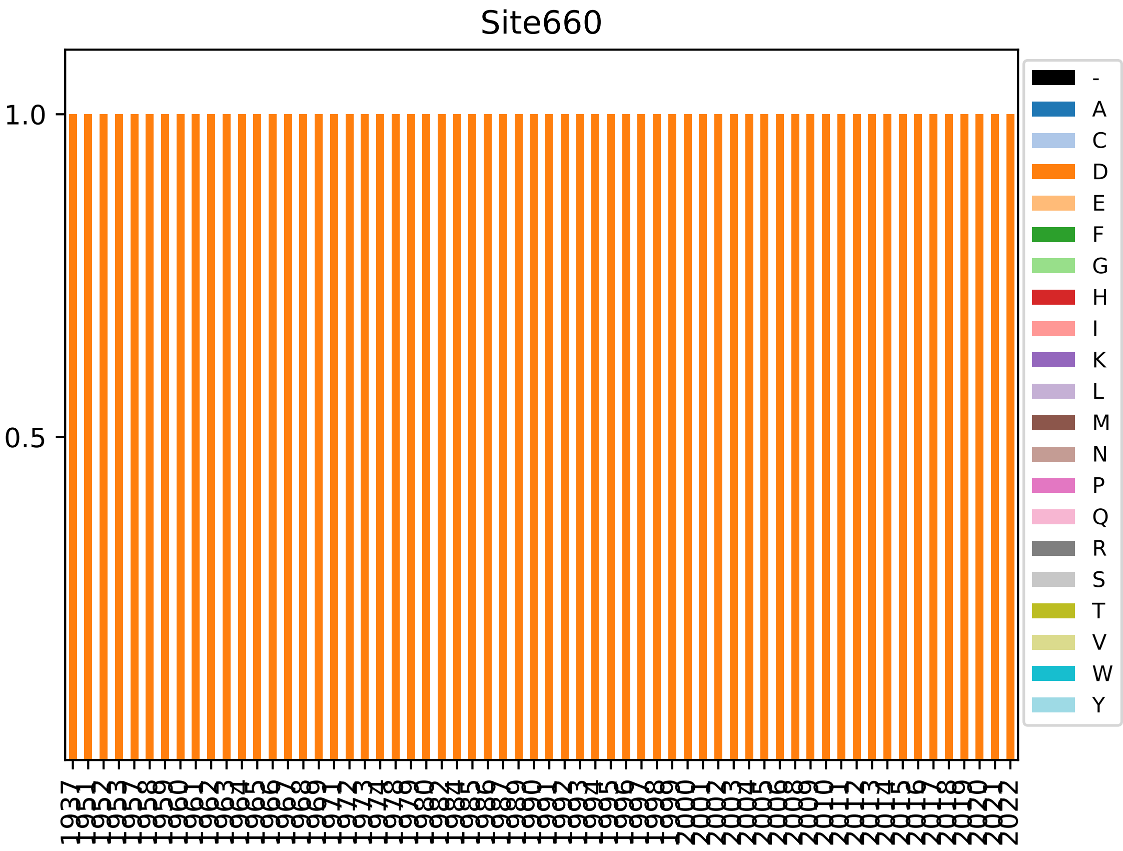
Site660
1.0
0.5
-
A
C
D
E
F
G
H
I
K
L
M
N
P
Q
R
S
T
V
W
Y

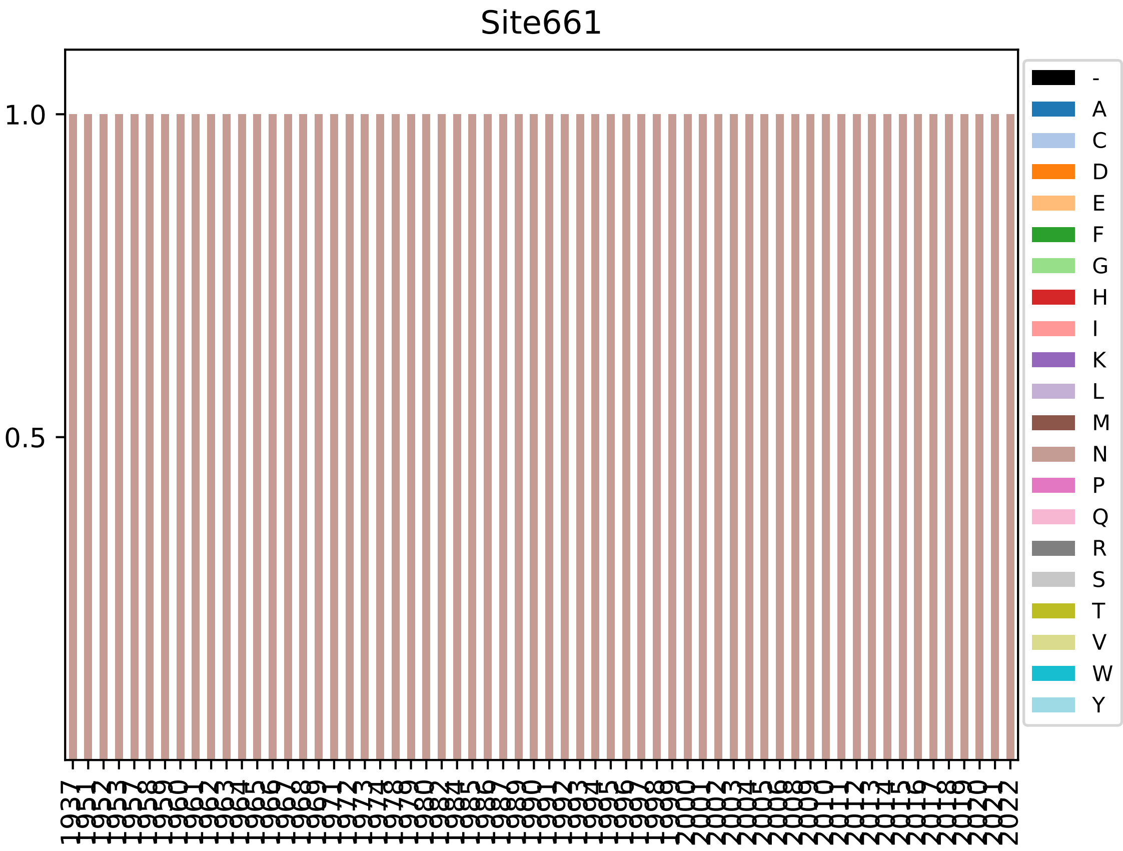
Site661
1.0
0.5
-
A
C
D
E
F
G
H
I
K
L
M
N
P
Q
R
S
T
V
W
Y

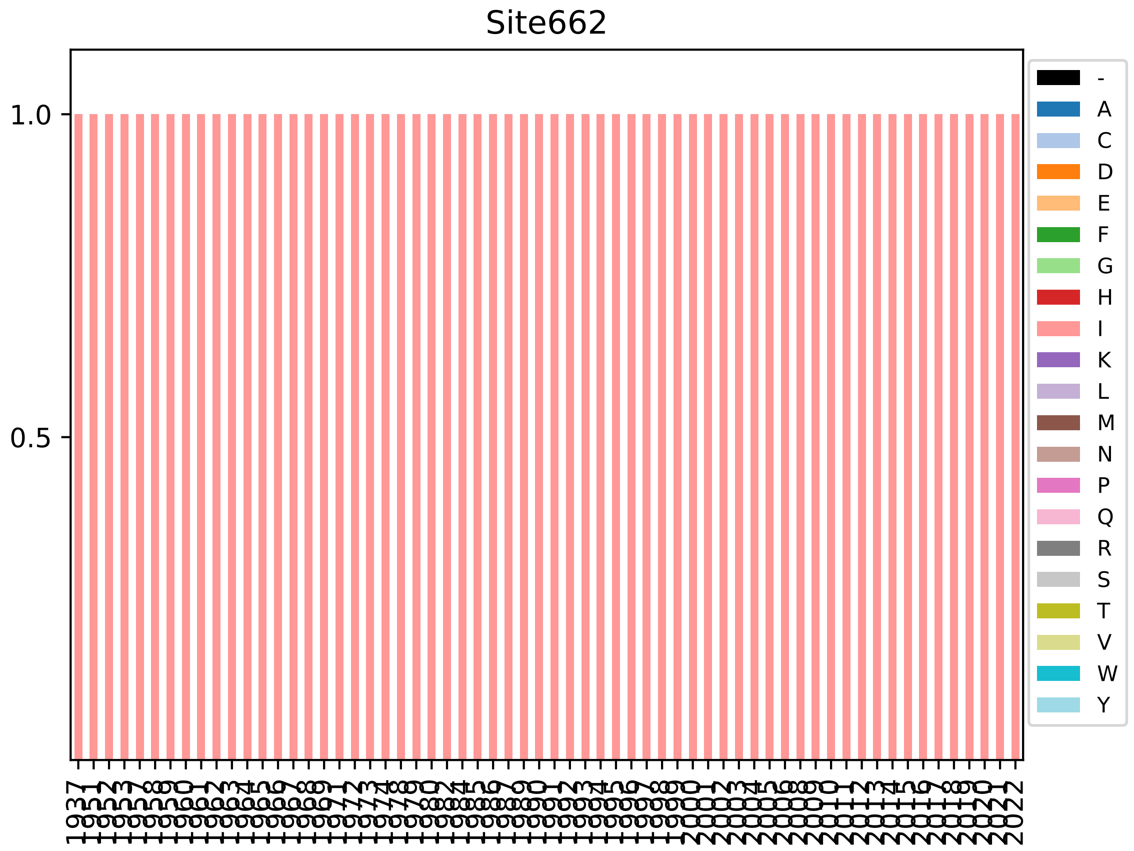
Site662
1.0
0.5
-
A
C
D
E
F
G
H
I
K
L
M
N
P
Q
R
S
T
V
W
Y

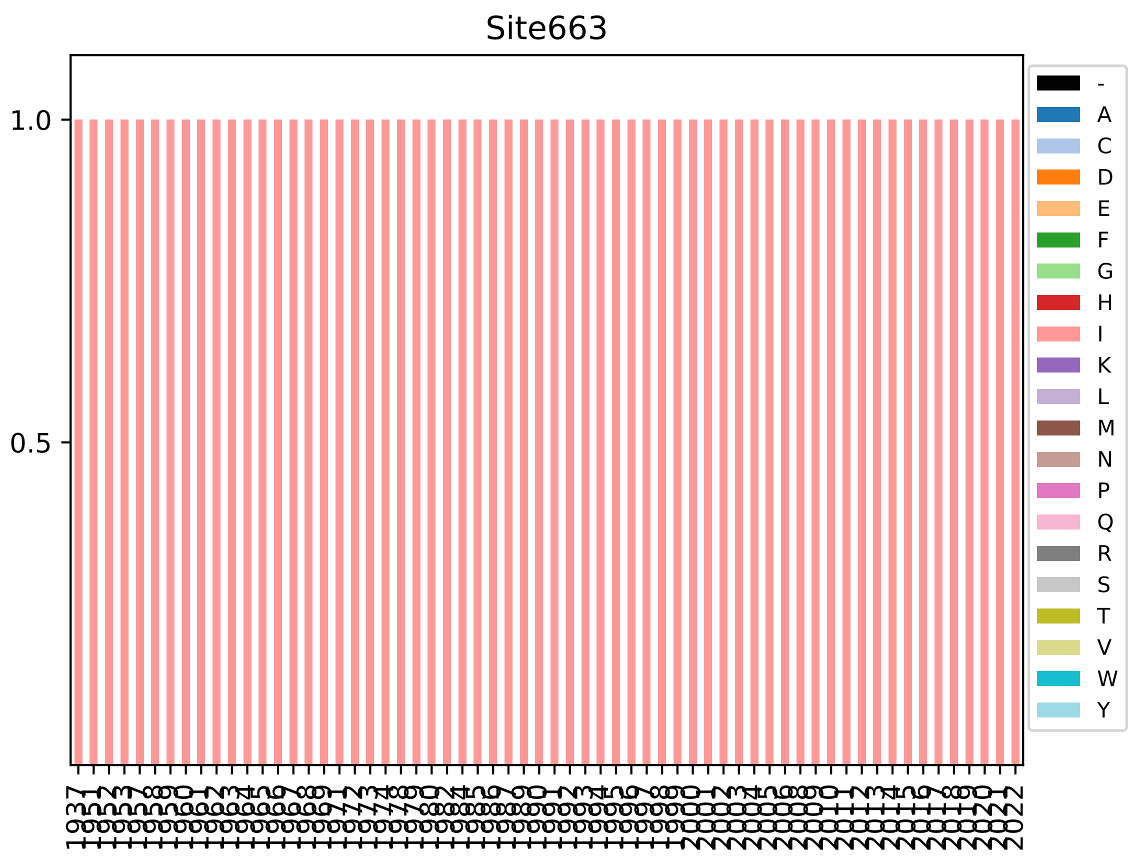

Site663
1.0
0.5
-
A
C
D
E
F
G
H
I
K
L
M
N
P
Q
R
S
T
V
W
Y

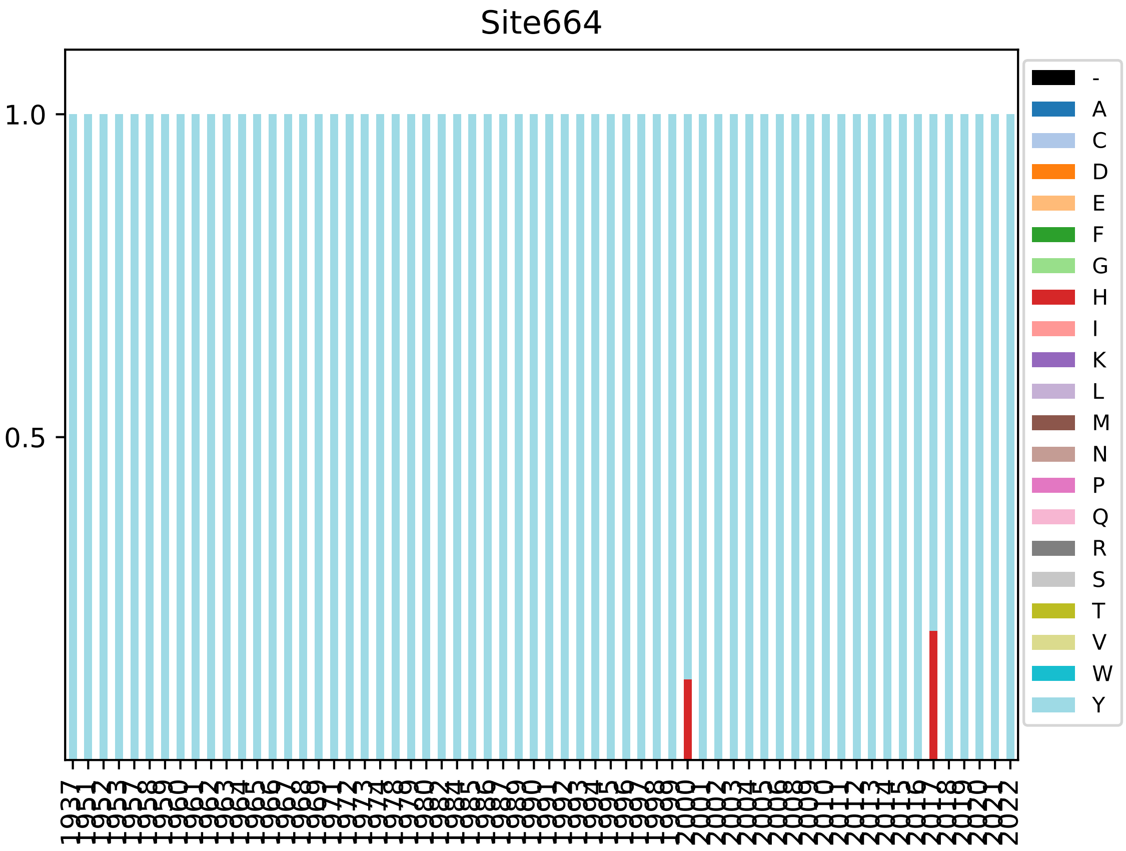

Site664
1.0
0.5
-
A
C
D
E
F
G
H
I
K
L
M
N
P
Q
R
S
T
V
W
Y

Site665

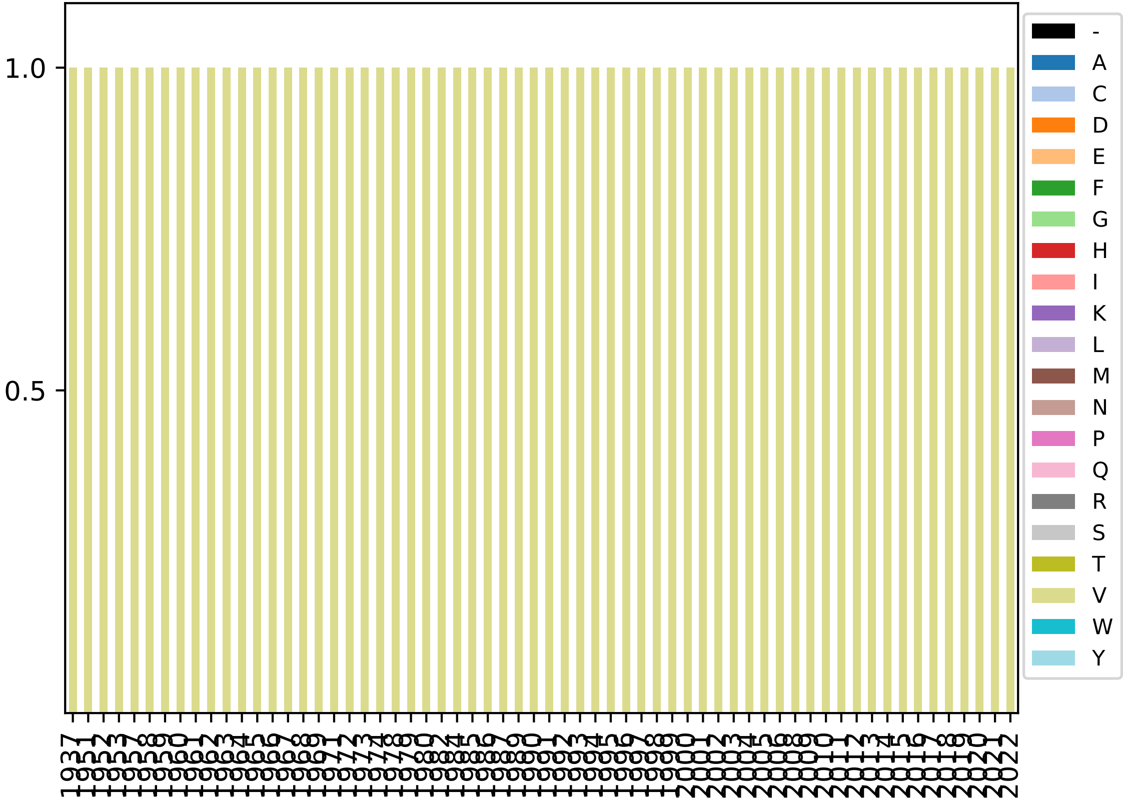

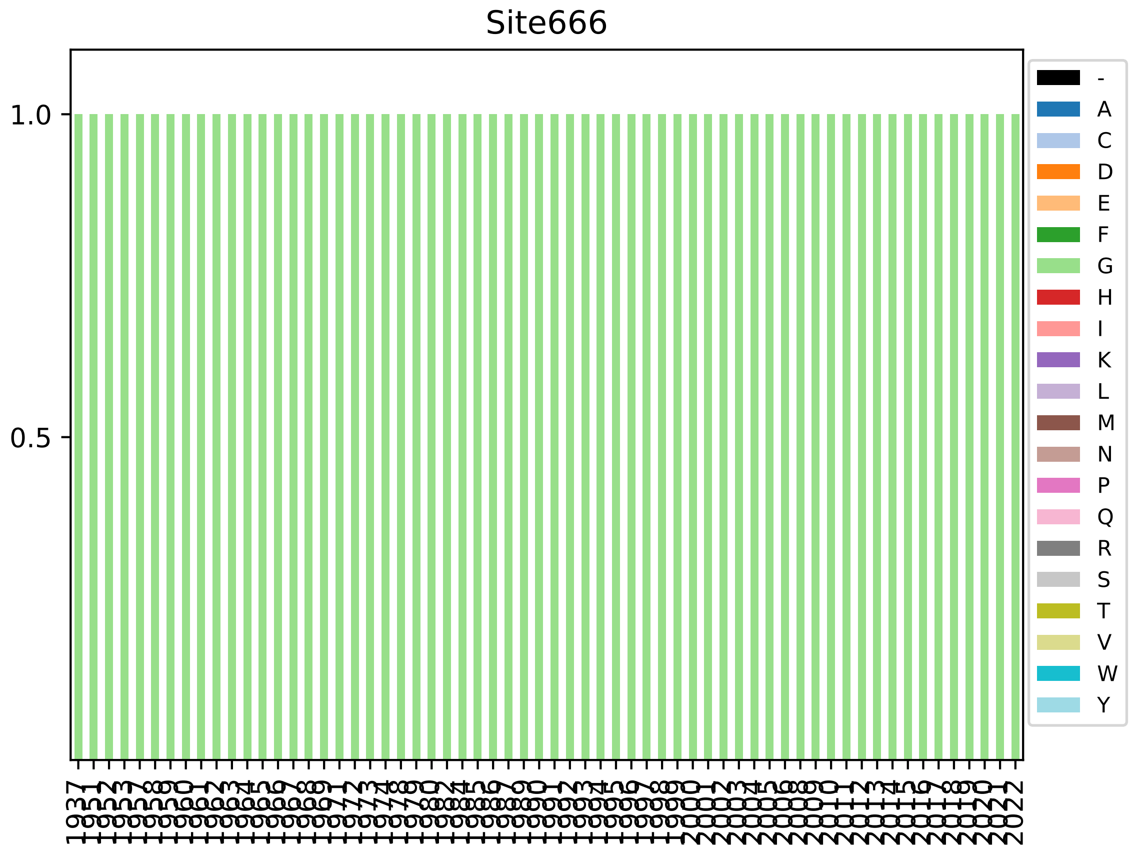
Site666
1.0
0.5
-
A
C
D
E
F
G
H
I
K
L
M
N
P
Q
R
S
T
V
W
Y

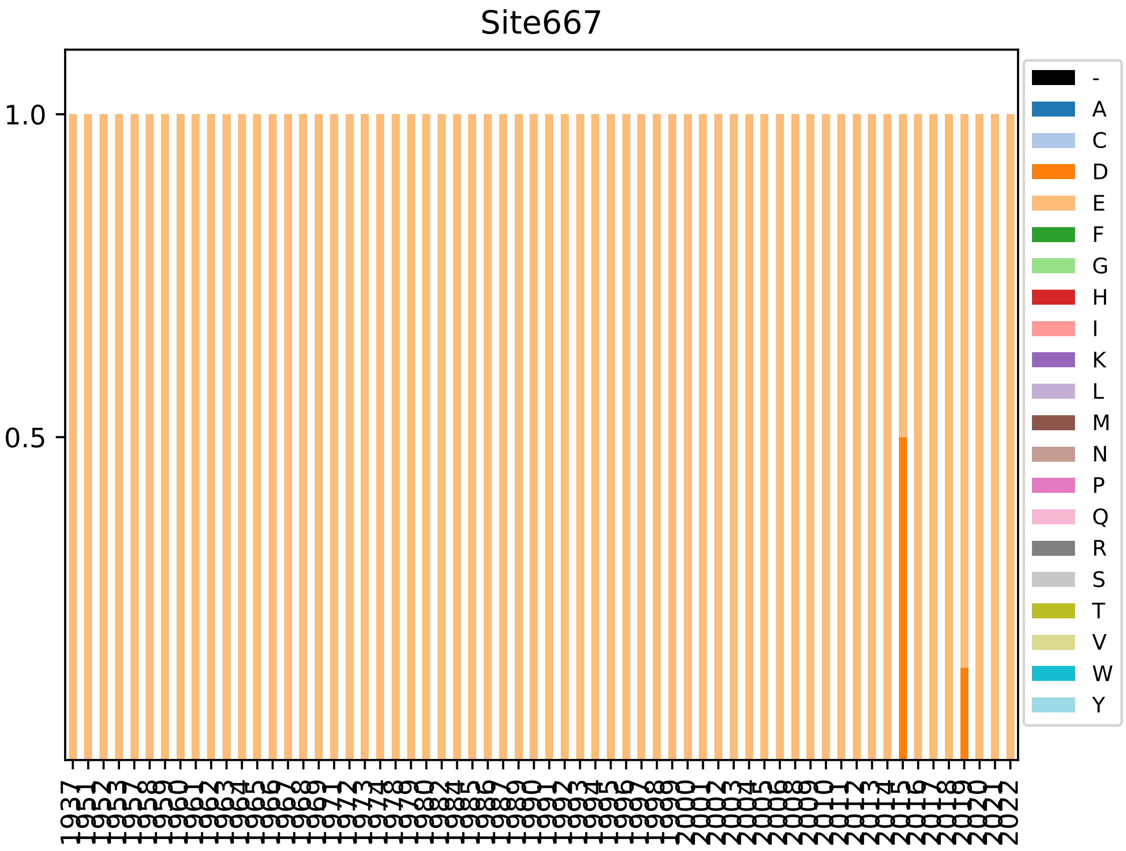
Site667
1.0
0.5
-
A
C
D
E
F
G
H
I
K
L
M
N
P
Q
R
S
T
V
W
Y

Site668

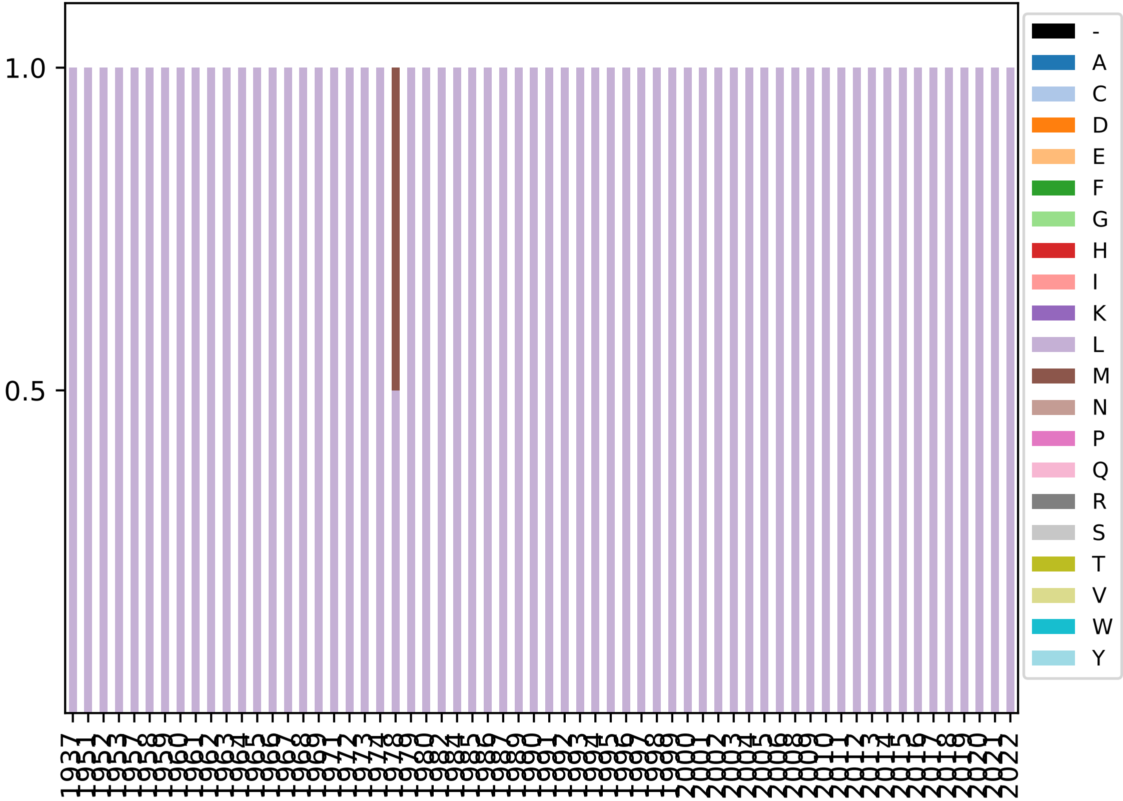

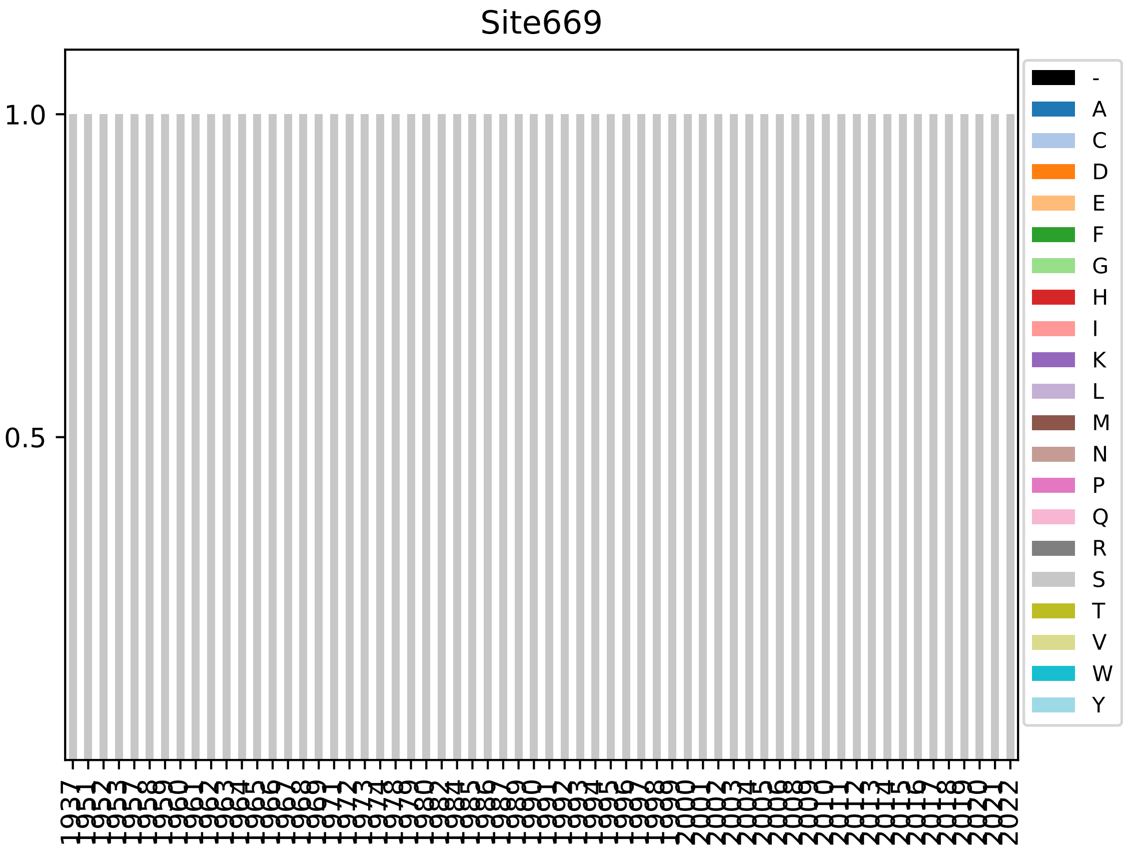
Site669
1.0
0.5
-
A
C
D
E
F
G
H
I
K
L
M
N
P
Q
R
S
T
V
W
Y

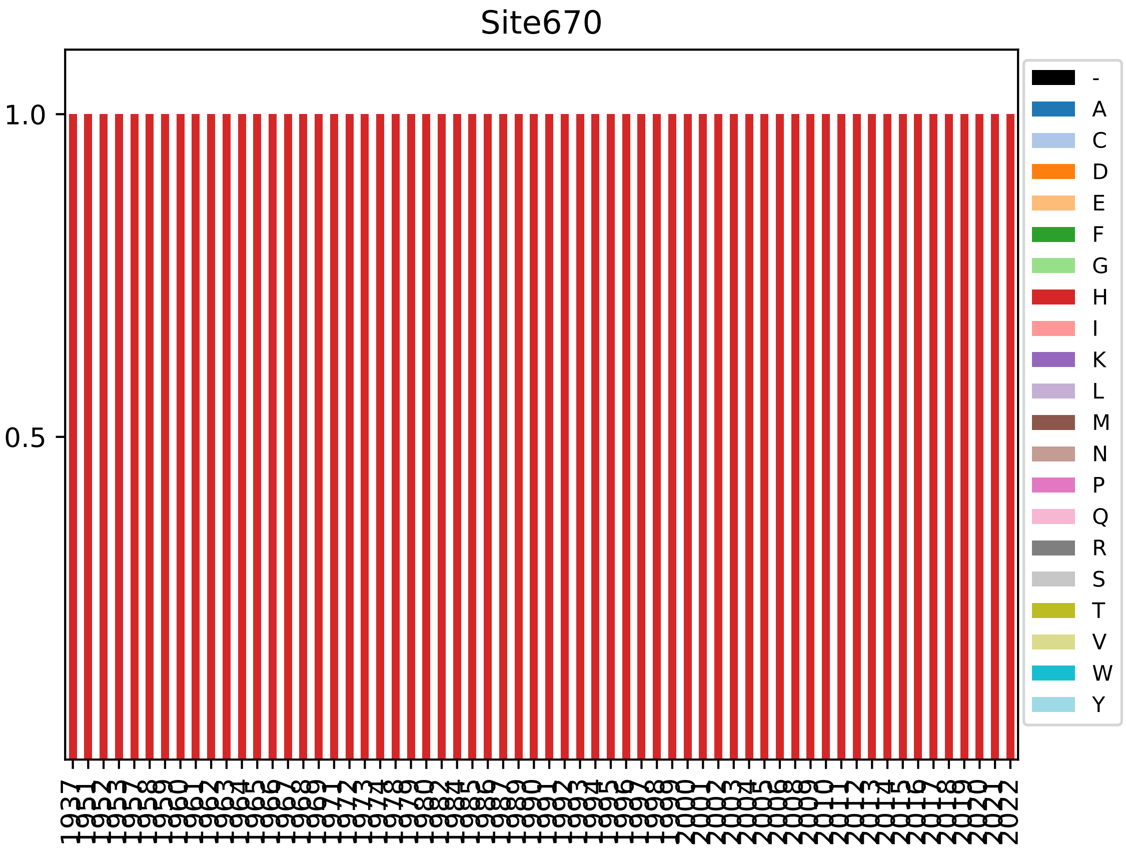
Site670
1.0
0.5
-
A
C
D
E
F
G
H
I
K
L
M
N
P
Q
R
S
T
V
W
Y

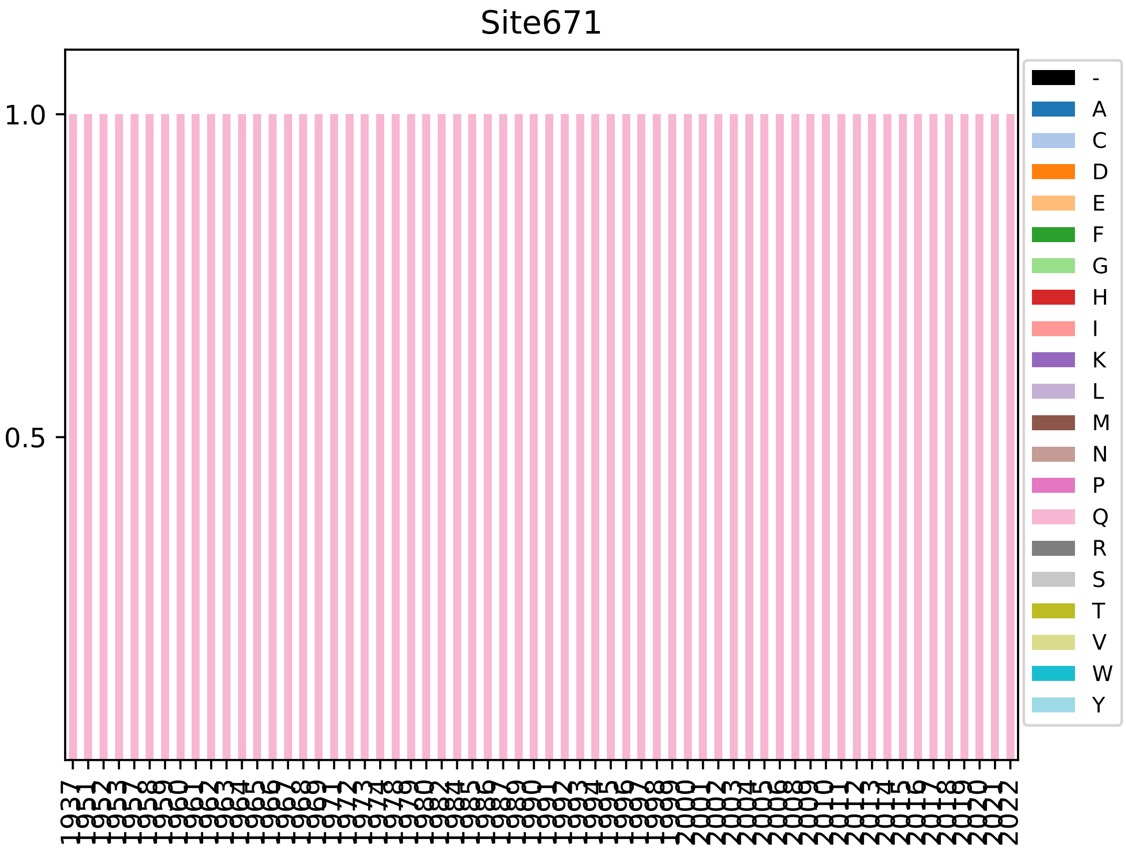

Site671
1.0
0.5
-
A
C
D
E
F
G
H
I
K
L
M
N
P
Q
R
S
T
V
W
Y

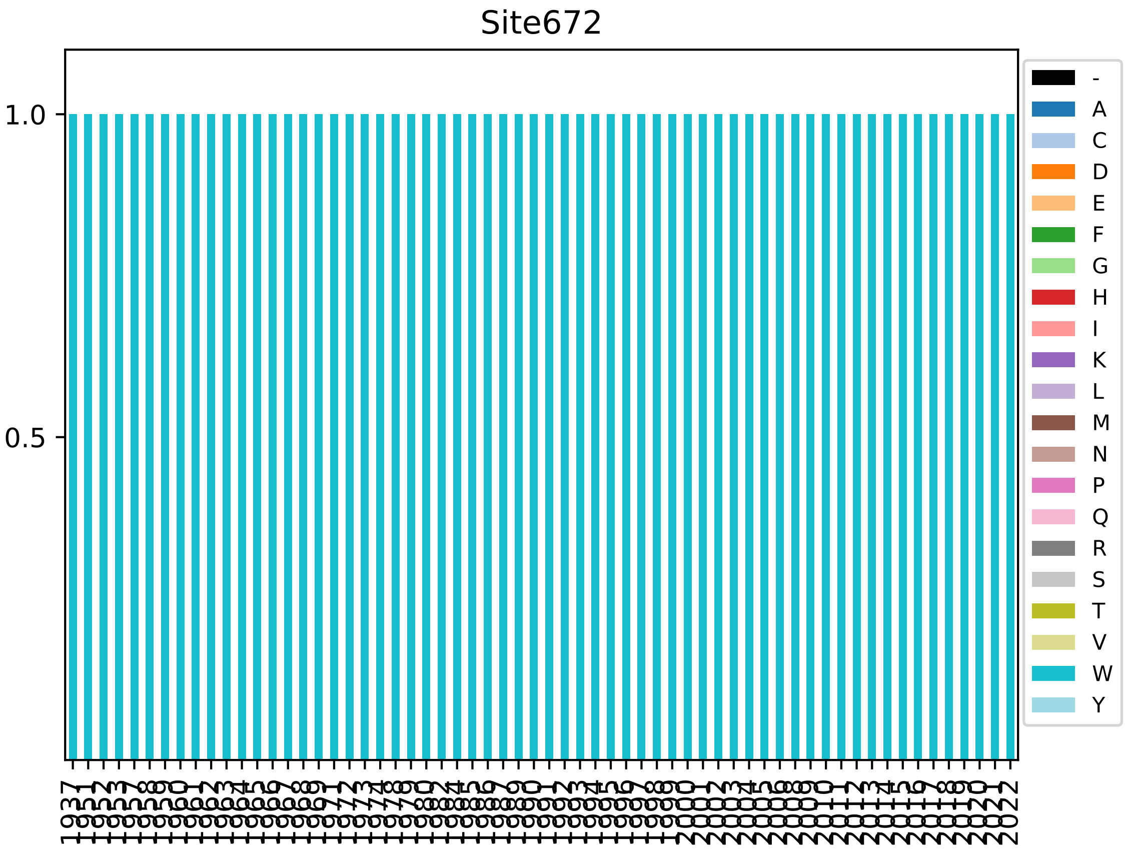
Site672
1.0
0.5
-
A
C
D
E
F
G
H
I
K
L
M
N
P
Q
R
S
T
V
W
Y

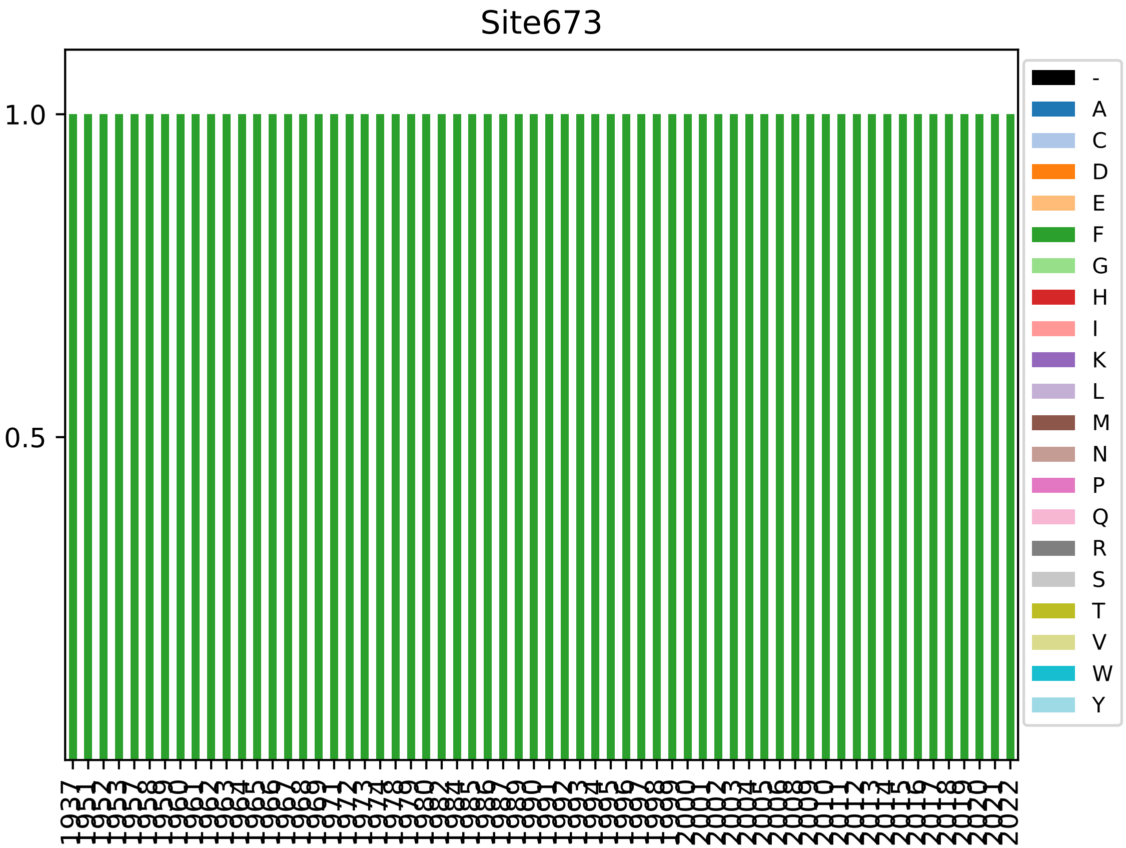
Site673
1.0
0.5
-
A
C
D
E
F
G
H
I
K
L
M
N
P
Q
R
S
T
V
W
Y

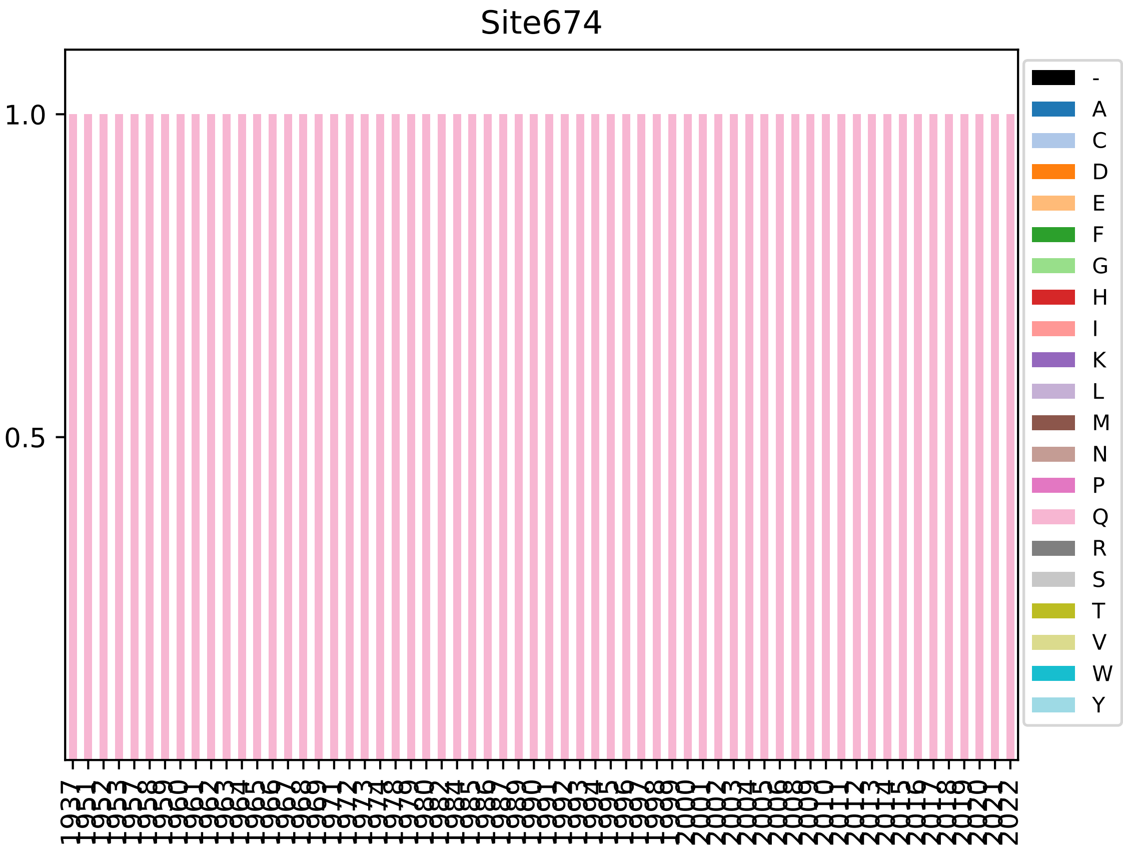
Site674
1.0
0.5
-
A
C
D
E
F
G
H
I
K
L
M
N
P
Q
R
S
T
V
W
Y

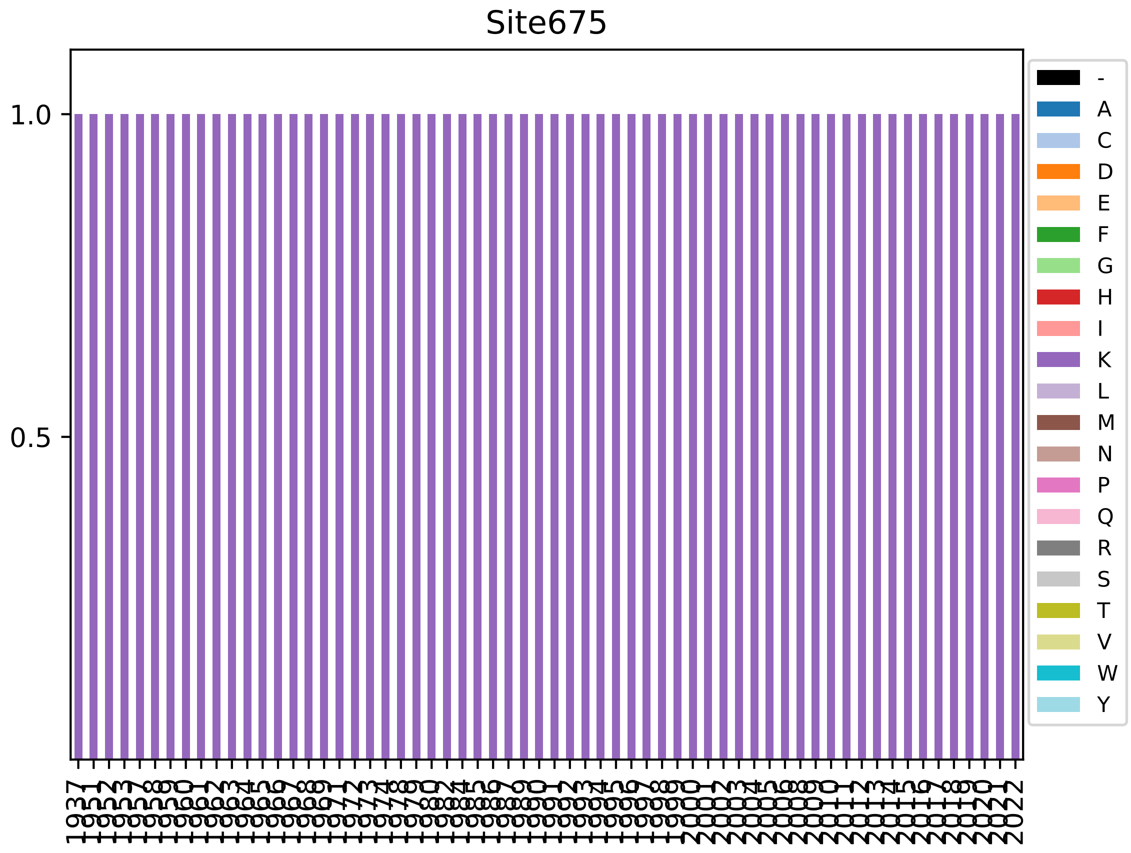
Site675
1.0
0.5
-
A
C
D
E
F
G
H
I
K
L
M
N
P
Q
R
S
T
V
W
Y

Site676

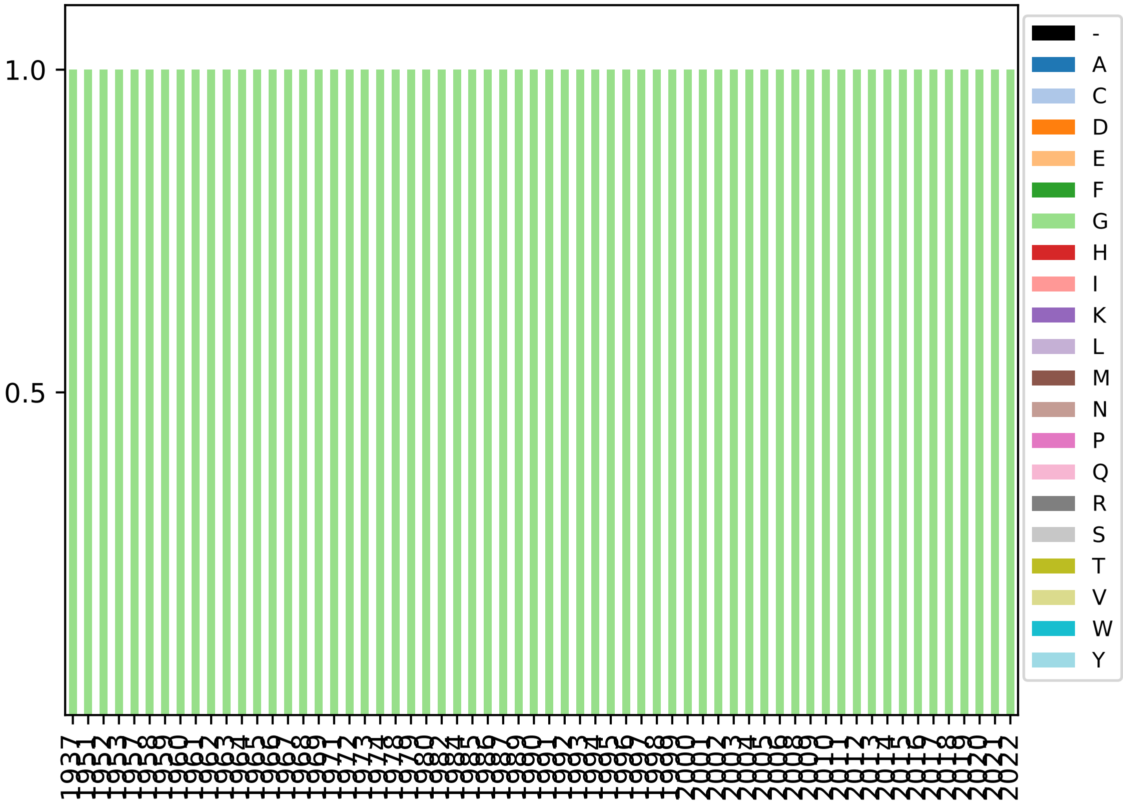

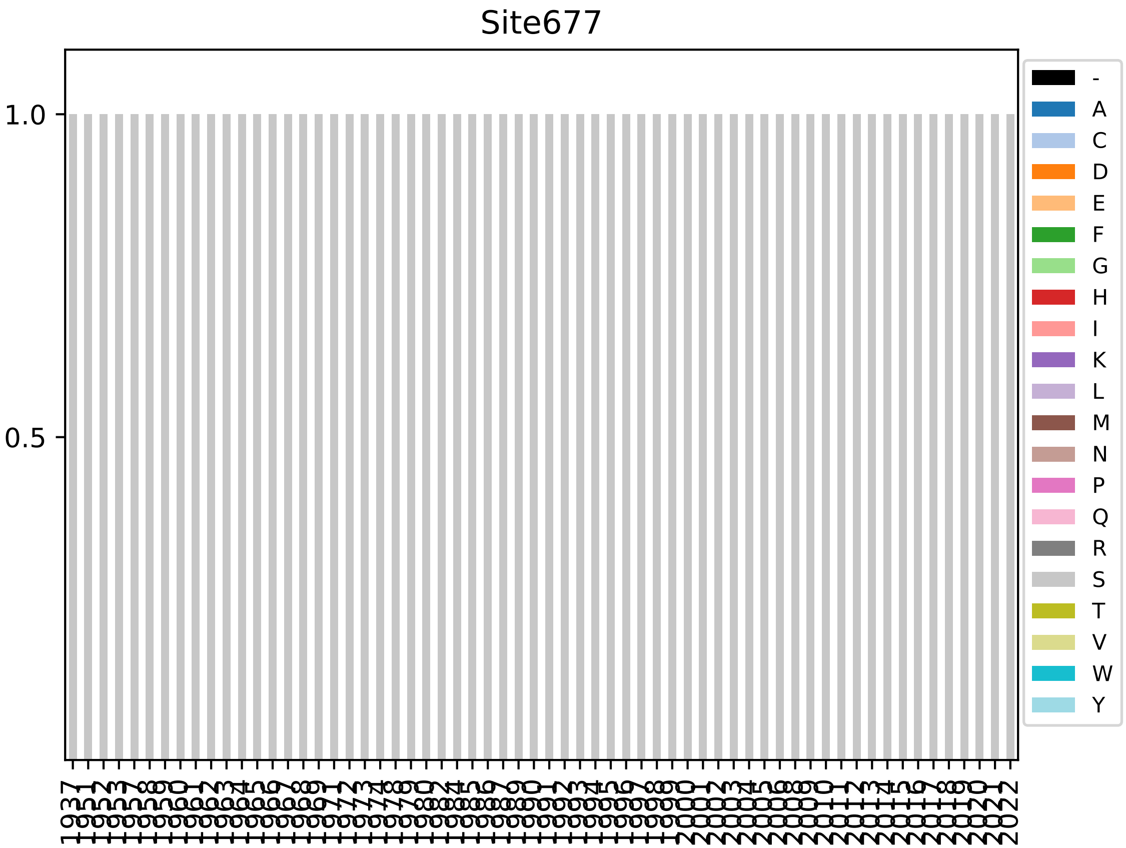
Site677
1.0
0.5
-
A
C
D
E
F
G
H
I
K
L
M
N
P
Q
R
S
T
V
W
Y

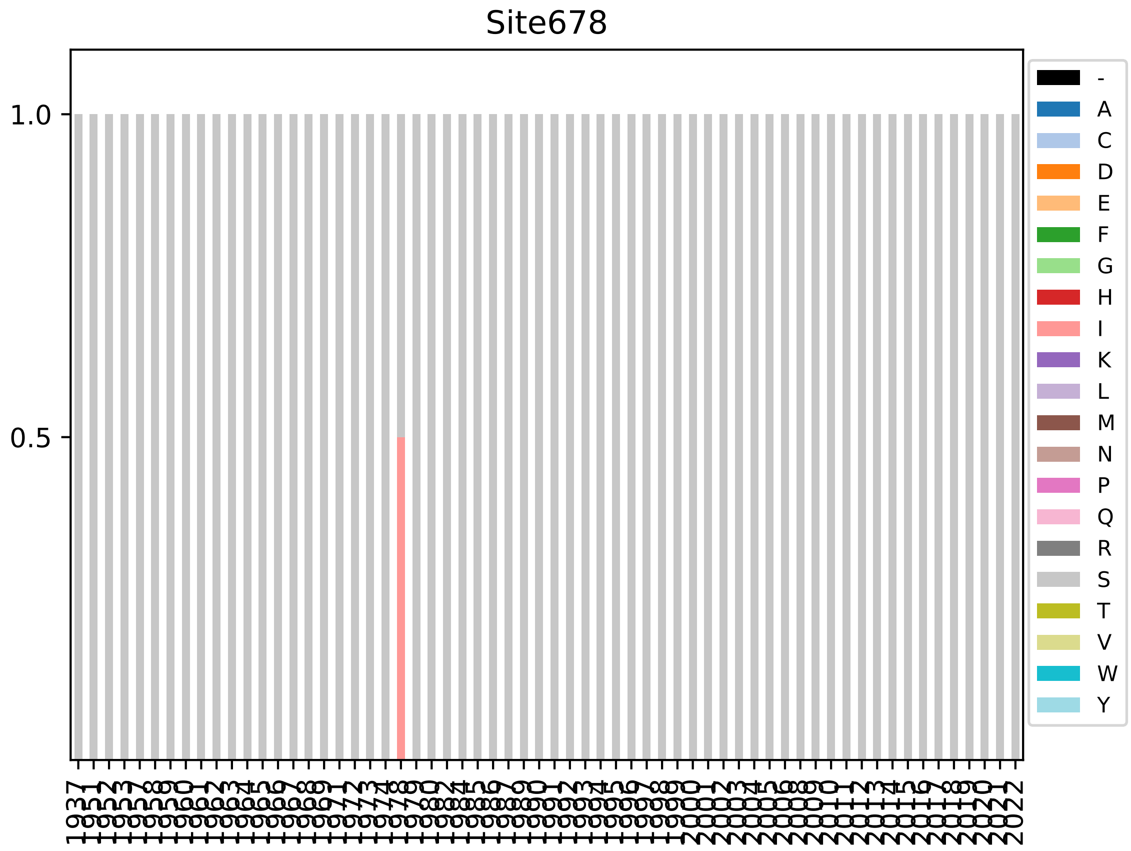

Site678
1.0
0.5
-
A
C
D
E
F
G
H
I
K
L
M
N
P
Q
R
S
T
V
W
Y

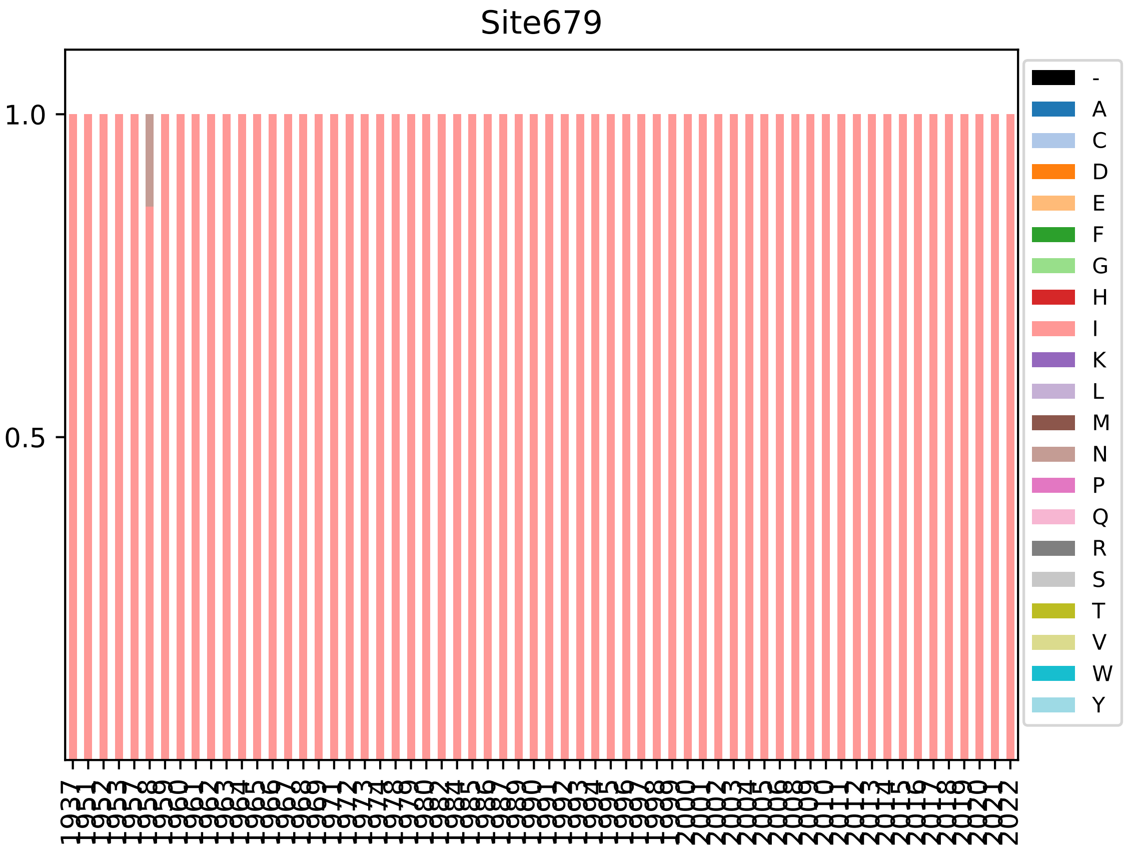
Site679
1.0
0.5
-
A
C
D
E
F
G
H
I
K
L
M
N
P
Q
R
S
T
V
W
Y

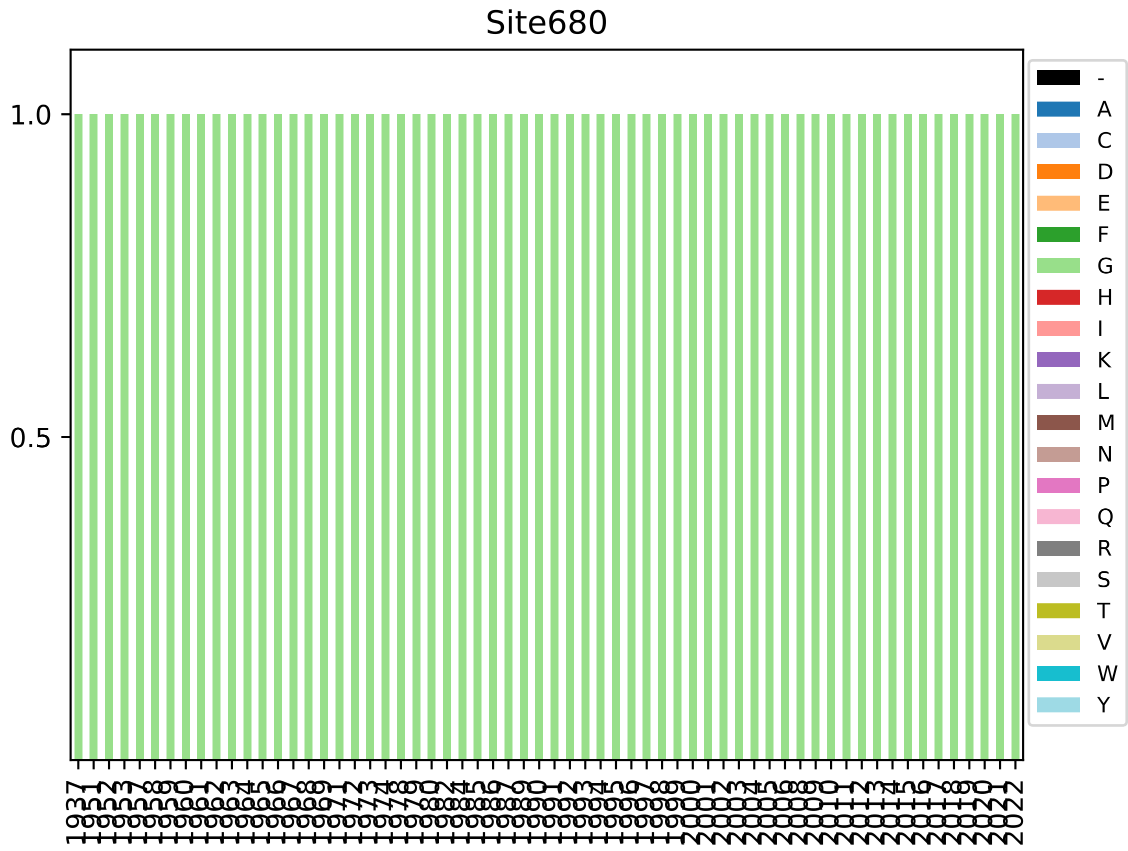
Site680
1.0
0.5
-
A
C
D
E
F
G
H
I
K
L
M
N
P
Q
R
S
T
V
W
Y

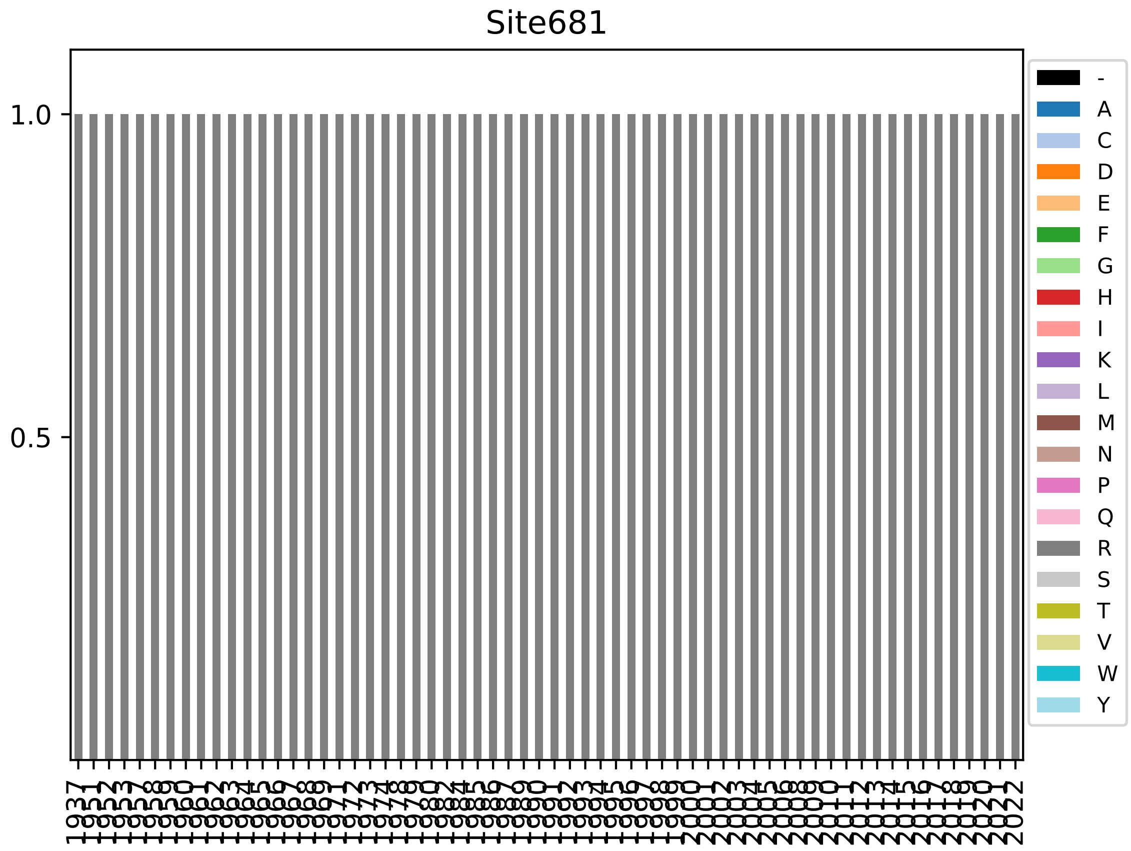
Site681
1.0
0.5
-
A
C
D
E
F
G
H
I
K
L
M
N
P
Q
R
S
T
V
W
Y

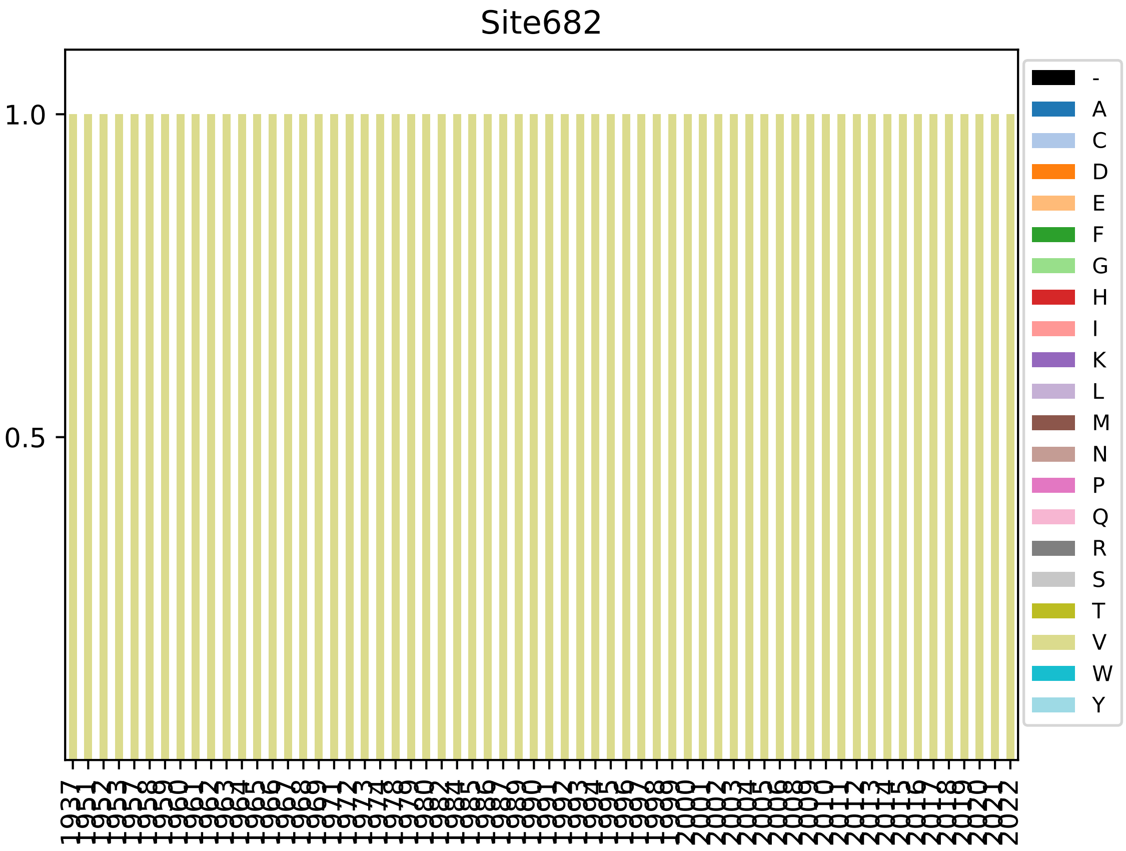
Site682
1.0
0.5
-
A
C
D
E
F
G
H
I
K
L
M
N
P
Q
R
S
T
V
W
Y

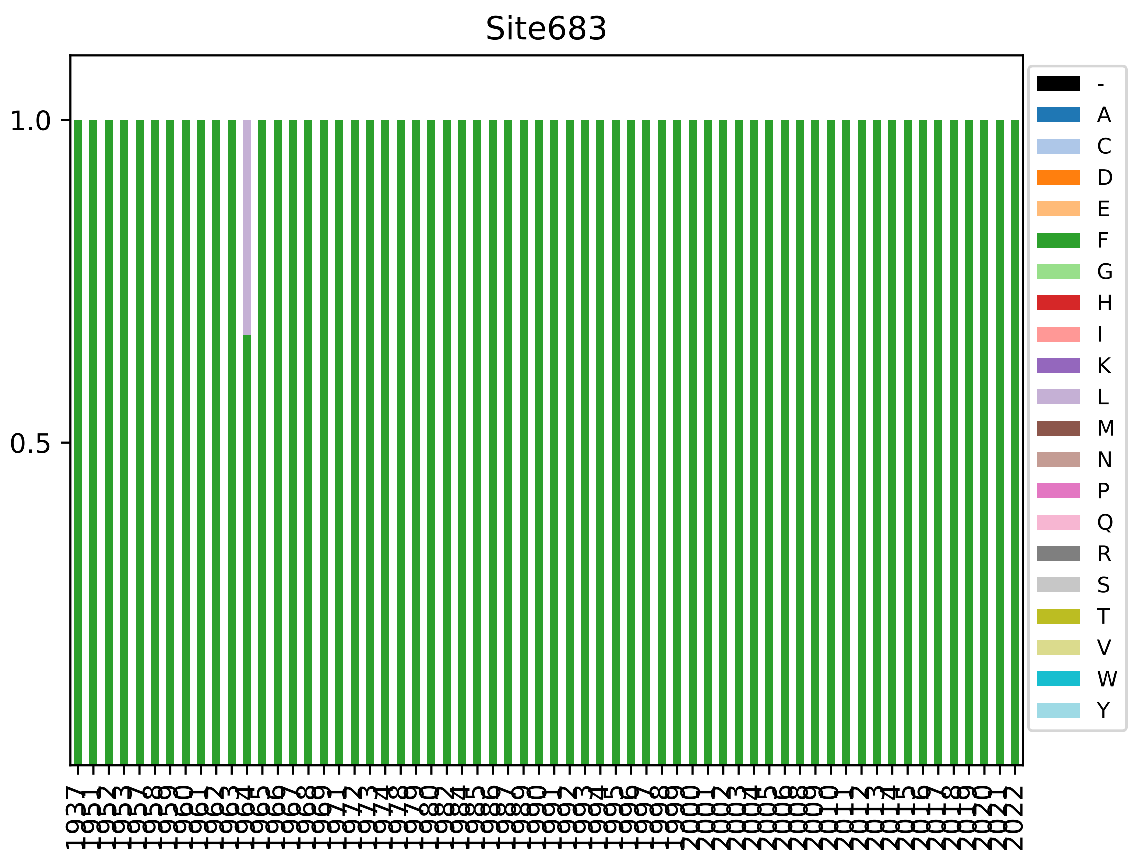
Site683
1.0
0.5
-
A
C
D
E
F
G
H
I
K
L
M
N
P
Q
R
S
T
V
W
Y

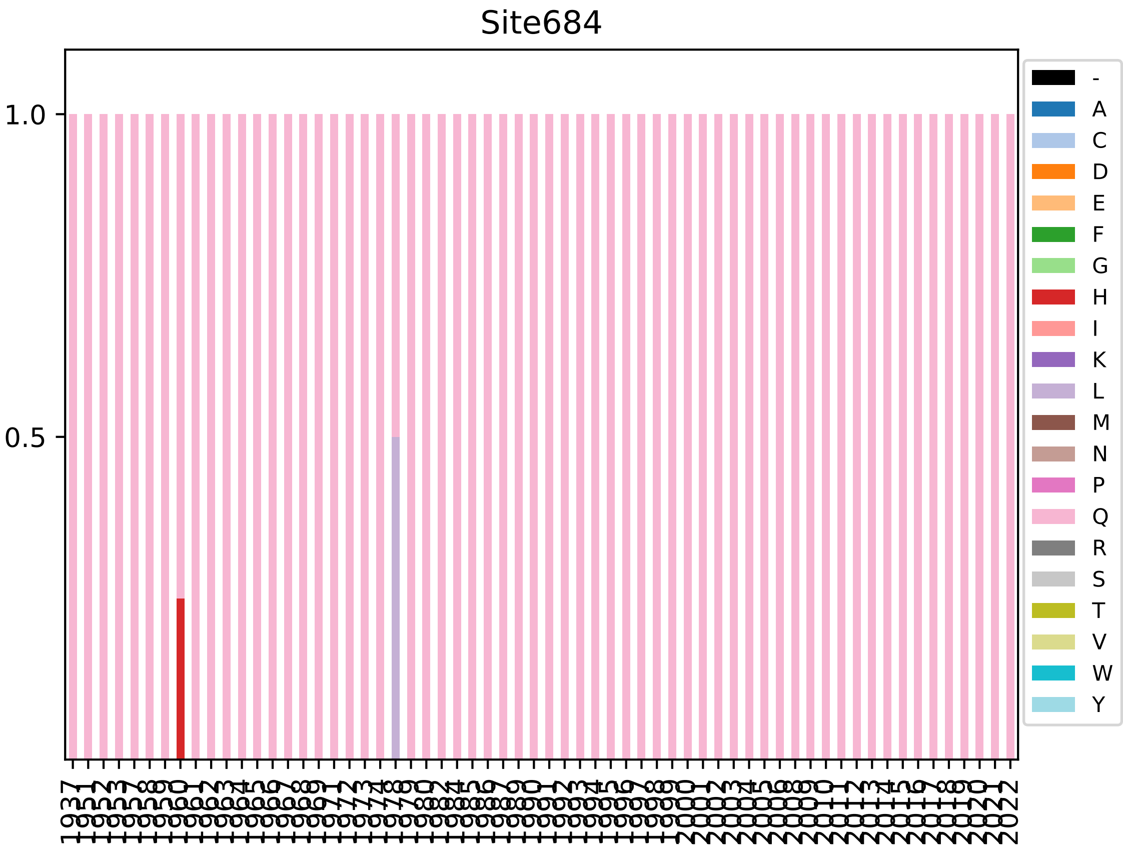
Site684
1.0
0.5
-
A
C
D
E
F
G
H
I
K
L
M
N
P
Q
R
S
T
V
W
Y

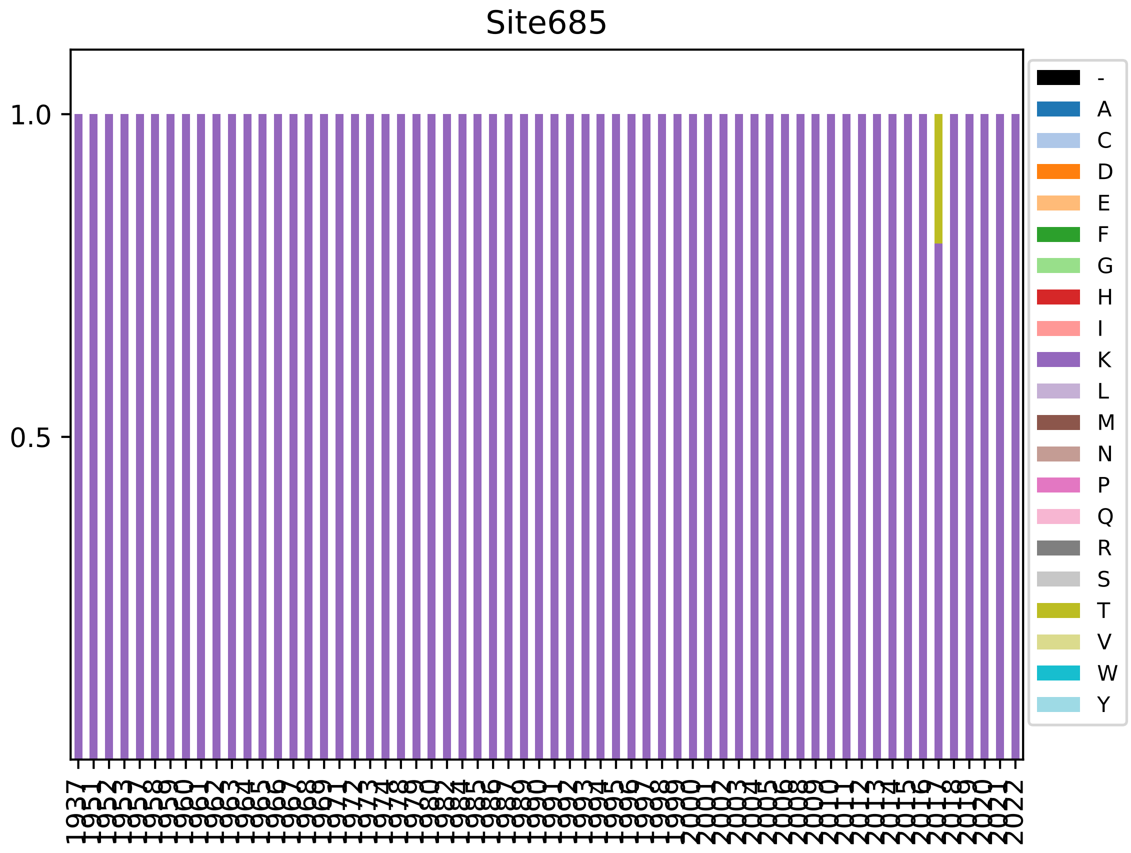
Site685
1.0
0.5
-
A
C
D
E
F
G
H
I
K
L
M
N
P
Q
R
S
T
V
W
Y

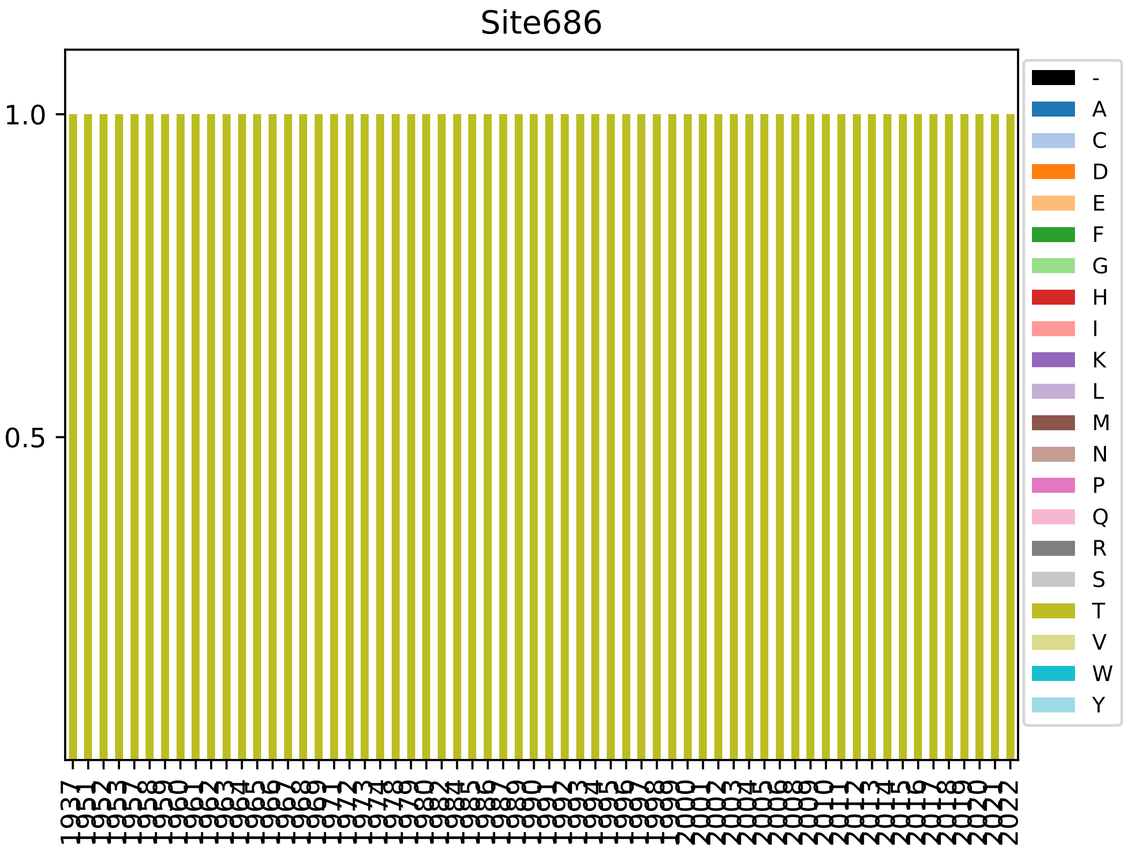
Site686
1.0
0.5
-
A
C
D
E
F
G
H
I
K
L
M
N
P
Q
R
S
T
V
W
Y

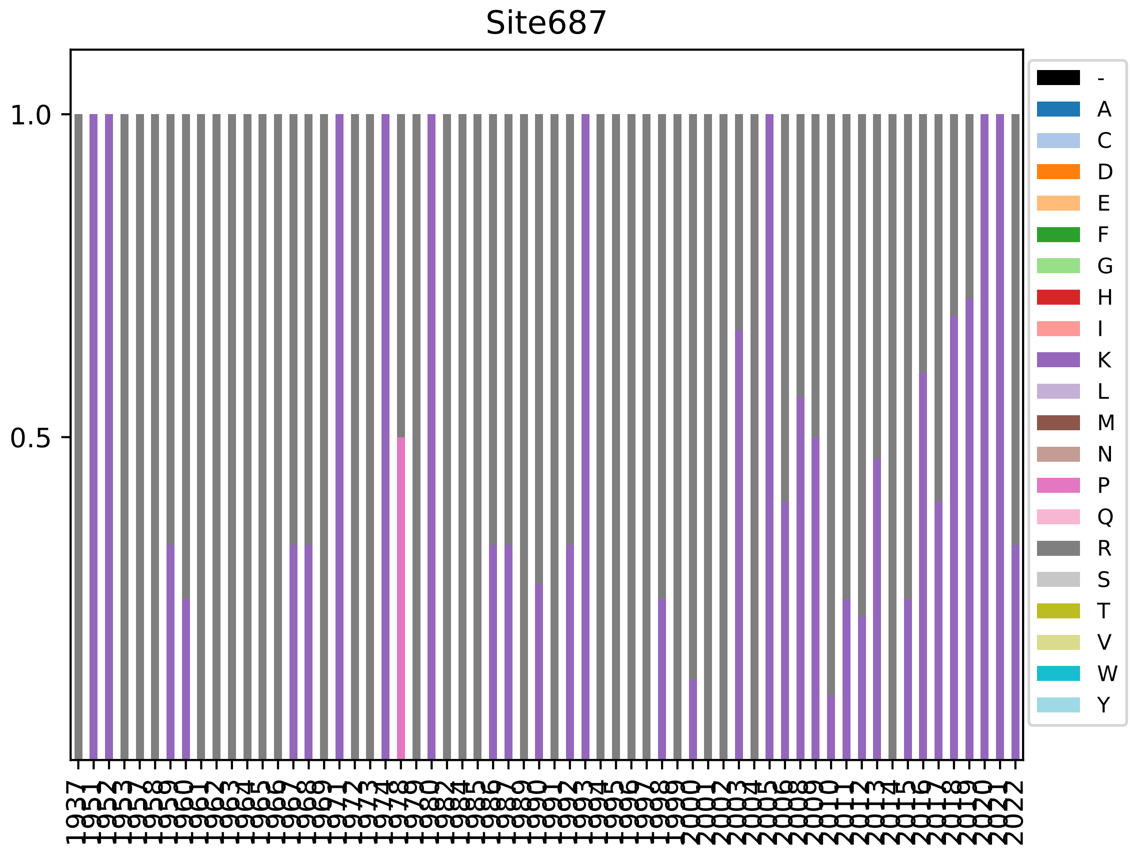
Site687
1.0
0.5
-
A
C
D
E
F
G
H
I
K
L
M
N
P
Q
R
S
T
V
W
Y

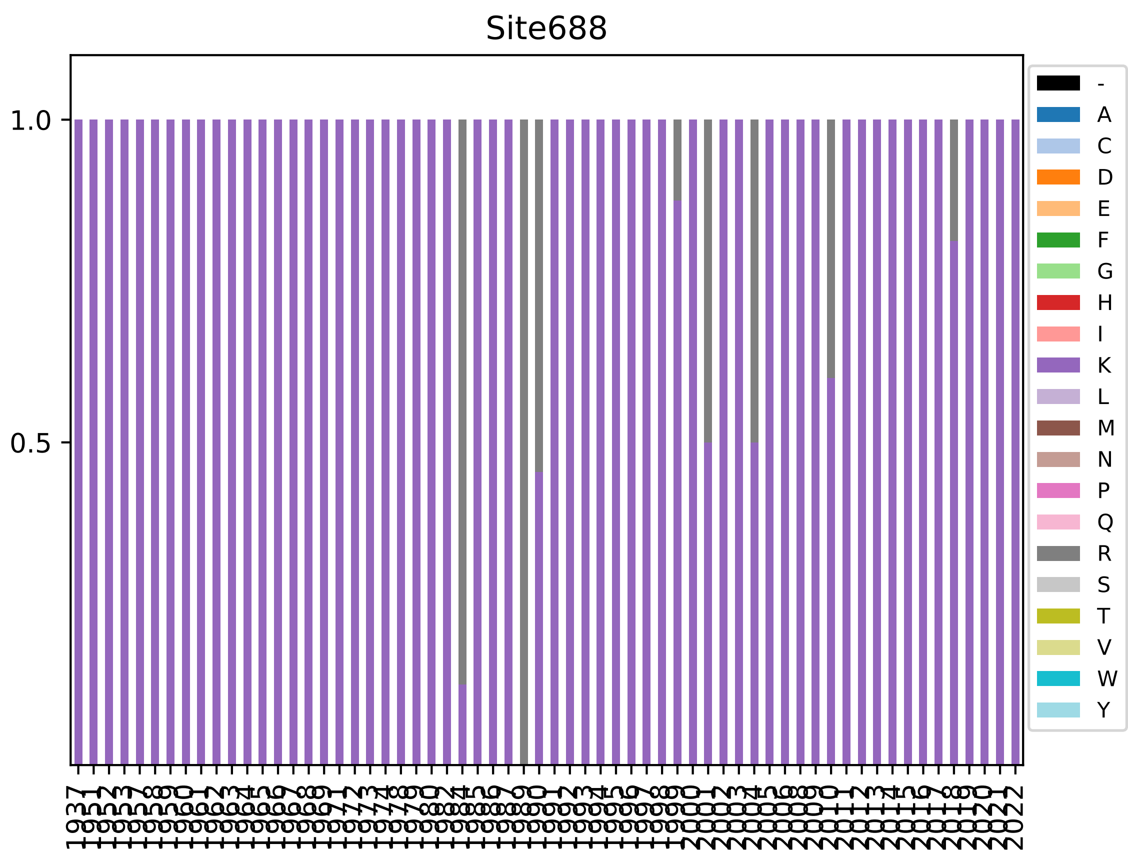
Site688
1.0
0.5
-
A
C
D
E
F
G
H
I
K
L
M
N
P
Q
R
S
T
V
W
Y

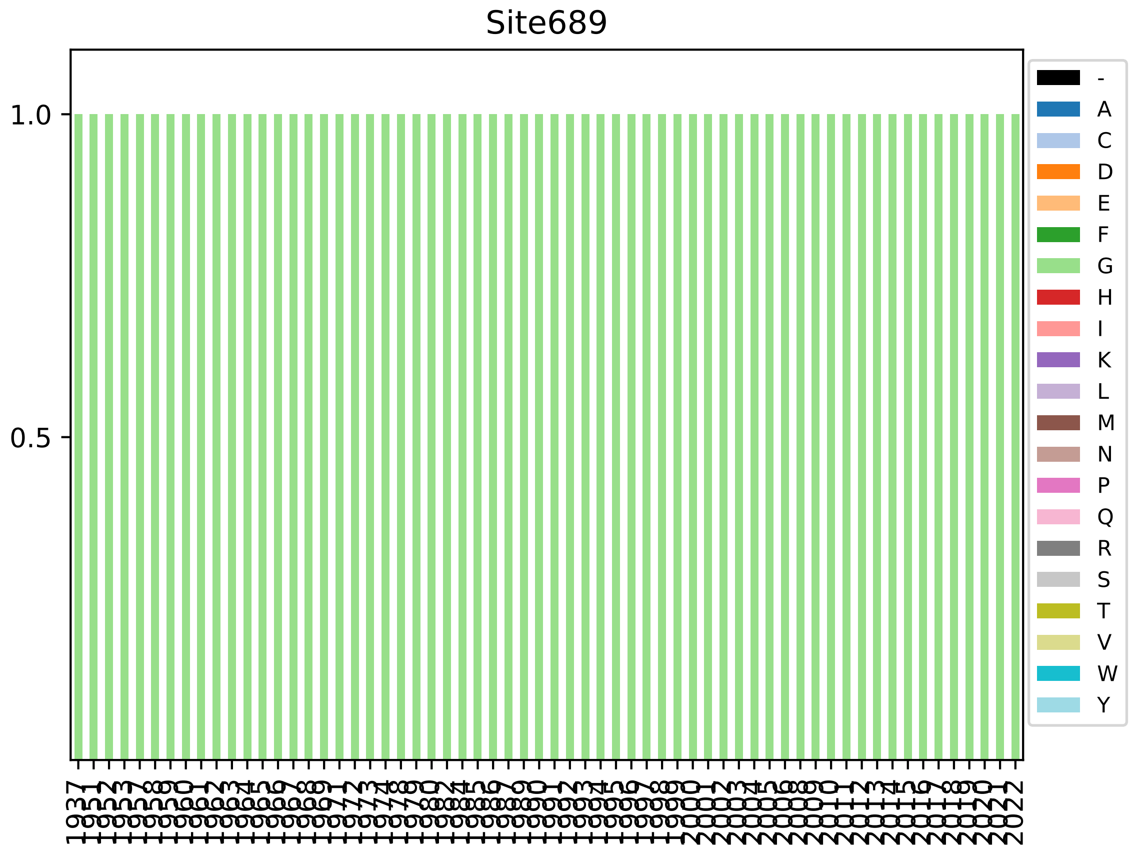
Site689
1.0
0.5
-
A
C
D
E
F
G
H
I
K
L
M
N
P
Q
R
S
T
V
W
Y

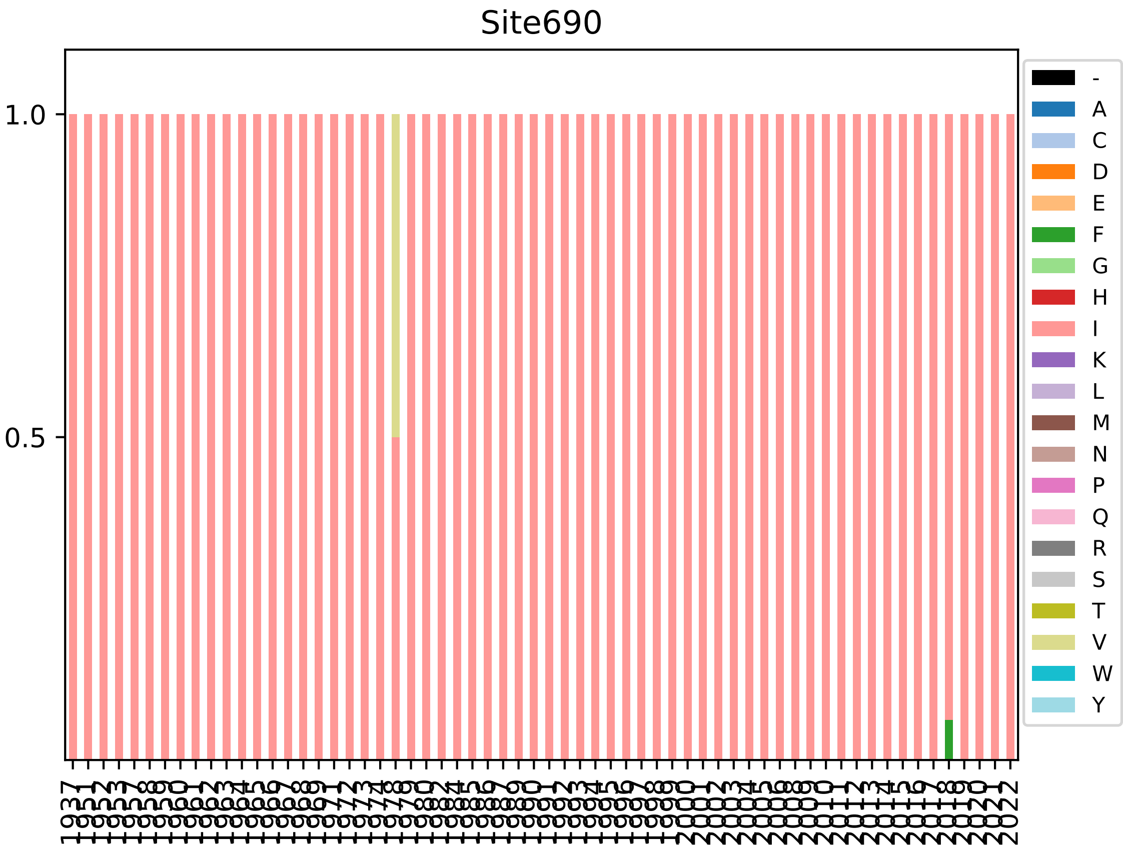
Site690
1.0
0.5
-
A
C
D
E
F
G
H
I
K
L
M
N
P
Q
R
S
T
V
W
Y

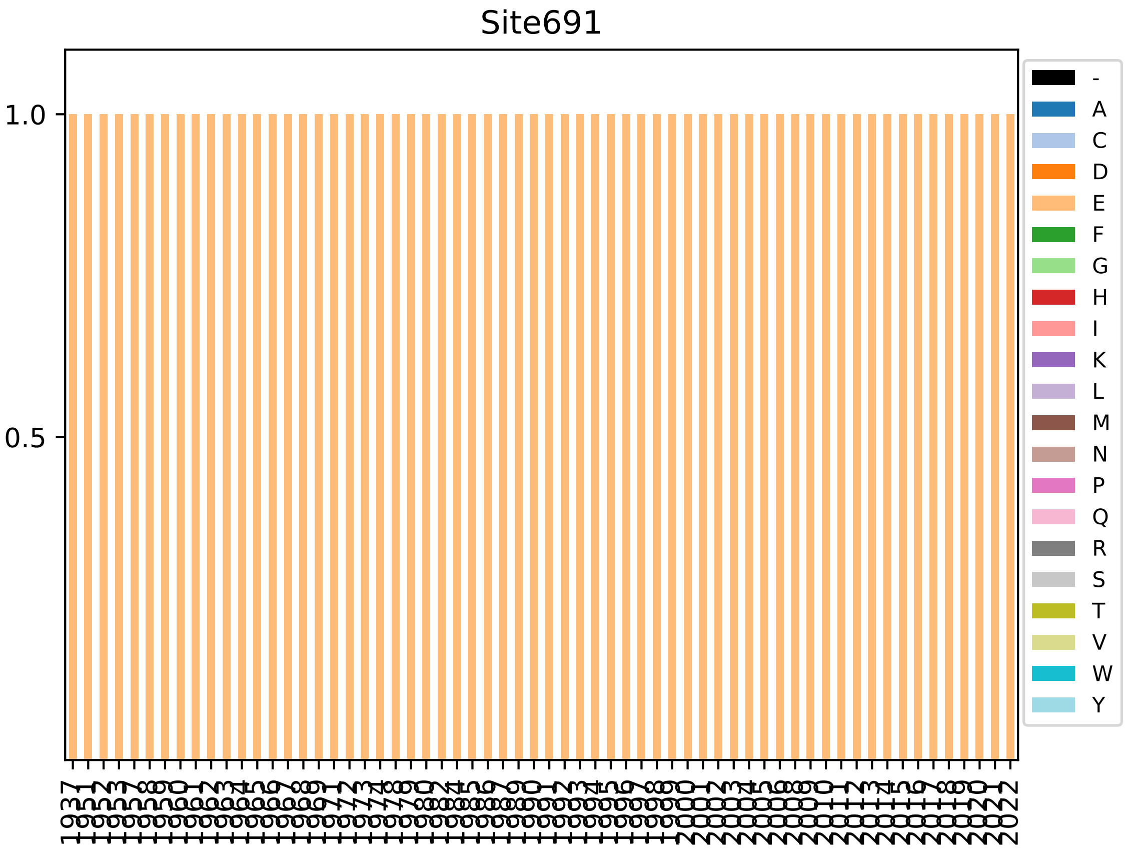
Site691
1.0
0.5
-
A
C
D
E
F
G
H
I
K
L
M
N
P
Q
R
S
T
V
W
Y

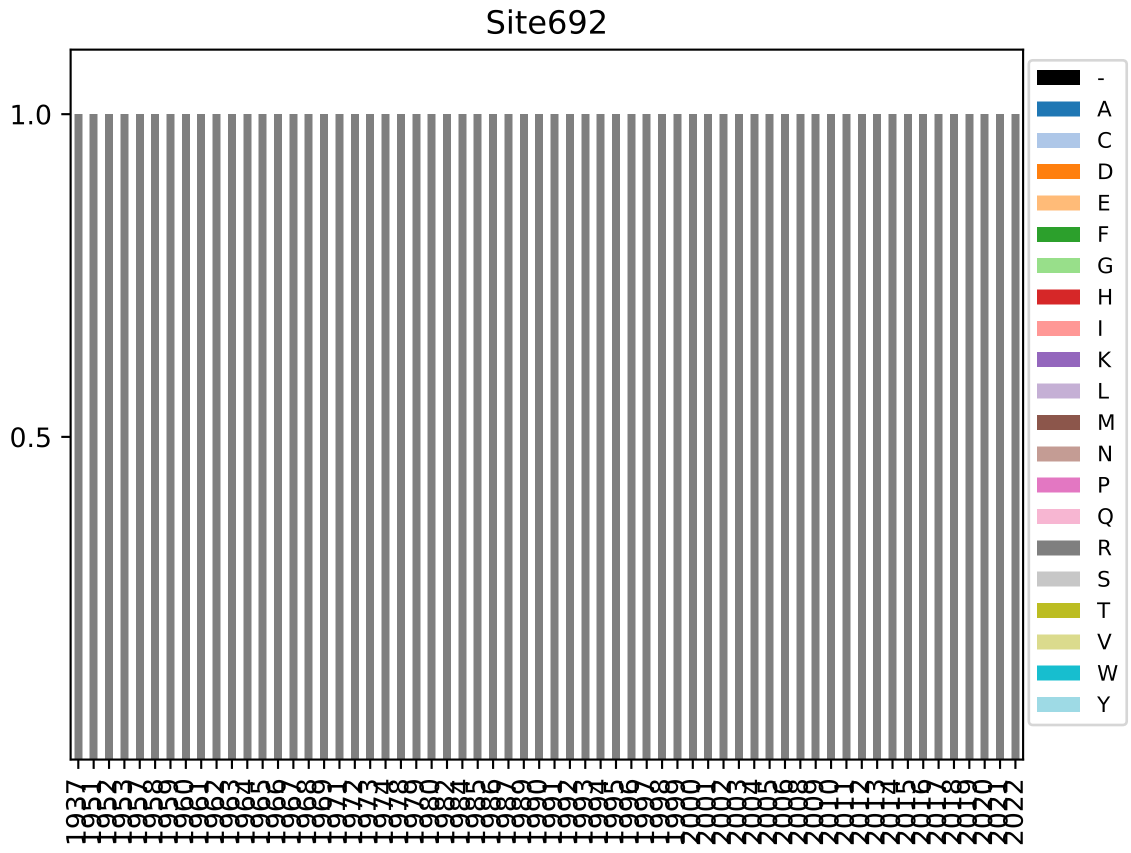
Site692
1.0
0.5
-
A
C
D
E
F
G
H
I
K
L
M
N
P
Q
R
S
T
V
W
Y

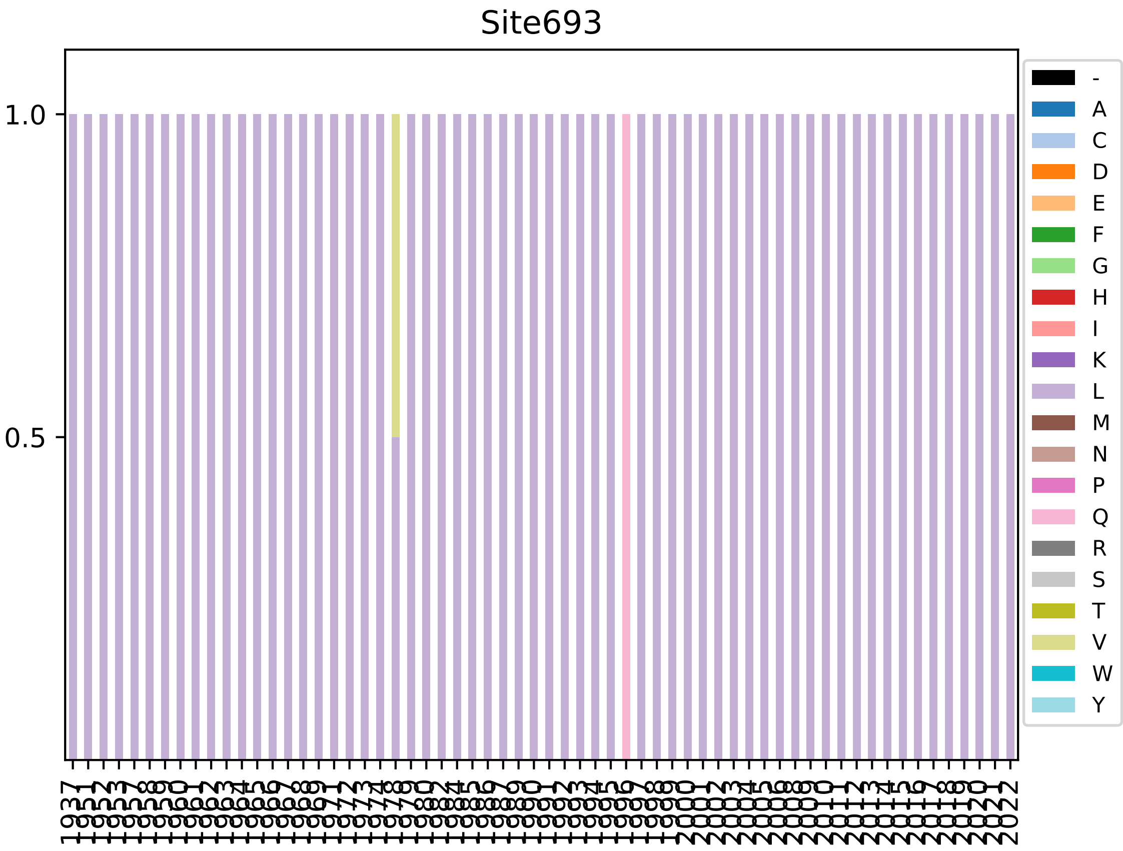
Site693
1.0
0.5
-
A
C
D
E
F
G
H
I
K
L
M
N
P
Q
R
S
T
V
W
Y

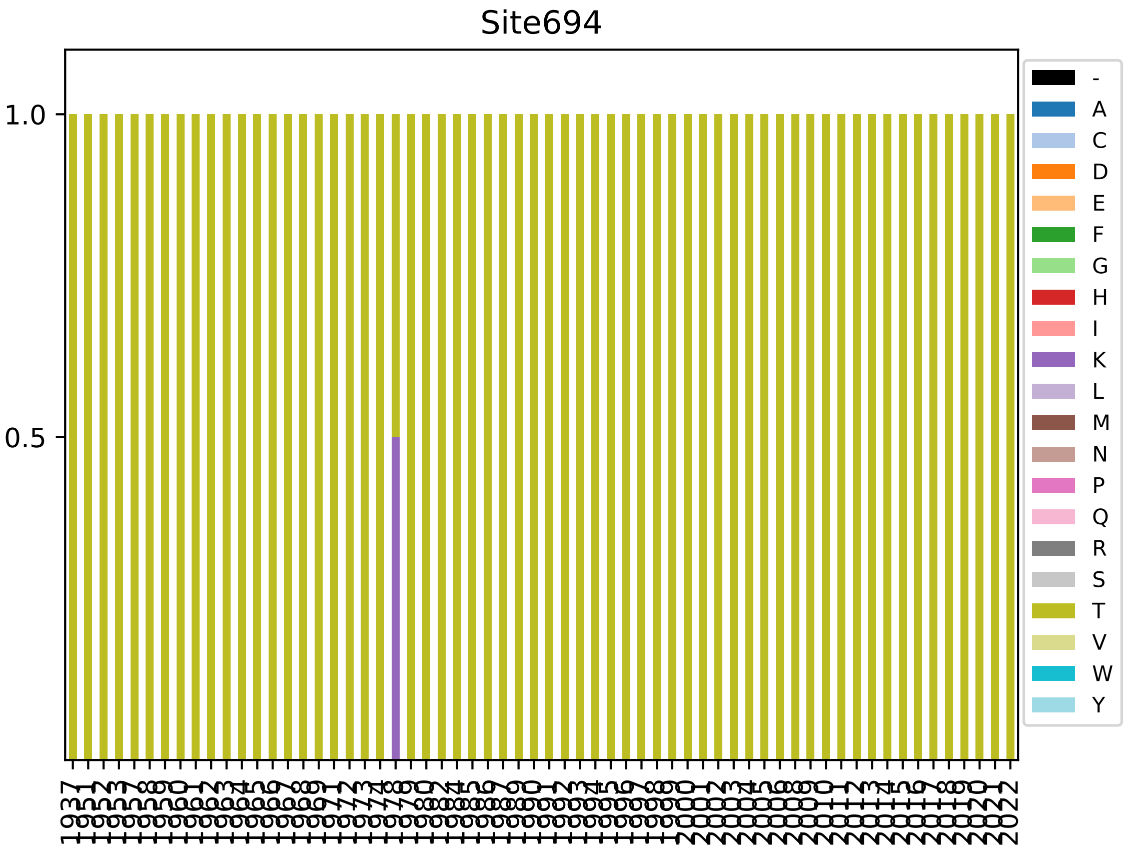
Site694
1.0
0.5
-
A
C
D
E
F
G
H
I
K
L
M
N
P
Q
R
S
T
V
W
Y

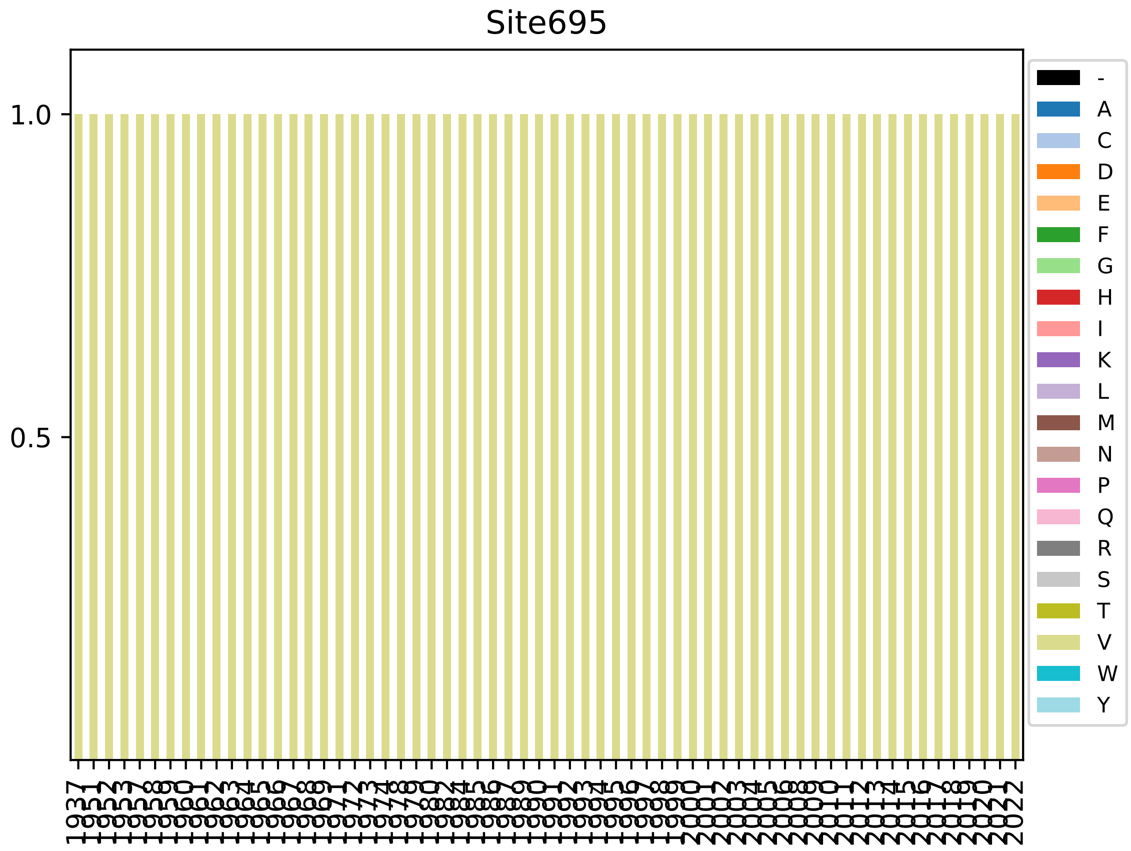
Site695
1.0
0.5
-
A
C
D
E
F
G
H
I
K
L
M
N
P
Q
R
S
T
V
W
Y

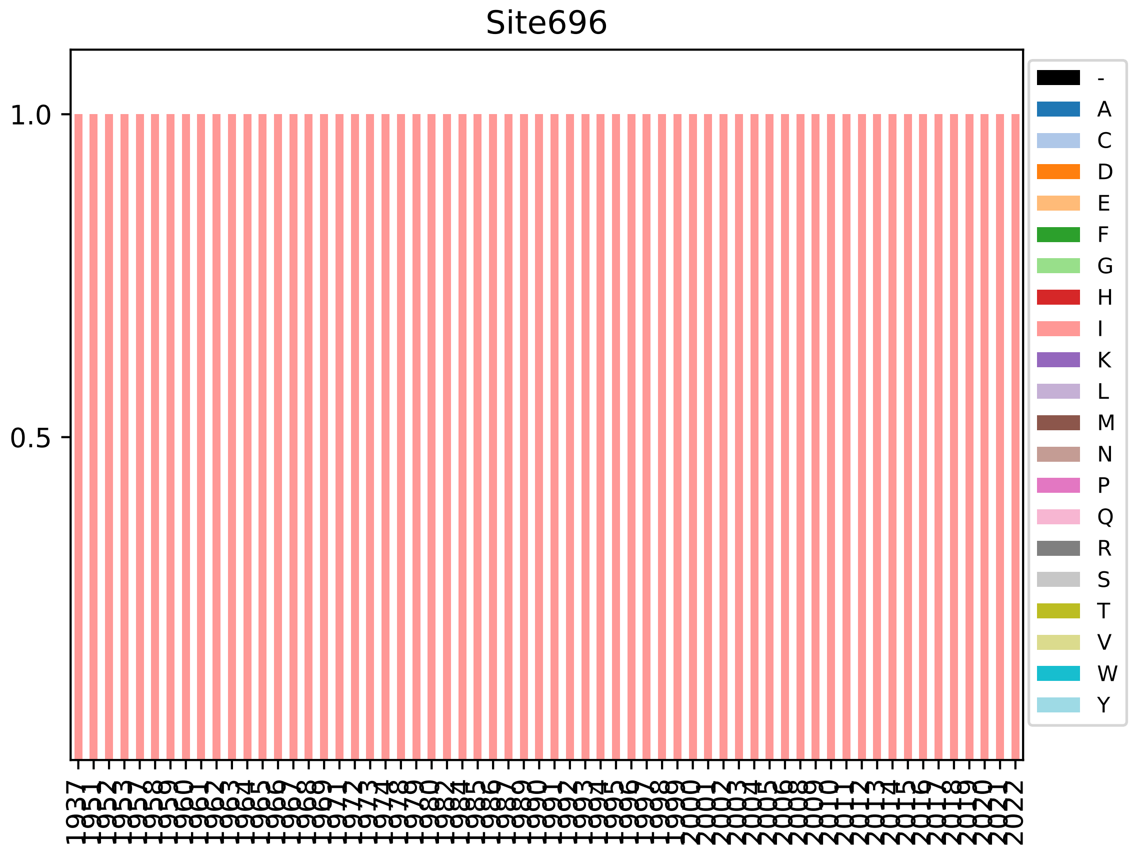
Site696
1.0
0.5
-
A
C
D
E
F
G
H
I
K
L
M
N
P
Q
R
S
T
V
W
Y

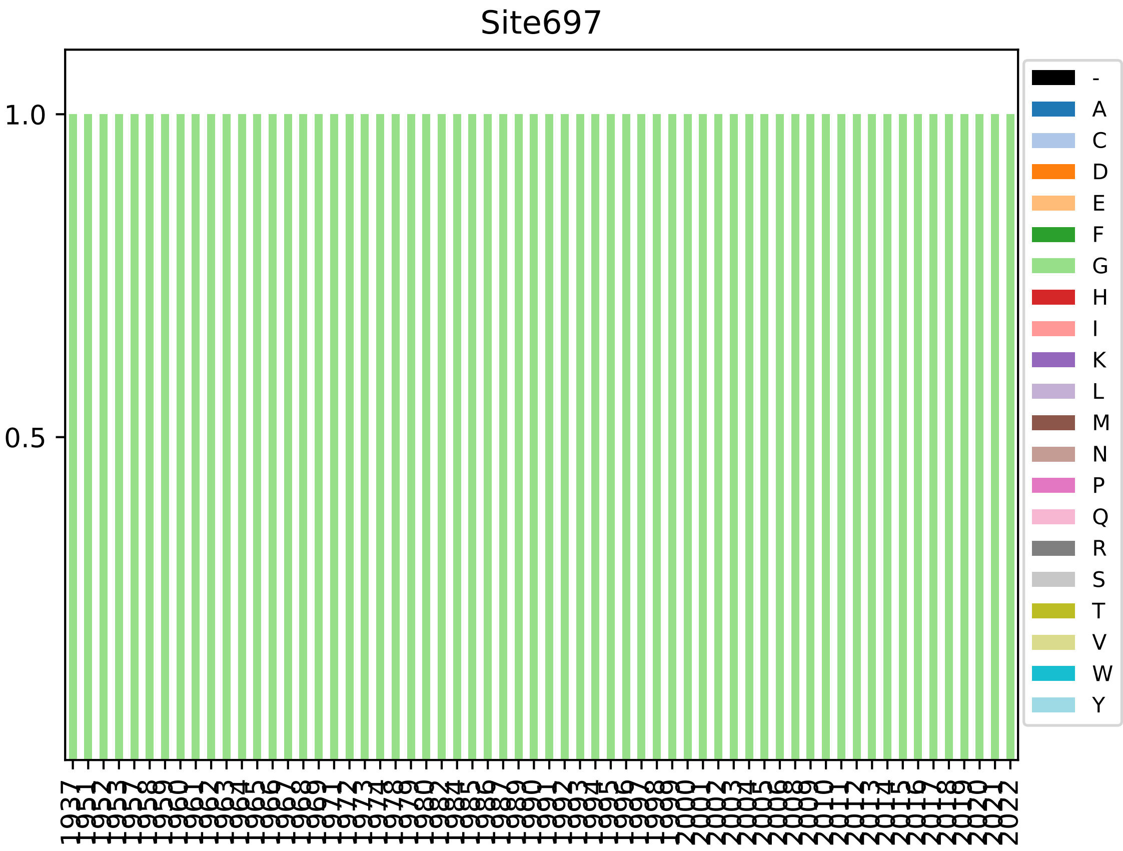
Site697
1.0
0.5
-
A
C
D
E
F
G
H
I
K
L
M
N
P
Q
R
S
T
V
W
Y

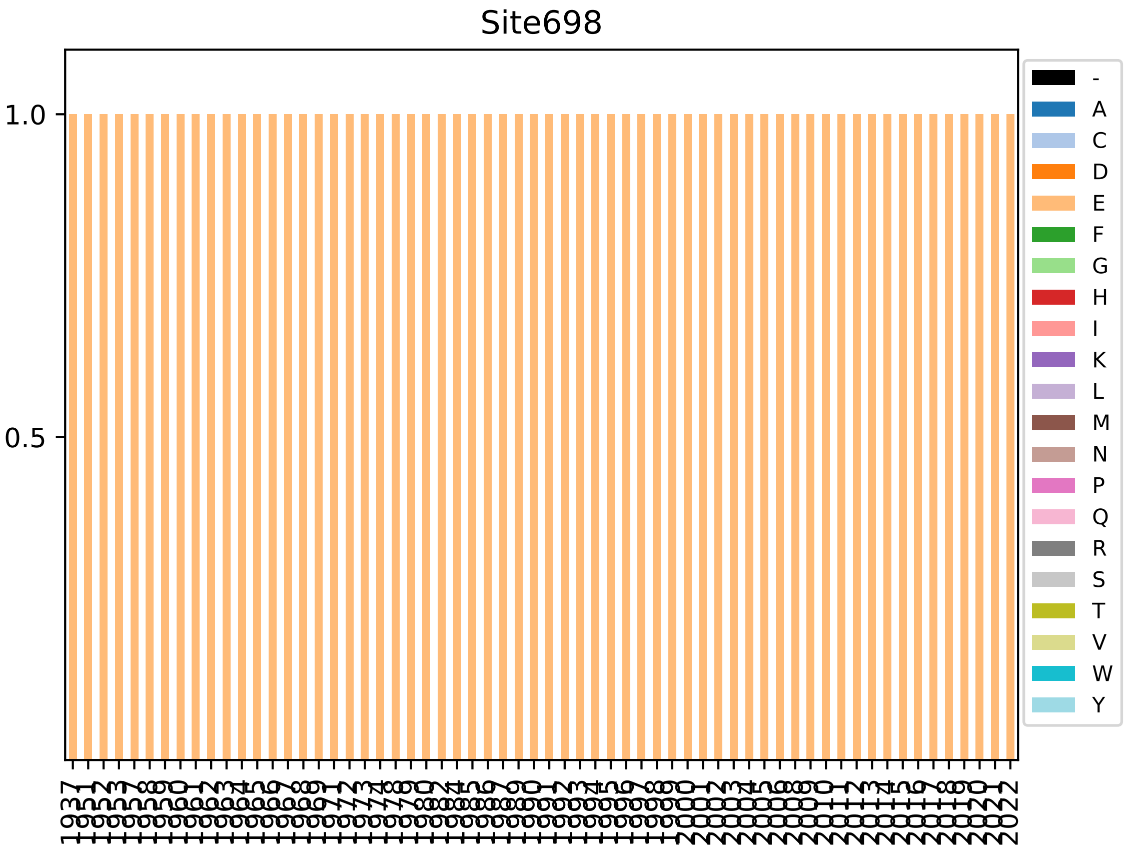
Site698
1.0
0.5
-
A
C
D
E
F
G
H
I
K
L
M
N
P
Q
R
S
T
V
W
Y

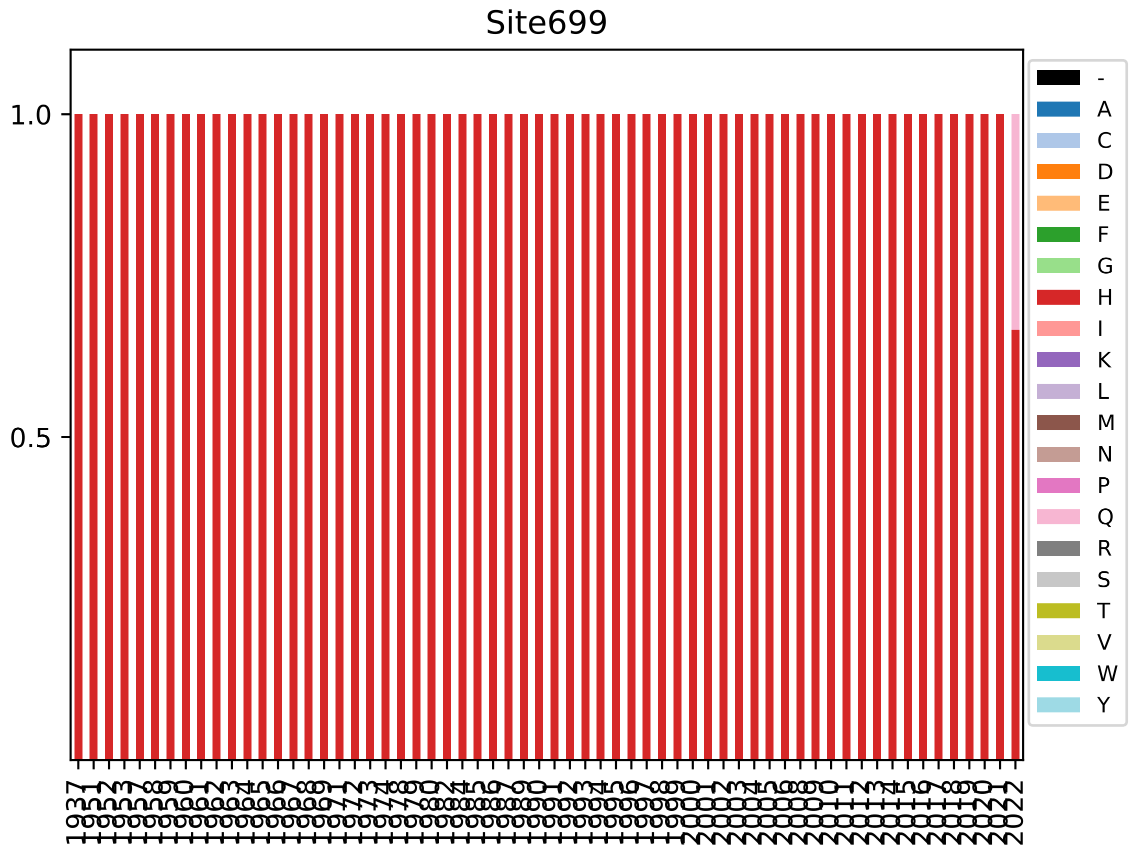
Site699
1.0
0.5
-
A
C
D
E
F
G
H
I
K
L
M
N
P
Q
R
S
T
V
W
Y

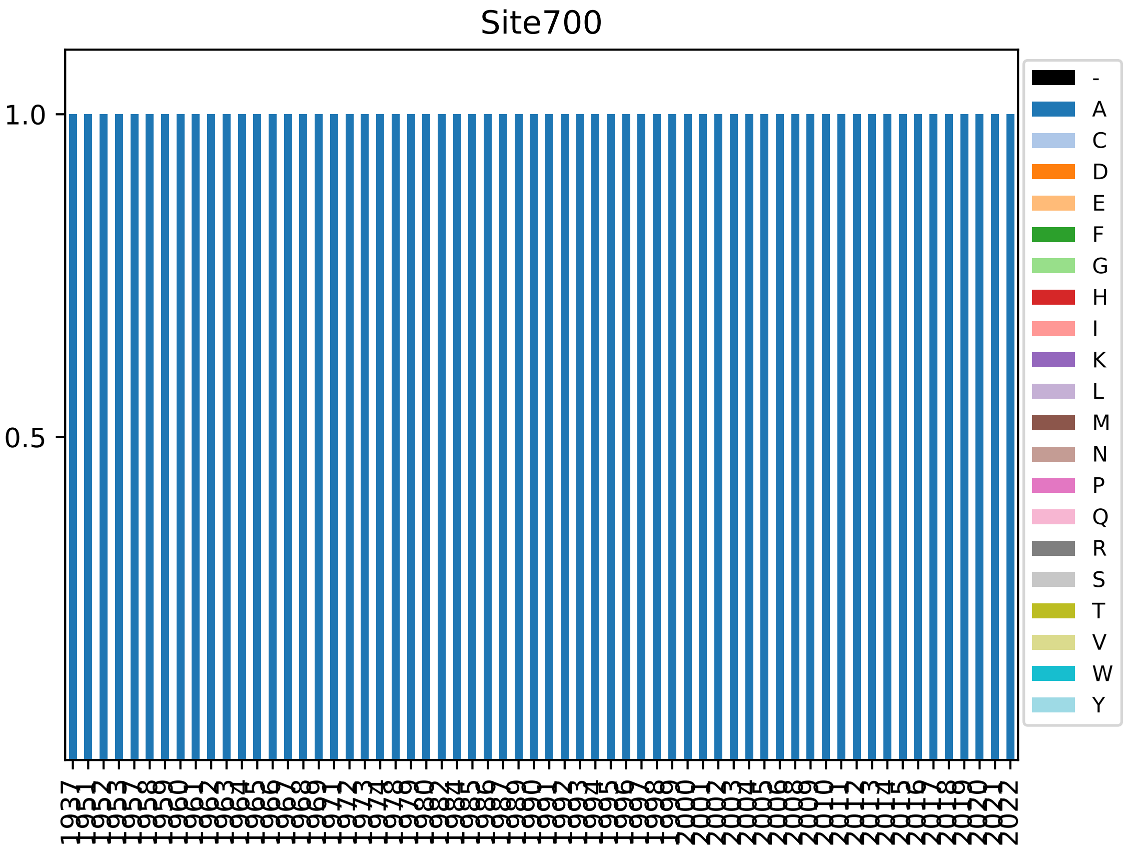
Site700
1.0
0.5
-
A
C
D
E
F
G
H
I
K
L
M
N
P
Q
R
S
T
V
W
Y

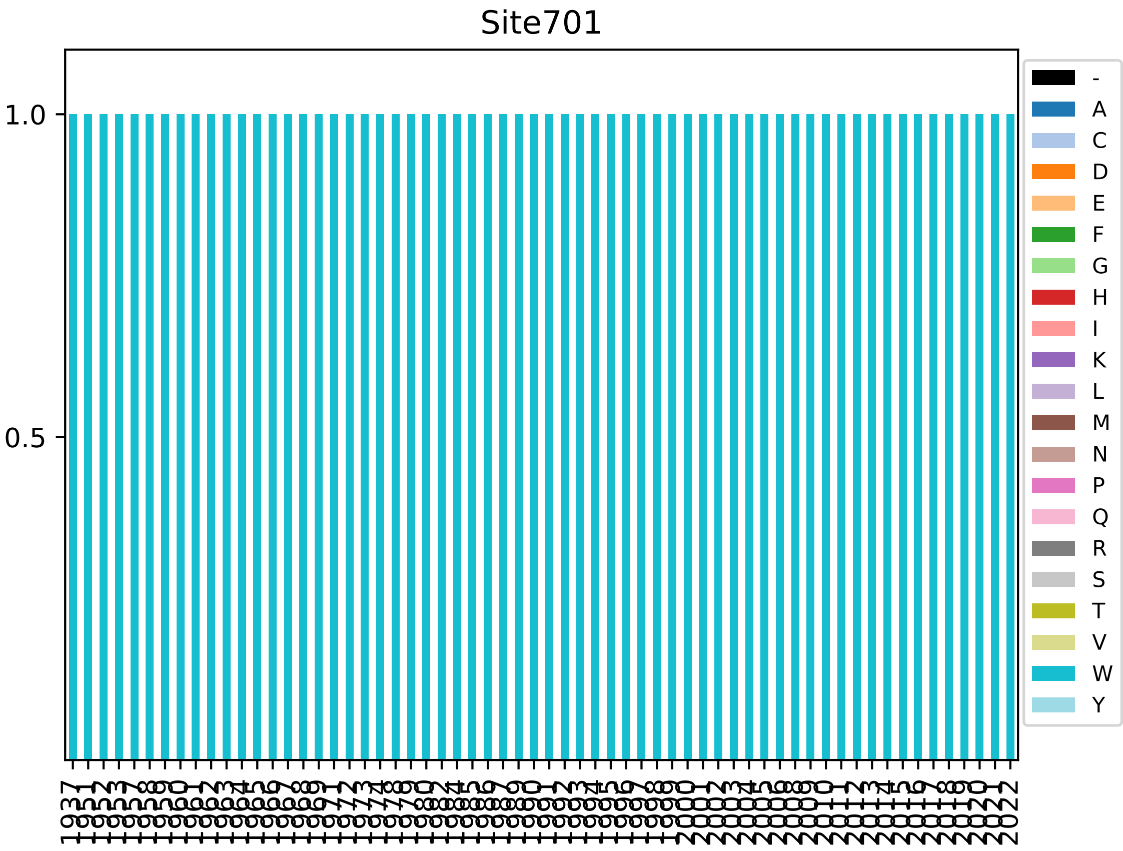
Site701
1.0
0.5
-
A
C
D
E
F
G
H
I
K
L
M
N
P
Q
R
S
T
V
W
Y

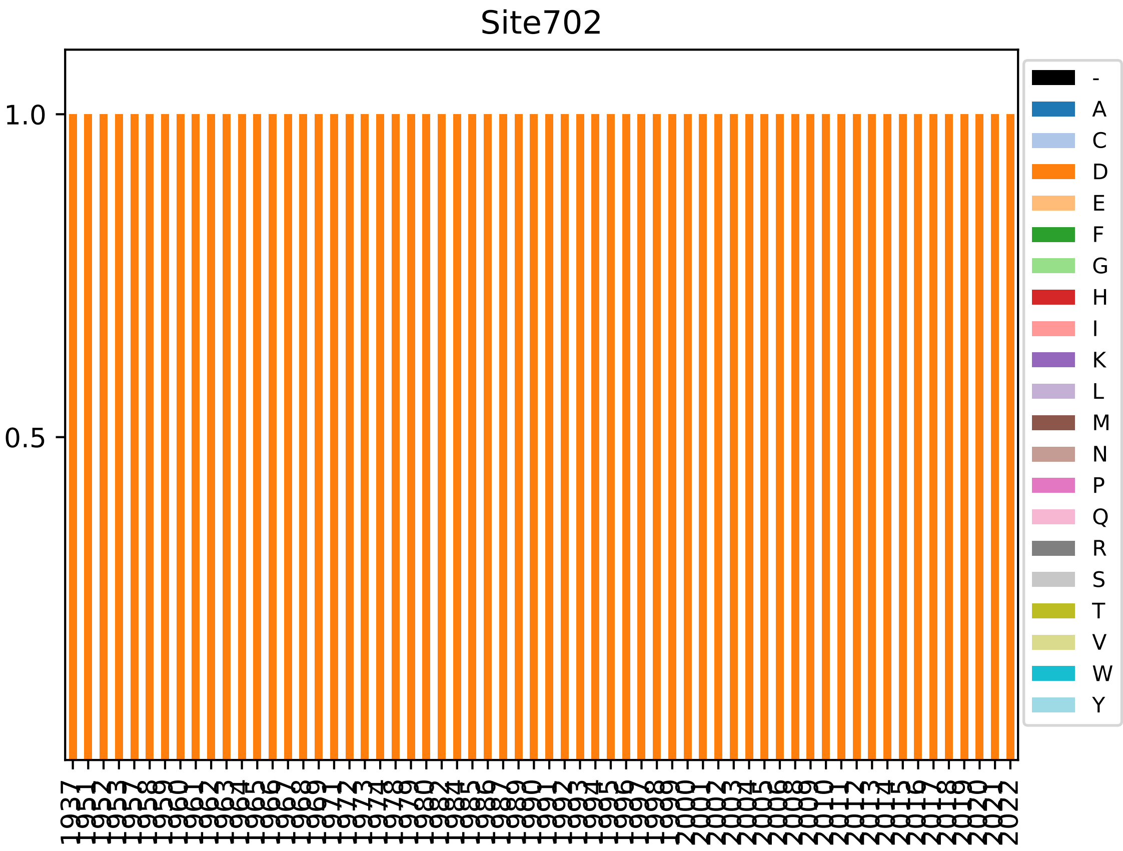
Site702
1.0
0.5
-
A
C
D
E
F
G
H
I
K
L
M
N
P
Q
R
S
T
V
W
Y

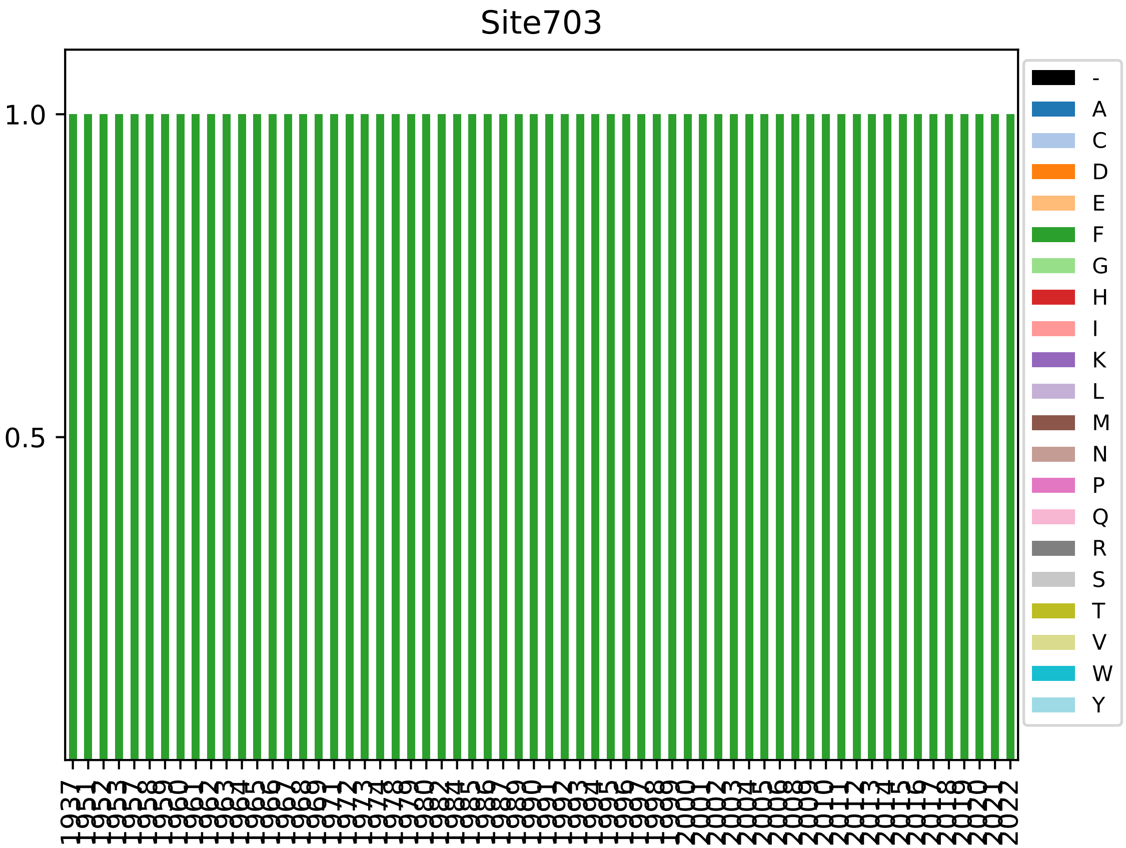
Site703
1.0
0.5
-
A
C
D
E
F
G
H
I
K
L
M
N
P
Q
R
S
T
V
W
Y

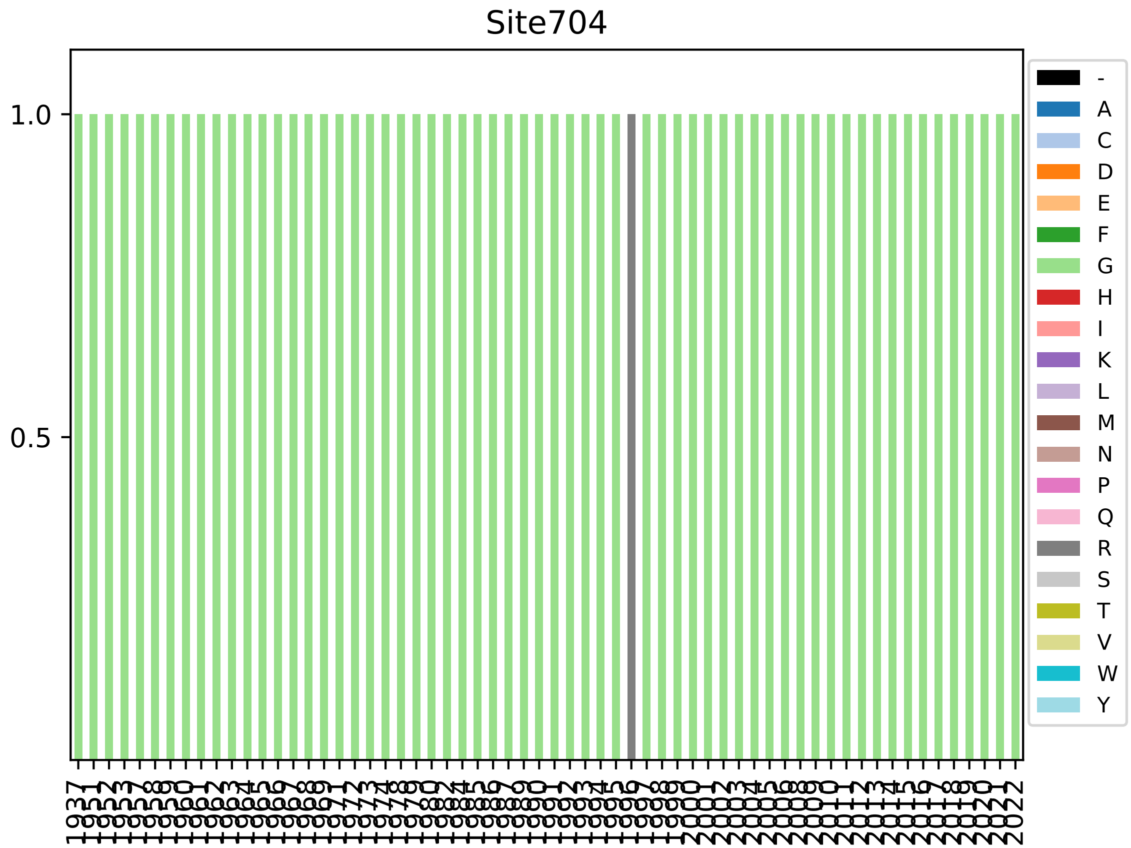
Site704
1.0
0.5
-
A
C
D
E
F
G
H
I
K
L
M
N
P
Q
R
S
T
V
W
Y

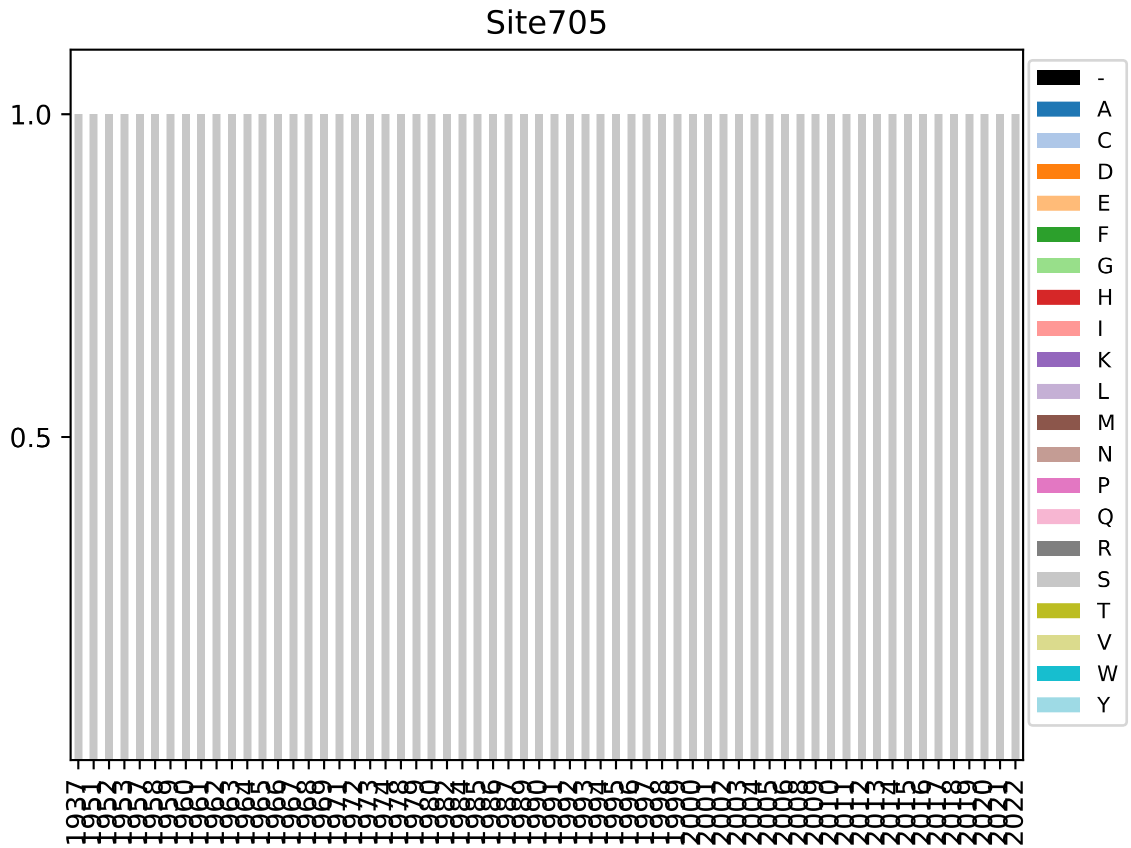

Site705
1.0
0.5
-
A
C
D
E
F
G
H
I
K
L
M
N
P
Q
R
S
T
V
W
Y

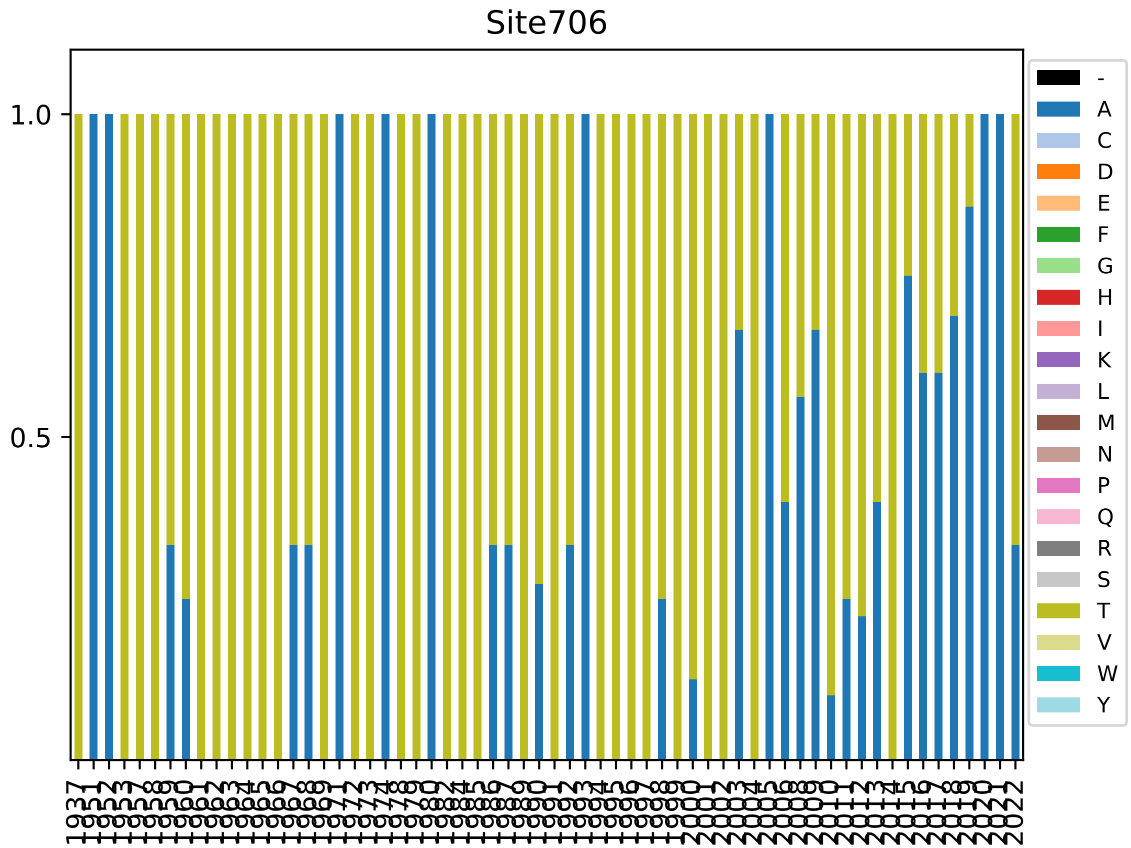

Site706
1.0
0.5
-
A
C
D
E
F
G
H
I
K
L
M
N
P
Q
R
S
T
V
W
Y

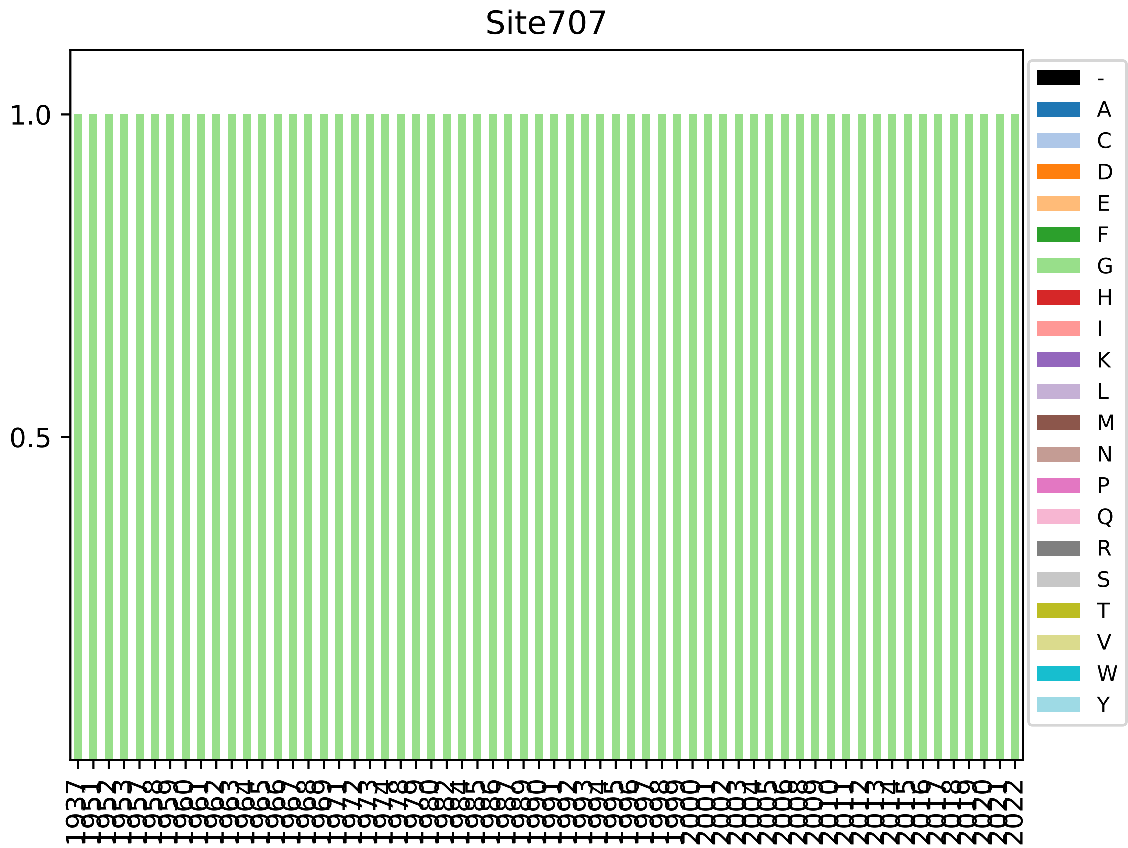
Site707
1.0
0.5
-
A
C
D
E
F
G
H
I
K
L
M
N
P
Q
R
S
T
V
W
Y

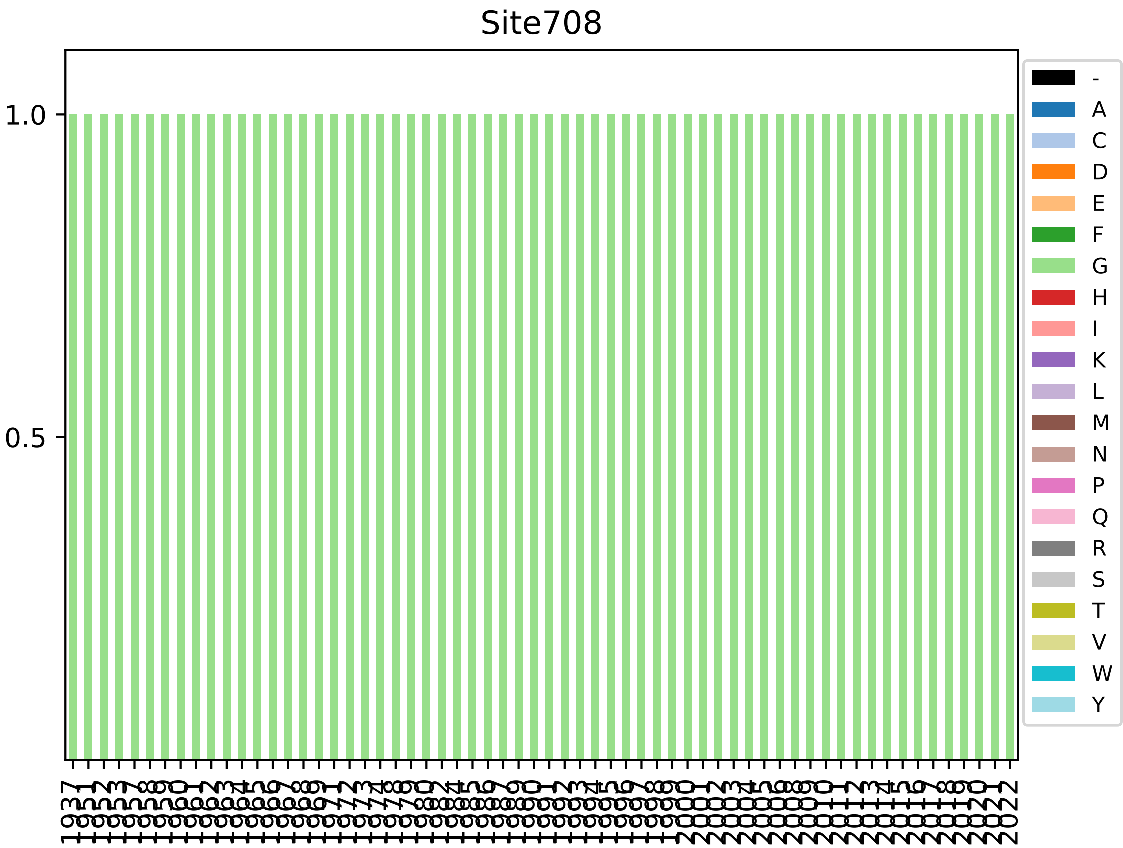
Site708
1.0
0.5
-
A
C
D
E
F
G
H
I
K
L
M
N
P
Q
R
S
T
V
W
Y

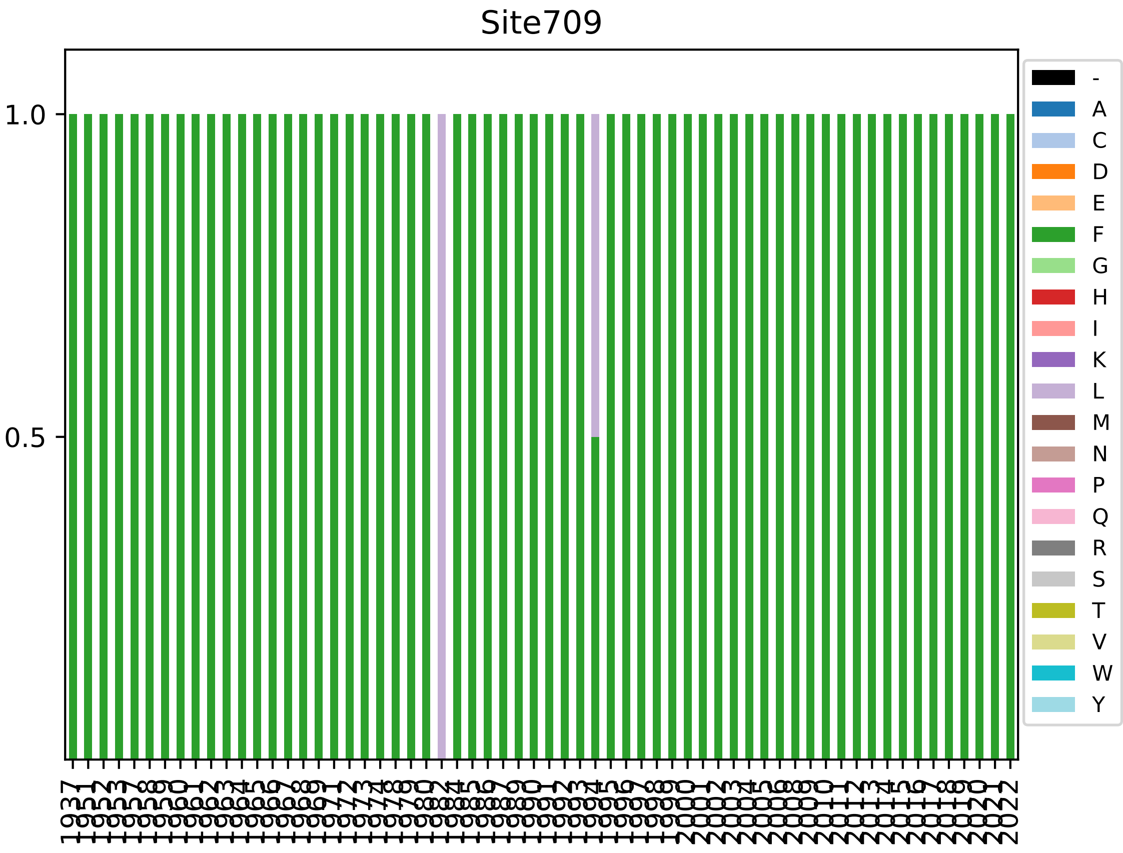
Site709
1.0
0.5
-
A
C
D
E
F
G
H
I
K
L
M
N
P
Q
R
S
T
V
W
Y

Site710

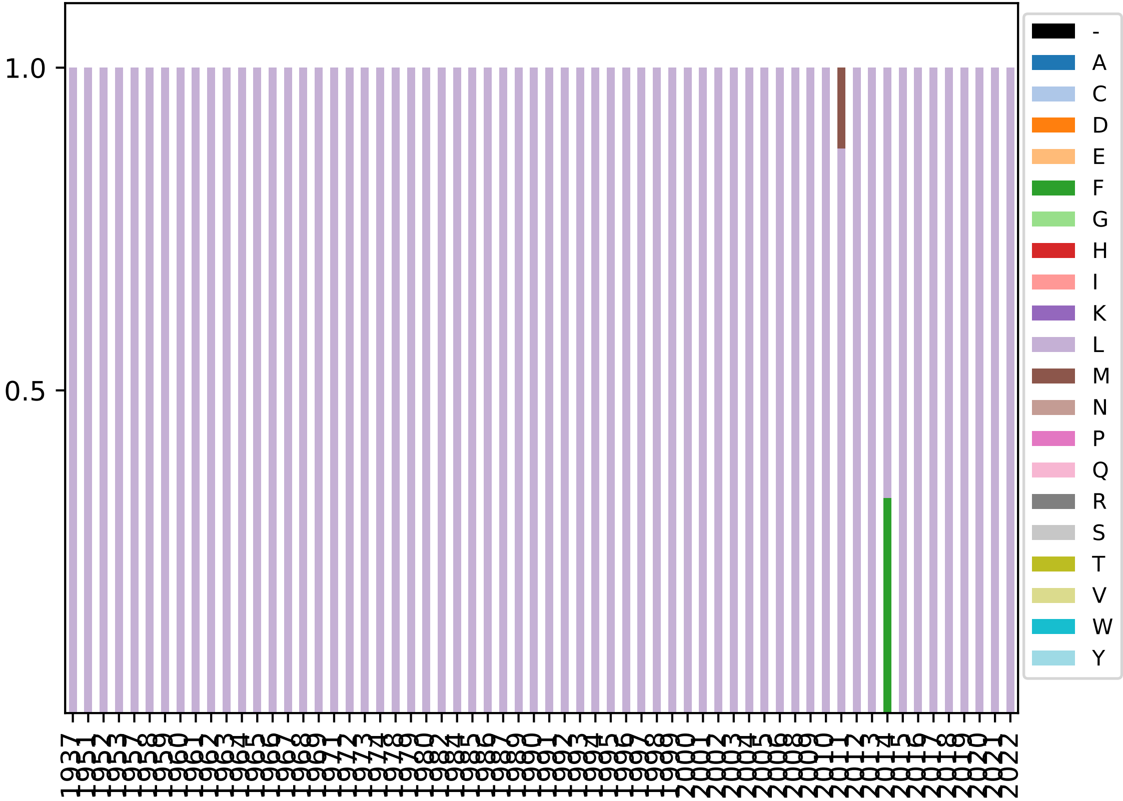

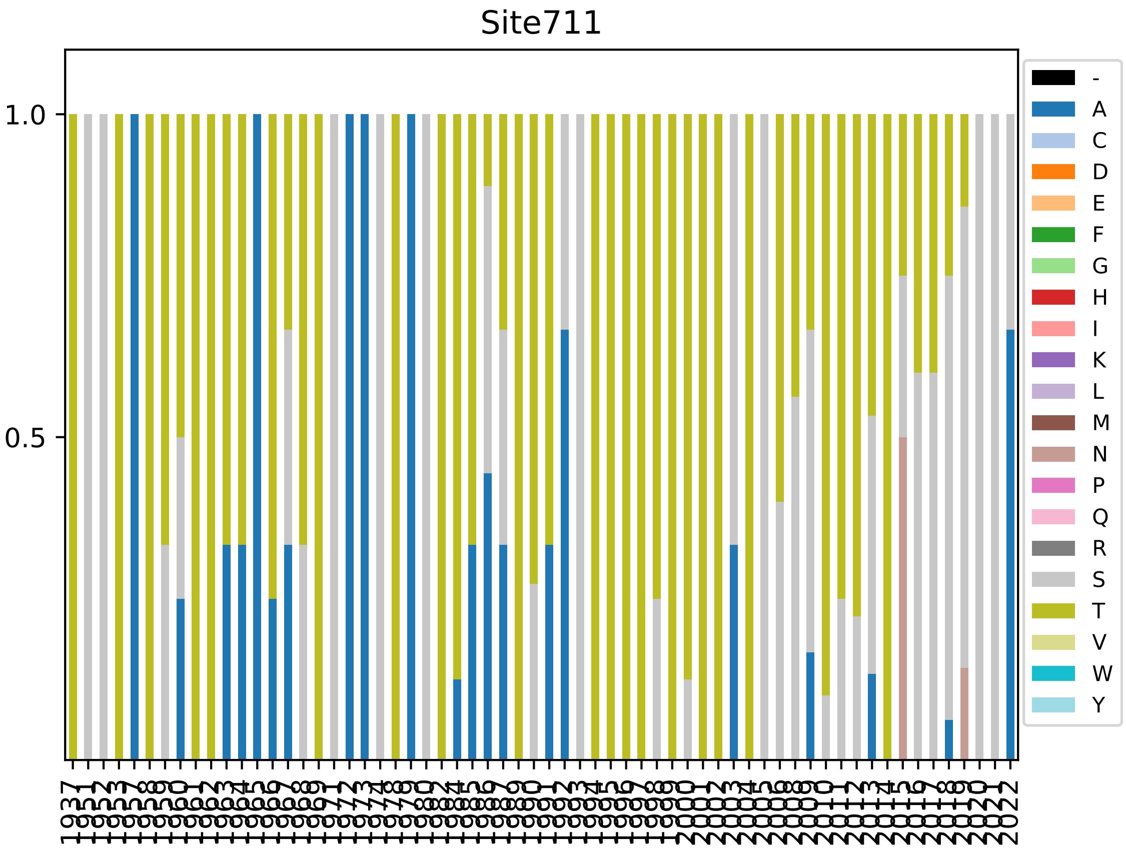
Site711
1.0
0.5
-
A
C
D
E
F
G
H
I
K
L
M
N
P
Q
R
S
T
V
W
Y

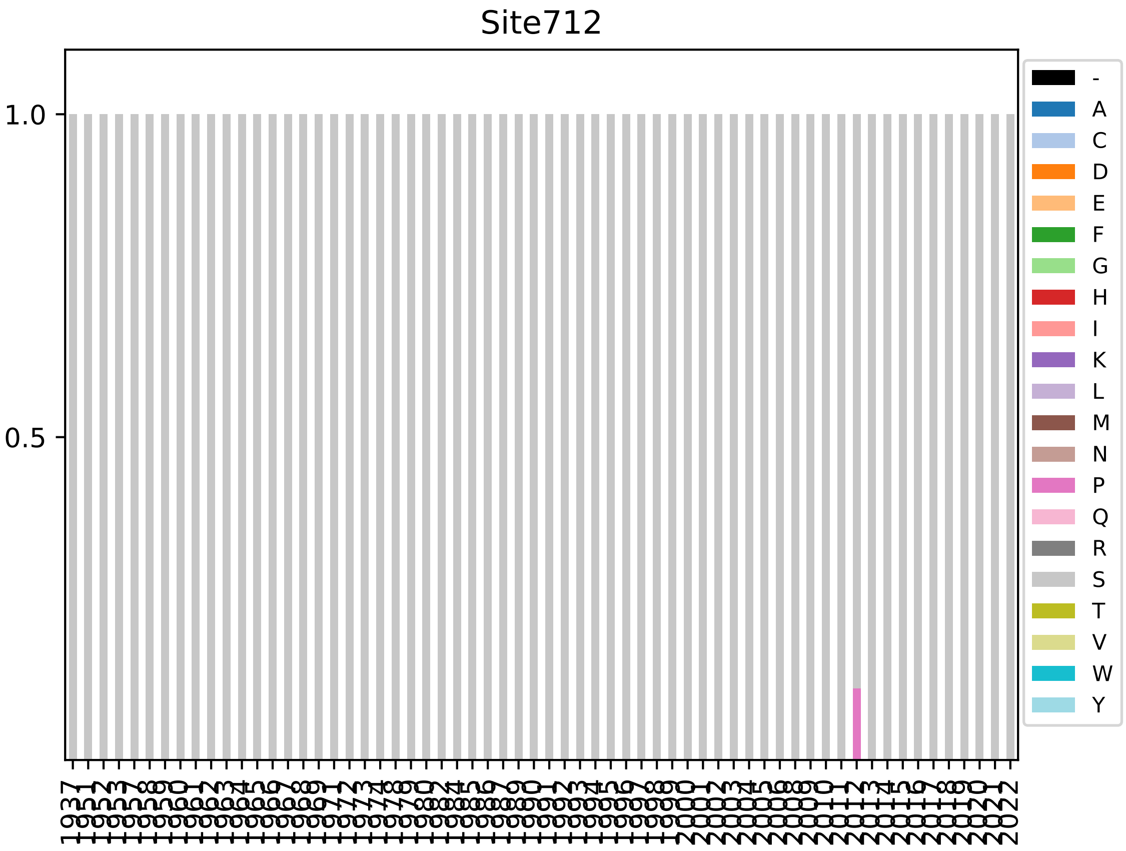
Site712
1.0
0.5
-
A
C
D
E
F
G
H
I
K
L
M
N
P
Q
R
S
T
V
W
Y

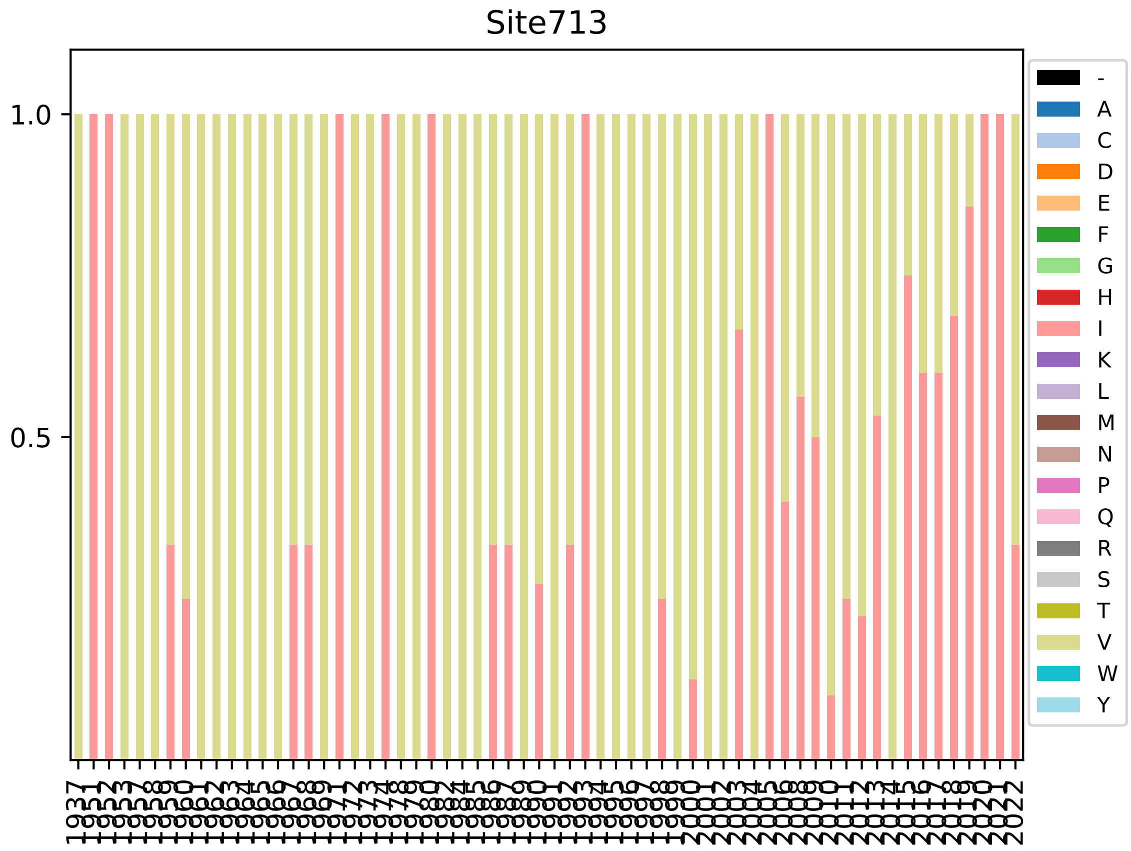
Site713
1.0
0.5
-
A
C
D
E
F
G
H
I
K
L
M
N
P
Q
R
S
T
V
W
Y

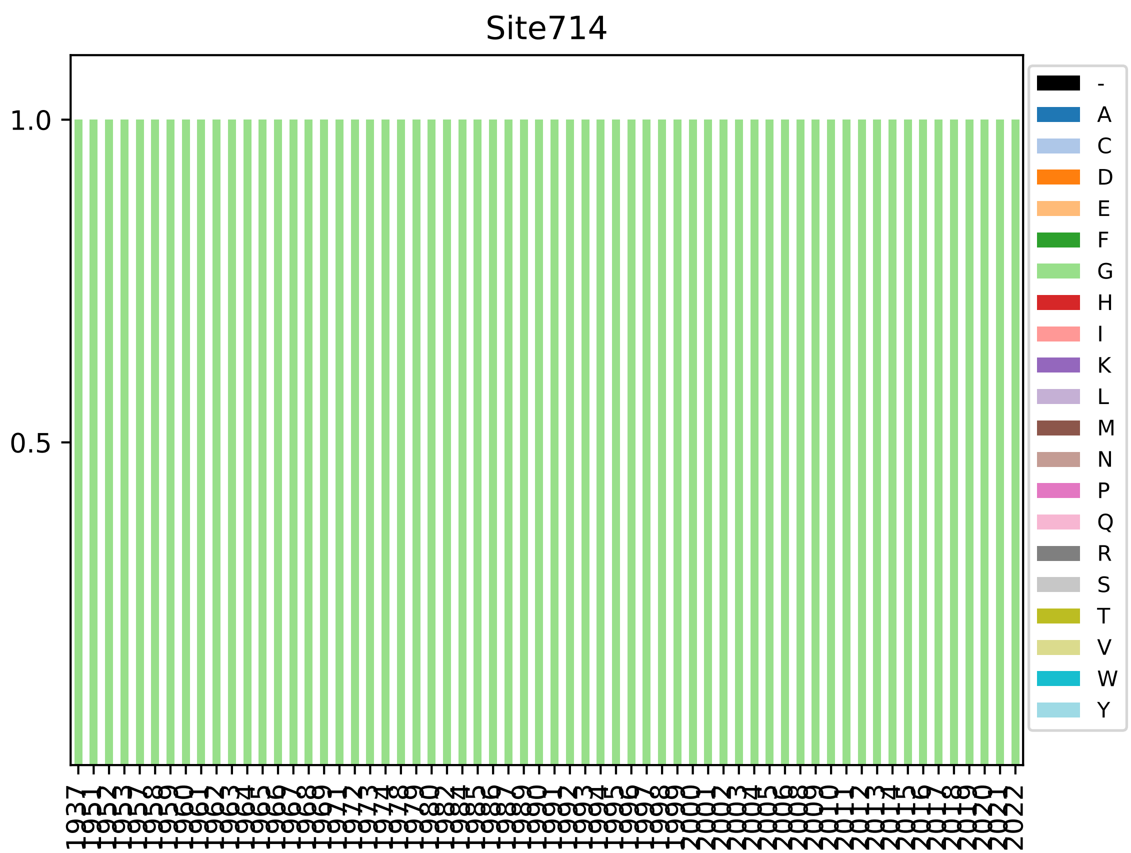
Site714
1.0
0.5
-
A
C
D
E
F
G
H
I
K
L
M
N
P
Q
R
S
T
V
W
Y

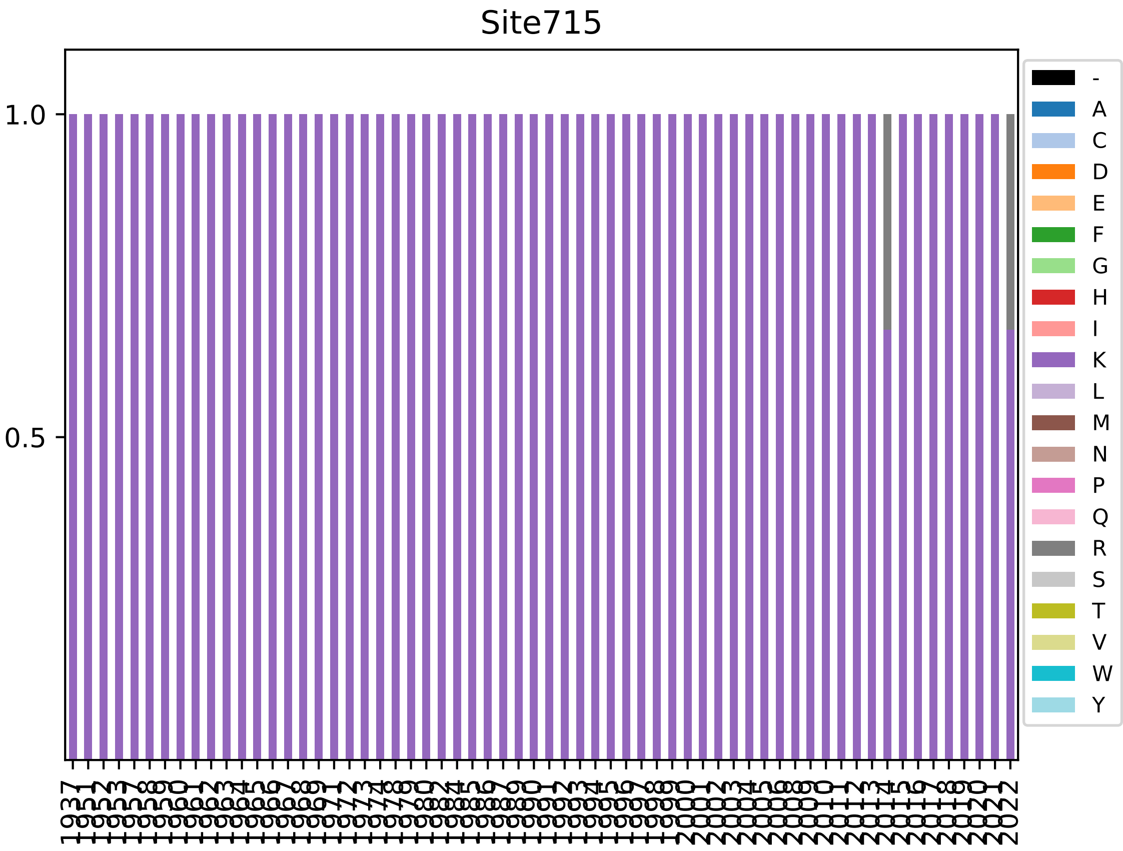
Site715
1.0
0.5
-
A
C
D
E
F
G
H
I
K
L
M
N
P
Q
R
S
T
V
W
Y

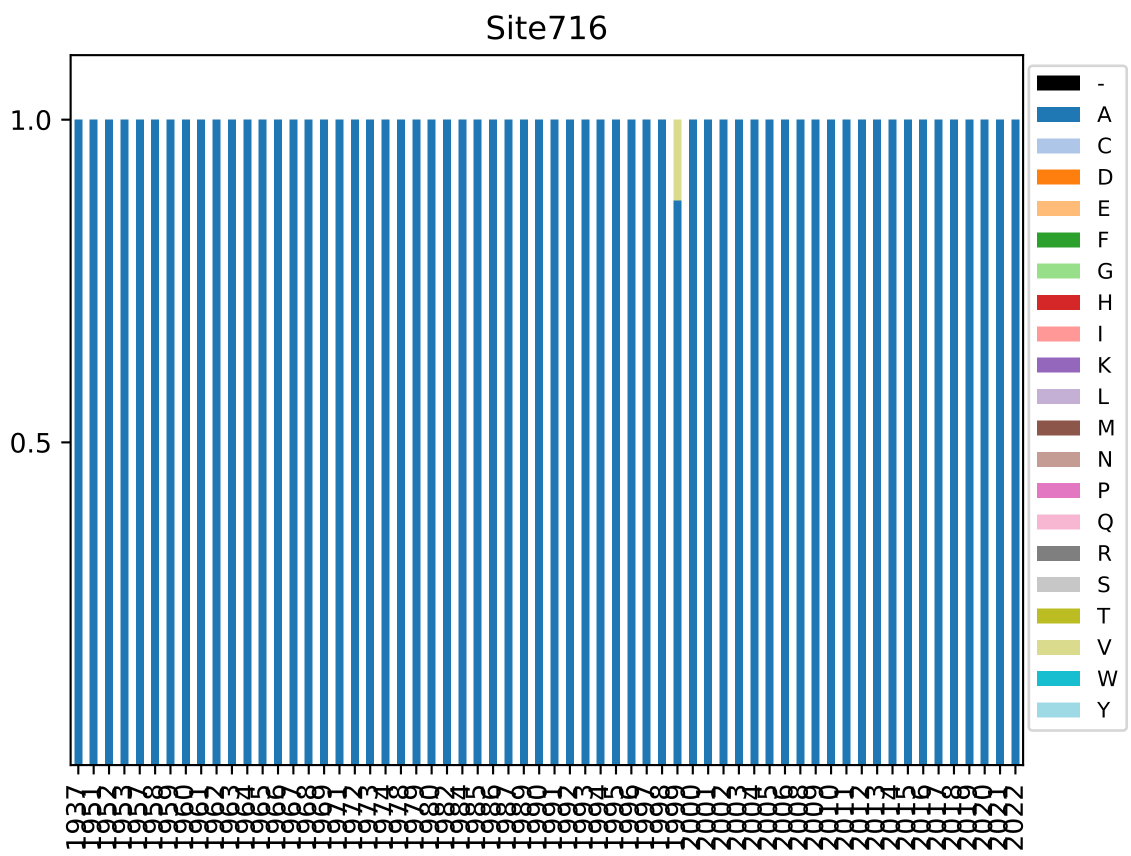
Site716
1.0
0.5
-
A
C
D
E
F
G
H
I
K
L
M
N
P
Q
R
S
T
V
W
Y

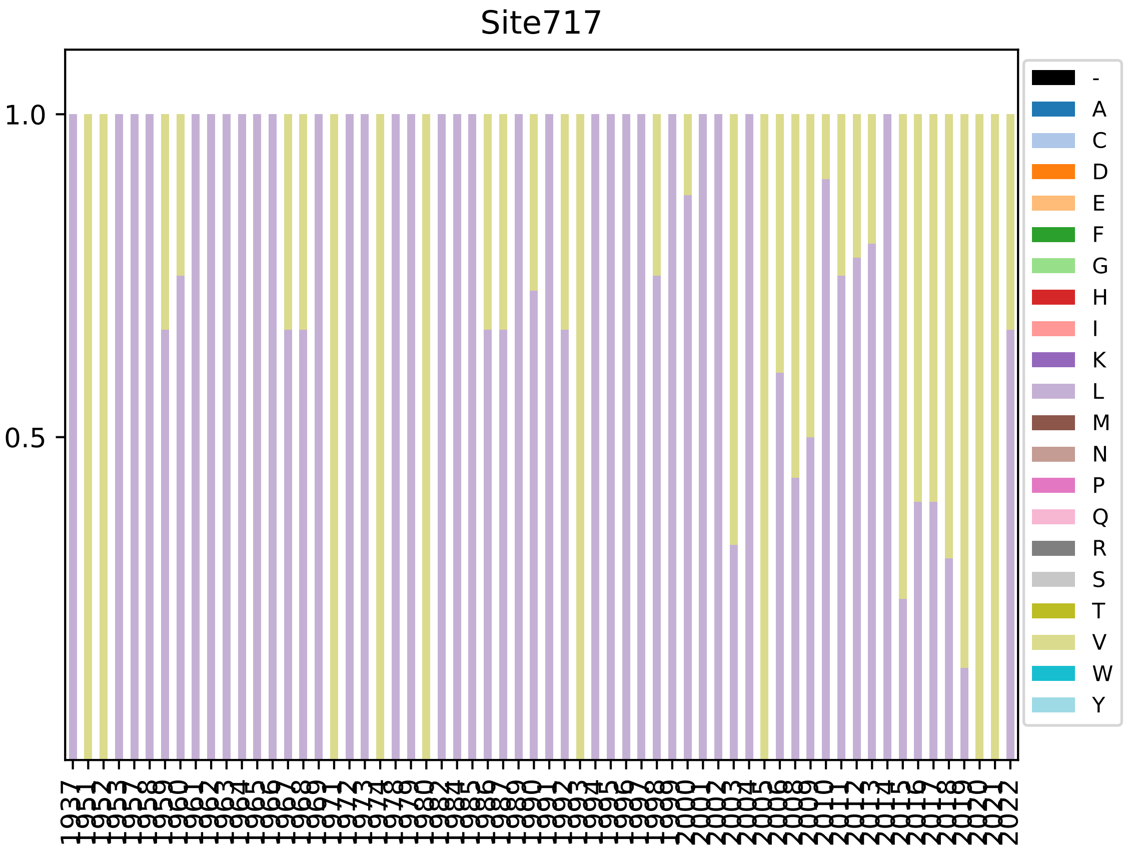

Site717
1.0
0.5
-
A
C
D
E
F
G
H
I
K
L
M
N
P
Q
R
S
T
V
W
Y

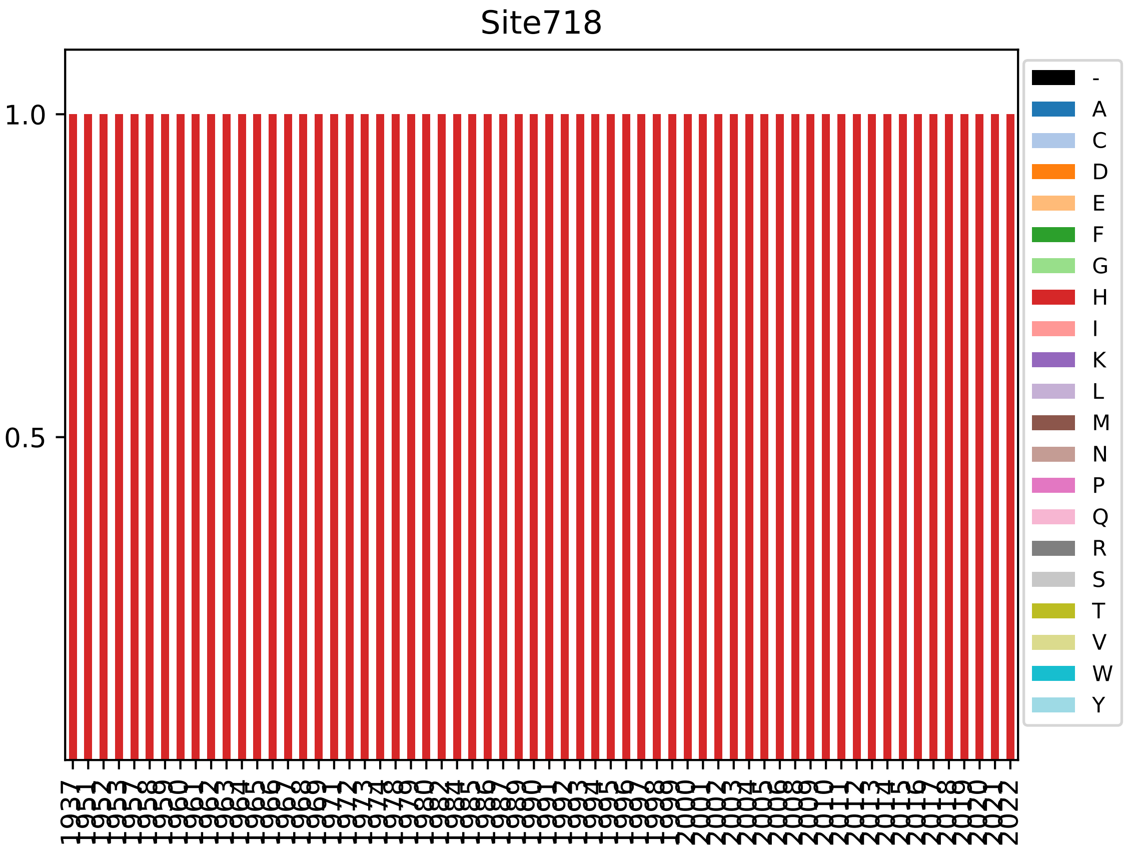
Site718
1.0
0.5
-
A
C
D
E
F
G
H
I
K
L
M
N
P
Q
R
S
T
V
W
Y

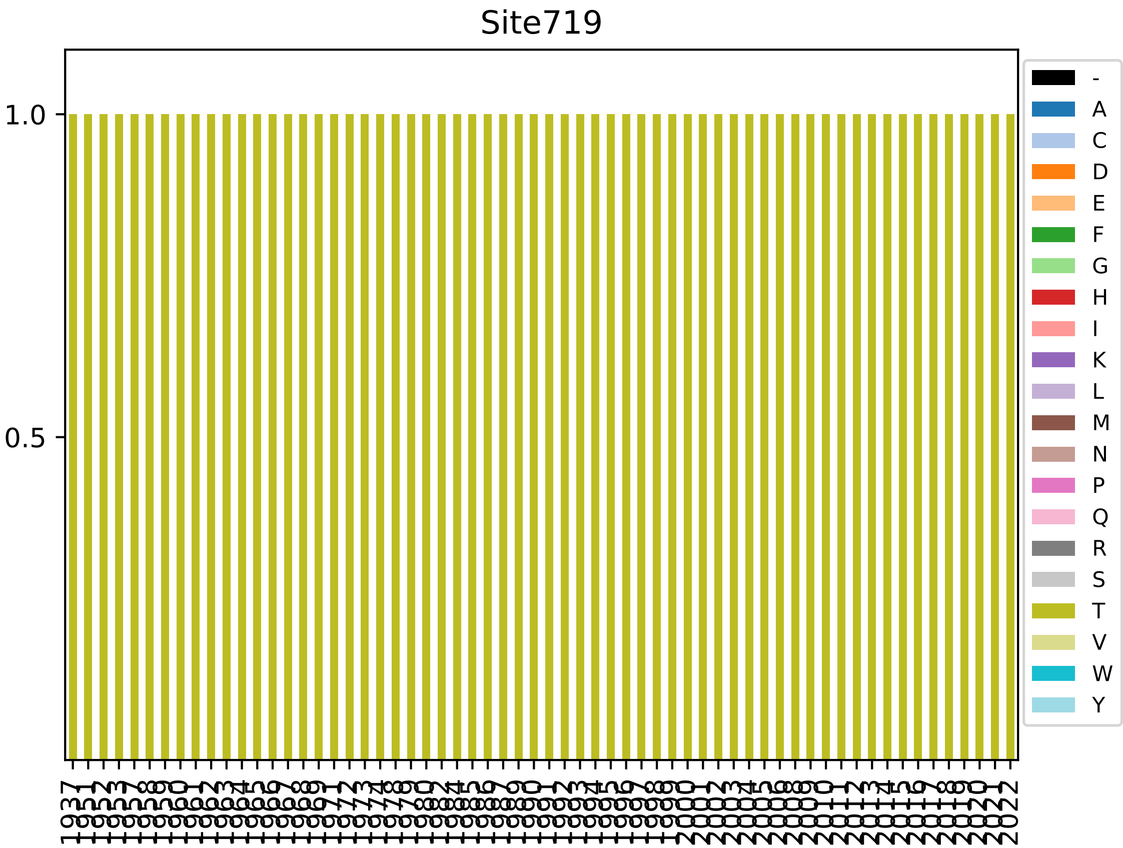
Site719
1.0
0.5
-
A
C
D
E
F
G
H
I
K
L
M
N
P
Q
R
S
T
V
W
Y

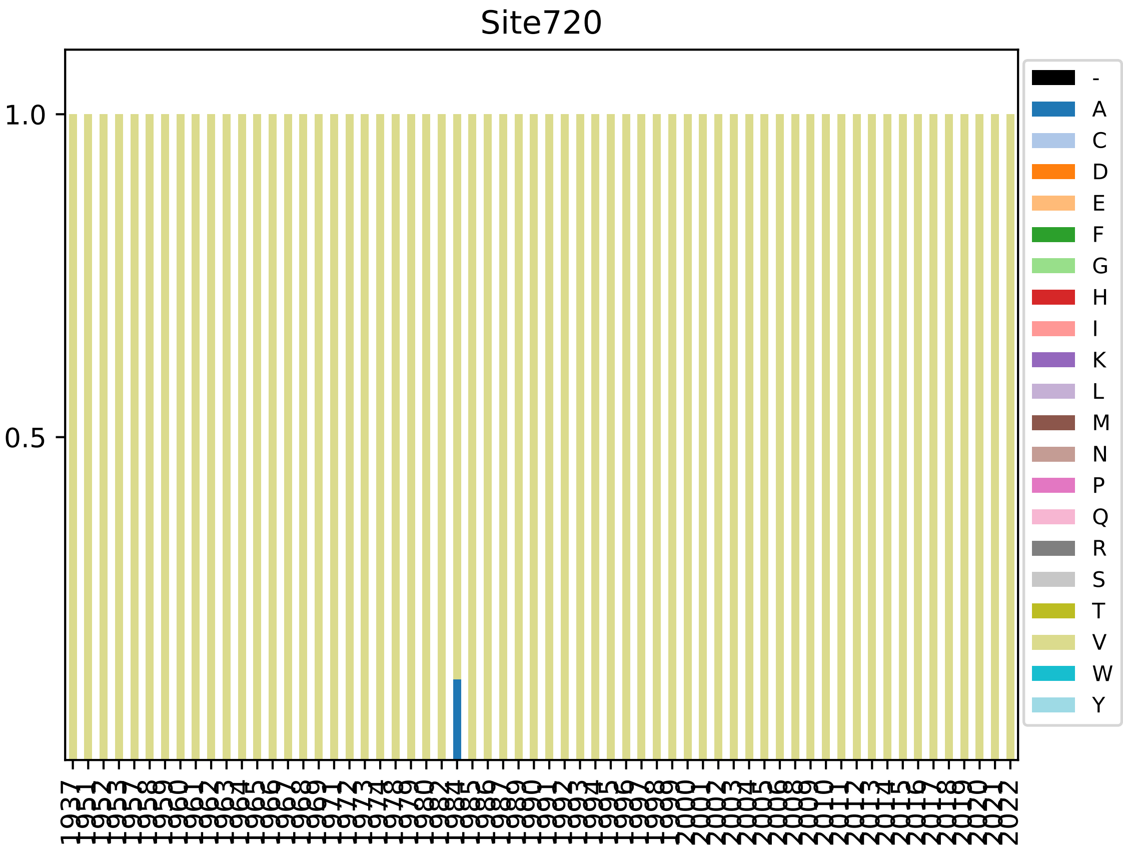
Site720
1.0
0.5
-
A
C
D
E
F
G
H
I
K
L
M
N
P
Q
R
S
T
V
W
Y

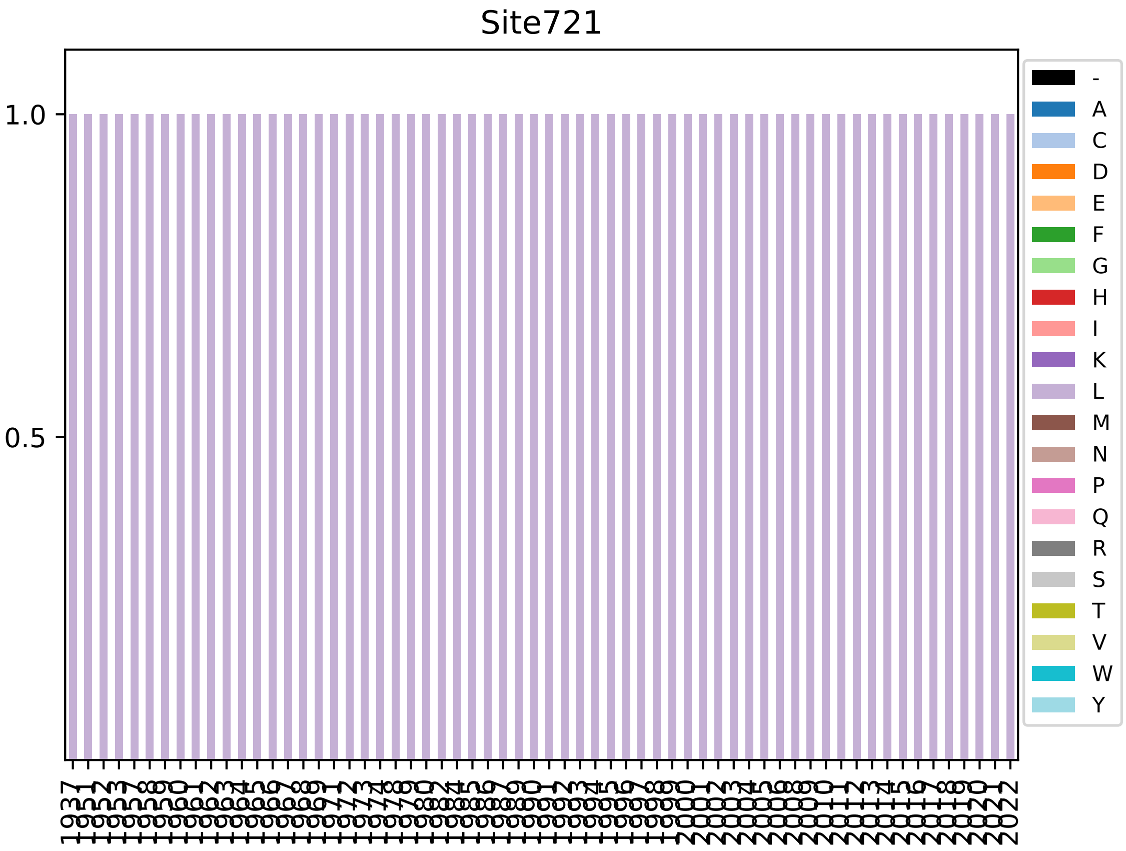
Site721
1.0
0.5
-
A
C
D
E
F
G
H
I
K
L
M
N
P
Q
R
S
T
V
W
Y

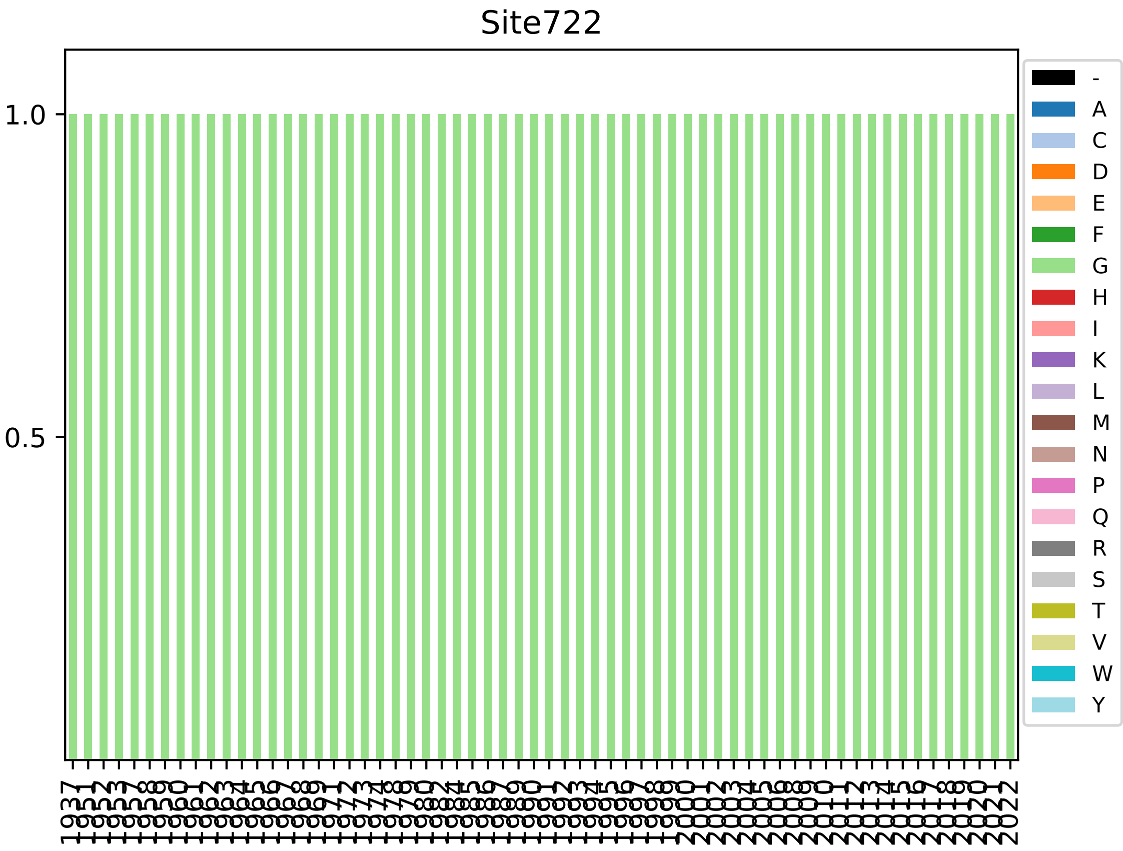
Site722
1.0
0.5
-
A
C
D
E
F
G
H
I
K
L
M
N
P
Q
R
S
T
V
W
Y

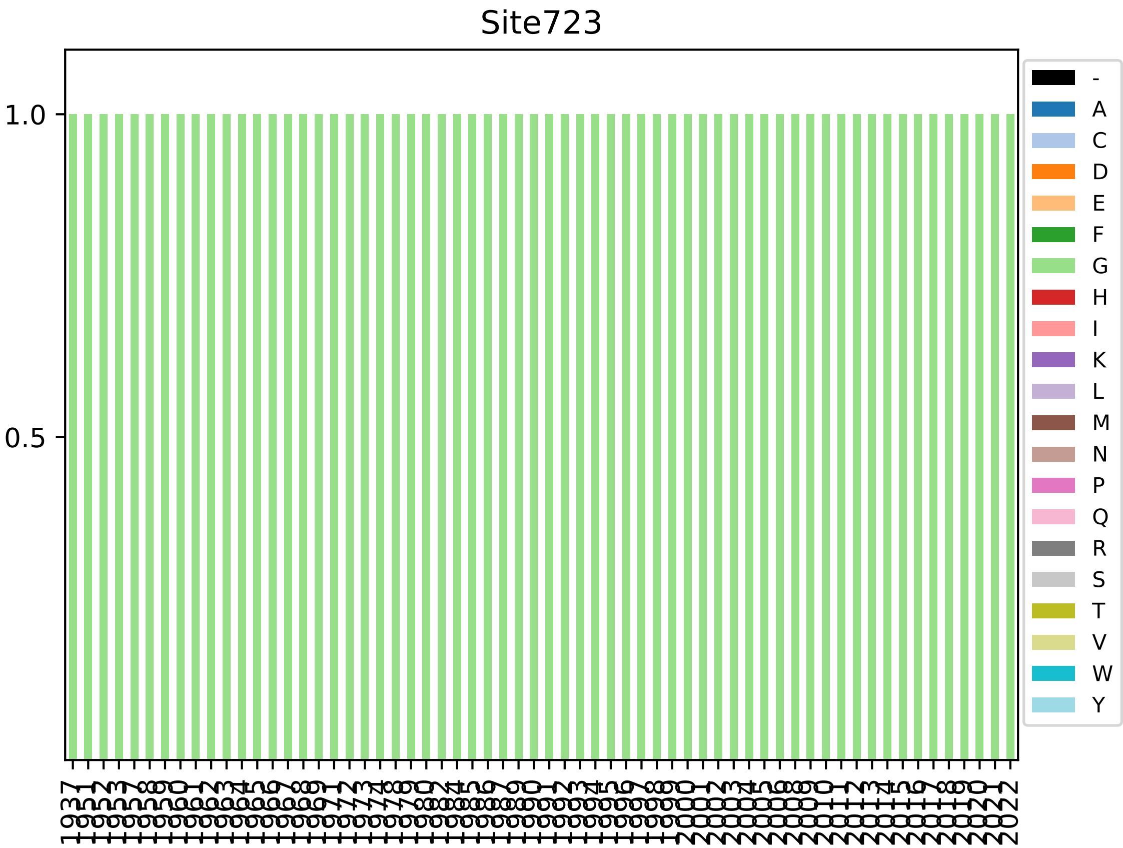
Site723
1.0
0.5
-
A
C
D
E
F
G
H
I
K
L
M
N
P
Q
R
S
T
V
W
Y

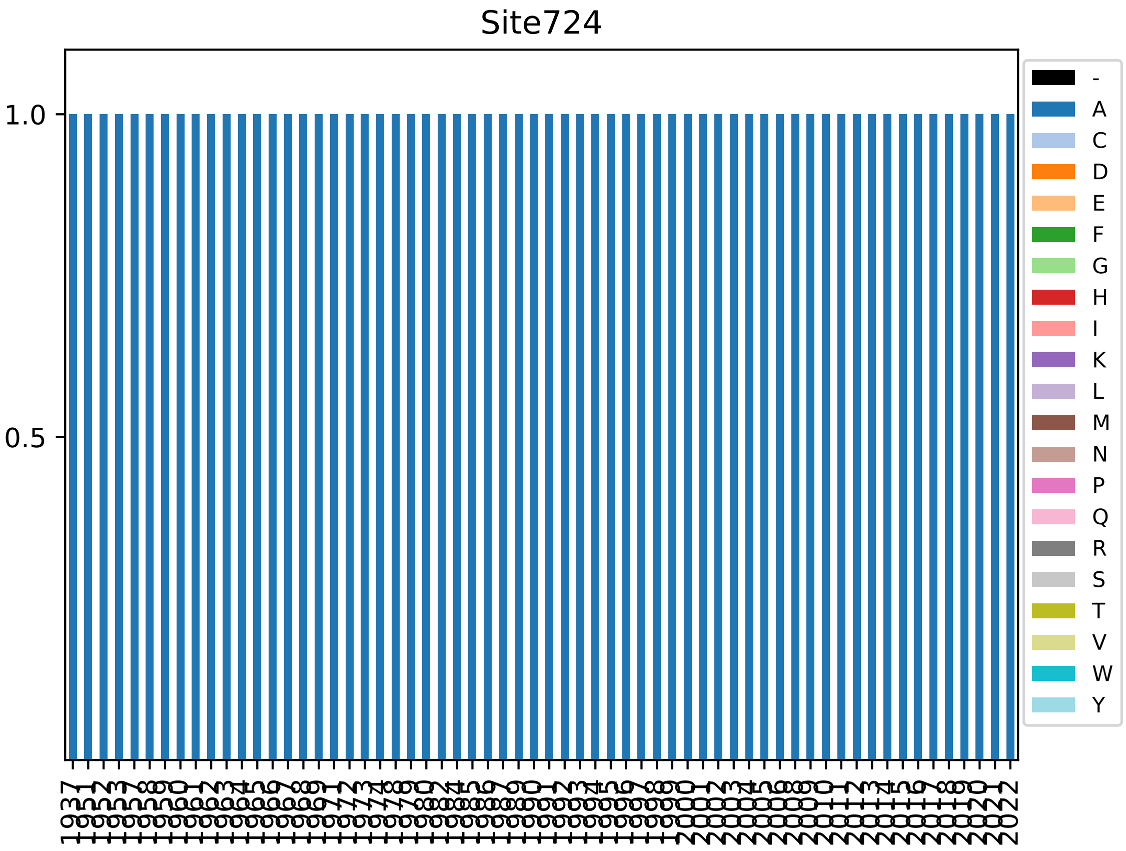
Site724
1.0
0.5
-
A
C
D
E
F
G
H
I
K
L
M
N
P
Q
R
S
T
V
W
Y

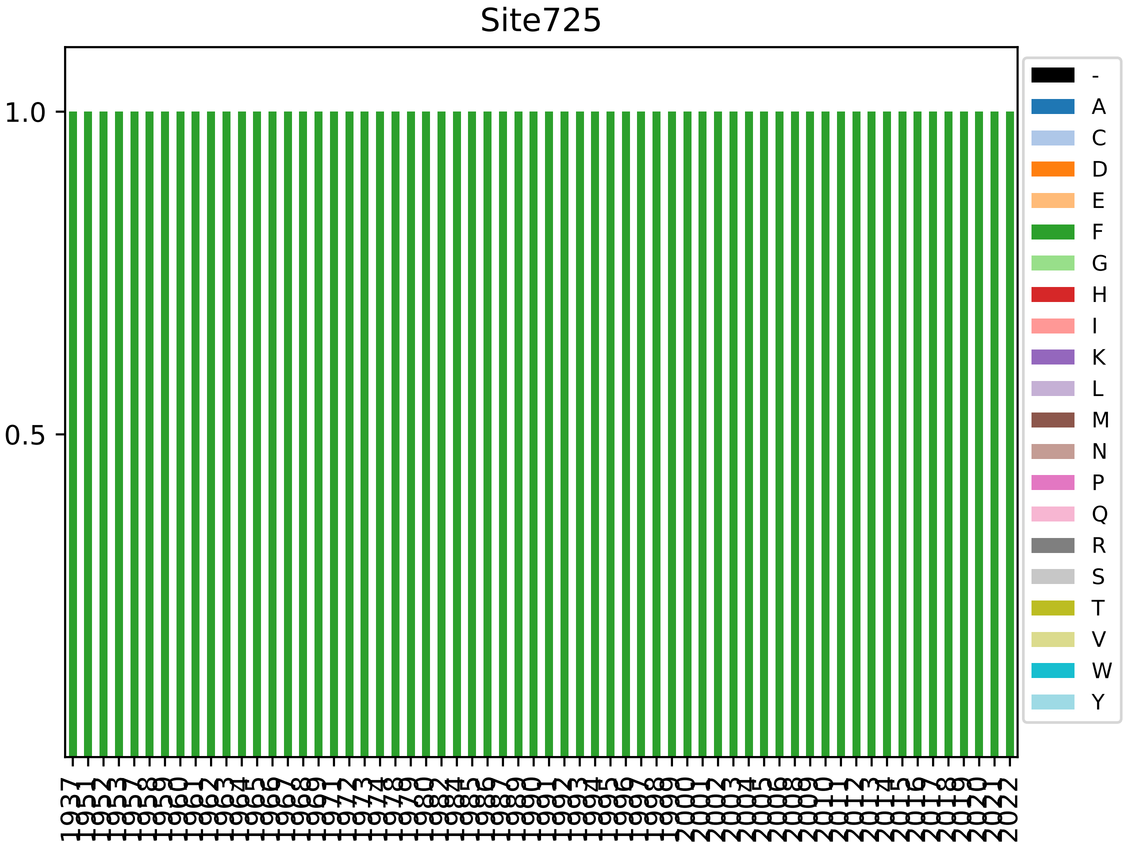
Site725
1.0
0.5
-
A
C
D
E
F
G
H
I
K
L
M
N
P
Q
R
S
T
V
W
Y

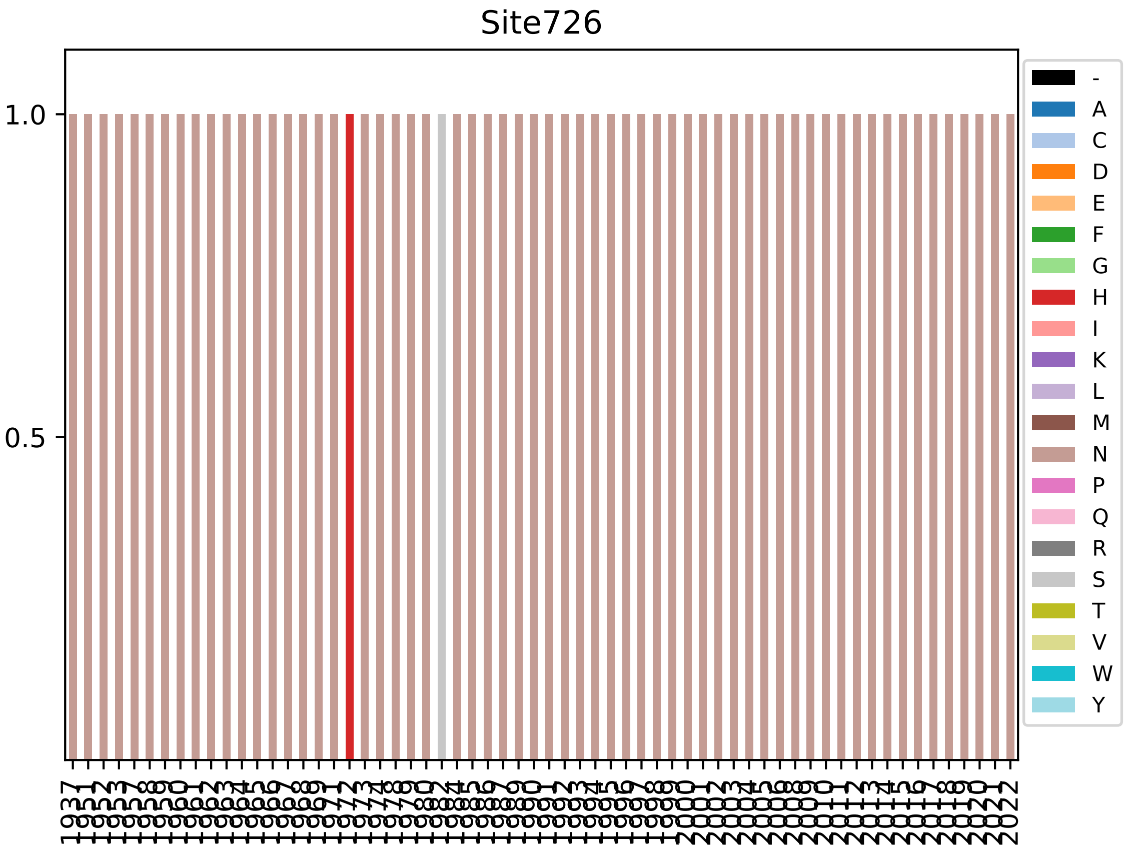
Site726
1.0
0.5
-
A
C
D
E
F
G
H
I
K
L
M
N
P
Q
R
S
T
V
W
Y

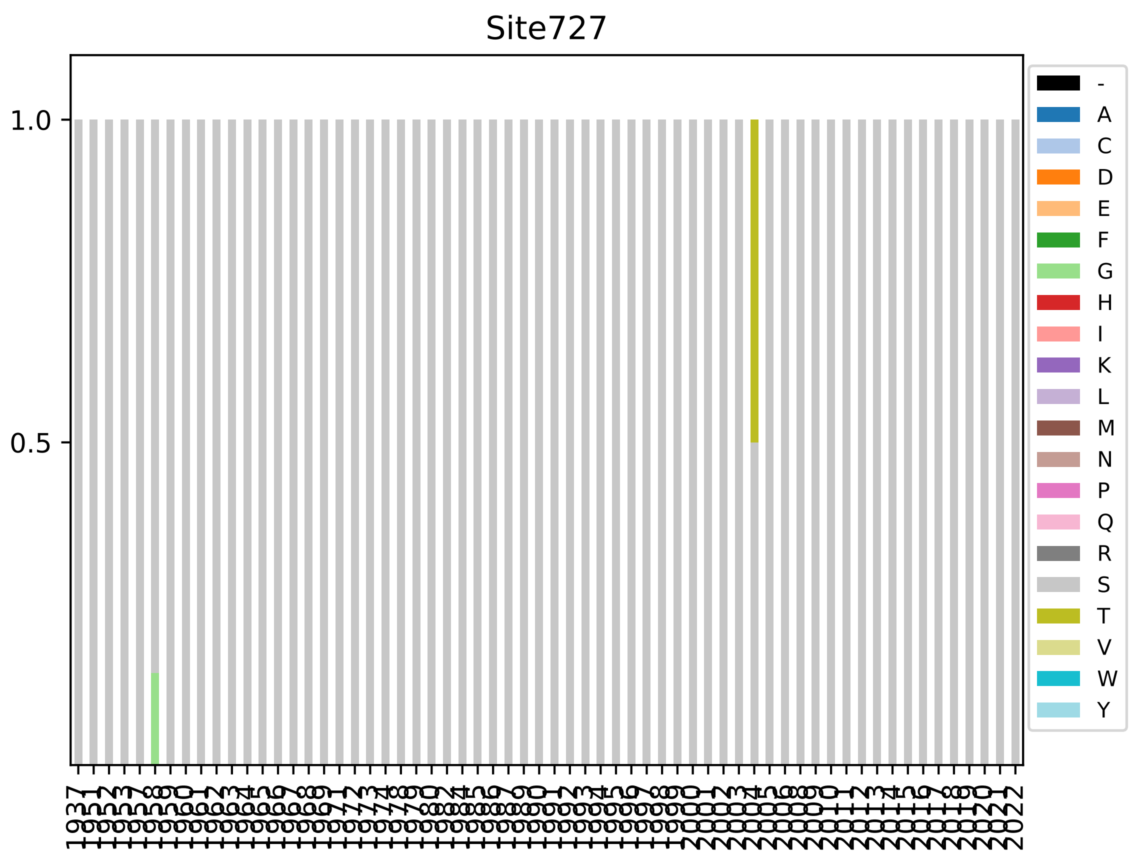

Site727
1.0
0.5
-
A
C
D
E
F
G
H
I
K
L
M
N
P
Q
R
S
T
V
W
Y

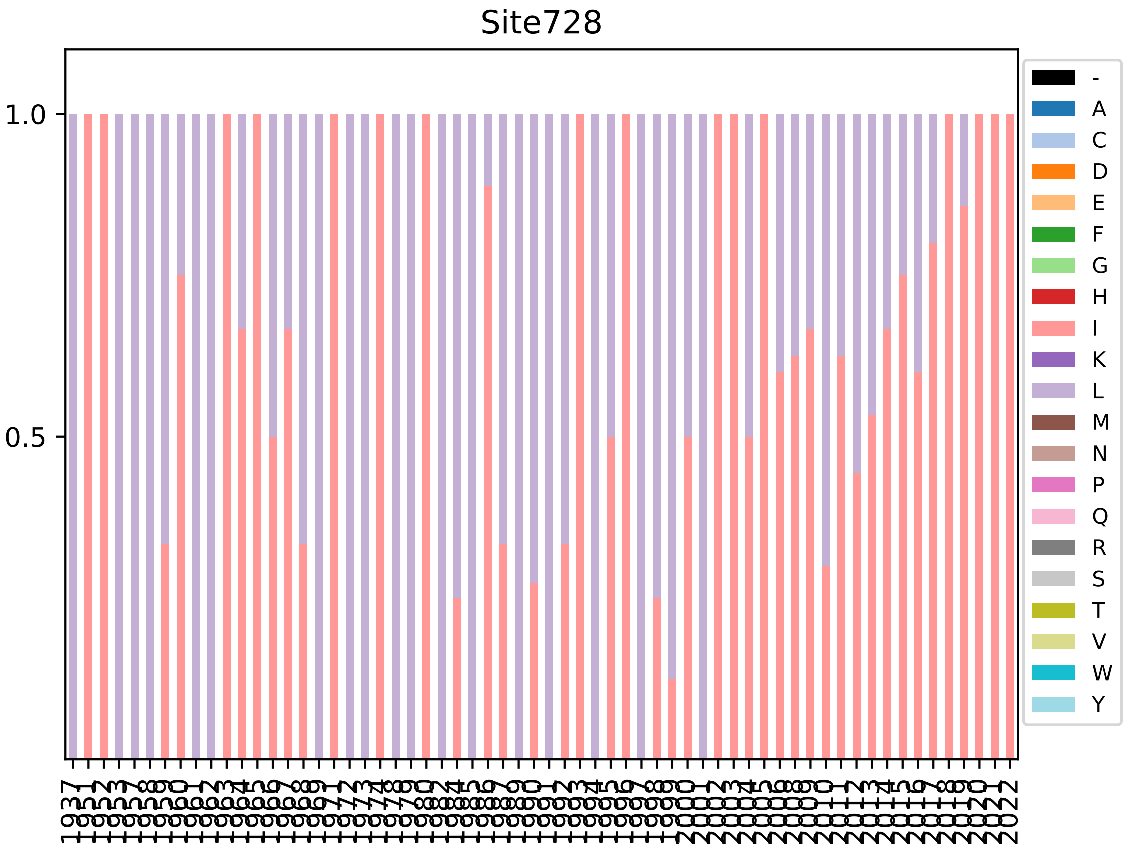
Site728
1.0
0.5
-
A
C
D
E
F
G
H
I
K
L
M
N
P
Q
R
S
T
V
W
Y

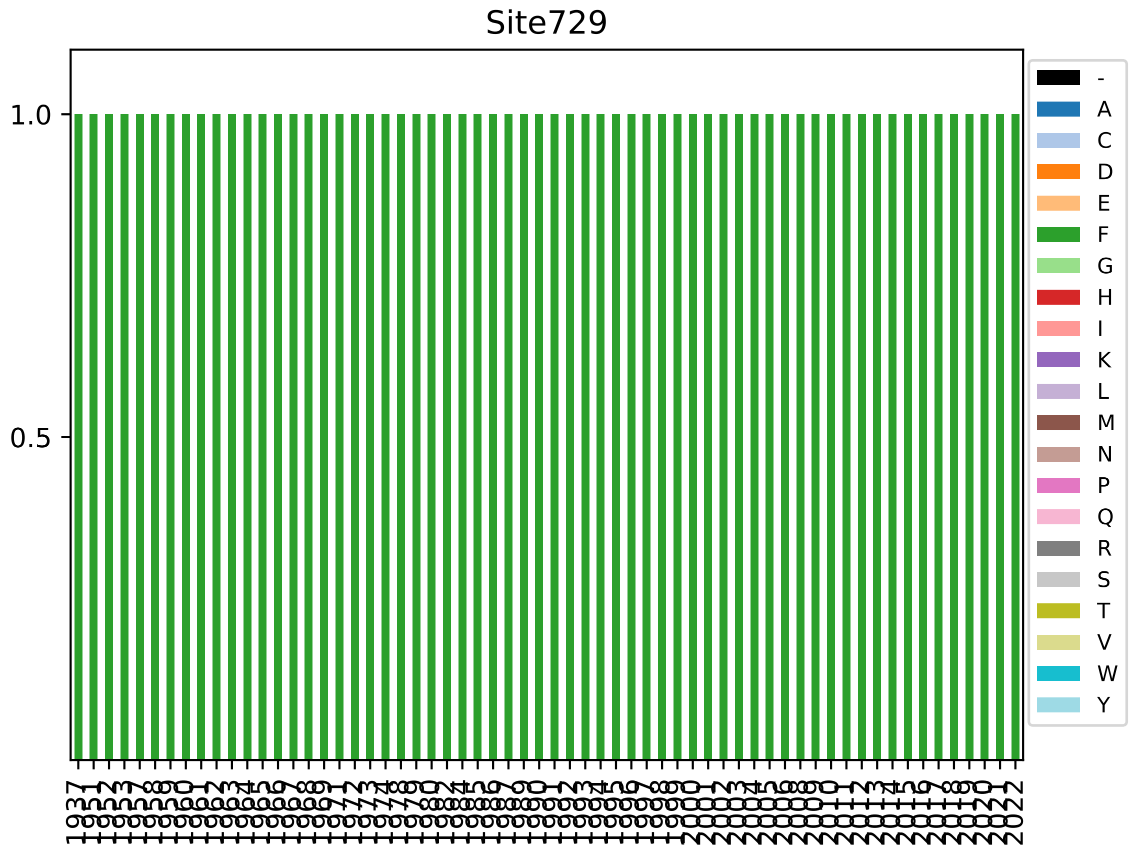
Site729
1.0
0.5
-
A
C
D
E
F
G
H
I
K
L
M
N
P
Q
R
S
T
V
W
Y

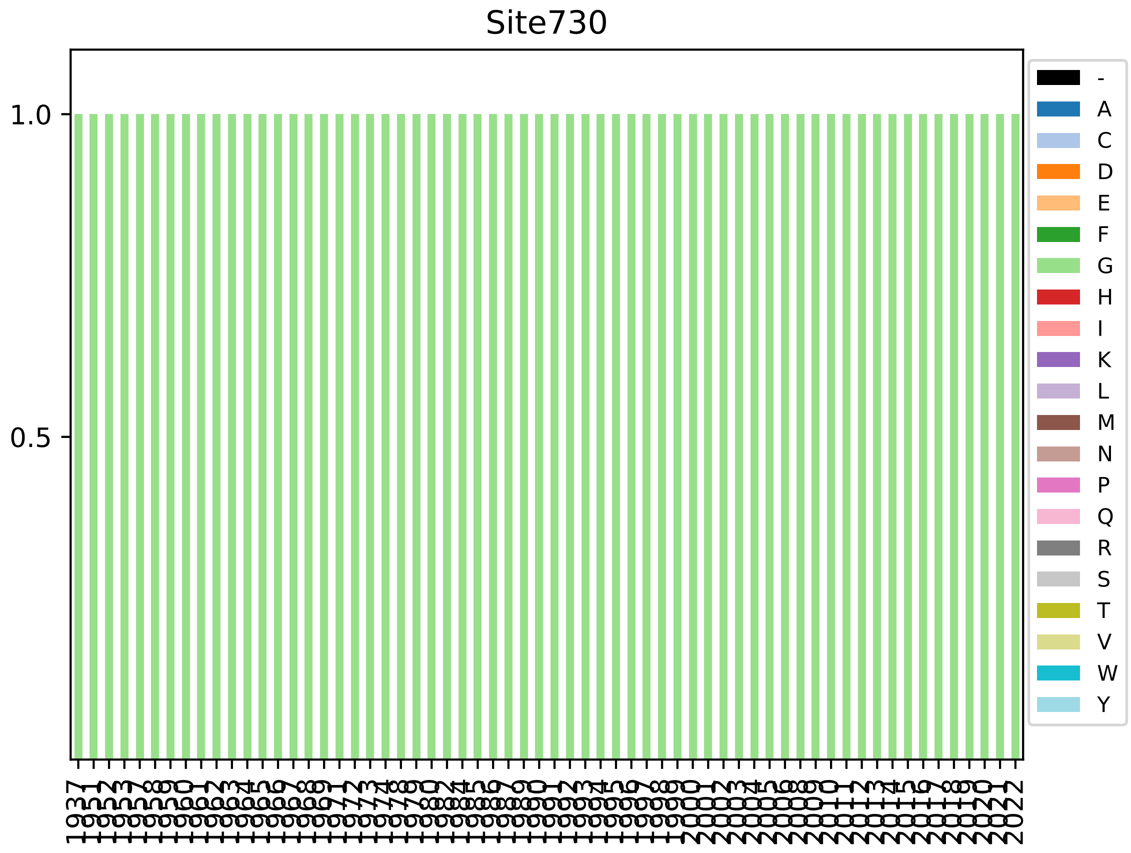
Site730
1.0
0.5
-
A
C
D
E
F
G
H
I
K
L
M
N
P
Q
R
S
T
V
W
Y

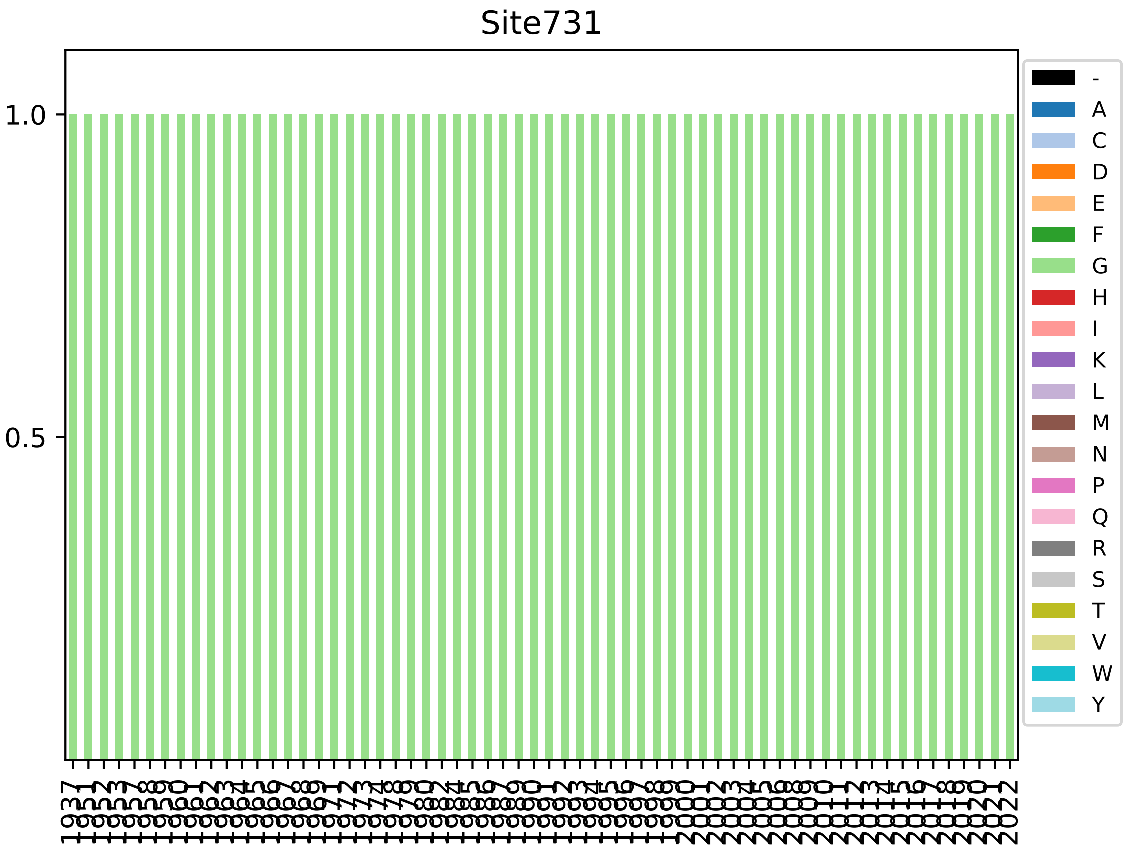
Site731
1.0
0.5
-
A
C
D
E
F
G
H
I
K
L
M
N
P
Q
R
S
T
V
W
Y

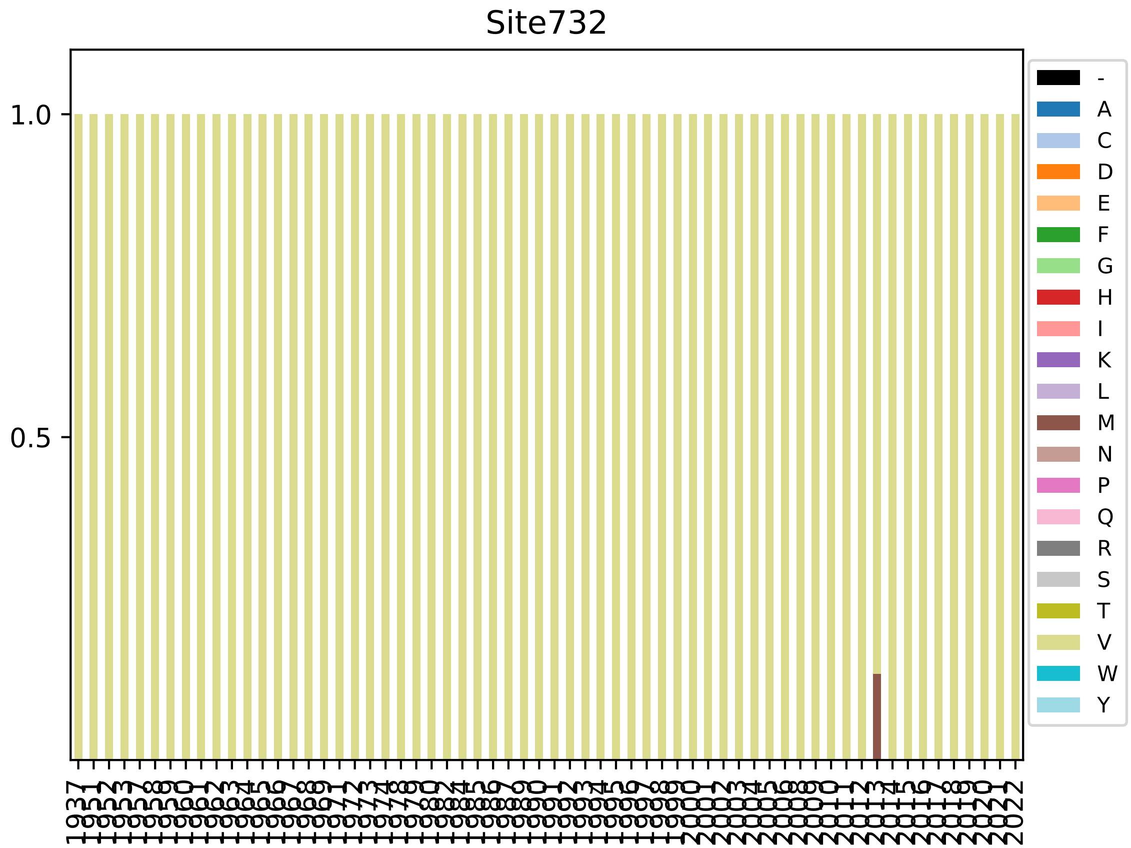
Site732
1.0
0.5
-
A
C
D
E
F
G
H
I
K
L
M
N
P
Q
R
S
T
V
W
Y

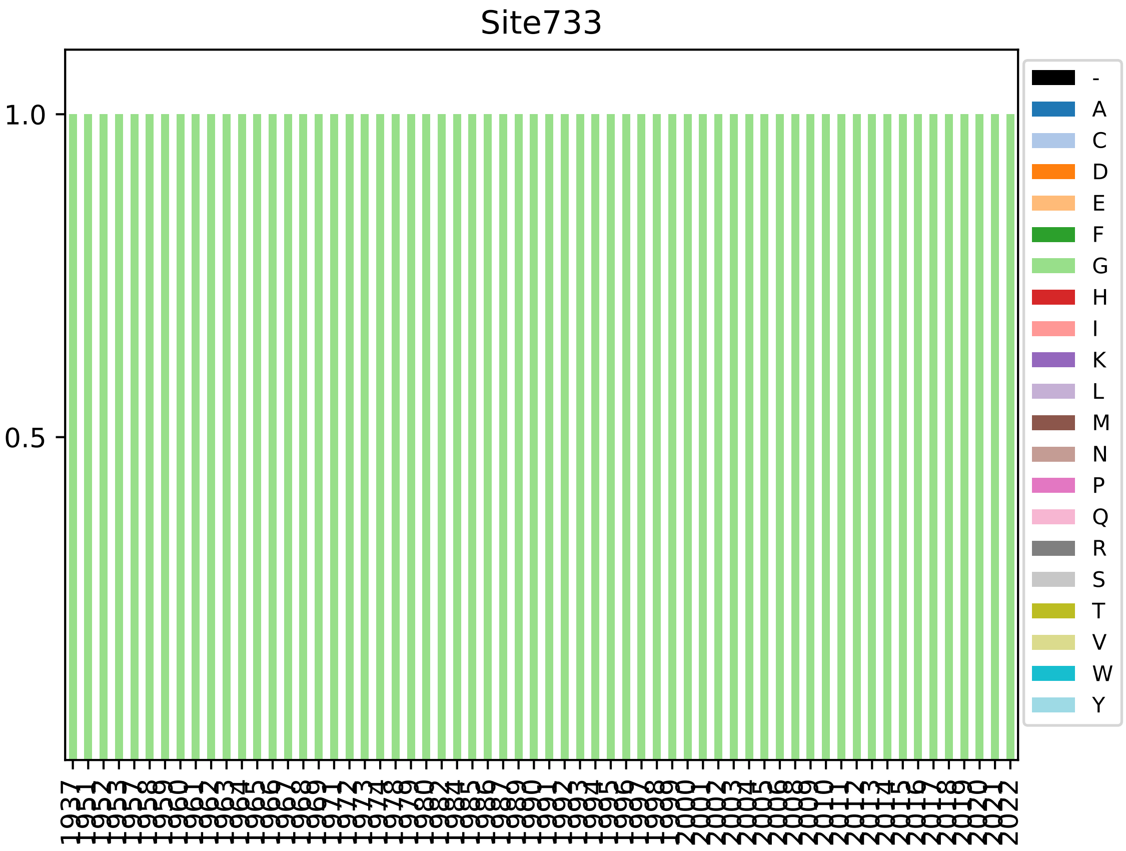
Site733
1.0
0.5
-
A
C
D
E
F
G
H
I
K
L
M
N
P
Q
R
S
T
V
W
Y

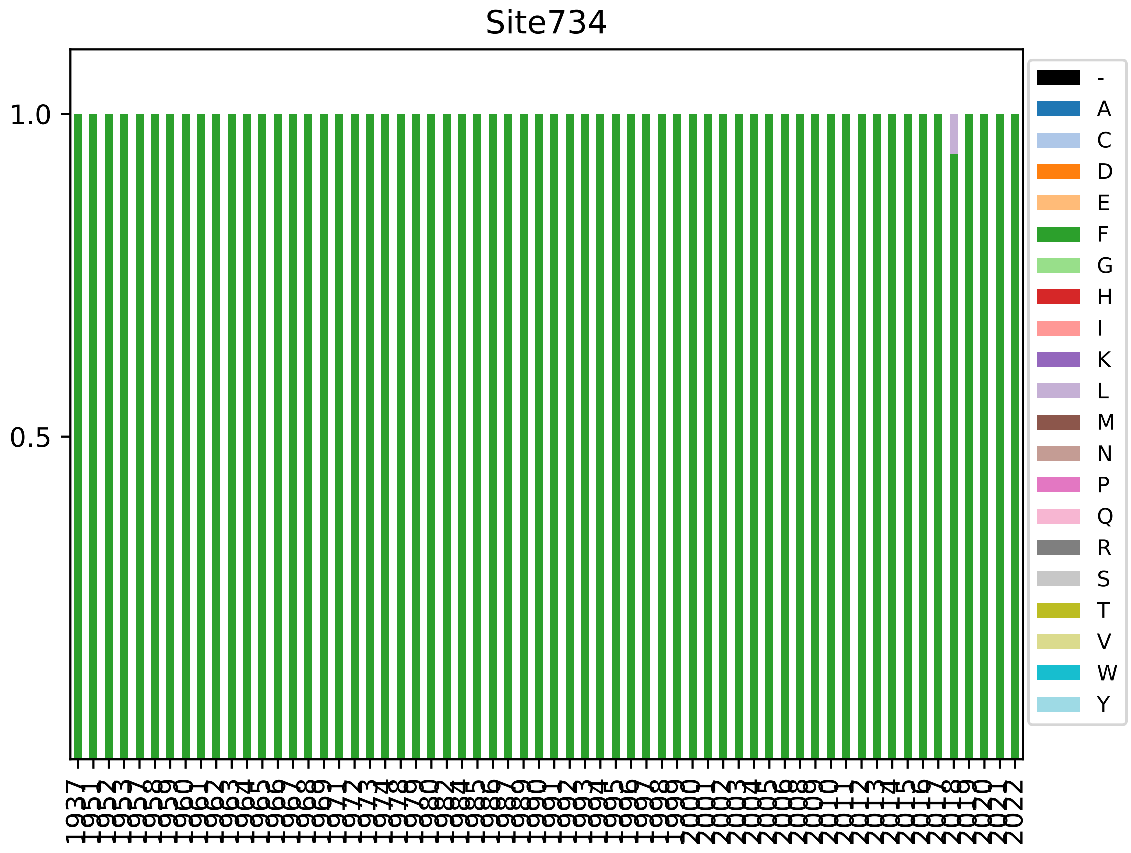
Site734
1.0
0.5
-
A
C
D
E
F
G
H
I
K
L
M
N
P
Q
R
S
T
V
W
Y

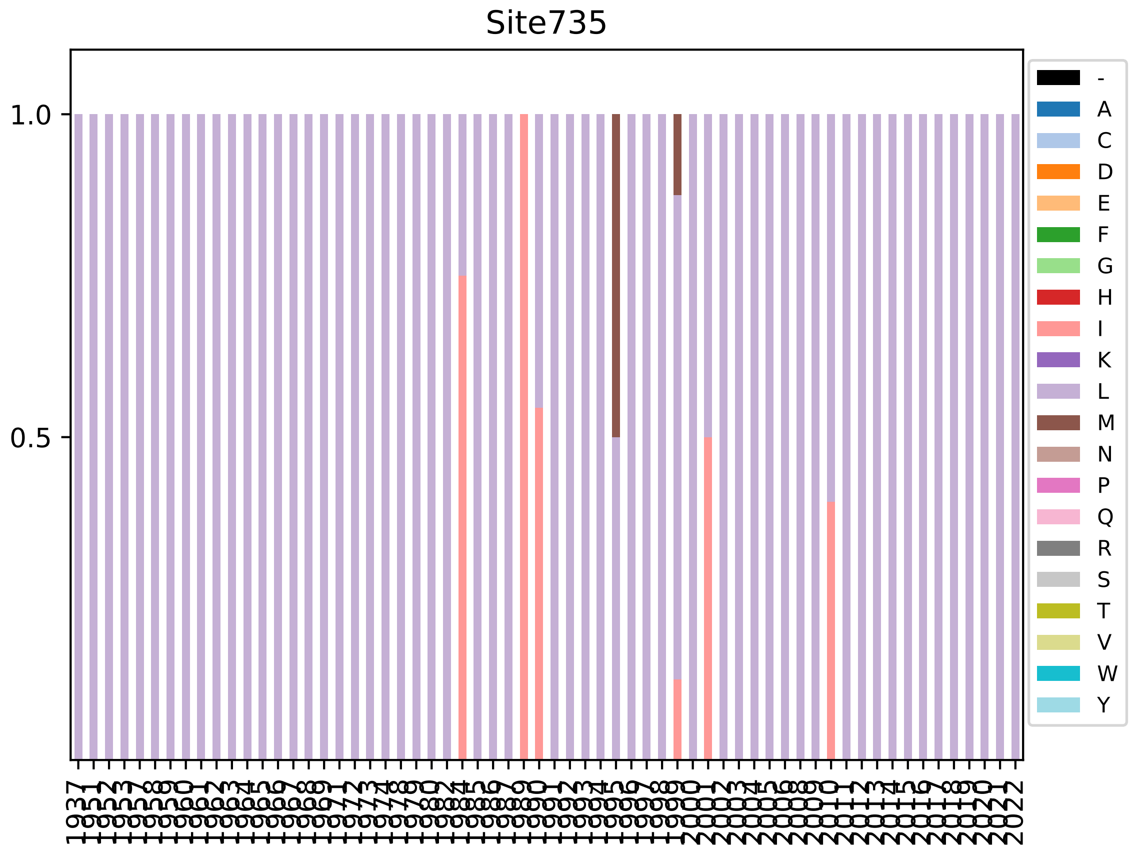
Site735
1.0
0.5
-
A
C
D
E
F
G
H
I
K
L
M
N
P
Q
R
S
T
V
W
Y

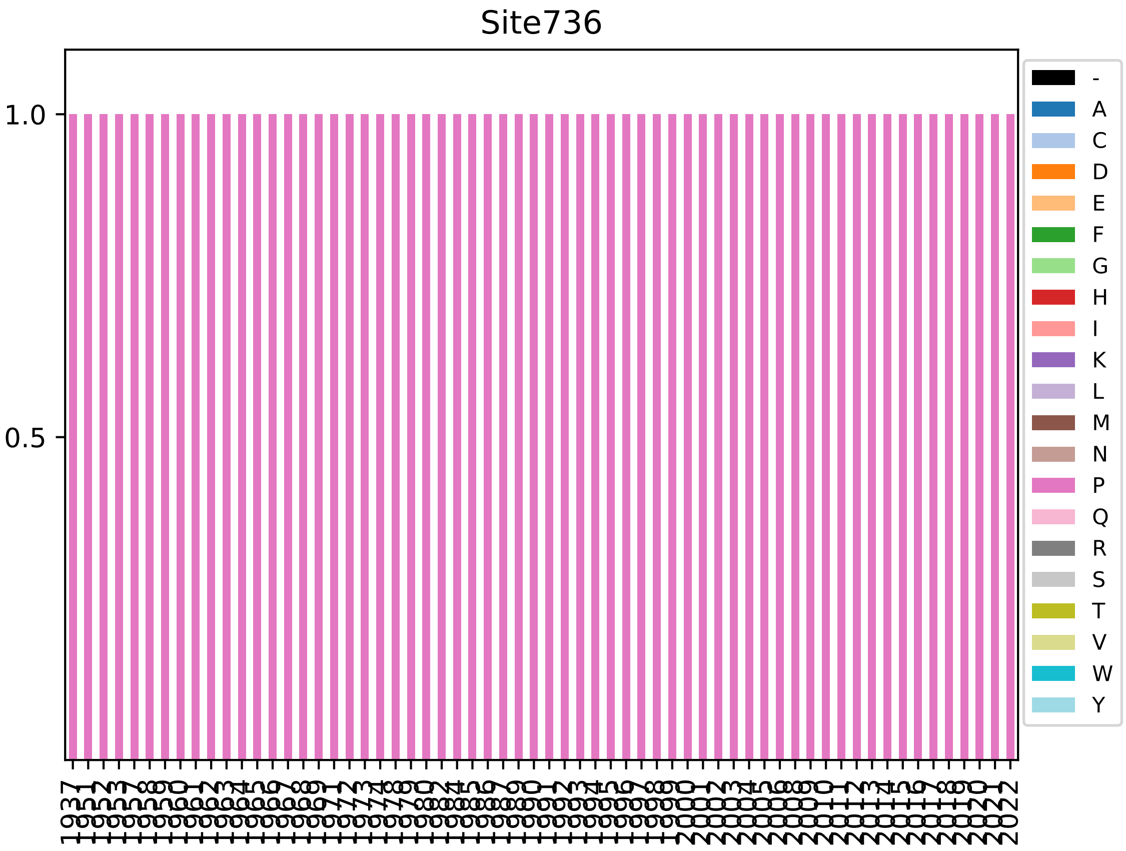

Site736
1.0
0.5
-
A
C
D
E
F
G
H
I
K
L
M
N
P
Q
R
S
T
V
W
Y

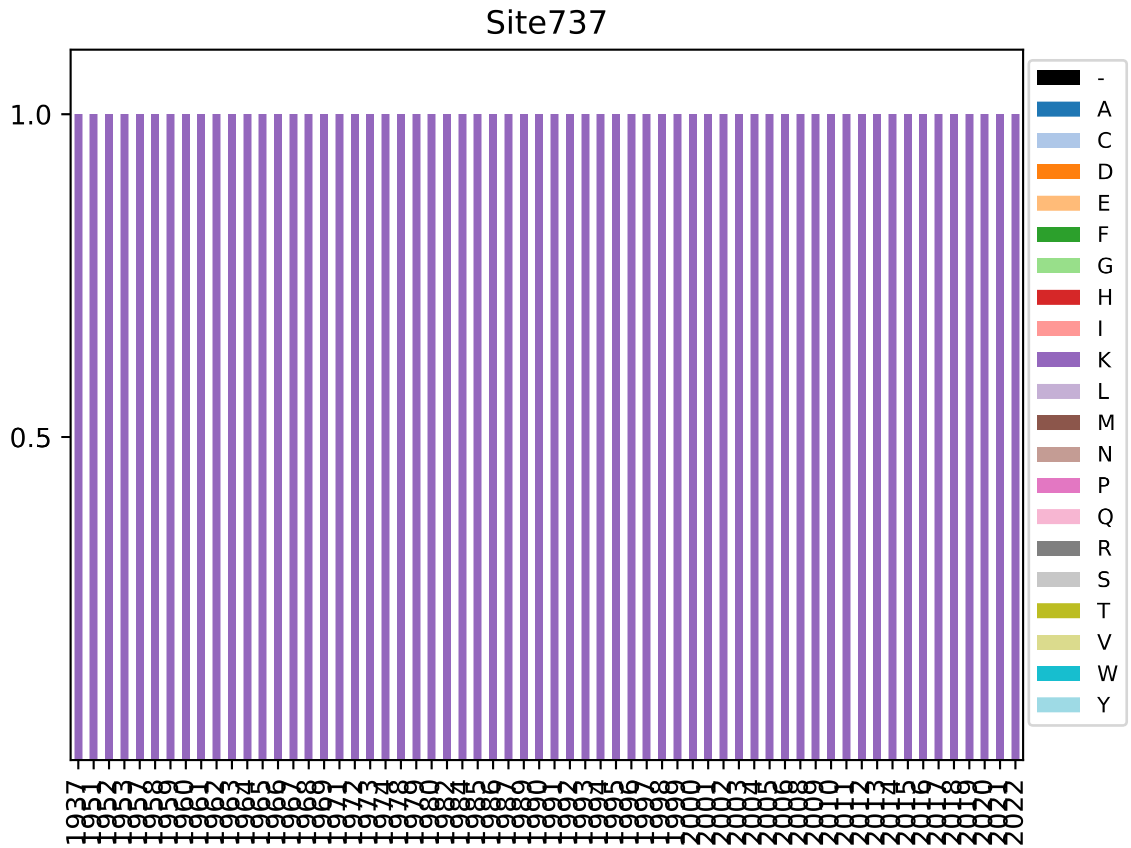
Site737
1.0
0.5
-
A
C
D
E
F
G
H
I
K
L
M
N
P
Q
R
S
T
V
W
Y

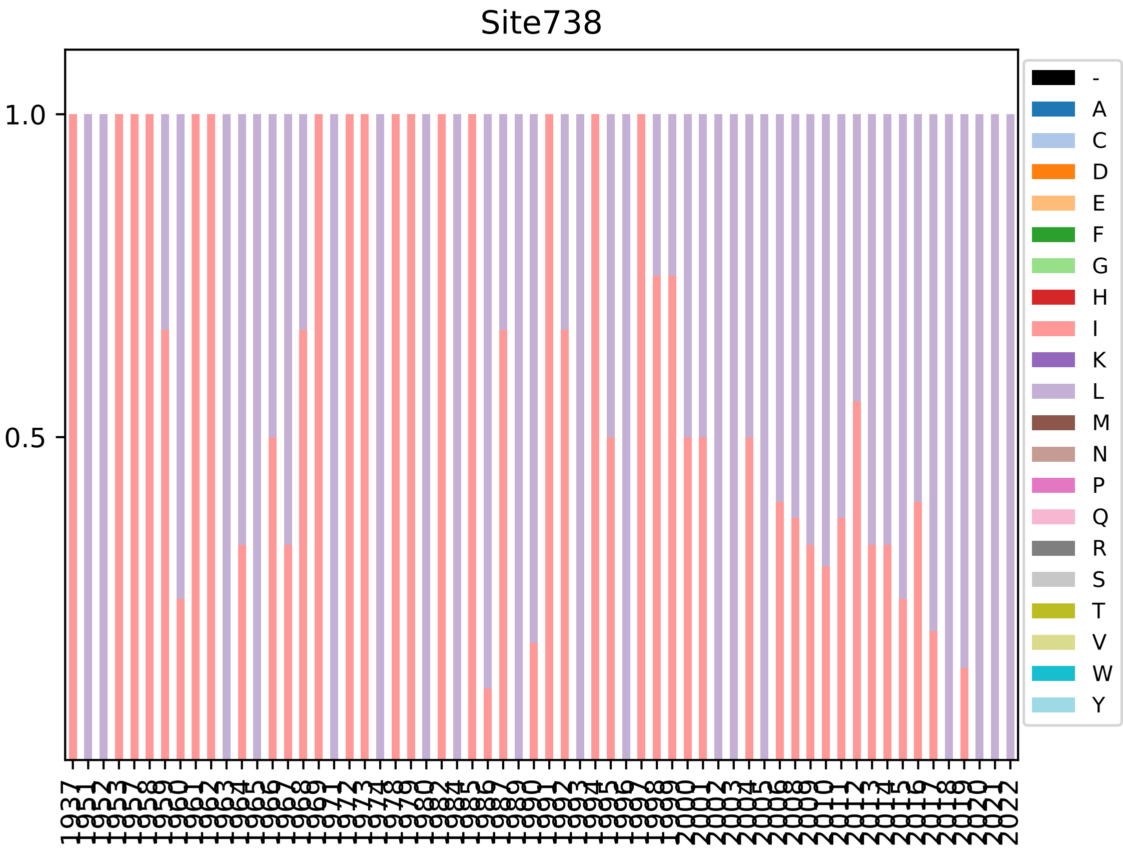
Site738
1.0
0.5
-
A
C
D
E
F
G
H
I
K
L
M
N
P
Q
R
S
T
V
W
Y

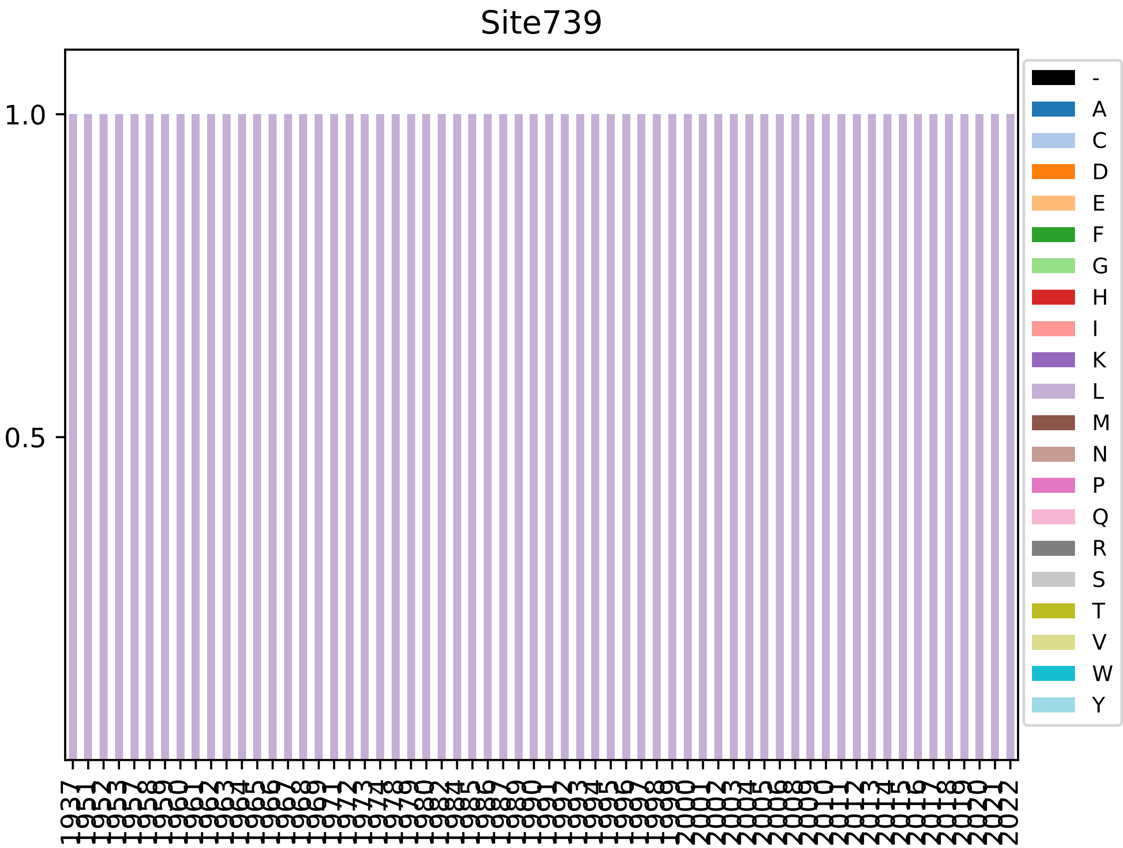
Site739
1.0
0.5
-
A
C
D
E
F
G
H
I
K
L
M
N
P
Q
R
S
T
V
W
Y

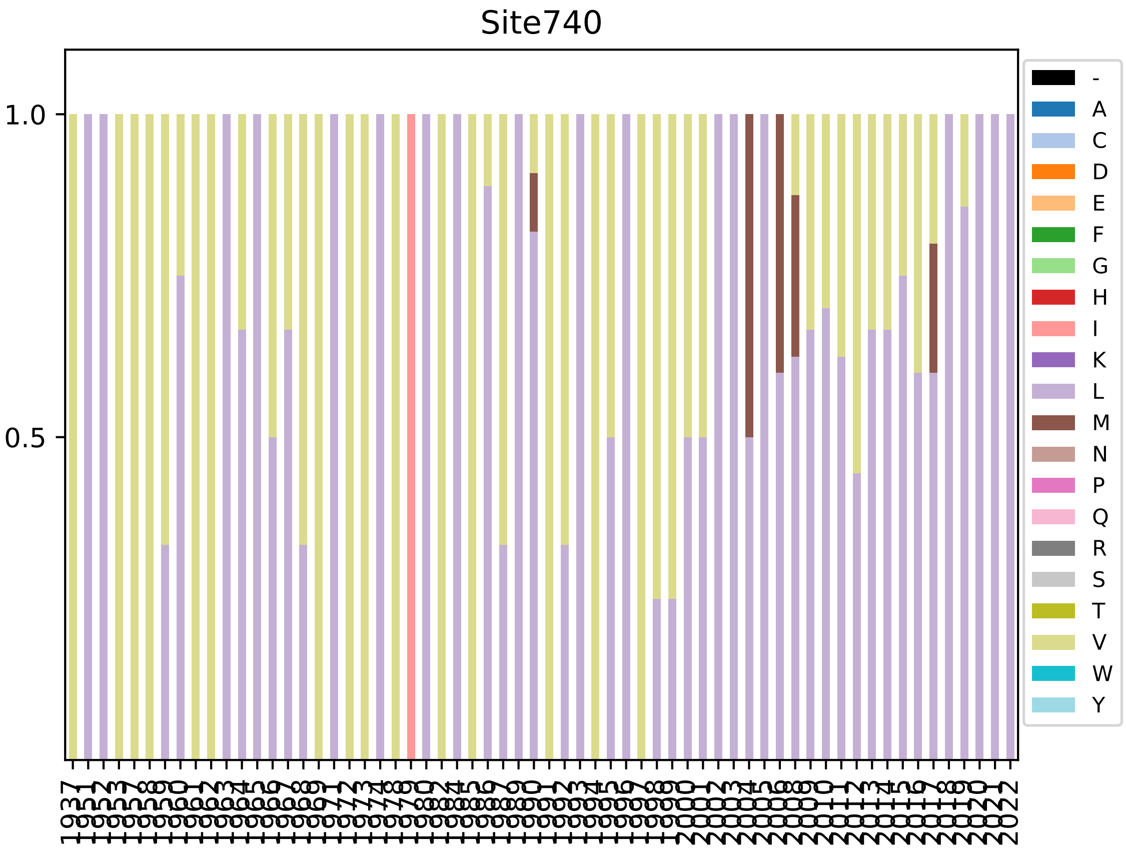
Site740
1.0
0.5
-
A
C
D
E
F
G
H
I
K
L
M
N
P
Q
R
S
T
V
W
Y

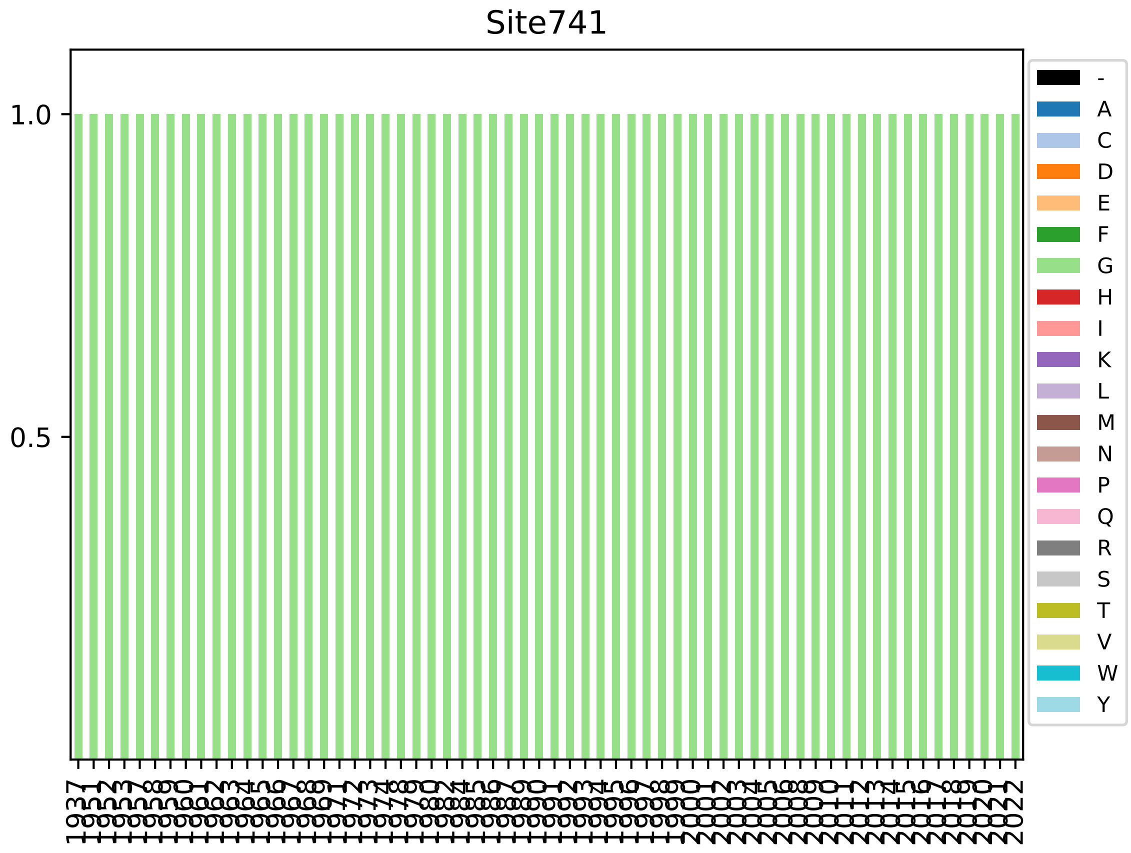
Site741
1.0
0.5
-
A
C
D
E
F
G
H
I
K
L
M
N
P
Q
R
S
T
V
W
Y

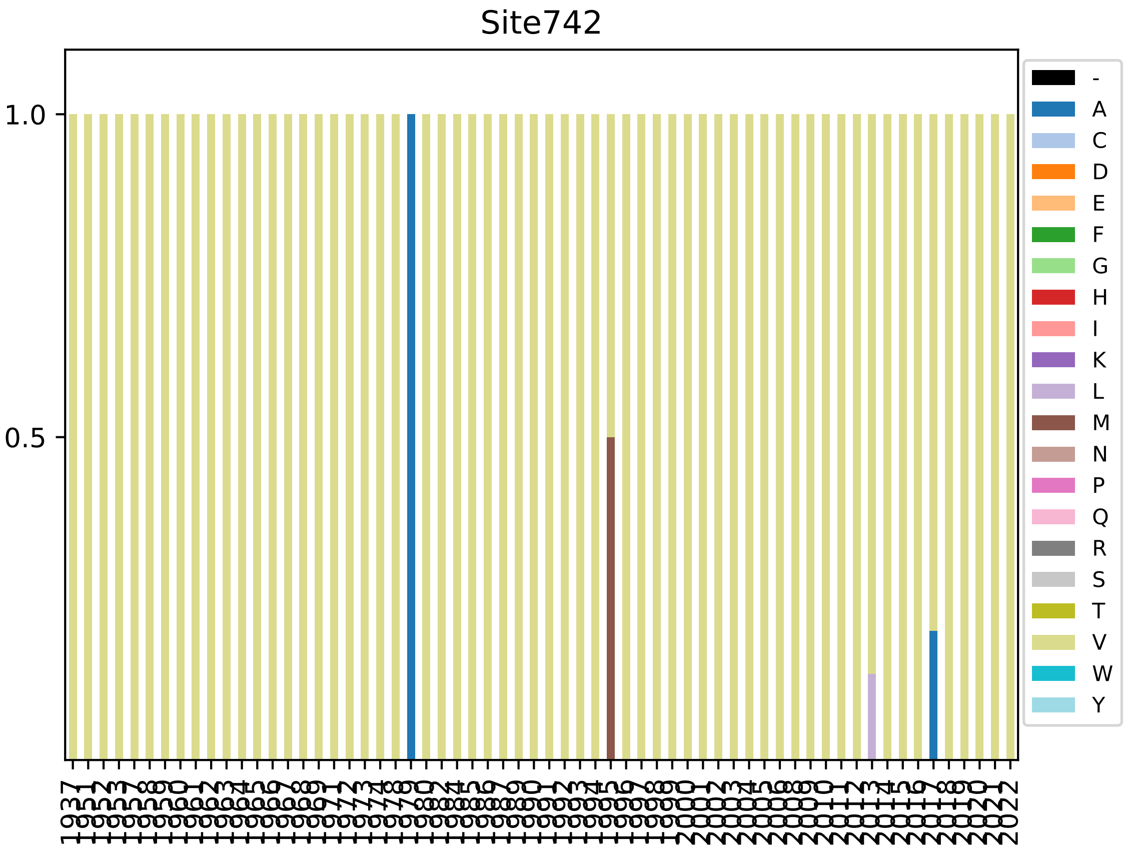
Site742
1.0
0.5
-
A
C
D
E
F
G
H
I
K
L
M
N
P
Q
R
S
T
V
W
Y

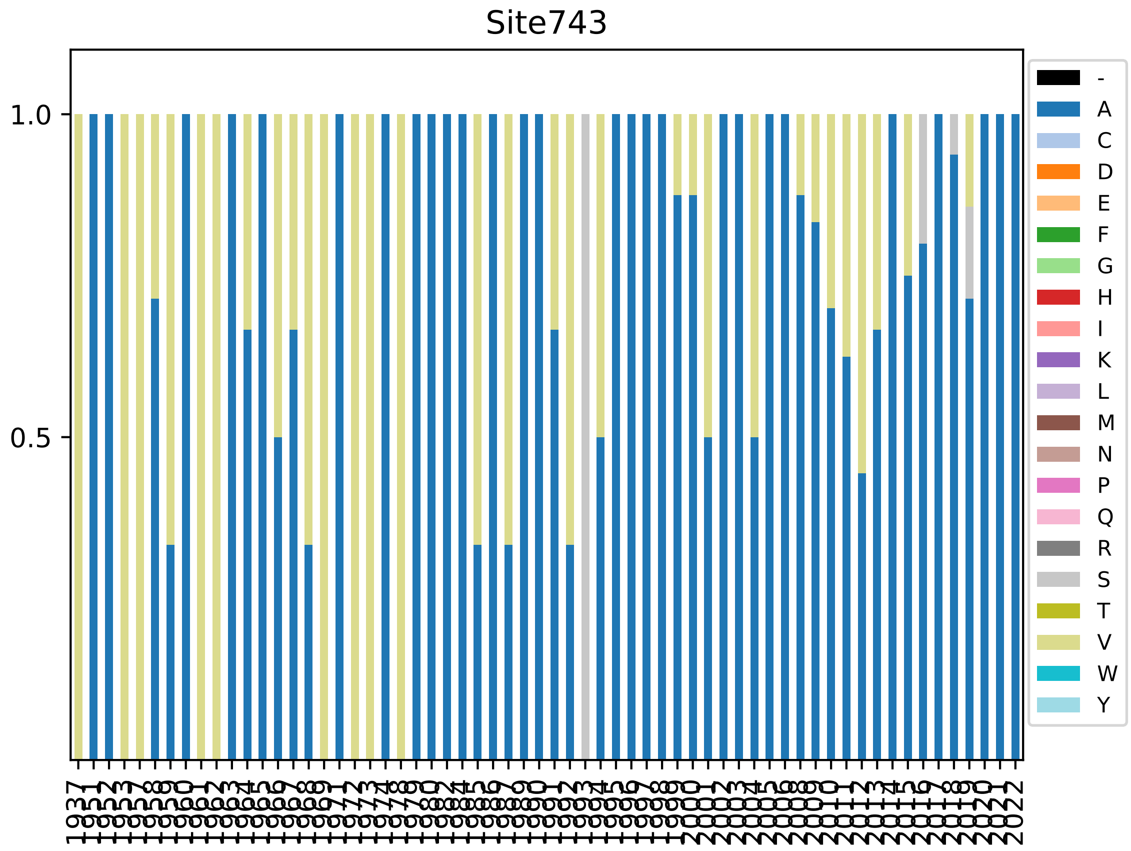
Site743
1.0
0.5
-
A
C
D
E
F
G
H
I
K
L
M
N
P
Q
R
S
T
V
W
Y

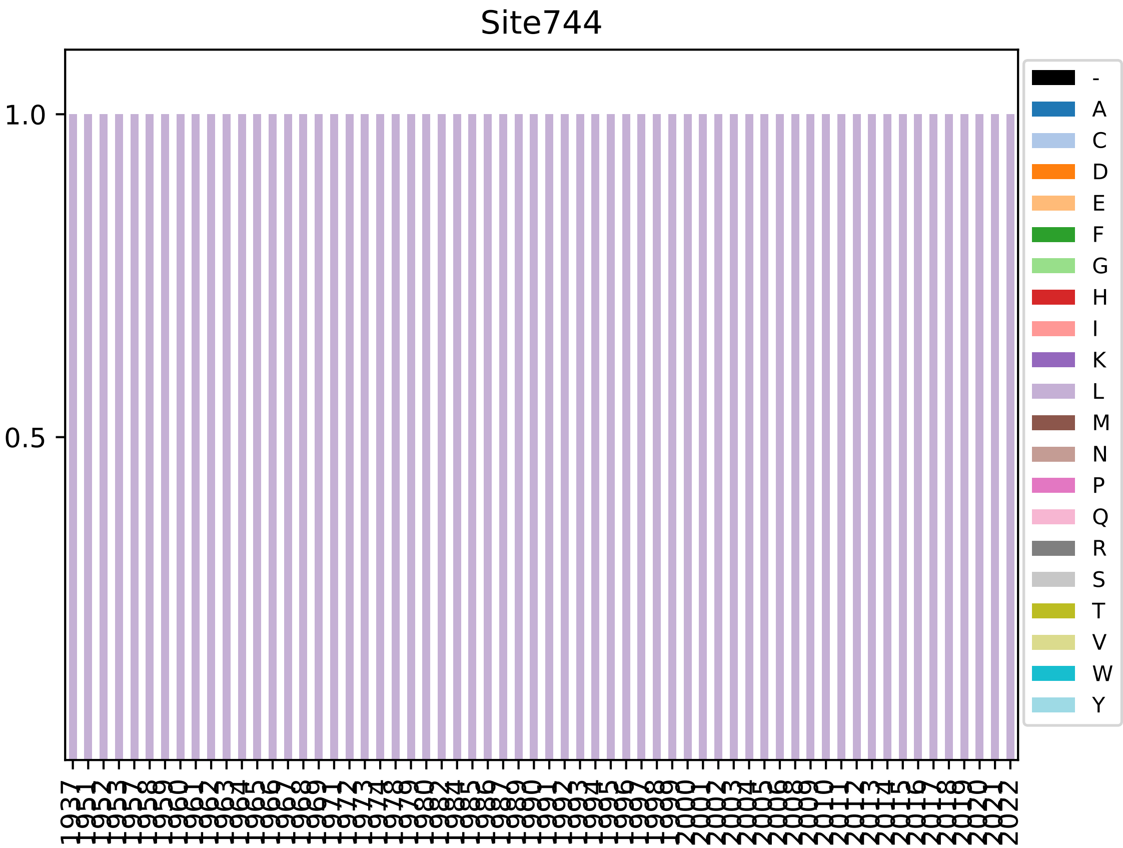
Site744
1.0
0.5
-
A
C
D
E
F
G
H
I
K
L
M
N
P
Q
R
S
T
V
W
Y

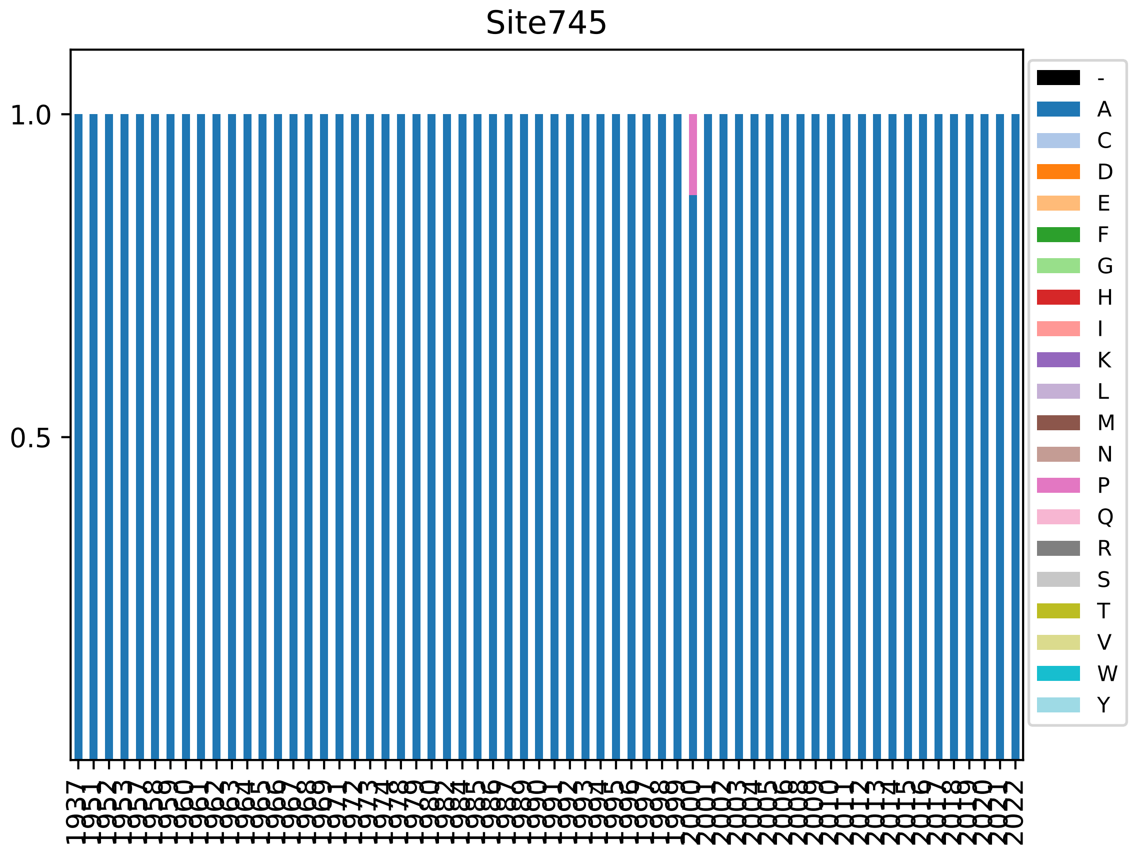

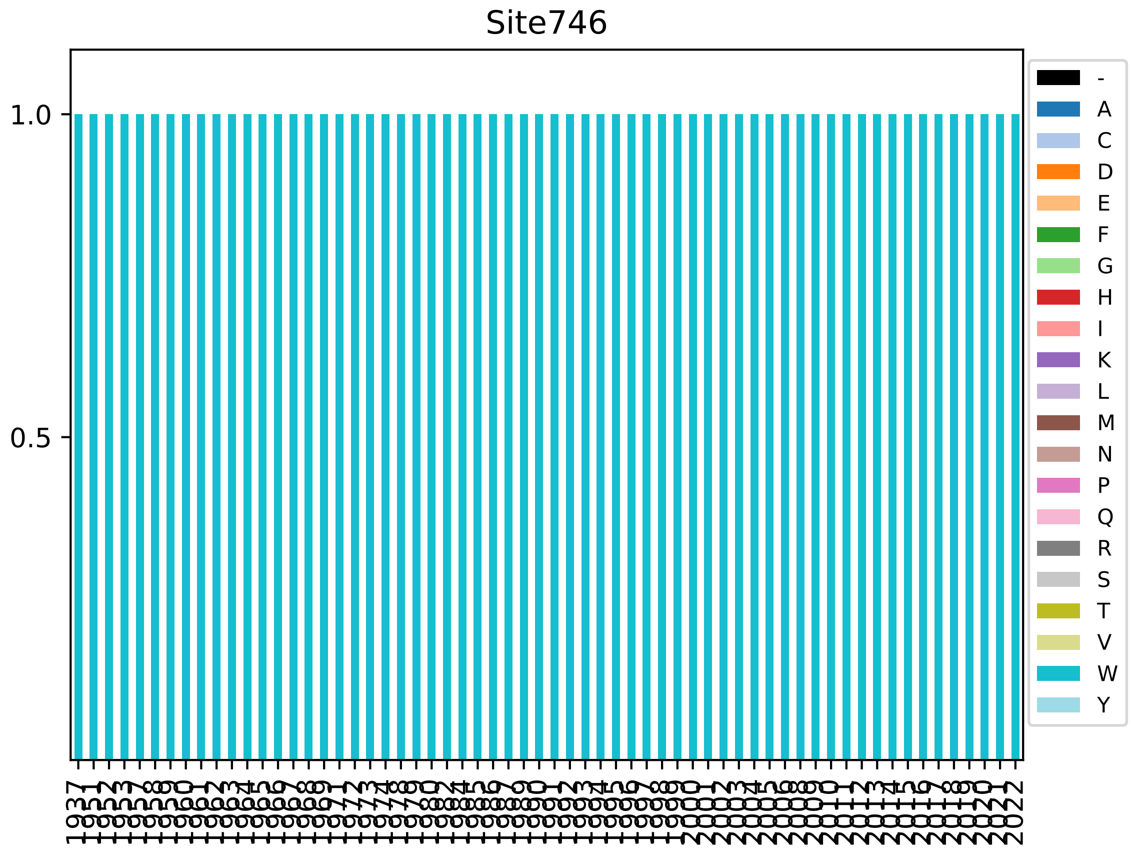
Site746
1.0
0.5
-
A
C
D
E
F
G
H
I
K
L
M
N
P
Q
R
S
T
V
W
Y

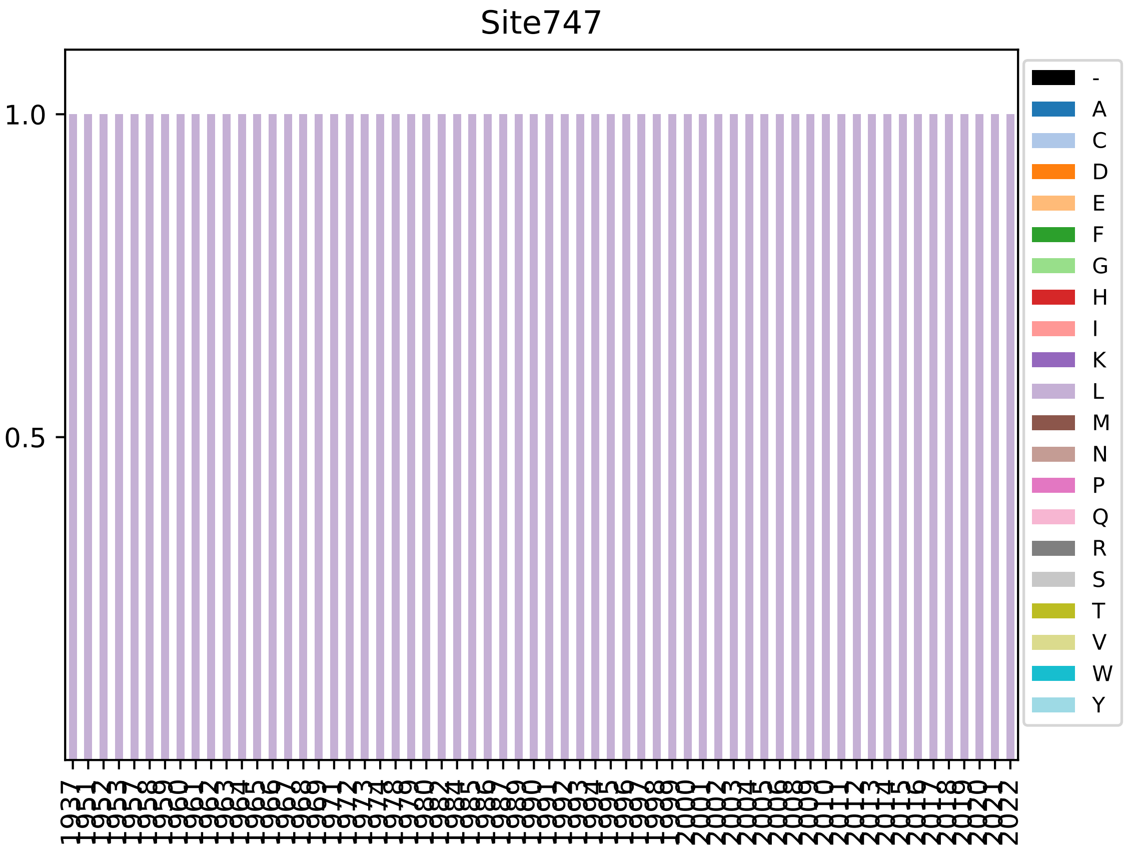
Site747
1.0
0.5
-
A
C
D
E
F
G
H
I
K
L
M
N
P
Q
R
S
T
V
W
Y

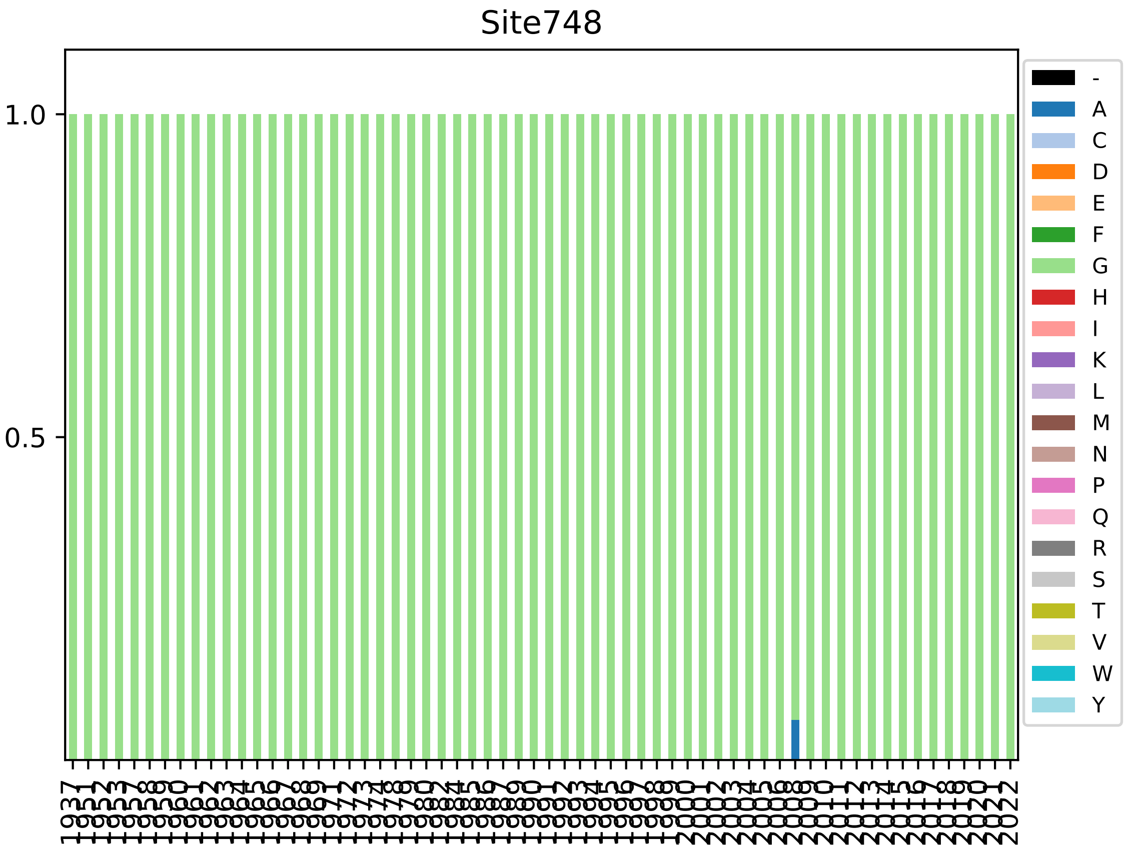
Site748
1.0
0.5
-
A
C
D
E
F
G
H
I
K
L
M
N
P
Q
R
S
T
V
W
Y

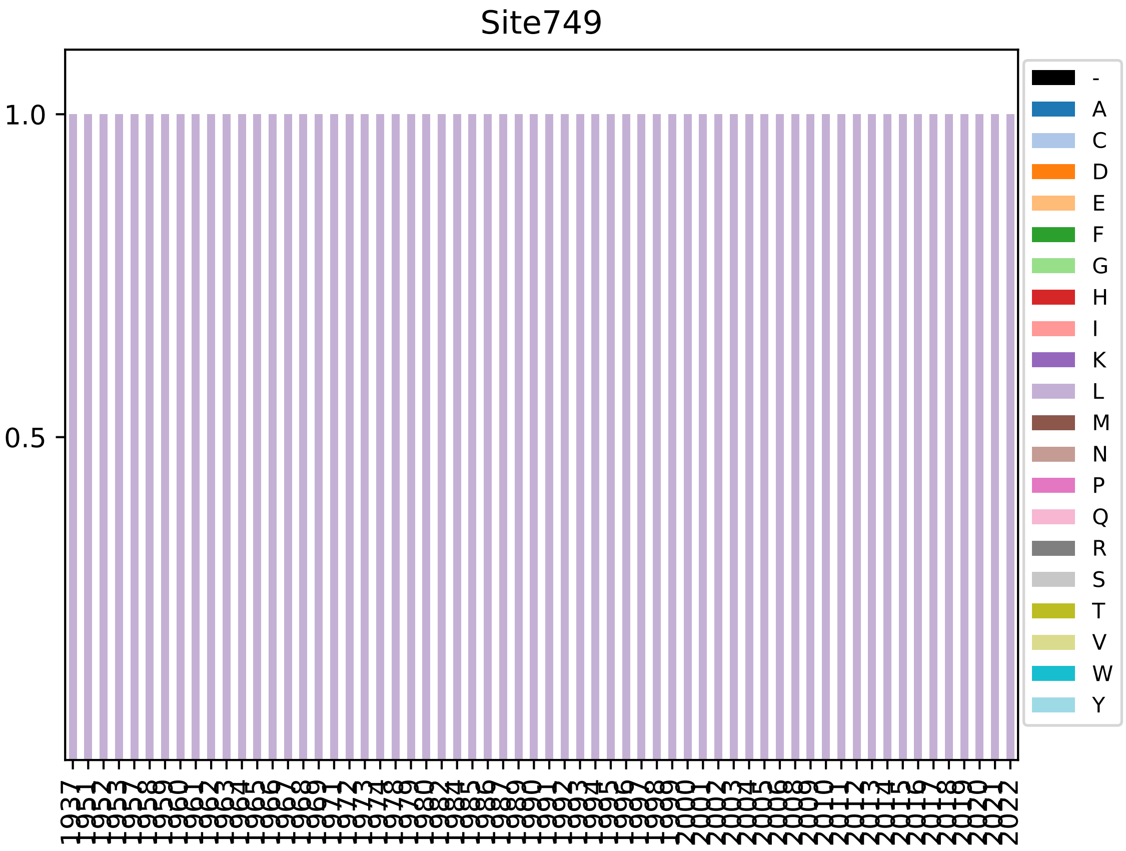
Site749
1.0
0.5
-
A
C
D
E
F
G
H
I
K
L
M
N
P
Q
R
S
T
V
W
Y

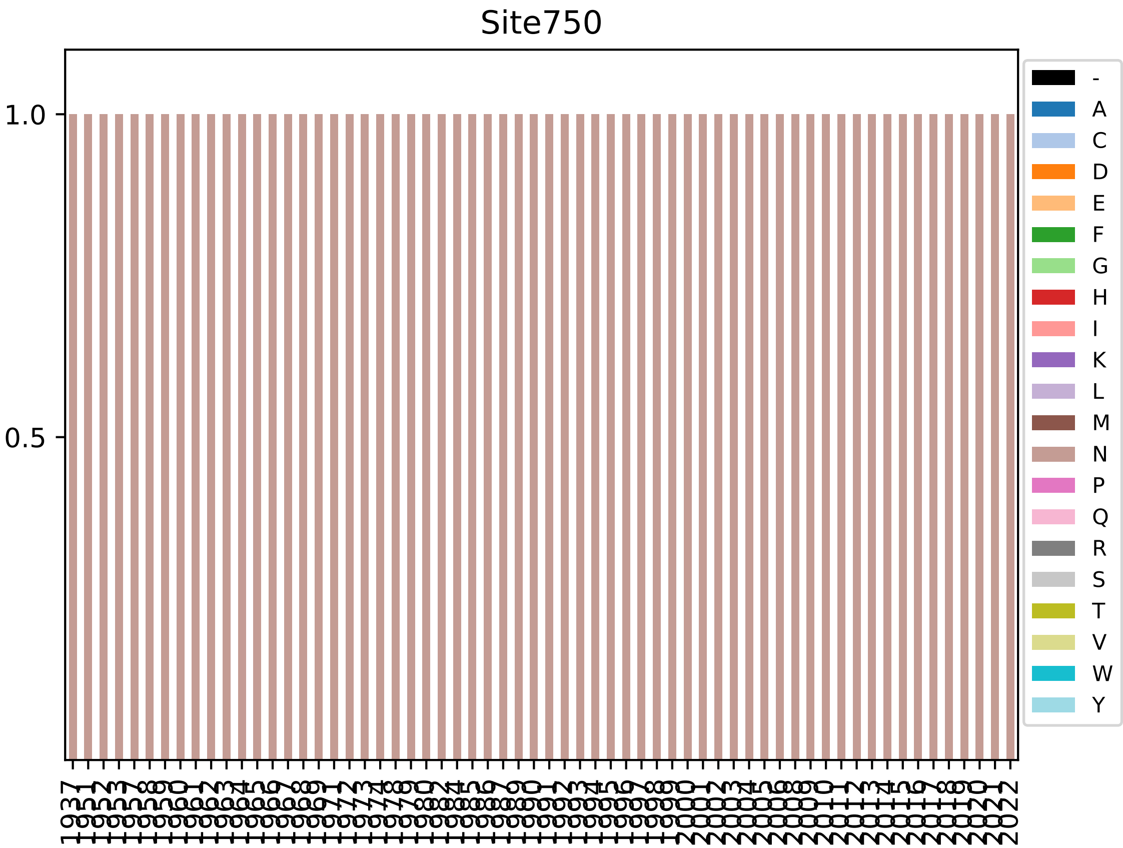
Site750
1.0
0.5
-
A
C
D
E
F
G
H
I
K
L
M
N
P
Q
R
S
T
V
W
Y

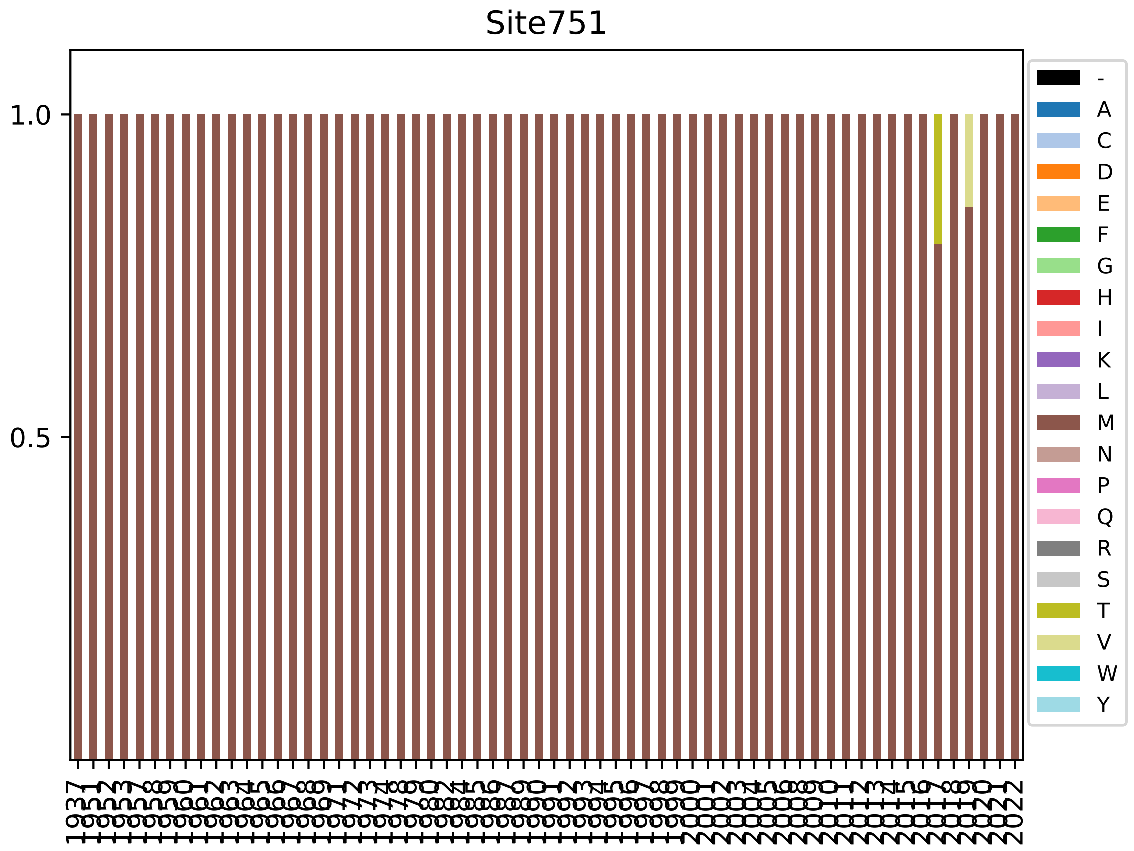
Site751
1.0
0.5
-
A
C
D
E
F
G
H
I
K
L
M
N
P
Q
R
S
T
V
W
Y

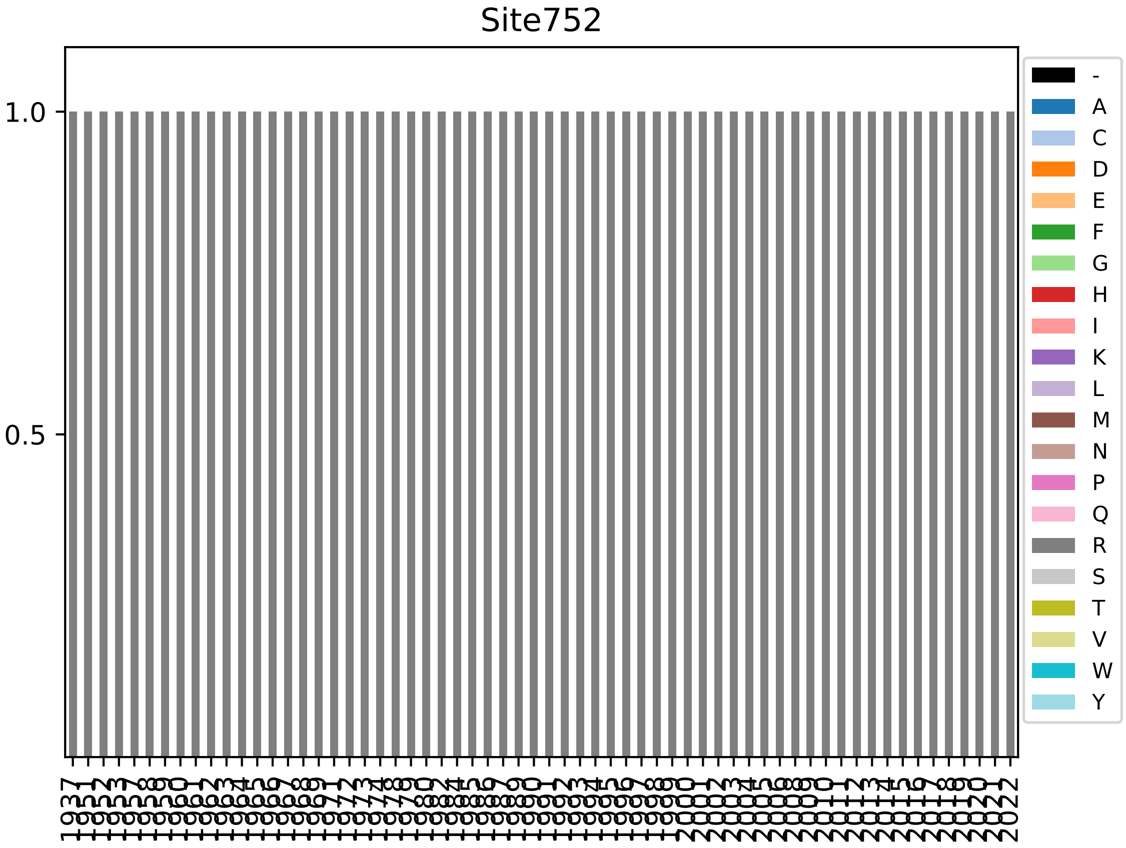
Site752
1.0
0.5
-
A
C
D
E
F
G
H
I
K
L
M
N
P
Q
R
S
T
V
W
Y

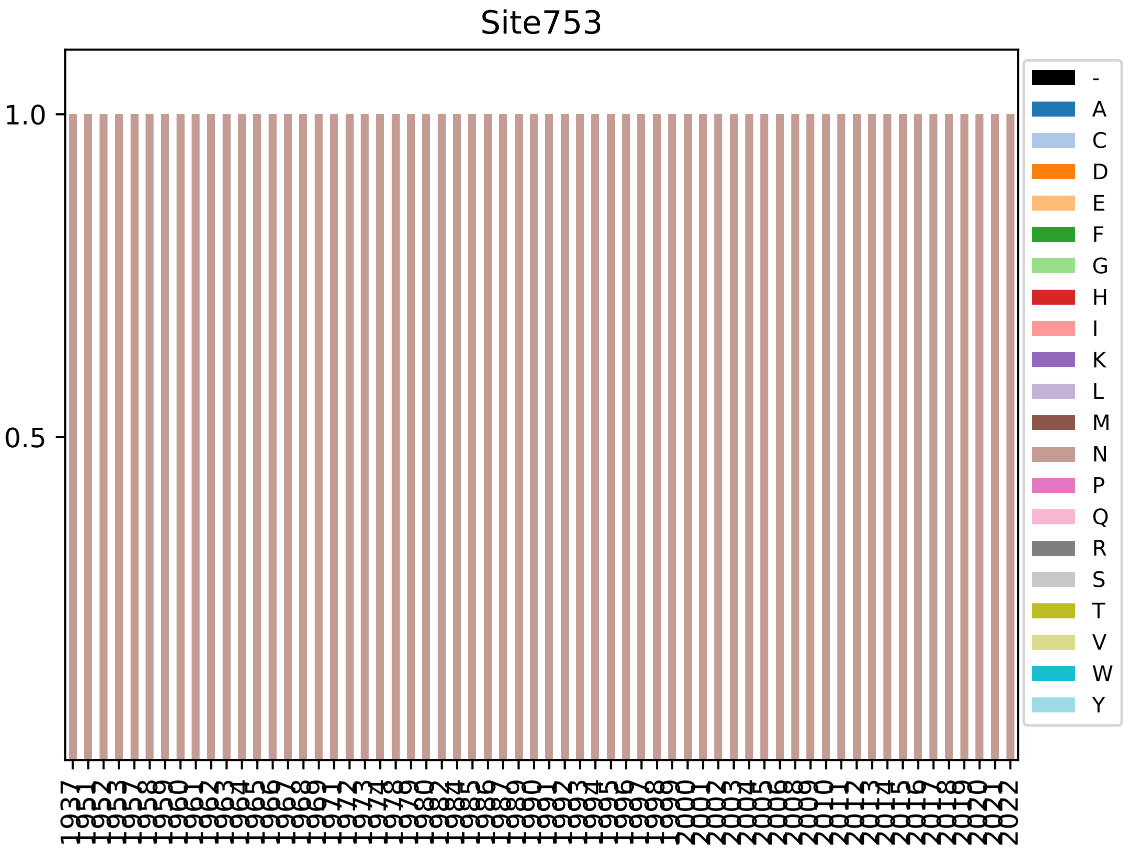
Site753
1.0
0.5
-
A
C
D
E
F
G
H
I
K
L
M
N
P
Q
R
S
T
V
W
Y

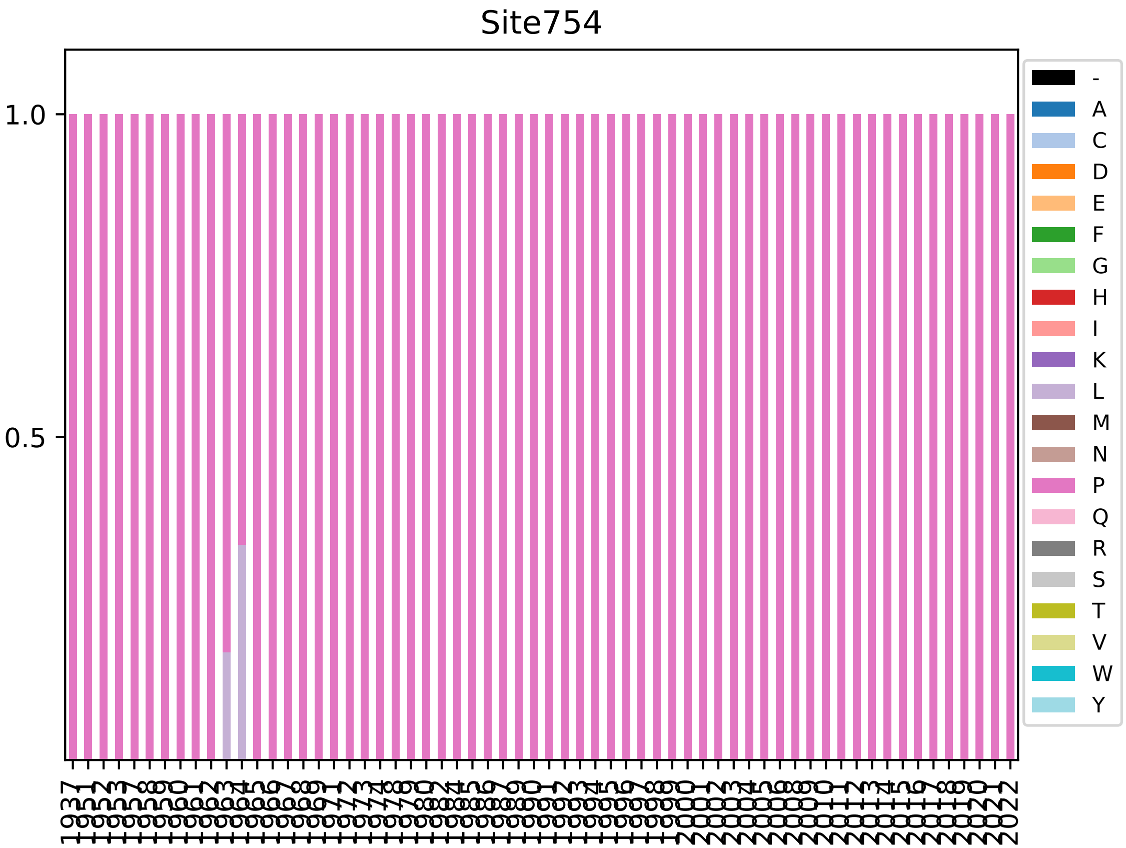
Site754
1.0
0.5
-
A
C
D
E
F
G
H
I
K
L
M
N
P
Q
R
S
T
V
W
Y

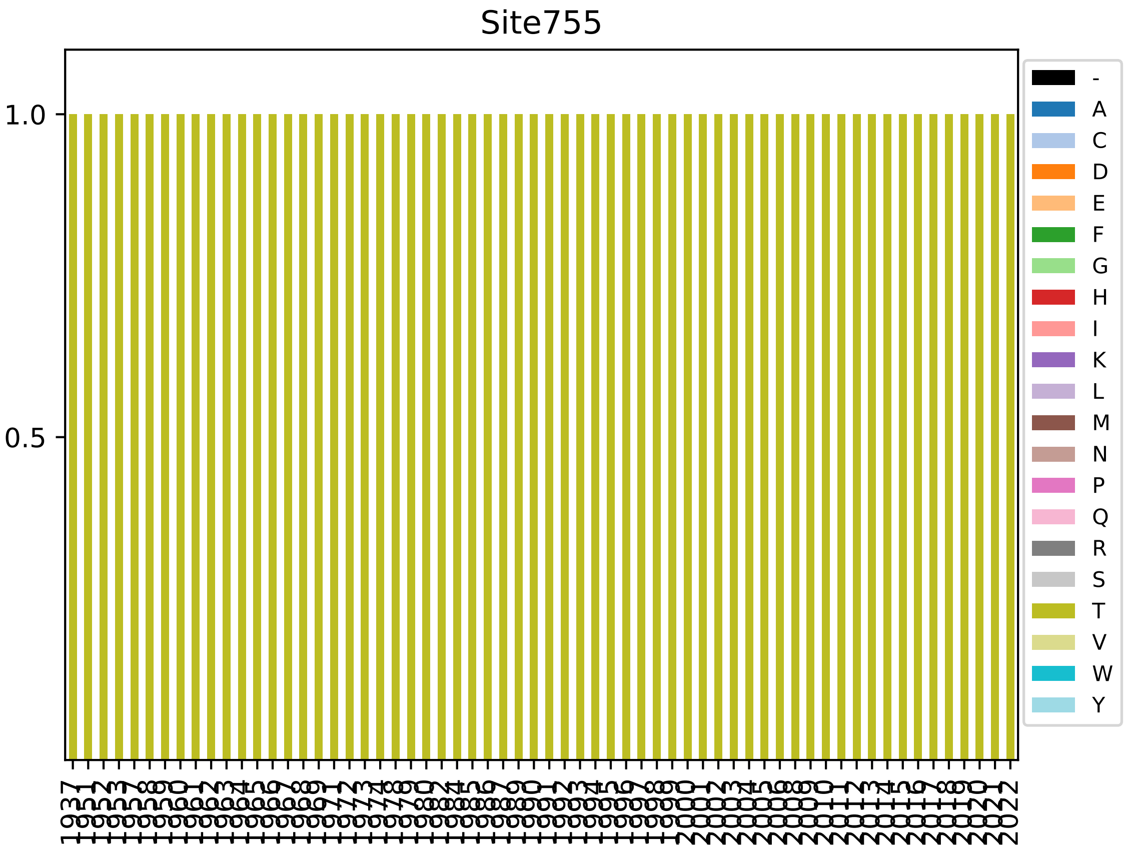
Site755
1.0
0.5
-
A
C
D
E
F
G
H
I
K
L
M
N
P
Q
R
S
T
V
W
Y

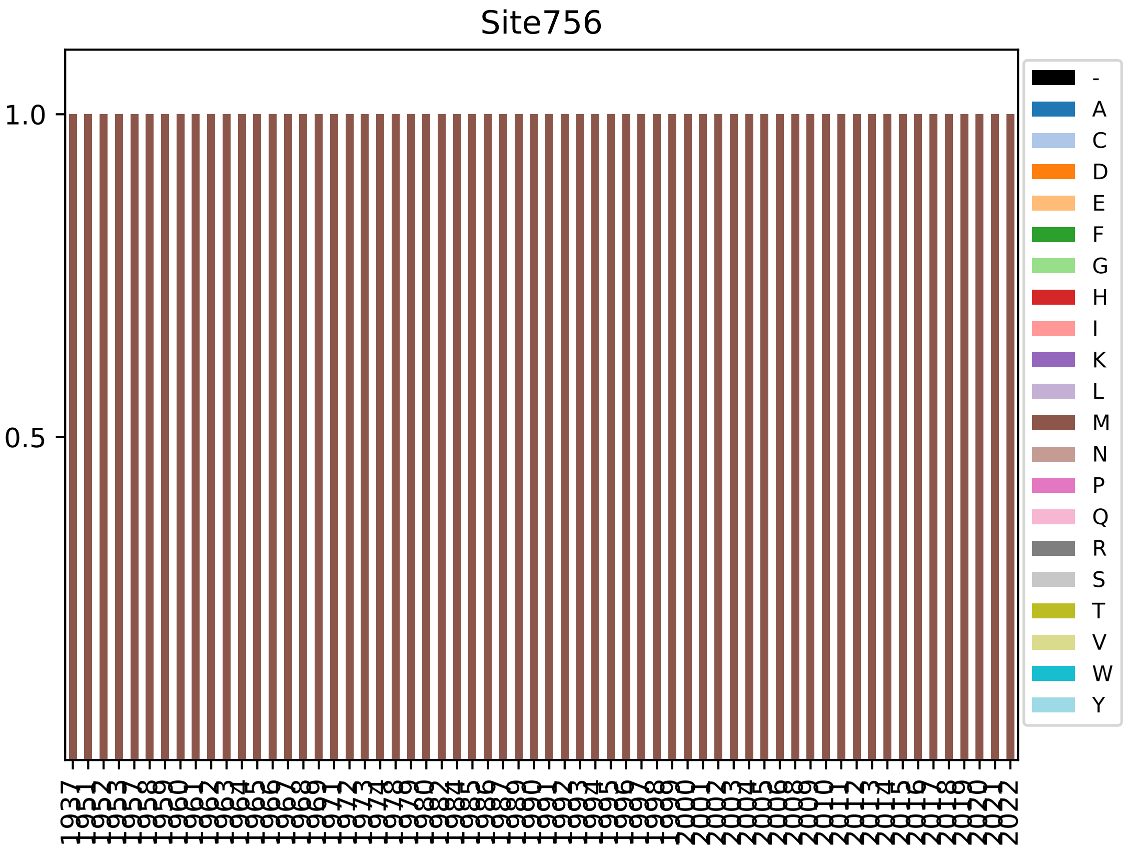

Site756
1.0
0.5
-
A
C
D
E
F
G
H
I
K
L
M
N
P
Q
R
S
T
V
W
Y

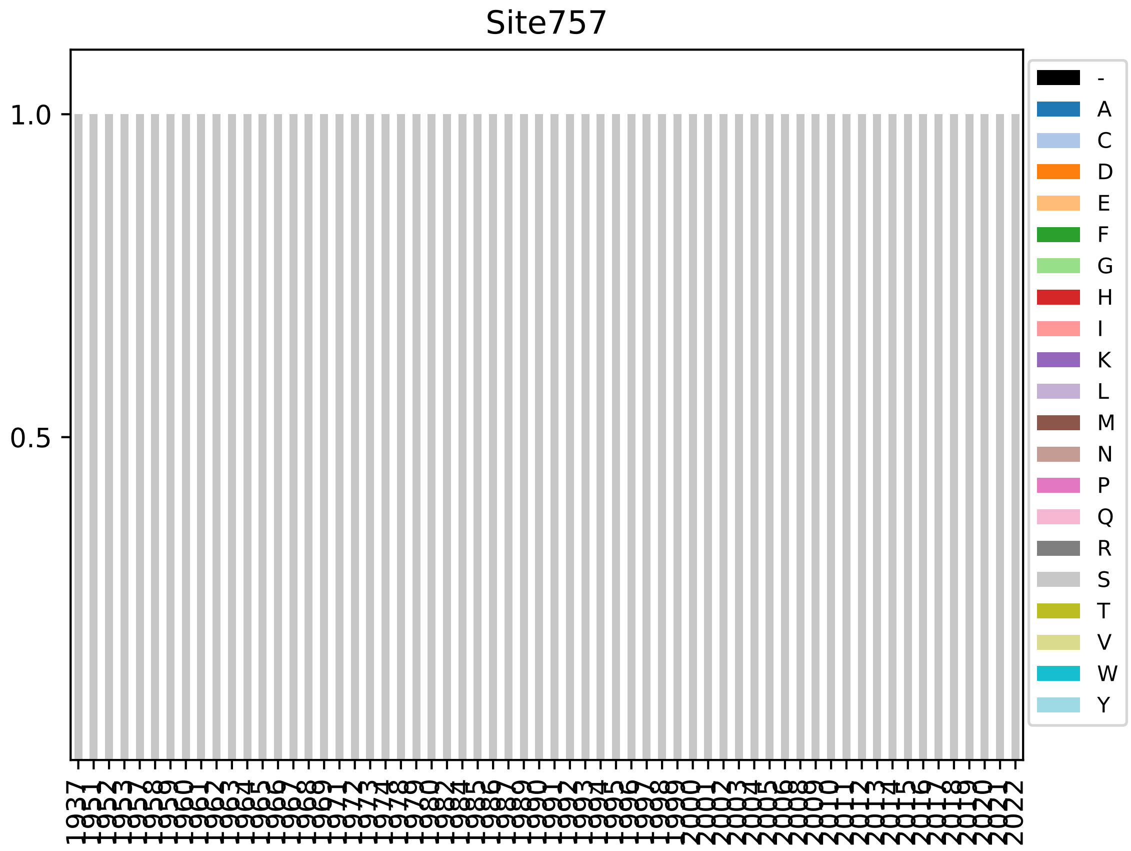

Site757
1.0
0.5
-
A
C
D
E
F
G
H
I
K
L
M
N
P
Q
R
S
T
V
W
Y

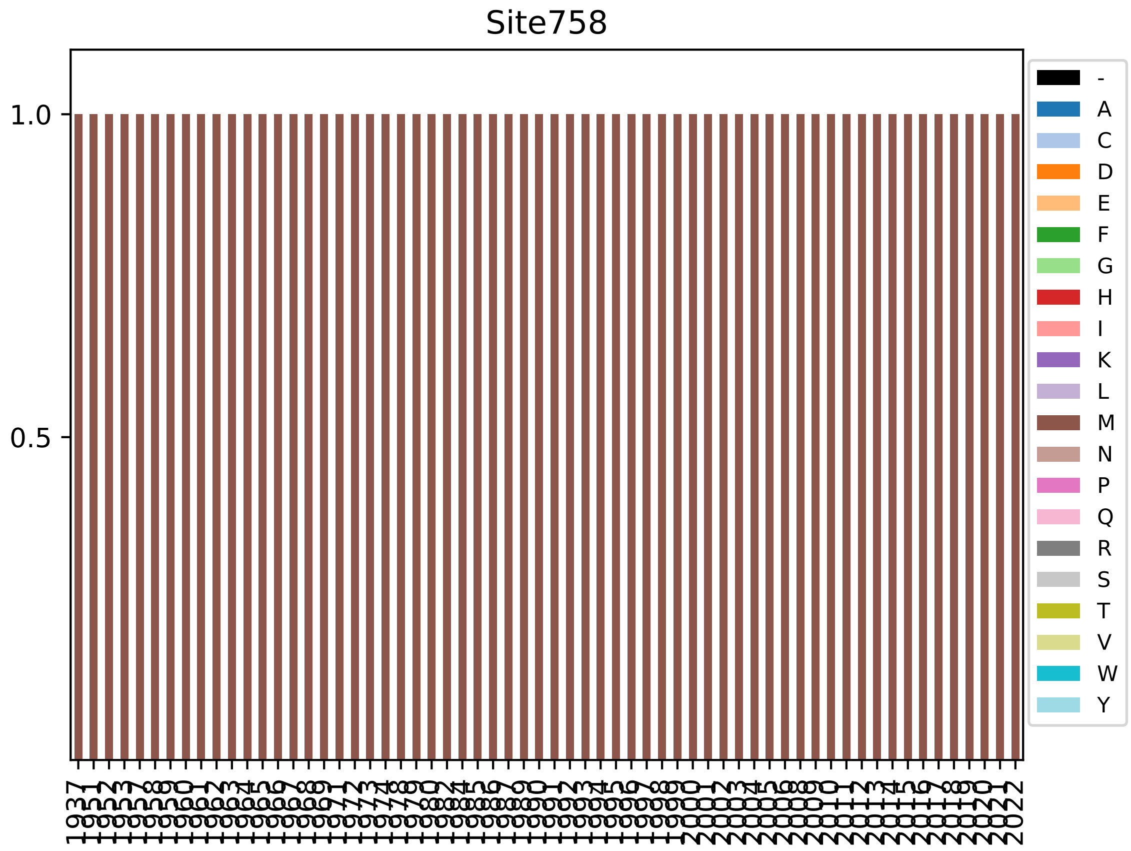
Site758
1.0
0.5
-
A
C
D
E
F
G
H
I
K
L
M
N
P
Q
R
S
T
V
W
Y

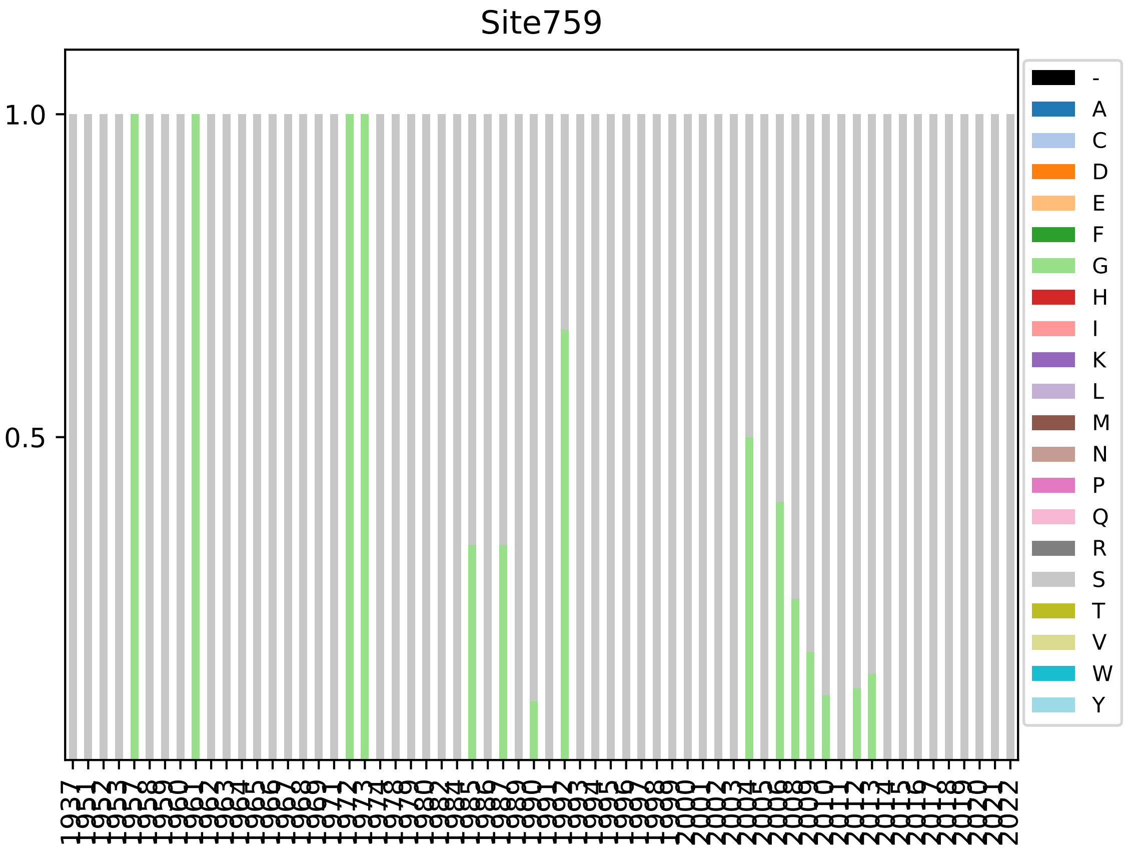
Site759
1.0
0.5
-
A
C
D
E
F
G
H
I
K
L
M
N
P
Q
R
S
T
V
W
Y

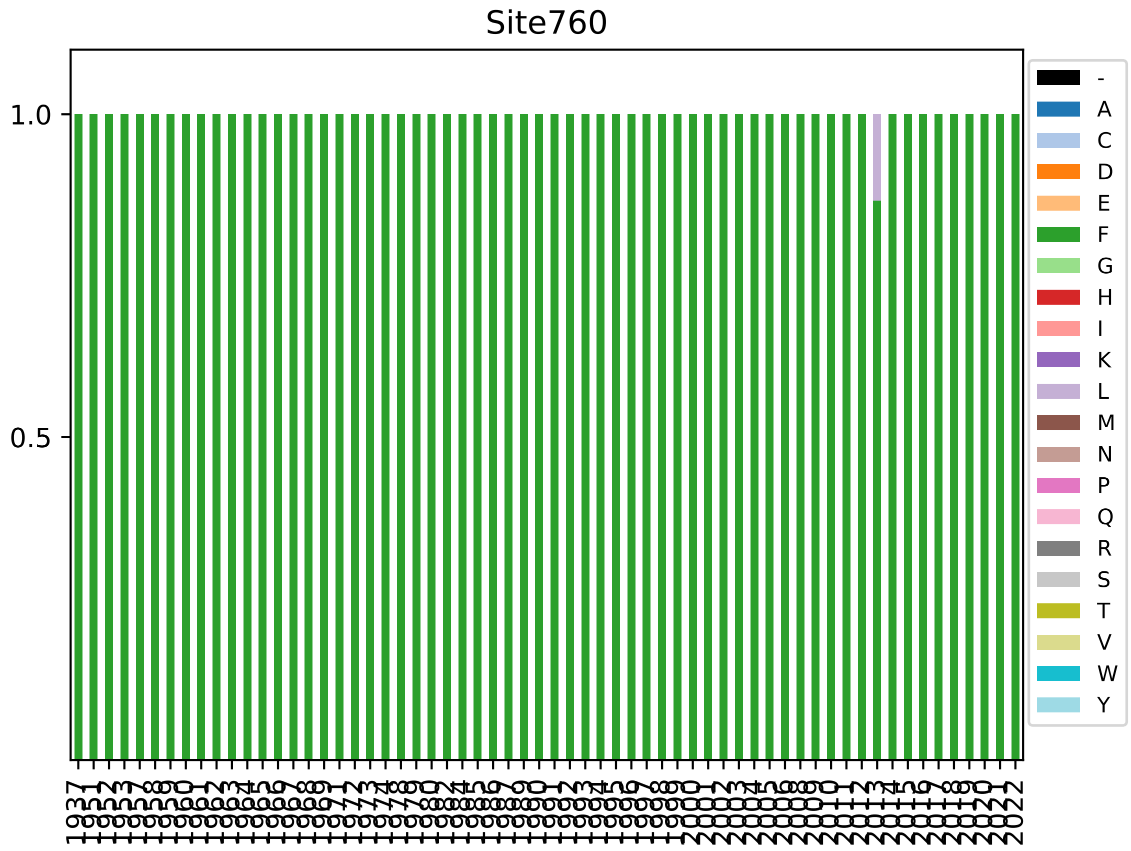
Site760
1.0
0.5
-
A
C
D
E
F
G
H
I
K
L
M
N
P
Q
R
S
T
V
W
Y

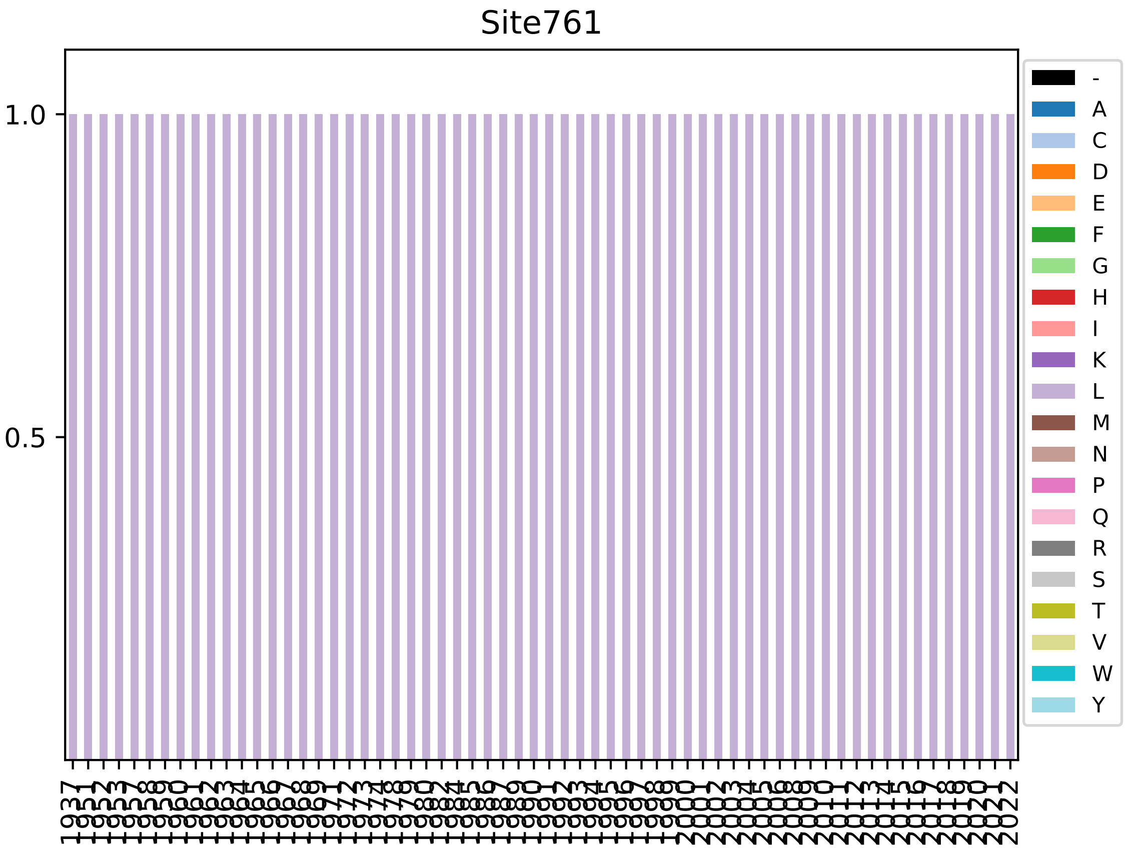
Site761
1.0
0.5
-
A
C
D
E
F
G
H
I
K
L
M
N
P
Q
R
S
T
V
W
Y

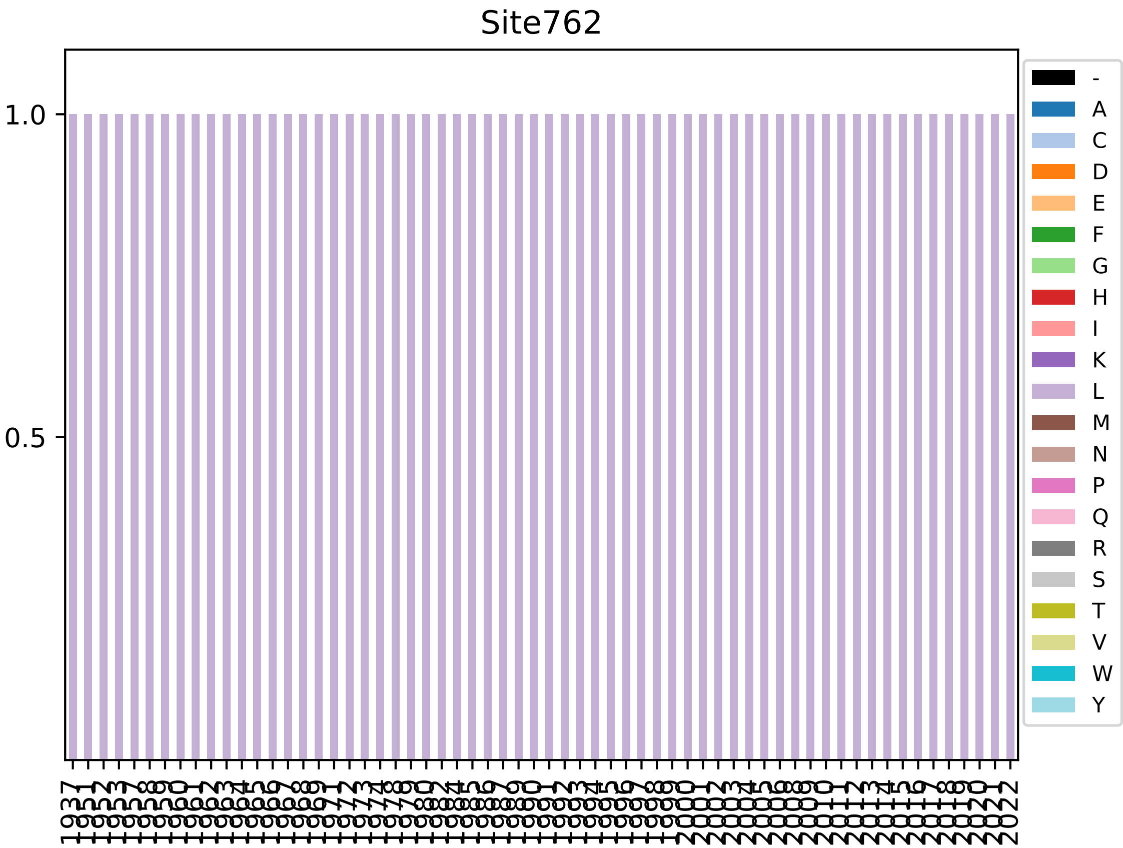
Site762
1.0
0.5
-
A
C
D
E
F
G
H
I
K
L
M
N
P
Q
R
S
T
V
W
Y

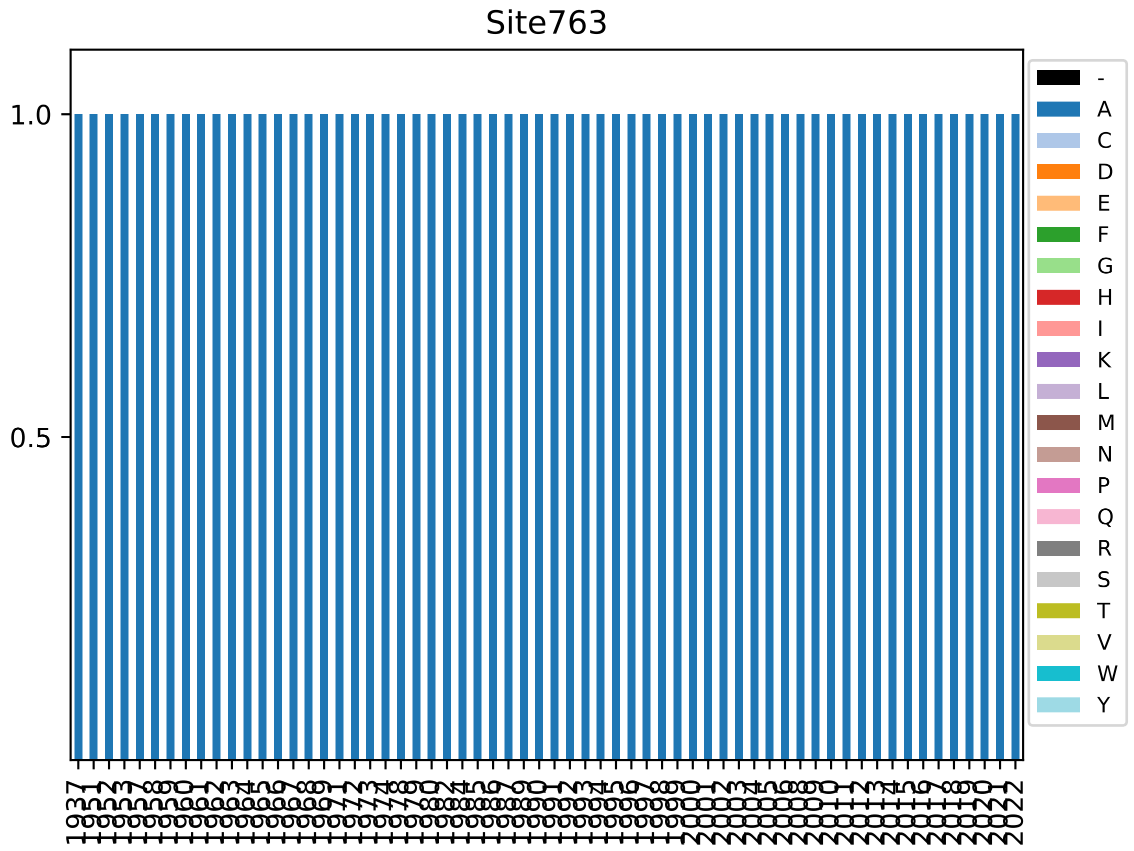
Site763
1.0
0.5
-
A
C
D
E
F
G
H
I
K
L
M
N
P
Q
R
S
T
V
W
Y

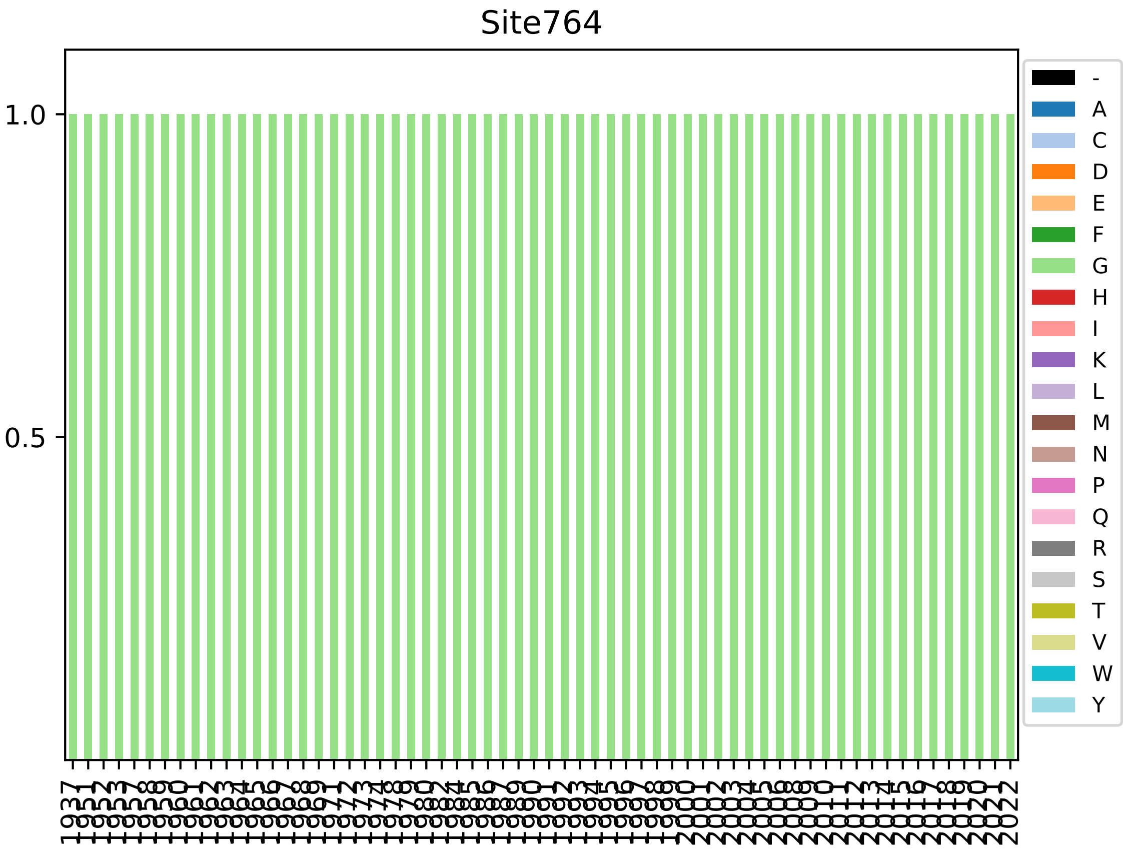
Site764
1.0
0.5
-
A
C
D
E
F
G
H
I
K
L
M
N
P
Q
R
S
T
V
W
Y

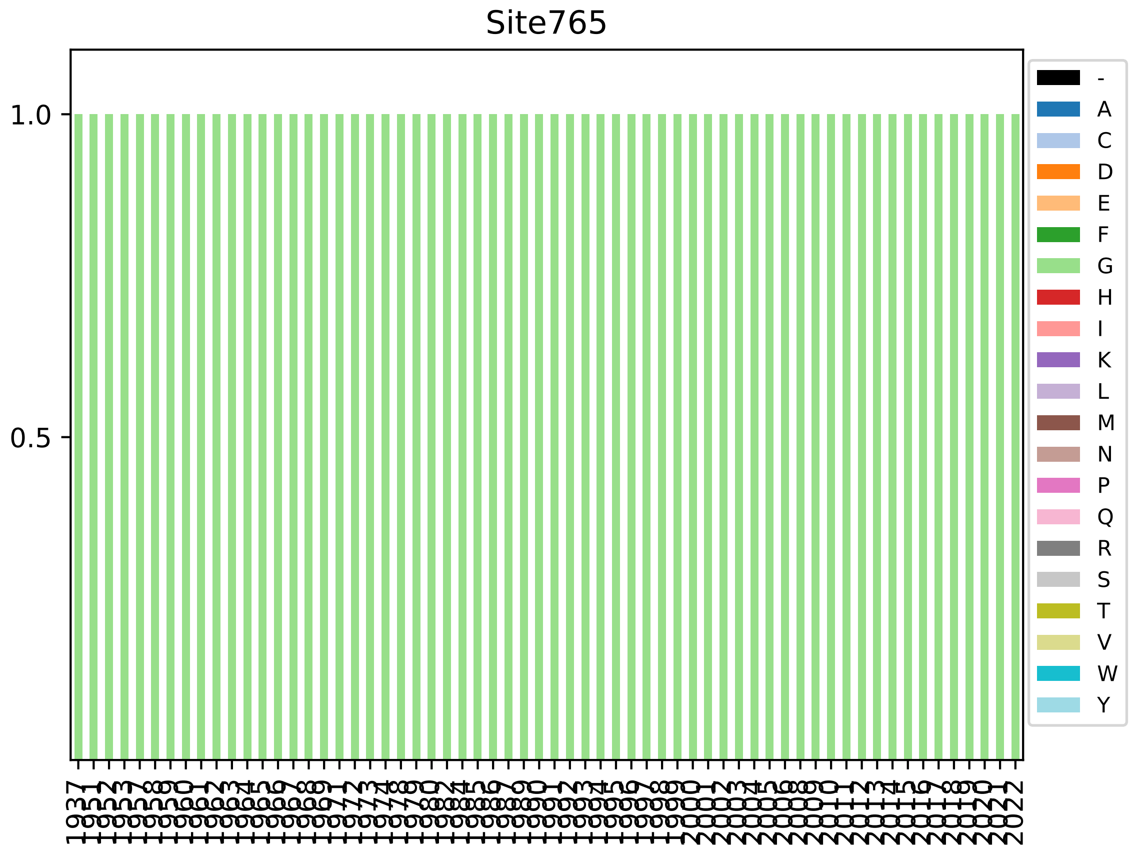
Site765
1.0
0.5
-
A
C
D
E
F
G
H
I
K
L
M
N
P
Q
R
S
T
V
W
Y

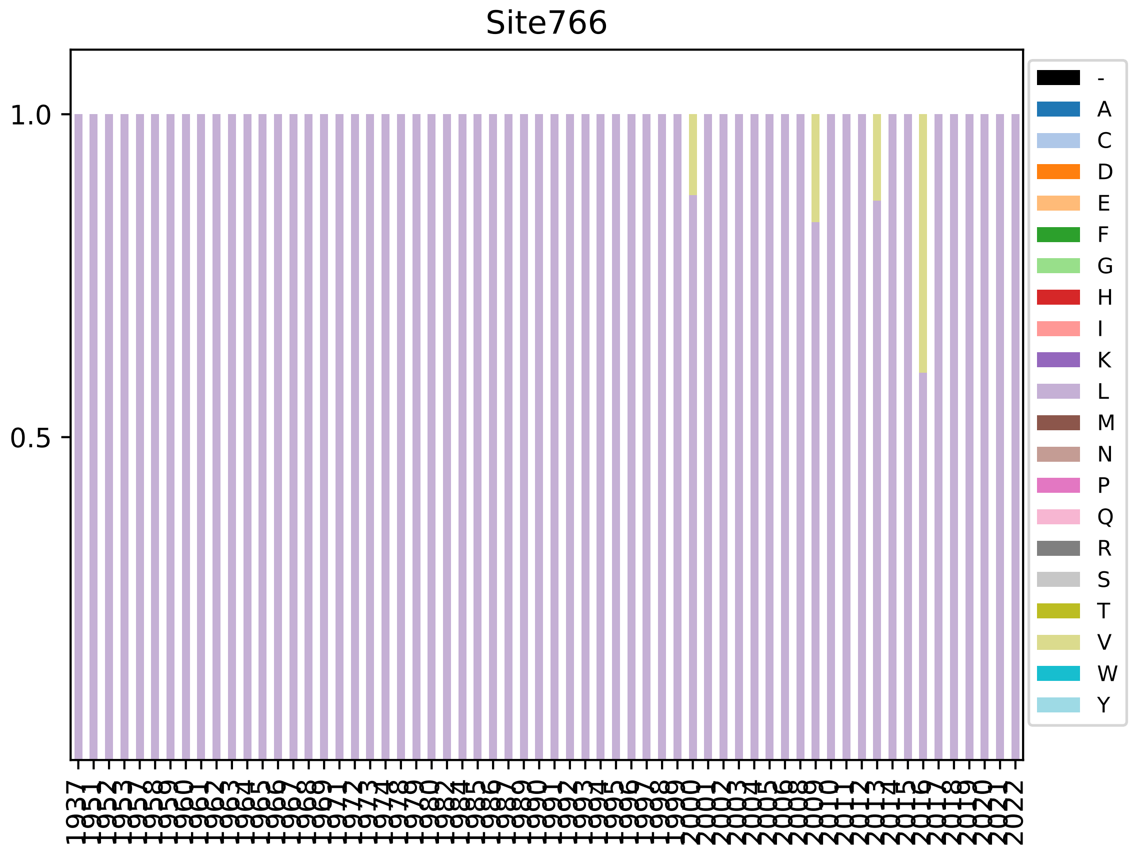
Site766
1.0
0.5
-
A
C
D
E
F
G
H
I
K
L
M
N
P
Q
R
S
T
V
W
Y

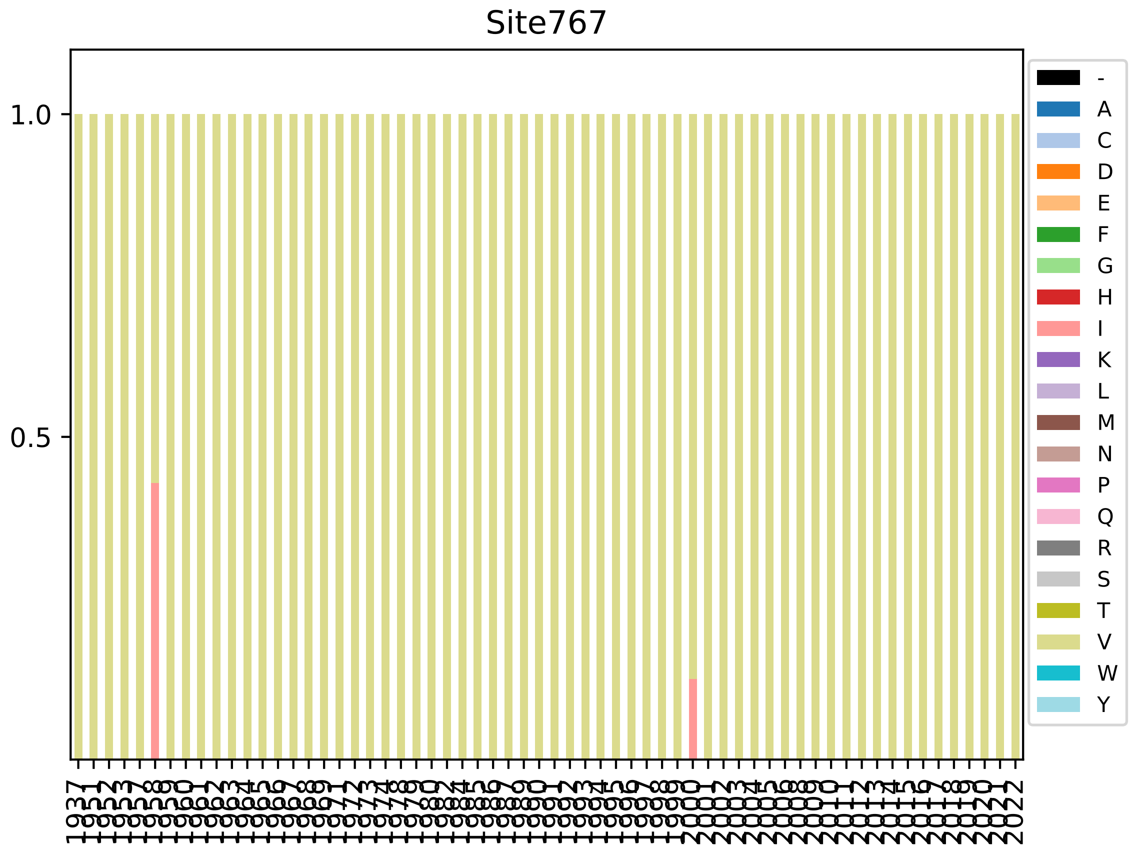
Site767
1.0
0.5
-
A
C
D
E
F
G
H
I
K
L
M
N
P
Q
R
S
T
V
W
Y

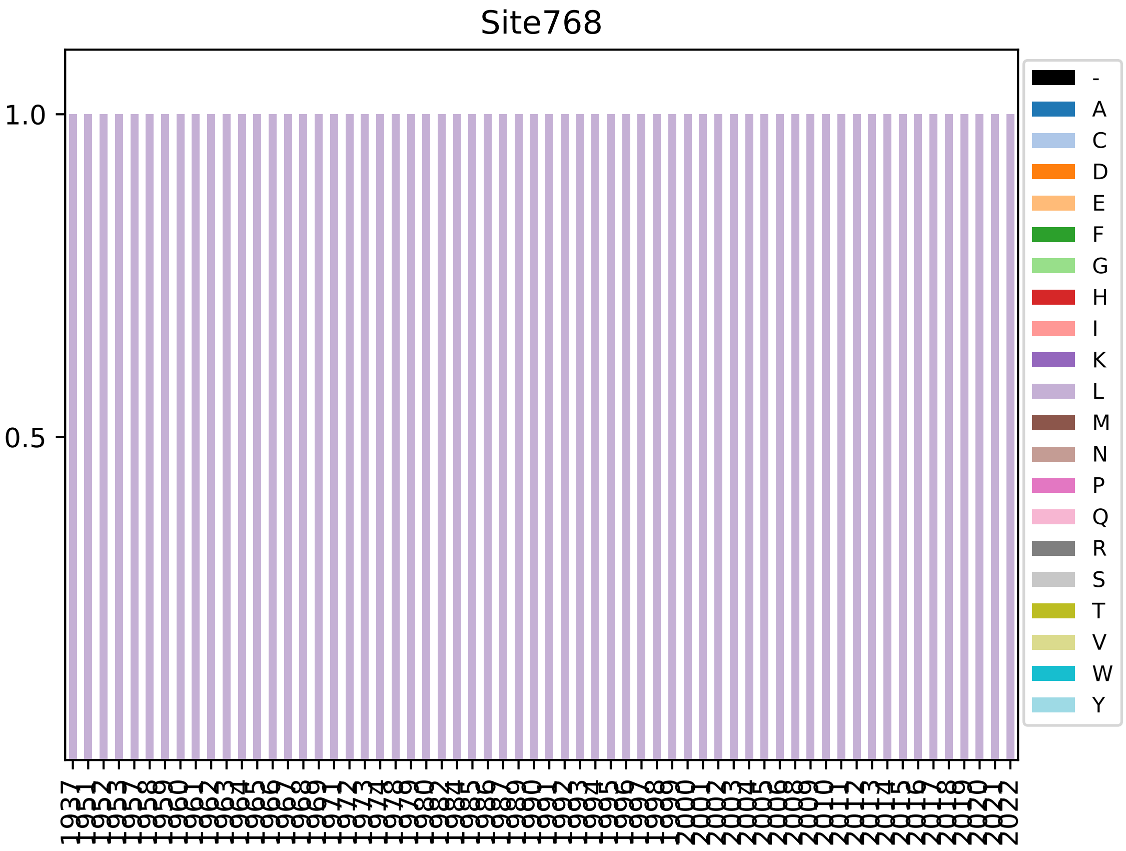
Site768
1.0
0.5
-
A
C
D
E
F
G
H
I
K
L
M
N
P
Q
R
S
T
V
W
Y

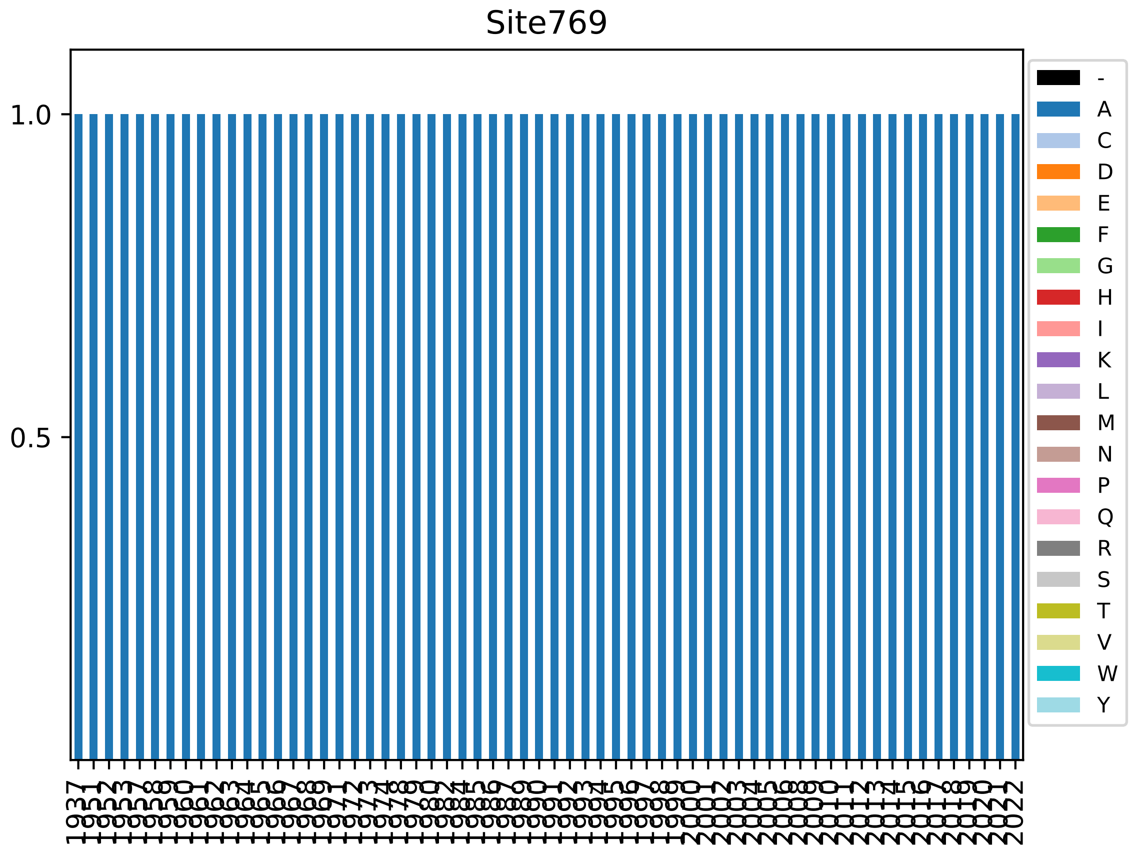

Site769
1.0
0.5
-
A
C
D
E
F
G
H
I
K
L
M
N
P
Q
R
S
T
V
W
Y

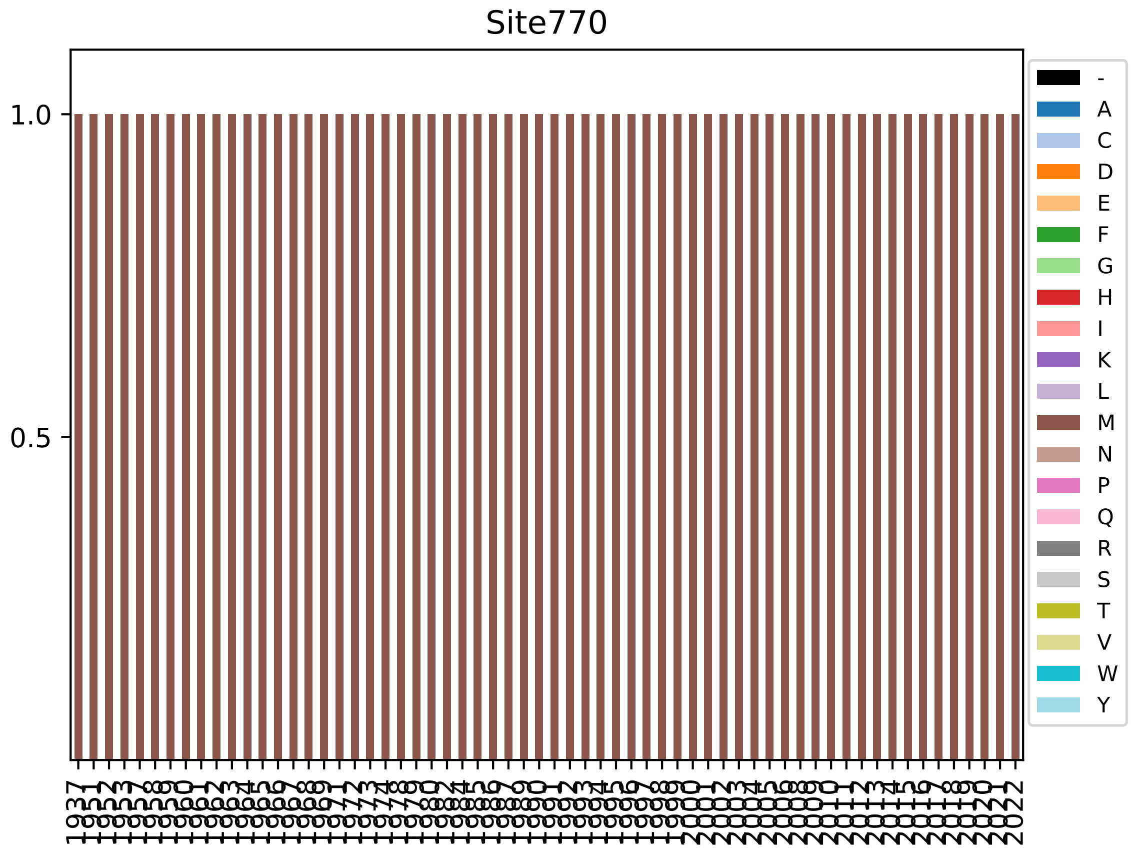
Site770
1.0
0.5
-
A
C
D
E
F
G
H
I
K
L
M
N
P
Q
R
S
T
V
W
Y

Site771

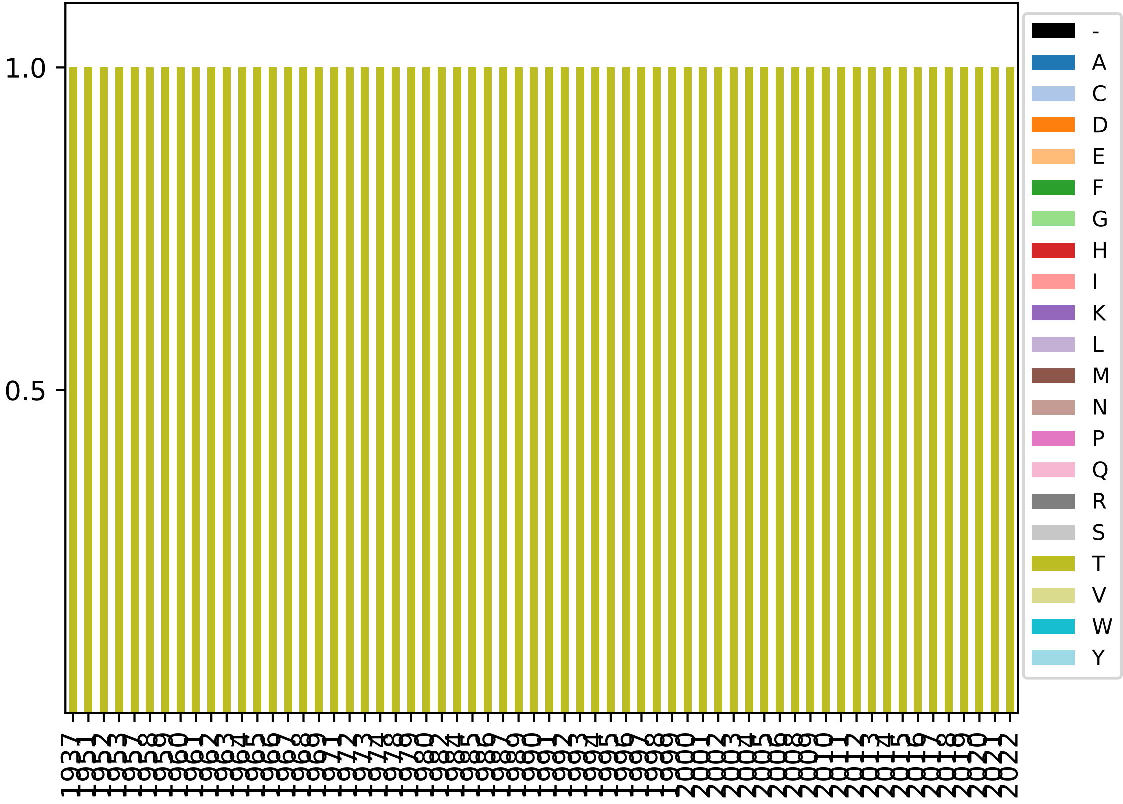

Site772

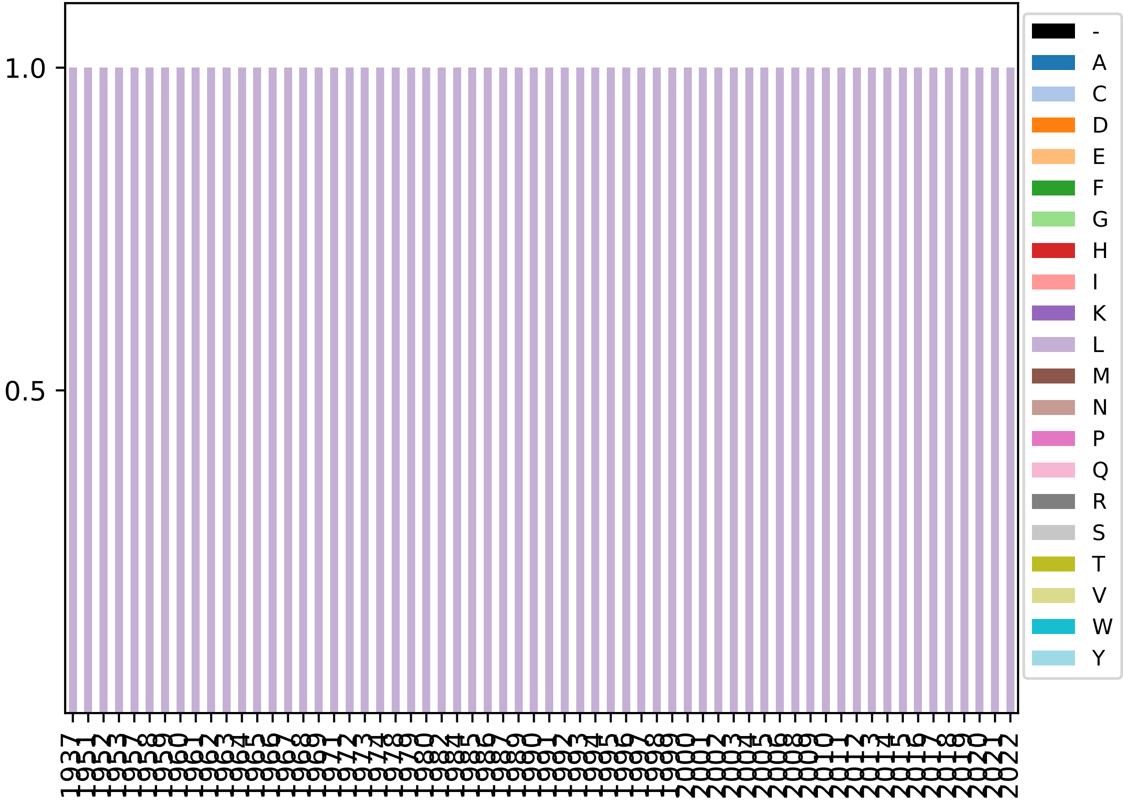

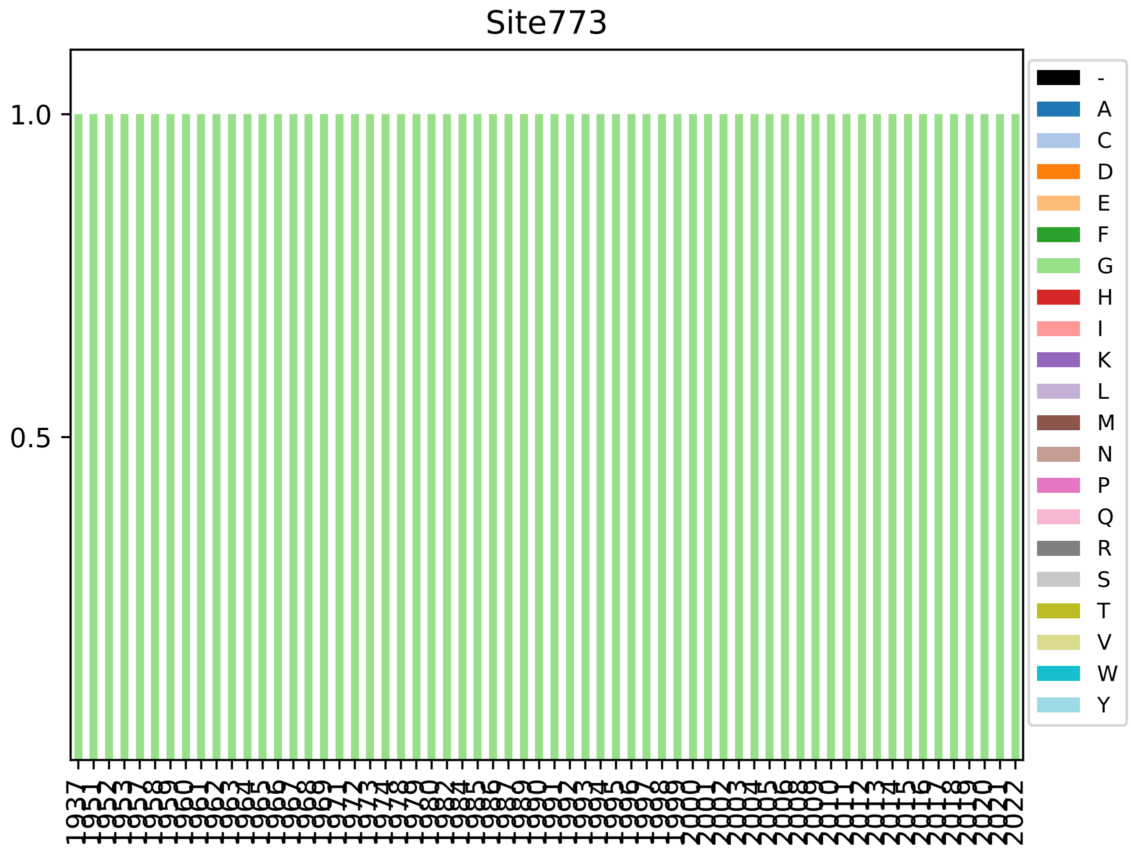
Site773
1.0
0.5
-
A
C
D
E
F
G
H
I
K
L
M
N
P
Q
R
S
T
V
W
Y

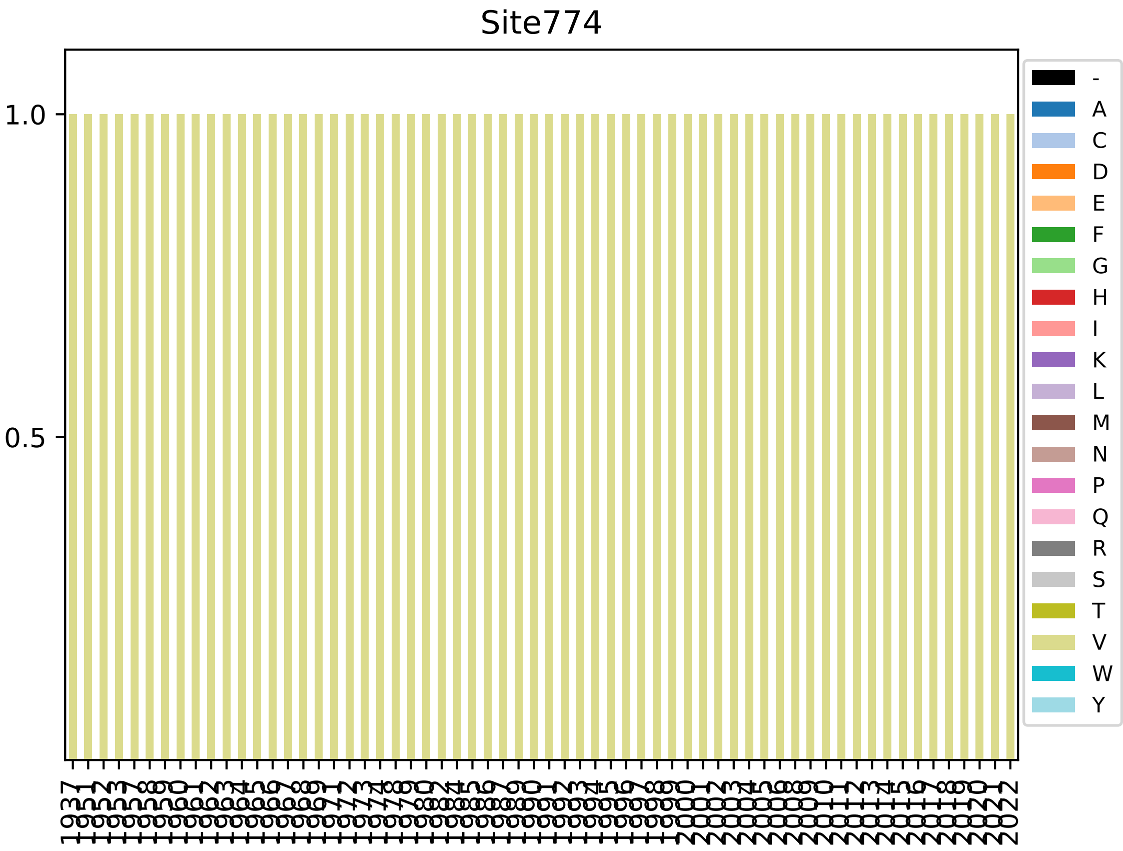
Site774
1.0
0.5
-
A
C
D
E
F
G
H
I
K
L
M
N
P
Q
R
S
T
V
W
Y

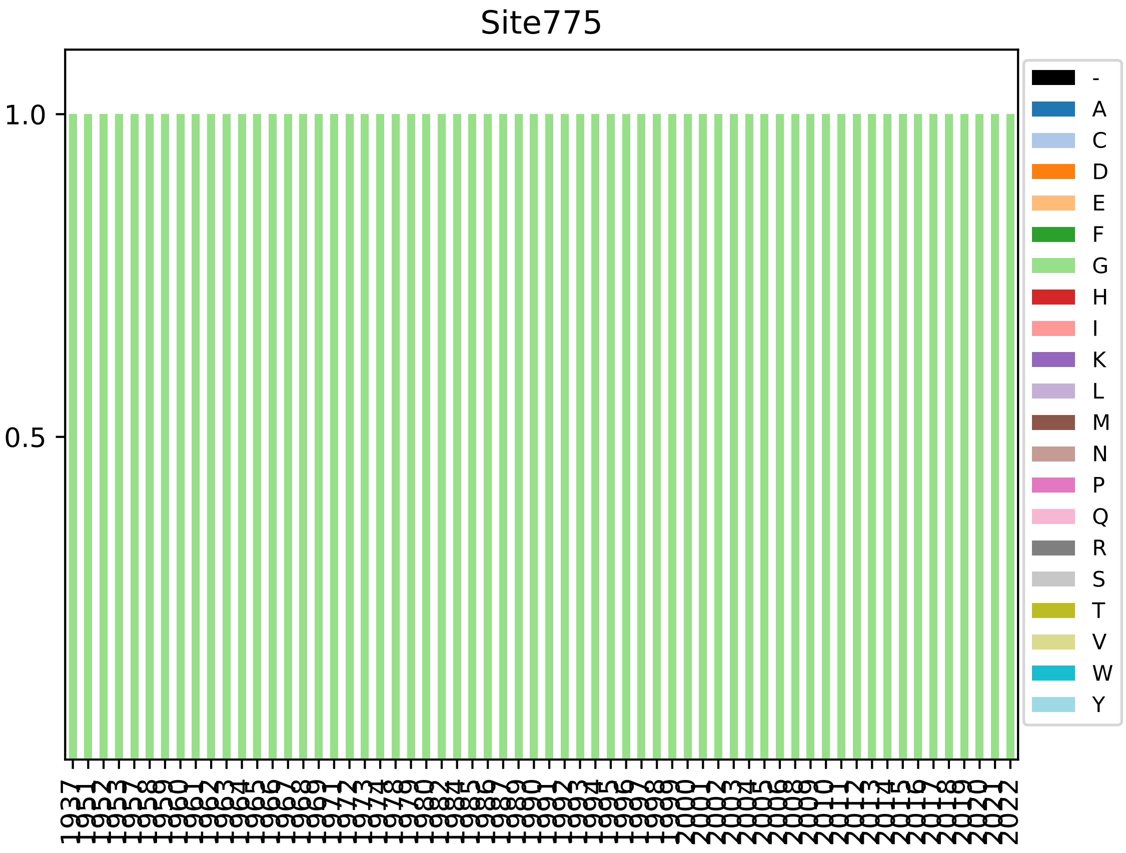
Site775
1.0
0.5
-
A
C
D
E
F
G
H
I
K
L
M
N
P
Q
R
S
T
V
W
Y

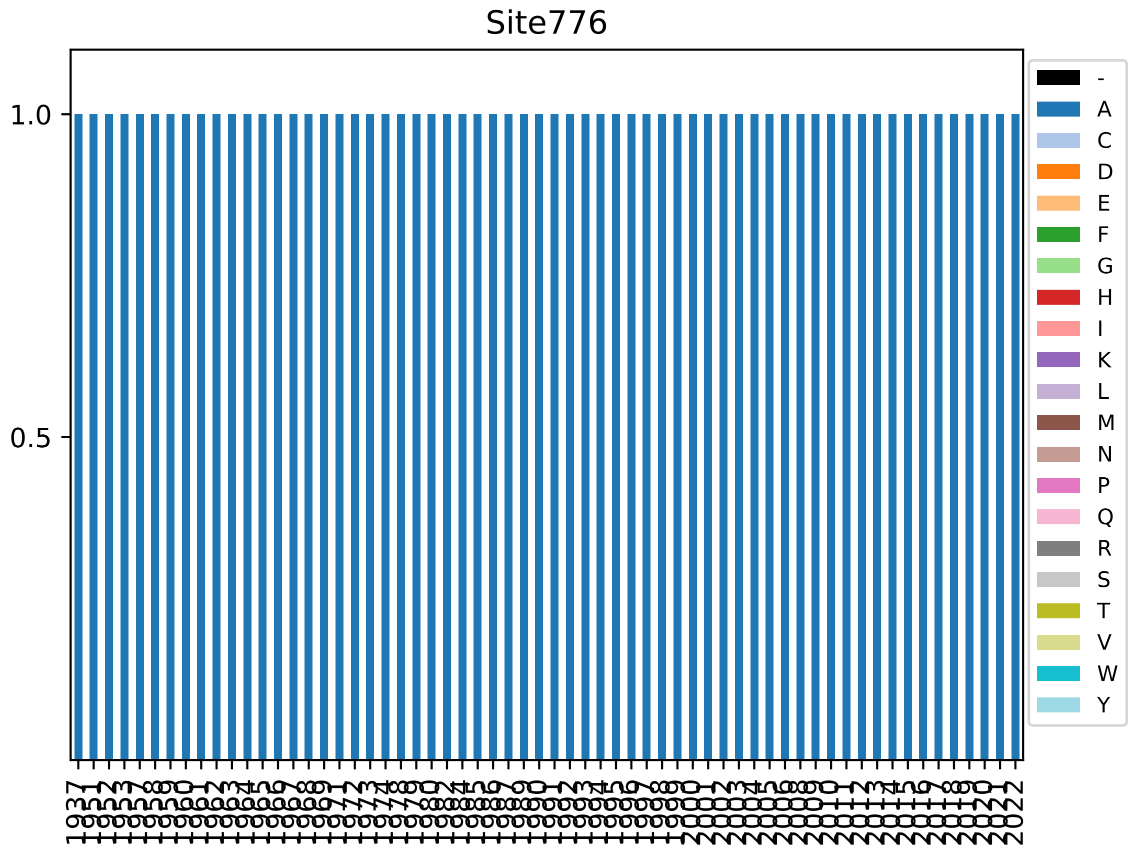
Site776
1.0
0.5
-
A
C
D
E
F
G
H
I
K
L
M
N
P
Q
R
S
T
V
W
Y

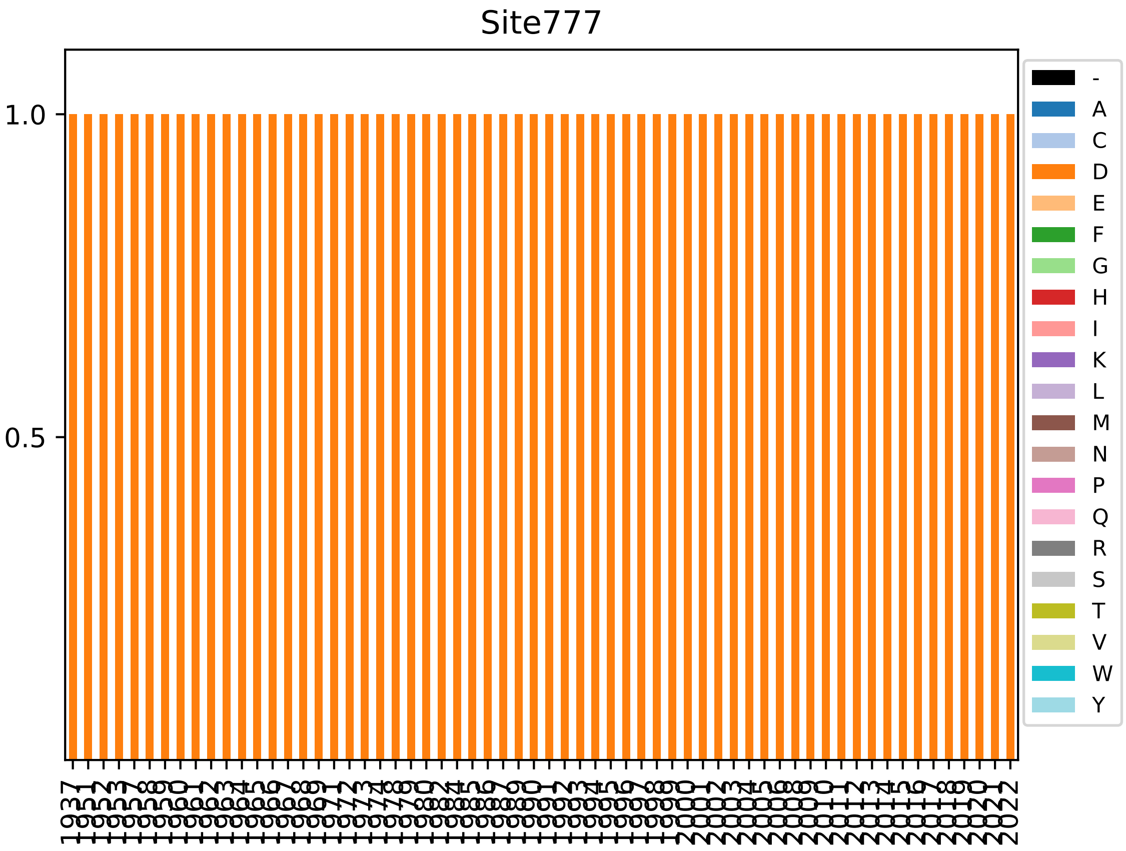

Site777
1.0
0.5
-
A
C
D
E
F
G
H
I
K
L
M
N
P
Q
R
S
T
V
W
Y

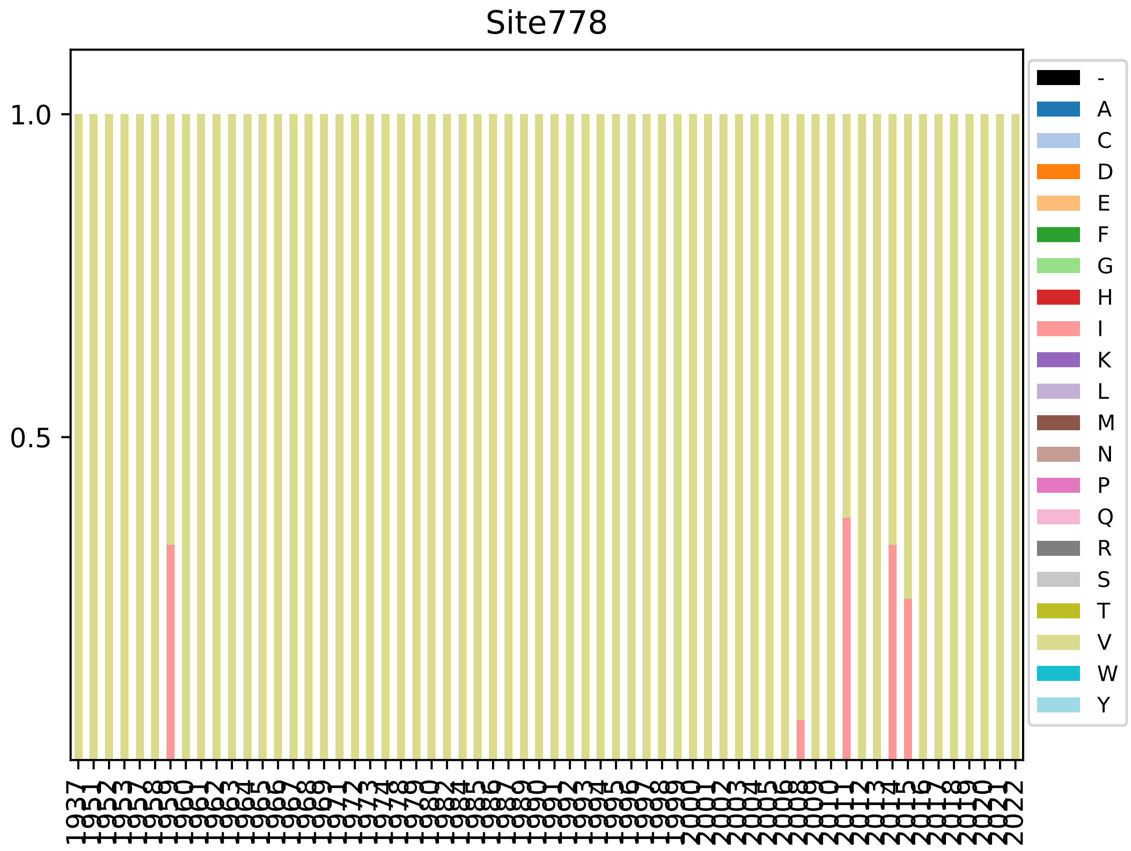
Site778
1.0
0.5
-
A
C
D
E
F
G
H
I
K
L
M
N
P
Q
R
S
T
V
W
Y

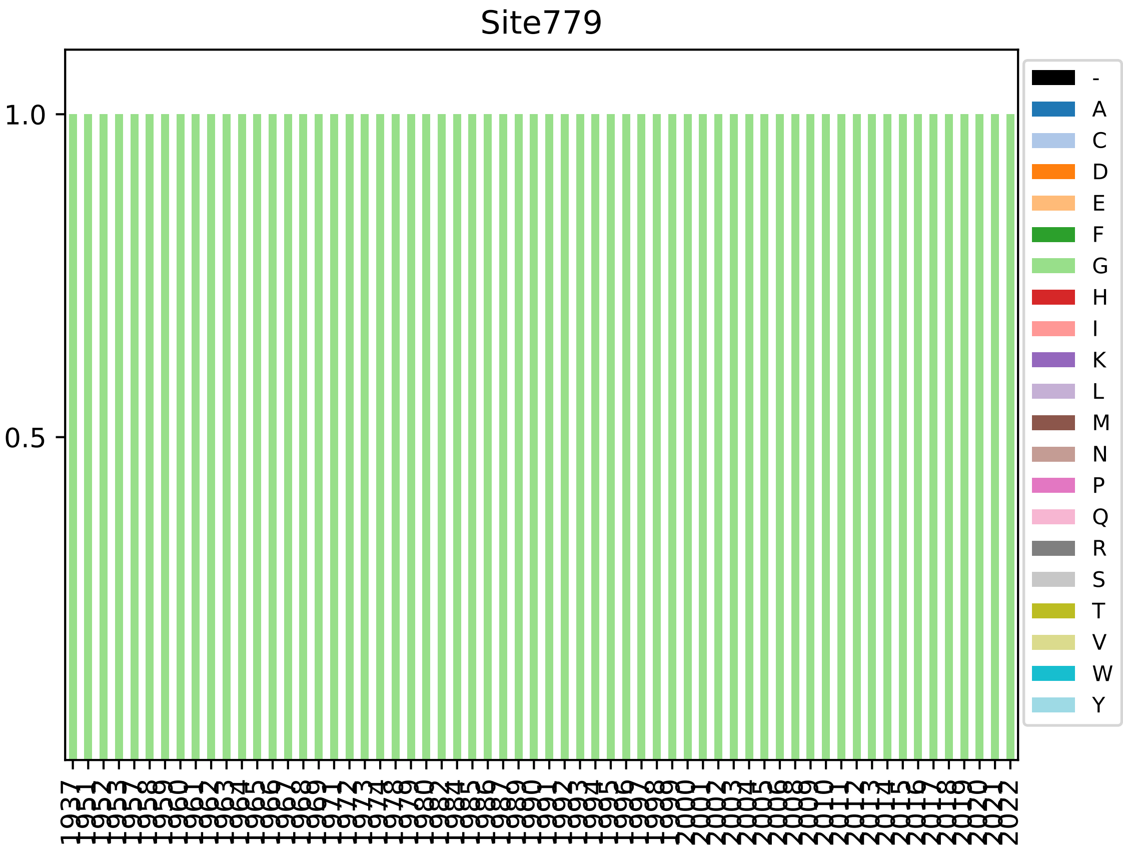
Site779
1.0
0.5
-
A
C
D
E
F
G
H
I
K
L
M
N
P
Q
R
S
T
V
W
Y

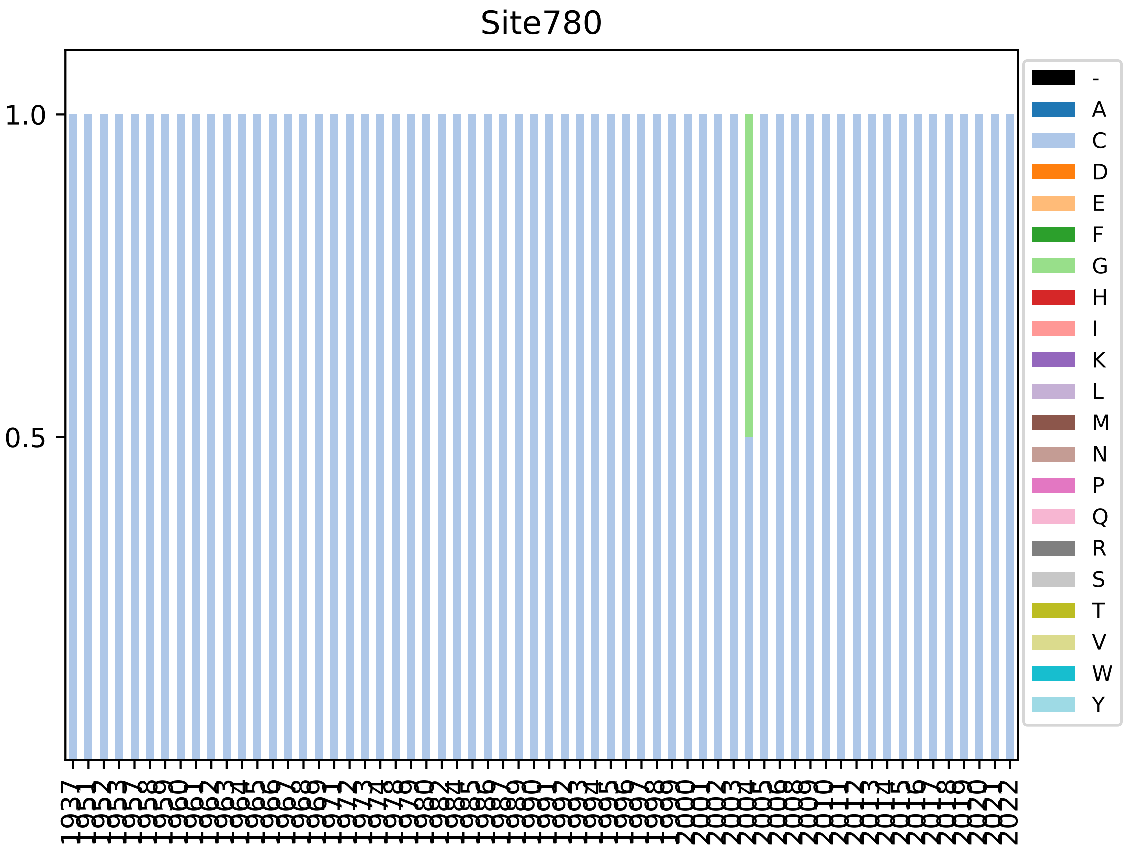
Site780
1.0
0.5
-
A
C
D
E
F
G
H
I
K
L
M
N
P
Q
R
S
T
V
W
Y

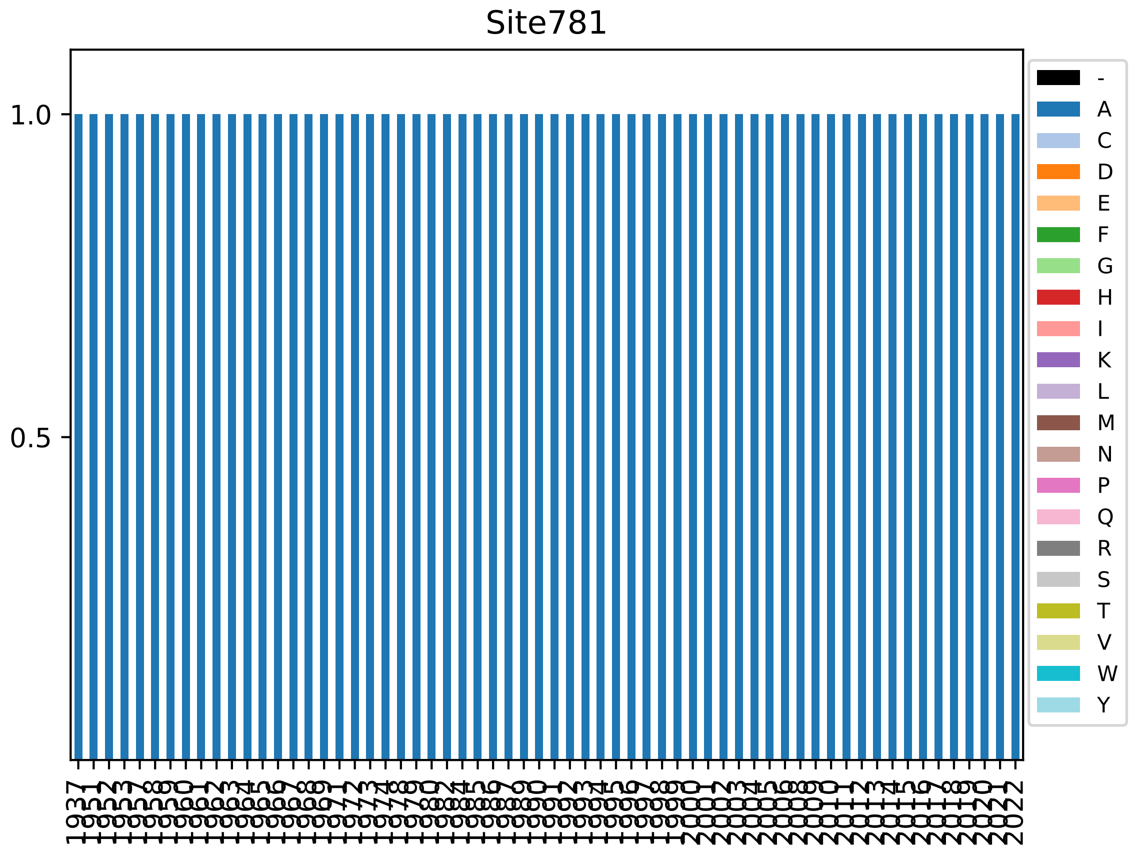

Site781
1.0
0.5
-
A
C
D
E
F
G
H
I
K
L
M
N
P
Q
R
S
T
V
W
Y

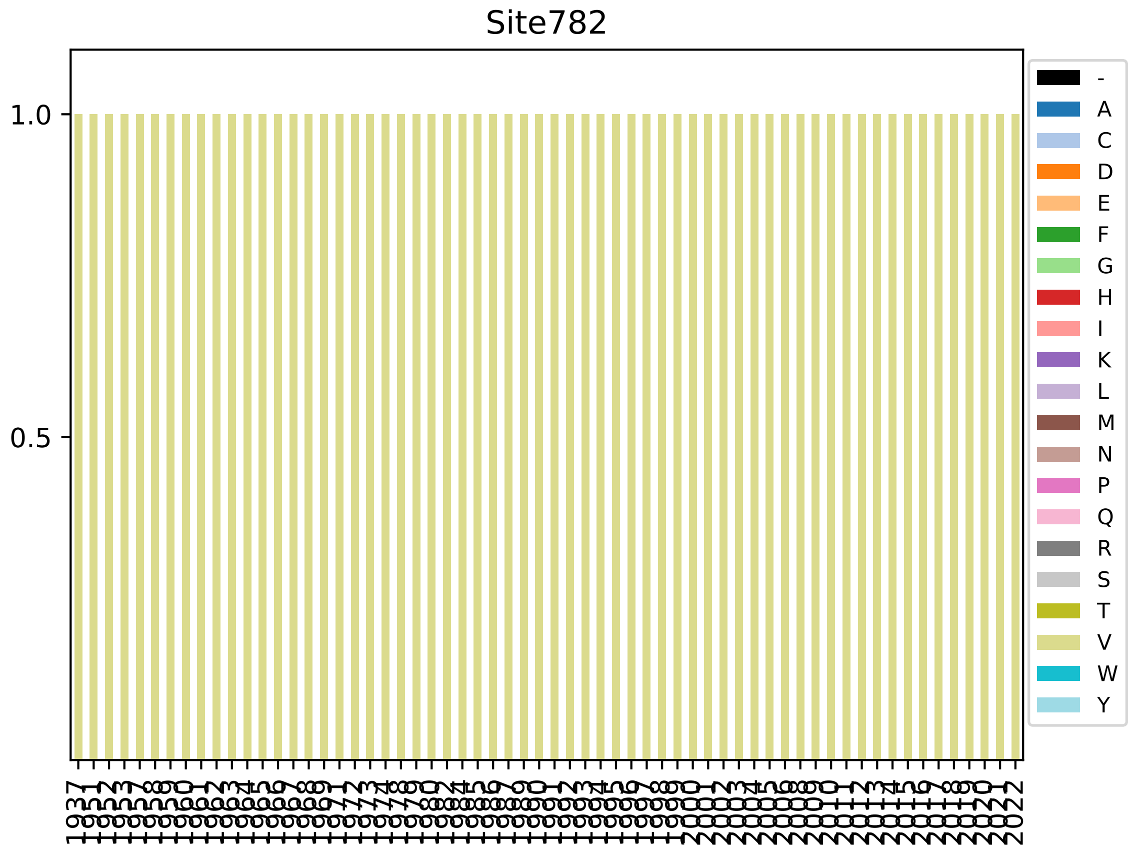
Site782
1.0
0.5
-
A
C
D
E
F
G
H
I
K
L
M
N
P
Q
R
S
T
V
W
Y

Site783

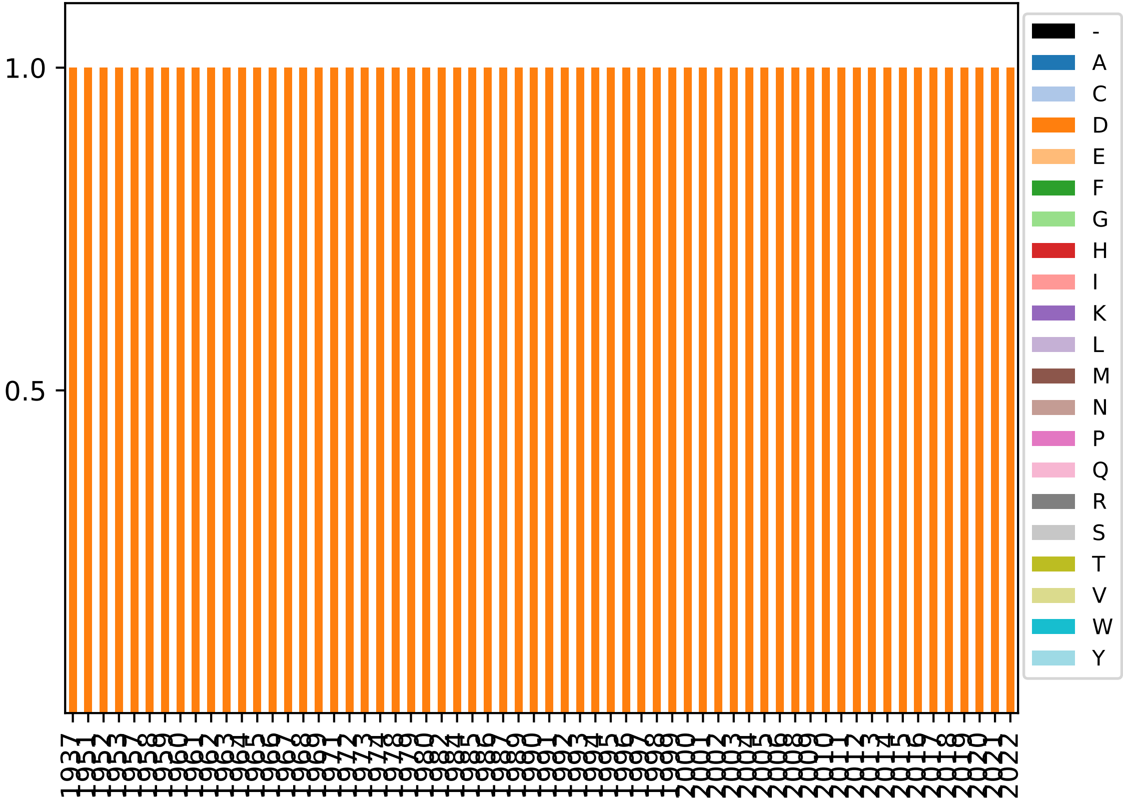

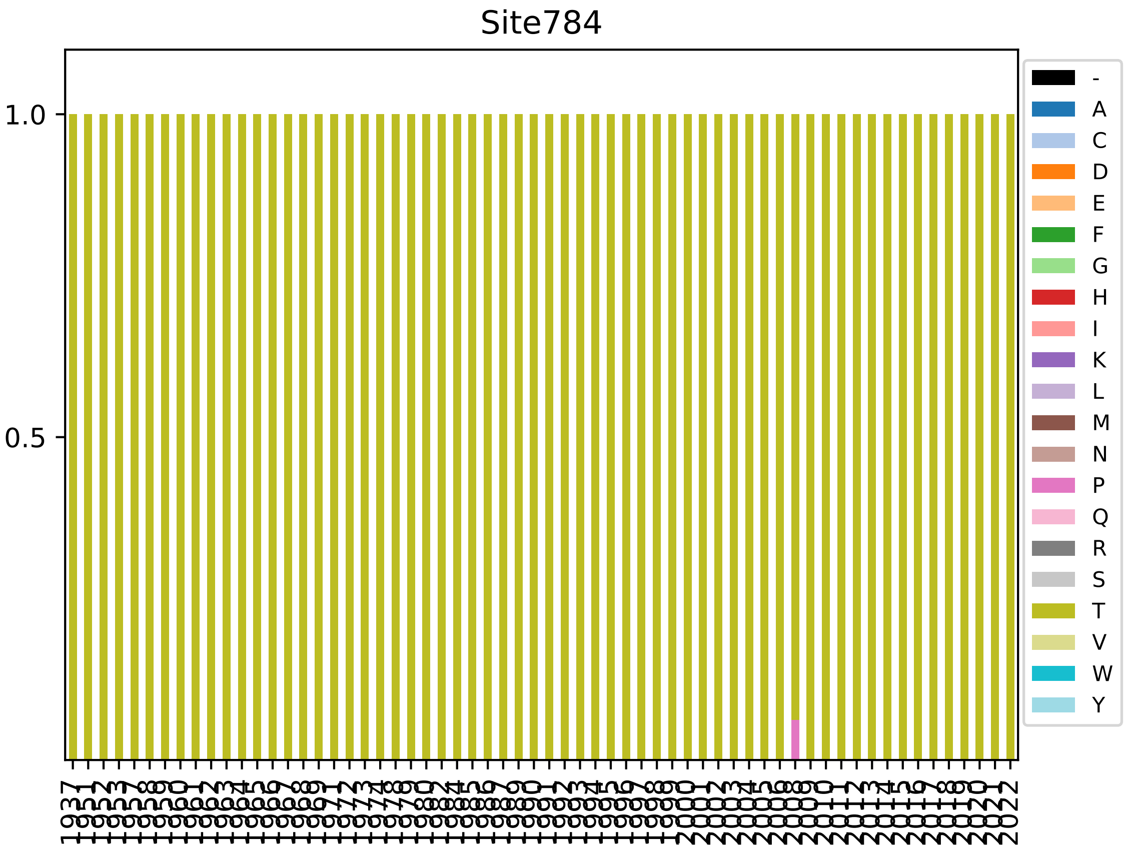

Site784
1.0
0.5
-
A
C
D
E
F
G
H
I
K
L
M
N
P
Q
R
S
T
V
W
Y

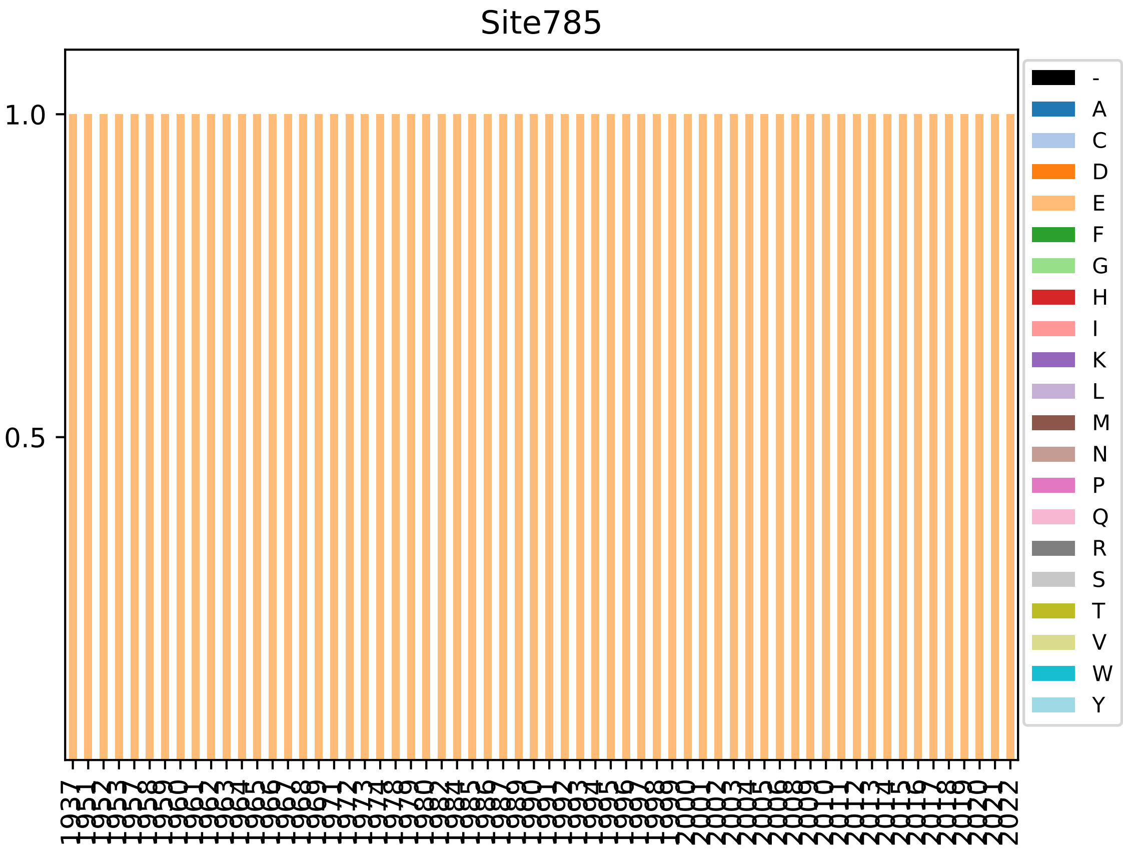
Site785
1.0
0.5
-
A
C
D
E
F
G
H
I
K
L
M
N
P
Q
R
S
T
V
W
Y

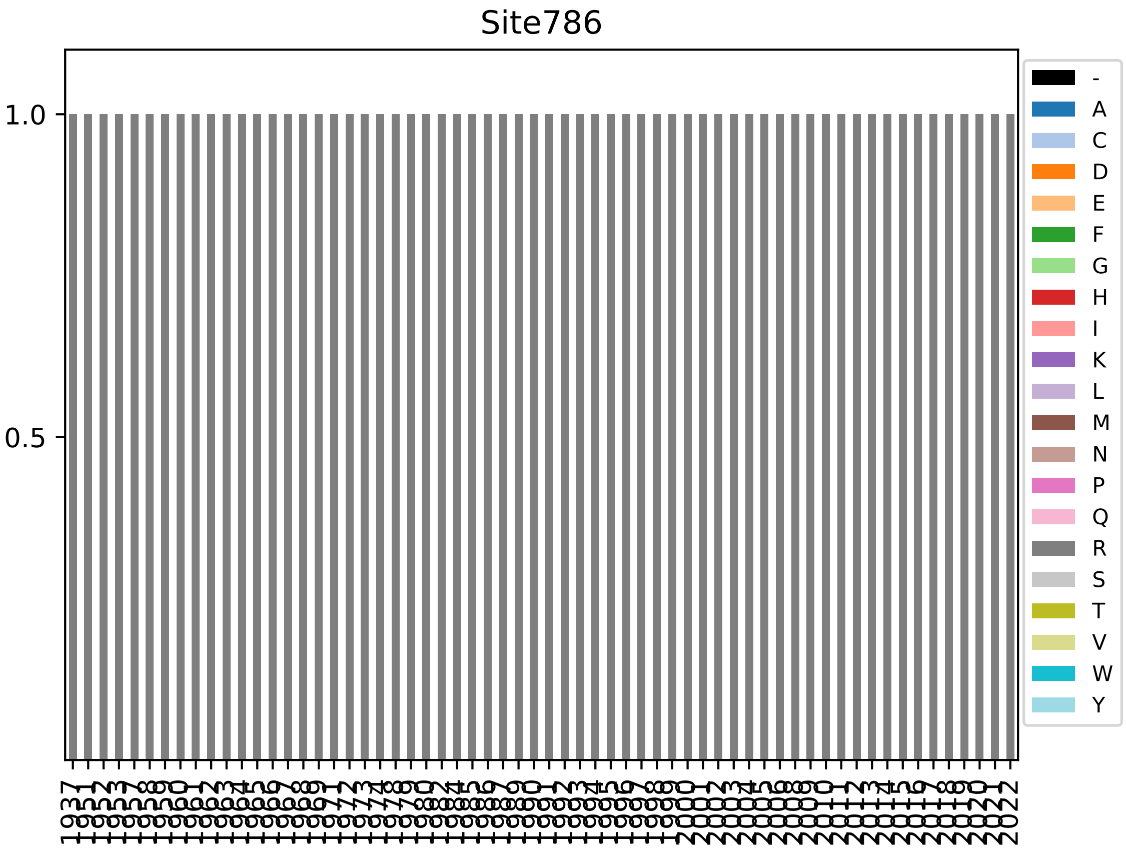
Site786
1.0
0.5
-
A
C
D
E
F
G
H
I
K
L
M
N
P
Q
R
S
T
V
W
Y

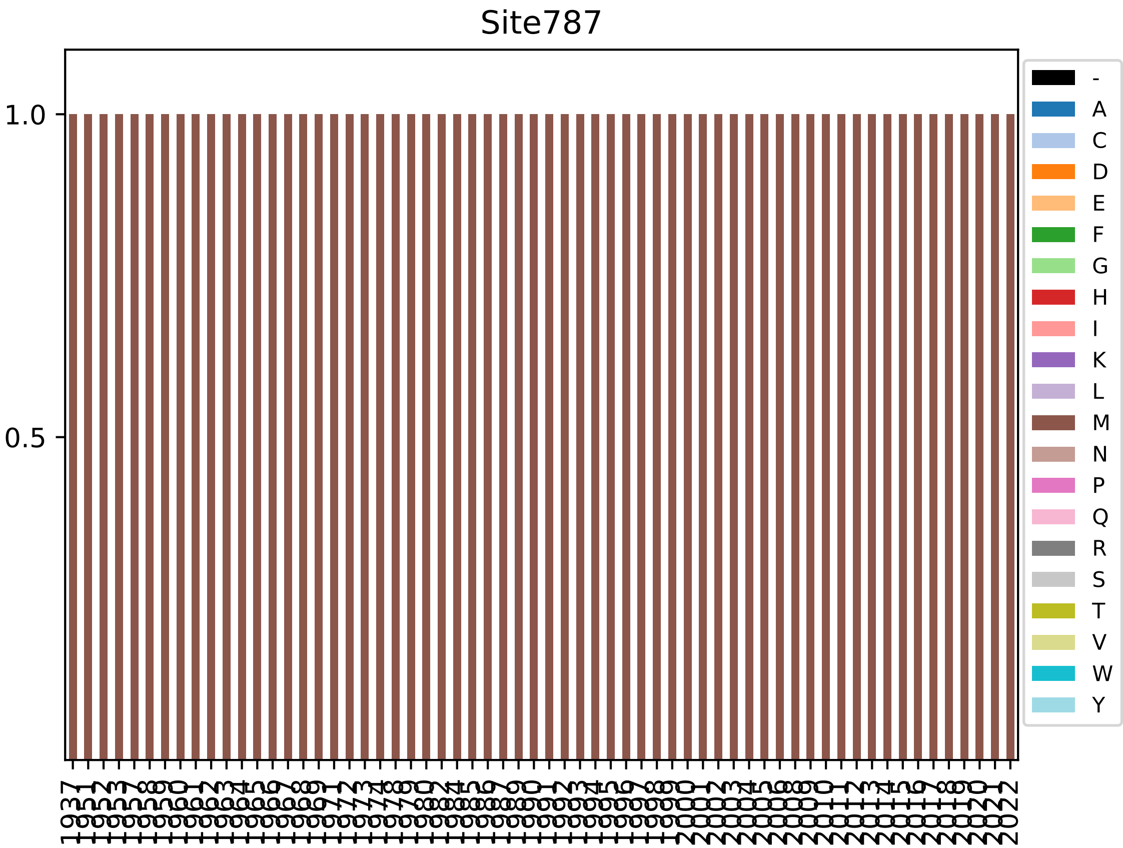
Site787
1.0
0.5
-
A
C
D
E
F
G
H
I
K
L
M
N
P
Q
R
S
T
V
W
Y

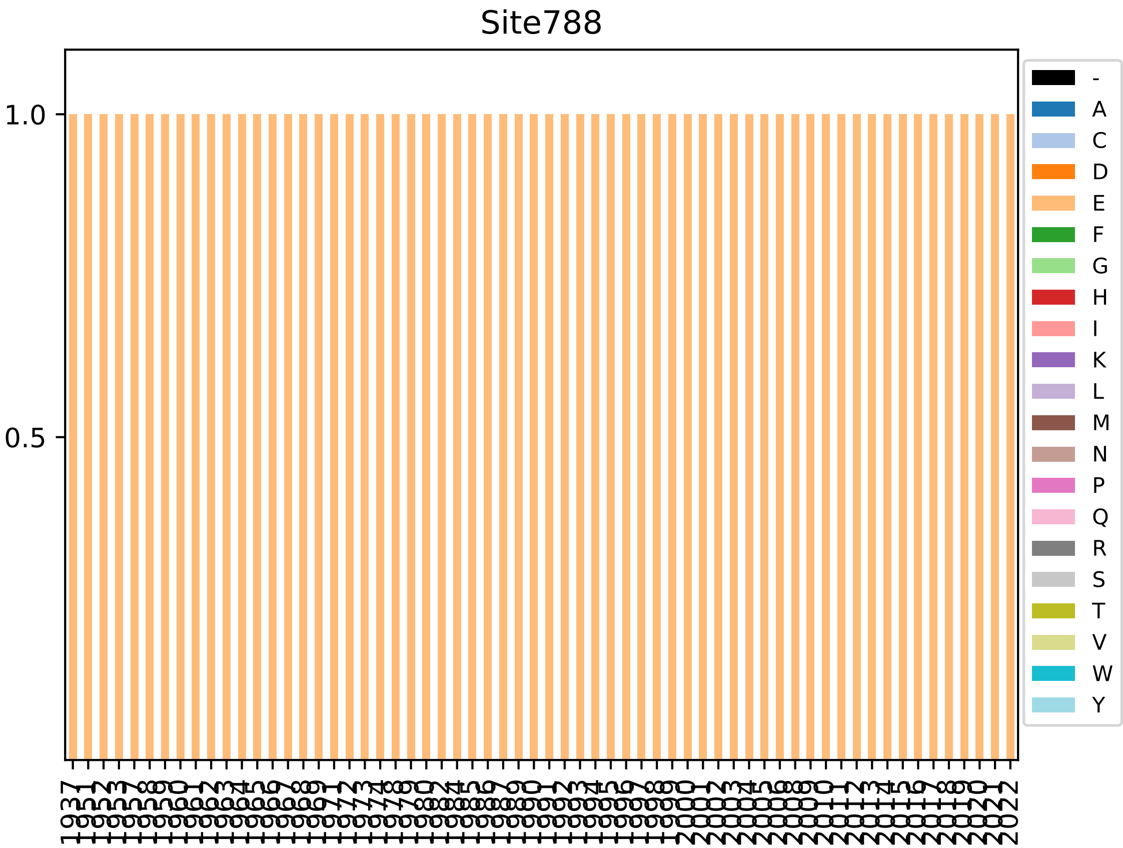
Site788
1.0
0.5
-
A
C
D
E
F
G
H
I
K
L
M
N
P
Q
R
S
T
V
W
Y

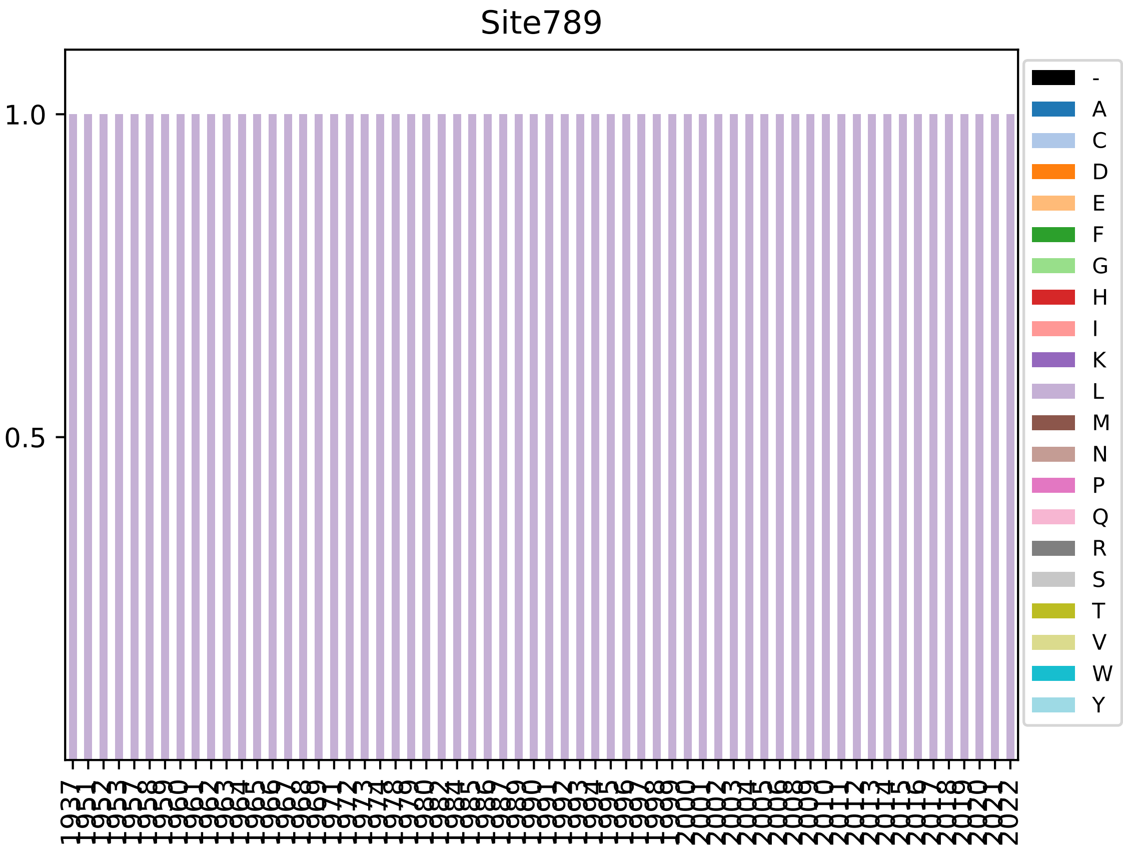
Site789
1.0
0.5
-
A
C
D
E
F
G
H
I
K
L
M
N
P
Q
R
S
T
V
W
Y

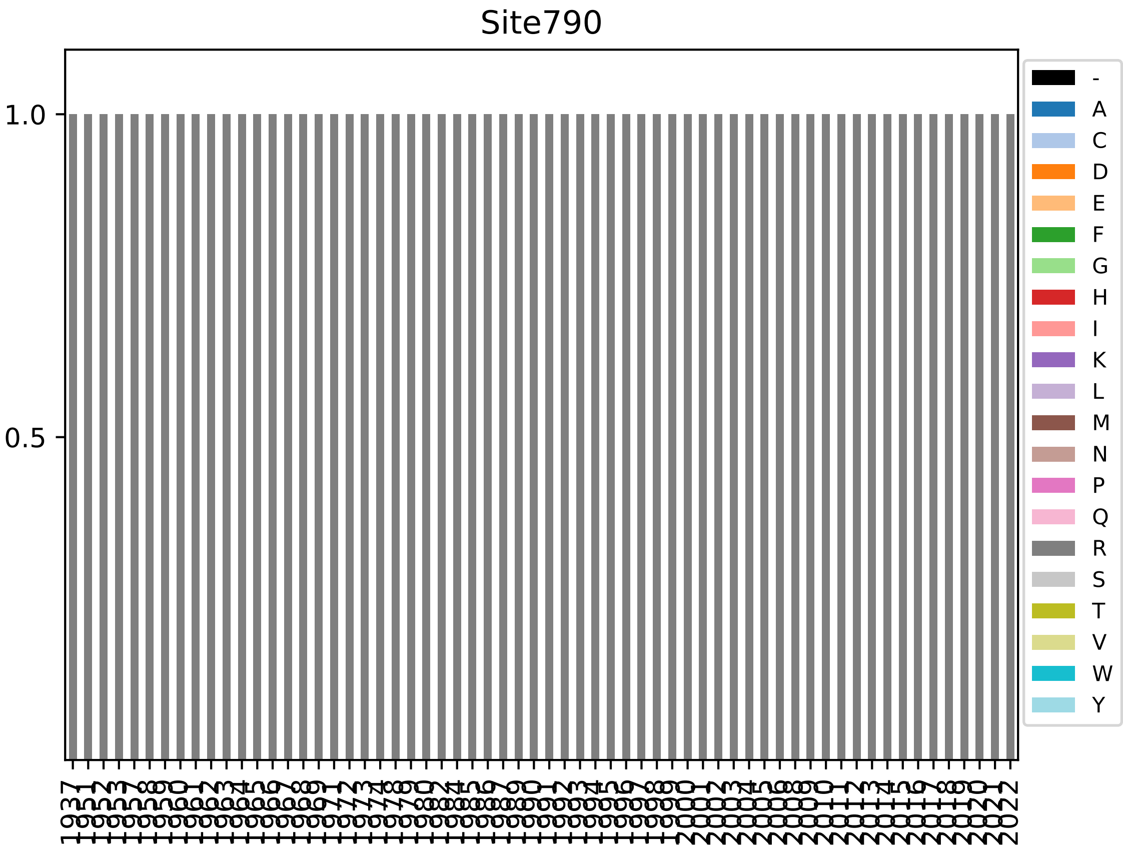
Site790
1.0
0.5
-
A
C
D
E
F
G
H
I
K
L
M
N
P
Q
R
S
T
V
W
Y

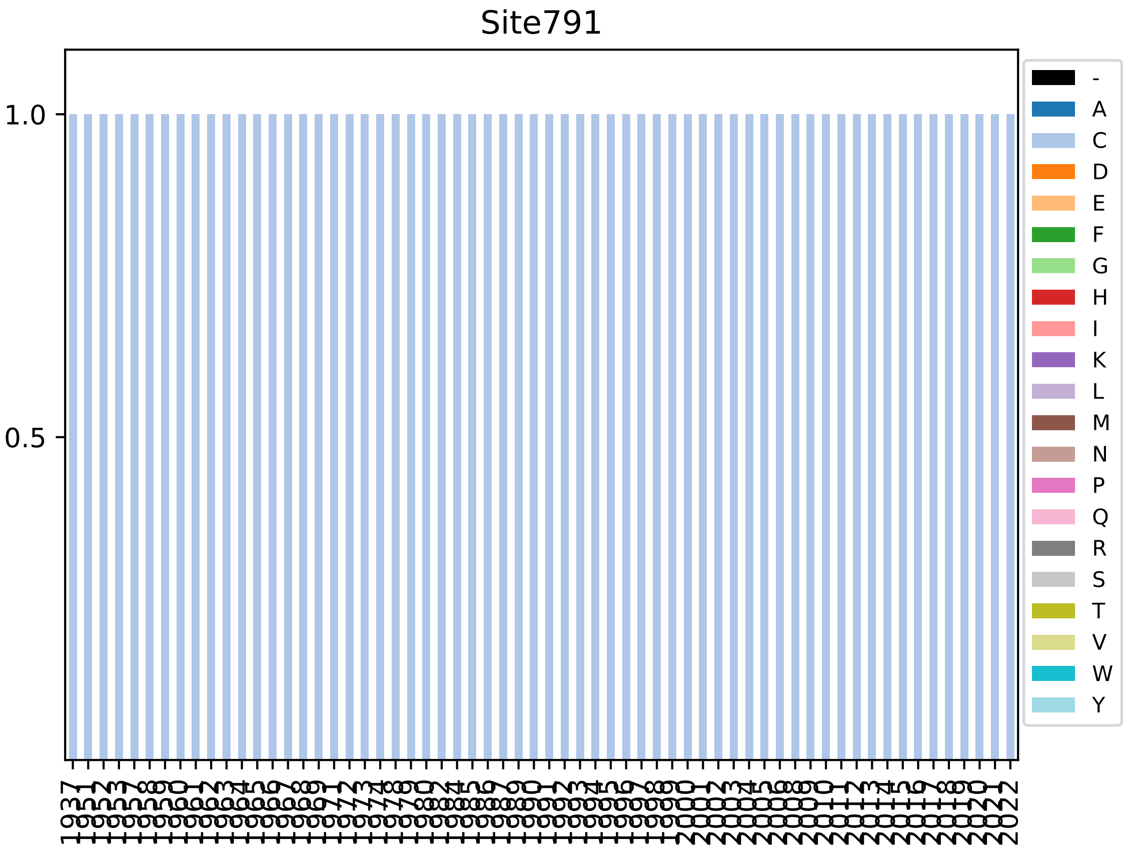
Site791
1.0
0.5
-
A
C
D
E
F
G
H
I
K
L
M
N
P
Q
R
S
T
V
W
Y

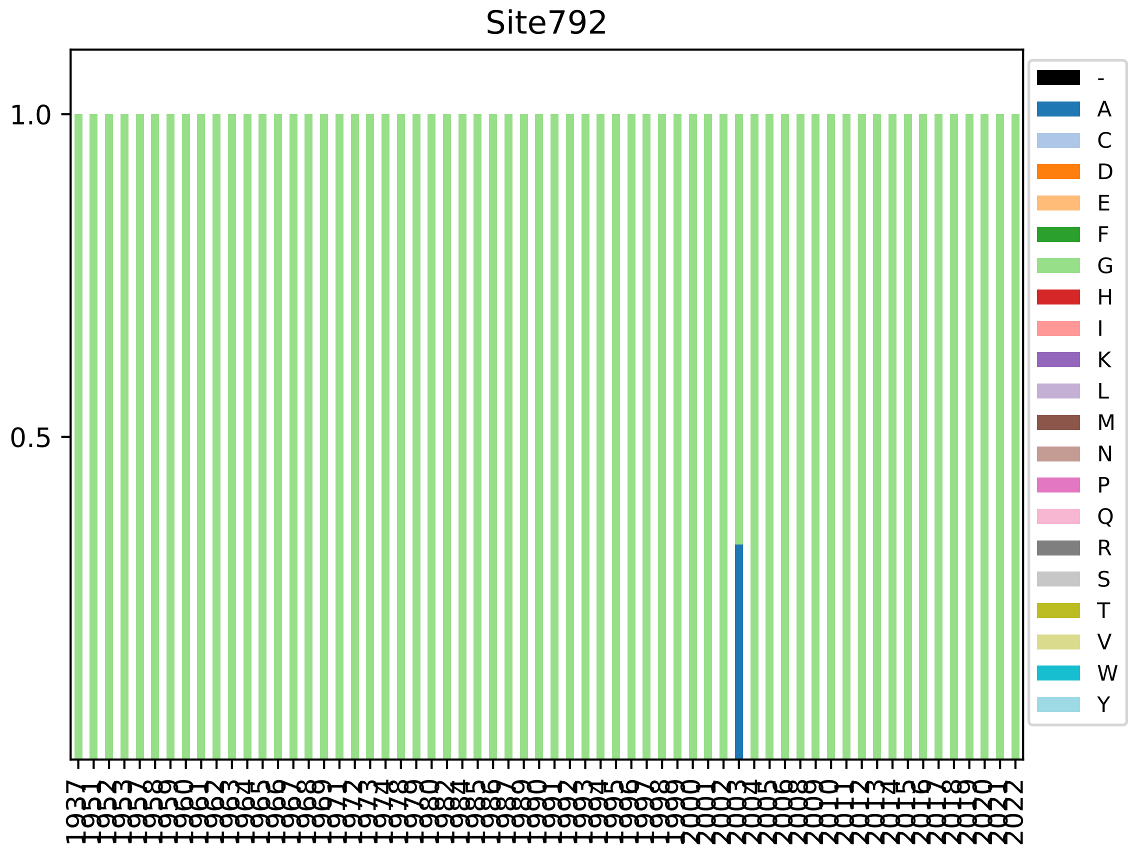
Site792
1.0
0.5
-
A
C
D
E
F
G
H
I
K
L
M
N
P
Q
R
S
T
V
W
Y

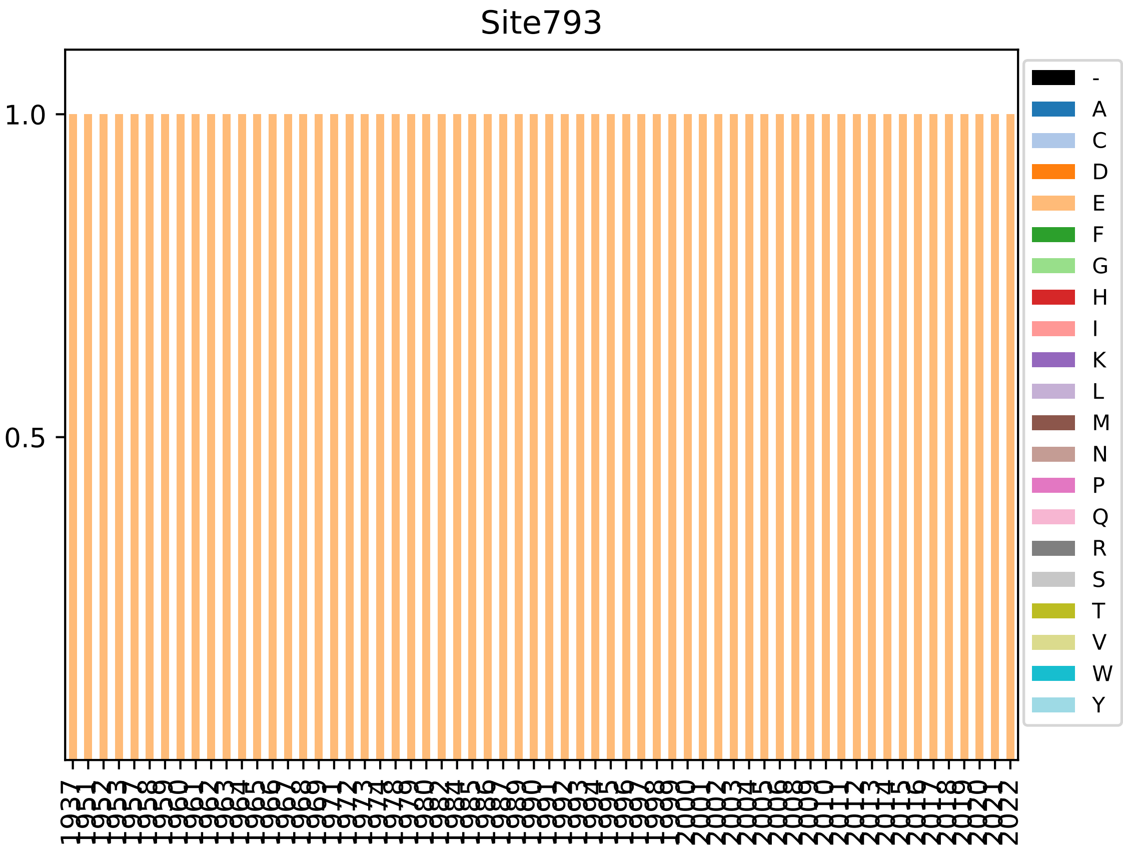
Site793
1.0
0.5
-
A
C
D
E
F
G
H
I
K
L
M
N
P
Q
R
S
T
V
W
Y

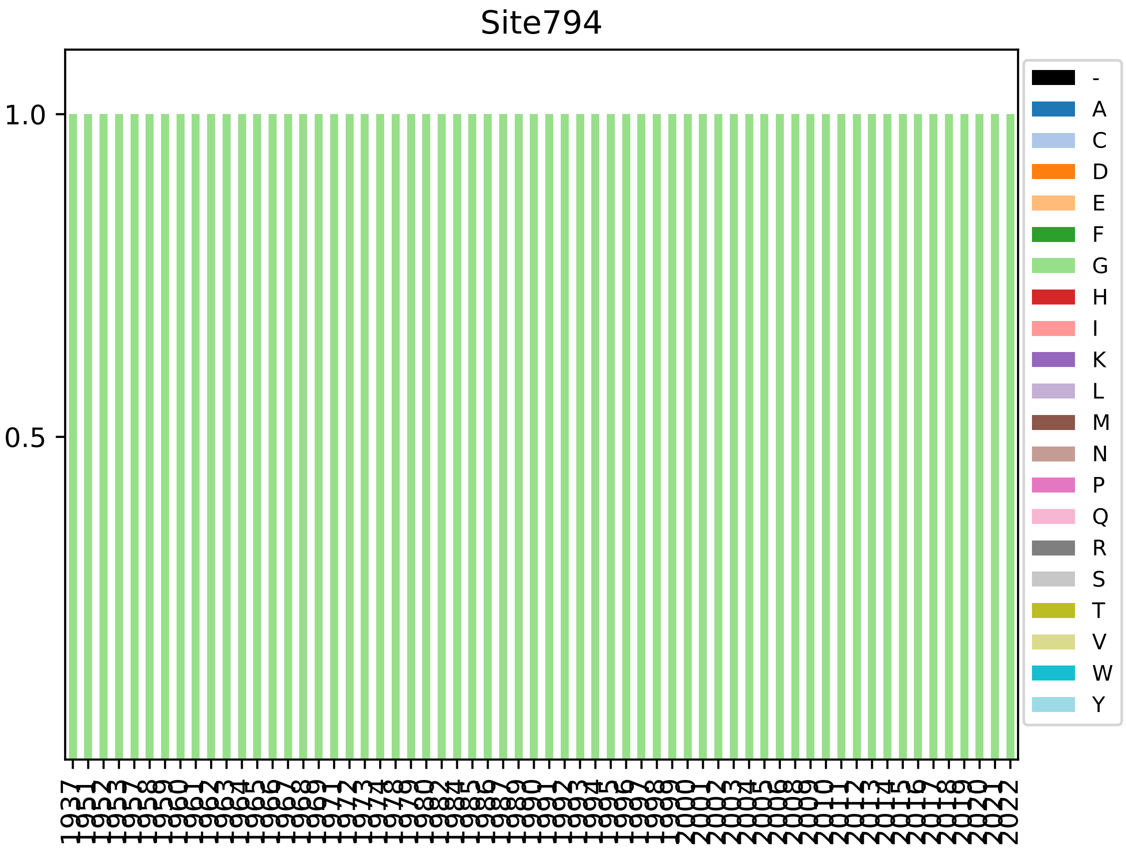
Site794
1.0
0.5
-
A
C
D
E
F
G
H
I
K
L
M
N
P
Q
R
S
T
V
W
Y

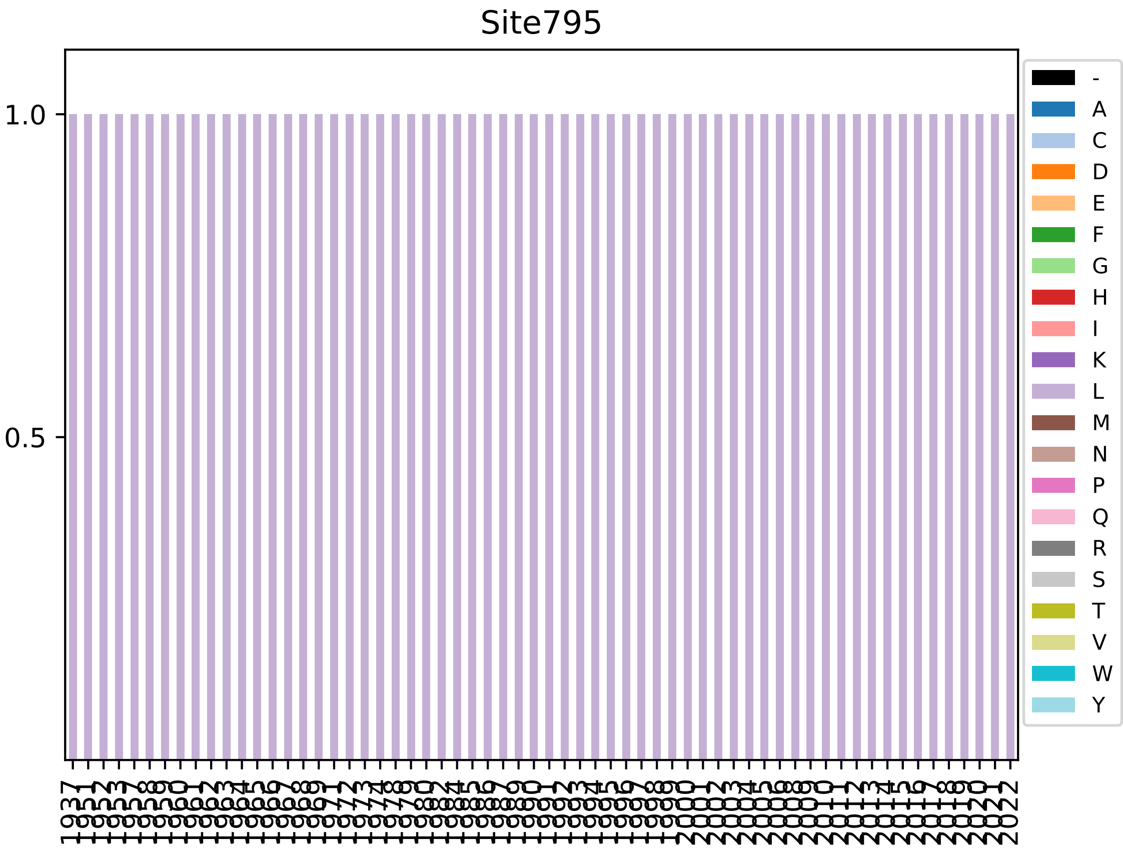

Site795
1.0
0.5
-
A
C
D
E
F
G
H
I
K
L
M
N
P
Q
R
S
T
V
W
Y

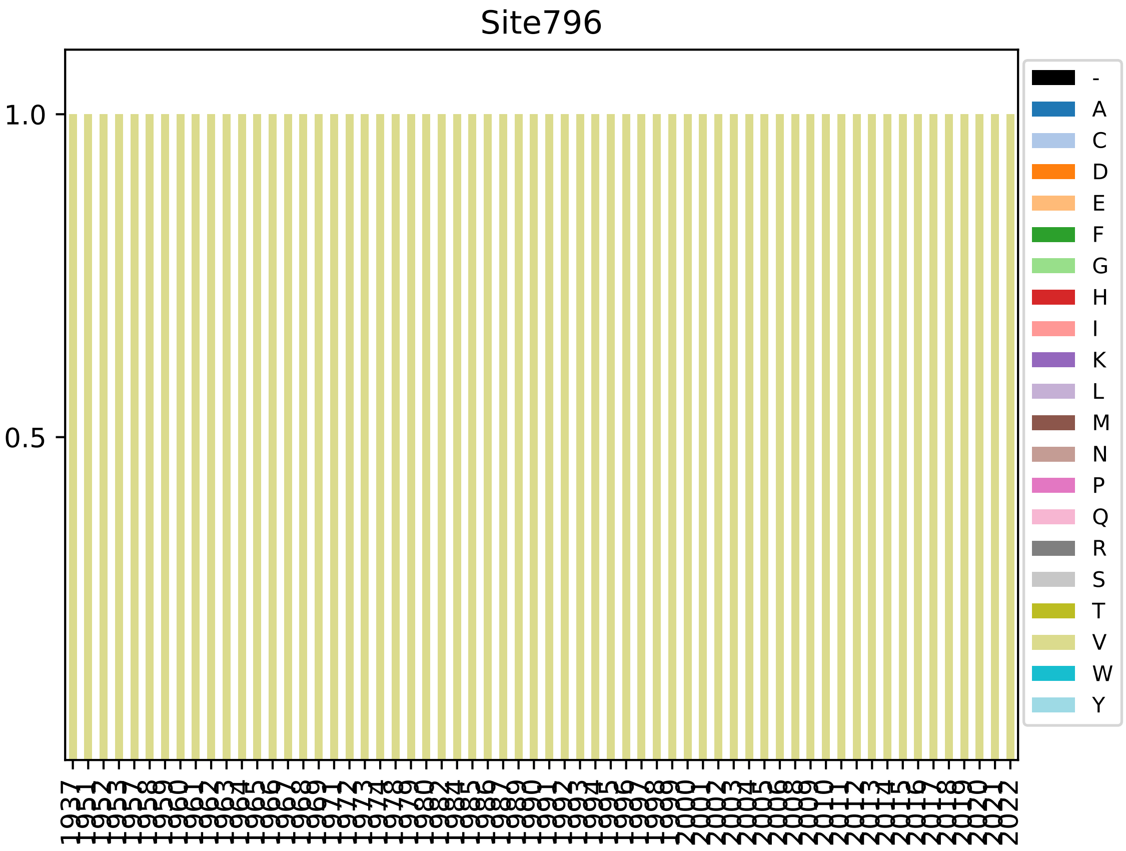

Site796
1.0
0.5
-
A
C
D
E
F
G
H
I
K
L
M
N
P
Q
R
S
T
V
W
Y

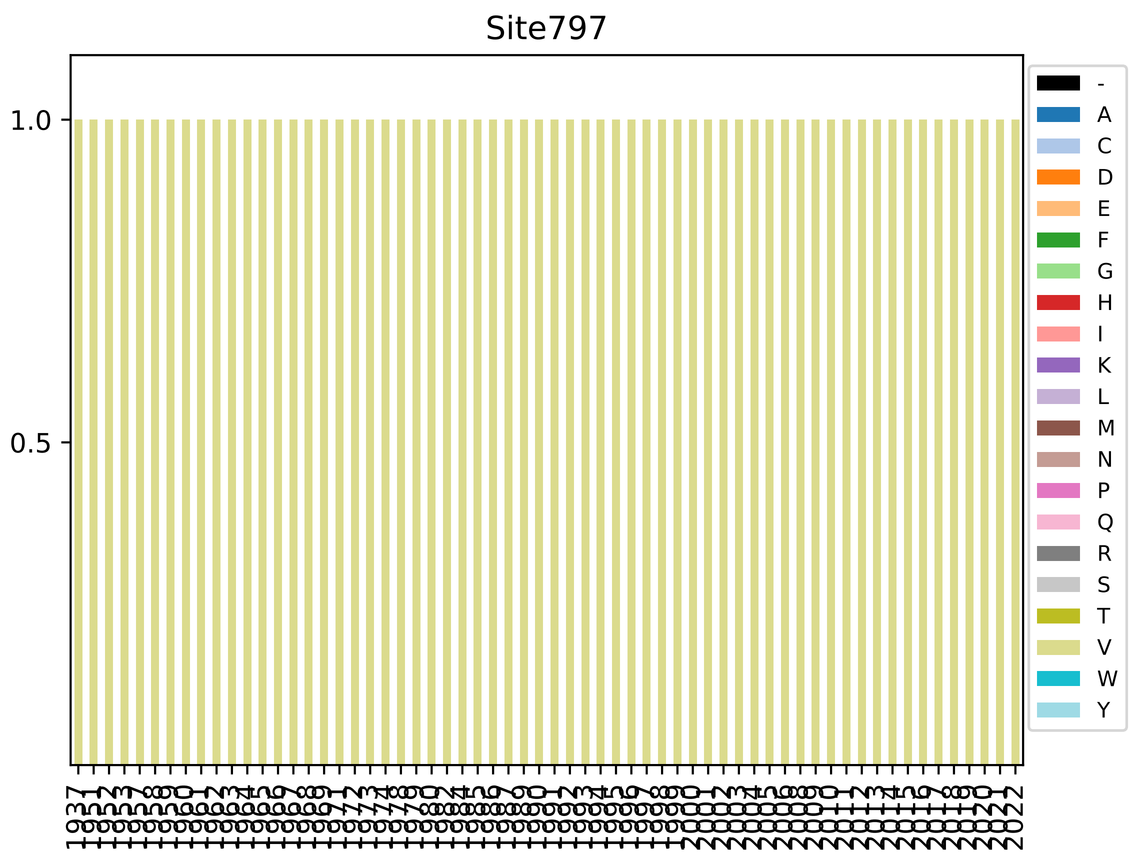
Site797
1.0
0.5
-
A
C
D
E
F
G
H
I
K
L
M
N
P
Q
R
S
T
V
W
Y

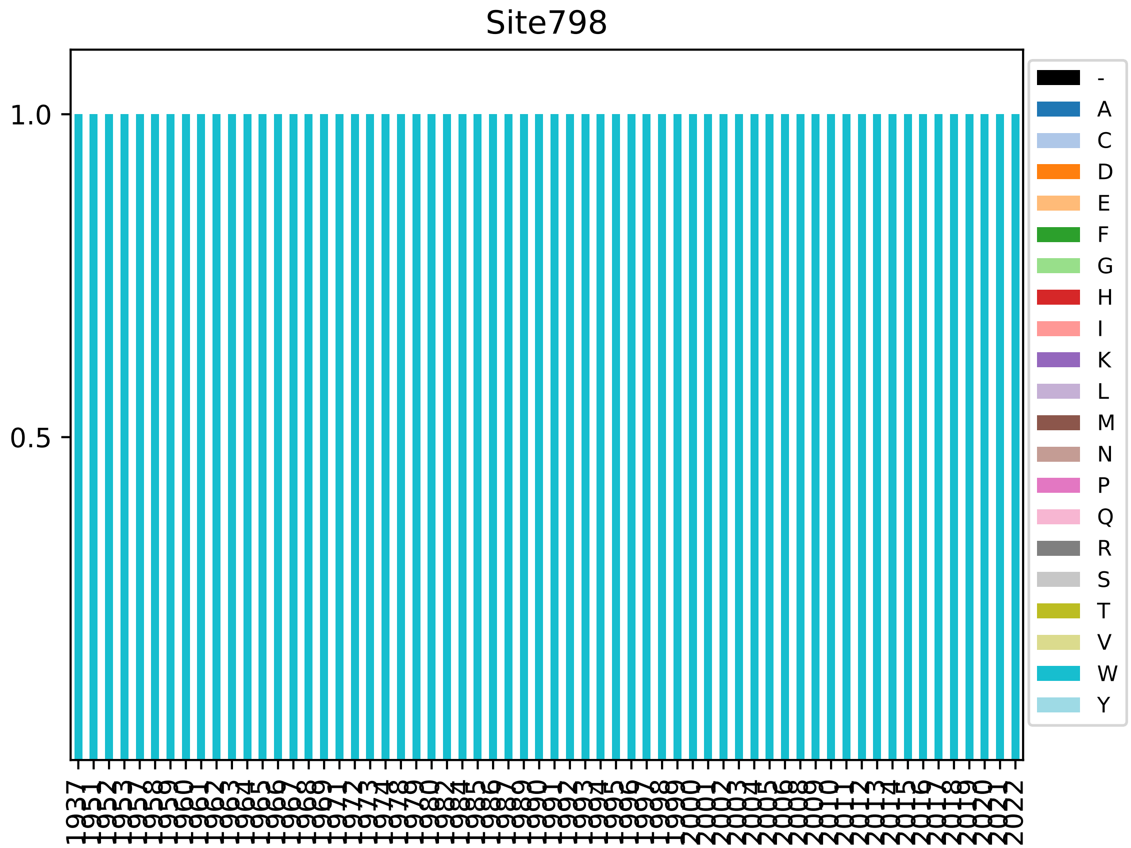

Site798
1.0
0.5
-
A
C
D
E
F
G
H
I
K
L
M
N
P
Q
R
S
T
V
W
Y

Site799

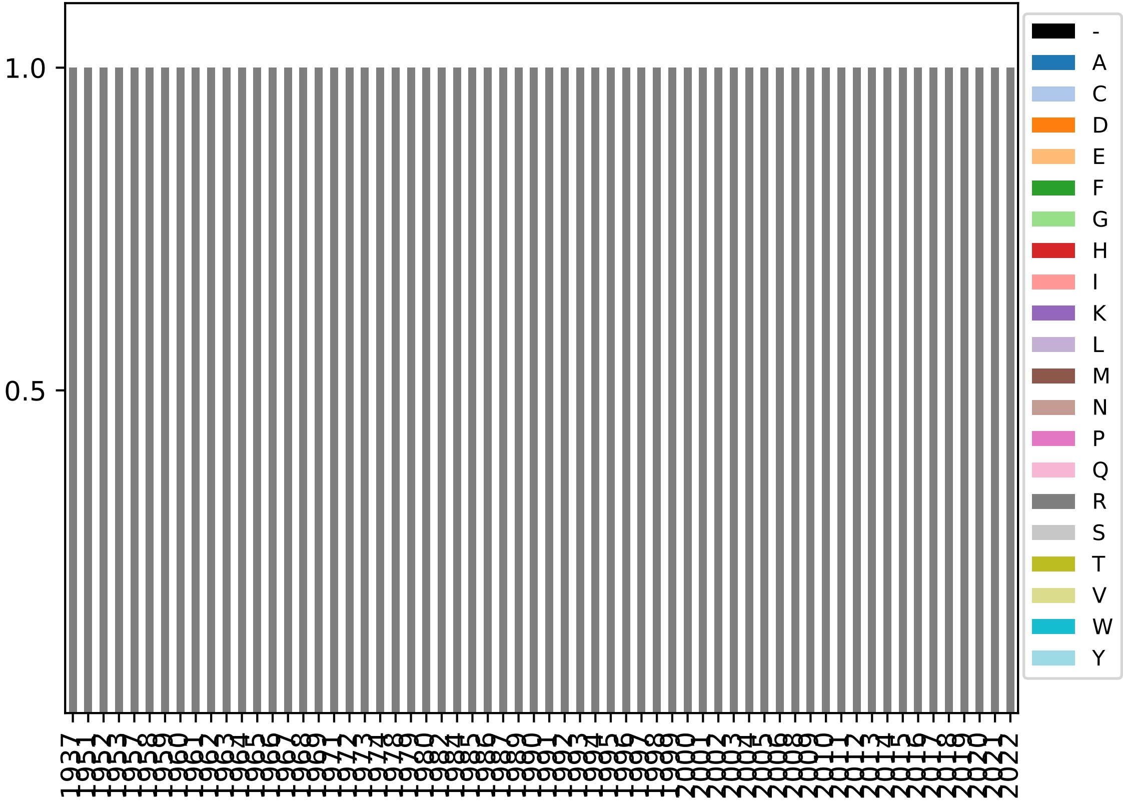

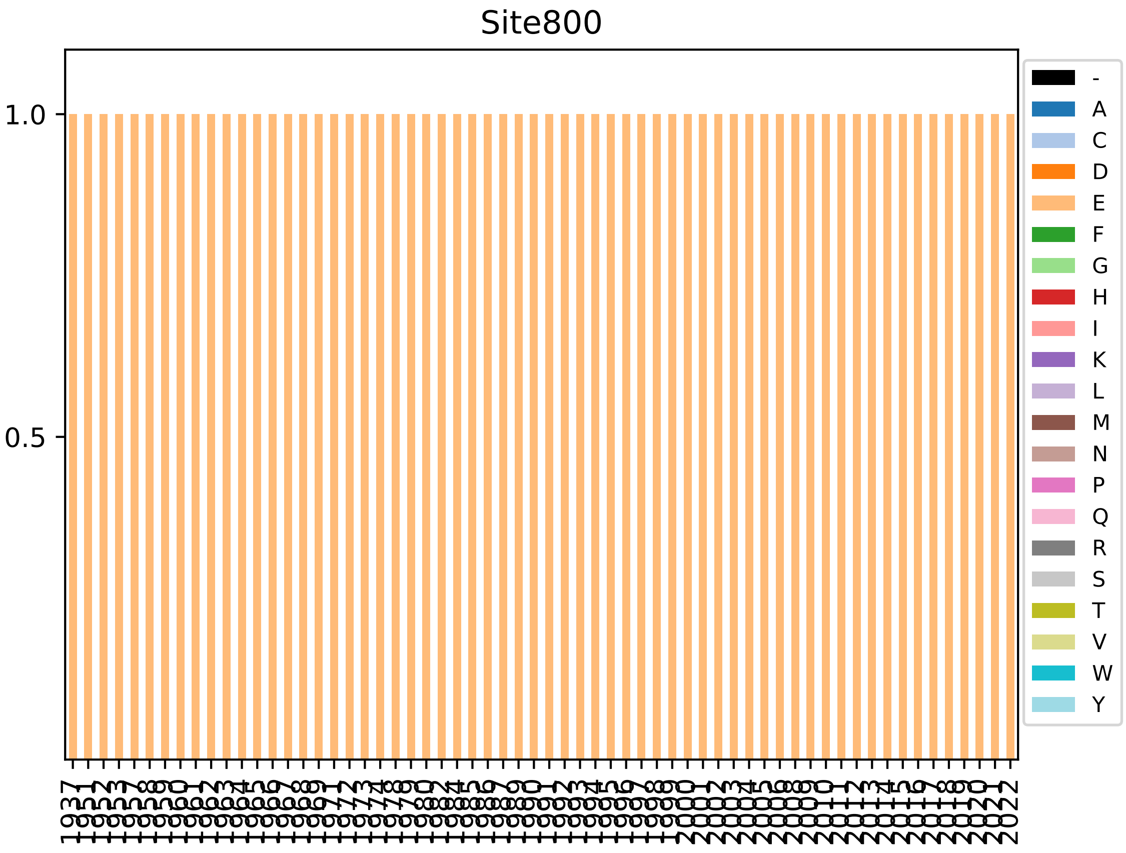

Site800
1.0
0.5
-
A
C
D
E
F
G
H
I
K
L
M
N
P
Q
R
S
T
V
W
Y

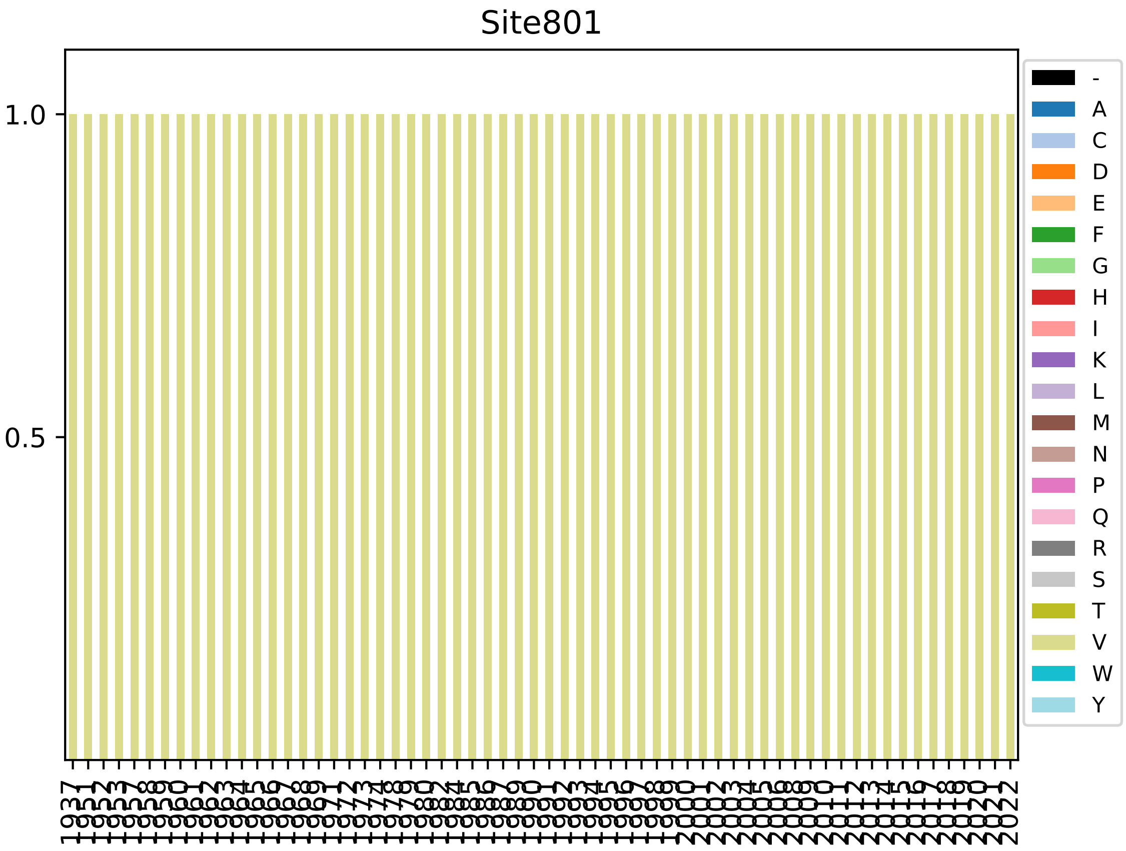
Site801
1.0
0.5
-
A
C
D
E
F
G
H
I
K
L
M
N
P
Q
R
S
T
V
W
Y

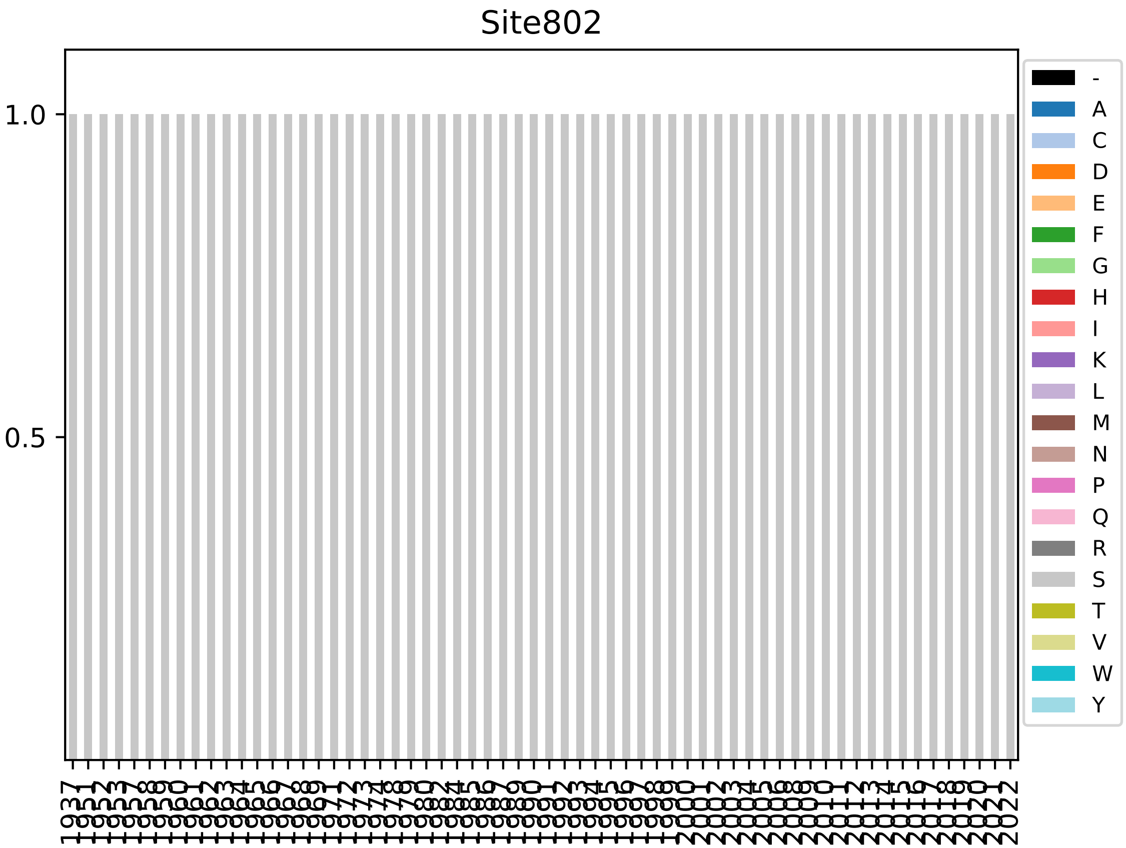

Site802
1.0
0.5
-
A
C
D
E
F
G
H
I
K
L
M
N
P
Q
R
S
T
V
W
Y

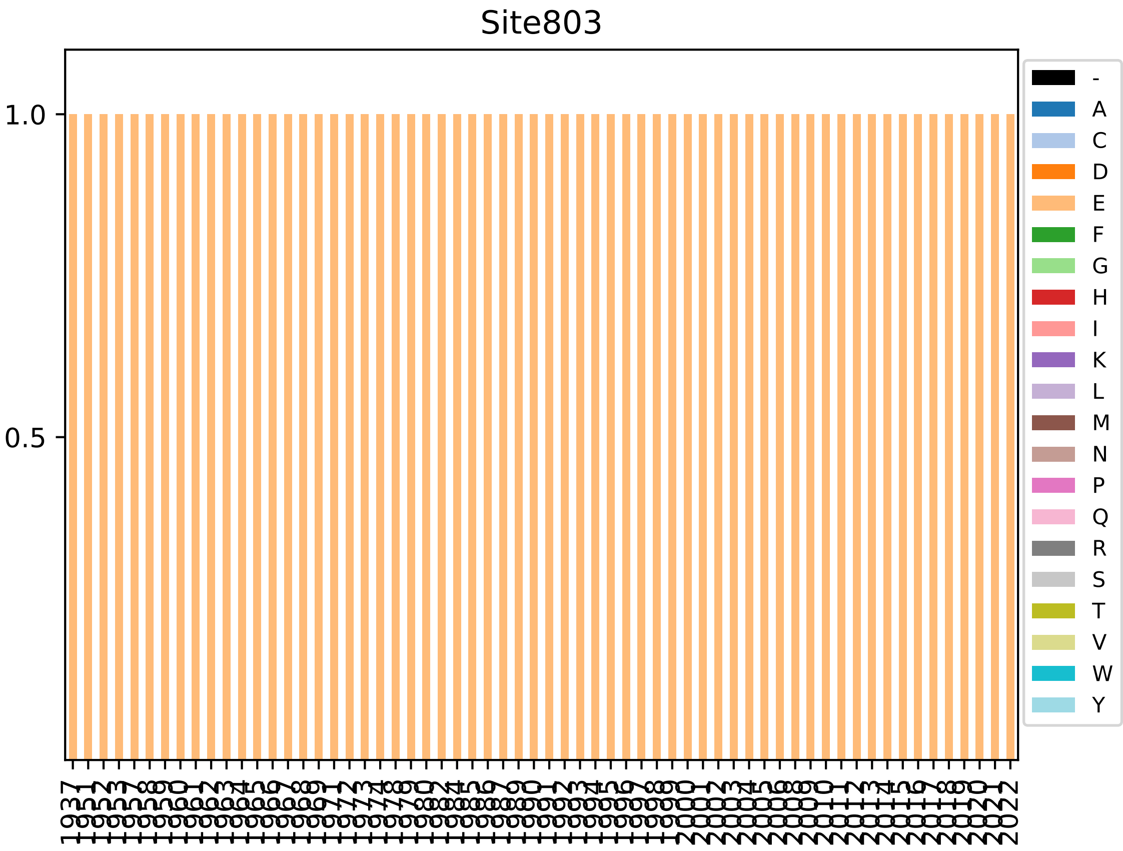
Site803
1.0
0.5
-
A
C
D
E
F
G
H
I
K
L
M
N
P
Q
R
S
T
V
W
Y

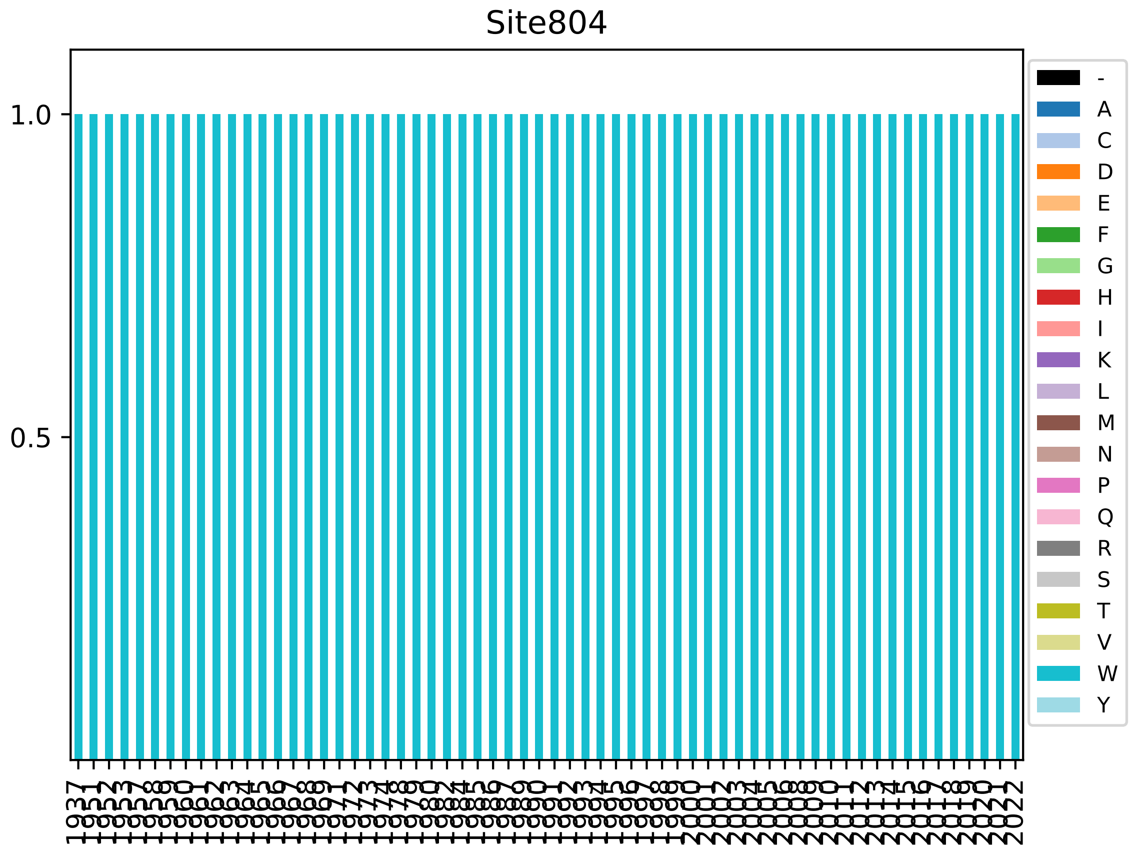
Site804
1.0
0.5
-
A
C
D
E
F
G
H
I
K
L
M
N
P
Q
R
S
T
V
W
Y

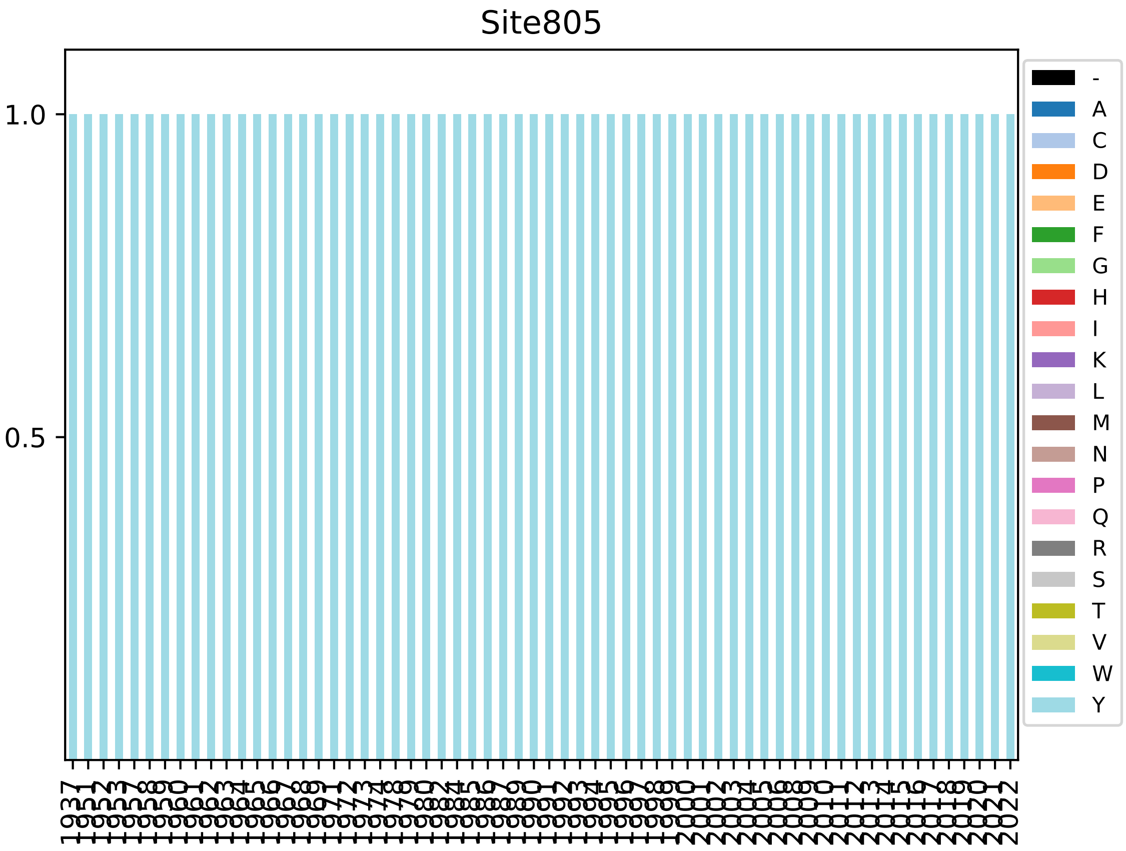
Site805
1.0
0.5
-
A
C
D
E
F
G
H
I
K
L
M
N
P
Q
R
S
T
V
W
Y

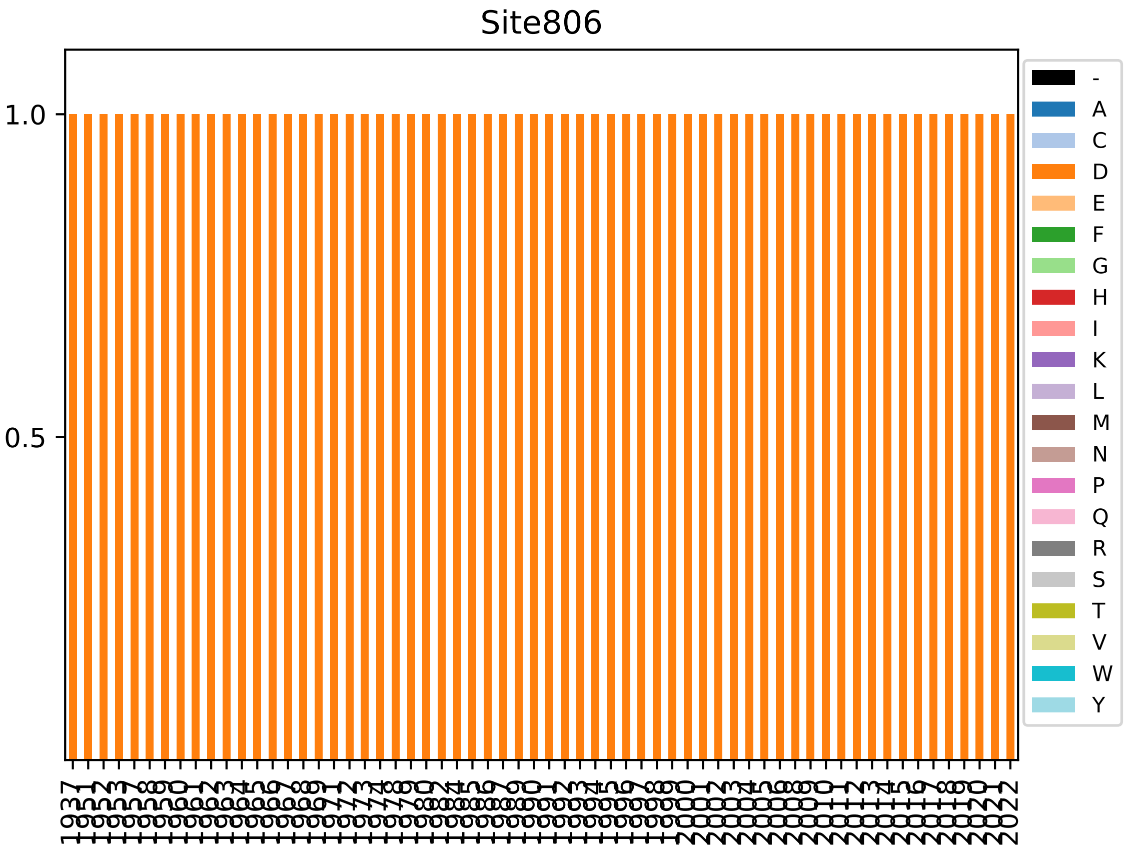
Site806
1.0
0.5
-
A
C
D
E
F
G
H
I
K
L
M
N
P
Q
R
S
T
V
W
Y

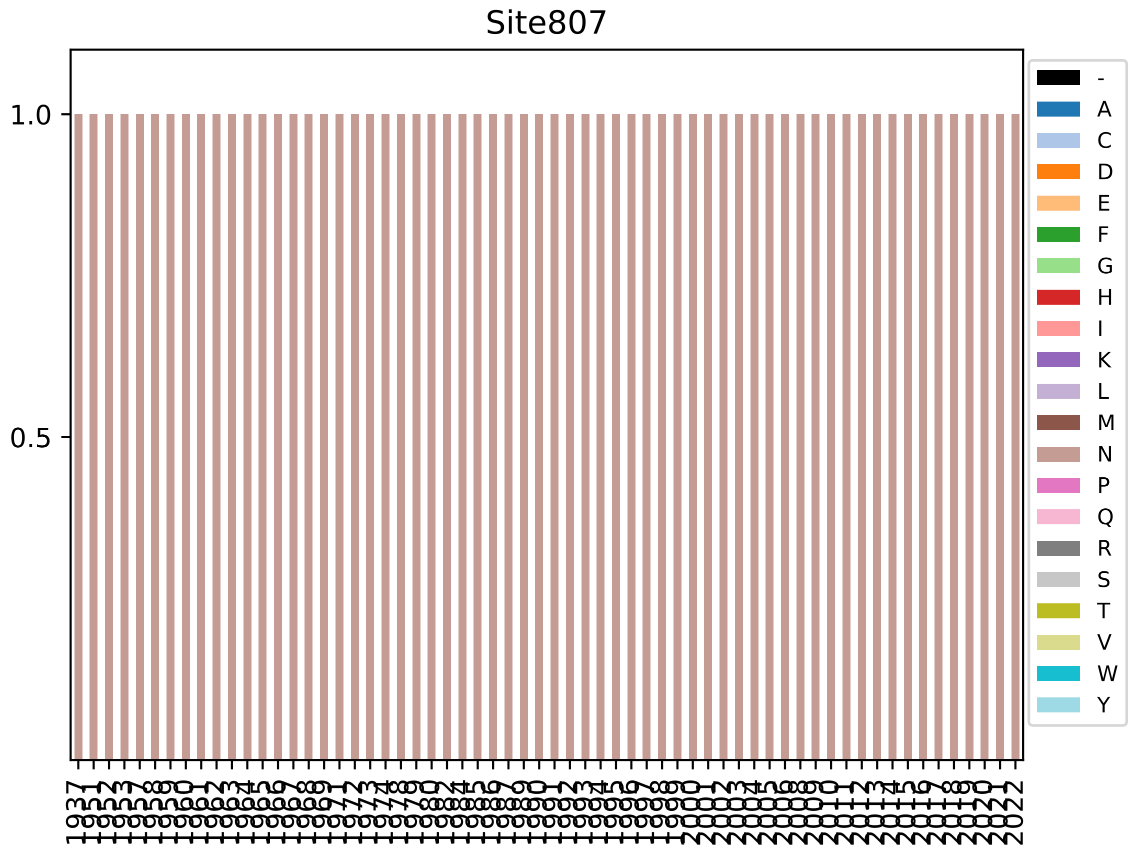
Site807
1.0
0.5
-
A
C
D
E
F
G
H
I
K
L
M
N
P
Q
R
S
T
V
W
Y

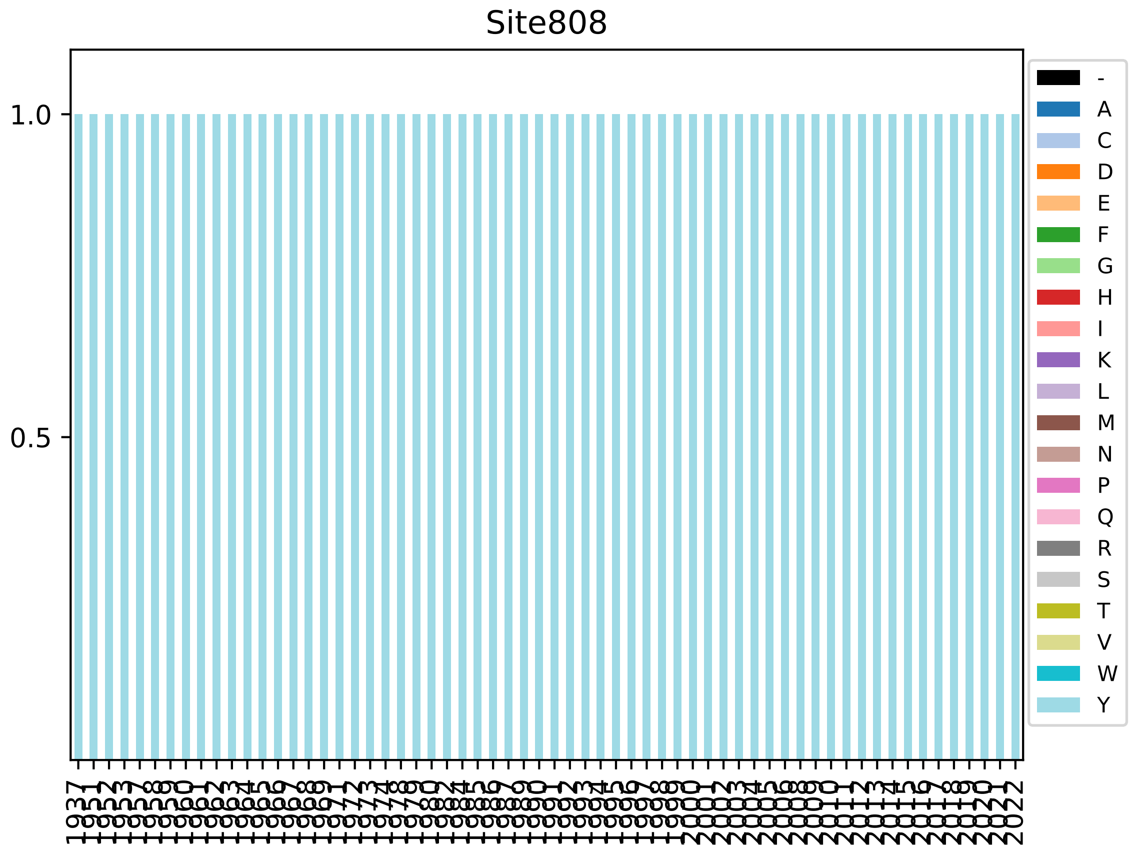

Site808
1.0
0.5
-
A
C
D
E
F
G
H
I
K
L
M
N
P
Q
R
S
T
V
W
Y

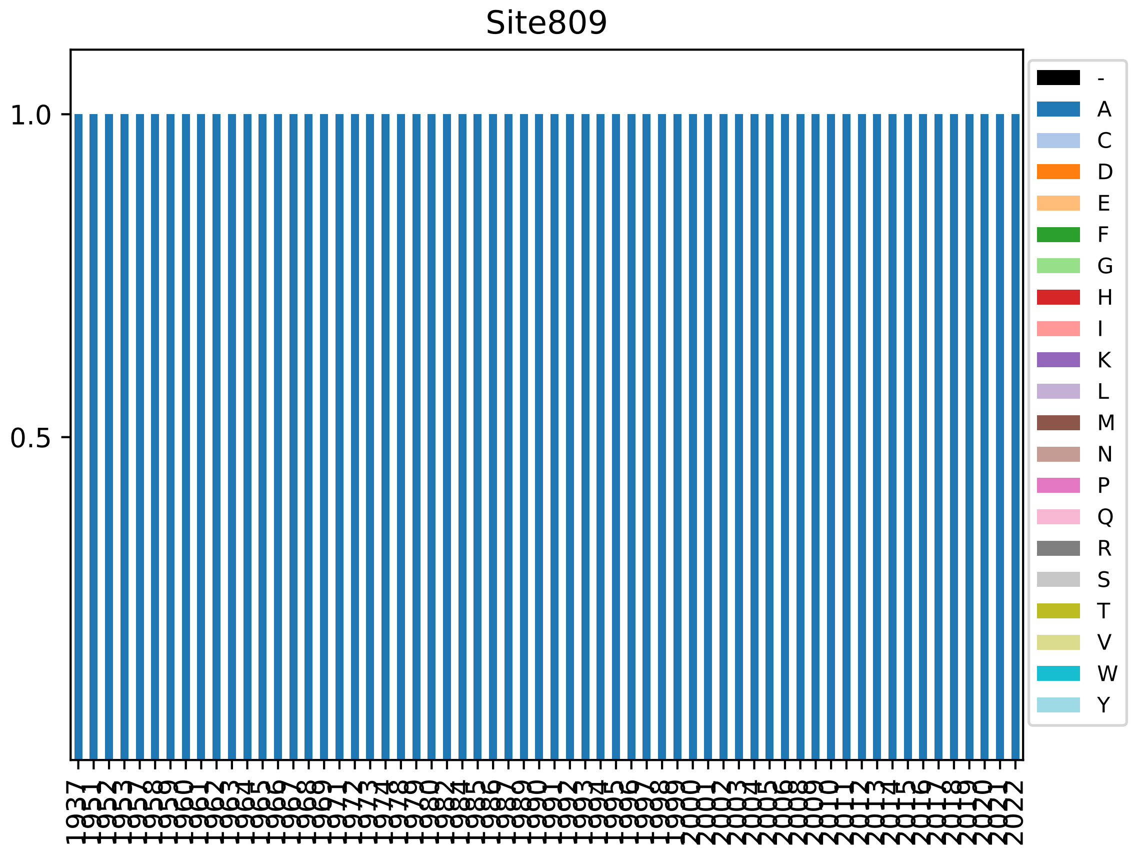
Site809
1.0
0.5
-
A
C
D
E
F
G
H
I
K
L
M
N
P
Q
R
S
T
V
W
Y

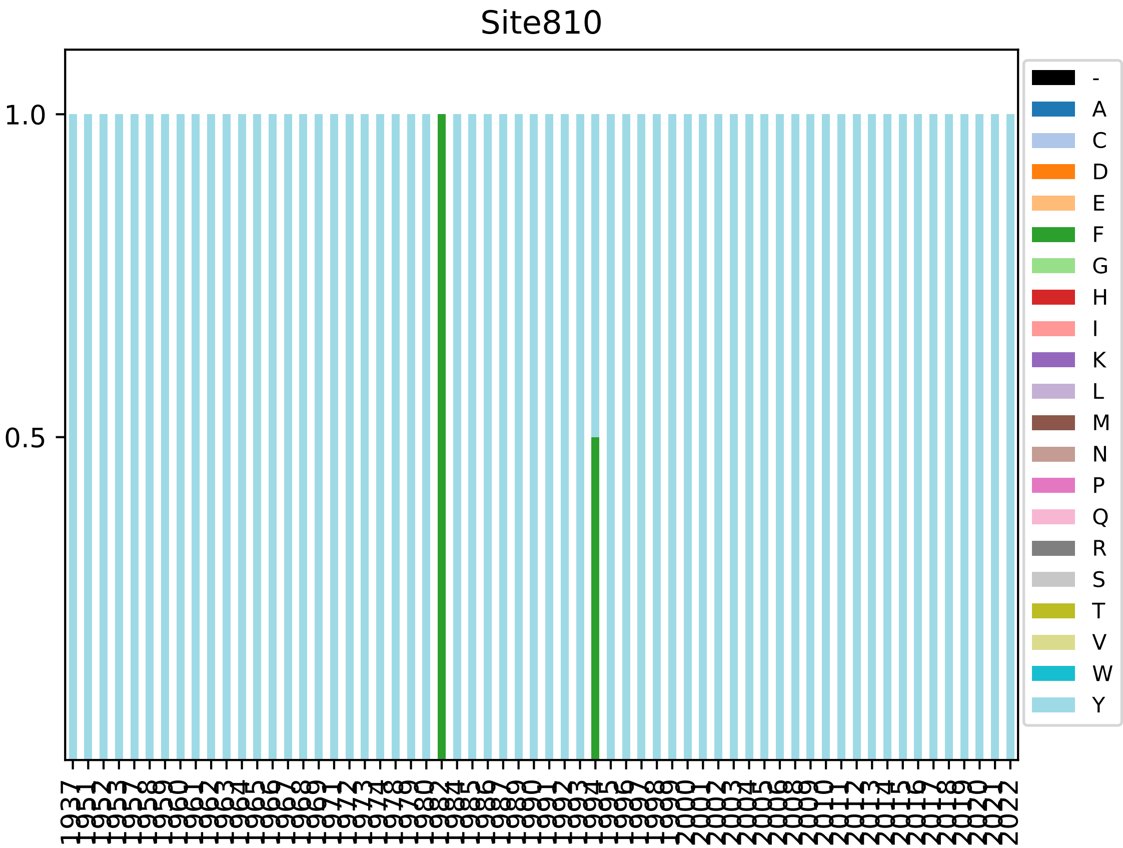
Site810
1.0
0.5
-
A
C
D
E
F
G
H
I
K
L
M
N
P
Q
R
S
T
V
W
Y

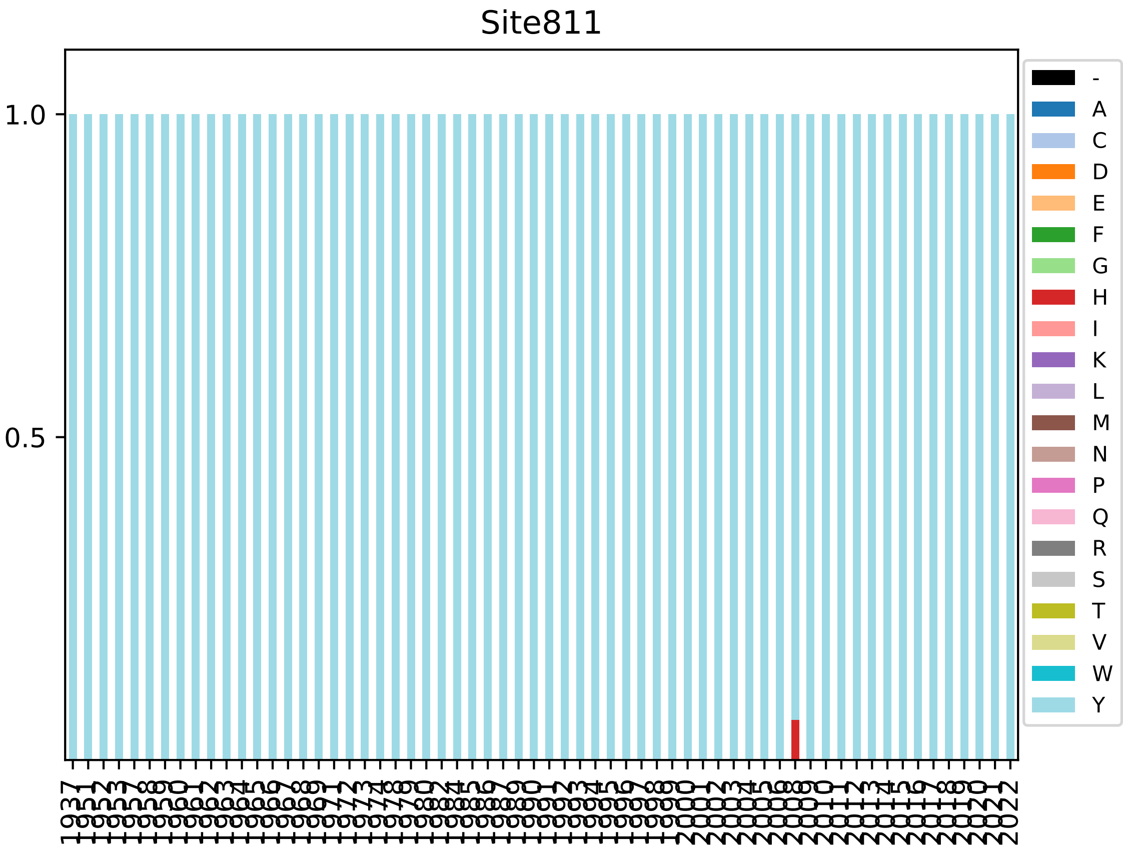
Site811
1.0
0.5
-
A
C
D
E
F
G
H
I
K
L
M
N
P
Q
R
S
T
V
W
Y

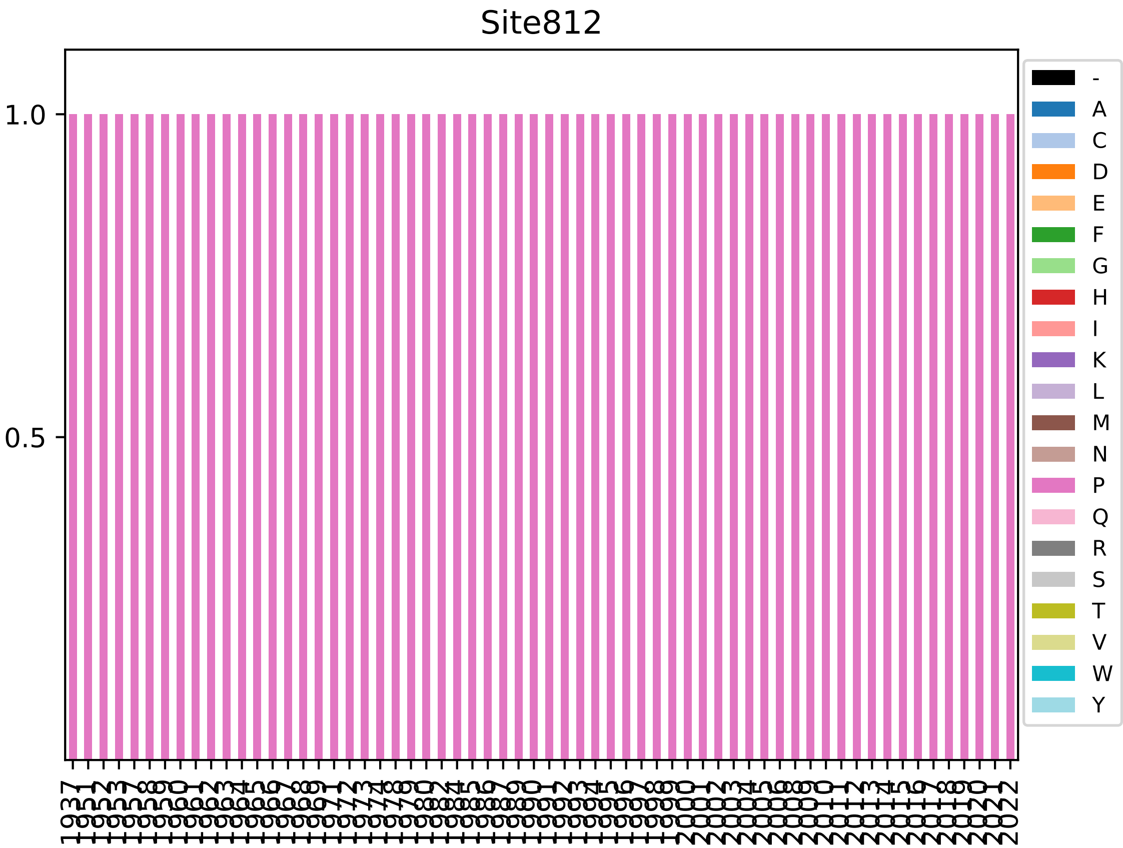
Site812
1.0
0.5
-
A
C
D
E
F
G
H
I
K
L
M
N
P
Q
R
S
T
V
W
Y

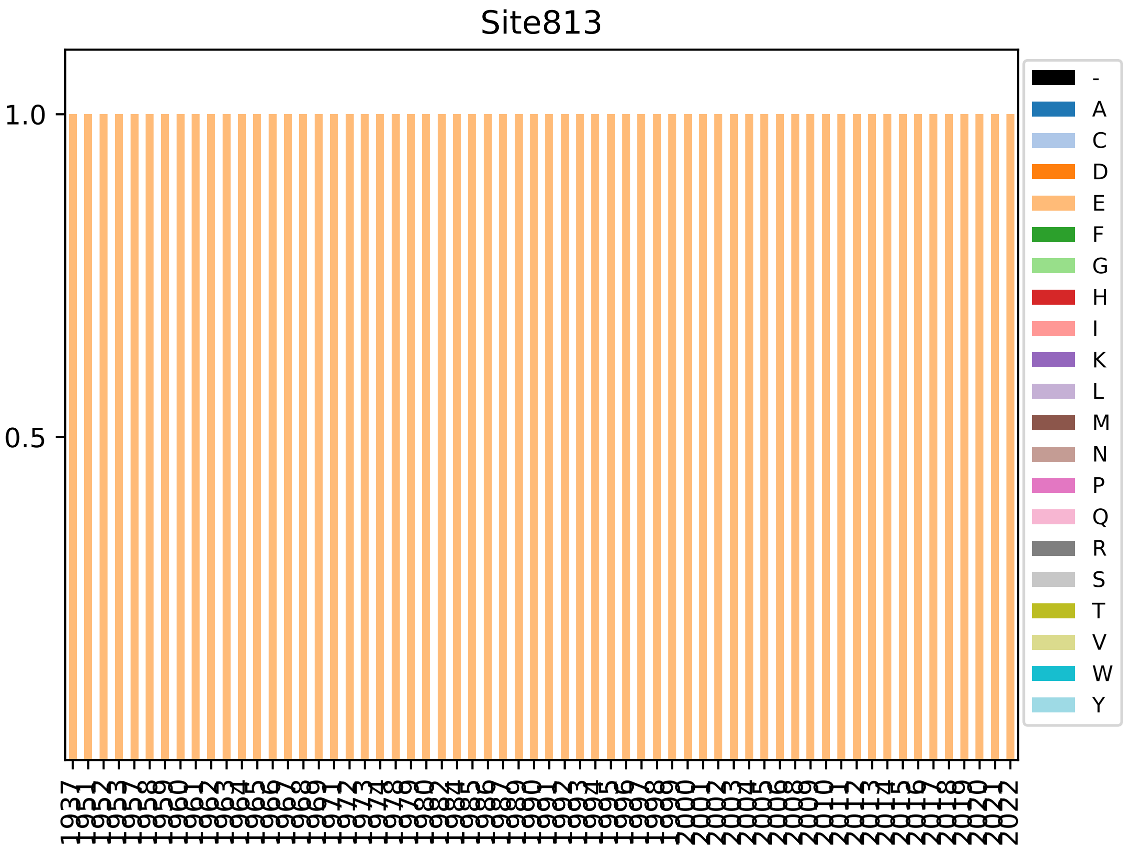
Site813
1.0
0.5
-
A
C
D
E
F
G
H
I
K
L
M
N
P
Q
R
S
T
V
W
Y

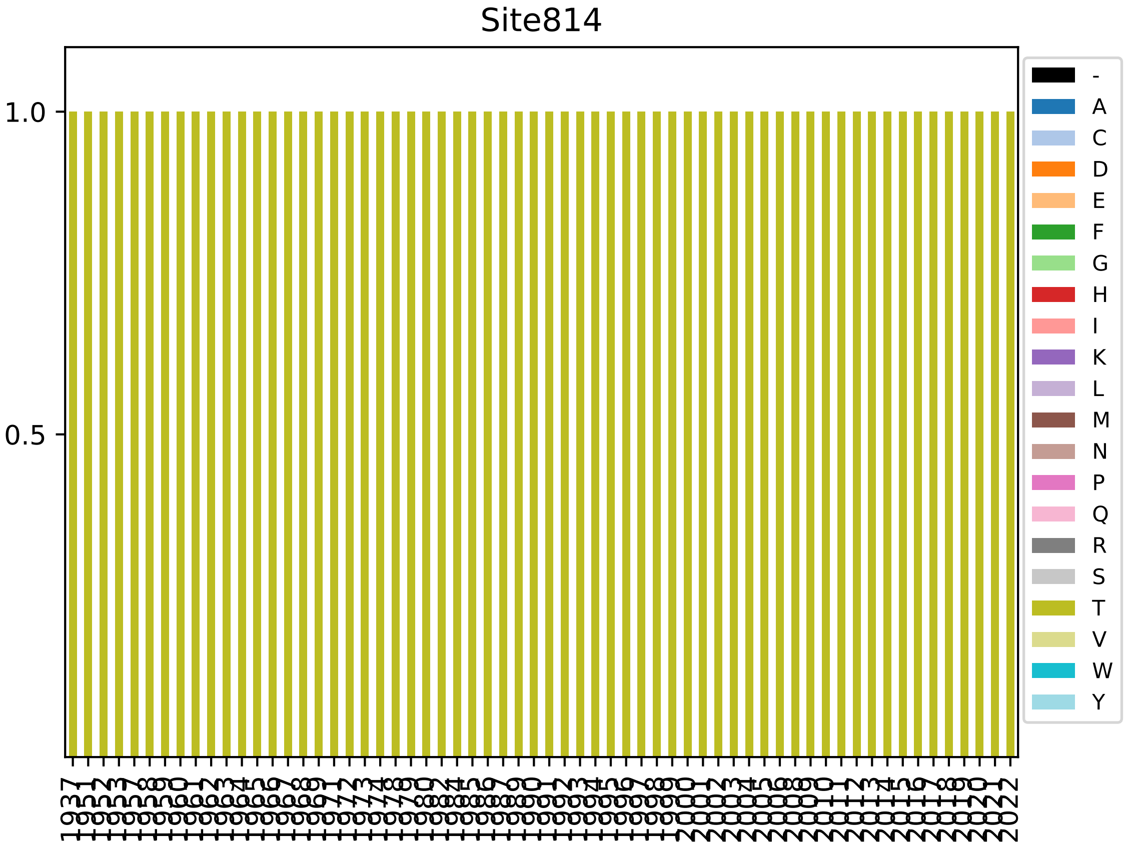
Site814
1.0
0.5
-
A
C
D
E
F
G
H
I
K
L
M
N
P
Q
R
S
T
V
W
Y

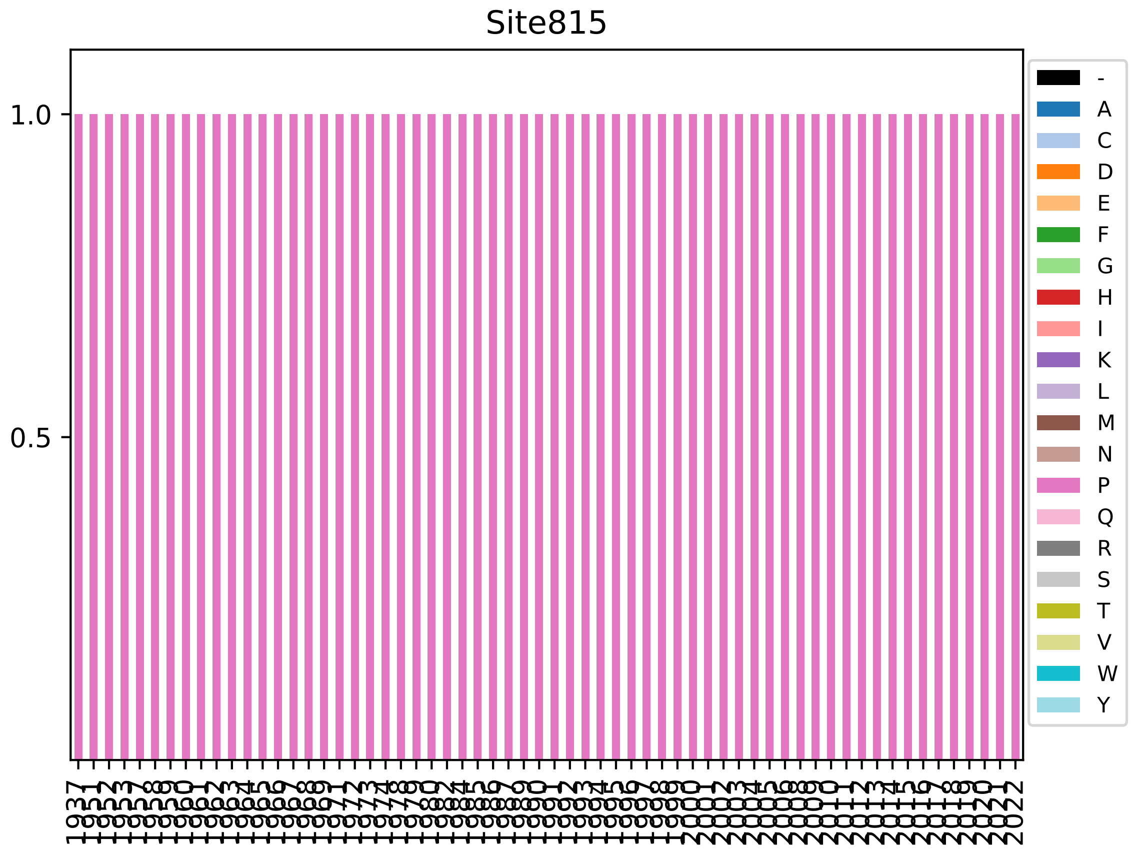
Site815
1.0
0.5
-
A
C
D
E
F
G
H
I
K
L
M
N
P
Q
R
S
T
V
W
Y

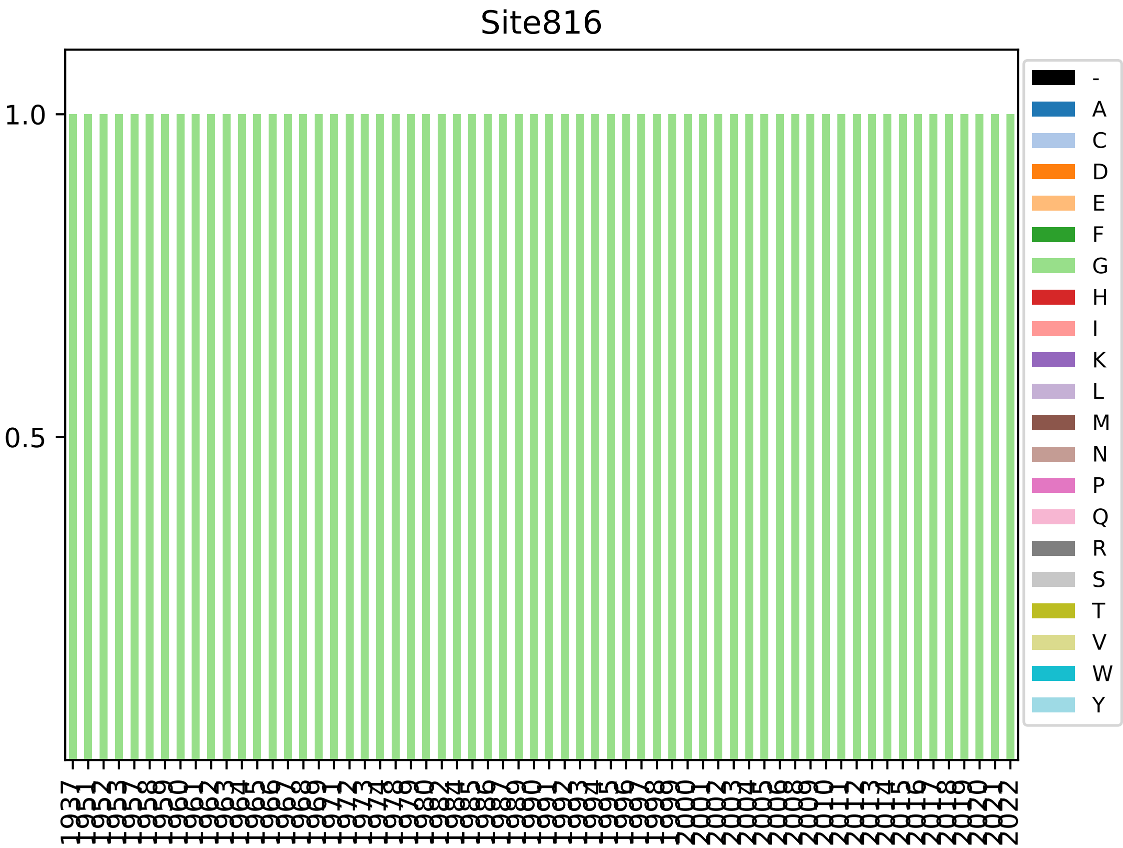
Site816
1.0
0.5
-
A
C
D
E
F
G
H
I
K
L
M
N
P
Q
R
S
T
V
W
Y

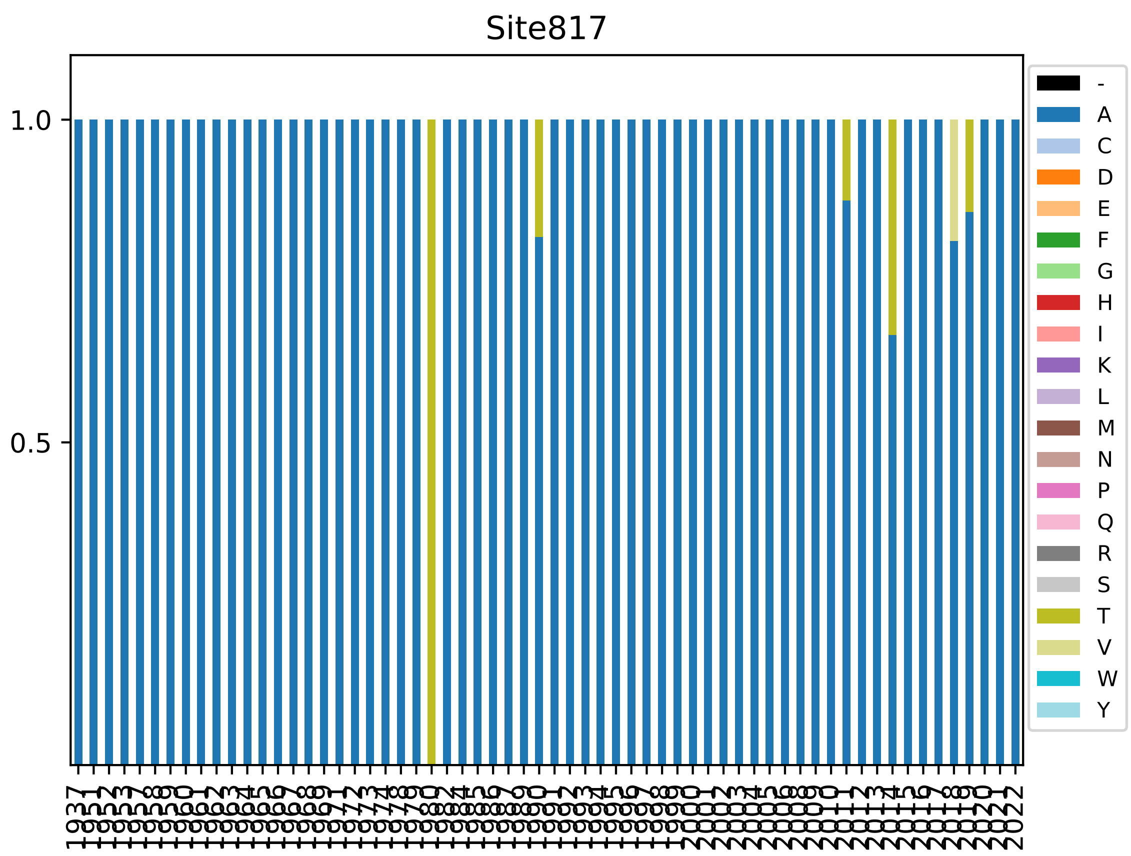
Site817
1.0
0.5
-
A
C
D
E
F
G
H
I
K
L
M
N
P
Q
R
S
T
V
W
Y

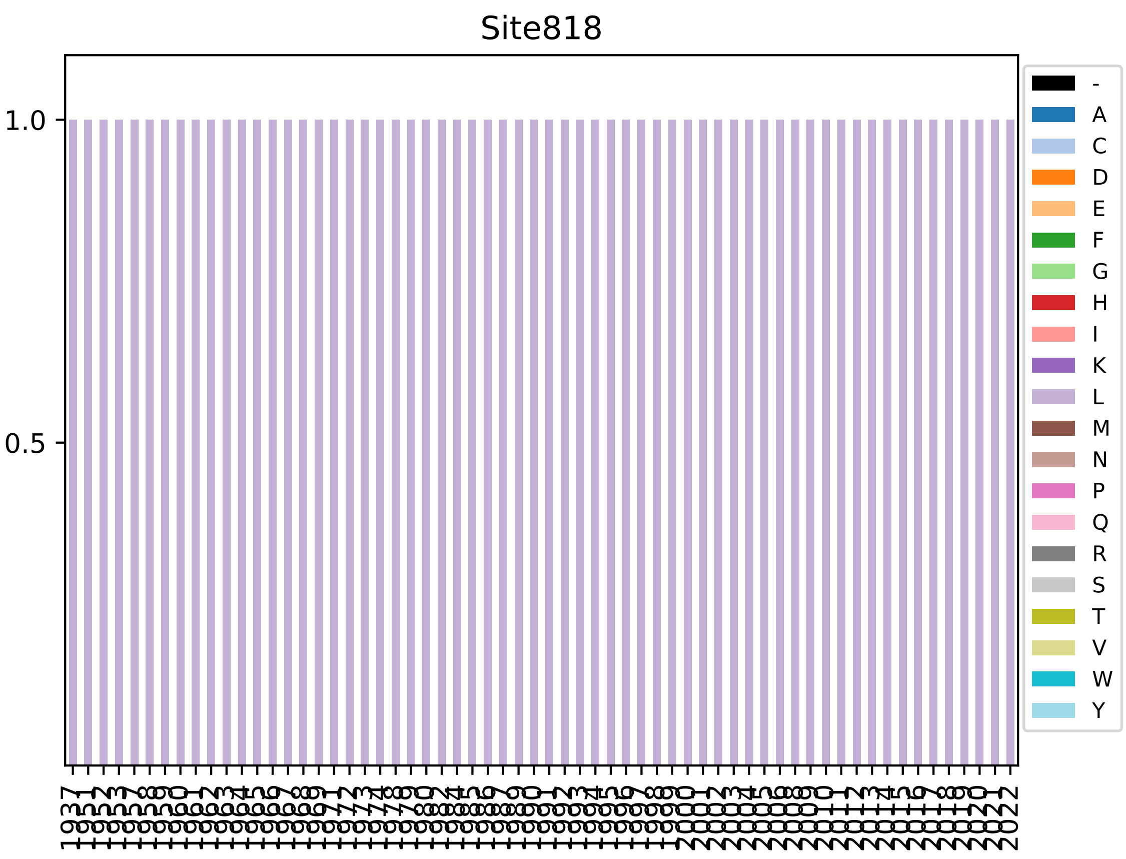
Site818
1.0
0.5
-
A
C
D
E
F
G
H
I
K
L
M
N
P
Q
R
S
T
V
W
Y

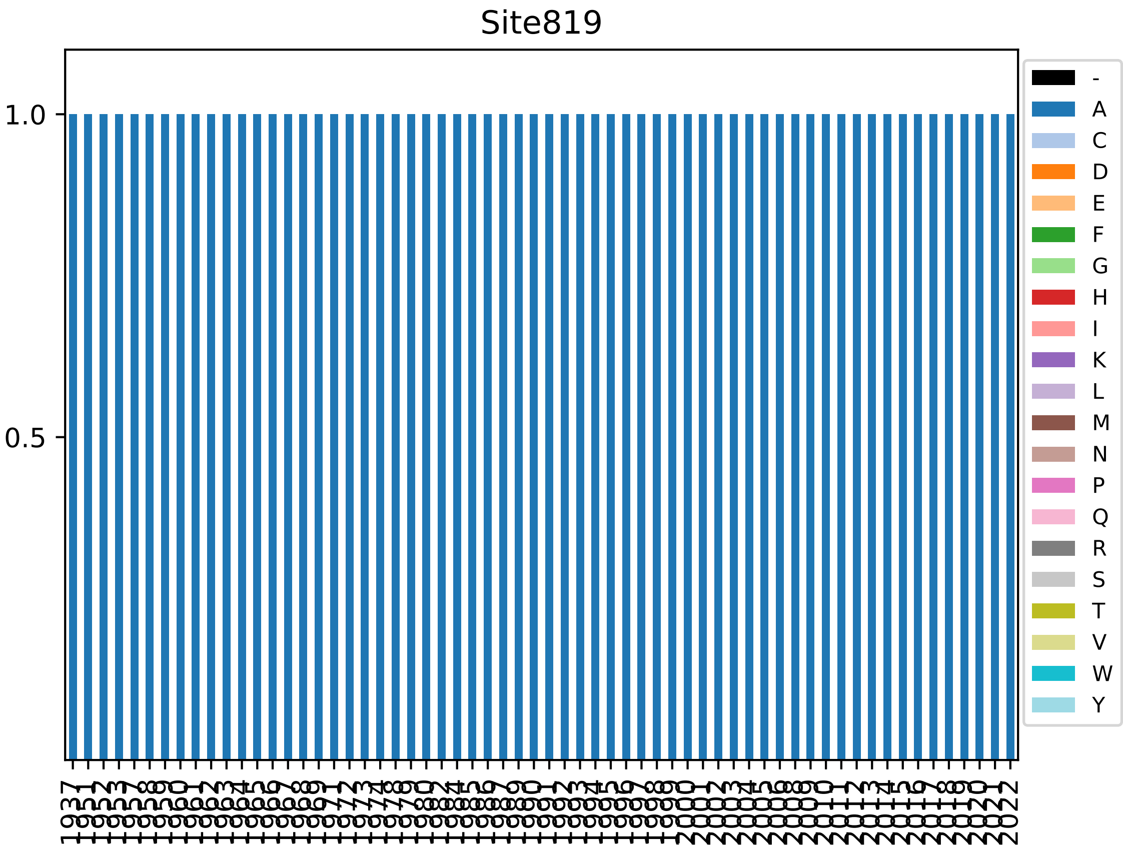
Site819
1.0
0.5
-
A
C
D
E
F
G
H
I
K
L
M
N
P
Q
R
S
T
V
W
Y

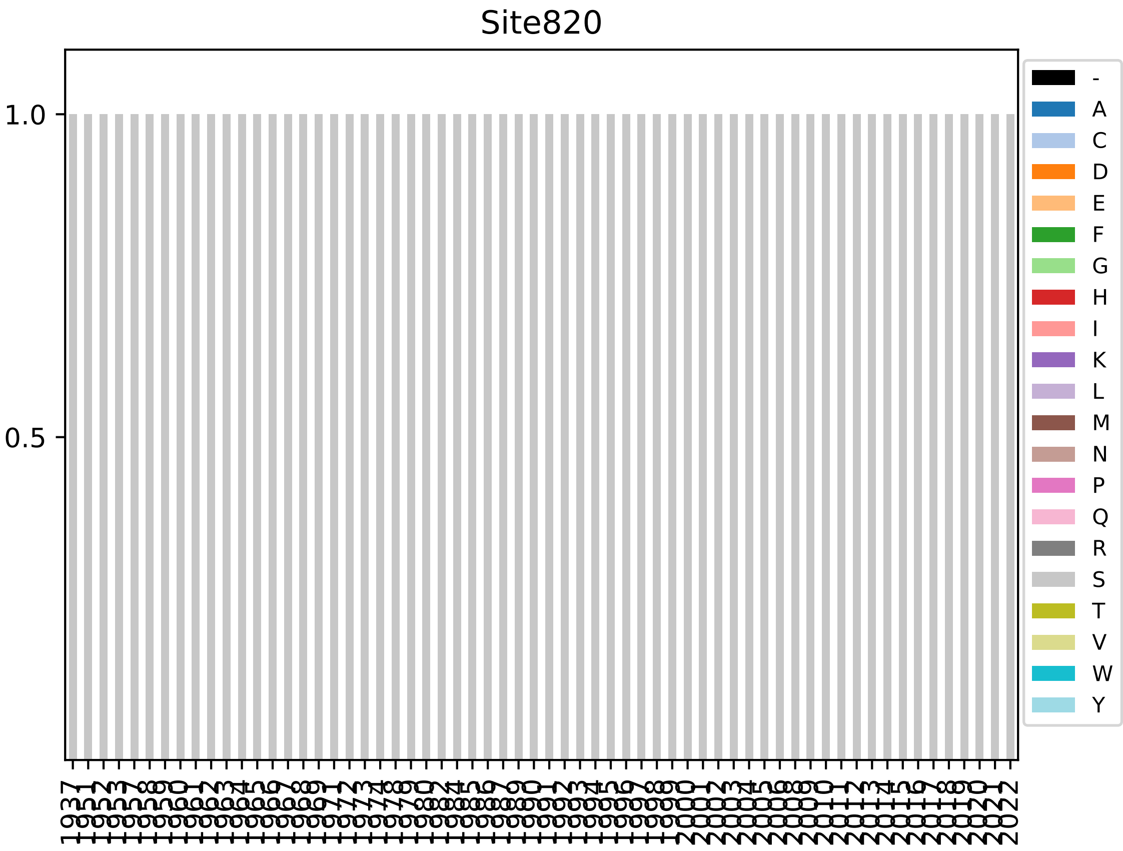
Site820
1.0
0.5
-
A
C
D
E
F
G
H
I
K
L
M
N
P
Q
R
S
T
V
W
Y

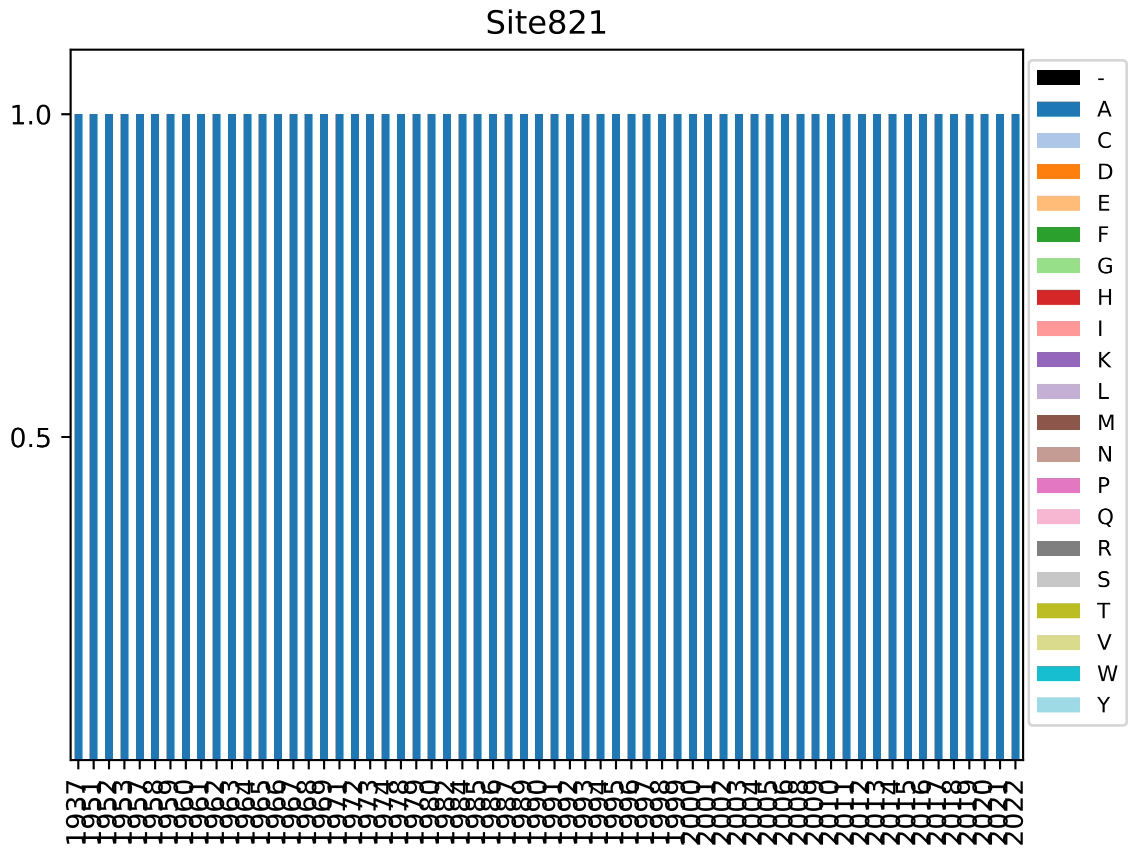
Site821
1.0
0.5
-
A
C
D
E
F
G
H
I
K
L
M
N
P
Q
R
S
T
V
W
Y

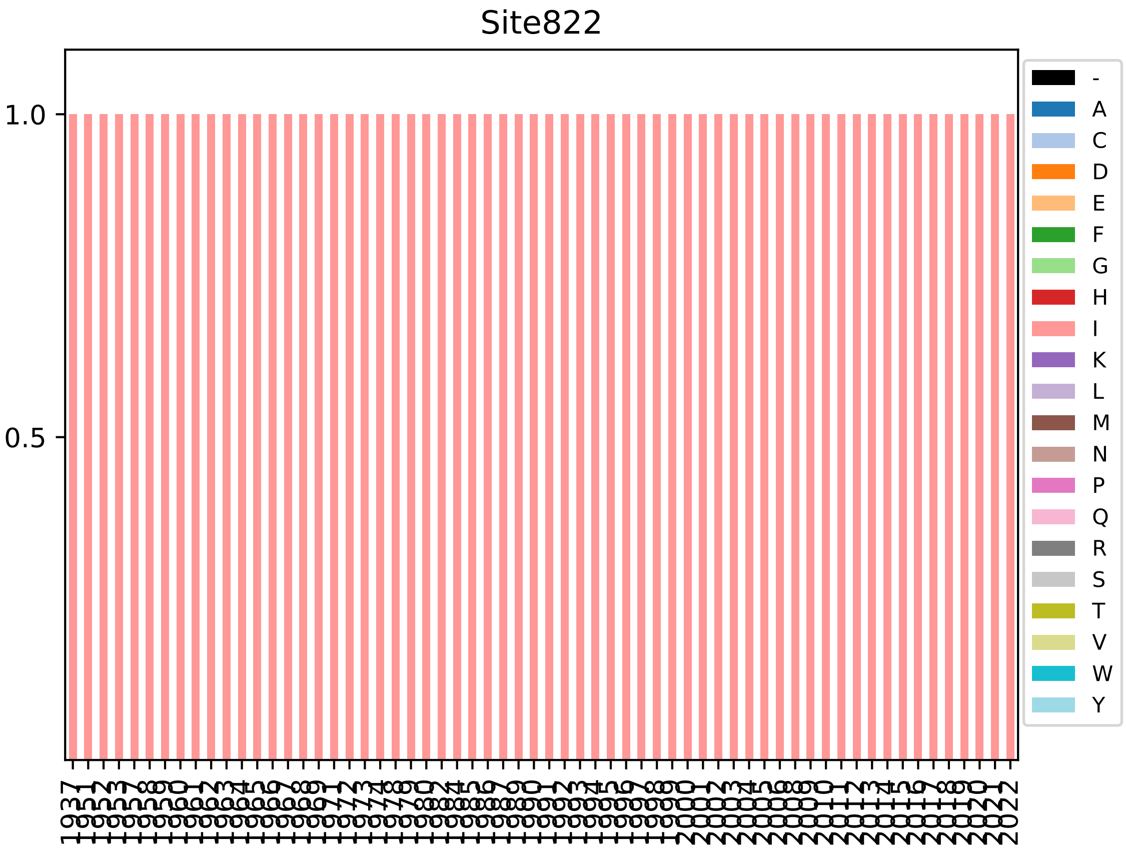
Site822
1.0
0.5
-
A
C
D
E
F
G
H
I
K
L
M
N
P
Q
R
S
T
V
W
Y

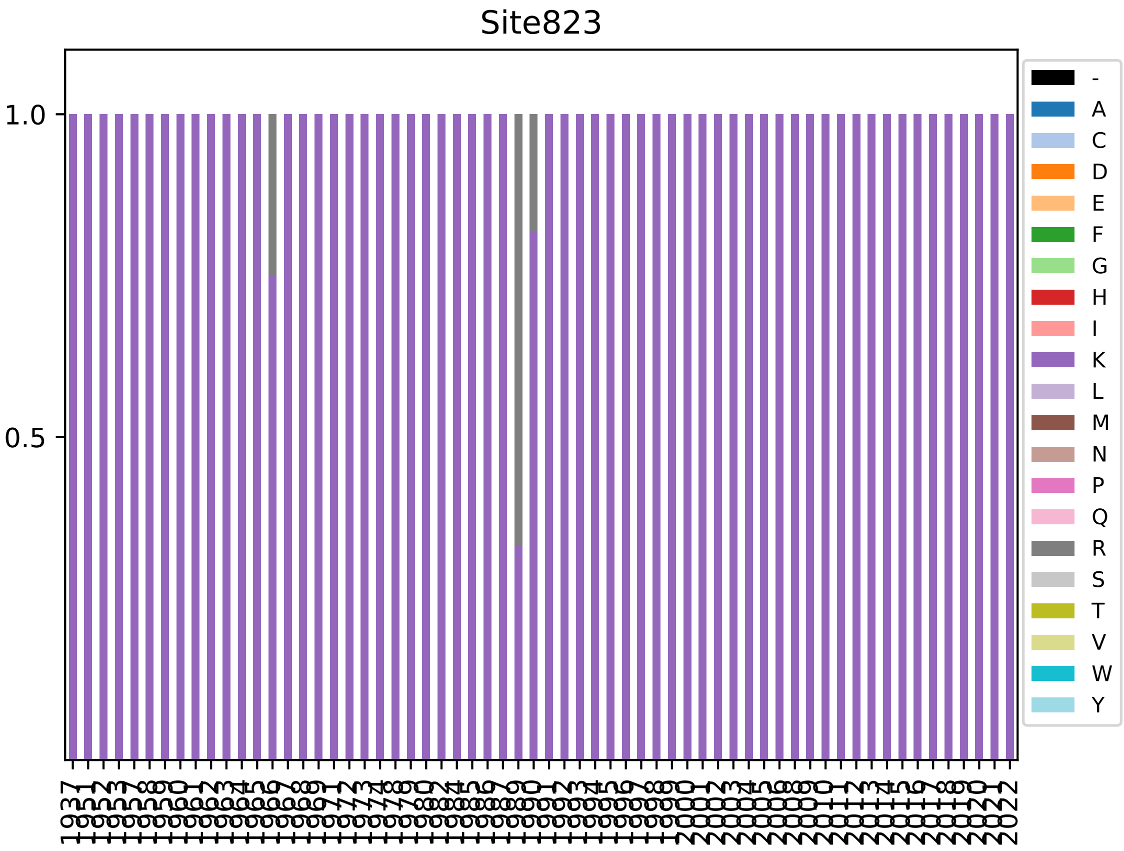
Site823
1.0
0.5
-
A
C
D
E
F
G
H
I
K
L
M
N
P
Q
R
S
T
V
W
Y

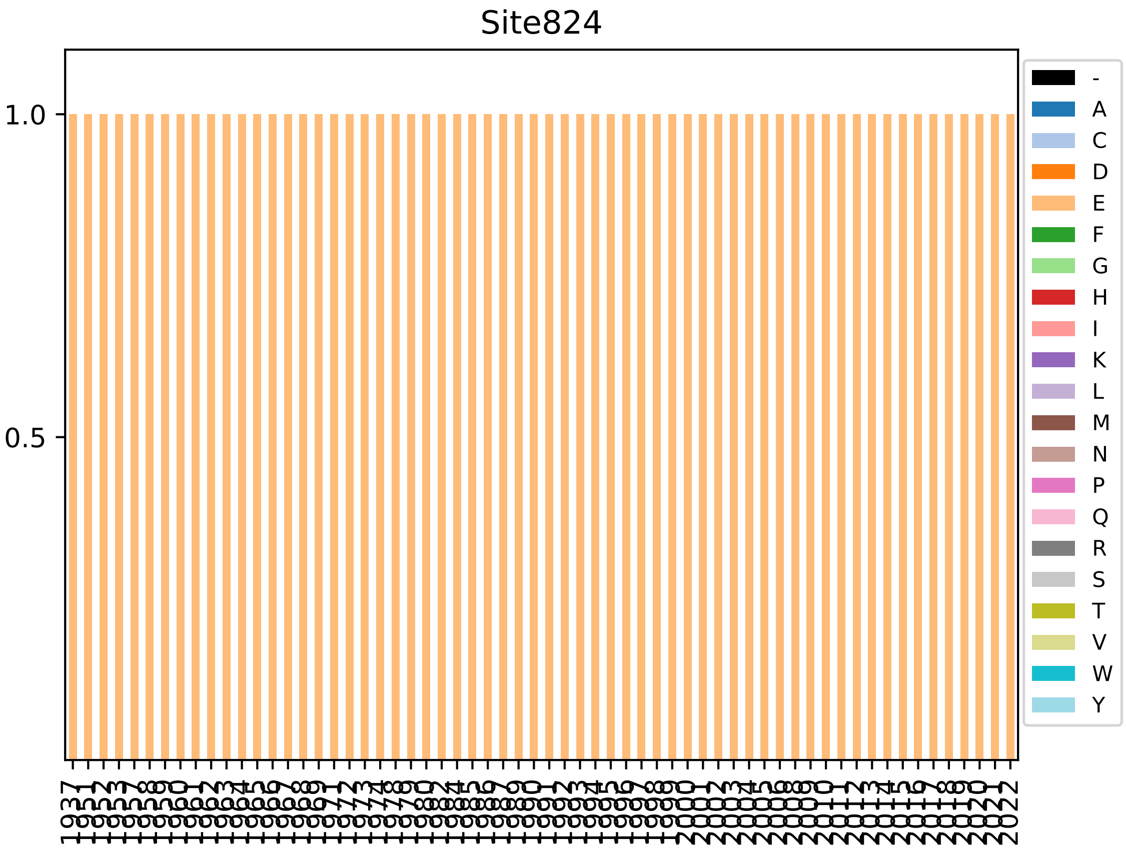
Site824
1.0
0.5
-
A
C
D
E
F
G
H
I
K
L
M
N
P
Q
R
S
T
V
W
Y

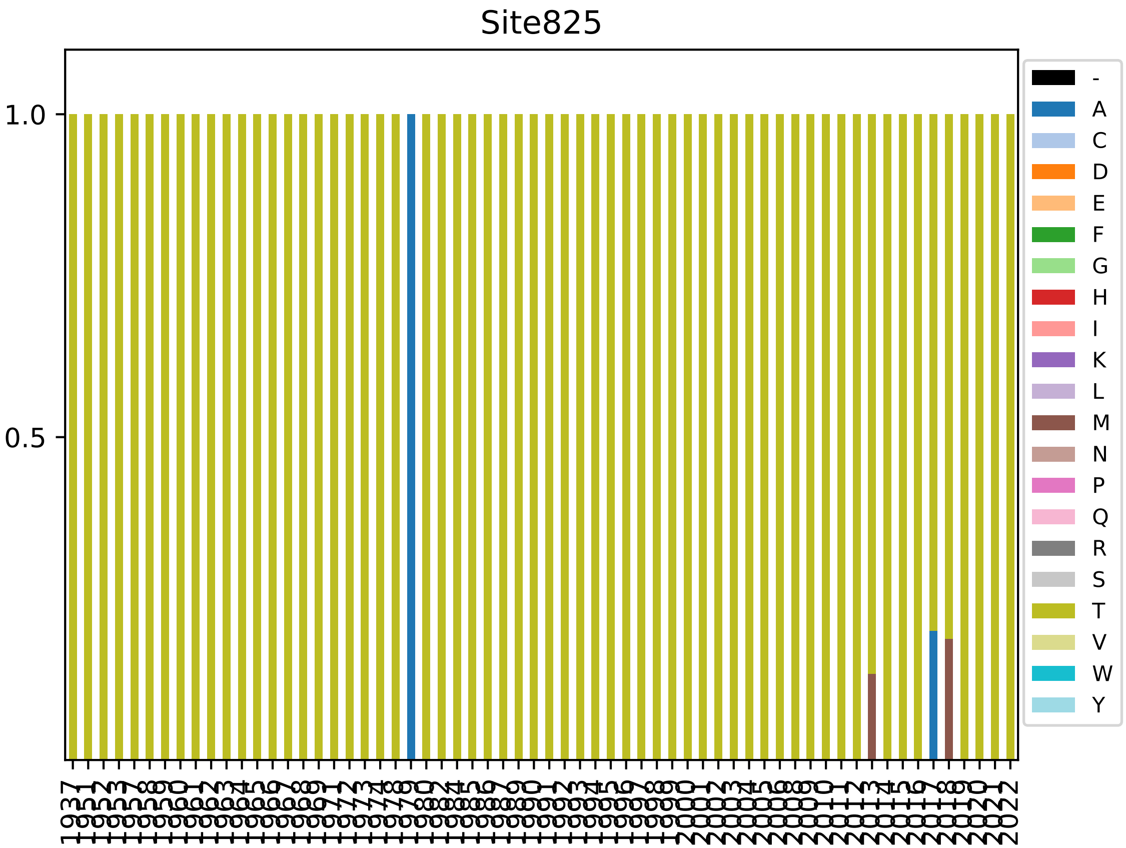
Site825
1.0
0.5
-
A
C
D
E
F
G
H
I
K
L
M
N
P
Q
R
S
T
V
W
Y

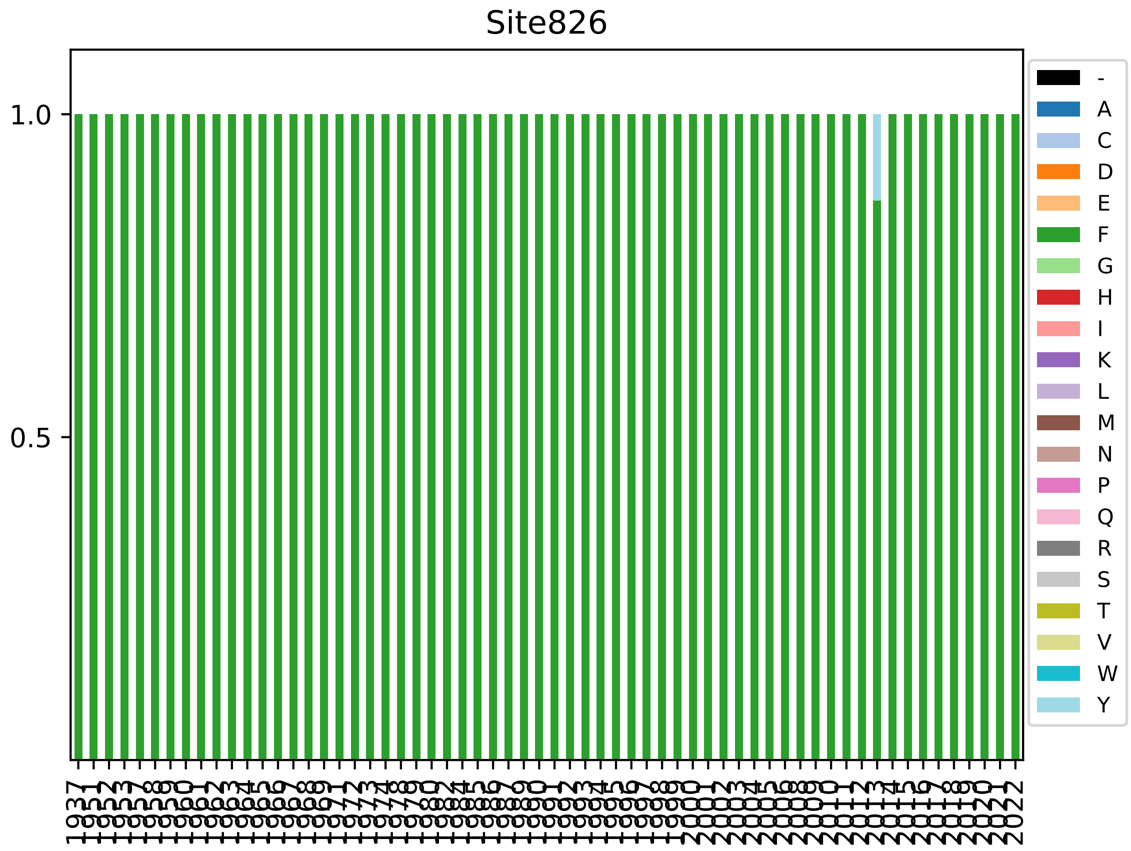
Site826
1.0
0.5
-
A
C
D
E
F
G
H
I
K
L
M
N
P
Q
R
S
T
V
W
Y

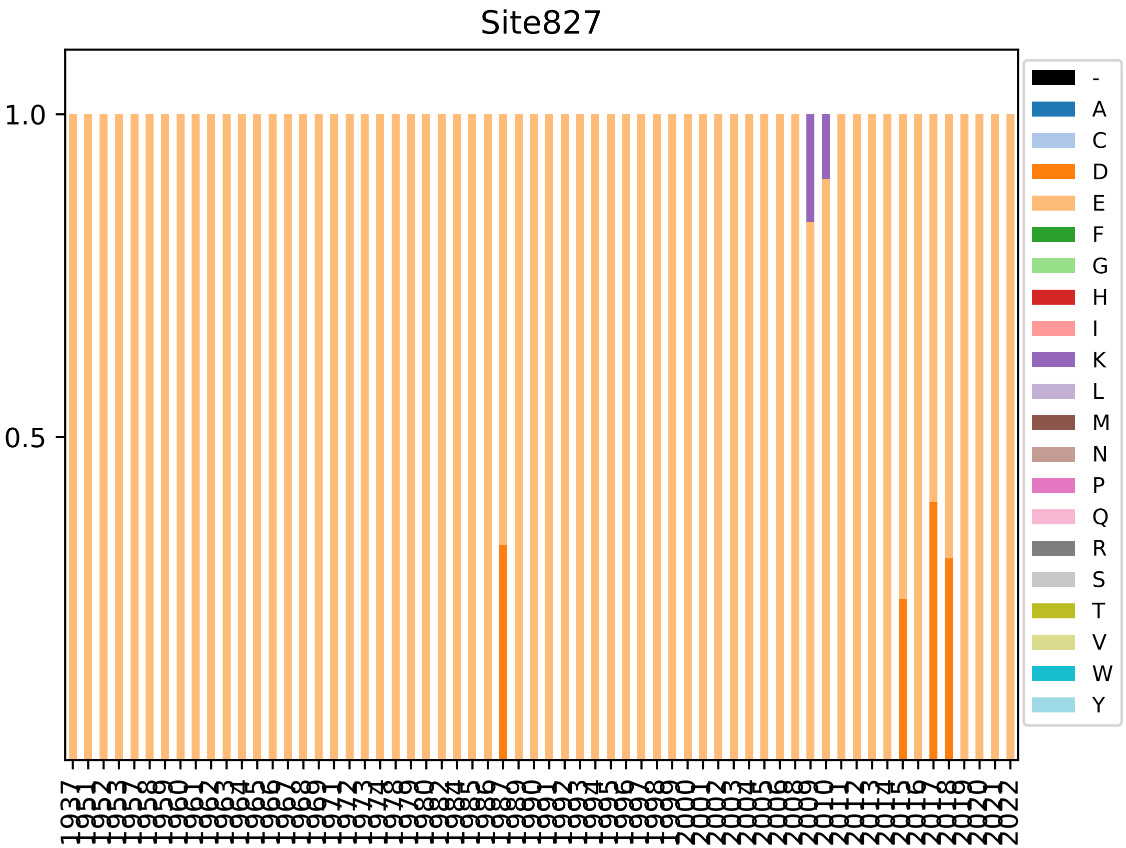

Site827
1.0
0.5
-
A
C
D
E
F
G
H
I
K
L
M
N
P
Q
R
S
T
V
W
Y

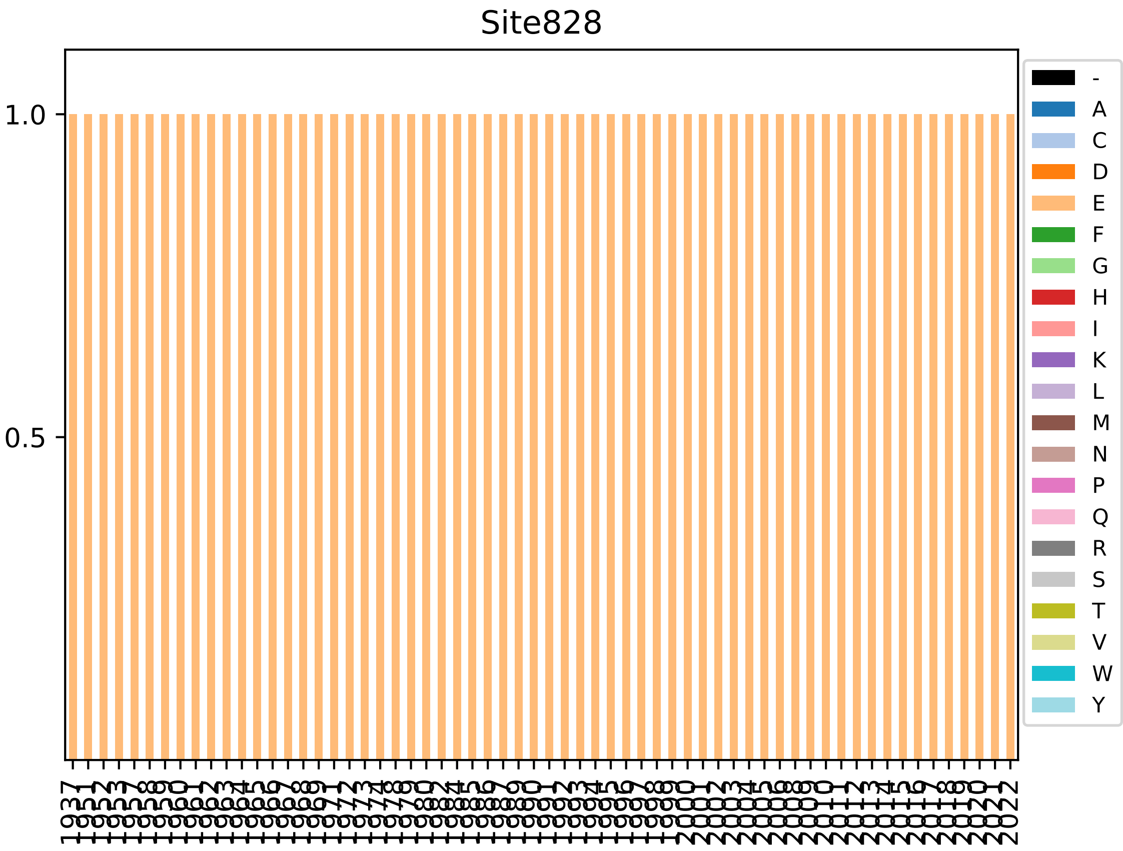
Site828
1.0
0.5
-
A
C
D
E
F
G
H
I
K
L
M
N
P
Q
R
S
T
V
W
Y

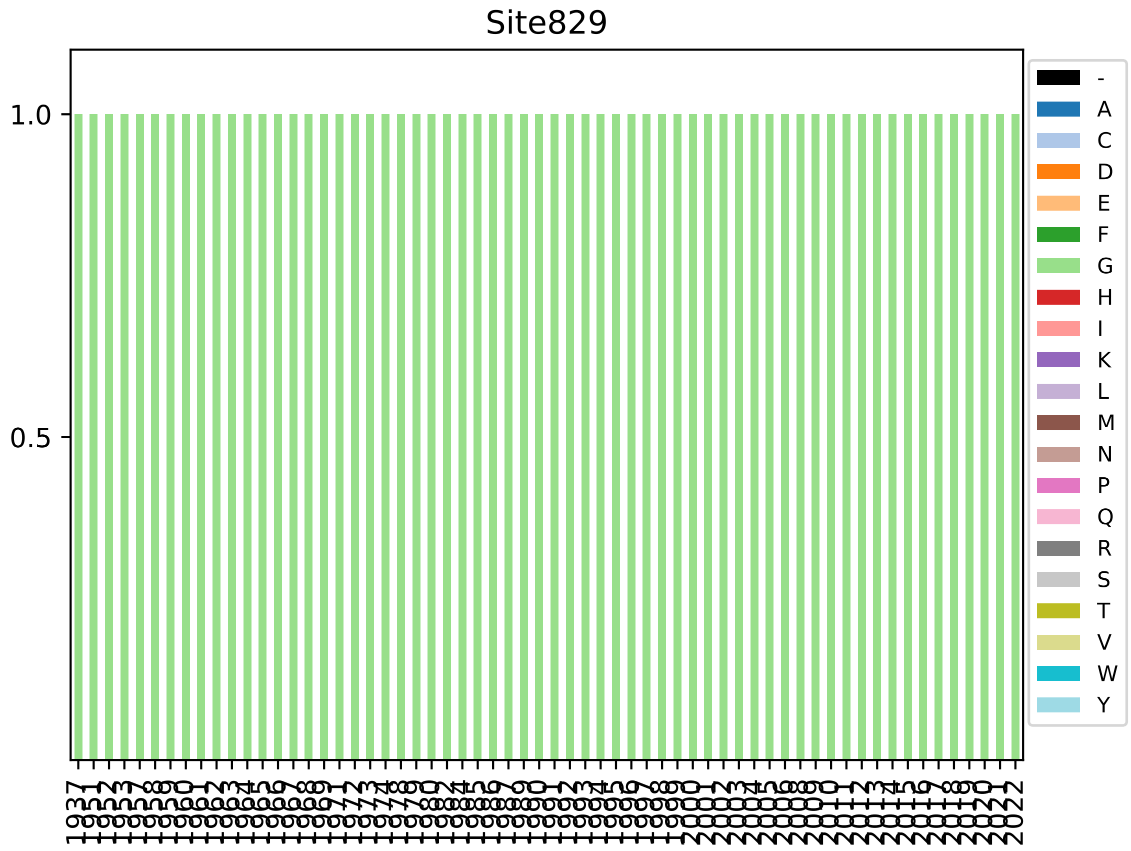
Site829
1.0
0.5
-
A
C
D
E
F
G
H
I
K
L
M
N
P
Q
R
S
T
V
W
Y

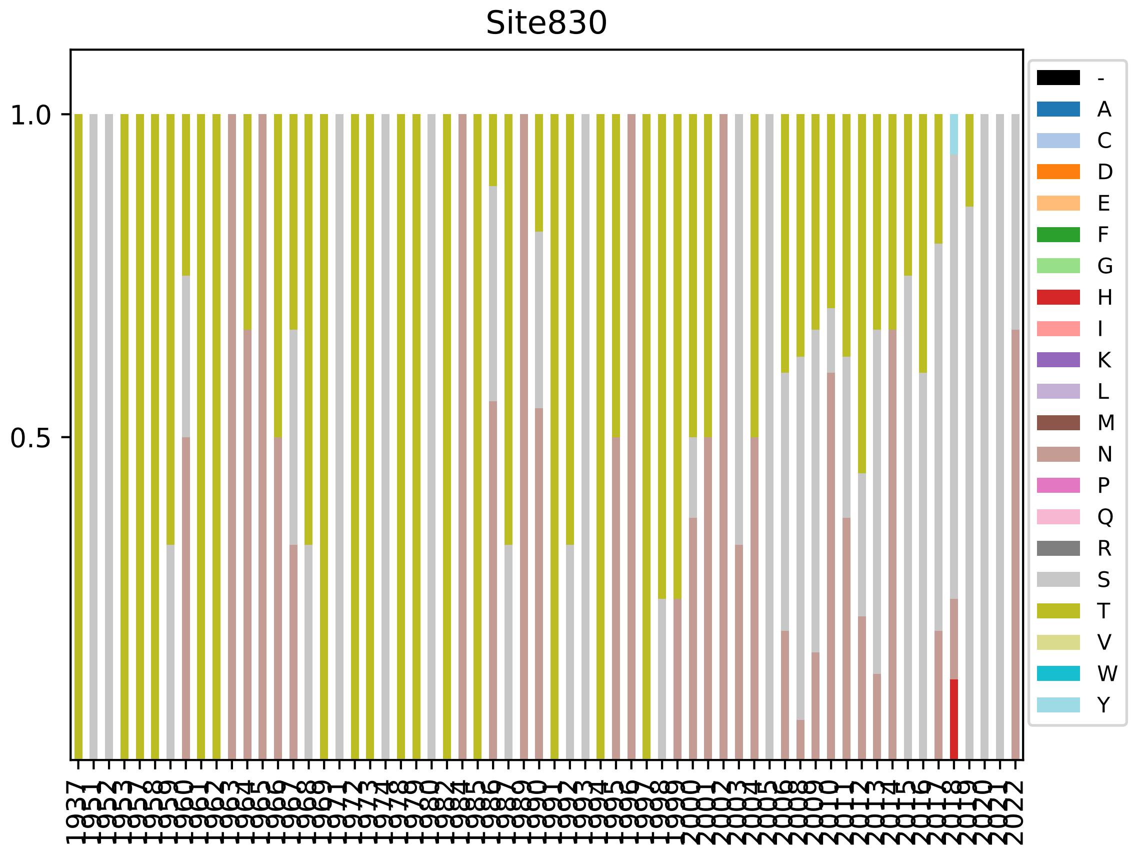
Site830
1.0
0.5
-
A
C
D
E
F
G
H
I
K
L
M
N
P
Q
R
S
T
V
W
Y

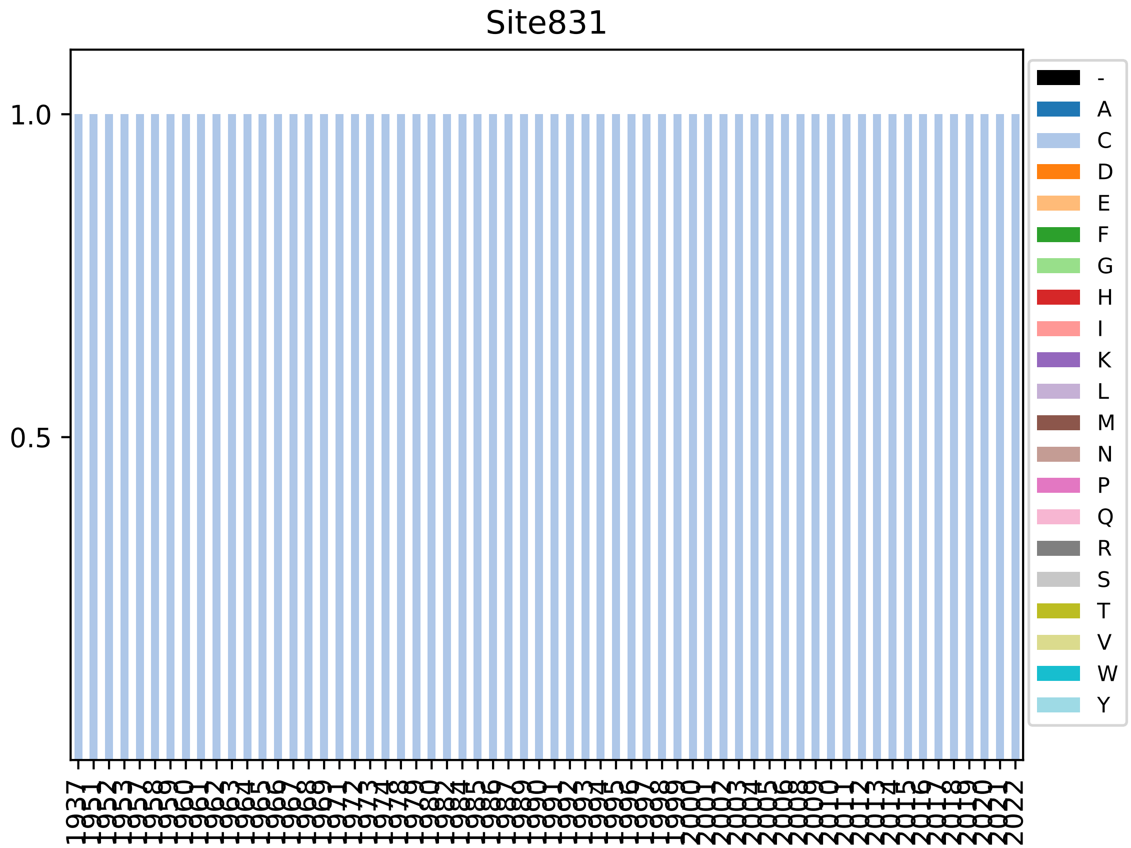

Site831
1.0
0.5
-
A
C
D
E
F
G
H
I
K
L
M
N
P
Q
R
S
T
V
W
Y

Site832

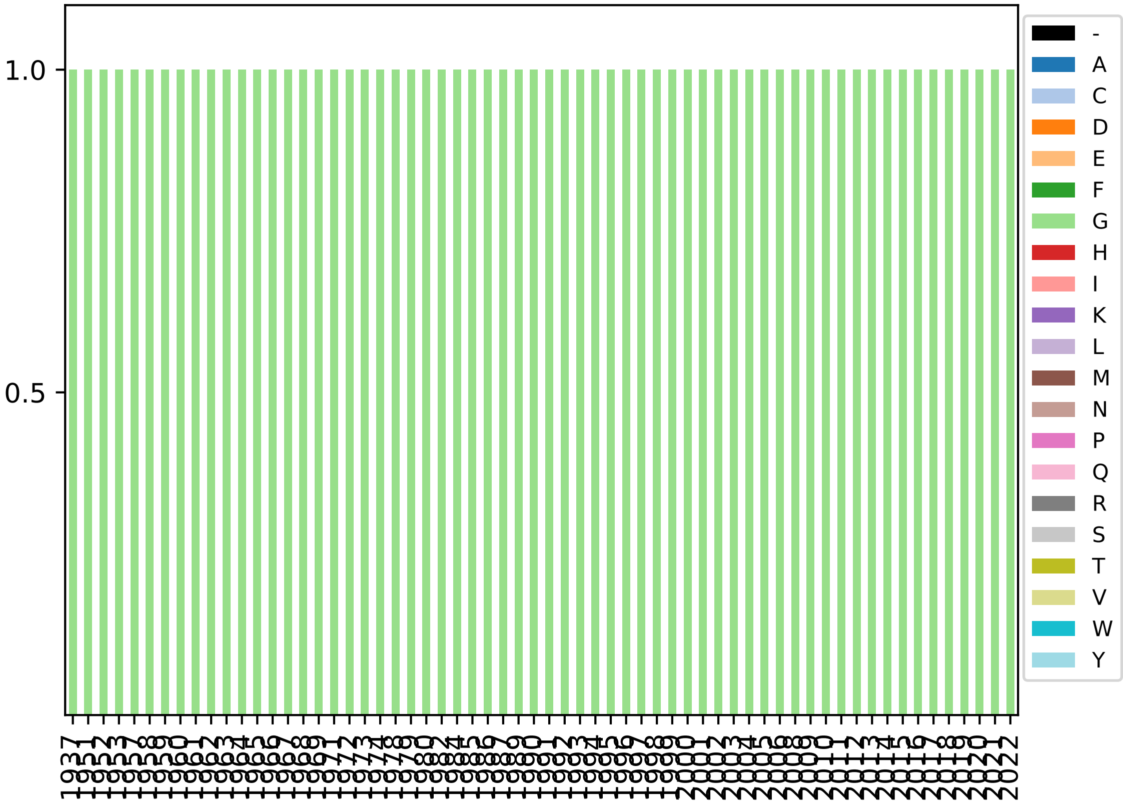

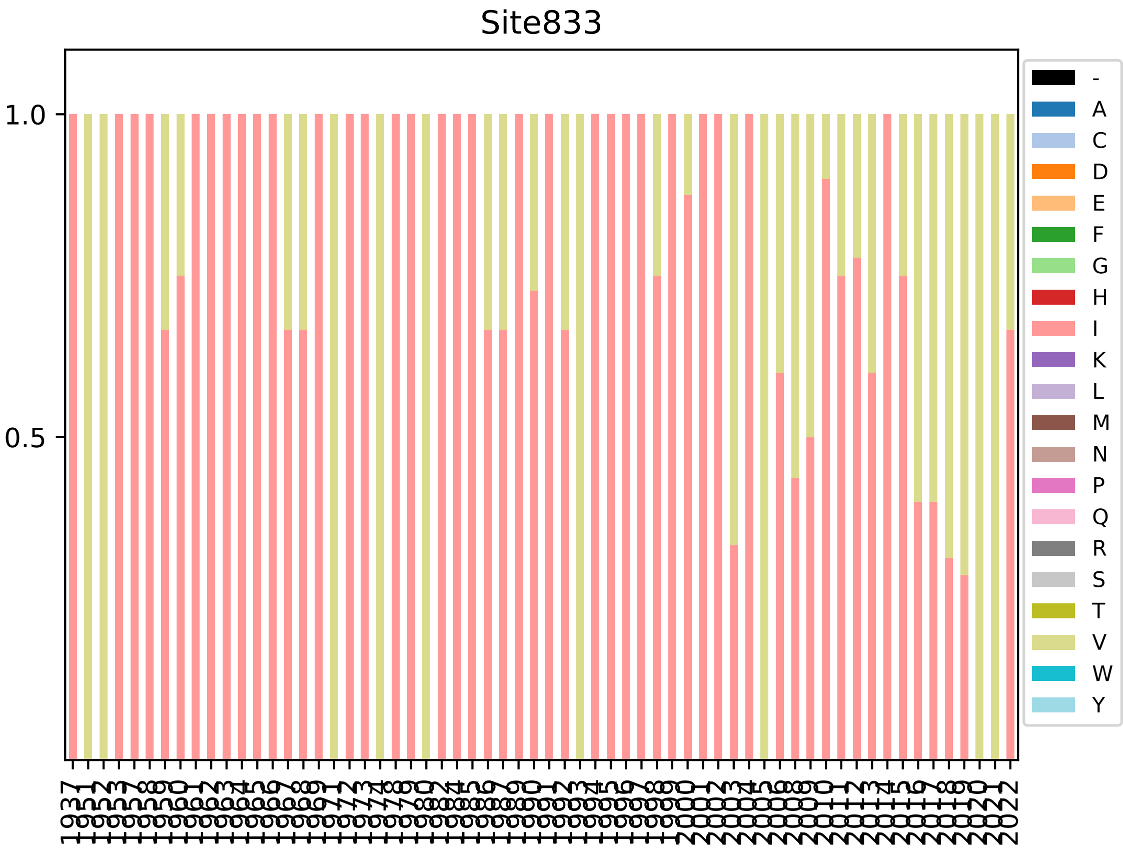
Site833
1.0
0.5
-
A
C
D
E
F
G
H
I
K
L
M
N
P
Q
R
S
T
V
W
Y

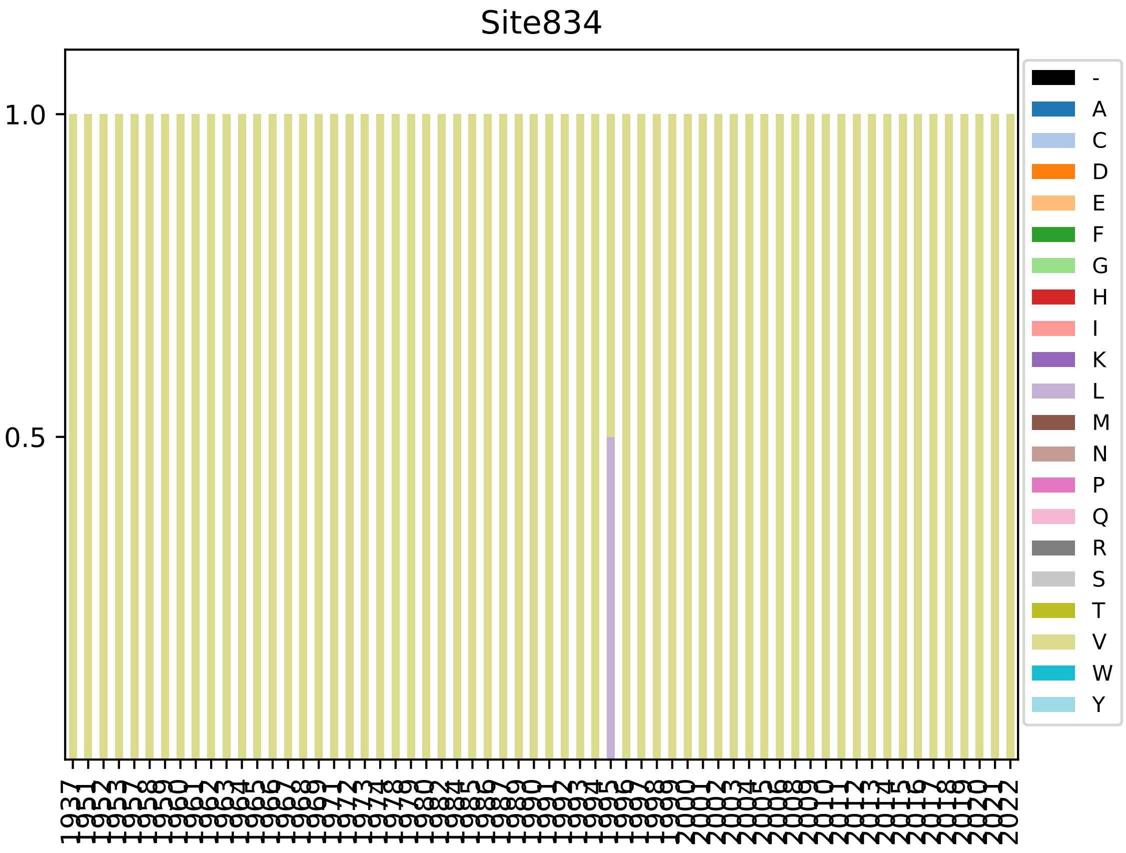
Site834
1.0
0.5
-
A
C
D
E
F
G
H
I
K
L
M
N
P
Q
R
S
T
V
W
Y

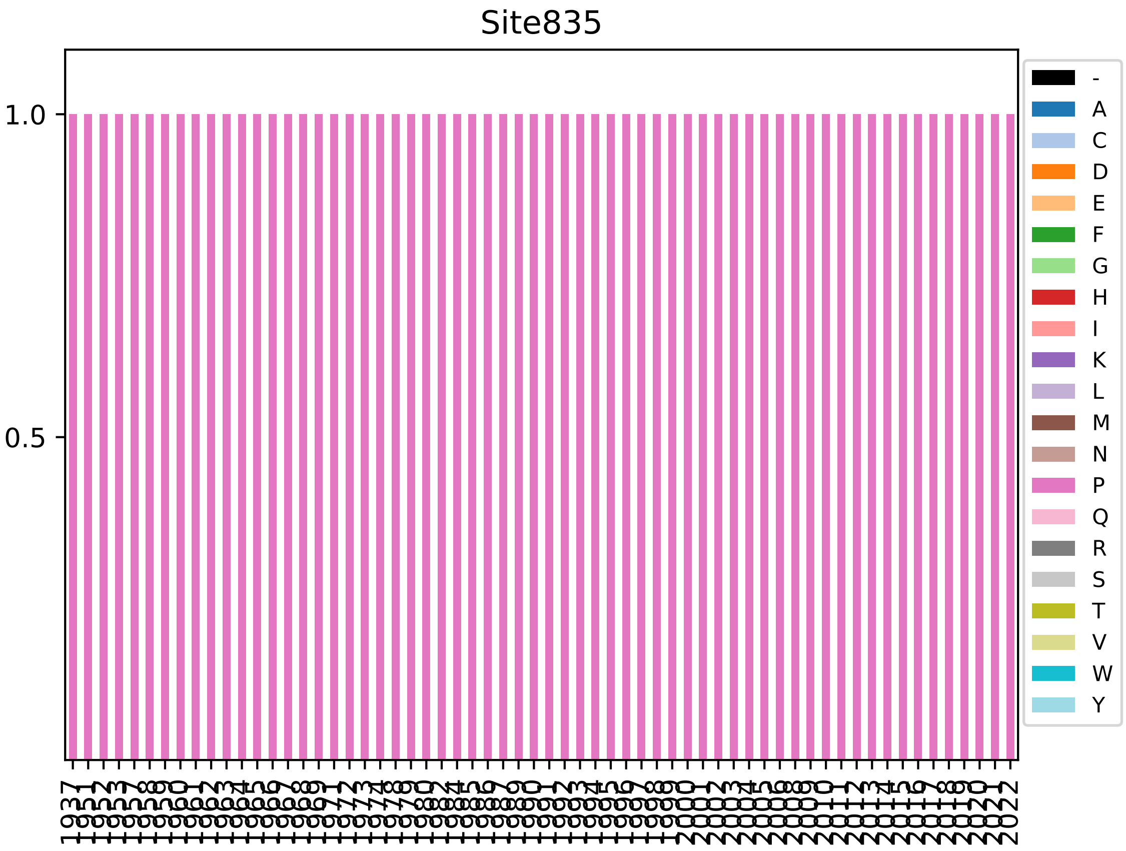
Site835
1.0
0.5
-
A
C
D
E
F
G
H
I
K
L
M
N
P
Q
R
S
T
V
W
Y

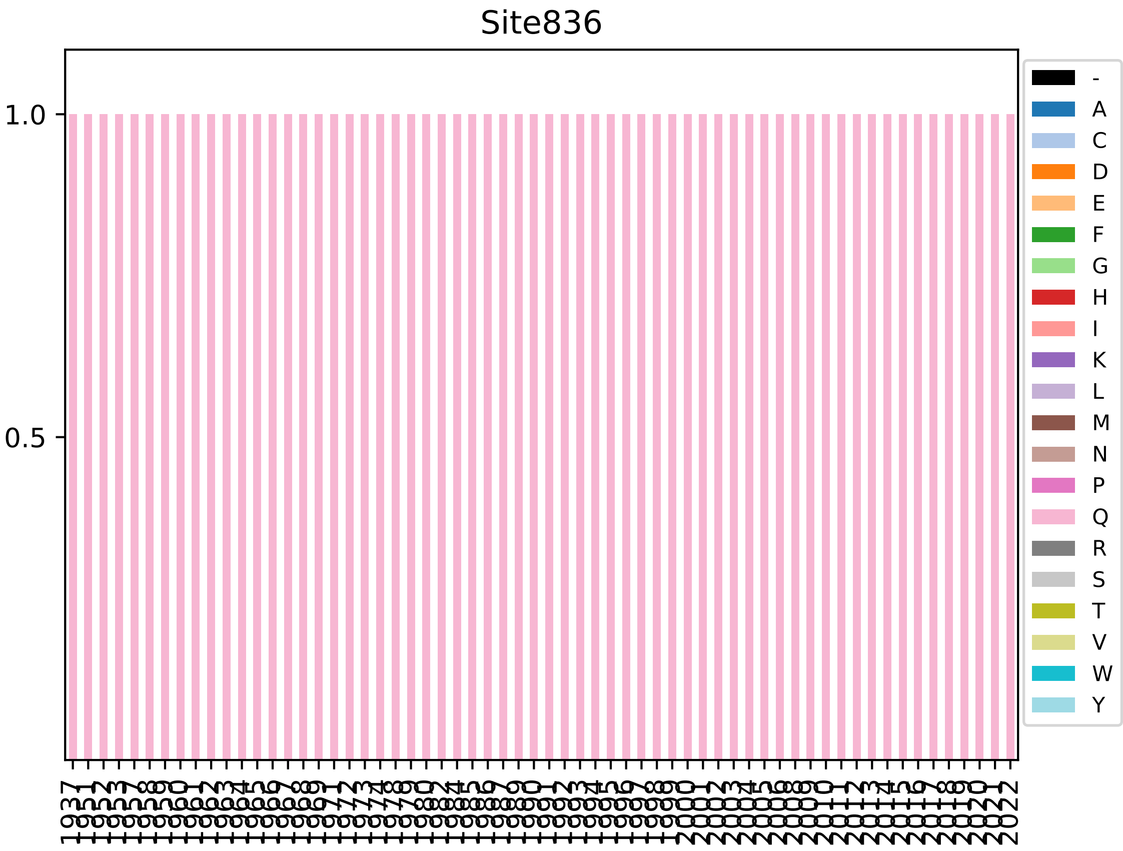
Site836
1.0
0.5
-
A
C
D
E
F
G
H
I
K
L
M
N
P
Q
R
S
T
V
W
Y

Site837

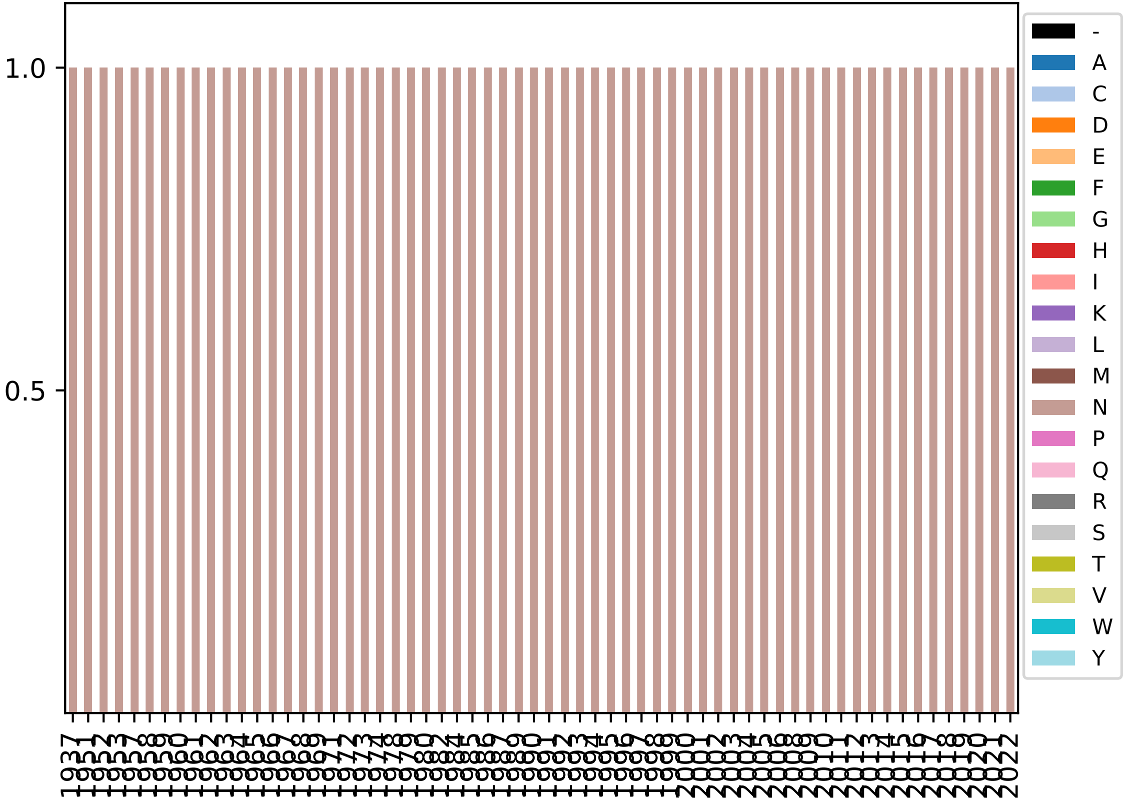

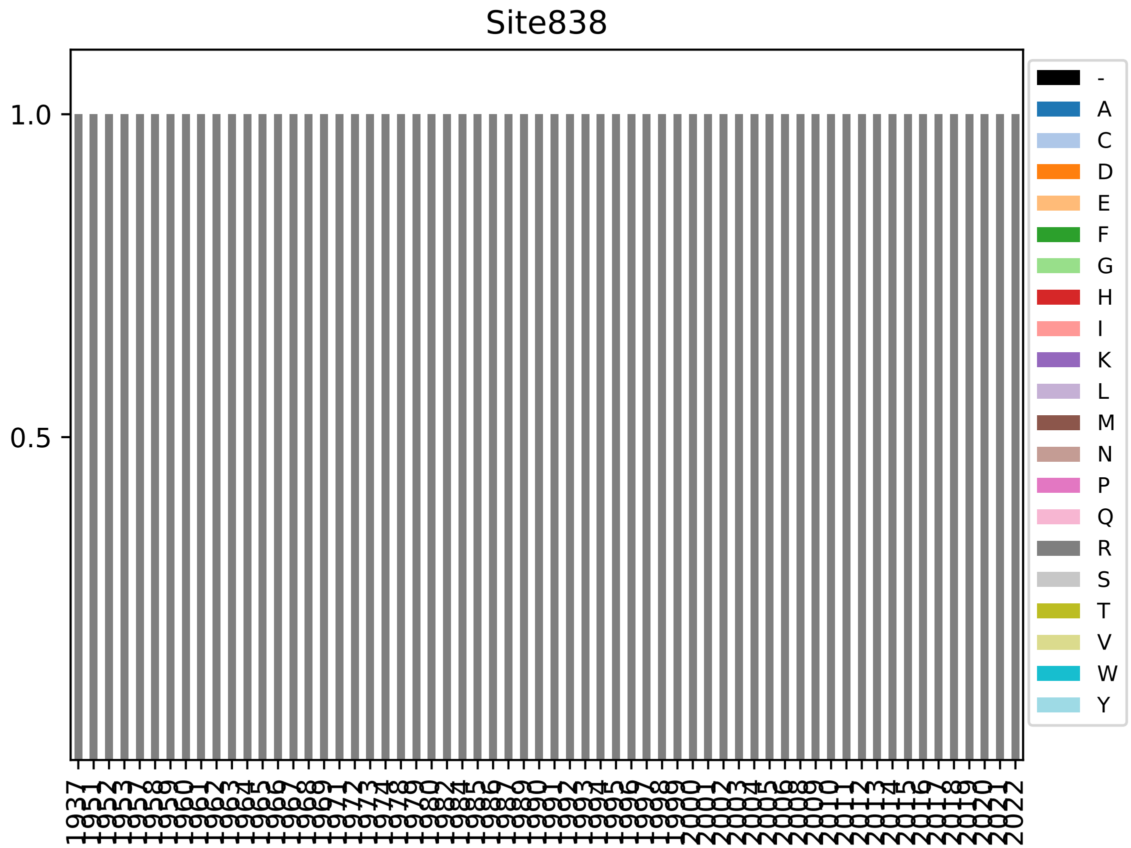
Site838
1.0
0.5
-
A
C
D
E
F
G
H
I
K
L
M
N
P
Q
R
S
T
V
W
Y

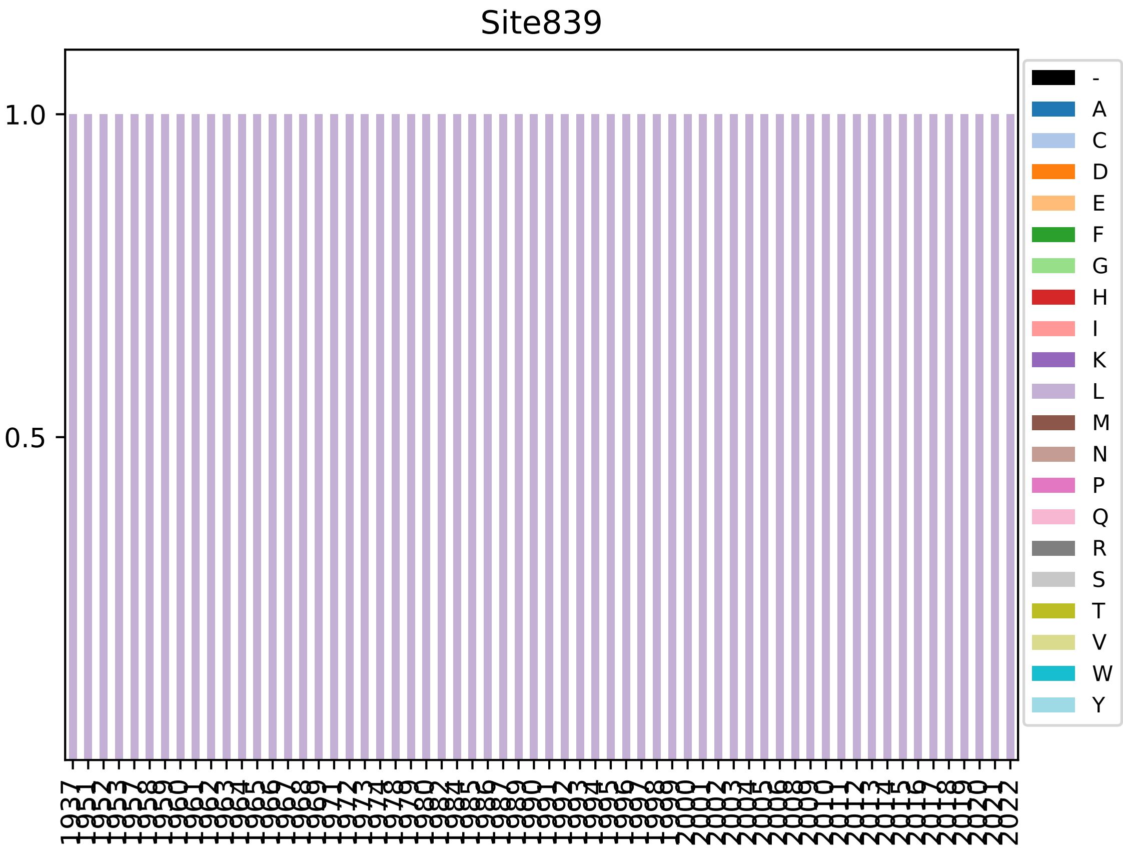
Site839
1.0
0.5
-
A
C
D
E
F
G
H
I
K
L
M
N
P
Q
R
S
T
V
W
Y

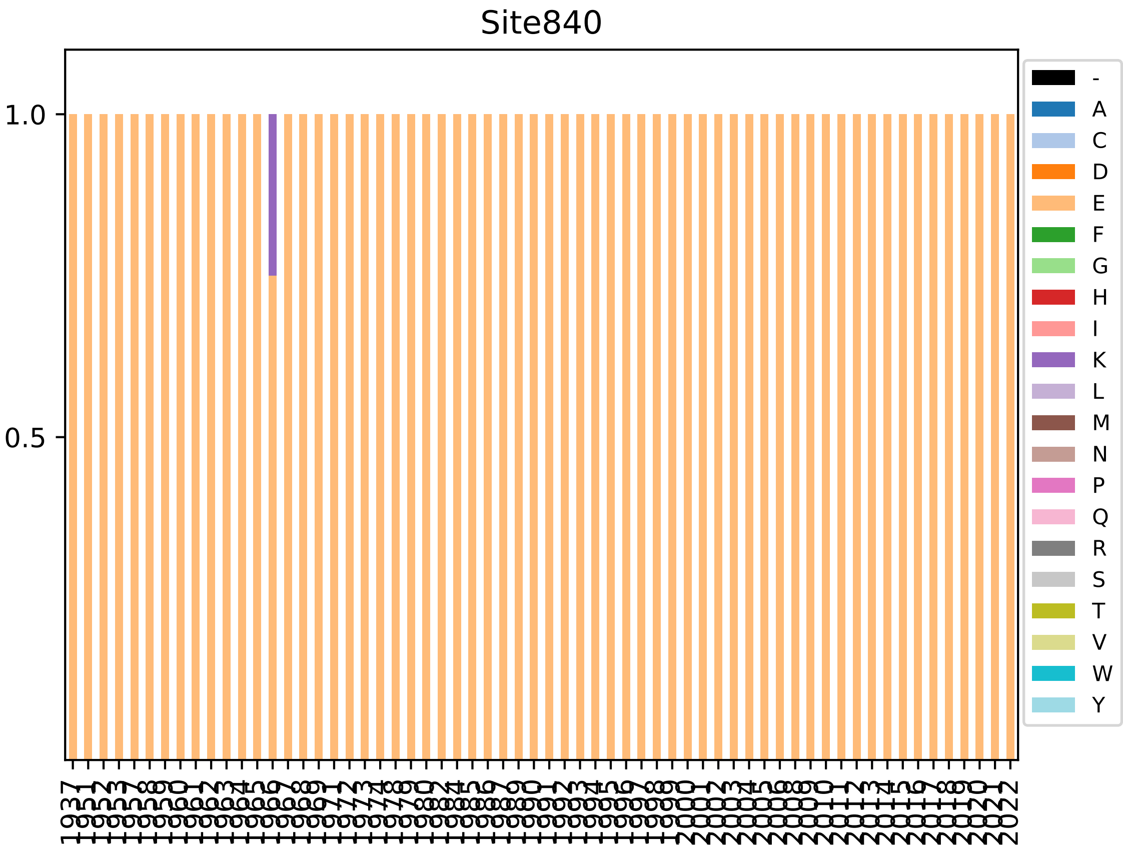
Site840
1.0
0.5
-
A
C
D
E
F
G
H
I
K
L
M
N
P
Q
R
S
T
V
W
Y

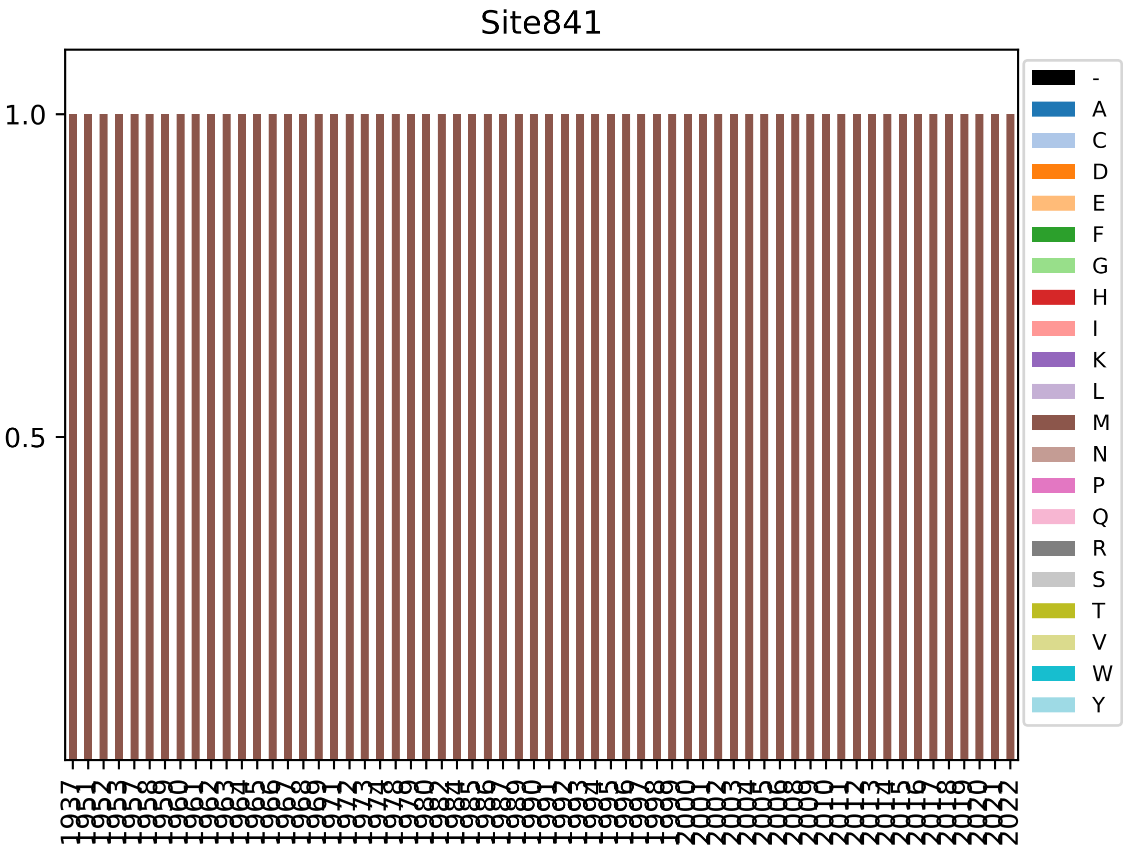
Site841
1.0
0.5
-
A
C
D
E
F
G
H
I
K
L
M
N
P
Q
R
S
T
V
W
Y

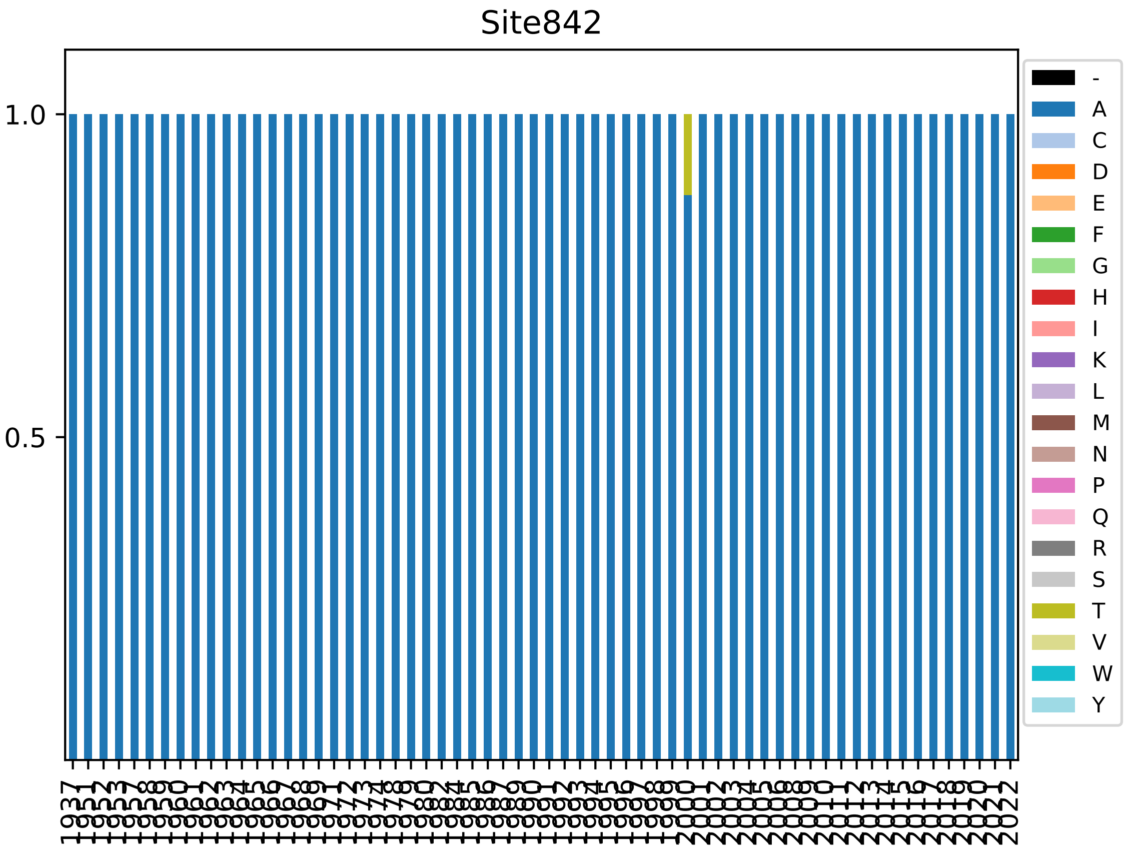

Site842
1.0
0.5
-
A
C
D
E
F
G
H
I
K
L
M
N
P
Q
R
S
T
V
W
Y

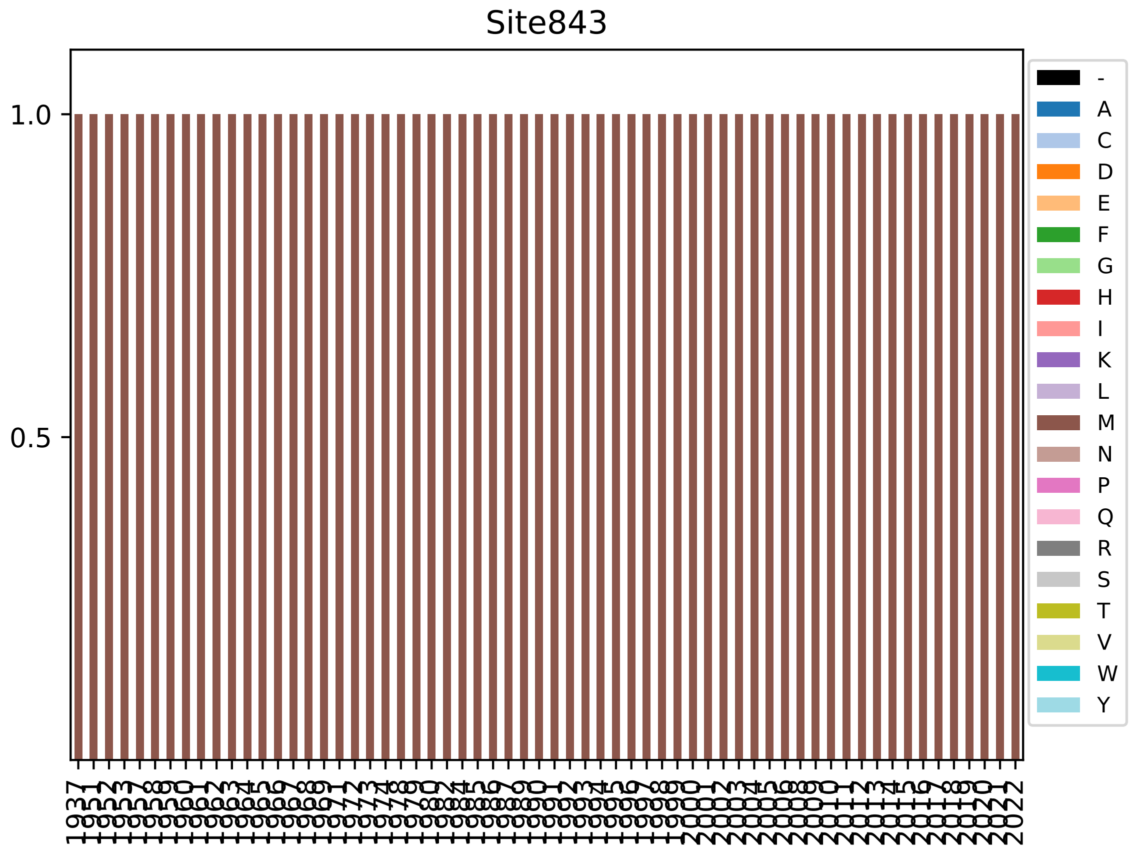
Site843
1.0
0.5
-
A
C
D
E
F
G
H
I
K
L
M
N
P
Q
R
S
T
V
W
Y

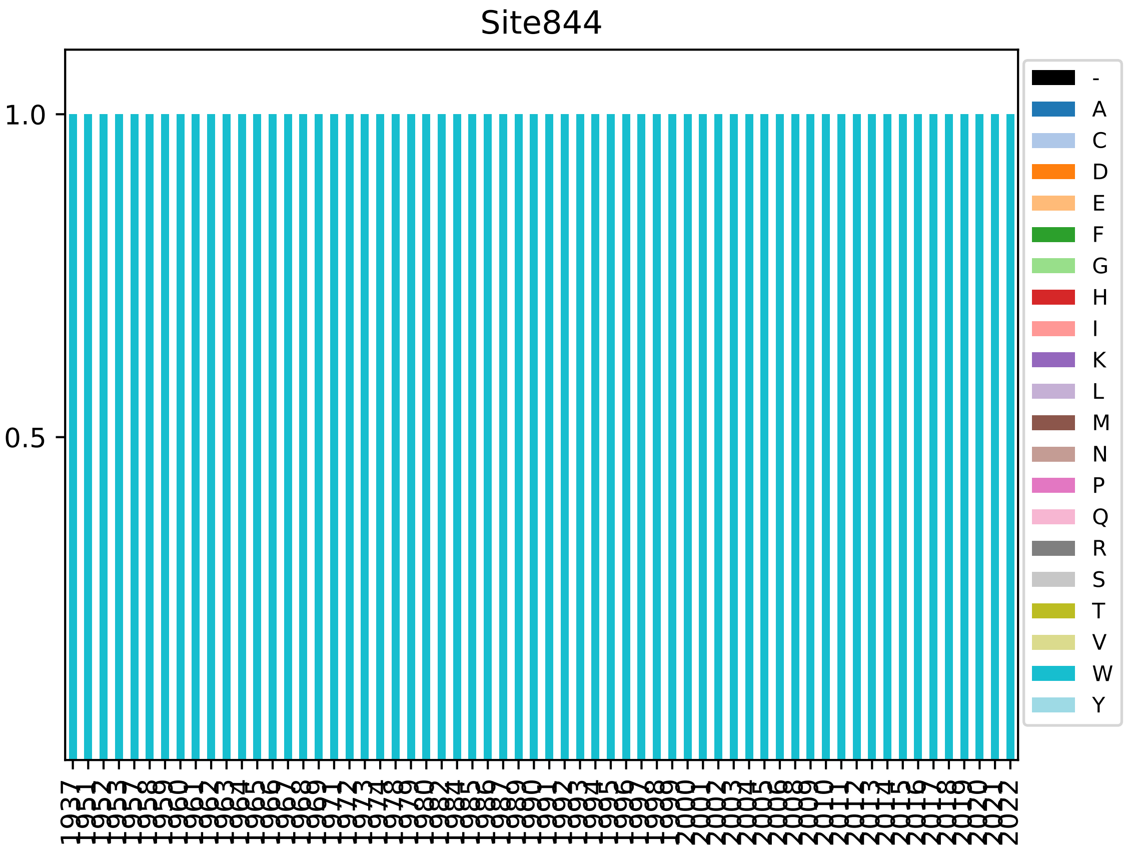
Site844
1.0
0.5
-
A
C
D
E
F
G
H
I
K
L
M
N
P
Q
R
S
T
V
W
Y

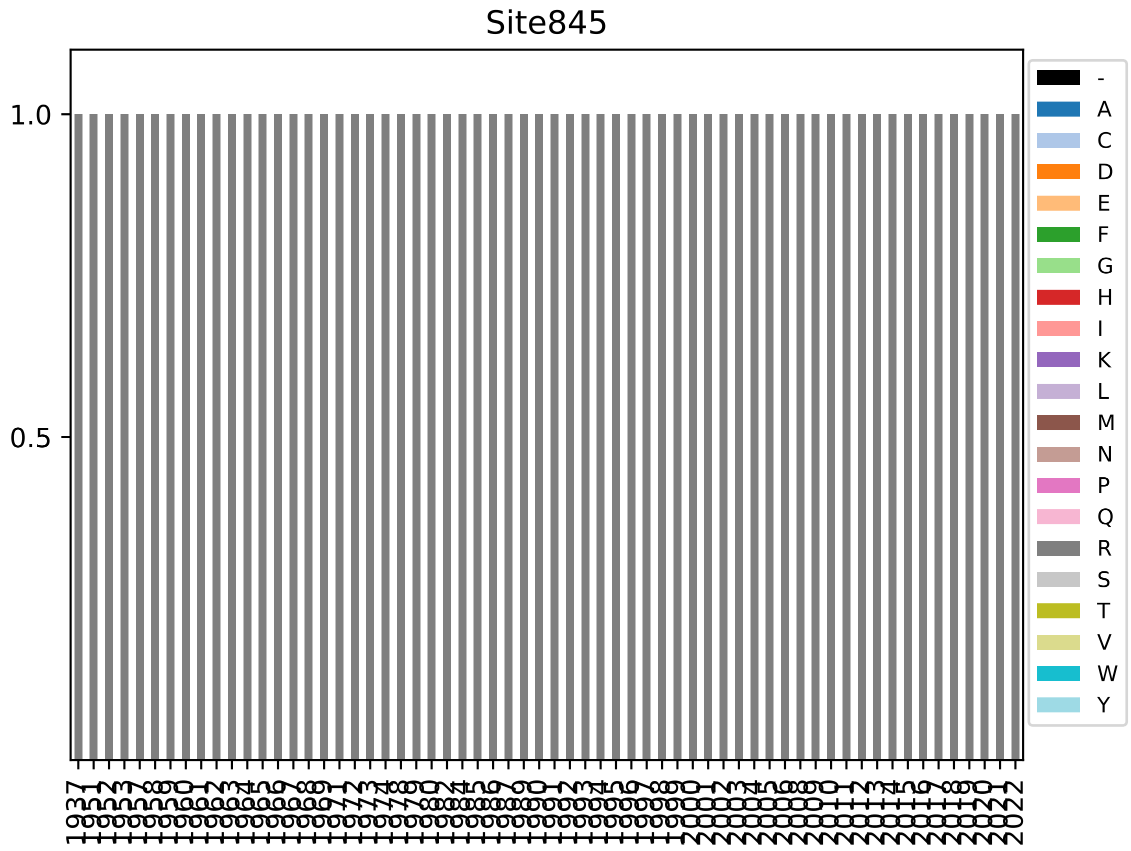
Site845
1.0
0.5
-
A
C
D
E
F
G
H
I
K
L
M
N
P
Q
R
S
T
V
W
Y

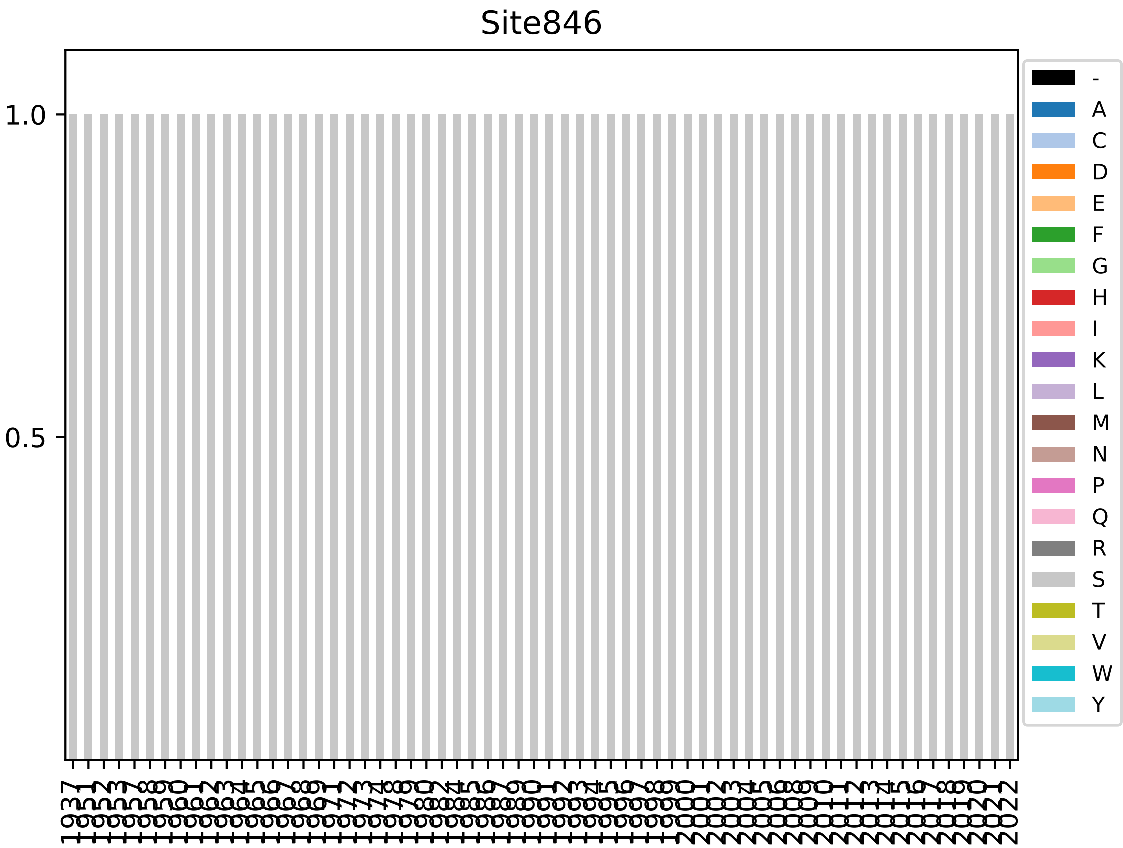
Site846
1.0
0.5
-
A
C
D
E
F
G
H
I
K
L
M
N
P
Q
R
S
T
V
W
Y

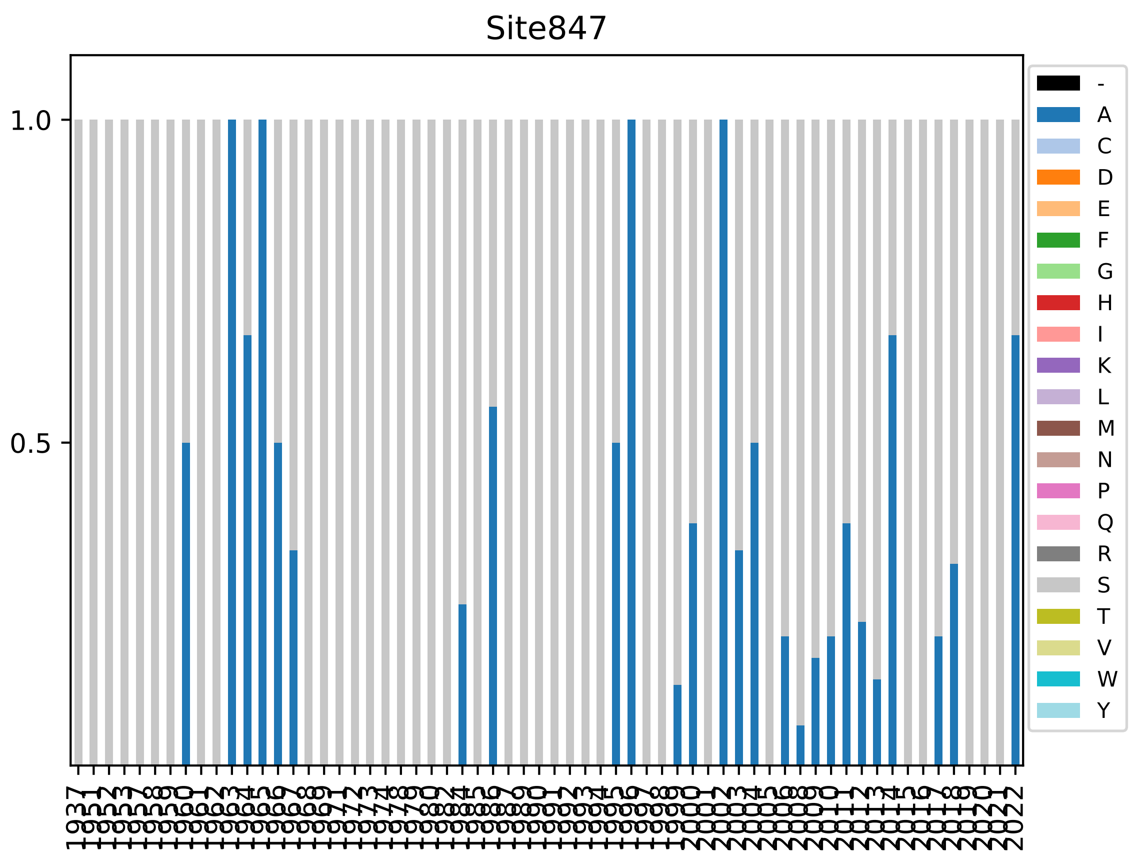
Site847
1.0
0.5
-
A
C
D
E
F
G
H
I
K
L
M
N
P
Q
R
S
T
V
W
Y

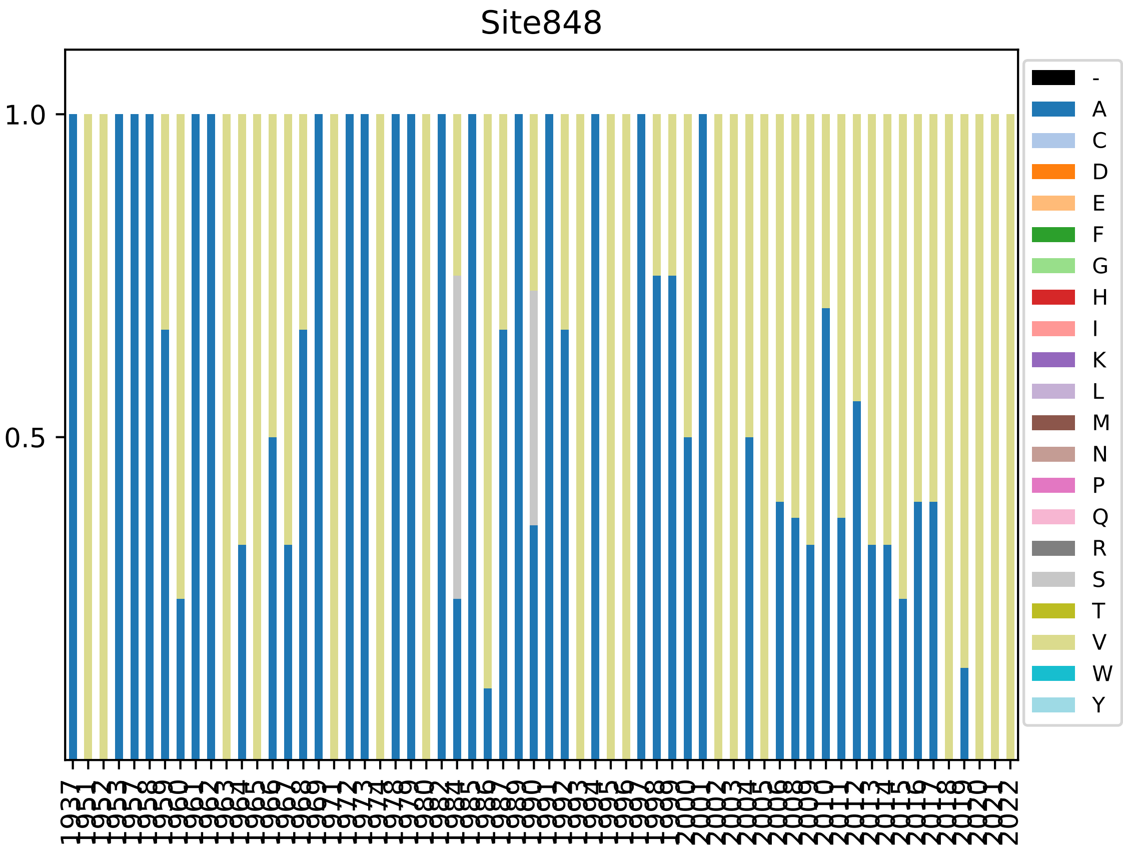
Site848
1.0
0.5
-
A
C
D
E
F
G
H
I
K
L
M
N
P
Q
R
S
T
V
W
Y

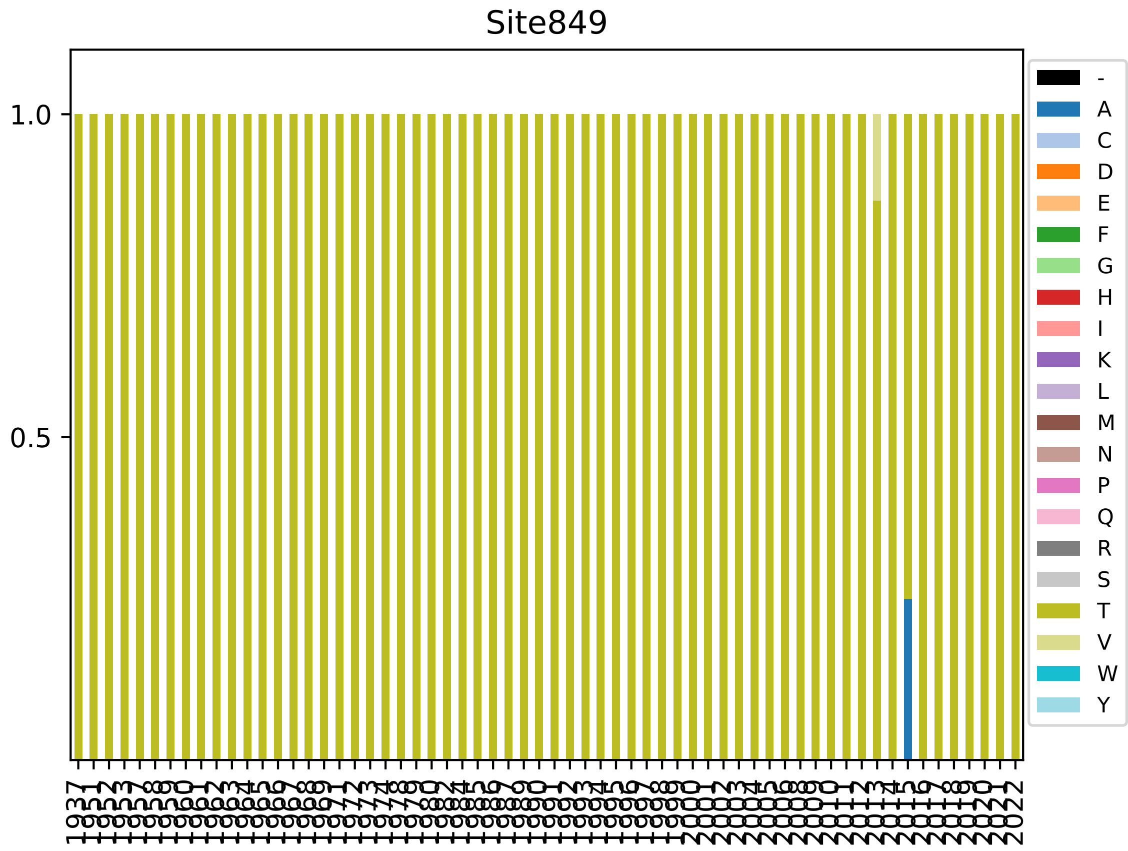
Site849
1.0
0.5
-
A
C
D
E
F
G
H
I
K
L
M
N
P
Q
R
S
T
V
W
Y

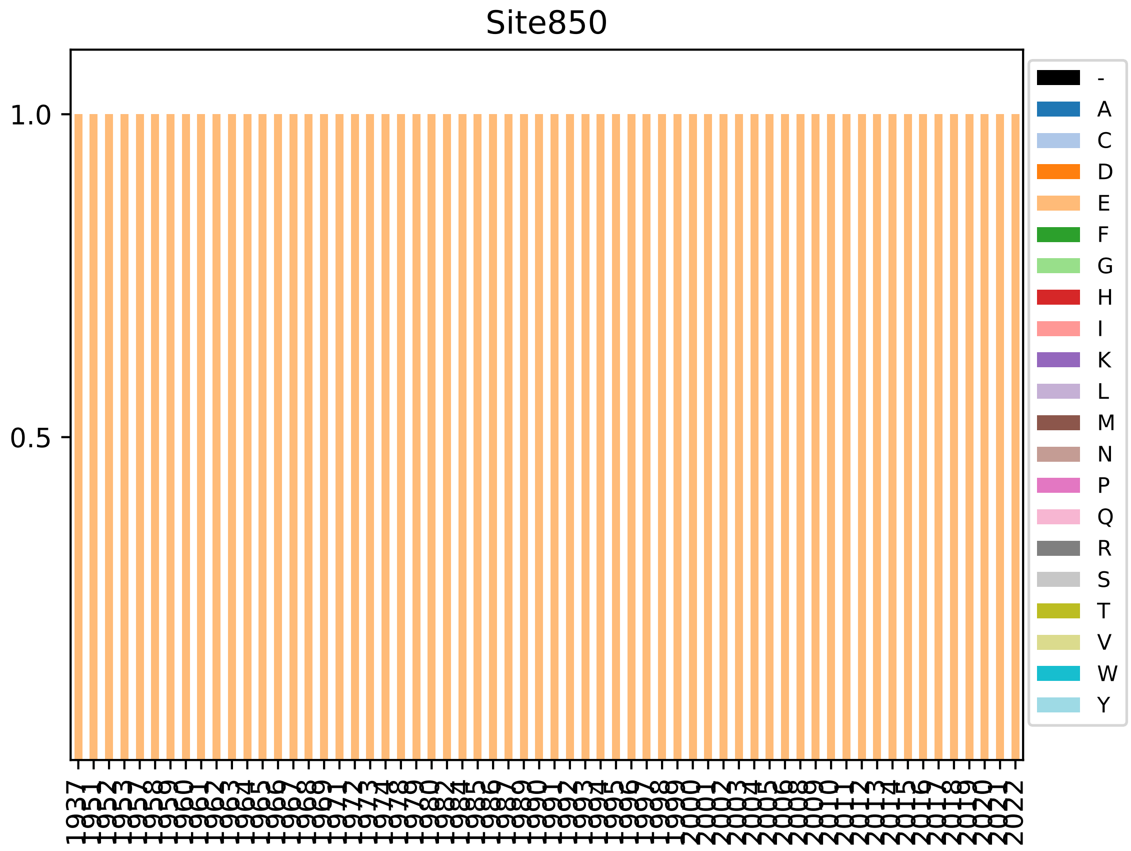
Site850
1.0
0.5
-
A
C
D
E
F
G
H
I
K
L
M
N
P
Q
R
S
T
V
W
Y

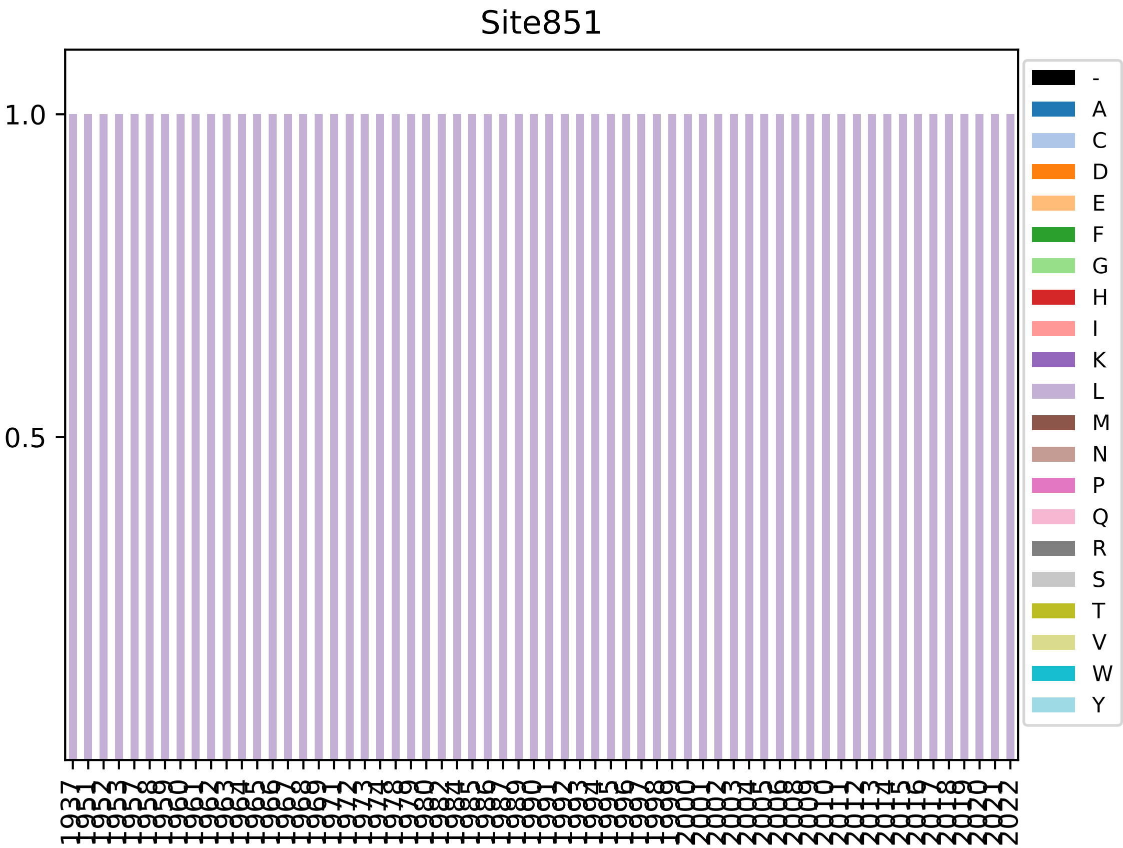
Site851
1.0
0.5
-
A
C
D
E
F
G
H
I
K
L
M
N
P
Q
R
S
T
V
W
Y

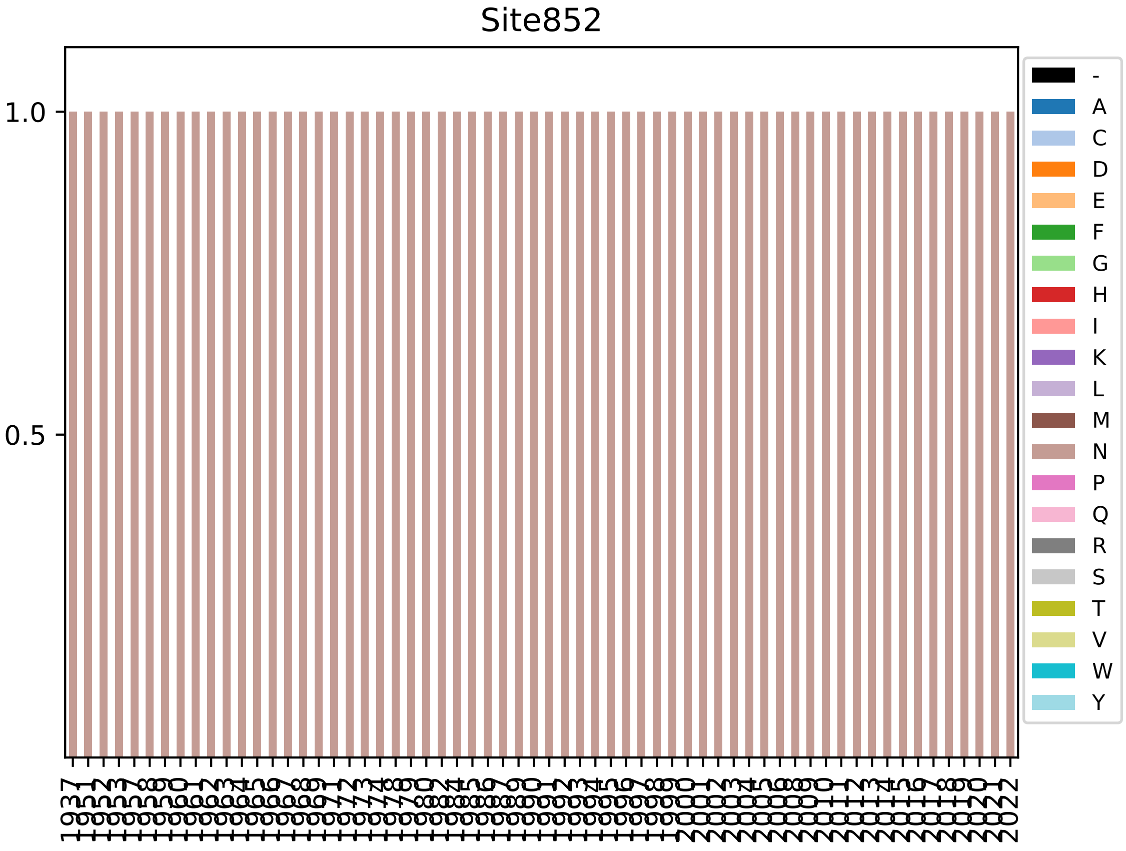
Site852
1.0
0.5
-
A
C
D
E
F
G
H
I
K
L
M
N
P
Q
R
S
T
V
W
Y

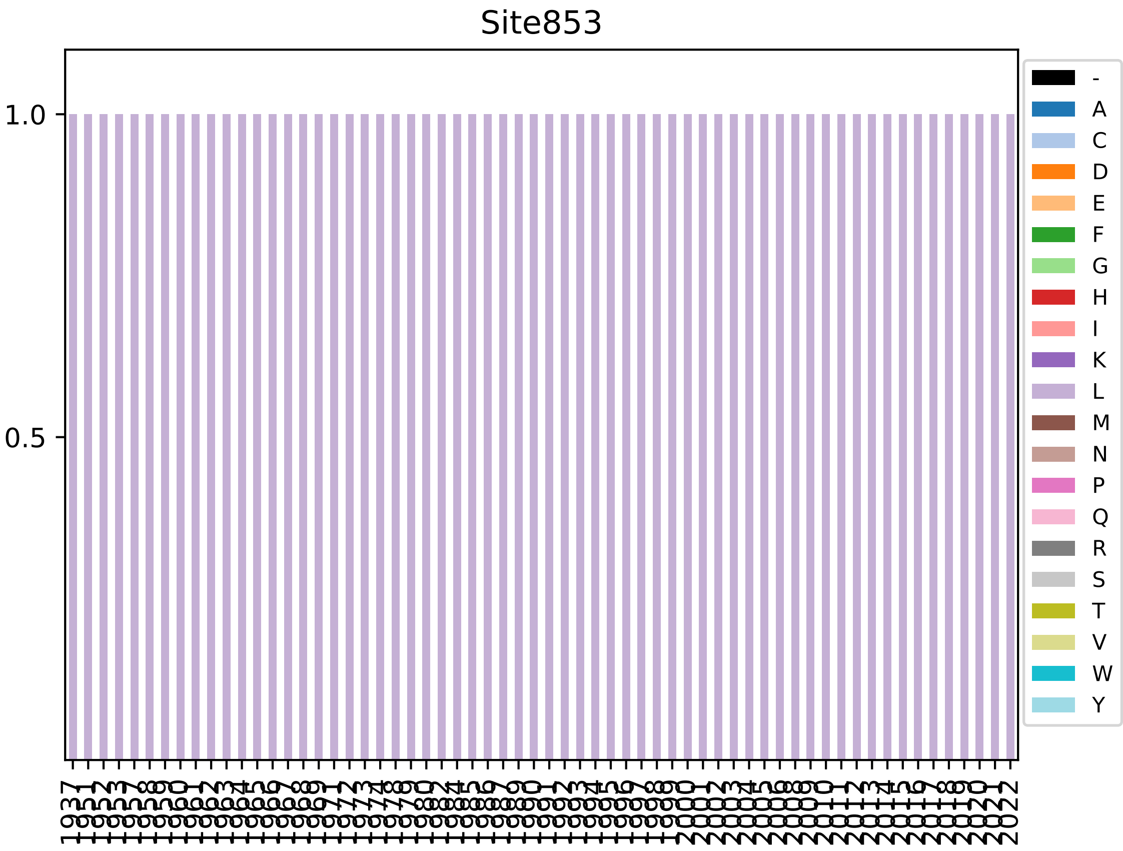
Site853
1.0
0.5
-
A
C
D
E
F
G
H
I
K
L
M
N
P
Q
R
S
T
V
W
Y

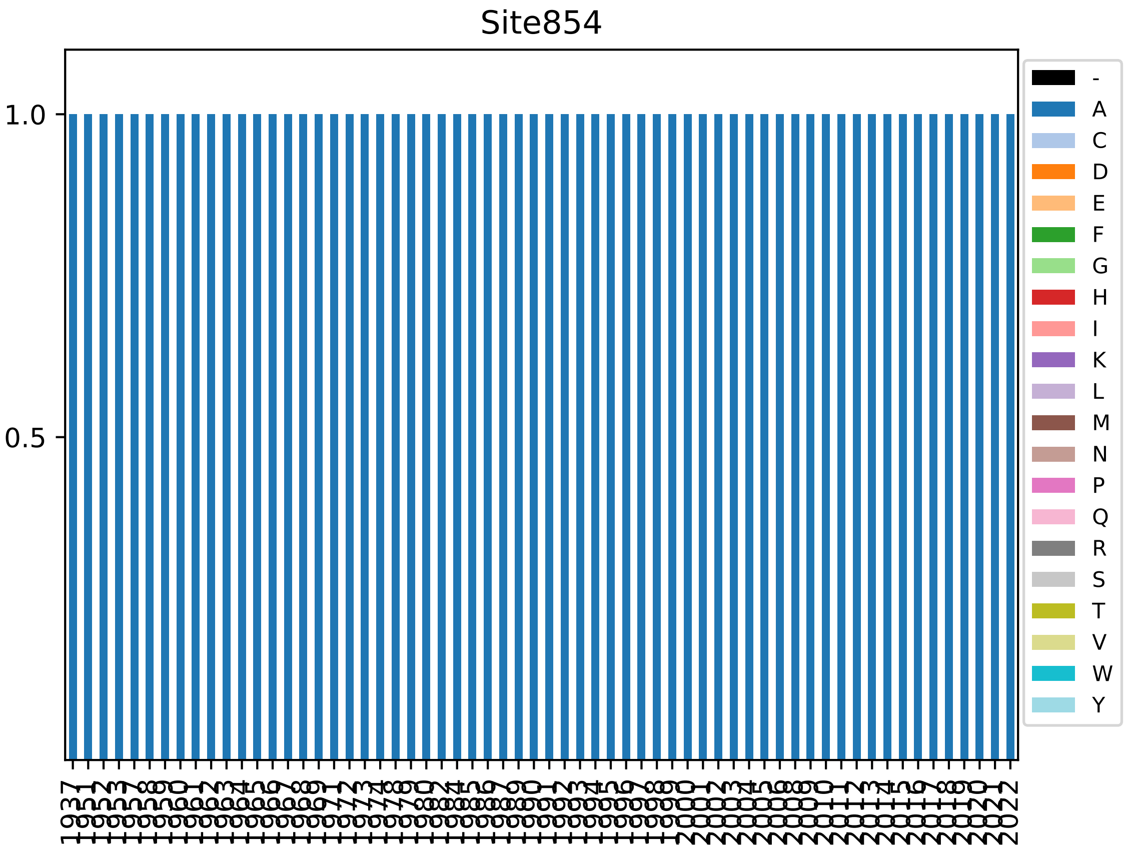
Site854
1.0
0.5
-
A
C
D
E
F
G
H
I
K
L
M
N
P
Q
R
S
T
V
W
Y

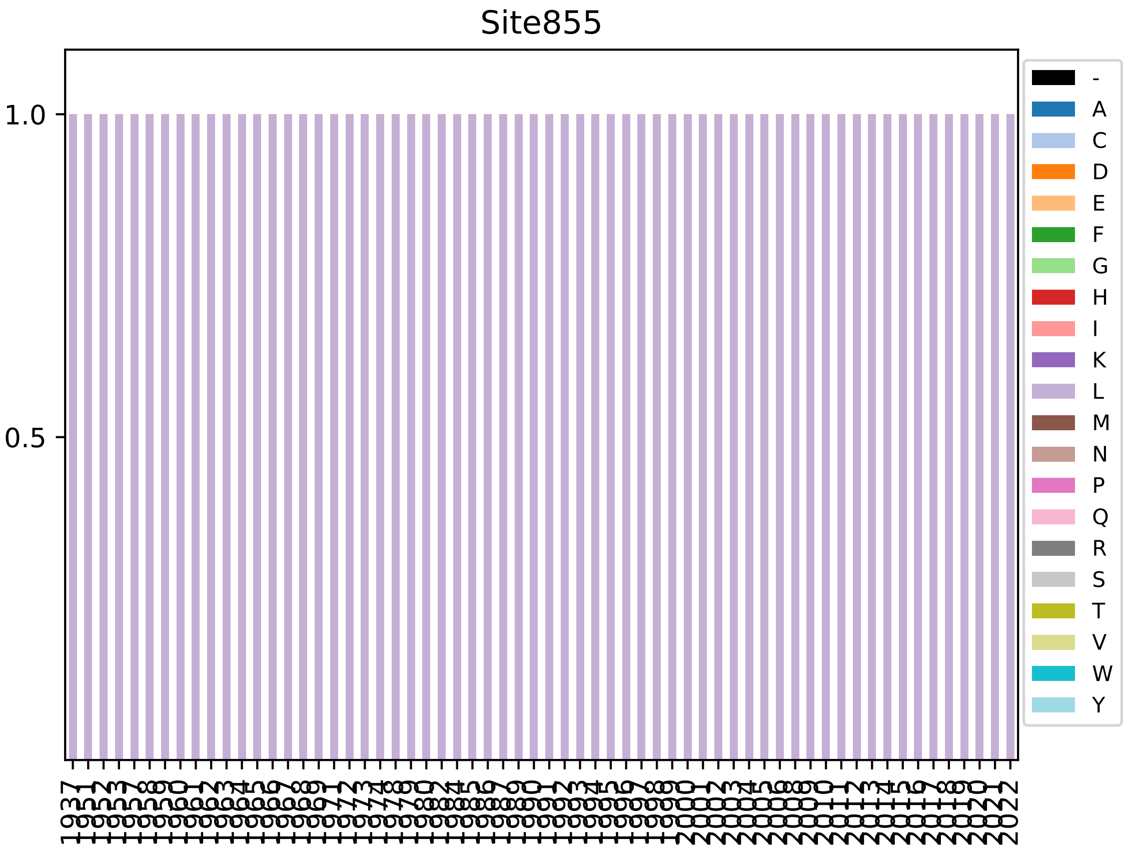
Site855
1.0
0.5
-
A
C
D
E
F
G
H
I
K
L
M
N
P
Q
R
S
T
V
W
Y

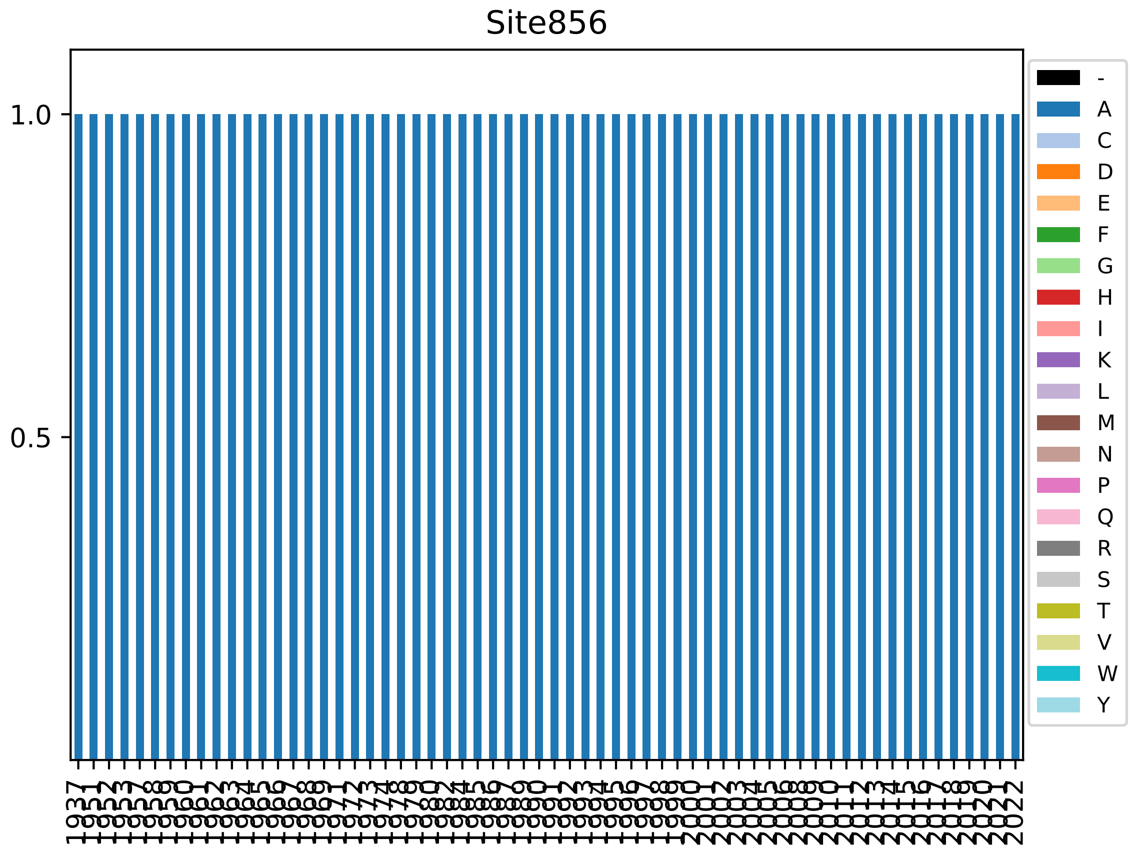

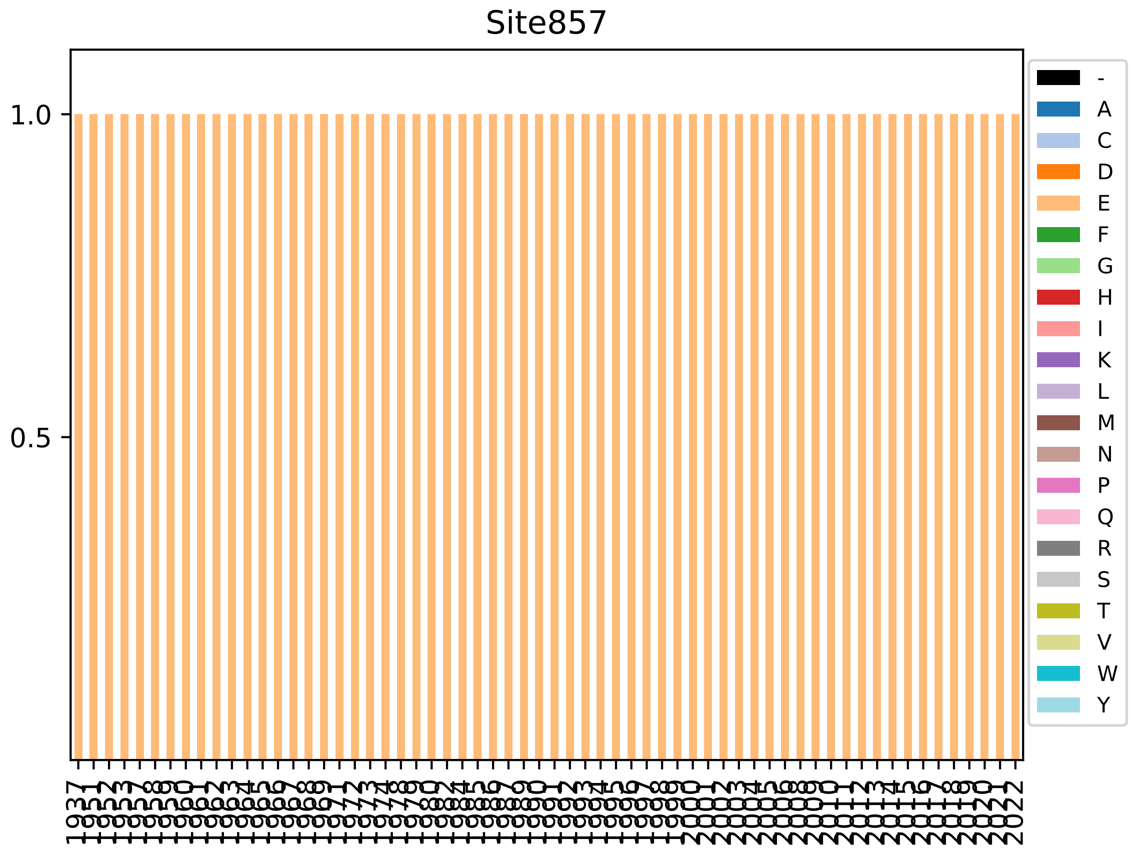

Site857
1.0
0.5
-
A
C
D
E
F
G
H
I
K
L
M
N
P
Q
R
S
T
V
W
Y

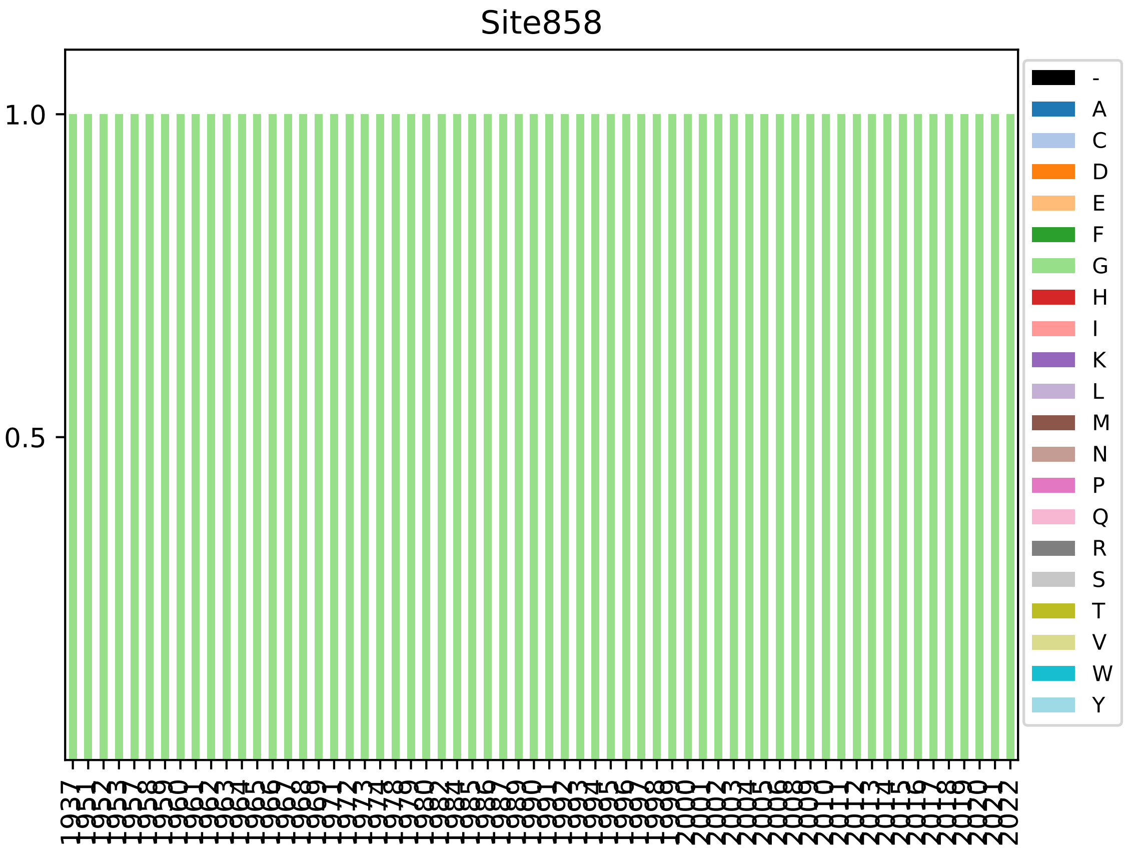
Site858
1.0
0.5
-
A
C
D
E
F
G
H
I
K
L
M
N
P
Q
R
S
T
V
W
Y

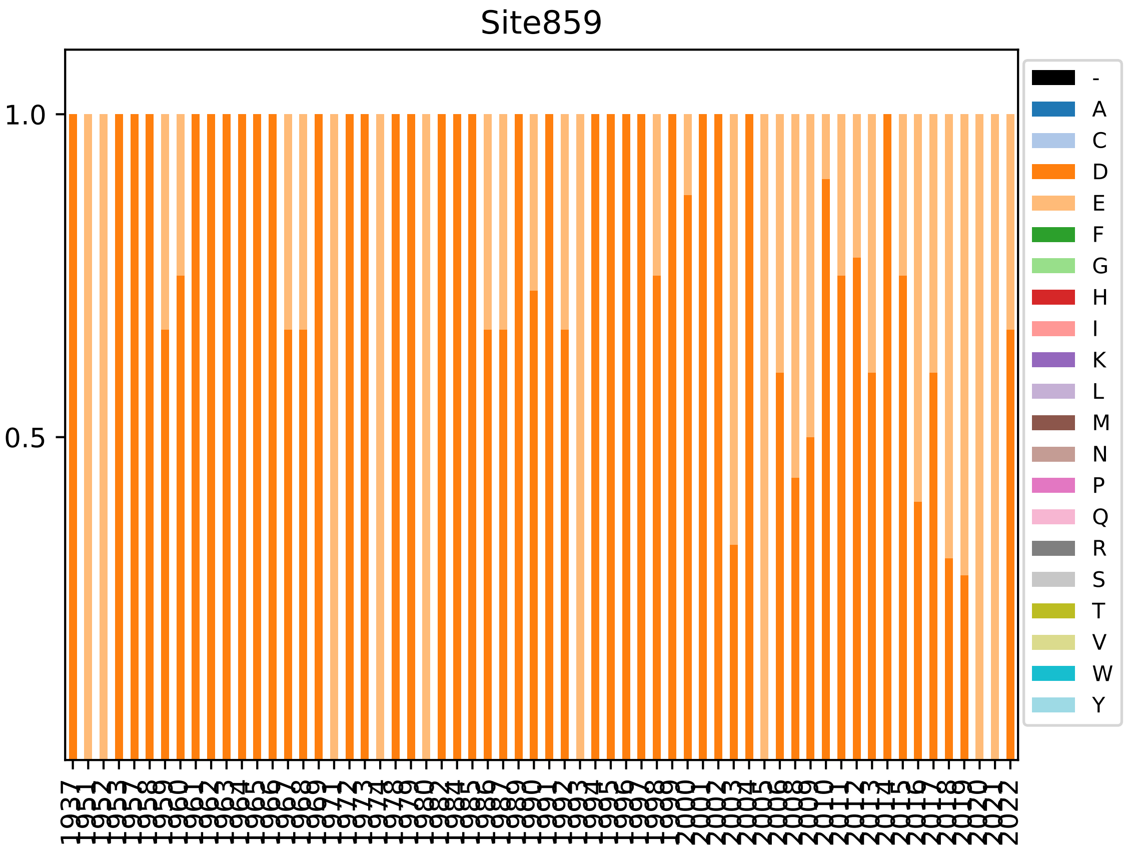
Site859
1.0
0.5
-
A
C
D
E
F
G
H
I
K
L
M
N
P
Q
R
S
T
V
W
Y

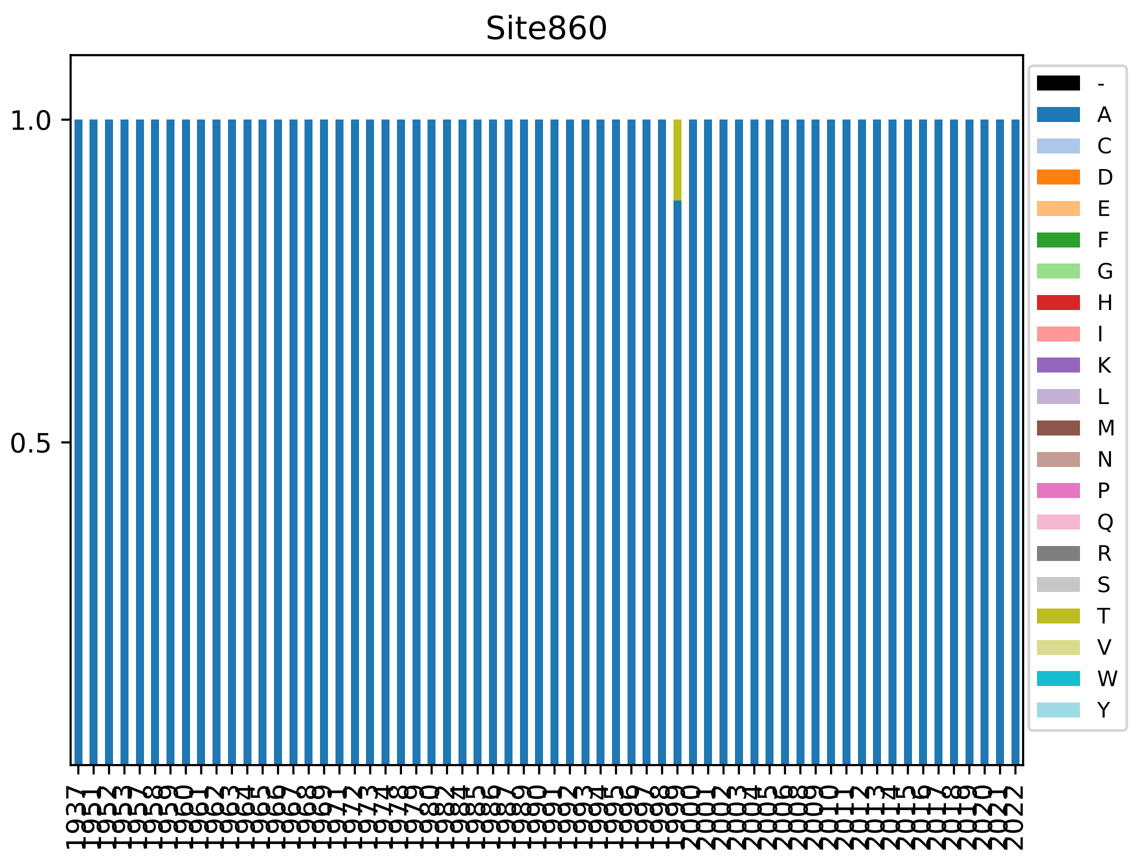
Site860
1.0
0.5
-
A
C
D
E
F
G
H
I
K
L
M
N
P
Q
R
S
T
V
W
Y

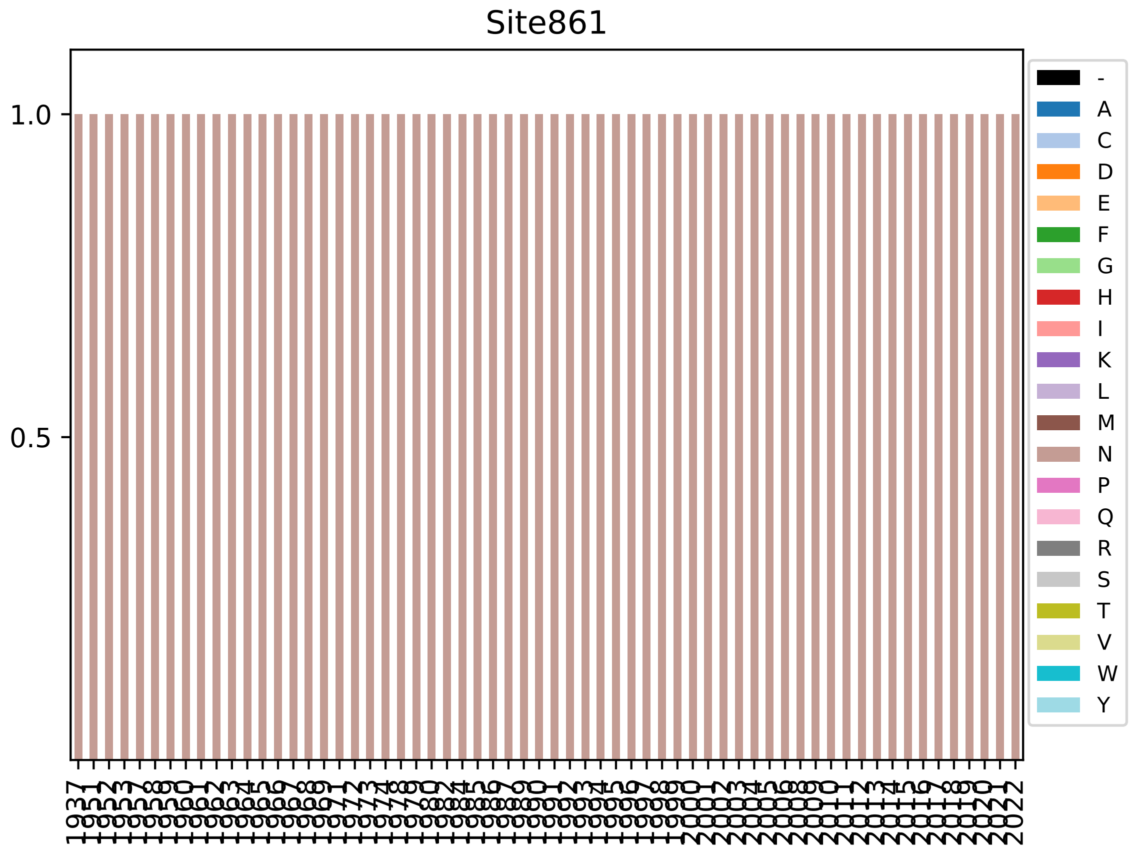
Site861
1.0
0.5
-
A
C
D
E
F
G
H
I
K
L
M
N
P
Q
R
S
T
V
W
Y

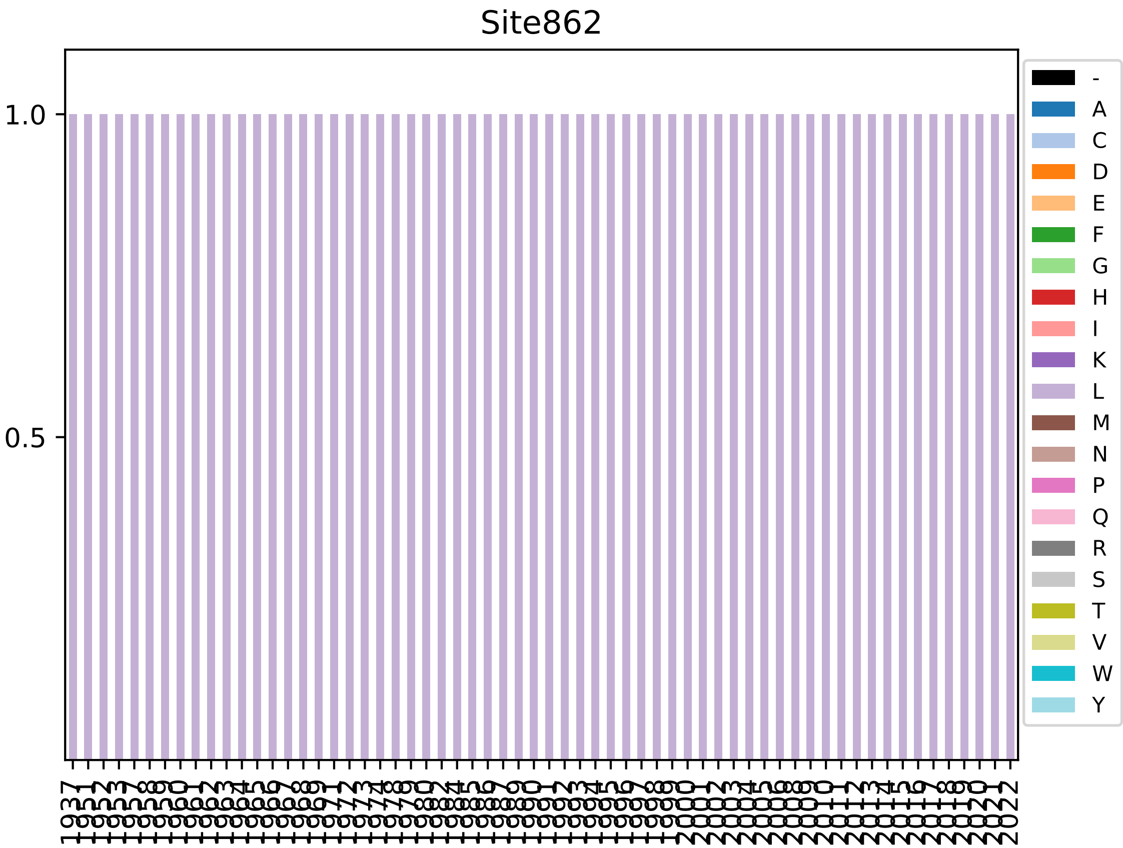
Site862
1.0
0.5
-
A
C
D
E
F
G
H
I
K
L
M
N
P
Q
R
S
T
V
W
Y

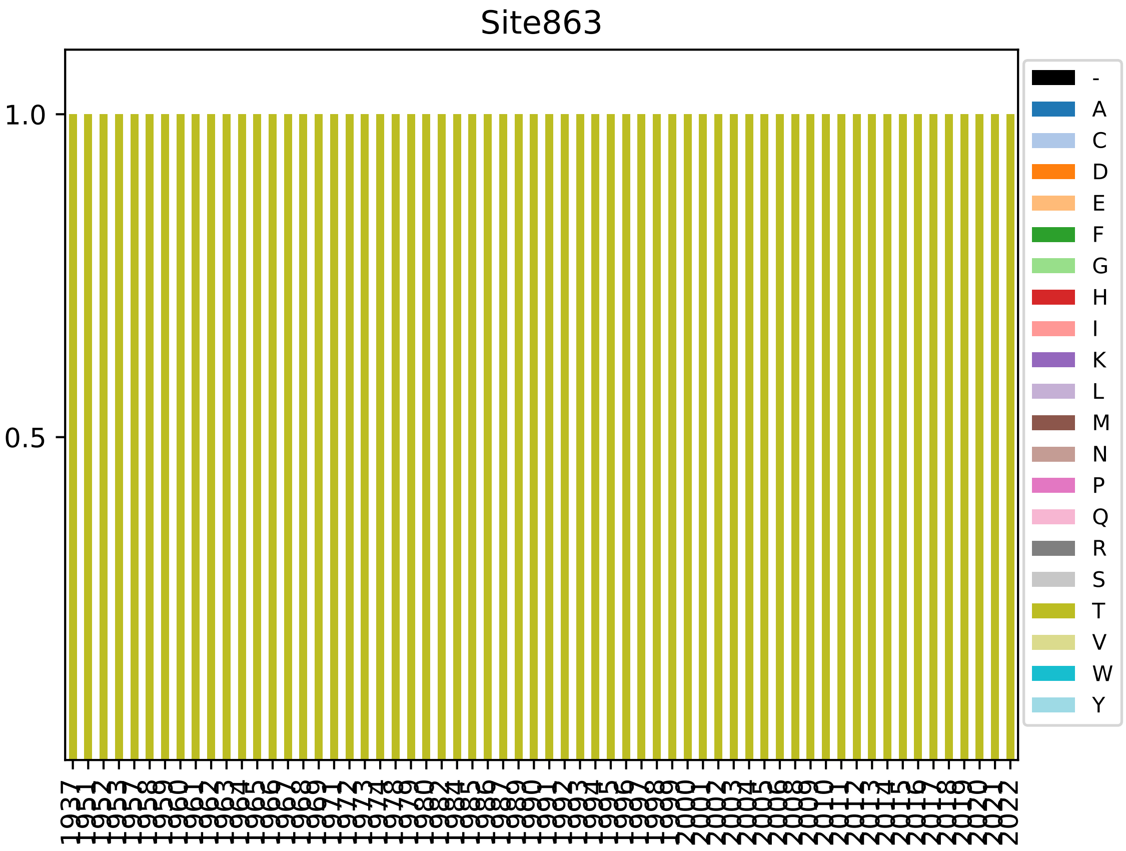
Site863
1.0
0.5
-
A
C
D
E
F
G
H
I
K
L
M
N
P
Q
R
S
T
V
W
Y

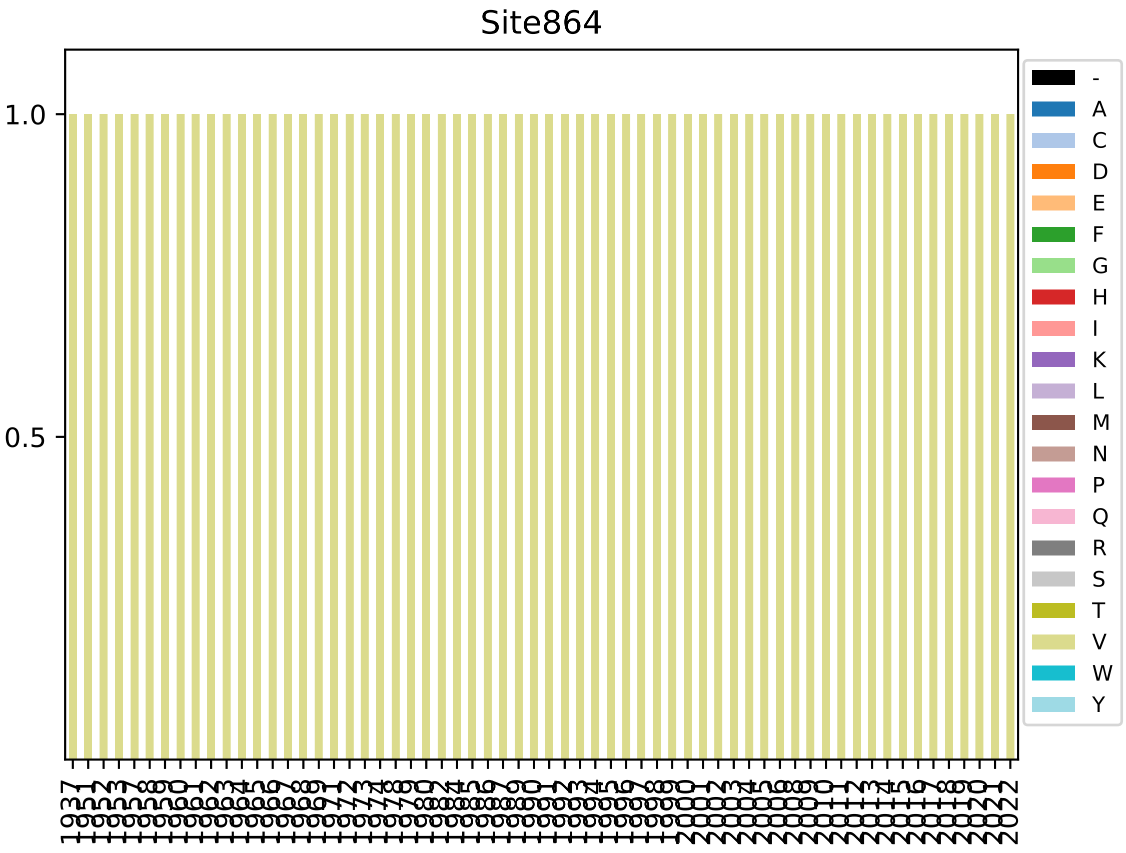

Site864
1.0
0.5
-
A
C
D
E
F
G
H
I
K
L
M
N
P
Q
R
S
T
V
W
Y

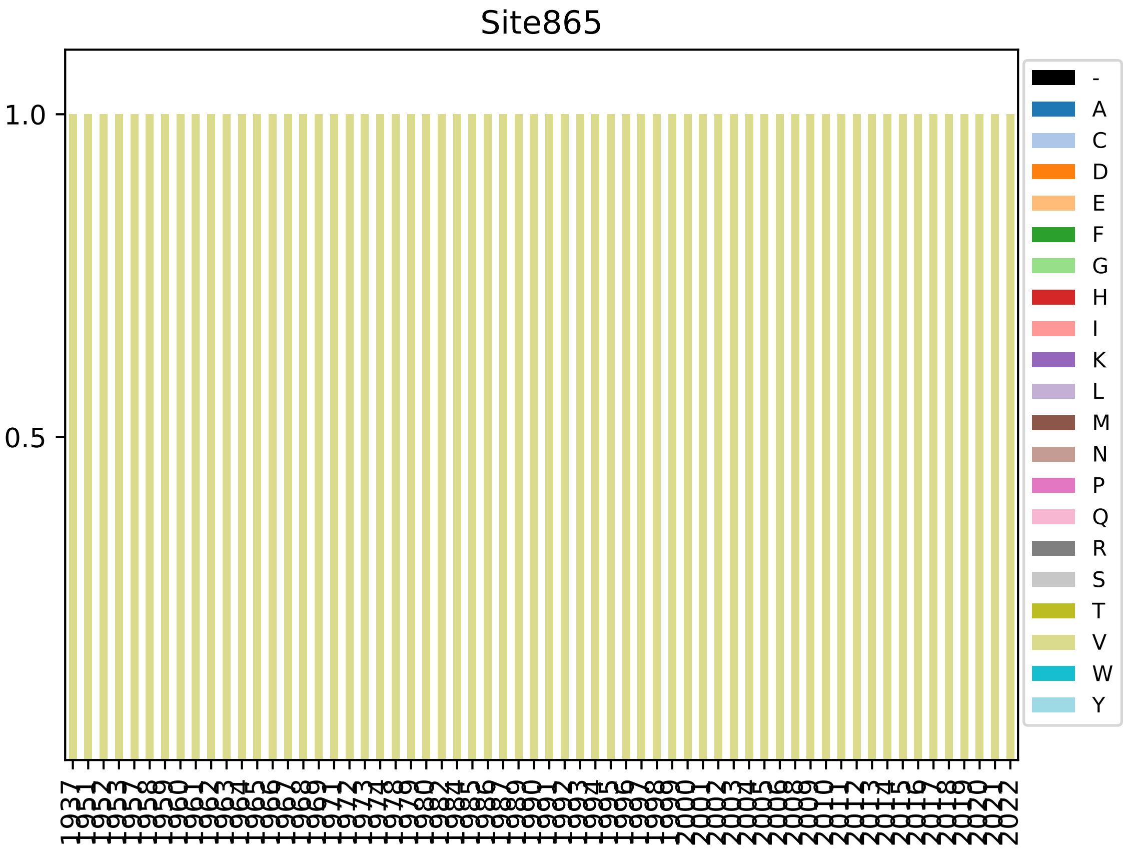

Site865
1.0
0.5
-
A
C
D
E
F
G
H
I
K
L
M
N
P
Q
R
S
T
V
W
Y

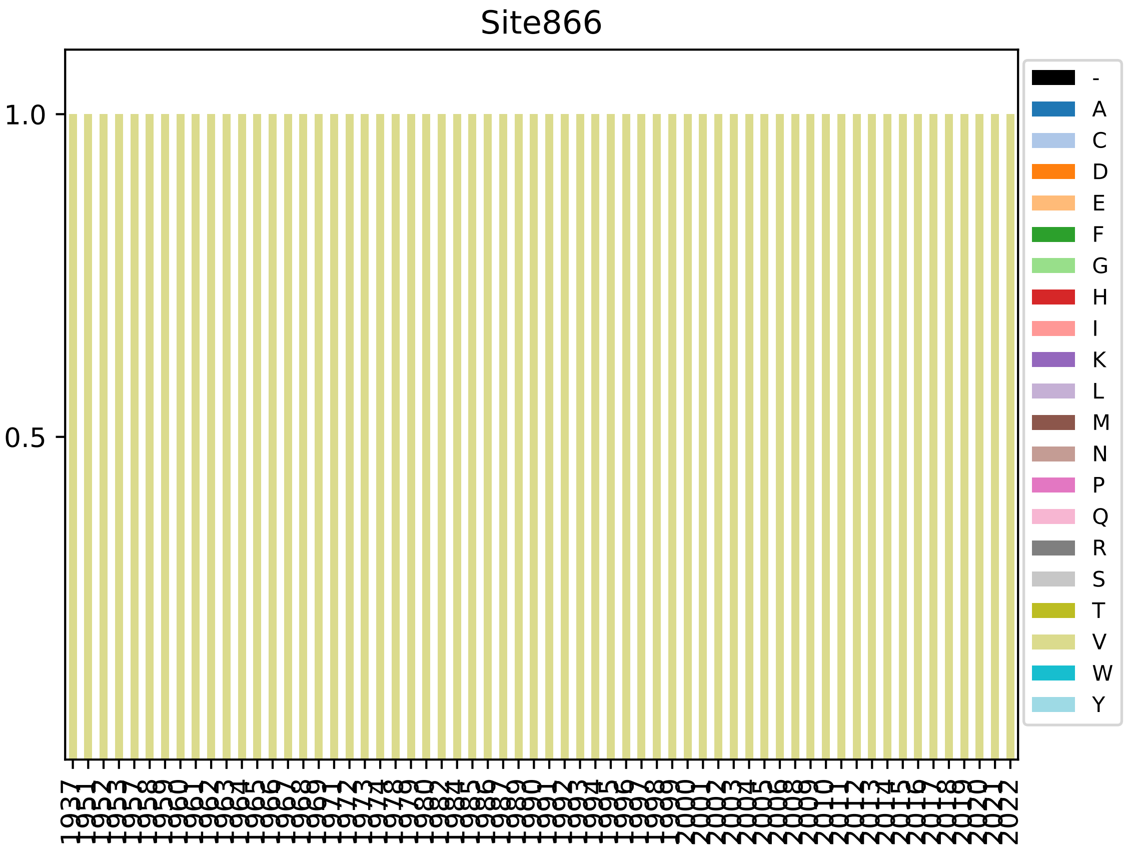
Site866
1.0
0.5
-
A
C
D
E
F
G
H
I
K
L
M
N
P
Q
R
S
T
V
W
Y

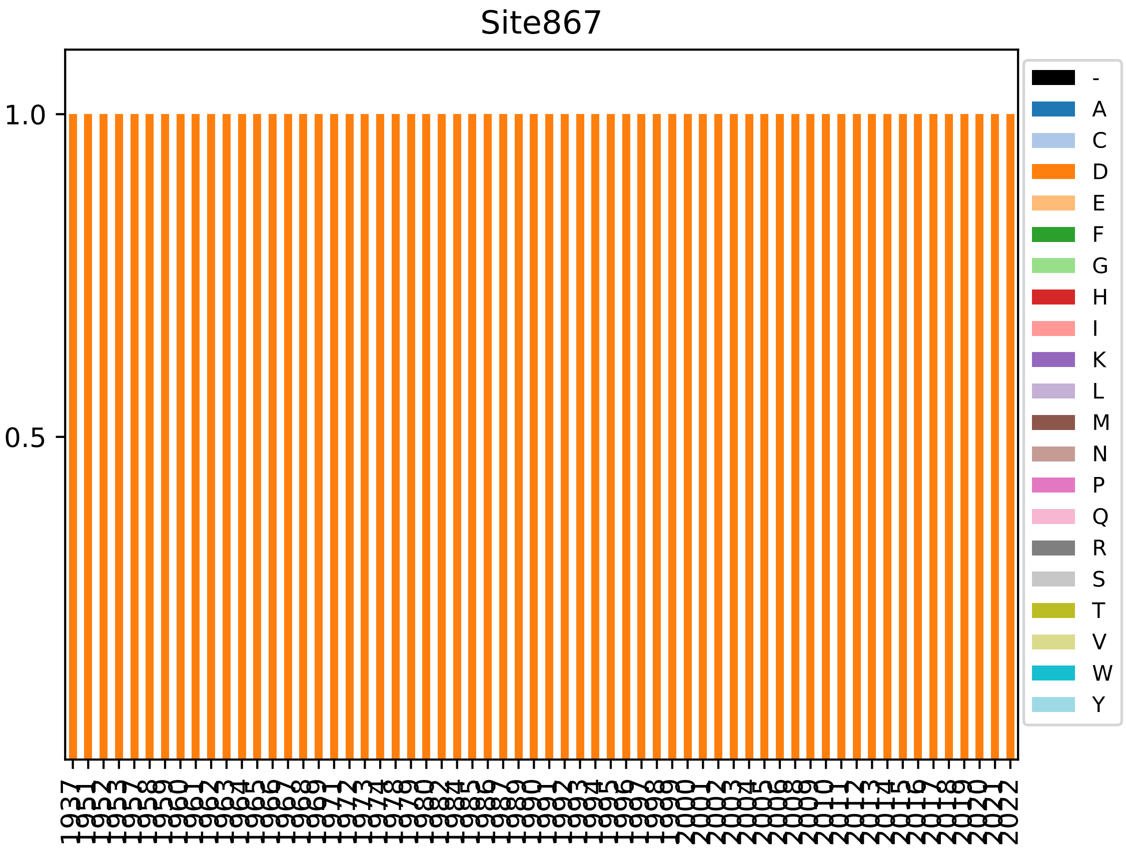
Site867
1.0
0.5
-
A
C
D
E
F
G
H
I
K
L
M
N
P
Q
R
S
T
V
W
Y

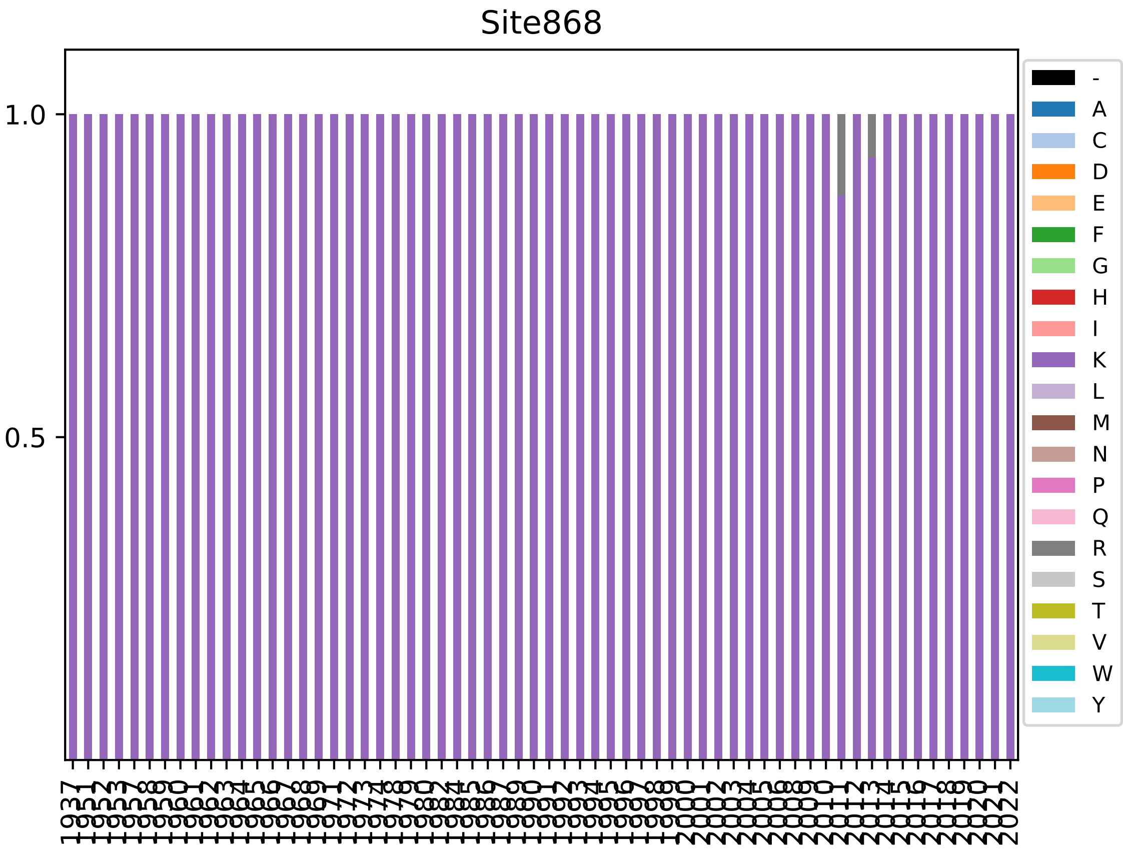

Site868
1.0
0.5
-
A
C
D
E
F
G
H
I
K
L
M
N
P
Q
R
S
T
V
W
Y

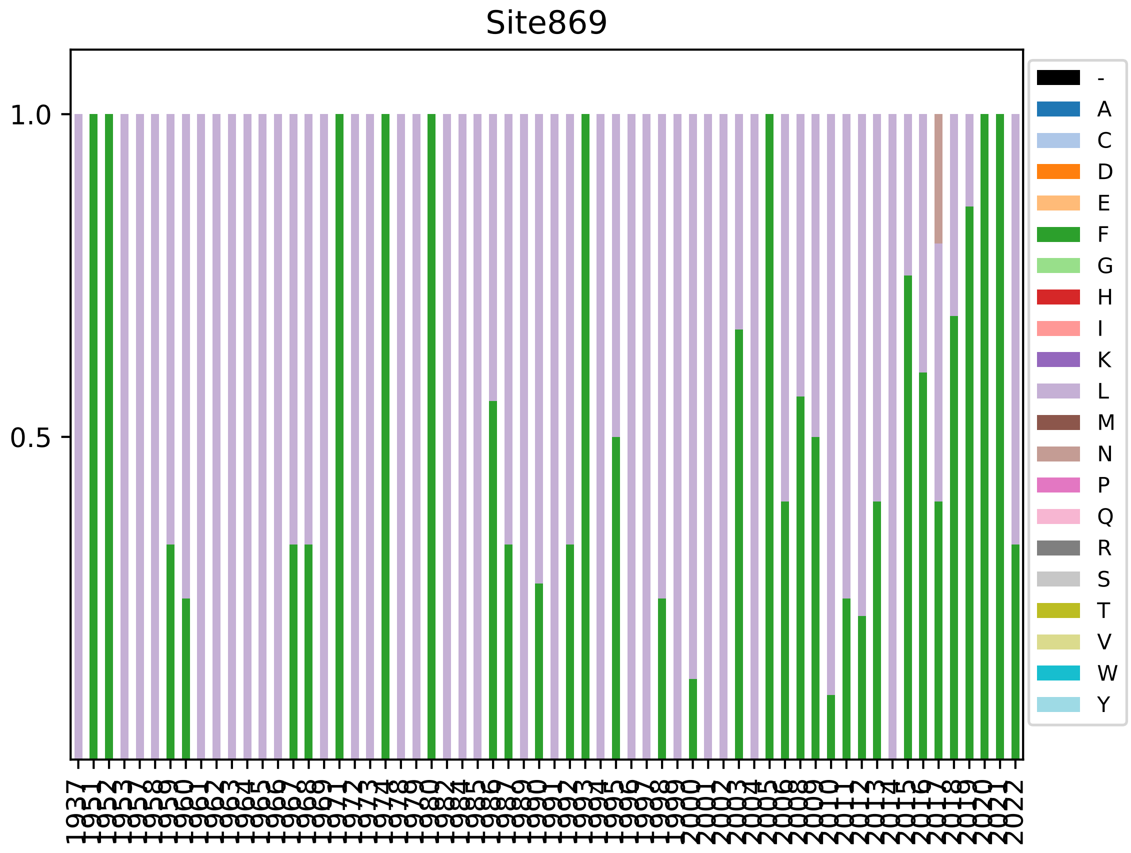
Site869
1.0
0.5
-
A
C
D
E
F
G
H
I
K
L
M
N
P
Q
R
S
T
V
W
Y

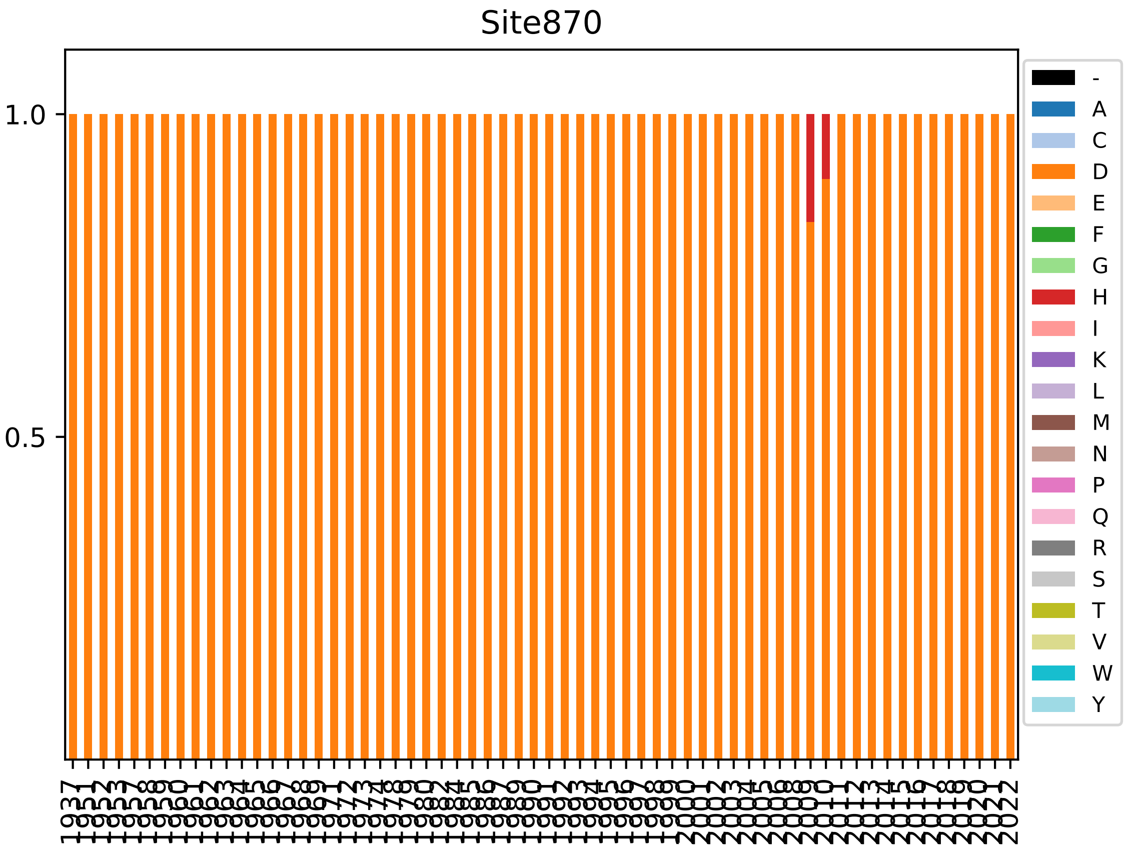
Site870
1.0
0.5
-
A
C
D
E
F
G
H
I
K
L
M
N
P
Q
R
S
T
V
W
Y

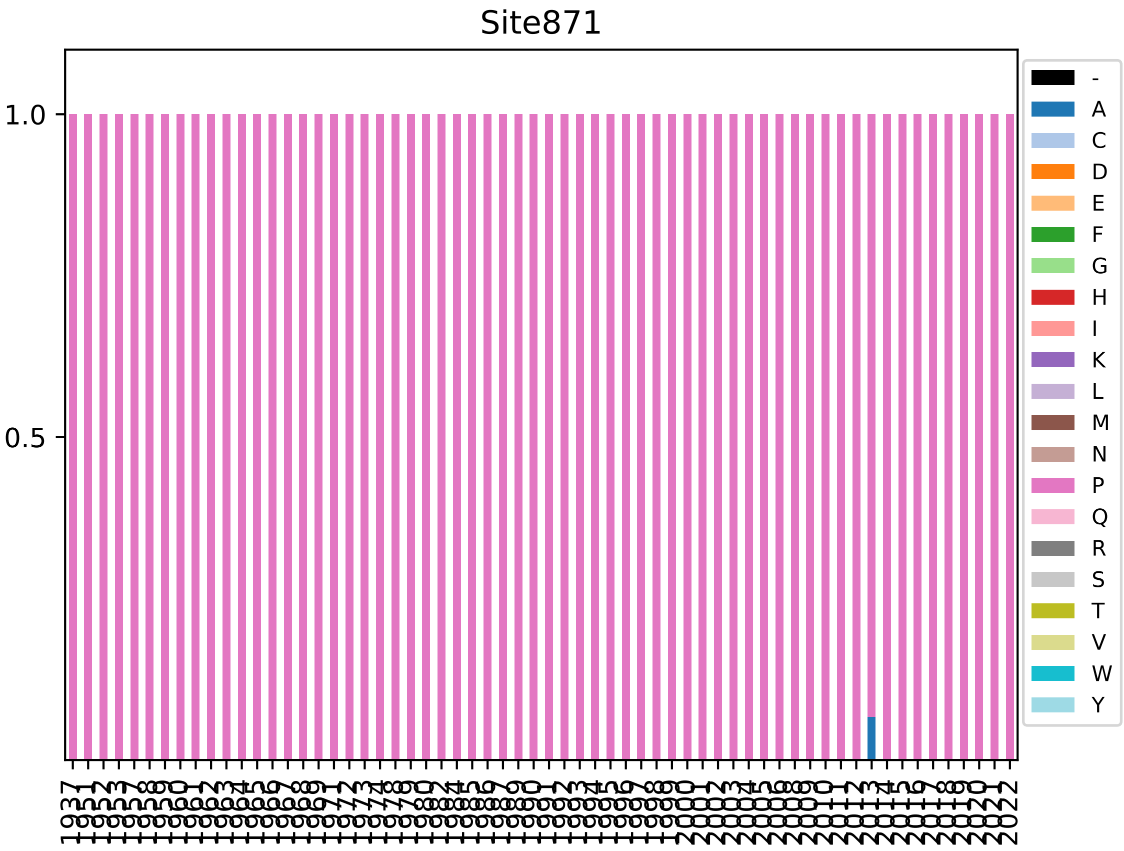

Site871
1.0
0.5
-
A
C
D
E
F
G
H
I
K
L
M
N
P
Q
R
S
T
V
W
Y

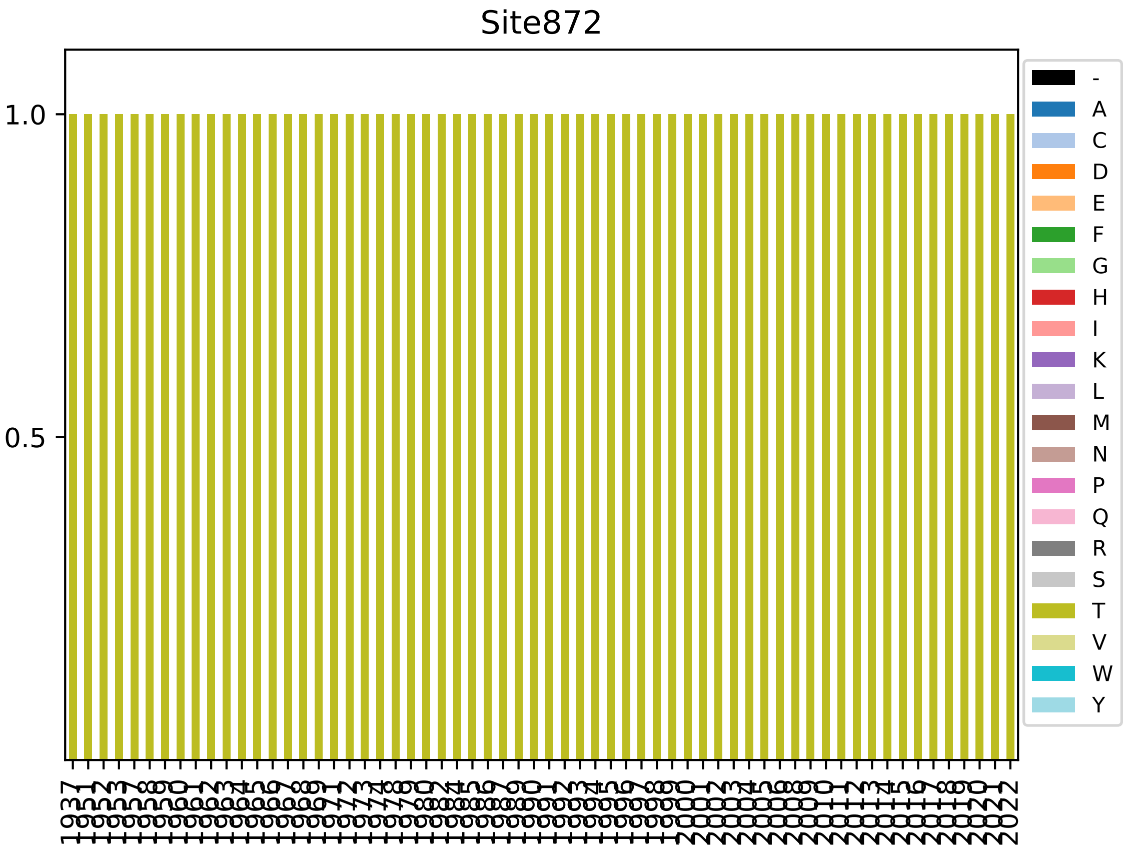

Site872
1.0
0.5
-
A
C
D
E
F
G
H
I
K
L
M
N
P
Q
R
S
T
V
W
Y

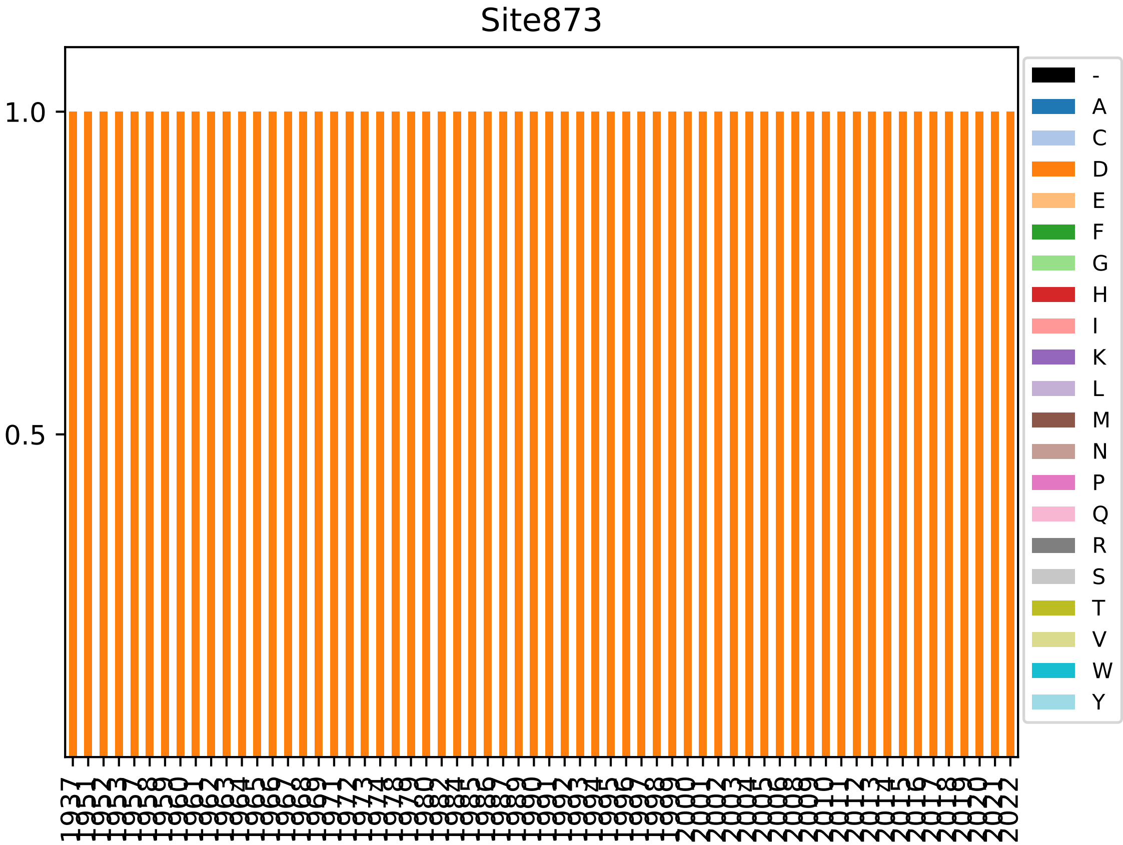
Site873
1.0
0.5
-
A
C
D
E
F
G
H
I
K
L
M
N
P
Q
R
S
T
V
W
Y

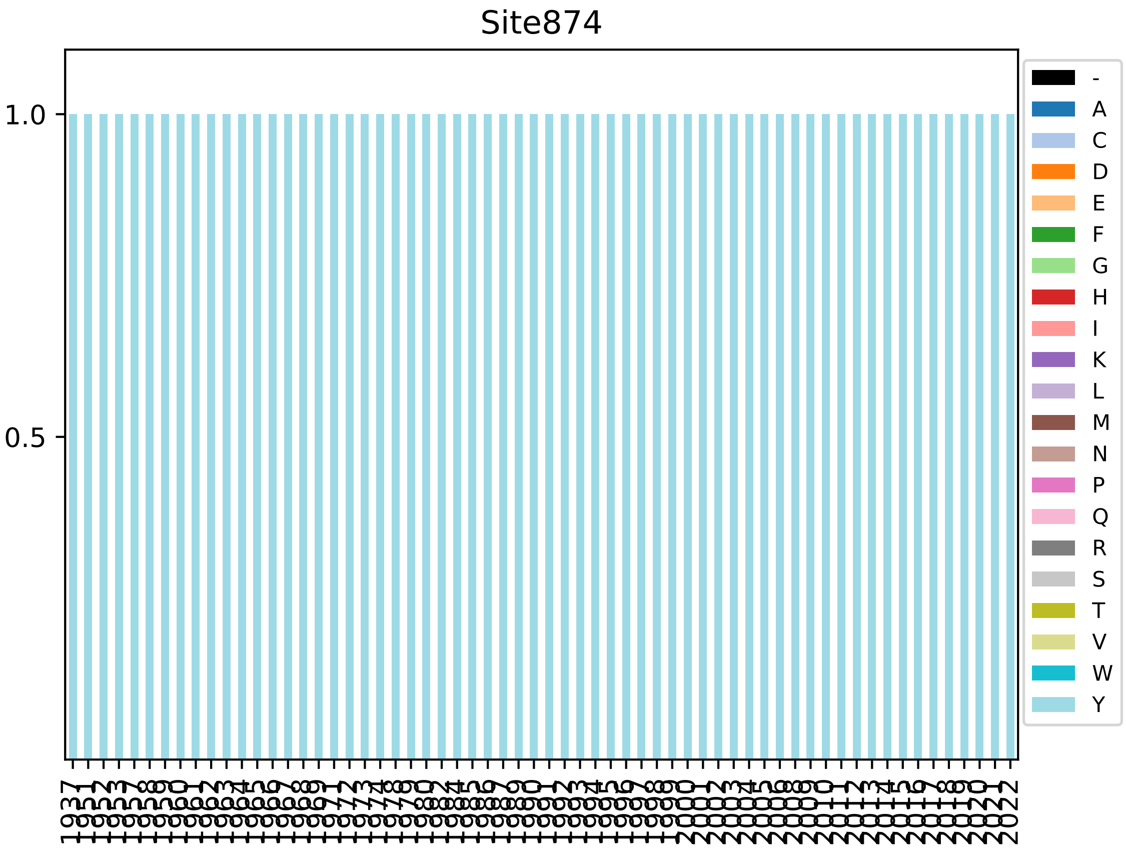
Site874
1.0
0.5
-
A
C
D
E
F
G
H
I
K
L
M
N
P
Q
R
S
T
V
W
Y

Site875

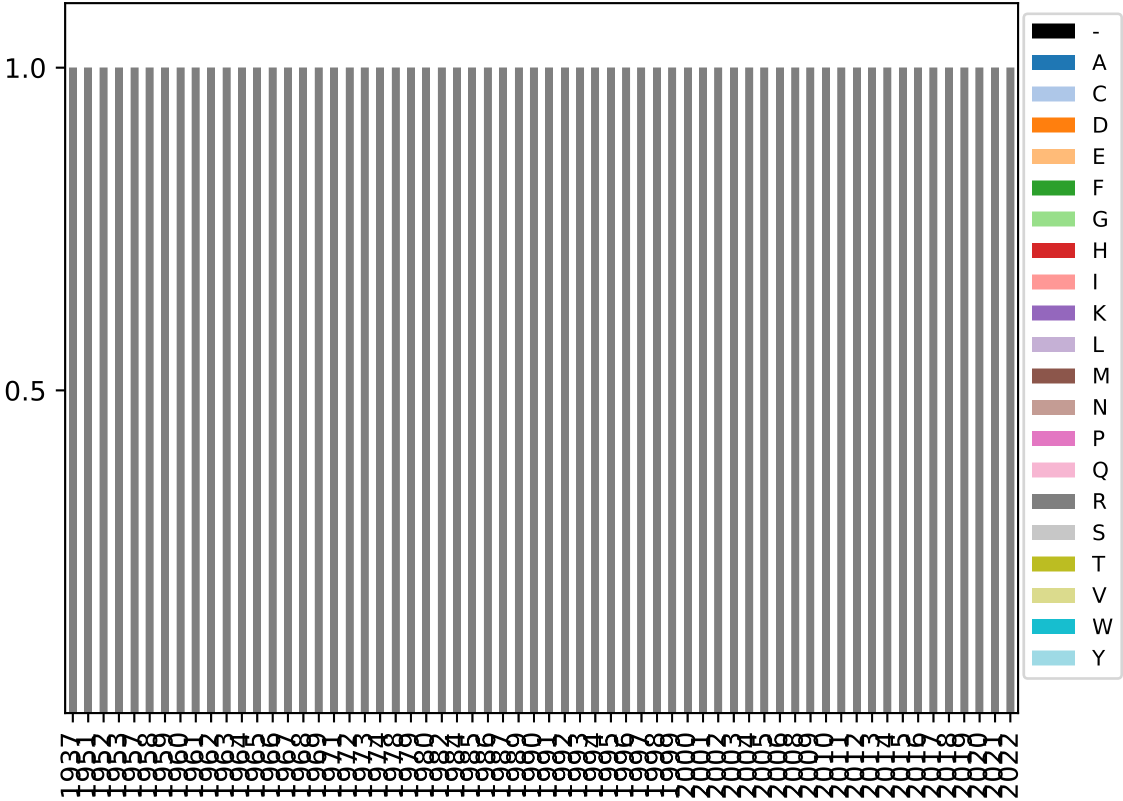

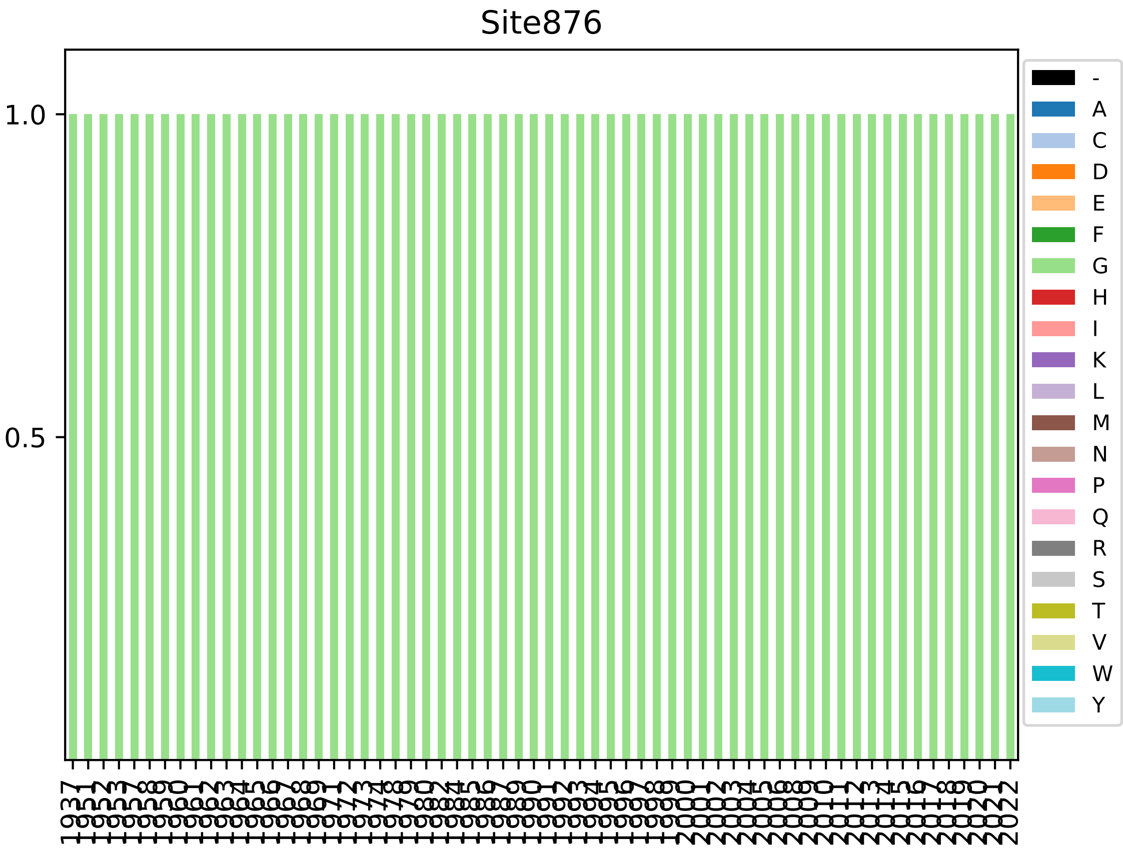

Site876
1.0
0.5
-
A
C
D
E
F
G
H
I
K
L
M
N
P
Q
R
S
T
V
W
Y

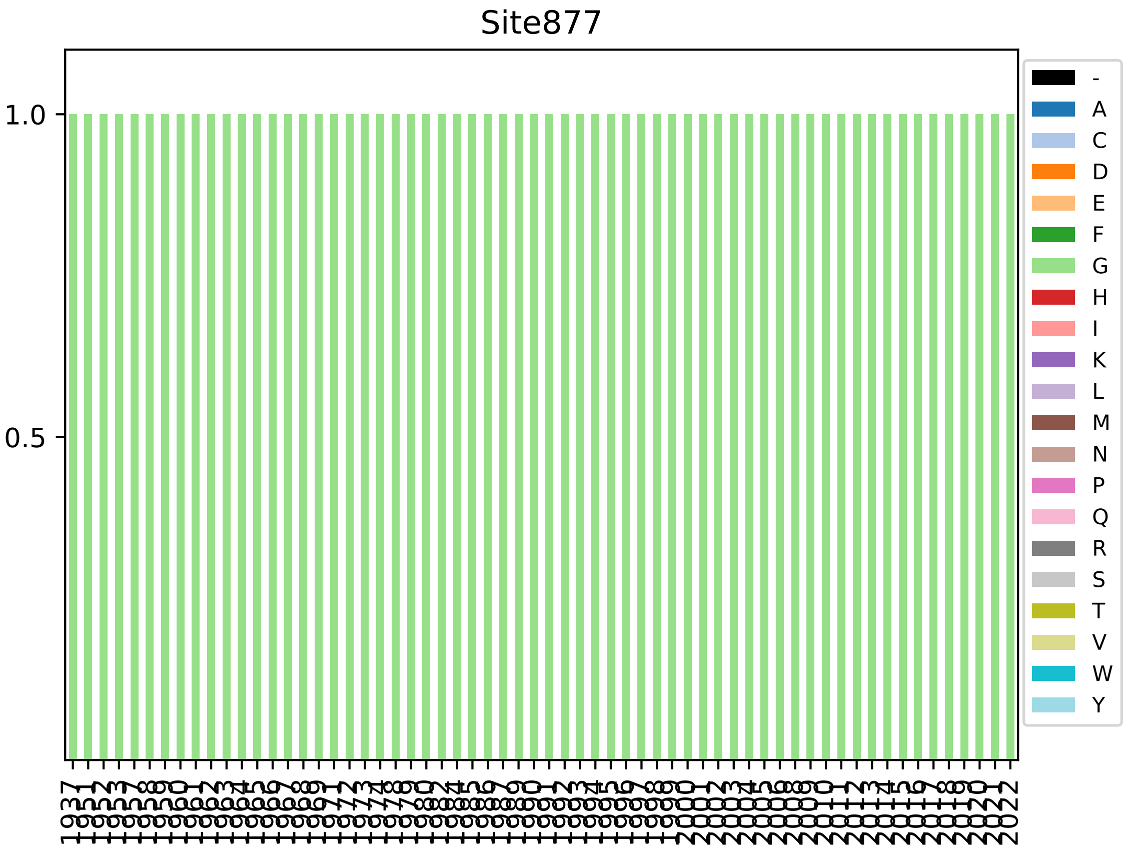
Site877
1.0
0.5
-
A
C
D
E
F
G
H
I
K
L
M
N
P
Q
R
S
T
V
W
Y

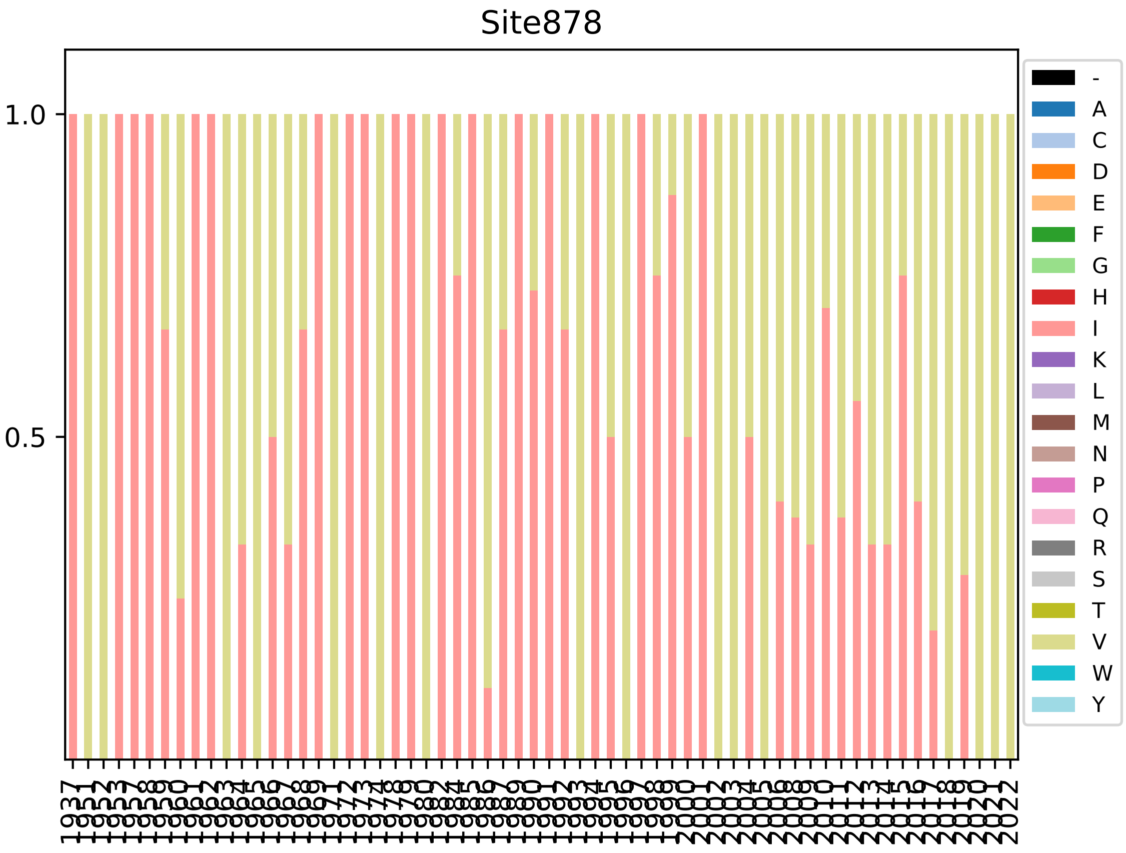
Site878
1.0
0.5
-
A
C
D
E
F
G
H
I
K
L
M
N
P
Q
R
S
T
V
W
Y

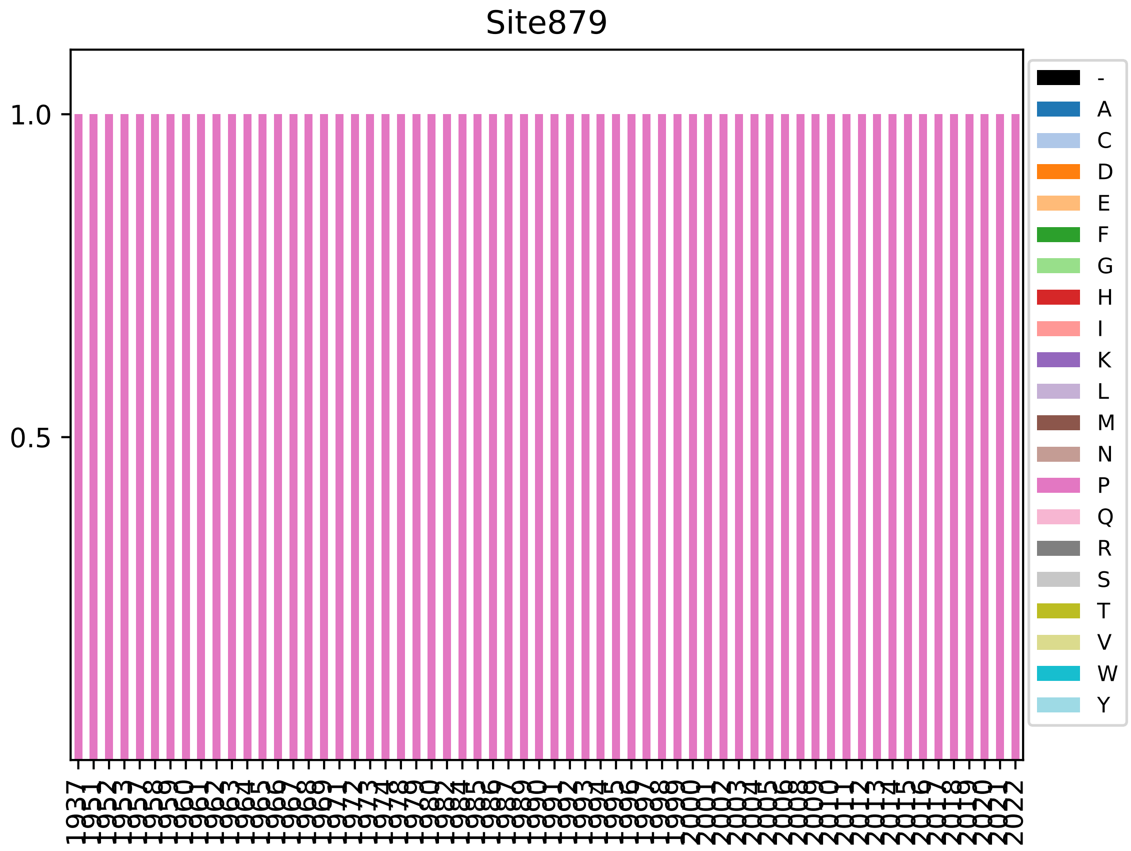
Site879
1.0
0.5
-
A
C
D
E
F
G
H
I
K
L
M
N
P
Q
R
S
T
V
W
Y

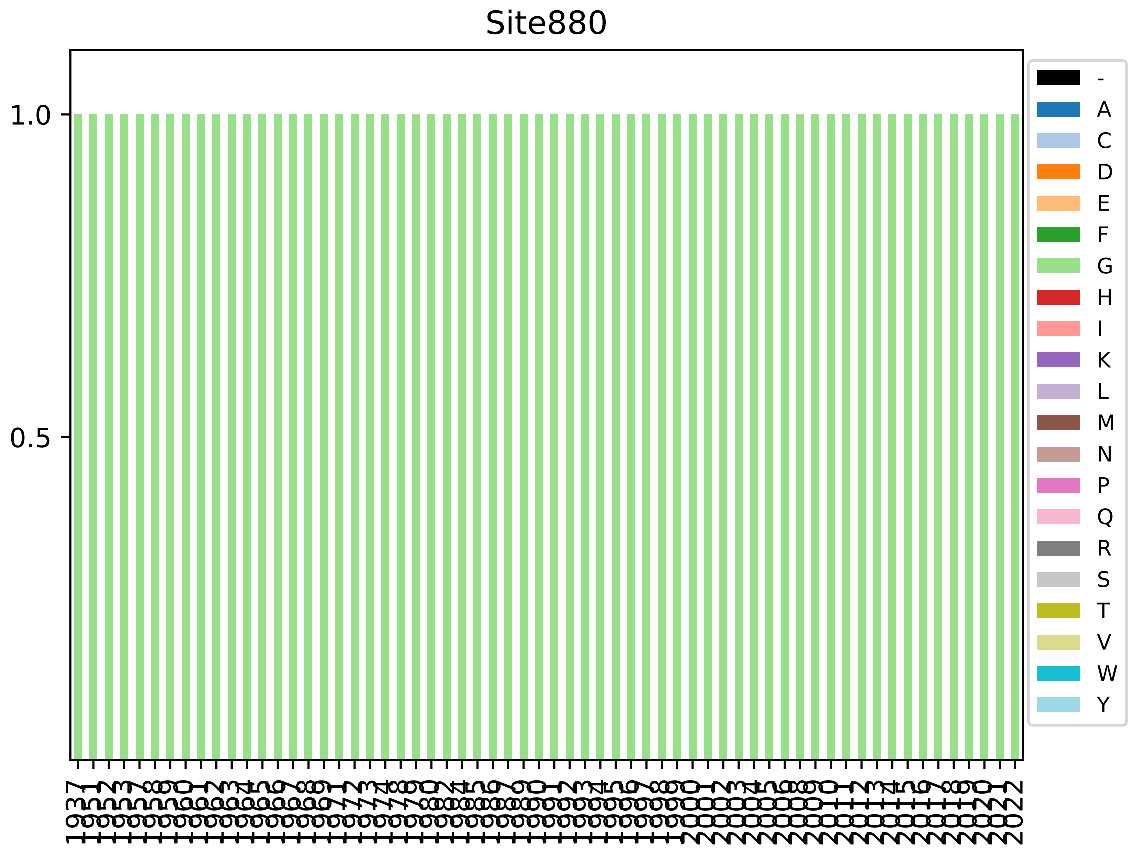
Site880
1.0
0.5
-
A
C
D
E
F
G
H
I
K
L
M
N
P
Q
R
S
T
V
W
Y

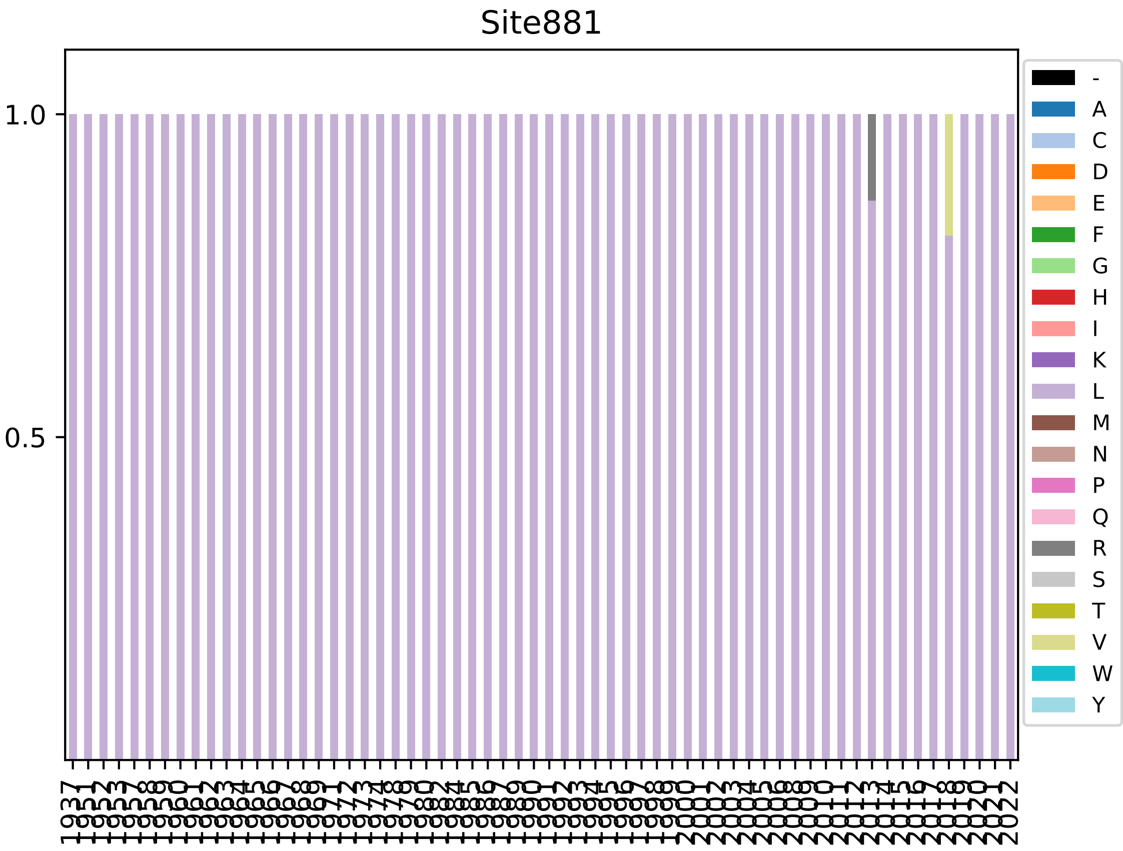
Site881
1.0
0.5
-
A
C
D
E
F
G
H
I
K
L
M
N
P
Q
R
S
T
V
W
Y

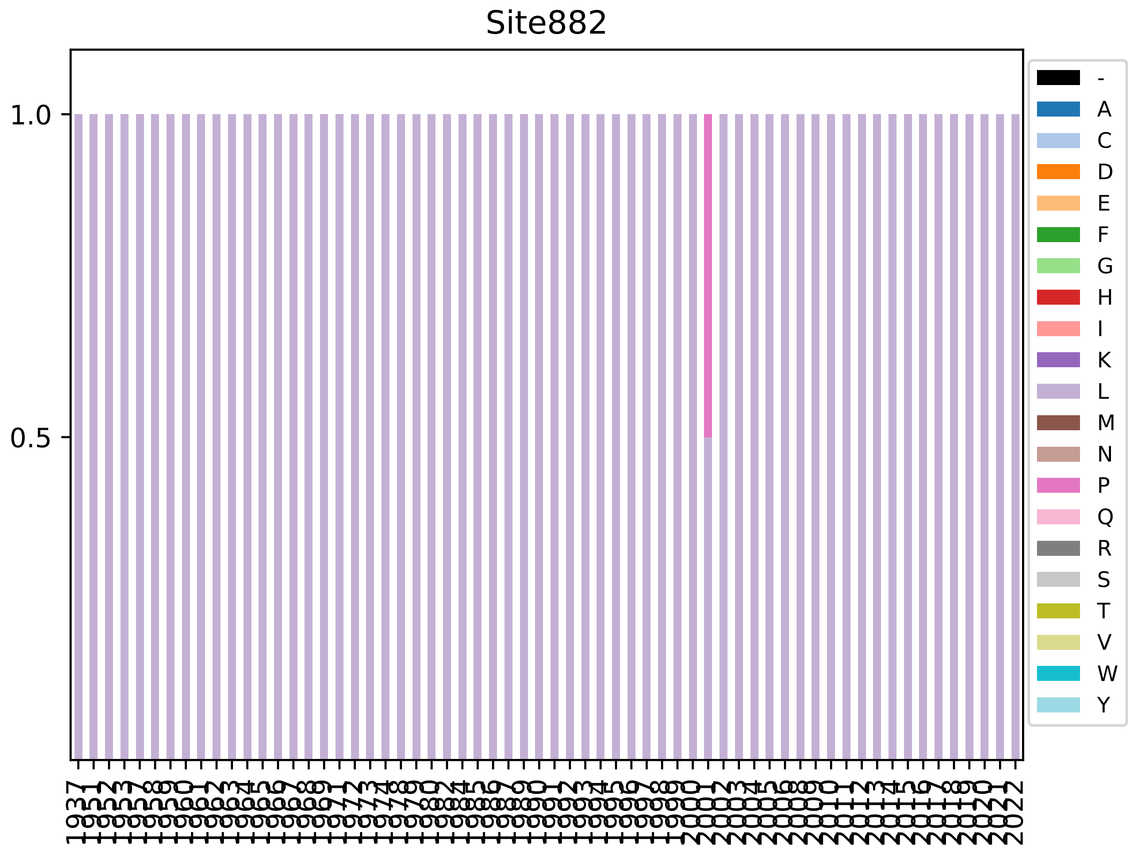
Site882
1.0
0.5
-
A
C
D
E
F
G
H
I
K
L
M
N
P
Q
R
S
T
V
W
Y

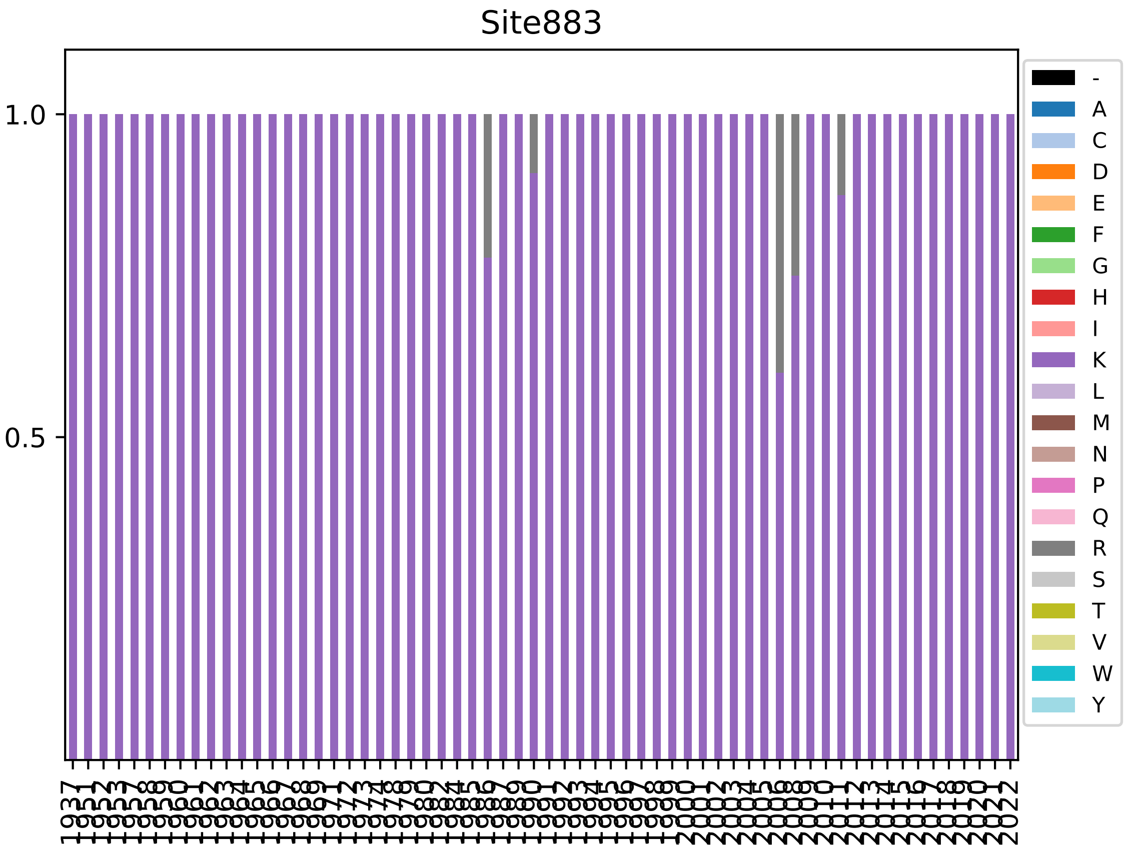
Site883
1.0
0.5
-
A
C
D
E
F
G
H
I
K
L
M
N
P
Q
R
S
T
V
W
Y

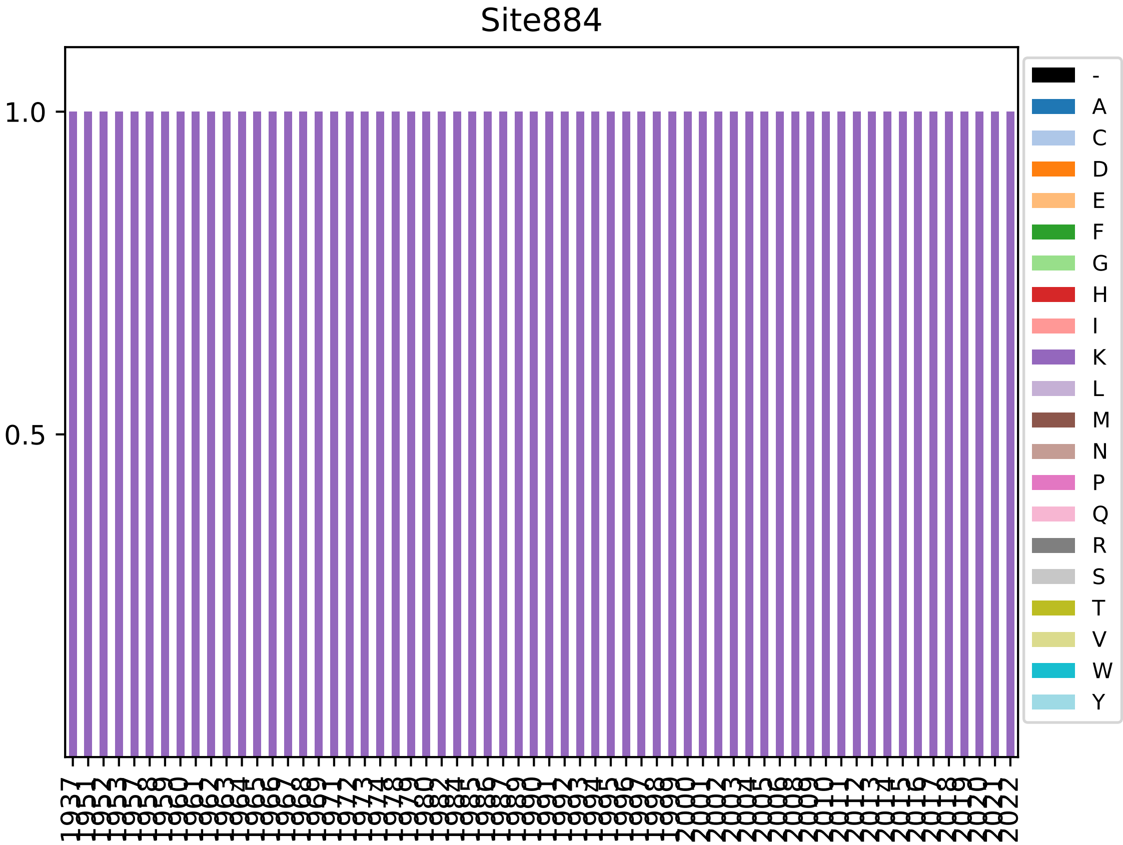
Site884
1.0
0.5
-
A
C
D
E
F
G
H
I
K
L
M
N
P
Q
R
S
T
V
W
Y

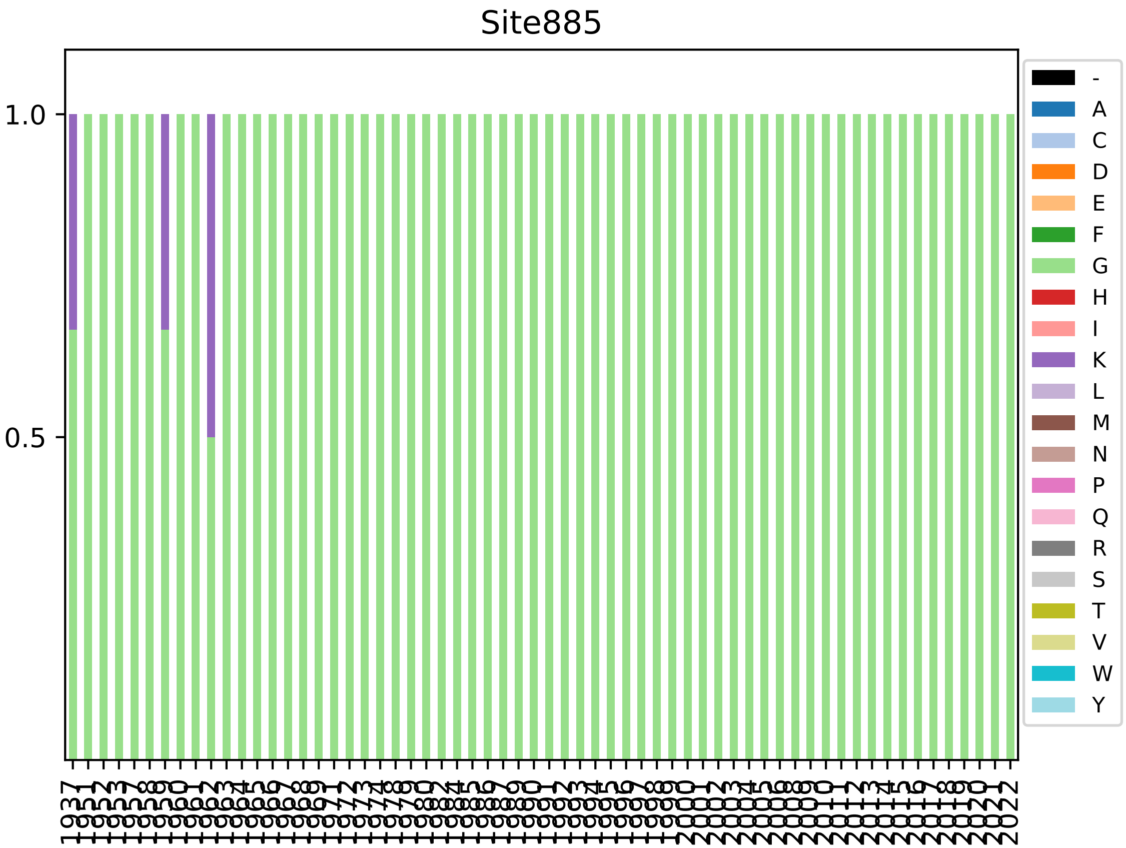
Site885
1.0
0.5
-
A
C
D
E
F
G
H
I
K
L
M
N
P
Q
R
S
T
V
W
Y

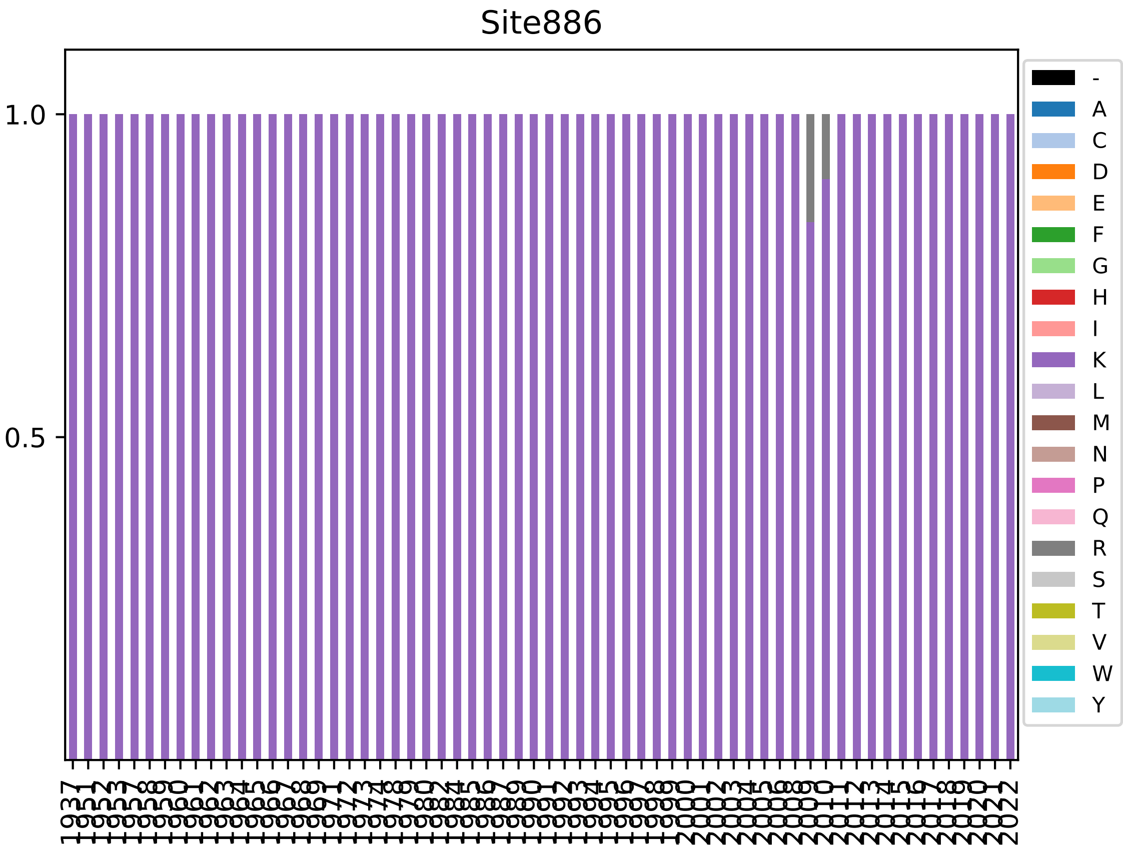
Site886
1.0
0.5
-
A
C
D
E
F
G
H
I
K
L
M
N
P
Q
R
S
T
V
W
Y

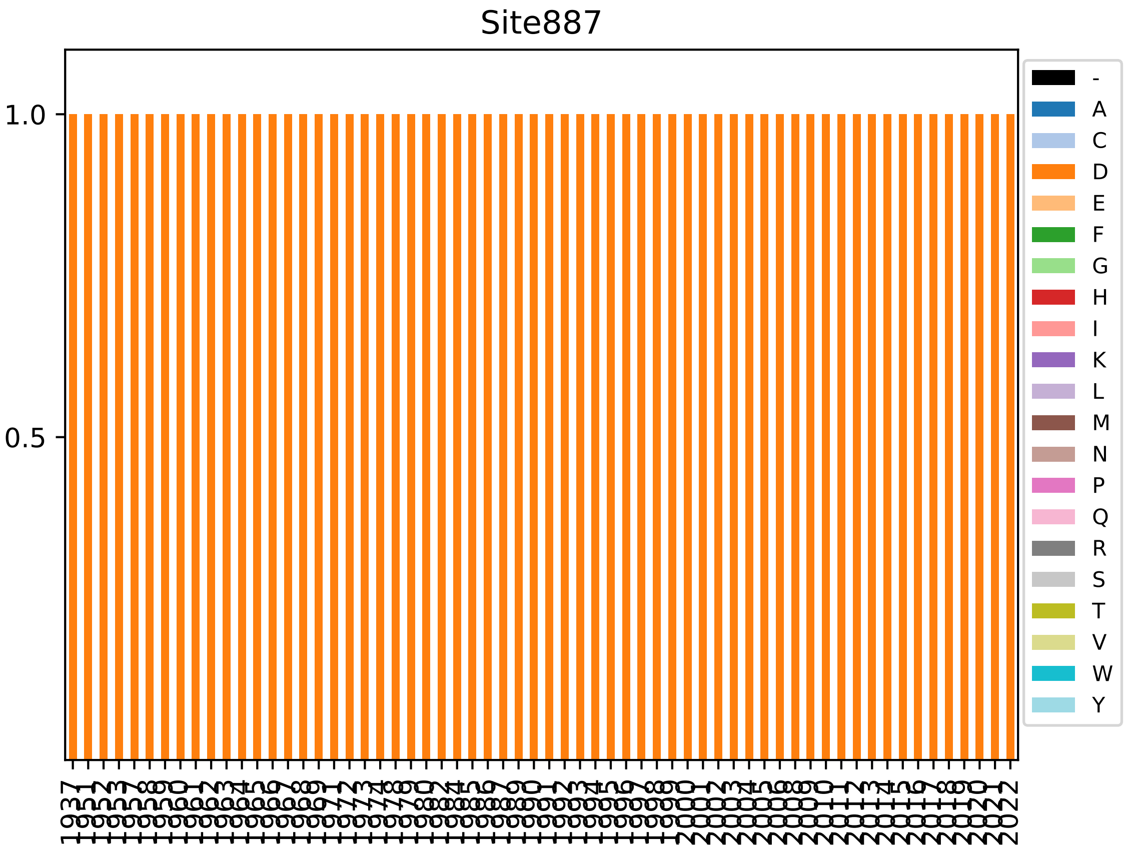
Site887
1.0
0.5
-
A
C
D
E
F
G
H
I
K
L
M
N
P
Q
R
S
T
V
W
Y

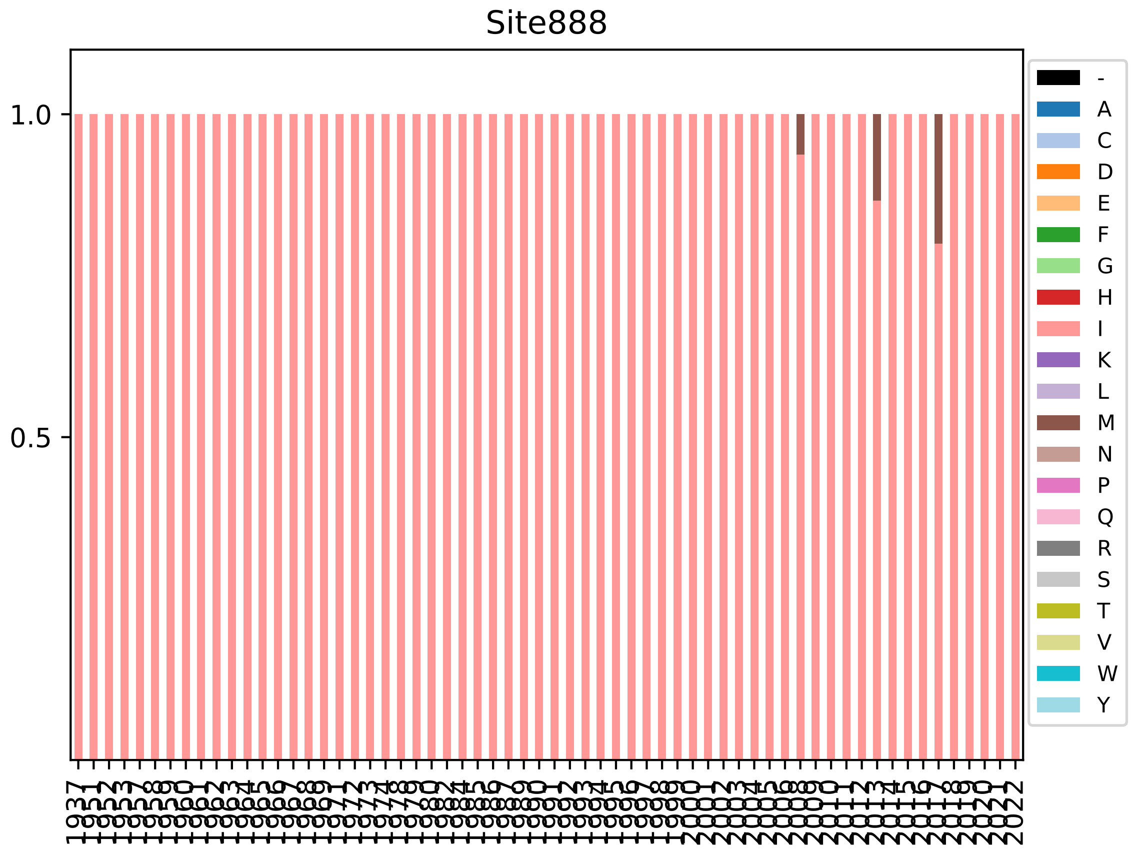
Site888
1.0
0.5
-
A
C
D
E
F
G
H
I
K
L
M
N
P
Q
R
S
T
V
W
Y

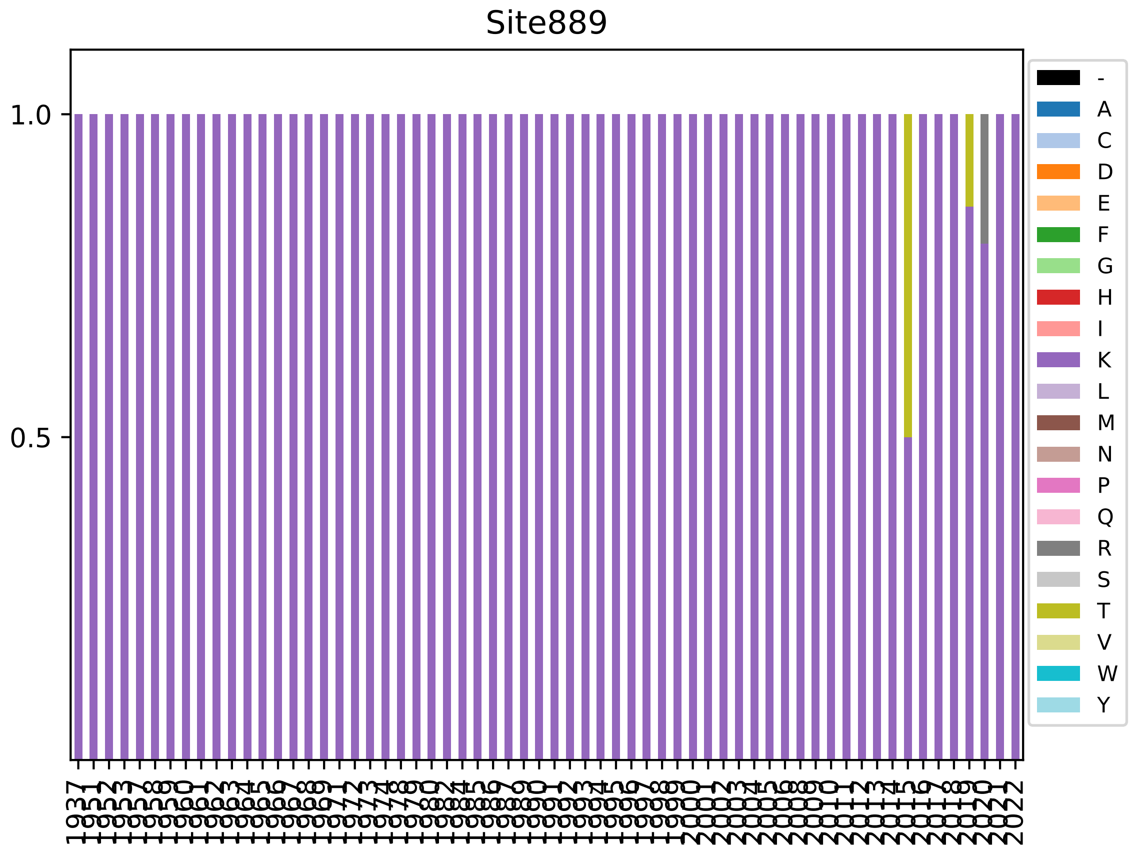
Site889
1.0
0.5
-
A
C
D
E
F
G
H
I
K
L
M
N
P
Q
R
S
T
V
W
Y

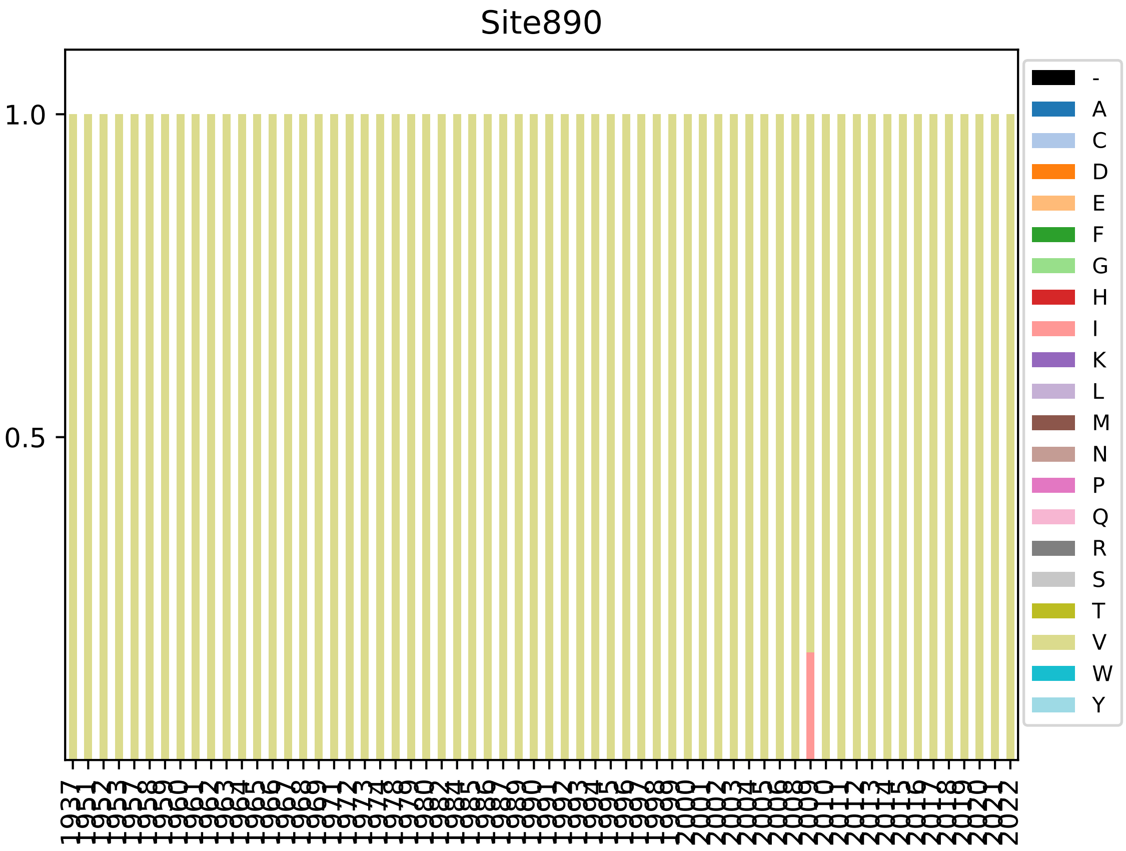
Site890
1.0
0.5
-
A
C
D
E
F
G
H
I
K
L
M
N
P
Q
R
S
T
V
W
Y

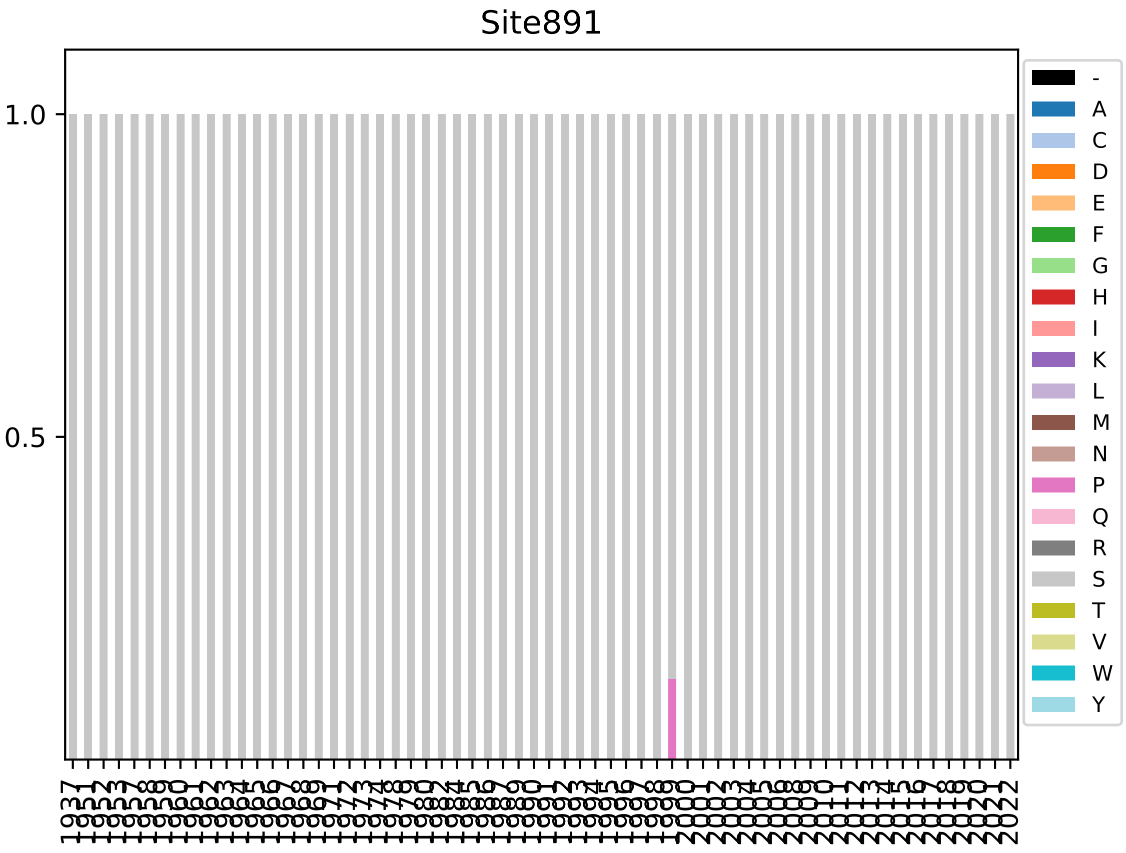
Site891
1.0
0.5
-
A
C
D
E
F
G
H
I
K
L
M
N
P
Q
R
S
T
V
W
Y

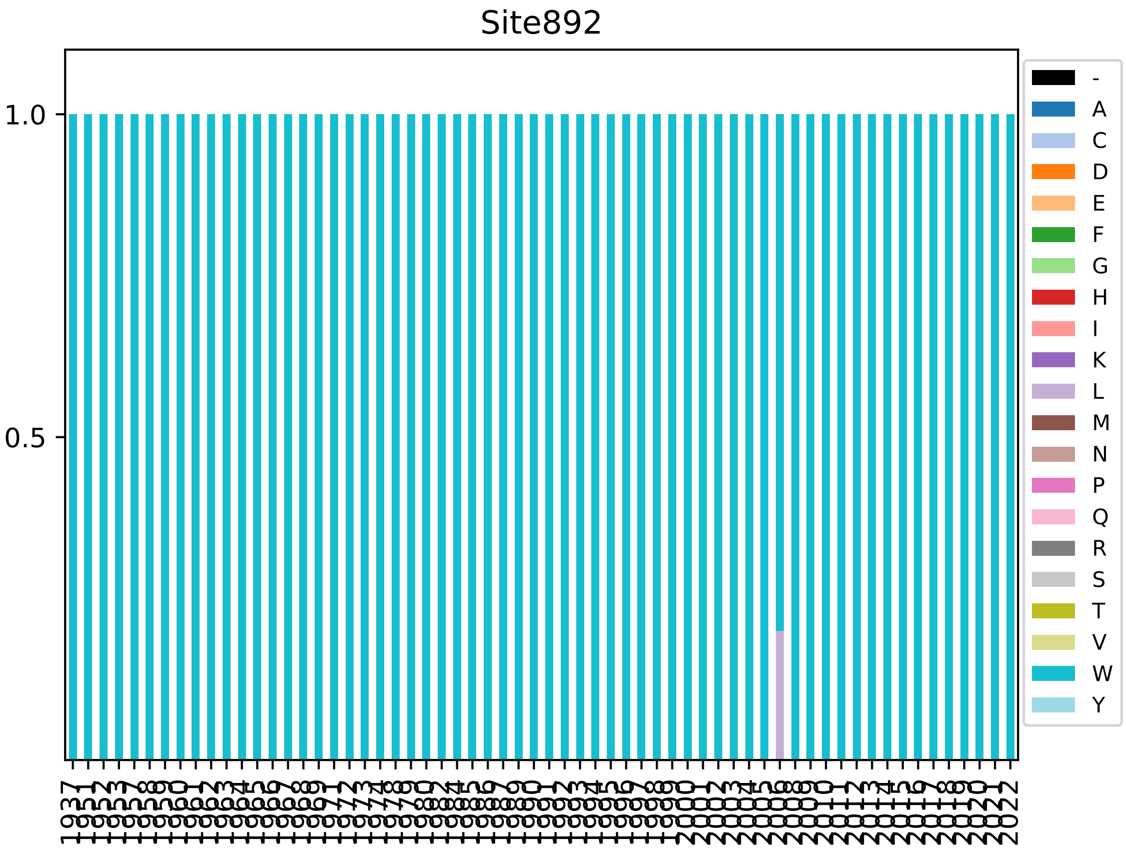
Site892
1.0
0.5
-
A
C
D
E
F
G
H
I
K
L
M
N
P
Q
R
S
T
V
W
Y

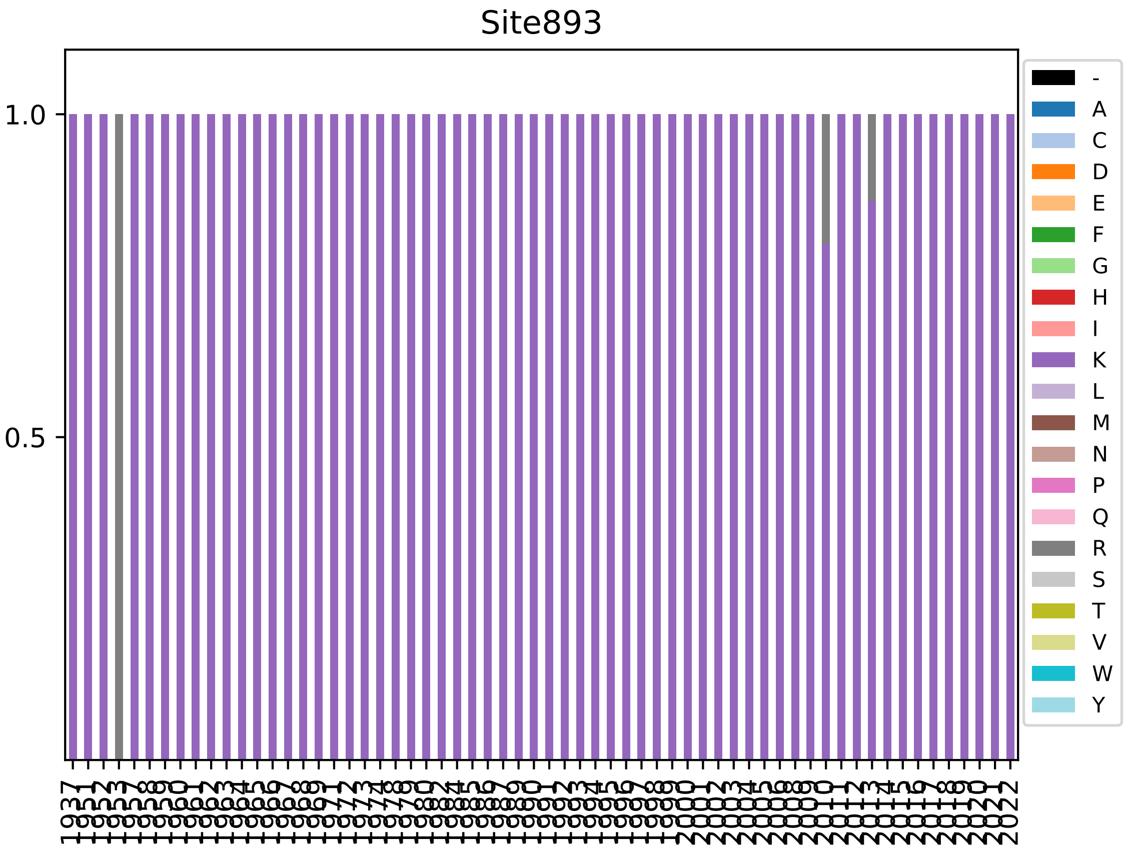
Site893
1.0
0.5
-
A
C
D
E
F
G
H
I
K
L
M
N
P
Q
R
S
T
V
W
Y

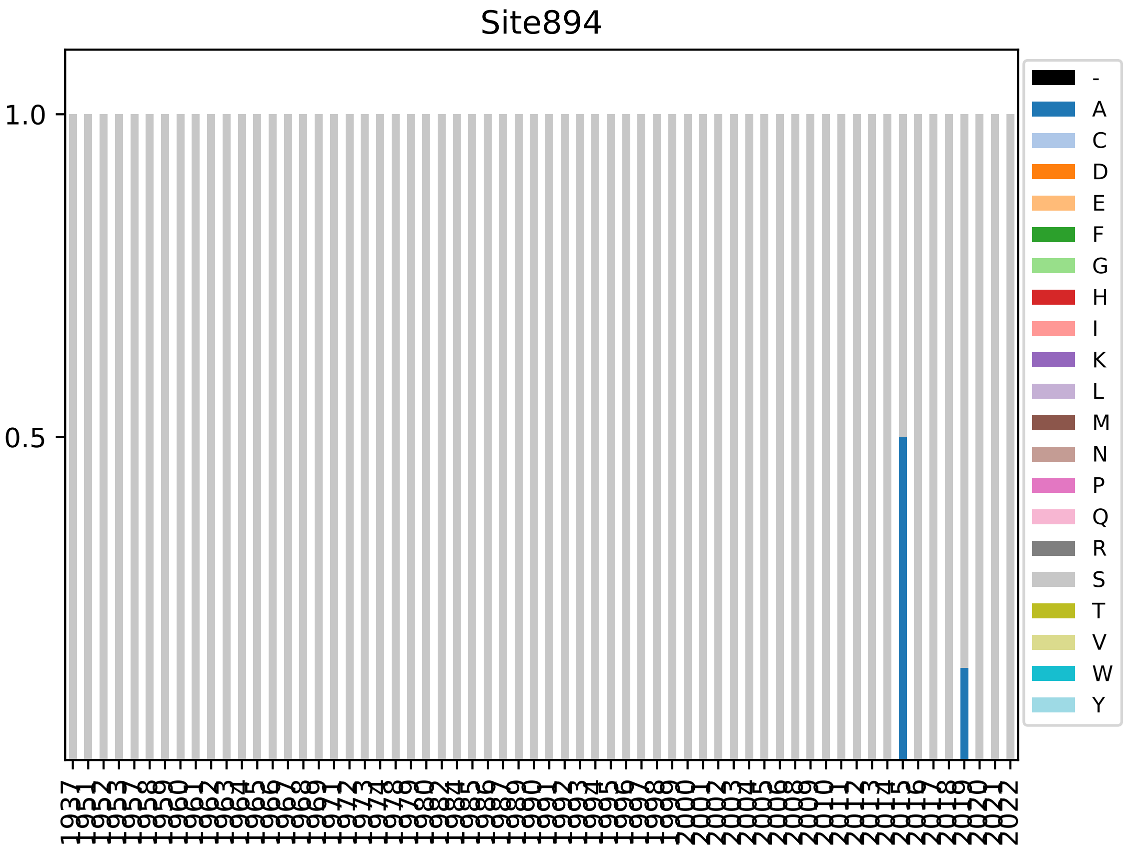
Site894
1.0
0.5
-
A
C
D
E
F
G
H
I
K
L
M
N
P
Q
R
S
T
V
W
Y

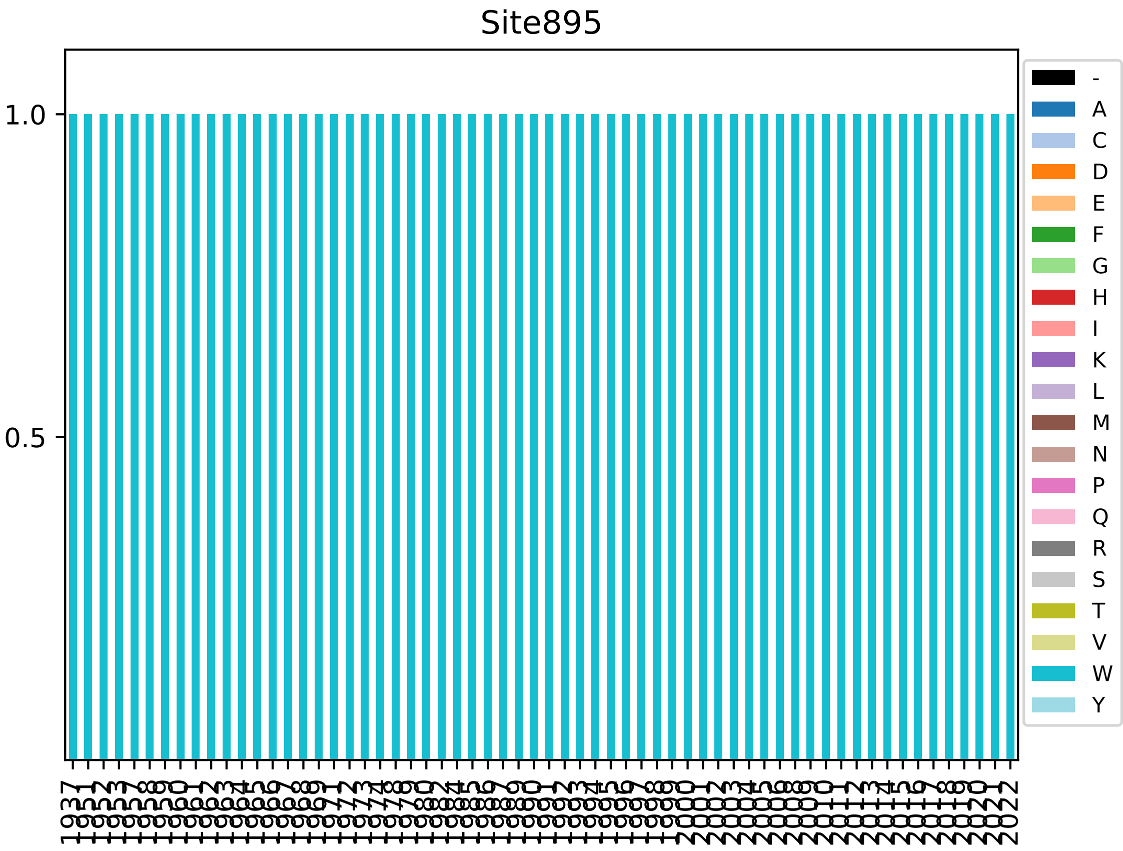
Site895
1.0
0.5
-
A
C
D
E
F
G
H
I
K
L
M
N
P
Q
R
S
T
V
W
Y

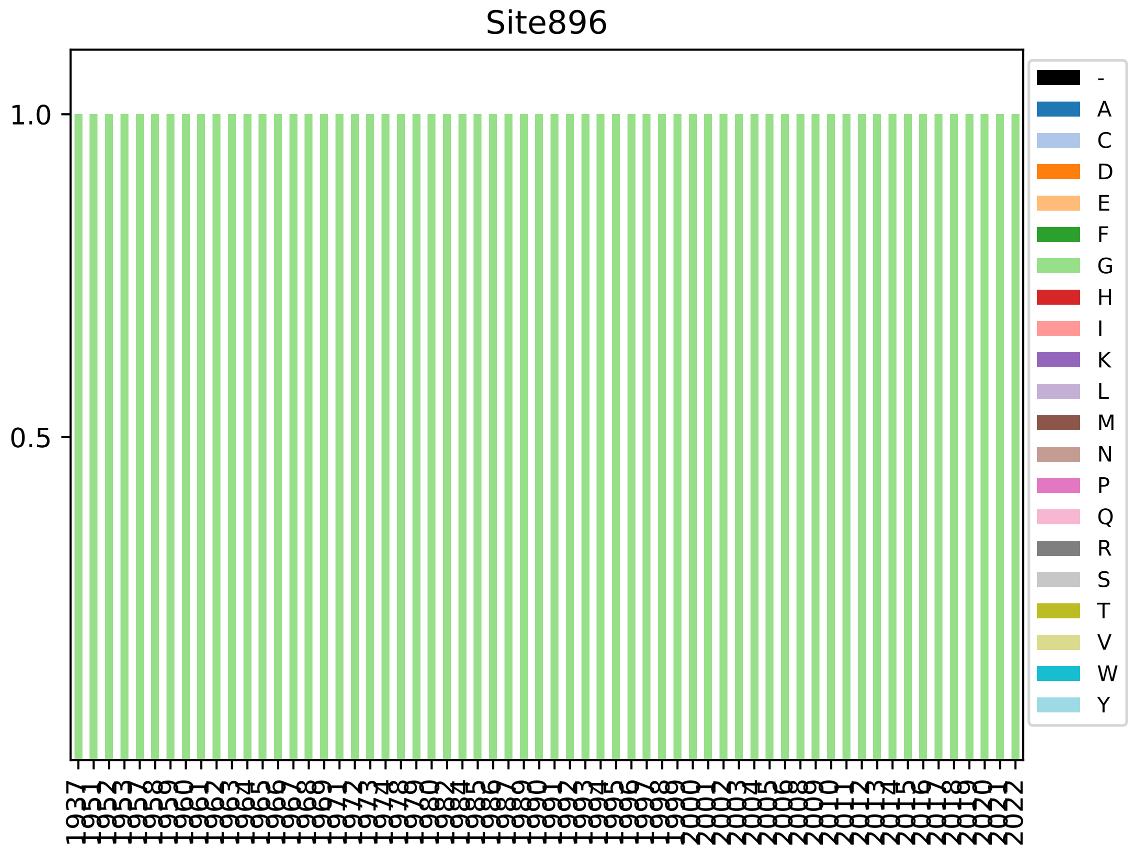
Site896
1.0
0.5
-
A
C
D
E
F
G
H
I
K
L
M
N
P
Q
R
S
T
V
W
Y

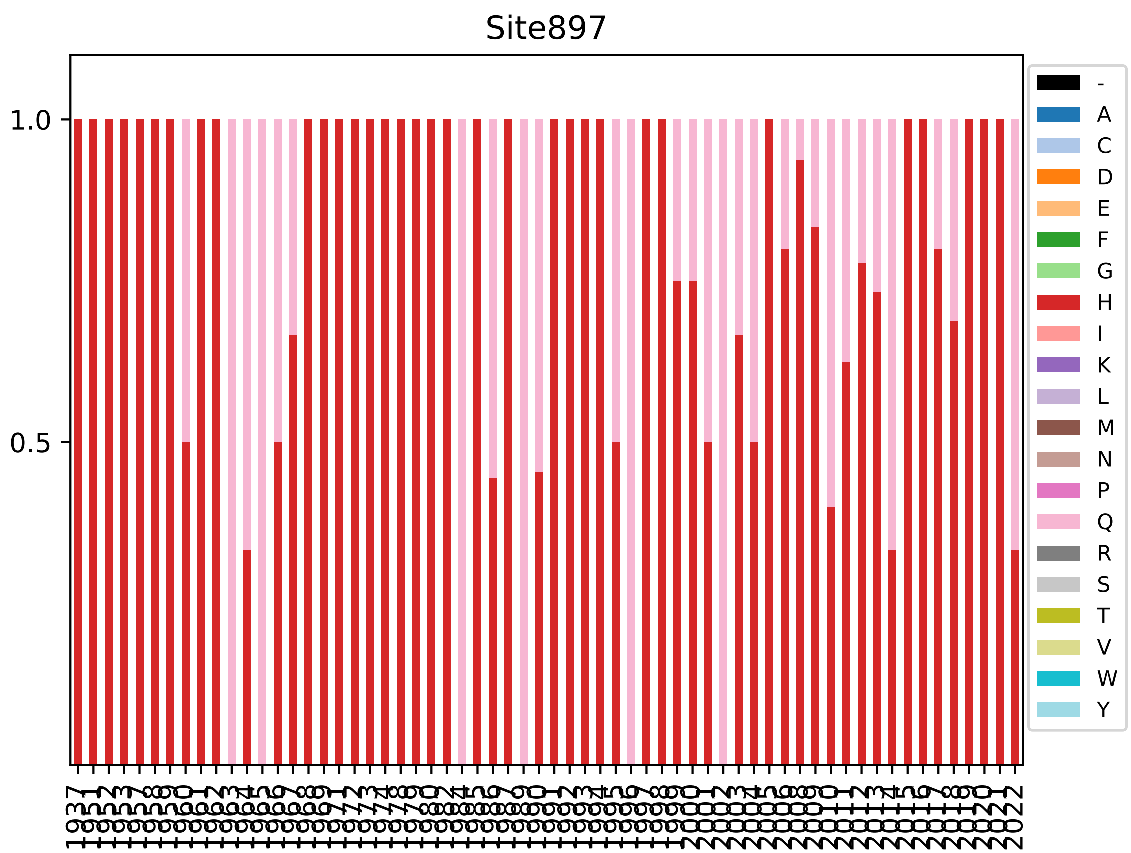
Site897
1.0
0.5
-
A
C
D
E
F
G
H
I
K
L
M
N
P
Q
R
S
T
V
W
Y

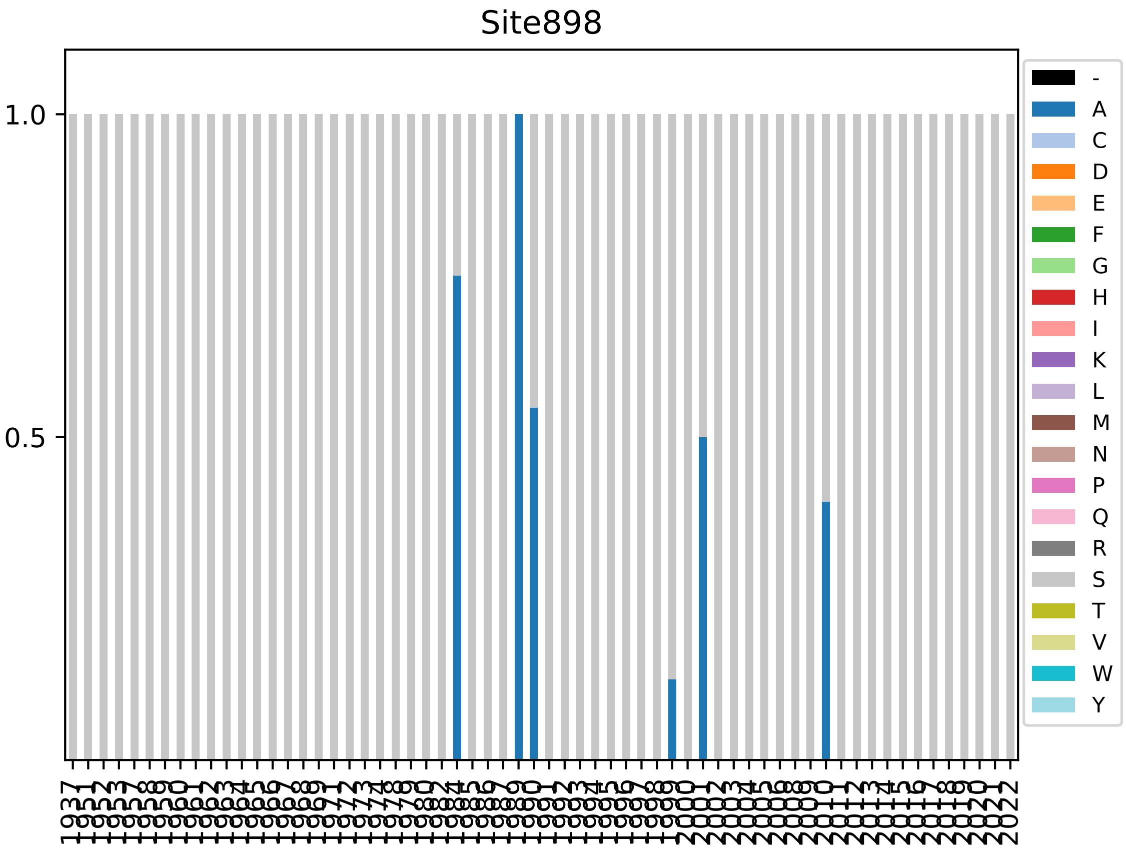
Site898
1.0
0.5
-
A
C
D
E
F
G
H
I
K
L
M
N
P
Q
R
S
T
V
W
Y

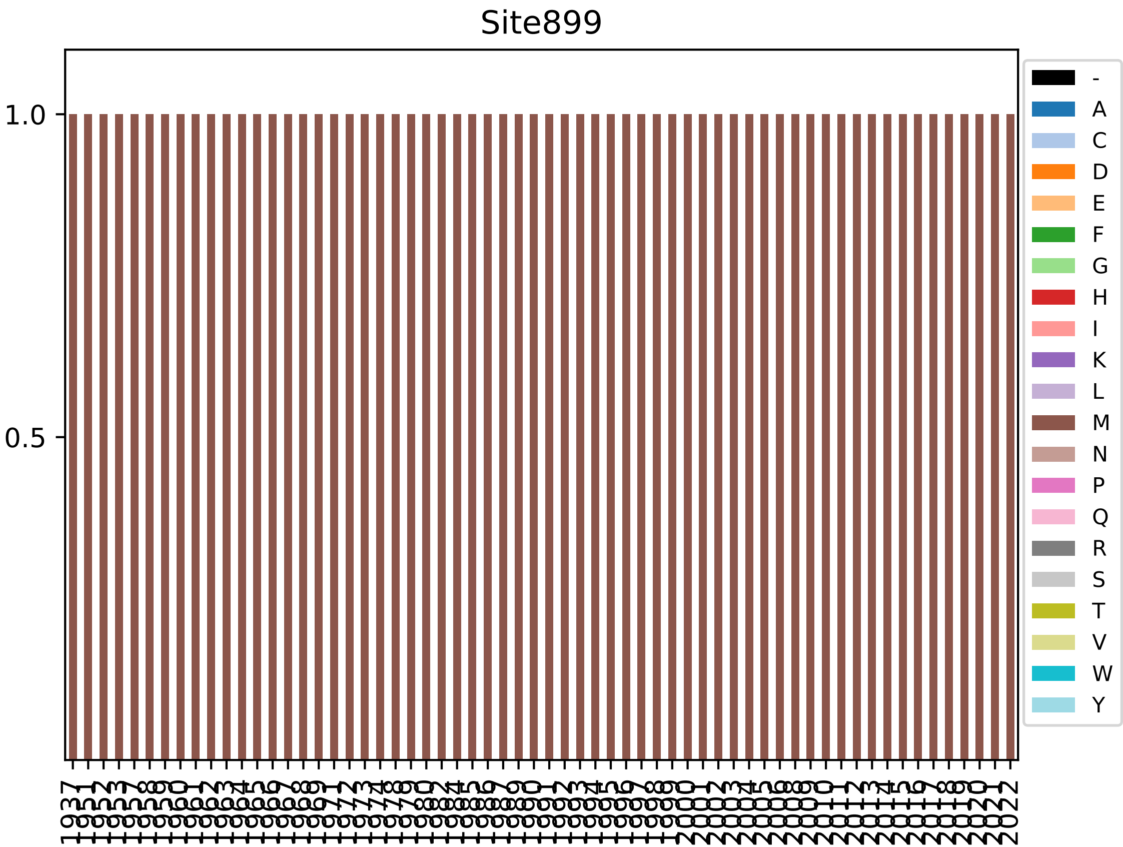
Site899
1.0
0.5
-
A
C
D
E
F
G
H
I
K
L
M
N
P
Q
R
S
T
V
W
Y

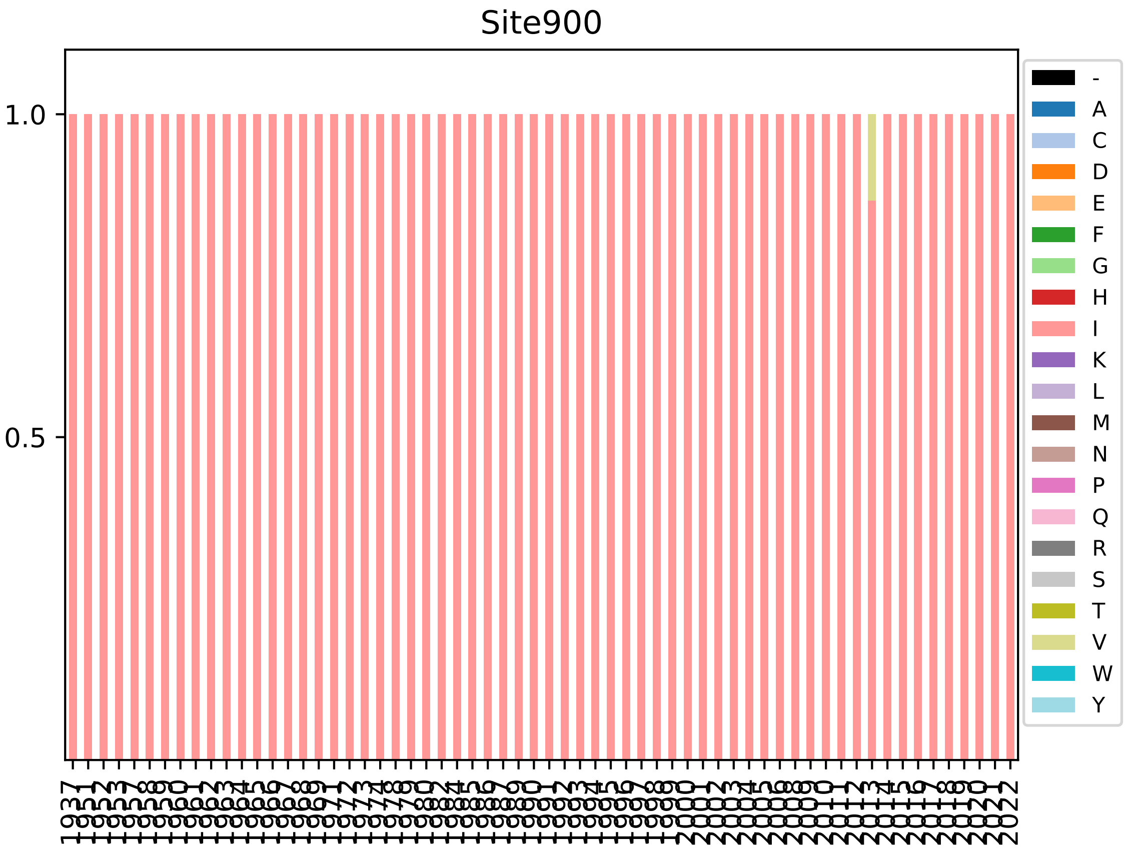
Site900
1.0
0.5
-
A
C
D
E
F
G
H
I
K
L
M
N
P
Q
R
S
T
V
W
Y

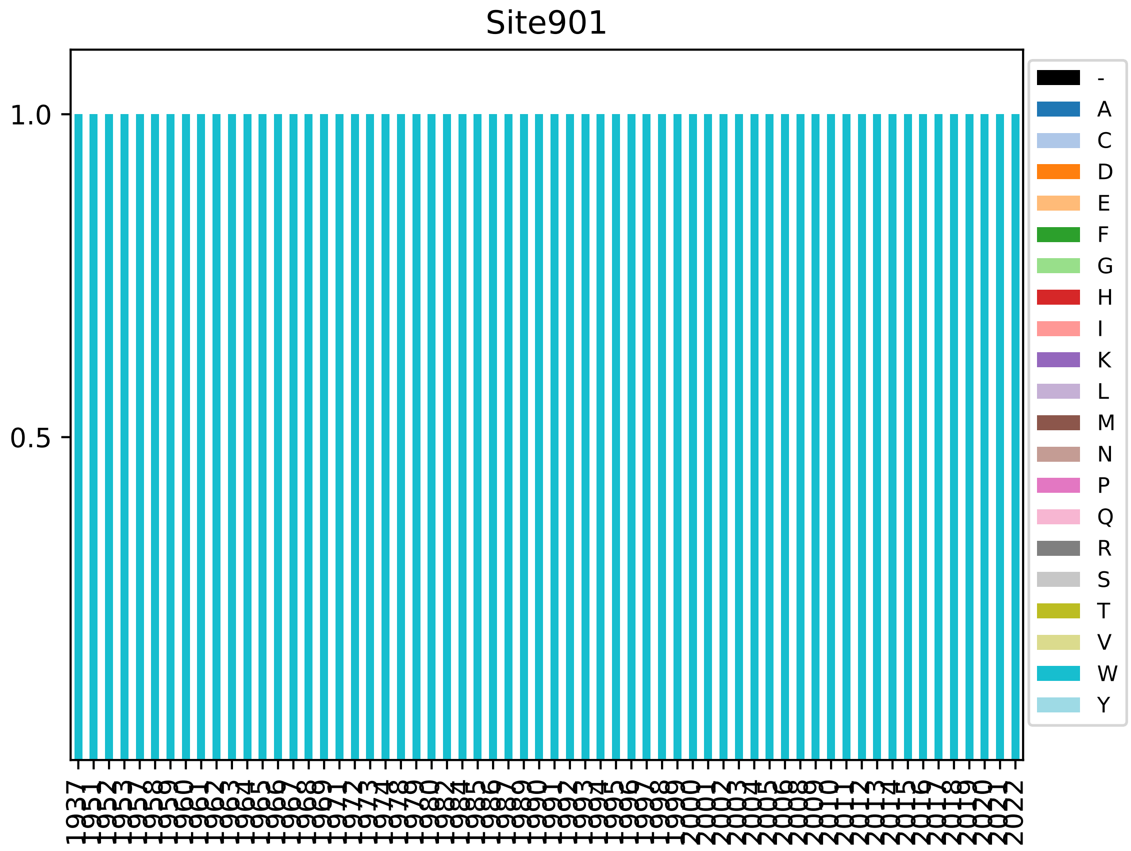
Site901
1.0
0.5
-
A
C
D
E
F
G
H
I
K
L
M
N
P
Q
R
S
T
V
W
Y

Site902

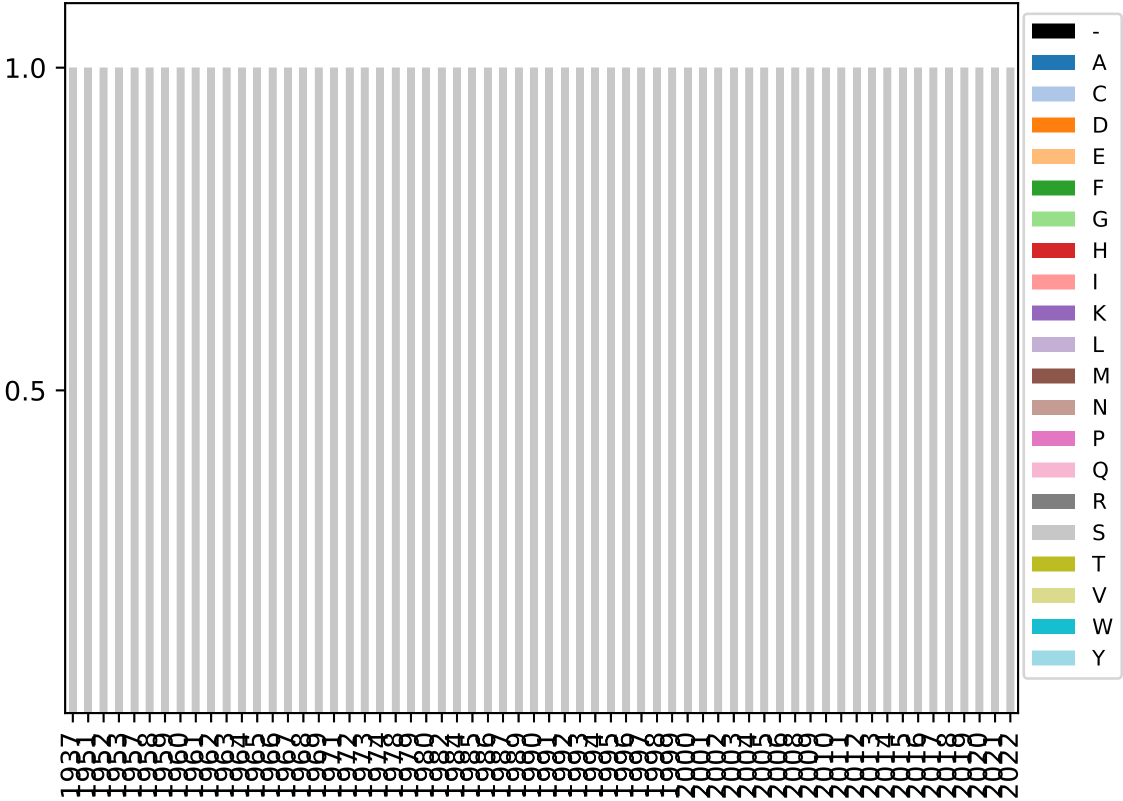

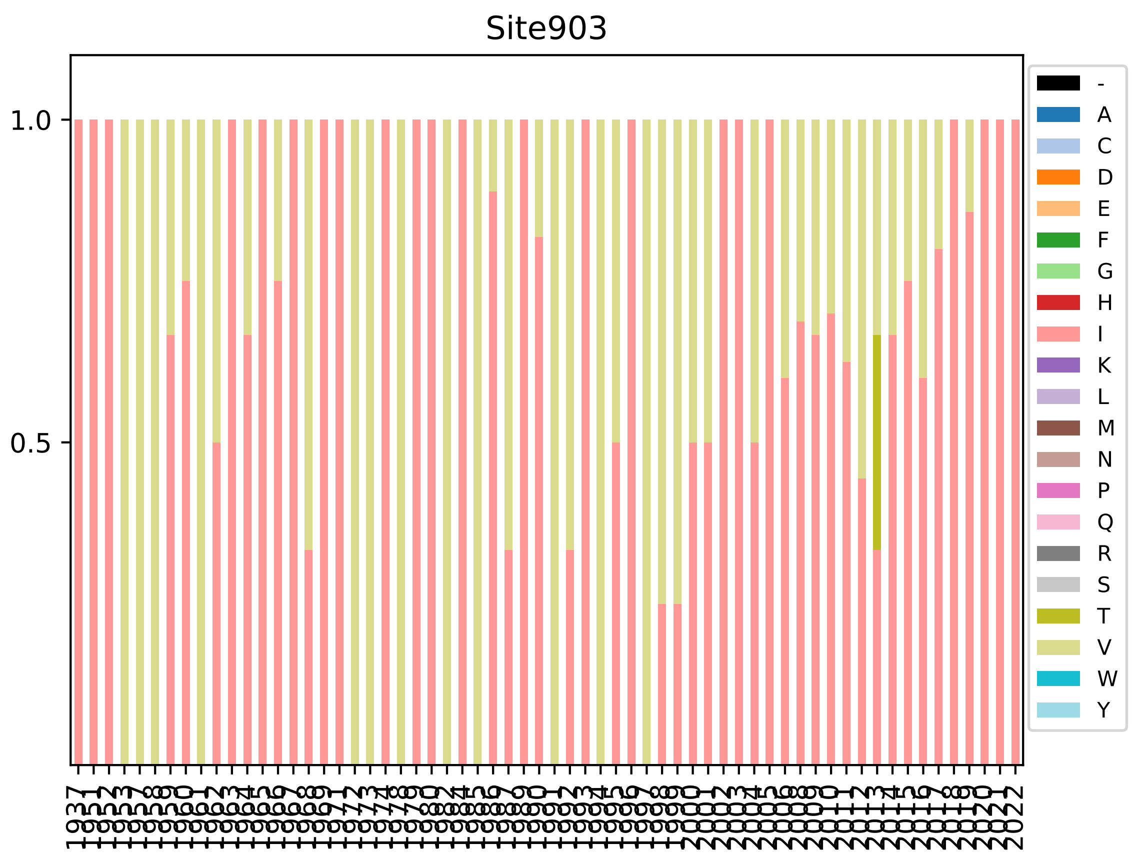
Site903
1.0
0.5
-
A
C
D
E
F
G
H
I
K
L
M
N
P
Q
R
S
T
V
W
Y

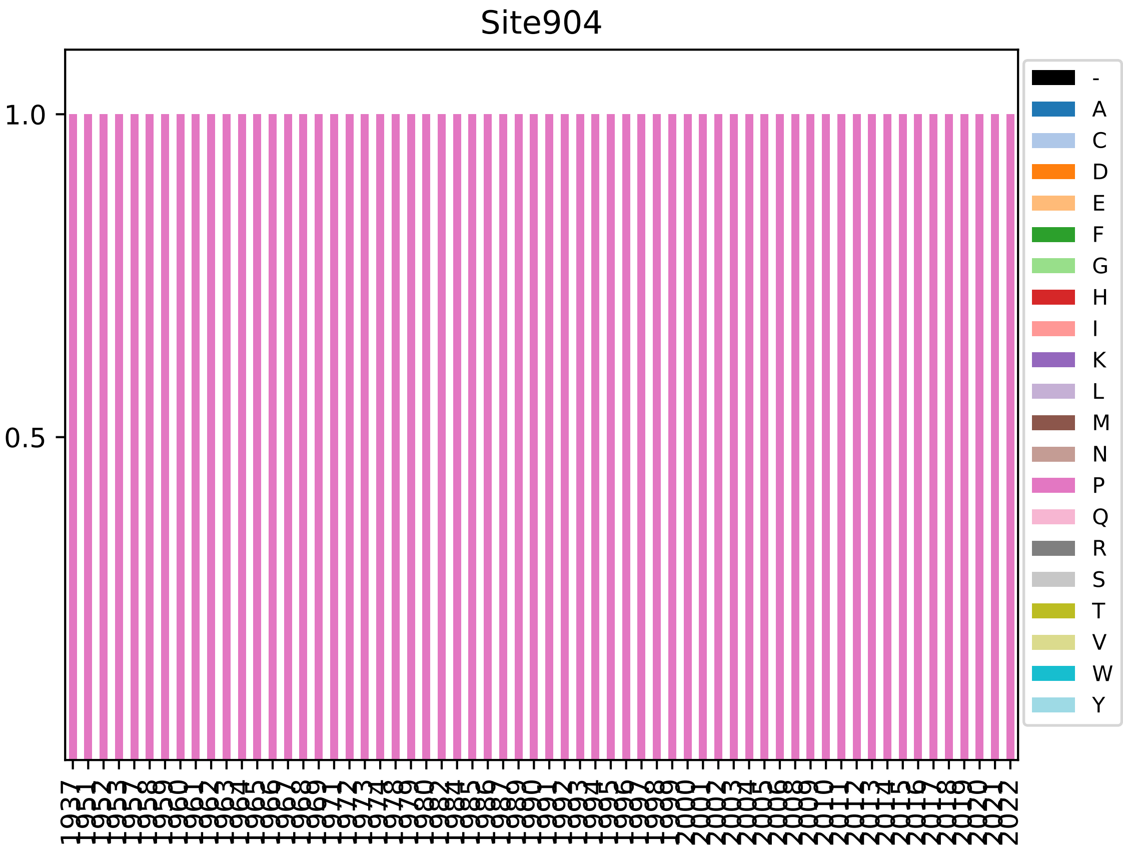
Site904
1.0
0.5
-
A
C
D
E
F
G
H
I
K
L
M
N
P
Q
R
S
T
V
W
Y

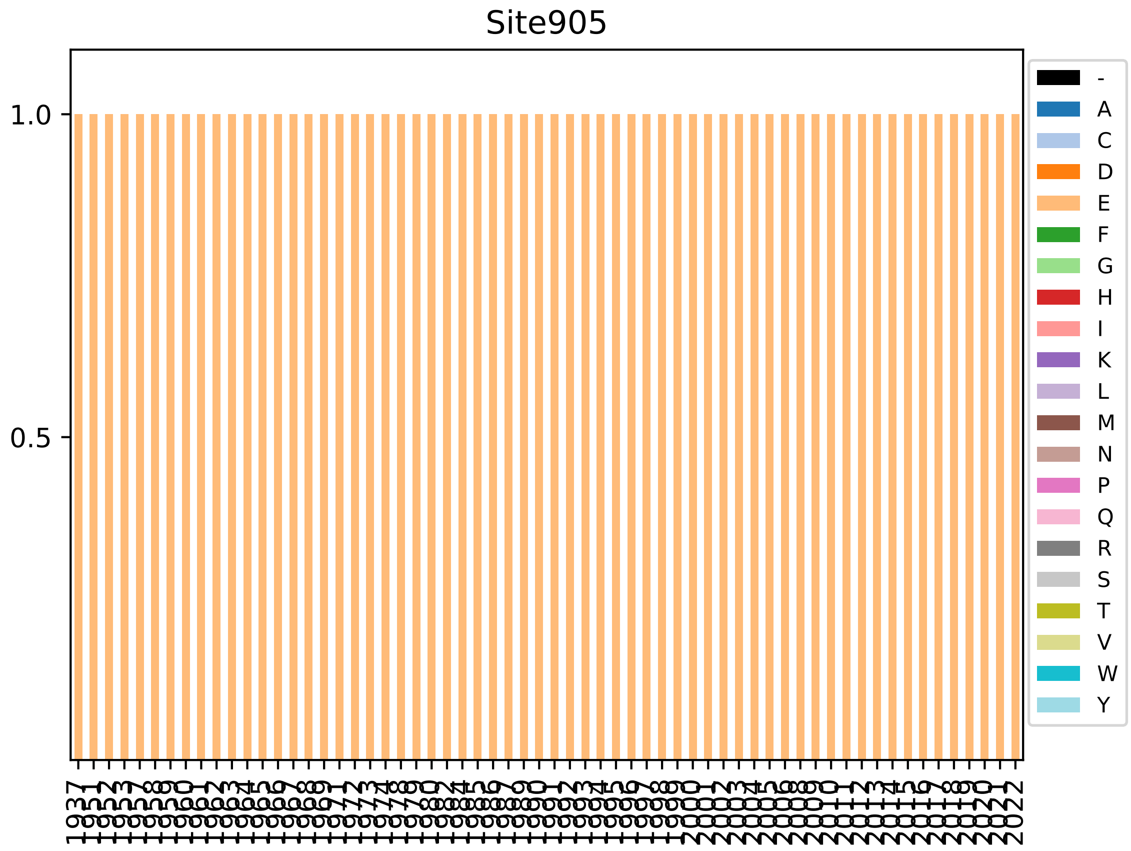
Site905
1.0
0.5
-
A
C
D
E
F
G
H
I
K
L
M
N
P
Q
R
S
T
V
W
Y

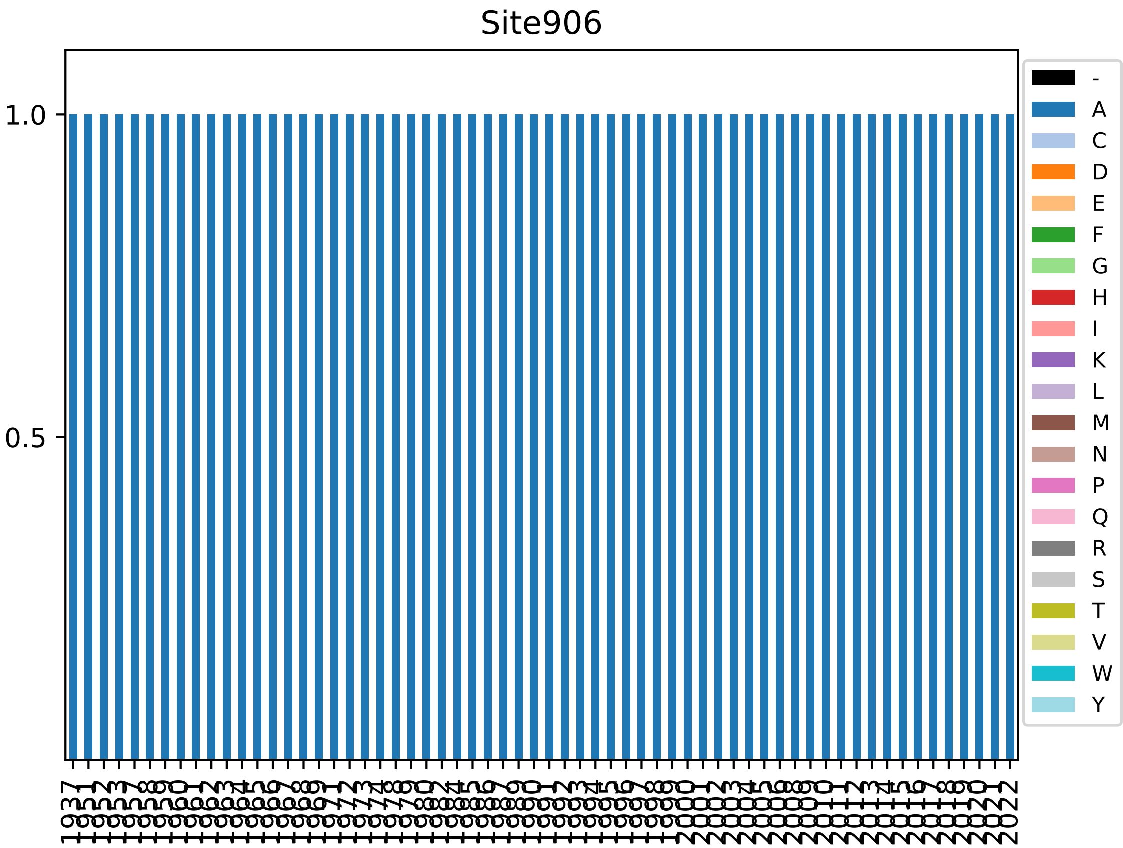
Site906
1.0
0.5
-
A
C
D
E
F
G
H
I
K
L
M
N
P
Q
R
S
T
V
W
Y

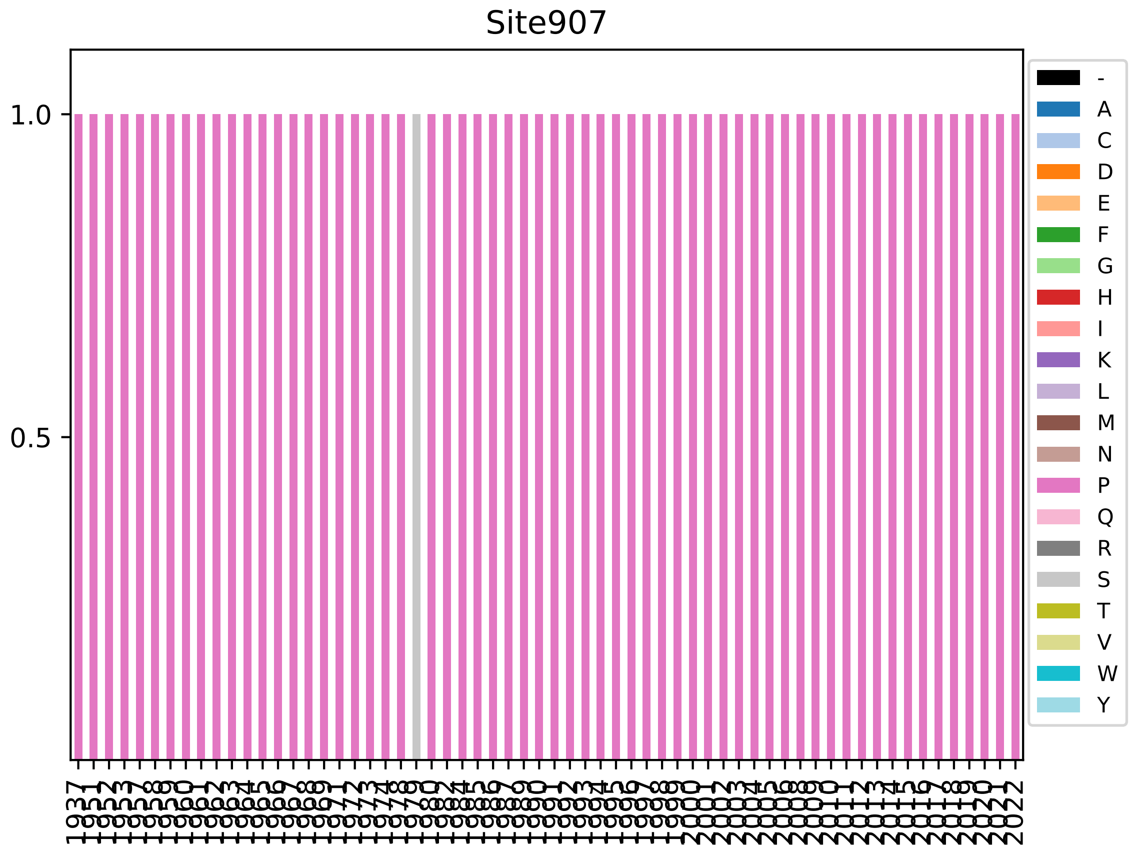
Site907
1.0
0.5
-
A
C
D
E
F
G
H
I
K
L
M
N
P
Q
R
S
T
V
W
Y

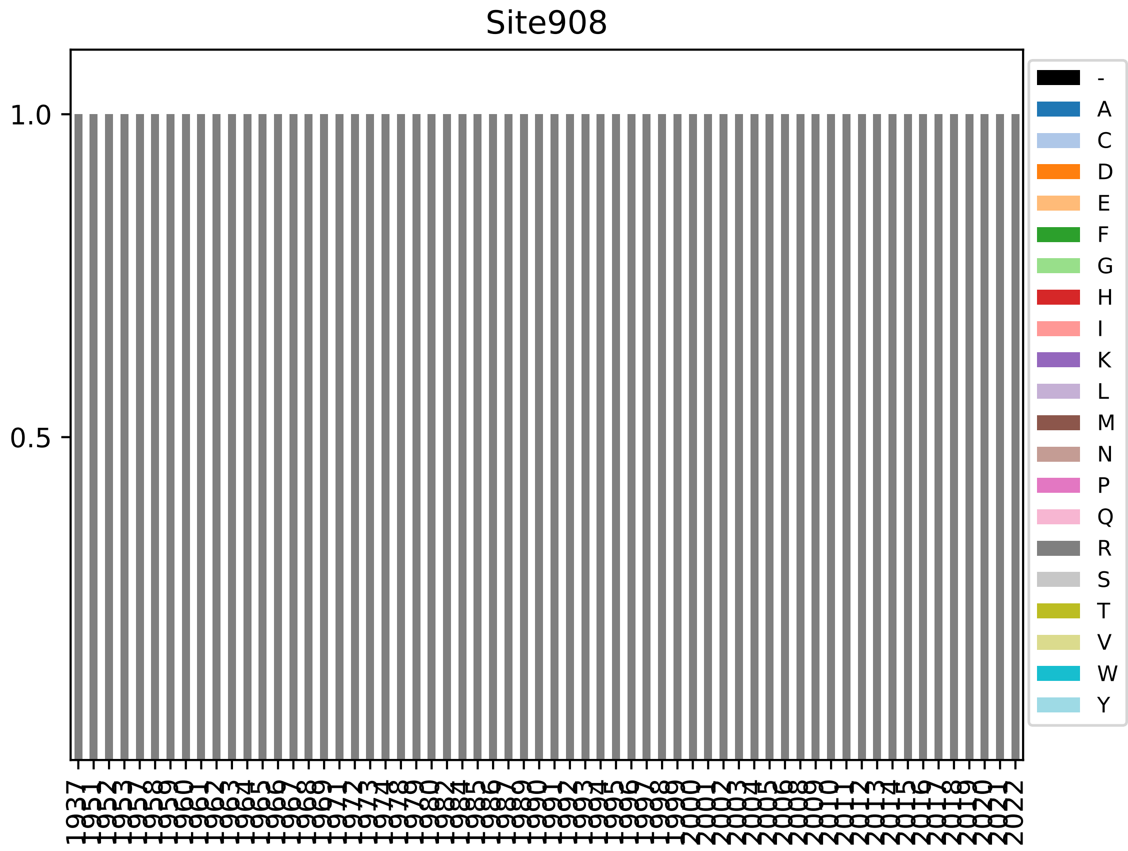
Site908
1.0
0.5
-
A
C
D
E
F
G
H
I
K
L
M
N
P
Q
R
S
T
V
W
Y

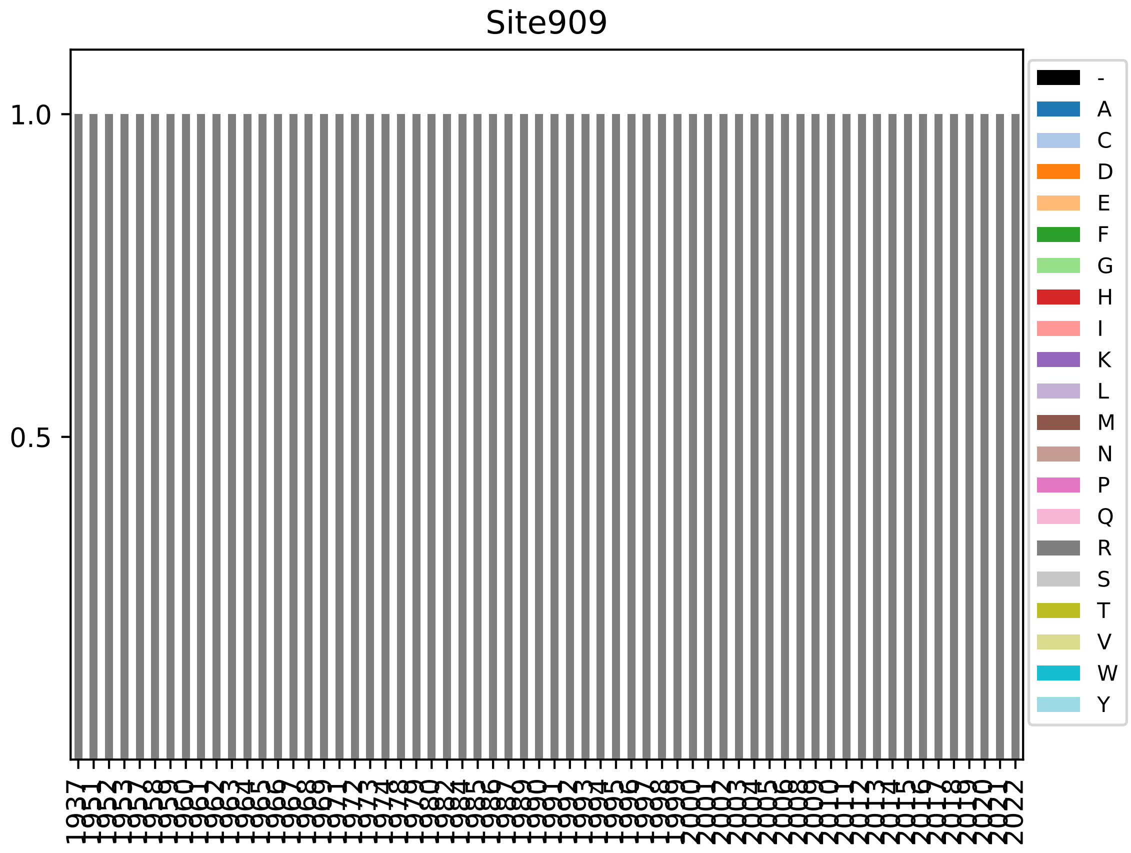
Site909
1.0
0.5
-
A
C
D
E
F
G
H
I
K
L
M
N
P
Q
R
S
T
V
W
Y

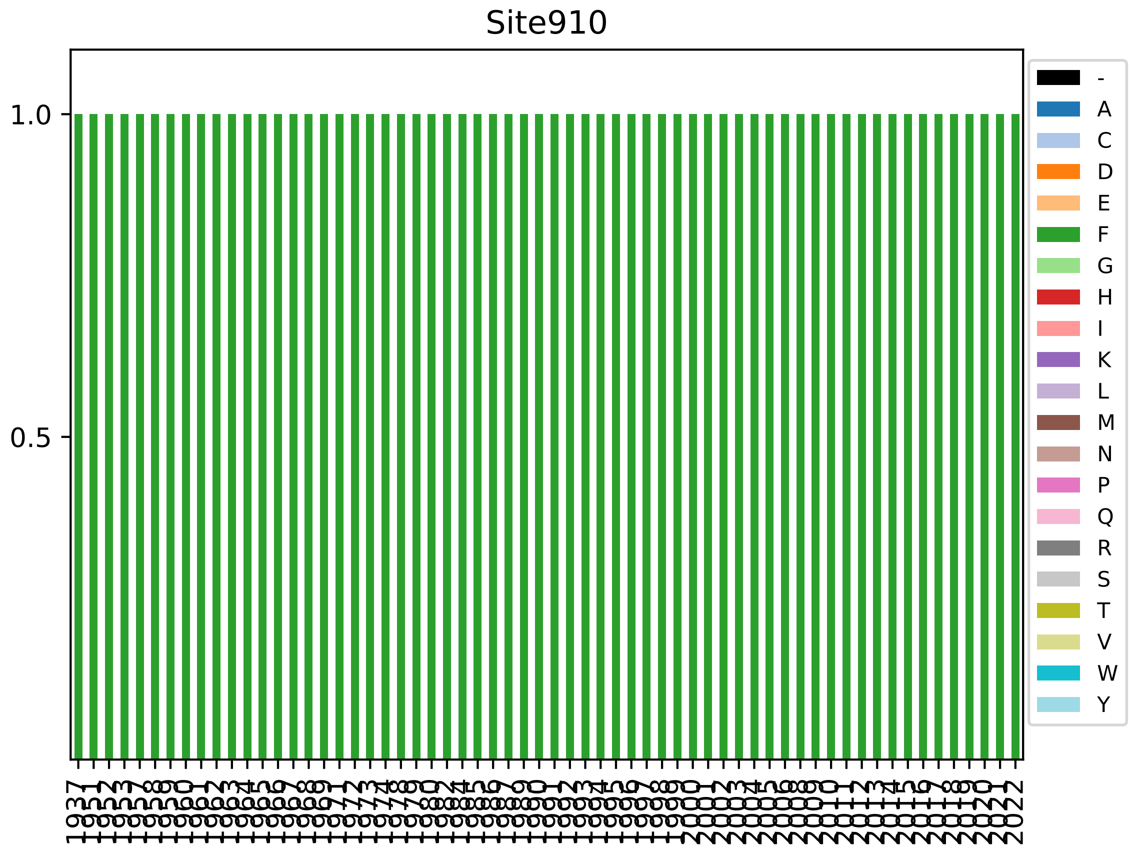
Site910
1.0
0.5
-
A
C
D
E
F
G
H
I
K
L
M
N
P
Q
R
S
T
V
W
Y

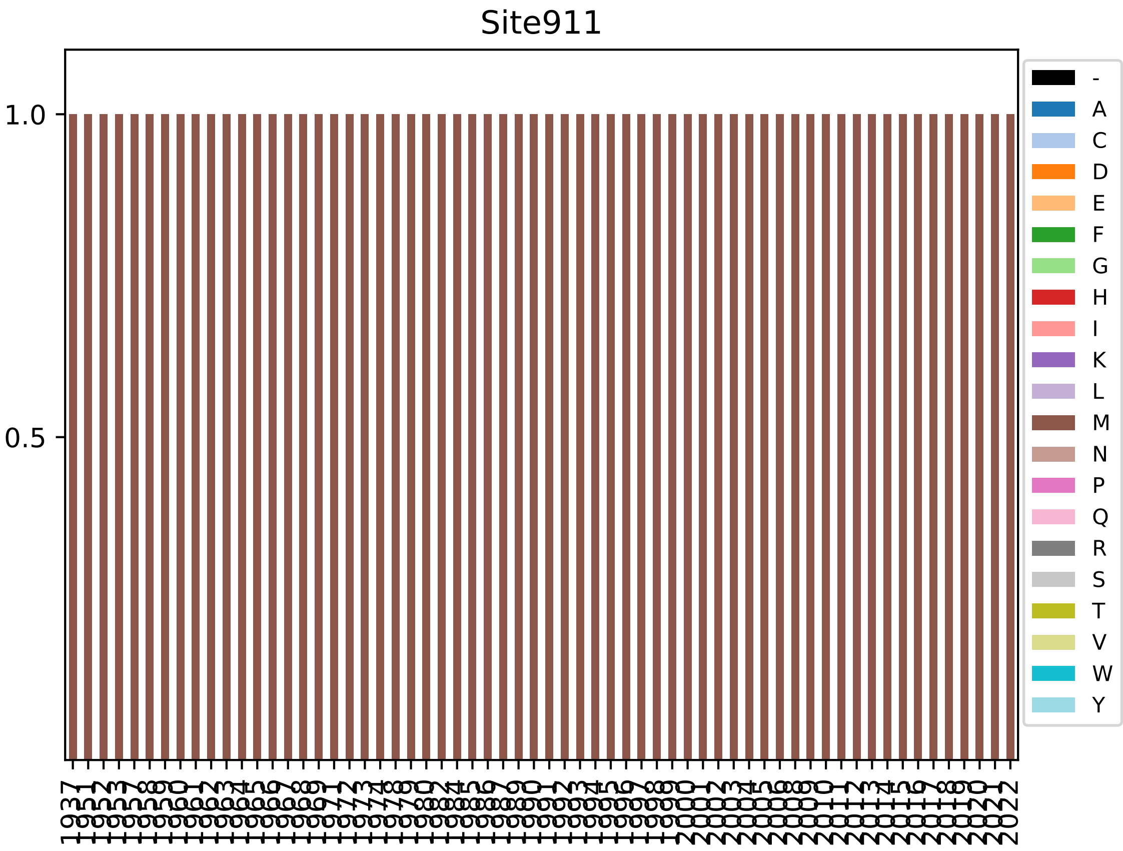
Site911
1.0
0.5
-
A
C
D
E
F
G
H
I
K
L
M
N
P
Q
R
S
T
V
W
Y

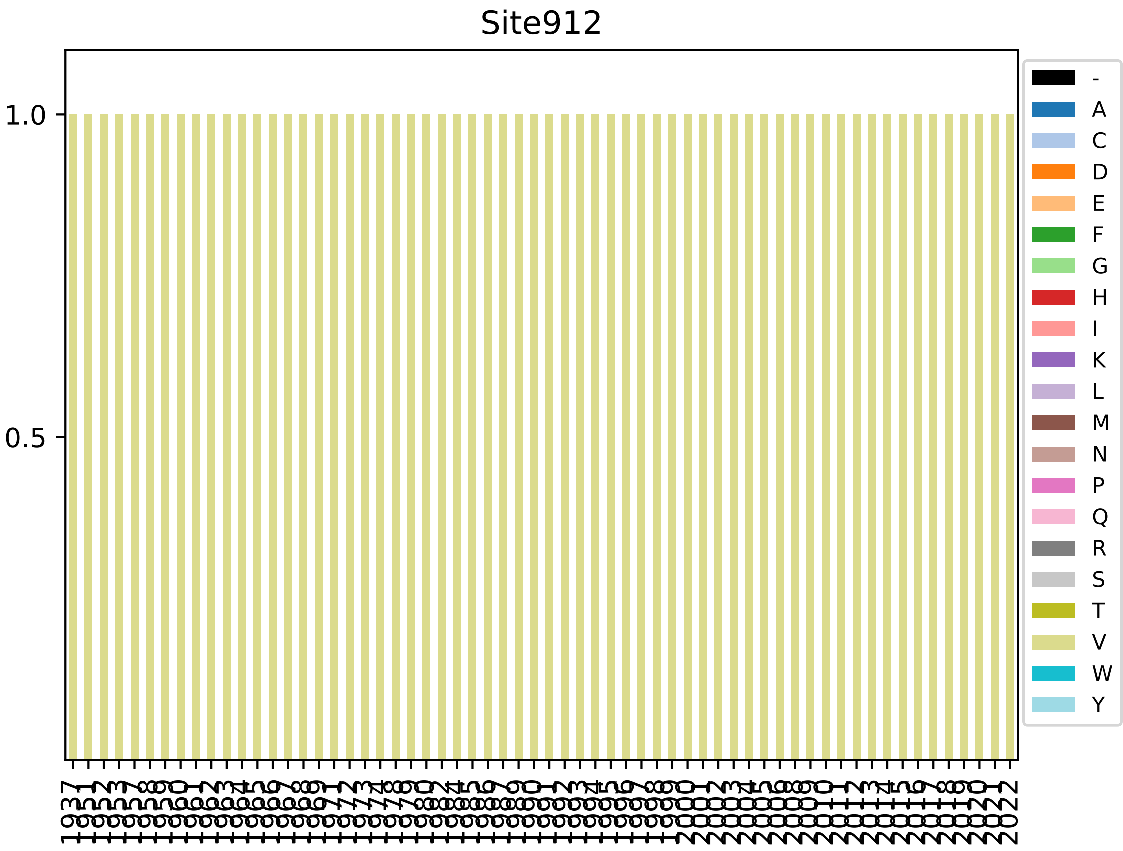

Site912
1.0
0.5
-
A
C
D
E
F
G
H
I
K
L
M
N
P
Q
R
S
T
V
W
Y

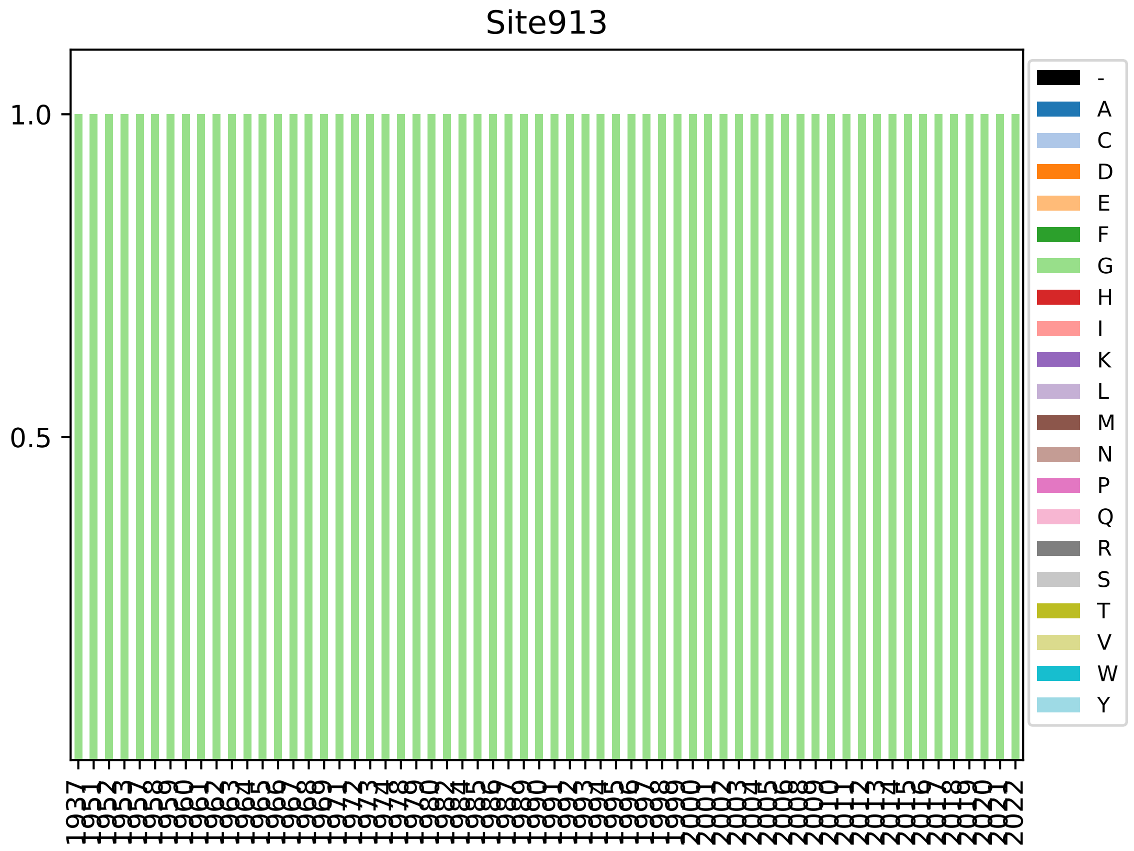
Site913
1.0
0.5
-
A
C
D
E
F
G
H
I
K
L
M
N
P
Q
R
S
T
V
W
Y

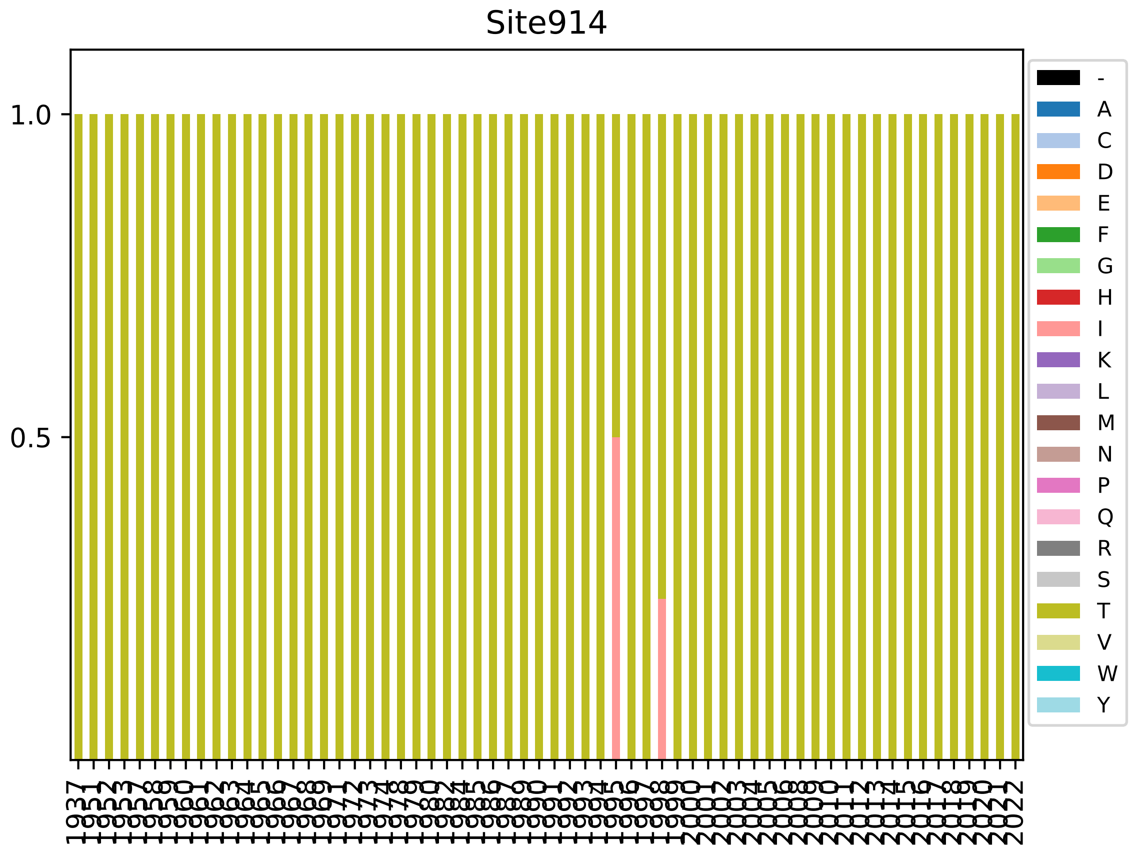
Site914
1.0
0.5
-
A
C
D
E
F
G
H
I
K
L
M
N
P
Q
R
S
T
V
W
Y

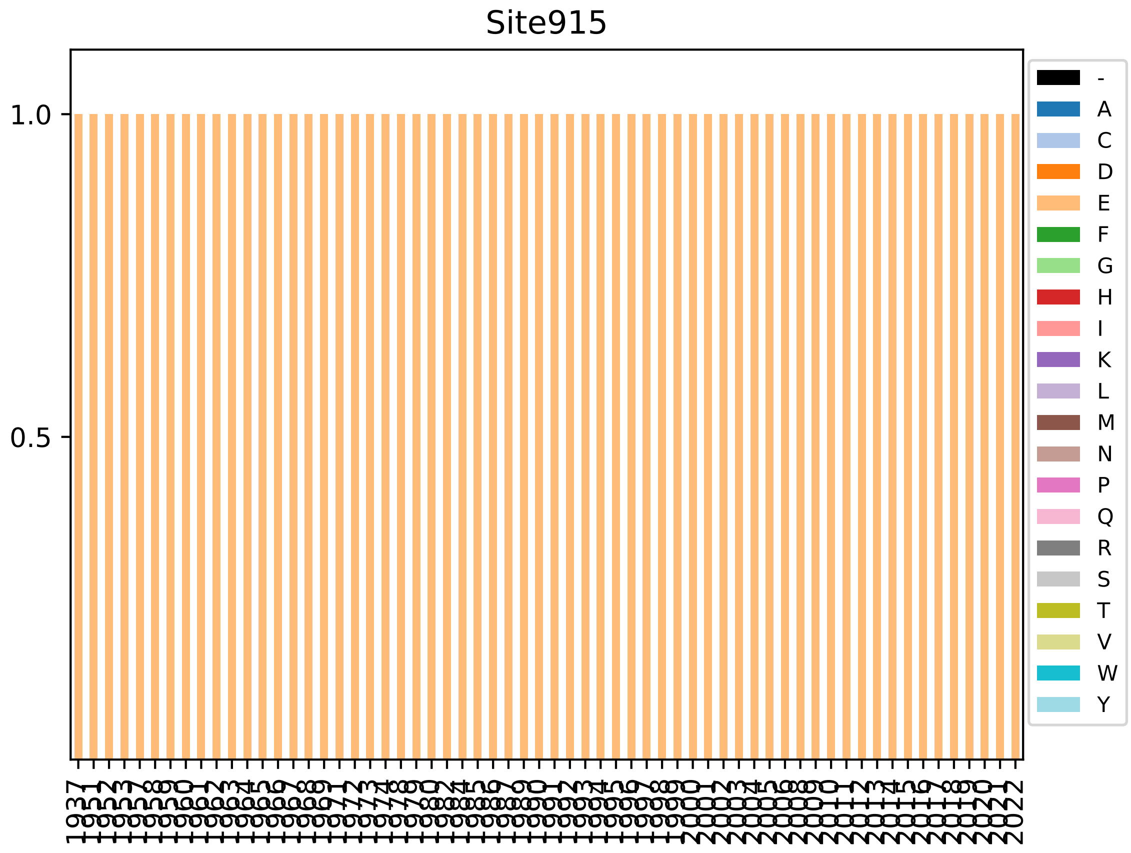

Site915
1.0
0.5
-
A
C
D
E
F
G
H
I
K
L
M
N
P
Q
R
S
T
V
W
Y

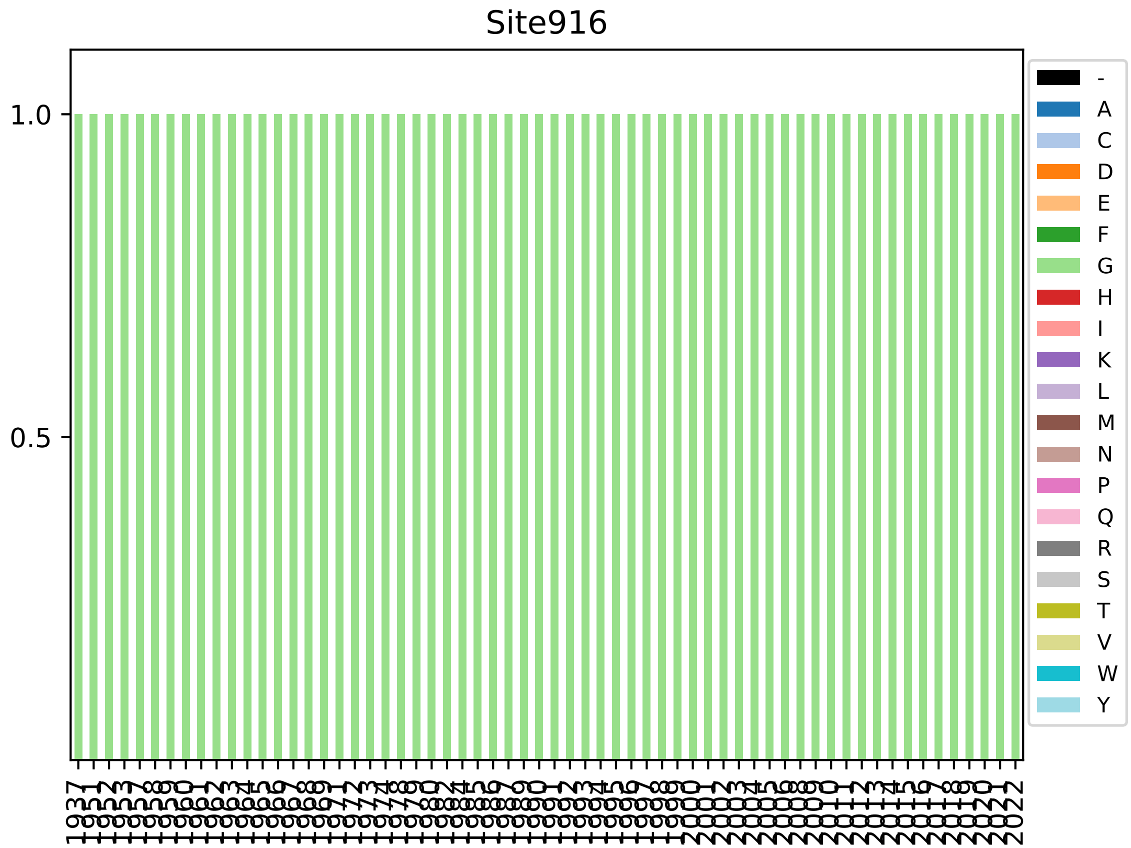
Site916
1.0
0.5
-
A
C
D
E
F
G
H
I
K
L
M
N
P
Q
R
S
T
V
W
Y

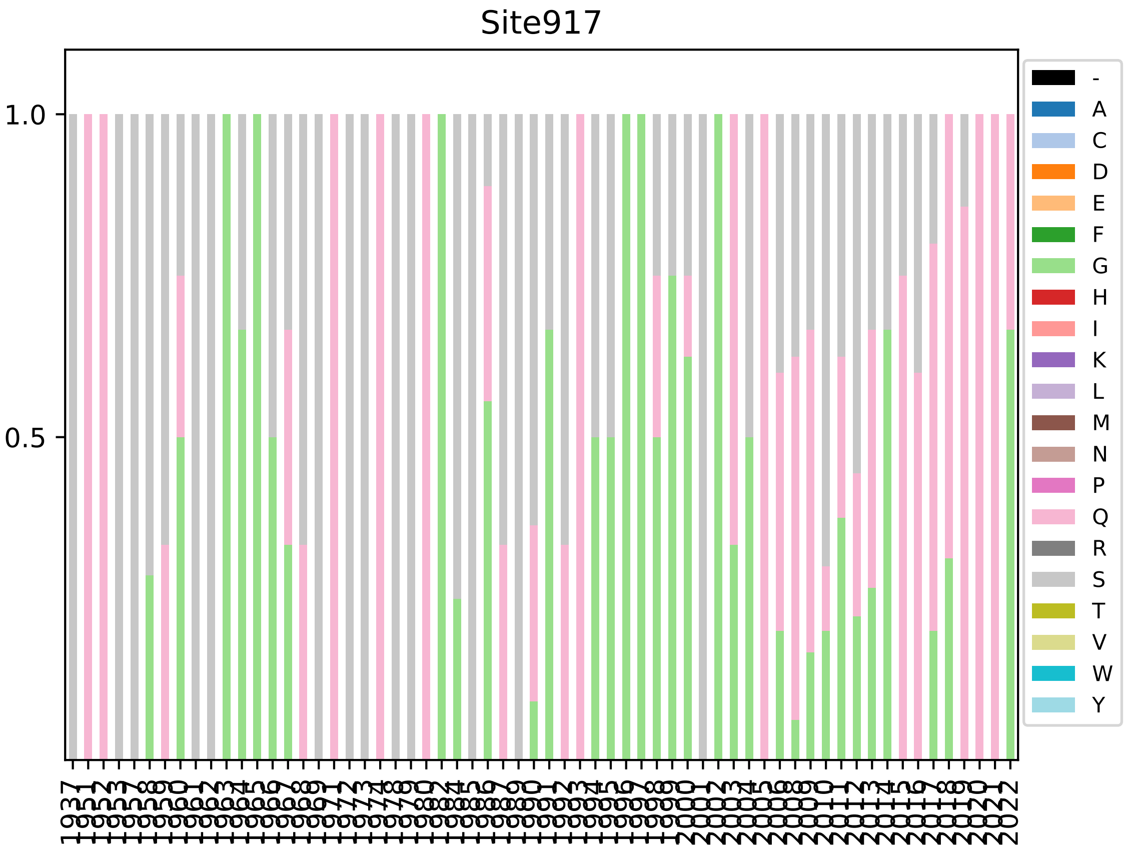
Site917
1.0
0.5
-
A
C
D
E
F
G
H
I
K
L
M
N
P
Q
R
S
T
V
W
Y

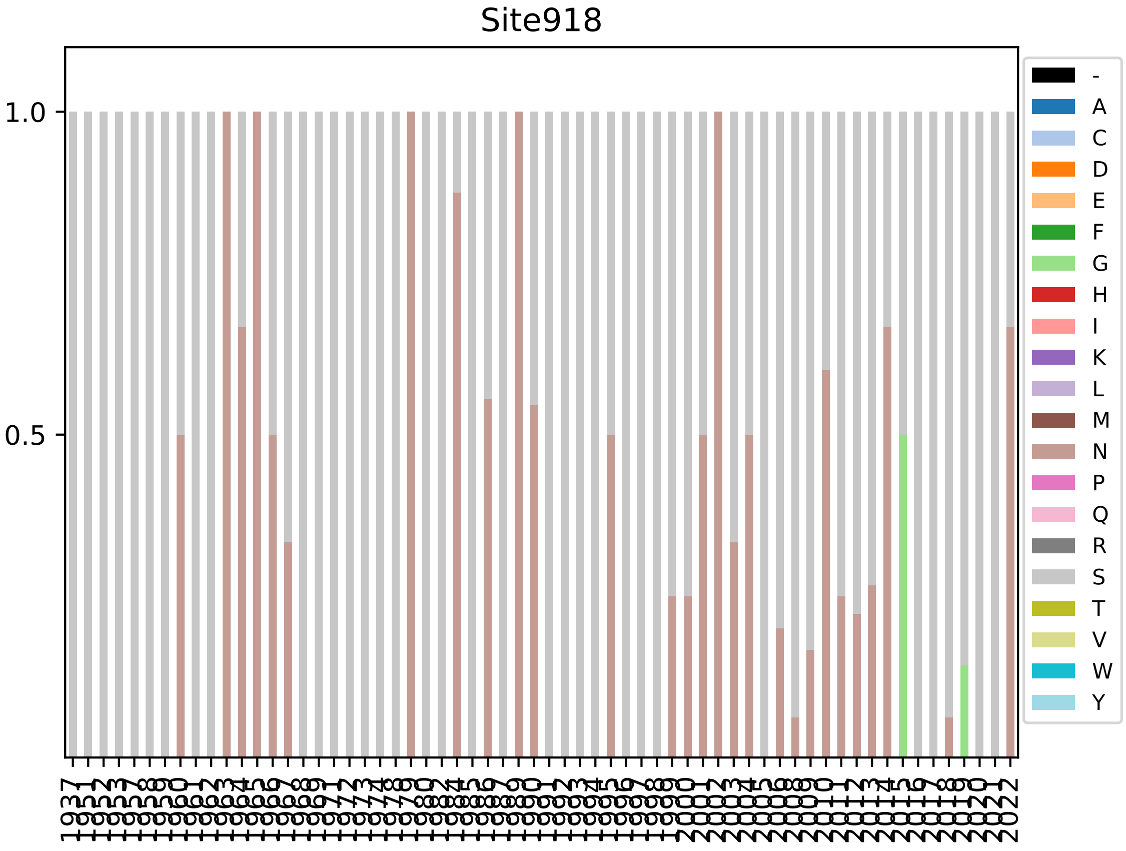
Site918
1.0
0.5
-
A
C
D
E
F
G
H
I
K
L
M
N
P
Q
R
S
T
V
W
Y

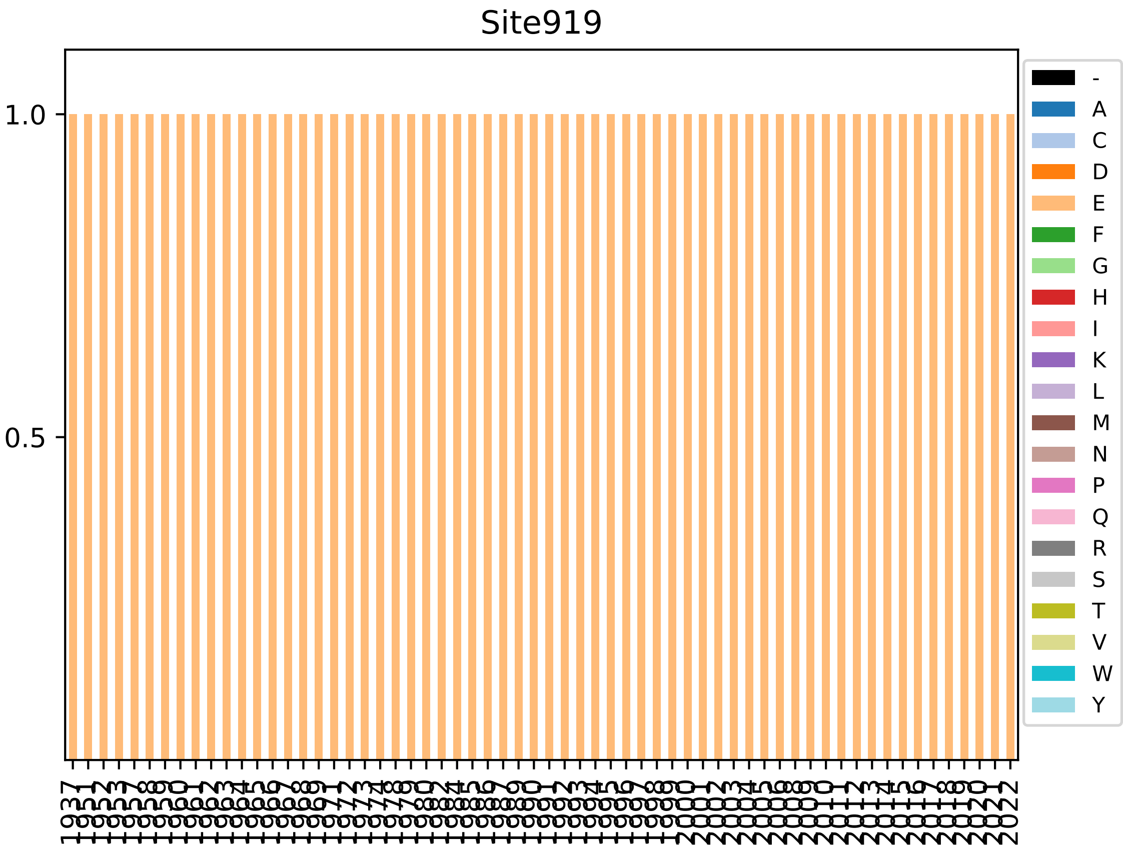
Site919
1.0
0.5
-
A
C
D
E
F
G
H
I
K
L
M
N
P
Q
R
S
T
V
W
Y

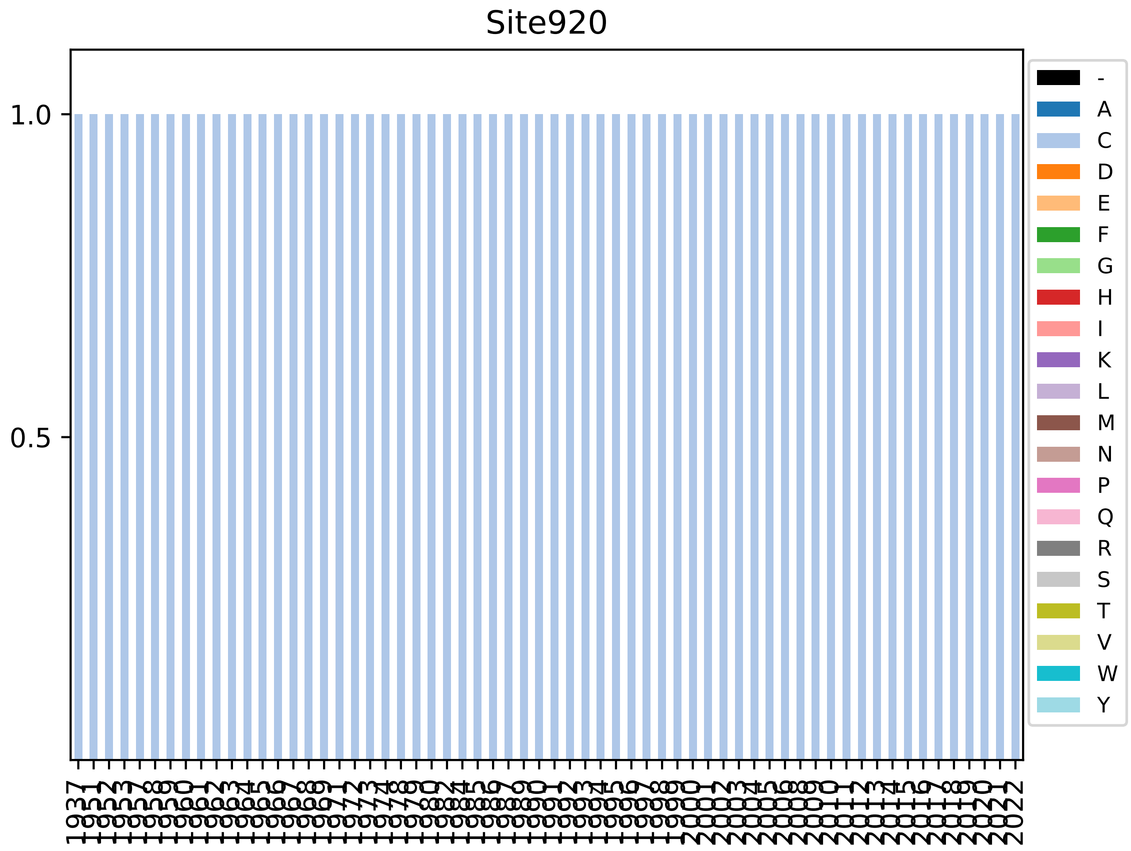
Site920
1.0
0.5
-
A
C
D
E
F
G
H
I
K
L
M
N
P
Q
R
S
T
V
W
Y

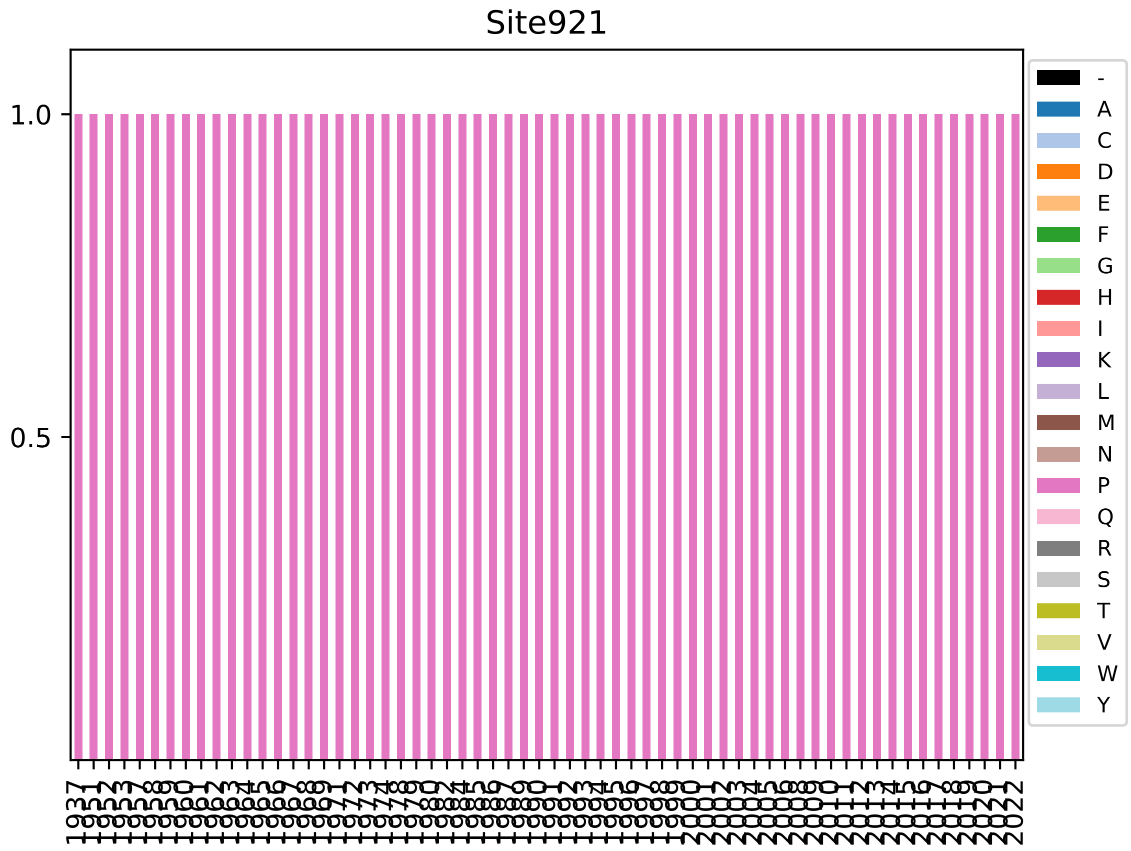

Site921
1.0
0.5
-
A
C
D
E
F
G
H
I
K
L
M
N
P
Q
R
S
T
V
W
Y

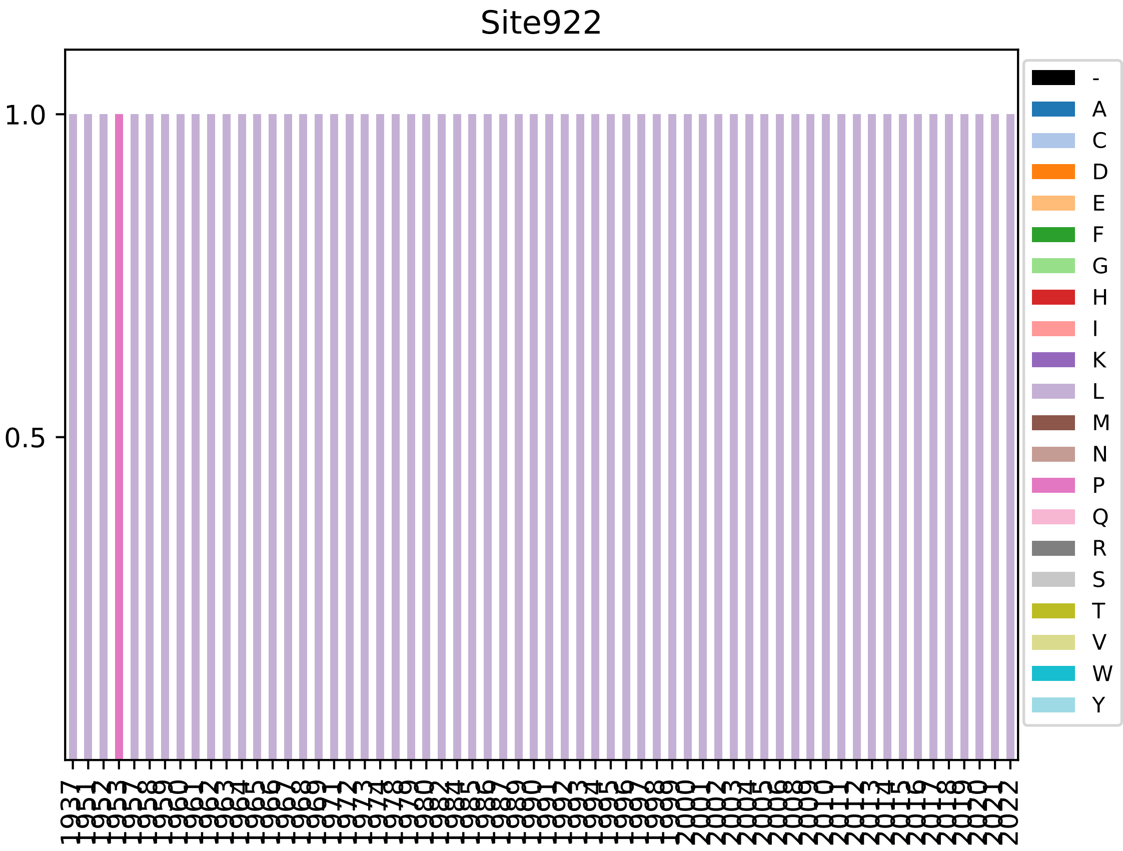
Site922
1.0
0.5
-
A
C
D
E
F
G
H
I
K
L
M
N
P
Q
R
S
T
V
W
Y

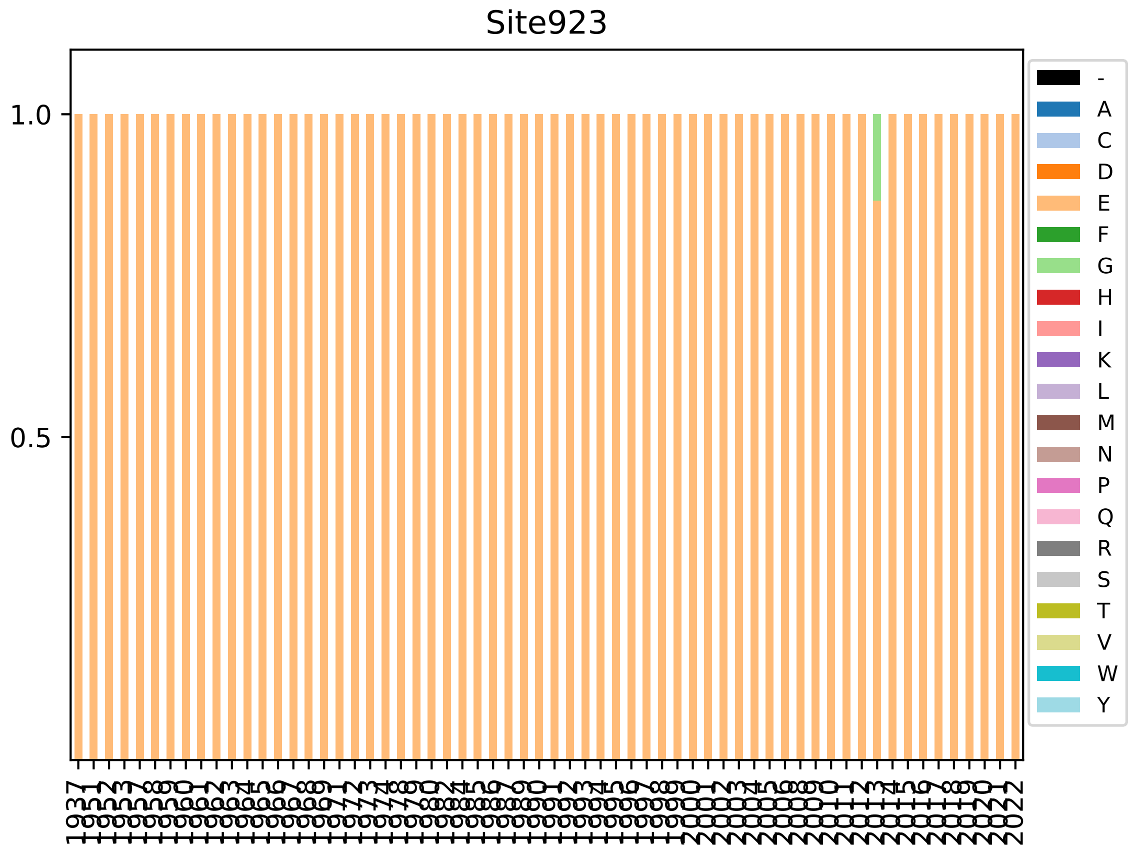
Site923
1.0
0.5
-
A
C
D
E
F
G
H
I
K
L
M
N
P
Q
R
S
T
V
W
Y

Site924

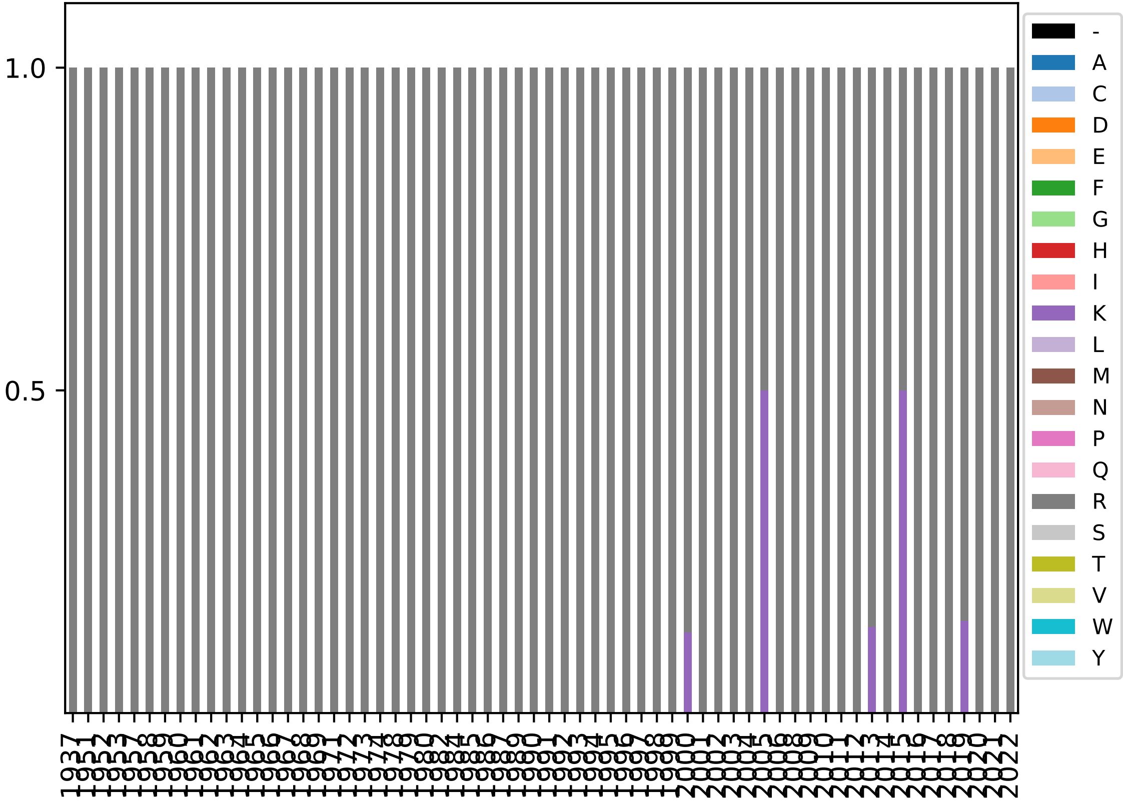

Site925

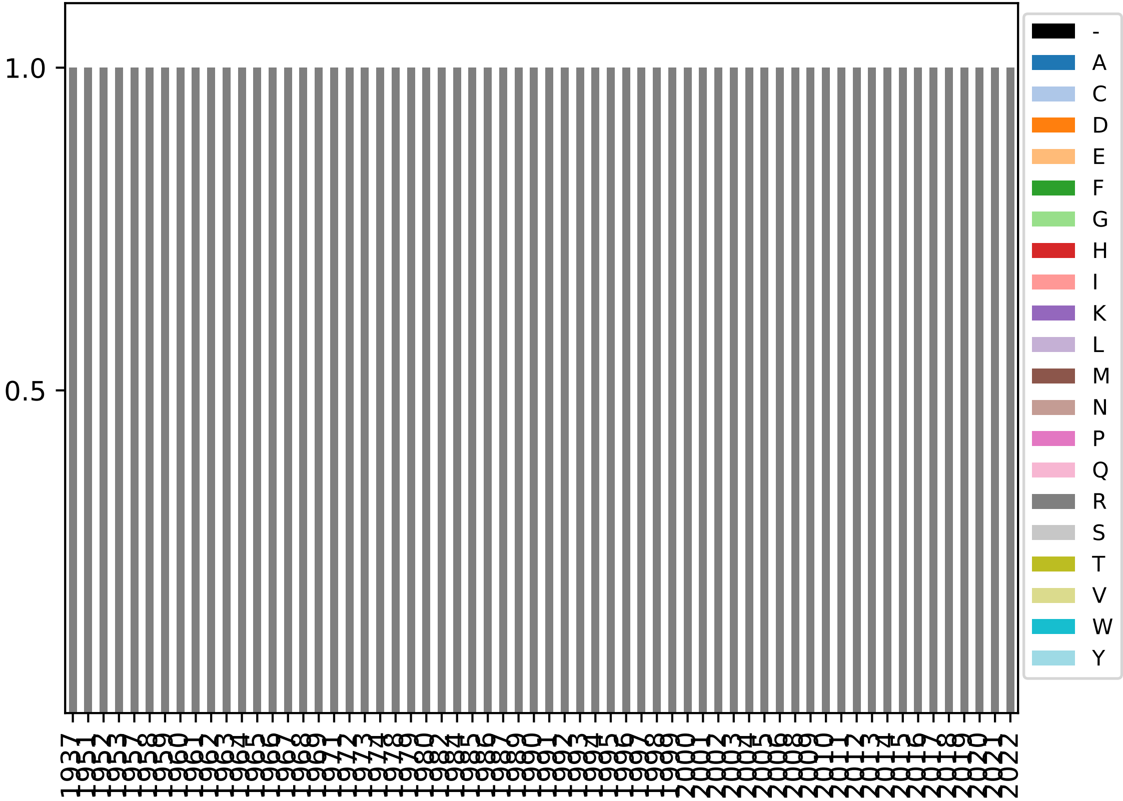

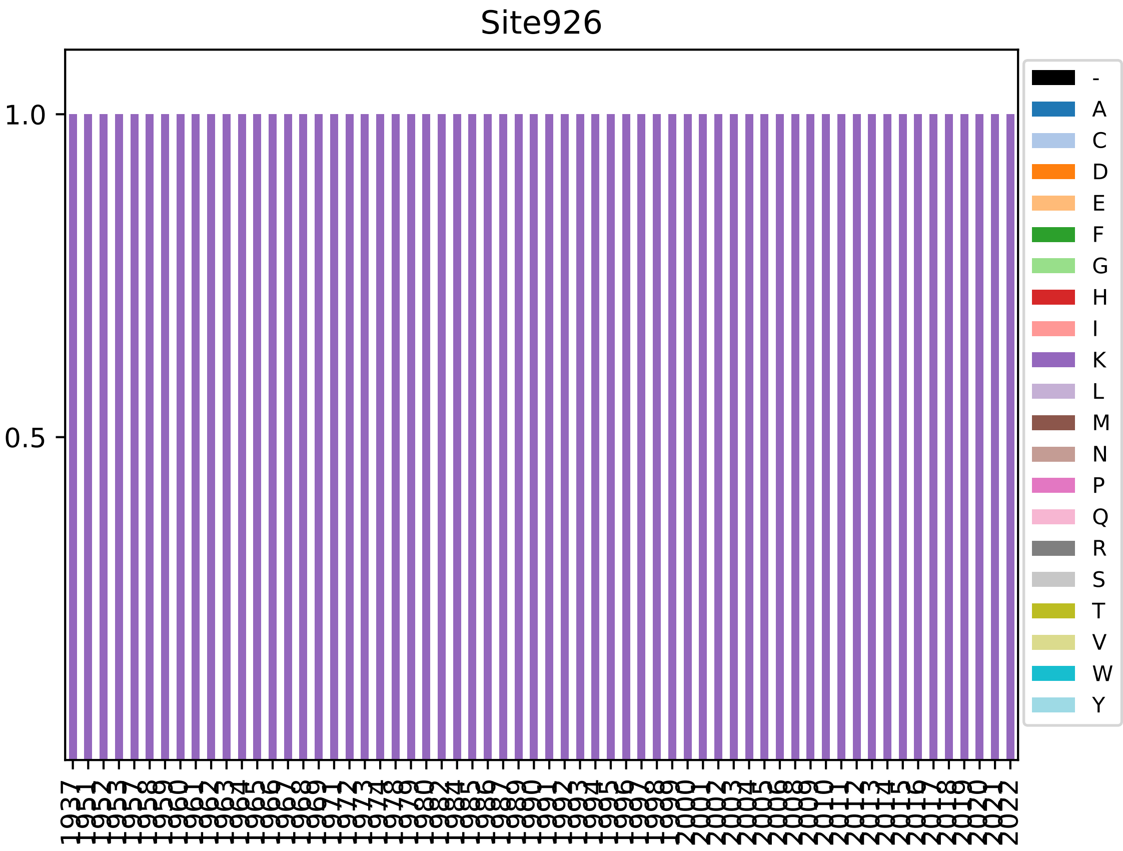
Site926
1.0
0.5
-
A
C
D
E
F
G
H
I
K
L
M
N
P
Q
R
S
T
V
W
Y

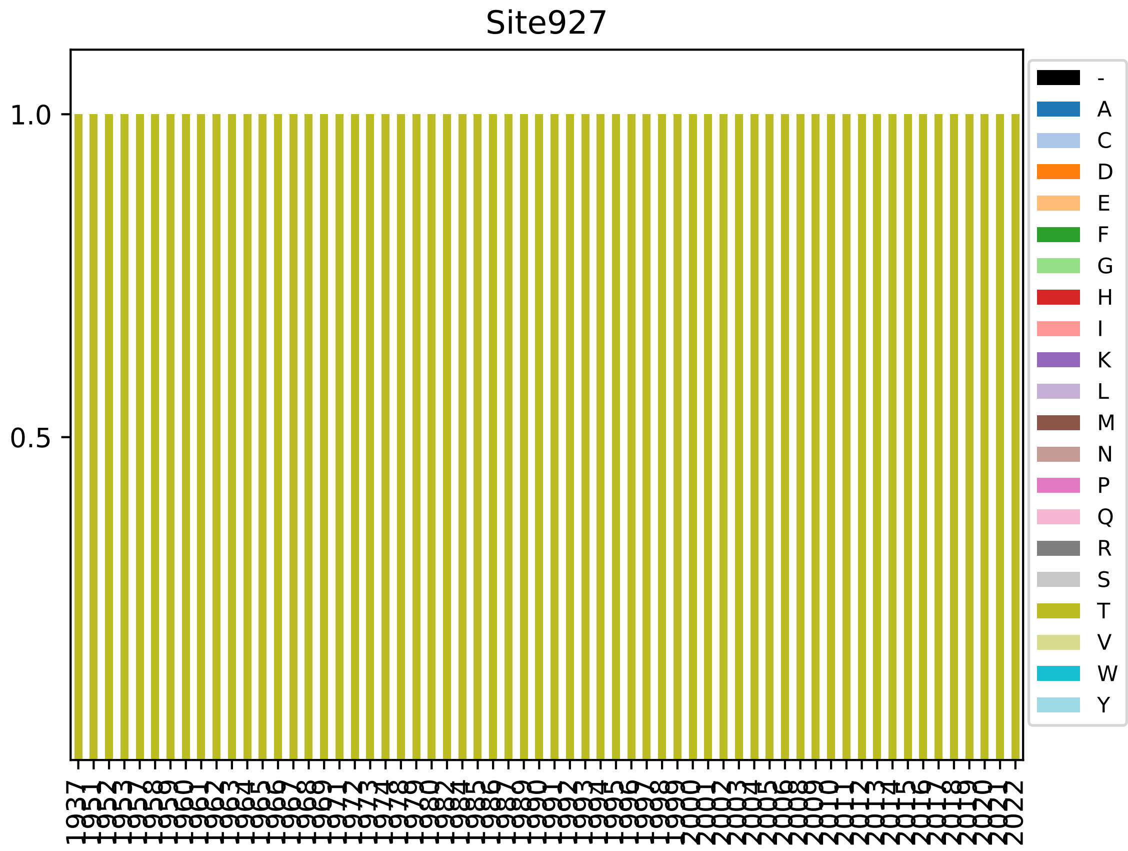
Site927
1.0
0.5
-
A
C
D
E
F
G
H
I
K
L
M
N
P
Q
R
S
T
V
W
Y

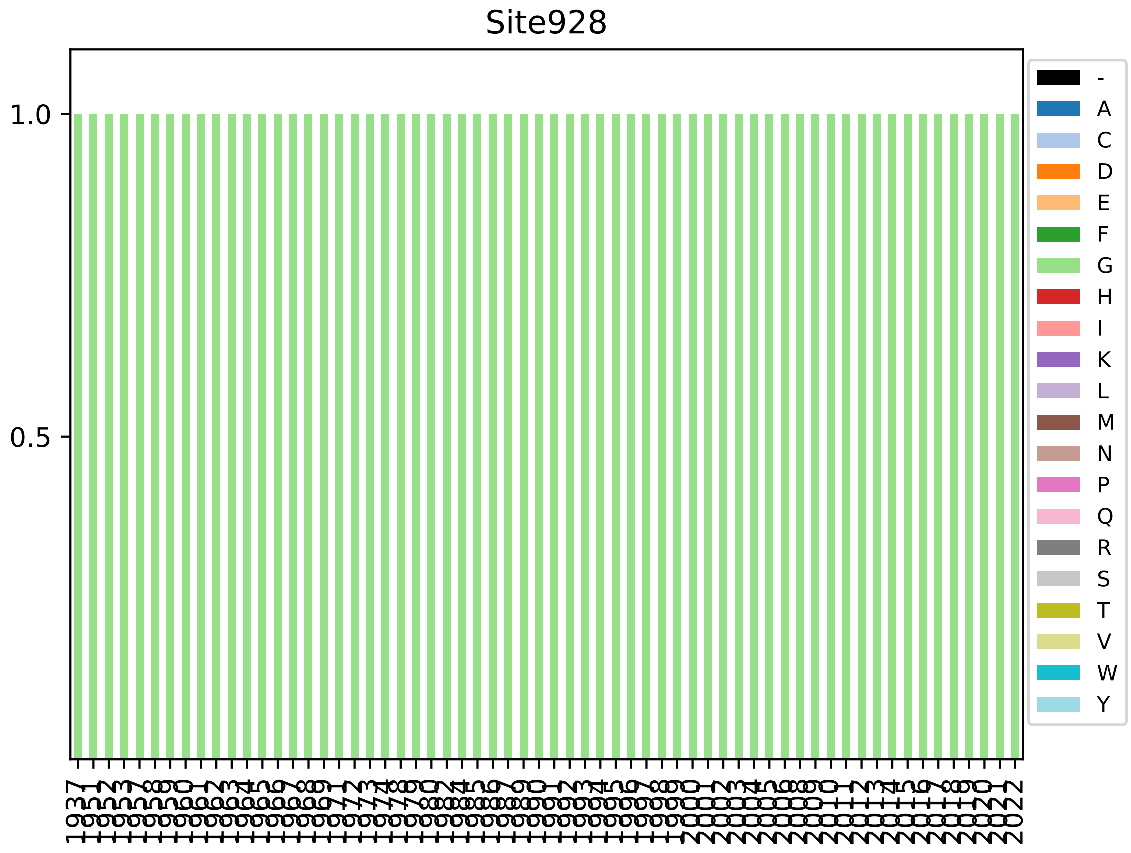
Site928
1.0
0.5
-
A
C
D
E
F
G
H
I
K
L
M
N
P
Q
R
S
T
V
W
Y

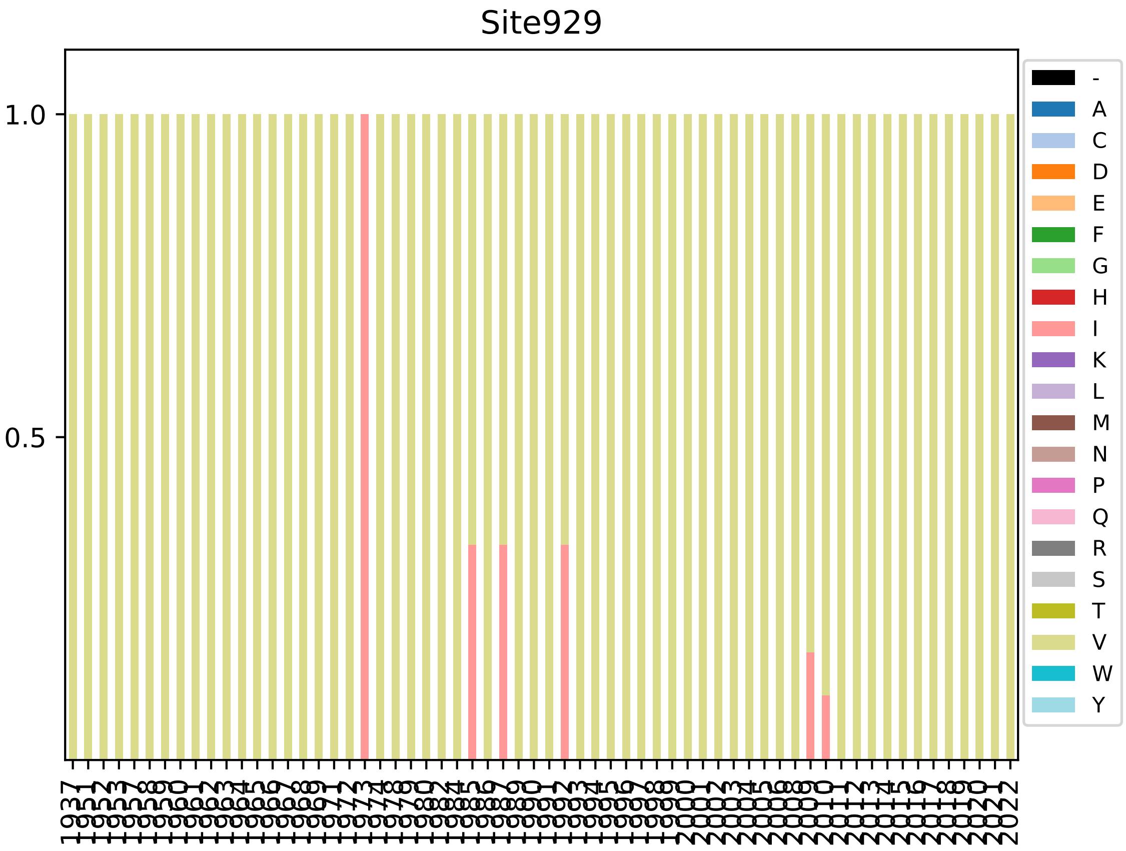
Site929
1.0
0.5
-
A
C
D
E
F
G
H
I
K
L
M
N
P
Q
R
S
T
V
W
Y

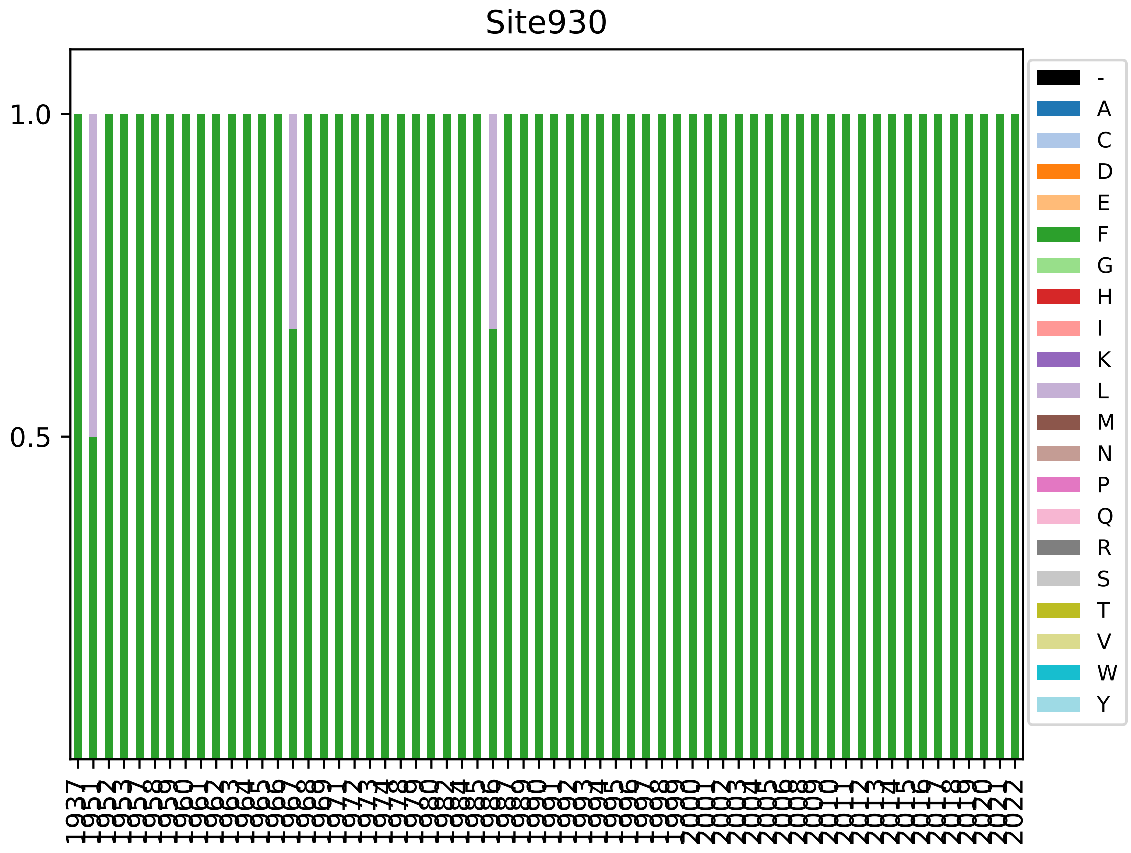
Site930
1.0
0.5
-
A
C
D
E
F
G
H
I
K
L
M
N
P
Q
R
S
T
V
W
Y

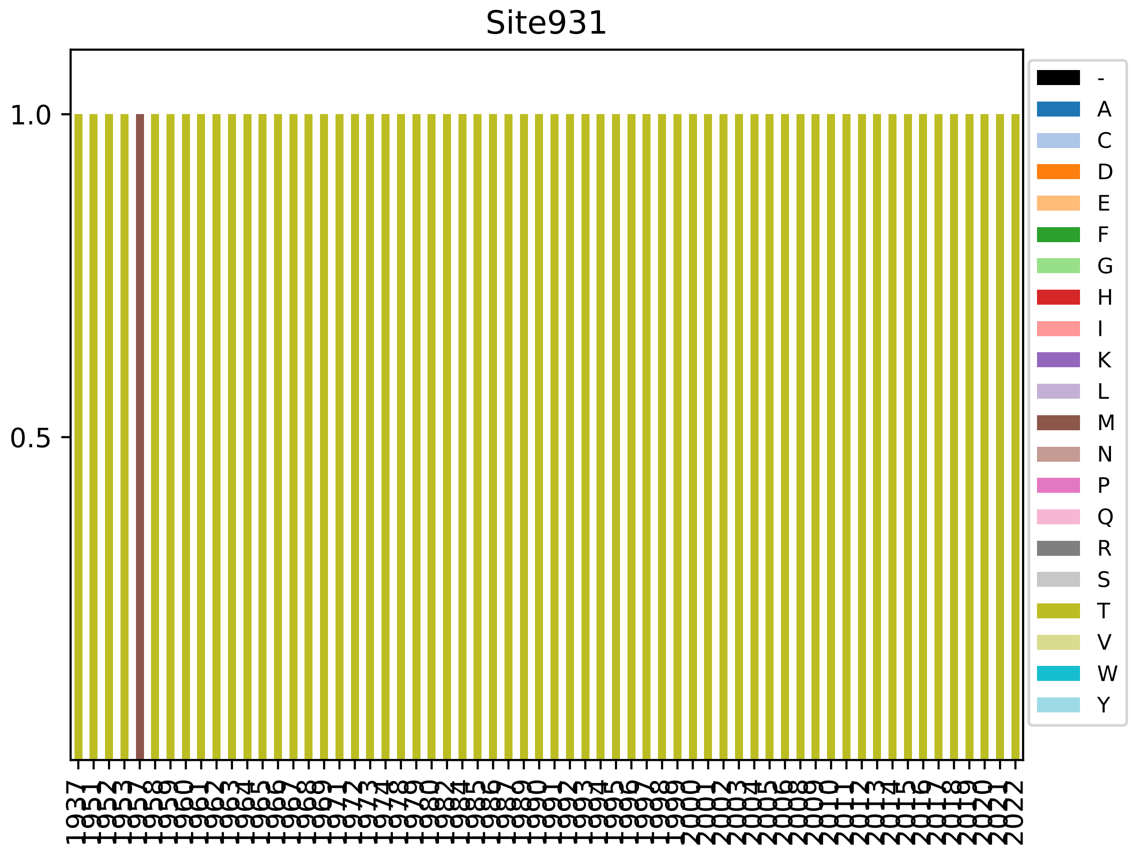

Site931
1.0
0.5
-
A
C
D
E
F
G
H
I
K
L
M
N
P
Q
R
S
T
V
W
Y

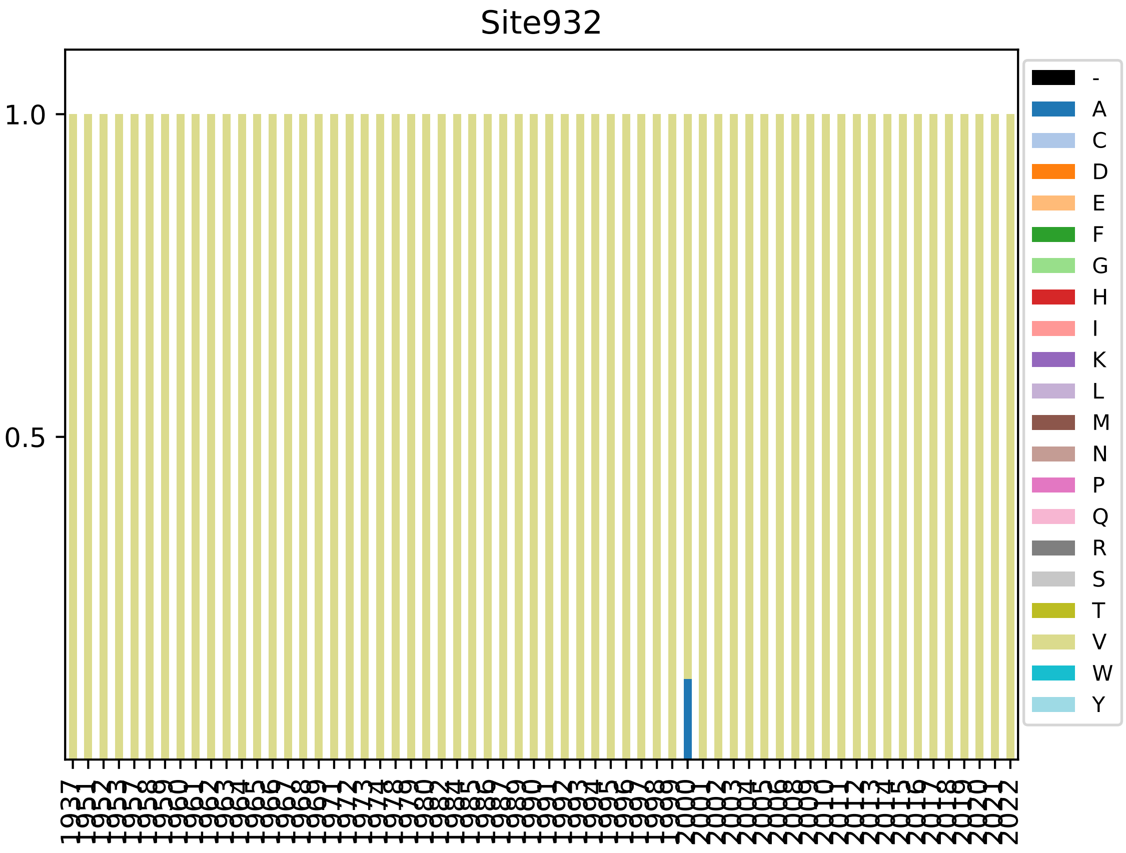
Site932
1.0
0.5
-
A
C
D
E
F
G
H
I
K
L
M
N
P
Q
R
S
T
V
W
Y

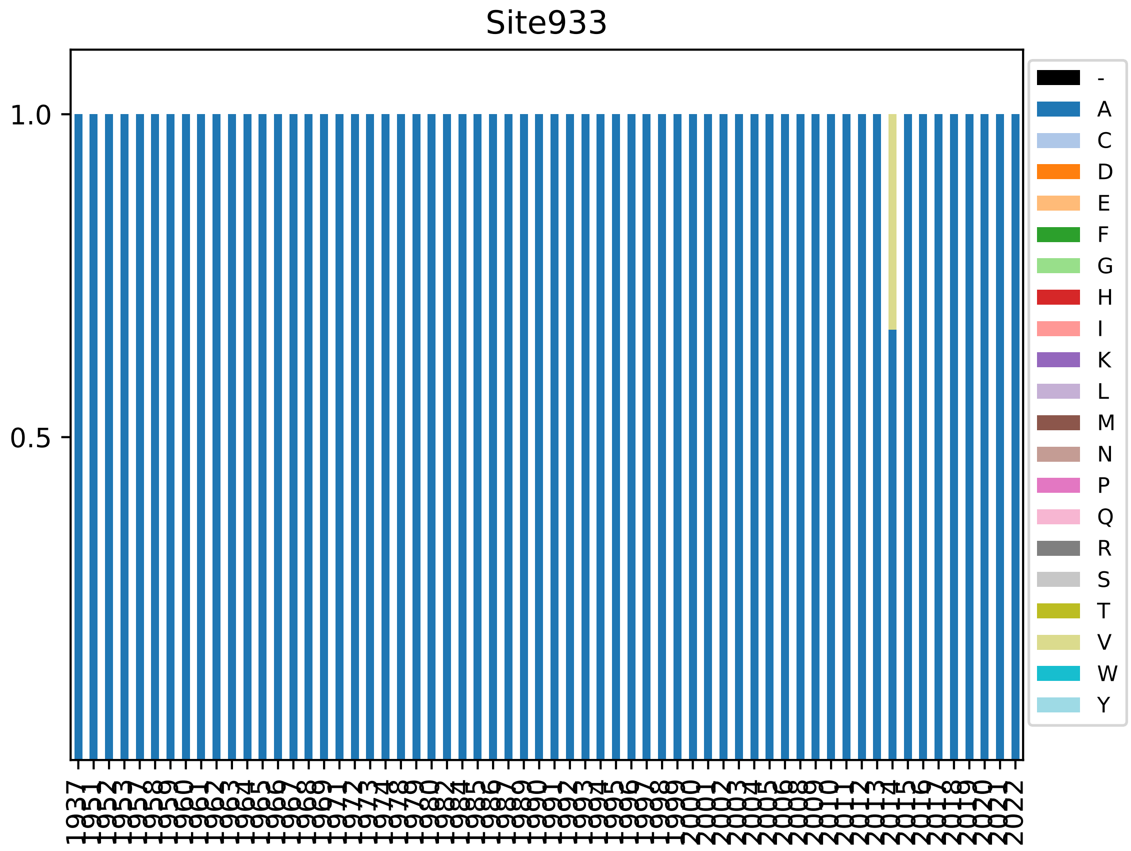

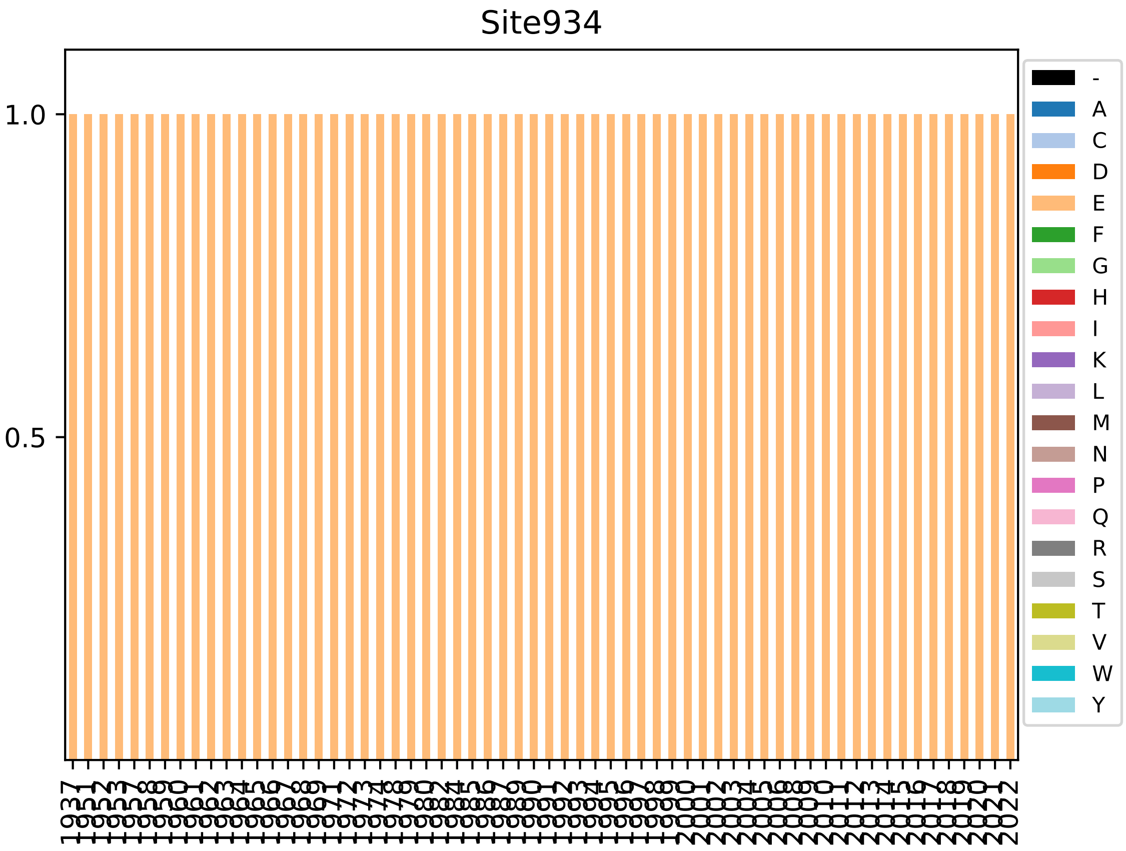
Site934
1.0
0.5
-
A
C
D
E
F
G
H
I
K
L
M
N
P
Q
R
S
T
V
W
Y

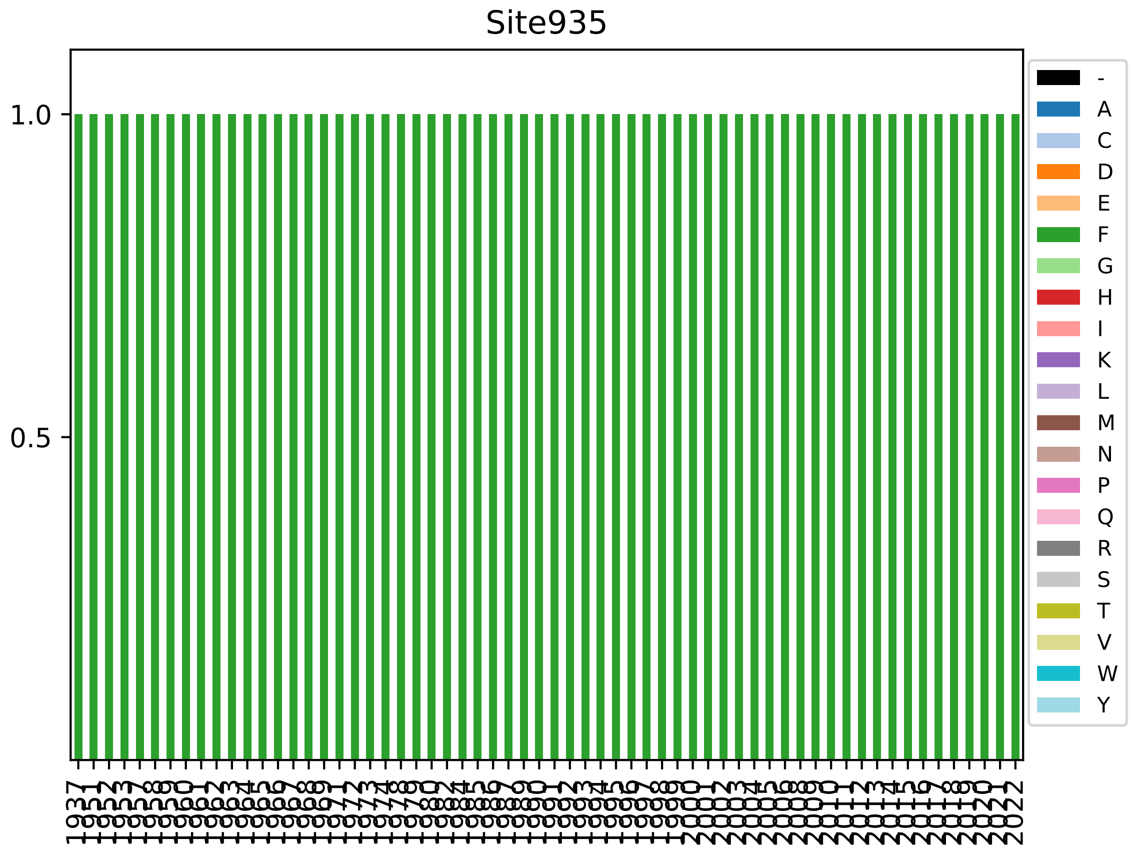
Site935
1.0
0.5
-
A
C
D
E
F
G
H
I
K
L
M
N
P
Q
R
S
T
V
W
Y

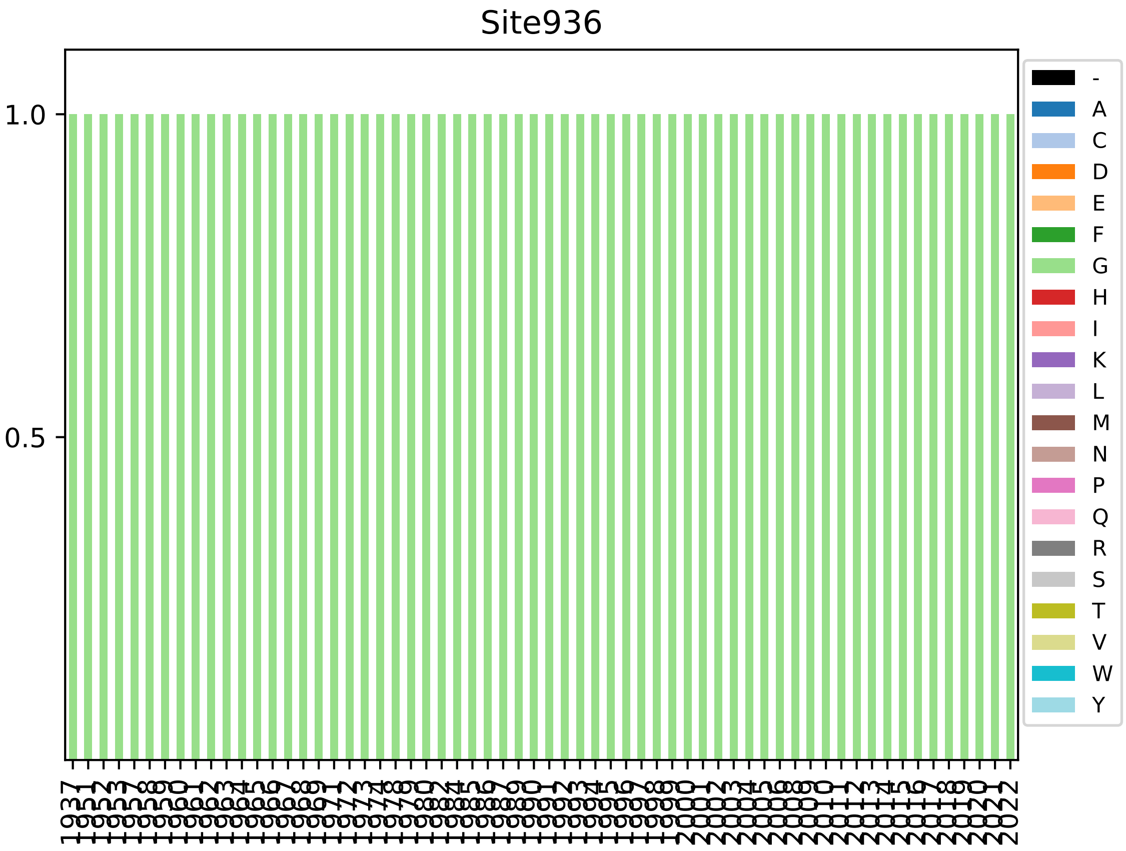
Site936
1.0
0.5
-
A
C
D
E
F
G
H
I
K
L
M
N
P
Q
R
S
T
V
W
Y

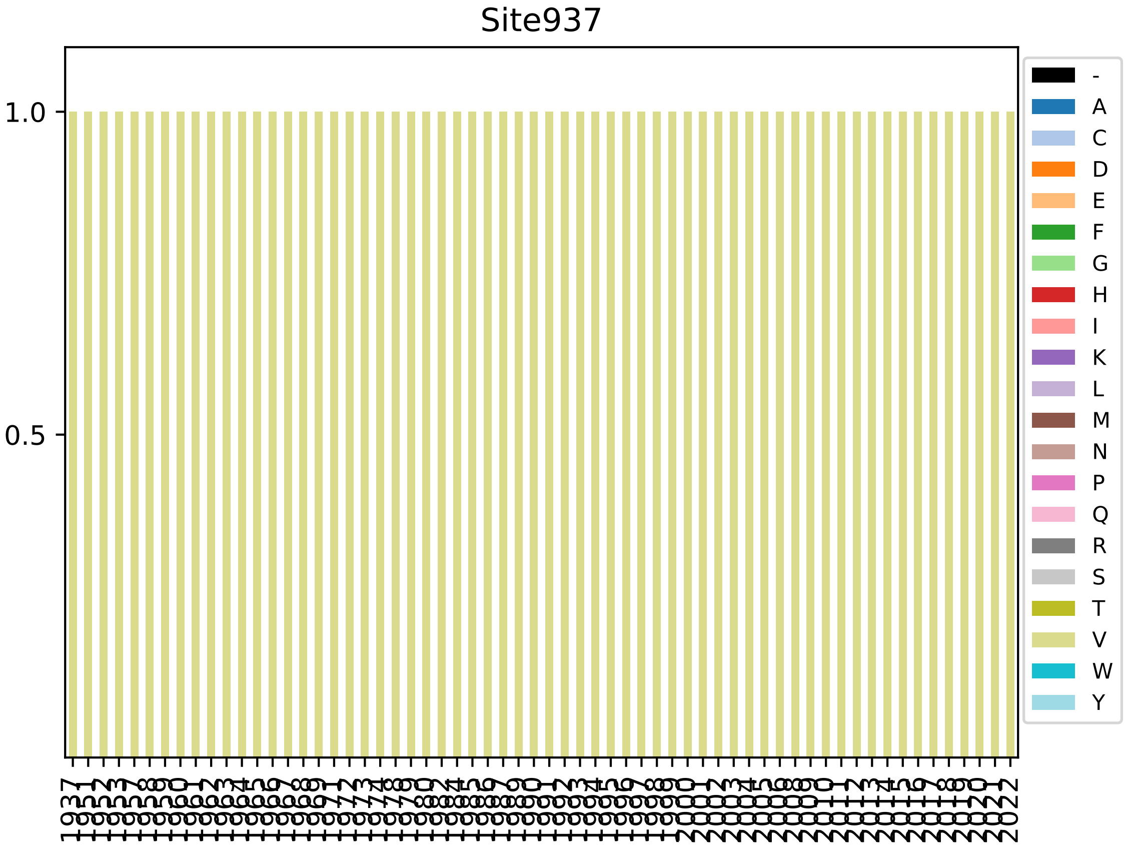
Site937
1.0
0.5
-
A
C
D
E
F
G
H
I
K
L
M
N
P
Q
R
S
T
V
W
Y

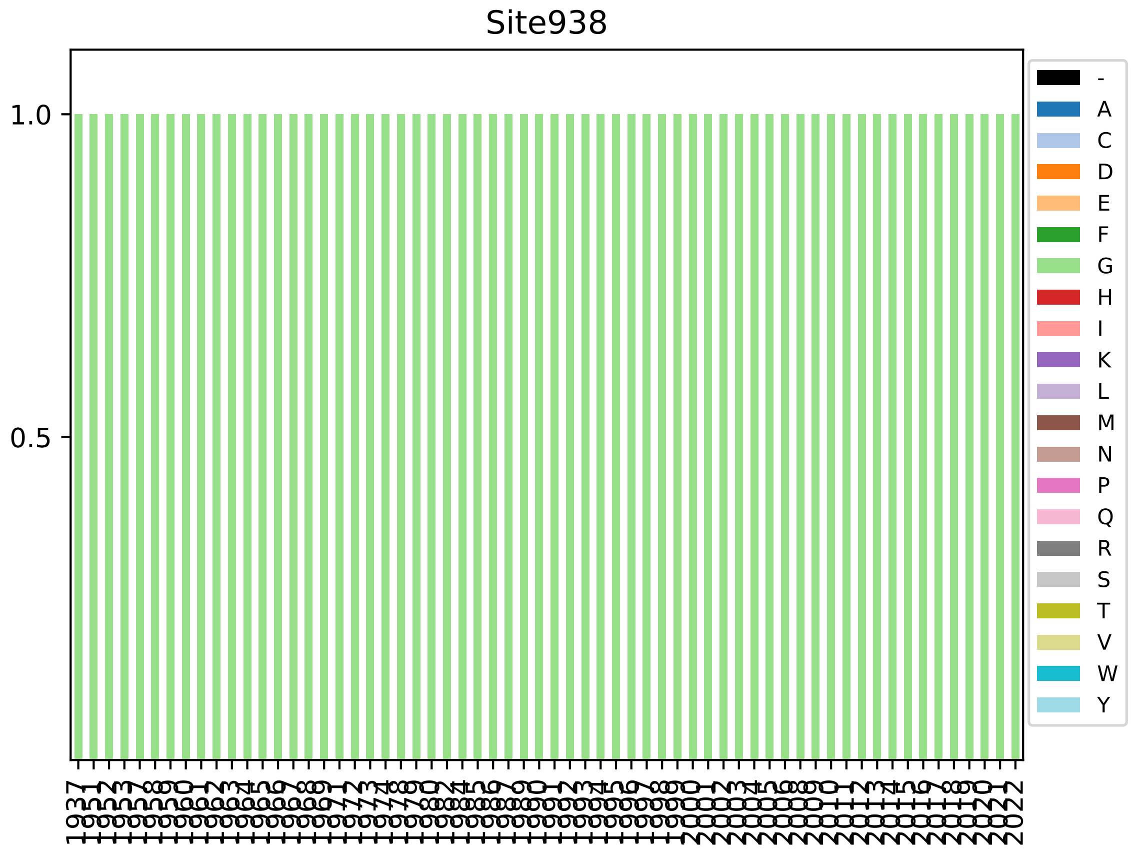
Site938
1.0
0.5
-
A
C
D
E
F
G
H
I
K
L
M
N
P
Q
R
S
T
V
W
Y

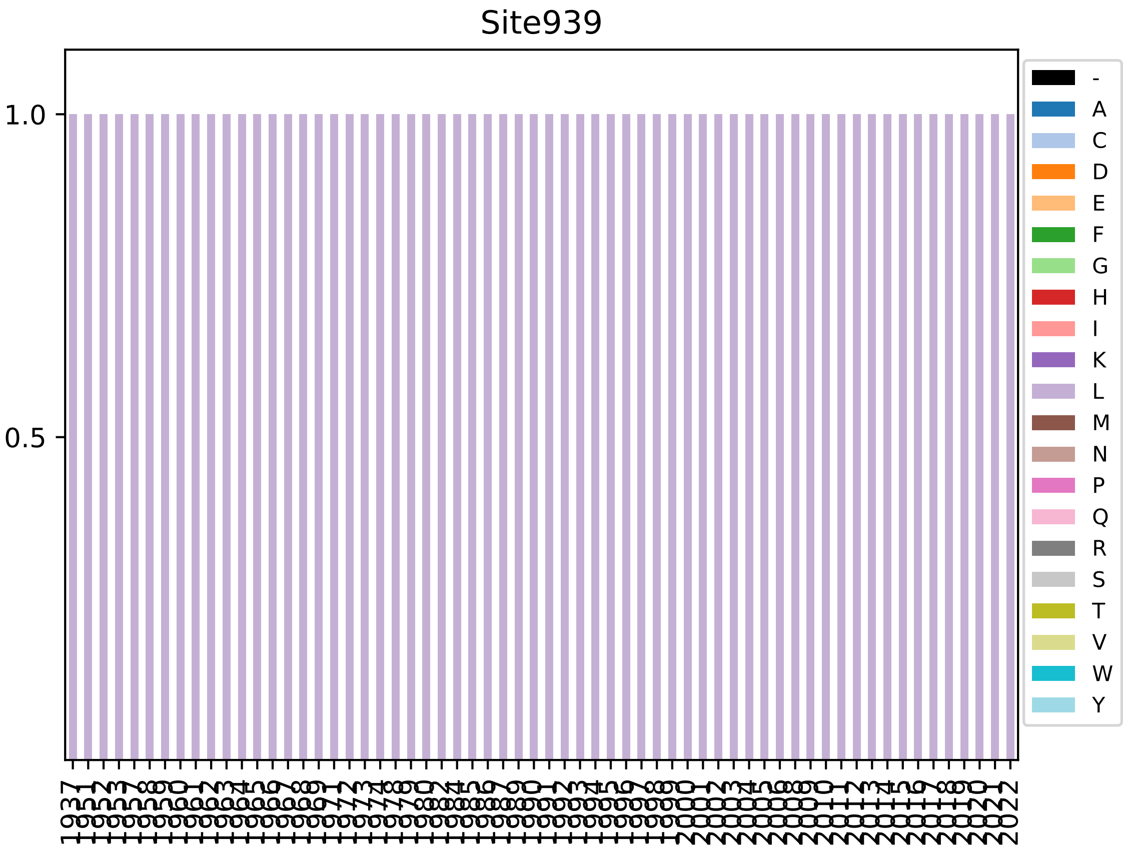
Site939
1.0
0.5
-
A
C
D
E
F
G
H
I
K
L
M
N
P
Q
R
S
T
V
W
Y

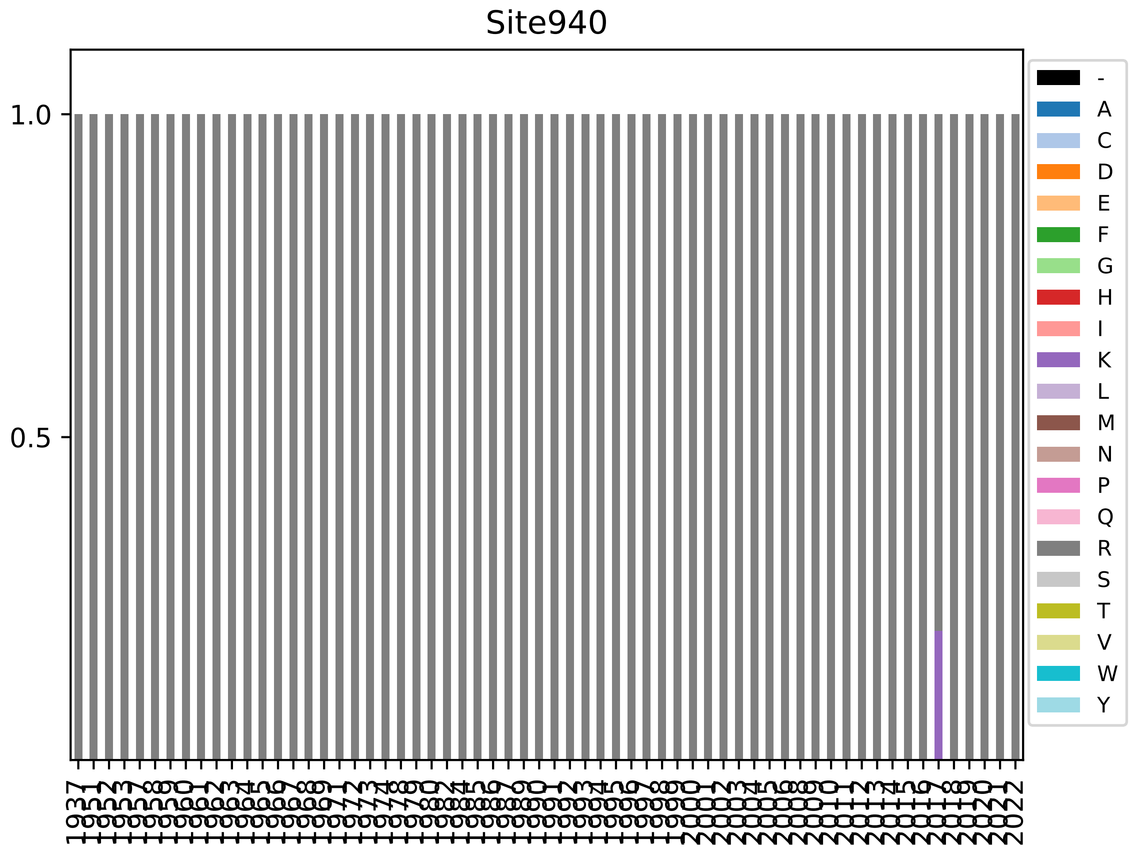
Site940
1.0
0.5
-
A
C
D
E
F
G
H
I
K
L
M
N
P
Q
R
S
T
V
W
Y

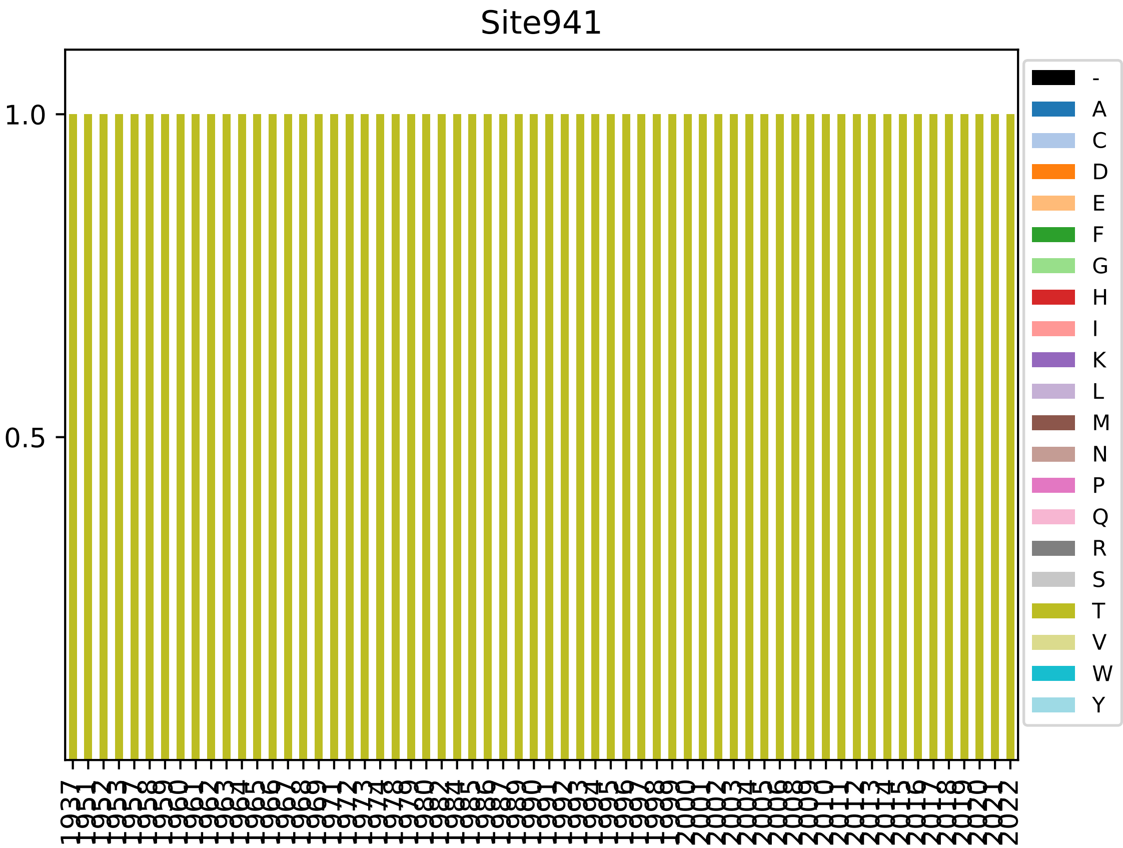
Site941
1.0
0.5
-
A
C
D
E
F
G
H
I
K
L
M
N
P
Q
R
S
T
V
W
Y

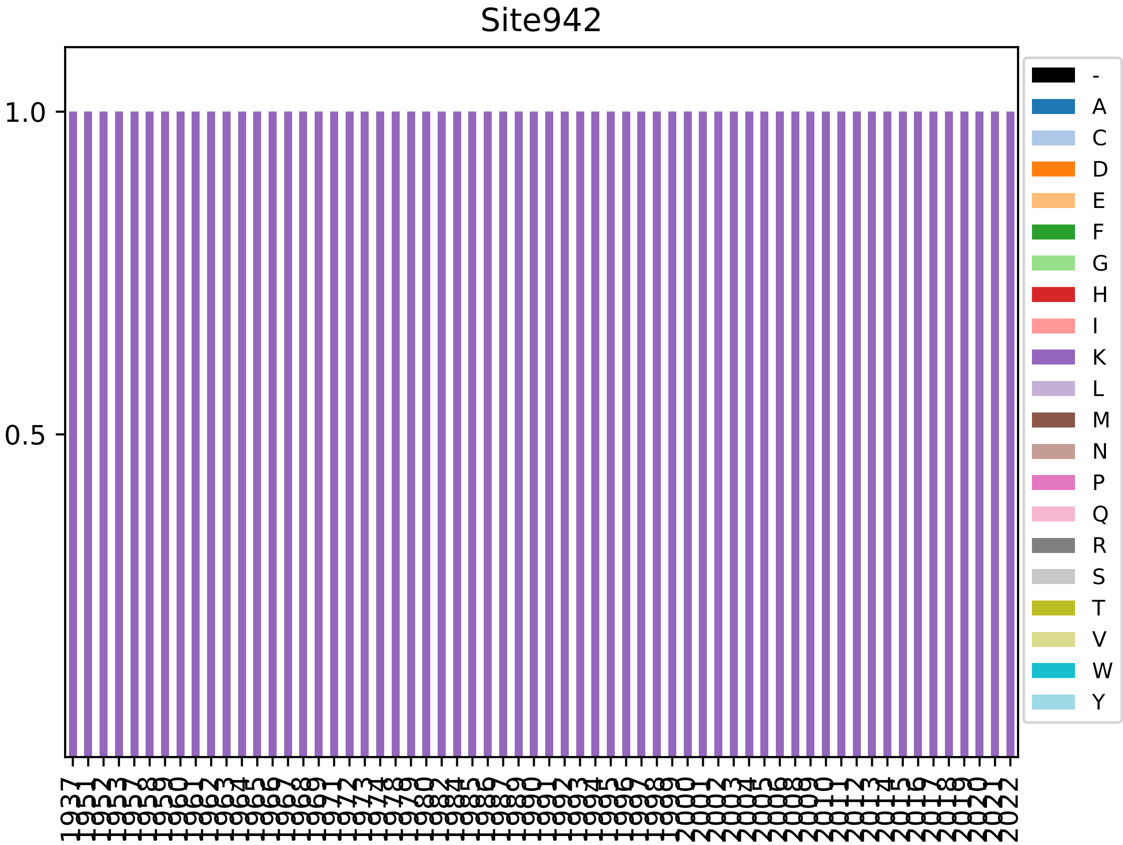
Site942
1.0
0.5
-
A
C
D
E
F
G
H
I
K
L
M
N
P
Q
R
S
T
V
W
Y

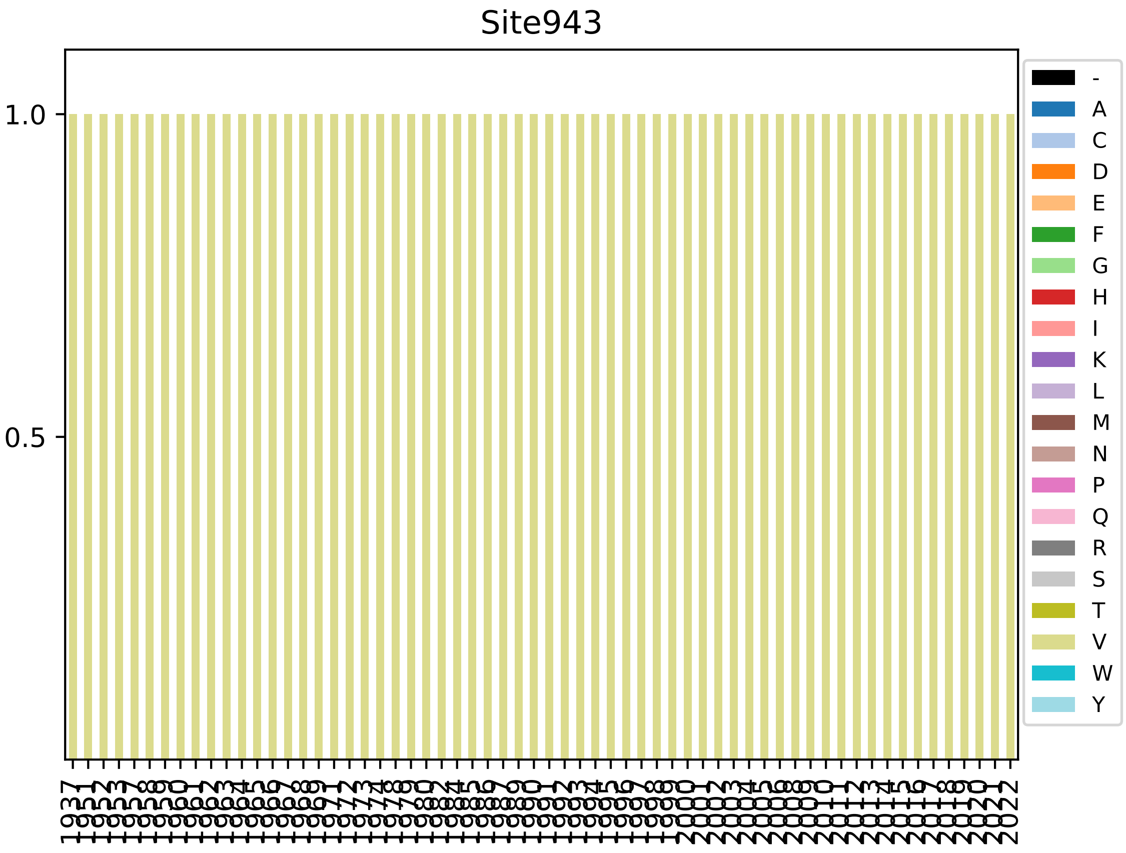
Site943
1.0
0.5
-
A
C
D
E
F
G
H
I
K
L
M
N
P
Q
R
S
T
V
W
Y

Site944

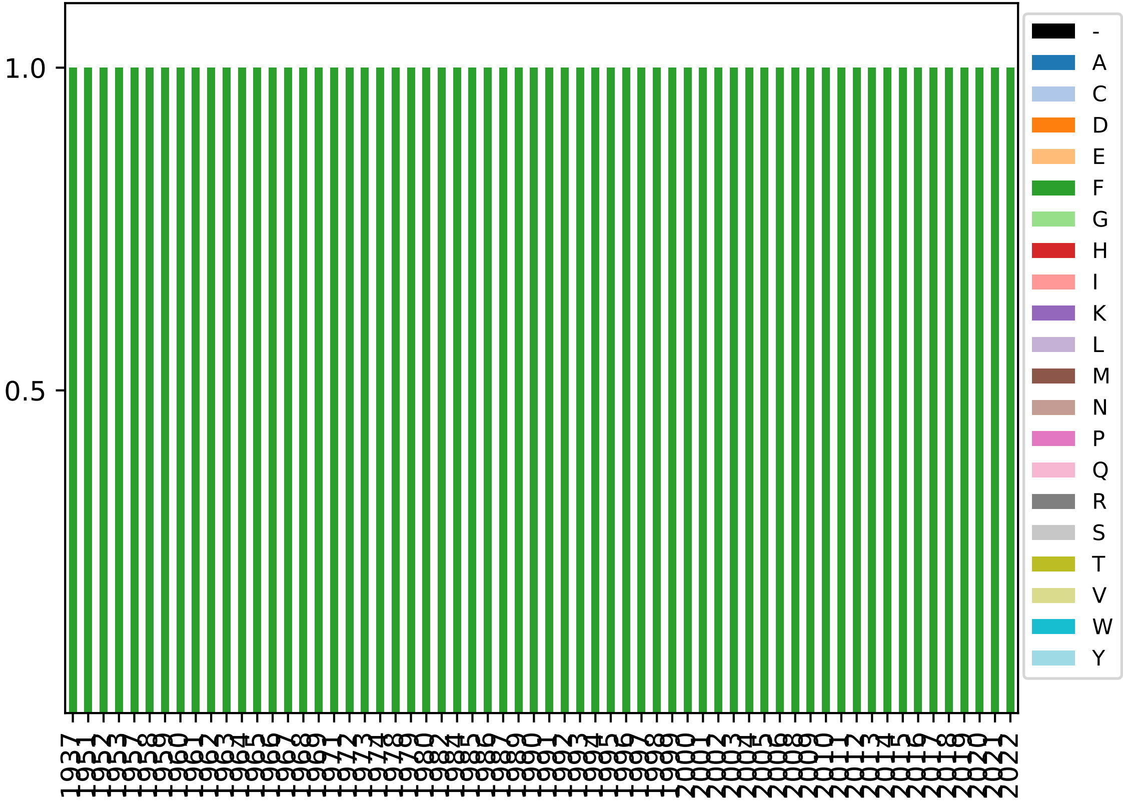

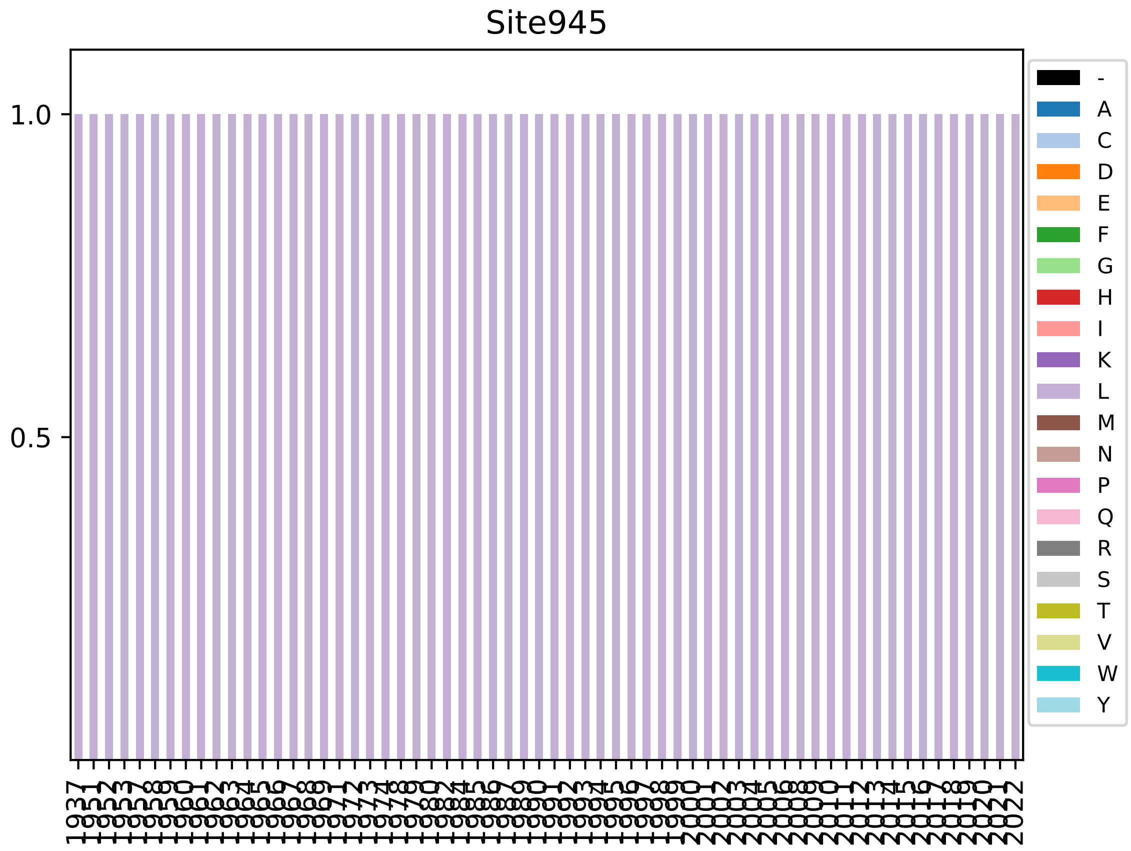
Site945
1.0
0.5
-
A
C
D
E
F
G
H
I
K
L
M
N
P
Q
R
S
T
V
W
Y

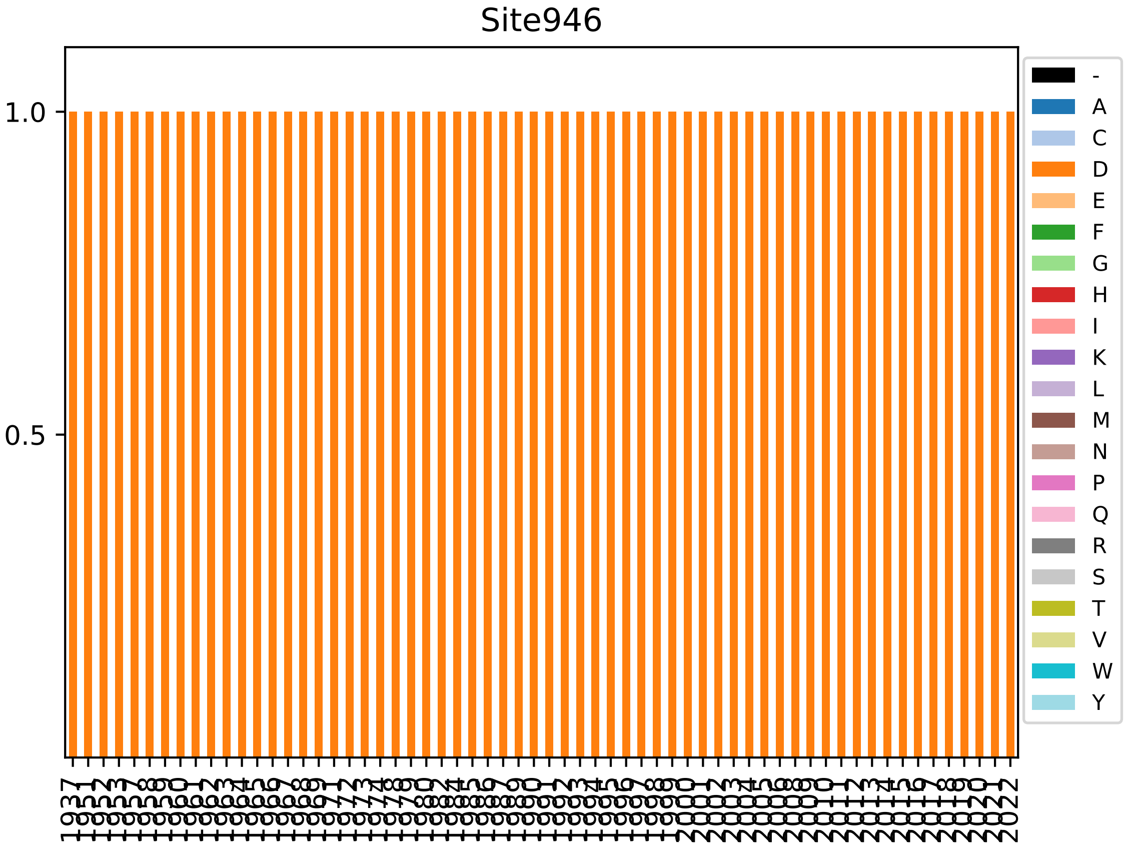
Site946
1.0
0.5
-
A
C
D
E
F
G
H
I
K
L
M
N
P
Q
R
S
T
V
W
Y

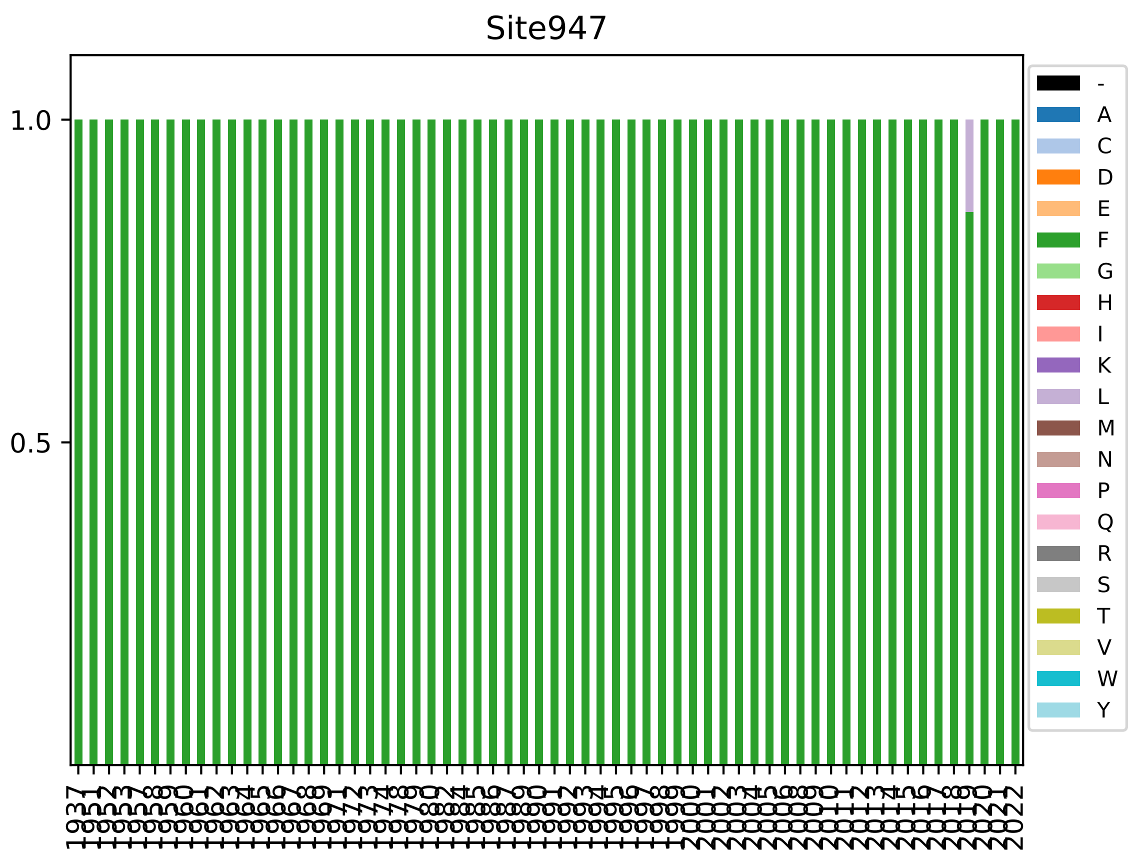
Site947
1.0
0.5
-
A
C
D
E
F
G
H
I
K
L
M
N
P
Q
R
S
T
V
W
Y

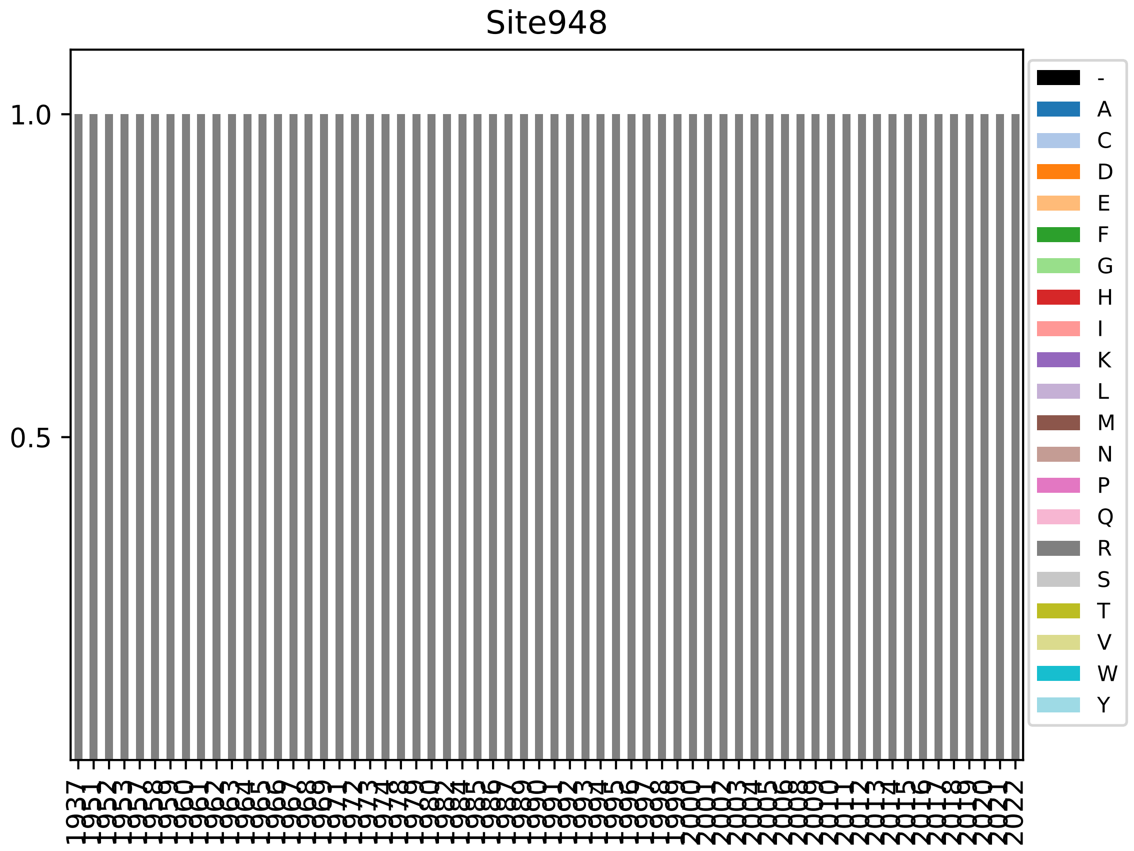
Site948
1.0
0.5
-
A
C
D
E
F
G
H
I
K
L
M
N
P
Q
R
S
T
V
W
Y

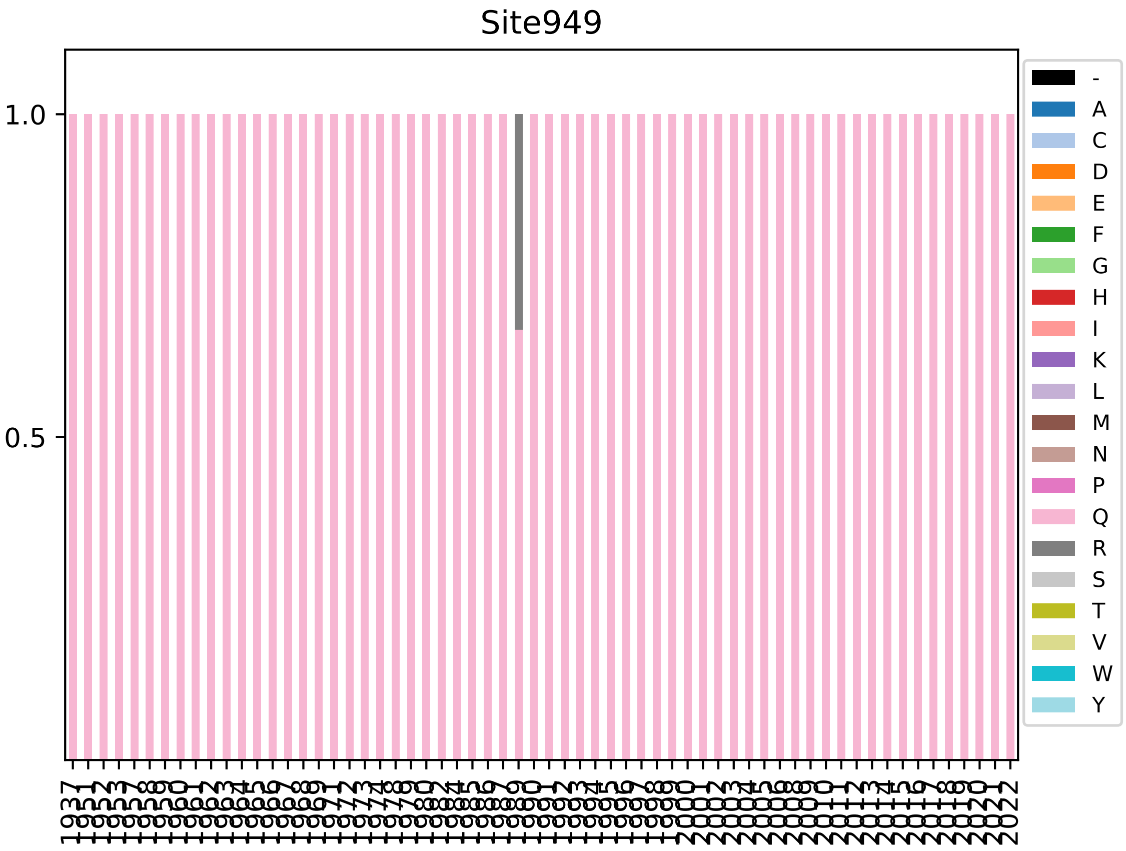
Site949
1.0
0.5
-
A
C
D
E
F
G
H
I
K
L
M
N
P
Q
R
S
T
V
W
Y

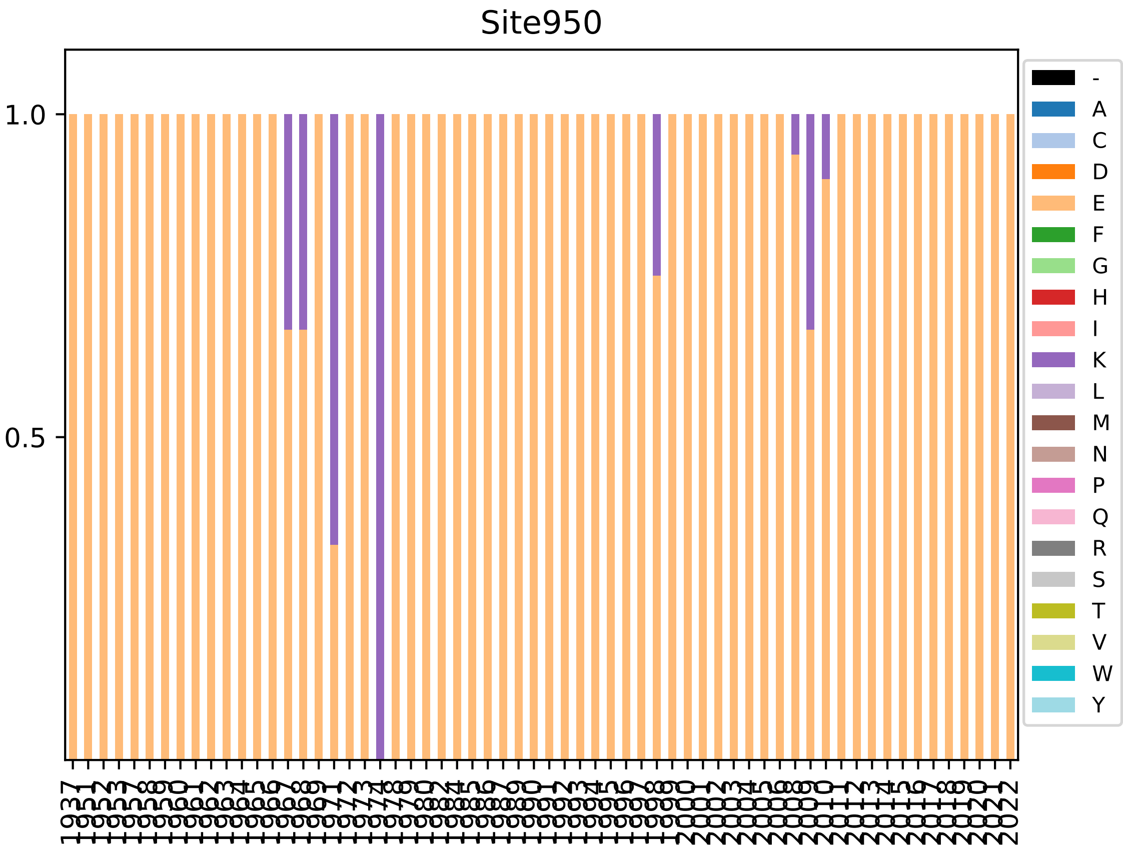
Site950
1.0
0.5
-
A
C
D
E
F
G
H
I
K
L
M
N
P
Q
R
S
T
V
W
Y

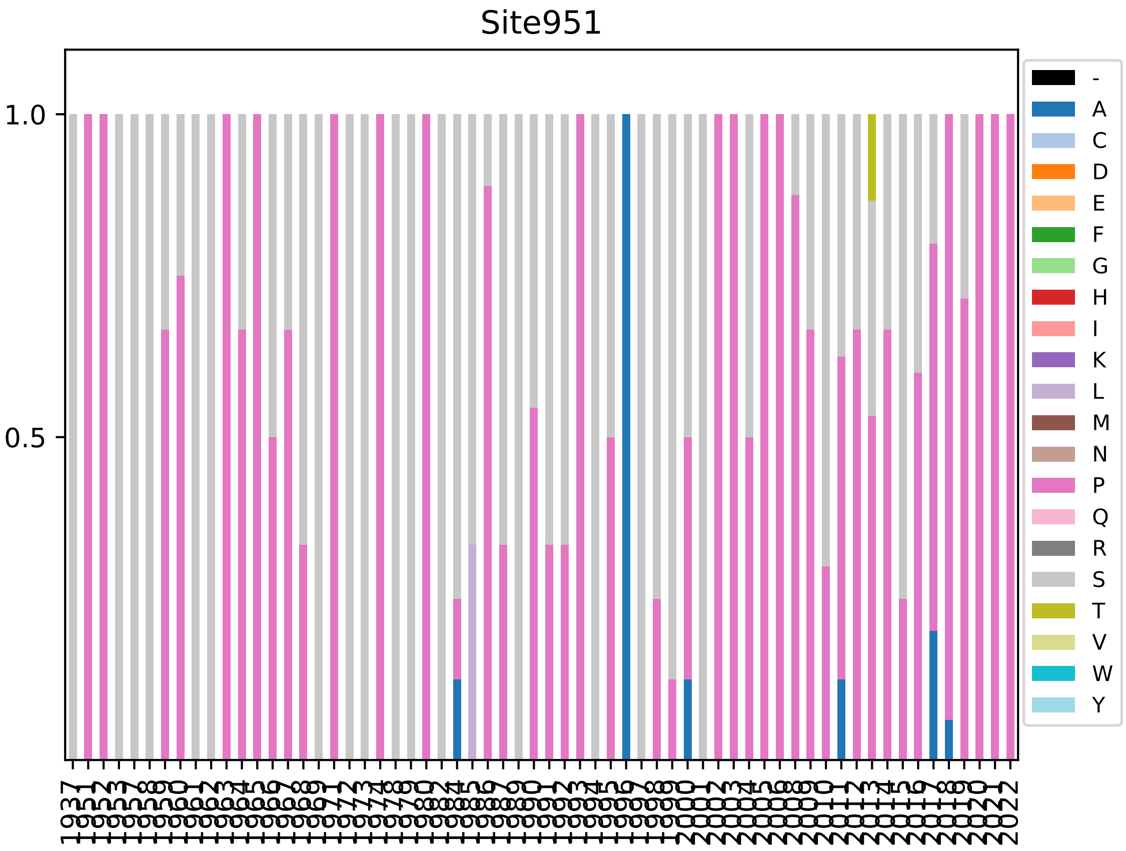
Site951
1.0
0.5
-
A
C
D
E
F
G
H
I
K
L
M
N
P
Q
R
S
T
V
W
Y

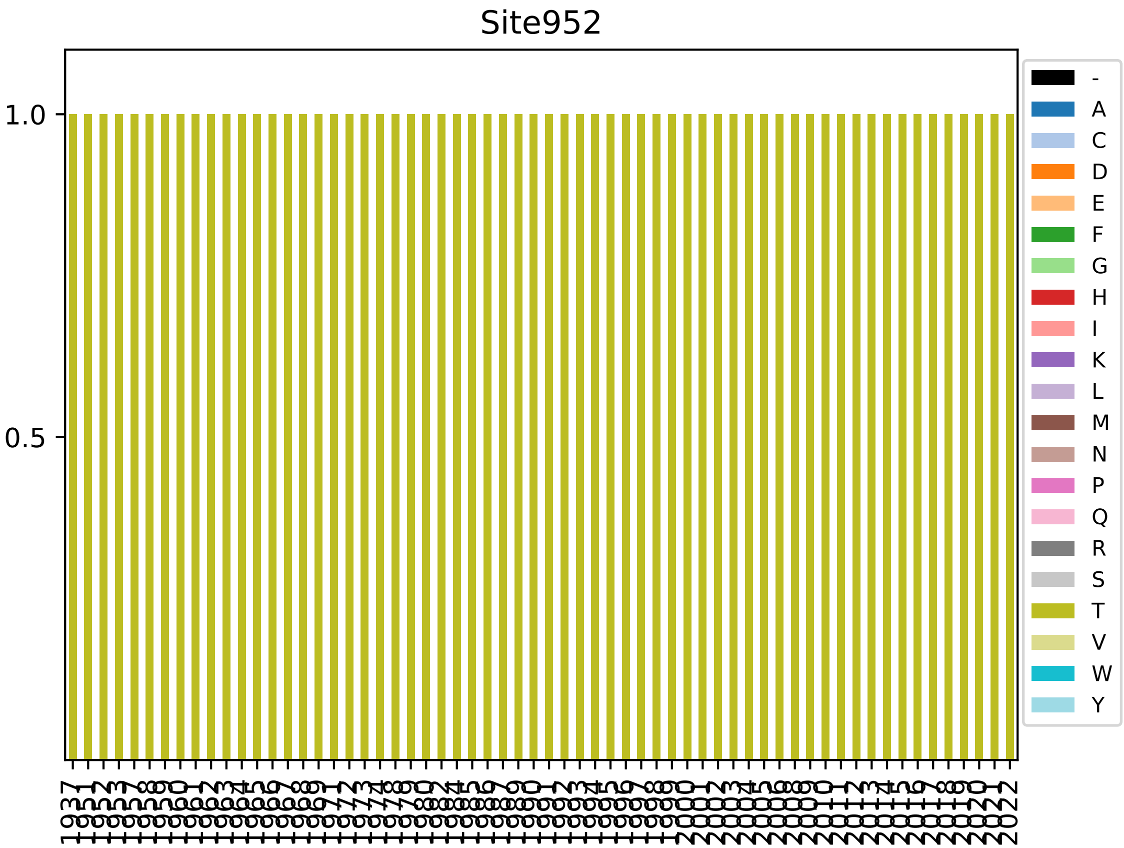
Site952
1.0
0.5
-
A
C
D
E
F
G
H
I
K
L
M
N
P
Q
R
S
T
V
W
Y

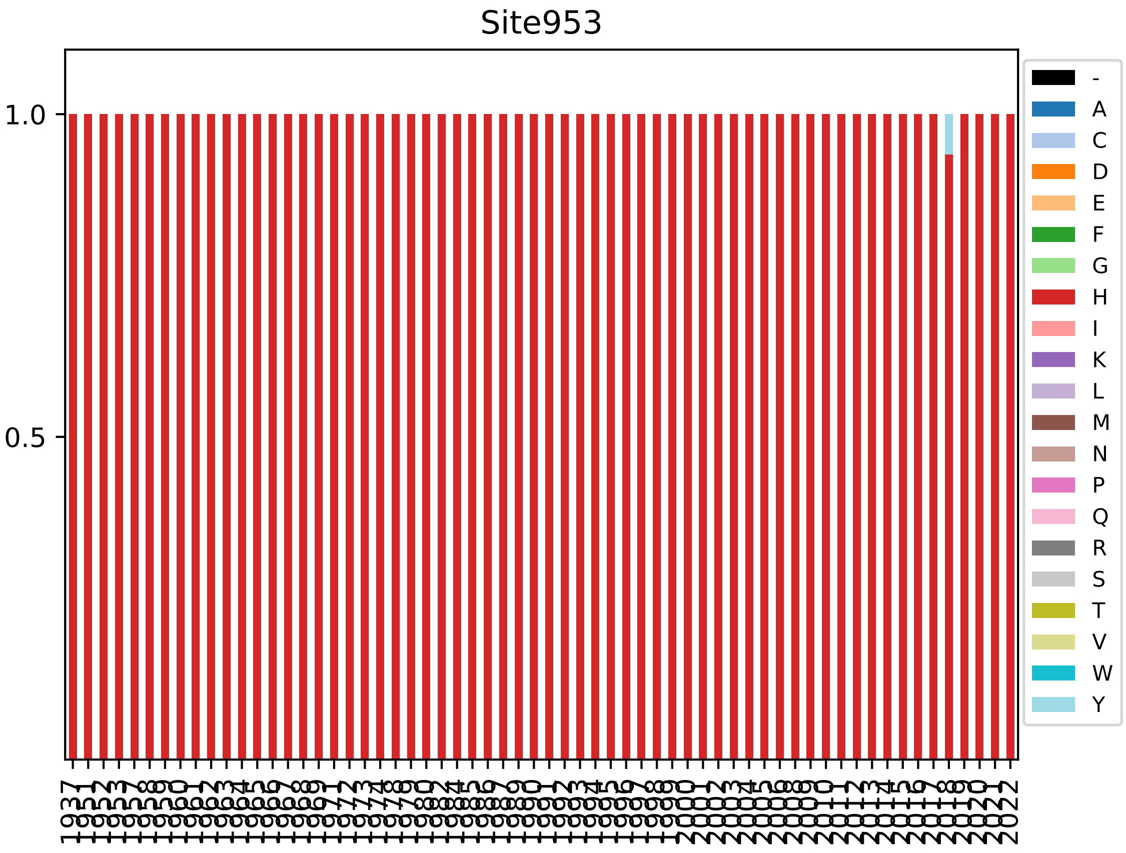
Site953
1.0
0.5
-
A
C
D
E
F
G
H
I
K
L
M
N
P
Q
R
S
T
V
W
Y

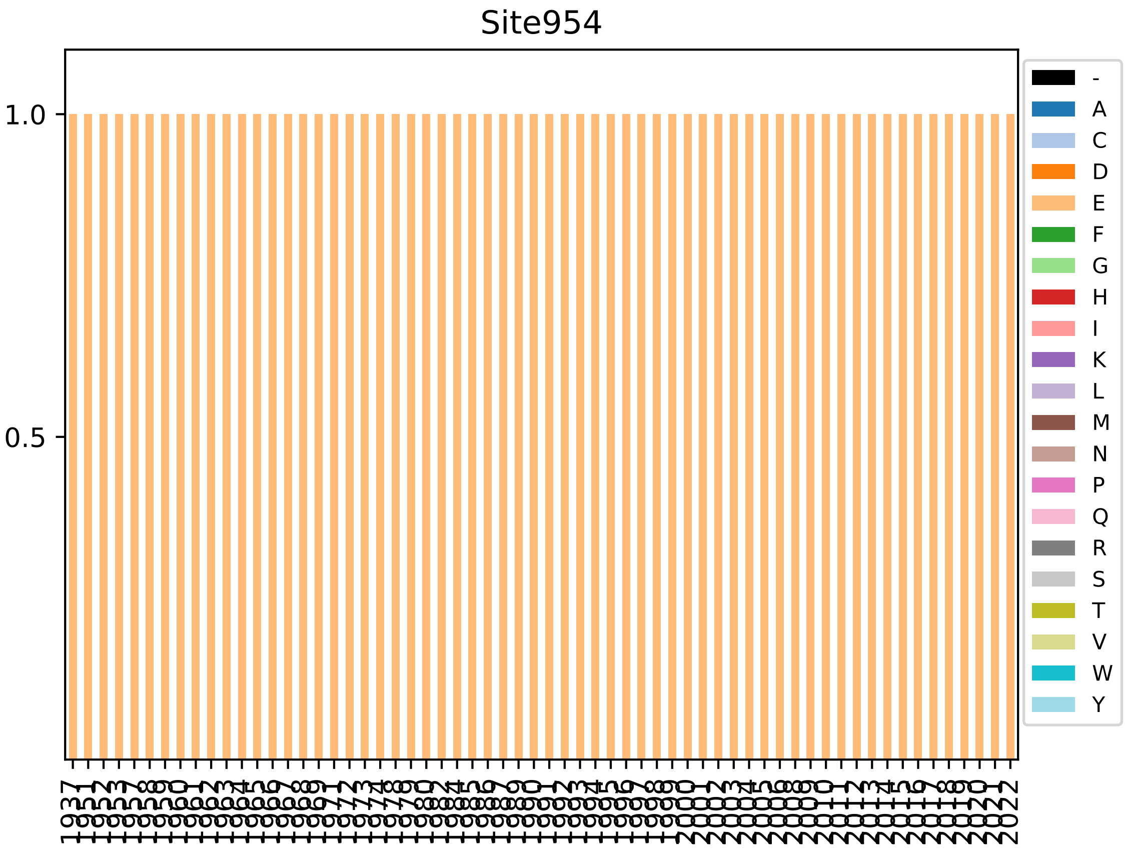
Site954
1.0
0.5
-
A
C
D
E
F
G
H
I
K
L
M
N
P
Q
R
S
T
V
W
Y

Site955

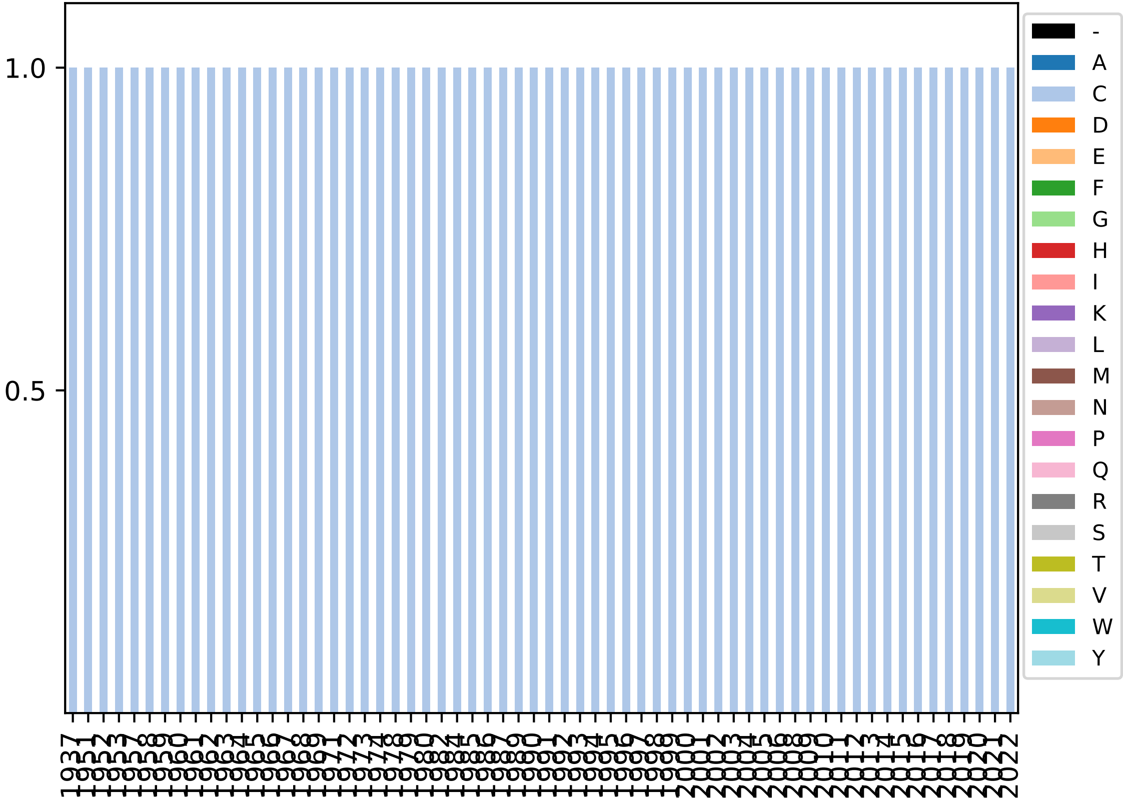

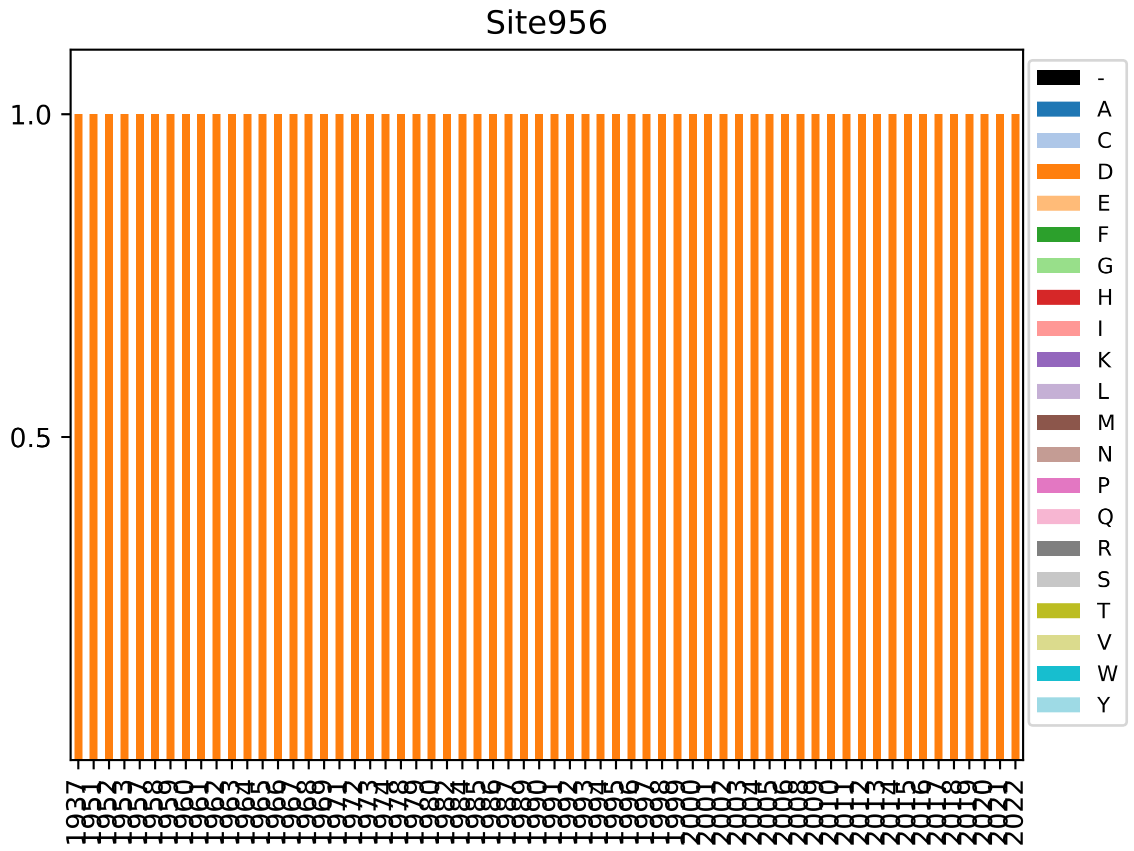
Site956
1.0
0.5
-
A
C
D
E
F
G
H
I
K
L
M
N
P
Q
R
S
T
V
W
Y

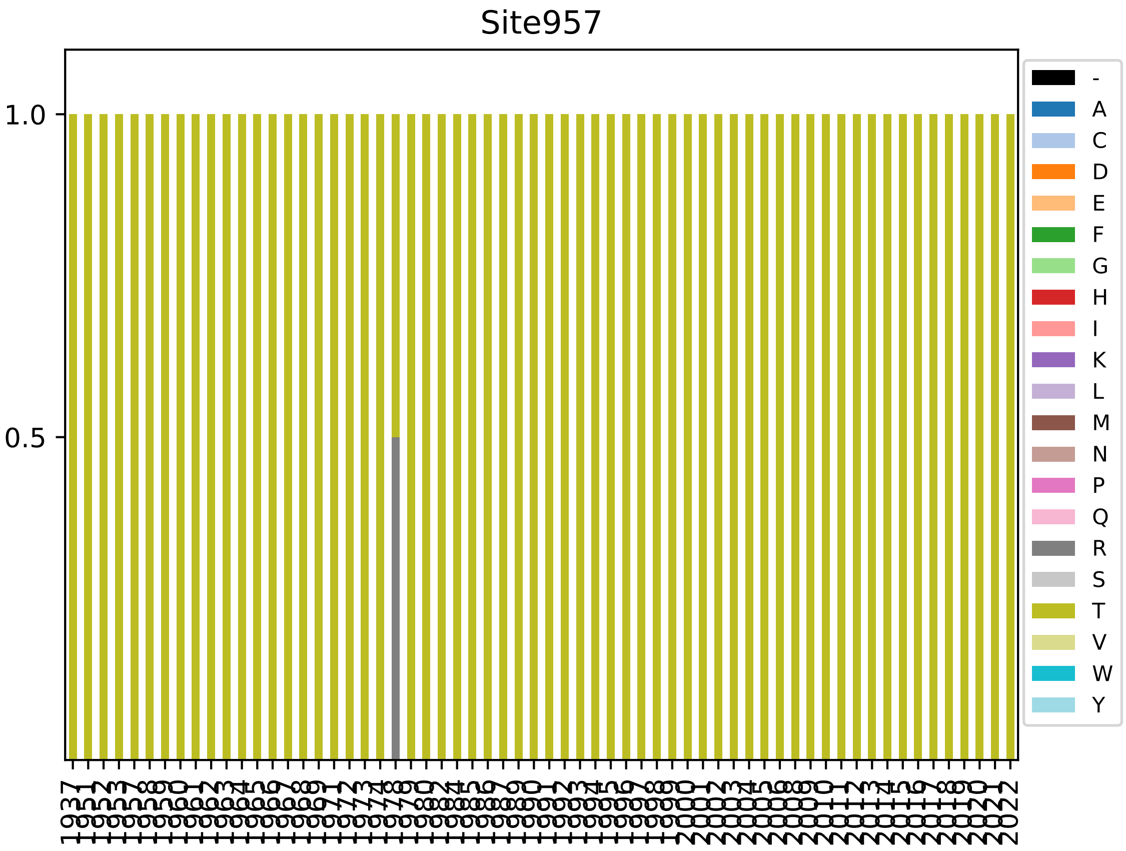
Site957
1.0
0.5
-
A
C
D
E
F
G
H
I
K
L
M
N
P
Q
R
S
T
V
W
Y

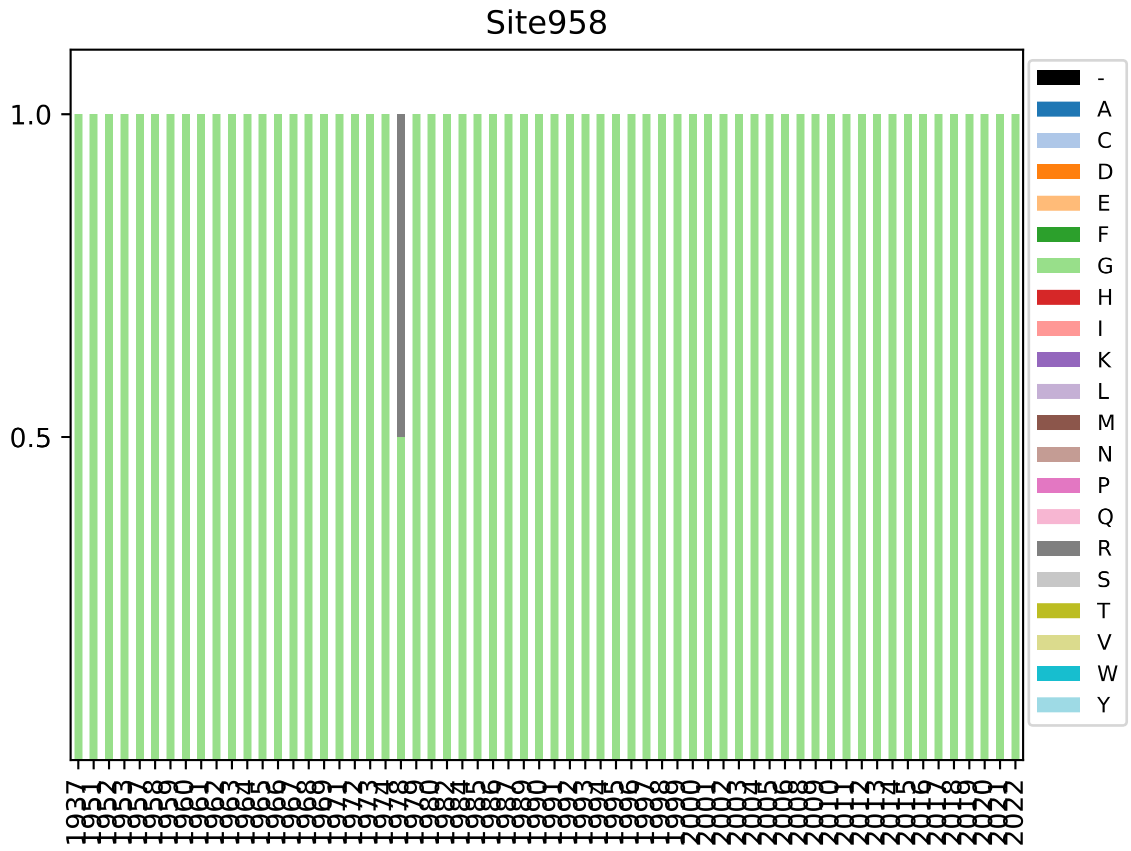
Site958
1.0
0.5
-
A
C
D
E
F
G
H
I
K
L
M
N
P
Q
R
S
T
V
W
Y

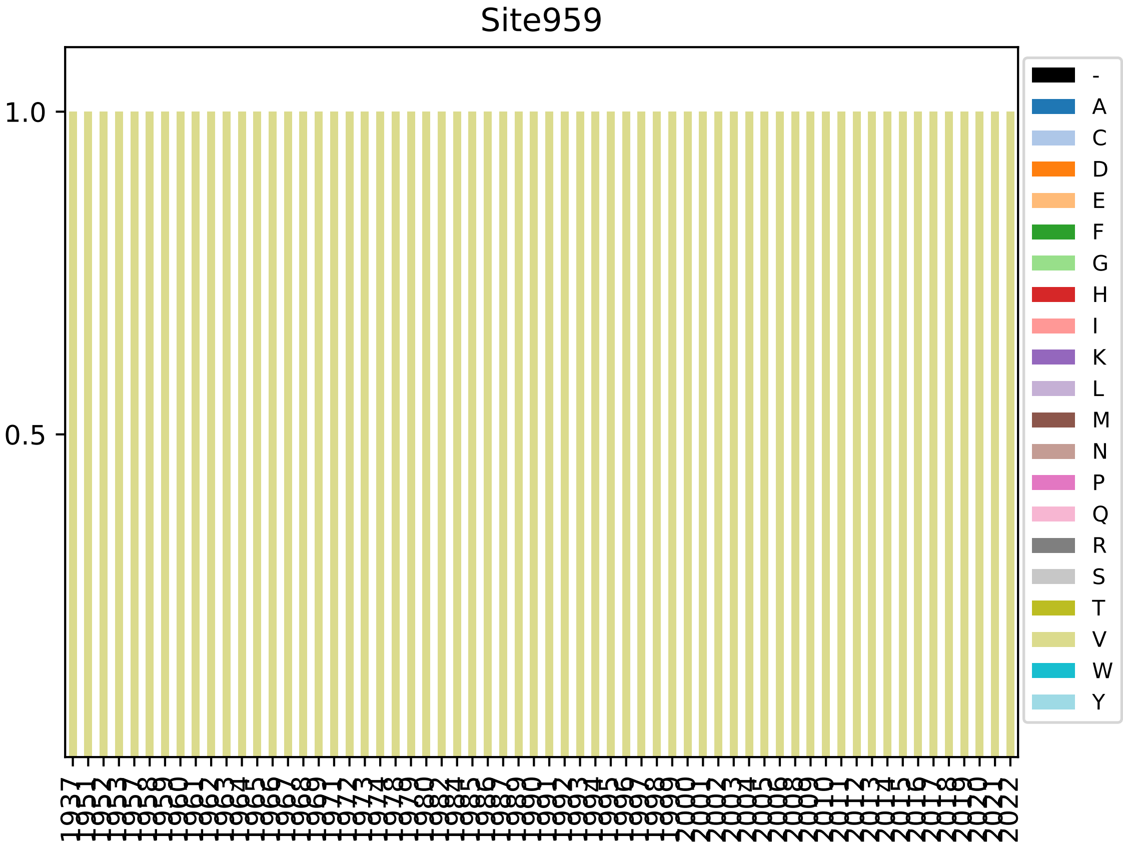
Site959
1.0
0.5
-
A
C
D
E
F
G
H
I
K
L
M
N
P
Q
R
S
T
V
W
Y

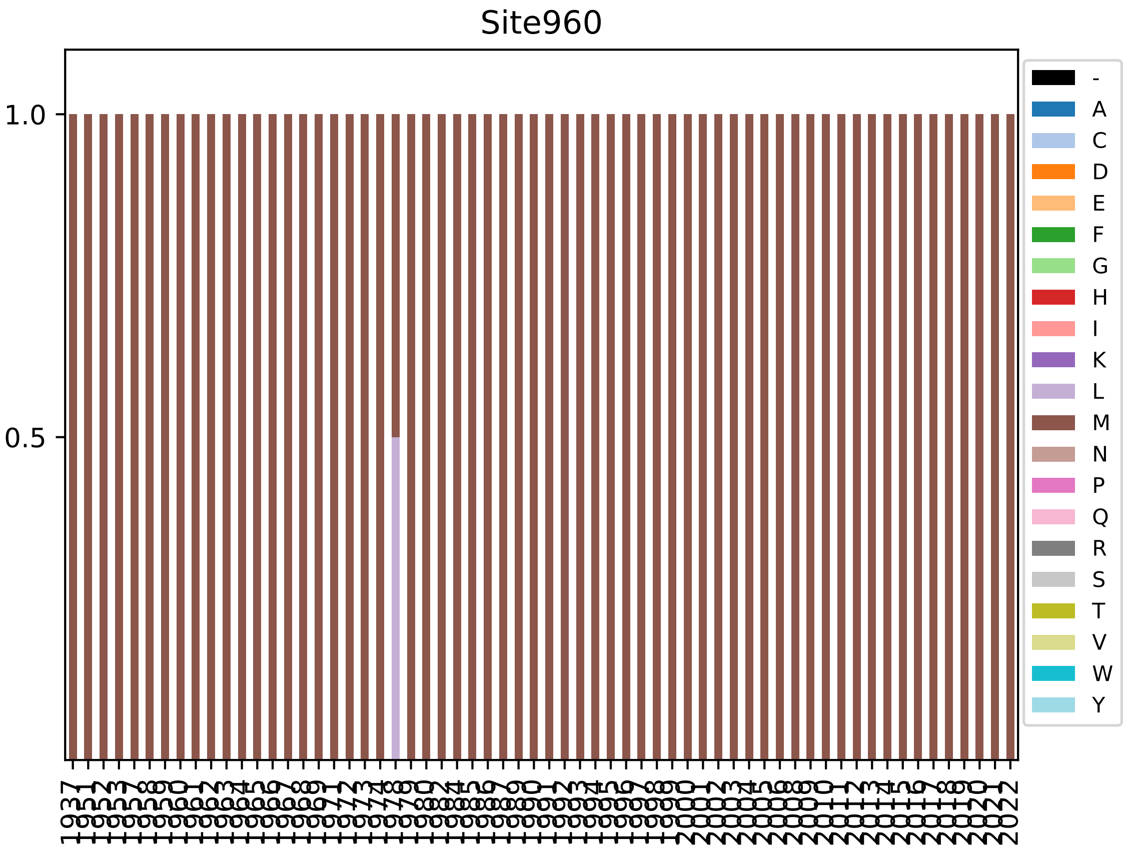
Site960
1.0
0.5
-
A
C
D
E
F
G
H
I
K
L
M
N
P
Q
R
S
T
V
W
Y

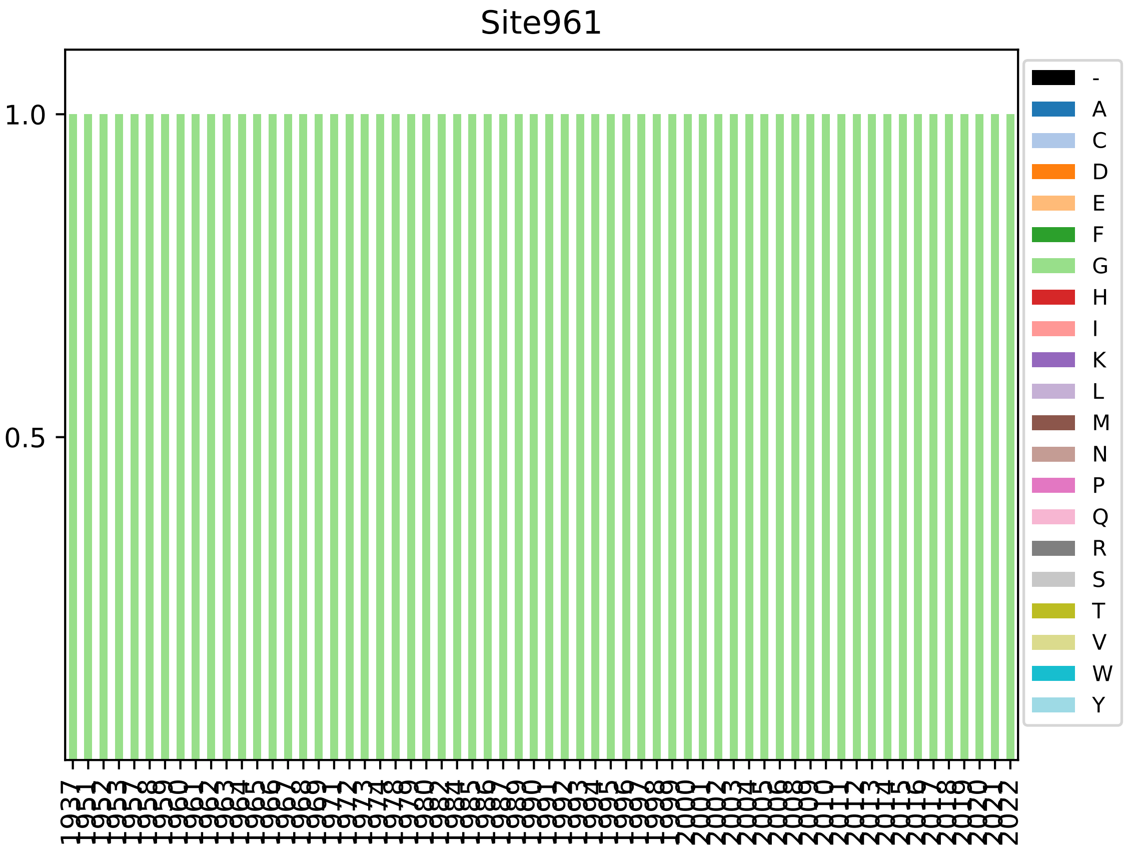

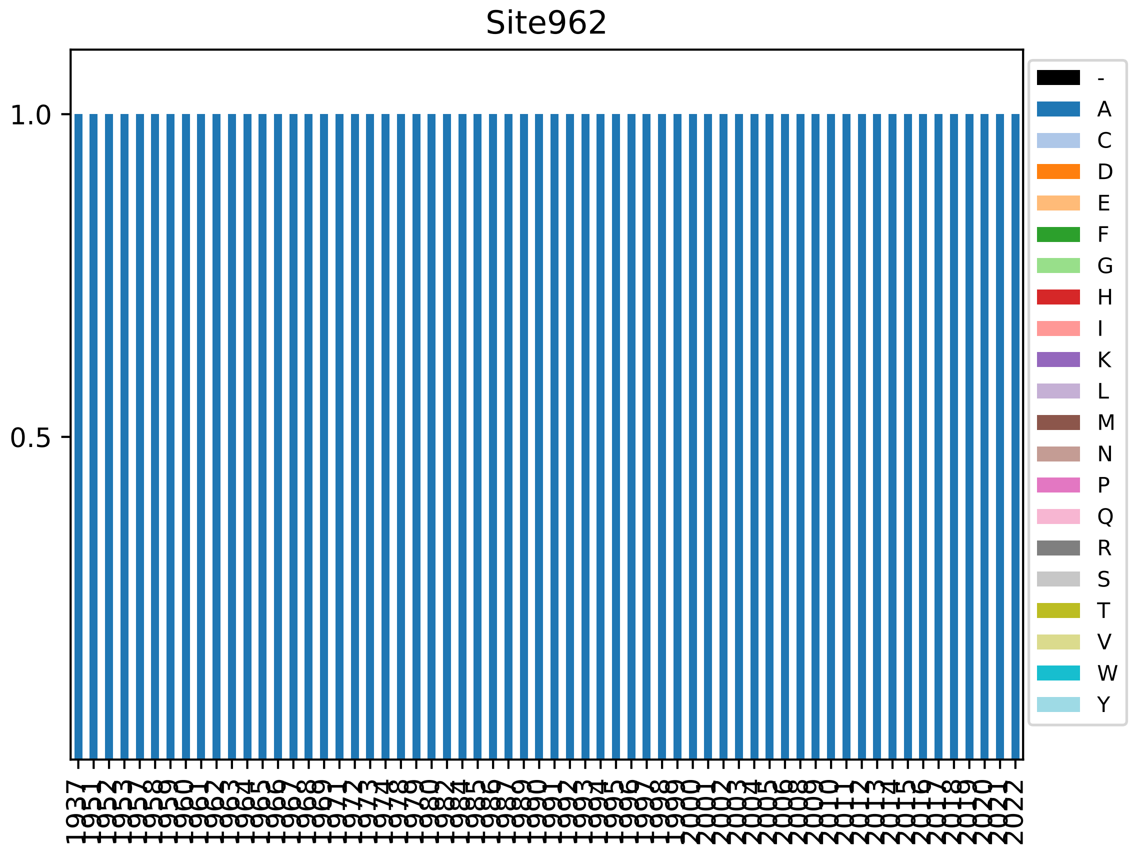
Site962
1.0
0.5
-
A
C
D
E
F
G
H
I
K
L
M
N
P
Q
R
S
T
V
W
Y

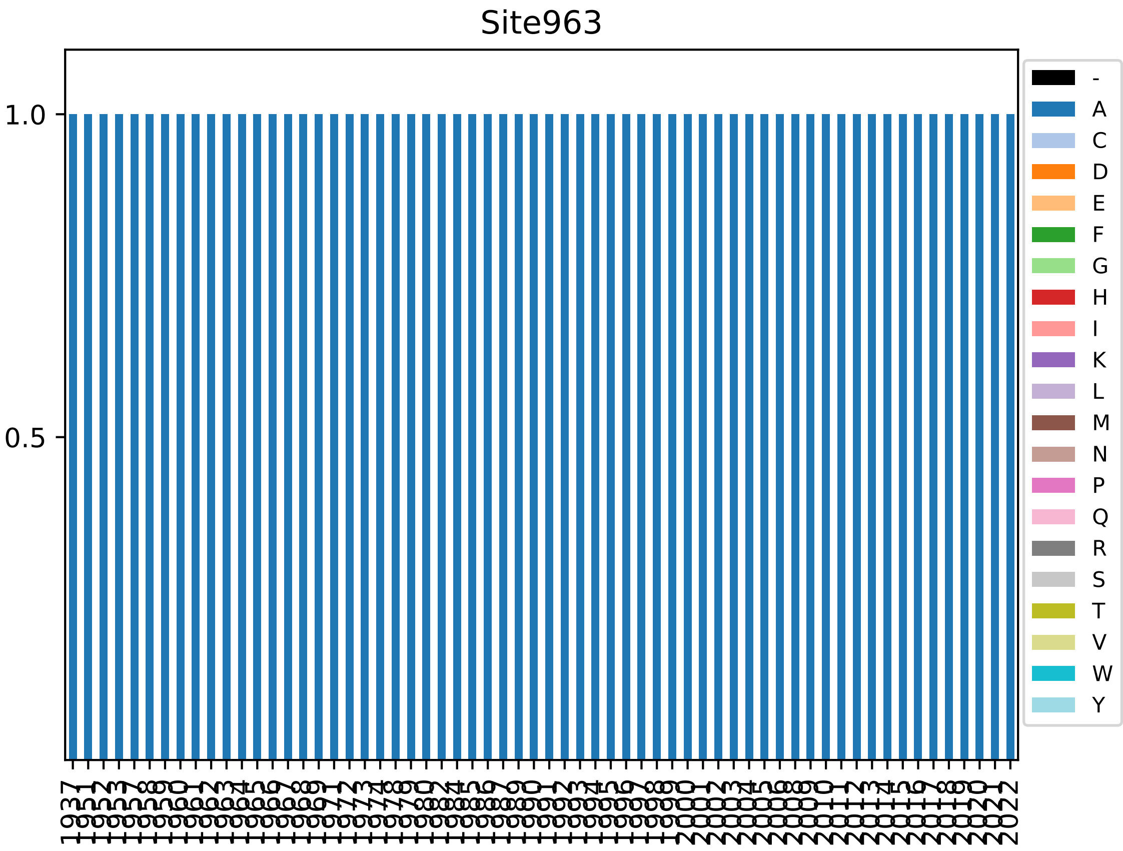

Site963
1.0
0.5
-
A
C
D
E
F
G
H
I
K
L
M
N
P
Q
R
S
T
V
W
Y

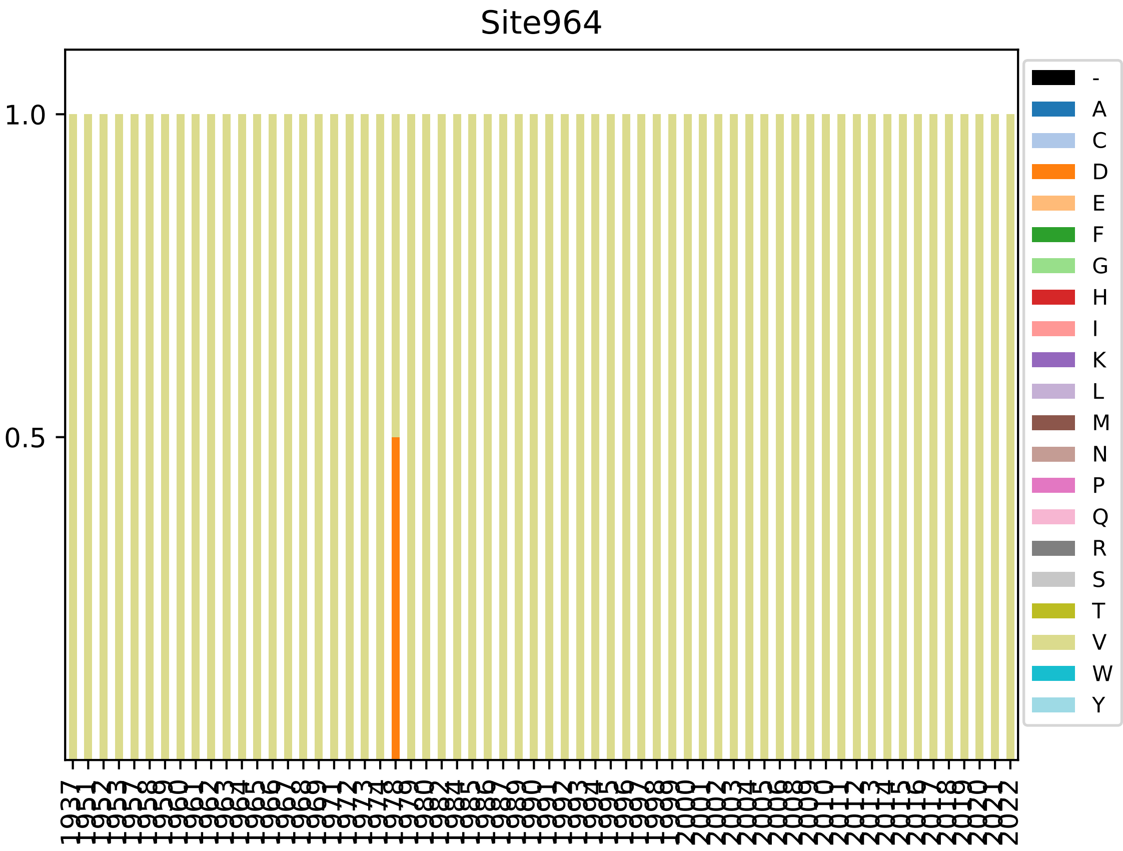
Site964
1.0
0.5
-
A
C
D
E
F
G
H
I
K
L
M
N
P
Q
R
S
T
V
W
Y

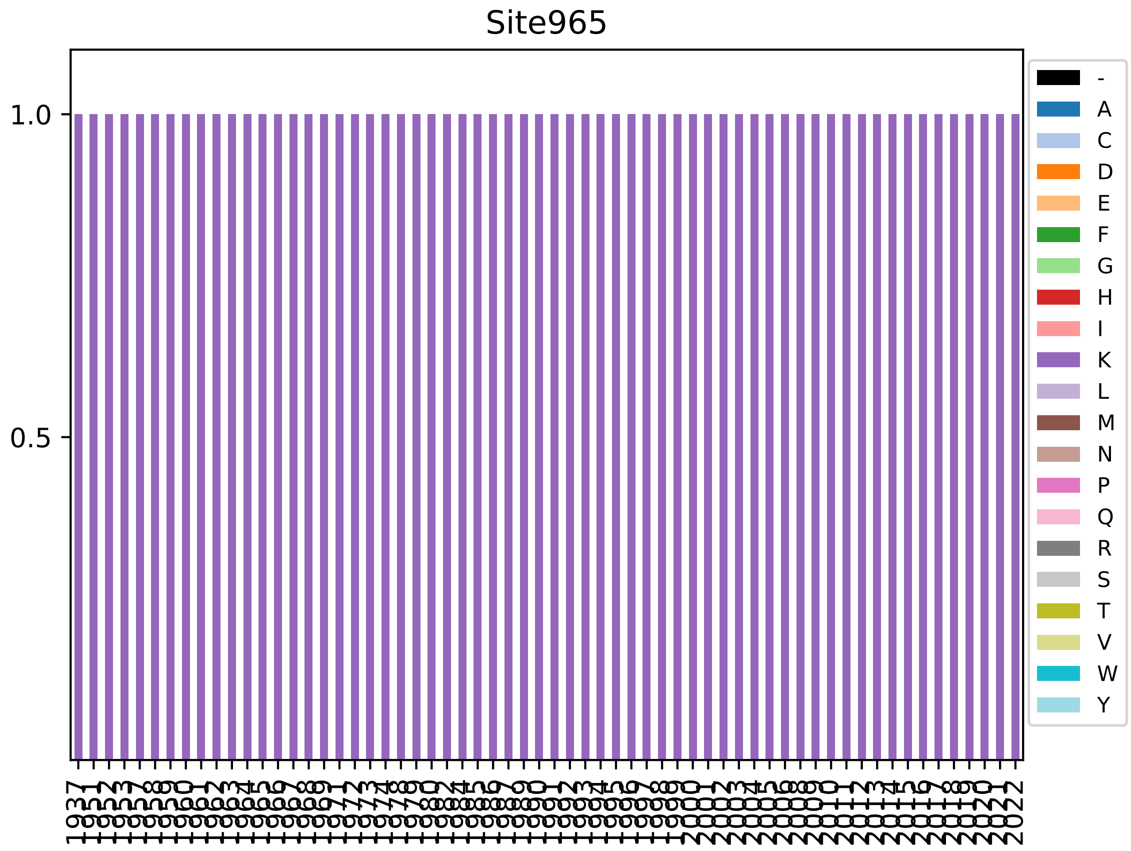
Site965
1.0
0.5
-
A
C
D
E
F
G
H
I
K
L
M
N
P
Q
R
S
T
V
W
Y

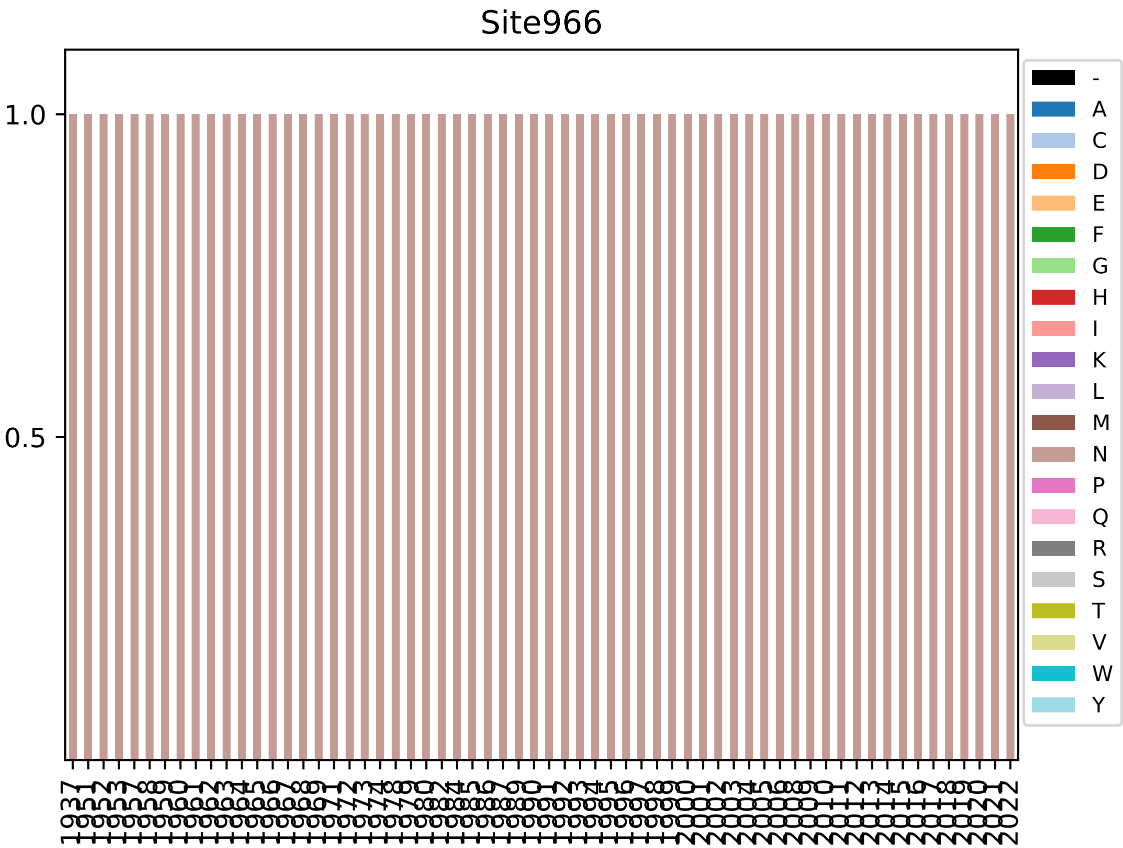
Site966
1.0
0.5
-
A
C
D
E
F
G
H
I
K
L
M
N
P
Q
R
S
T
V
W
Y

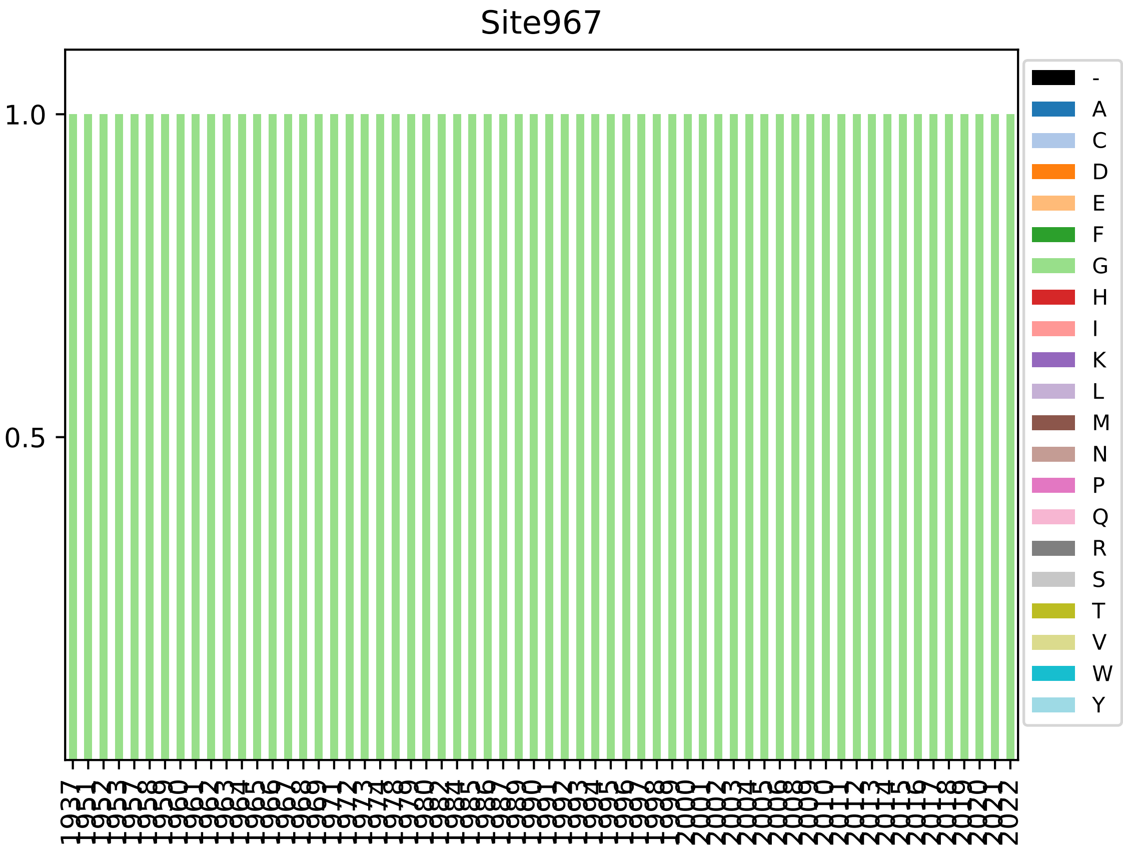
Site967
1.0
0.5
-
A
C
D
E
F
G
H
I
K
L
M
N
P
Q
R
S
T
V
W
Y

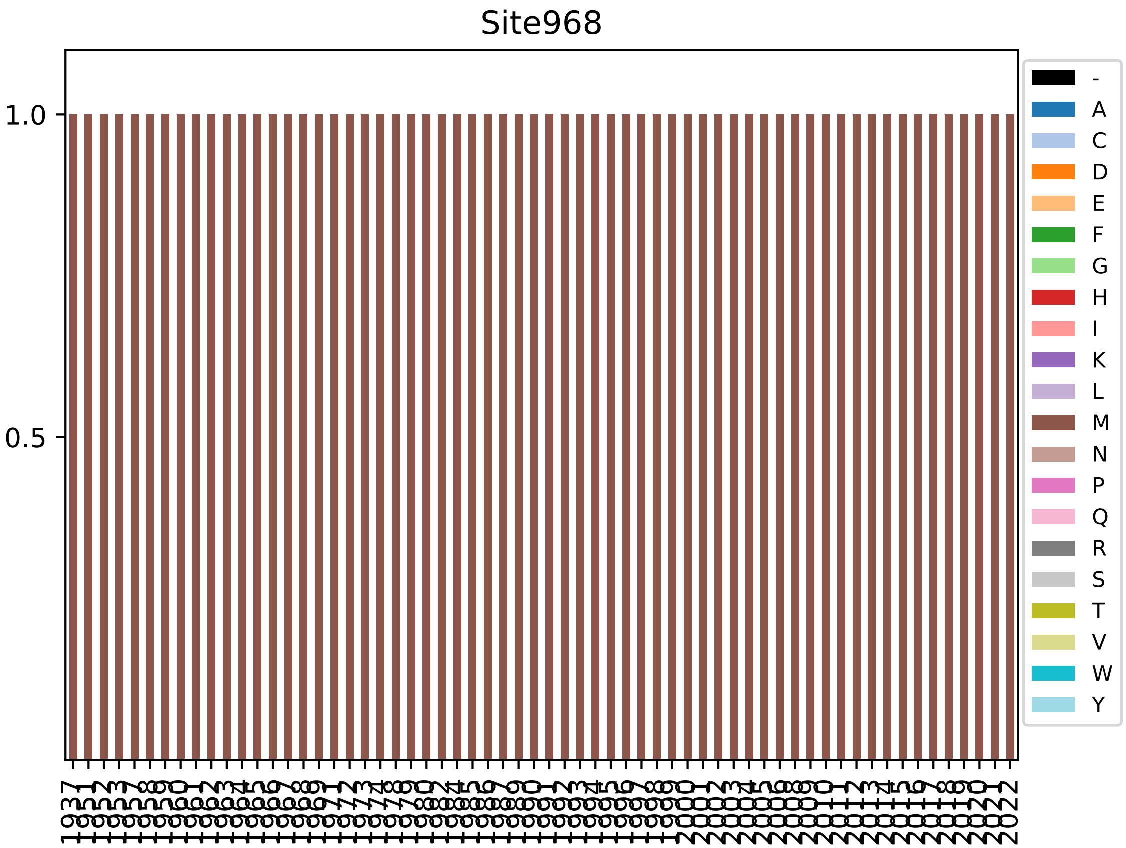

Site968
1.0
0.5
-
A
C
D
E
F
G
H
I
K
L
M
N
P
Q
R
S
T
V
W
Y

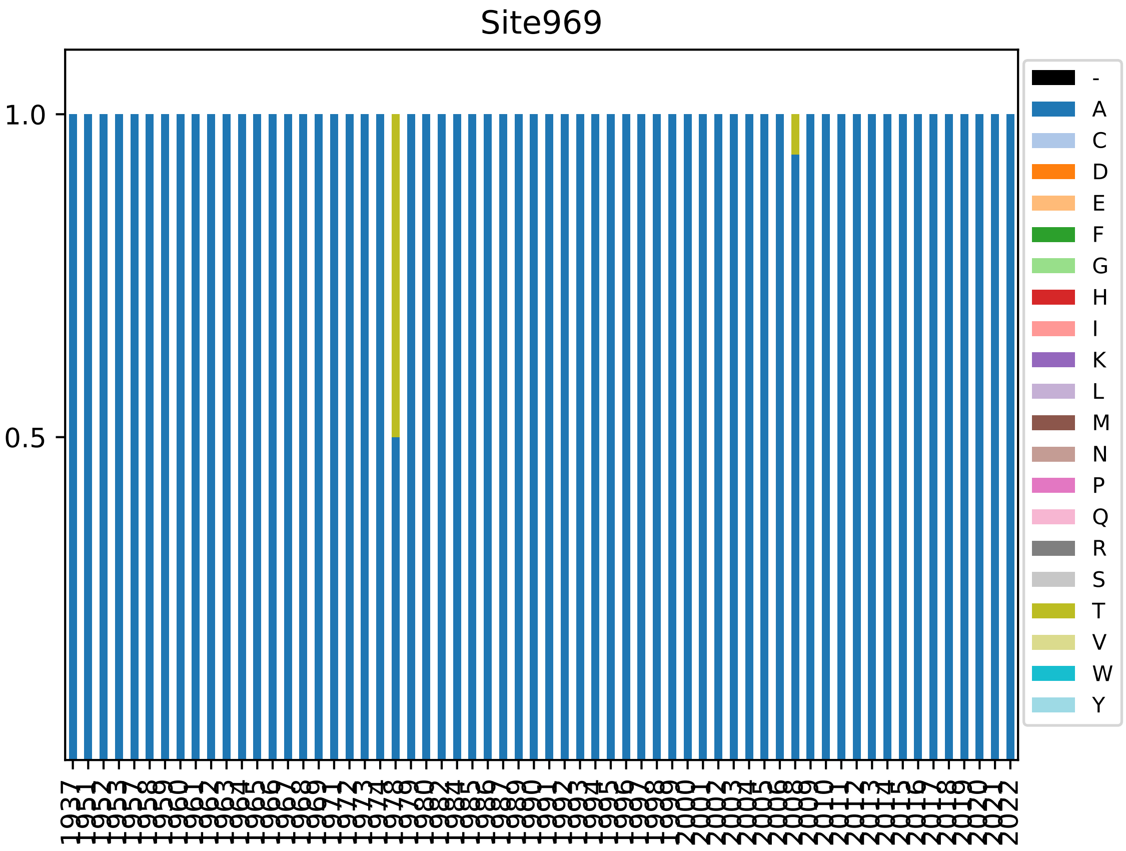
Site969
1.0
0.5
-
A
C
D
E
F
G
H
I
K
L
M
N
P
Q
R
S
T
V
W
Y

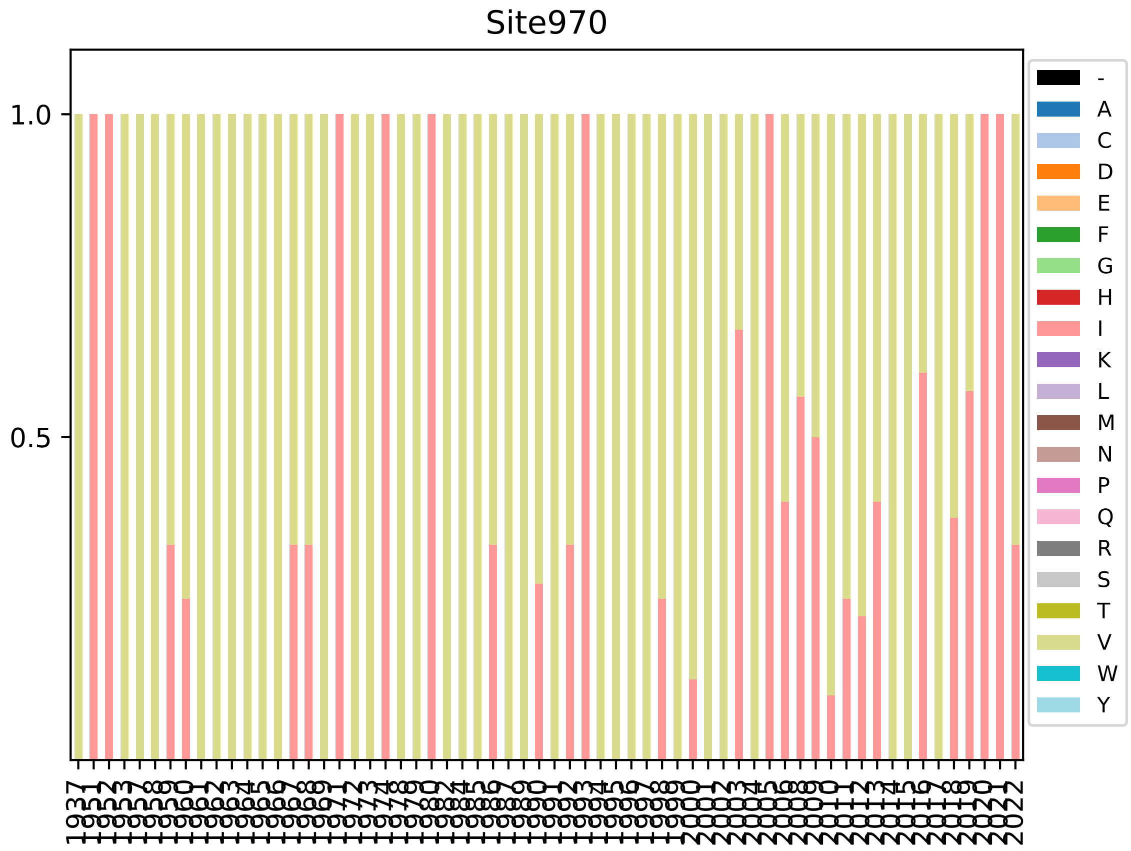
Site970
1.0
0.5
-
A
C
D
E
F
G
H
I
K
L
M
N
P
Q
R
S
T
V
W
Y

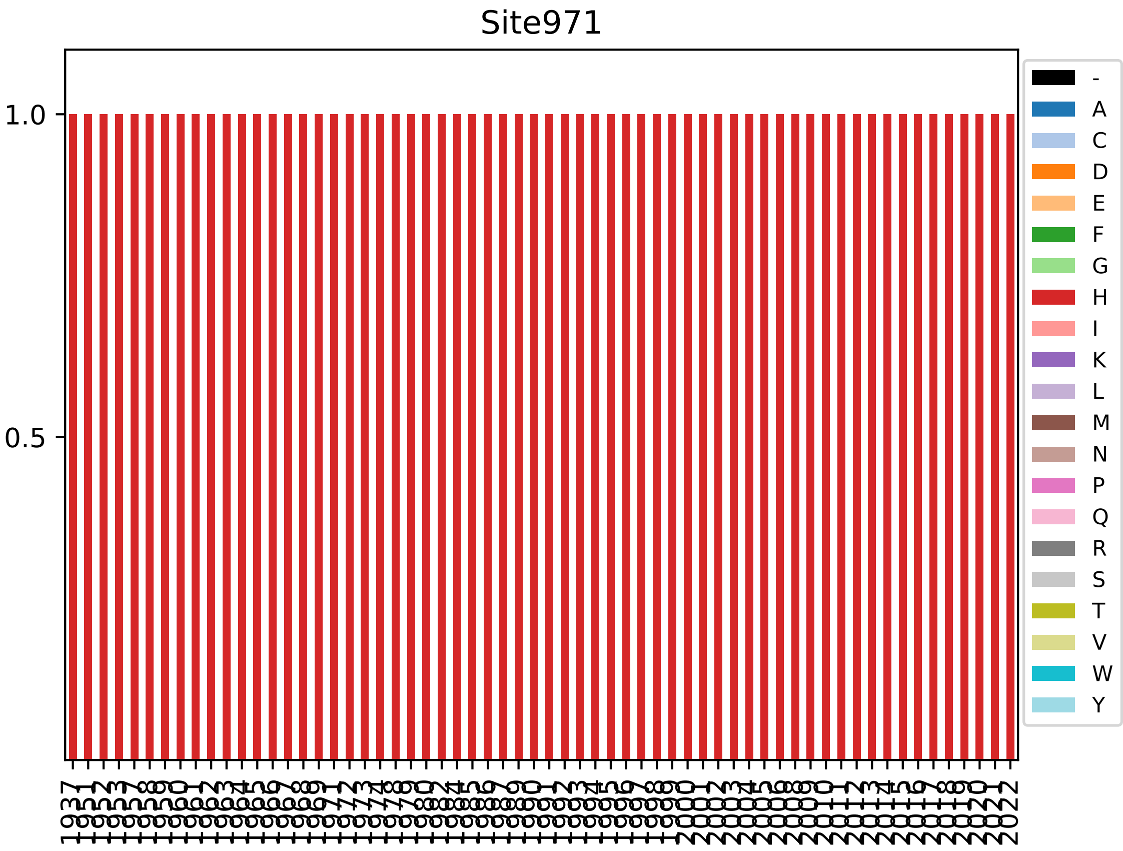
Site971
1.0
0.5
-
A
C
D
E
F
G
H
I
K
L
M
N
P
Q
R
S
T
V
W
Y

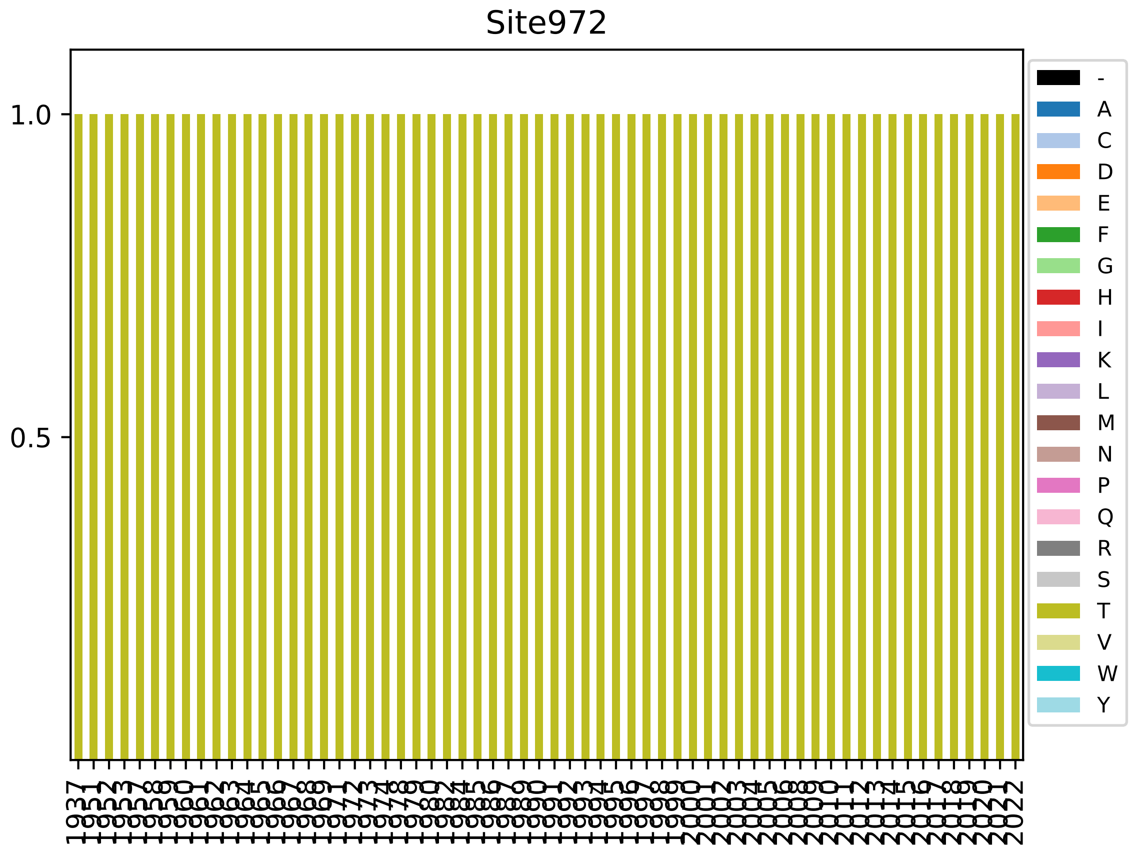
Site972
1.0
0.5
-
A
C
D
E
F
G
H
I
K
L
M
N
P
Q
R
S
T
V
W
Y

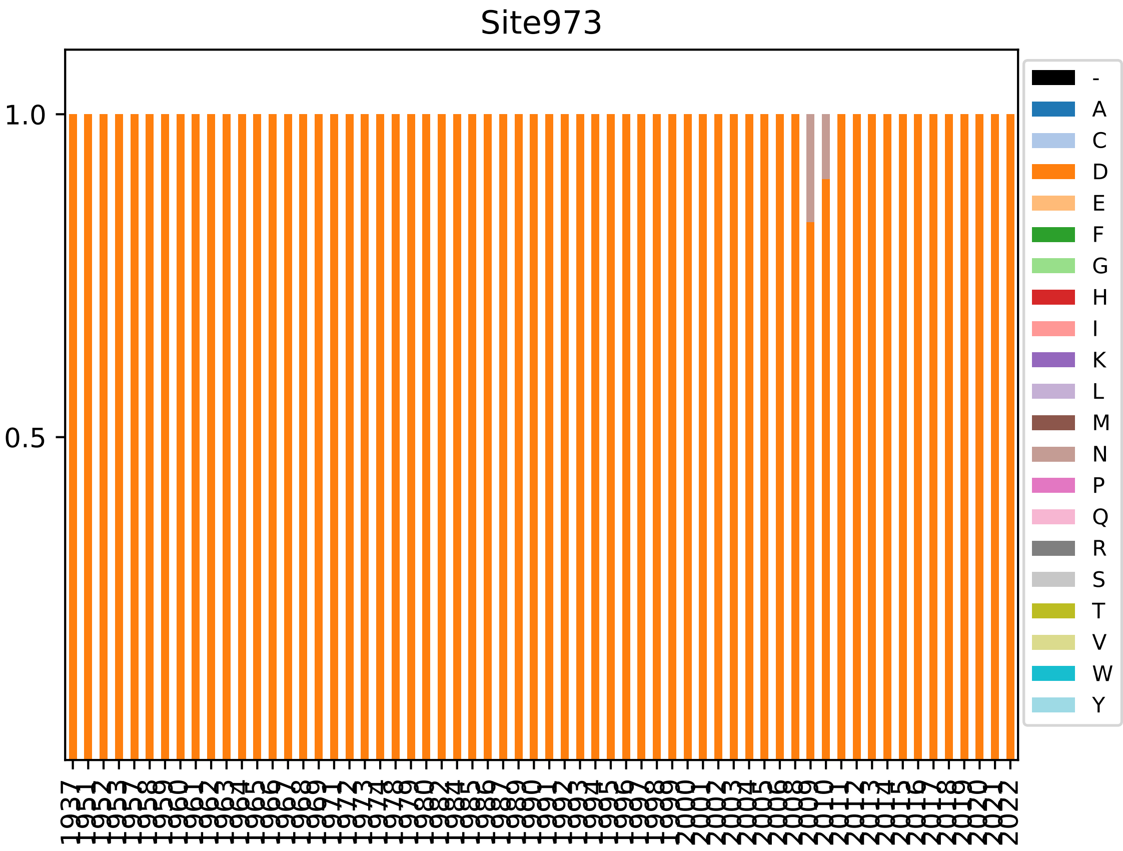
Site973
1.0
0.5
-
A
C
D
E
F
G
H
I
K
L
M
N
P
Q
R
S
T
V
W
Y

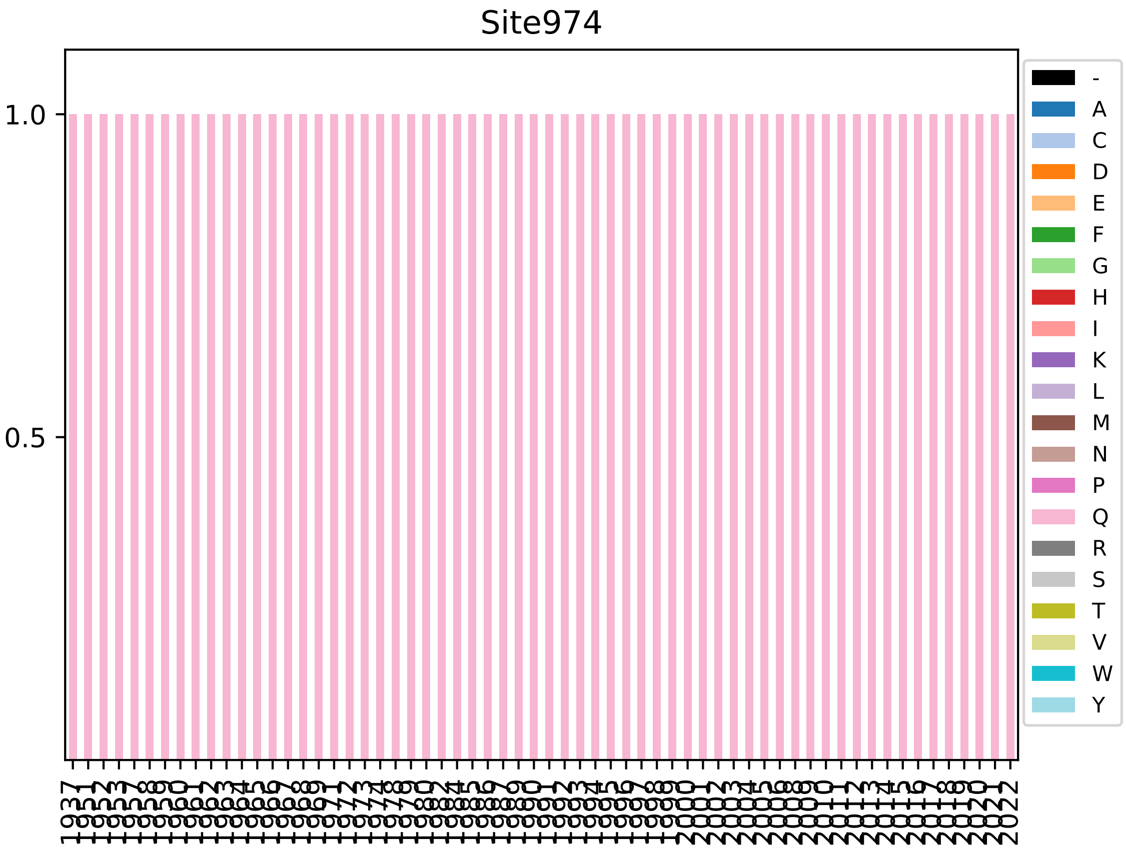
Site974
1.0
0.5
-
A
C
D
E
F
G
H
I
K
L
M
N
P
Q
R
S
T
V
W
Y

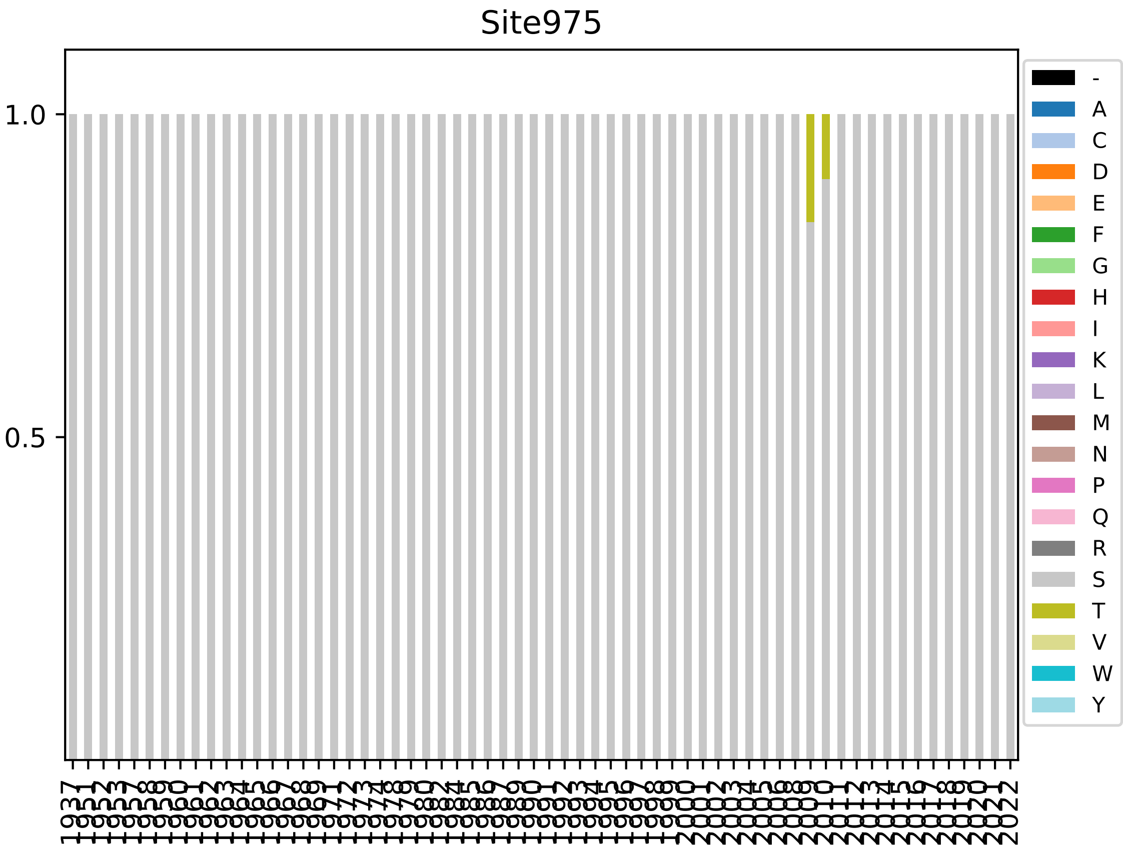
Site975
1.0
0.5
-
A
C
D
E
F
G
H
I
K
L
M
N
P
Q
R
S
T
V
W
Y

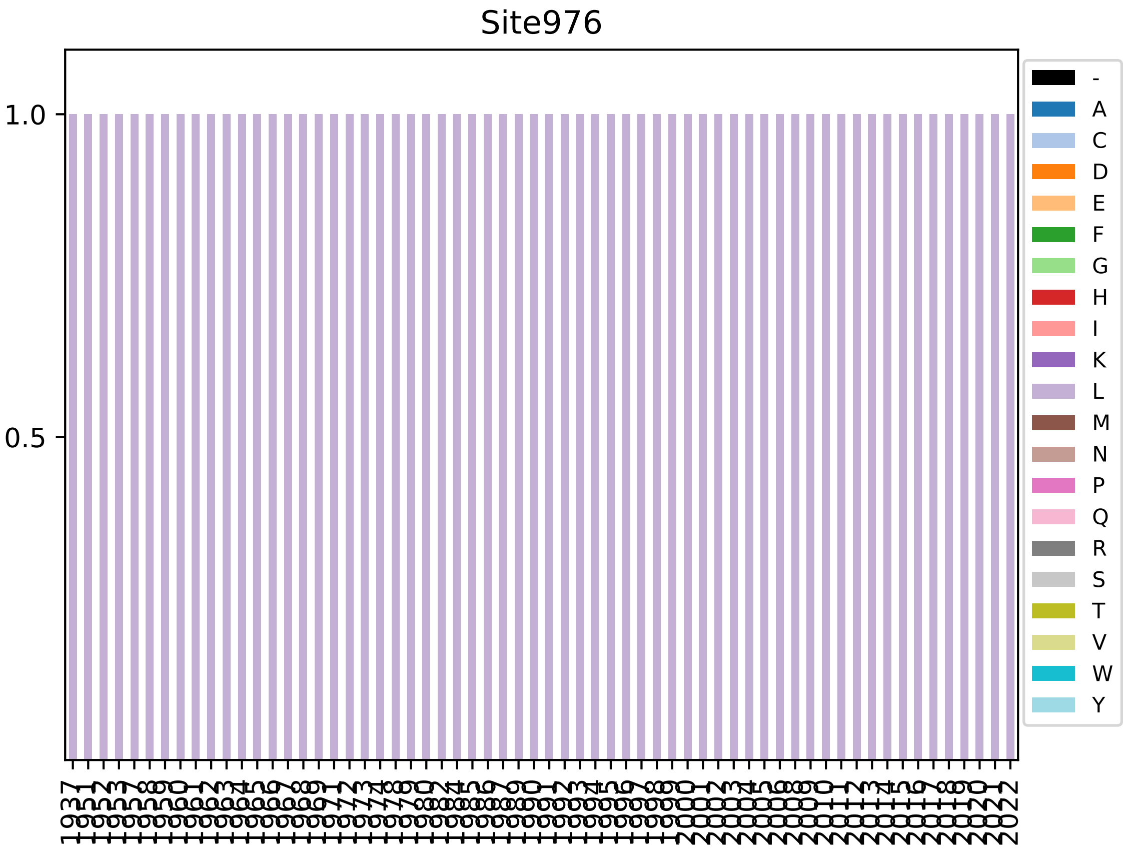
Site976
1.0
0.5
-
A
C
D
E
F
G
H
I
K
L
M
N
P
Q
R
S
T
V
W
Y

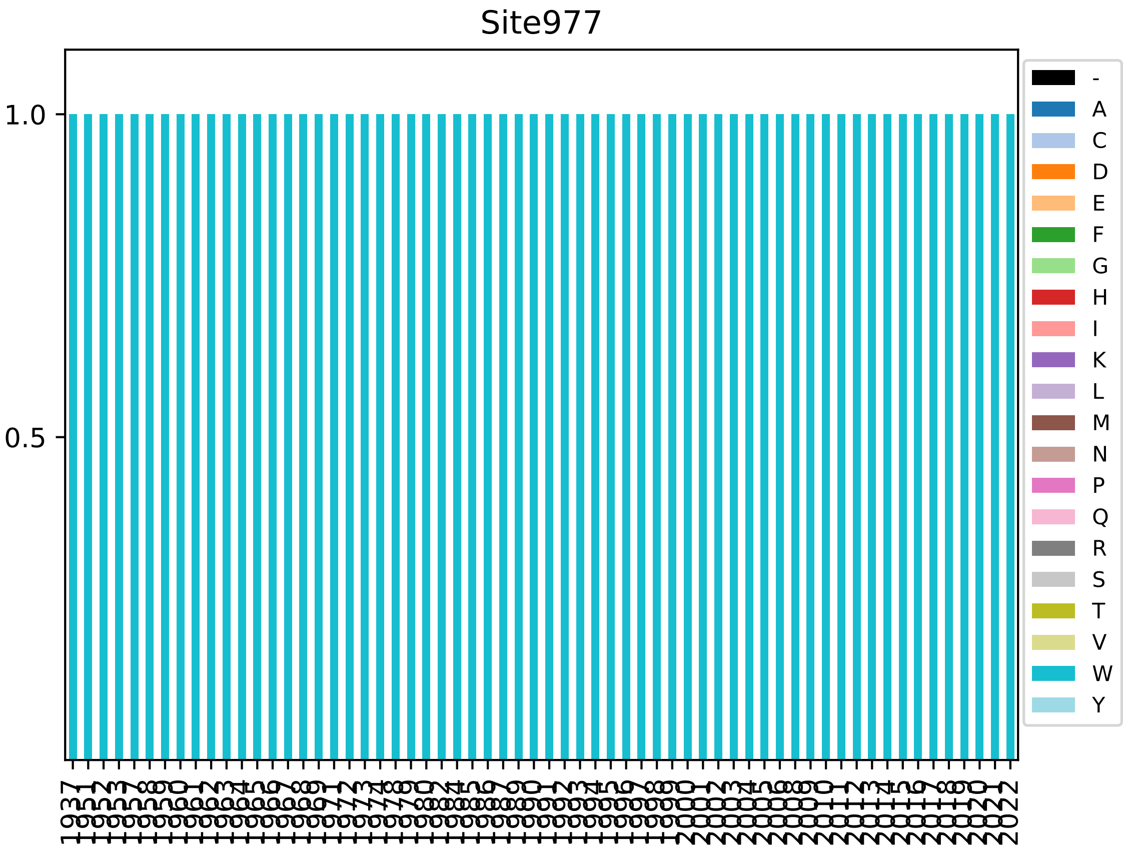
Site977
1.0
0.5
-
A
C
D
E
F
G
H
I
K
L
M
N
P
Q
R
S
T
V
W
Y

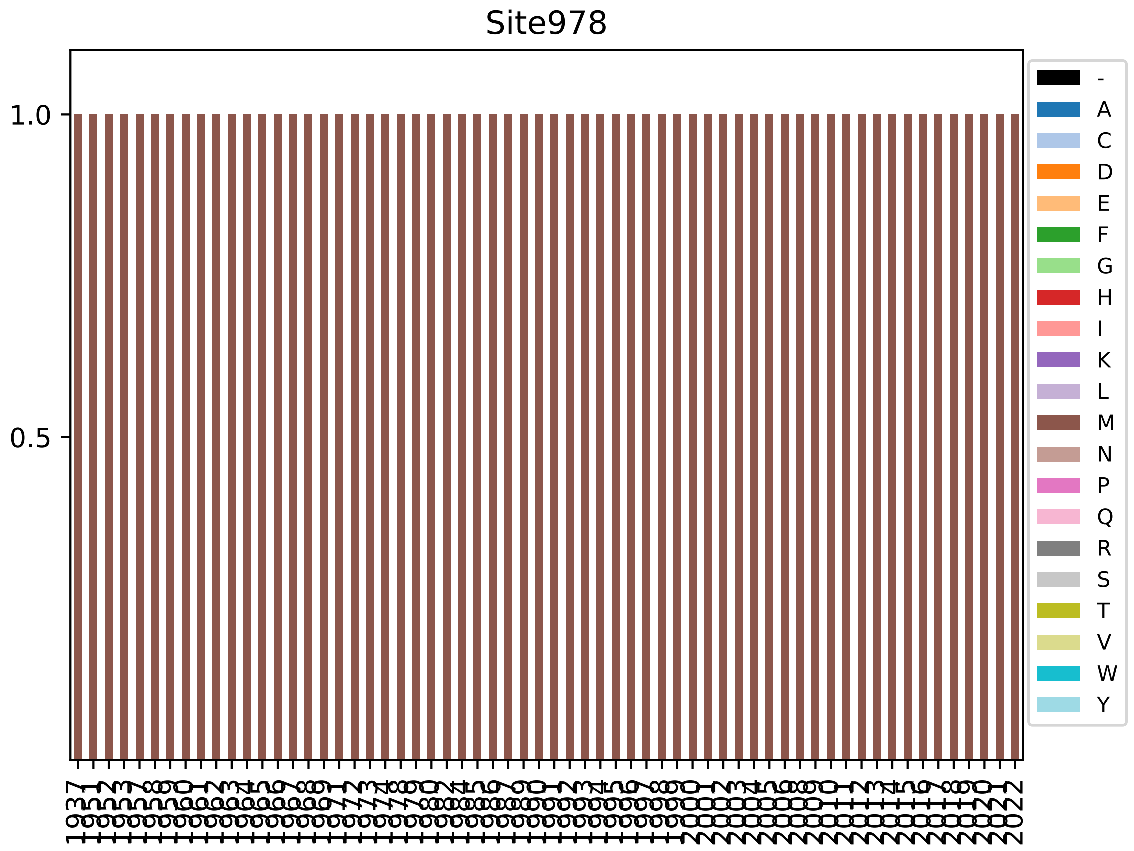
Site978
1.0
0.5
-
A
C
D
E
F
G
H
I
K
L
M
N
P
Q
R
S
T
V
W
Y

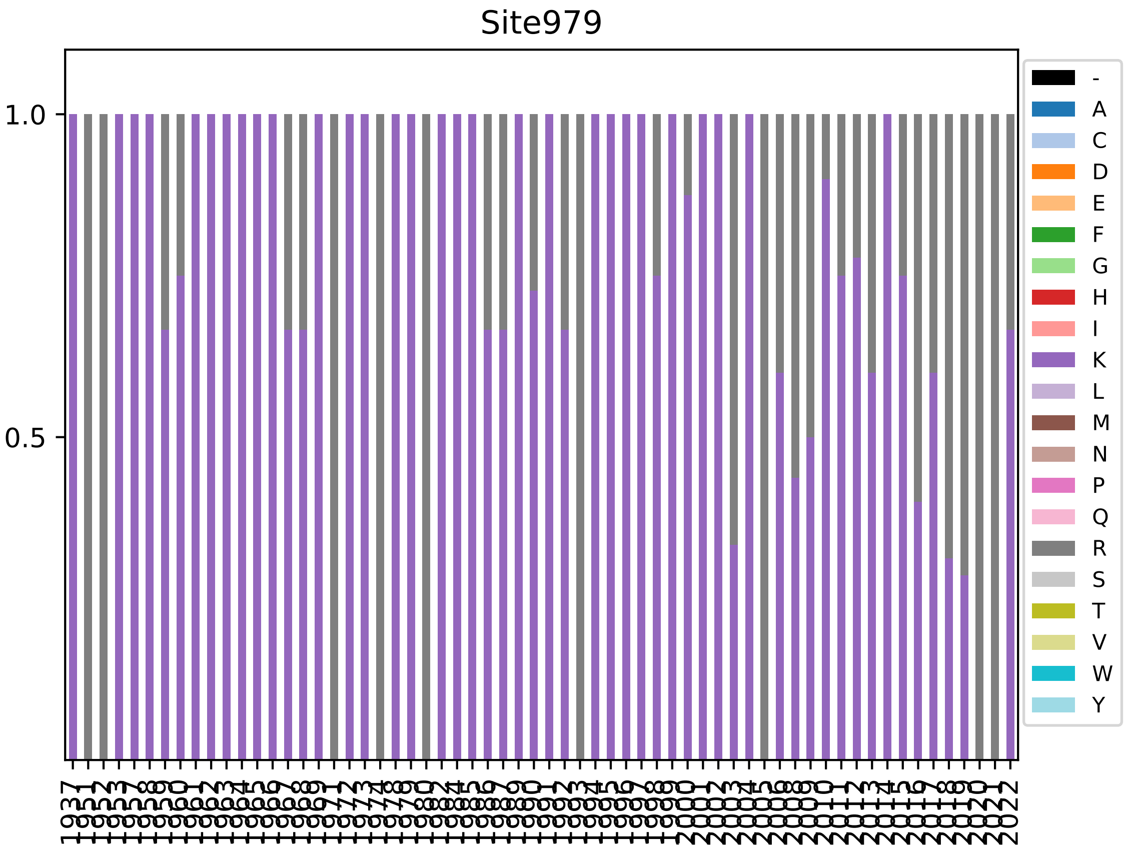
Site979
1.0
0.5
-
A
C
D
E
F
G
H
I
K
L
M
N
P
Q
R
S
T
V
W
Y

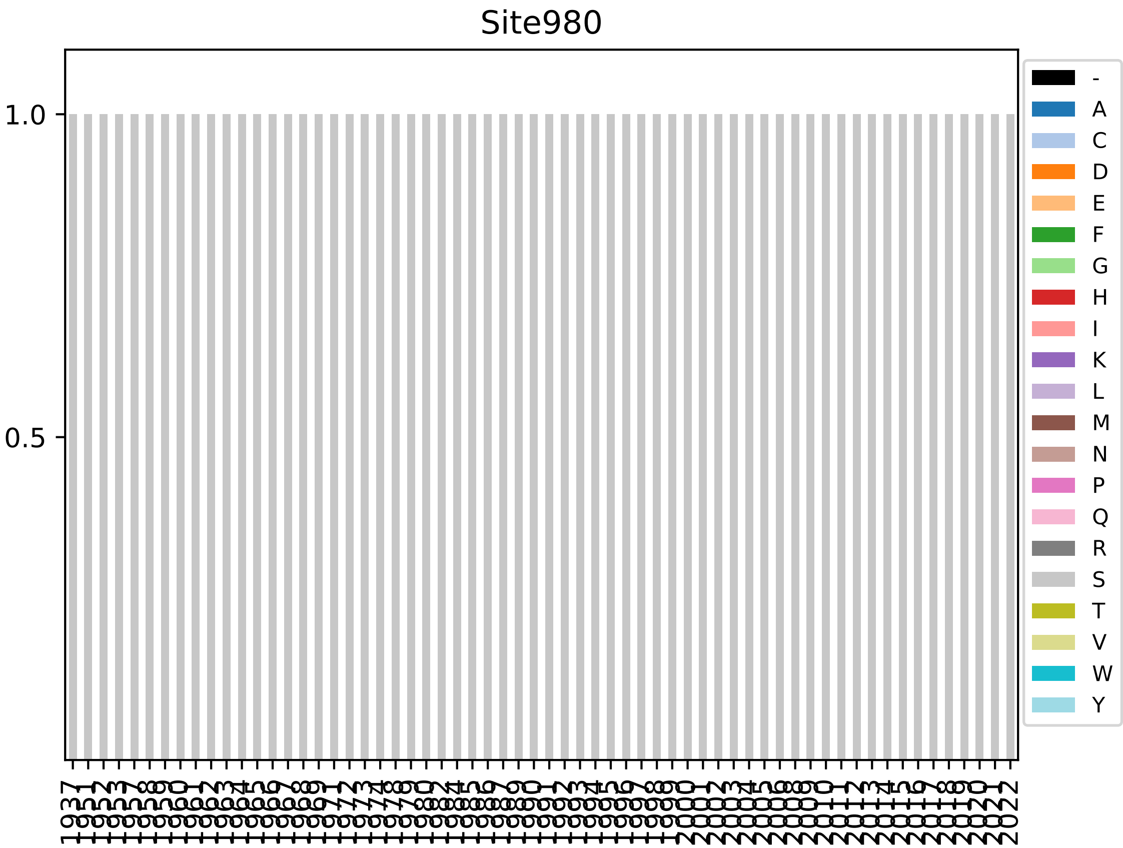
Site980
1.0
0.5
-
A
C
D
E
F
G
H
I
K
L
M
N
P
Q
R
S
T
V
W
Y

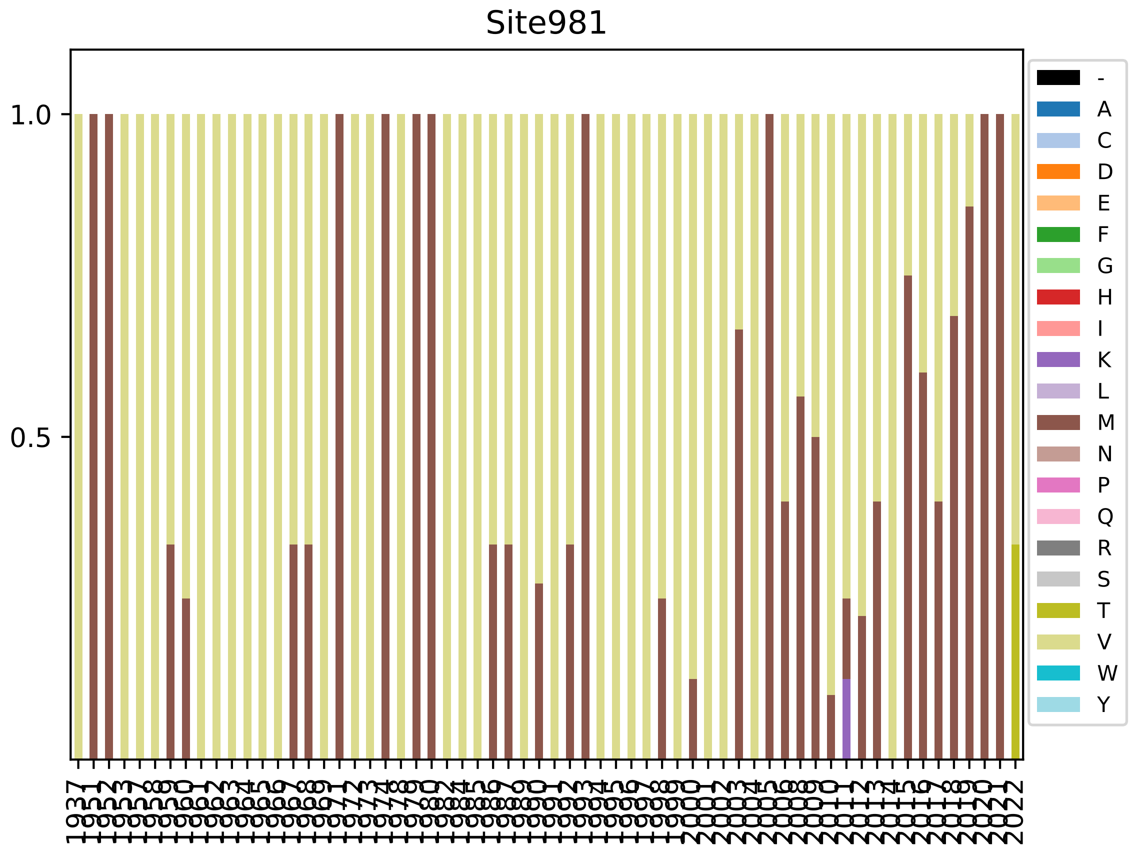
Site981
1.0
0.5
-
A
C
D
E
F
G
H
I
K
L
M
N
P
Q
R
S
T
V
W
Y

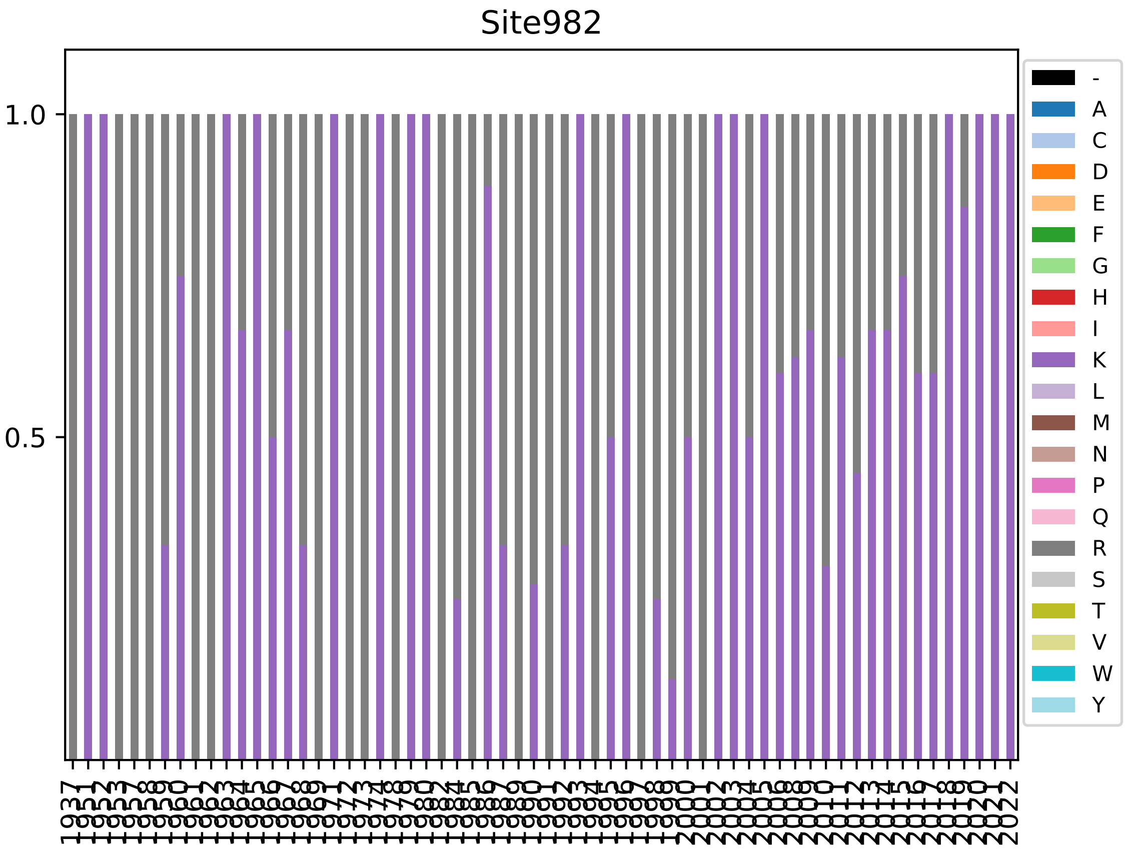

Site982
1.0
0.5
-
A
C
D
E
F
G
H
I
K
L
M
N
P
Q
R
S
T
V
W
Y

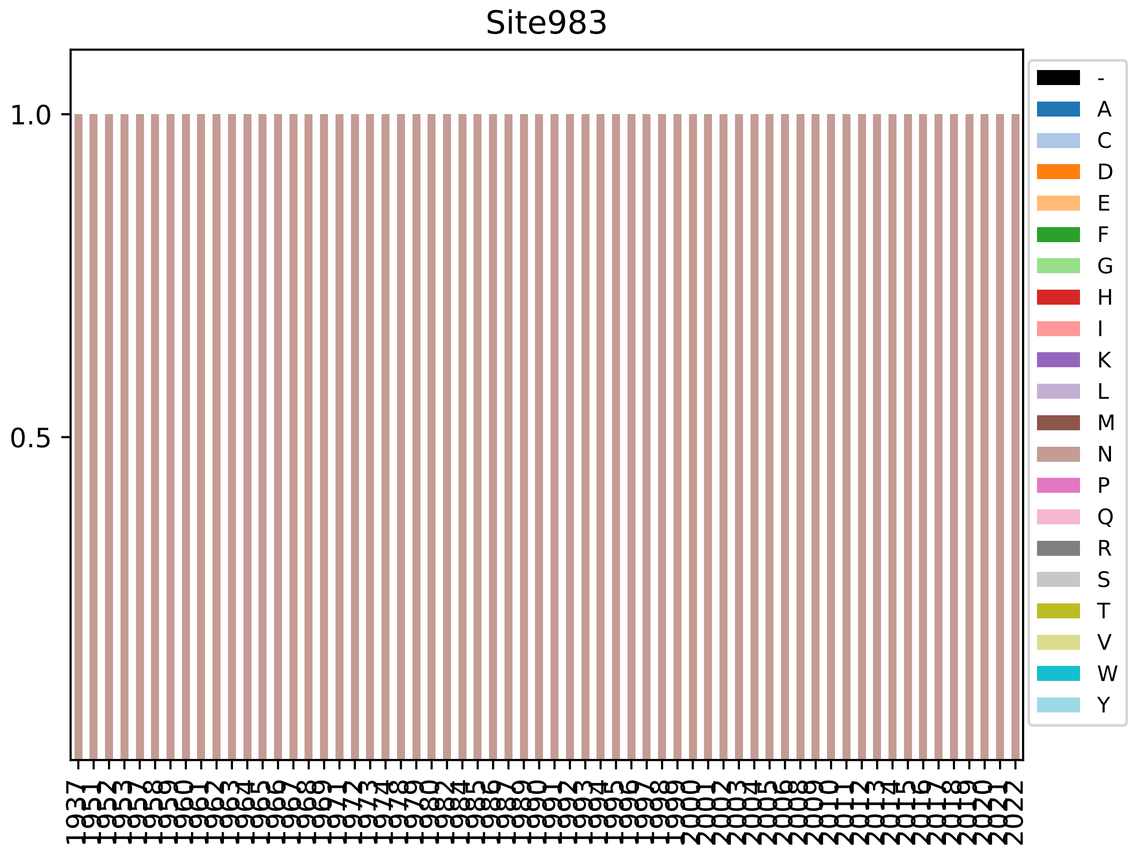
Site983
1.0
0.5
-
A
C
D
E
F
G
H
I
K
L
M
N
P
Q
R
S
T
V
W
Y

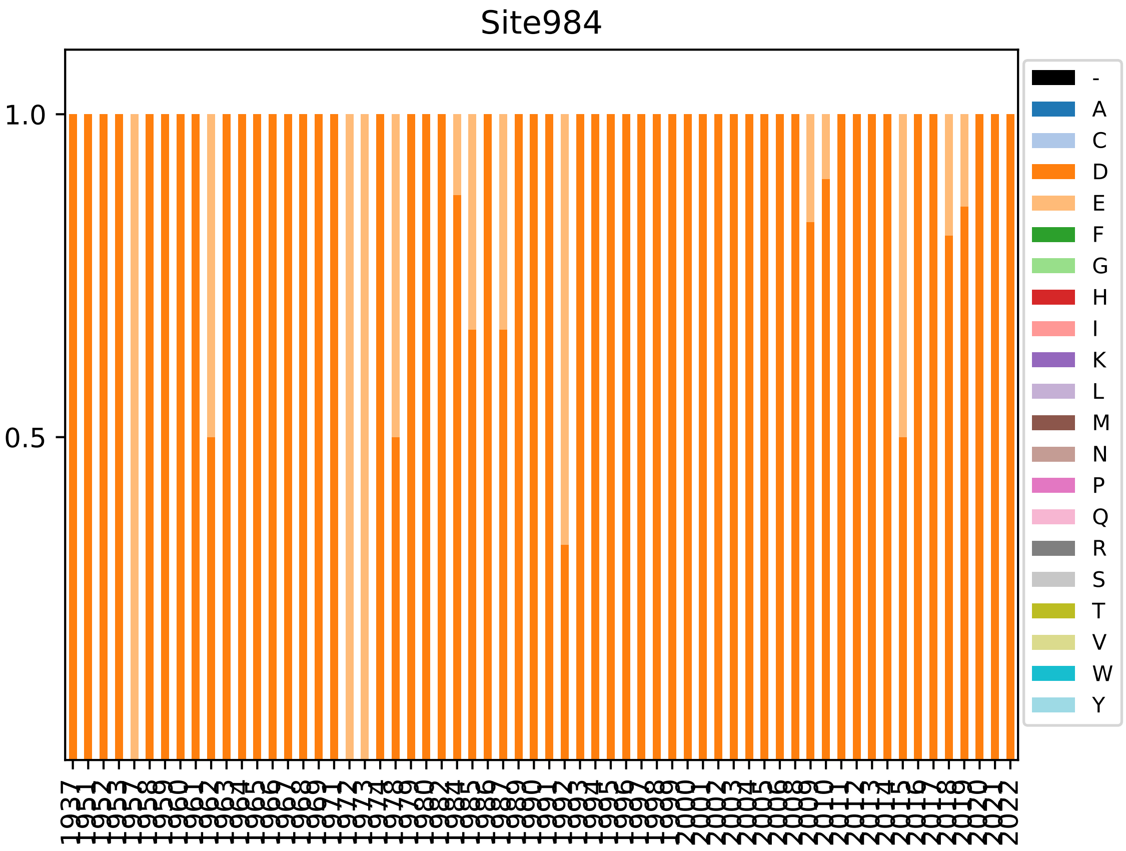

Site984
1.0
0.5
-
A
C
D
E
F
G
H
I
K
L
M
N
P
Q
R
S
T
V
W
Y

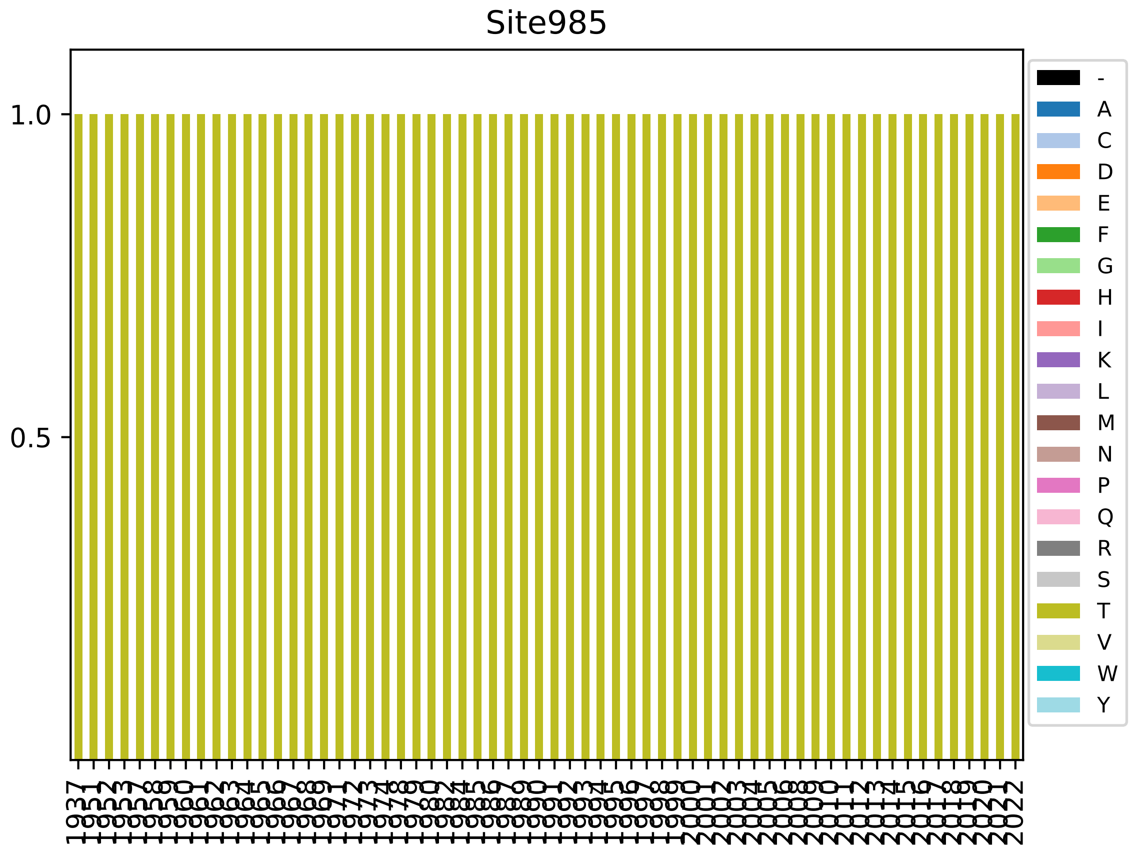
Site985
1.0
0.5
-
A
C
D
E
F
G
H
I
K
L
M
N
P
Q
R
S
T
V
W
Y

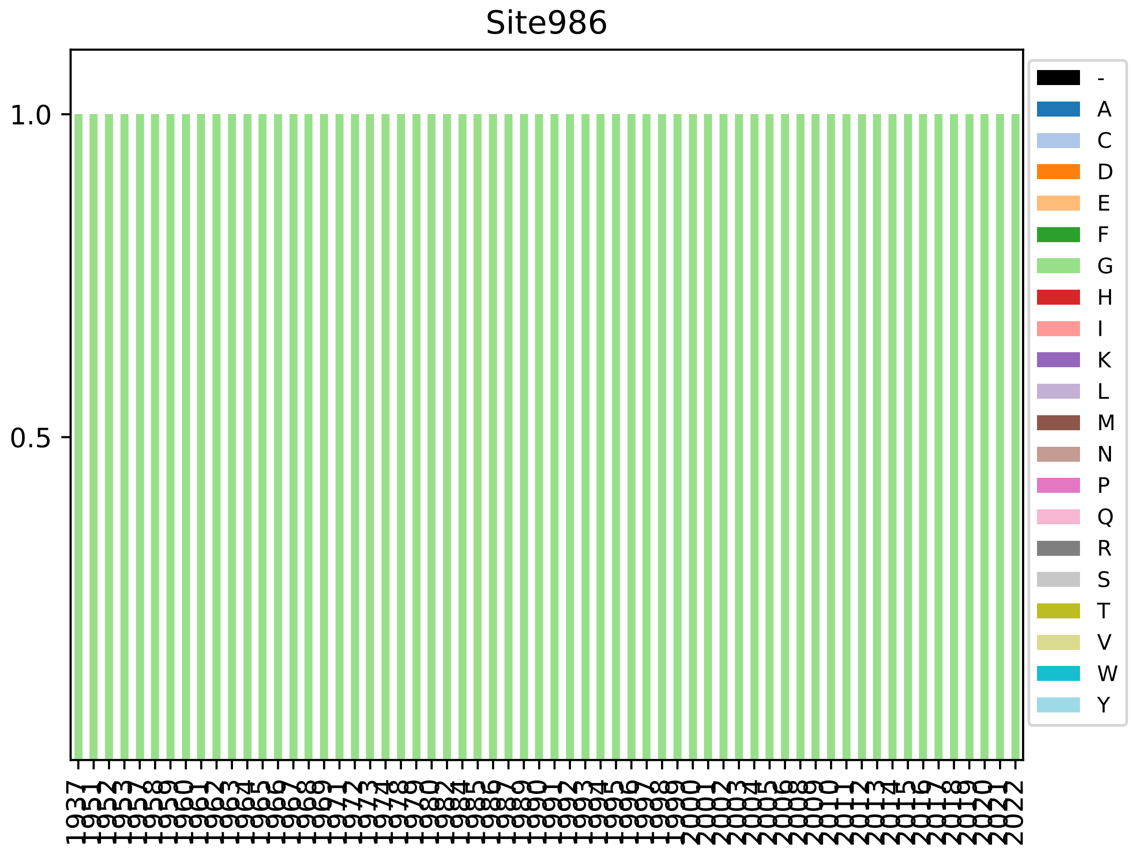
Site986
1.0
0.5
-
A
C
D
E
F
G
H
I
K
L
M
N
P
Q
R
S
T
V
W
Y

Site987

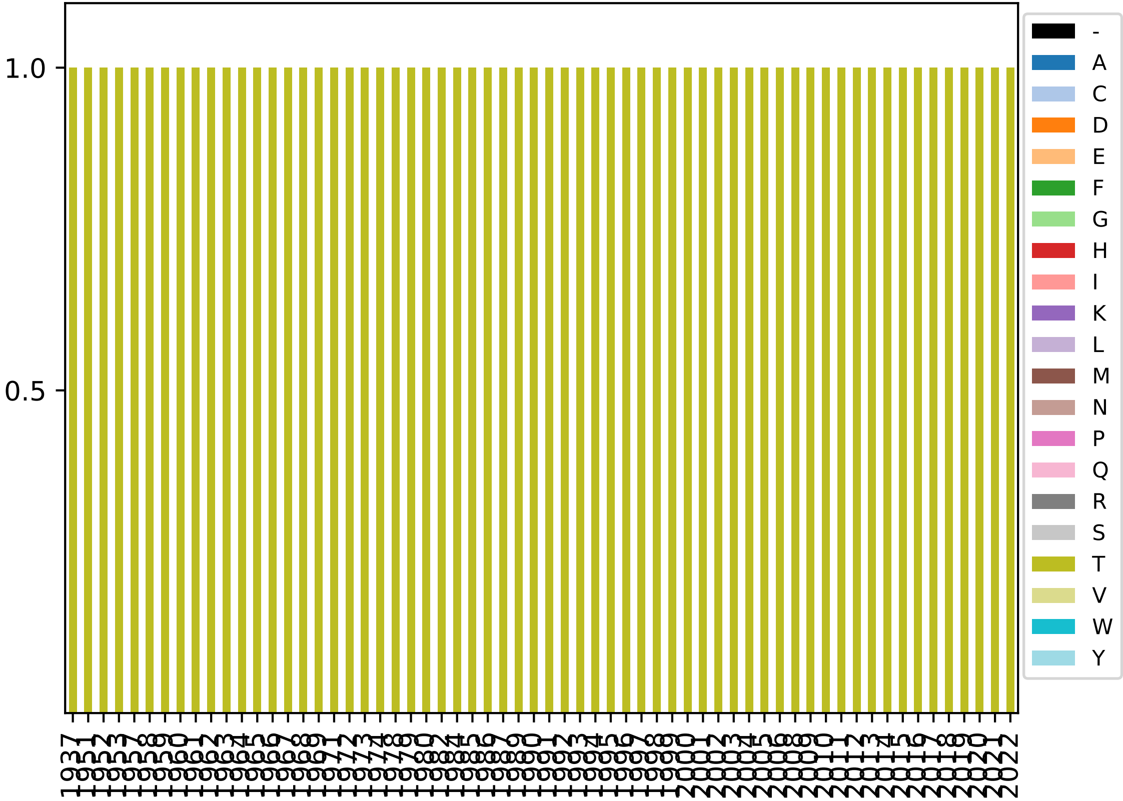

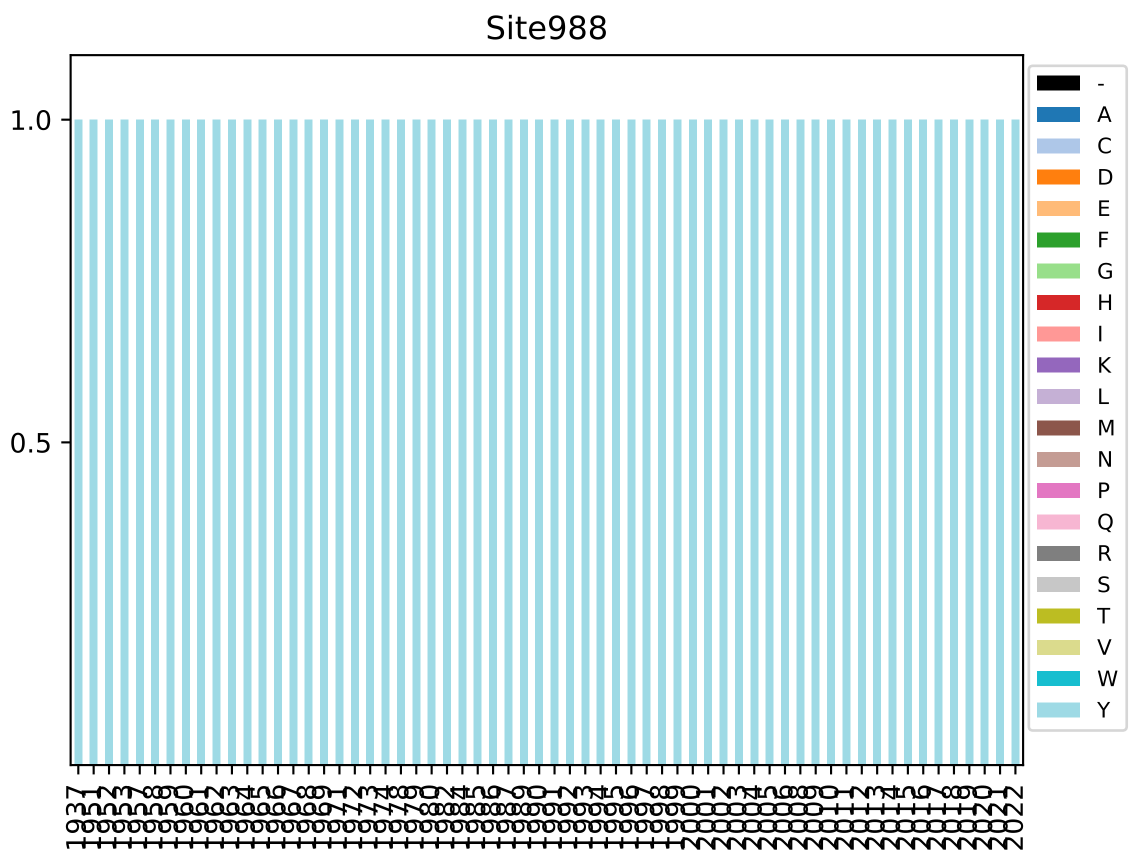
Site988
1.0
0.5
-
A
C
D
E
F
G
H
I
K
L
M
N
P
Q
R
S
T
V
W
Y

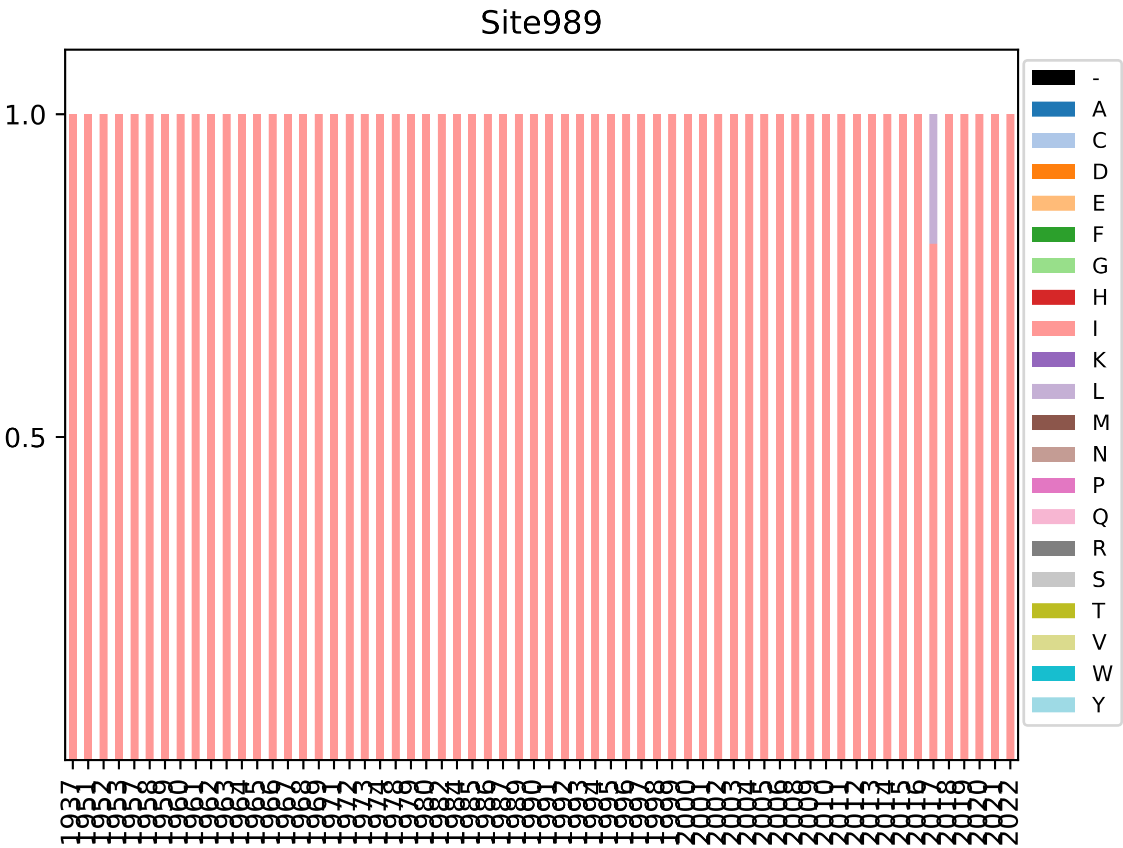
Site989
1.0
0.5
-
A
C
D
E
F
G
H
I
K
L
M
N
P
Q
R
S
T
V
W
Y

Site990

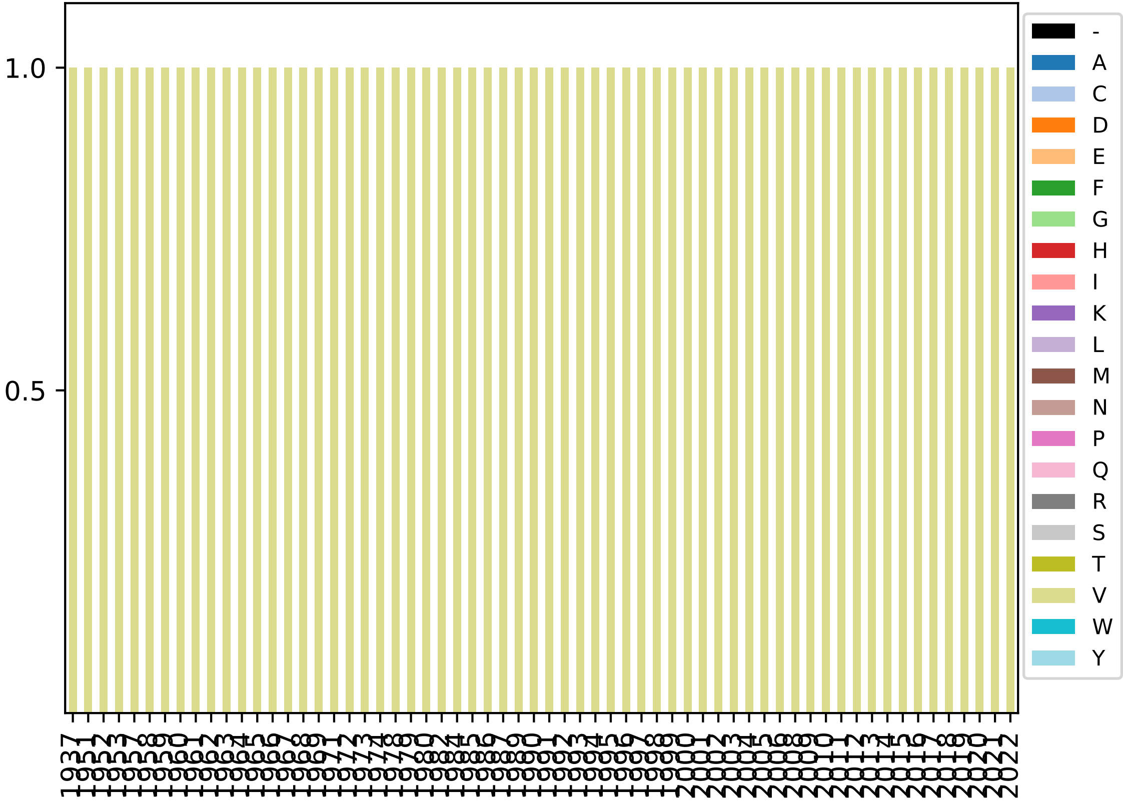

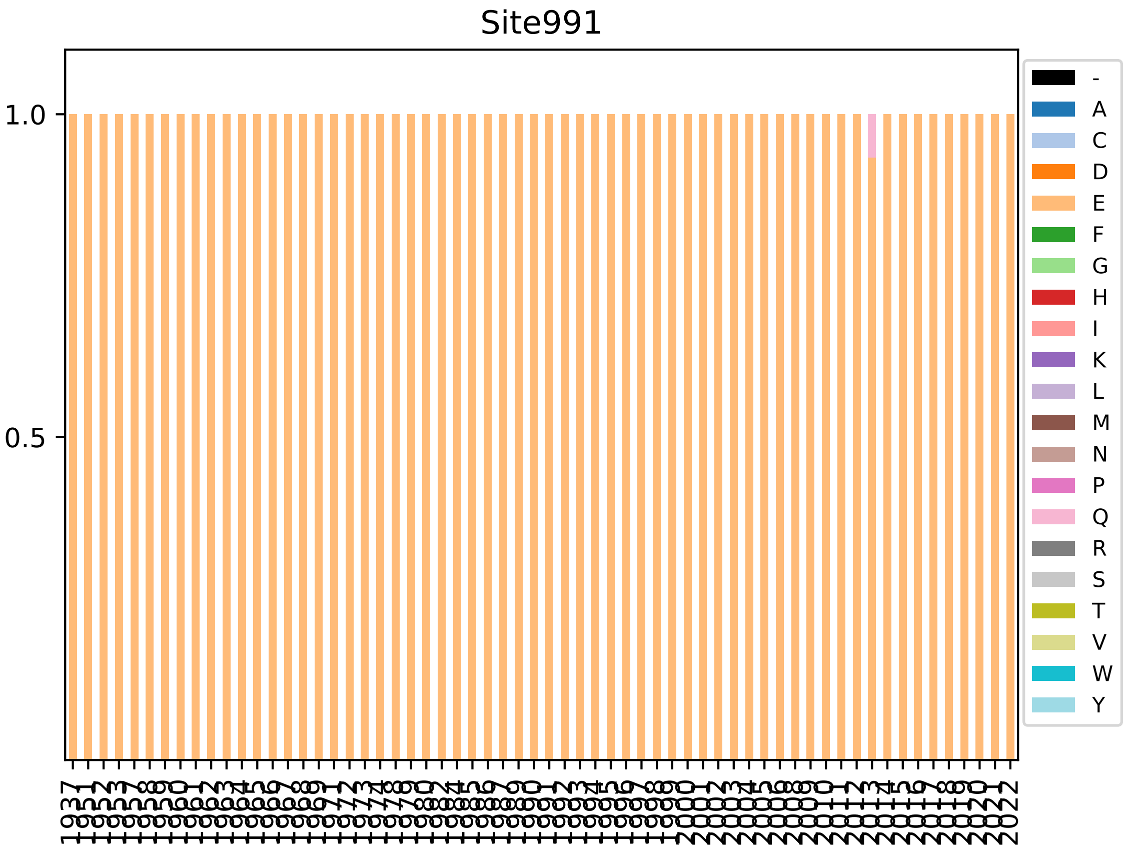
Site991
1.0
0.5
-
A
C
D
E
F
G
H
I
K
L
M
N
P
Q
R
S
T
V
W
Y

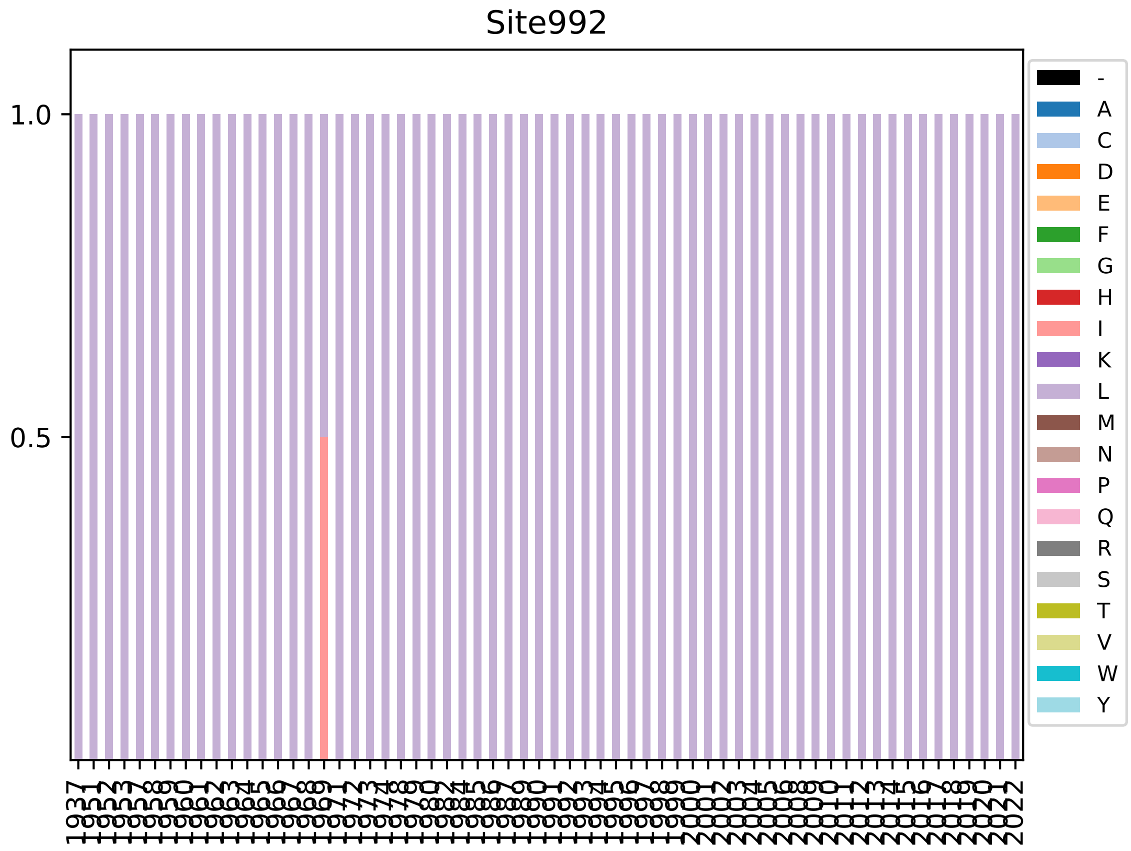
Site992
1.0
0.5
-
A
C
D
E
F
G
H
I
K
L
M
N
P
Q
R
S
T
V
W
Y

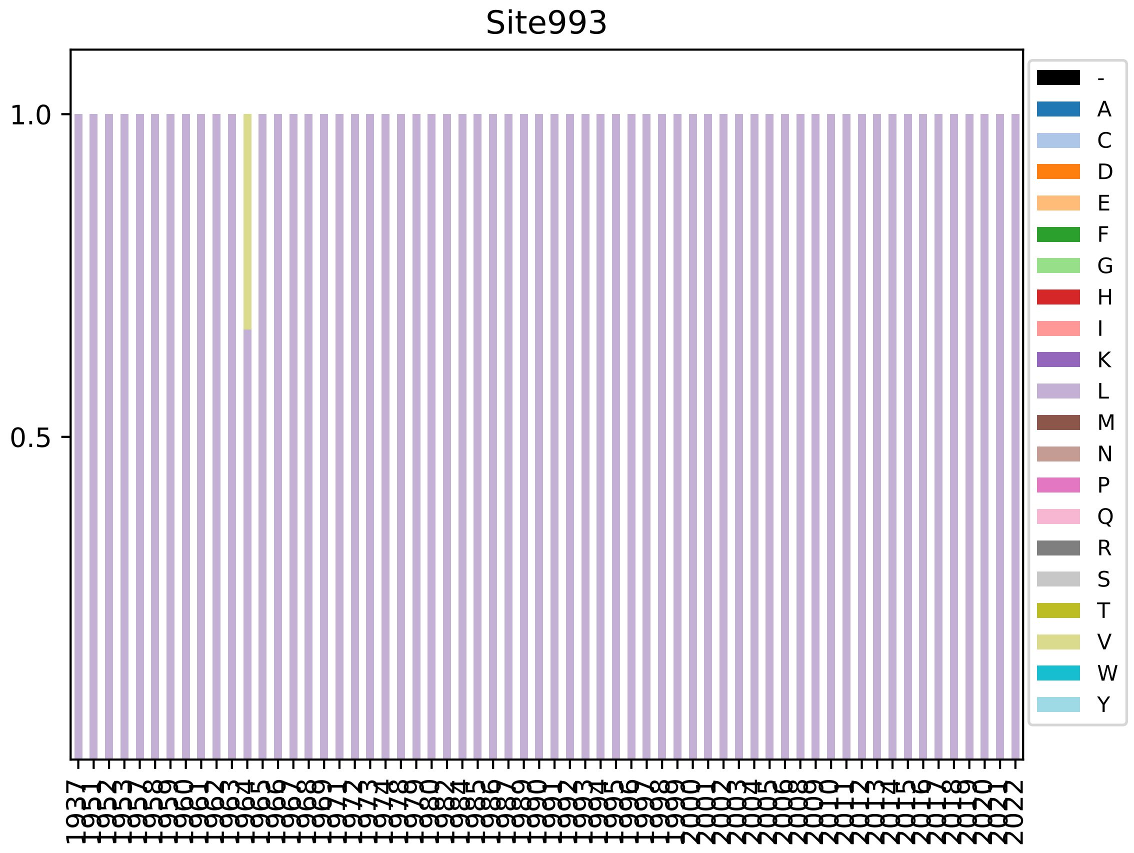
Site993
1.0
0.5
-
A
C
D
E
F
G
H
I
K
L
M
N
P
Q
R
S
T
V
W
Y

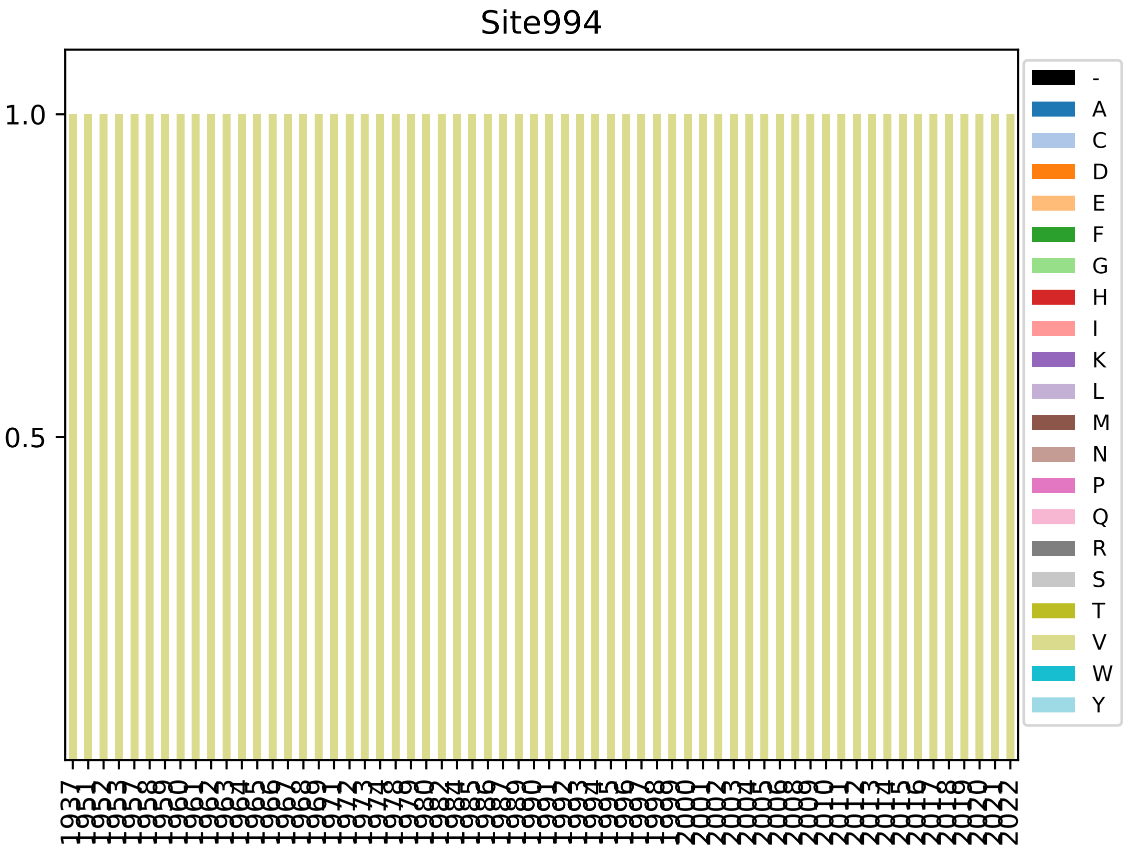
Site994
1.0
0.5
-
A
C
D
E
F
G
H
I
K
L
M
N
P
Q
R
S
T
V
W
Y

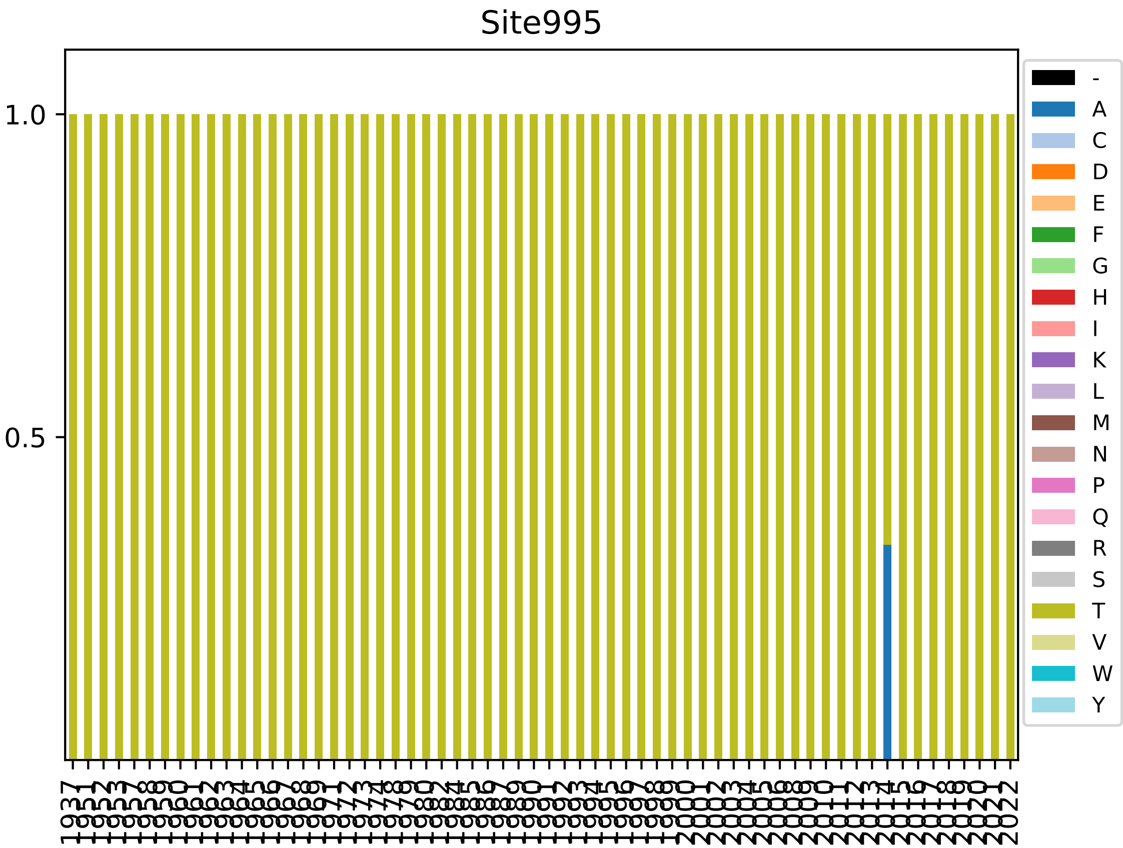
Site995
1.0
0.5
-
A
C
D
E
F
G
H
I
K
L
M
N
P
Q
R
S
T
V
W
Y

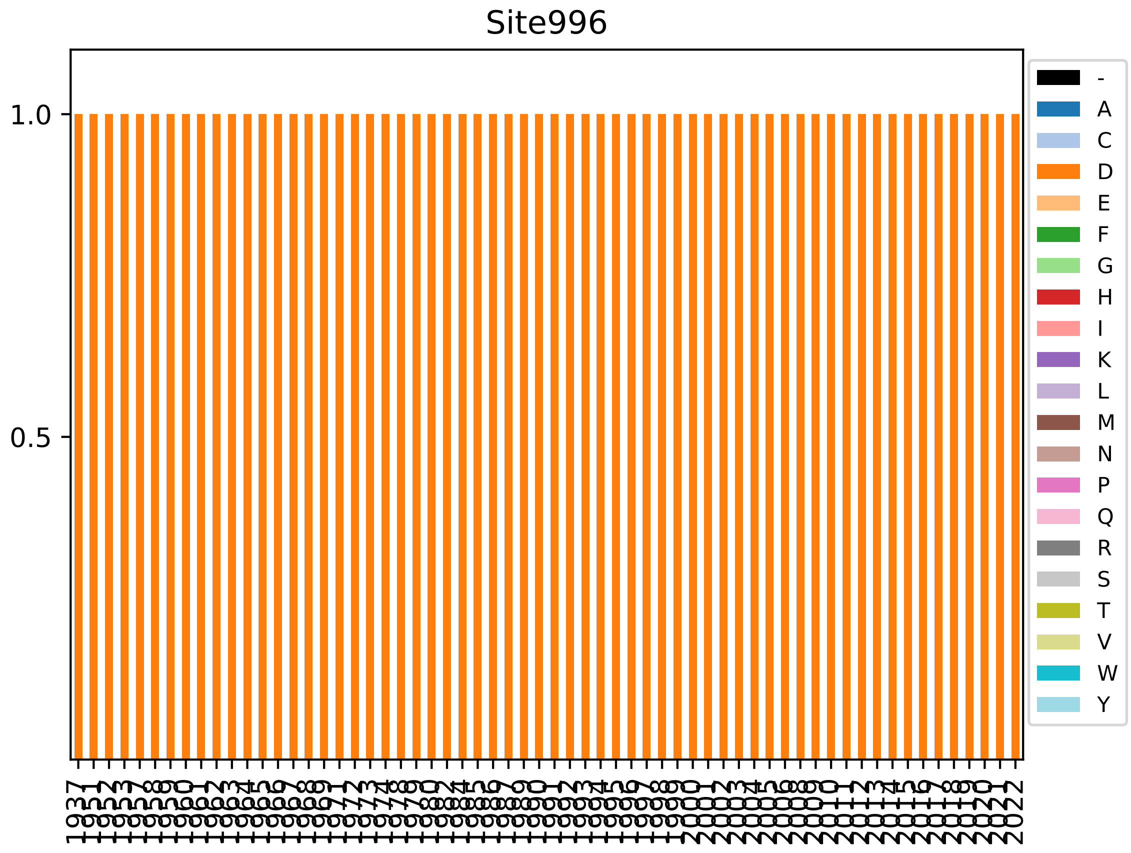
Site996
1.0
0.5
-
A
C
D
E
F
G
H
I
K
L
M
N
P
Q
R
S
T
V
W
Y

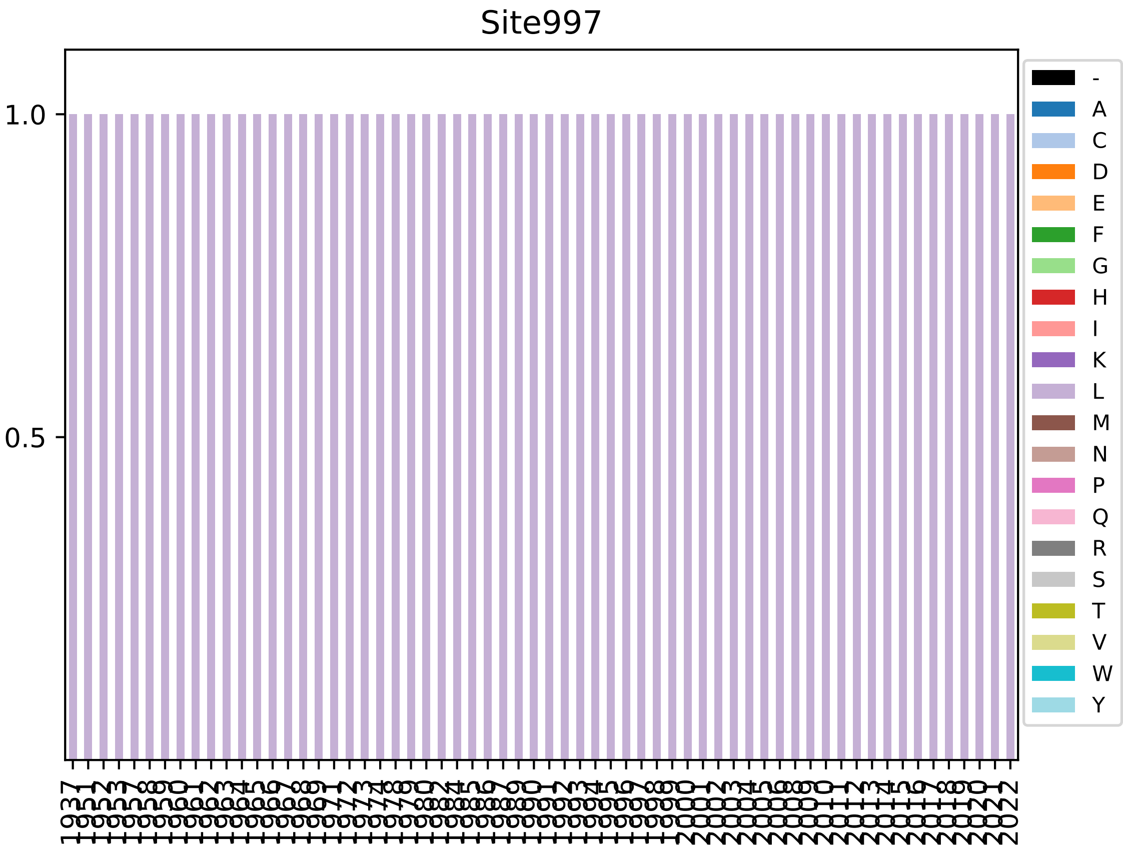
Site997
1.0
0.5
-
A
C
D
E
F
G
H
I
K
L
M
N
P
Q
R
S
T
V
W
Y

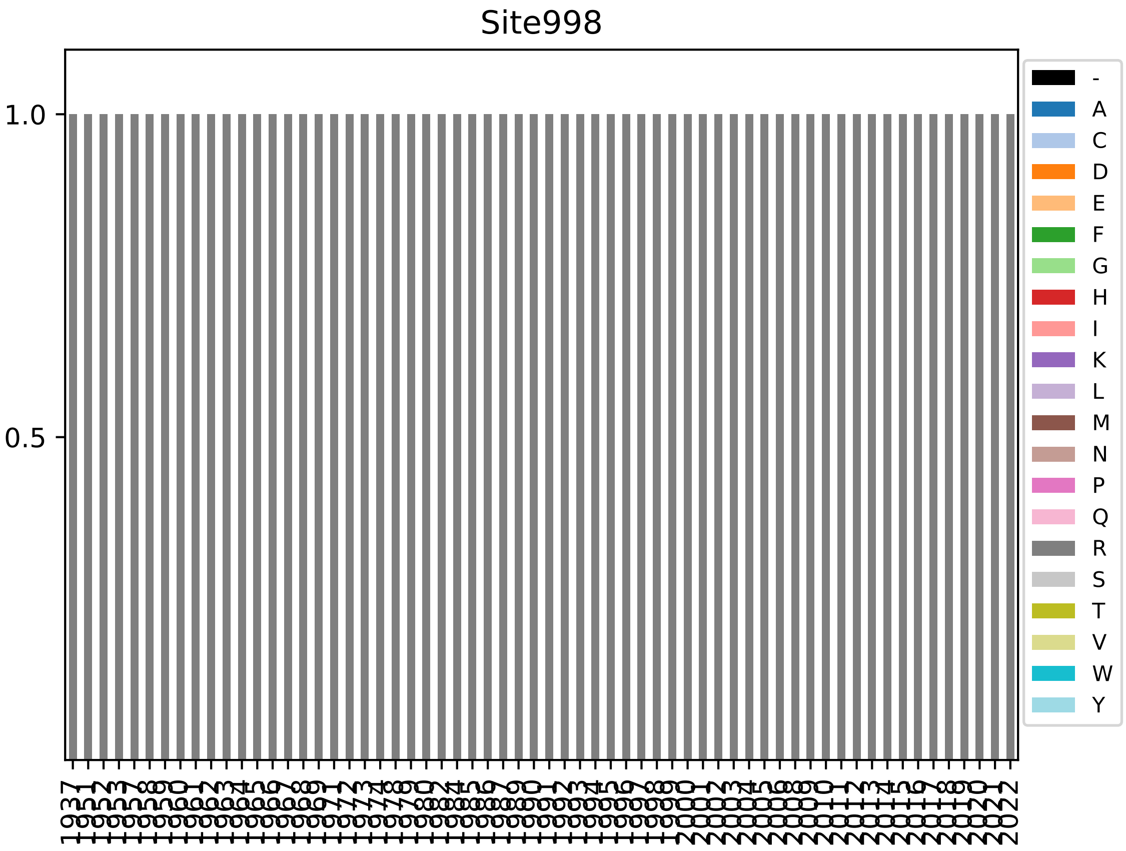
Site998
1.0
0.5
-
A
C
D
E
F
G
H
I
K
L
M
N
P
Q
R
S
T
V
W
Y

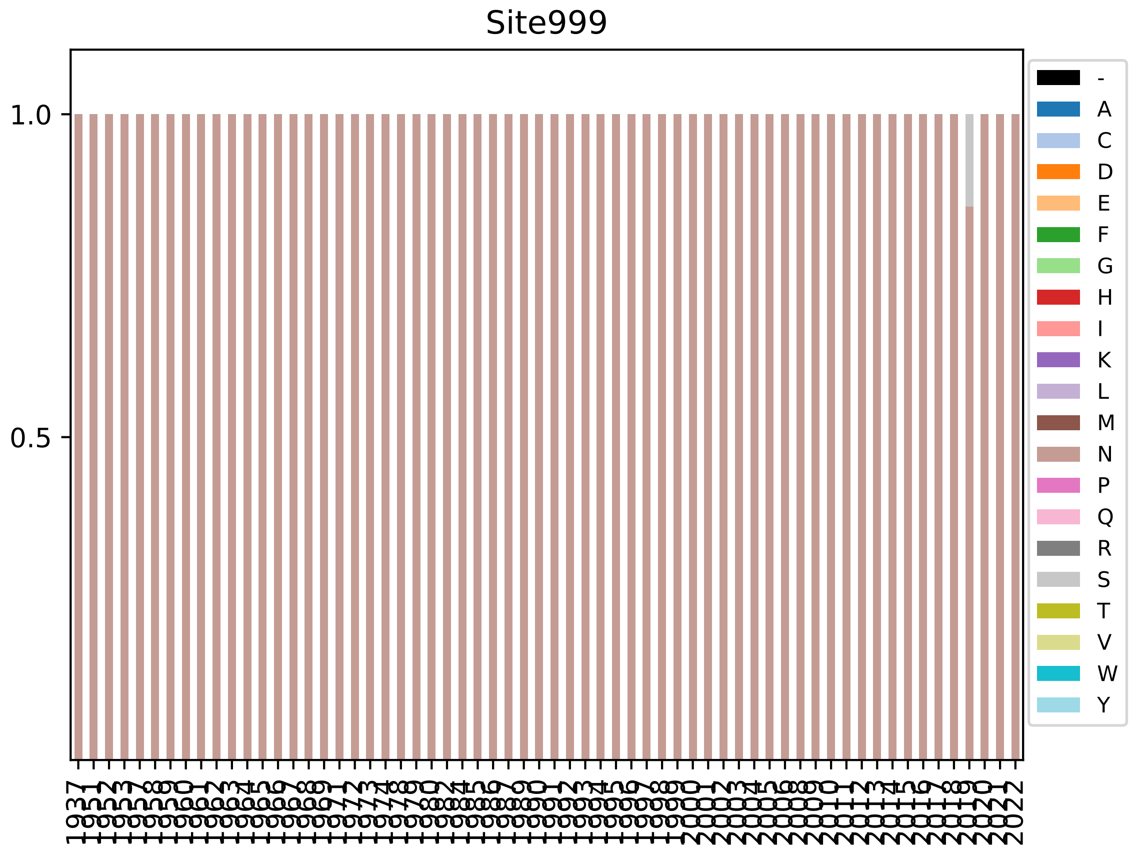
Site999
1.0
0.5
-
A
C
D
E
F
G
H
I
K
L
M
N
P
Q
R
S
T
V
W
Y

Site1000

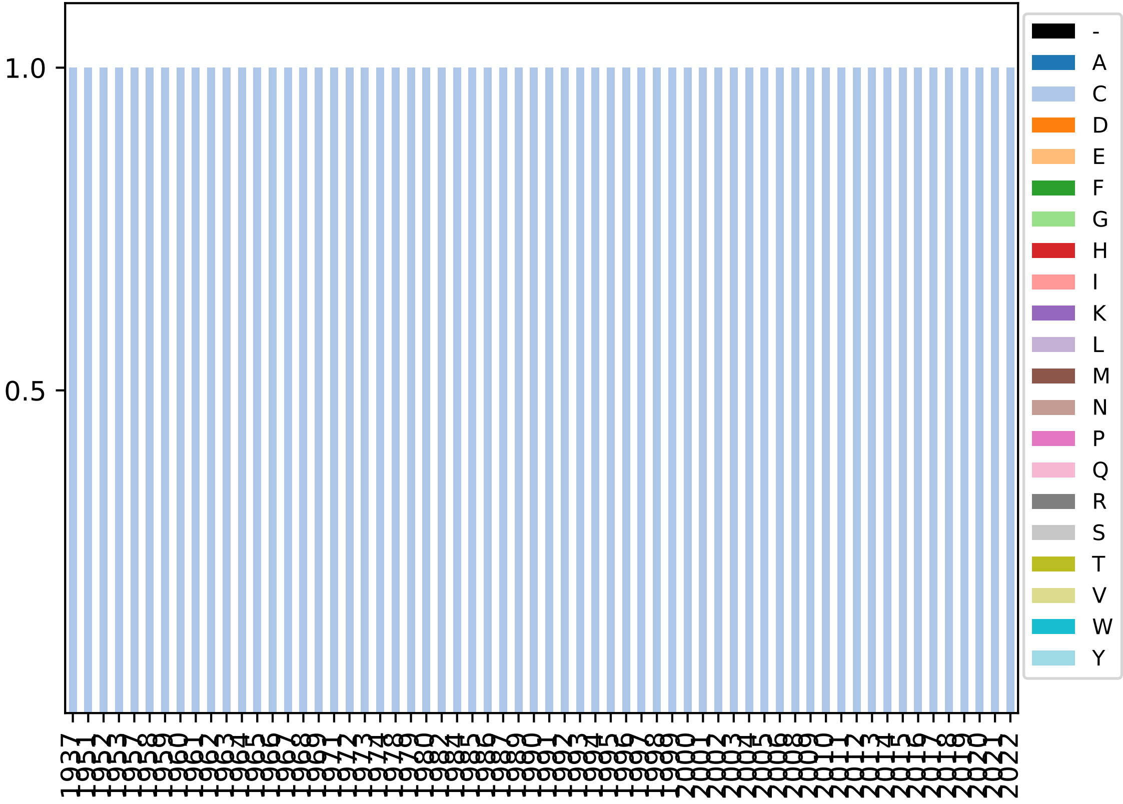

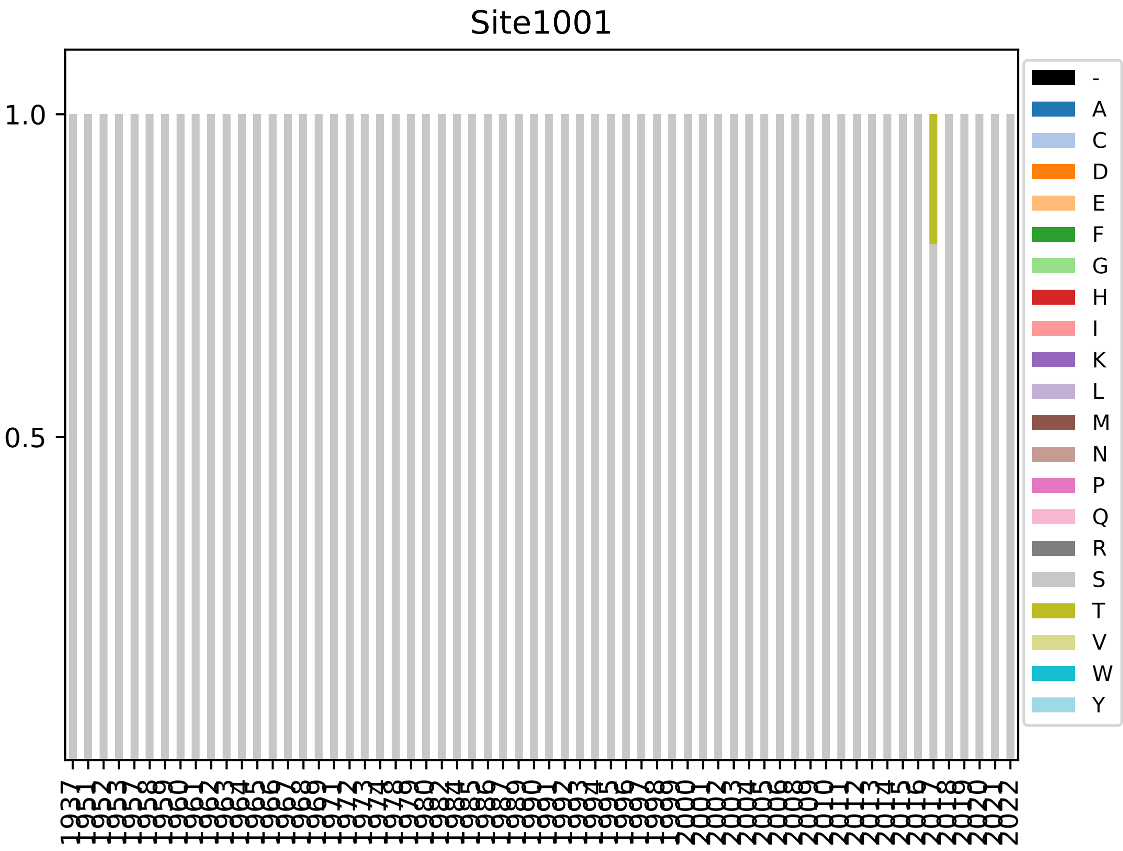

Site1001
1.0
0.5
-
A
C
D
E
F
G
H
I
K
L
M
N
P
Q
R
S
T
V
W
Y

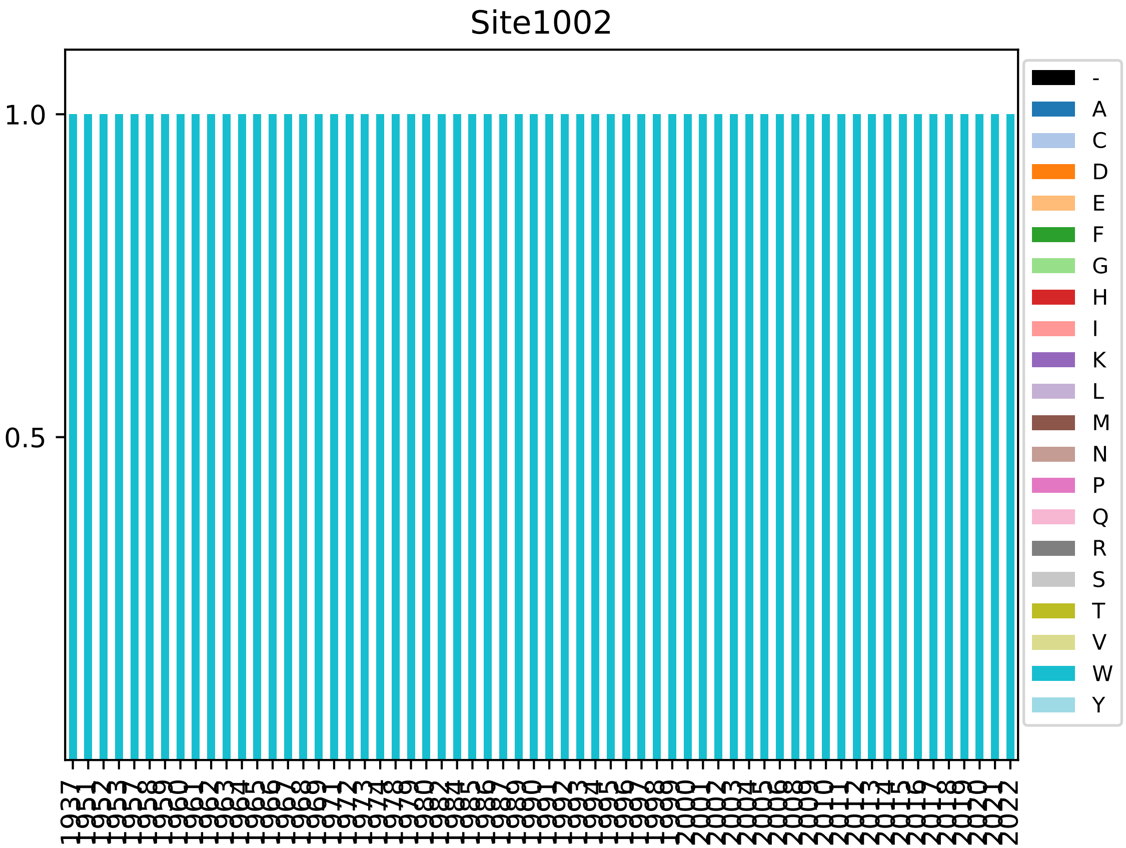
Site1002
1.0
0.5
-
A
C
D
E
F
G
H
I
K
L
M
N
P
Q
R
S
T
V
W
Y

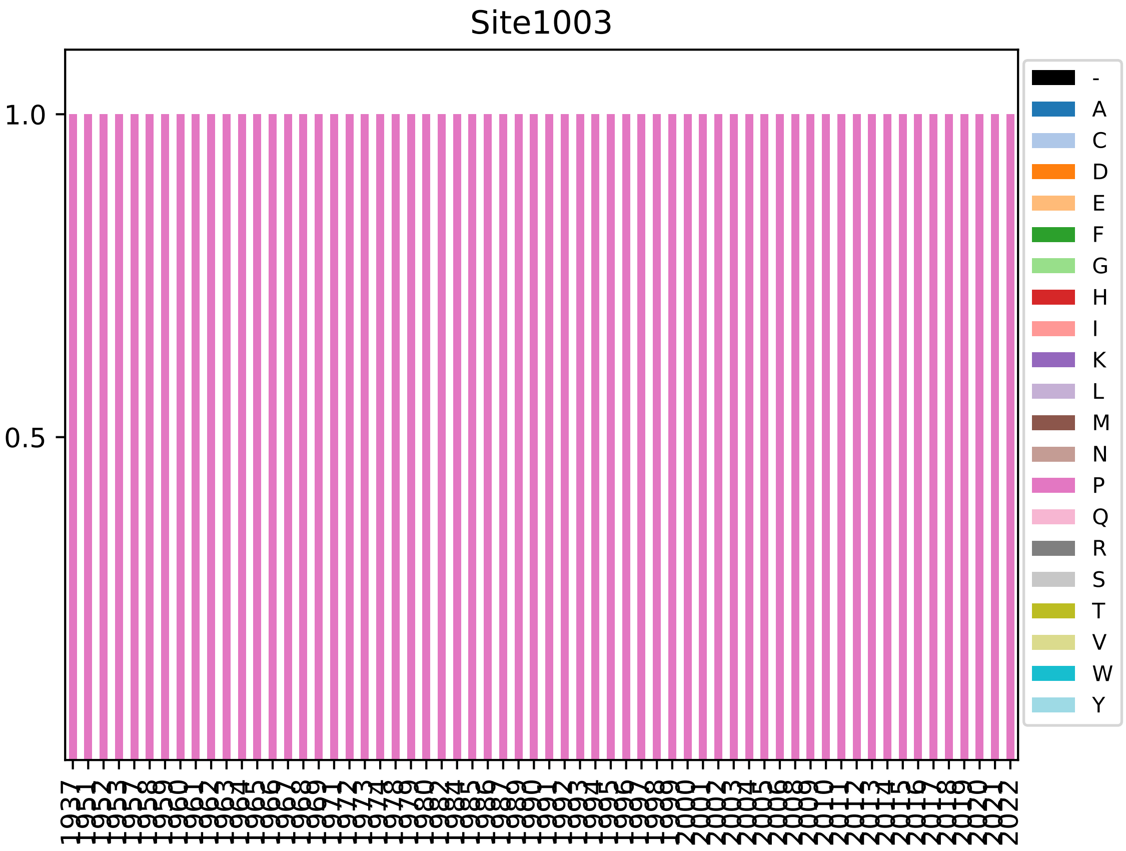
Site1003
1.0
0.5
-
A
C
D
E
F
G
H
I
K
L
M
N
P
Q
R
S
T
V
W
Y

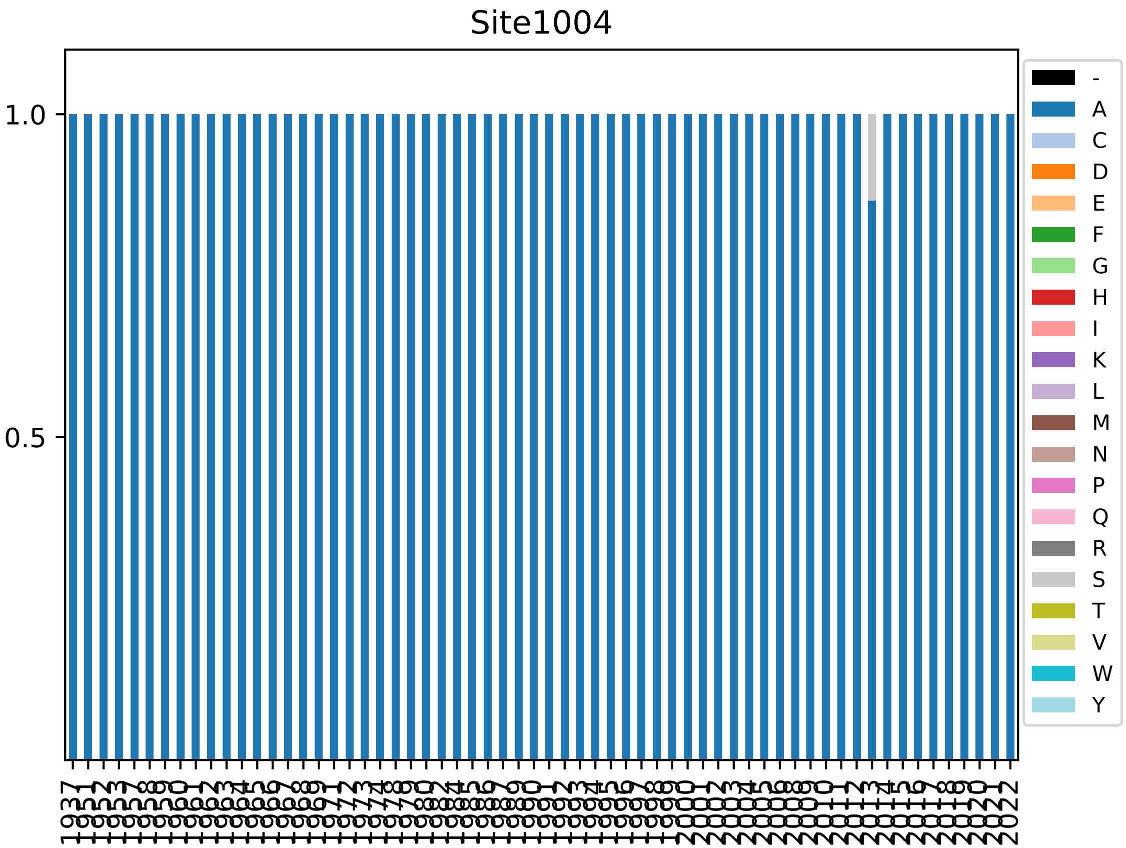
Site1004
1.0
0.5
-
A
C
D
E
F
G
H
I
K
L
M
N
P
Q
R
S
T
V
W
Y

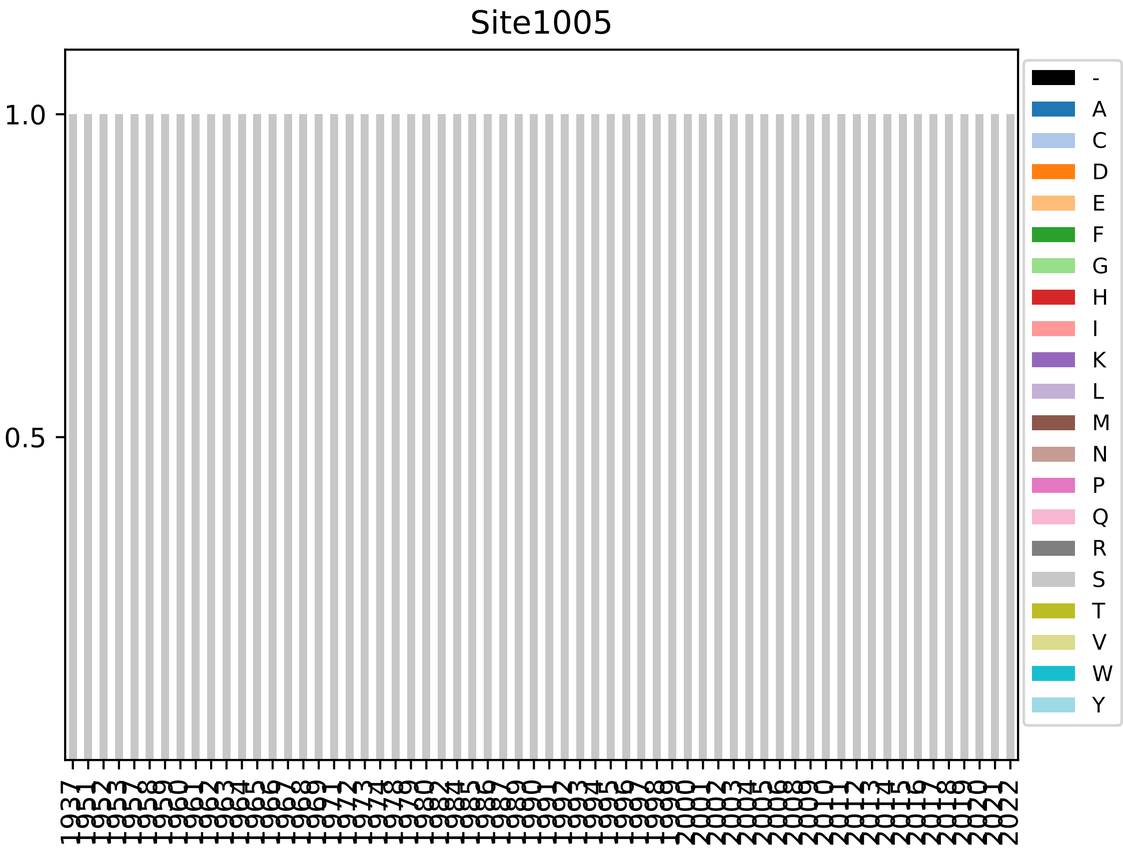
Site1005
1.0
0.5
-
A
C
D
E
F
G
H
I
K
L
M
N
P
Q
R
S
T
V
W
Y

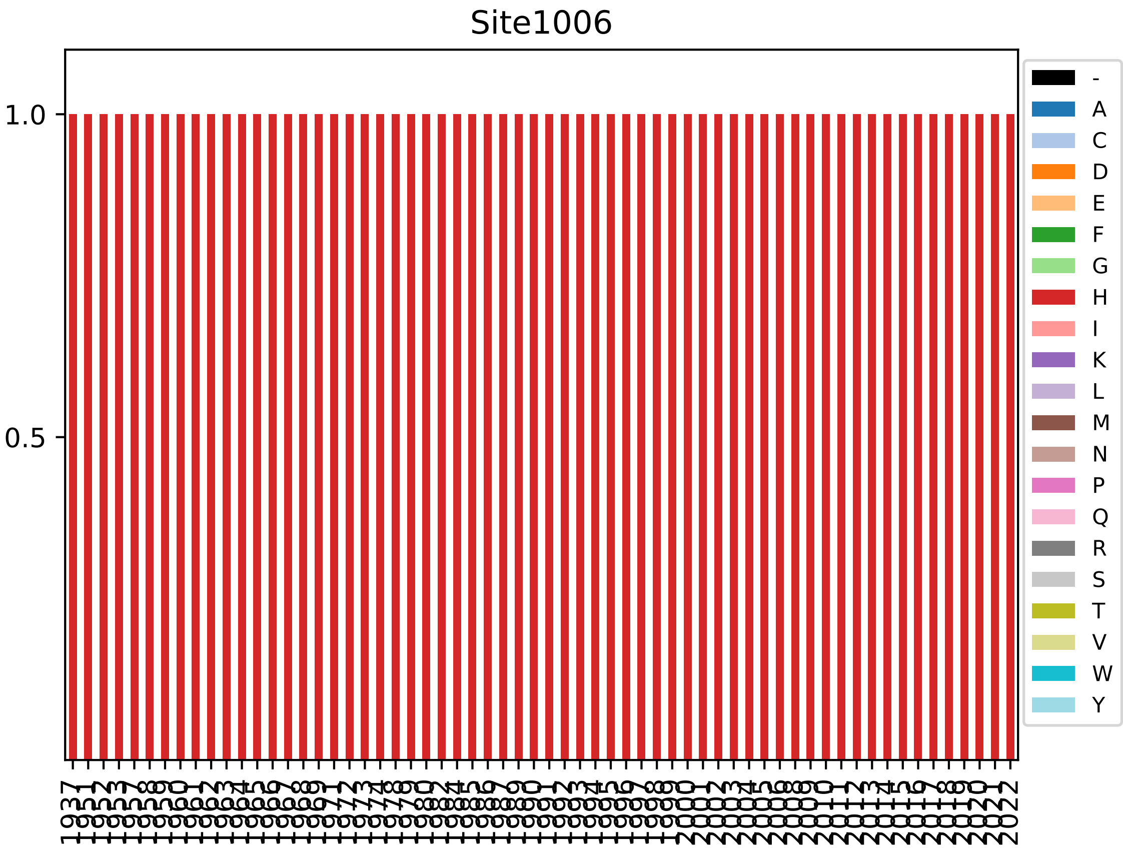
Site1006
1.0
0.5
-
A
C
D
E
F
G
H
I
K
L
M
N
P
Q
R
S
T
V
W
Y

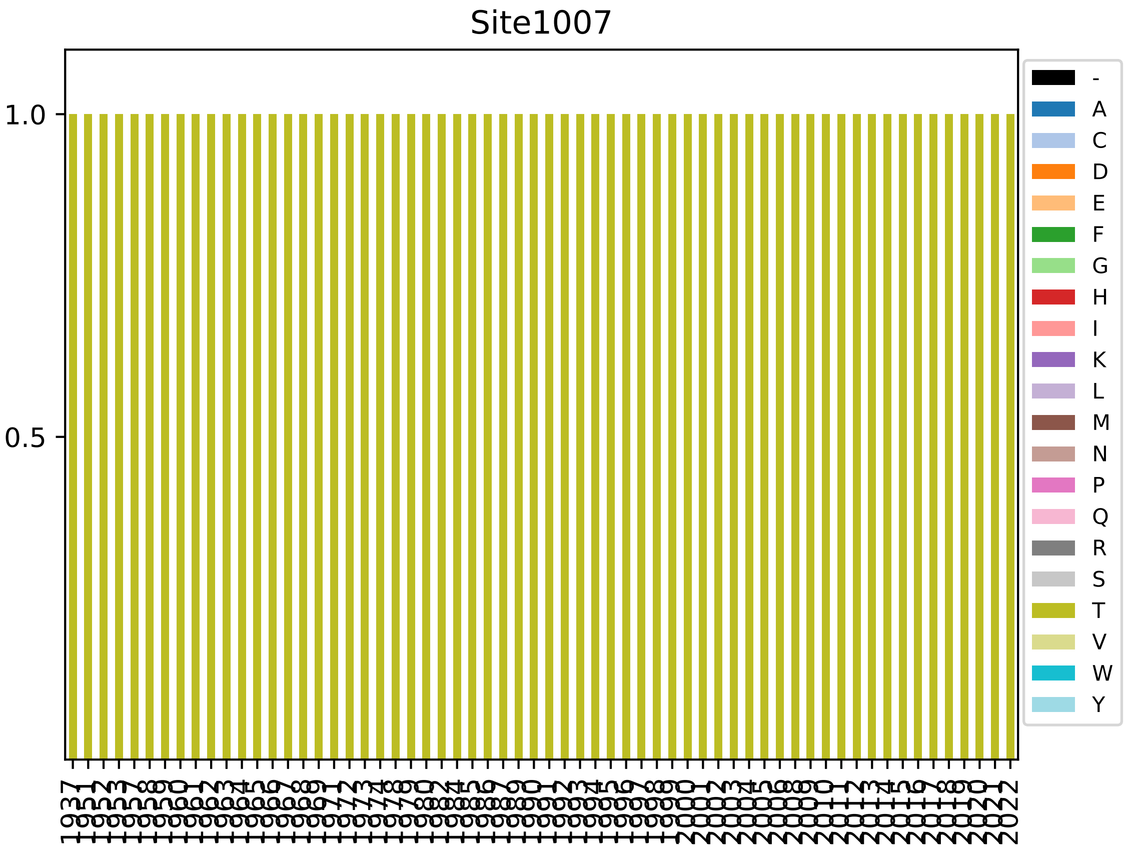

Site1007
1.0
0.5
-
A
C
D
E
F
G
H
I
K
L
M
N
P
Q
R
S
T
V
W
Y

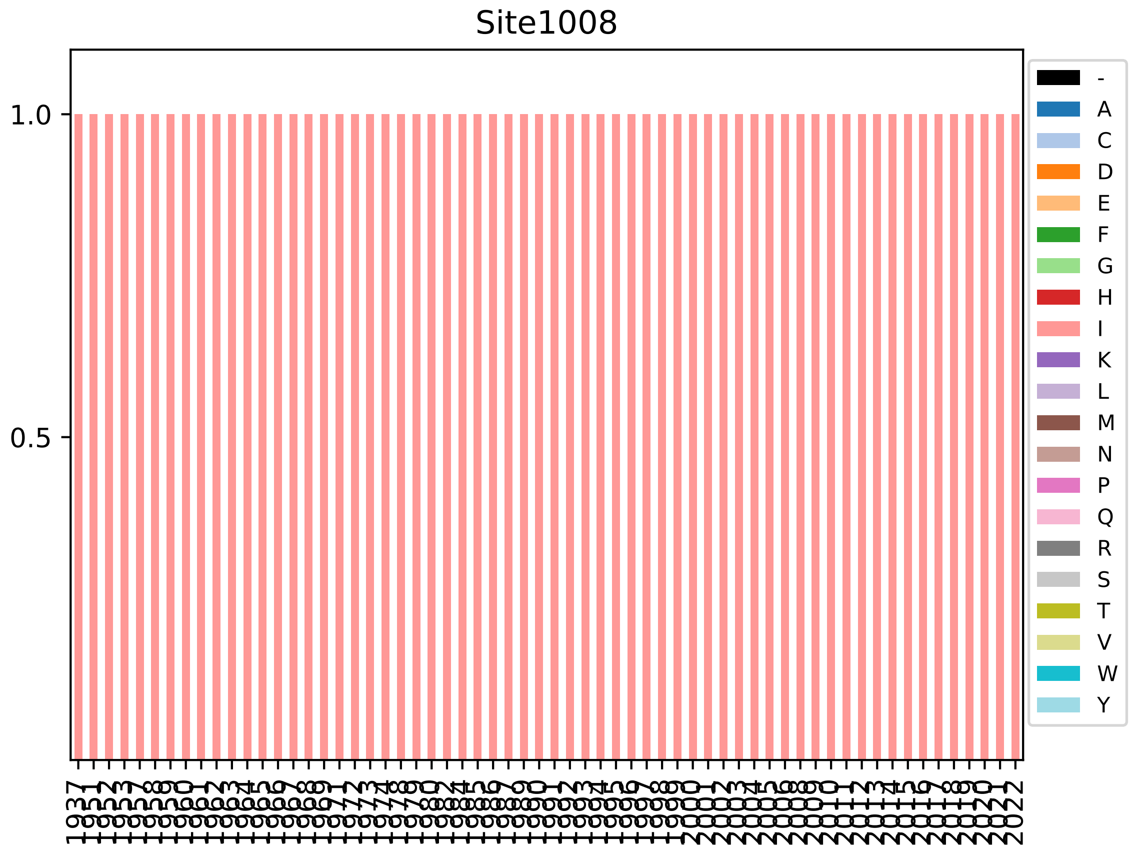

Site1008
1.0
0.5
-
A
C
D
E
F
G
H
I
K
L
M
N
P
Q
R
S
T
V
W
Y

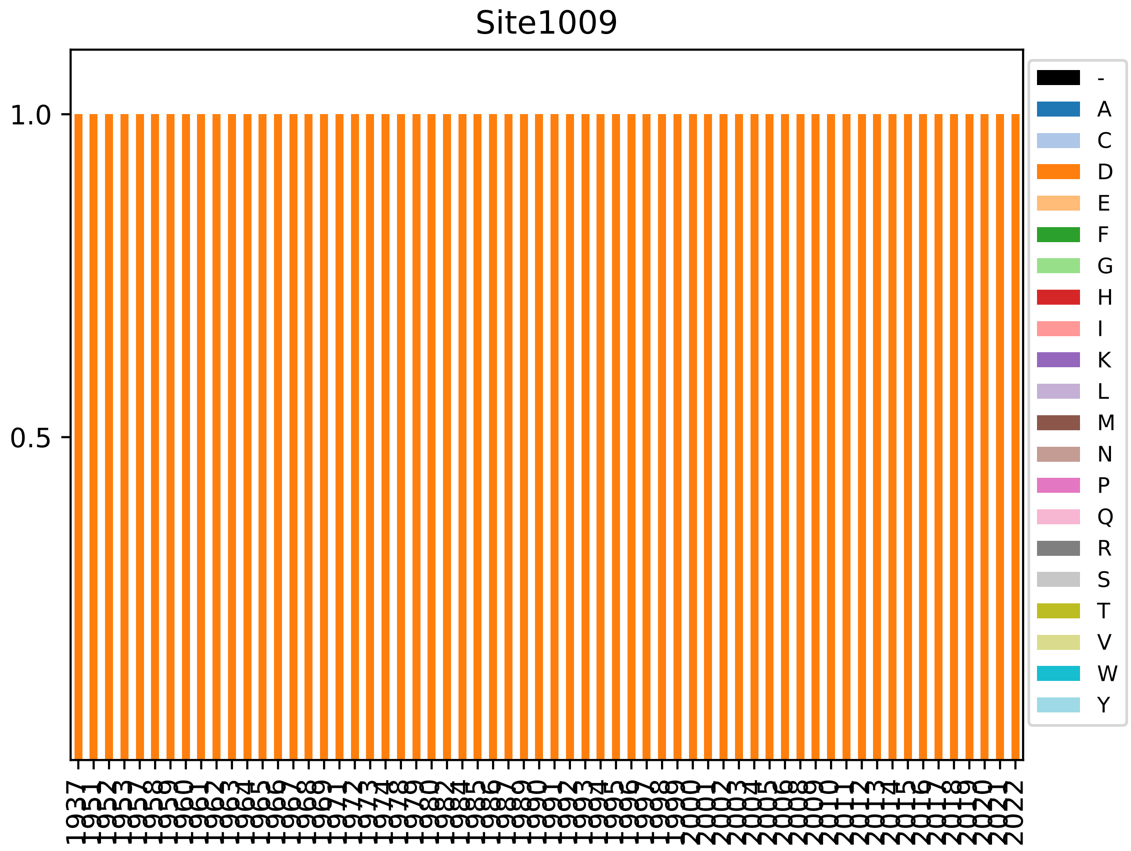
Site1009
1.0
0.5
-
A
C
D
E
F
G
H
I
K
L
M
N
P
Q
R
S
T
V
W
Y

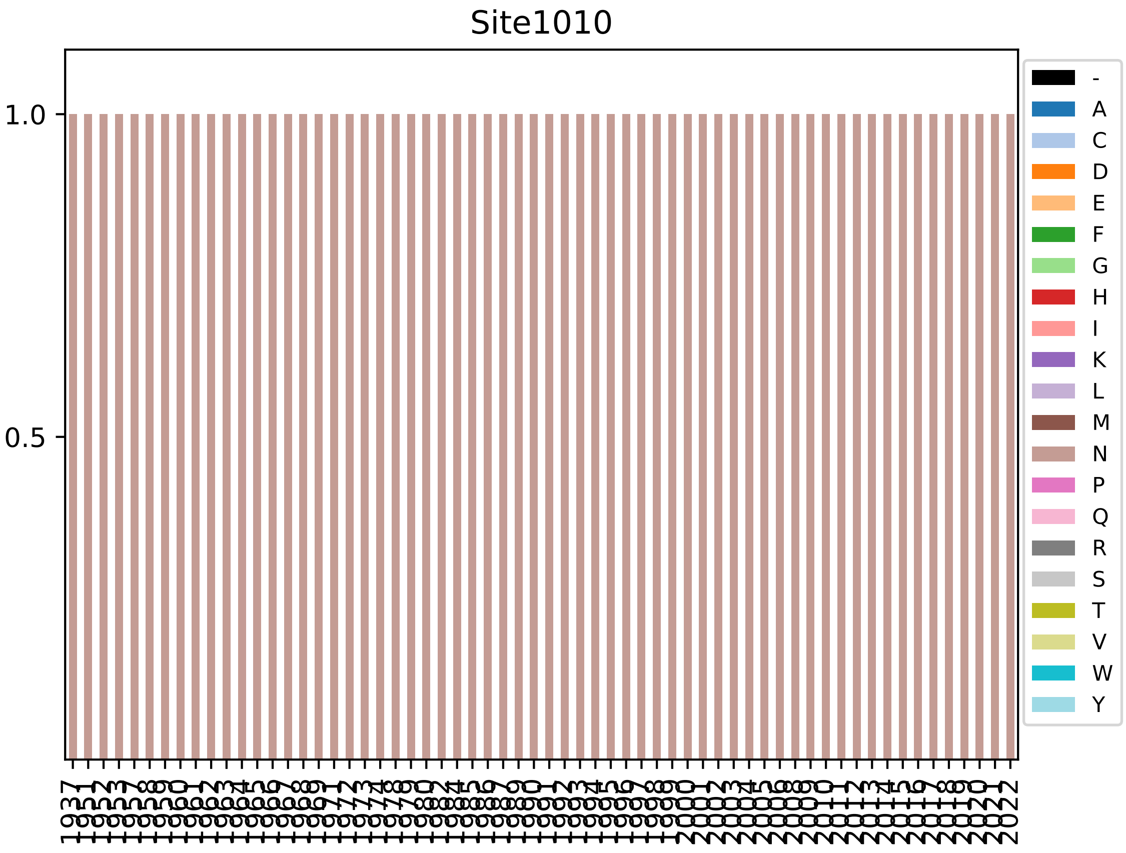
Site1010
1.0
0.5
-
A
C
D
E
F
G
H
I
K
L
M
N
P
Q
R
S
T
V
W
Y

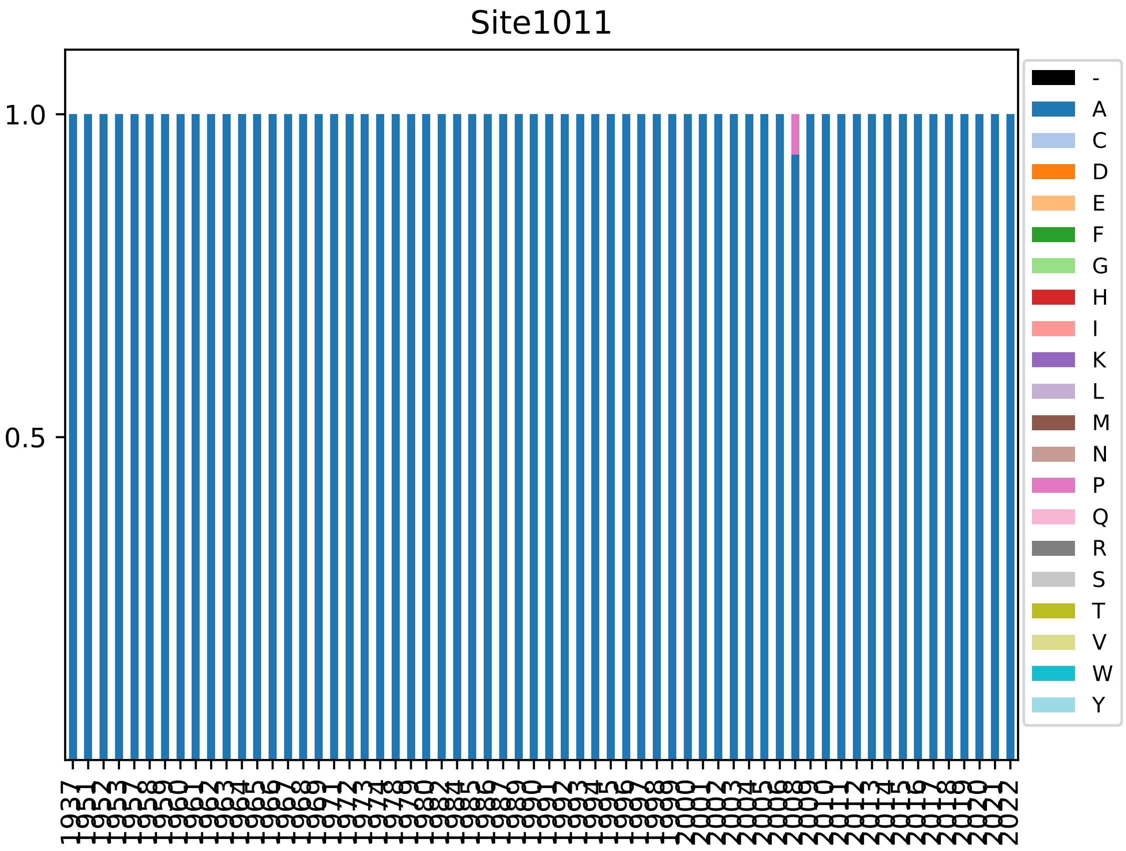
Site1011
1.0
0.5
-
A
C
D
E
F
G
H
I
K
L
M
N
P
Q
R
S
T
V
W
Y

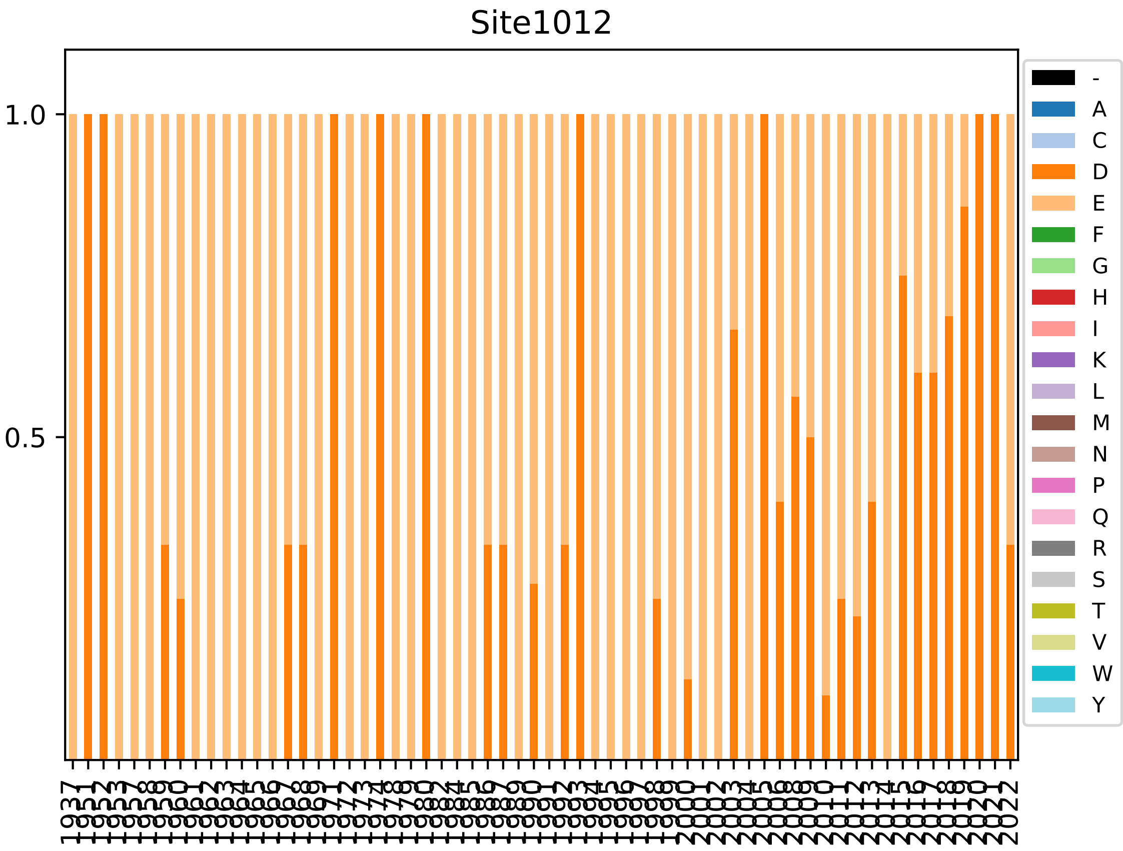
Site1012
1.0
0.5
-
A
C
D
E
F
G
H
I
K
L
M
N
P
Q
R
S
T
V
W
Y

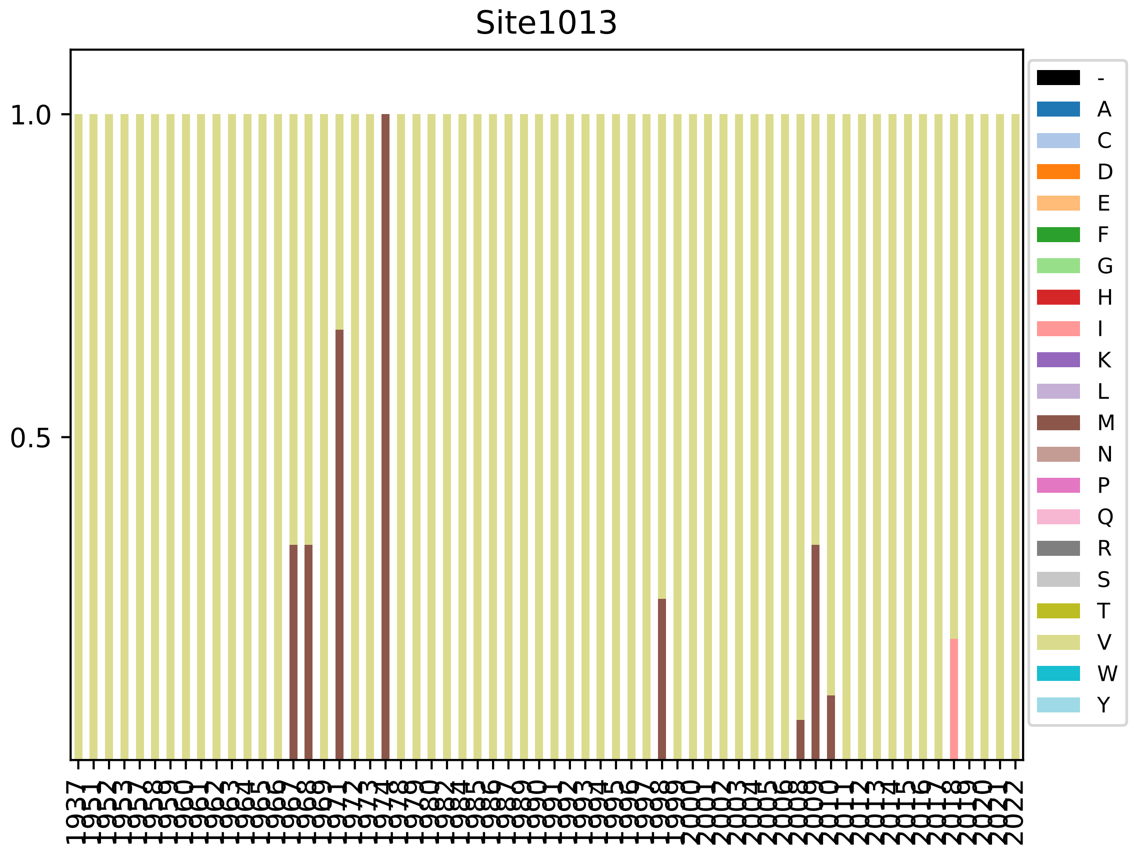
Site1013
1.0
0.5
-
A
C
D
E
F
G
H
I
K
L
M
N
P
Q
R
S
T
V
W
Y

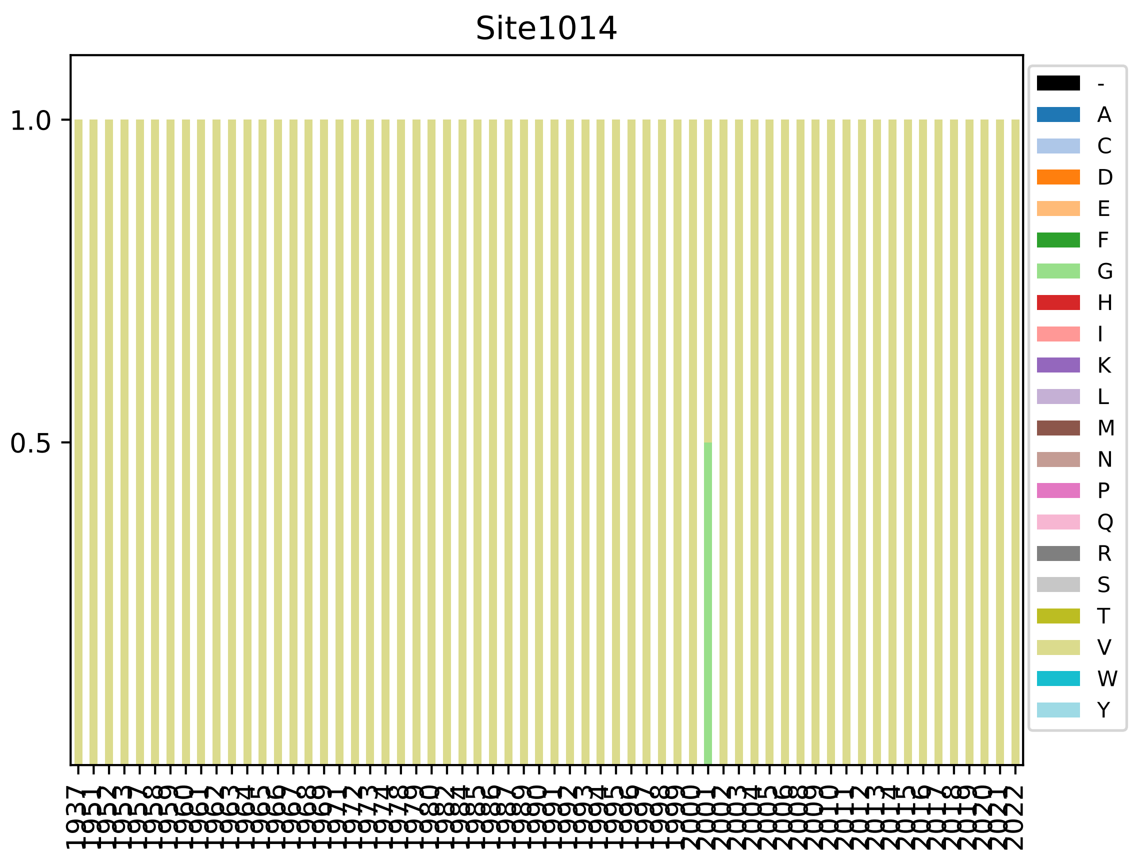
Site1014
1.0
0.5
-
A
C
D
E
F
G
H
I
K
L
M
N
P
Q
R
S
T
V
W
Y

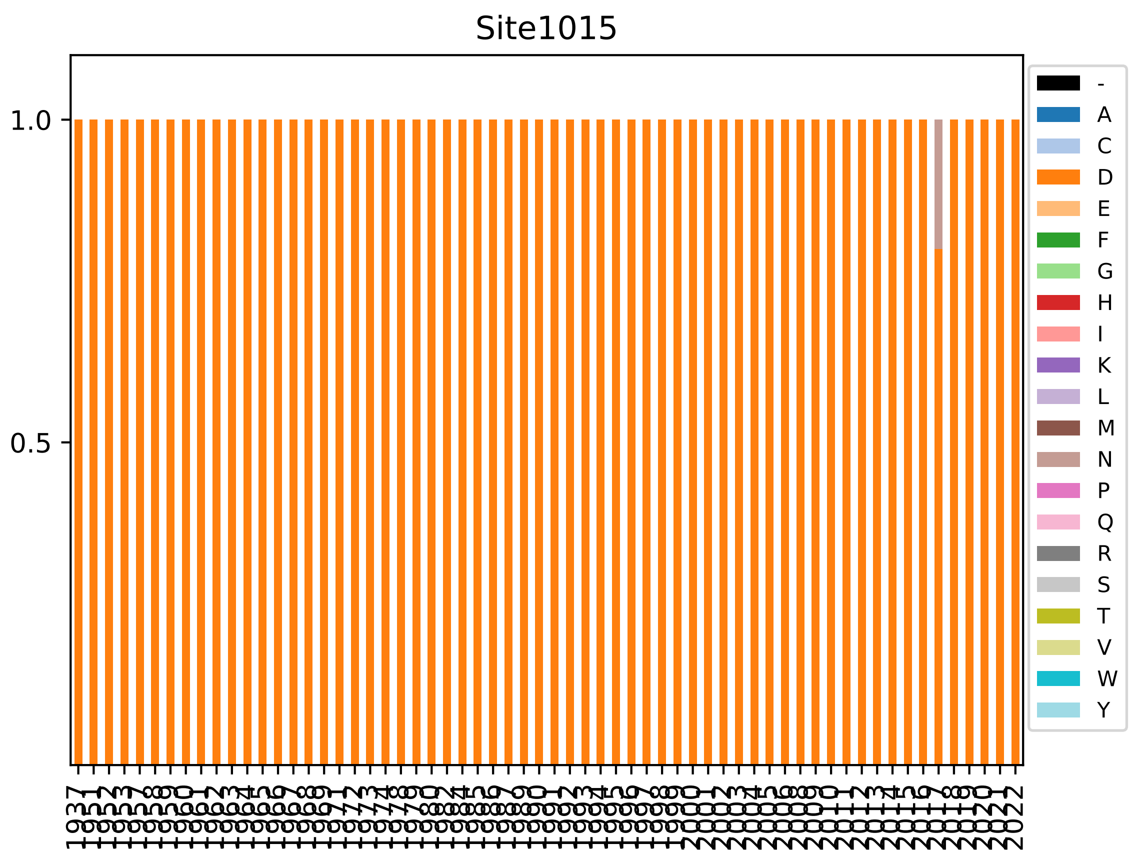
Site1015
1.0
0.5
-
A
C
D
E
F
G
H
I
K
L
M
N
P
Q
R
S
T
V
W
Y

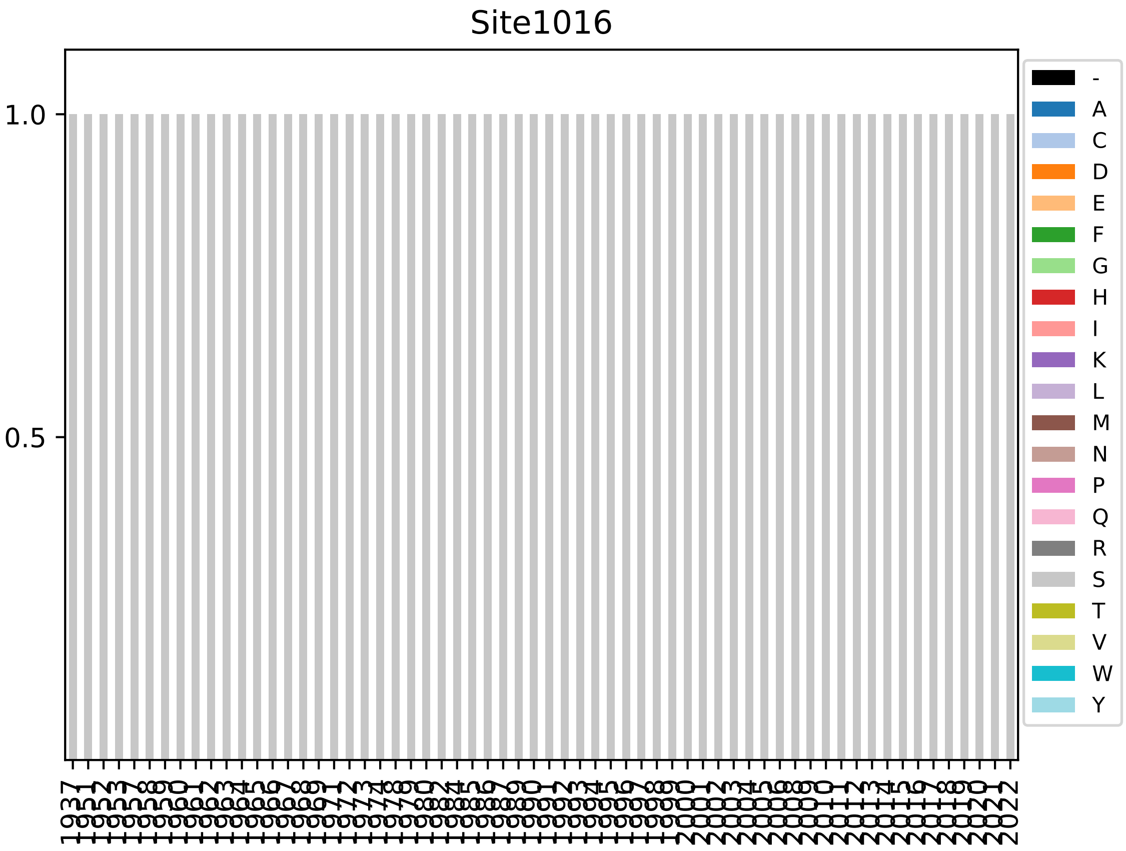
Site1016
1.0
0.5
-
A
C
D
E
F
G
H
I
K
L
M
N
P
Q
R
S
T
V
W
Y

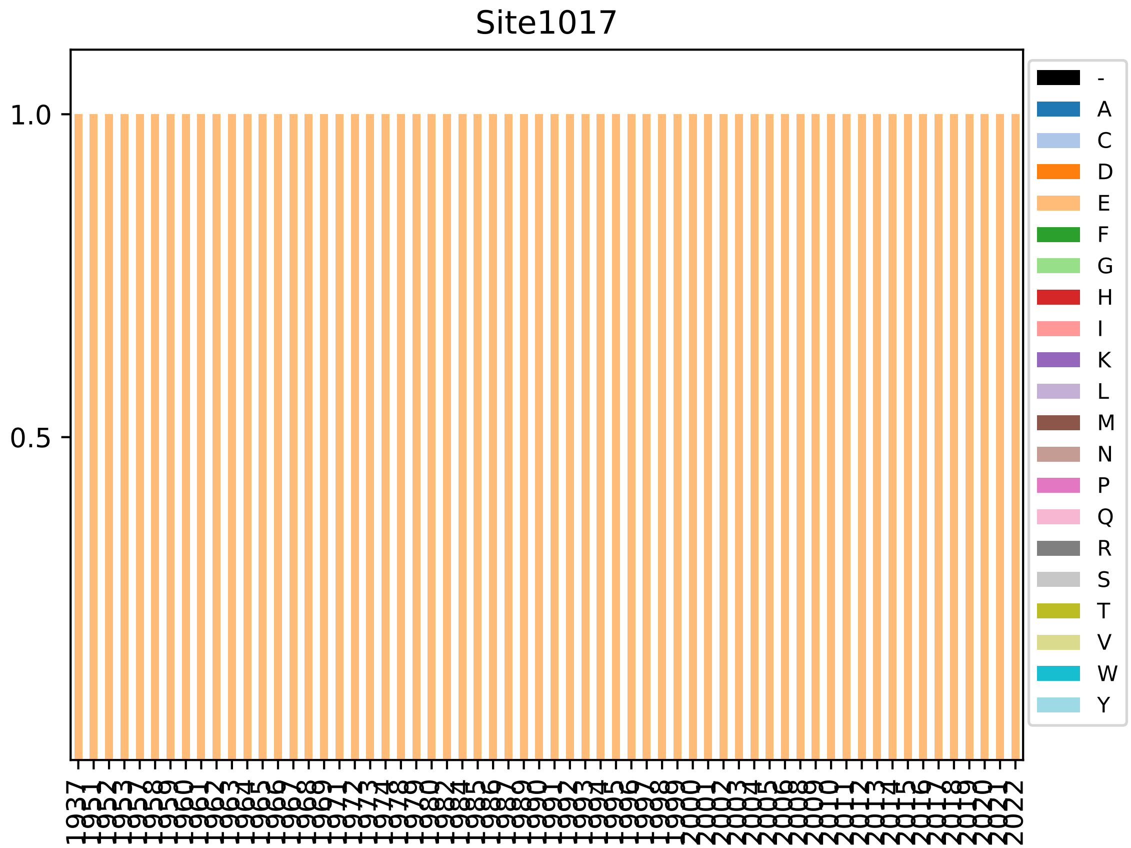
Site1017
1.0
0.5
-
A
C
D
E
F
G
H
I
K
L
M
N
P
Q
R
S
T
V
W
Y

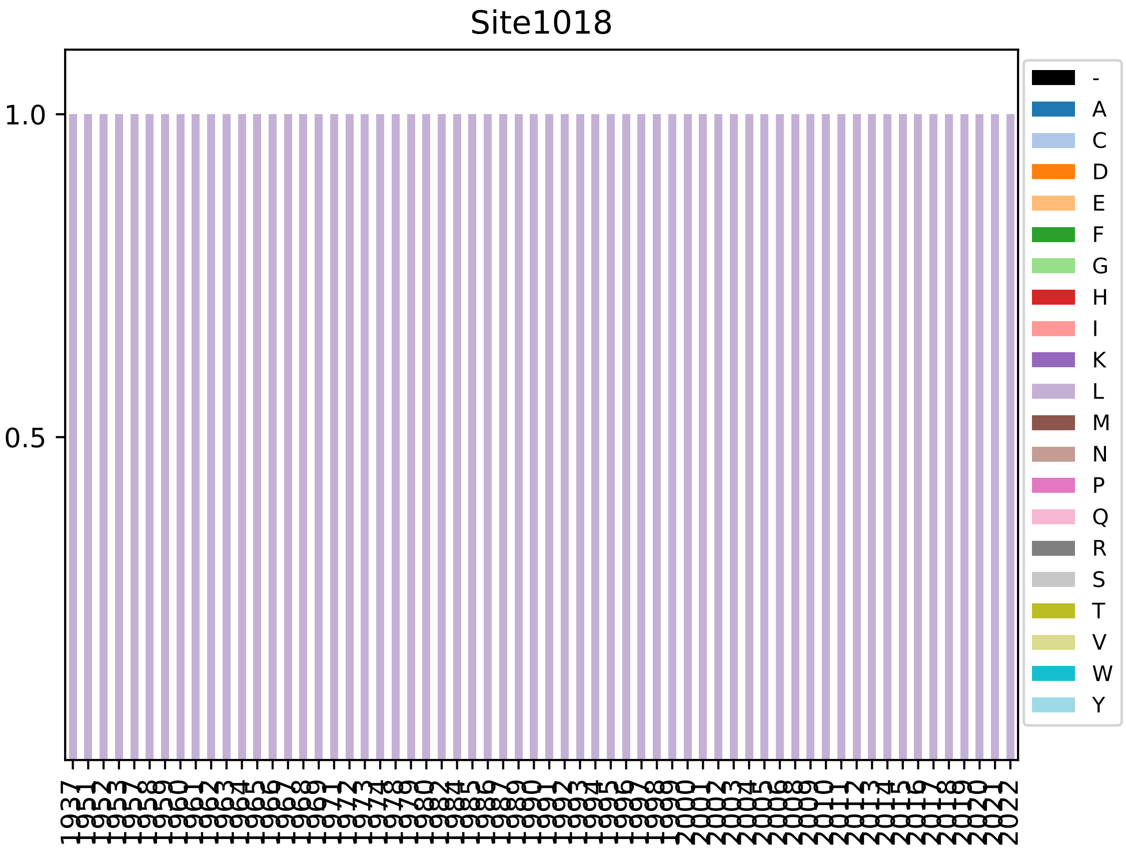
Site1018
1.0
0.5
-
A
C
D
E
F
G
H
I
K
L
M
N
P
Q
R
S
T
V
W
Y

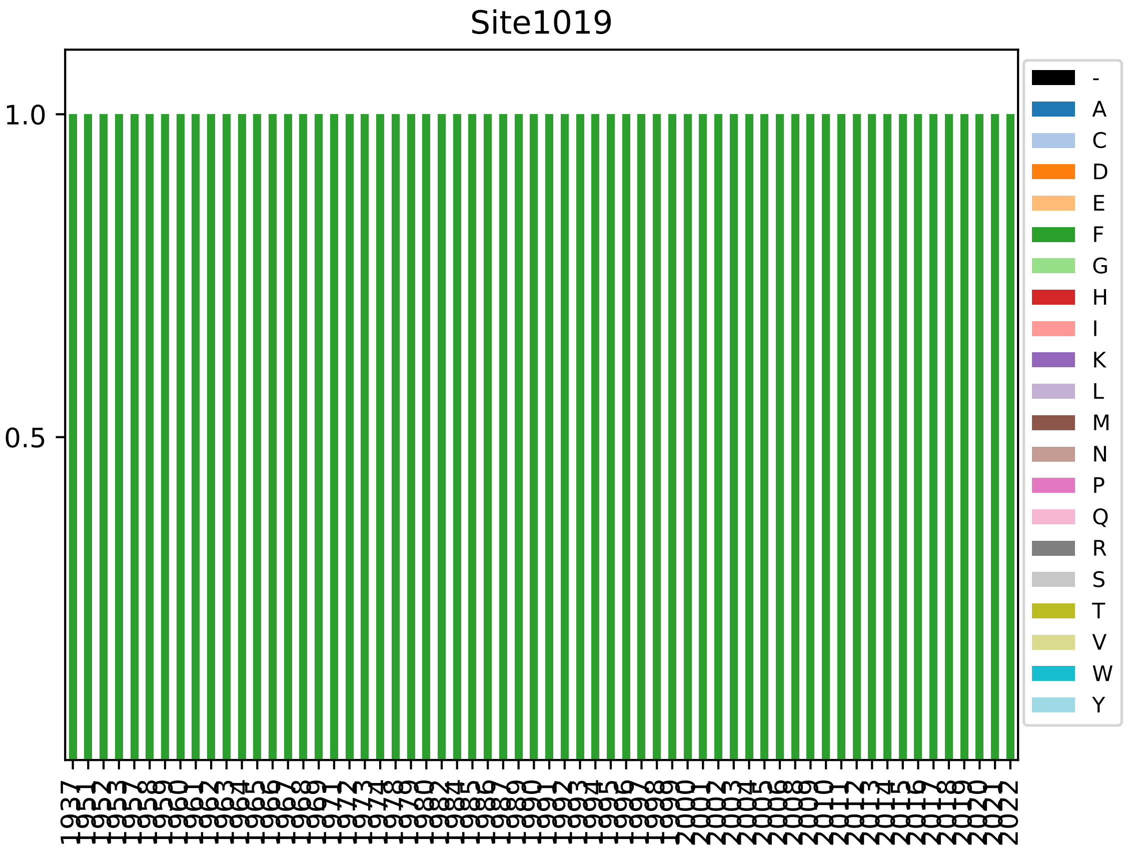
Site1019
1.0
0.5
-
A
C
D
E
F
G
H
I
K
L
M
N
P
Q
R
S
T
V
W
Y

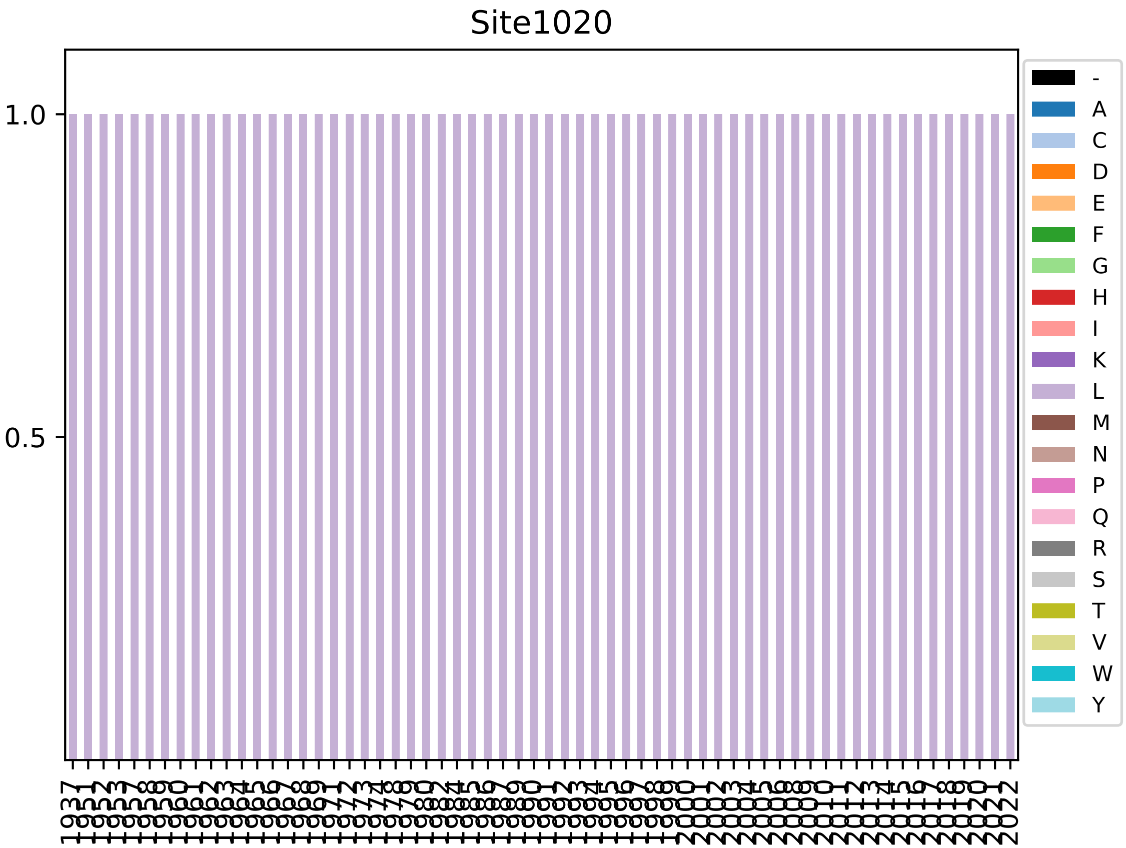
Site1020
1.0
0.5
-
A
C
D
E
F
G
H
I
K
L
M
N
P
Q
R
S
T
V
W
Y

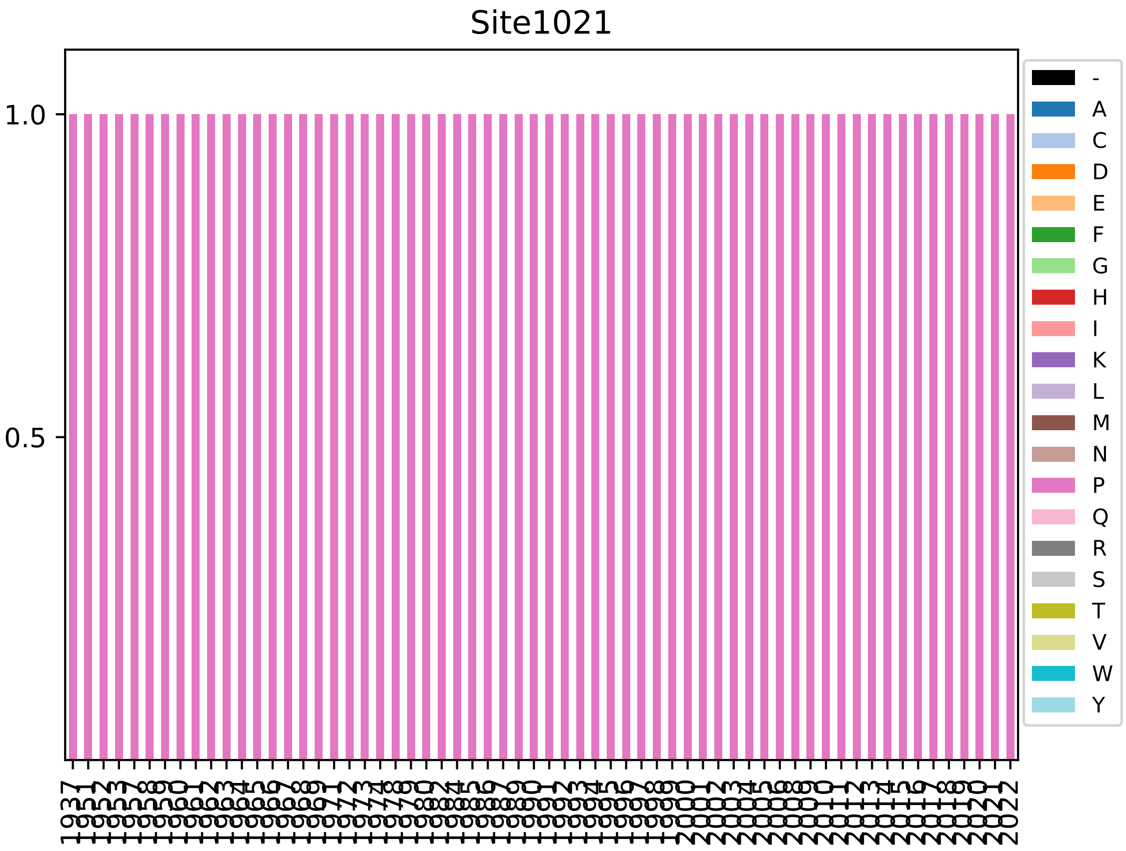
Site1021
1.0
0.5
-
A
C
D
E
F
G
H
I
K
L
M
N
P
Q
R
S
T
V
W
Y

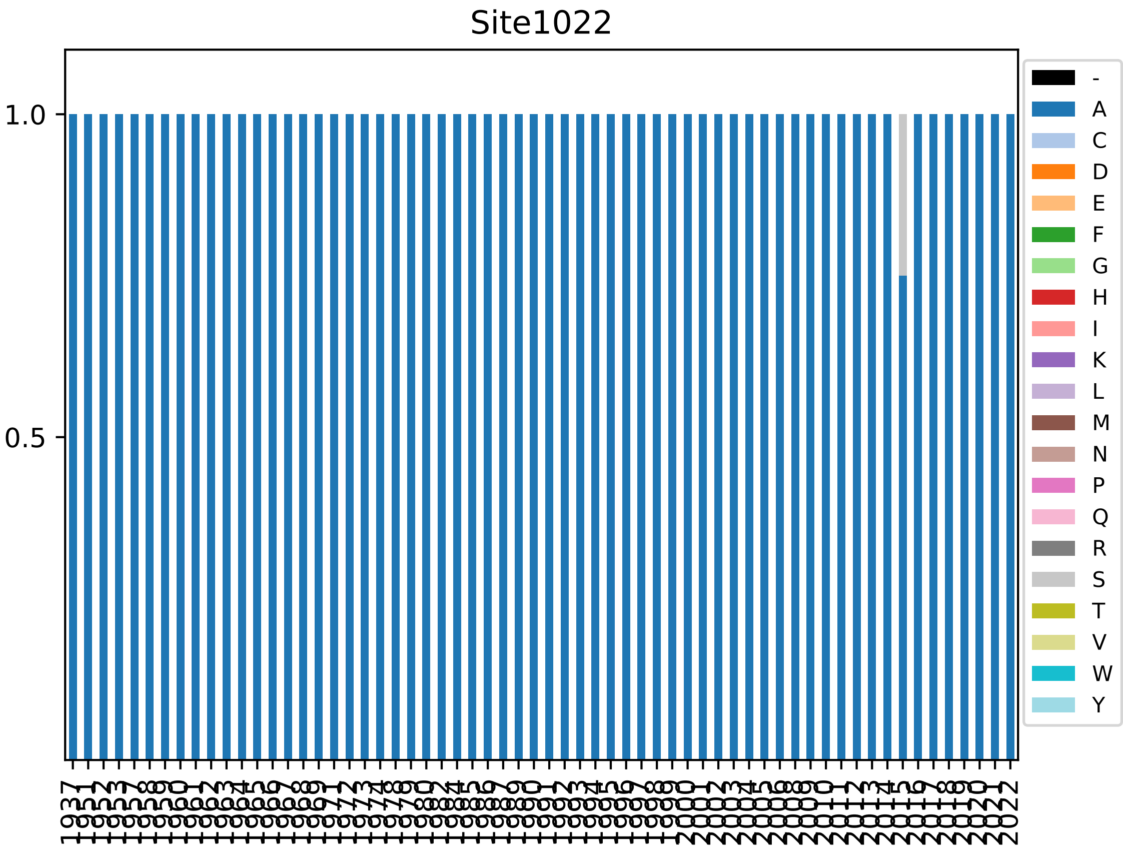
Site1022
1.0
0.5
-
A
C
D
E
F
G
H
I
K
L
M
N
P
Q
R
S
T
V
W
Y

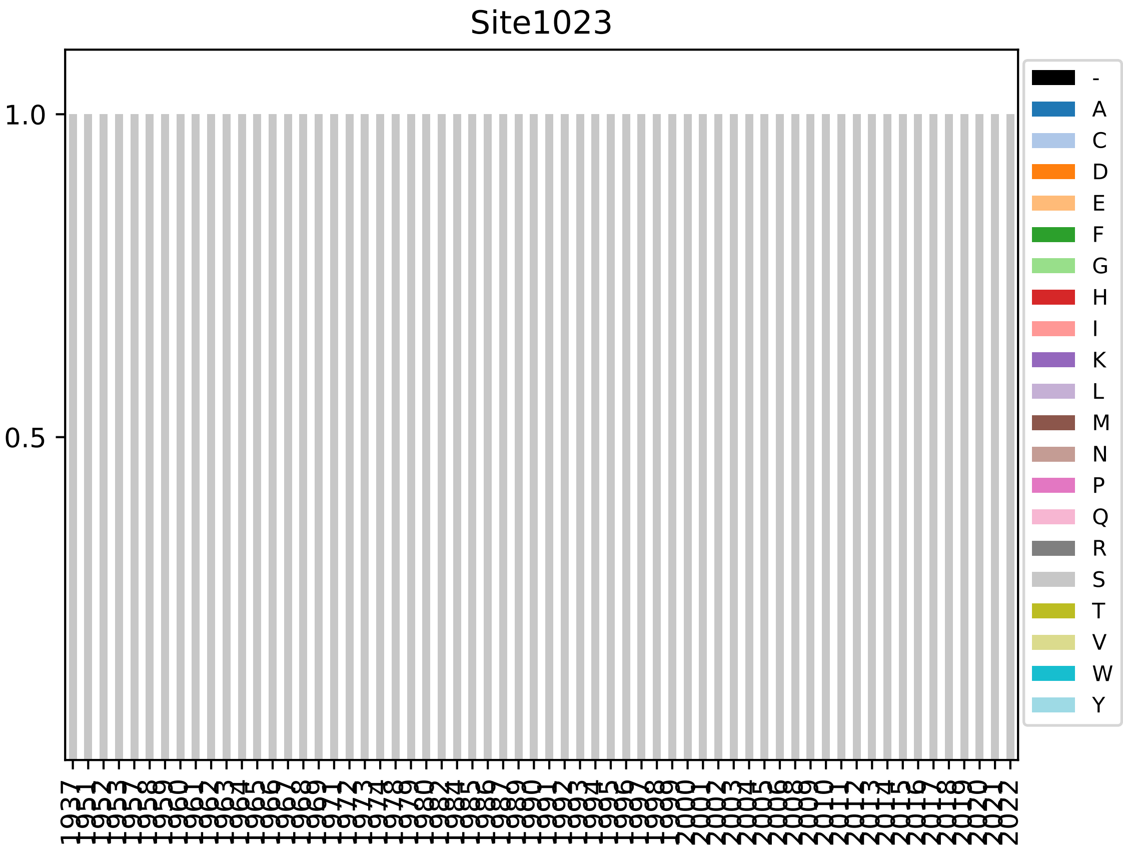
Site1023
1.0
0.5
-
A
C
D
E
F
G
H
I
K
L
M
N
P
Q
R
S
T
V
W
Y

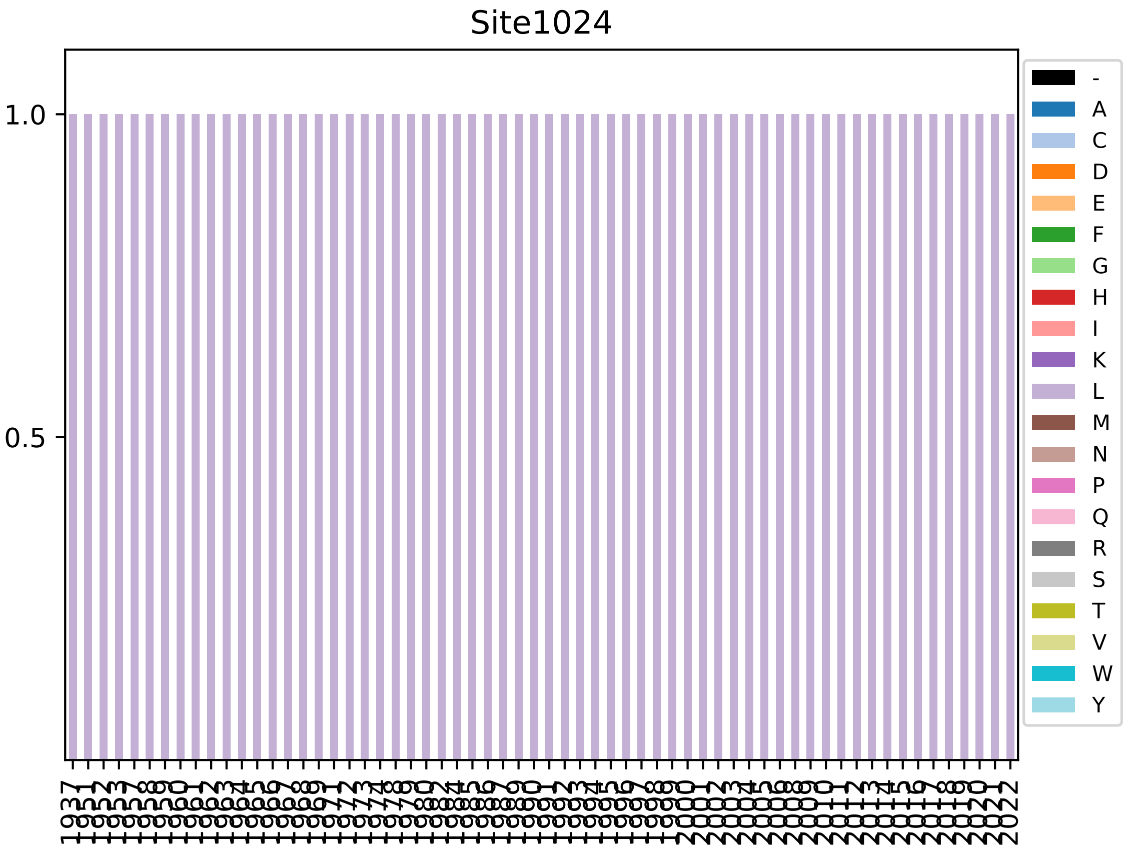
Site1024
1.0
0.5
-
A
C
D
E
F
G
H
I
K
L
M
N
P
Q
R
S
T
V
W
Y

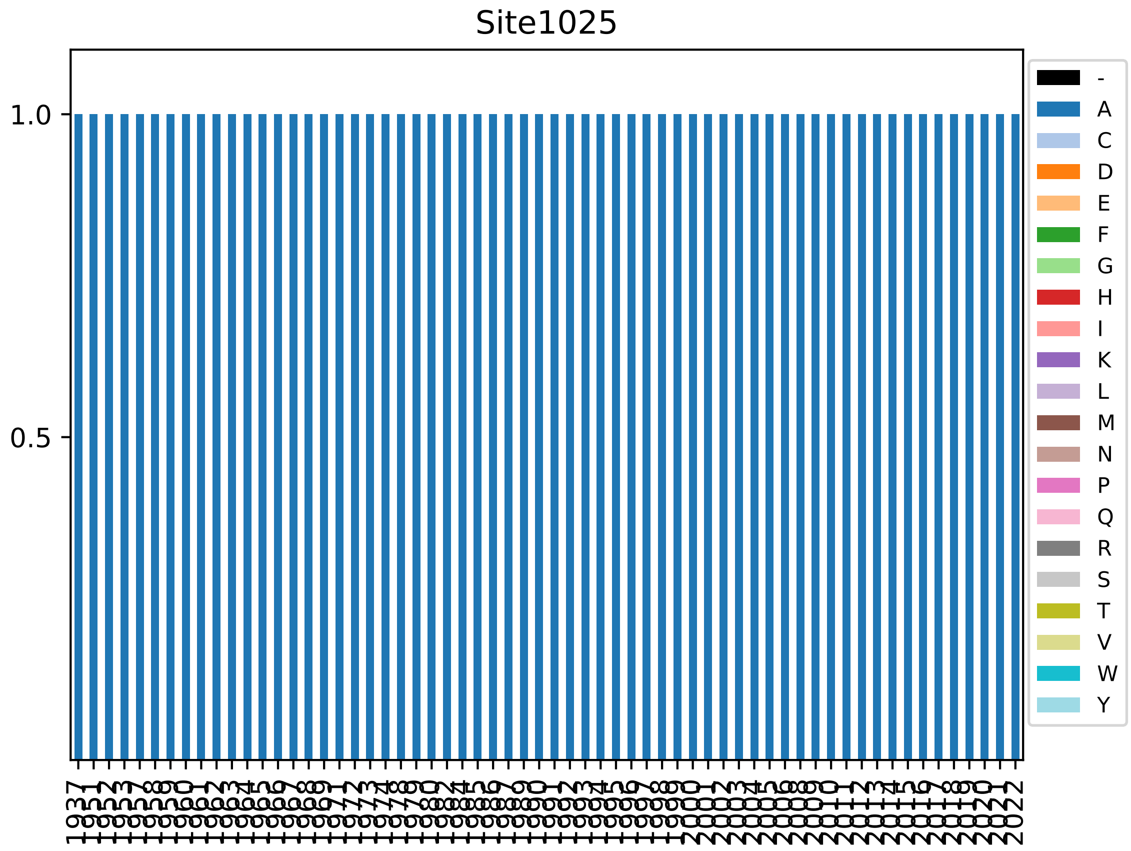
Site1025
1.0
0.5
-
A
C
D
E
F
G
H
I
K
L
M
N
P
Q
R
S
T
V
W
Y

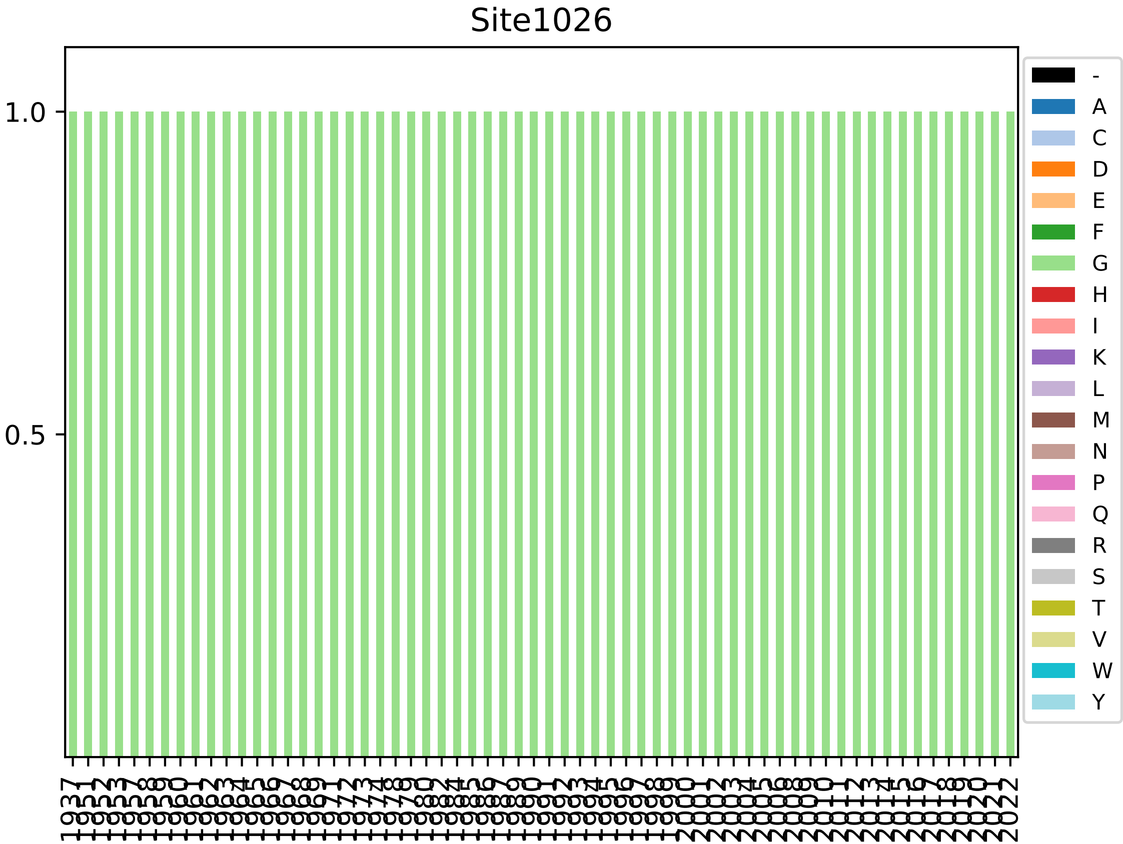
Site1026
1.0
0.5
-
A
C
D
E
F
G
H
I
K
L
M
N
P
Q
R
S
T
V
W
Y

Site1027

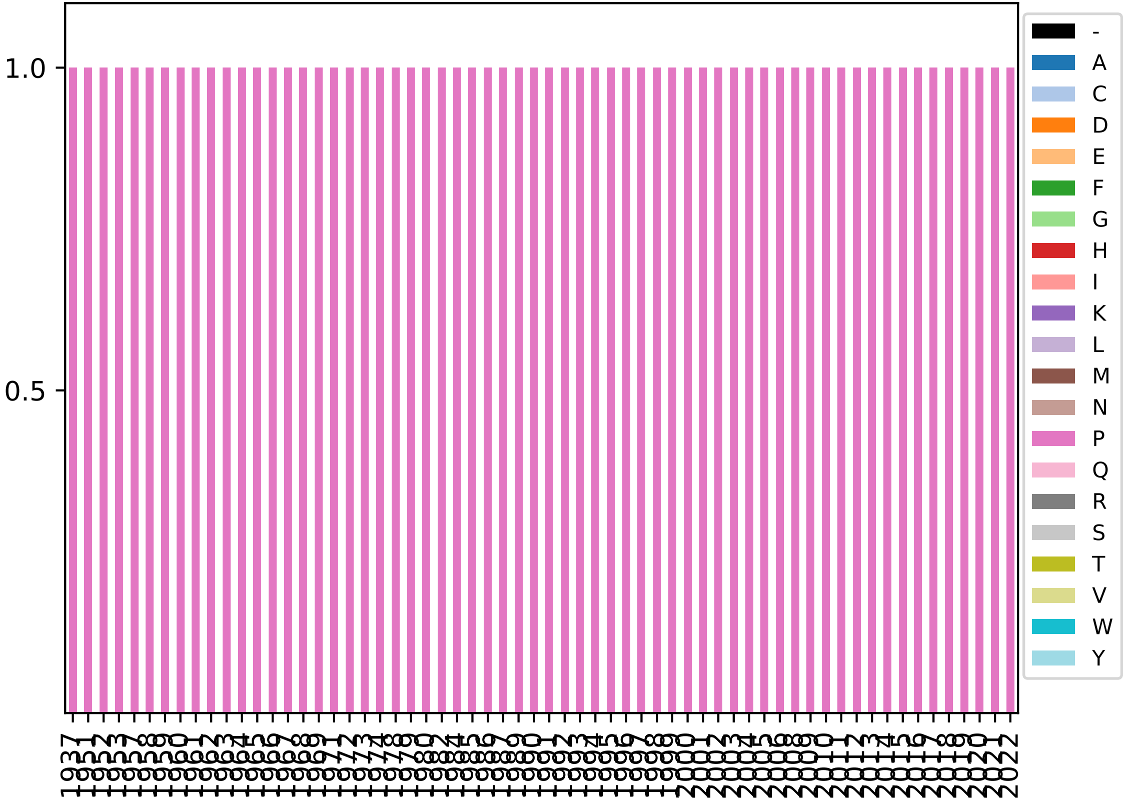

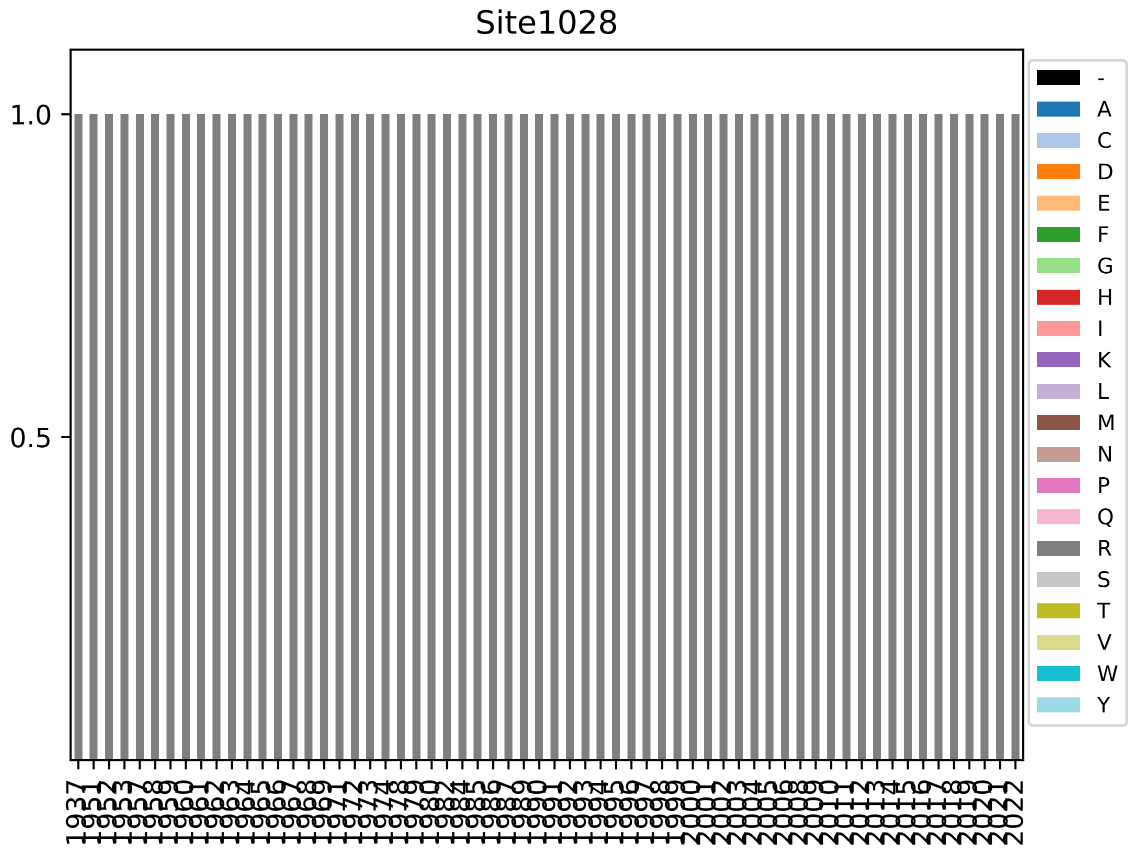
Site1028
1.0
0.5
-
A
C
D
E
F
G
H
I
K
L
M
N
P
Q
R
S
T
V
W
Y

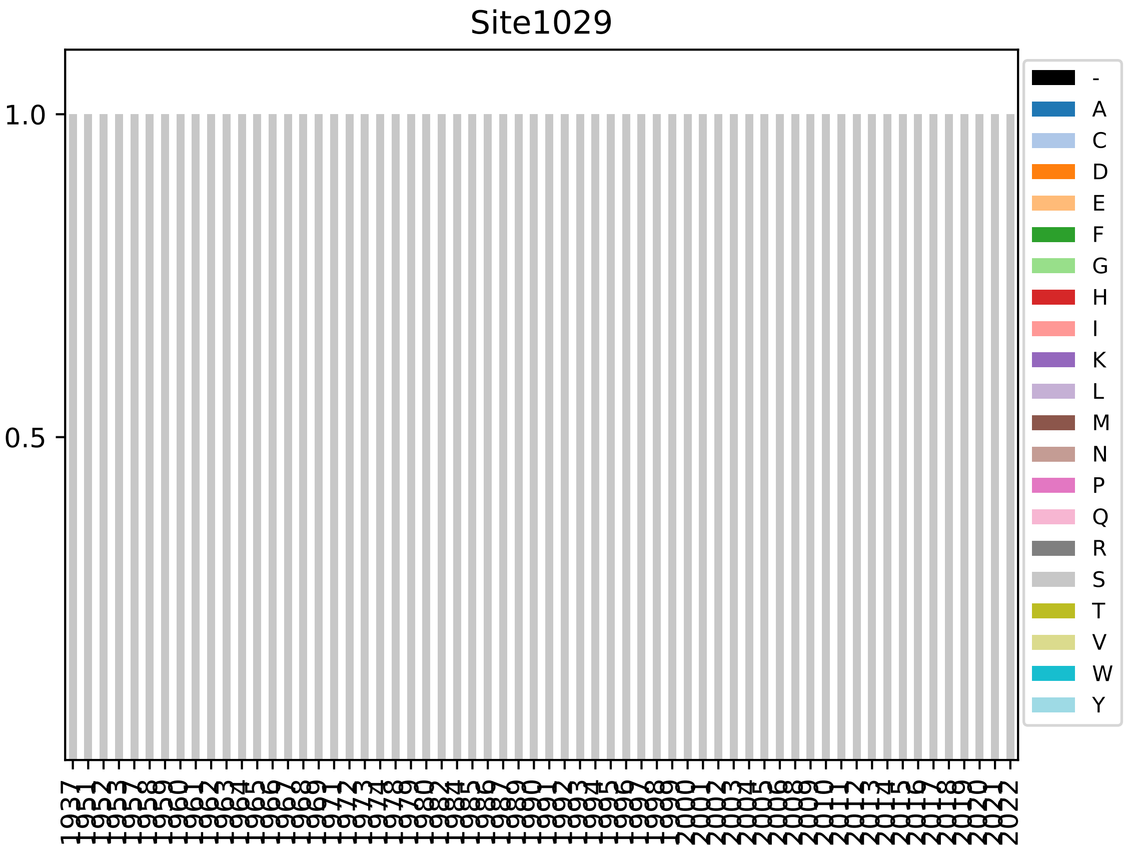
Site1029
1.0
0.5
-
A
C
D
E
F
G
H
I
K
L
M
N
P
Q
R
S
T
V
W
Y

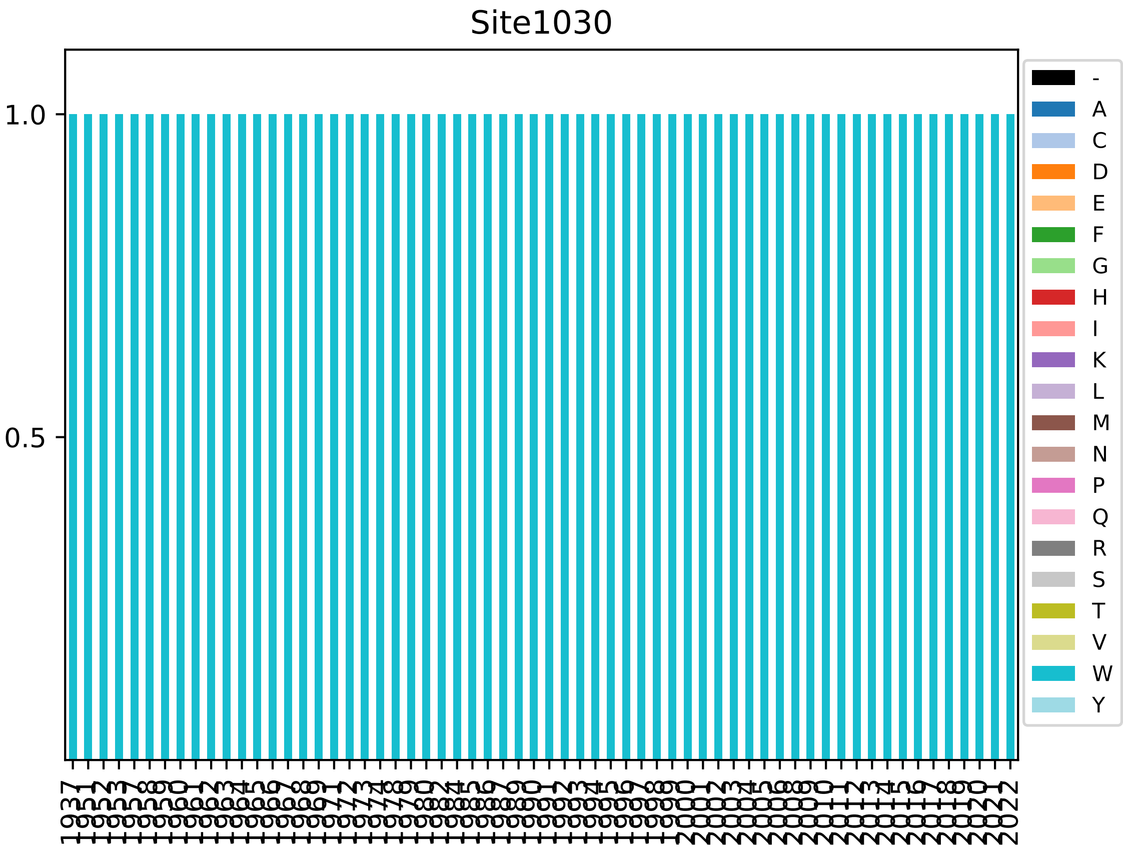

Site1030
1.0
0.5
-
A
C
D
E
F
G
H
I
K
L
M
N
P
Q
R
S
T
V
W
Y

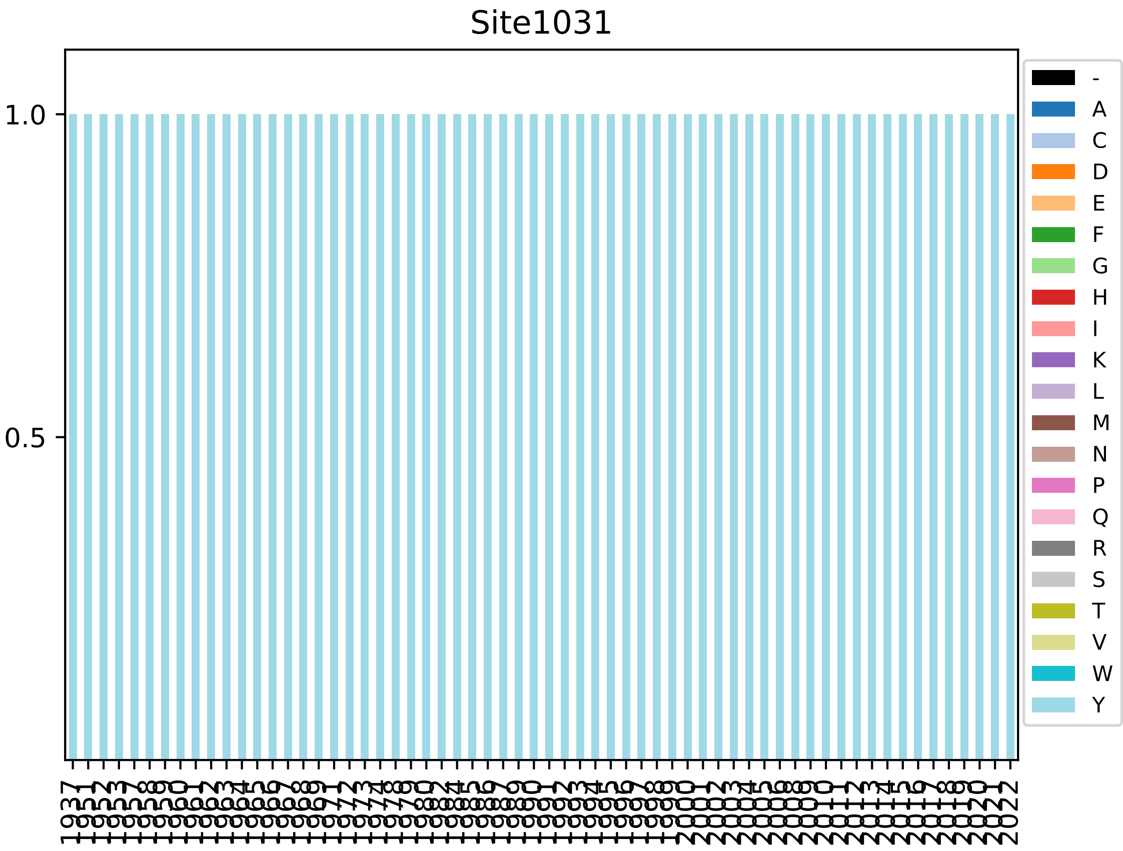
Site1031
1.0
0.5
-
A
C
D
E
F
G
H
I
K
L
M
N
P
Q
R
S
T
V
W
Y

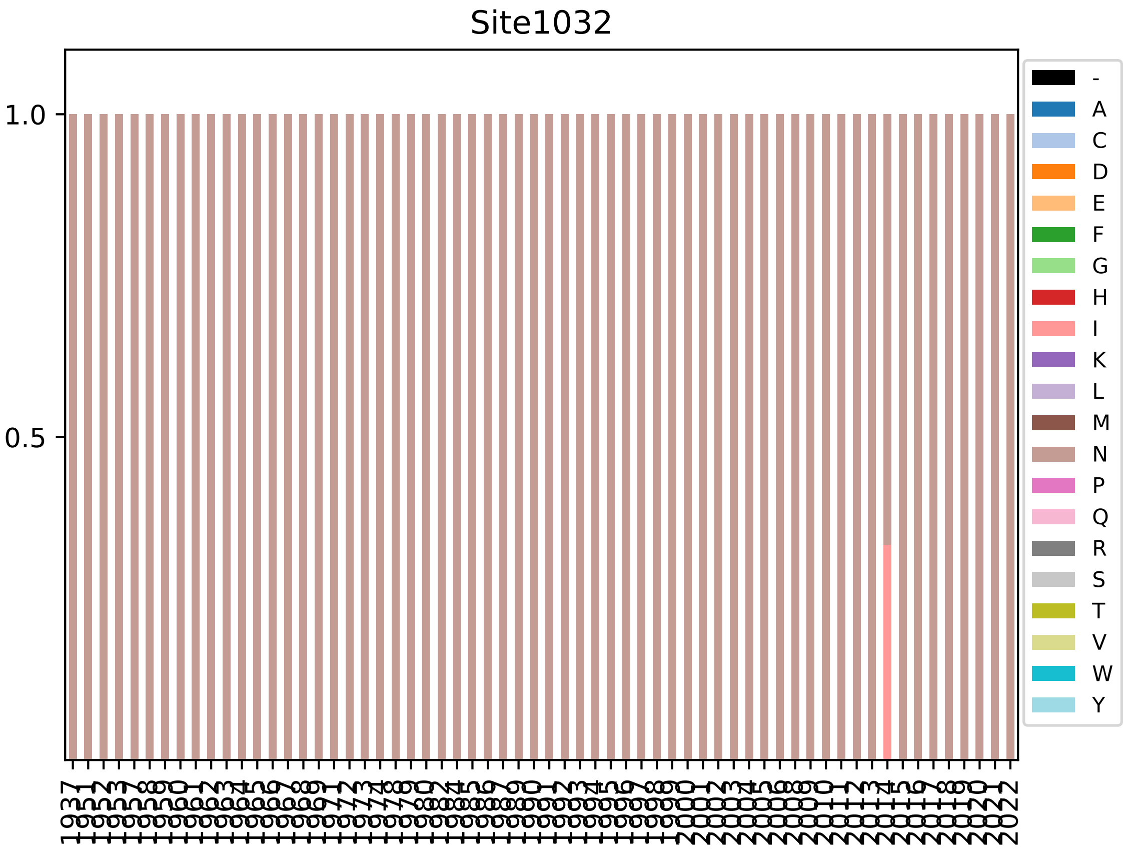
Site1032
1.0
0.5
-
A
C
D
E
F
G
H
I
K
L
M
N
P
Q
R
S
T
V
W
Y

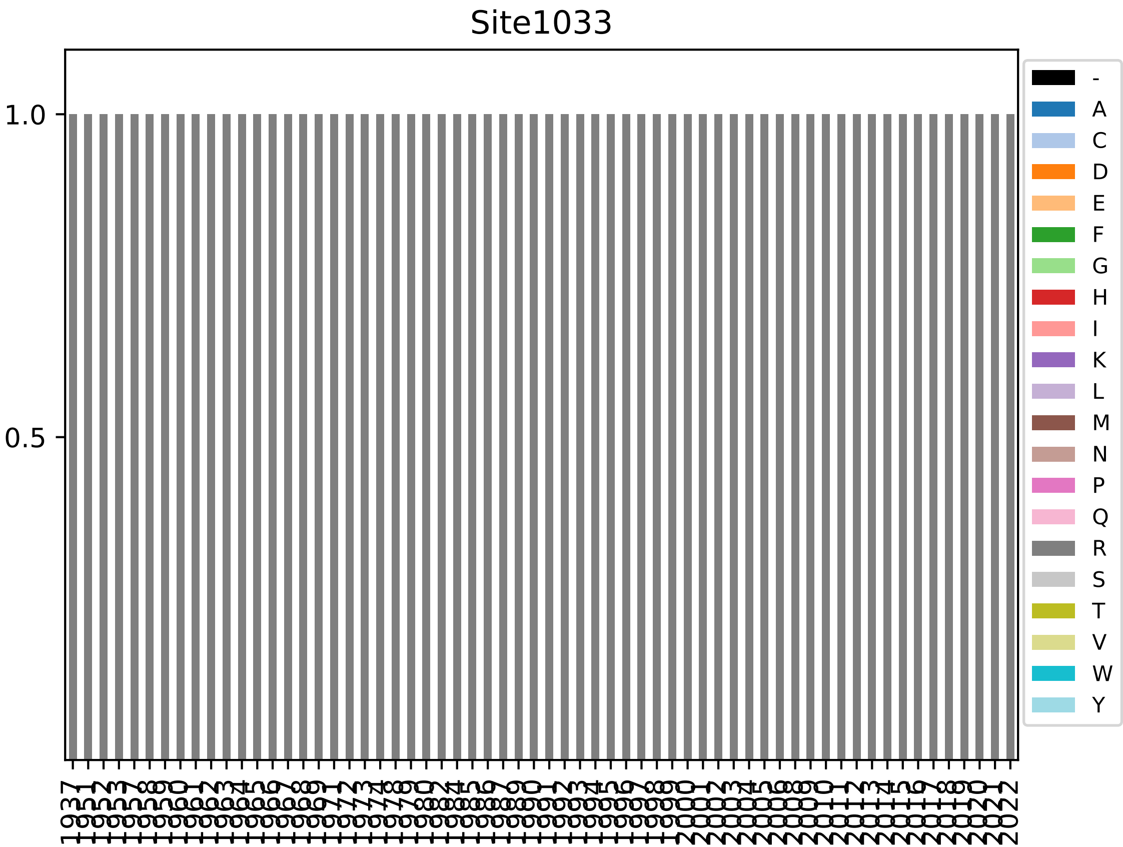
Site1033
1.0
0.5
-
A
C
D
E
F
G
H
I
K
L
M
N
P
Q
R
S
T
V
W
Y

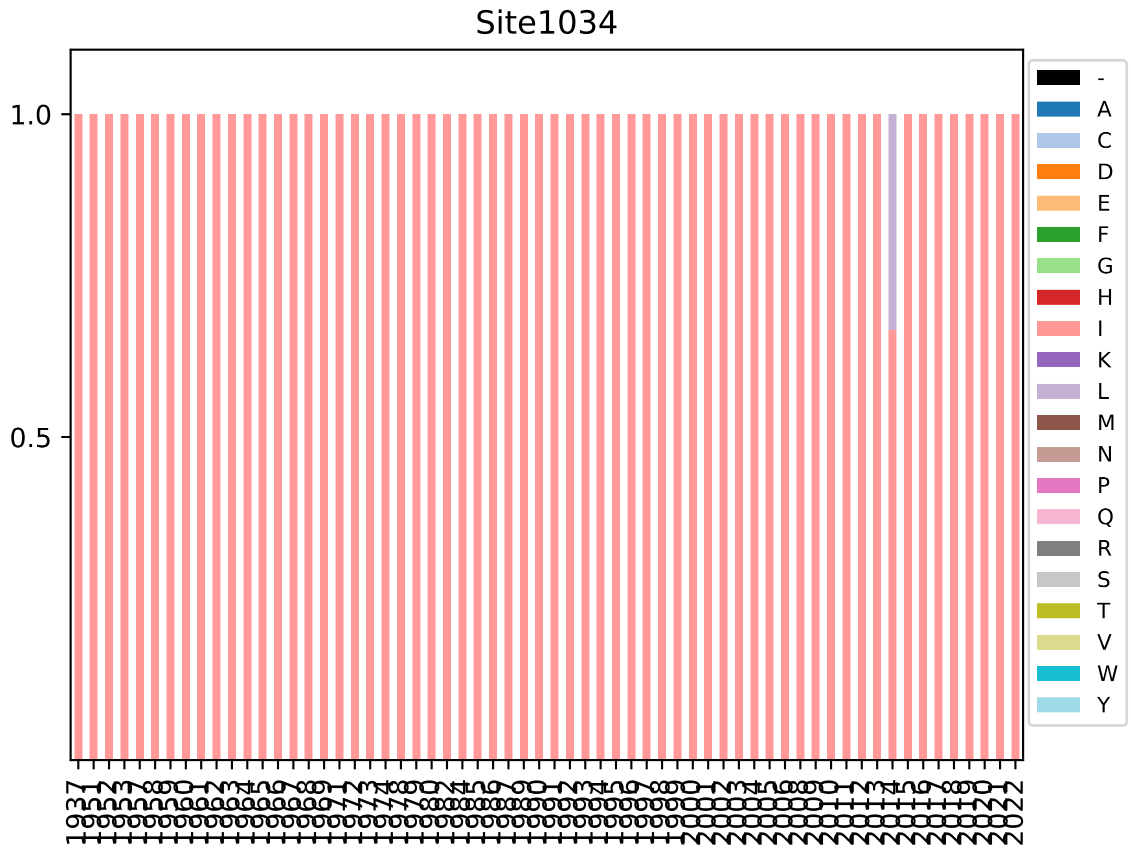
Site1034
1.0
0.5
-
A
C
D
E
F
G
H
I
K
L
M
N
P
Q
R
S
T
V
W
Y

Site1035

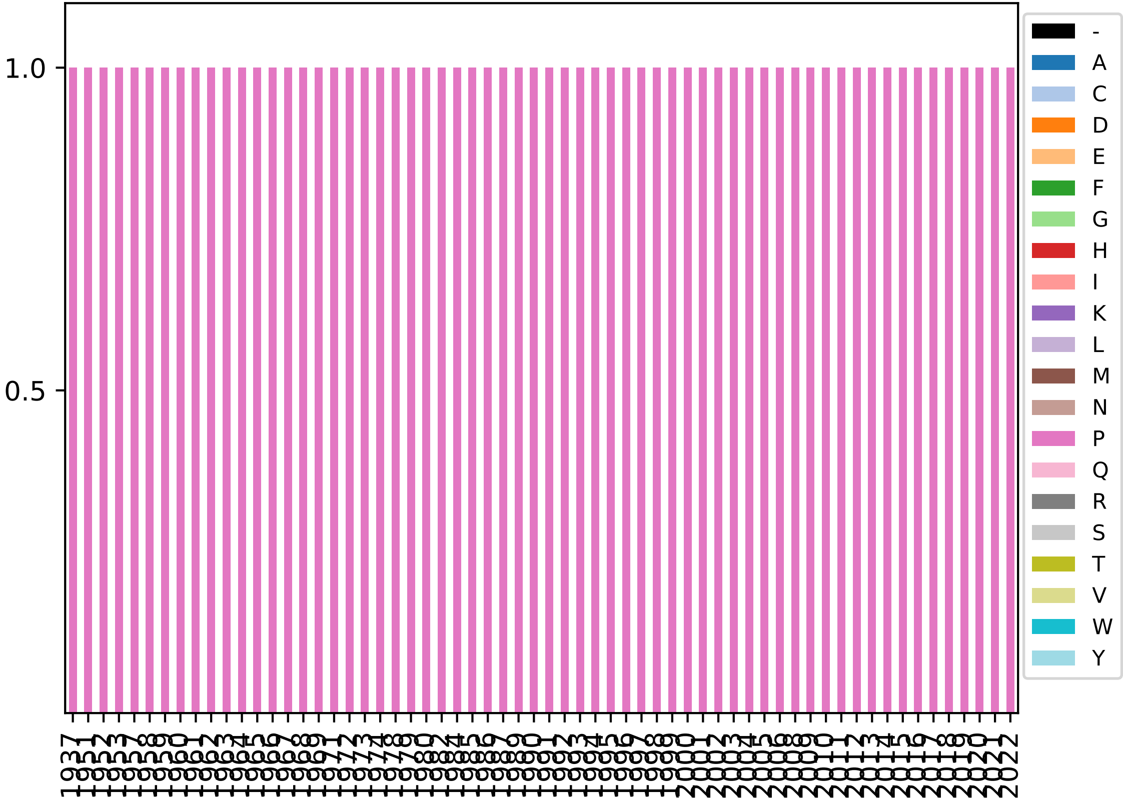

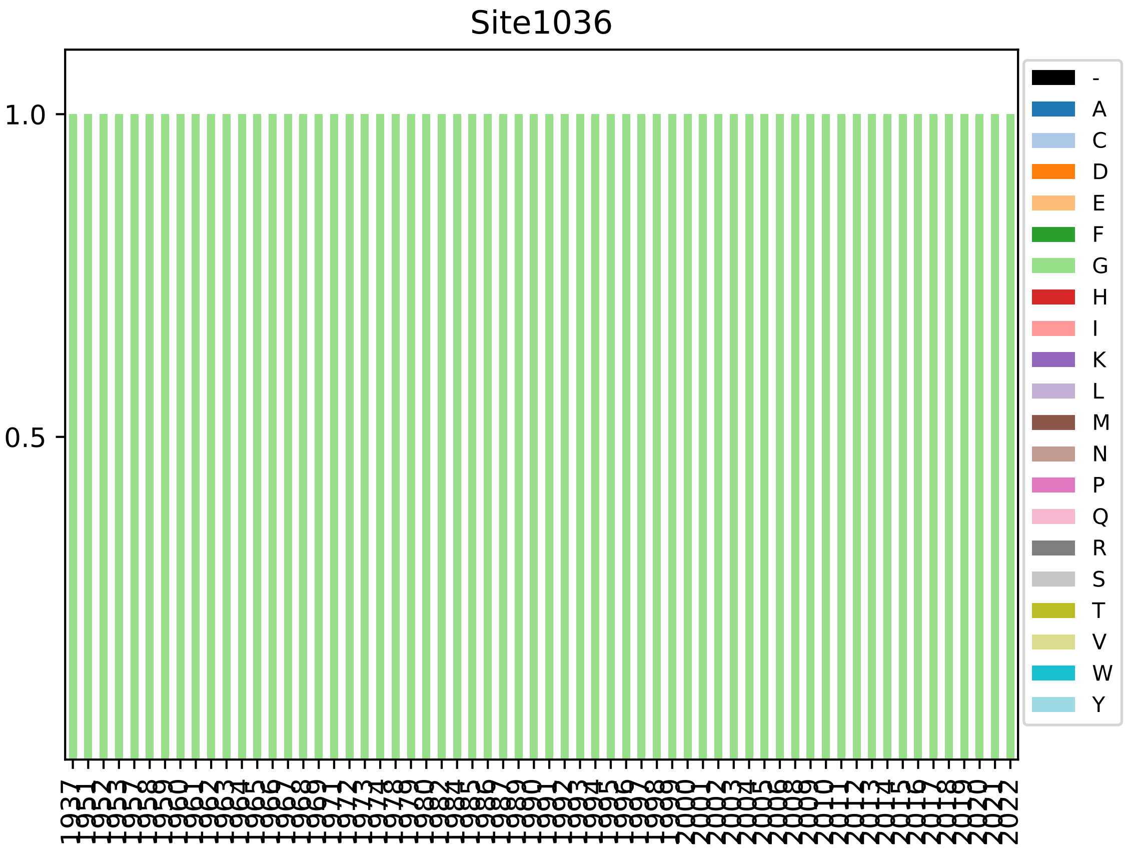
Site1036
1.0
0.5
-
A
C
D
E
F
G
H
I
K
L
M
N
P
Q
R
S
T
V
W
Y

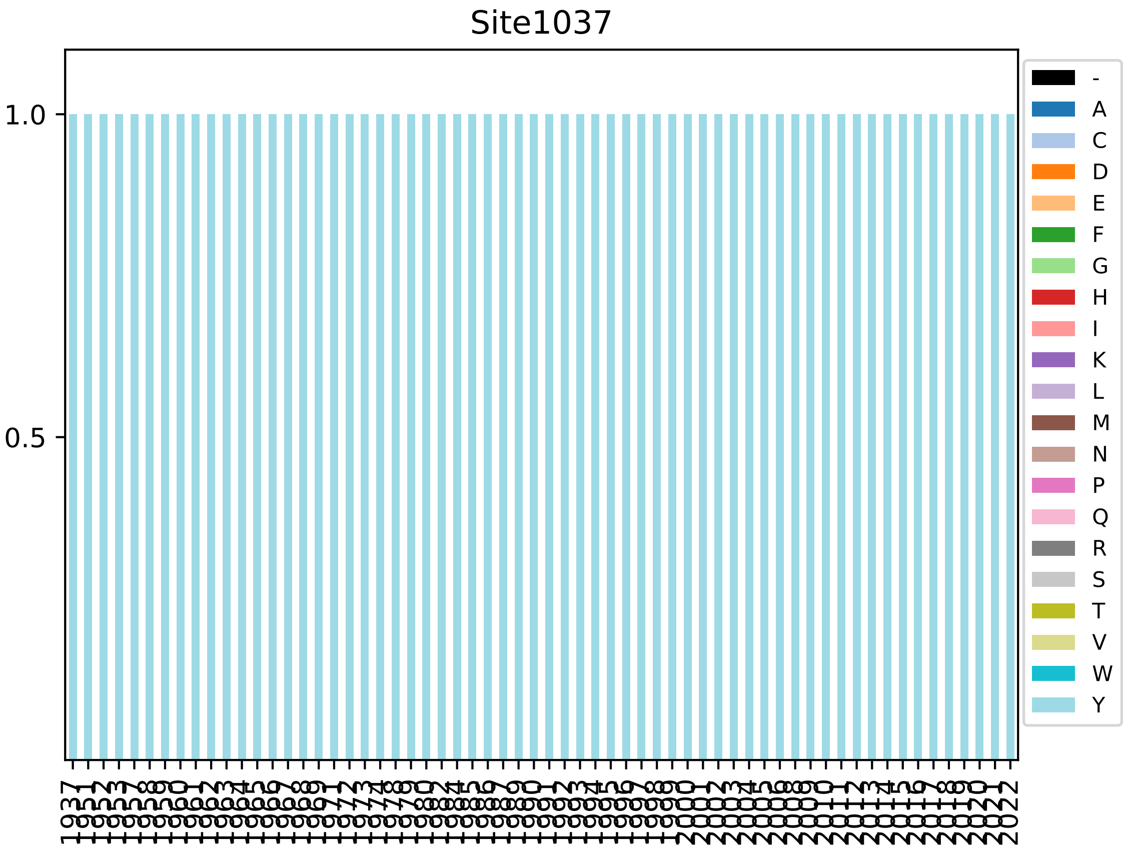
Site1037
1.0
0.5
-
A
C
D
E
F
G
H
I
K
L
M
N
P
Q
R
S
T
V
W
Y

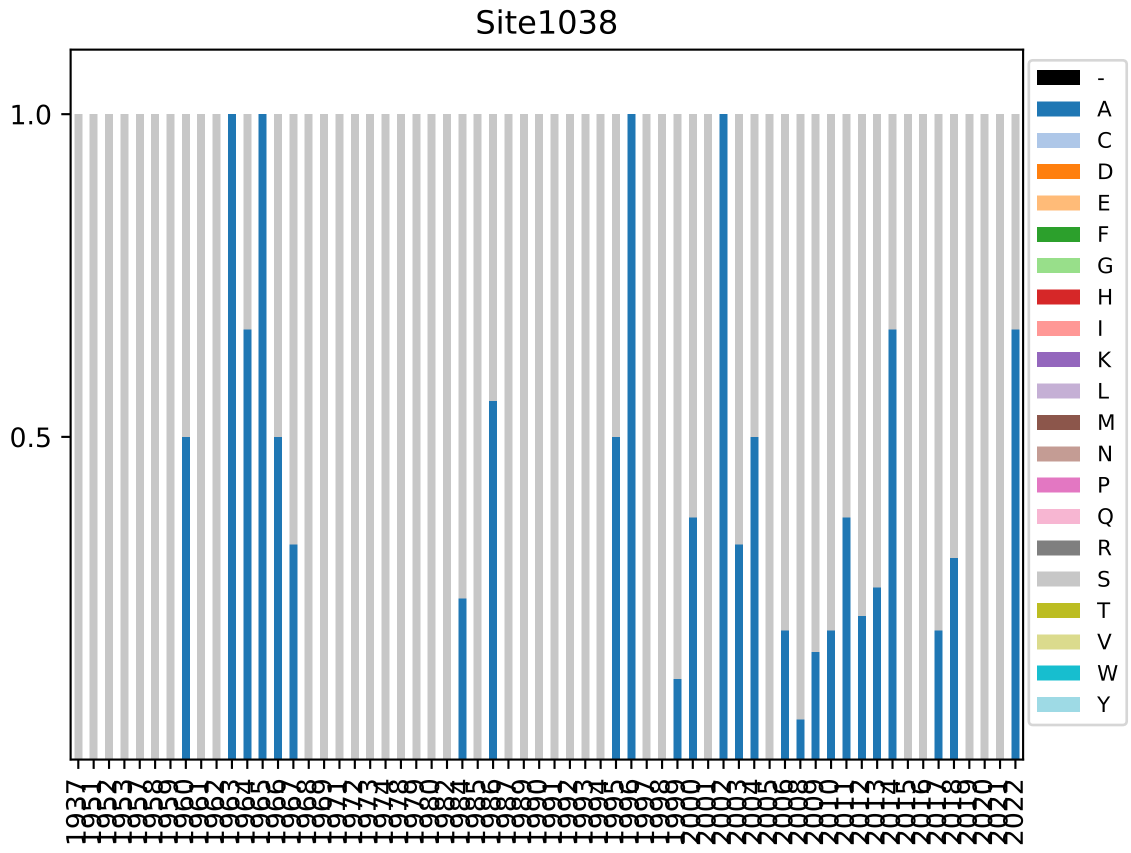
Site1038
1.0
0.5
-
A
C
D
E
F
G
H
I
K
L
M
N
P
Q
R
S
T
V
W
Y

Site1039

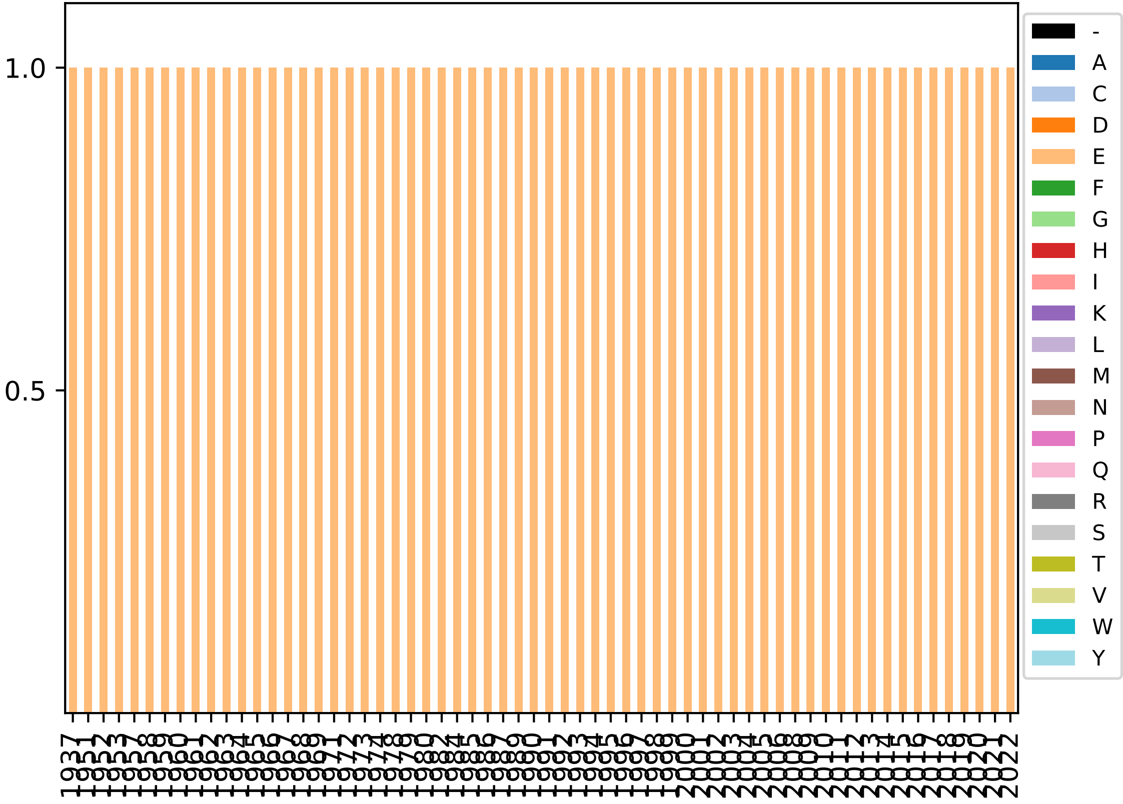

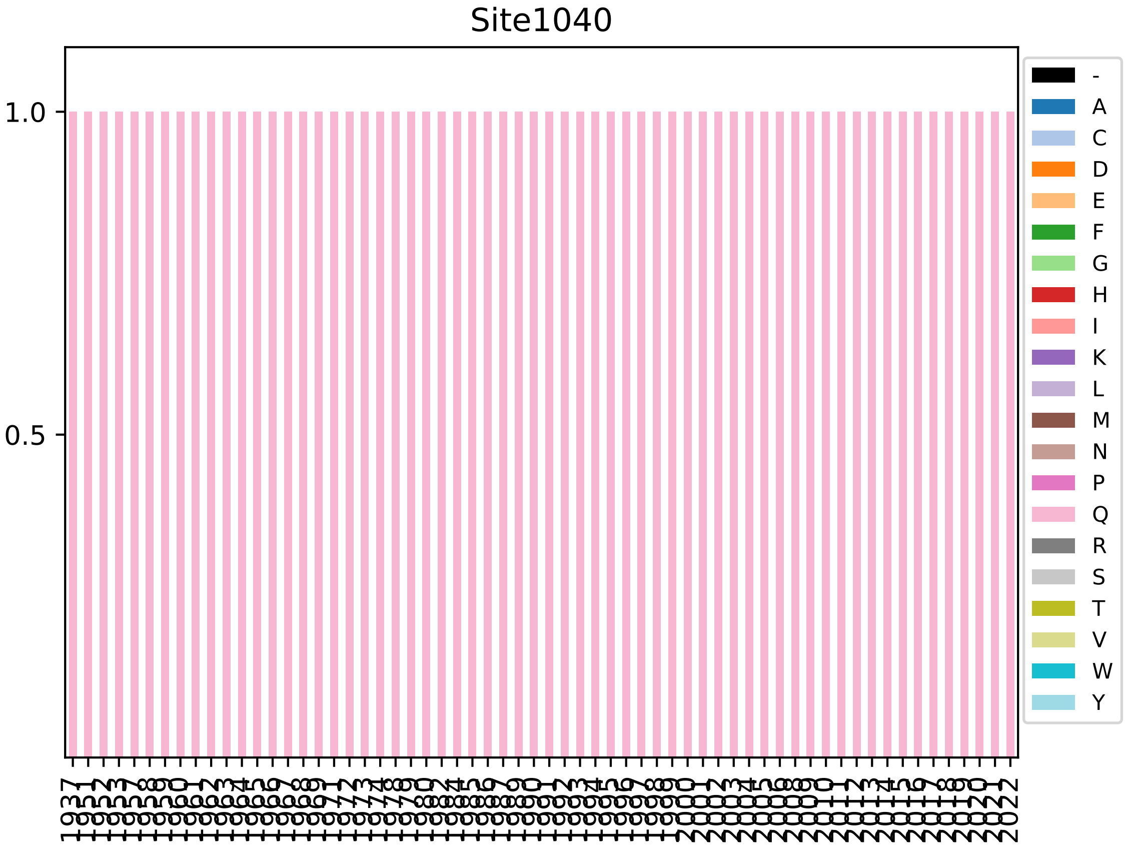
Site1040
1.0
0.5
-
A
C
D
E
F
G
H
I
K
L
M
N
P
Q
R
S
T
V
W
Y

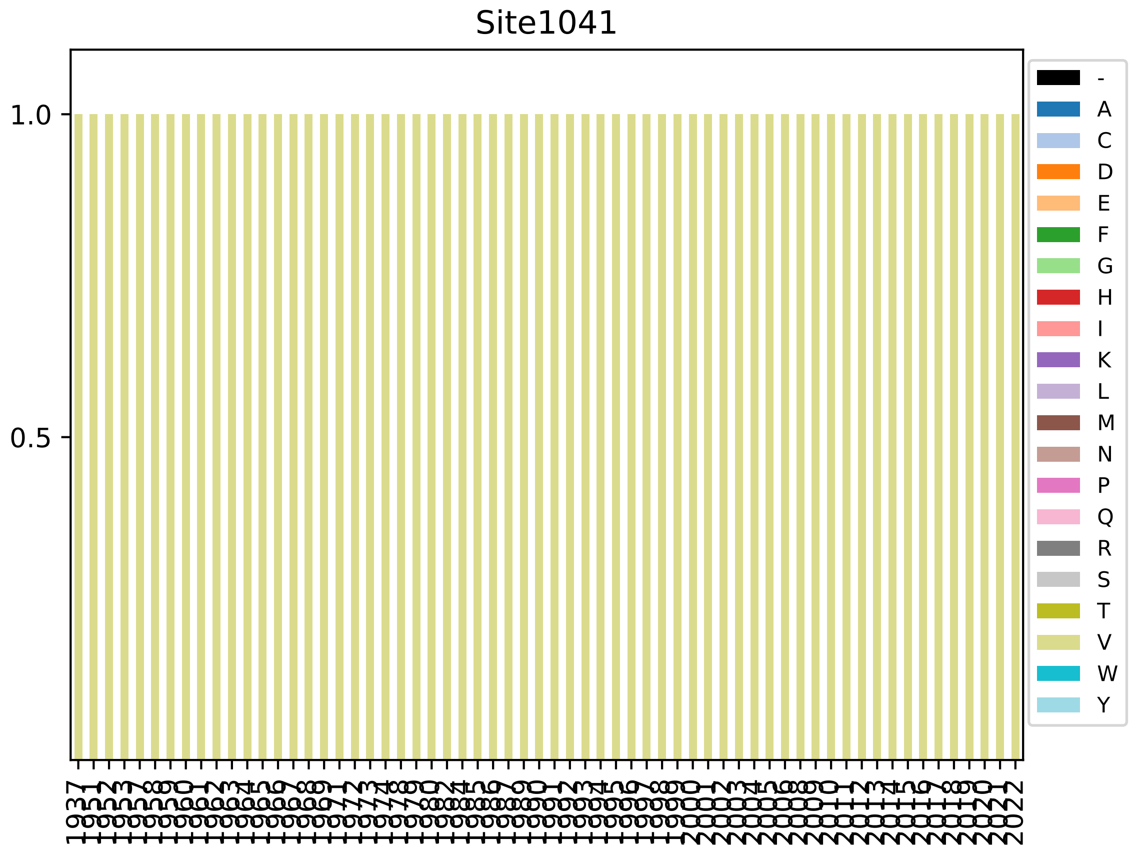
Site1041
1.0
0.5
-
A
C
D
E
F
G
H
I
K
L
M
N
P
Q
R
S
T
V
W
Y

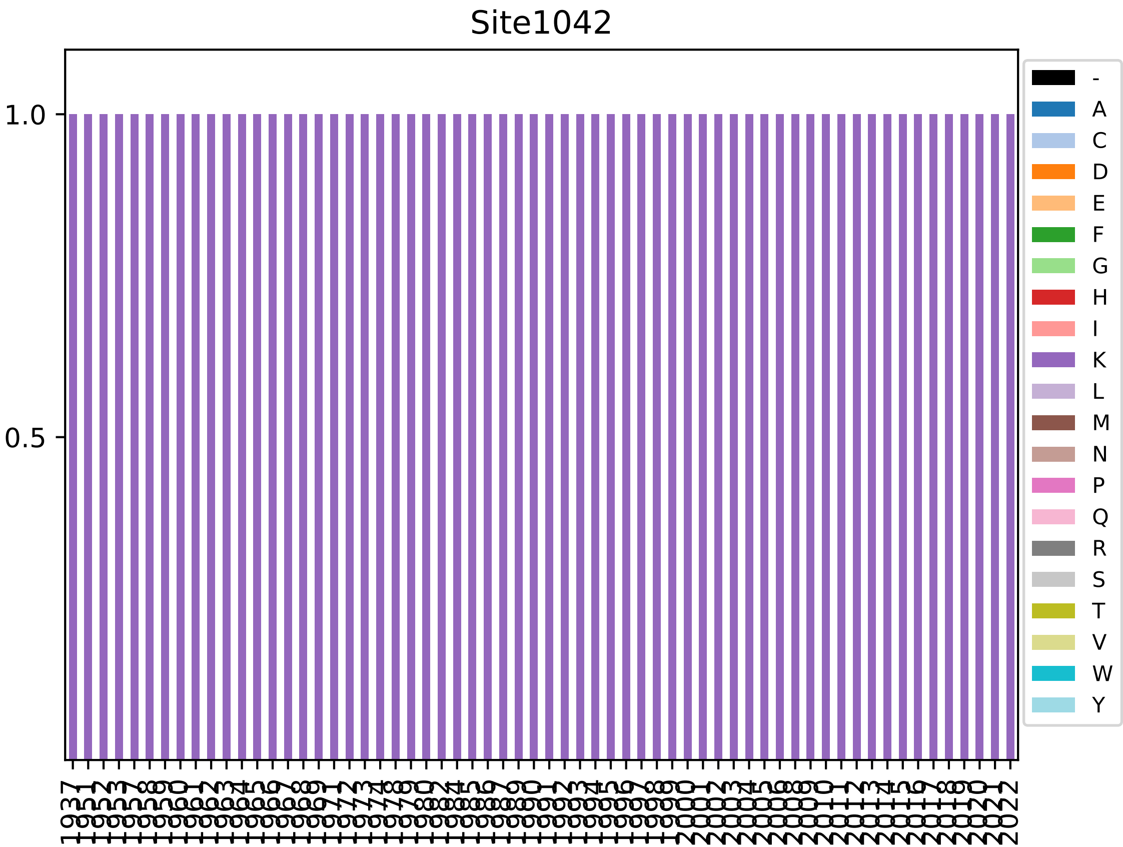
Site1042
1.0
0.5
-
A
C
D
E
F
G
H
I
K
L
M
N
P
Q
R
S
T
V
W
Y

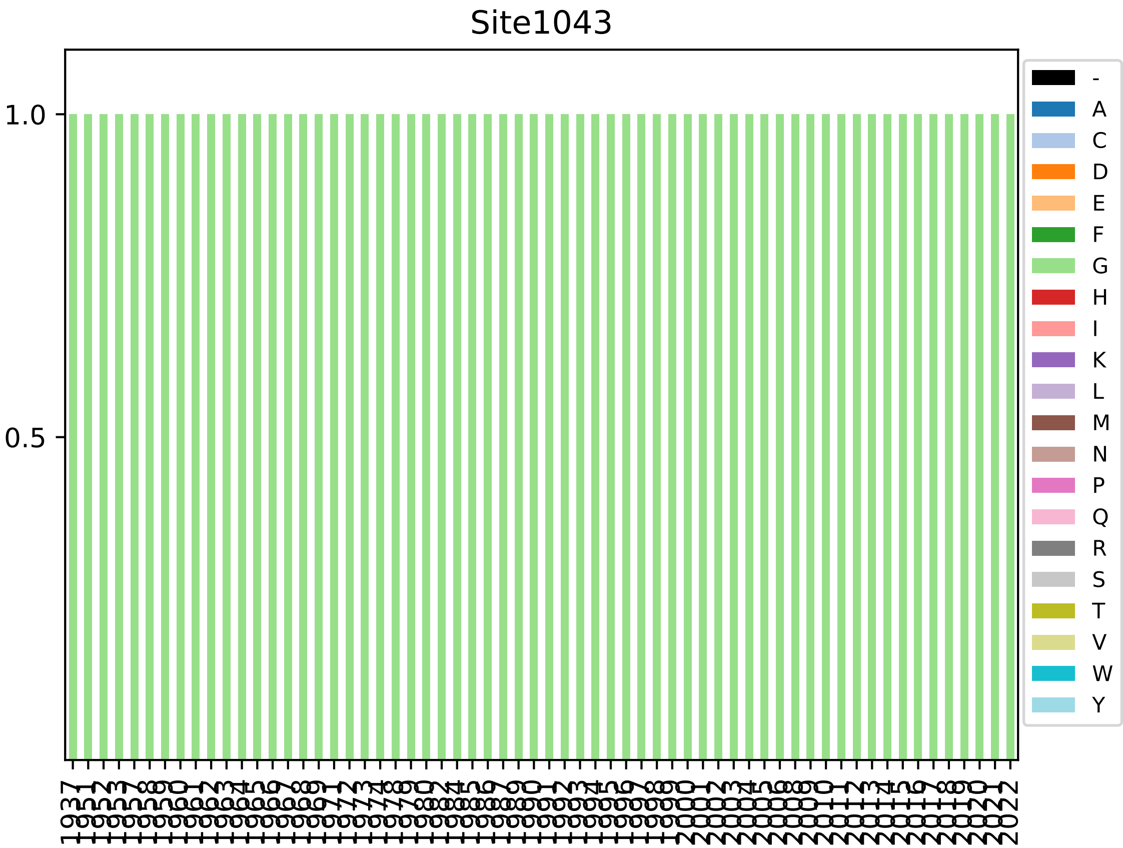
Site1043
1.0
0.5
-
A
C
D
E
F
G
H
I
K
L
M
N
P
Q
R
S
T
V
W
Y

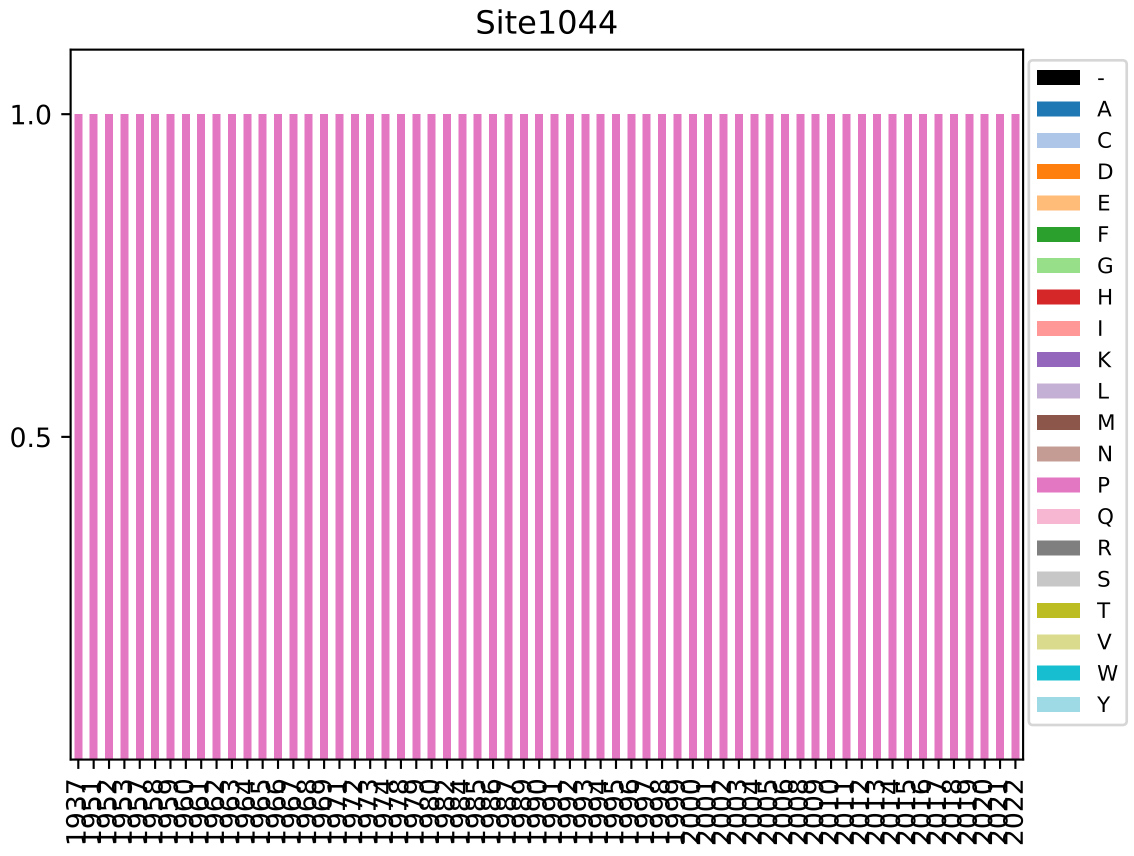
Site1044
1.0
0.5
-
A
C
D
E
F
G
H
I
K
L
M
N
P
Q
R
S
T
V
W
Y

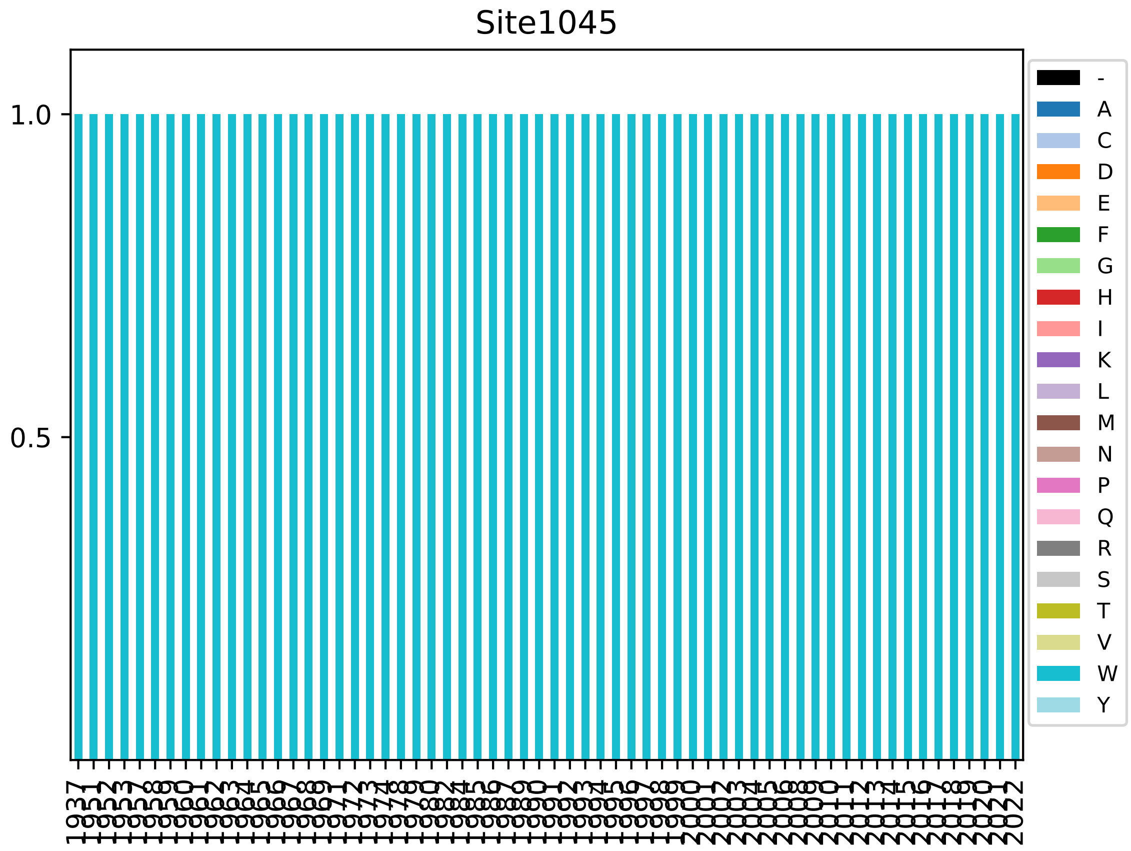
Site1045
1.0
0.5
-
A
C
D
E
F
G
H
I
K
L
M
N
P
Q
R
S
T
V
W
Y

Site1046

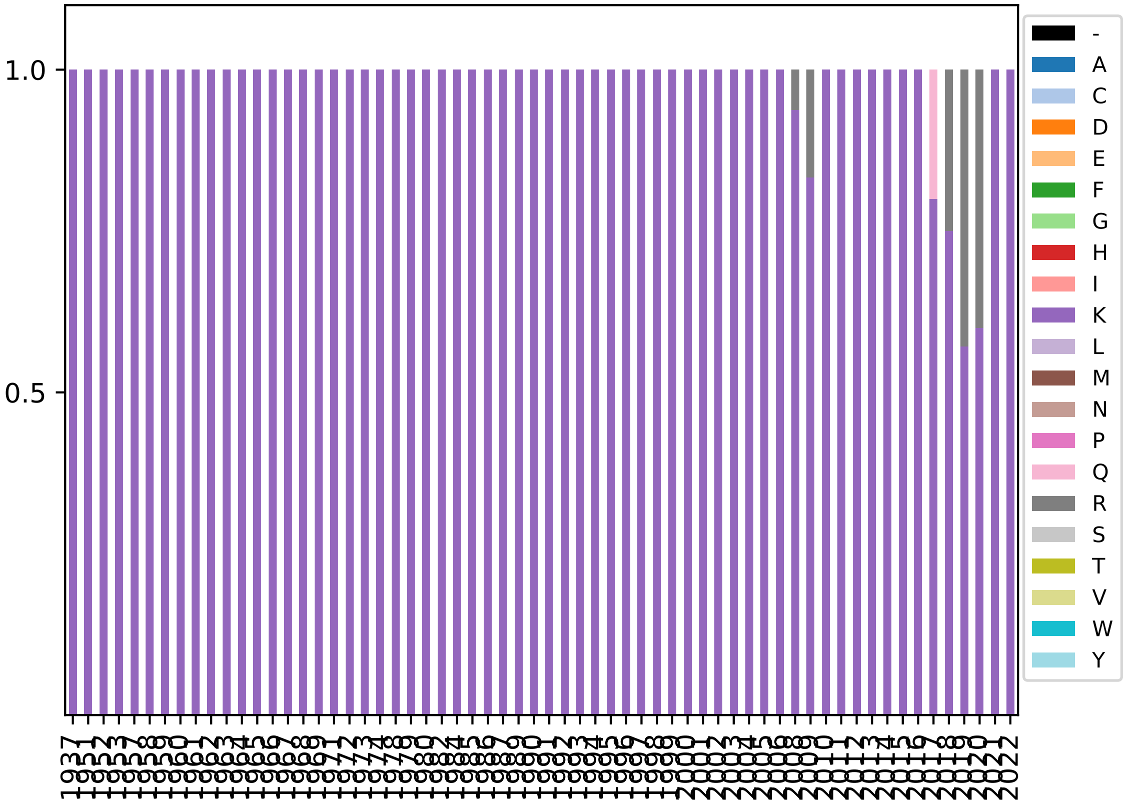

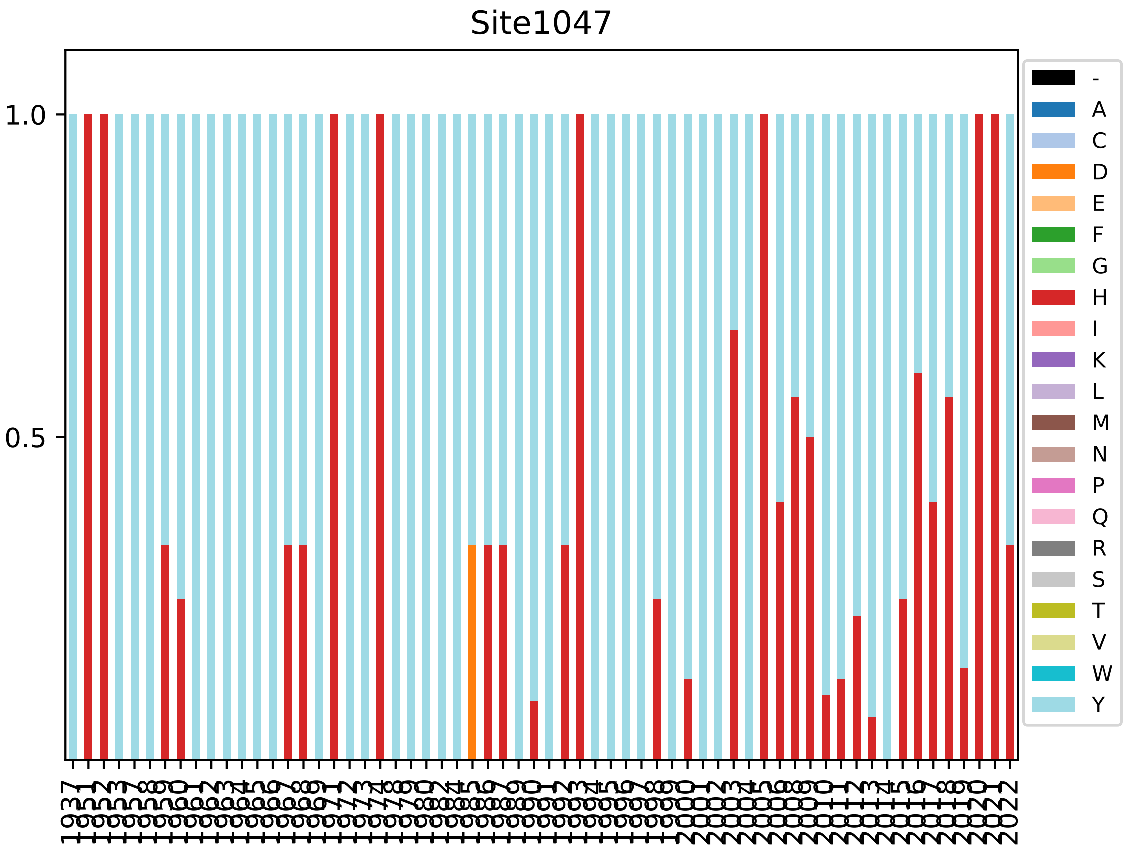
Site1047
1.0
0.5
-
A
C
D
E
F
G
H
I
K
L
M
N
P
Q
R
S
T
V
W
Y

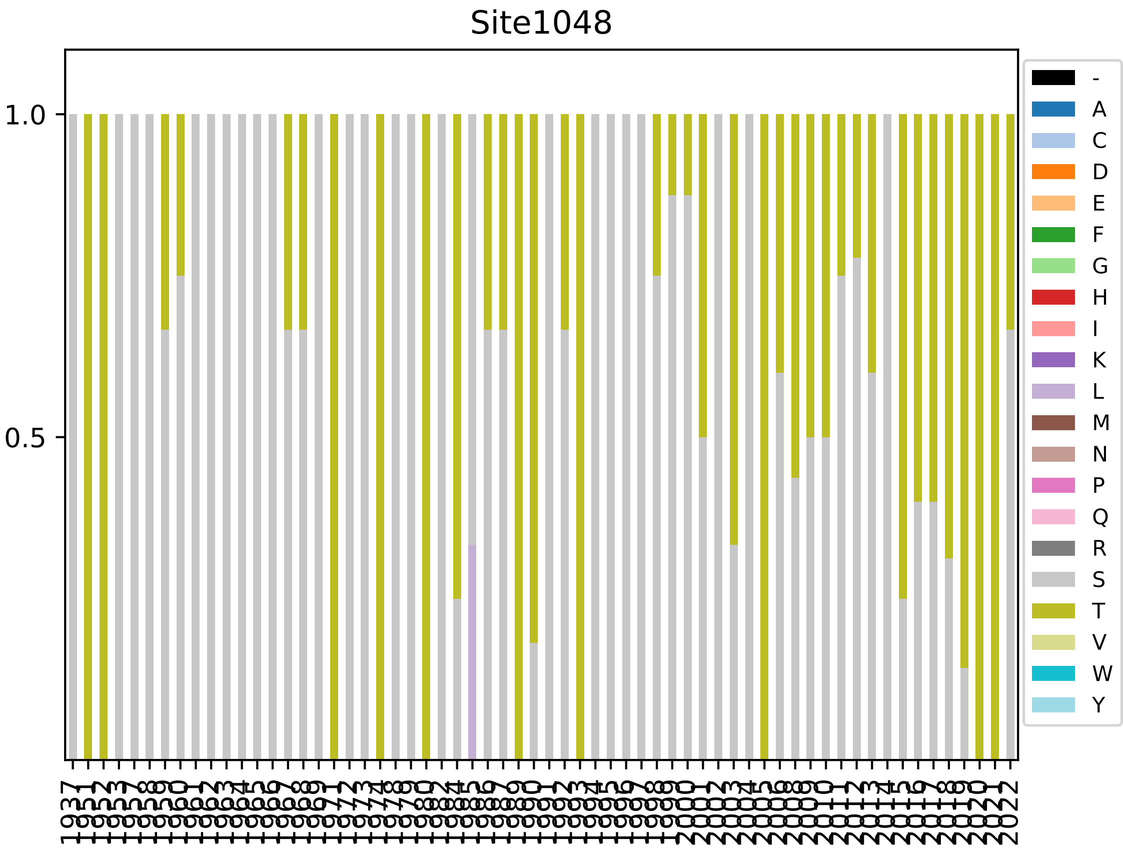
Site1048
1.0
0.5
-
A
C
D
E
F
G
H
I
K
L
M
N
P
Q
R
S
T
V
W
Y

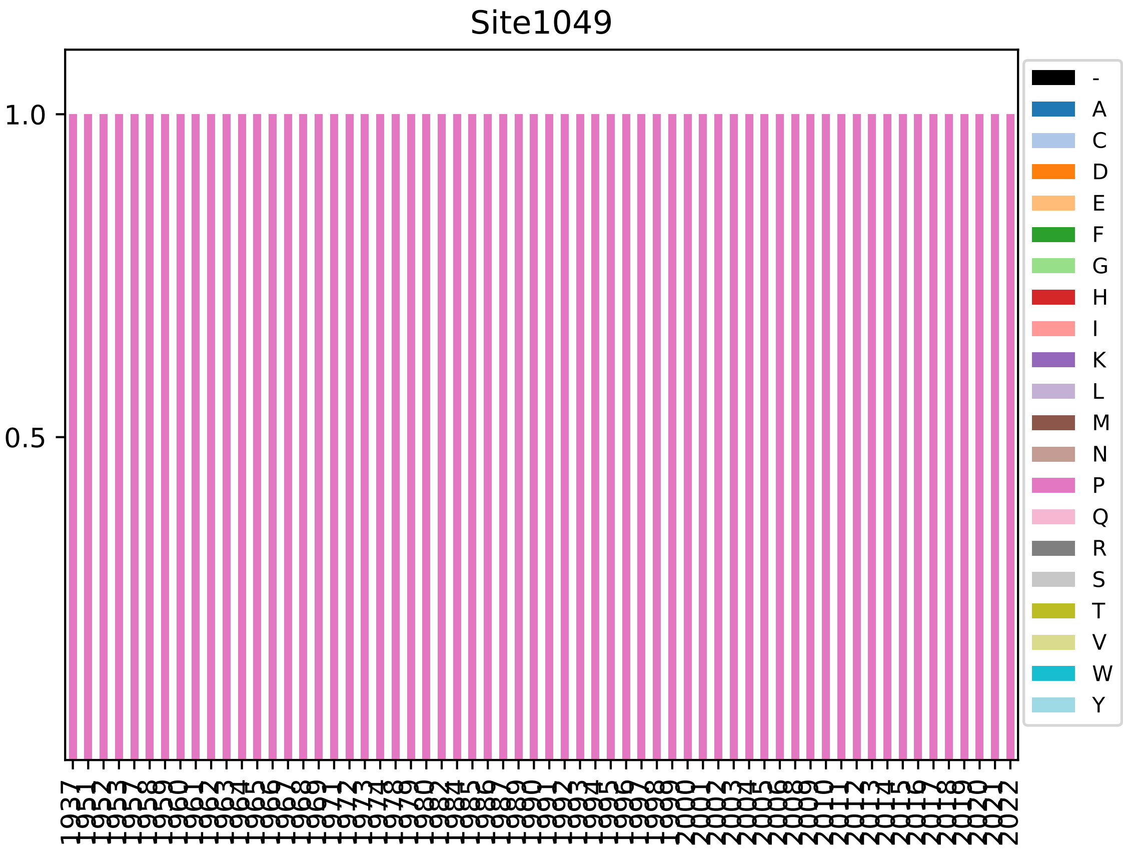
Site1049
1.0
0.5
-
A
C
D
E
F
G
H
I
K
L
M
N
P
Q
R
S
T
V
W
Y

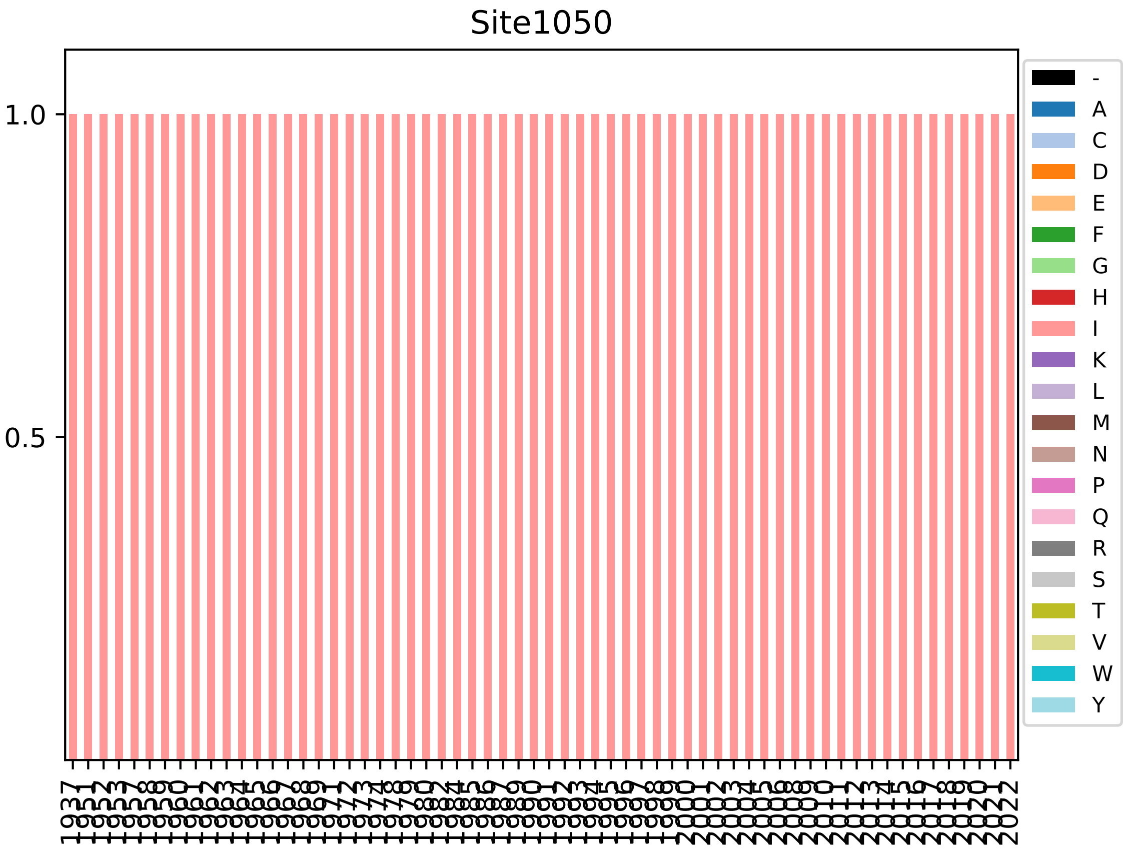
Site1050
1.0
0.5
-
A
C
D
E
F
G
H
I
K
L
M
N
P
Q
R
S
T
V
W
Y

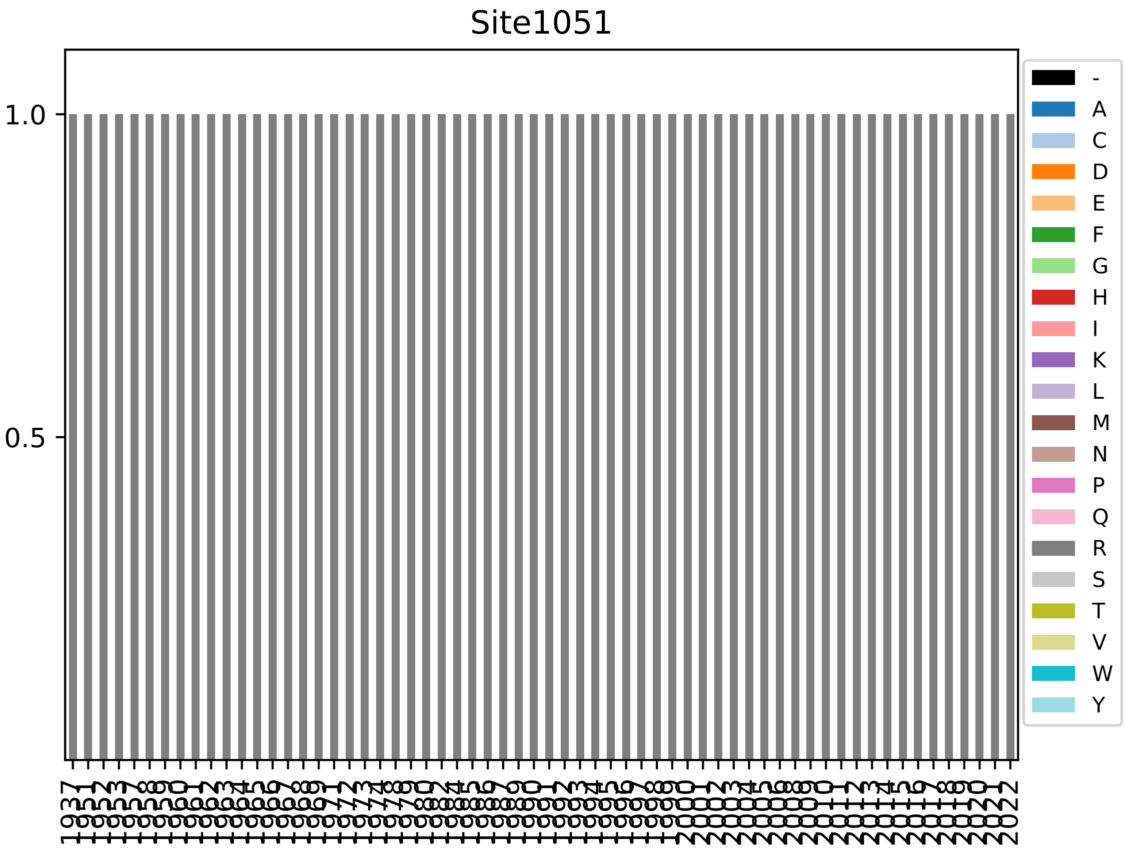
Site1051
1.0
0.5
-
A
C
D
E
F
G
H
I
K
L
M
N
P
Q
R
S
T
V
W
Y

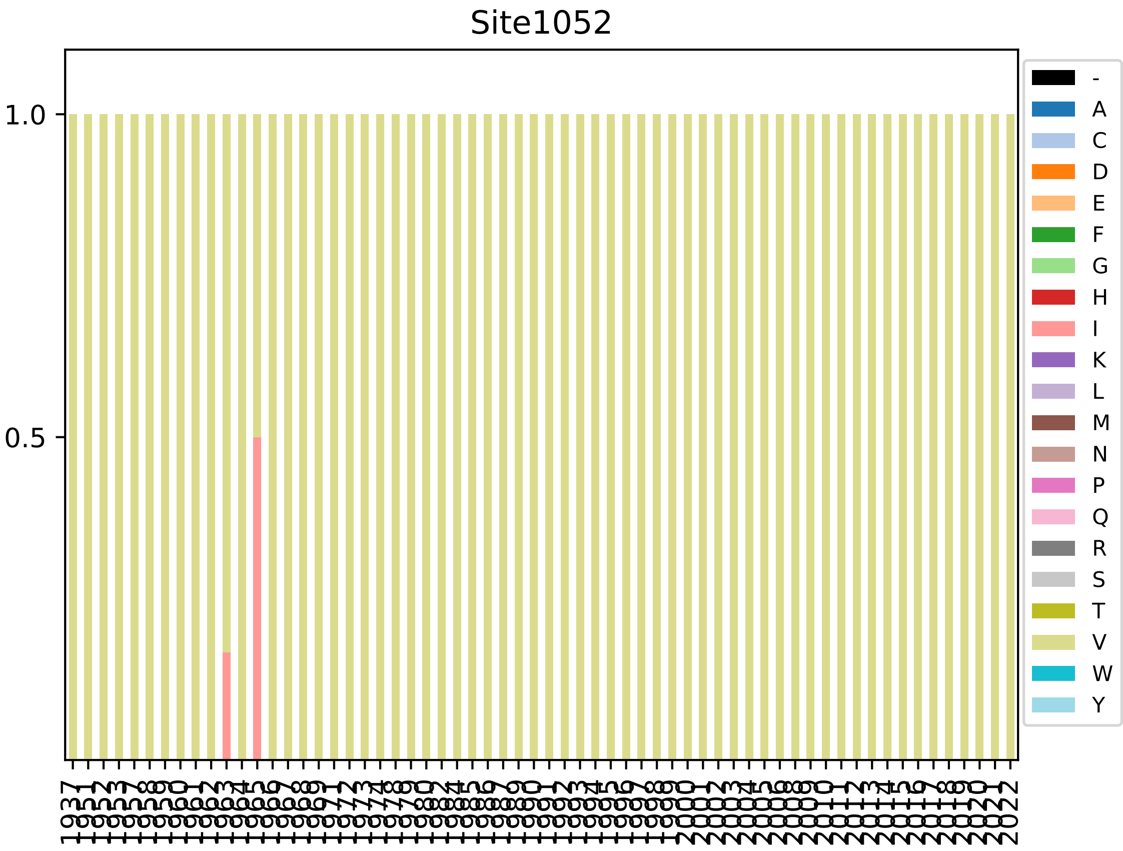
Site1052
1.0
0.5
-
A
C
D
E
F
G
H
I
K
L
M
N
P
Q
R
S
T
V
W
Y

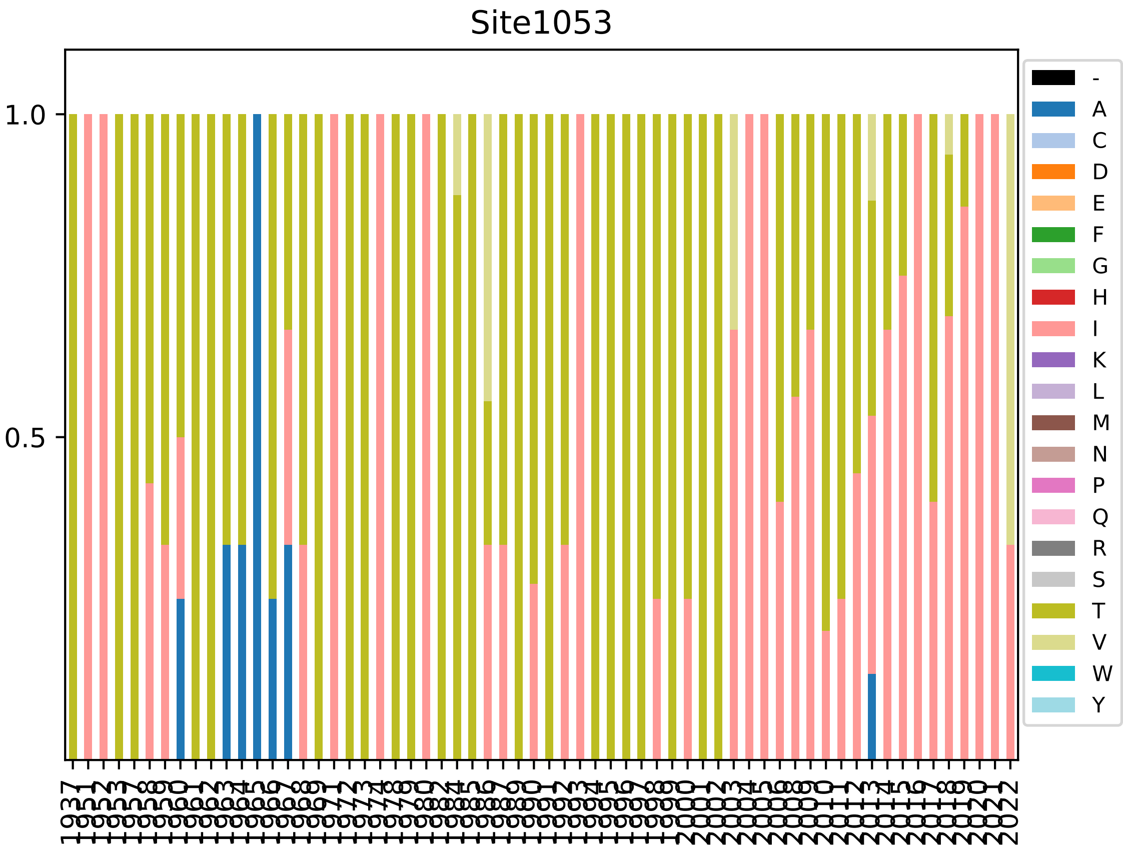
Site1053
1.0
0.5
-
A
C
D
E
F
G
H
I
K
L
M
N
P
Q
R
S
T
V
W
Y

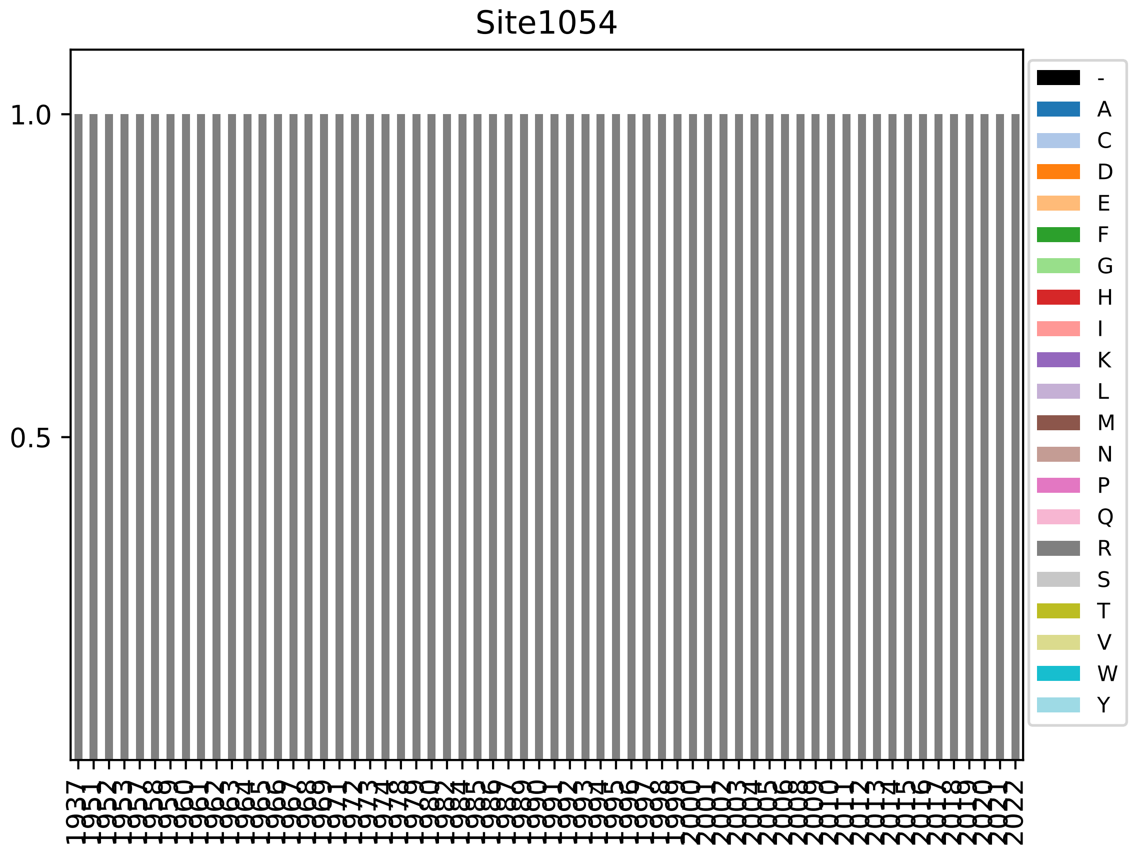

Site1054
1.0
0.5
-
A
C
D
E
F
G
H
I
K
L
M
N
P
Q
R
S
T
V
W
Y

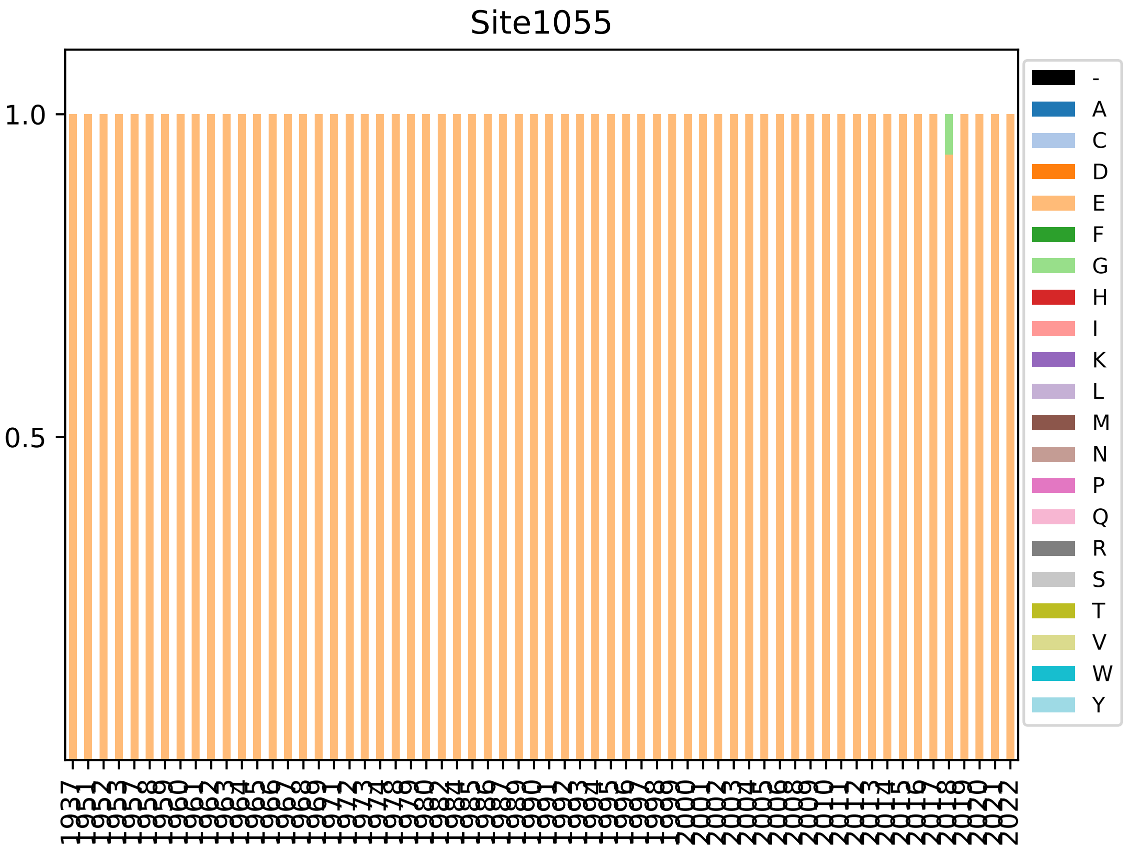
Site1055
1.0
0.5
-
A
C
D
E
F
G
H
I
K
L
M
N
P
Q
R
S
T
V
W
Y

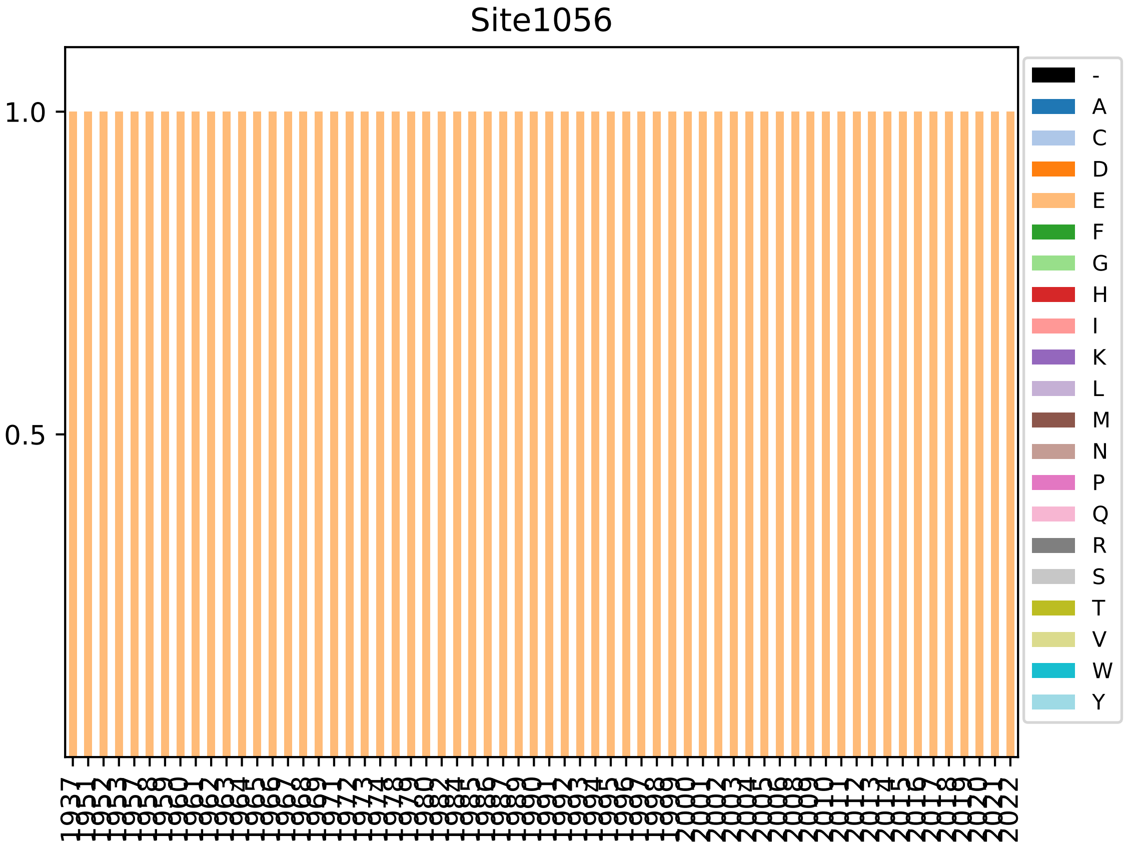
Site1056
1.0
0.5
-
A
C
D
E
F
G
H
I
K
L
M
N
P
Q
R
S
T
V
W
Y

Site1057

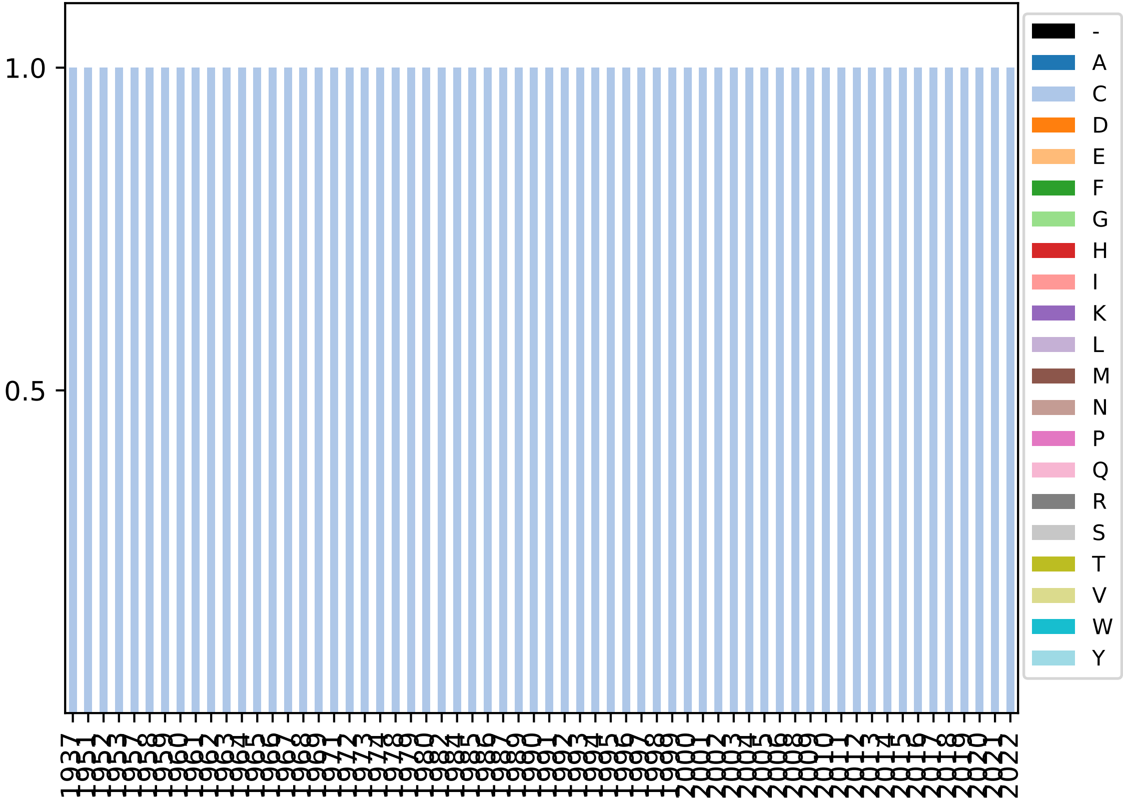

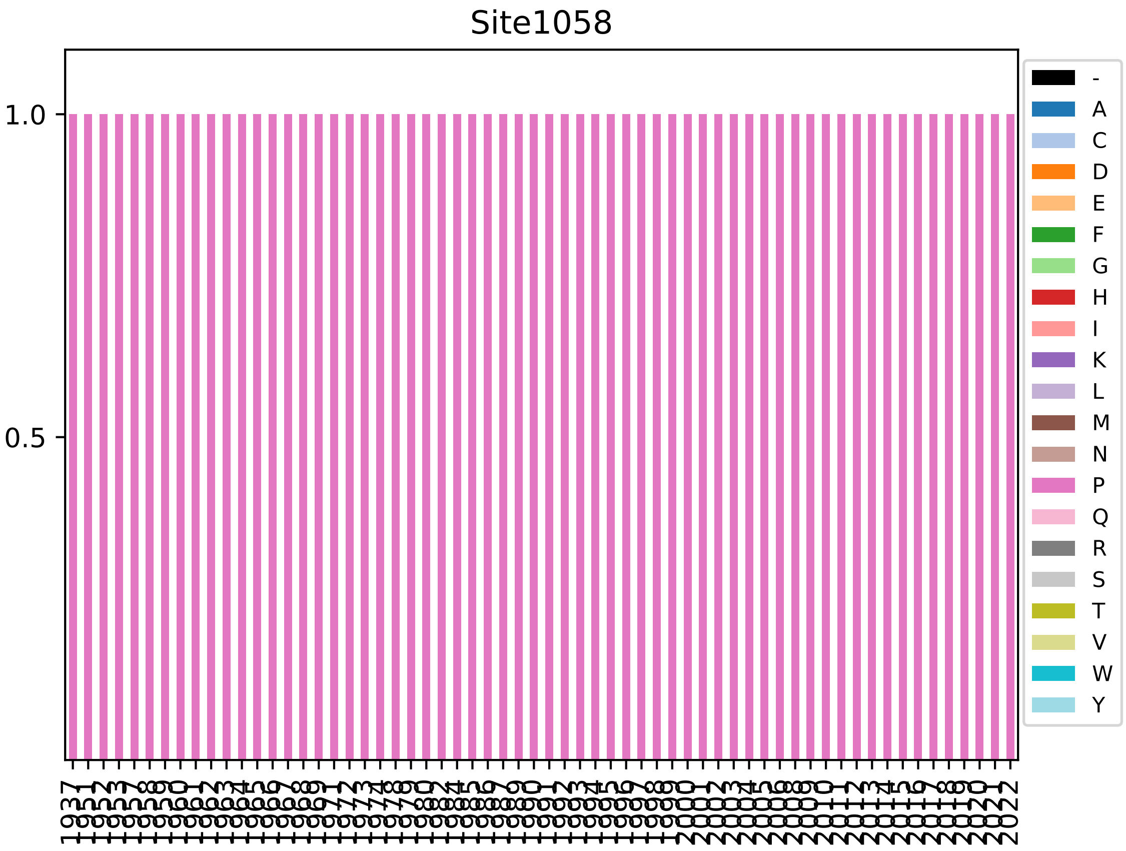

Site1058
1.0
0.5
-
A
C
D
E
F
G
H
I
K
L
M
N
P
Q
R
S
T
V
W
Y

Site1059

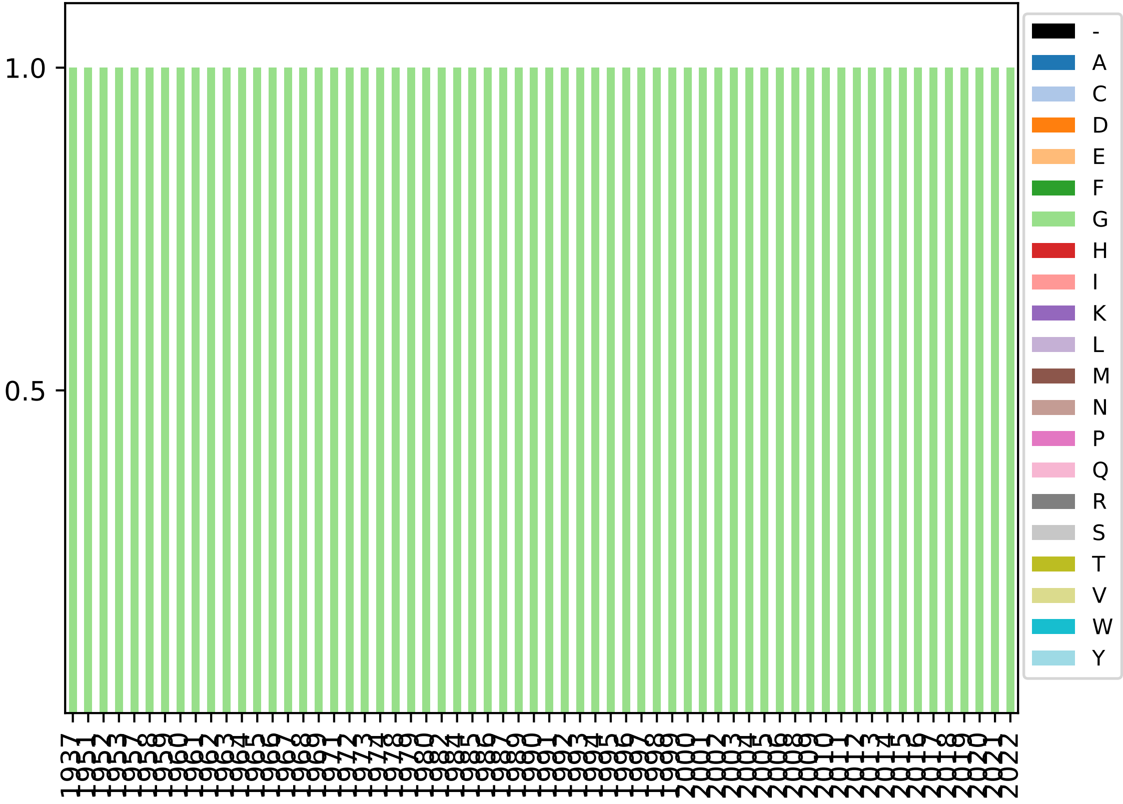

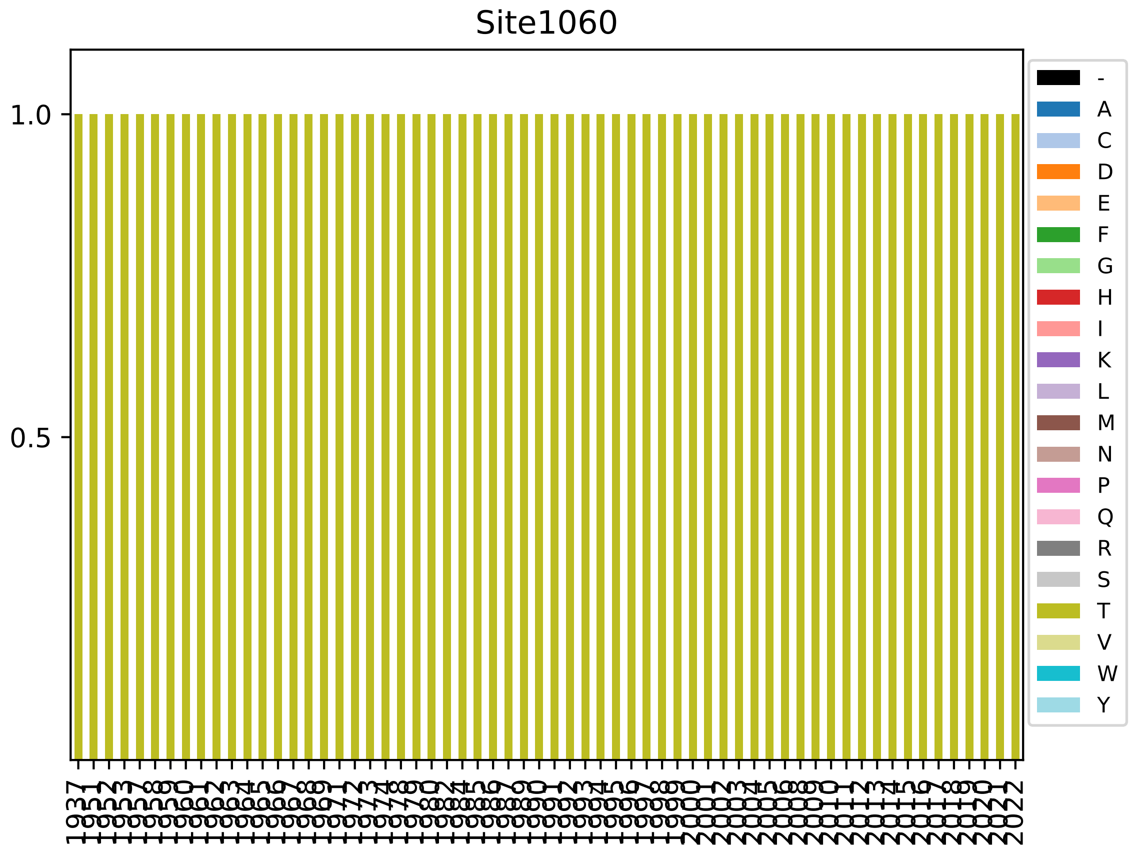
Site1060
1.0
0.5
-
A
C
D
E
F
G
H
I
K
L
M
N
P
Q
R
S
T
V
W
Y

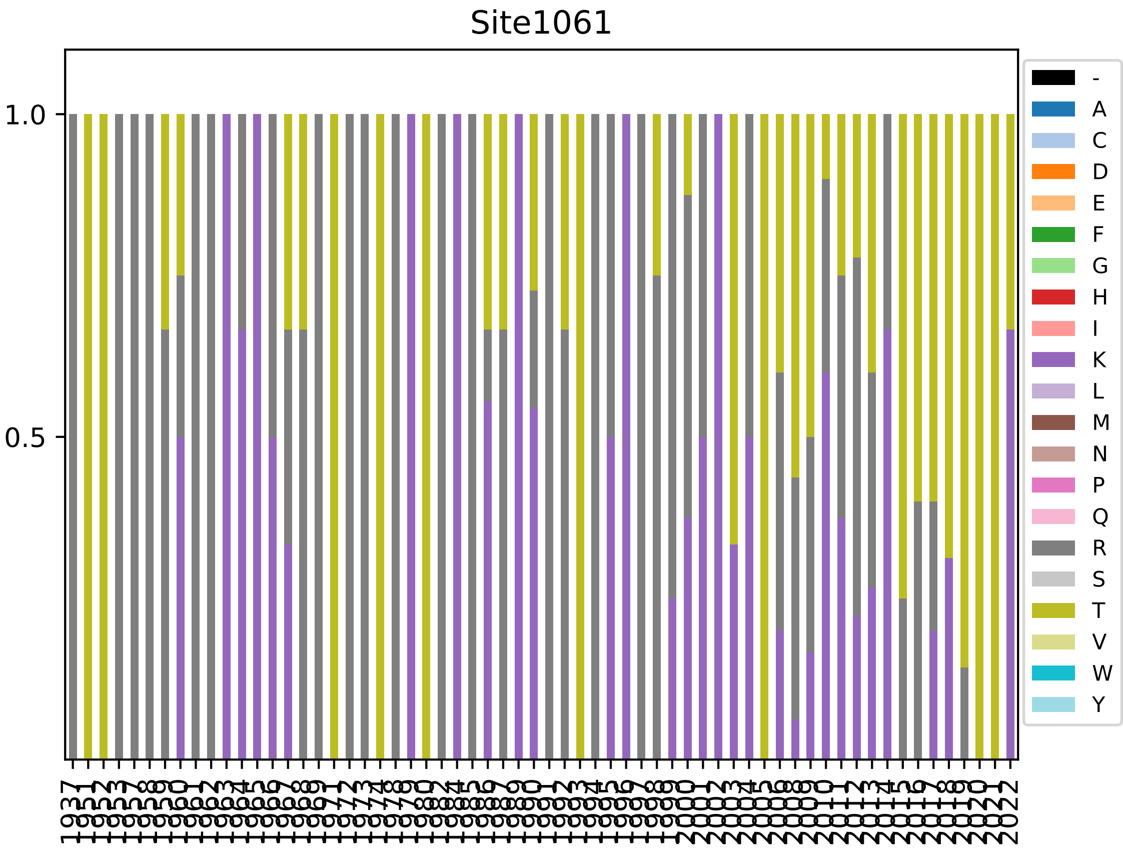
Site1061
1.0
0.5
-
A
C
D
E
F
G
H
I
K
L
M
N
P
Q
R
S
T
V
W
Y

Site1062

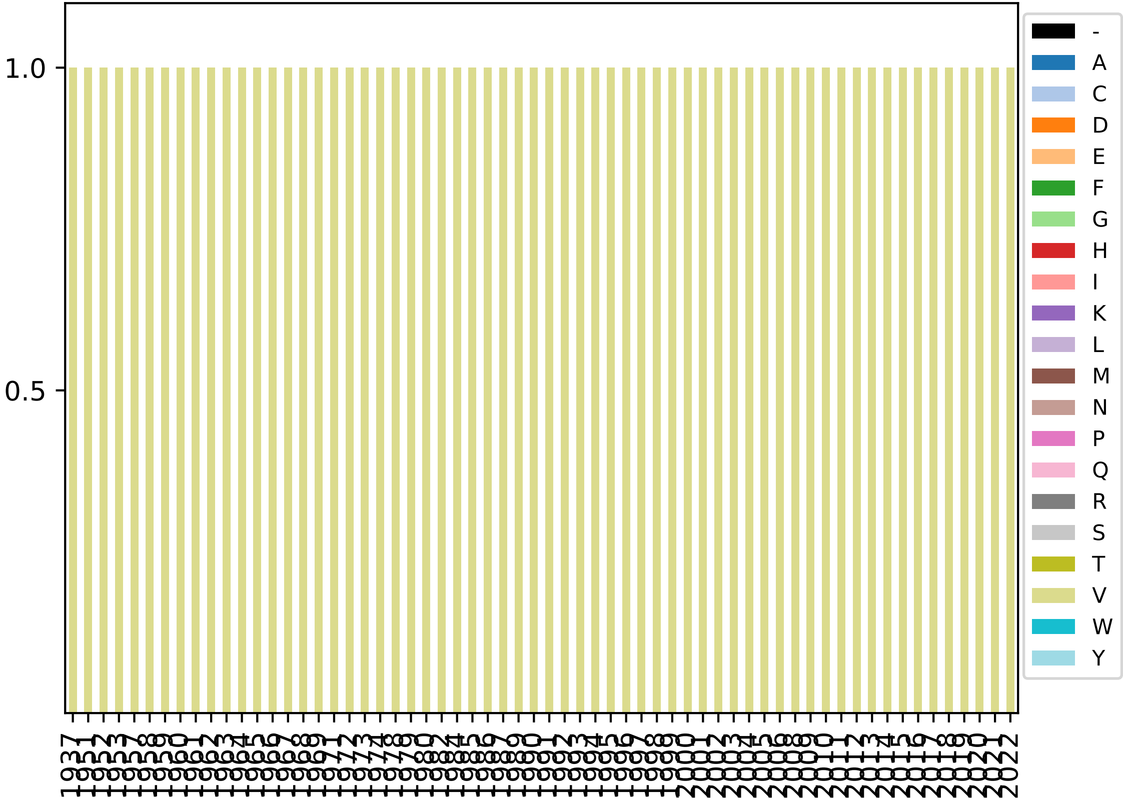

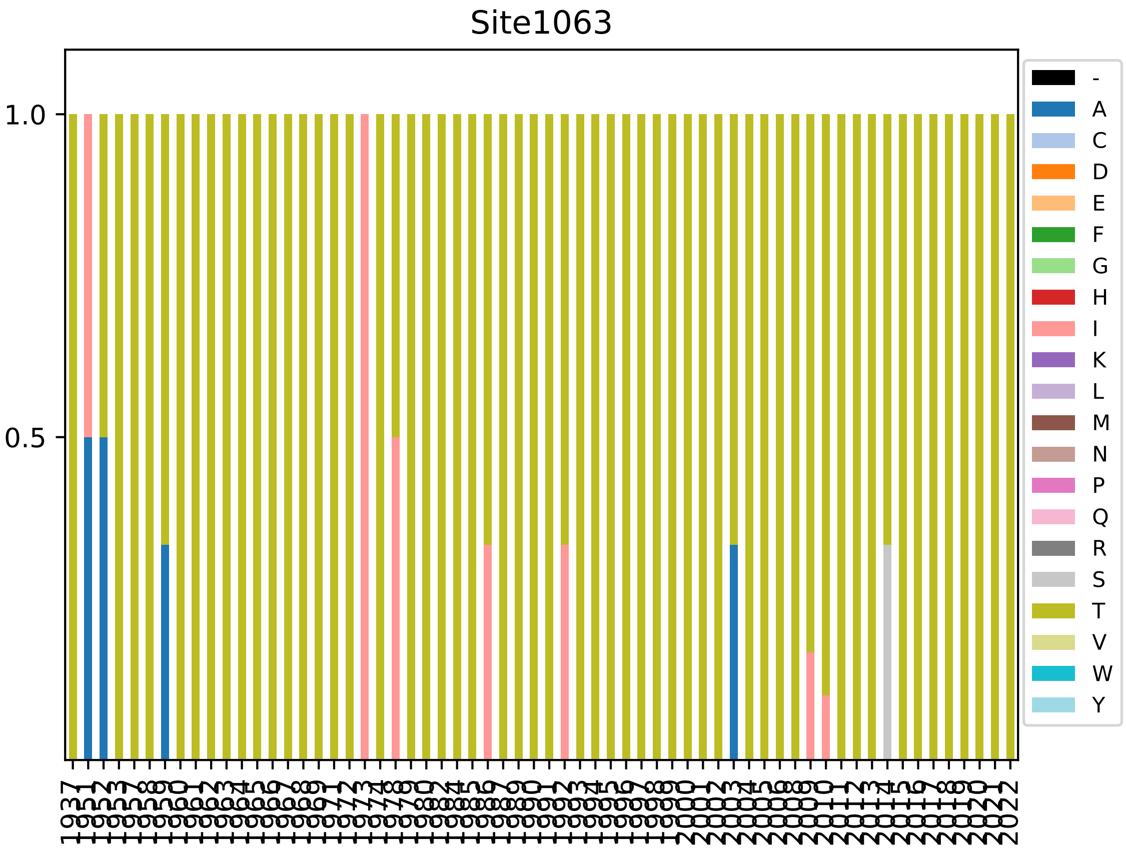
Site1063
1.0
0.5
-
A
C
D
E
F
G
H
I
K
L
M
N
P
Q
R
S
T
V
W
Y

Site1064

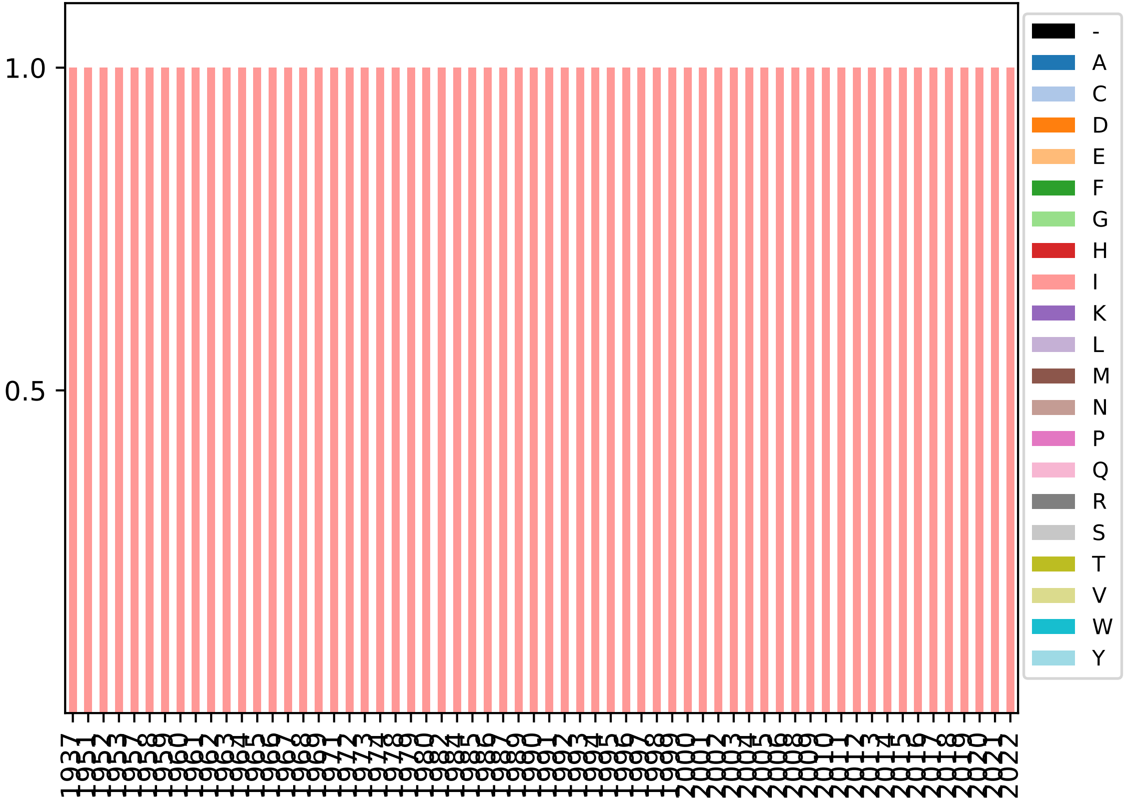

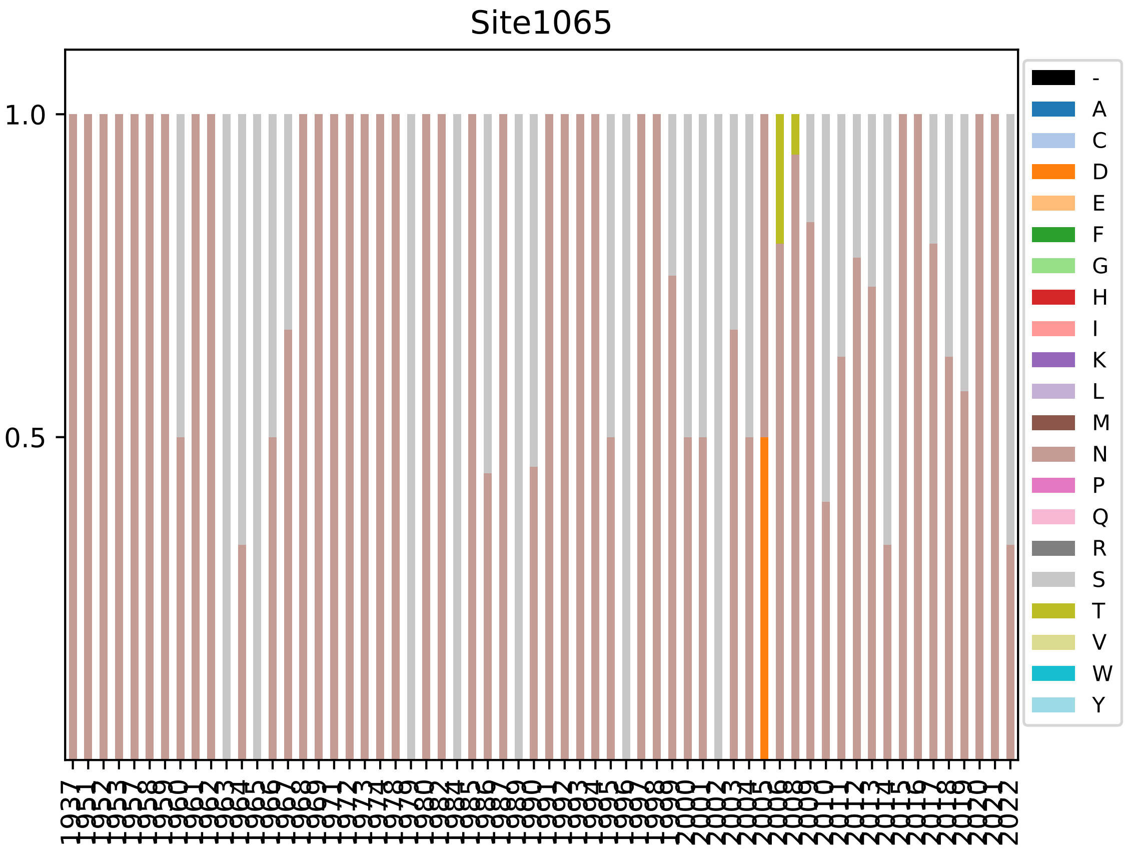
Site1065
1.0
0.5
-
A
C
D
E
F
G
H
I
K
L
M
N
P
Q
R
S
T
V
W
Y

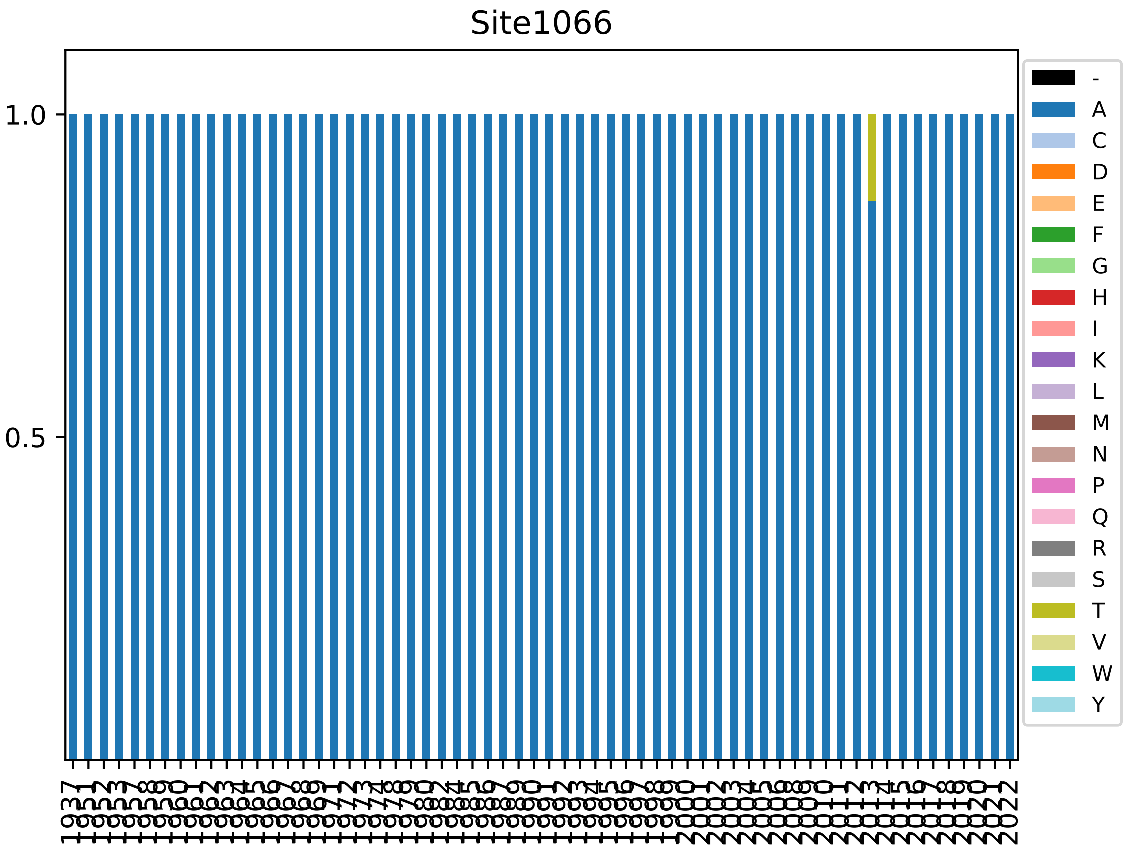
Site1066
1.0
0.5
-
A
C
D
E
F
G
H
I
K
L
M
N
P
Q
R
S
T
V
W
Y

Site1067

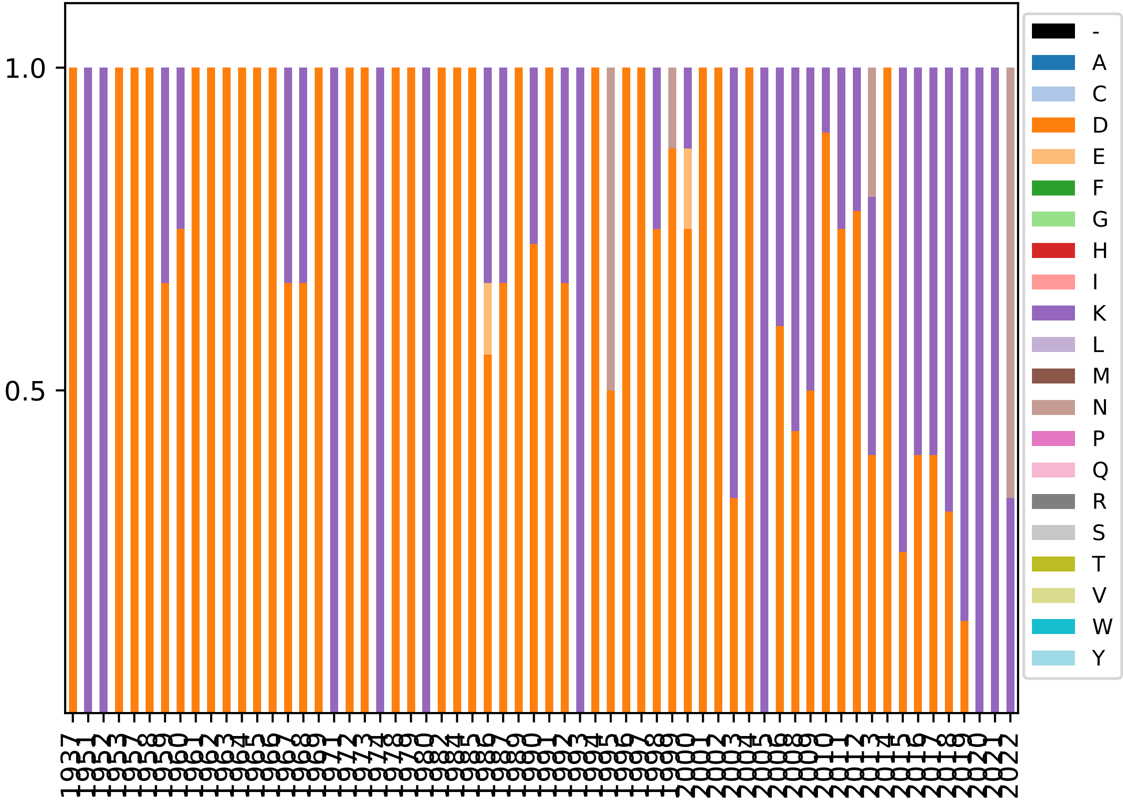

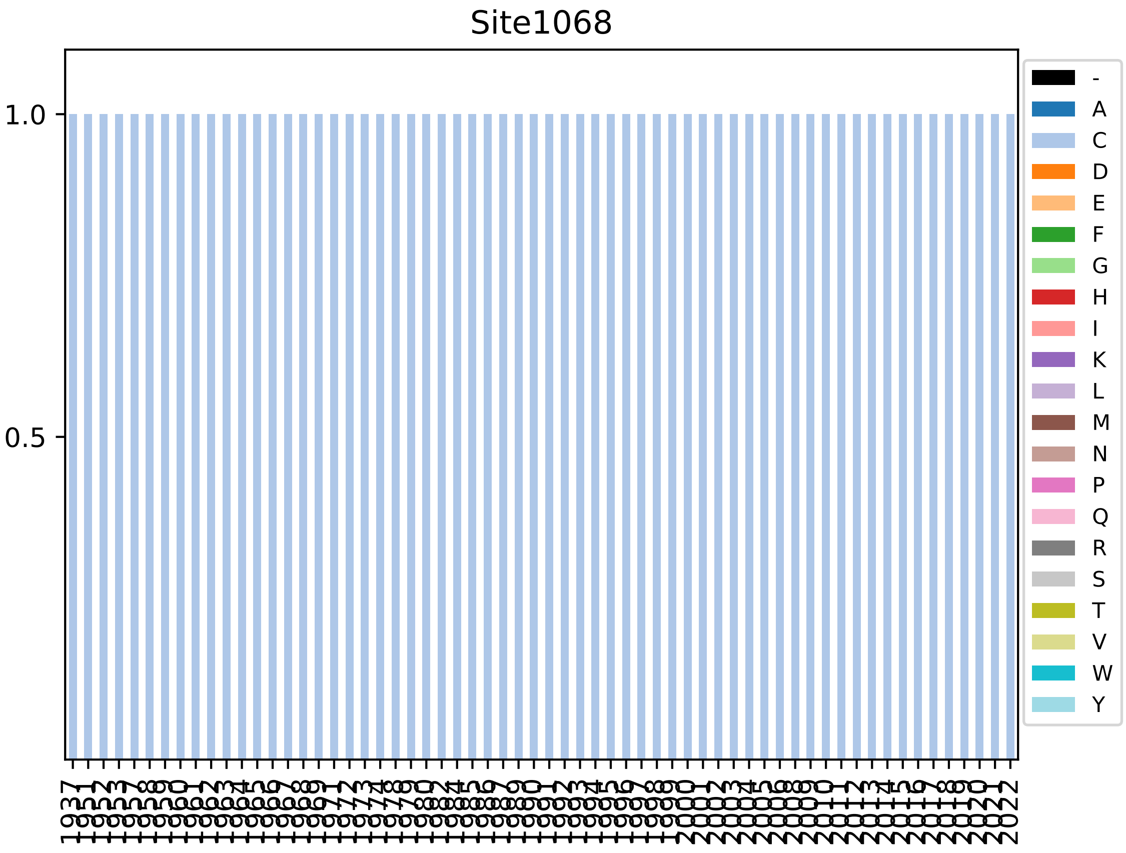
Site1068
1.0
0.5
-
A
C
D
E
F
G
H
I
K
L
M
N
P
Q
R
S
T
V
W
Y

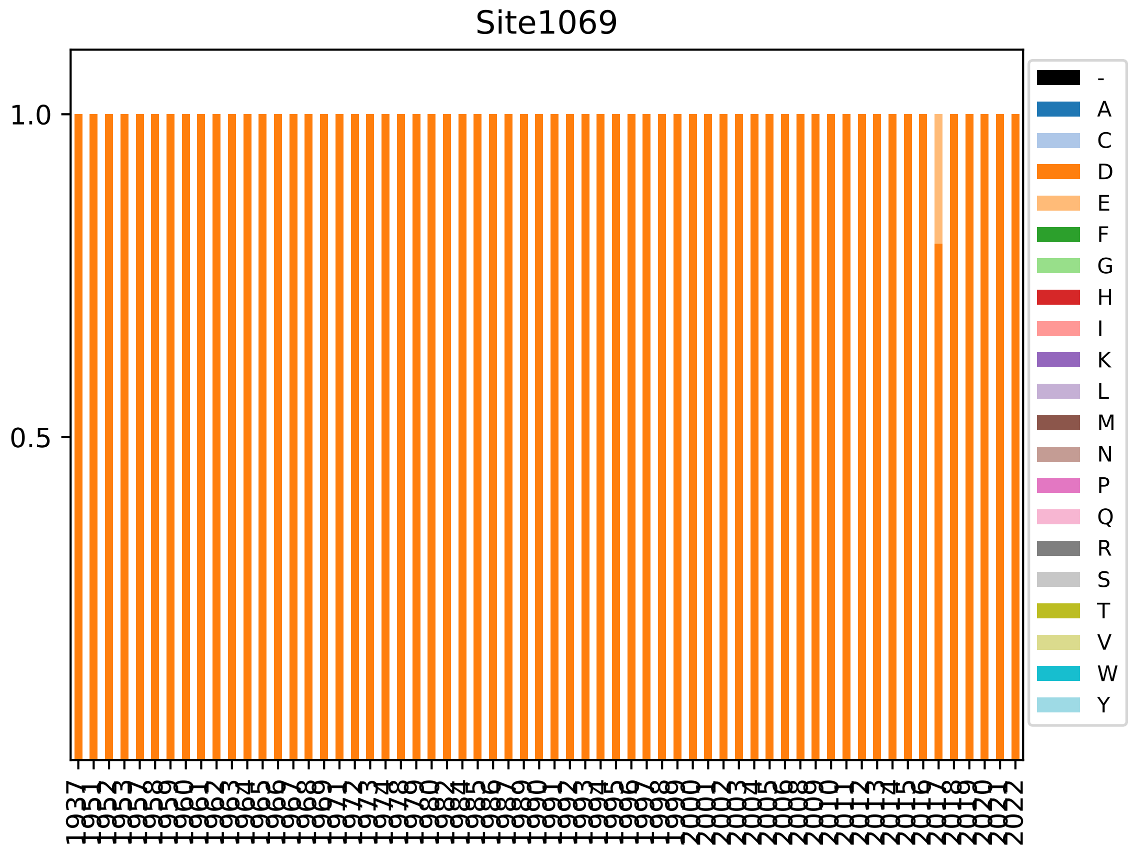

Site1069
1.0
0.5
-
A
C
D
E
F
G
H
I
K
L
M
N
P
Q
R
S
T
V
W
Y

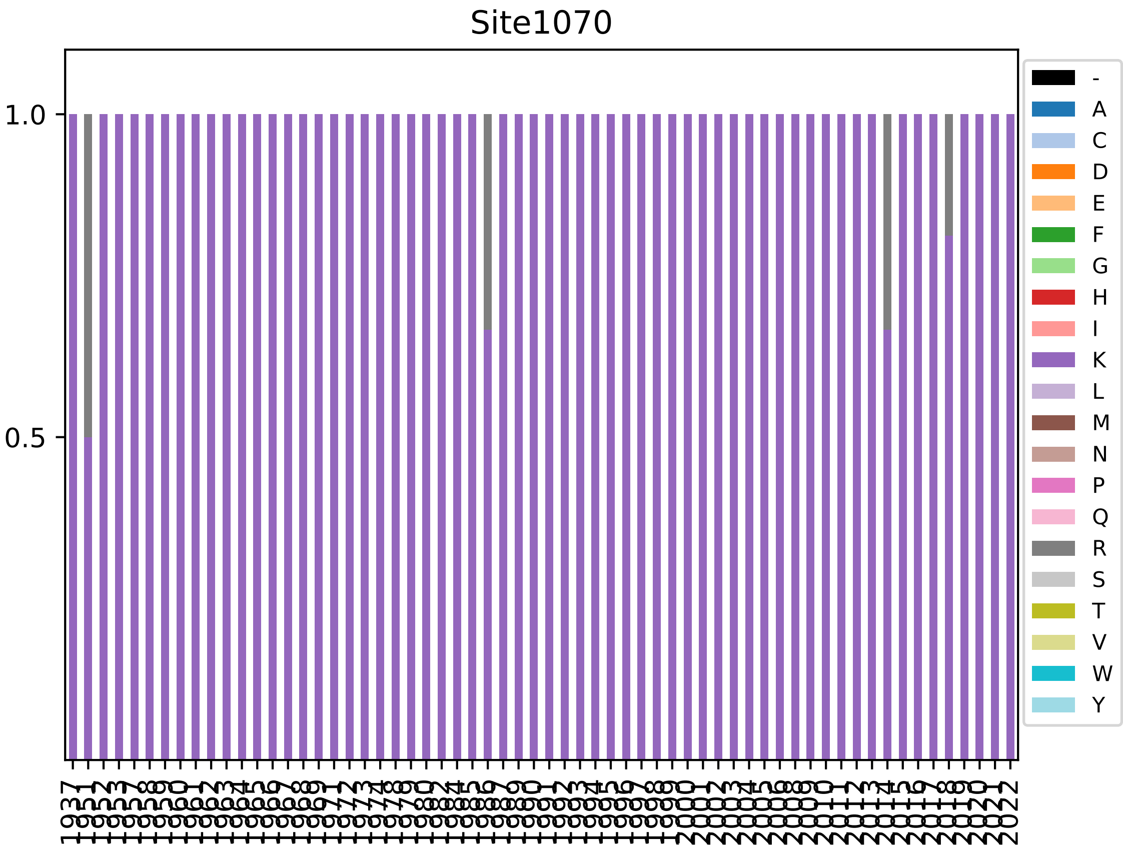
Site1070
1.0
0.5
-
A
C
D
E
F
G
H
I
K
L
M
N
P
Q
R
S
T
V
W
Y

Site1071

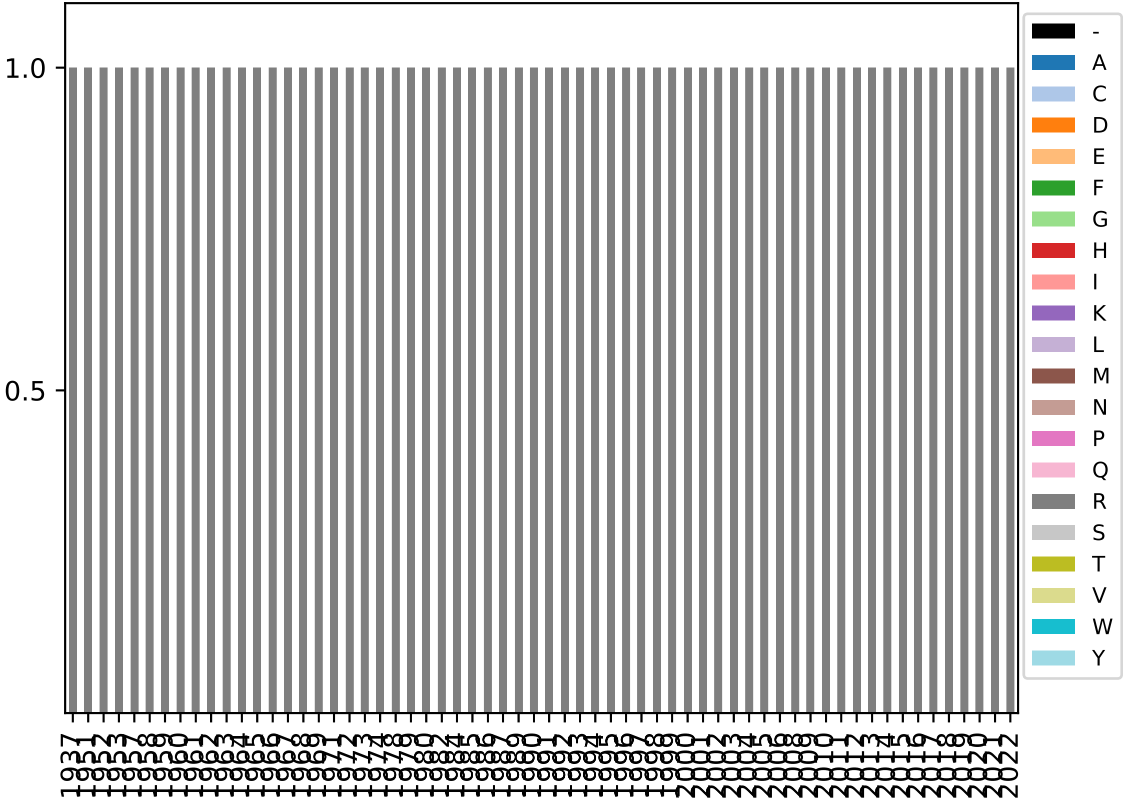

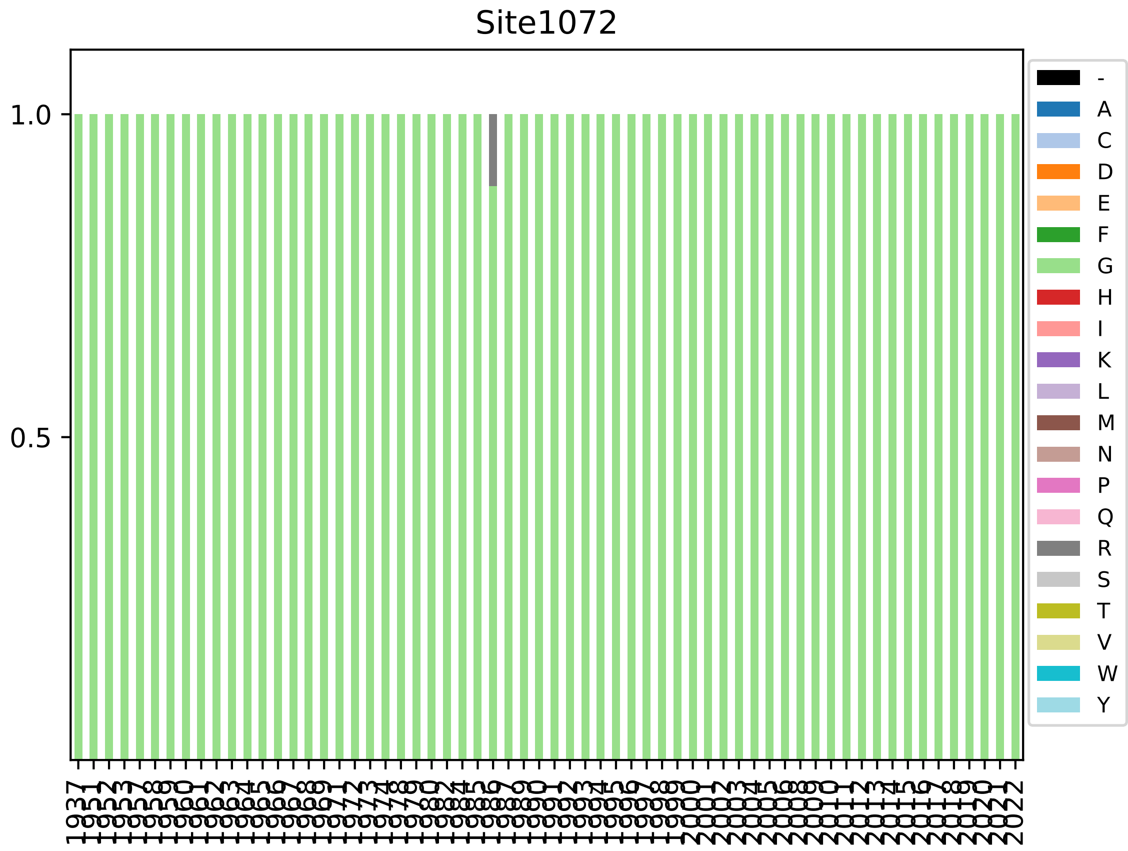

Site1072
1.0
0.5
-
A
C
D
E
F
G
H
I
K
L
M
N
P
Q
R
S
T
V
W
Y

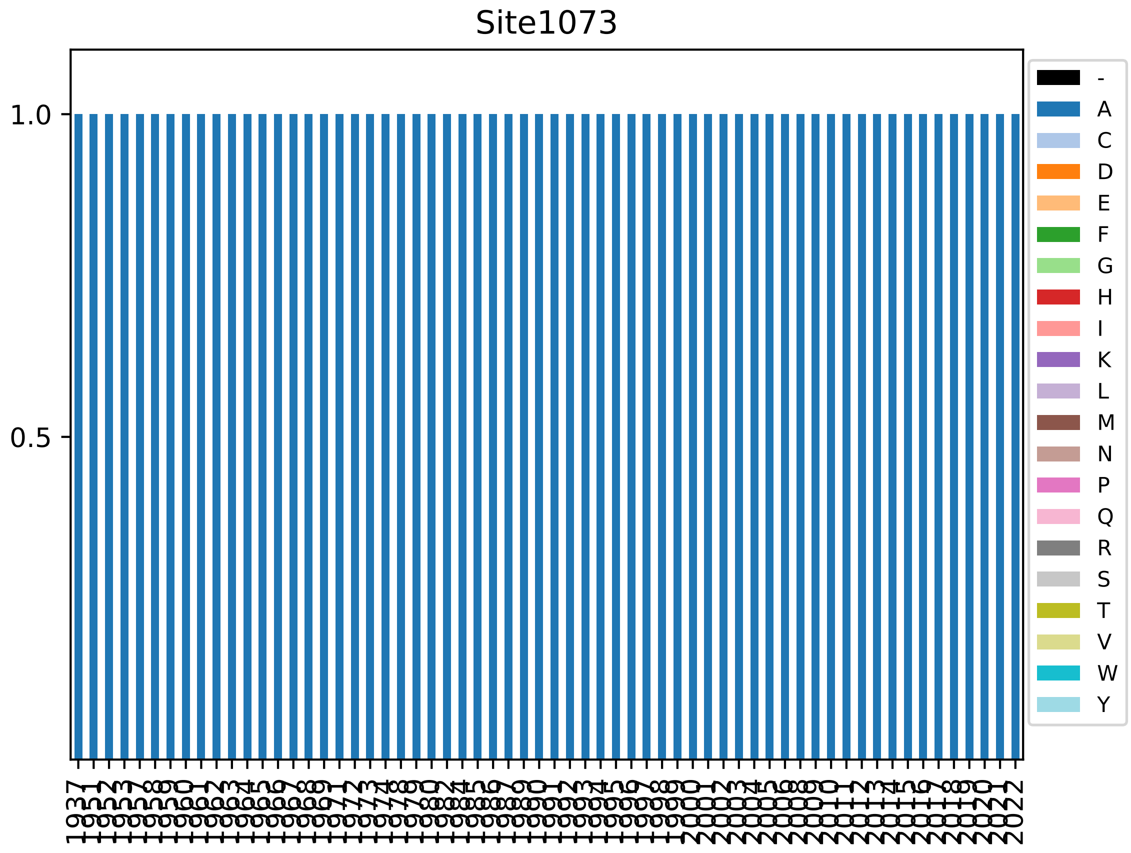
Site1073
1.0
0.5
-
A
C
D
E
F
G
H
I
K
L
M
N
P
Q
R
S
T
V
W
Y

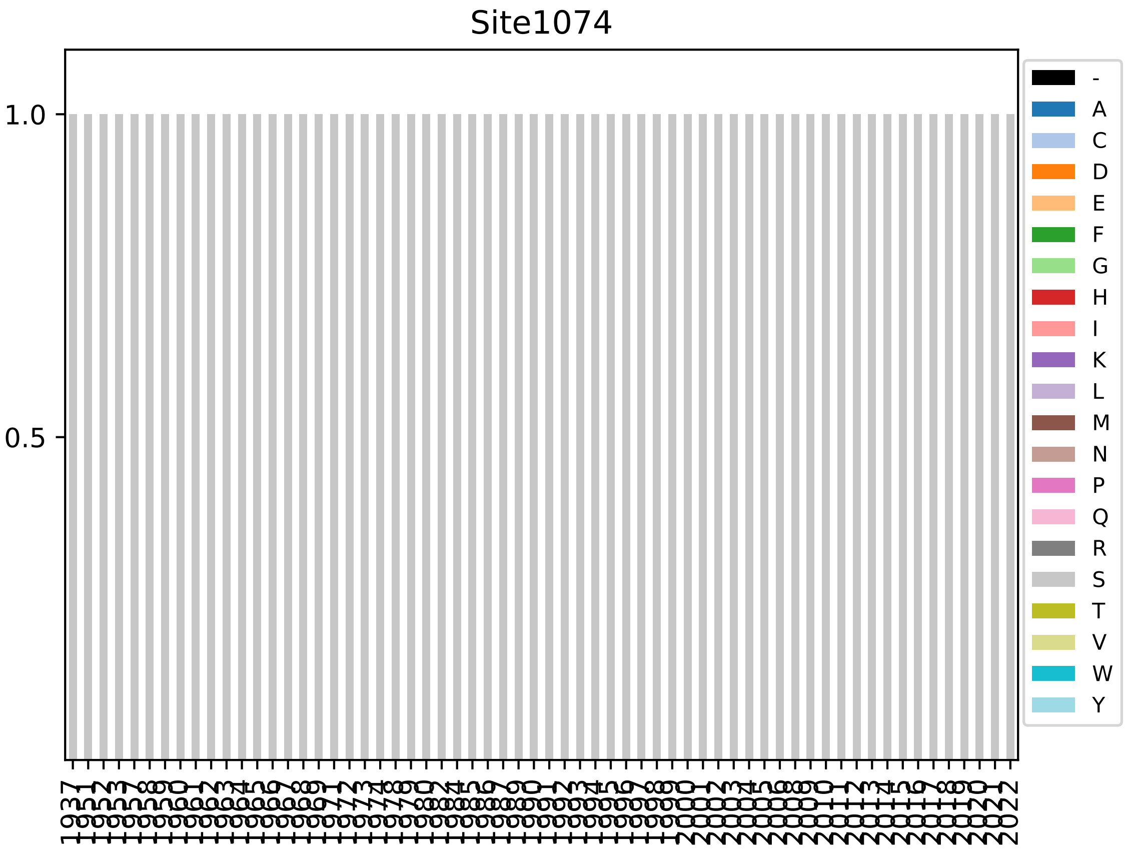
Site1074
1.0
0.5
-
A
C
D
E
F
G
H
I
K
L
M
N
P
Q
R
S
T
V
W
Y

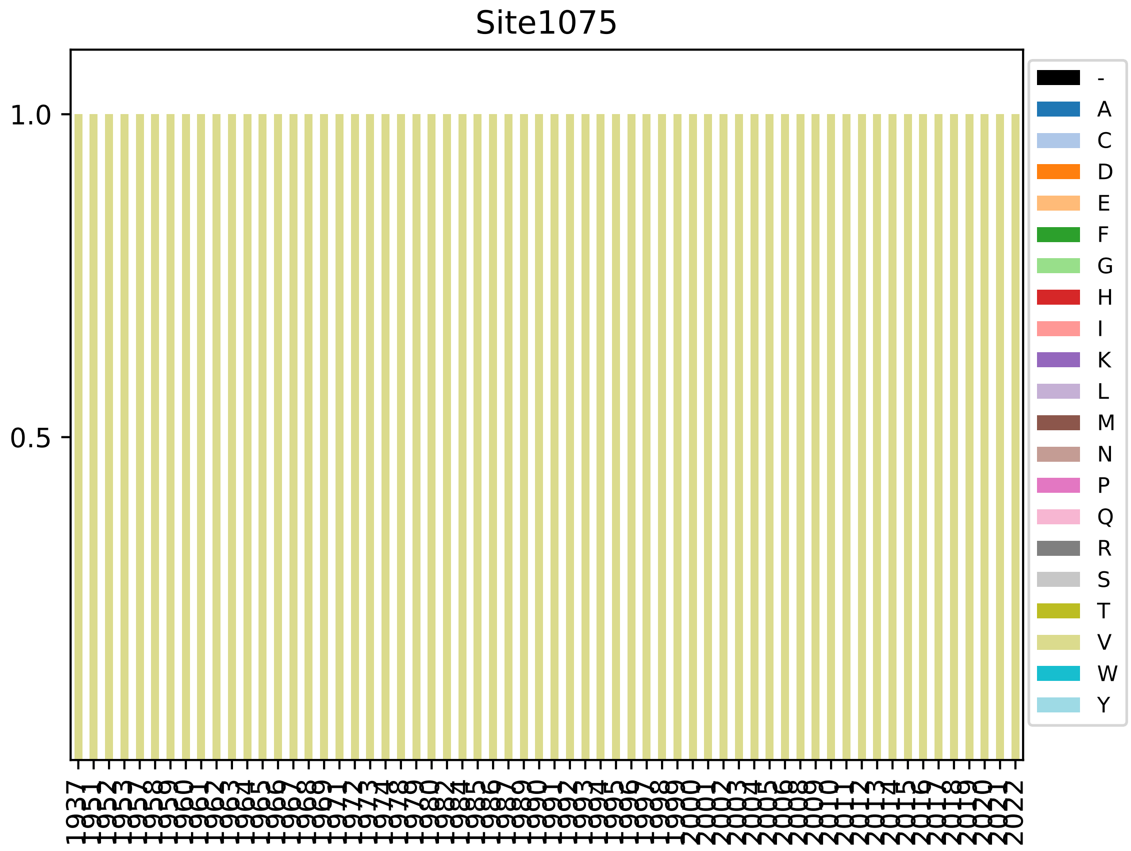
Site1075
1.0
0.5
-
A
C
D
E
F
G
H
I
K
L
M
N
P
Q
R
S
T
V
W
Y

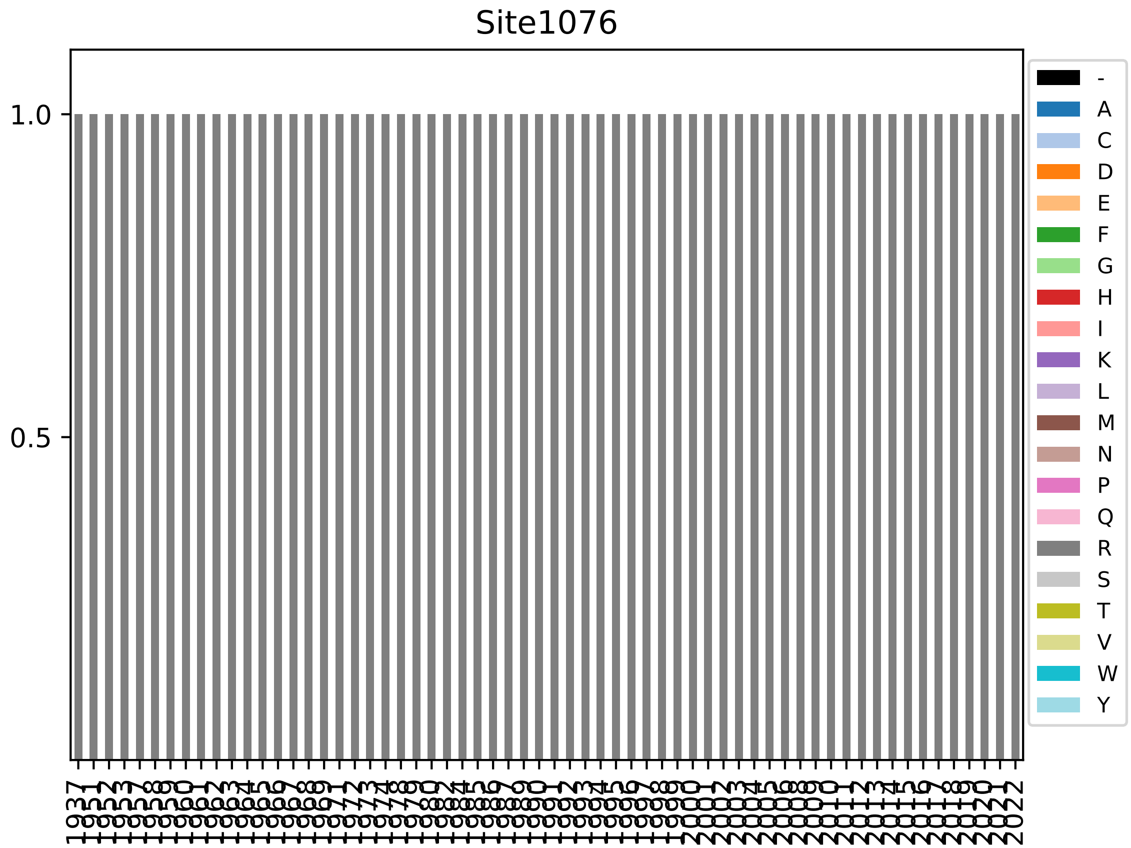
Site1076
1.0
0.5
-
A
C
D
E
F
G
H
I
K
L
M
N
P
Q
R
S
T
V
W
Y

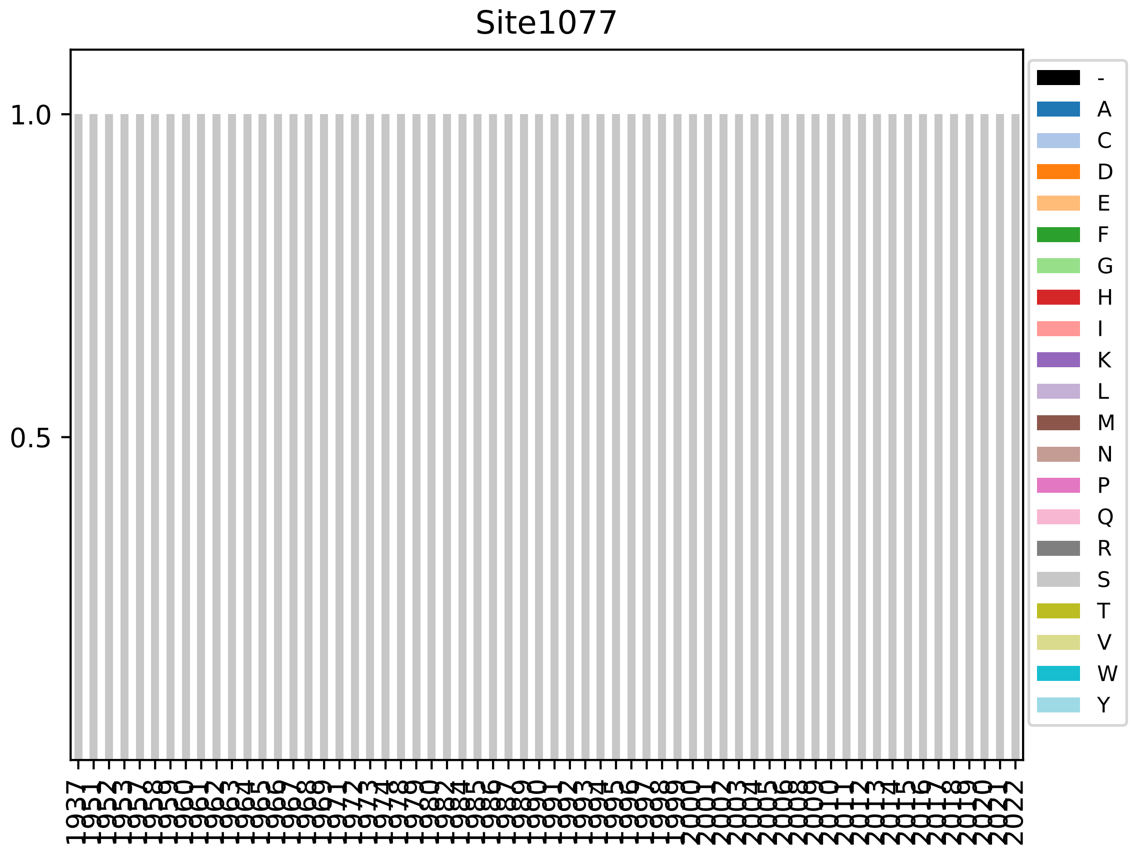
Site1077
1.0
0.5
-
A
C
D
E
F
G
H
I
K
L
M
N
P
Q
R
S
T
V
W
Y

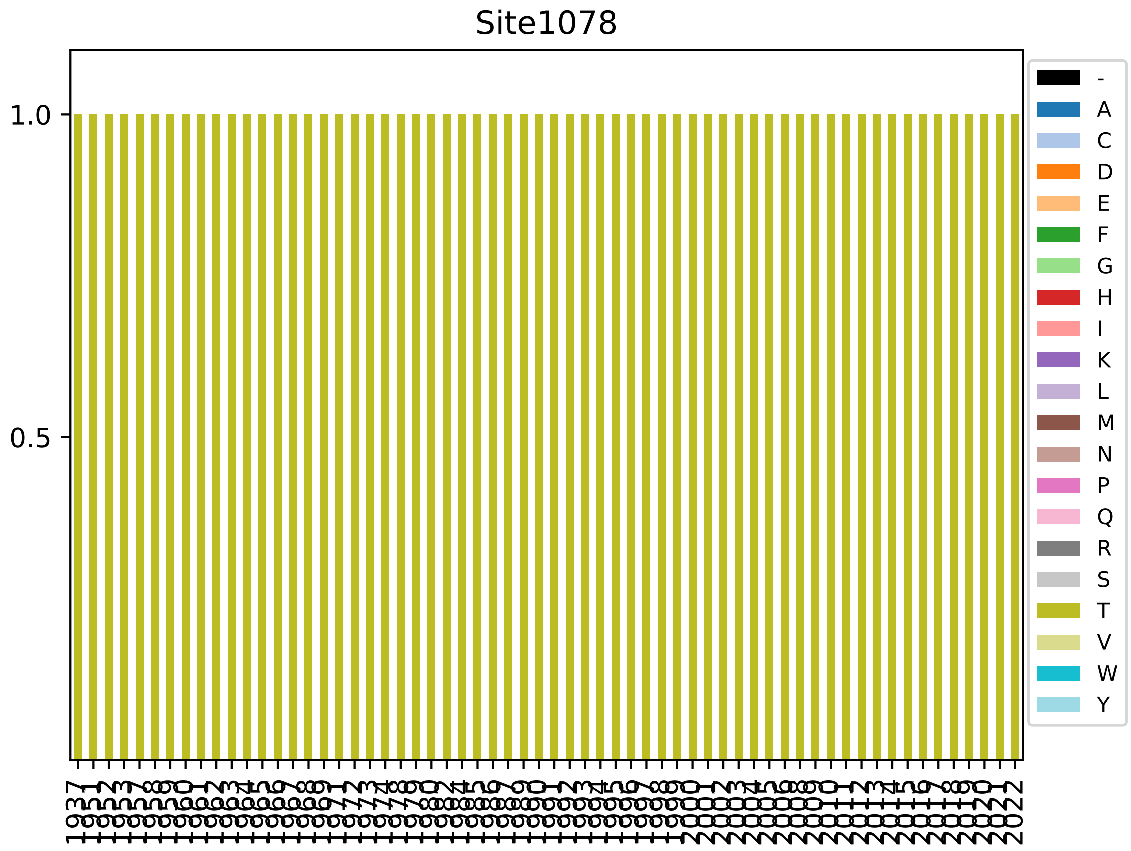

Site1078
1.0
0.5
-
A
C
D
E
F
G
H
I
K
L
M
N
P
Q
R
S
T
V
W
Y

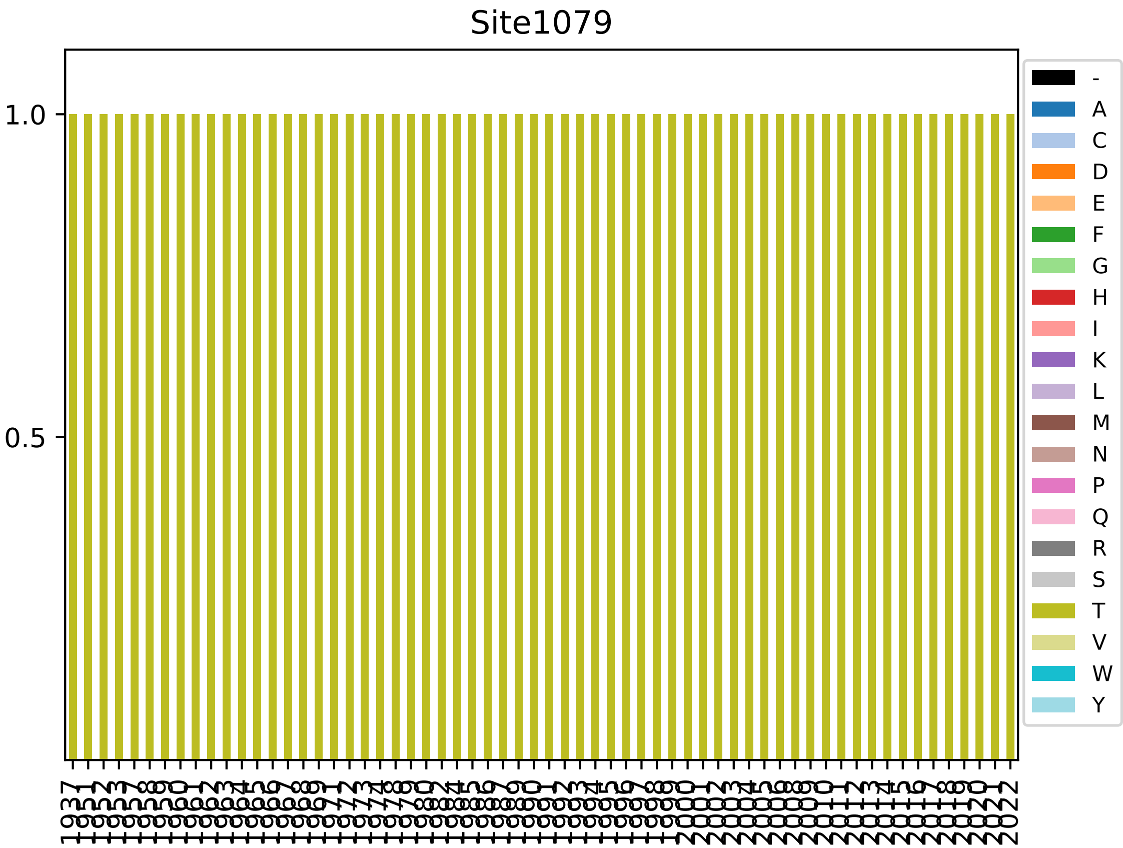
Site1079
1.0
0.5
-
A
C
D
E
F
G
H
I
K
L
M
N
P
Q
R
S
T
V
W
Y

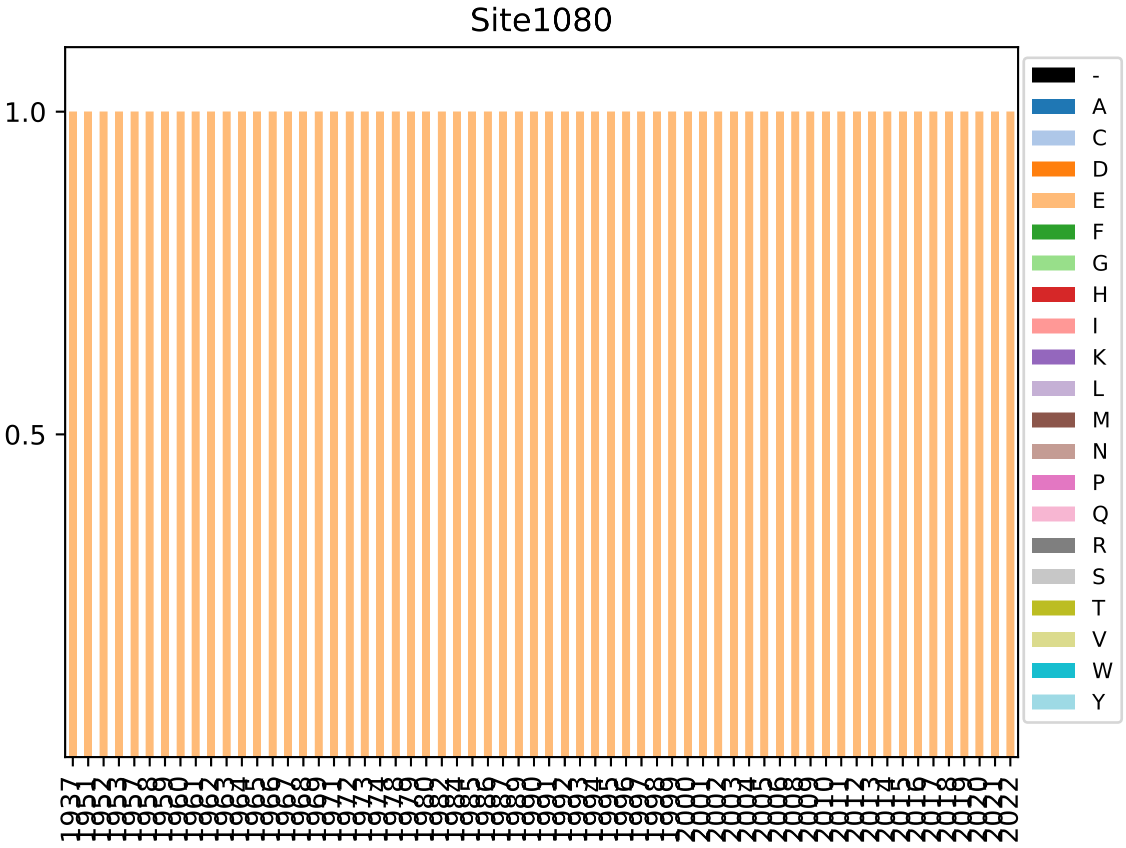
Site1080
1.0
0.5
-
A
C
D
E
F
G
H
I
K
L
M
N
P
Q
R
S
T
V
W
Y

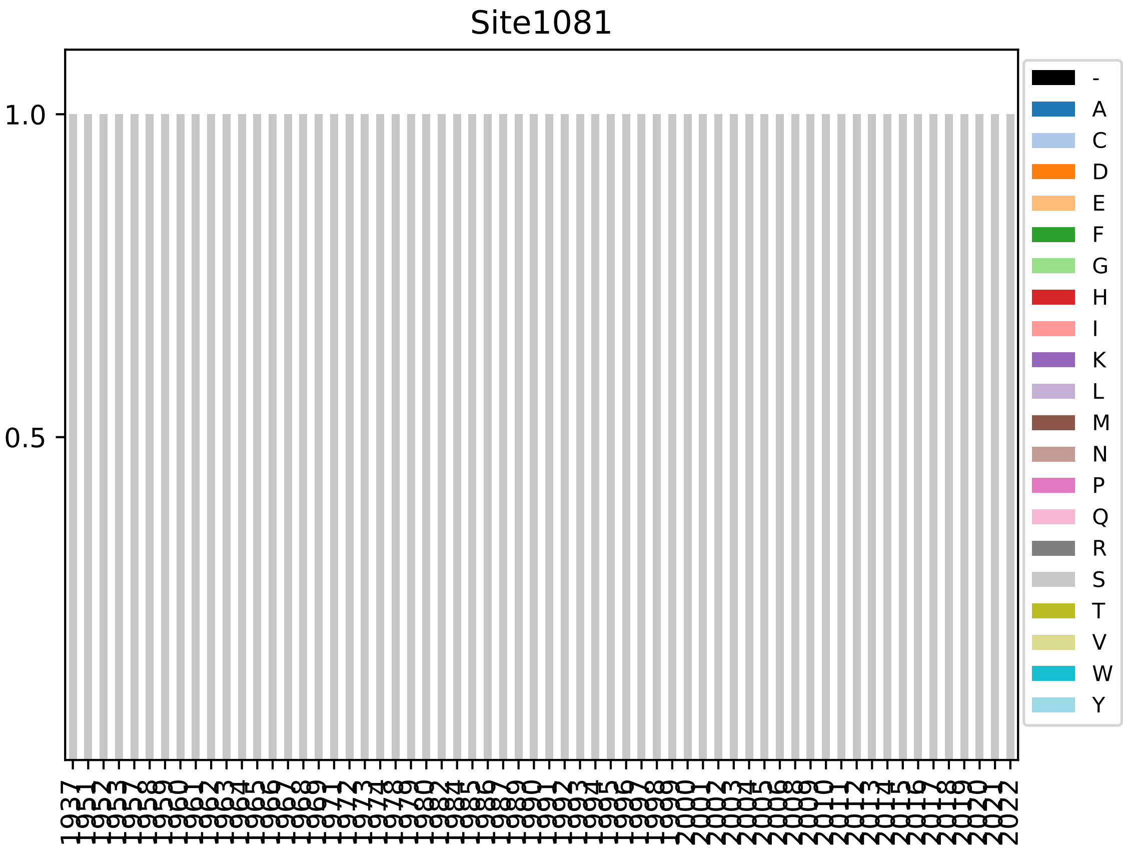
Site1081
1.0
0.5
-
A
C
D
E
F
G
H
I
K
L
M
N
P
Q
R
S
T
V
W
Y

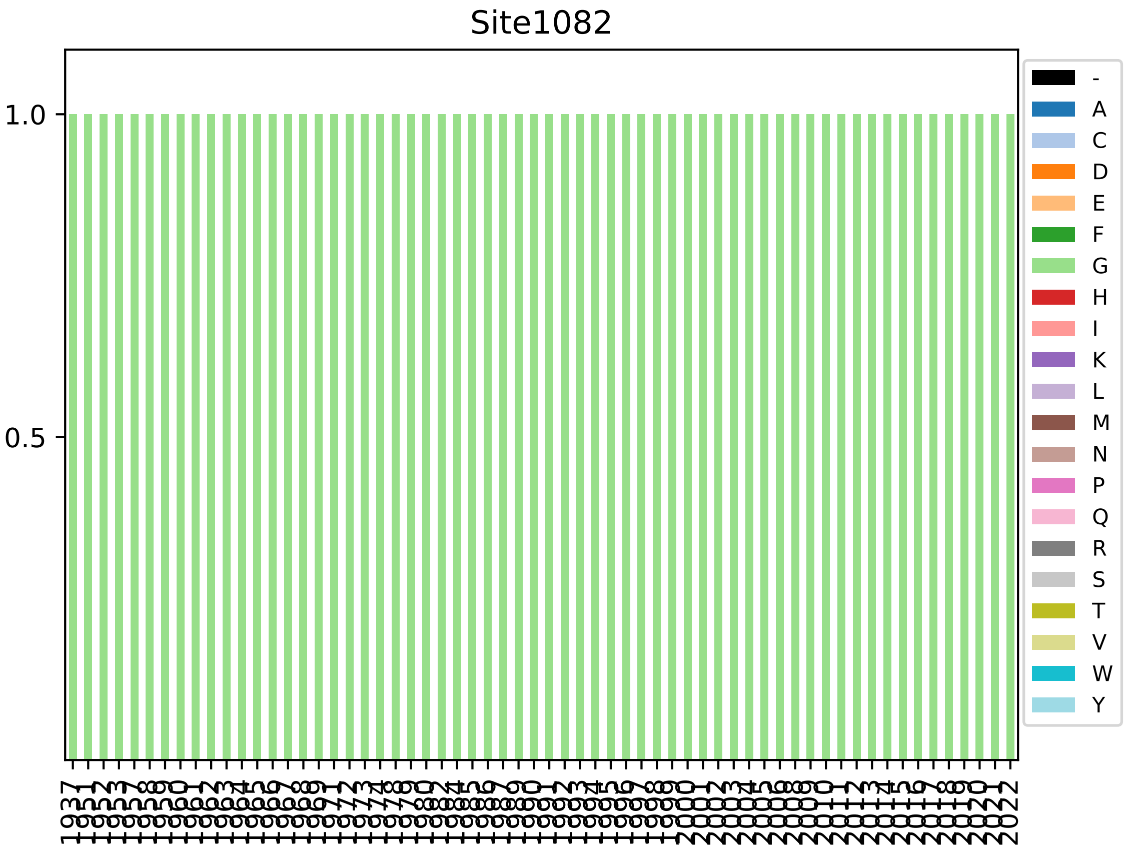

Site1082
1.0
0.5
-
A
C
D
E
F
G
H
I
K
L
M
N
P
Q
R
S
T
V
W
Y

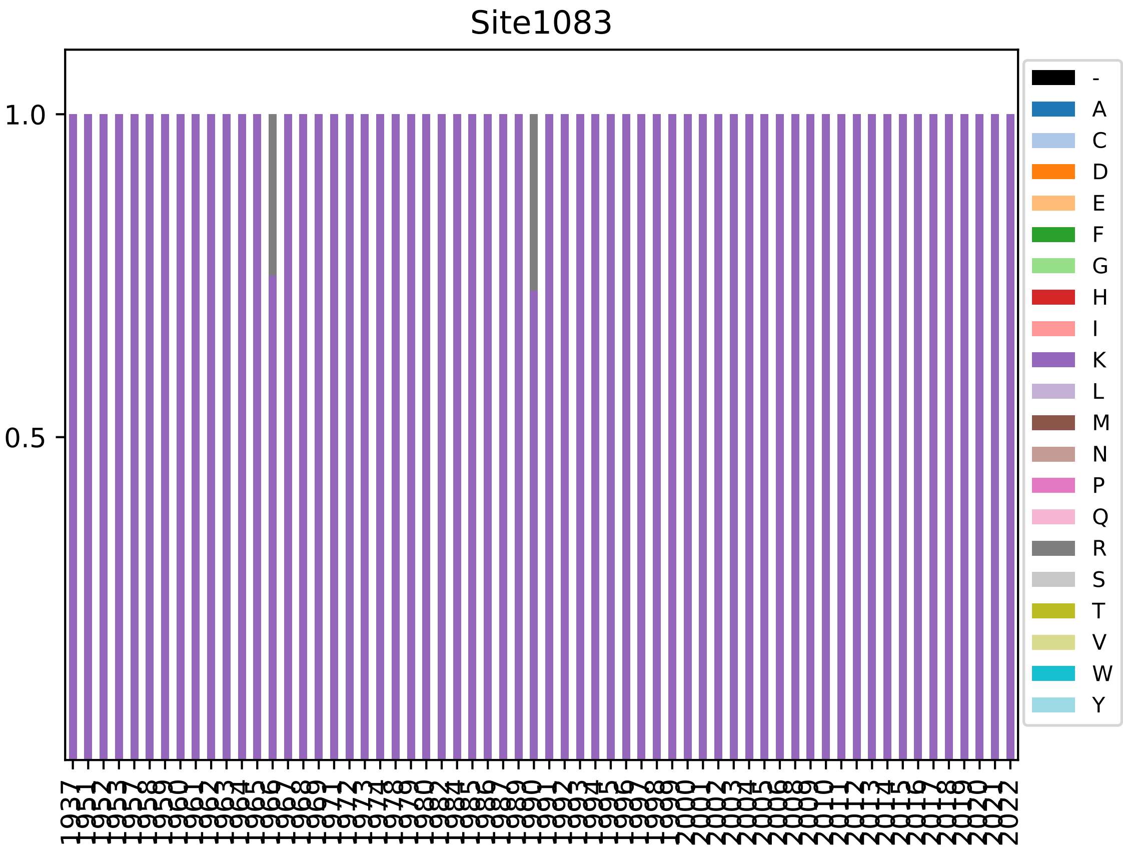
Site1083
1.0
0.5
-
A
C
D
E
F
G
H
I
K
L
M
N
P
Q
R
S
T
V
W
Y

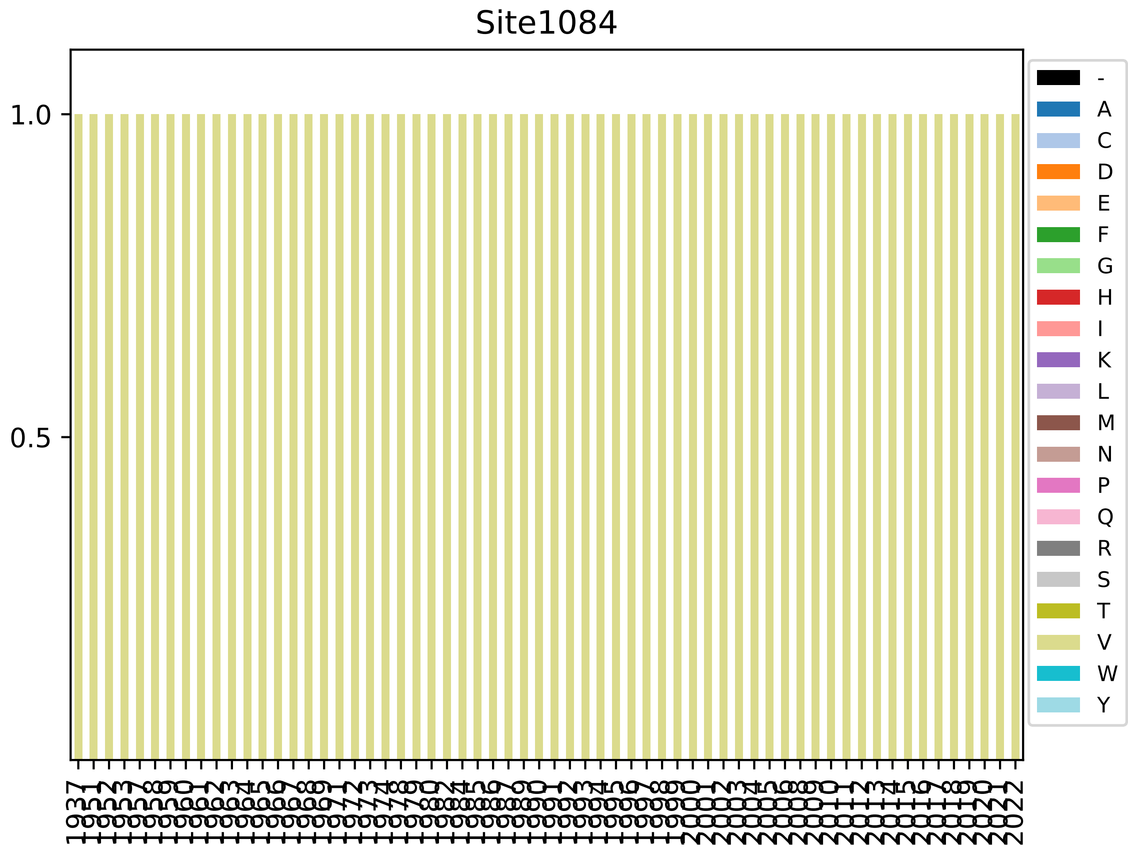
Site1084
1.0
0.5
-
A
C
D
E
F
G
H
I
K
L
M
N
P
Q
R
S
T
V
W
Y

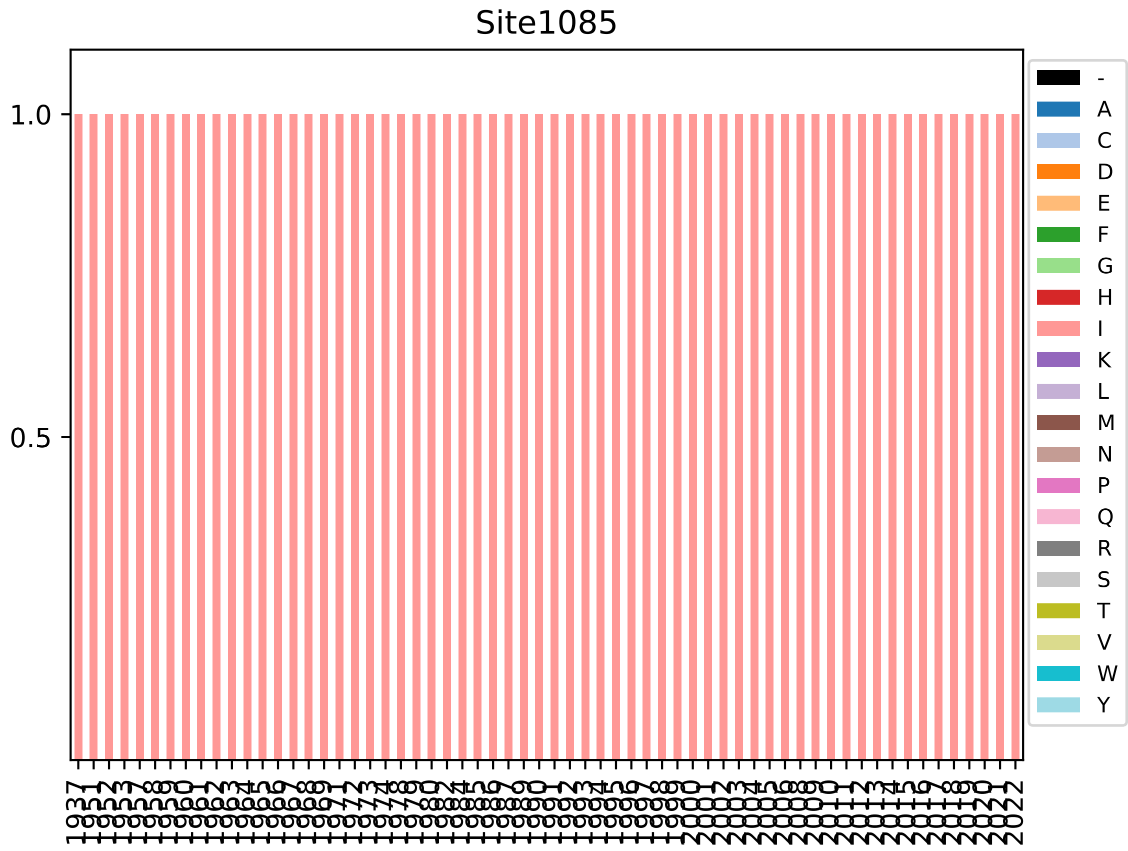
Site1085
1.0
0.5
-
A
C
D
E
F
G
H
I
K
L
M
N
P
Q
R
S
T
V
W
Y

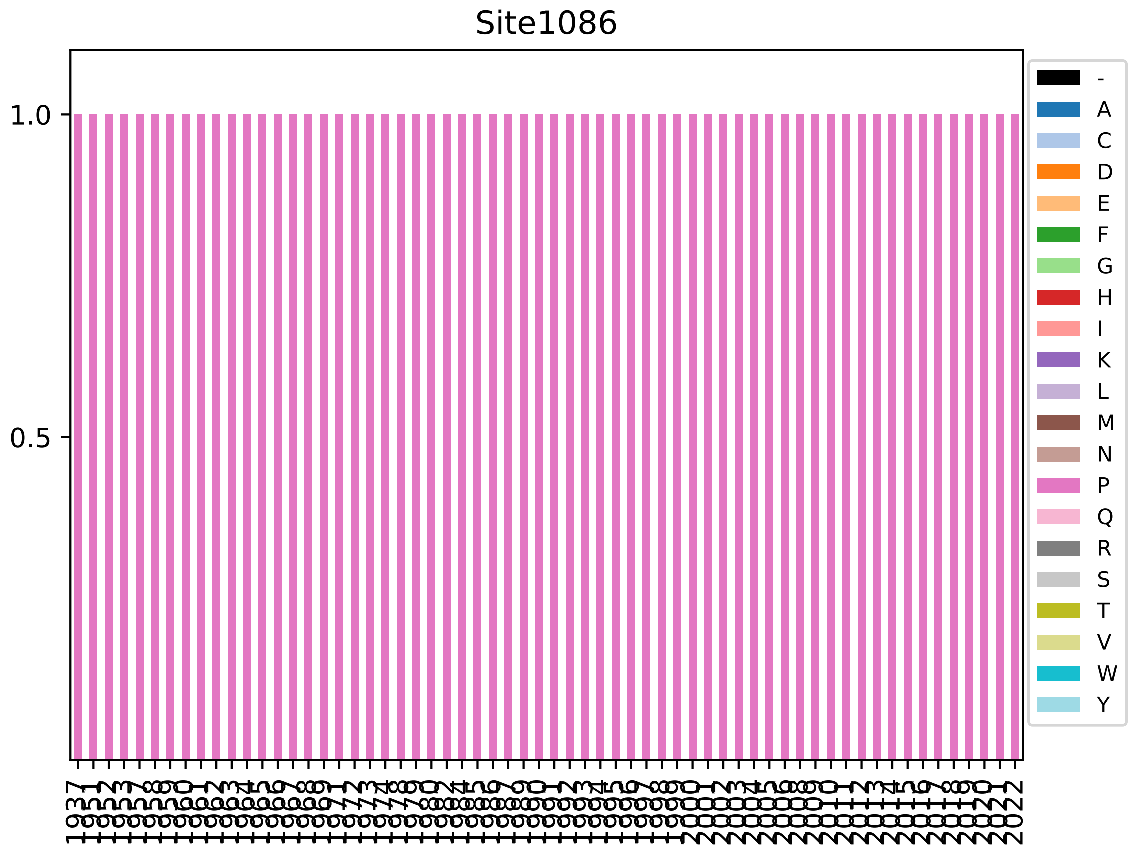
Site1086
1.0
0.5
-
A
C
D
E
F
G
H
I
K
L
M
N
P
Q
R
S
T
V
W
Y

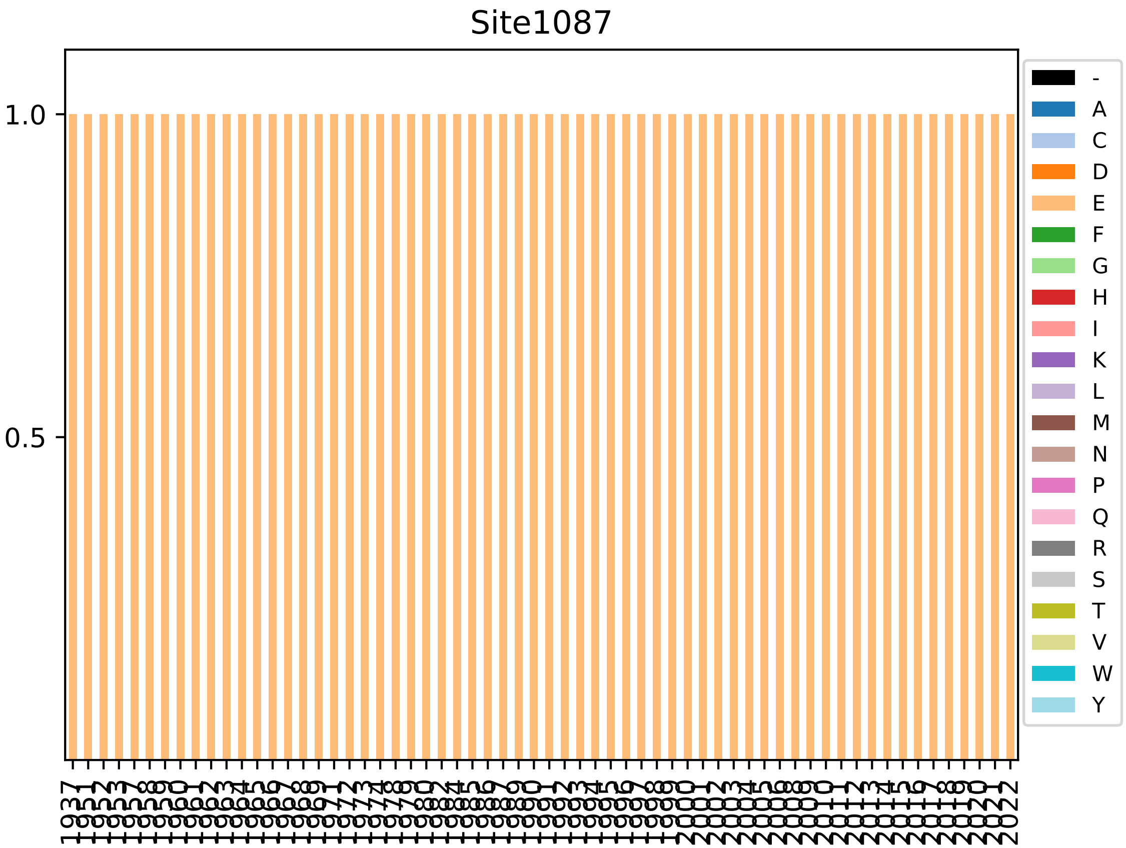
Site1087
1.0
0.5
-
A
C
D
E
F
G
H
I
K
L
M
N
P
Q
R
S
T
V
W
Y

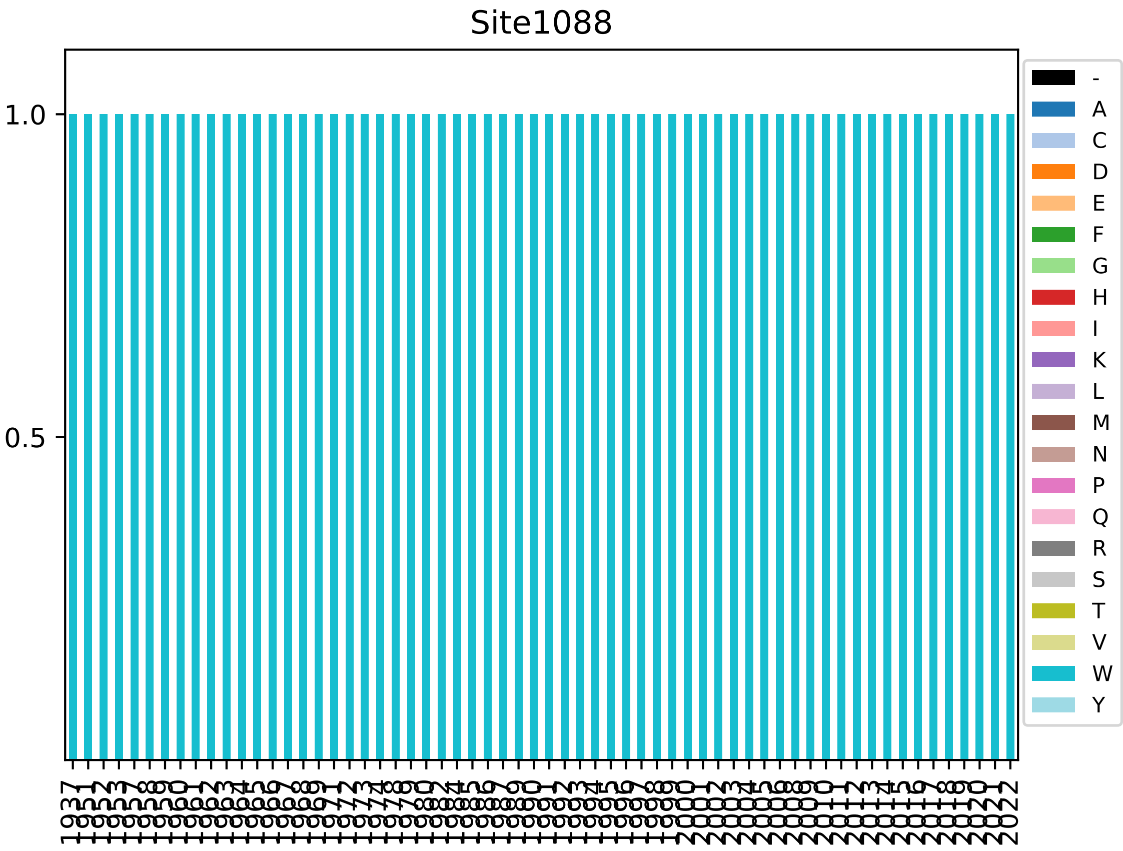
Site1088
1.0
0.5
-
A
C
D
E
F
G
H
I
K
L
M
N
P
Q
R
S
T
V
W
Y

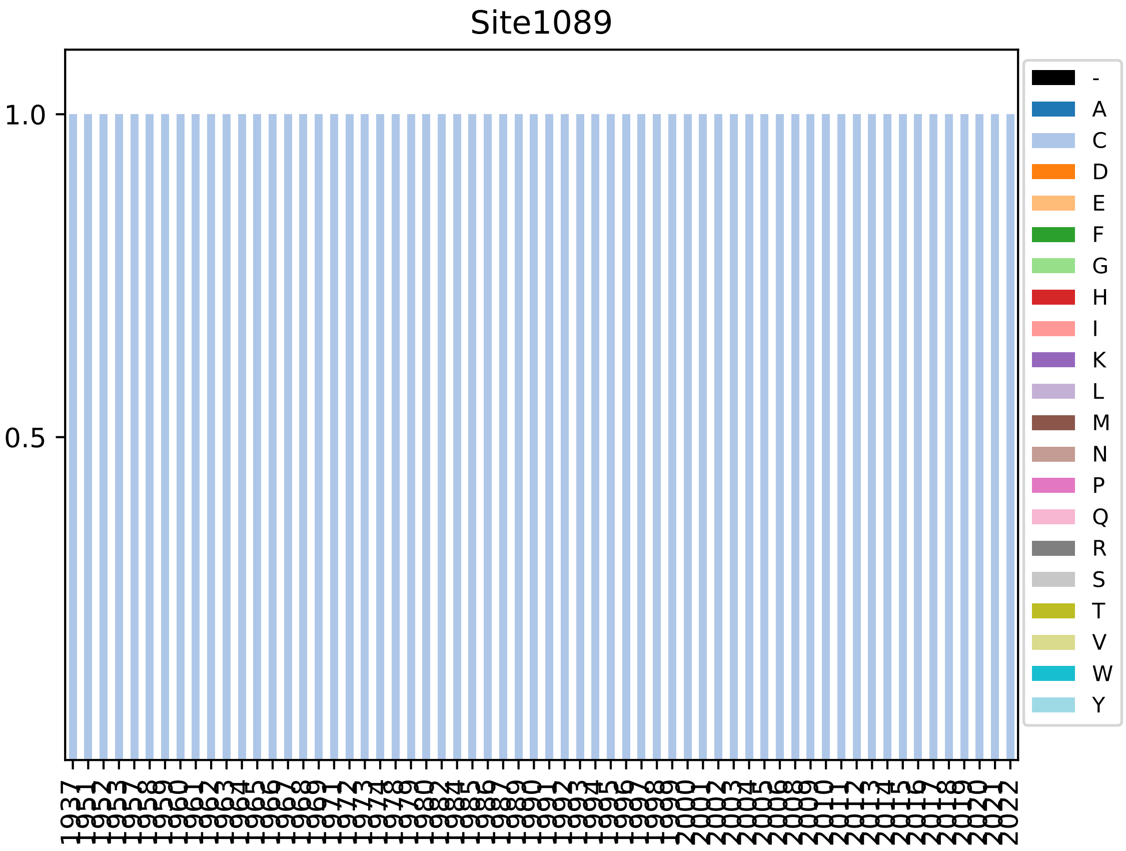
Site1089
1.0
0.5
-
A
C
D
E
F
G
H
I
K
L
M
N
P
Q
R
S
T
V
W
Y

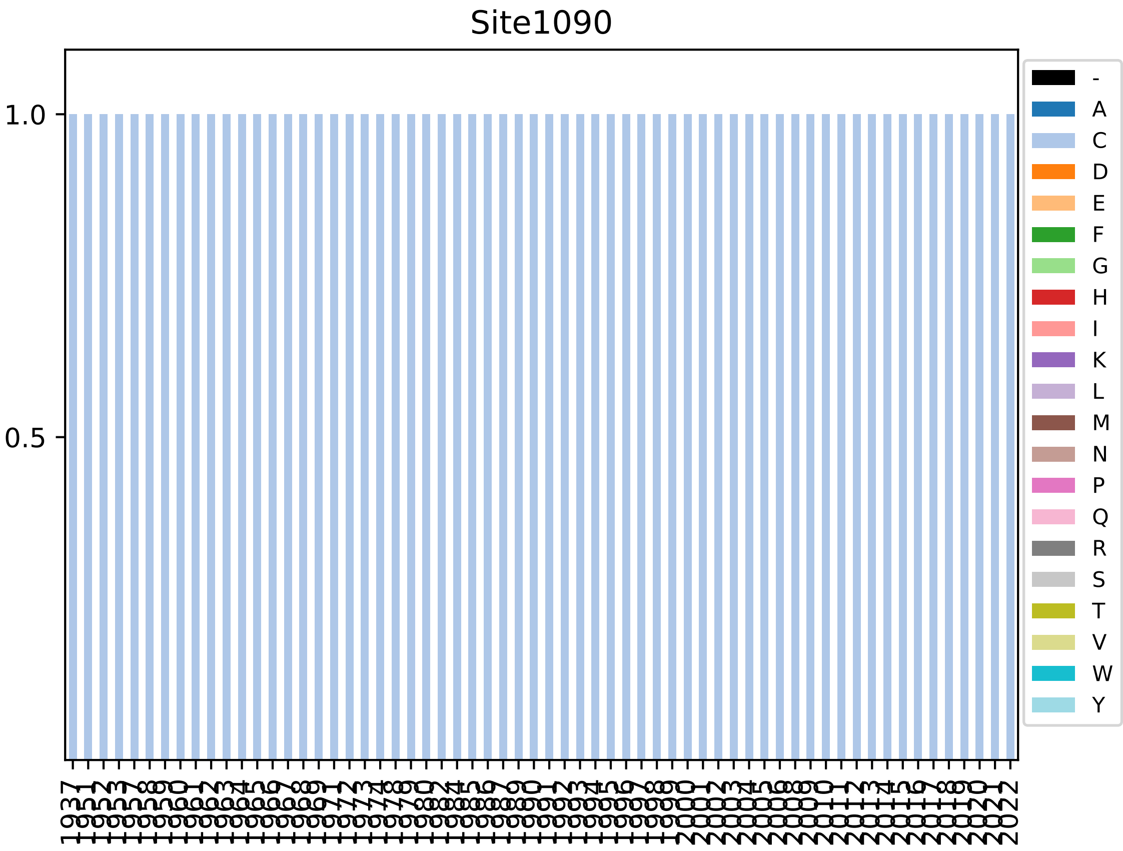
Site1090
1.0
0.5
-
A
C
D
E
F
G
H
I
K
L
M
N
P
Q
R
S
T
V
W
Y

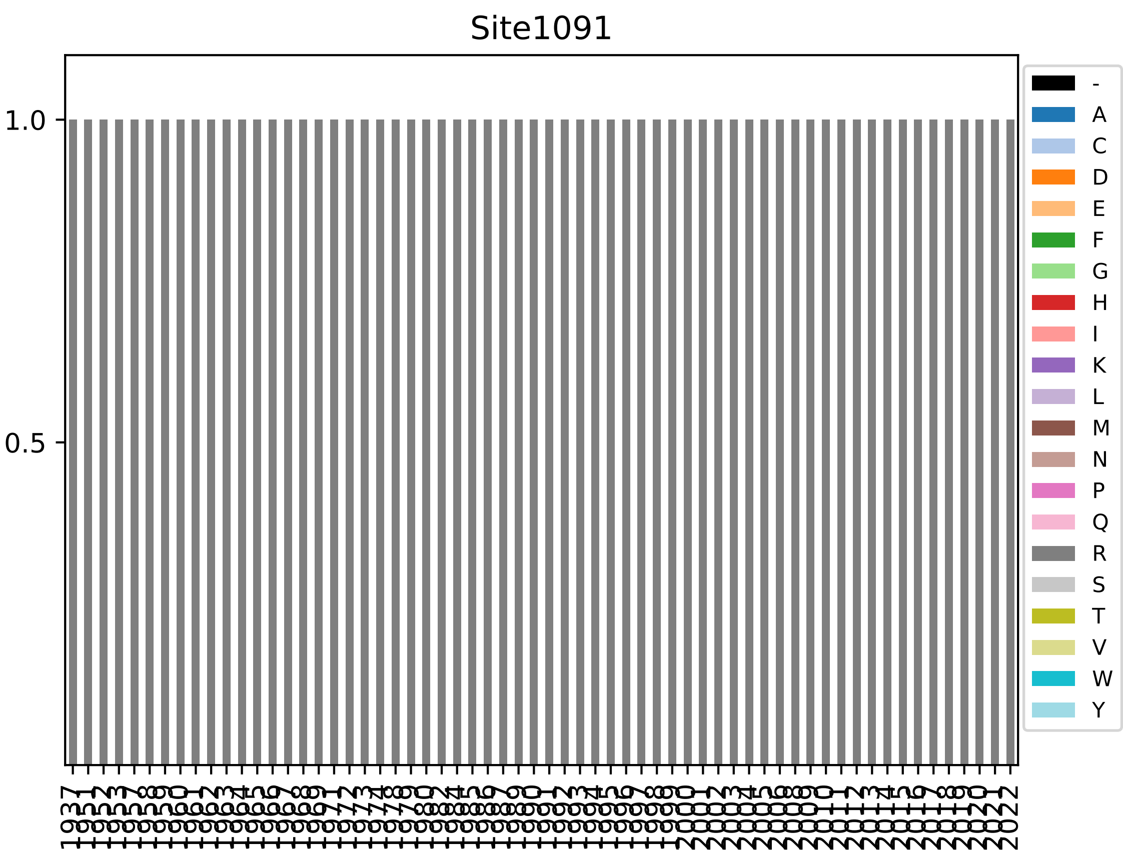
Site1091
1.0
0.5
-
A
C
D
E
F
G
H
I
K
L
M
N
P
Q
R
S
T
V
W
Y

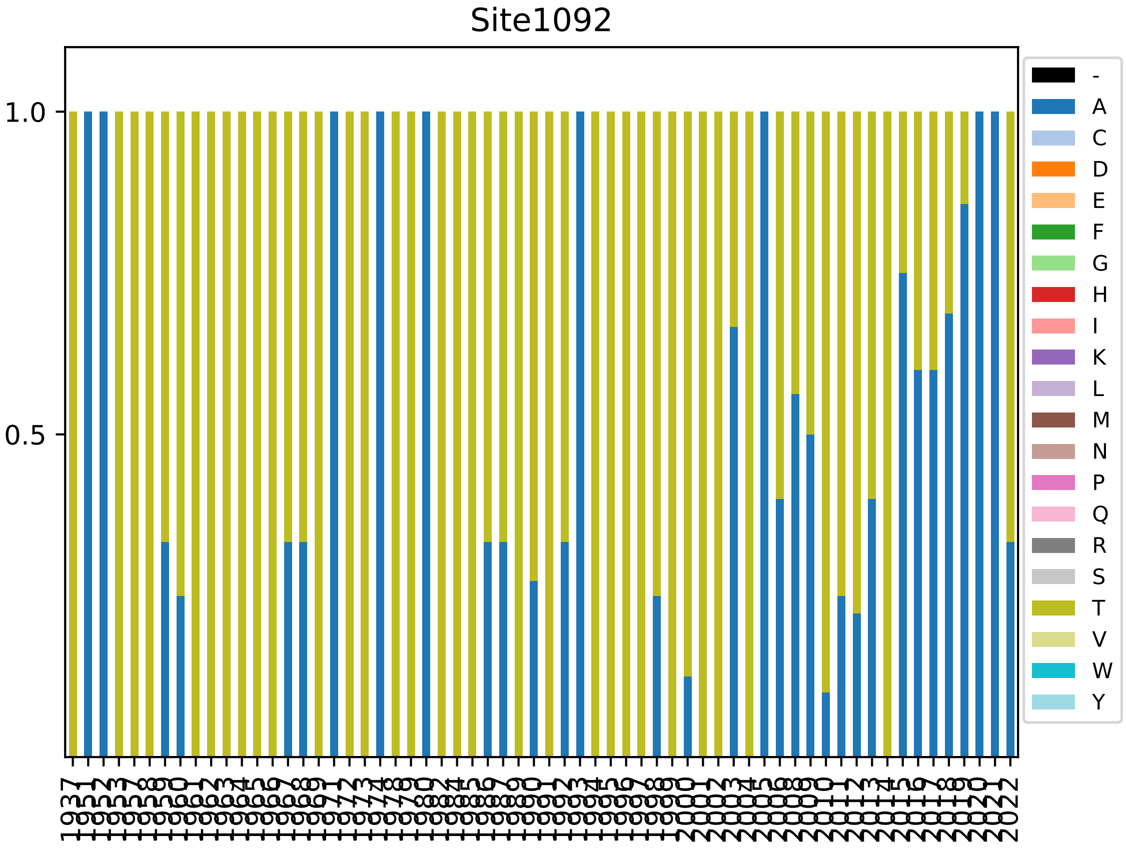
Site1092
1.0
0.5
-
A
C
D
E
F
G
H
I
K
L
M
N
P
Q
R
S
T
V
W
Y

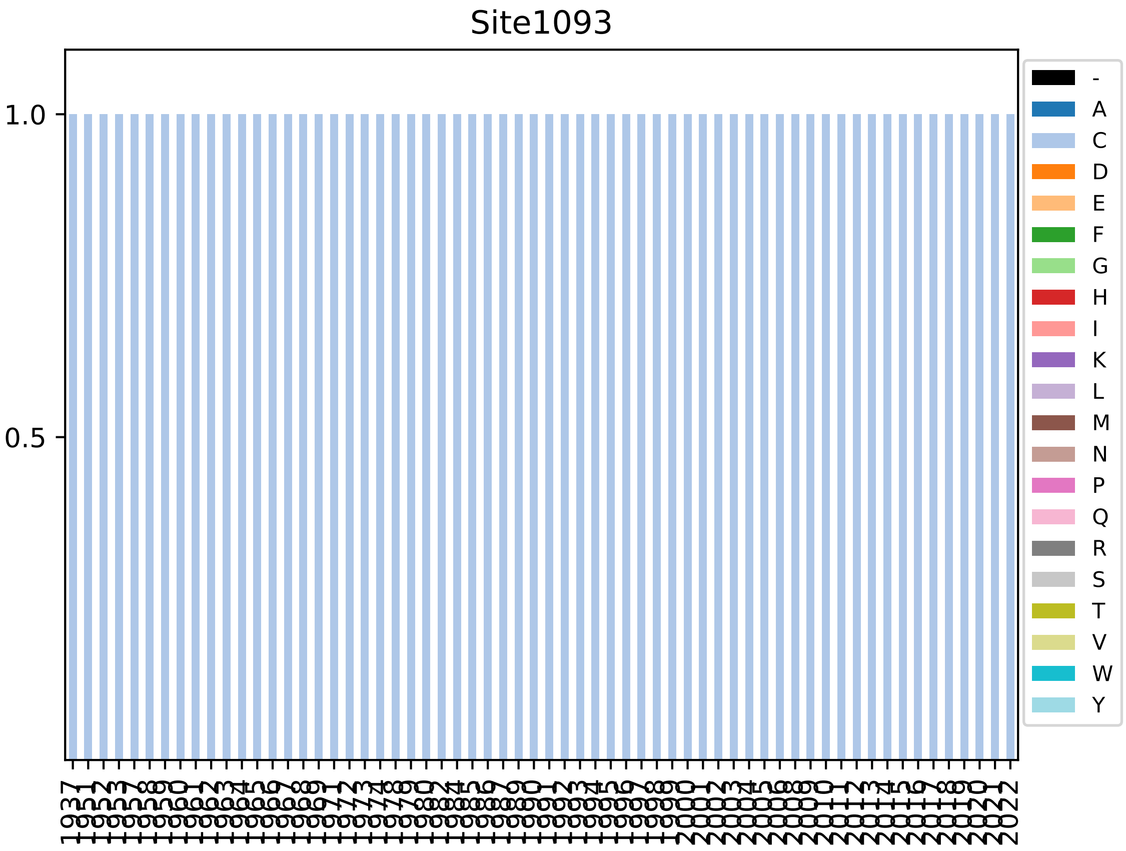
Site1093
1.0
0.5
-
A
C
D
E
F
G
H
I
K
L
M
N
P
Q
R
S
T
V
W
Y

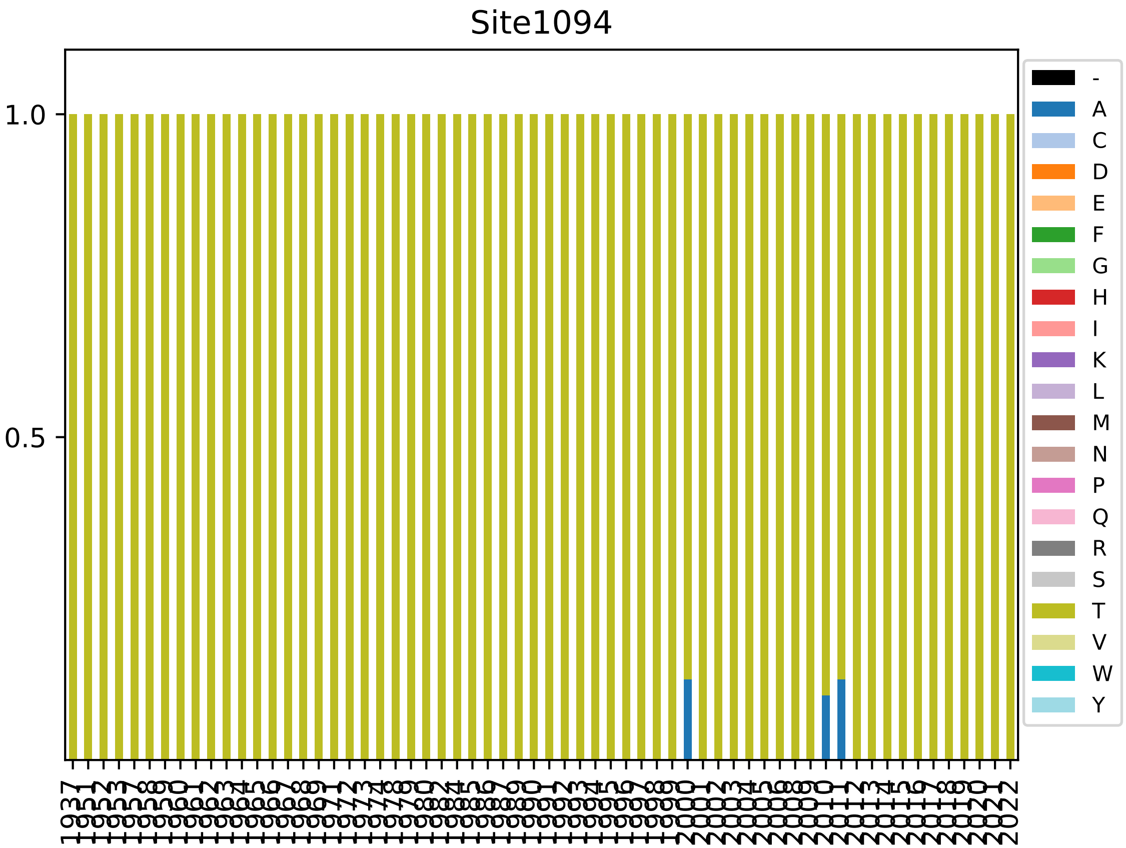

Site1094
1.0
0.5
-
A
C
D
E
F
G
H
I
K
L
M
N
P
Q
R
S
T
V
W
Y

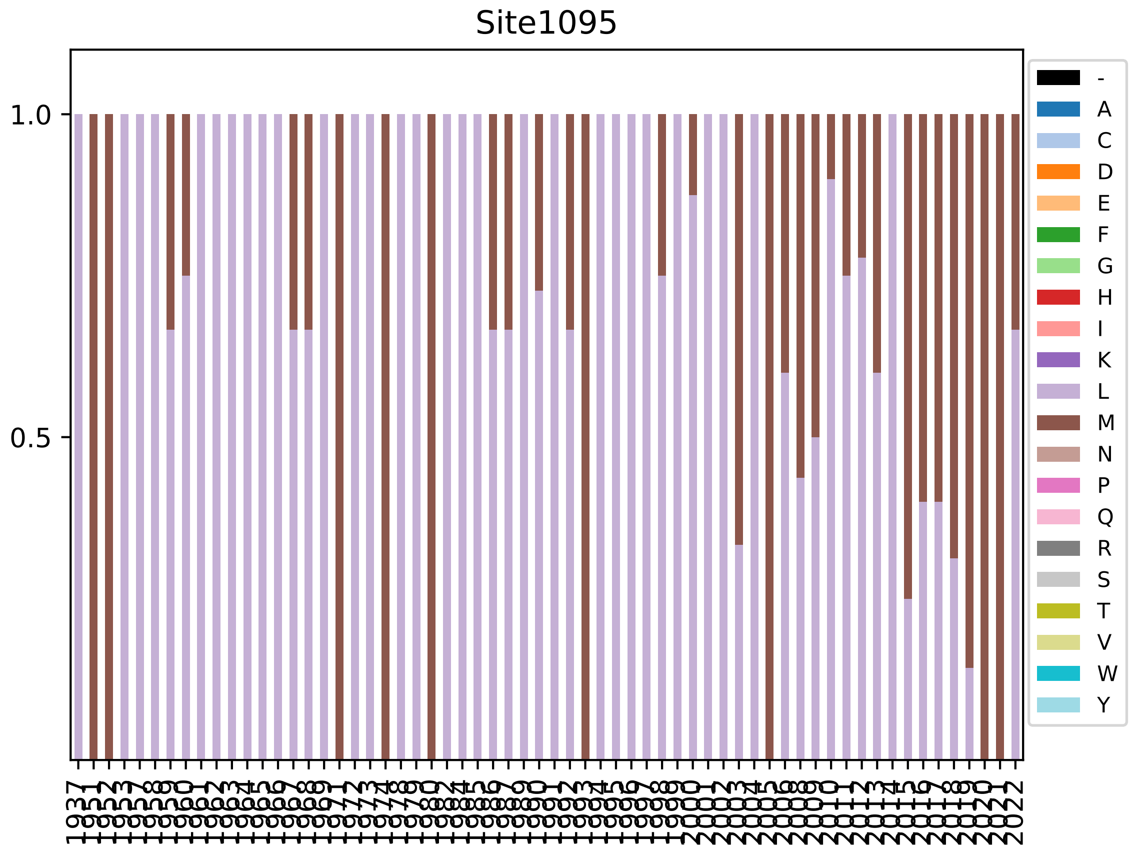
Site1095
1.0
0.5
-
A
C
D
E
F
G
H
I
K
L
M
N
P
Q
R
S
T
V
W
Y

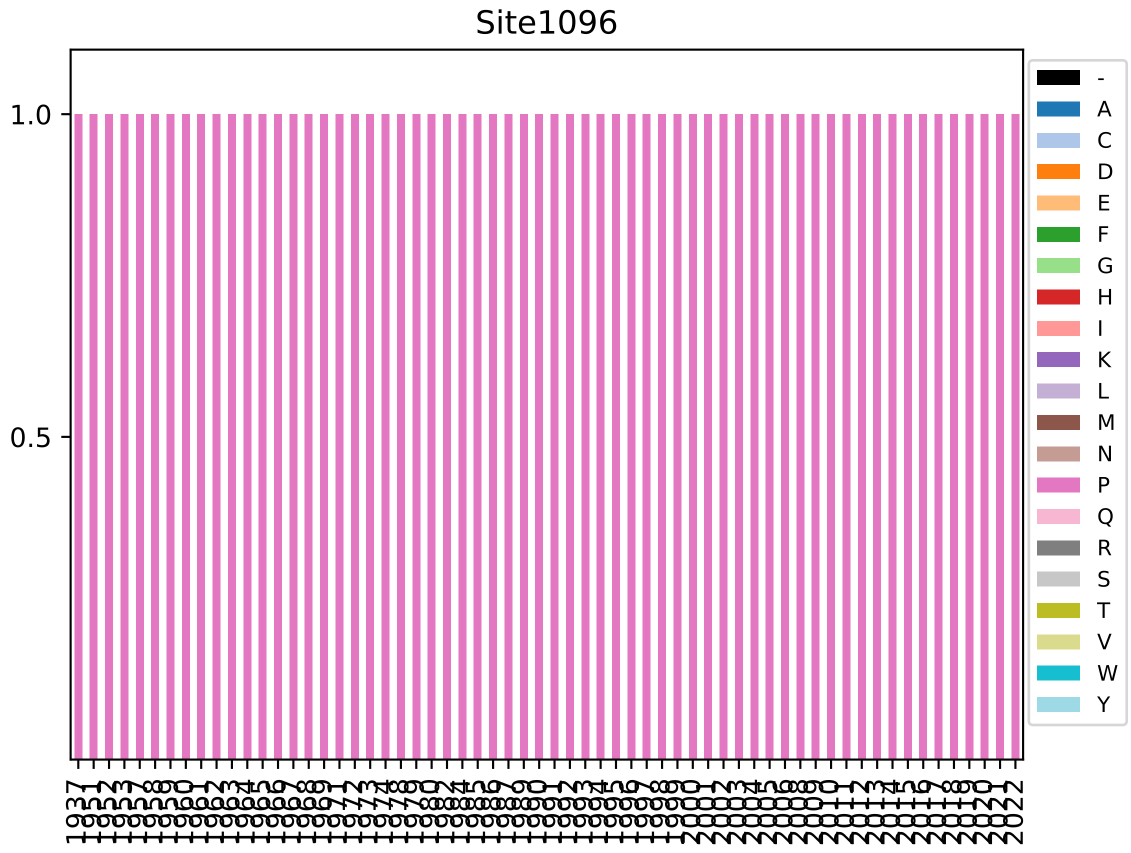

Site1096
1.0
0.5
-
A
C
D
E
F
G
H
I
K
L
M
N
P
Q
R
S
T
V
W
Y

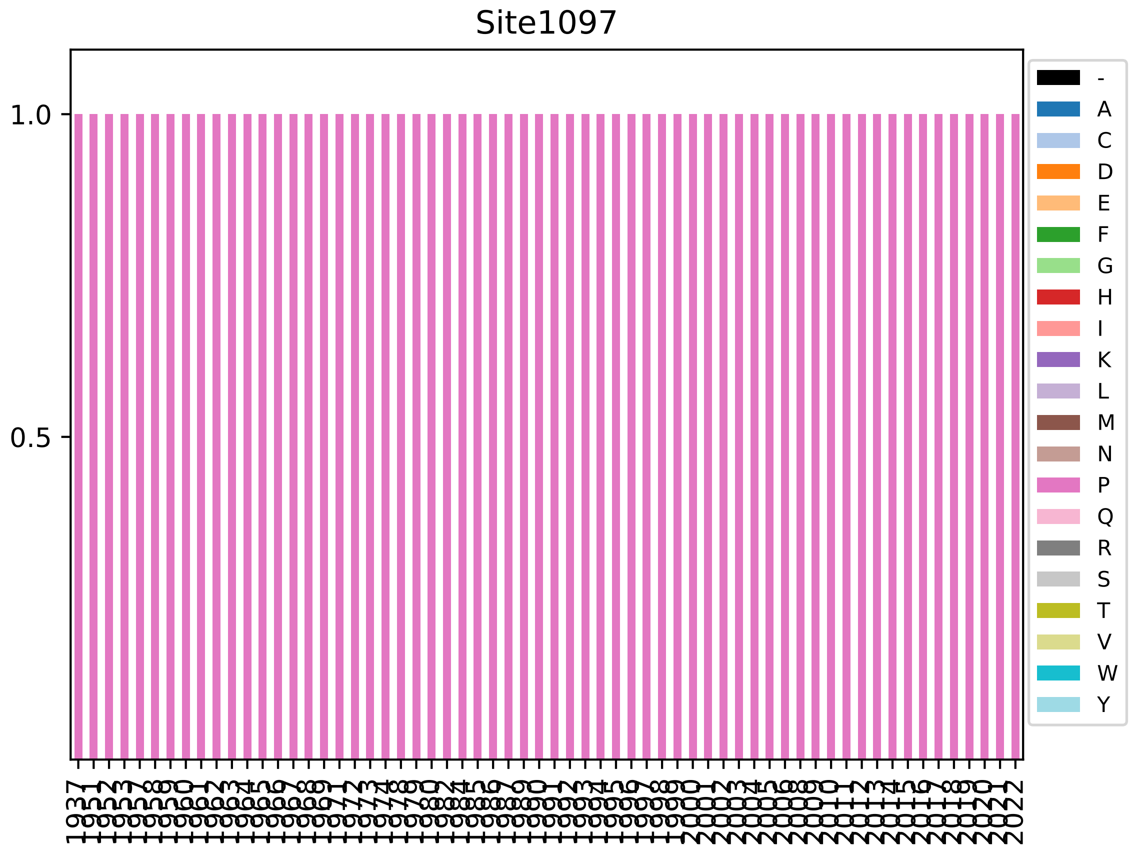
Site1097
1.0
0.5
-
A
C
D
E
F
G
H
I
K
L
M
N
P
Q
R
S
T
V
W
Y

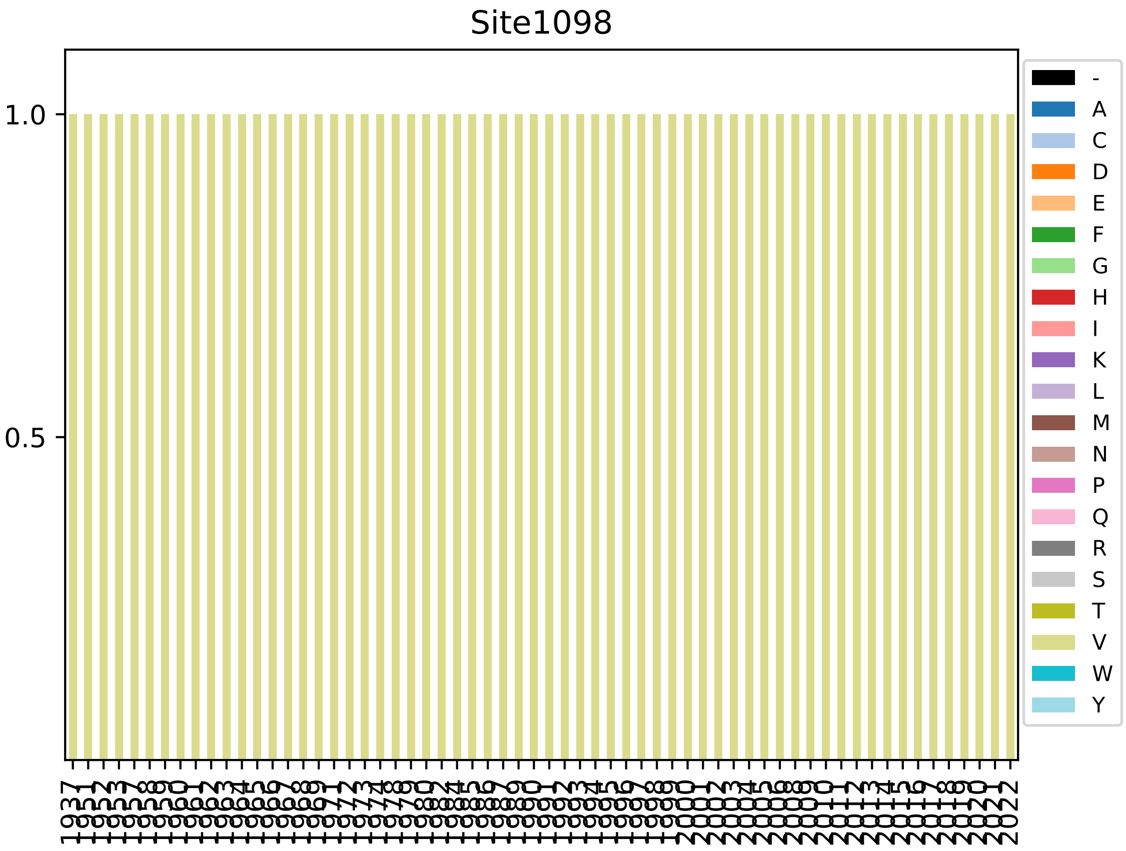
Site1098
1.0
0.5
-
A
C
D
E
F
G
H
I
K
L
M
N
P
Q
R
S
T
V
W
Y

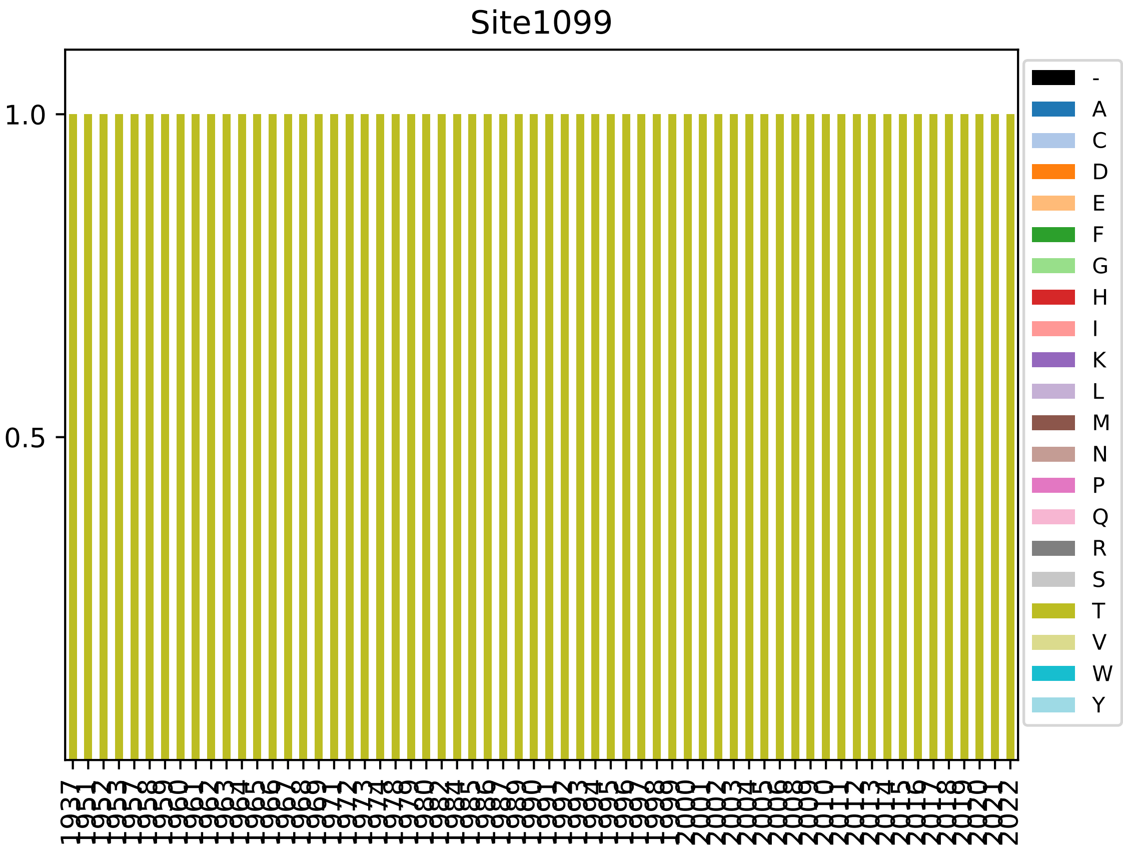
Site1099
1.0
0.5
-
A
C
D
E
F
G
H
I
K
L
M
N
P
Q
R
S
T
V
W
Y

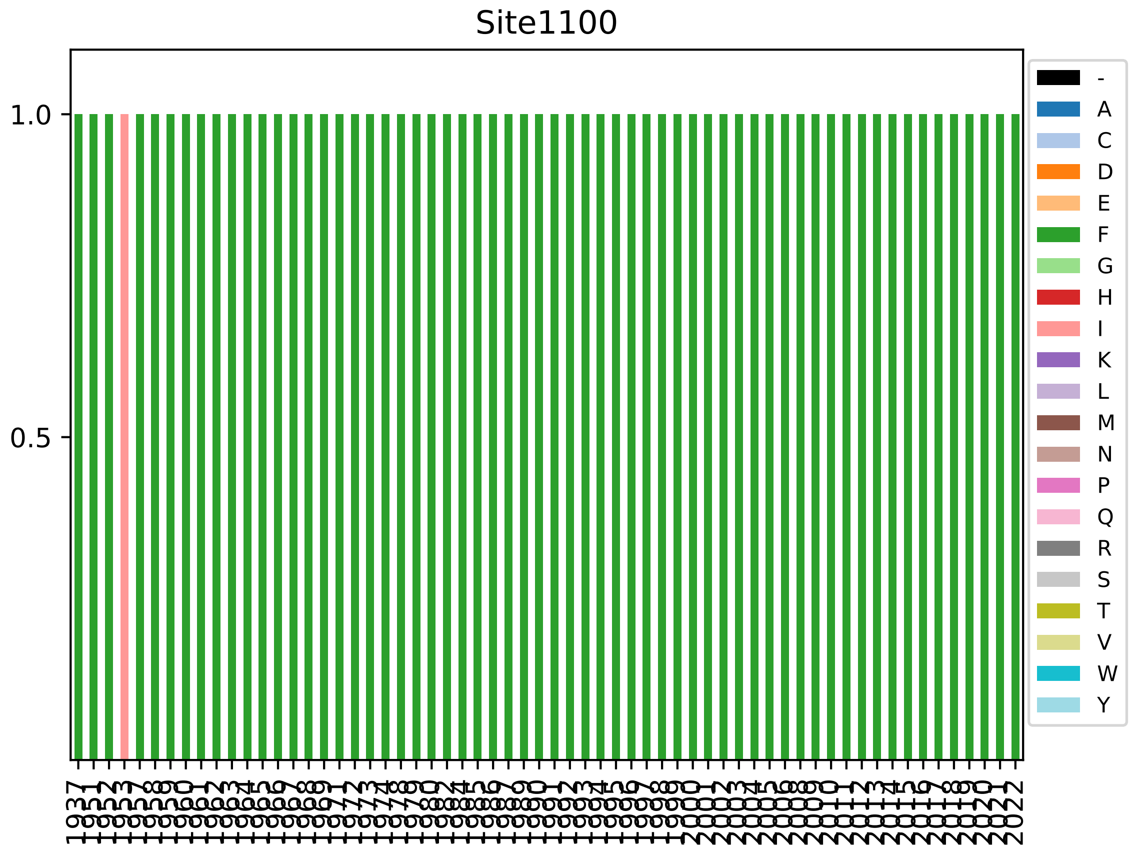
Site1100
1.0
0.5
-
A
C
D
E
F
G
H
I
K
L
M
N
P
Q
R
S
T
V
W
Y

Site1101

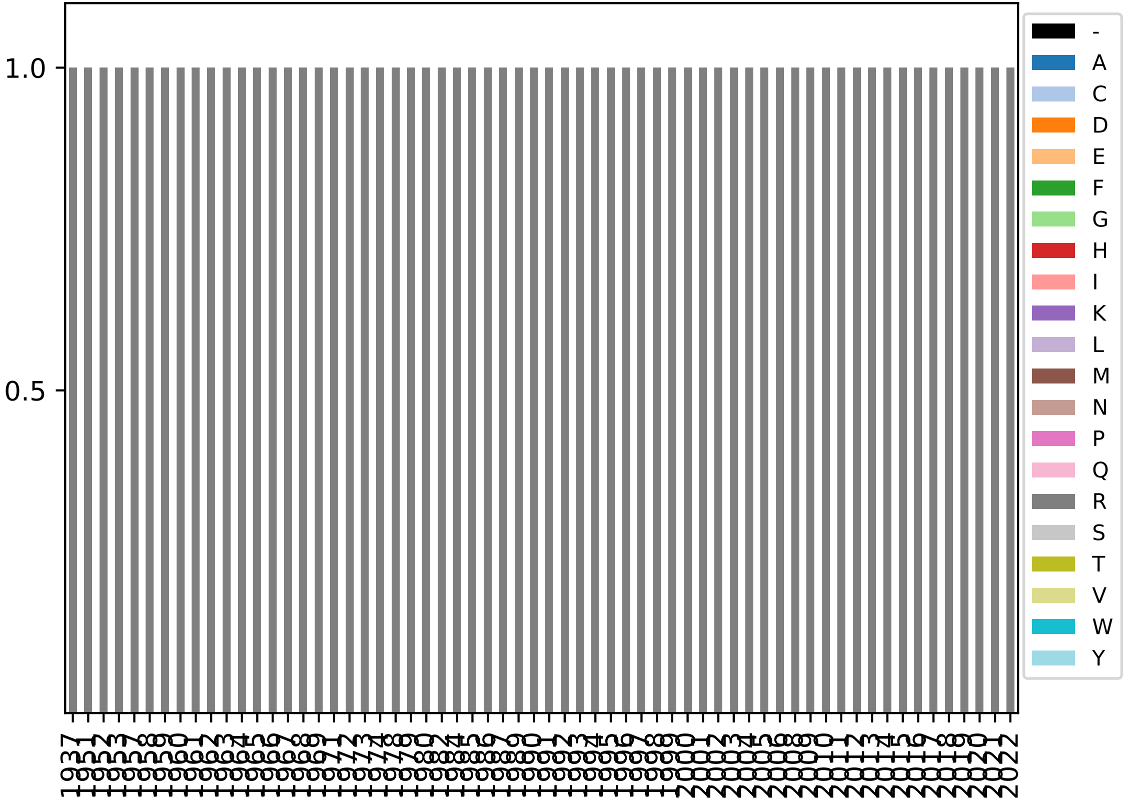

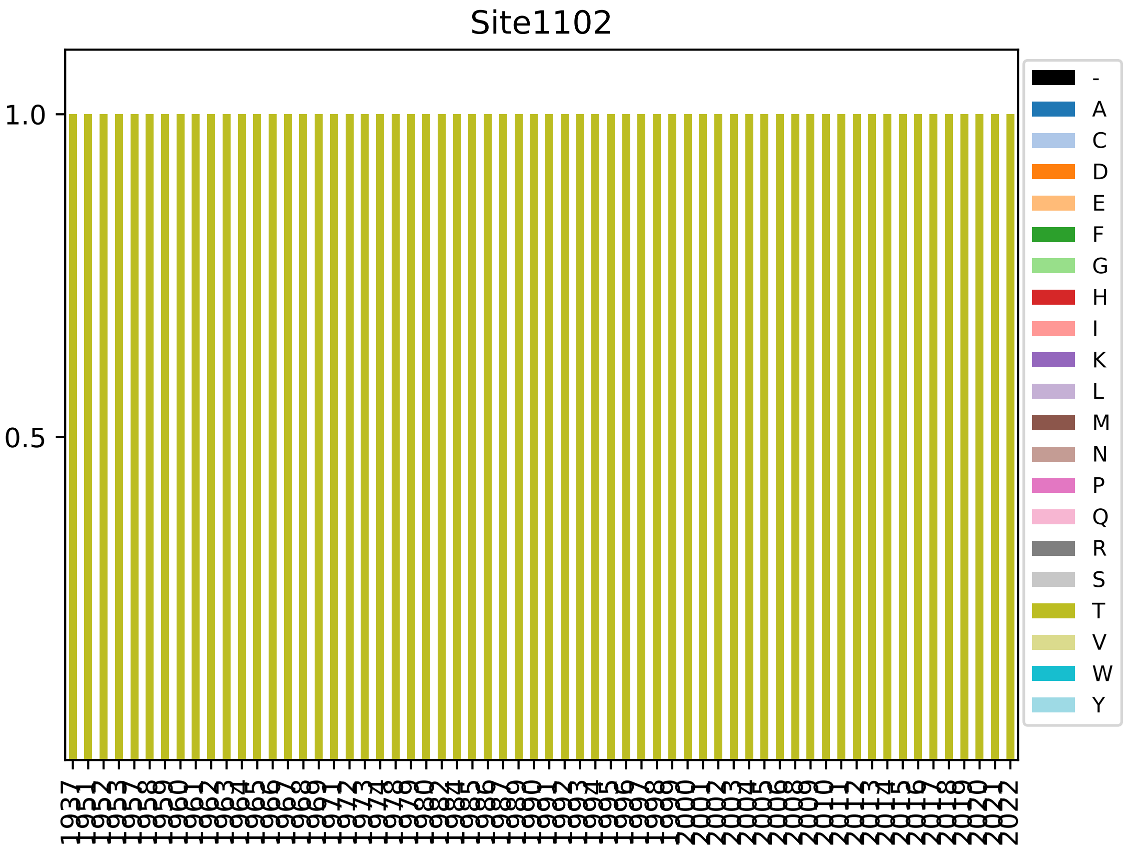
Site1102
1.0
0.5
-
A
C
D
E
F
G
H
I
K
L
M
N
P
Q
R
S
T
V
W
Y

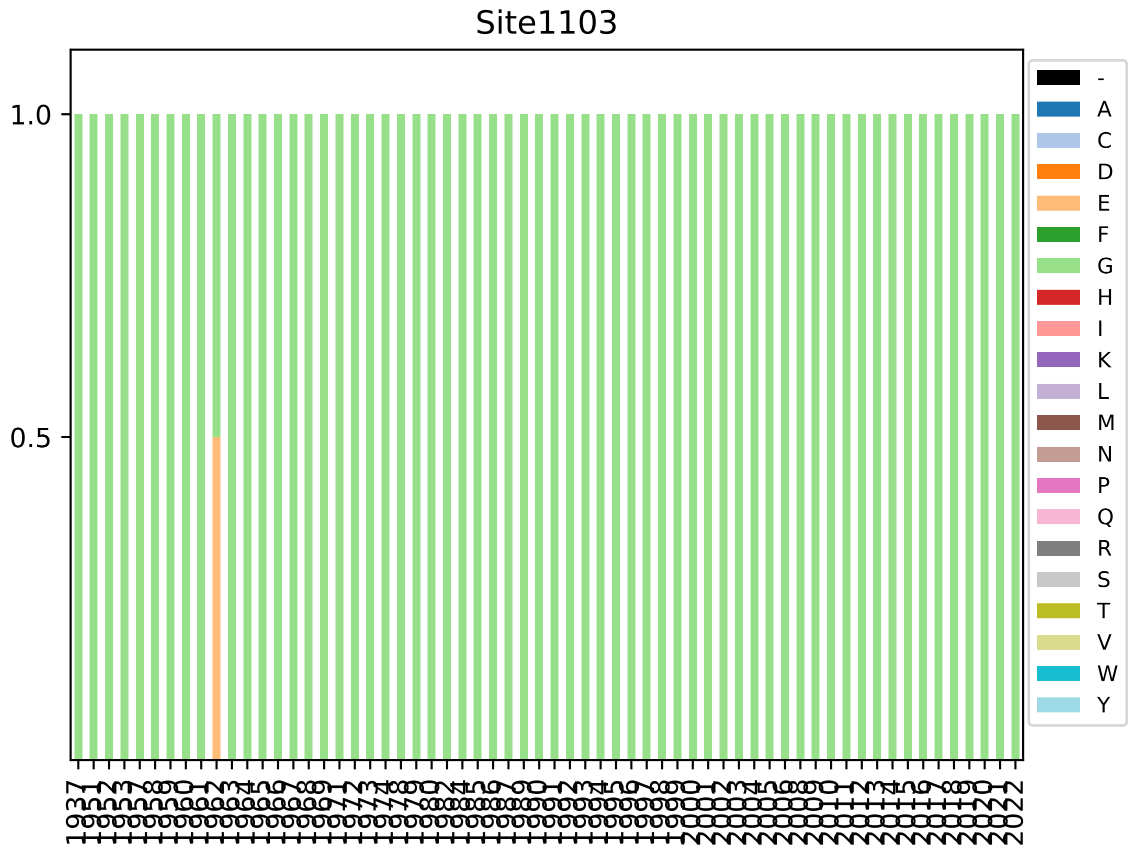
Site1103
1.0
0.5
-
A
C
D
E
F
G
H
I
K
L
M
N
P
Q
R
S
T
V
W
Y

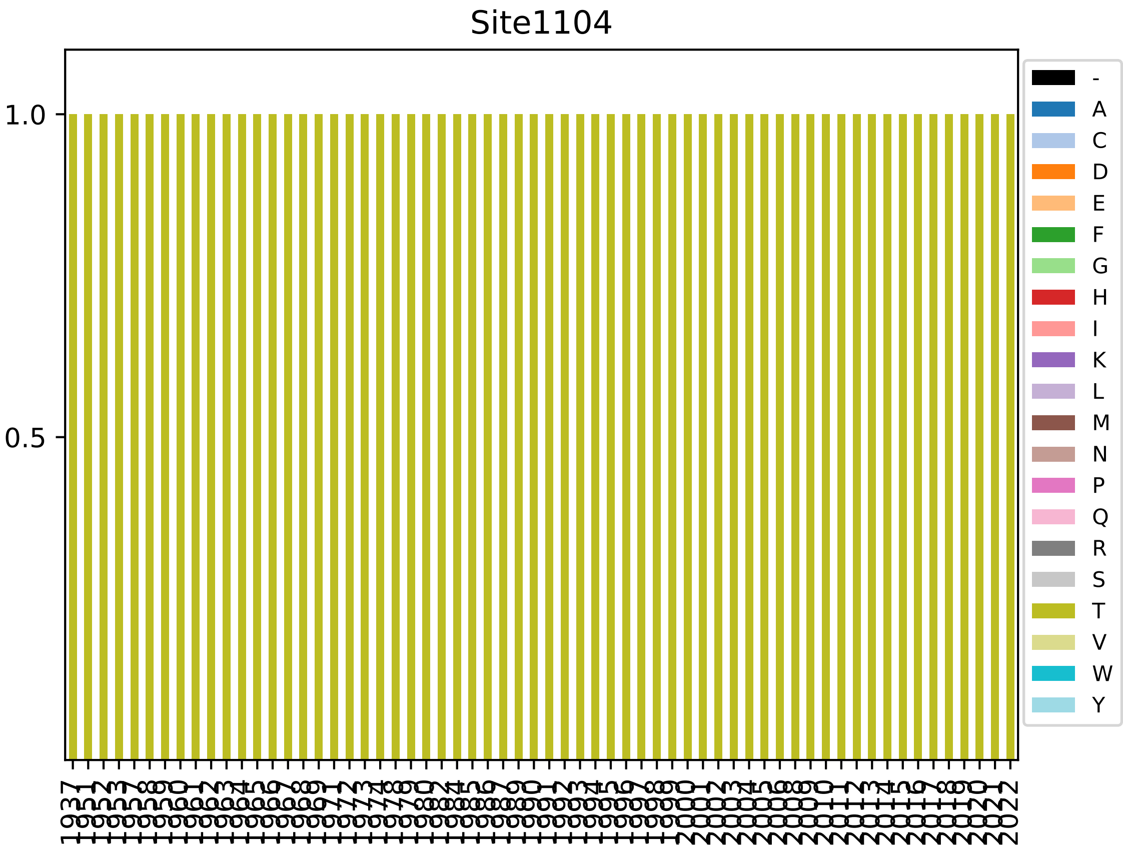
Site1104
1.0
0.5
-
A
C
D
E
F
G
H
I
K
L
M
N
P
Q
R
S
T
V
W
Y

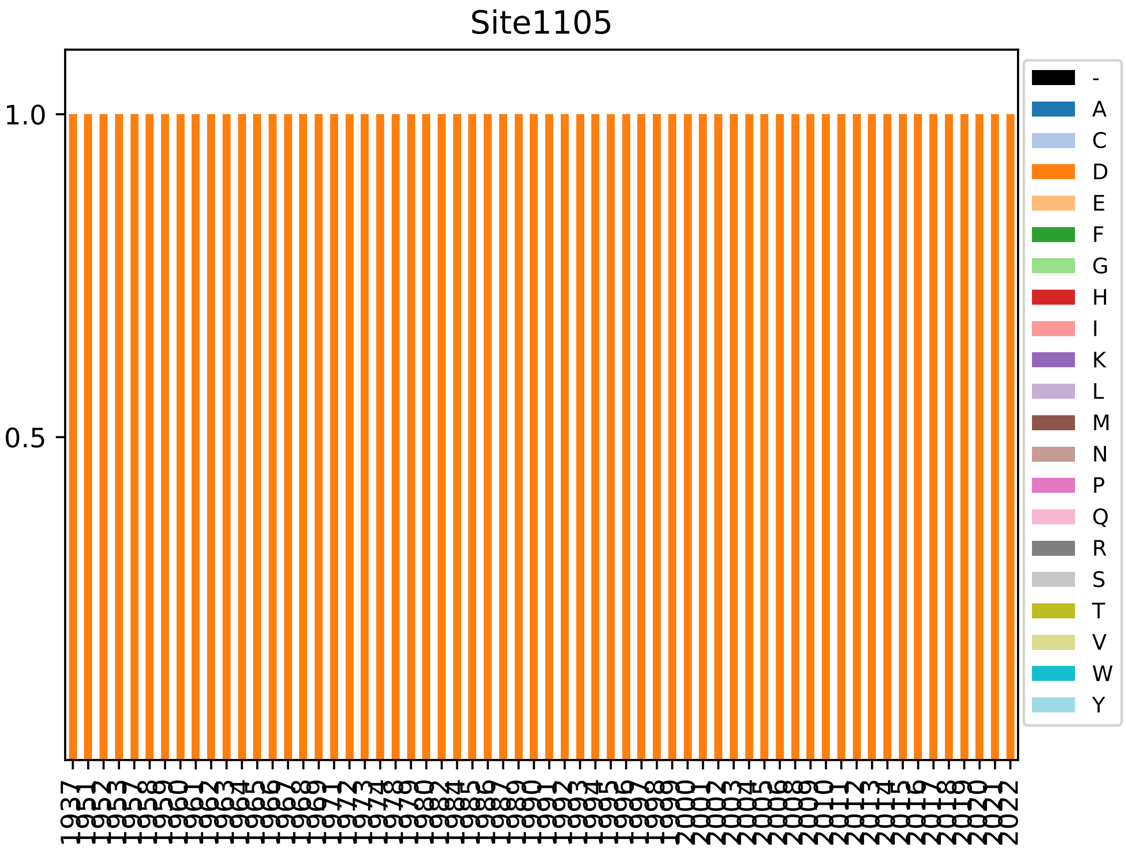
Site1105
1.0
0.5
-
A
C
D
E
F
G
H
I
K
L
M
N
P
Q
R
S
T
V
W
Y

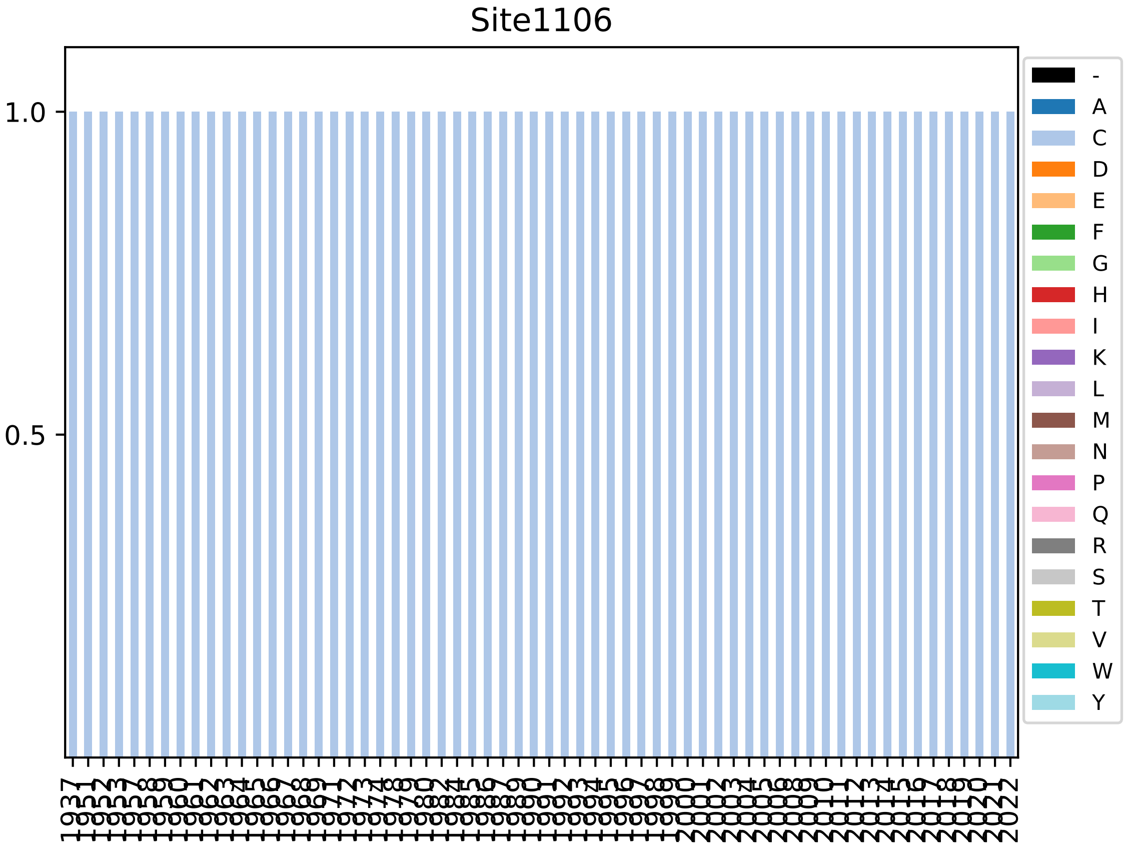
Site1106
1.0
0.5
-
A
C
D
E
F
G
H
I
K
L
M
N
P
Q
R
S
T
V
W
Y

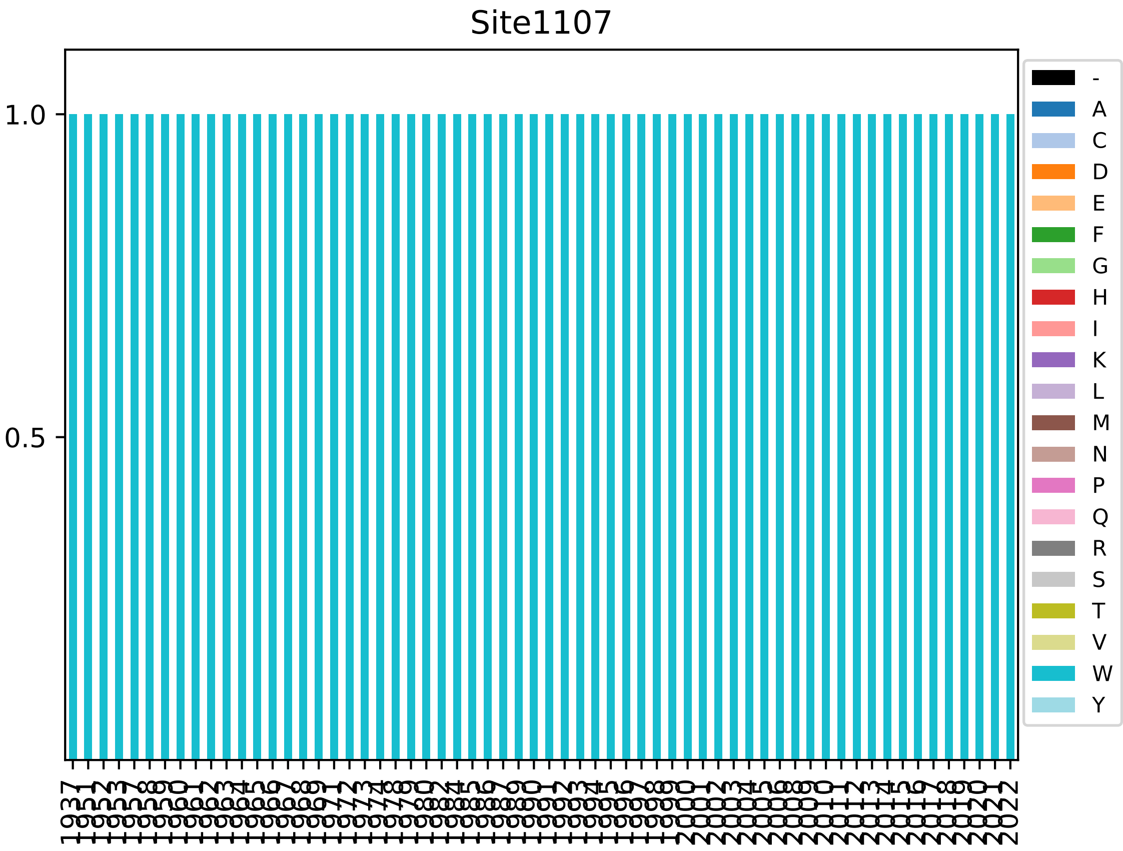
Site1107
1.0
0.5
-
A
C
D
E
F
G
H
I
K
L
M
N
P
Q
R
S
T
V
W
Y

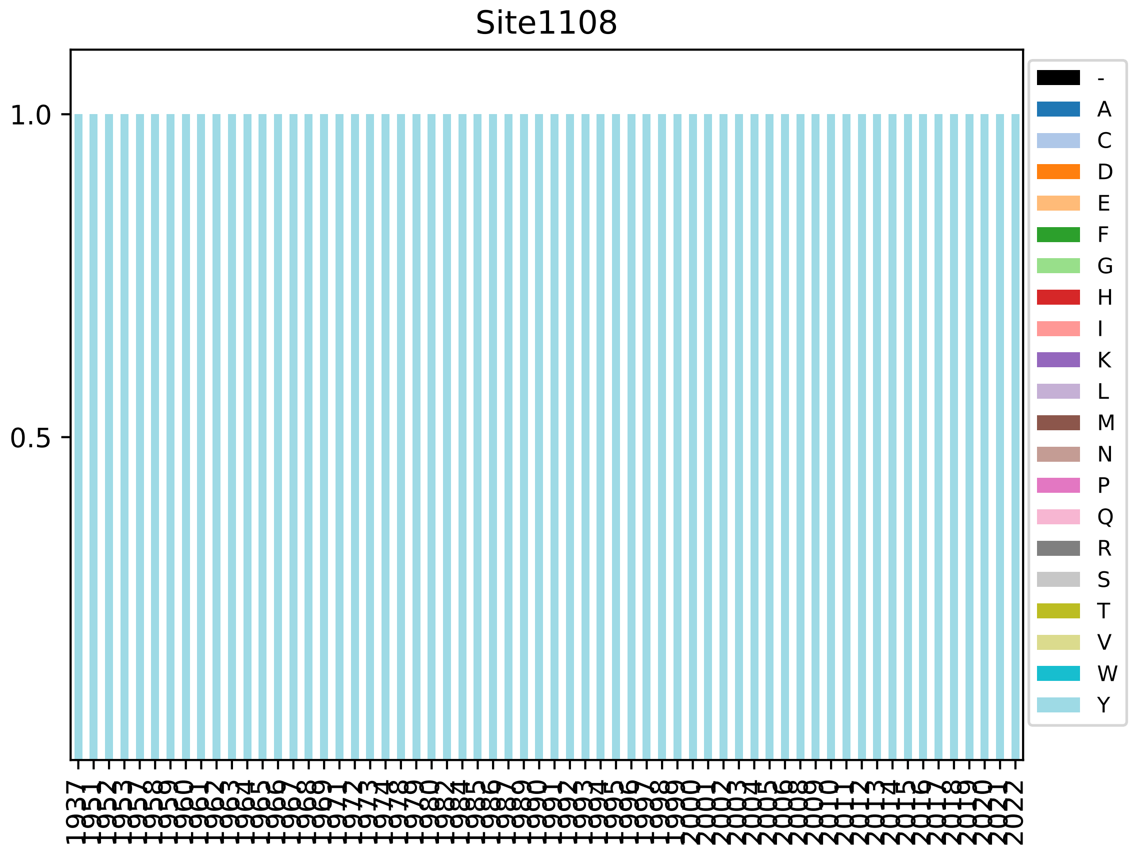
Site1108
1.0
0.5
-
A
C
D
E
F
G
H
I
K
L
M
N
P
Q
R
S
T
V
W
Y

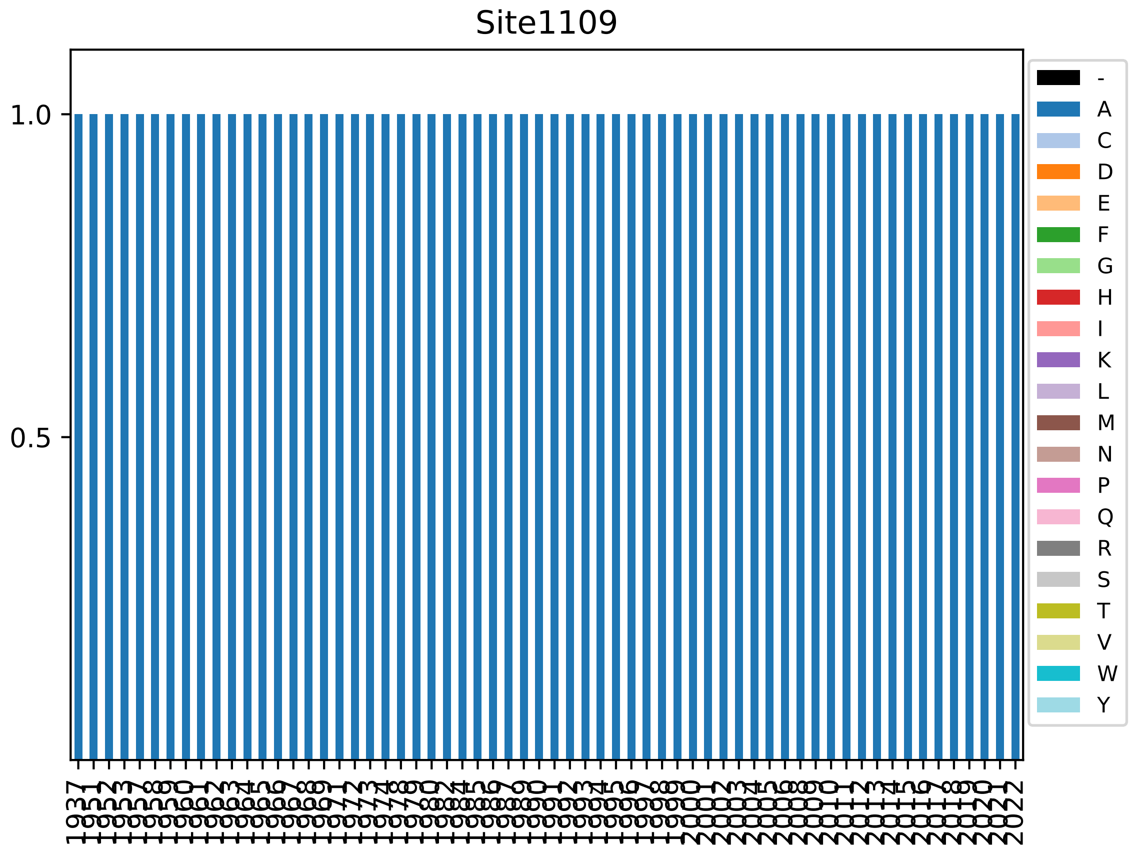
Site1109
1.0
0.5
-
A
C
D
E
F
G
H
I
K
L
M
N
P
Q
R
S
T
V
W
Y

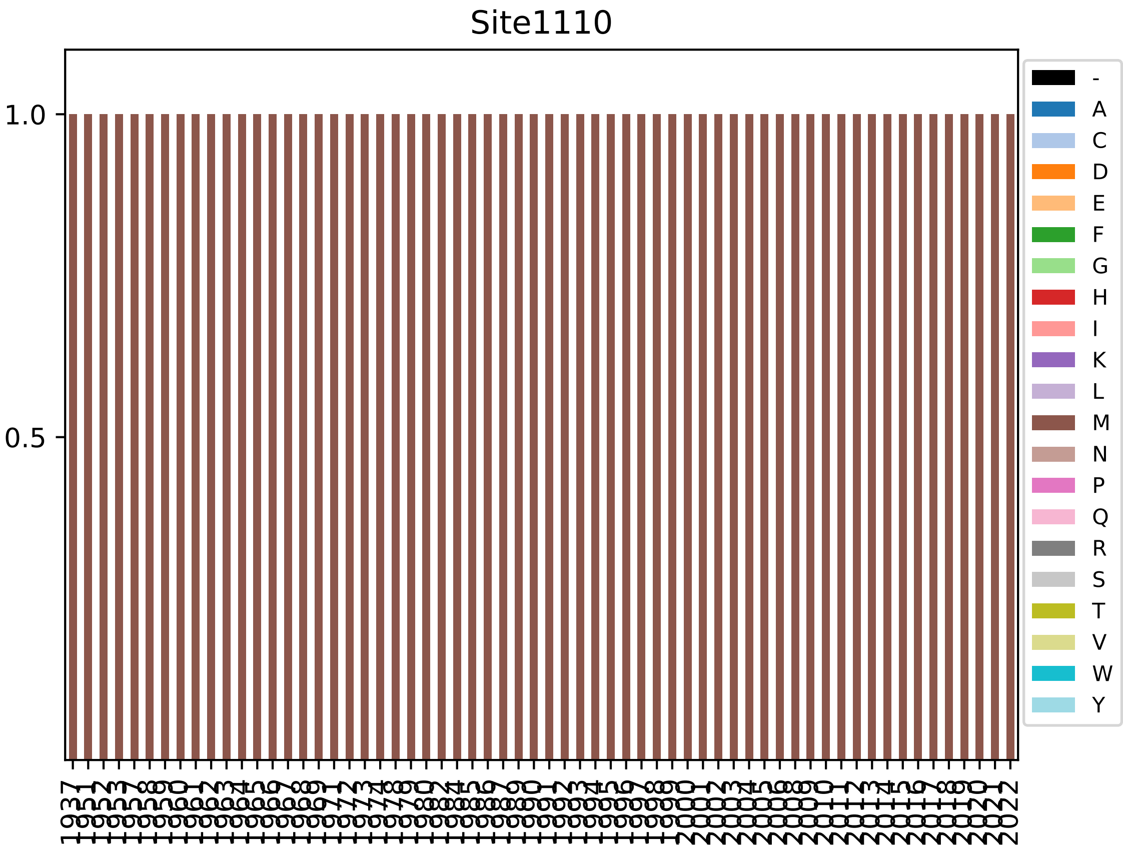
Site1110
1.0
0.5
-
A
C
D
E
F
G
H
I
K
L
M
N
P
Q
R
S
T
V
W
Y

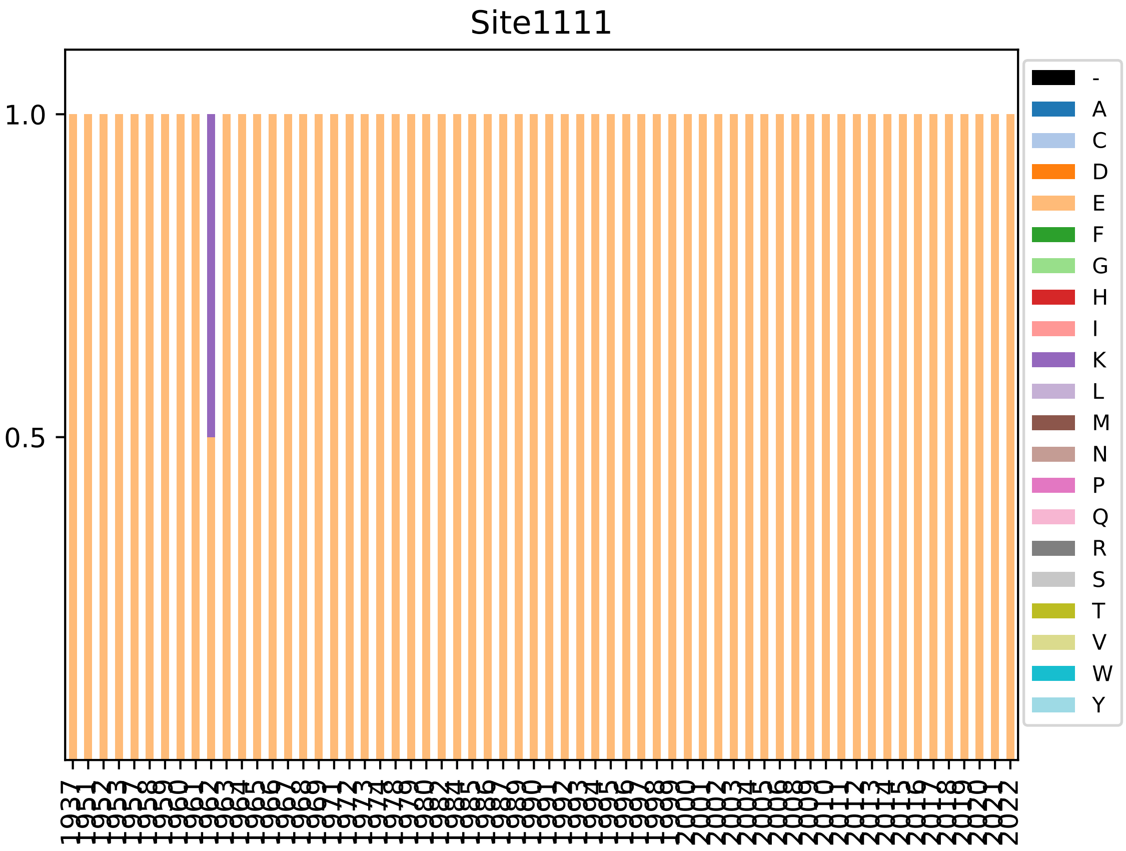
Site1111
1.0
0.5
-
A
C
D
E
F
G
H
I
K
L
M
N
P
Q
R
S
T
V
W
Y

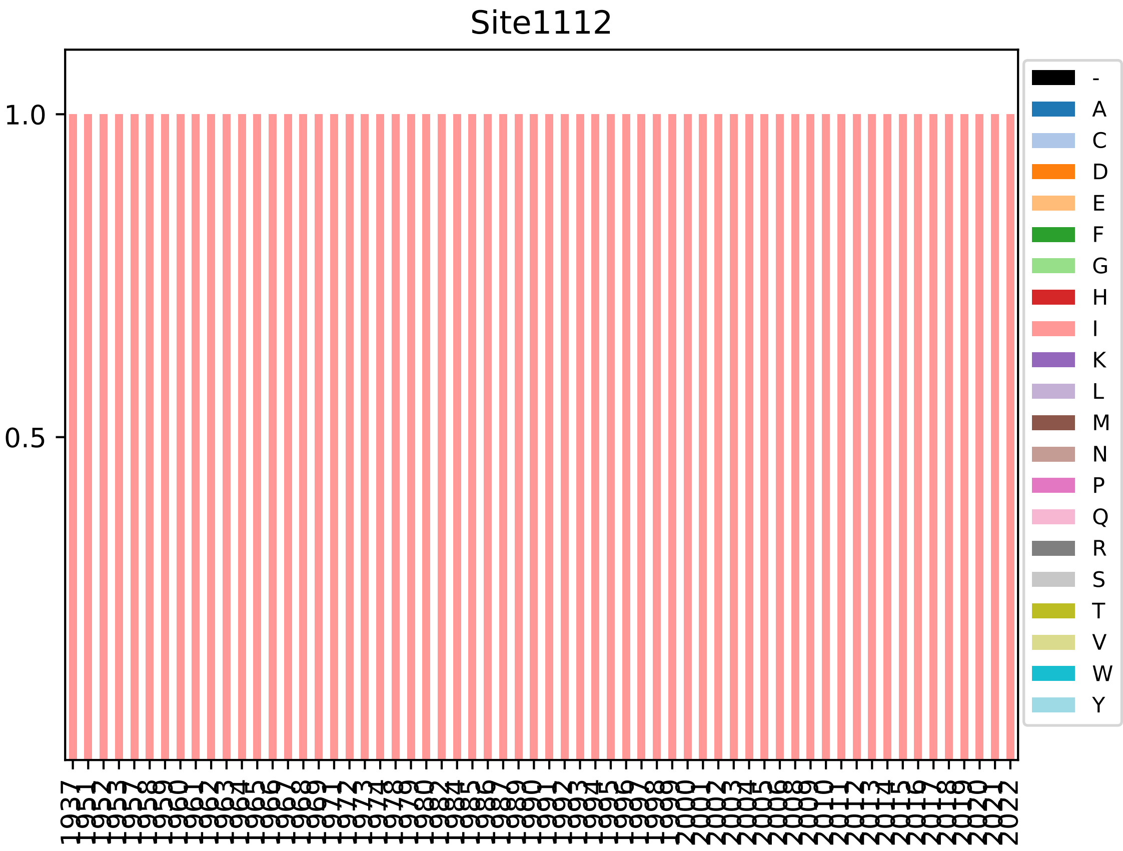

Site1112
1.0
0.5
-
A
C
D
E
F
G
H
I
K
L
M
N
P
Q
R
S
T
V
W
Y

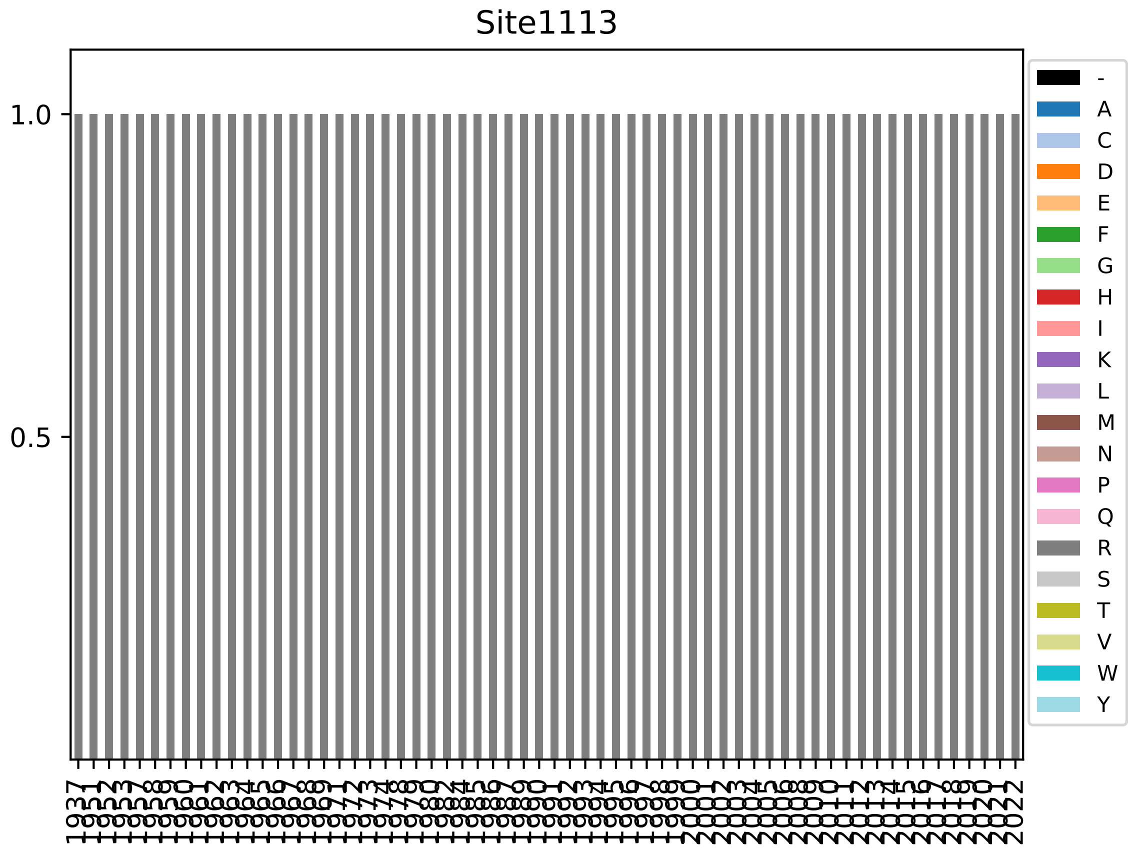
Site1113
1.0
0.5
-
A
C
D
E
F
G
H
I
K
L
M
N
P
Q
R
S
T
V
W
Y

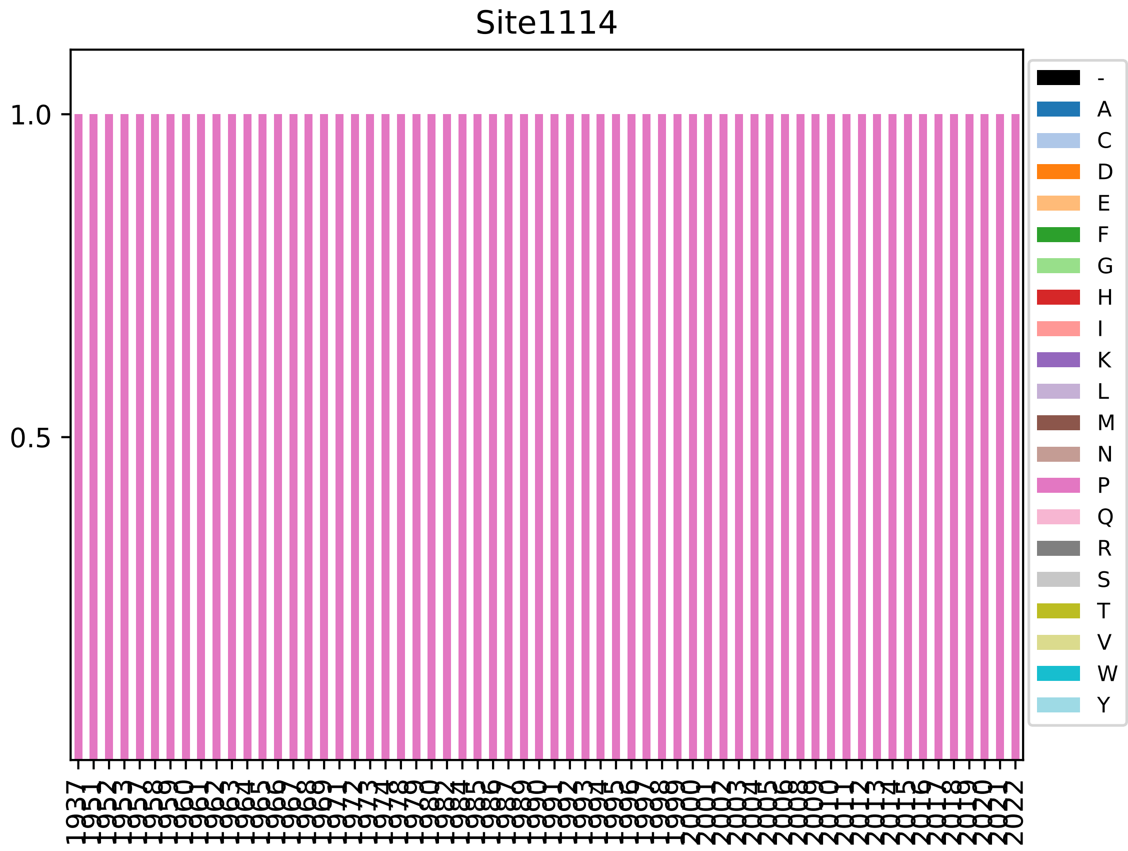
Site1114
1.0
0.5
-
A
C
D
E
F
G
H
I
K
L
M
N
P
Q
R
S
T
V
W
Y

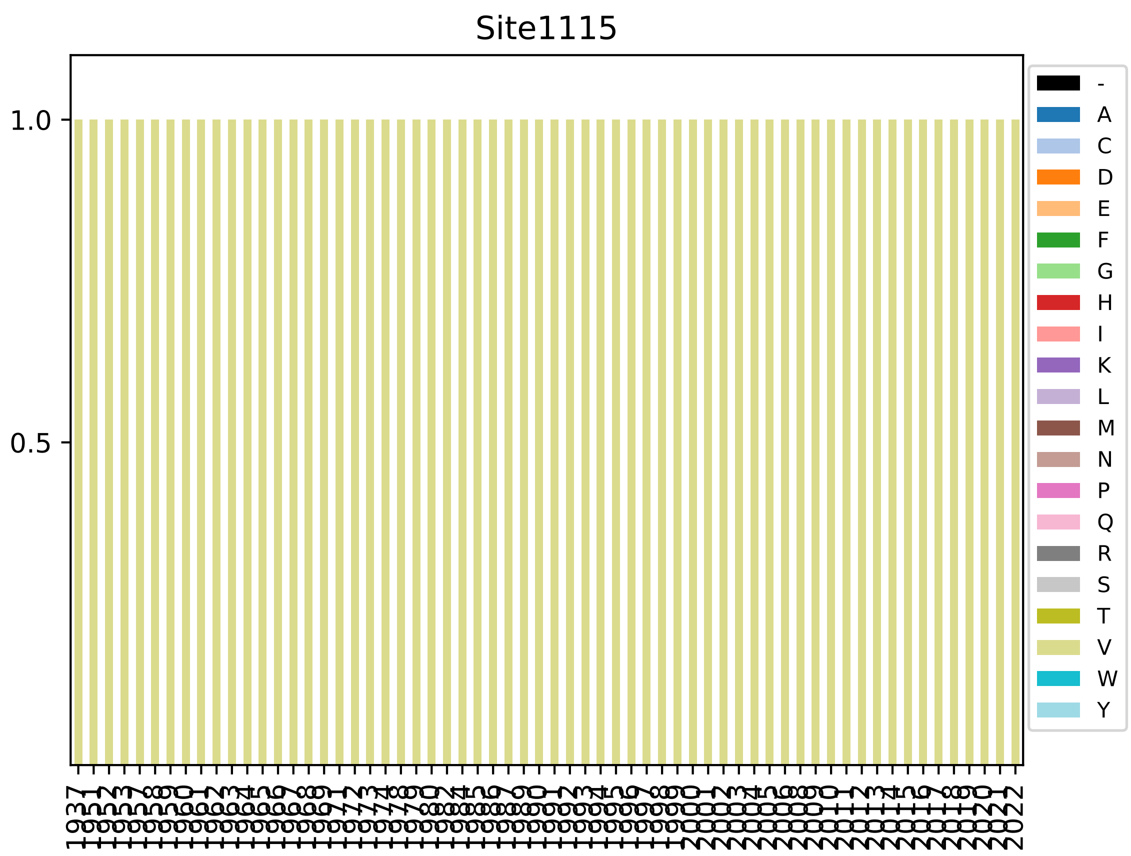
Site1115
1.0
0.5
-
A
C
D
E
F
G
H
I
K
L
M
N
P
Q
R
S
T
V
W
Y

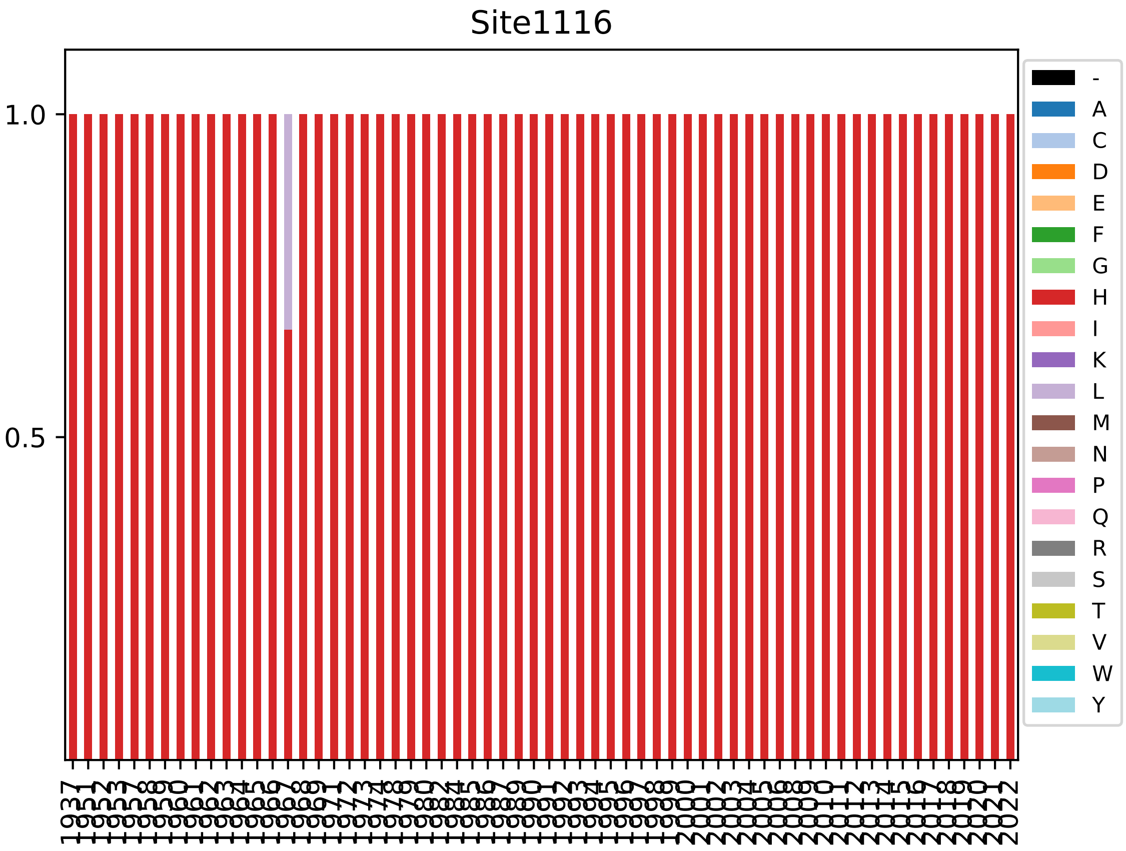
Site1116
1.0
0.5
-
A
C
D
E
F
G
H
I
K
L
M
N
P
Q
R
S
T
V
W
Y

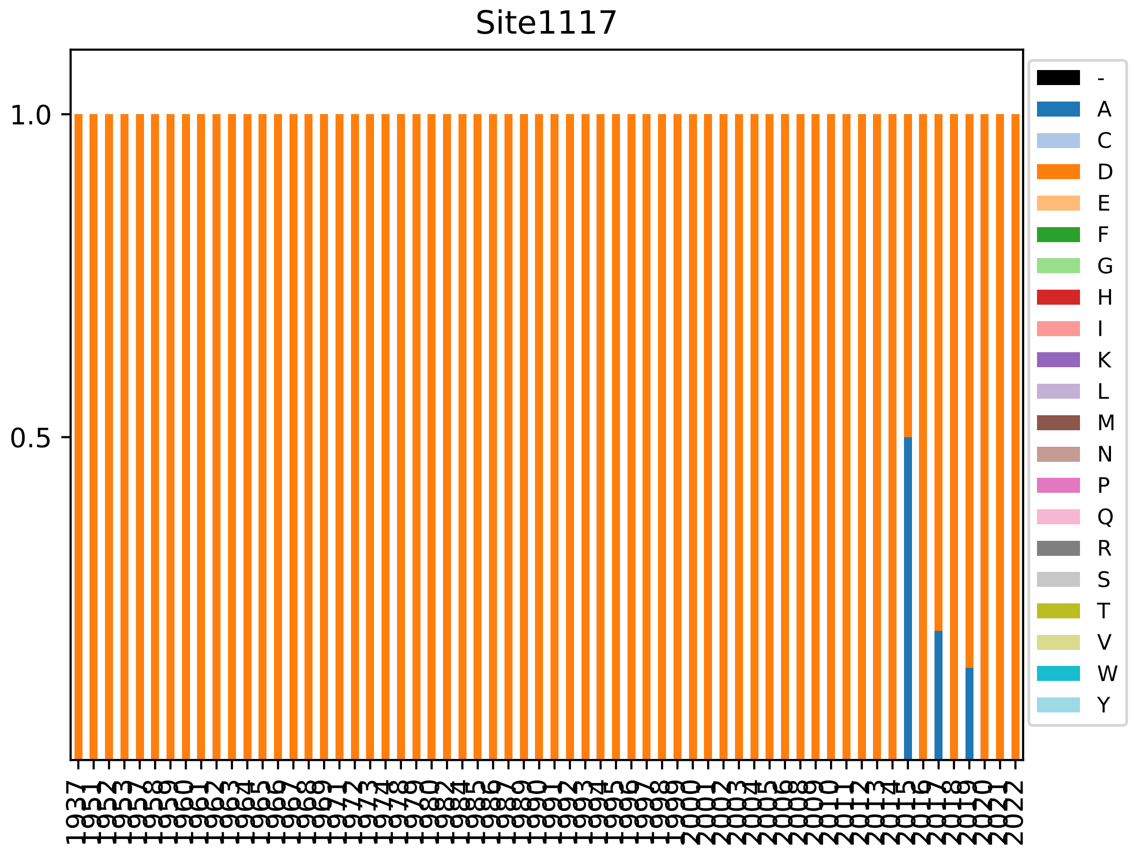
Site1117
1.0
0.5
-
A
C
D
E
F
G
H
I
K
L
M
N
P
Q
R
S
T
V
W
Y

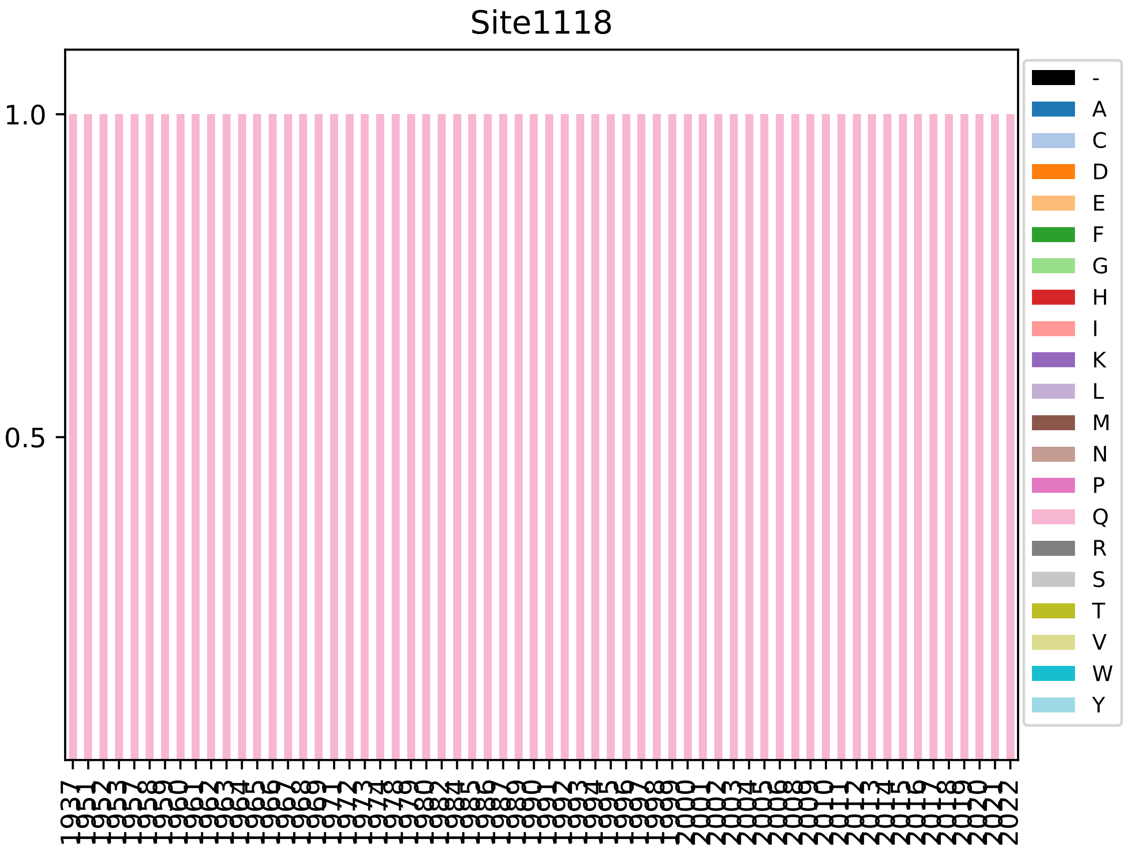
Site1118
1.0
0.5
-
A
C
D
E
F
G
H
I
K
L
M
N
P
Q
R
S
T
V
W
Y

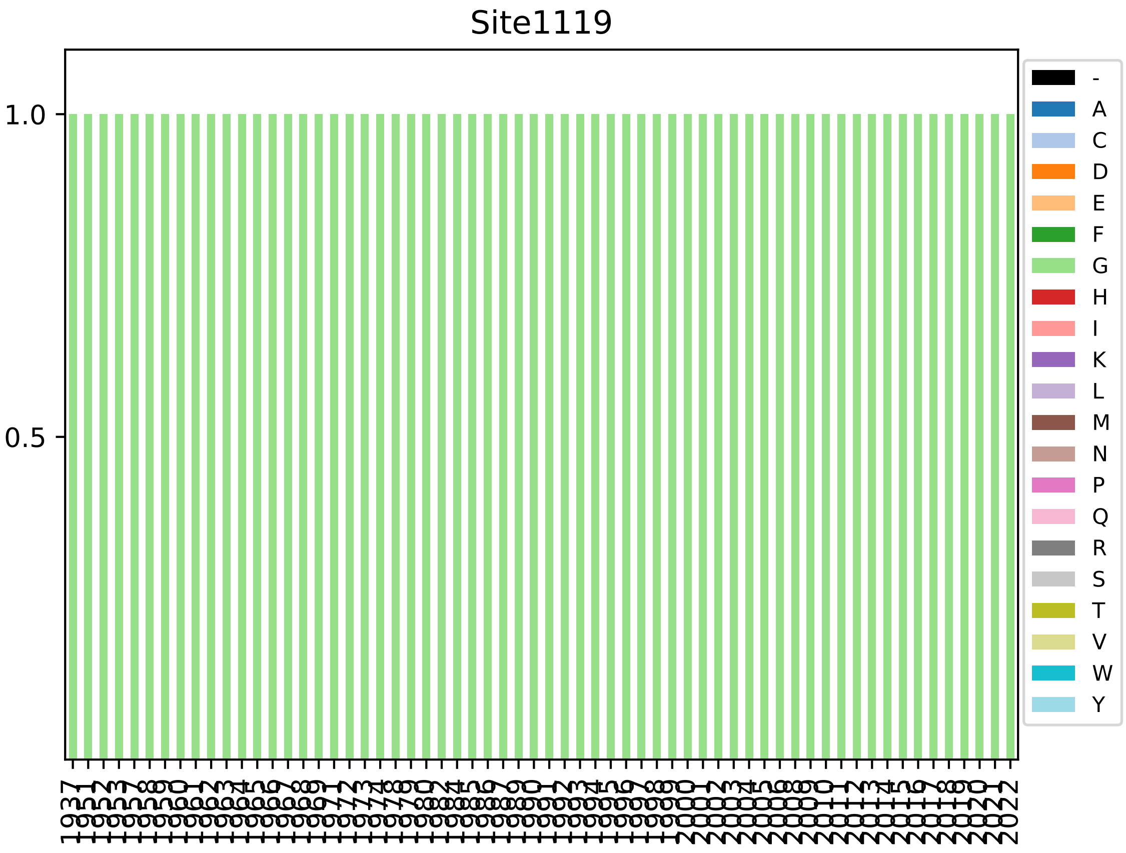
Site1119
1.0
0.5
-
A
C
D
E
F
G
H
I
K
L
M
N
P
Q
R
S
T
V
W
Y

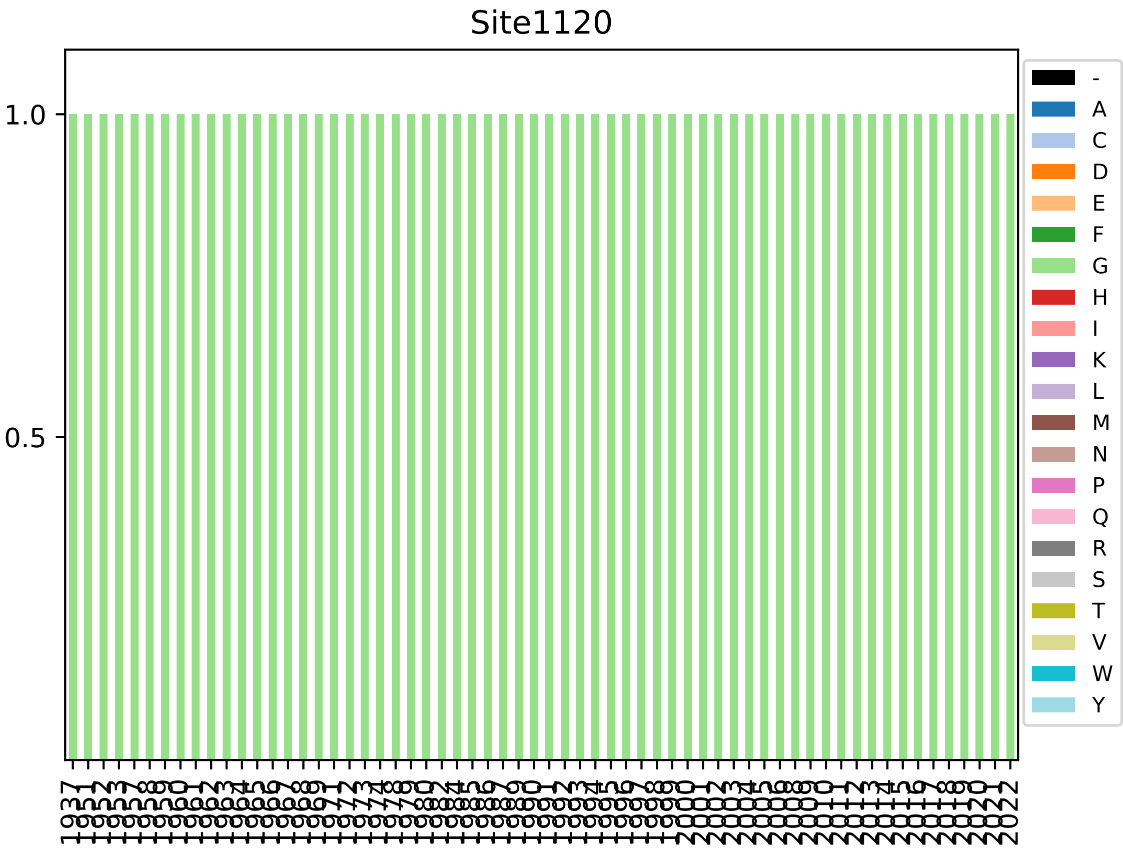
Site1120
1.0
0.5
-
A
C
D
E
F
G
H
I
K
L
M
N
P
Q
R
S
T
V
W
Y

Site1121

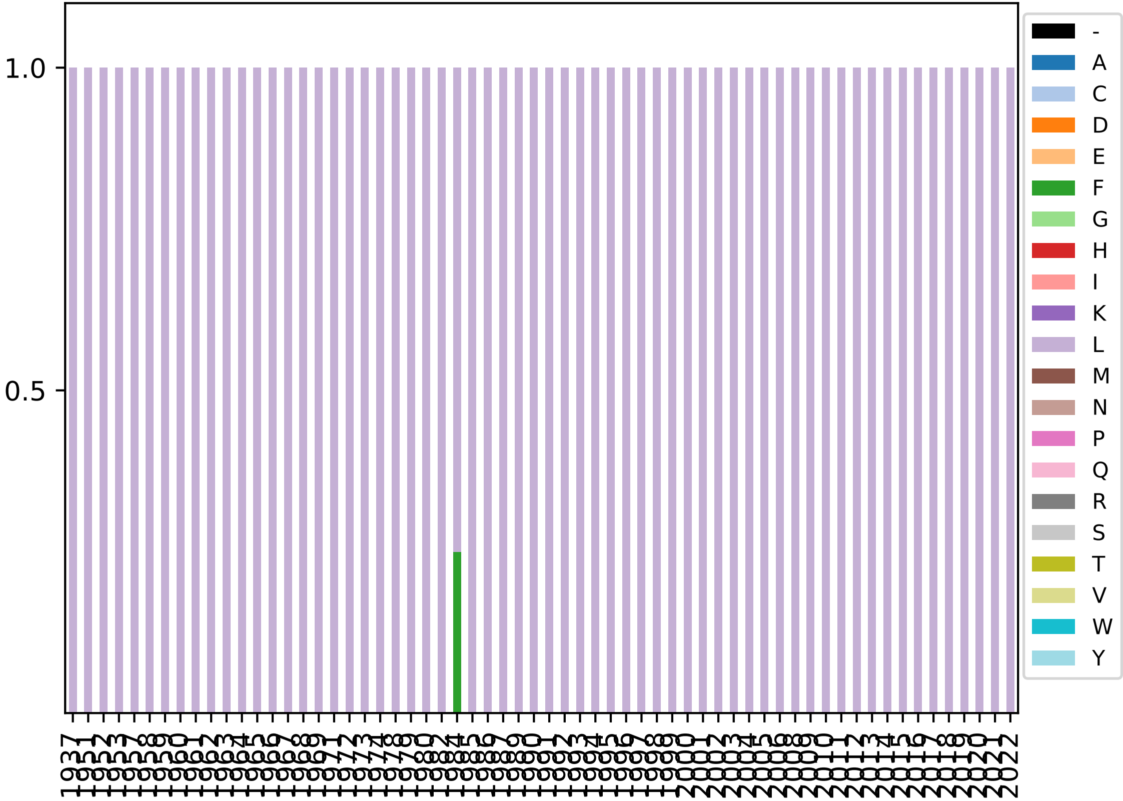

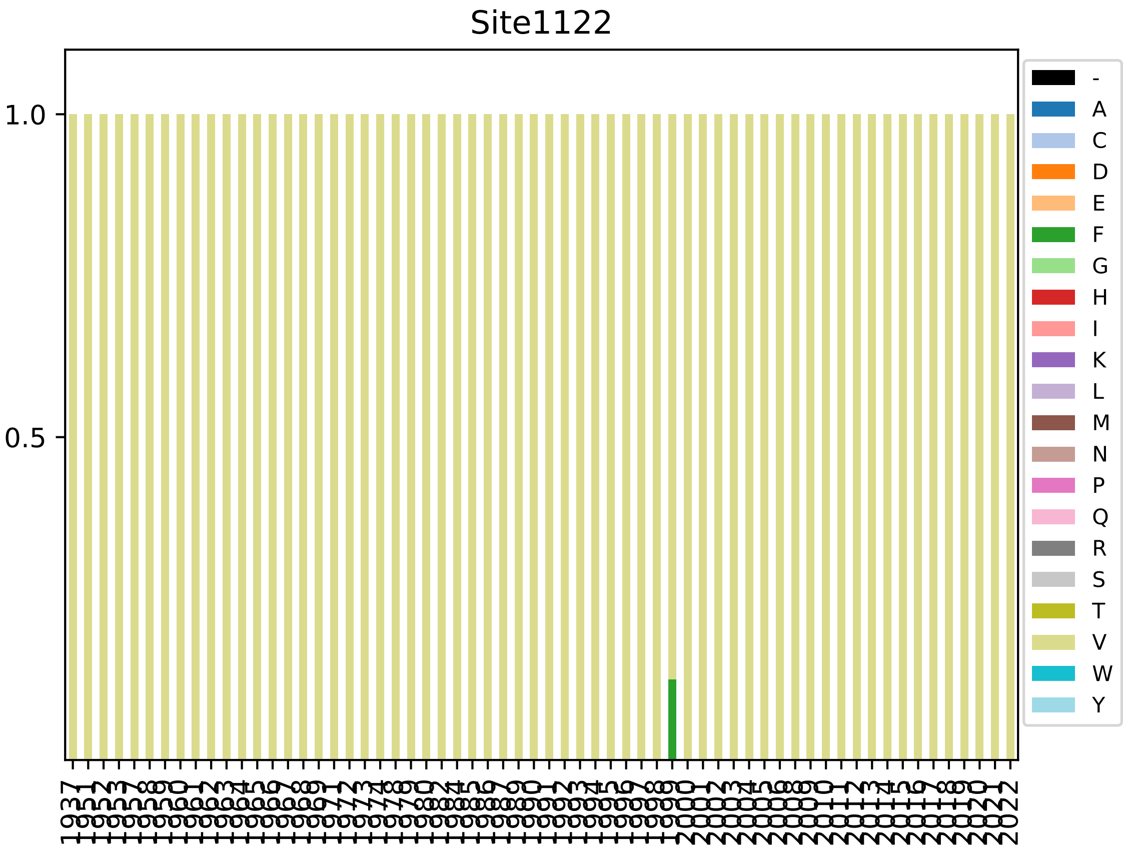
Site1122
1.0
0.5
-
A
C
D
E
F
G
H
I
K
L
M
N
P
Q
R
S
T
V
W
Y

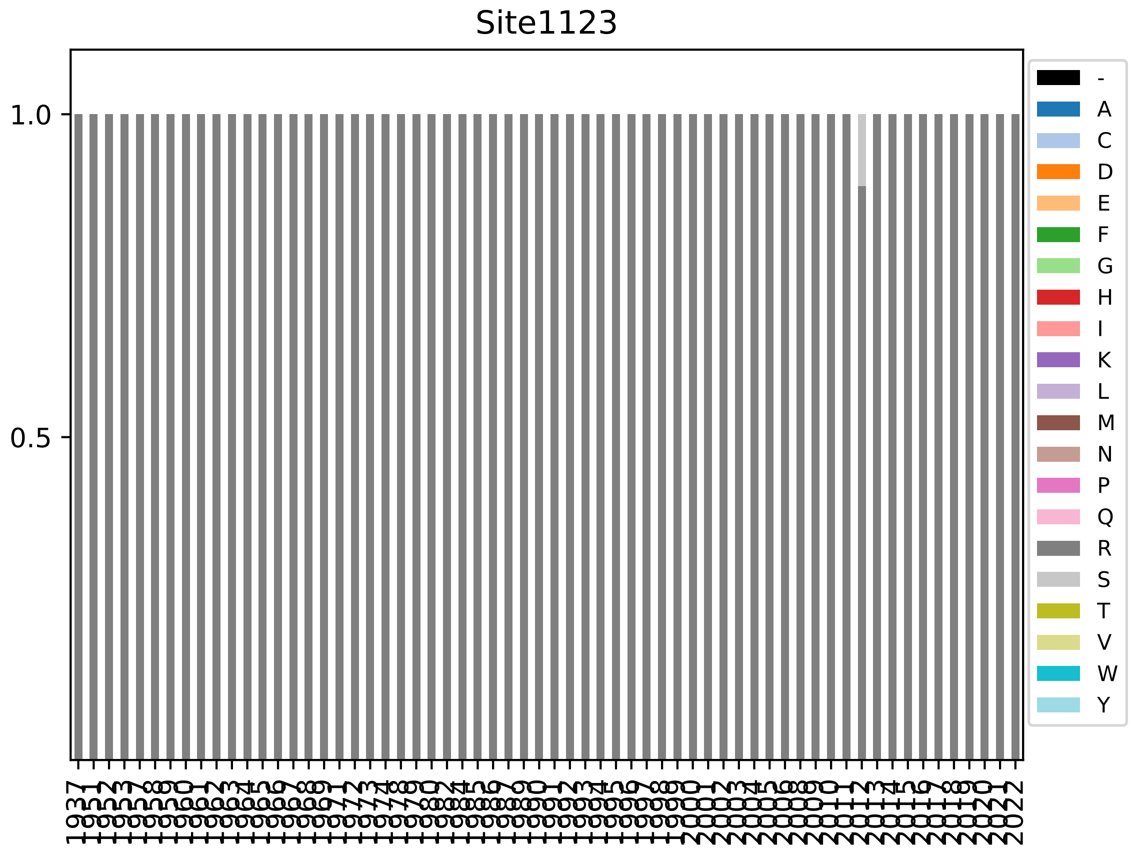
Site1123
1.0
0.5
-
A
C
D
E
F
G
H
I
K
L
M
N
P
Q
R
S
T
V
W
Y

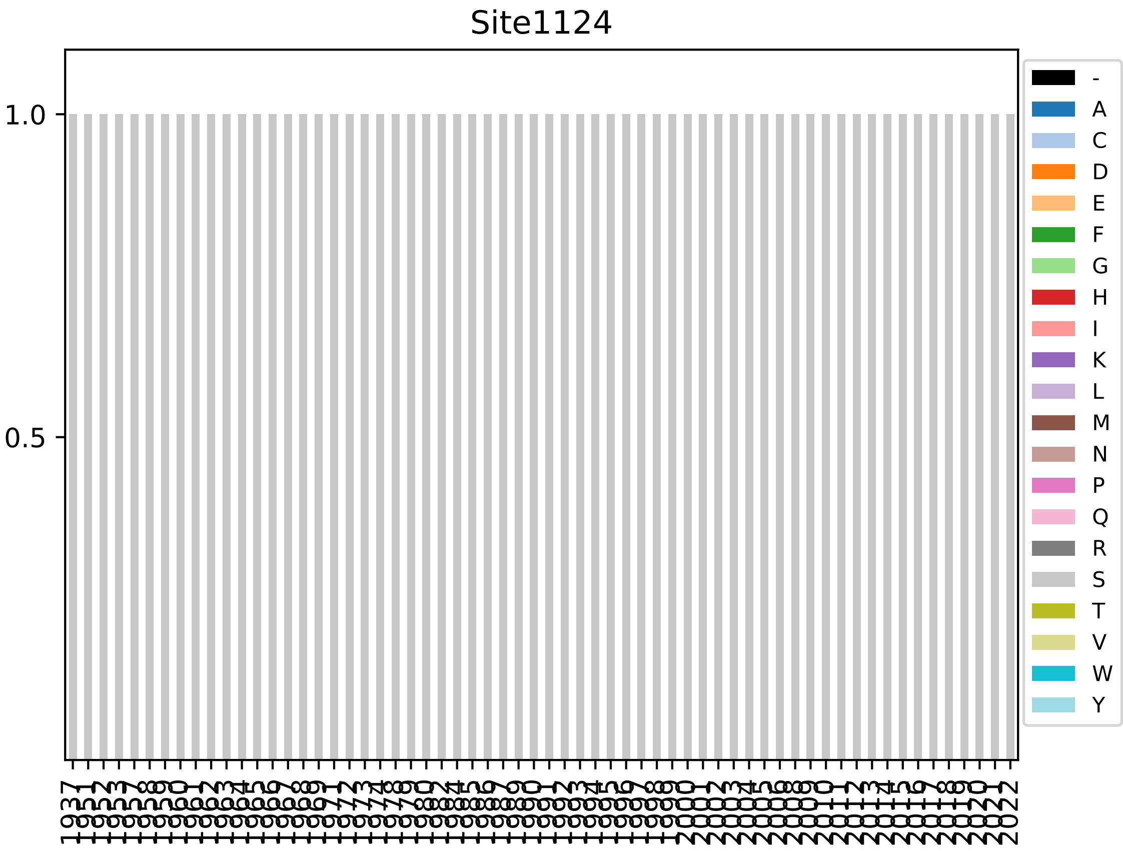
Site1124
1.0
0.5
-
A
C
D
E
F
G
H
I
K
L
M
N
P
Q
R
S
T
V
W
Y

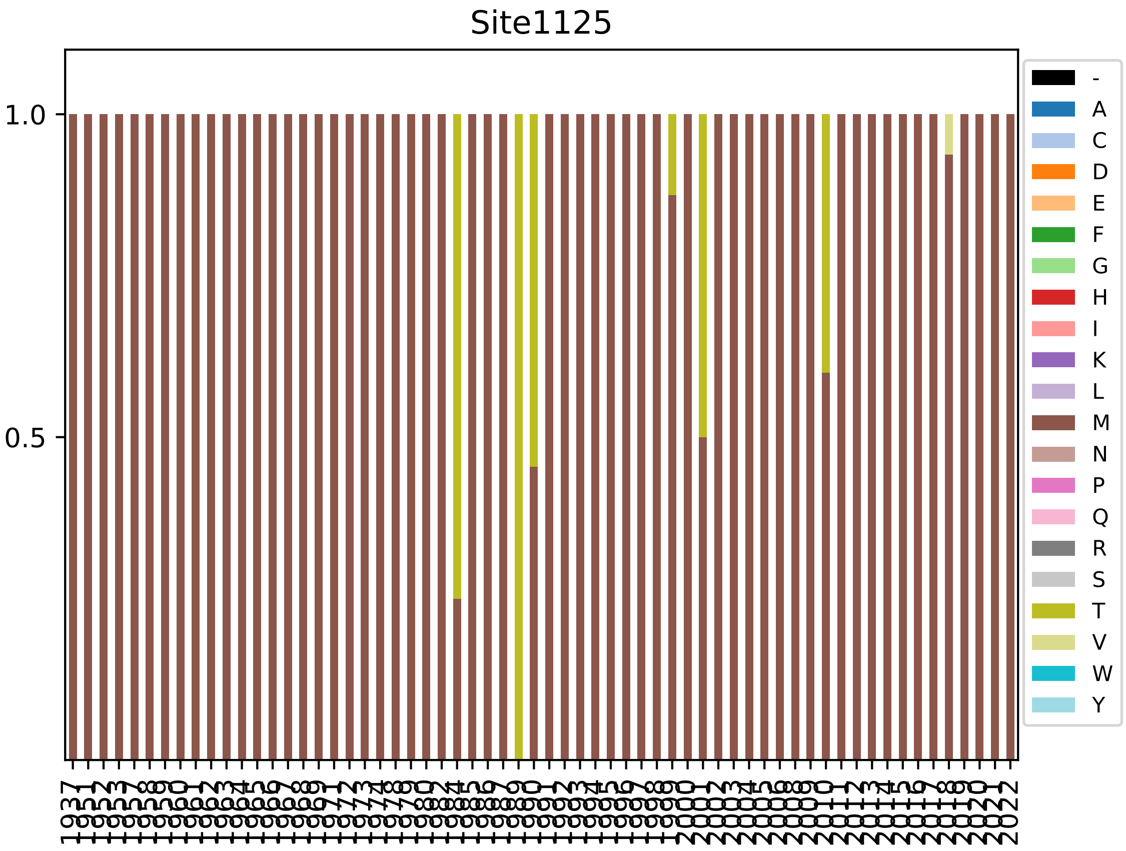
Site1125
1.0
0.5
-
A
C
D
E
F
G
H
I
K
L
M
N
P
Q
R
S
T
V
W
Y

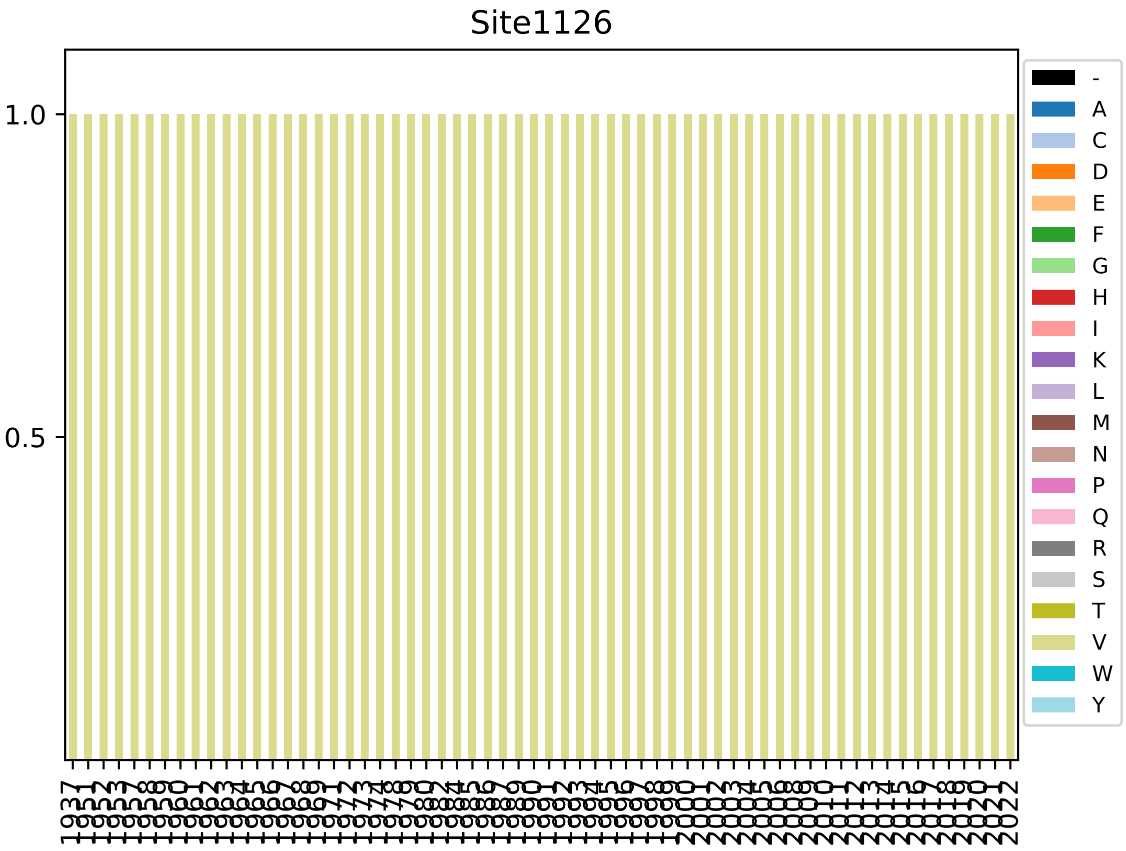
Site1126
1.0
0.5
-
A
C
D
E
F
G
H
I
K
L
M
N
P
Q
R
S
T
V
W
Y

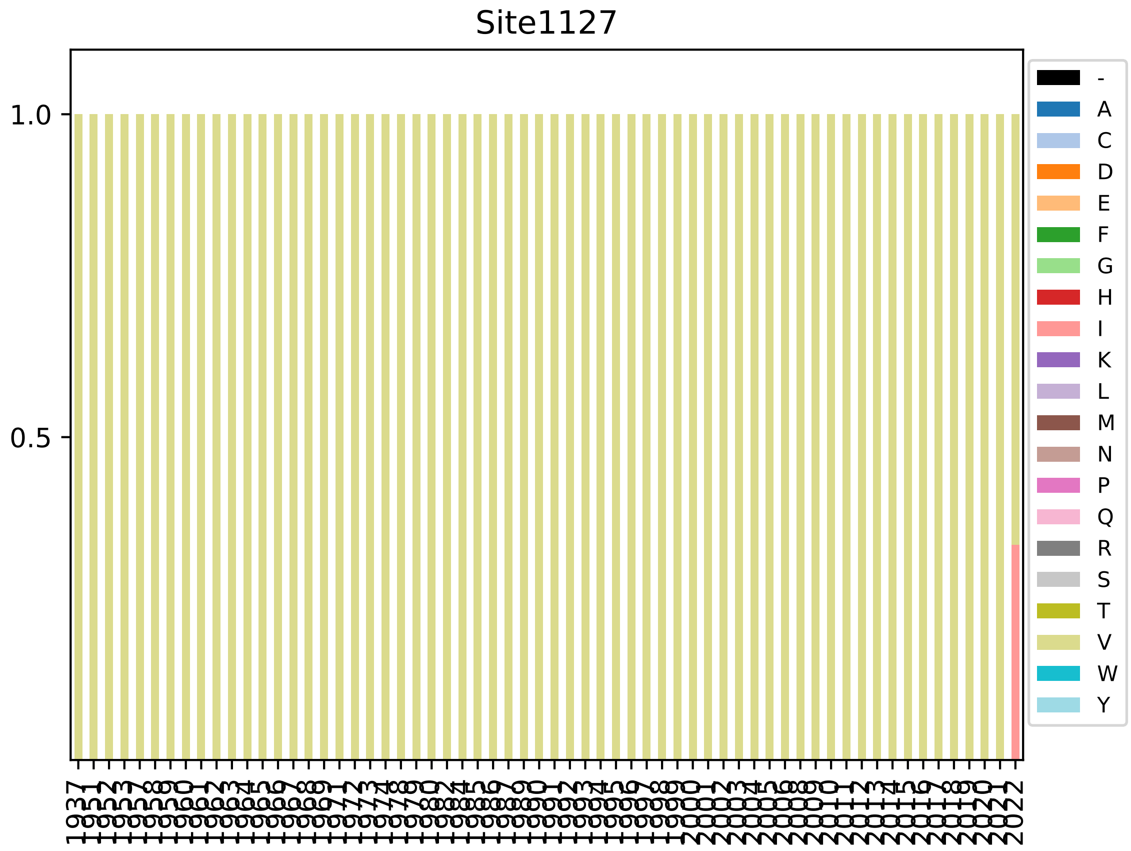
Site1127
1.0
0.5
-
A
C
D
E
F
G
H
I
K
L
M
N
P
Q
R
S
T
V
W
Y

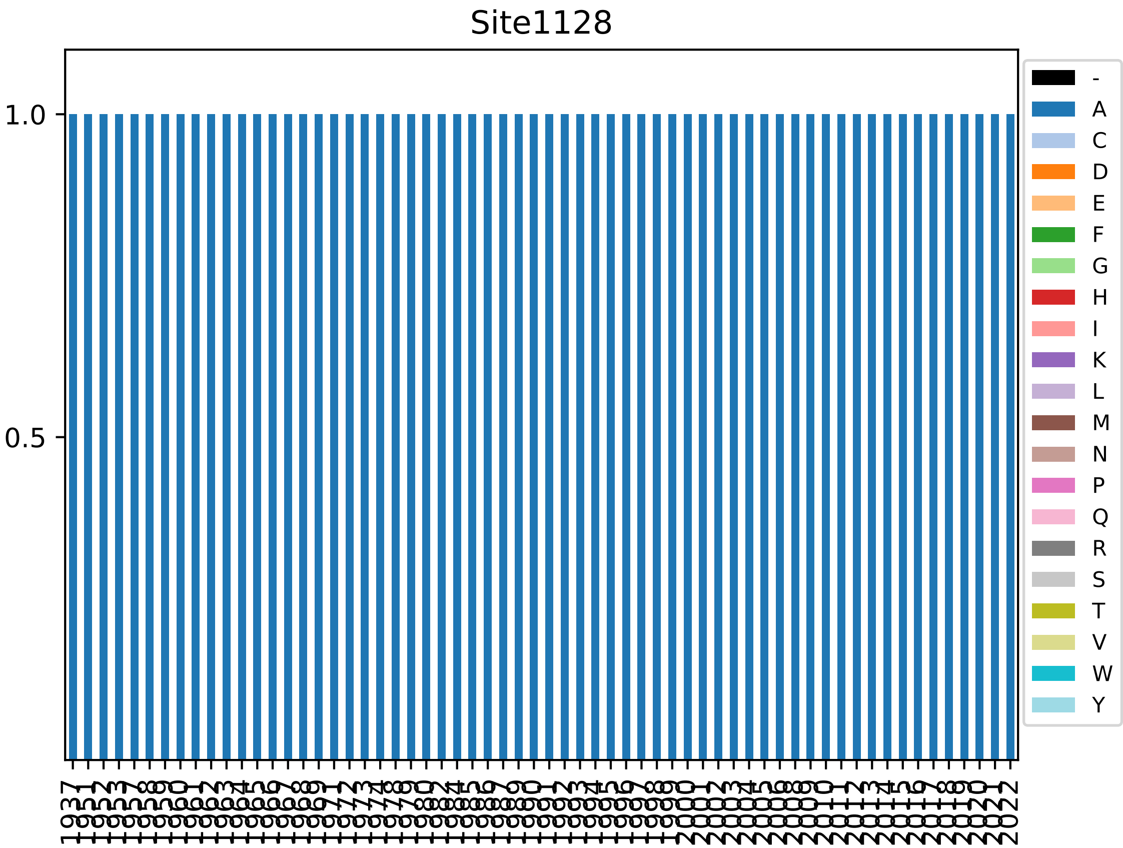
Site1128
1.0
0.5
-
A
C
D
E
F
G
H
I
K
L
M
N
P
Q
R
S
T
V
W
Y

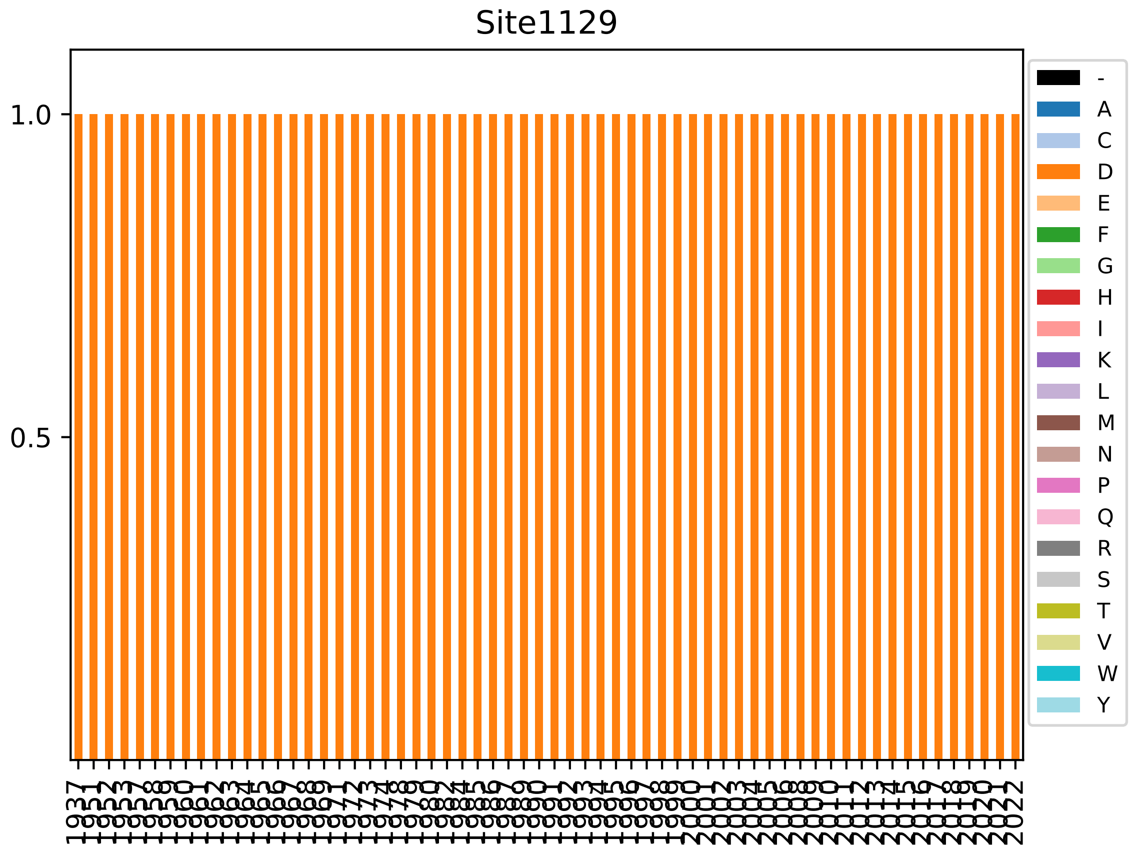

Site1129
1.0
0.5
-
A
C
D
E
F
G
H
I
K
L
M
N
P
Q
R
S
T
V
W
Y

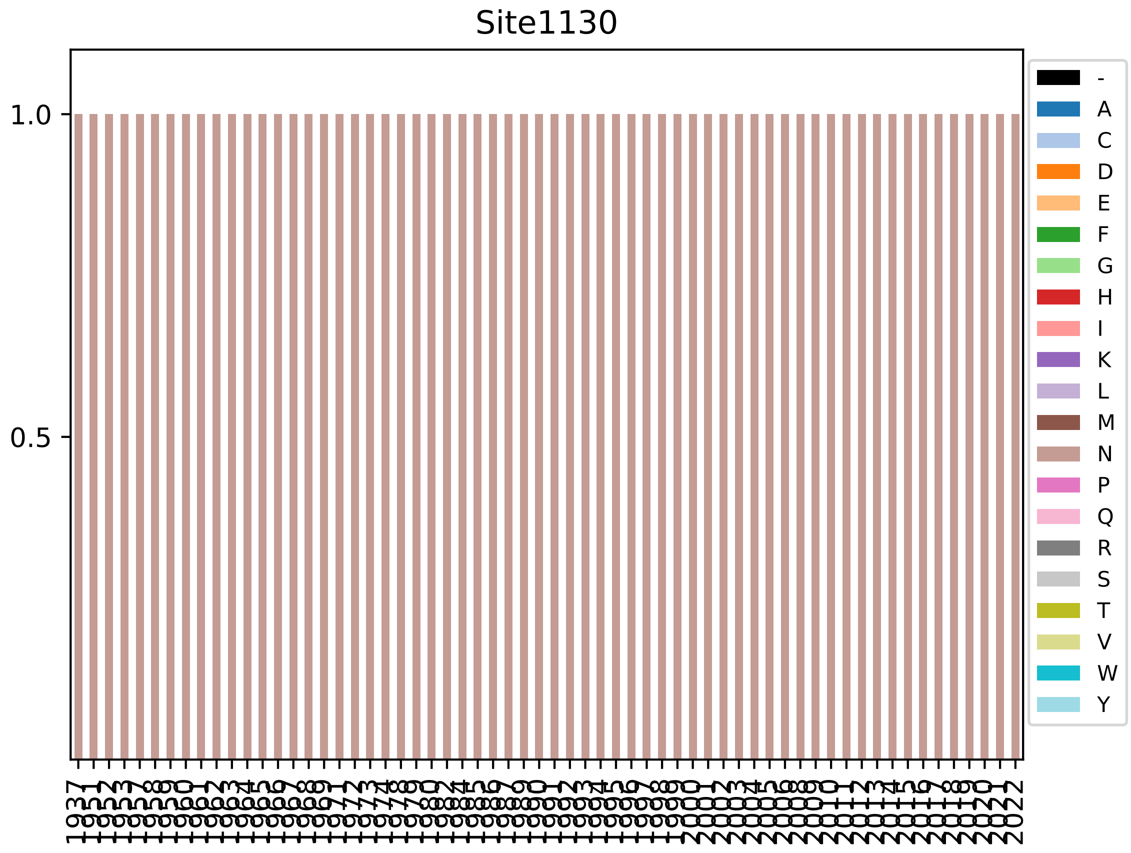
Site1130
1.0
0.5
-
A
C
D
E
F
G
H
I
K
L
M
N
P
Q
R
S
T
V
W
Y

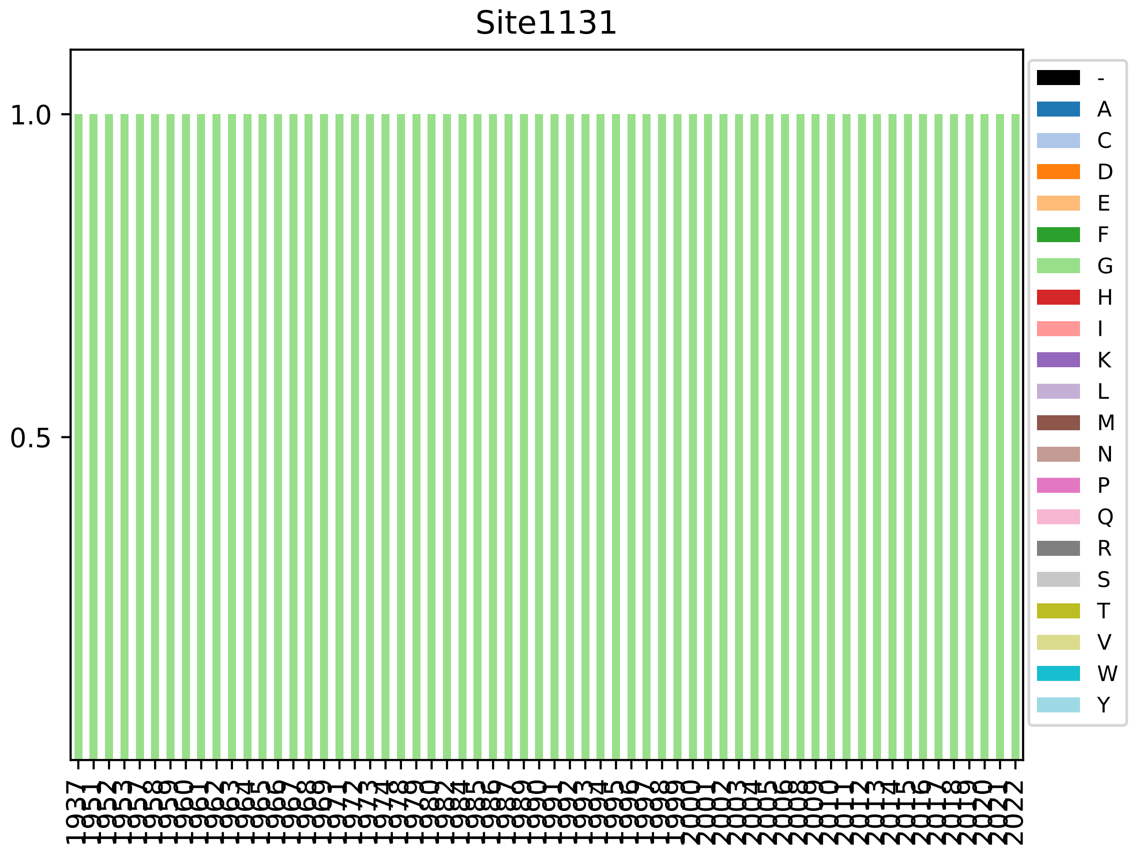
Site1131
1.0
0.5
-
A
C
D
E
F
G
H
I
K
L
M
N
P
Q
R
S
T
V
W
Y

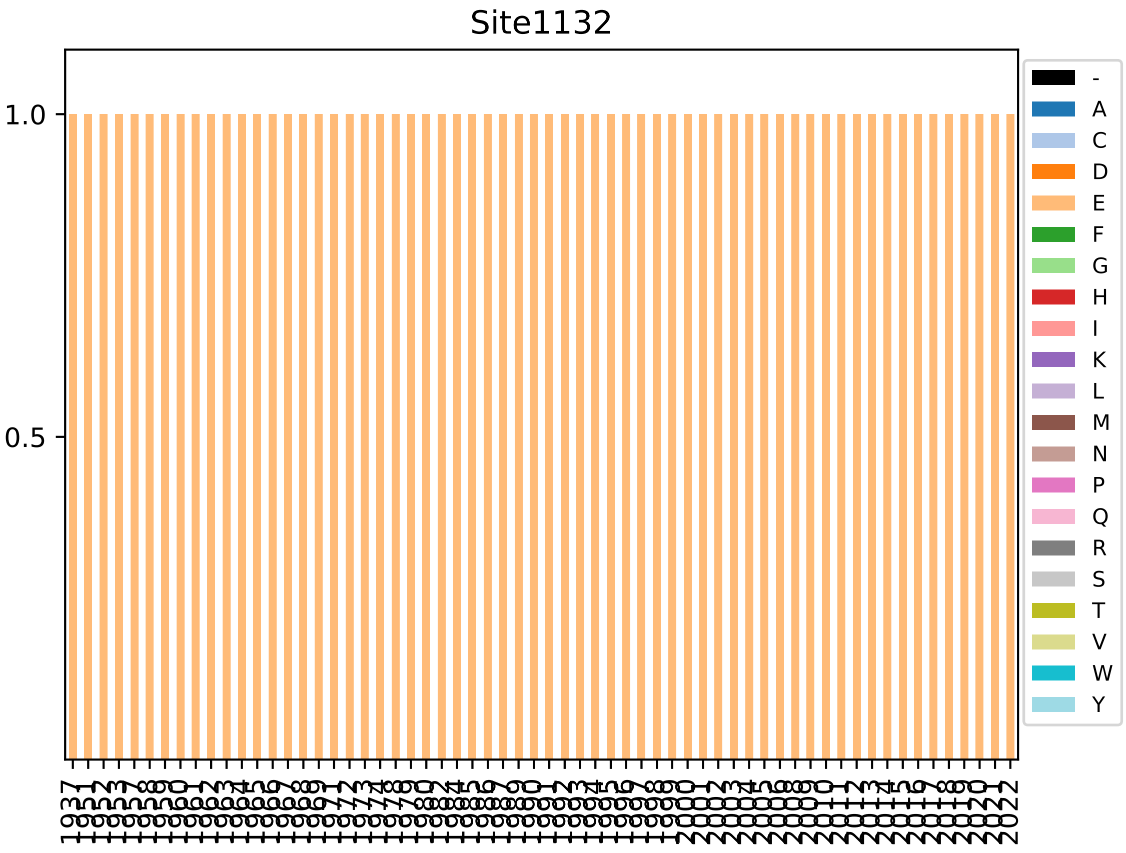
Site1132
1.0
0.5
-
A
C
D
E
F
G
H
I
K
L
M
N
P
Q
R
S
T
V
W
Y

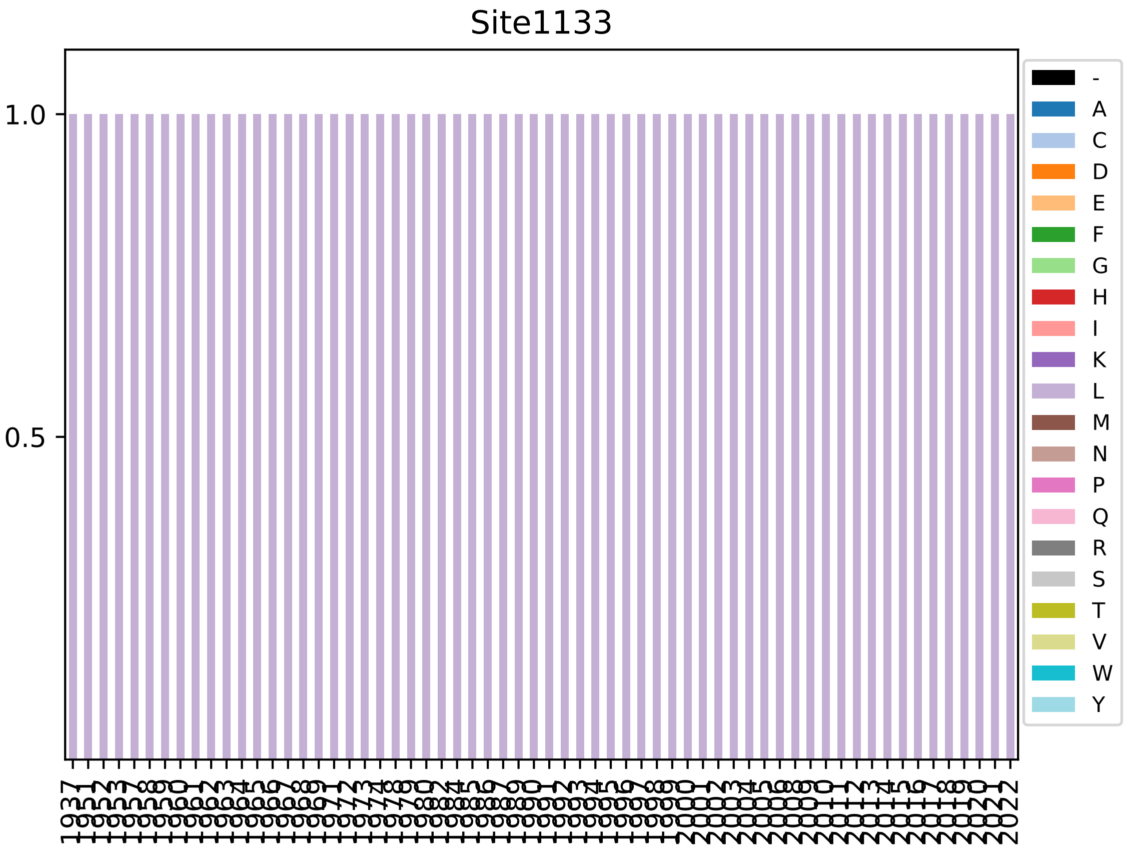

Site1133
1.0
0.5
-
A
C
D
E
F
G
H
I
K
L
M
N
P
Q
R
S
T
V
W
Y

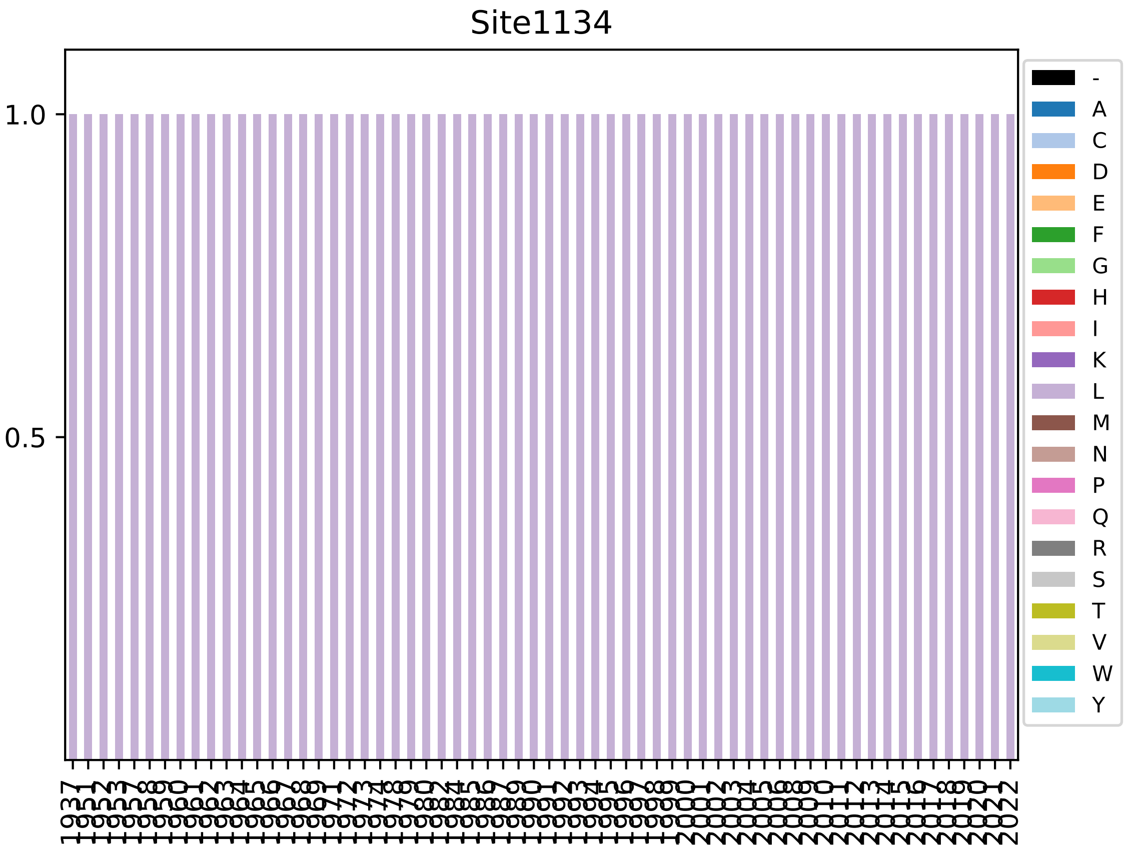
Site1134
1.0
0.5
-
A
C
D
E
F
G
H
I
K
L
M
N
P
Q
R
S
T
V
W
Y

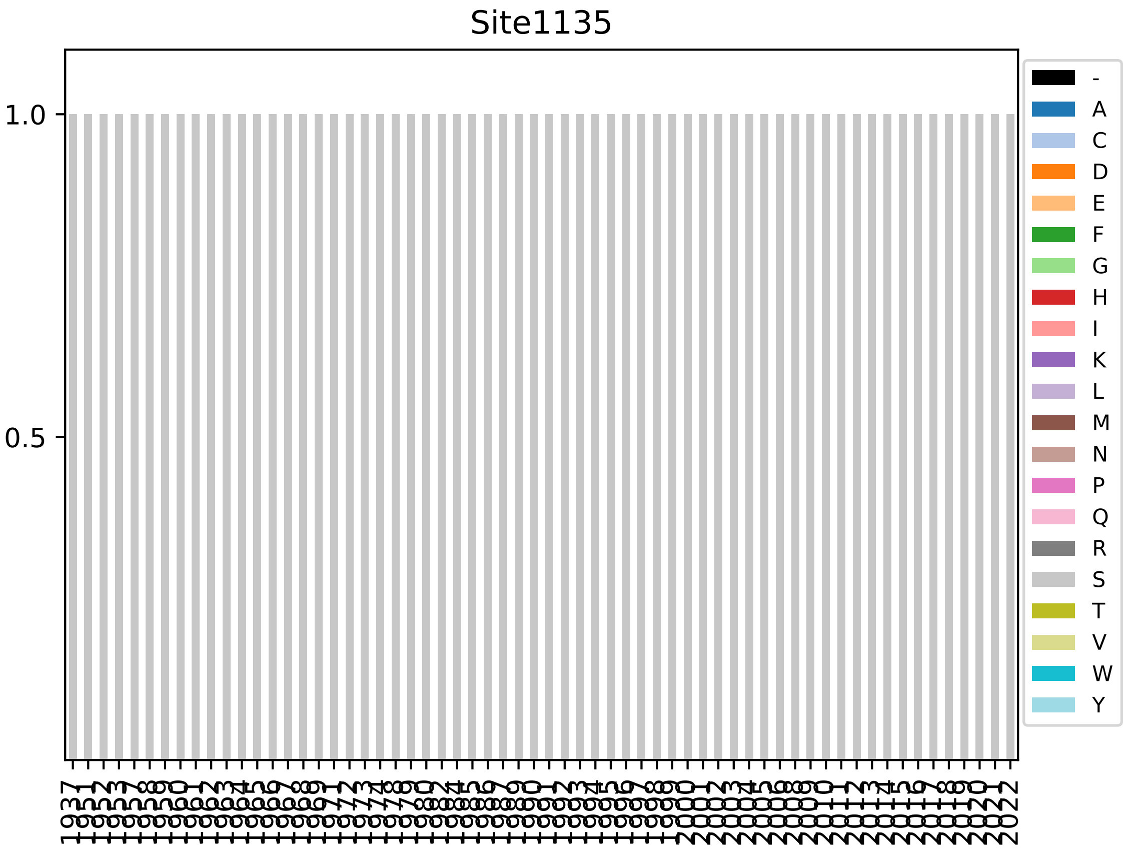
Site1135
1.0
0.5
-
A
C
D
E
F
G
H
I
K
L
M
N
P
Q
R
S
T
V
W
Y

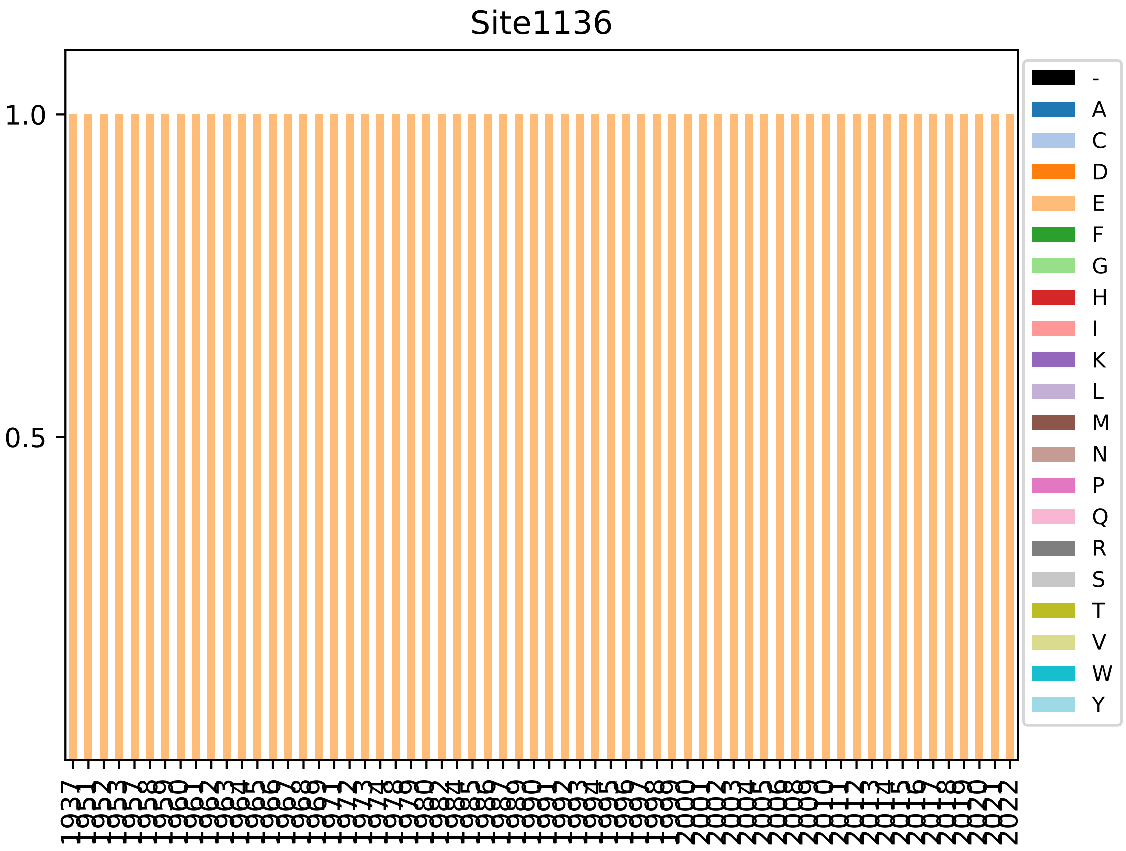

Site1136
1.0
0.5
-
A
C
D
E
F
G
H
I
K
L
M
N
P
Q
R
S
T
V
W
Y

Site1137

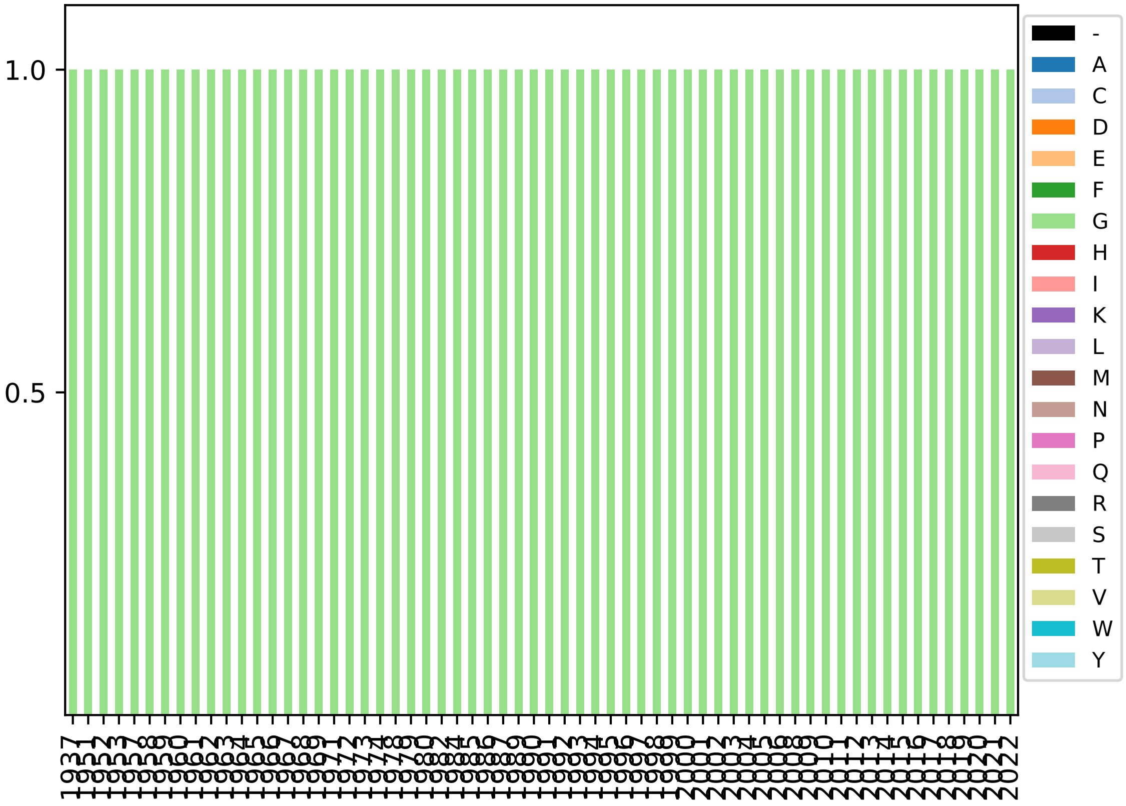

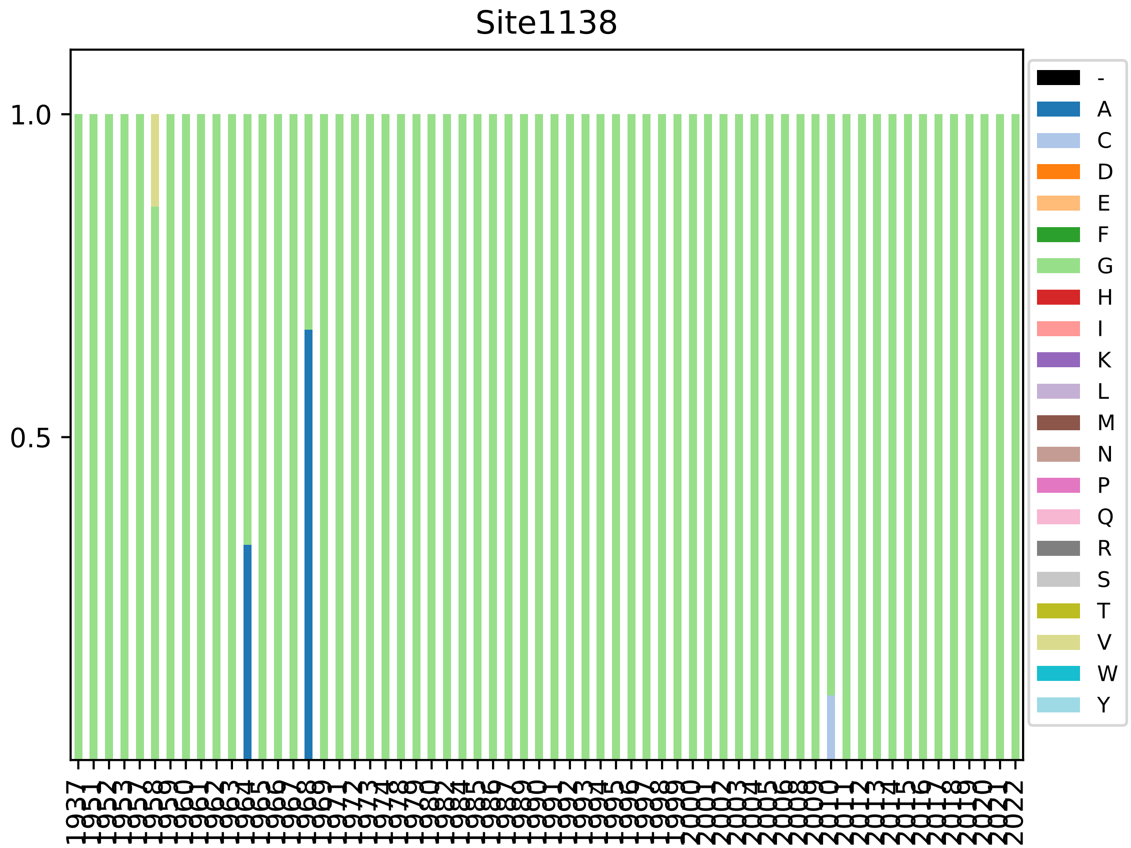
Site1138
1.0
0.5
-
A
C
D
E
F
G
H
I
K
L
M
N
P
Q
R
S
T
V
W
Y

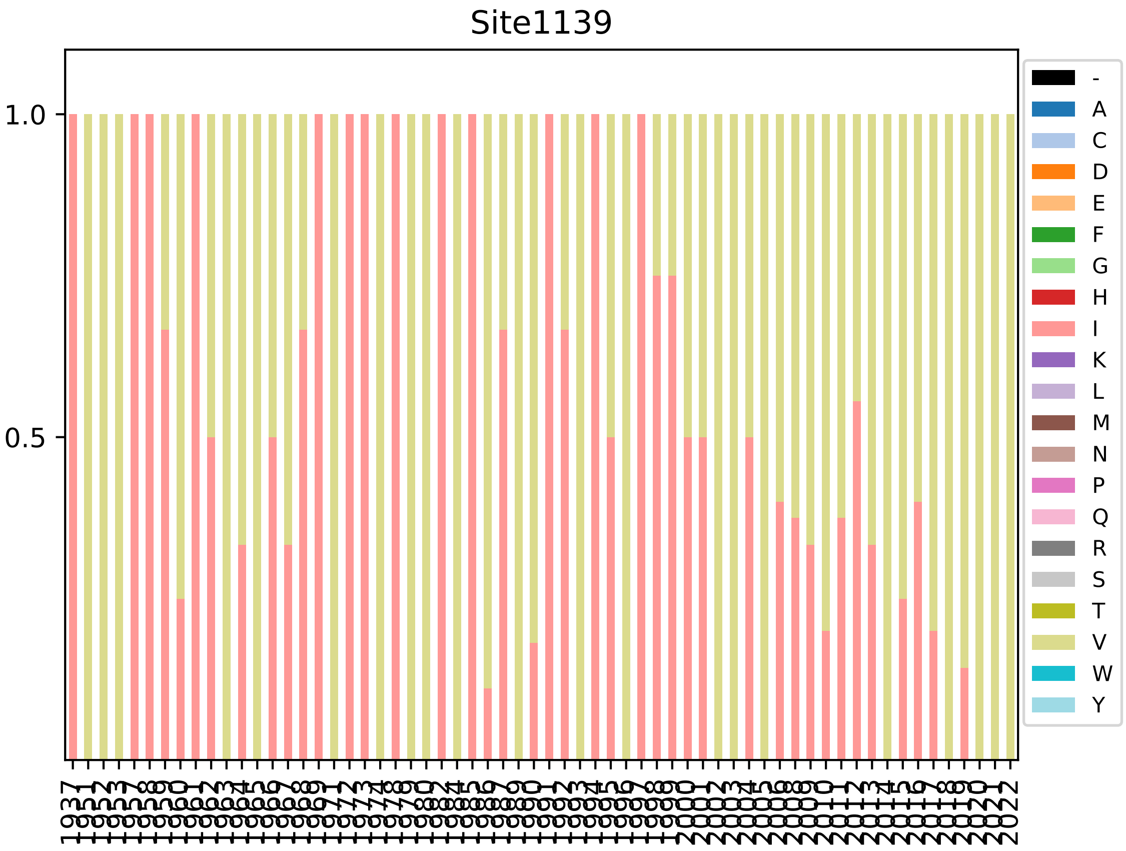
Site1139
1.0
0.5
-
A
C
D
E
F
G
H
I
K
L
M
N
P
Q
R
S
T
V
W
Y

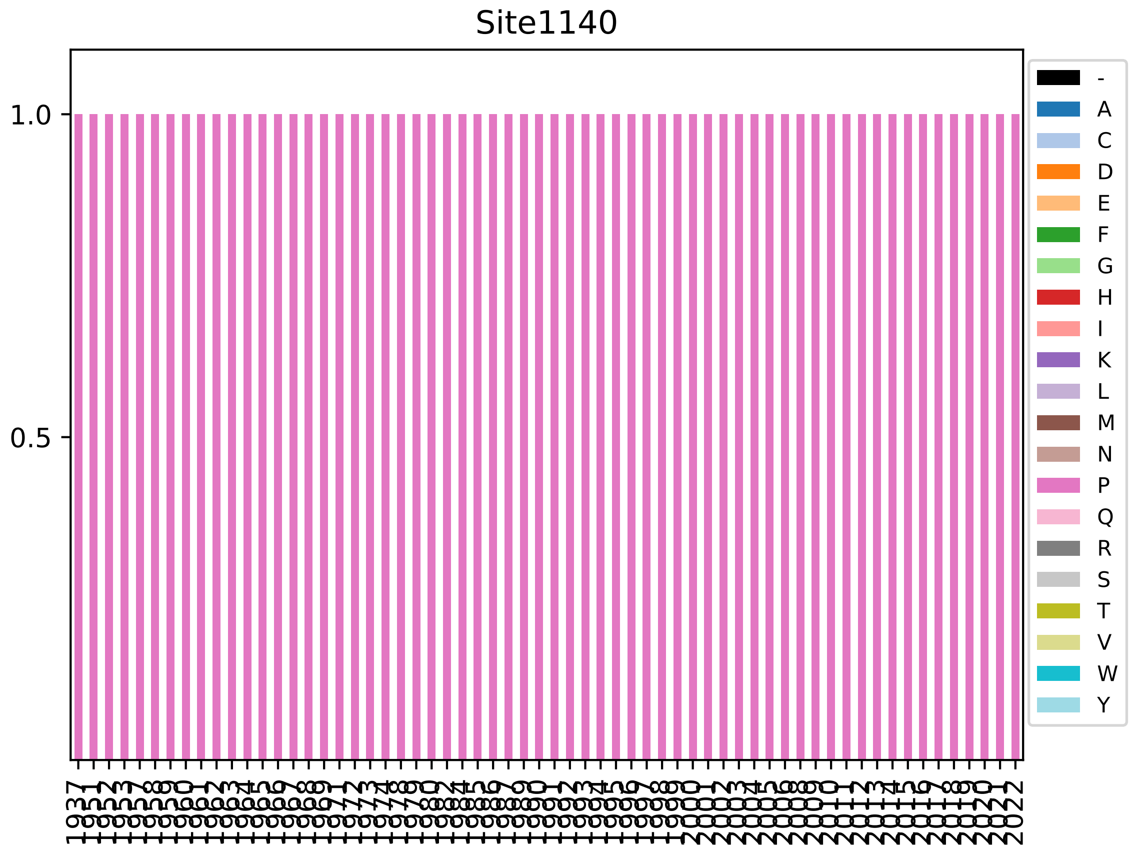
Site1140
1.0
0.5
-
A
C
D
E
F
G
H
I
K
L
M
N
P
Q
R
S
T
V
W
Y

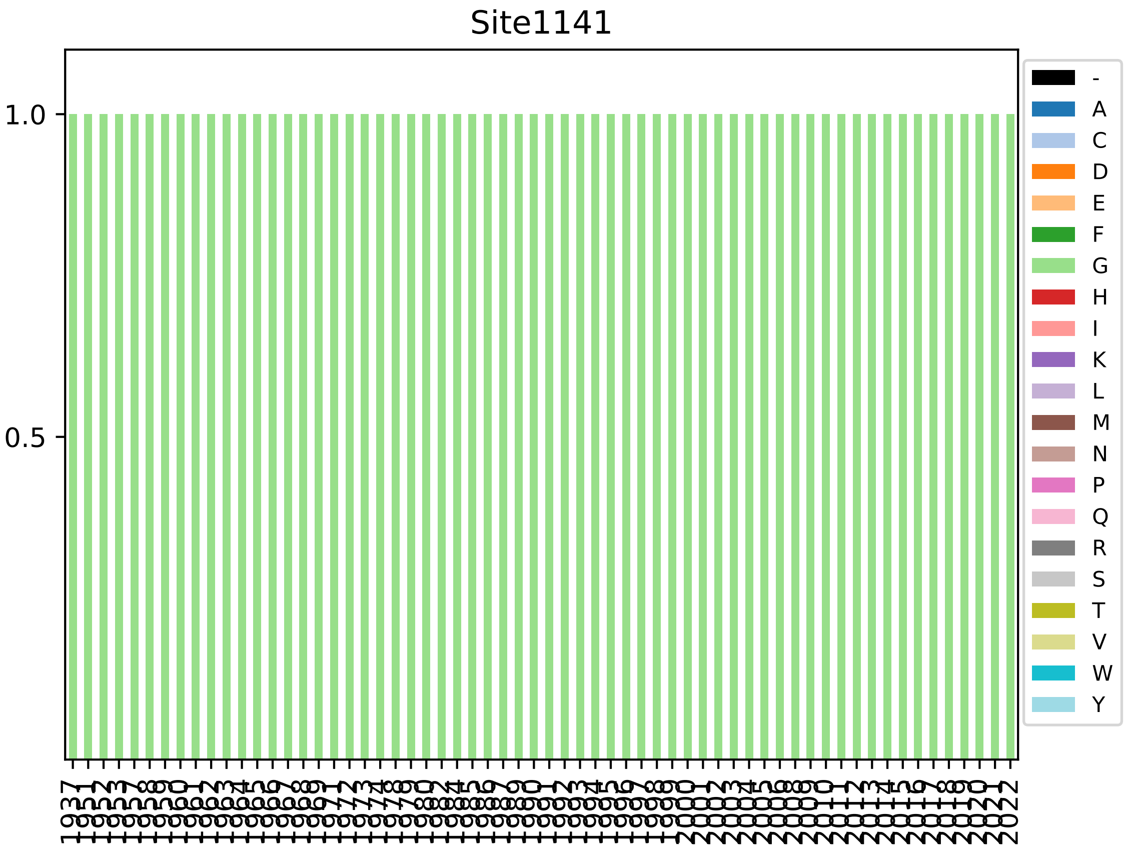
Site1141
1.0
0.5
-
A
C
D
E
F
G
H
I
K
L
M
N
P
Q
R
S
T
V
W
Y

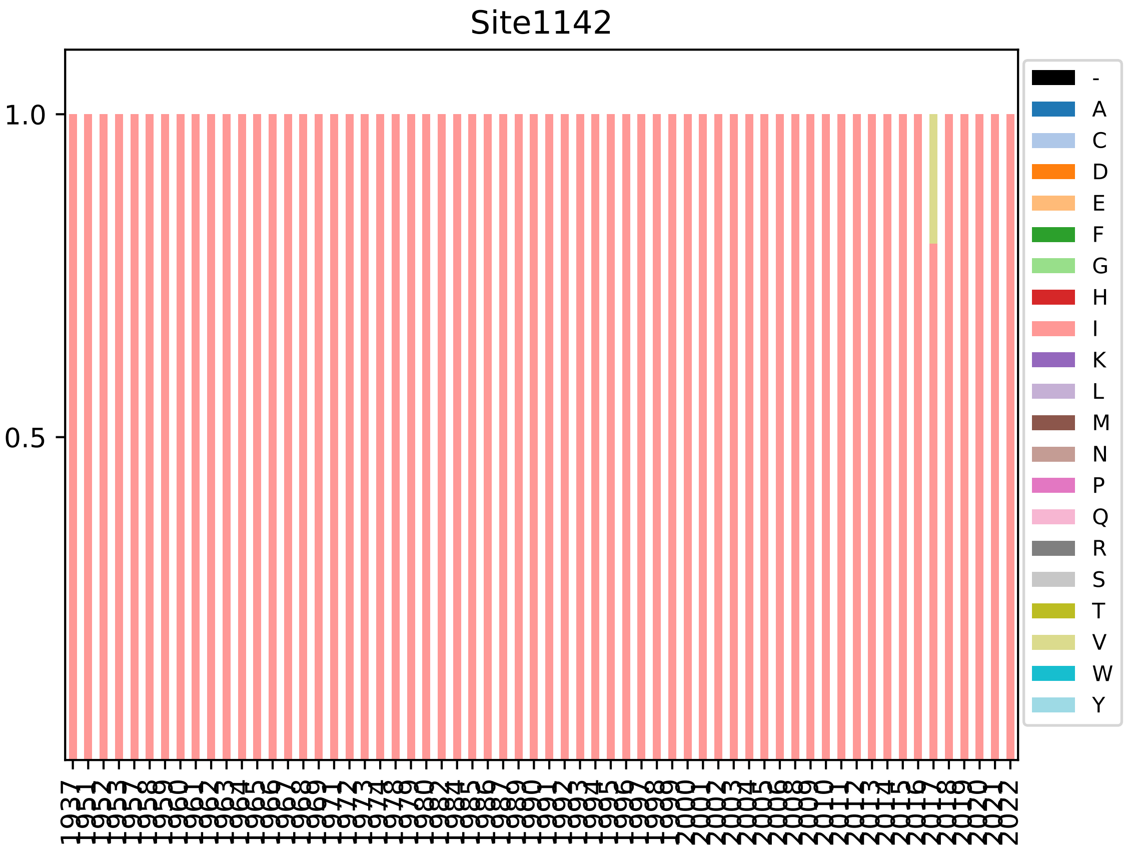
Site1142
1.0
0.5
-
A
C
D
E
F
G
H
I
K
L
M
N
P
Q
R
S
T
V
W
Y

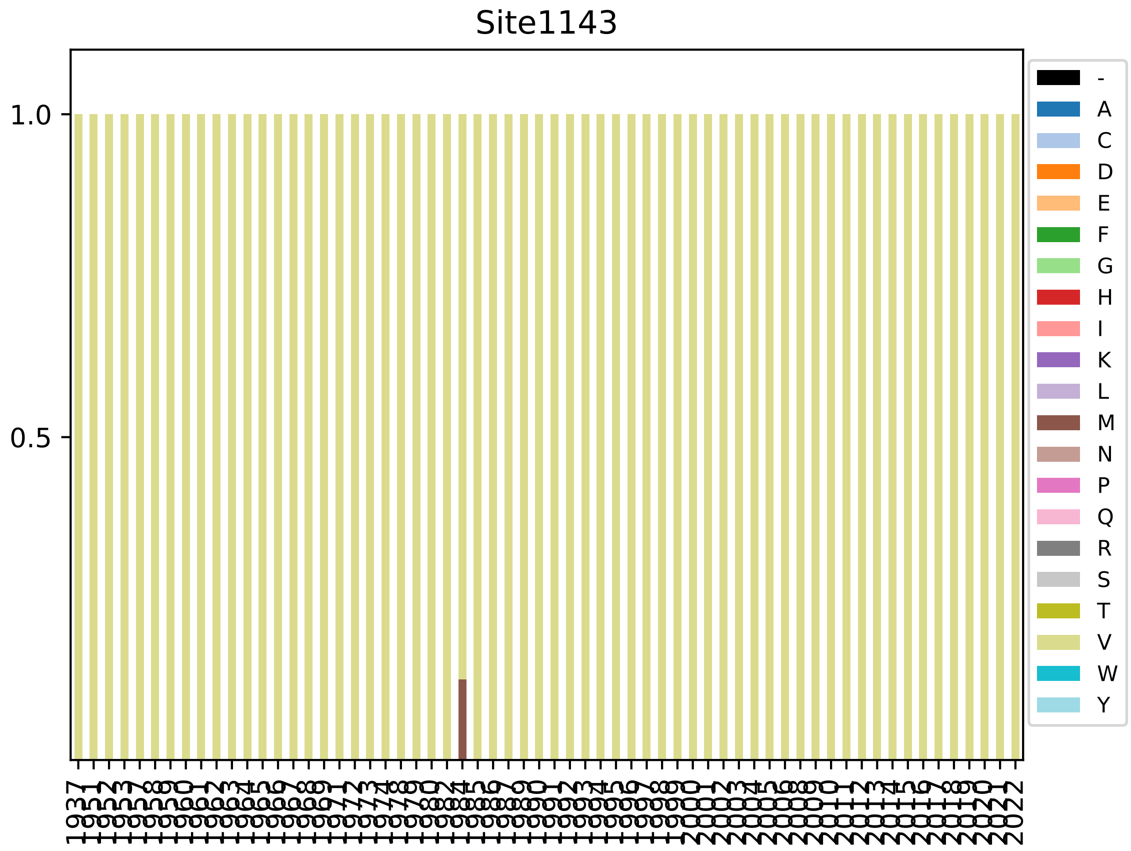
Site1143
1.0
0.5
-
A
C
D
E
F
G
H
I
K
L
M
N
P
Q
R
S
T
V
W
Y

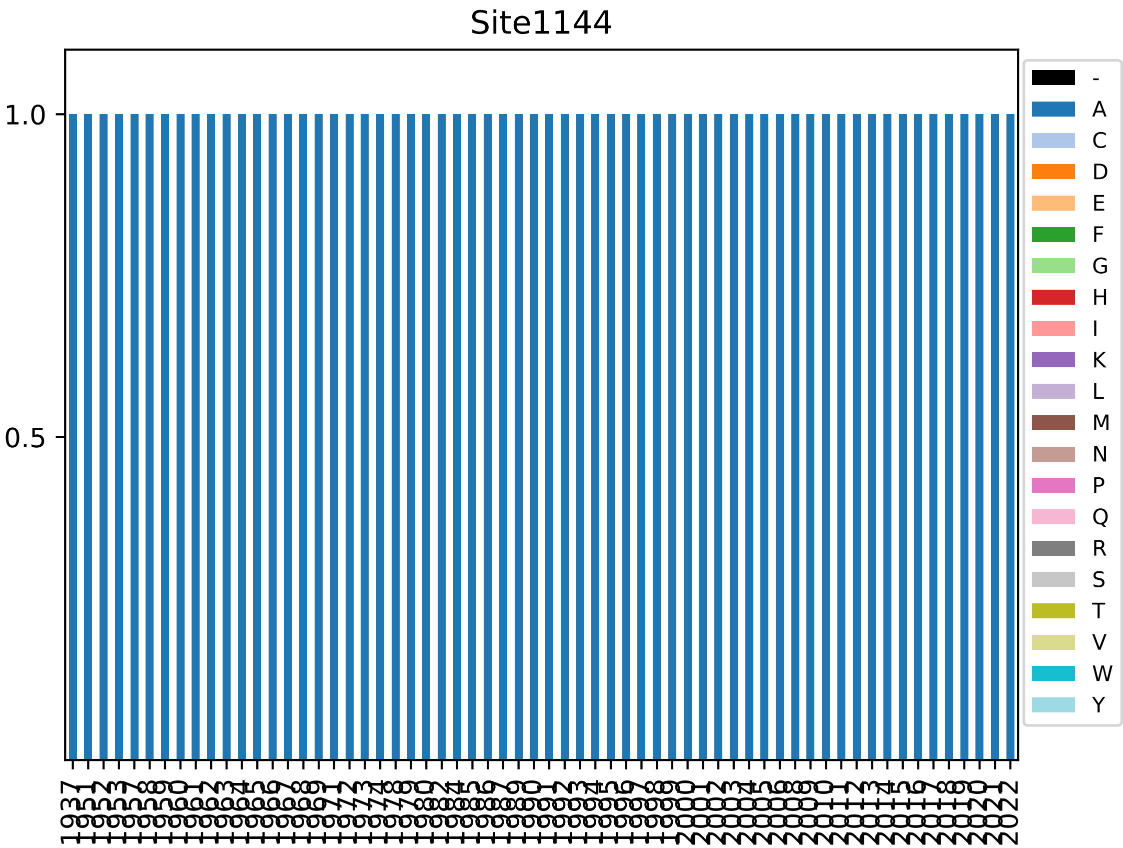
Site1144
1.0
0.5
-
A
C
D
E
F
G
H
I
K
L
M
N
P
Q
R
S
T
V
W
Y

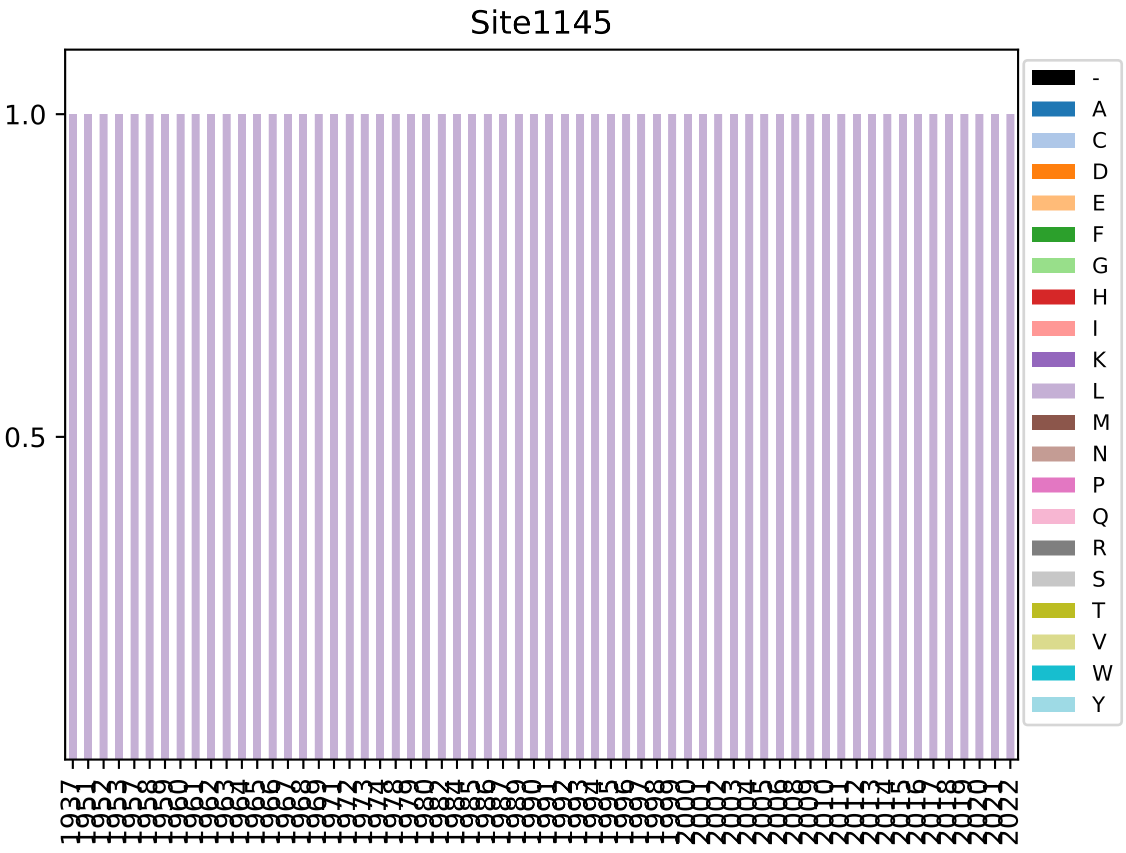
Site1145
1.0
0.5
-
A
C
D
E
F
G
H
I
K
L
M
N
P
Q
R
S
T
V
W
Y

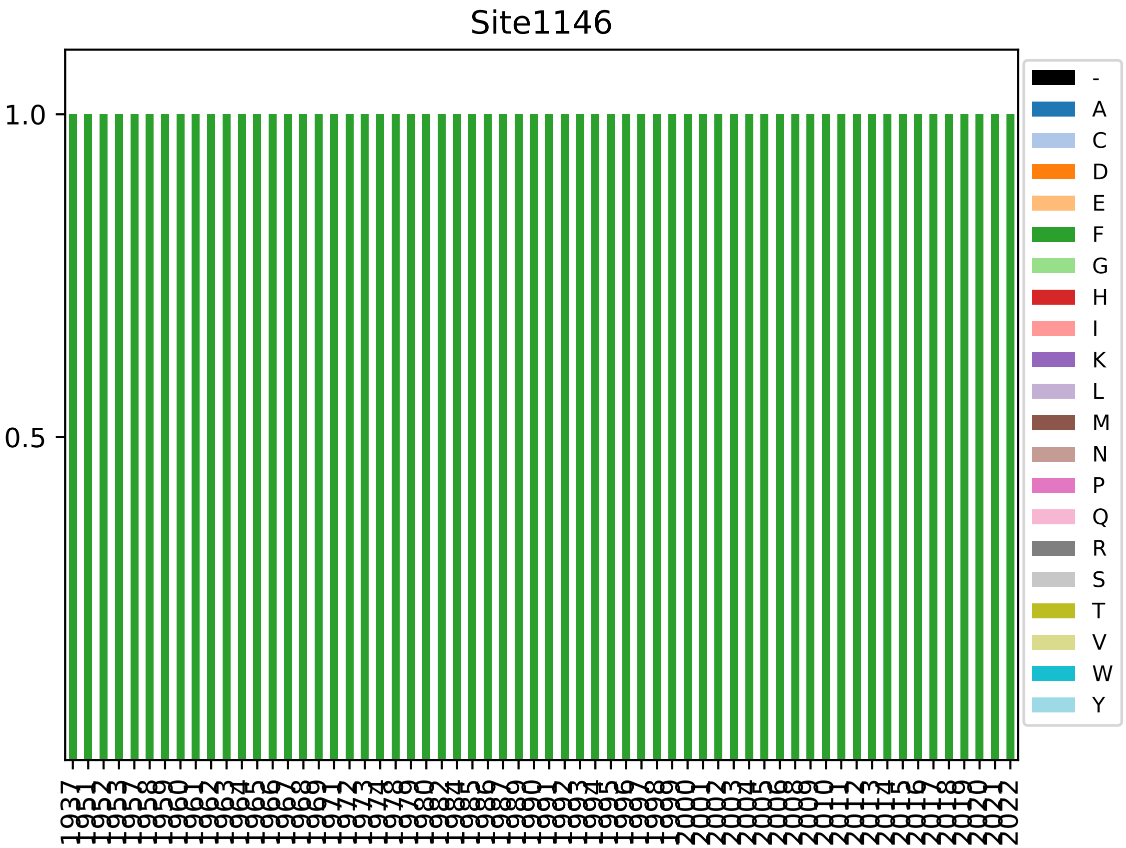
Site1146
1.0
0.5
-
A
C
D
E
F
G
H
I
K
L
M
N
P
Q
R
S
T
V
W
Y

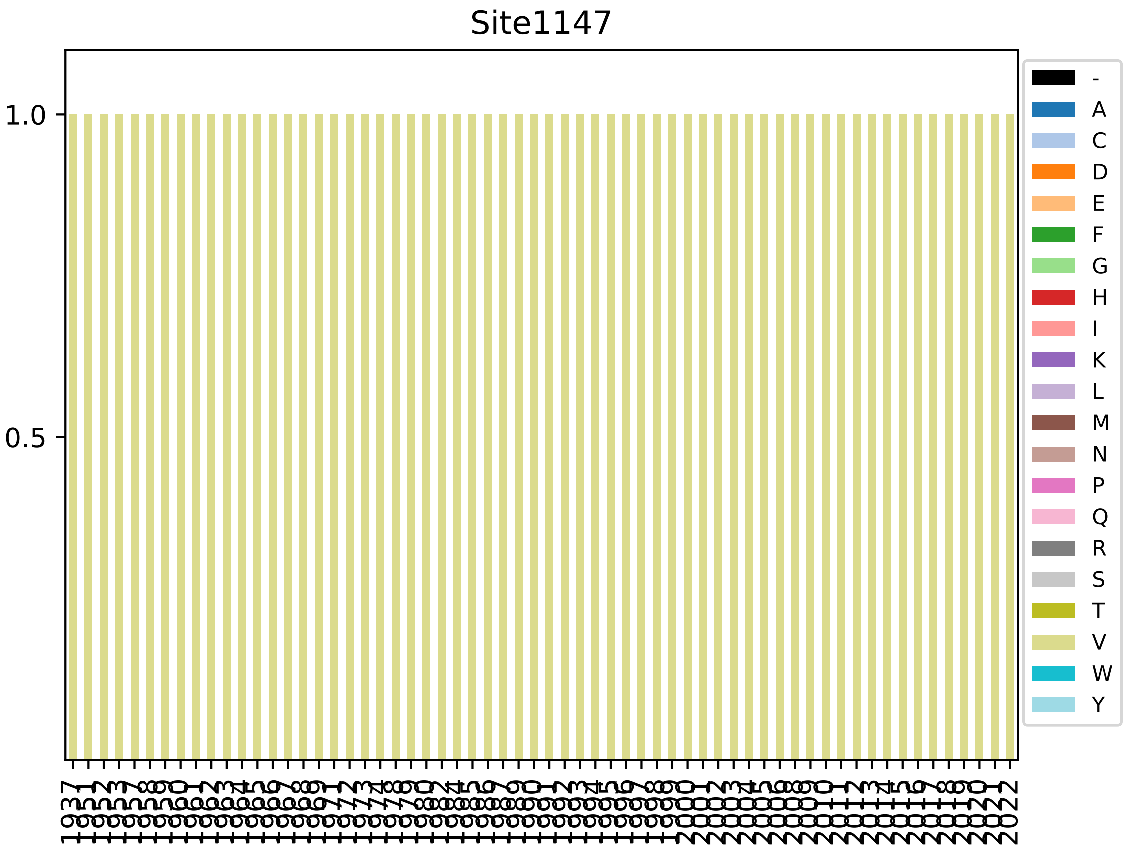
Site1147
1.0
0.5
-
A
C
D
E
F
G
H
I
K
L
M
N
P
Q
R
S
T
V
W
Y

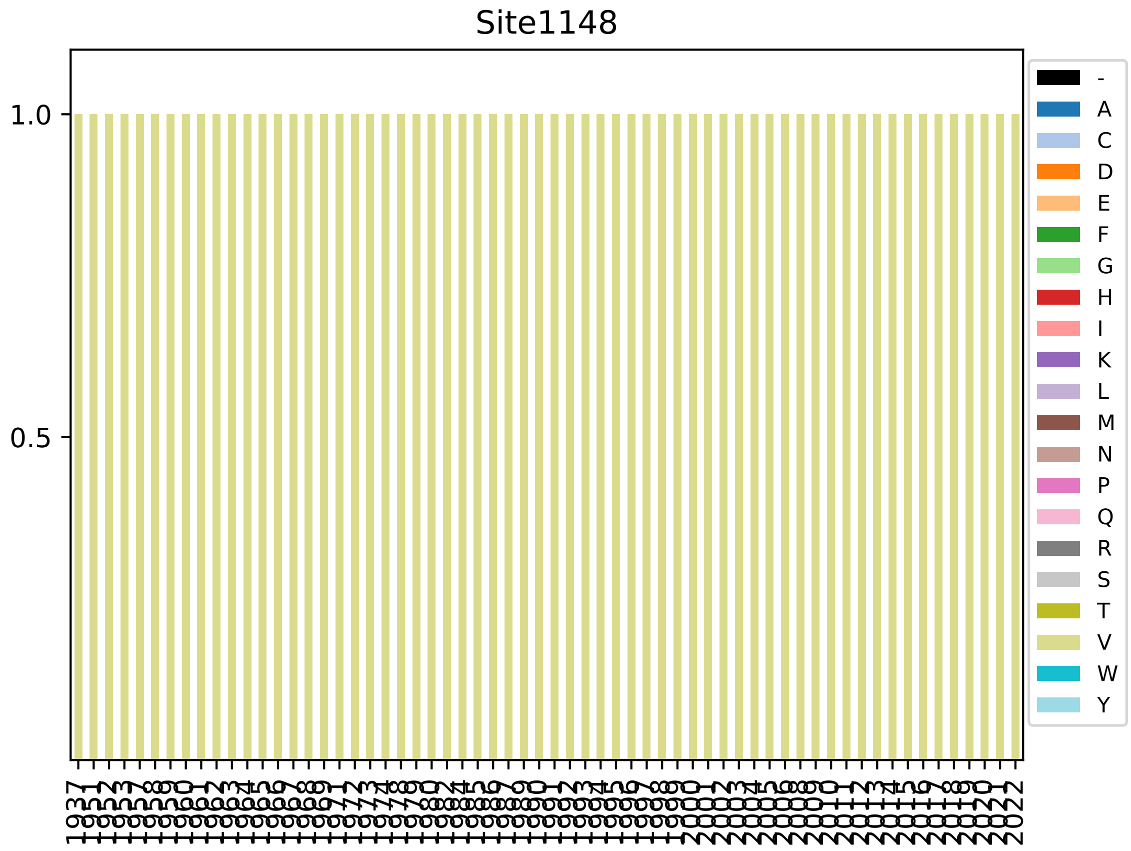
Site1148
1.0
0.5
-
A
C
D
E
F
G
H
I
K
L
M
N
P
Q
R
S
T
V
W
Y

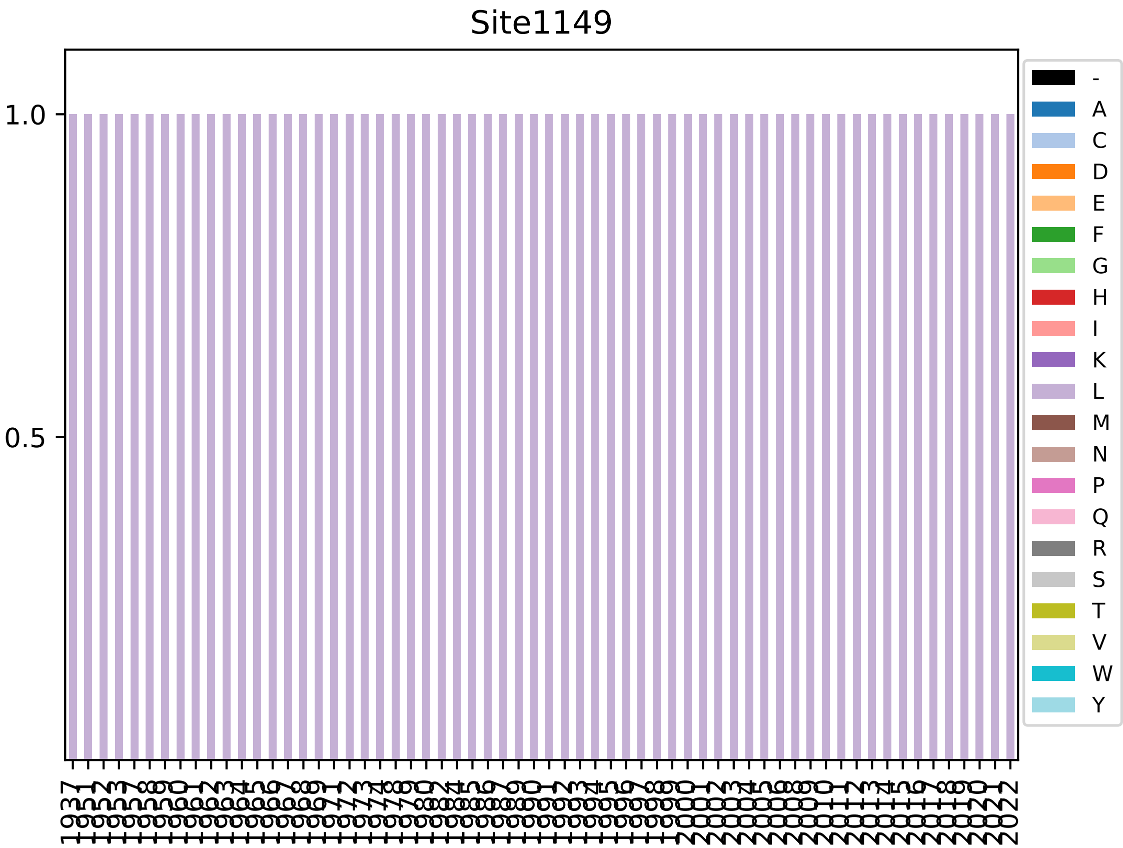
Site1149
1.0
0.5
-
A
C
D
E
F
G
H
I
K
L
M
N
P
Q
R
S
T
V
W
Y

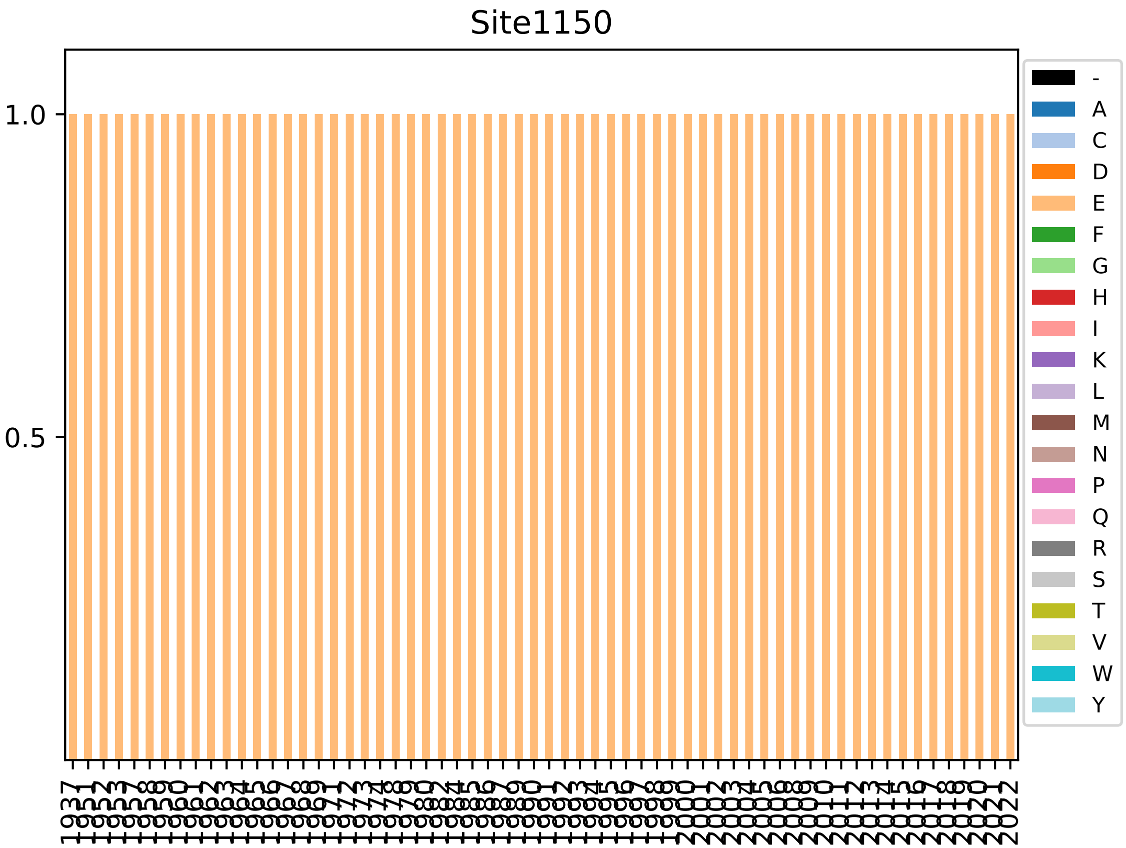

Site1150
1.0
0.5
-
A
C
D
E
F
G
H
I
K
L
M
N
P
Q
R
S
T
V
W
Y

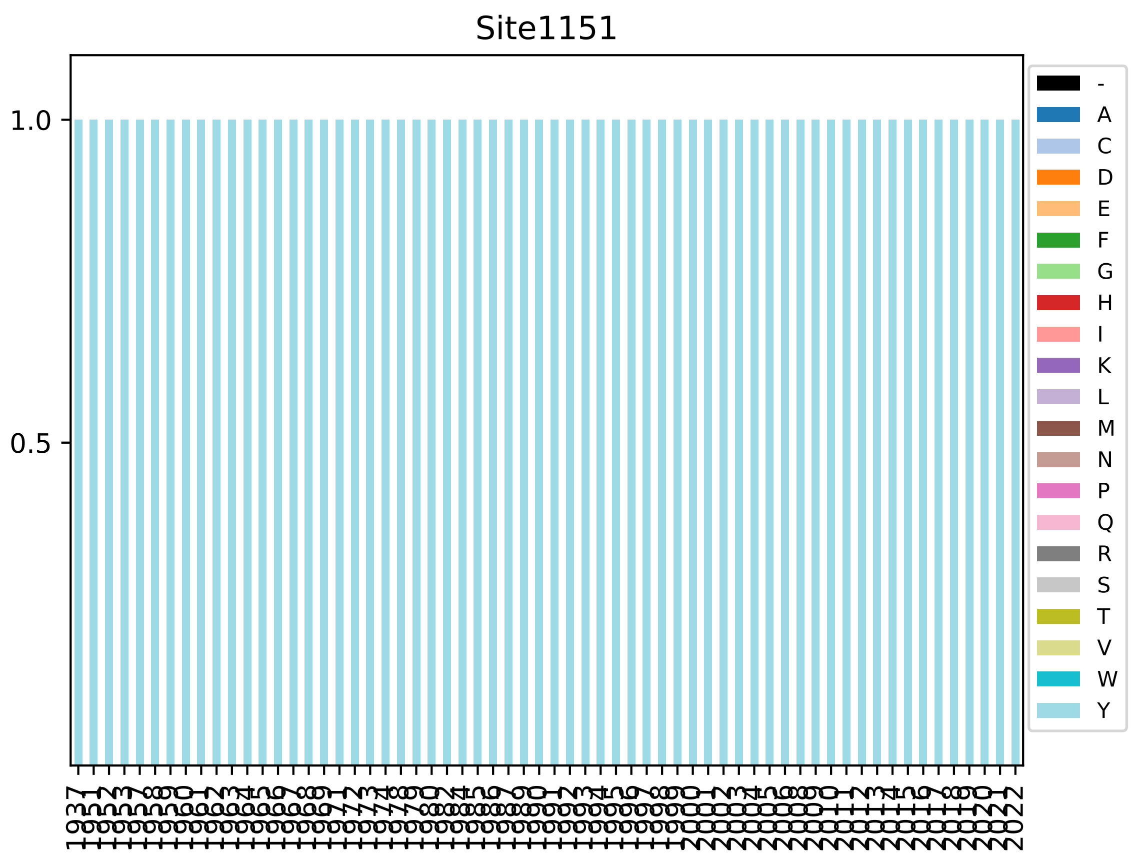
Site1151
1.0
0.5
-
A
C
D
E
F
G
H
I
K
L
M
N
P
Q
R
S
T
V
W
Y

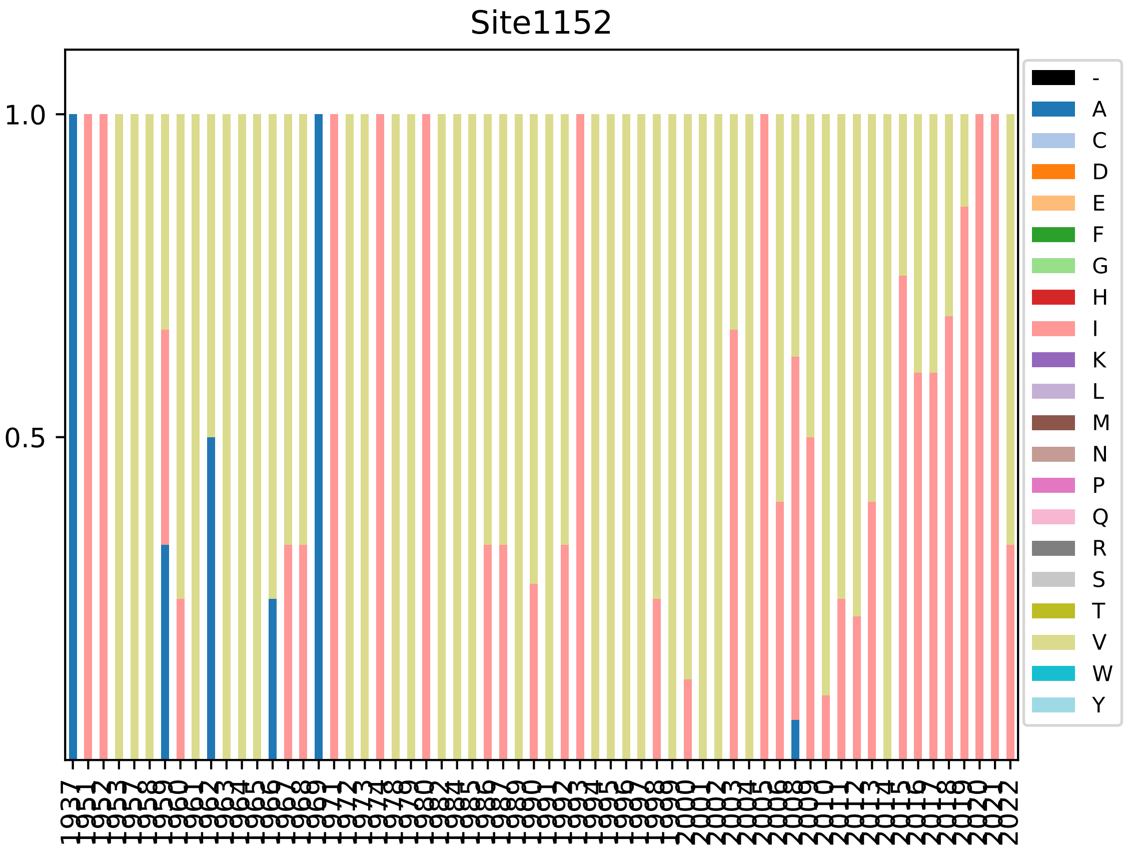
Site1152
1.0
0.5
-
A
C
D
E
F
G
H
I
K
L
M
N
P
Q
R
S
T
V
W
Y

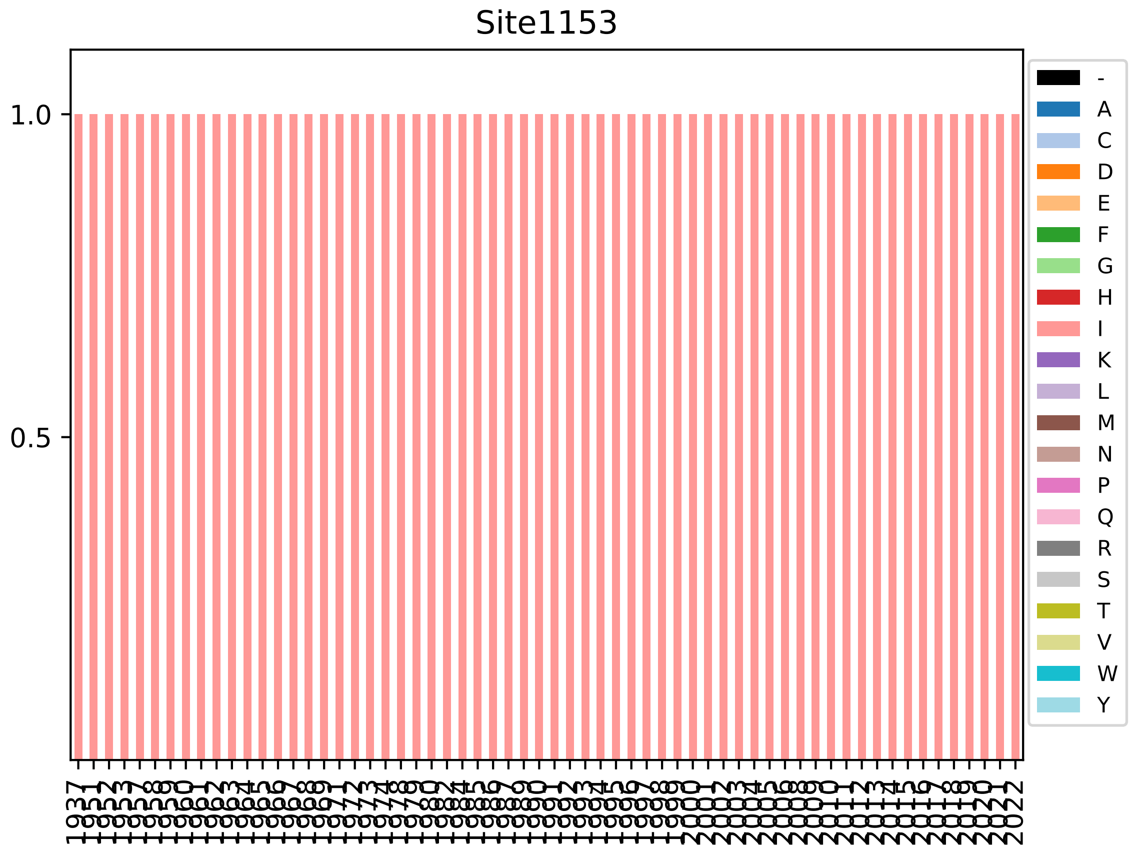

Site1153
1.0
0.5
-
A
C
D
E
F
G
H
I
K
L
M
N
P
Q
R
S
T
V
W
Y

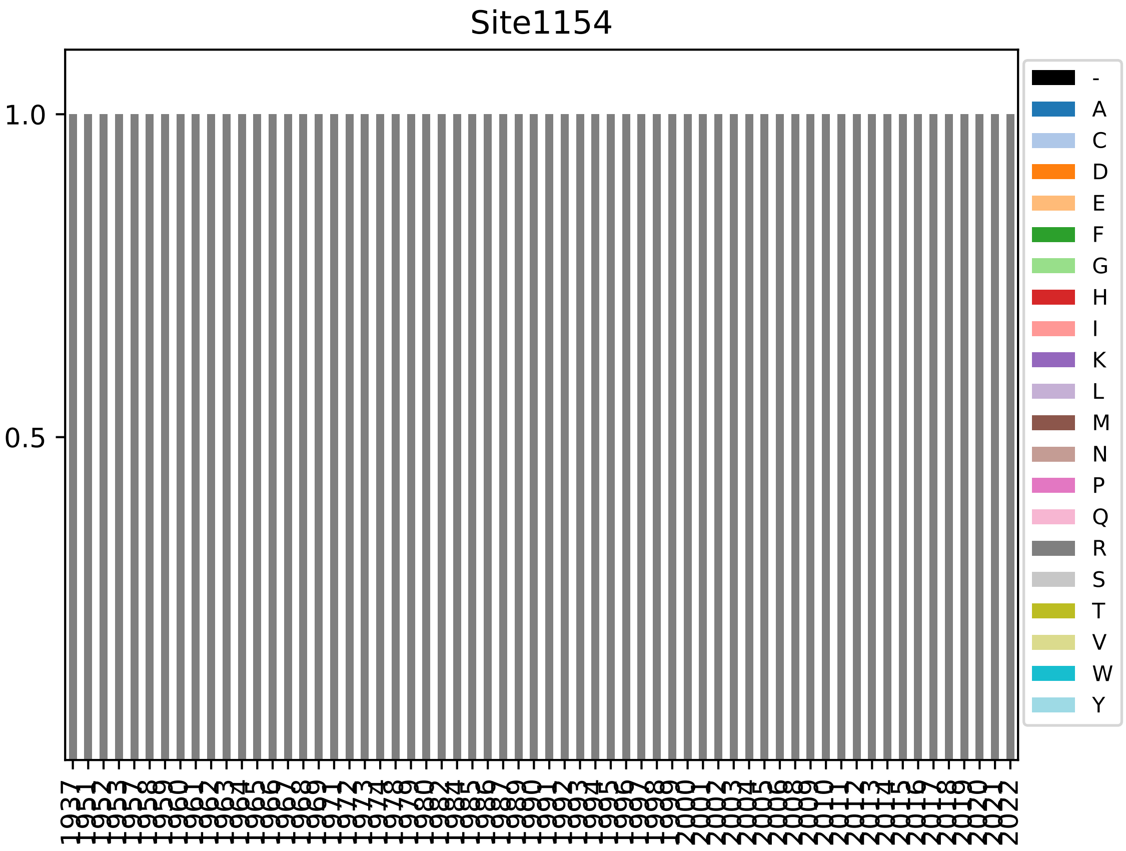
Site1154
1.0
0.5
-
A
C
D
E
F
G
H
I
K
L
M
N
P
Q
R
S
T
V
W
Y

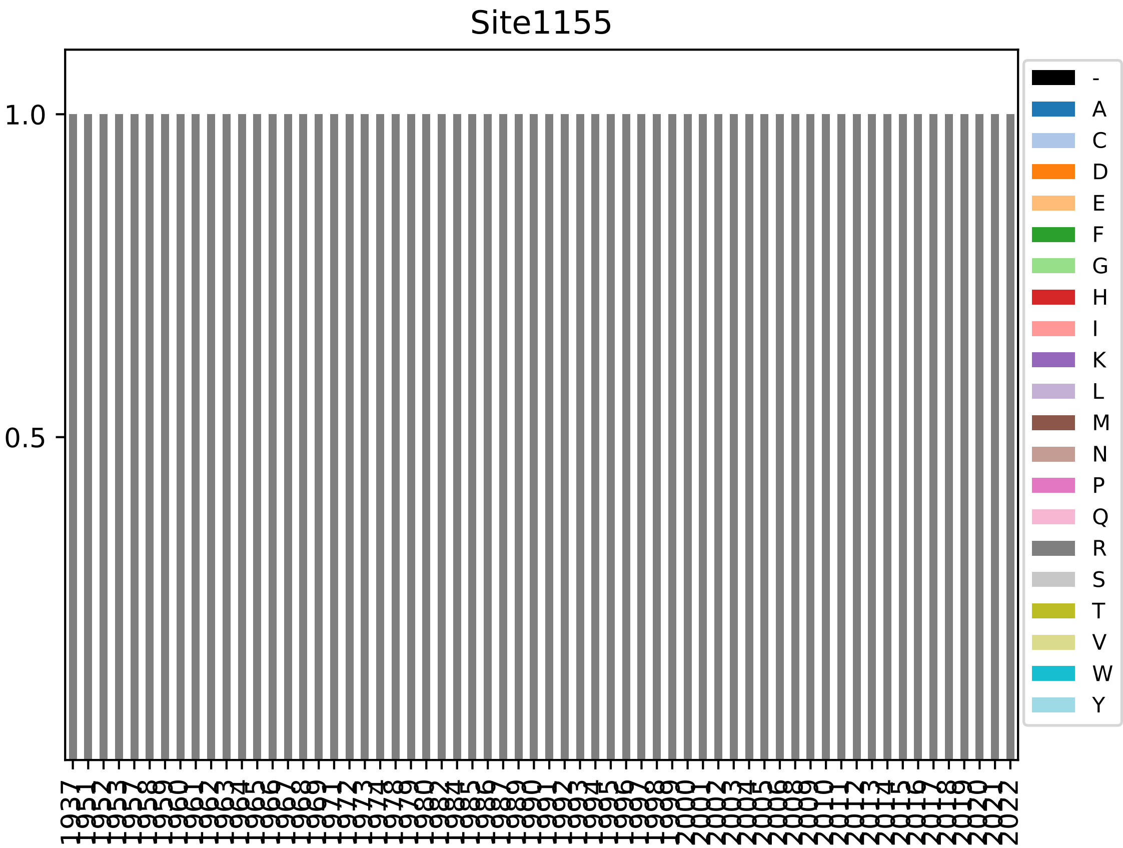
Site1155
1.0
0.5
-
A
C
D
E
F
G
H
I
K
L
M
N
P
Q
R
S
T
V
W
Y

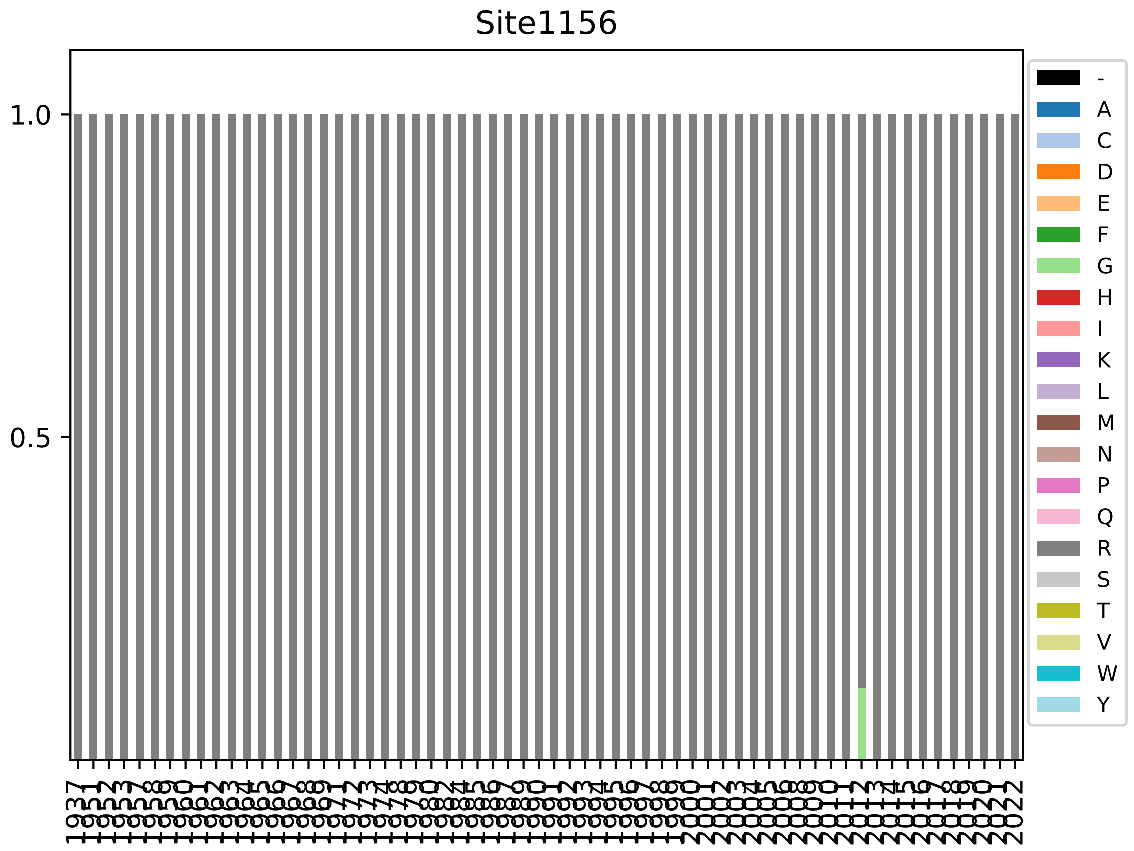

Site1156
1.0
0.5
-
A
C
D
E
F
G
H
I
K
L
M
N
P
Q
R
S
T
V
W
Y

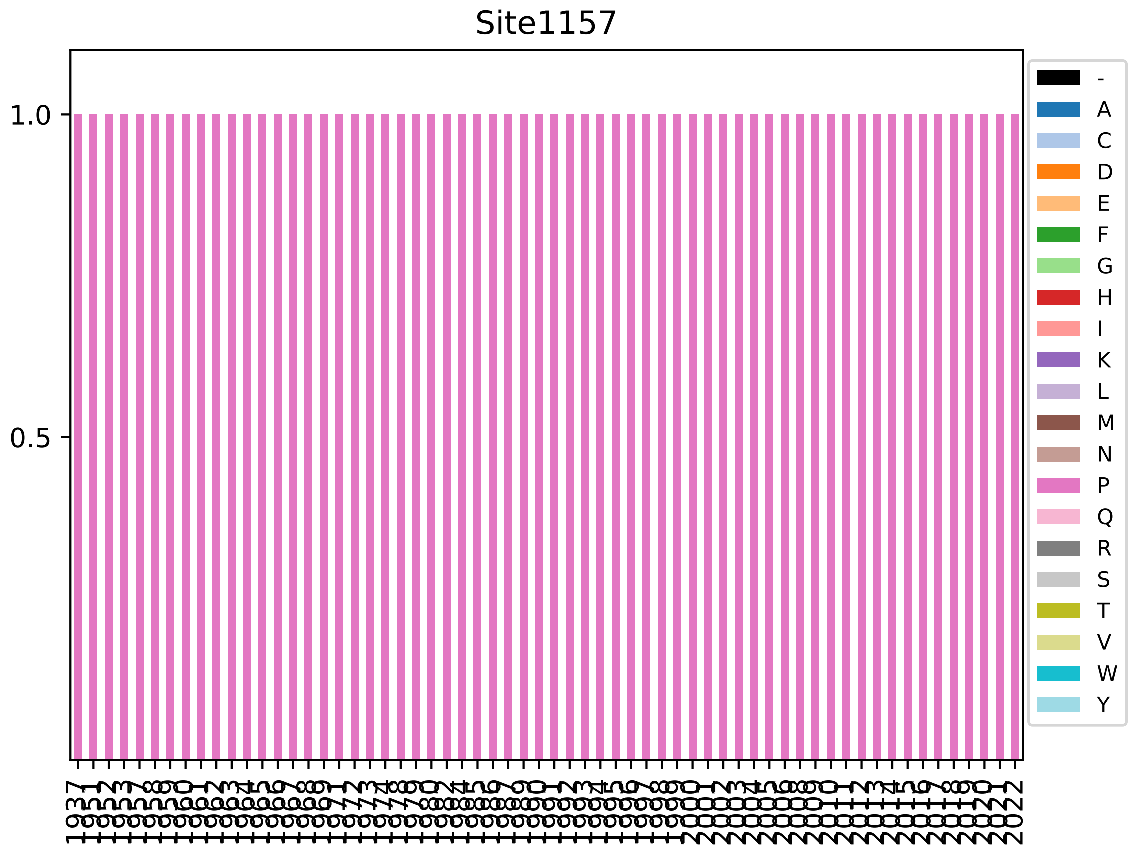
Site1157
1.0
0.5
-
A
C
D
E
F
G
H
I
K
L
M
N
P
Q
R
S
T
V
W
Y

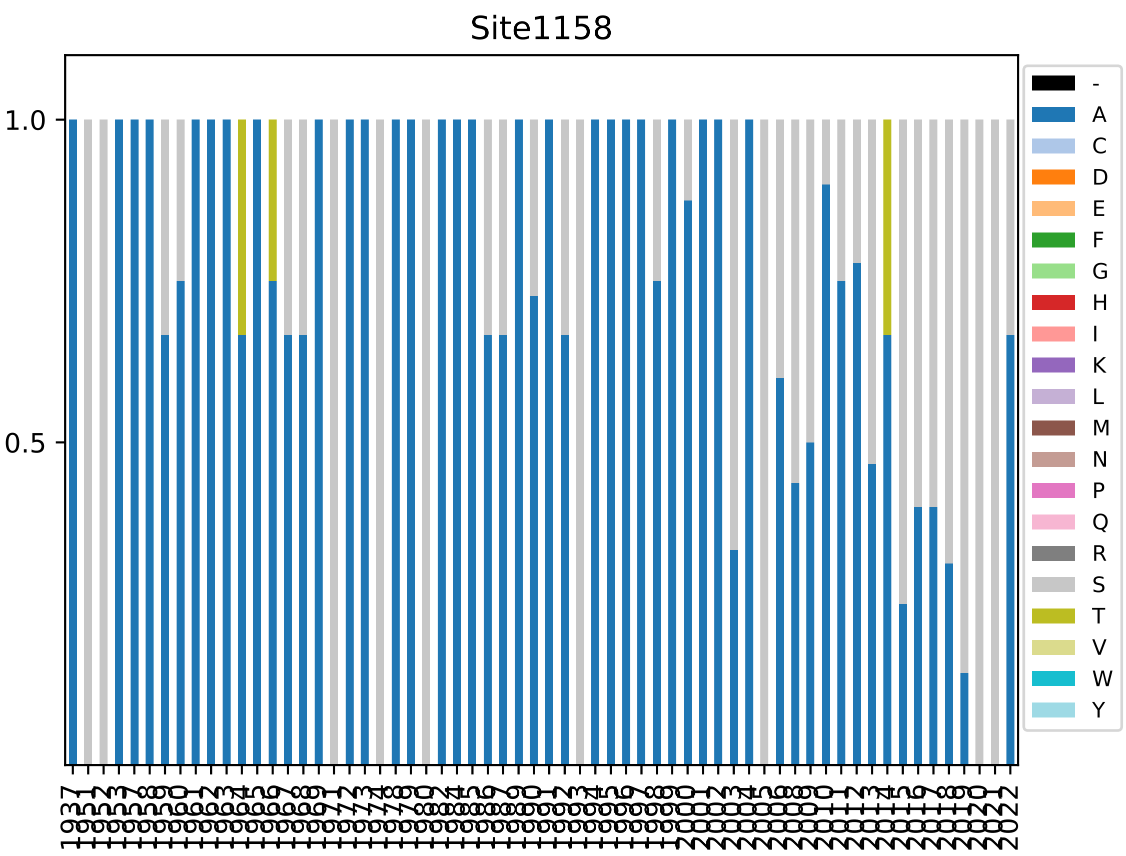

Site1158
1.0
0.5
-
A
C
D
E
F
G
H
I
K
L
M
N
P
Q
R
S
T
V
W
Y

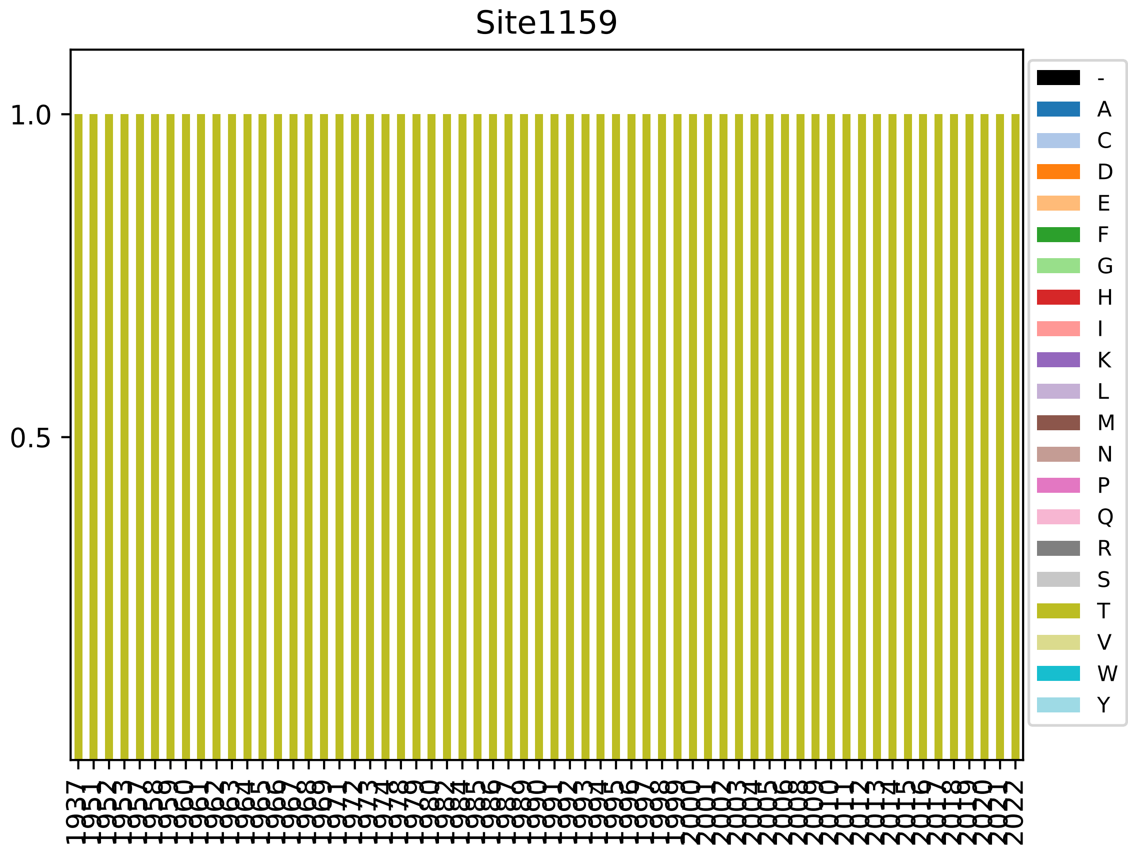
Site1159
1.0
0.5
-
A
C
D
E
F
G
H
I
K
L
M
N
P
Q
R
S
T
V
W
Y

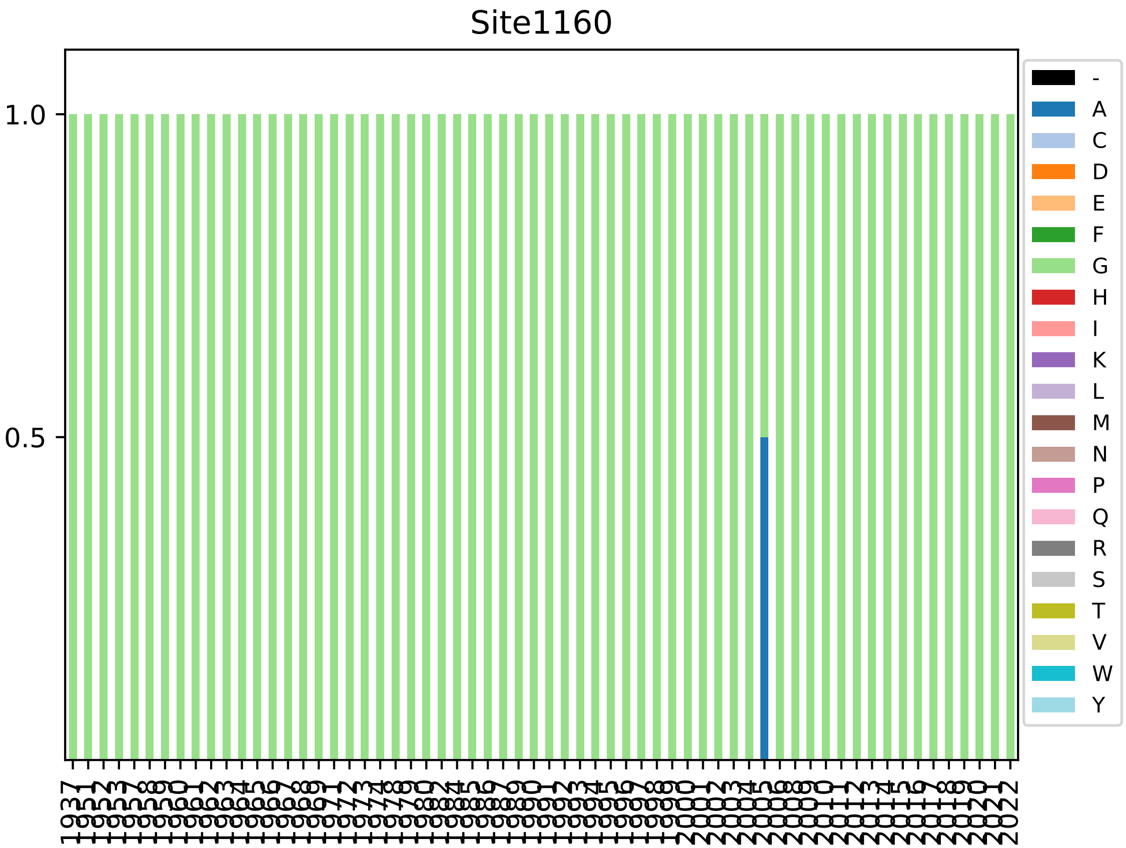
Site1160
1.0
0.5
-
A
C
D
E
F
G
H
I
K
L
M
N
P
Q
R
S
T
V
W
Y

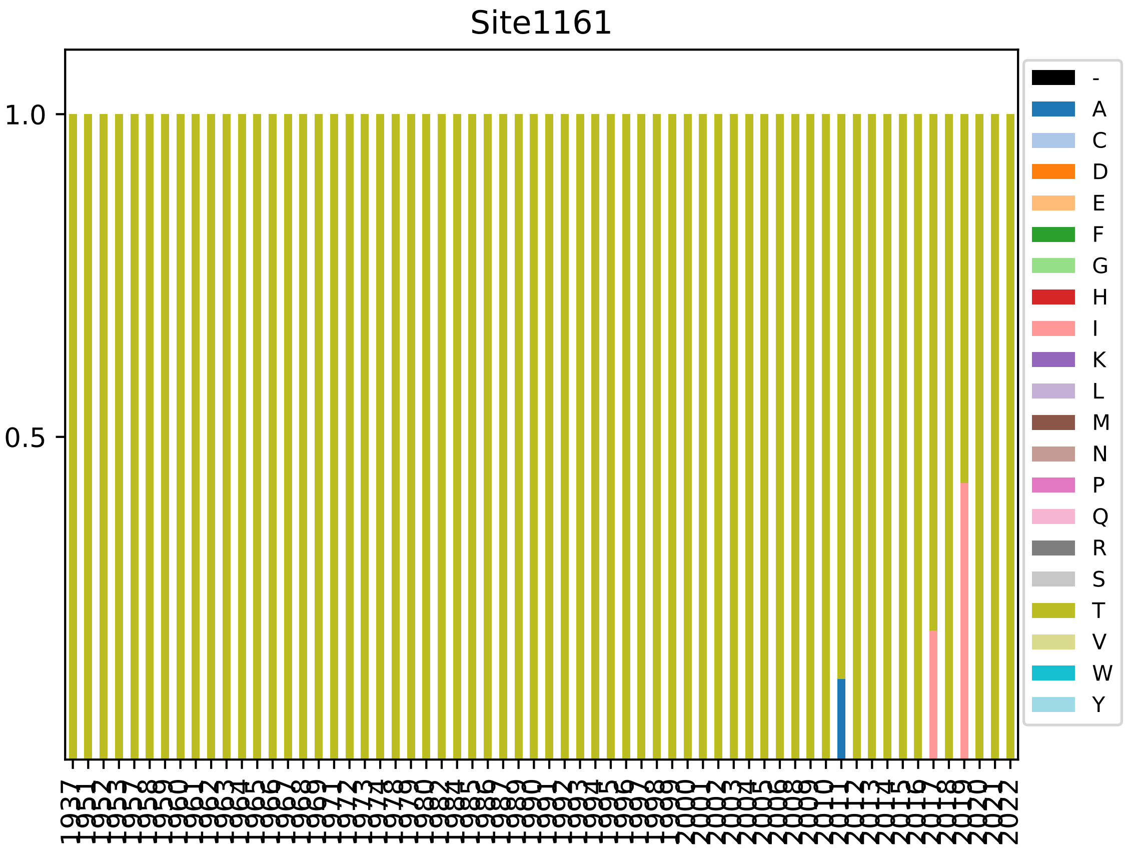
Site1161
1.0
0.5
-
A
C
D
E
F
G
H
I
K
L
M
N
P
Q
R
S
T
V
W
Y

Site1162

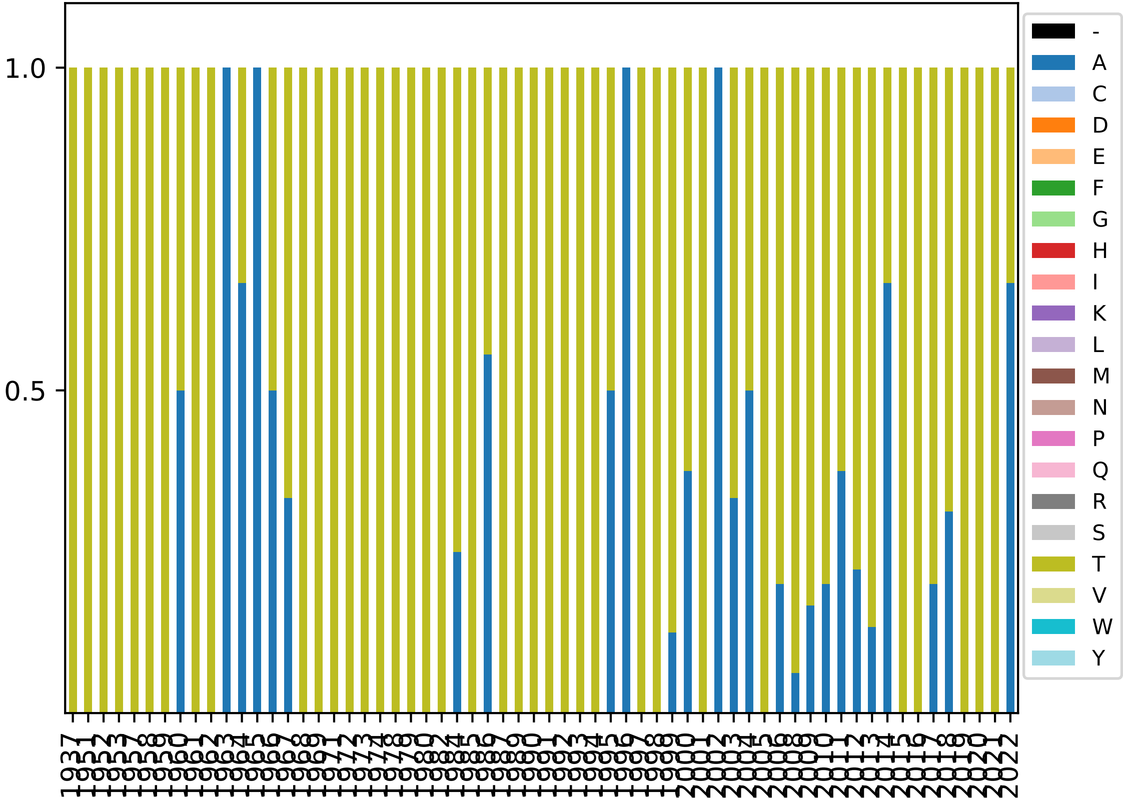

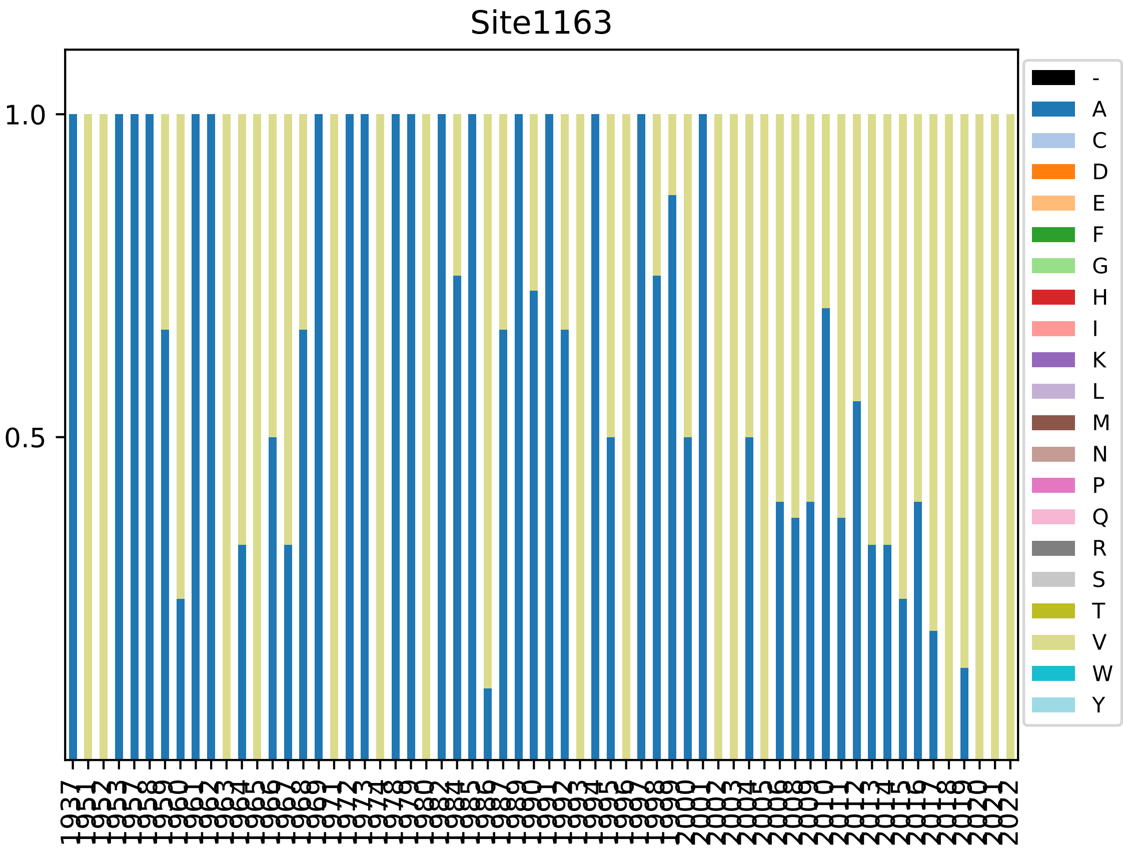
Site1163
1.0
0.5
-
A
C
D
E
F
G
H
I
K
L
M
N
P
Q
R
S
T
V
W
Y

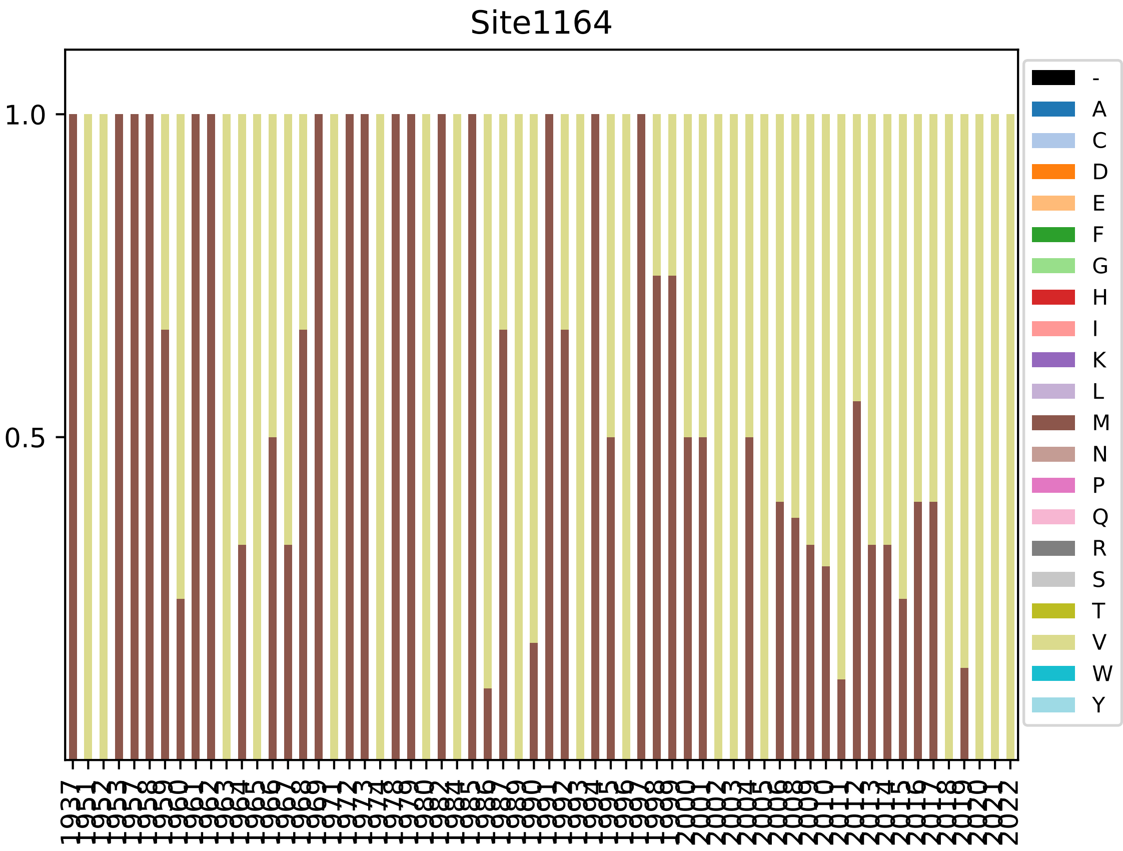
Site1164
1.0
0.5
-
A
C
D
E
F
G
H
I
K
L
M
N
P
Q
R
S
T
V
W
Y

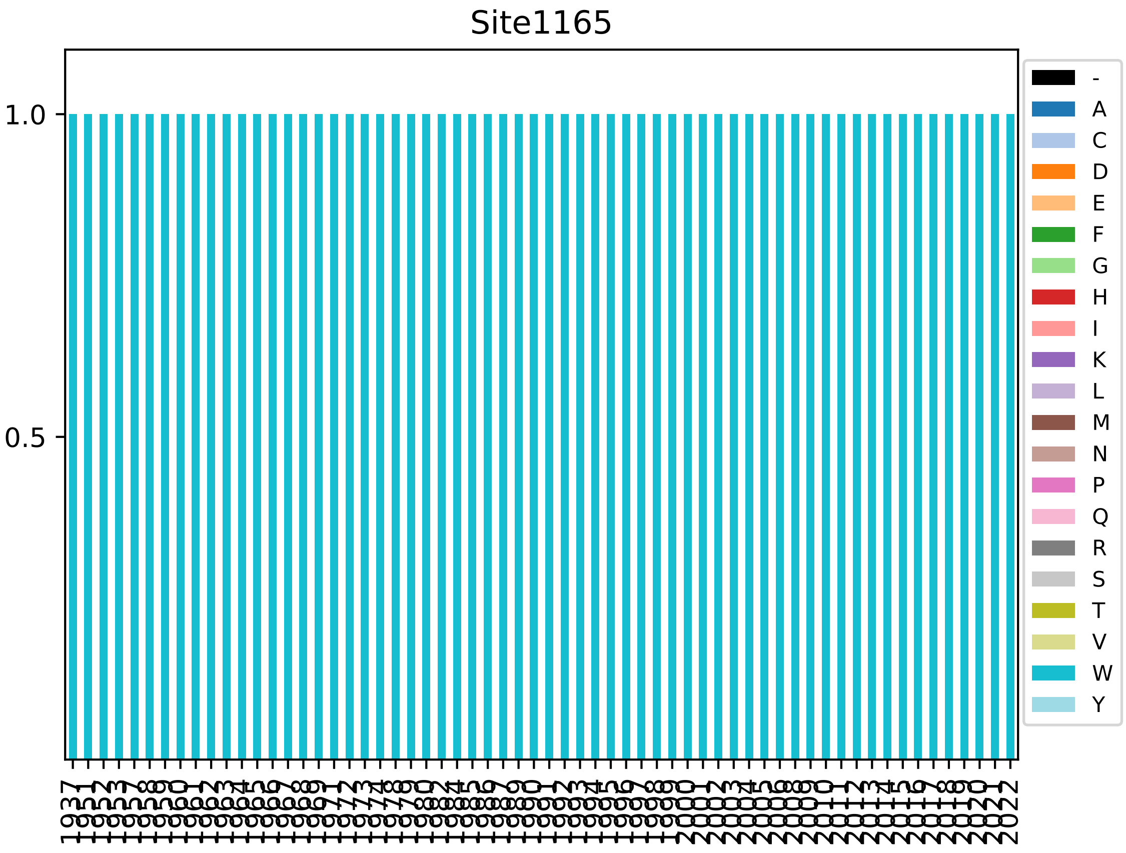
Site1165
1.0
0.5
-
A
C
D
E
F
G
H
I
K
L
M
N
P
Q
R
S
T
V
W
Y

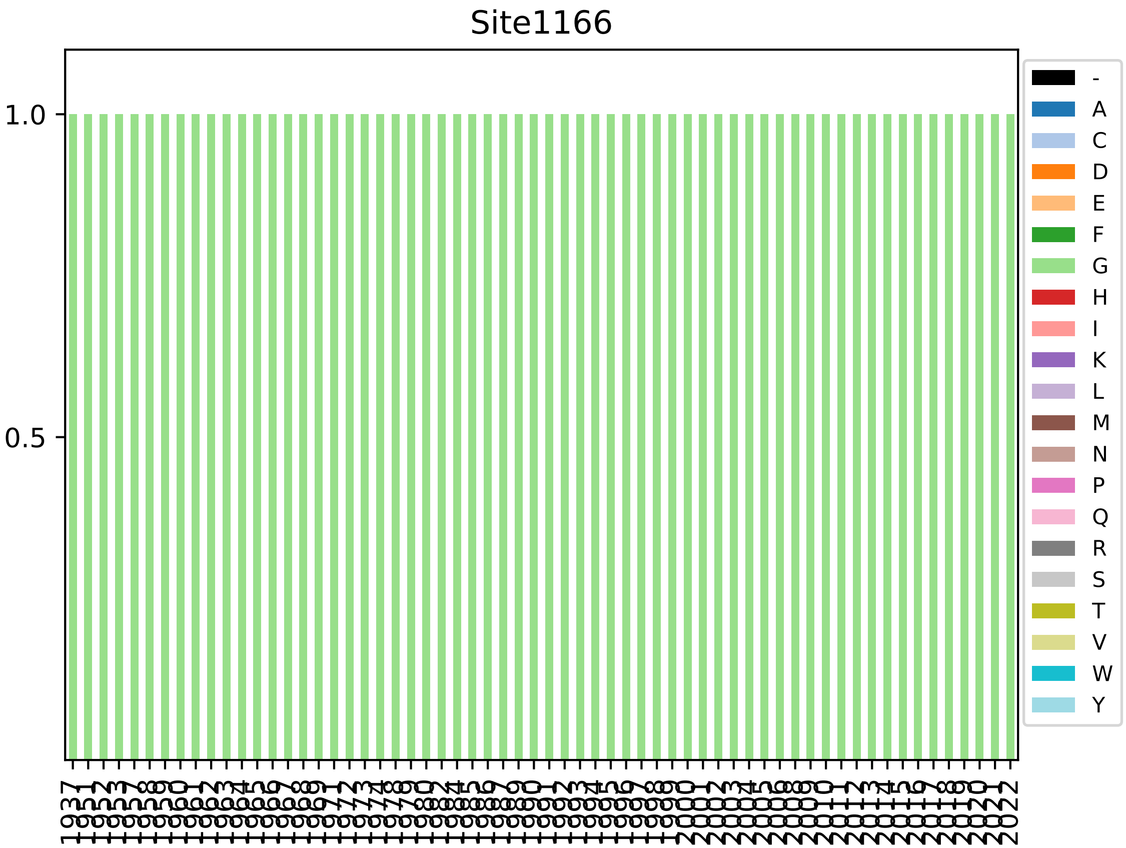
Site1166
1.0
0.5
-
A
C
D
E
F
G
H
I
K
L
M
N
P
Q
R
S
T
V
W
Y

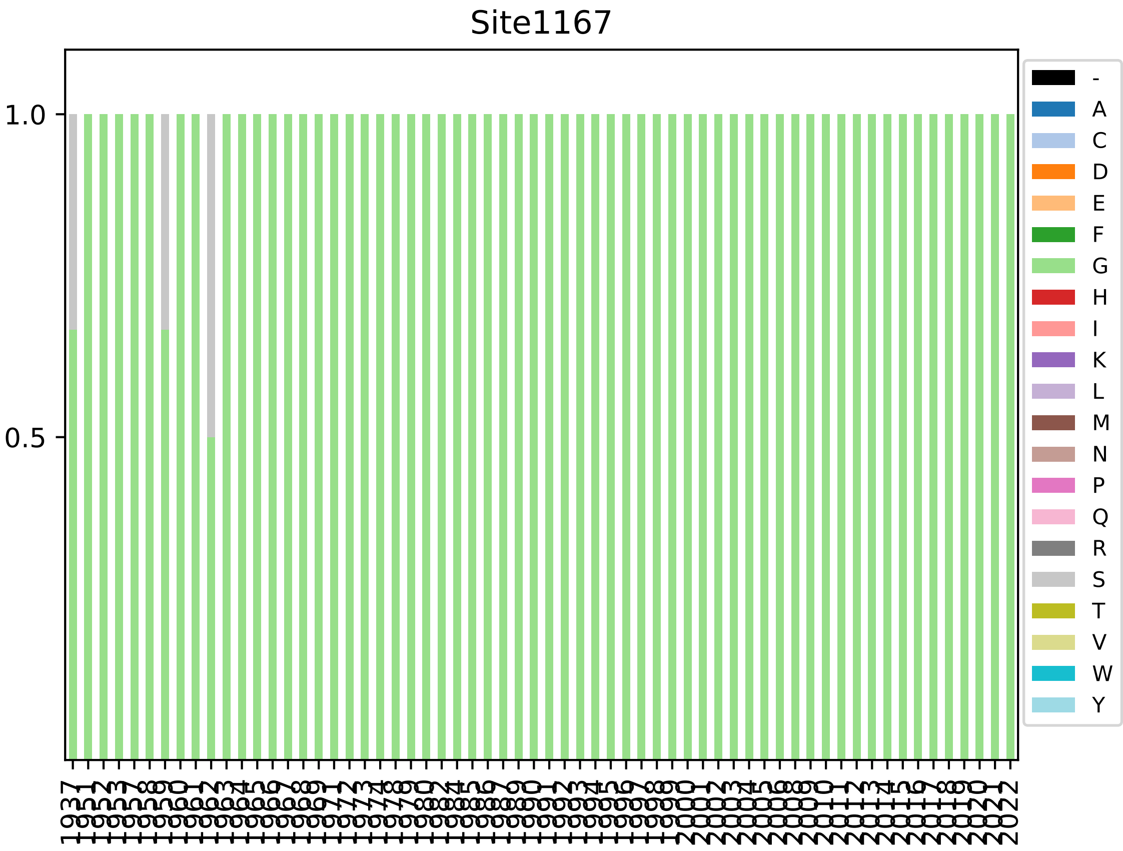
Site1167
1.0
0.5
-
A
C
D
E
F
G
H
I
K
L
M
N
P
Q
R
S
T
V
W
Y

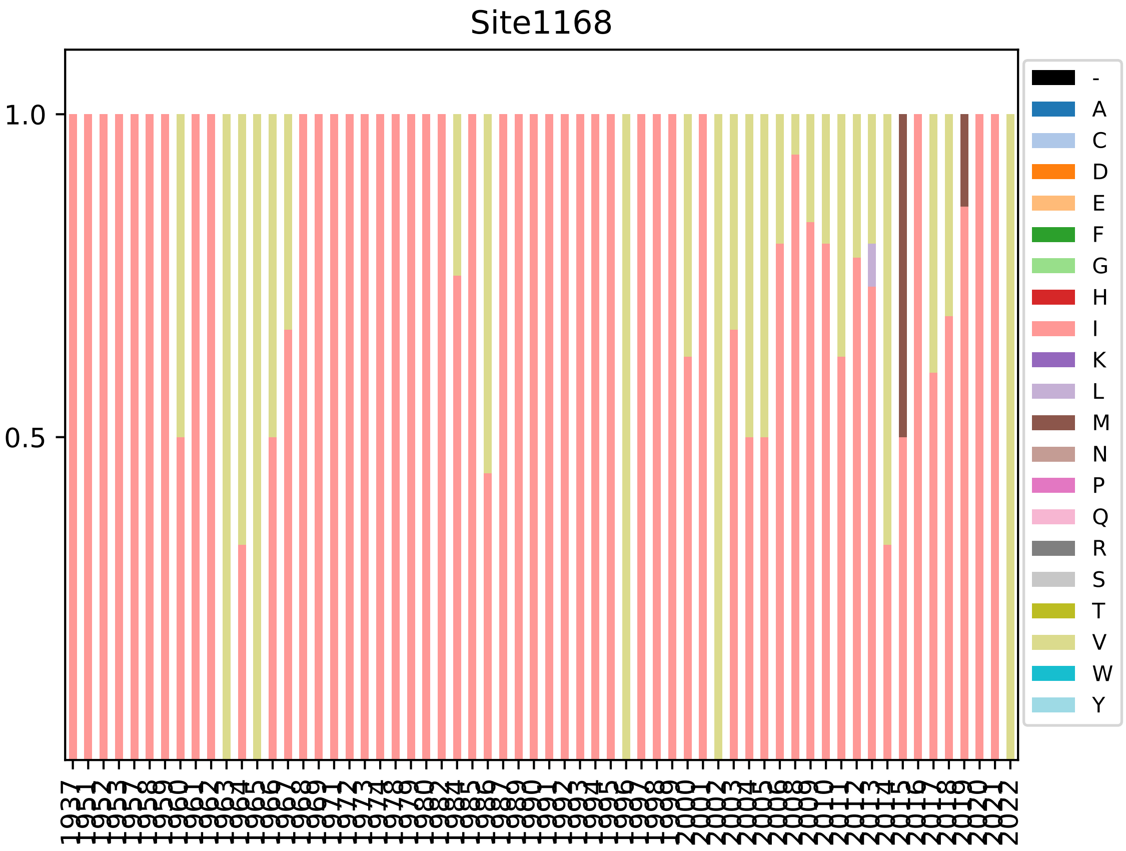
Site1168
1.0
0.5
-
A
C
D
E
F
G
H
I
K
L
M
N
P
Q
R
S
T
V
W
Y

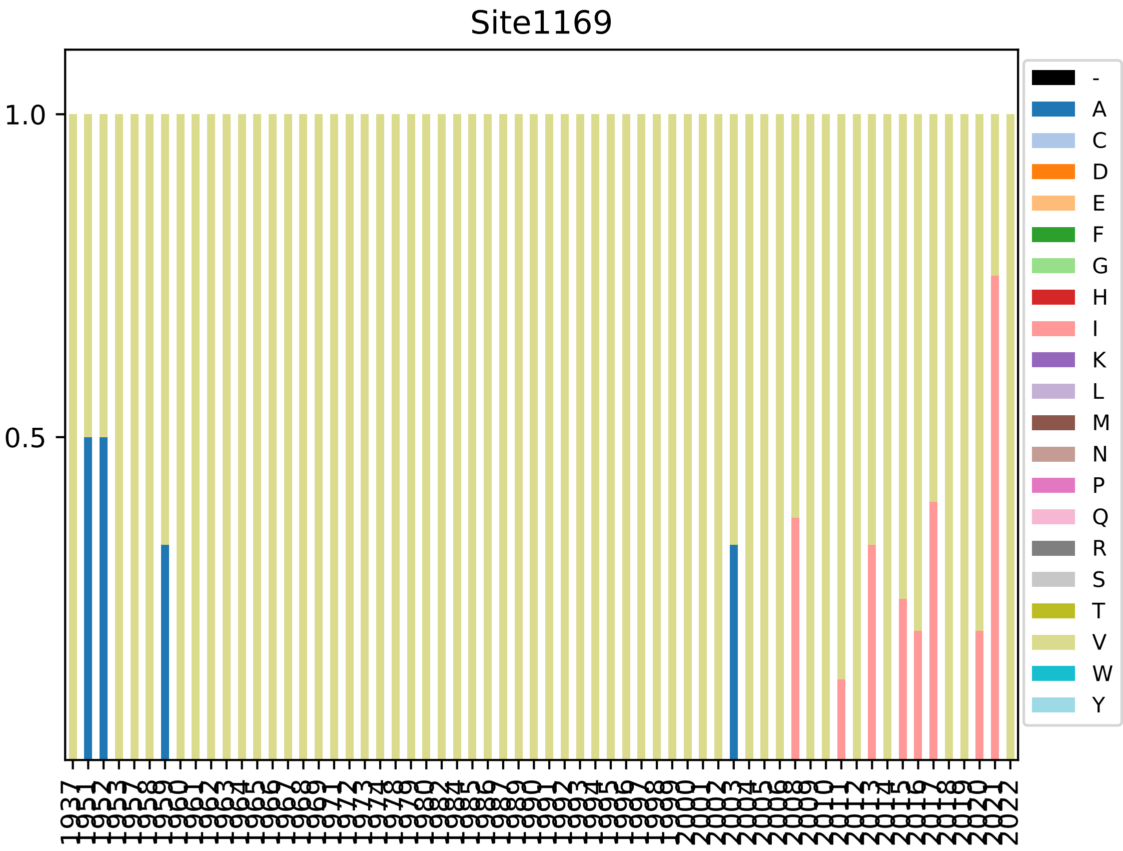
Site1169
1.0
0.5
-
A
C
D
E
F
G
H
I
K
L
M
N
P
Q
R
S
T
V
W
Y

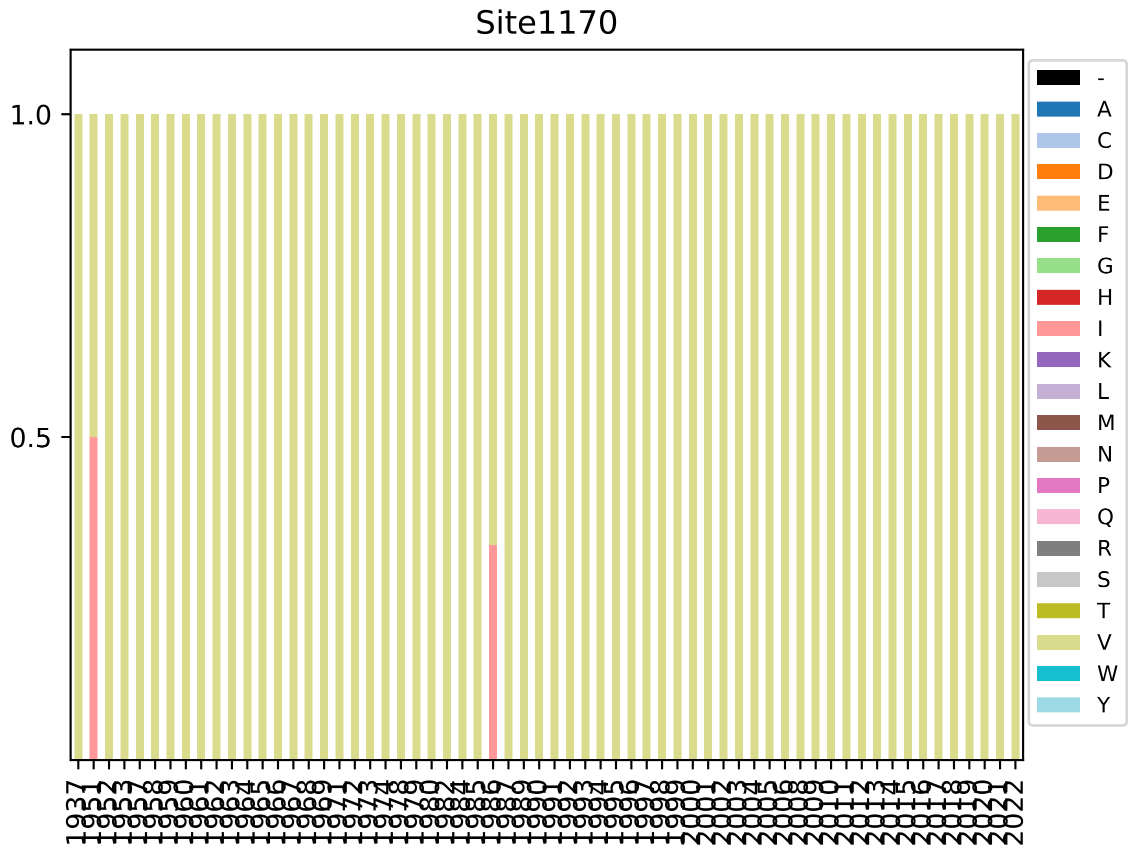
Site1170
1.0
0.5
-
A
C
D
E
F
G
H
I
K
L
M
N
P
Q
R
S
T
V
W
Y

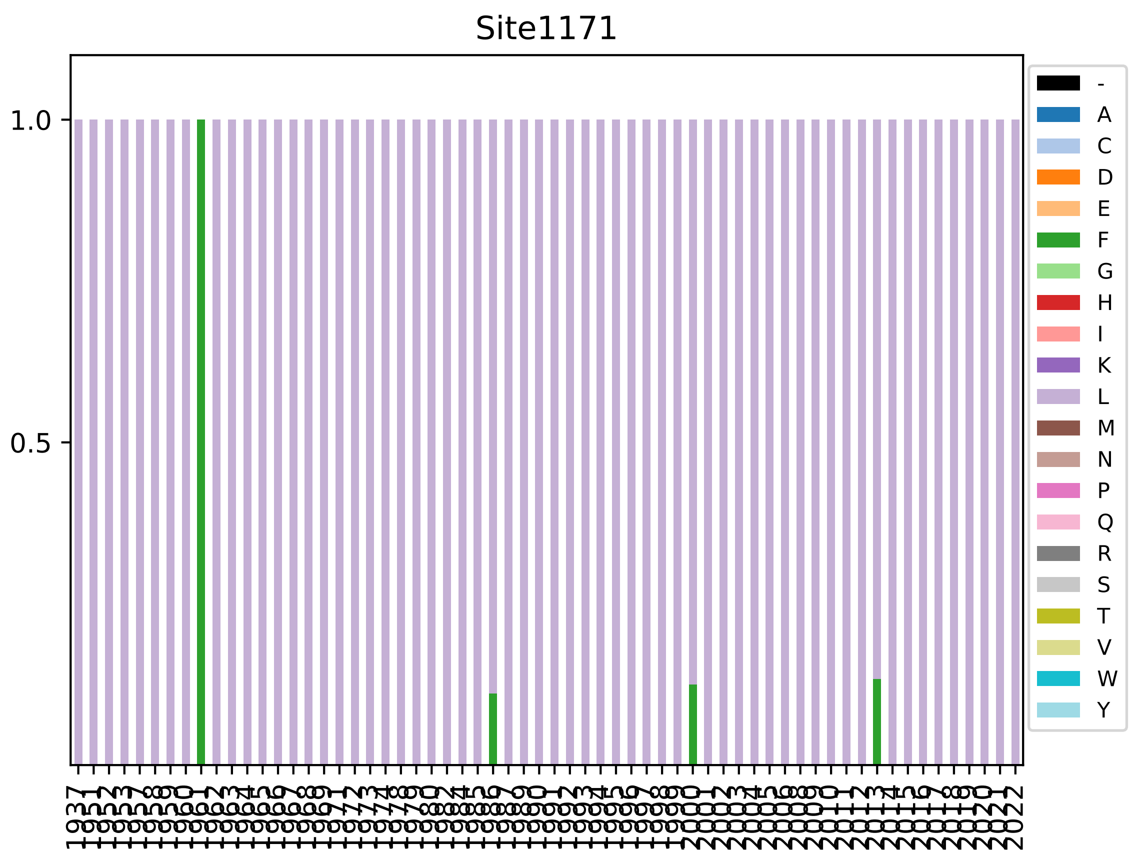
Site1171
1.0
0.5
-
A
C
D
E
F
G
H
I
K
L
M
N
P
Q
R
S
T
V
W
Y

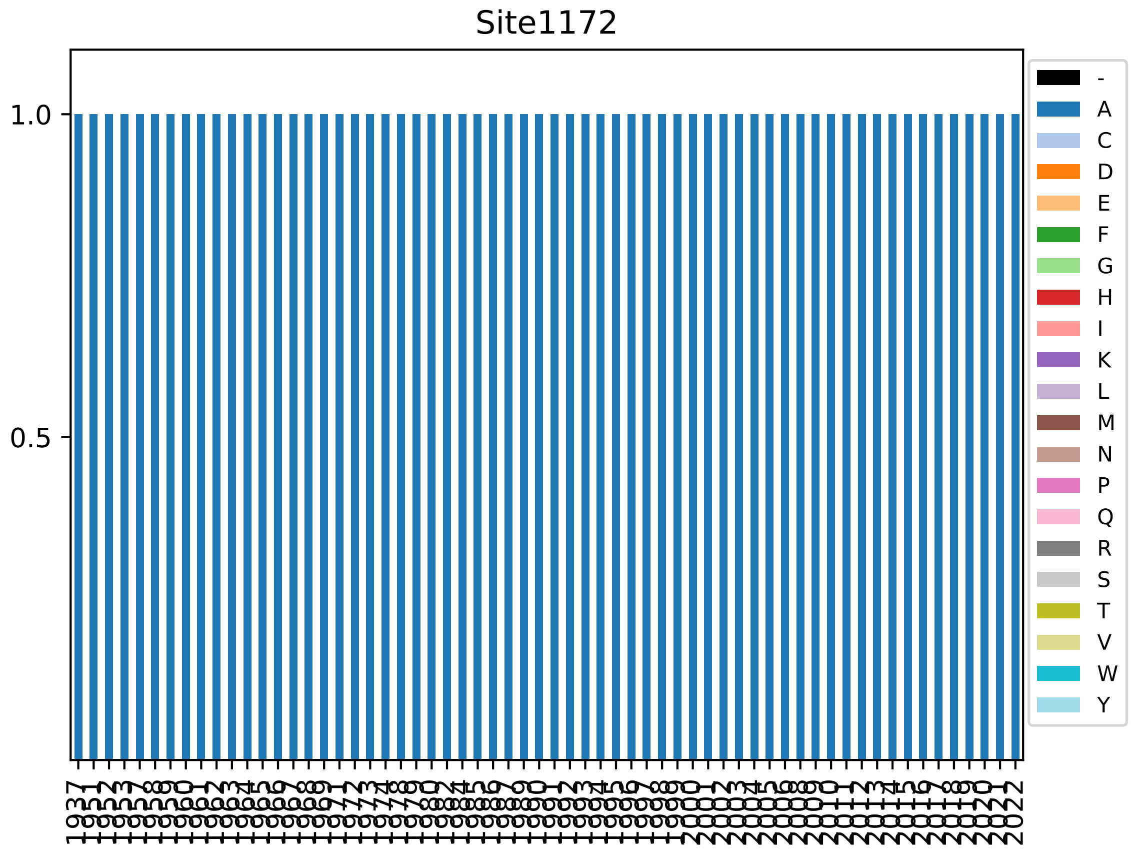
Site1172
1.0
0.5
-
A
C
D
E
F
G
H
I
K
L
M
N
P
Q
R
S
T
V
W
Y

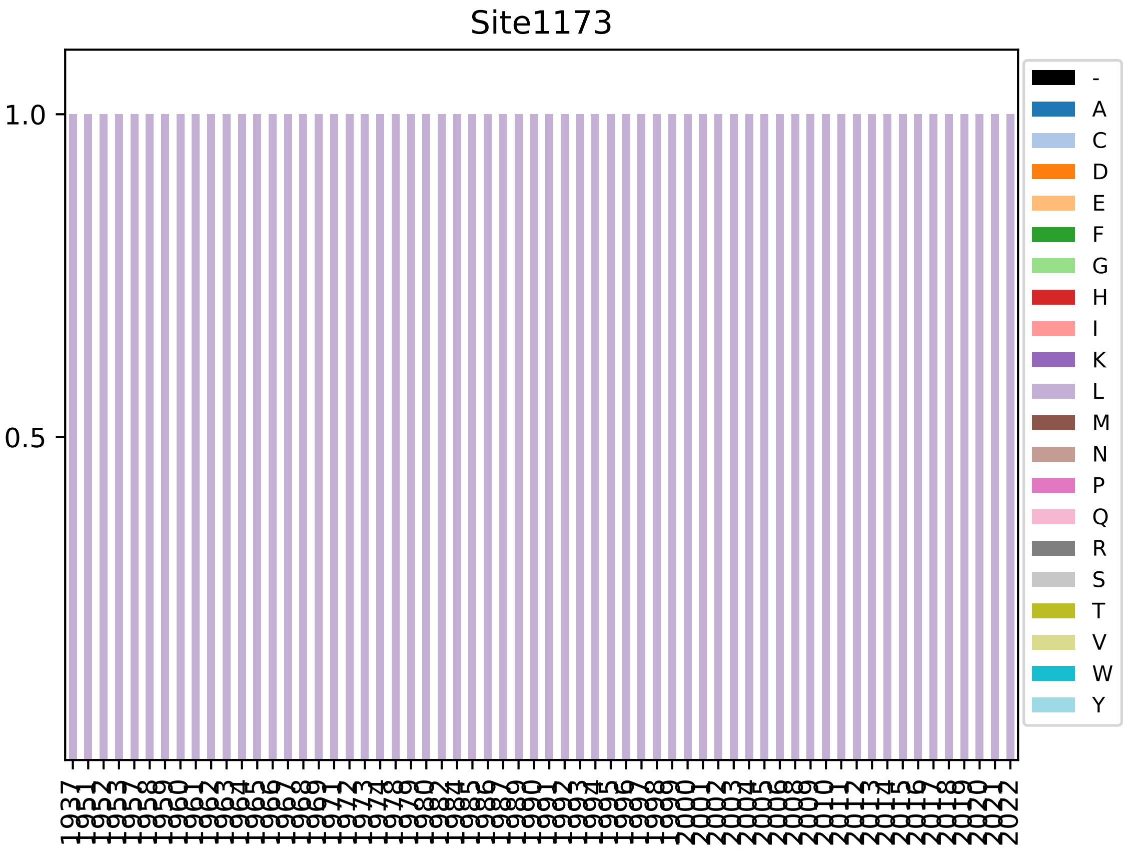
Site1173
1.0
0.5
-
A
C
D
E
F
G
H
I
K
L
M
N
P
Q
R
S
T
V
W
Y

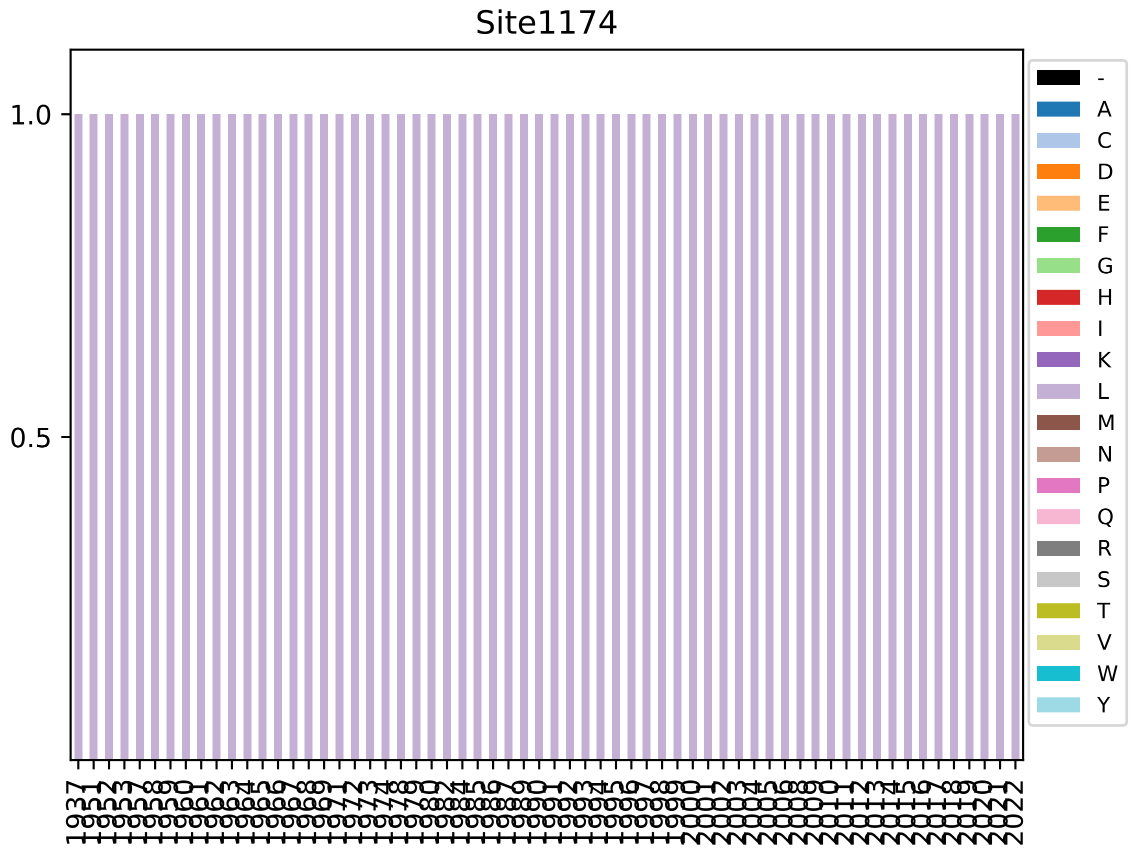
Site1174
1.0
0.5
-
A
C
D
E
F
G
H
I
K
L
M
N
P
Q
R
S
T
V
W
Y

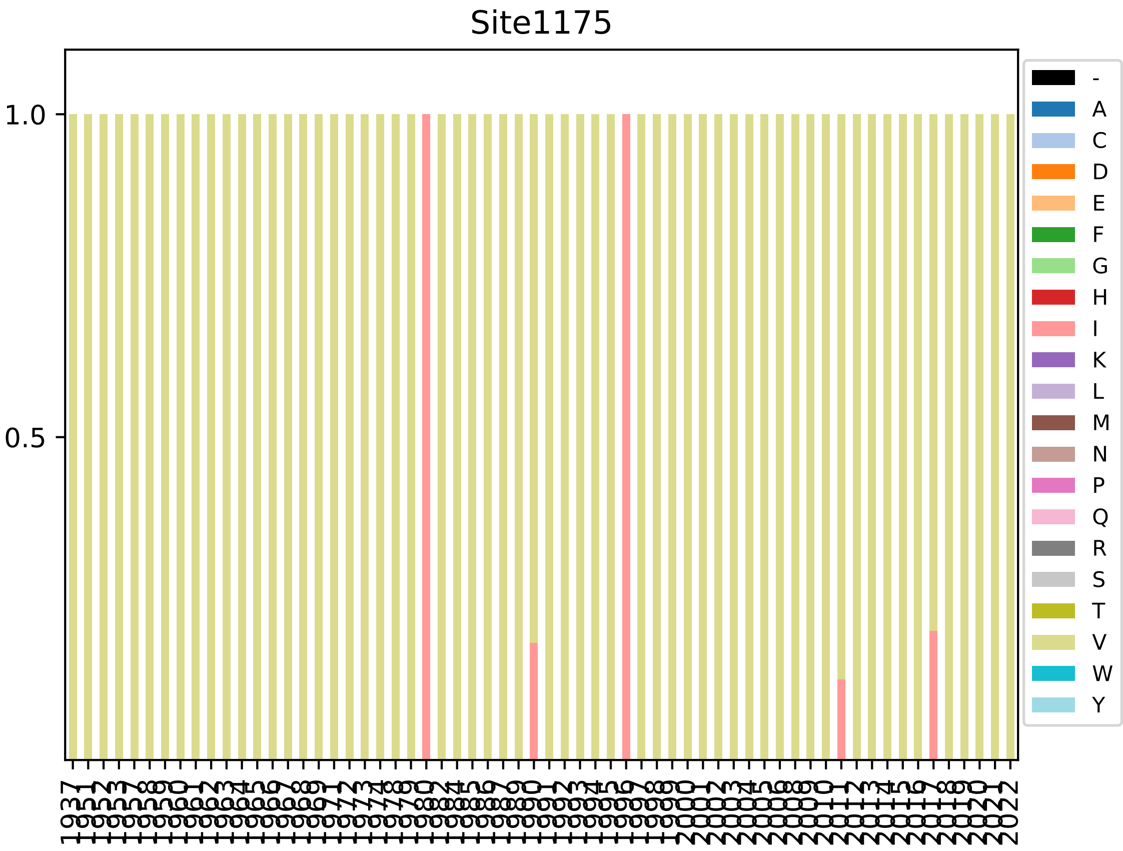
Site1175
1.0
0.5
-
A
C
D
E
F
G
H
I
K
L
M
N
P
Q
R
S
T
V
W
Y

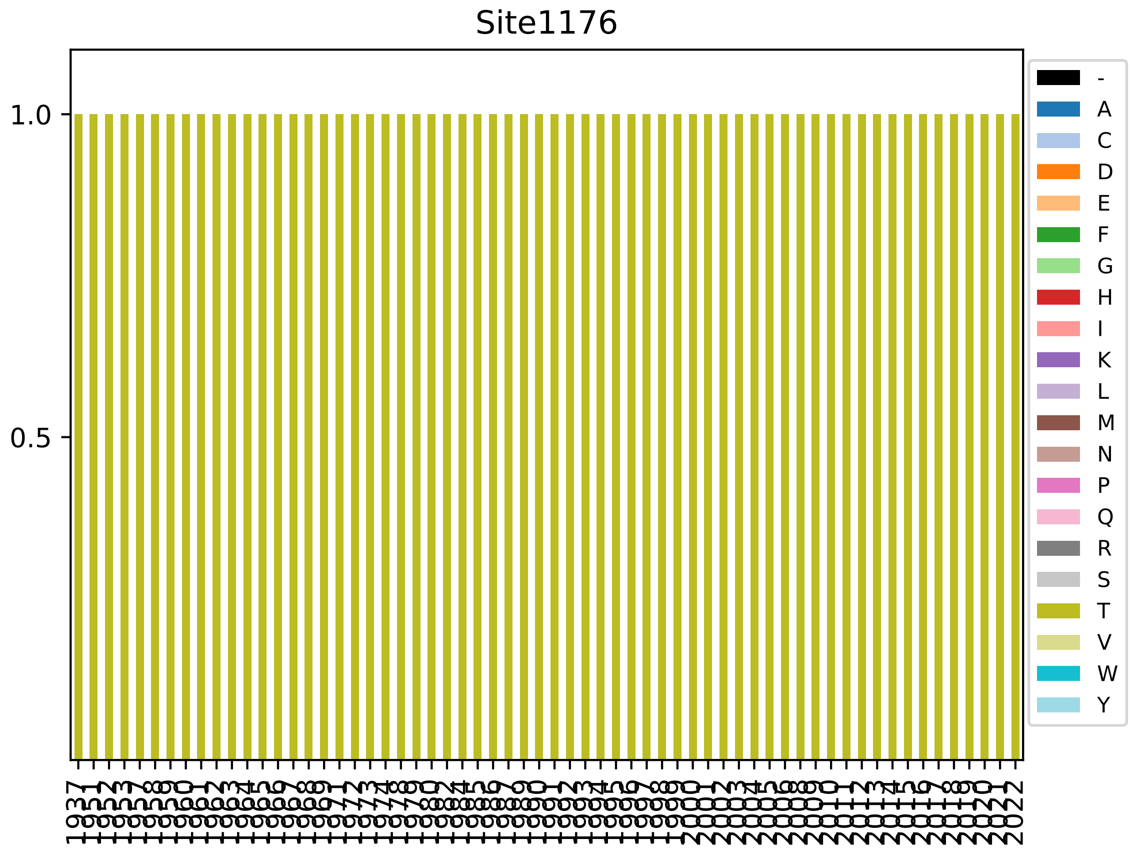
Site1176
1.0
0.5
-
A
C
D
E
F
G
H
I
K
L
M
N
P
Q
R
S
T
V
W
Y

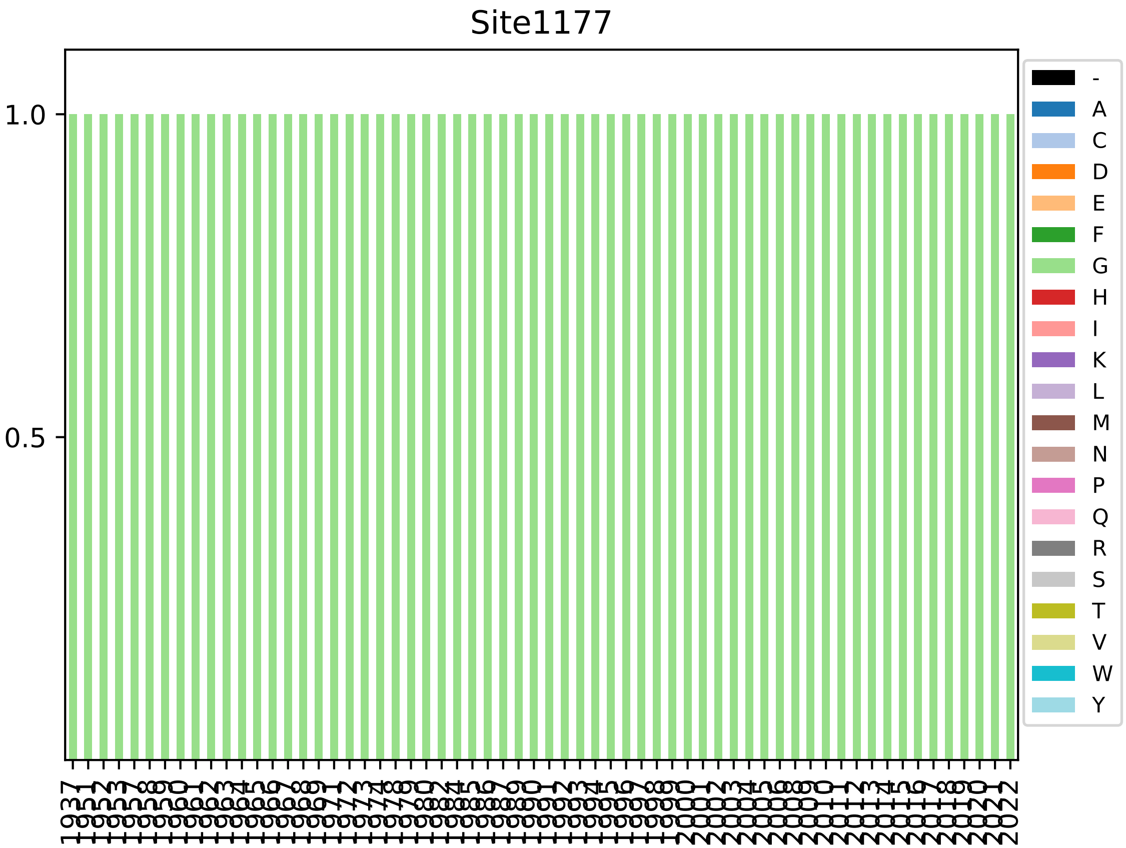
Site1177
1.0
0.5
-
A
C
D
E
F
G
H
I
K
L
M
N
P
Q
R
S
T
V
W
Y

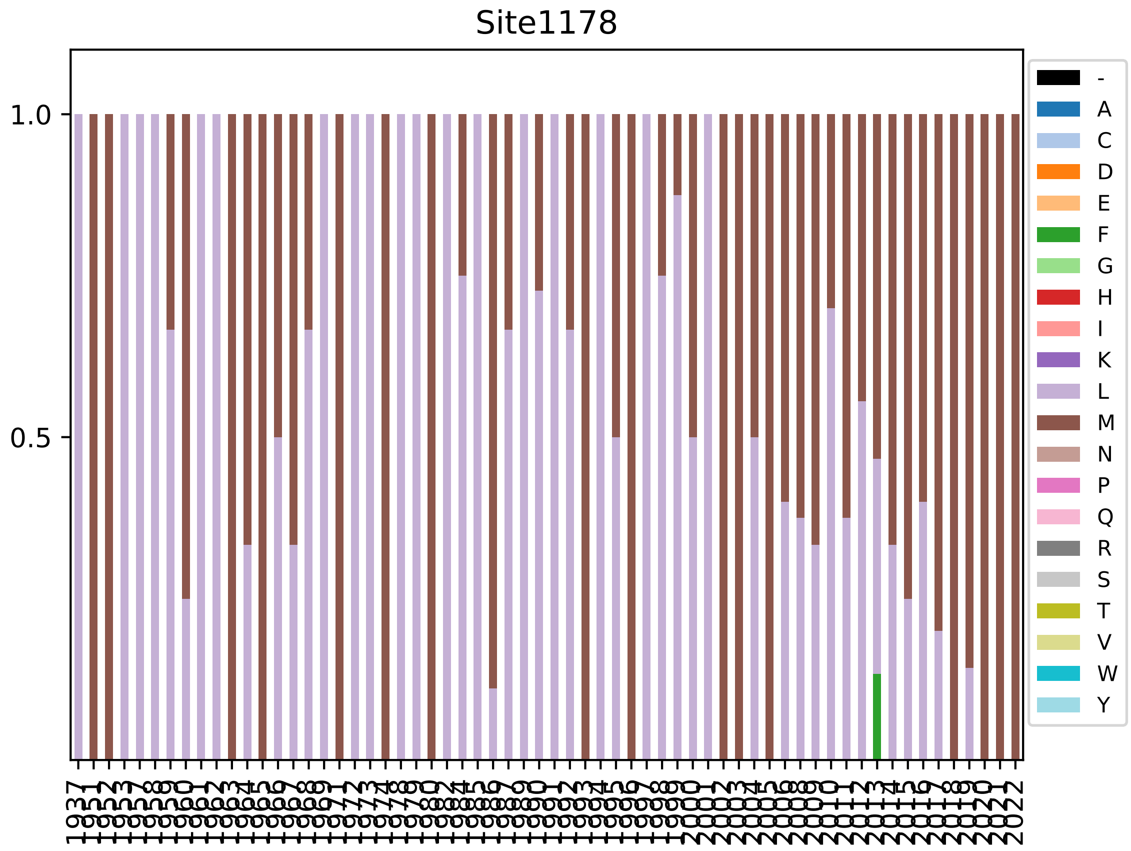
Site1178
1.0
0.5
-
A
C
D
E
F
G
H
I
K
L
M
N
P
Q
R
S
T
V
W
Y

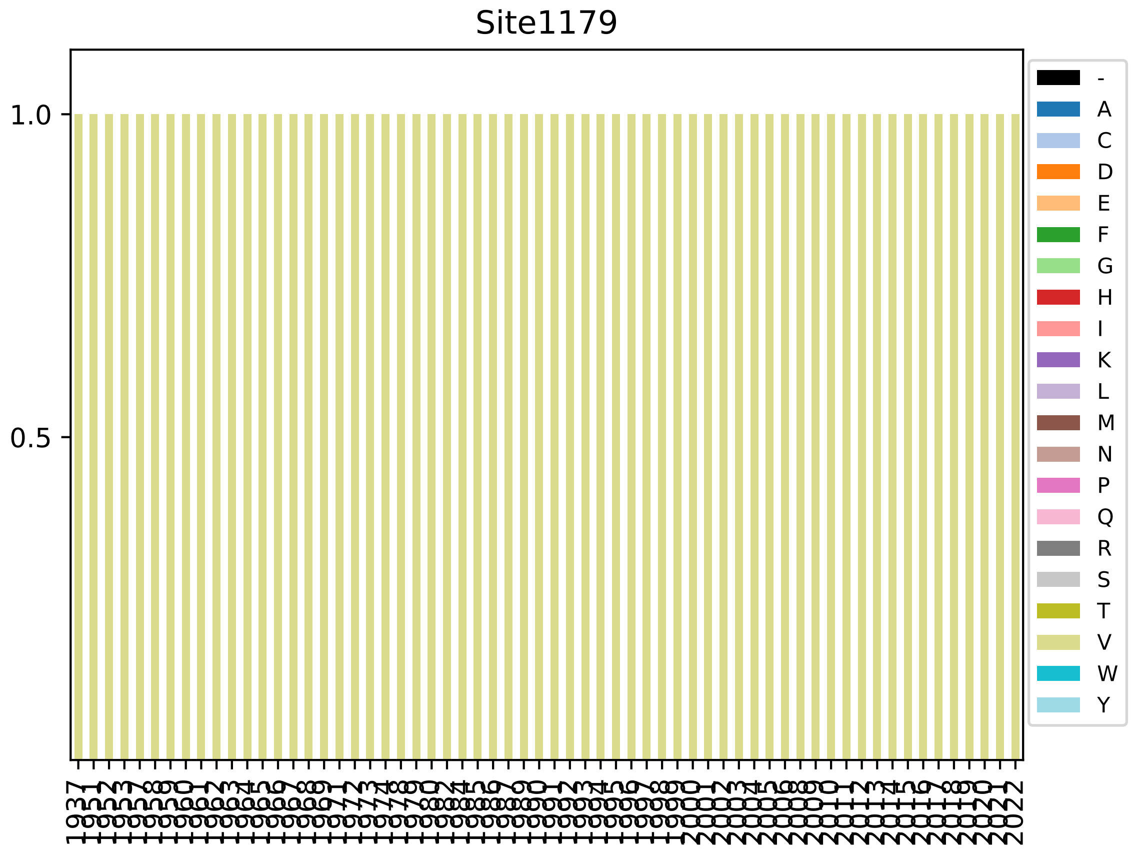

Site1179
1.0
0.5
-
A
C
D
E
F
G
H
I
K
L
M
N
P
Q
R
S
T
V
W
Y

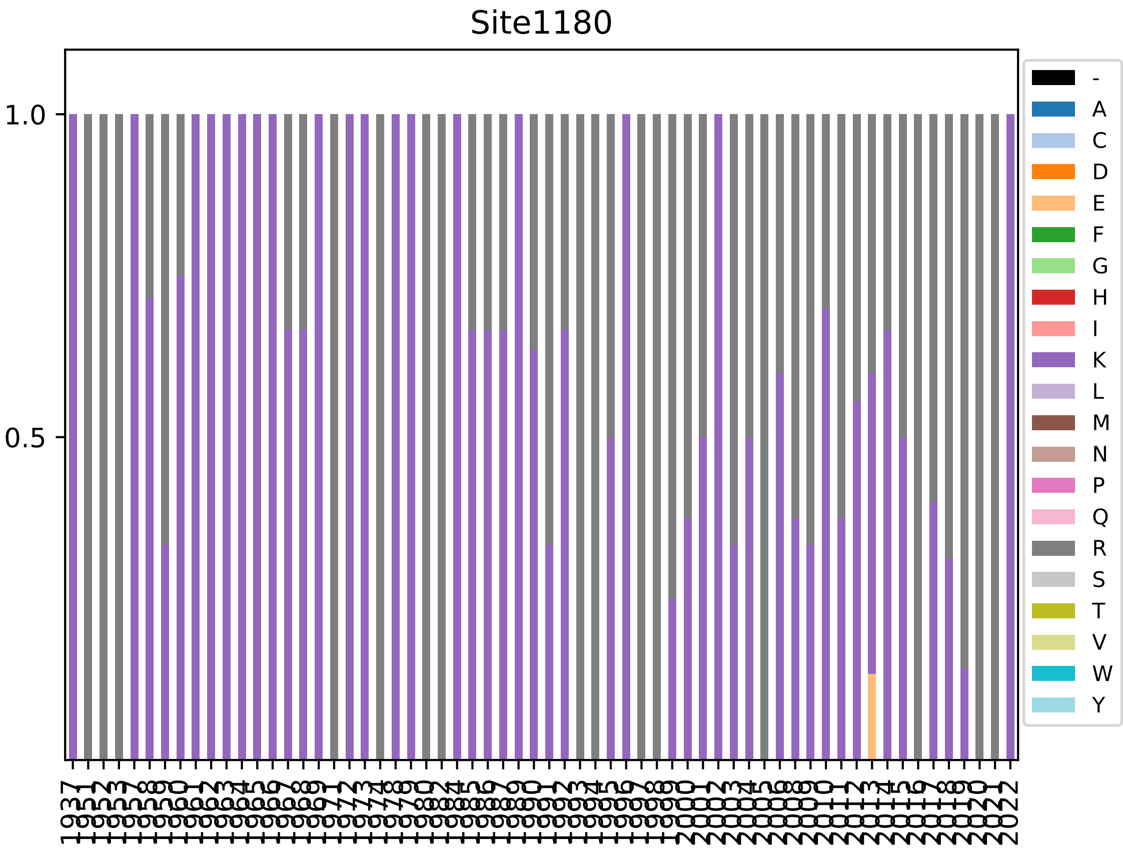
Site1180
1.0
0.5
-
A
C
D
E
F
G
H
I
K
L
M
N
P
Q
R
S
T
V
W
Y

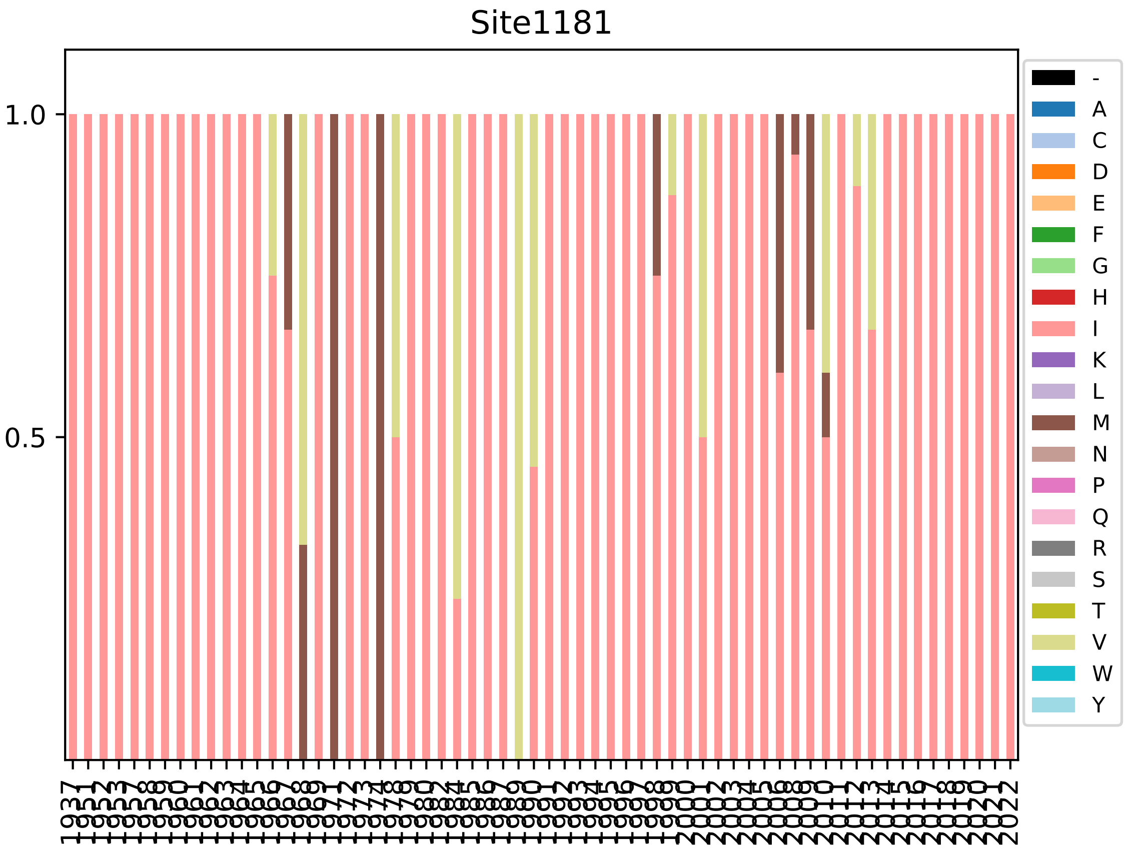
Site1181
1.0
0.5
-
A
C
D
E
F
G
H
I
K
L
M
N
P
Q
R
S
T
V
W
Y

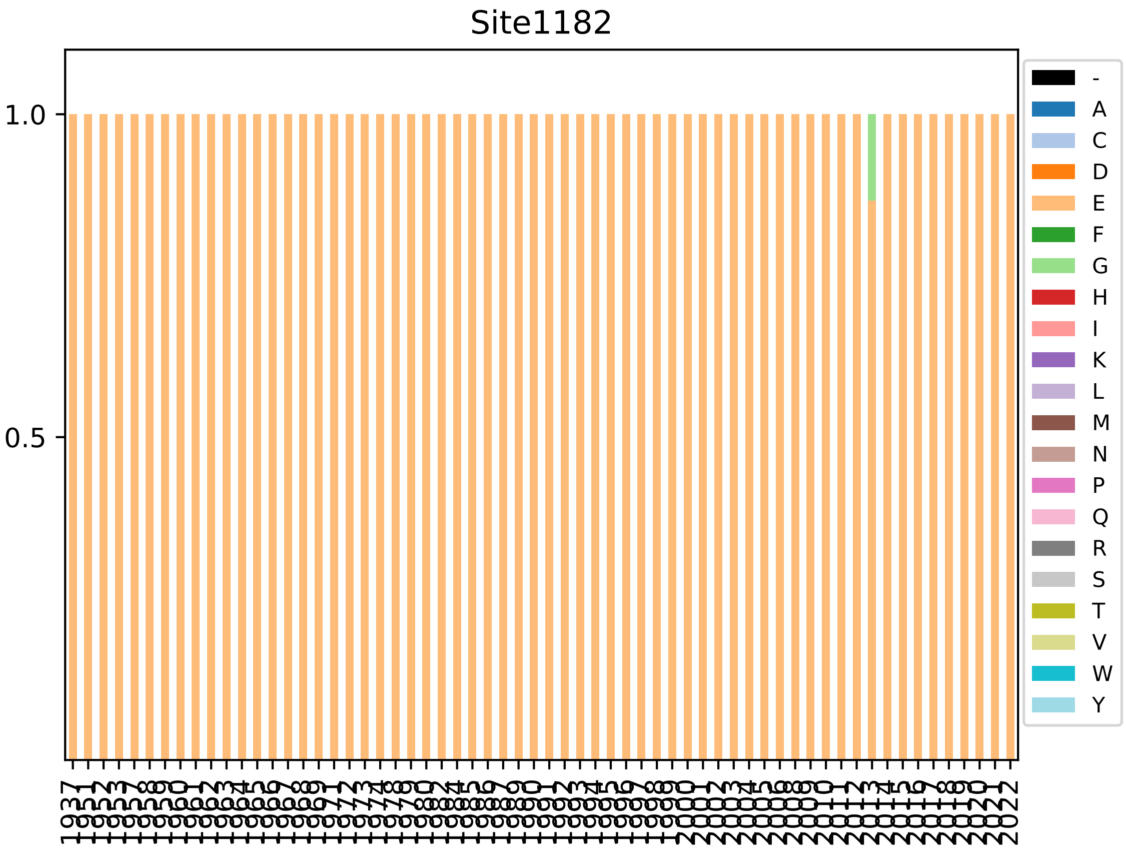

Site1182
1.0
0.5
-
A
C
D
E
F
G
H
I
K
L
M
N
P
Q
R
S
T
V
W
Y

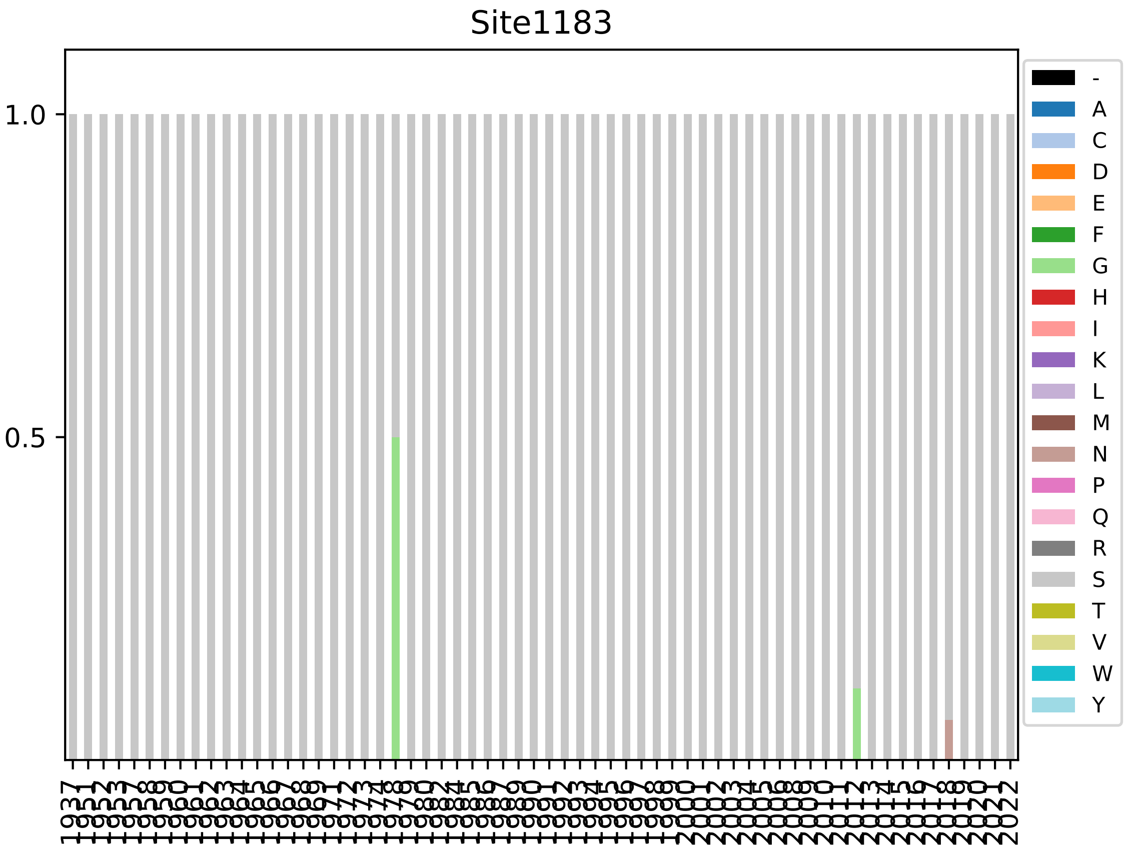

Site1183
1.0
0.5
-
A
C
D
E
F
G
H
I
K
L
M
N
P
Q
R
S
T
V
W
Y

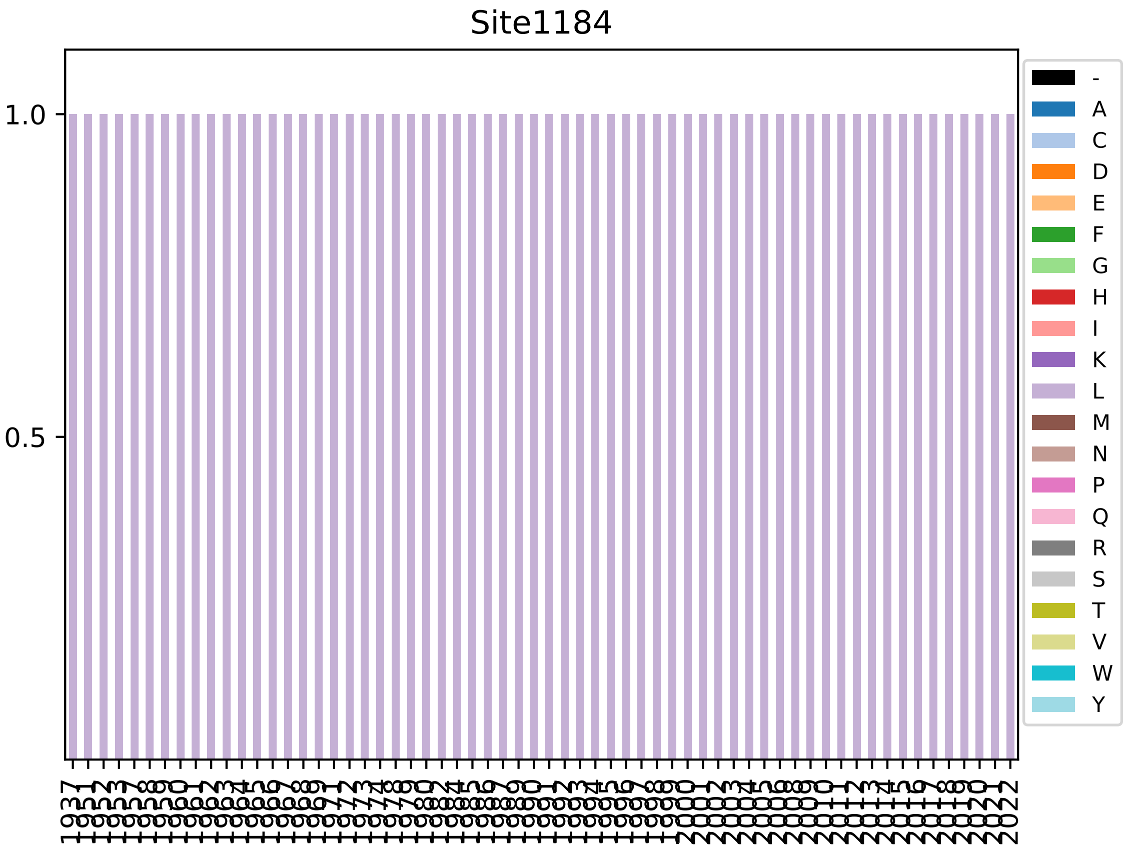
Site1184
1.0
0.5
-
A
C
D
E
F
G
H
I
K
L
M
N
P
Q
R
S
T
V
W
Y

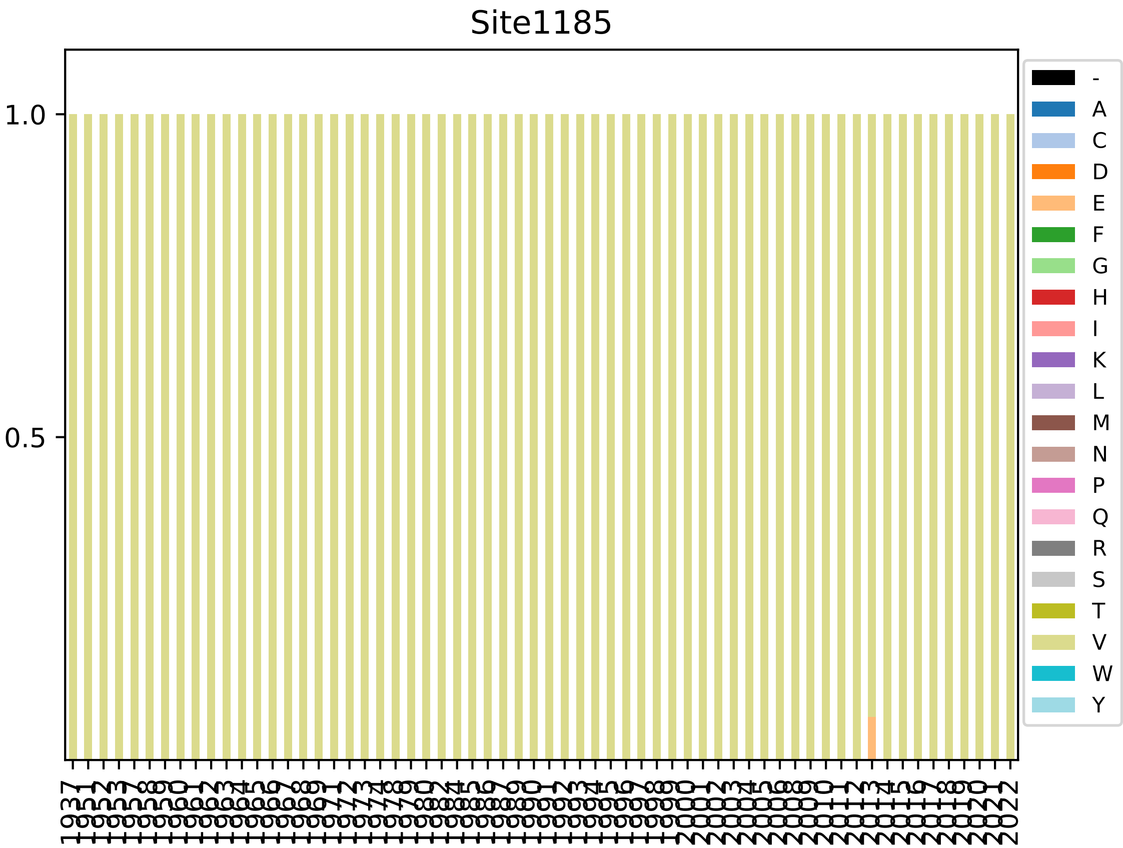
Site1185
1.0
0.5
-
A
C
D
E
F
G
H
I
K
L
M
N
P
Q
R
S
T
V
W
Y

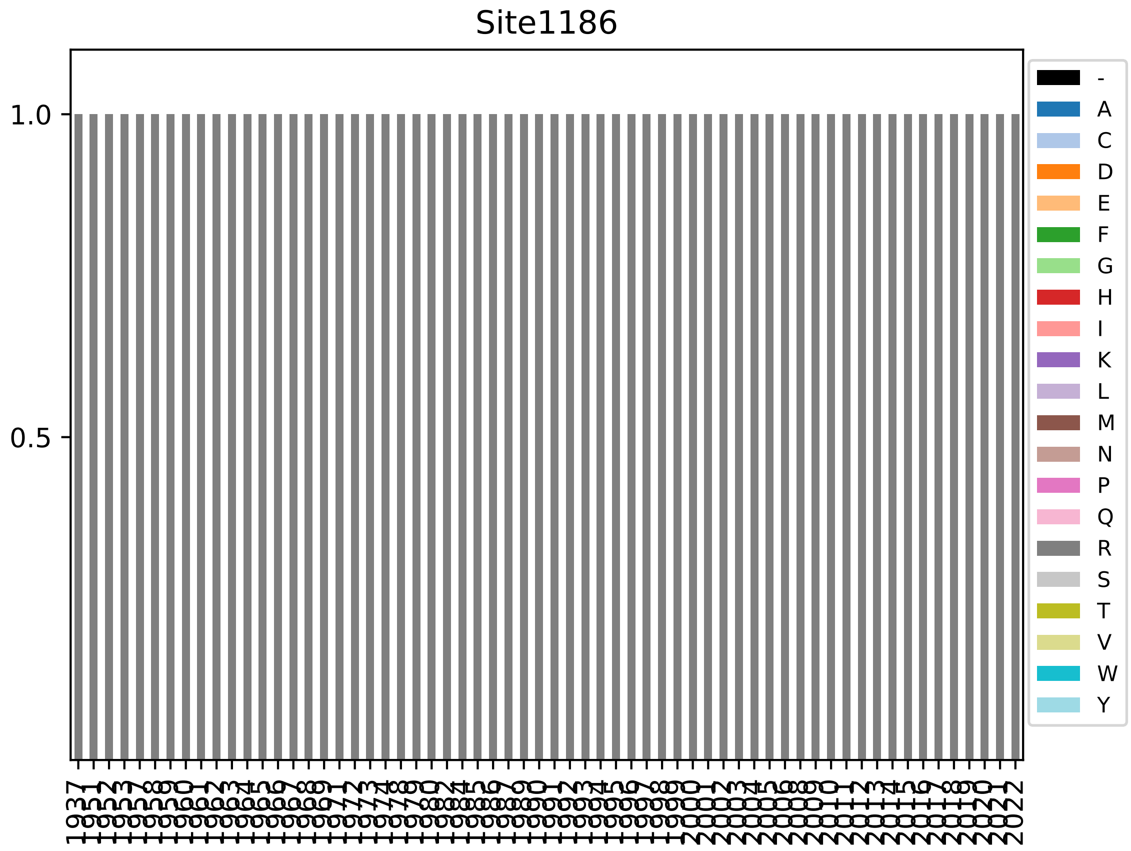
Site1186
1.0
0.5
-
A
C
D
E
F
G
H
I
K
L
M
N
P
Q
R
S
T
V
W
Y

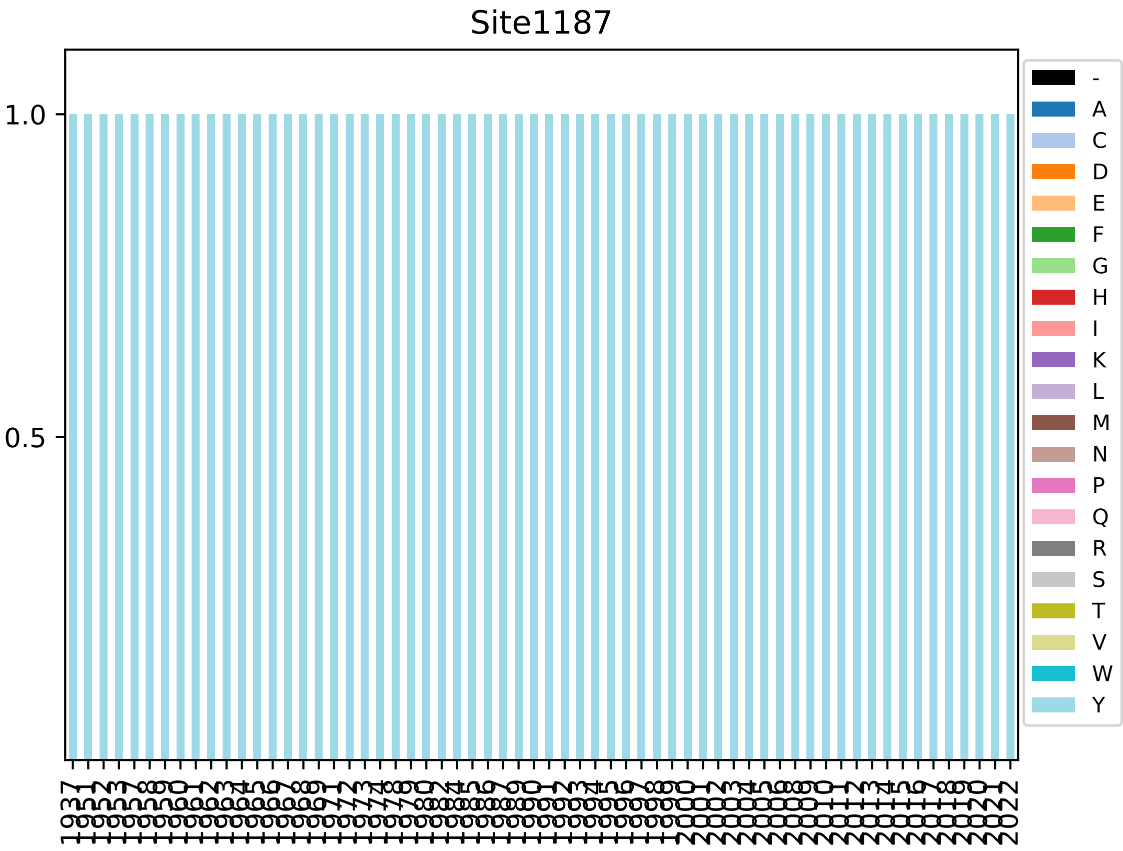
Site1187
1.0
0.5
-
A
C
D
E
F
G
H
I
K
L
M
N
P
Q
R
S
T
V
W
Y

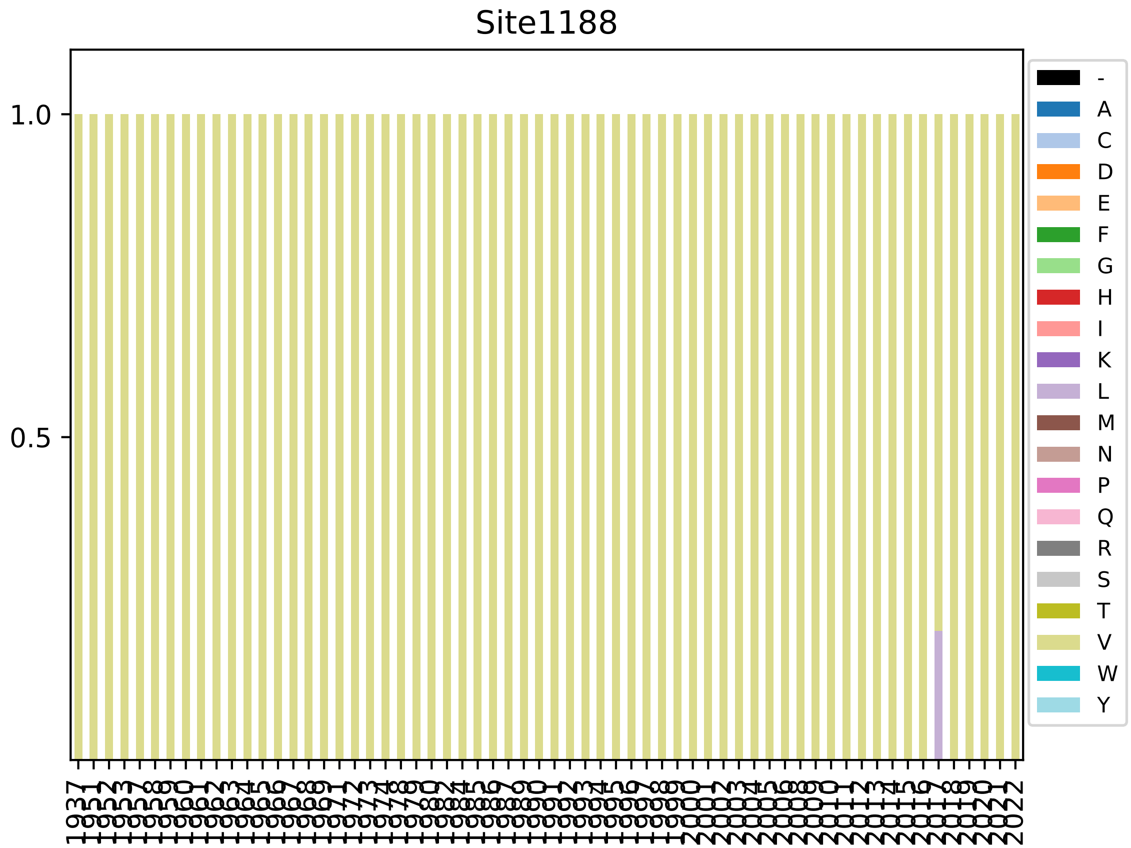
Site1188
1.0
0.5
-
A
C
D
E
F
G
H
I
K
L
M
N
P
Q
R
S
T
V
W
Y

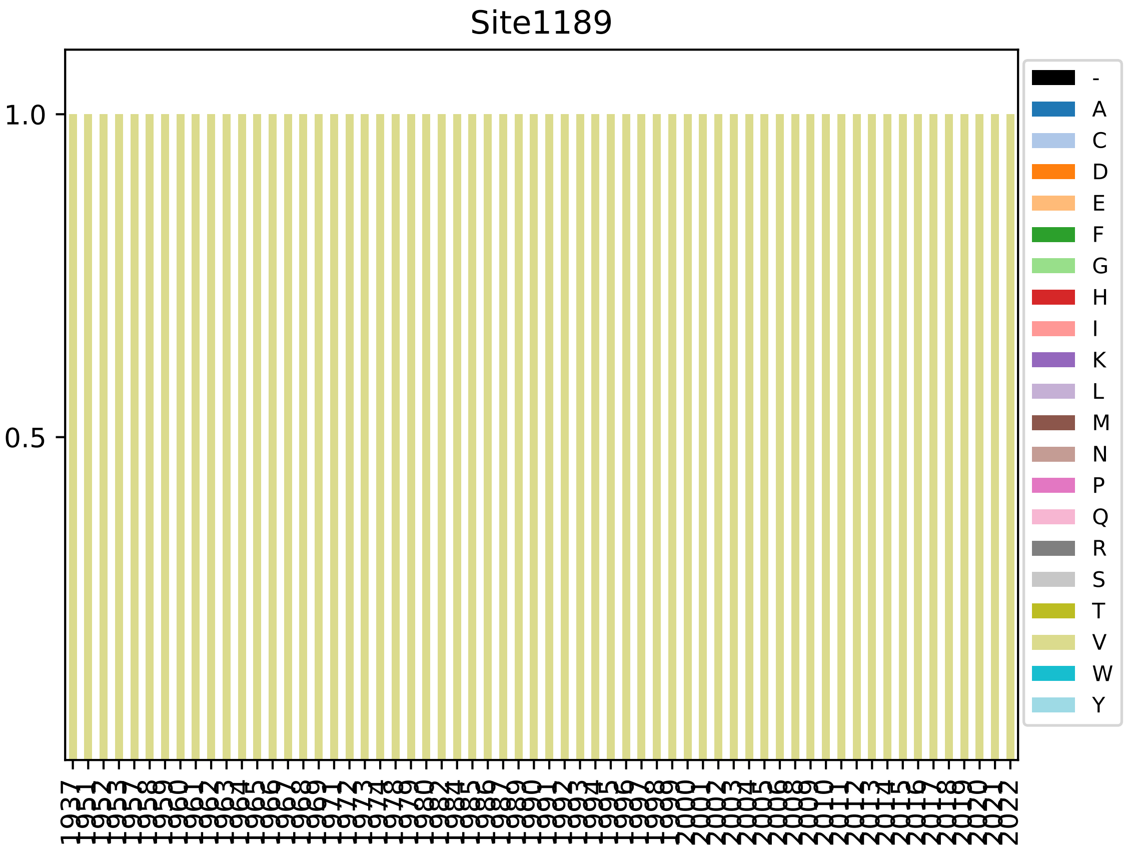
Site1189
1.0
0.5
-
A
C
D
E
F
G
H
I
K
L
M
N
P
Q
R
S
T
V
W
Y

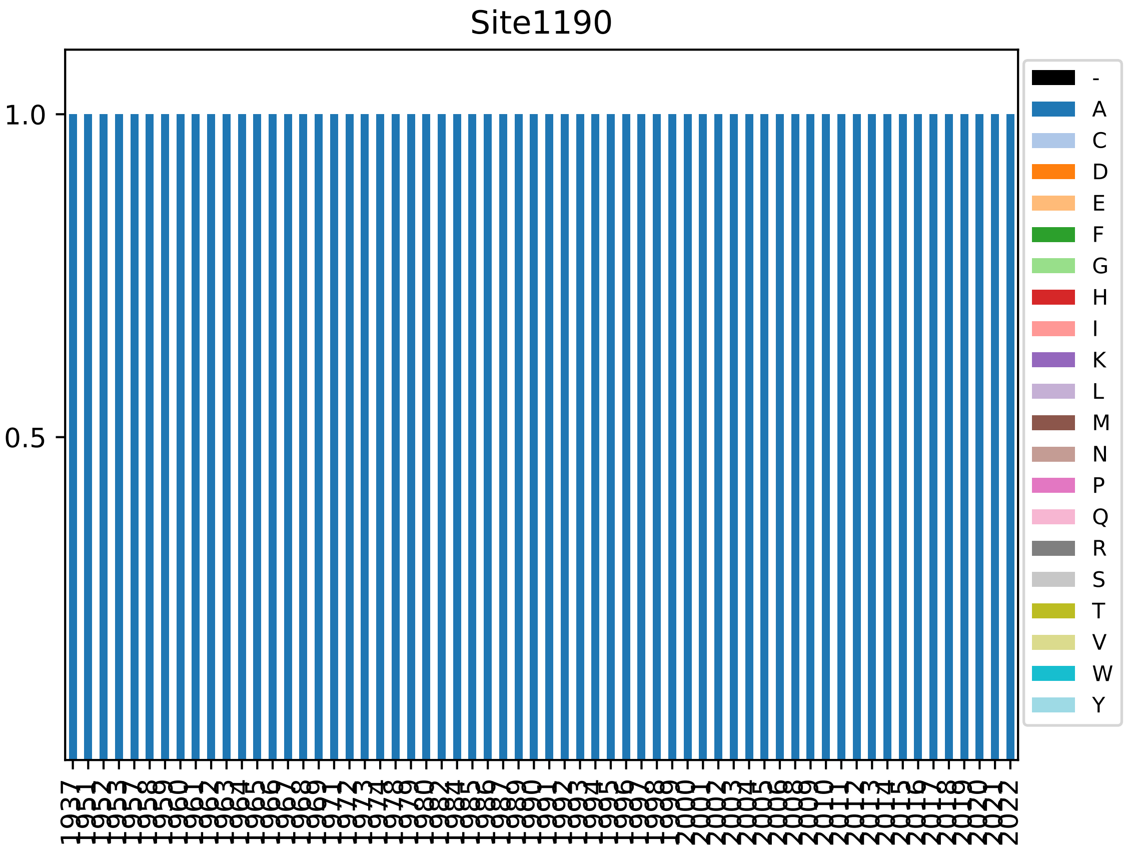
Site1190
1.0
0.5
-
A
C
D
E
F
G
H
I
K
L
M
N
P
Q
R
S
T
V
W
Y

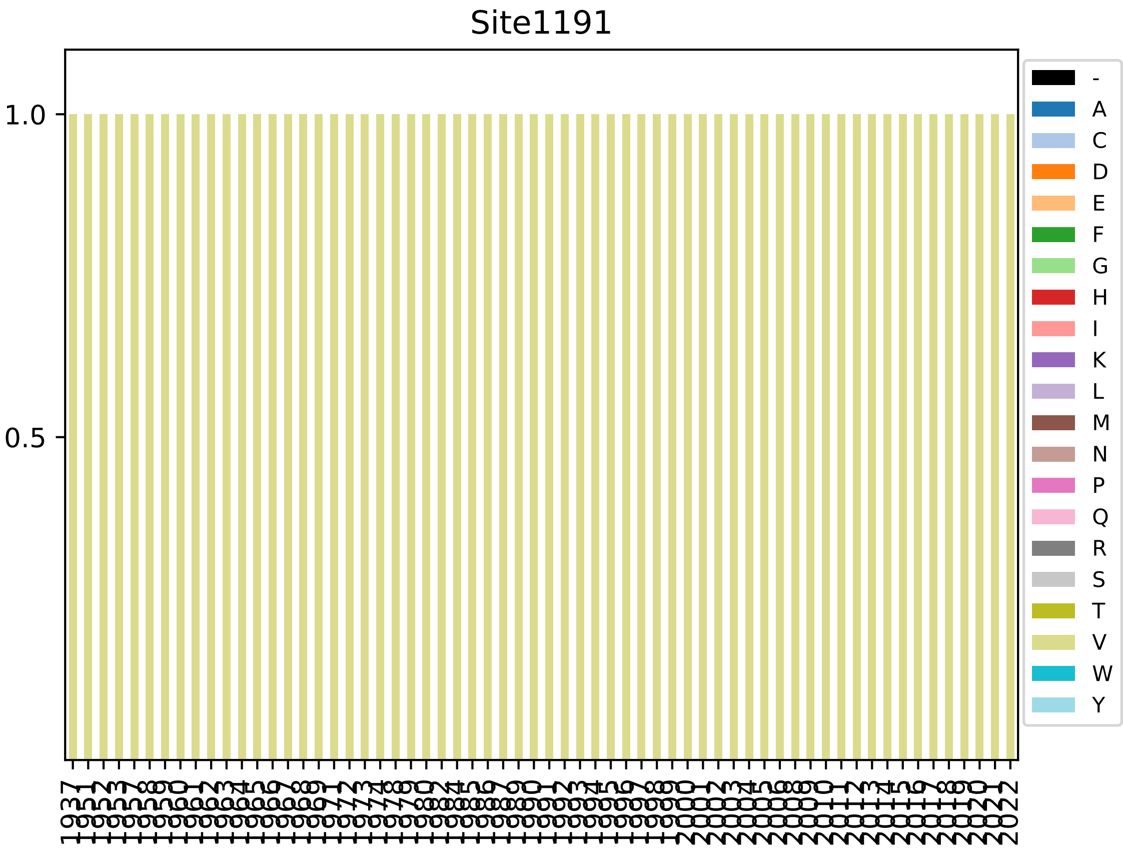
Site1191
1.0
0.5
-
A
C
D
E
F
G
H
I
K
L
M
N
P
Q
R
S
T
V
W
Y

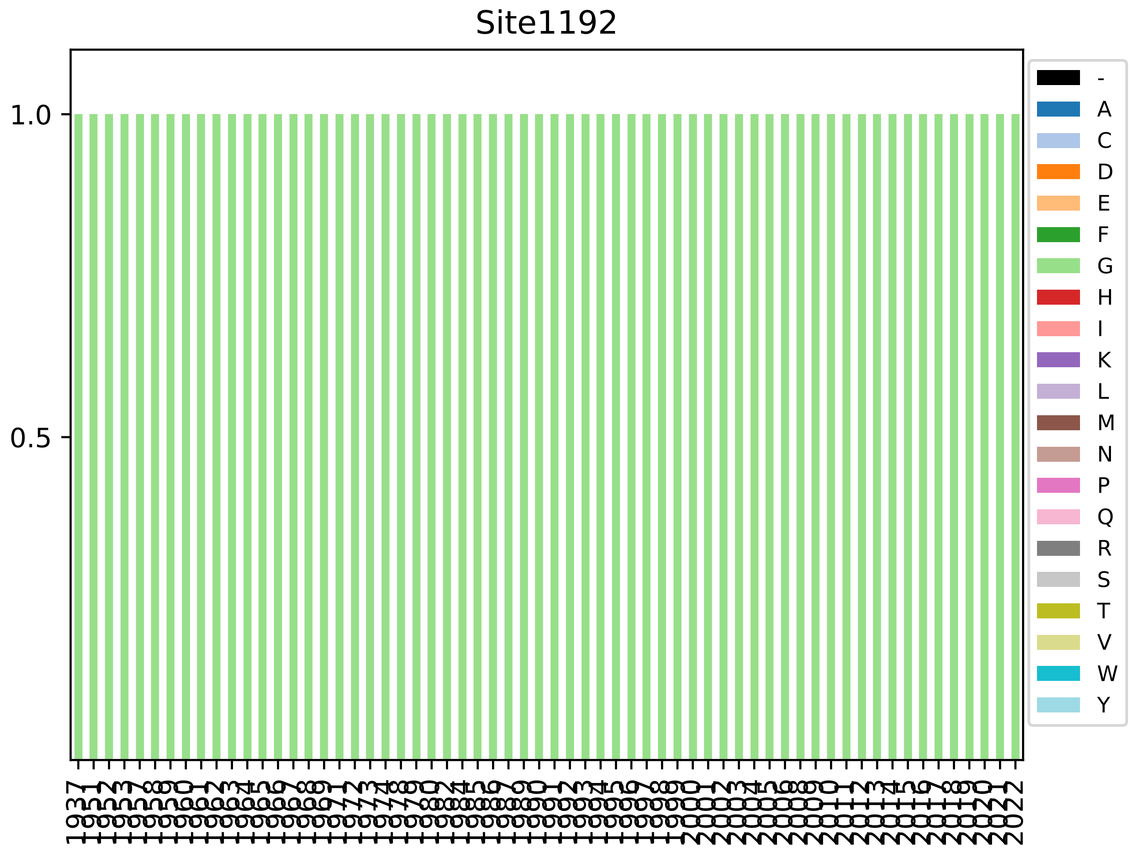
Site1192
1.0
0.5
-
A
C
D
E
F
G
H
I
K
L
M
N
P
Q
R
S
T
V
W
Y

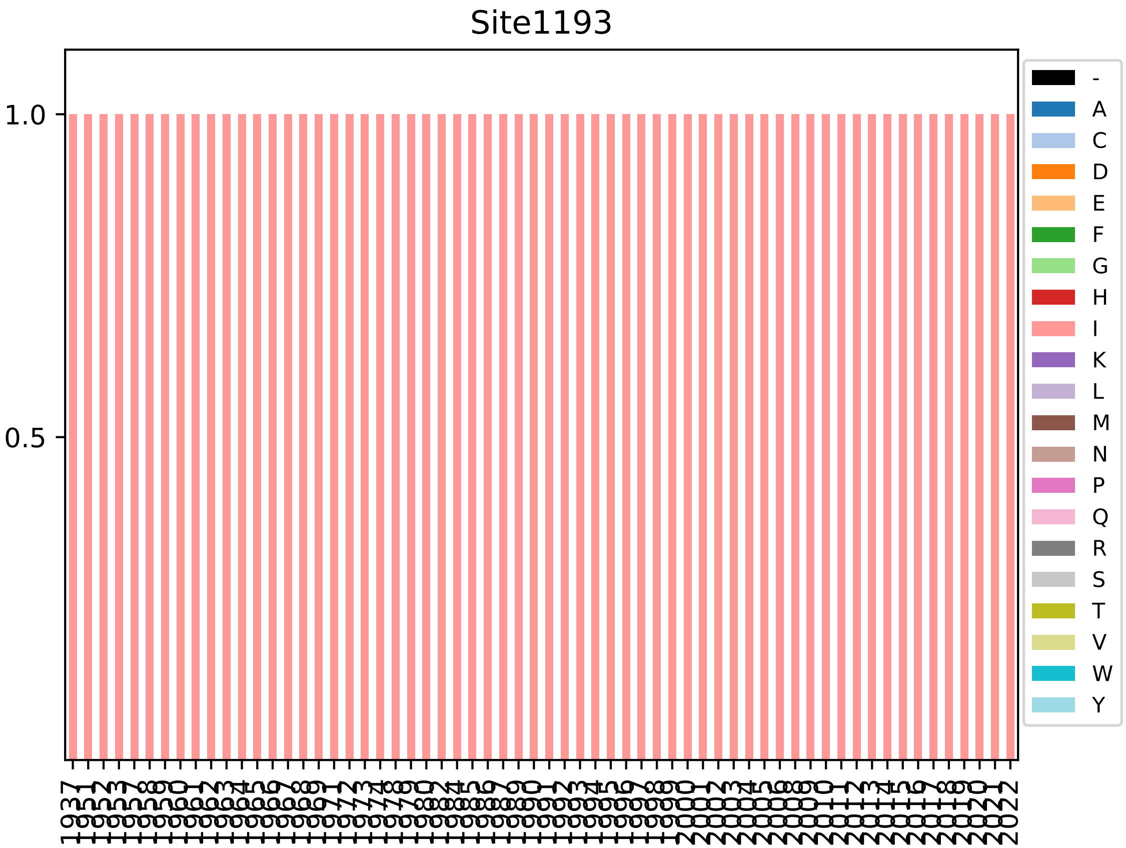
Site1193
1.0
0.5
-
A
C
D
E
F
G
H
I
K
L
M
N
P
Q
R
S
T
V
W
Y

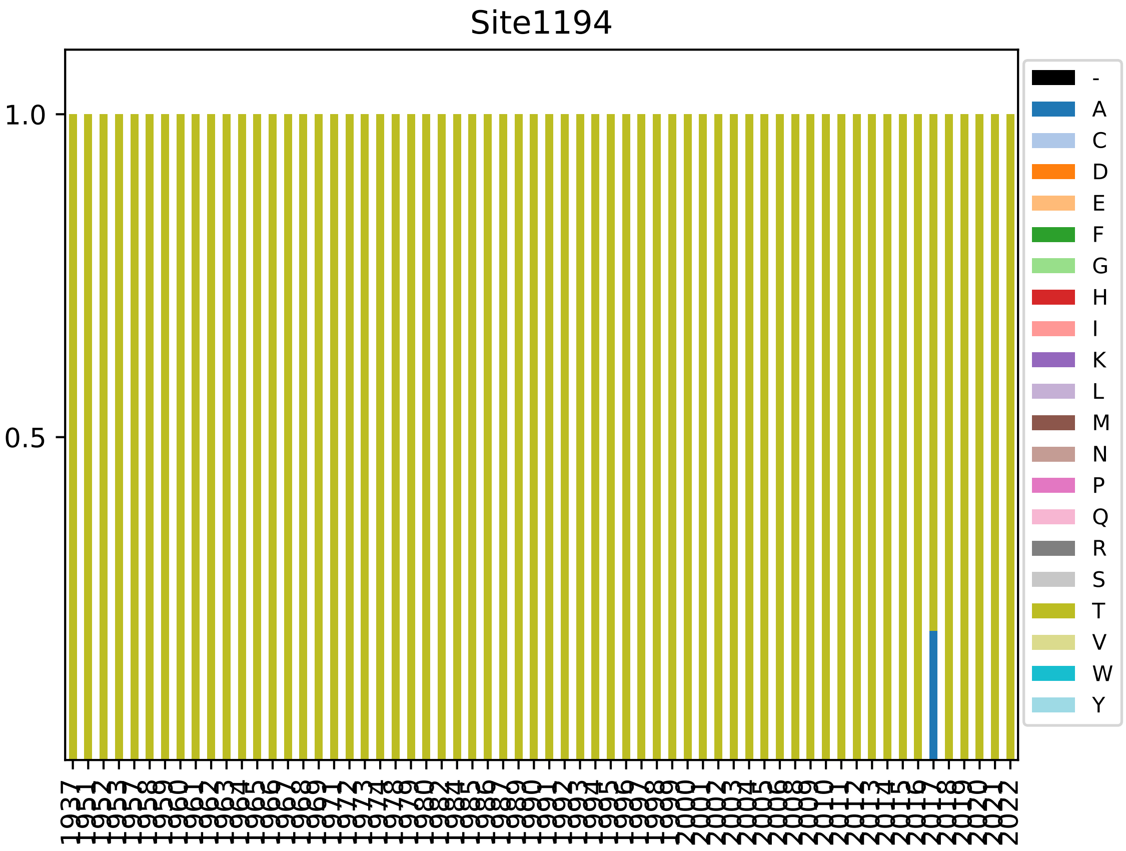
Site1194
1.0
0.5
-
A
C
D
E
F
G
H
I
K
L
M
N
P
Q
R
S
T
V
W
Y

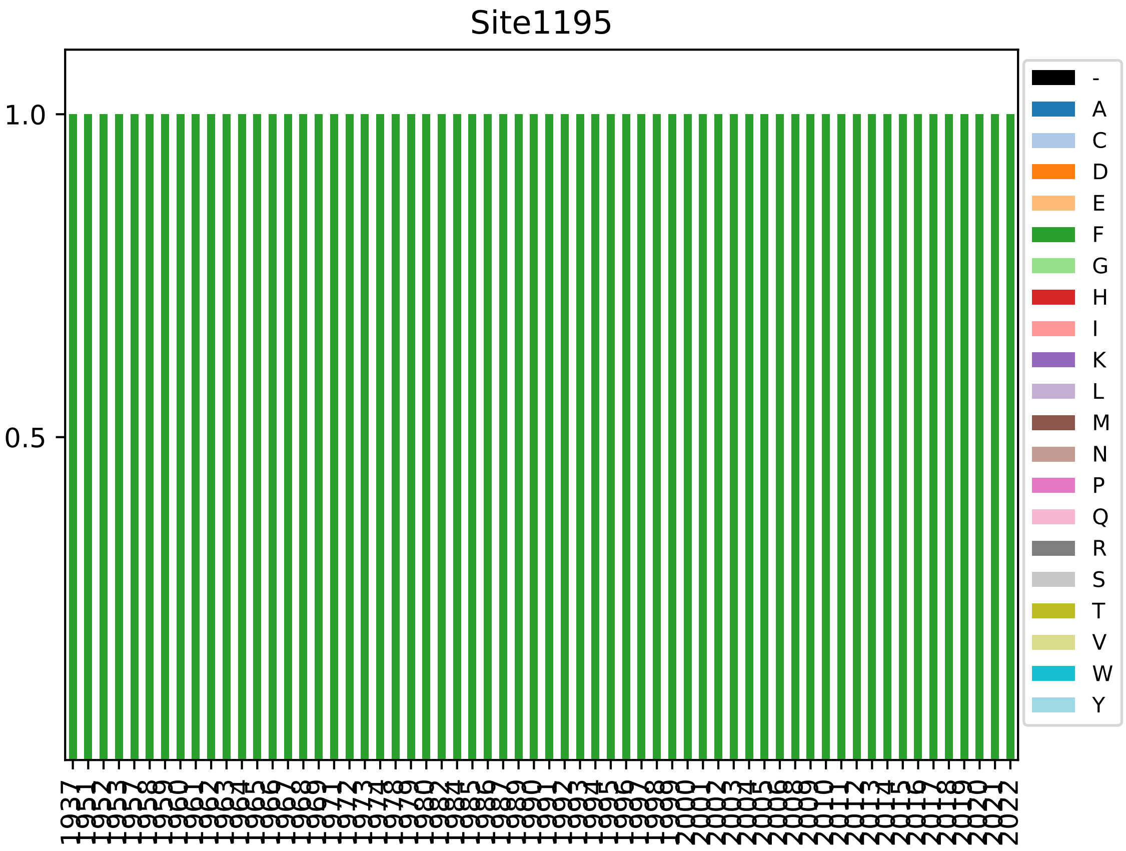
Site1195
1.0
0.5
-
A
C
D
E
F
G
H
I
K
L
M
N
P
Q
R
S
T
V
W
Y

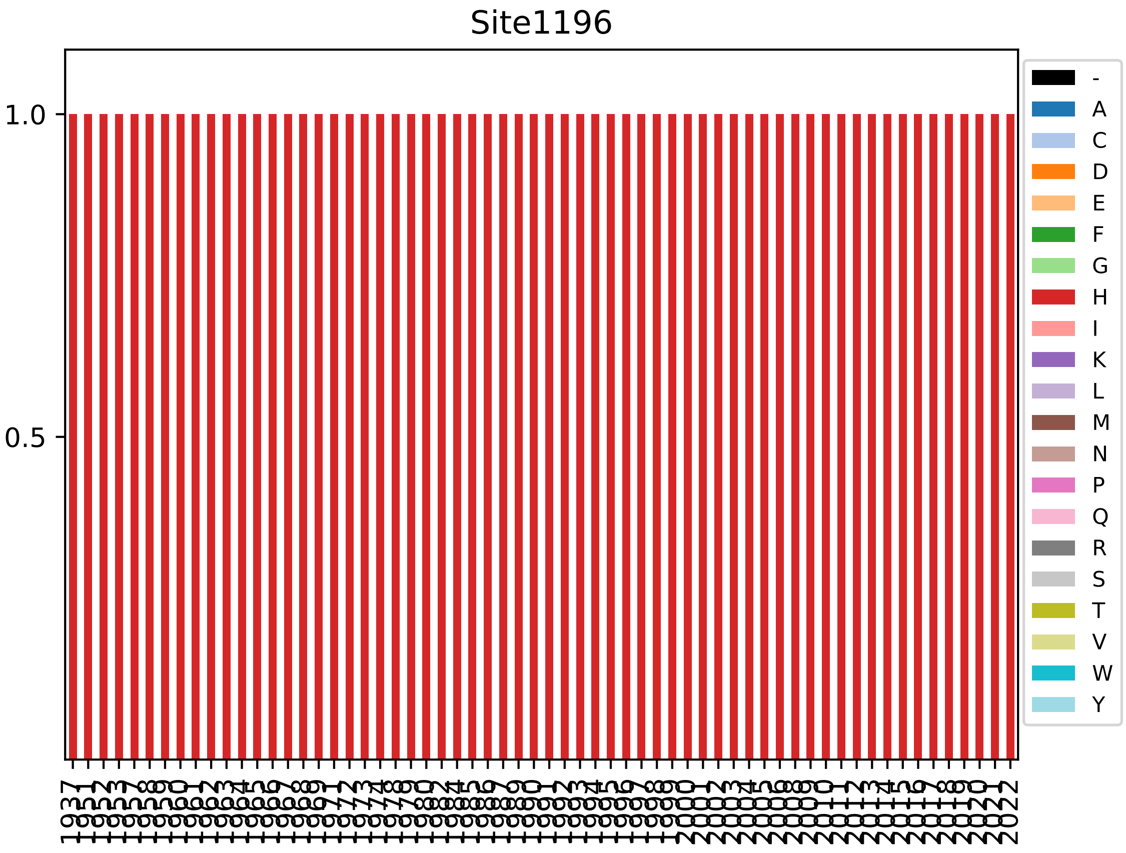
Site1196
1.0
0.5
-
A
C
D
E
F
G
H
I
K
L
M
N
P
Q
R
S
T
V
W
Y

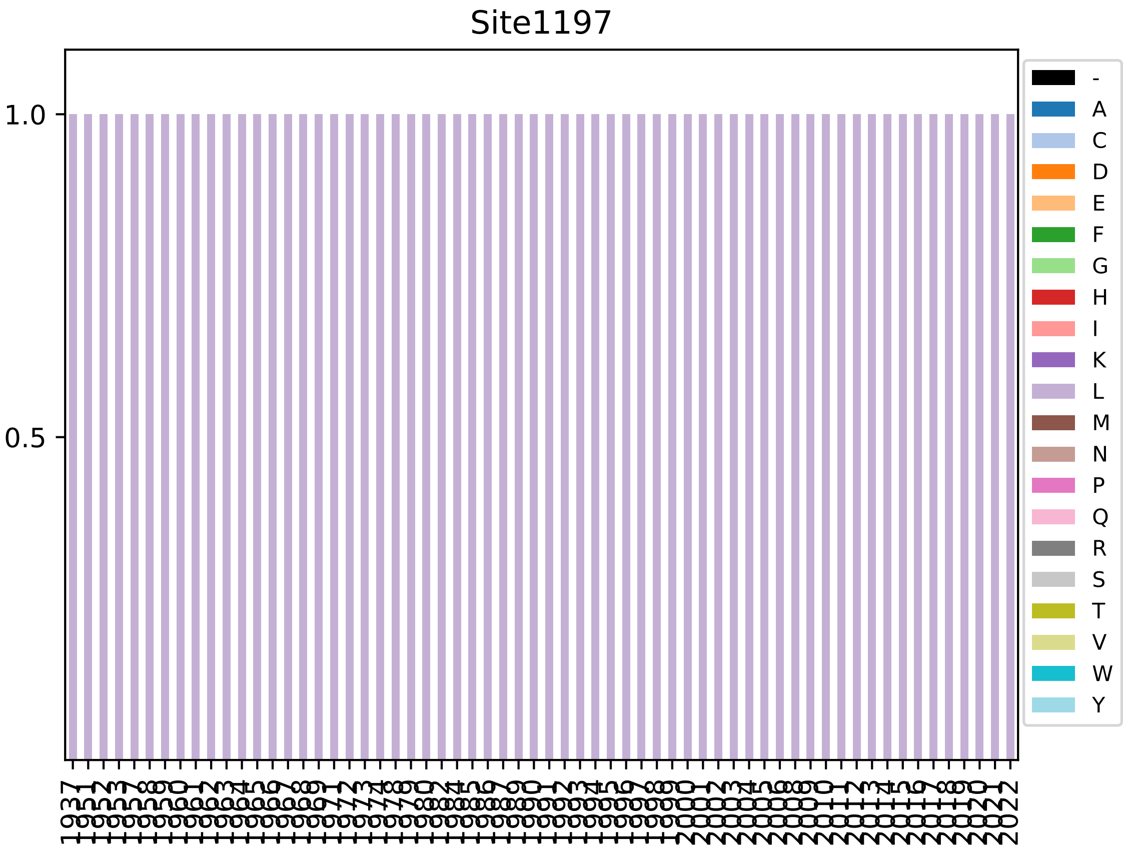
Site1197
1.0
0.5
-
A
C
D
E
F
G
H
I
K
L
M
N
P
Q
R
S
T
V
W
Y

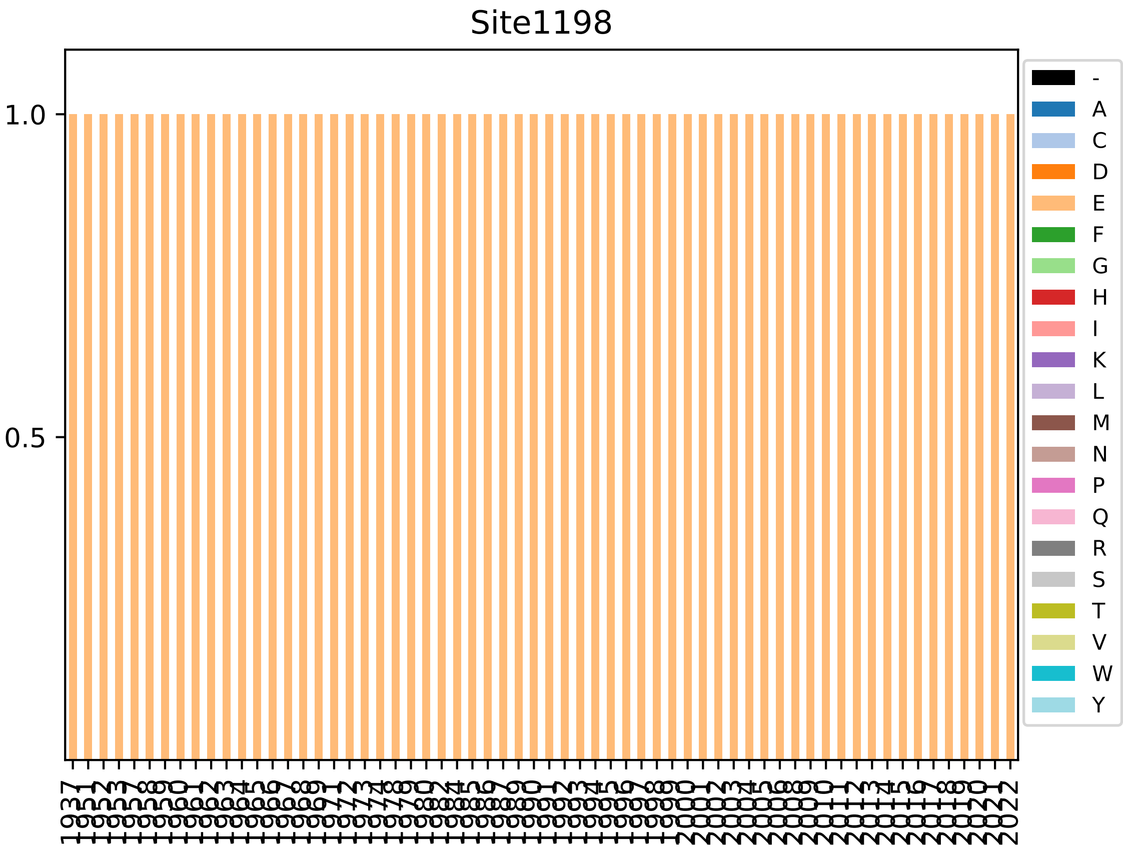
Site1198
1.0
0.5
-
A
C
D
E
F
G
H
I
K
L
M
N
P
Q
R
S
T
V
W
Y

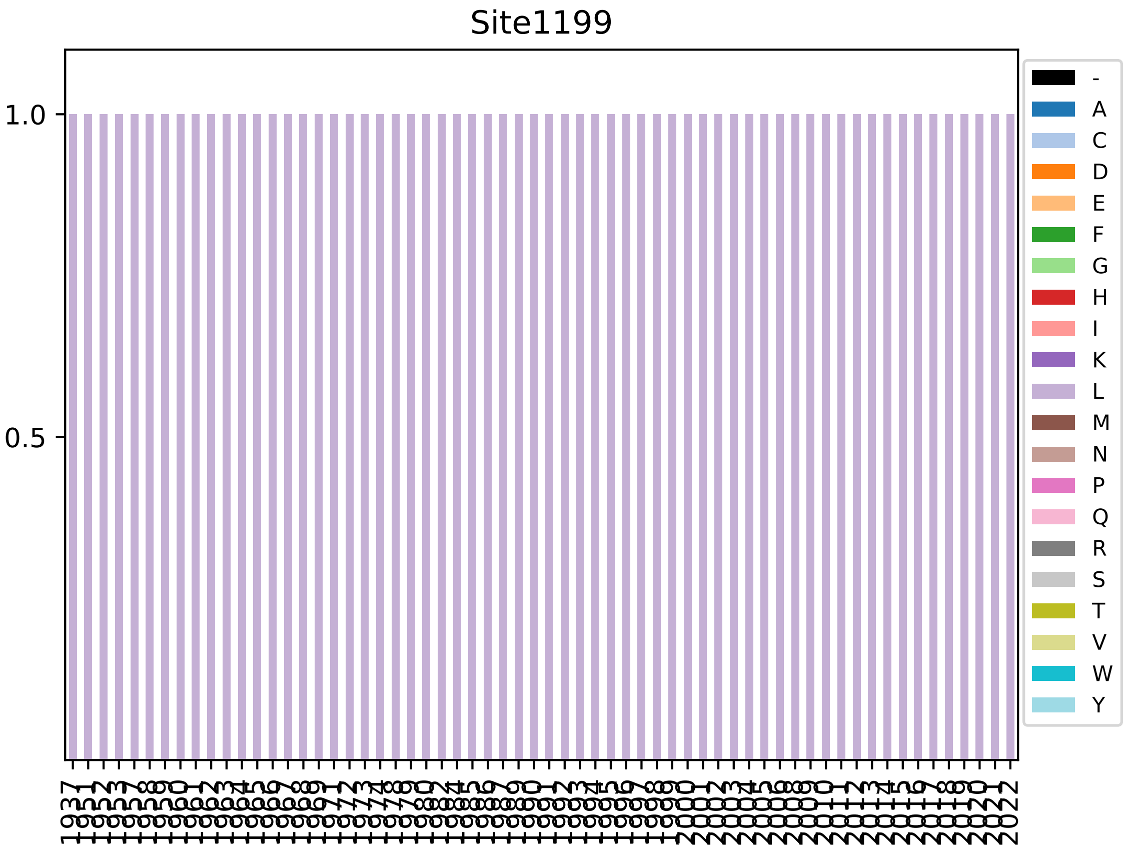
Site1199
1.0
0.5
-
A
C
D
E
F
G
H
I
K
L
M
N
P
Q
R
S
T
V
W
Y

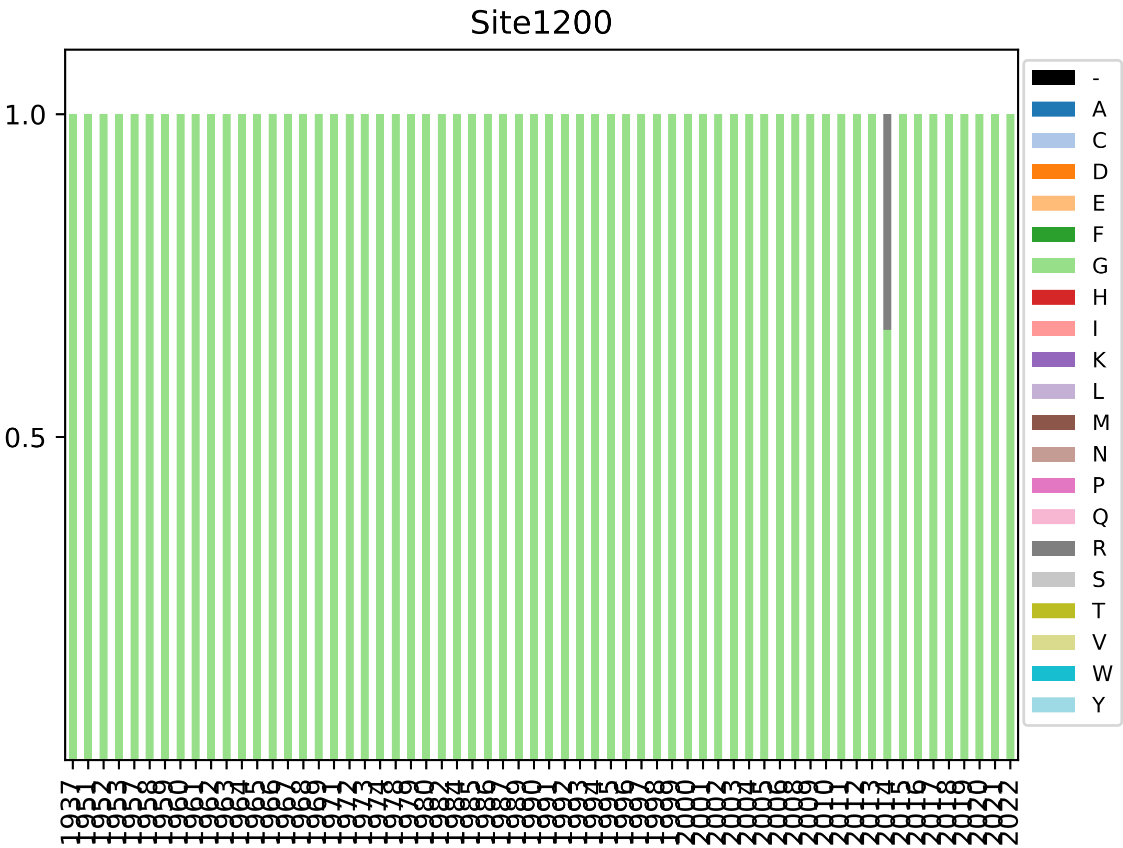
Site1200
1.0
0.5
-
A
C
D
E
F
G
H
I
K
L
M
N
P
Q
R
S
T
V
W
Y

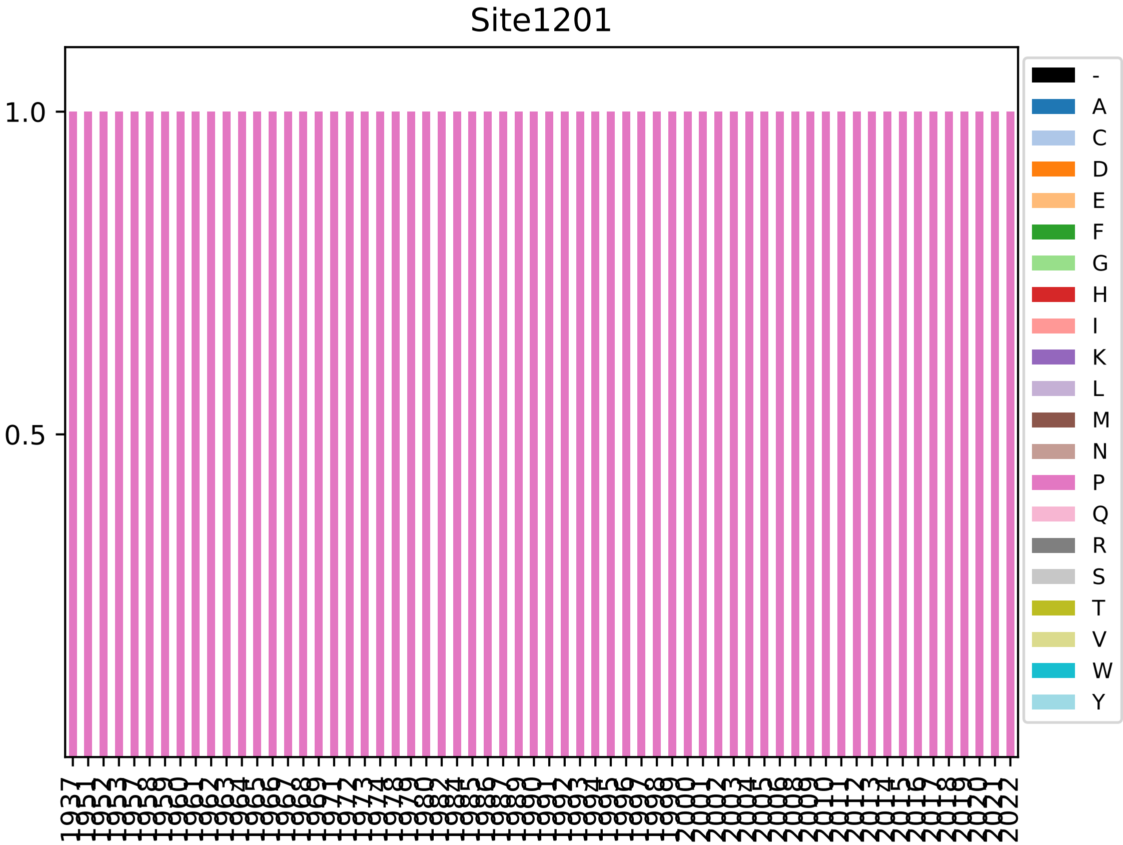
Site1201
1.0
0.5
-
A
C
D
E
F
G
H
I
K
L
M
N
P
Q
R
S
T
V
W
Y

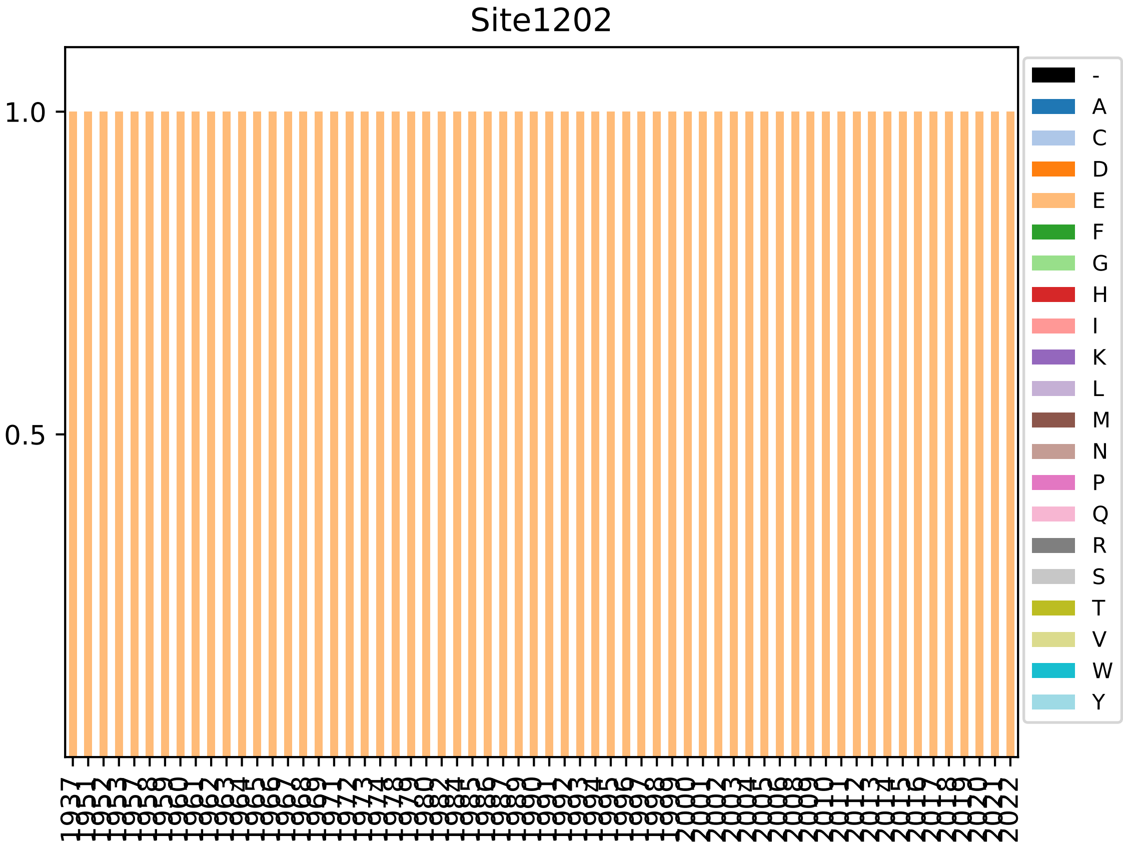
Site1202
1.0
0.5
-
A
C
D
E
F
G
H
I
K
L
M
N
P
Q
R
S
T
V
W
Y

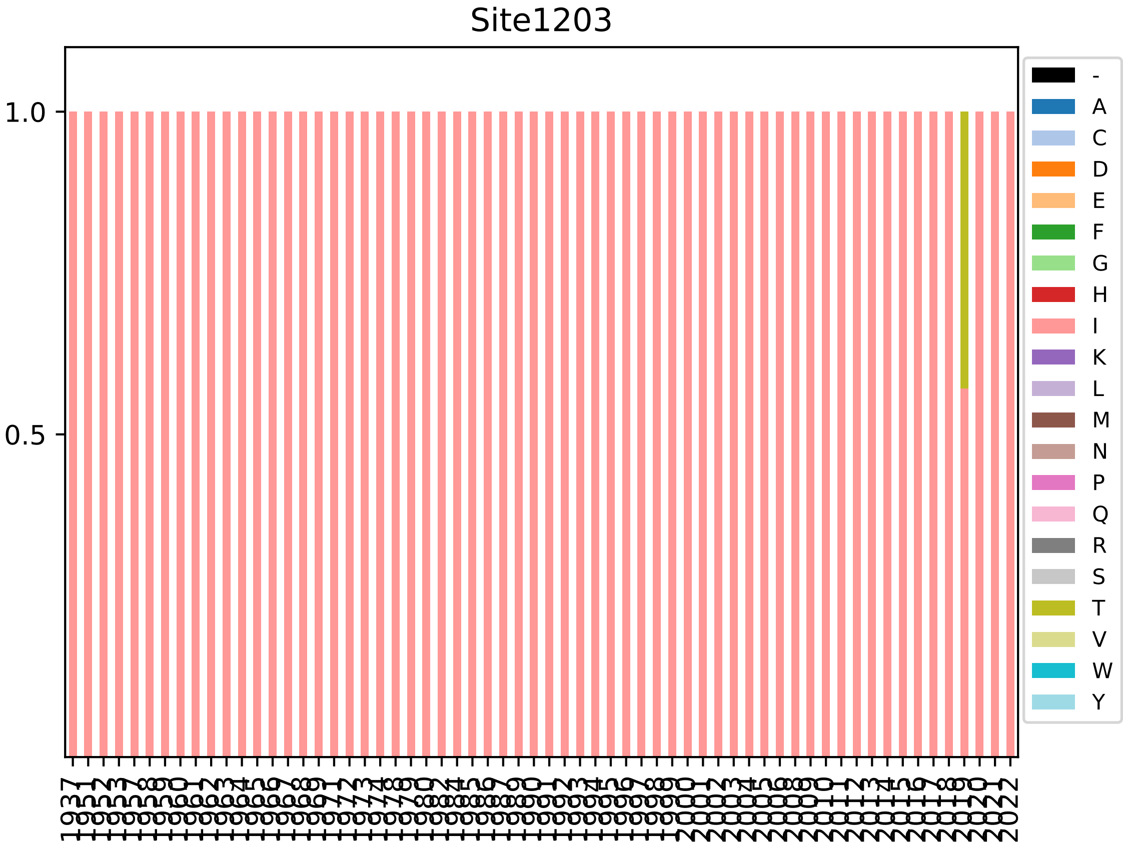
Site1203
1.0
0.5
-
A
C
D
E
F
G
H
I
K
L
M
N
P
Q
R
S
T
V
W
Y

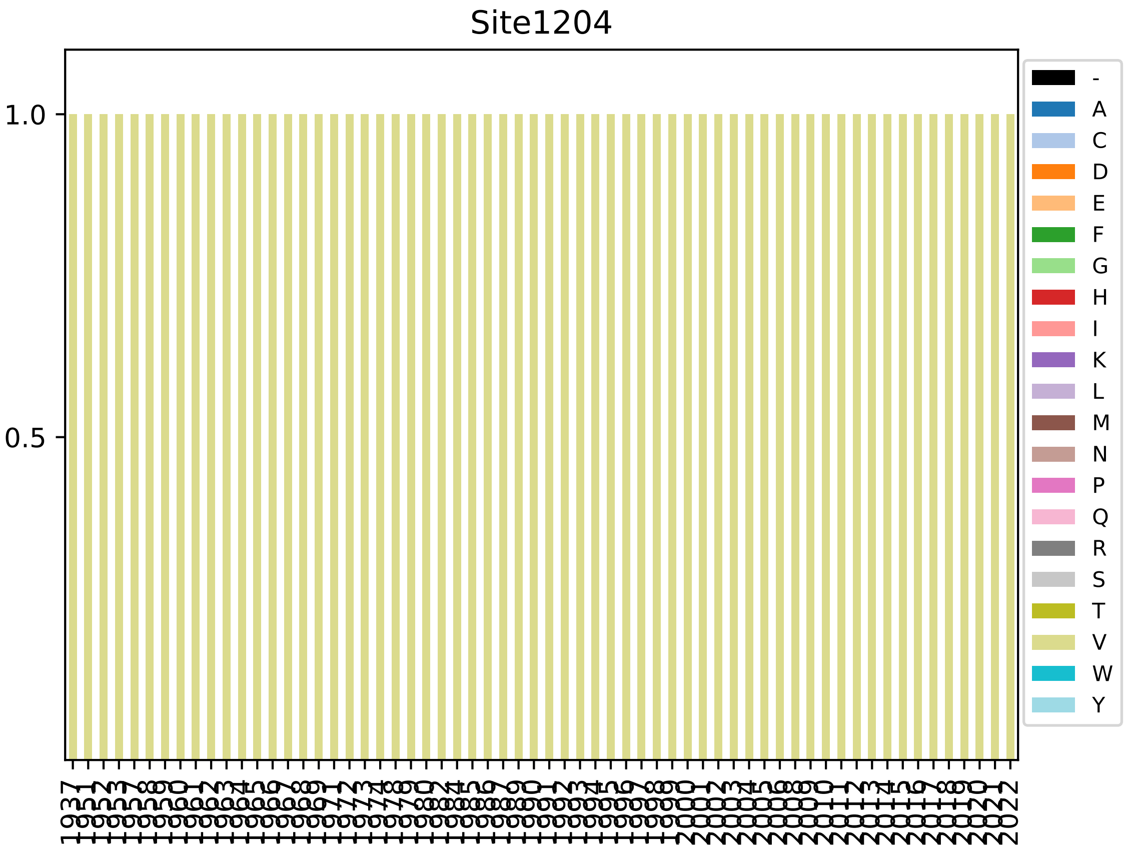
Site1204
1.0
0.5
-
A
C
D
E
F
G
H
I
K
L
M
N
P
Q
R
S
T
V
W
Y

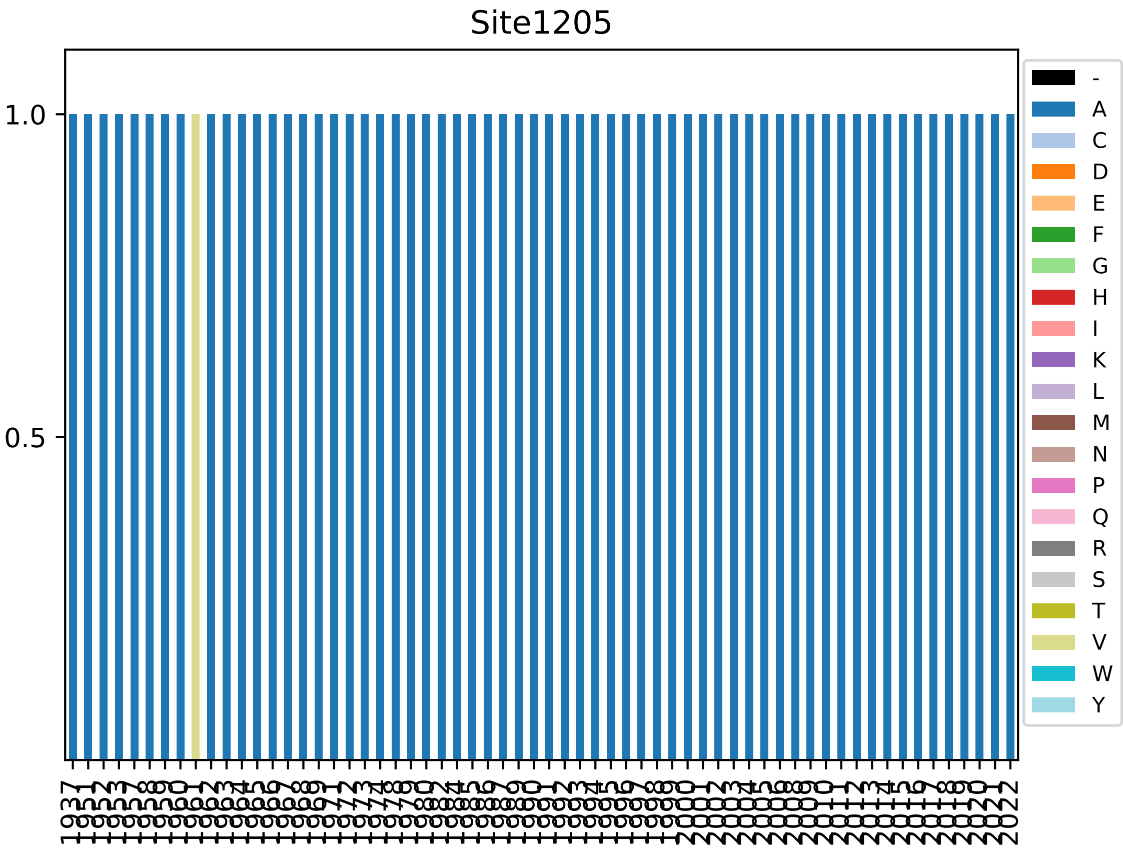
Site1205
1.0
0.5
-
A
C
D
E
F
G
H
I
K
L
M
N
P
Q
R
S
T
V
W
Y

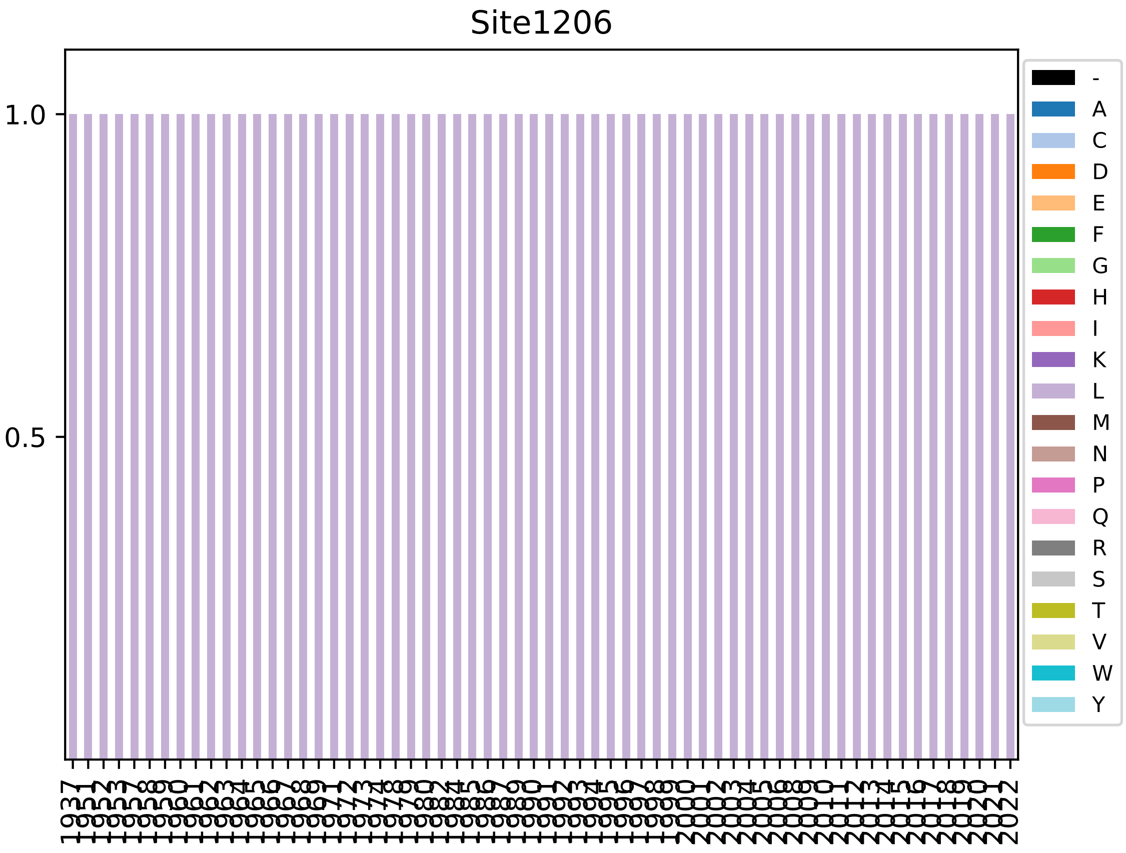
Site1206
1.0
0.5
-
A
C
D
E
F
G
H
I
K
L
M
N
P
Q
R
S
T
V
W
Y

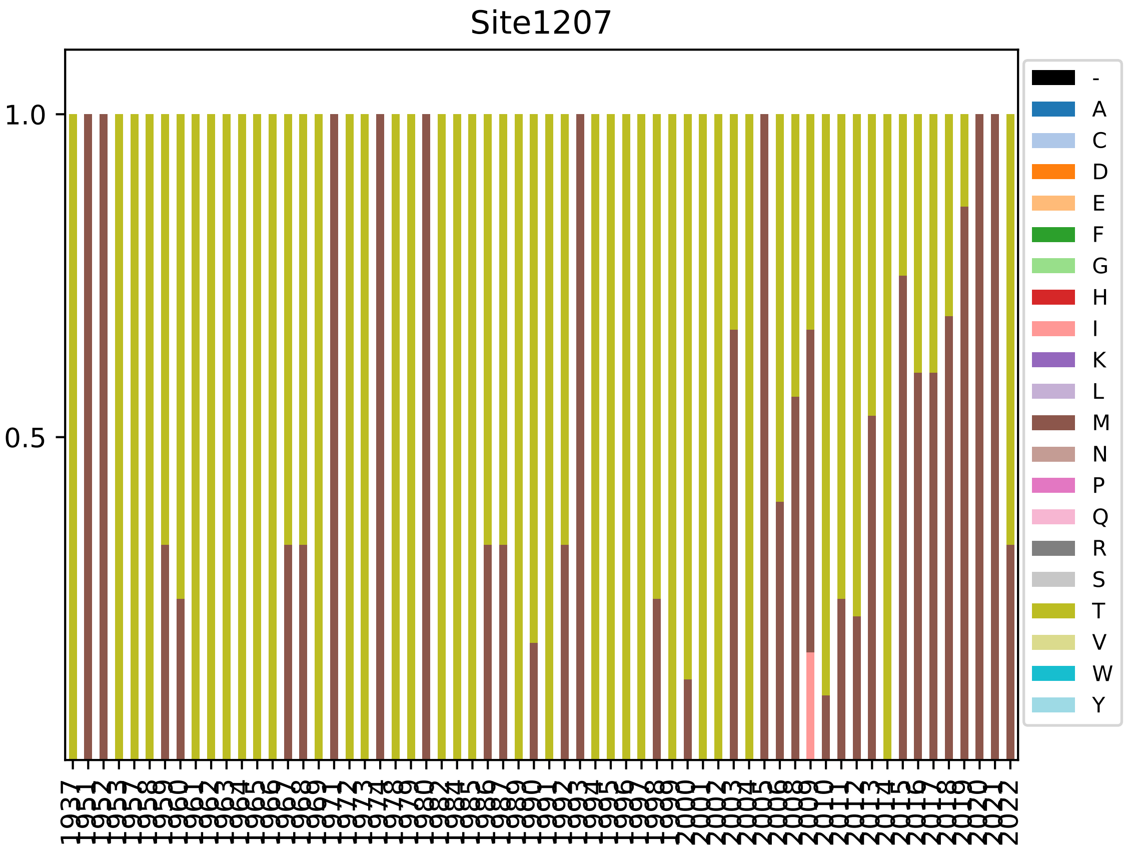
Site1207
1.0
0.5
-
A
C
D
E
F
G
H
I
K
L
M
N
P
Q
R
S
T
V
W
Y

Site1208

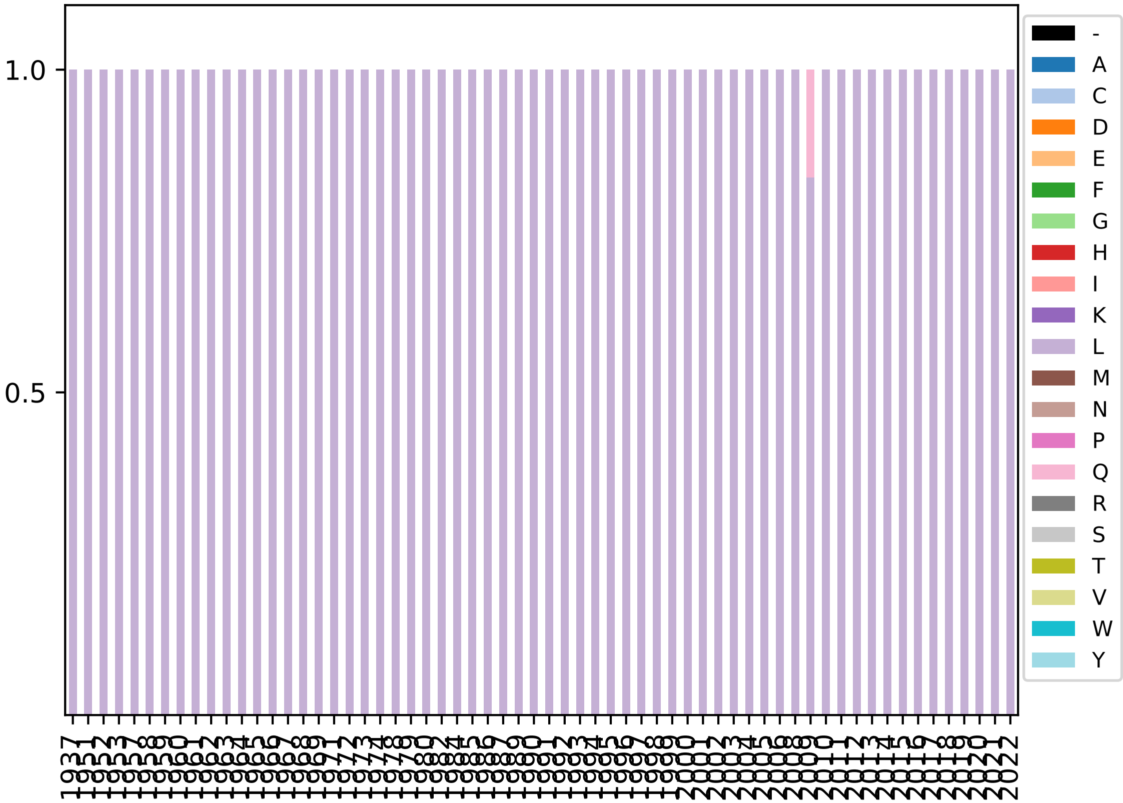

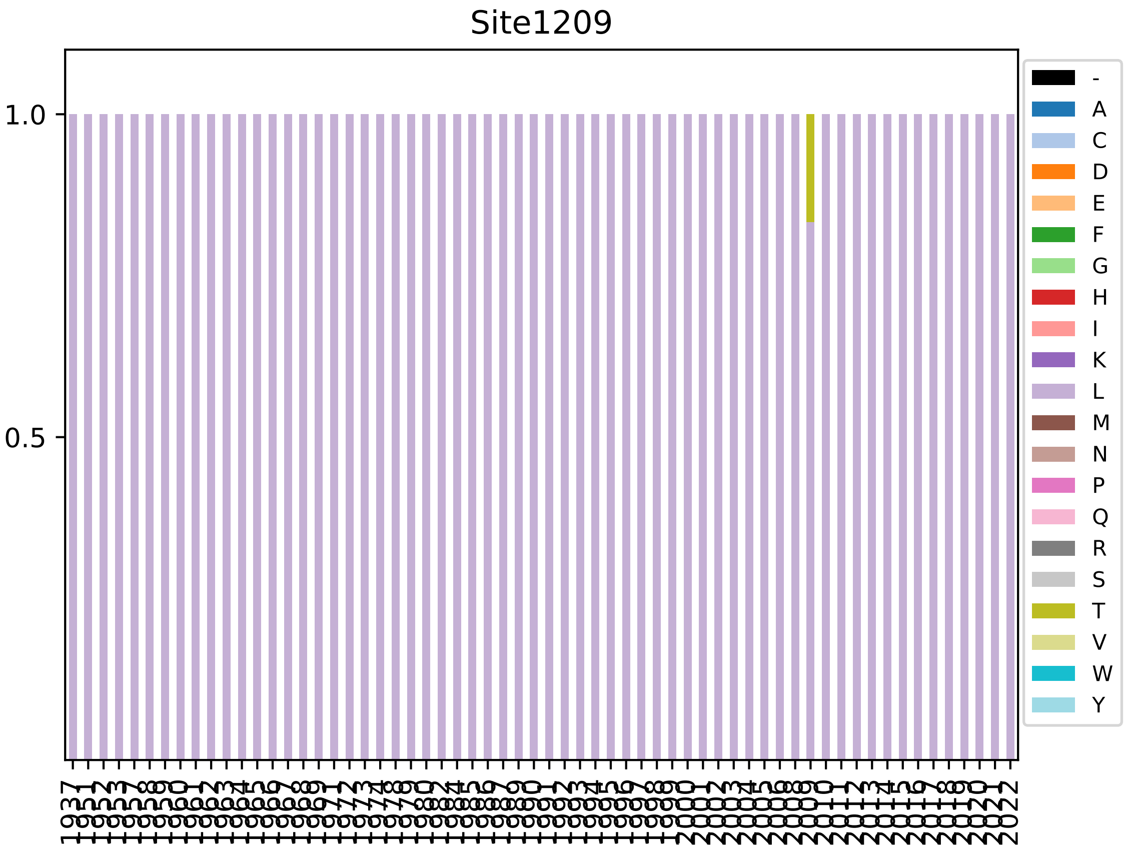

Site1209
1.0
0.5
-
A
C
D
E
F
G
H
I
K
L
M
N
P
Q
R
S
T
V
W
Y

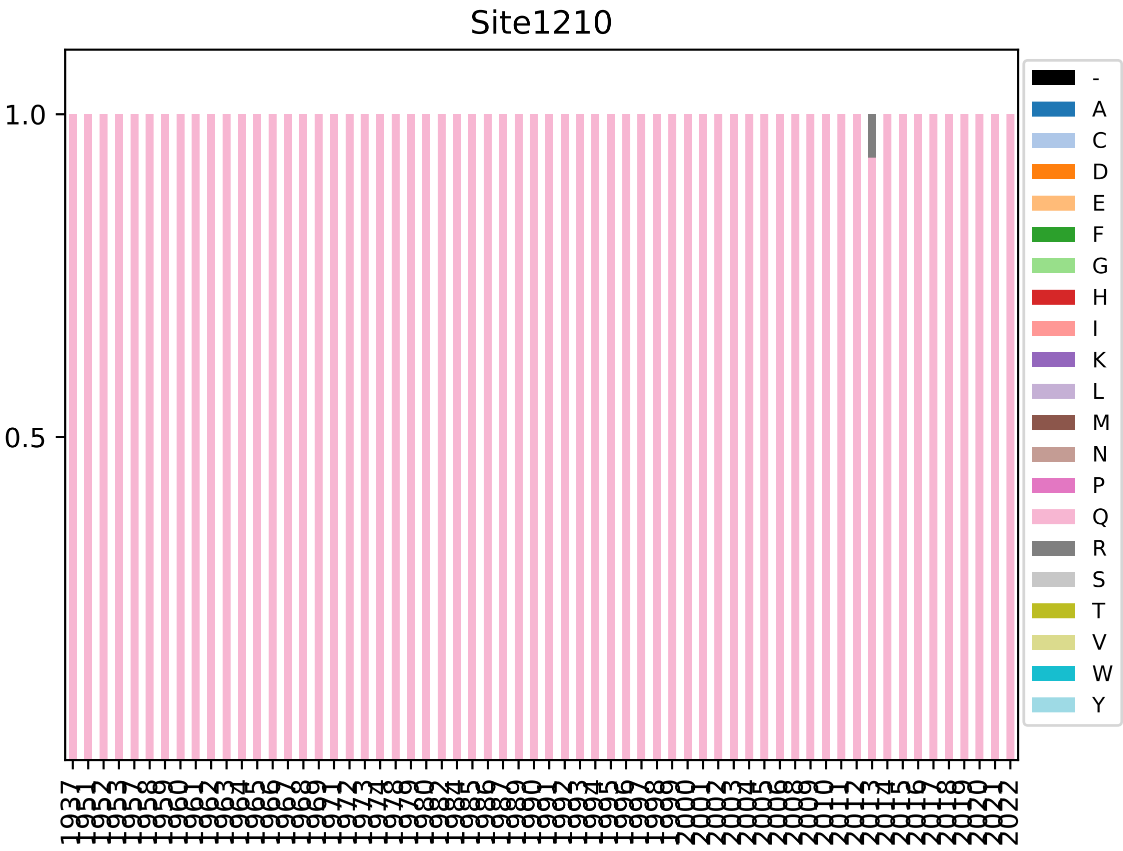
Site1210
1.0
0.5
-
A
C
D
E
F
G
H
I
K
L
M
N
P
Q
R
S
T
V
W
Y

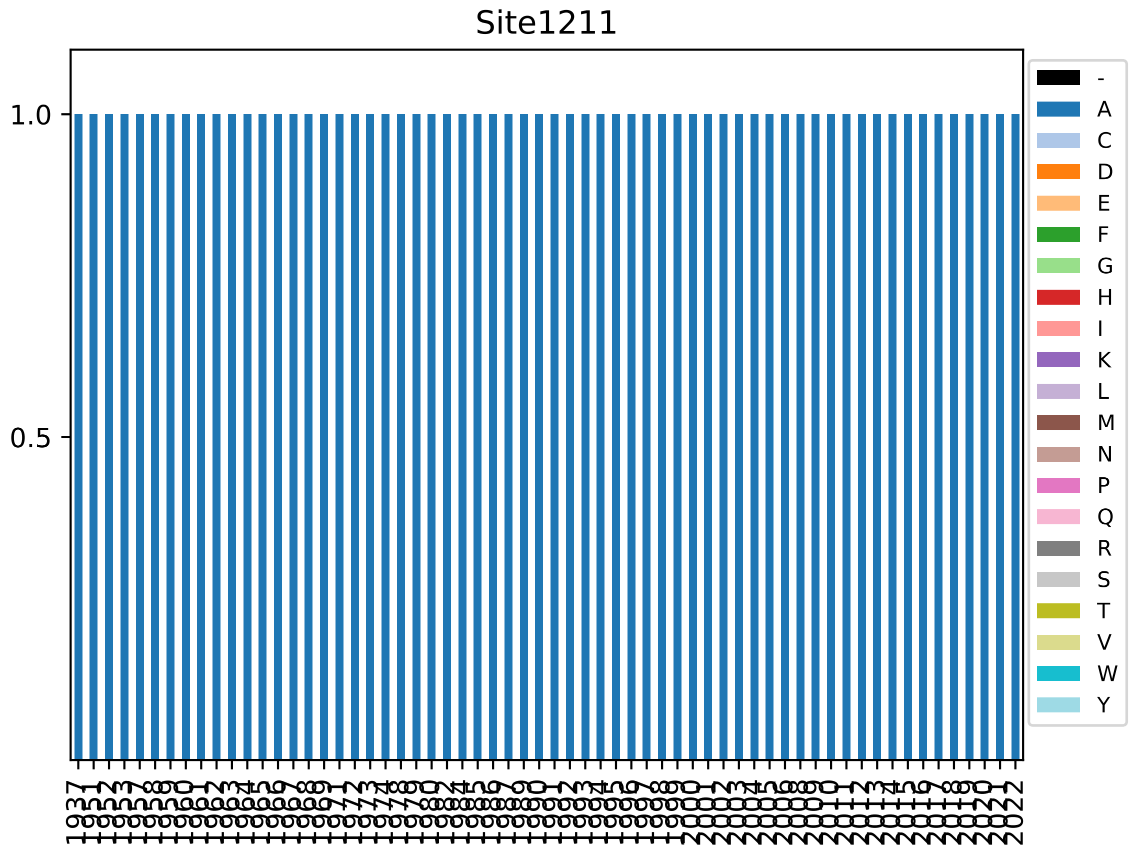
Site1211
1.0
0.5
-
A
C
D
E
F
G
H
I
K
L
M
N
P
Q
R
S
T
V
W
Y

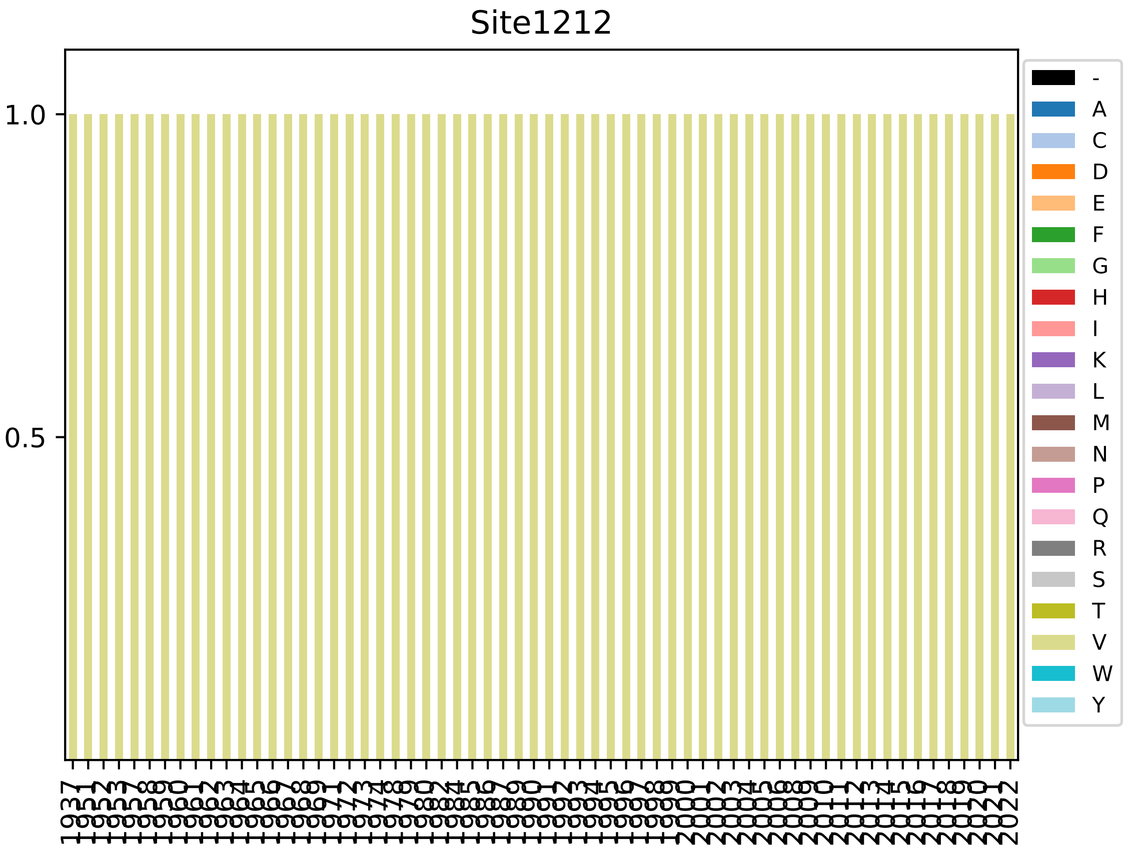
Site1212
1.0
0.5
-
A
C
D
E
F
G
H
I
K
L
M
N
P
Q
R
S
T
V
W
Y

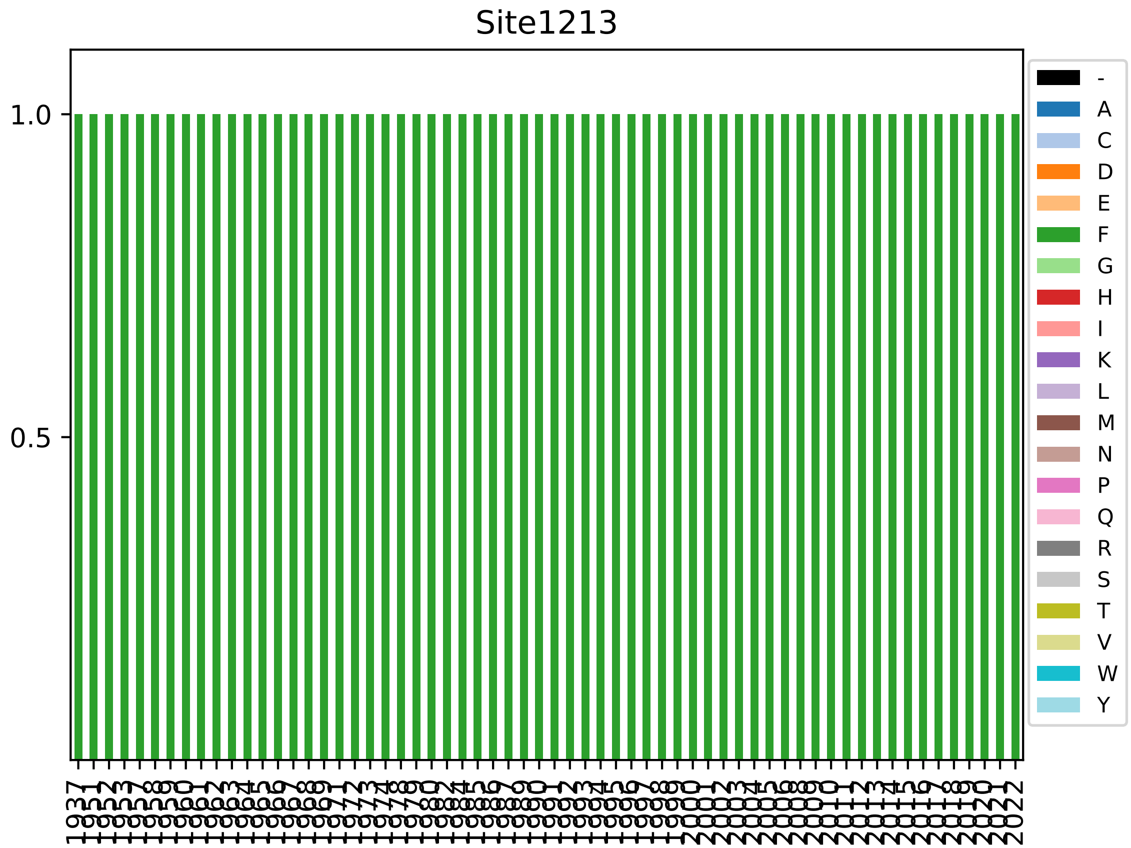

Site1213
1.0
0.5
-
A
C
D
E
F
G
H
I
K
L
M
N
P
Q
R
S
T
V
W
Y

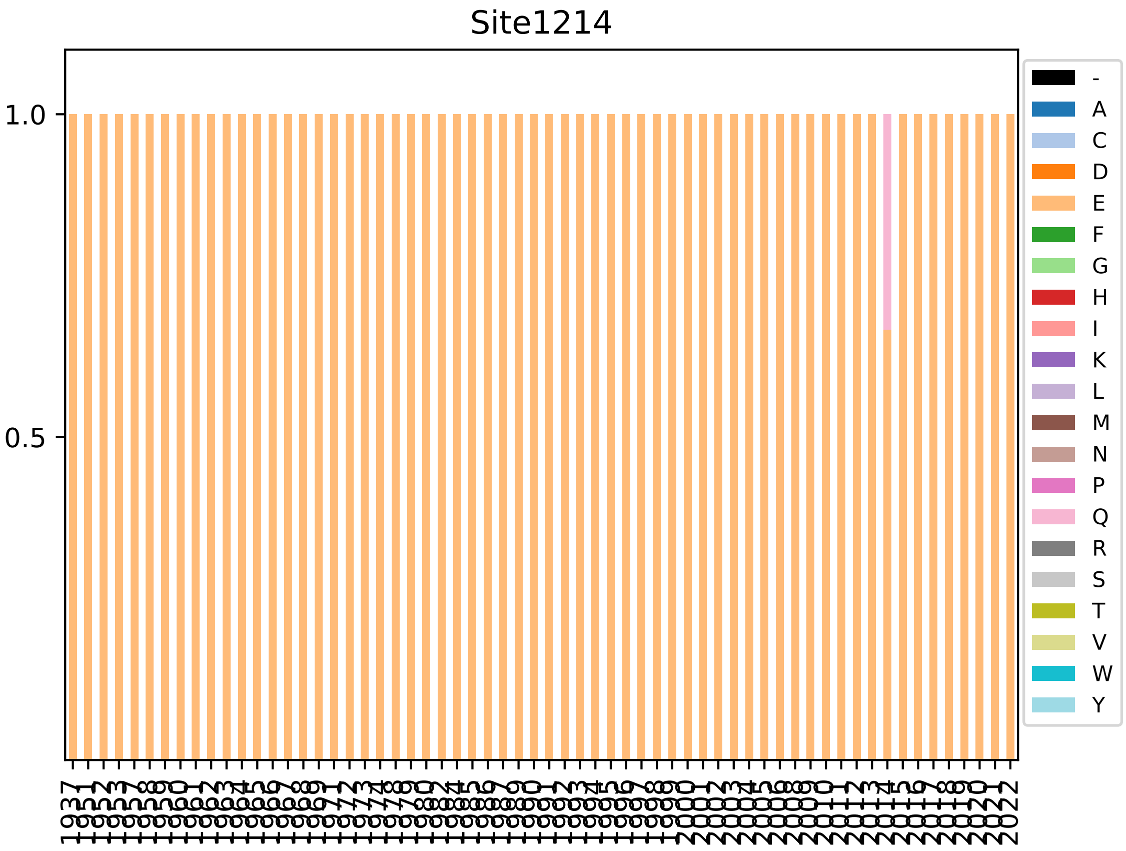
Site1214
1.0
0.5
-
A
C
D
E
F
G
H
I
K
L
M
N
P
Q
R
S
T
V
W
Y

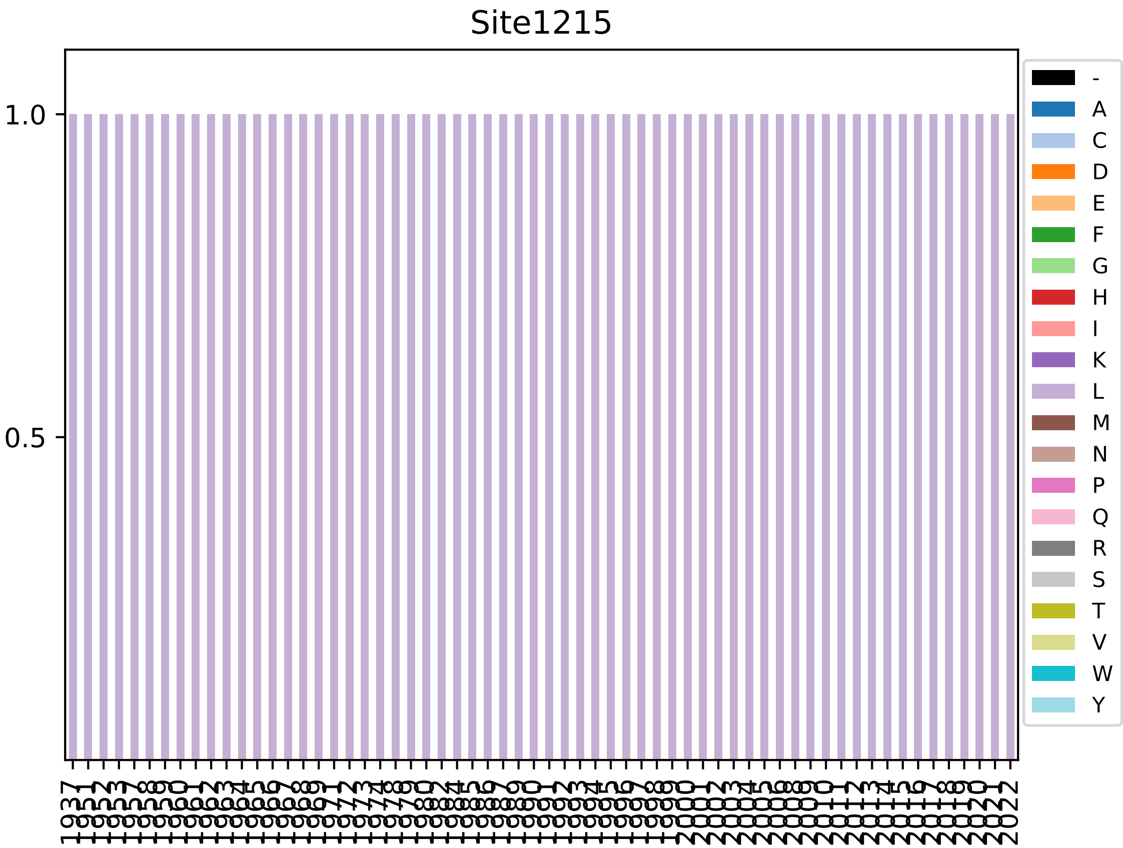
Site1215
1.0
0.5
-
A
C
D
E
F
G
H
I
K
L
M
N
P
Q
R
S
T
V
W
Y

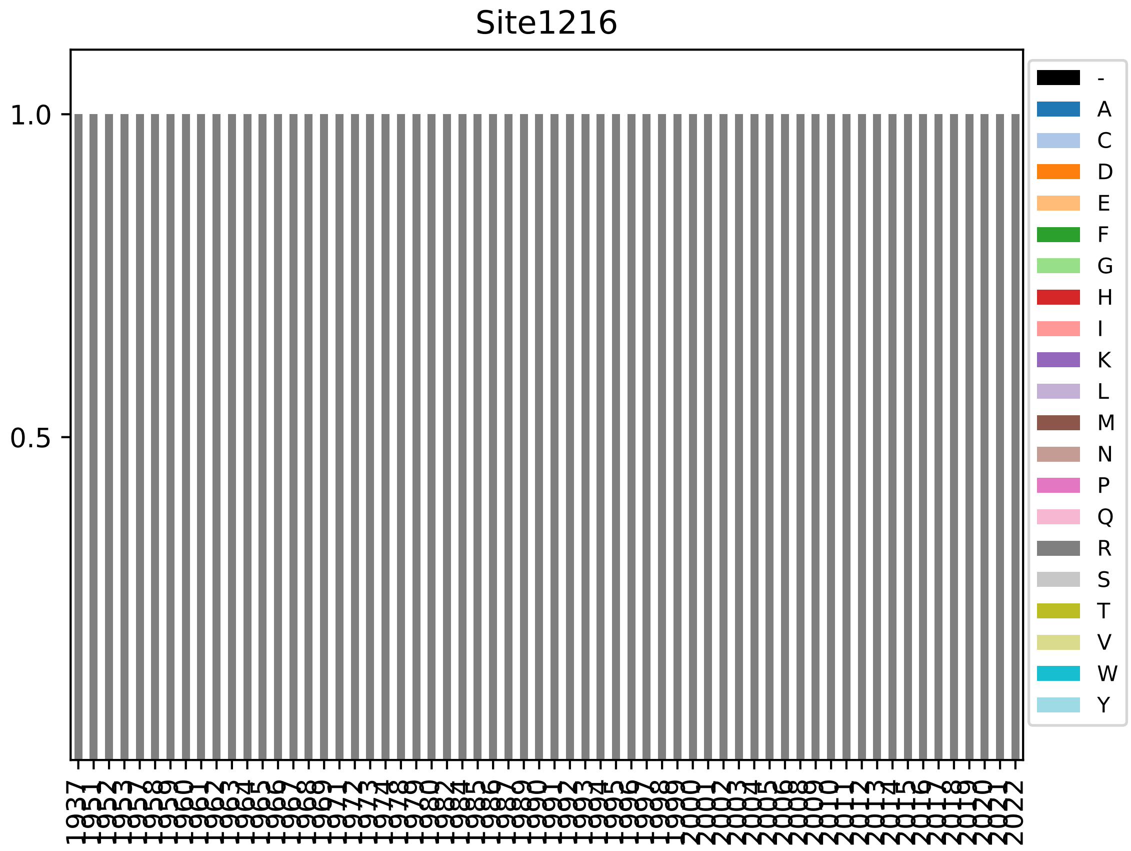
Site1216
1.0
0.5
-
A
C
D
E
F
G
H
I
K
L
M
N
P
Q
R
S
T
V
W
Y

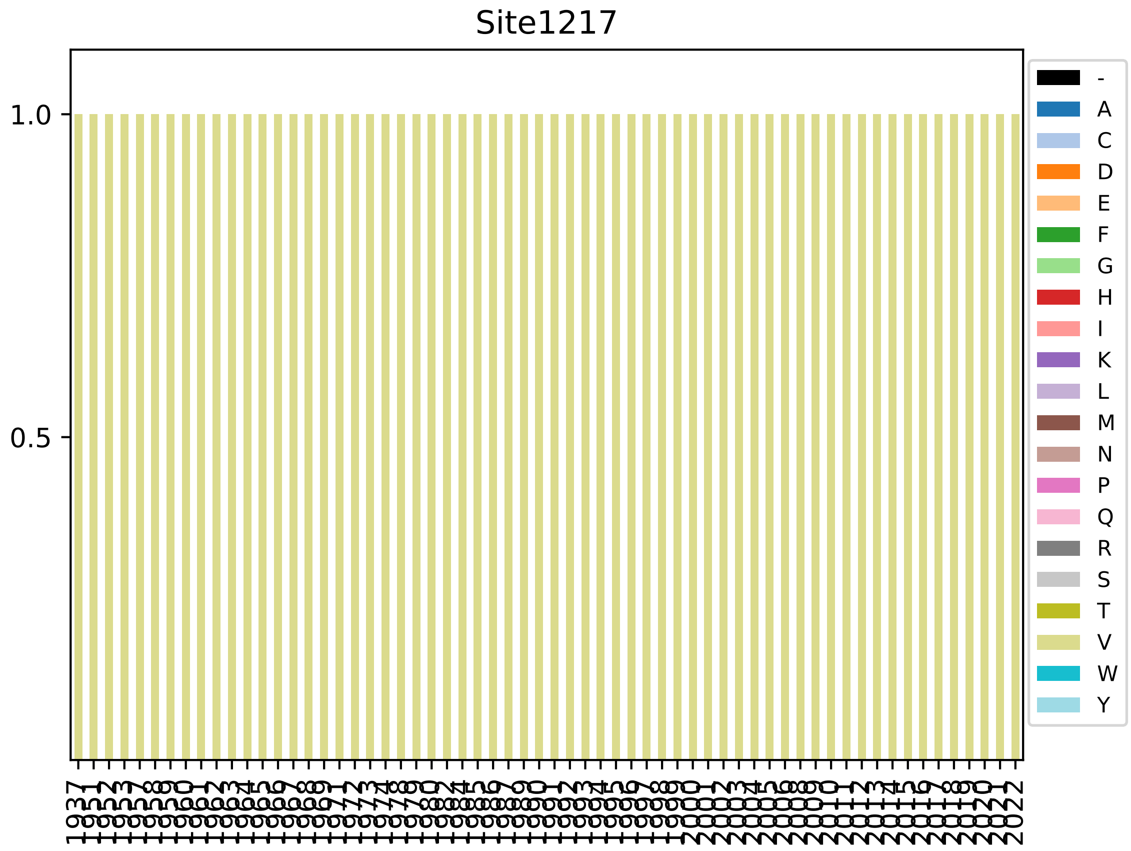
Site1217
1.0
0.5
-
A
C
D
E
F
G
H
I
K
L
M
N
P
Q
R
S
T
V
W
Y

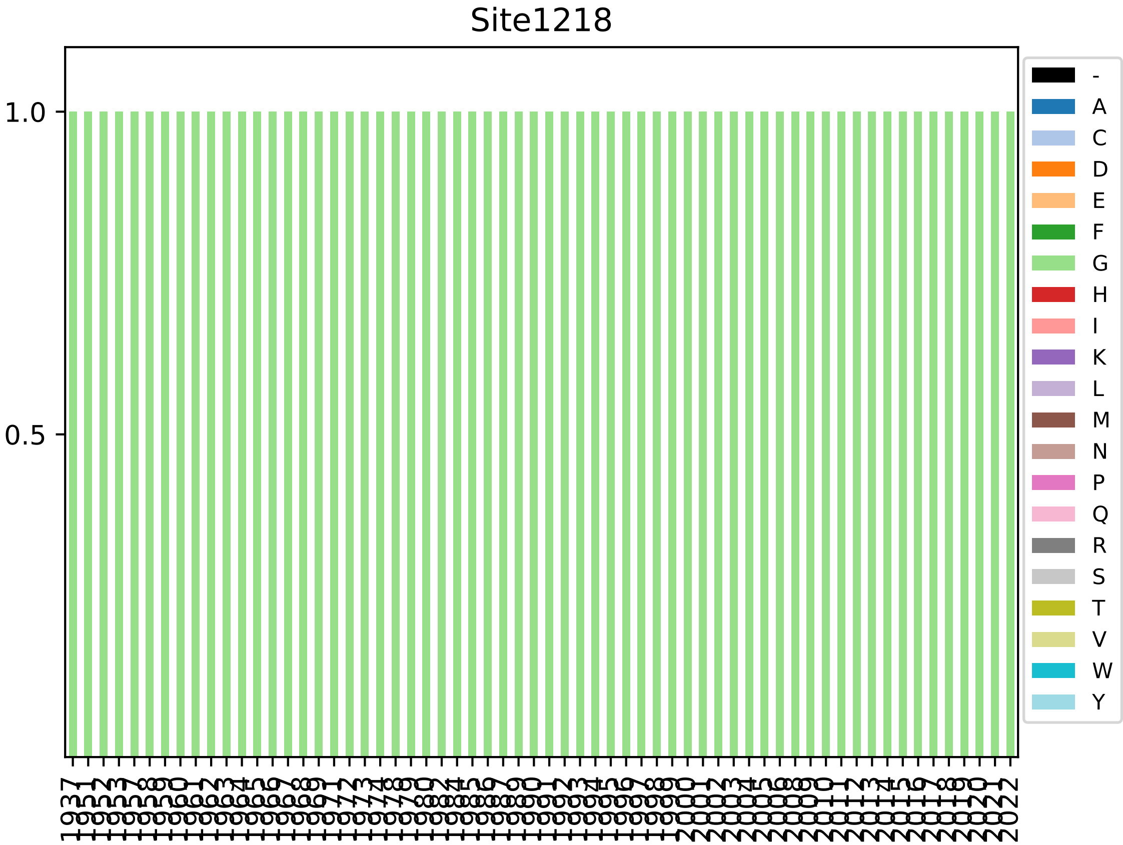
Site1218
1.0
0.5
-
A
C
D
E
F
G
H
I
K
L
M
N
P
Q
R
S
T
V
W
Y

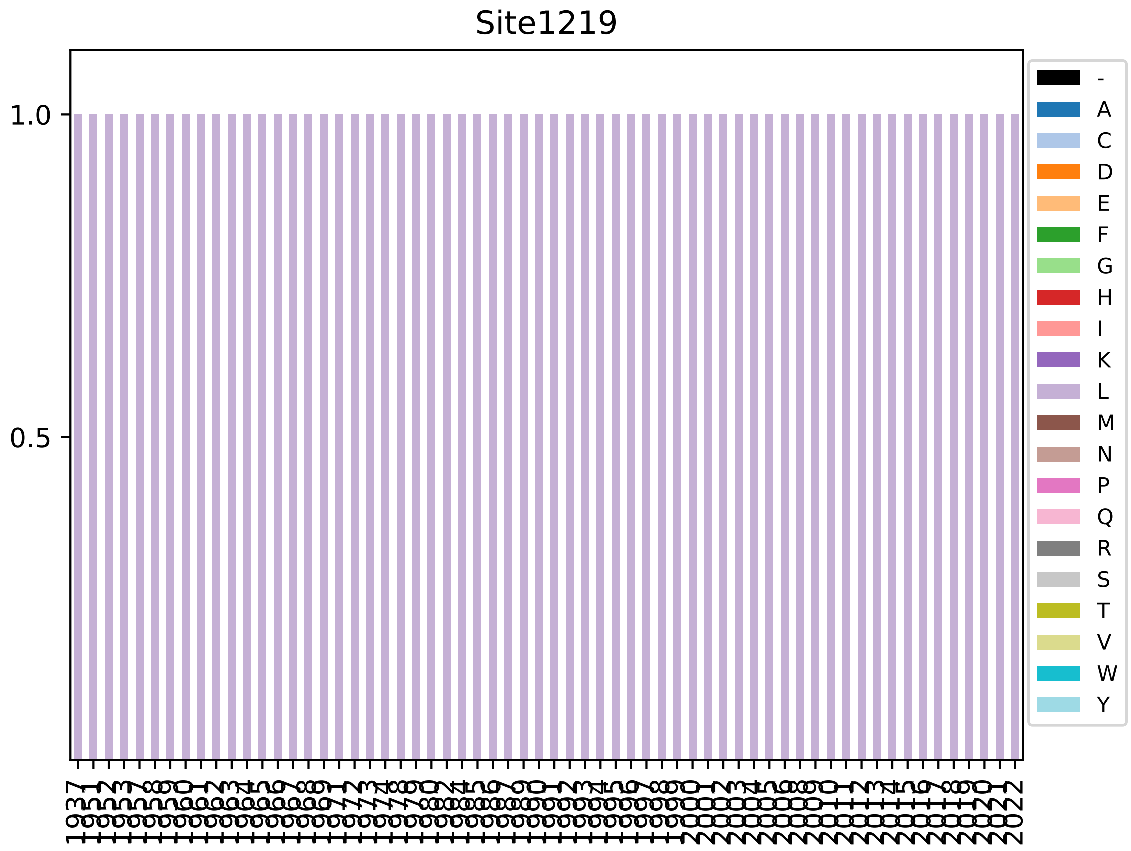
Site1219
1.0
0.5
-
A
C
D
E
F
G
H
I
K
L
M
N
P
Q
R
S
T
V
W
Y

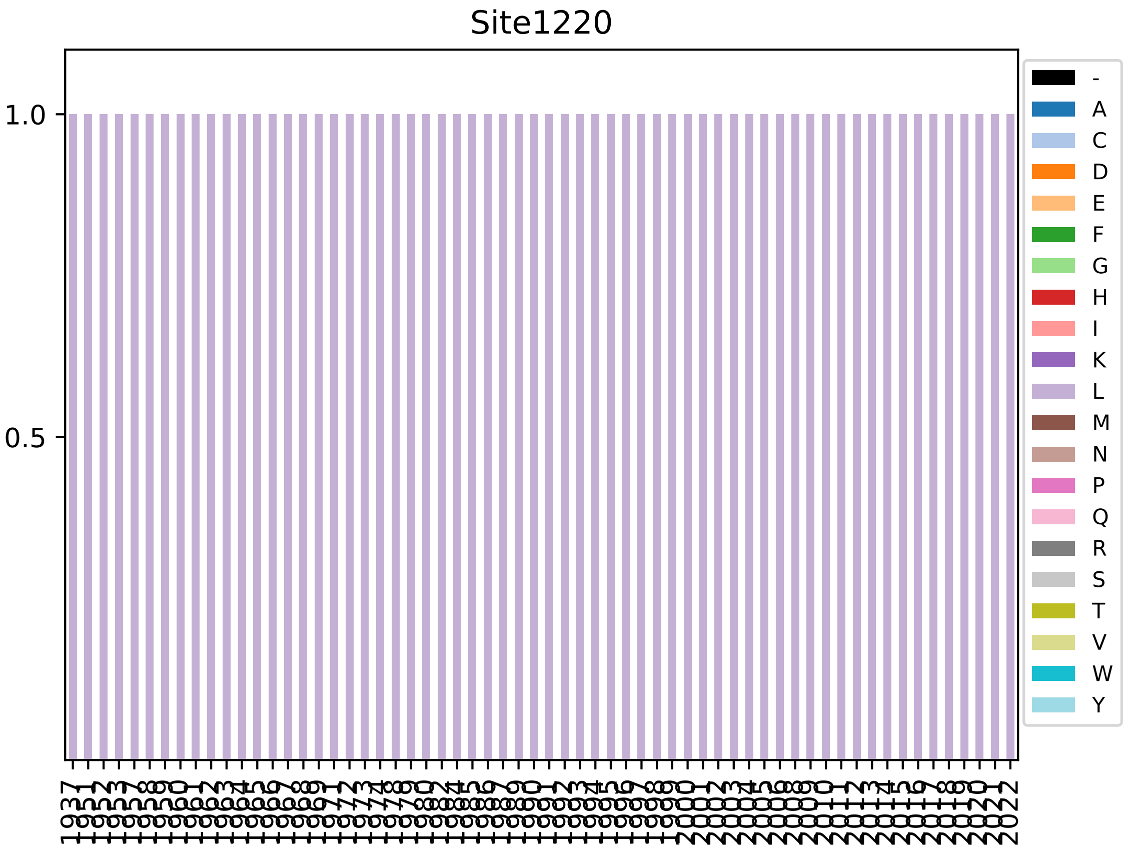
Site1220
1.0
0.5
-
A
C
D
E
F
G
H
I
K
L
M
N
P
Q
R
S
T
V
W
Y

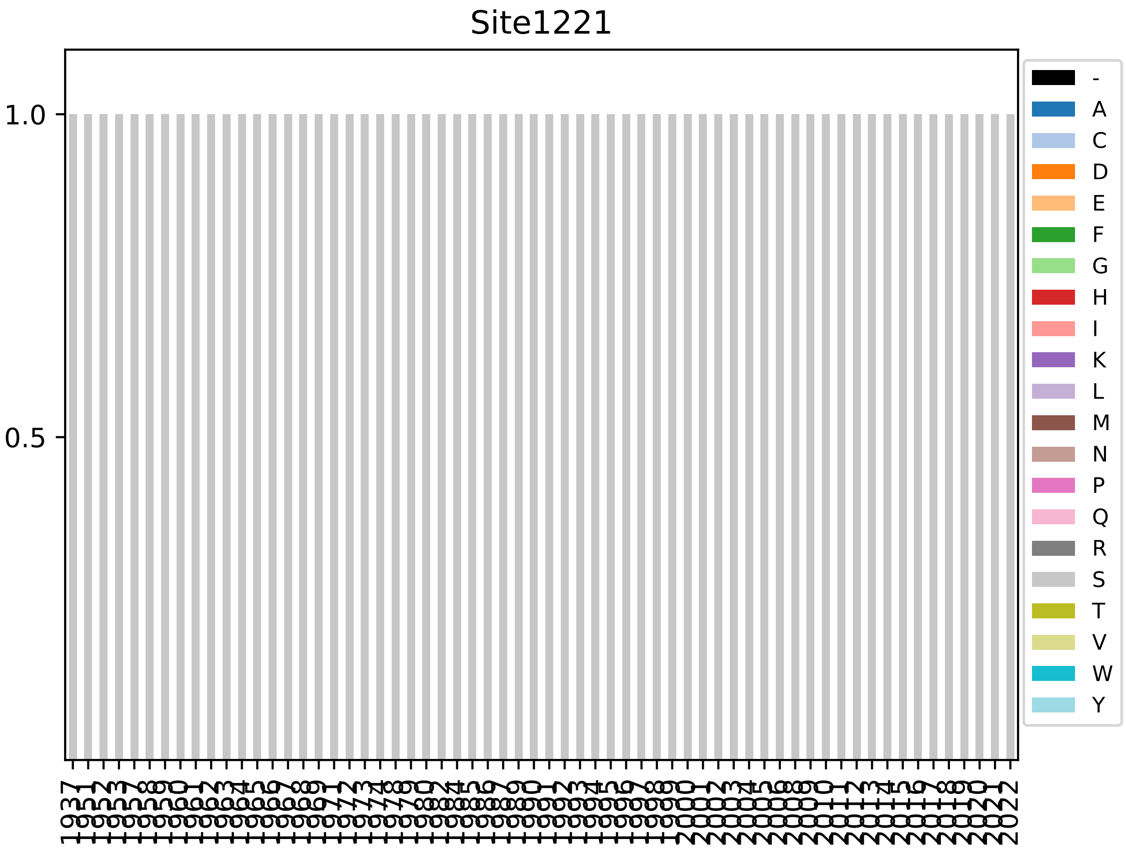

Site1221
1.0
0.5
-
A
C
D
E
F
G
H
I
K
L
M
N
P
Q
R
S
T
V
W
Y

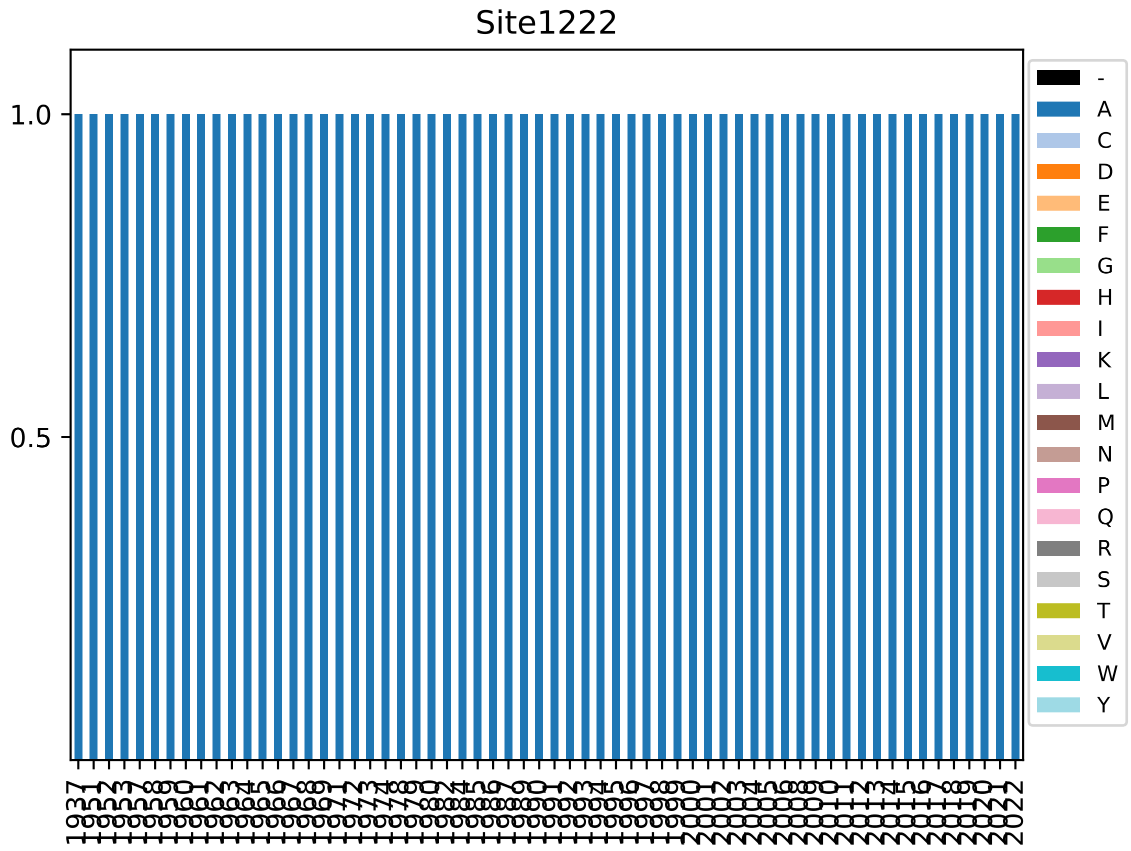

Site1222
1.0
0.5
-
A
C
D
E
F
G
H
I
K
L
M
N
P
Q
R
S
T
V
W
Y

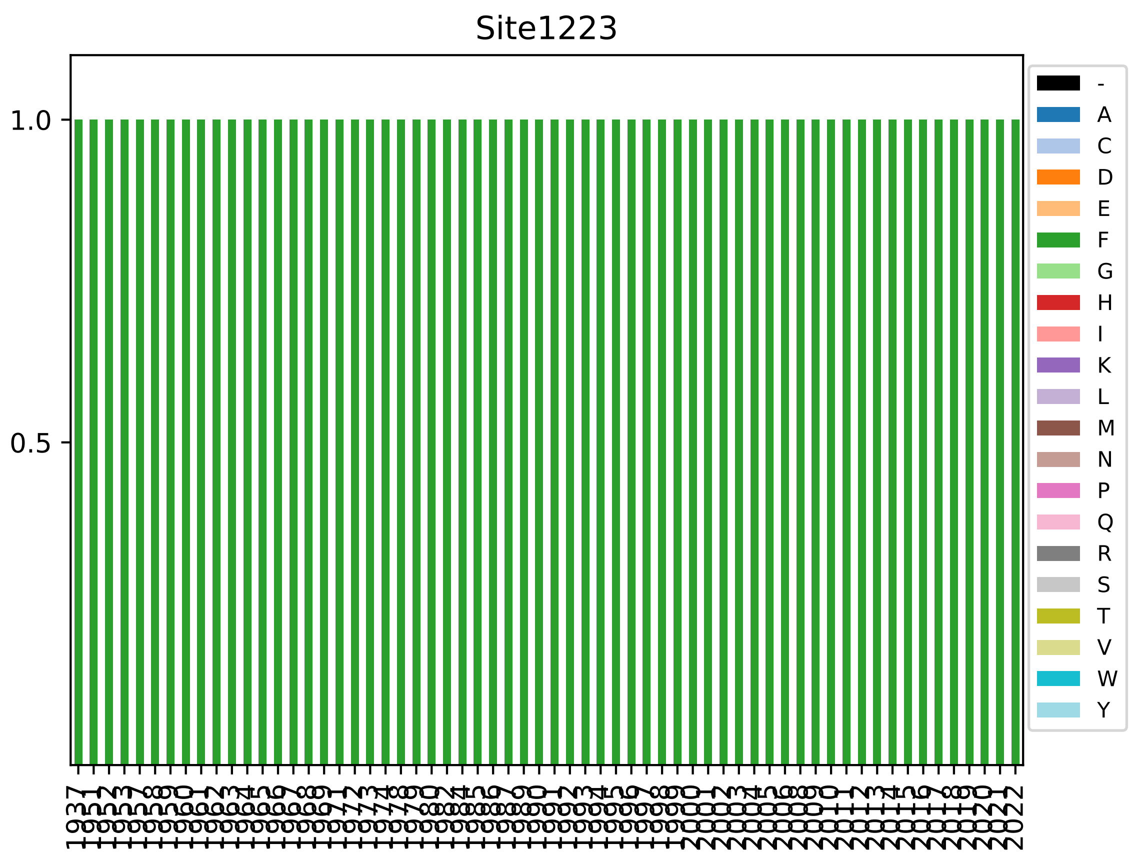
Site1223
1.0
0.5
-
A
C
D
E
F
G
H
I
K
L
M
N
P
Q
R
S
T
V
W
Y

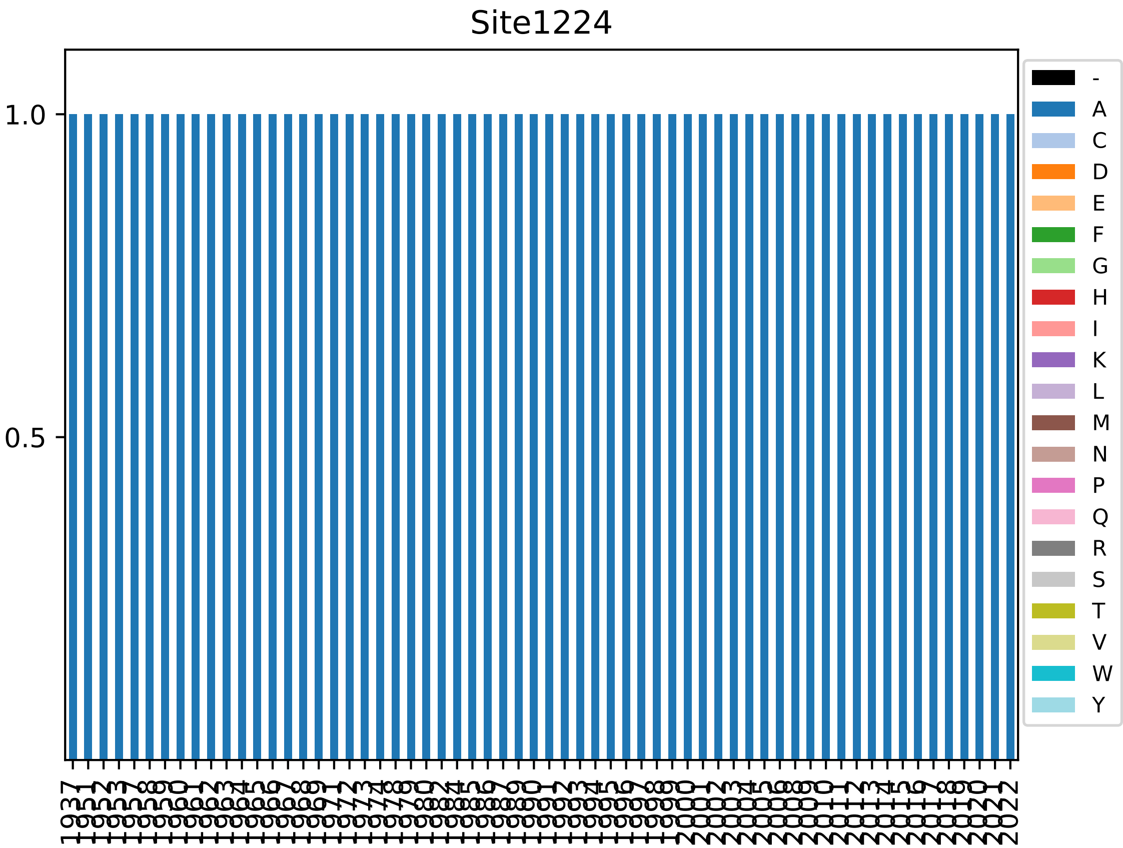
Site1224
1.0
0.5
-
A
C
D
E
F
G
H
I
K
L
M
N
P
Q
R
S
T
V
W
Y

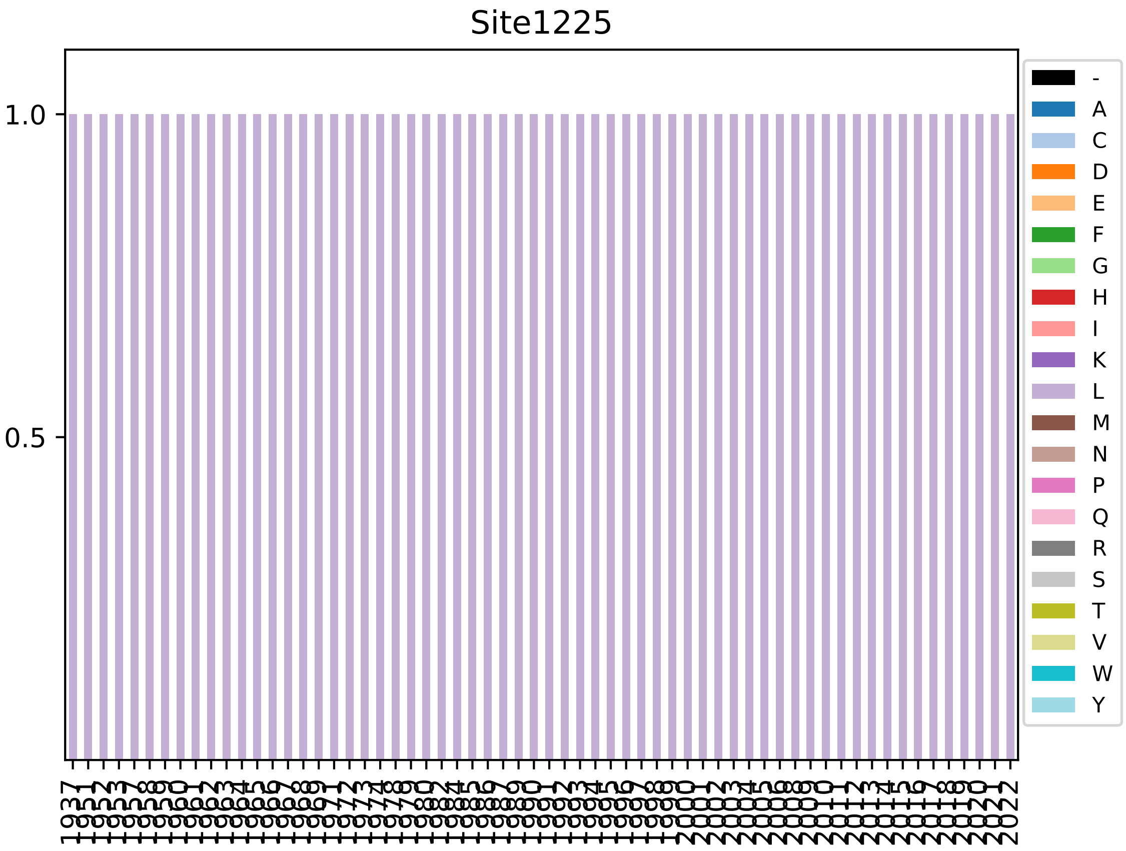
Site1225
1.0
0.5
-
A
C
D
E
F
G
H
I
K
L
M
N
P
Q
R
S
T
V
W
Y

Site1226

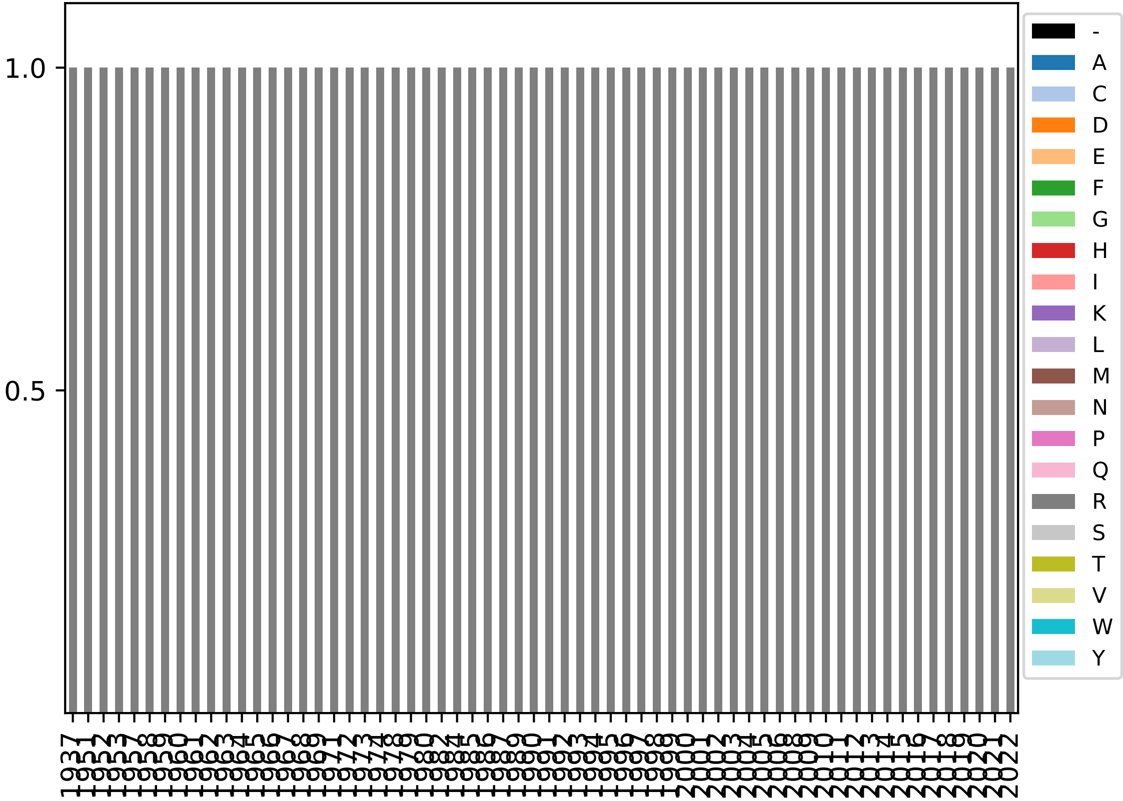

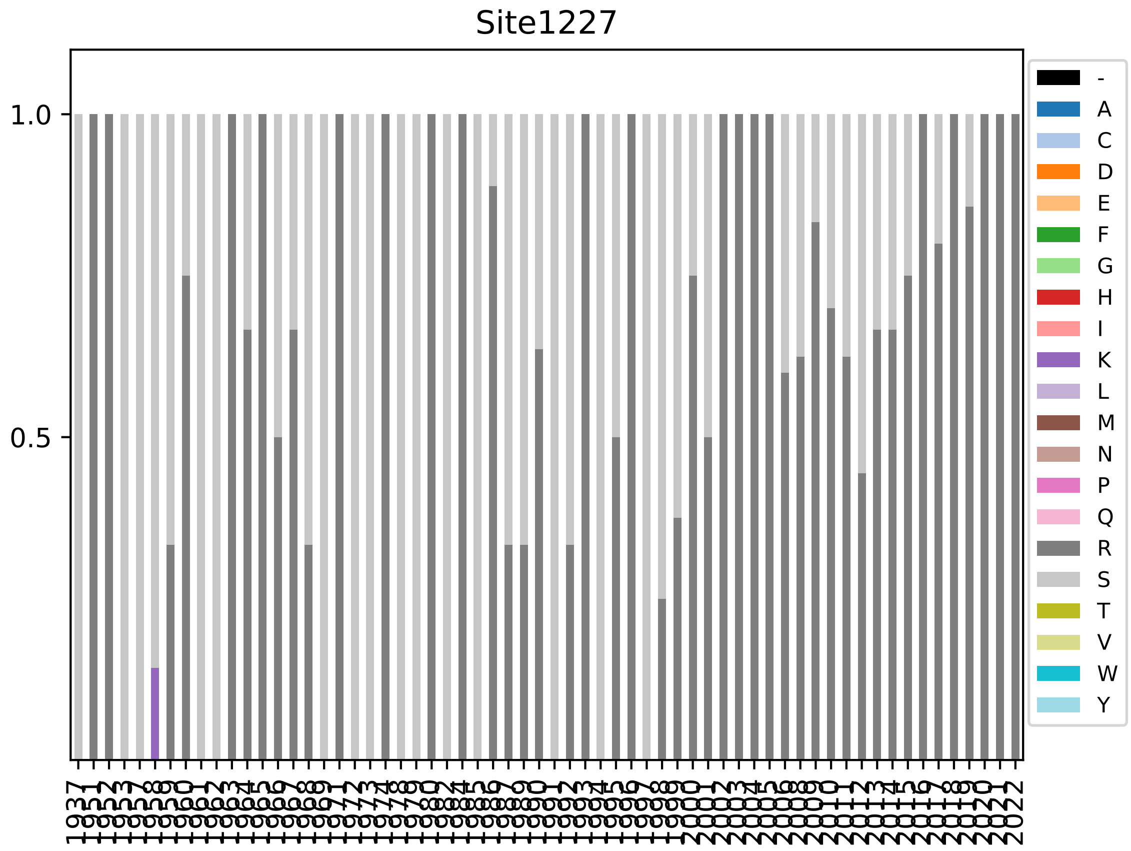
Site1227
1.0
0.5
-
A
C
D
E
F
G
H
I
K
L
M
N
P
Q
R
S
T
V
W
Y

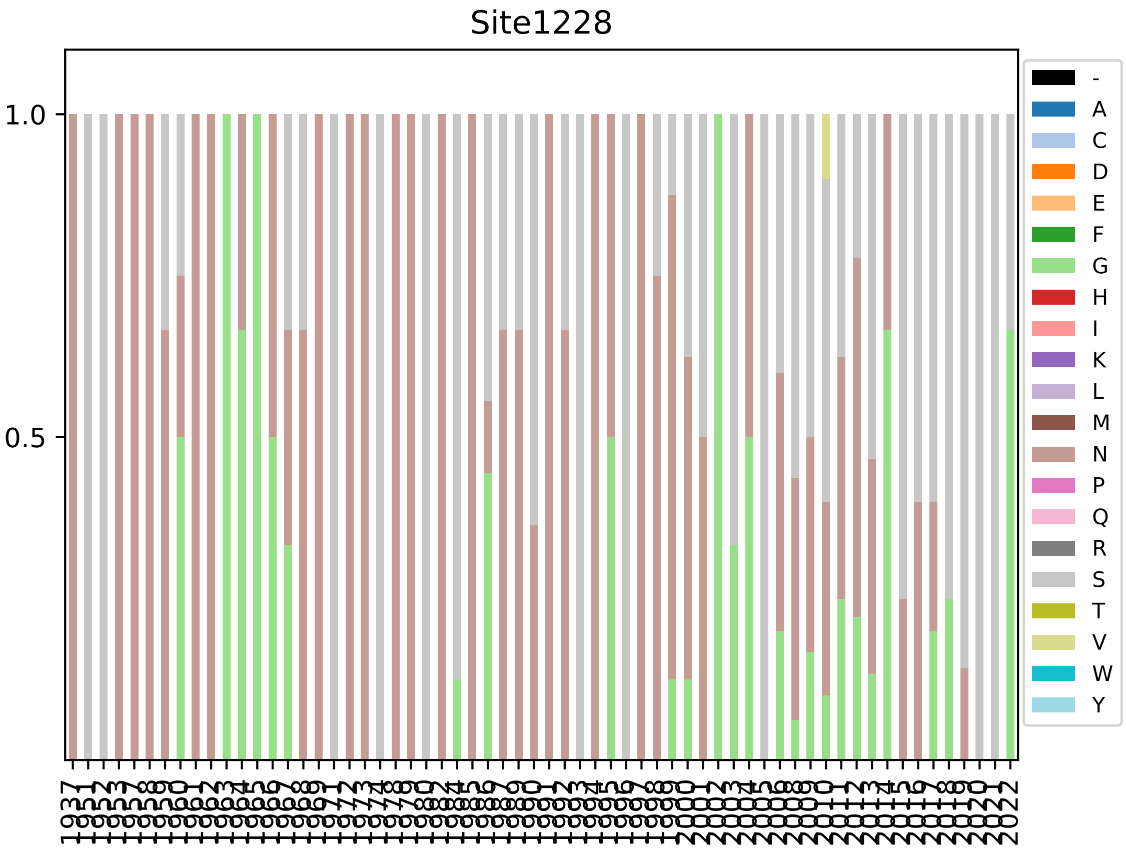
Site1228
1.0
0.5
-
A
C
D
E
F
G
H
I
K
L
M
N
P
Q
R
S
T
V
W
Y

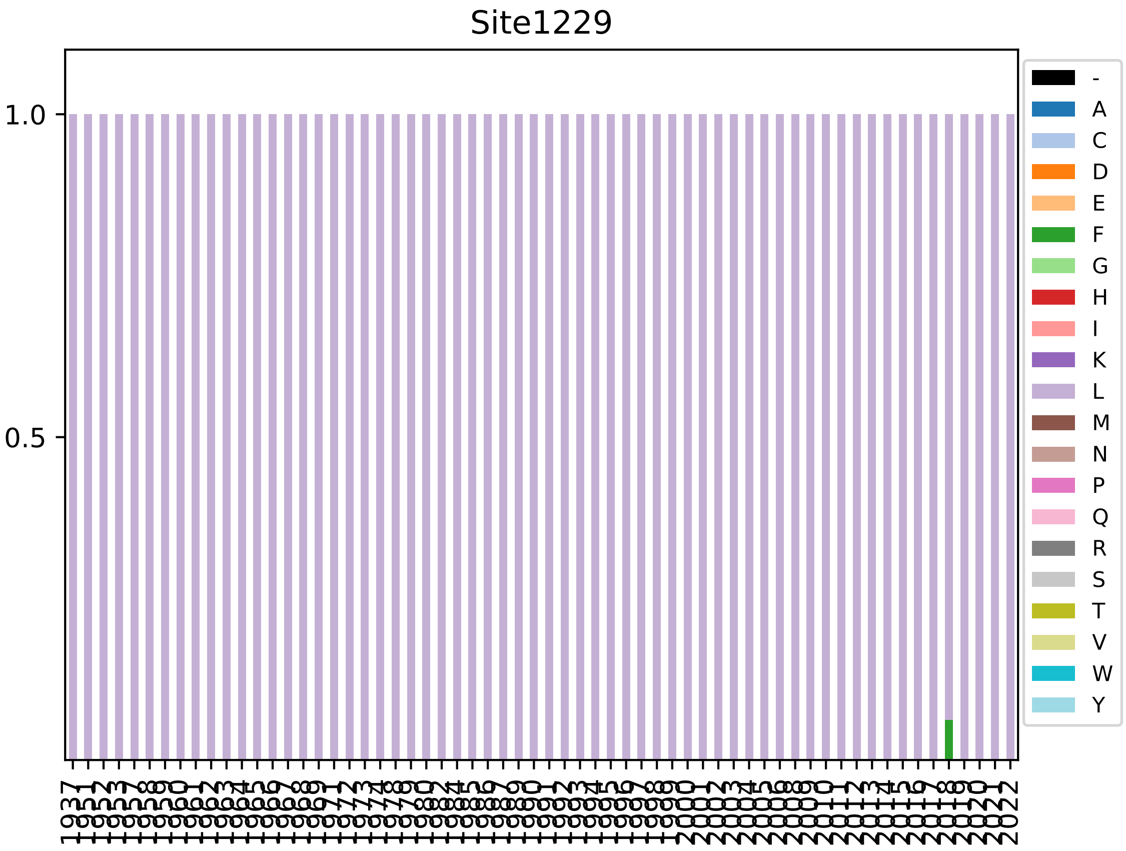

Site1229
1.0
0.5
-
A
C
D
E
F
G
H
I
K
L
M
N
P
Q
R
S
T
V
W
Y

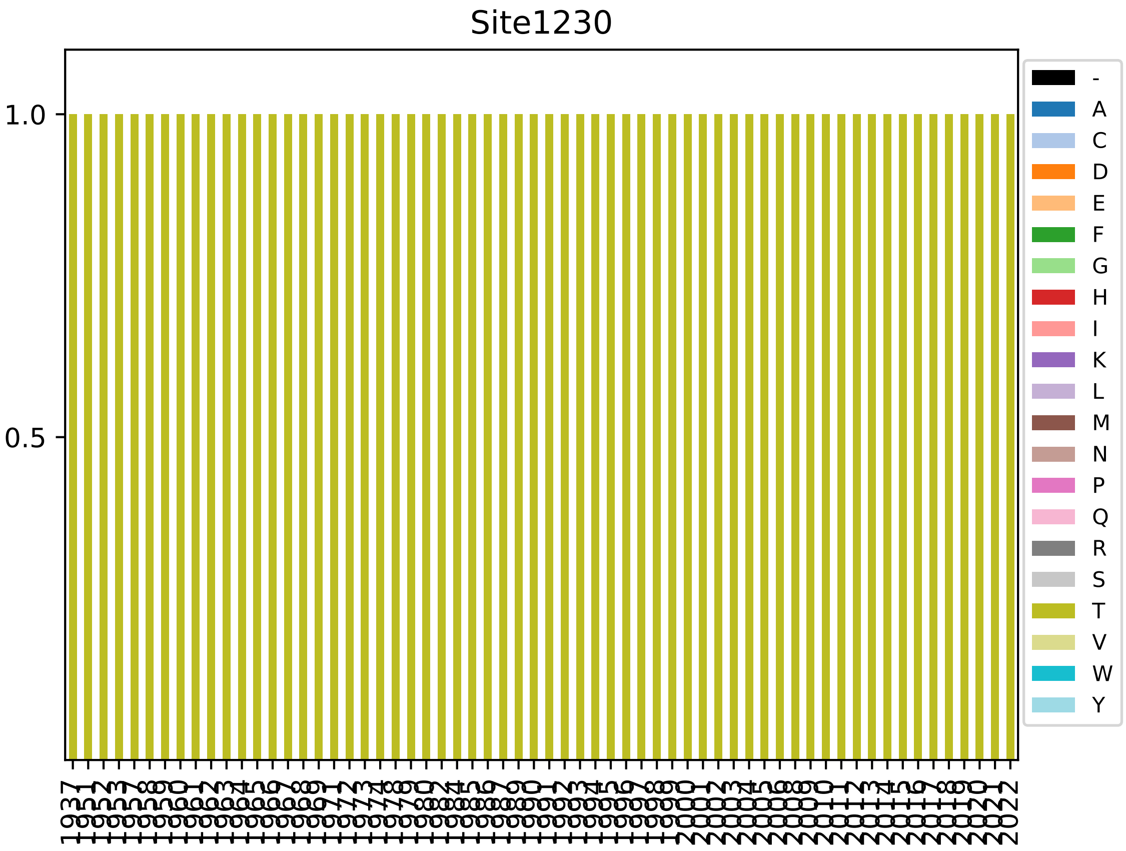
Site1230
1.0
0.5
-
A
C
D
E
F
G
H
I
K
L
M
N
P
Q
R
S
T
V
W
Y

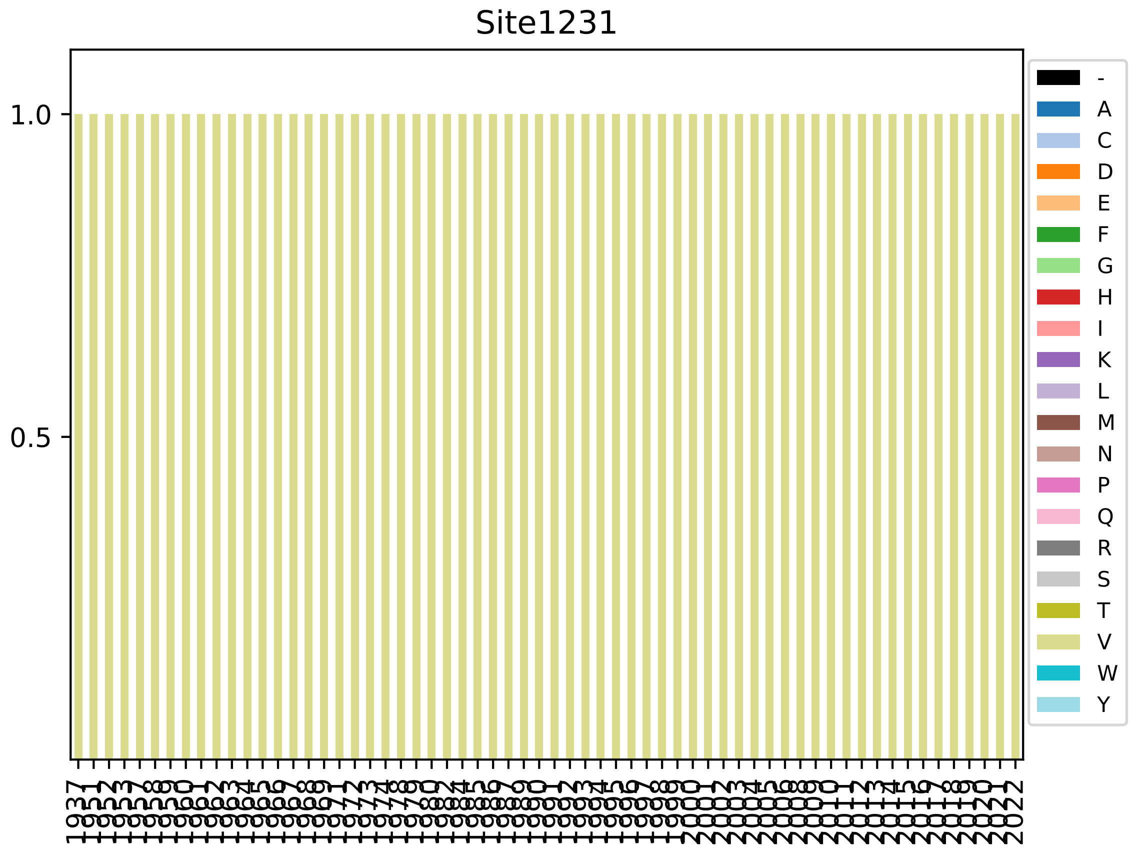
Site1231
1.0
0.5
-
A
C
D
E
F
G
H
I
K
L
M
N
P
Q
R
S
T
V
W
Y

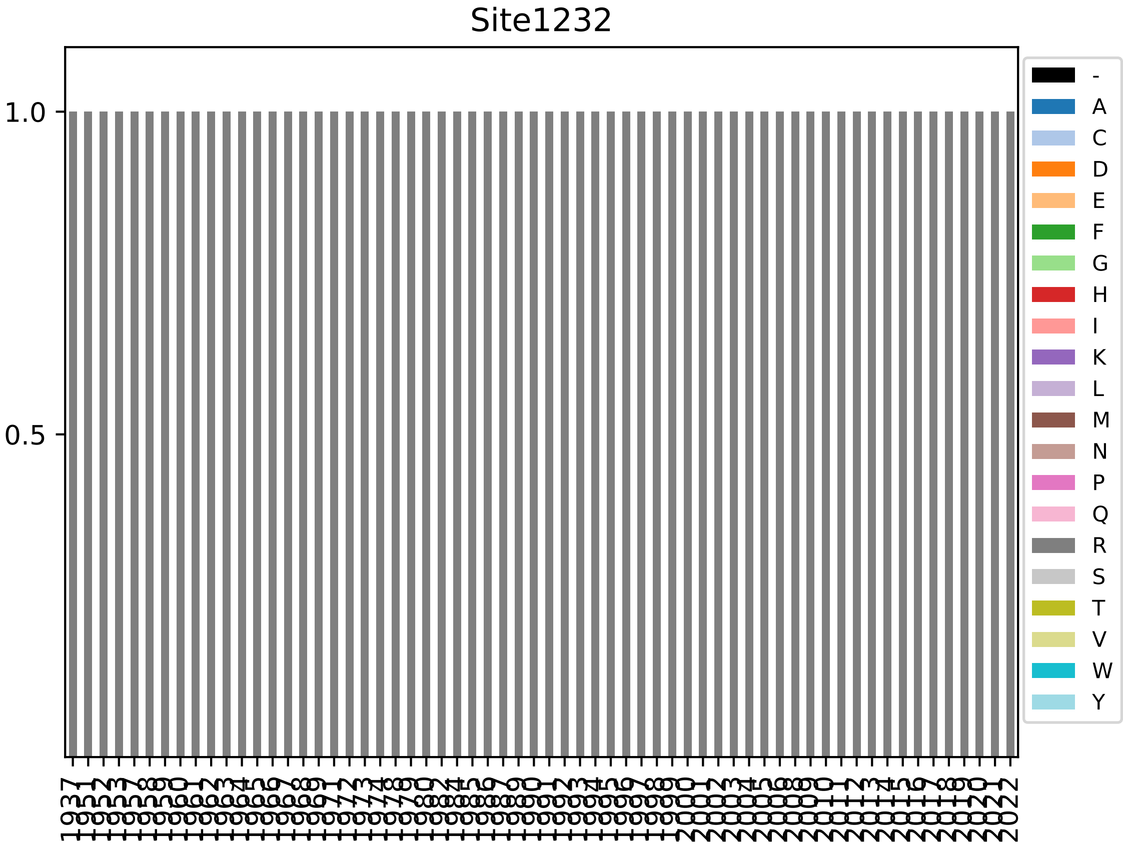
Site1232
1.0
0.5
-
A
C
D
E
F
G
H
I
K
L
M
N
P
Q
R
S
T
V
W
Y

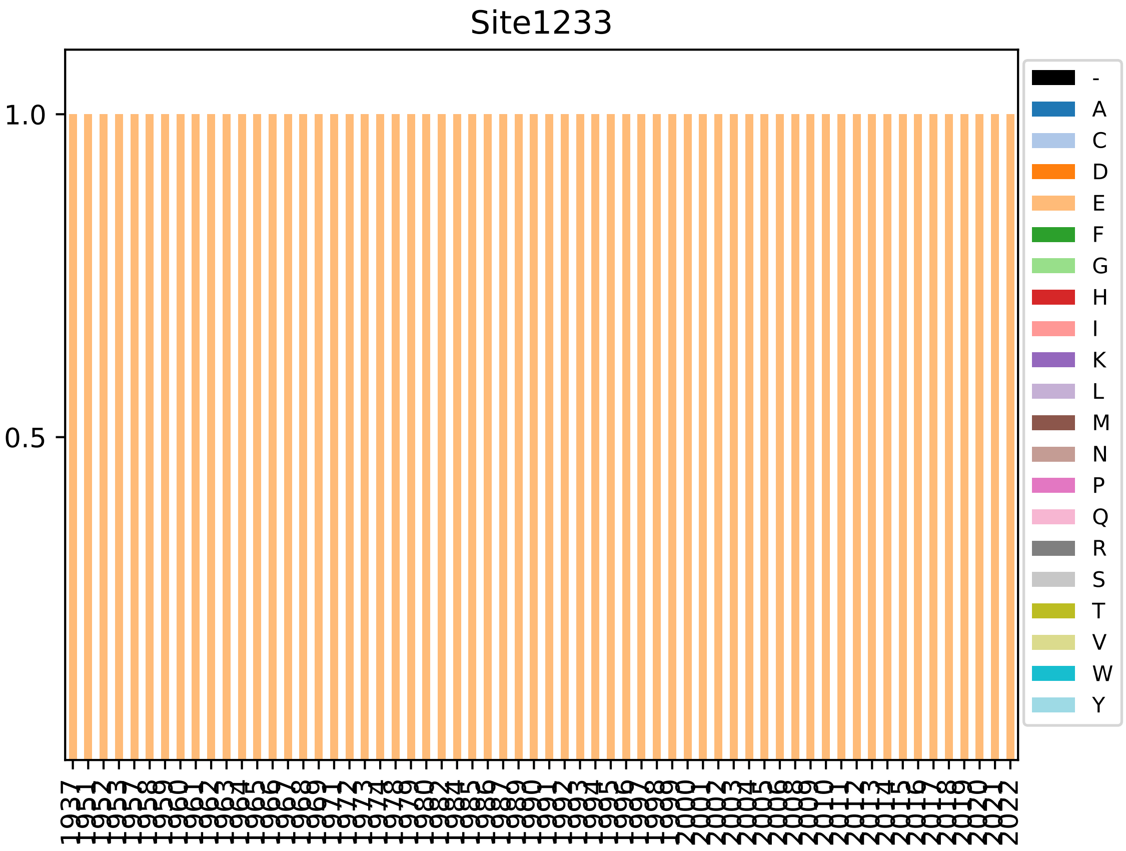

Site1233
1.0
0.5
-
A
C
D
E
F
G
H
I
K
L
M
N
P
Q
R
S
T
V
W
Y

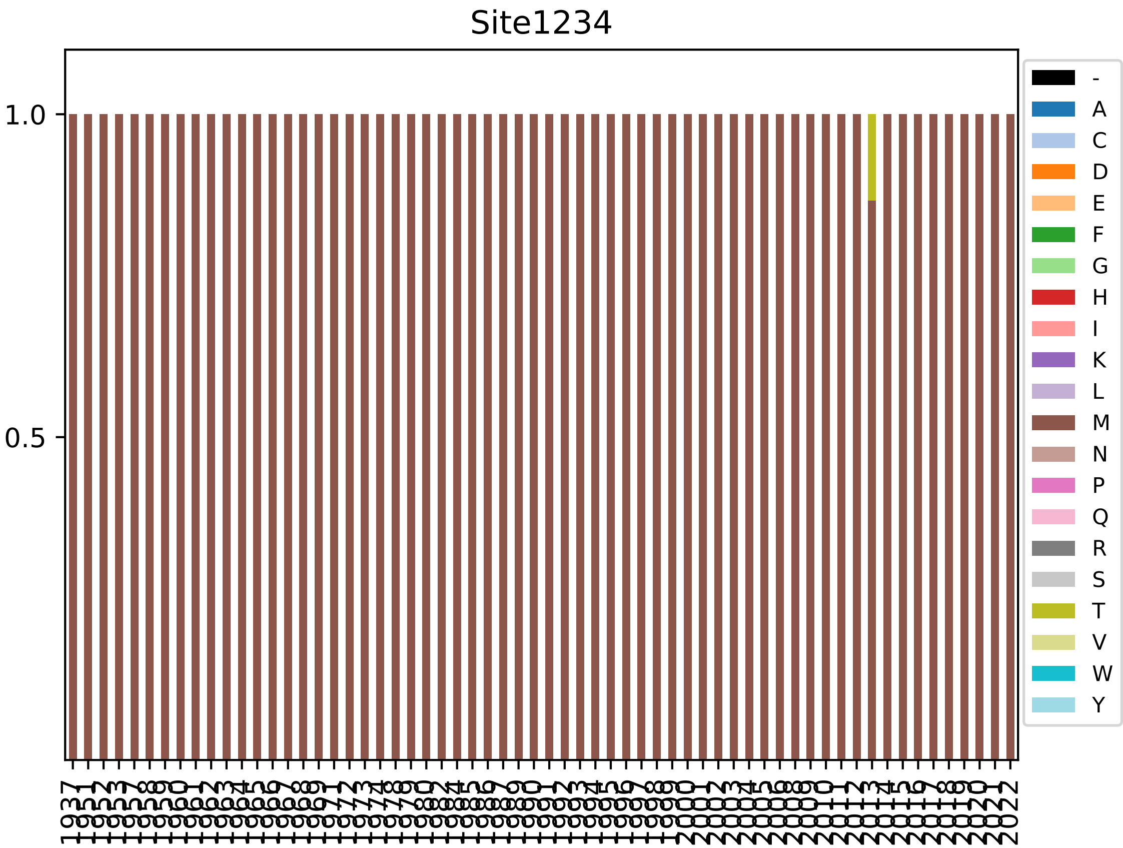
Site1234
1.0
0.5
-
A
C
D
E
F
G
H
I
K
L
M
N
P
Q
R
S
T
V
W
Y

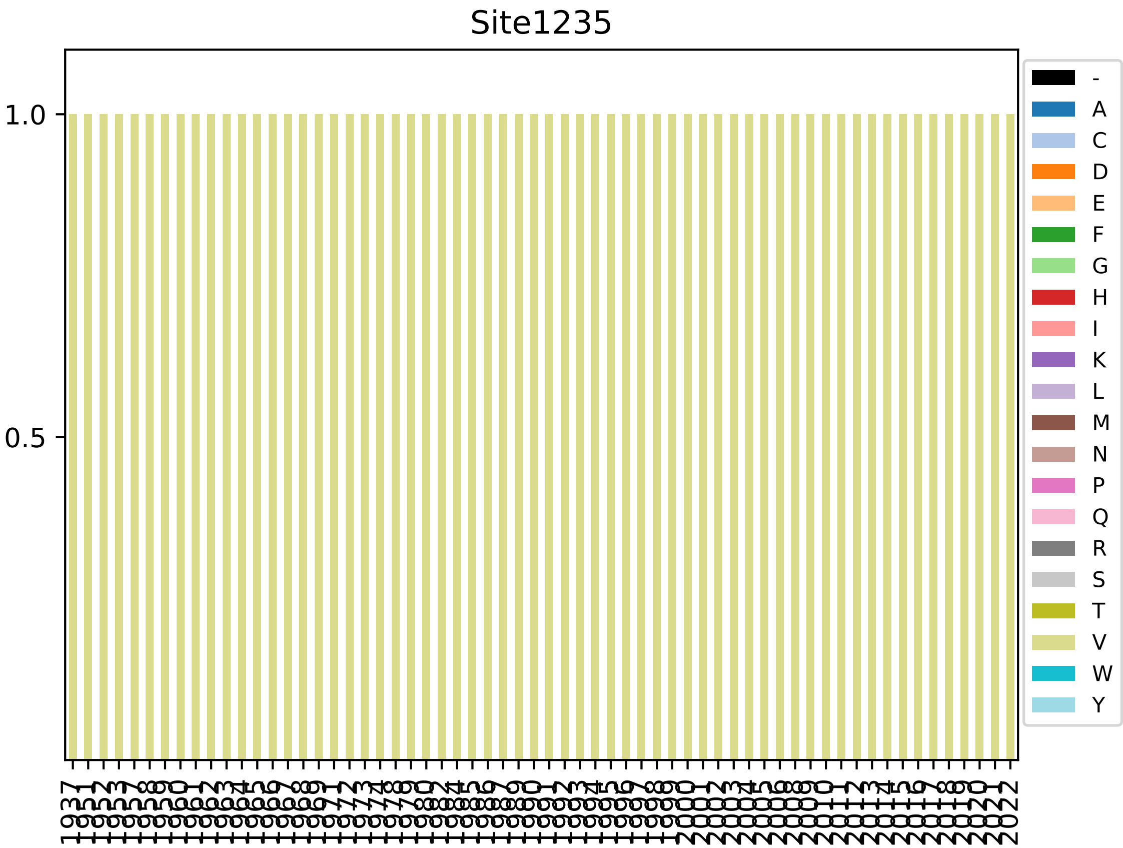
Site1235
1.0
0.5
-
A
C
D
E
F
G
H
I
K
L
M
N
P
Q
R
S
T
V
W
Y

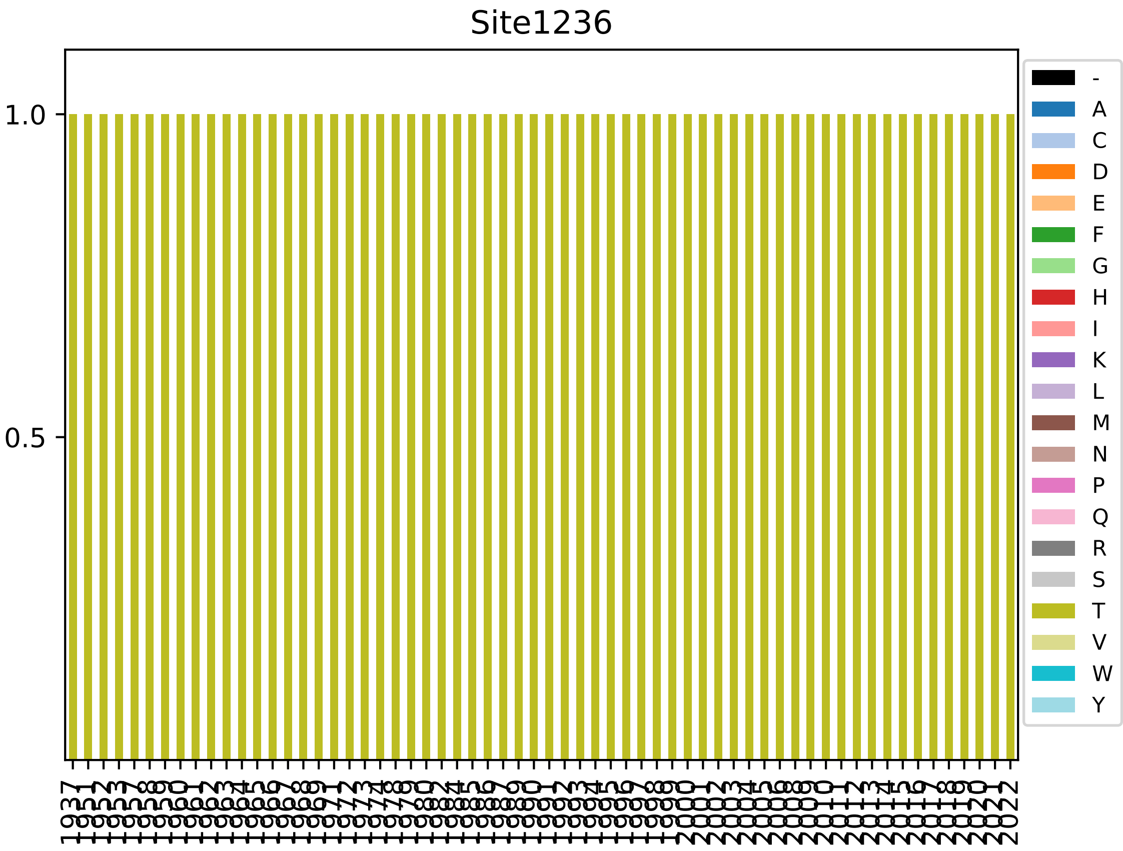

Site1236
1.0
0.5
-
A
C
D
E
F
G
H
I
K
L
M
N
P
Q
R
S
T
V
W
Y

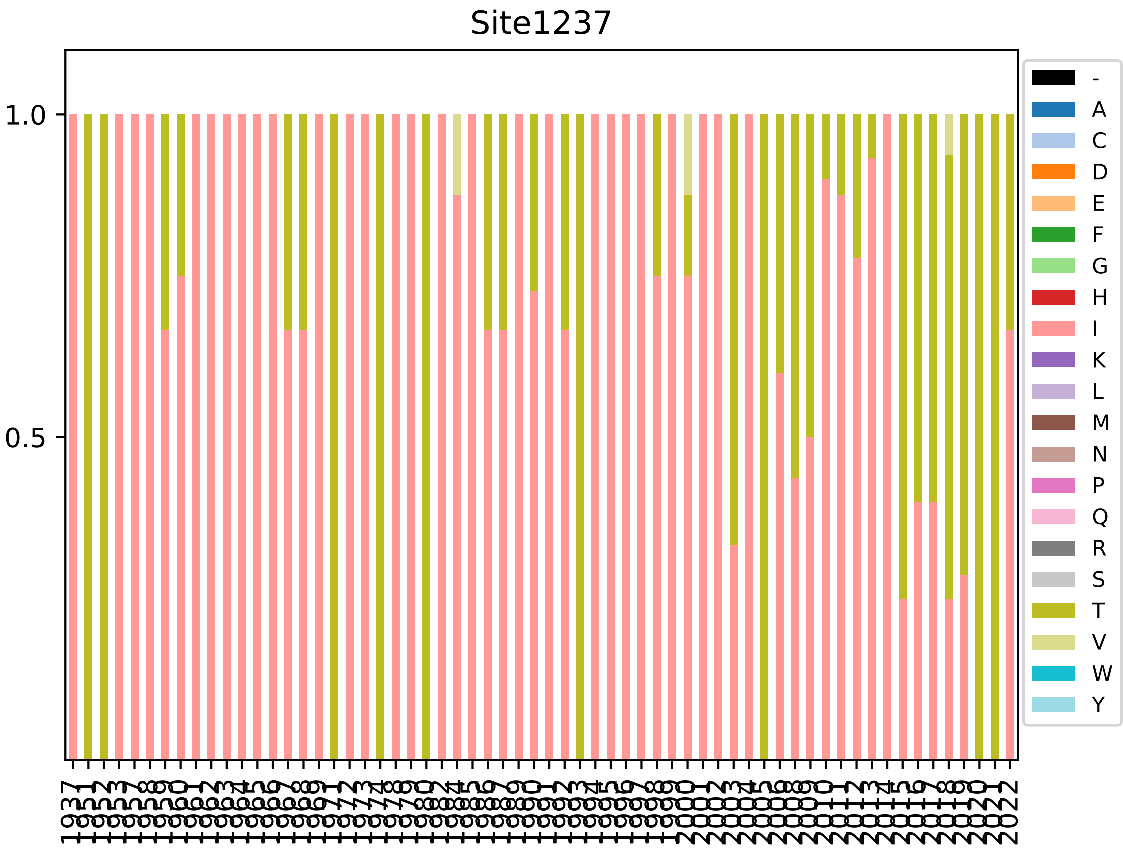

Site1237
1.0
0.5
-
A
C
D
E
F
G
H
I
K
L
M
N
P
Q
R
S
T
V
W
Y

Site1238

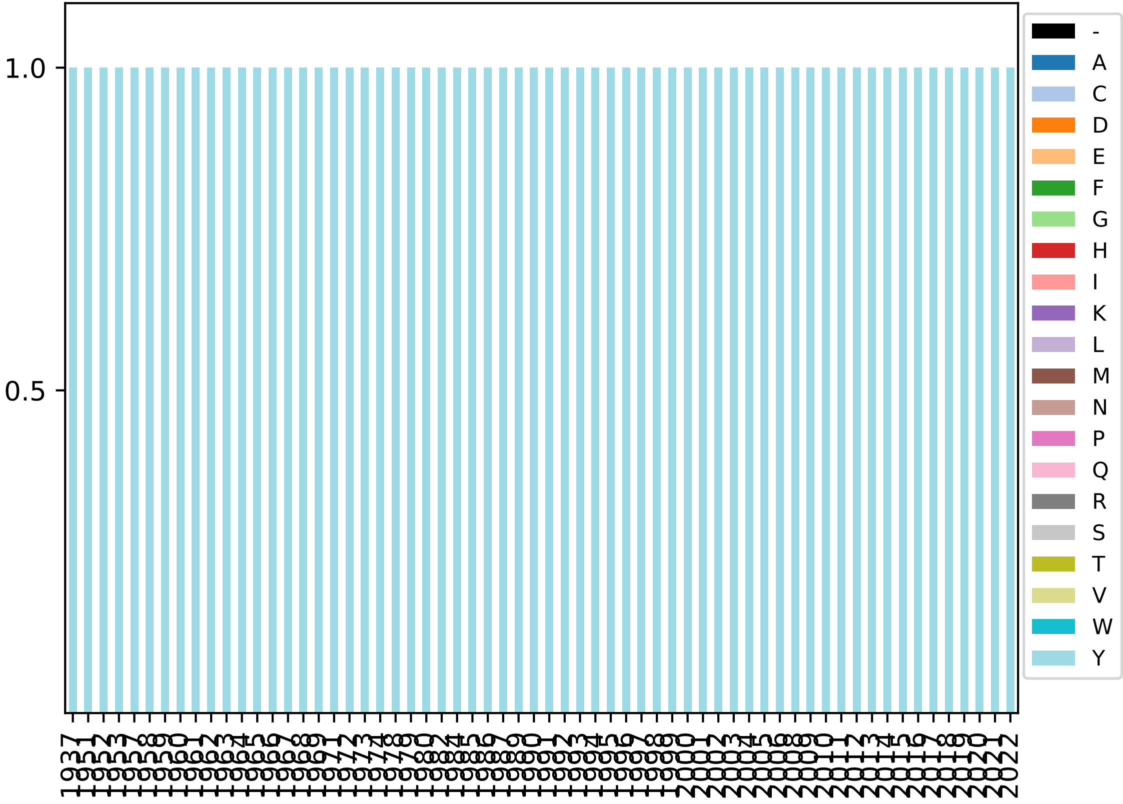

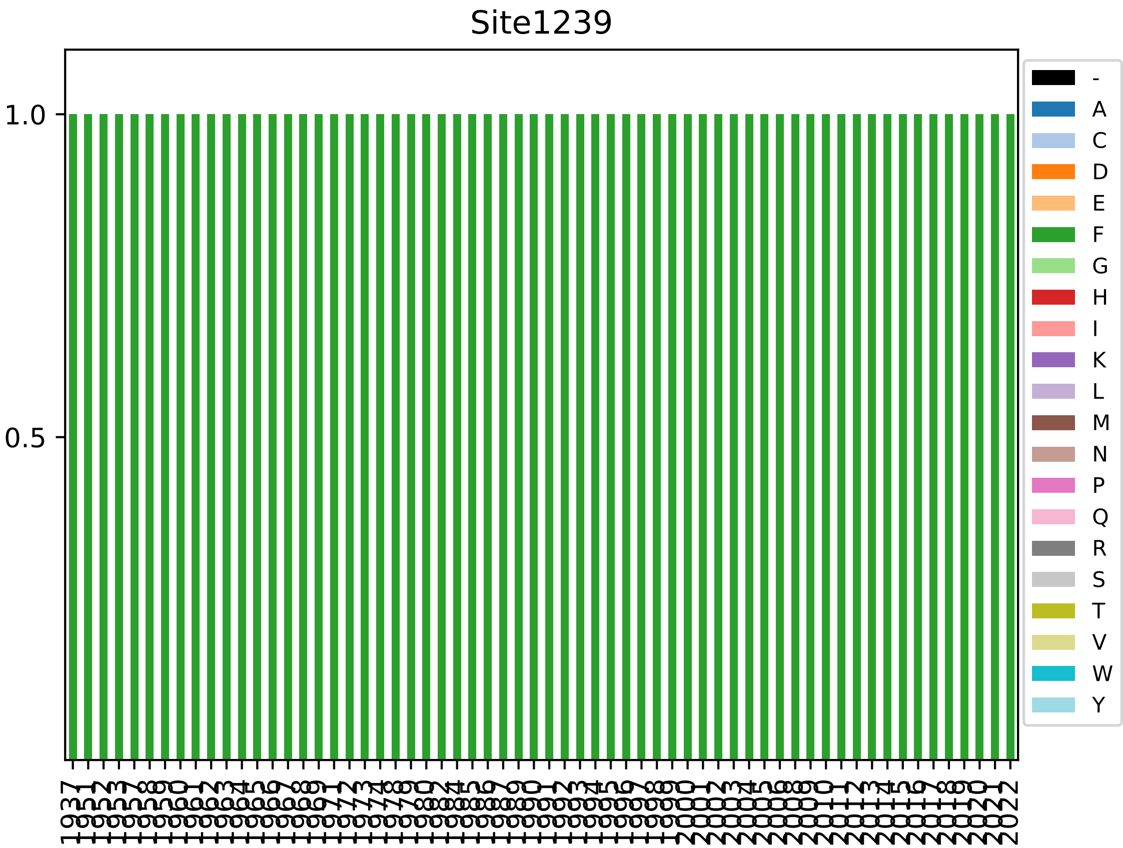
Site1239
1.0
0.5
-
A
C
D
E
F
G
H
I
K
L
M
N
P
Q
R
S
T
V
W
Y

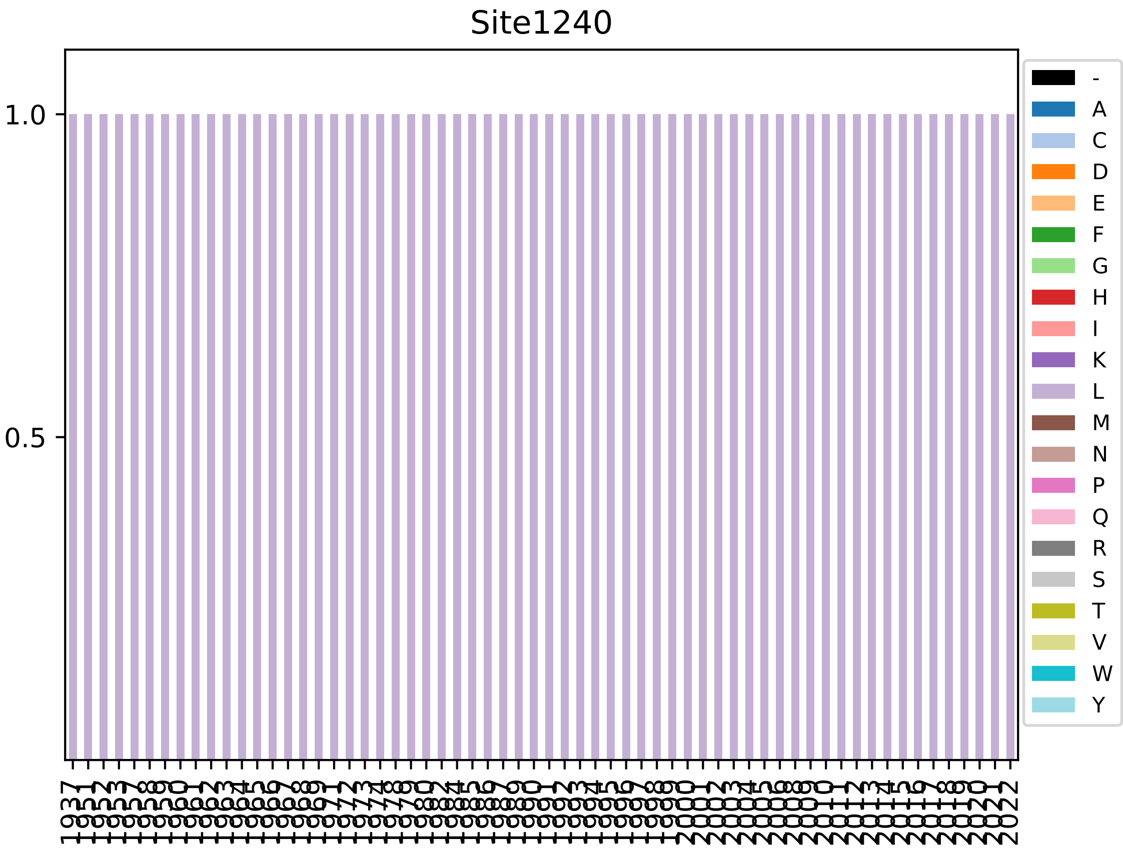
Site1240
1.0
0.5
-
A
C
D
E
F
G
H
I
K
L
M
N
P
Q
R
S
T
V
W
Y

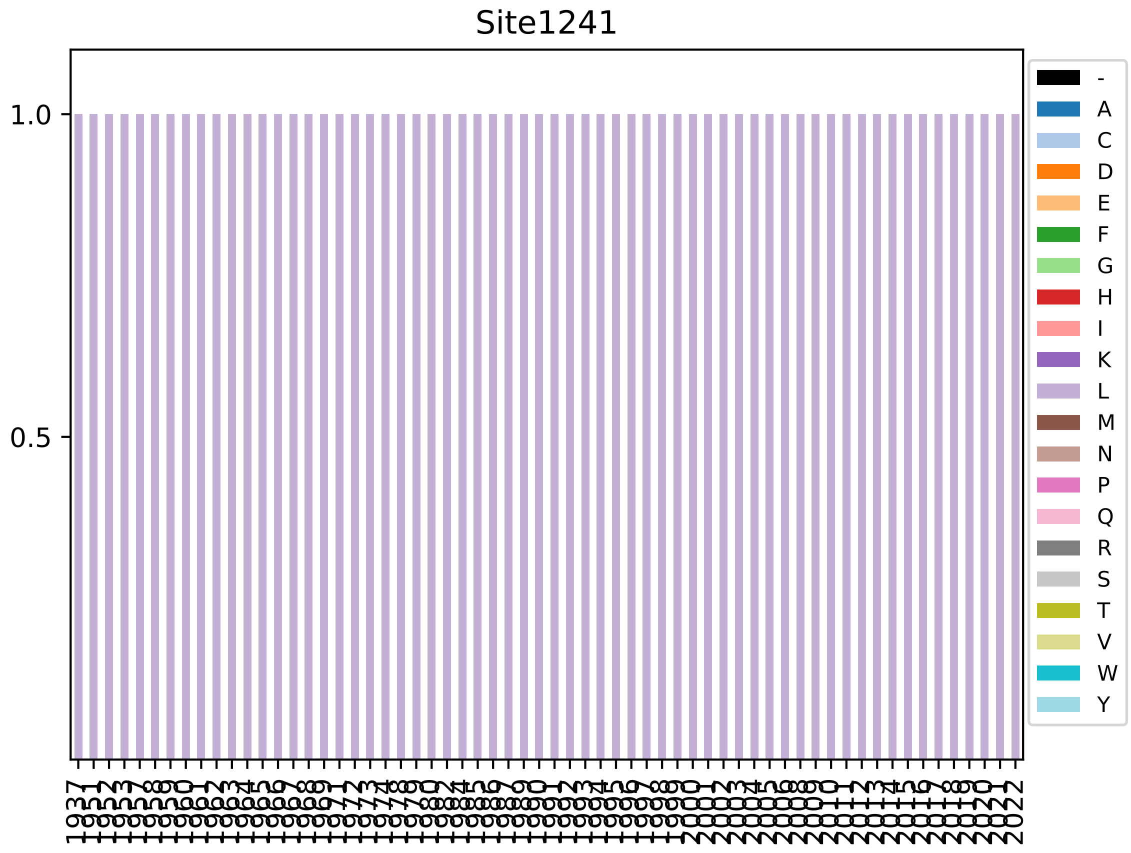
Site1241
1.0
0.5
-
A
C
D
E
F
G
H
I
K
L
M
N
P
Q
R
S
T
V
W
Y

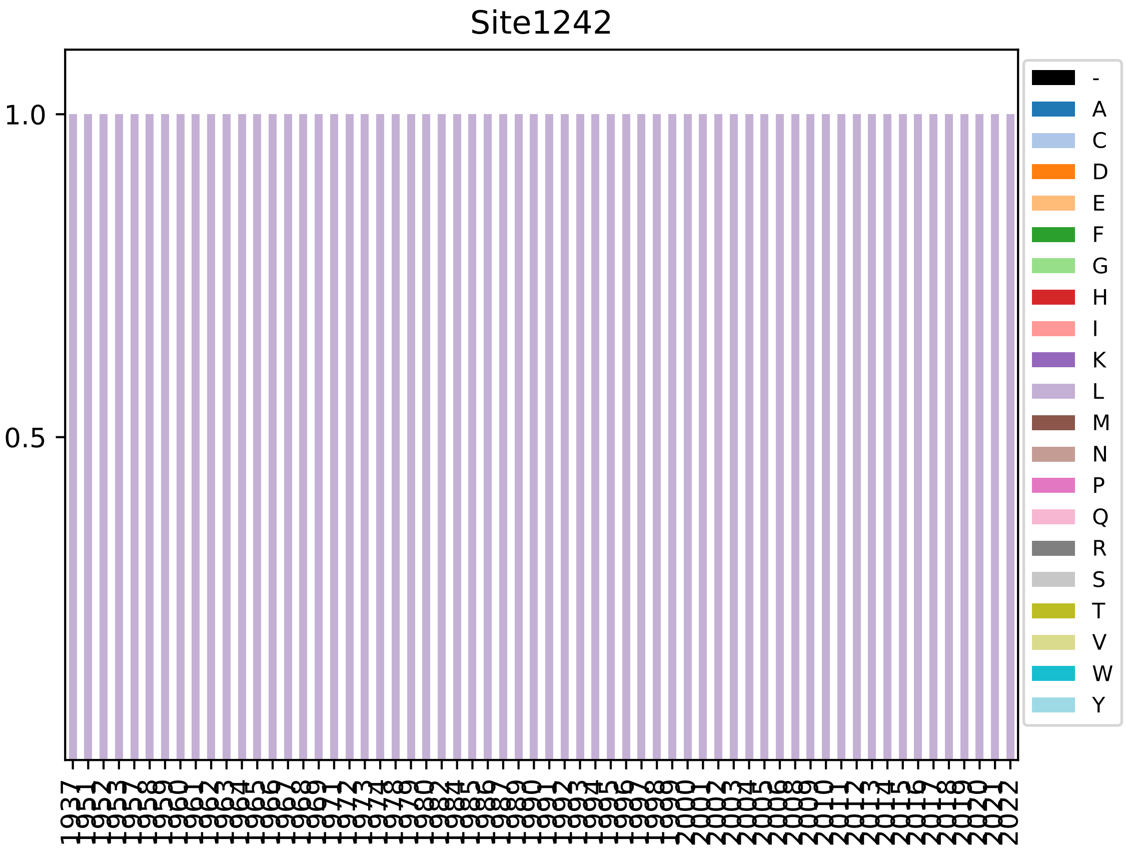
Site1242
1.0
0.5
-
A
C
D
E
F
G
H
I
K
L
M
N
P
Q
R
S
T
V
W
Y

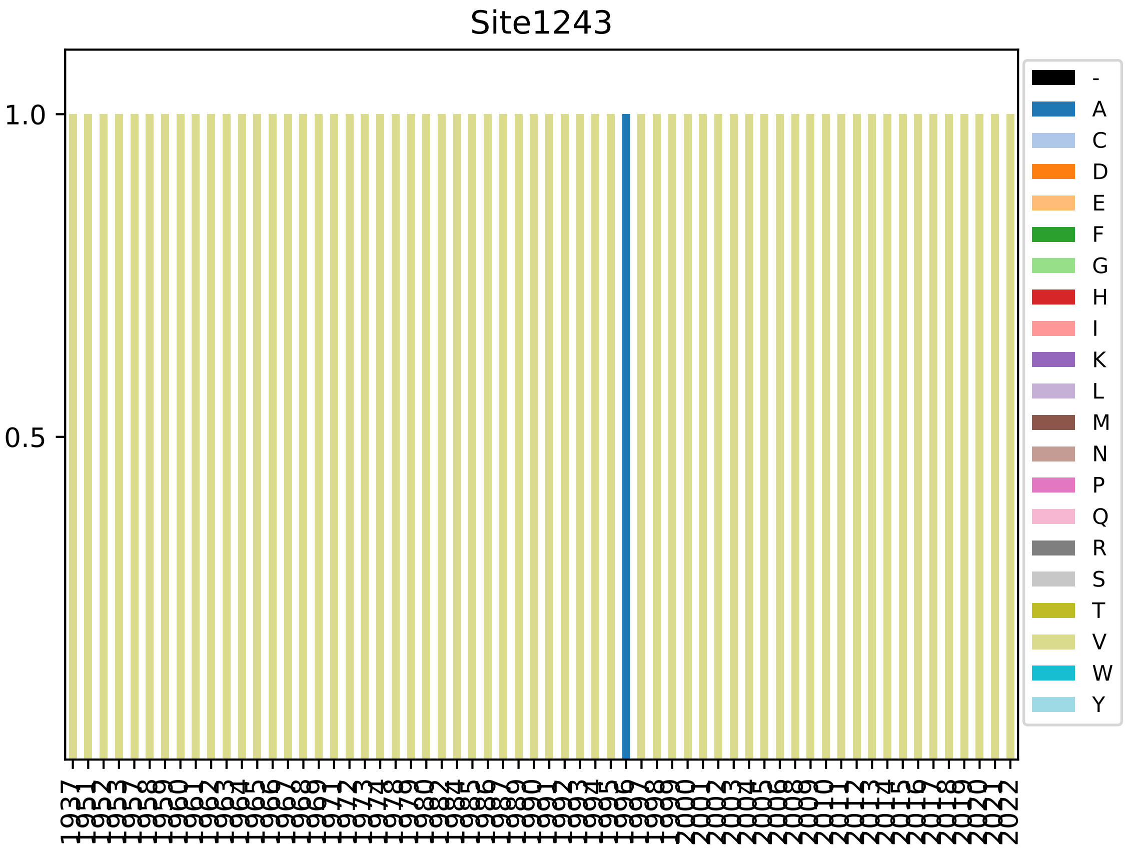
Site1243
1.0
0.5
-
A
C
D
E
F
G
H
I
K
L
M
N
P
Q
R
S
T
V
W
Y

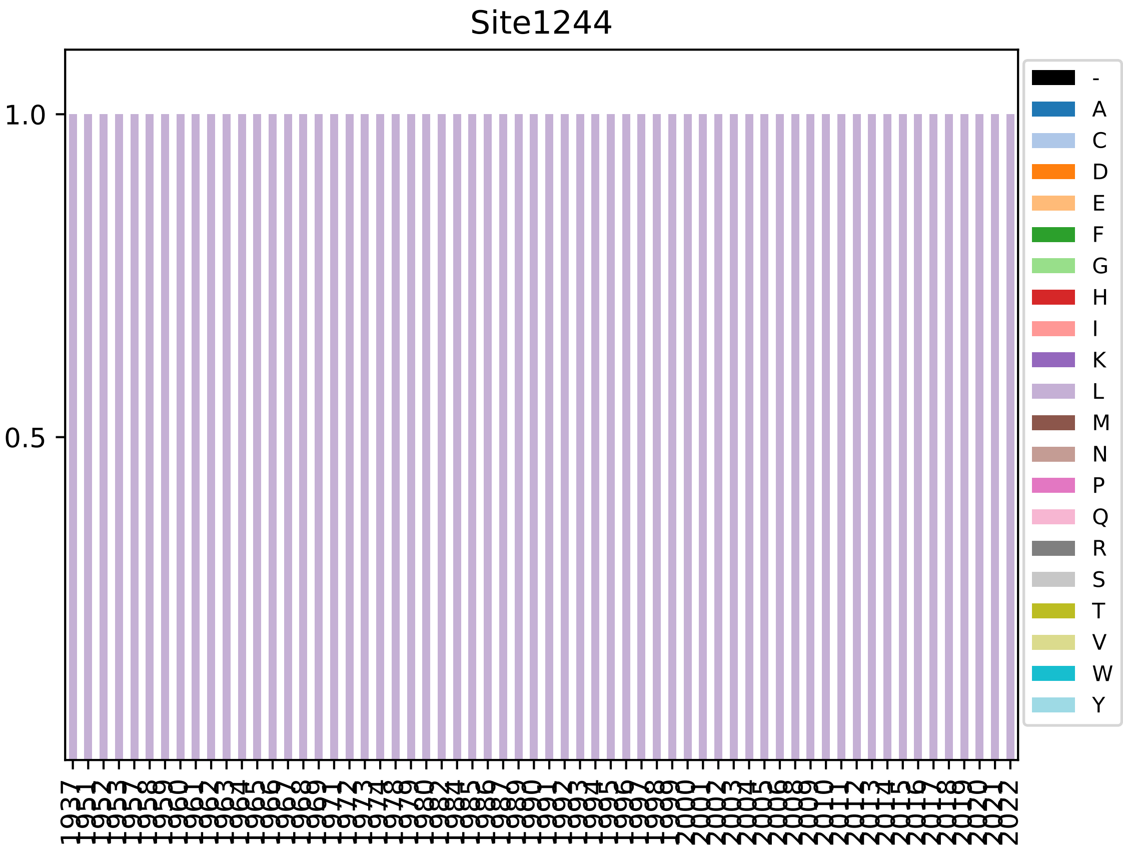
Site1244
1.0
0.5
-
A
C
D
E
F
G
H
I
K
L
M
N
P
Q
R
S
T
V
W
Y

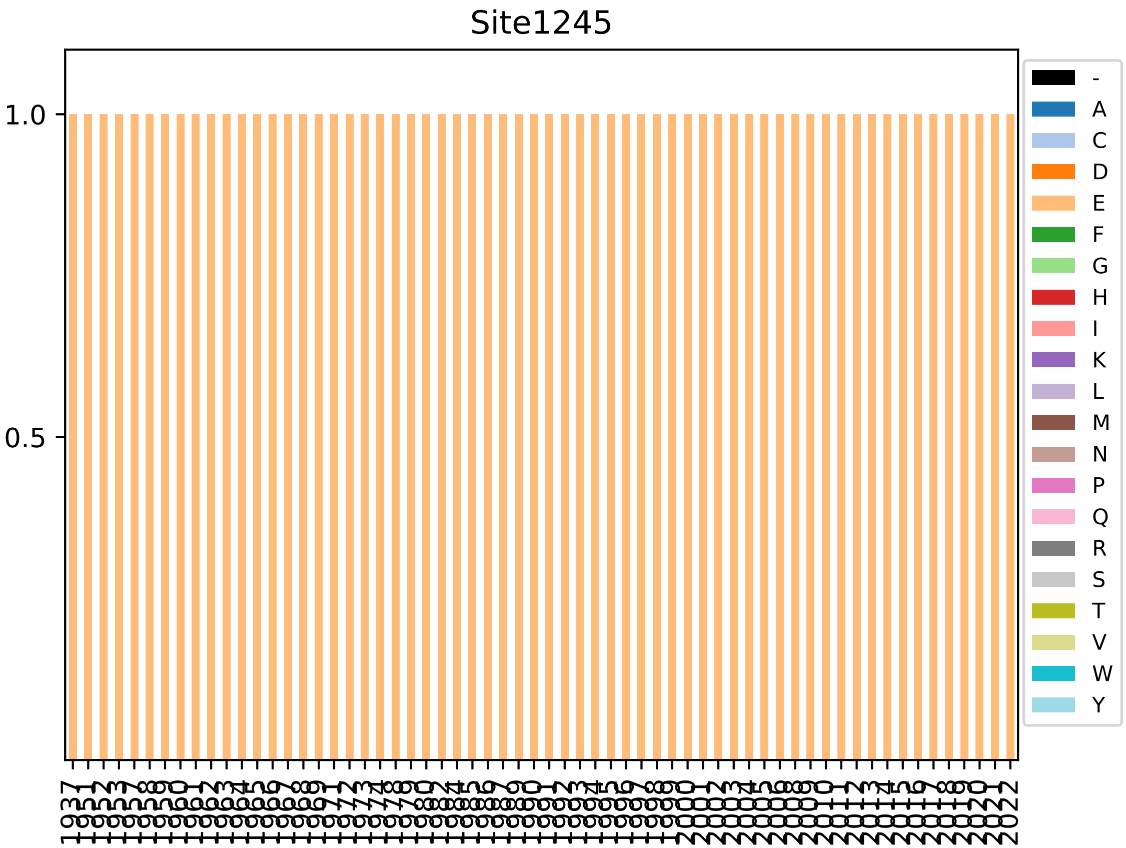
Site1245
1.0
0.5
-
A
C
D
E
F
G
H
I
K
L
M
N
P
Q
R
S
T
V
W
Y

Site1246

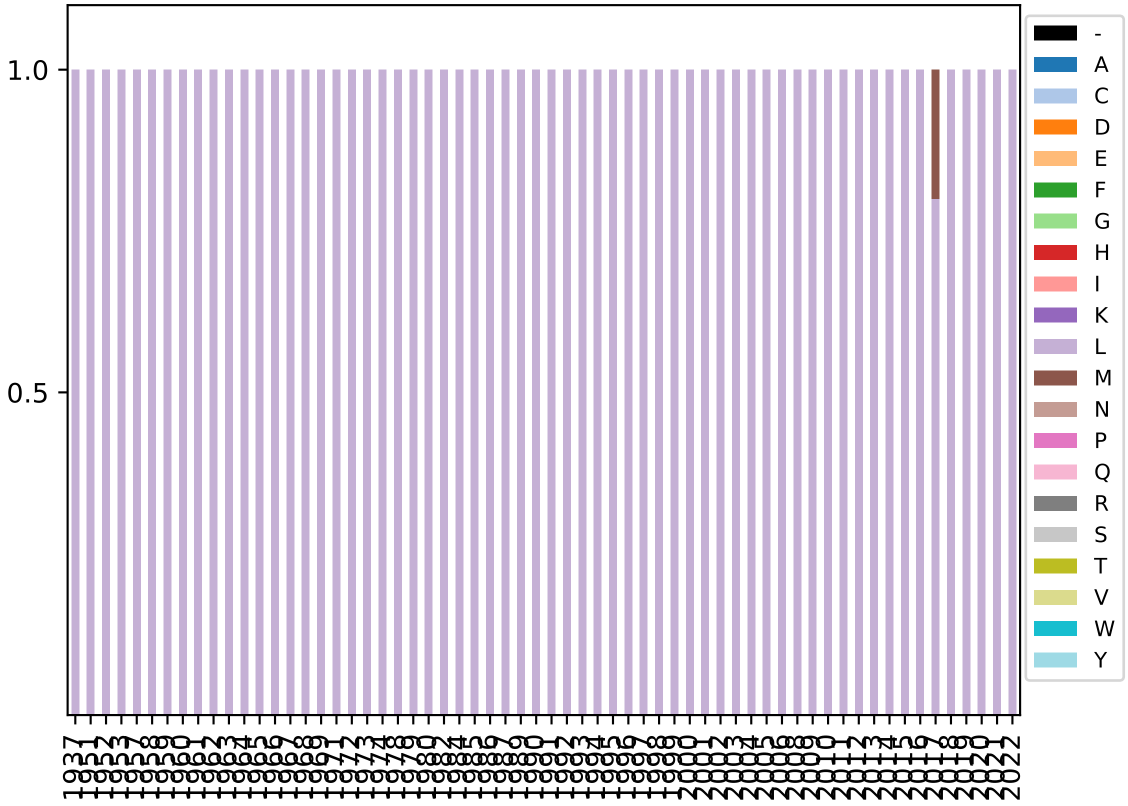

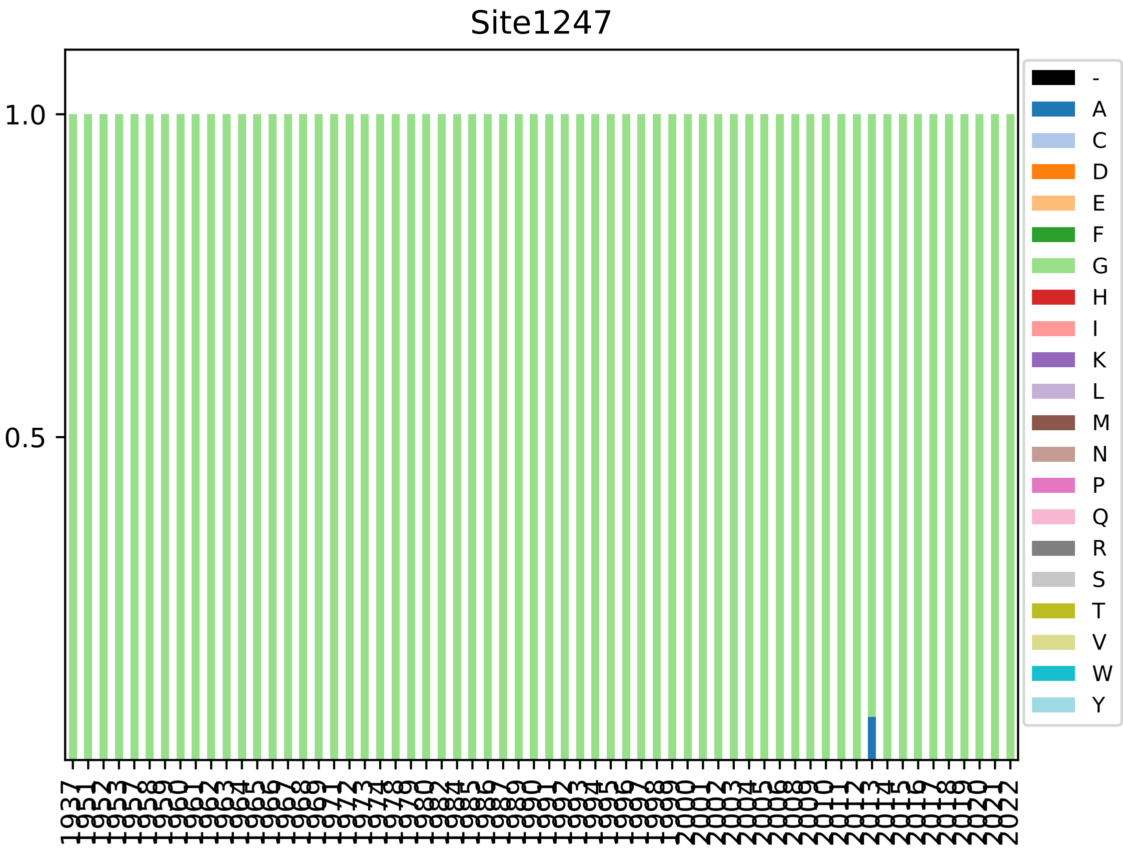
Site1247
1.0
0.5
-
A
C
D
E
F
G
H
I
K
L
M
N
P
Q
R
S
T
V
W
Y

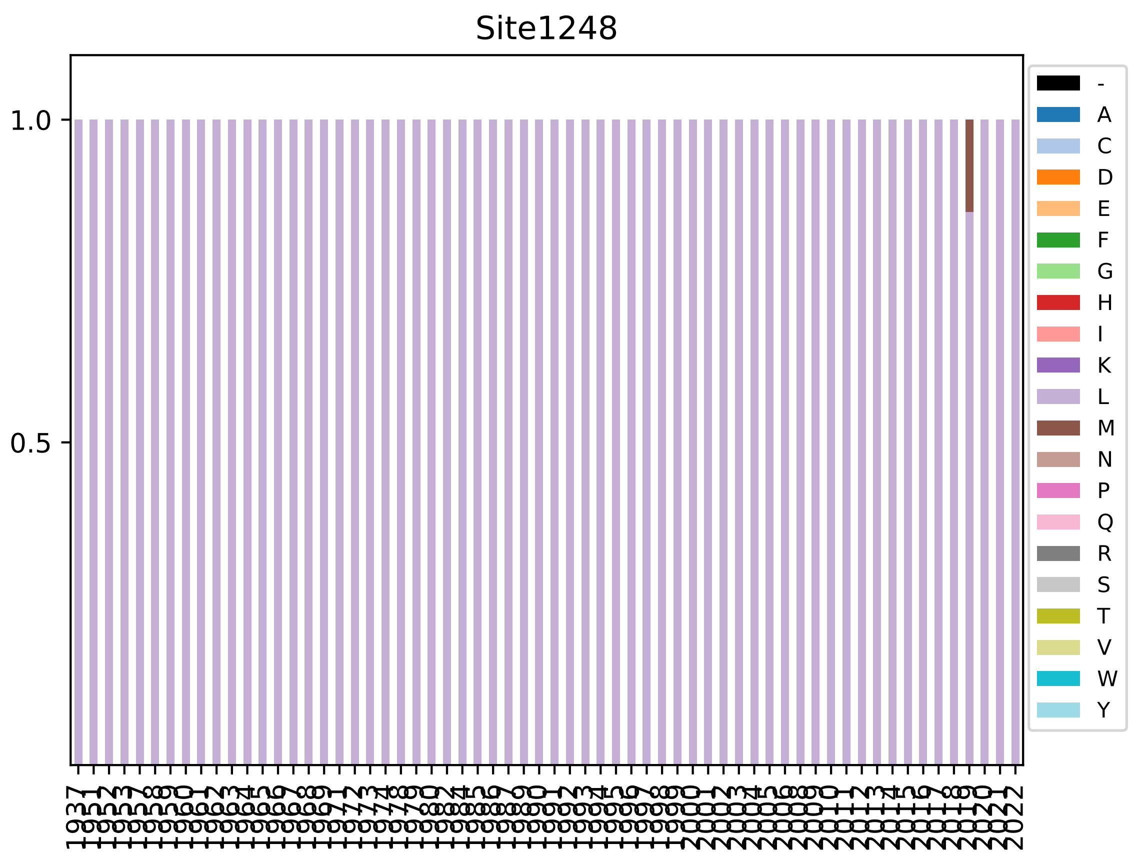

Site1248
1.0
0.5
-
A
C
D
E
F
G
H
I
K
L
M
N
P
Q
R
S
T
V
W
Y

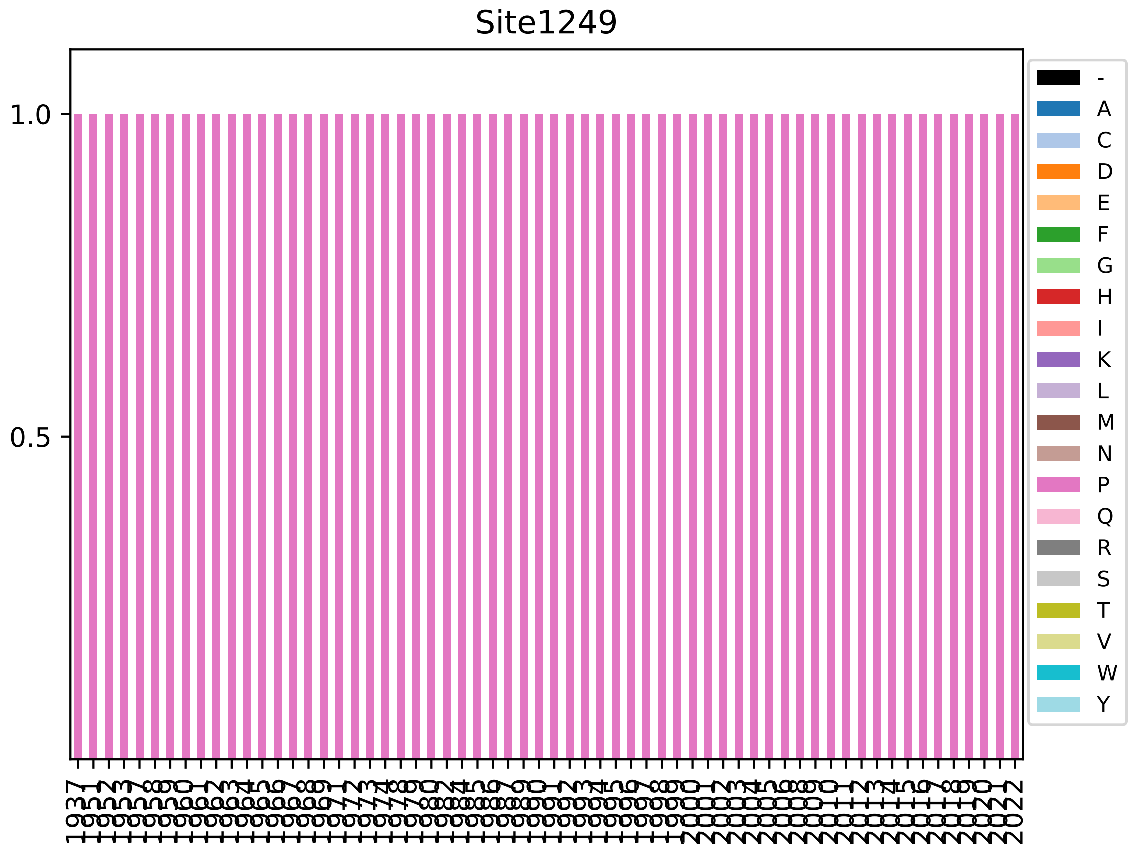
Site1249
1.0
0.5
-
A
C
D
E
F
G
H
I
K
L
M
N
P
Q
R
S
T
V
W
Y

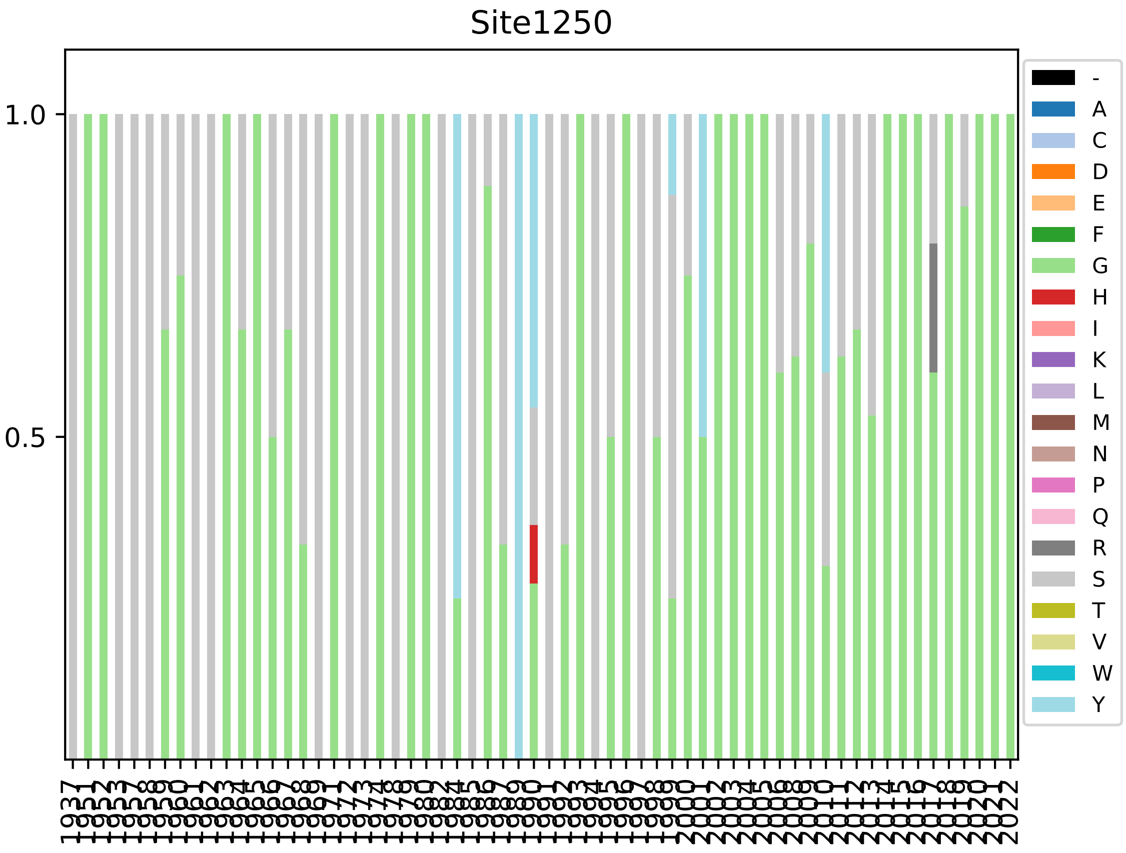
Site1250
1.0
0.5
-
A
C
D
E
F
G
H
I
K
L
M
N
P
Q
R
S
T
V
W
Y

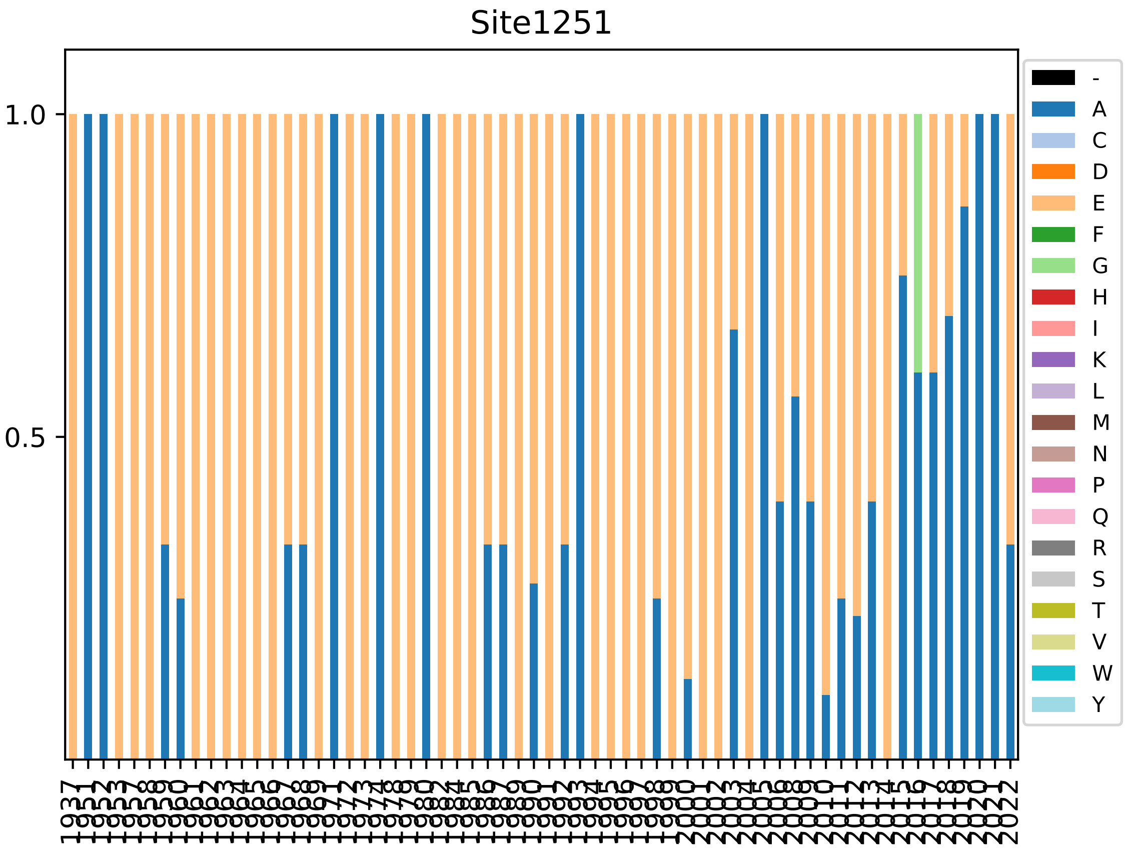
Site1251
1.0
0.5
-
A
C
D
E
F
G
H
I
K
L
M
N
P
Q
R
S
T
V
W
Y

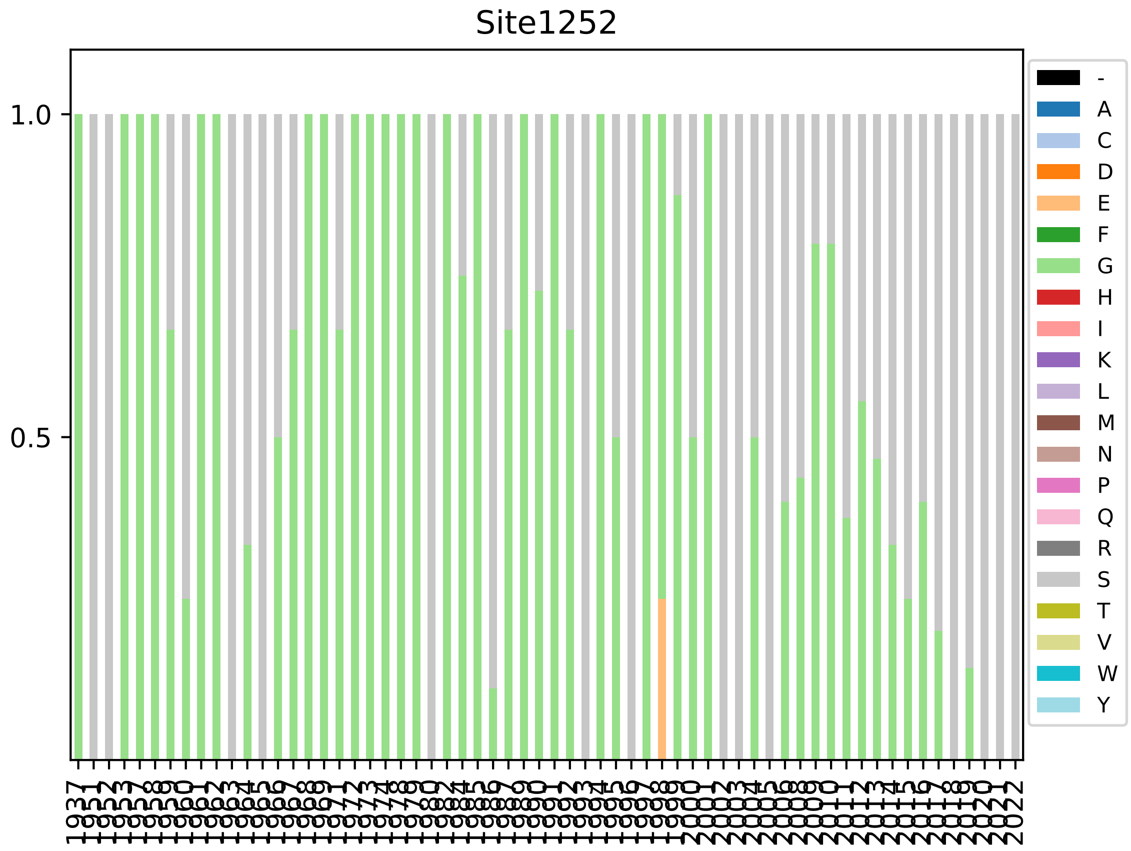
Site1252
1.0
0.5
-
A
C
D
E
F
G
H
I
K
L
M
N
P
Q
R
S
T
V
W
Y

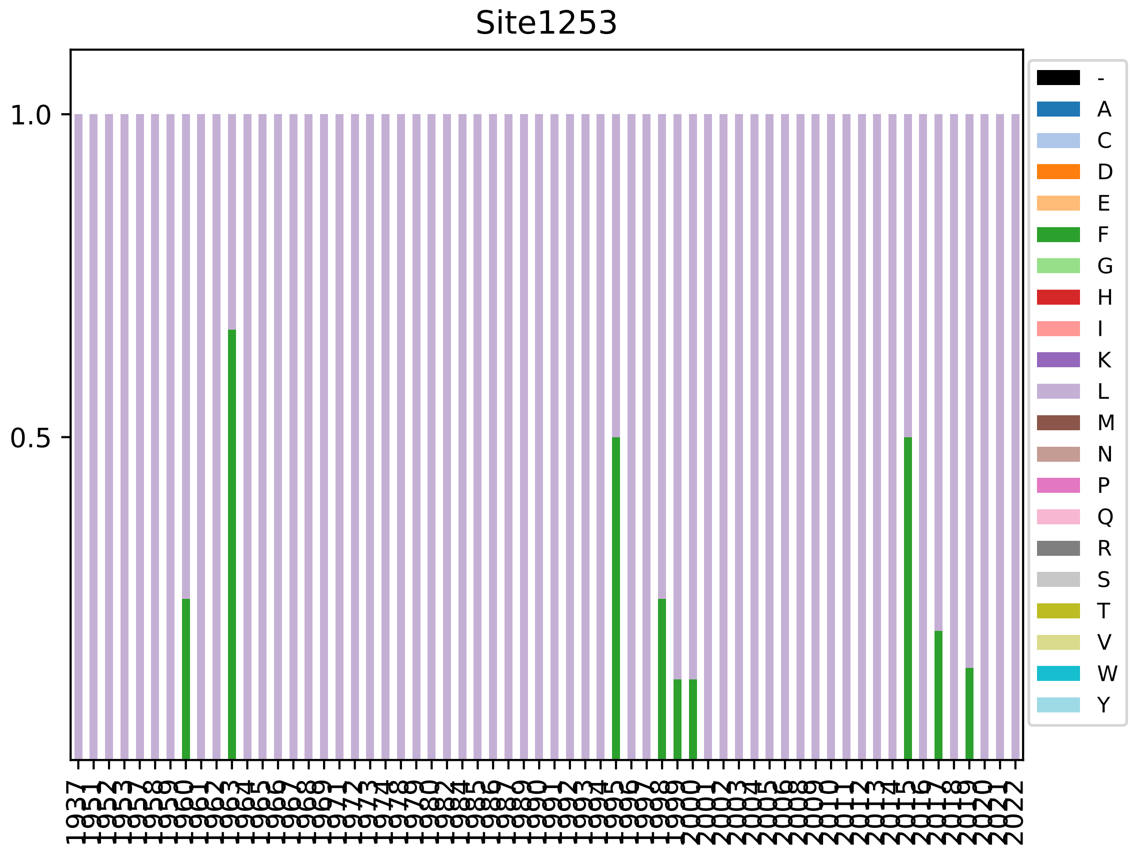
Site1253
1.0
0.5
-
A
C
D
E
F
G
H
I
K
L
M
N
P
Q
R
S
T
V
W
Y

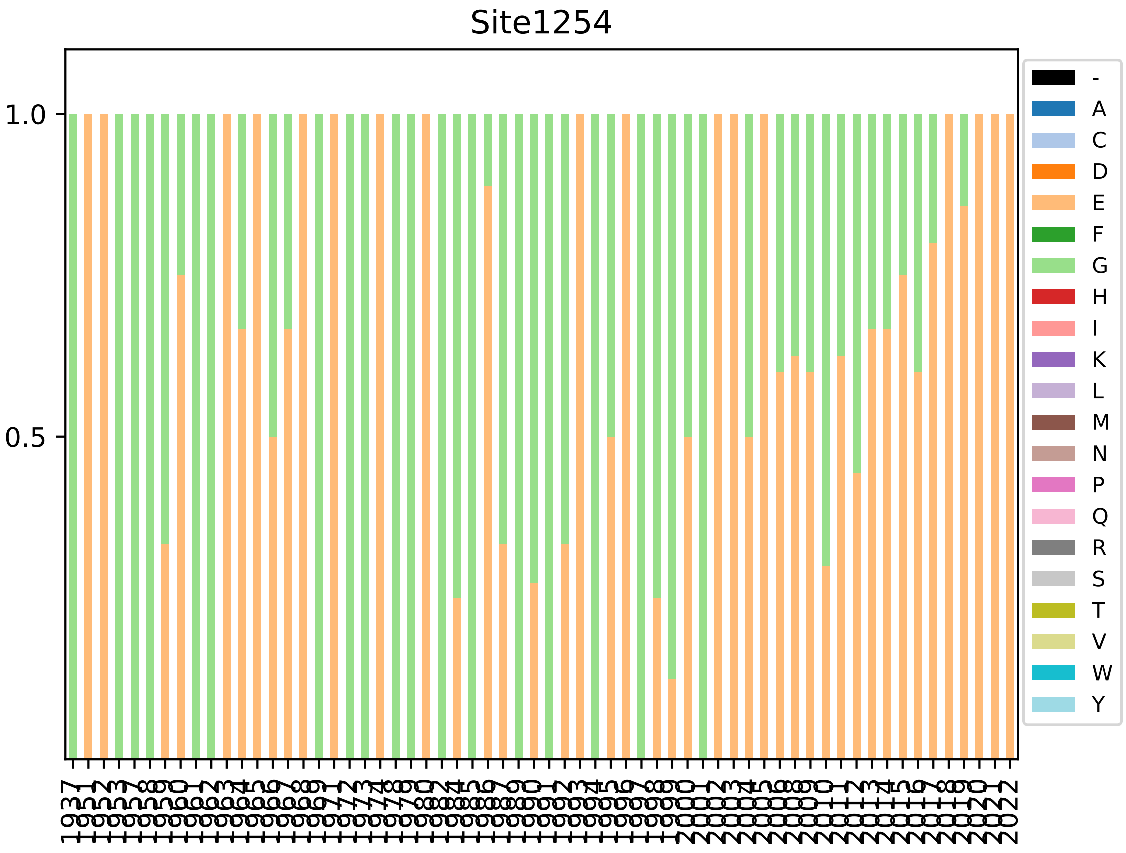
Site1254
1.0
0.5
-
A
C
D
E
F
G
H
I
K
L
M
N
P
Q
R
S
T
V
W
Y

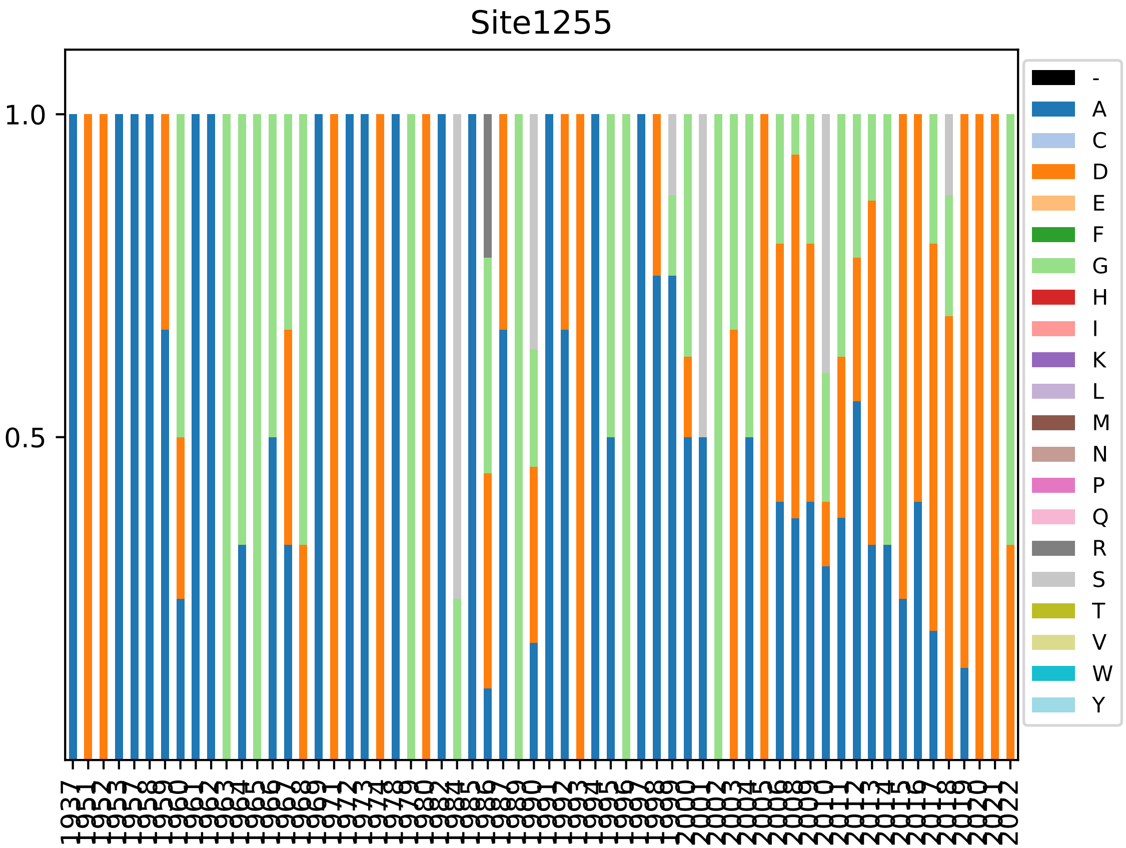

Site1255
1.0
0.5
-
A
C
D
E
F
G
H
I
K
L
M
N
P
Q
R
S
T
V
W
Y

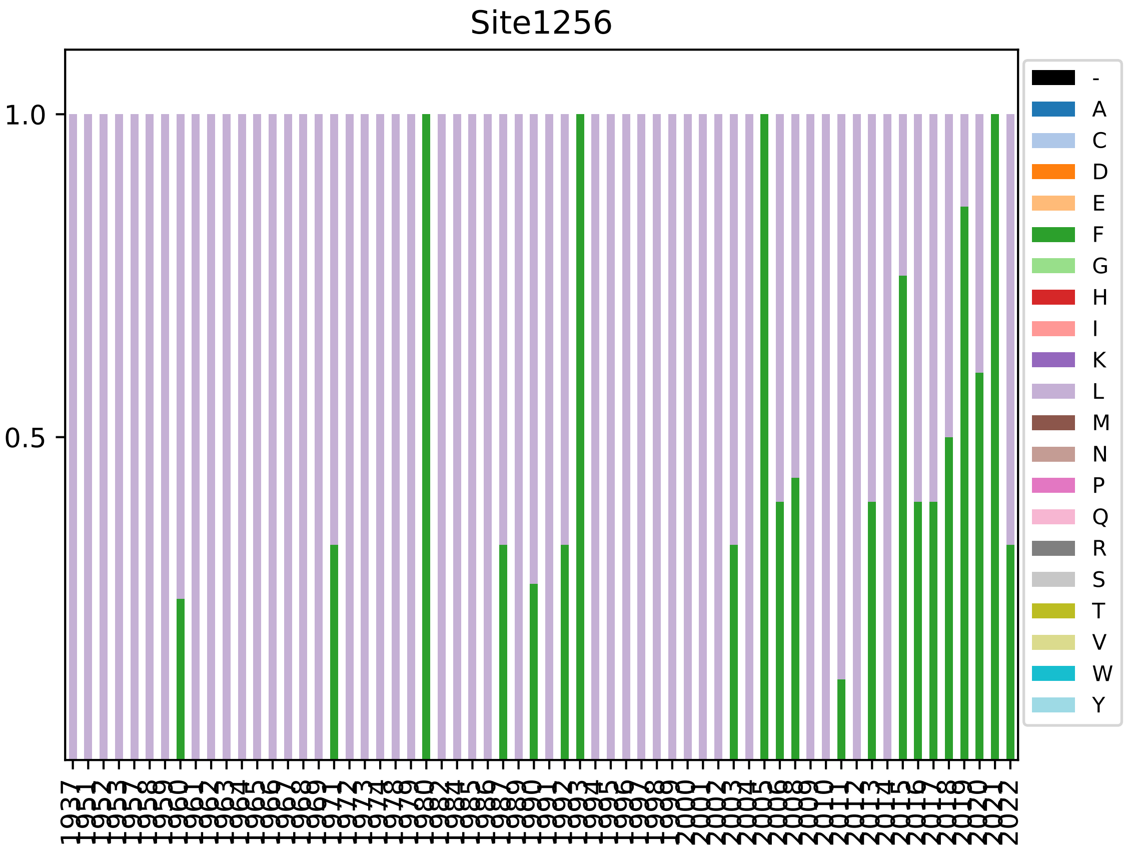
Site1256
1.0
0.5
-
A
C
D
E
F
G
H
I
K
L
M
N
P
Q
R
S
T
V
W
Y

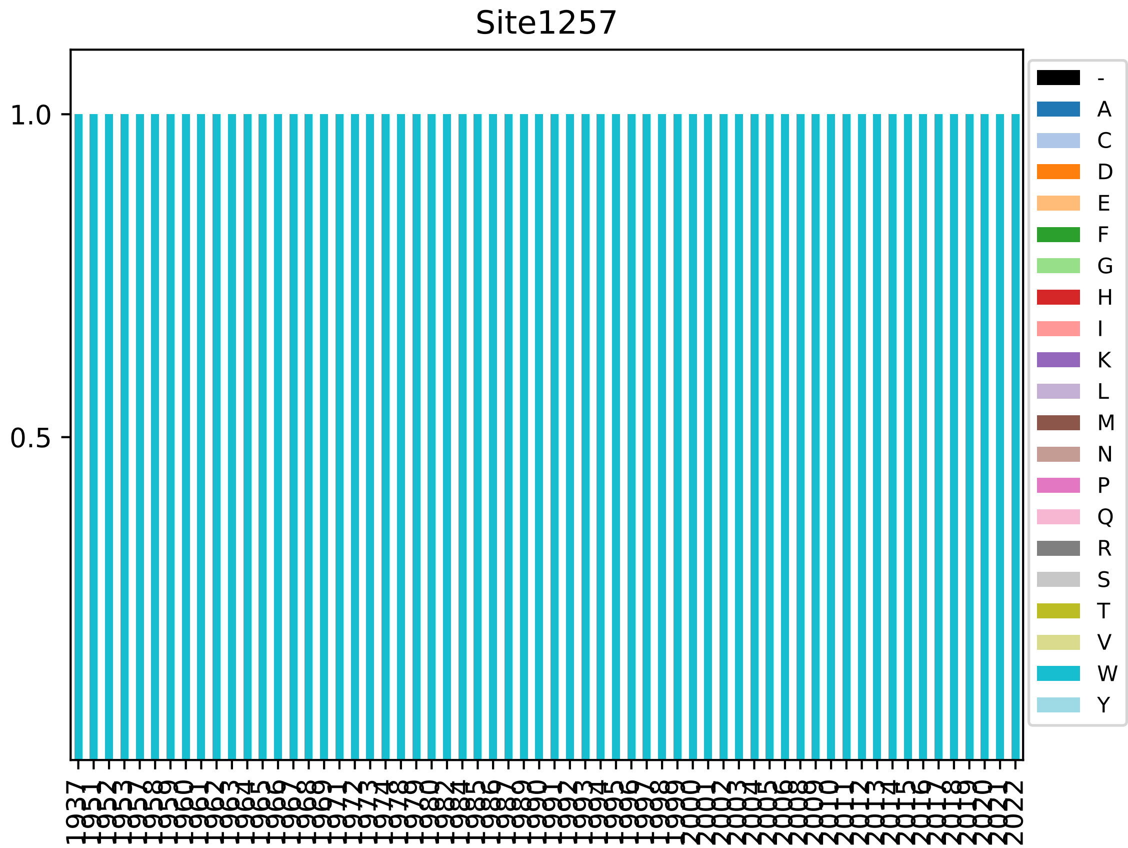
Site1257
1.0
0.5
-
A
C
D
E
F
G
H
I
K
L
M
N
P
Q
R
S
T
V
W
Y

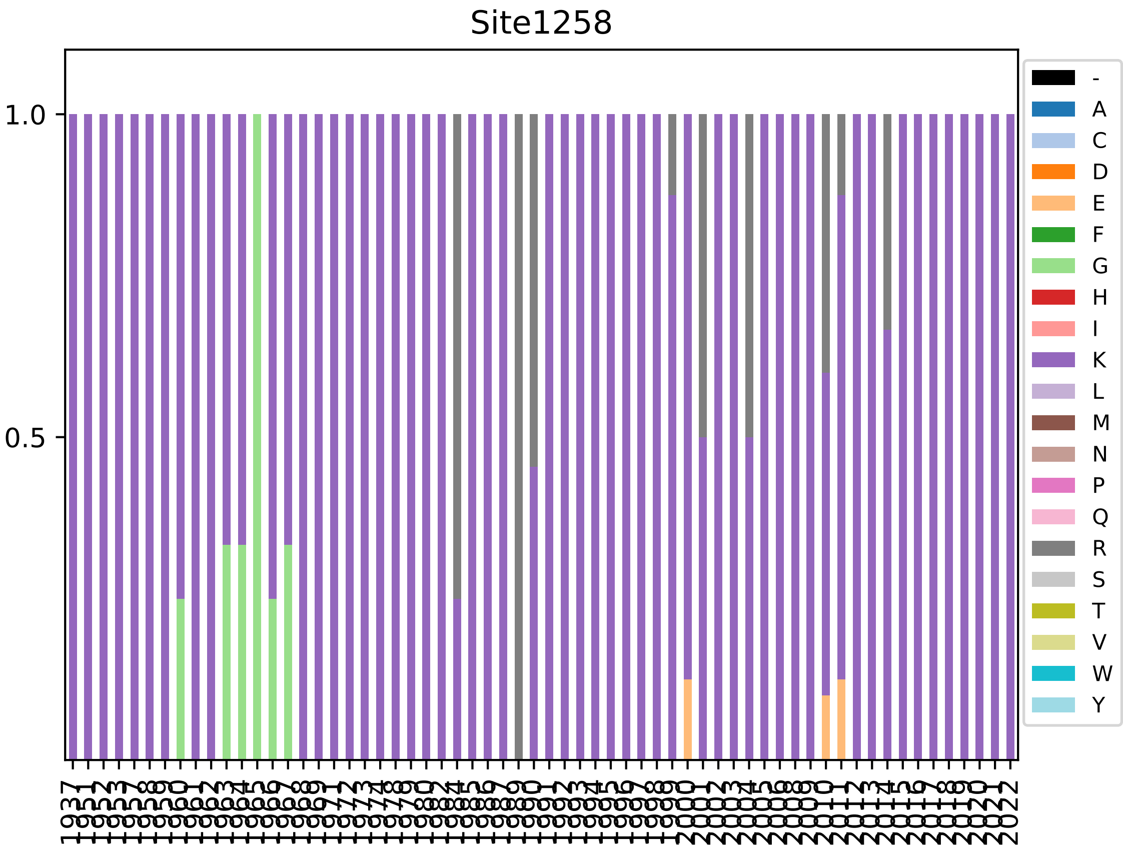

Site1258
1.0
0.5
-
A
C
D
E
F
G
H
I
K
L
M
N
P
Q
R
S
T
V
W
Y

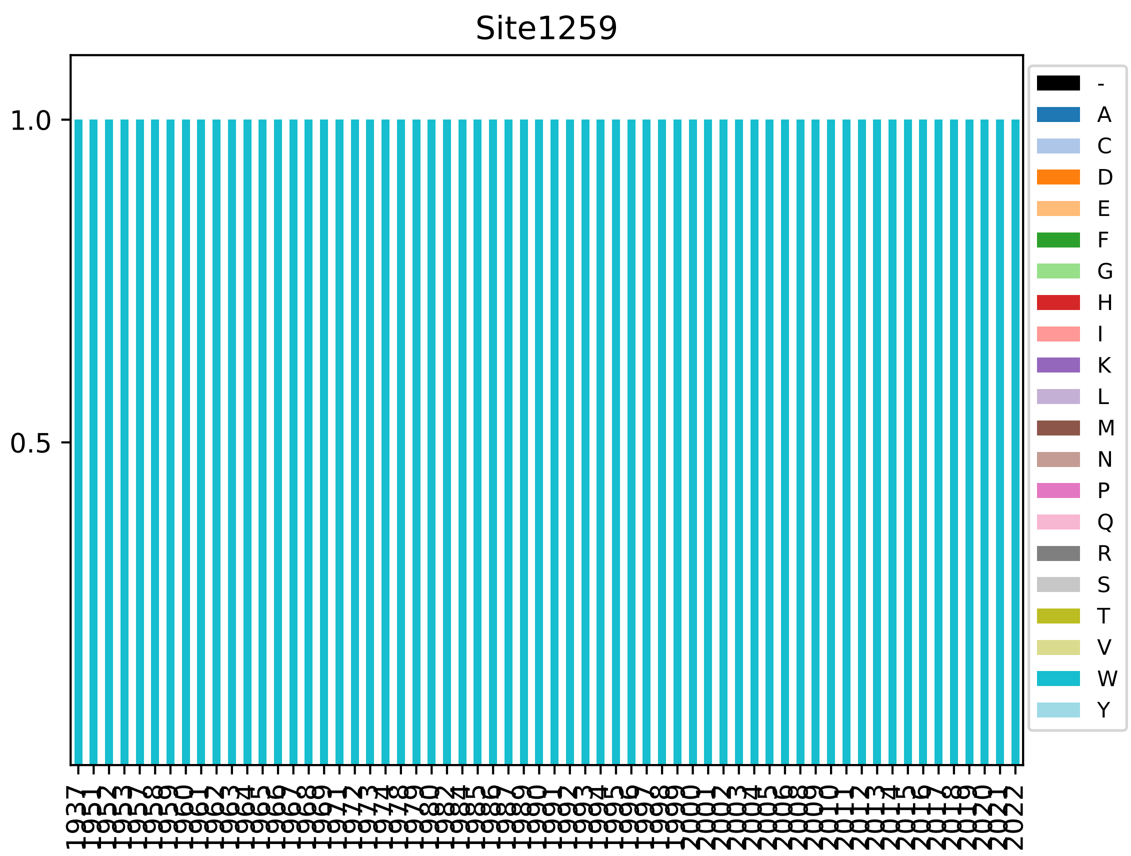
Site1259
1.0
0.5
-
A
C
D
E
F
G
H
I
K
L
M
N
P
Q
R
S
T
V
W
Y

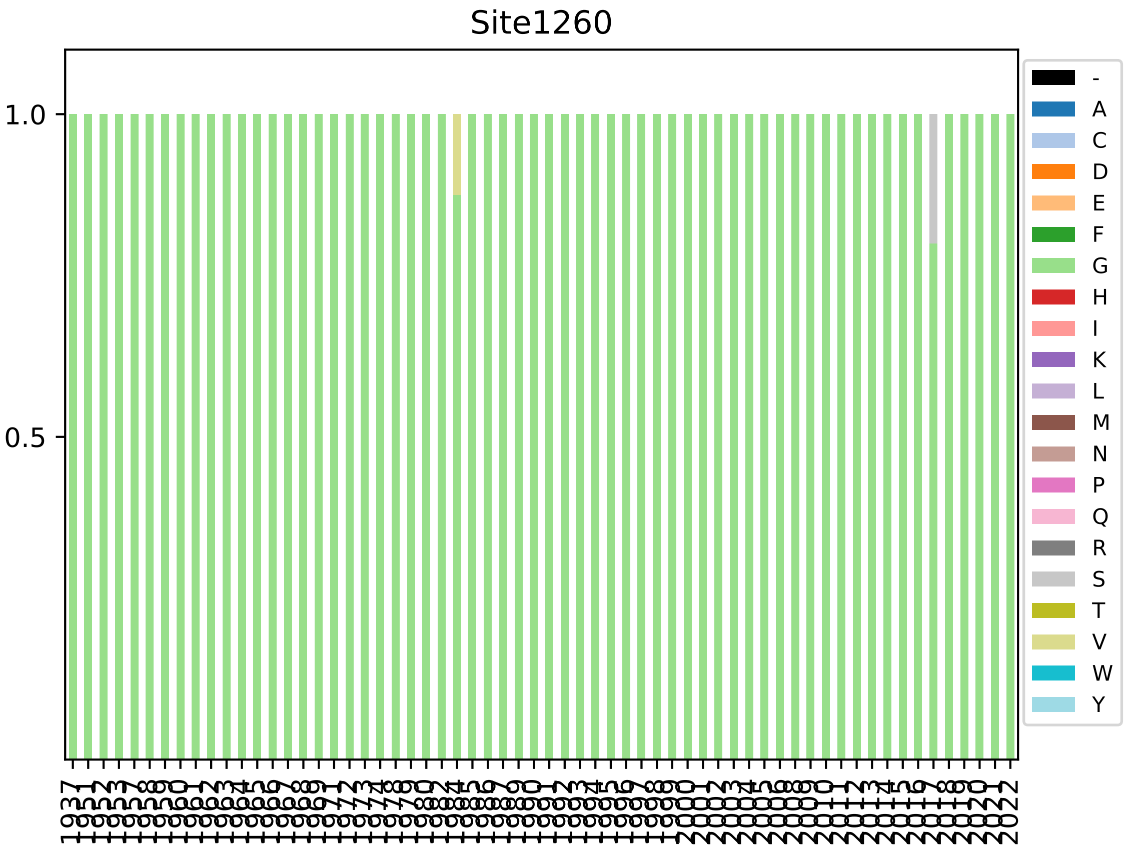
Site1260
1.0
0.5
-
A
C
D
E
F
G
H
I
K
L
M
N
P
Q
R
S
T
V
W
Y

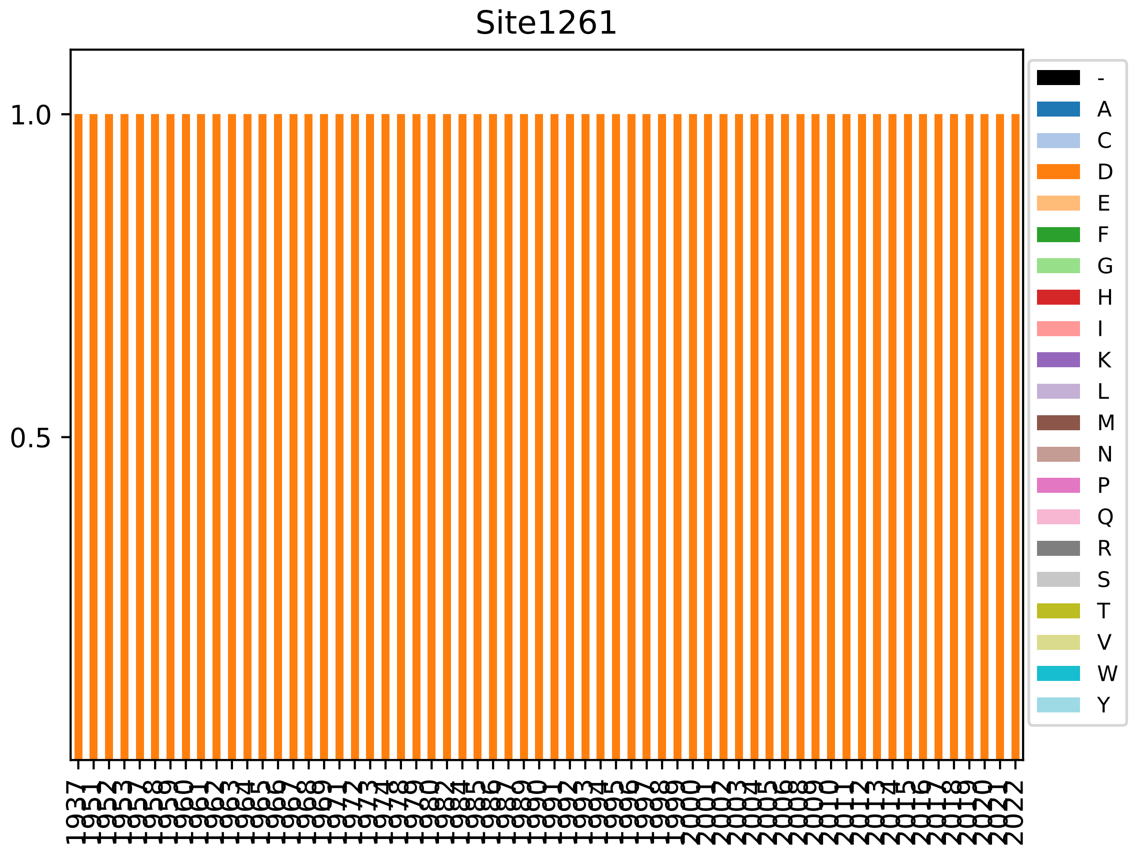
Site1261
1.0
0.5
-
A
C
D
E
F
G
H
I
K
L
M
N
P
Q
R
S
T
V
W
Y

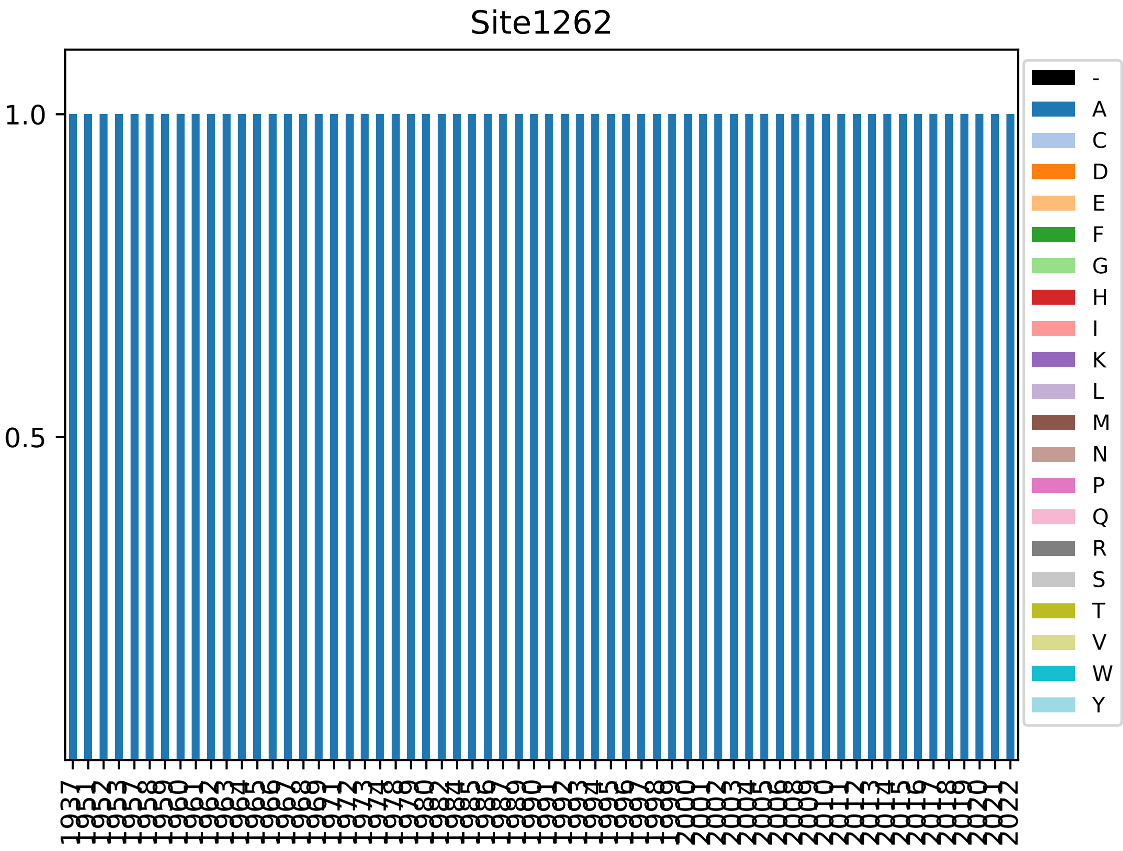
Site1262
1.0
0.5
-
A
C
D
E
F
G
H
I
K
L
M
N
P
Q
R
S
T
V
W
Y

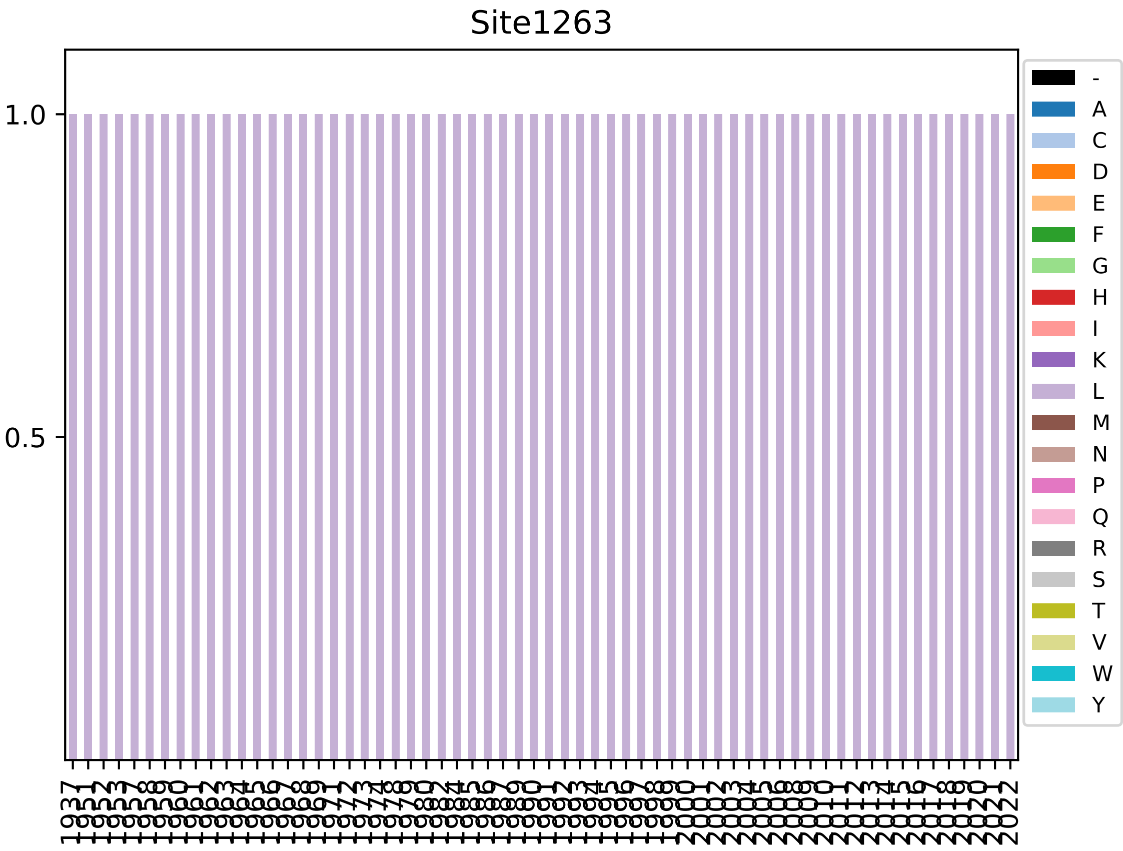
Site1263
1.0
0.5
-
A
C
D
E
F
G
H
I
K
L
M
N
P
Q
R
S
T
V
W
Y

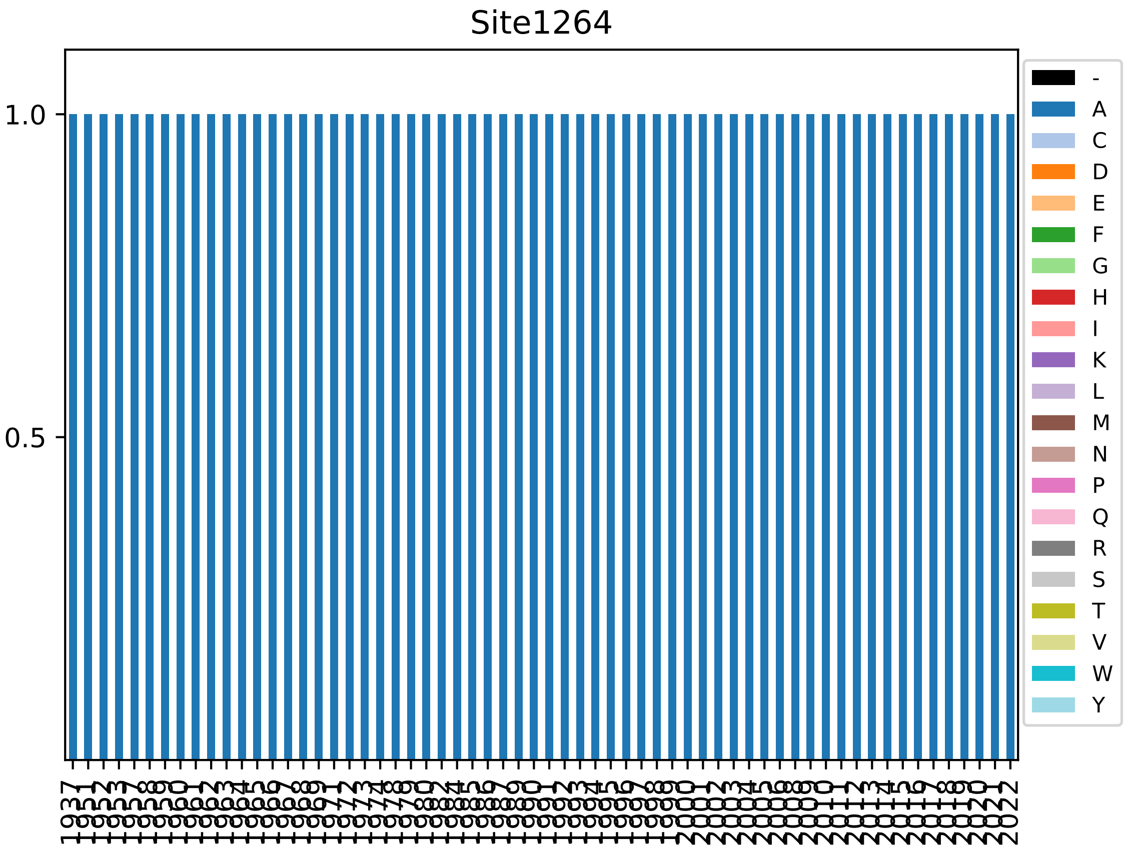
Site1264
1.0
0.5
-
A
C
D
E
F
G
H
I
K
L
M
N
P
Q
R
S
T
V
W
Y

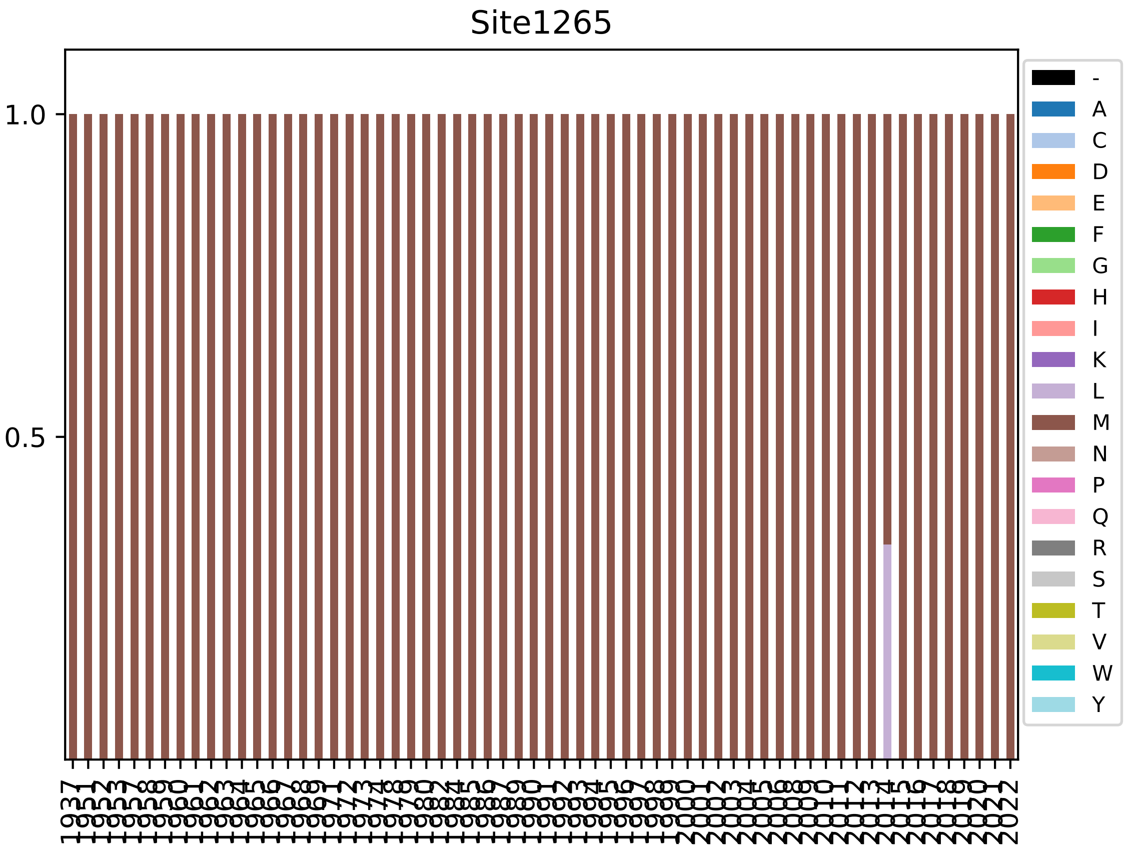
Site1265
1.0
0.5
-
A
C
D
E
F
G
H
I
K
L
M
N
P
Q
R
S
T
V
W
Y

Site1266

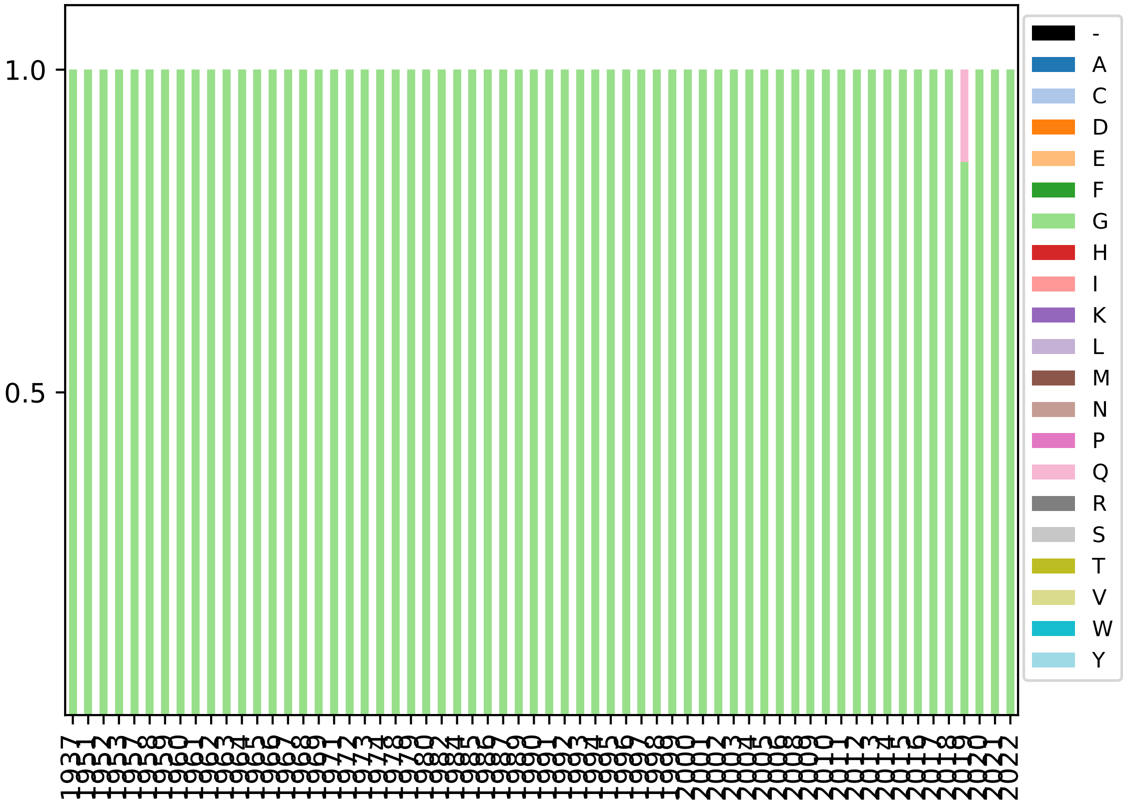

Site1267

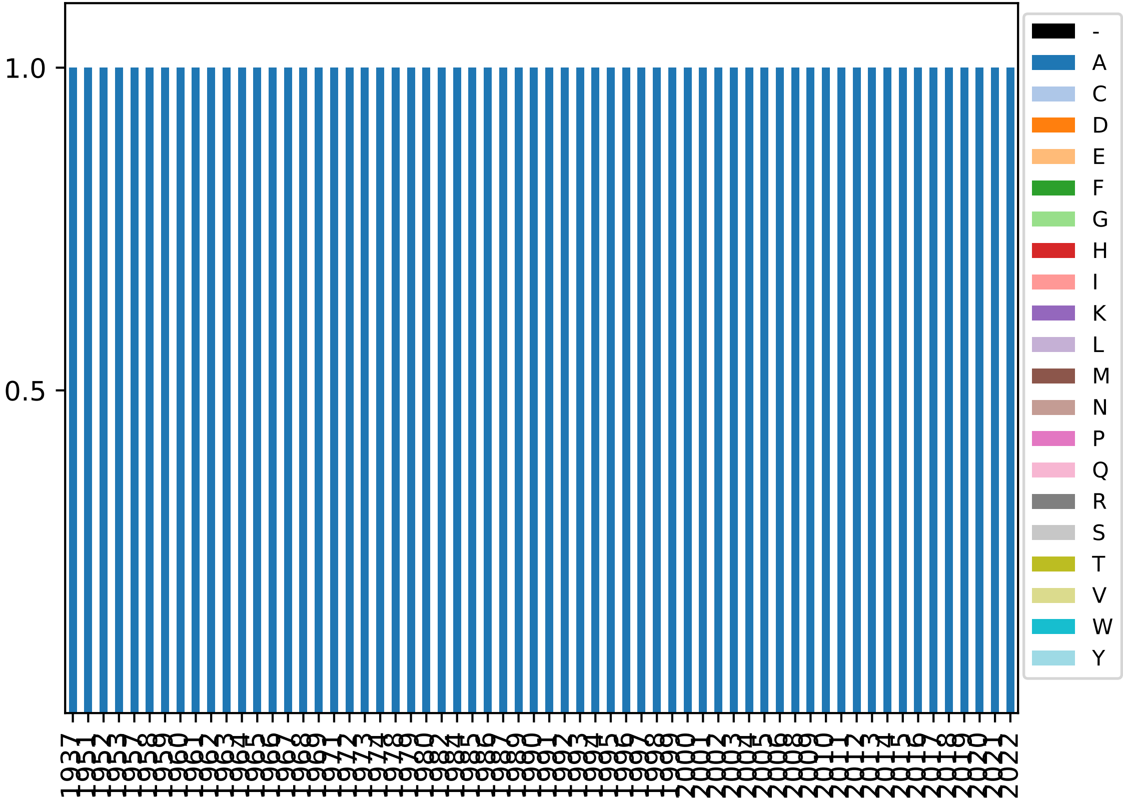

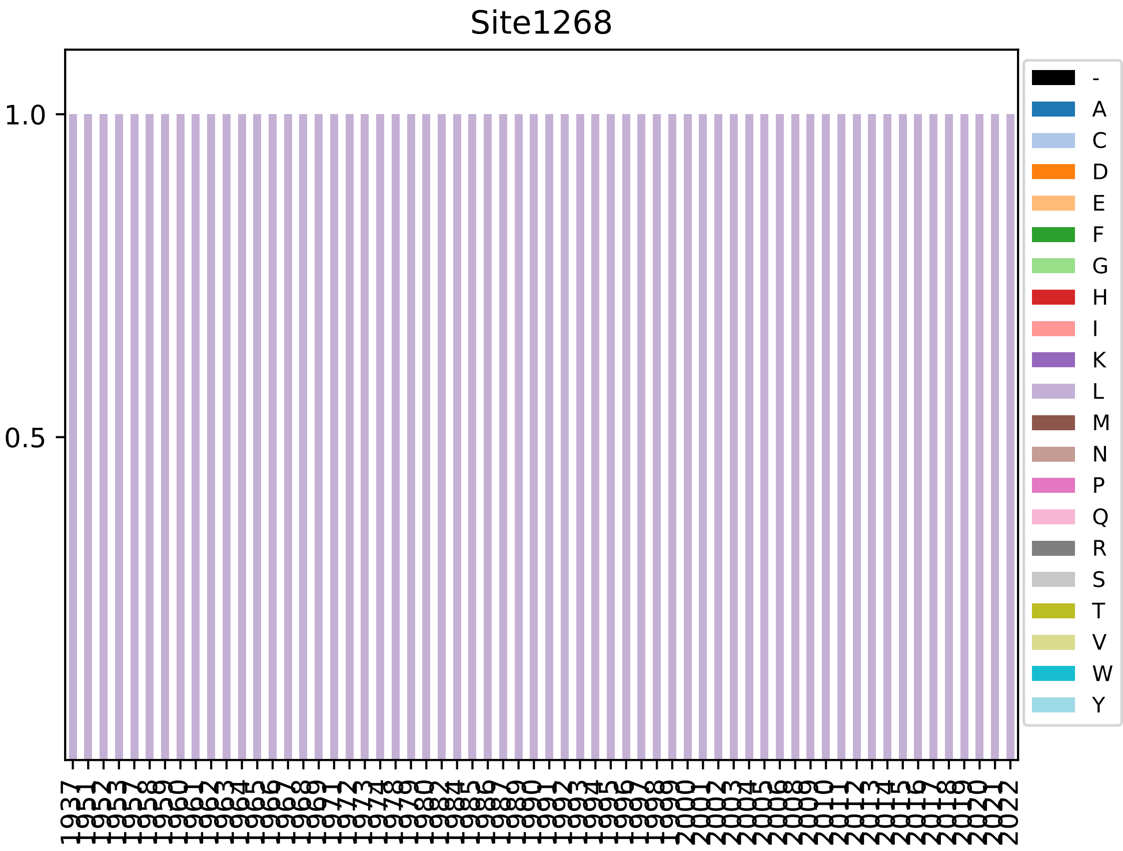
Site1268
1.0
0.5
-
A
C
D
E
F
G
H
I
K
L
M
N
P
Q
R
S
T
V
W
Y

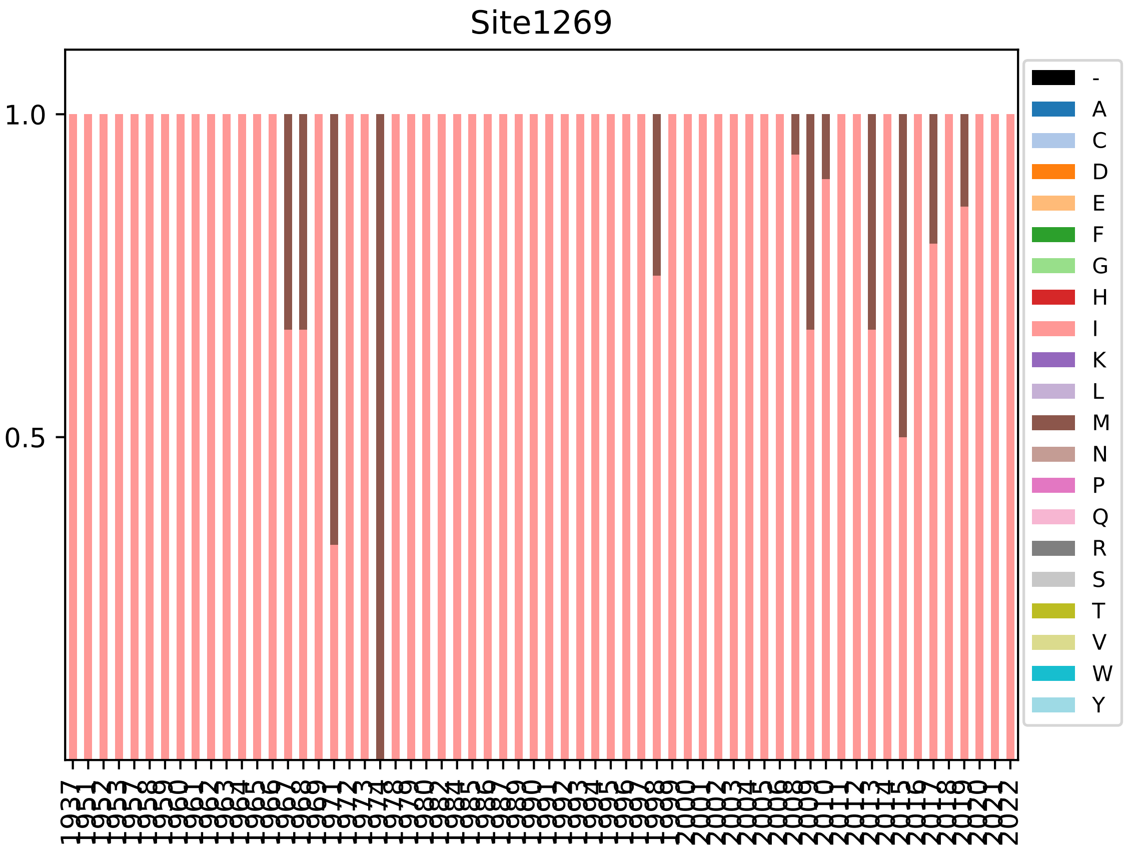
Site1269
1.0
0.5
-
A
C
D
E
F
G
H
I
K
L
M
N
P
Q
R
S
T
V
W
Y

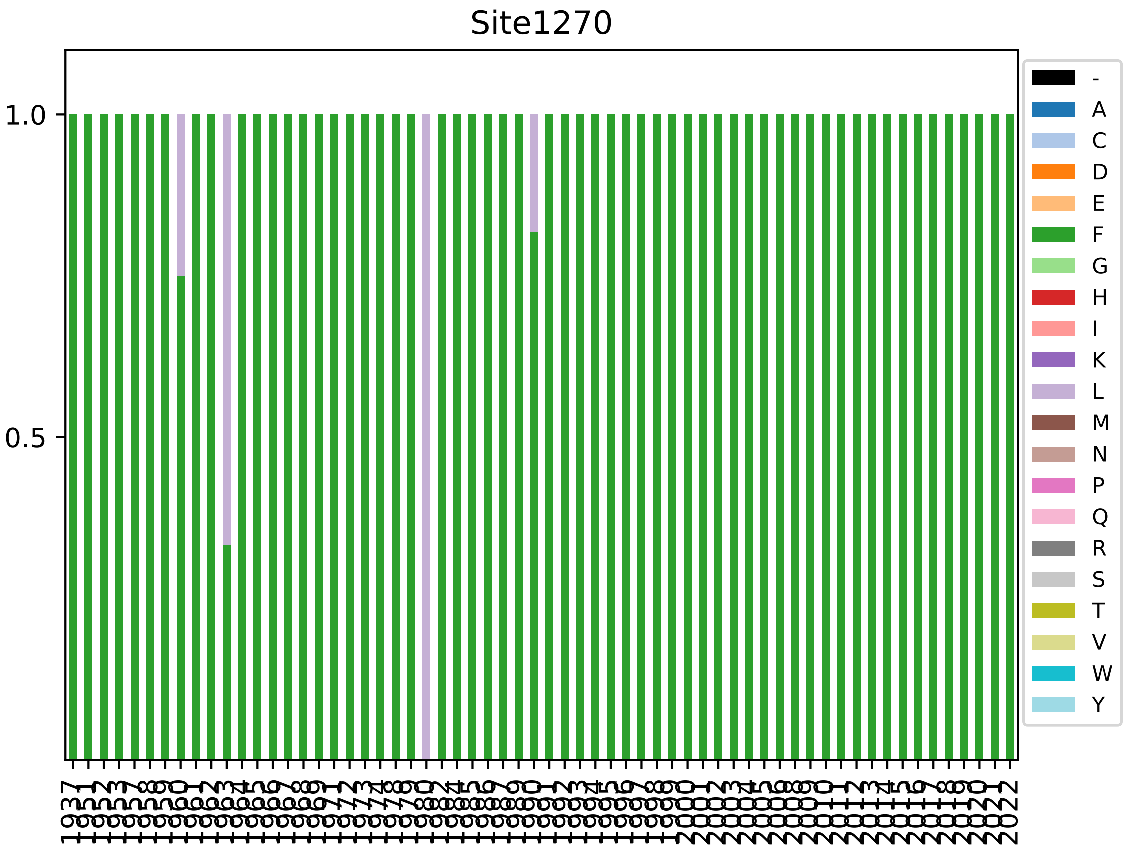
Site1270
1.0
0.5
-
A
C
D
E
F
G
H
I
K
L
M
N
P
Q
R
S
T
V
W
Y

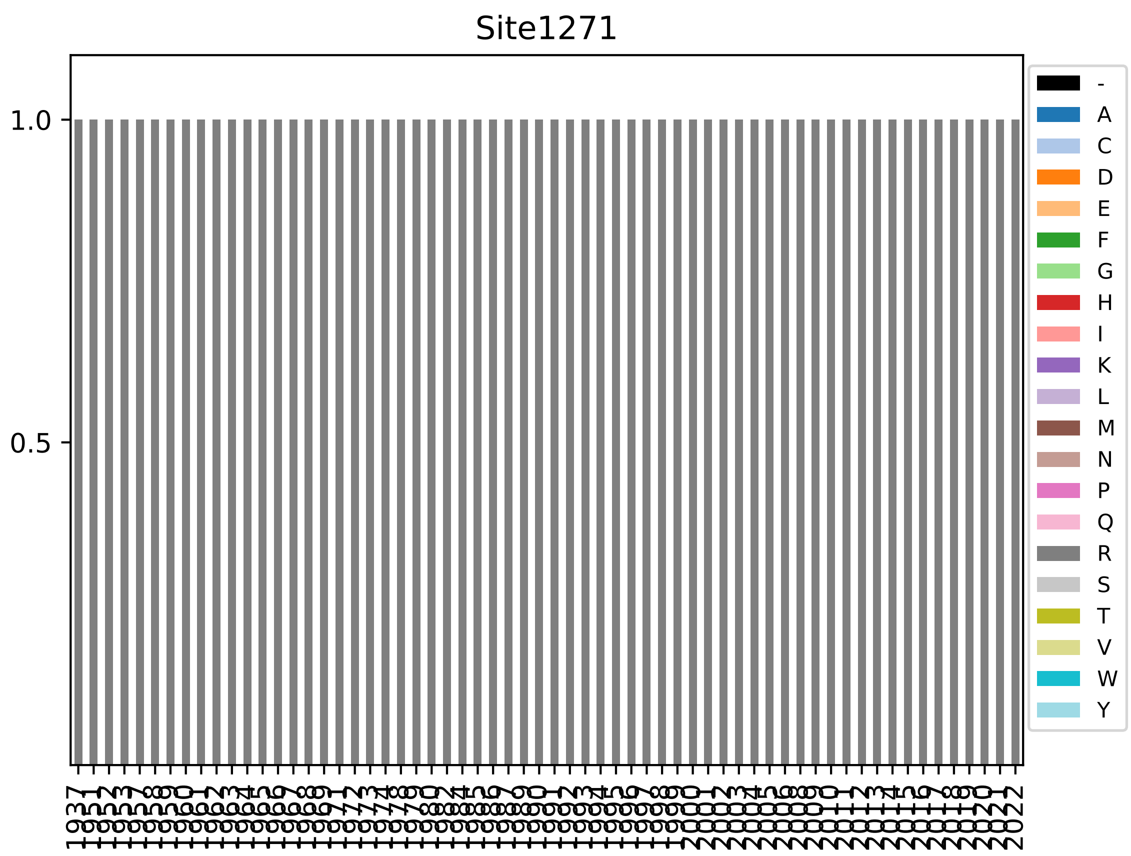
Site1271
1.0
0.5
-
A
C
D
E
F
G
H
I
K
L
M
N
P
Q
R
S
T
V
W
Y

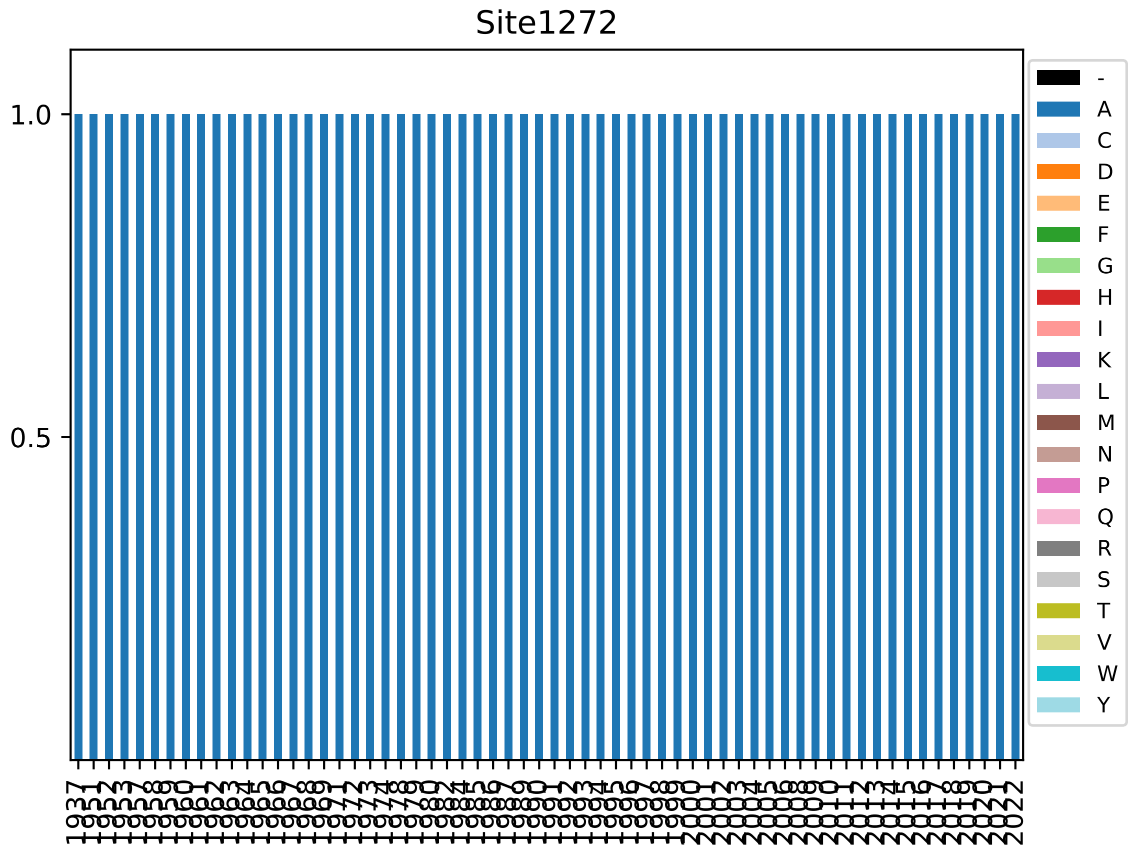
Site1272
1.0
0.5
-
A
C
D
E
F
G
H
I
K
L
M
N
P
Q
R
S
T
V
W
Y

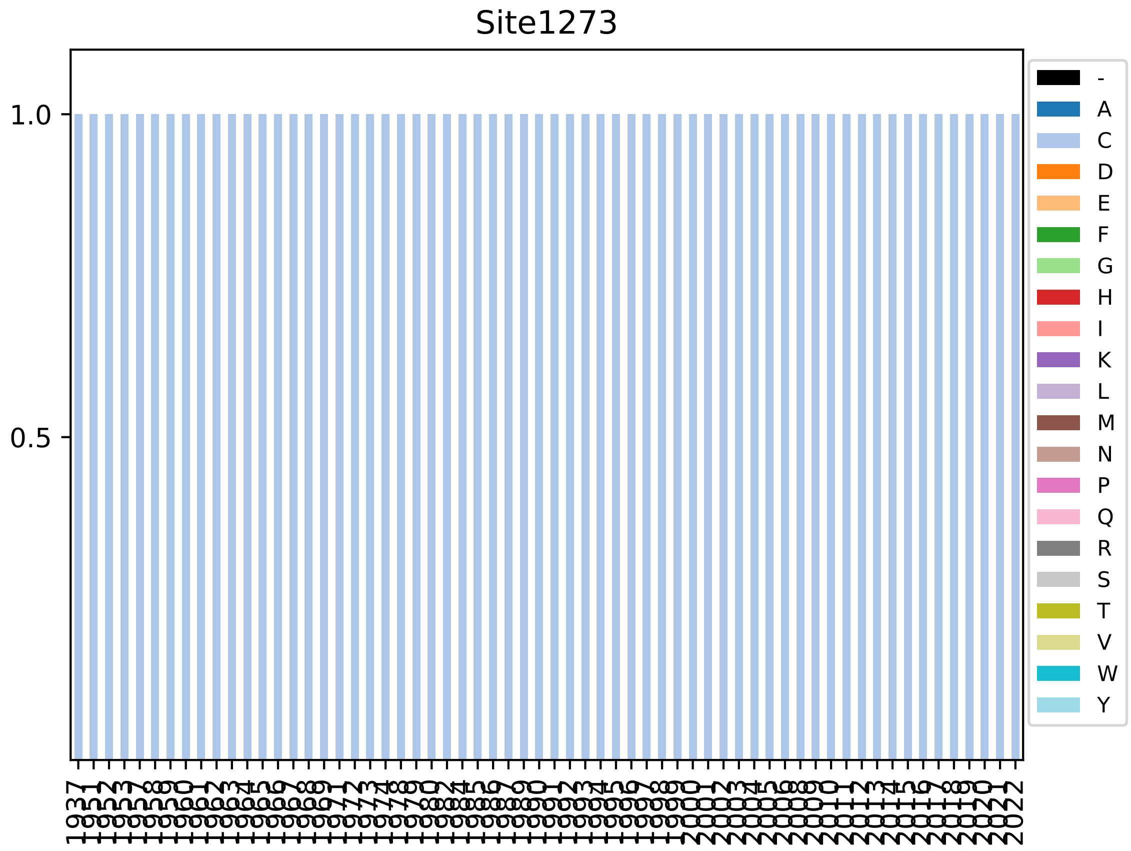
Site1273
1.0
0.5
-
A
C
D
E
F
G
H
I
K
L
M
N
P
Q
R
S
T
V
W
Y

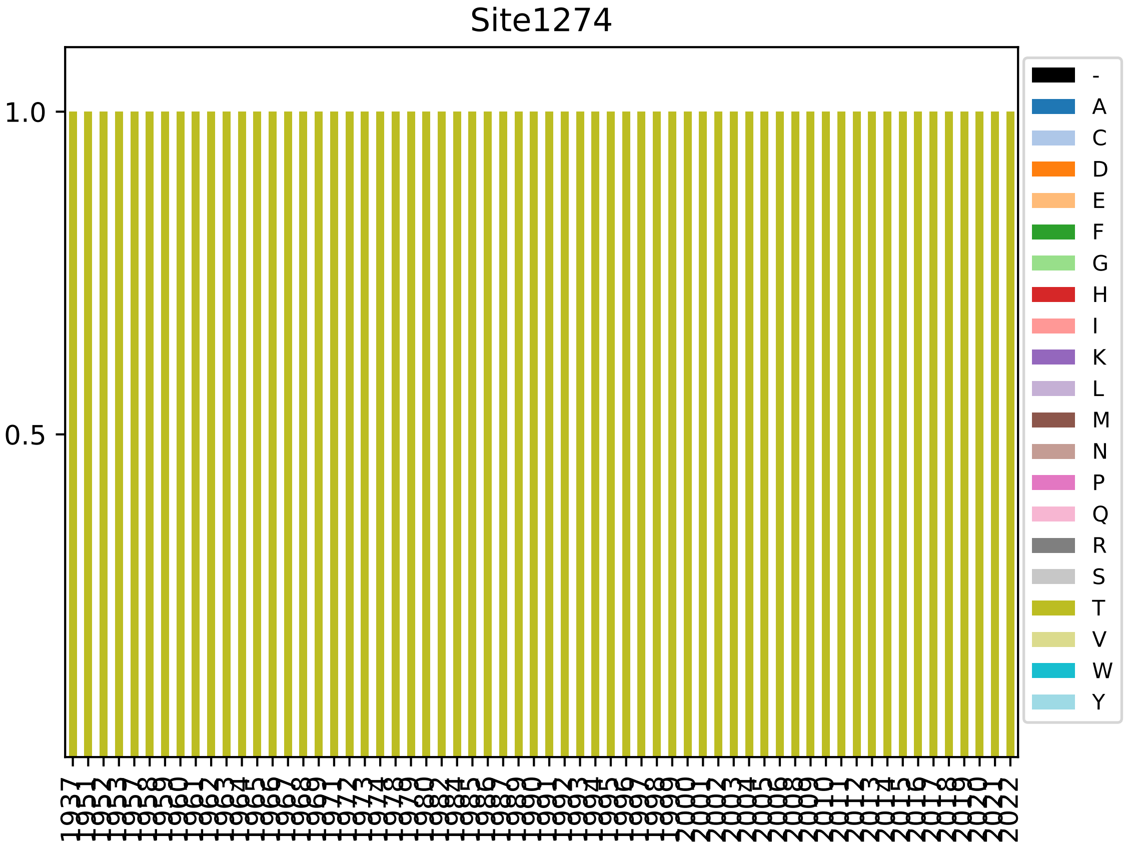
Site1274
1.0
0.5
-
A
C
D
E
F
G
H
I
K
L
M
N
P
Q
R
S
T
V
W
Y

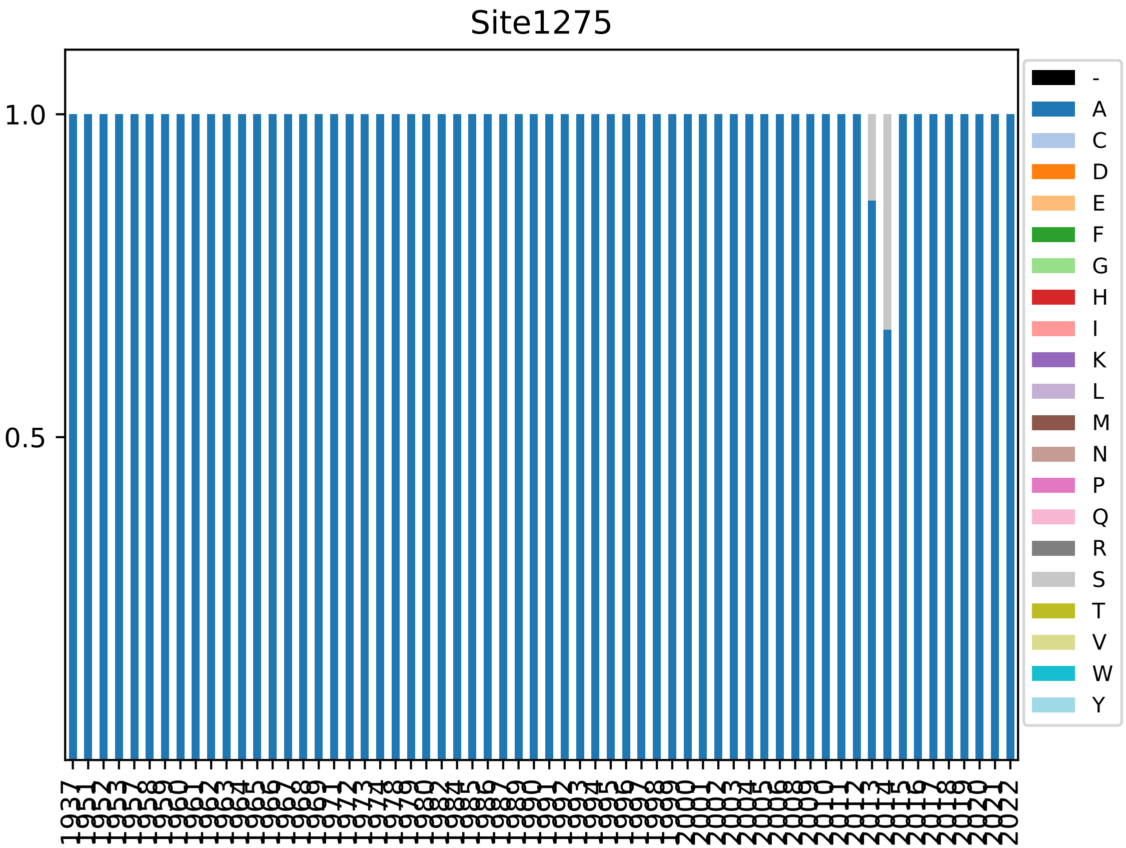
Site1275
1.0
0.5
-
A
C
D
E
F
G
H
I
K
L
M
N
P
Q
R
S
T
V
W
Y

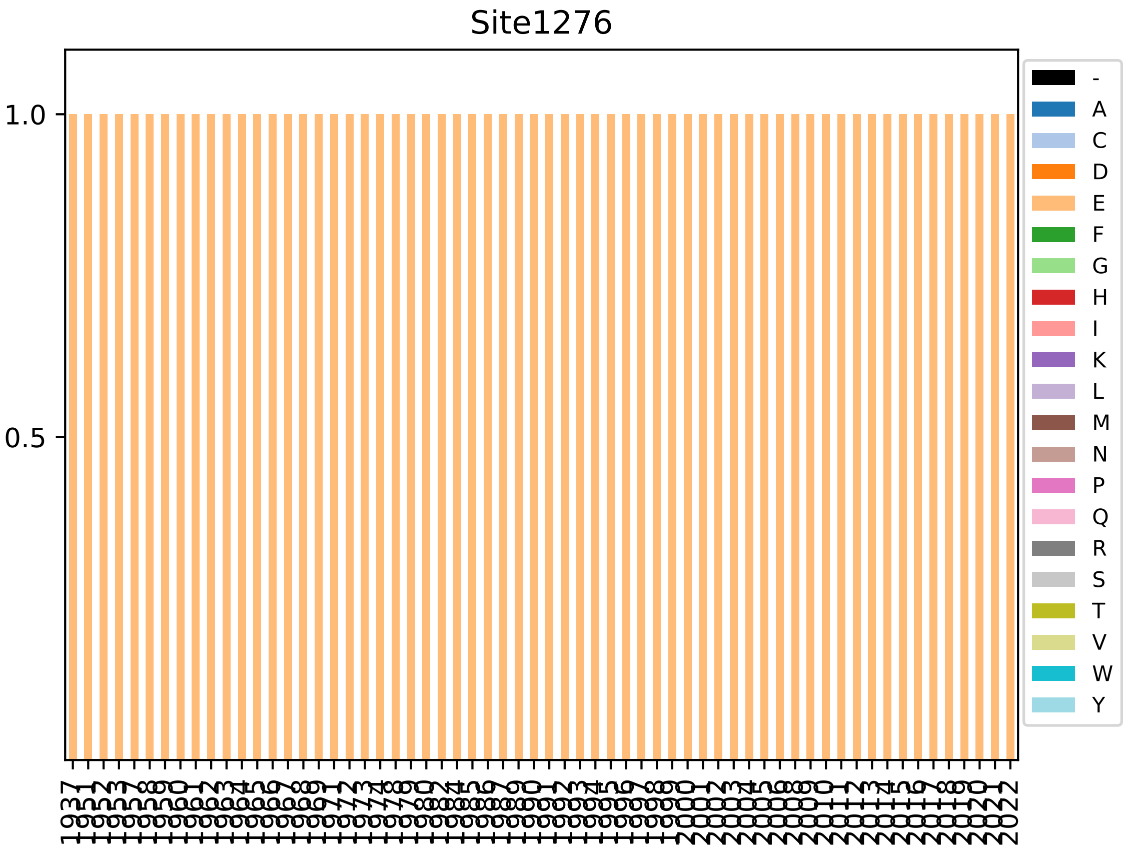
Site1276
1.0
0.5
-
A
C
D
E
F
G
H
I
K
L
M
N
P
Q
R
S
T
V
W
Y

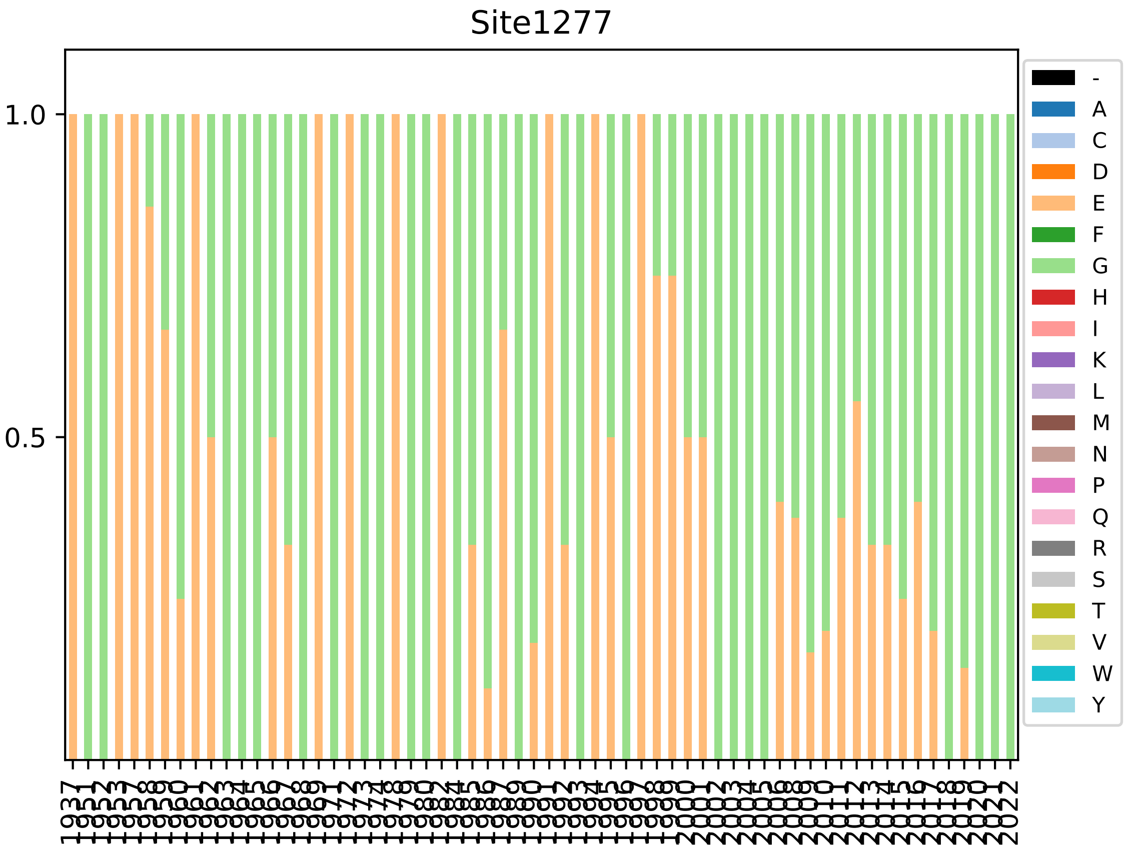
Site1277
1.0
0.5
-
A
C
D
E
F
G
H
I
K
L
M
N
P
Q
R
S
T
V
W
Y

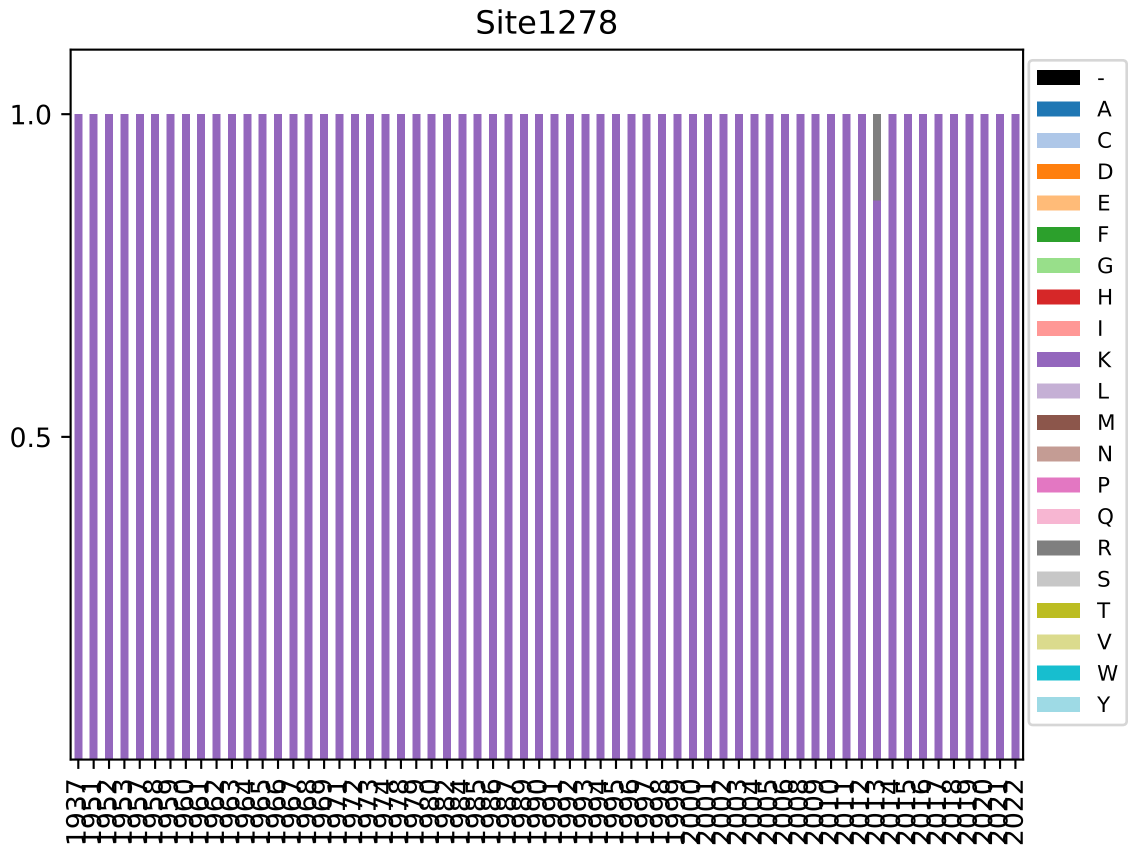
Site1278
1.0
0.5
-
A
C
D
E
F
G
H
I
K
L
M
N
P
Q
R
S
T
V
W
Y

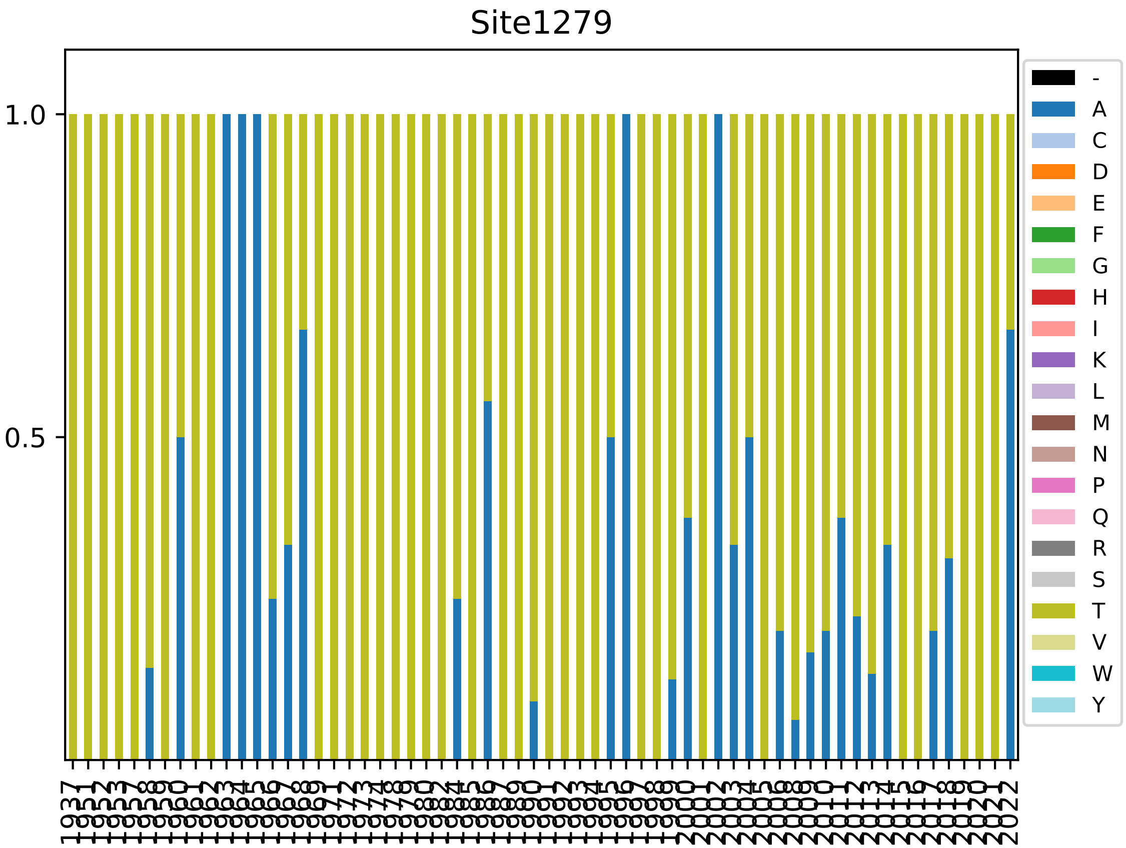
Site1279
1.0
0.5
-
A
C
D
E
F
G
H
I
K
L
M
N
P
Q
R
S
T
V
W
Y

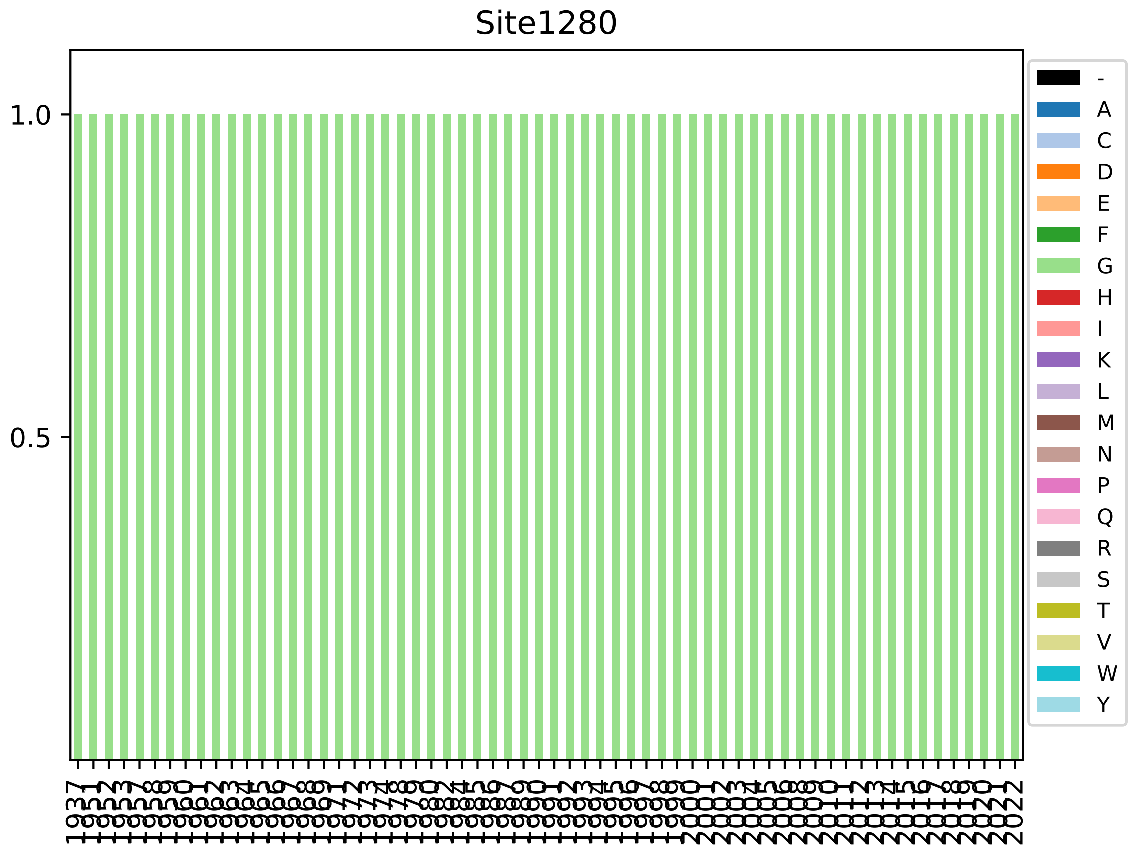
Site1280
1.0
0.5
-
A
C
D
E
F
G
H
I
K
L
M
N
P
Q
R
S
T
V
W
Y

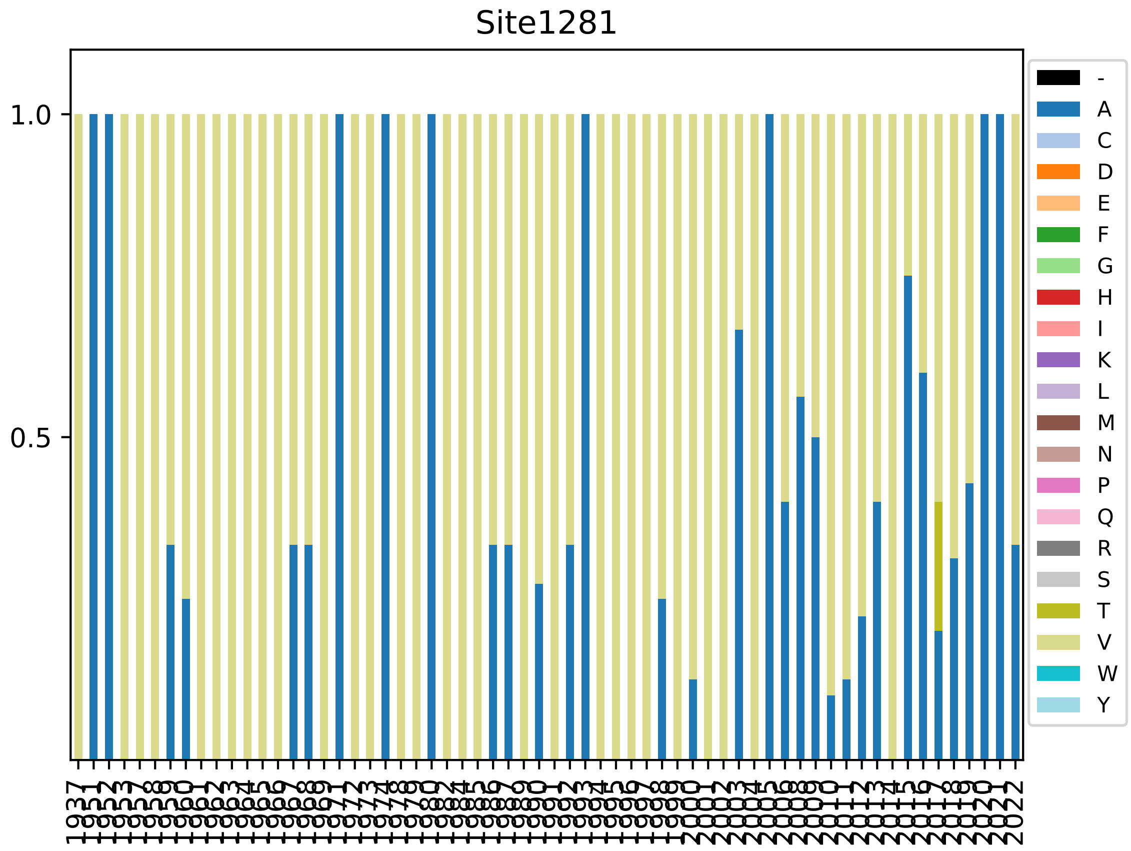
Site1281
1.0
0.5
-
A
C
D
E
F
G
H
I
K
L
M
N
P
Q
R
S
T
V
W
Y

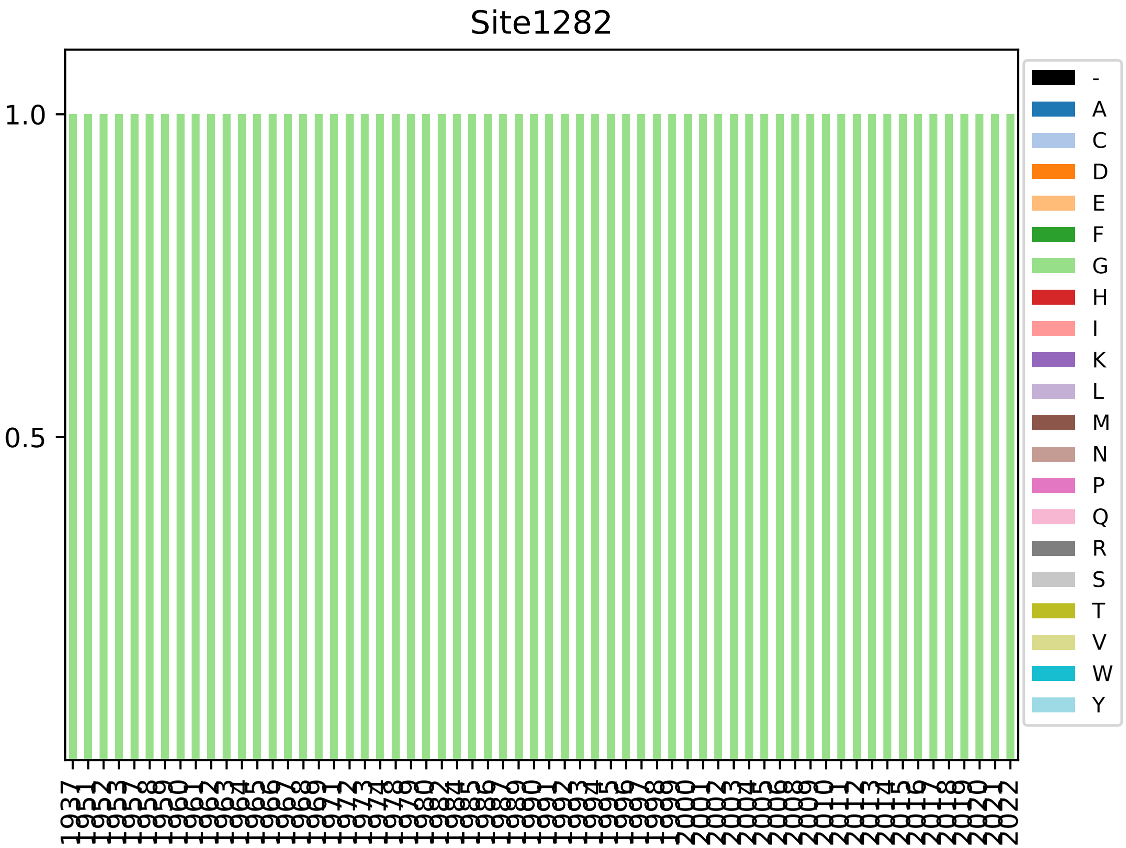
Site1282
1.0
0.5
-
A
C
D
E
F
G
H
I
K
L
M
N
P
Q
R
S
T
V
W
Y

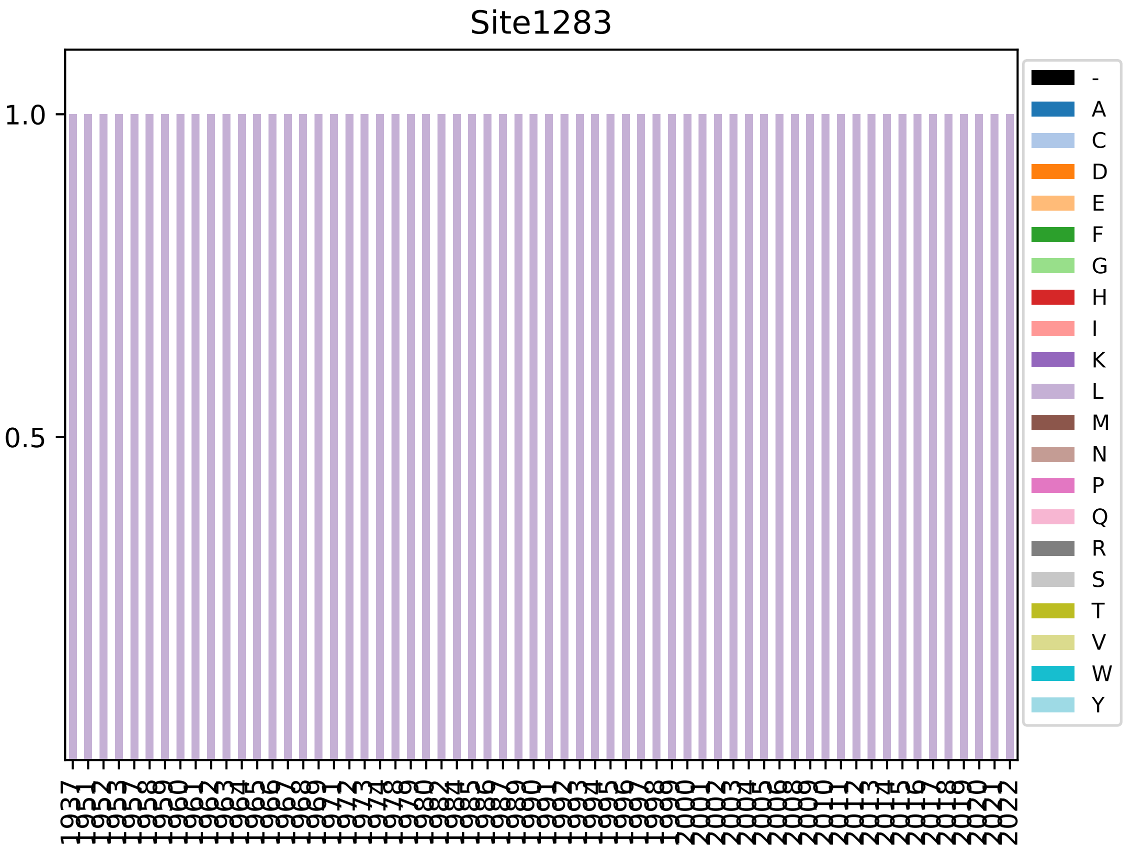

Site1283
1.0
0.5
-
A
C
D
E
F
G
H
I
K
L
M
N
P
Q
R
S
T
V
W
Y

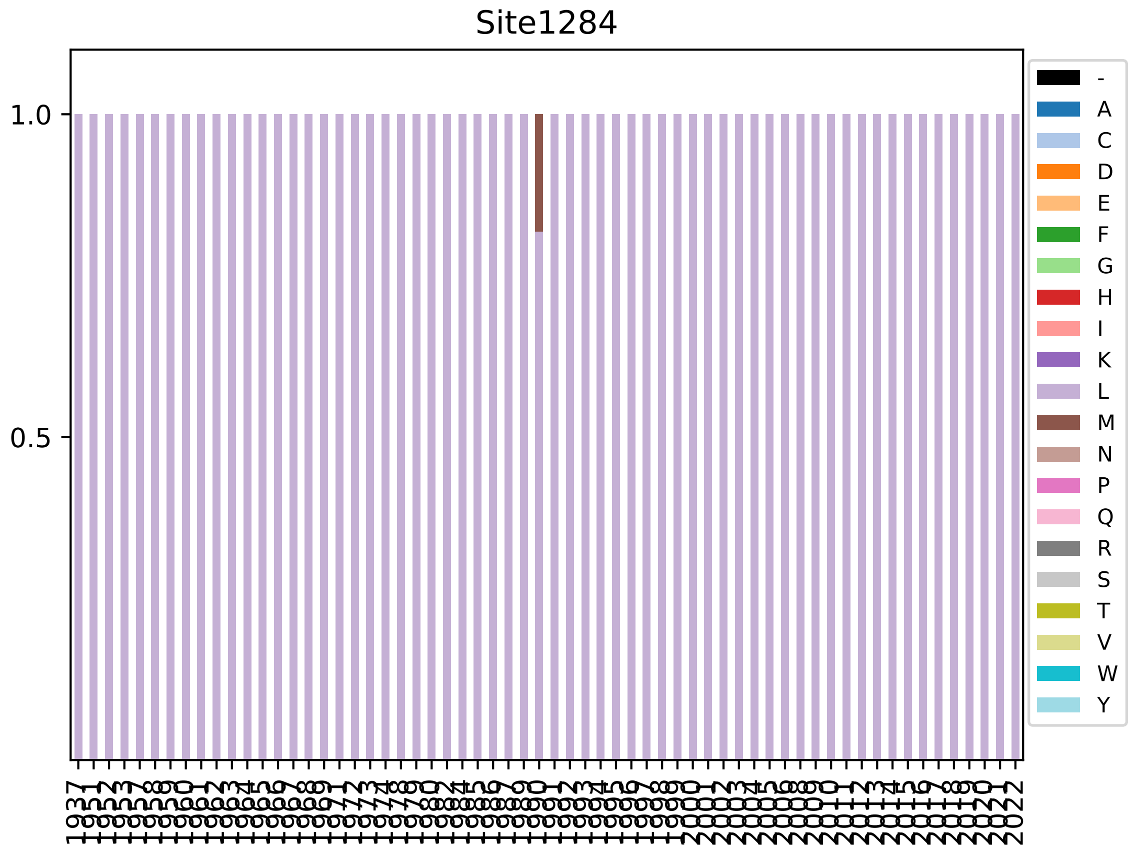
Site1284
1.0
0.5
-
A
C
D
E
F
G
H
I
K
L
M
N
P
Q
R
S
T
V
W
Y

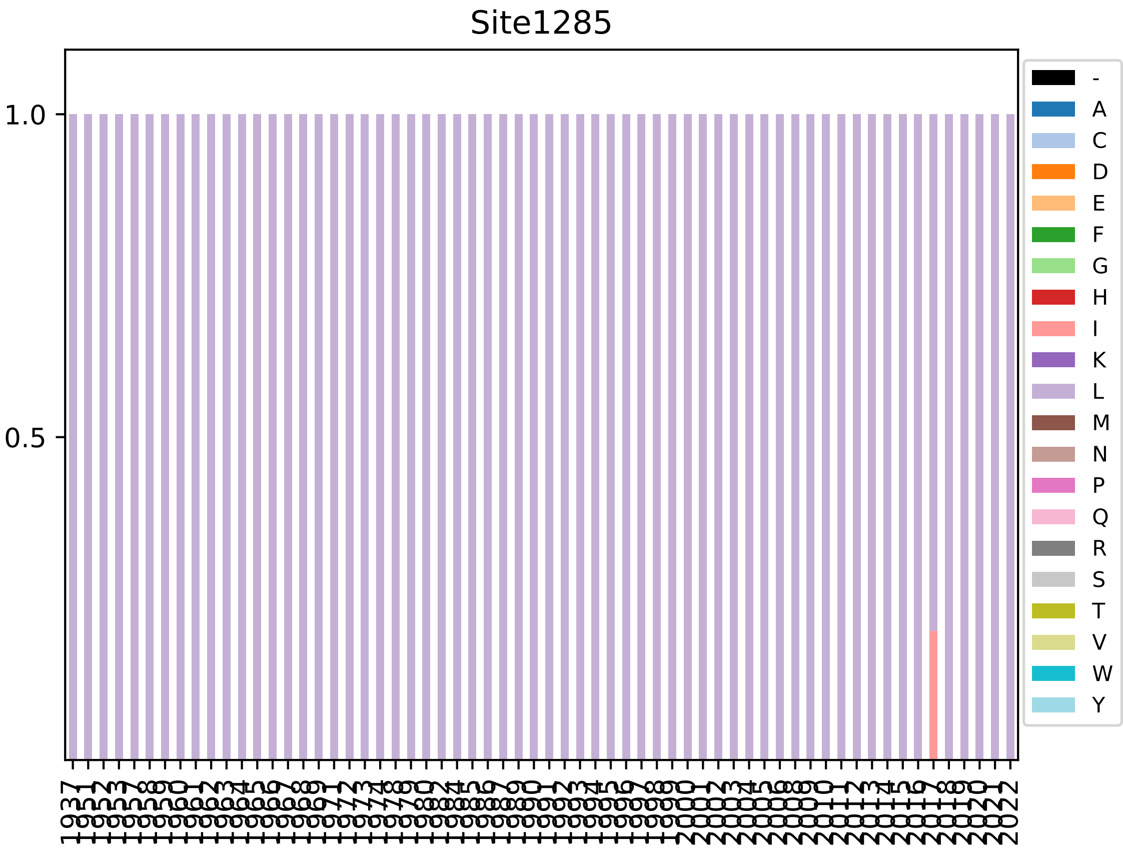
Site1285
1.0
0.5
-
A
C
D
E
F
G
H
I
K
L
M
N
P
Q
R
S
T
V
W
Y

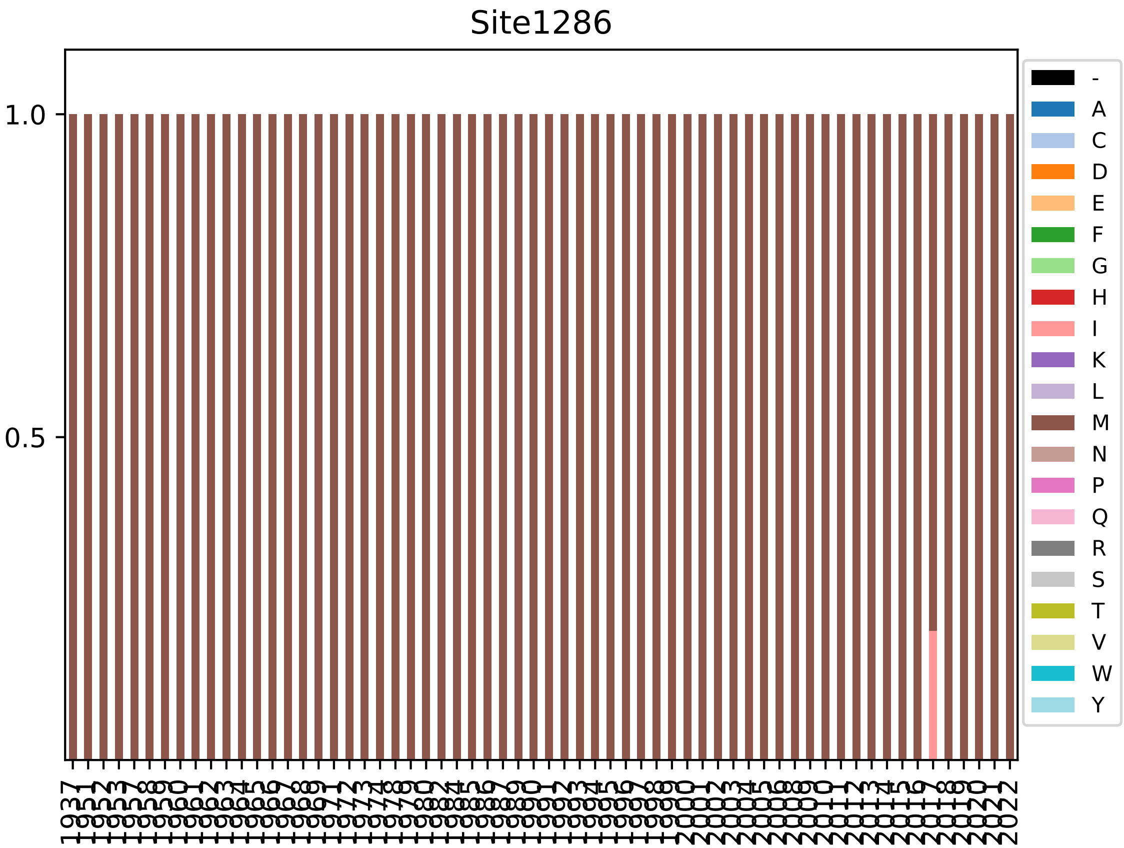
Site1286
1.0
0.5
-
A
C
D
E
F
G
H
I
K
L
M
N
P
Q
R
S
T
V
W
Y

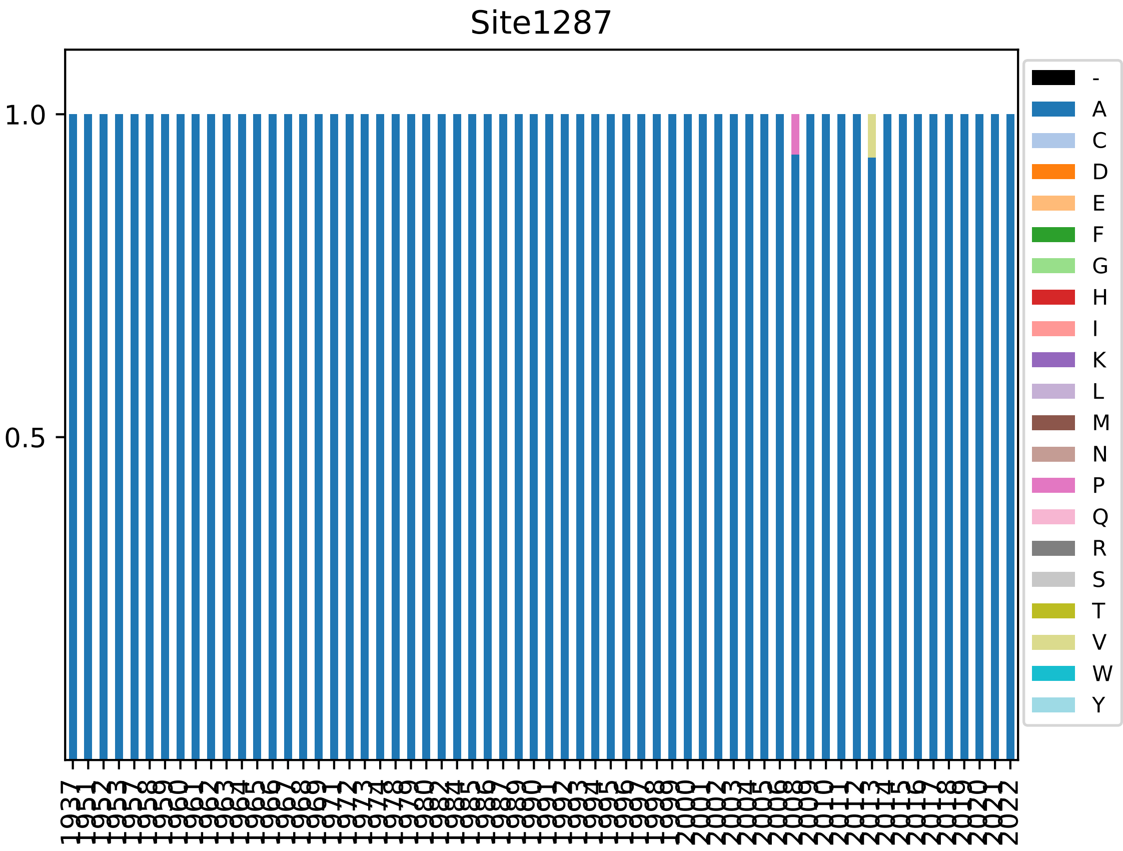
Site1287
1.0
0.5
-
A
C
D
E
F
G
H
I
K
L
M
N
P
Q
R
S
T
V
W
Y

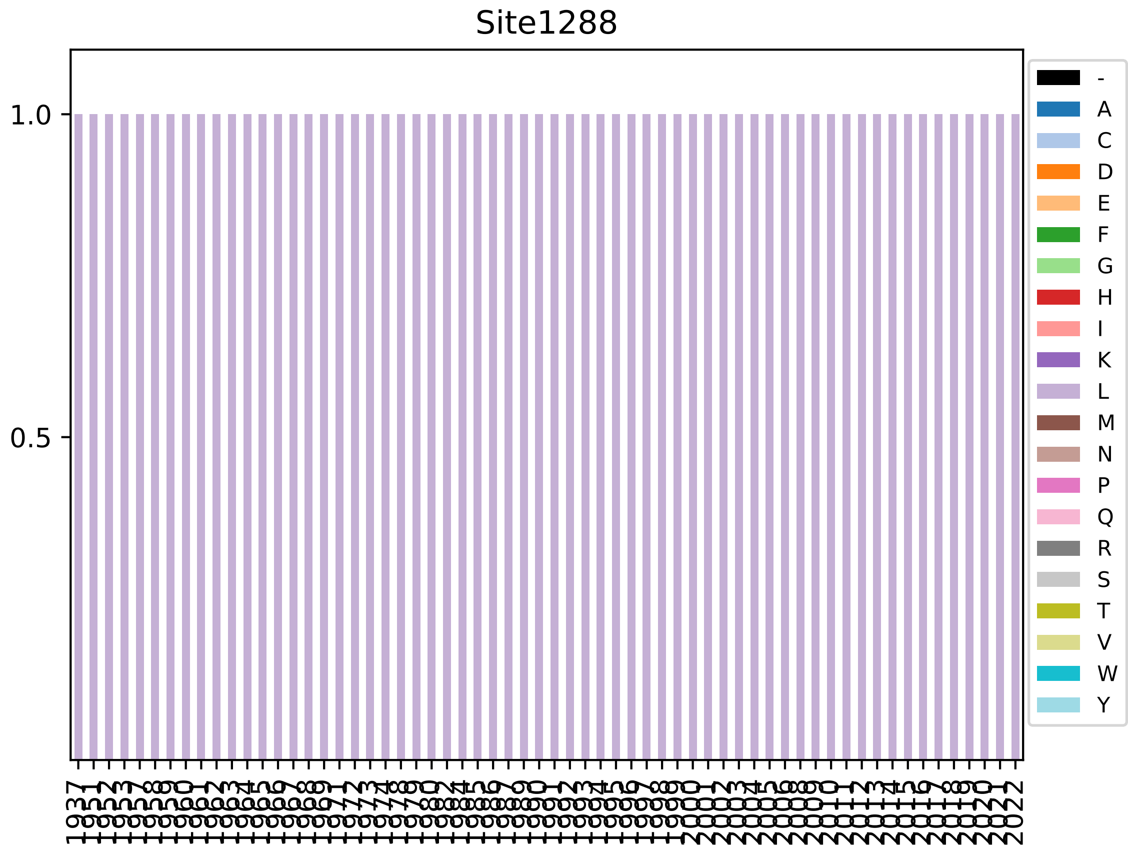
Site1288
1.0
0.5
-
A
C
D
E
F
G
H
I
K
L
M
N
P
Q
R
S
T
V
W
Y

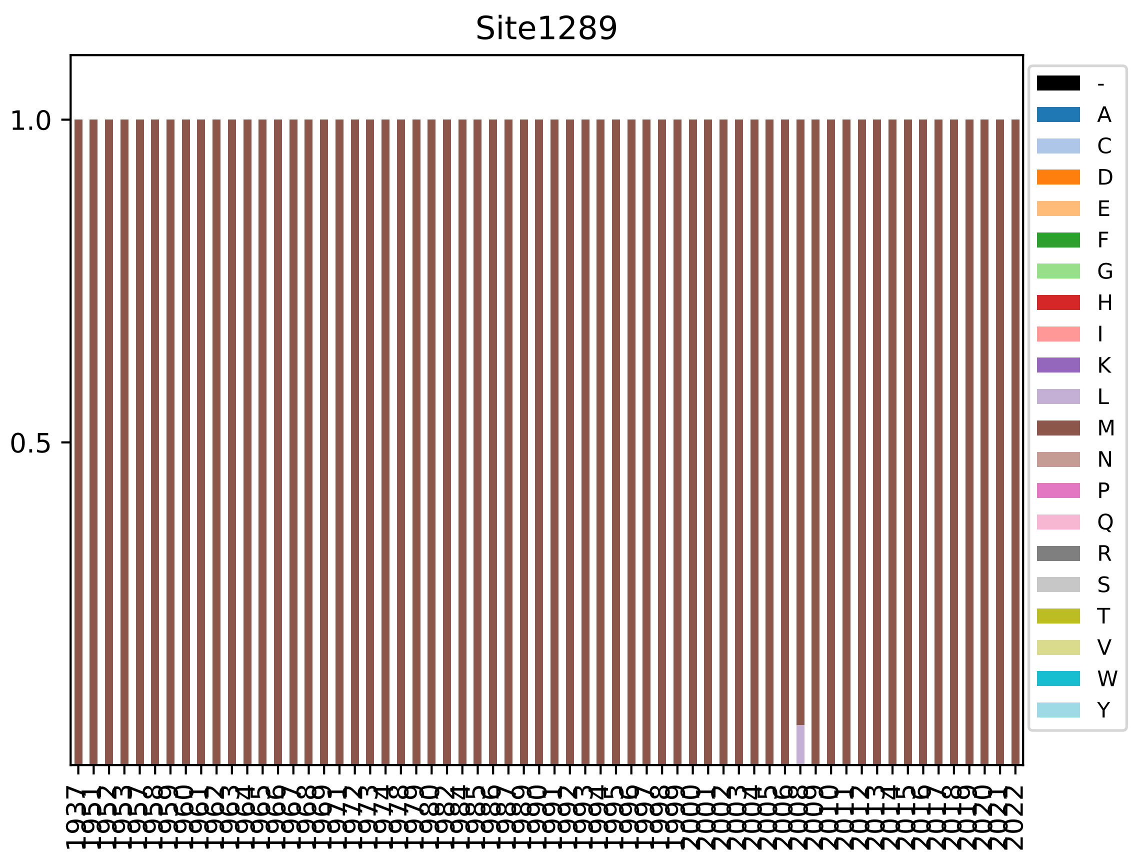
Site1289
1.0
0.5
-
A
C
D
E
F
G
H
I
K
L
M
N
P
Q
R
S
T
V
W
Y

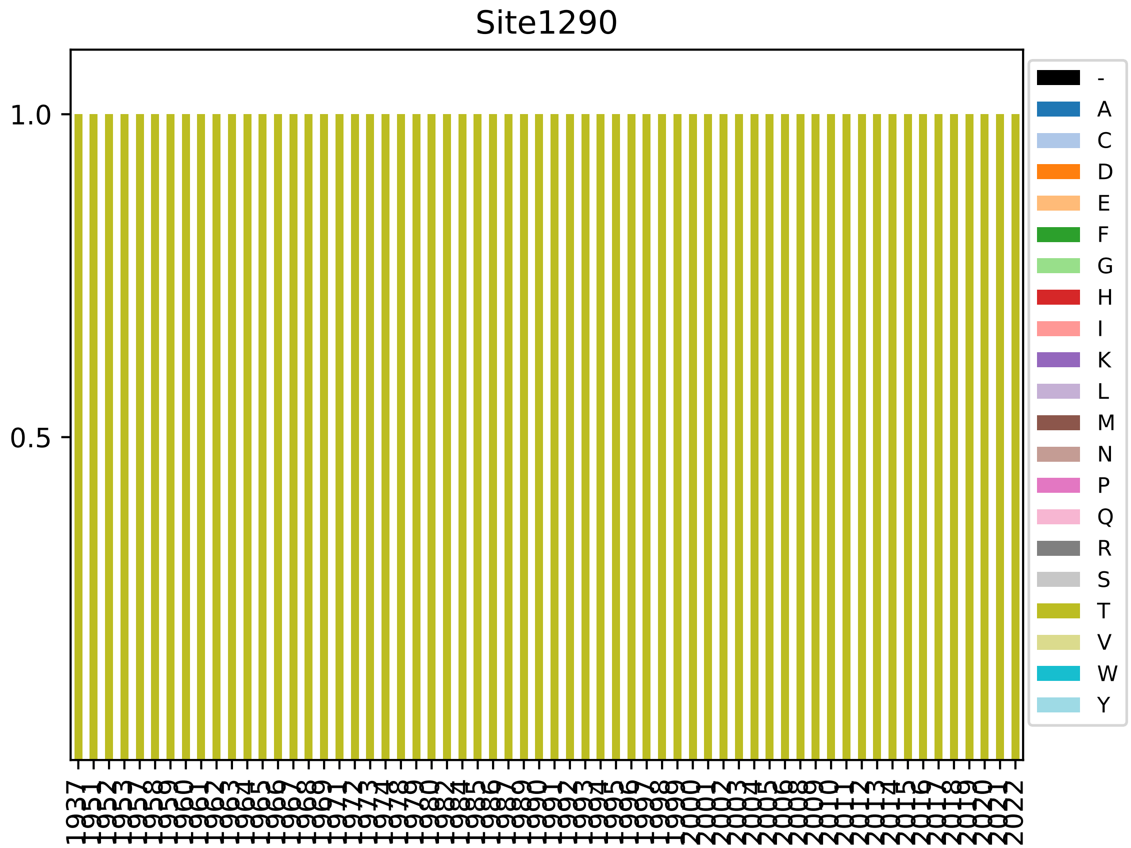
Site1290
1.0
0.5
-
A
C
D
E
F
G
H
I
K
L
M
N
P
Q
R
S
T
V
W
Y

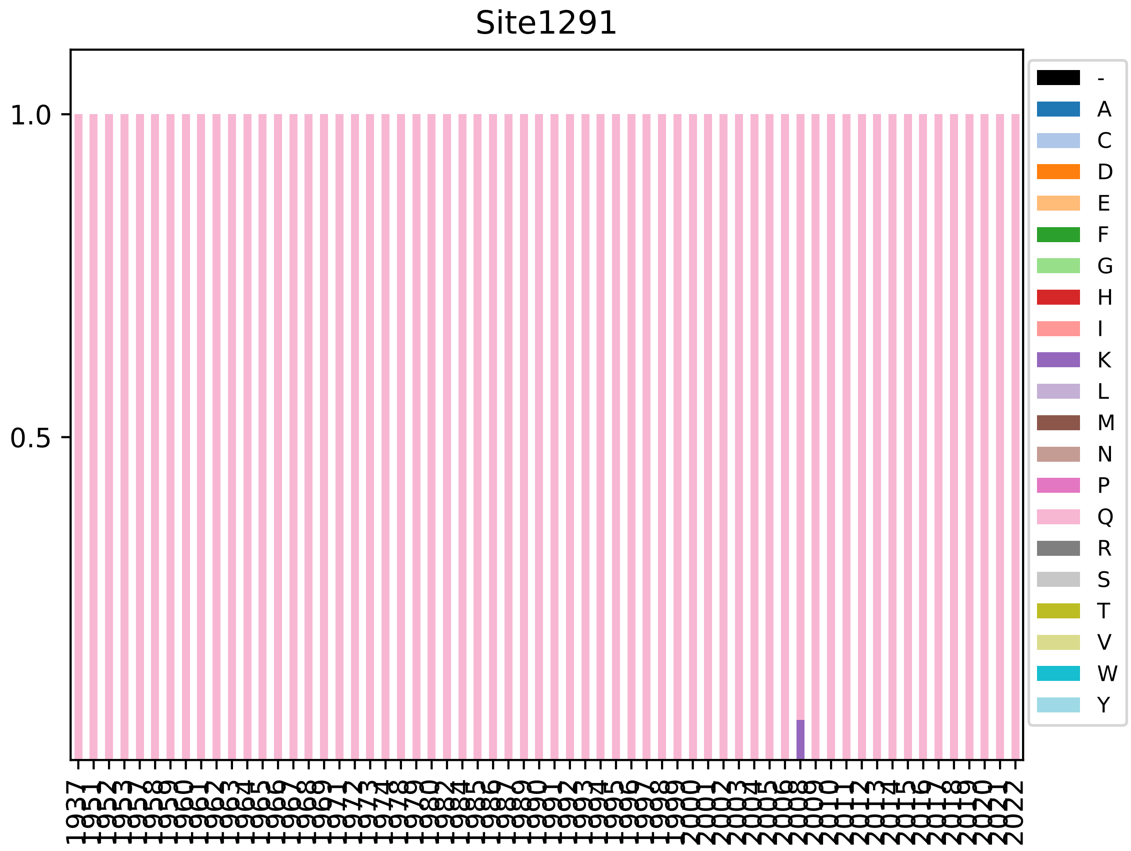

Site1291
1.0
0.5
-
A
C
D
E
F
G
H
I
K
L
M
N
P
Q
R
S
T
V
W
Y

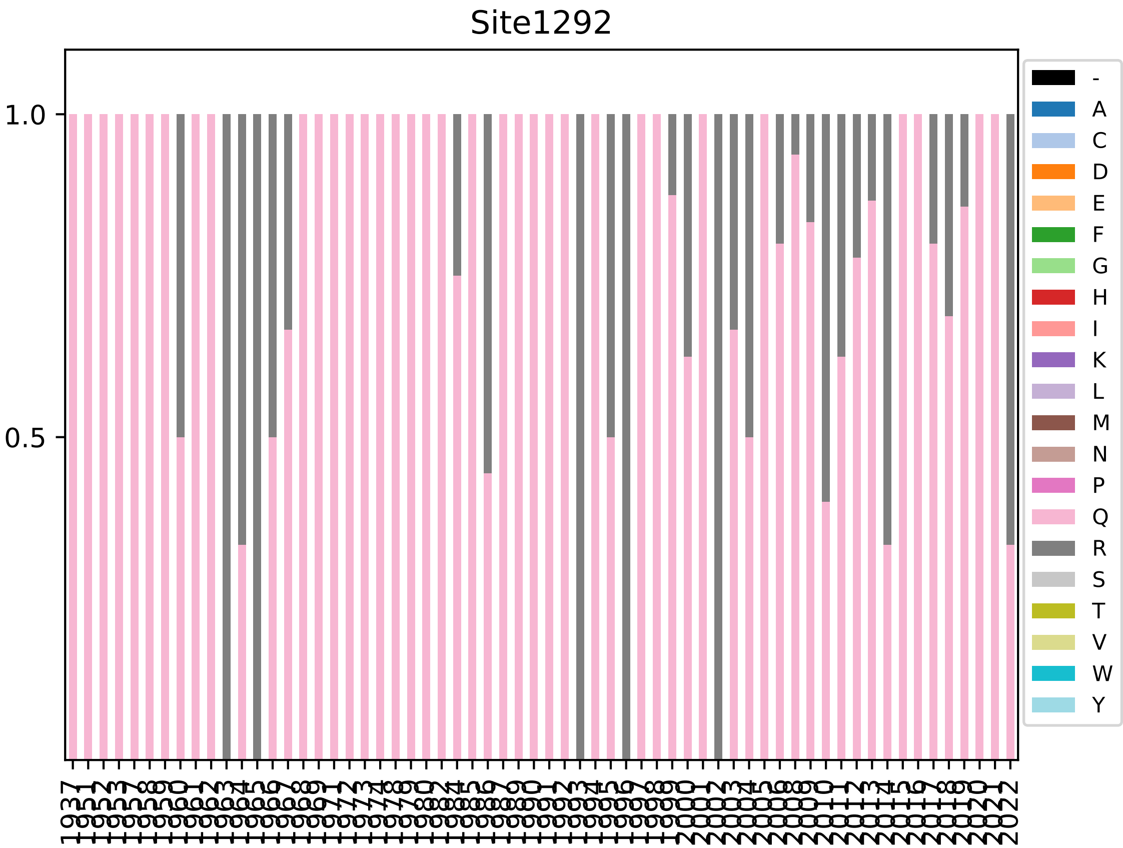
Site1292
1.0
0.5
-
A
C
D
E
F
G
H
I
K
L
M
N
P
Q
R
S
T
V
W
Y

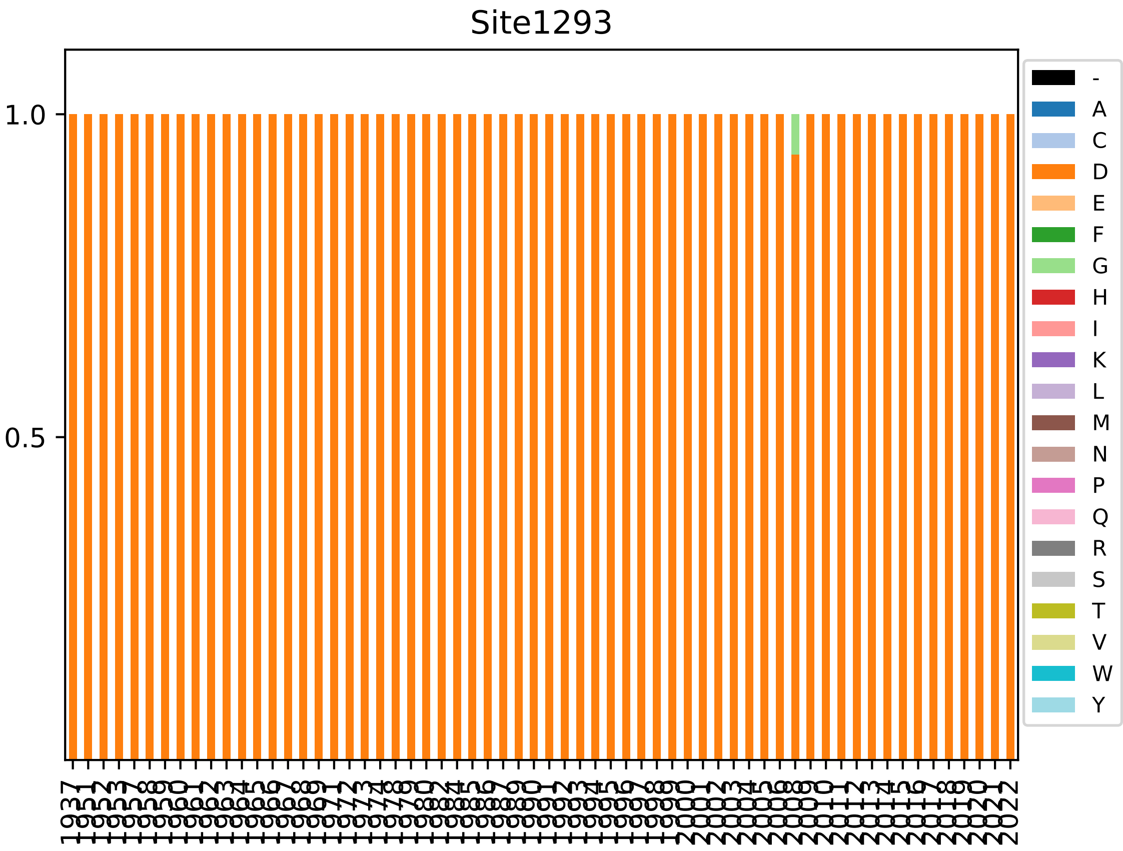
Site1293
1.0
0.5
-
A
C
D
E
F
G
H
I
K
L
M
N
P
Q
R
S
T
V
W
Y

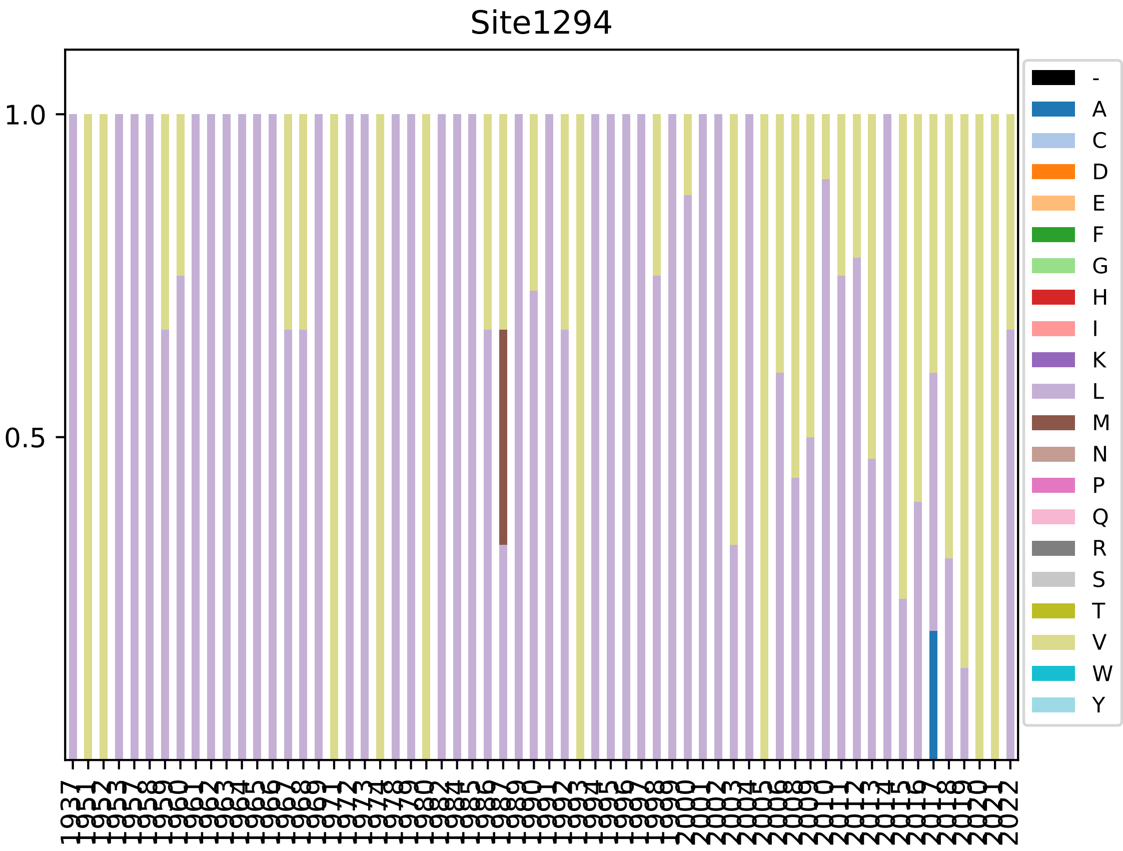
Site1294
1.0
0.5
-
A
C
D
E
F
G
H
I
K
L
M
N
P
Q
R
S
T
V
W
Y

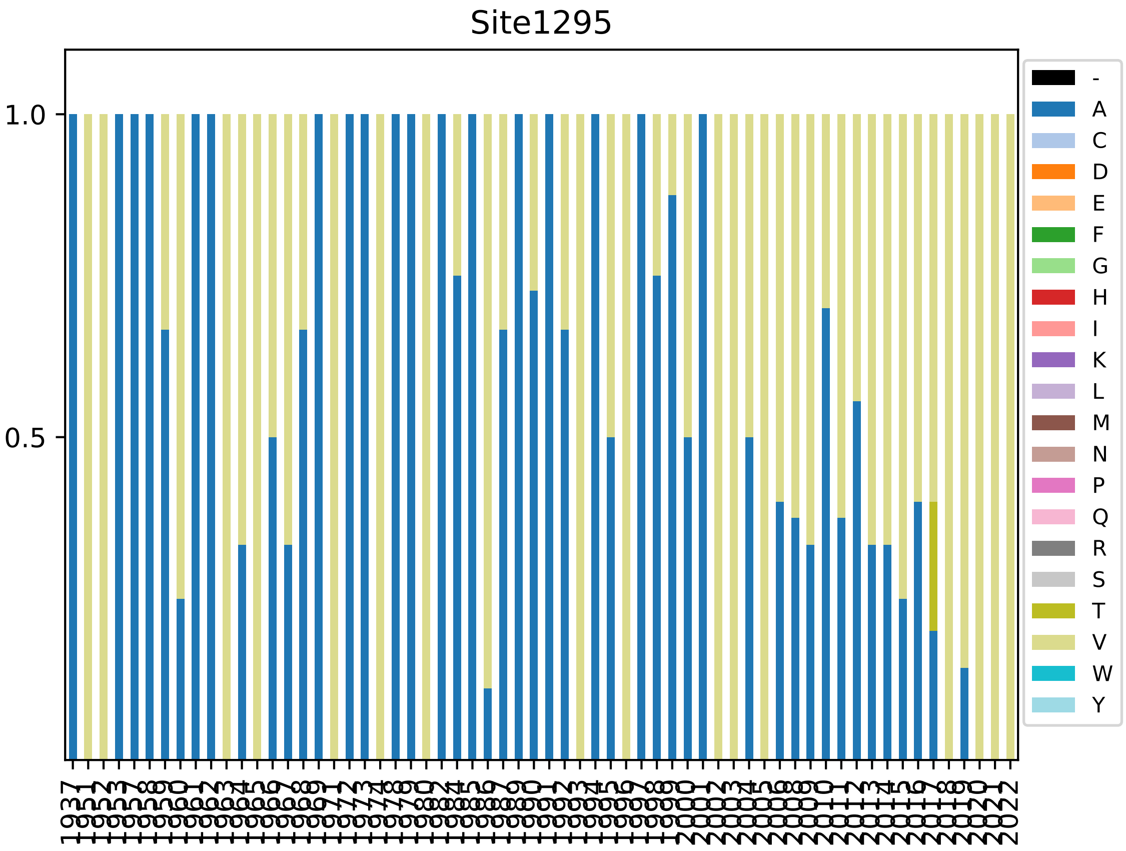
Site1295
1.0
0.5
-
A
C
D
E
F
G
H
I
K
L
M
N
P
Q
R
S
T
V
W
Y

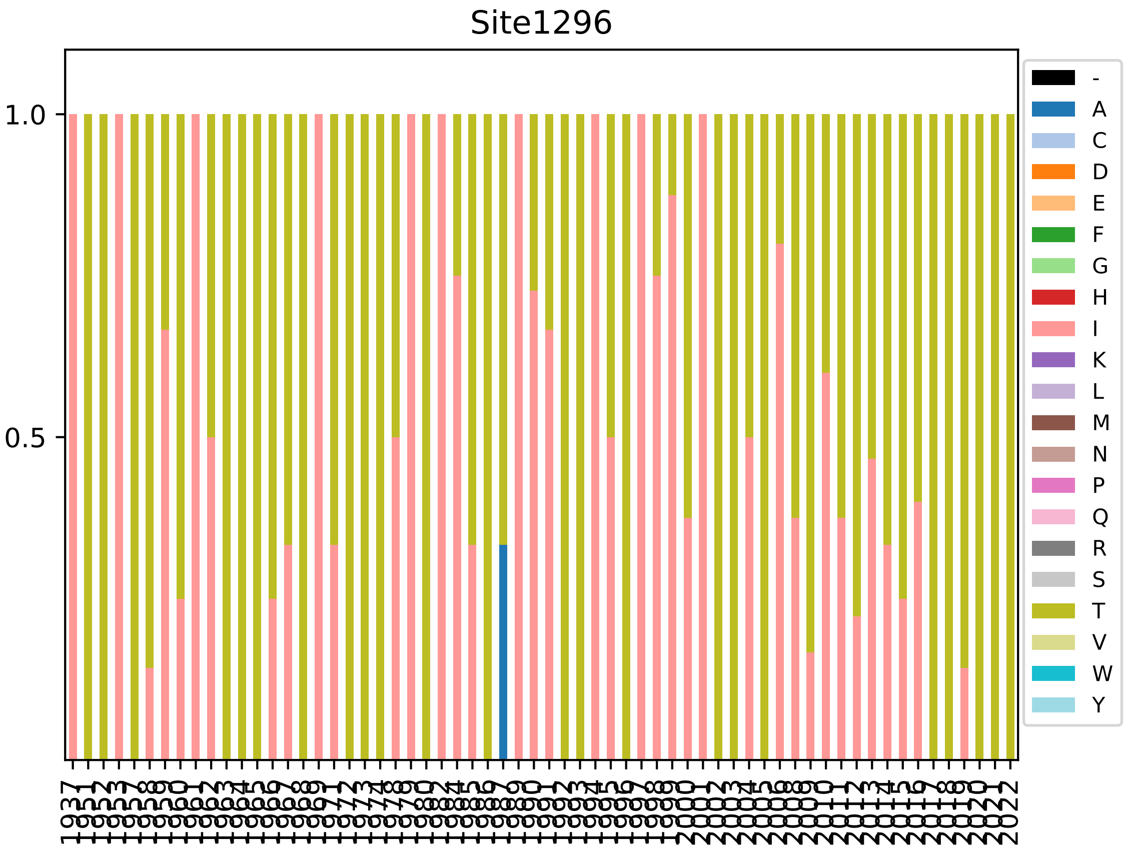
Site1296
1.0
0.5
-
A
C
D
E
F
G
H
I
K
L
M
N
P
Q
R
S
T
V
W
Y

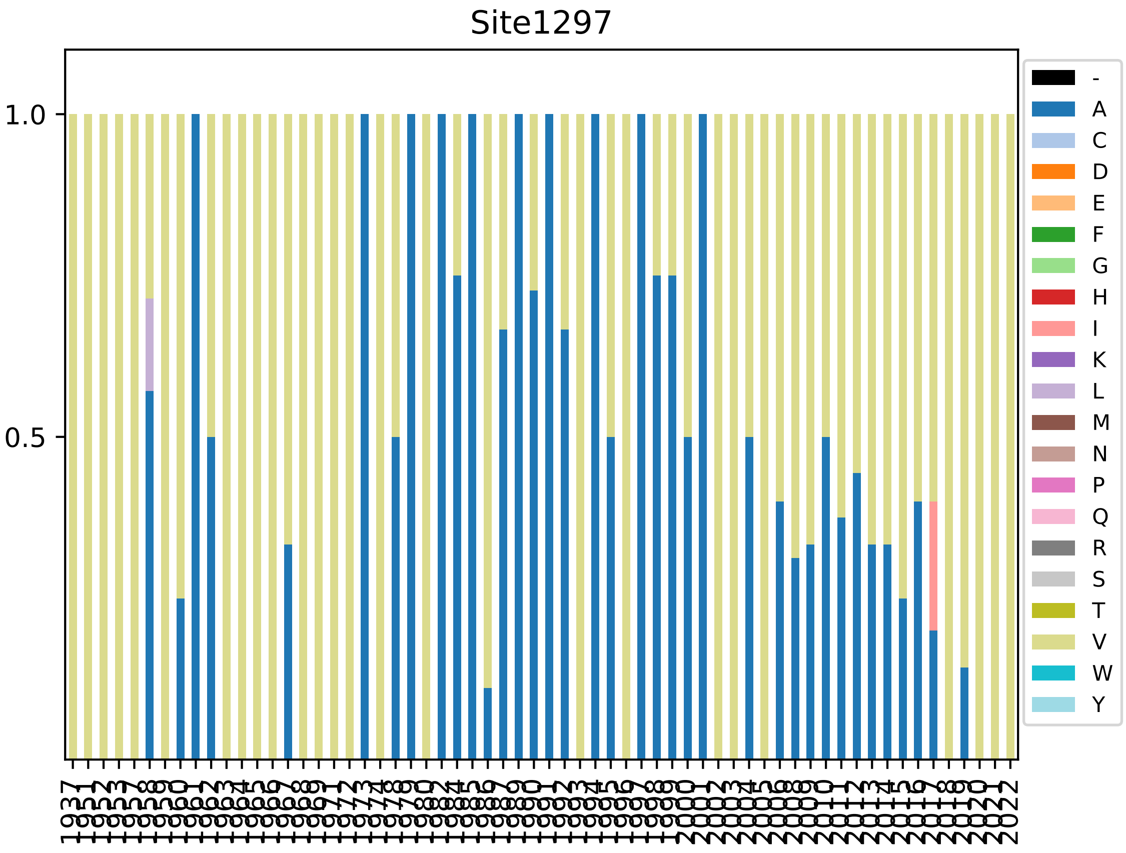

Site1297
1.0
0.5
-
A
C
D
E
F
G
H
I
K
L
M
N
P
Q
R
S
T
V
W
Y

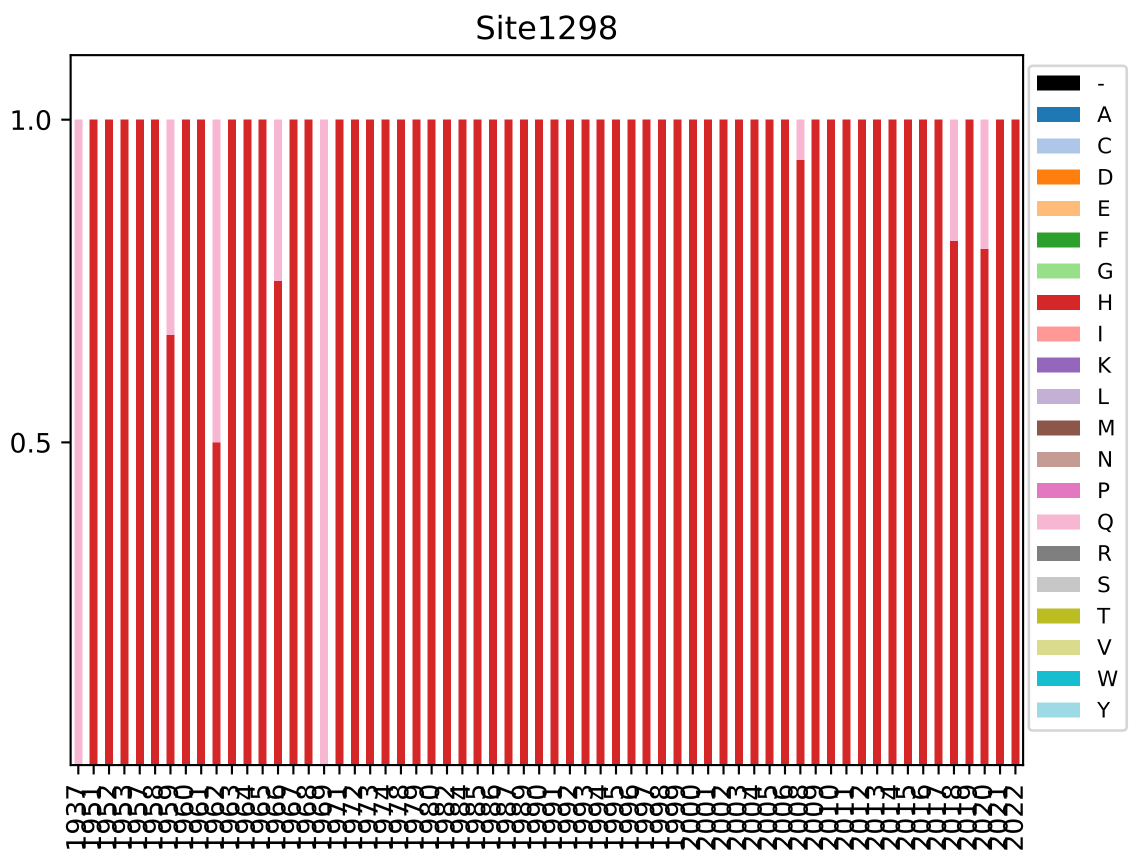
Site1298
1.0
0.5
-
A
C
D
E
F
G
H
I
K
L
M
N
P
Q
R
S
T
V
W
Y

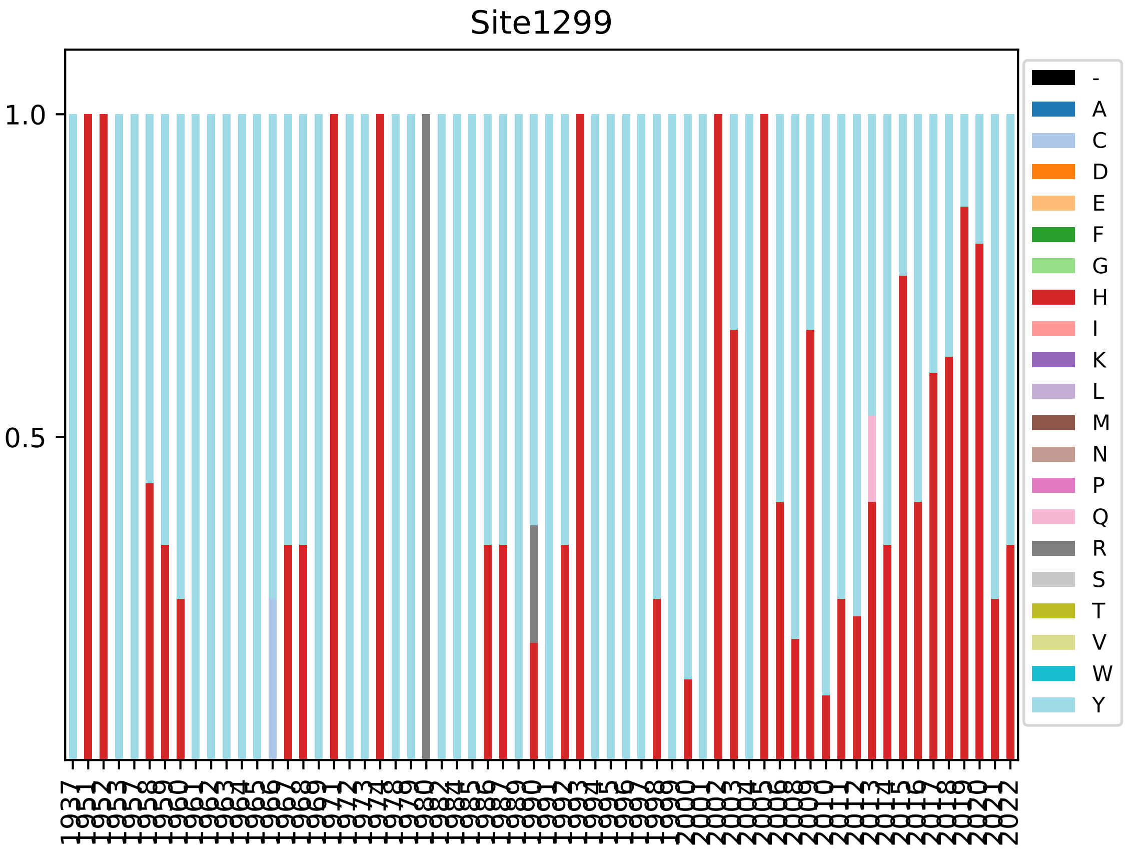
Site1299
1.0
0.5
-
A
C
D
E
F
G
H
I
K
L
M
N
P
Q
R
S
T
V
W
Y

Site1300

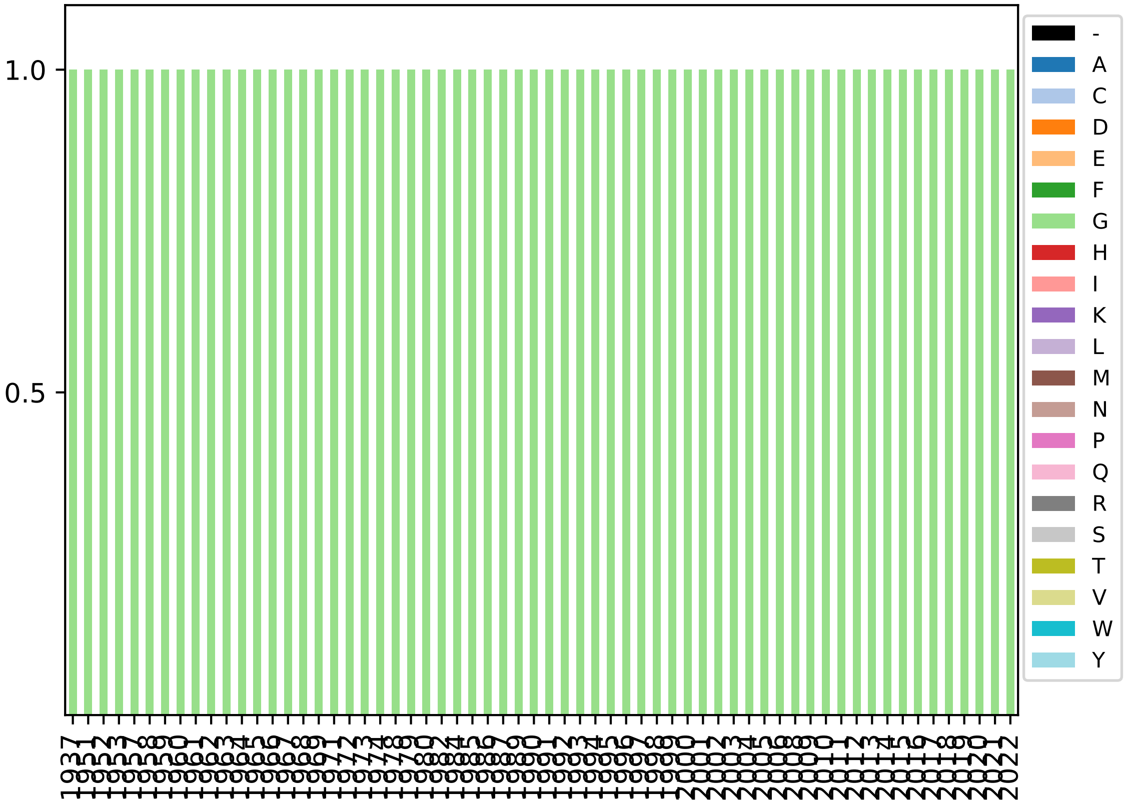

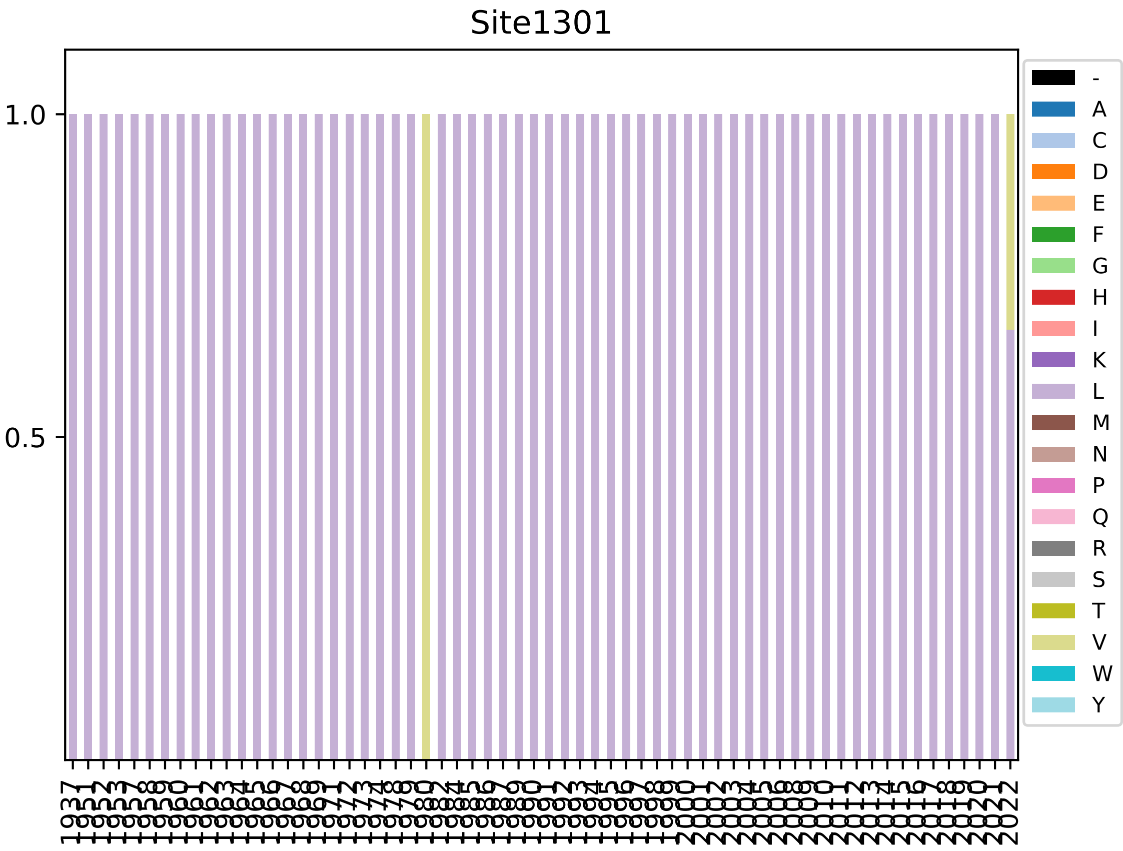

Site1301
1.0
0.5
-
A
C
D
E
F
G
H
I
K
L
M
N
P
Q
R
S
T
V
W
Y

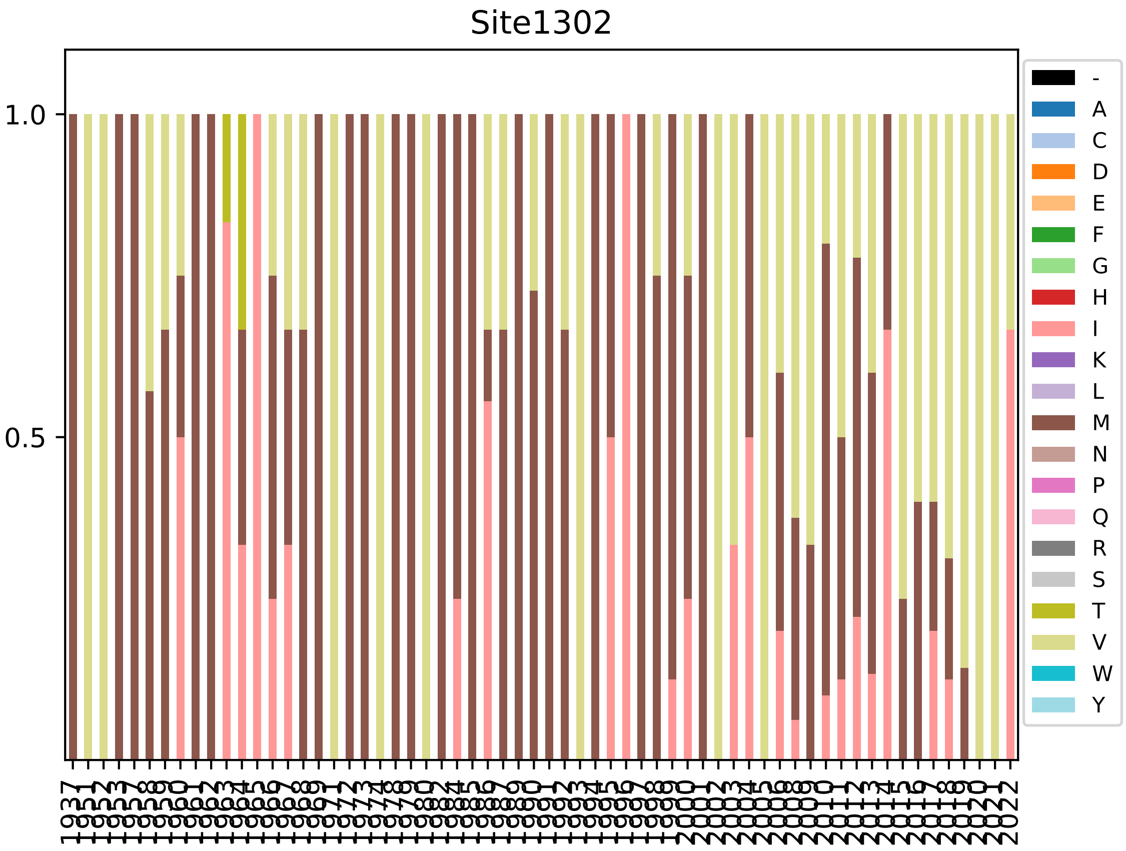
Site1302
1.0
0.5
-
A
C
D
E
F
G
H
I
K
L
M
N
P
Q
R
S
T
V
W
Y

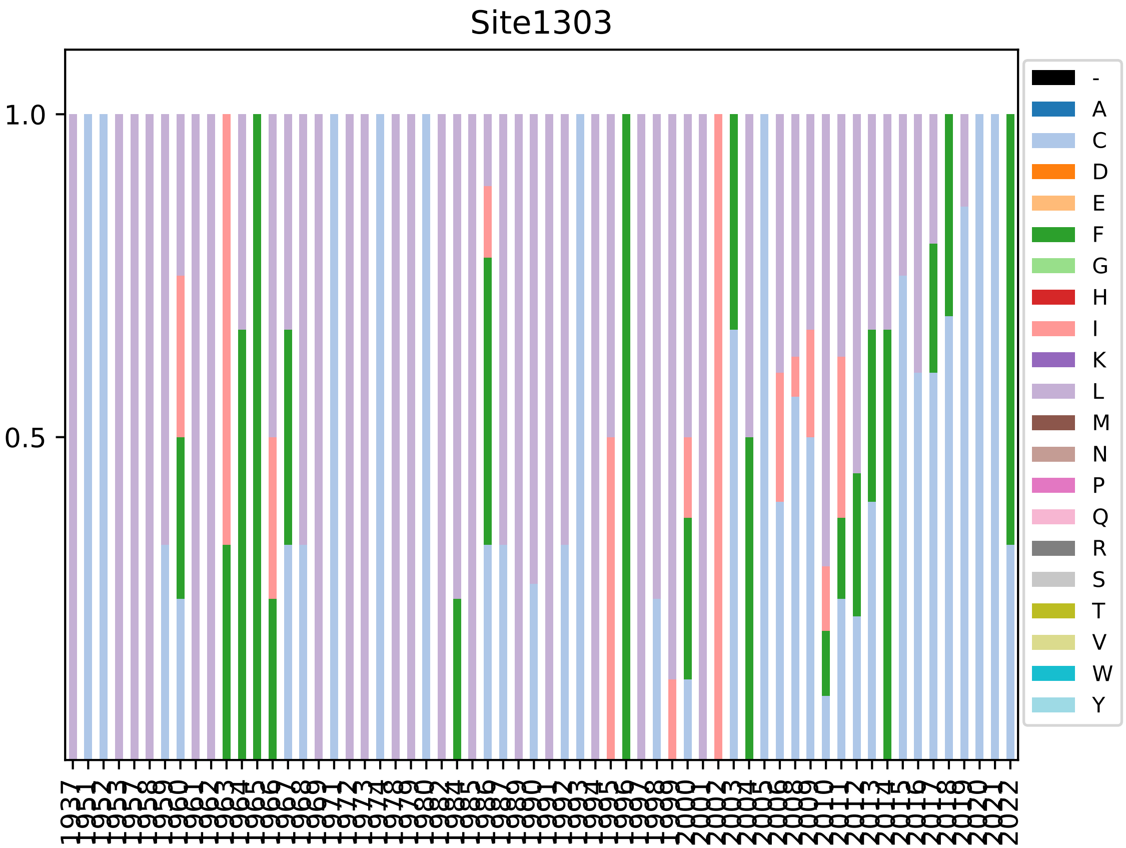

Site1303
1.0
0.5
-
A
C
D
E
F
G
H
I
K
L
M
N
P
Q
R
S
T
V
W
Y

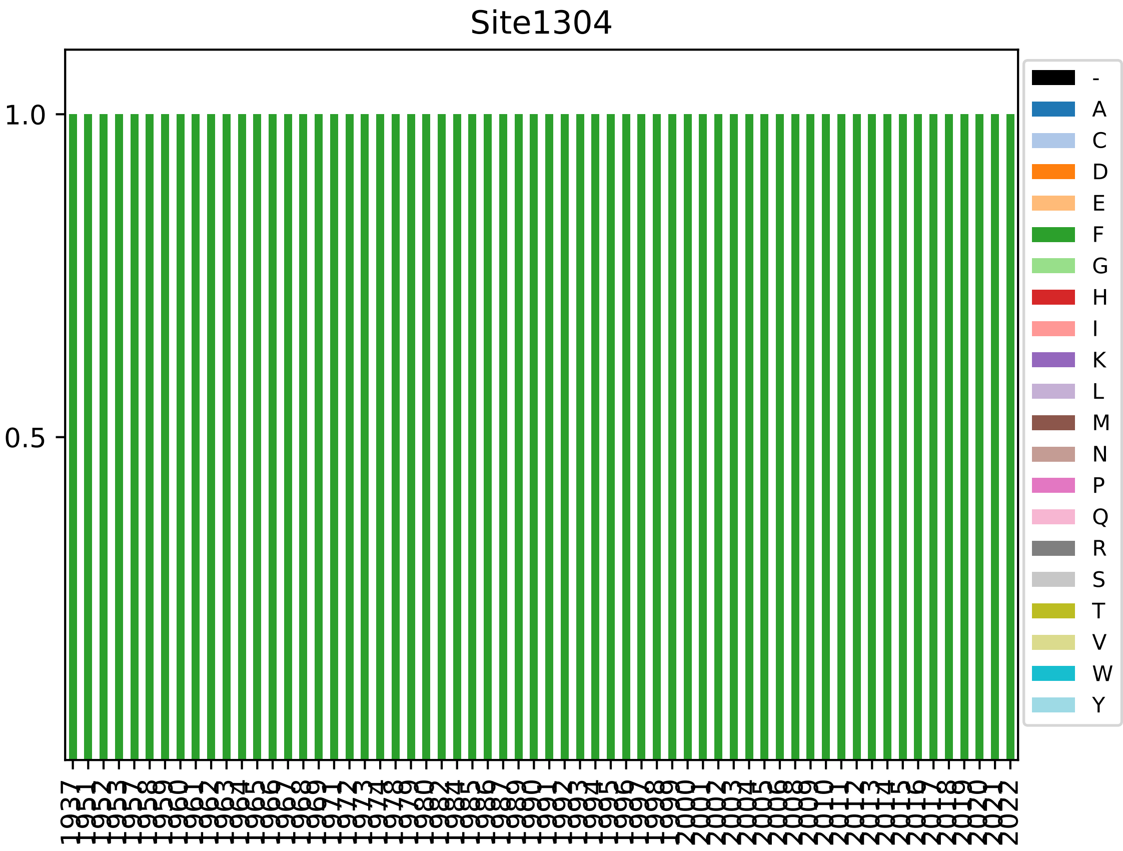
Site1304
1.0
0.5
-
A
C
D
E
F
G
H
I
K
L
M
N
P
Q
R
S
T
V
W
Y

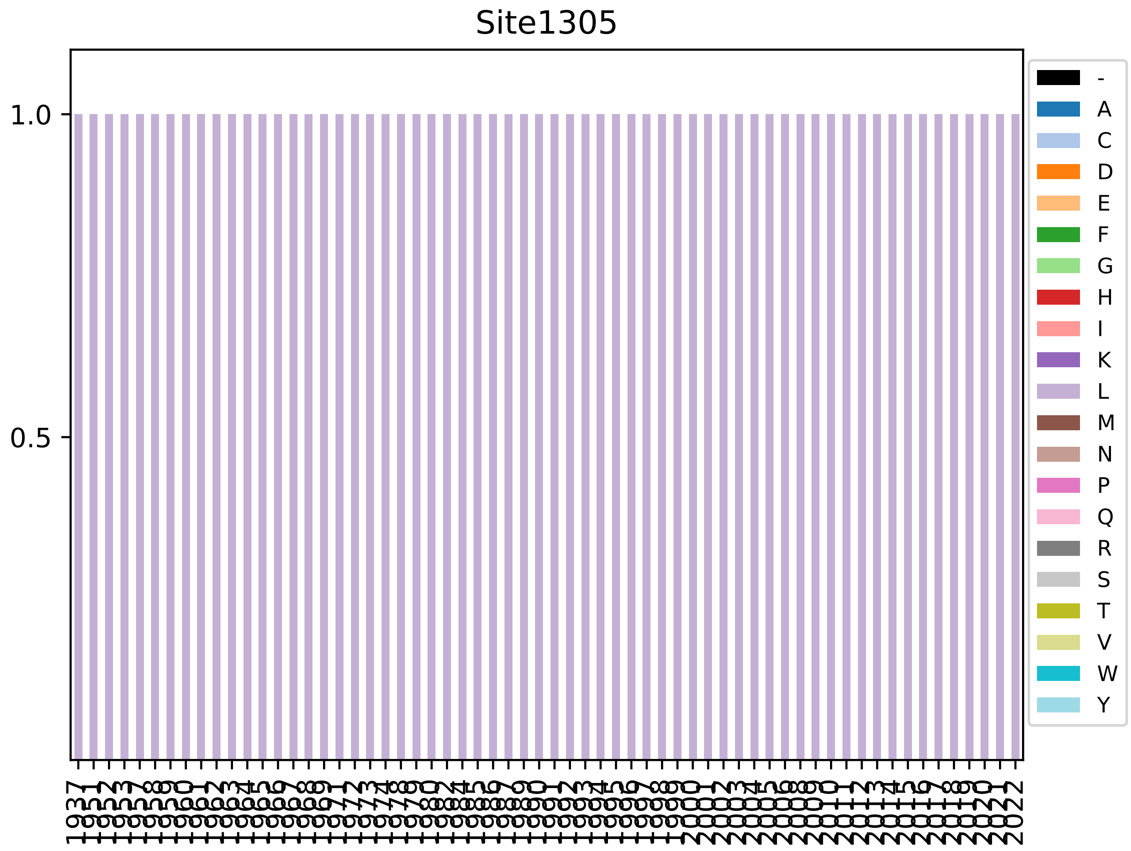
Site1305
1.0
0.5
-
A
C
D
E
F
G
H
I
K
L
M
N
P
Q
R
S
T
V
W
Y

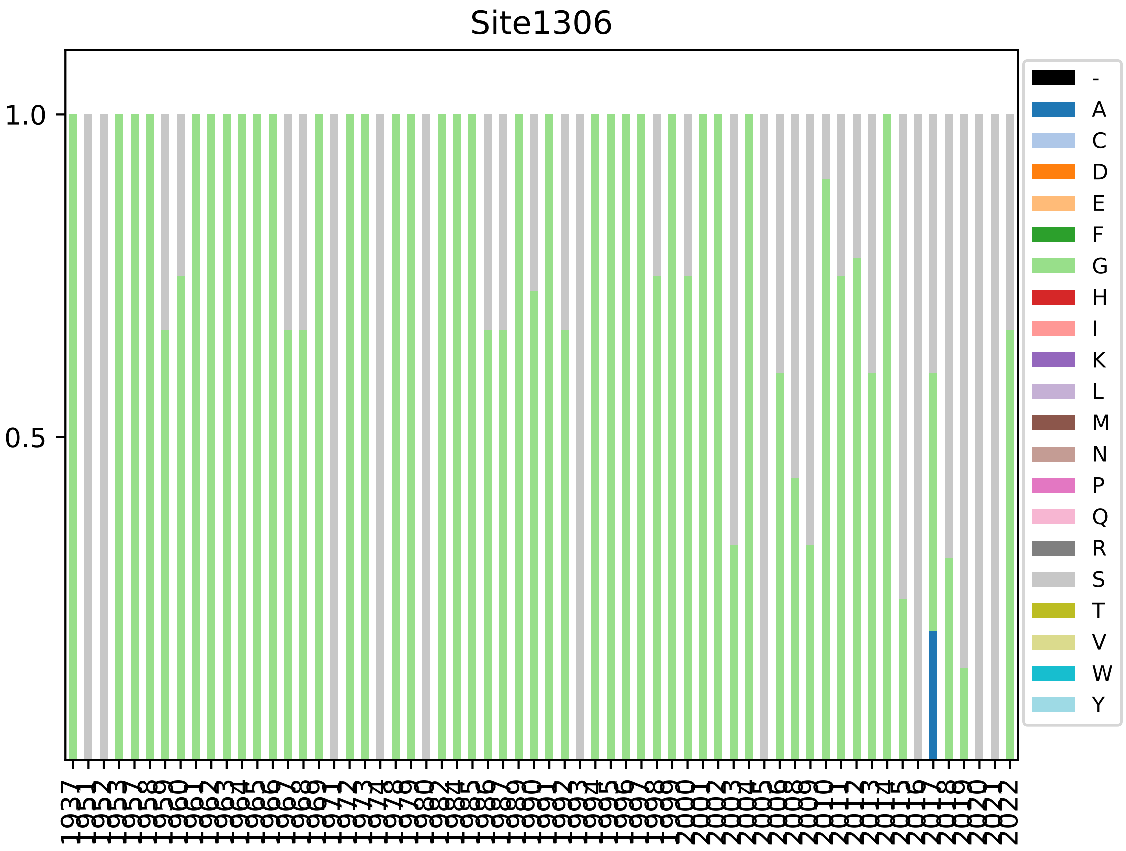
Site1306
1.0
0.5
-
A
C
D
E
F
G
H
I
K
L
M
N
P
Q
R
S
T
V
W
Y

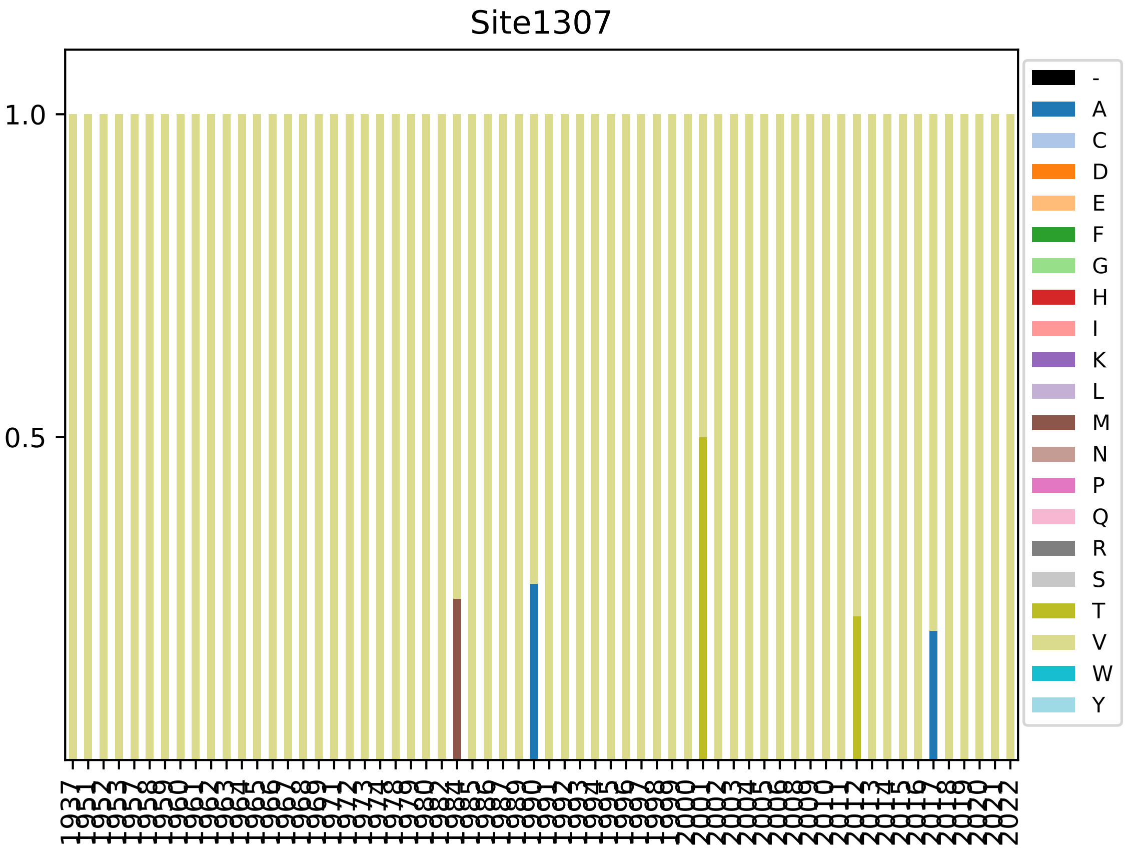
Site1307
1.0
0.5
-
A
C
D
E
F
G
H
I
K
L
M
N
P
Q
R
S
T
V
W
Y

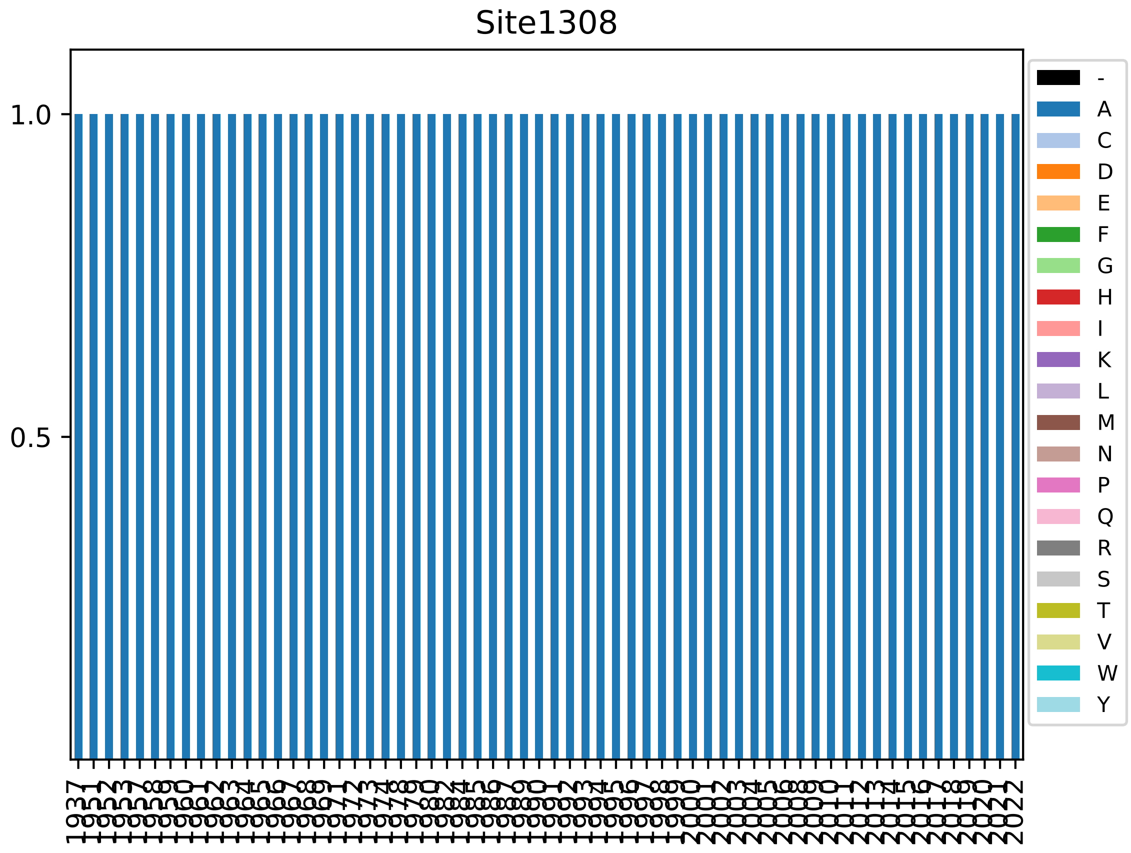
Site1308
1.0
0.5
-
A
C
D
E
F
G
H
I
K
L
M
N
P
Q
R
S
T
V
W
Y

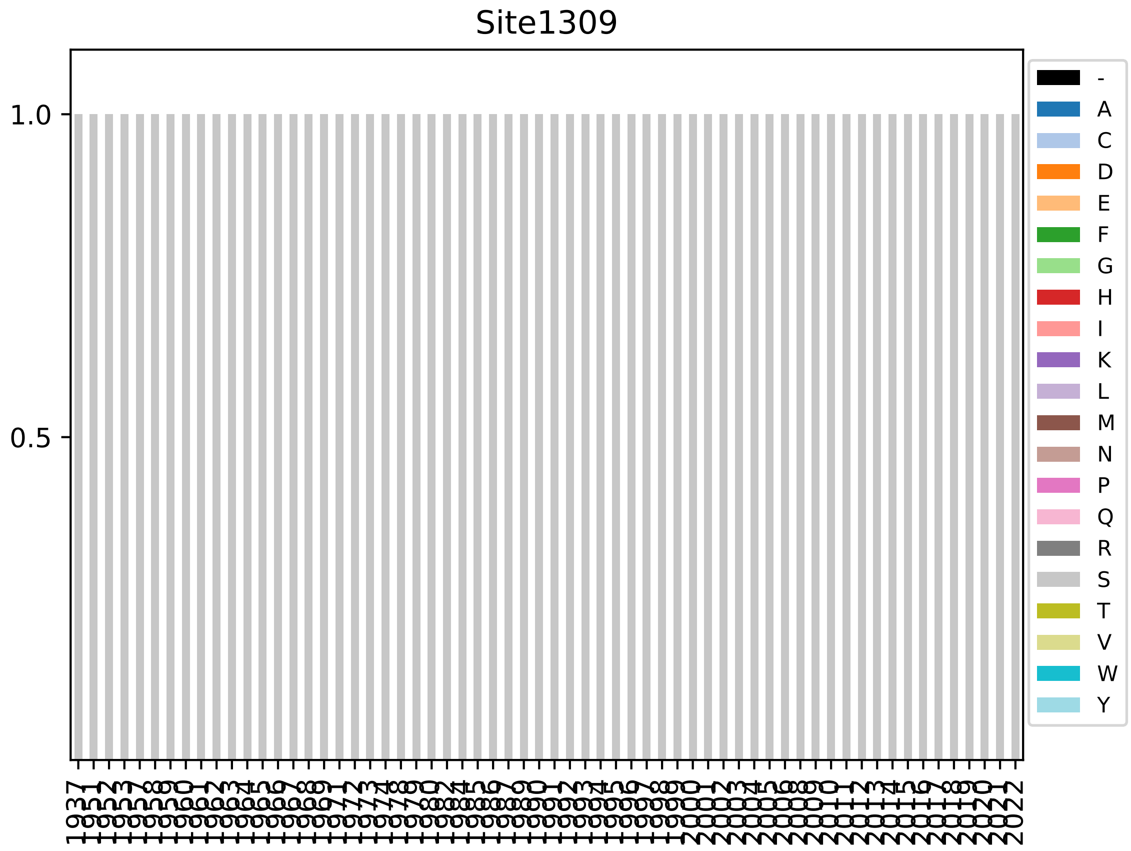
Site1309
1.0
0.5
-
A
C
D
E
F
G
H
I
K
L
M
N
P
Q
R
S
T
V
W
Y

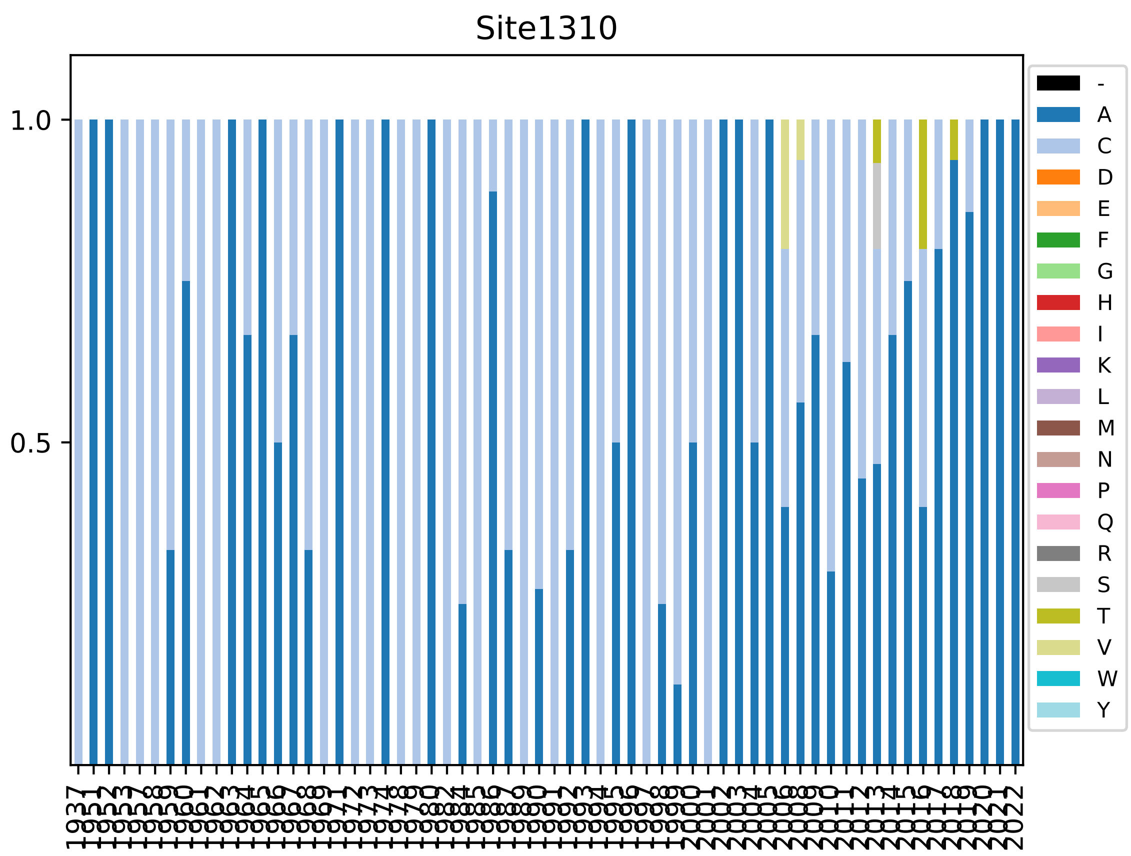
Site1310
1.0
0.5
-
A
C
D
E
F
G
H
I
K
L
M
N
P
Q
R
S
T
V
W
Y

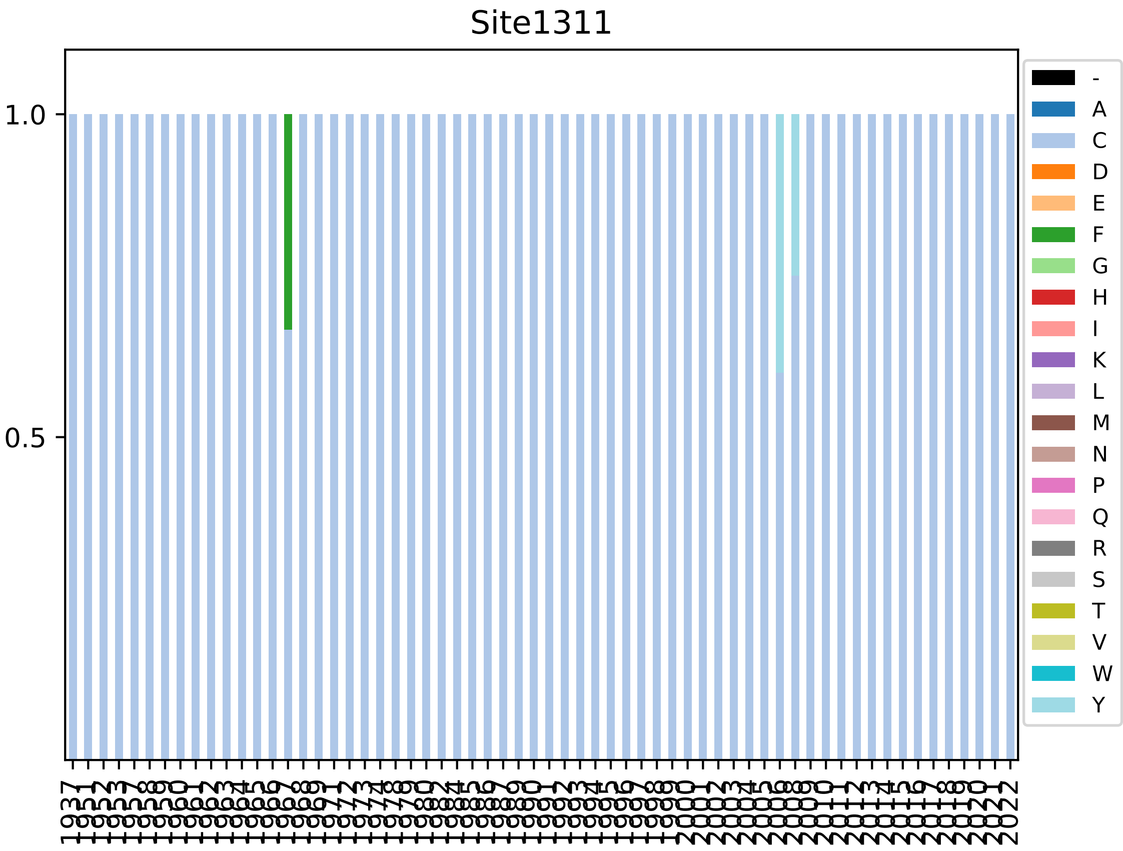
Site1311
1.0
0.5
-
A
C
D
E
F
G
H
I
K
L
M
N
P
Q
R
S
T
V
W
Y

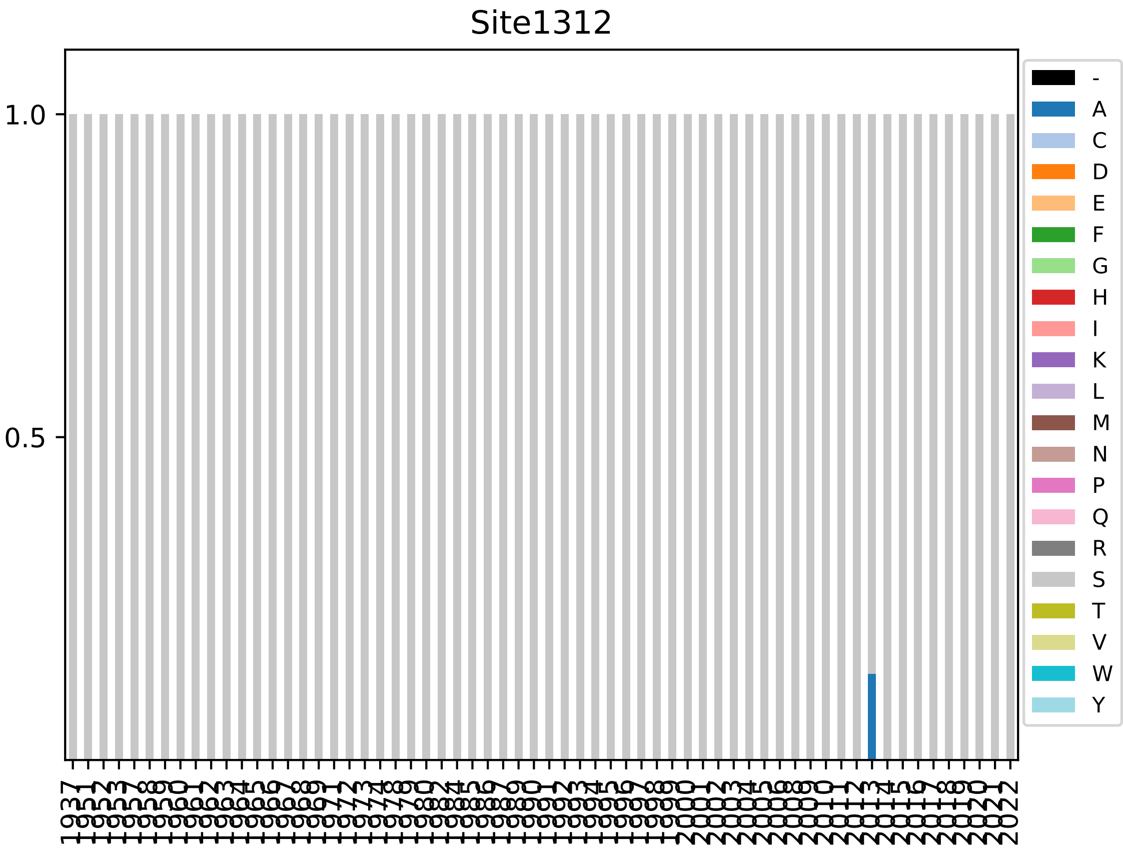

Site1312
1.0
0.5
-
A
C
D
E
F
G
H
I
K
L
M
N
P
Q
R
S
T
V
W
Y

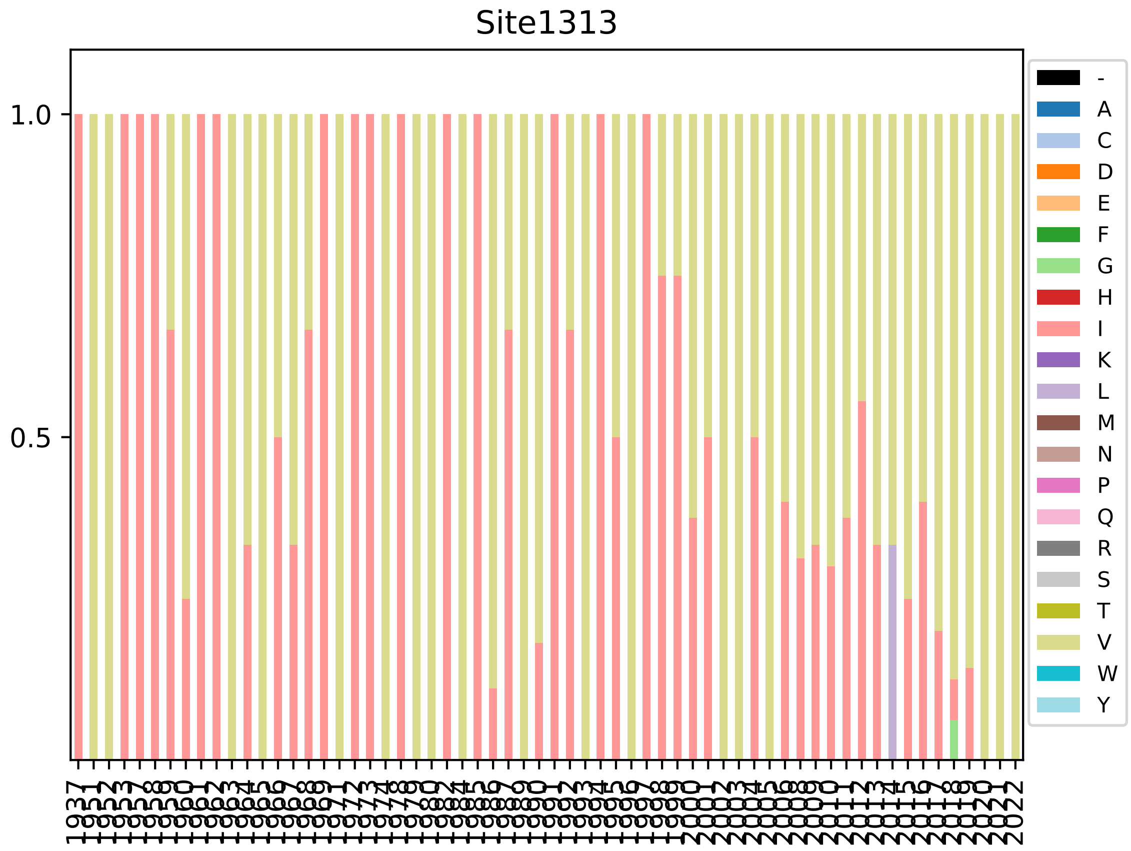
Site1313
1.0
0.5
-
A
C
D
E
F
G
H
I
K
L
M
N
P
Q
R
S
T
V
W
Y

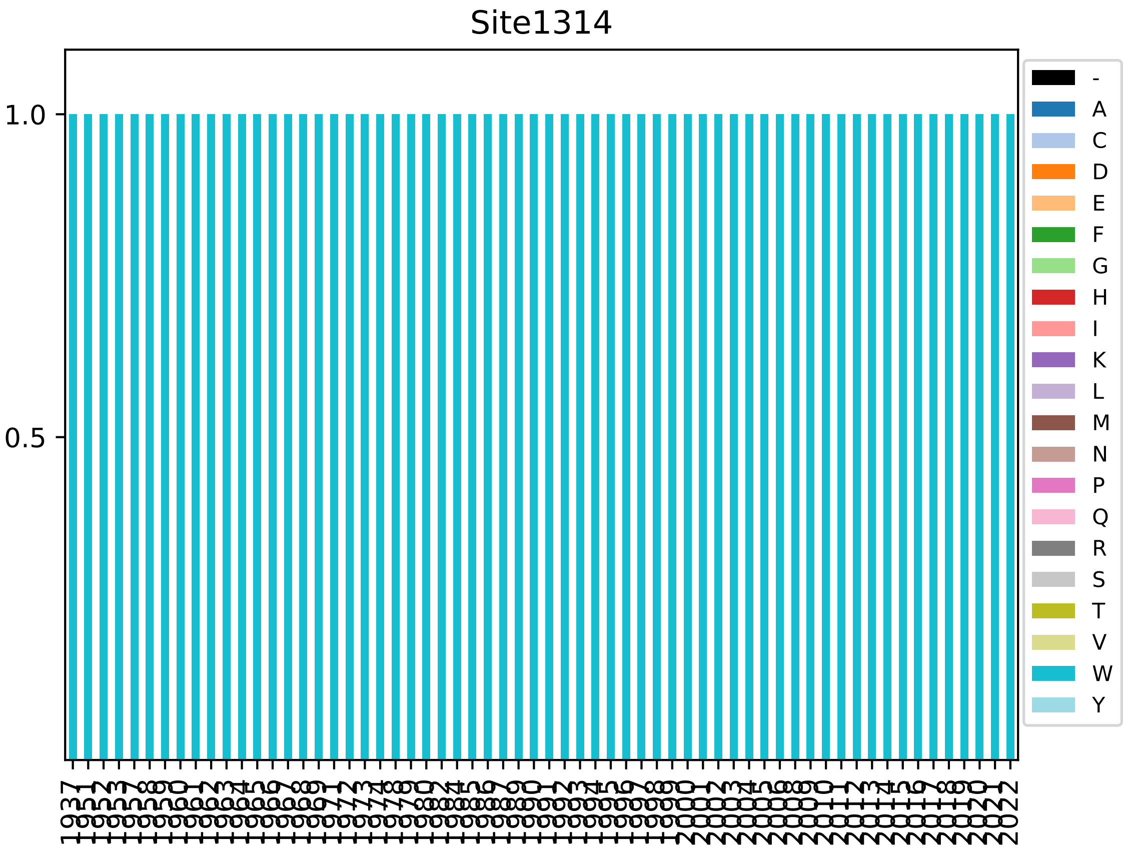
Site1314
1.0
0.5
-
A
C
D
E
F
G
H
I
K
L
M
N
P
Q
R
S
T
V
W
Y

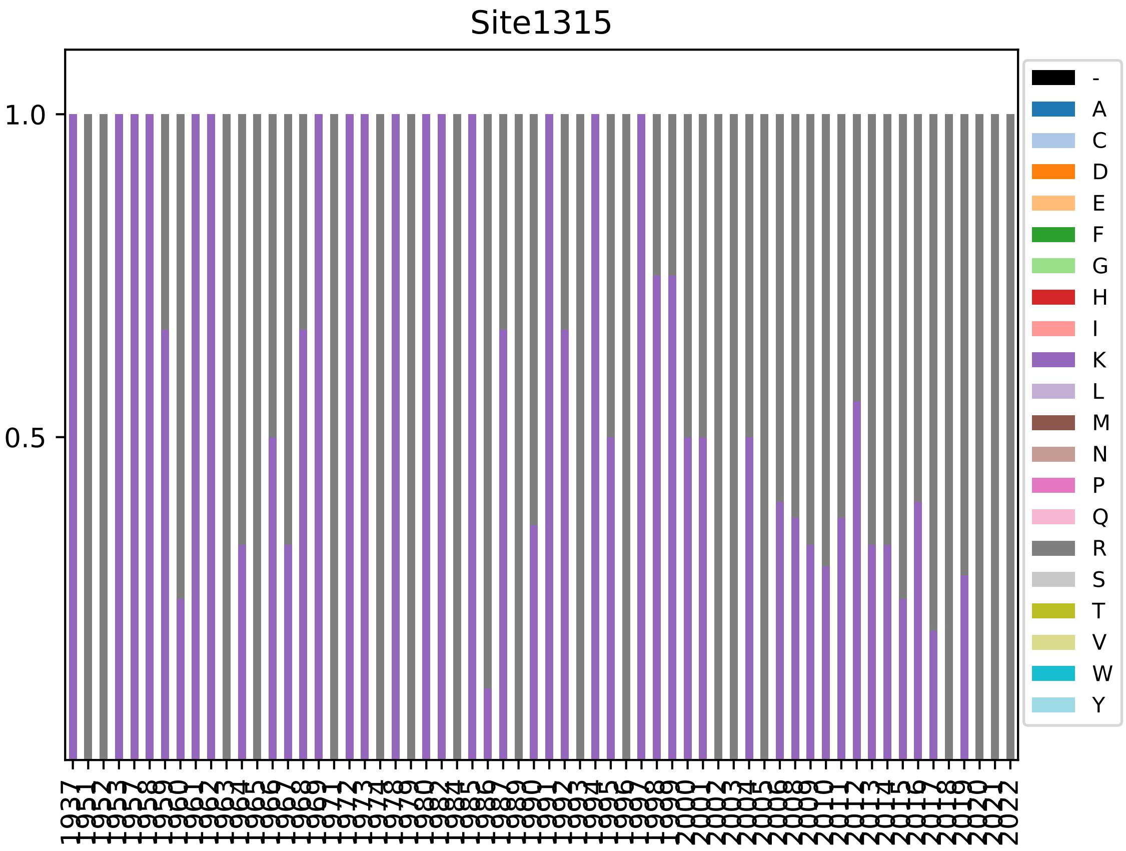
Site1315
1.0
0.5
-
A
C
D
E
F
G
H
I
K
L
M
N
P
Q
R
S
T
V
W
Y

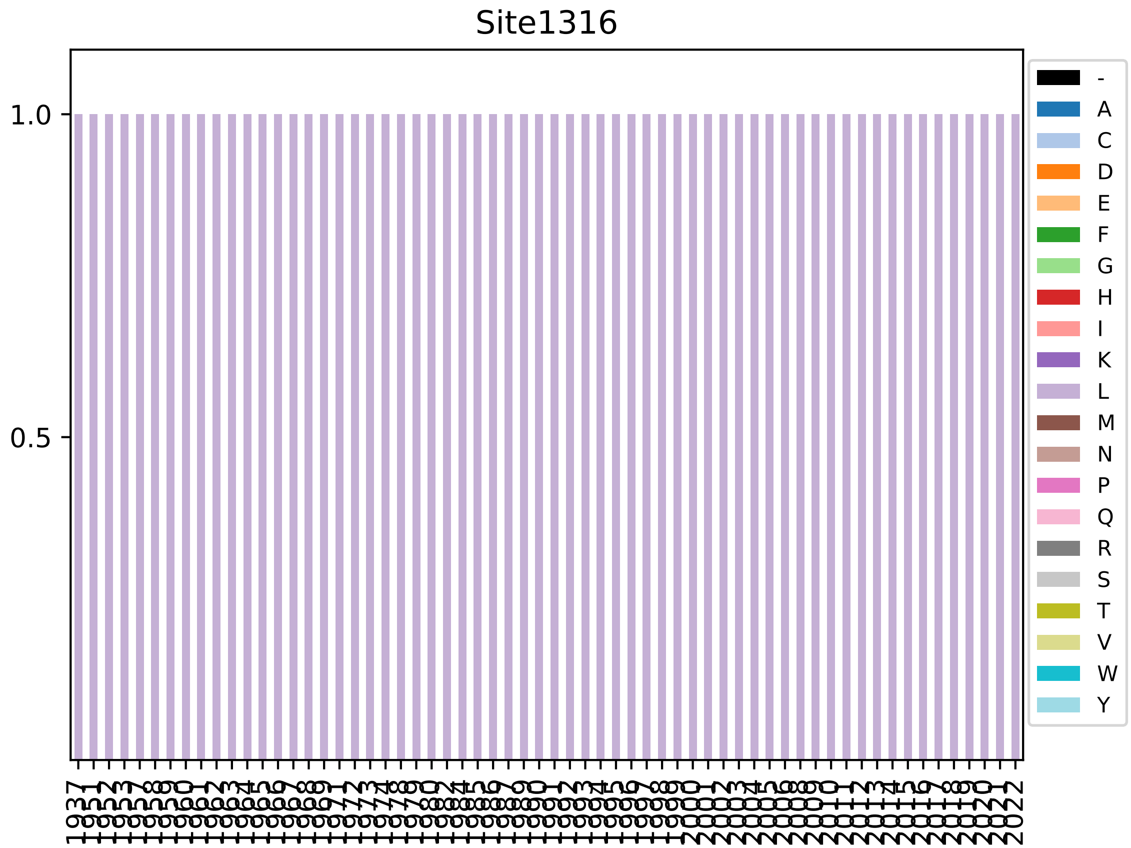
Site1316
1.0
0.5
-
A
C
D
E
F
G
H
I
K
L
M
N
P
Q
R
S
T
V
W
Y

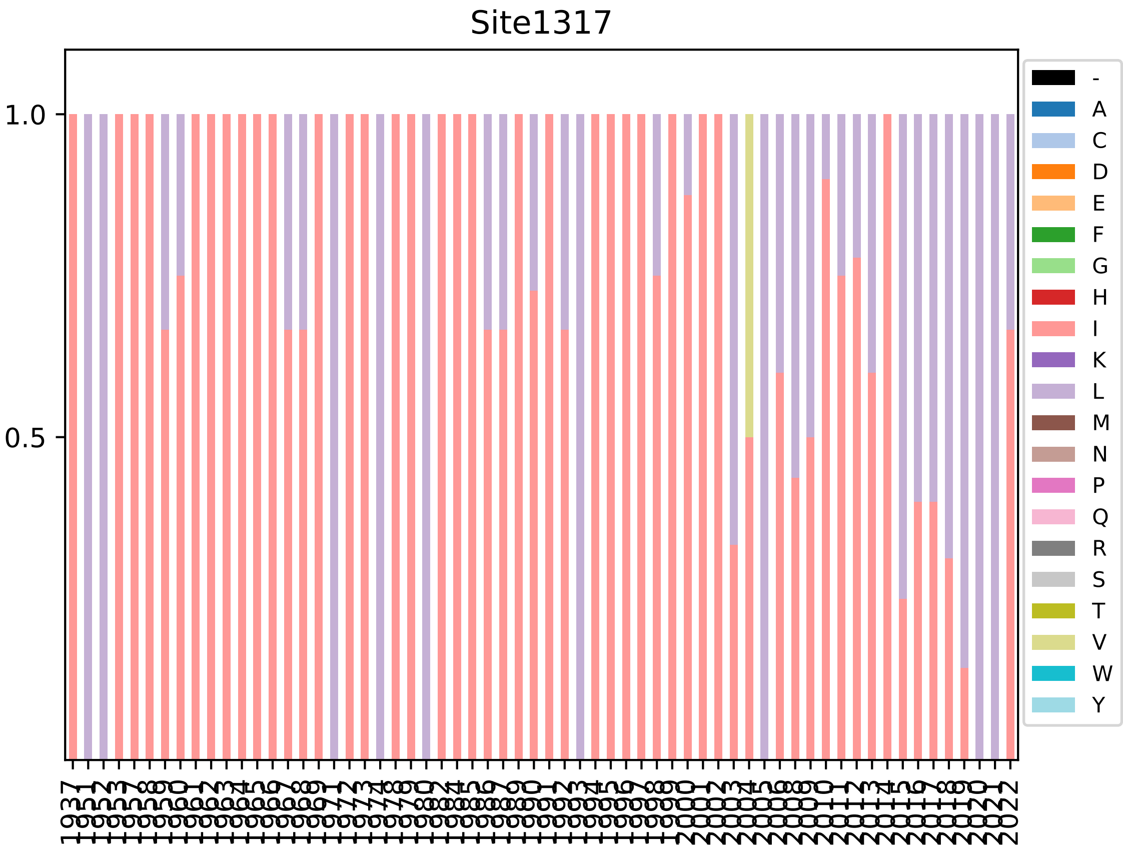
Site1317
1.0
0.5
-
A
C
D
E
F
G
H
I
K
L
M
N
P
Q
R
S
T
V
W
Y

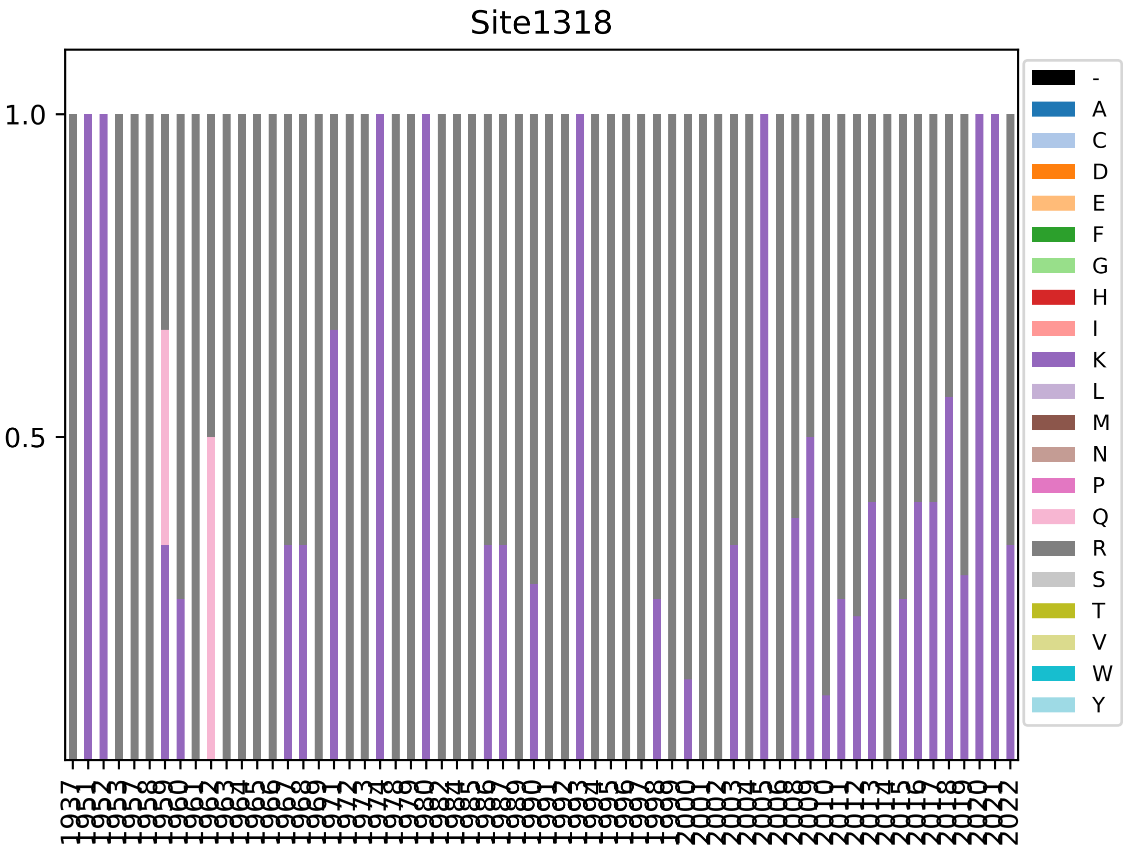

Site1318
1.0
0.5
-
A
C
D
E
F
G
H
I
K
L
M
N
P
Q
R
S
T
V
W
Y

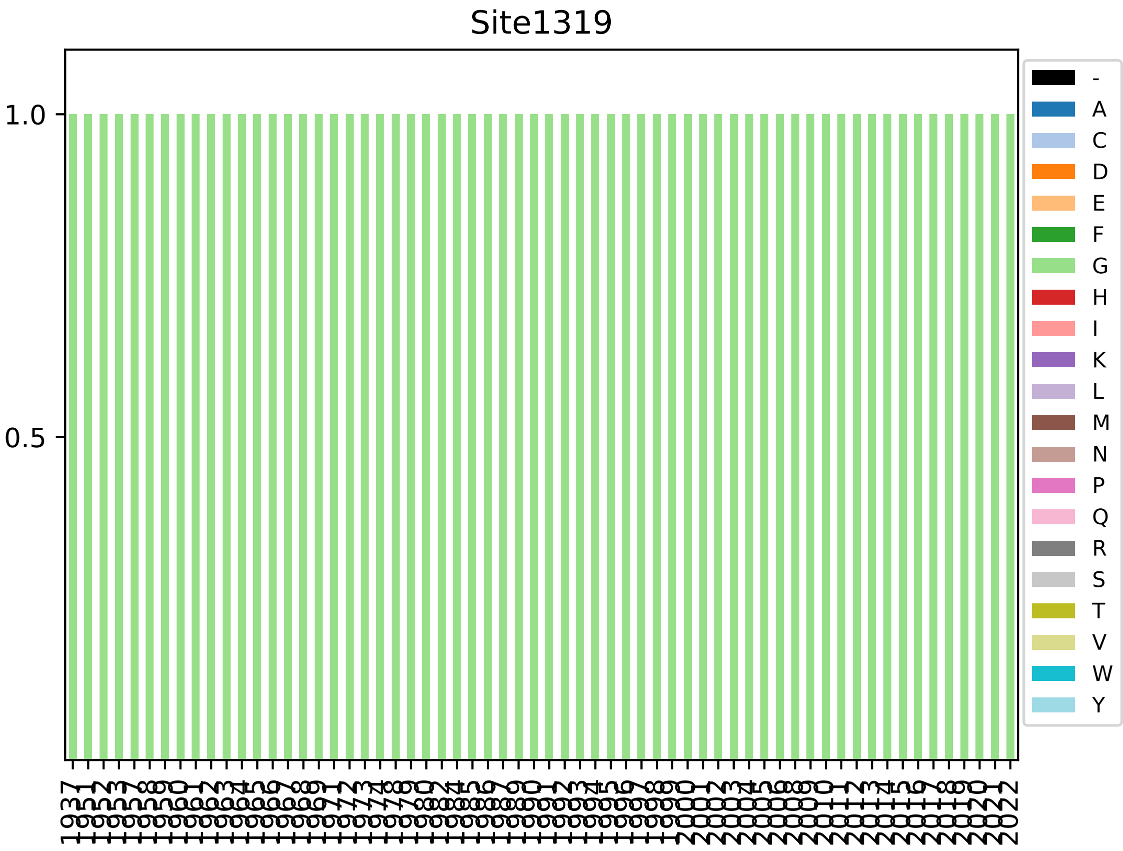
Site1319
1.0
0.5
-
A
C
D
E
F
G
H
I
K
L
M
N
P
Q
R
S
T
V
W
Y

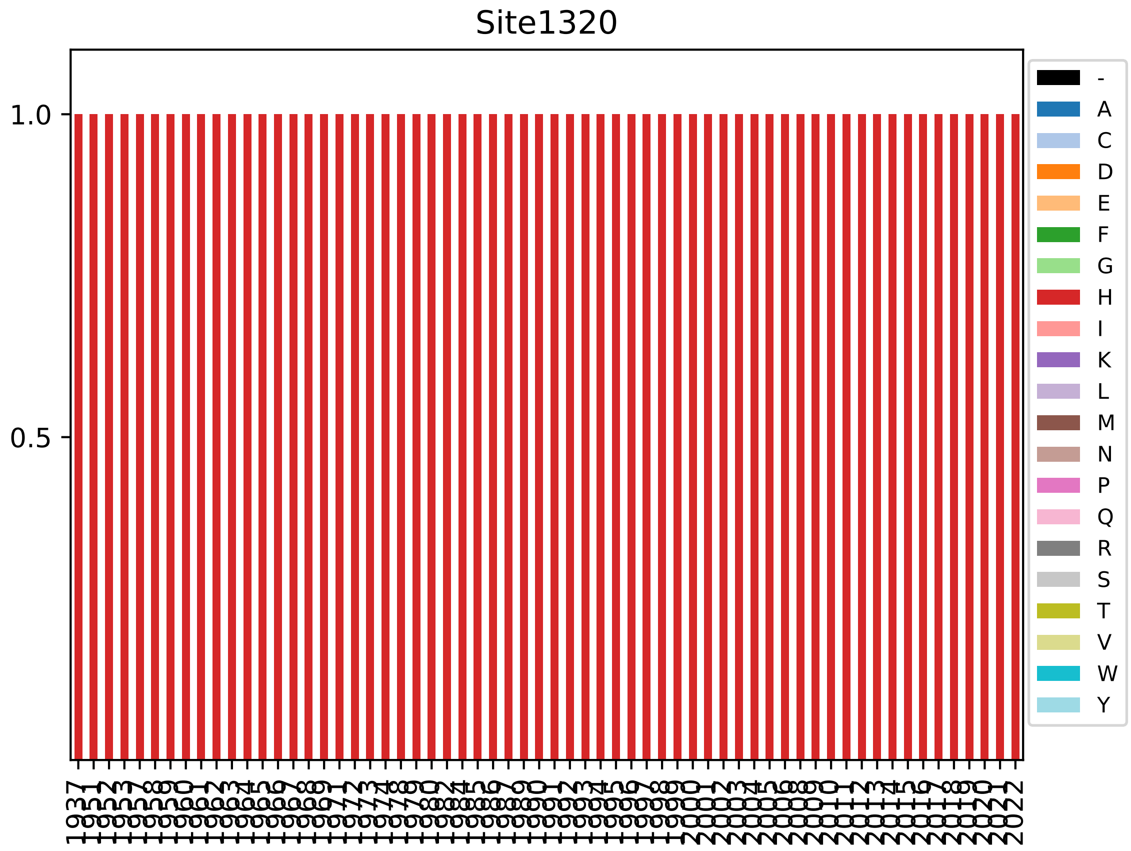

Site1320
1.0
0.5
-
A
C
D
E
F
G
H
I
K
L
M
N
P
Q
R
S
T
V
W
Y

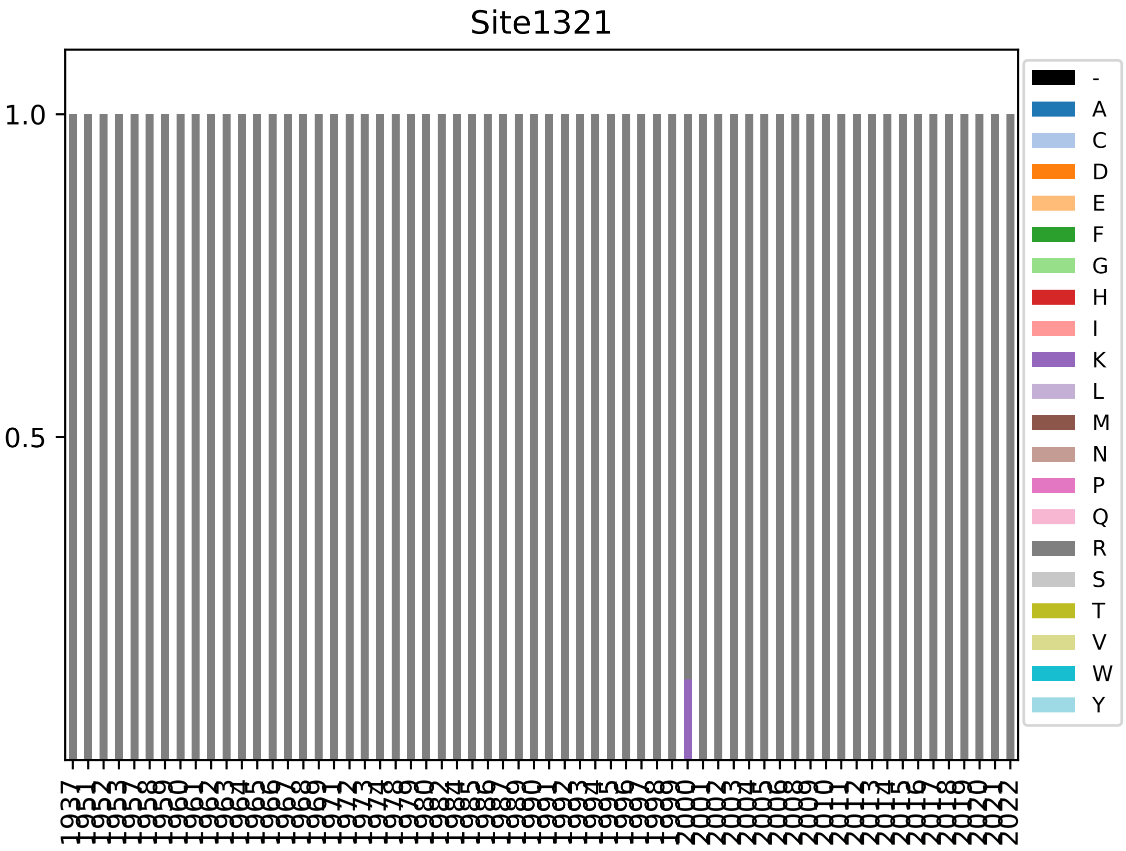
Site1321
1.0
0.5
-
A
C
D
E
F
G
H
I
K
L
M
N
P
Q
R
S
T
V
W
Y

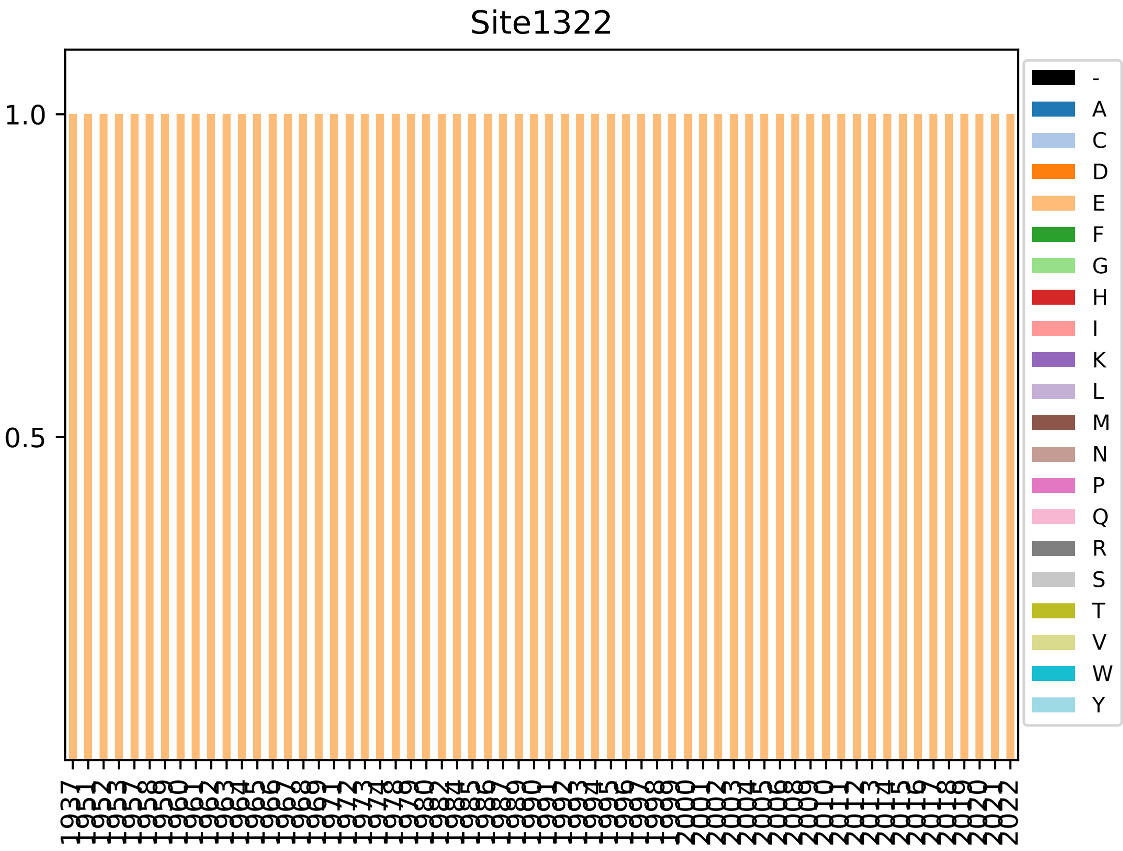
Site1322
1.0
0.5
-
A
C
D
E
F
G
H
I
K
L
M
N
P
Q
R
S
T
V
W
Y

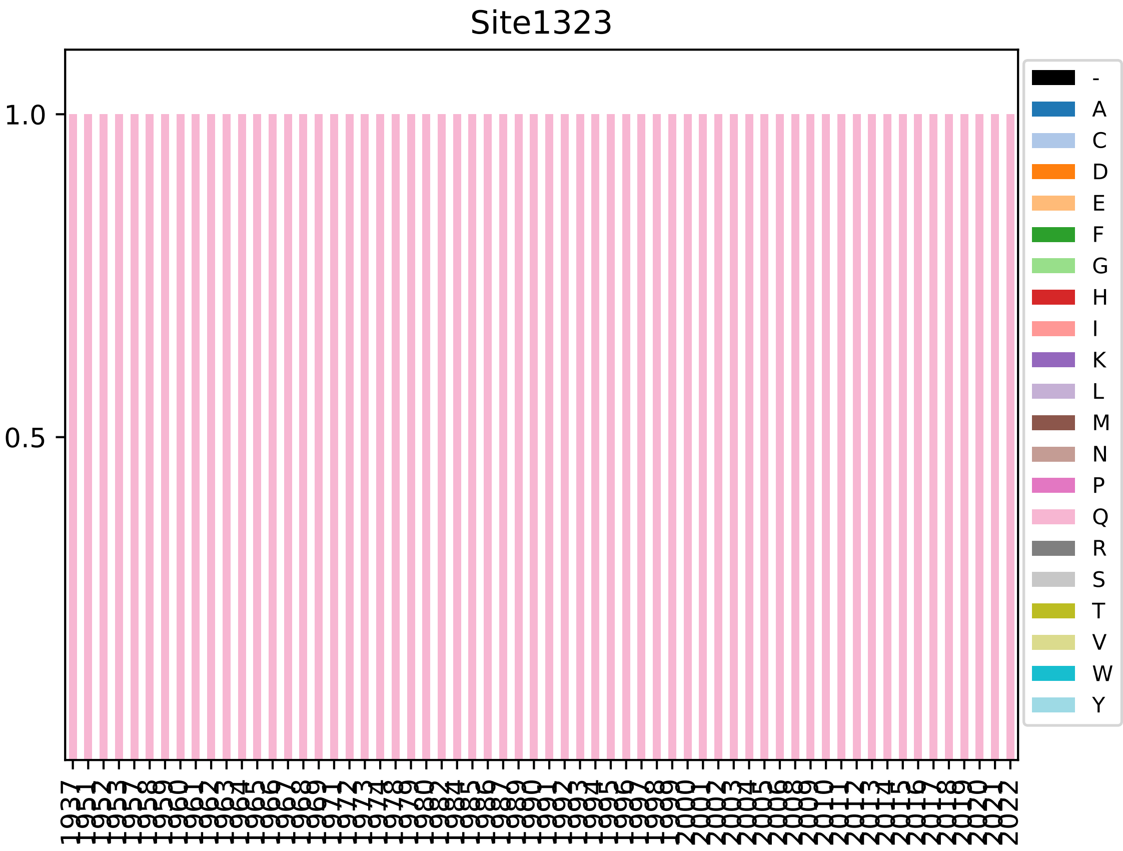
Site1323
1.0
0.5
-
A
C
D
E
F
G
H
I
K
L
M
N
P
Q
R
S
T
V
W
Y

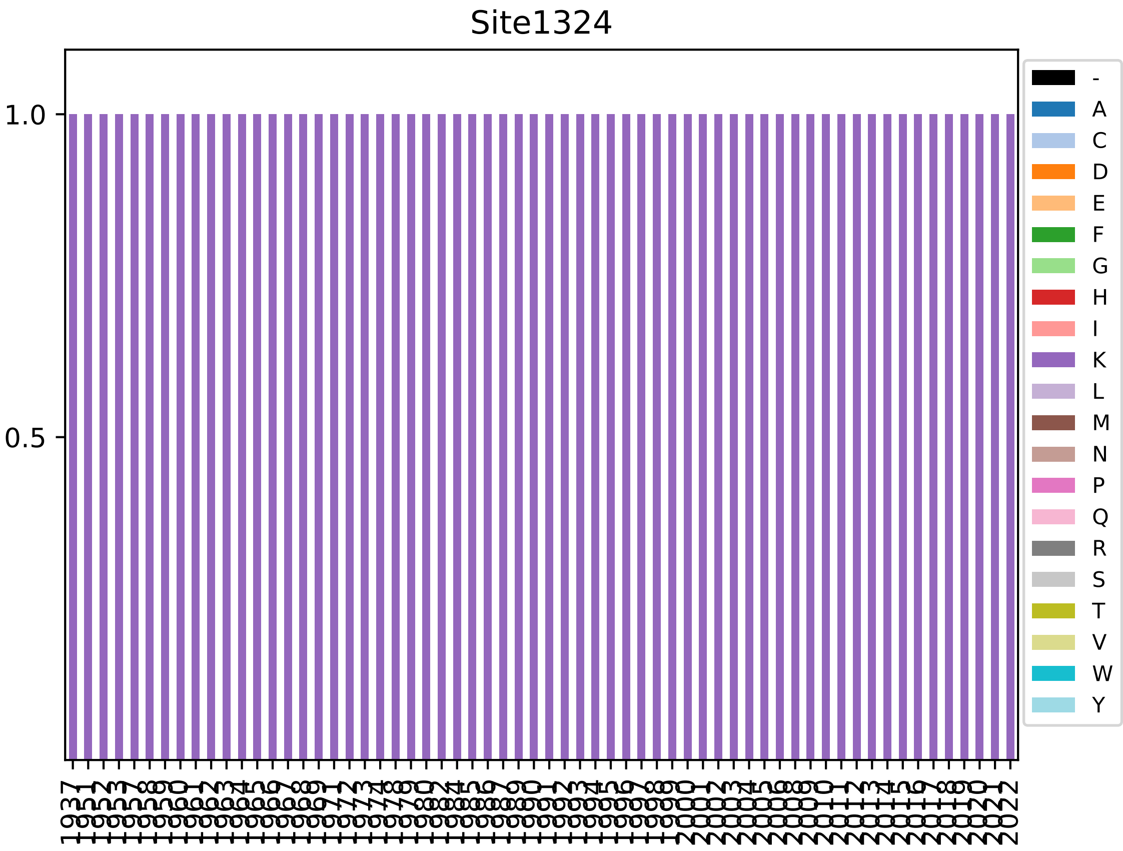
Site1324
1.0
0.5
-
A
C
D
E
F
G
H
I
K
L
M
N
P
Q
R
S
T
V
W
Y

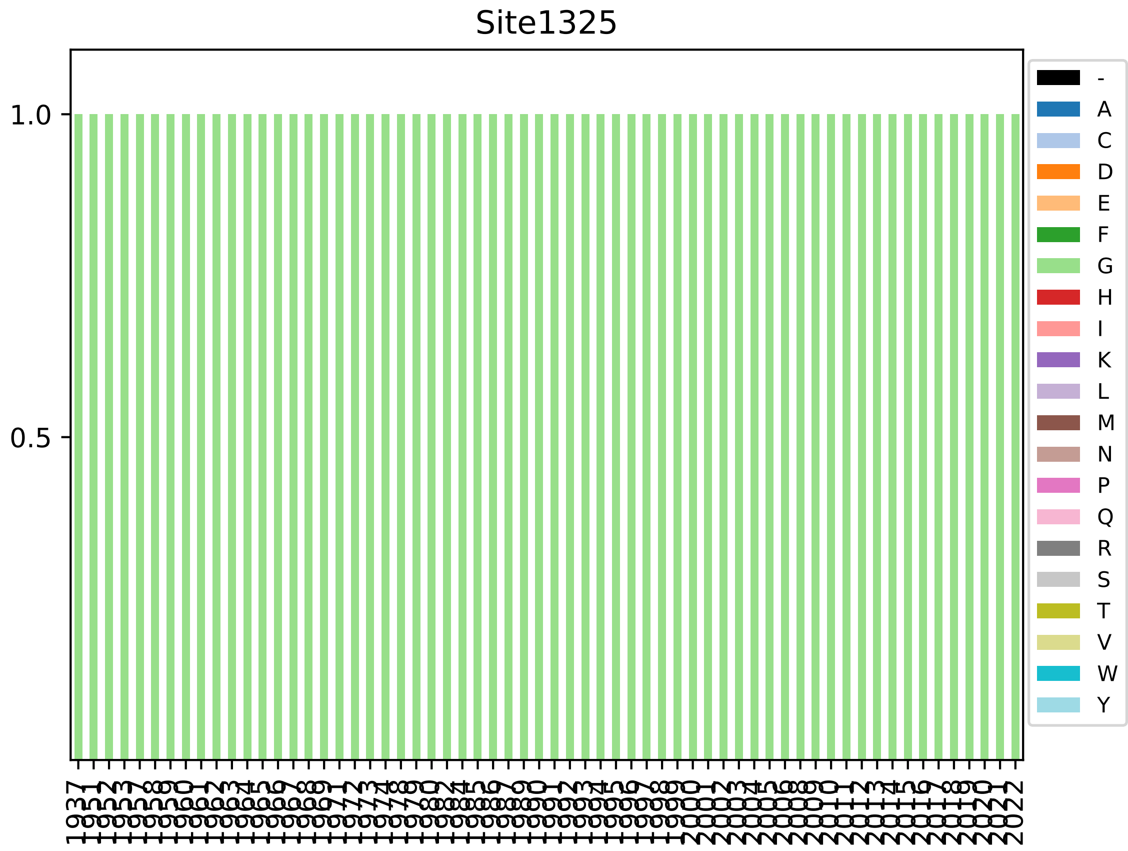
Site1325
1.0
0.5
-
A
C
D
E
F
G
H
I
K
L
M
N
P
Q
R
S
T
V
W
Y

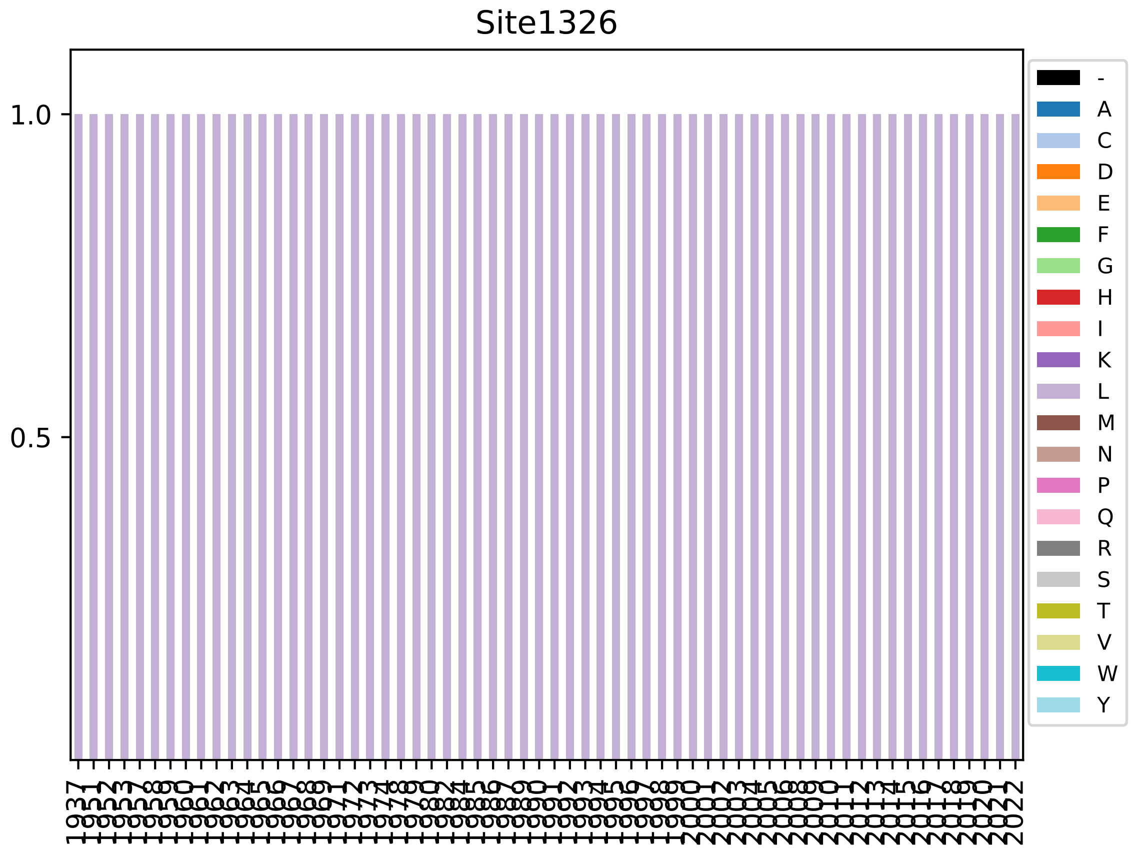
Site1326
1.0
0.5
-
A
C
D
E
F
G
H
I
K
L
M
N
P
Q
R
S
T
V
W
Y

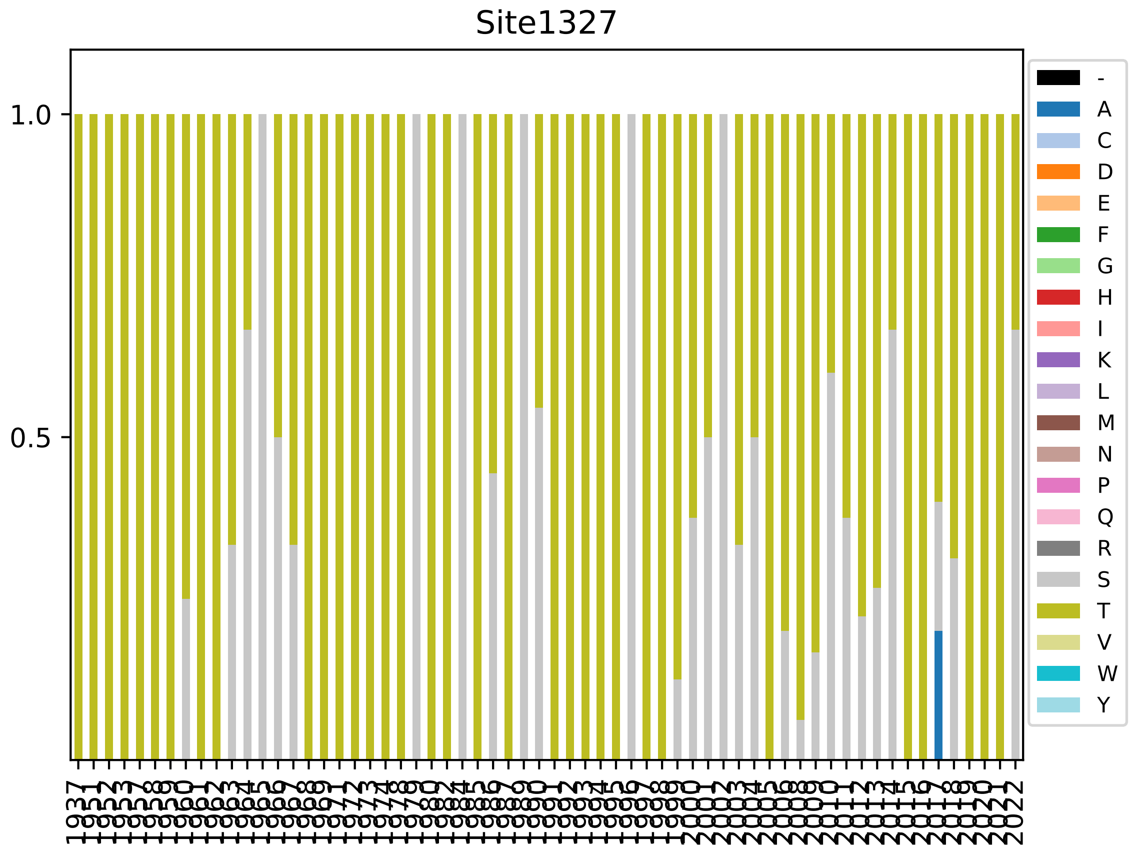
Site1327
1.0
0.5
-
A
C
D
E
F
G
H
I
K
L
M
N
P
Q
R
S
T
V
W
Y

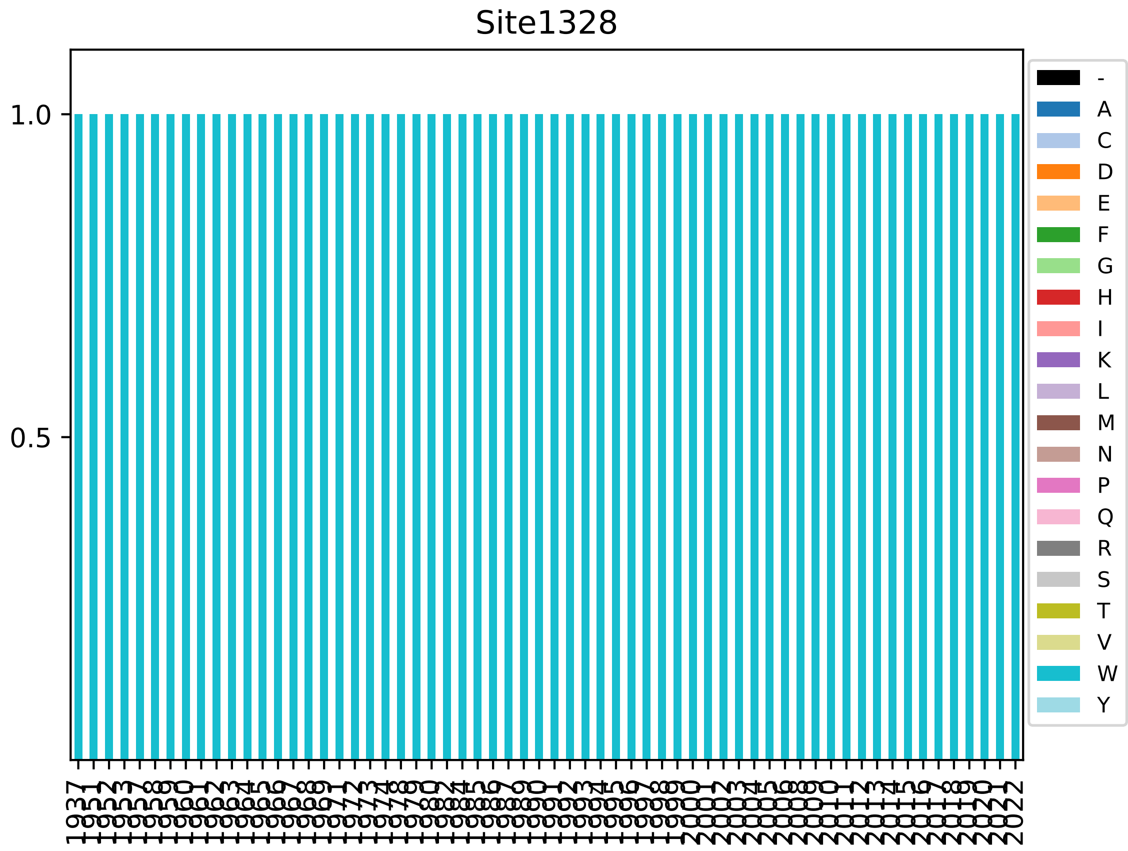
Site1328
1.0
0.5
-
A
C
D
E
F
G
H
I
K
L
M
N
P
Q
R
S
T
V
W
Y

Site1329

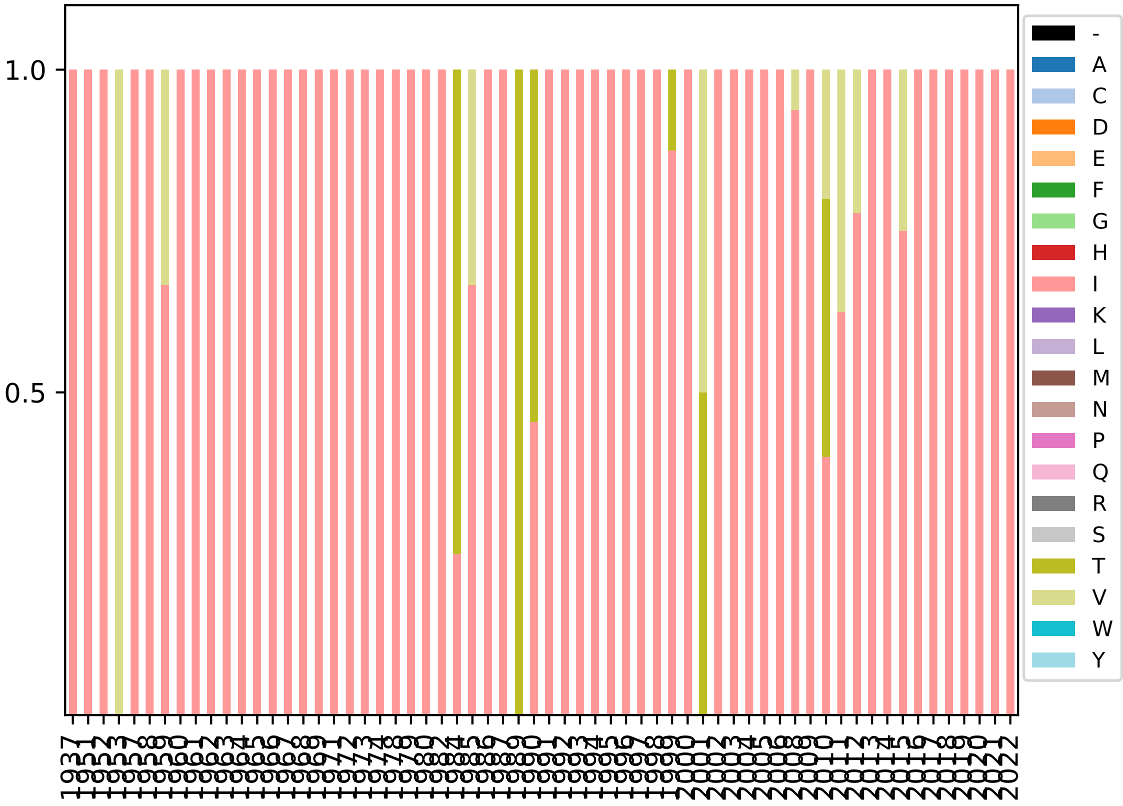

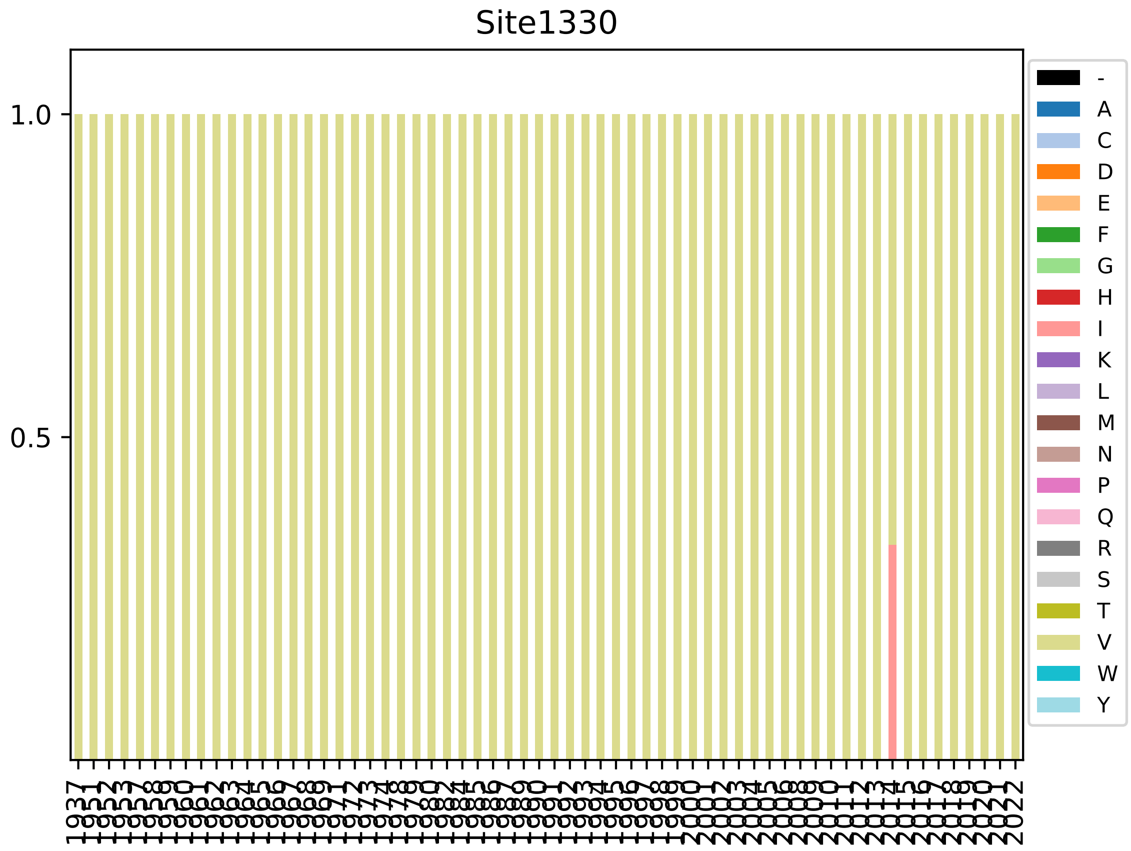

Site1330
1.0
0.5
-
A
C
D
E
F
G
H
I
K
L
M
N
P
Q
R
S
T
V
W
Y

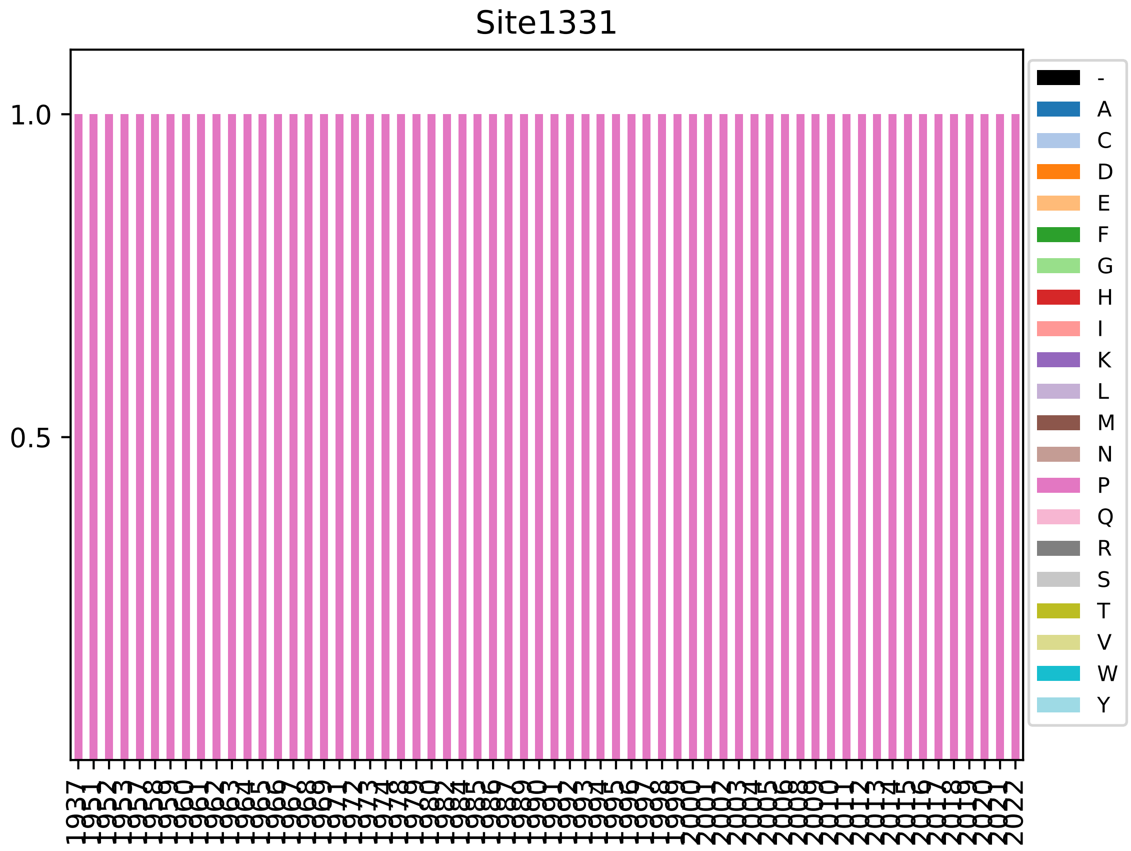
Site1331
1.0
0.5
-
A
C
D
E
F
G
H
I
K
L
M
N
P
Q
R
S
T
V
W
Y

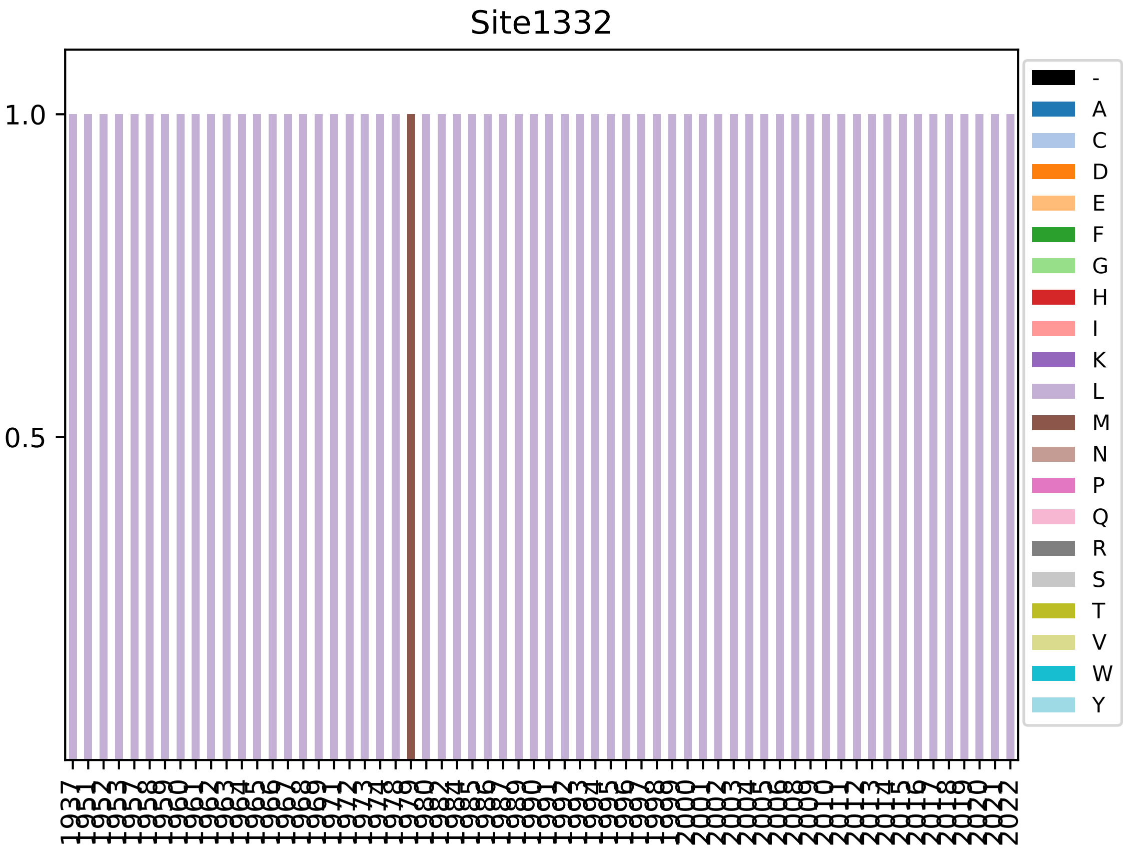
Site1332
1.0
0.5
-
A
C
D
E
F
G
H
I
K
L
M
N
P
Q
R
S
T
V
W
Y

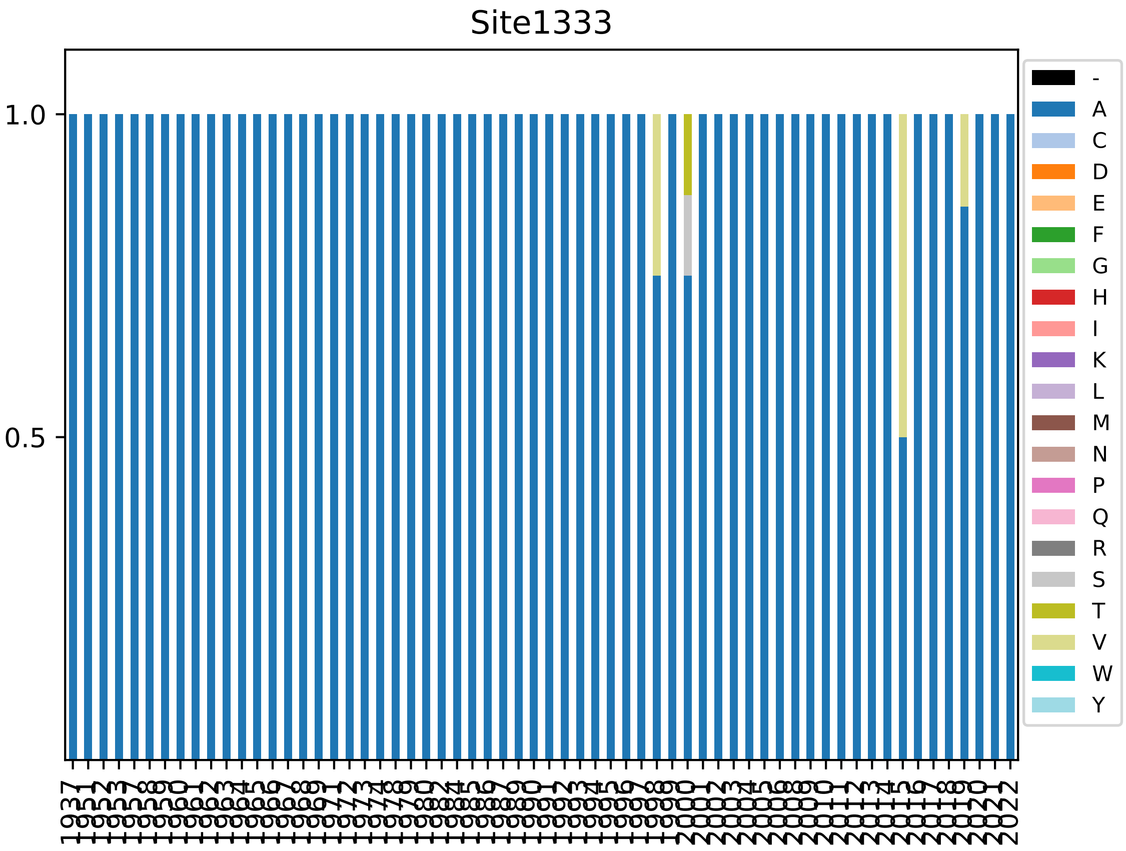

Site1333
1.0
0.5
-
A
C
D
E
F
G
H
I
K
L
M
N
P
Q
R
S
T
V
W
Y

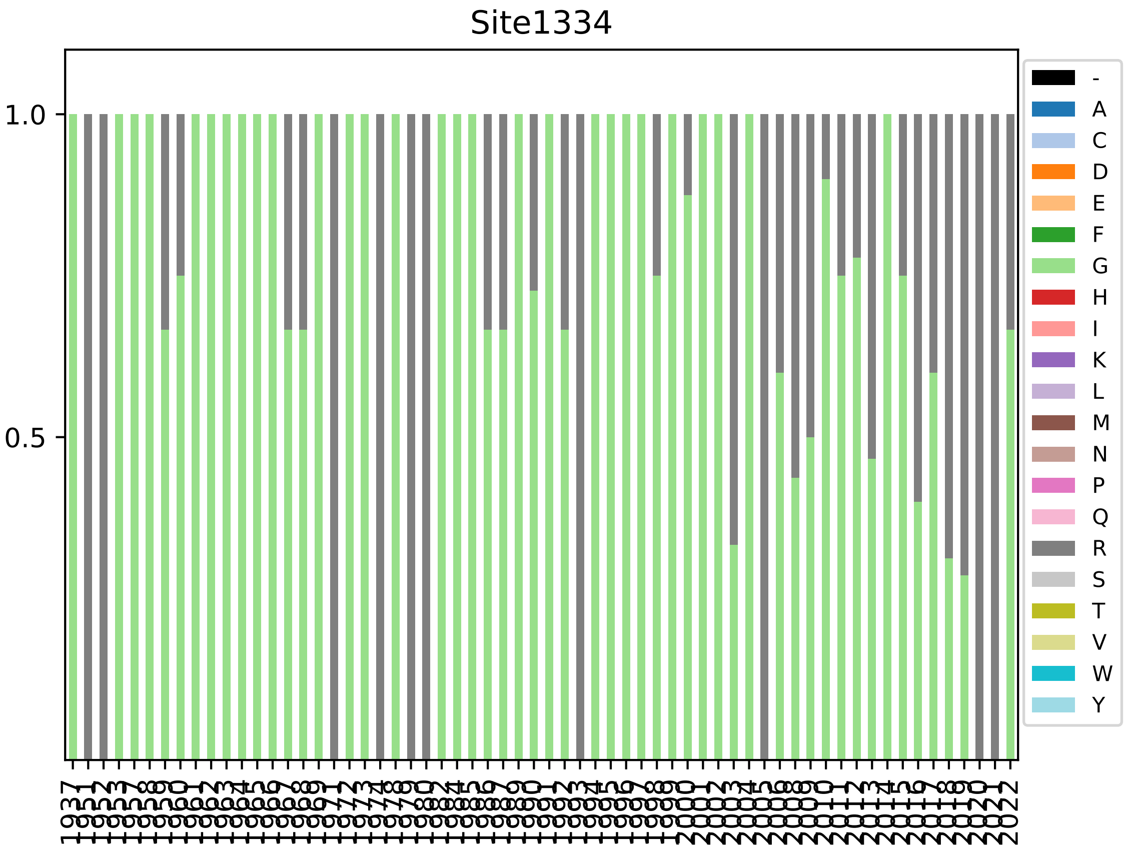

Site1334
1.0
0.5
-
A
C
D
E
F
G
H
I
K
L
M
N
P
Q
R
S
T
V
W
Y

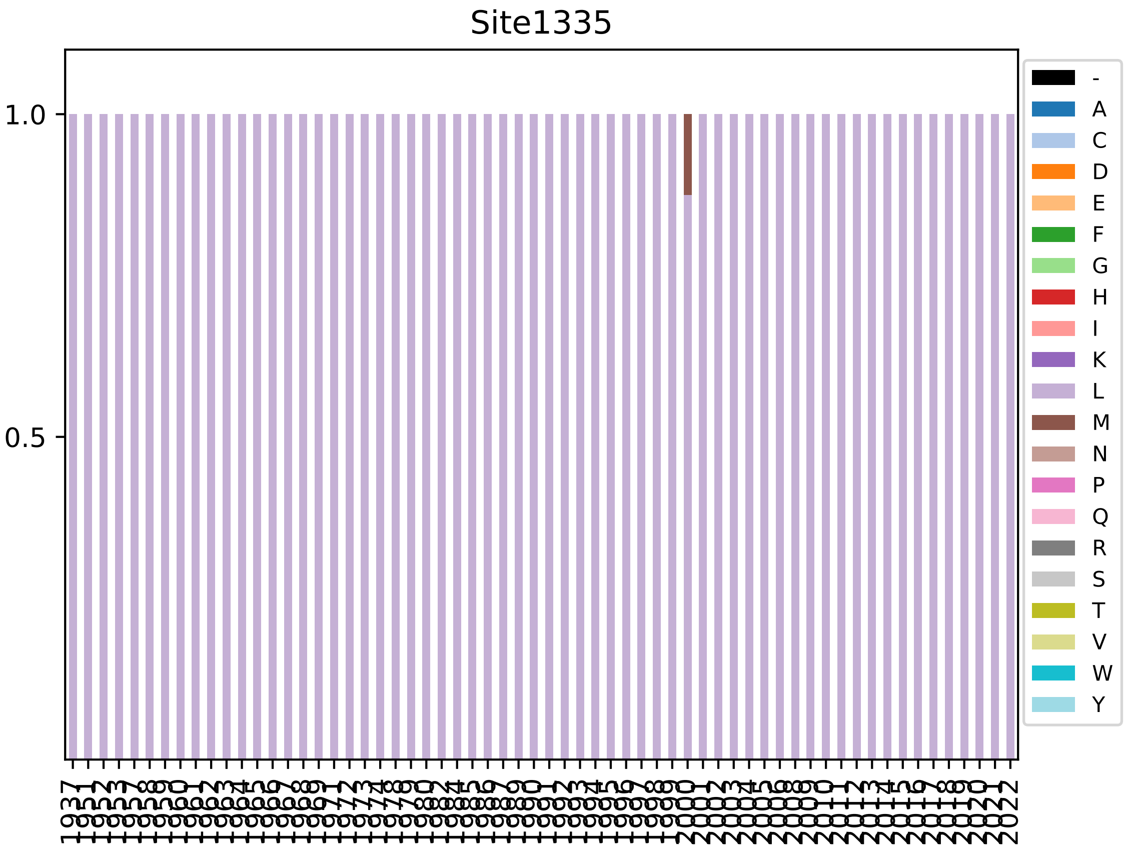

Site1335
1.0
0.5
-
A
C
D
E
F
G
H
I
K
L
M
N
P
Q
R
S
T
V
W
Y

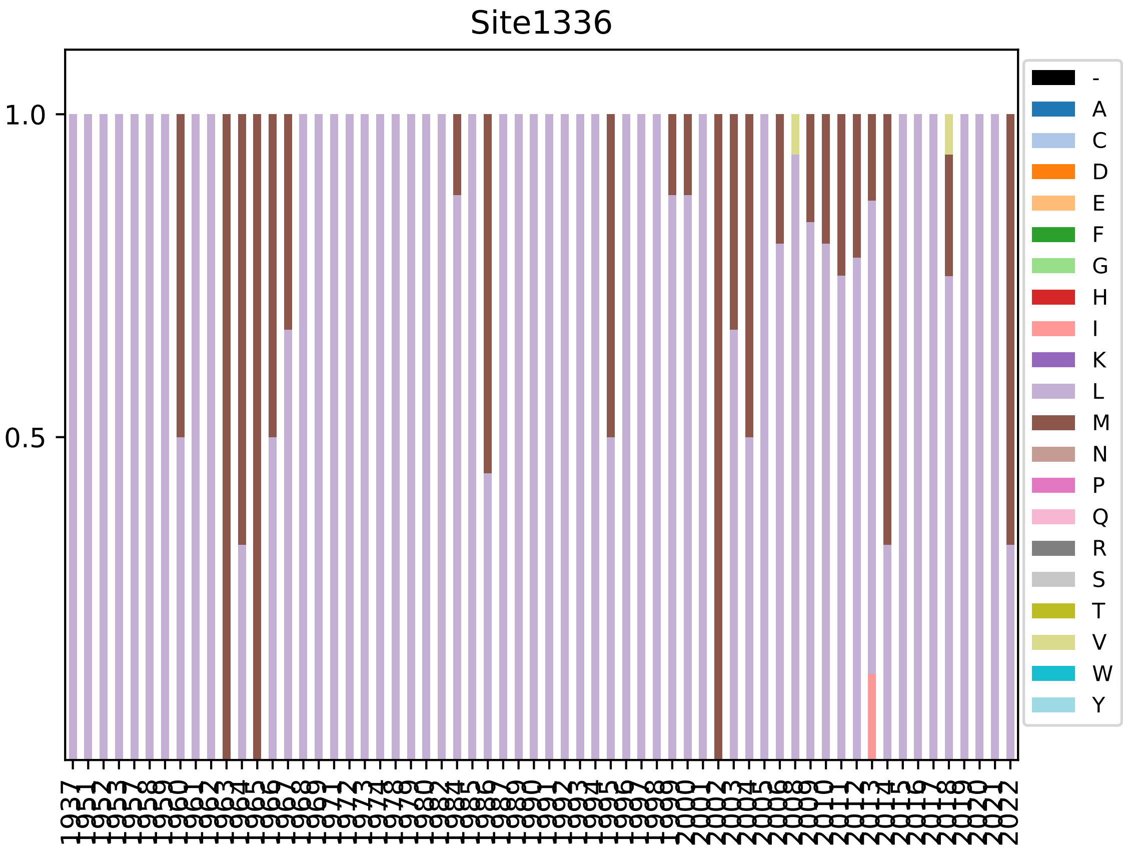
Site1336
1.0
0.5
-
A
C
D
E
F
G
H
I
K
L
M
N
P
Q
R
S
T
V
W
Y

Site1337

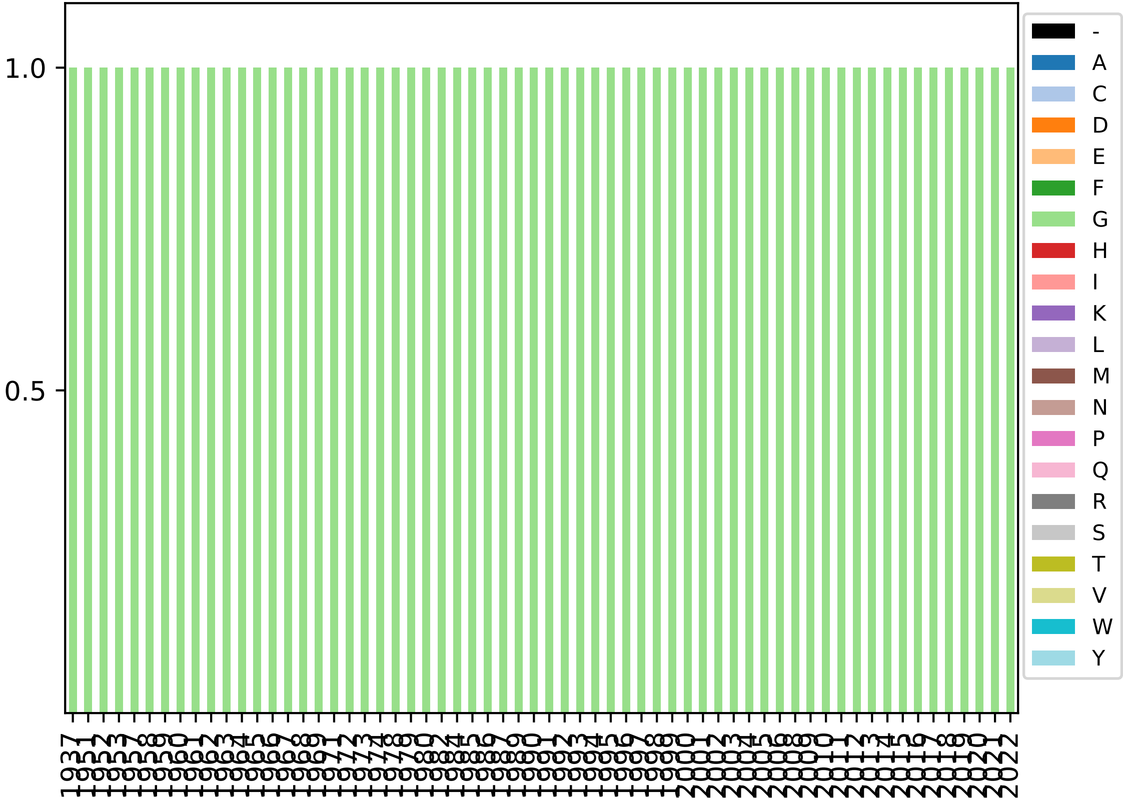

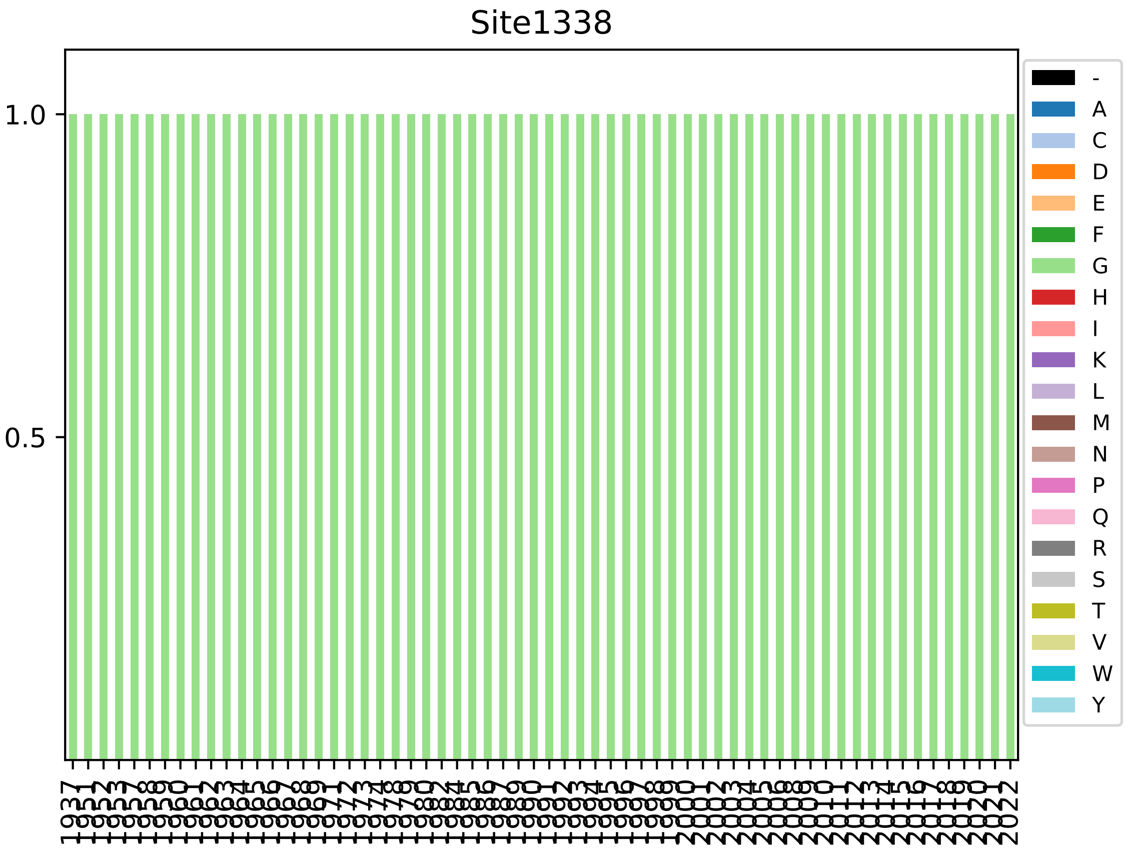
Site1338
1.0
0.5
-
A
C
D
E
F
G
H
I
K
L
M
N
P
Q
R
S
T
V
W
Y

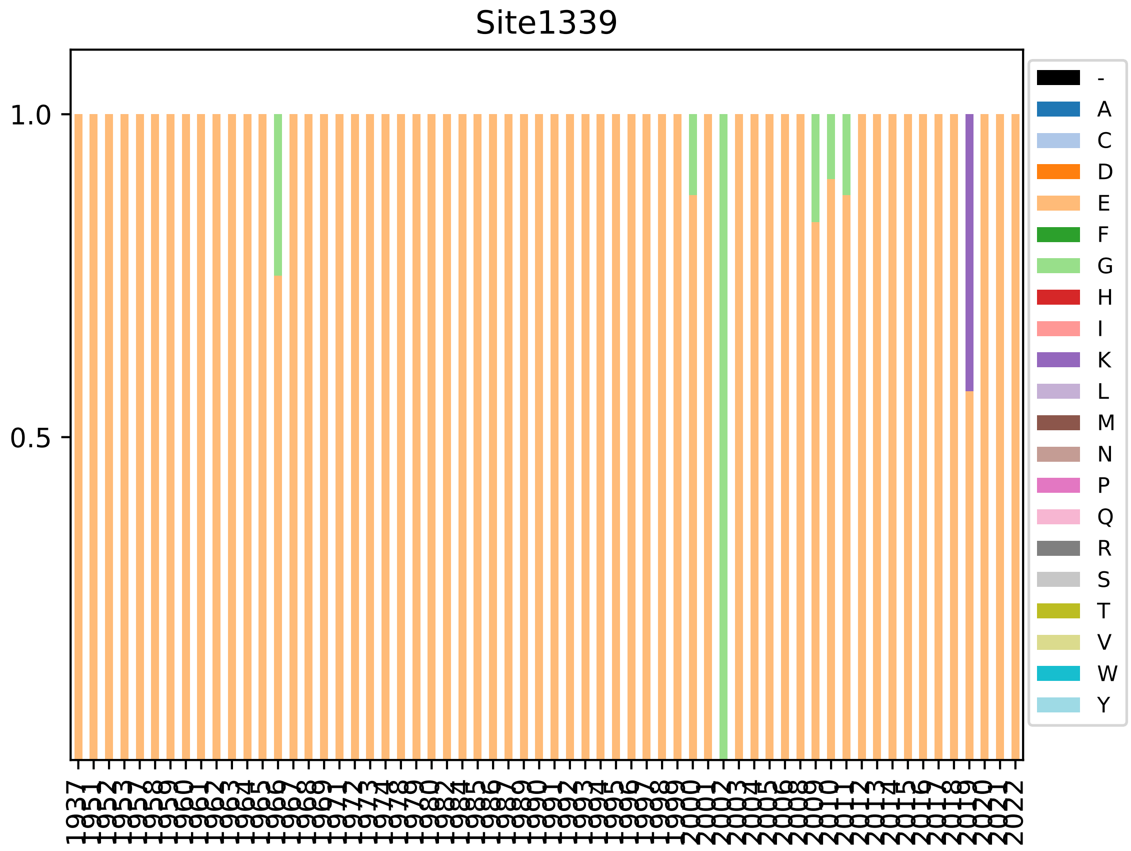
Site1339
1.0
0.5
-
A
C
D
E
F
G
H
I
K
L
M
N
P
Q
R
S
T
V
W
Y

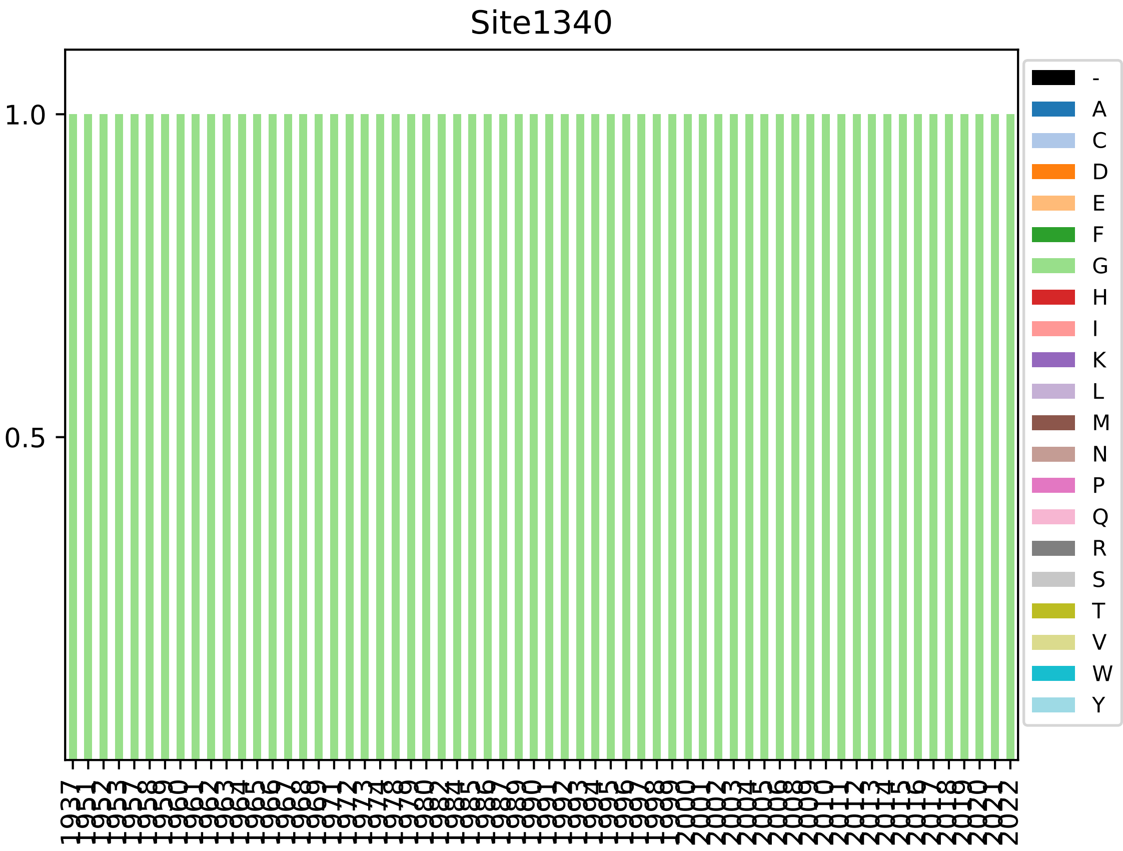
Site1340
1.0
0.5
-
A
C
D
E
F
G
H
I
K
L
M
N
P
Q
R
S
T
V
W
Y

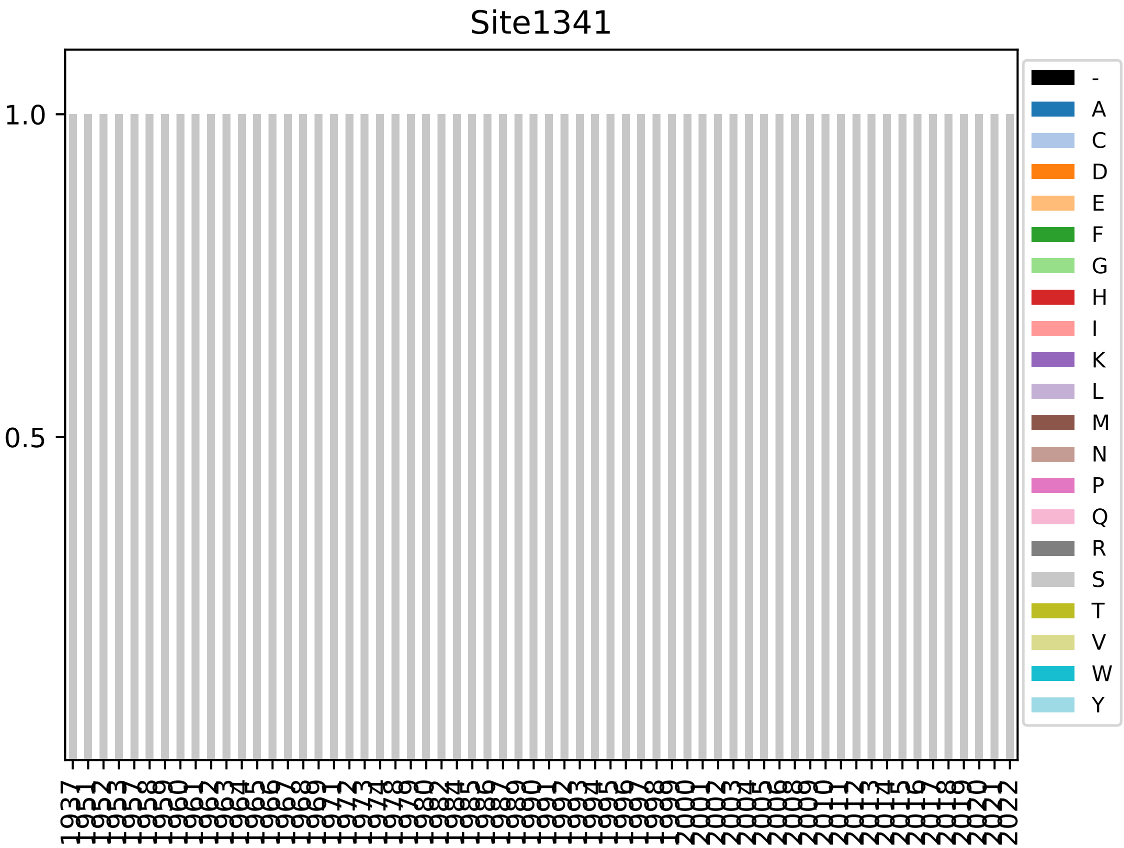

Site1341
1.0
0.5
-
A
C
D
E
F
G
H
I
K
L
M
N
P
Q
R
S
T
V
W
Y

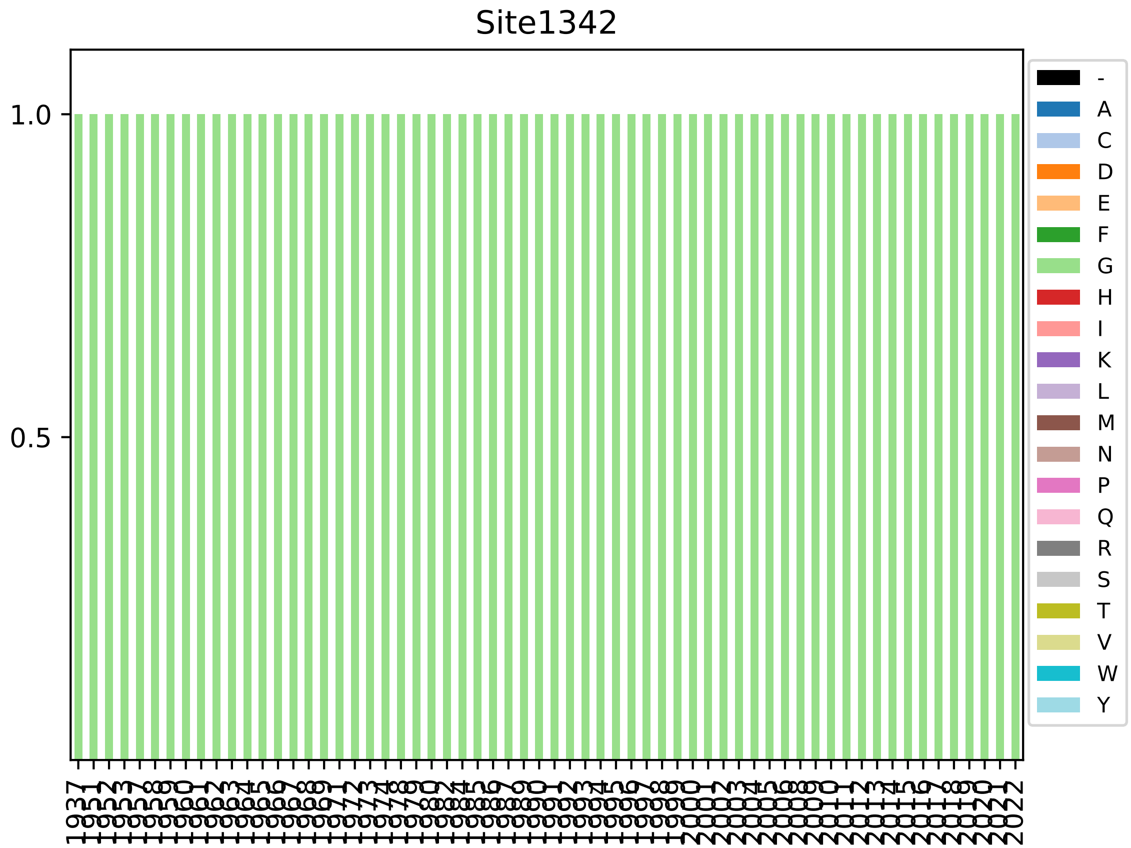

Site1342
1.0
0.5
-
A
C
D
E
F
G
H
I
K
L
M
N
P
Q
R
S
T
V
W
Y

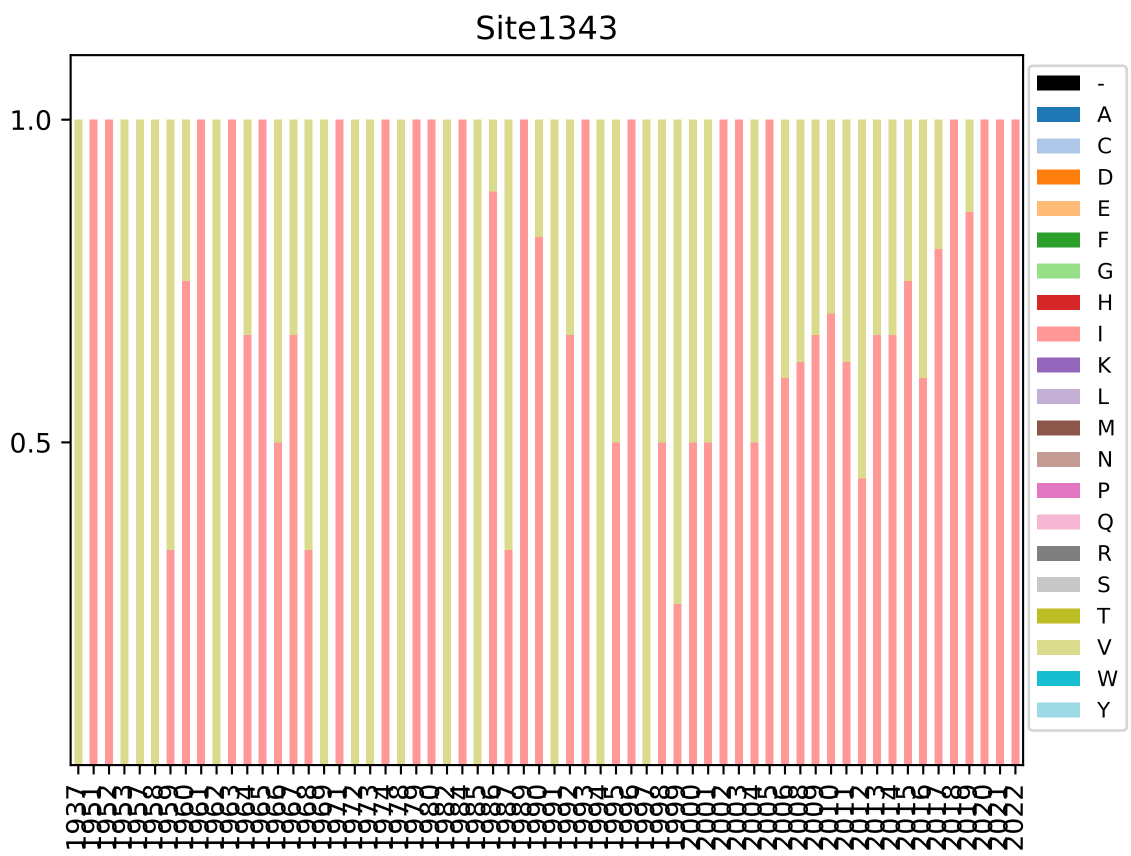
Site1343
1.0
0.5
-
A
C
D
E
F
G
H
I
K
L
M
N
P
Q
R
S
T
V
W
Y

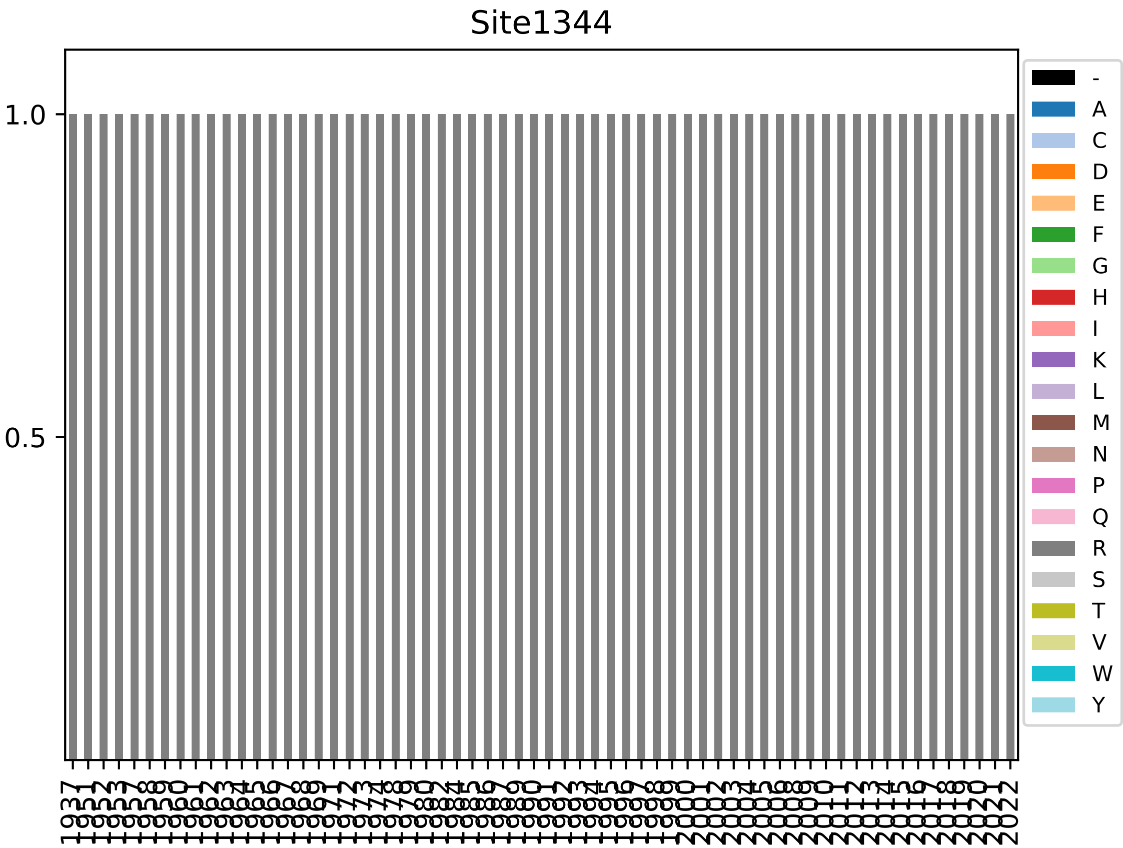
Site1344
1.0
0.5
-
A
C
D
E
F
G
H
I
K
L
M
N
P
Q
R
S
T
V
W
Y

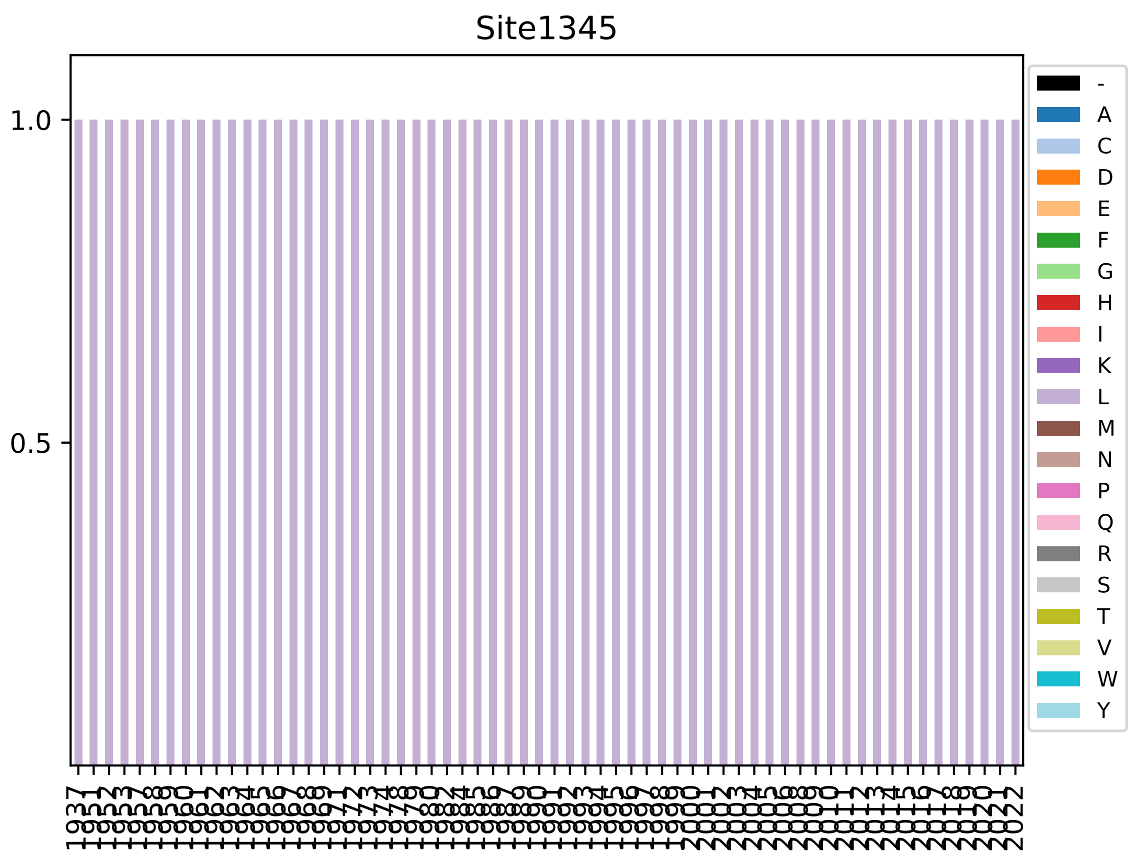

Site1345
1.0
0.5
-
A
C
D
E
F
G
H
I
K
L
M
N
P
Q
R
S
T
V
W
Y

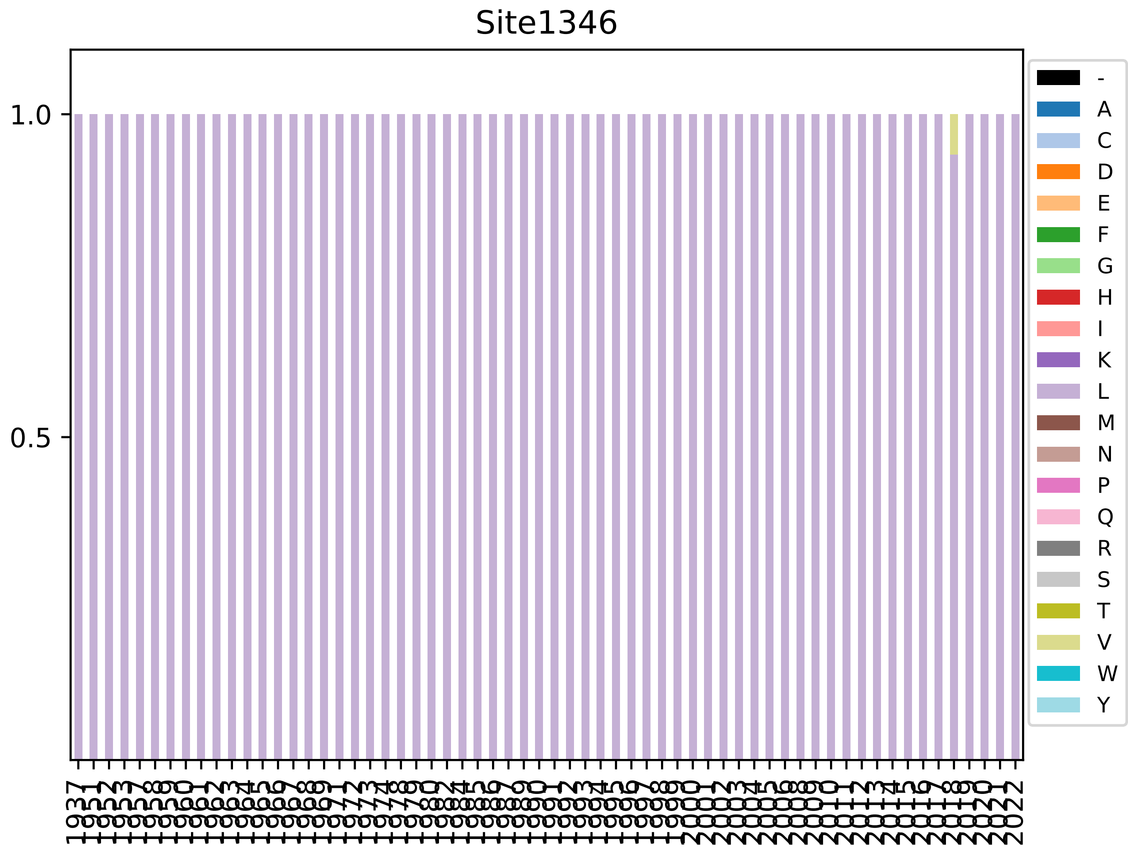
Site1346
1.0
0.5
-
A
C
D
E
F
G
H
I
K
L
M
N
P
Q
R
S
T
V
W
Y

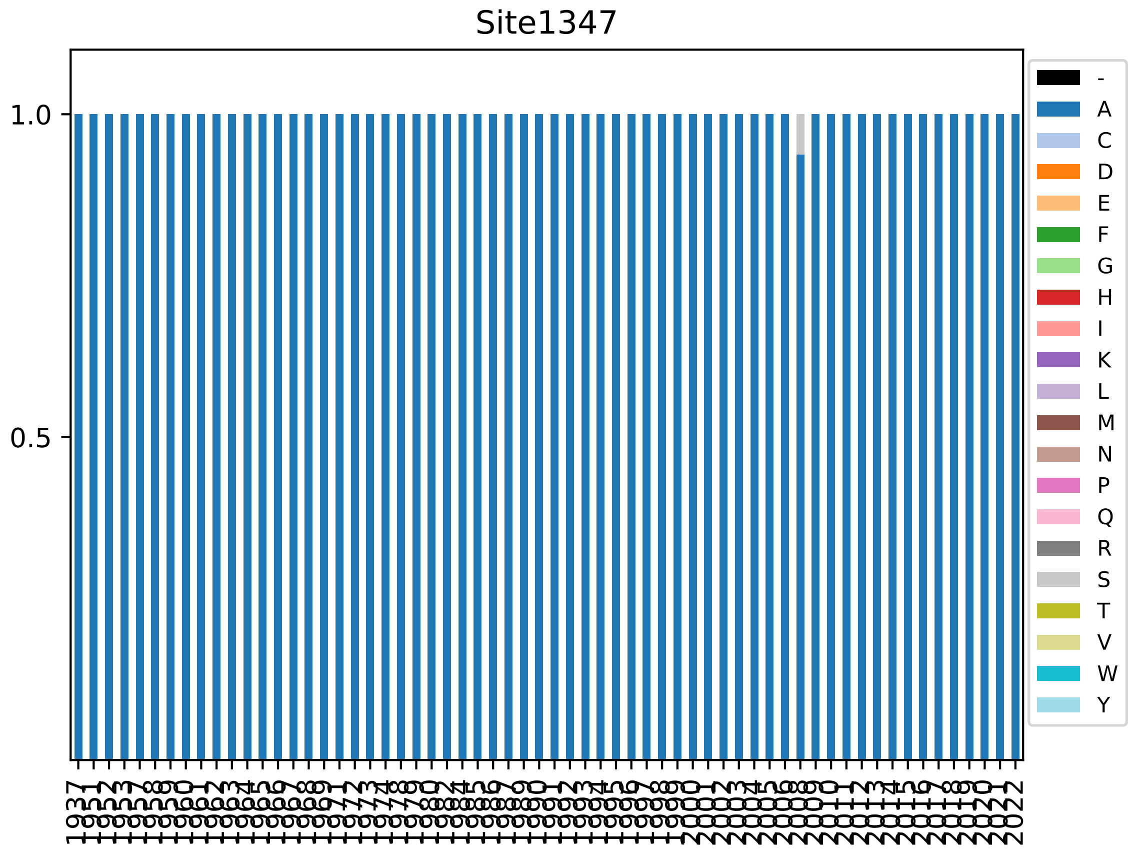

Site1347
1.0
0.5
-
A
C
D
E
F
G
H
I
K
L
M
N
P
Q
R
S
T
V
W
Y

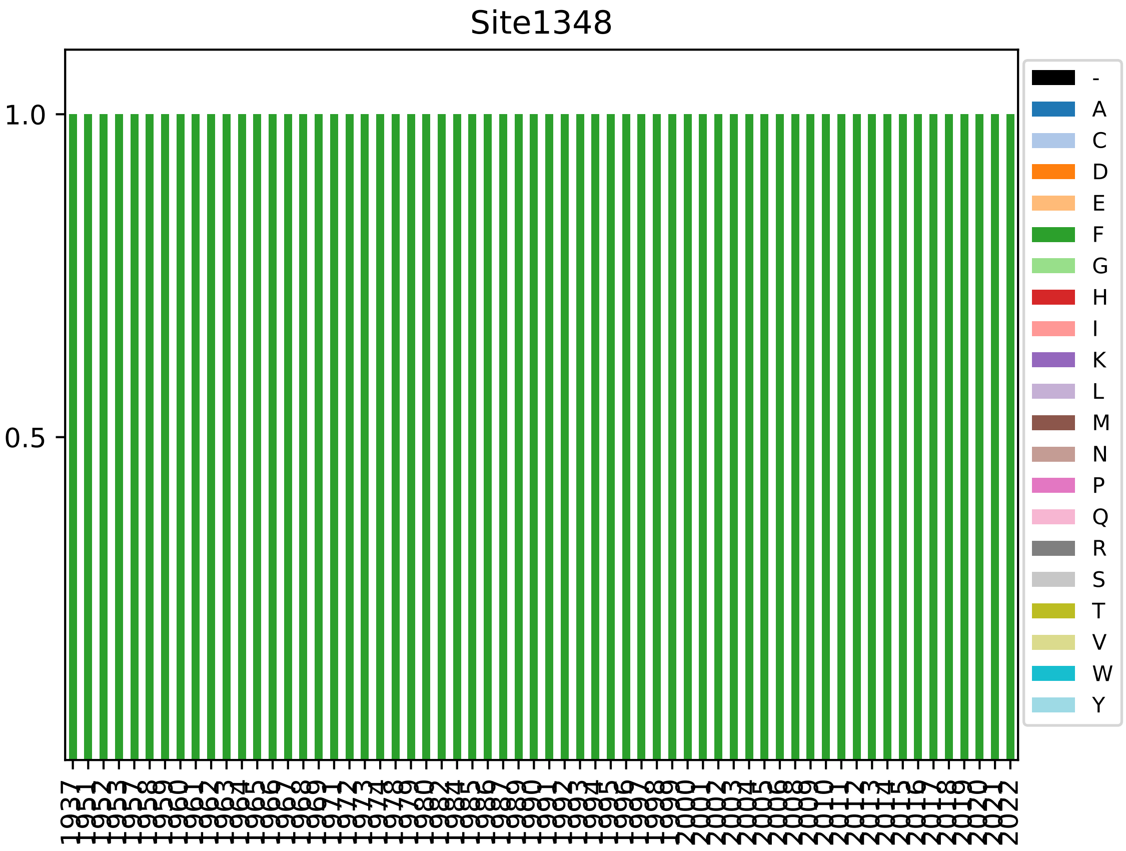
Site1348
1.0
0.5
-
A
C
D
E
F
G
H
I
K
L
M
N
P
Q
R
S
T
V
W
Y

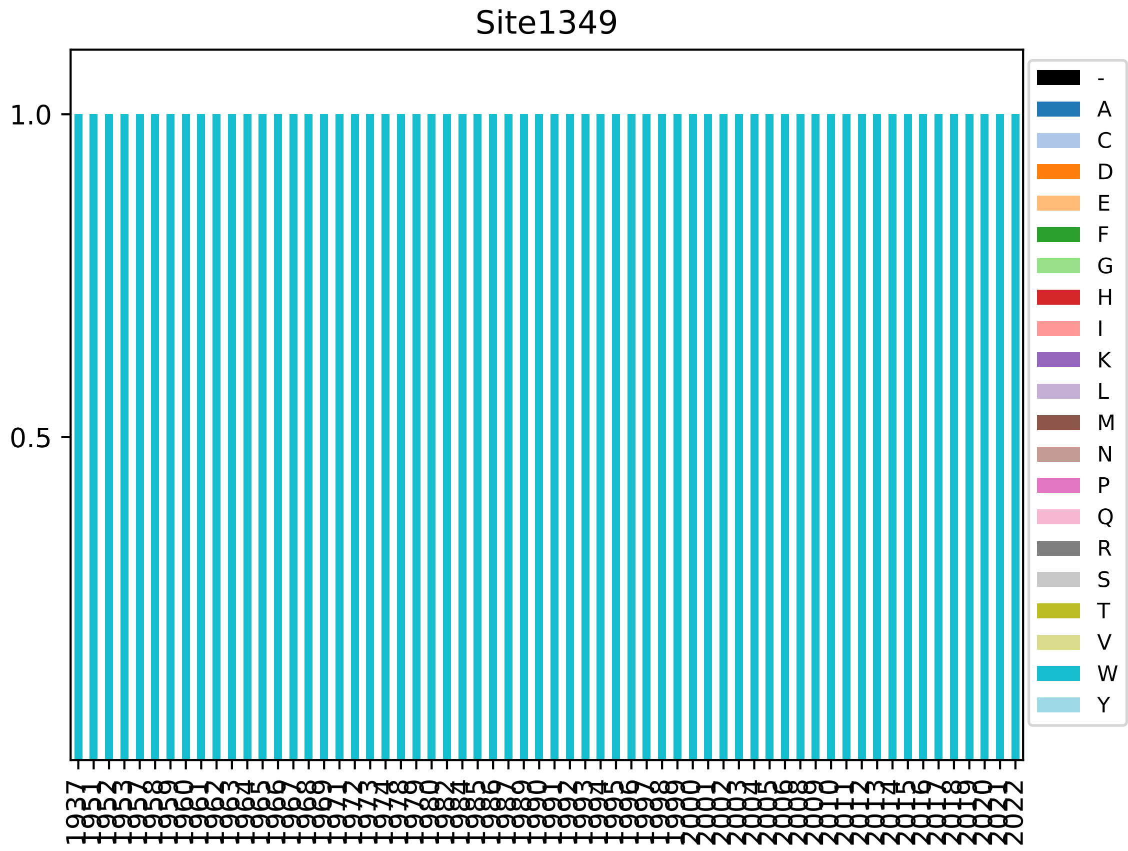
Site1349
1.0
0.5
-
A
C
D
E
F
G
H
I
K
L
M
N
P
Q
R
S
T
V
W
Y

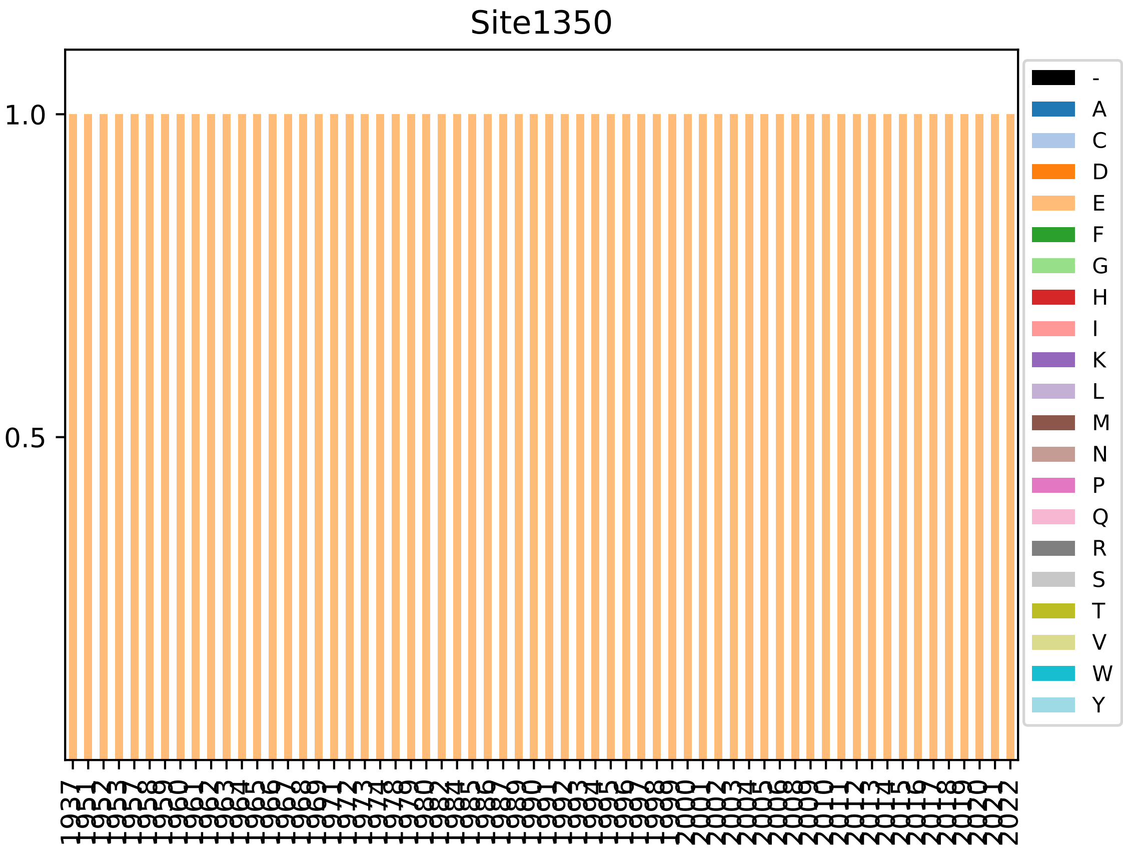

Site1350
1.0
0.5
-
A
C
D
E
F
G
H
I
K
L
M
N
P
Q
R
S
T
V
W
Y

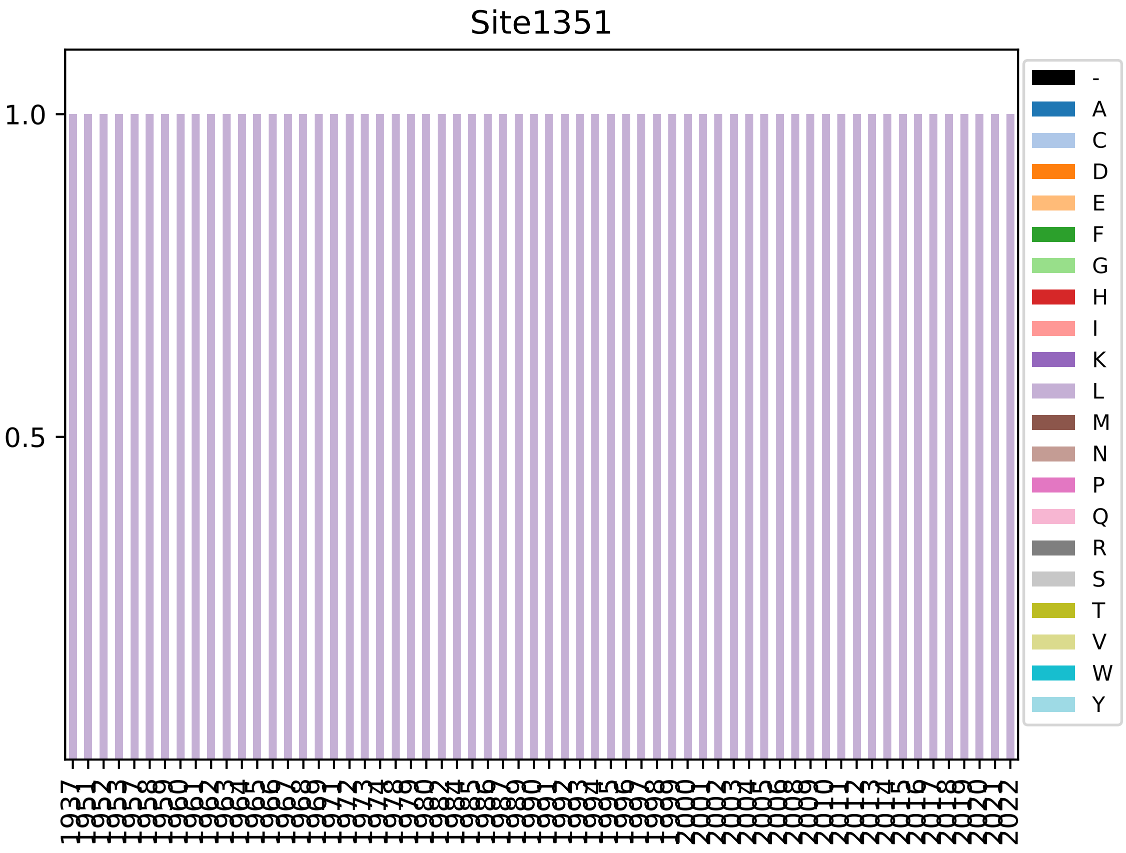
Site1351
1.0
0.5
-
A
C
D
E
F
G
H
I
K
L
M
N
P
Q
R
S
T
V
W
Y

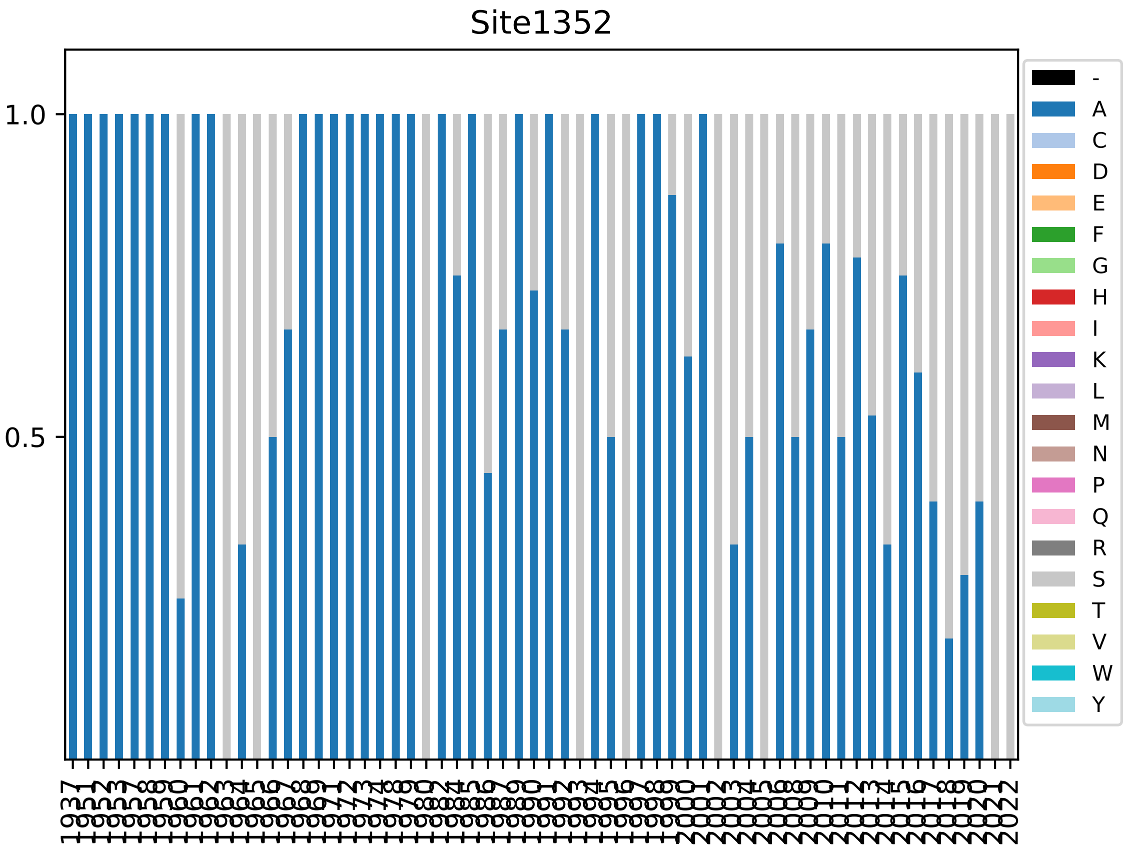

Site1352
1.0
0.5
-
A
C
D
E
F
G
H
I
K
L
M
N
P
Q
R
S
T
V
W
Y

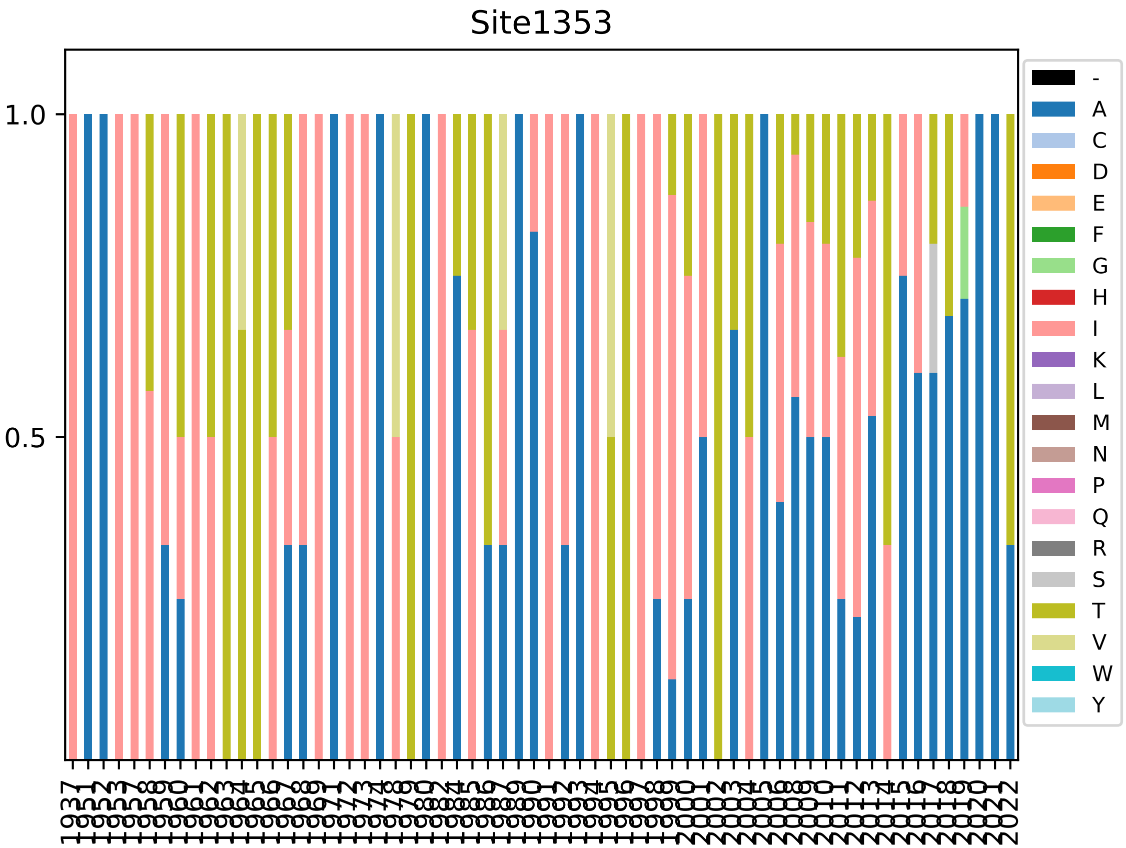
Site1353
1.0
0.5
-
A
C
D
E
F
G
H
I
K
L
M
N
P
Q
R
S
T
V
W
Y

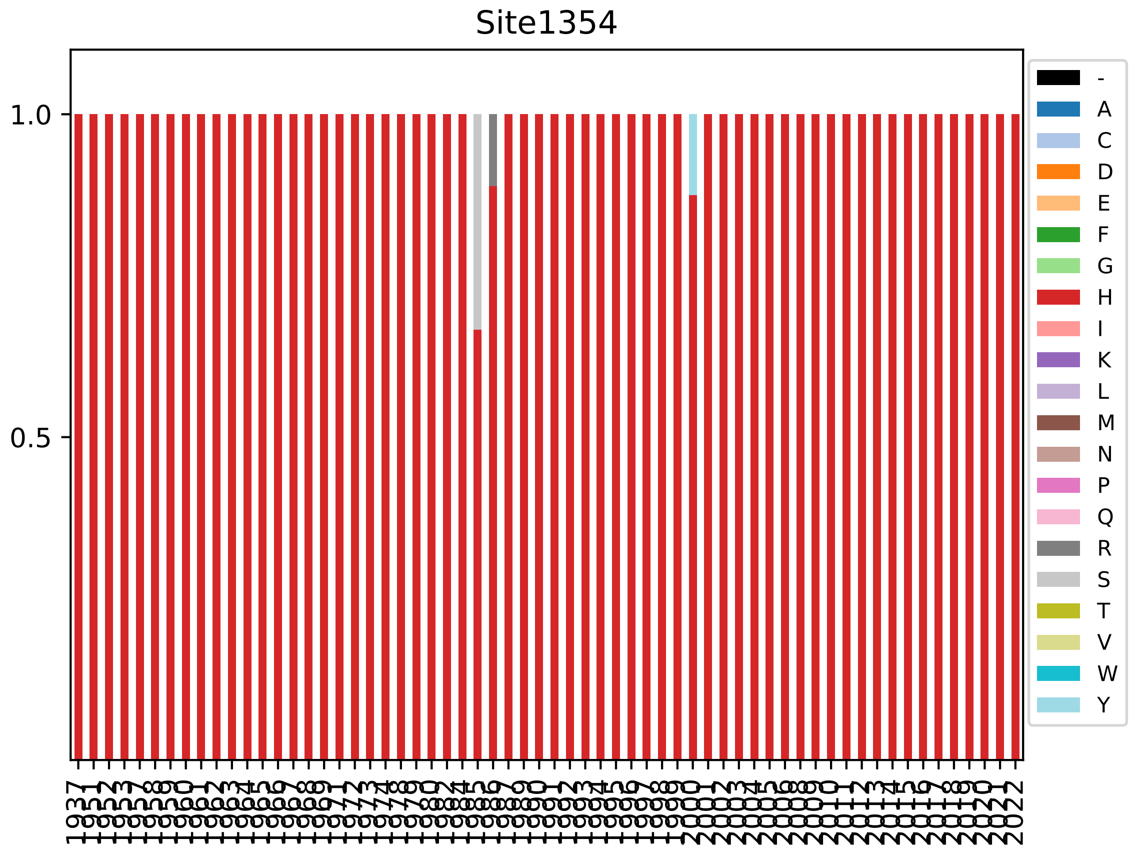
Site1354
1.0
0.5
-
A
C
D
E
F
G
H
I
K
L
M
N
P
Q
R
S
T
V
W
Y

Site1355

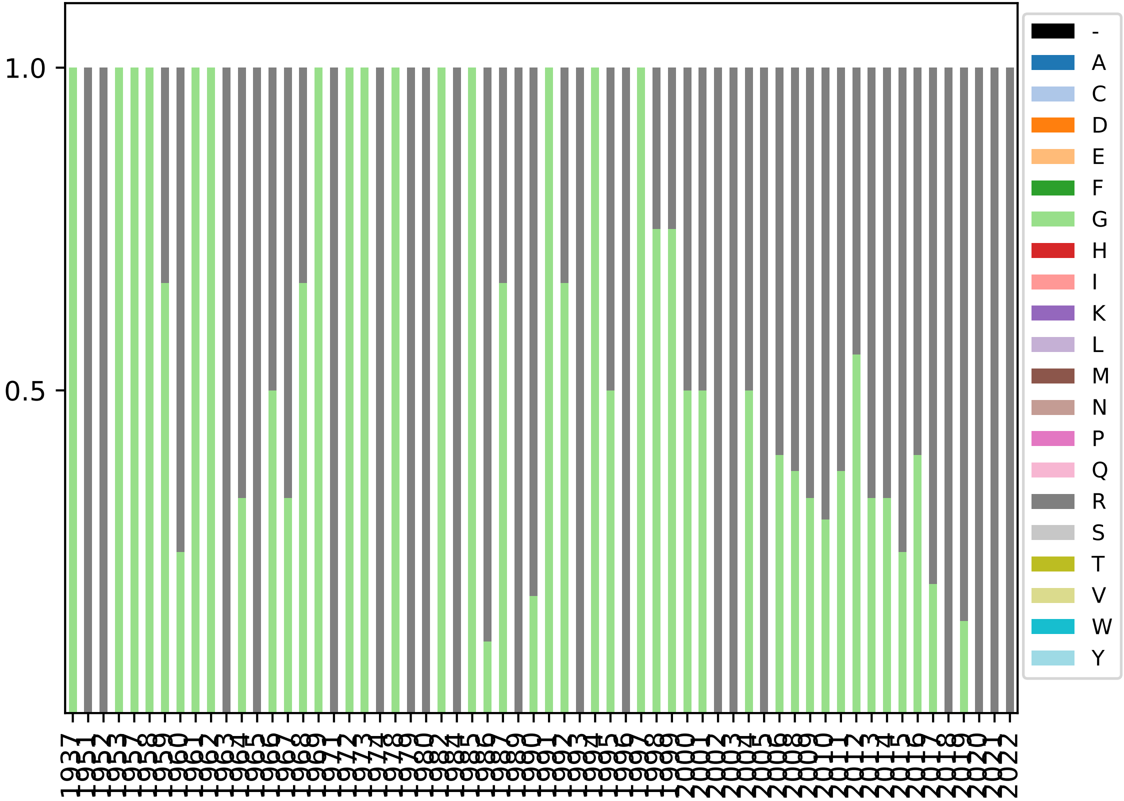

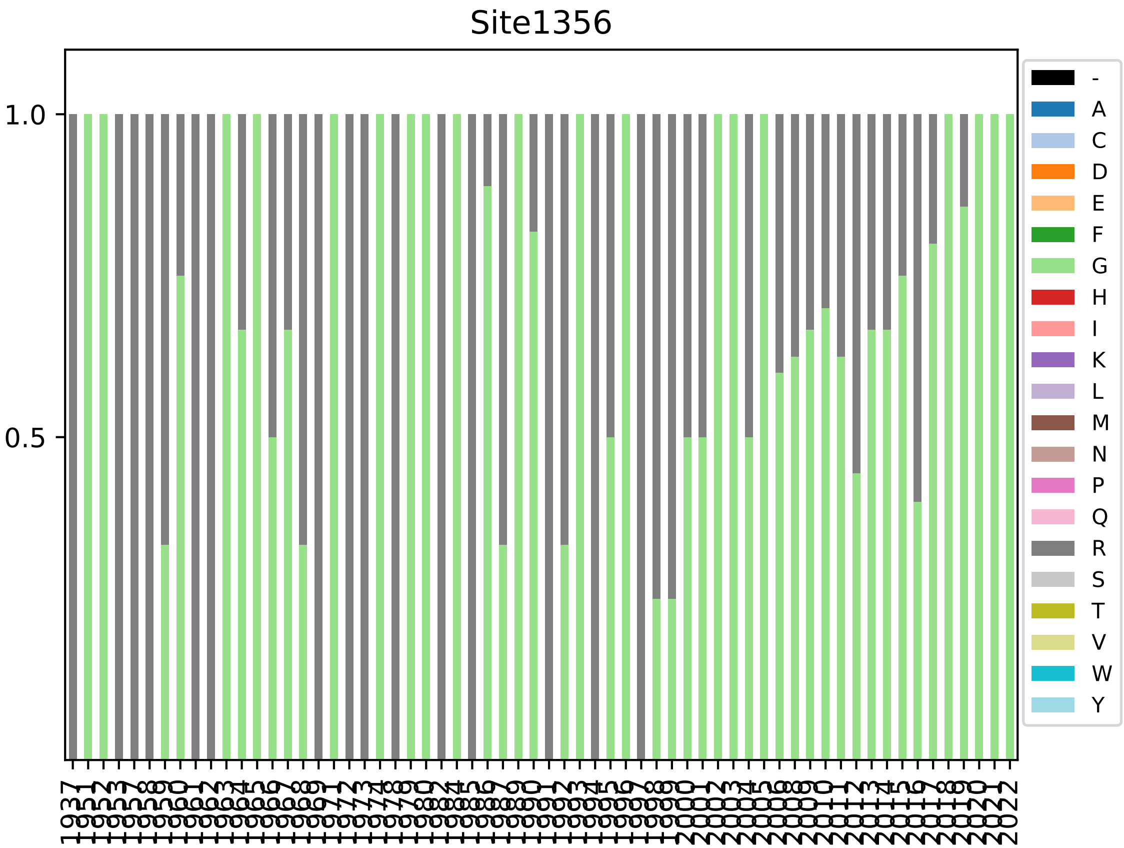
Site1356
1.0
0.5
-
A
C
D
E
F
G
H
I
K
L
M
N
P
Q
R
S
T
V
W
Y

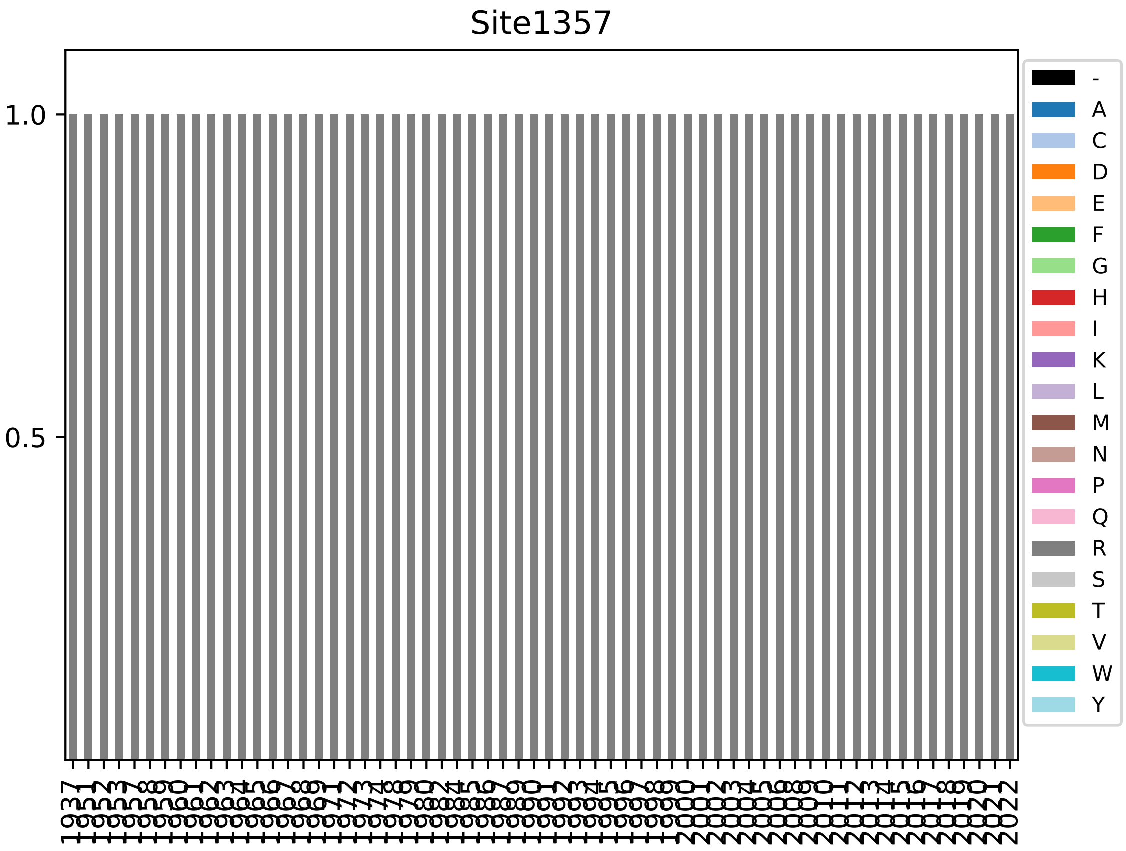
Site1357
1.0
0.5
-
A
C
D
E
F
G
H
I
K
L
M
N
P
Q
R
S
T
V
W
Y

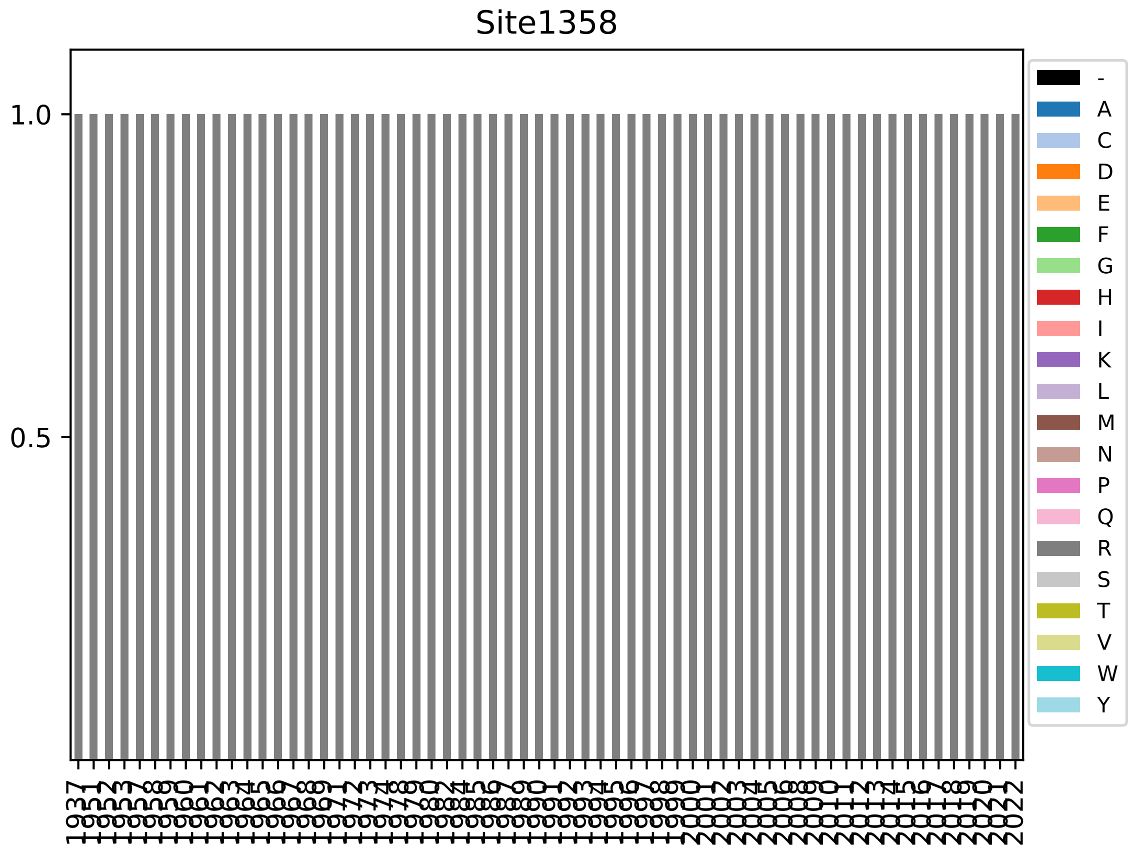

Site1358
1.0
0.5
-
A
C
D
E
F
G
H
I
K
L
M
N
P
Q
R
S
T
V
W
Y

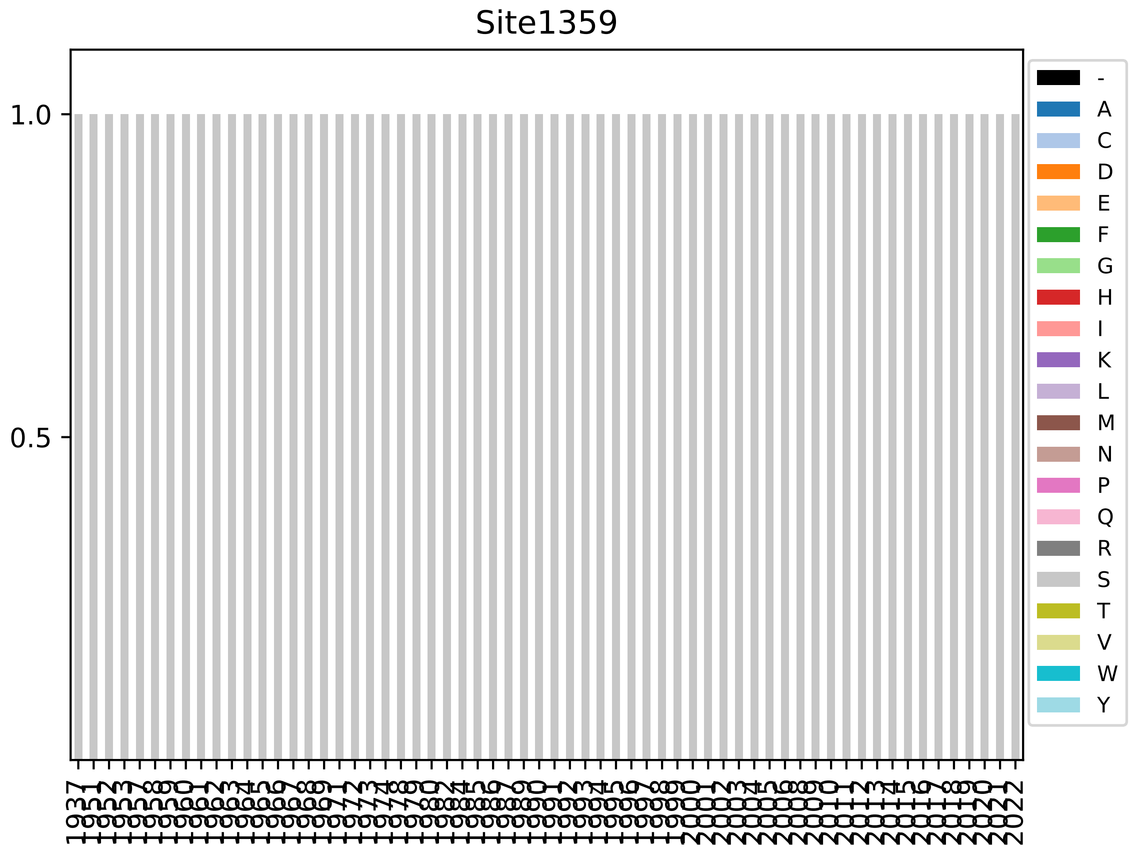
Site1359
1.0
0.5
-
A
C
D
E
F
G
H
I
K
L
M
N
P
Q
R
S
T
V
W
Y

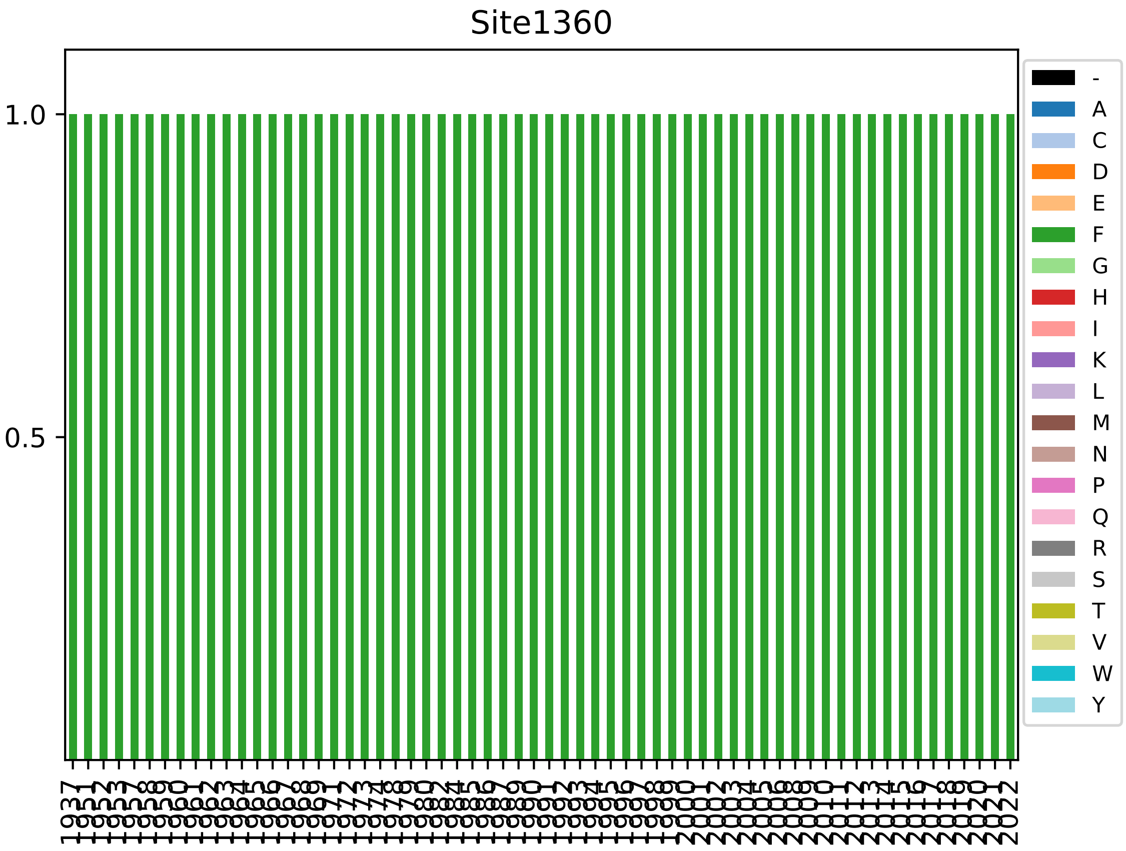
Site1360
1.0
0.5
-
A
C
D
E
F
G
H
I
K
L
M
N
P
Q
R
S
T
V
W
Y

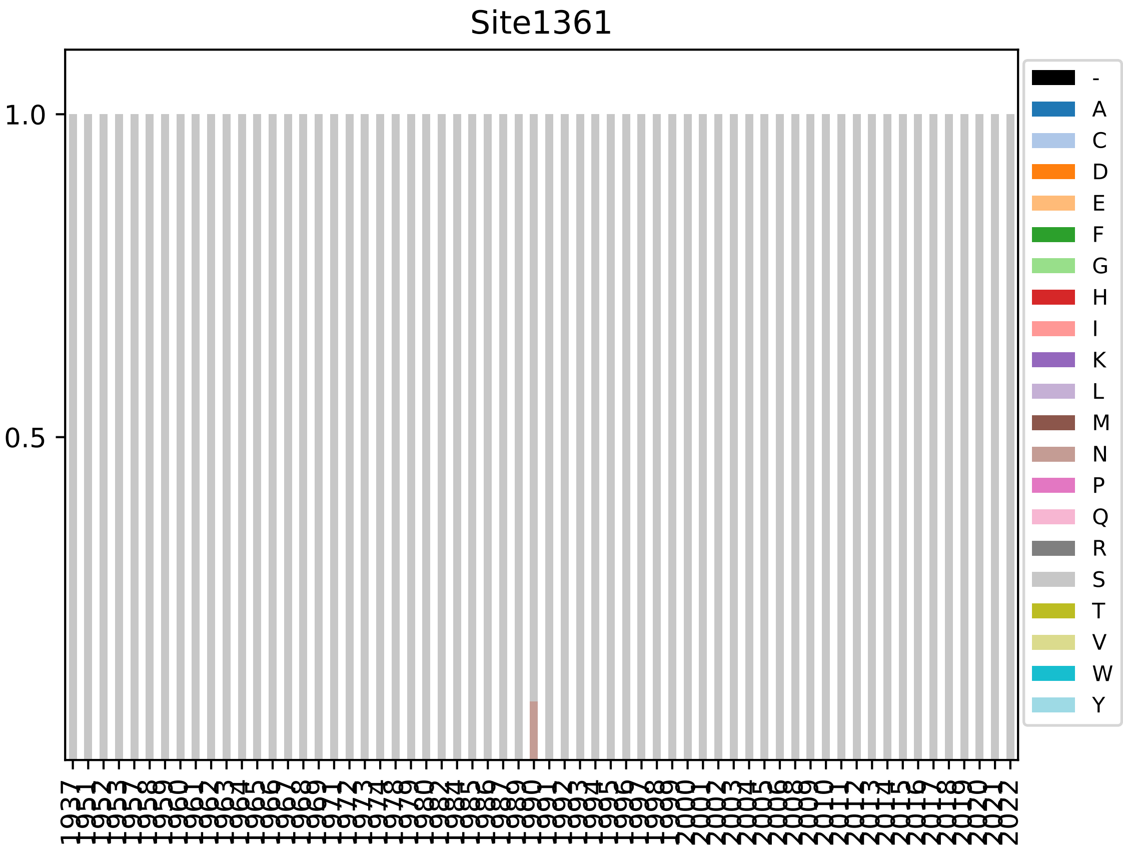
Site1361
1.0
0.5
-
A
C
D
E
F
G
H
I
K
L
M
N
P
Q
R
S
T
V
W
Y

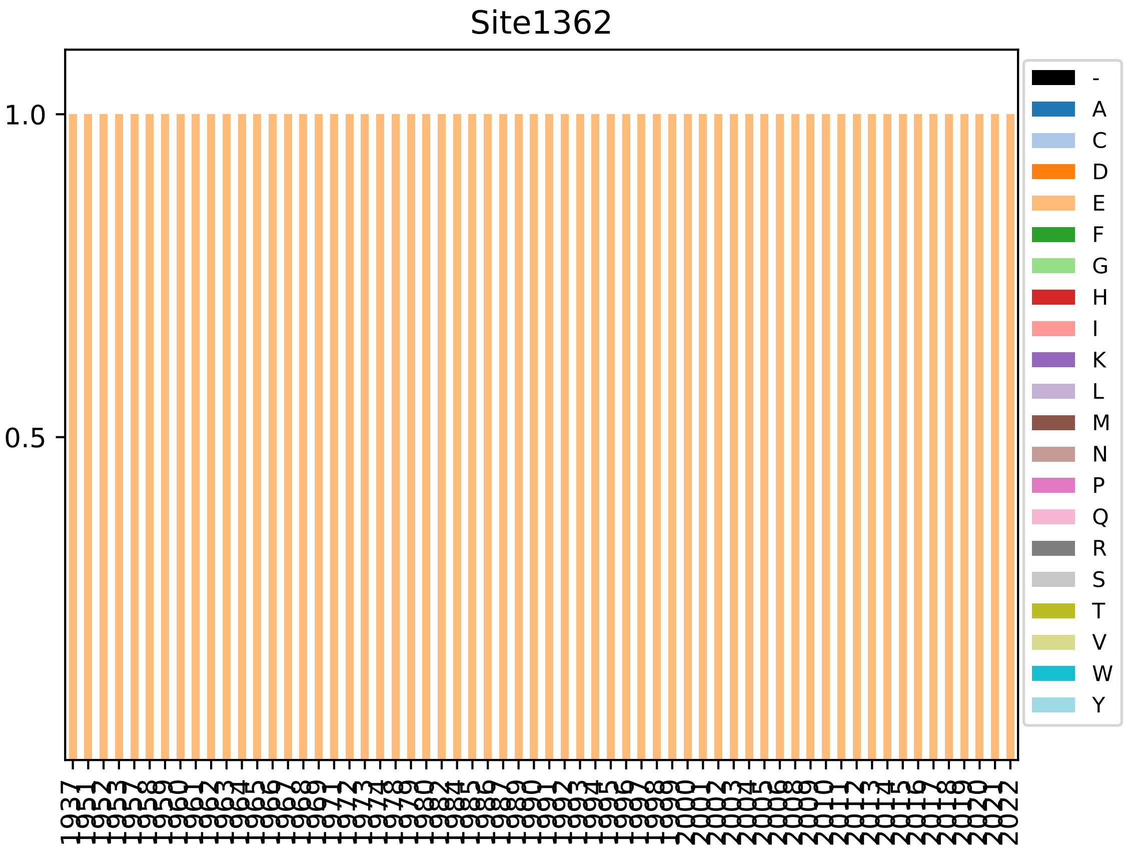
Site1362
1.0
0.5
-
A
C
D
E
F
G
H
I
K
L
M
N
P
Q
R
S
T
V
W
Y

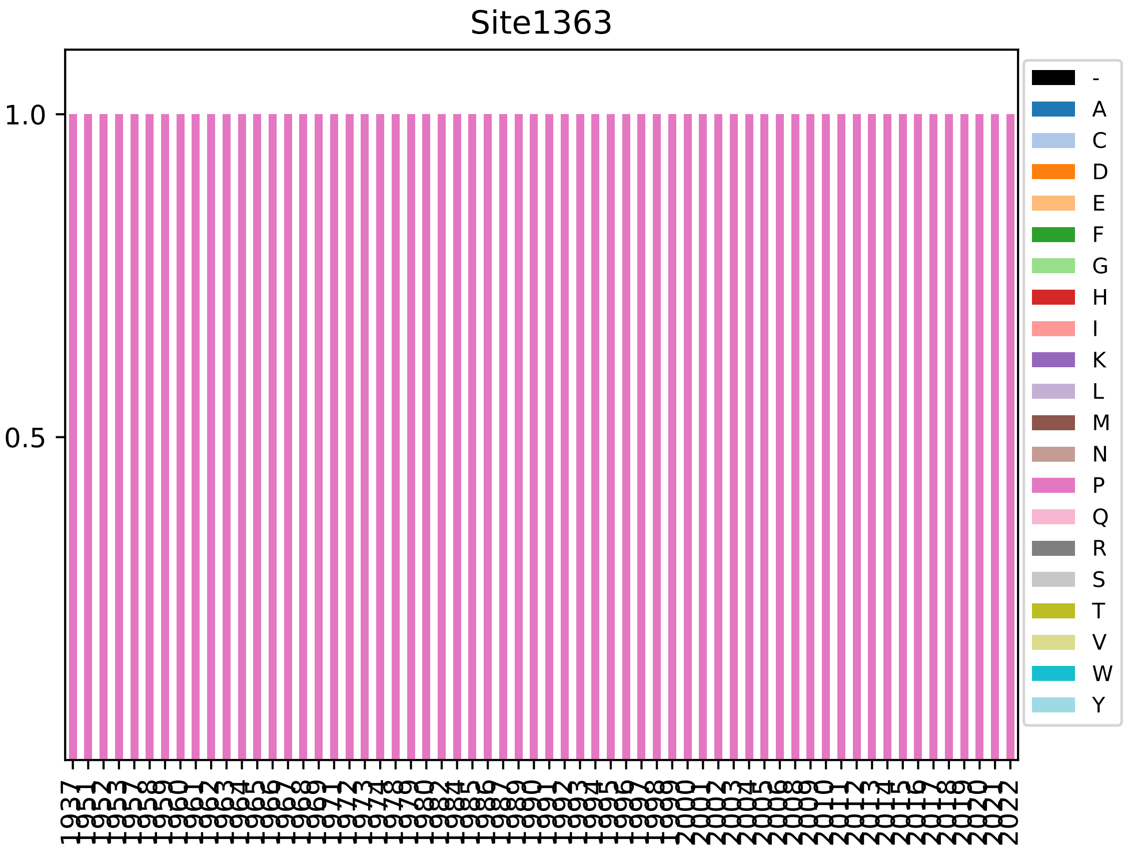
Site1363
1.0
0.5
-
A
C
D
E
F
G
H
I
K
L
M
N
P
Q
R
S
T
V
W
Y

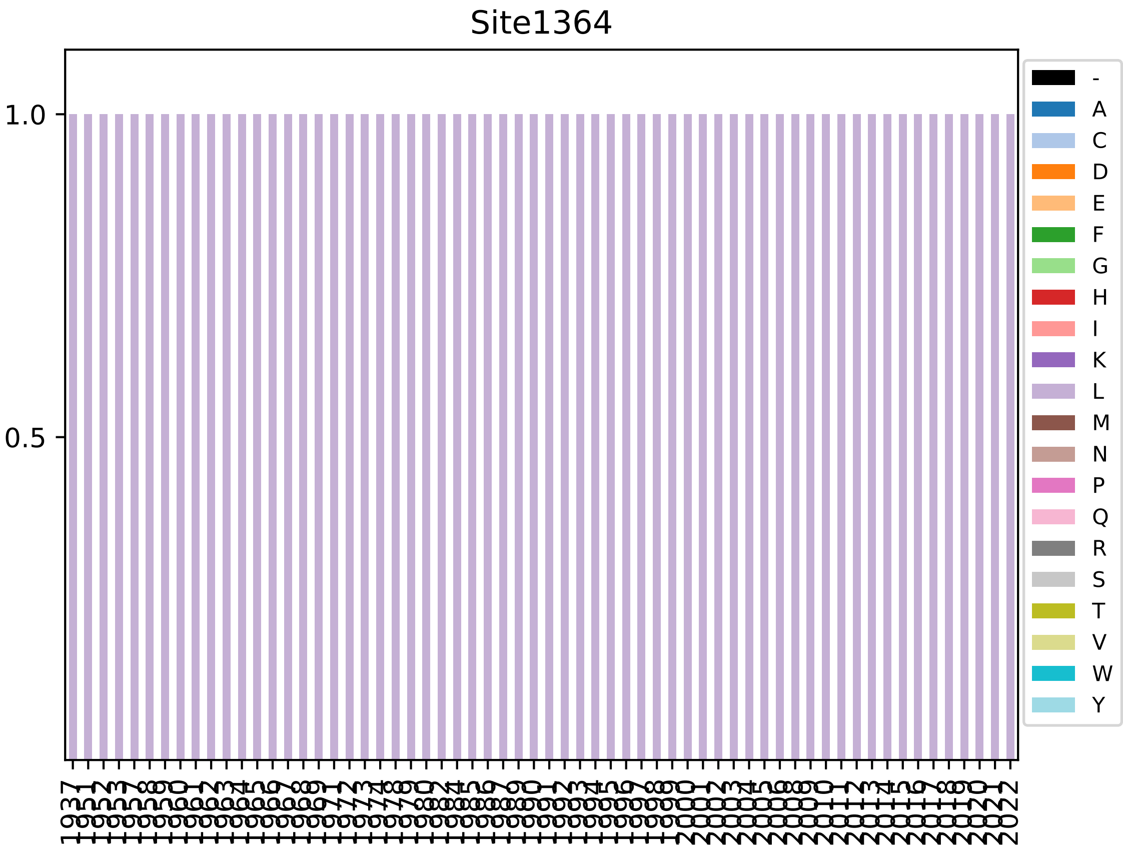
Site1364
1.0
0.5
-
A
C
D
E
F
G
H
I
K
L
M
N
P
Q
R
S
T
V
W
Y

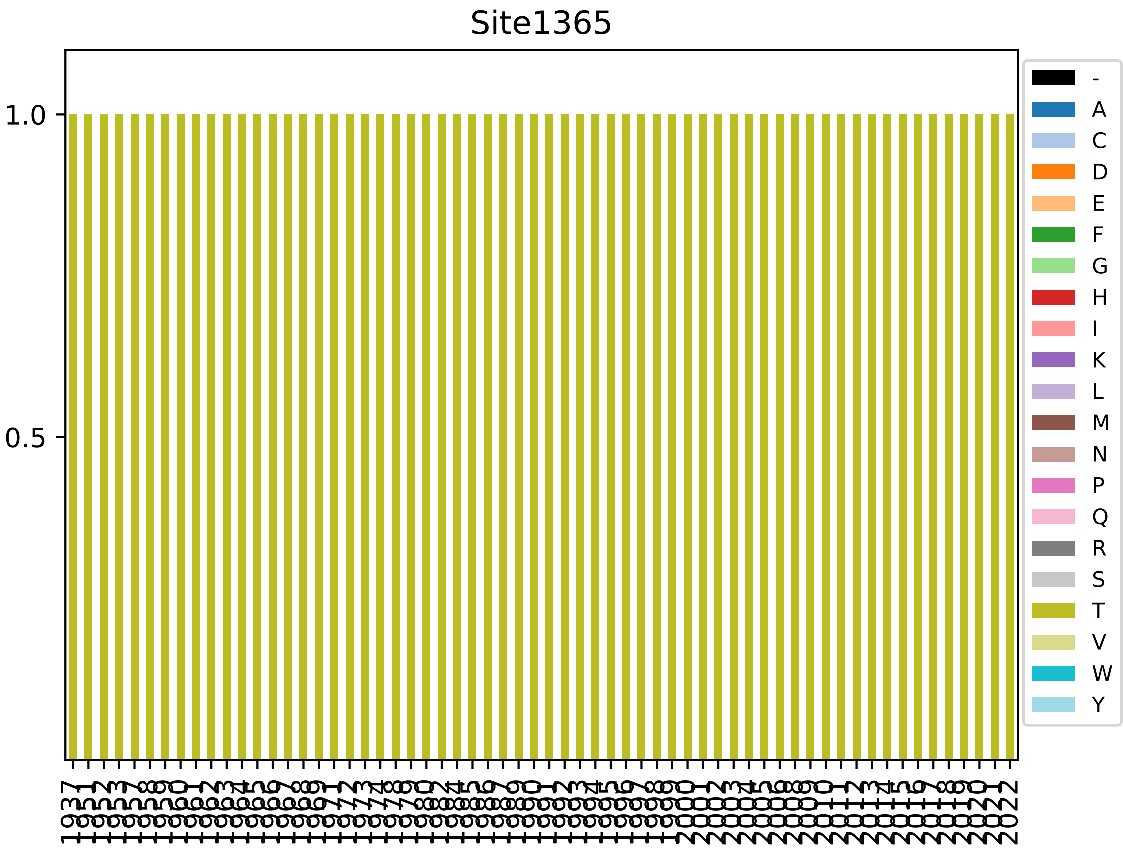

Site1365
1.0
0.5
-
A
C
D
E
F
G
H
I
K
L
M
N
P
Q
R
S
T
V
W
Y

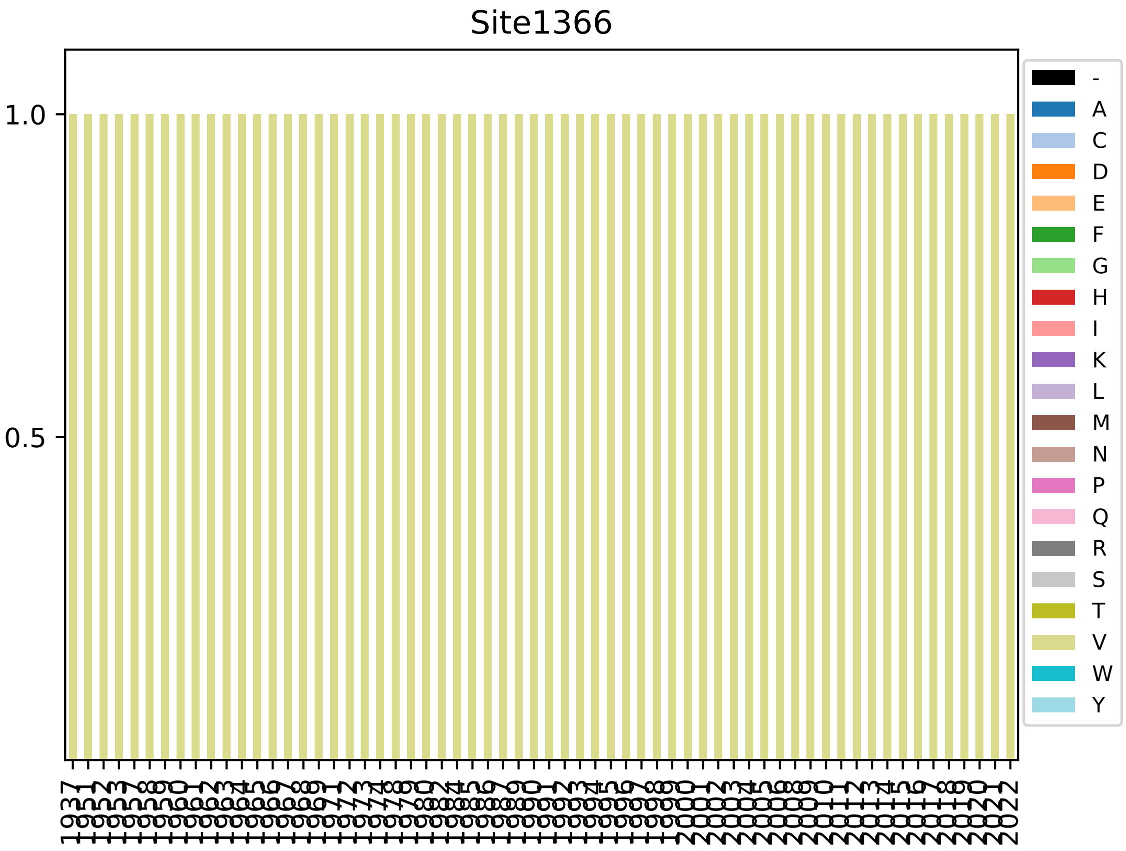
Site1366
1.0
0.5
-
A
C
D
E
F
G
H
I
K
L
M
N
P
Q
R
S
T
V
W
Y

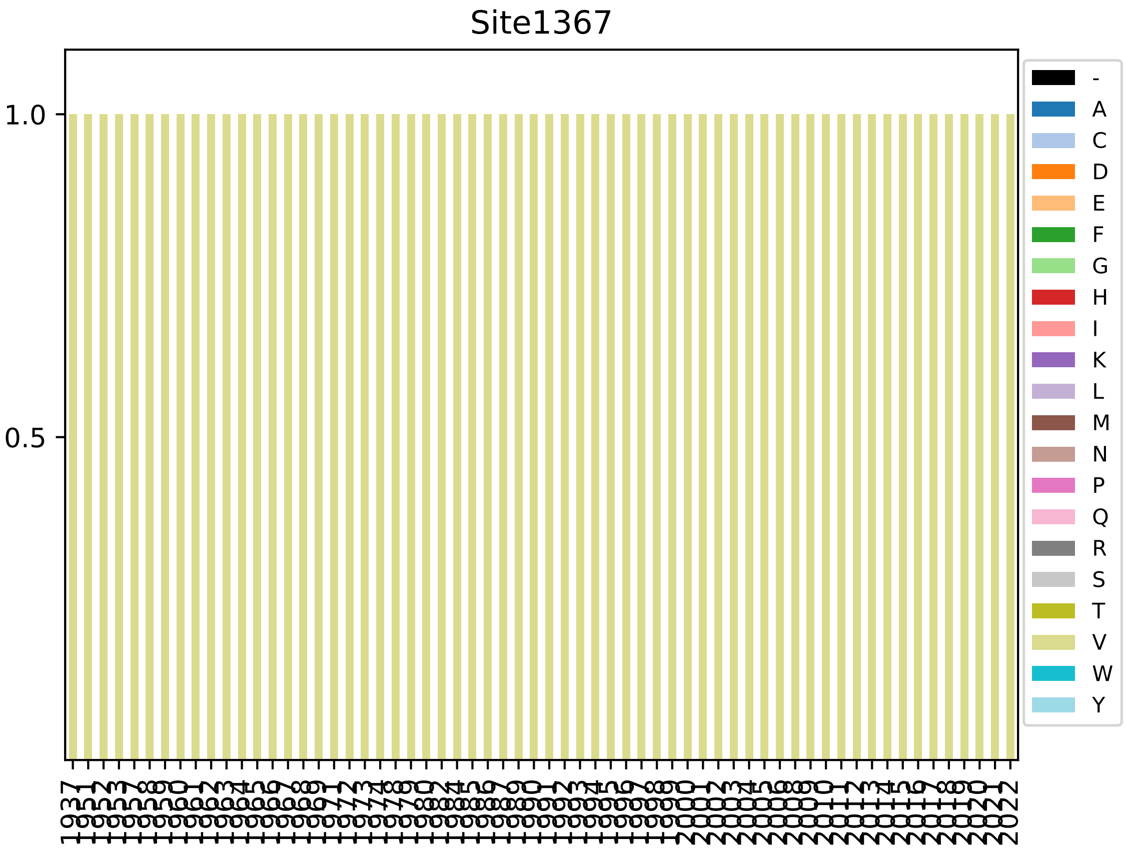
Site1367
1.0
0.5
-
A
C
D
E
F
G
H
I
K
L
M
N
P
Q
R
S
T
V
W
Y

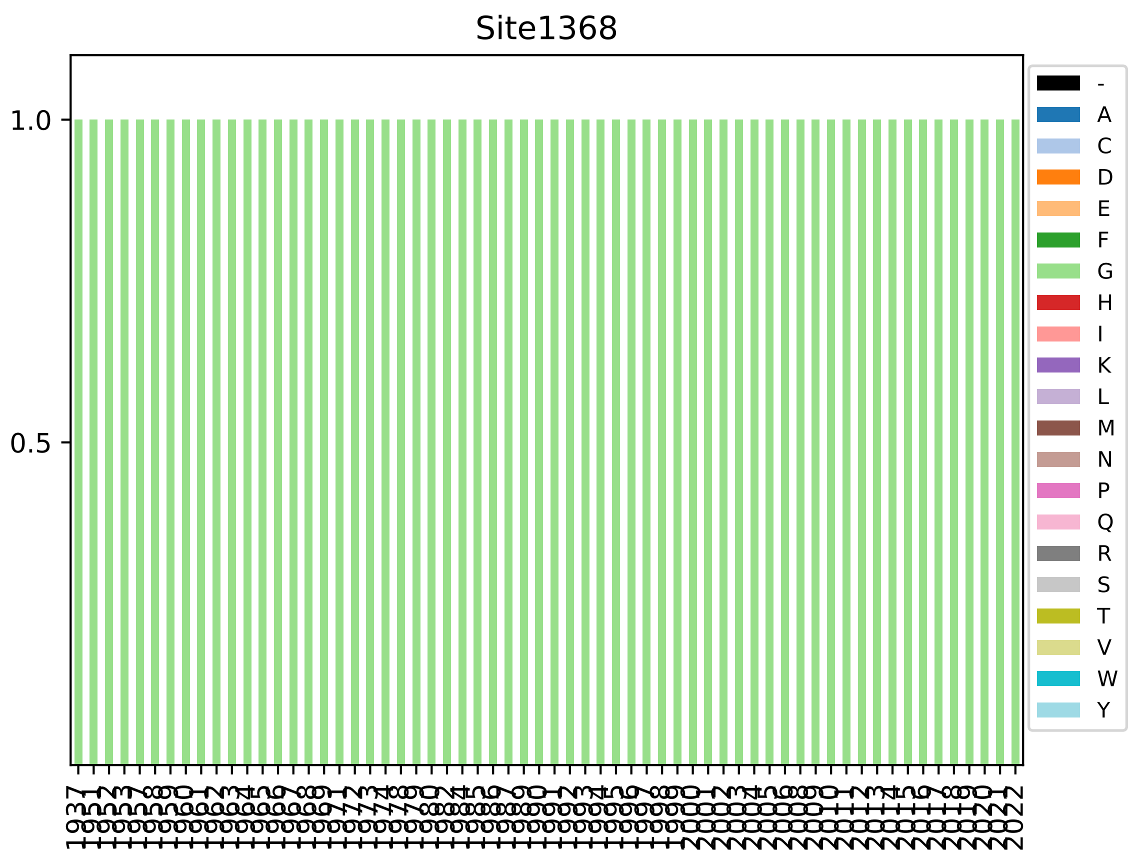
Site1368
1.0
0.5
-
A
C
D
E
F
G
H
I
K
L
M
N
P
Q
R
S
T
V
W
Y

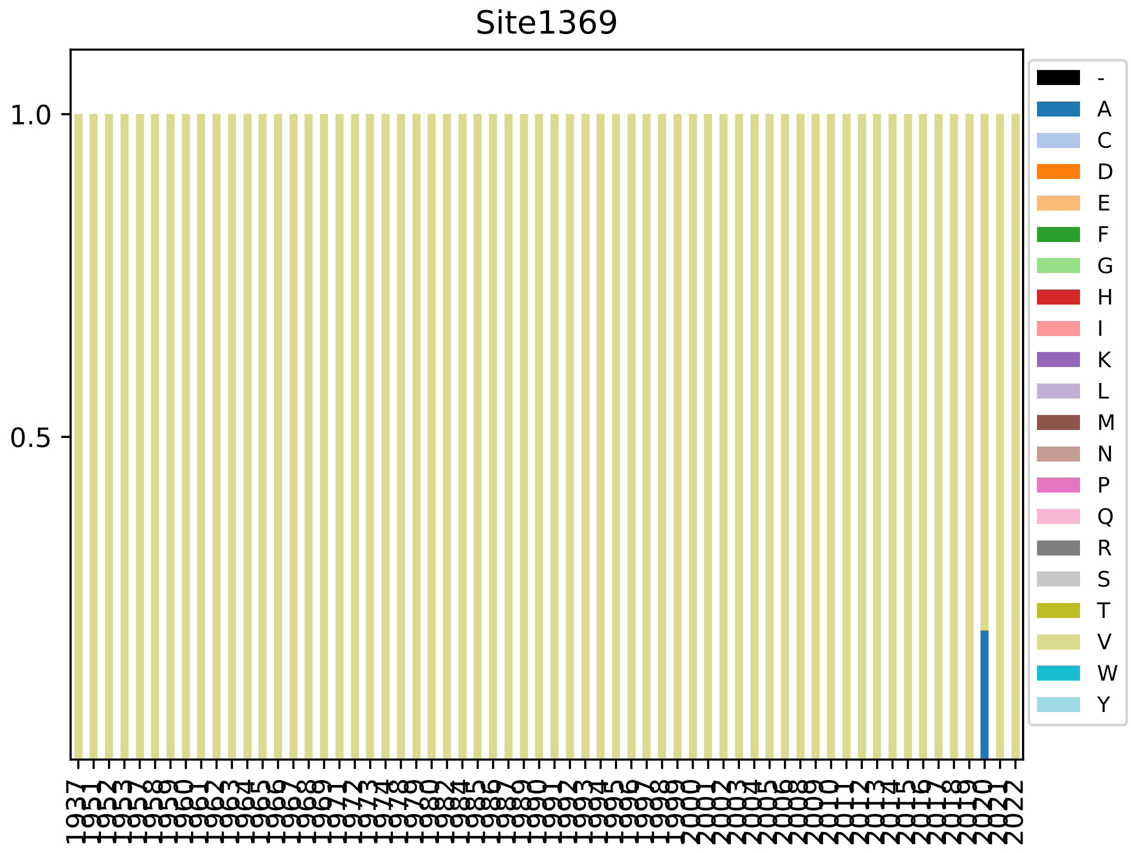

Site1369
1.0
0.5
-
A
C
D
E
F
G
H
I
K
L
M
N
P
Q
R
S
T
V
W
Y

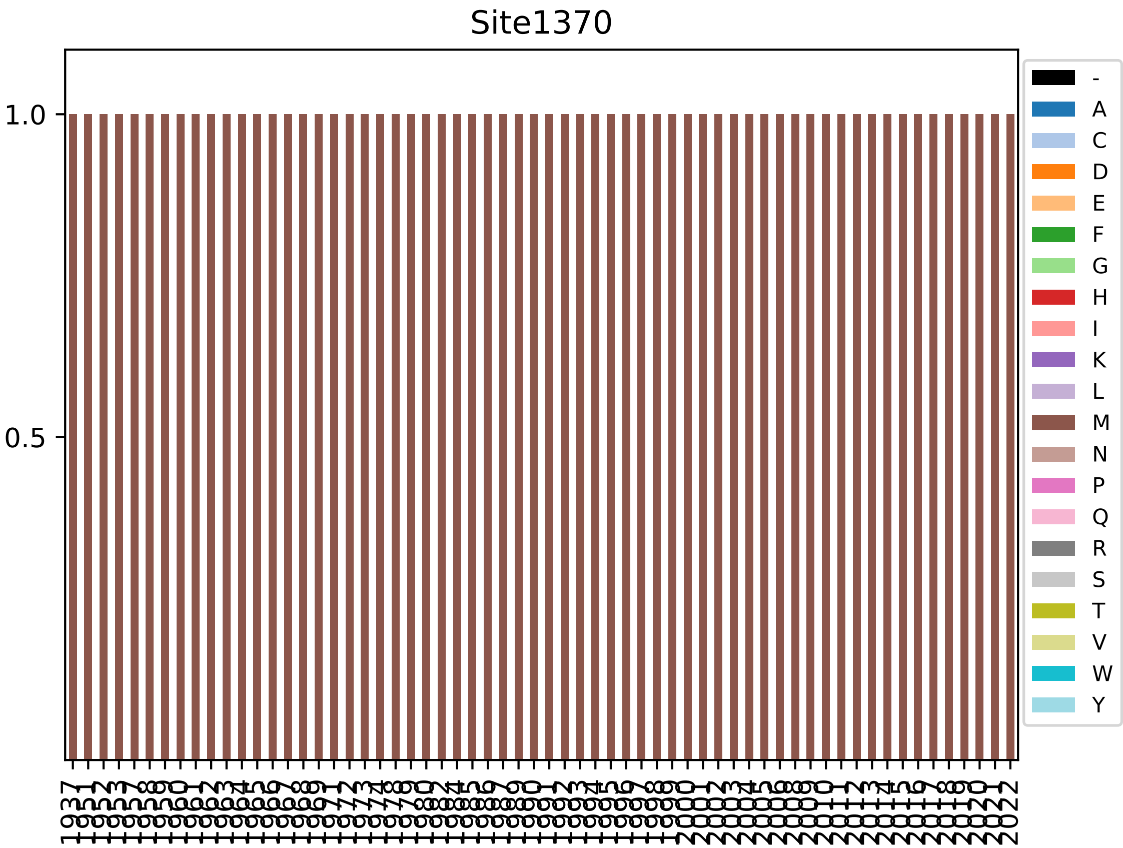
Site1370
1.0
0.5
-
A
C
D
E
F
G
H
I
K
L
M
N
P
Q
R
S
T
V
W
Y

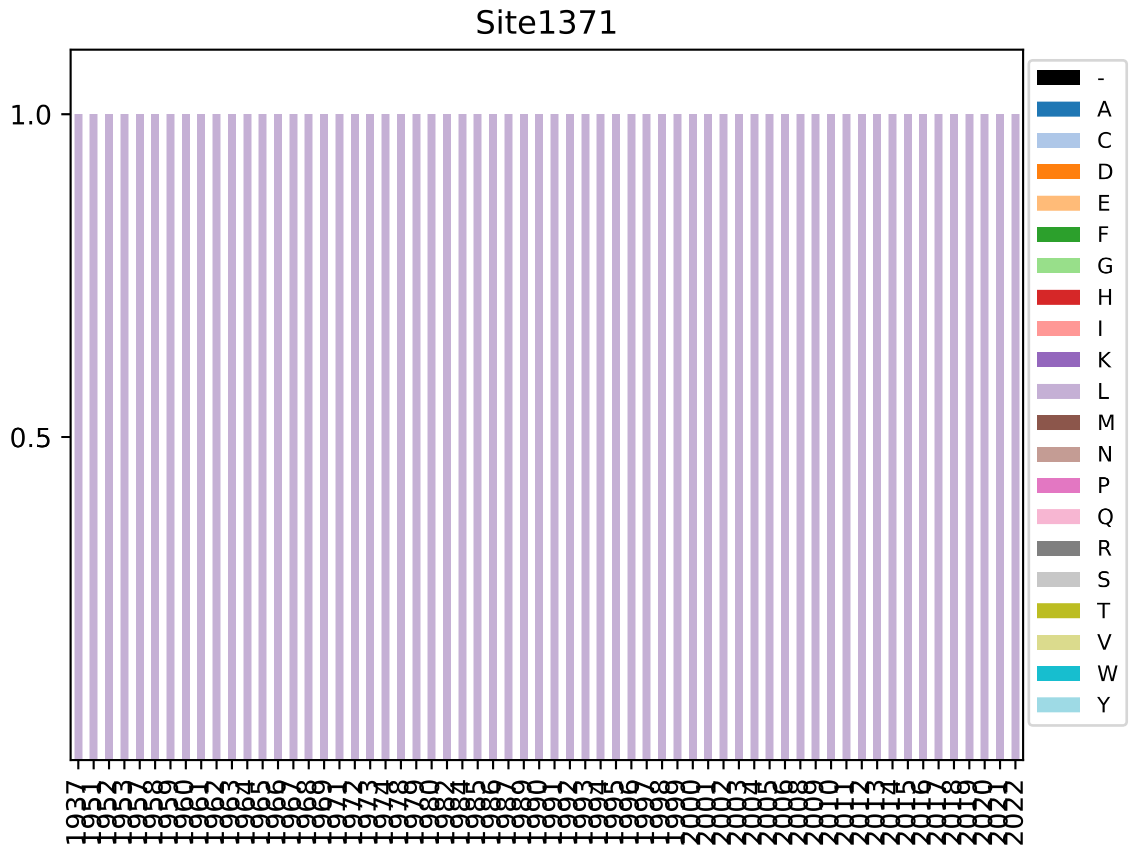
Site1371
1.0
0.5
-
A
C
D
E
F
G
H
I
K
L
M
N
P
Q
R
S
T
V
W
Y

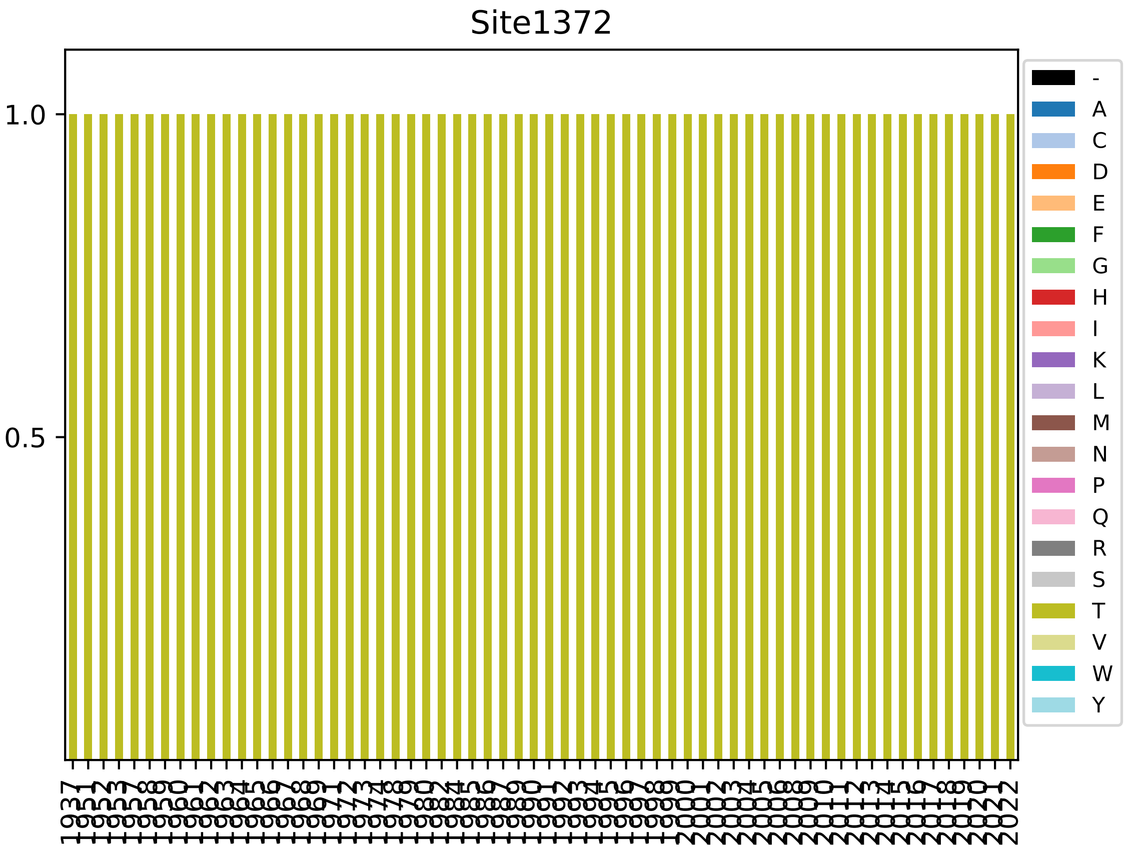
Site1372
1.0
0.5
-
A
C
D
E
F
G
H
I
K
L
M
N
P
Q
R
S
T
V
W
Y

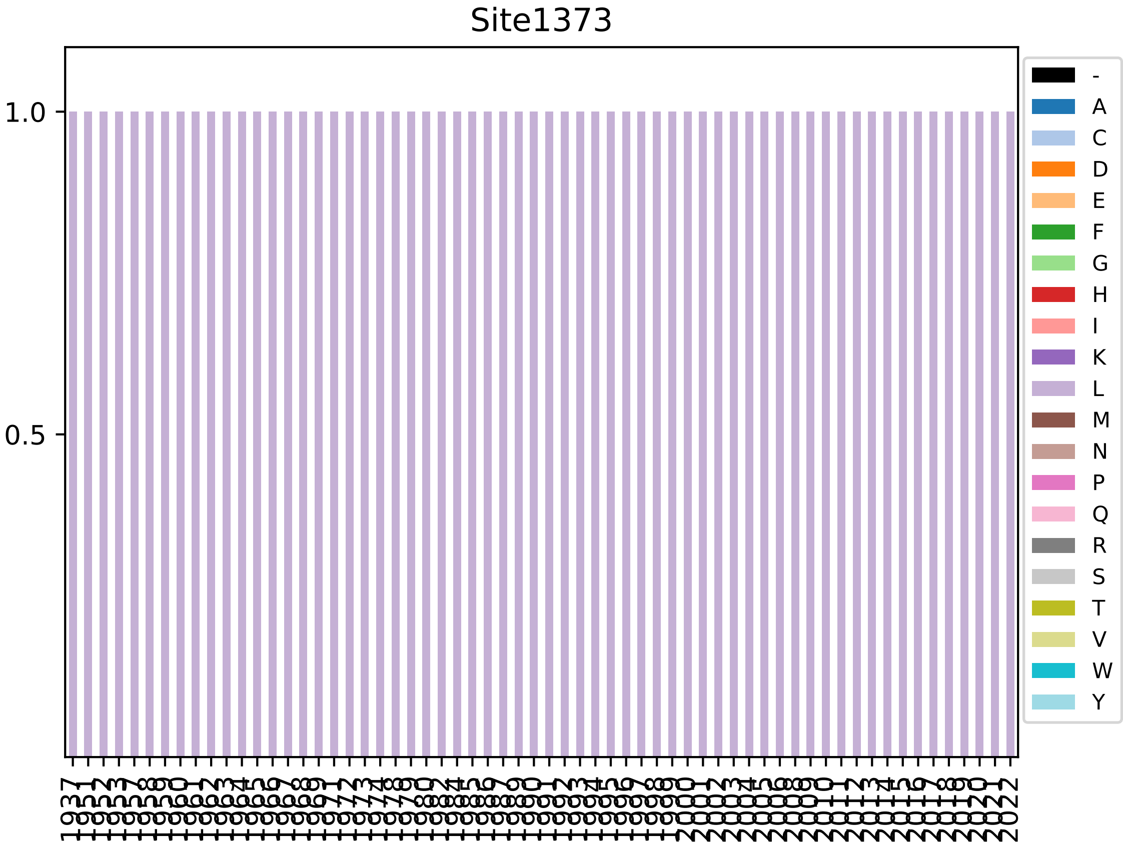
Site1373
1.0
0.5
-
A
C
D
E
F
G
H
I
K
L
M
N
P
Q
R
S
T
V
W
Y

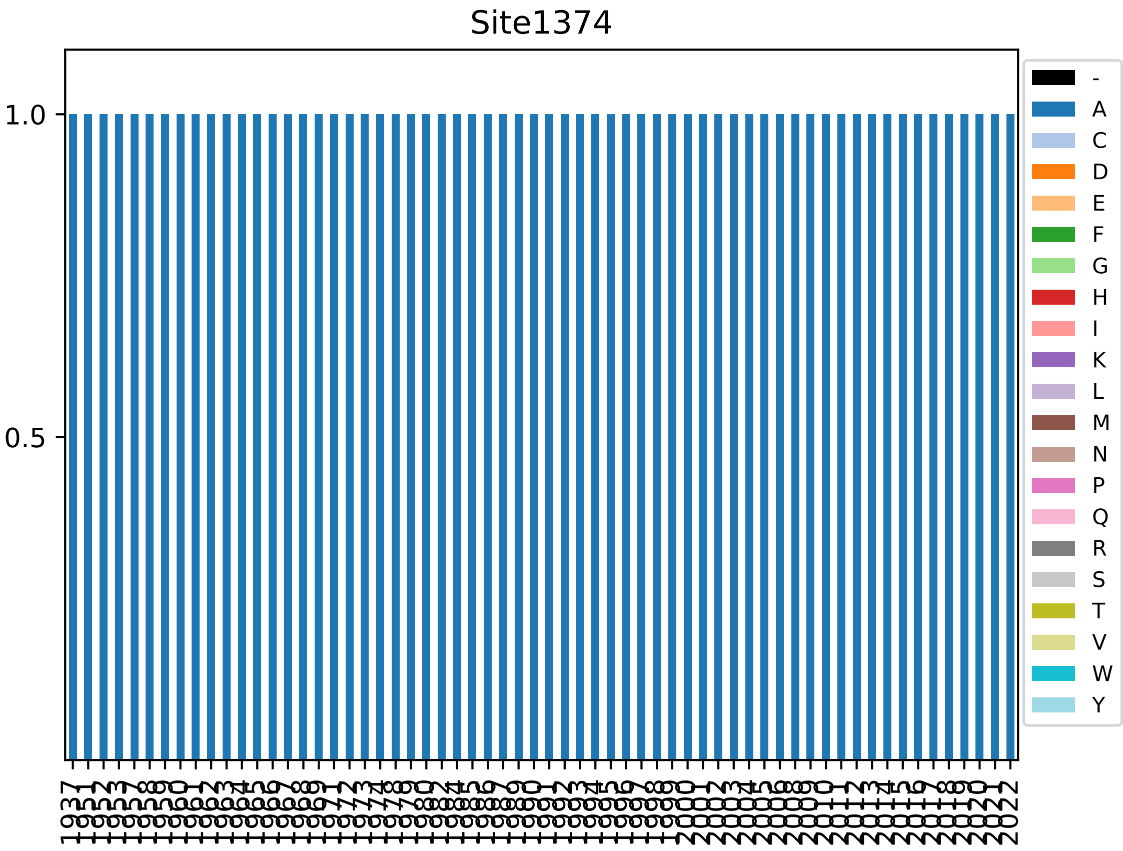
Site1374
1.0
0.5
-
A
C
D
E
F
G
H
I
K
L
M
N
P
Q
R
S
T
V
W
Y

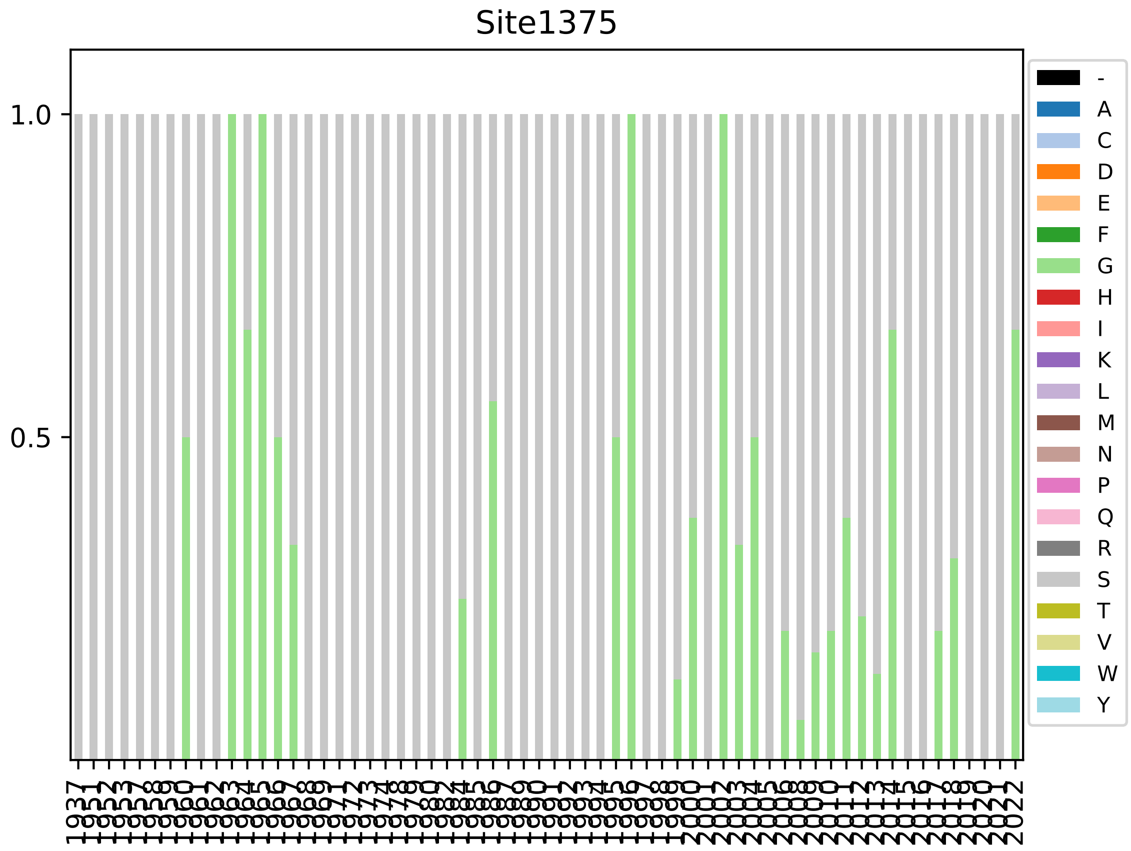
Site1375
1.0
0.5
-
A
C
D
E
F
G
H
I
K
L
M
N
P
Q
R
S
T
V
W
Y

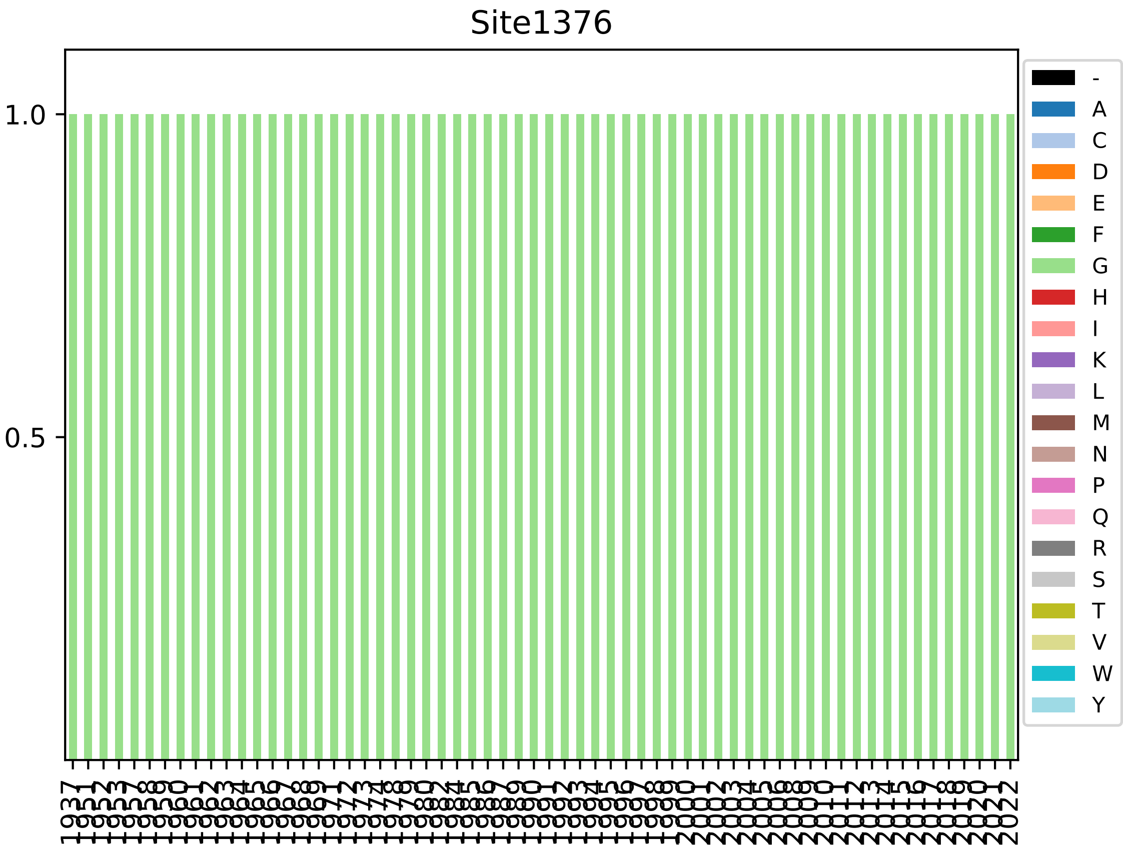
Site1376
1.0
0.5
-
A
C
D
E
F
G
H
I
K
L
M
N
P
Q
R
S
T
V
W
Y

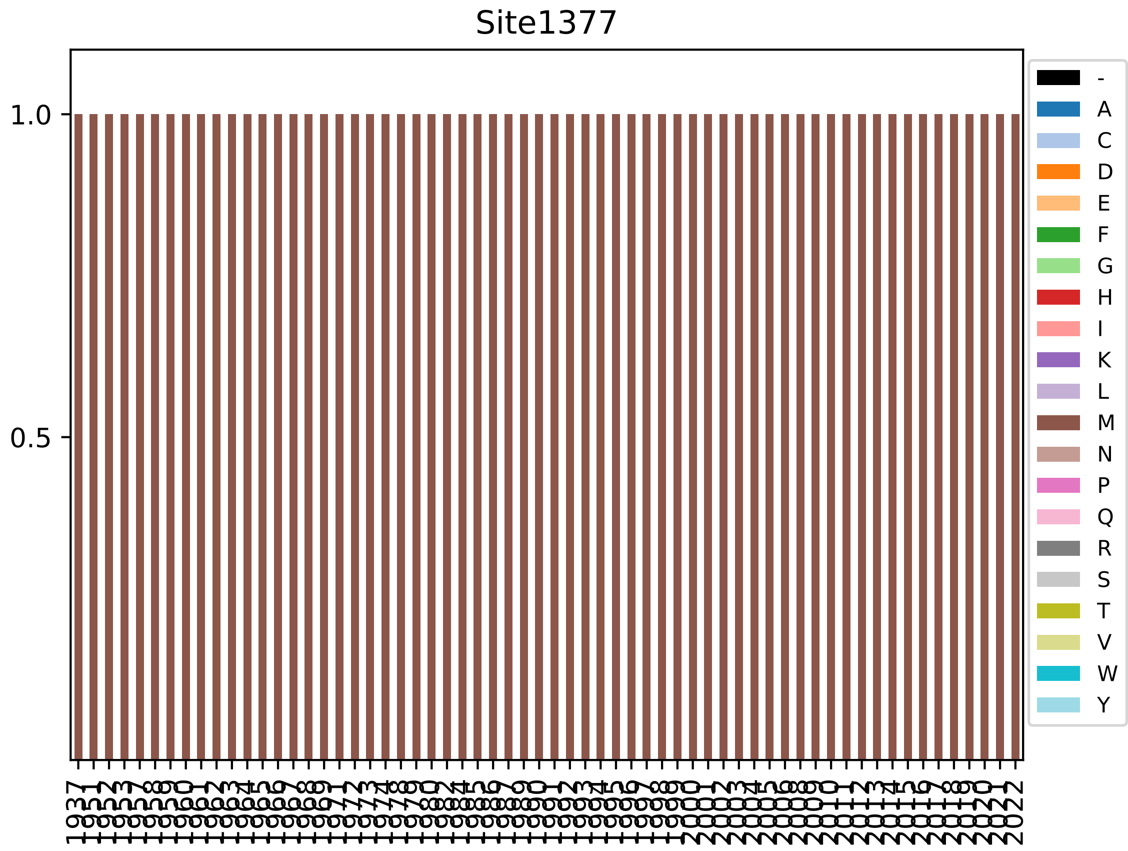
Site1377
1.0
0.5
-
A
C
D
E
F
G
H
I
K
L
M
N
P
Q
R
S
T
V
W
Y

Site1378

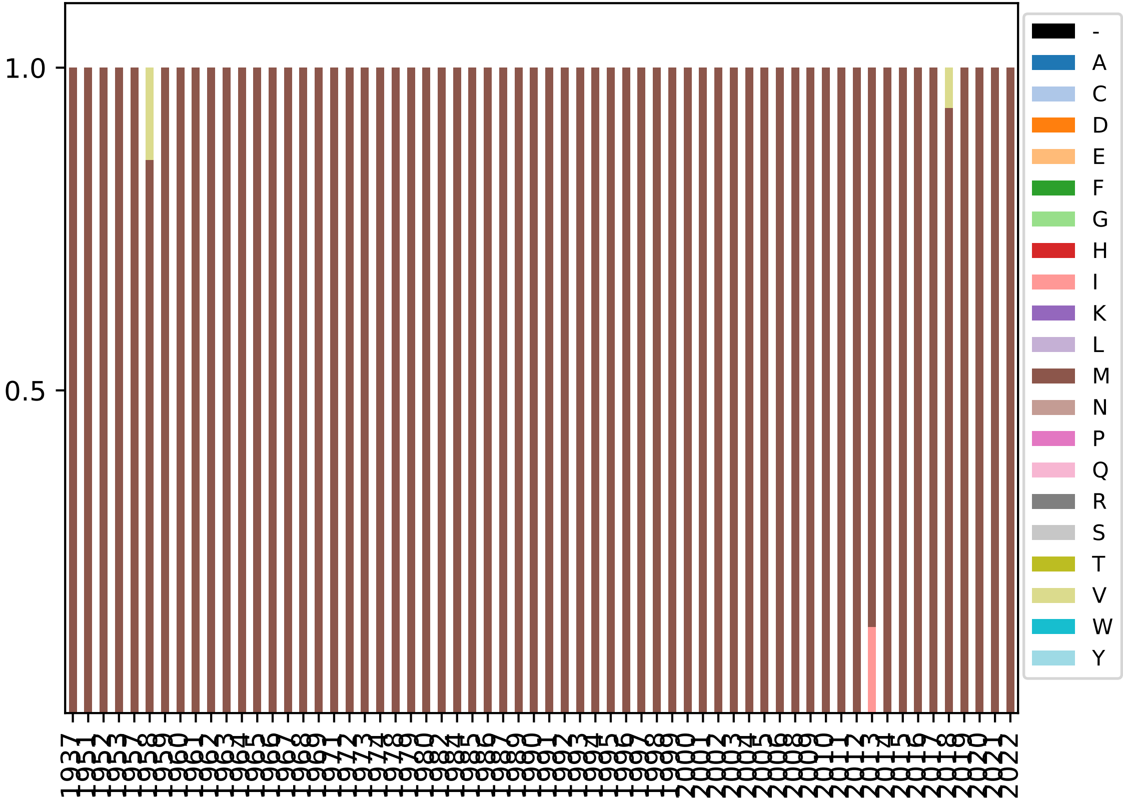

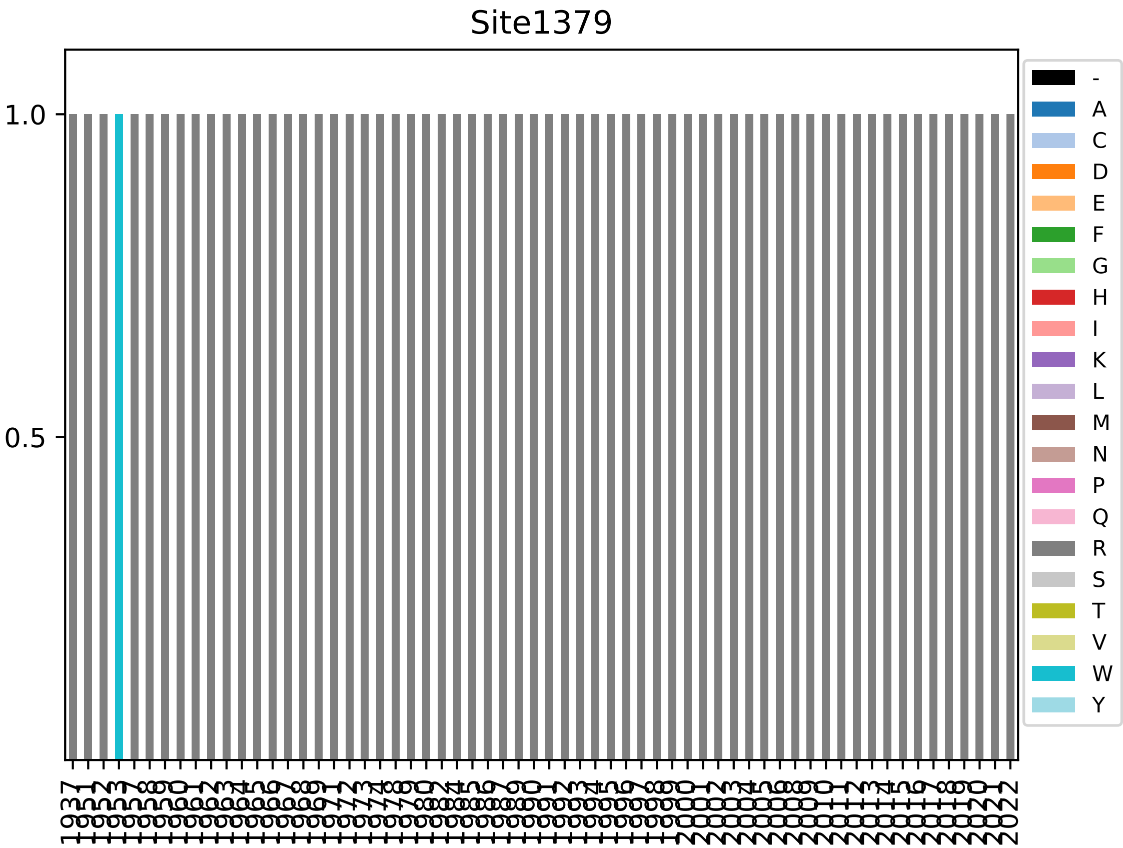
Site1379
1.0
0.5
-
A
C
D
E
F
G
H
I
K
L
M
N
P
Q
R
S
T
V
W
Y

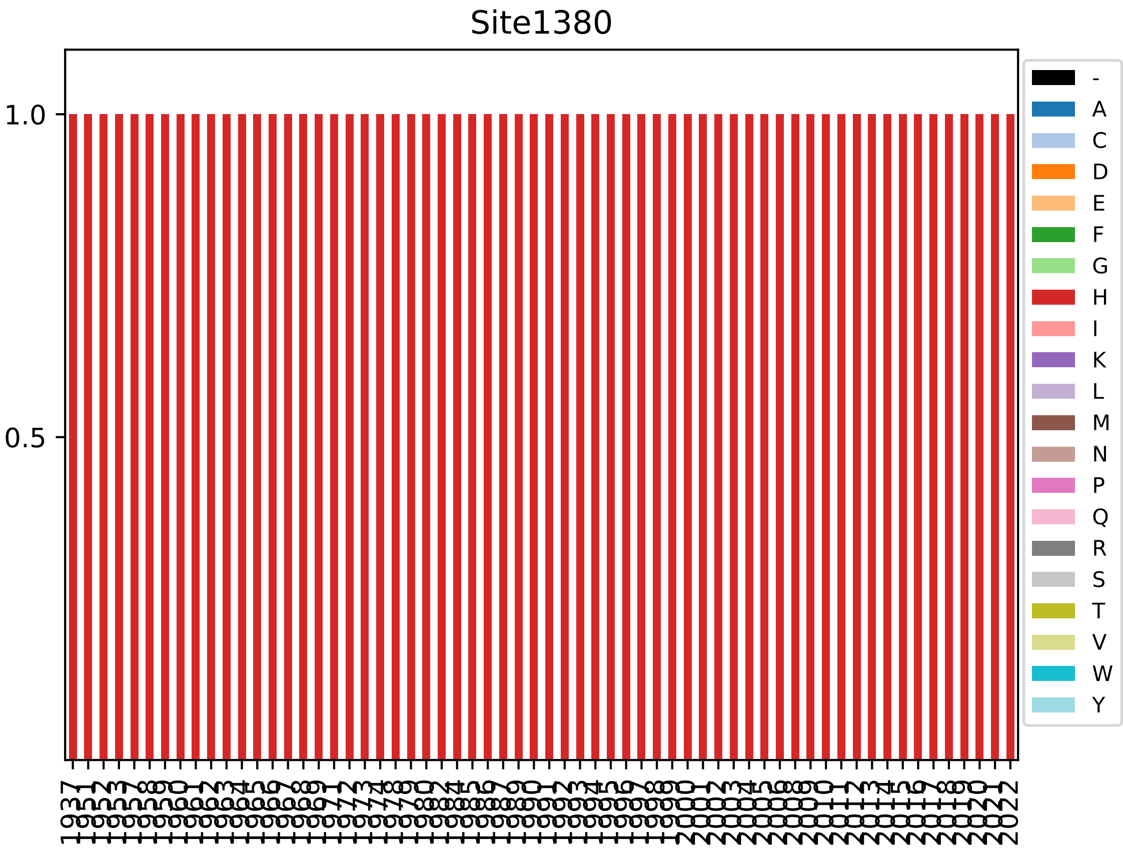

Site1380
1.0
0.5
-
A
C
D
E
F
G
H
I
K
L
M
N
P
Q
R
S
T
V
W
Y

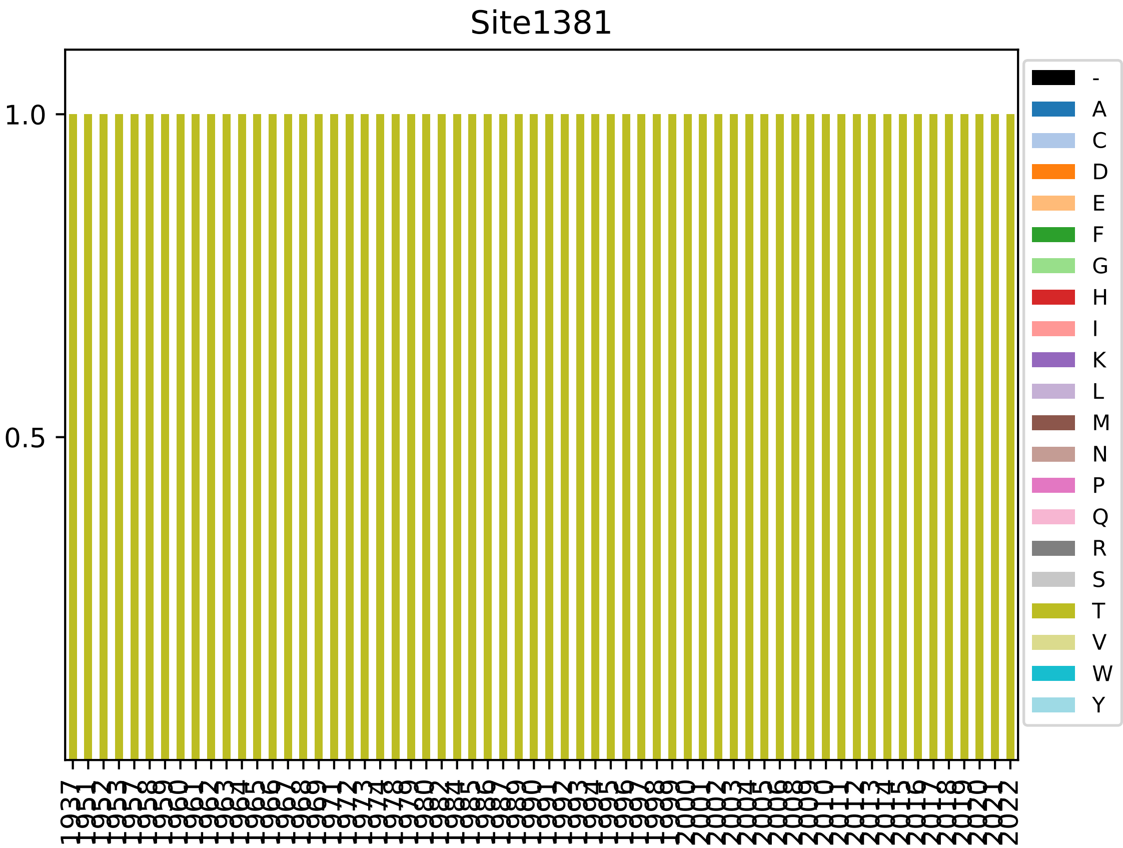
Site1381
1.0
0.5
-
A
C
D
E
F
G
H
I
K
L
M
N
P
Q
R
S
T
V
W
Y

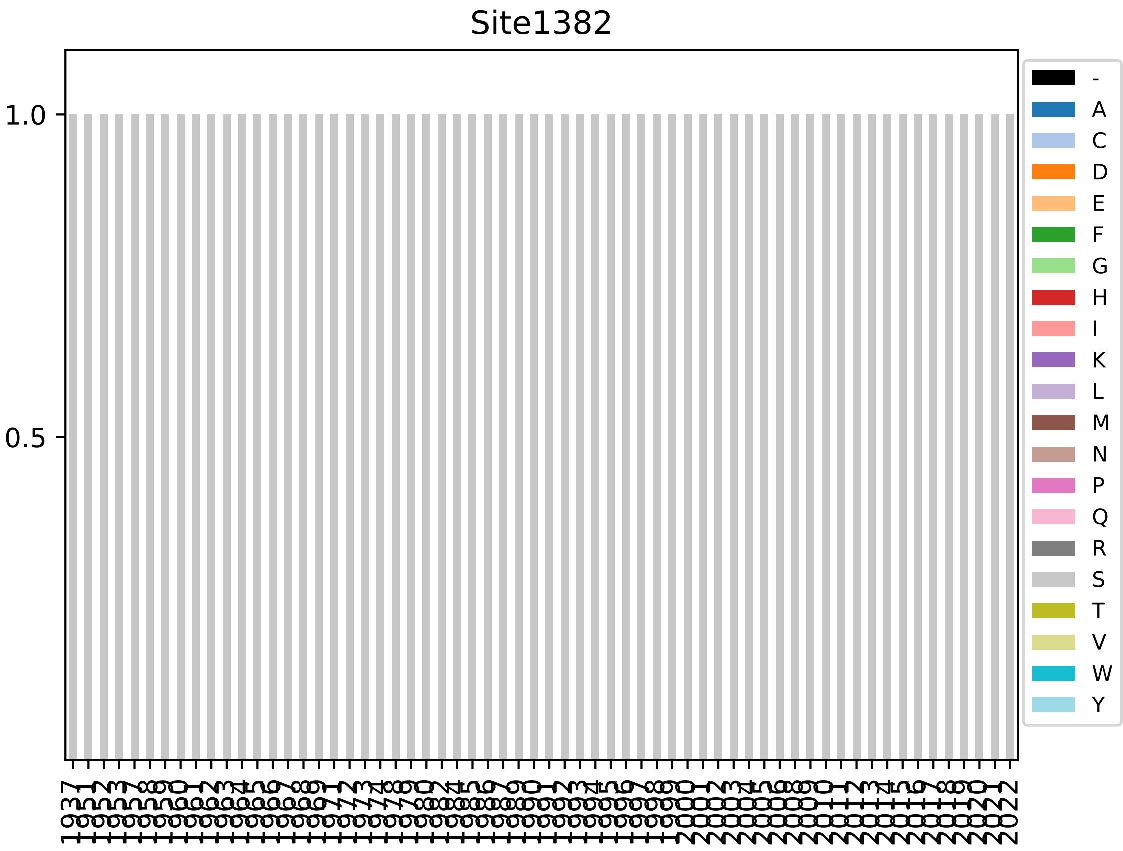
Site1382
1.0
0.5
-
A
C
D
E
F
G
H
I
K
L
M
N
P
Q
R
S
T
V
W
Y

Site1383

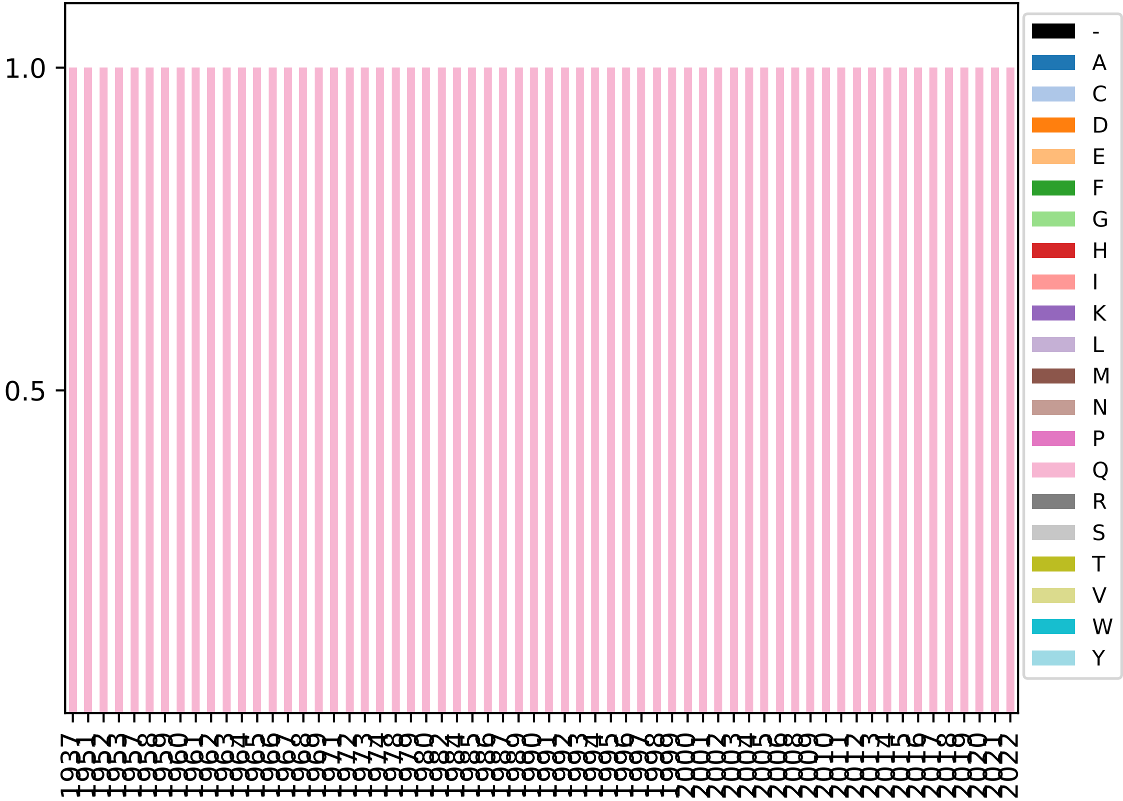

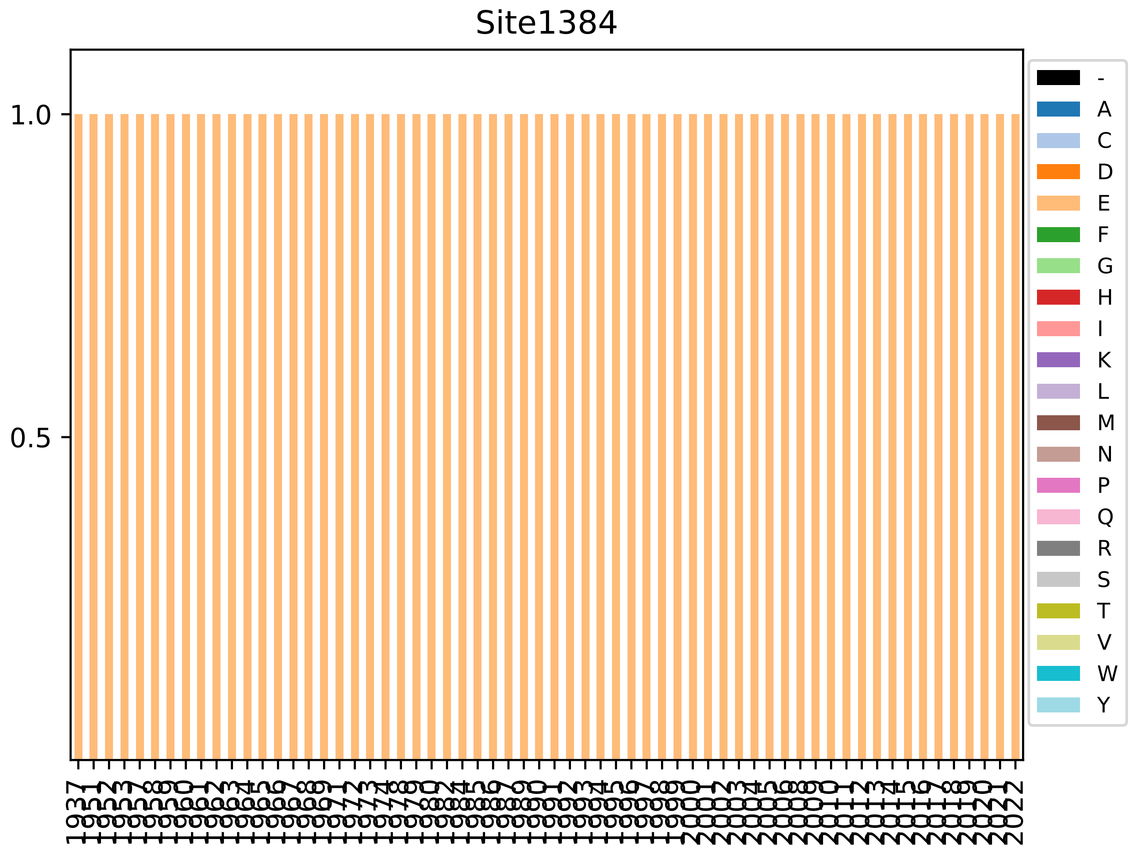
Site1384
1.0
0.5
-
A
C
D
E
F
G
H
I
K
L
M
N
P
Q
R
S
T
V
W
Y

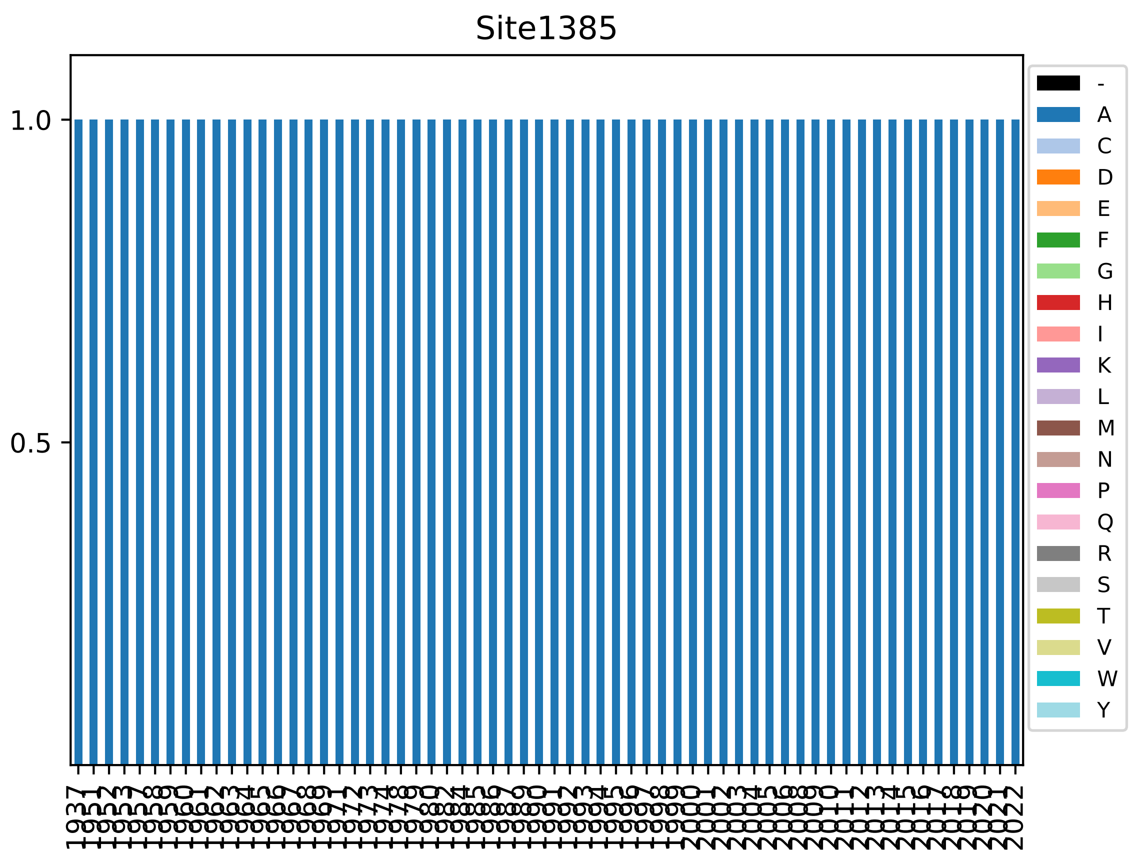
Site1385
1.0
0.5
-
A
C
D
E
F
G
H
I
K
L
M
N
P
Q
R
S
T
V
W
Y

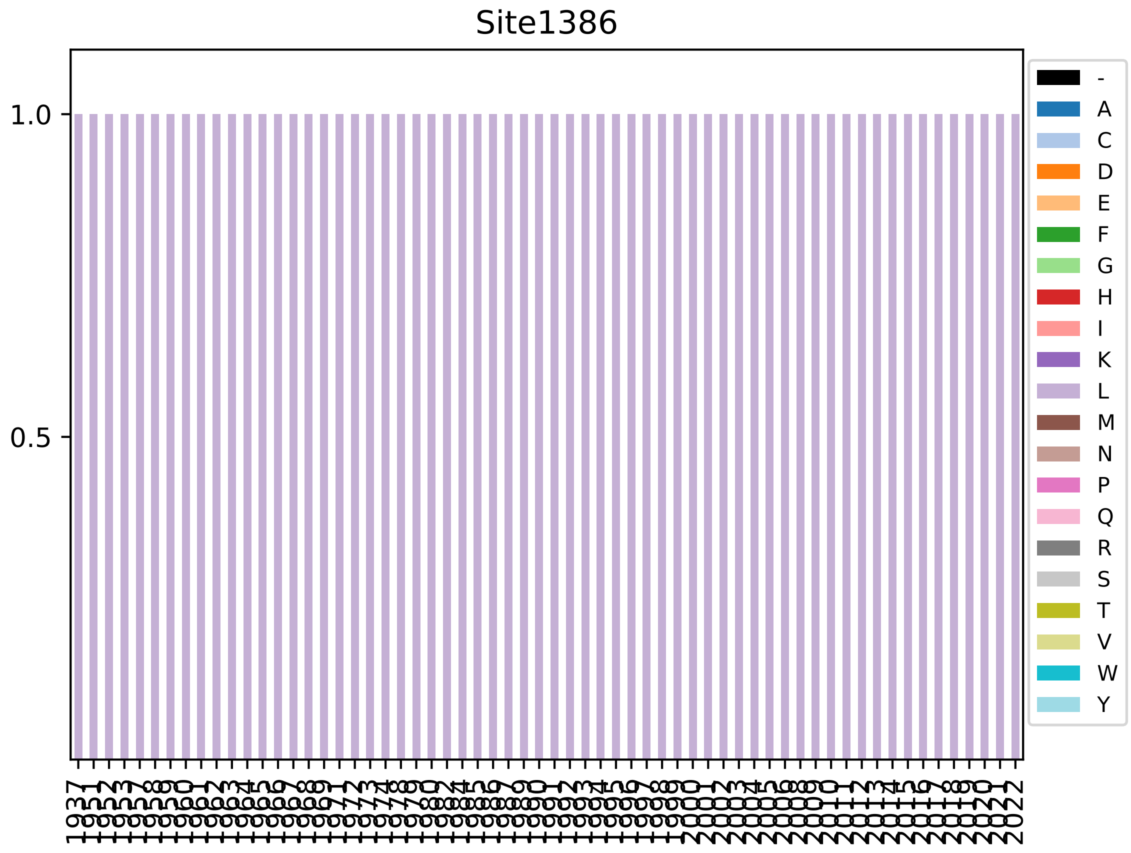
Site1386
1.0
0.5
-
A
C
D
E
F
G
H
I
K
L
M
N
P
Q
R
S
T
V
W
Y

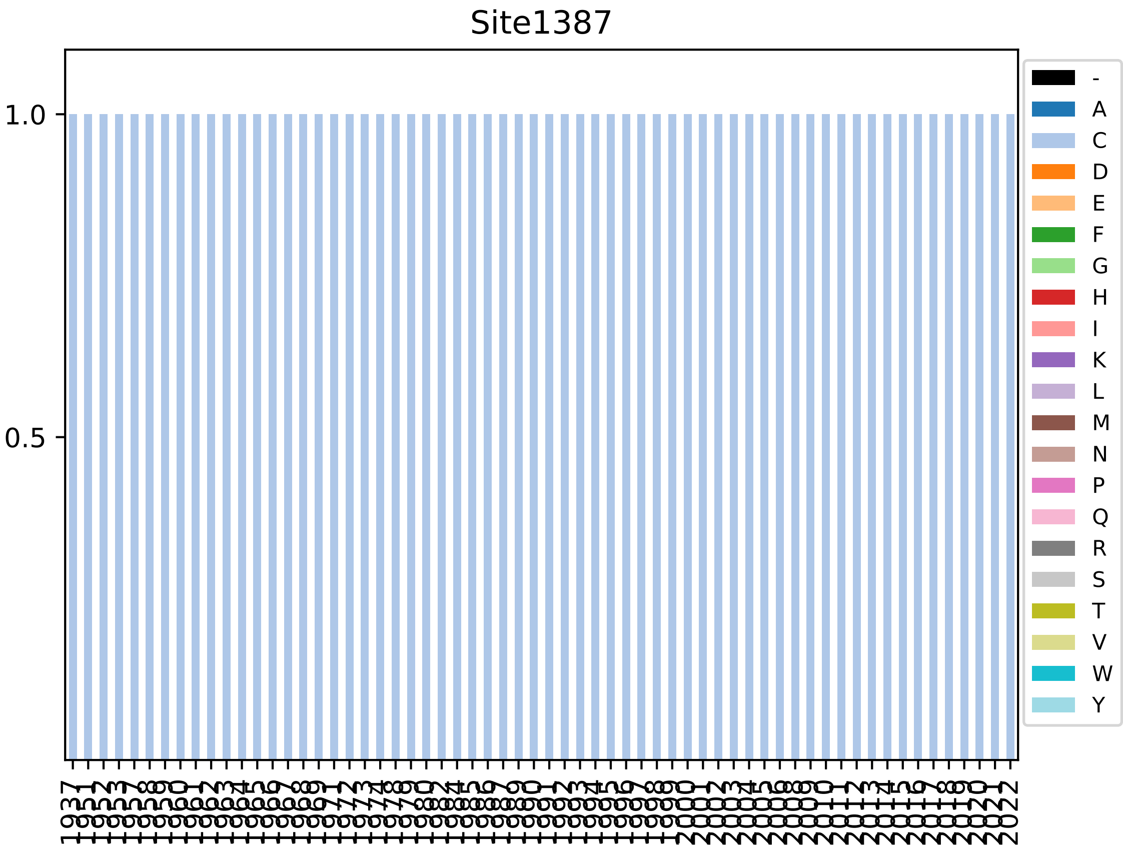

Site1387
1.0
0.5
-
A
C
D
E
F
G
H
I
K
L
M
N
P
Q
R
S
T
V
W
Y

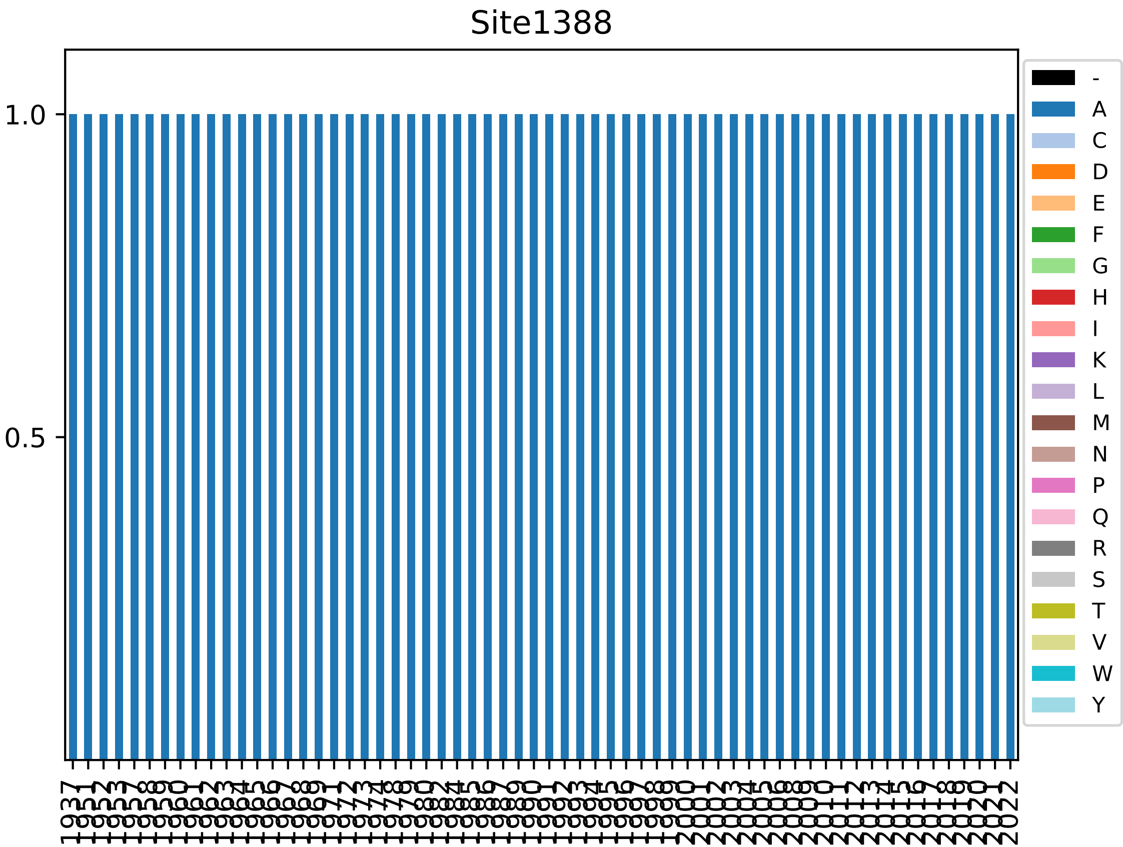
Site1388
1.0
0.5
-
A
C
D
E
F
G
H
I
K
L
M
N
P
Q
R
S
T
V
W
Y

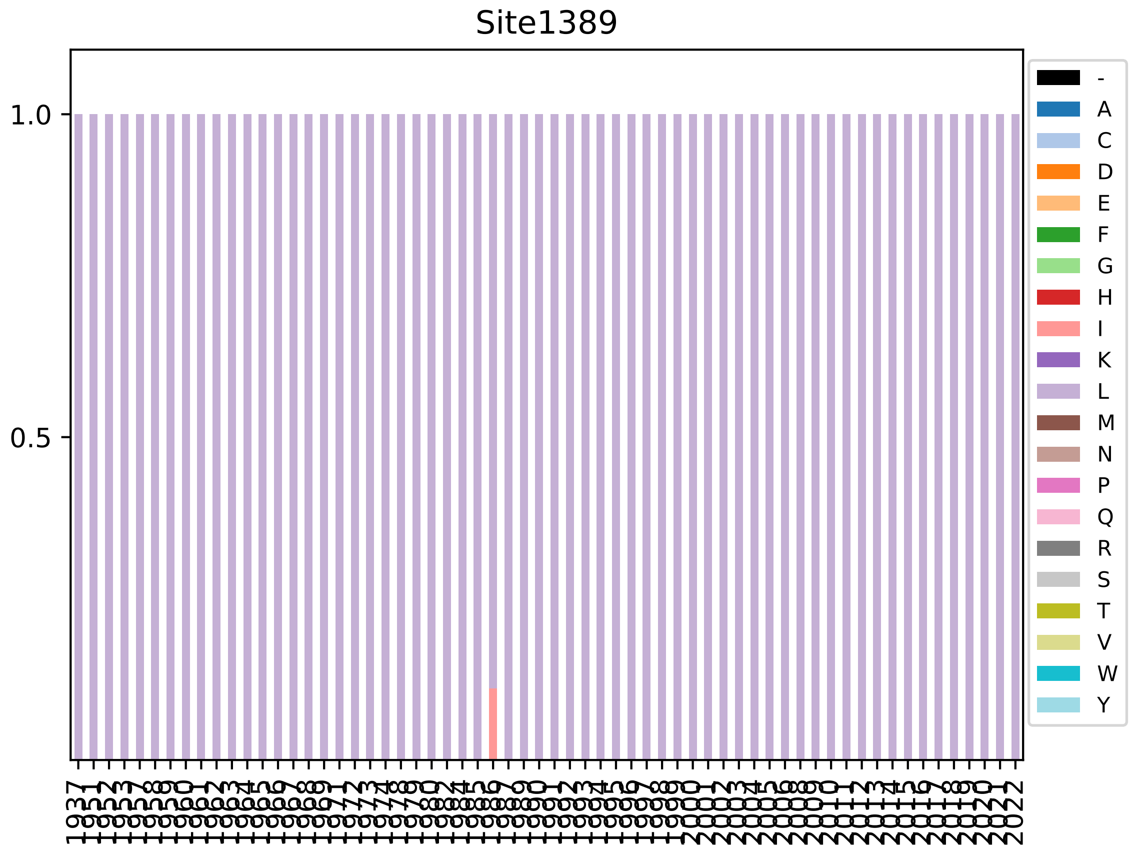
Site1389
1.0
0.5
-
A
C
D
E
F
G
H
I
K
L
M
N
P
Q
R
S
T
V
W
Y

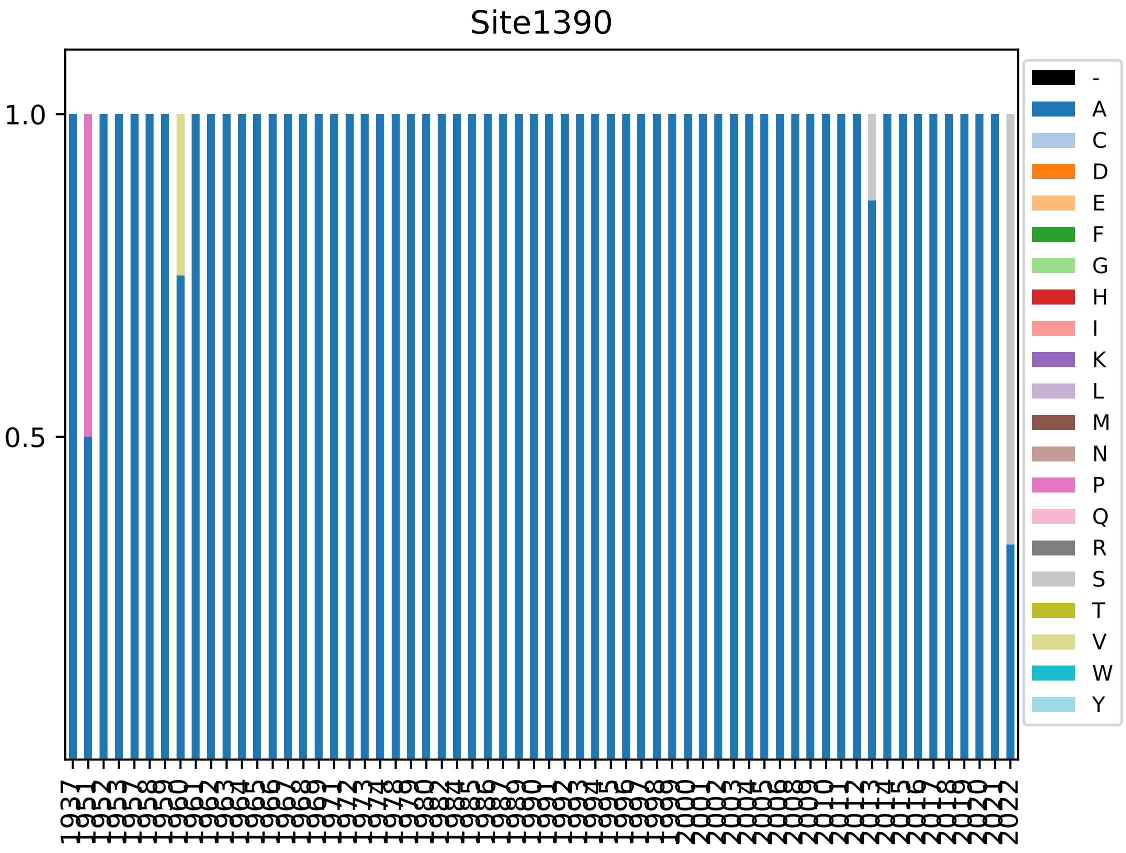
Site1390
1.0
0.5
-
A
C
D
E
F
G
H
I
K
L
M
N
P
Q
R
S
T
V
W
Y

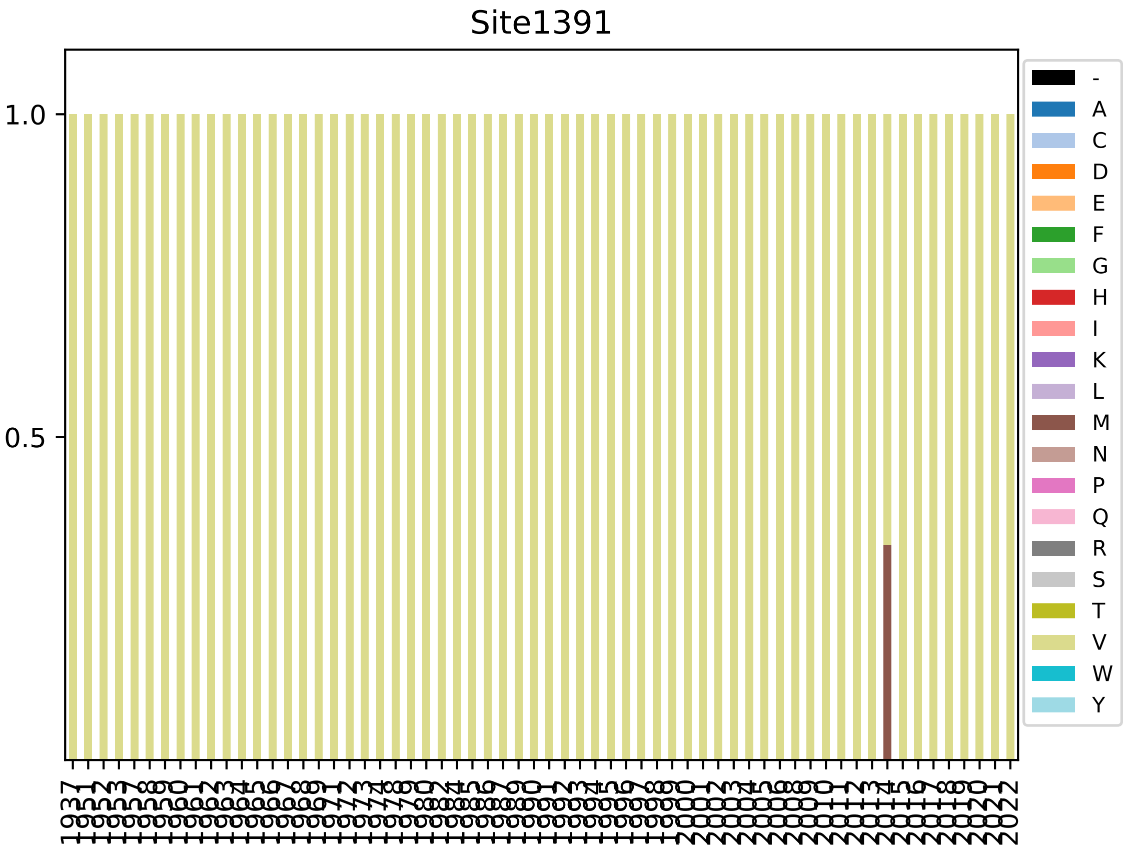
Site1391
1.0
0.5
-
A
C
D
E
F
G
H
I
K
L
M
N
P
Q
R
S
T
V
W
Y

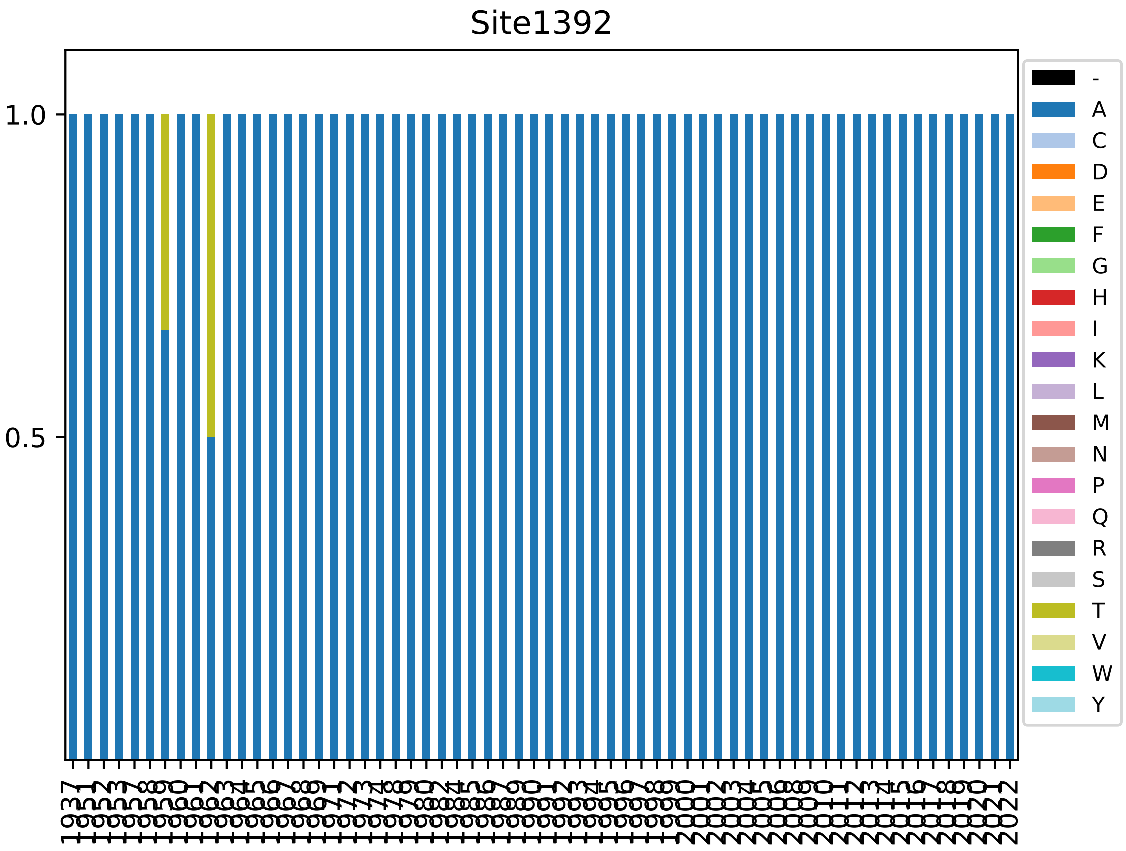

Site1392
1.0
0.5
-
A
C
D
E
F
G
H
I
K
L
M
N
P
Q
R
S
T
V
W
Y

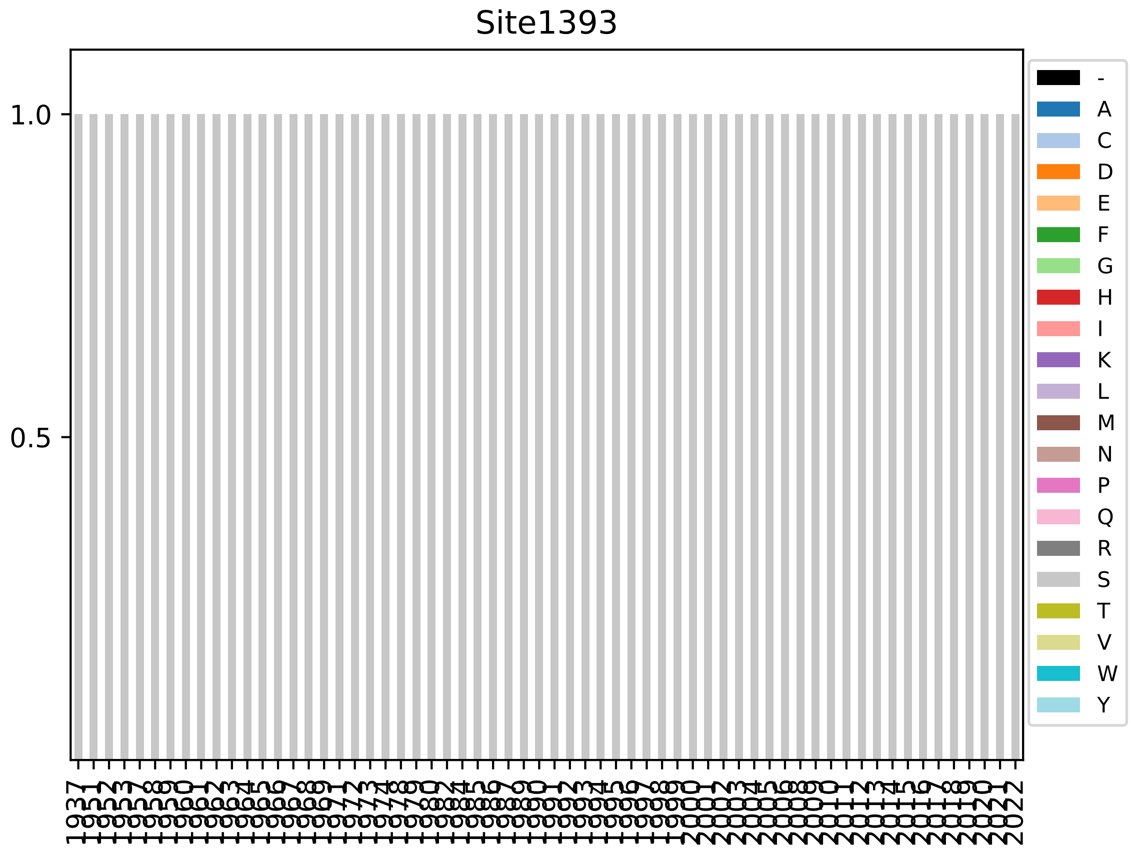
Site1393
1.0
0.5
-
A
C
D
E
F
G
H
I
K
L
M
N
P
Q
R
S
T
V
W
Y

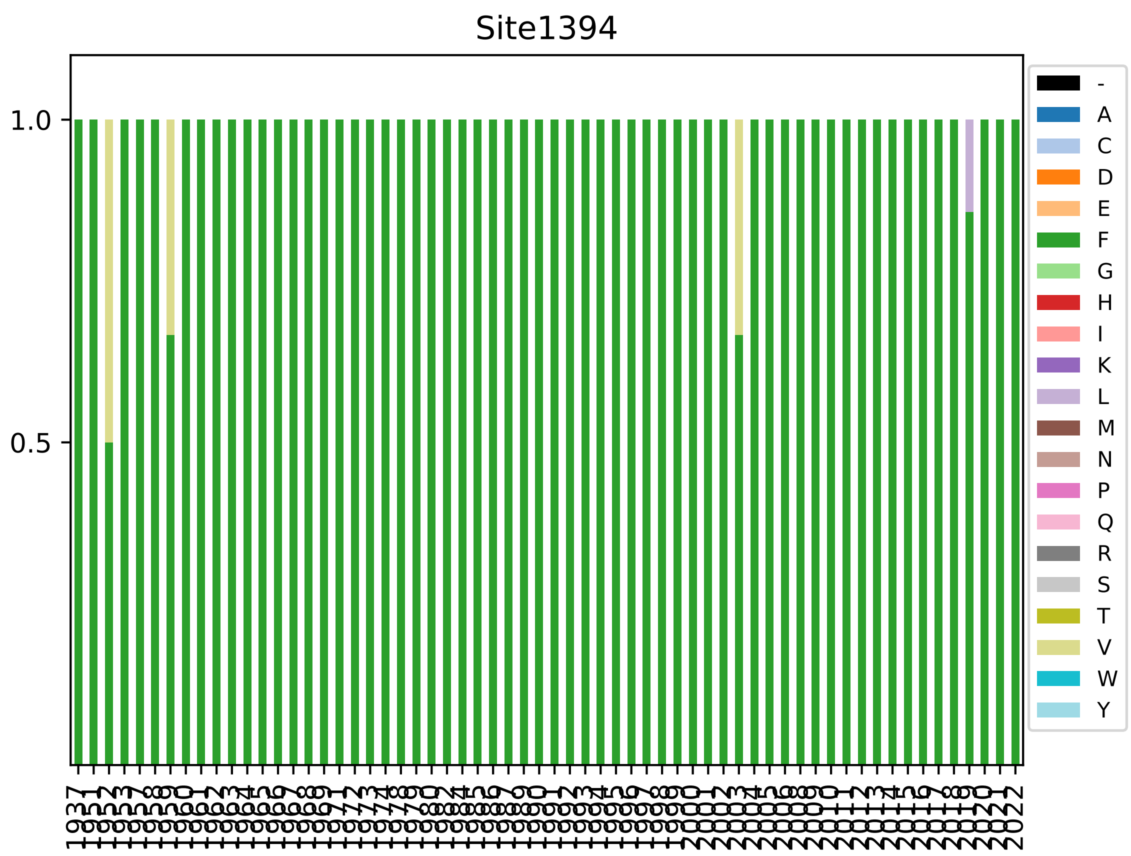
Site1394
1.0
0.5
-
A
C
D
E
F
G
H
I
K
L
M
N
P
Q
R
S
T
V
W
Y

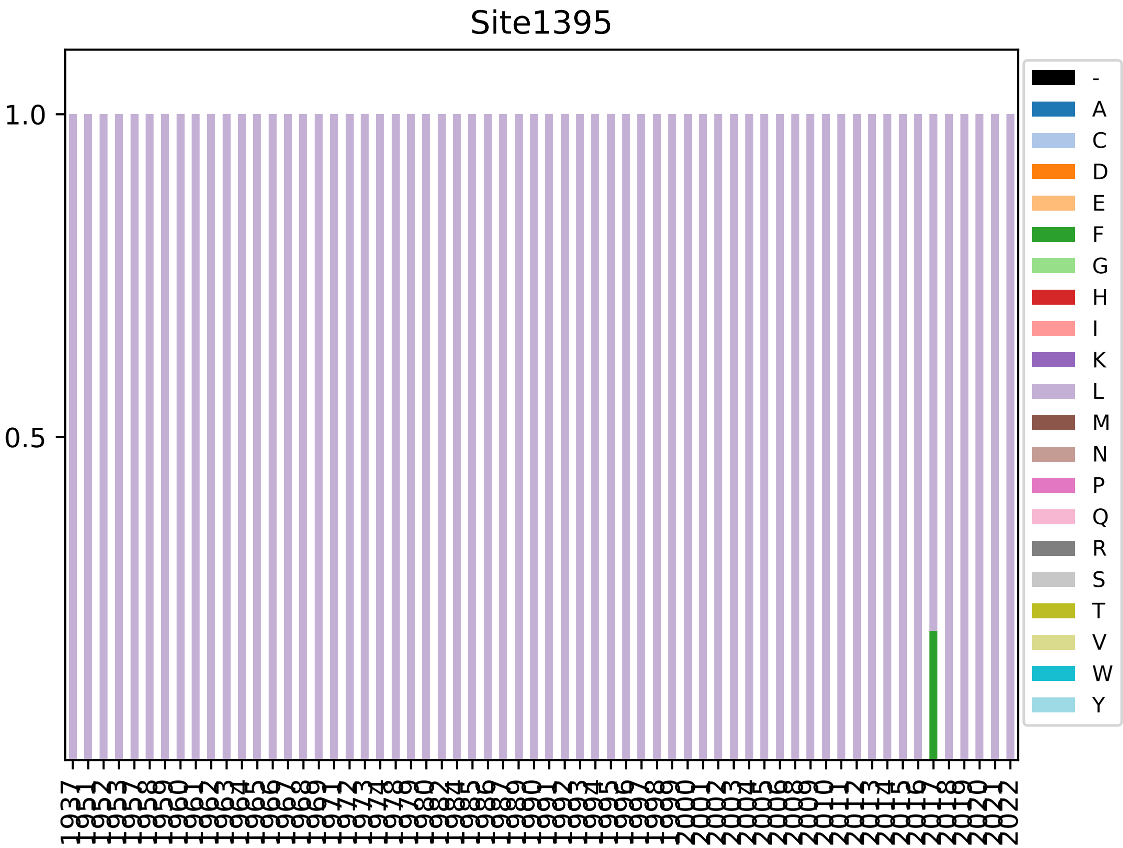
Site1395
1.0
0.5
-
A
C
D
E
F
G
H
I
K
L
M
N
P
Q
R
S
T
V
W
Y

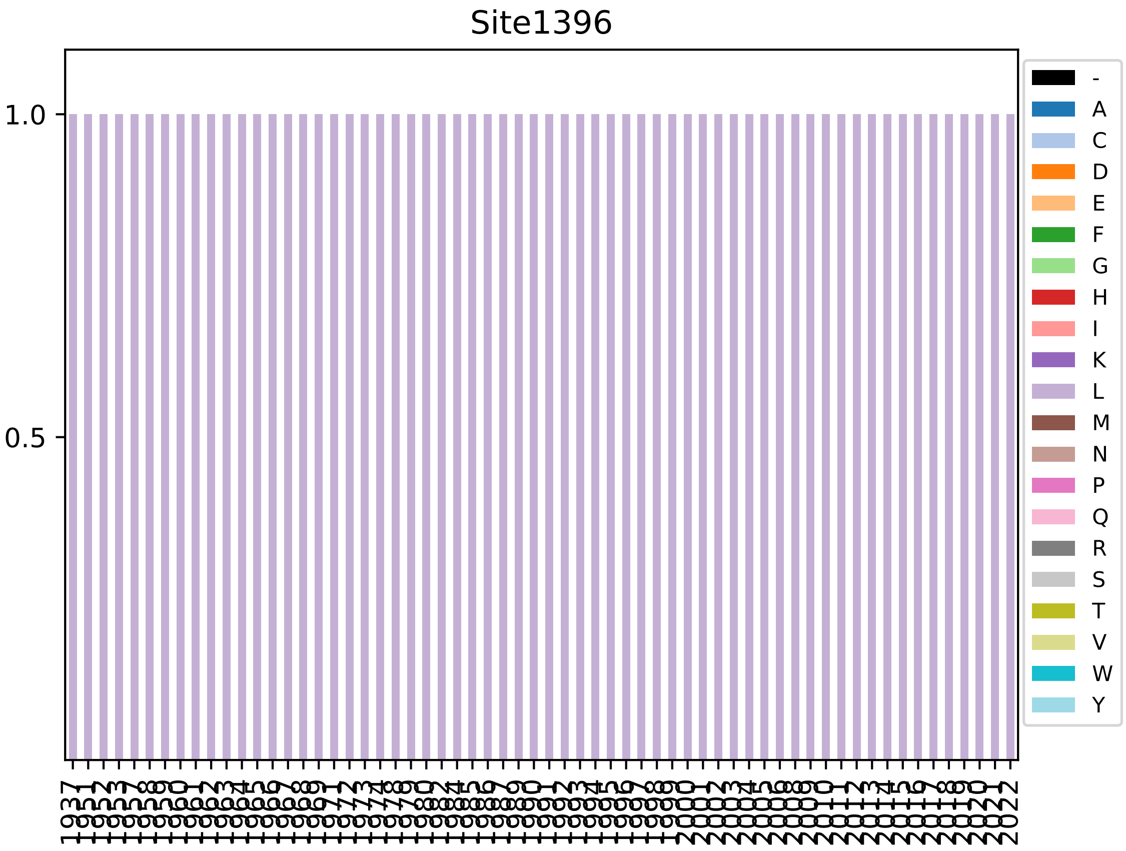

Site1396
1.0
0.5
-
A
C
D
E
F
G
H
I
K
L
M
N
P
Q
R
S
T
V
W
Y

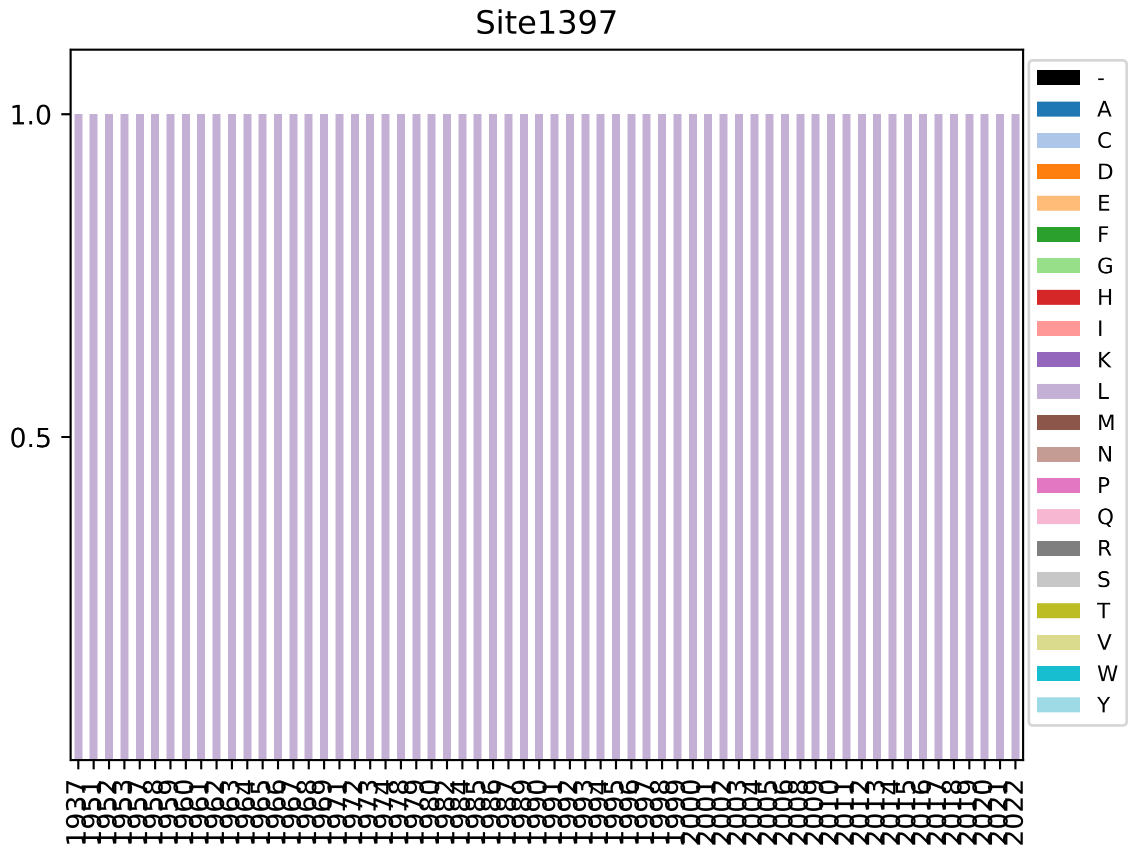
Site1397
1.0
0.5
-
A
C
D
E
F
G
H
I
K
L
M
N
P
Q
R
S
T
V
W
Y

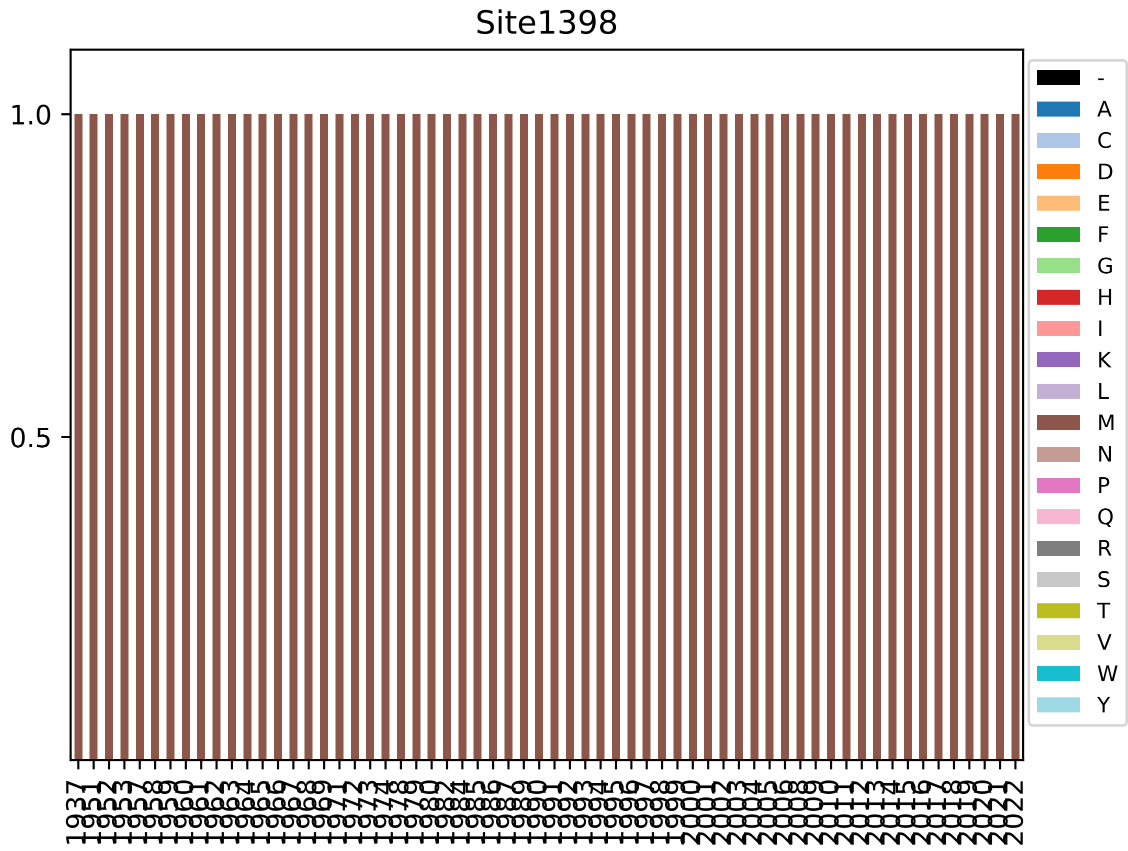
Site1398
1.0
0.5
-
A
C
D
E
F
G
H
I
K
L
M
N
P
Q
R
S
T
V
W
Y

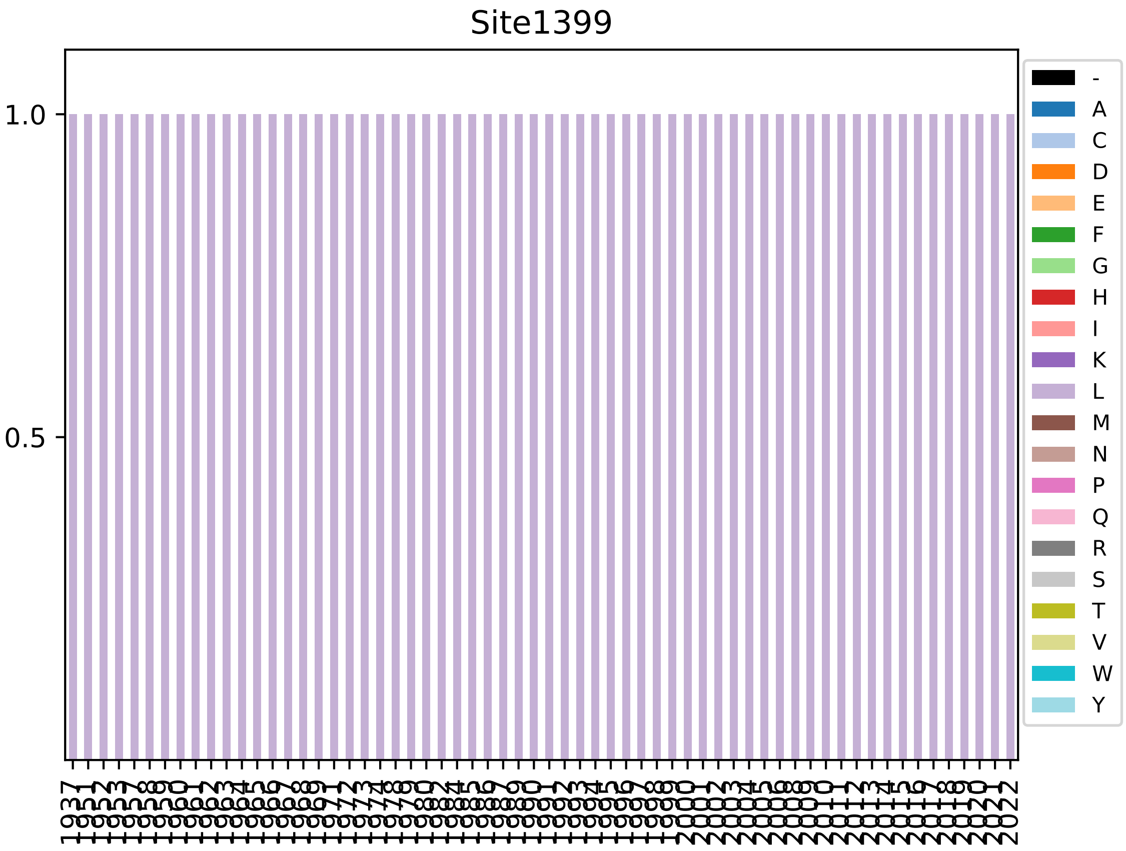
Site1399
1.0
0.5
-
A
C
D
E
F
G
H
I
K
L
M
N
P
Q
R
S
T
V
W
Y

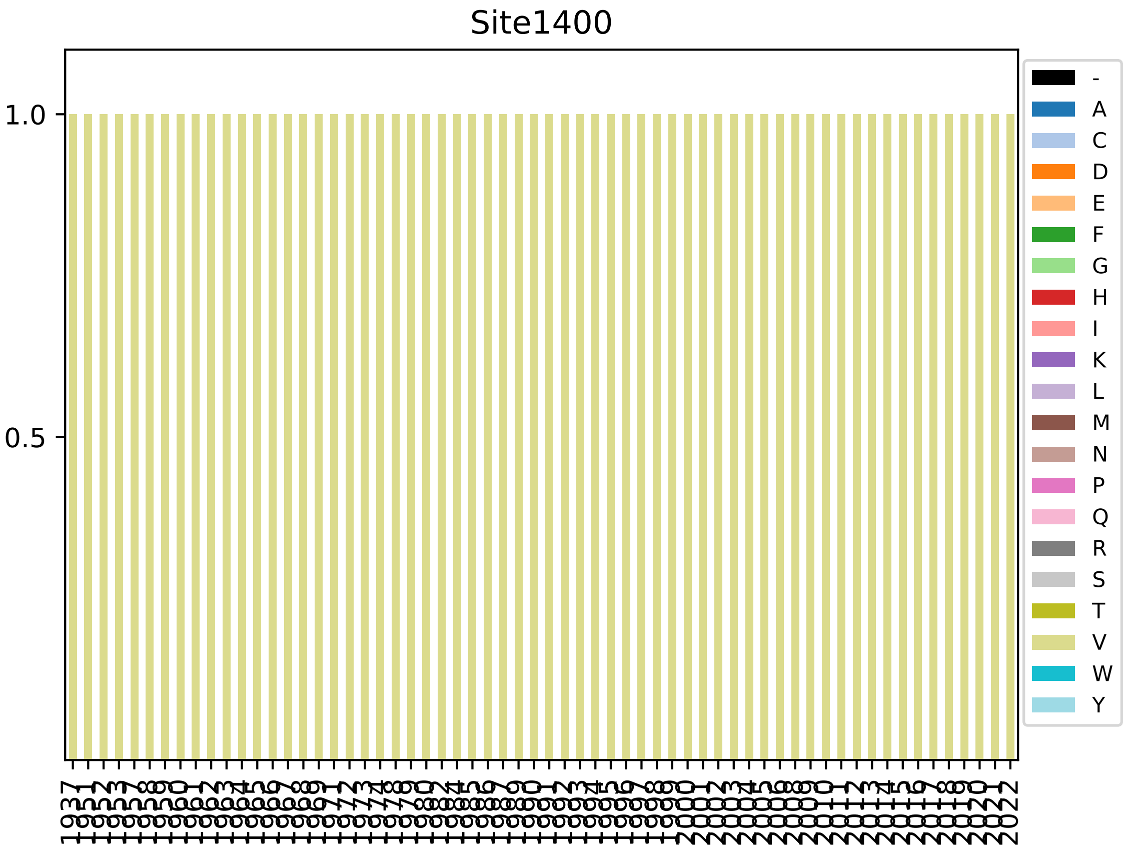
Site1400
1.0
0.5
-
A
C
D
E
F
G
H
I
K
L
M
N
P
Q
R
S
T
V
W
Y

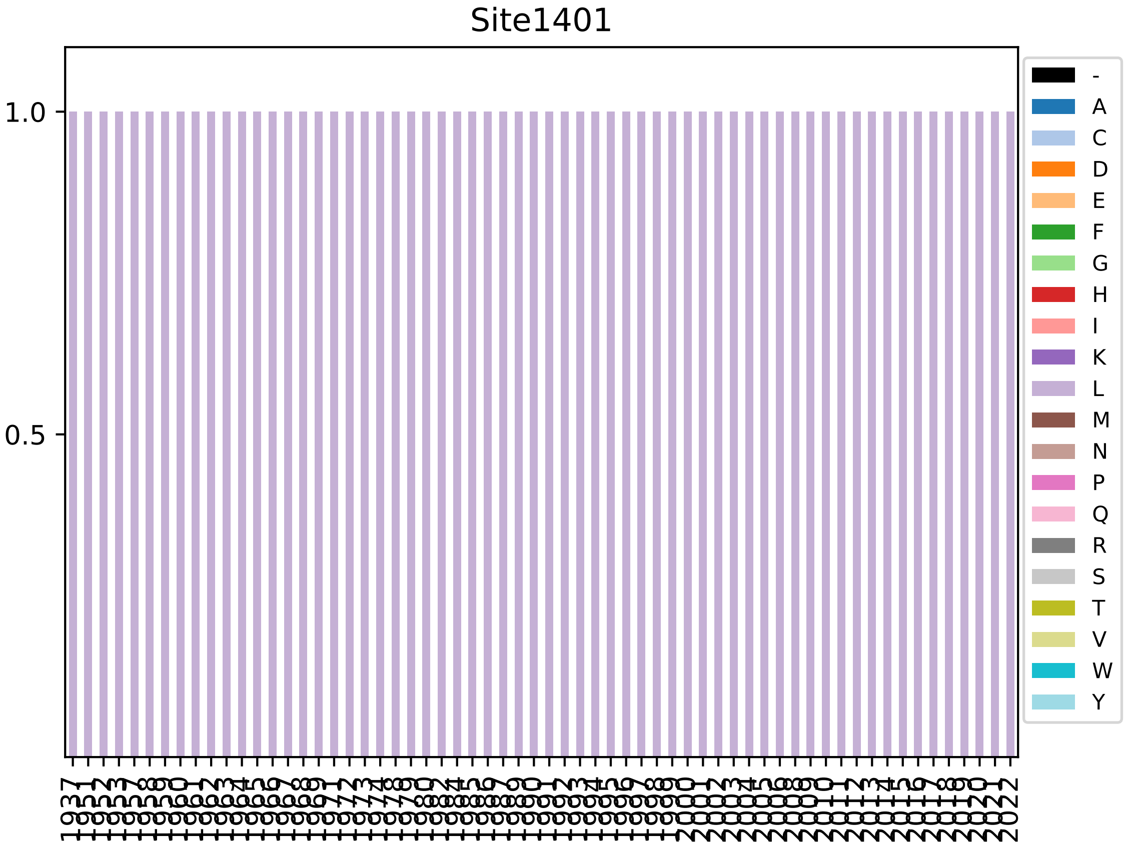
Site1401
1.0
0.5
-
A
C
D
E
F
G
H
I
K
L
M
N
P
Q
R
S
T
V
W
Y

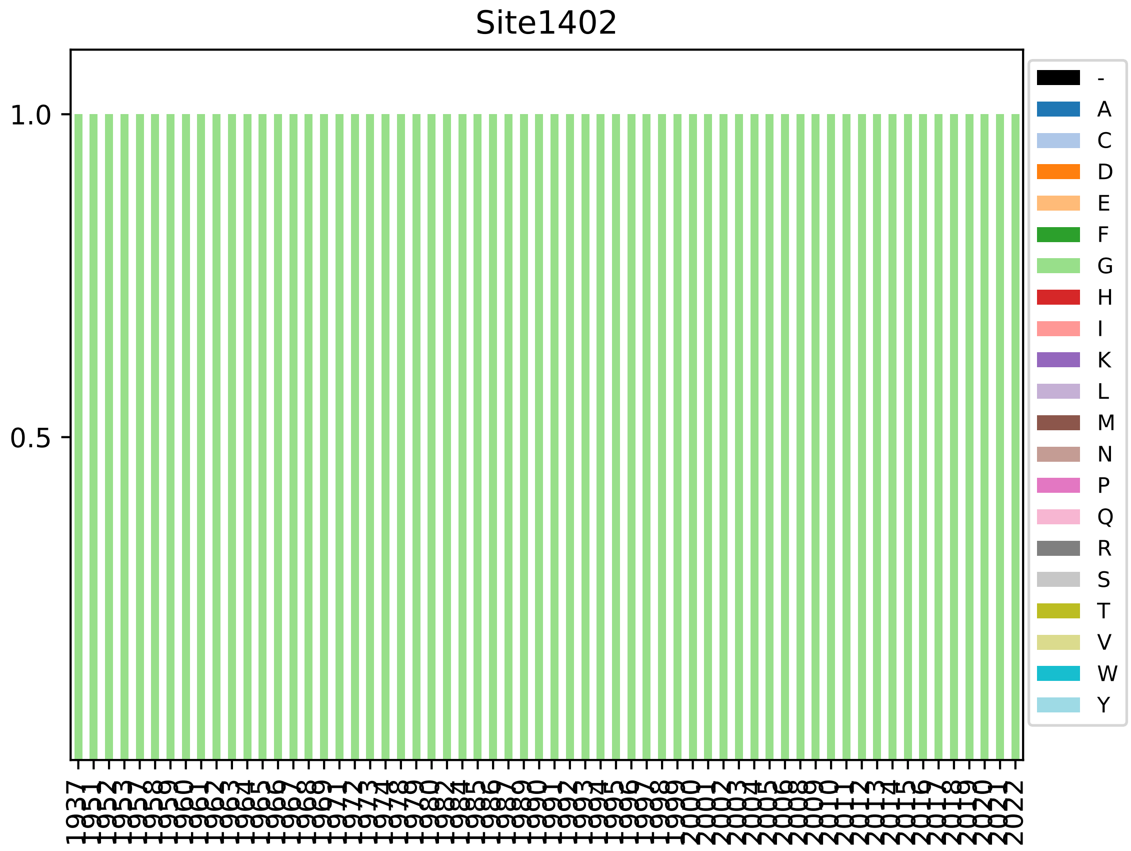
Site1402
1.0
0.5
-
A
C
D
E
F
G
H
I
K
L
M
N
P
Q
R
S
T
V
W
Y

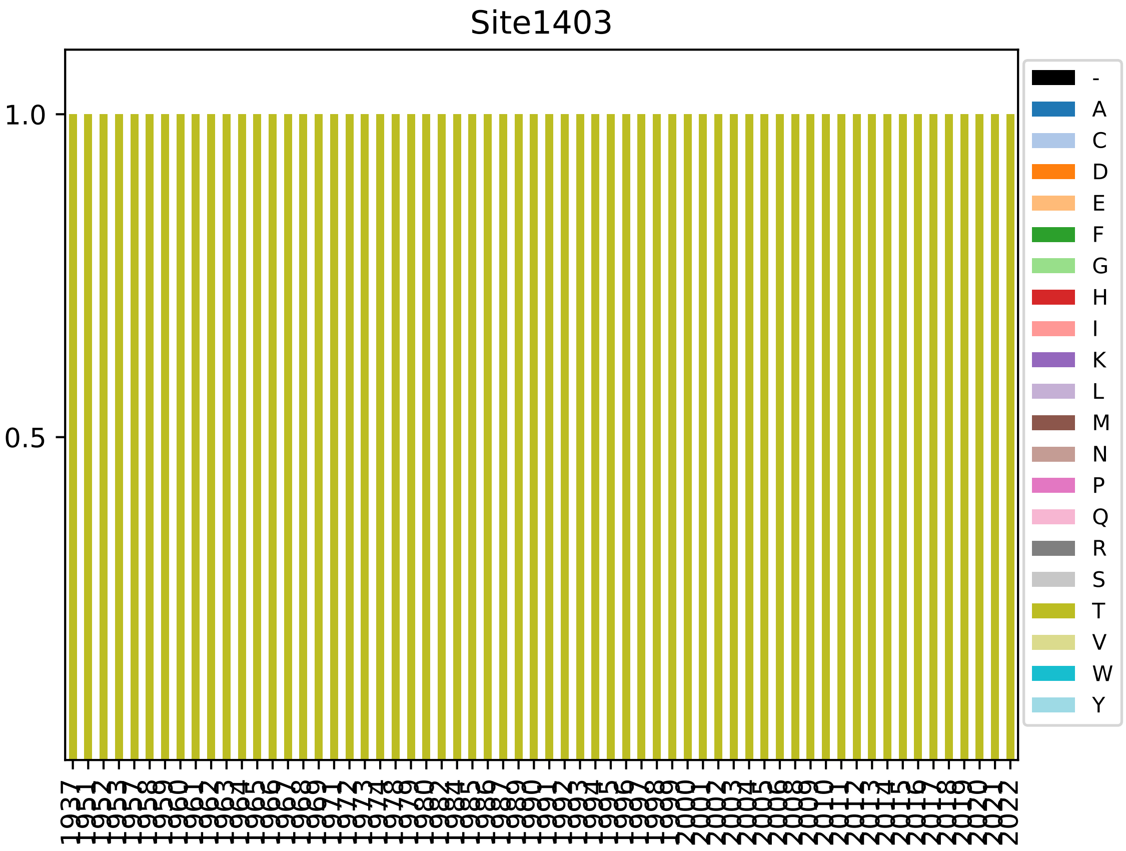
Site1403
1.0
0.5
-
A
C
D
E
F
G
H
I
K
L
M
N
P
Q
R
S
T
V
W
Y

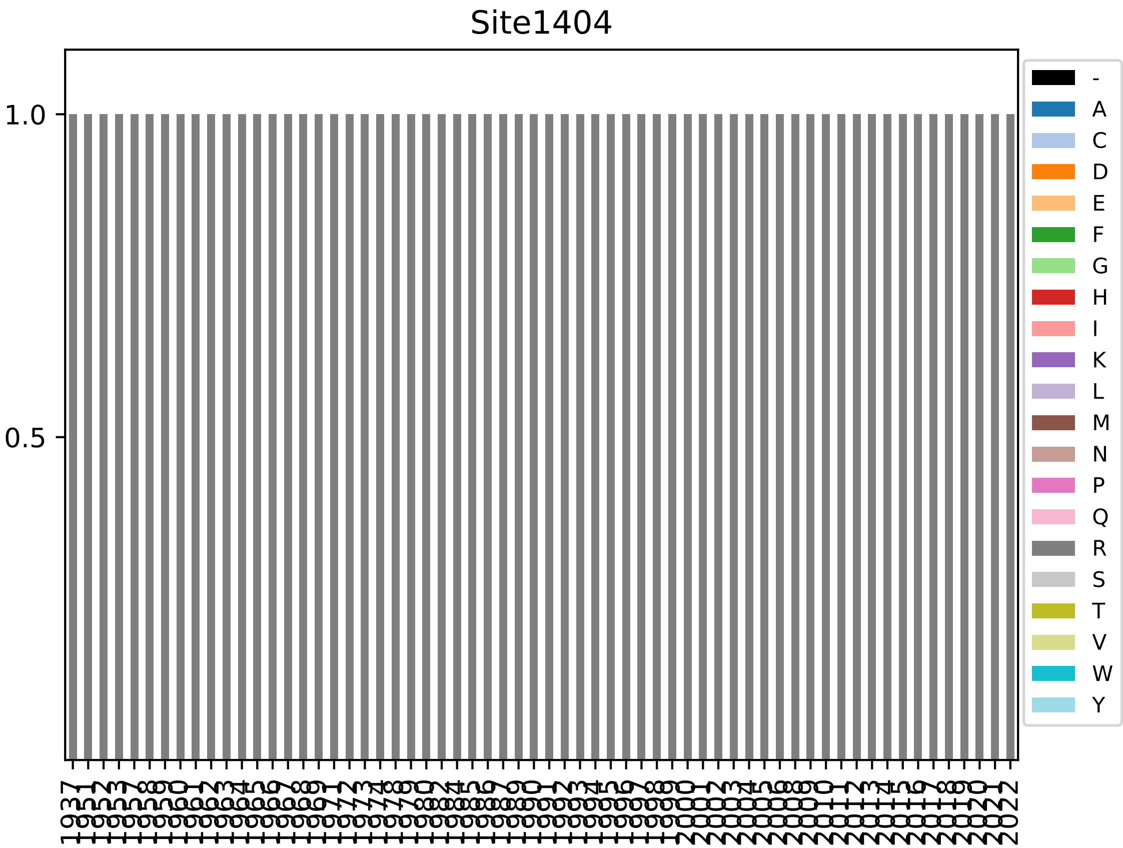
Site1404
1.0
0.5
-
A
C
D
E
F
G
H
I
K
L
M
N
P
Q
R
S
T
V
W
Y

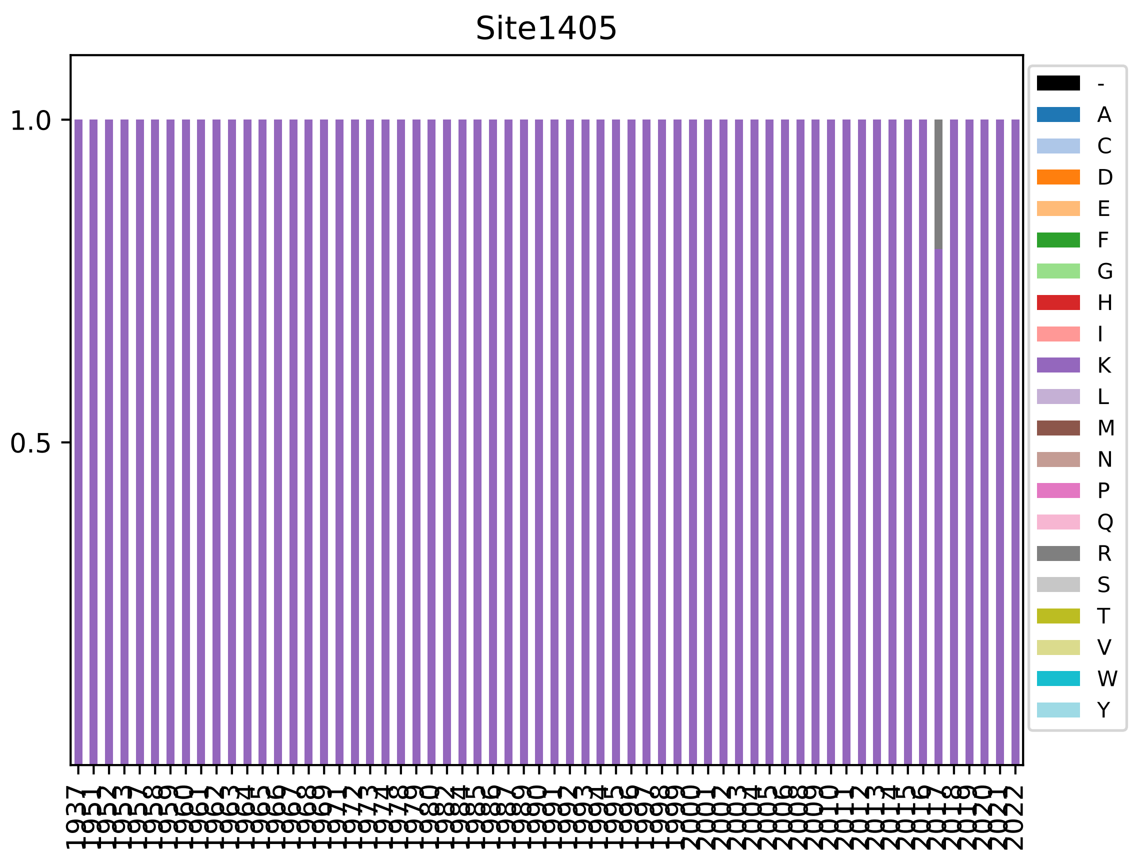
Site1405
1.0
0.5
-
A
C
D
E
F
G
H
I
K
L
M
N
P
Q
R
S
T
V
W
Y

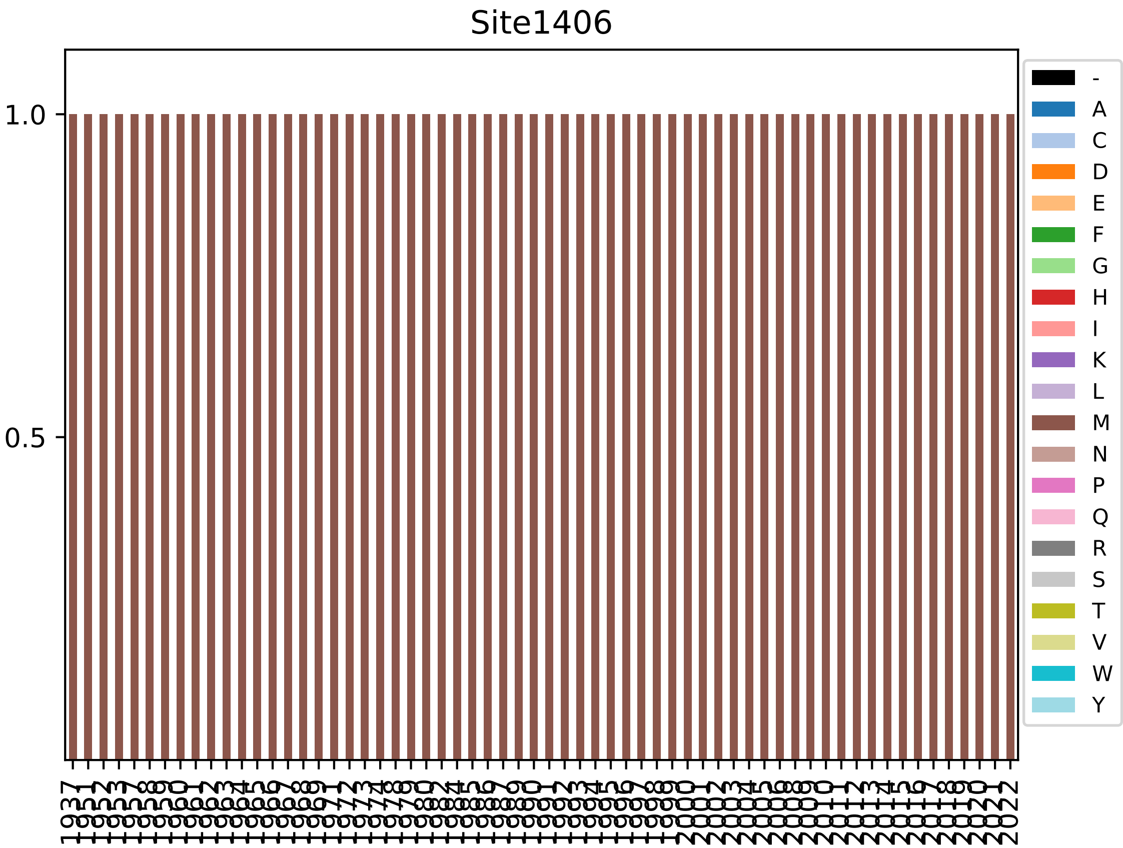
Site1406
1.0
0.5
-
A
C
D
E
F
G
H
I
K
L
M
N
P
Q
R
S
T
V
W
Y

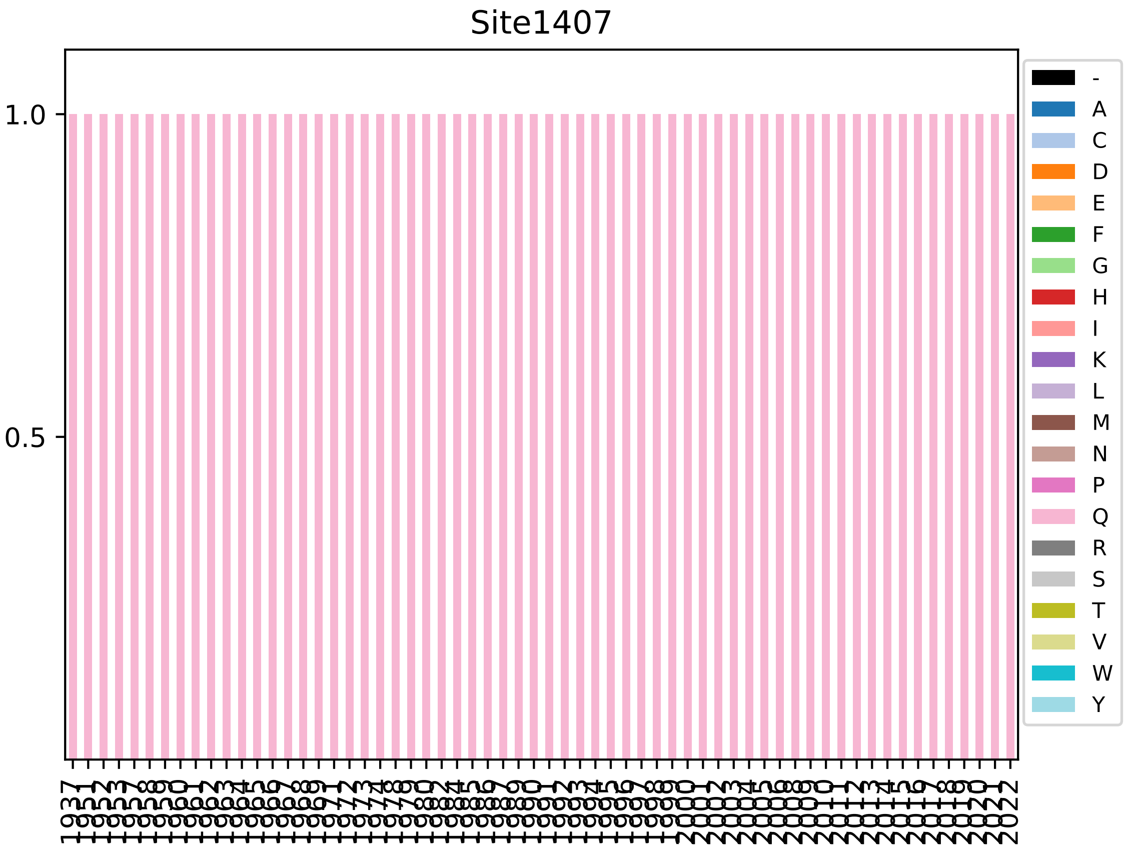
Site1407
1.0
0.5
-
A
C
D
E
F
G
H
I
K
L
M
N
P
Q
R
S
T
V
W
Y

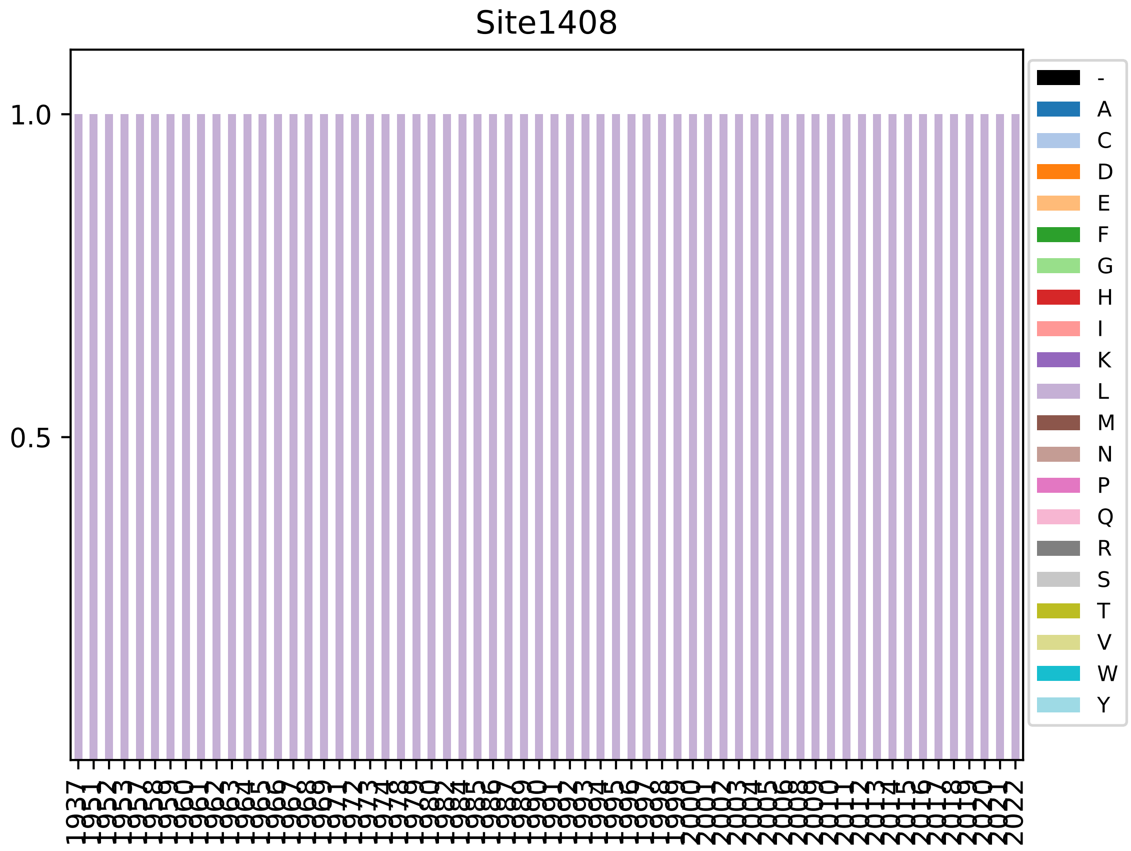
Site1408
1.0
0.5
-
A
C
D
E
F
G
H
I
K
L
M
N
P
Q
R
S
T
V
W
Y

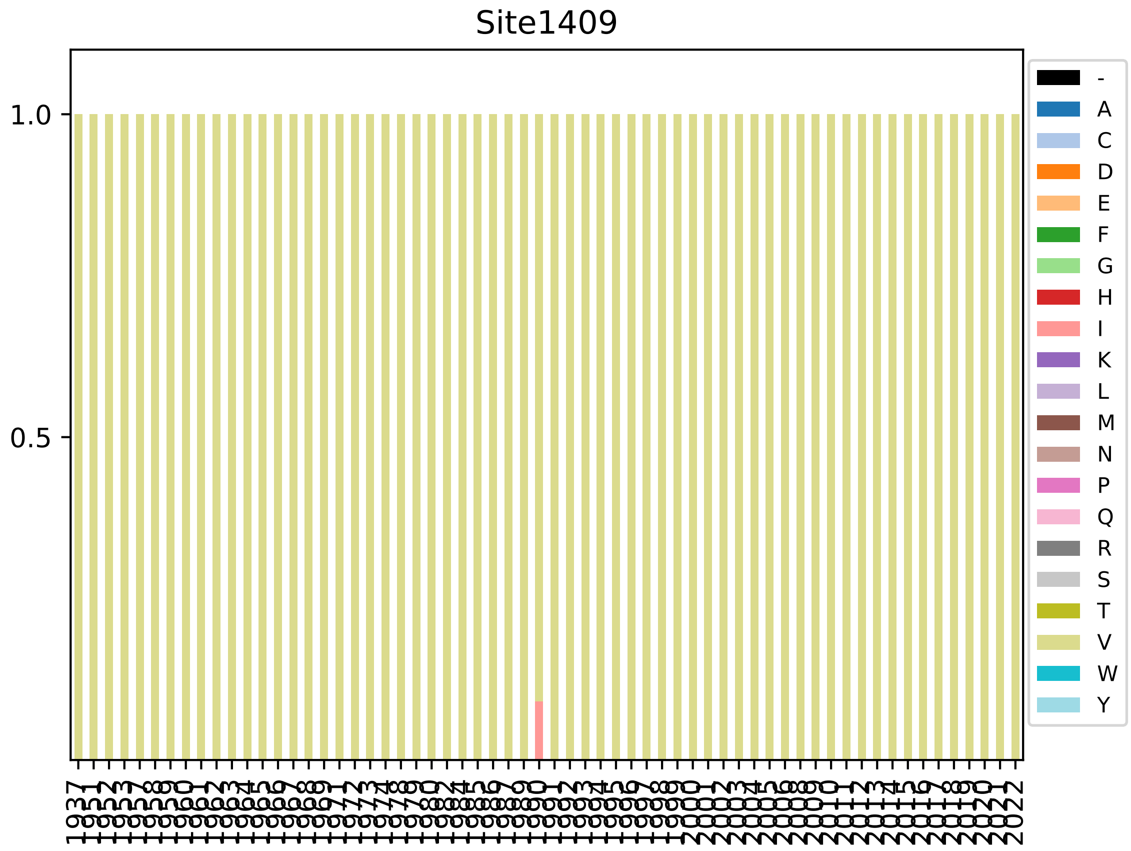
Site1409
1.0
0.5
-
A
C
D
E
F
G
H
I
K
L
M
N
P
Q
R
S
T
V
W
Y

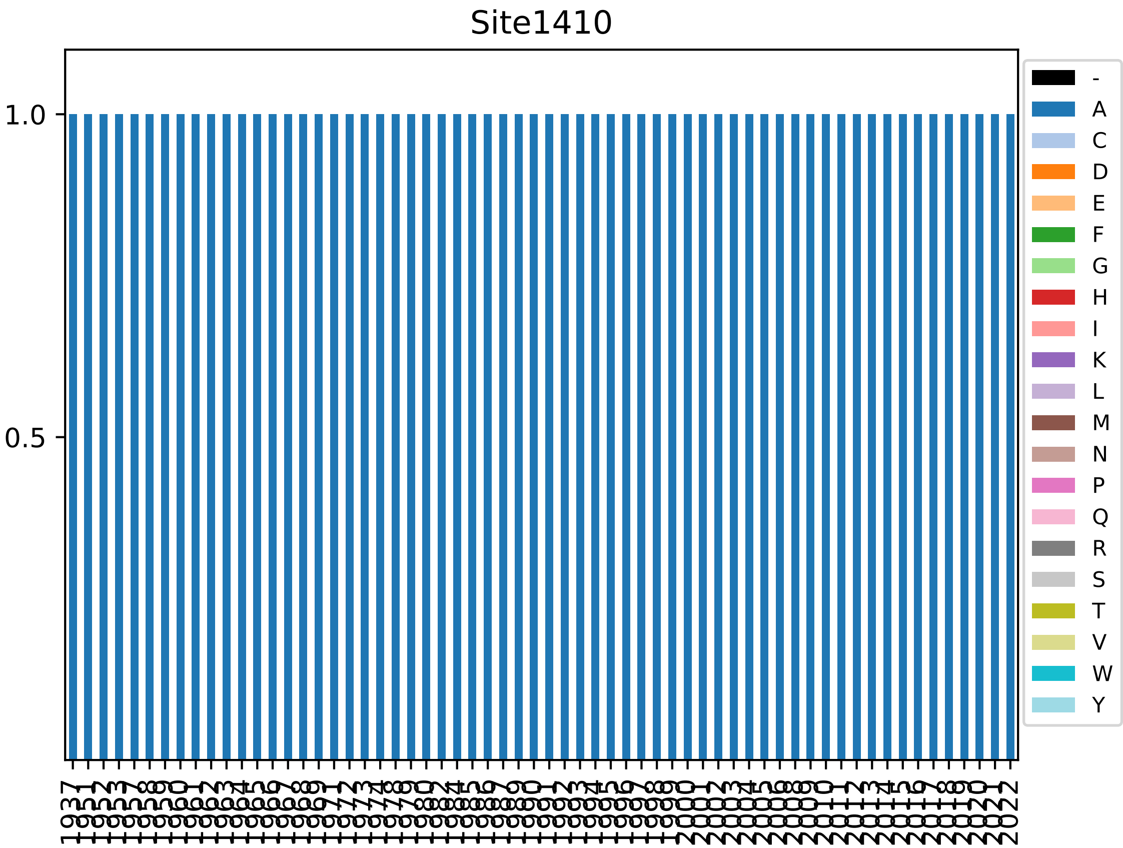
Site1410
1.0
0.5
-
A
C
D
E
F
G
H
I
K
L
M
N
P
Q
R
S
T
V
W
Y

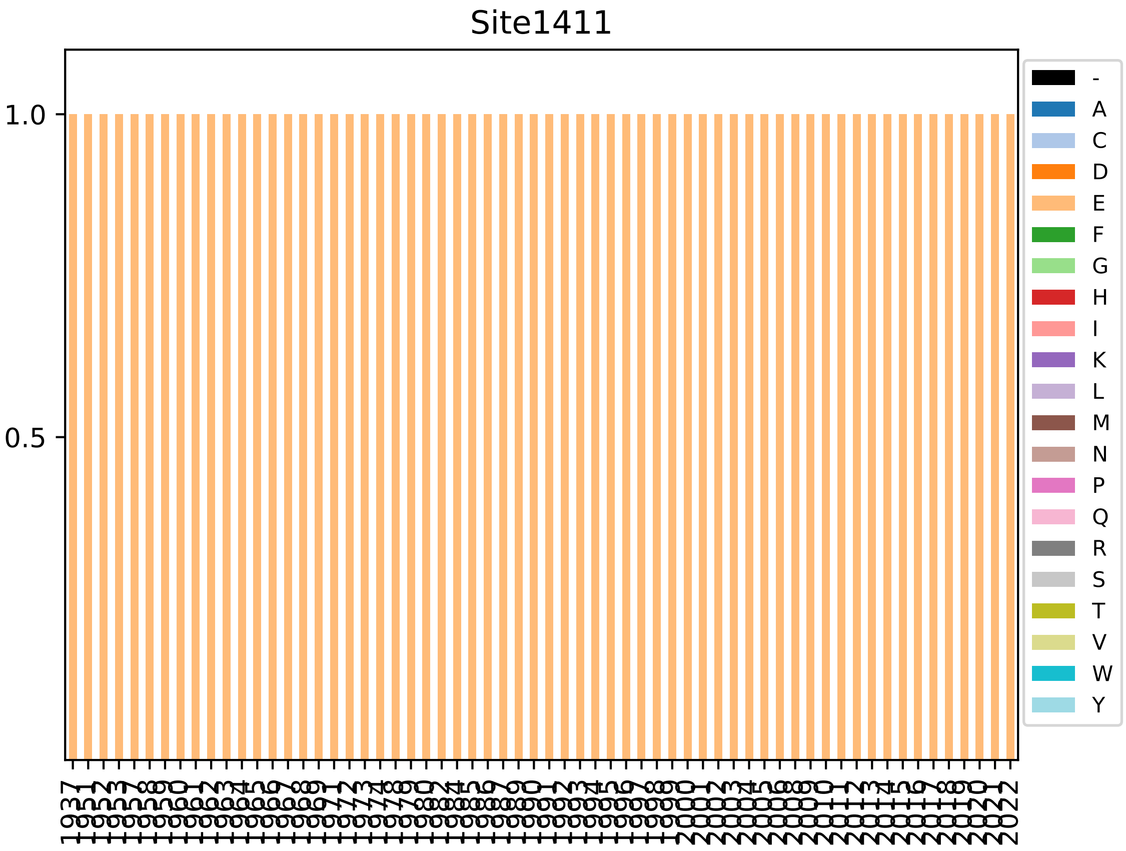
Site1411
1.0
0.5
-
A
C
D
E
F
G
H
I
K
L
M
N
P
Q
R
S
T
V
W
Y

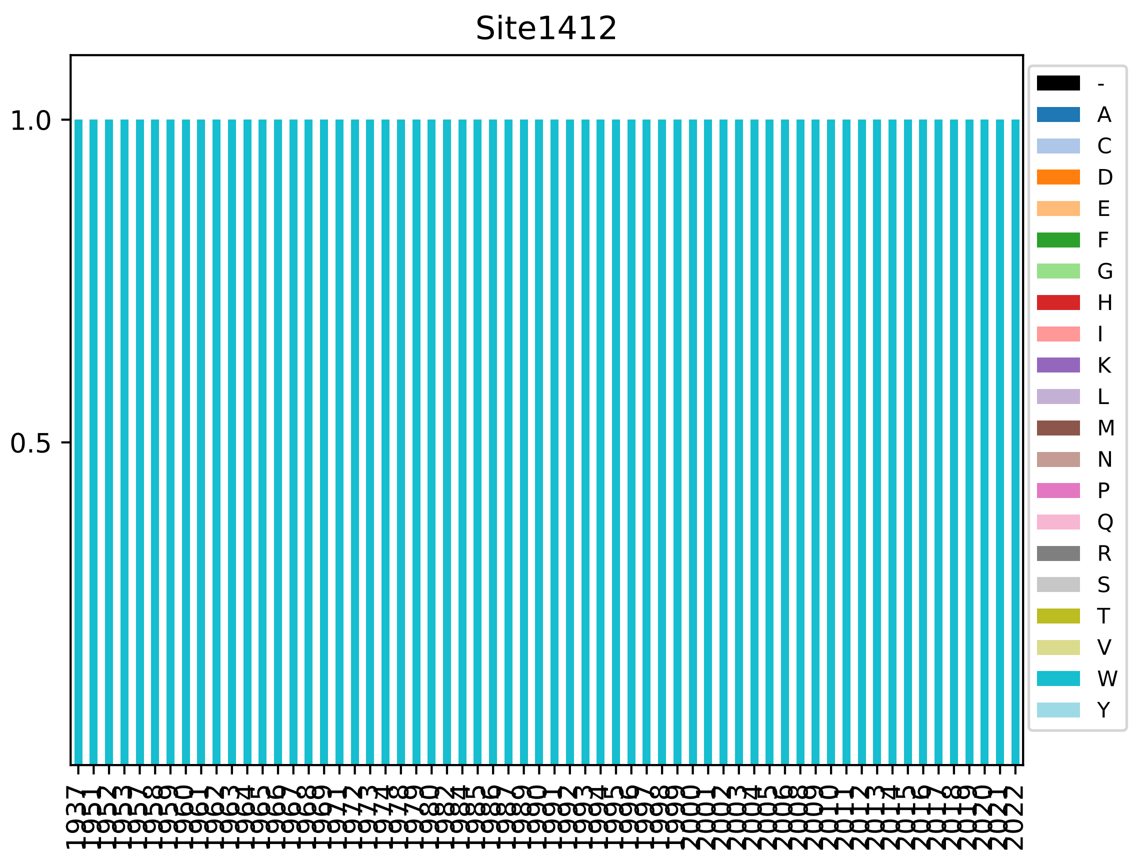

Site1412
1.0
0.5
-
A
C
D
E
F
G
H
I
K
L
M
N
P
Q
R
S
T
V
W
Y

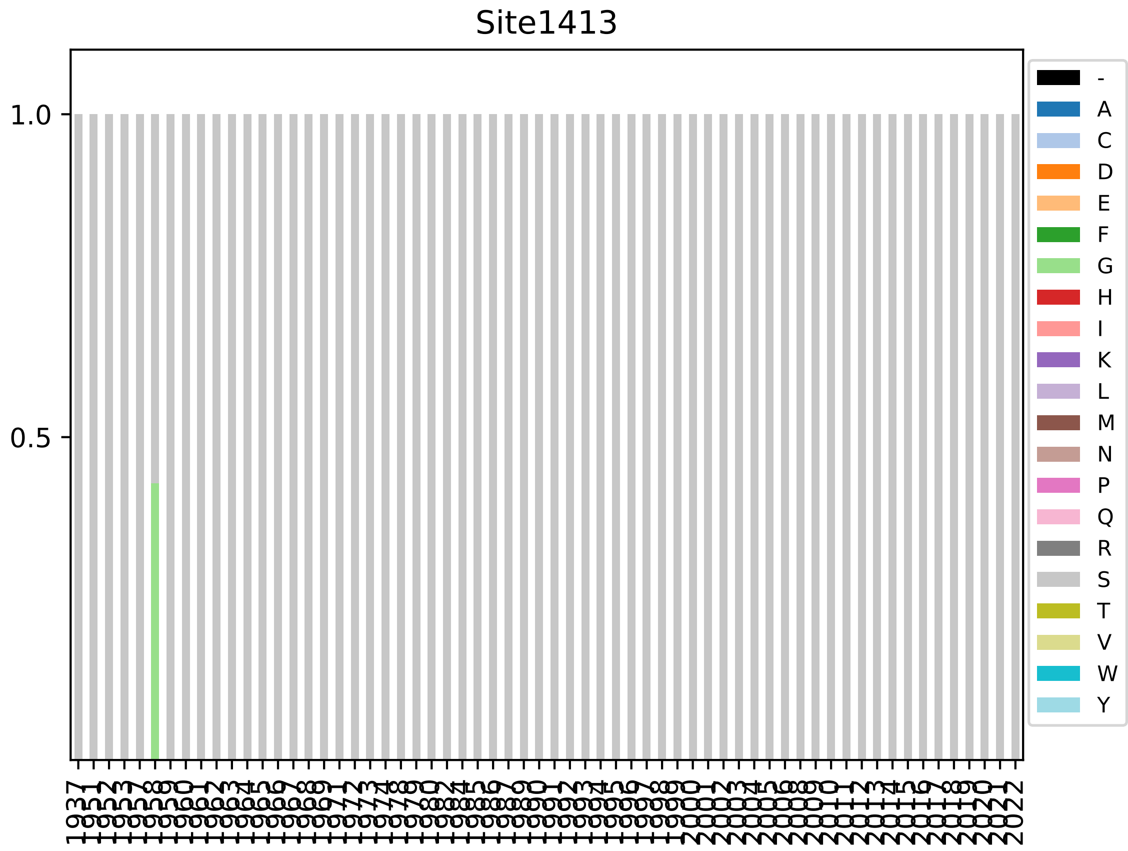
Site1413
1.0
0.5
-
A
C
D
E
F
G
H
I
K
L
M
N
P
Q
R
S
T
V
W
Y

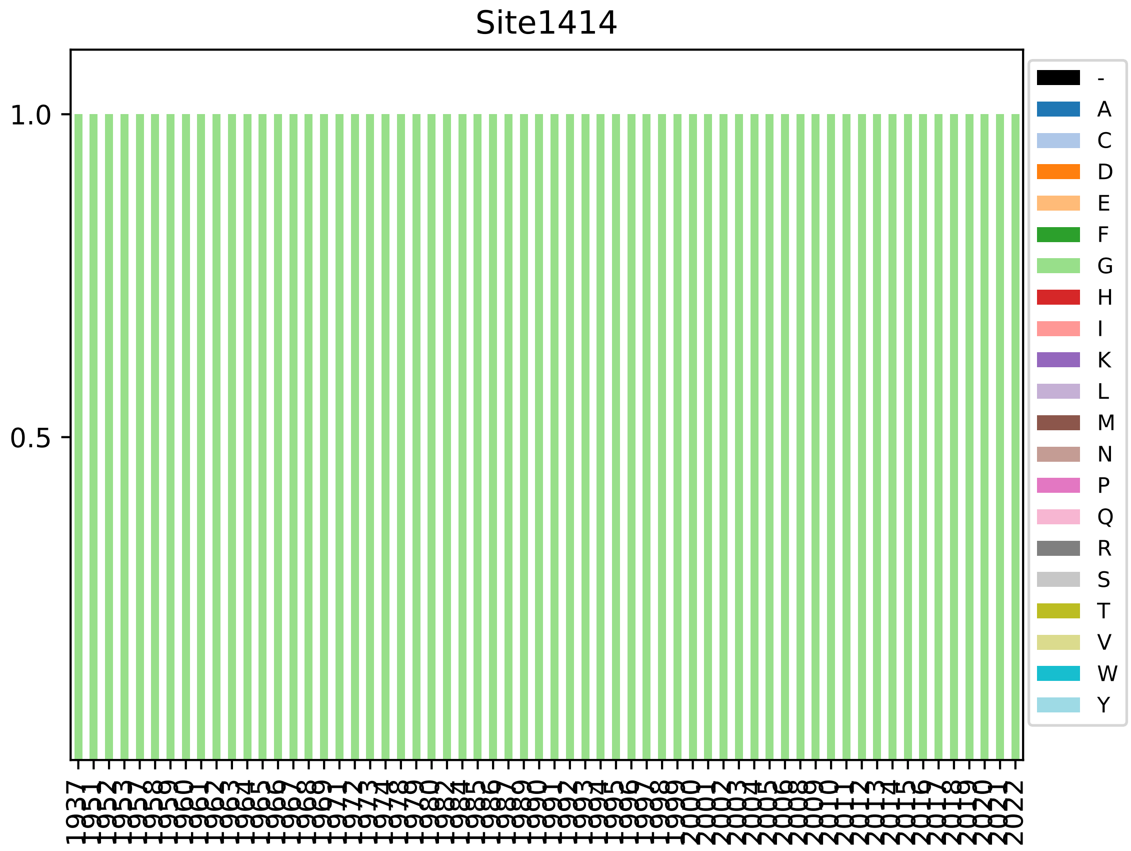
Site1414
1.0
0.5
-
A
C
D
E
F
G
H
I
K
L
M
N
P
Q
R
S
T
V
W
Y

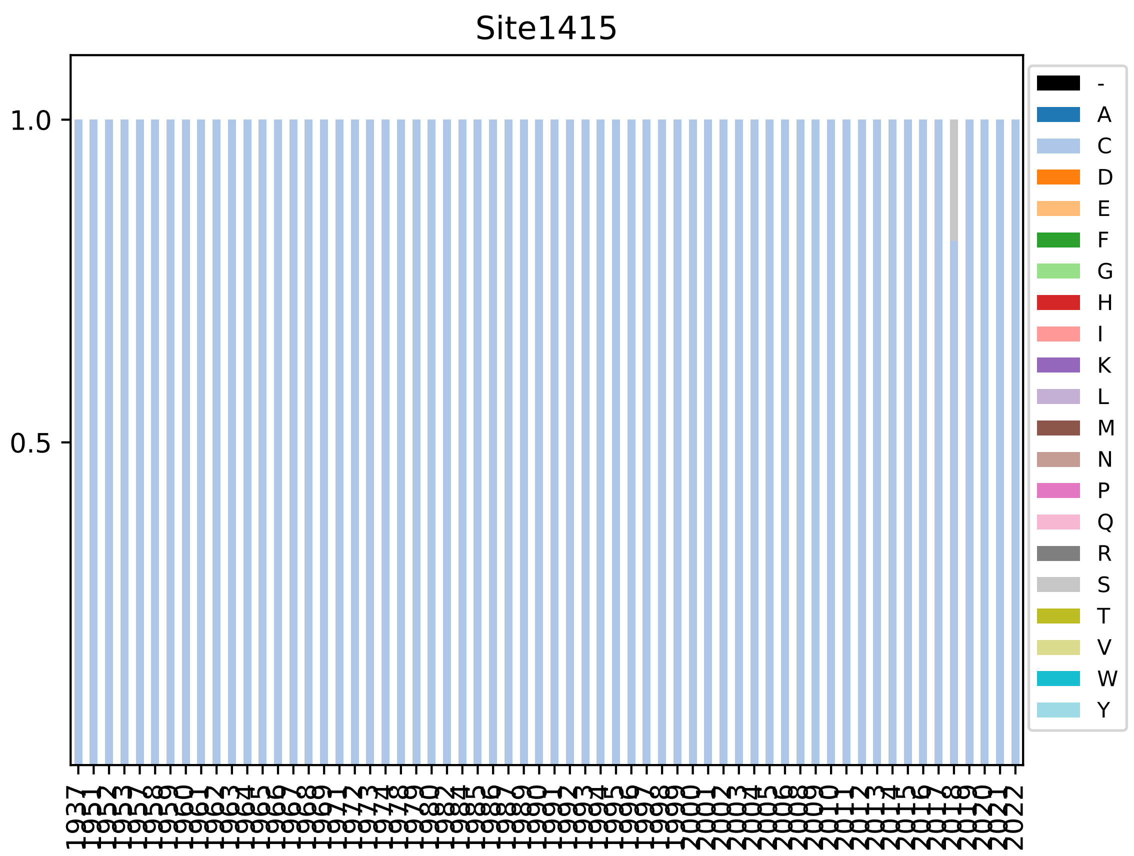
Site1415
1.0
0.5
-
A
C
D
E
F
G
H
I
K
L
M
N
P
Q
R
S
T
V
W
Y

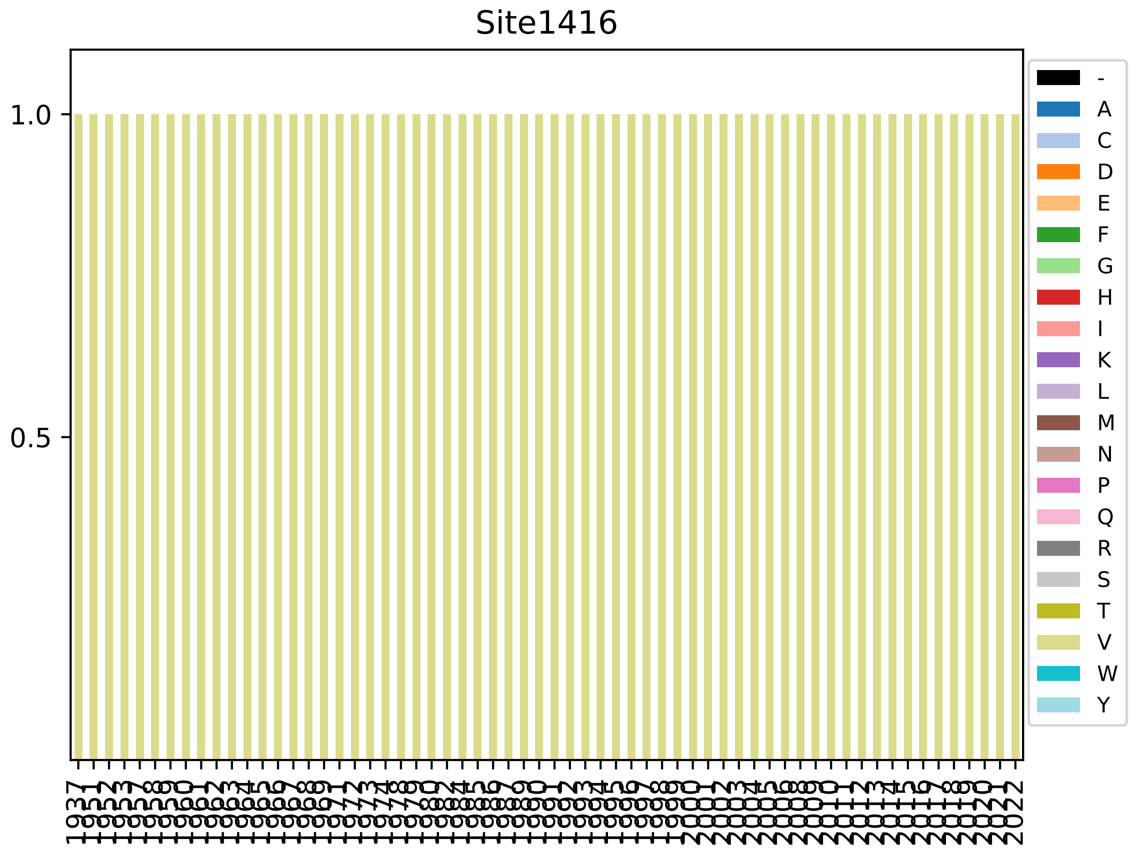
Site1416
1.0
0.5
-
A
C
D
E
F
G
H
I
K
L
M
N
P
Q
R
S
T
V
W
Y

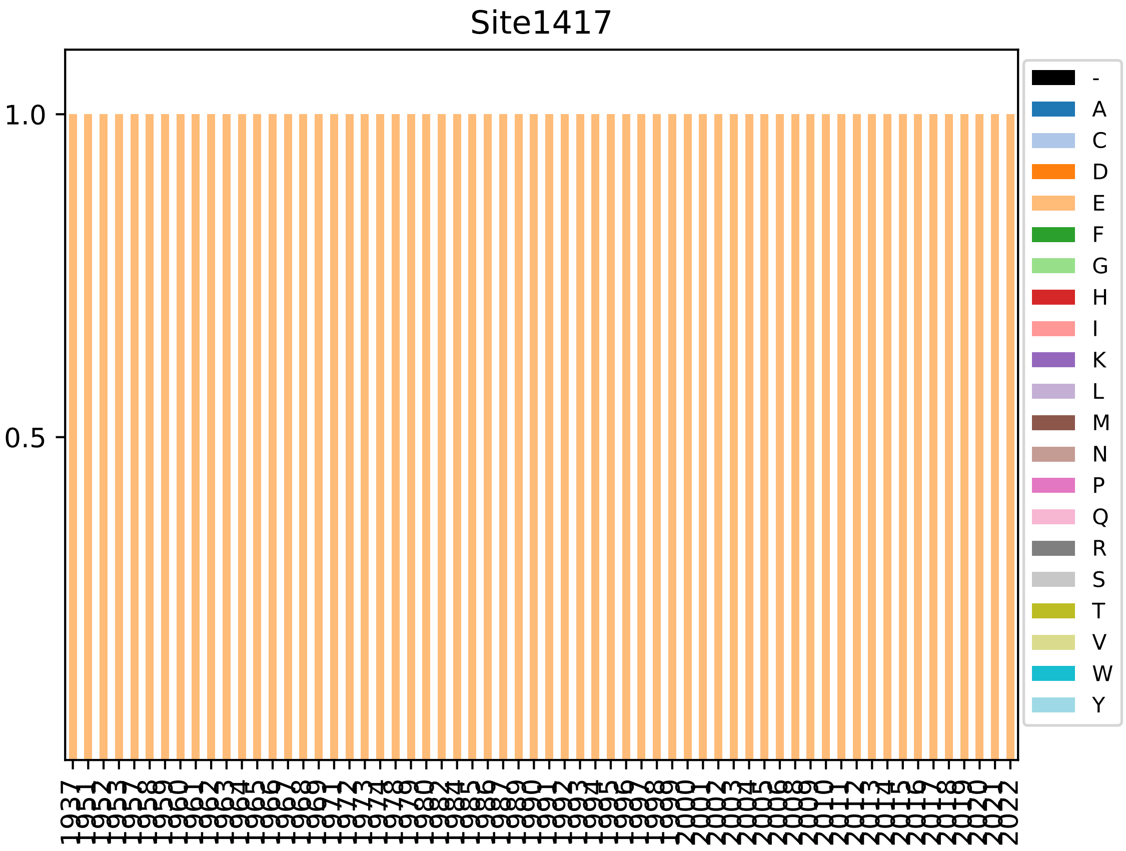

Site1417
1.0
0.5
-
A
C
D
E
F
G
H
I
K
L
M
N
P
Q
R
S
T
V
W
Y

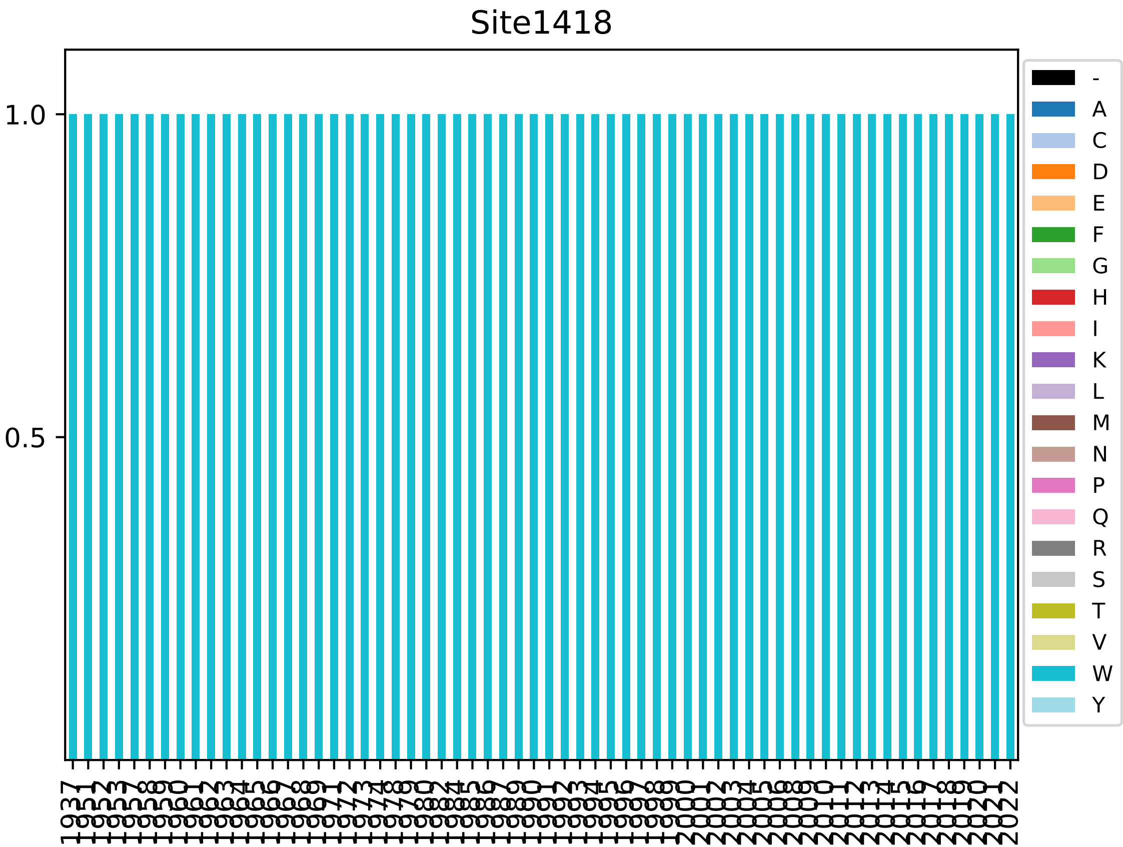
Site1418
1.0
0.5
-
A
C
D
E
F
G
H
I
K
L
M
N
P
Q
R
S
T
V
W
Y

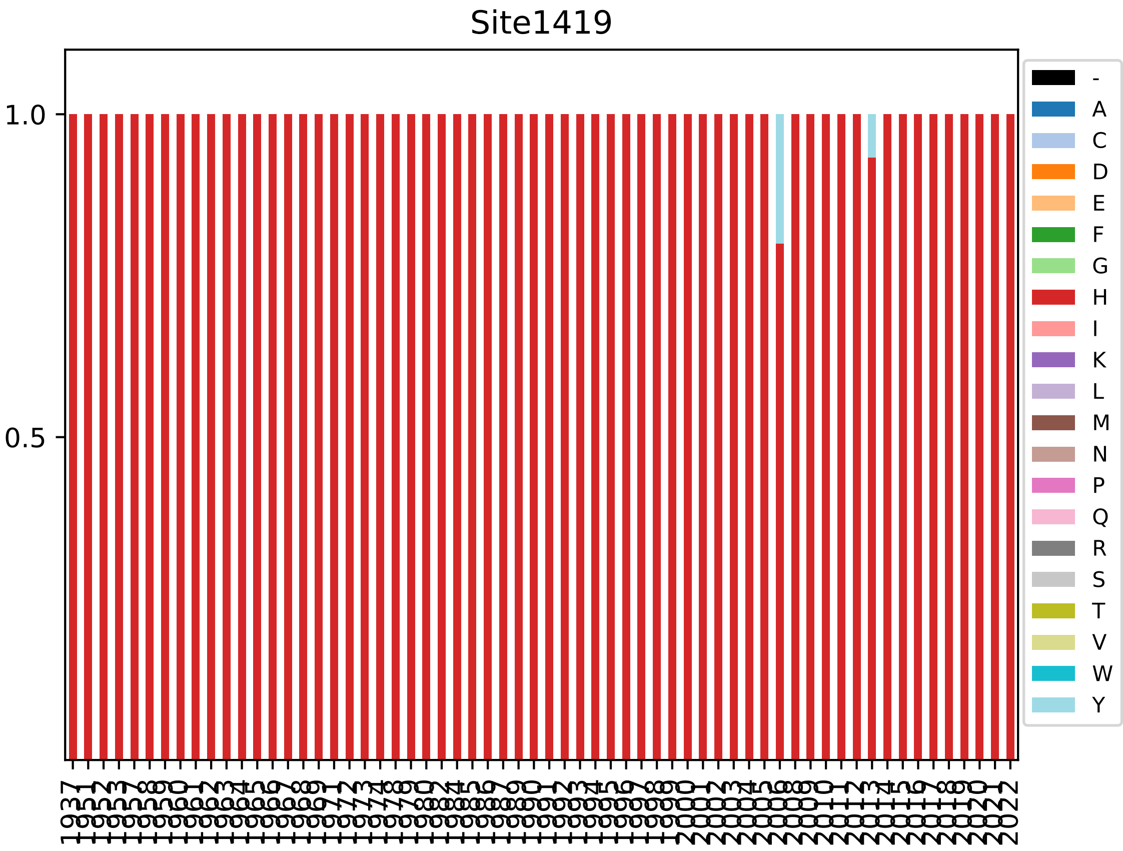
Site1419
1.0
0.5
-
A
C
D
E
F
G
H
I
K
L
M
N
P
Q
R
S
T
V
W
Y

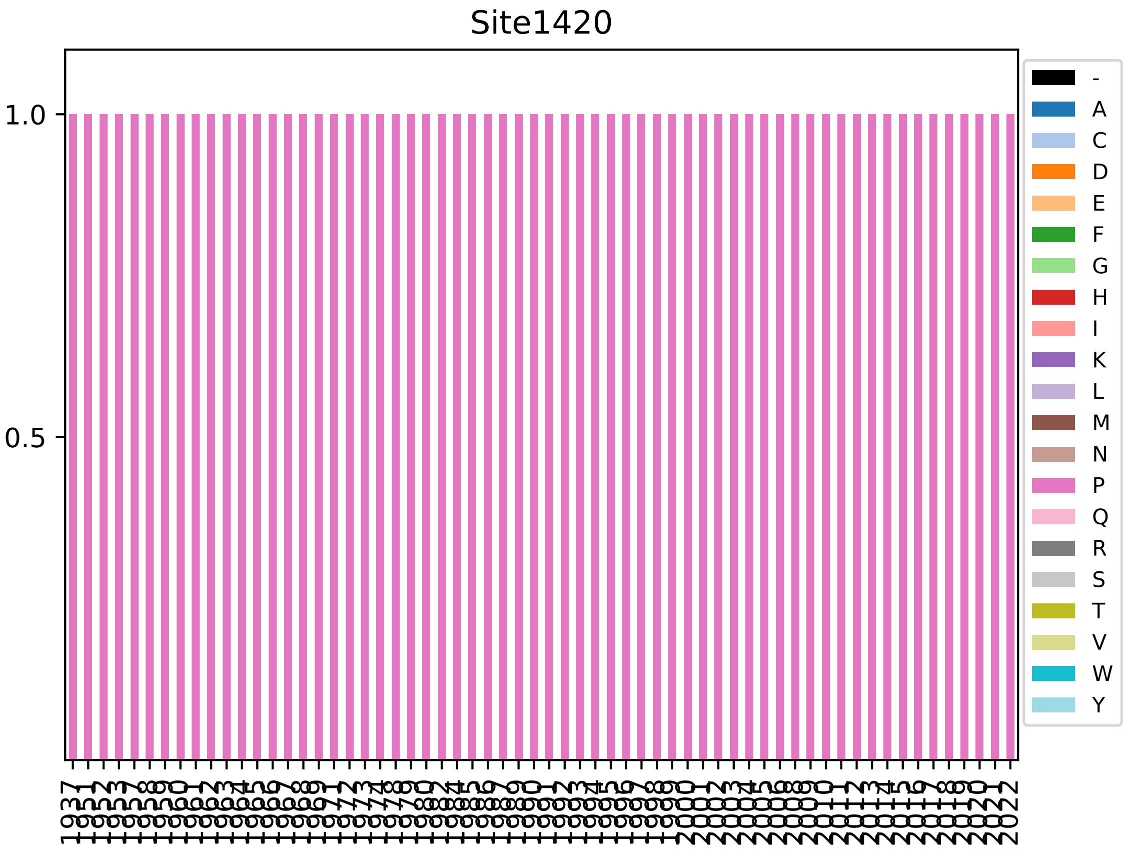

Site1420
1.0
0.5
-
A
C
D
E
F
G
H
I
K
L
M
N
P
Q
R
S
T
V
W
Y

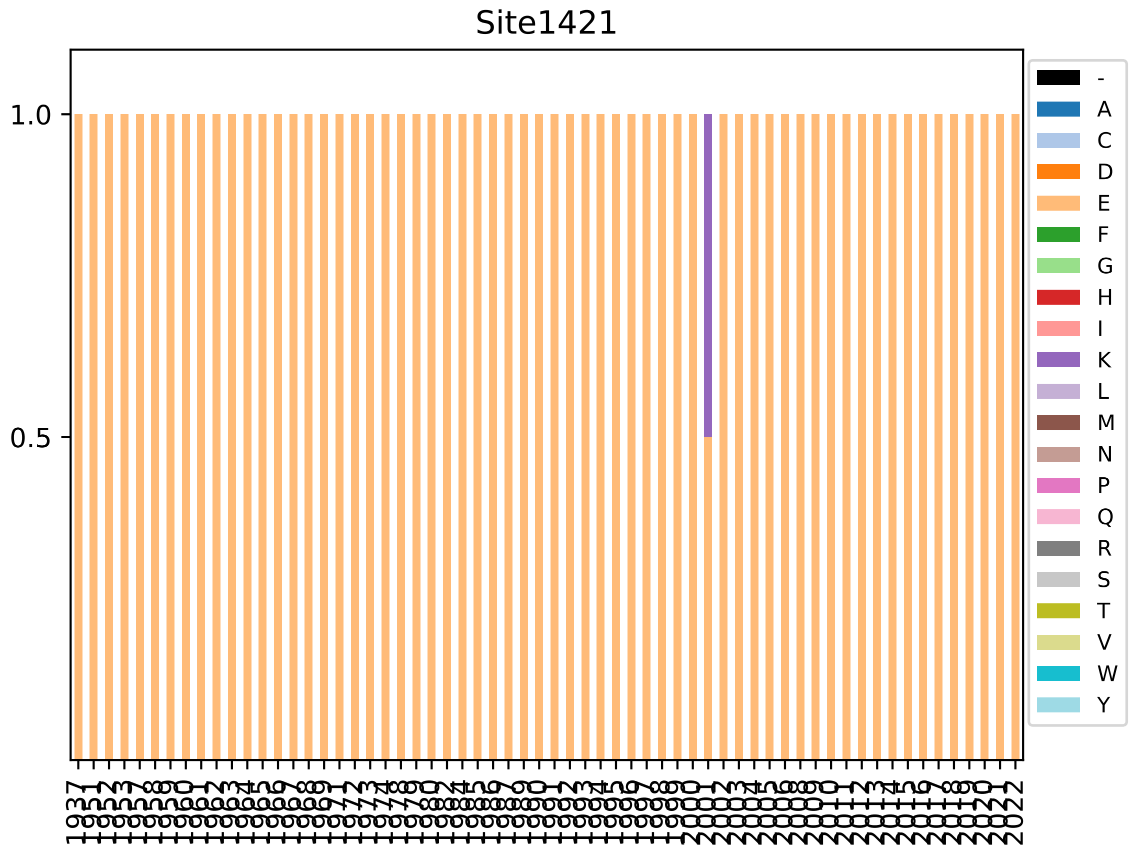
Site1421
1.0
0.5
-
A
C
D
E
F
G
H
I
K
L
M
N
P
Q
R
S
T
V
W
Y

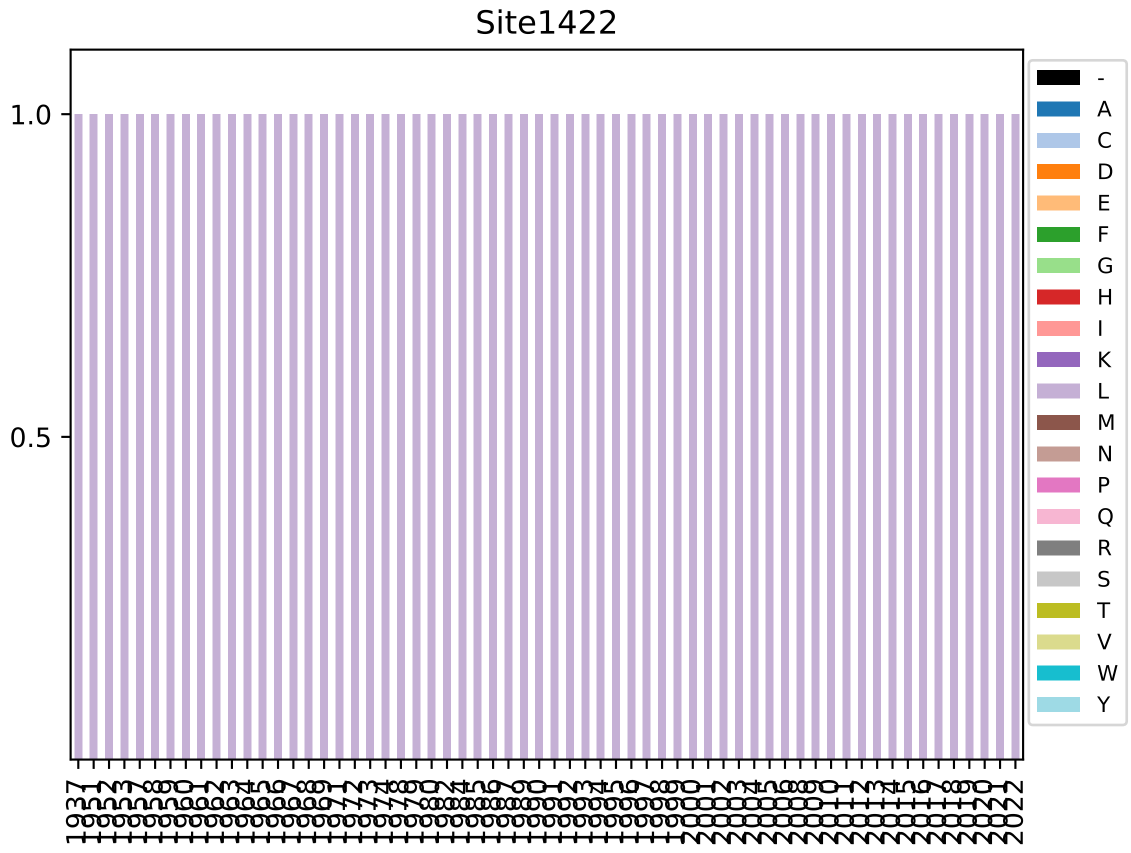
Site1422
1.0
0.5
-
A
C
D
E
F
G
H
I
K
L
M
N
P
Q
R
S
T
V
W
Y

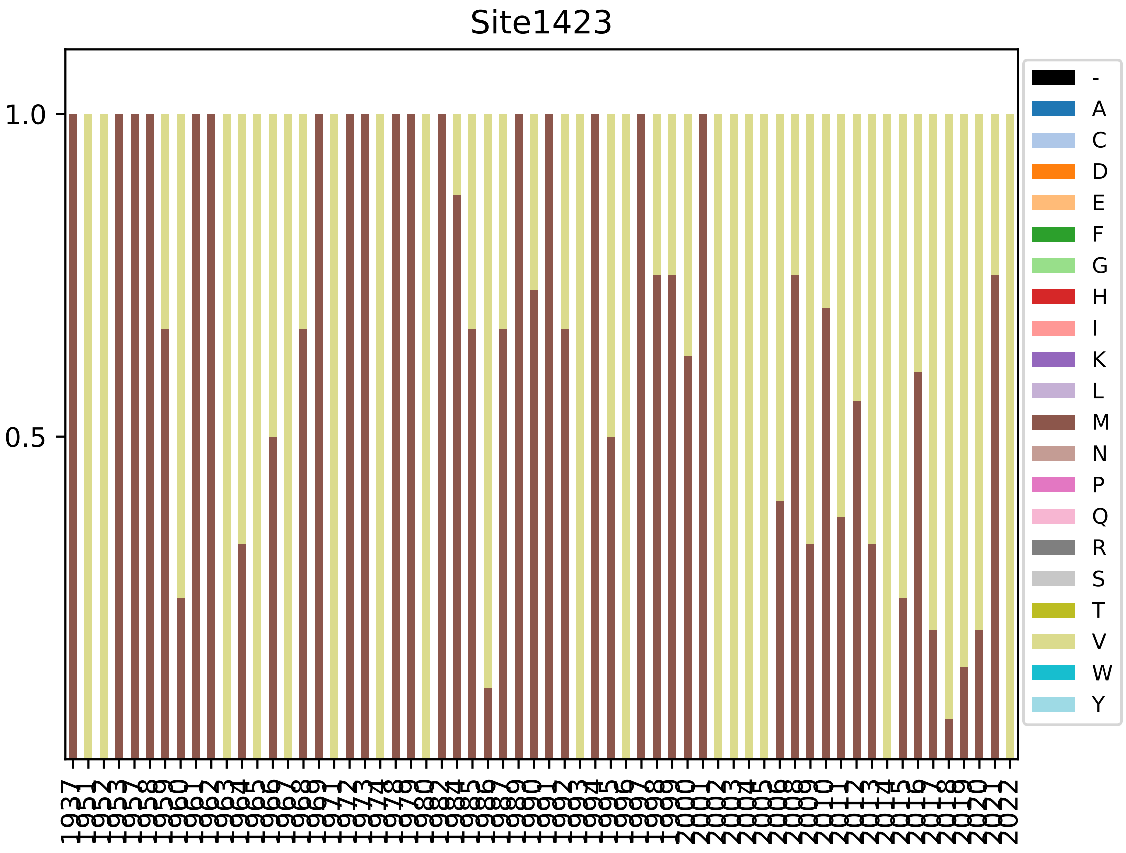
Site1423
1.0
0.5
-
A
C
D
E
F
G
H
I
K
L
M
N
P
Q
R
S
T
V
W
Y

Site1424

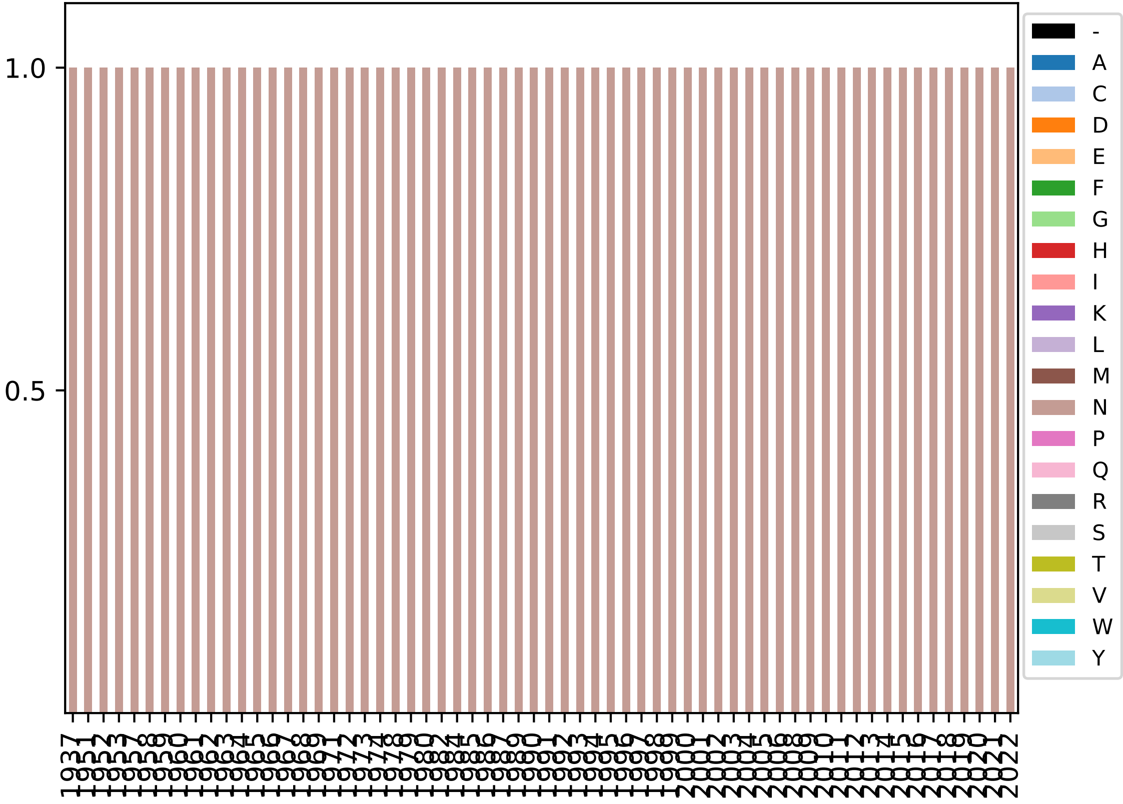

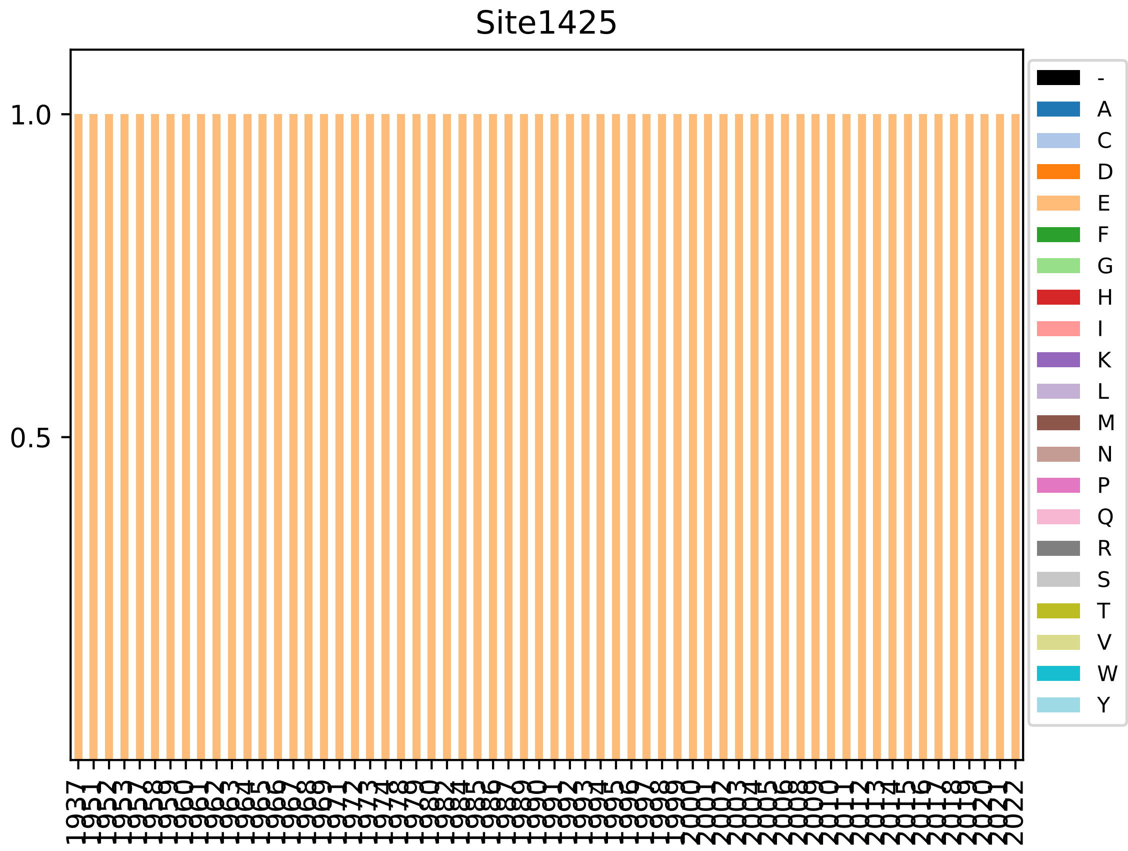
Site1425
1.0
0.5
-
A
C
D
E
F
G
H
I
K
L
M
N
P
Q
R
S
T
V
W
Y

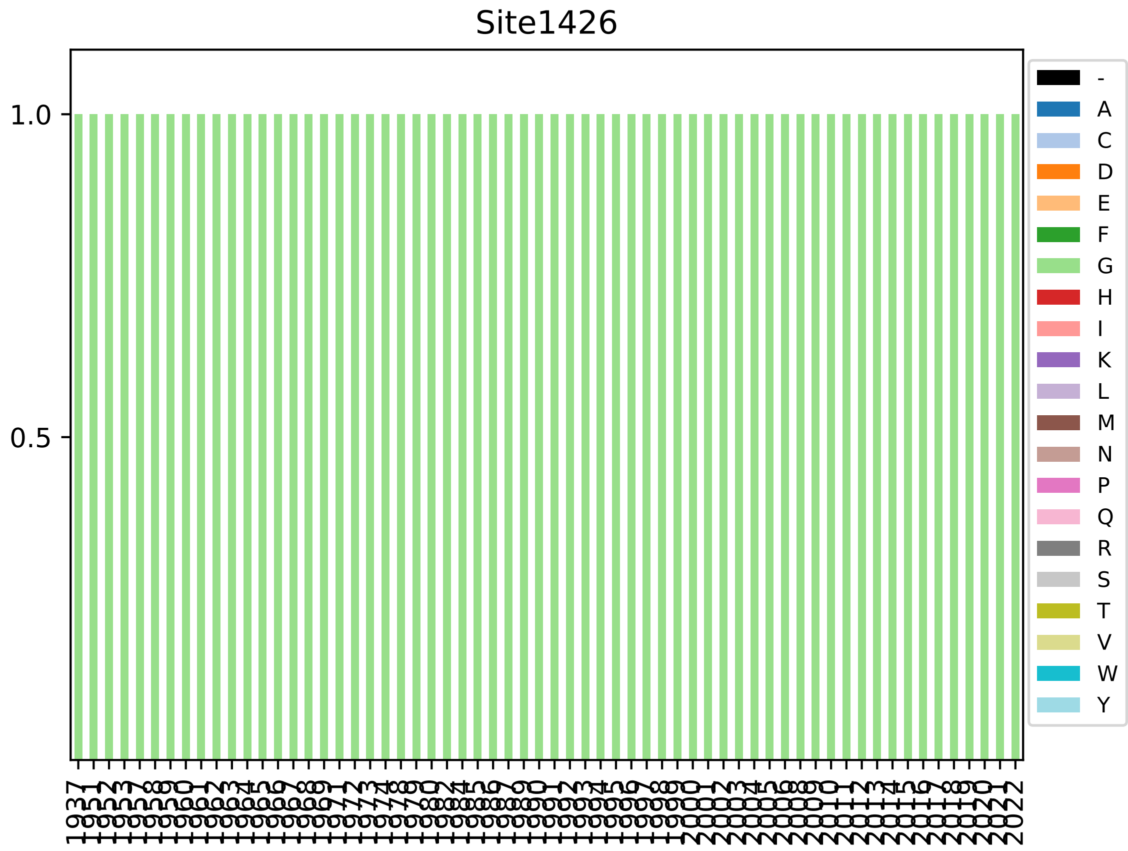
Site1426
1.0
0.5
-
A
C
D
E
F
G
H
I
K
L
M
N
P
Q
R
S
T
V
W
Y

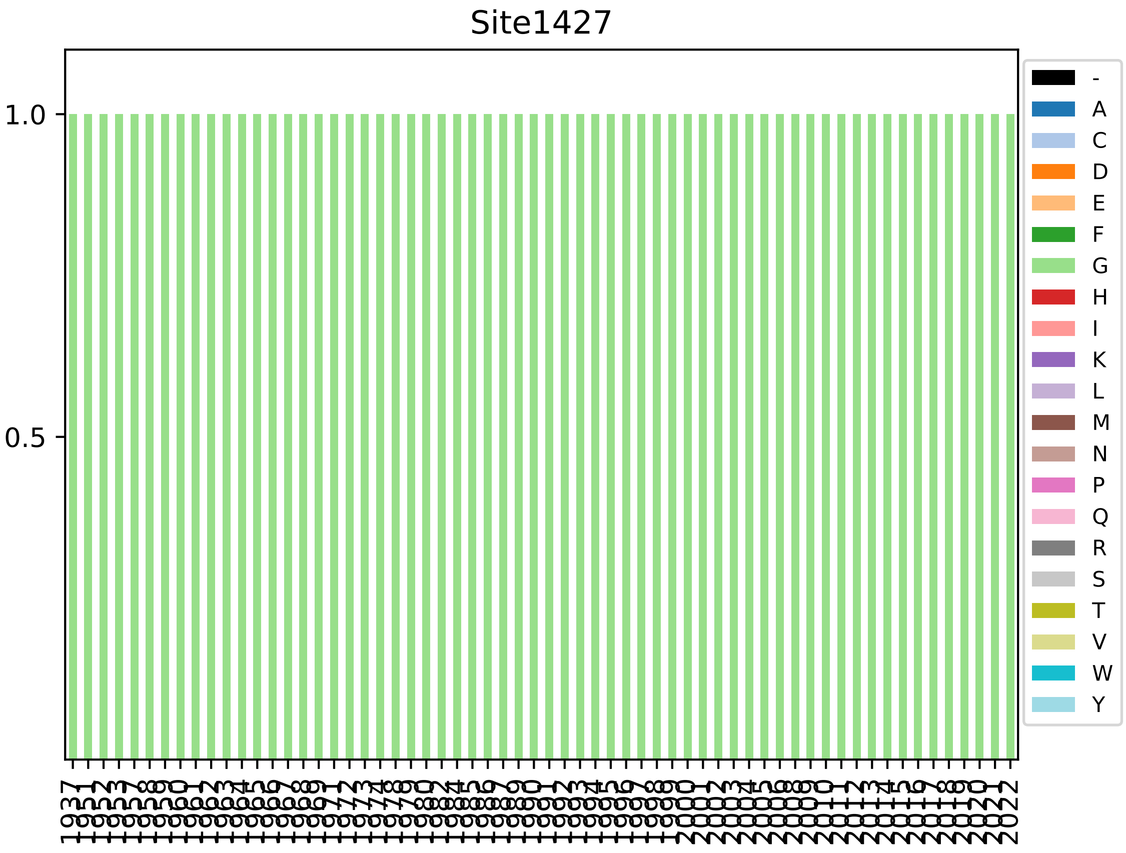

Site1427
1.0
0.5
-
A
C
D
E
F
G
H
I
K
L
M
N
P
Q
R
S
T
V
W
Y

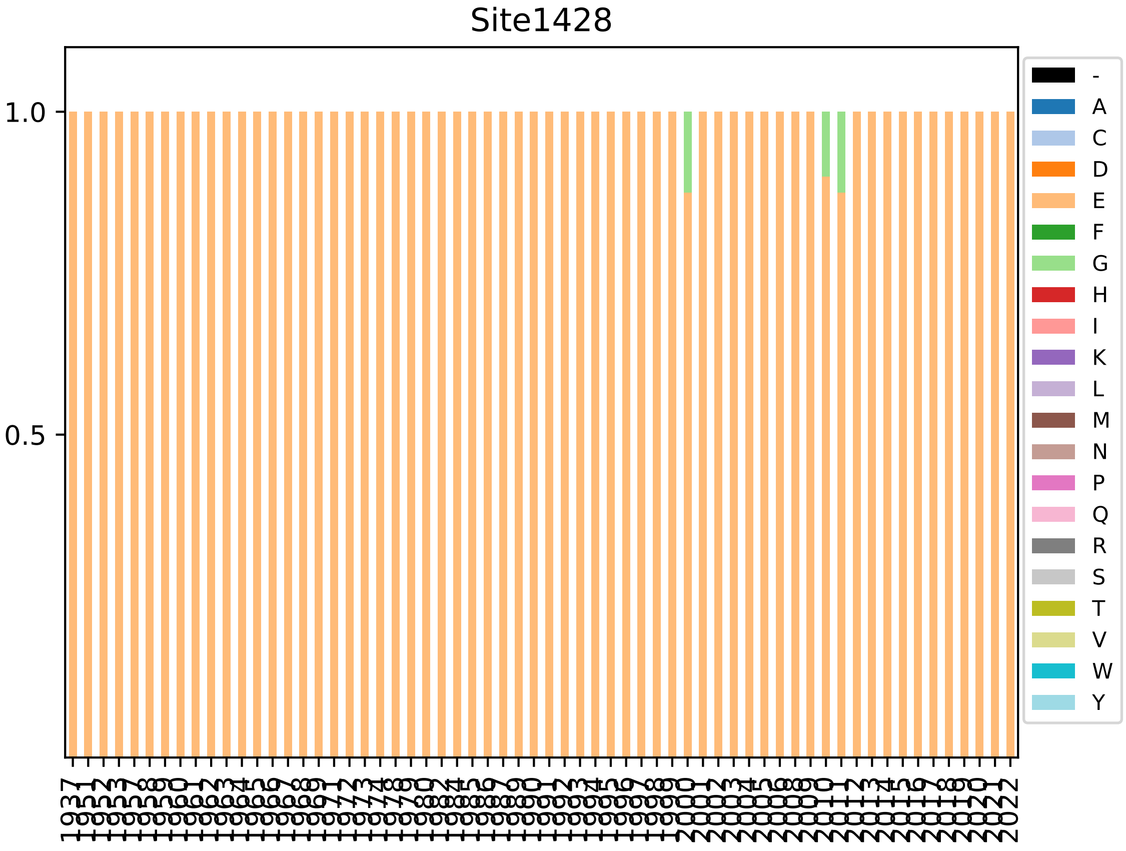
Site1428
1.0
0.5
-
A
C
D
E
F
G
H
I
K
L
M
N
P
Q
R
S
T
V
W
Y

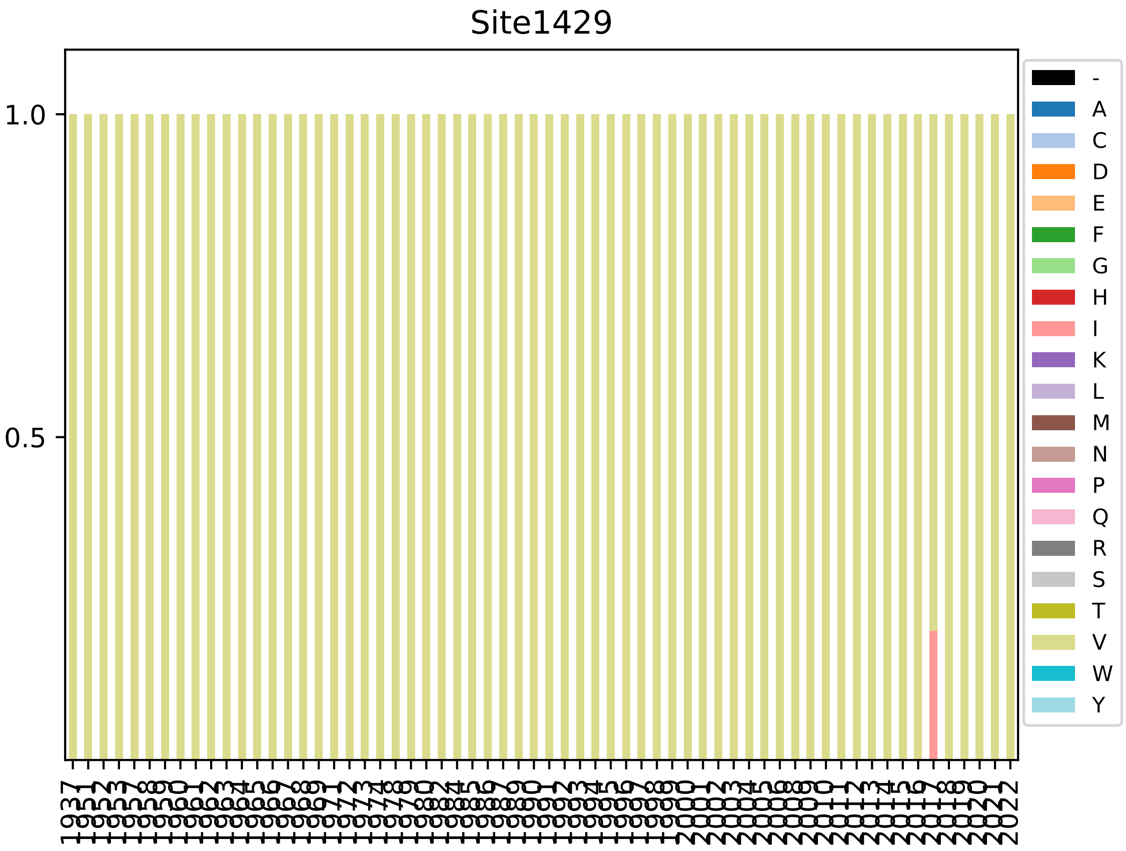
Site1429
1.0
0.5
-
A
C
D
E
F
G
H
I
K
L
M
N
P
Q
R
S
T
V
W
Y

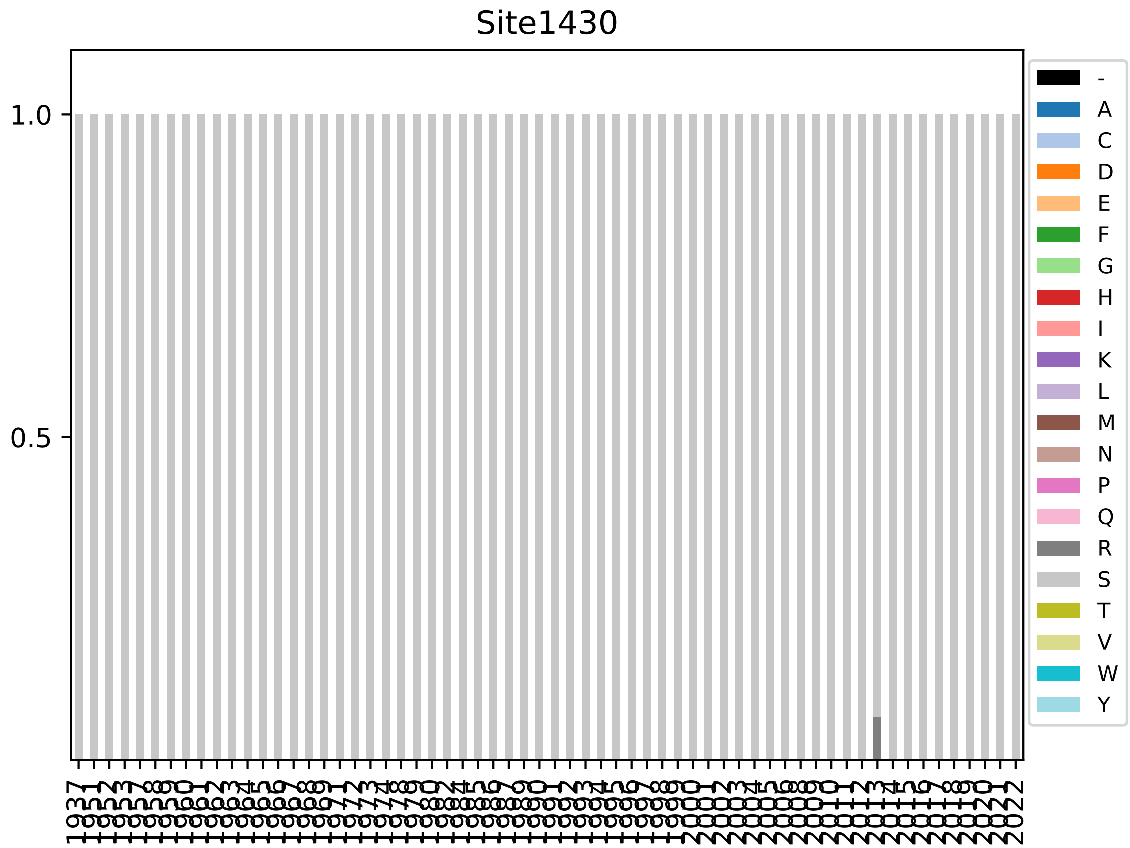
Site1430
1.0
0.5
-
A
C
D
E
F
G
H
I
K
L
M
N
P
Q
R
S
T
V
W
Y

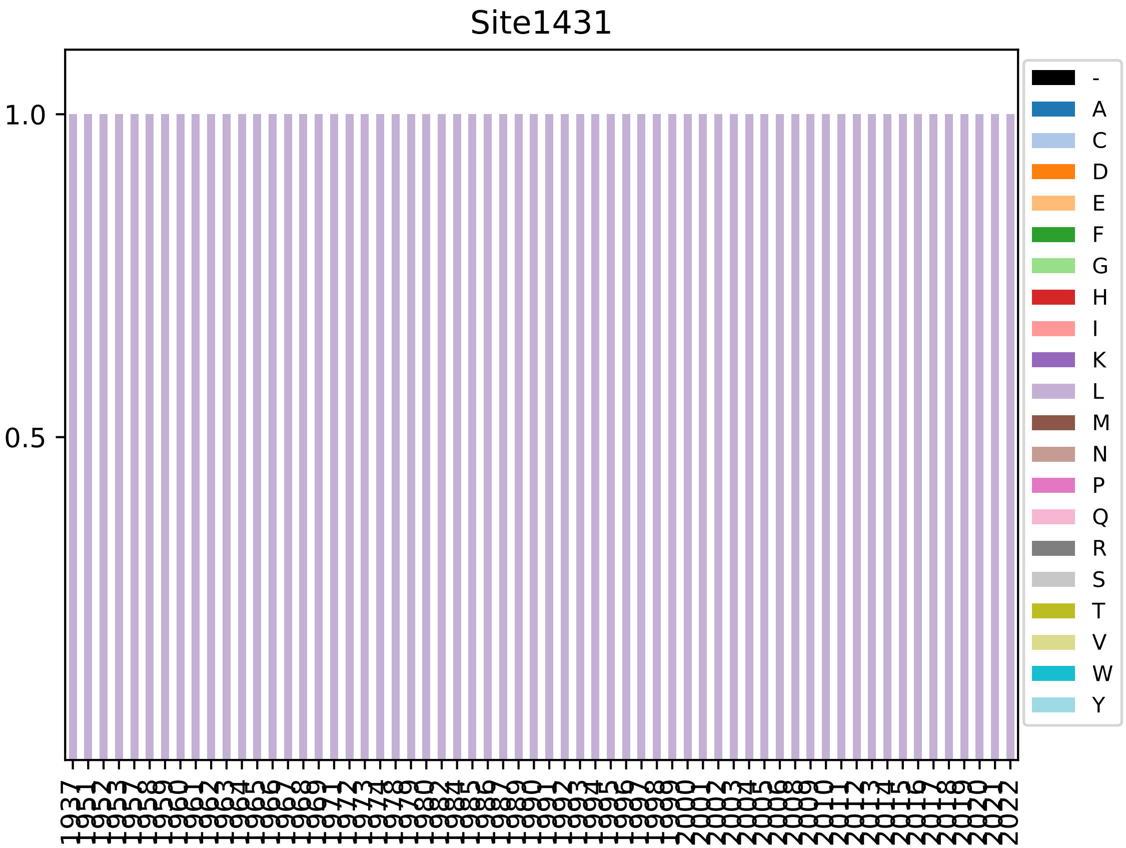
Site1431
1.0
0.5
-
A
C
D
E
F
G
H
I
K
L
M
N
P
Q
R
S
T
V
W
Y

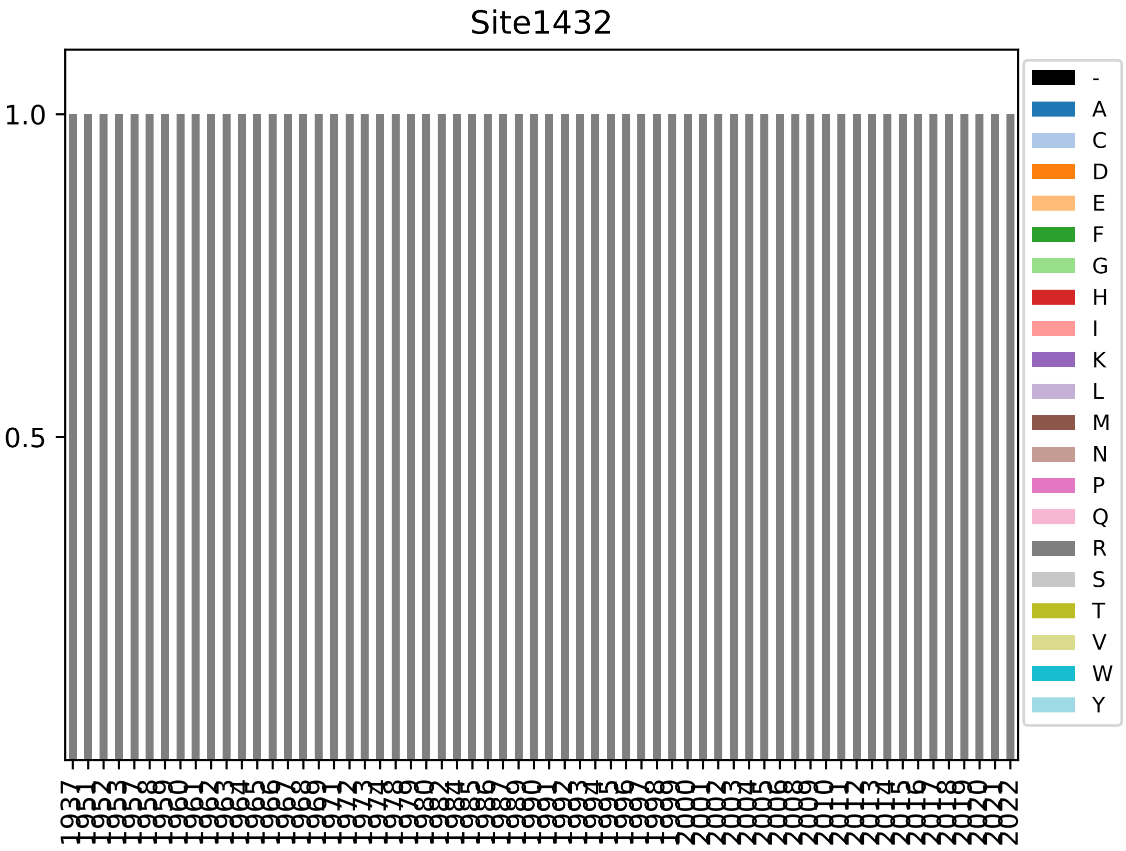
Site1432
1.0
0.5
-
A
C
D
E
F
G
H
I
K
L
M
N
P
Q
R
S
T
V
W
Y

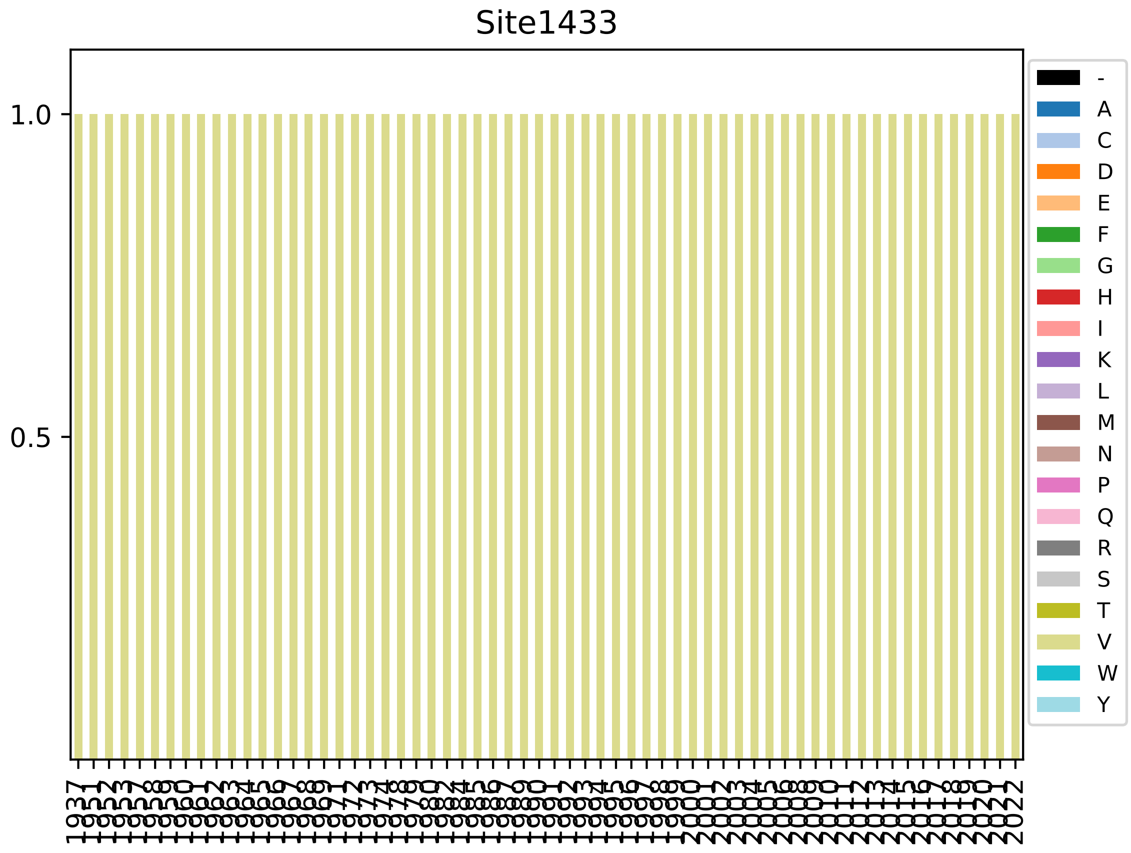
Site1433
1.0
0.5
-
A
C
D
E
F
G
H
I
K
L
M
N
P
Q
R
S
T
V
W
Y

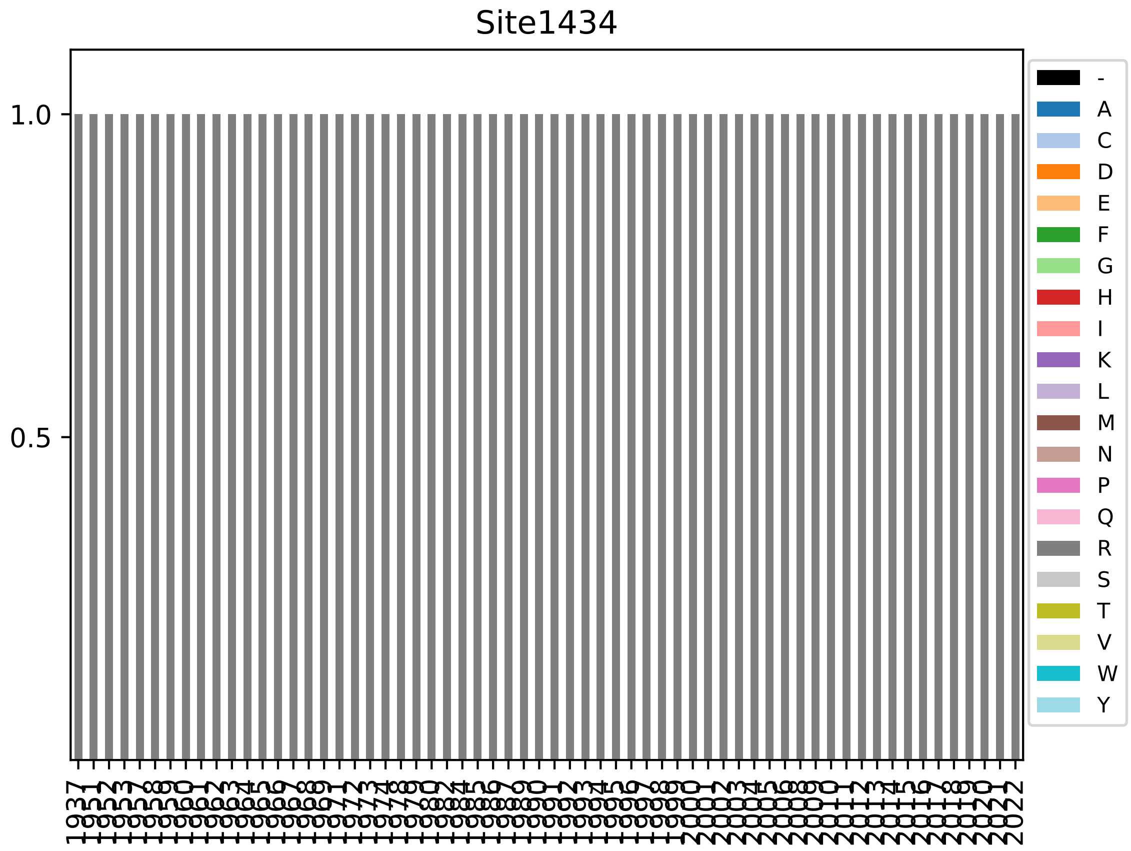
Site1434
1.0
0.5
-
A
C
D
E
F
G
H
I
K
L
M
N
P
Q
R
S
T
V
W
Y

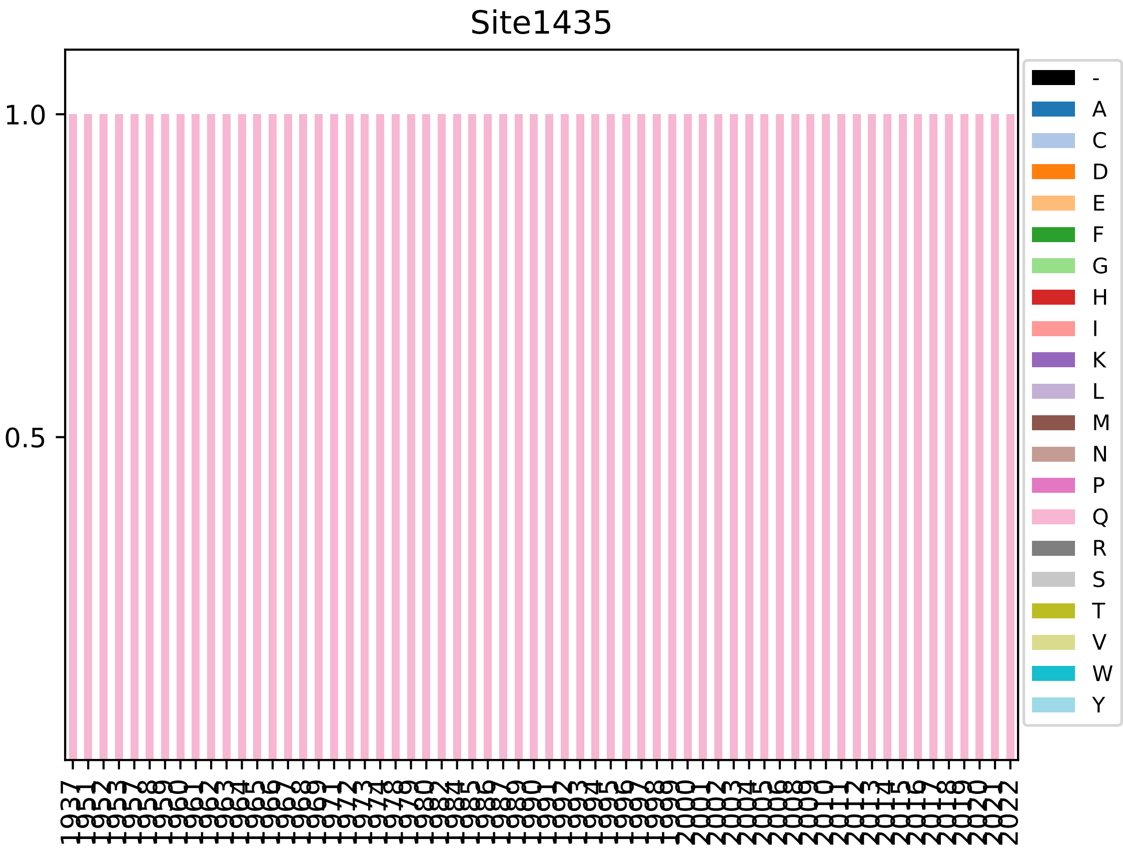
Site1435
1.0
0.5
-
A
C
D
E
F
G
H
I
K
L
M
N
P
Q
R
S
T
V
W
Y

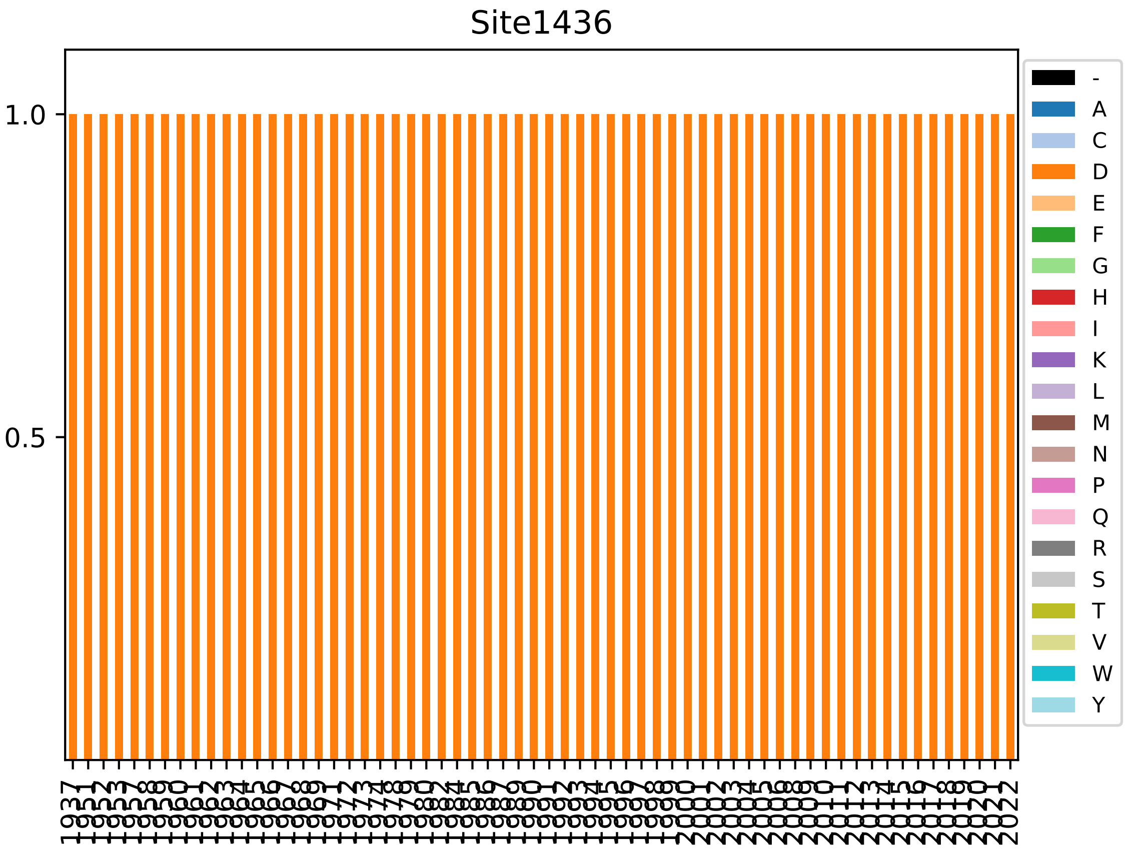
Site1436
1.0
0.5
-
A
C
D
E
F
G
H
I
K
L
M
N
P
Q
R
S
T
V
W
Y

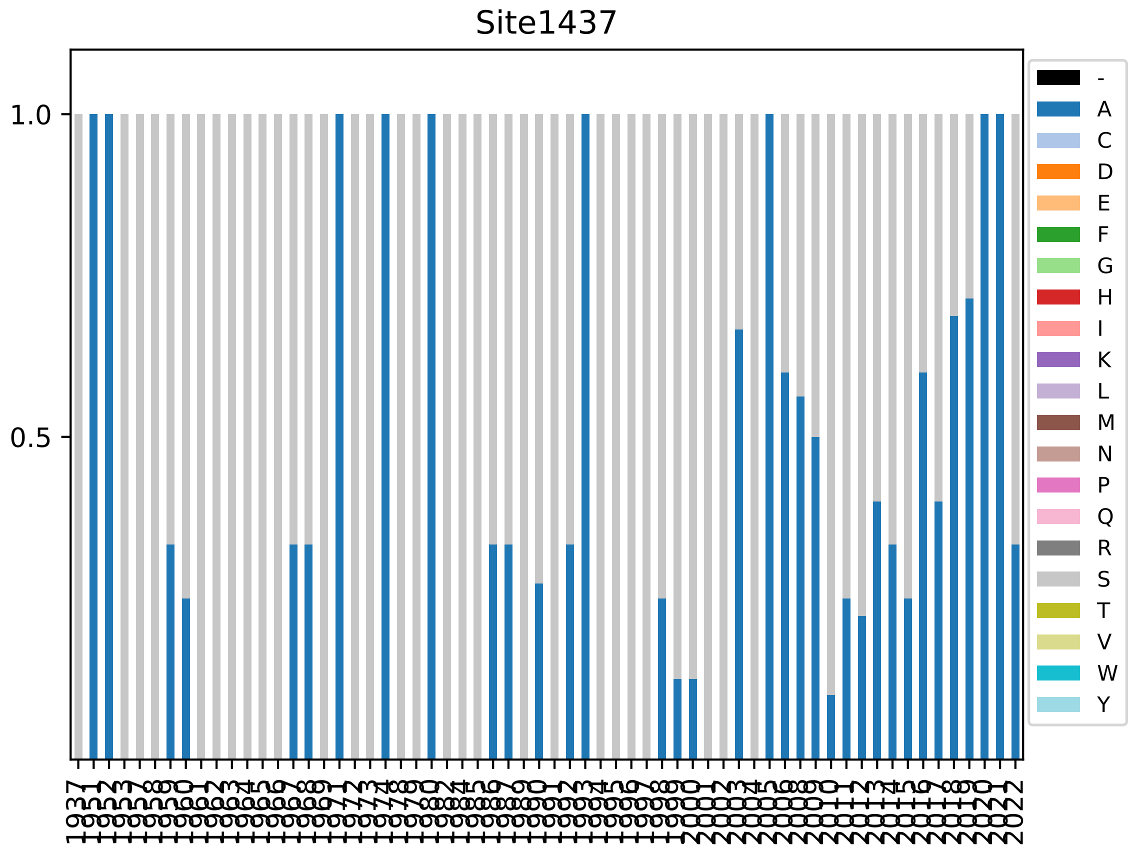

Site1437
1.0
0.5
-
A
C
D
E
F
G
H
I
K
L
M
N
P
Q
R
S
T
V
W
Y

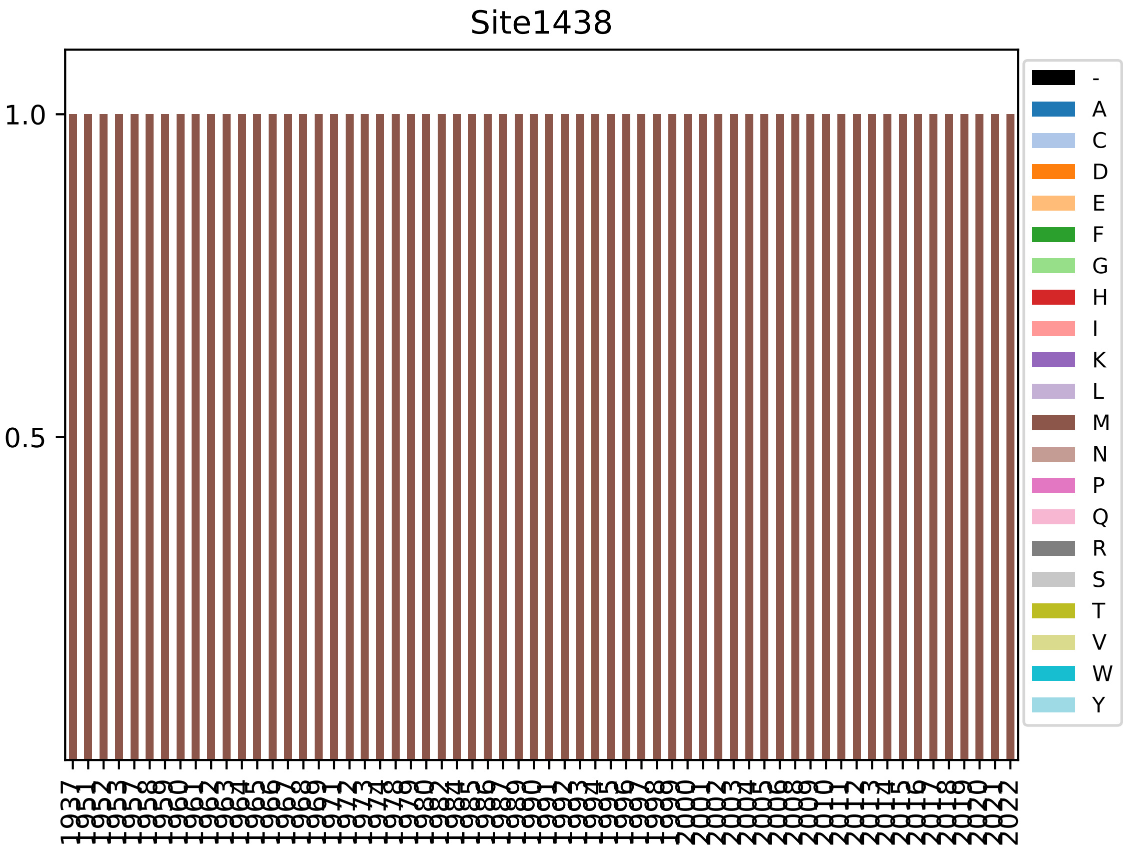

Site1438
1.0
0.5
-
A
C
D
E
F
G
H
I
K
L
M
N
P
Q
R
S
T
V
W
Y

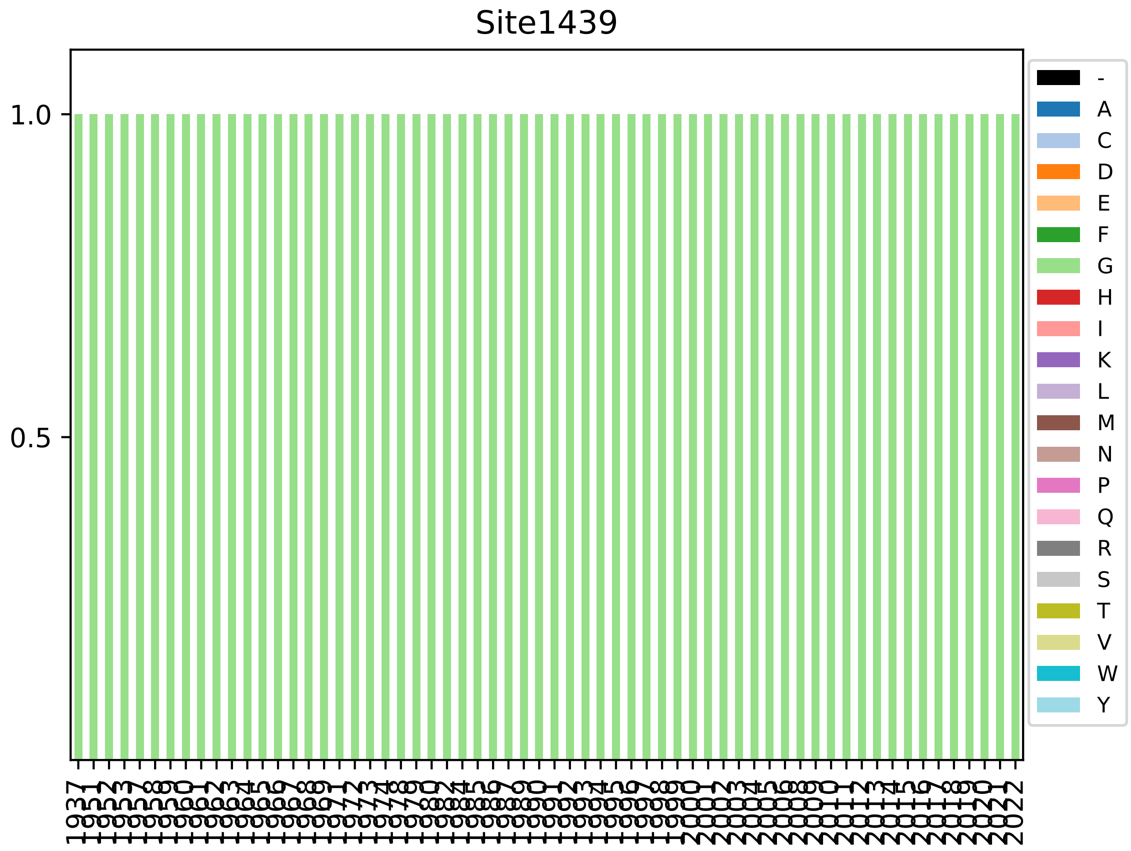
Site1439
1.0
0.5
-
A
C
D
E
F
G
H
I
K
L
M
N
P
Q
R
S
T
V
W
Y

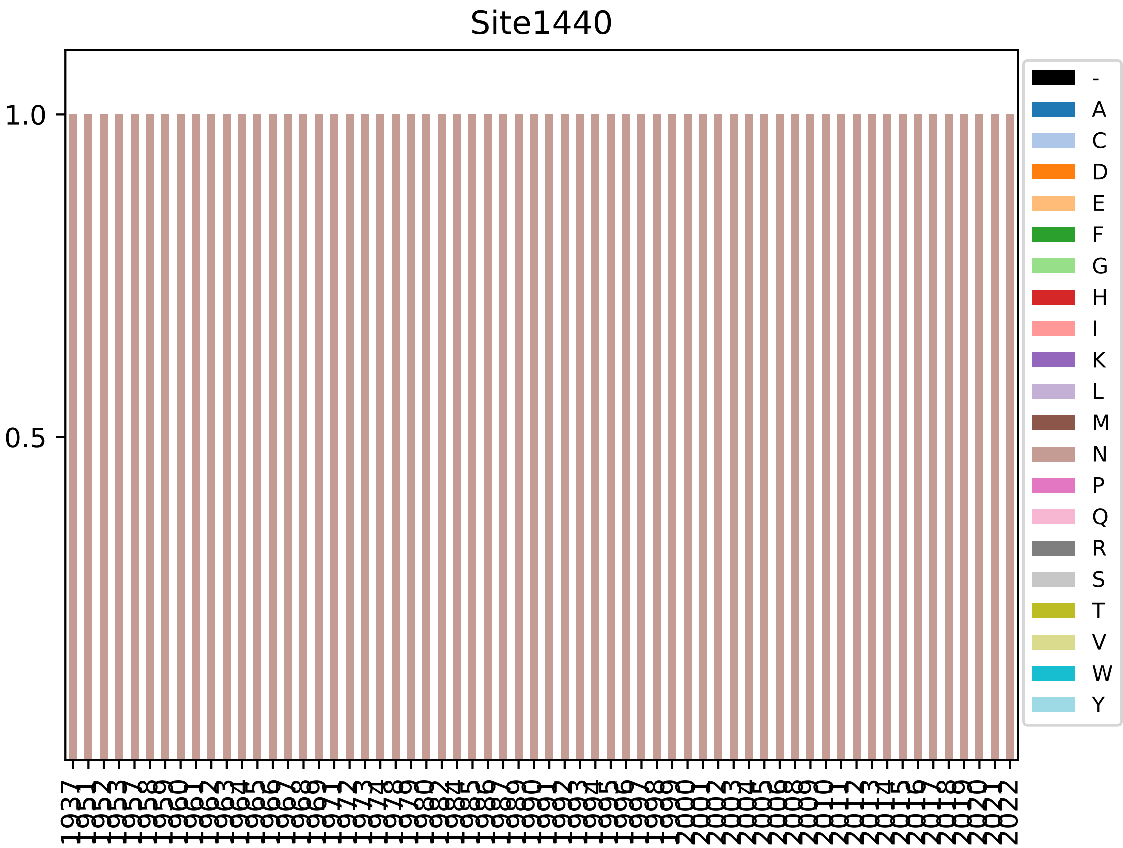
Site1440
1.0
0.5
-
A
C
D
E
F
G
H
I
K
L
M
N
P
Q
R
S
T
V
W
Y

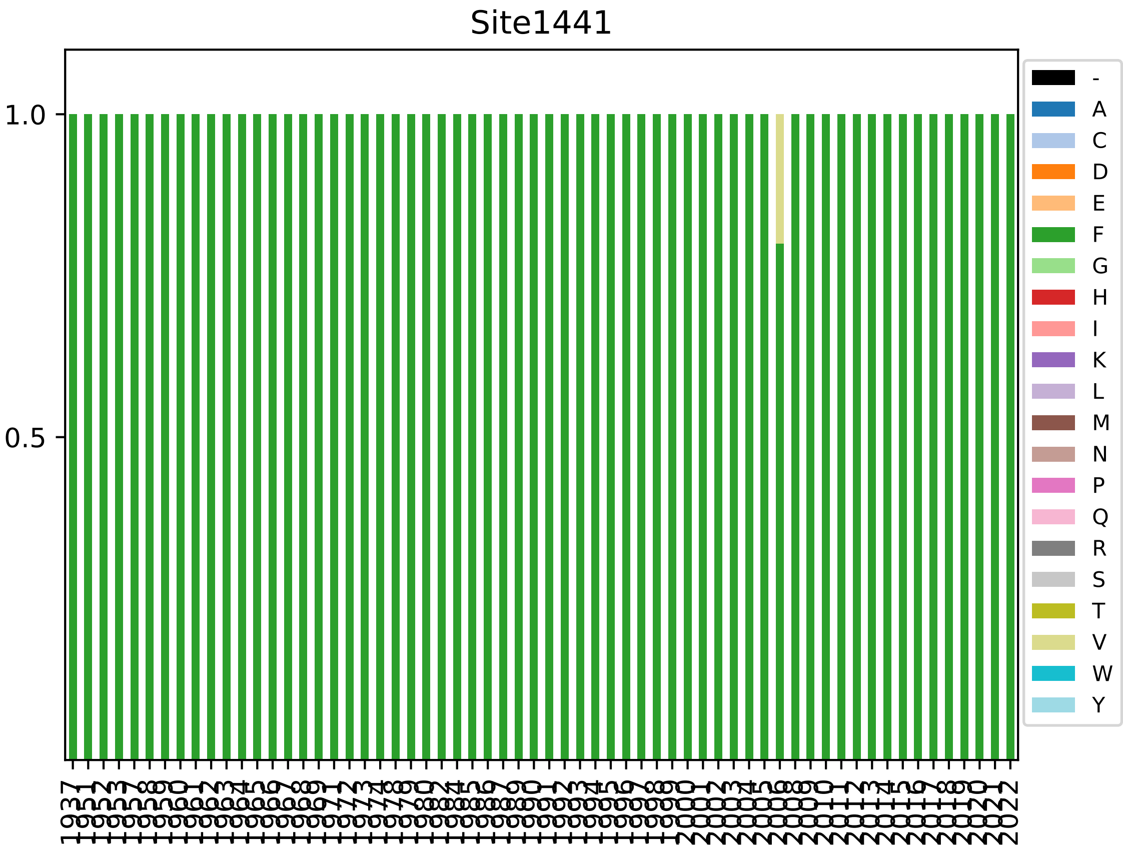
Site1441
1.0
0.5
-
A
C
D
E
F
G
H
I
K
L
M
N
P
Q
R
S
T
V
W
Y

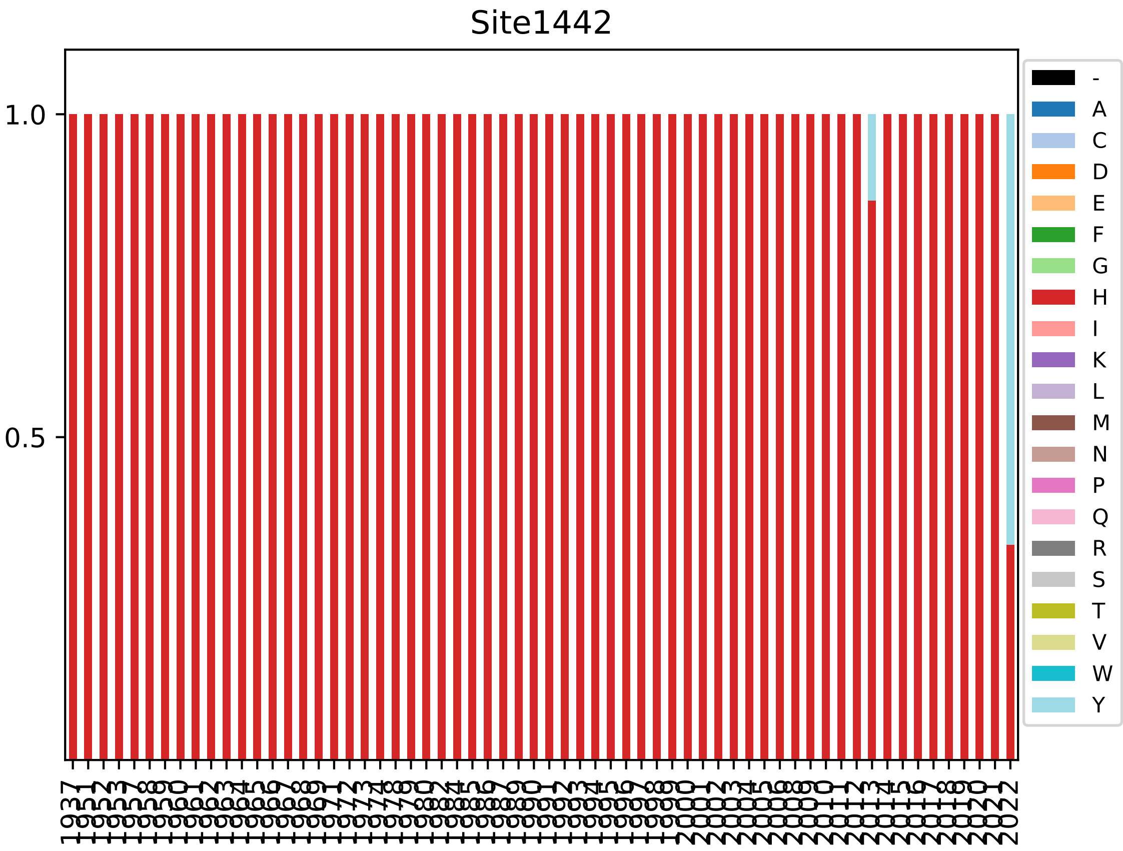
Site1442
1.0
0.5
-
A
C
D
E
F
G
H
I
K
L
M
N
P
Q
R
S
T
V
W
Y

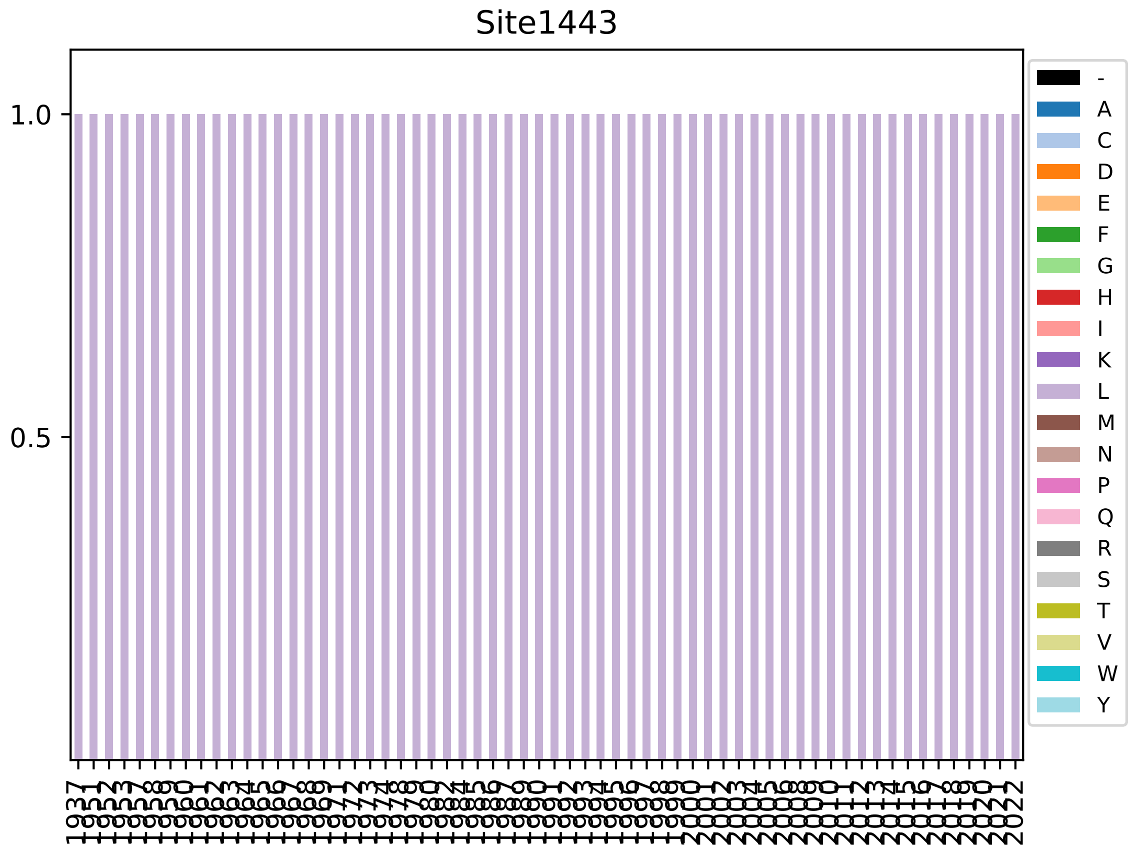
Site1443
1.0
0.5
-
A
C
D
E
F
G
H
I
K
L
M
N
P
Q
R
S
T
V
W
Y

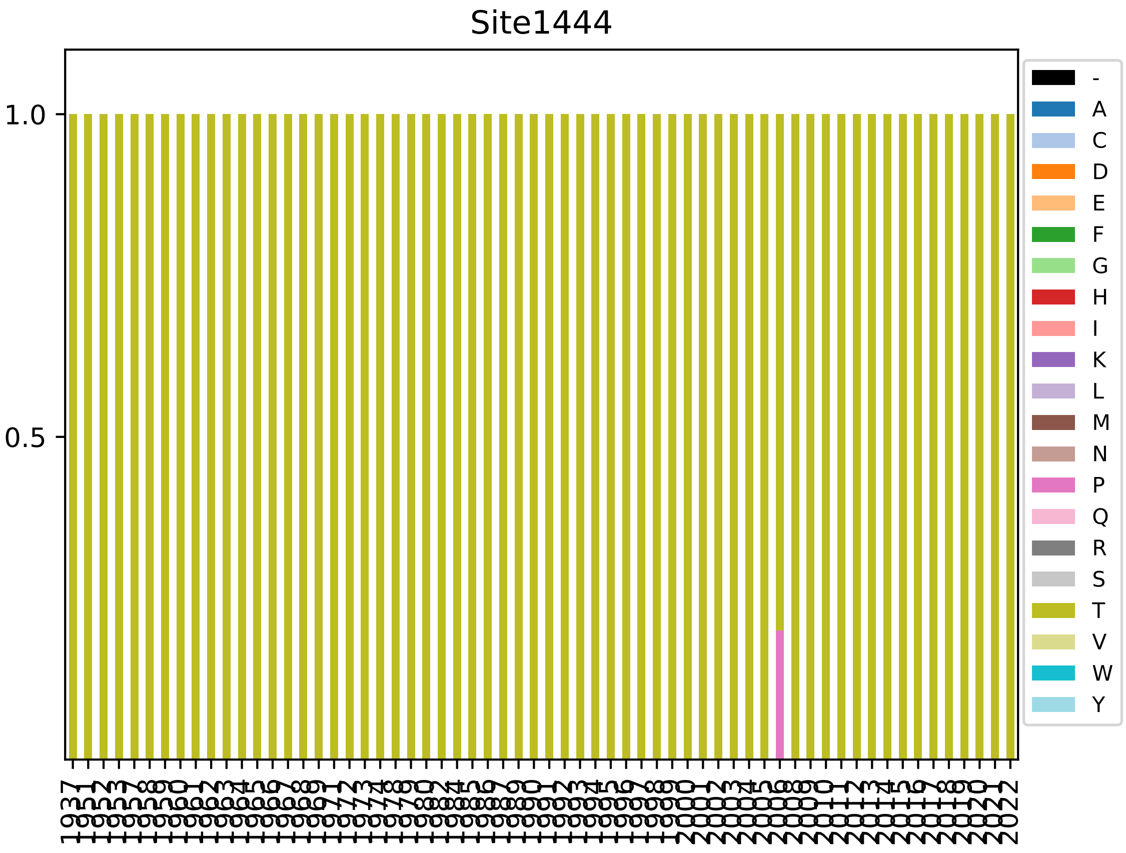
Site1444
1.0
0.5
-
A
C
D
E
F
G
H
I
K
L
M
N
P
Q
R
S
T
V
W
Y

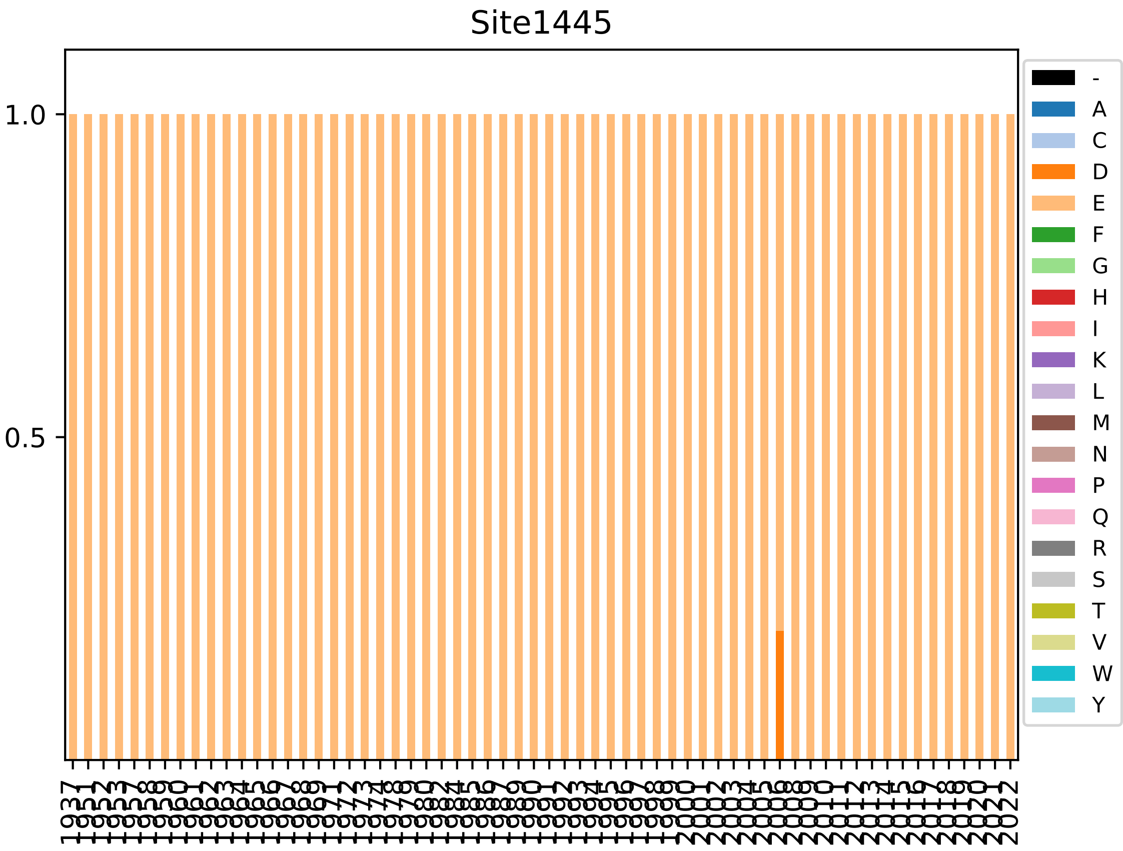
Site1445
1.0
0.5
-
A
C
D
E
F
G
H
I
K
L
M
N
P
Q
R
S
T
V
W
Y

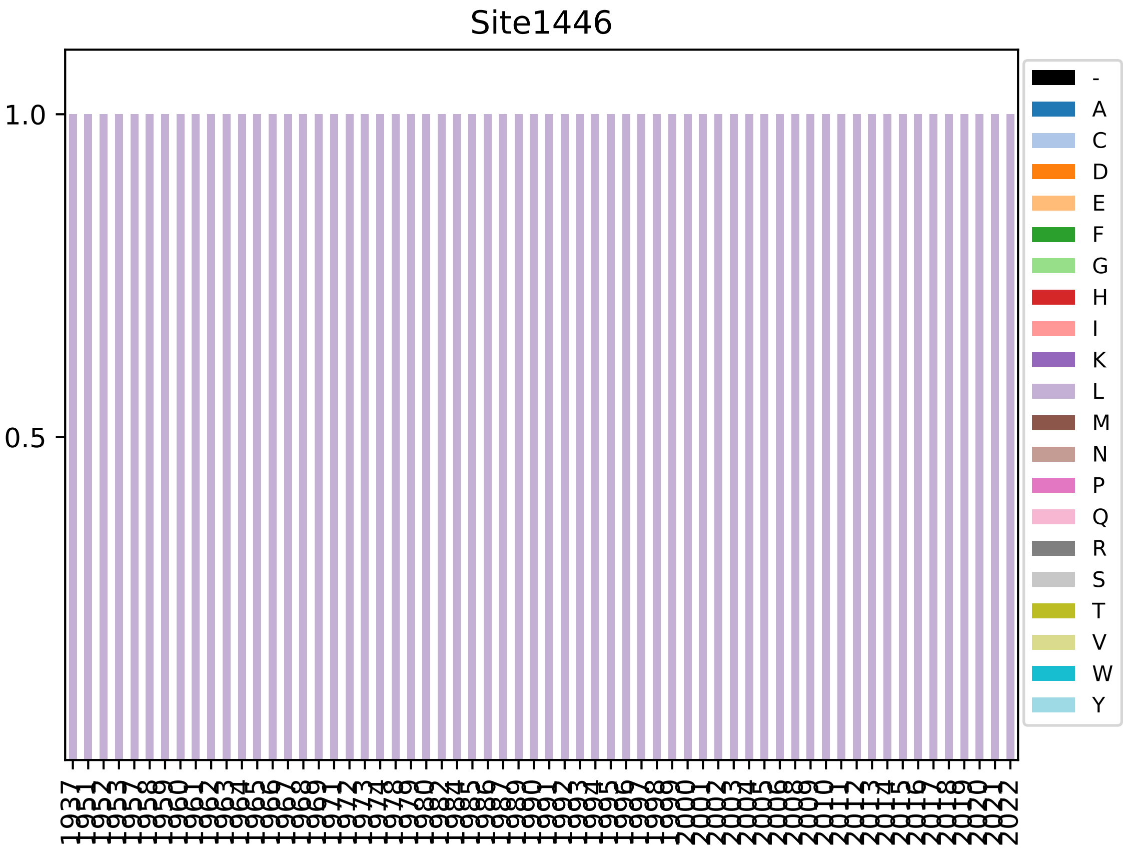
Site1446
1.0
0.5
-
A
C
D
E
F
G
H
I
K
L
M
N
P
Q
R
S
T
V
W
Y

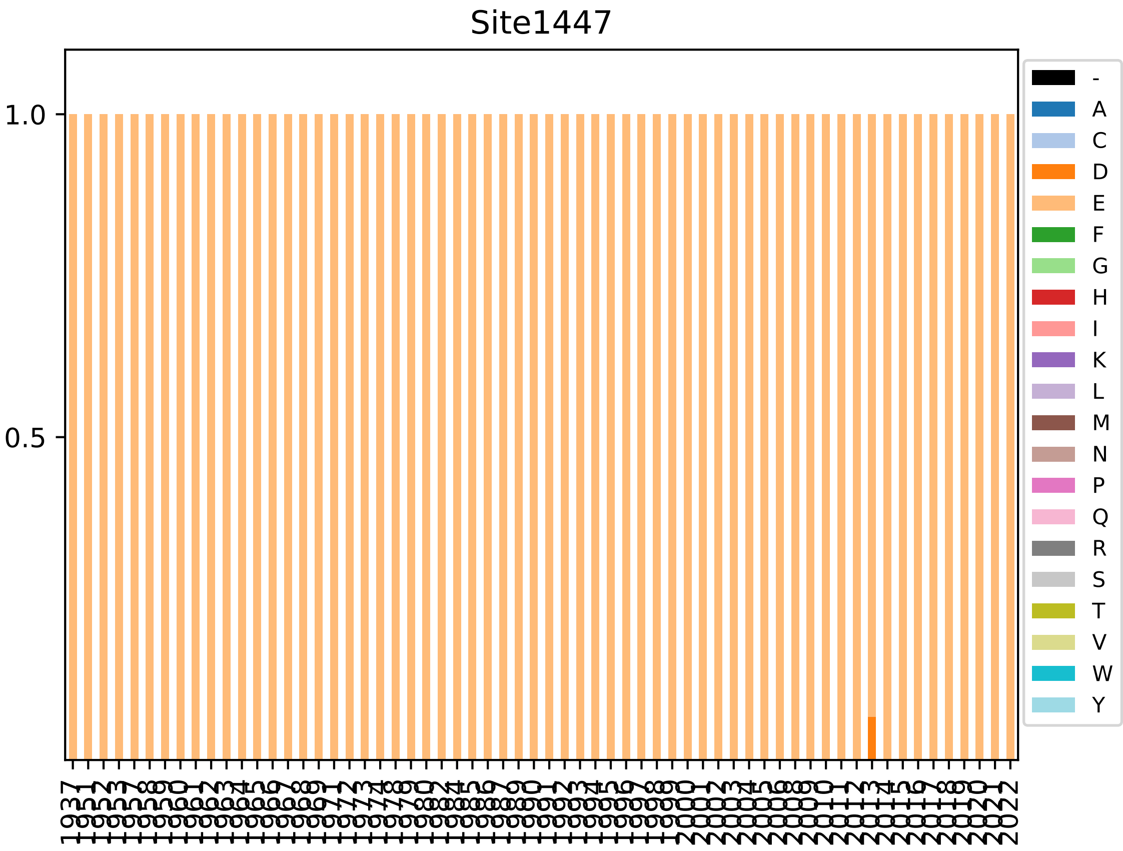

Site1447
1.0
0.5
-
A
C
D
E
F
G
H
I
K
L
M
N
P
Q
R
S
T
V
W
Y

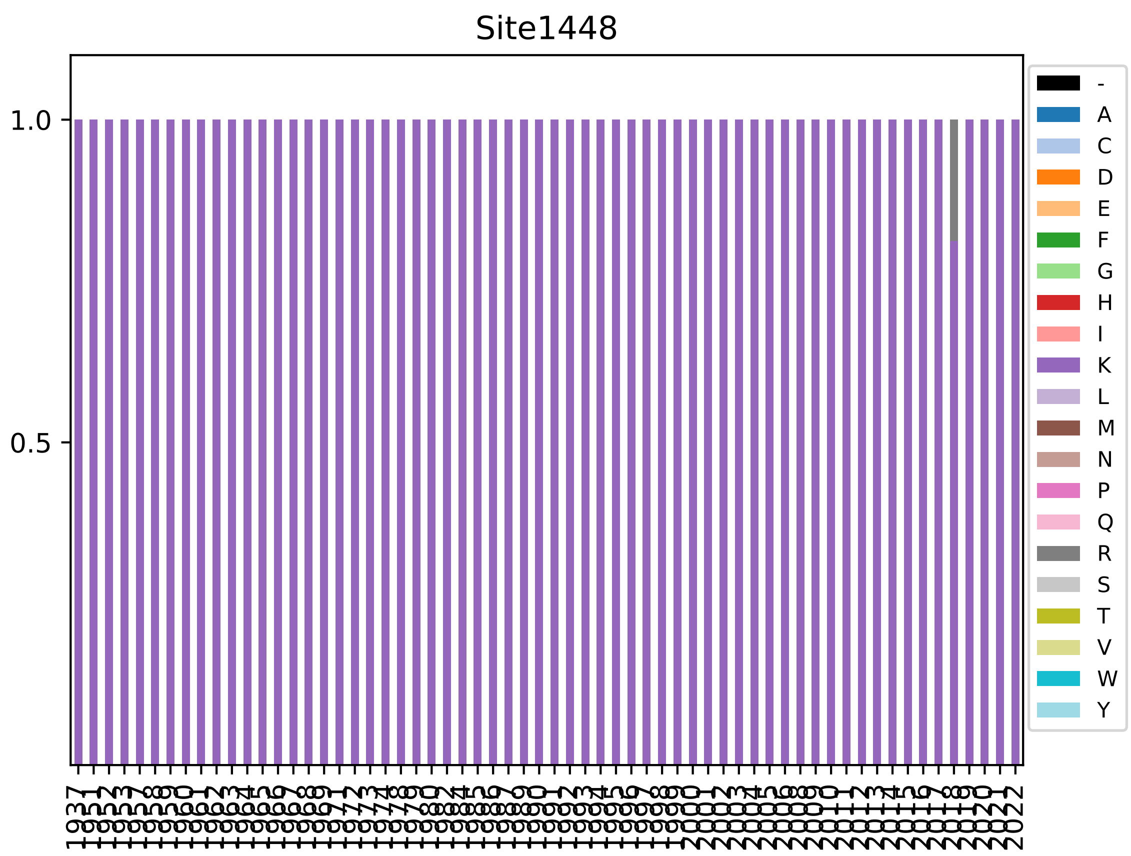
Site1448
1.0
0.5
-
A
C
D
E
F
G
H
I
K
L
M
N
P
Q
R
S
T
V
W
Y

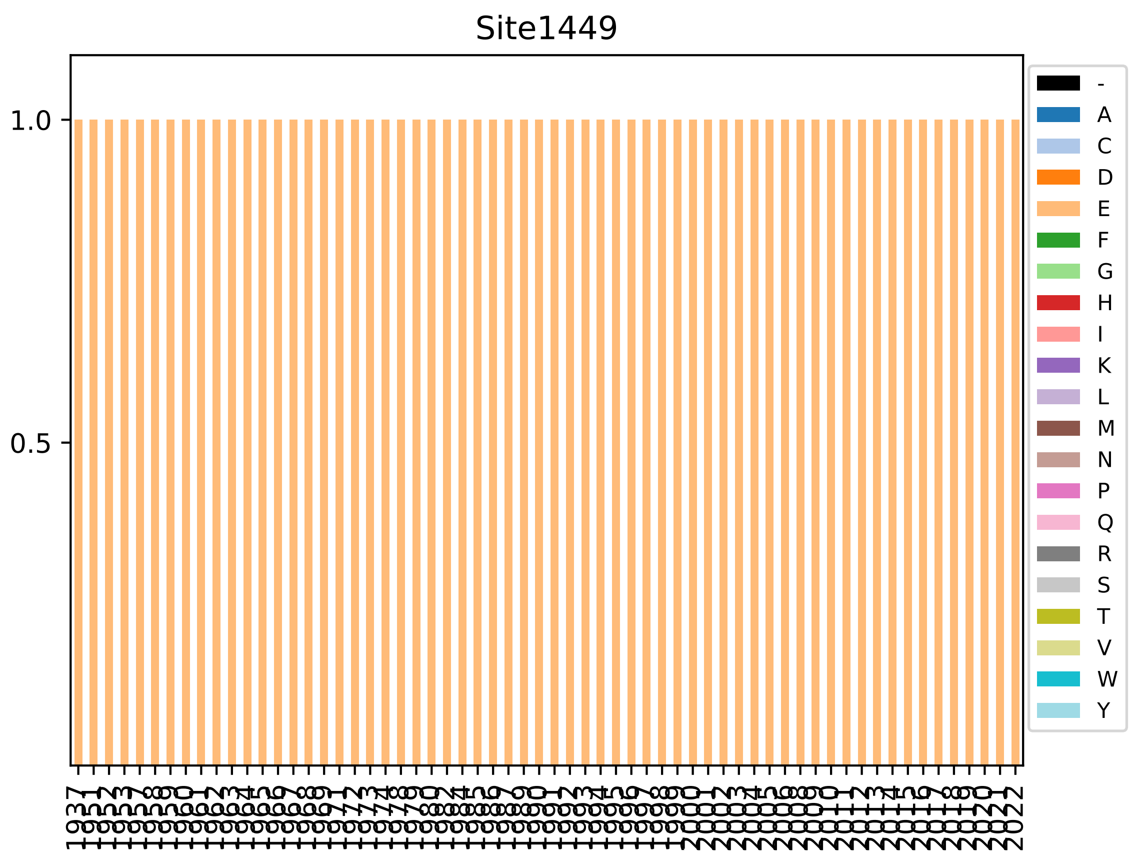
Site1449
1.0
0.5
-
A
C
D
E
F
G
H
I
K
L
M
N
P
Q
R
S
T
V
W
Y

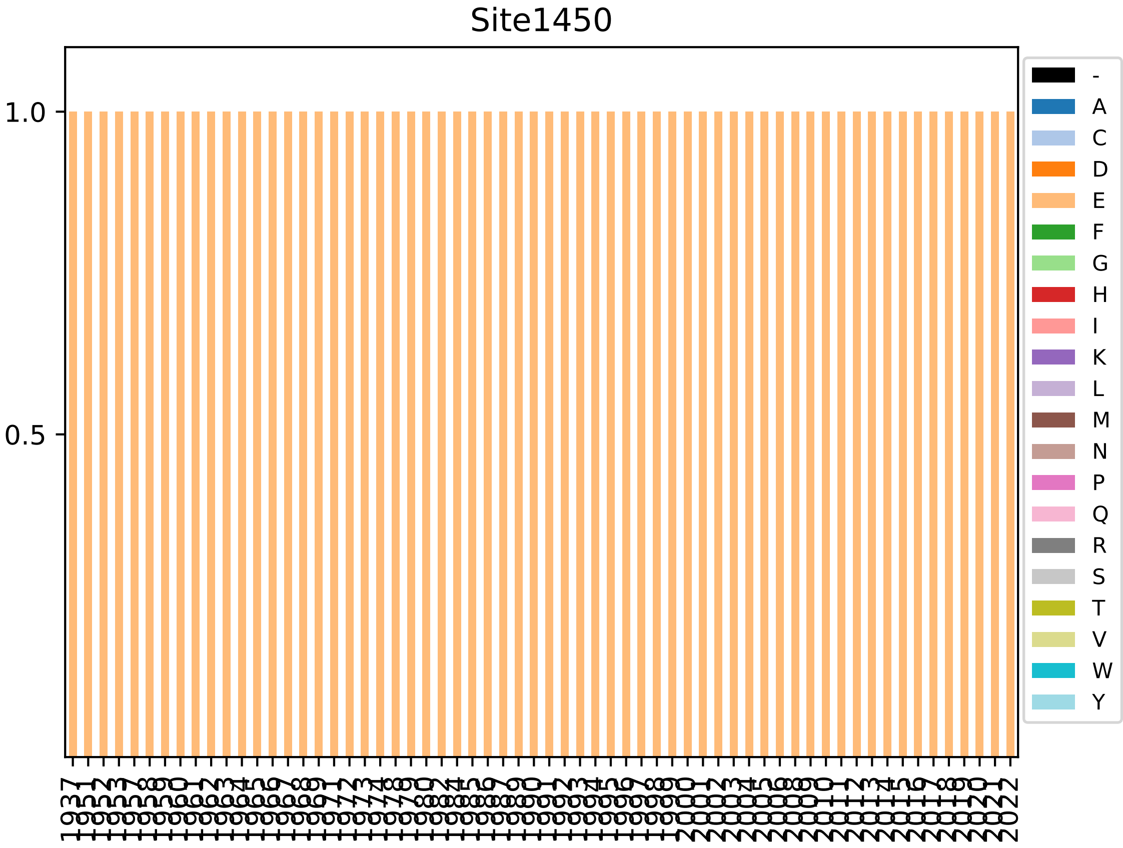

Site1450
1.0
0.5
-
A
C
D
E
F
G
H
I
K
L
M
N
P
Q
R
S
T
V
W
Y

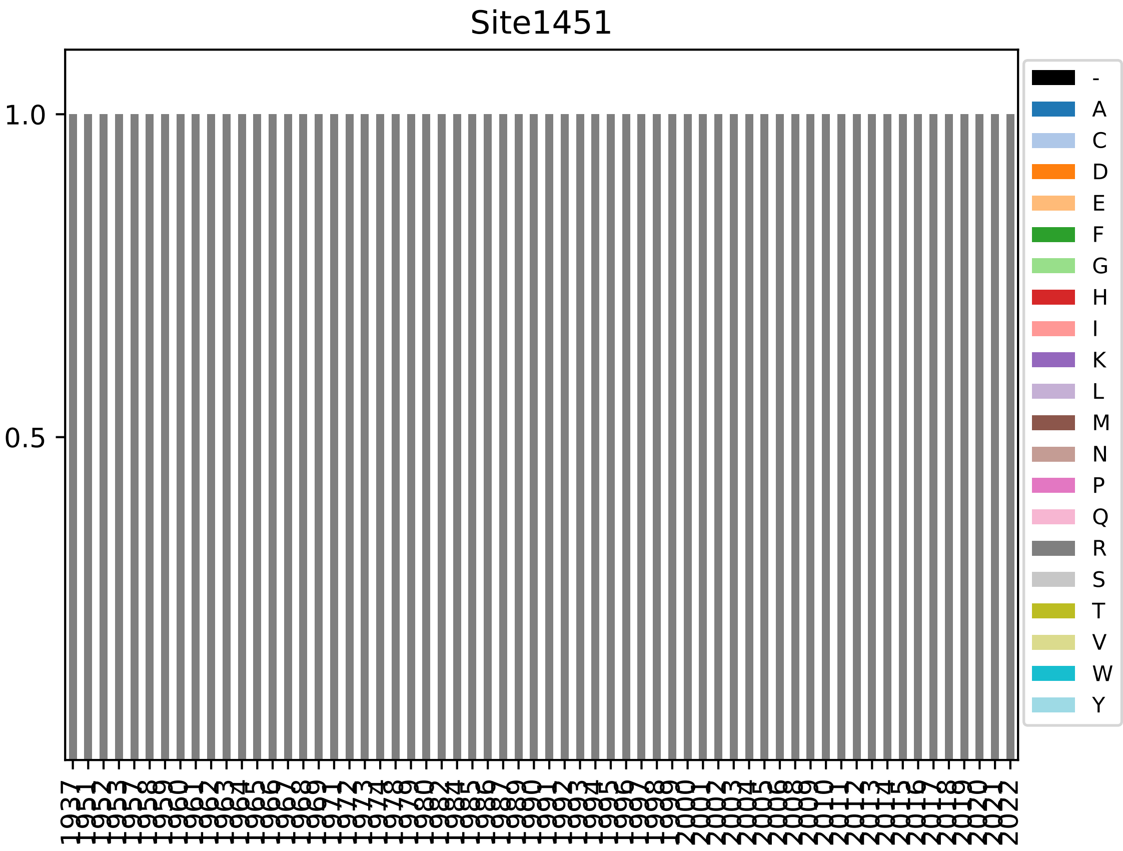
Site1451
1.0
0.5
-
A
C
D
E
F
G
H
I
K
L
M
N
P
Q
R
S
T
V
W
Y

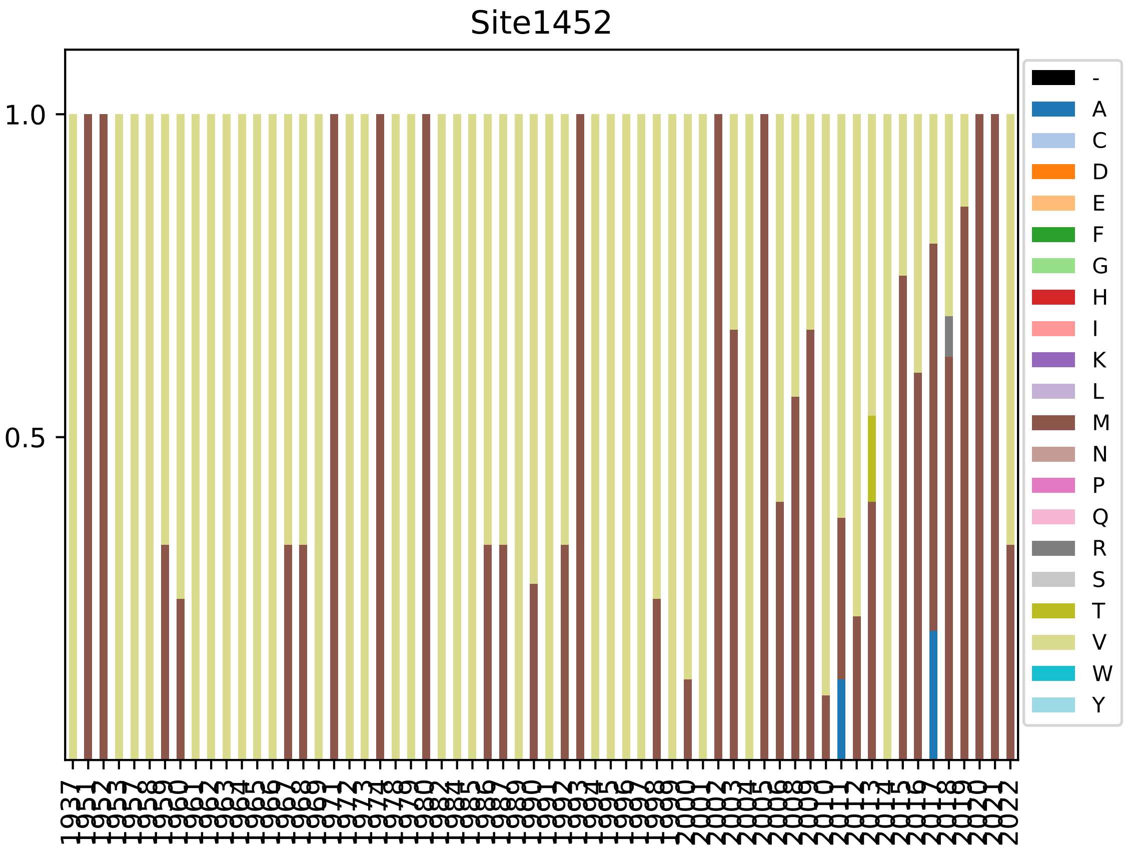
Site1452
1.0
0.5
-
A
C
D
E
F
G
H
I
K
L
M
N
P
Q
R
S
T
V
W
Y

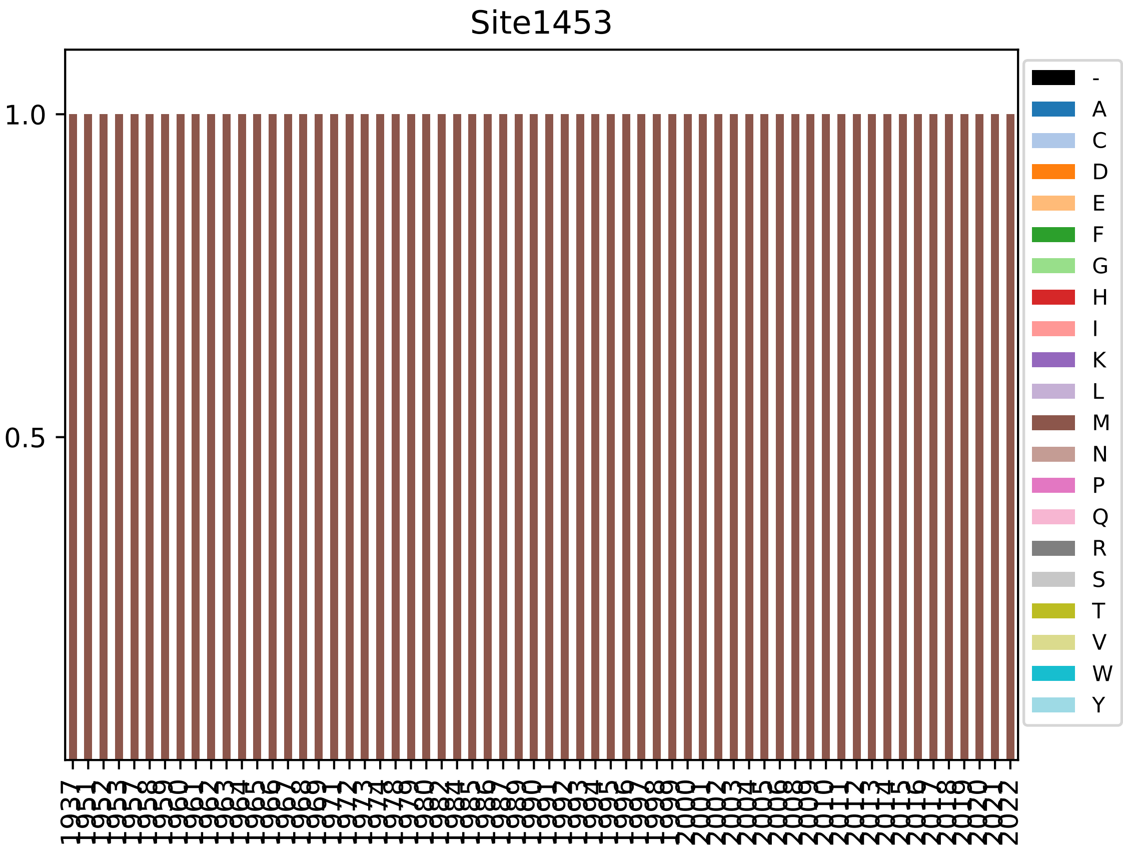

Site1453
1.0
0.5
-
A
C
D
E
F
G
H
I
K
L
M
N
P
Q
R
S
T
V
W
Y

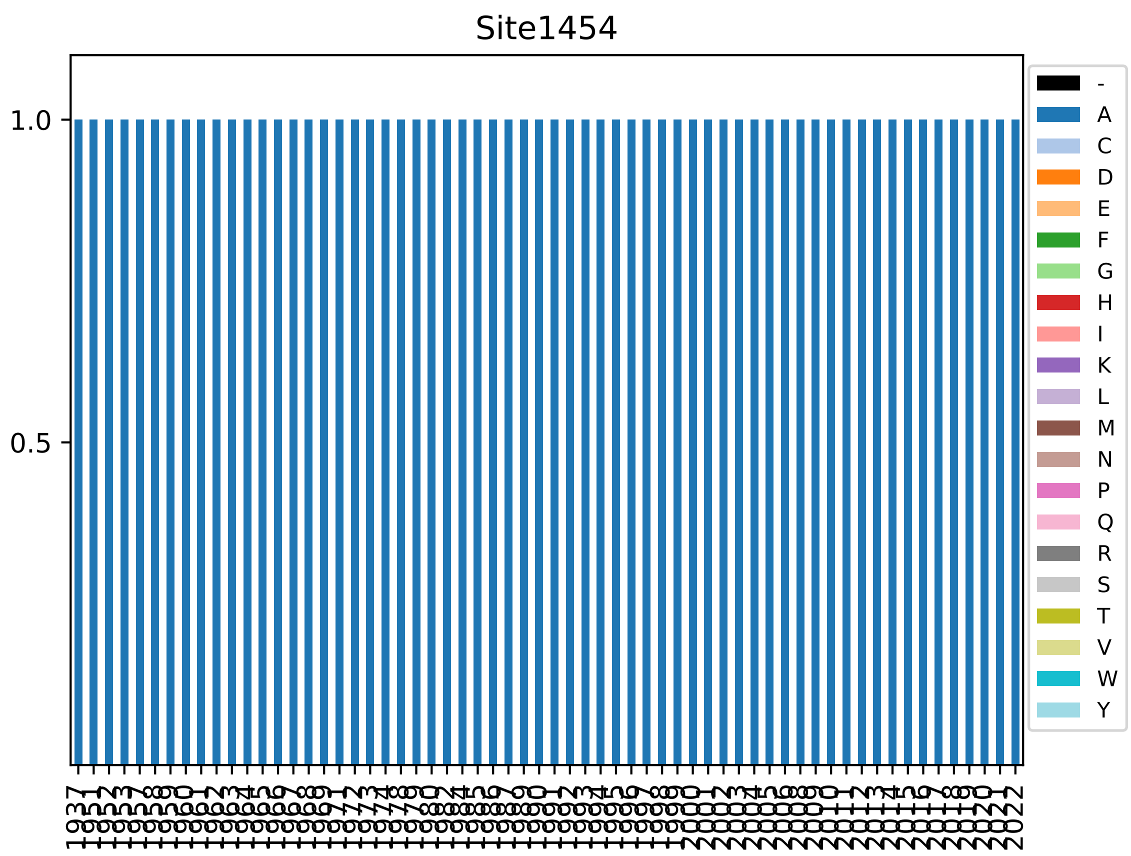
Site1454
1.0
0.5
-
A
C
D
E
F
G
H
I
K
L
M
N
P
Q
R
S
T
V
W
Y

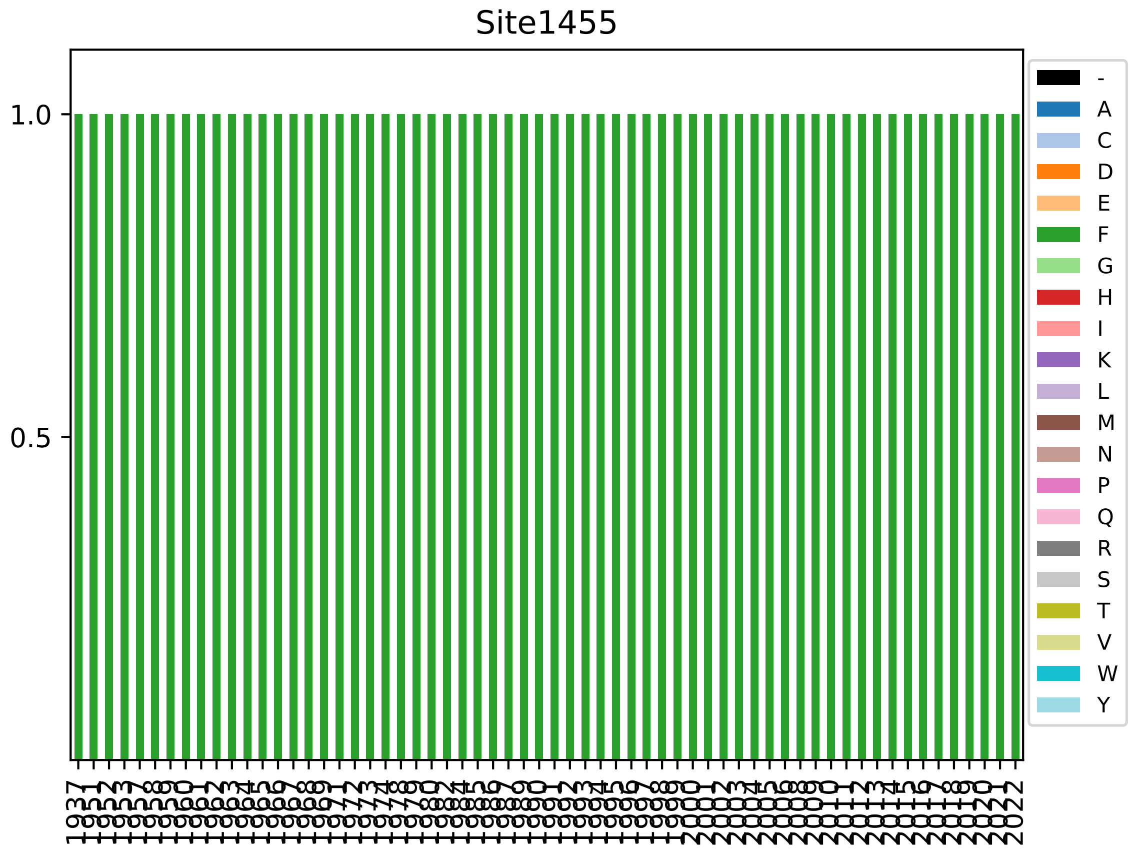
Site1455
1.0
0.5
-
A
C
D
E
F
G
H
I
K
L
M
N
P
Q
R
S
T
V
W
Y

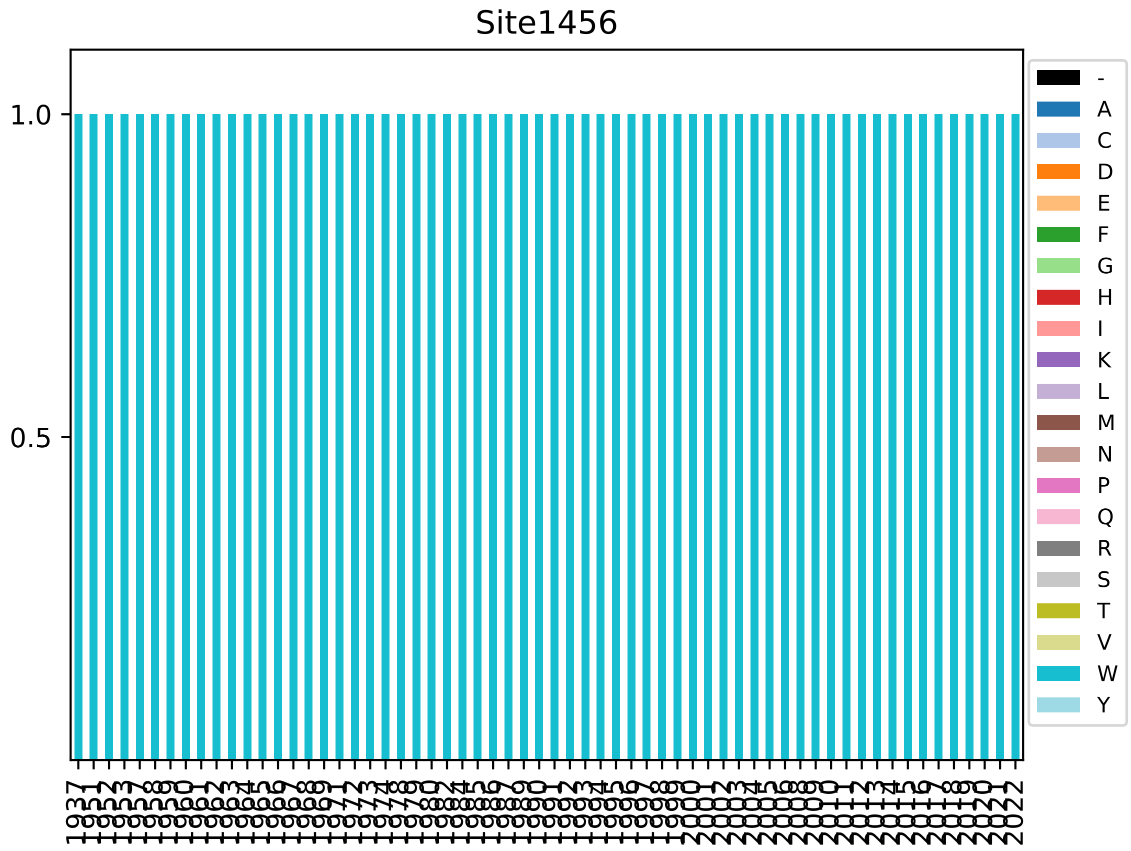

Site1456
1.0
0.5
-
A
C
D
E
F
G
H
I
K
L
M
N
P
Q
R
S
T
V
W
Y

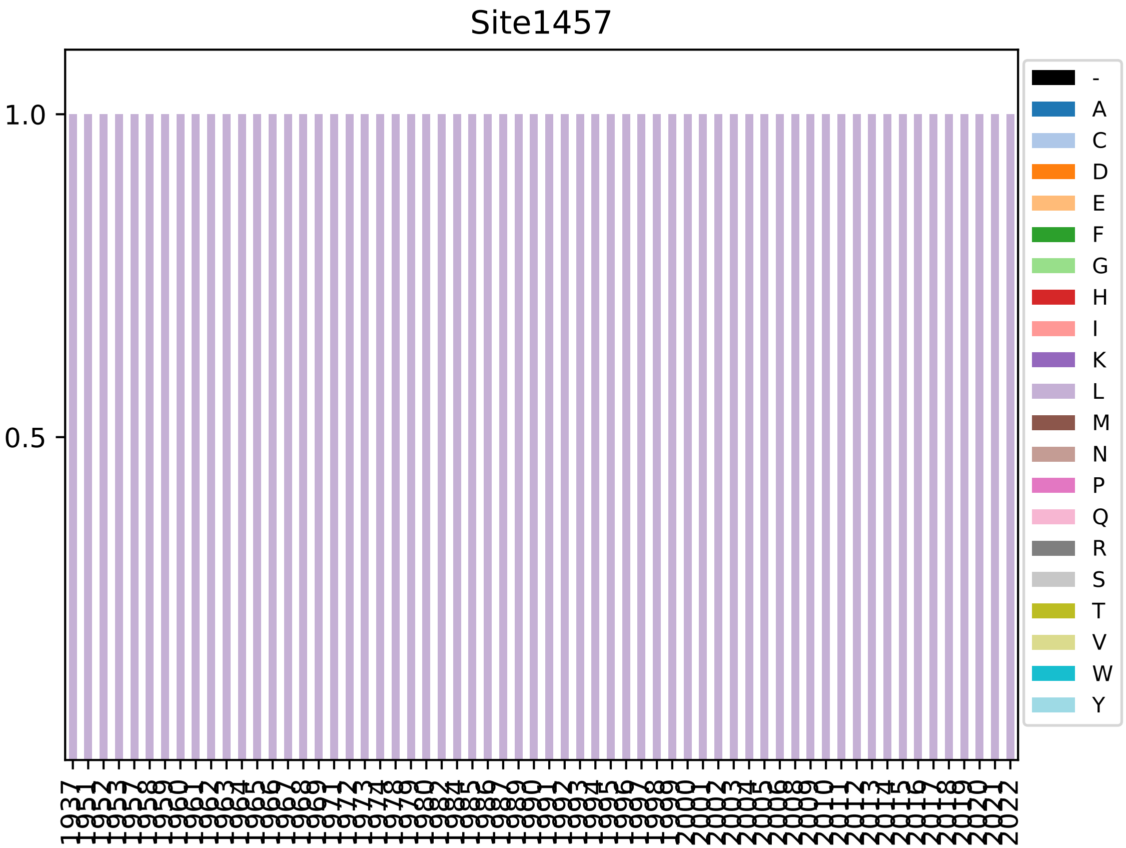
Site1457
1.0
0.5
-
A
C
D
E
F
G
H
I
K
L
M
N
P
Q
R
S
T
V
W
Y

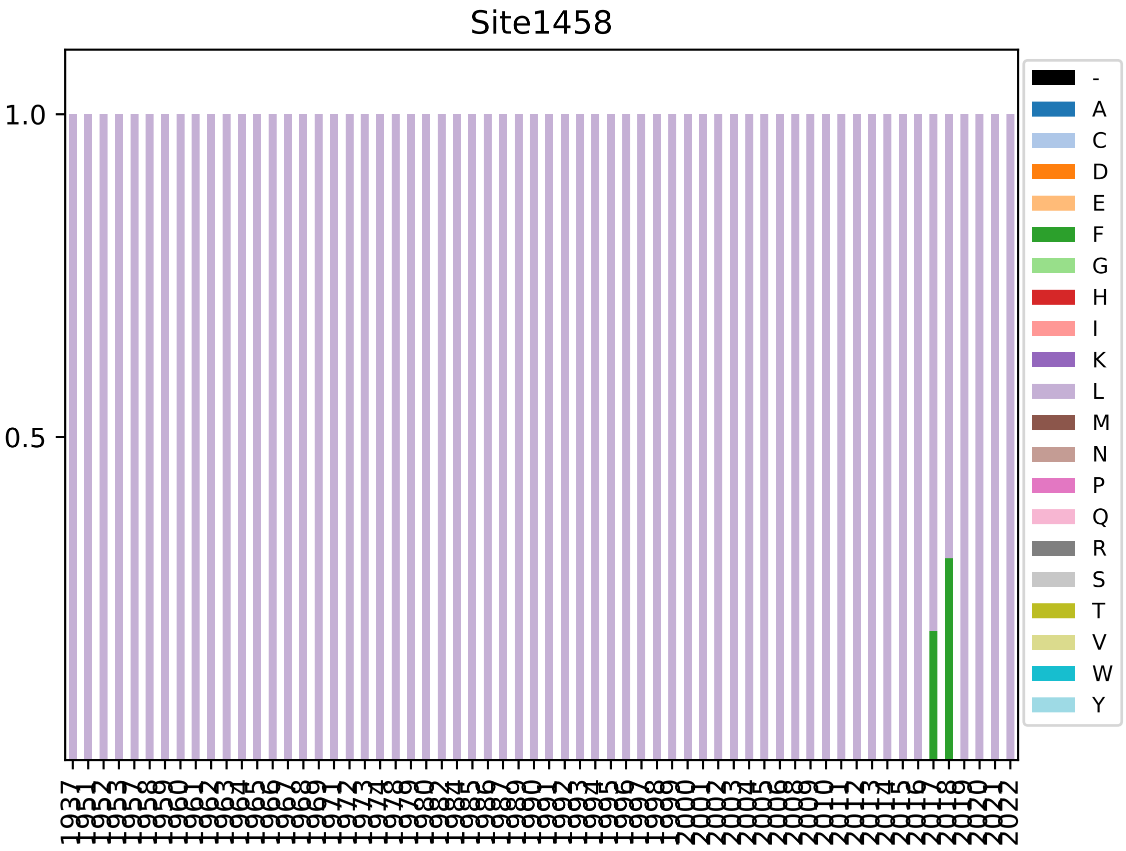
Site1458
1.0
0.5
-
A
C
D
E
F
G
H
I
K
L
M
N
P
Q
R
S
T
V
W
Y

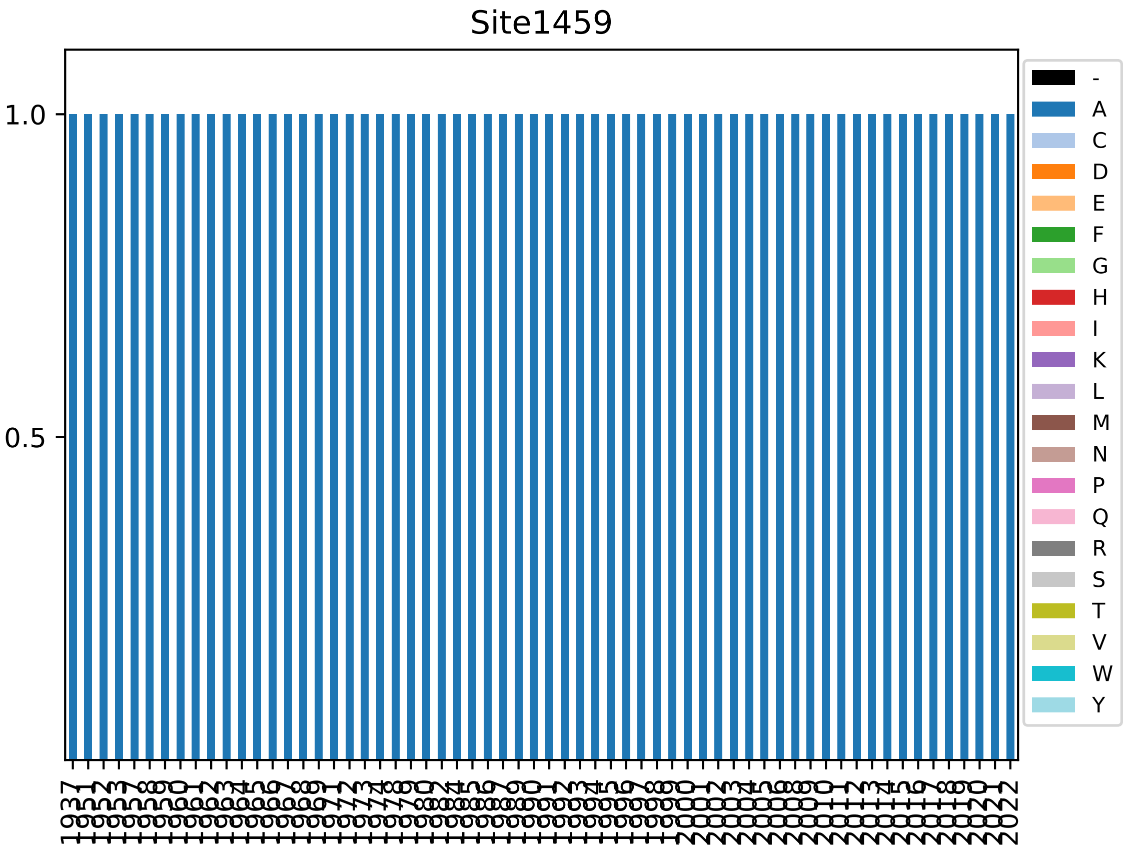
Site1459
1.0
0.5
-
A
C
D
E
F
G
H
I
K
L
M
N
P
Q
R
S
T
V
W
Y

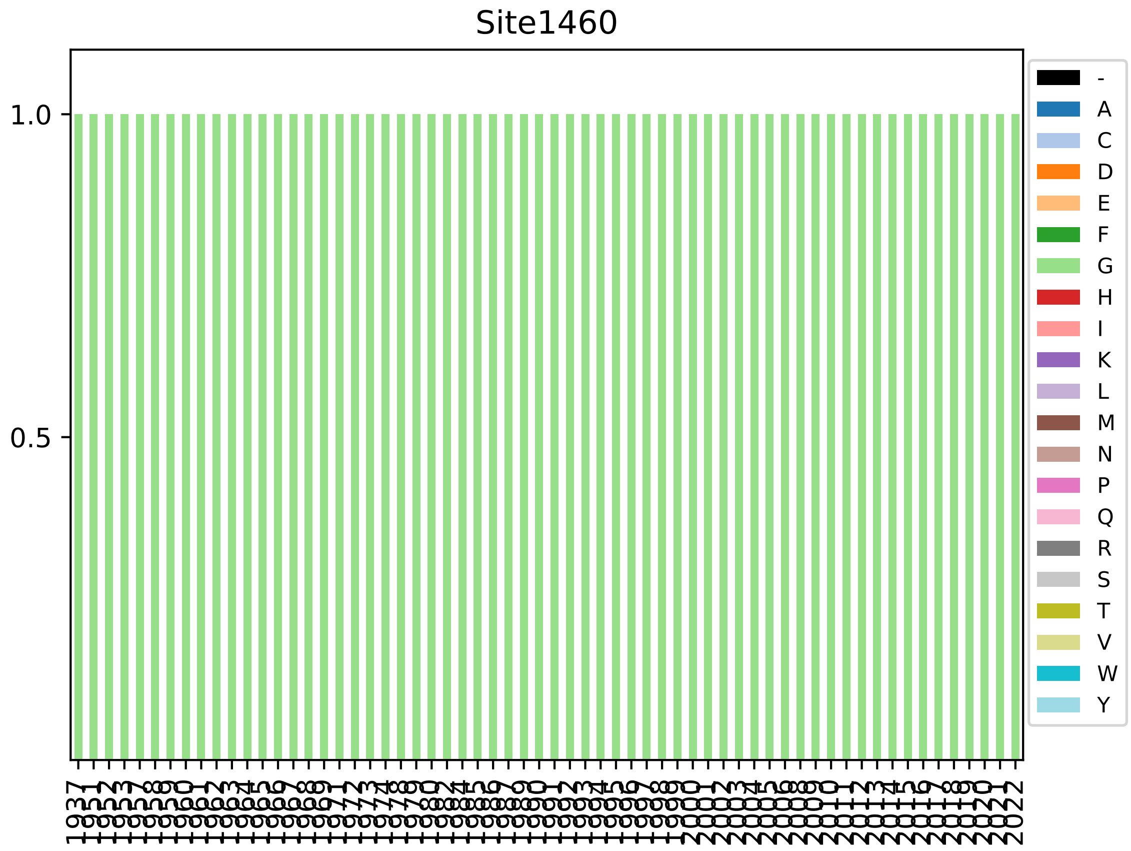
Site1460
1.0
0.5
-
A
C
D
E
F
G
H
I
K
L
M
N
P
Q
R
S
T
V
W
Y

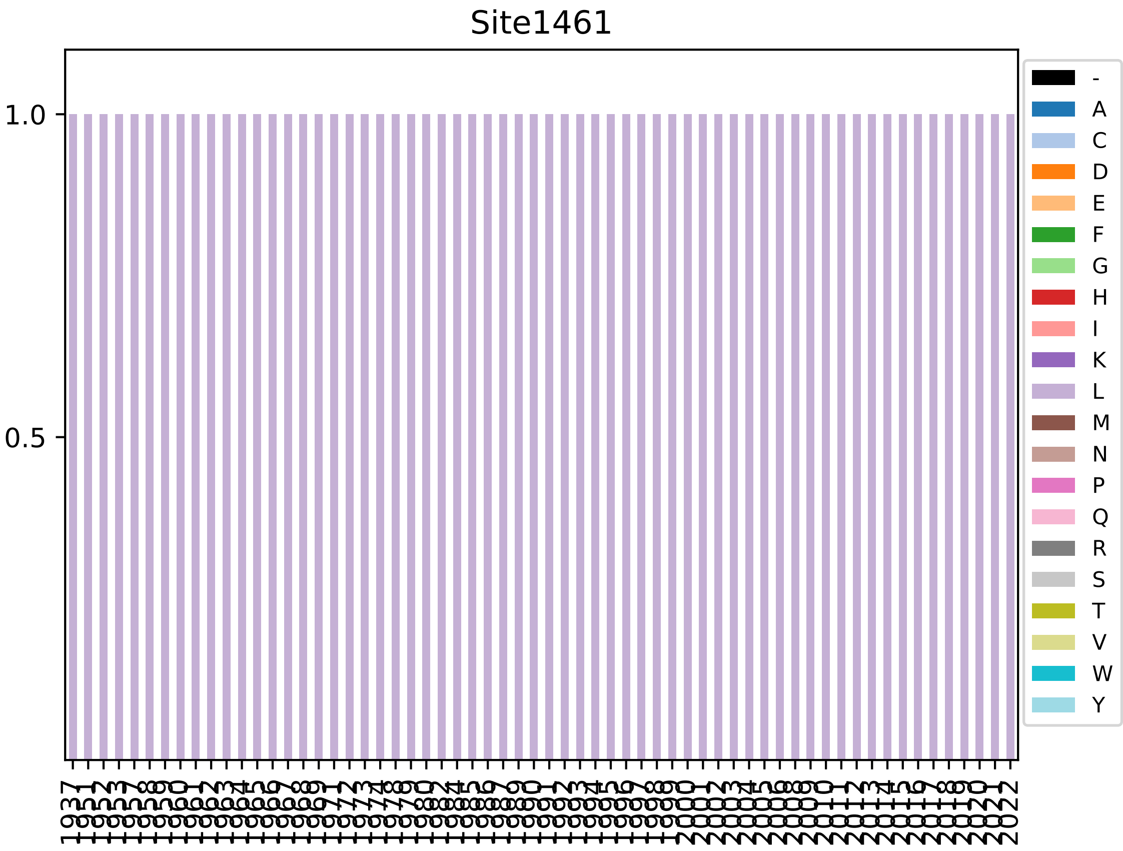
Site1461
1.0
0.5
-
A
C
D
E
F
G
H
I
K
L
M
N
P
Q
R
S
T
V
W
Y

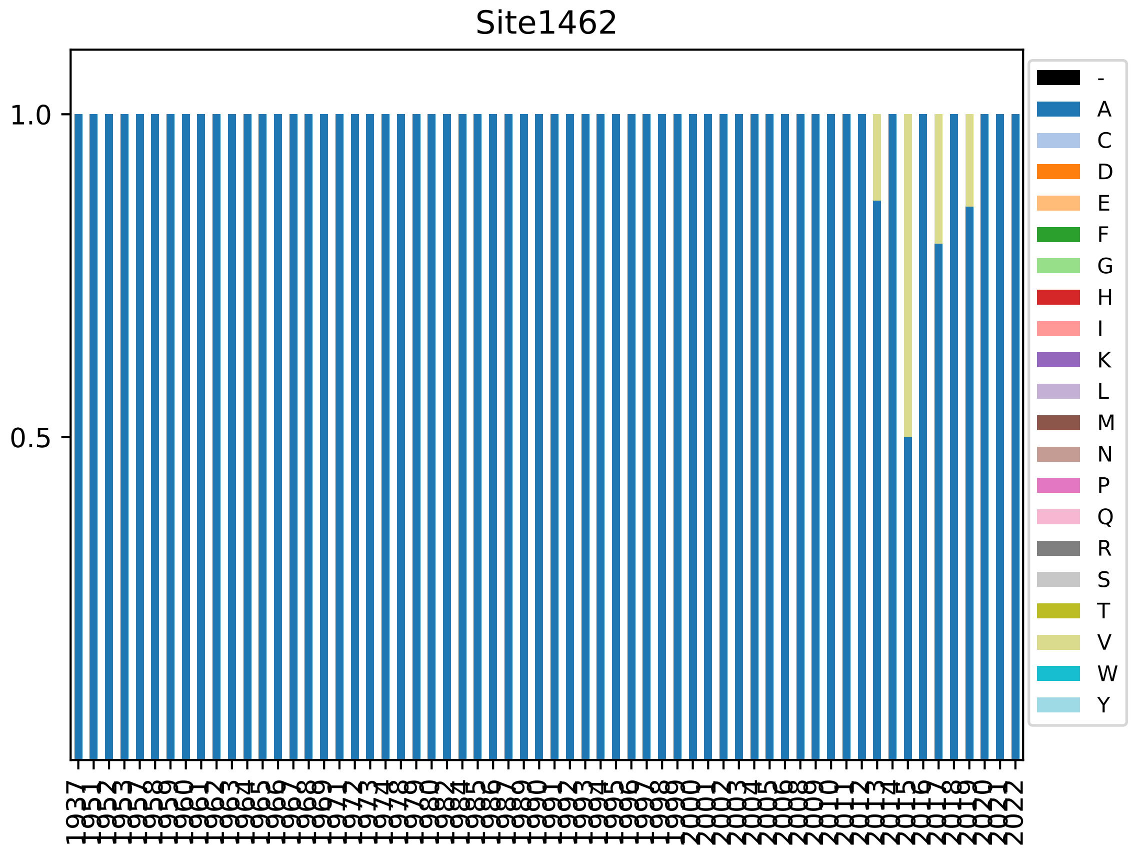
Site1462
1.0
0.5
-
A
C
D
E
F
G
H
I
K
L
M
N
P
Q
R
S
T
V
W
Y

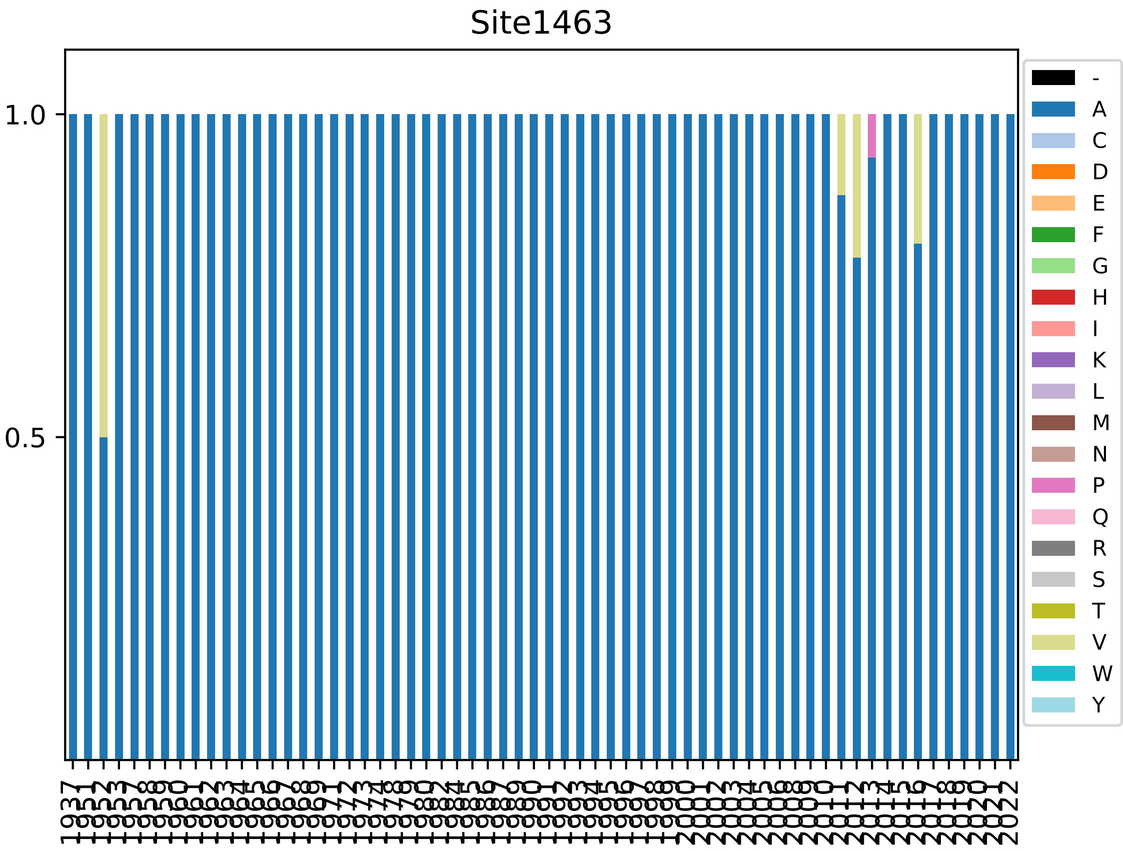
Site1463
1.0
0.5
-
A
C
D
E
F
G
H
I
K
L
M
N
P
Q
R
S
T
V
W
Y

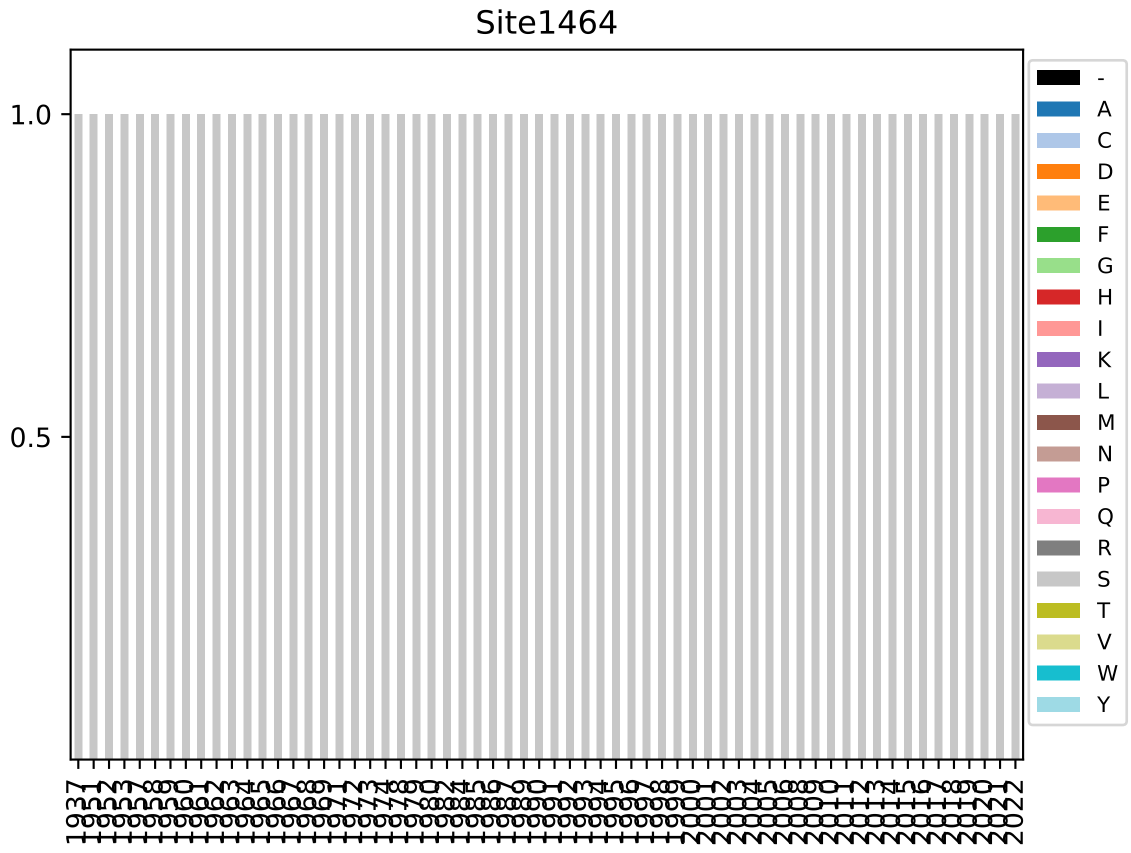

Site1464
1.0
0.5
-
A
C
D
E
F
G
H
I
K
L
M
N
P
Q
R
S
T
V
W
Y

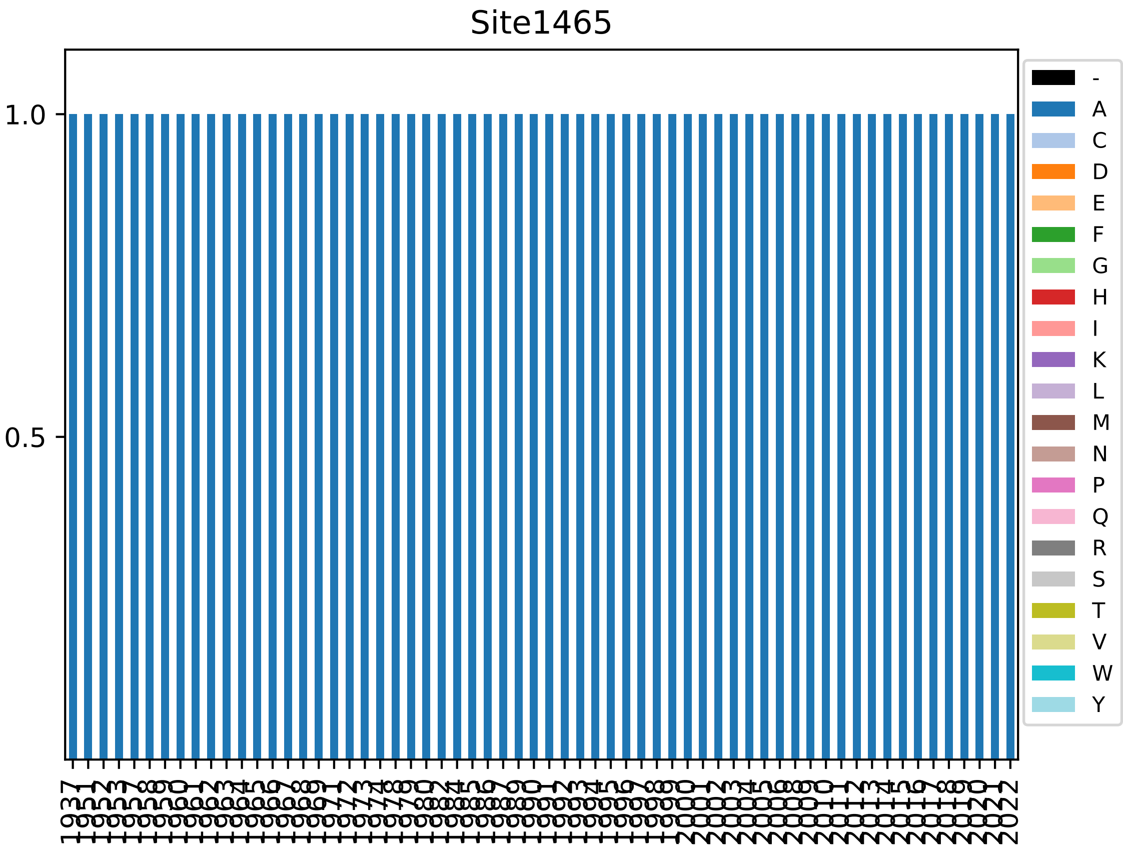
Site1465
1.0
0.5
-
A
C
D
E
F
G
H
I
K
L
M
N
P
Q
R
S
T
V
W
Y

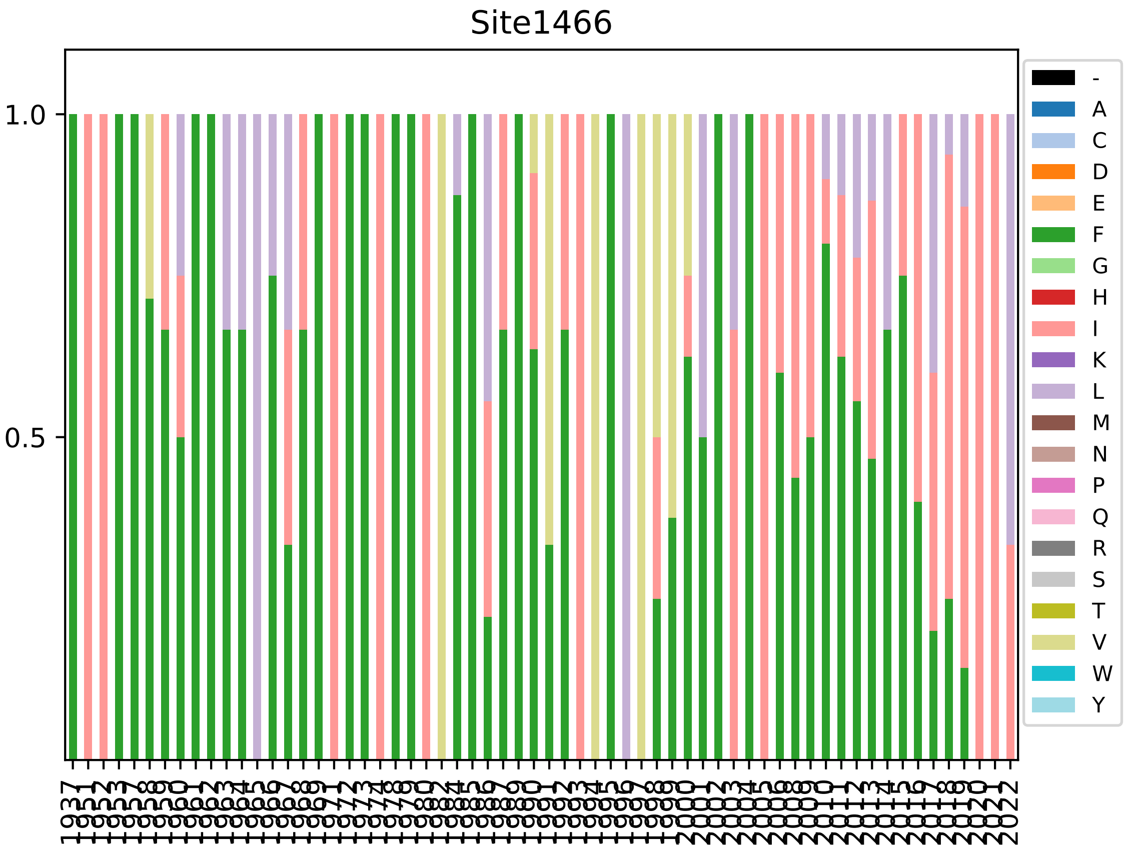
Site1466
1.0
0.5
-
A
C
D
E
F
G
H
I
K
L
M
N
P
Q
R
S
T
V
W
Y

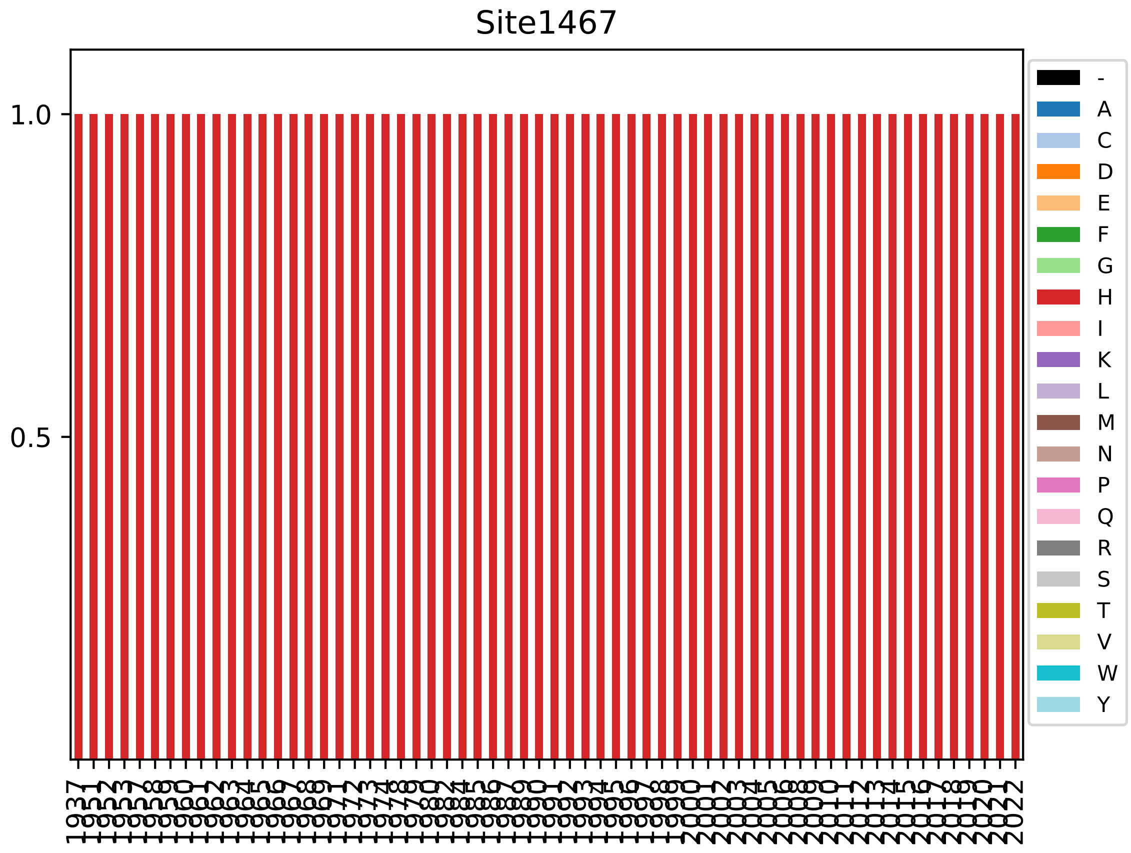
Site1467
1.0
0.5
-
A
C
D
E
F
G
H
I
K
L
M
N
P
Q
R
S
T
V
W
Y

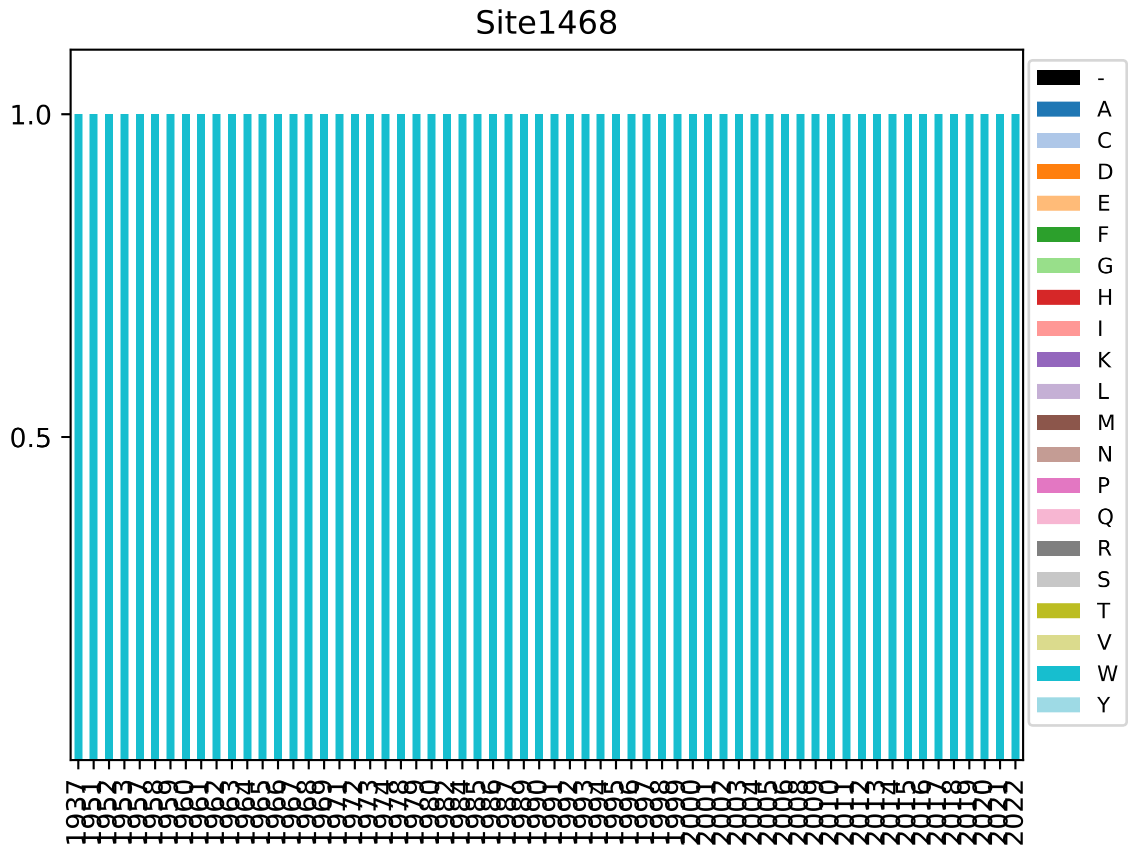
Site1468
1.0
0.5
-
A
C
D
E
F
G
H
I
K
L
M
N
P
Q
R
S
T
V
W
Y

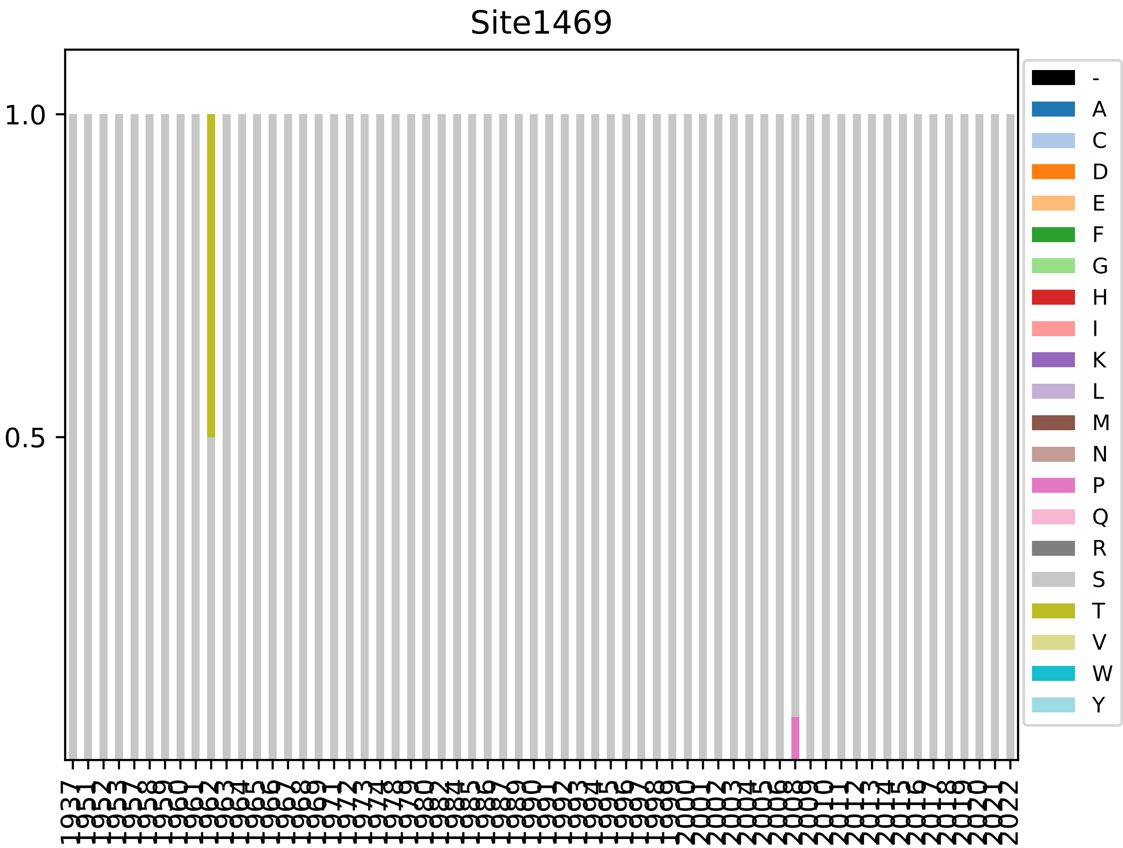
Site1469
1.0
0.5
-
A
C
D
E
F
G
H
I
K
L
M
N
P
Q
R
S
T
V
W
Y

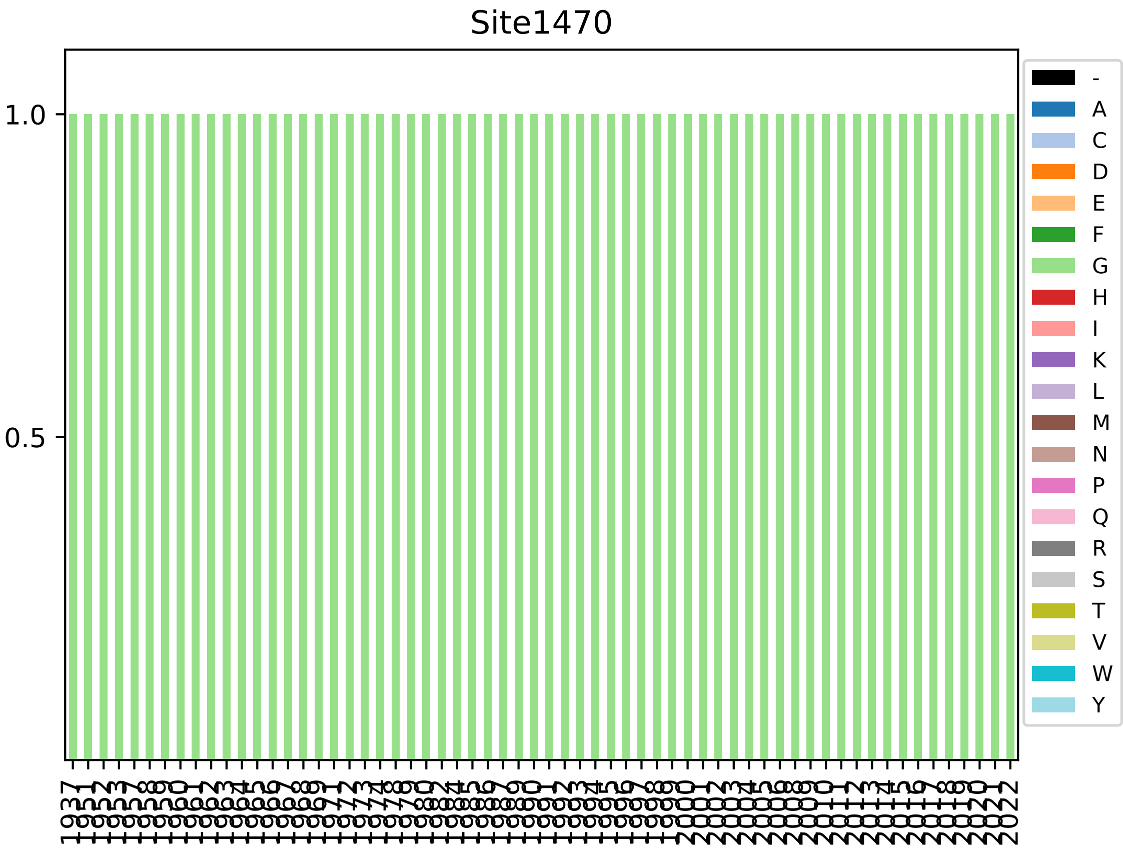
Site1470
1.0
0.5
-
A
C
D
E
F
G
H
I
K
L
M
N
P
Q
R
S
T
V
W
Y

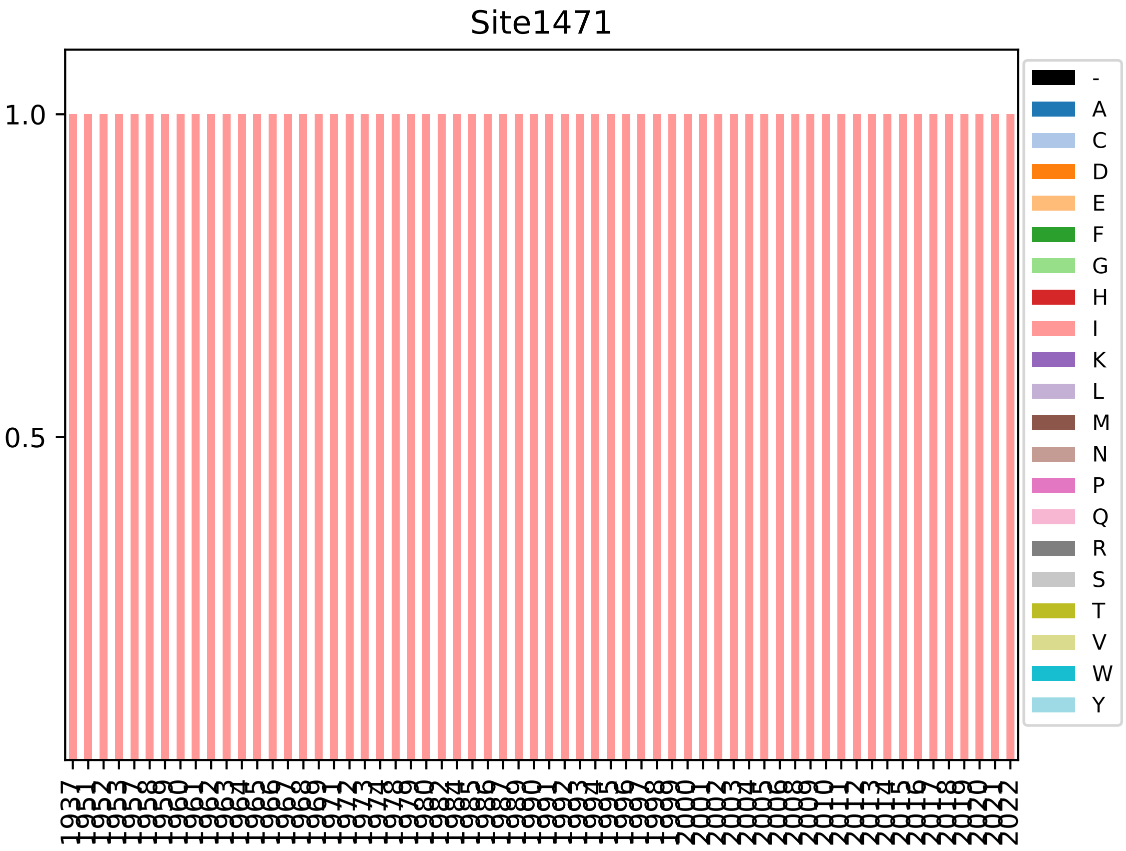
Site1471
1.0
0.5
-
A
C
D
E
F
G
H
I
K
L
M
N
P
Q
R
S
T
V
W
Y

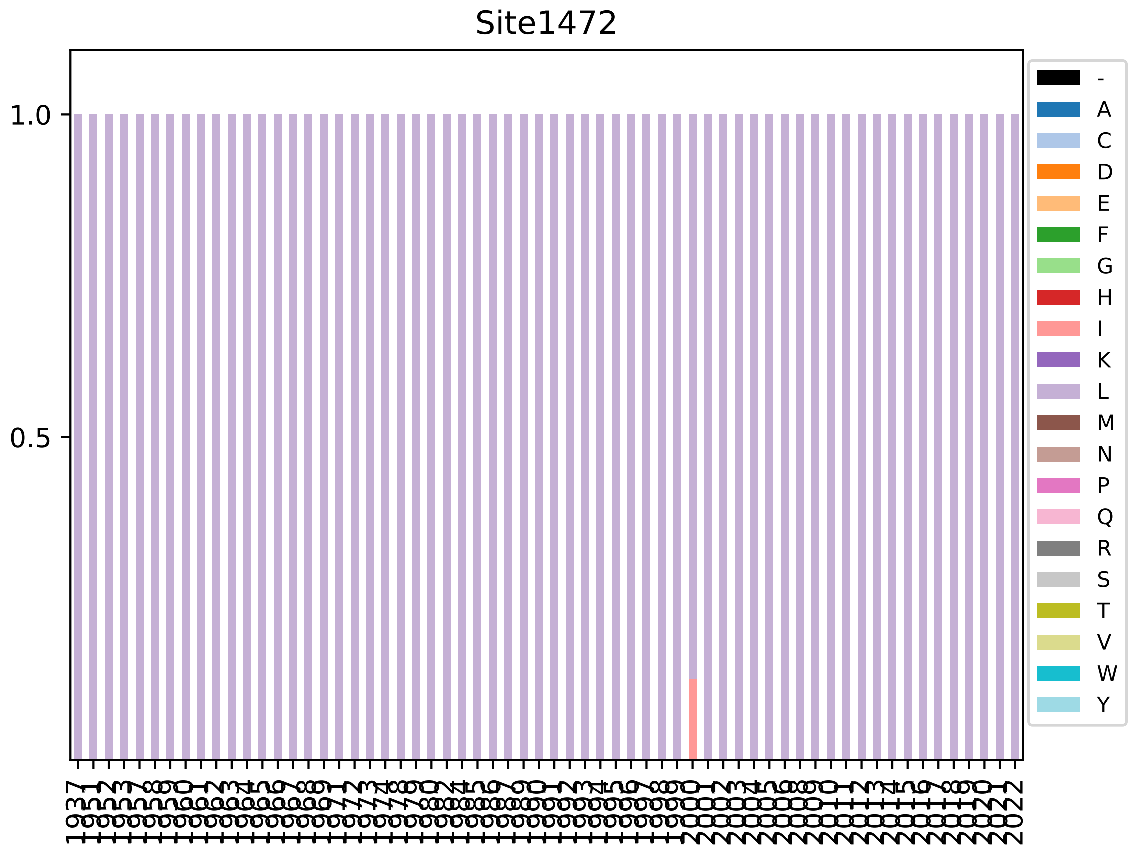

Site1472
1.0
0.5
-
A
C
D
E
F
G
H
I
K
L
M
N
P
Q
R
S
T
V
W
Y

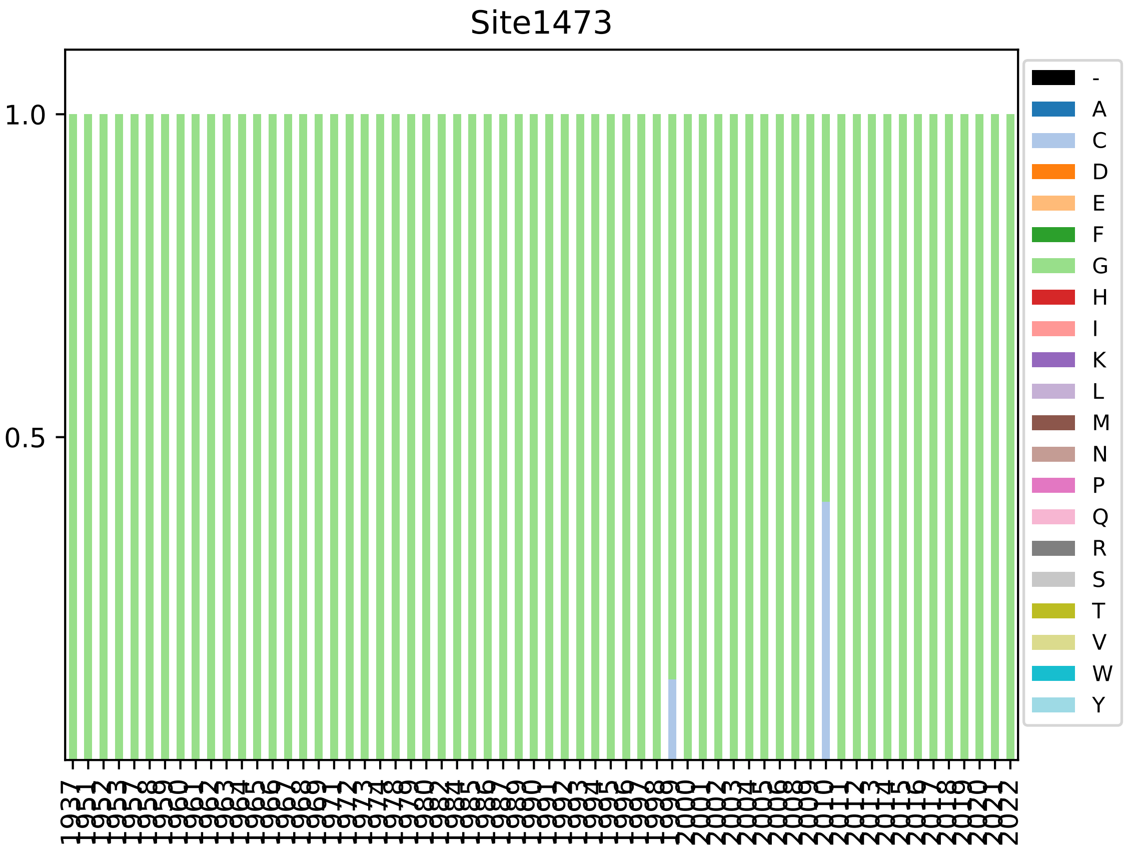
Site1473
1.0
0.5
-
A
C
D
E
F
G
H
I
K
L
M
N
P
Q
R
S
T
V
W
Y

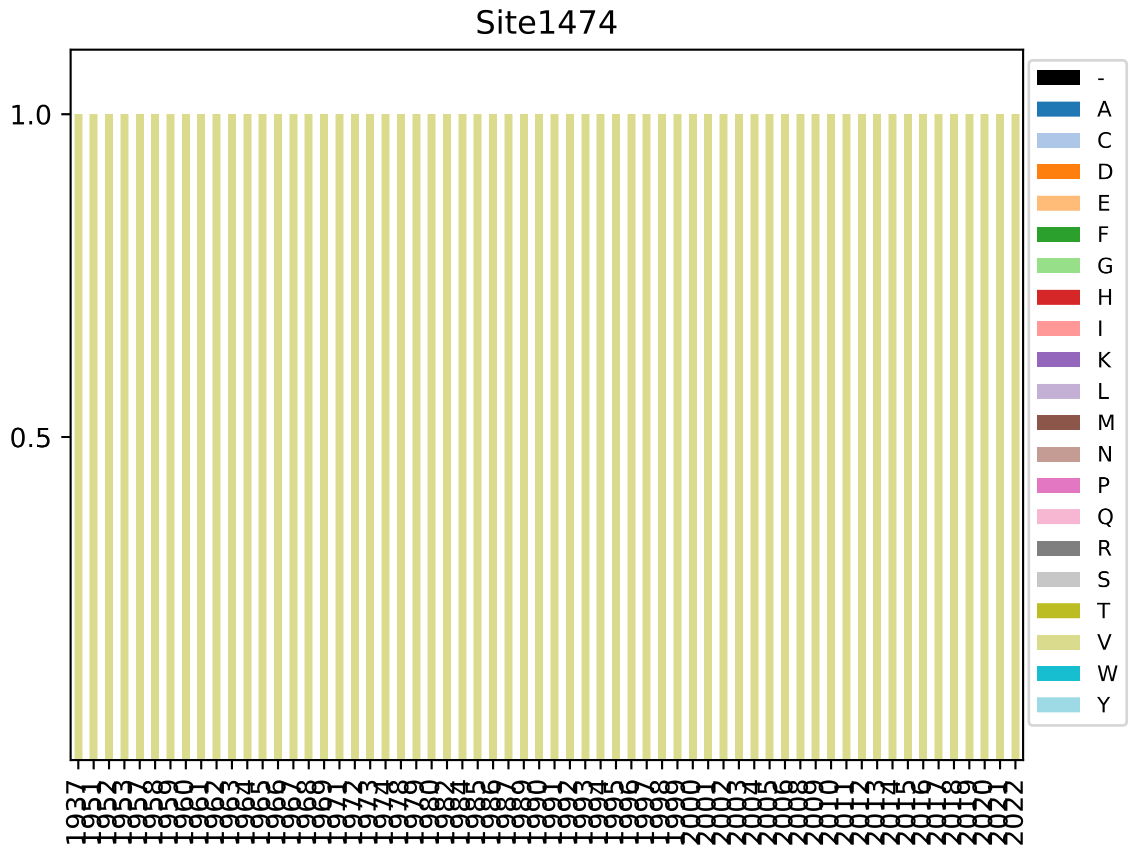
Site1474
1.0
0.5
-
A
C
D
E
F
G
H
I
K
L
M
N
P
Q
R
S
T
V
W
Y

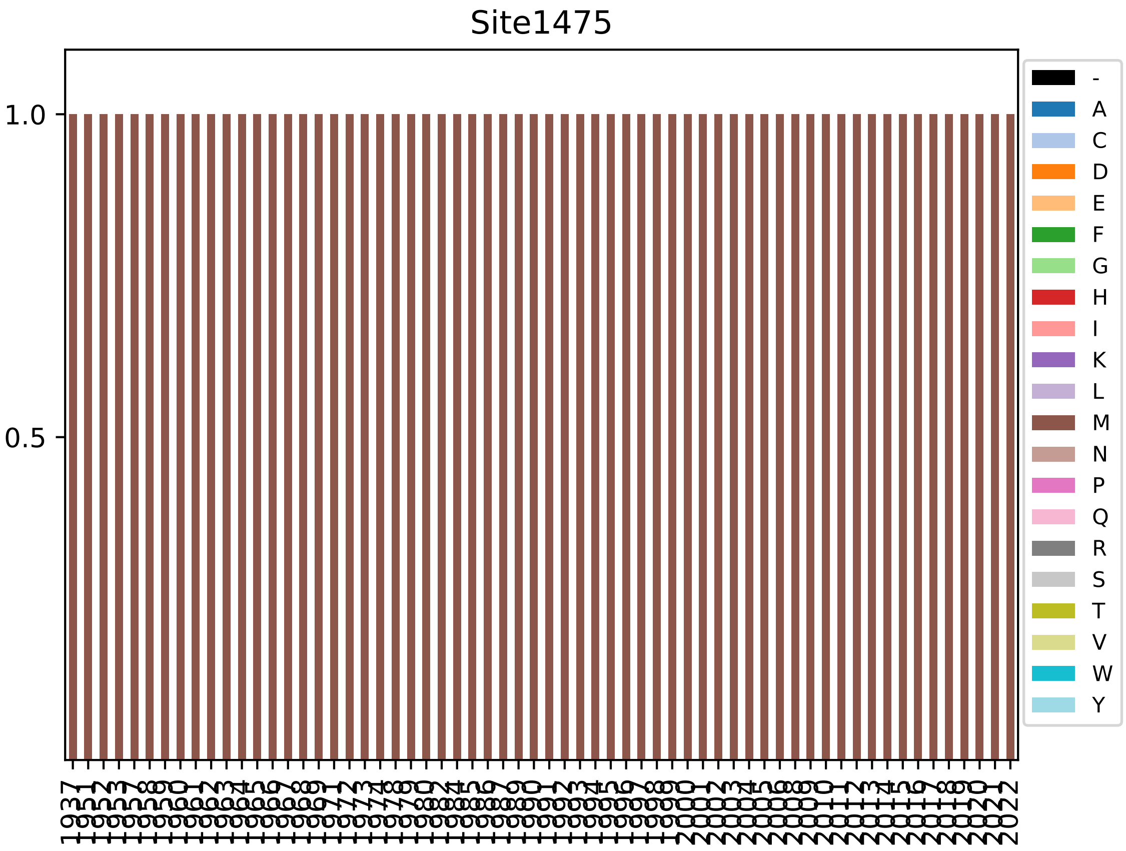
Site1475
1.0
0.5
-
A
C
D
E
F
G
H
I
K
L
M
N
P
Q
R
S
T
V
W
Y

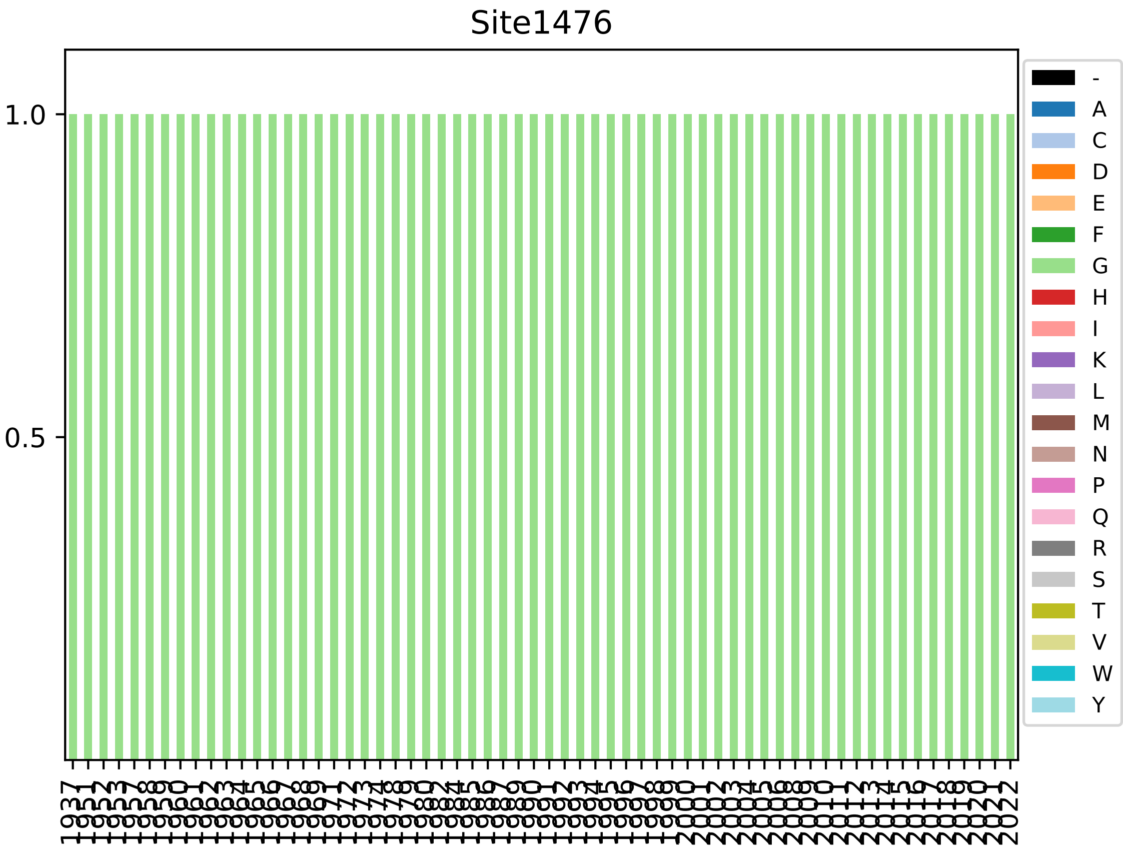

Site1476
1.0
0.5
-
A
C
D
E
F
G
H
I
K
L
M
N
P
Q
R
S
T
V
W
Y

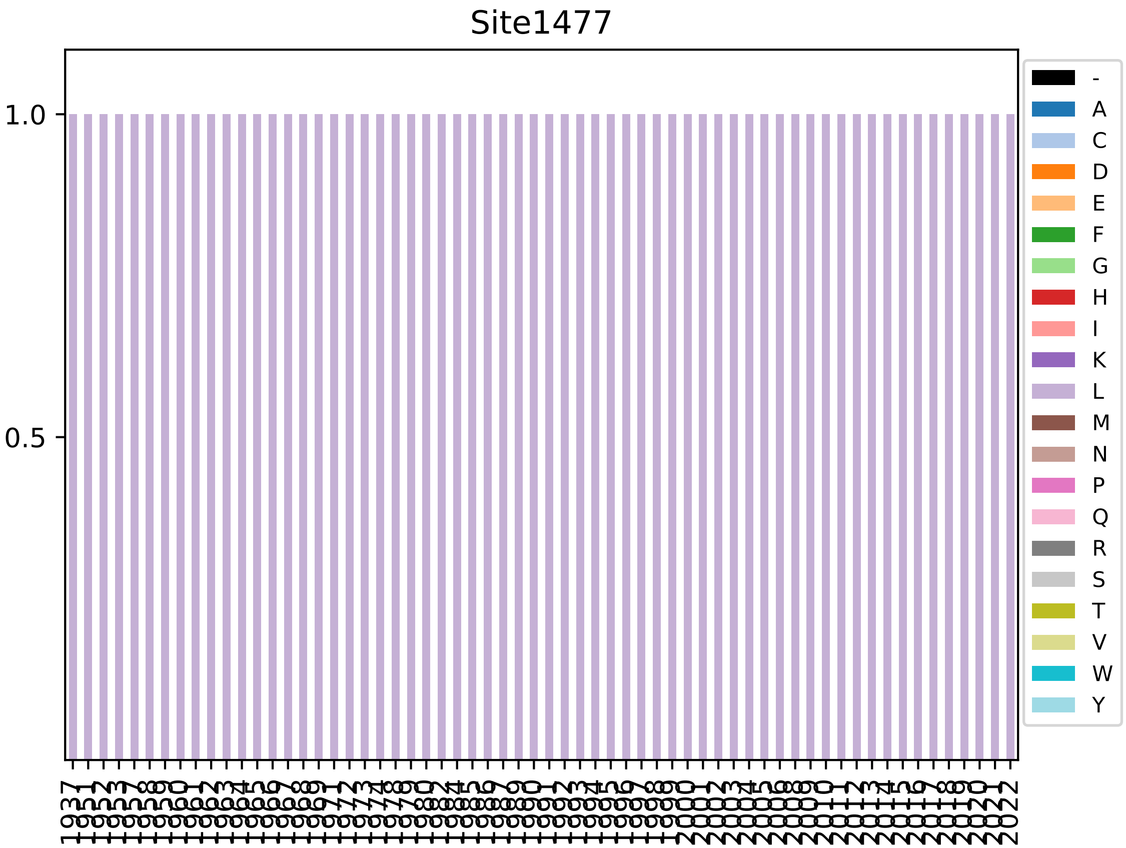
Site1477
1.0
0.5
-
A
C
D
E
F
G
H
I
K
L
M
N
P
Q
R
S
T
V
W
Y

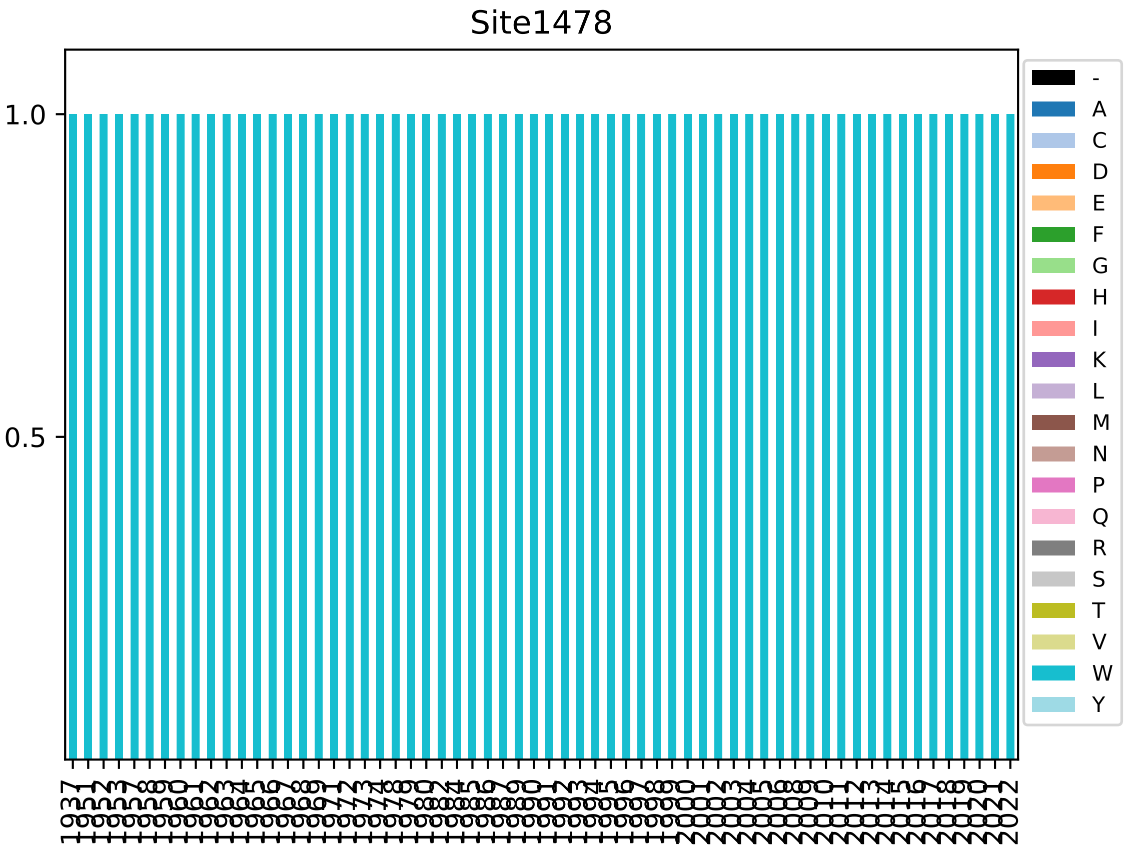

Site1478
1.0
0.5
-
A
C
D
E
F
G
H
I
K
L
M
N
P
Q
R
S
T
V
W
Y

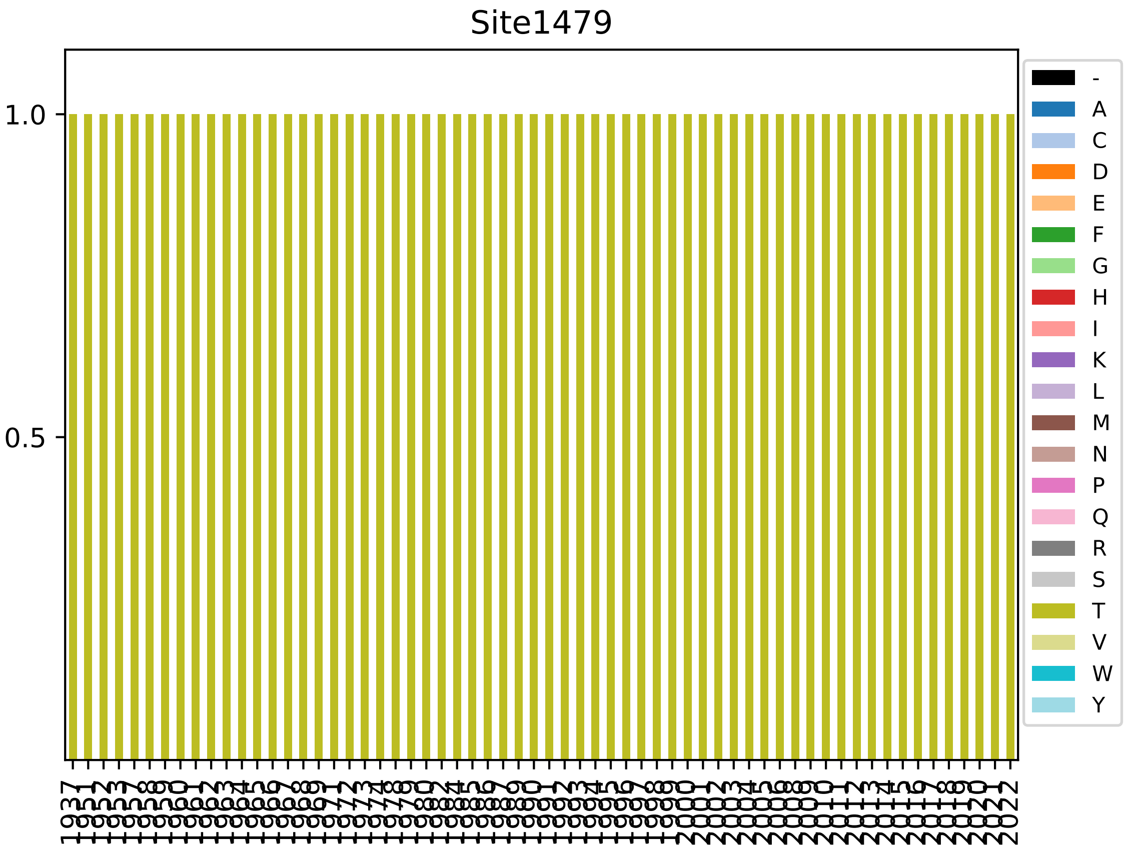
Site1479
1.0
0.5
-
A
C
D
E
F
G
H
I
K
L
M
N
P
Q
R
S
T
V
W
Y

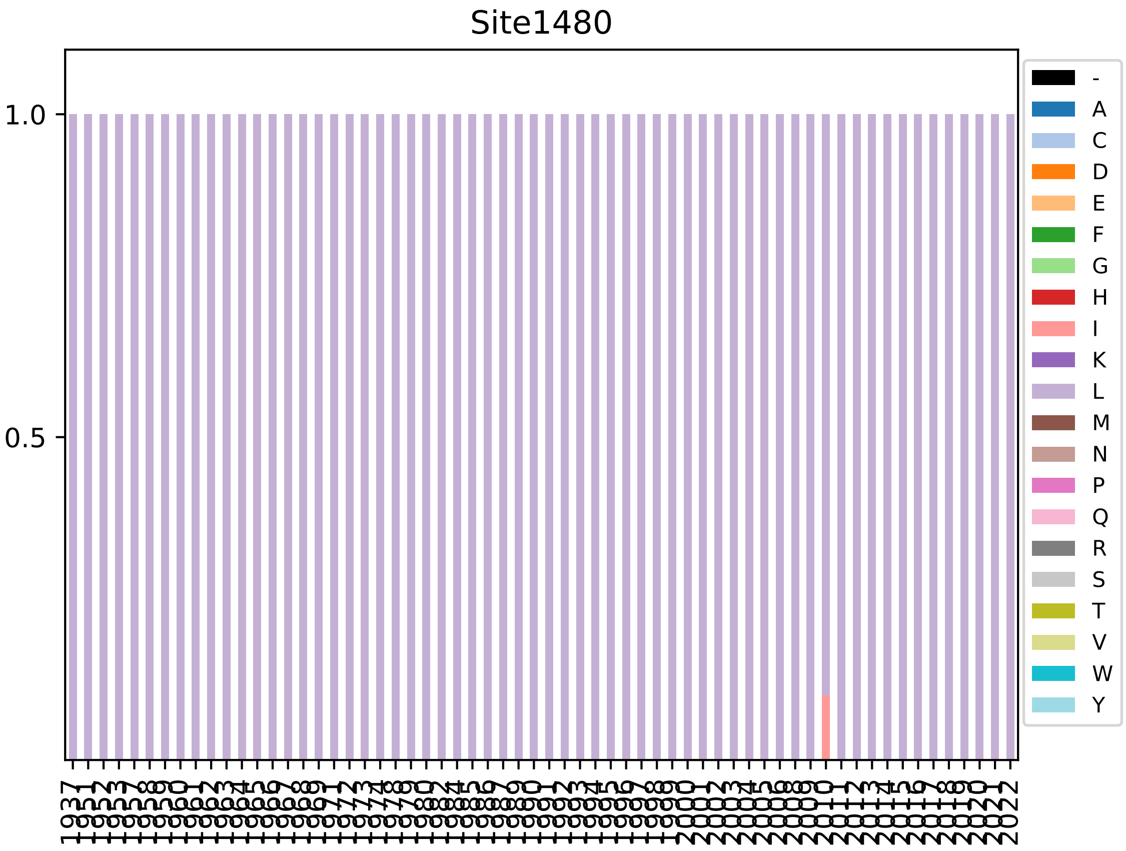
Site1480
1.0
0.5
-
A
C
D
E
F
G
H
I
K
L
M
N
P
Q
R
S
T
V
W
Y

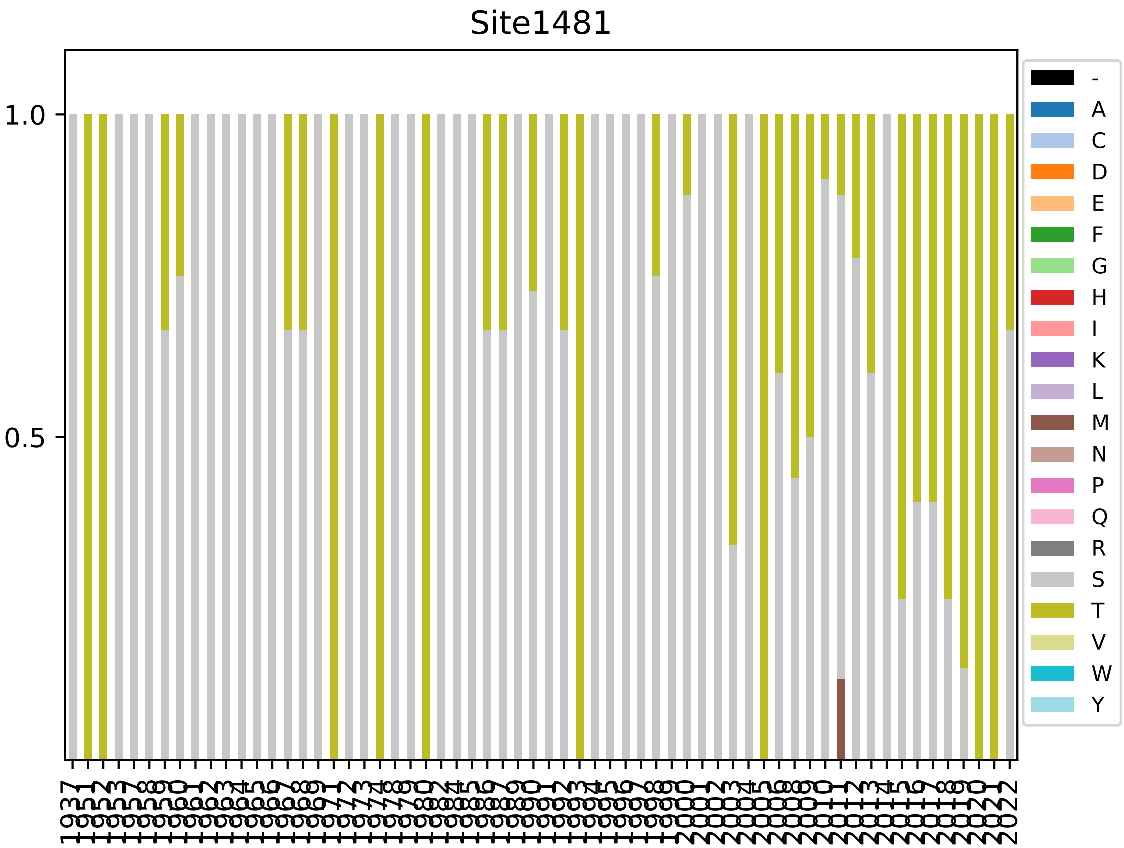
Site1481
1.0
0.5
-
A
C
D
E
F
G
H
I
K
L
M
N
P
Q
R
S
T
V
W
Y

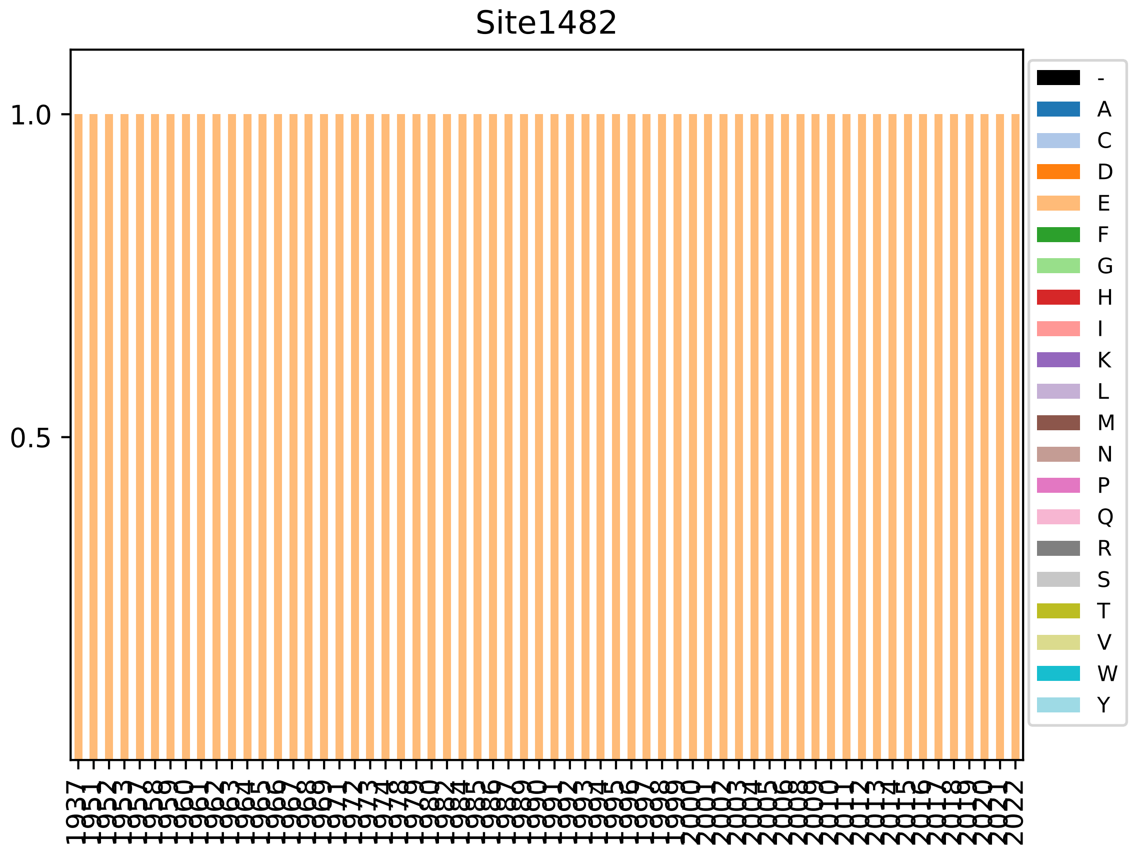
Site1482
1.0
0.5
-
A
C
D
E
F
G
H
I
K
L
M
N
P
Q
R
S
T
V
W
Y

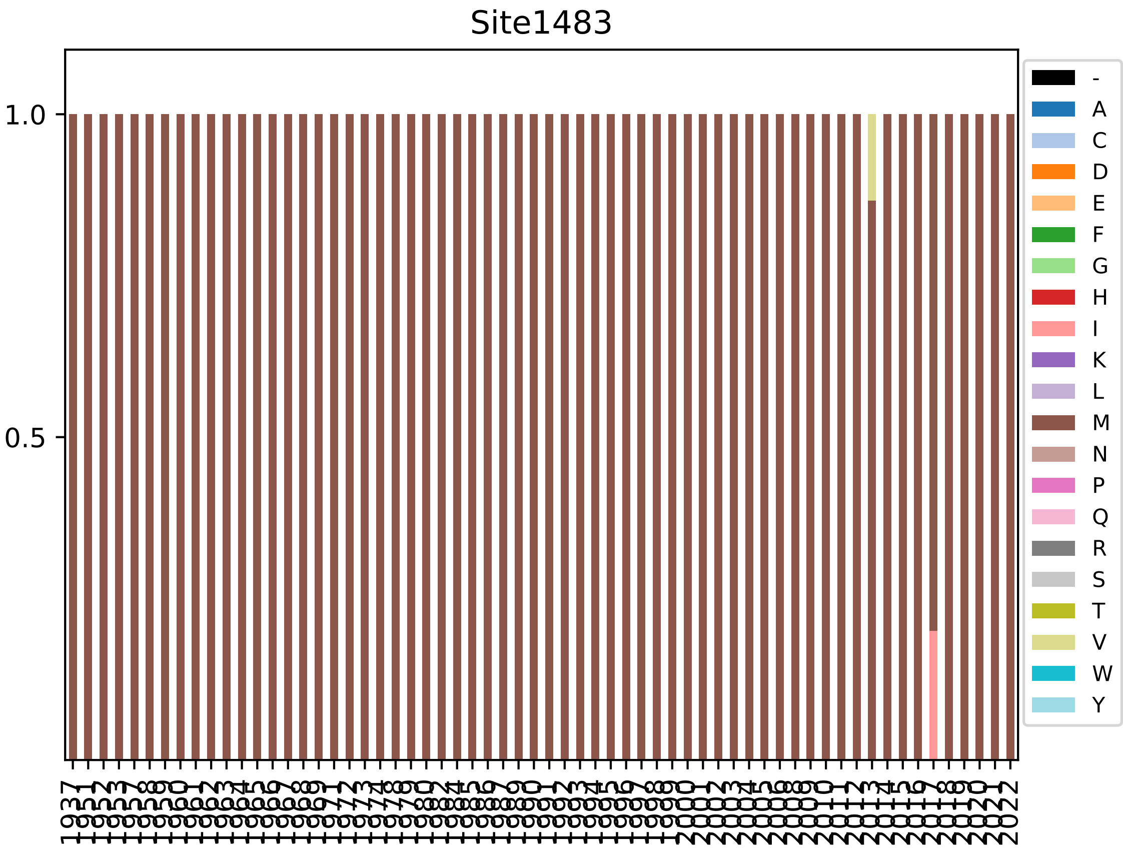
Site1483
1.0
0.5
-
A
C
D
E
F
G
H
I
K
L
M
N
P
Q
R
S
T
V
W
Y

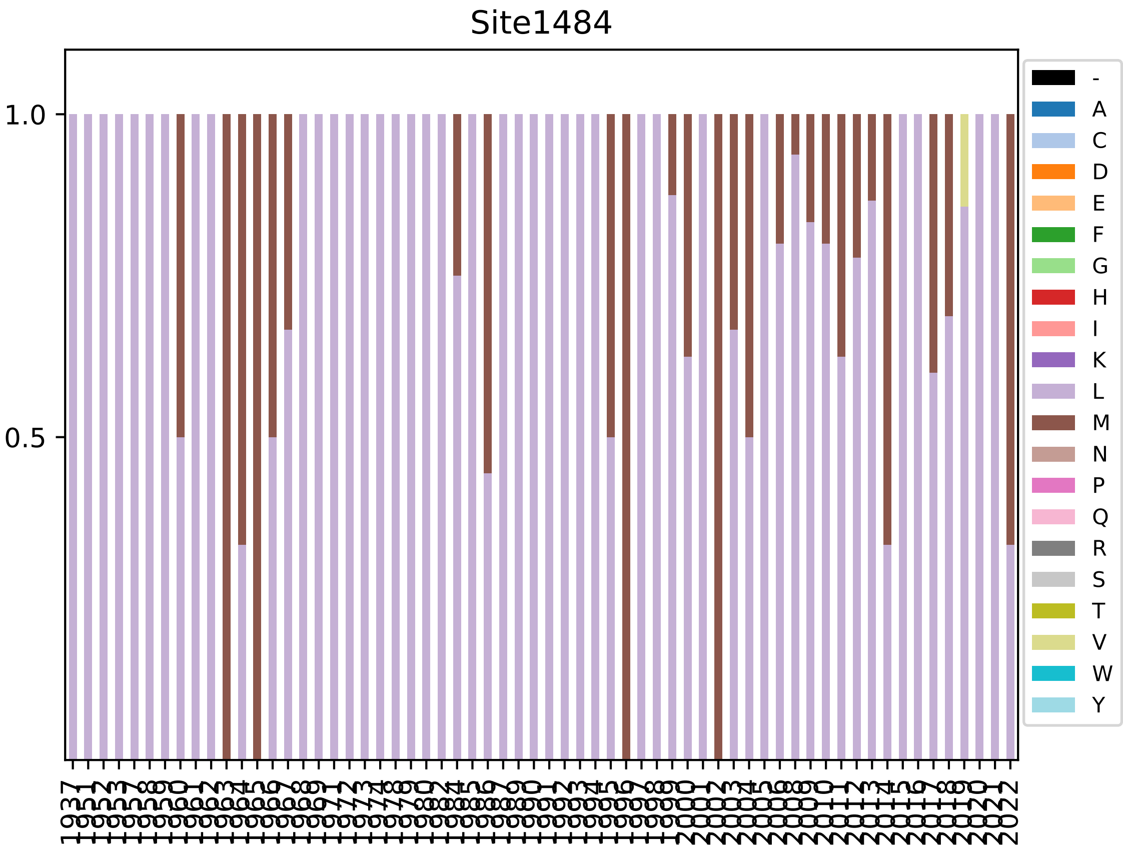
Site1484
1.0
0.5
-
A
C
D
E
F
G
H
I
K
L
M
N
P
Q
R
S
T
V
W
Y

Site1485

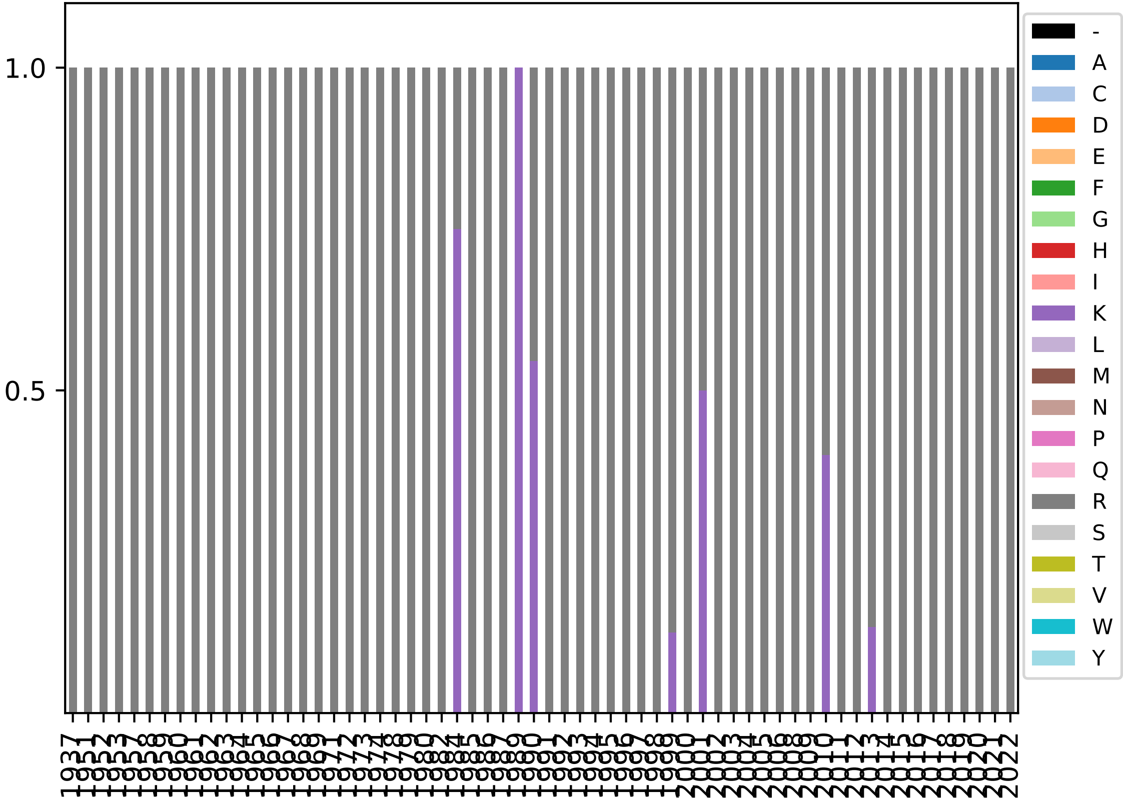

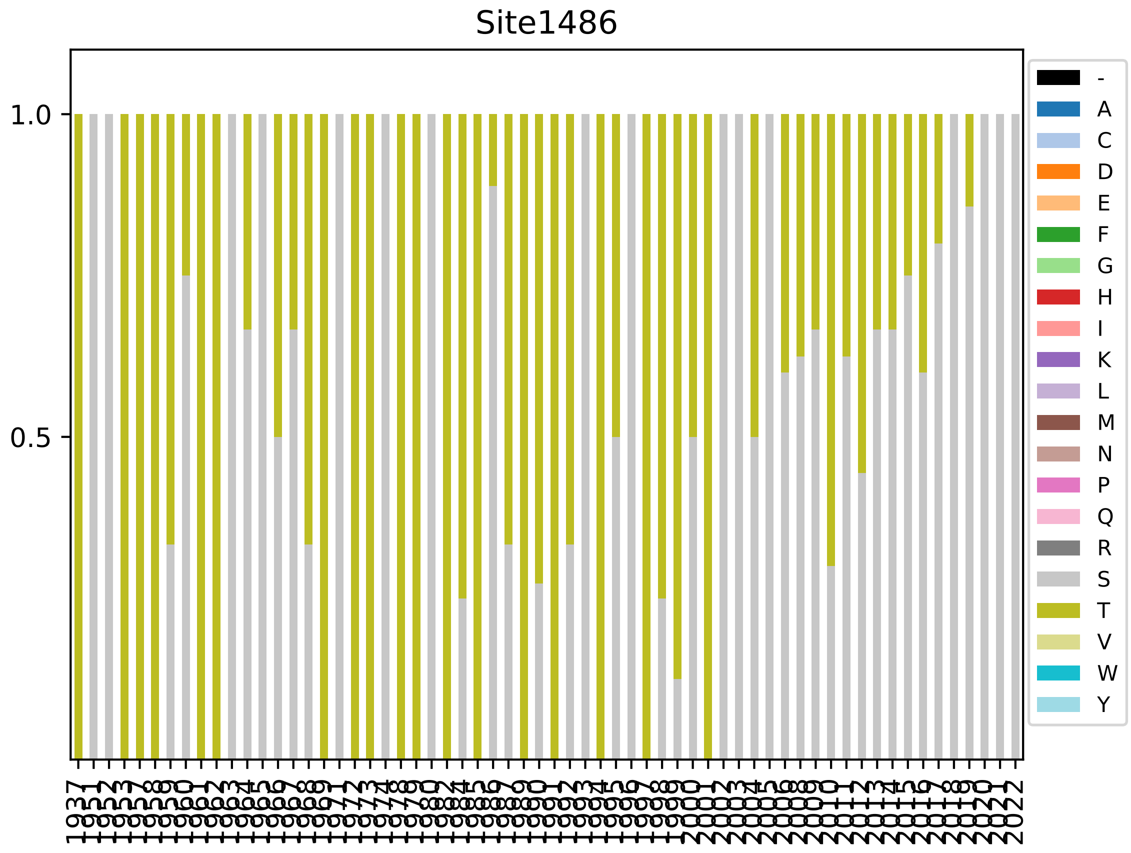
Site1486
1.0
0.5
-
A
C
D
E
F
G
H
I
K
L
M
N
P
Q
R
S
T
V
W
Y

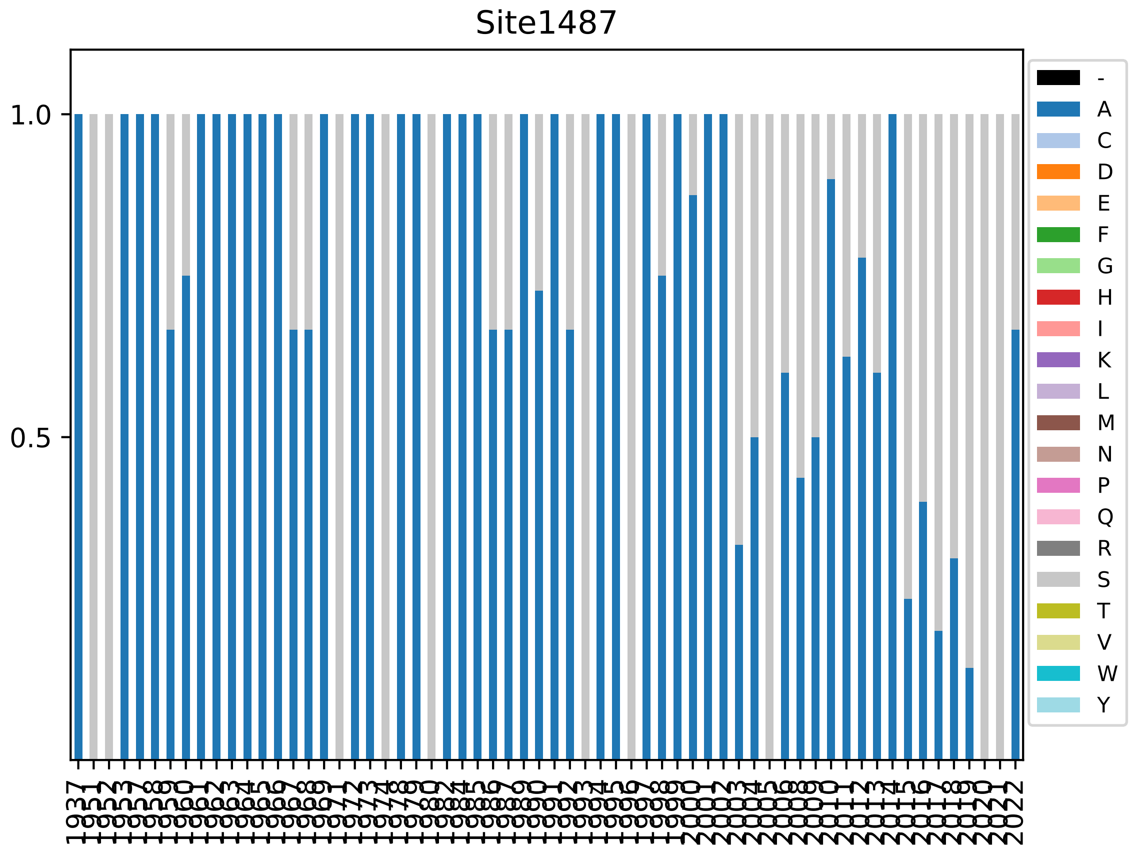
Site1487
1.0
0.5
-
A
C
D
E
F
G
H
I
K
L
M
N
P
Q
R
S
T
V
W
Y

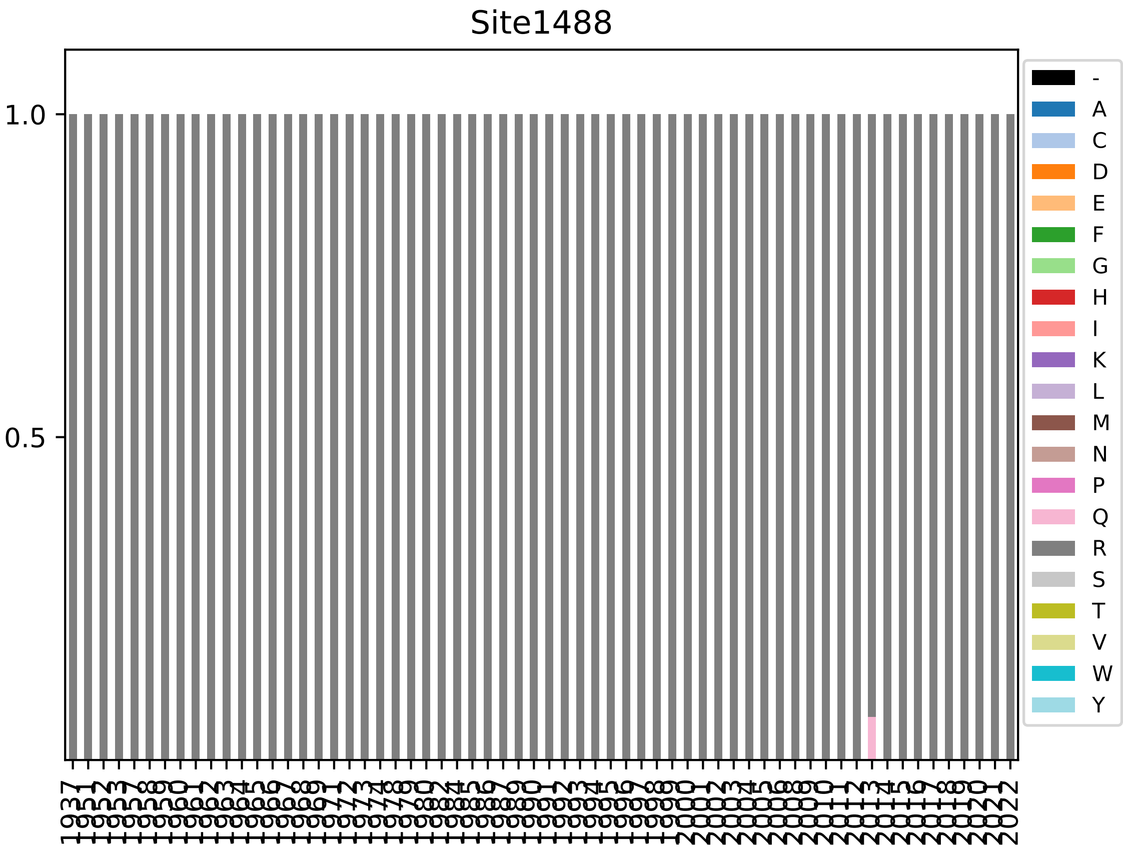
Site1488
1.0
0.5
-
A
C
D
E
F
G
H
I
K
L
M
N
P
Q
R
S
T
V
W
Y

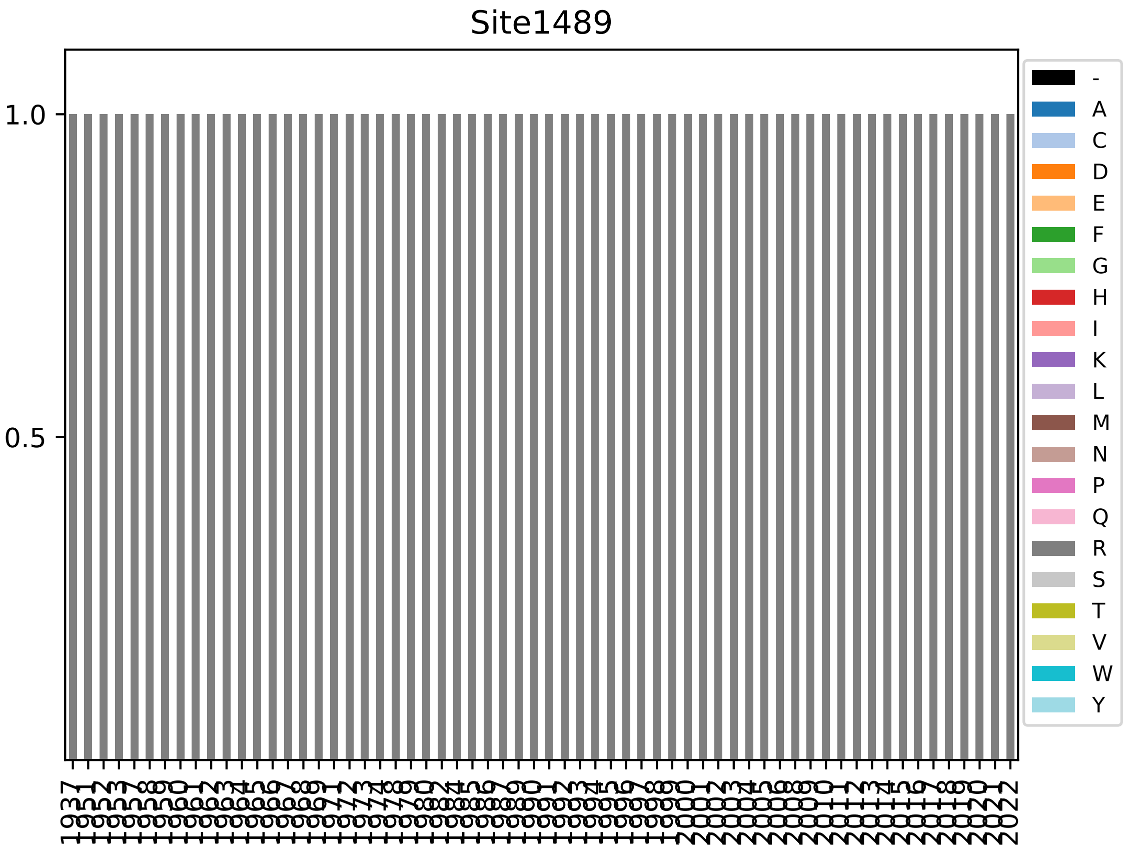
Site1489
1.0
0.5
-
A
C
D
E
F
G
H
I
K
L
M
N
P
Q
R
S
T
V
W
Y

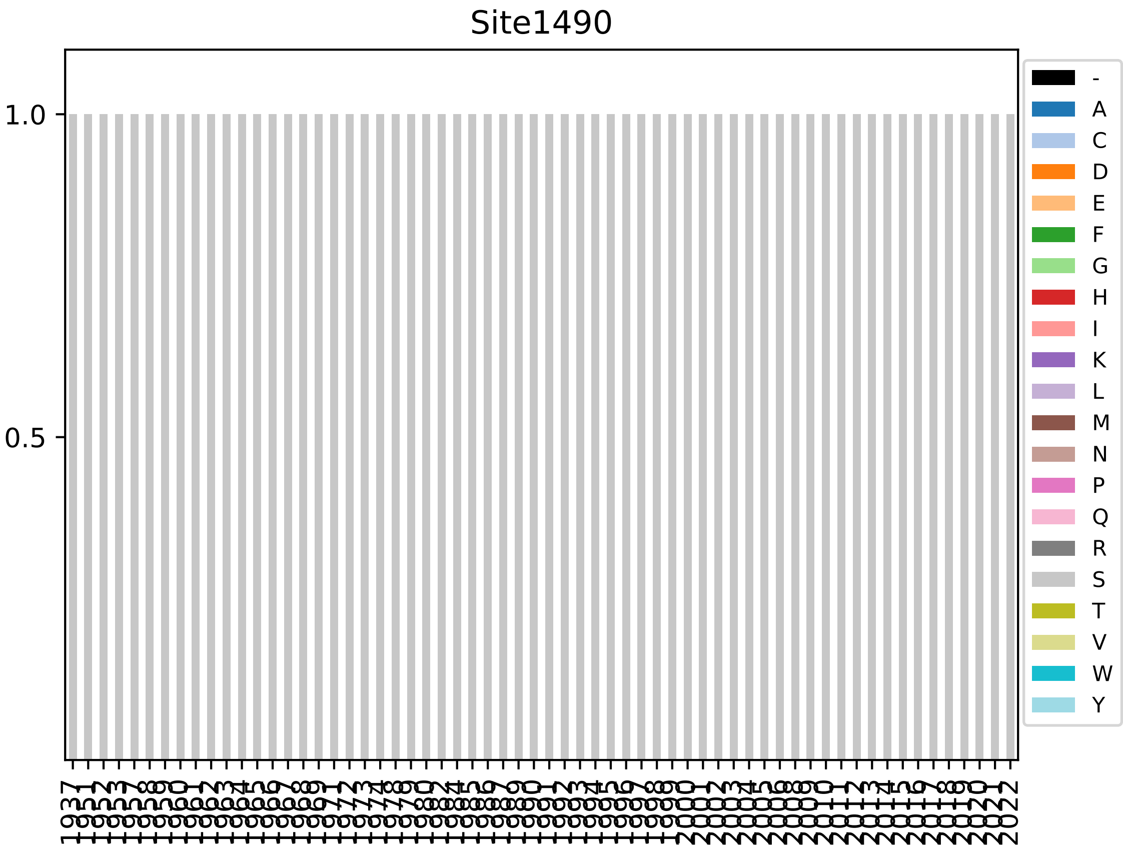
Site1490
1.0
0.5
-
A
C
D
E
F
G
H
I
K
L
M
N
P
Q
R
S
T
V
W
Y

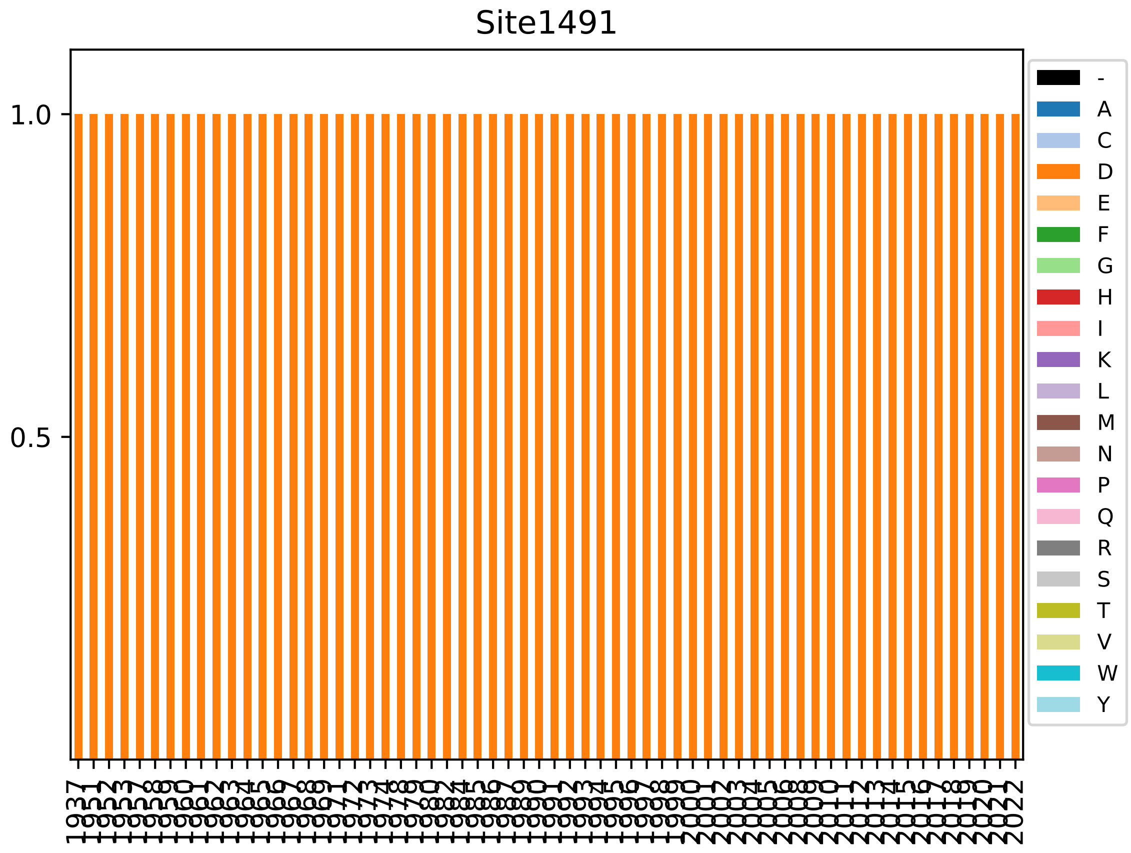
Site1491
1.0
0.5
-
A
C
D
E
F
G
H
I
K
L
M
N
P
Q
R
S
T
V
W
Y

Site1492

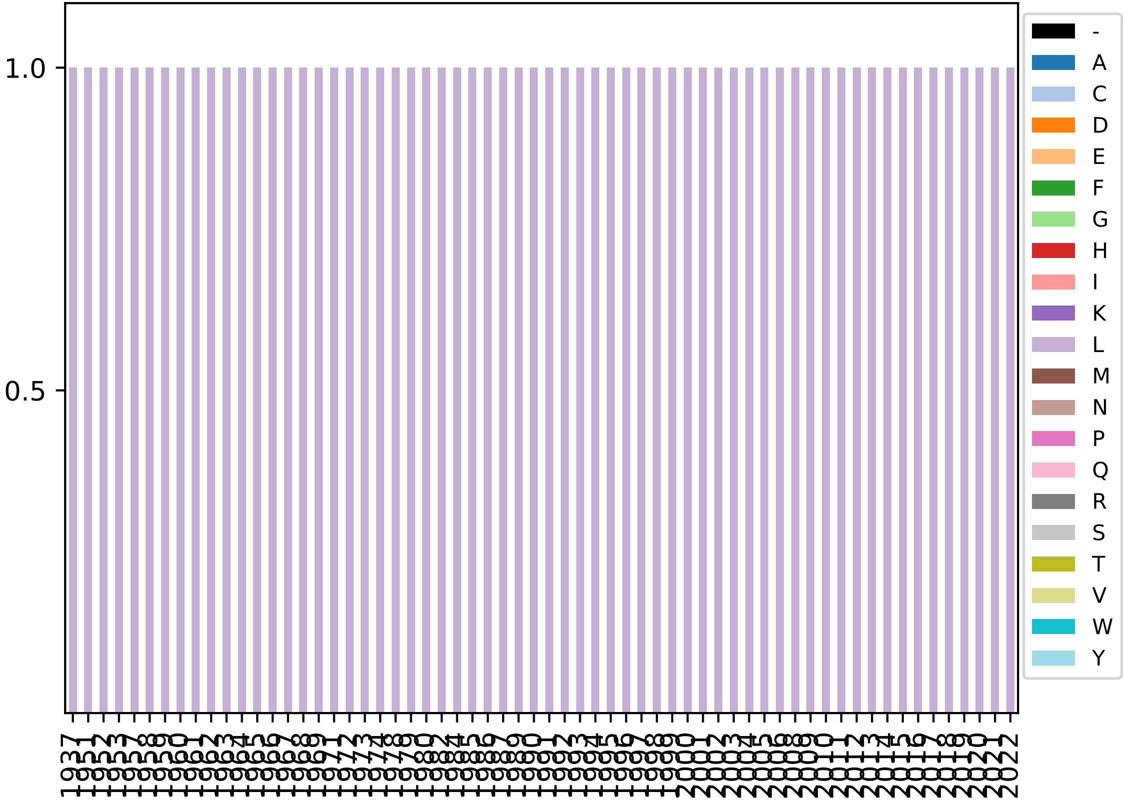

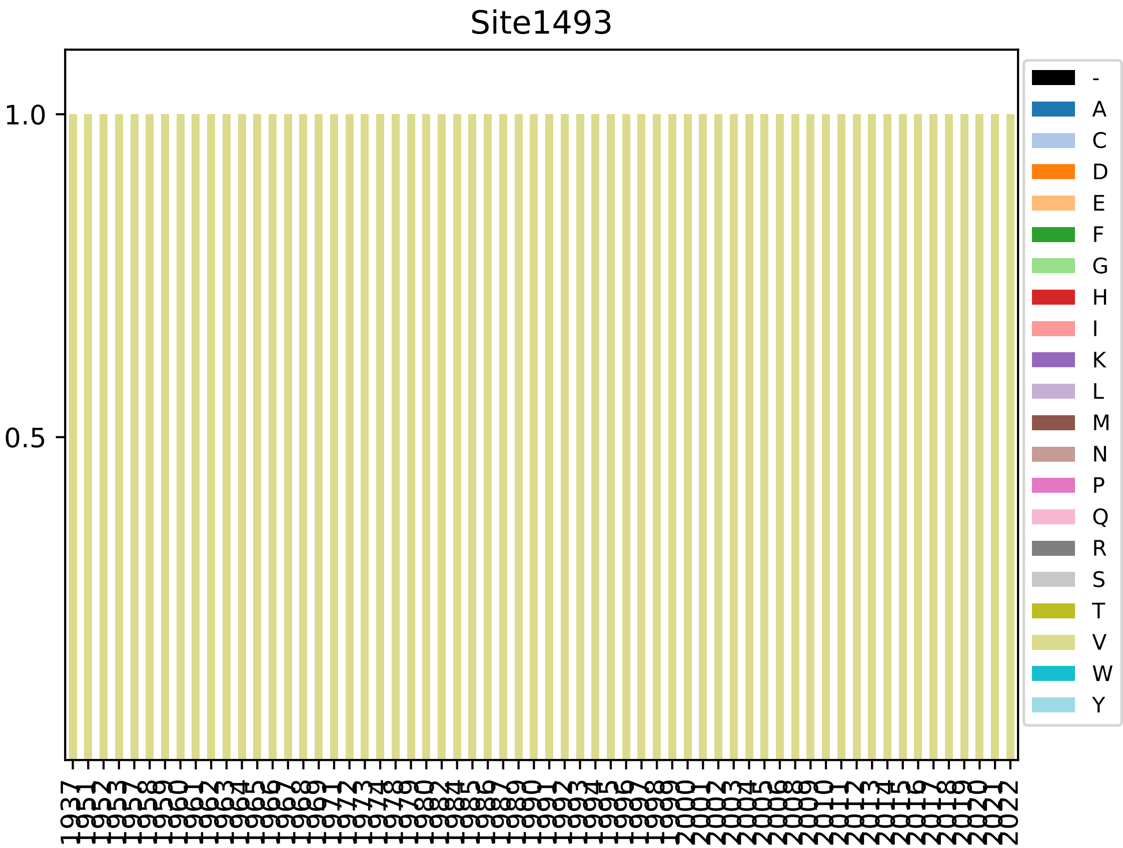
Site1493
1.0
0.5
-
A
C
D
E
F
G
H
I
K
L
M
N
P
Q
R
S
T
V
W
Y

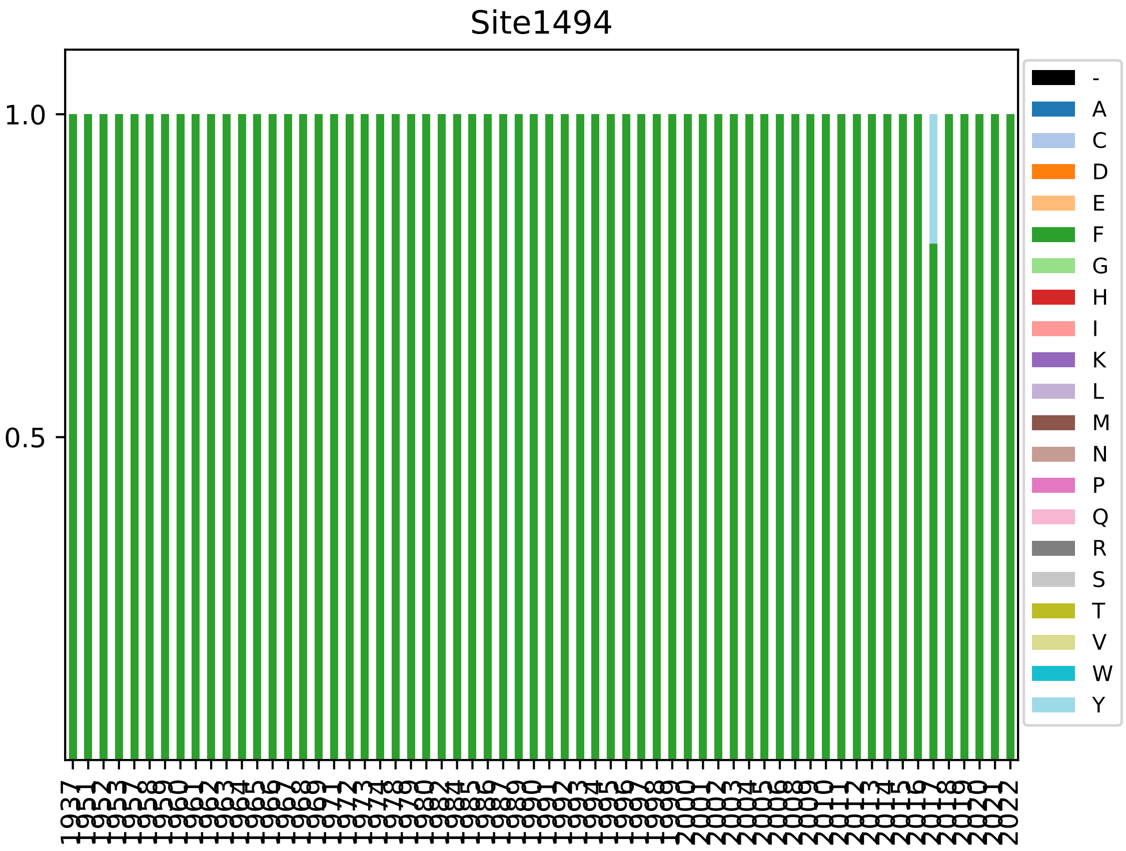

Site1494
1.0
0.5
-
A
C
D
E
F
G
H
I
K
L
M
N
P
Q
R
S
T
V
W
Y

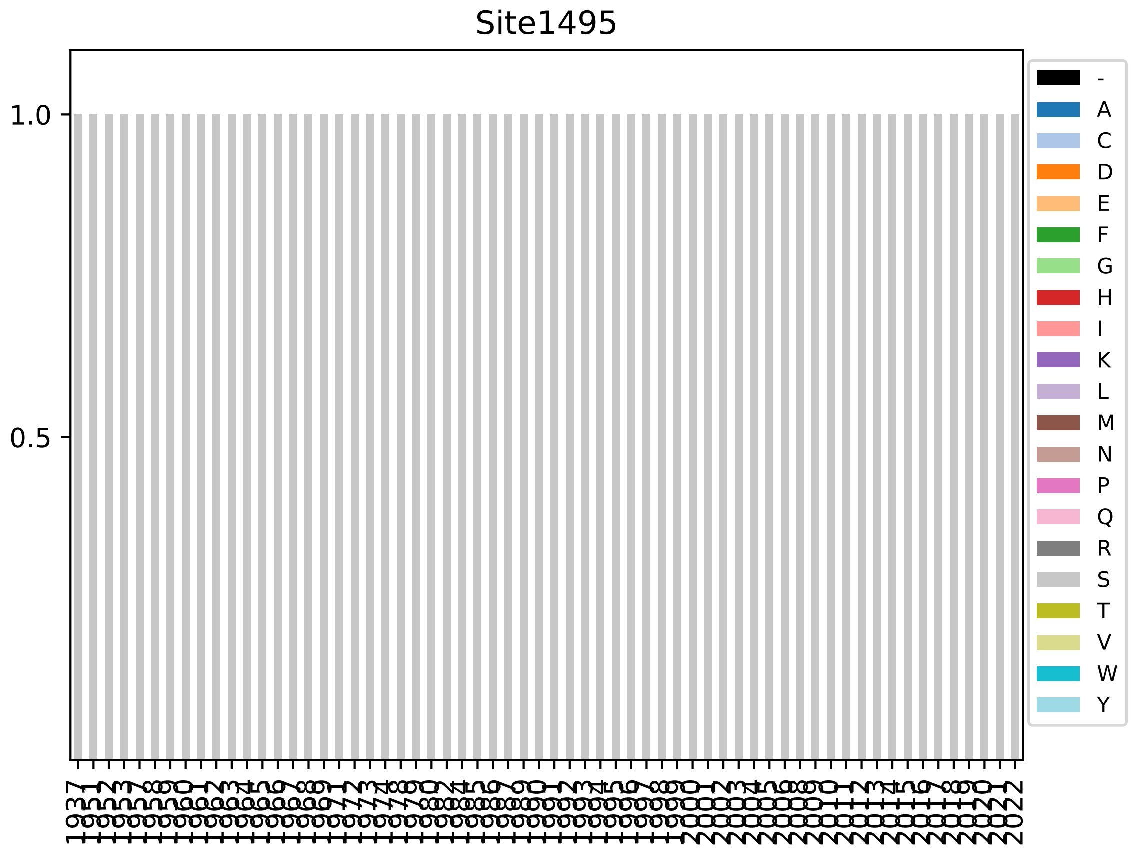
Site1495
1.0
0.5
-
A
C
D
E
F
G
H
I
K
L
M
N
P
Q
R
S
T
V
W
Y

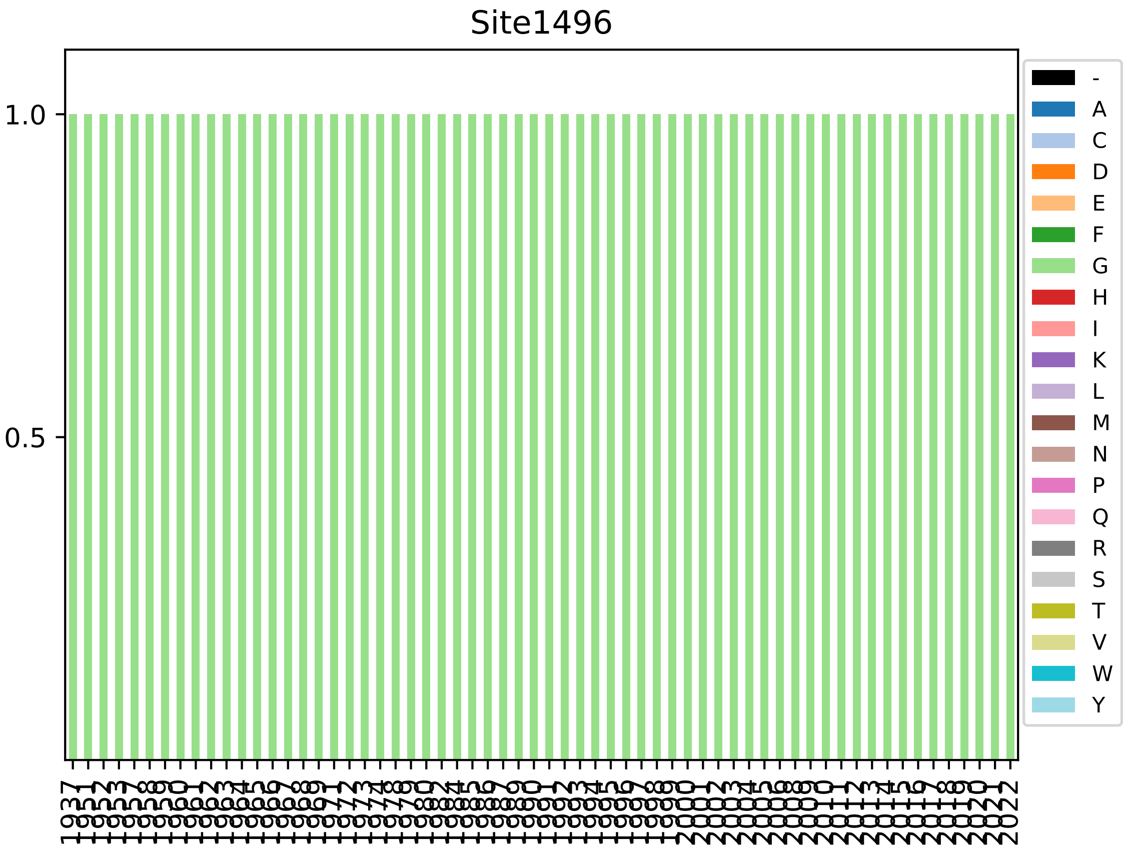
Site1496
1.0
0.5
-
A
C
D
E
F
G
H
I
K
L
M
N
P
Q
R
S
T
V
W
Y

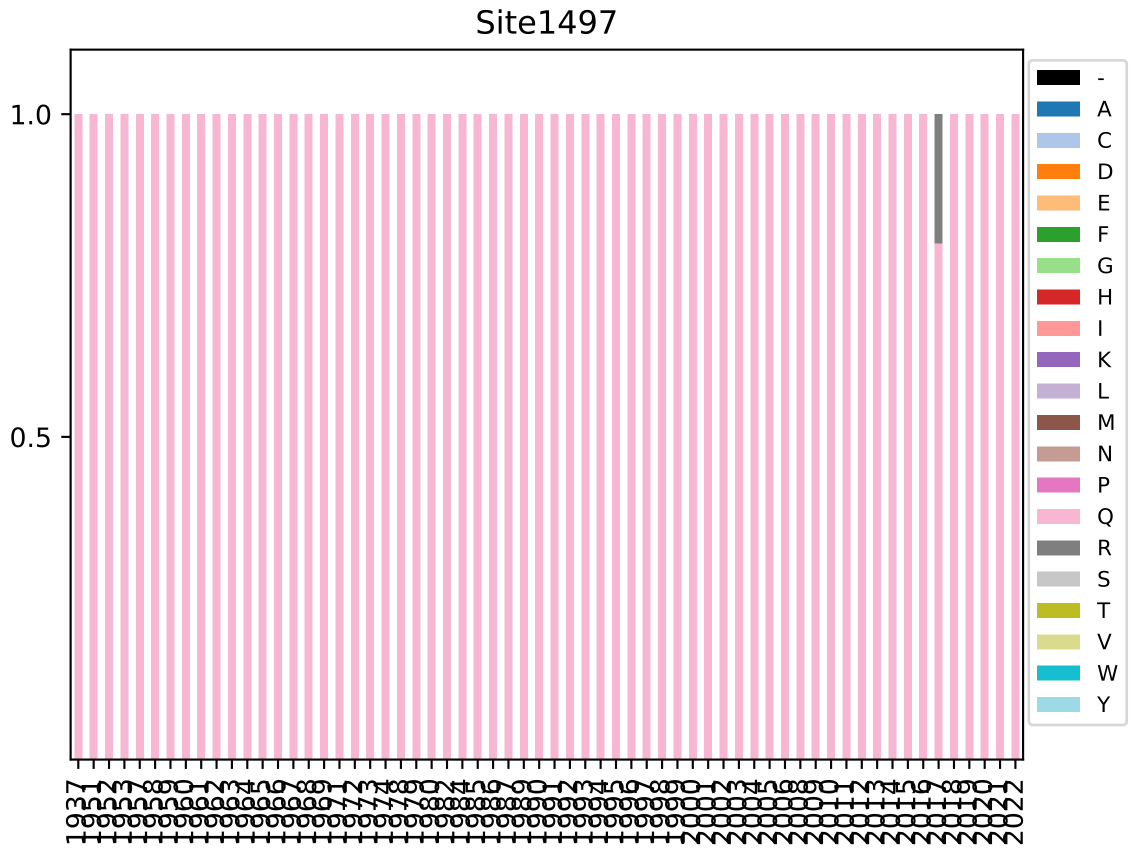
Site1497
1.0
0.5
-
A
C
D
E
F
G
H
I
K
L
M
N
P
Q
R
S
T
V
W
Y

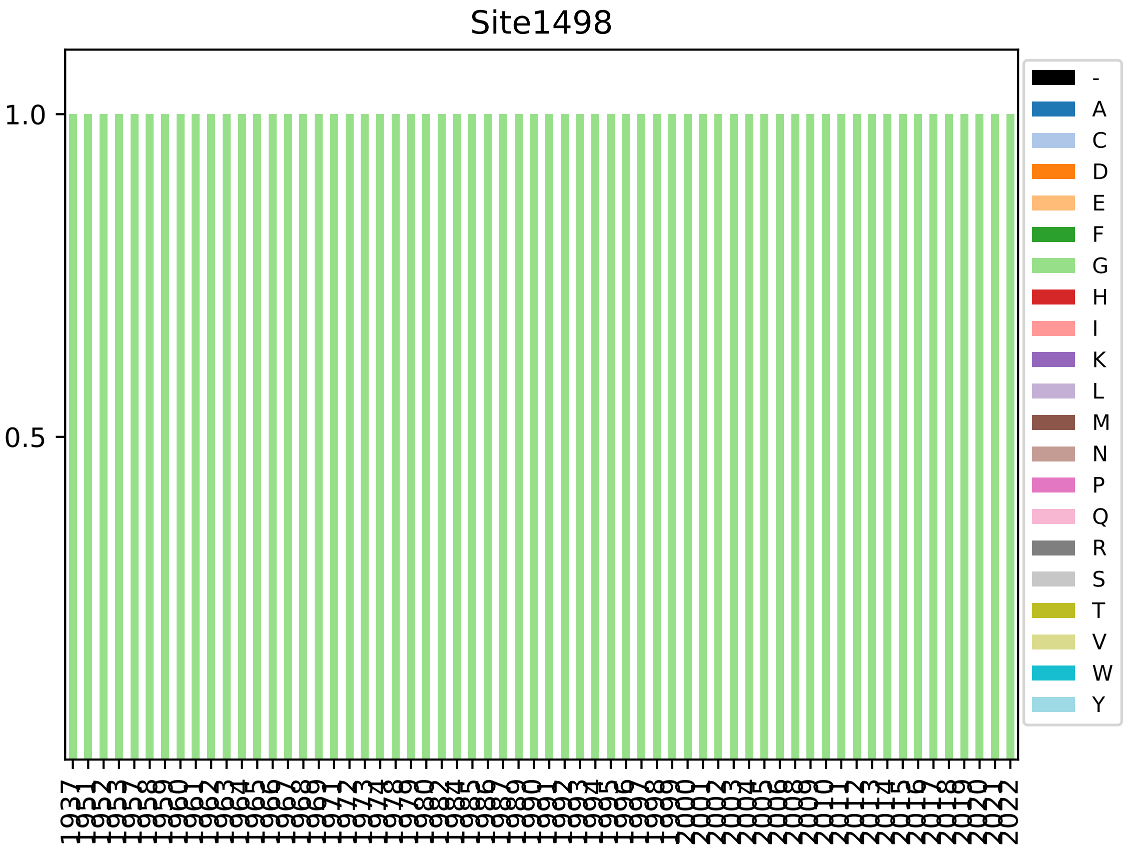
Site1498
1.0
0.5
-
A
C
D
E
F
G
H
I
K
L
M
N
P
Q
R
S
T
V
W
Y

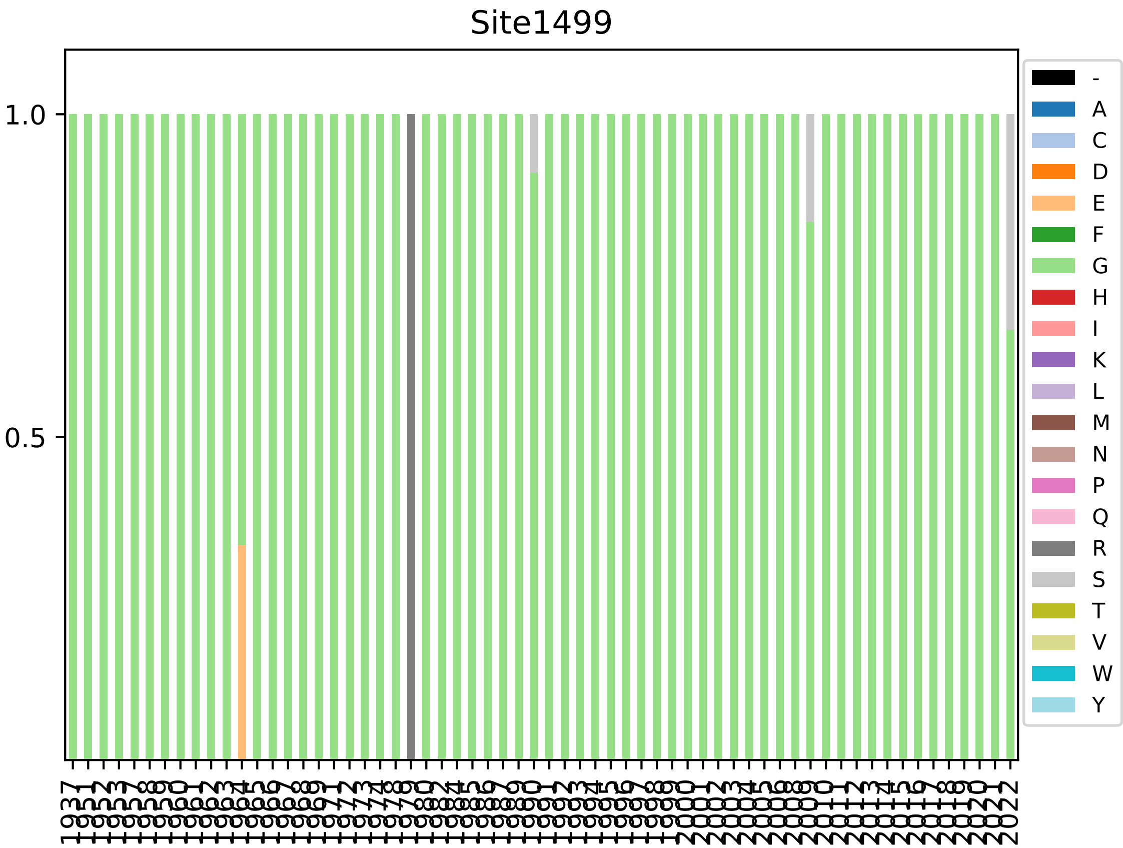
Site1499
1.0
0.5
-
A
C
D
E
F
G
H
I
K
L
M
N
P
Q
R
S
T
V
W
Y

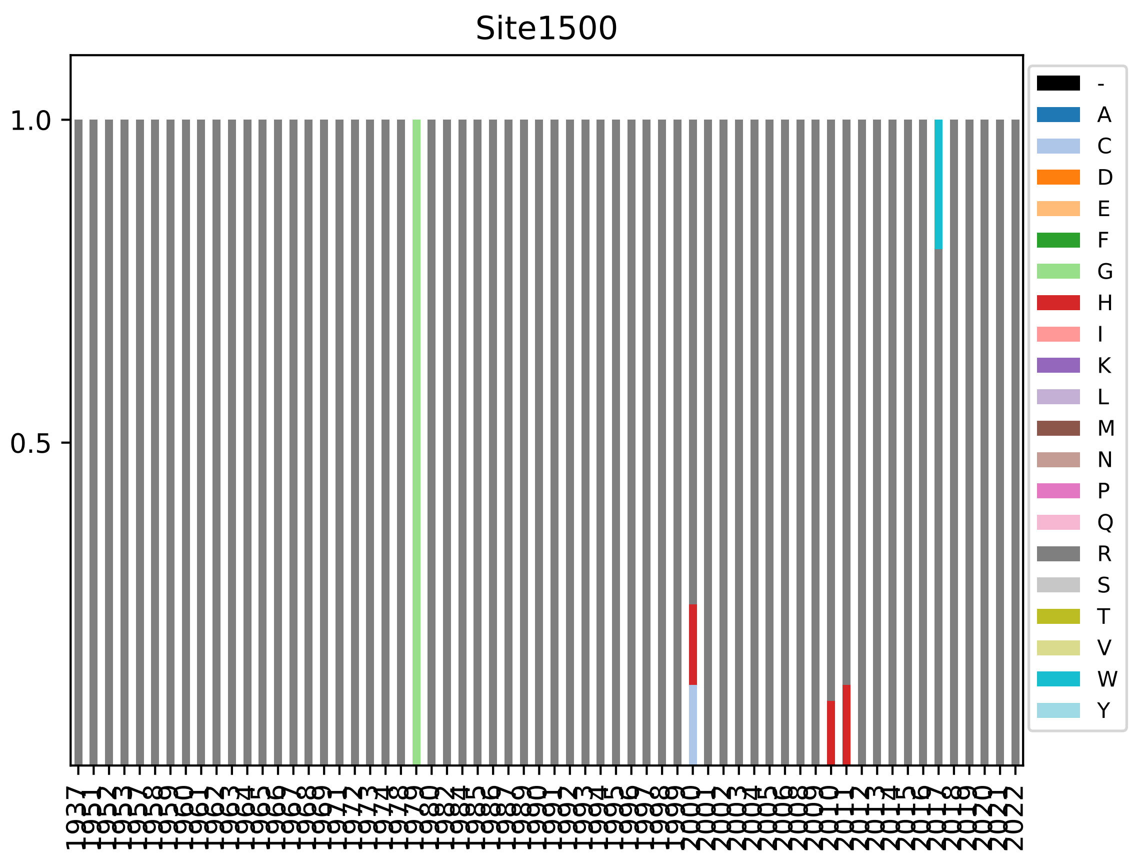

Site1500
1.0
0.5
-
A
C
D
E
F
G
H
I
K
L
M
N
P
Q
R
S
T
V
W
Y

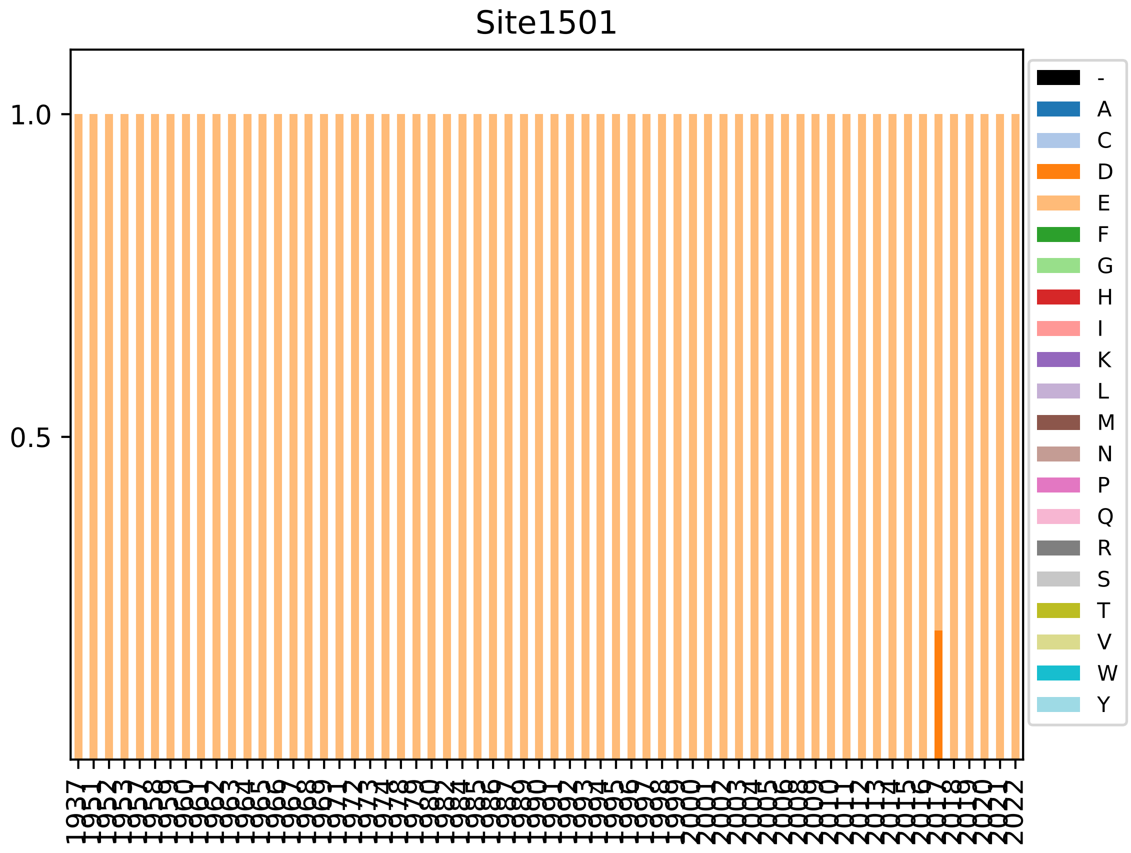
Site1501
1.0
0.5
-
A
C
D
E
F
G
H
I
K
L
M
N
P
Q
R
S
T
V
W
Y

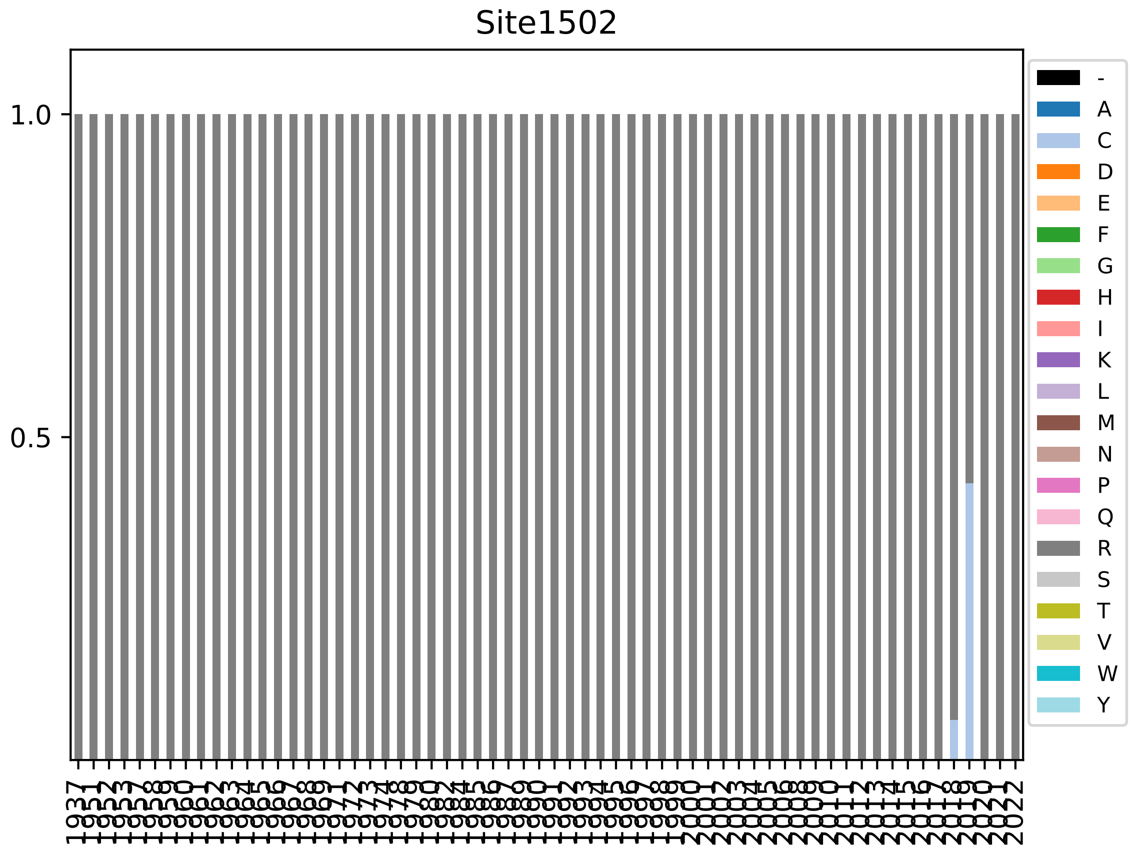
Site1502
1.0
0.5
-
A
C
D
E
F
G
H
I
K
L
M
N
P
Q
R
S
T
V
W
Y

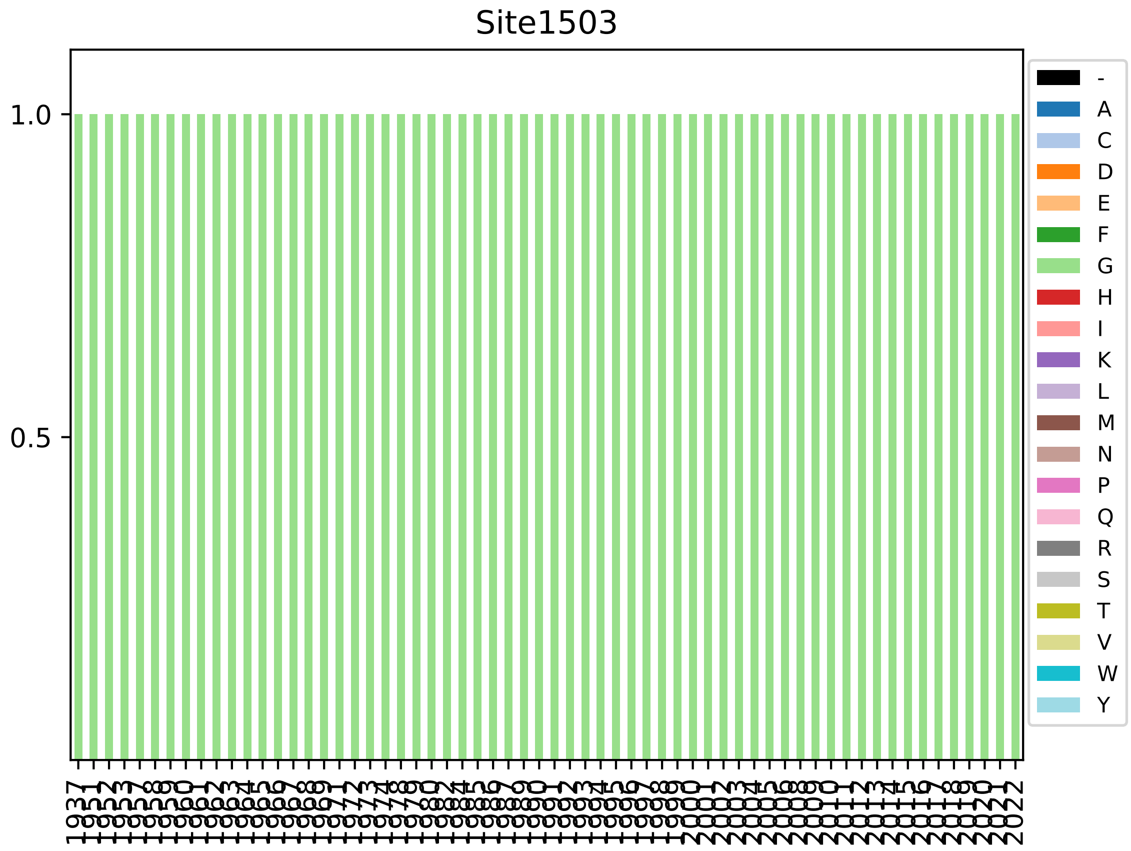

Site1503
1.0
0.5
-
A
C
D
E
F
G
H
I
K
L
M
N
P
Q
R
S
T
V
W
Y

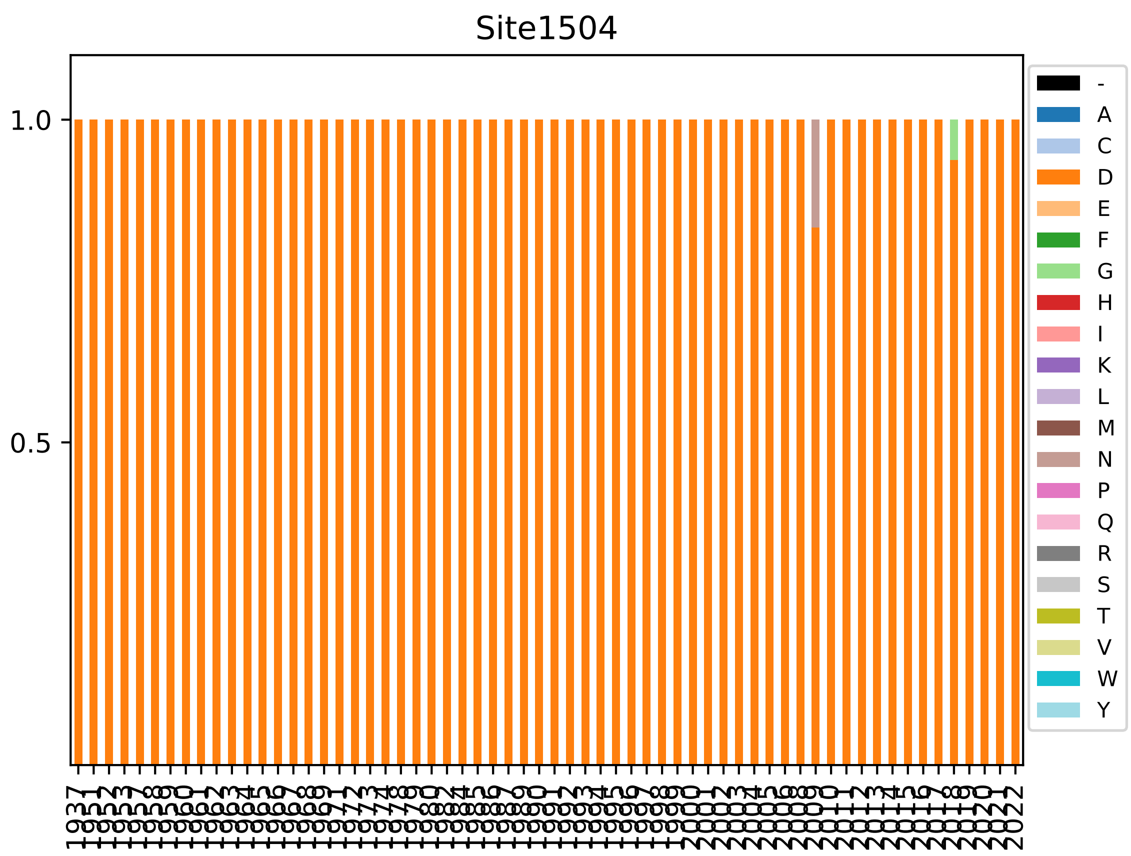
Site1504
1.0
0.5
-
A
C
D
E
F
G
H
I
K
L
M
N
P
Q
R
S
T
V
W
Y

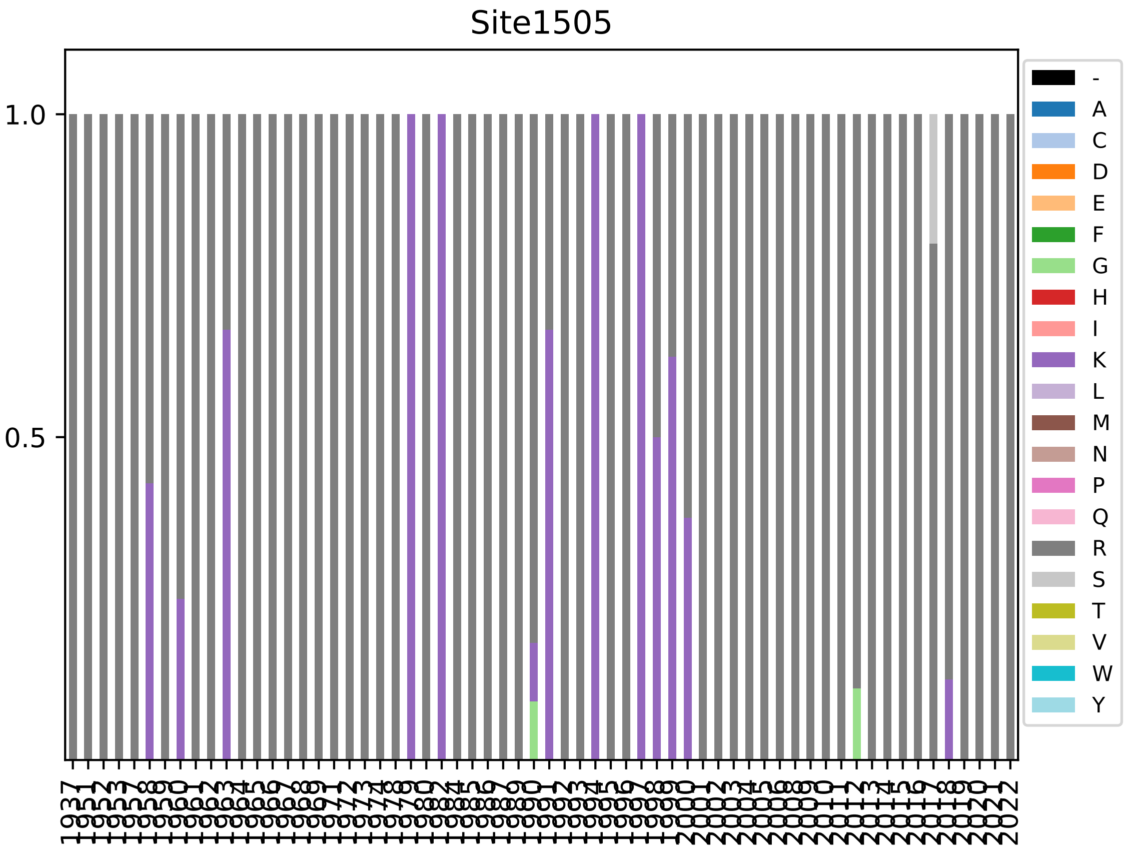
Site1505
1.0
0.5
-
A
C
D
E
F
G
H
I
K
L
M
N
P
Q
R
S
T
V
W
Y

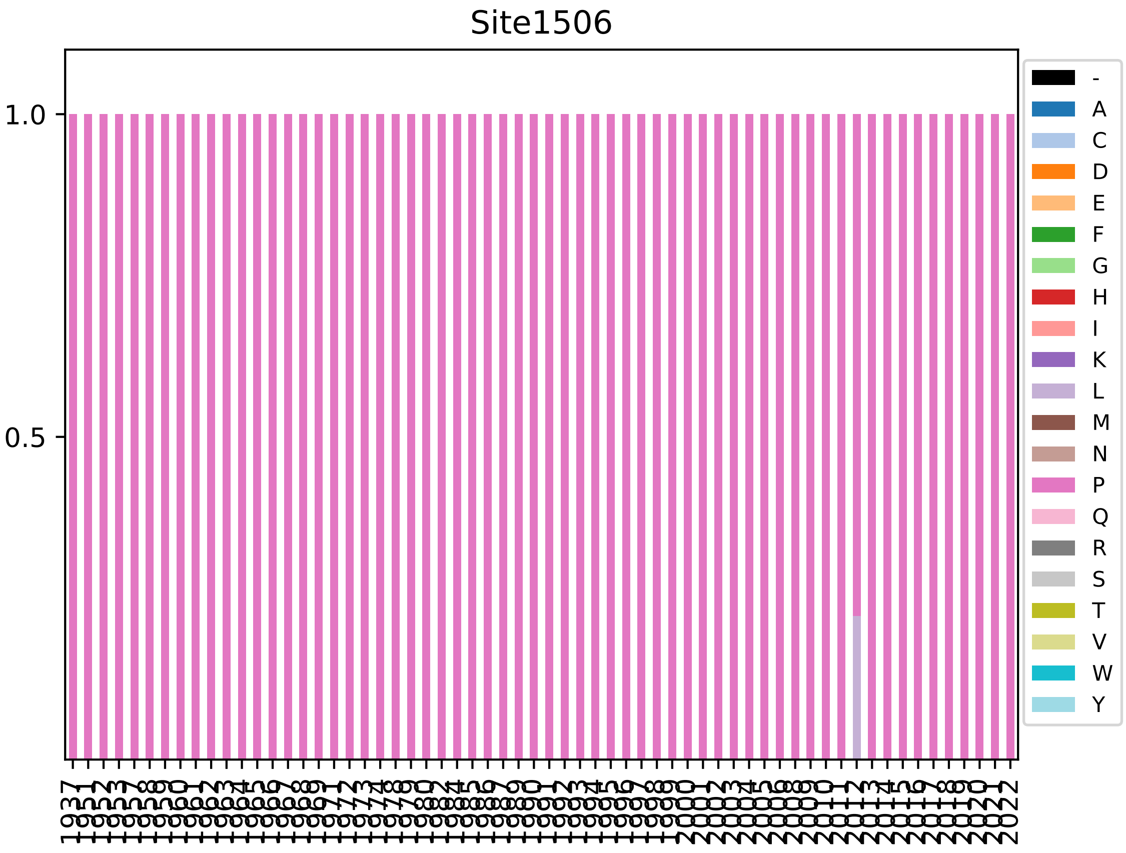
Site1506
1.0
0.5
-
A
C
D
E
F
G
H
I
K
L
M
N
P
Q
R
S
T
V
W
Y

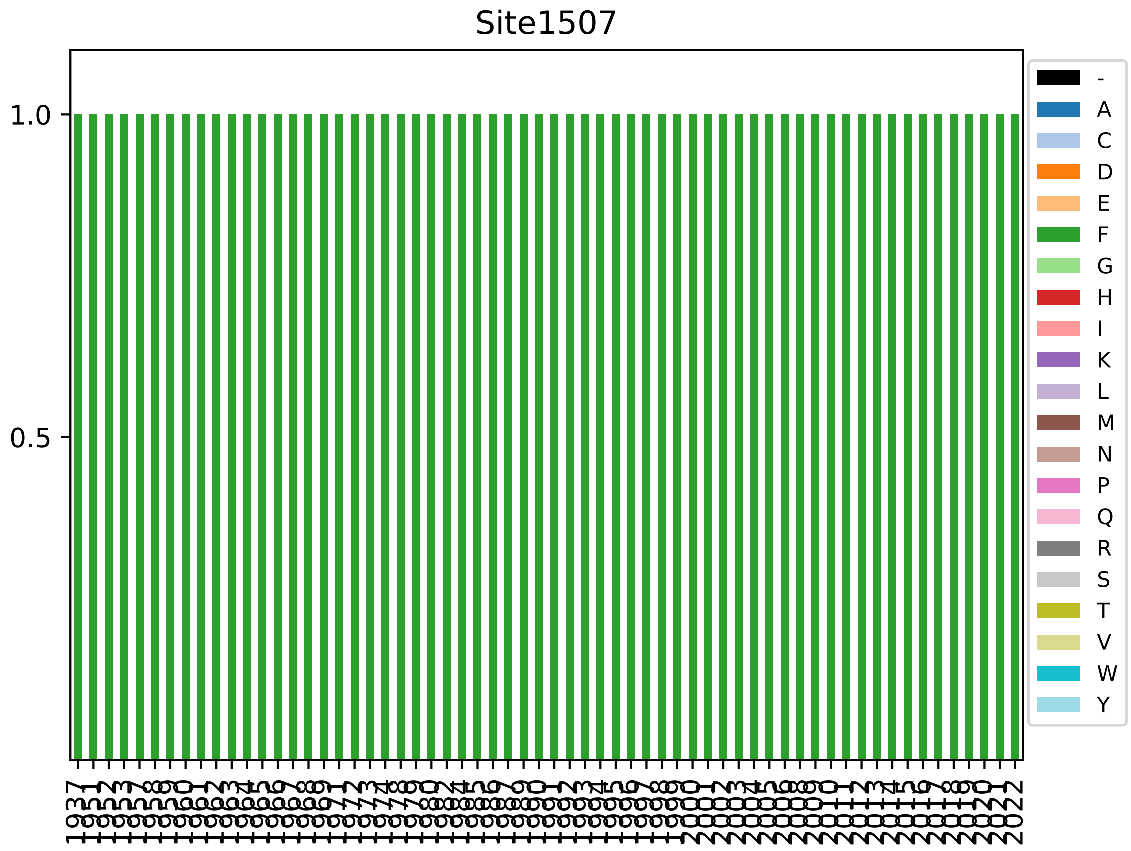
Site1507
1.0
0.5
-
A
C
D
E
F
G
H
I
K
L
M
N
P
Q
R
S
T
V
W
Y

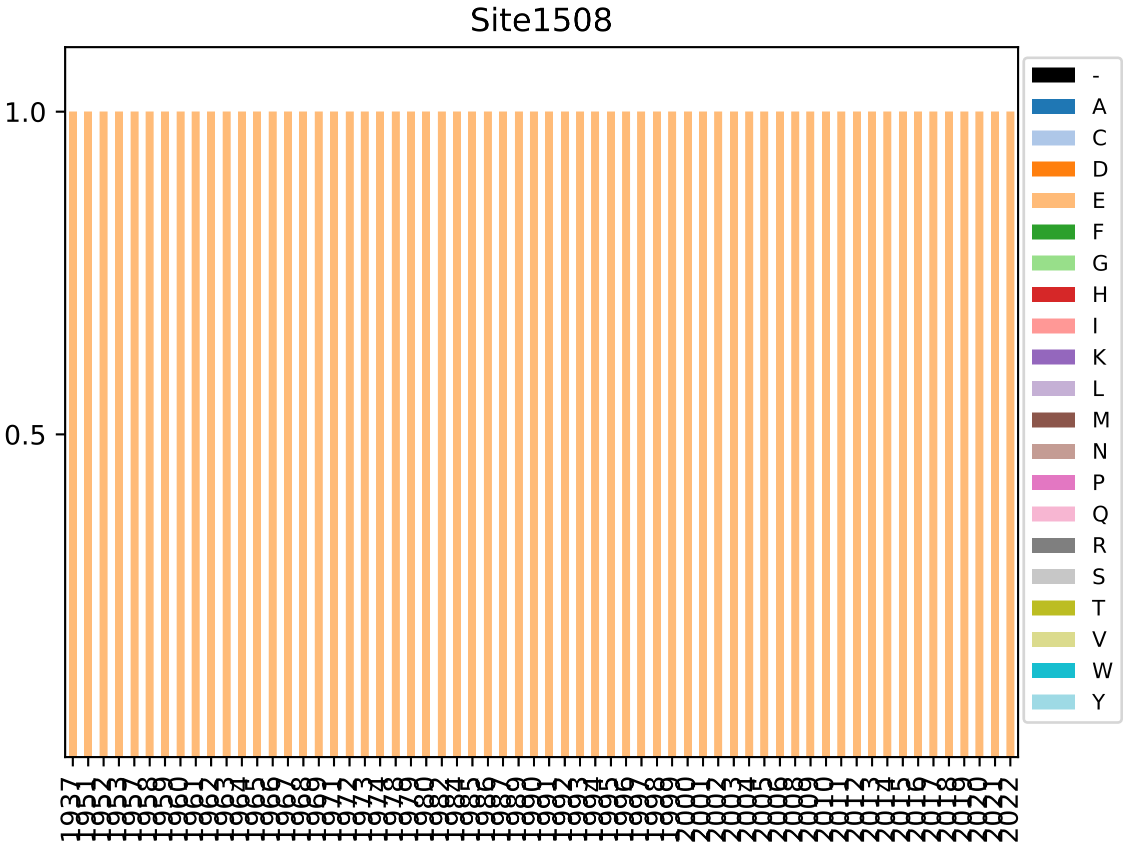
Site1508
1.0
0.5
-
A
C
D
E
F
G
H
I
K
L
M
N
P
Q
R
S
T
V
W
Y

Site1509

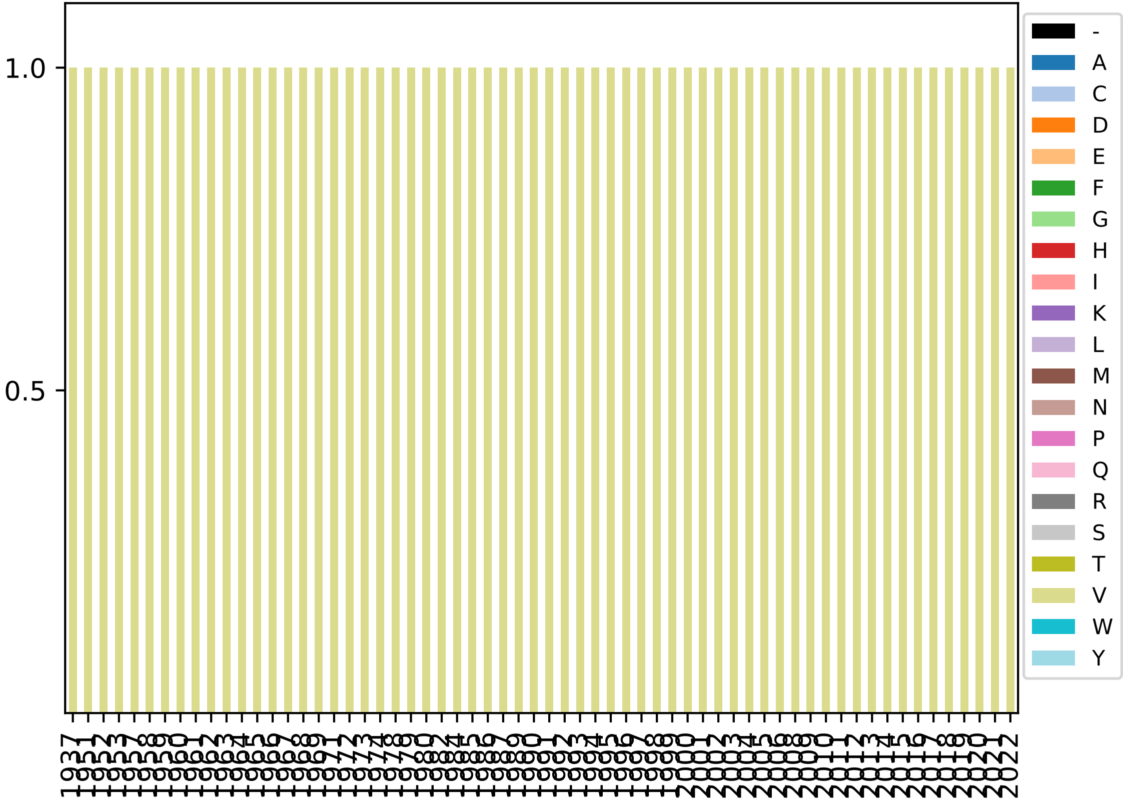

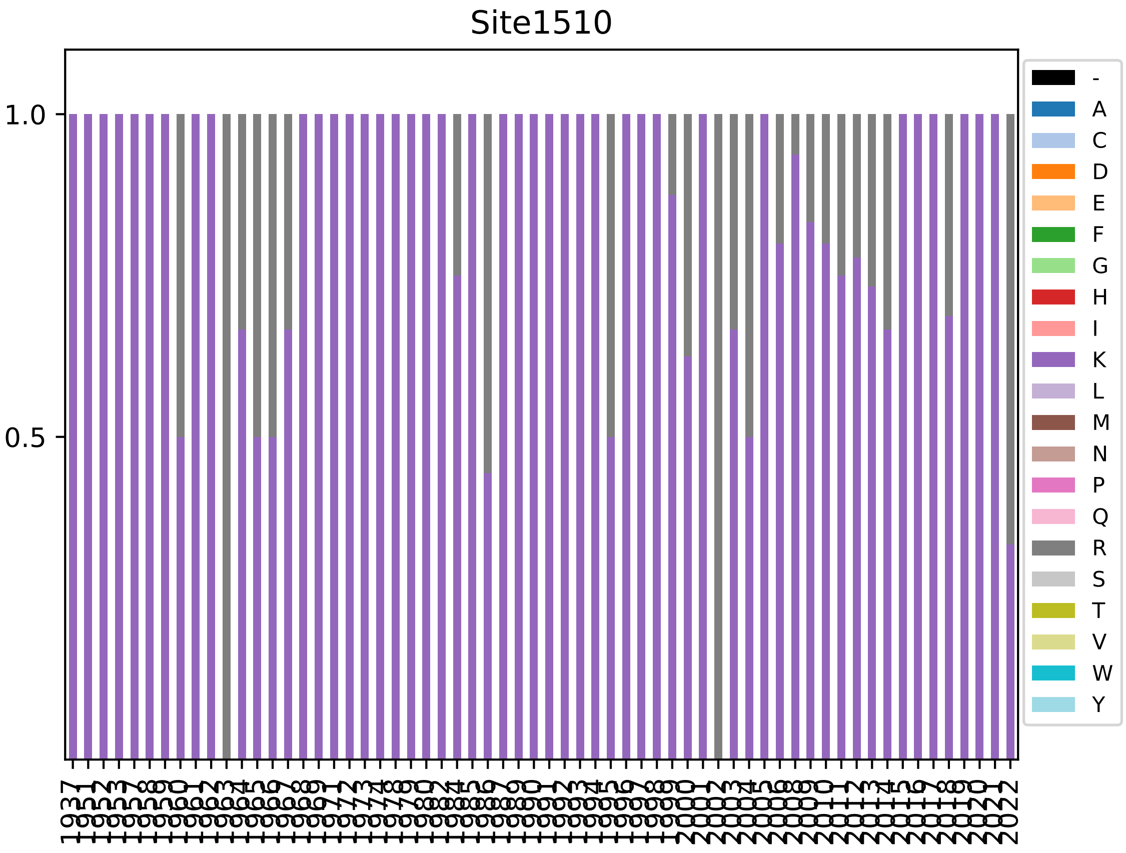
Site1510
1.0
0.5
-
A
C
D
E
F
G
H
I
K
L
M
N
P
Q
R
S
T
V
W
Y

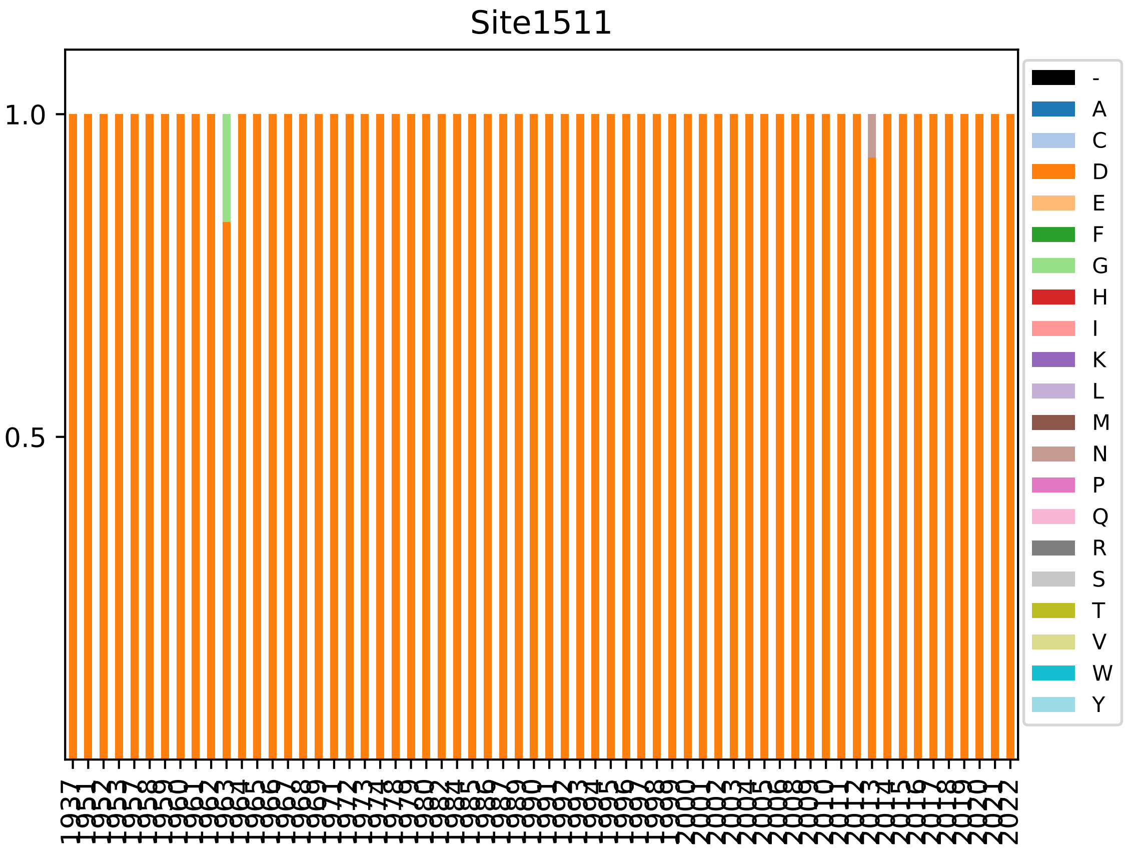
Site1511
1.0
0.5
-
A
C
D
E
F
G
H
I
K
L
M
N
P
Q
R
S
T
V
W
Y

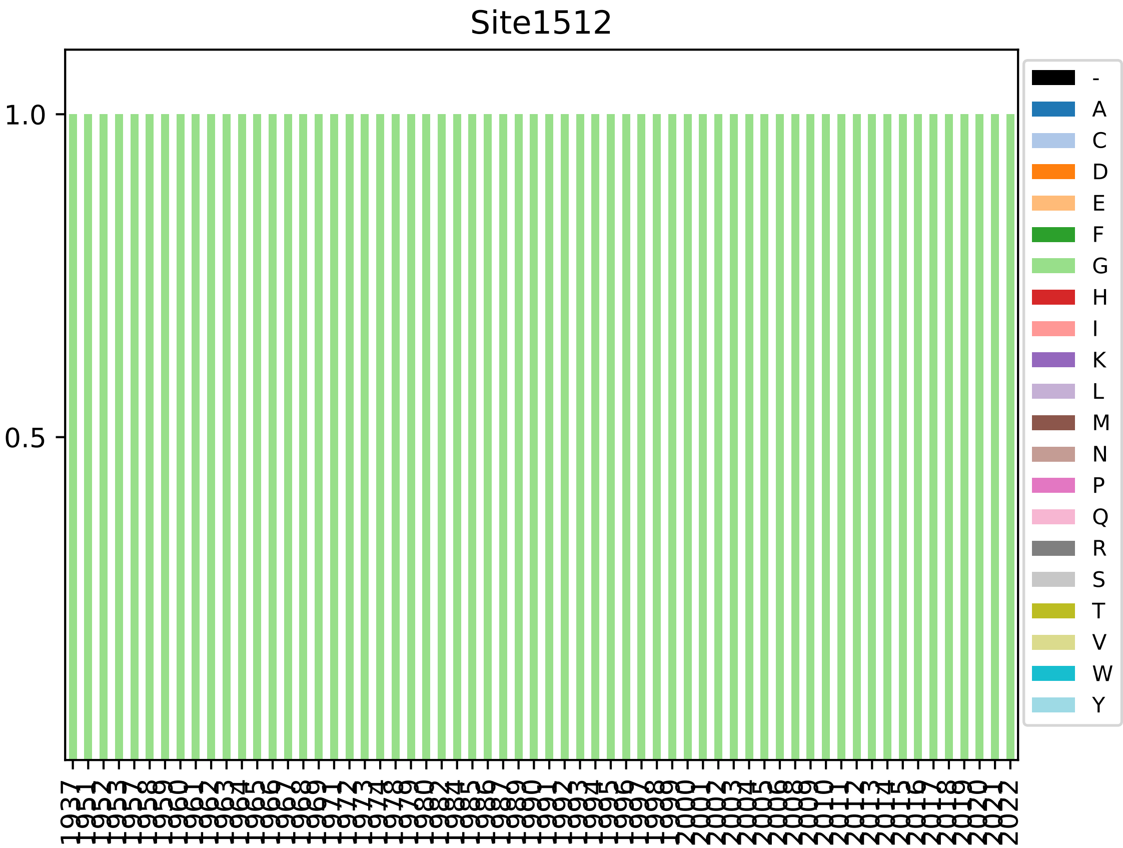
Site1512
1.0
0.5
-
A
C
D
E
F
G
H
I
K
L
M
N
P
Q
R
S
T
V
W
Y

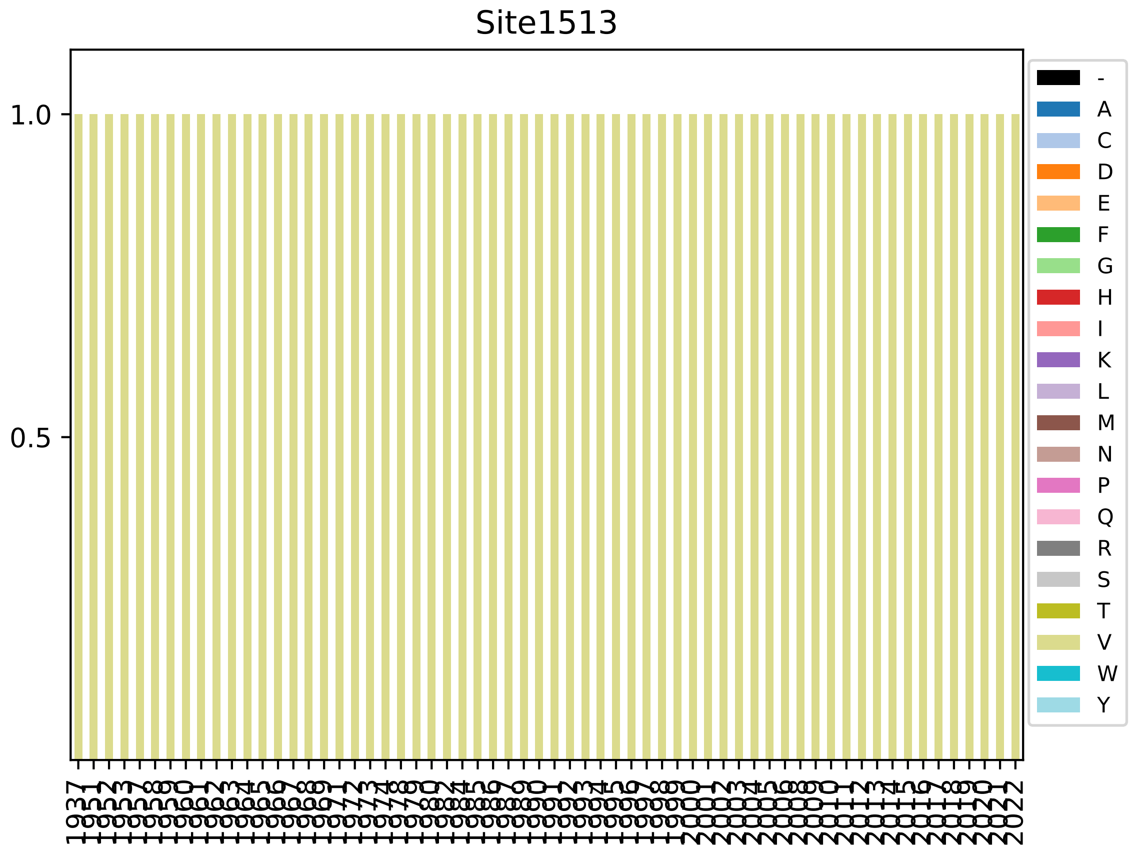
Site1513
1.0
0.5
-
A
C
D
E
F
G
H
I
K
L
M
N
P
Q
R
S
T
V
W
Y

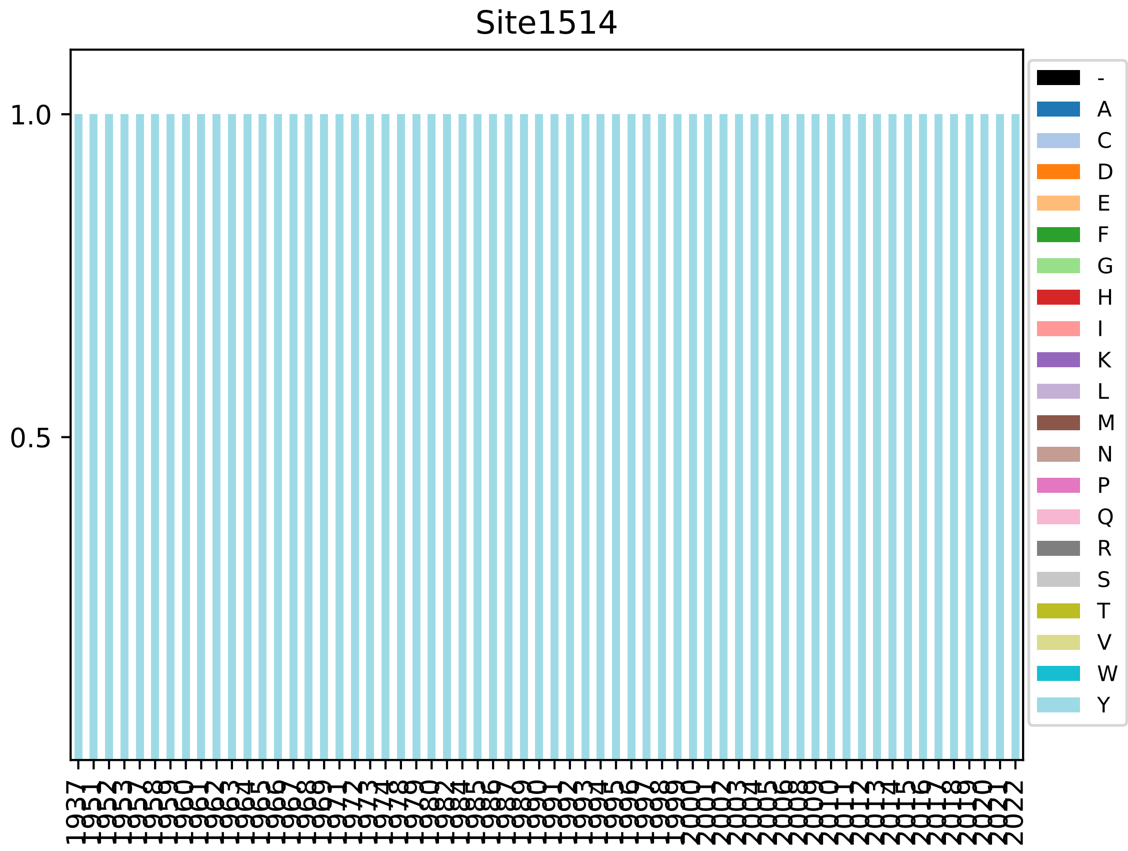
Site1514
1.0
0.5
-
A
C
D
E
F
G
H
I
K
L
M
N
P
Q
R
S
T
V
W
Y

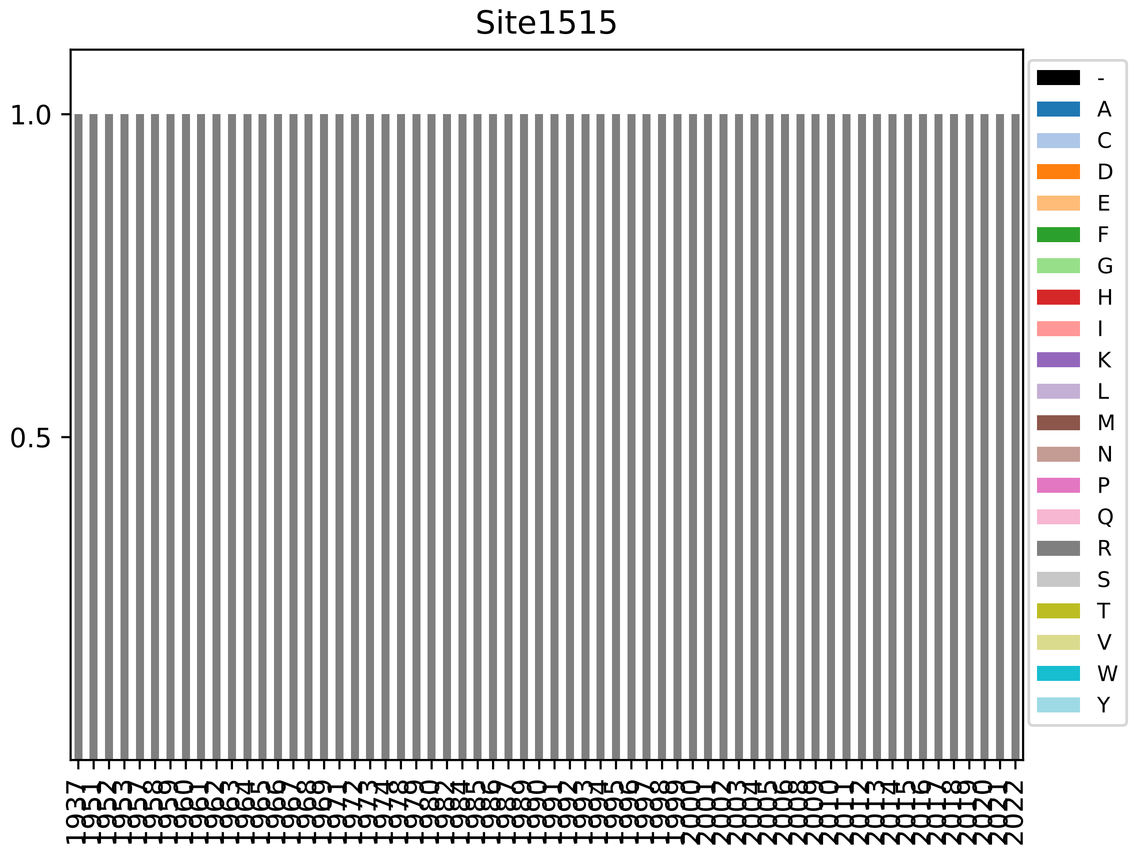
Site1515
1.0
0.5
-
A
C
D
E
F
G
H
I
K
L
M
N
P
Q
R
S
T
V
W
Y

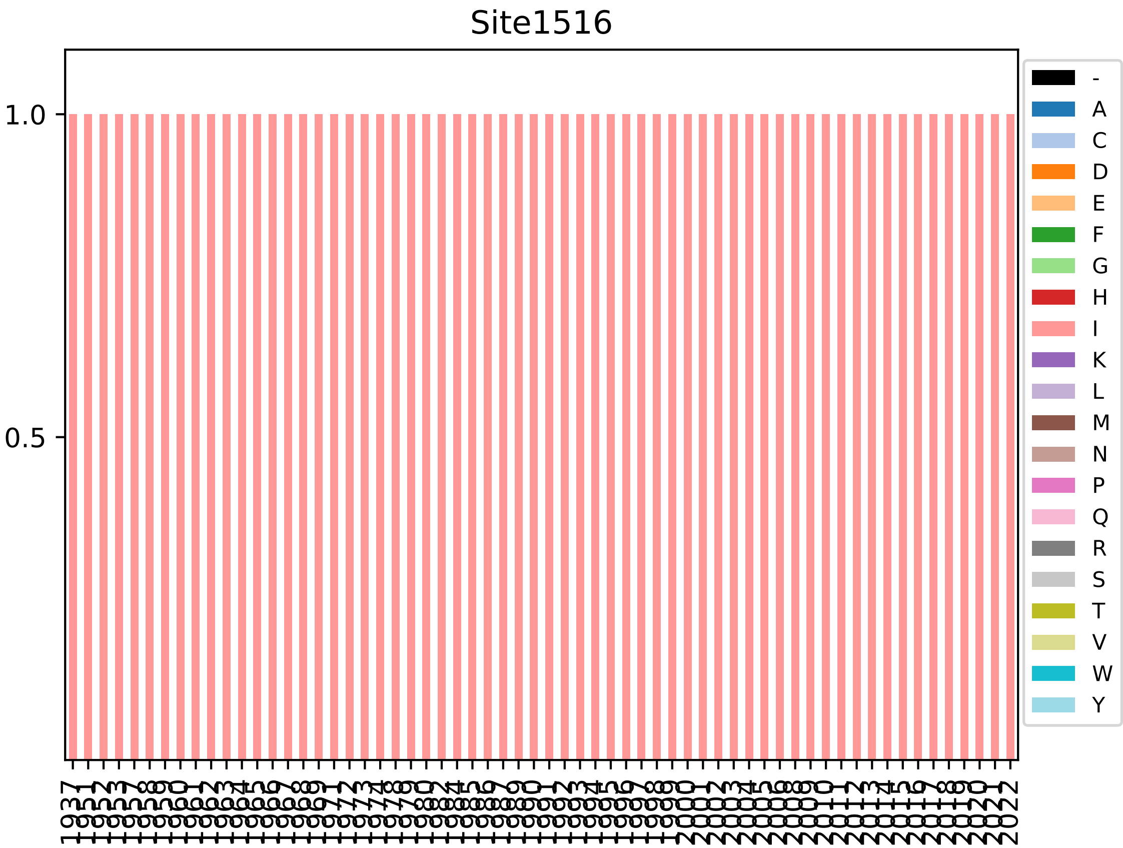
Site1516
1.0
0.5
-
A
C
D
E
F
G
H
I
K
L
M
N
P
Q
R
S
T
V
W
Y

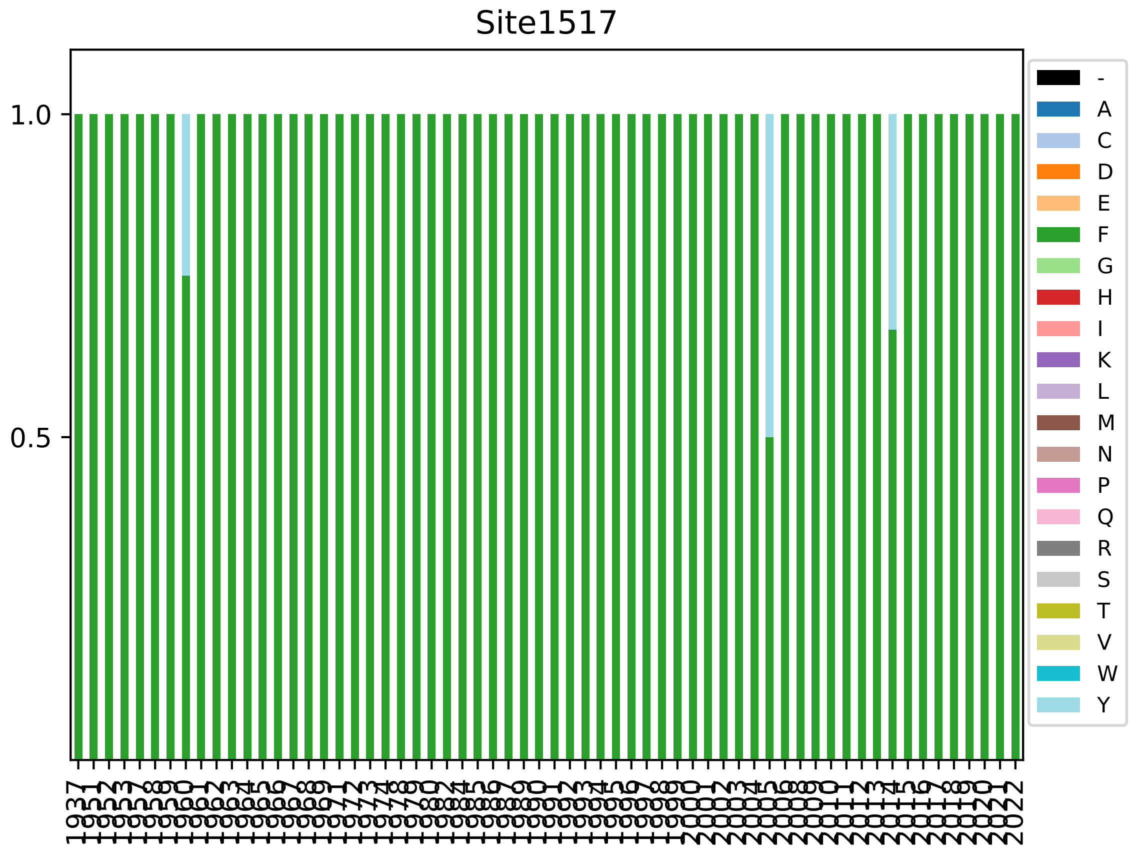
Site1517
1.0
0.5
-
A
C
D
E
F
G
H
I
K
L
M
N
P
Q
R
S
T
V
W
Y

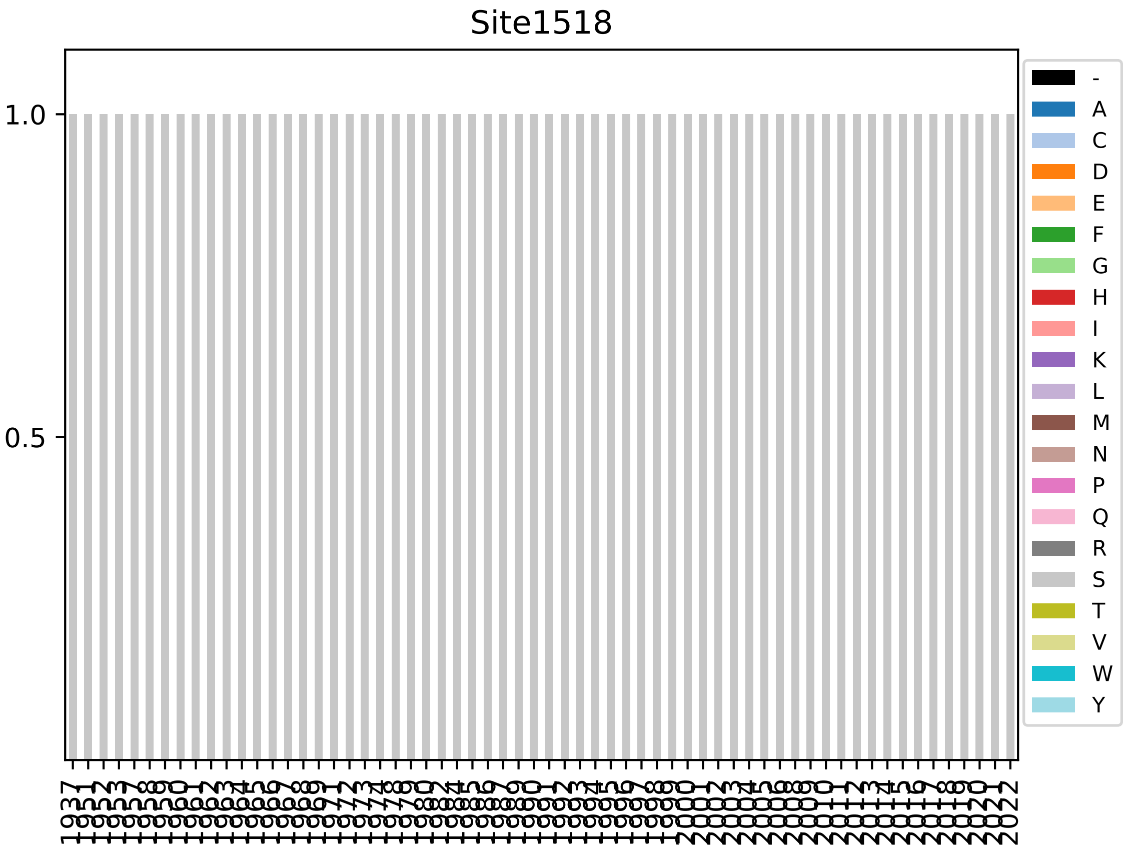

Site1518
1.0
0.5
-
A
C
D
E
F
G
H
I
K
L
M
N
P
Q
R
S
T
V
W
Y

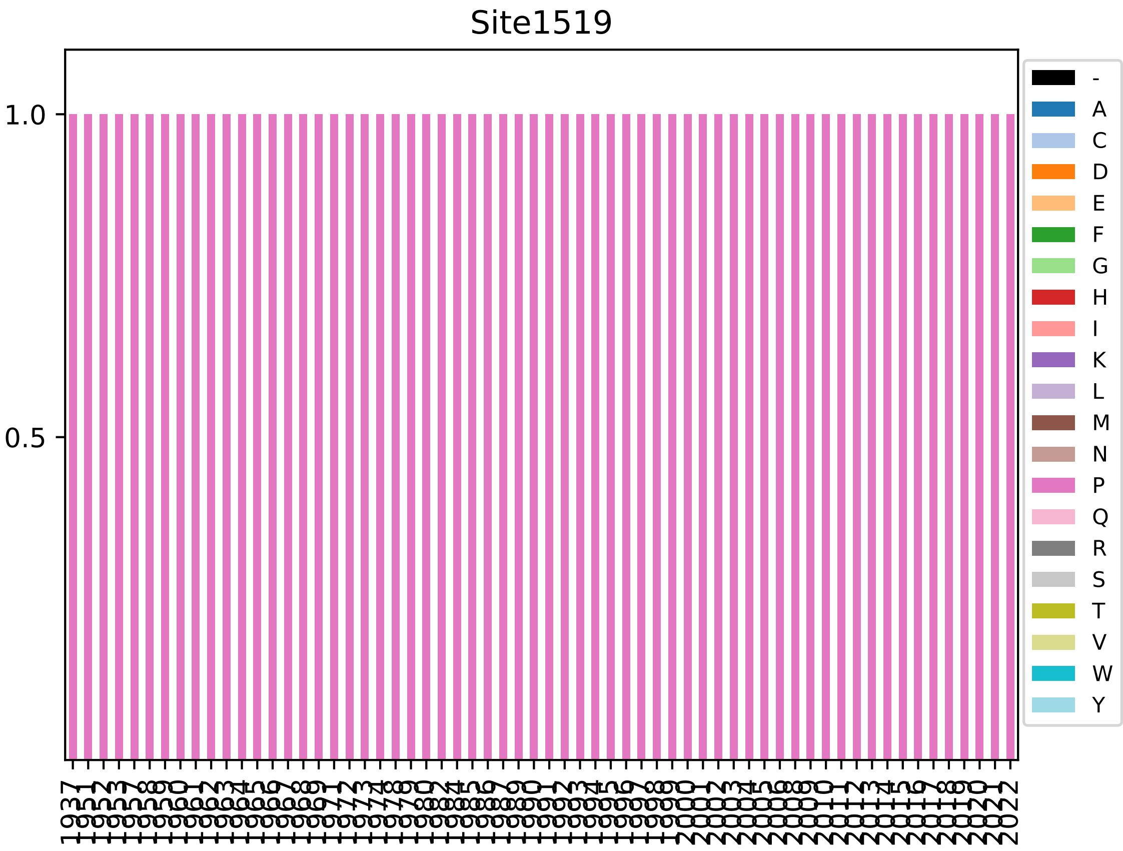

Site1519
1.0
0.5
-
A
C
D
E
F
G
H
I
K
L
M
N
P
Q
R
S
T
V
W
Y

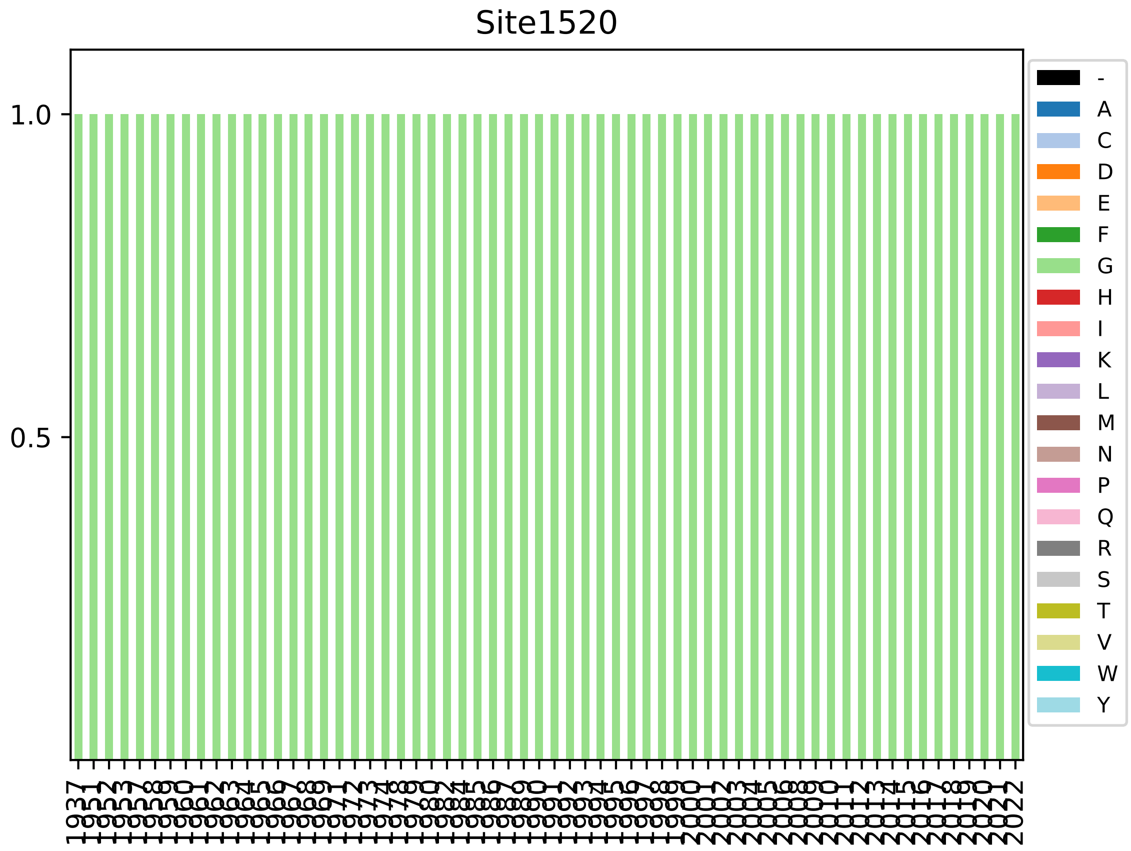

Site1520
1.0
0.5
-
A
C
D
E
F
G
H
I
K
L
M
N
P
Q
R
S
T
V
W
Y

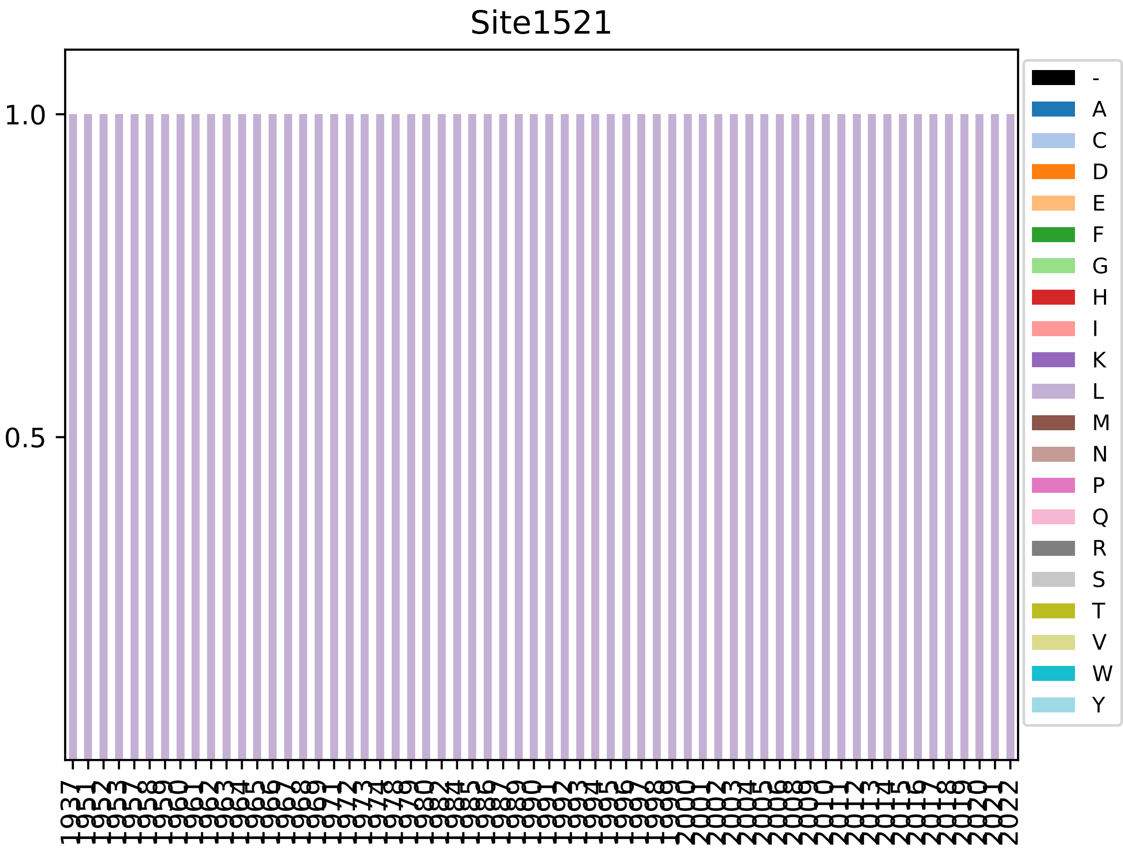
Site1521
1.0
0.5
-
A
C
D
E
F
G
H
I
K
L
M
N
P
Q
R
S
T
V
W
Y

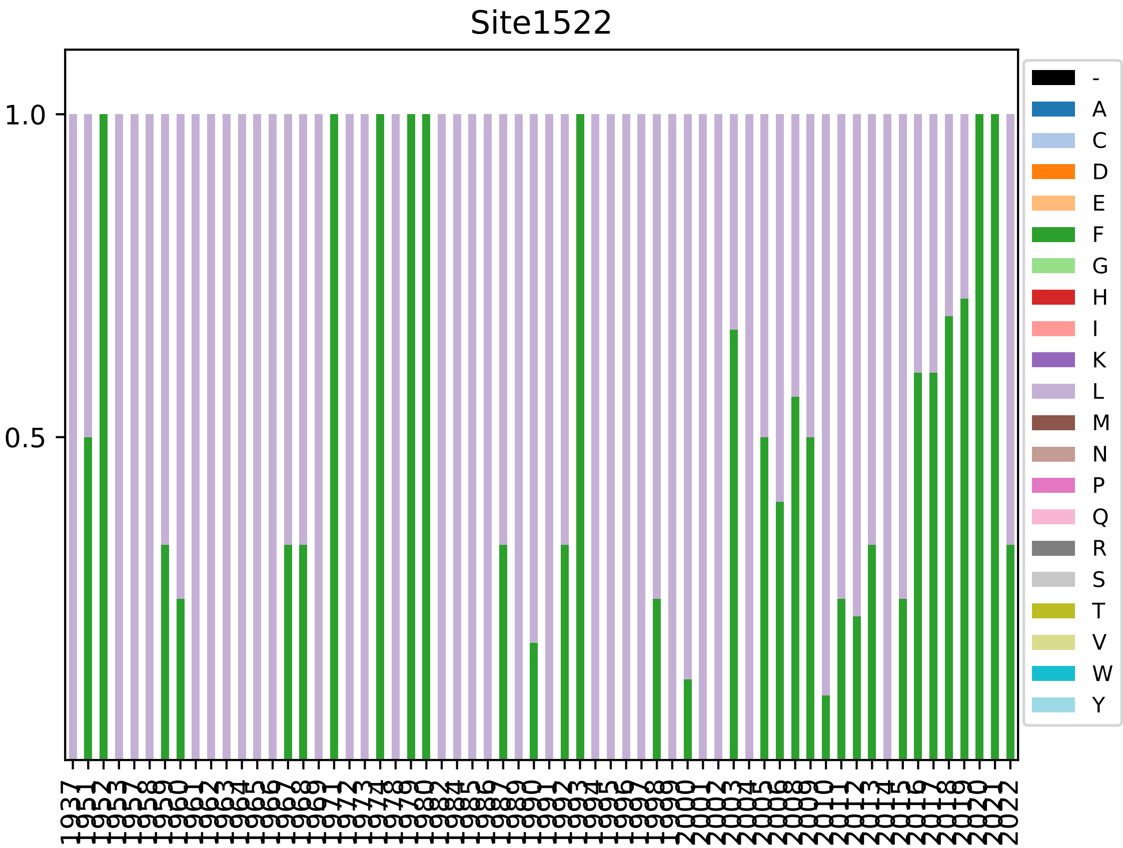
Site1522
1.0
0.5
-
A
C
D
E
F
G
H
I
K
L
M
N
P
Q
R
S
T
V
W
Y

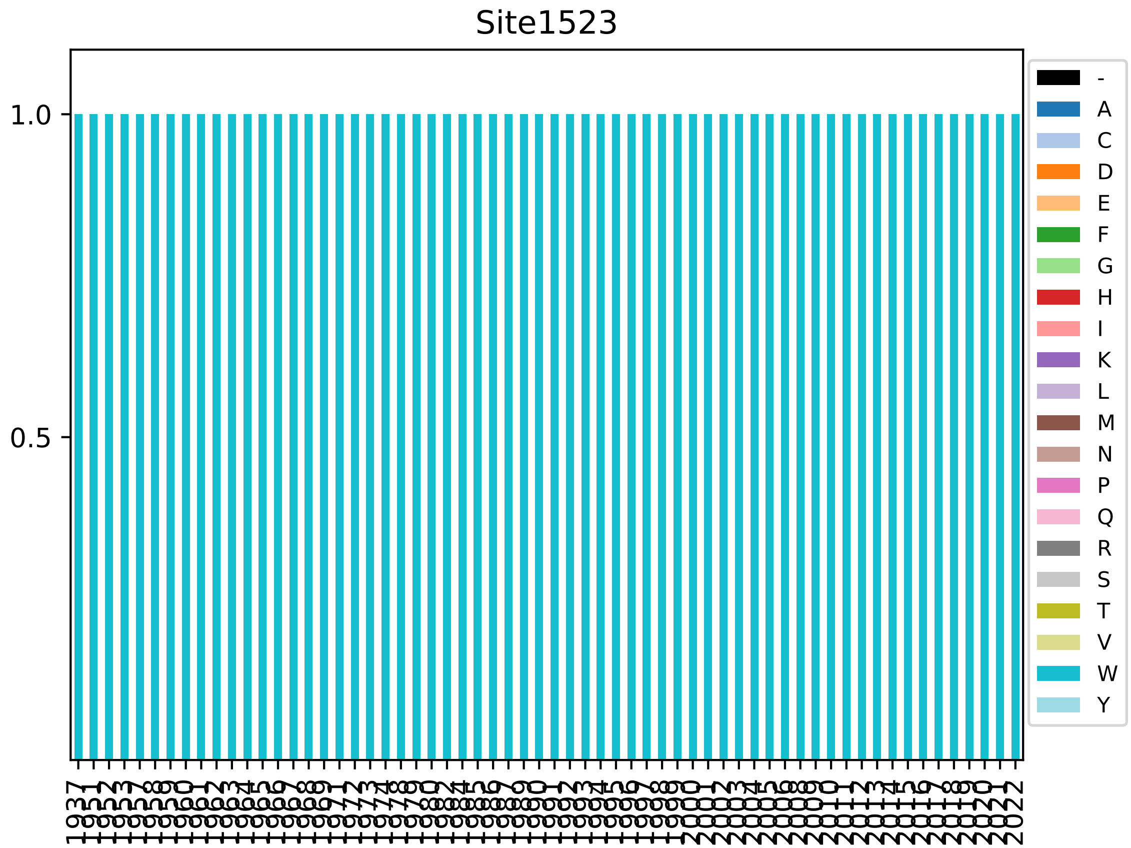
Site1523
1.0
0.5
-
A
C
D
E
F
G
H
I
K
L
M
N
P
Q
R
S
T
V
W
Y

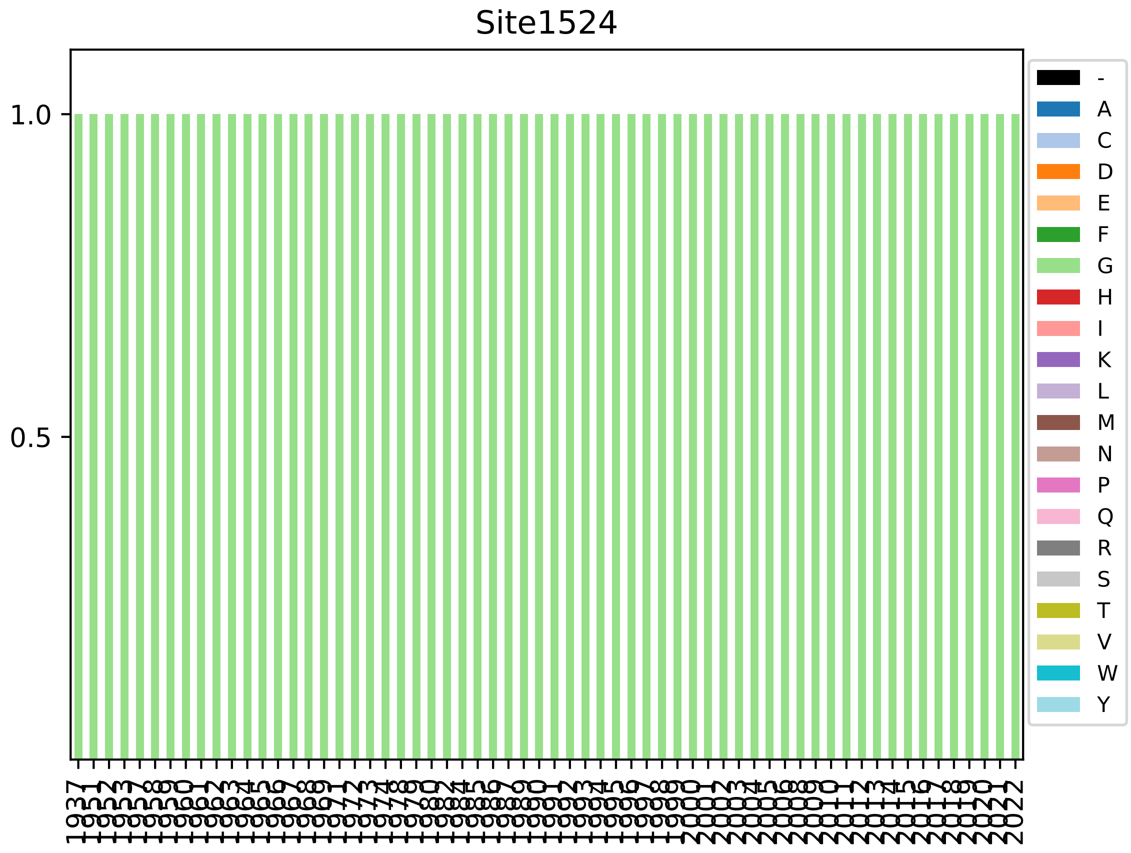
Site1524
1.0
0.5
-
A
C
D
E
F
G
H
I
K
L
M
N
P
Q
R
S
T
V
W
Y

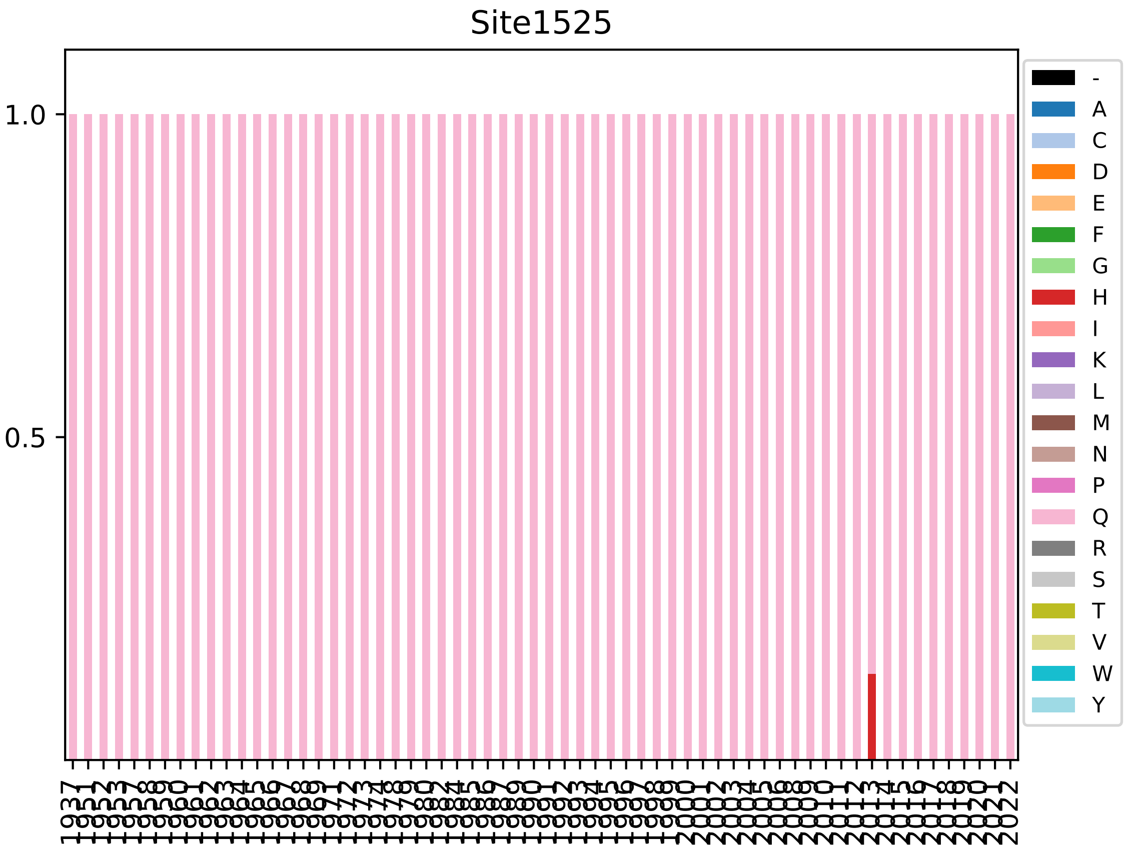
Site1525
1.0
0.5
-
A
C
D
E
F
G
H
I
K
L
M
N
P
Q
R
S
T
V
W
Y

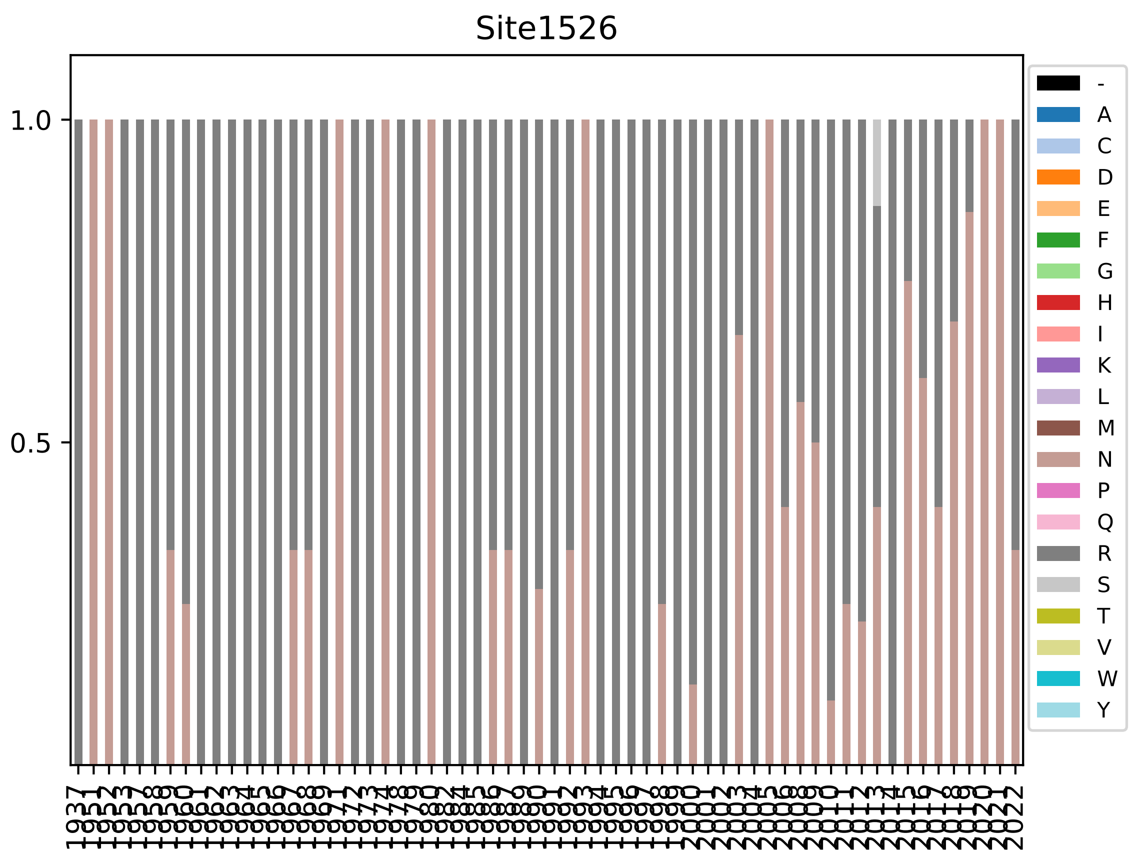
Site1526
1.0
0.5
-
A
C
D
E
F
G
H
I
K
L
M
N
P
Q
R
S
T
V
W
Y

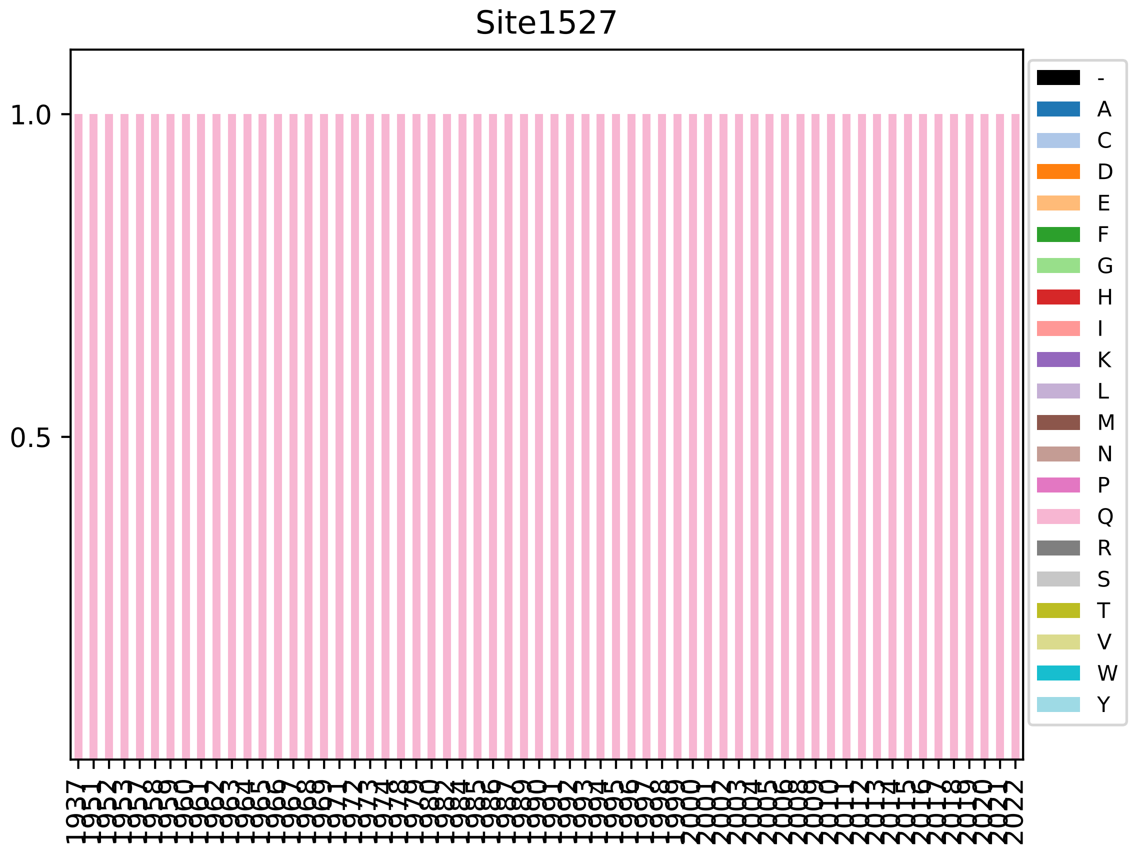

Site1527
1.0
0.5
-
A
C
D
E
F
G
H
I
K
L
M
N
P
Q
R
S
T
V
W
Y

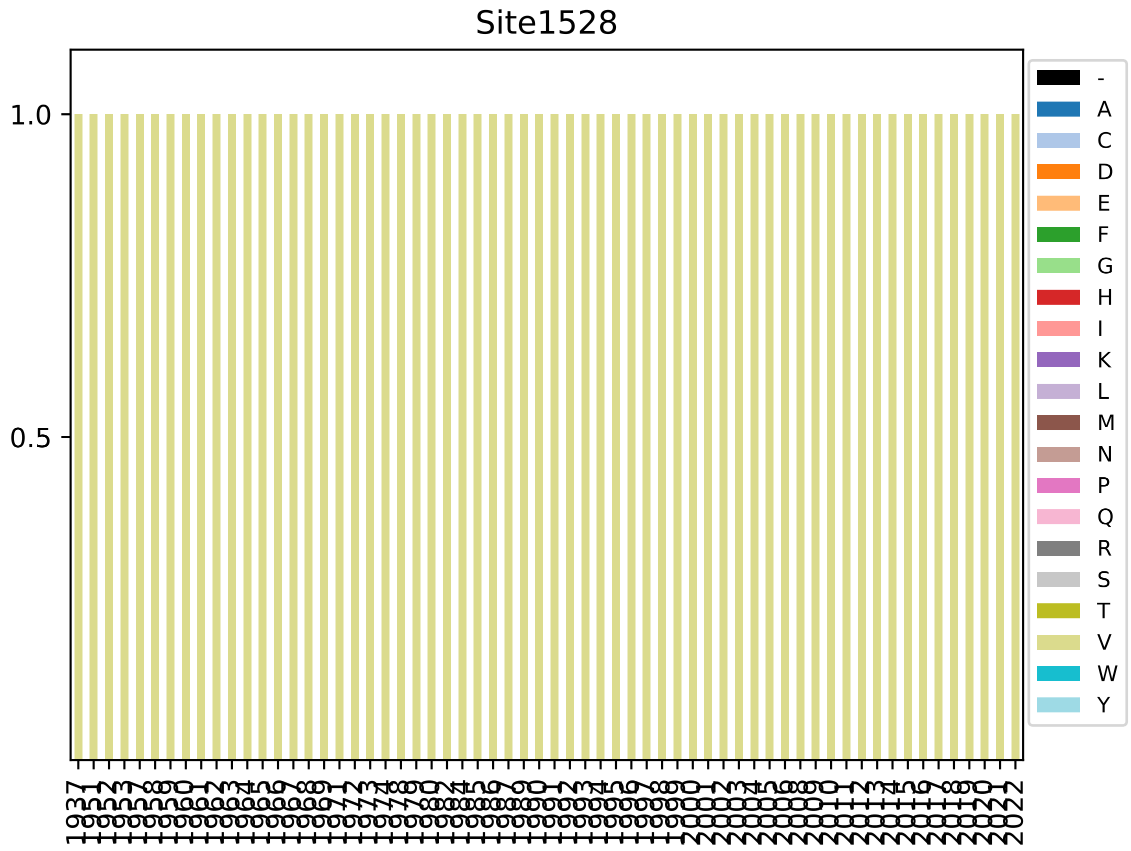
Site1528
1.0
0.5
-
A
C
D
E
F
G
H
I
K
L
M
N
P
Q
R
S
T
V
W
Y

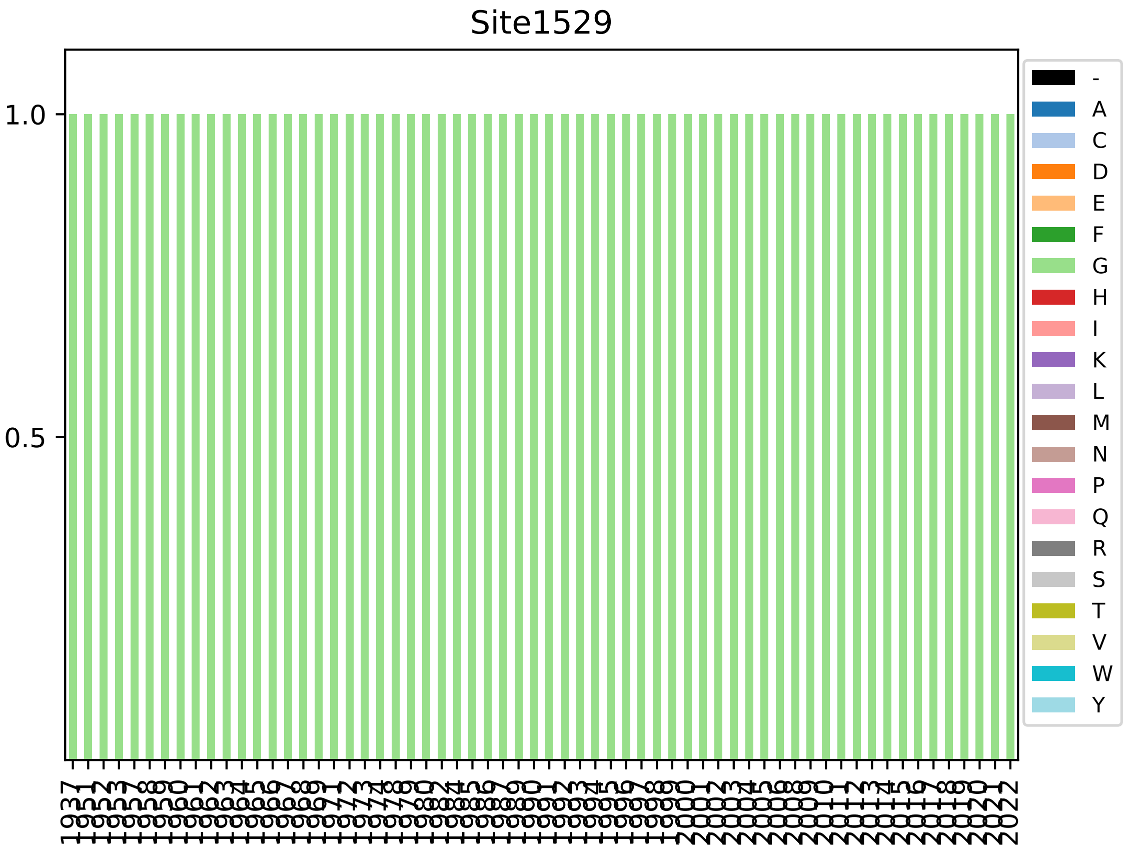
Site1529
1.0
0.5
-
A
C
D
E
F
G
H
I
K
L
M
N
P
Q
R
S
T
V
W
Y

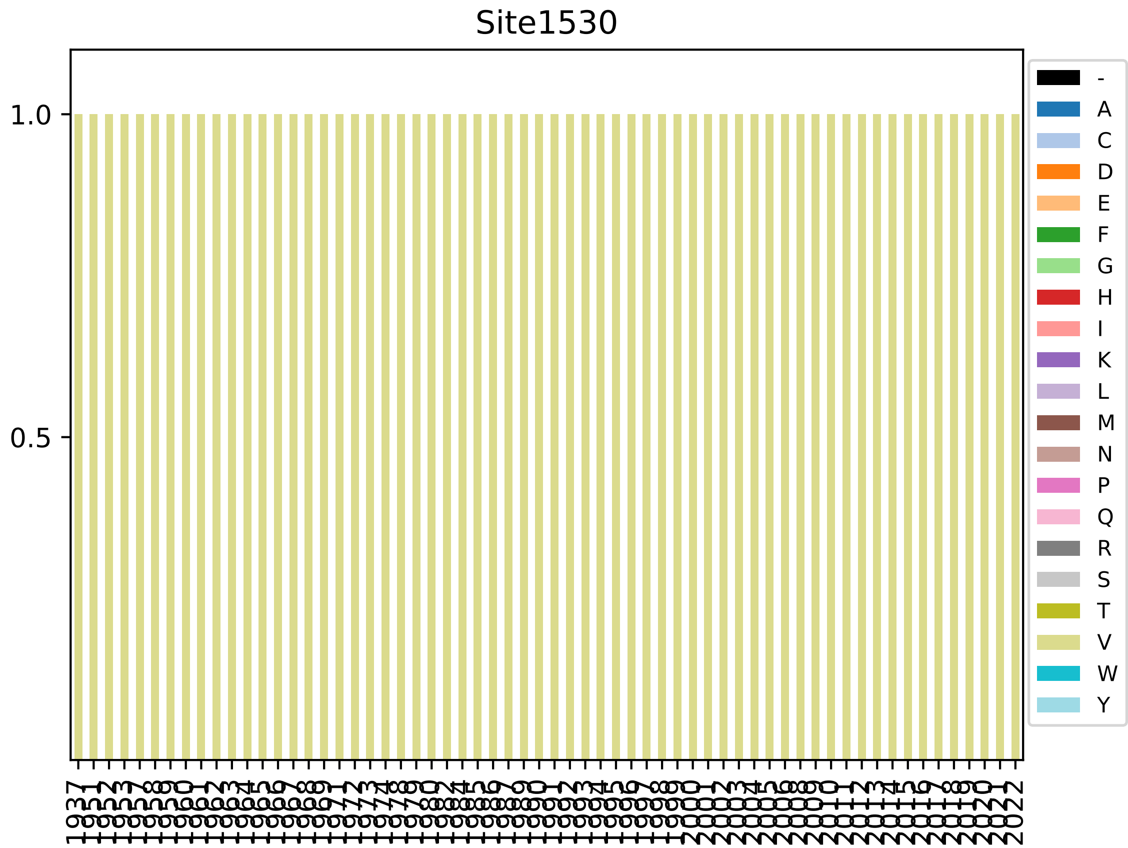
Site1530
1.0
0.5
-
A
C
D
E
F
G
H
I
K
L
M
N
P
Q
R
S
T
V
W
Y

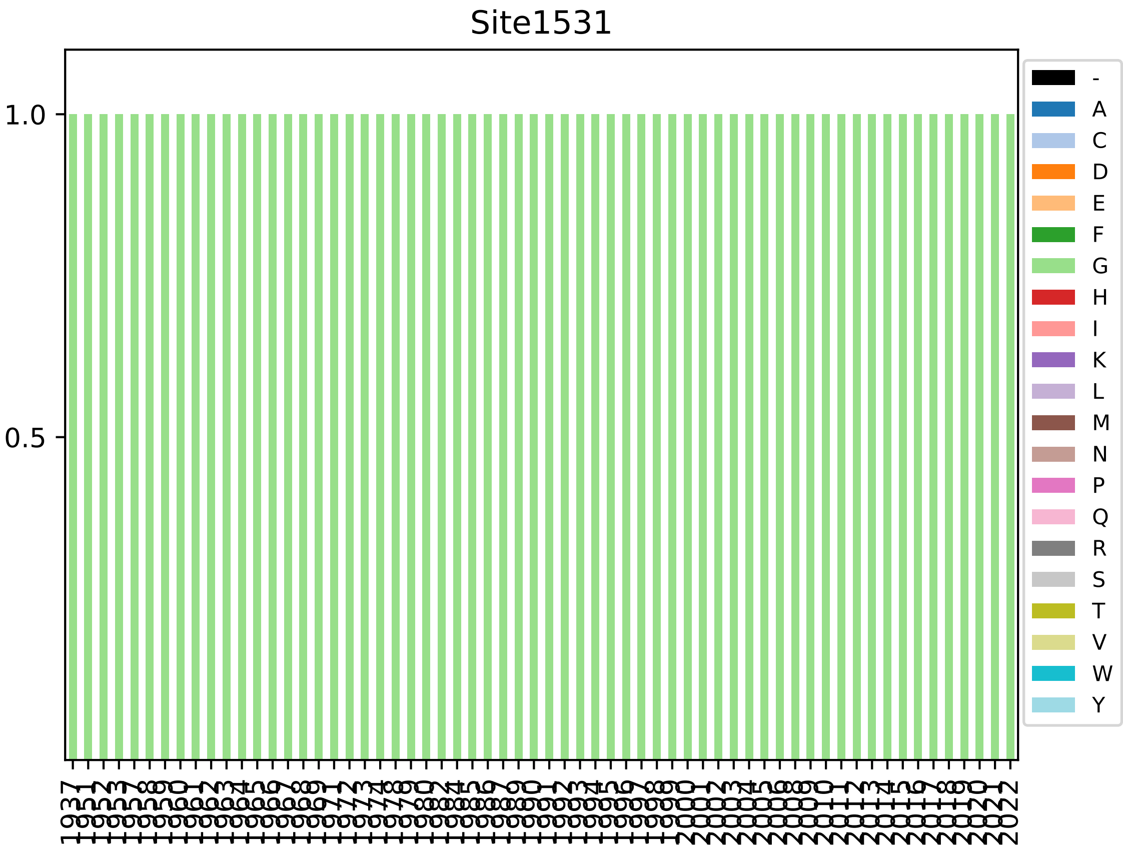

Site1531
1.0
0.5
-
A
C
D
E
F
G
H
I
K
L
M
N
P
Q
R
S
T
V
W
Y

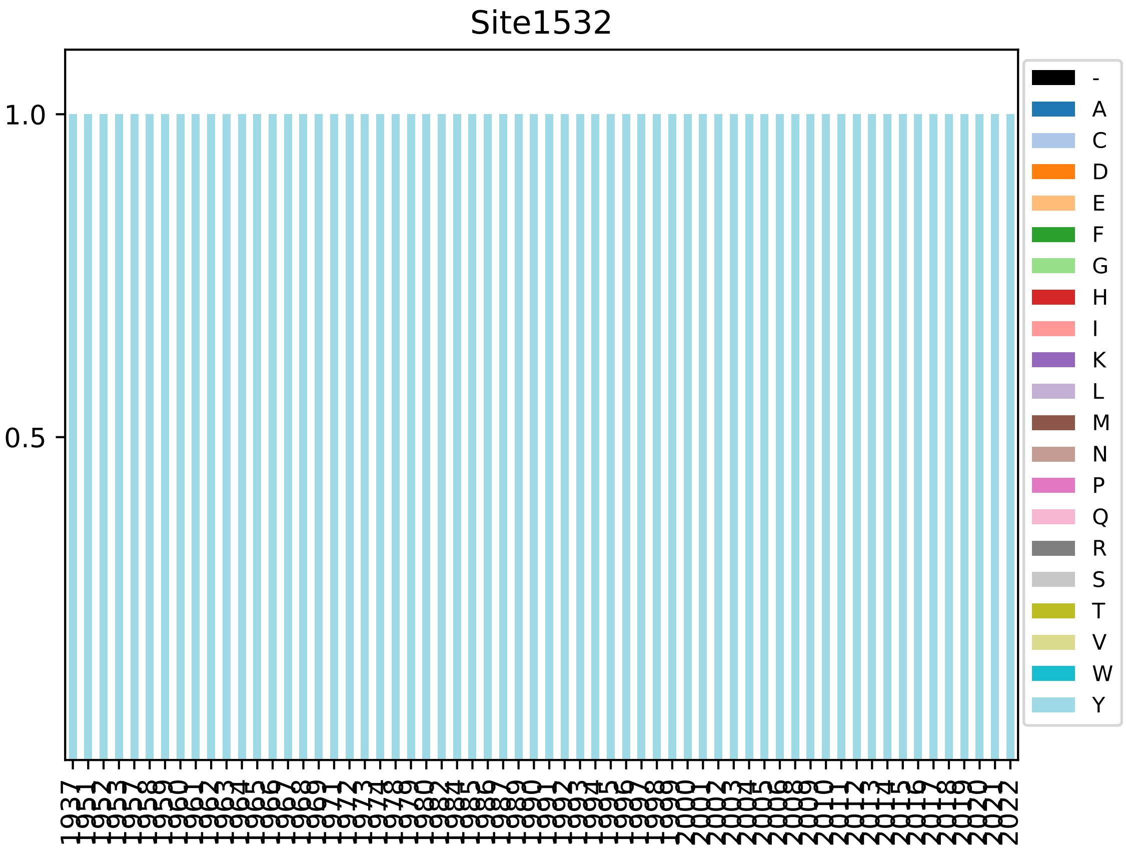
Site1532
1.0
0.5
-
A
C
D
E
F
G
H
I
K
L
M
N
P
Q
R
S
T
V
W
Y

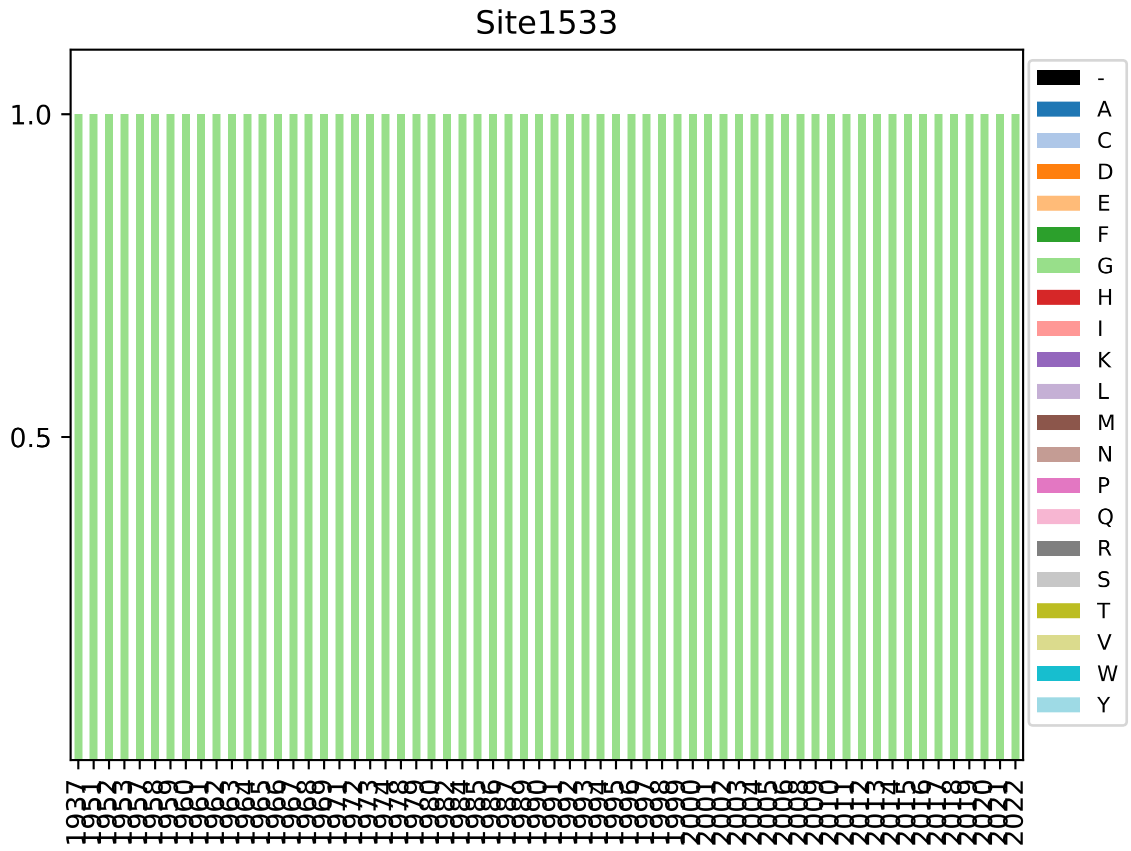
Site1533
1.0
0.5
-
A
C
D
E
F
G
H
I
K
L
M
N
P
Q
R
S
T
V
W
Y

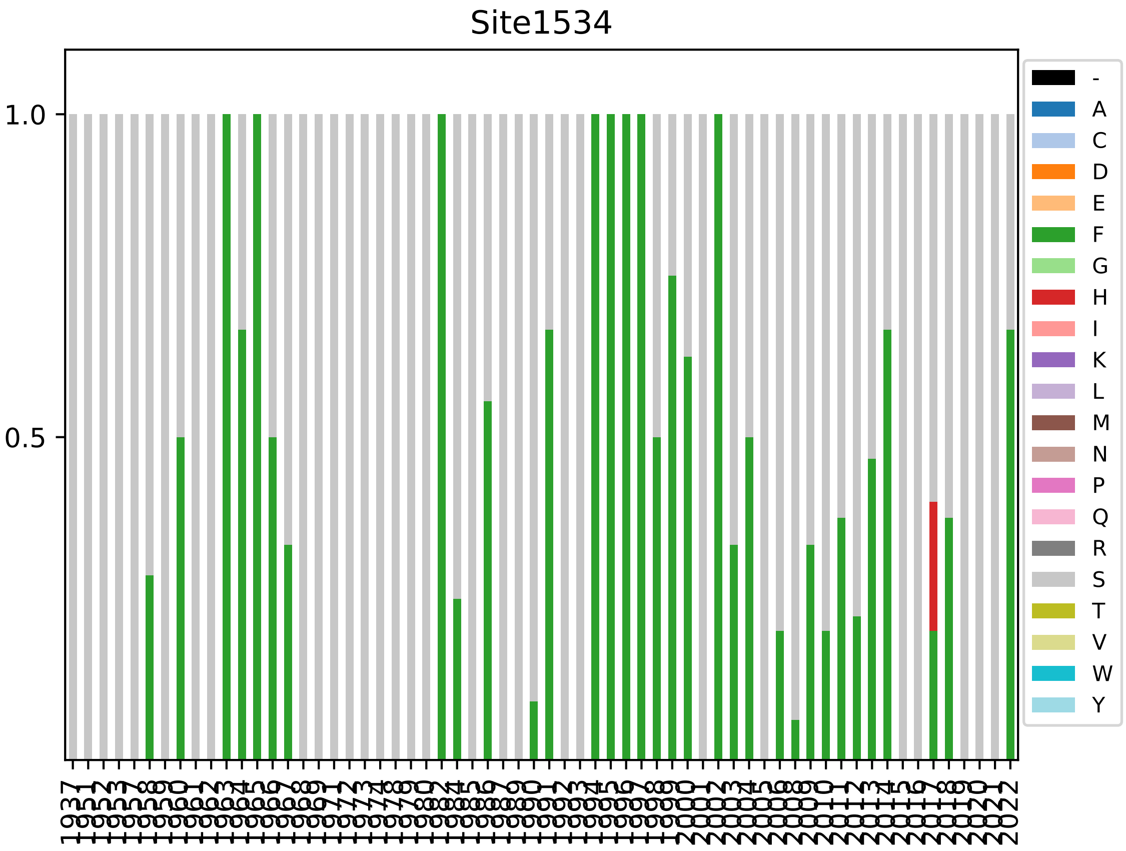

Site1534
1.0
0.5
-
A
C
D
E
F
G
H
I
K
L
M
N
P
Q
R
S
T
V
W
Y

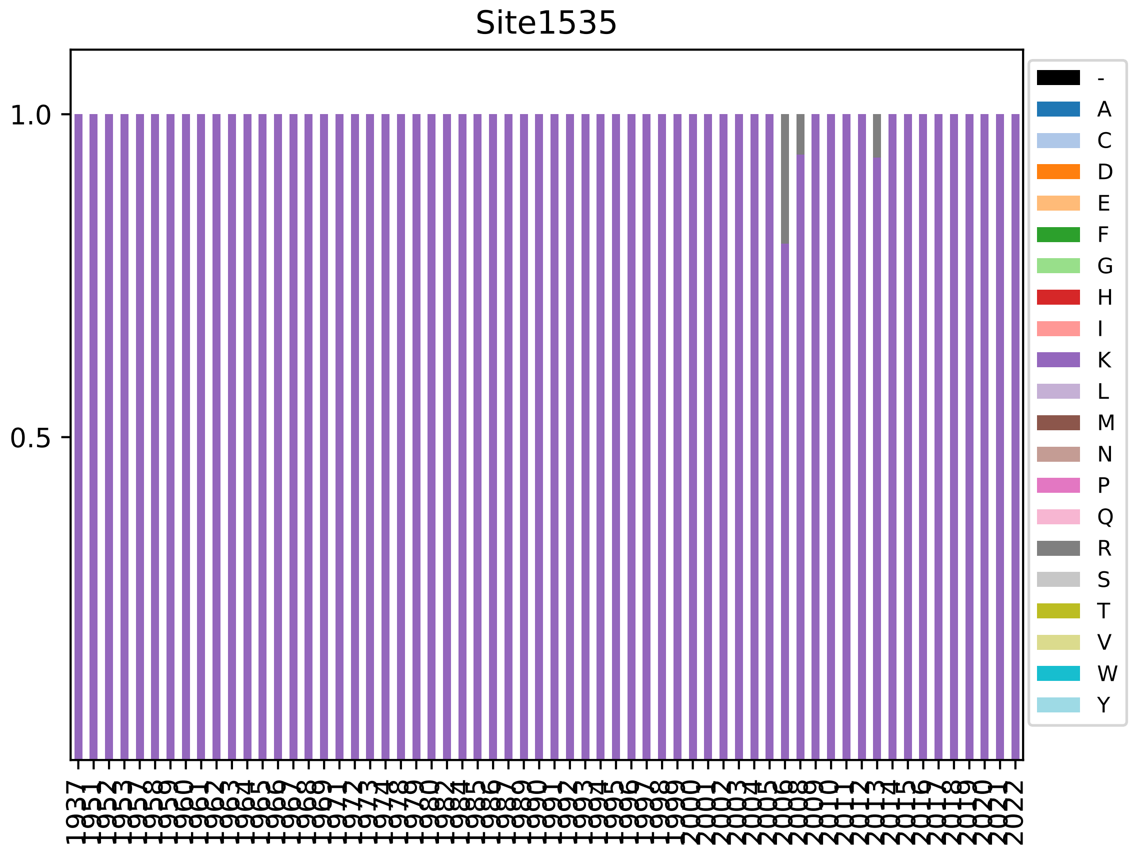
Site1535
1.0
0.5
-
A
C
D
E
F
G
H
I
K
L
M
N
P
Q
R
S
T
V
W
Y

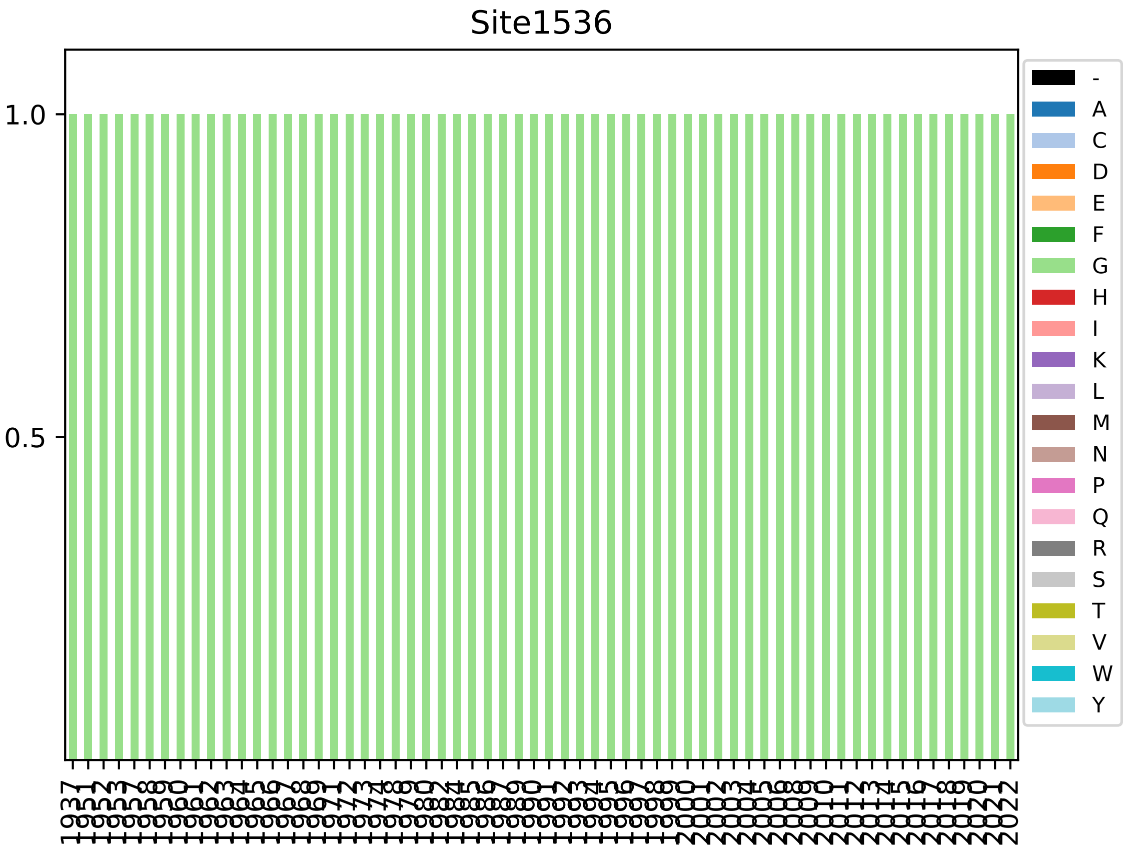
Site1536
1.0
0.5
-
A
C
D
E
F
G
H
I
K
L
M
N
P
Q
R
S
T
V
W
Y

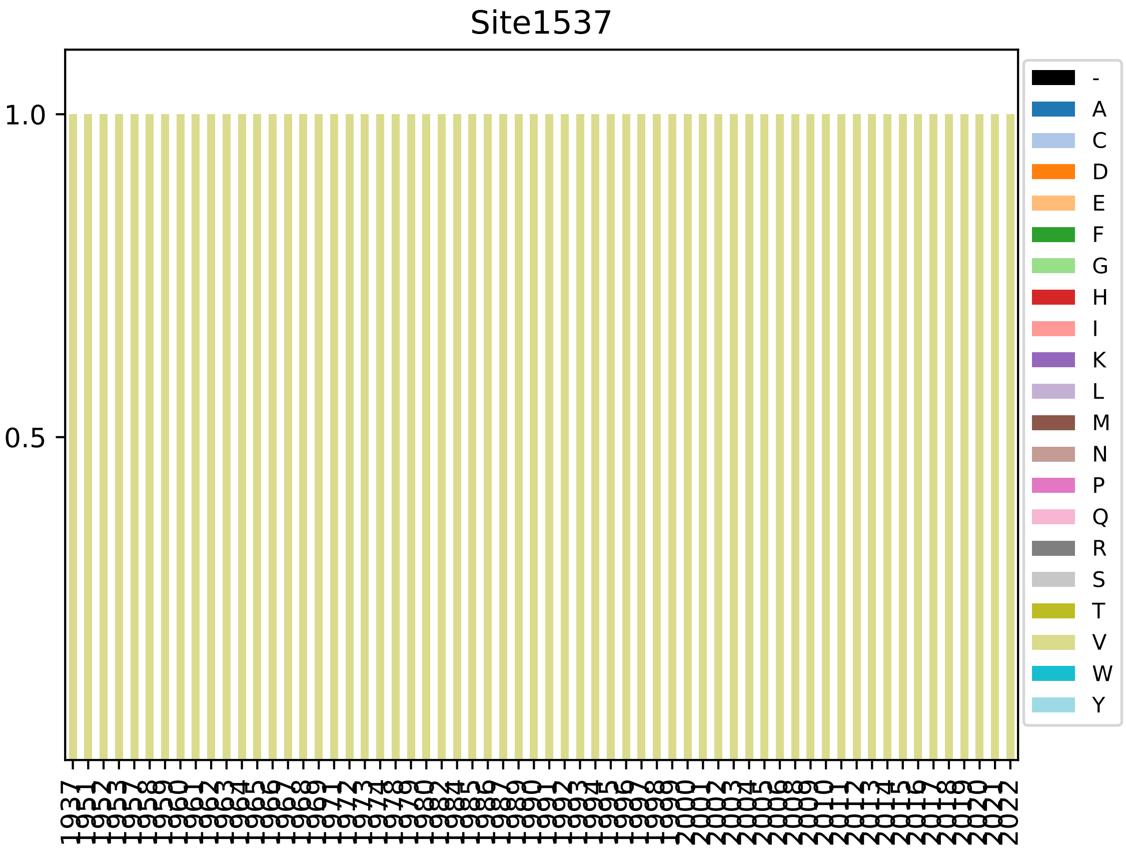
Site1537
1.0
0.5
-
A
C
D
E
F
G
H
I
K
L
M
N
P
Q
R
S
T
V
W
Y

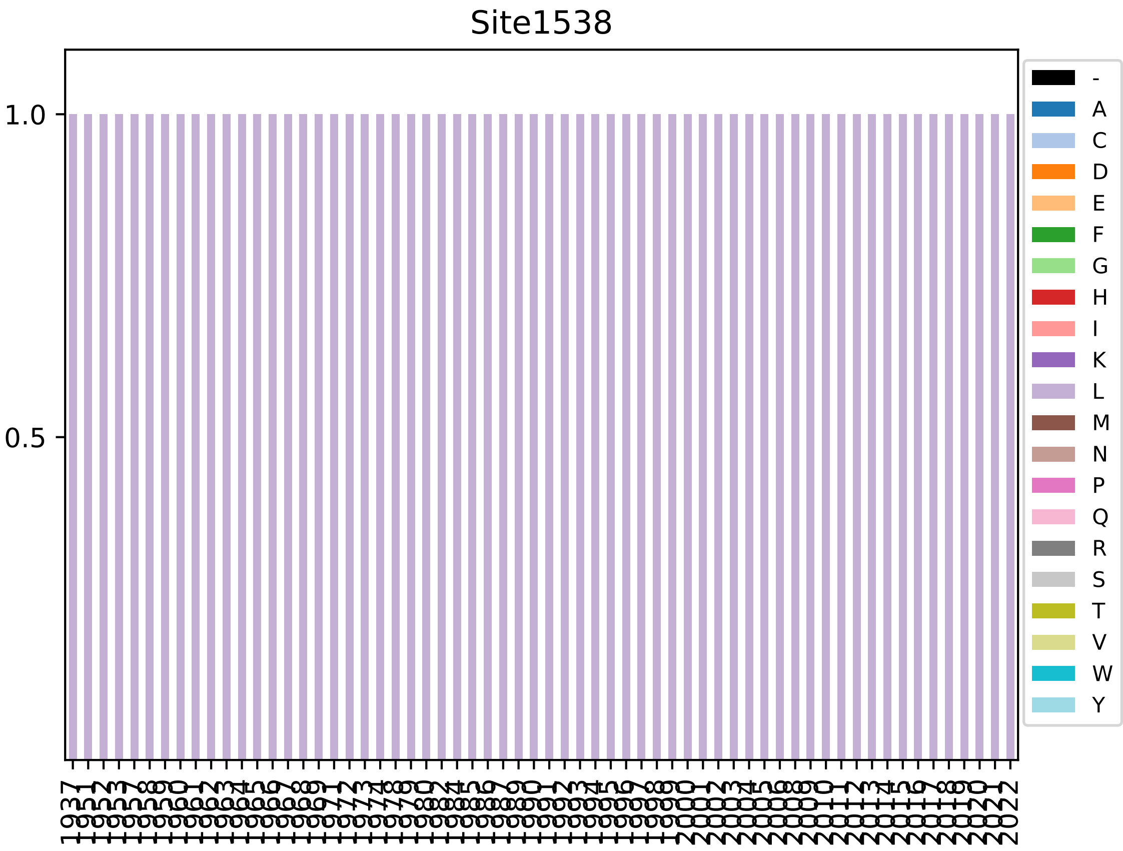

Site1538
1.0
0.5
-
A
C
D
E
F
G
H
I
K
L
M
N
P
Q
R
S
T
V
W
Y

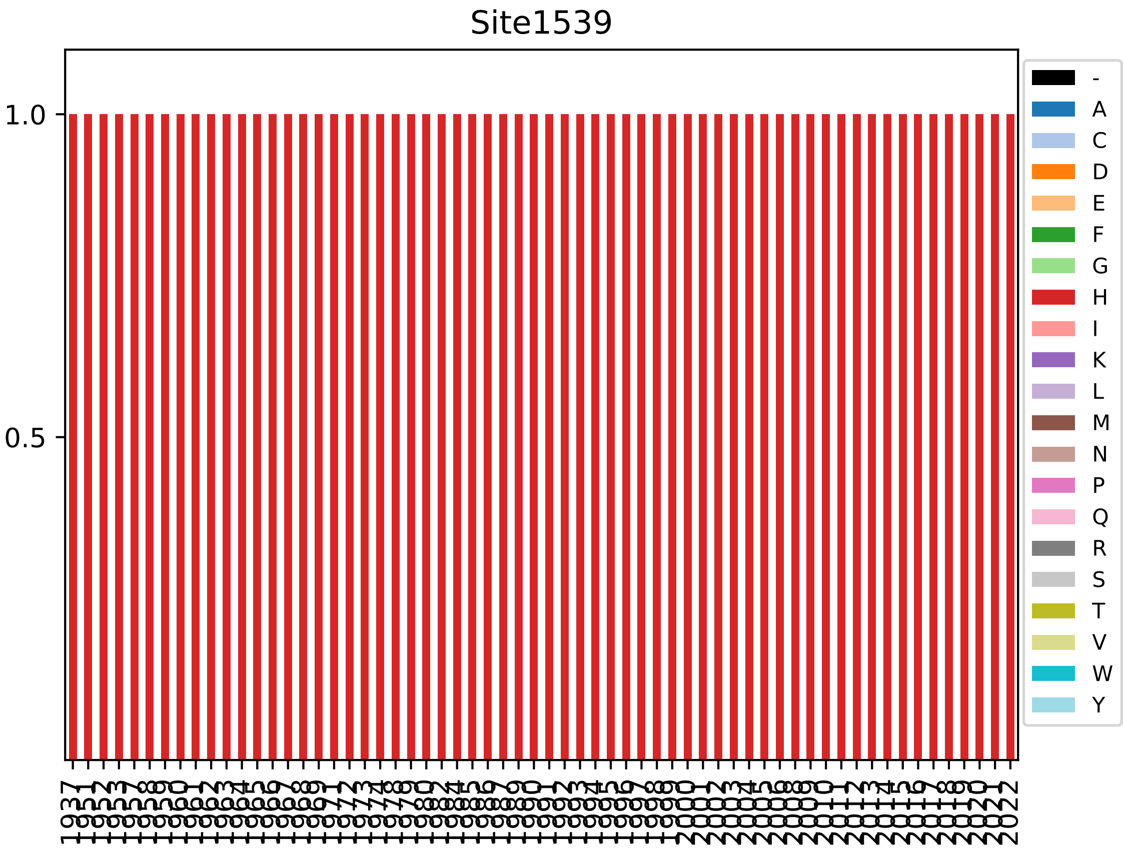

Site1539
1.0
0.5
-
A
C
D
E
F
G
H
I
K
L
M
N
P
Q
R
S
T
V
W
Y

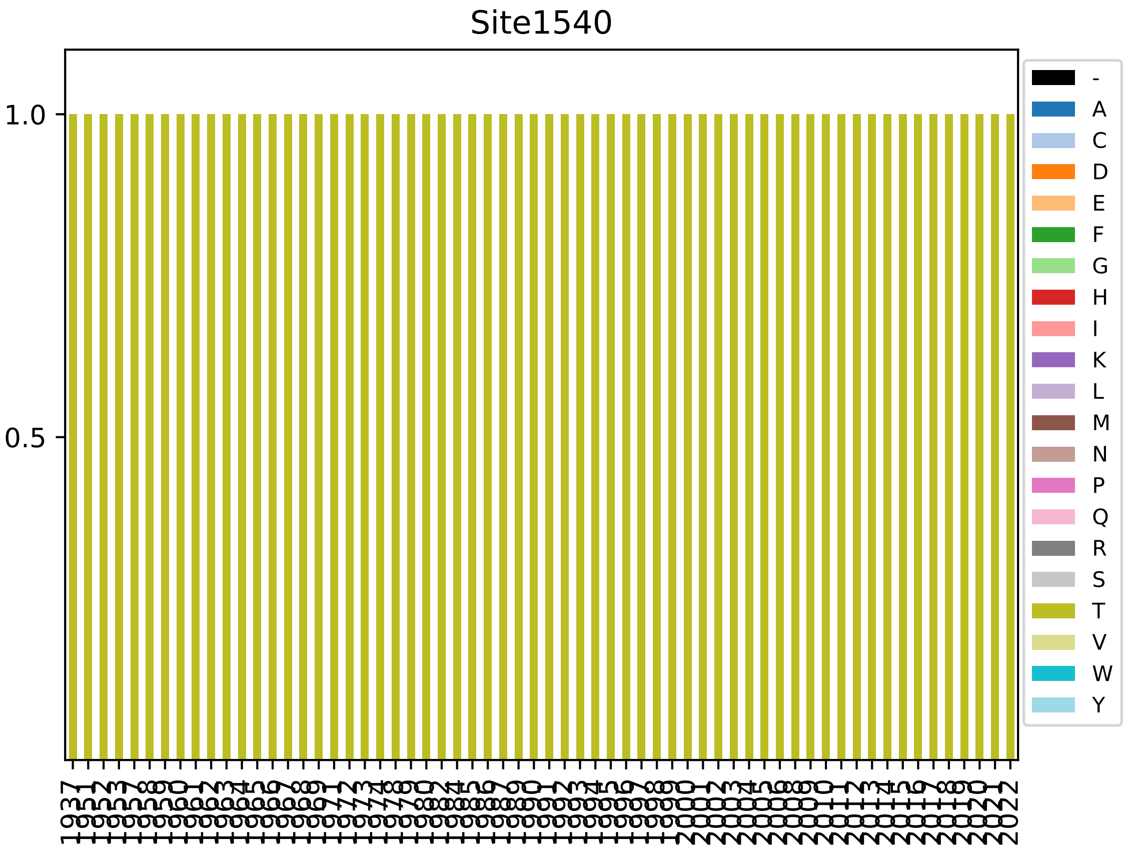
Site1540
1.0
0.5
-
A
C
D
E
F
G
H
I
K
L
M
N
P
Q
R
S
T
V
W
Y

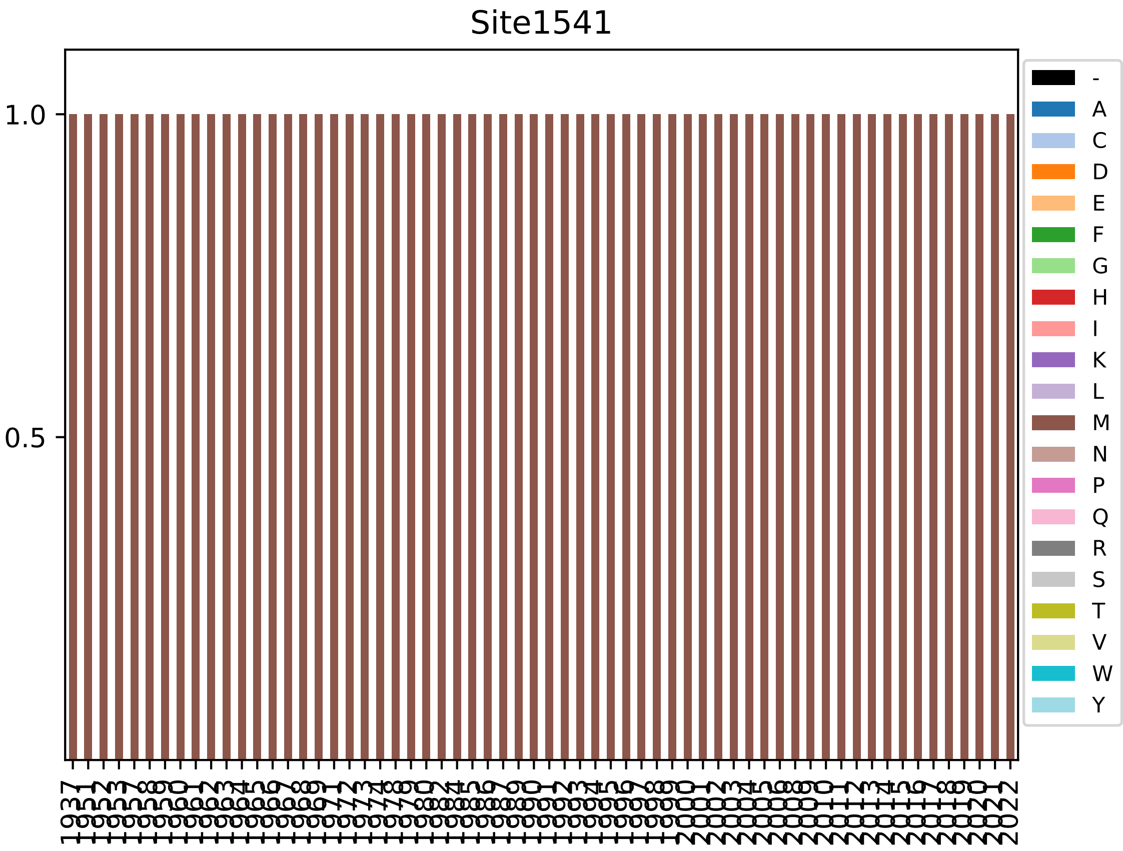
Site1541
1.0
0.5
-
A
C
D
E
F
G
H
I
K
L
M
N
P
Q
R
S
T
V
W
Y

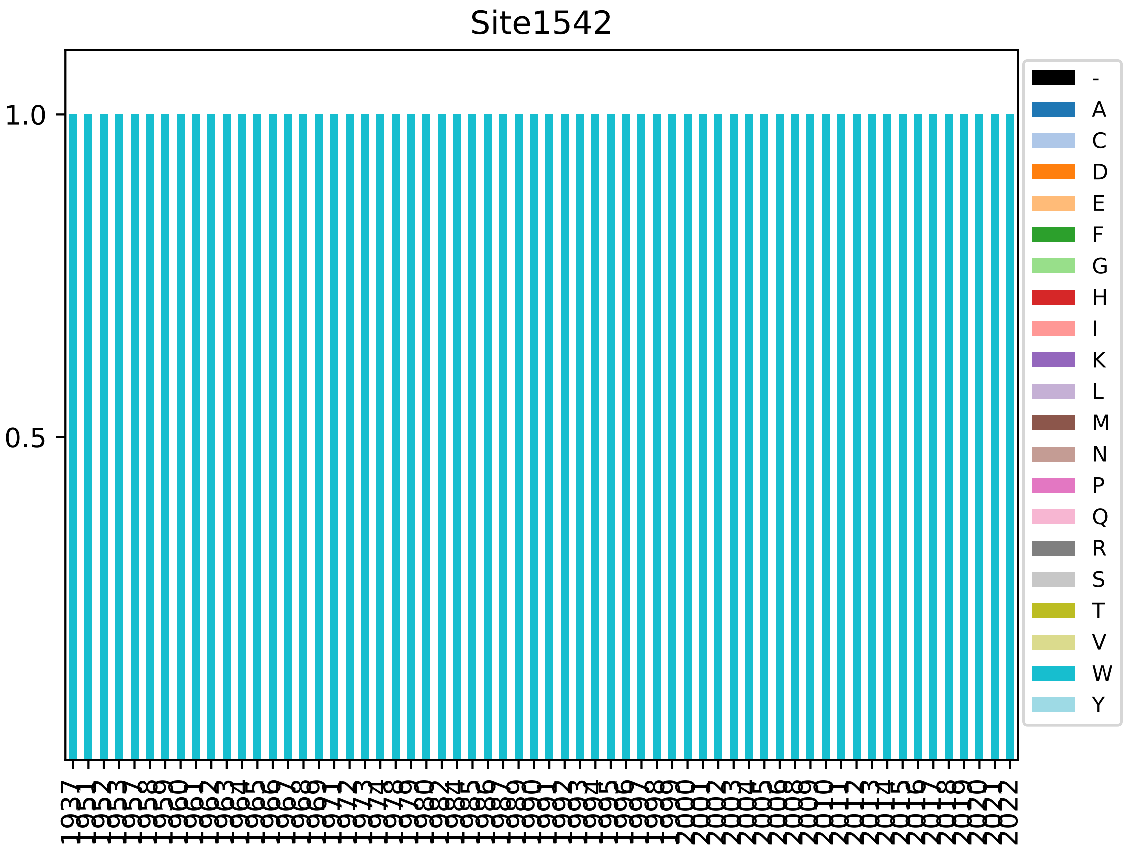
Site1542
1.0
0.5
-
A
C
D
E
F
G
H
I
K
L
M
N
P
Q
R
S
T
V
W
Y

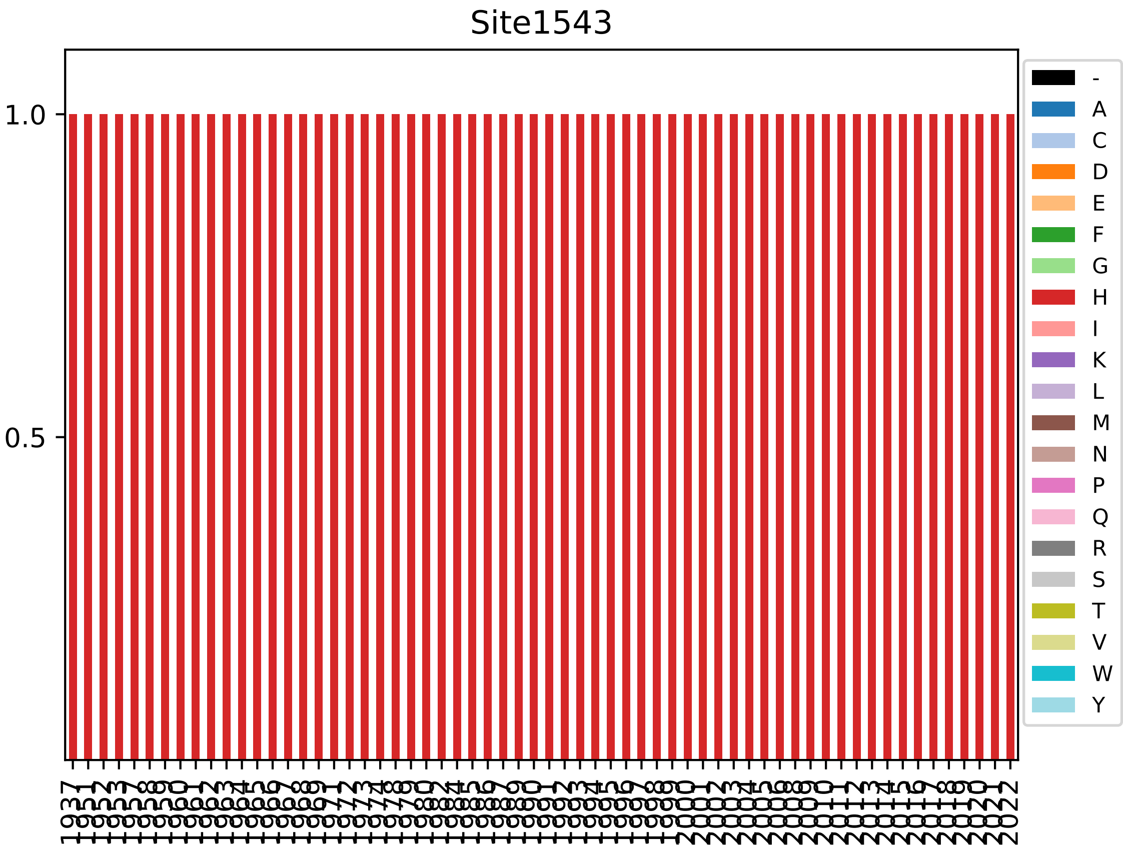
Site1543
1.0
0.5
-
A
C
D
E
F
G
H
I
K
L
M
N
P
Q
R
S
T
V
W
Y

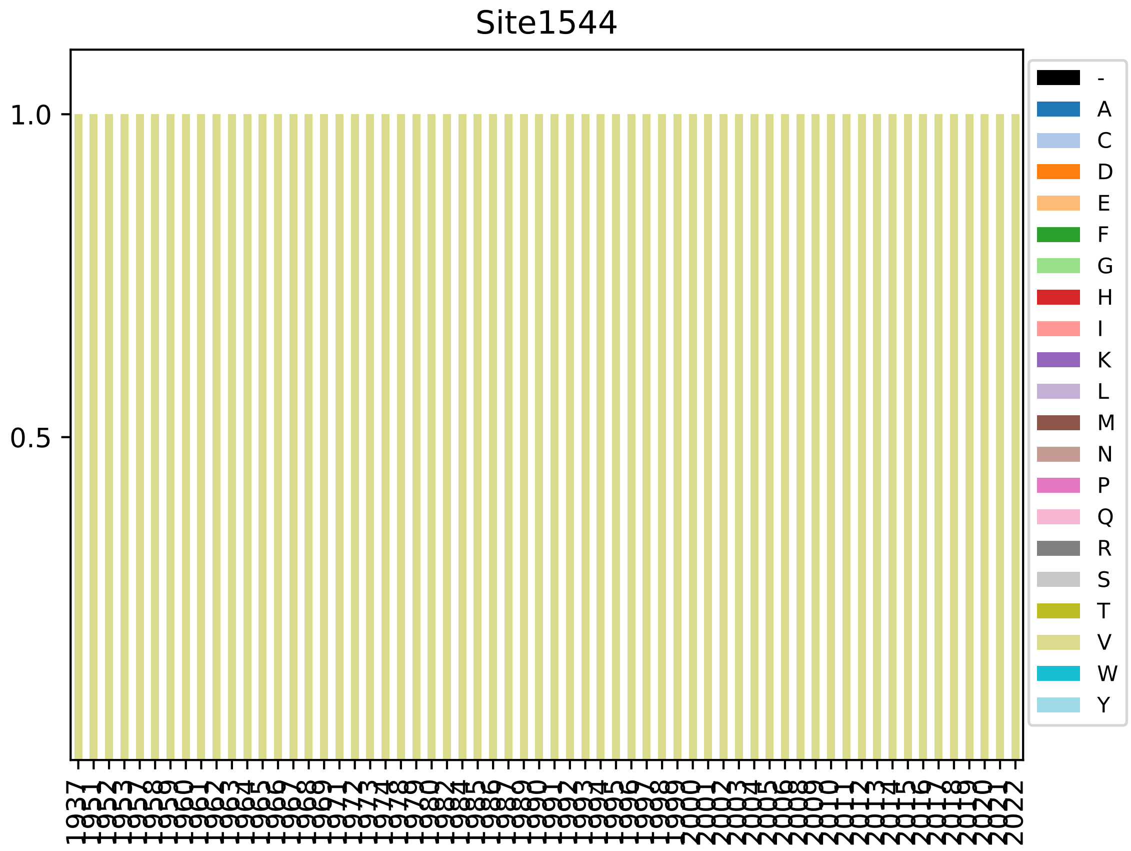
Site1544
1.0
0.5
-
A
C
D
E
F
G
H
I
K
L
M
N
P
Q
R
S
T
V
W
Y

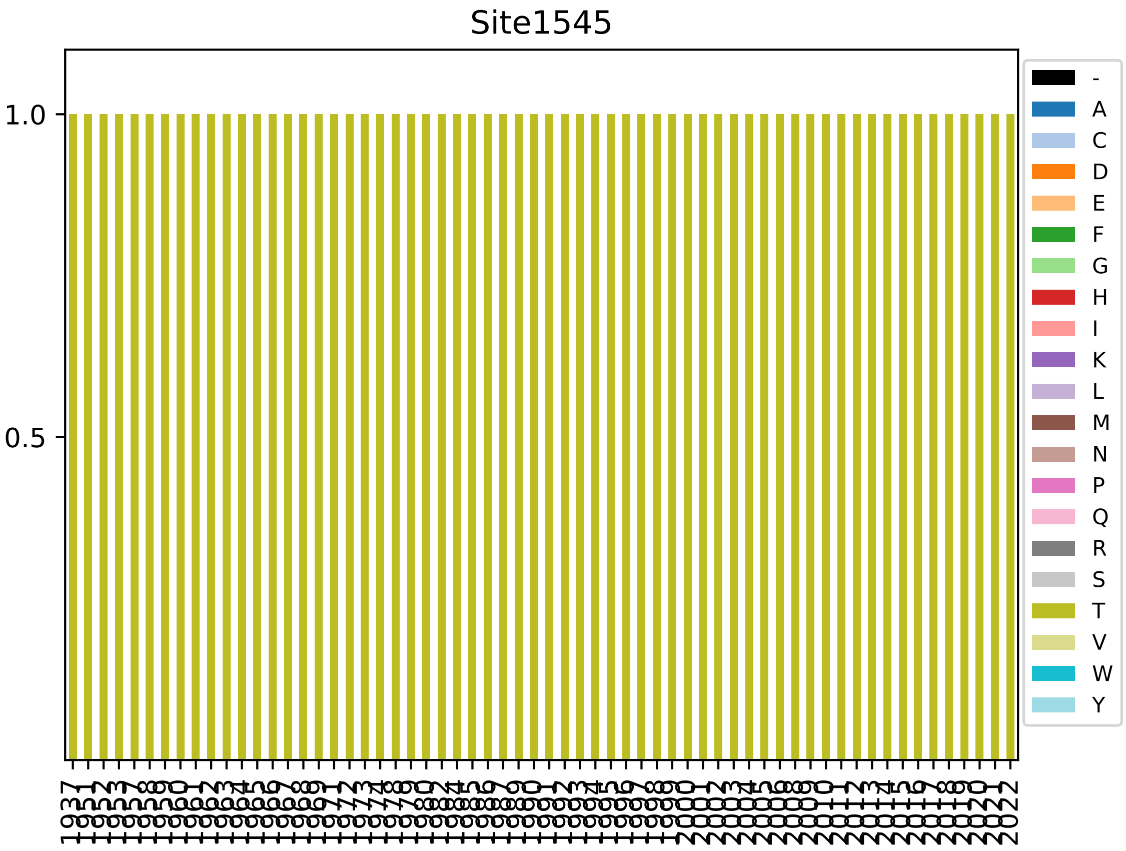
Site1545
1.0
0.5
-
A
C
D
E
F
G
H
I
K
L
M
N
P
Q
R
S
T
V
W
Y

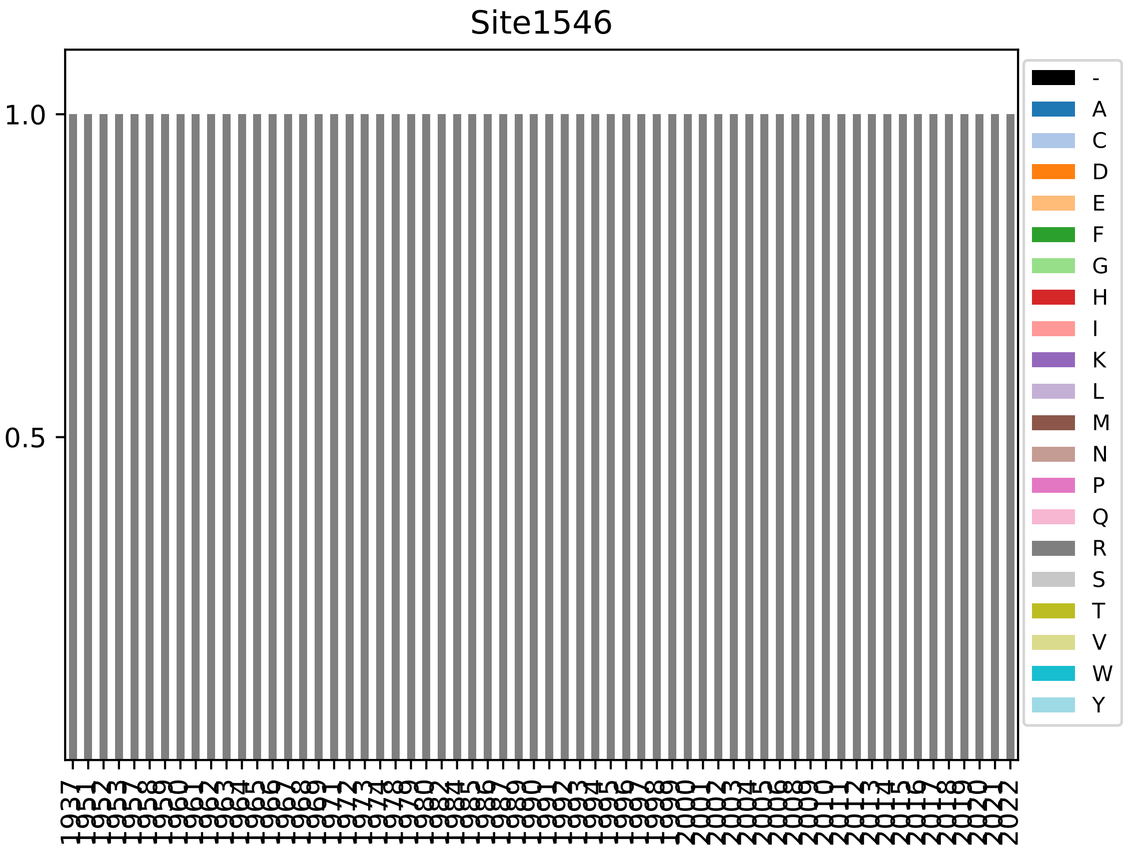
Site1546
1.0
0.5
-
A
C
D
E
F
G
H
I
K
L
M
N
P
Q
R
S
T
V
W
Y

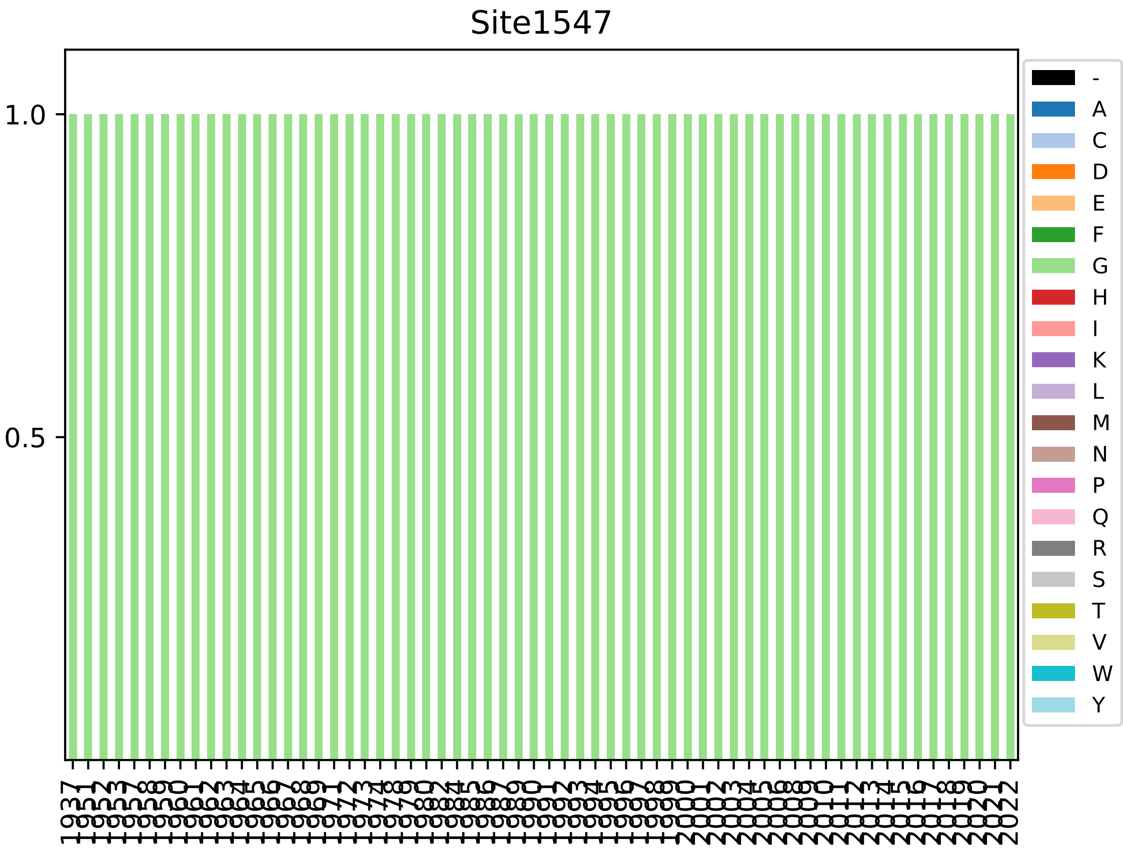

Site1547
1.0
0.5
-
A
C
D
E
F
G
H
I
K
L
M
N
P
Q
R
S
T
V
W
Y

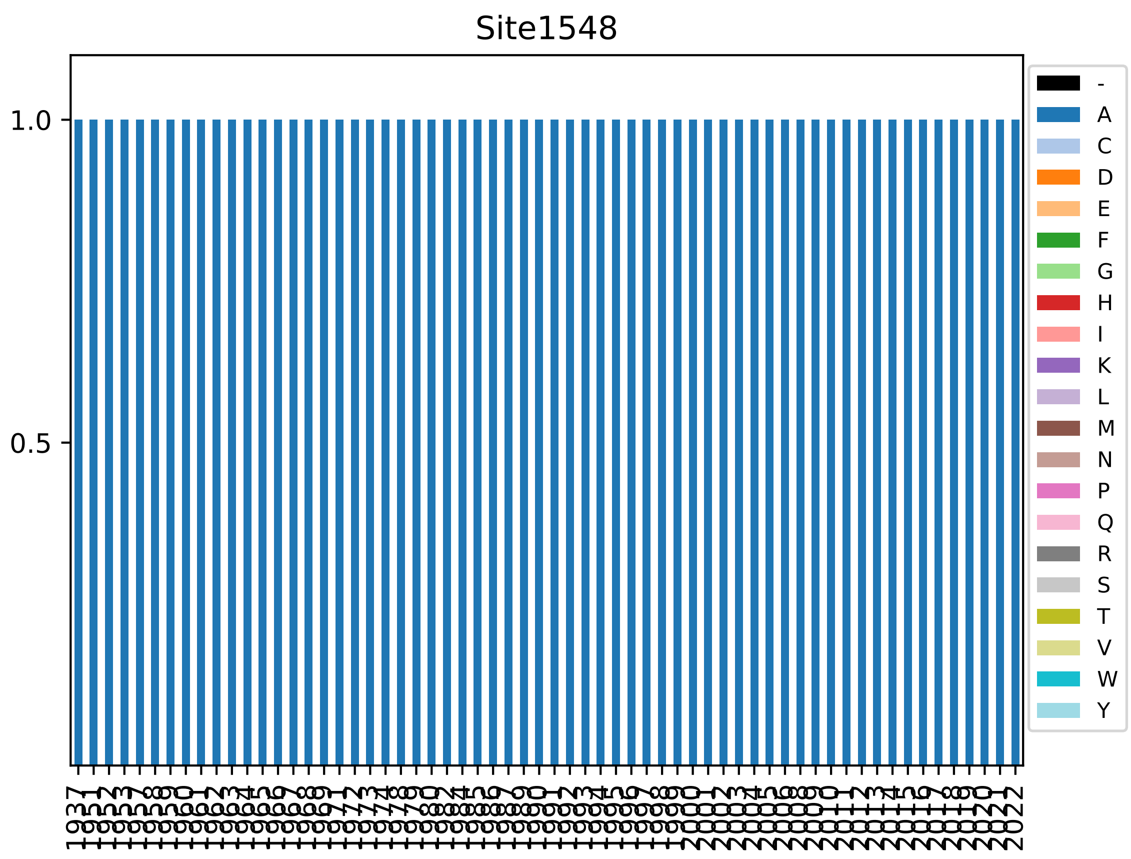
Site1548
1.0
0.5
-
A
C
D
E
F
G
H
I
K
L
M
N
P
Q
R
S
T
V
W
Y

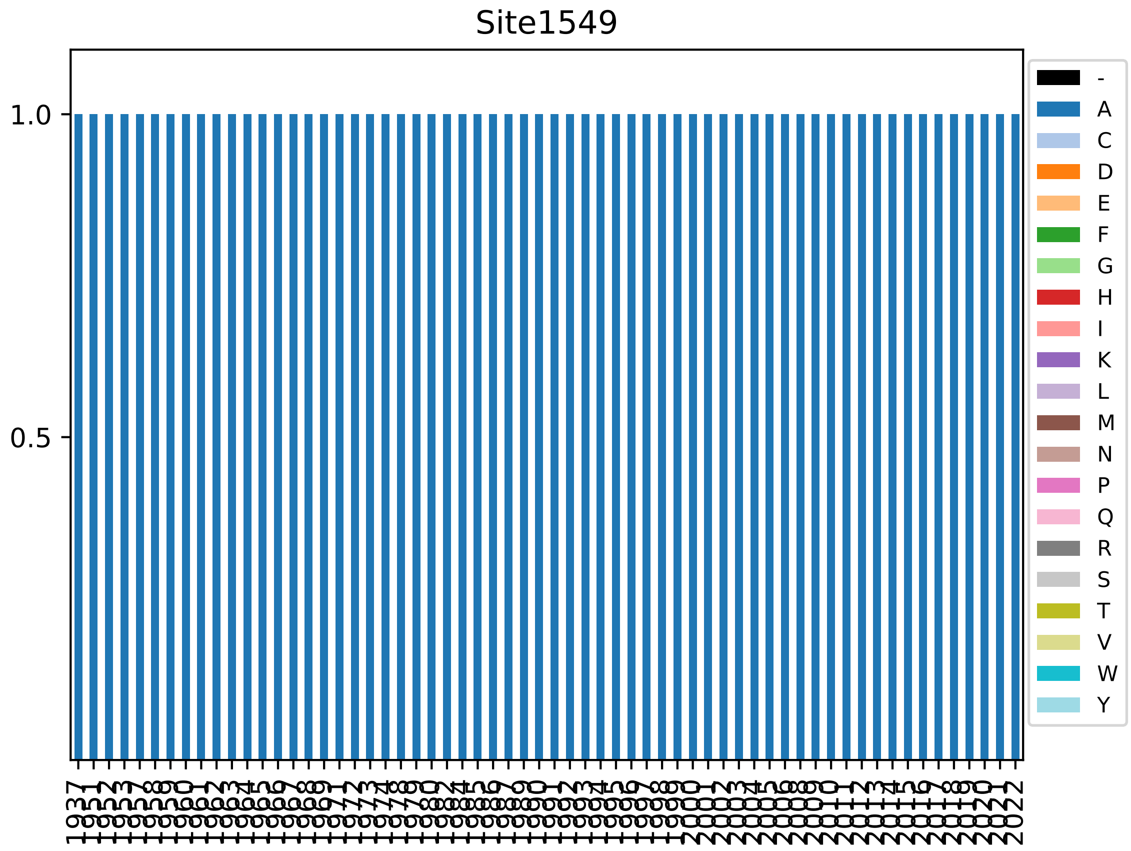
Site1549
1.0
0.5
-
A
C
D
E
F
G
H
I
K
L
M
N
P
Q
R
S
T
V
W
Y

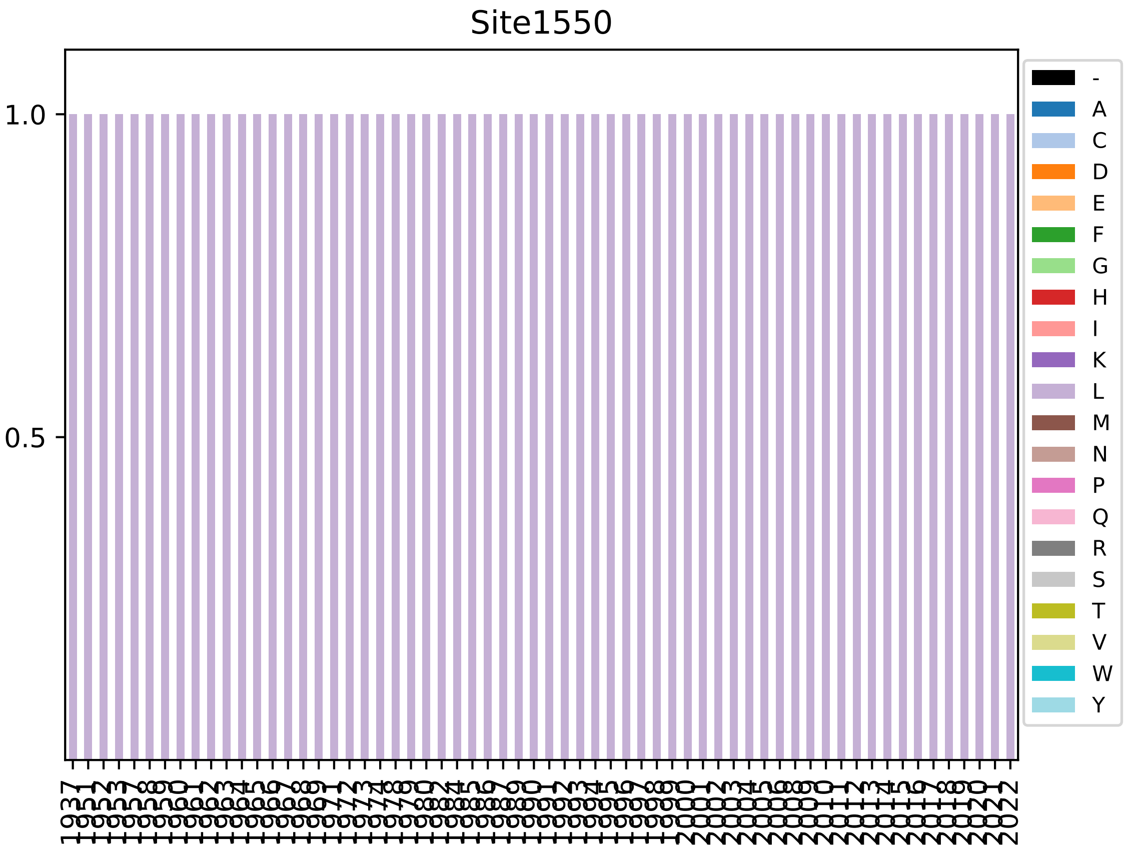
Site1550
1.0
0.5
-
A
C
D
E
F
G
H
I
K
L
M
N
P
Q
R
S
T
V
W
Y

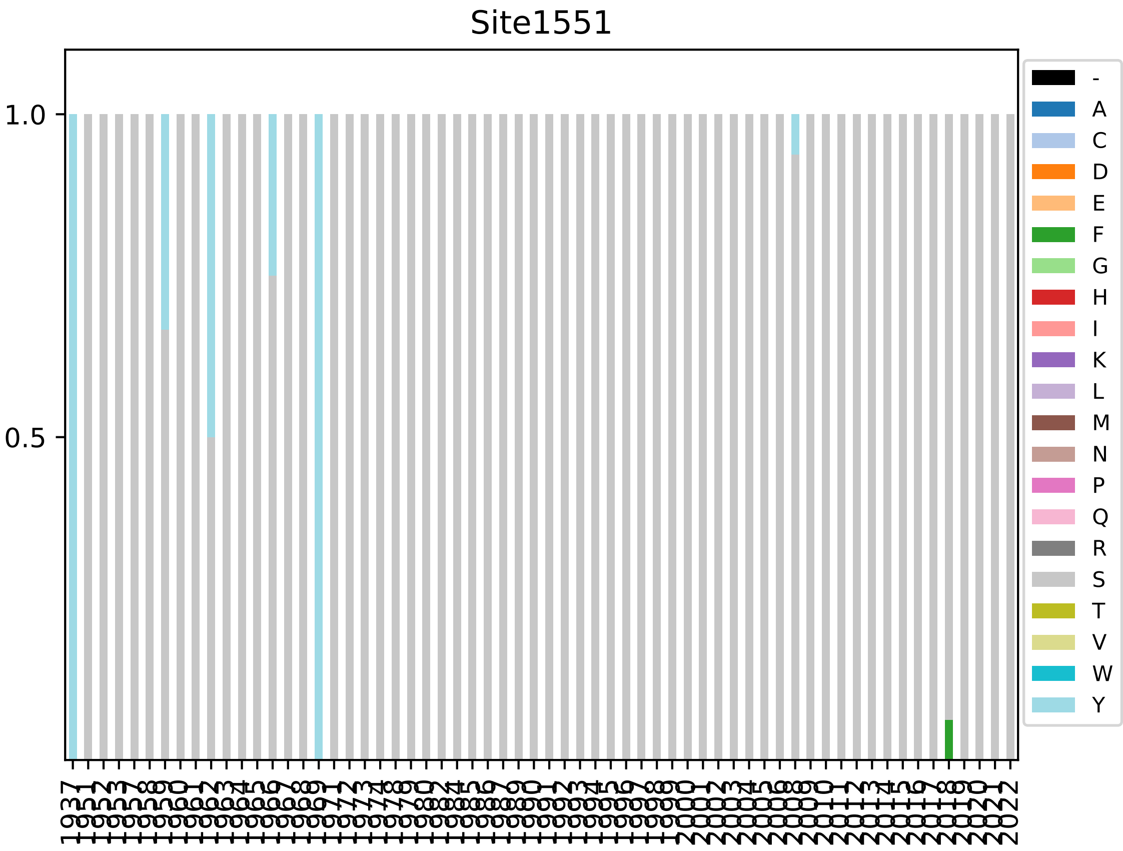
Site1551
1.0
0.5
-
A
C
D
E
F
G
H
I
K
L
M
N
P
Q
R
S
T
V
W
Y

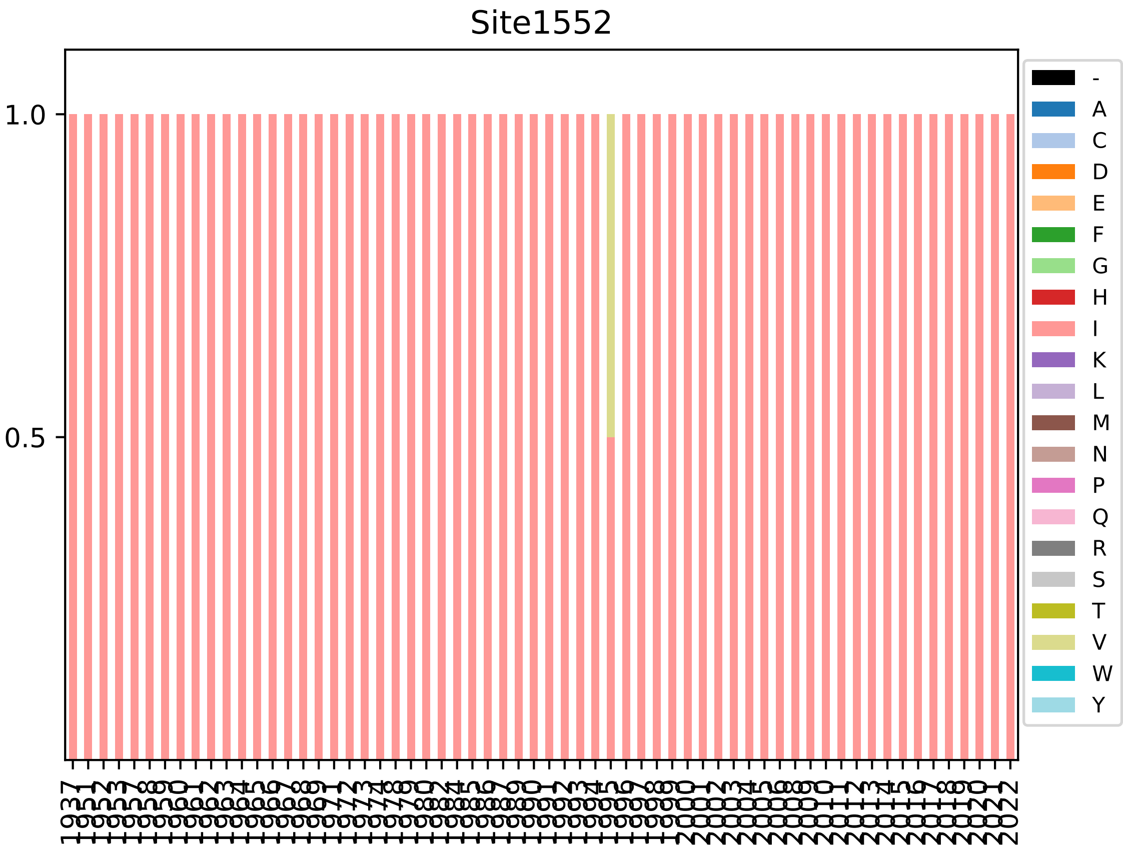
Site1552
1.0
0.5
-
A
C
D
E
F
G
H
I
K
L
M
N
P
Q
R
S
T
V
W
Y

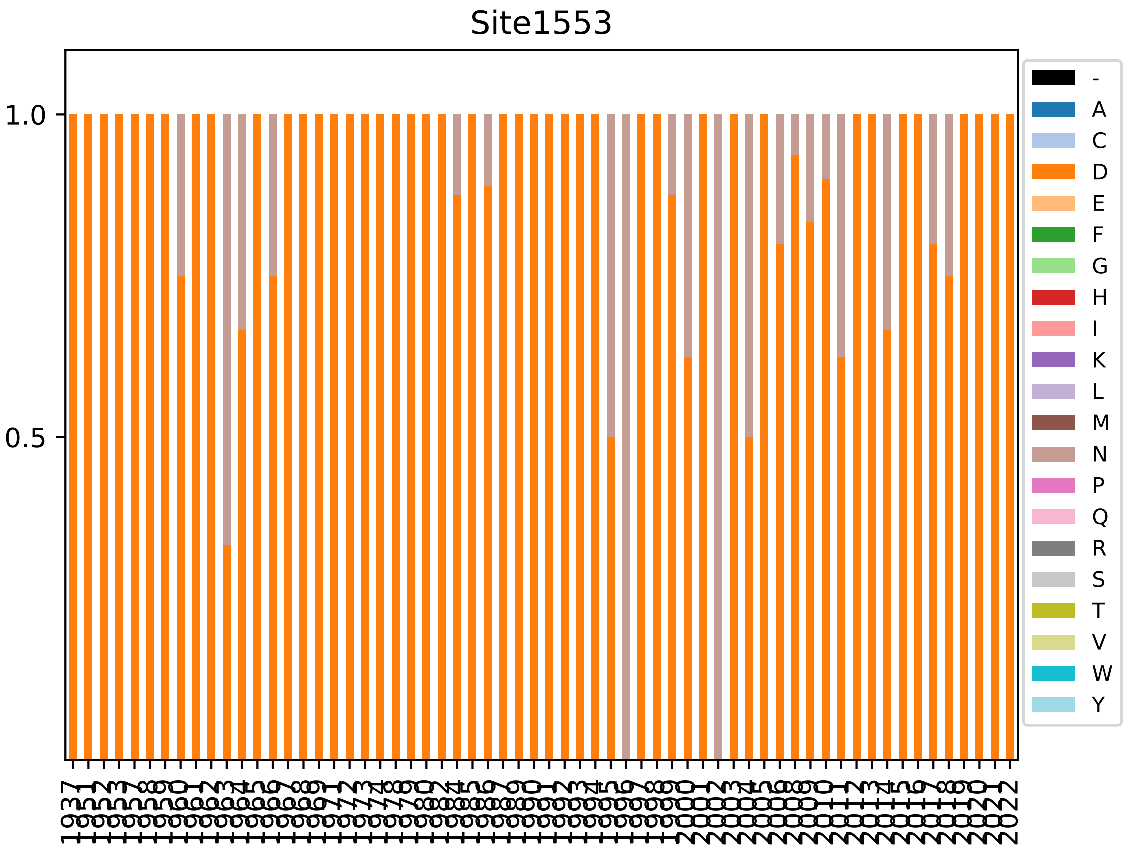
Site1553
1.0
0.5
-
A
C
D
E
F
G
H
I
K
L
M
N
P
Q
R
S
T
V
W
Y

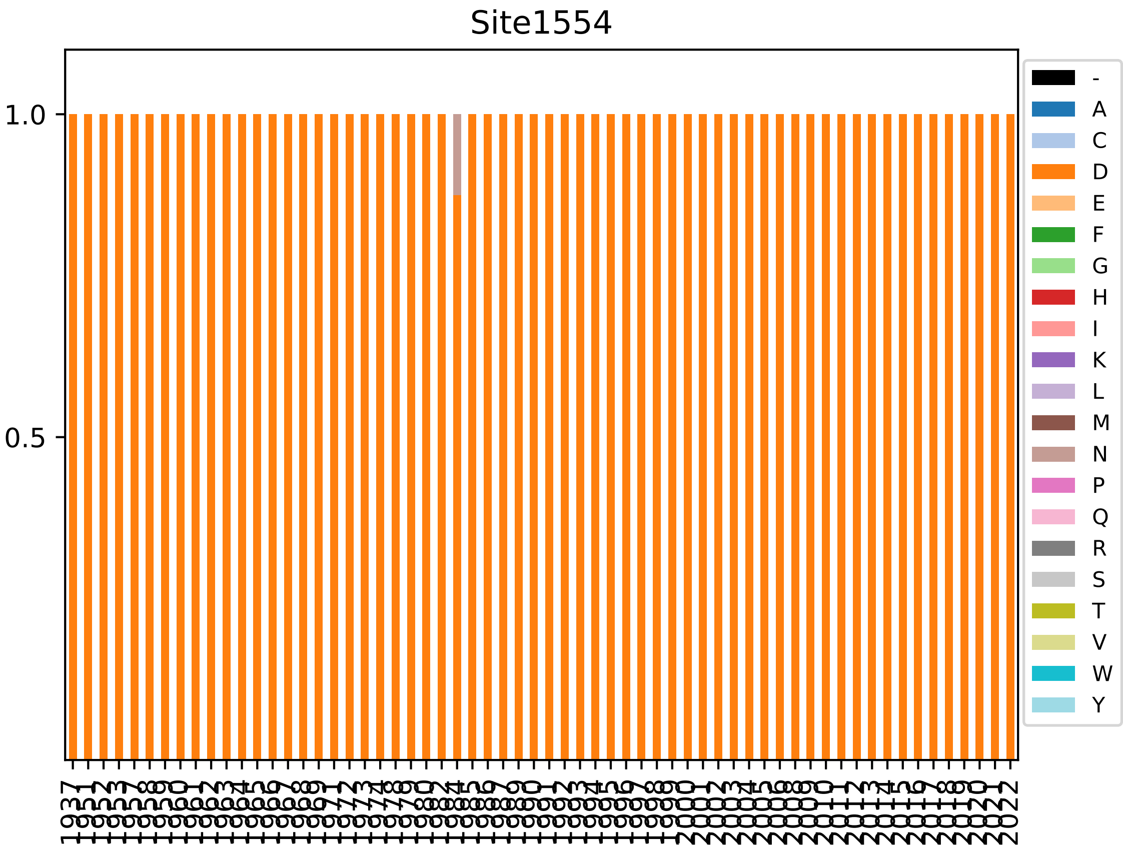
Site1554
1.0
0.5
-
A
C
D
E
F
G
H
I
K
L
M
N
P
Q
R
S
T
V
W
Y

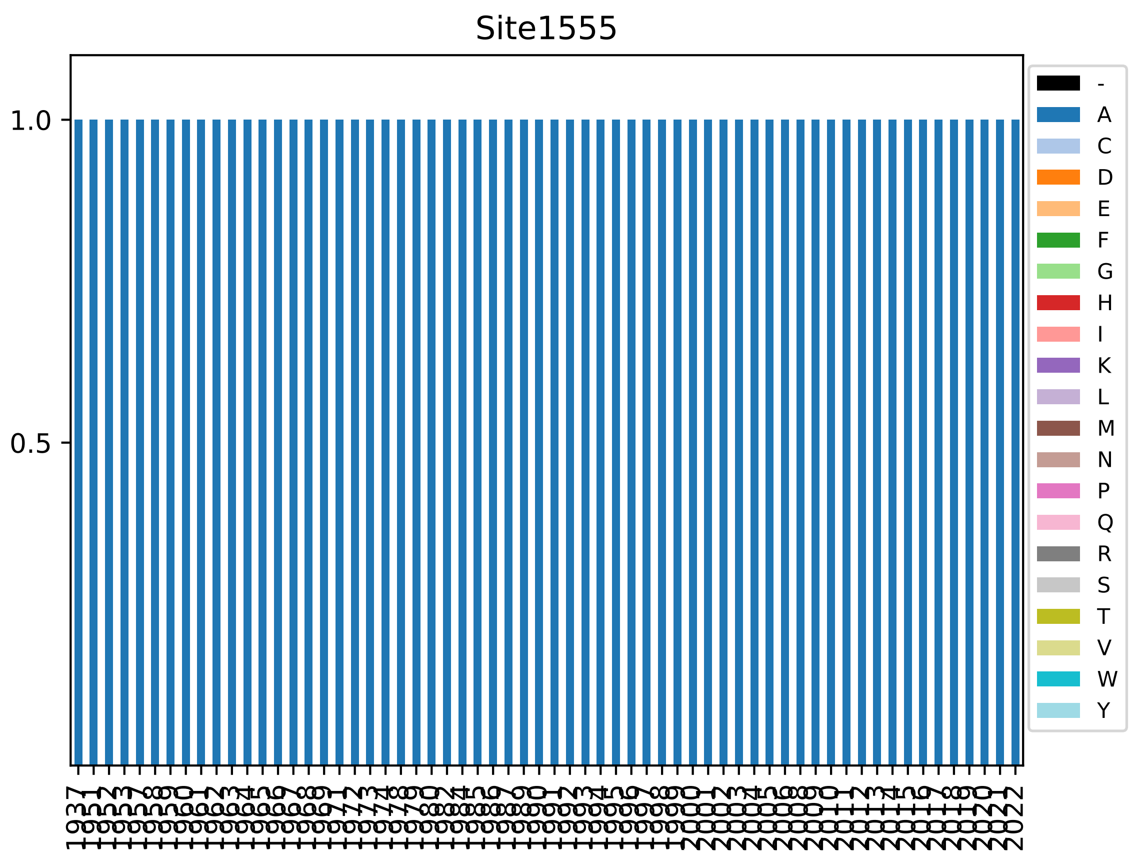
Site1555
1.0
0.5
-
A
C
D
E
F
G
H
I
K
L
M
N
P
Q
R
S
T
V
W
Y

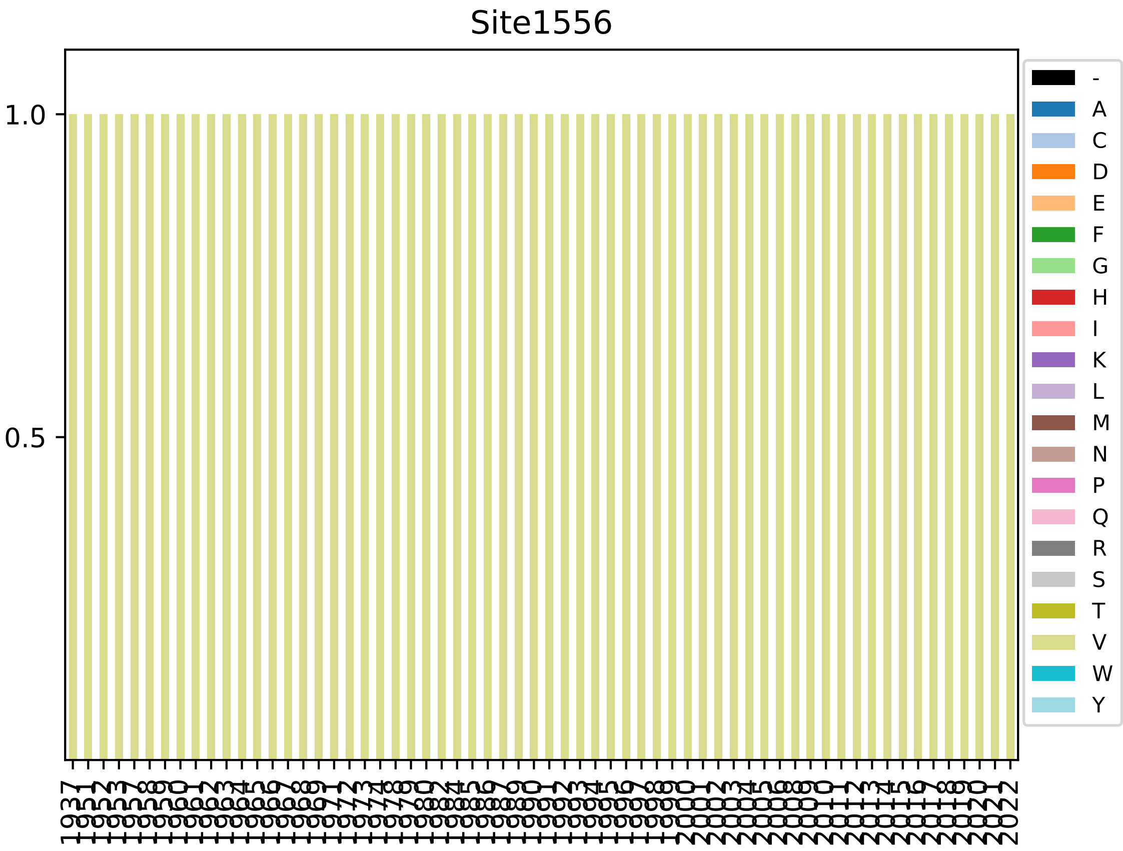

Site1556
1.0
0.5
-
A
C
D
E
F
G
H
I
K
L
M
N
P
Q
R
S
T
V
W
Y

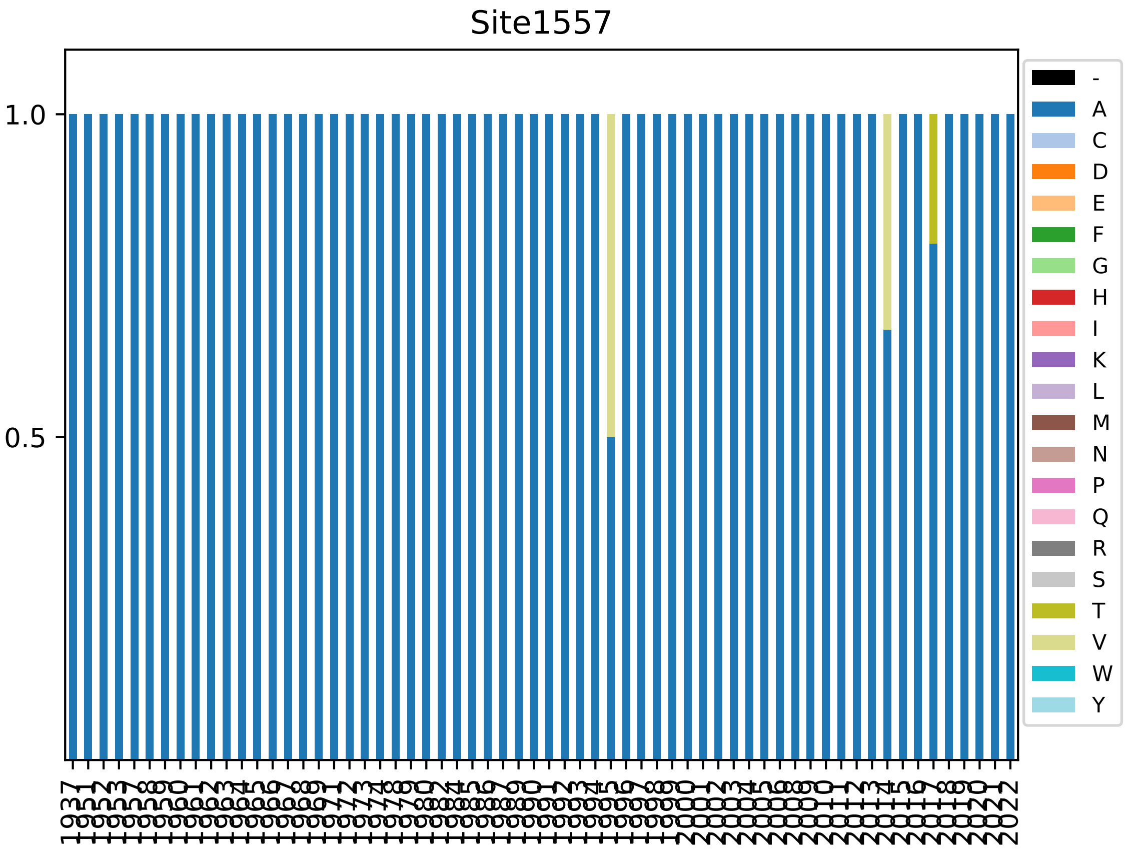
Site1557
1.0
0.5
-
A
C
D
E
F
G
H
I
K
L
M
N
P
Q
R
S
T
V
W
Y

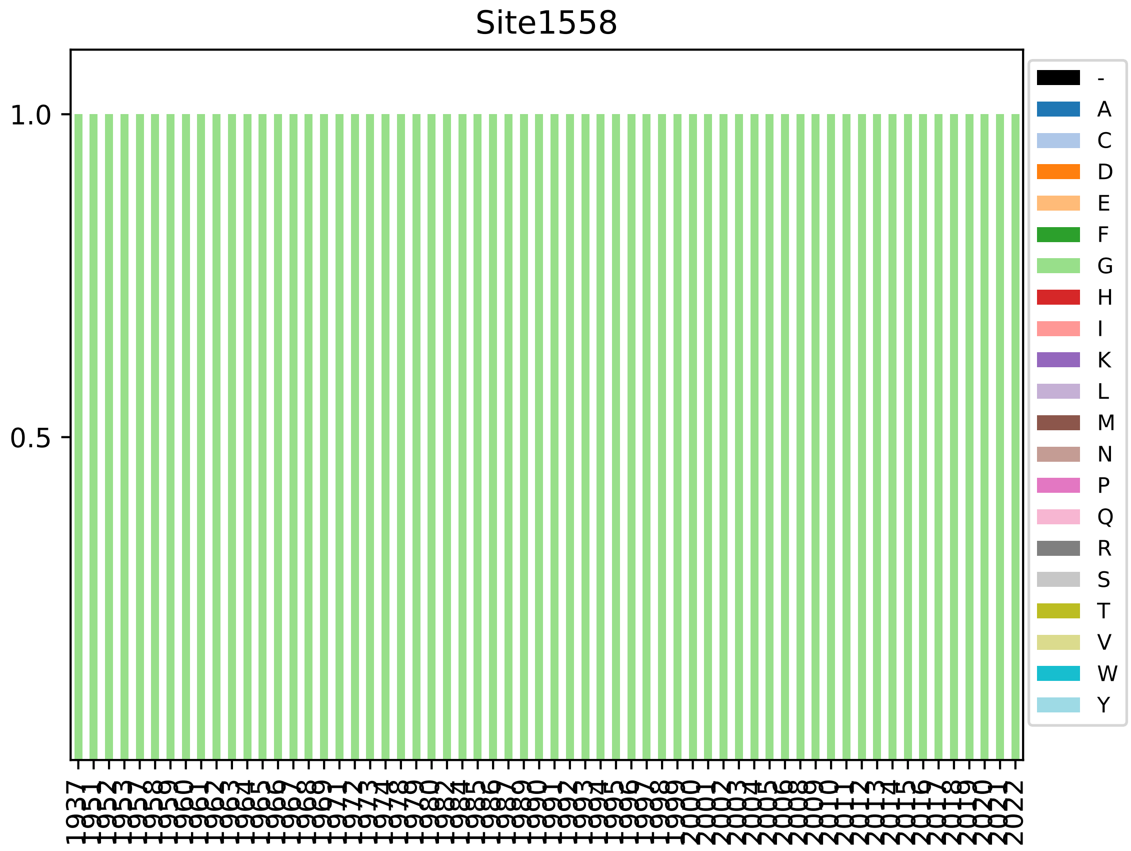
Site1558
1.0
0.5
-
A
C
D
E
F
G
H
I
K
L
M
N
P
Q
R
S
T
V
W
Y

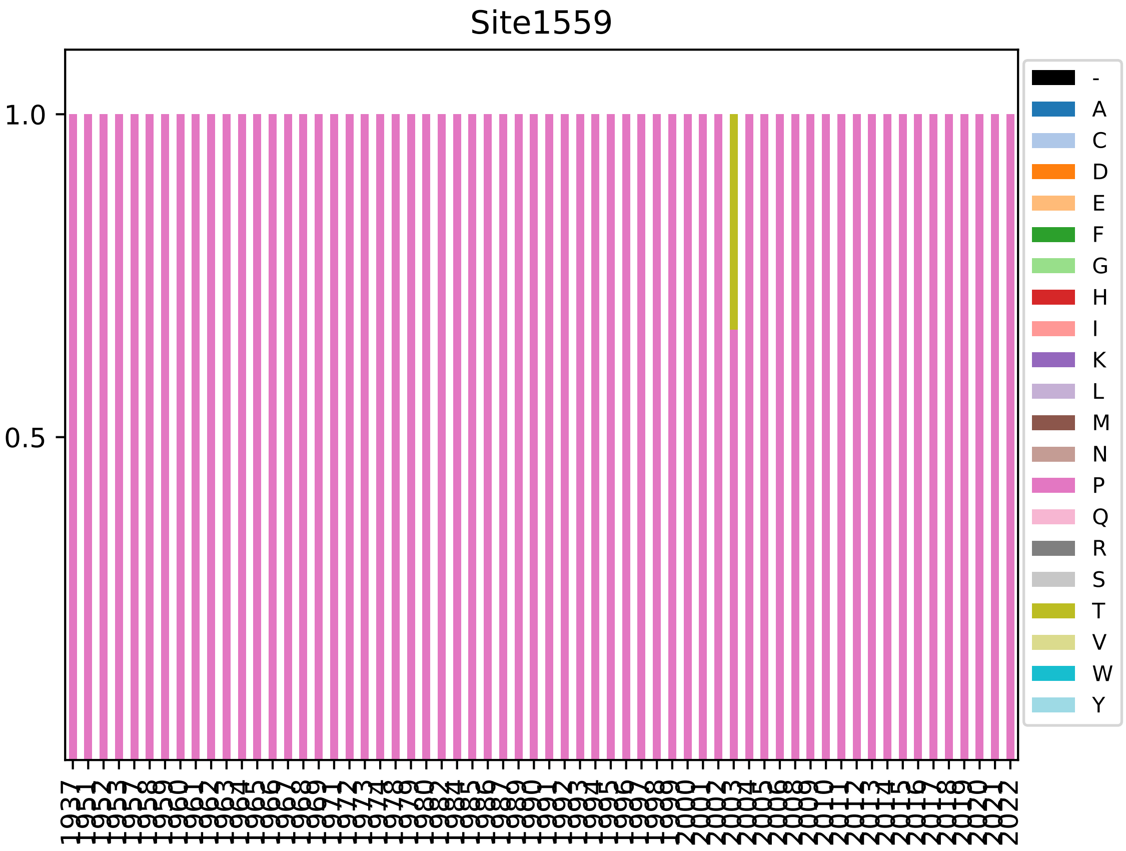
Site1559
1.0
0.5
-
A
C
D
E
F
G
H
I
K
L
M
N
P
Q
R
S
T
V
W
Y

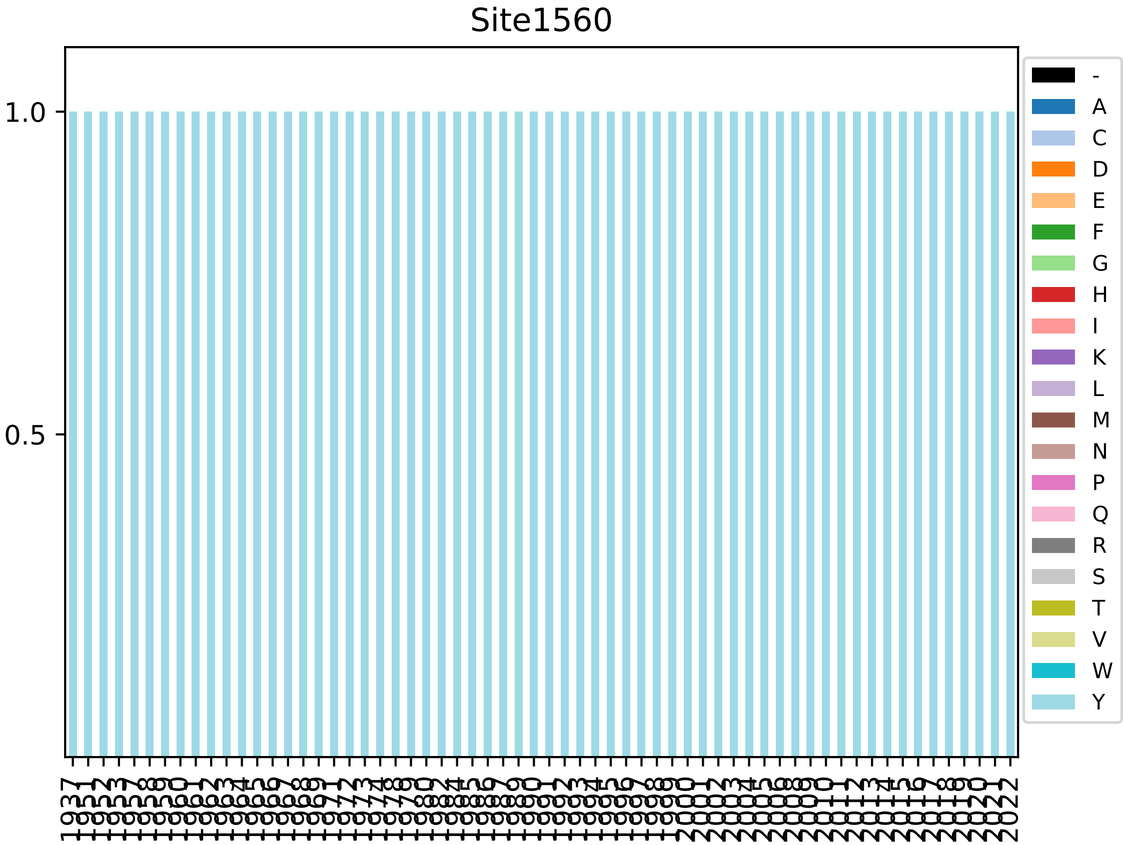
Site1560
1.0
0.5
-
A
C
D
E
F
G
H
I
K
L
M
N
P
Q
R
S
T
V
W
Y

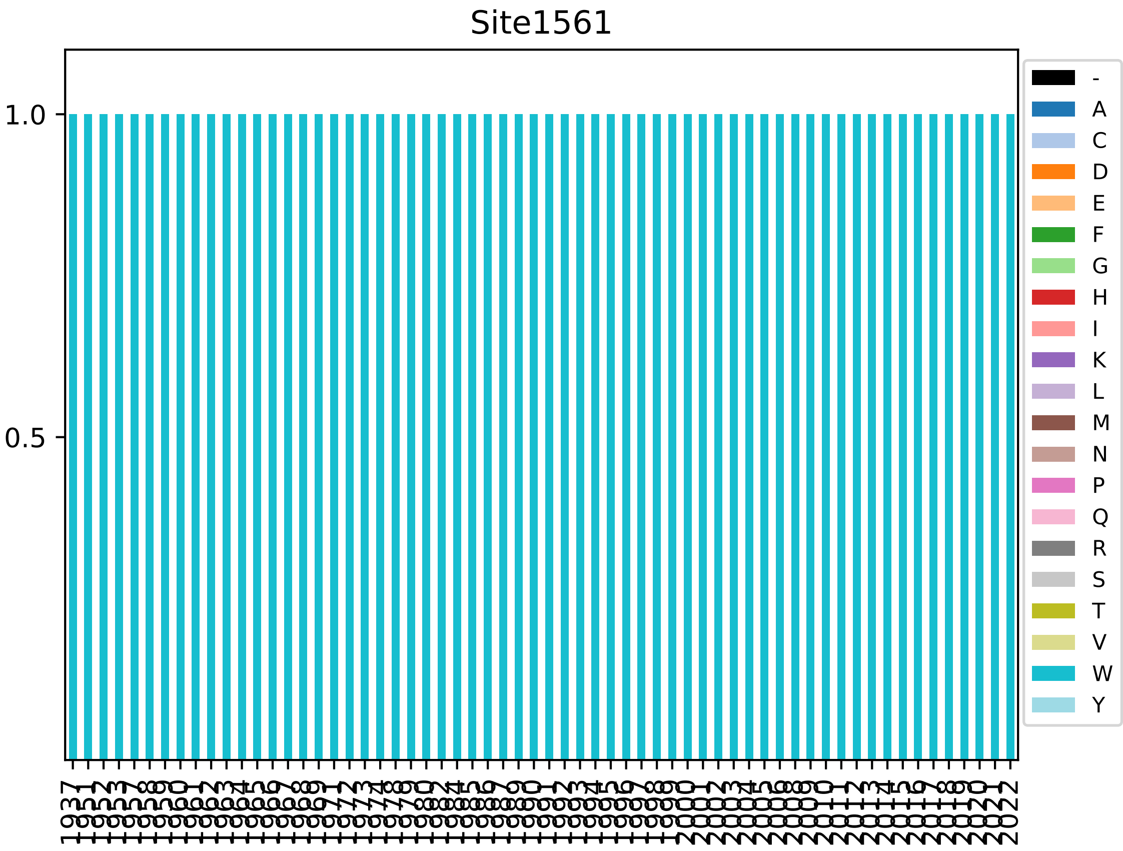
Site1561
1.0
0.5
-
A
C
D
E
F
G
H
I
K
L
M
N
P
Q
R
S
T
V
W
Y

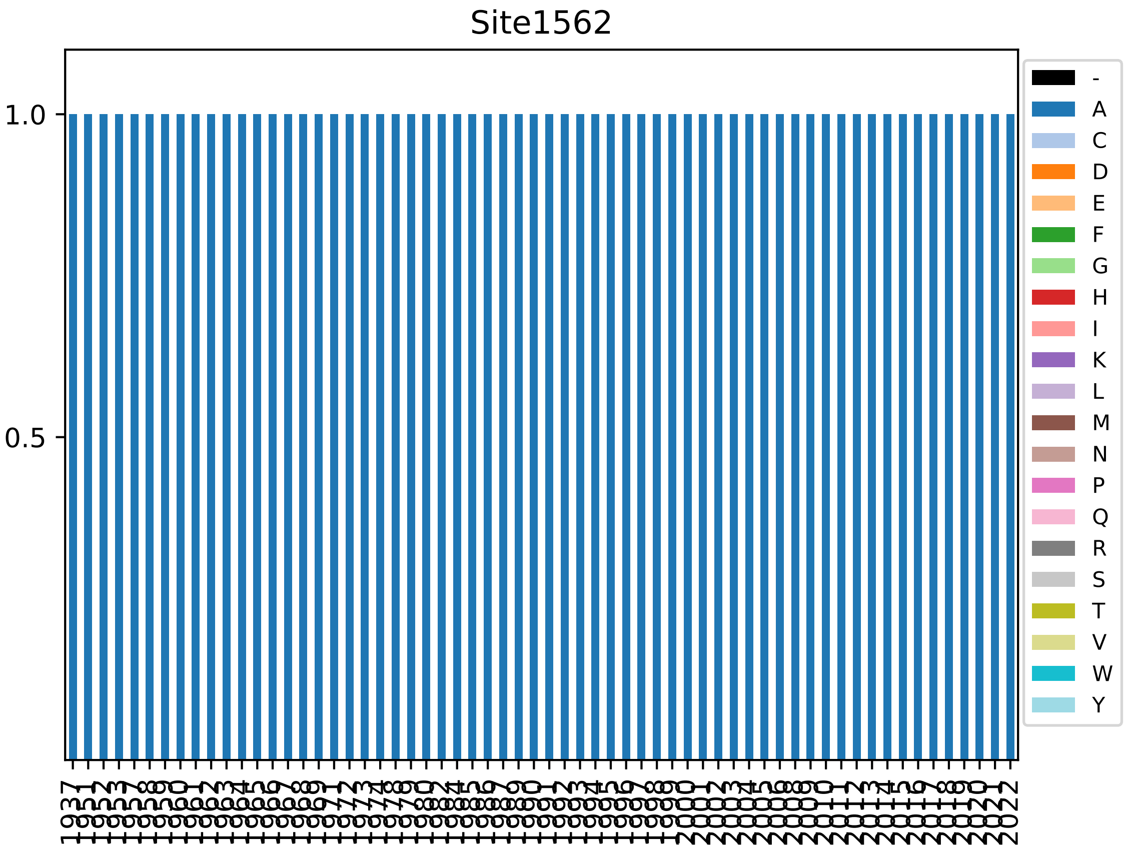
Site1562
1.0
0.5
-
A
C
D
E
F
G
H
I
K
L
M
N
P
Q
R
S
T
V
W
Y

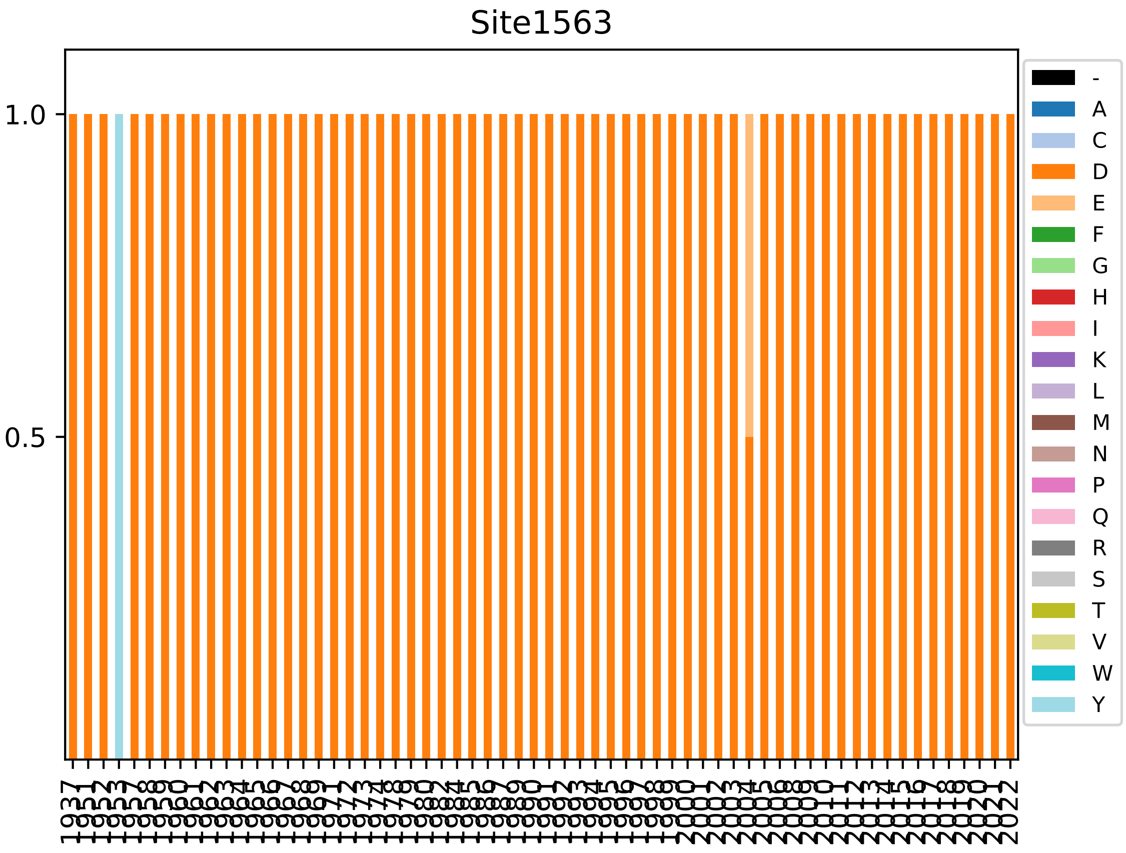
Site1563
1.0
0.5
-
A
C
D
E
F
G
H
I
K
L
M
N
P
Q
R
S
T
V
W
Y

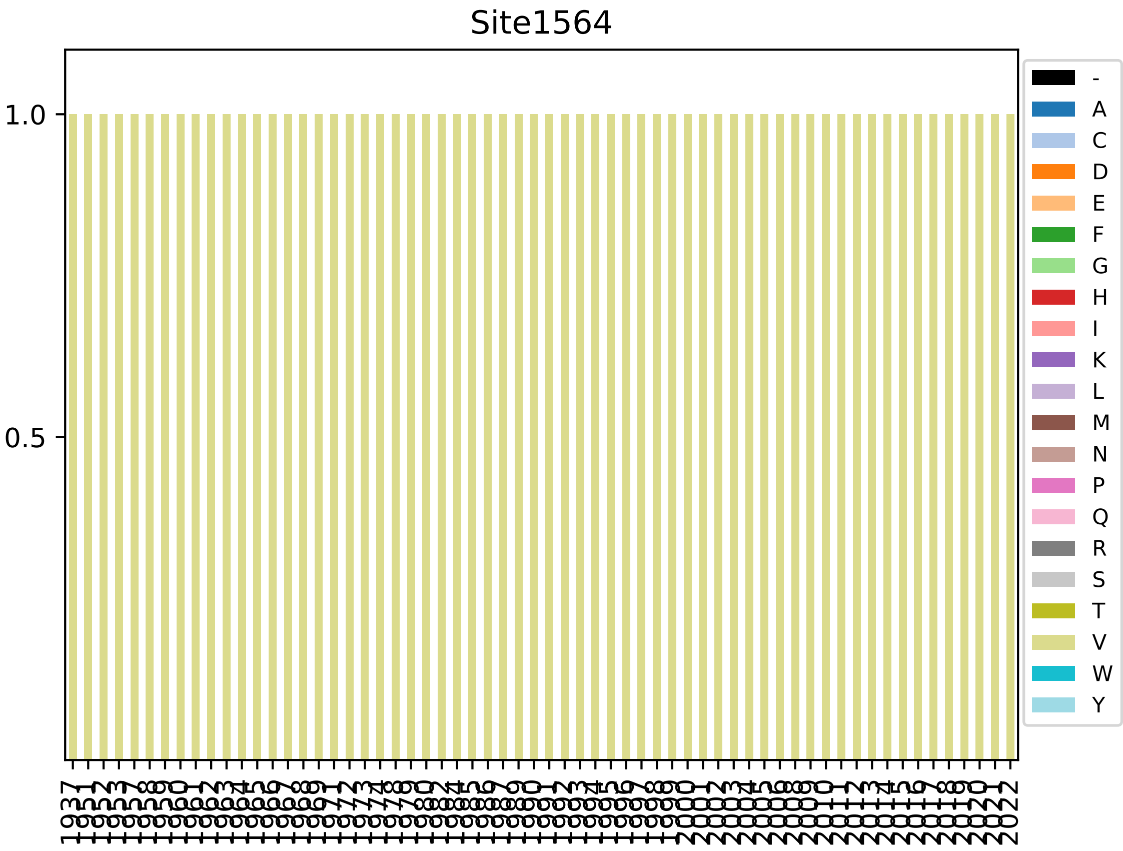
Site1564
1.0
0.5
-
A
C
D
E
F
G
H
I
K
L
M
N
P
Q
R
S
T
V
W
Y

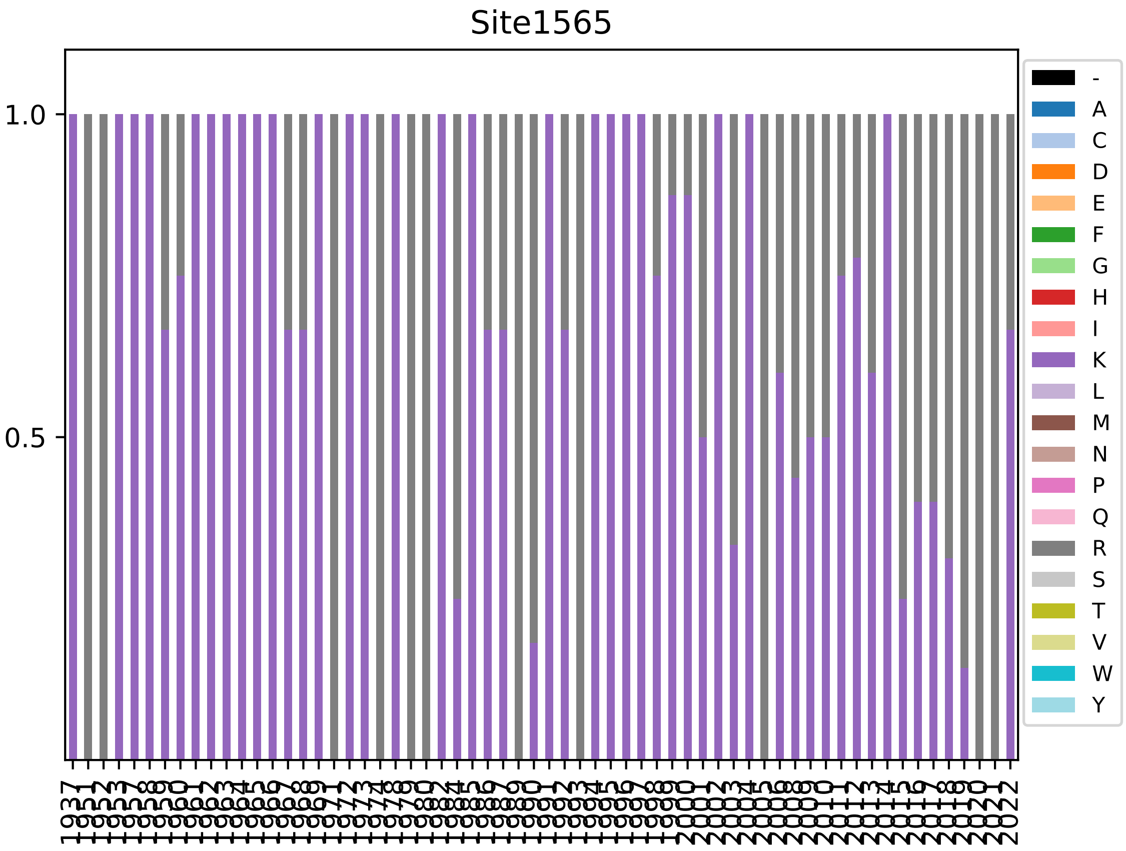
Site1565
1.0
0.5
-
A
C
D
E
F
G
H
I
K
L
M
N
P
Q
R
S
T
V
W
Y

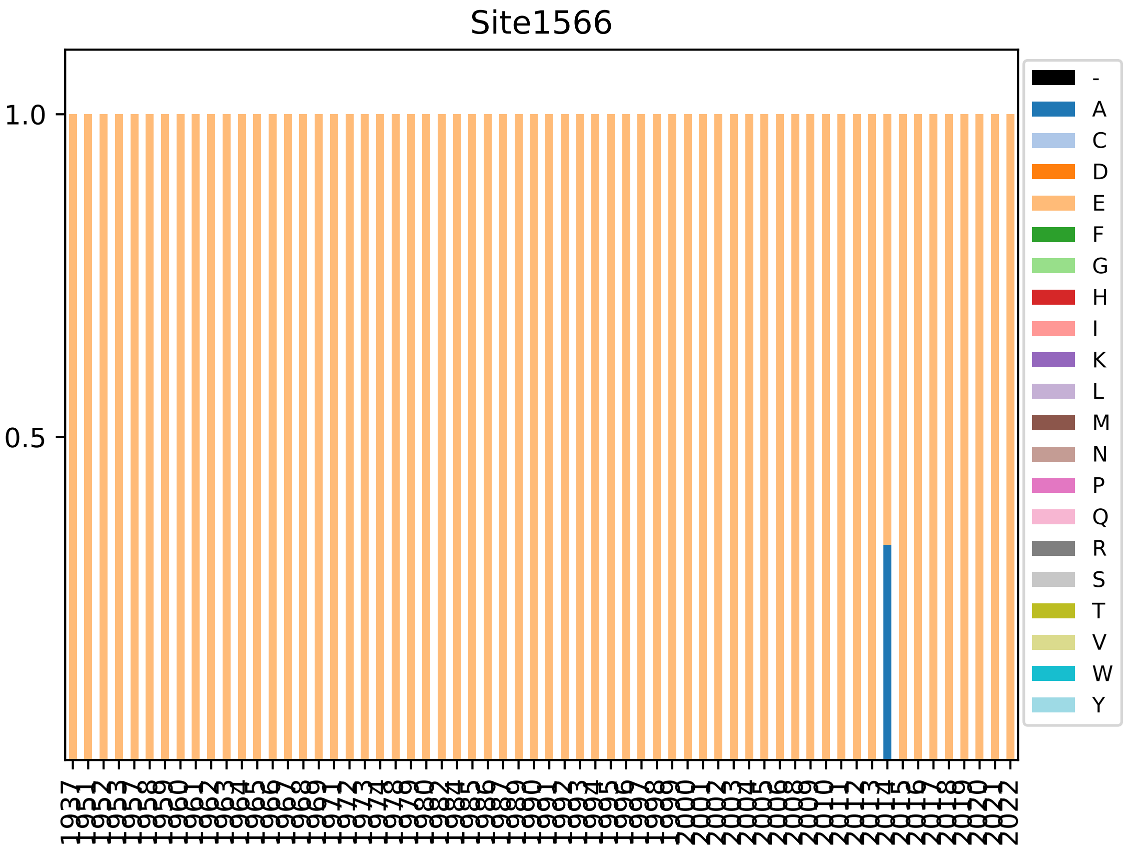
Site1566
1.0
0.5
-
A
C
D
E
F
G
H
I
K
L
M
N
P
Q
R
S
T
V
W
Y

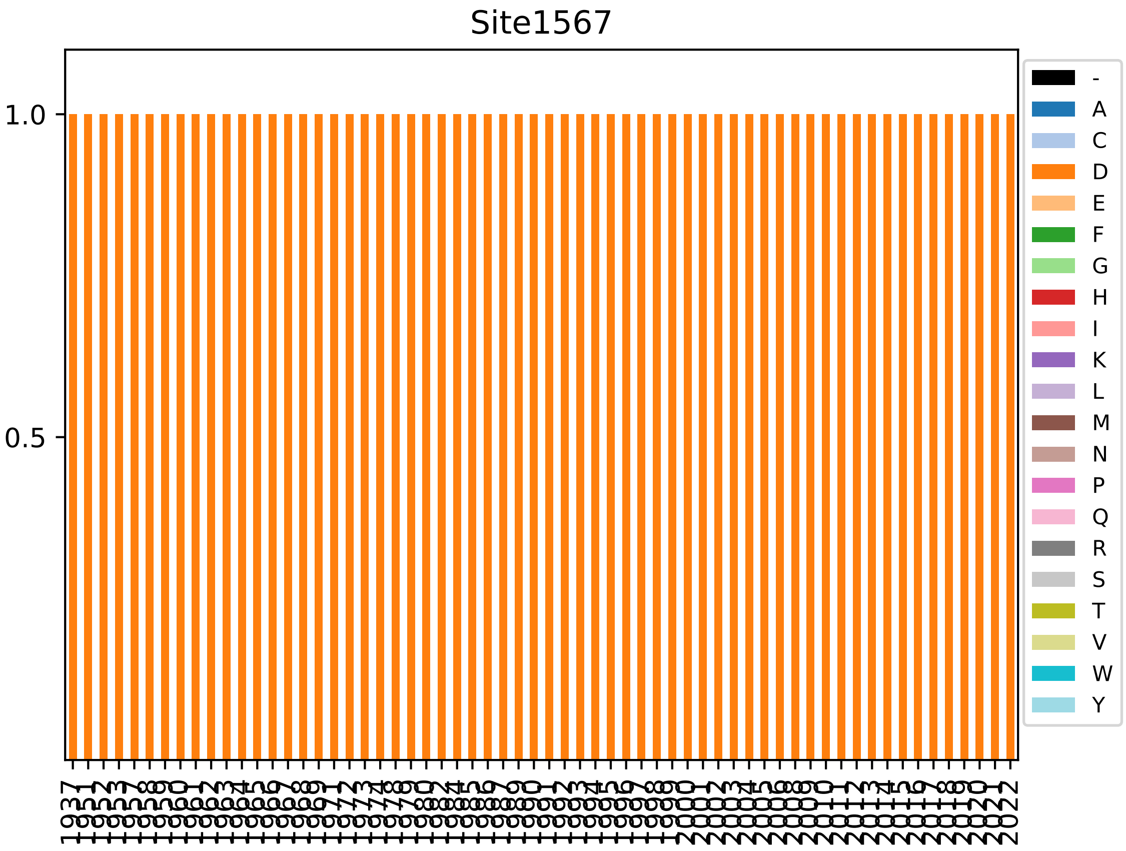
Site1567
1.0
0.5
-
A
C
D
E
F
G
H
I
K
L
M
N
P
Q
R
S
T
V
W
Y

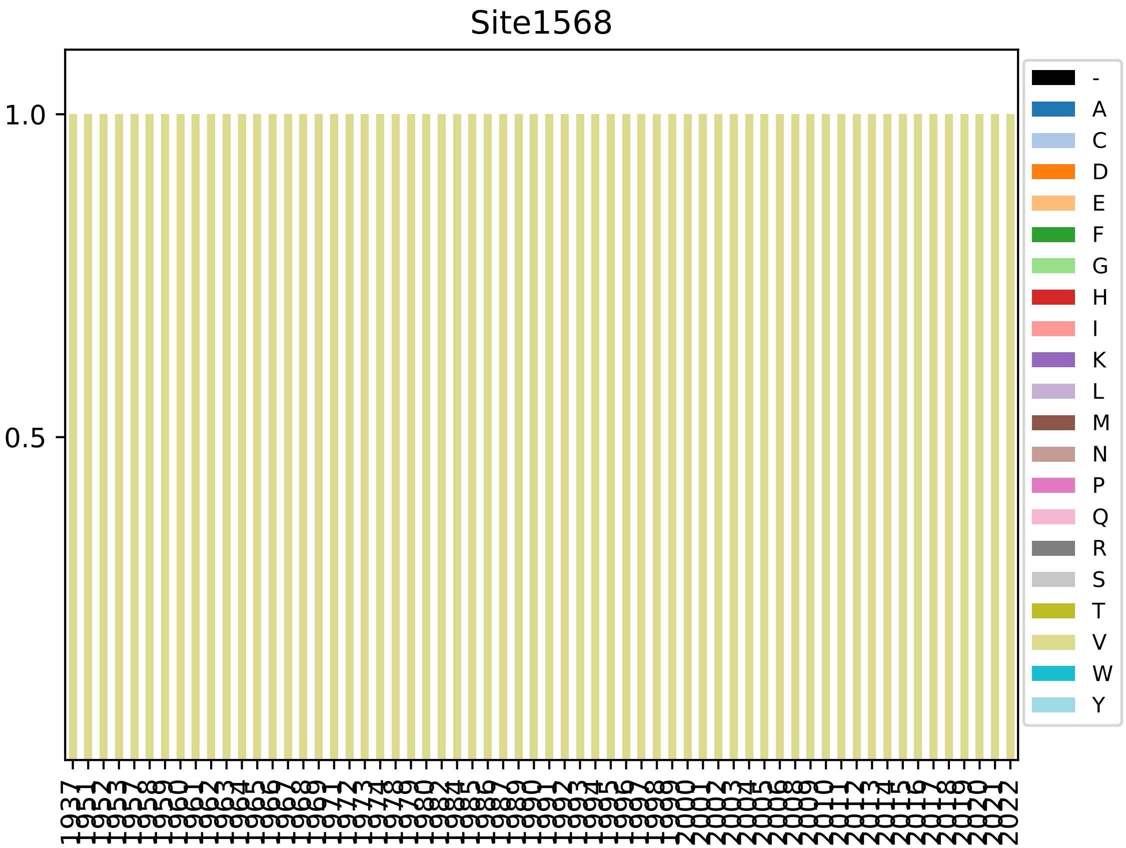
Site1568
1.0
0.5
-
A
C
D
E
F
G
H
I
K
L
M
N
P
Q
R
S
T
V
W
Y

Site1569

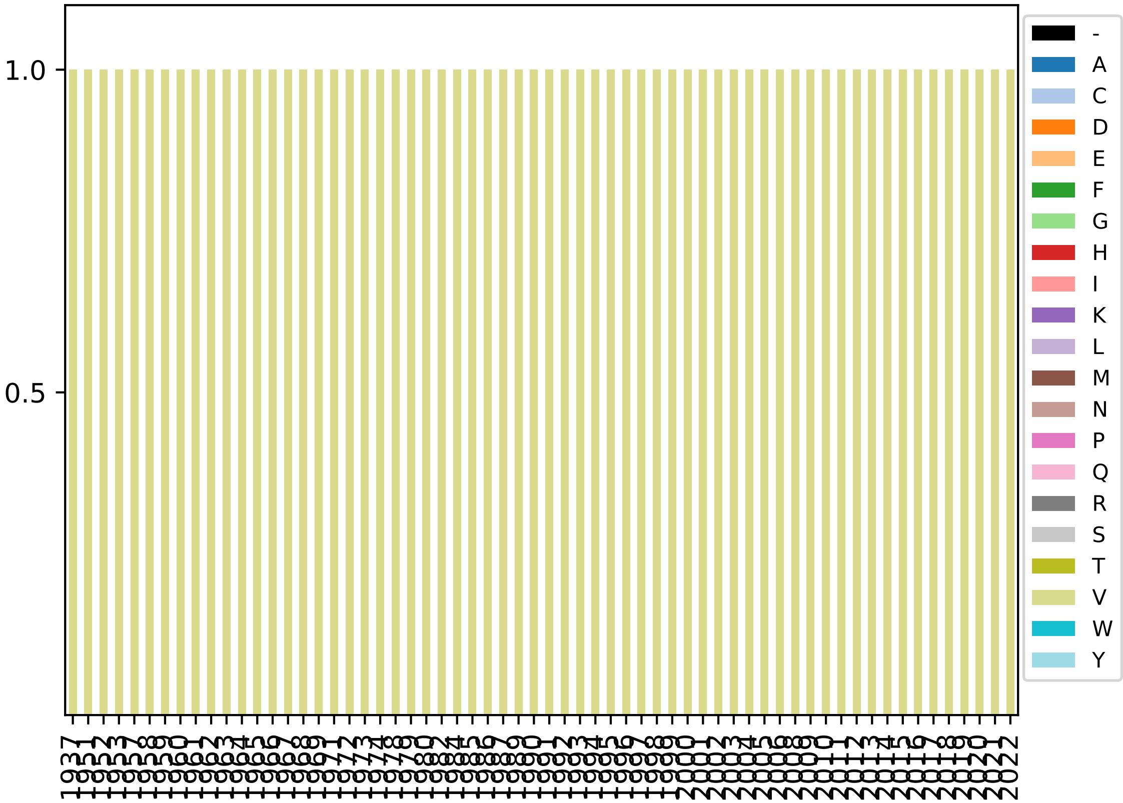

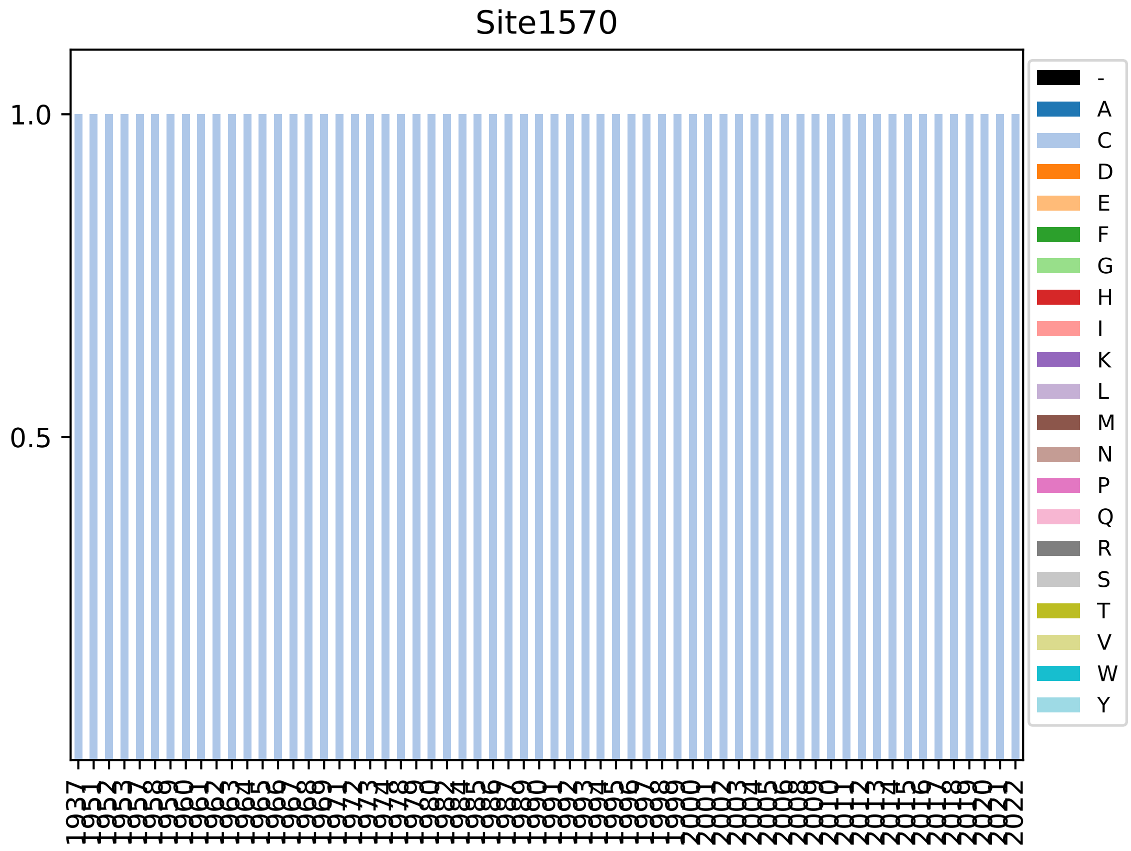
Site1570
1.0
0.5
-
A
C
D
E
F
G
H
I
K
L
M
N
P
Q
R
S
T
V
W
Y

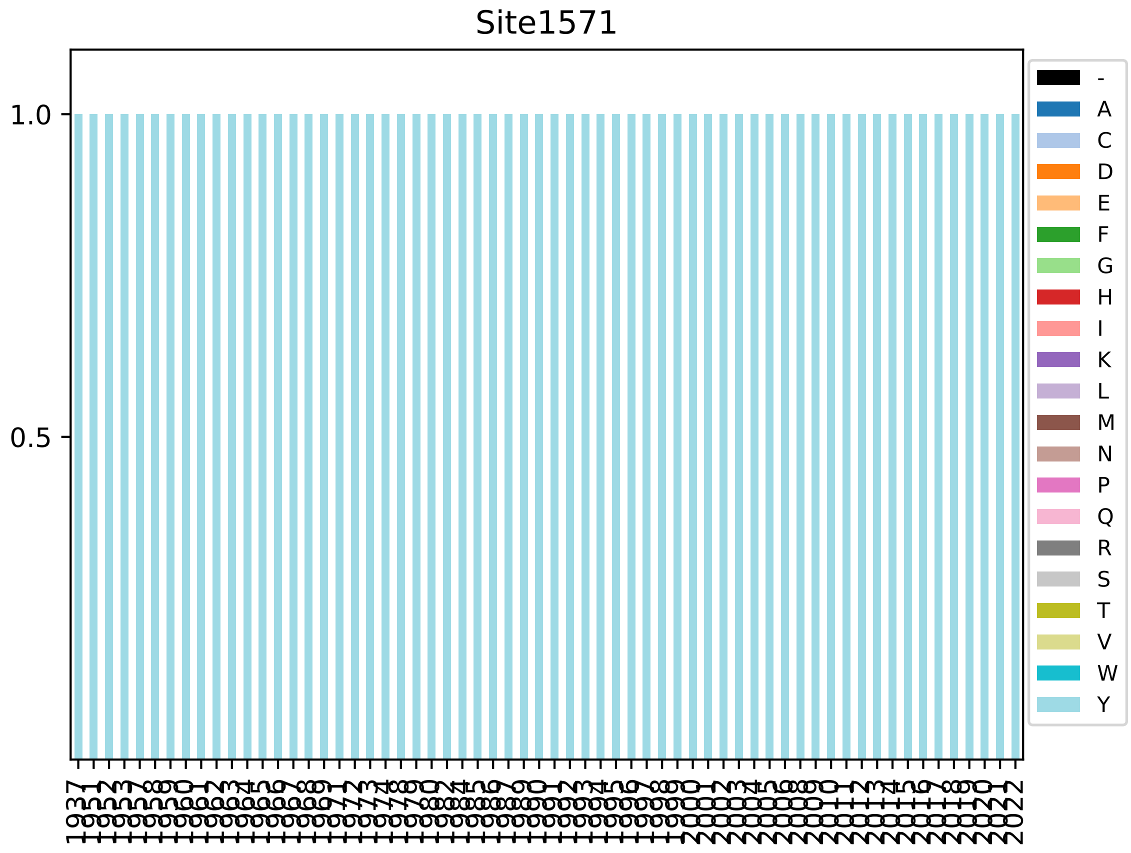
Site1571
1.0
0.5
-
A
C
D
E
F
G
H
I
K
L
M
N
P
Q
R
S
T
V
W
Y

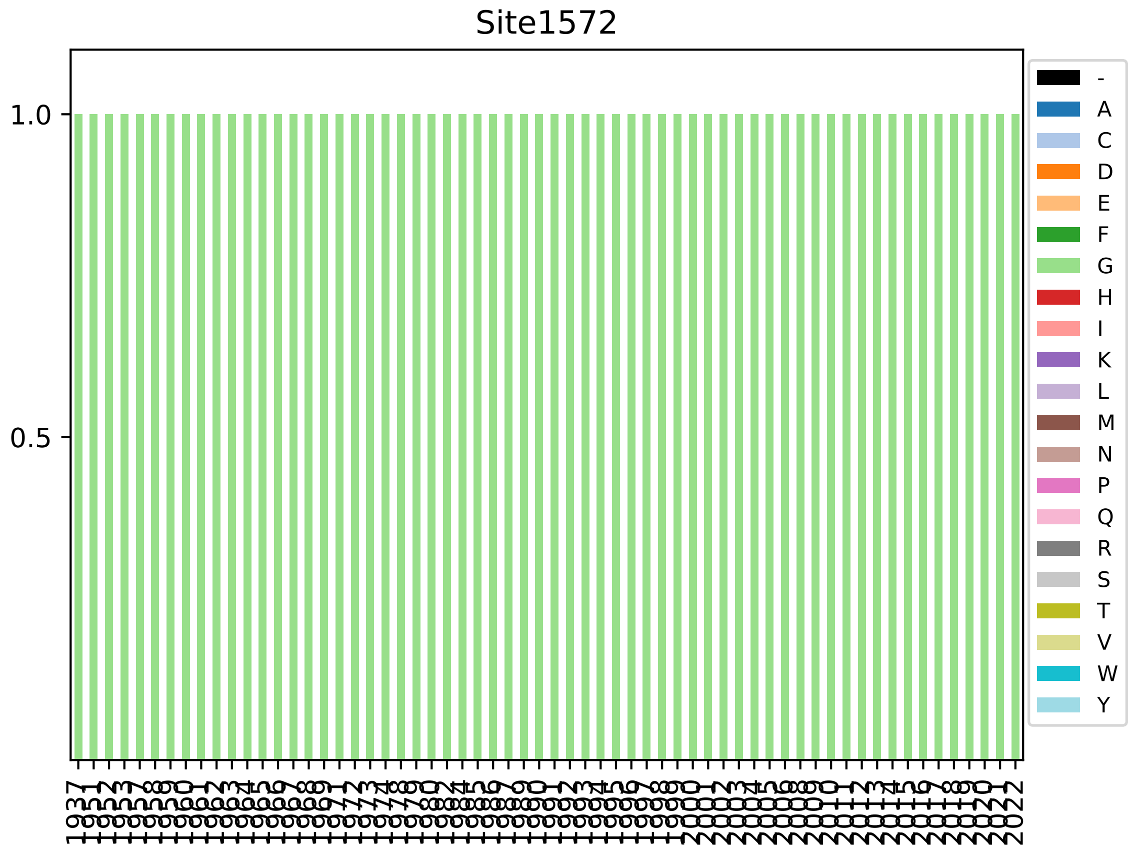
Site1572
1.0
0.5
-
A
C
D
E
F
G
H
I
K
L
M
N
P
Q
R
S
T
V
W
Y

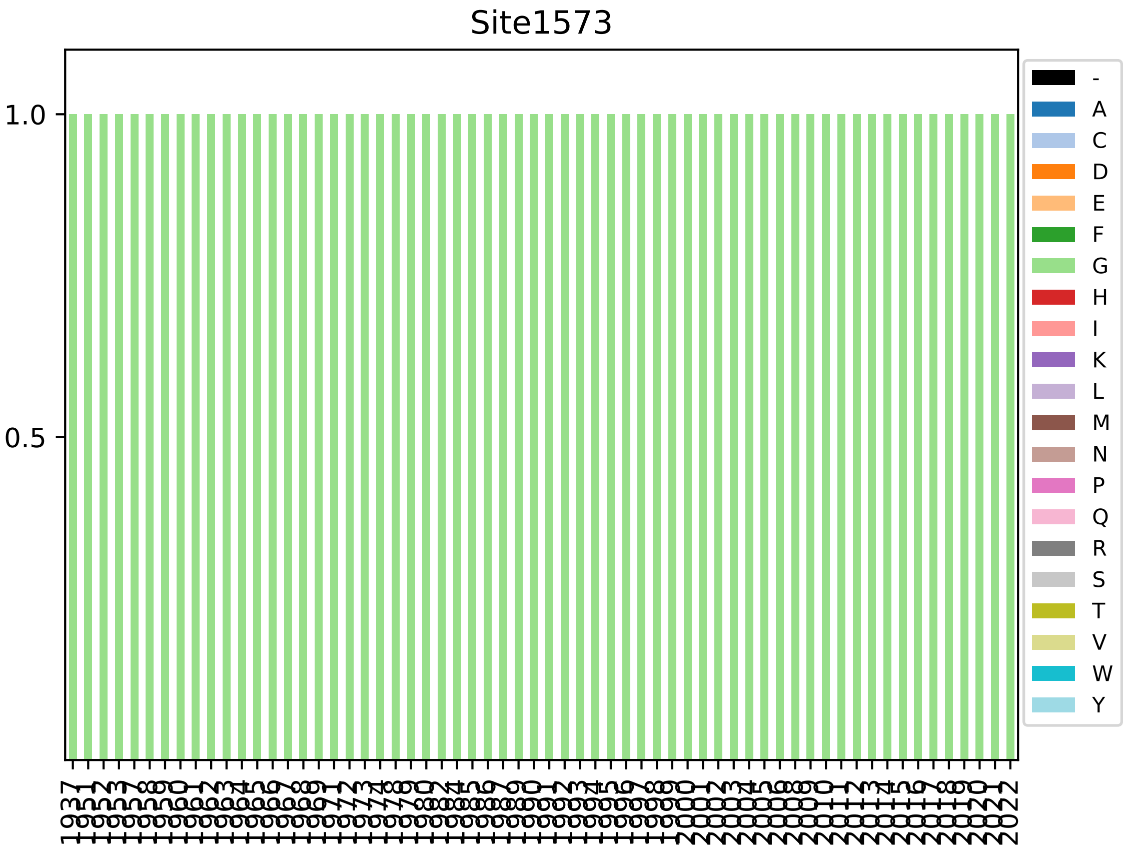
Site1573
1.0
0.5
-
A
C
D
E
F
G
H
I
K
L
M
N
P
Q
R
S
T
V
W
Y

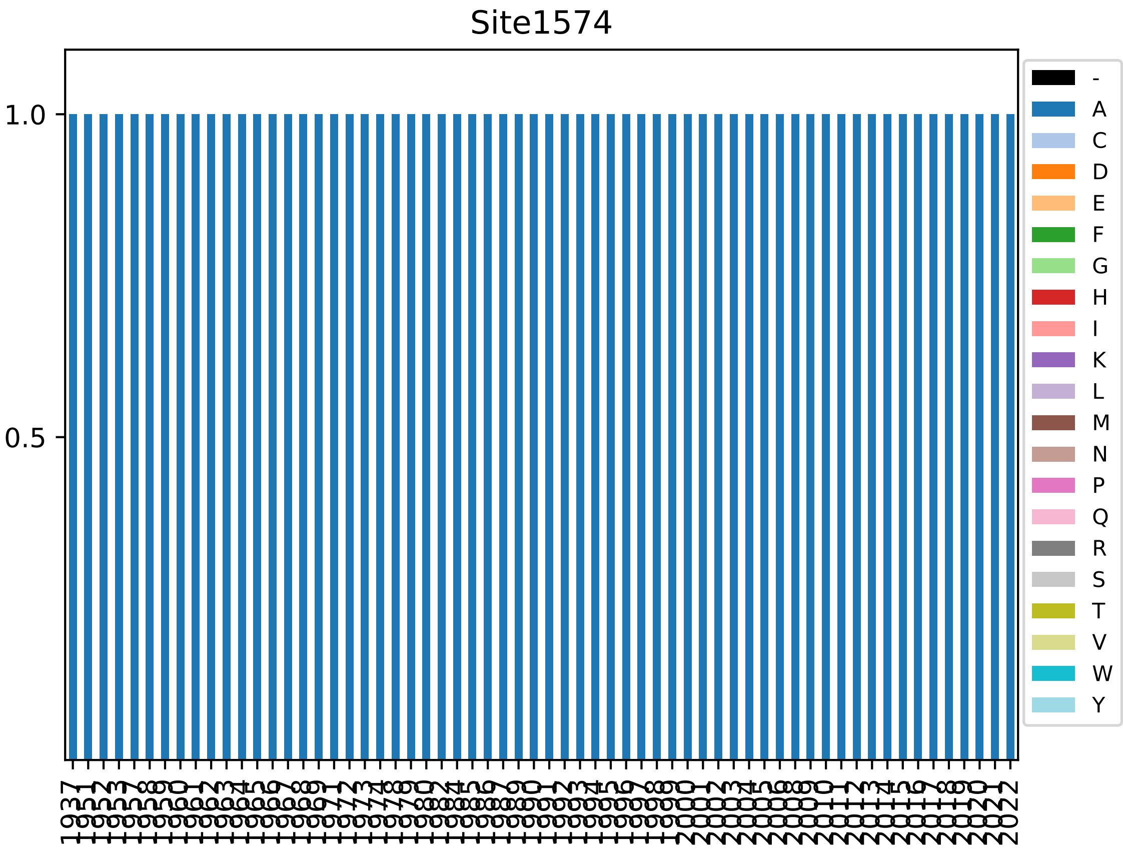
Site1574
1.0
0.5
-
A
C
D
E
F
G
H
I
K
L
M
N
P
Q
R
S
T
V
W
Y

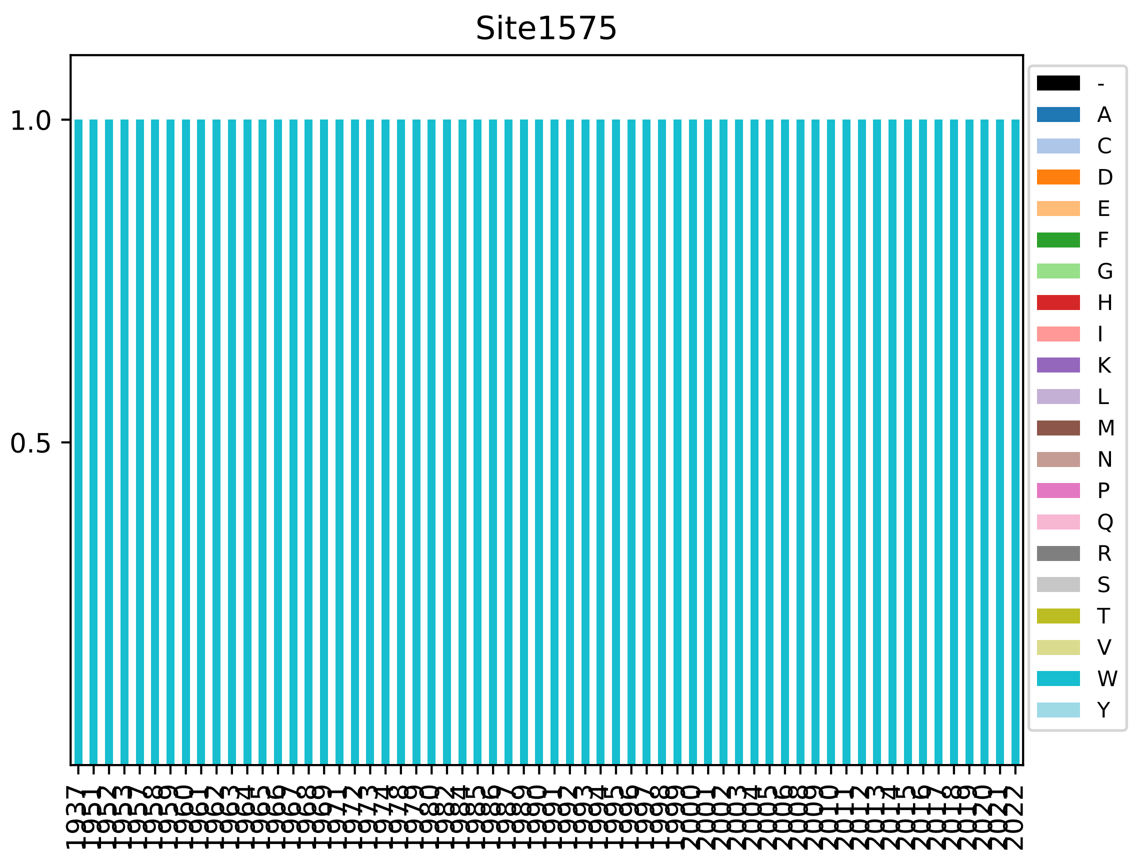
Site1575
1.0
0.5
-
A
C
D
E
F
G
H
I
K
L
M
N
P
Q
R
S
T
V
W
Y

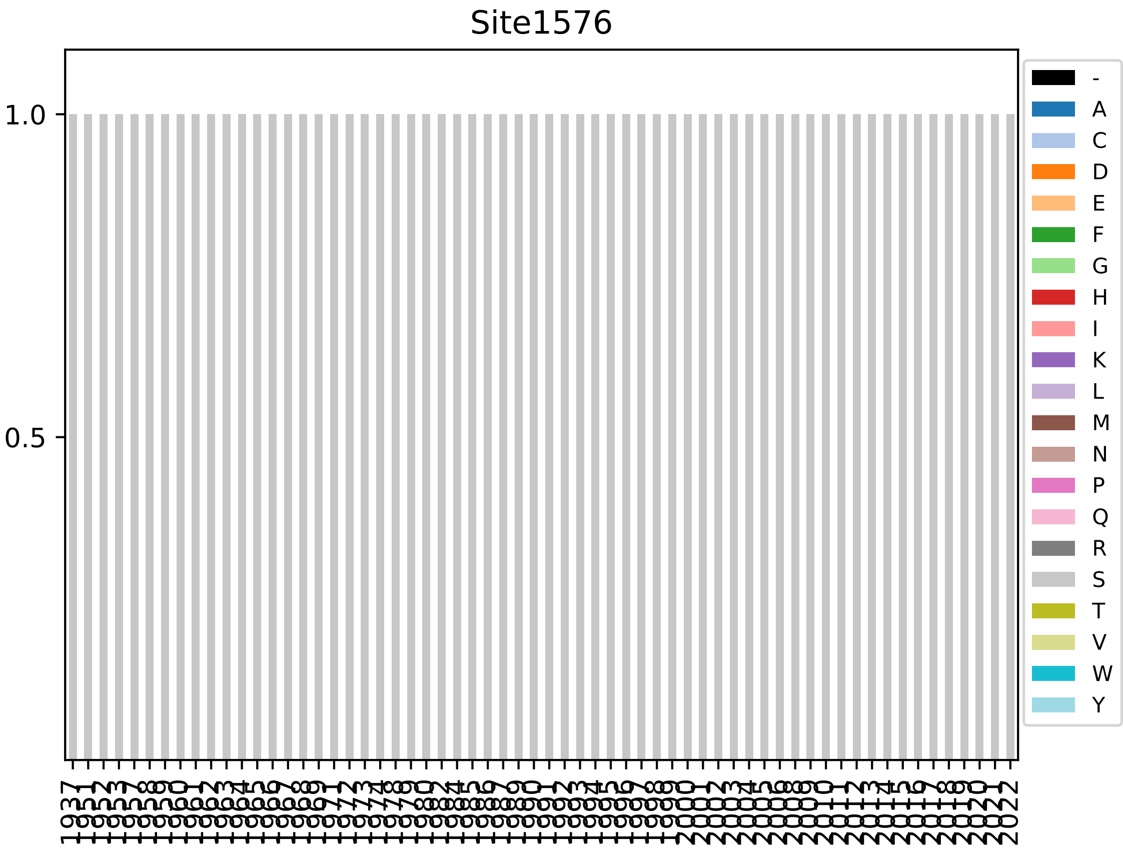
Site1576
1.0
0.5
-
A
C
D
E
F
G
H
I
K
L
M
N
P
Q
R
S
T
V
W
Y

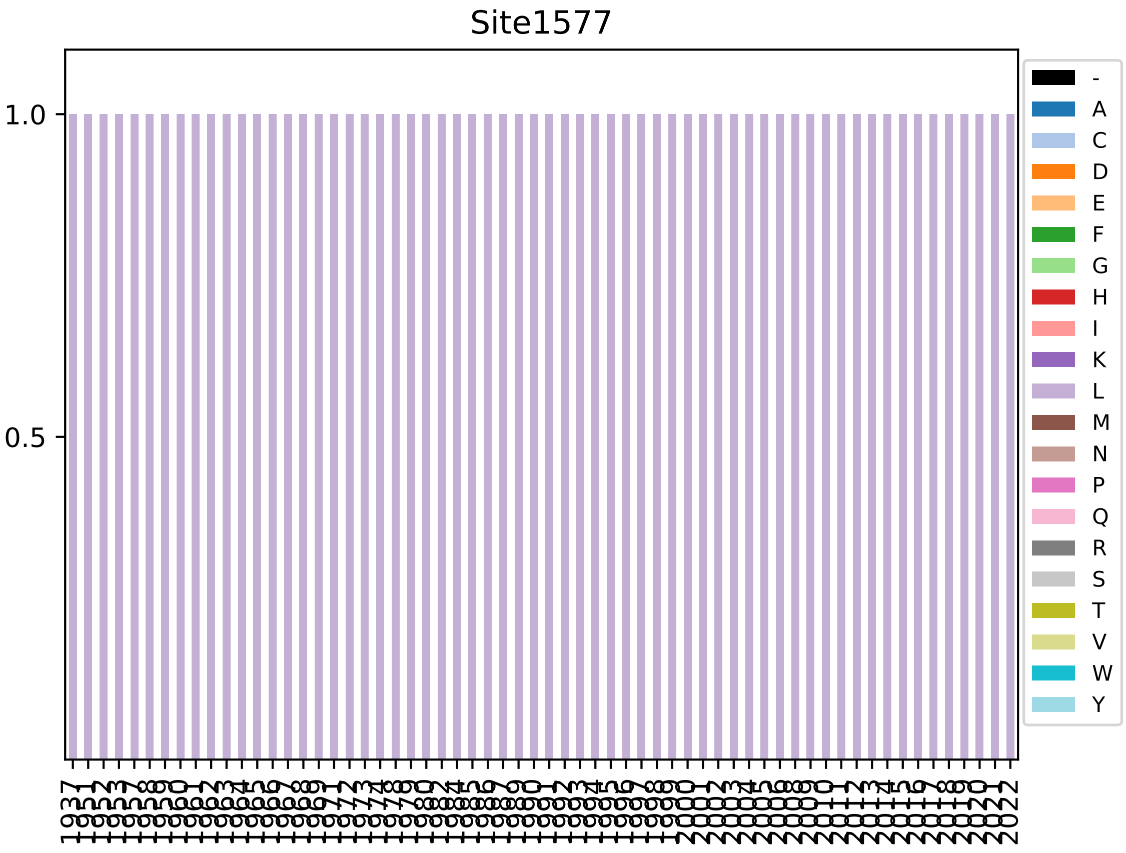
Site1577
1.0
0.5
-
A
C
D
E
F
G
H
I
K
L
M
N
P
Q
R
S
T
V
W
Y

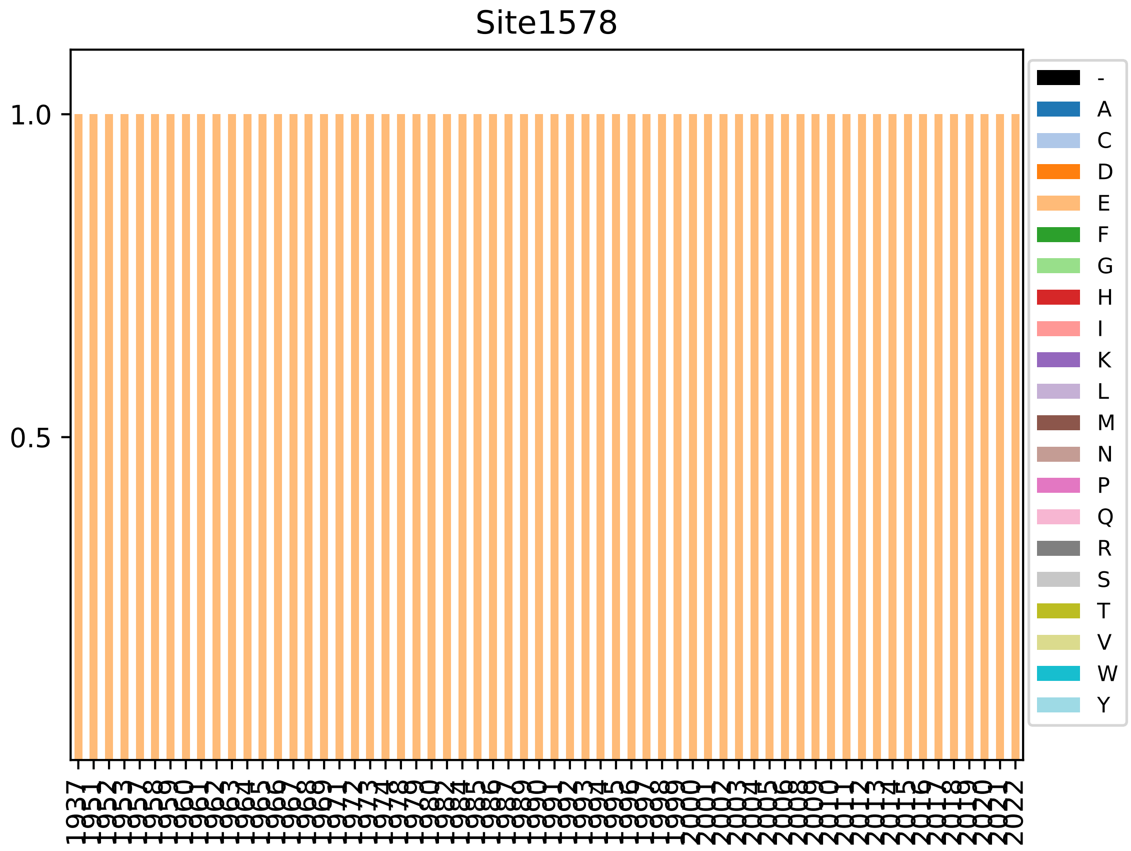
Site1578
1.0
0.5
-
A
C
D
E
F
G
H
I
K
L
M
N
P
Q
R
S
T
V
W
Y

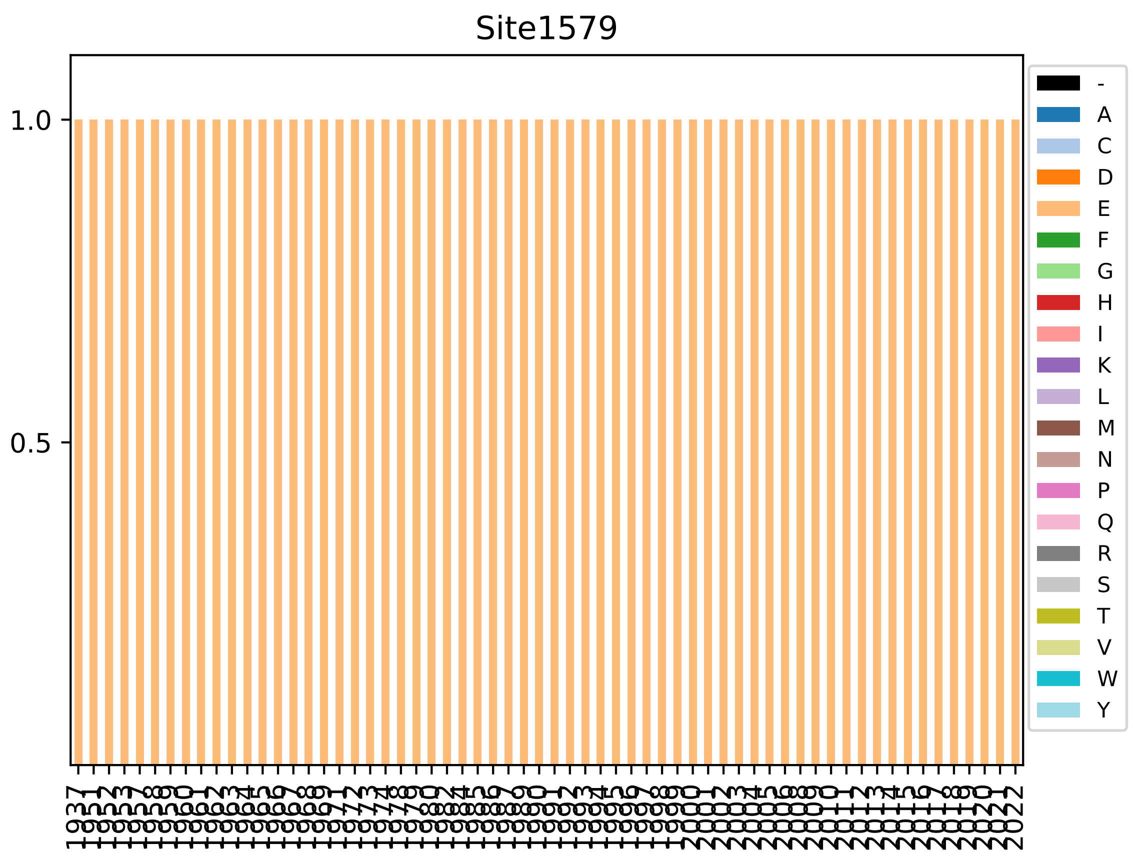

Site1579
1.0
0.5
-
A
C
D
E
F
G
H
I
K
L
M
N
P
Q
R
S
T
V
W
Y

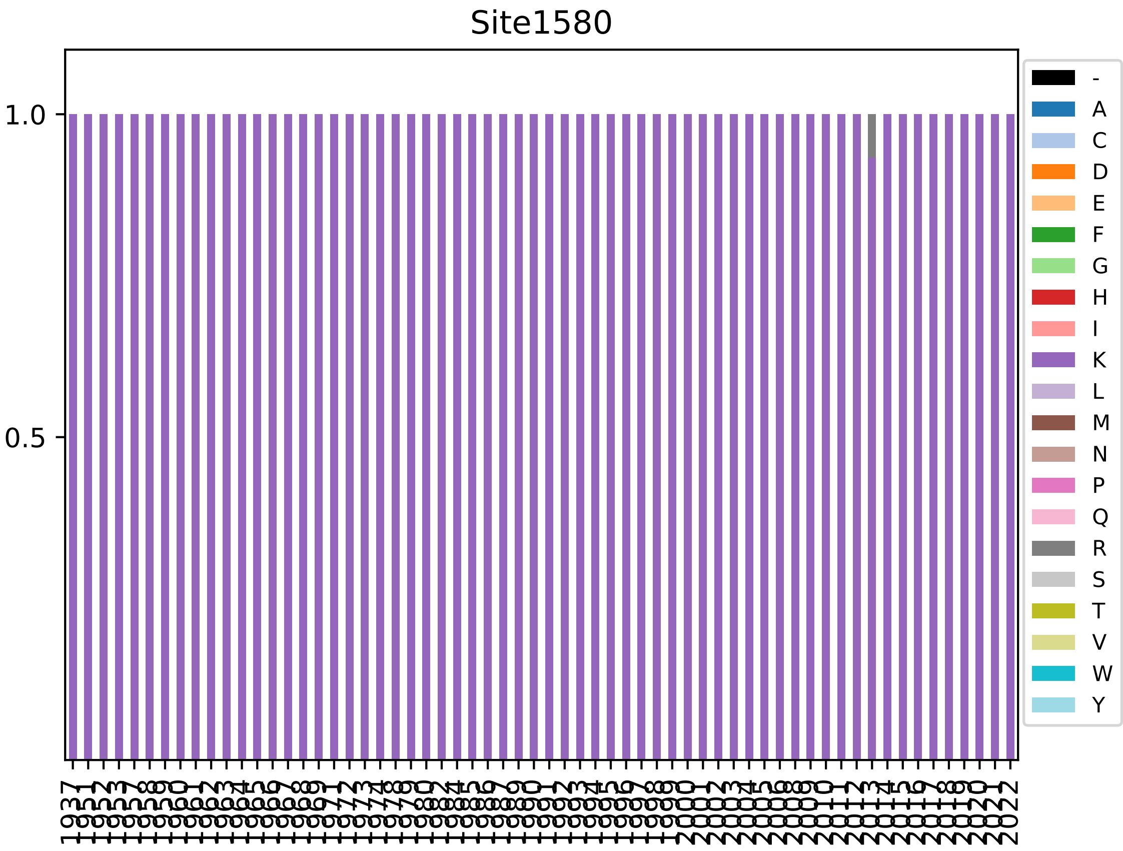
Site1580
1.0
0.5
-
A
C
D
E
F
G
H
I
K
L
M
N
P
Q
R
S
T
V
W
Y

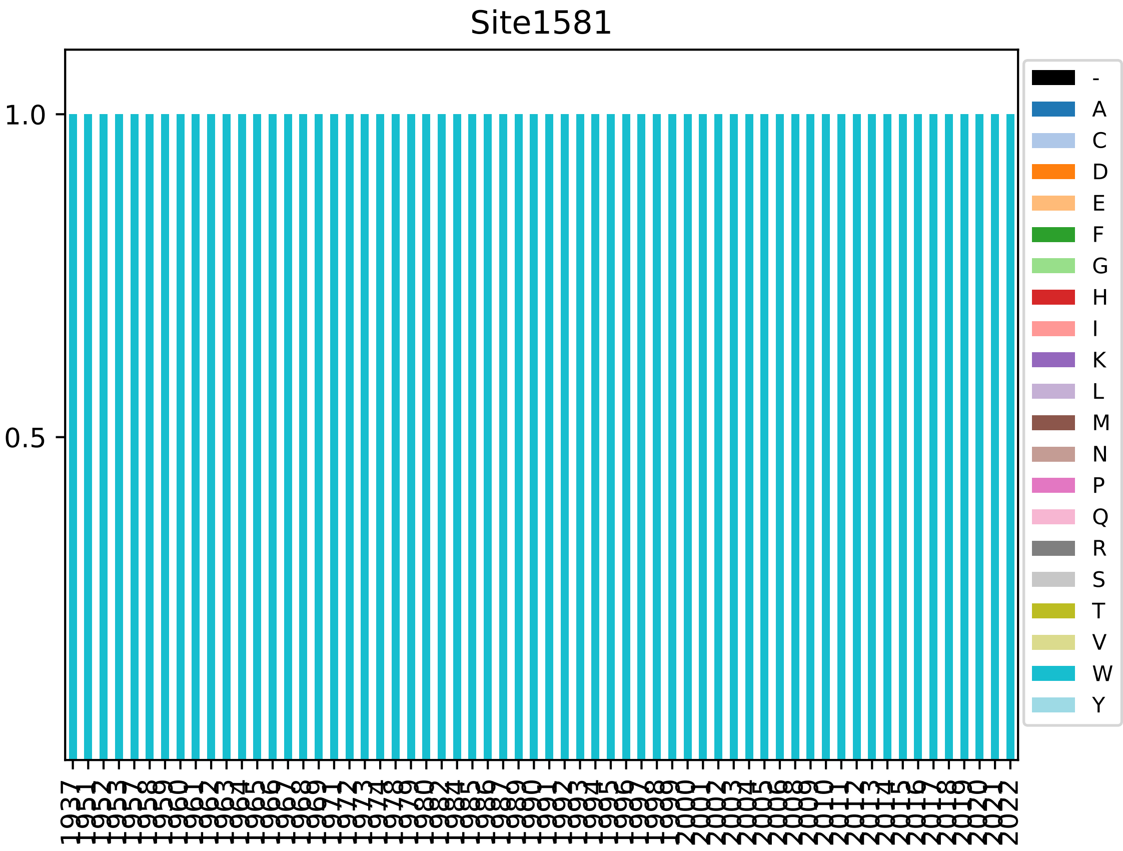
Site1581
1.0
0.5
-
A
C
D
E
F
G
H
I
K
L
M
N
P
Q
R
S
T
V
W
Y

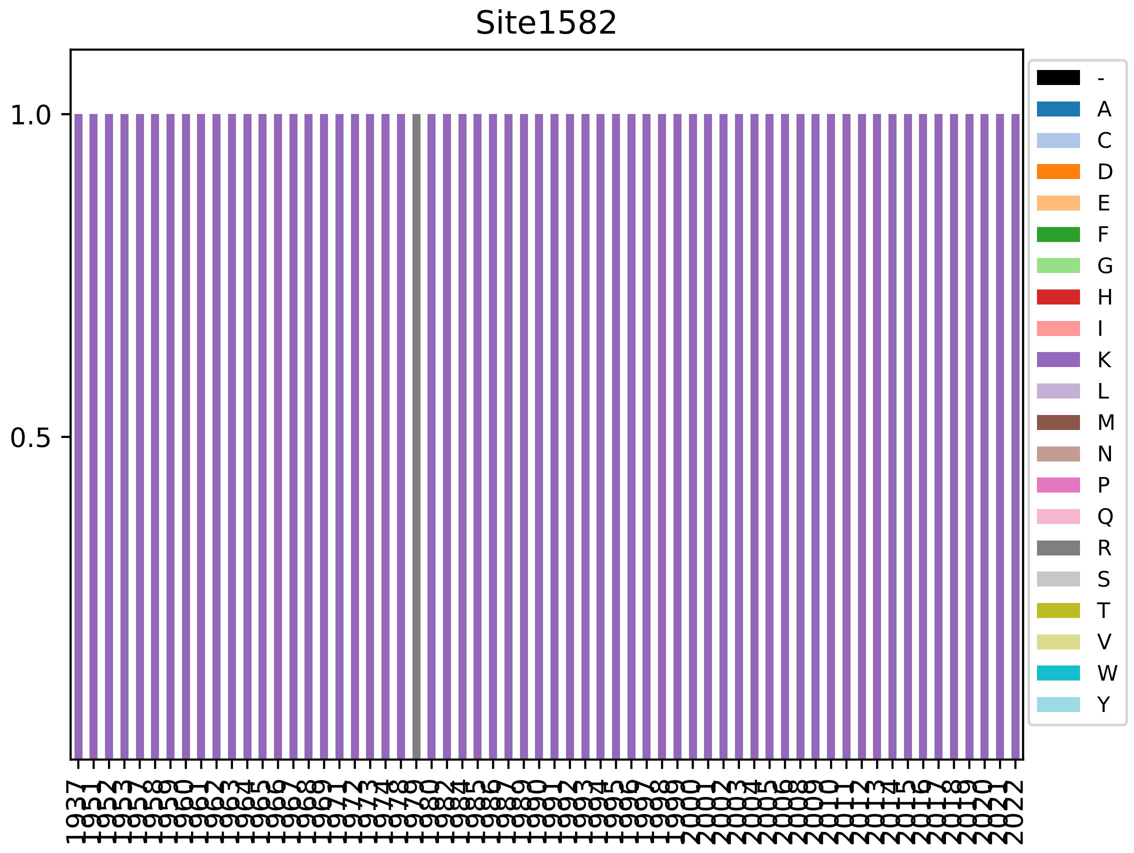
Site1582
1.0
0.5
-
A
C
D
E
F
G
H
I
K
L
M
N
P
Q
R
S
T
V
W
Y

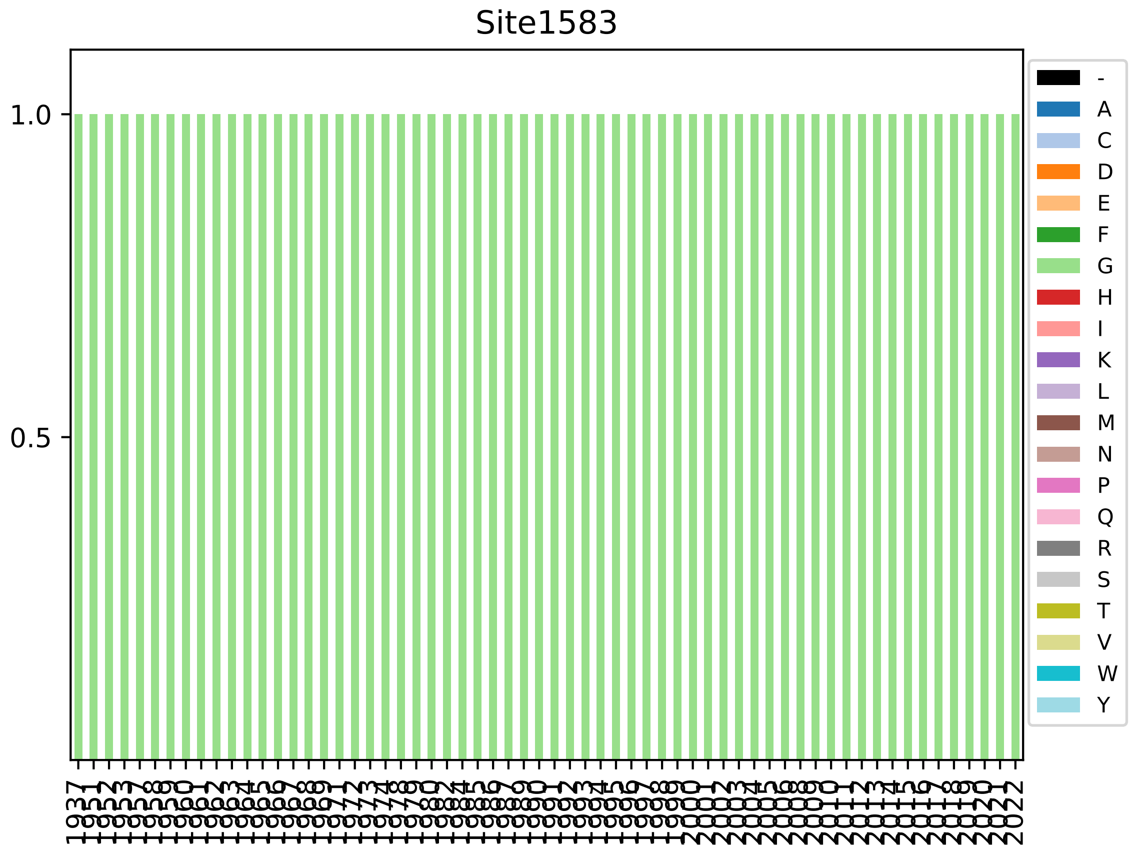
Site1583
1.0
0.5
-
A
C
D
E
F
G
H
I
K
L
M
N
P
Q
R
S
T
V
W
Y

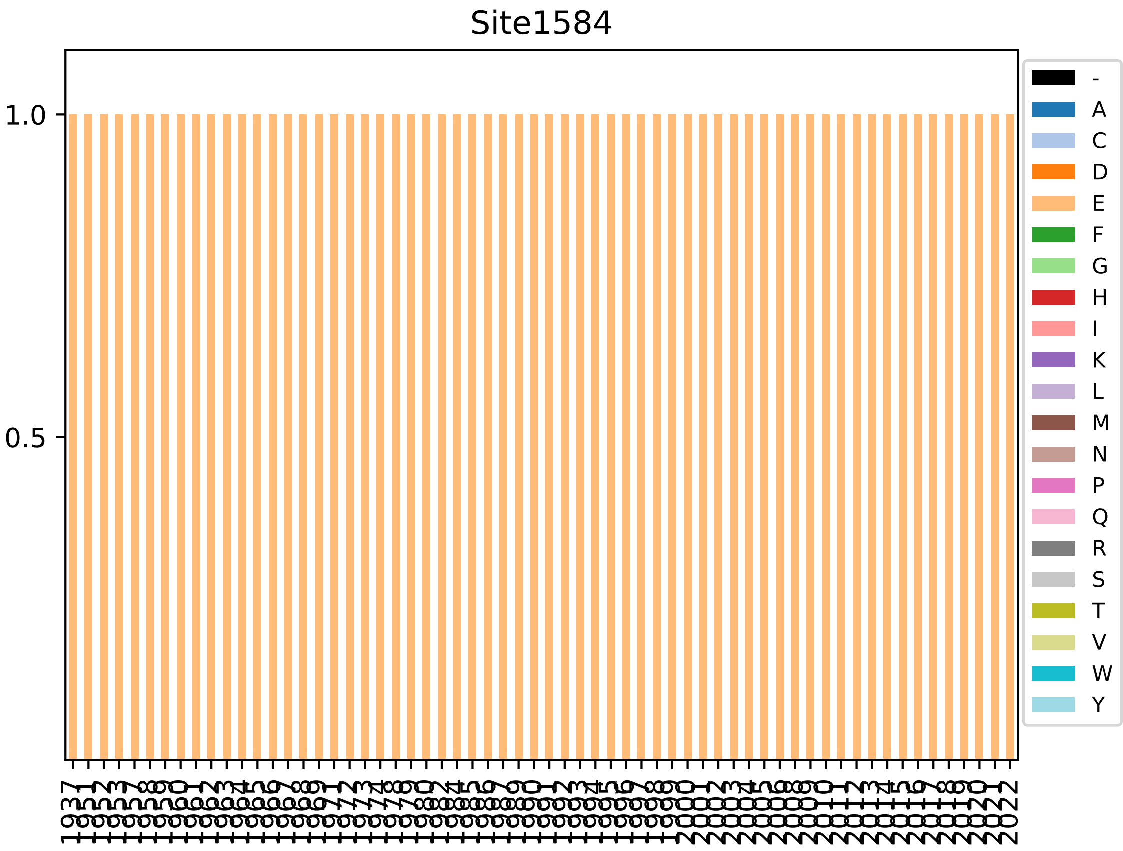
Site1584
1.0
0.5
-
A
C
D
E
F
G
H
I
K
L
M
N
P
Q
R
S
T
V
W
Y

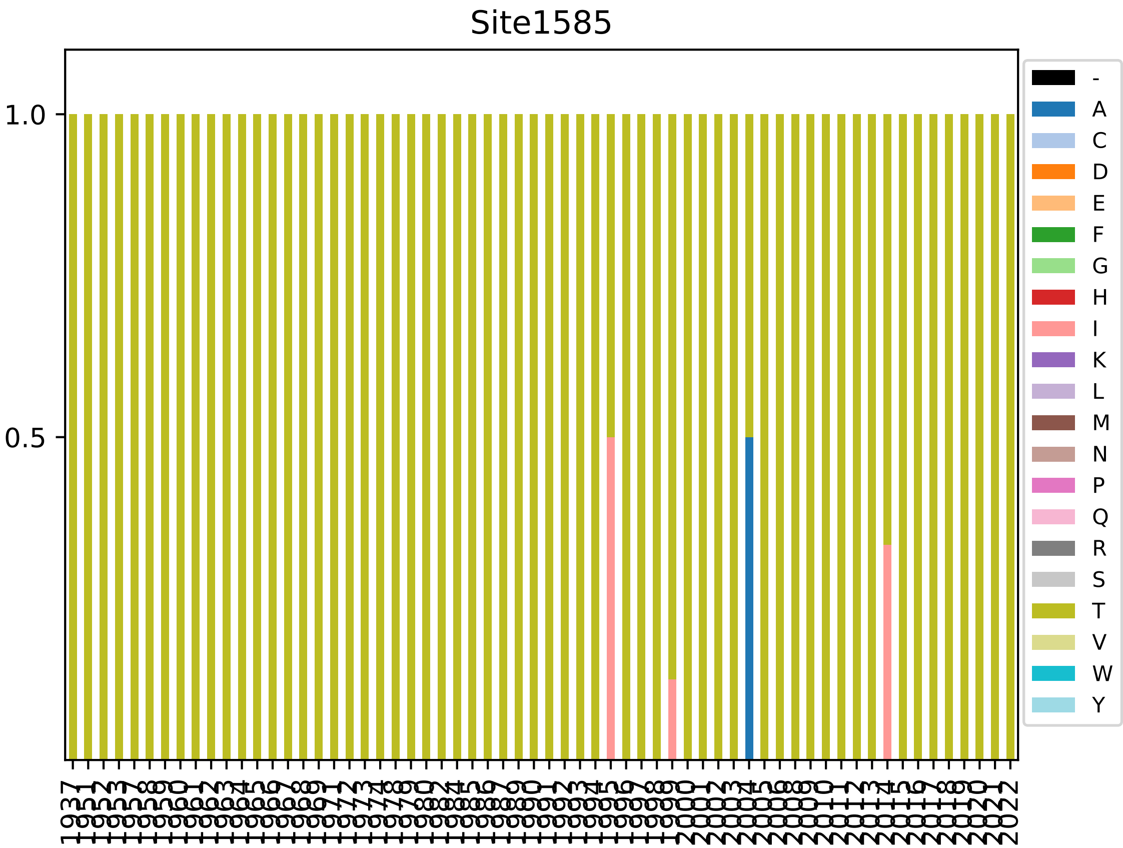
Site1585
1.0
0.5
-
A
C
D
E
F
G
H
I
K
L
M
N
P
Q
R
S
T
V
W
Y

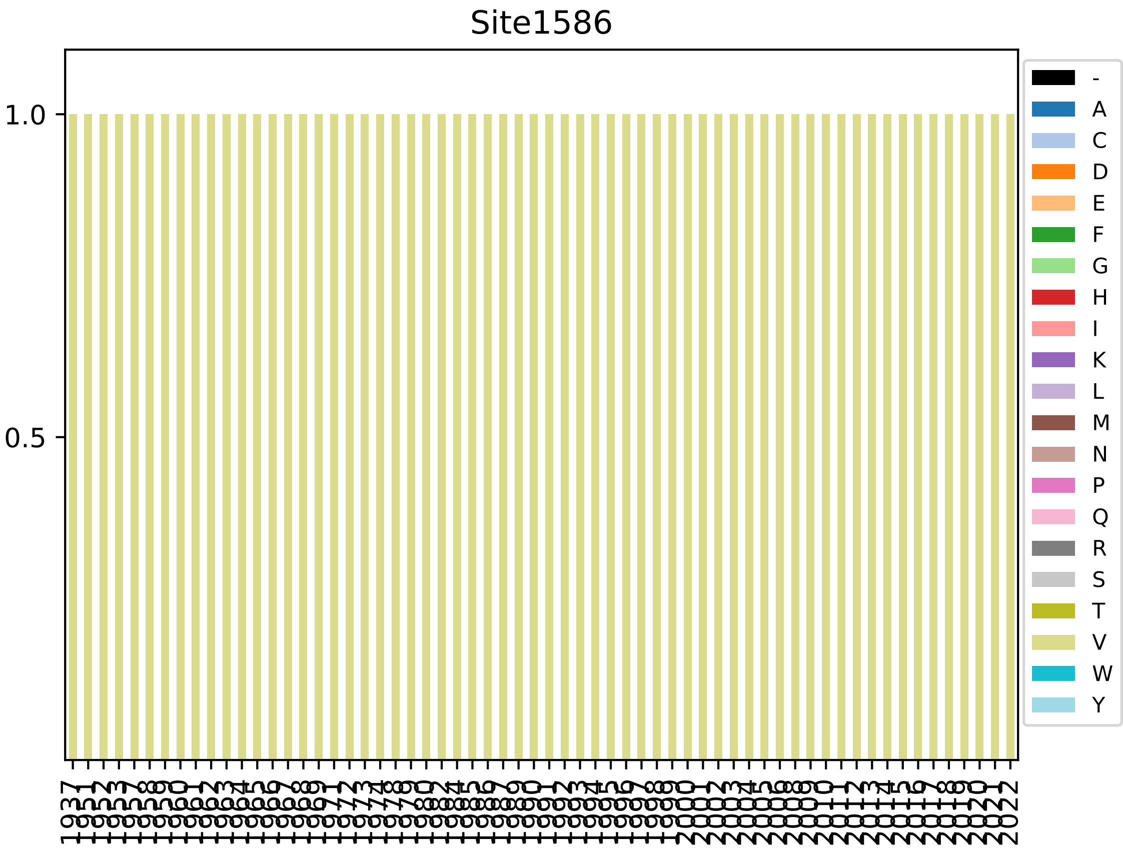

Site1586
1.0
0.5
-
A
C
D
E
F
G
H
I
K
L
M
N
P
Q
R
S
T
V
W
Y

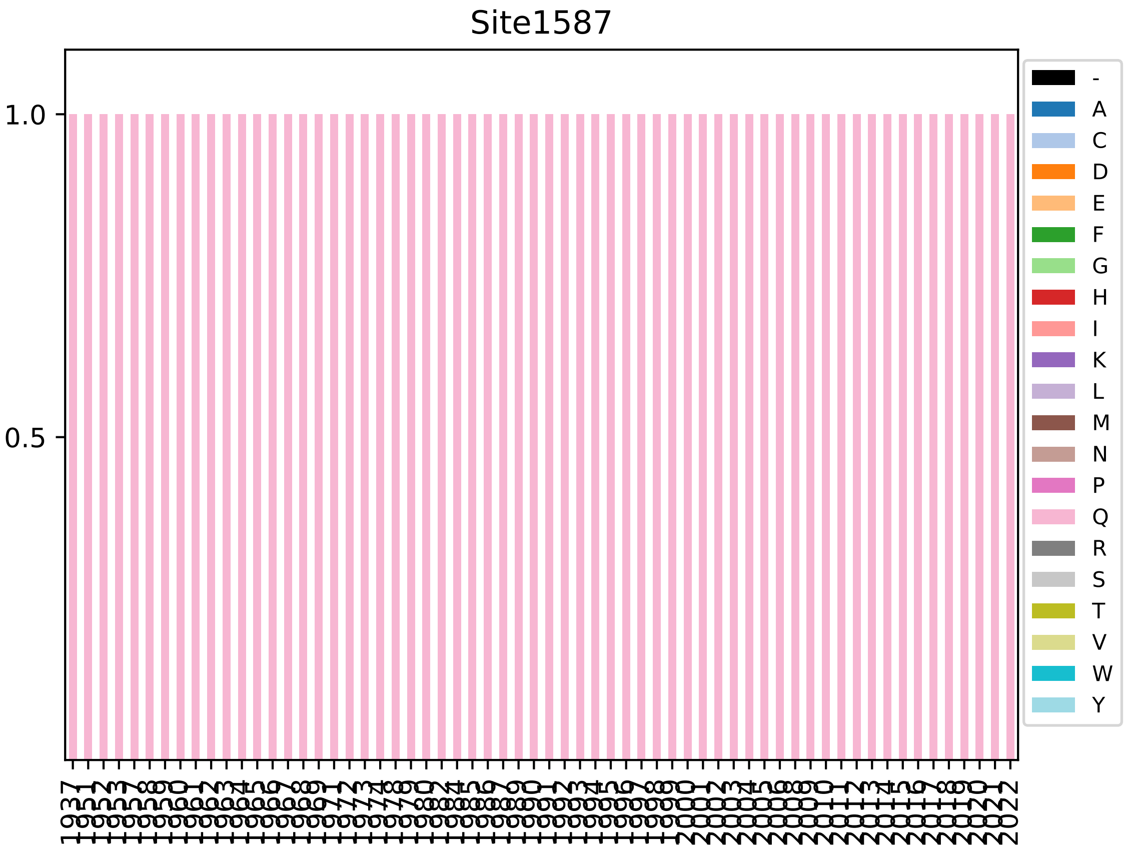
Site1587
1.0
0.5
-
A
C
D
E
F
G
H
I
K
L
M
N
P
Q
R
S
T
V
W
Y

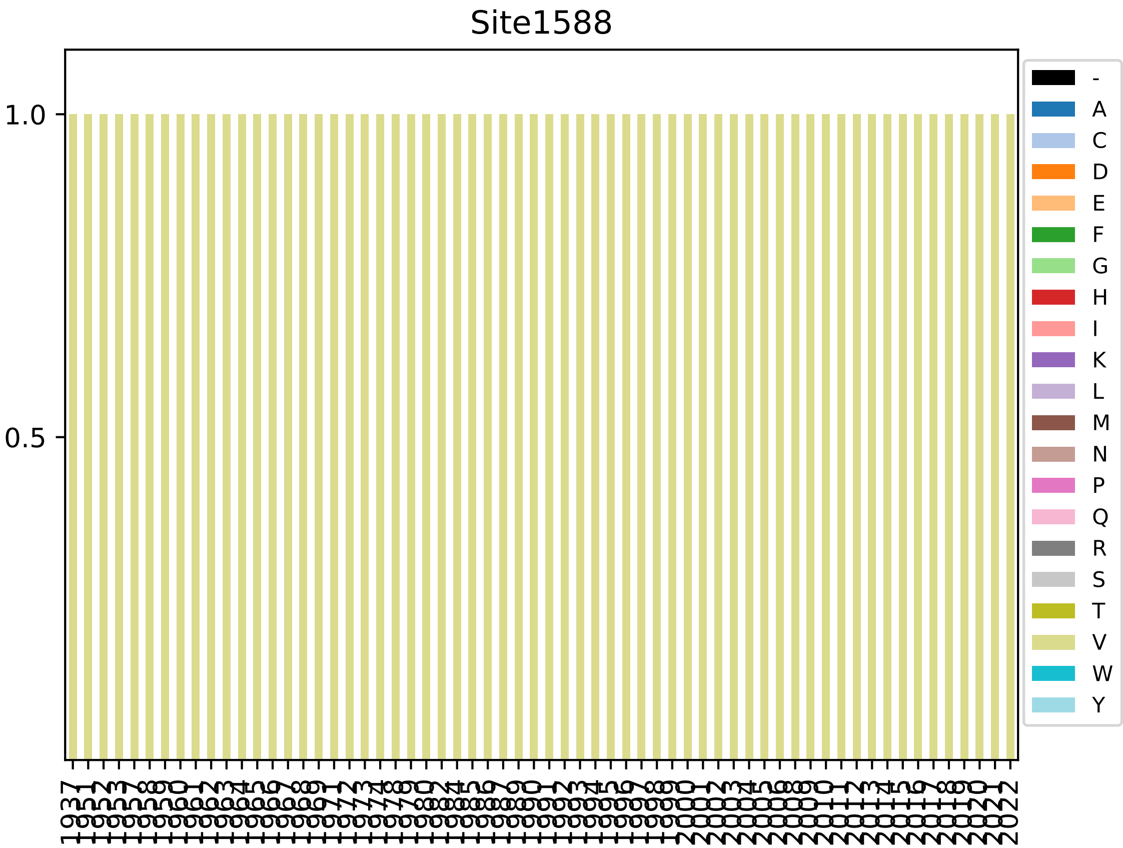
Site1588
1.0
0.5
-
A
C
D
E
F
G
H
I
K
L
M
N
P
Q
R
S
T
V
W
Y

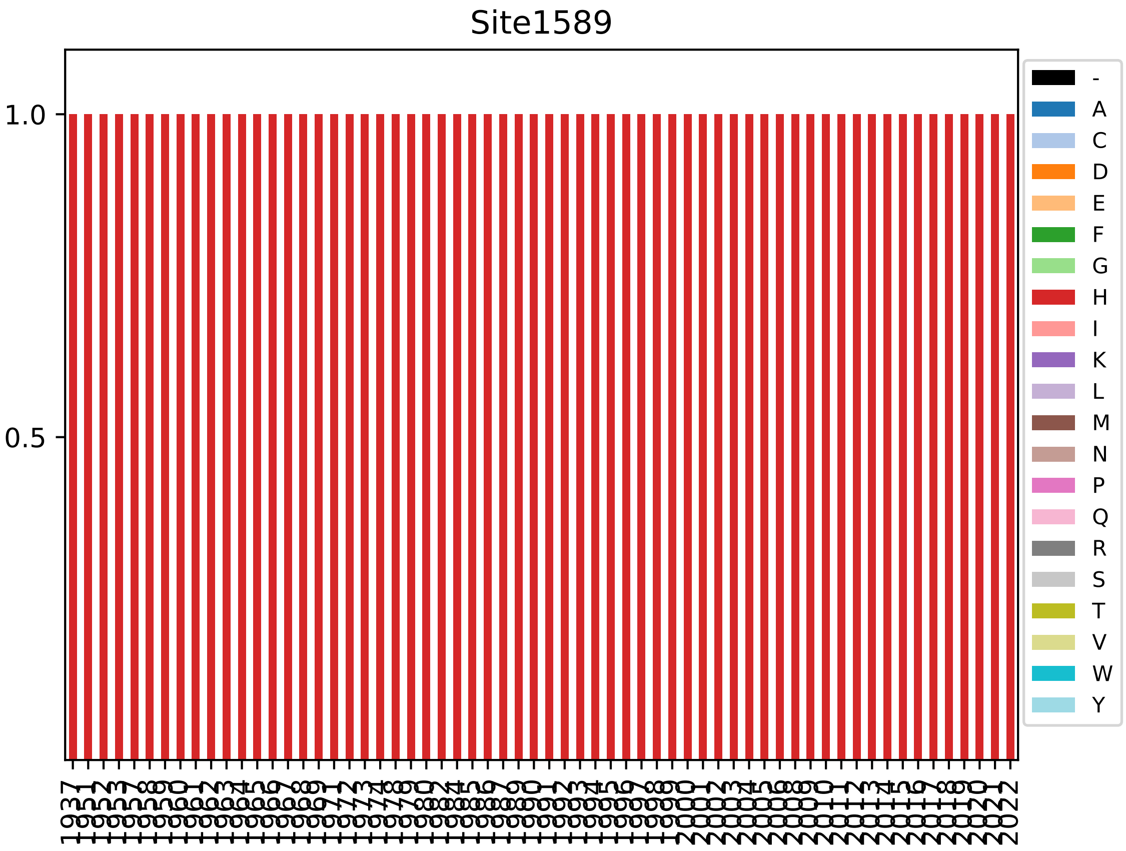
Site1589
1.0
0.5
-
A
C
D
E
F
G
H
I
K
L
M
N
P
Q
R
S
T
V
W
Y

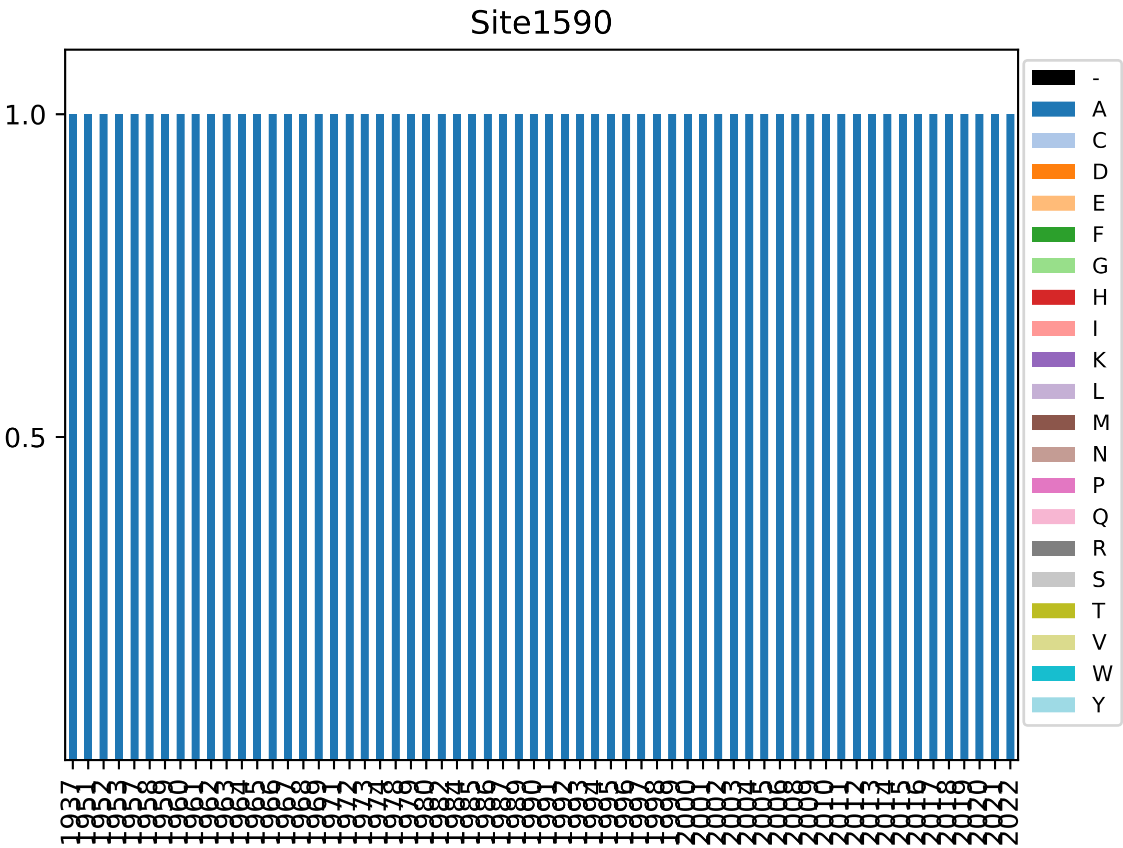
Site1590
1.0
0.5
-
A
C
D
E
F
G
H
I
K
L
M
N
P
Q
R
S
T
V
W
Y

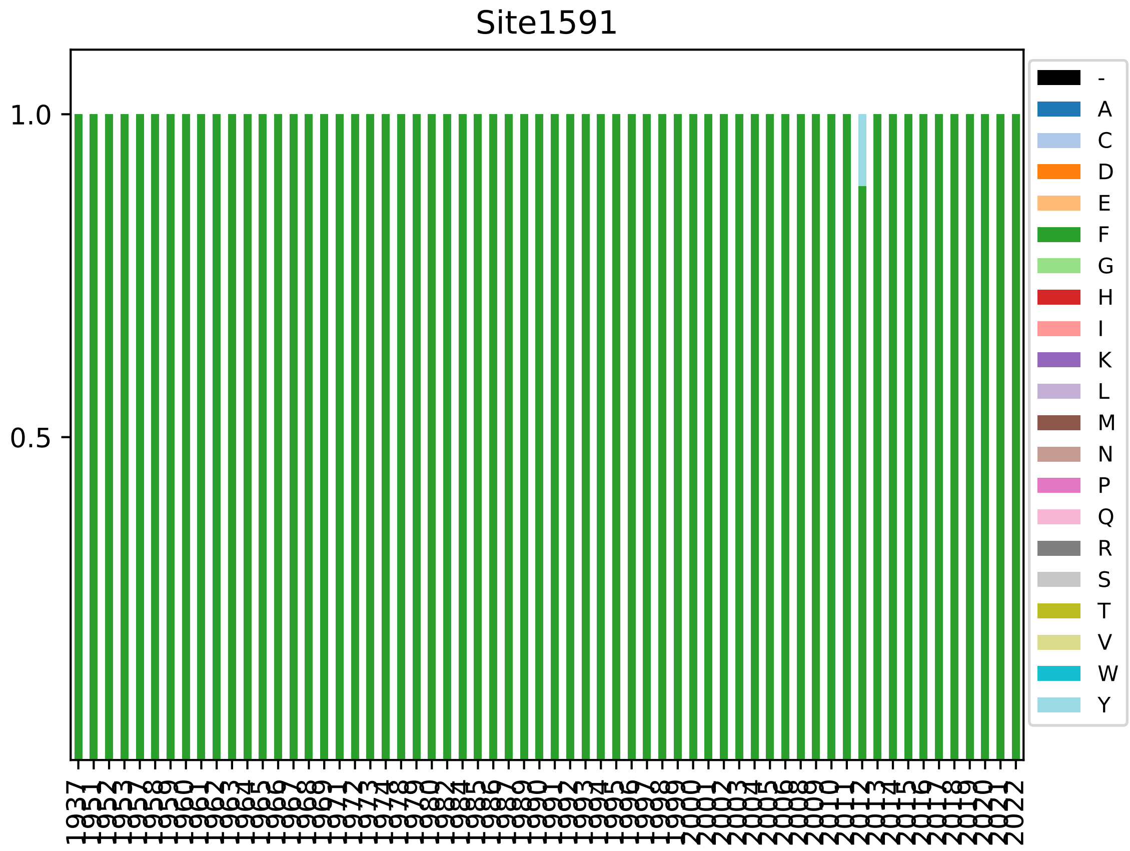
Site1591
1.0
0.5
-
A
C
D
E
F
G
H
I
K
L
M
N
P
Q
R
S
T
V
W
Y

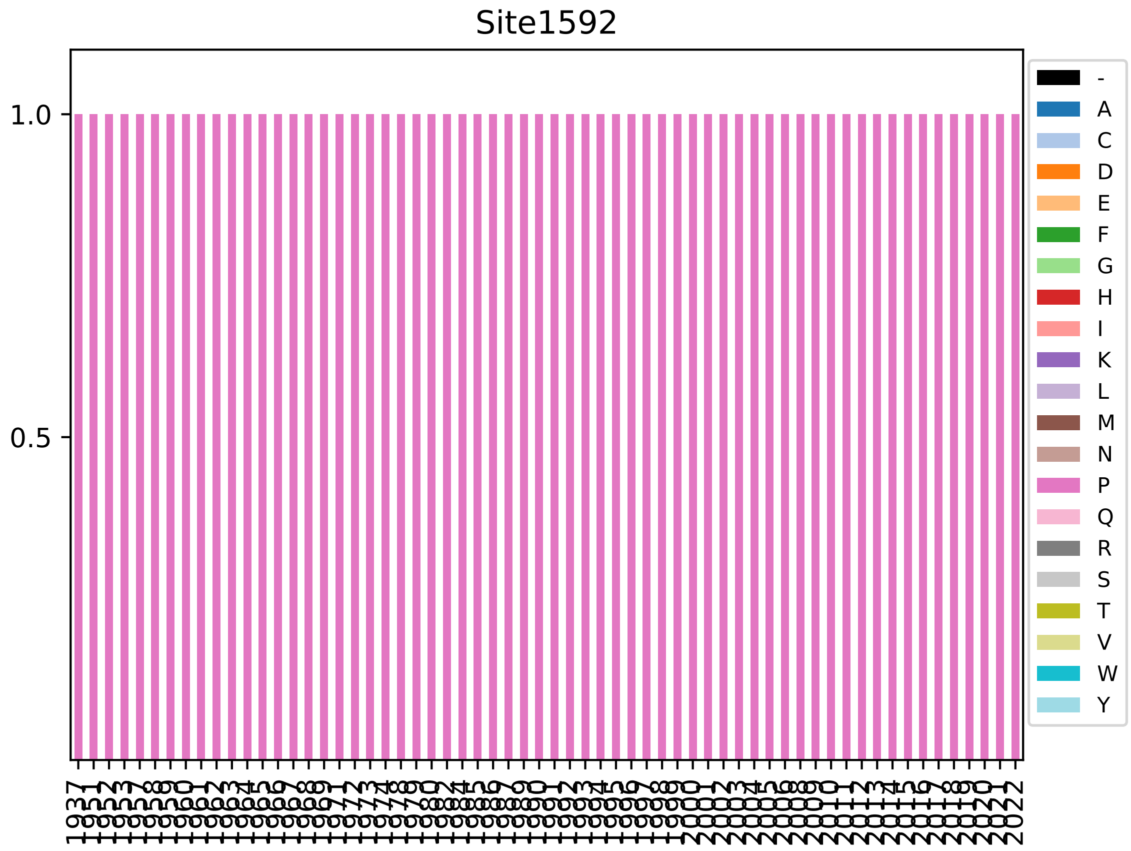
Site1592
1.0
0.5
-
A
C
D
E
F
G
H
I
K
L
M
N
P
Q
R
S
T
V
W
Y

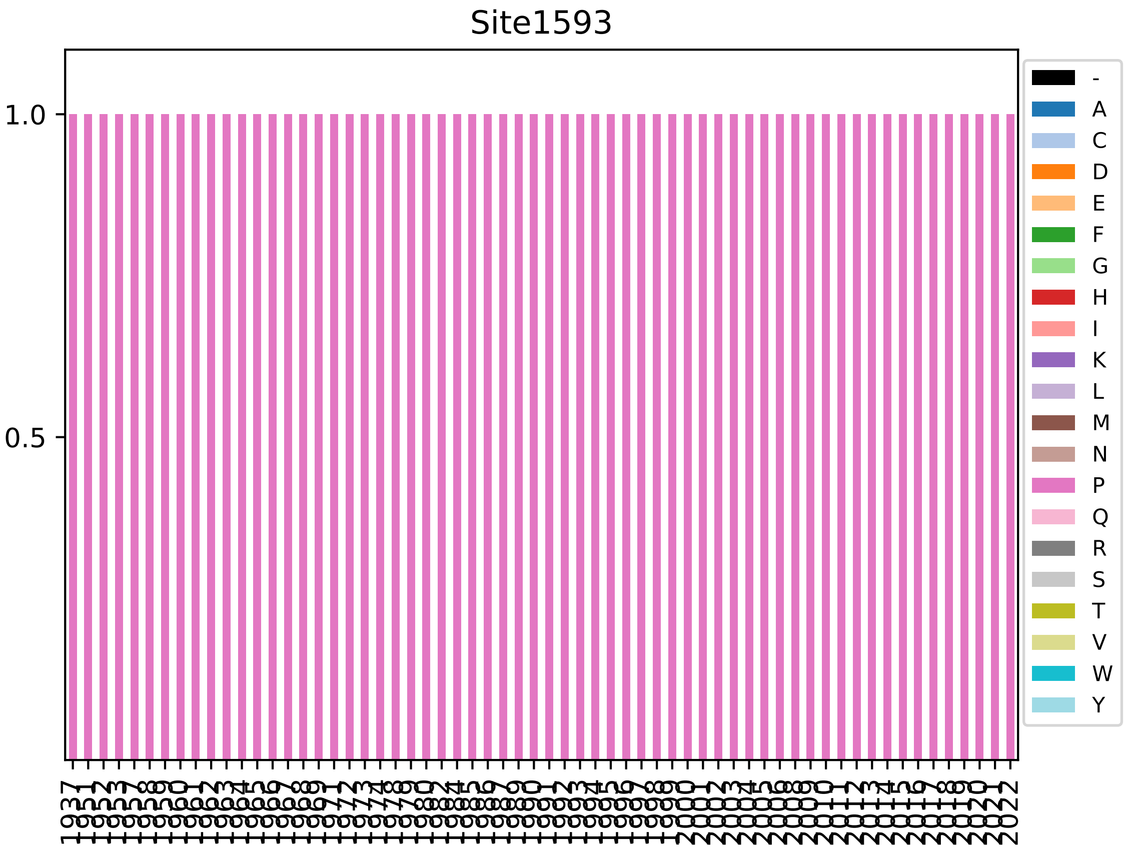
Site1593
1.0
0.5
-
A
C
D
E
F
G
H
I
K
L
M
N
P
Q
R
S
T
V
W
Y

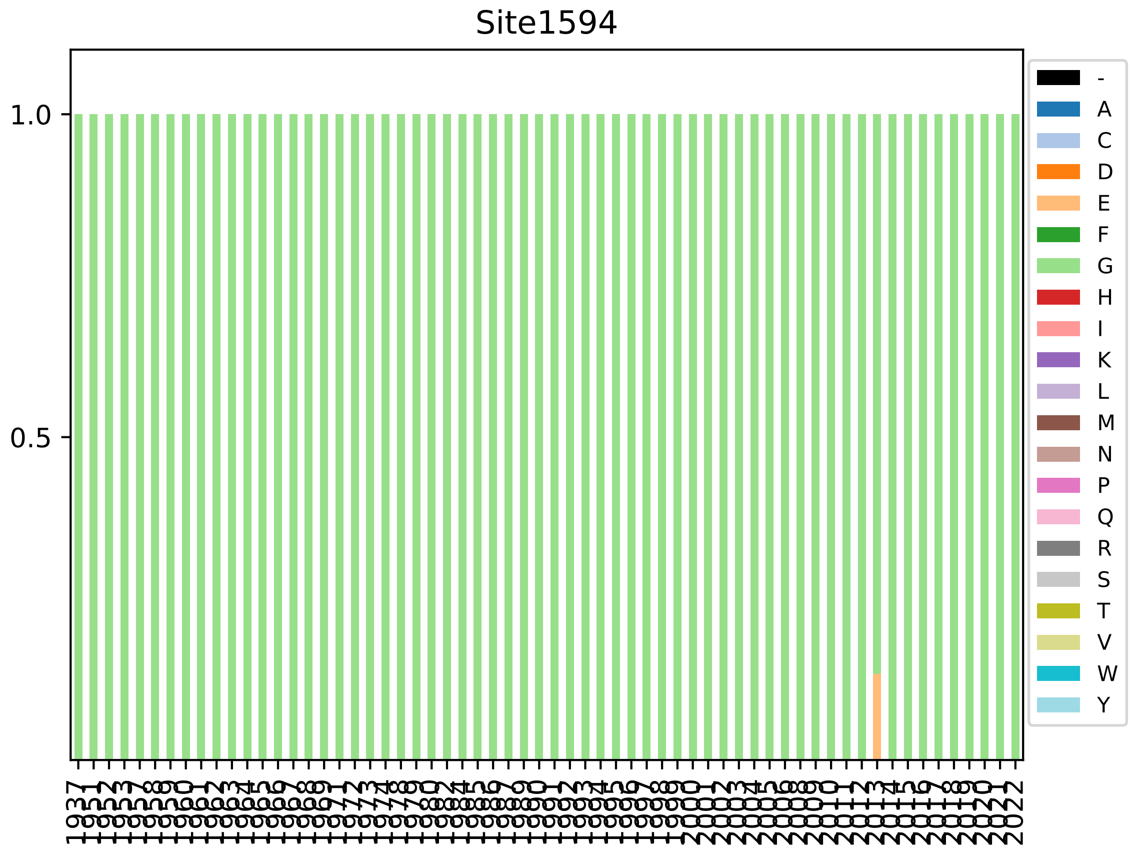

Site1594
1.0
0.5
-
A
C
D
E
F
G
H
I
K
L
M
N
P
Q
R
S
T
V
W
Y

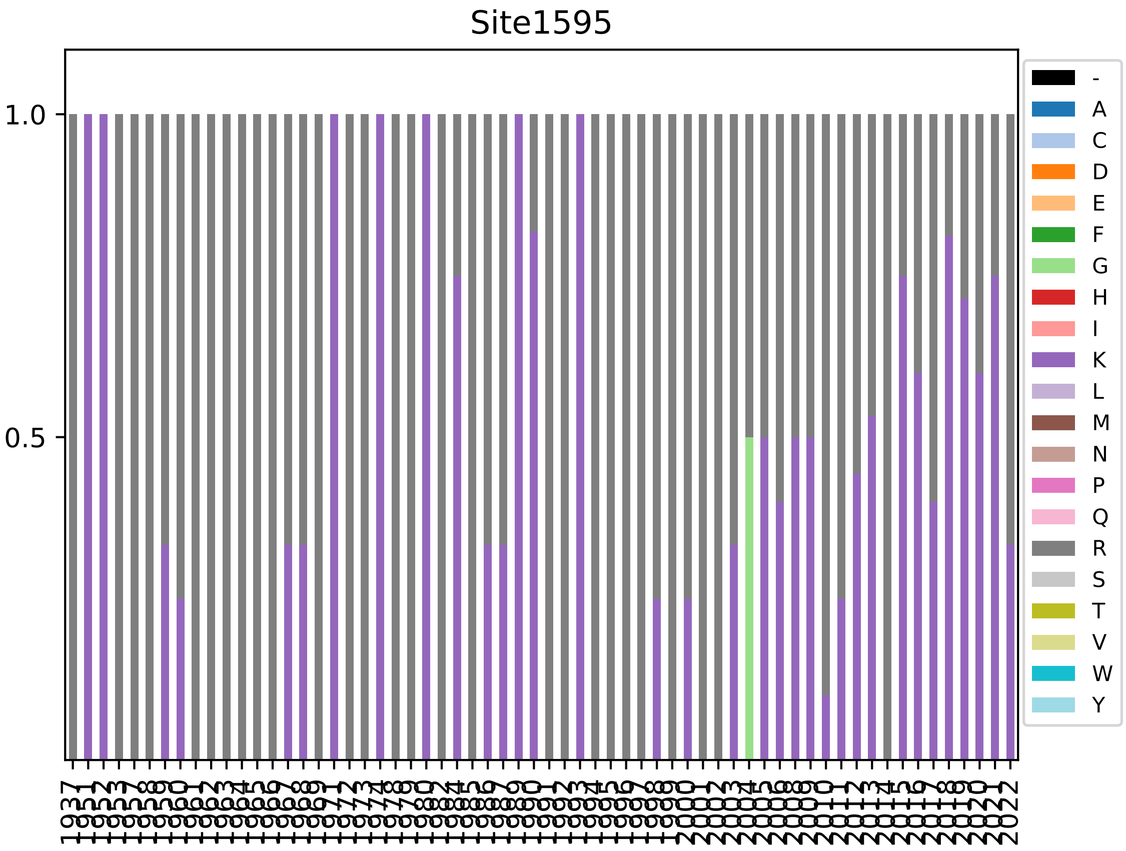

Site1595
1.0
0.5
-
A
C
D
E
F
G
H
I
K
L
M
N
P
Q
R
S
T
V
W
Y

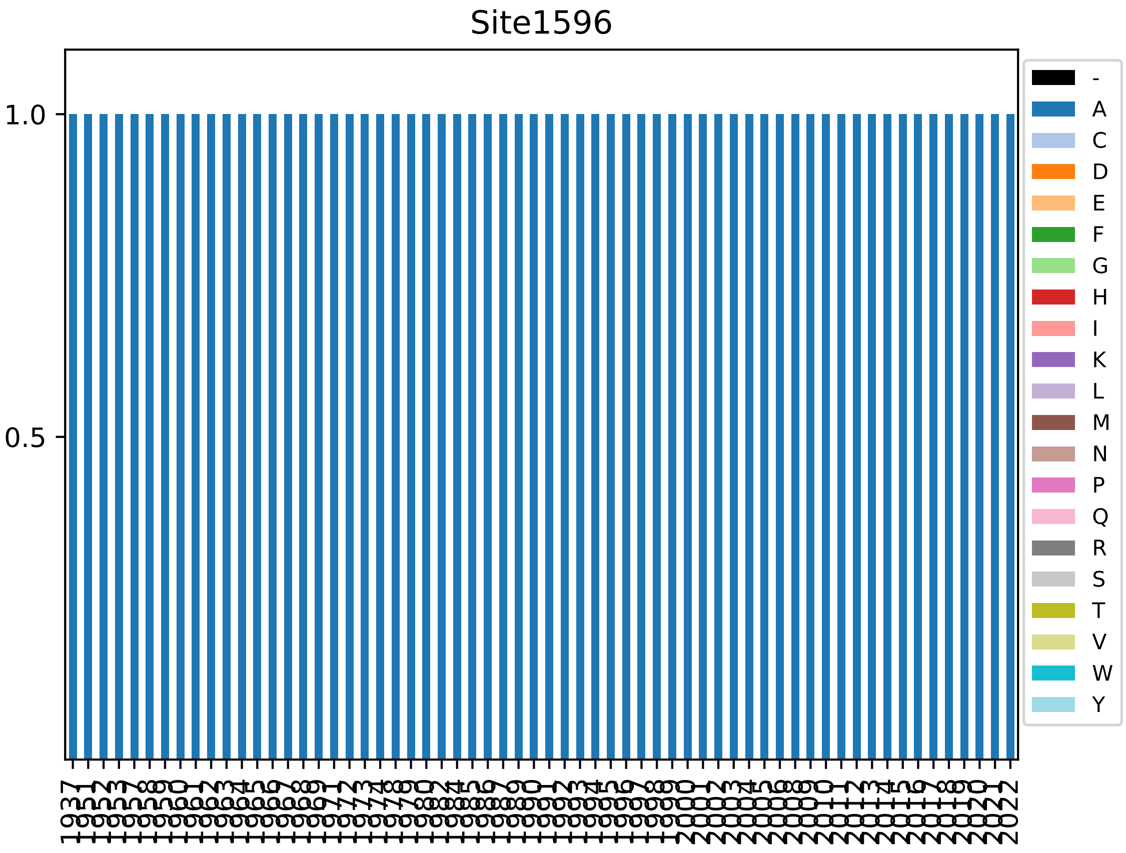
Site1596
1.0
0.5
-
A
C
D
E
F
G
H
I
K
L
M
N
P
Q
R
S
T
V
W
Y

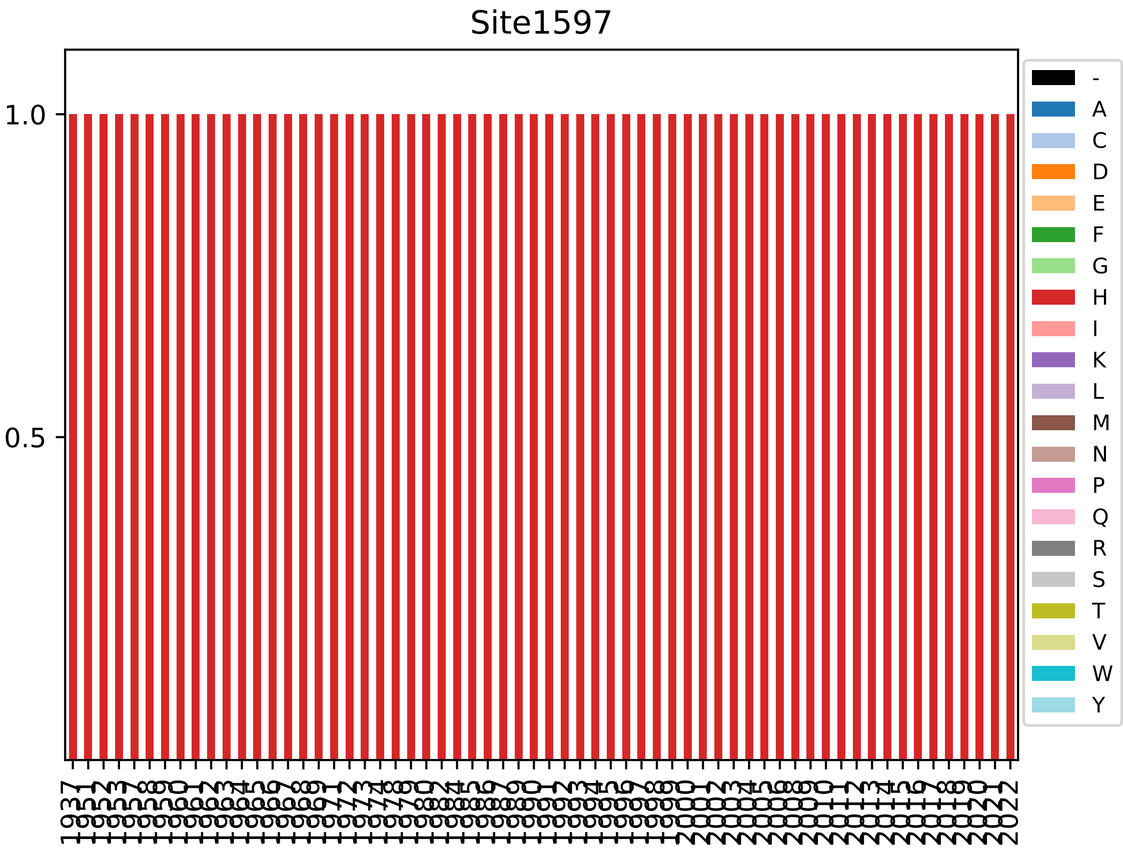
Site1597
1.0
0.5
-
A
C
D
E
F
G
H
I
K
L
M
N
P
Q
R
S
T
V
W
Y

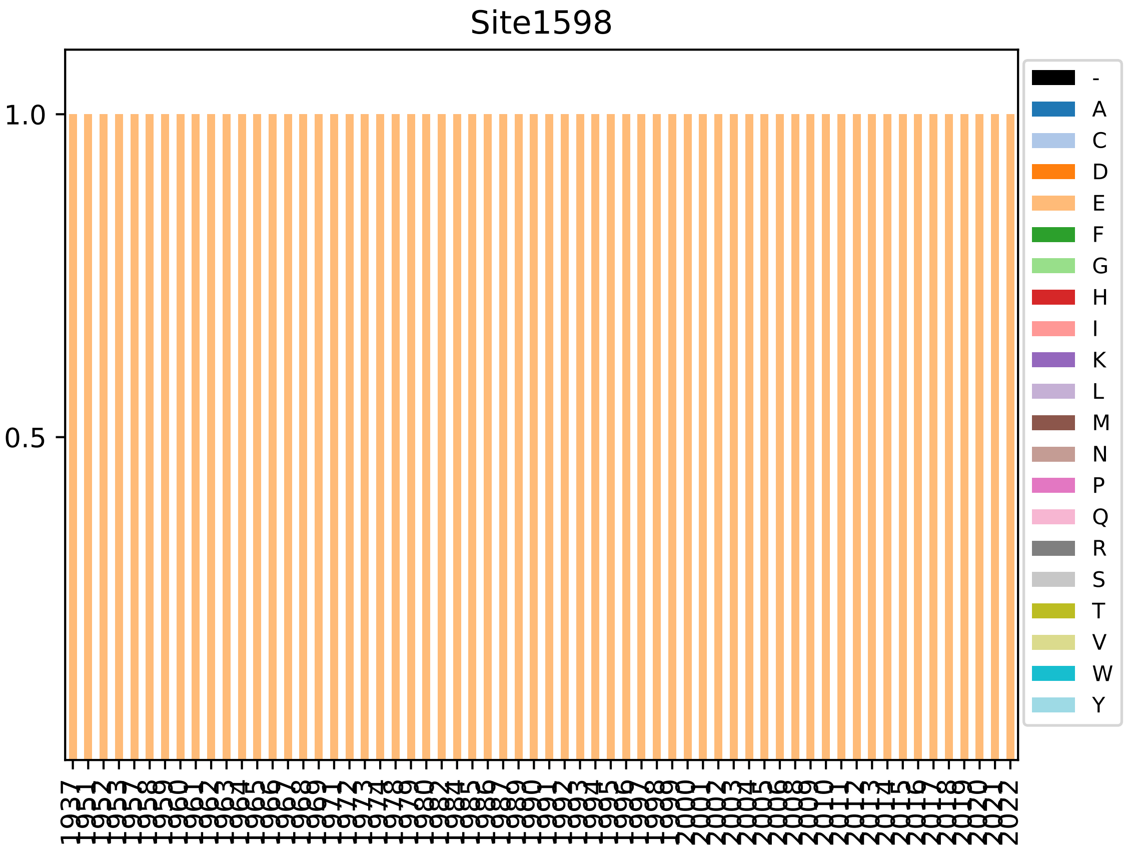
Site1598
1.0
0.5
-
A
C
D
E
F
G
H
I
K
L
M
N
P
Q
R
S
T
V
W
Y

Site1599

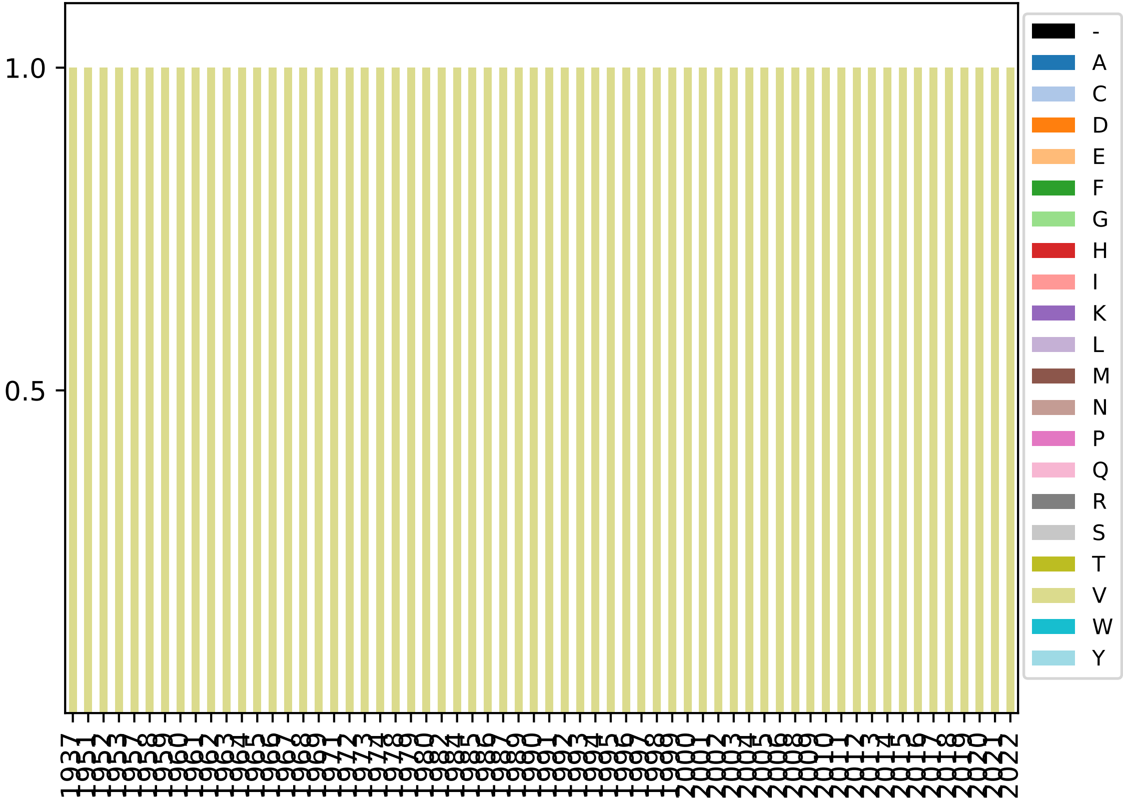

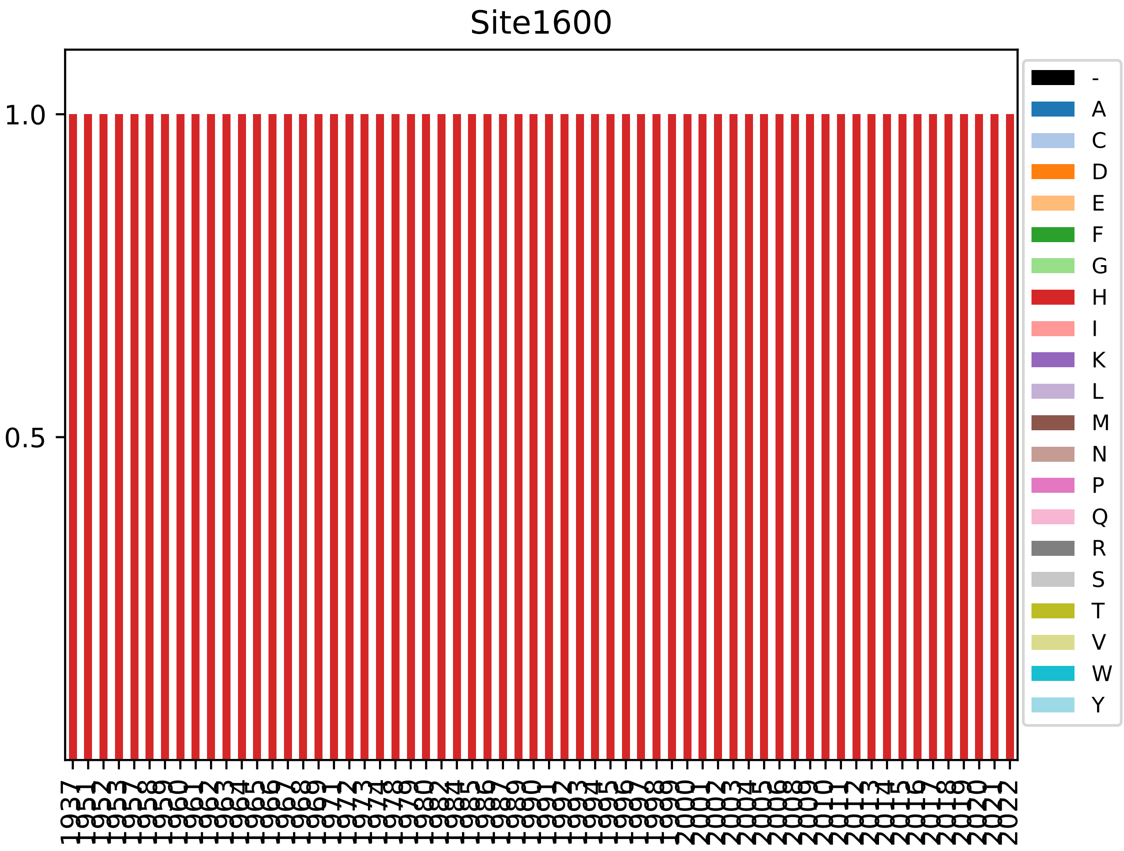
Site1600
1.0
0.5
-
A
C
D
E
F
G
H
I
K
L
M
N
P
Q
R
S
T
V
W
Y

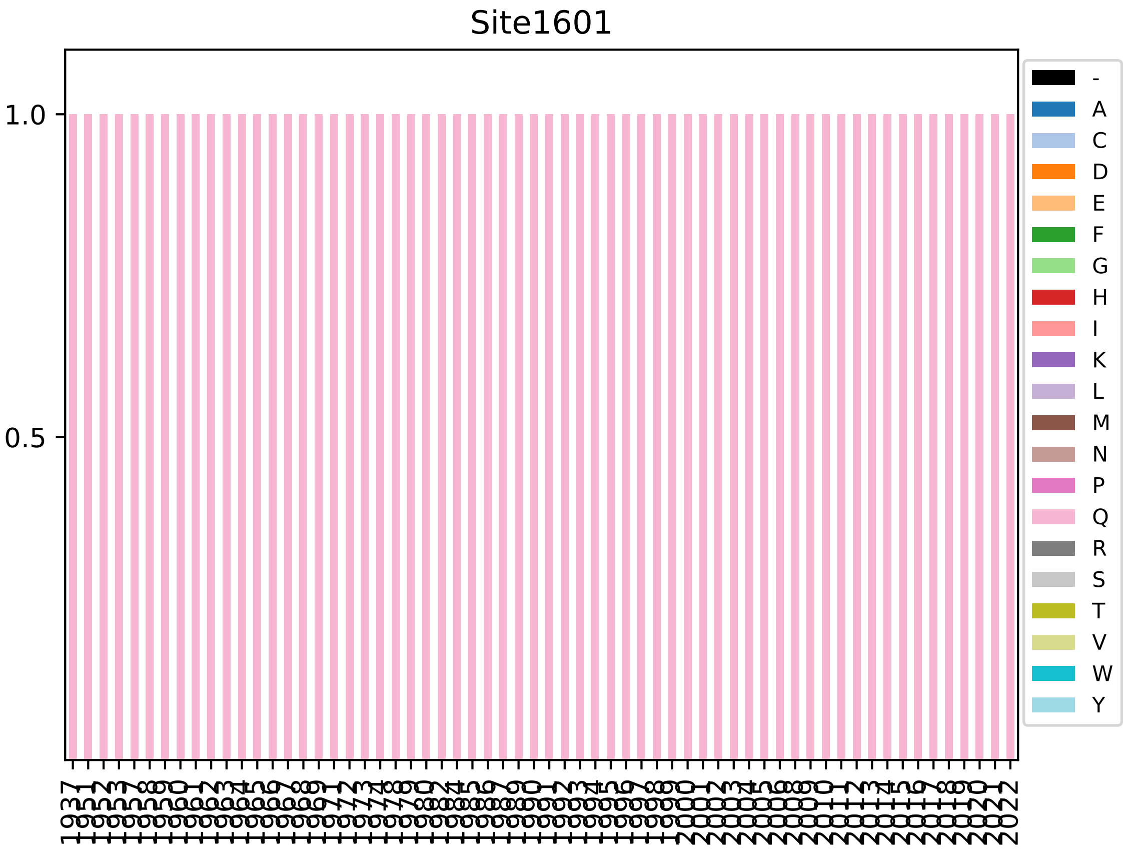

Site1601
1.0
0.5
-
A
C
D
E
F
G
H
I
K
L
M
N
P
Q
R
S
T
V
W
Y

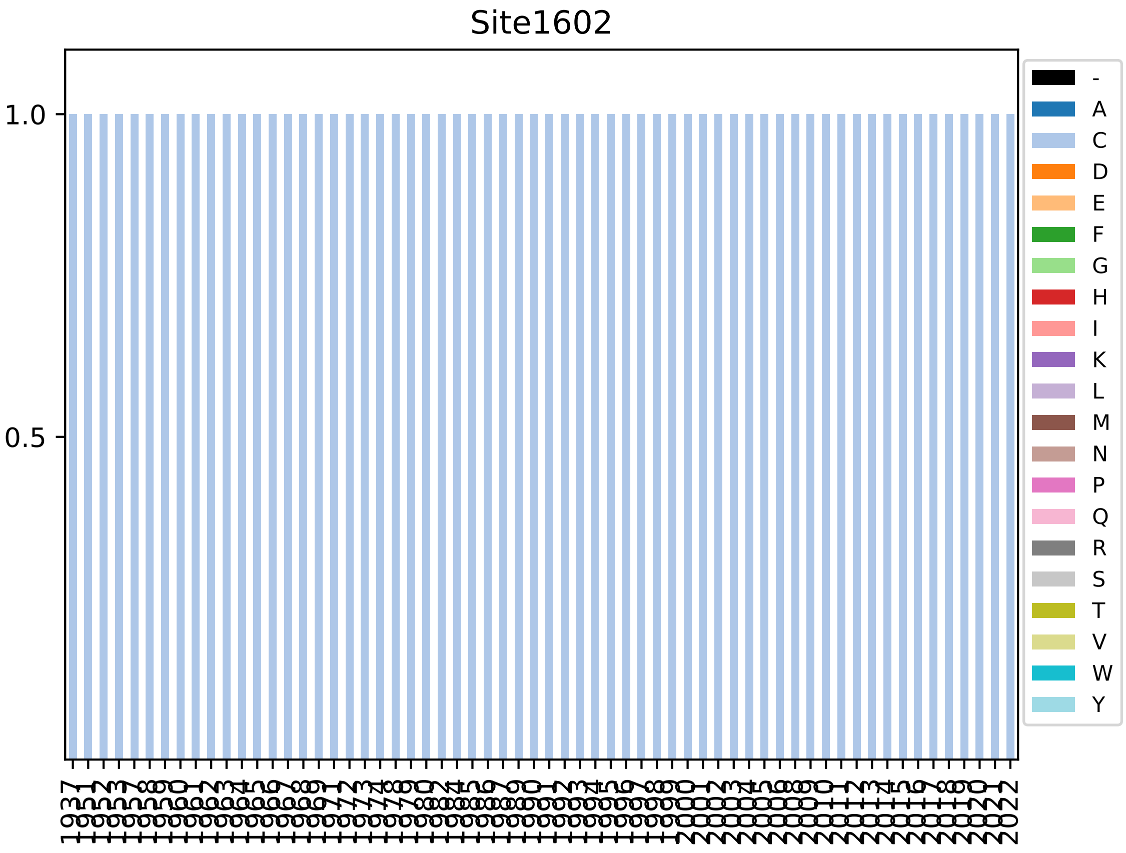
Site1602
1.0
0.5
-
A
C
D
E
F
G
H
I
K
L
M
N
P
Q
R
S
T
V
W
Y

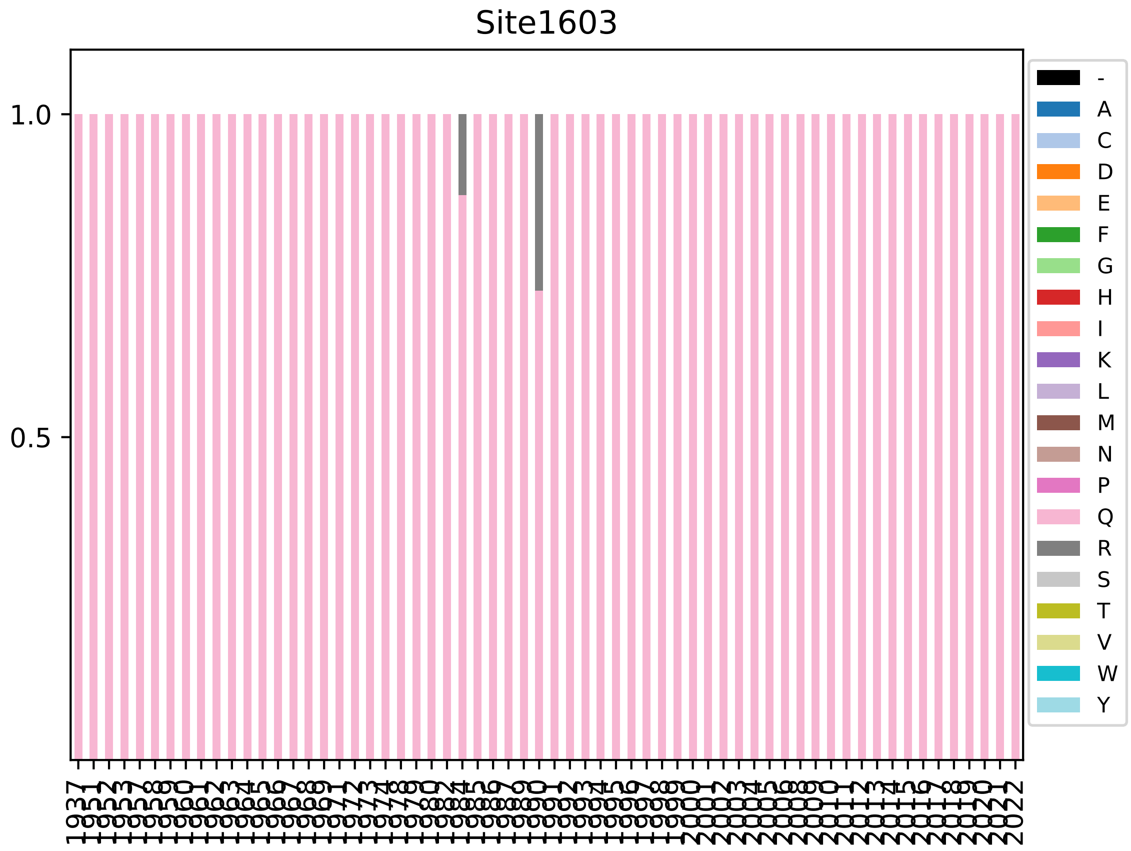
Site1603
1.0
0.5
-
A
C
D
E
F
G
H
I
K
L
M
N
P
Q
R
S
T
V
W
Y

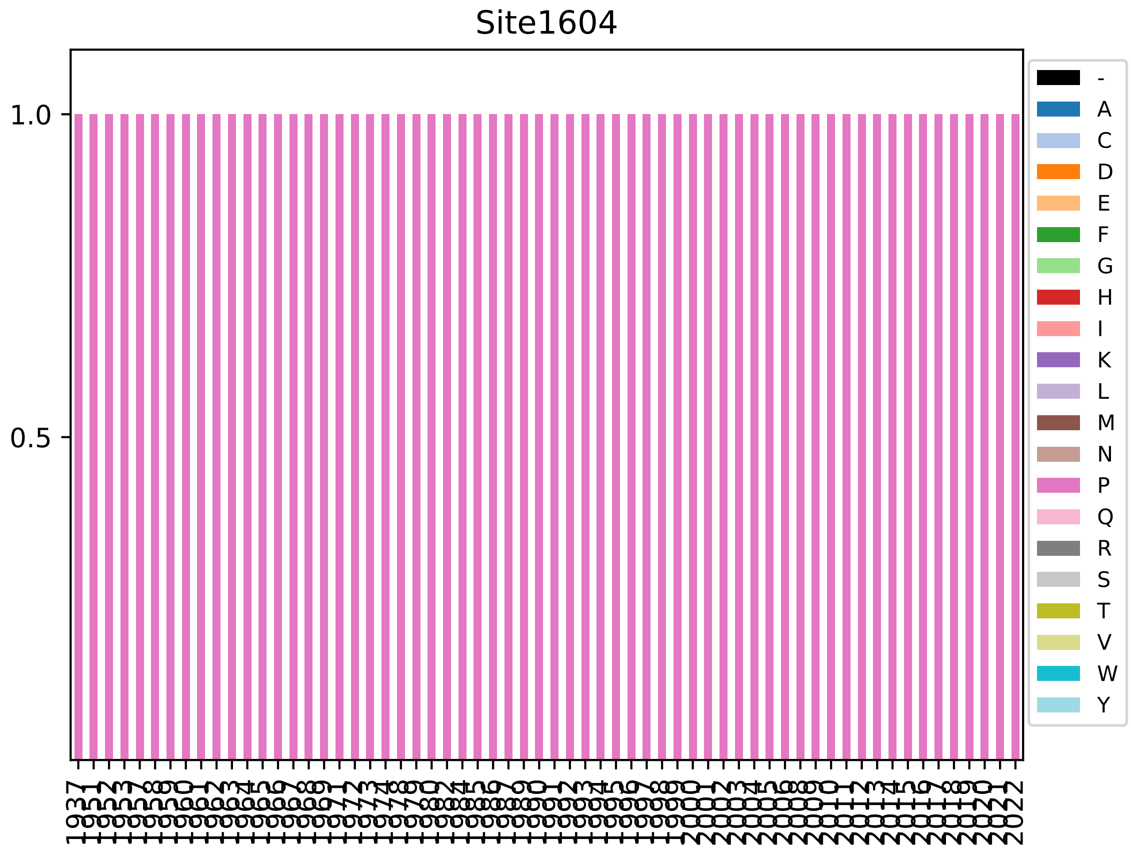
Site1604
1.0
0.5
-
A
C
D
E
F
G
H
I
K
L
M
N
P
Q
R
S
T
V
W
Y

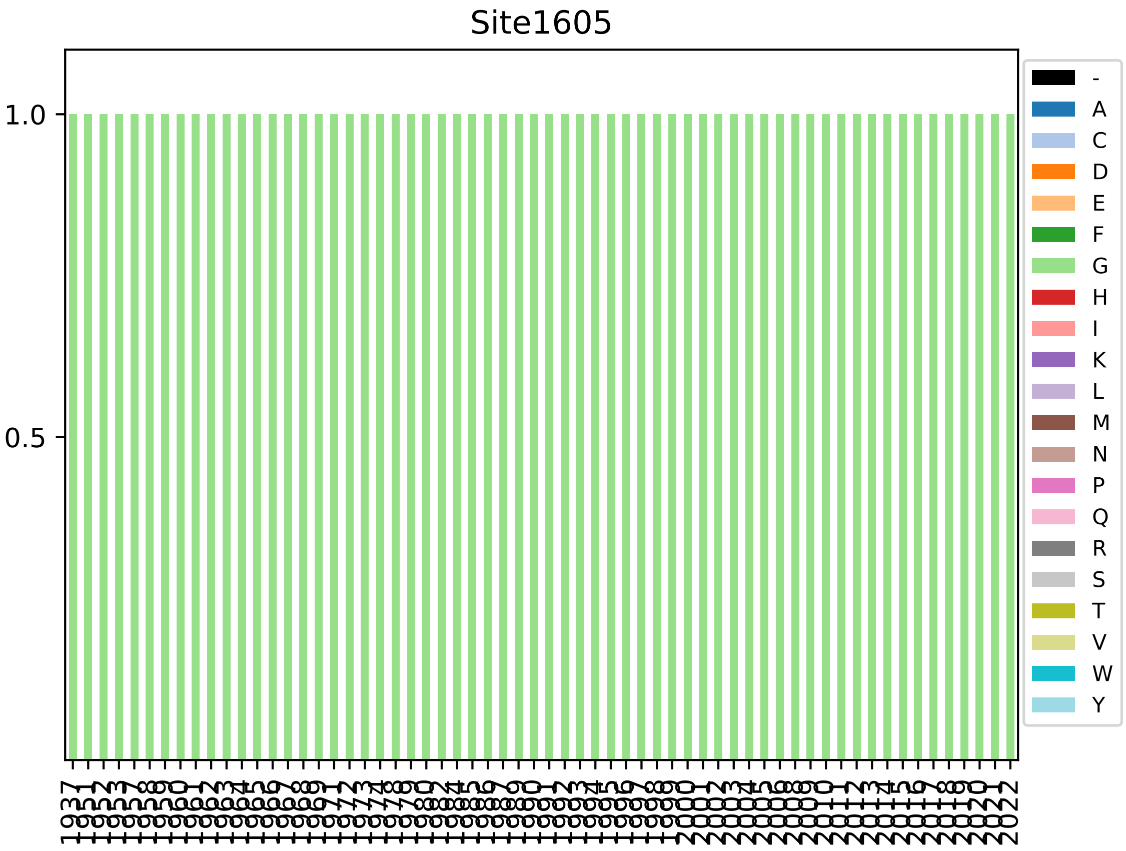
Site1605
1.0
0.5
-
A
C
D
E
F
G
H
I
K
L
M
N
P
Q
R
S
T
V
W
Y

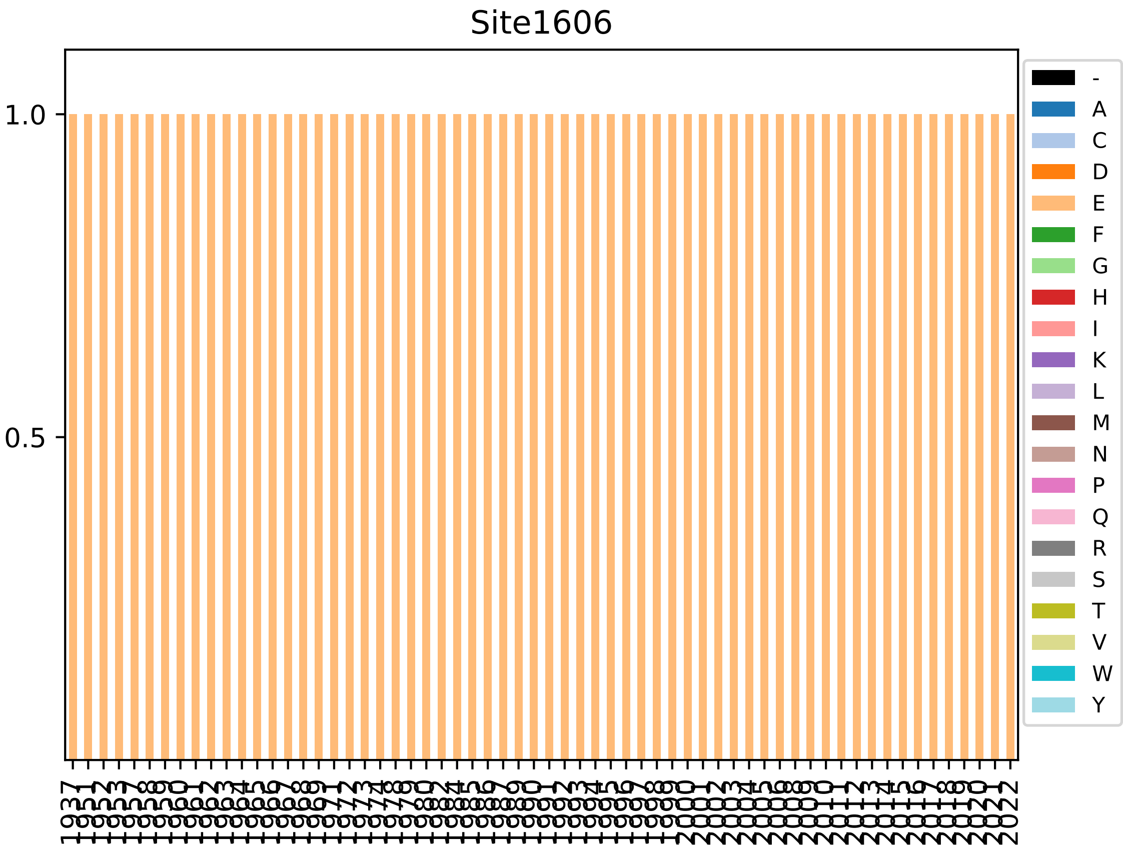
Site1606
1.0
0.5
-
A
C
D
E
F
G
H
I
K
L
M
N
P
Q
R
S
T
V
W
Y

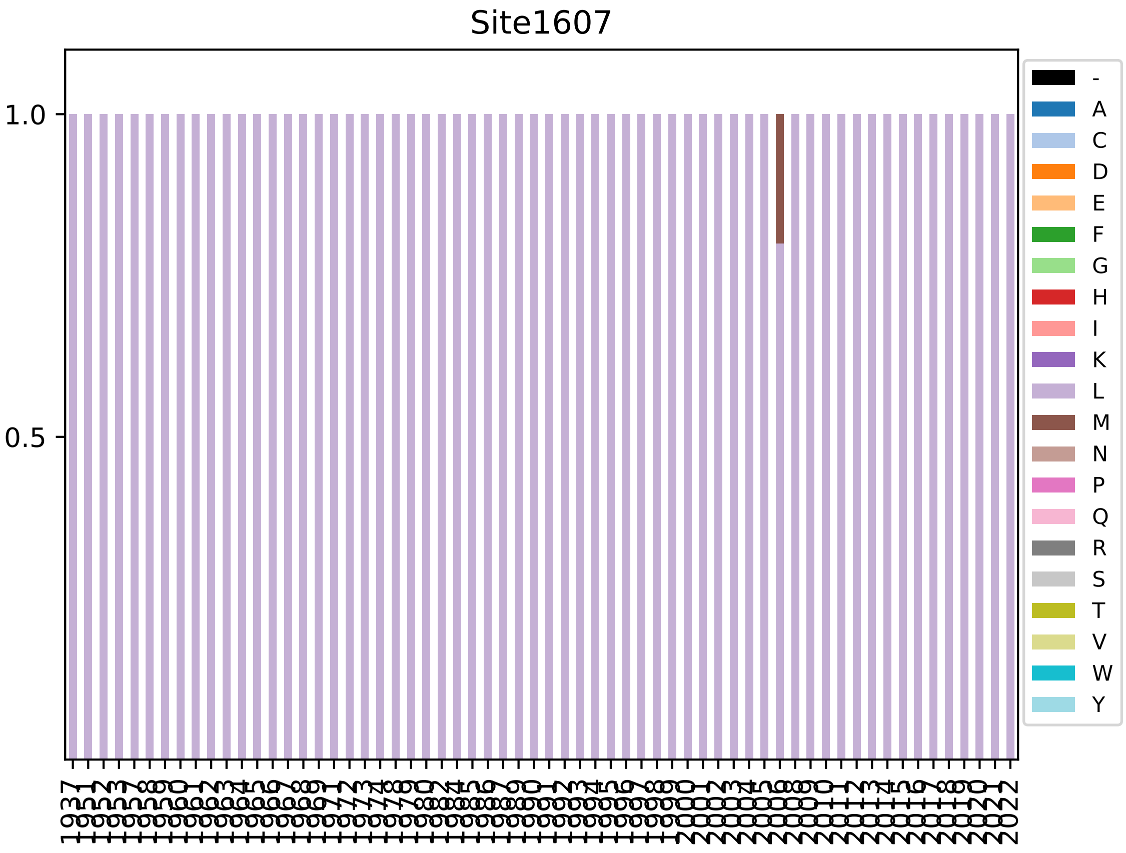
Site1607
1.0
0.5
-
A
C
D
E
F
G
H
I
K
L
M
N
P
Q
R
S
T
V
W
Y

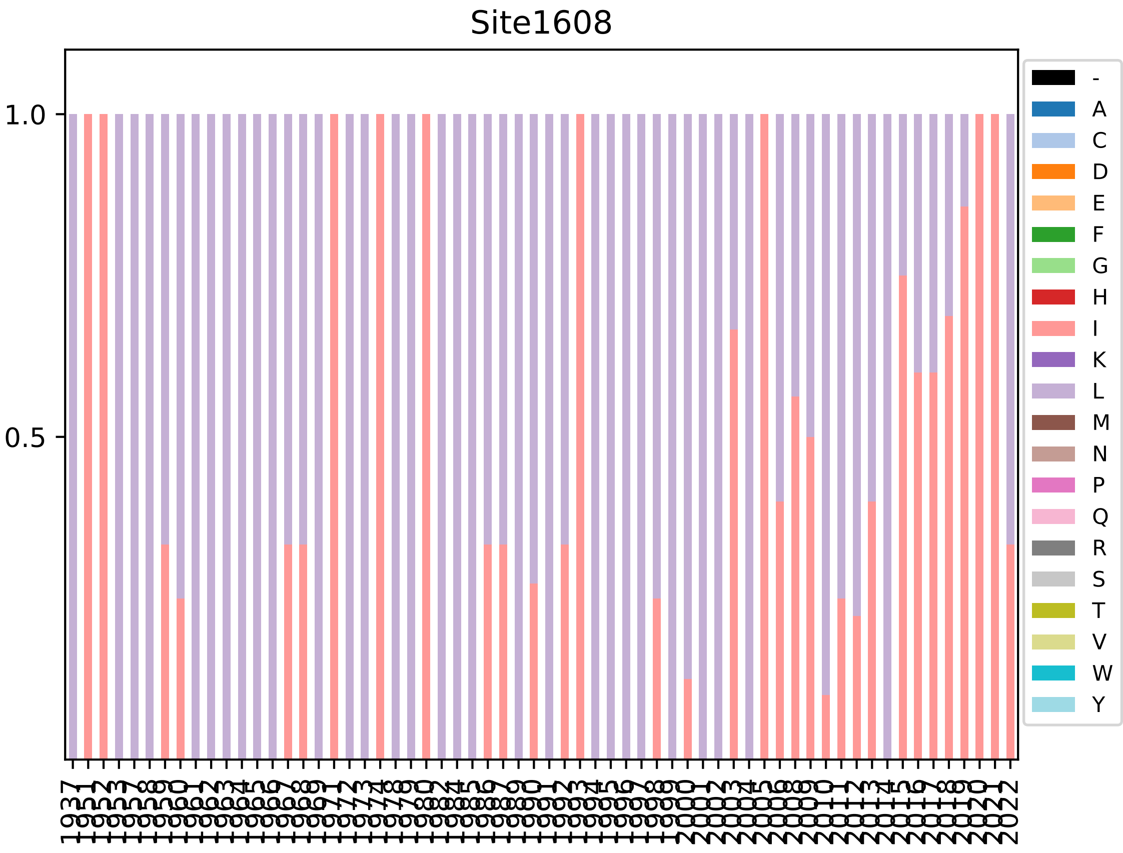
Site1608
1.0
0.5
-
A
C
D
E
F
G
H
I
K
L
M
N
P
Q
R
S
T
V
W
Y

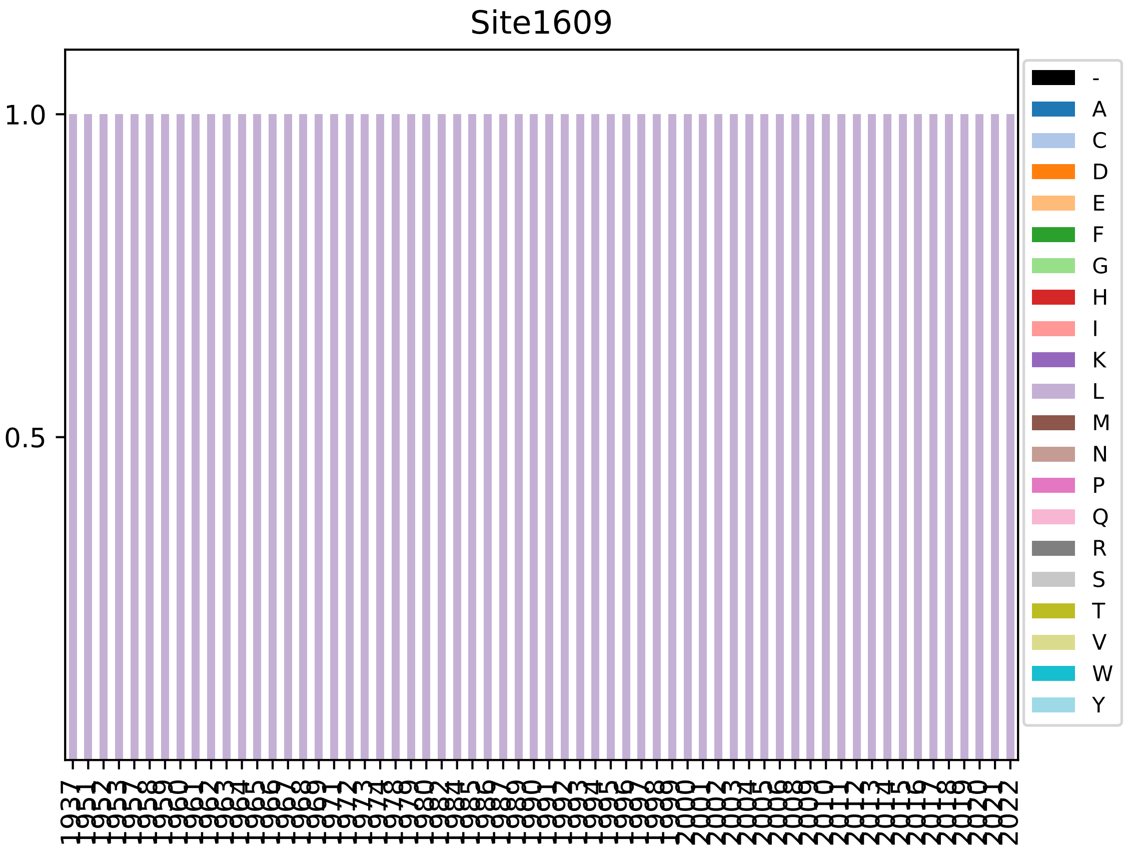
Site1609
1.0
0.5
-
A
C
D
E
F
G
H
I
K
L
M
N
P
Q
R
S
T
V
W
Y

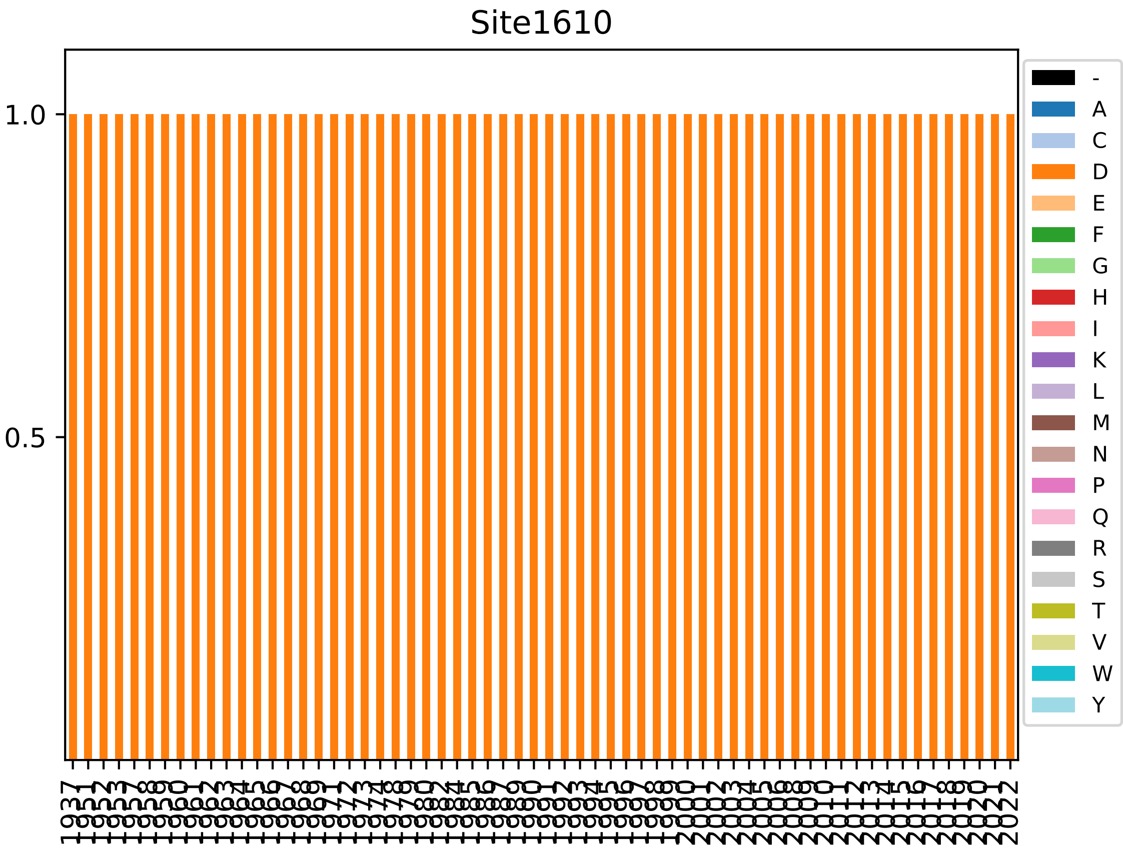
Site1610
1.0
0.5
-
A
C
D
E
F
G
H
I
K
L
M
N
P
Q
R
S
T
V
W
Y

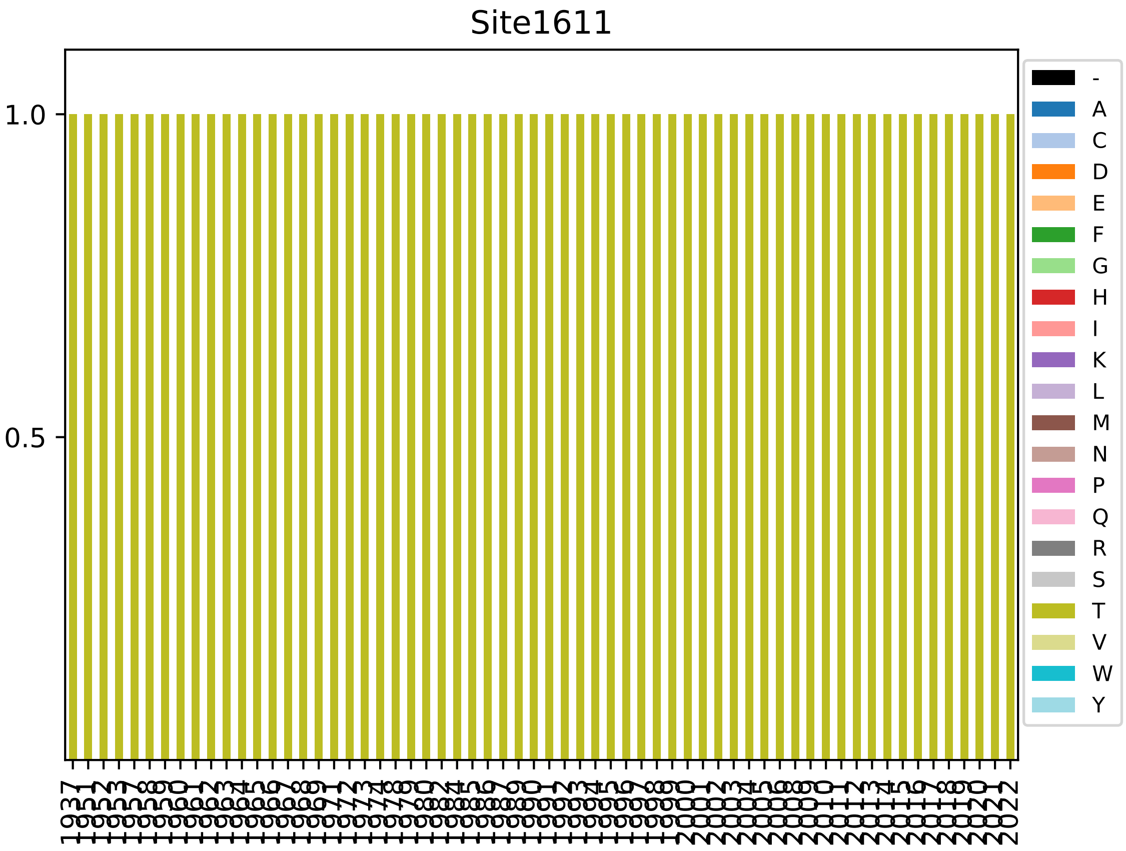
Site1611
1.0
0.5
-
A
C
D
E
F
G
H
I
K
L
M
N
P
Q
R
S
T
V
W
Y

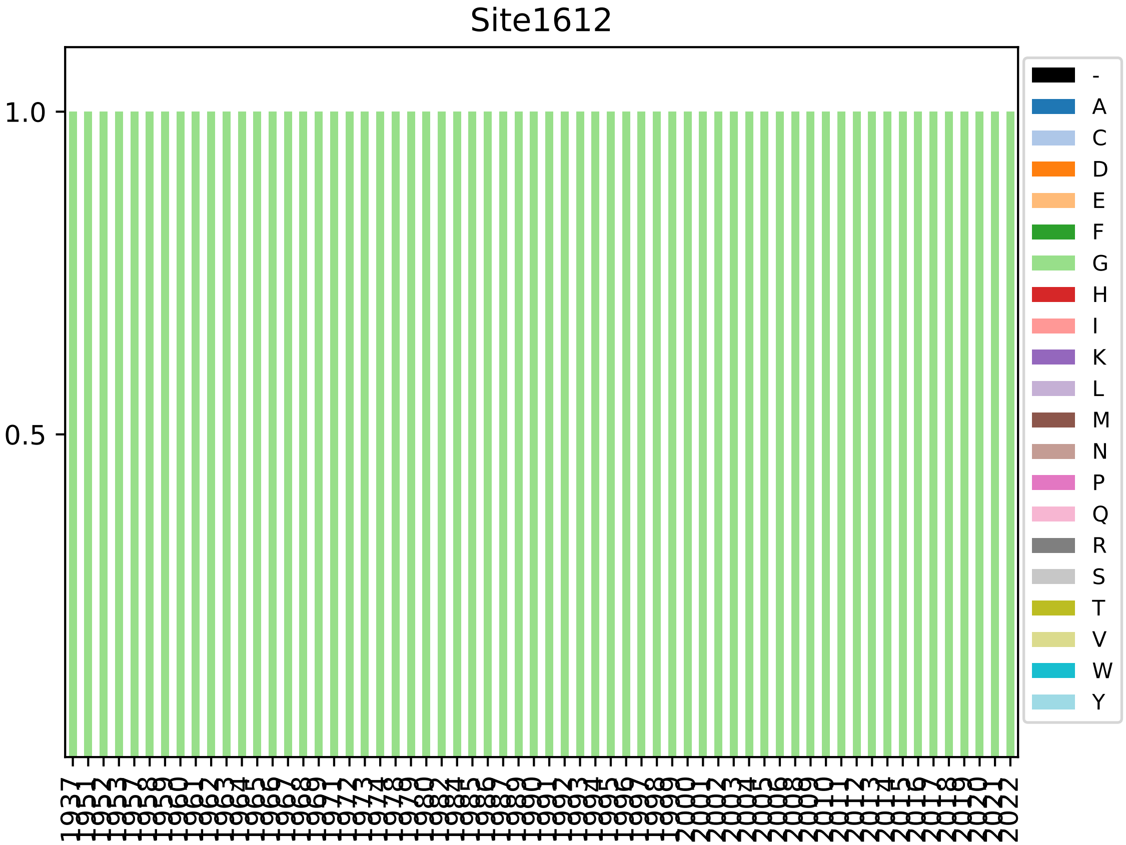
Site1612
1.0
0.5
-
A
C
D
E
F
G
H
I
K
L
M
N
P
Q
R
S
T
V
W
Y

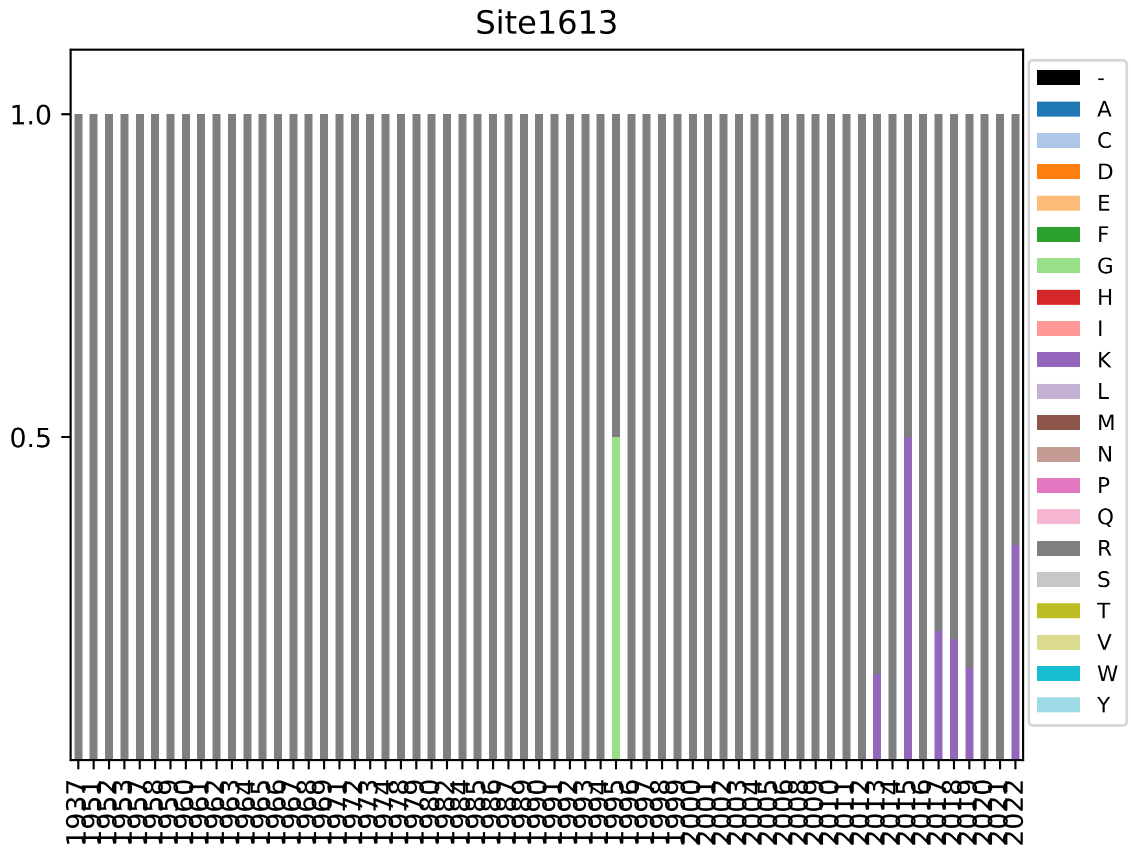
Site1613
1.0
0.5
-
A
C
D
E
F
G
H
I
K
L
M
N
P
Q
R
S
T
V
W
Y

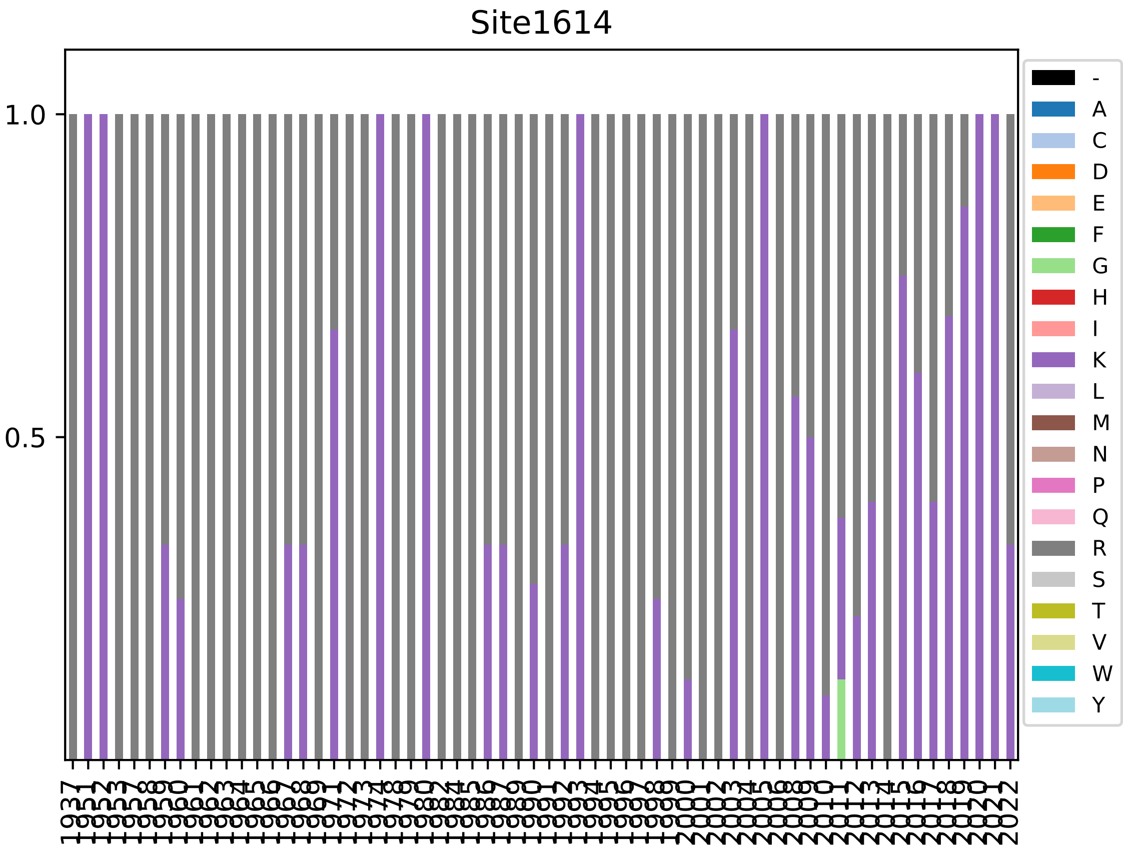
Site1614
1.0
0.5
-
A
C
D
E
F
G
H
I
K
L
M
N
P
Q
R
S
T
V
W
Y

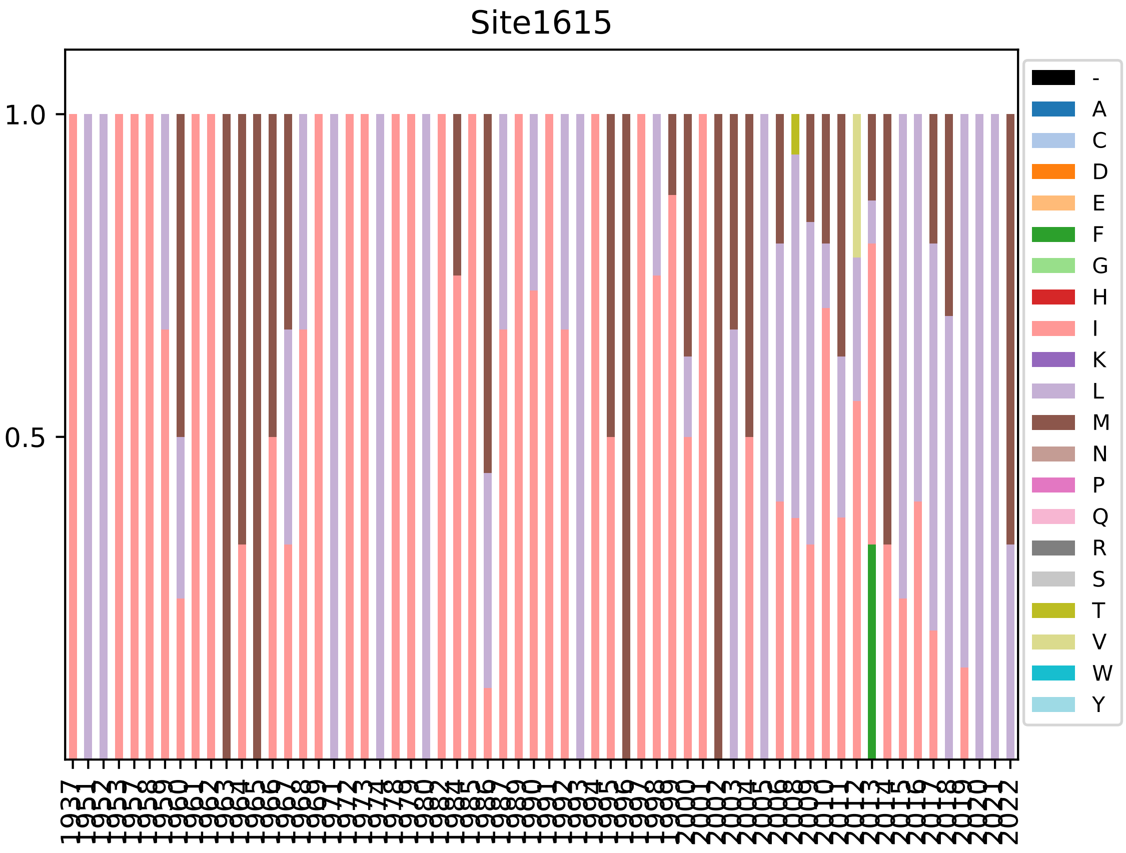
Site1615
1.0
0.5
-
A
C
D
E
F
G
H
I
K
L
M
N
P
Q
R
S
T
V
W
Y

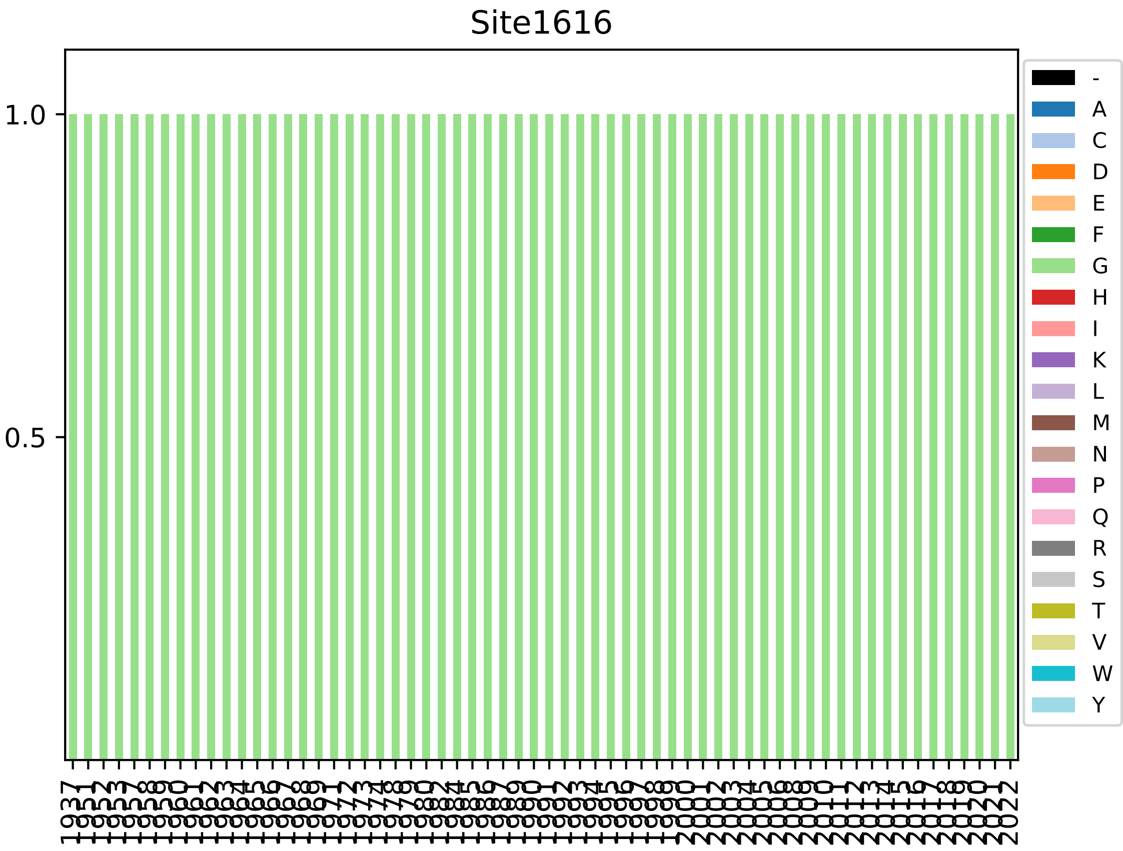
Site1616
1.0
0.5
-
A
C
D
E
F
G
H
I
K
L
M
N
P
Q
R
S
T
V
W
Y

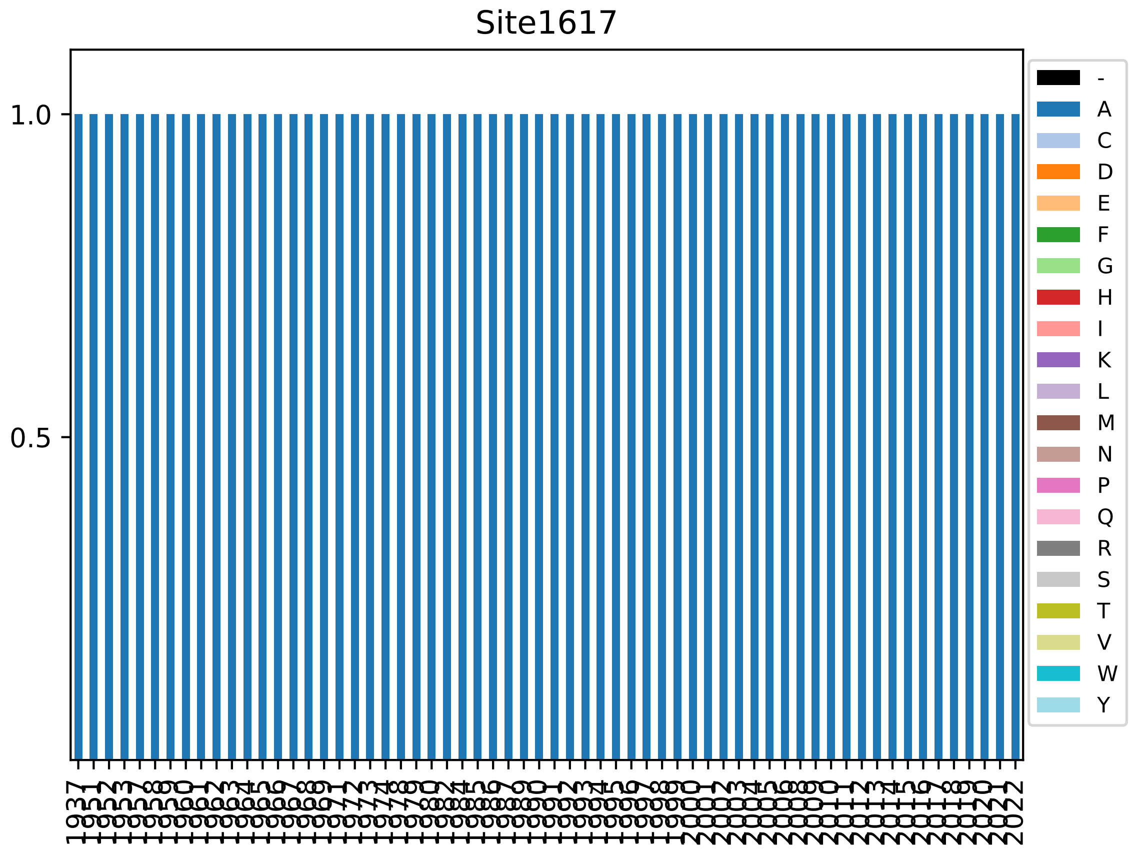
Site1617
1.0
0.5
-
A
C
D
E
F
G
H
I
K
L
M
N
P
Q
R
S
T
V
W
Y

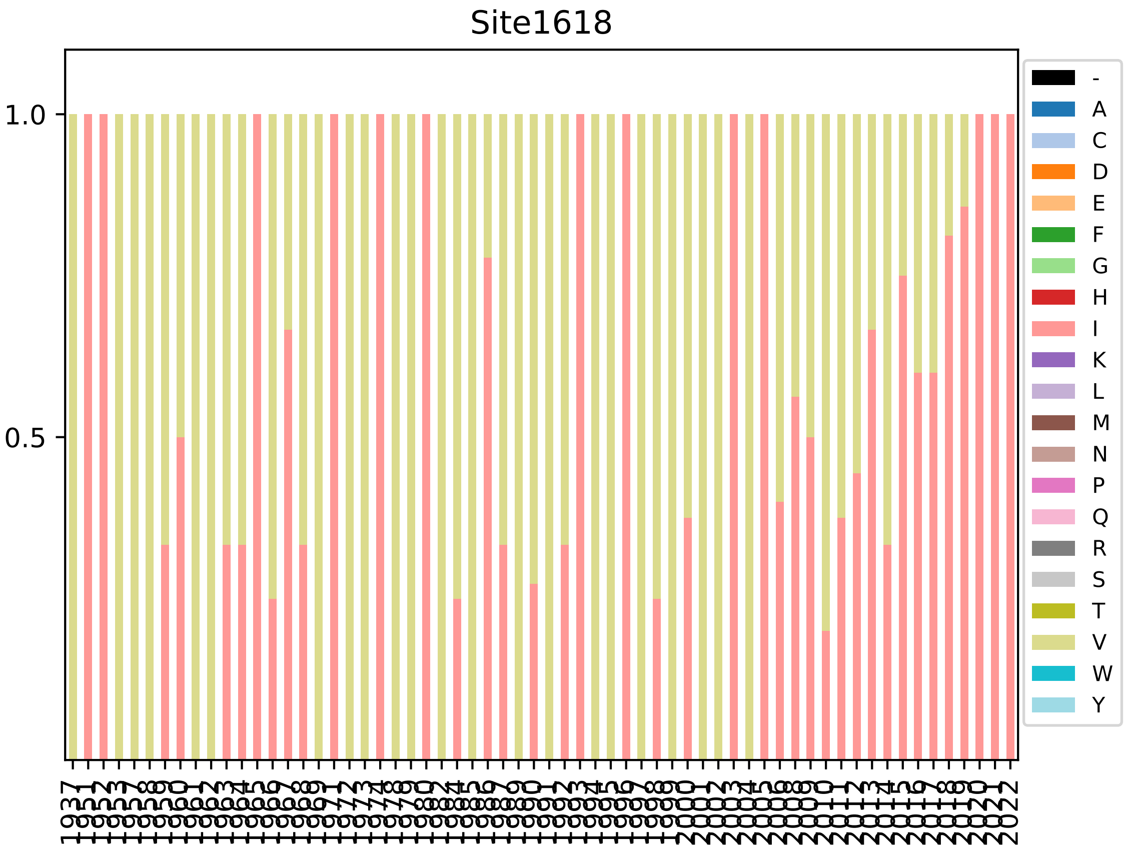
Site1618
1.0
0.5
-
A
C
D
E
F
G
H
I
K
L
M
N
P
Q
R
S
T
V
W
Y

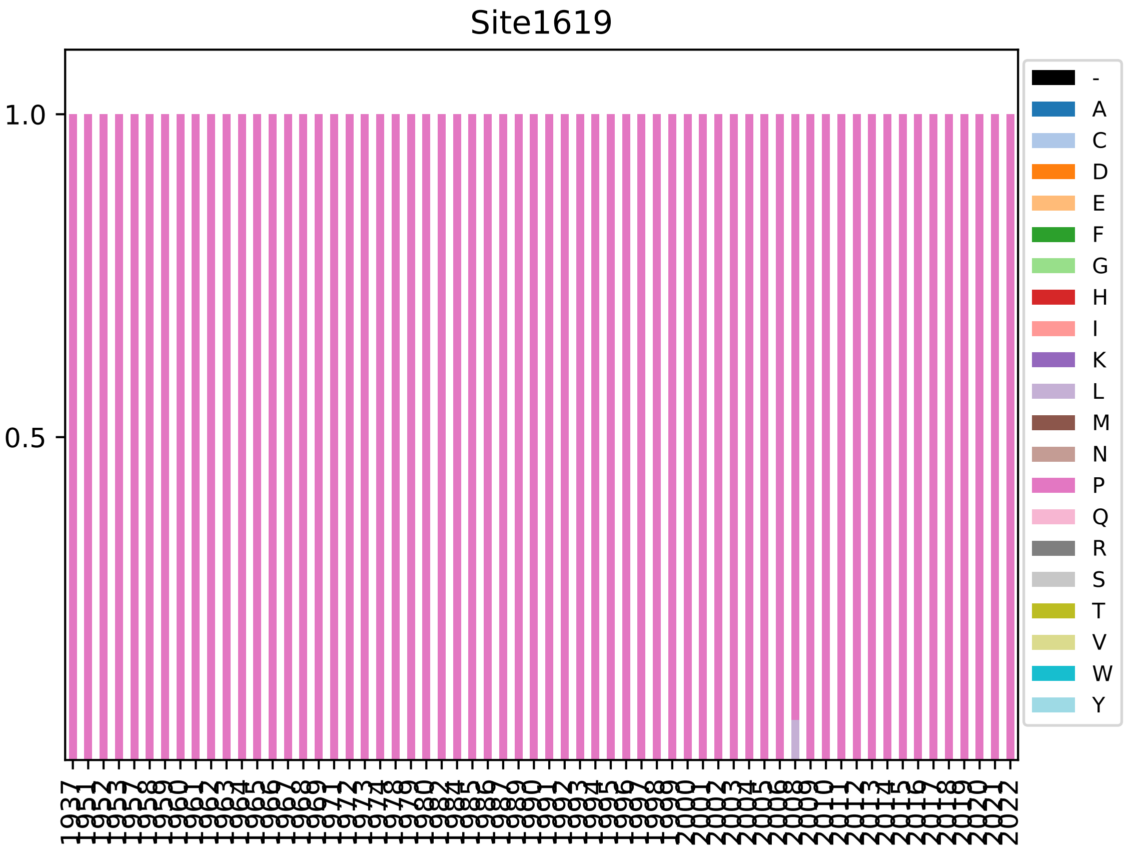

Site1619
1.0
0.5
-
A
C
D
E
F
G
H
I
K
L
M
N
P
Q
R
S
T
V
W
Y

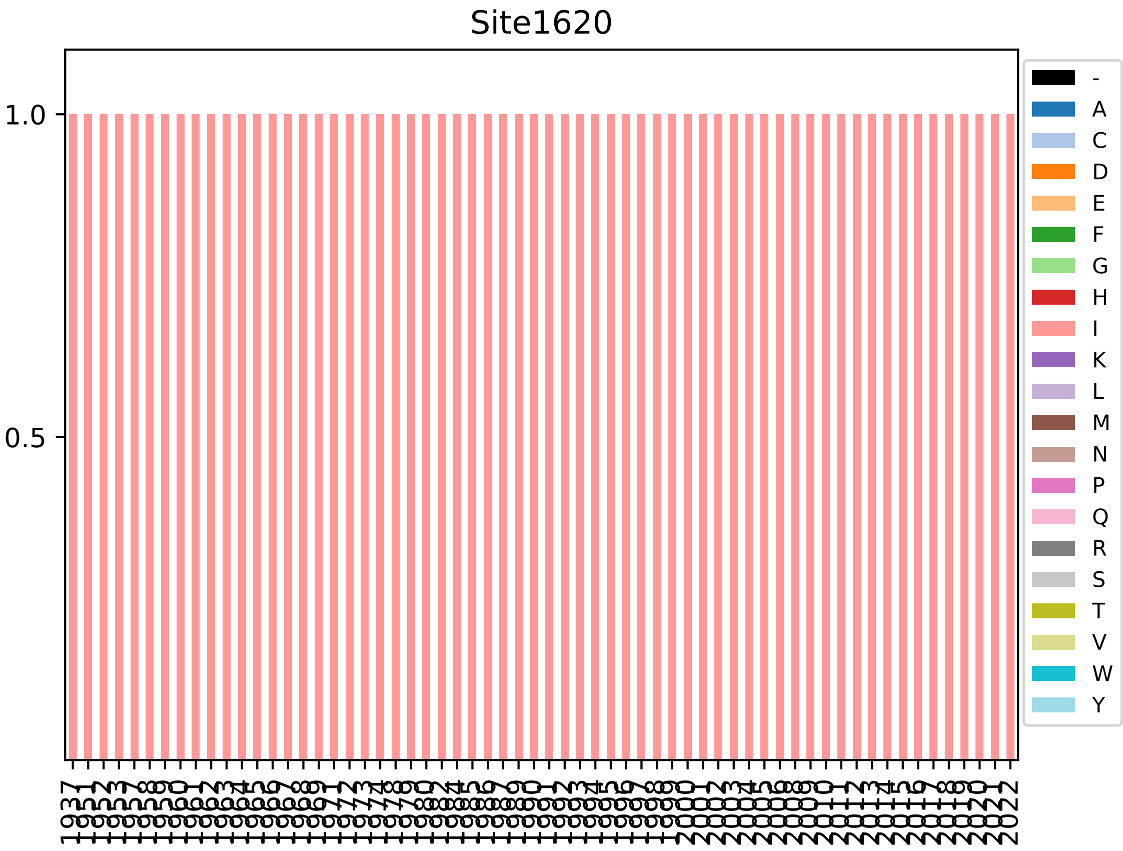
Site1620
1.0
0.5
-
A
C
D
E
F
G
H
I
K
L
M
N
P
Q
R
S
T
V
W
Y

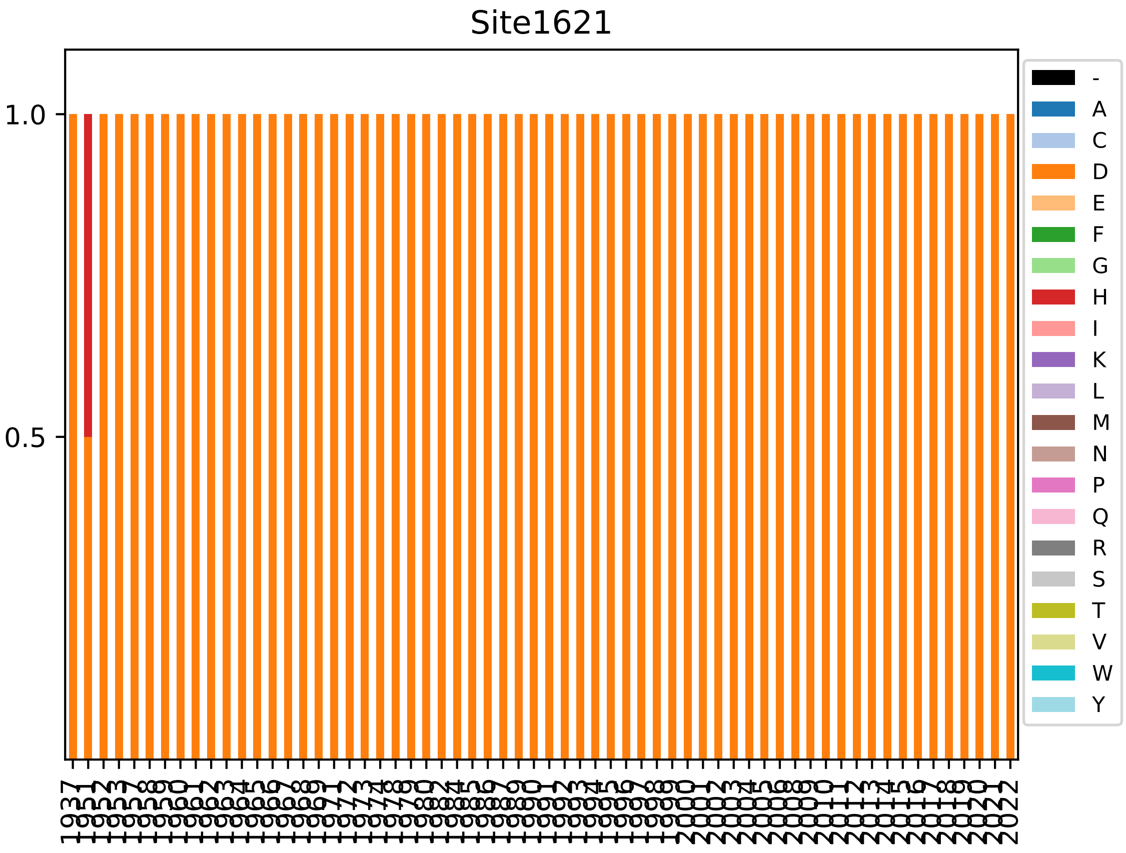
Site1621
1.0
0.5
-
A
C
D
E
F
G
H
I
K
L
M
N
P
Q
R
S
T
V
W
Y

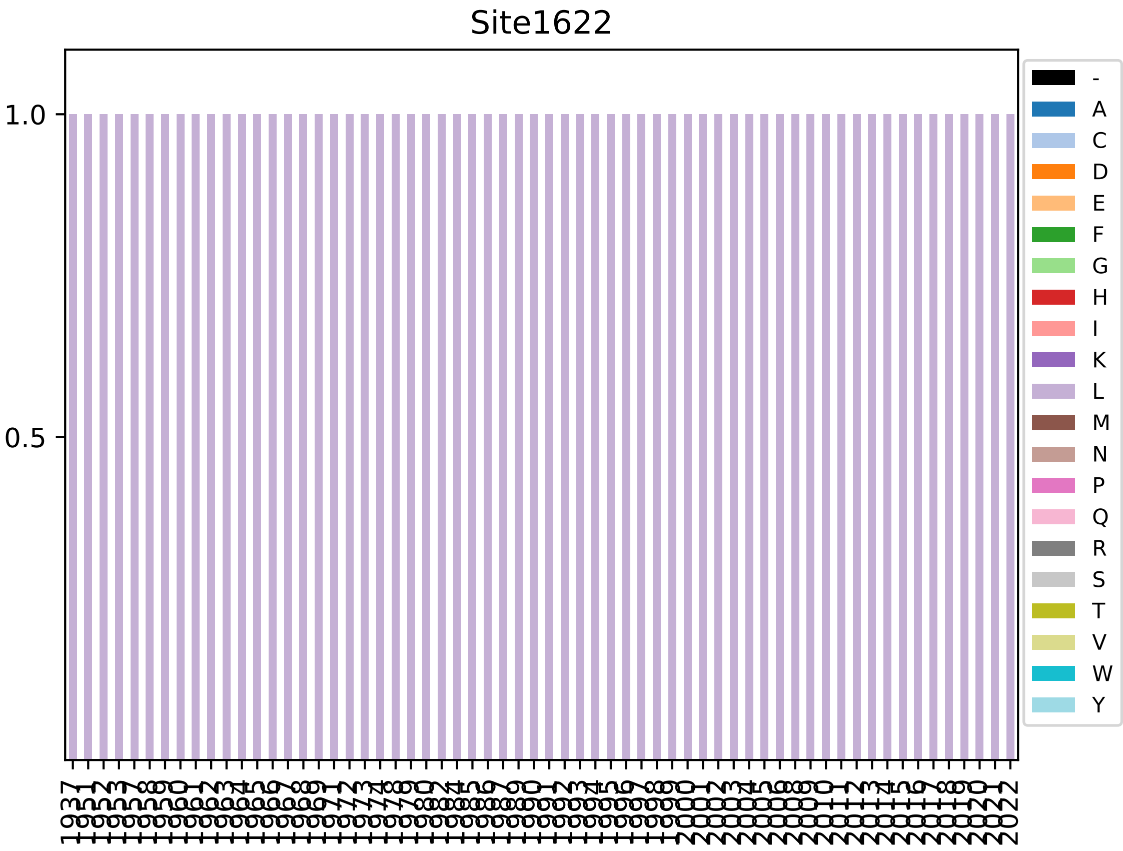
Site1622
1.0
0.5
-
A
C
D
E
F
G
H
I
K
L
M
N
P
Q
R
S
T
V
W
Y

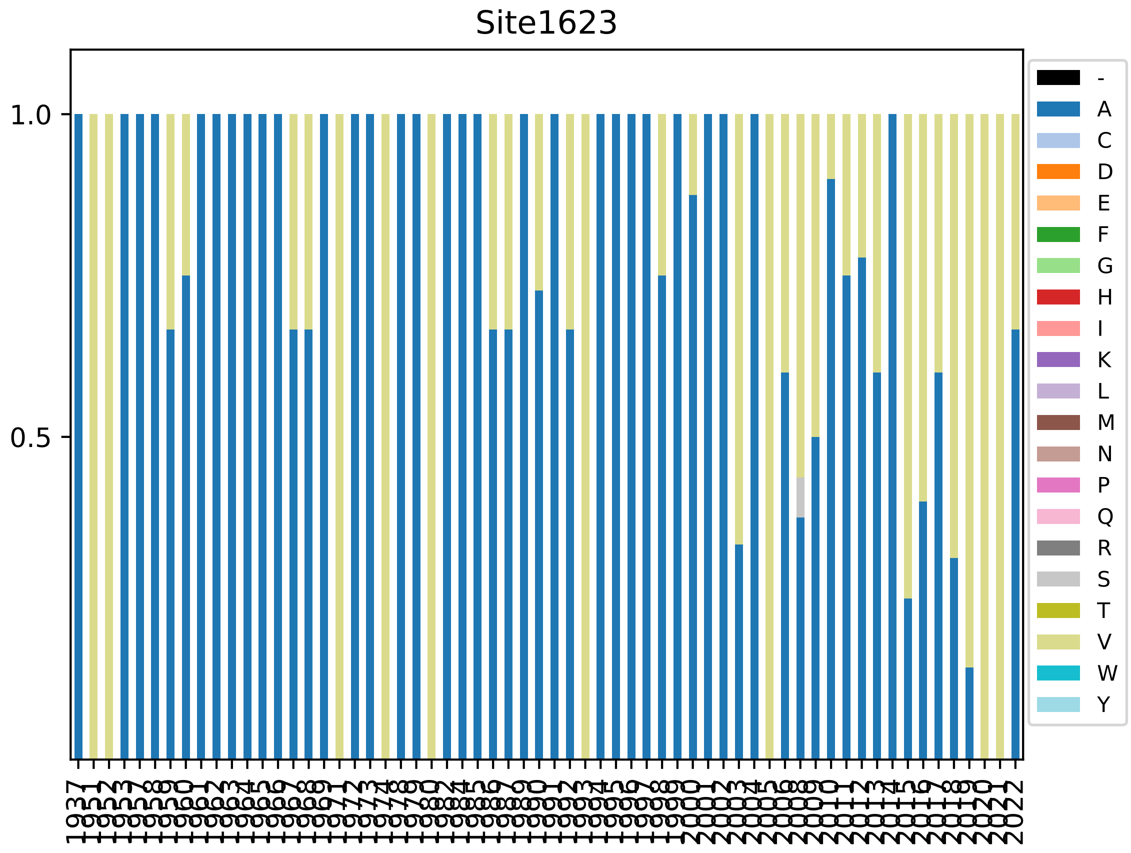
Site1623
1.0
0.5
-
A
C
D
E
F
G
H
I
K
L
M
N
P
Q
R
S
T
V
W
Y

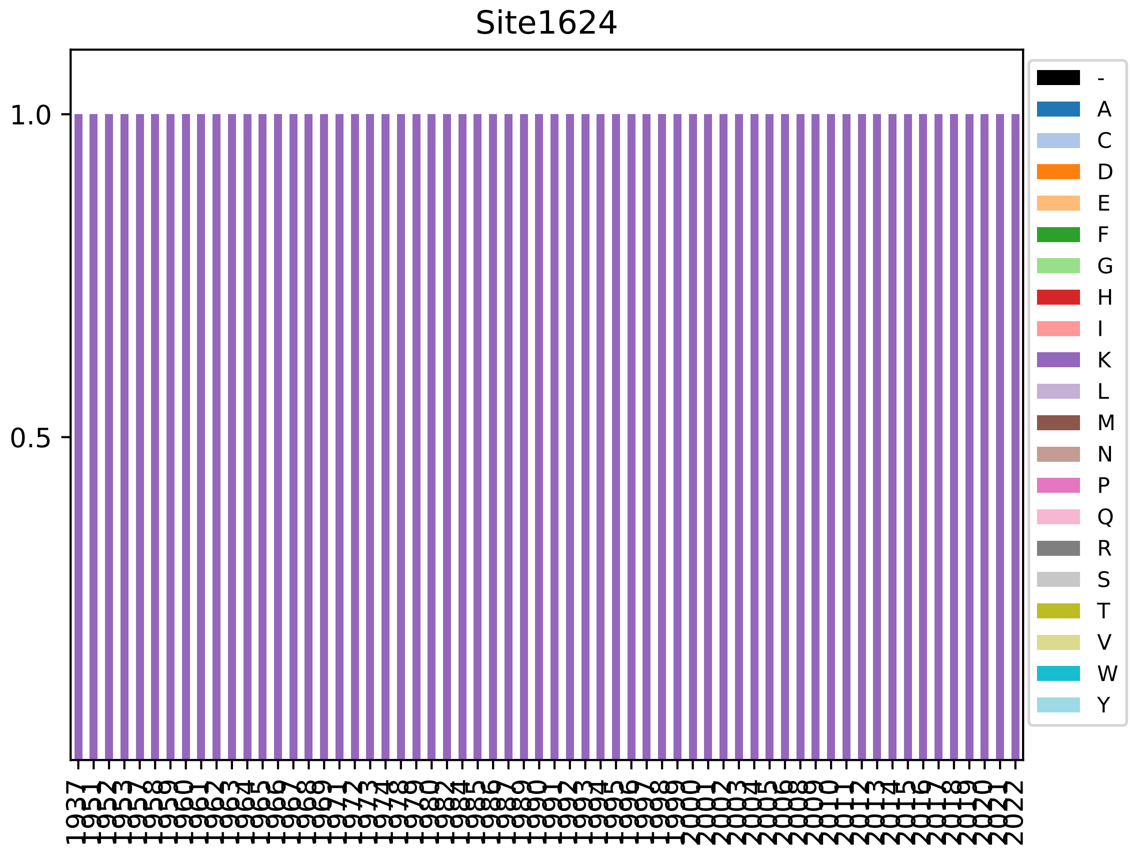

Site1624
1.0
0.5
-
A
C
D
E
F
G
H
I
K
L
M
N
P
Q
R
S
T
V
W
Y

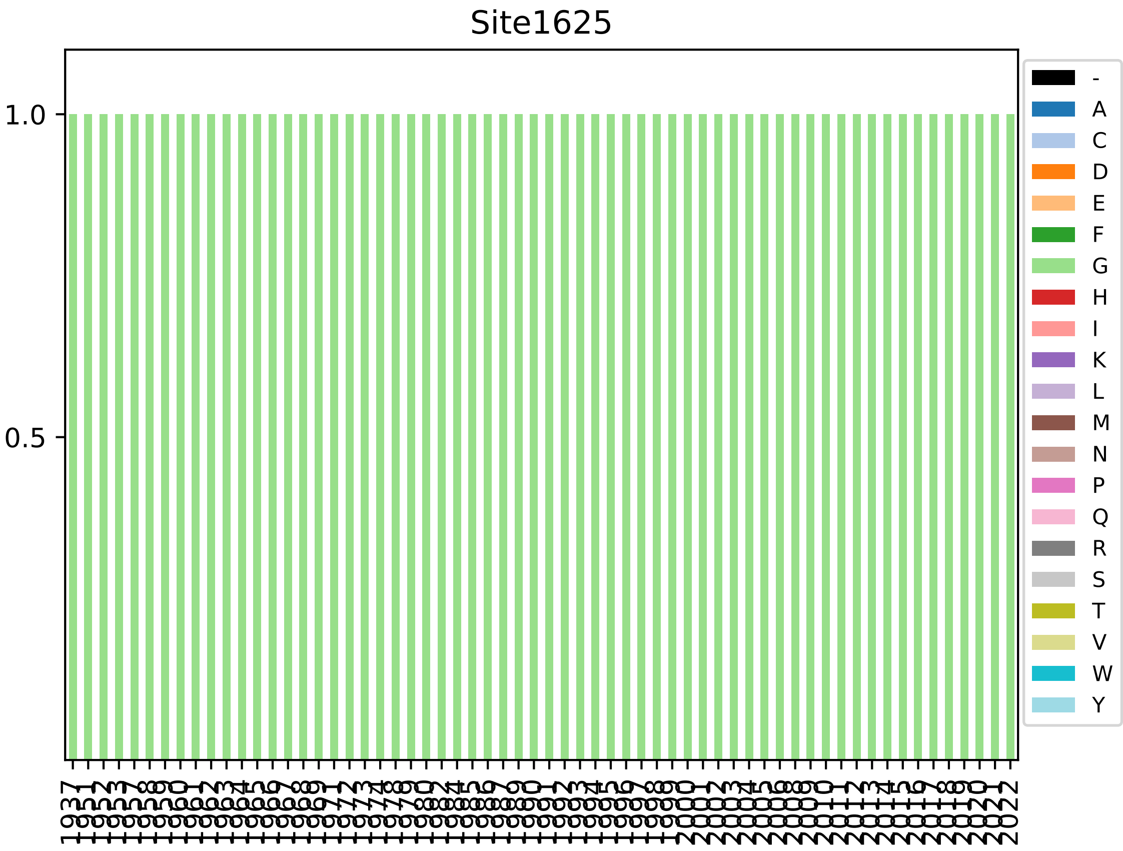
Site1625
1.0
0.5
-
A
C
D
E
F
G
H
I
K
L
M
N
P
Q
R
S
T
V
W
Y

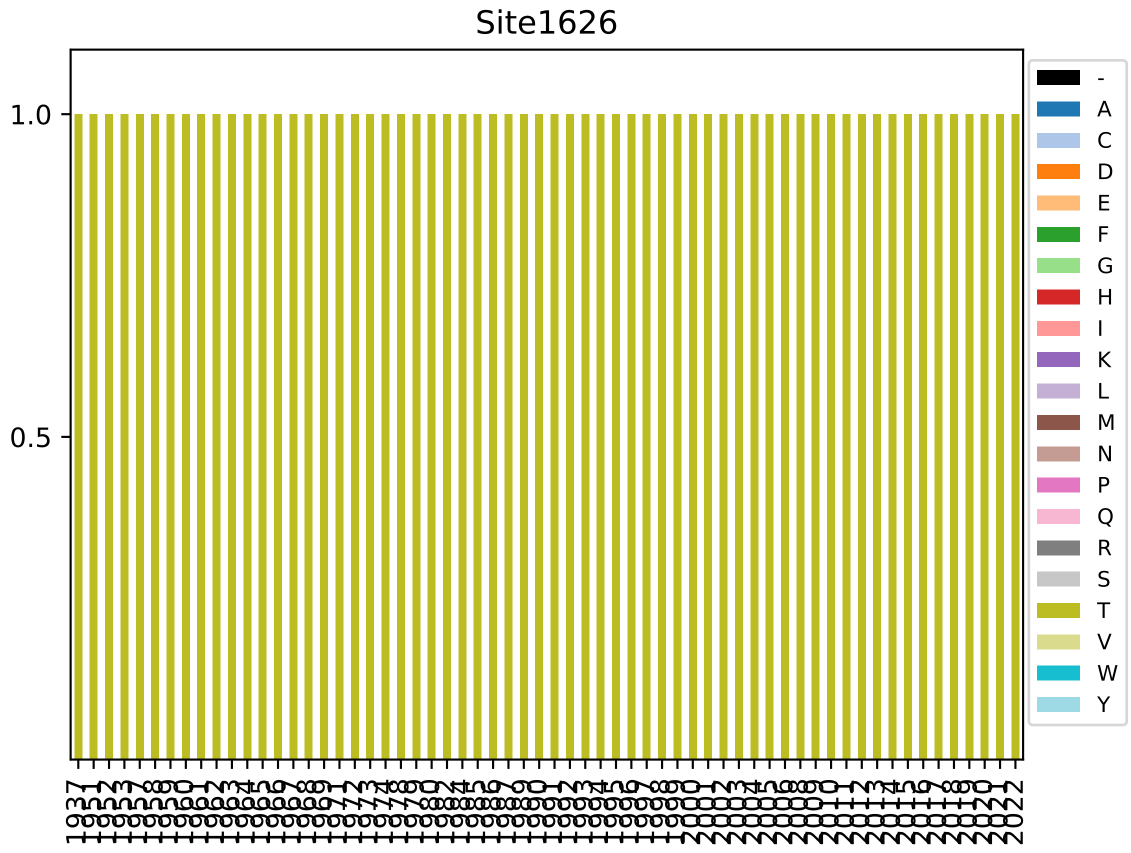
Site1626
1.0
0.5
-
A
C
D
E
F
G
H
I
K
L
M
N
P
Q
R
S
T
V
W
Y

Site1627

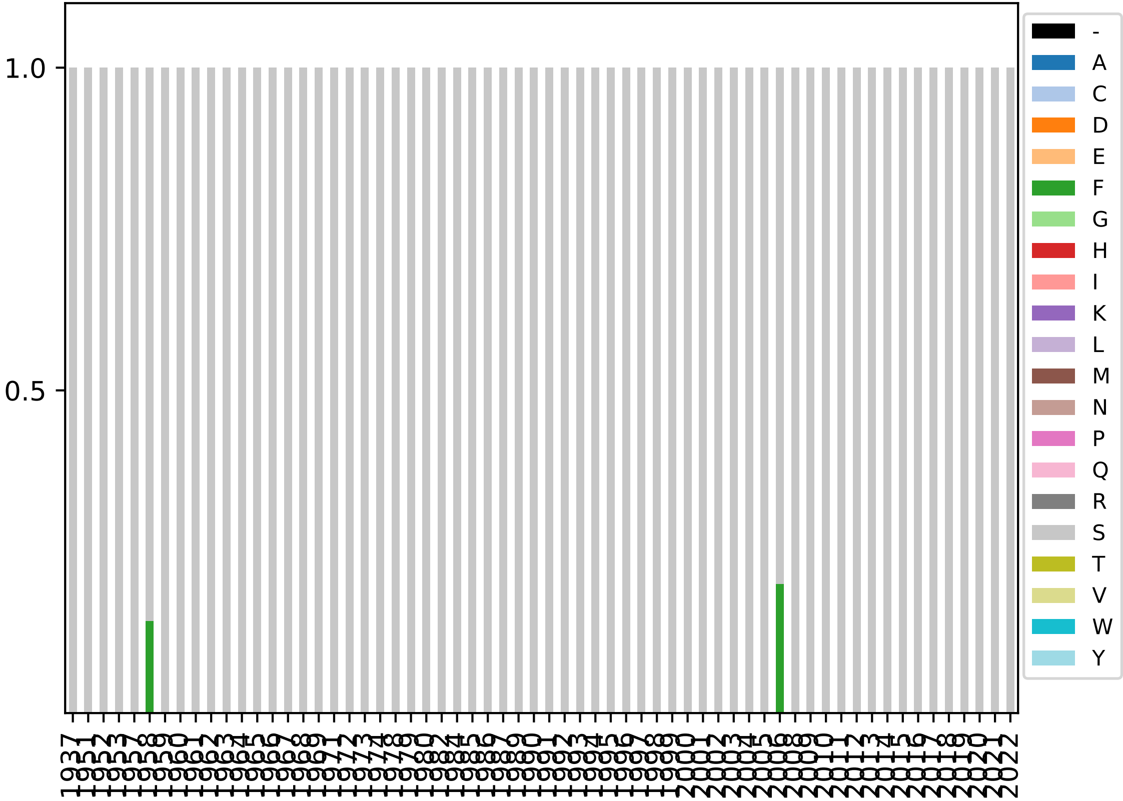

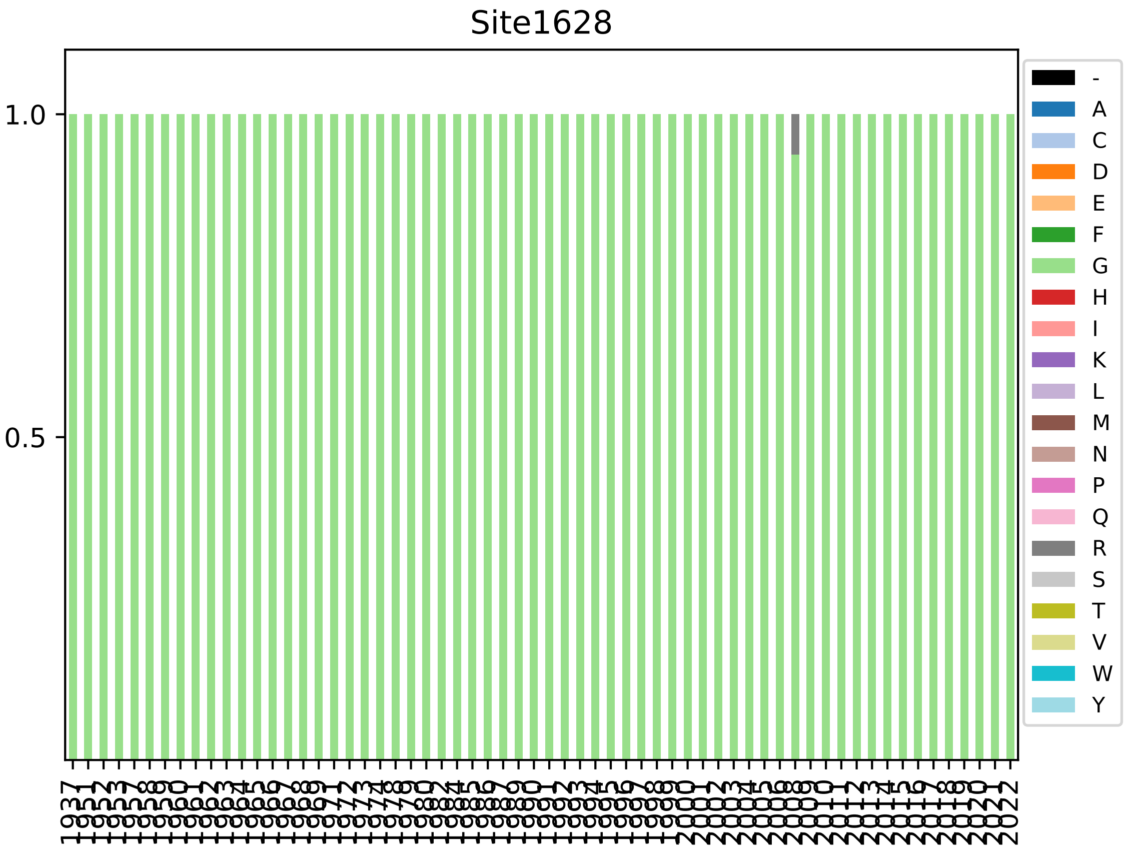
Site1628
1.0
0.5
-
A
C
D
E
F
G
H
I
K
L
M
N
P
Q
R
S
T
V
W
Y

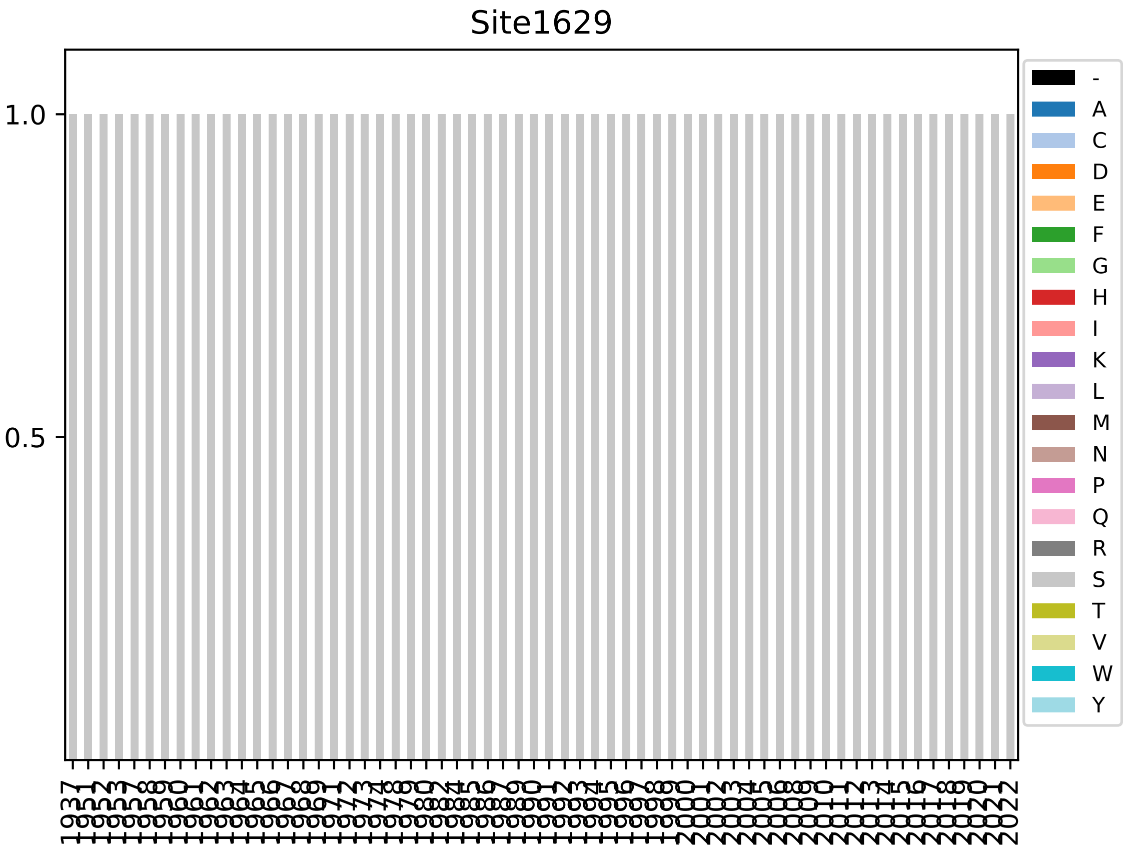
Site1629
1.0
0.5
-
A
C
D
E
F
G
H
I
K
L
M
N
P
Q
R
S
T
V
W
Y

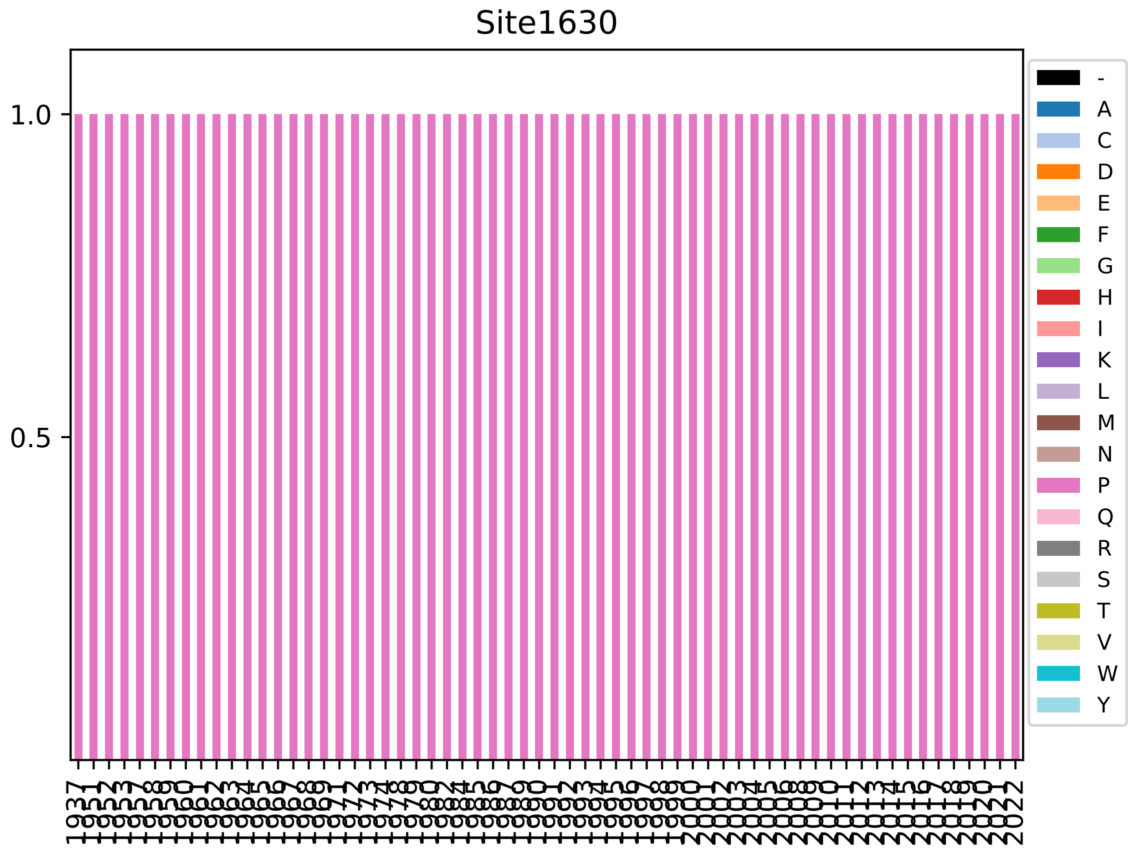
Site1630
1.0
0.5
-
A
C
D
E
F
G
H
I
K
L
M
N
P
Q
R
S
T
V
W
Y

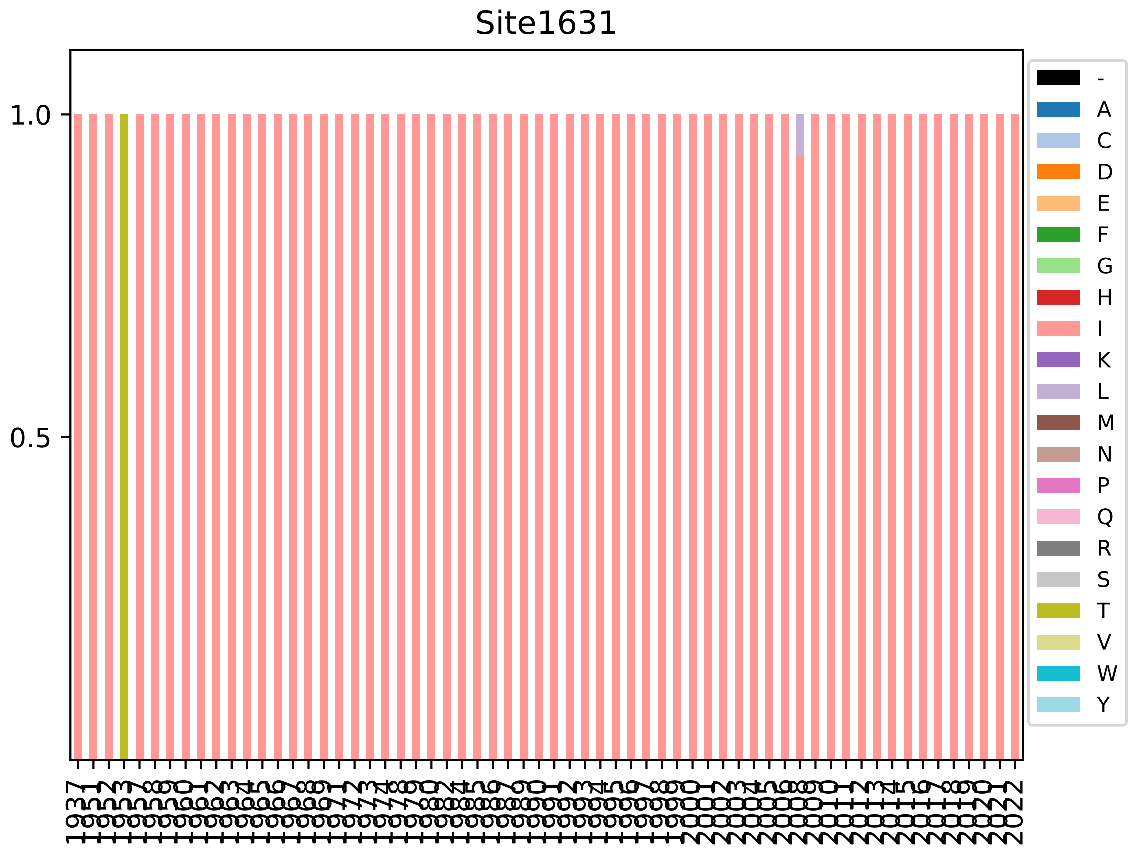
Site1631
1.0
0.5
-
A
C
D
E
F
G
H
I
K
L
M
N
P
Q
R
S
T
V
W
Y

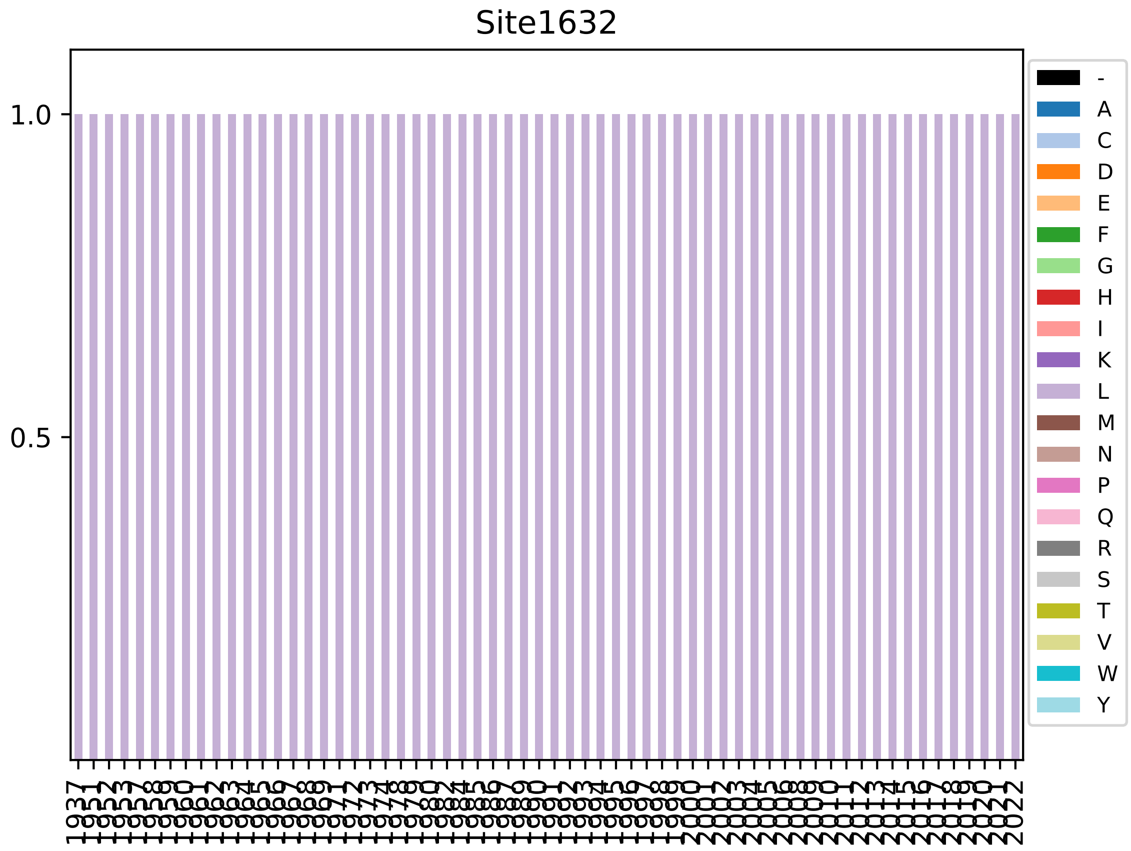
Site1632
1.0
0.5
-
A
C
D
E
F
G
H
I
K
L
M
N
P
Q
R
S
T
V
W
Y

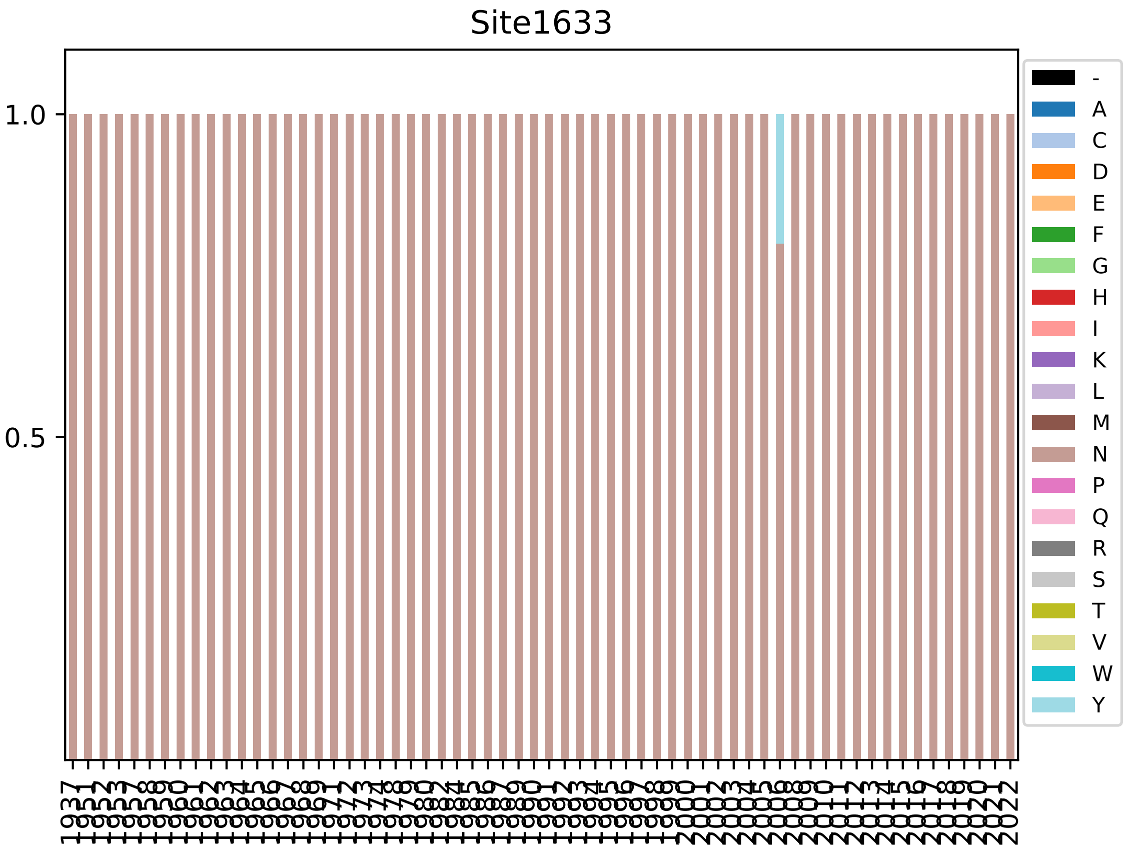
Site1633
1.0
0.5
-
A
C
D
E
F
G
H
I
K
L
M
N
P
Q
R
S
T
V
W
Y

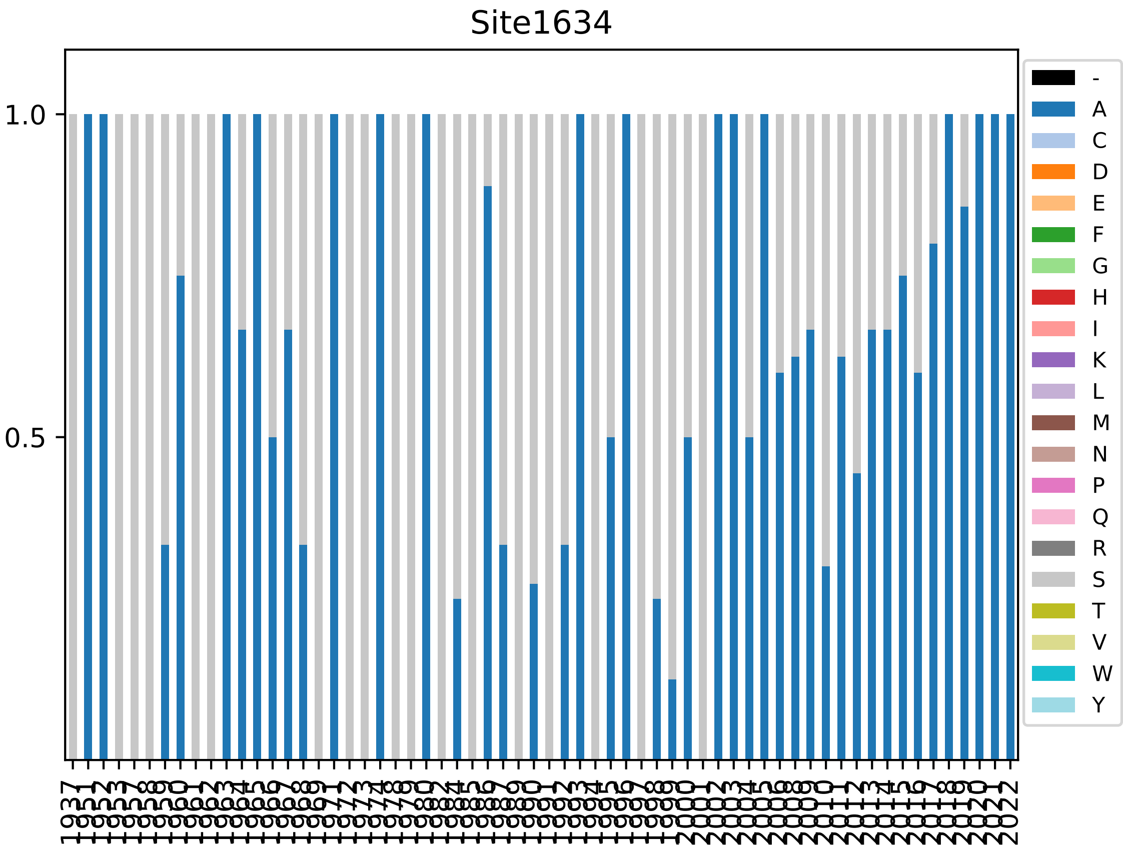
Site1634
1.0
0.5
-
A
C
D
E
F
G
H
I
K
L
M
N
P
Q
R
S
T
V
W
Y

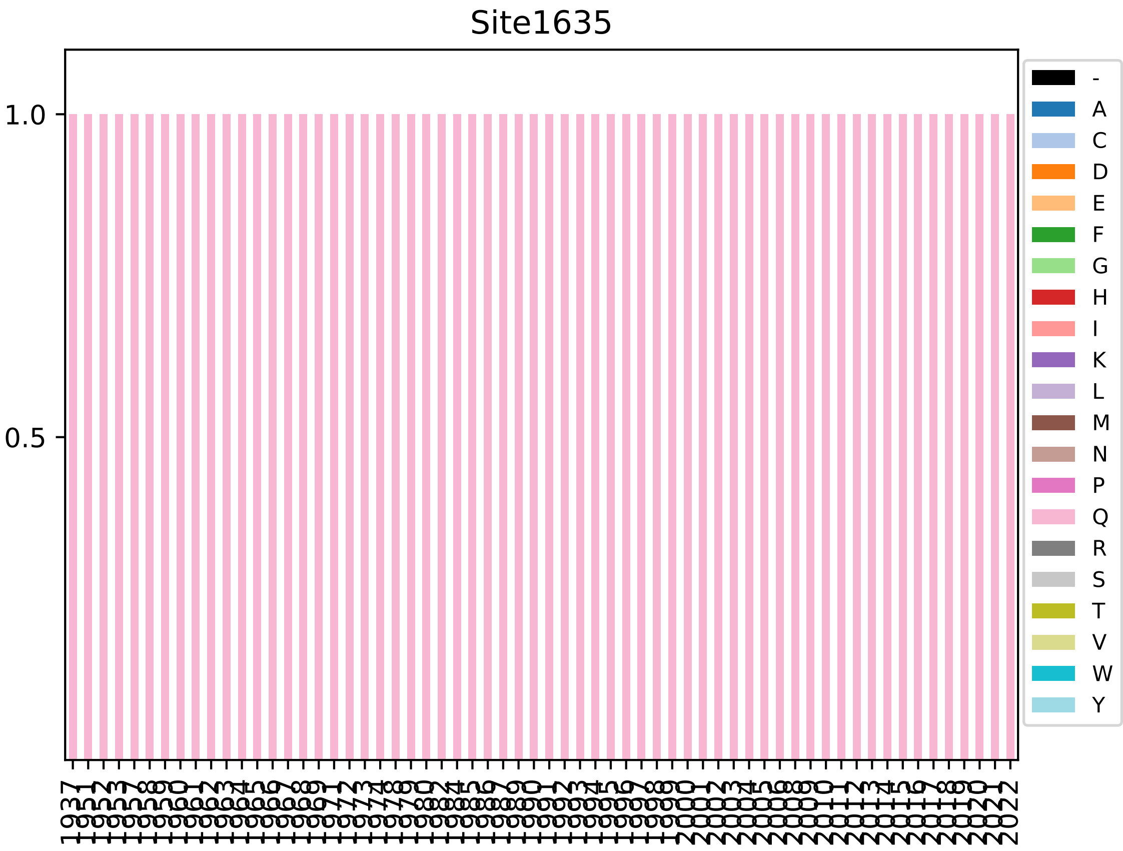
Site1635
1.0
0.5
-
A
C
D
E
F
G
H
I
K
L
M
N
P
Q
R
S
T
V
W
Y

Site1636

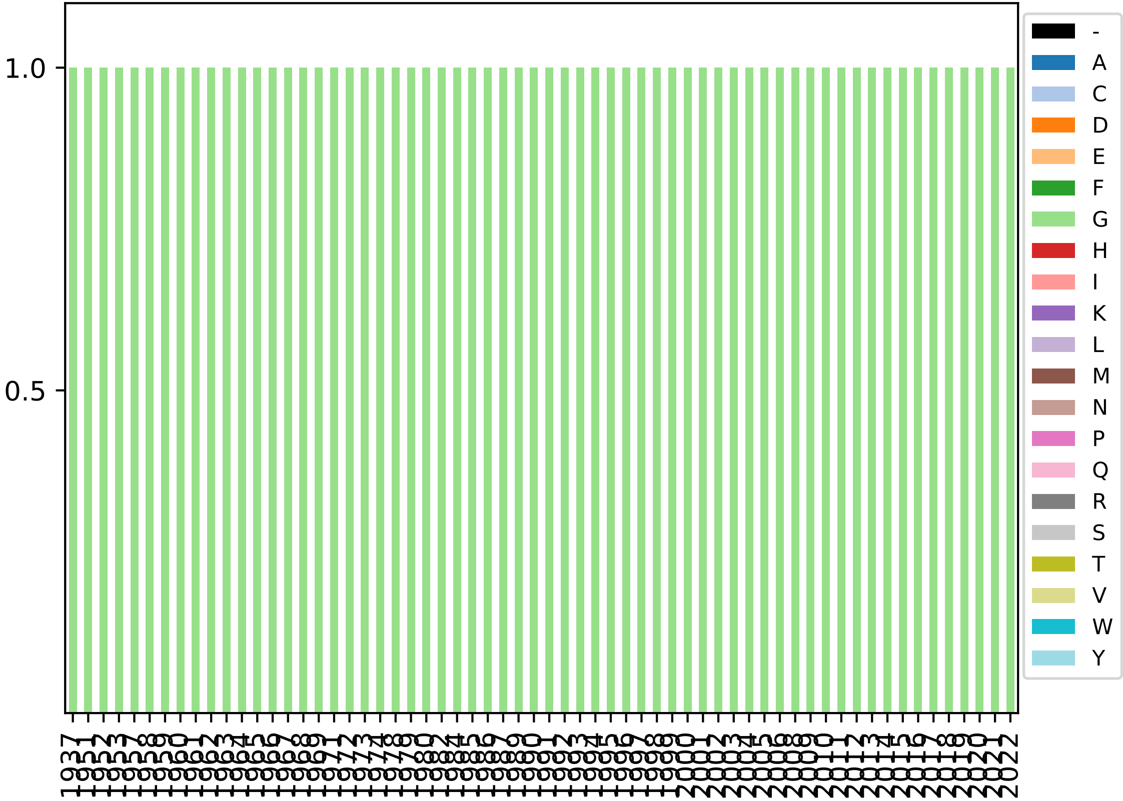

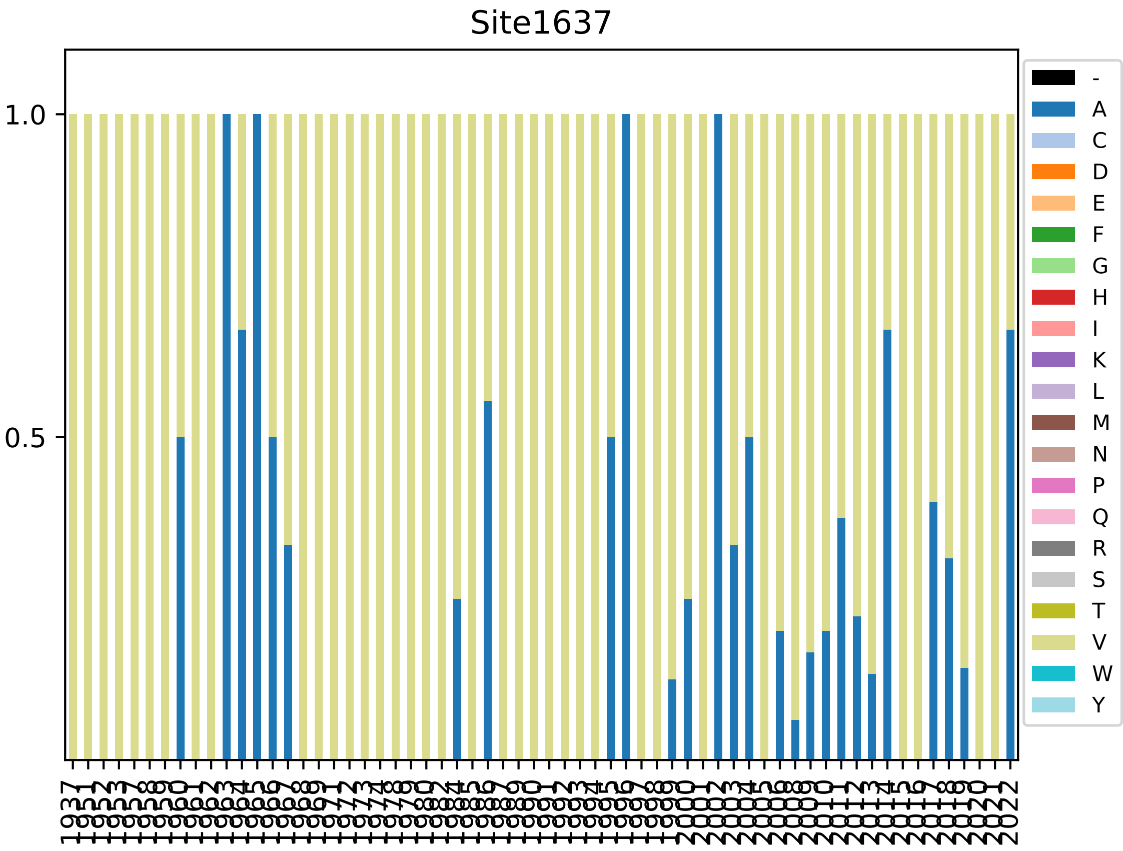
Site1637
1.0
0.5
-
A
C
D
E
F
G
H
I
K
L
M
N
P
Q
R
S
T
V
W
Y

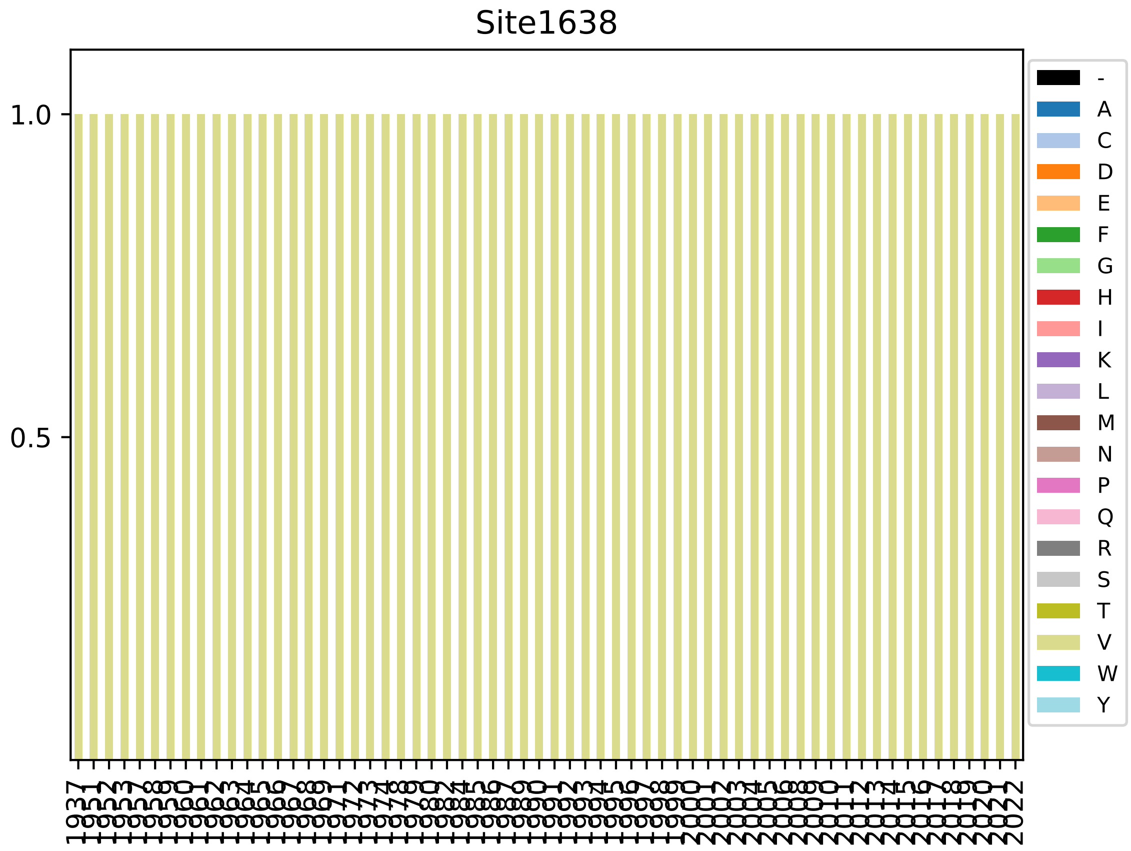
Site1638
1.0
0.5
-
A
C
D
E
F
G
H
I
K
L
M
N
P
Q
R
S
T
V
W
Y

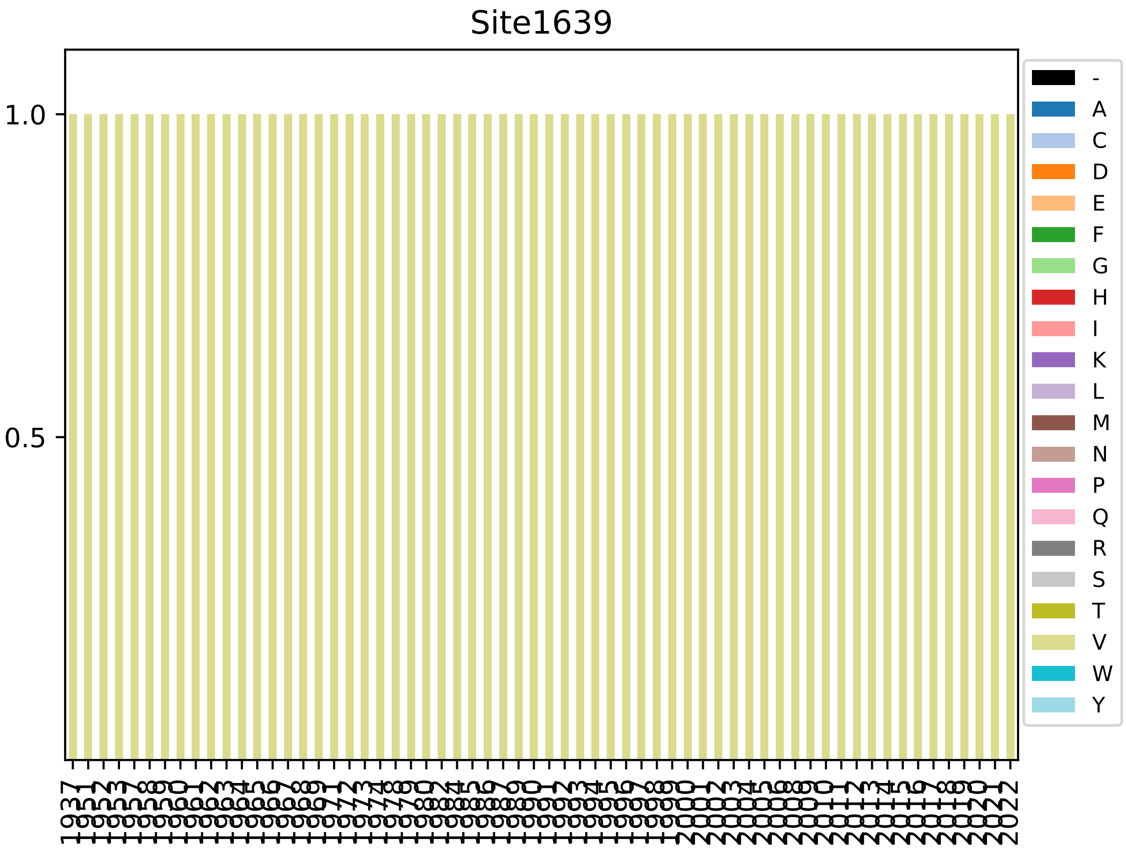
Site1639
1.0
0.5
-
A
C
D
E
F
G
H
I
K
L
M
N
P
Q
R
S
T
V
W
Y

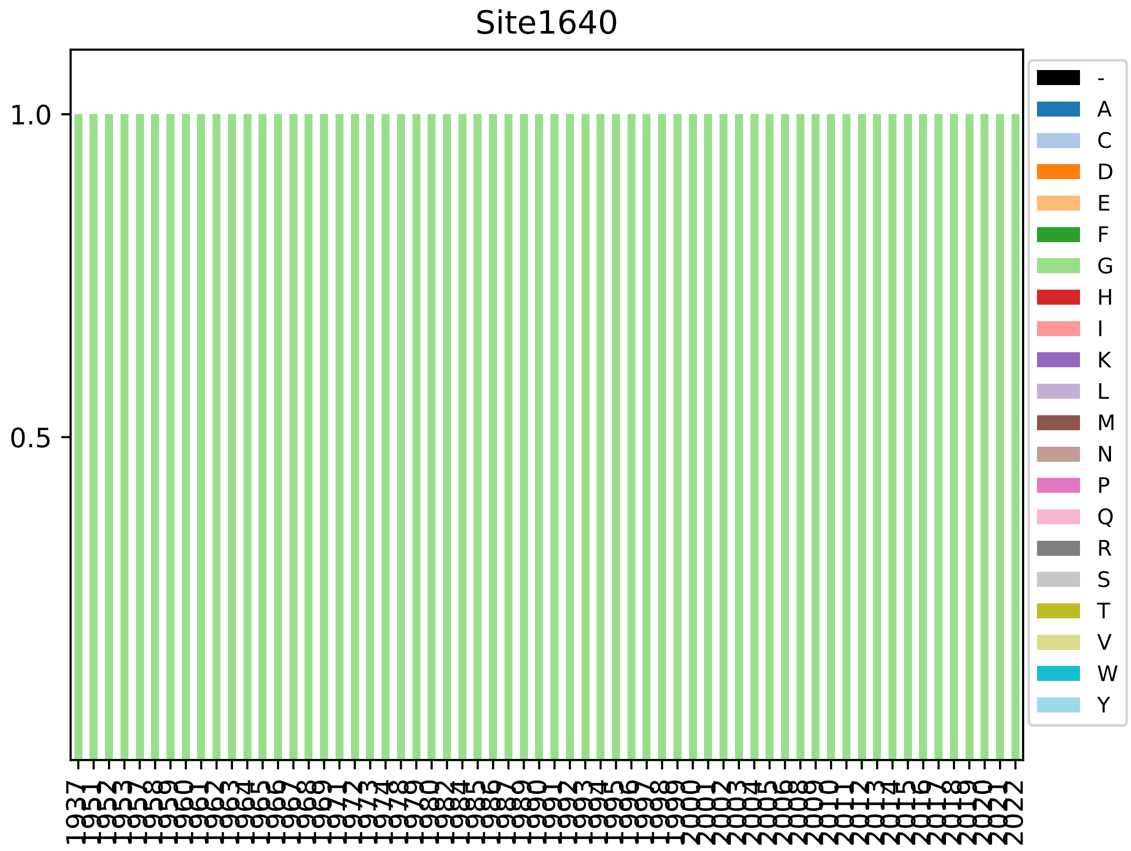
Site1640
1.0
0.5
-
A
C
D
E
F
G
H
I
K
L
M
N
P
Q
R
S
T
V
W
Y

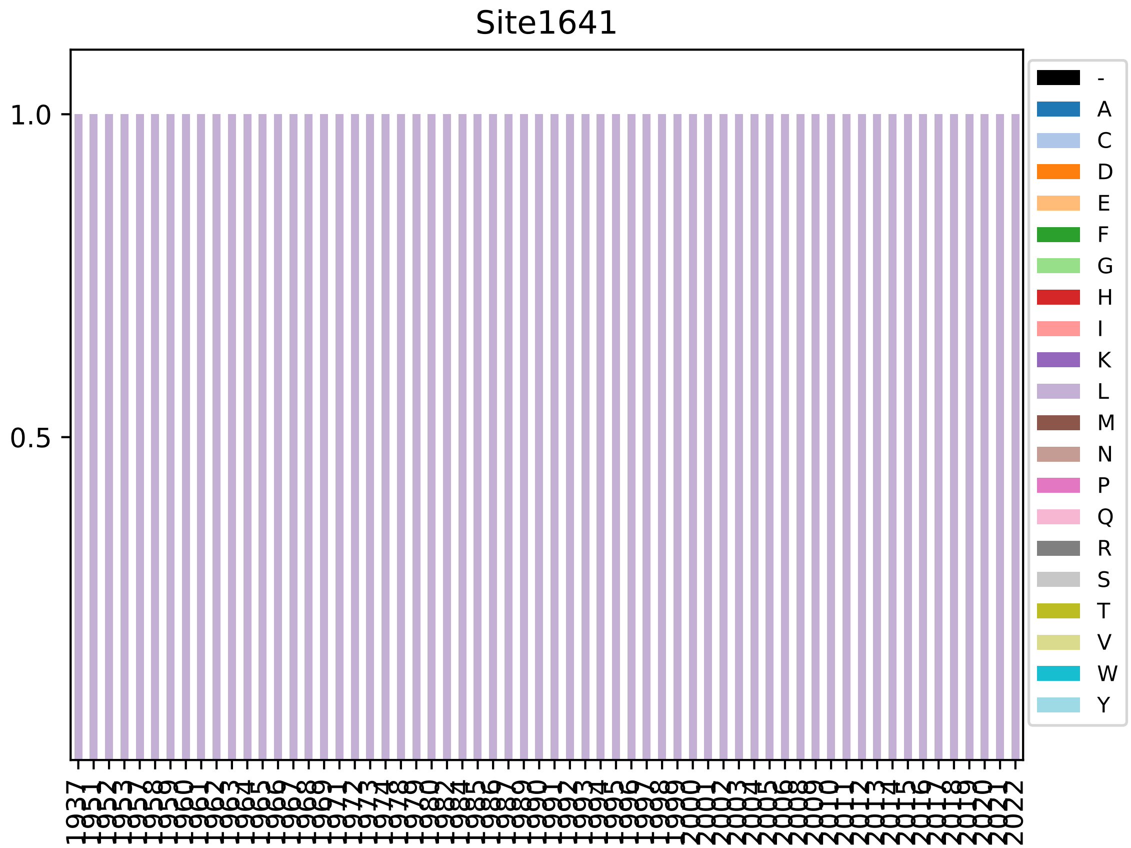
Site1641
1.0
0.5
-
A
C
D
E
F
G
H
I
K
L
M
N
P
Q
R
S
T
V
W
Y

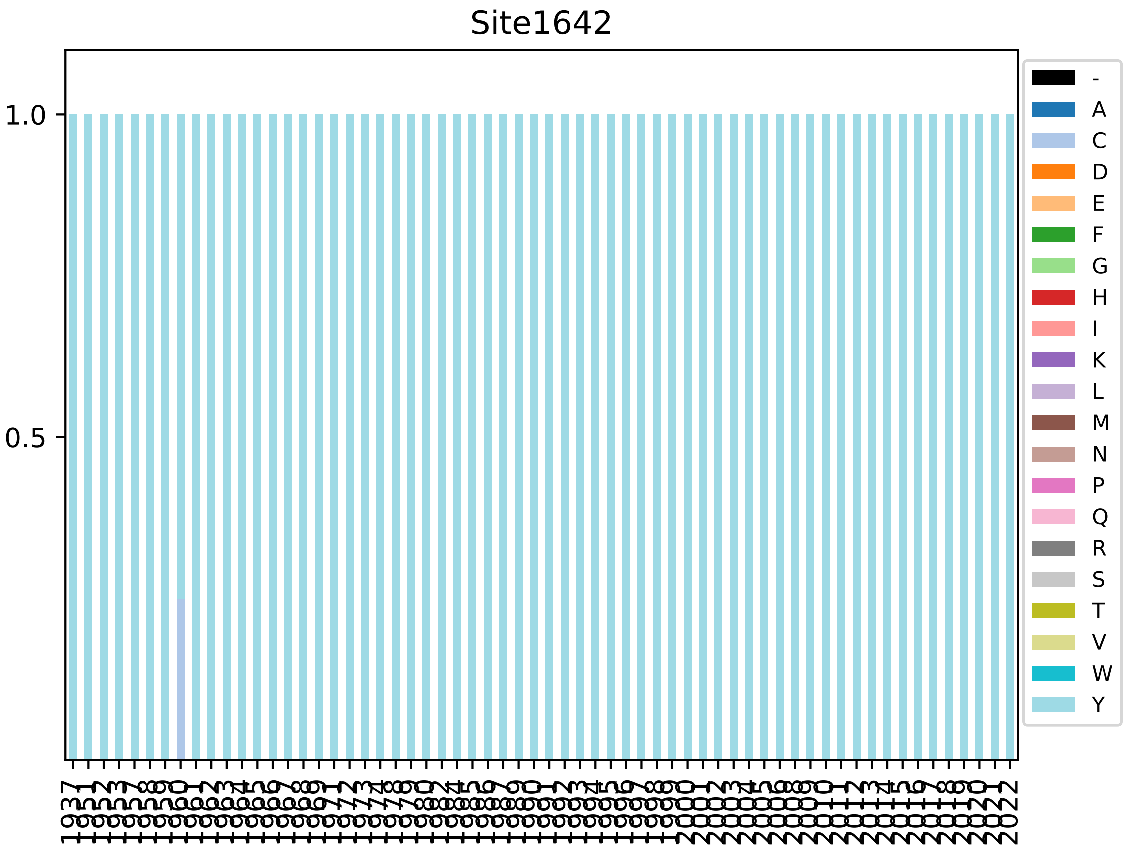

Site1642
1.0
0.5
-
A
C
D
E
F
G
H
I
K
L
M
N
P
Q
R
S
T
V
W
Y

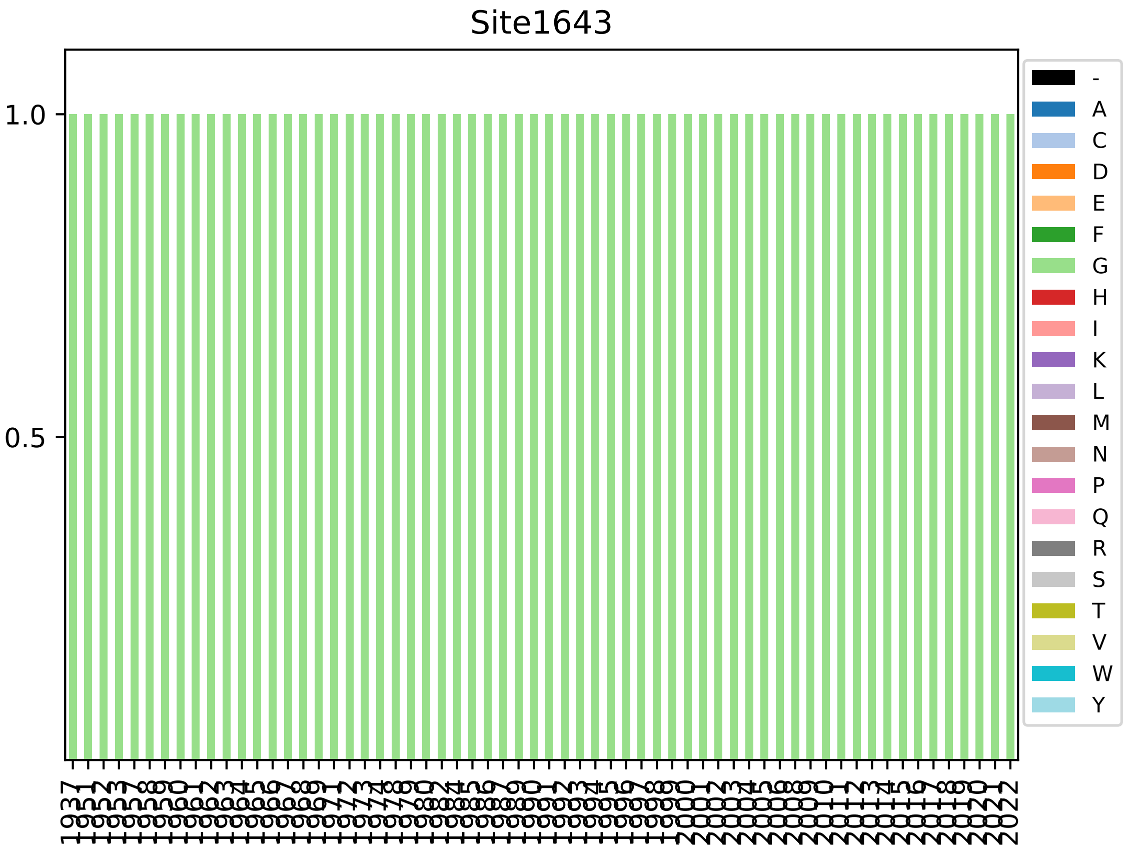
Site1643
1.0
0.5
-
A
C
D
E
F
G
H
I
K
L
M
N
P
Q
R
S
T
V
W
Y

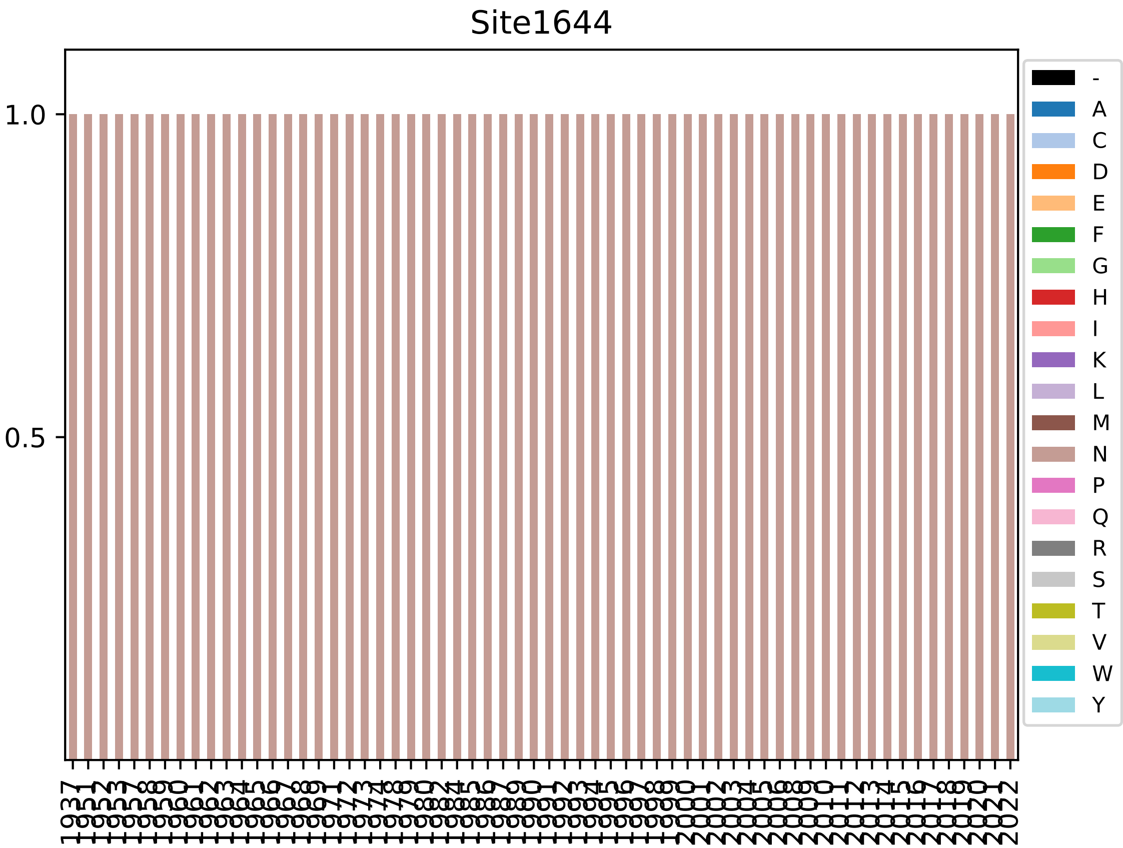
Site1644
1.0
0.5
-
A
C
D
E
F
G
H
I
K
L
M
N
P
Q
R
S
T
V
W
Y

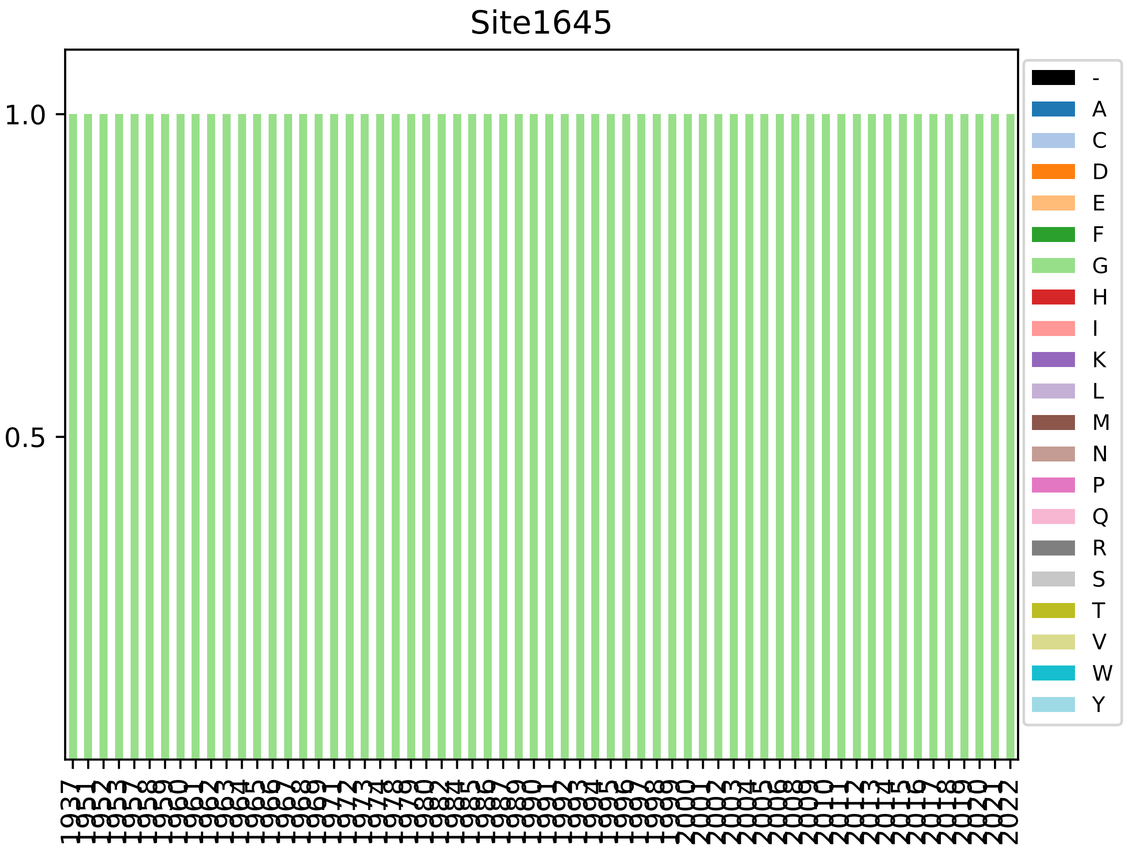
Site1645
1.0
0.5
-
A
C
D
E
F
G
H
I
K
L
M
N
P
Q
R
S
T
V
W
Y

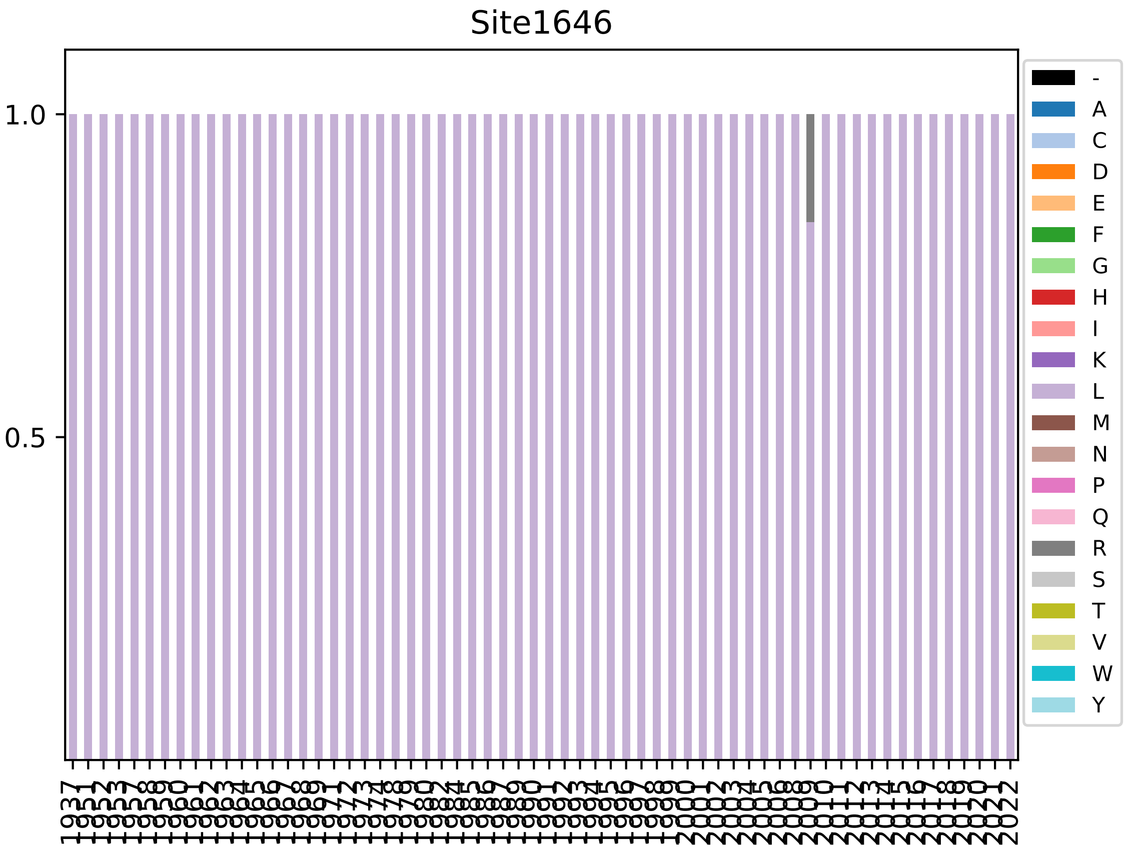
Site1646
1.0
0.5
-
A
C
D
E
F
G
H
I
K
L
M
N
P
Q
R
S
T
V
W
Y

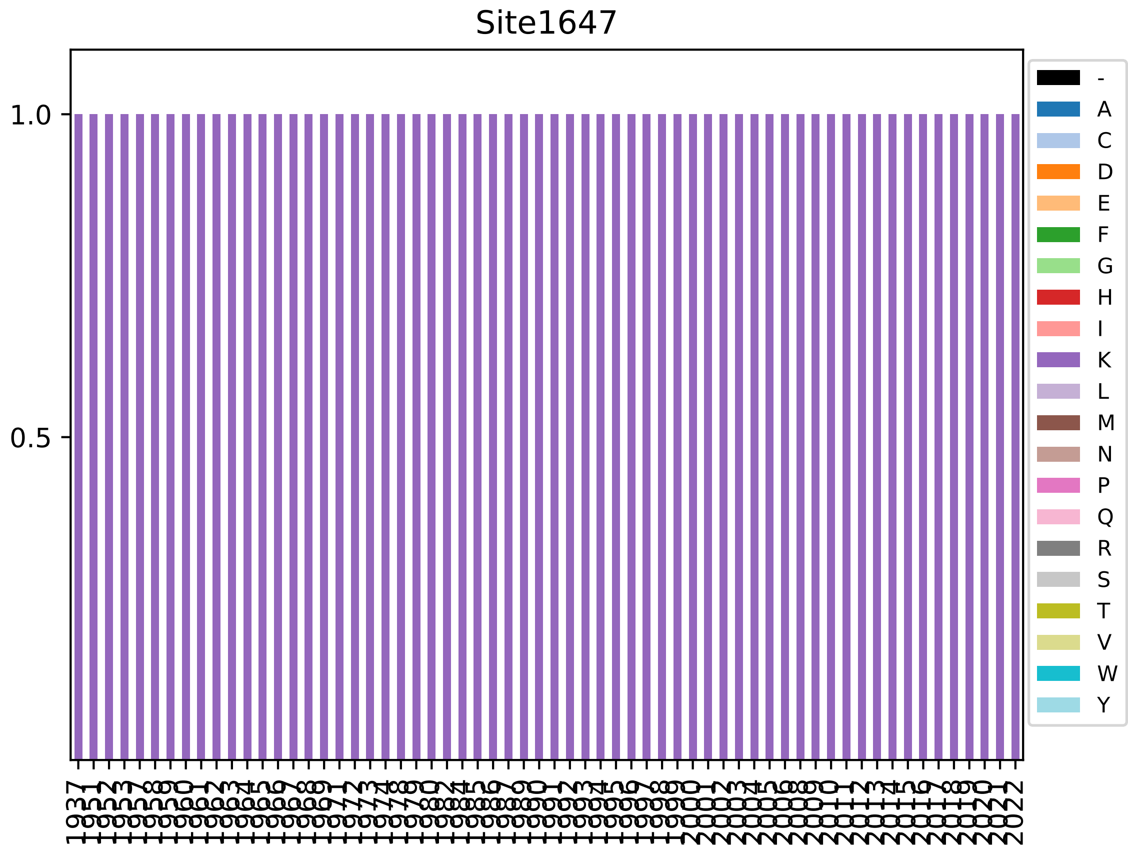

Site1647
1.0
0.5
-
A
C
D
E
F
G
H
I
K
L
M
N
P
Q
R
S
T
V
W
Y

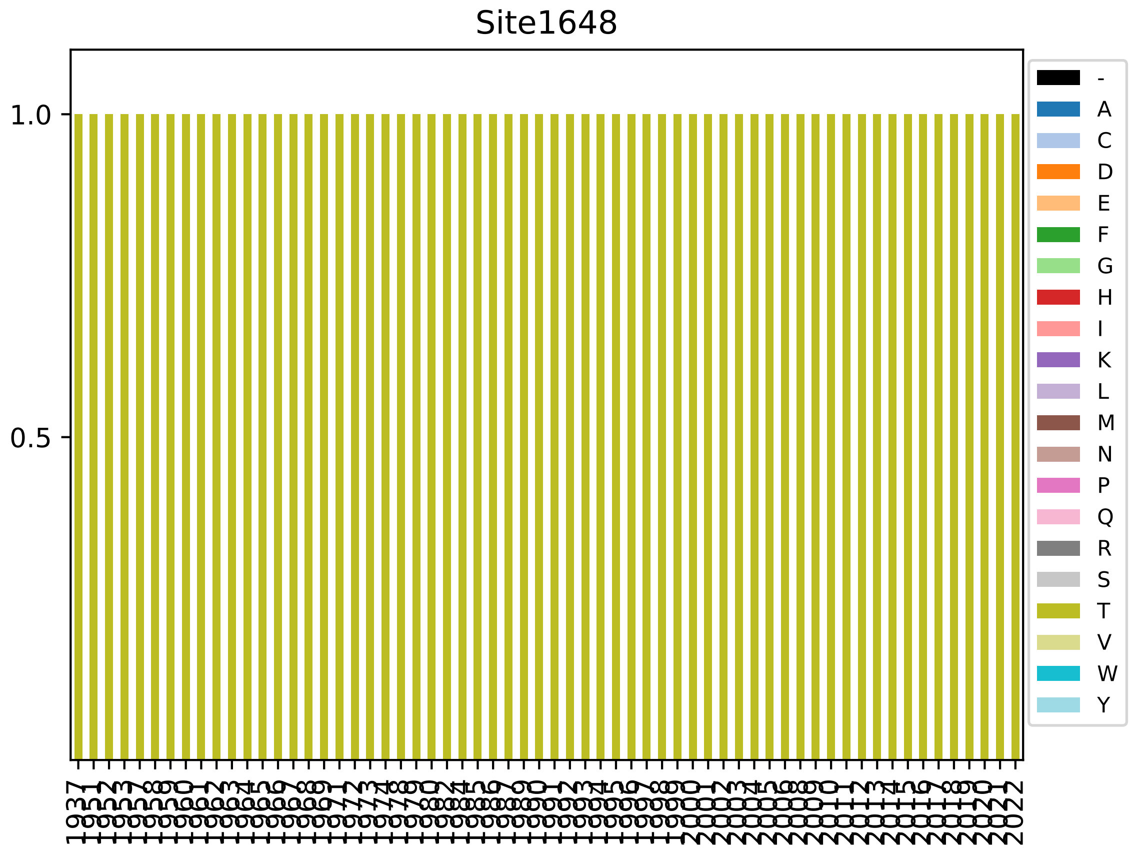
Site1648
1.0
0.5
-
A
C
D
E
F
G
H
I
K
L
M
N
P
Q
R
S
T
V
W
Y

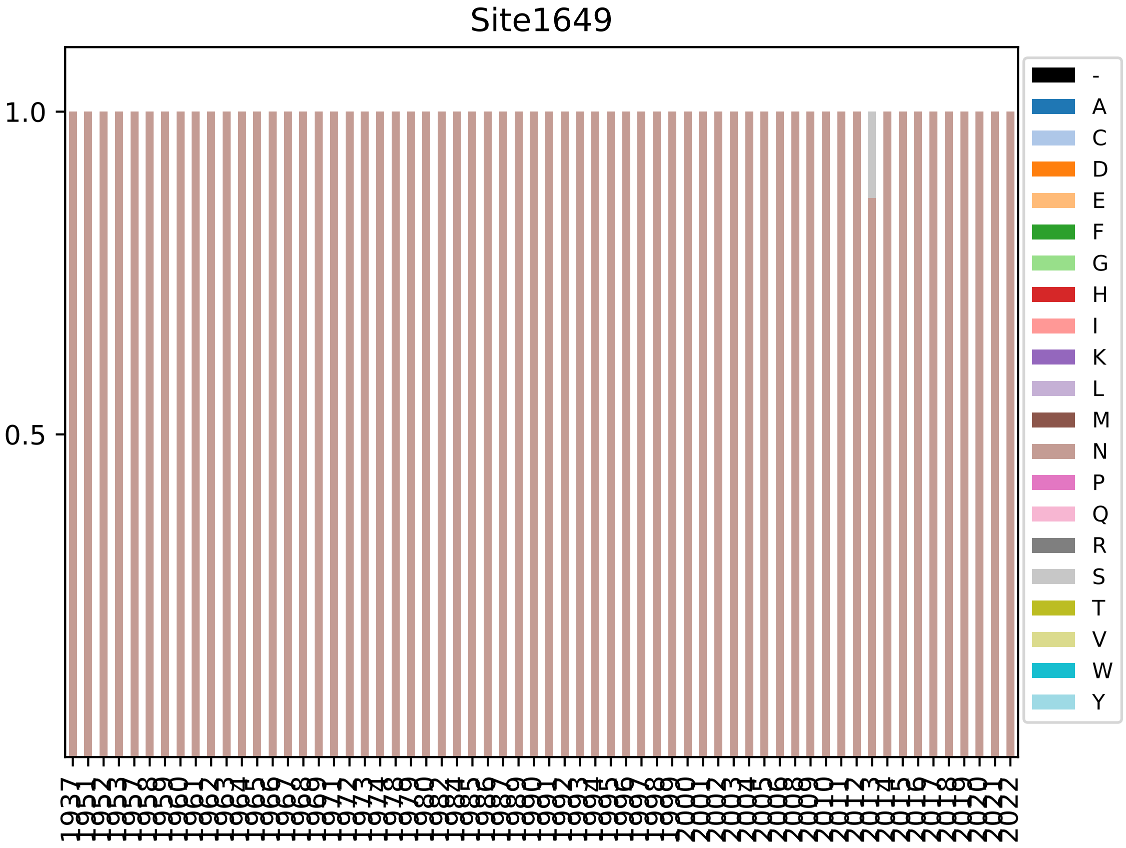
Site1649
1.0
0.5
-
A
C
D
E
F
G
H
I
K
L
M
N
P
Q
R
S
T
V
W
Y

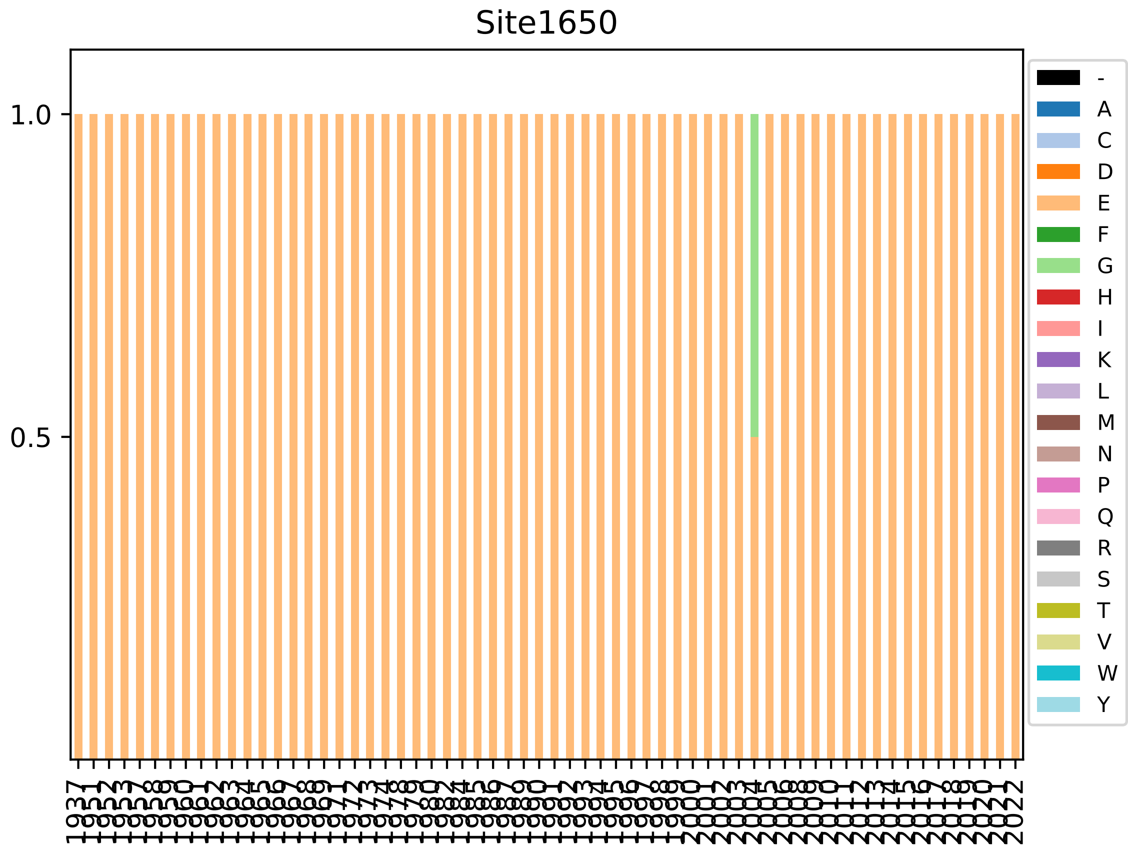
Site1650
1.0
0.5
-
A
C
D
E
F
G
H
I
K
L
M
N
P
Q
R
S
T
V
W
Y

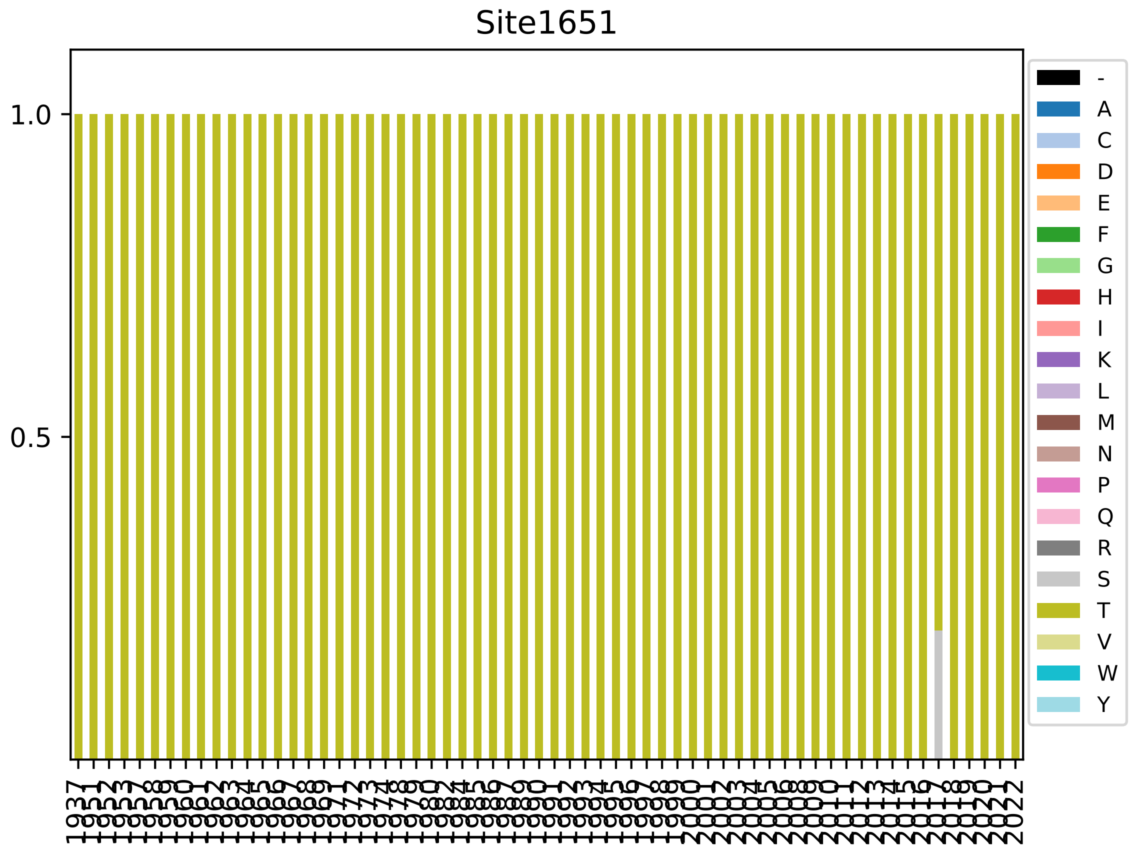
Site1651
1.0
0.5
-
A
C
D
E
F
G
H
I
K
L
M
N
P
Q
R
S
T
V
W
Y

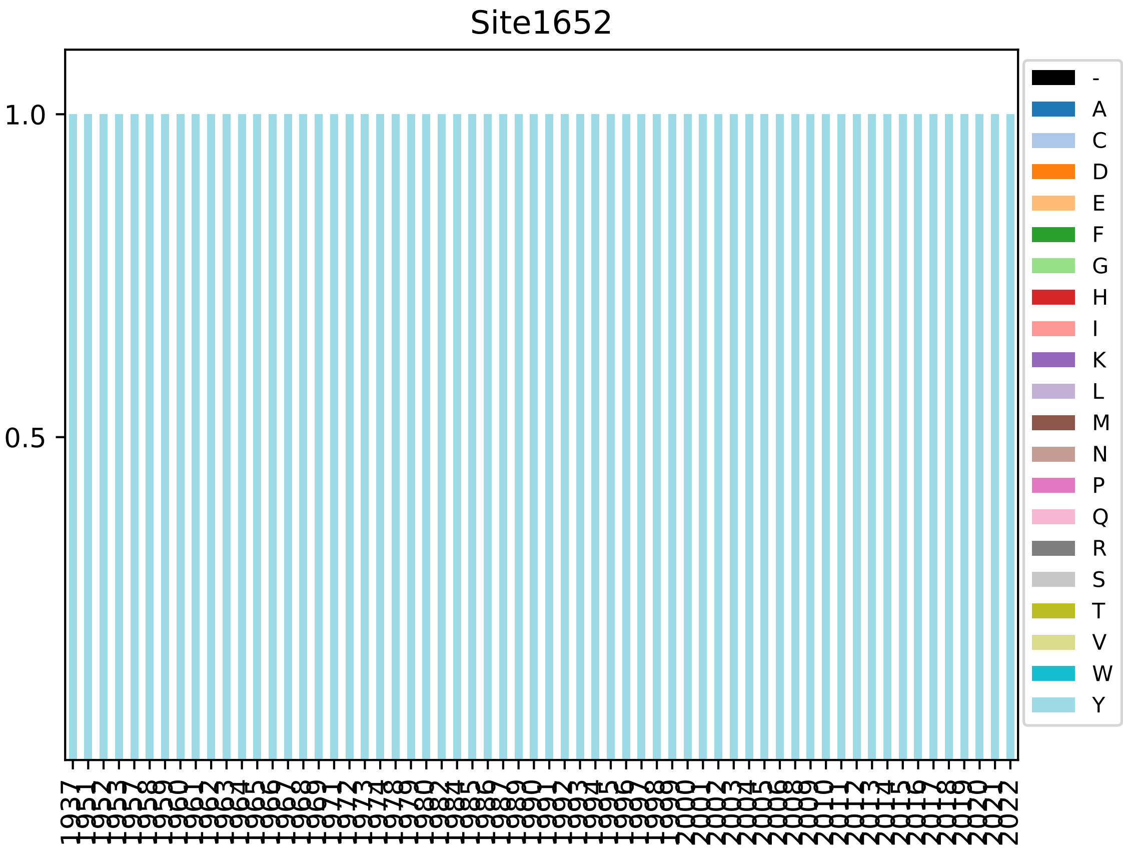
Site1652
1.0
0.5
-
A
C
D
E
F
G
H
I
K
L
M
N
P
Q
R
S
T
V
W
Y

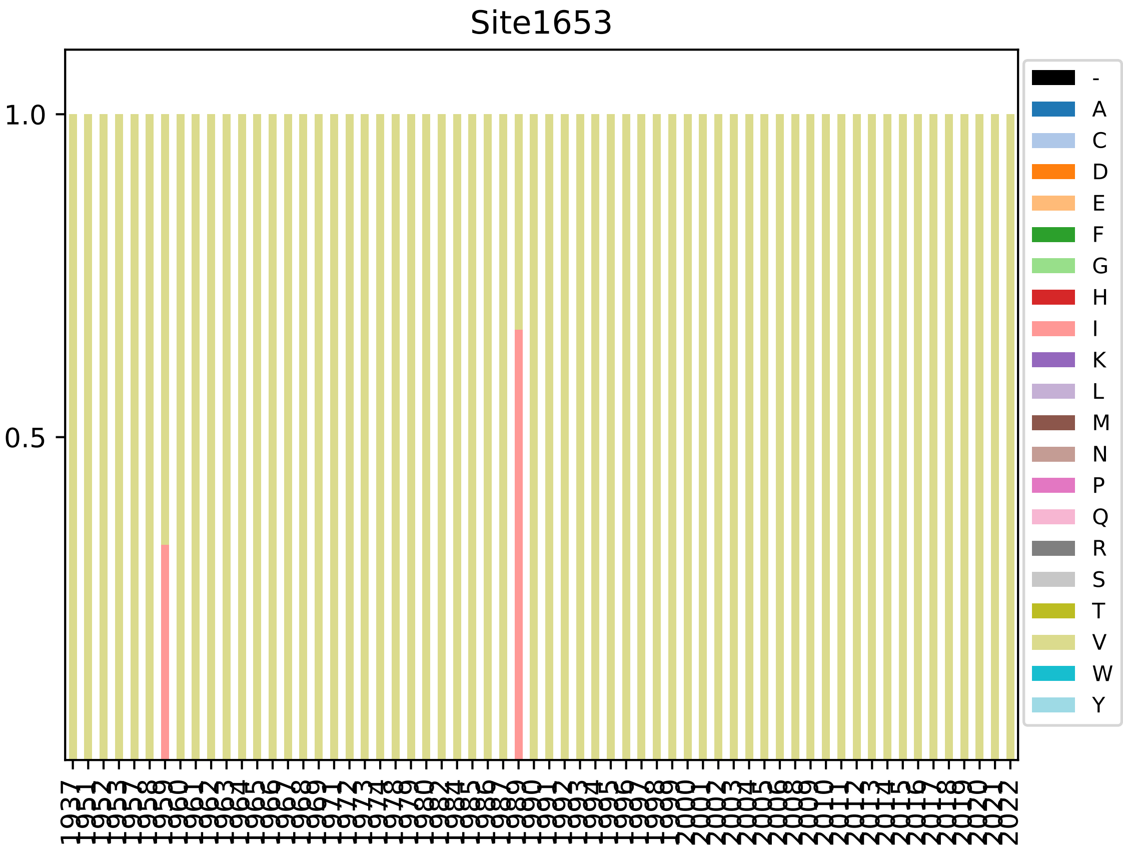

Site1653
1.0
0.5
-
A
C
D
E
F
G
H
I
K
L
M
N
P
Q
R
S
T
V
W
Y

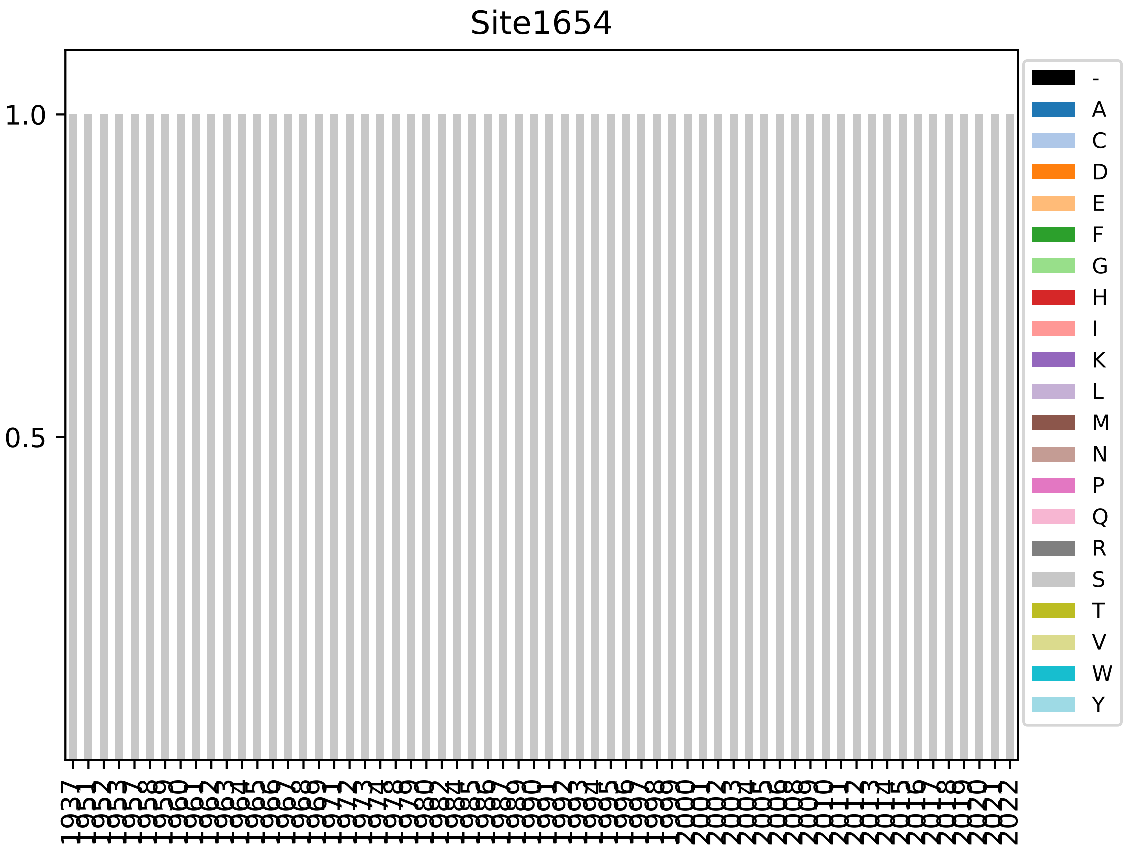

Site1654
1.0
0.5
-
A
C
D
E
F
G
H
I
K
L
M
N
P
Q
R
S
T
V
W
Y

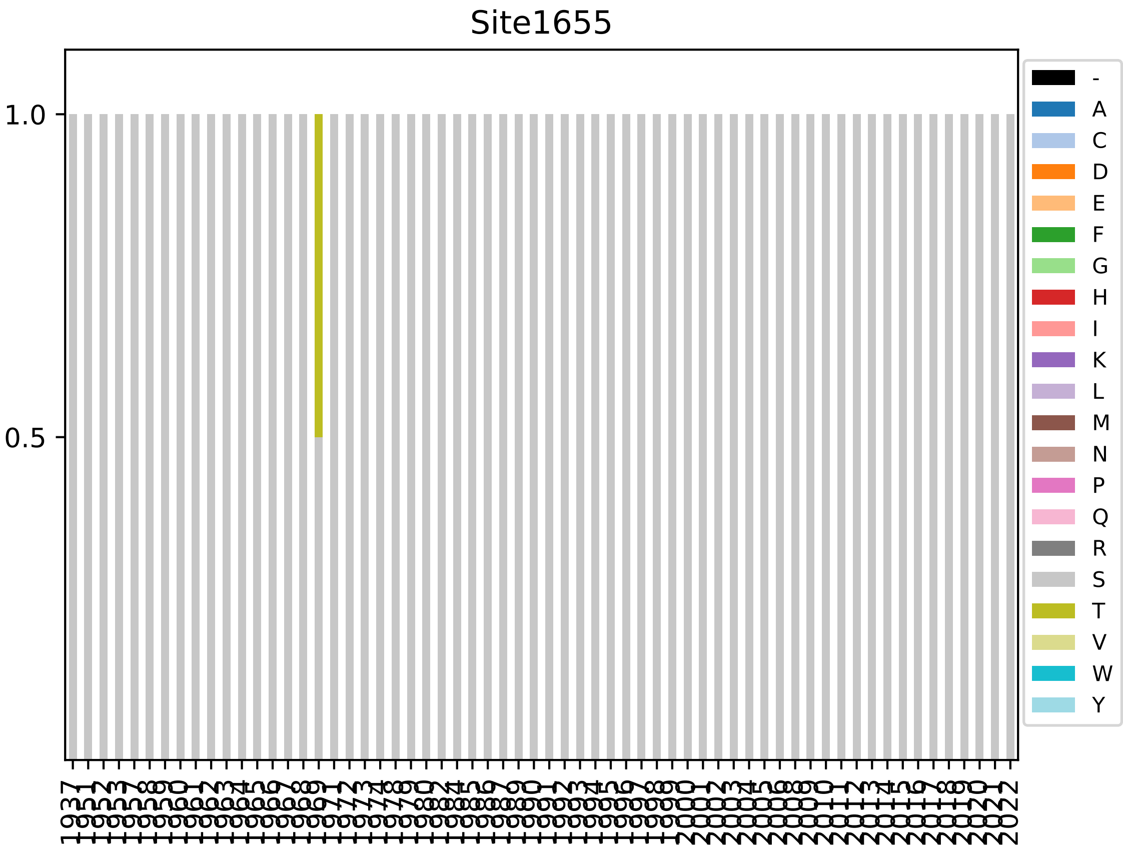
Site1655
1.0
0.5
-
A
C
D
E
F
G
H
I
K
L
M
N
P
Q
R
S
T
V
W
Y

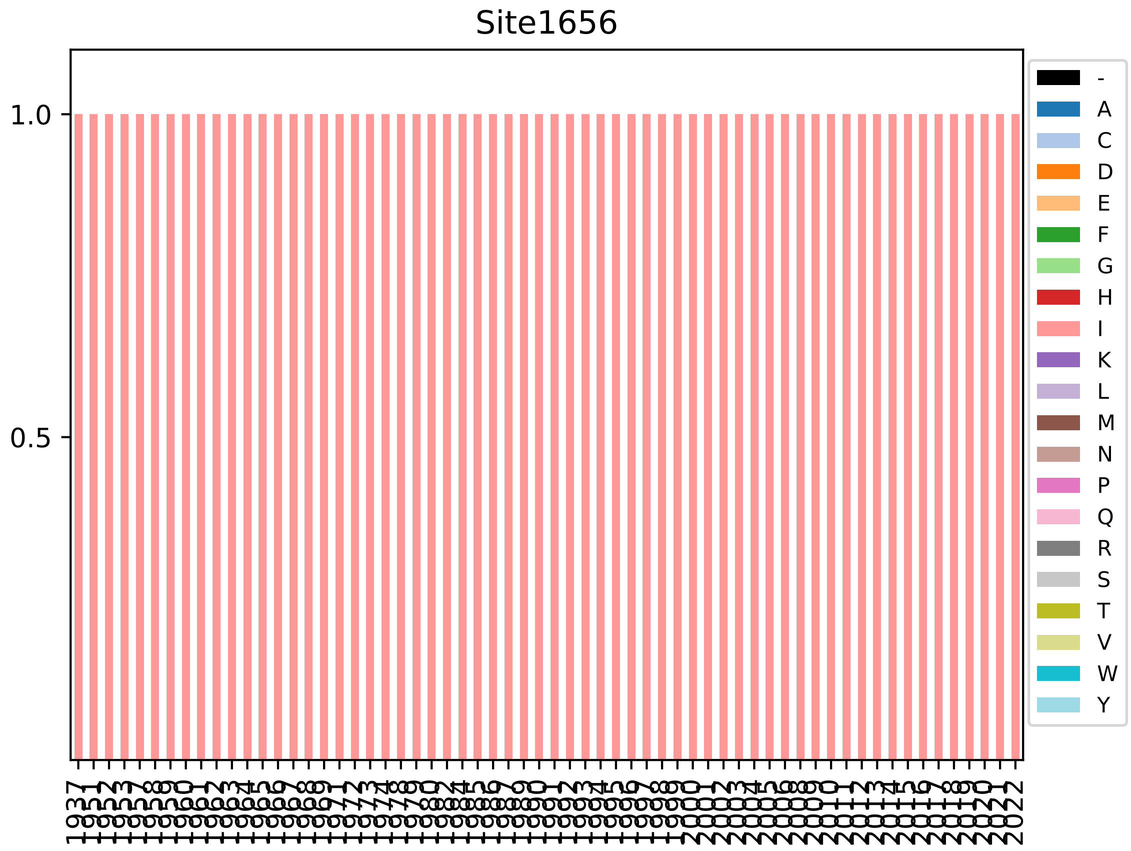

Site1656
1.0
0.5
-
A
C
D
E
F
G
H
I
K
L
M
N
P
Q
R
S
T
V
W
Y

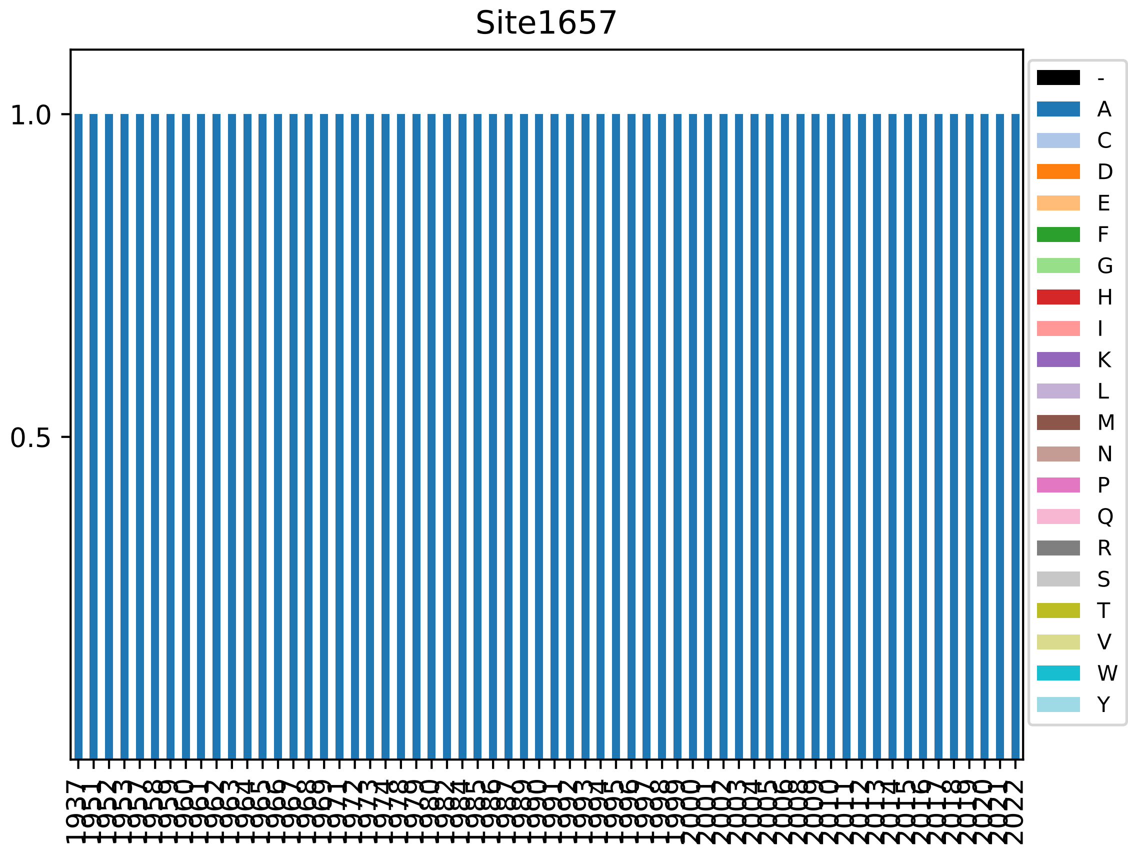
Site1657
1.0
0.5
-
A
C
D
E
F
G
H
I
K
L
M
N
P
Q
R
S
T
V
W
Y

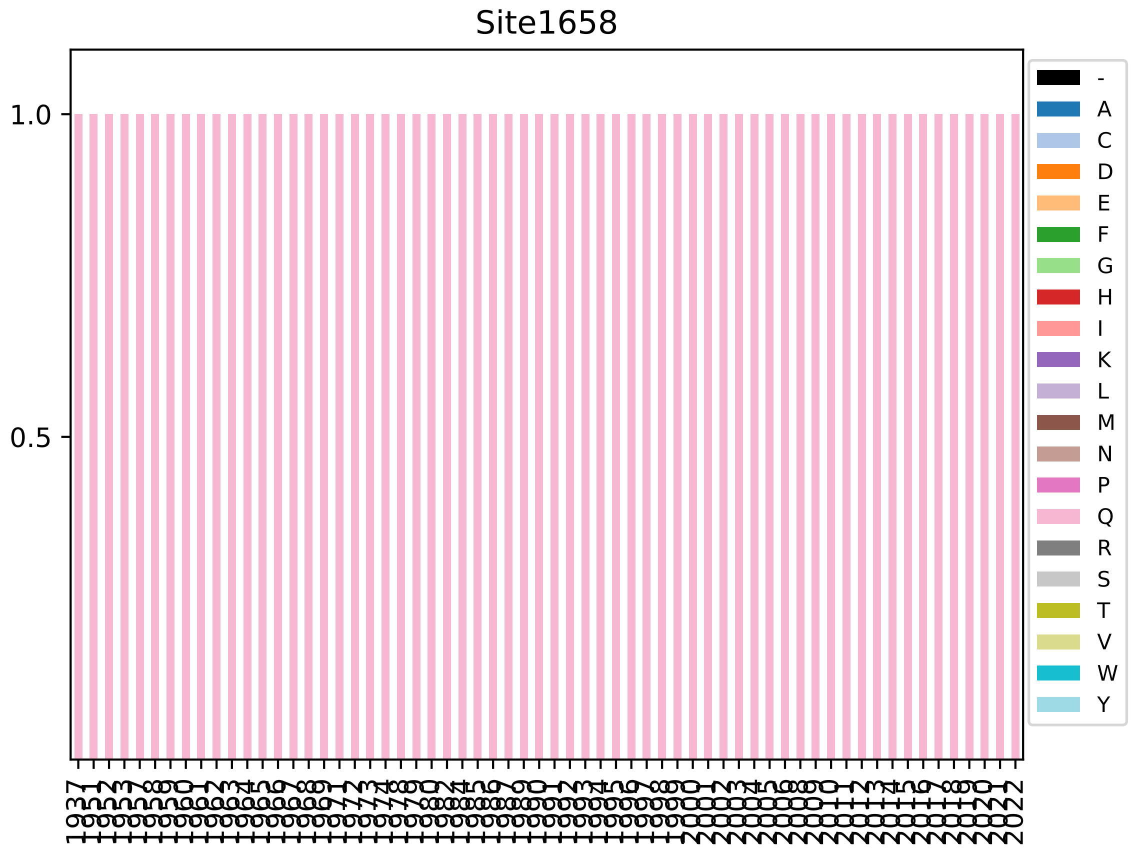
Site1658
1.0
0.5
-
A
C
D
E
F
G
H
I
K
L
M
N
P
Q
R
S
T
V
W
Y

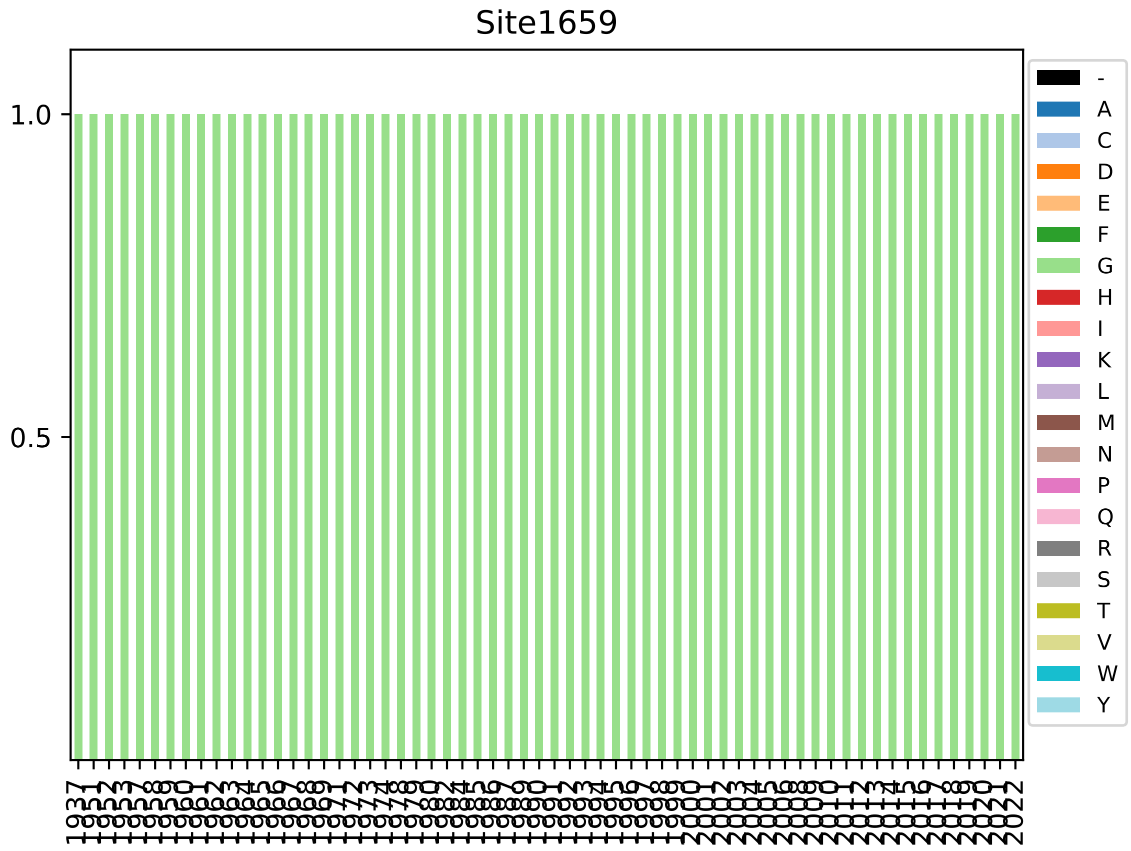
Site1659
1.0
0.5
-
A
C
D
E
F
G
H
I
K
L
M
N
P
Q
R
S
T
V
W
Y

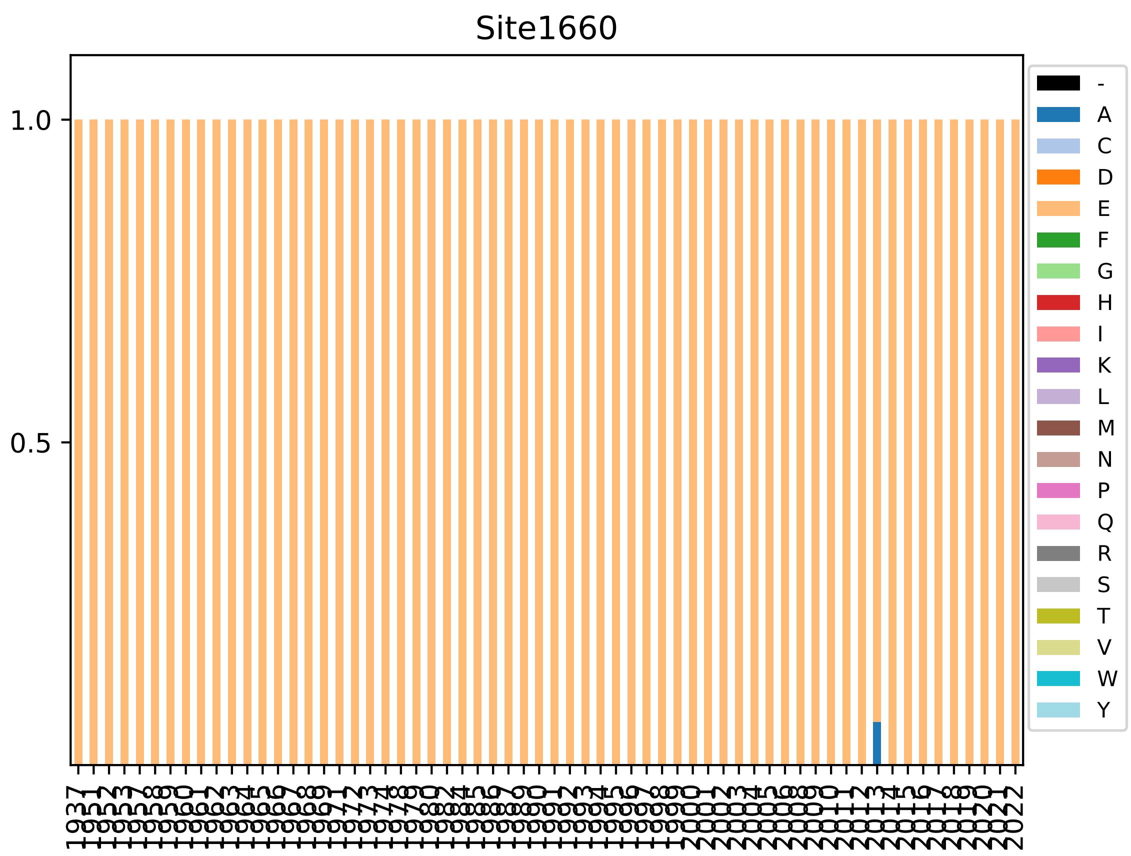
Site1660
1.0
0.5
-
A
C
D
E
F
G
H
I
K
L
M
N
P
Q
R
S
T
V
W
Y

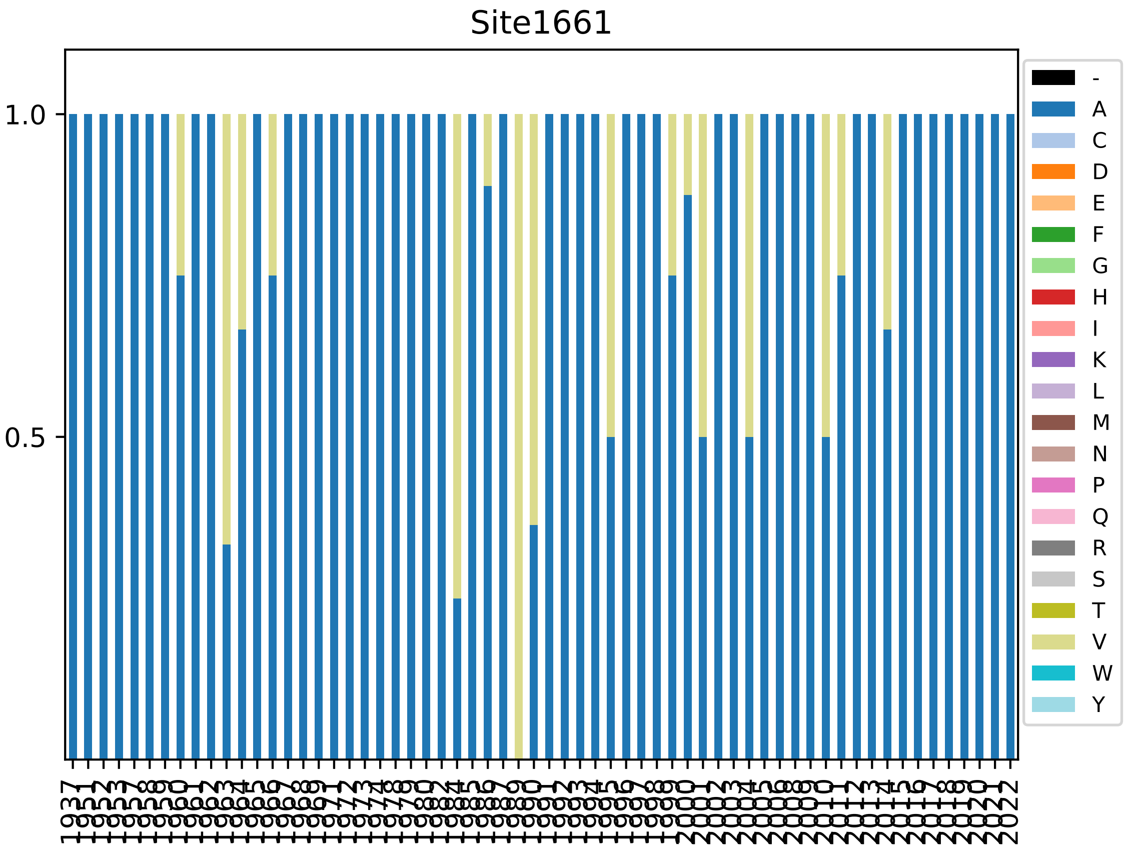
Site1661
1.0
0.5
-
A
C
D
E
F
G
H
I
K
L
M
N
P
Q
R
S
T
V
W
Y

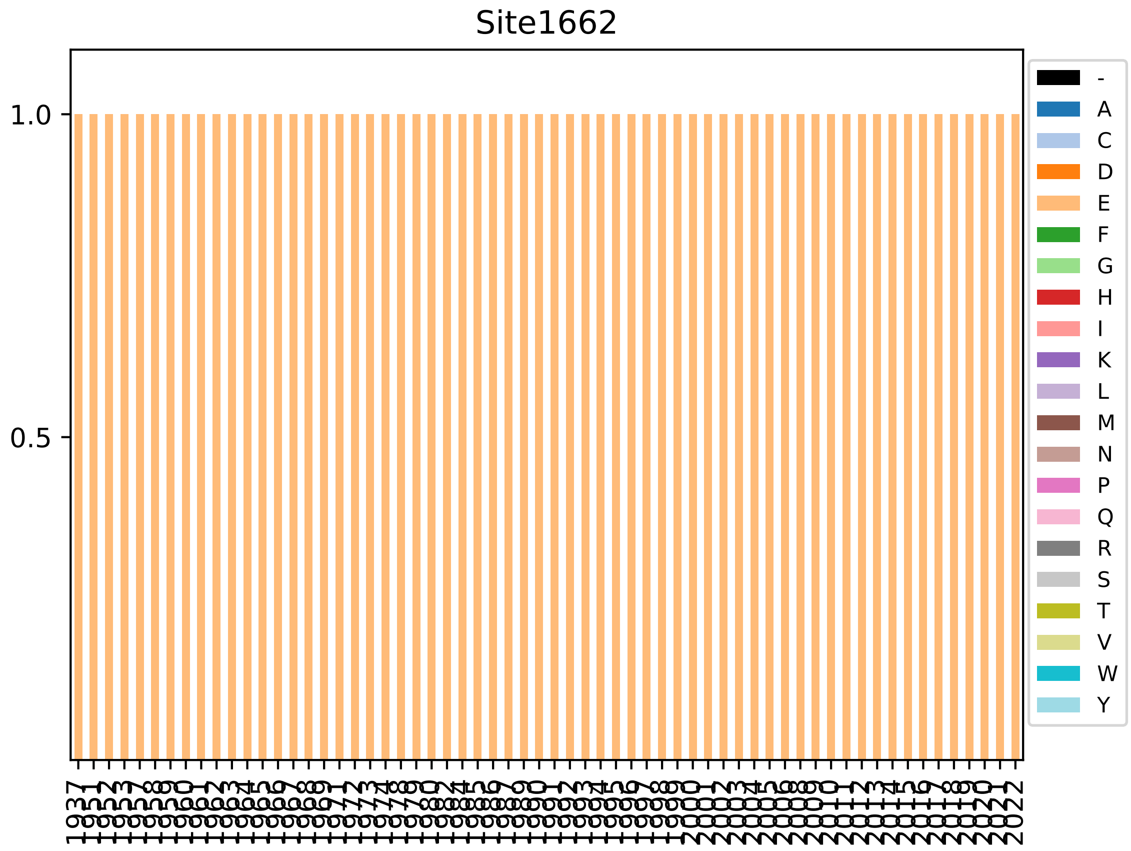
Site1662
1.0
0.5
-
A
C
D
E
F
G
H
I
K
L
M
N
P
Q
R
S
T
V
W
Y

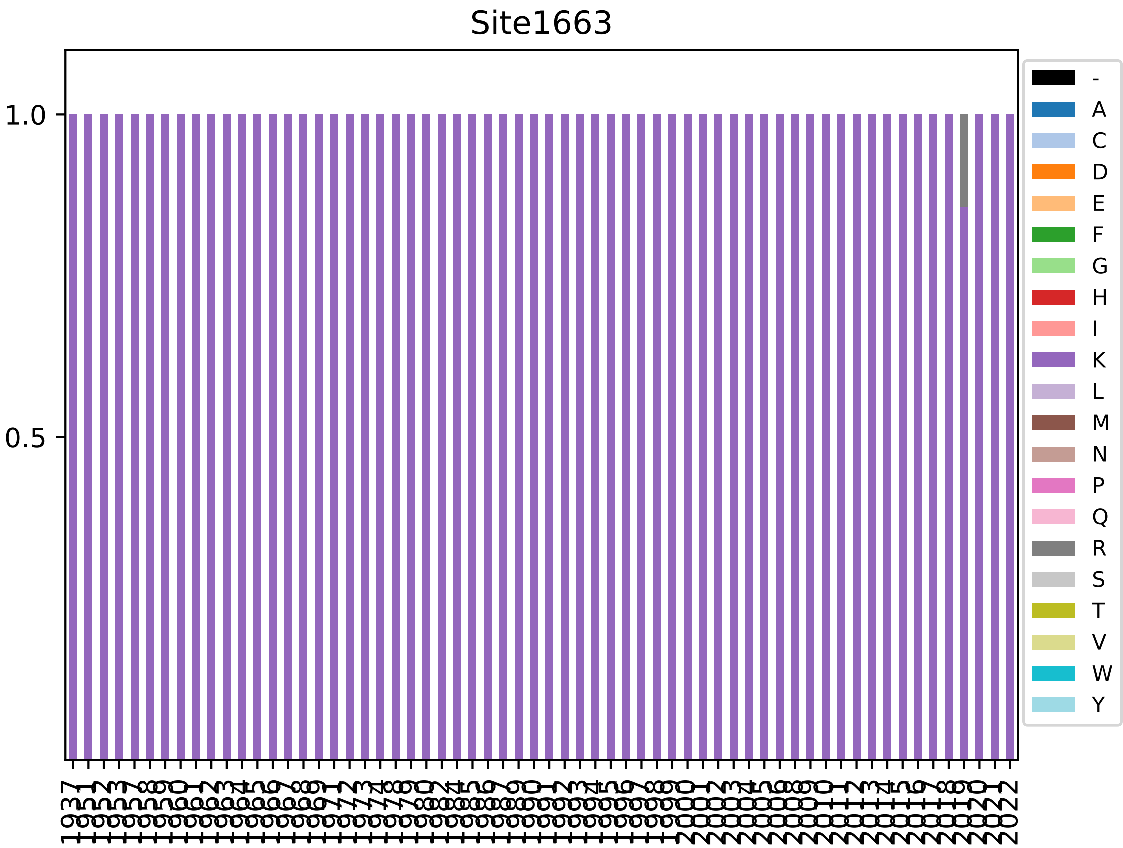

Site1663
1.0
0.5
-
A
C
D
E
F
G
H
I
K
L
M
N
P
Q
R
S
T
V
W
Y

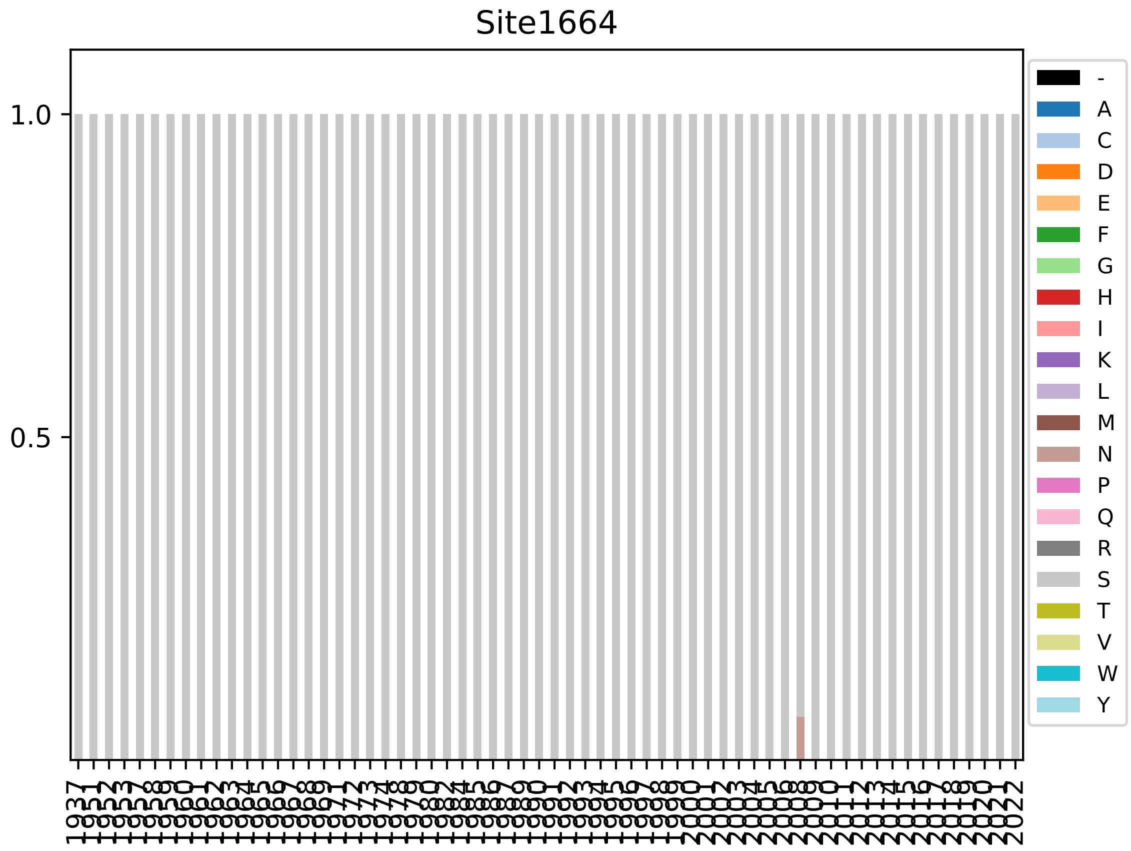
Site1664
1.0
0.5
-
A
C
D
E
F
G
H
I
K
L
M
N
P
Q
R
S
T
V
W
Y

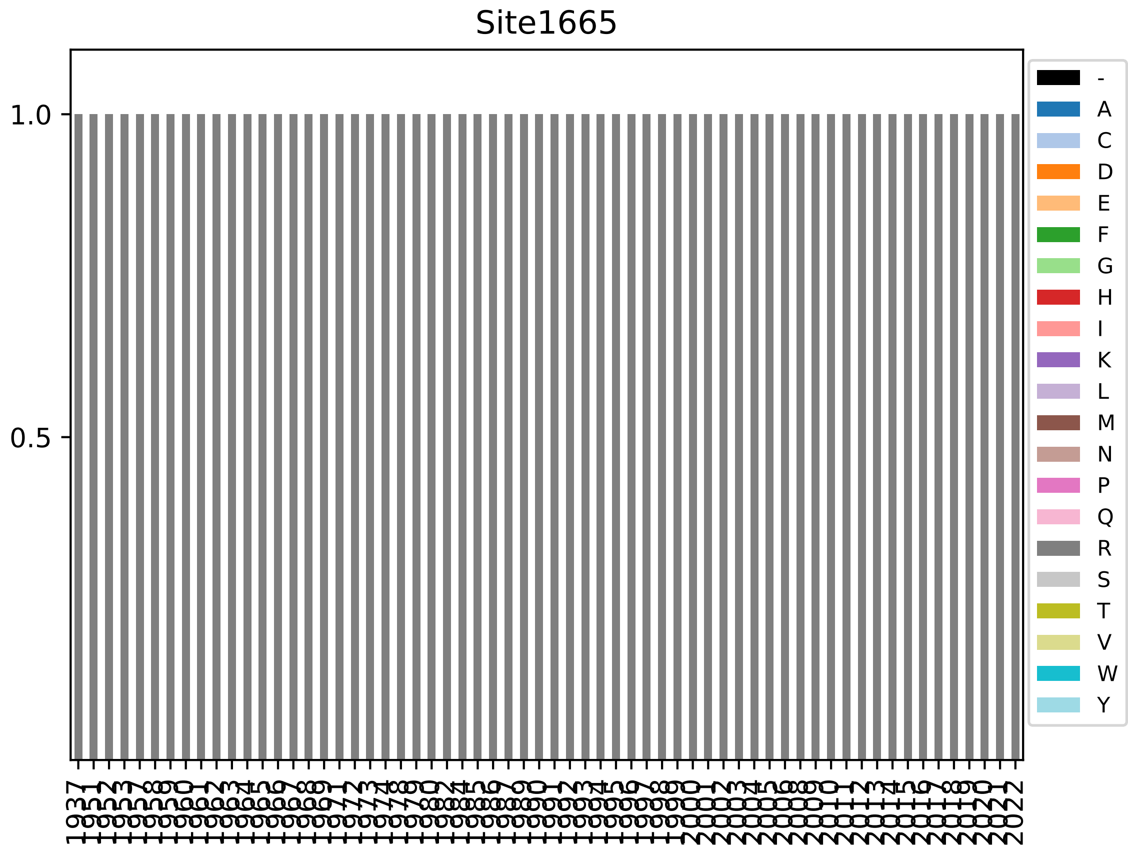
Site1665
1.0
0.5
-
A
C
D
E
F
G
H
I
K
L
M
N
P
Q
R
S
T
V
W
Y

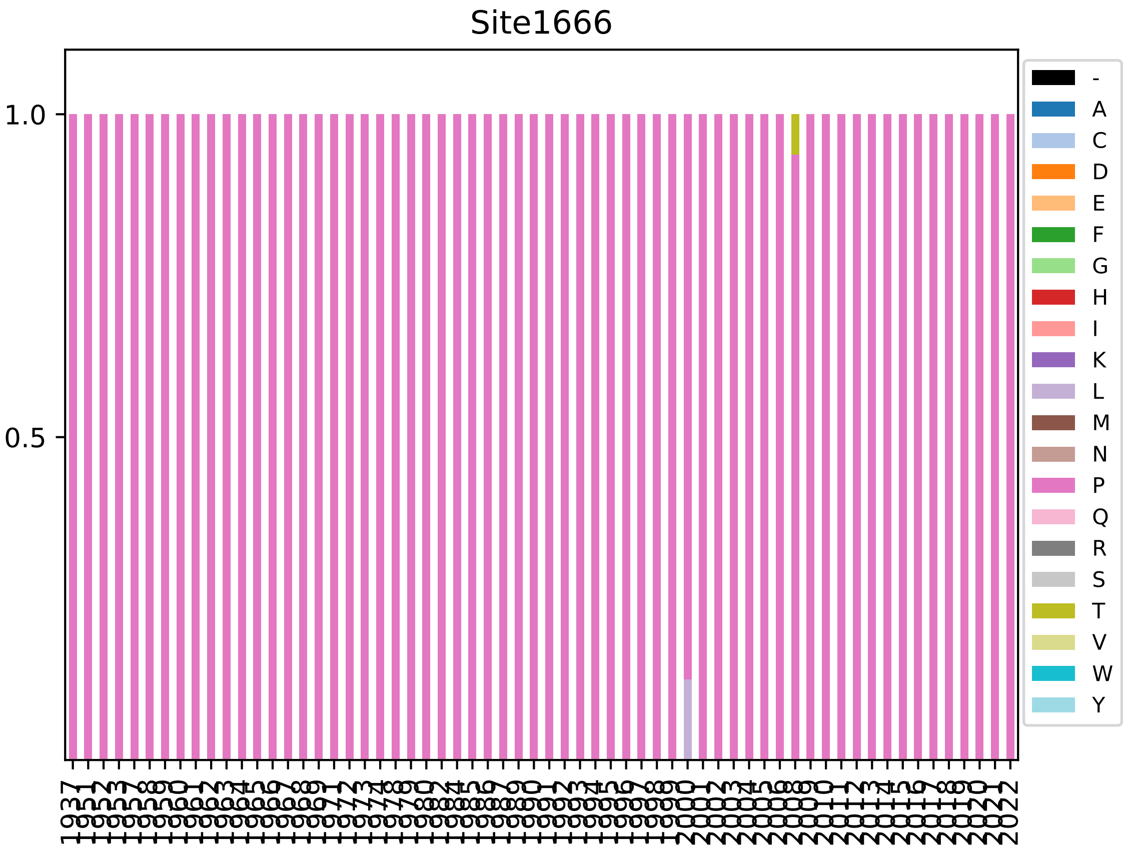
Site1666
1.0
0.5
-
A
C
D
E
F
G
H
I
K
L
M
N
P
Q
R
S
T
V
W
Y

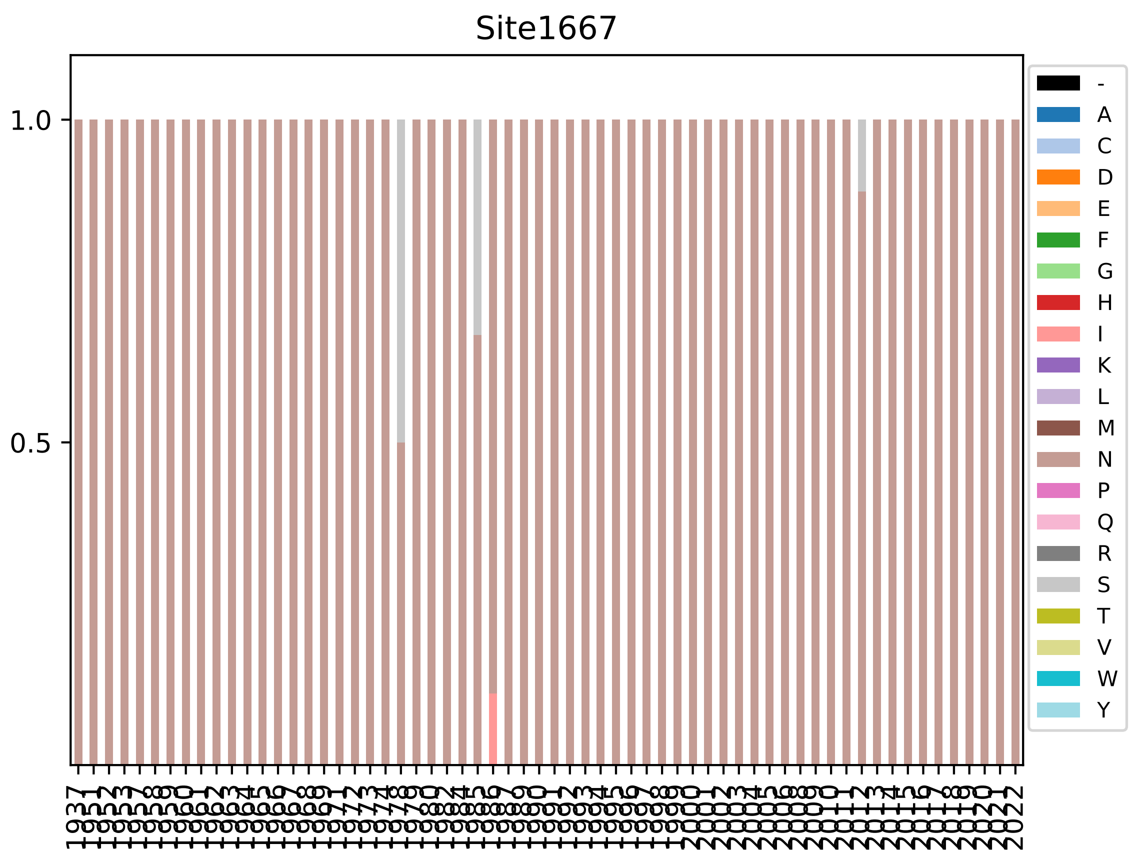
Site1667
1.0
0.5
-
A
C
D
E
F
G
H
I
K
L
M
N
P
Q
R
S
T
V
W
Y

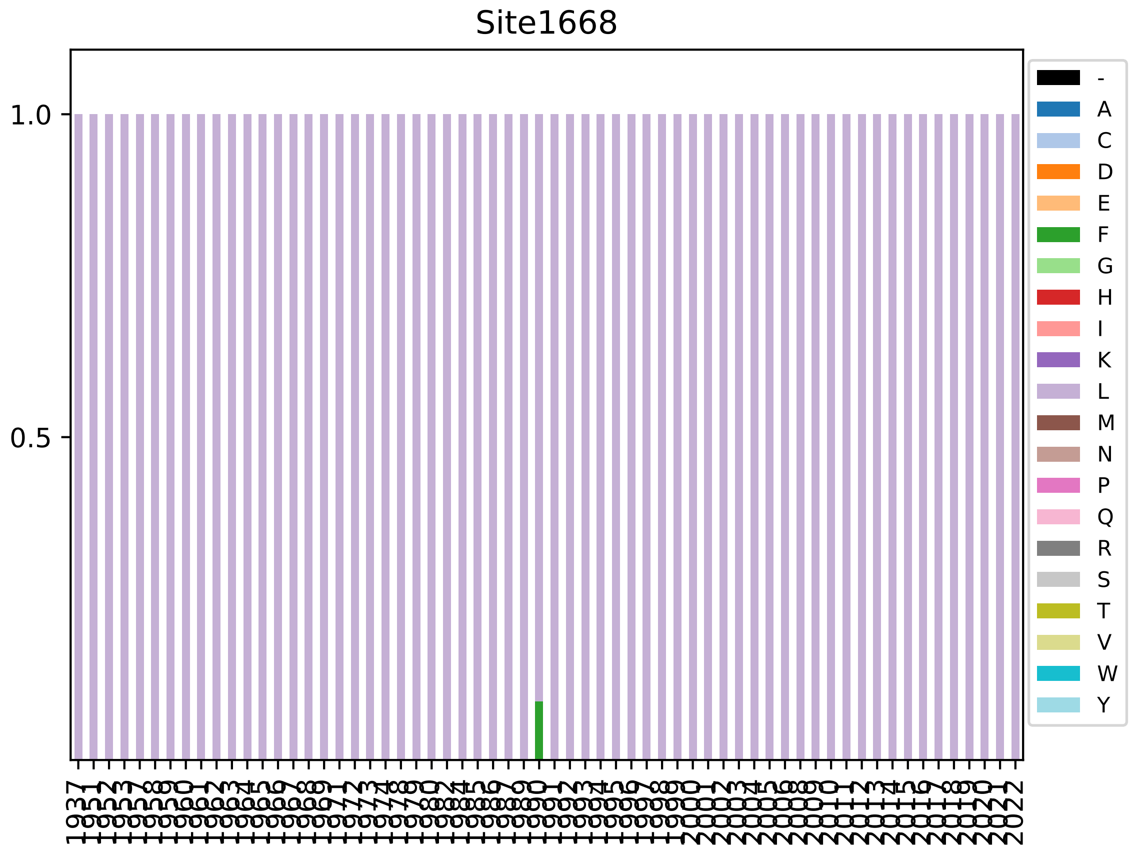
Site1668
1.0
0.5
-
A
C
D
E
F
G
H
I
K
L
M
N
P
Q
R
S
T
V
W
Y

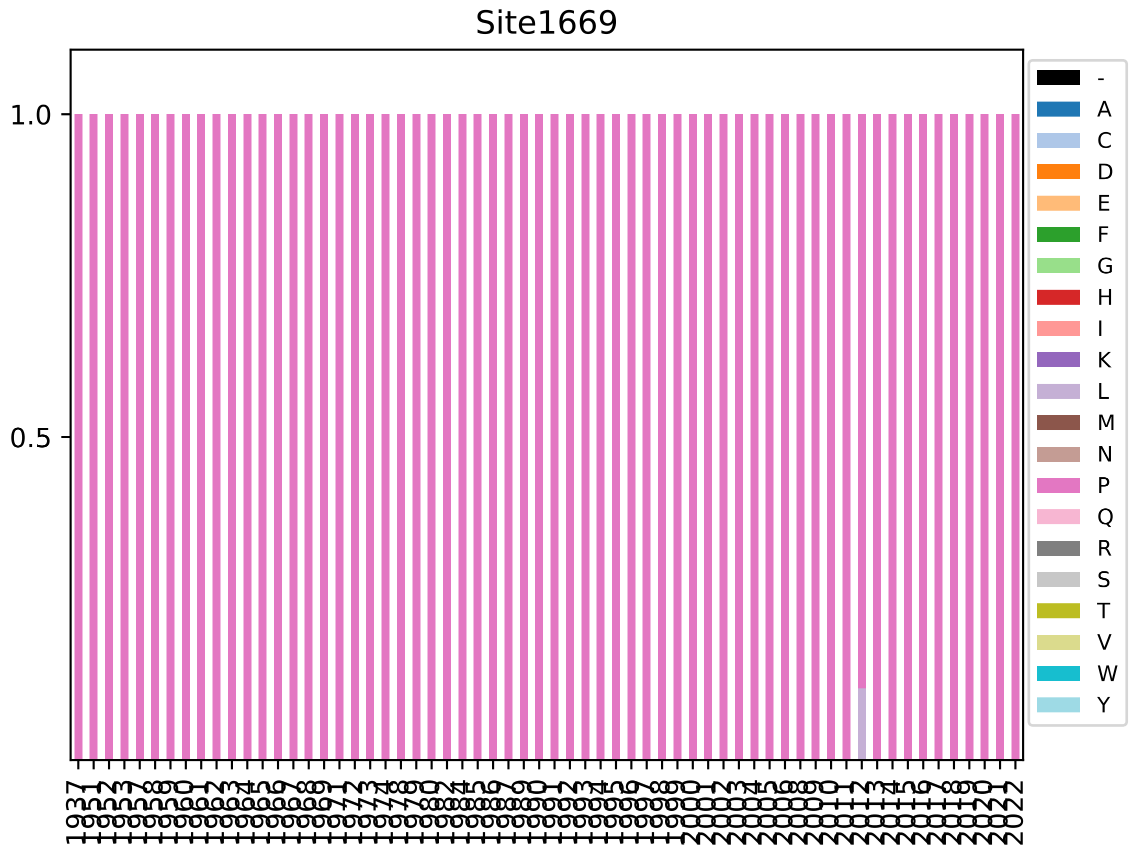
Site1669
1.0
0.5
-
A
C
D
E
F
G
H
I
K
L
M
N
P
Q
R
S
T
V
W
Y

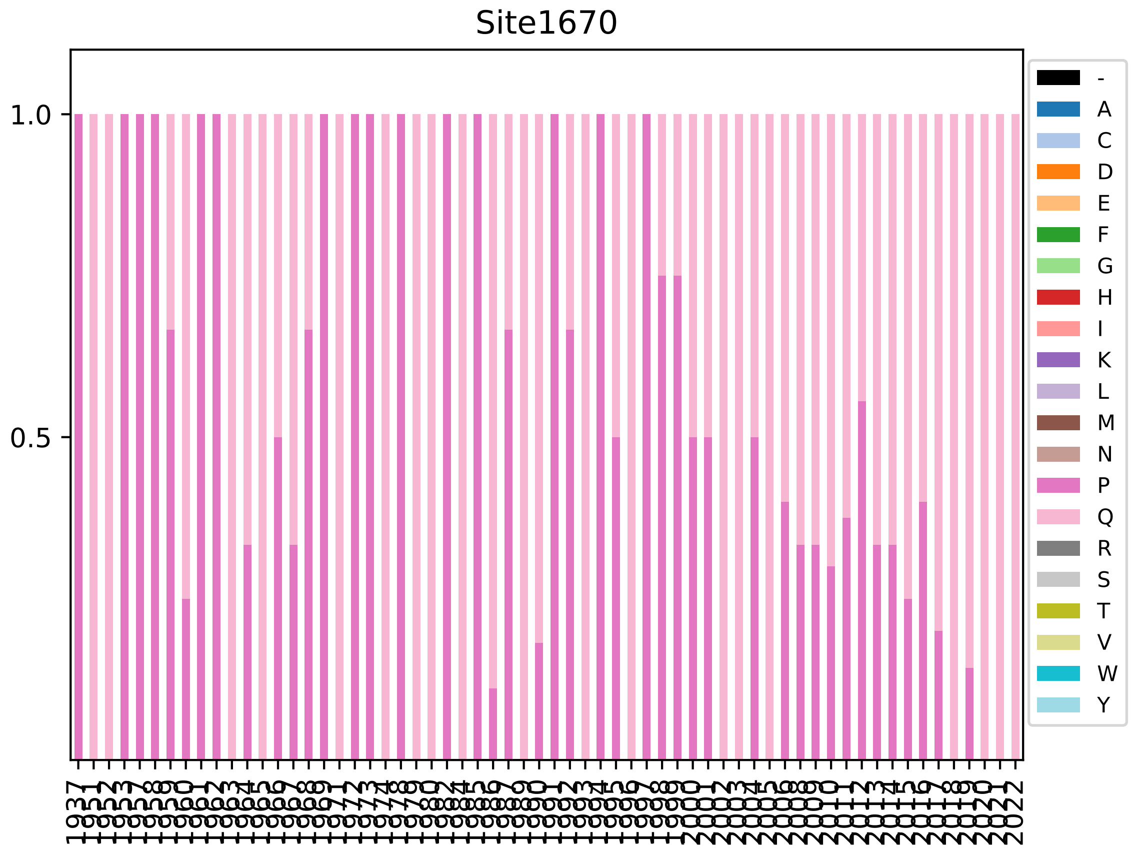

Site1670
1.0
0.5
-
A
C
D
E
F
G
H
I
K
L
M
N
P
Q
R
S
T
V
W
Y

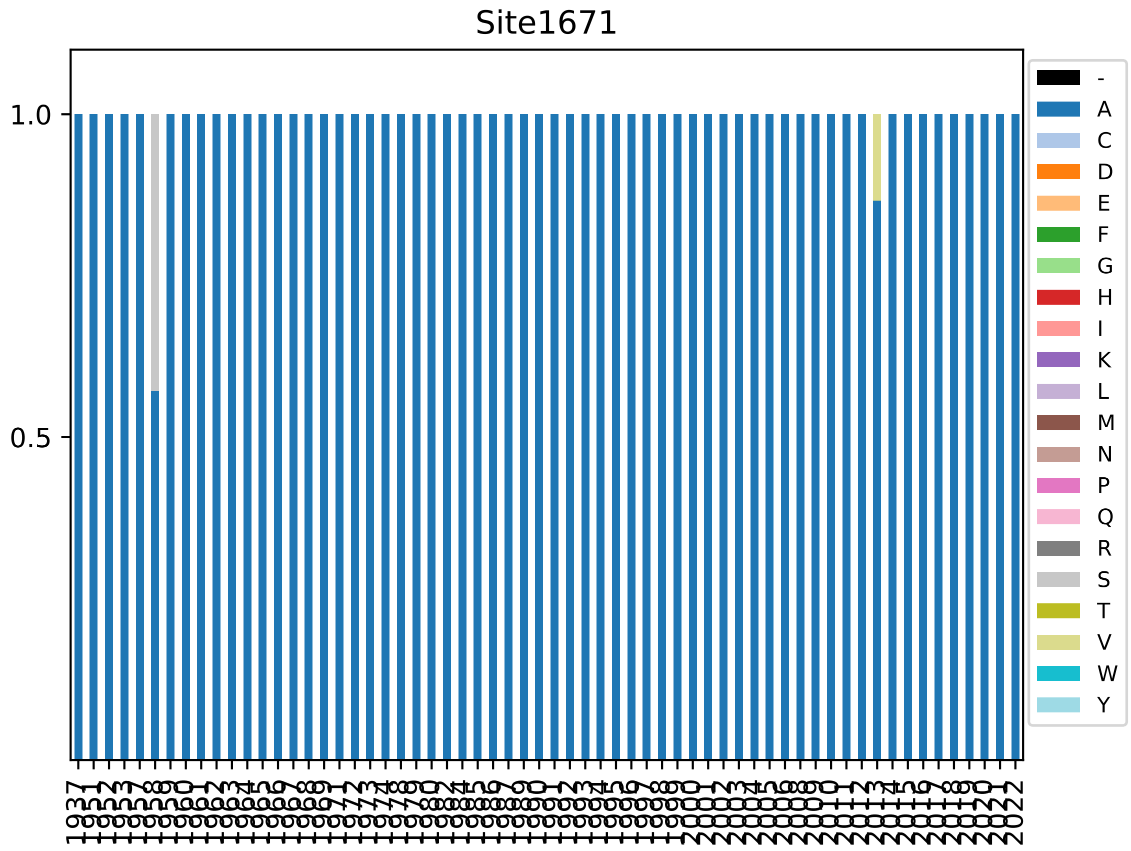
Site1671
1.0
0.5
-
A
C
D
E
F
G
H
I
K
L
M
N
P
Q
R
S
T
V
W
Y

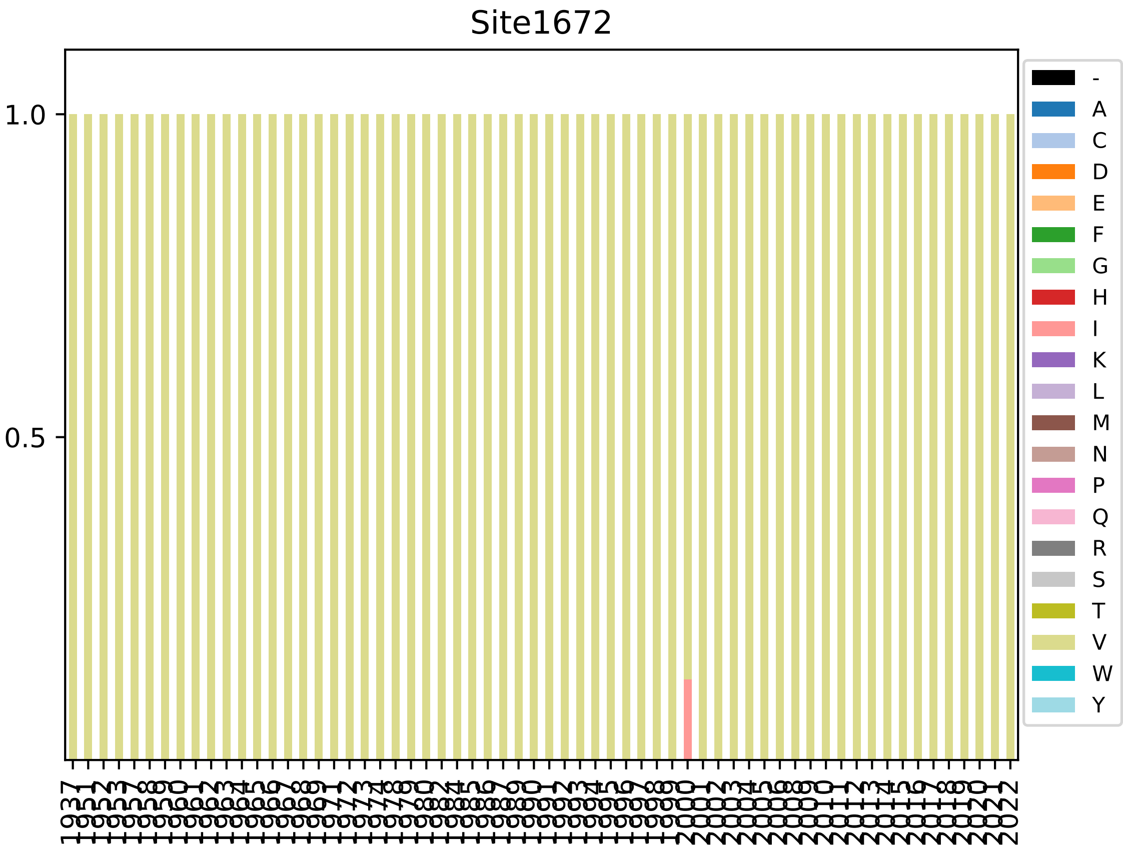
Site1672
1.0
0.5
-
A
C
D
E
F
G
H
I
K
L
M
N
P
Q
R
S
T
V
W
Y

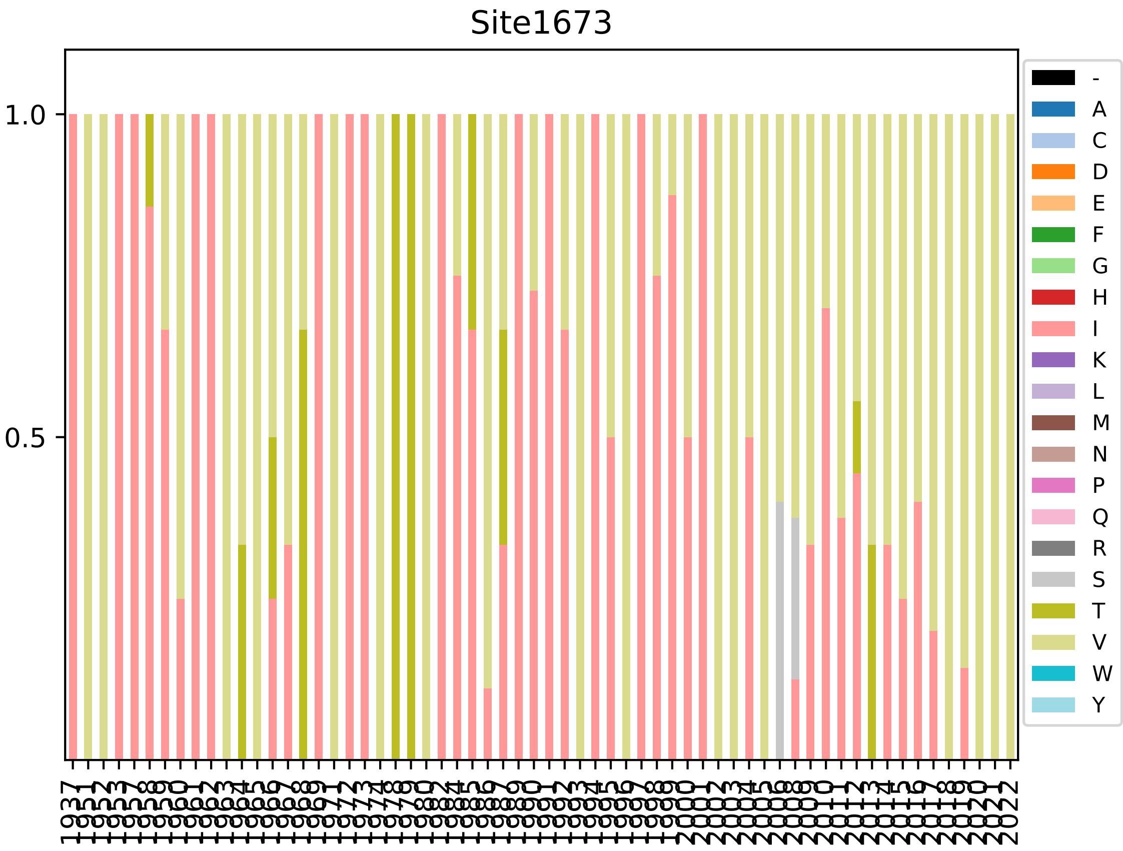
Site1673
1.0
0.5
-
A
C
D
E
F
G
H
I
K
L
M
N
P
Q
R
S
T
V
W
Y

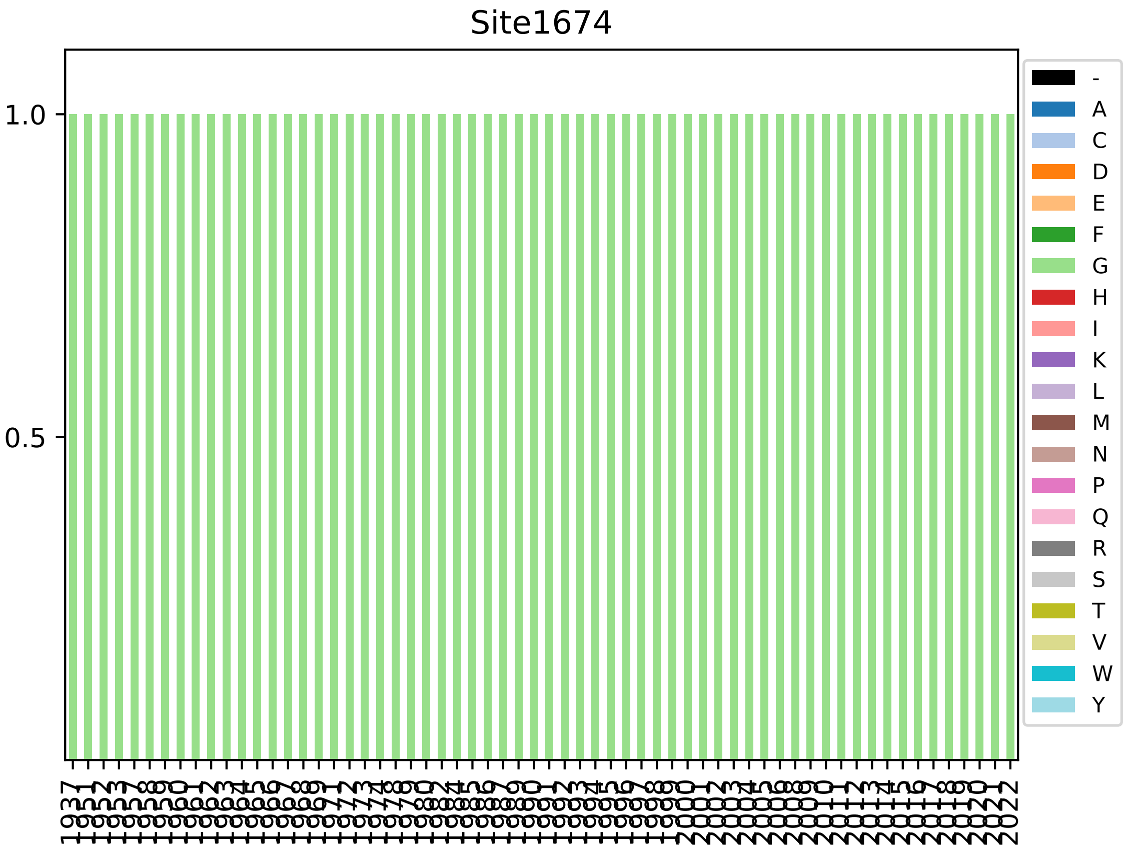
Site1674
1.0
0.5
-
A
C
D
E
F
G
H
I
K
L
M
N
P
Q
R
S
T
V
W
Y

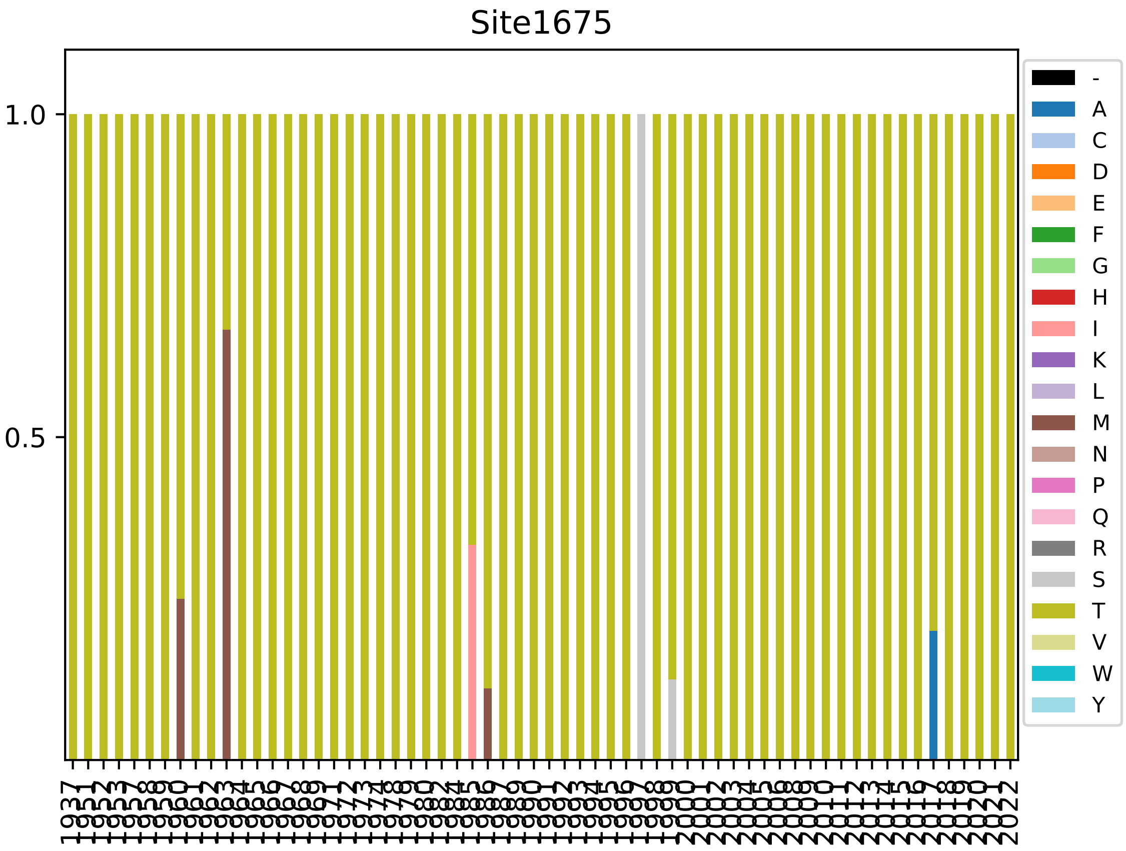
Site1675
1.0
0.5
-
A
C
D
E
F
G
H
I
K
L
M
N
P
Q
R
S
T
V
W
Y

Site1676

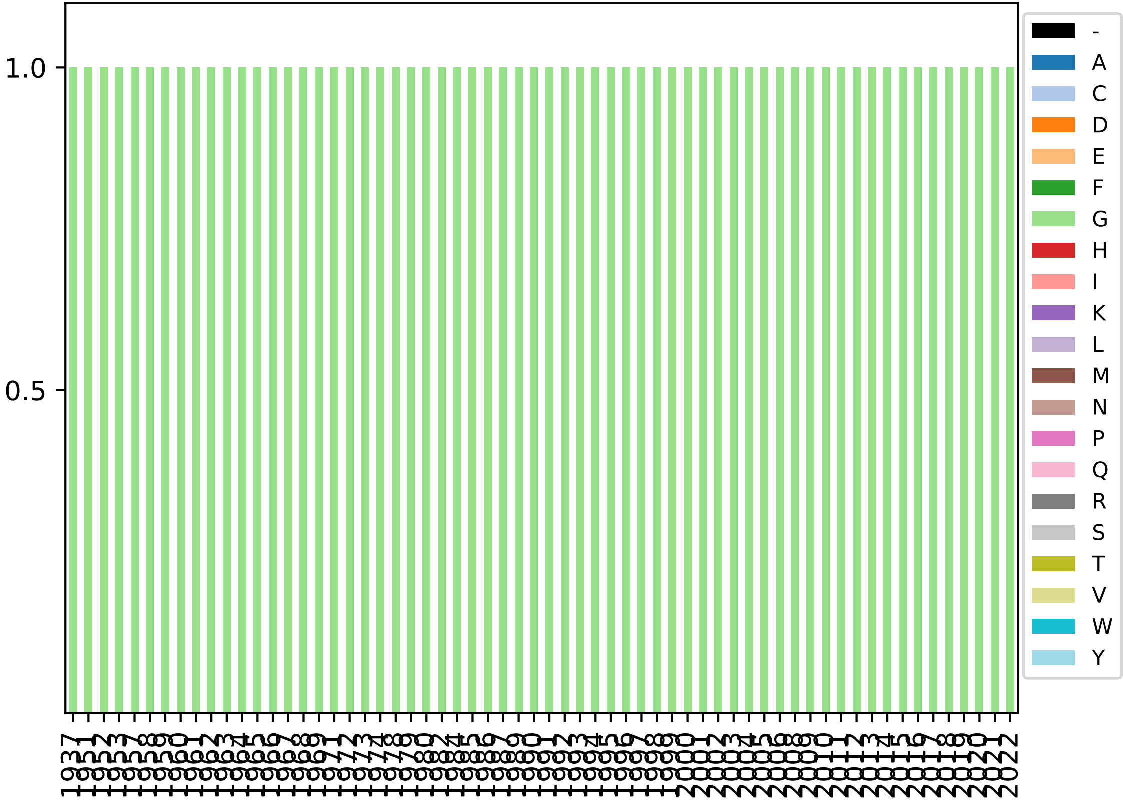

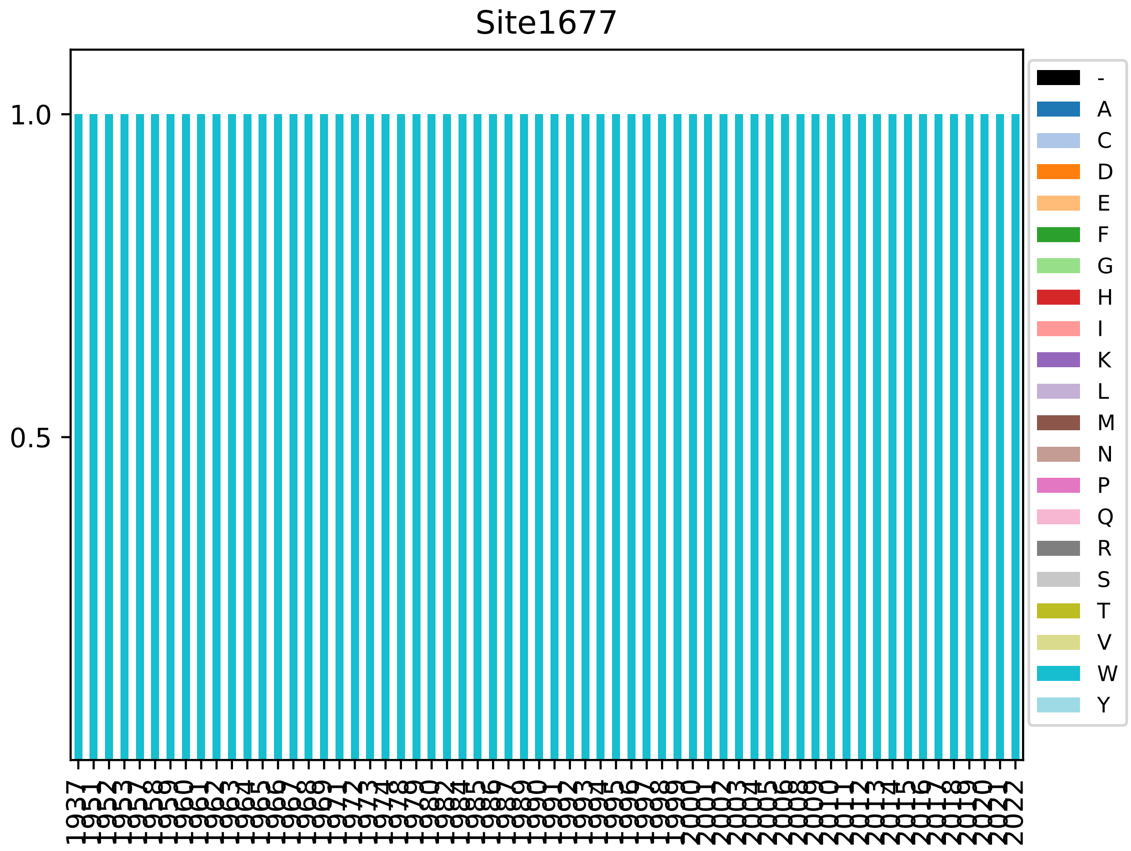
Site1677
1.0
0.5
-
A
C
D
E
F
G
H
I
K
L
M
N
P
Q
R
S
T
V
W
Y

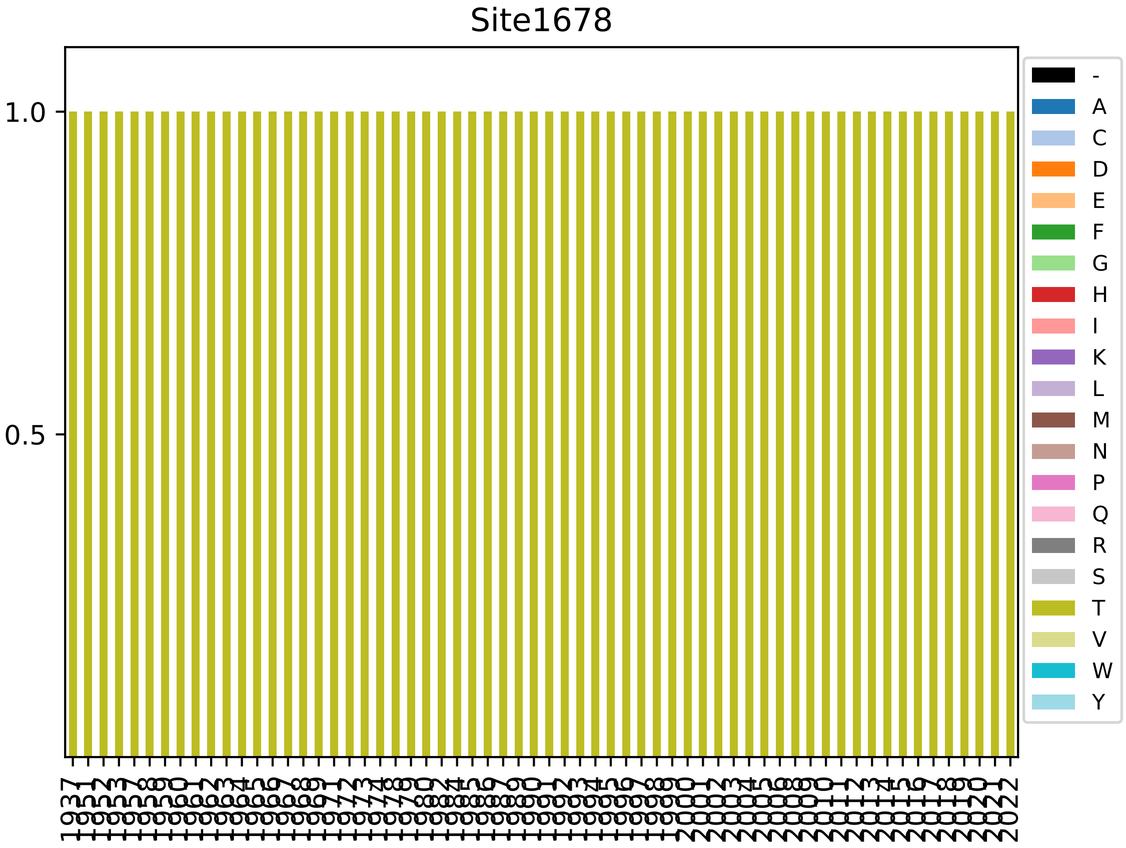
Site1678
1.0
0.5
-
A
C
D
E
F
G
H
I
K
L
M
N
P
Q
R
S
T
V
W
Y

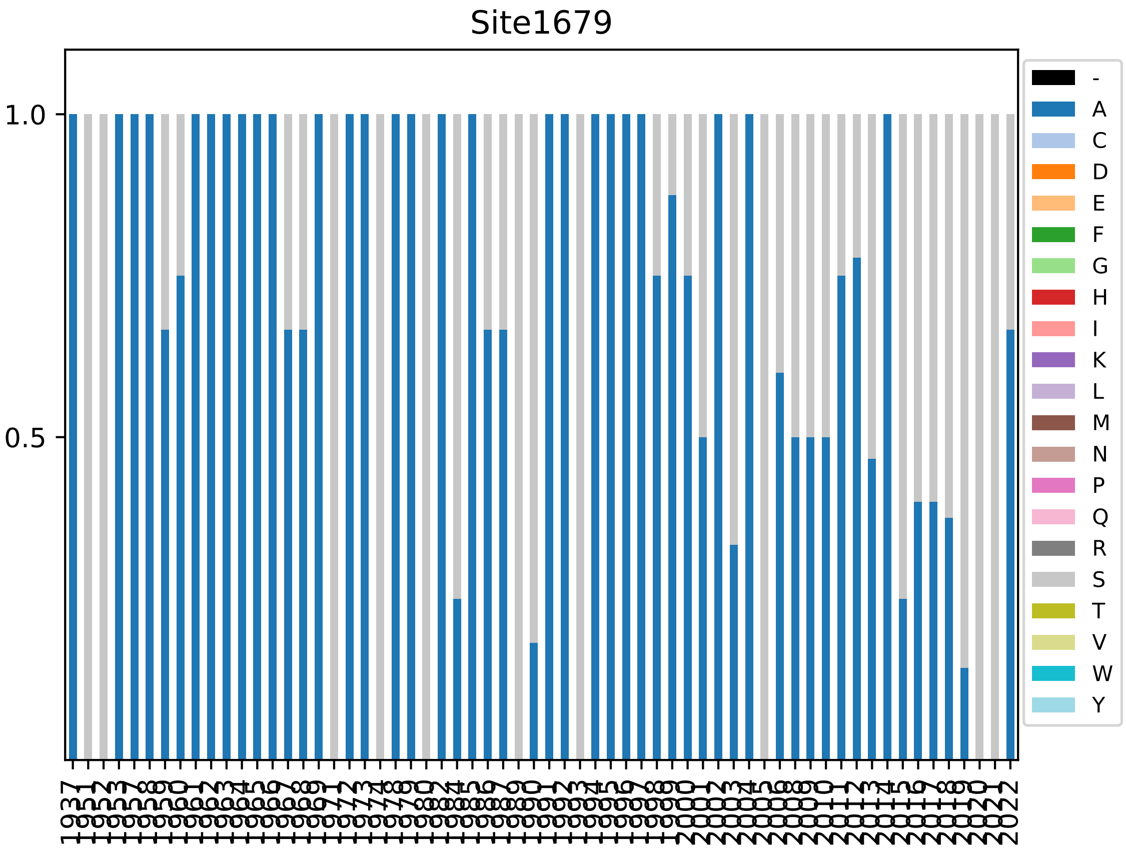
Site1679
1.0
0.5
-
A
C
D
E
F
G
H
I
K
L
M
N
P
Q
R
S
T
V
W
Y

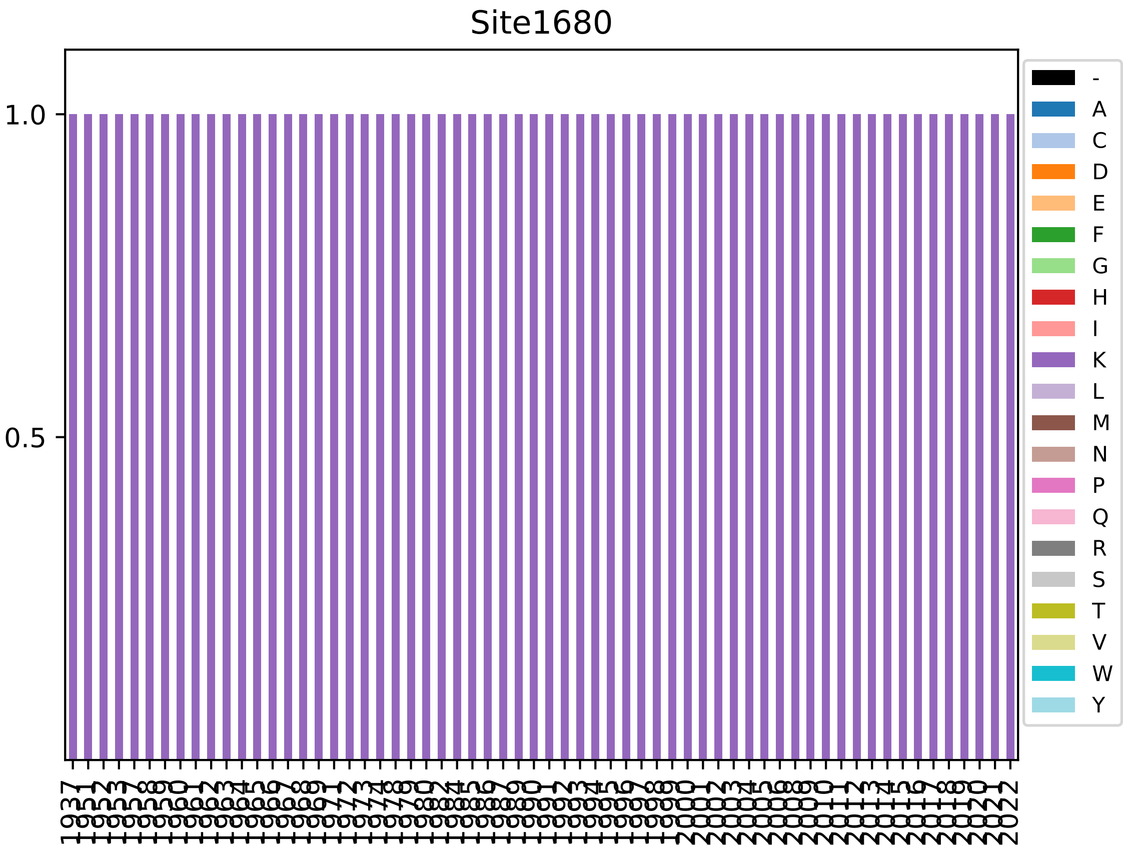
Site1680
1.0
0.5
-
A
C
D
E
F
G
H
I
K
L
M
N
P
Q
R
S
T
V
W
Y

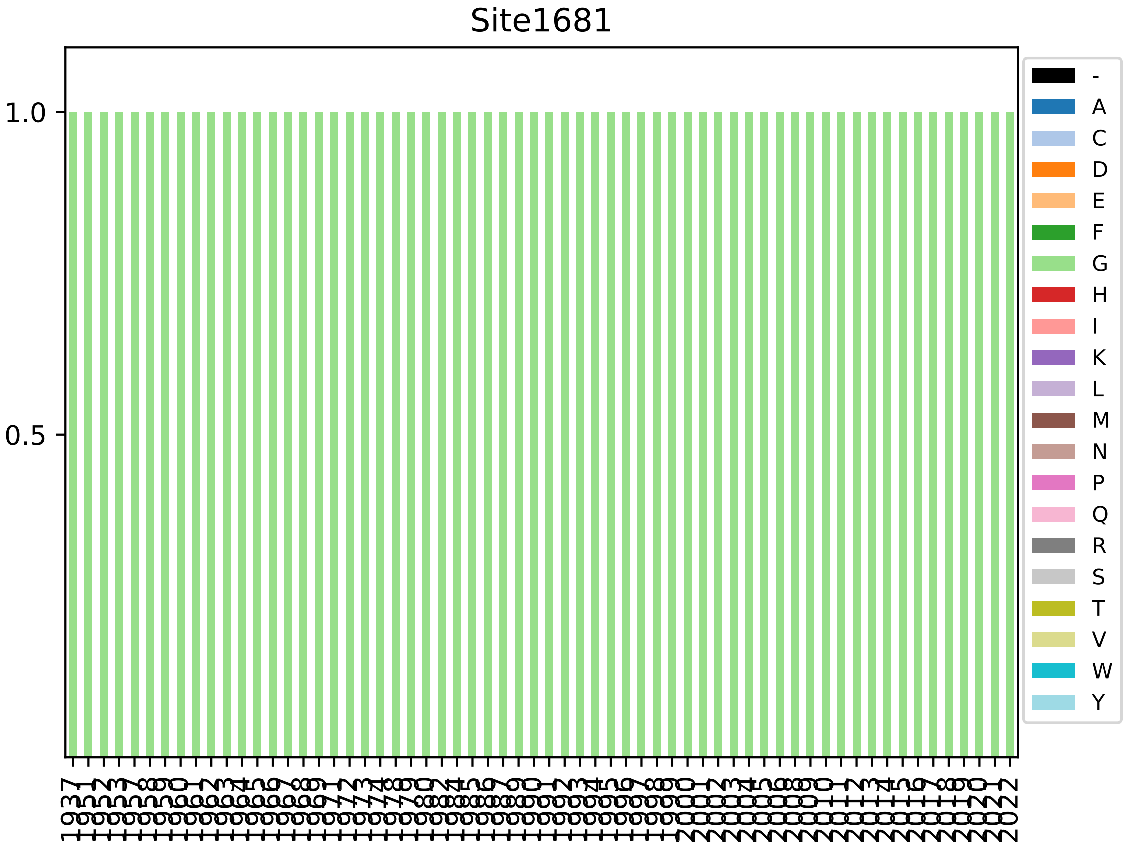
Site1681
1.0
0.5
-
A
C
D
E
F
G
H
I
K
L
M
N
P
Q
R
S
T
V
W
Y

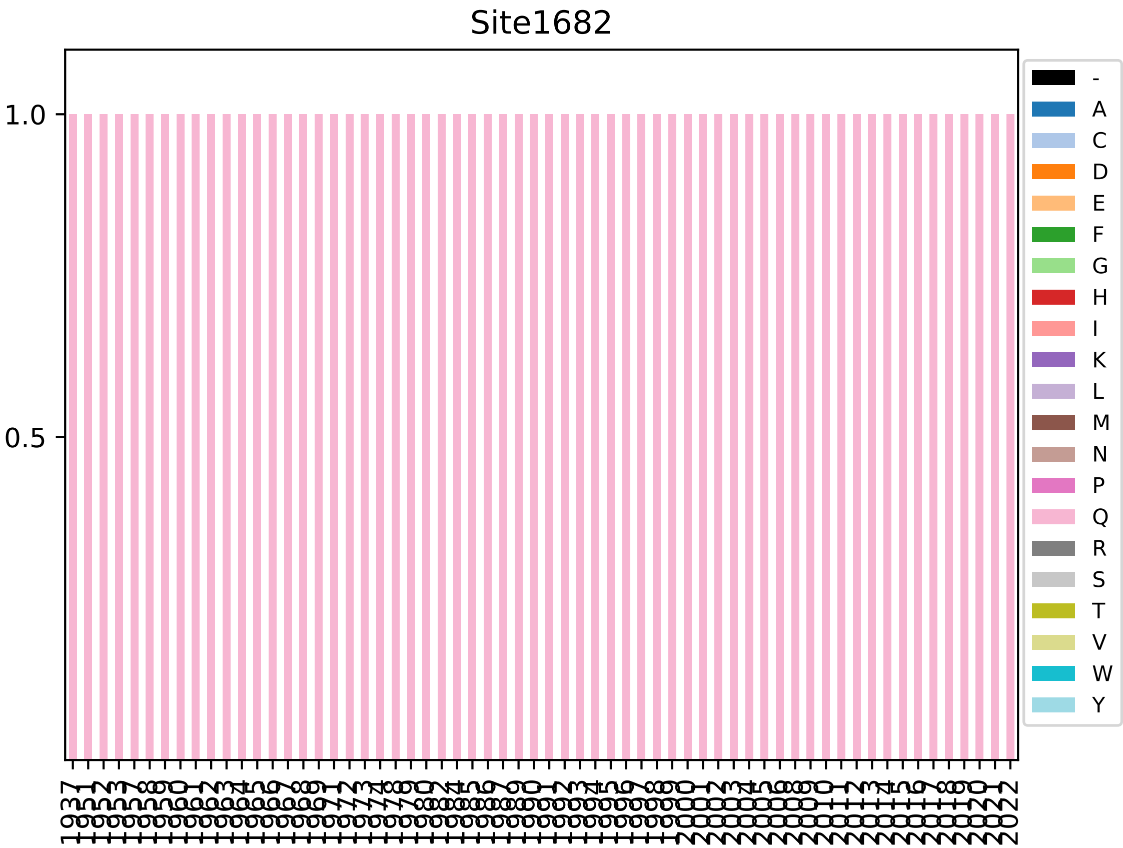

Site1682
1.0
0.5
-
A
C
D
E
F
G
H
I
K
L
M
N
P
Q
R
S
T
V
W
Y

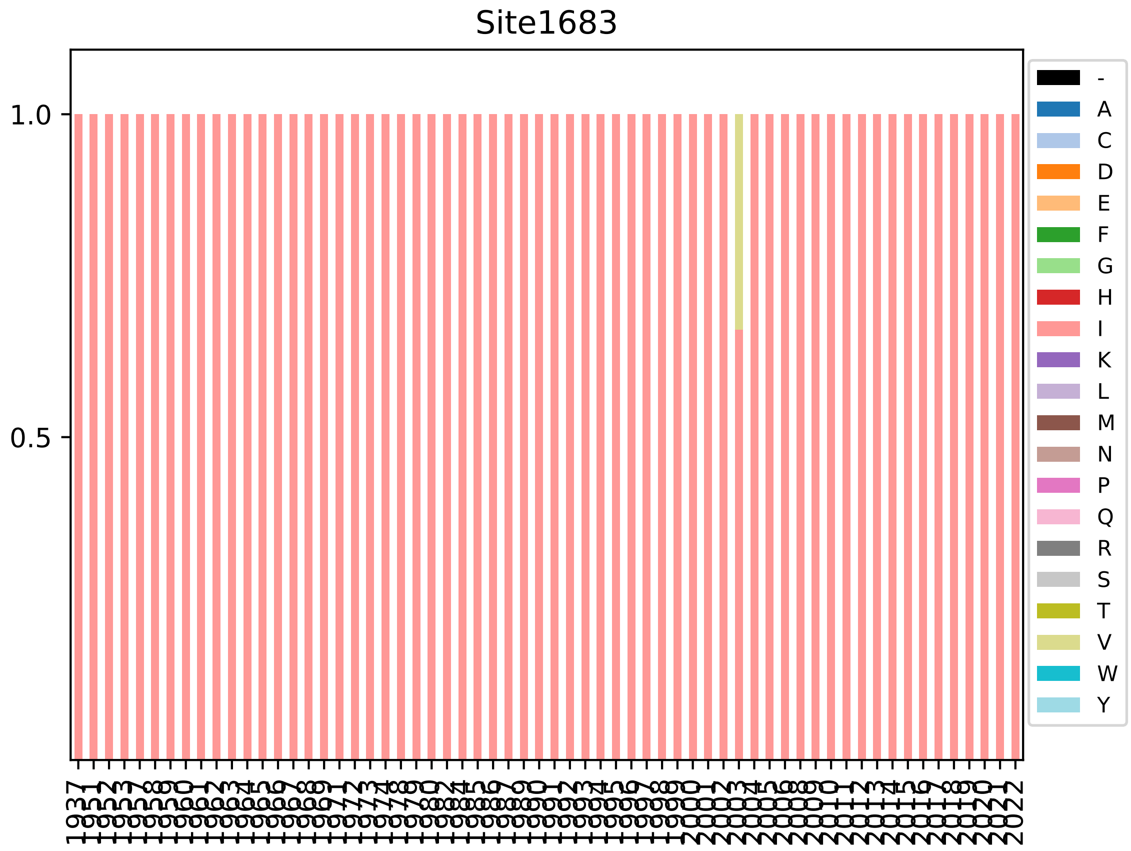
Site1683
1.0
0.5
-
A
C
D
E
F
G
H
I
K
L
M
N
P
Q
R
S
T
V
W
Y

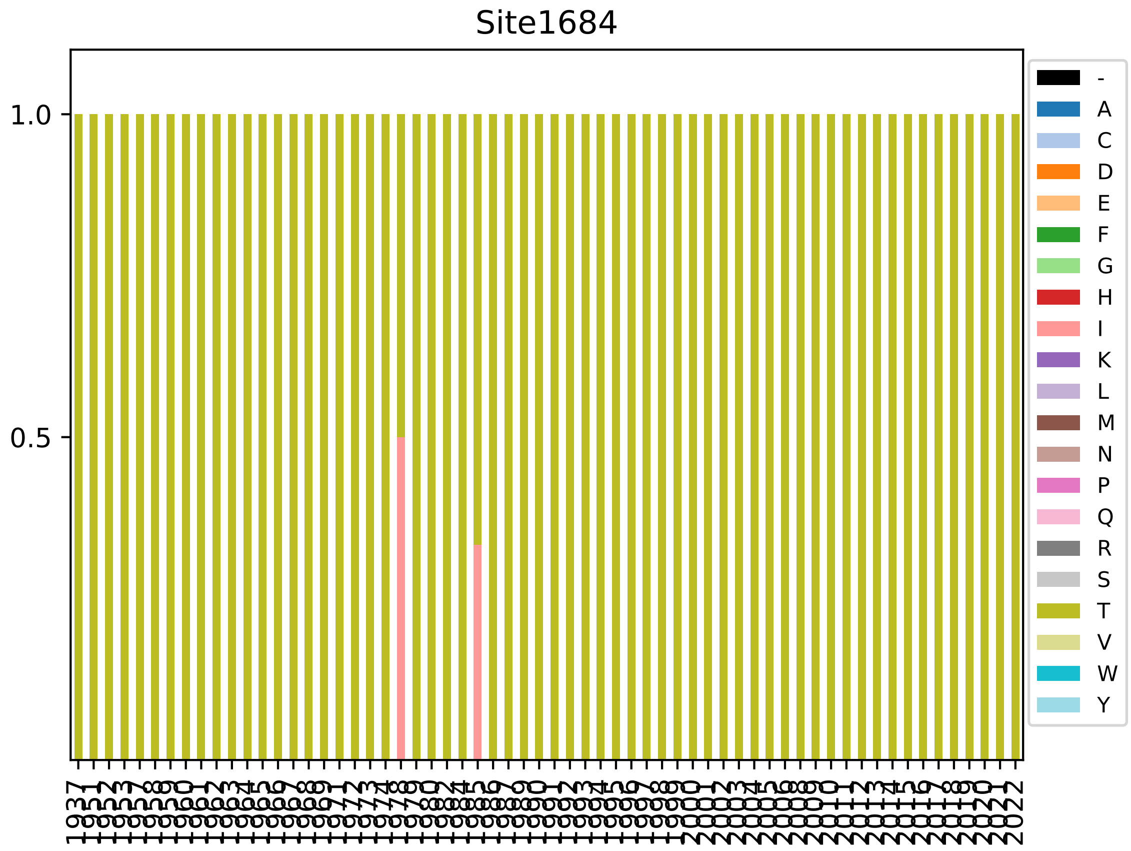
Site1684
1.0
0.5
-
A
C
D
E
F
G
H
I
K
L
M
N
P
Q
R
S
T
V
W
Y

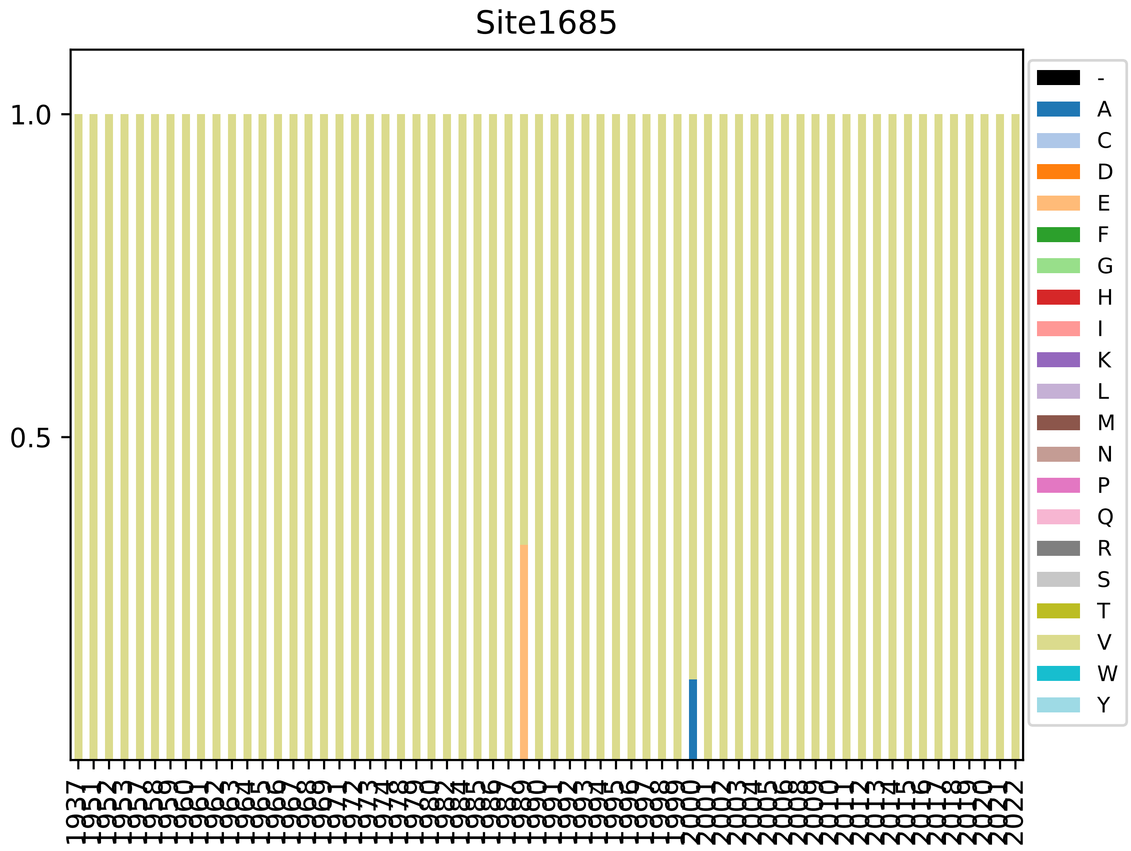
Site1685
1.0
0.5
-
A
C
D
E
F
G
H
I
K
L
M
N
P
Q
R
S
T
V
W
Y

Site1686

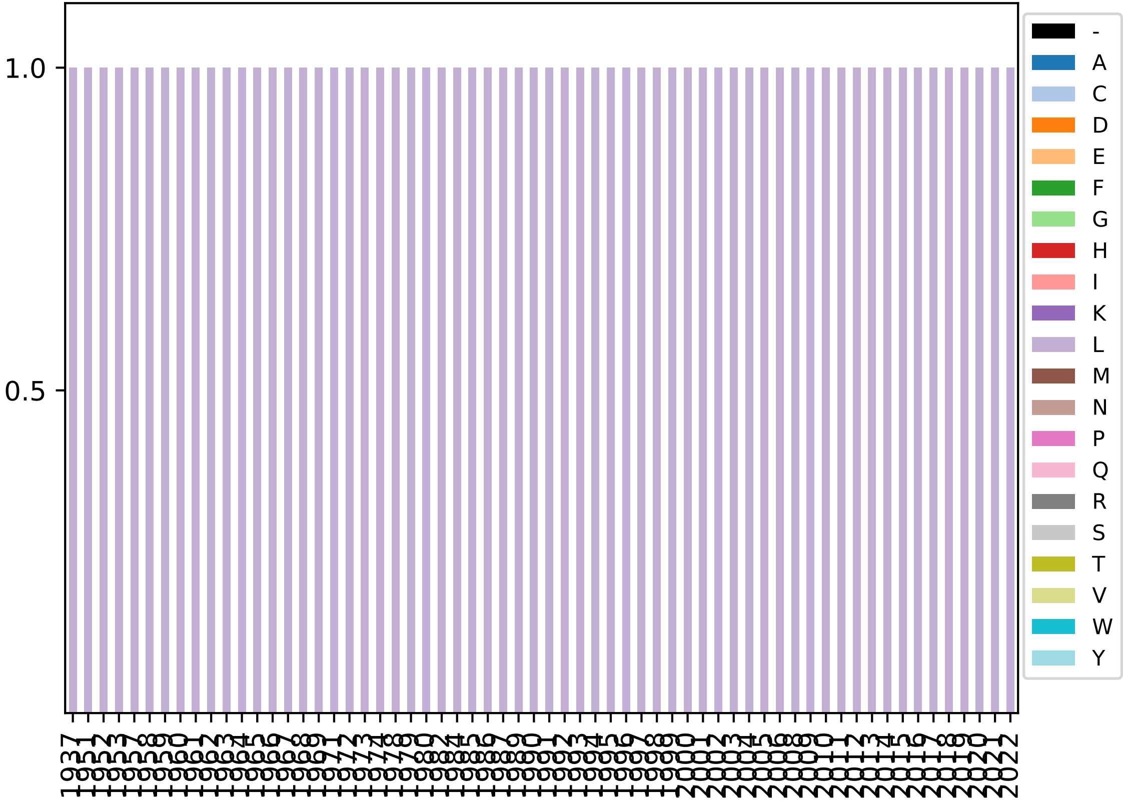

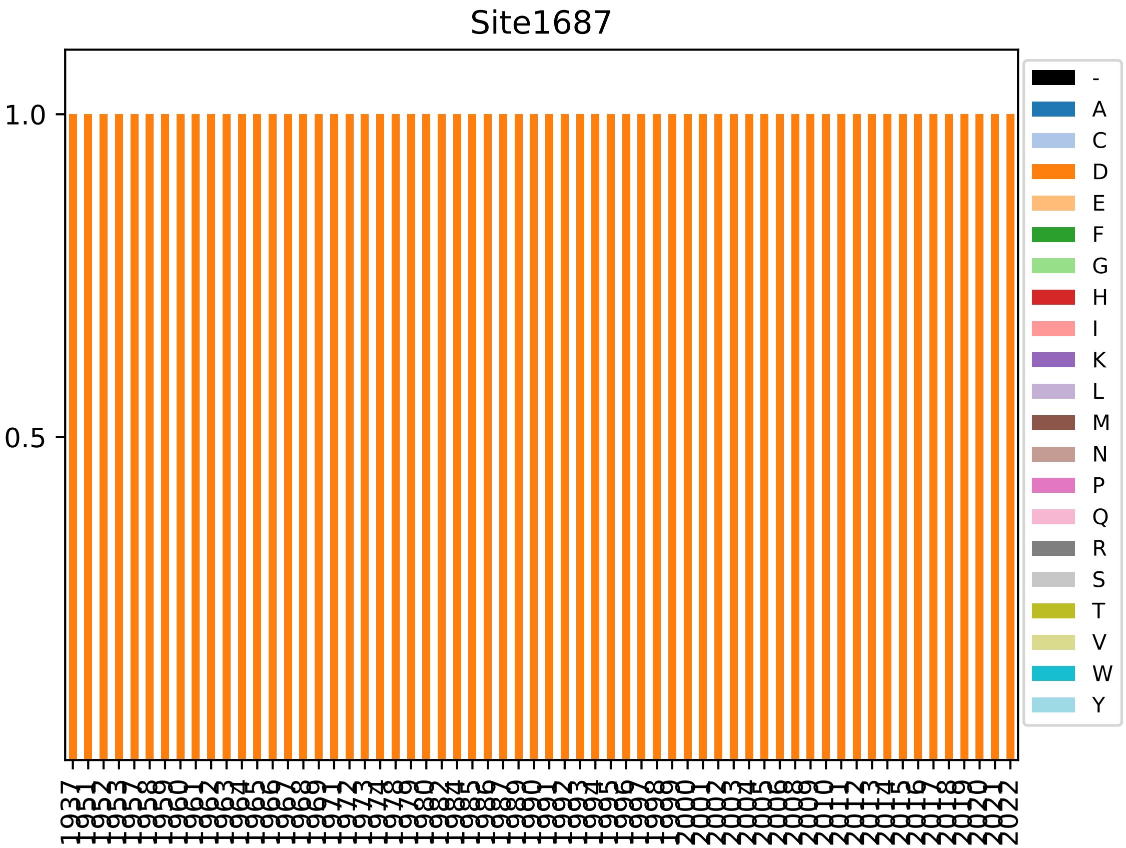
Site1687
1.0
0.5
-
A
C
D
E
F
G
H
I
K
L
M
N
P
Q
R
S
T
V
W
Y

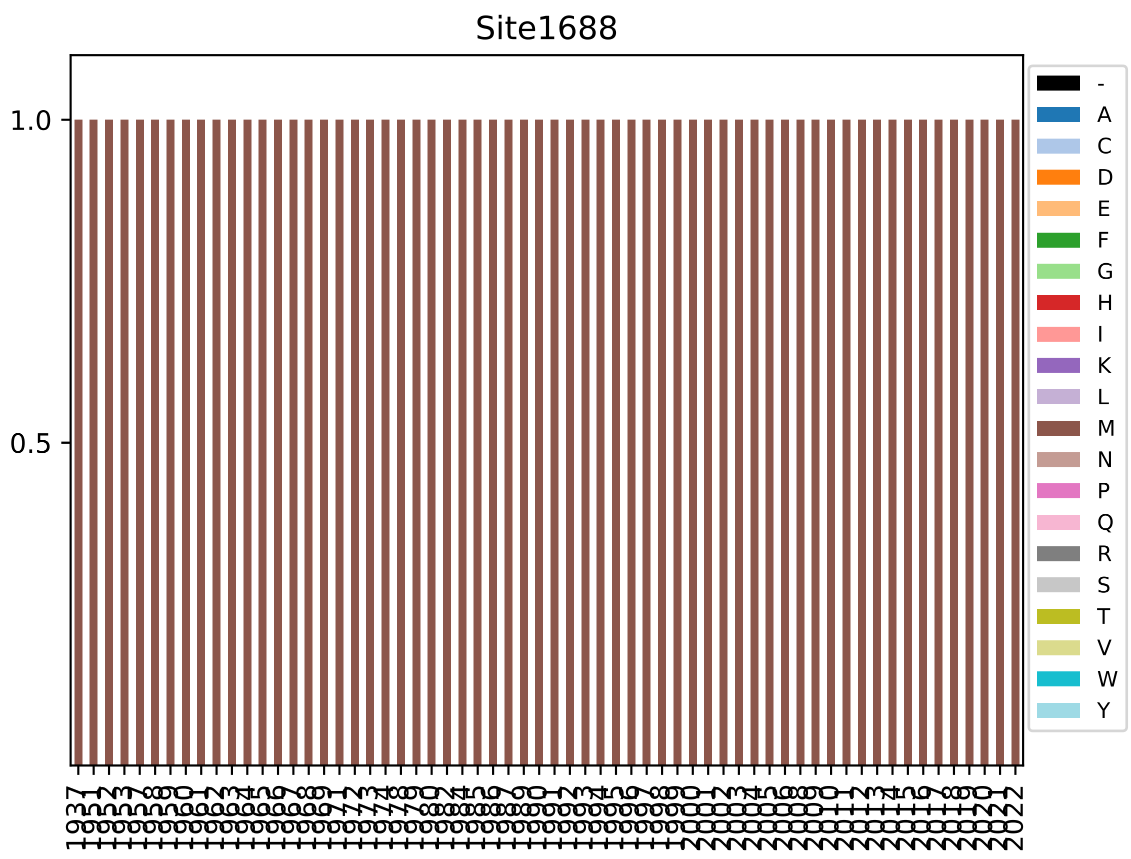
Site1688
1.0
0.5
-
A
C
D
E
F
G
H
I
K
L
M
N
P
Q
R
S
T
V
W
Y

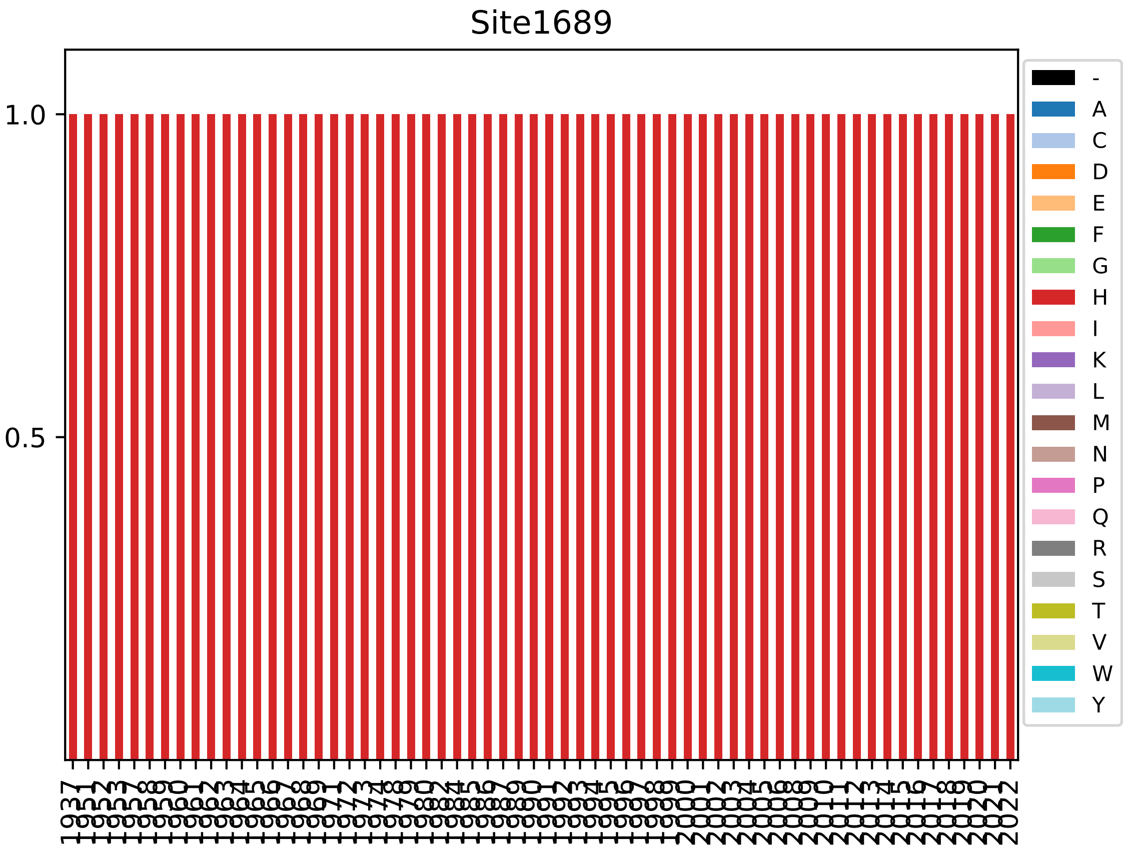
Site1689
1.0
0.5
-
A
C
D
E
F
G
H
I
K
L
M
N
P
Q
R
S
T
V
W
Y

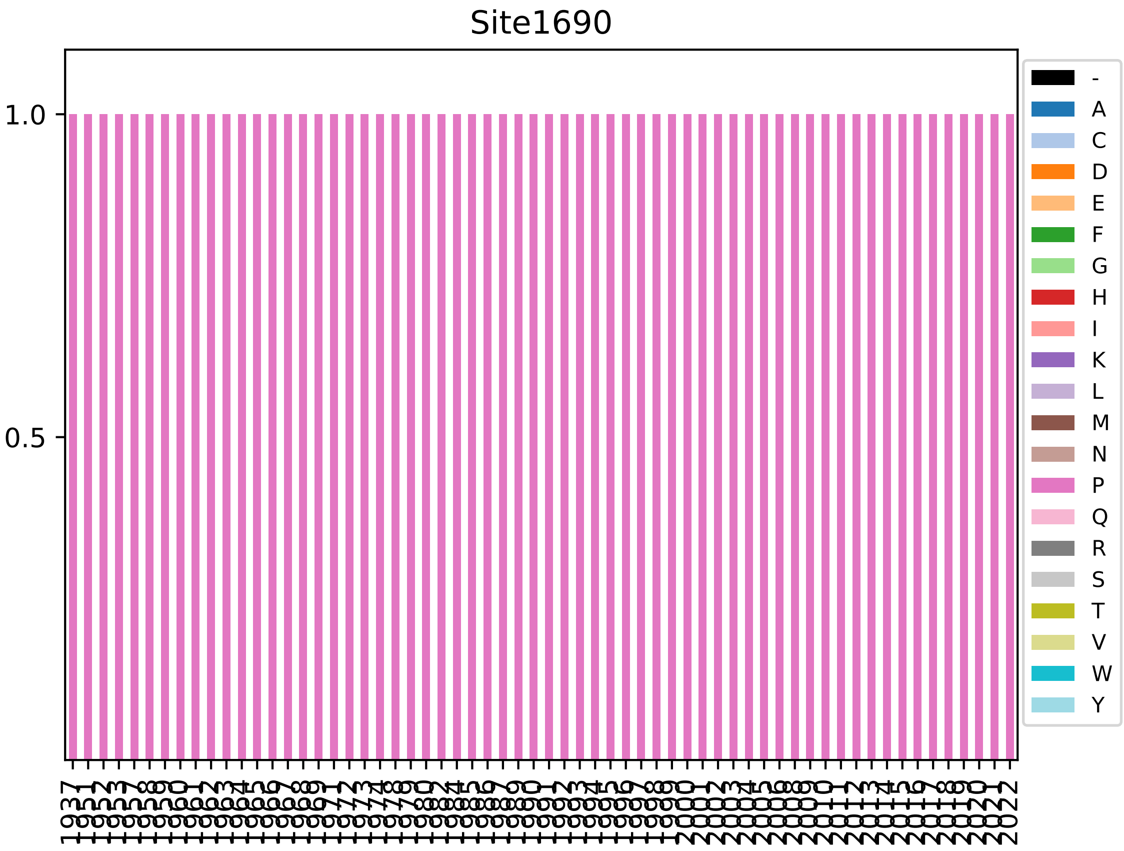
Site1690
1.0
0.5
-
A
C
D
E
F
G
H
I
K
L
M
N
P
Q
R
S
T
V
W
Y

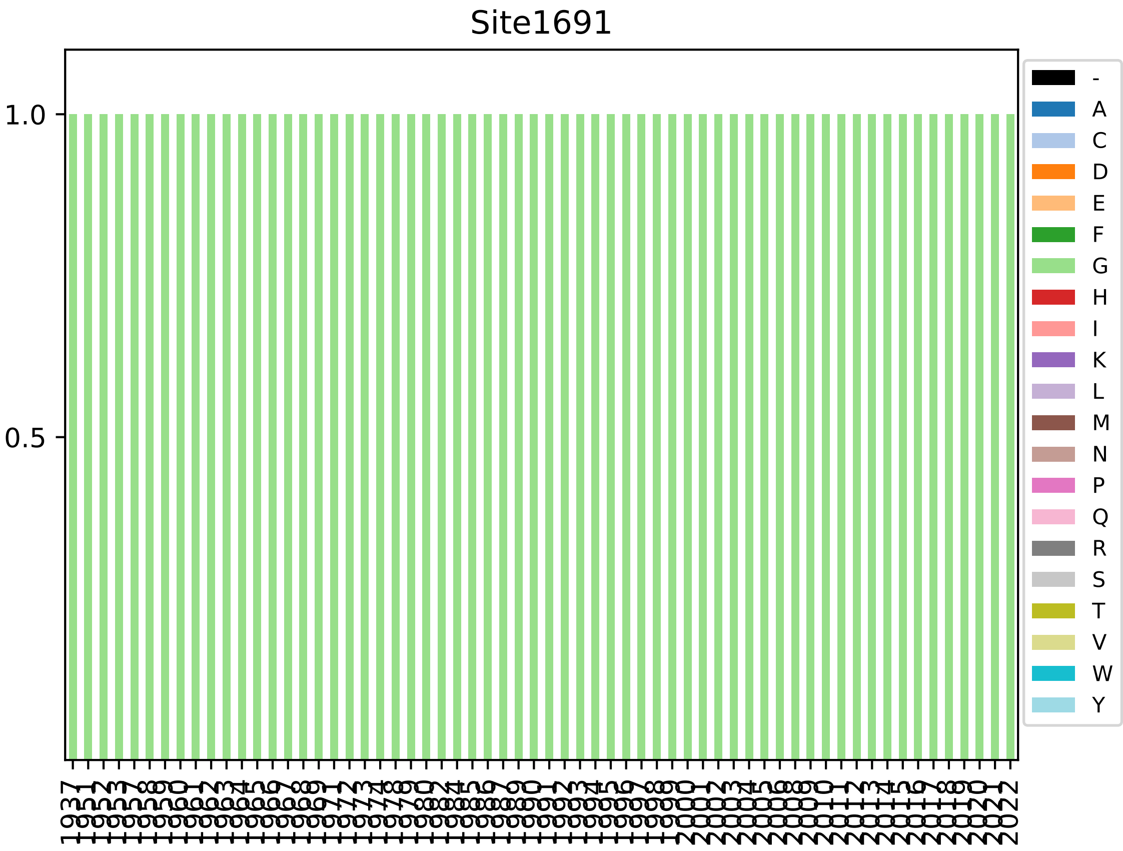
Site1691
1.0
0.5
-
A
C
D
E
F
G
H
I
K
L
M
N
P
Q
R
S
T
V
W
Y

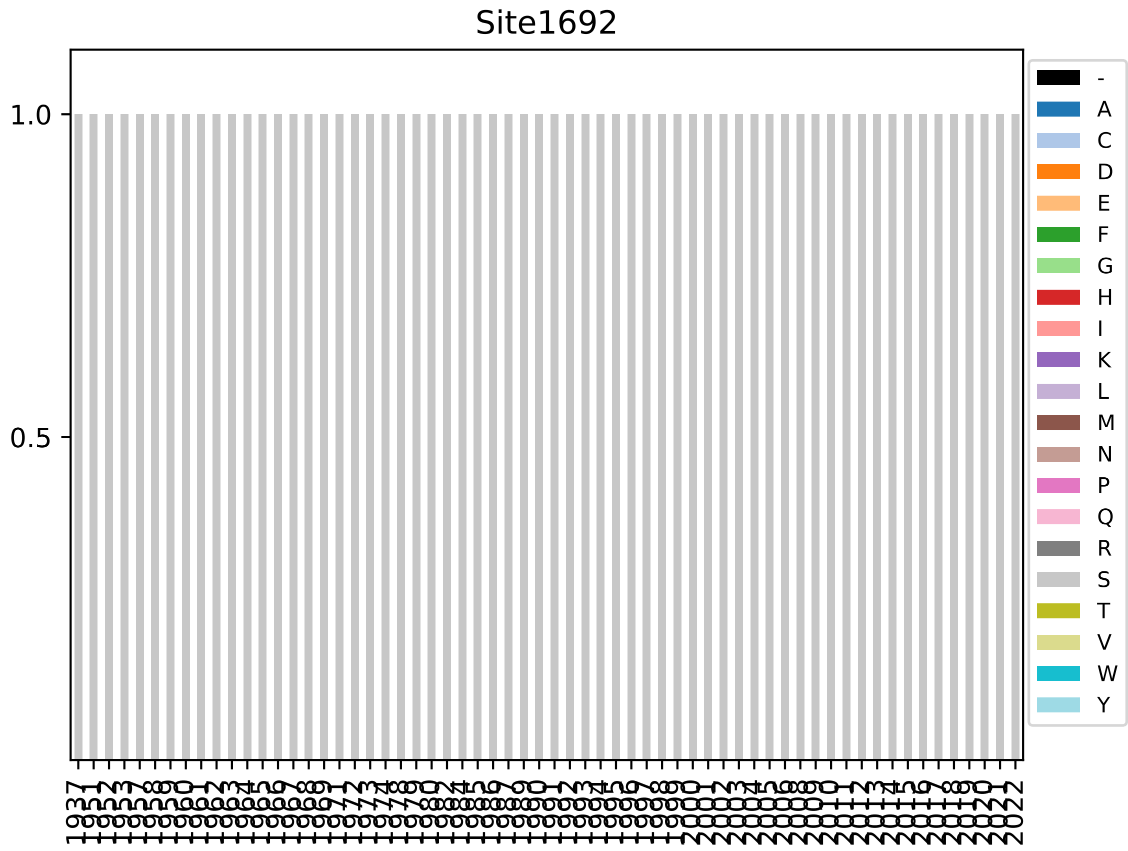
Site1692
1.0
0.5
-
A
C
D
E
F
G
H
I
K
L
M
N
P
Q
R
S
T
V
W
Y

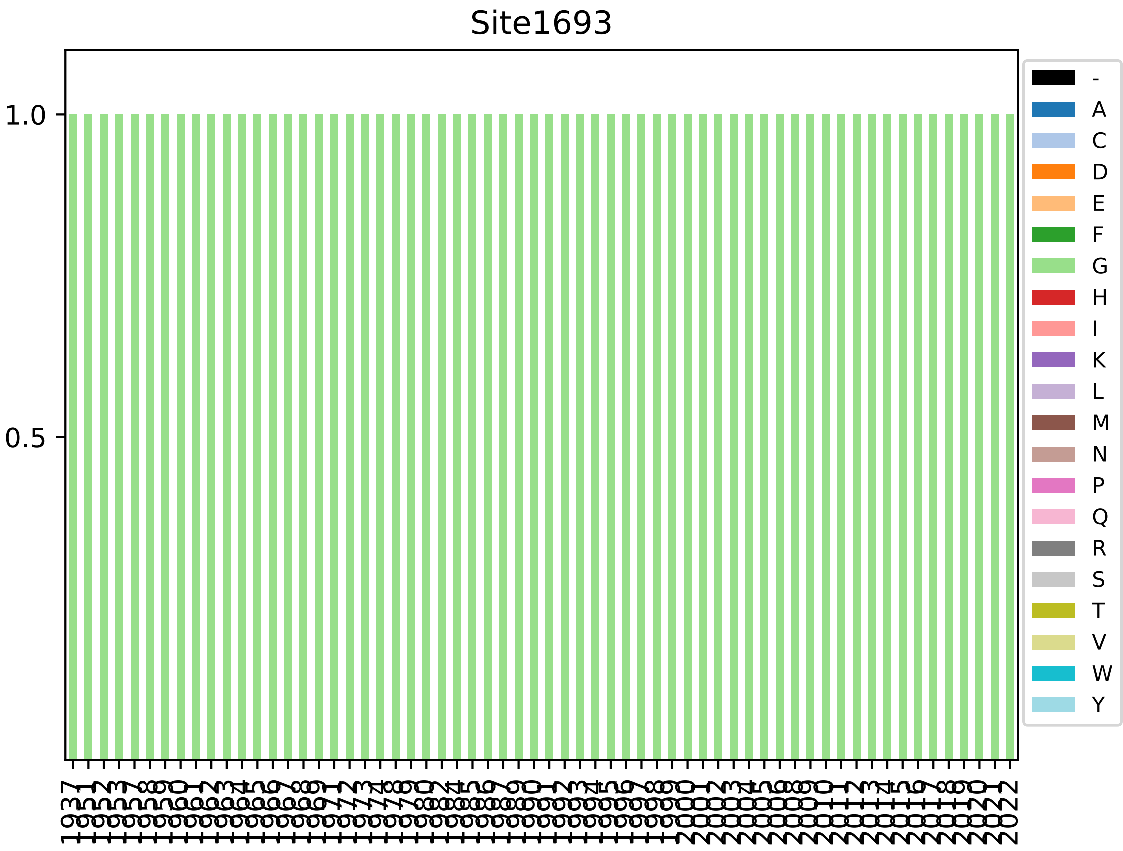
Site1693
1.0
0.5
-
A
C
D
E
F
G
H
I
K
L
M
N
P
Q
R
S
T
V
W
Y

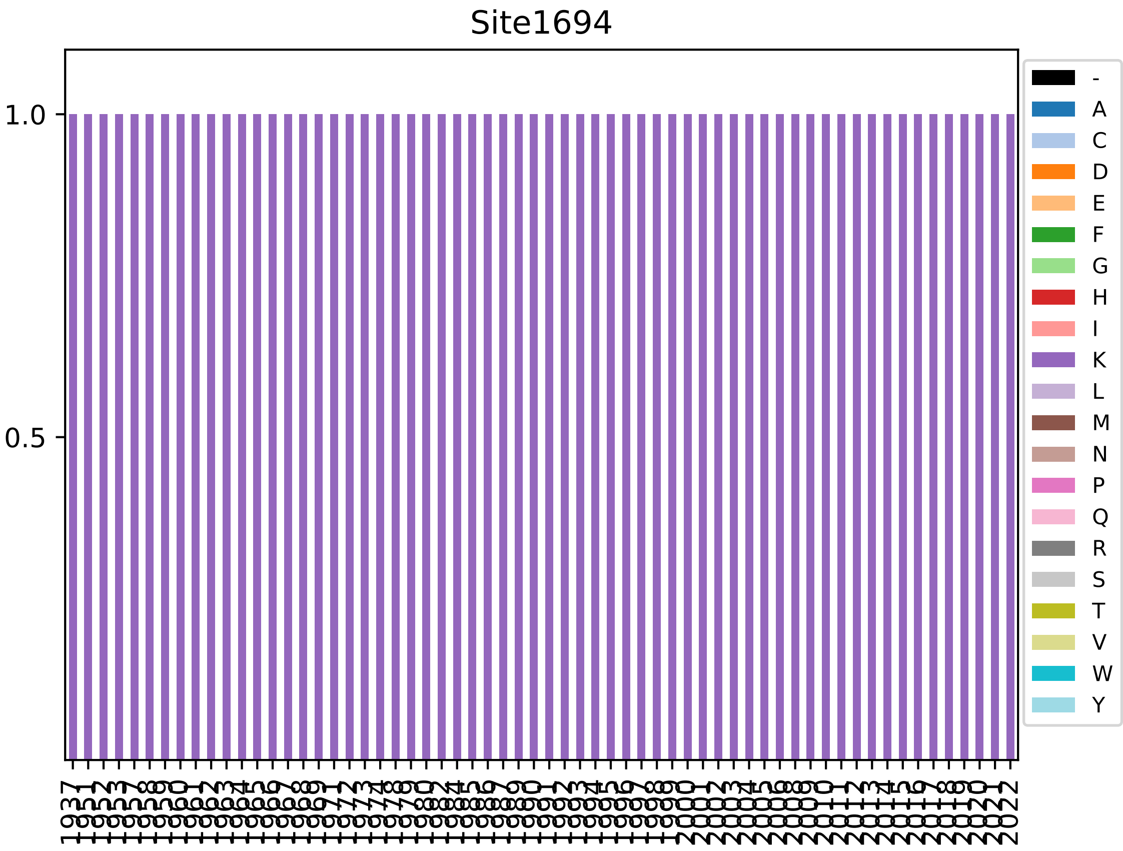
Site1694
1.0
0.5
-
A
C
D
E
F
G
H
I
K
L
M
N
P
Q
R
S
T
V
W
Y

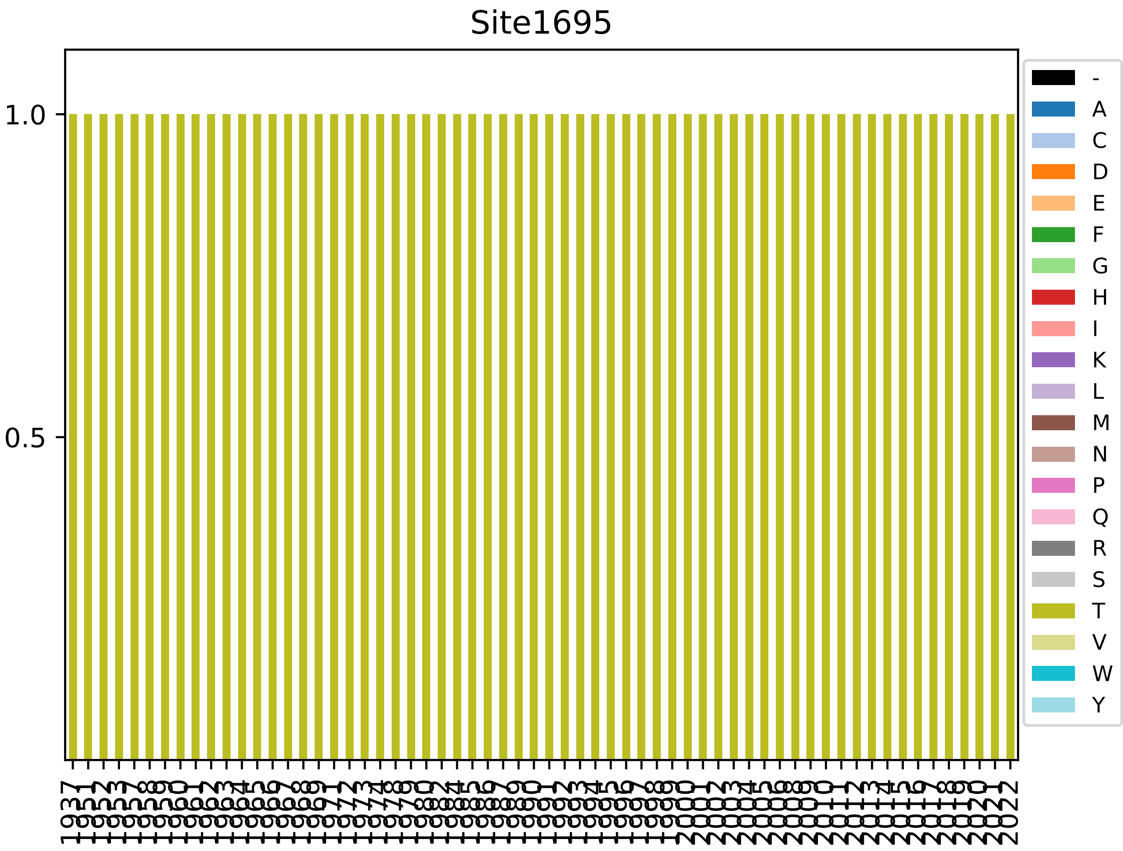
Site1695
1.0
0.5
-
A
C
D
E
F
G
H
I
K
L
M
N
P
Q
R
S
T
V
W
Y

Site1696

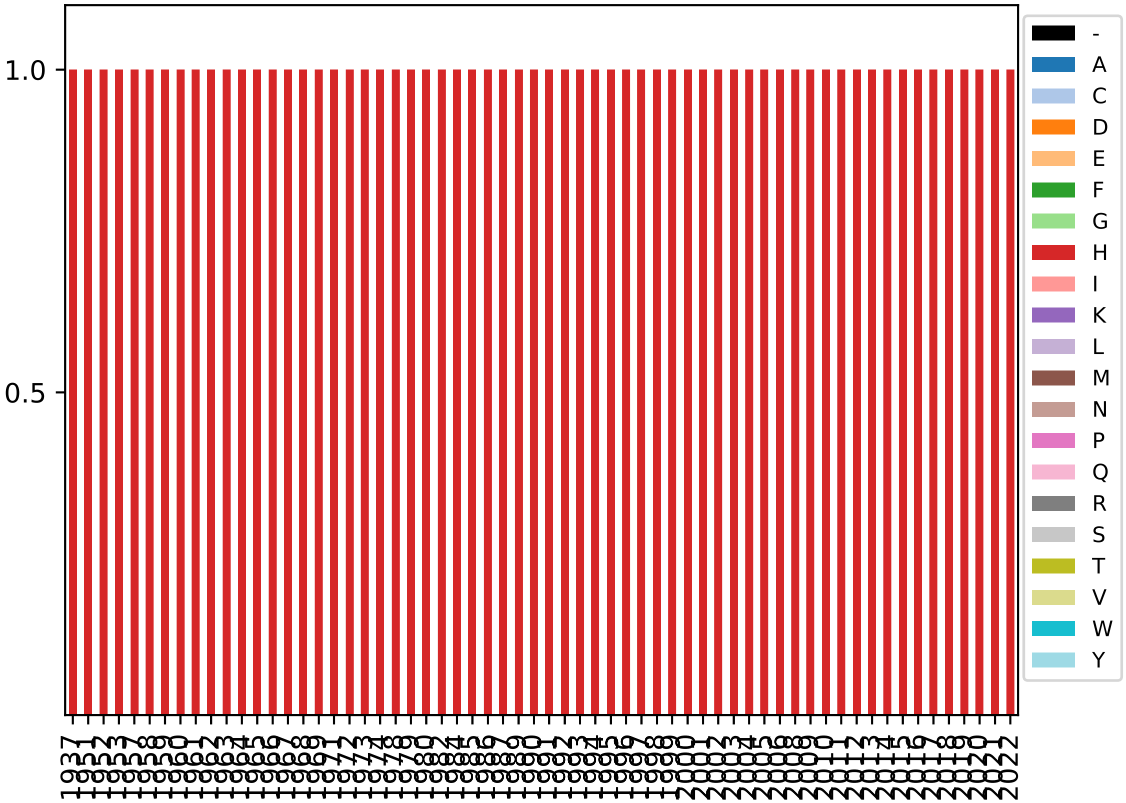

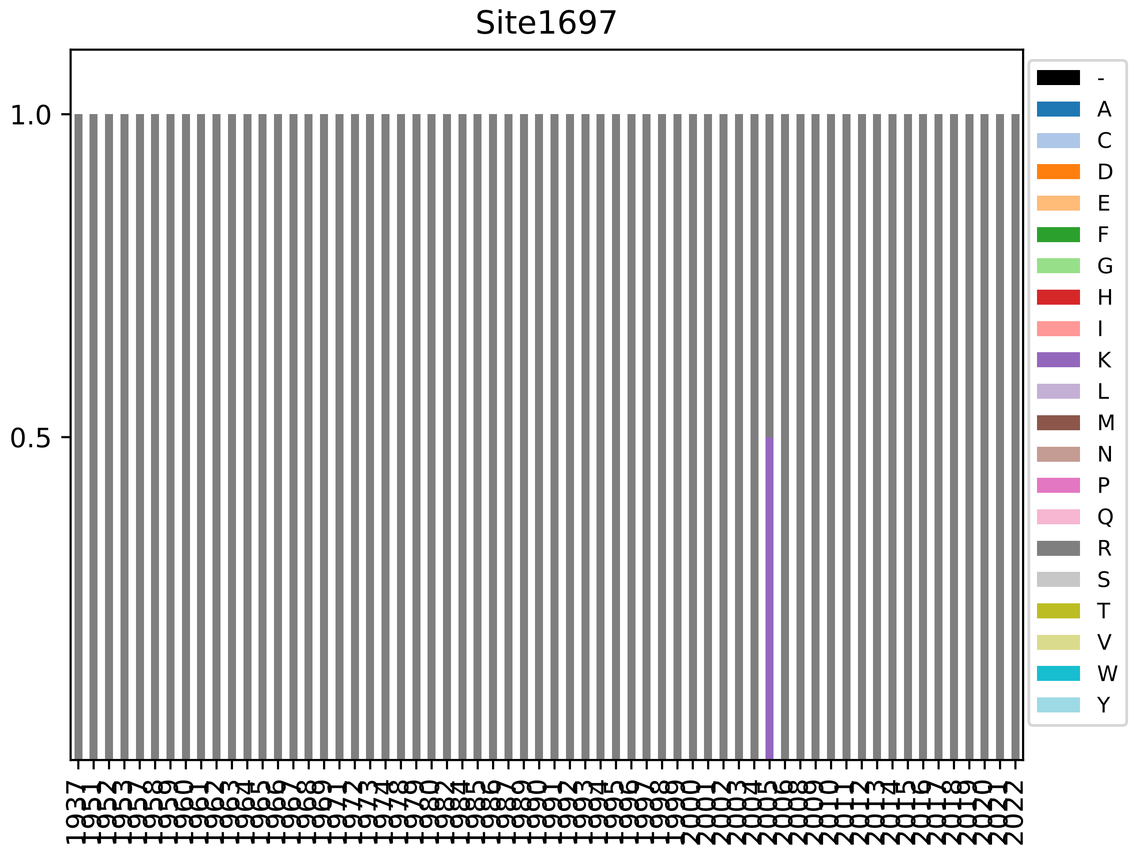
Site1697
1.0
0.5
-
A
C
D
E
F
G
H
I
K
L
M
N
P
Q
R
S
T
V
W
Y

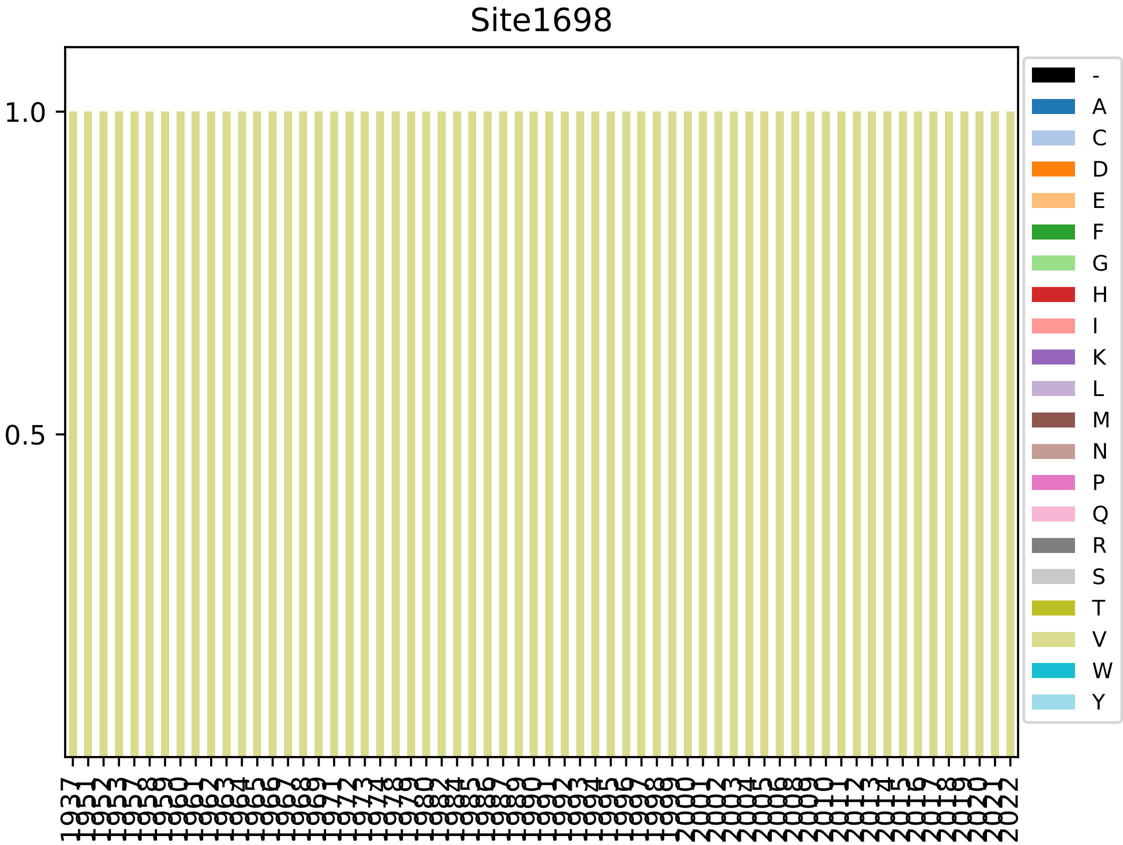
Site1698
1.0
0.5
-
A
C
D
E
F
G
H
I
K
L
M
N
P
Q
R
S
T
V
W
Y

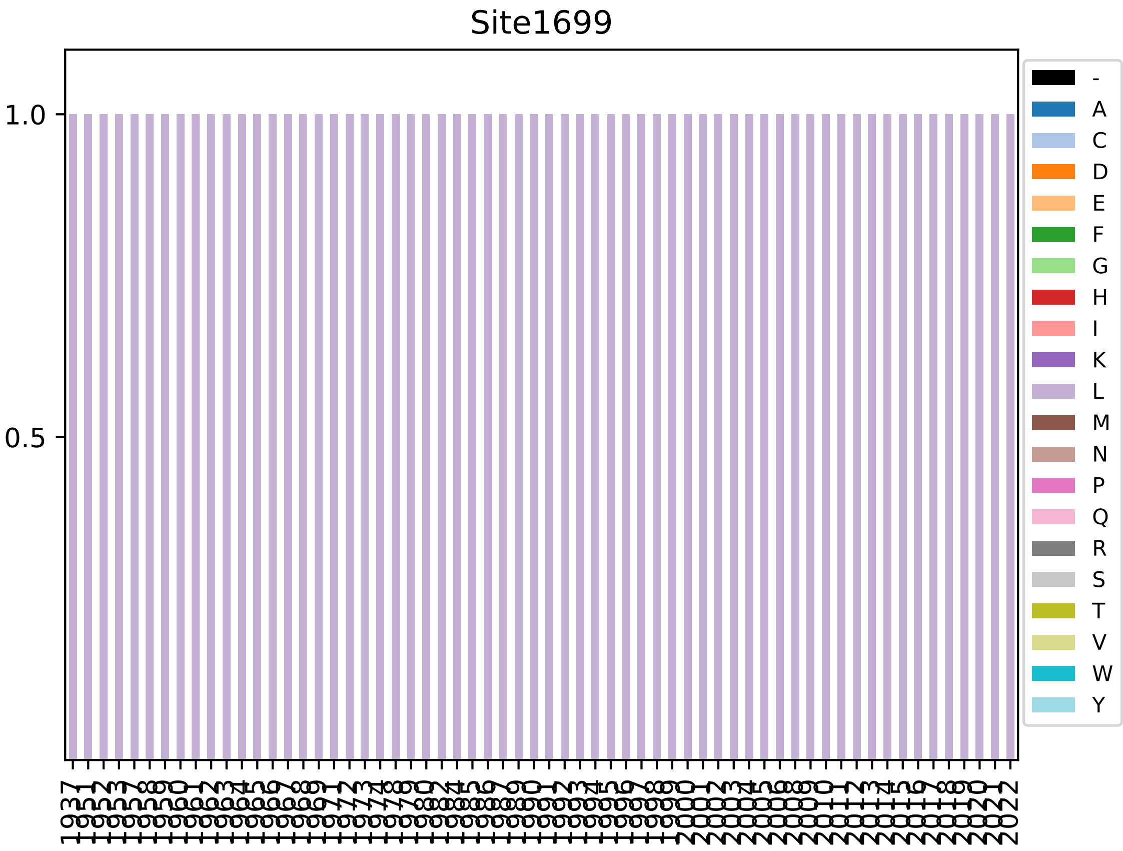
Site1699
1.0
0.5
-
A
C
D
E
F
G
H
I
K
L
M
N
P
Q
R
S
T
V
W
Y

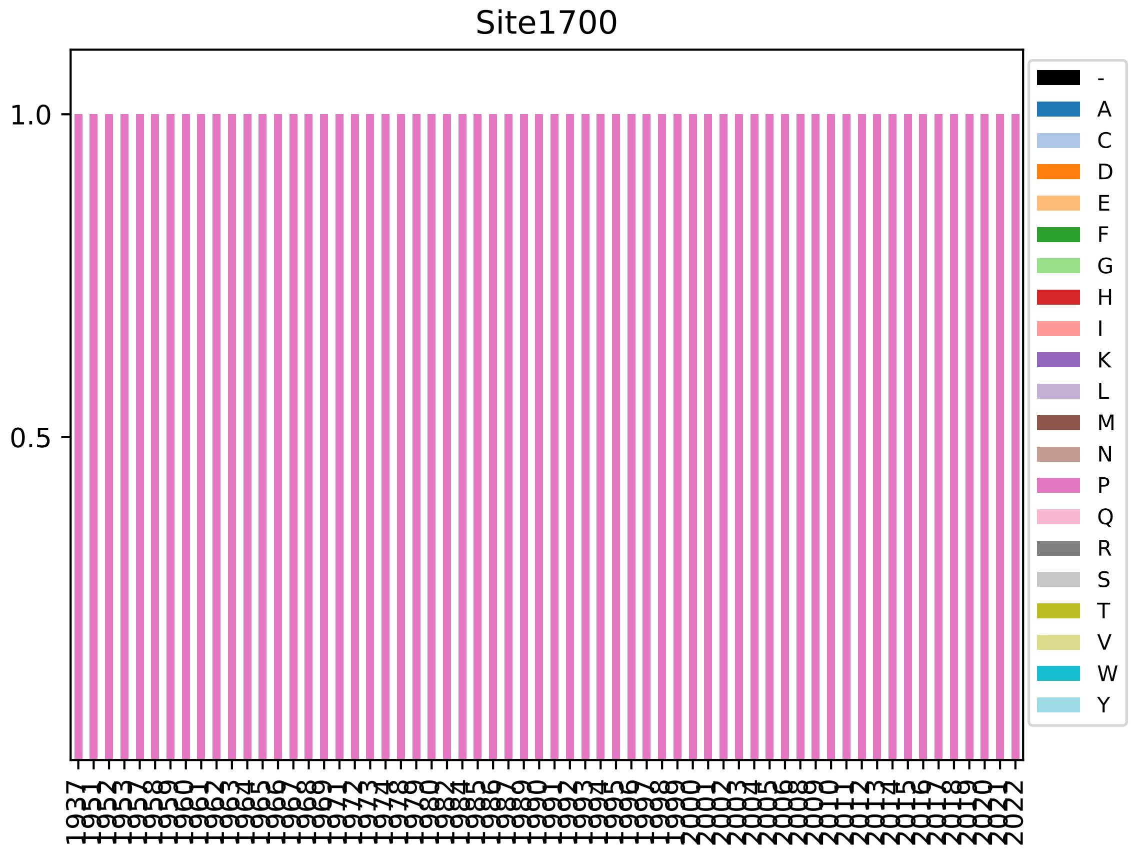
Site1700
1.0
0.5
-
A
C
D
E
F
G
H
I
K
L
M
N
P
Q
R
S
T
V
W
Y

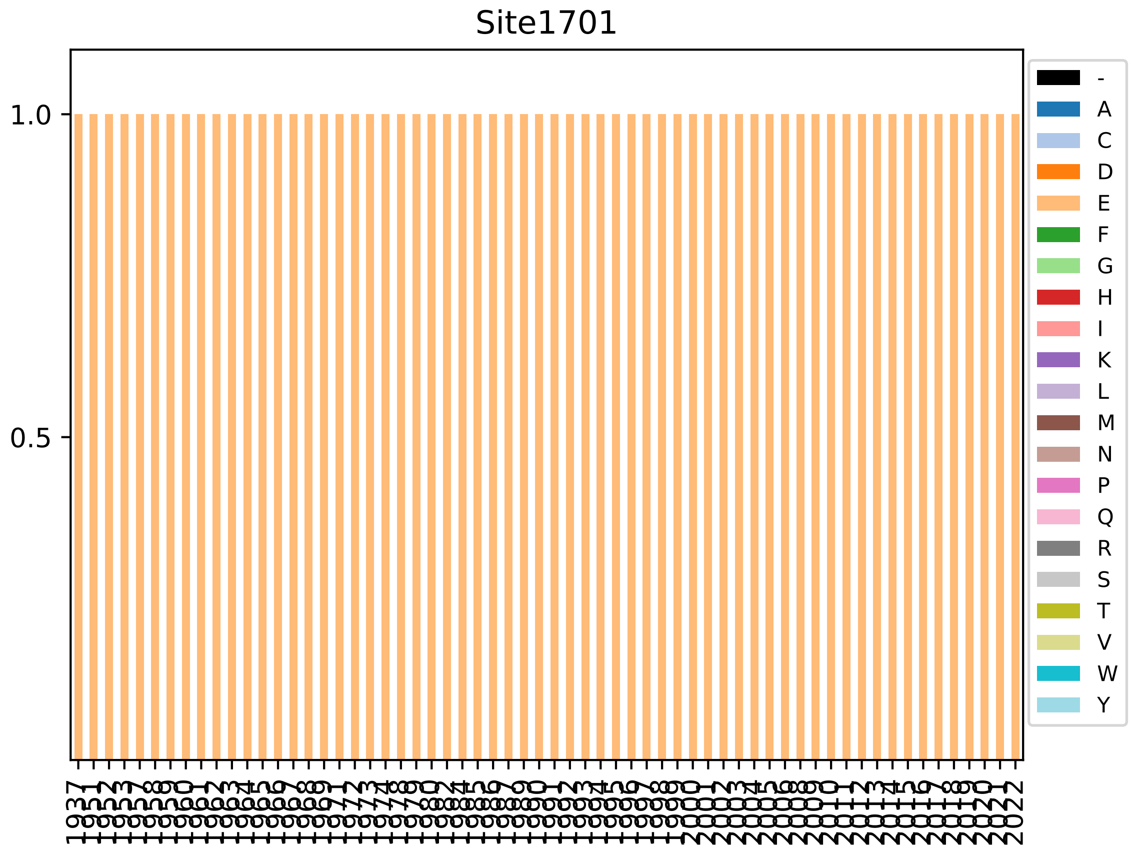
Site1701
1.0
0.5
-
A
C
D
E
F
G
H
I
K
L
M
N
P
Q
R
S
T
V
W
Y

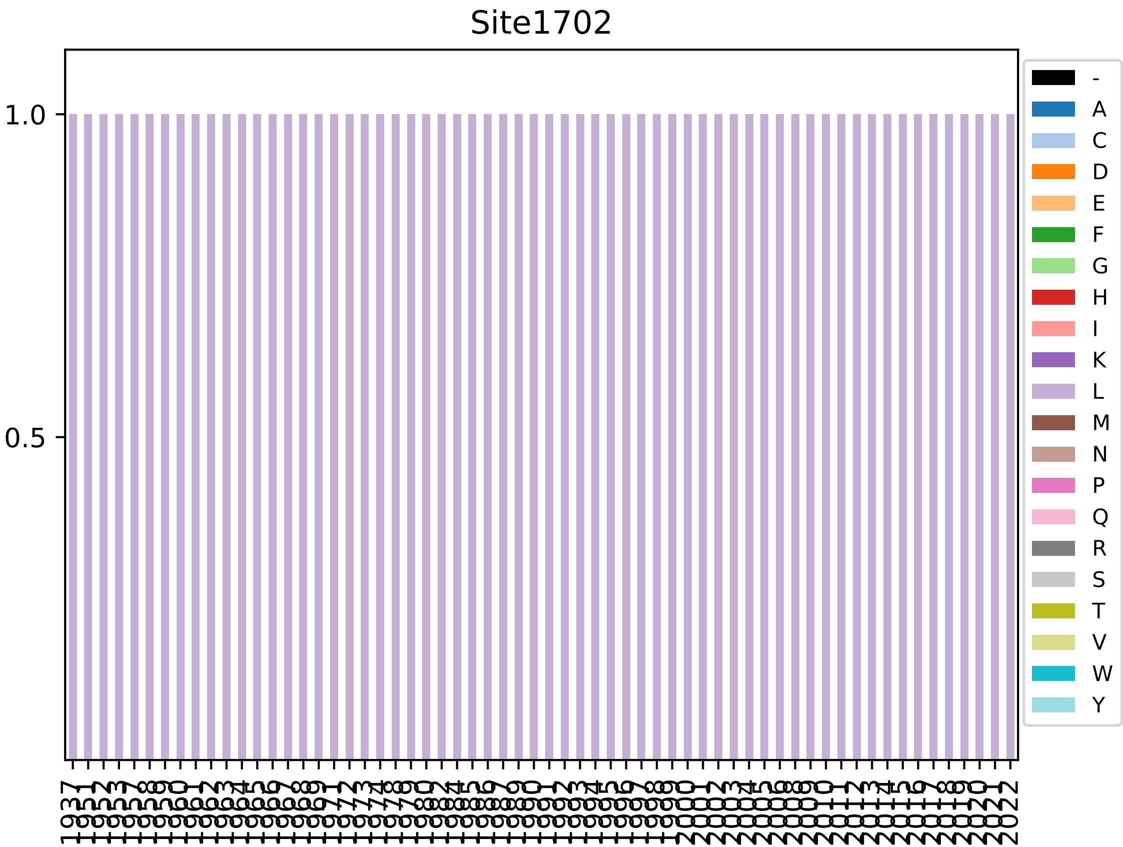
Site1702
1.0
0.5
-
A
C
D
E
F
G
H
I
K
L
M
N
P
Q
R
S
T
V
W
Y

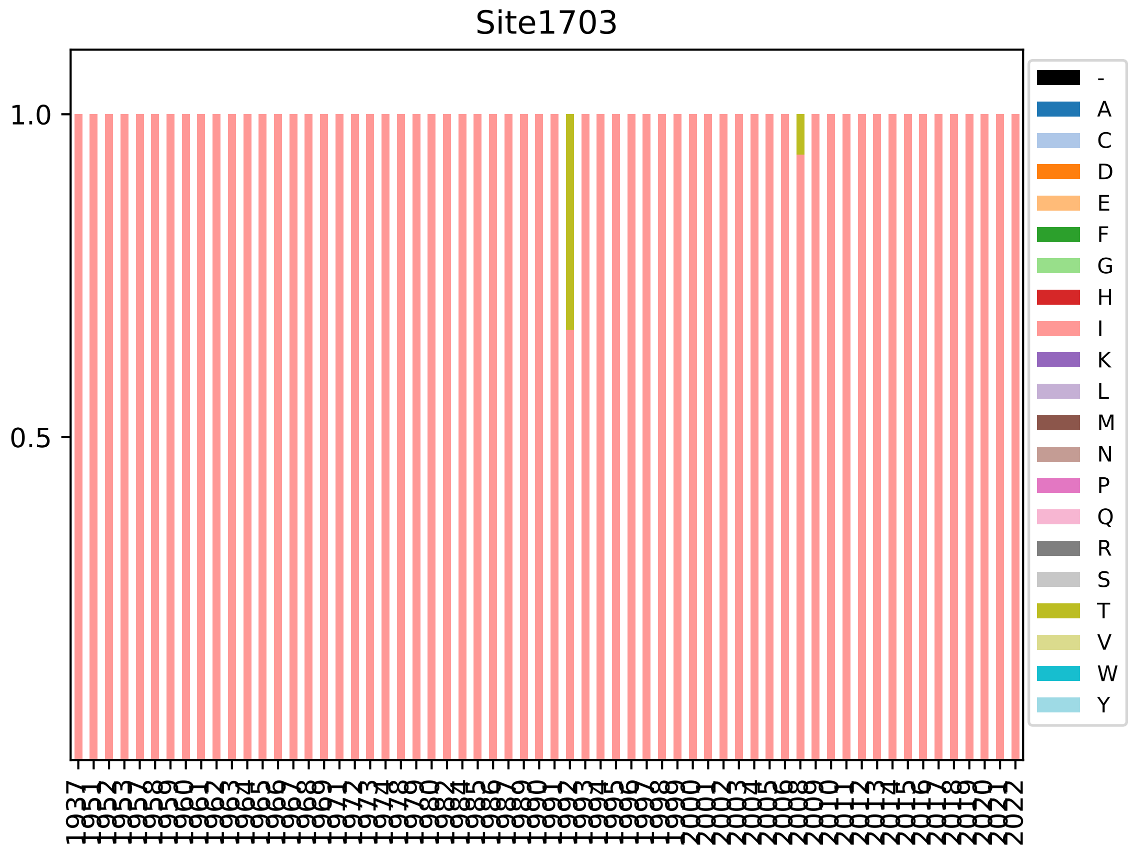
Site1703
1.0
0.5
-
A
C
D
E
F
G
H
I
K
L
M
N
P
Q
R
S
T
V
W
Y

Site1704

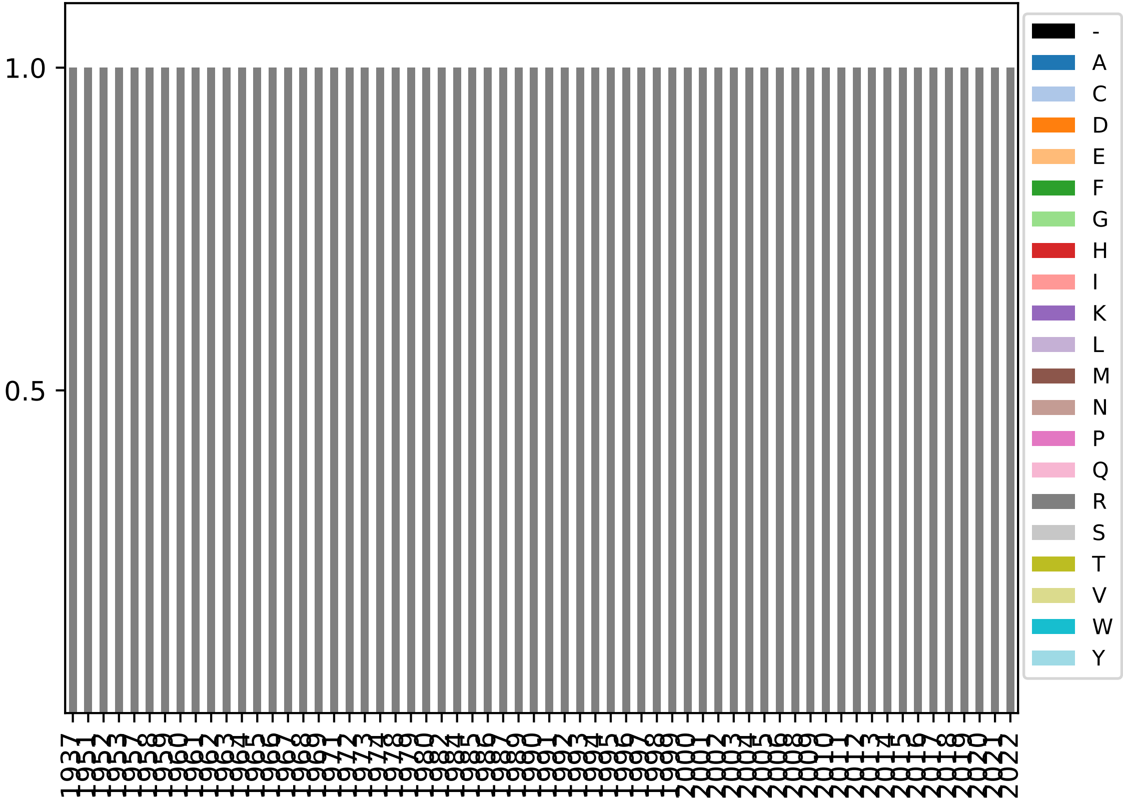

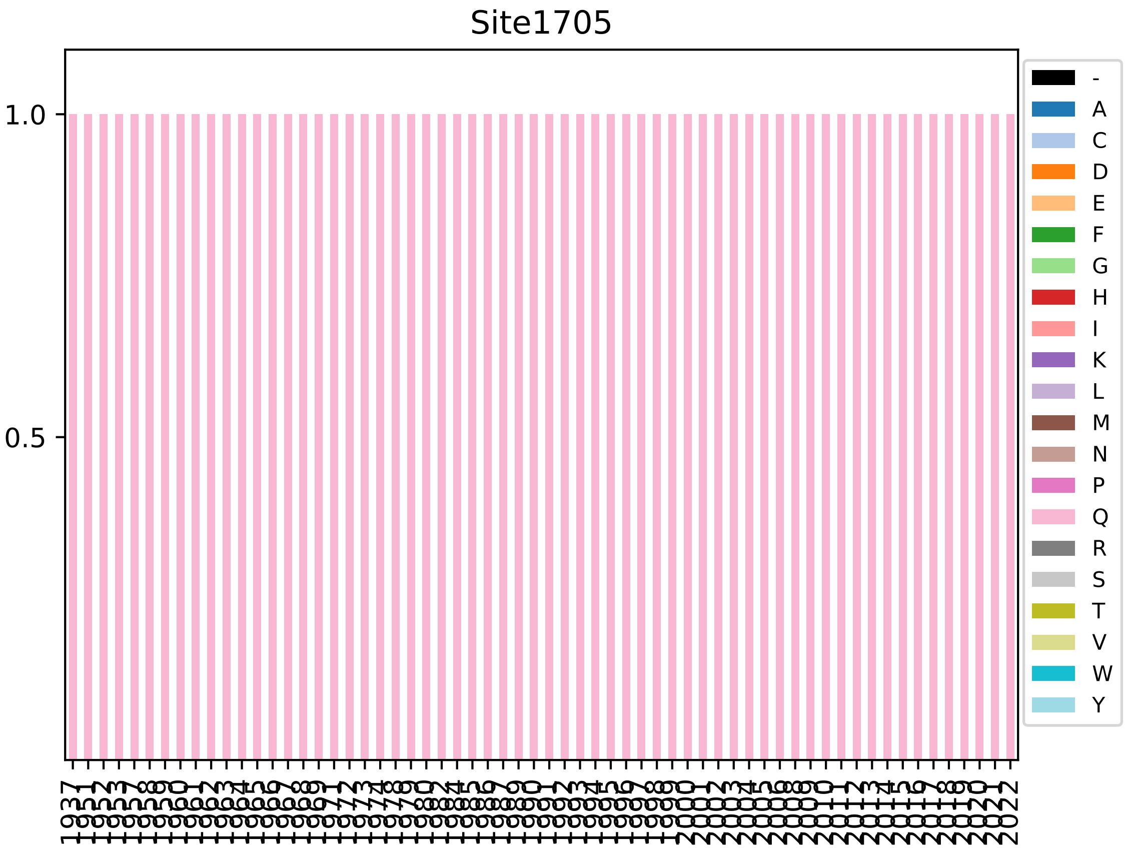
Site1705
1.0
0.5
-
A
C
D
E
F
G
H
I
K
L
M
N
P
Q
R
S
T
V
W
Y

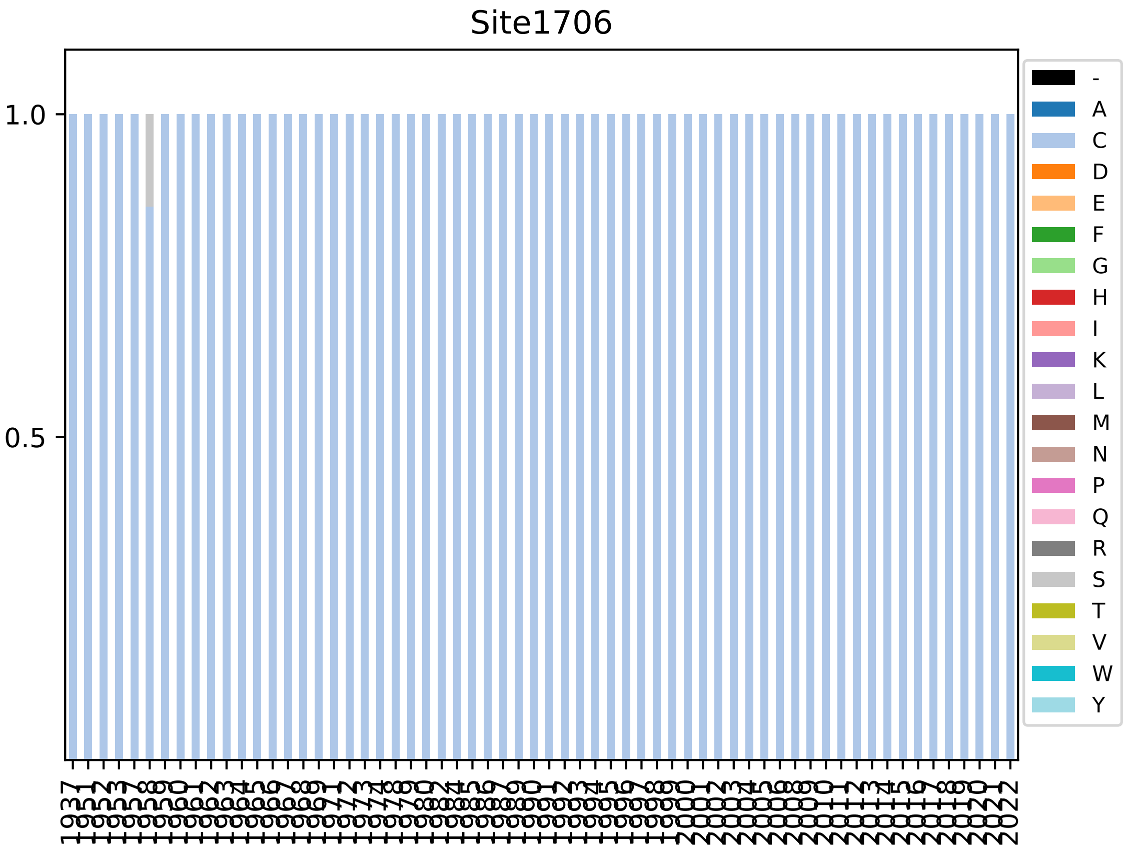
Site1706
1.0
0.5
-
A
C
D
E
F
G
H
I
K
L
M
N
P
Q
R
S
T
V
W
Y

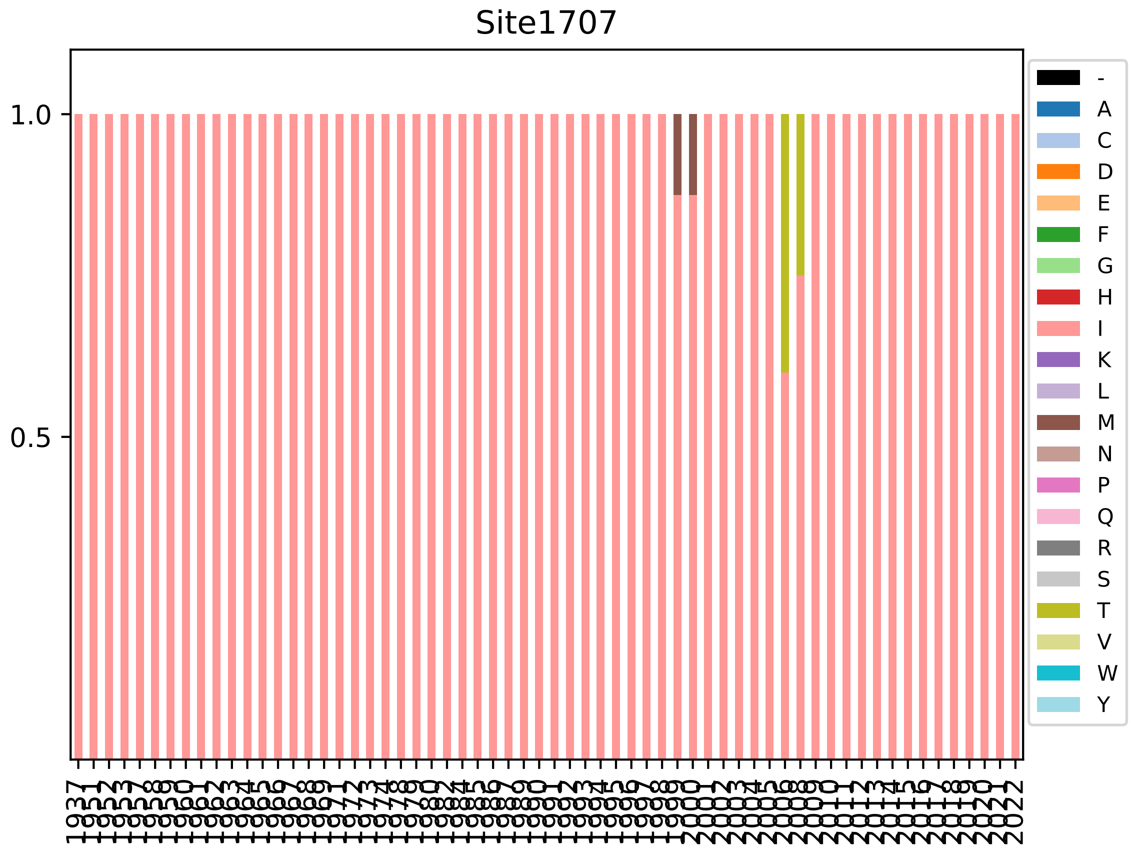
Site1707
1.0
0.5
-
A
C
D
E
F
G
H
I
K
L
M
N
P
Q
R
S
T
V
W
Y

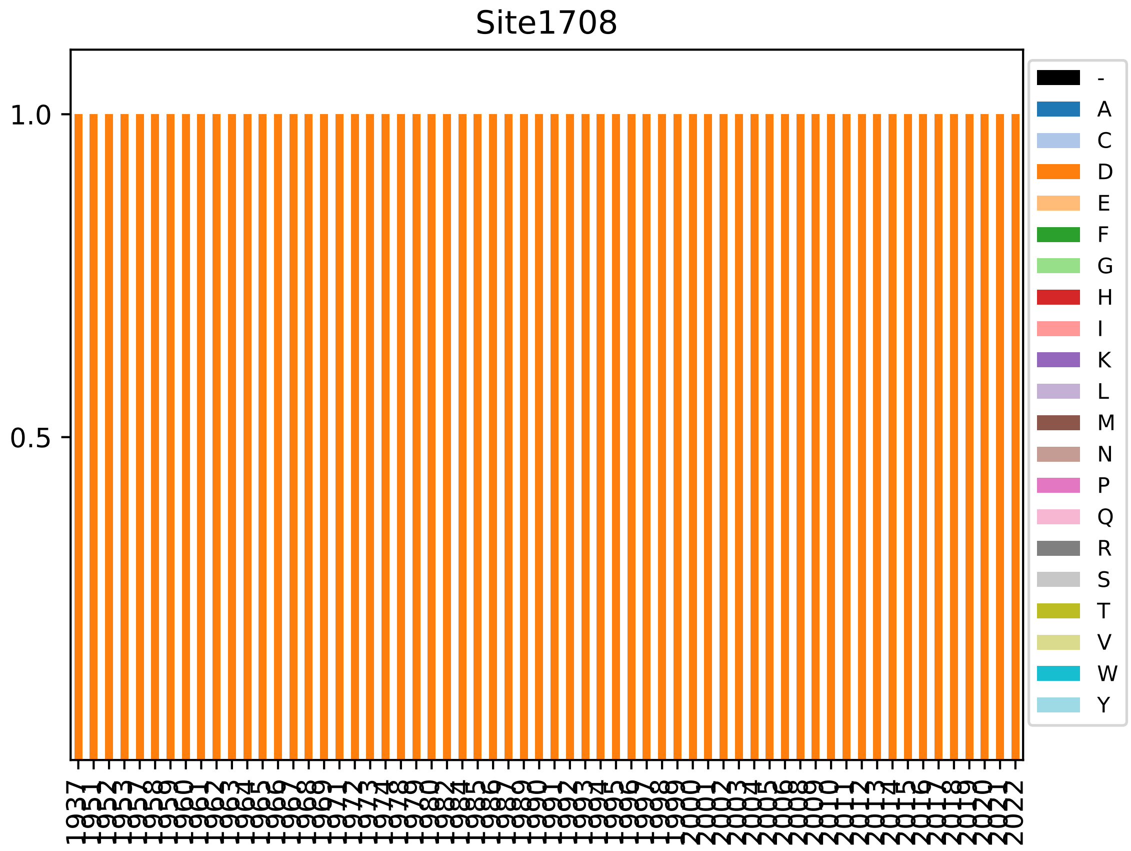
Site1708
1.0
0.5
-
A
C
D
E
F
G
H
I
K
L
M
N
P
Q
R
S
T
V
W
Y

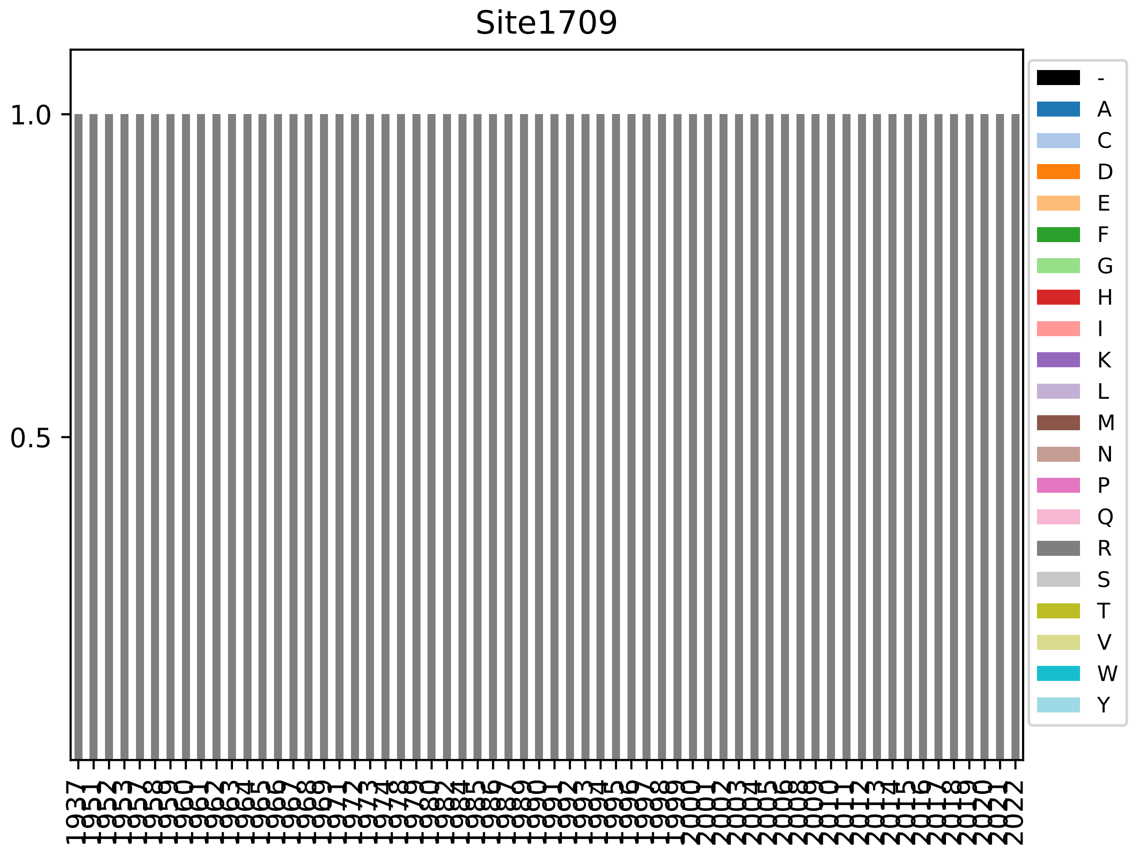
Site1709
1.0
0.5
-
A
C
D
E
F
G
H
I
K
L
M
N
P
Q
R
S
T
V
W
Y

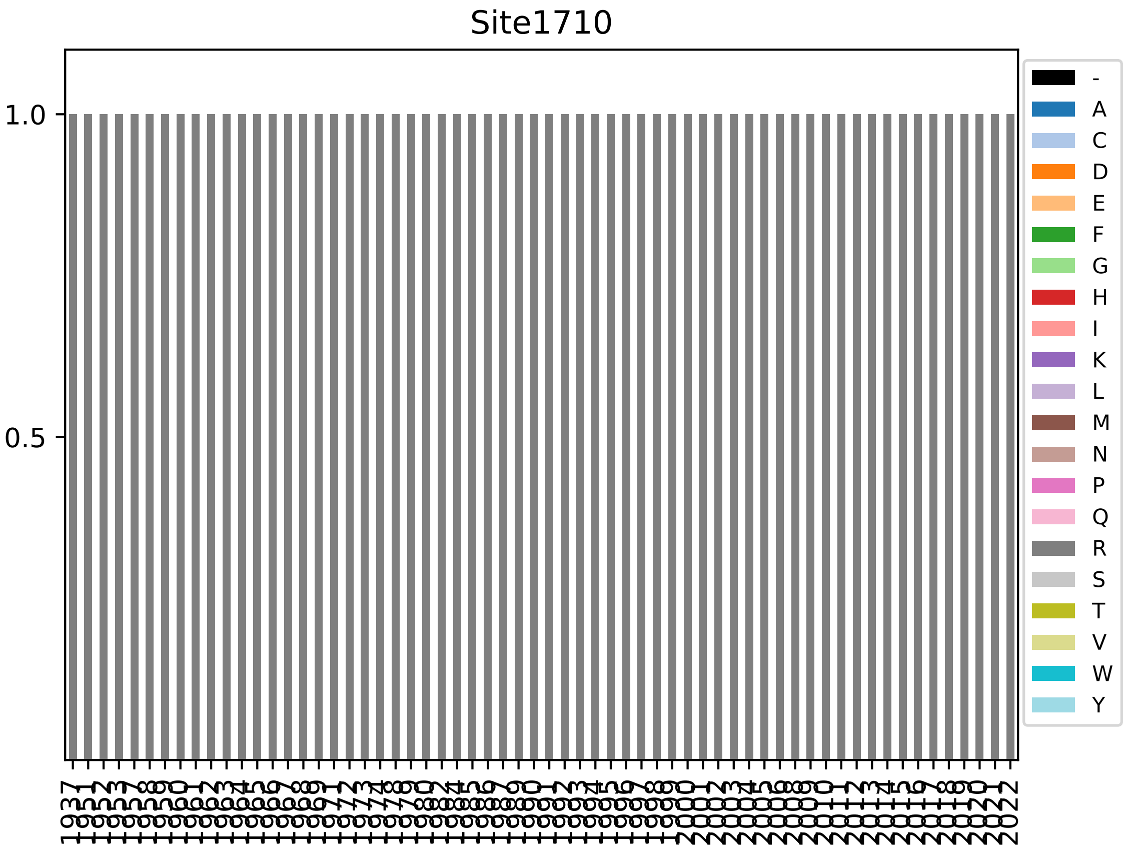
Site1710
1.0
0.5
-
A
C
D
E
F
G
H
I
K
L
M
N
P
Q
R
S
T
V
W
Y

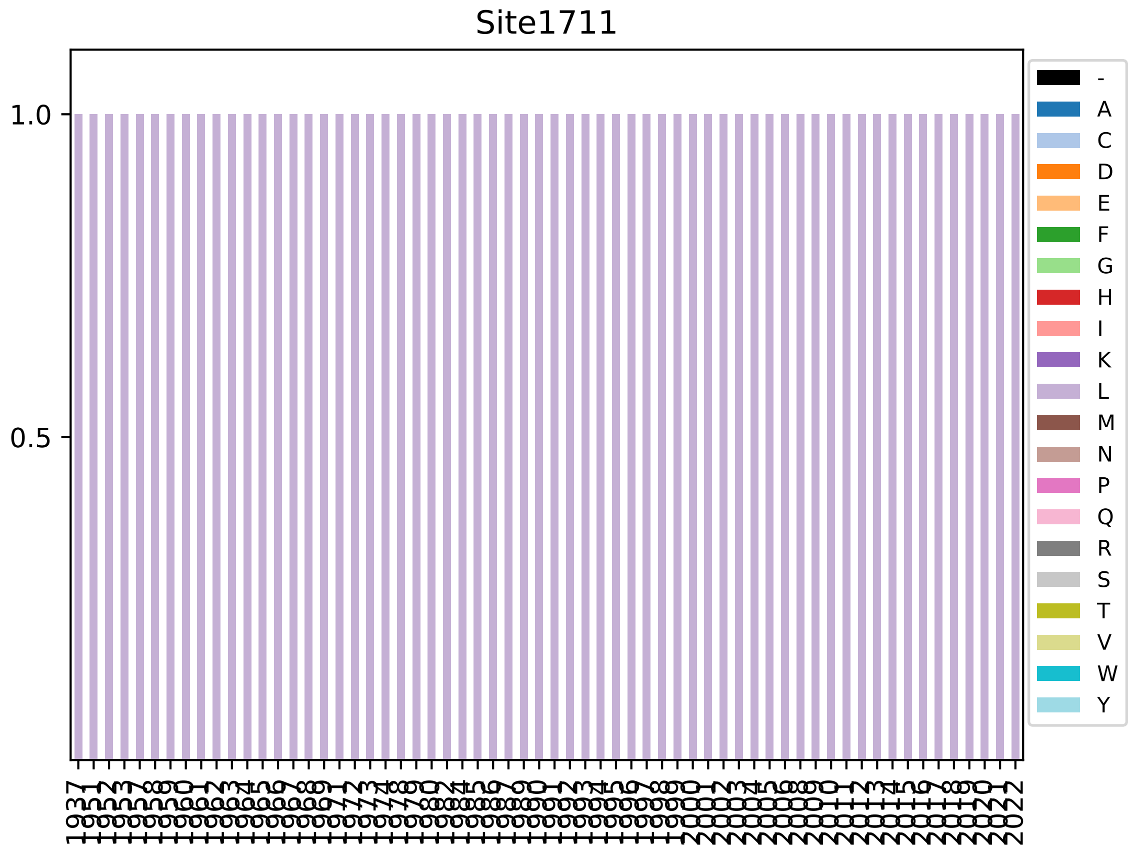
Site1711
1.0
0.5
-
A
C
D
E
F
G
H
I
K
L
M
N
P
Q
R
S
T
V
W
Y

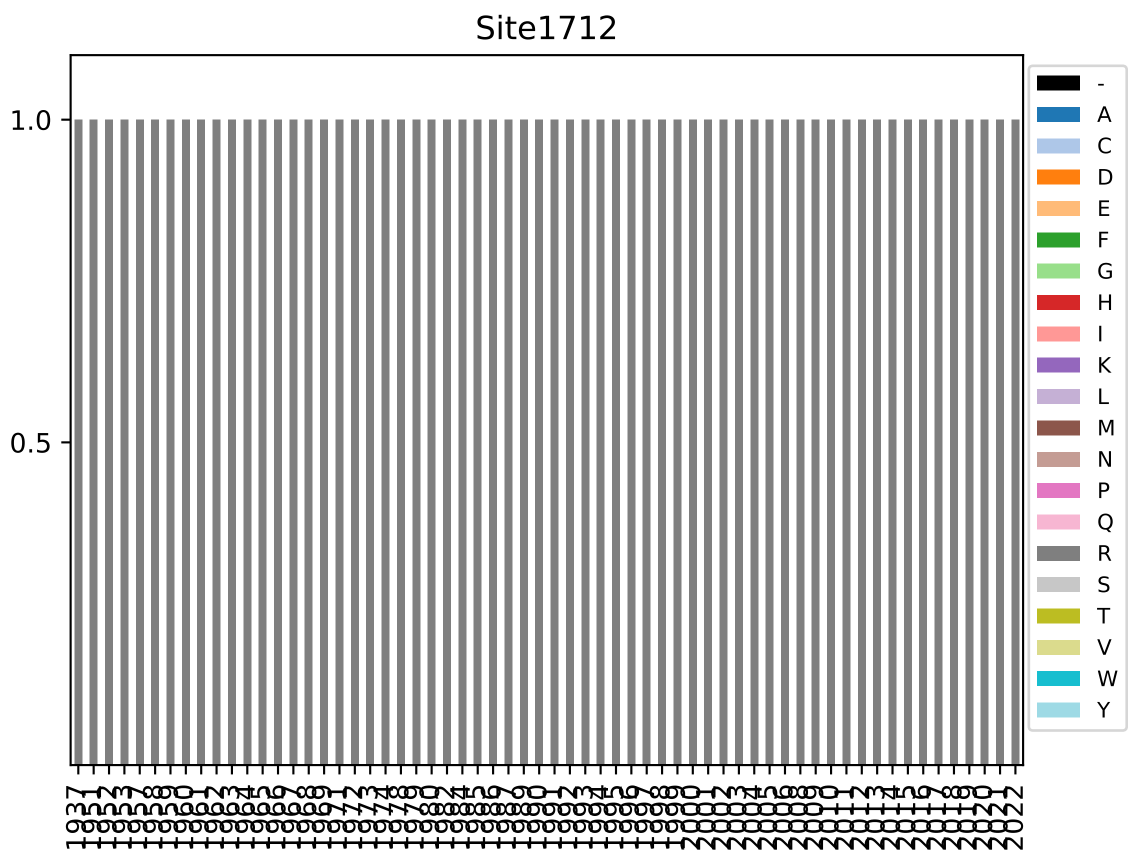
Site1712
1.0
0.5
-
A
C
D
E
F
G
H
I
K
L
M
N
P
Q
R
S
T
V
W
Y

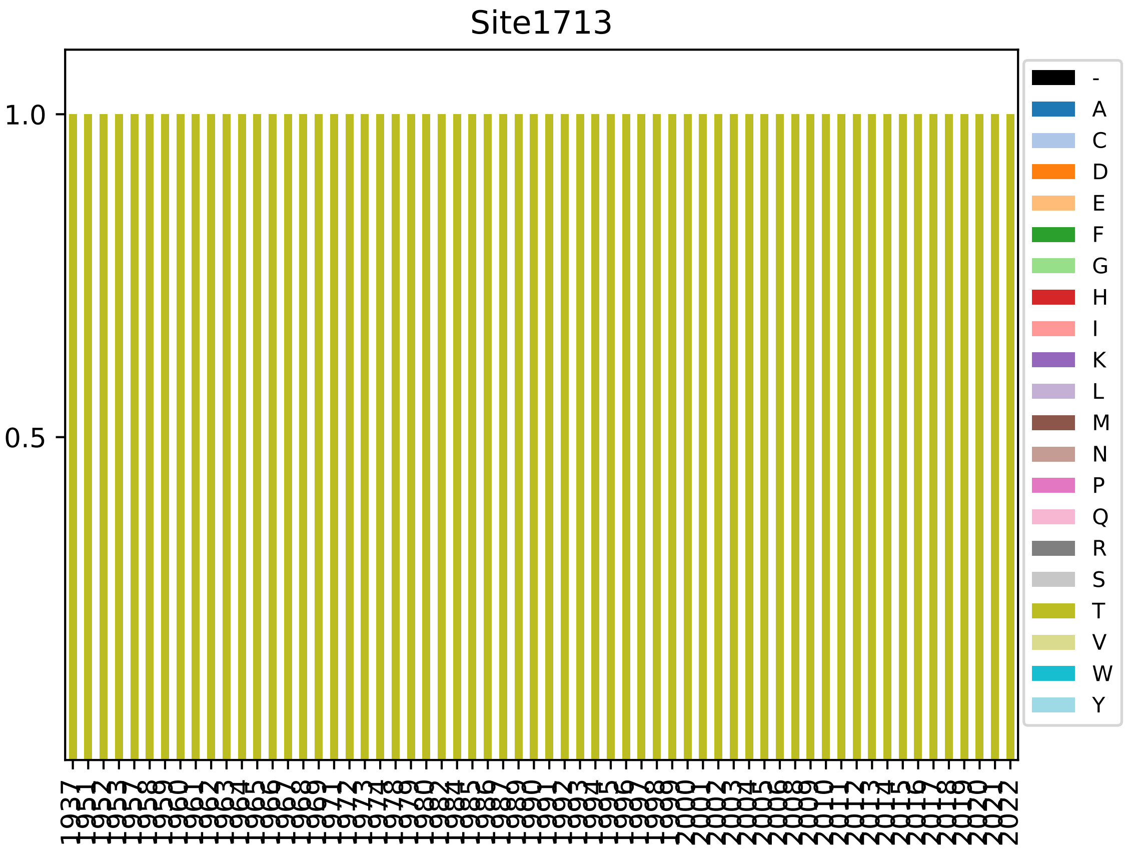
Site1713
1.0
0.5
-
A
C
D
E
F
G
H
I
K
L
M
N
P
Q
R
S
T
V
W
Y

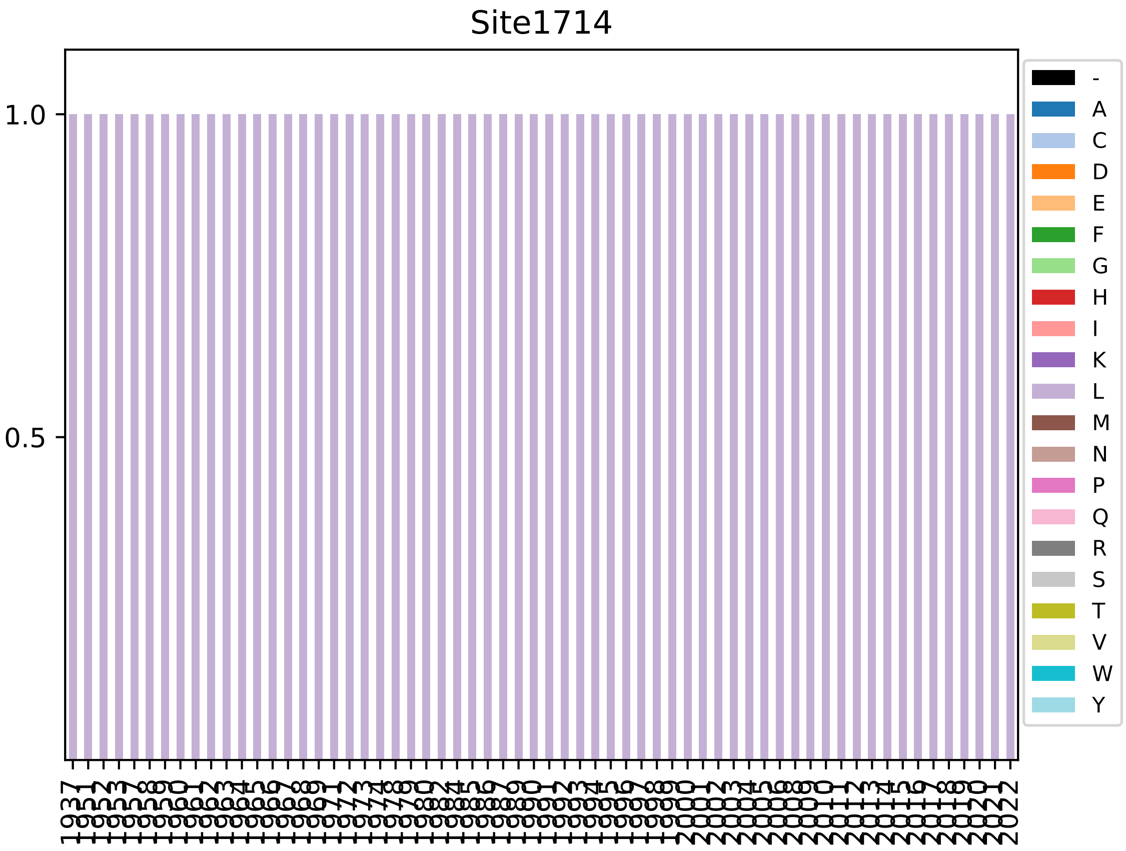
Site1714
1.0
0.5
-
A
C
D
E
F
G
H
I
K
L
M
N
P
Q
R
S
T
V
W
Y

Site1715

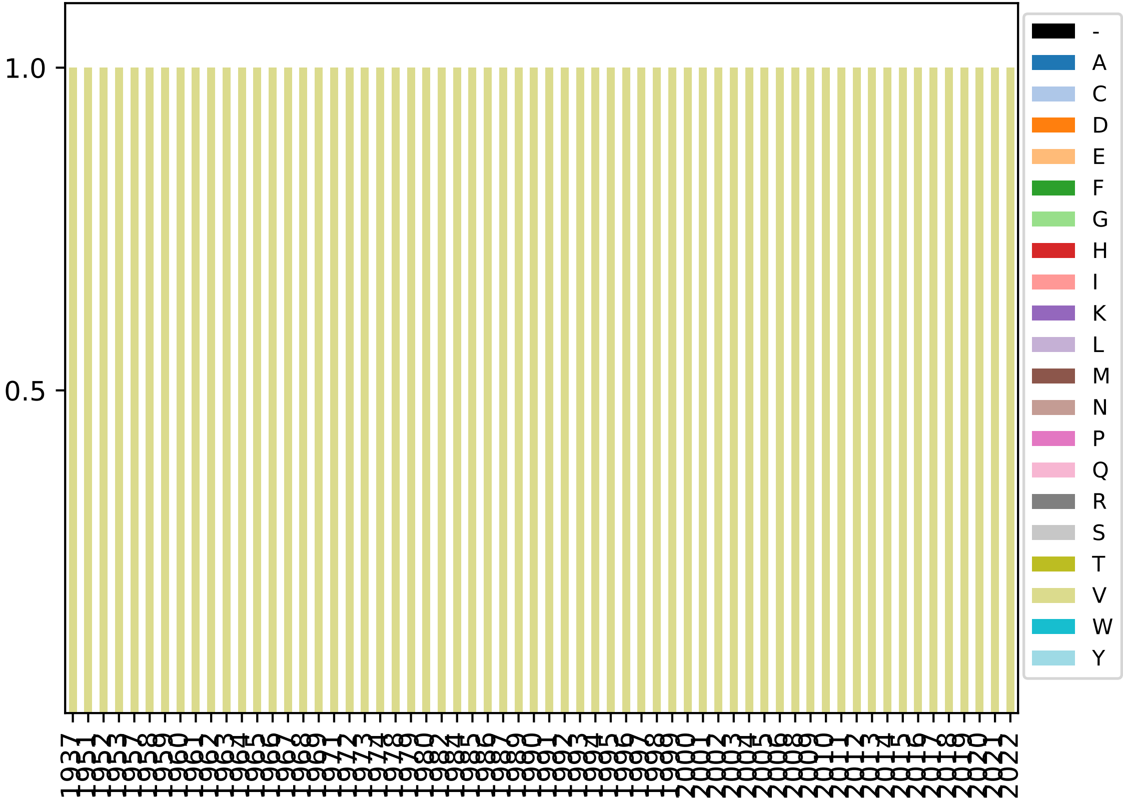

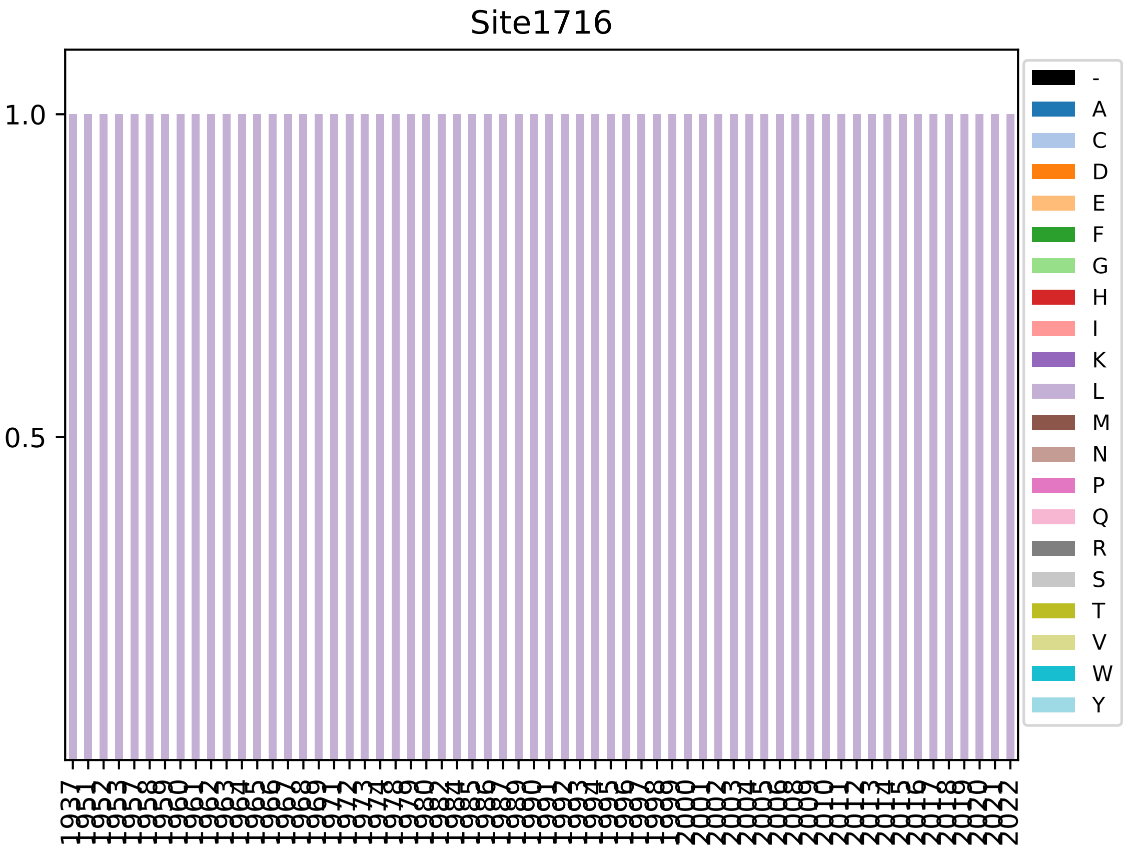

Site1716
1.0
0.5
-
A
C
D
E
F
G
H
I
K
L
M
N
P
Q
R
S
T
V
W
Y

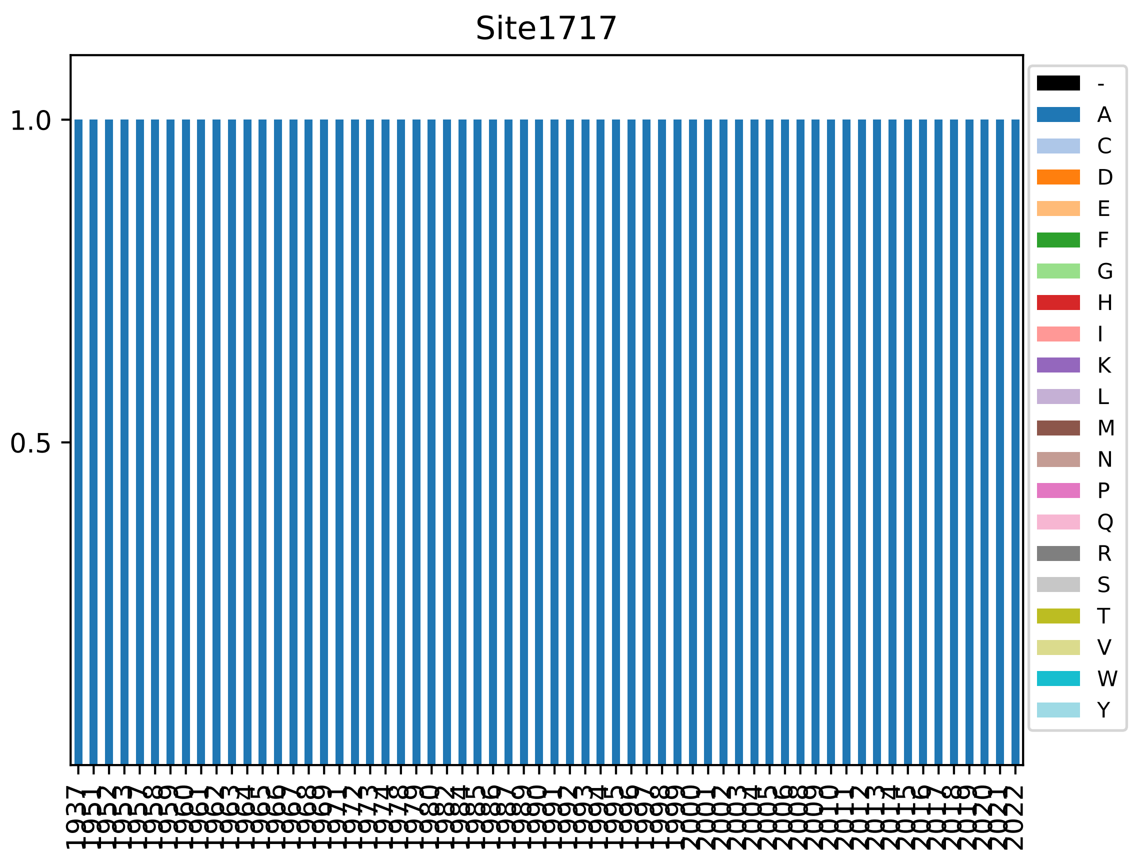
Site1717
1.0
0.5
-
A
C
D
E
F
G
H
I
K
L
M
N
P
Q
R
S
T
V
W
Y

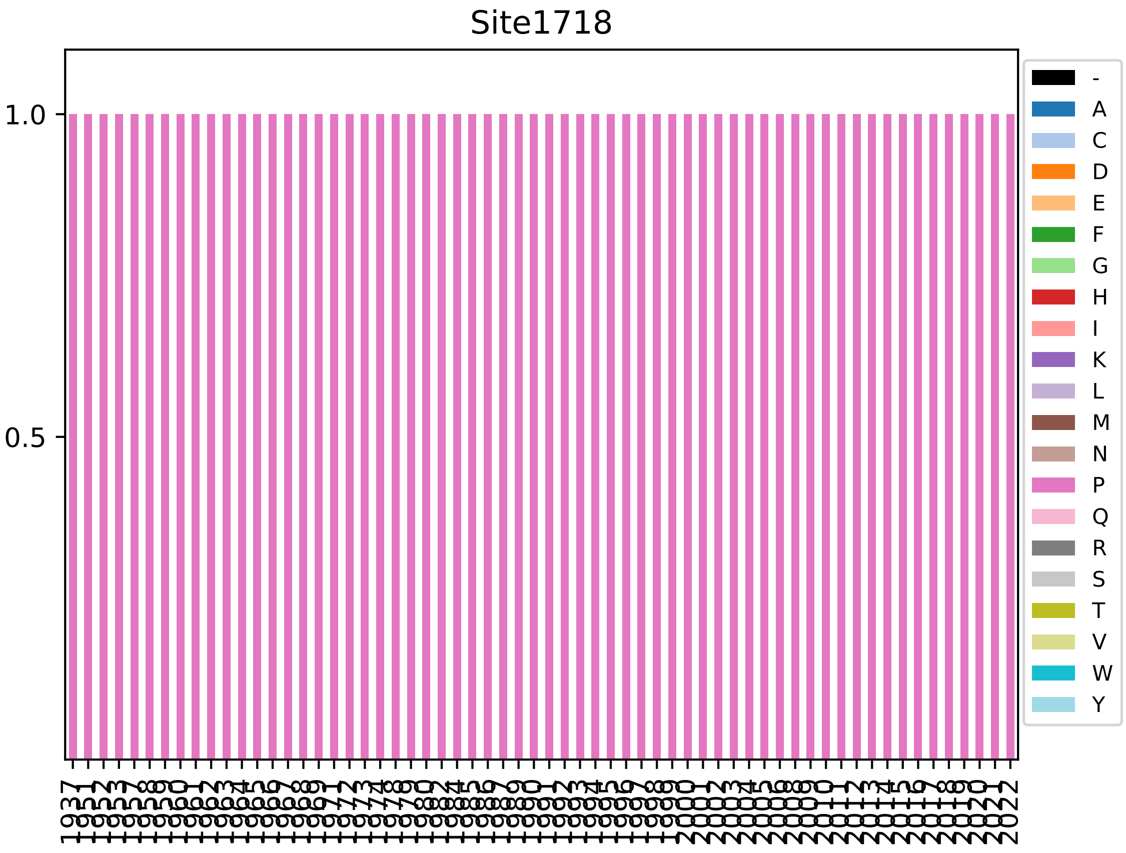
Site1718
1.0
0.5
-
A
C
D
E
F
G
H
I
K
L
M
N
P
Q
R
S
T
V
W
Y

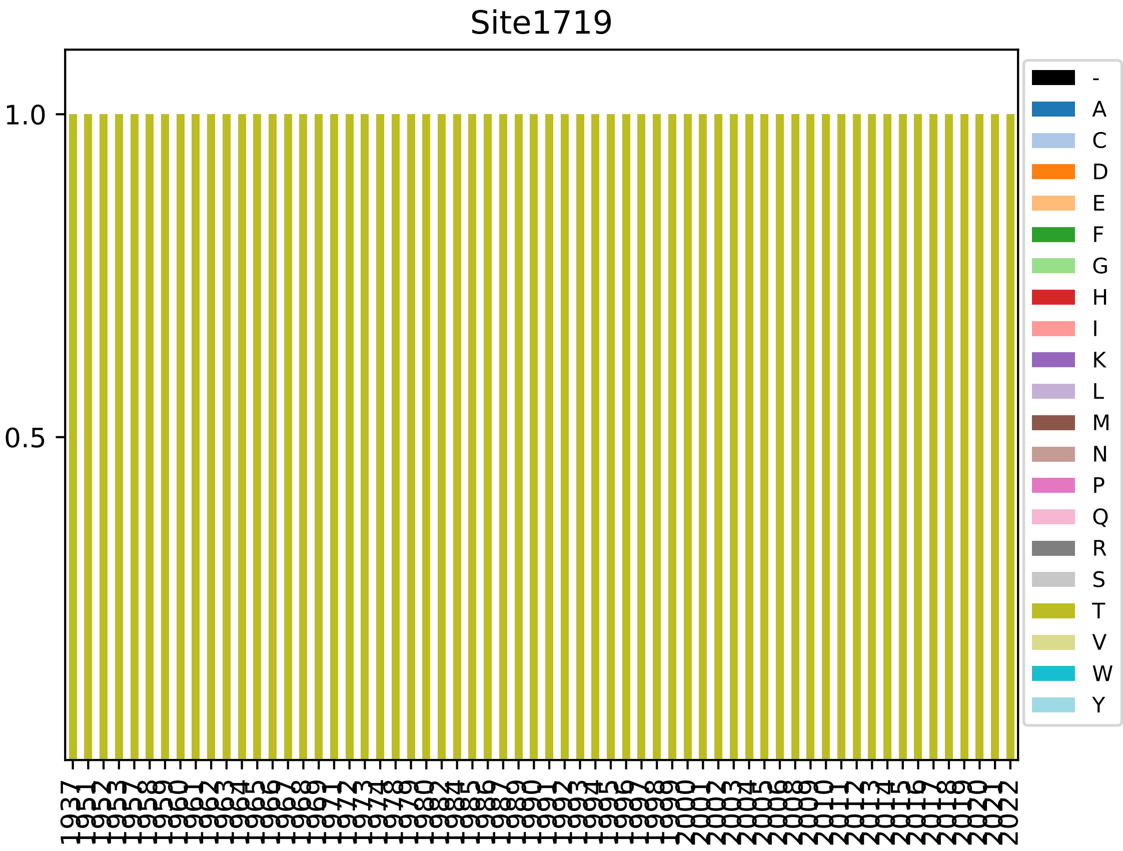
Site1719
1.0
0.5
-
A
C
D
E
F
G
H
I
K
L
M
N
P
Q
R
S
T
V
W
Y

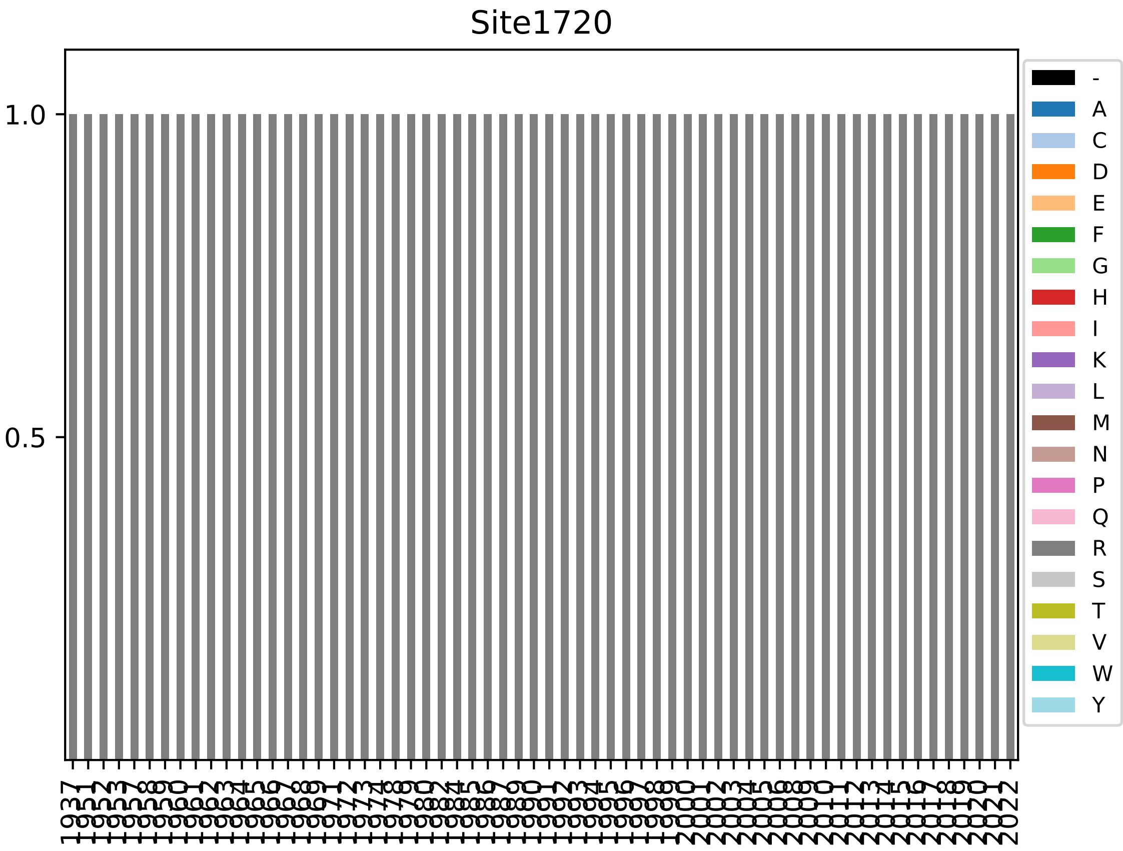
Site1720
1.0
0.5
-
A
C
D
E
F
G
H
I
K
L
M
N
P
Q
R
S
T
V
W
Y

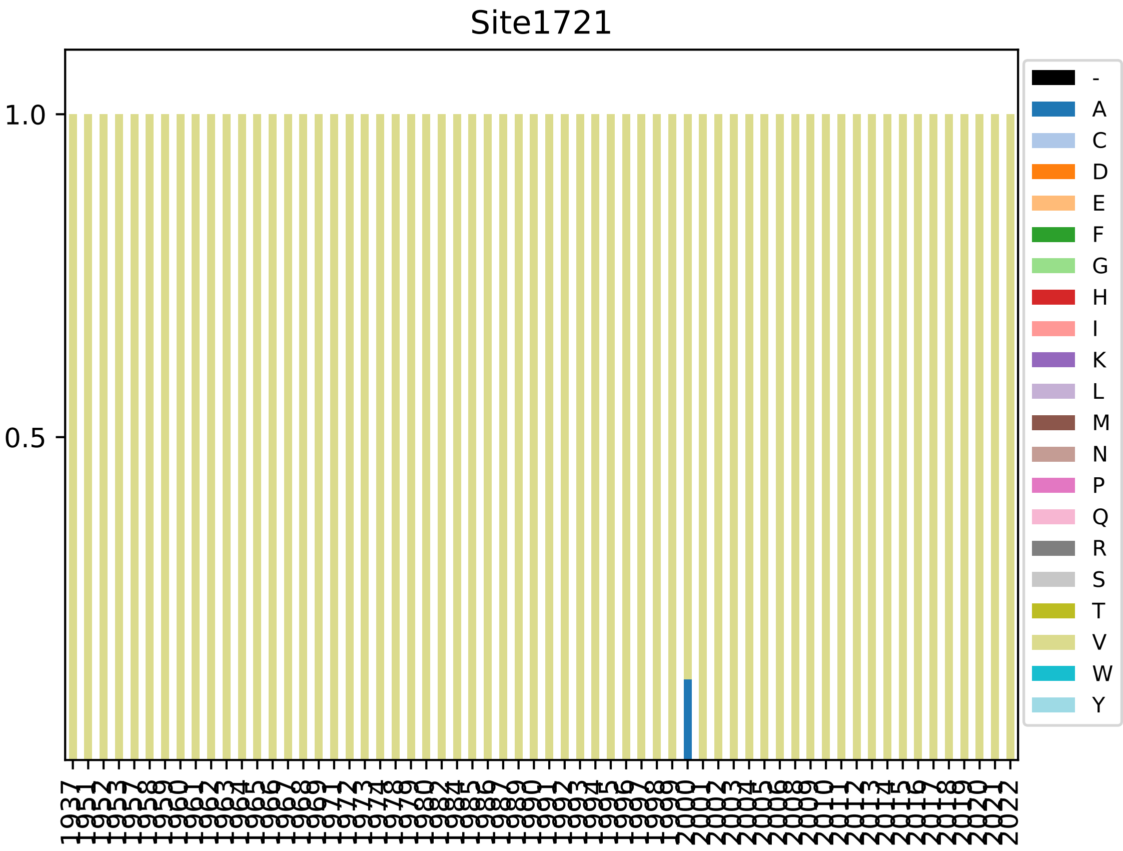
Site1721
1.0
0.5
-
A
C
D
E
F
G
H
I
K
L
M
N
P
Q
R
S
T
V
W
Y

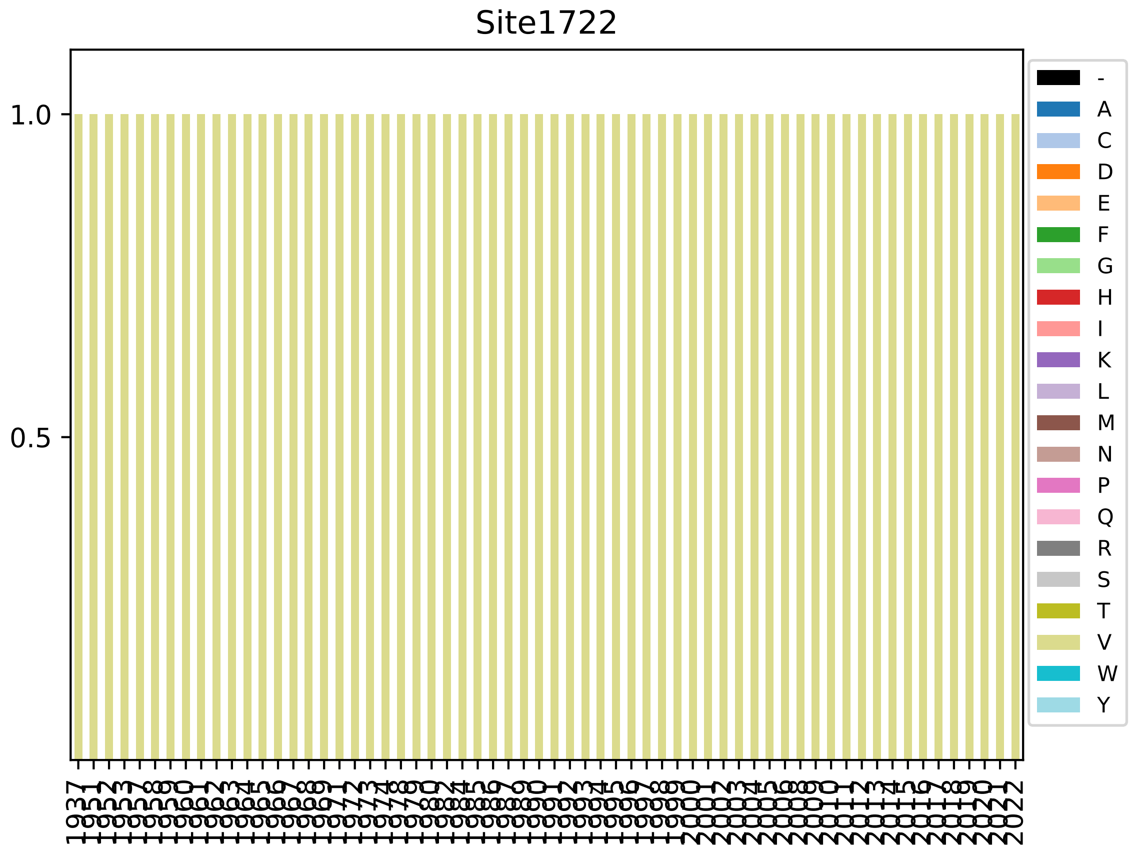
Site1722
1.0
0.5
-
A
C
D
E
F
G
H
I
K
L
M
N
P
Q
R
S
T
V
W
Y

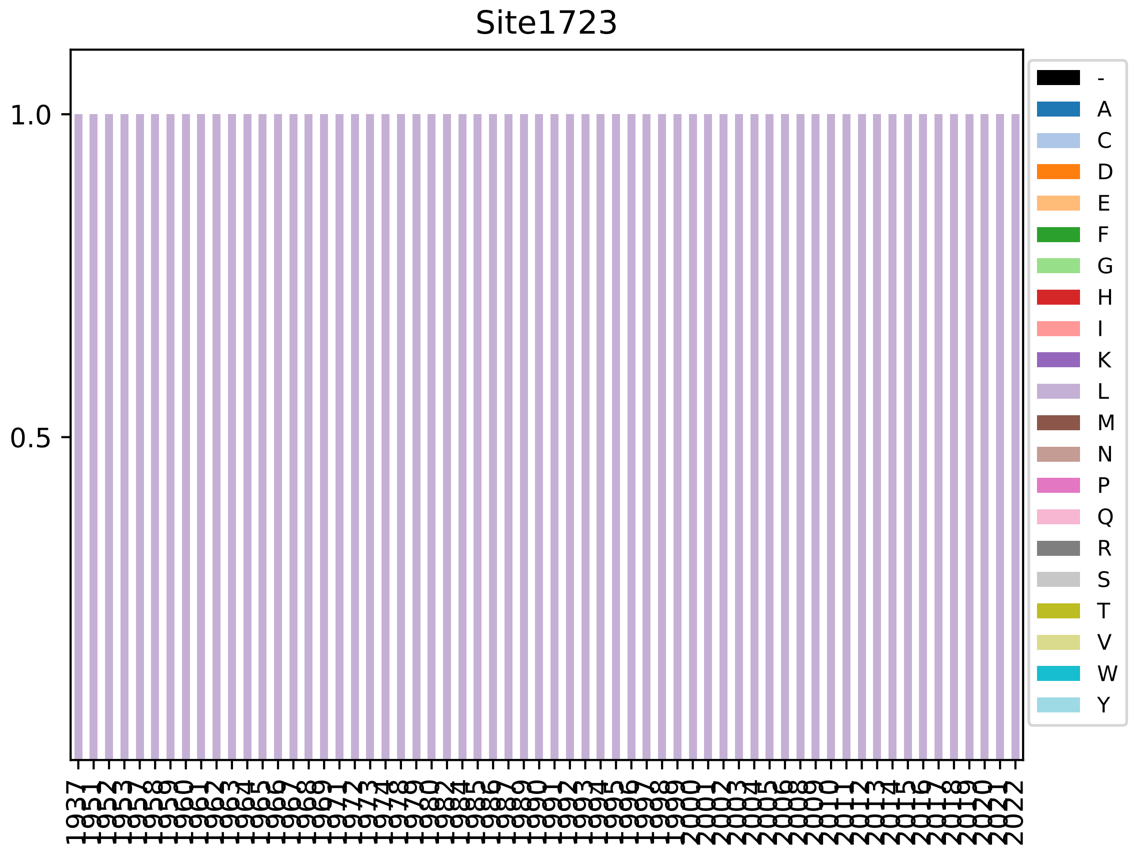
Site1723
1.0
0.5
-
A
C
D
E
F
G
H
I
K
L
M
N
P
Q
R
S
T
V
W
Y

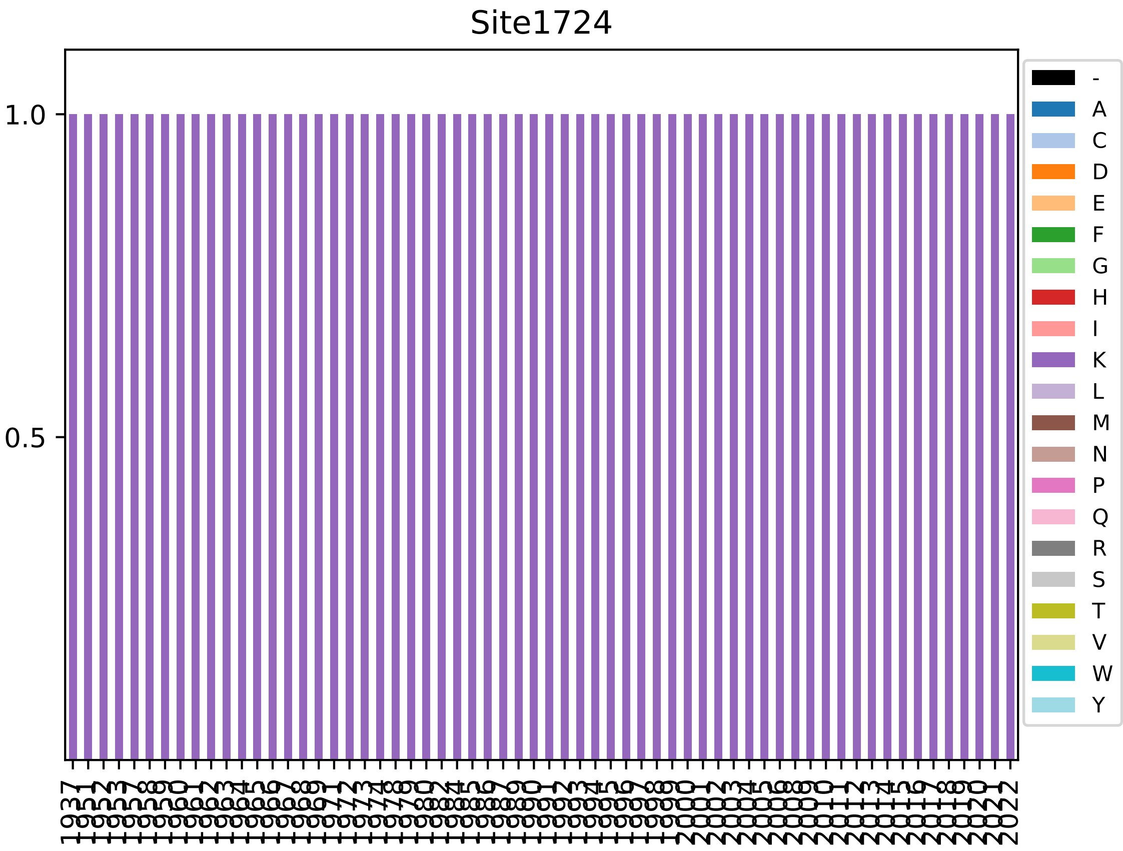

Site1724
1.0
0.5
-
A
C
D
E
F
G
H
I
K
L
M
N
P
Q
R
S
T
V
W
Y

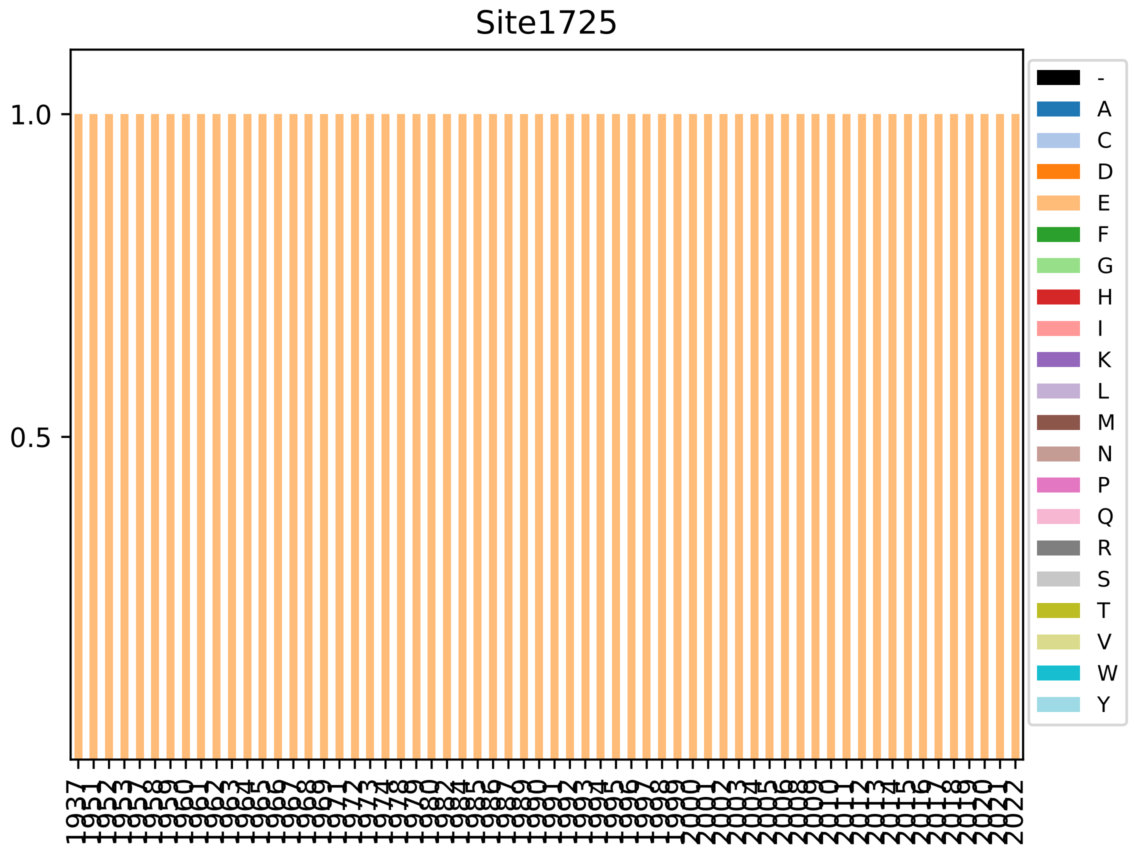
Site1725
1.0
0.5
-
A
C
D
E
F
G
H
I
K
L
M
N
P
Q
R
S
T
V
W
Y

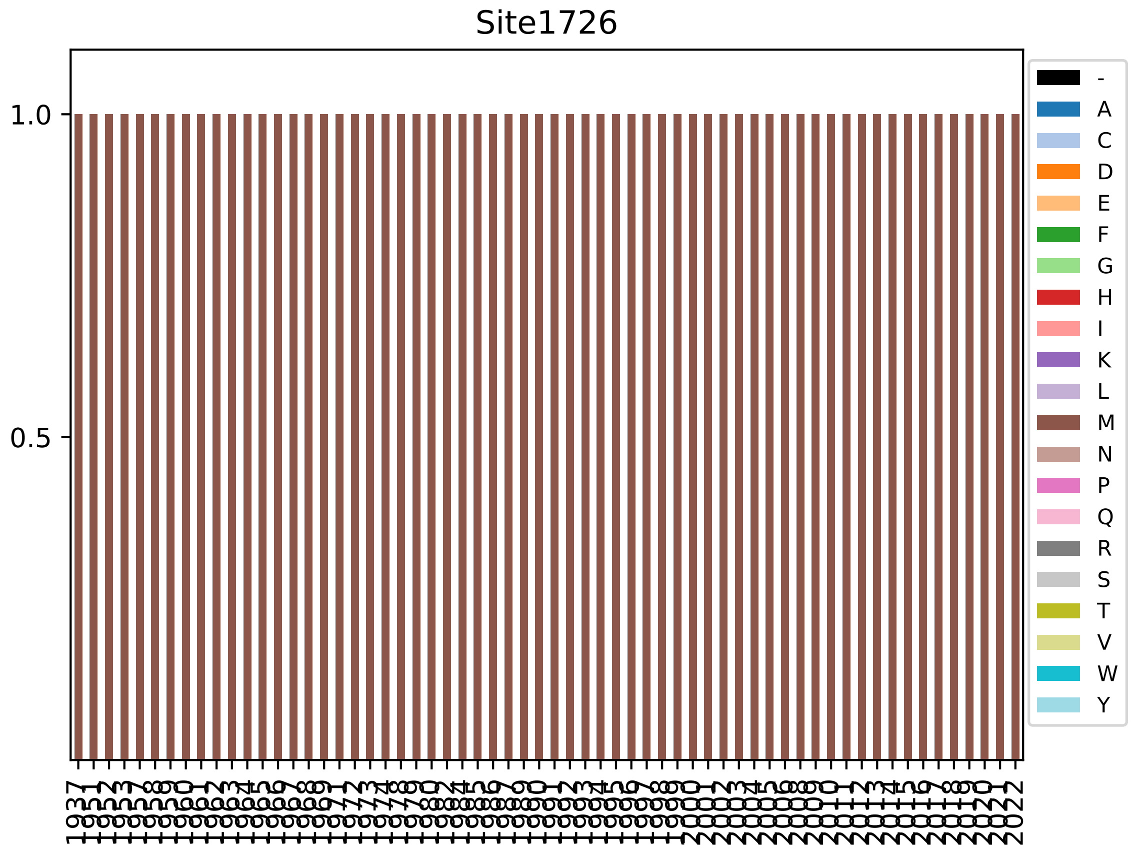
Site1726
1.0
0.5
-
A
C
D
E
F
G
H
I
K
L
M
N
P
Q
R
S
T
V
W
Y

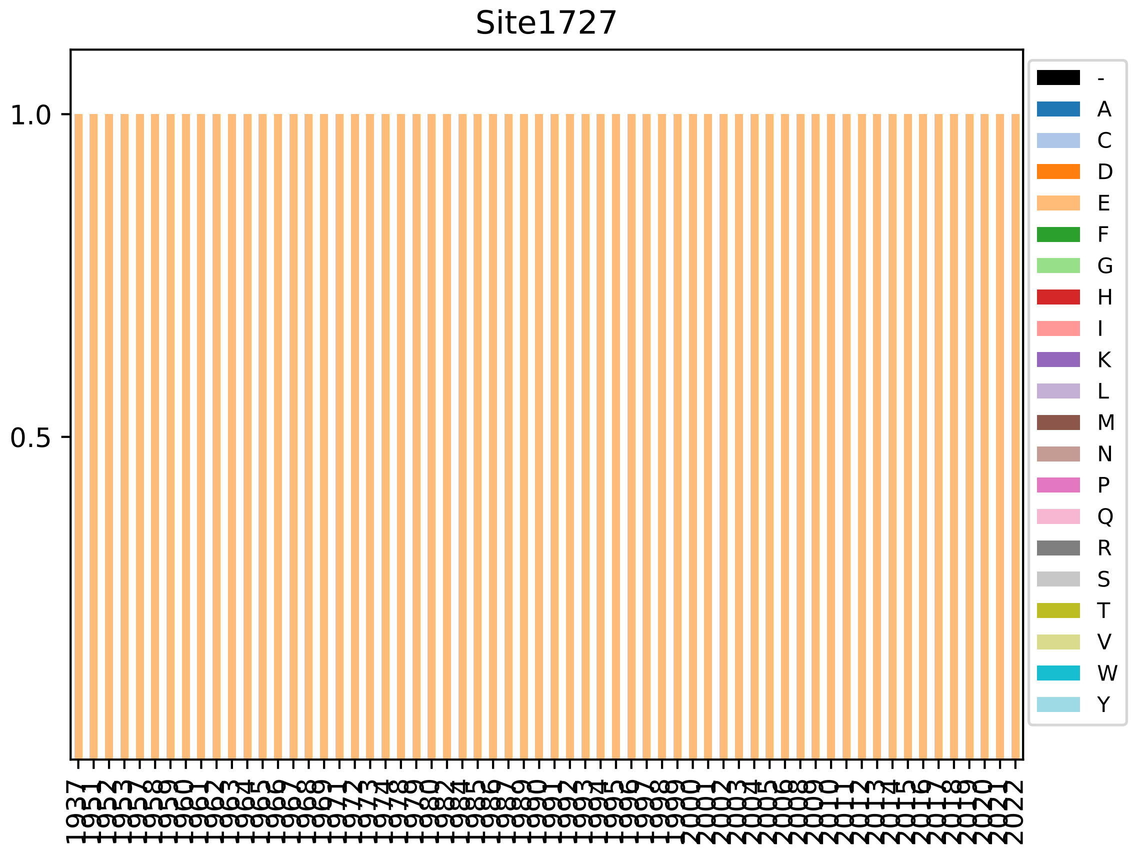
Site1727
1.0
0.5
-
A
C
D
E
F
G
H
I
K
L
M
N
P
Q
R
S
T
V
W
Y

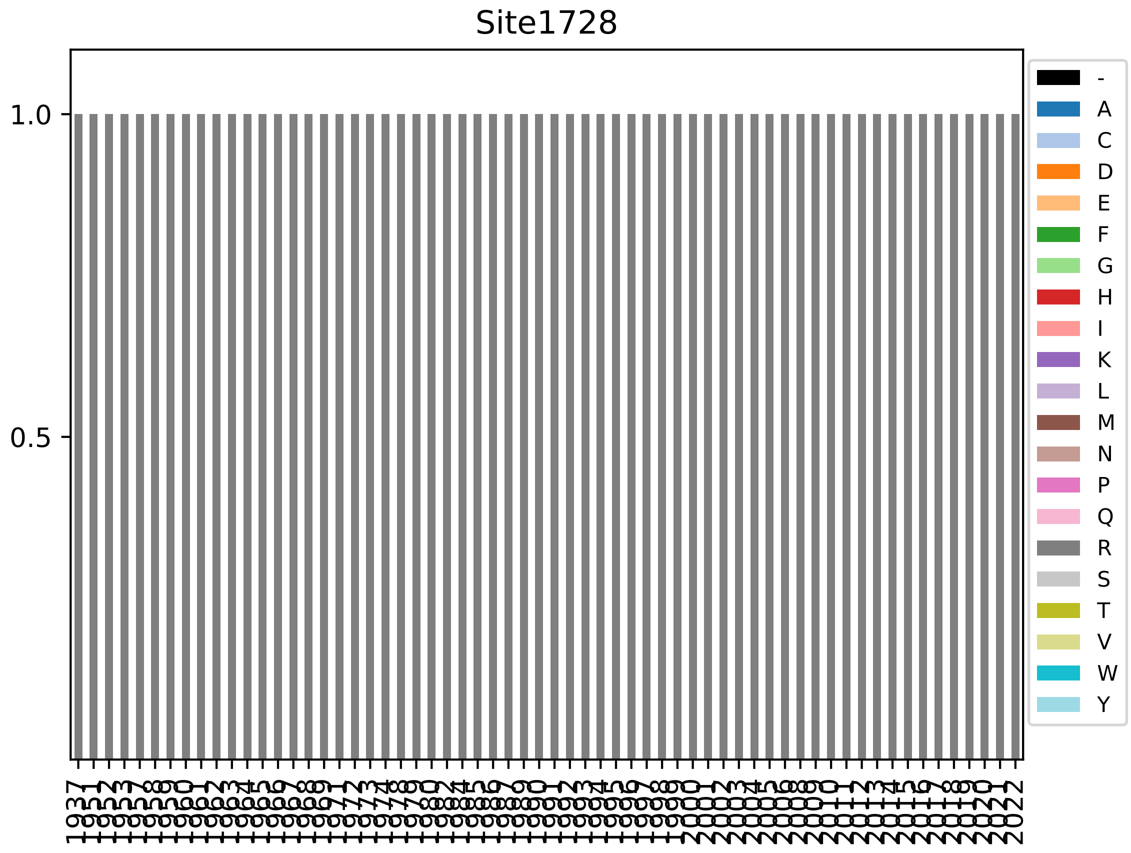
Site1728
1.0
0.5
-
A
C
D
E
F
G
H
I
K
L
M
N
P
Q
R
S
T
V
W
Y

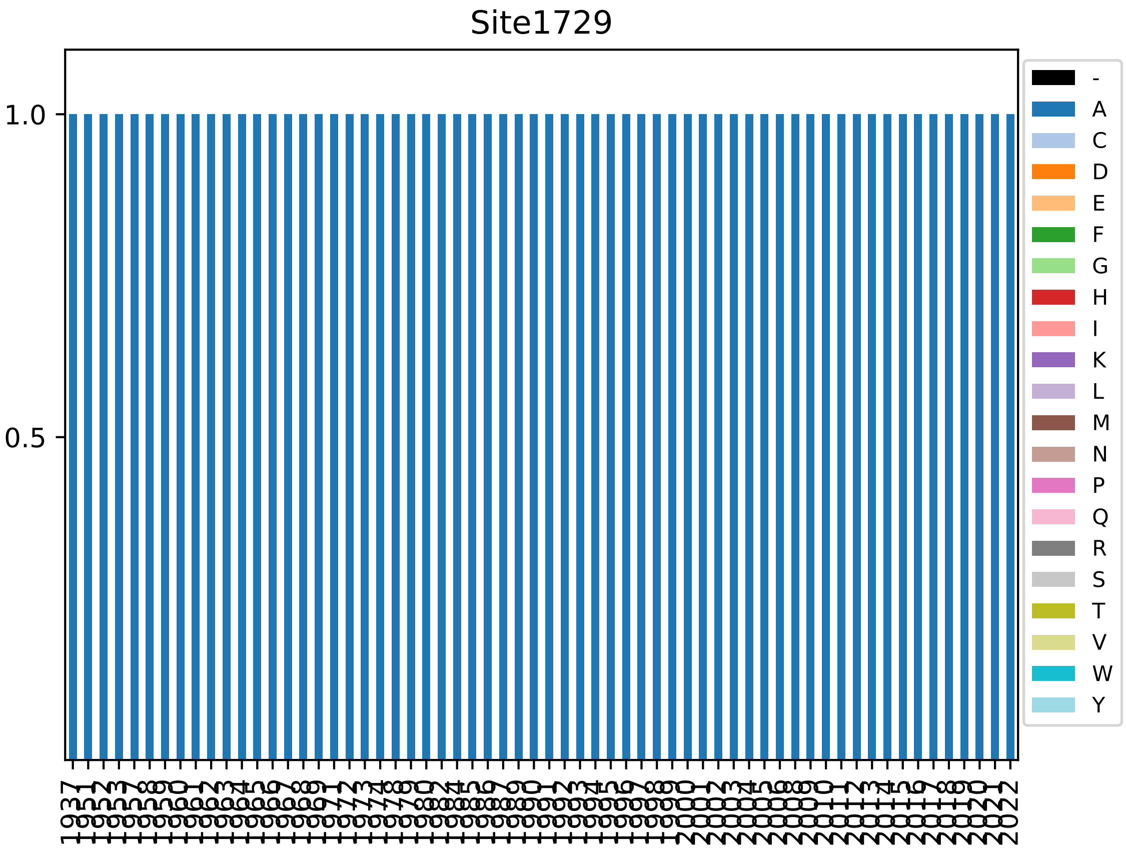
Site1729
1.0
0.5
-
A
C
D
E
F
G
H
I
K
L
M
N
P
Q
R
S
T
V
W
Y

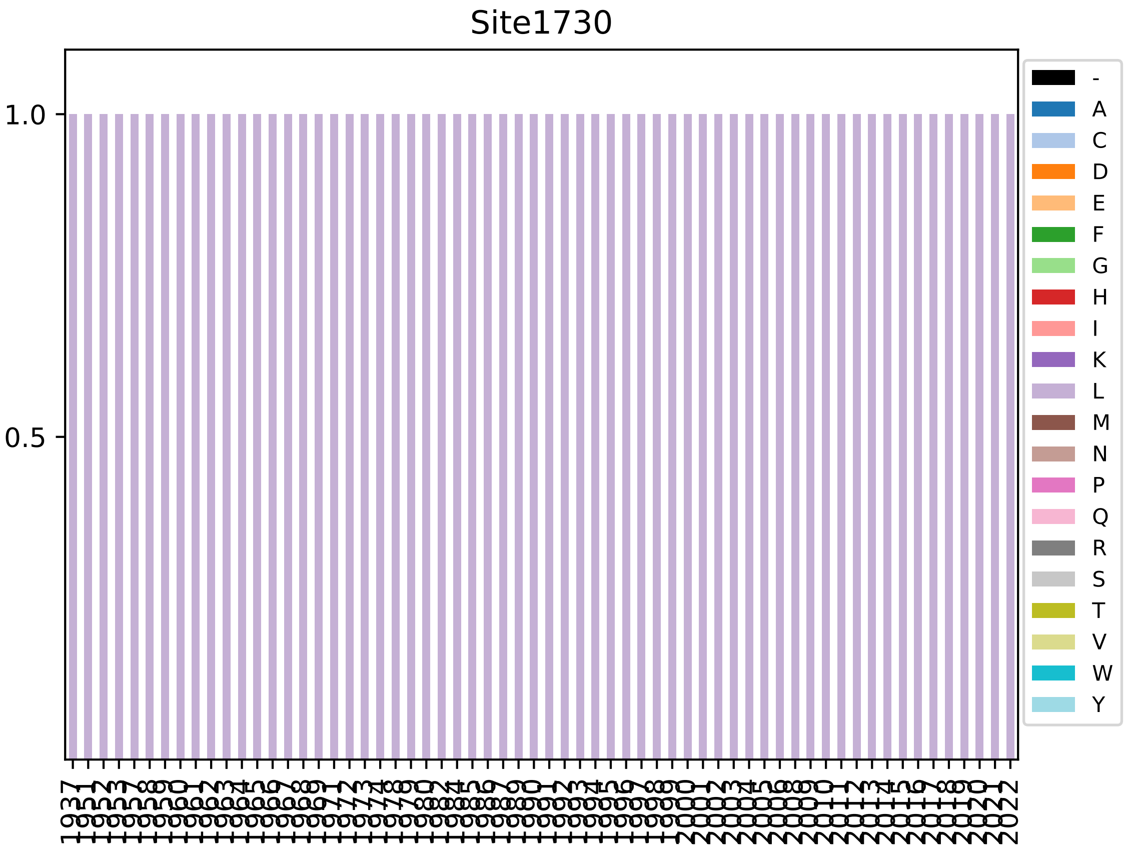
Site1730
1.0
0.5
-
A
C
D
E
F
G
H
I
K
L
M
N
P
Q
R
S
T
V
W
Y

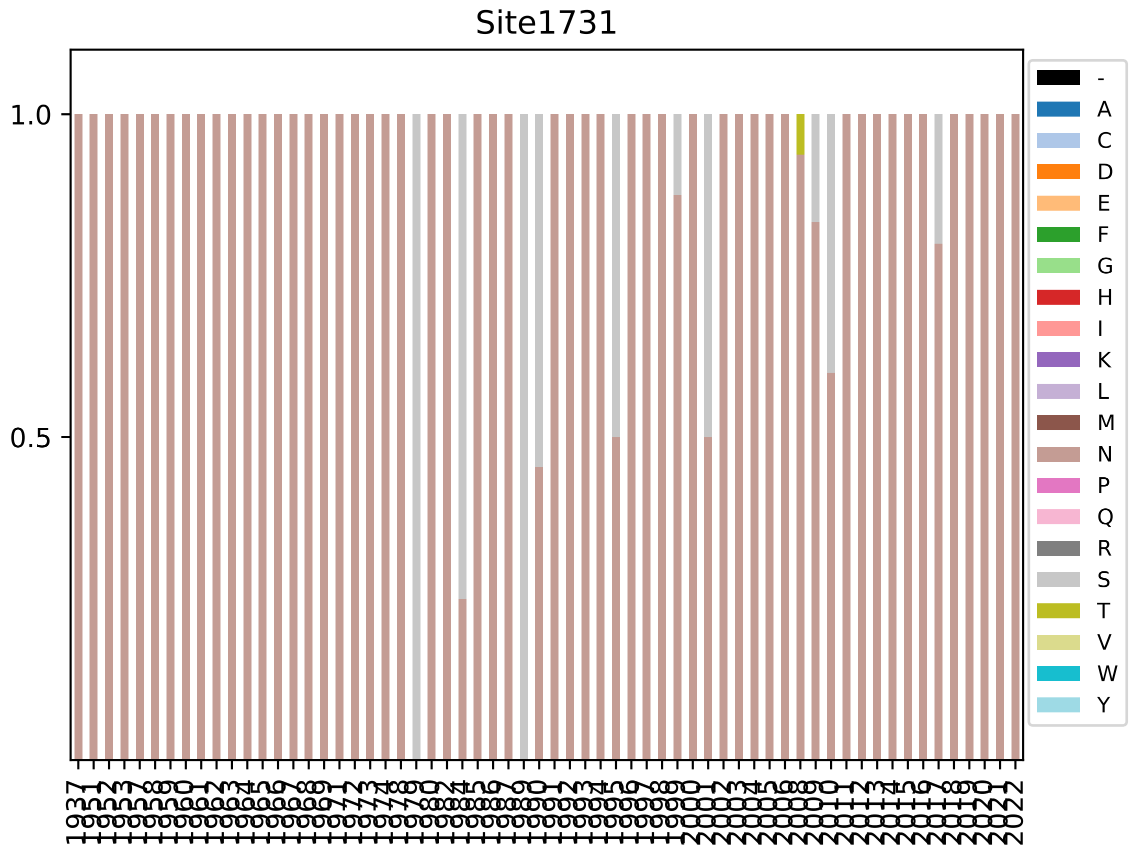
Site1731
1.0
0.5
-
A
C
D
E
F
G
H
I
K
L
M
N
P
Q
R
S
T
V
W
Y

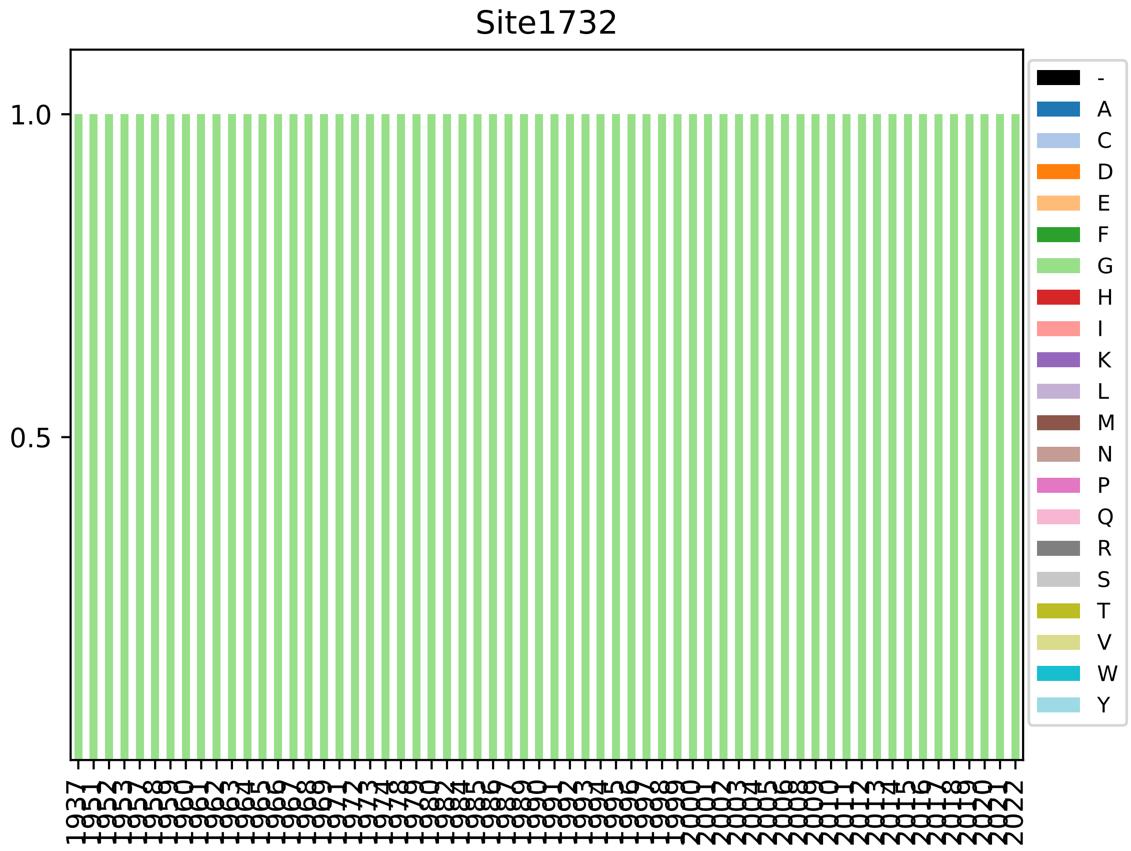
Site1732
1.0
0.5
-
A
C
D
E
F
G
H
I
K
L
M
N
P
Q
R
S
T
V
W
Y

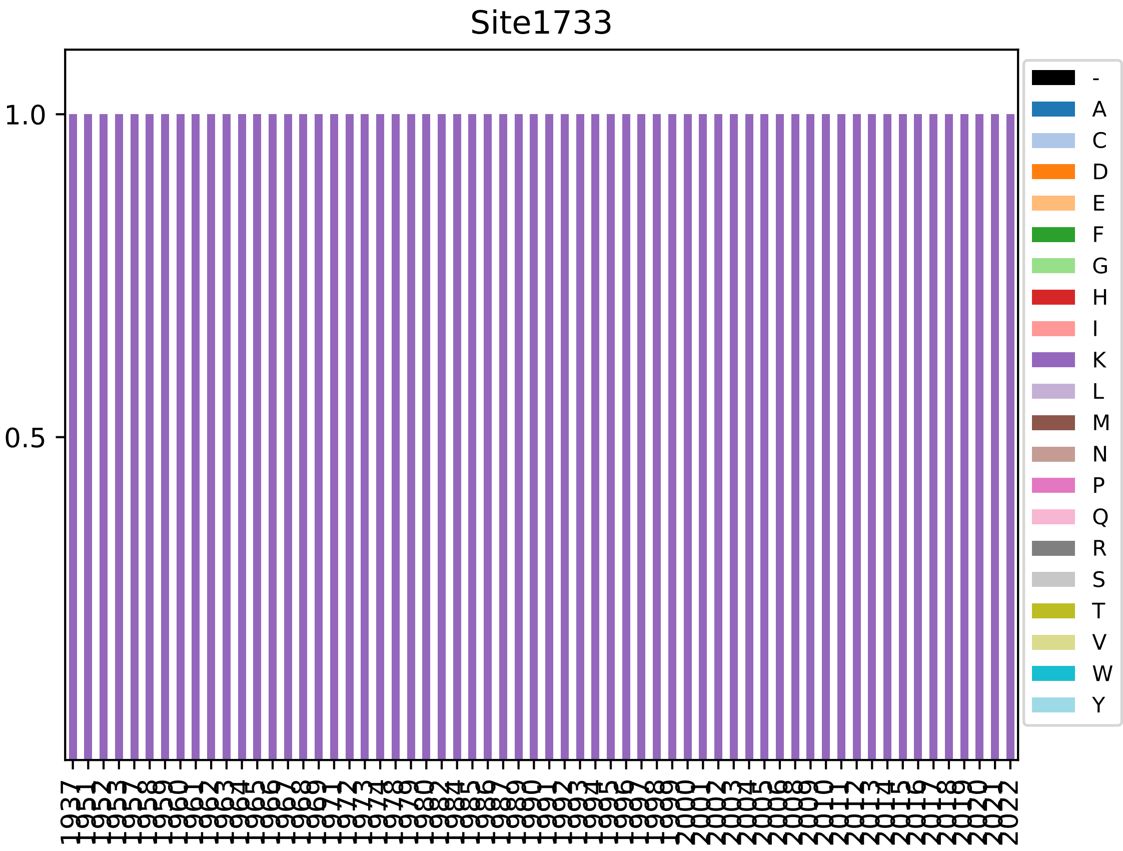
Site1733
1.0
0.5
-
A
C
D
E
F
G
H
I
K
L
M
N
P
Q
R
S
T
V
W
Y

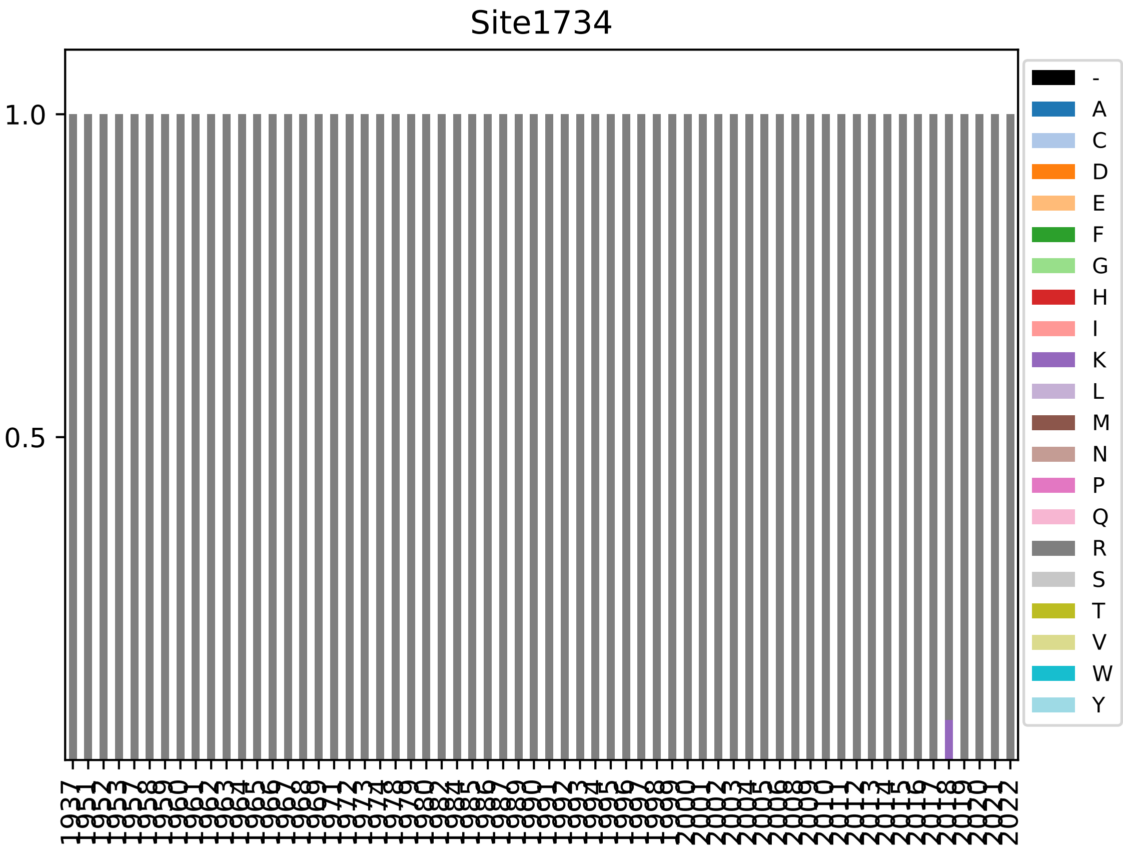
Site1734
1.0
0.5
-
A
C
D
E
F
G
H
I
K
L
M
N
P
Q
R
S
T
V
W
Y

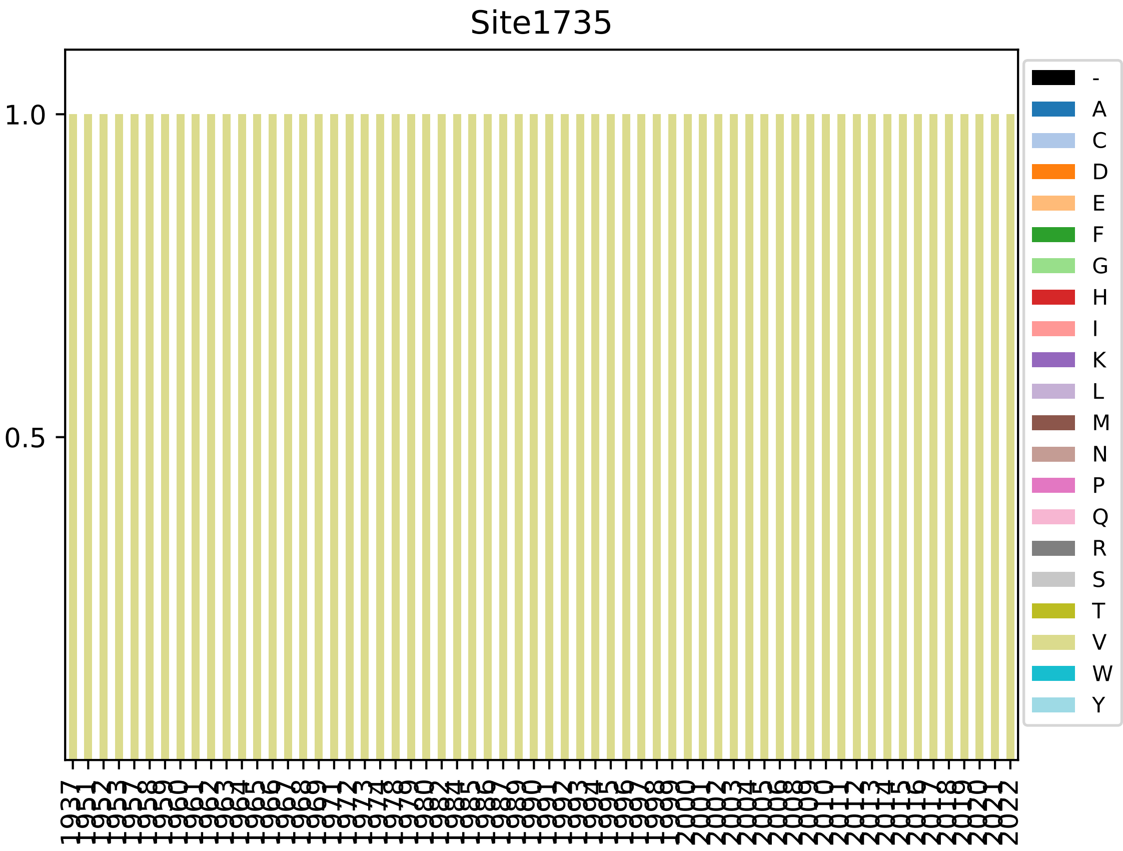
Site1735
1.0
0.5
-
A
C
D
E
F
G
H
I
K
L
M
N
P
Q
R
S
T
V
W
Y

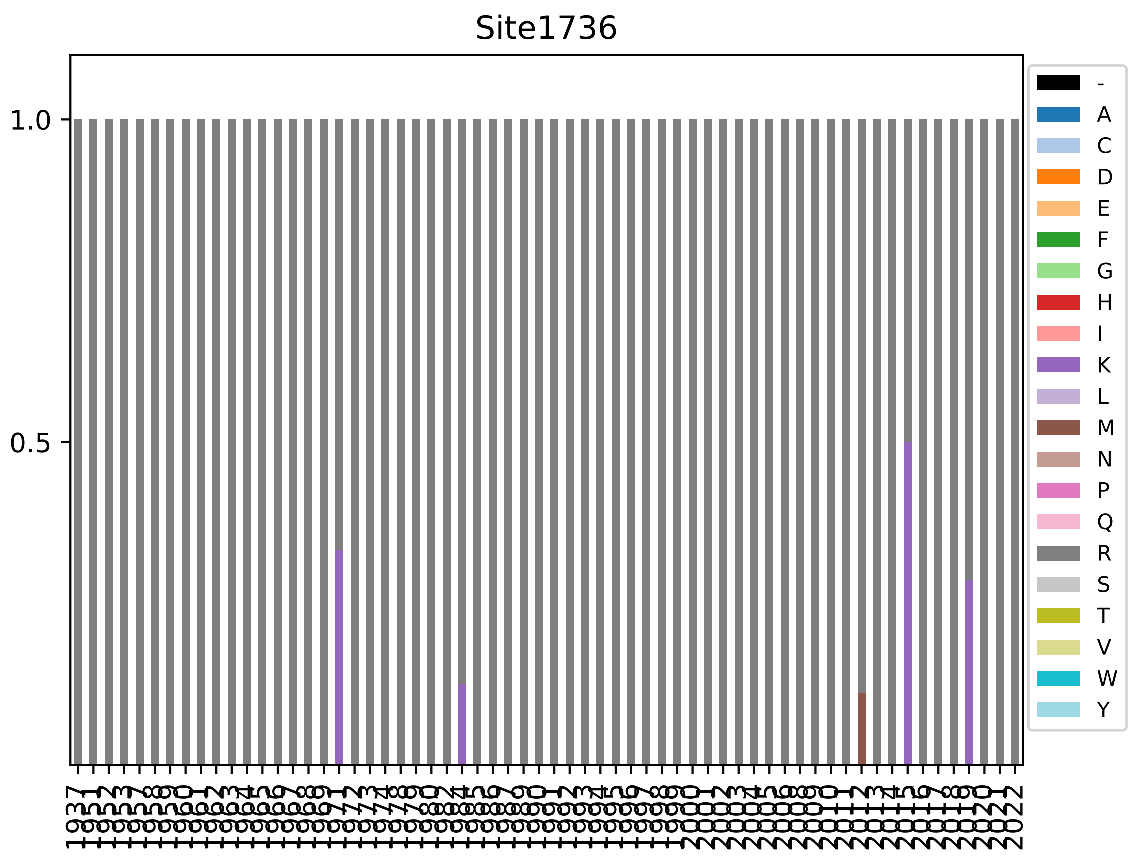
Site1736
1.0
0.5
-
A
C
D
E
F
G
H
I
K
L
M
N
P
Q
R
S
T
V
W
Y

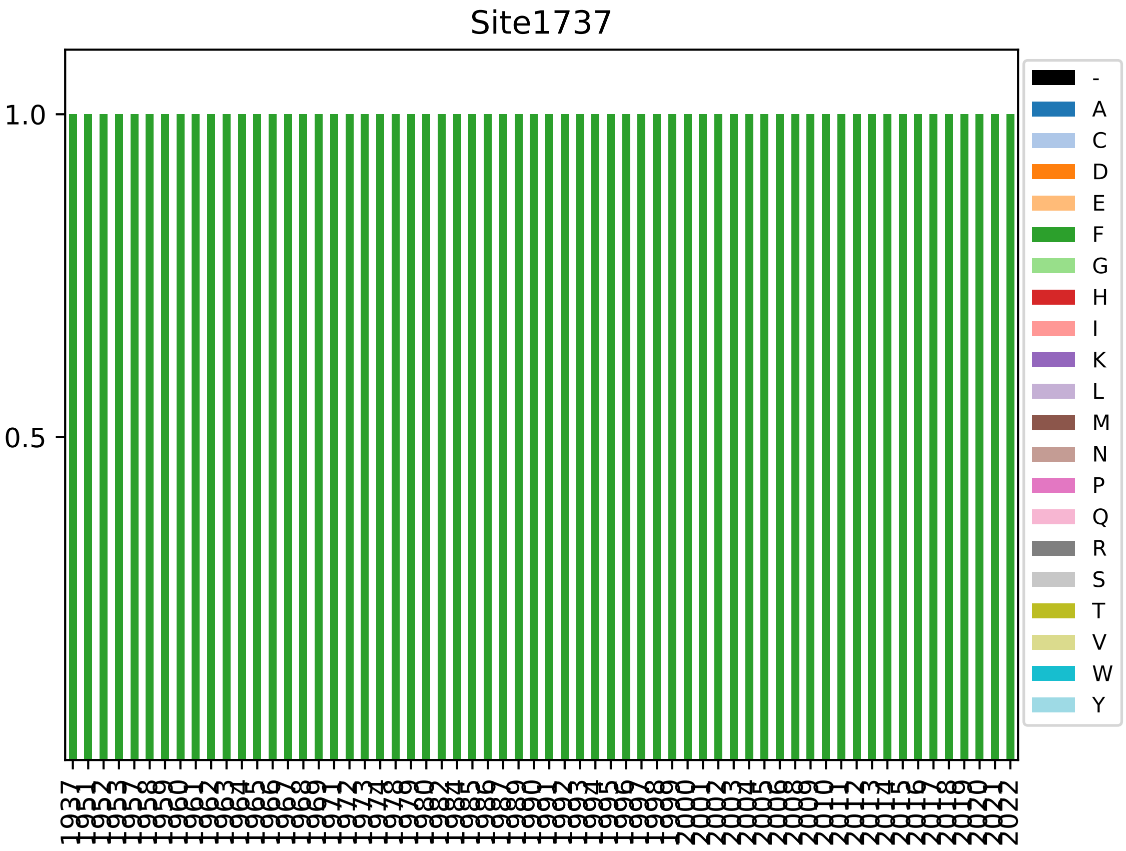
Site1737
1.0
0.5
-
A
C
D
E
F
G
H
I
K
L
M
N
P
Q
R
S
T
V
W
Y

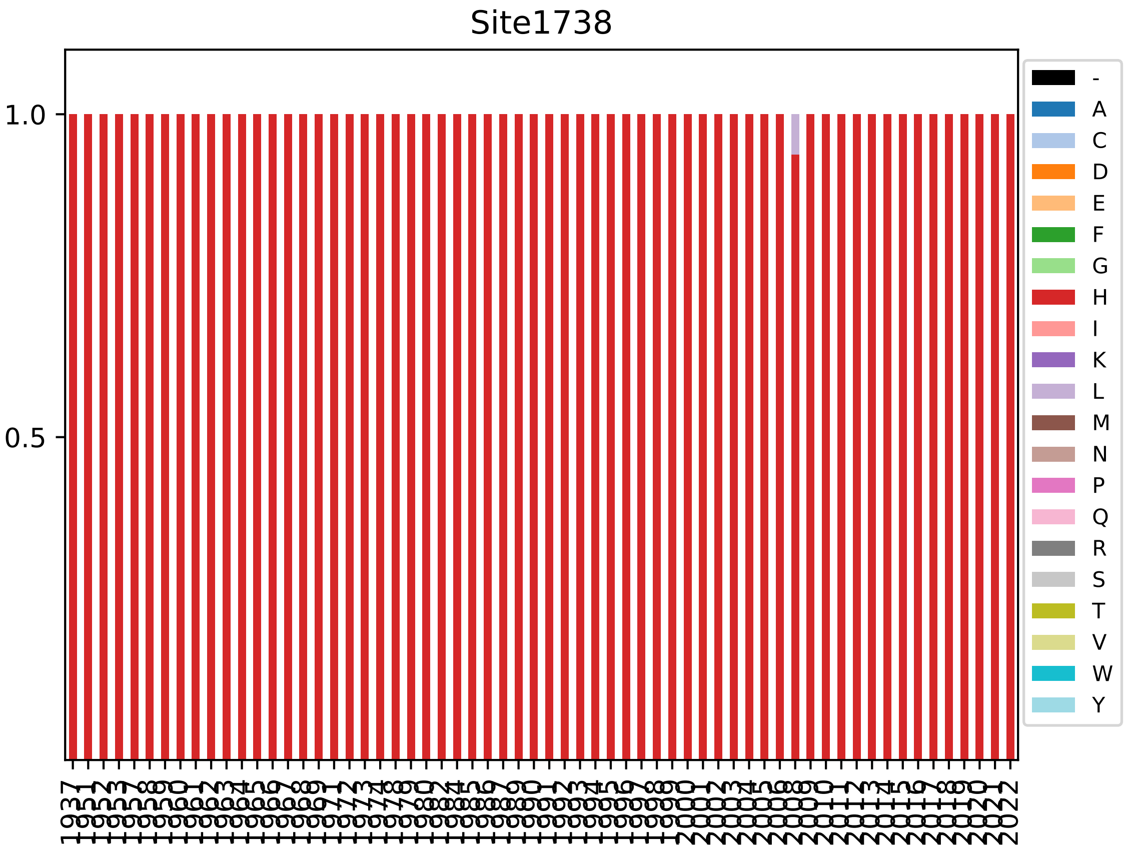

Site1738
1.0
0.5
-
A
C
D
E
F
G
H
I
K
L
M
N
P
Q
R
S
T
V
W
Y

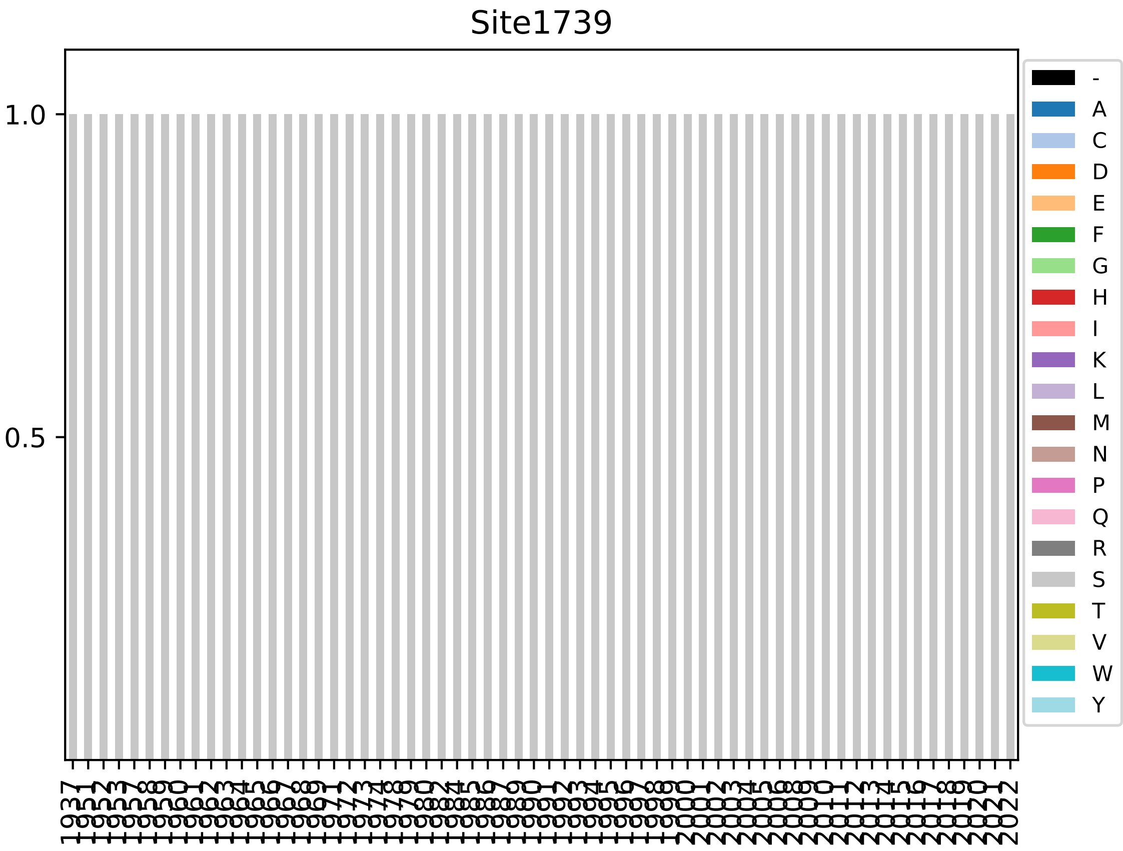
Site1739
1.0
0.5
-
A
C
D
E
F
G
H
I
K
L
M
N
P
Q
R
S
T
V
W
Y

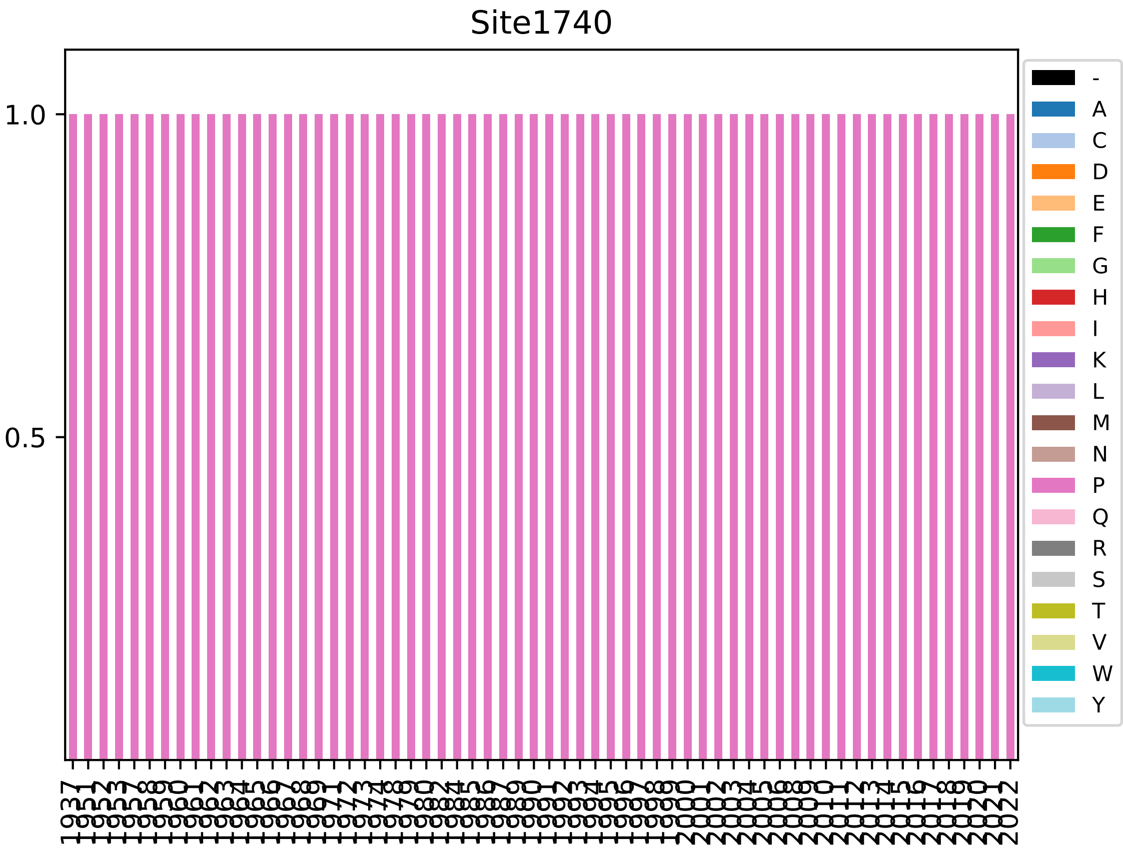

Site1740
1.0
0.5
-
A
C
D
E
F
G
H
I
K
L
M
N
P
Q
R
S
T
V
W
Y

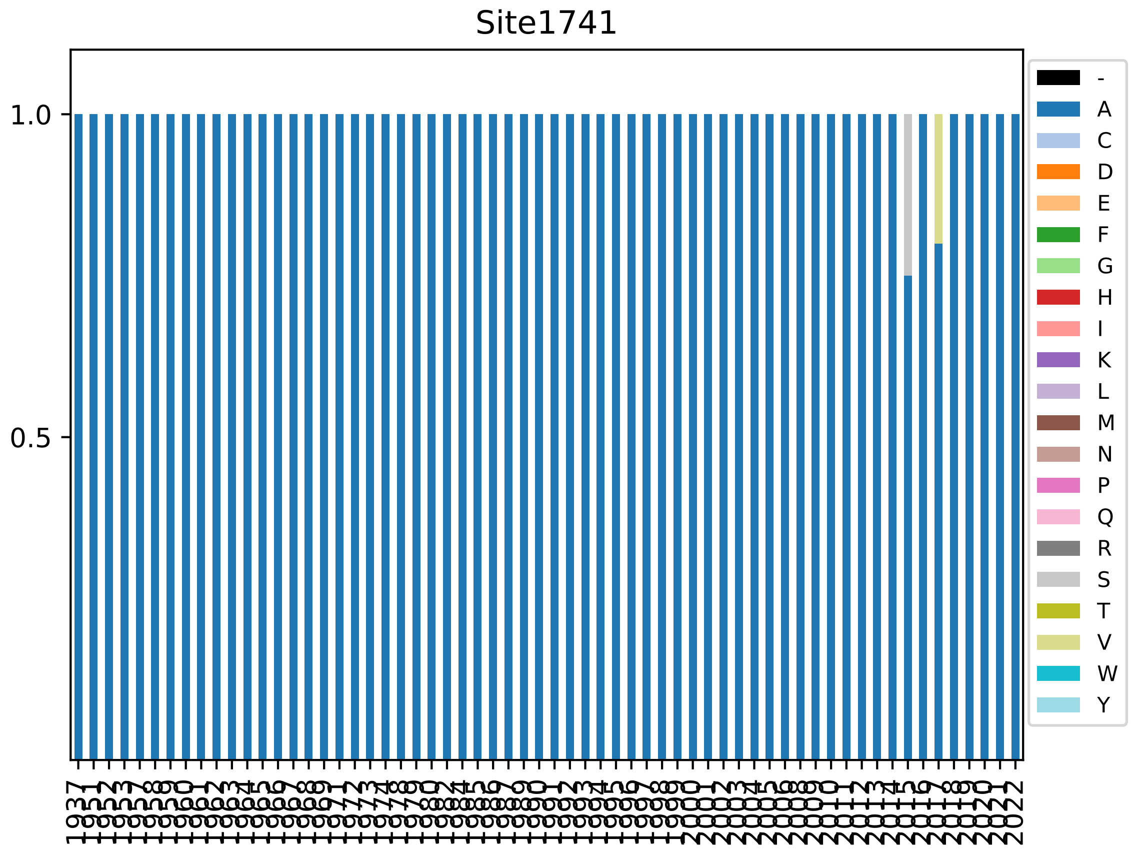

Site1741
1.0
0.5
-
A
C
D
E
F
G
H
I
K
L
M
N
P
Q
R
S
T
V
W
Y

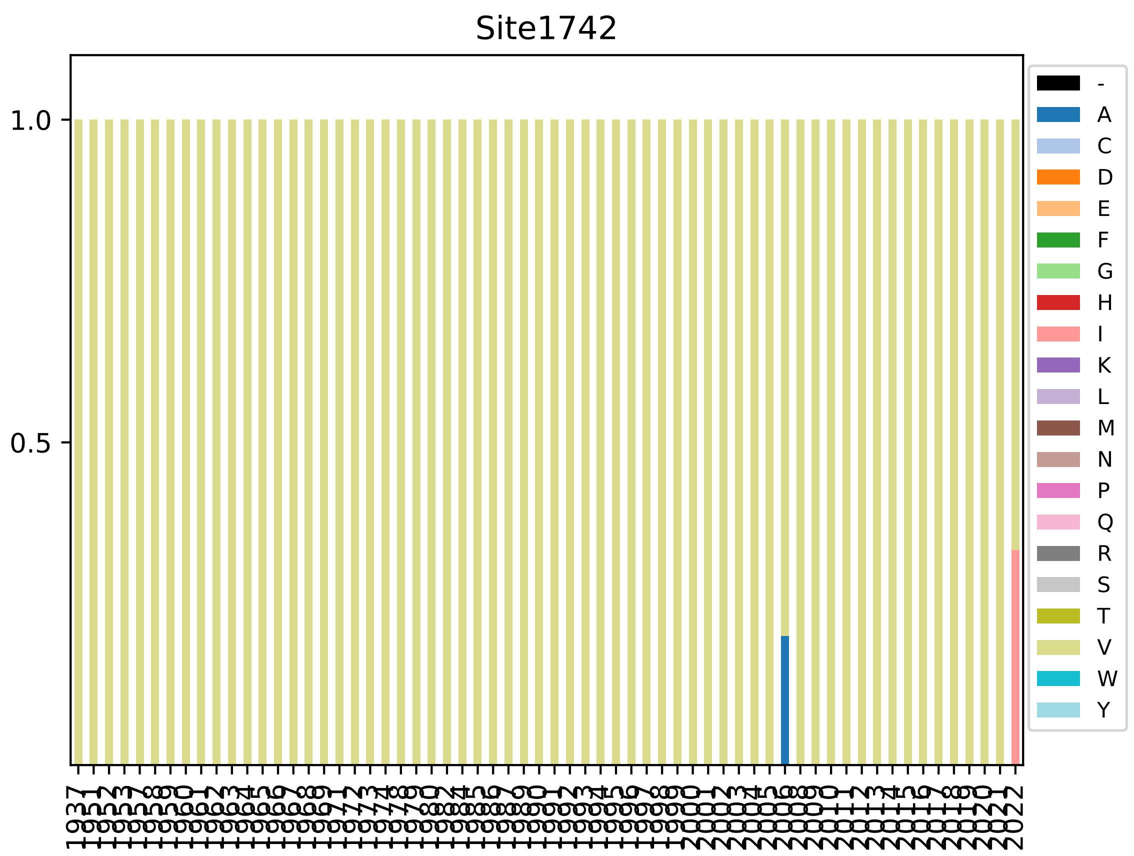
Site1742
1.0
0.5
-
A
C
D
E
F
G
H
I
K
L
M
N
P
Q
R
S
T
V
W
Y

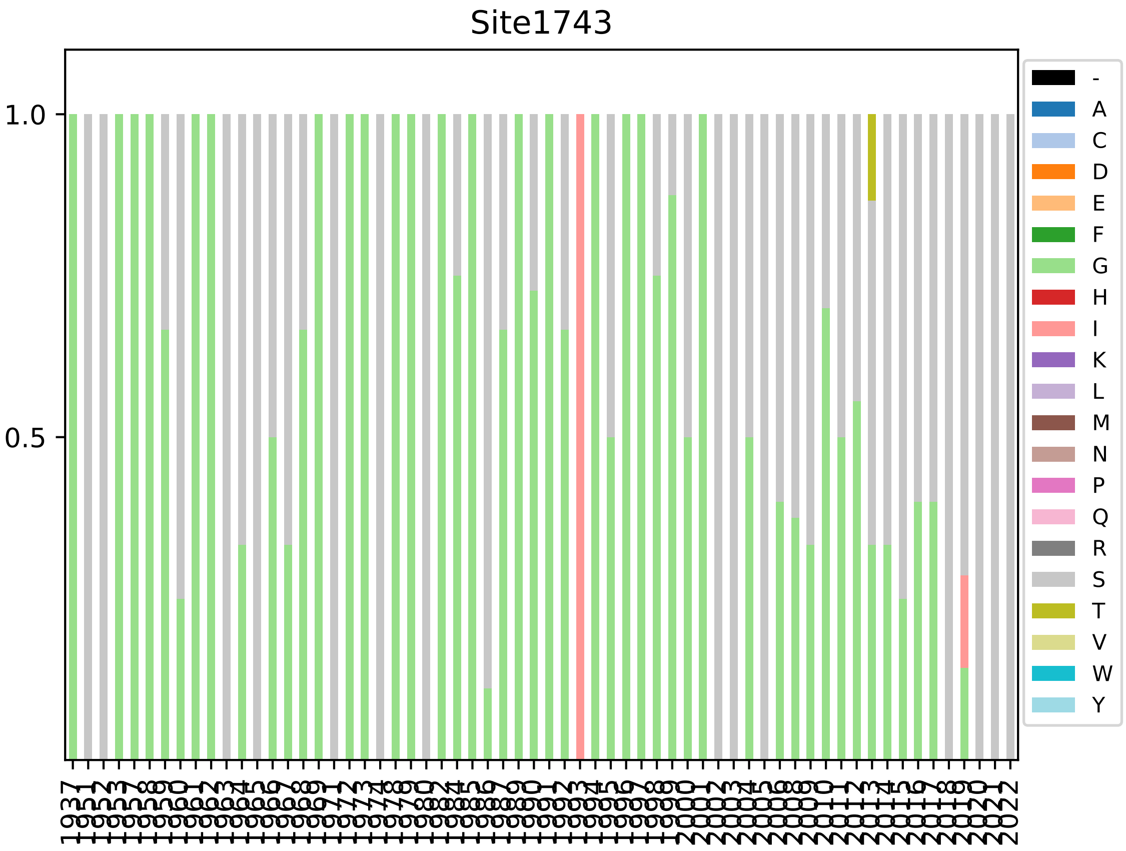
Site1743
1.0
0.5
-
A
C
D
E
F
G
H
I
K
L
M
N
P
Q
R
S
T
V
W
Y

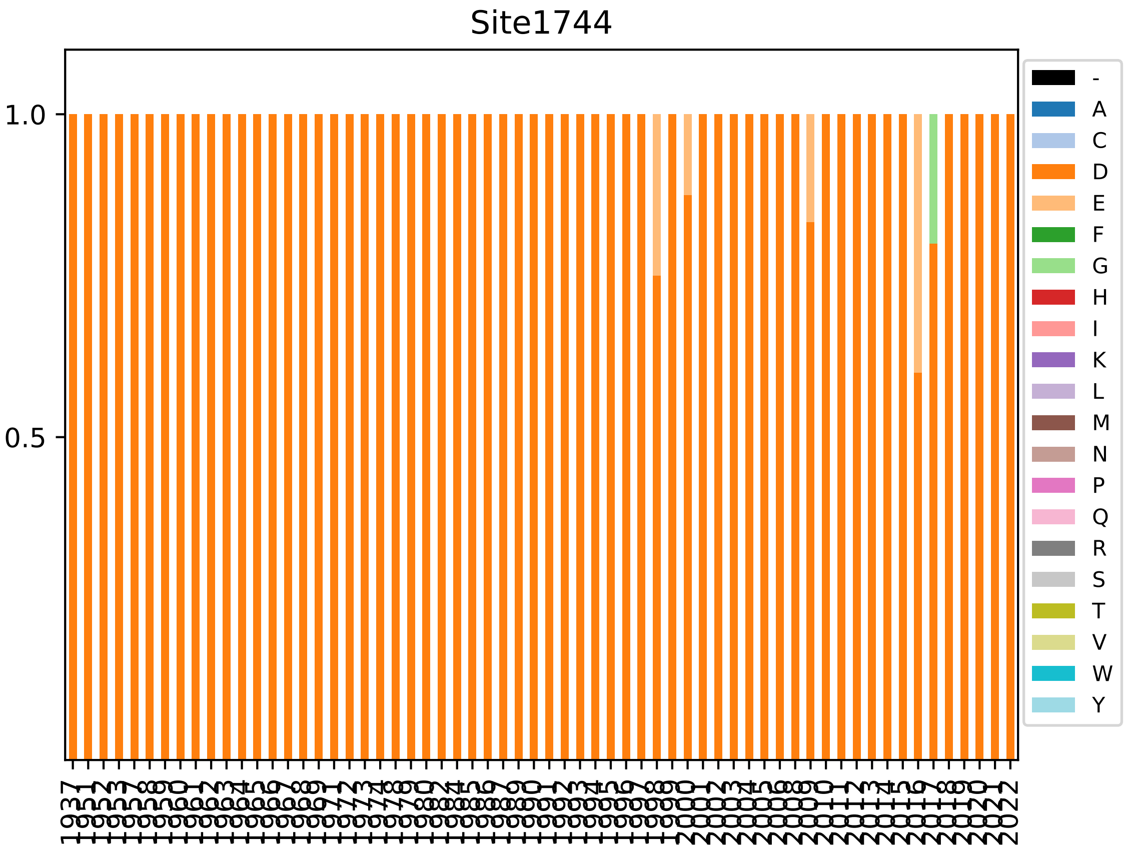
Site1744
1.0
0.5
-
A
C
D
E
F
G
H
I
K
L
M
N
P
Q
R
S
T
V
W
Y

Site1745

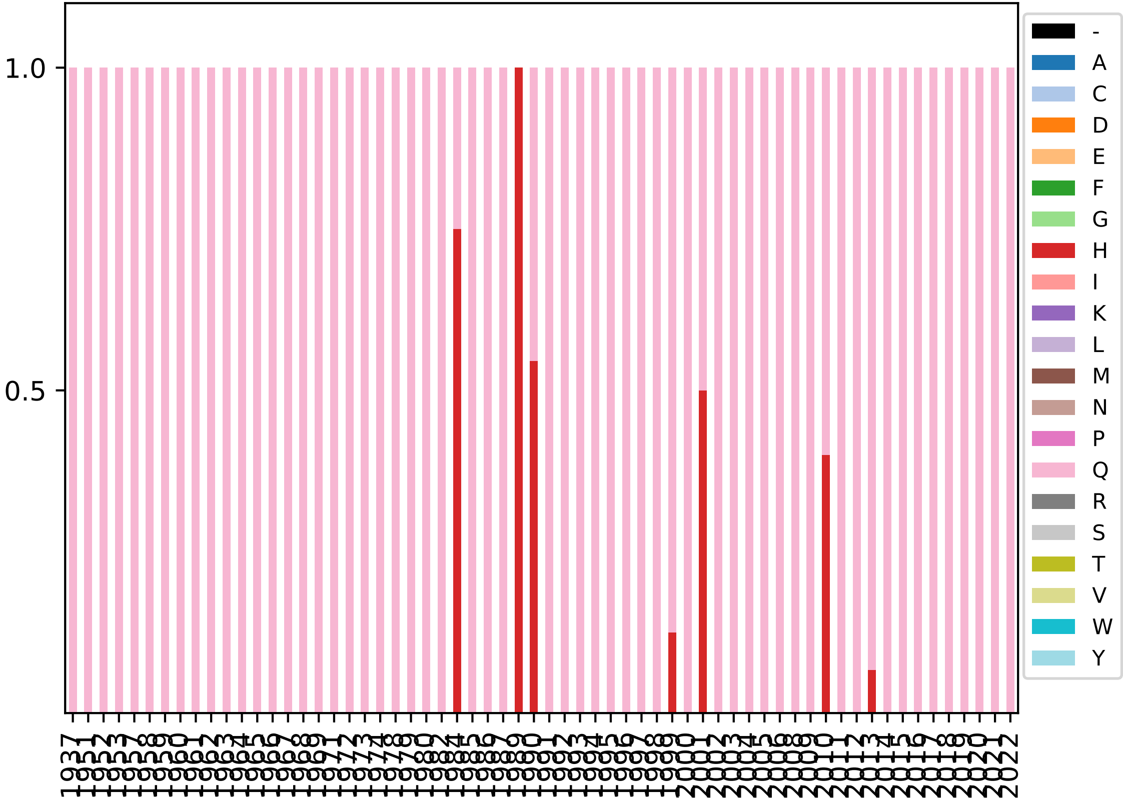

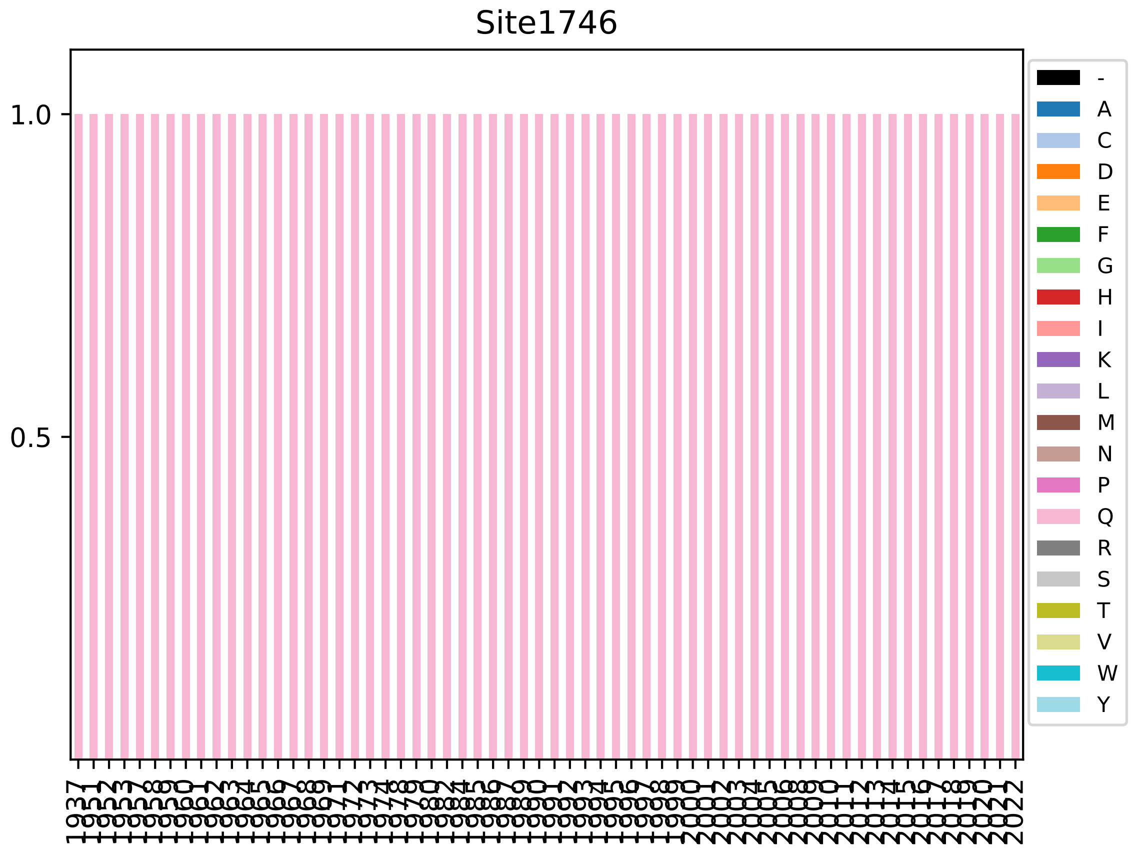
Site1746
1.0
0.5
-
A
C
D
E
F
G
H
I
K
L
M
N
P
Q
R
S
T
V
W
Y

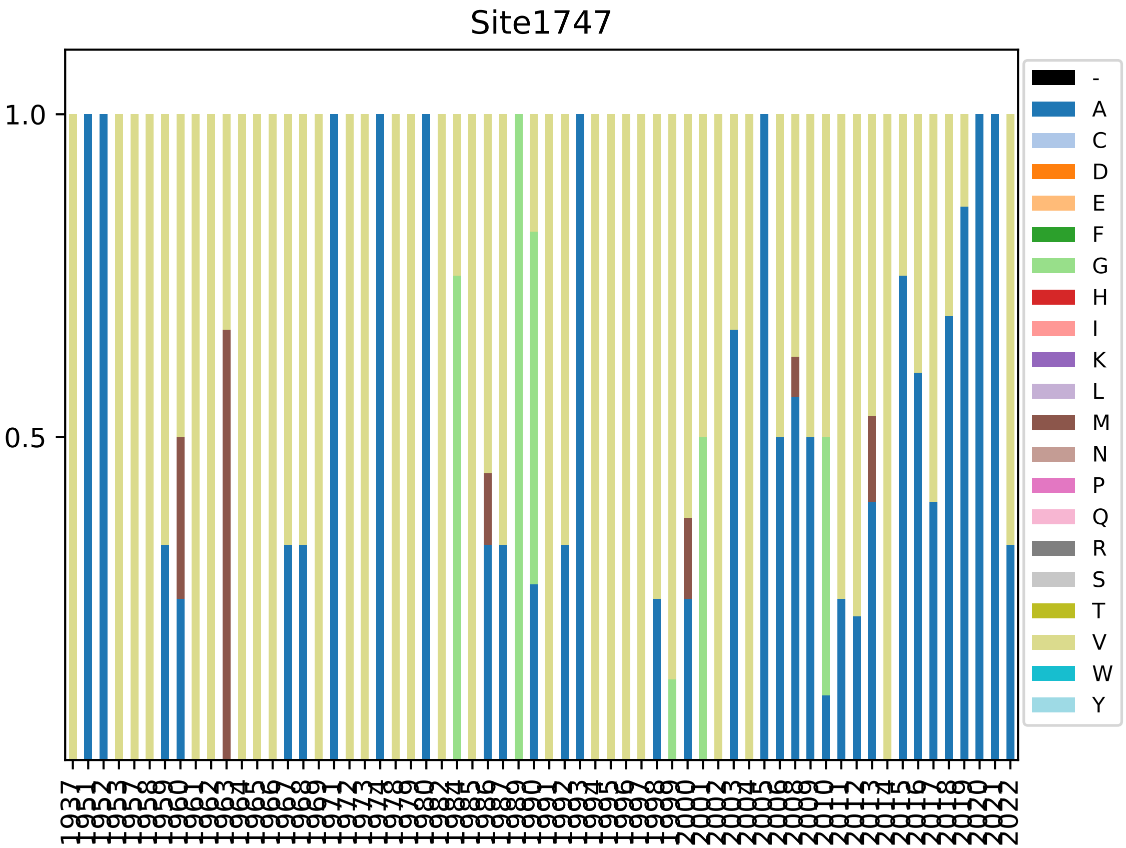
Site1747
1.0
0.5
-
A
C
D
E
F
G
H
I
K
L
M
N
P
Q
R
S
T
V
W
Y

Site1748

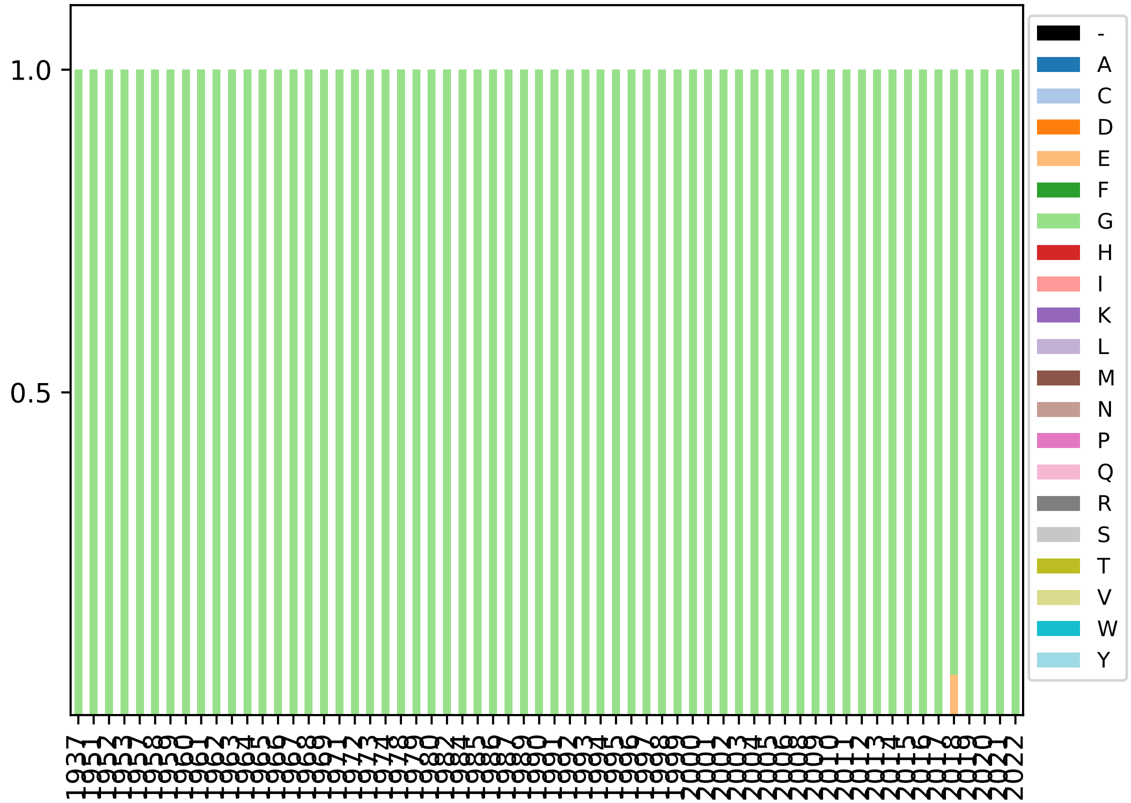

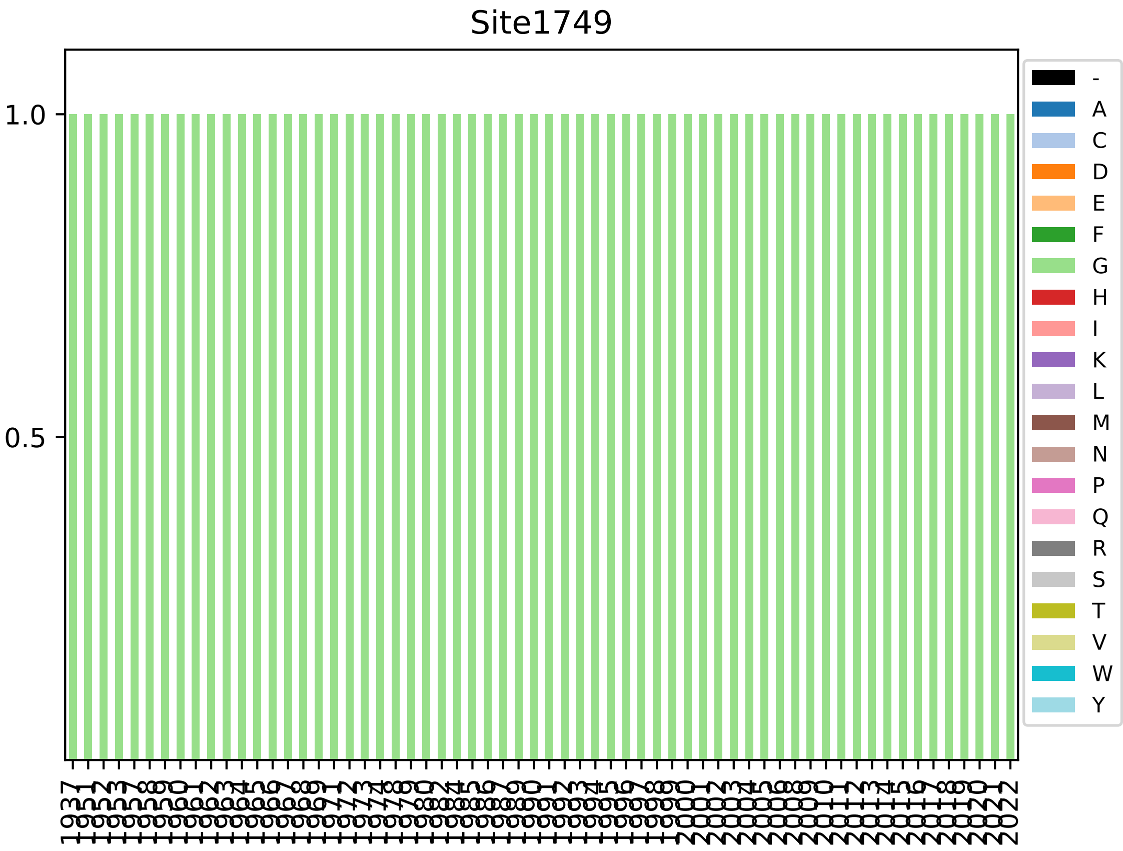
Site1749
1.0
0.5
-
A
C
D
E
F
G
H
I
K
L
M
N
P
Q
R
S
T
V
W
Y

Site1750

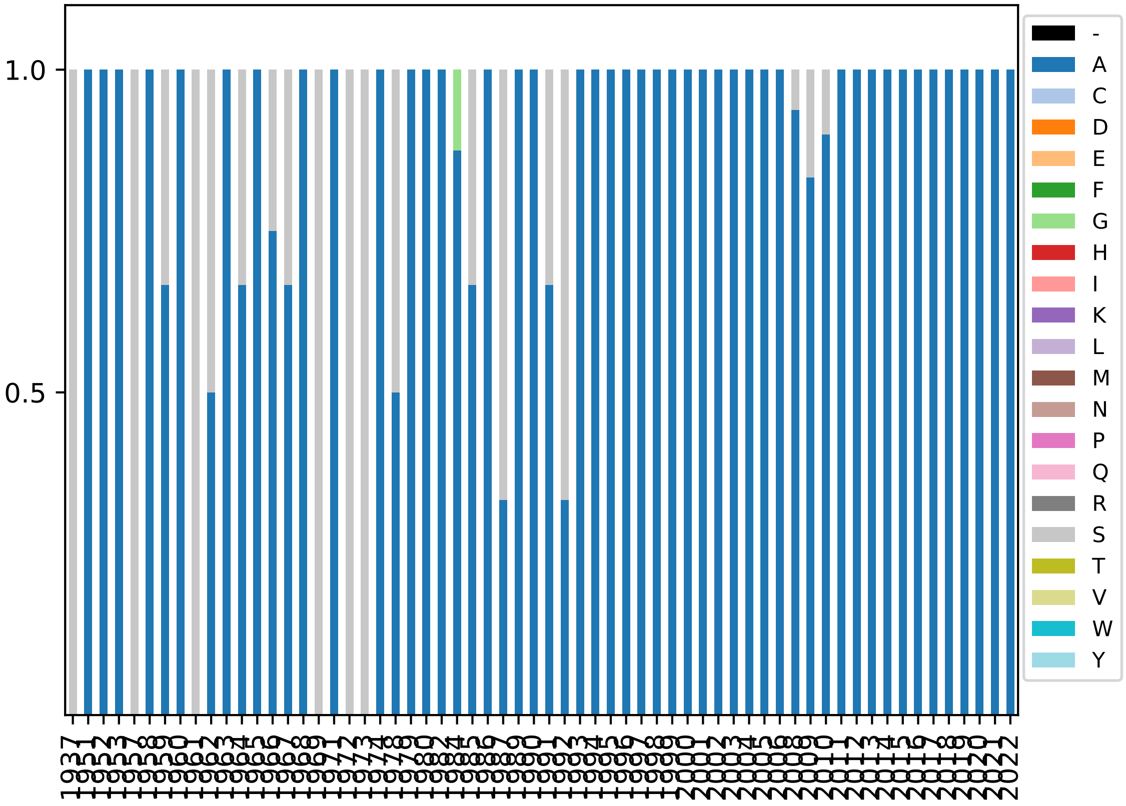

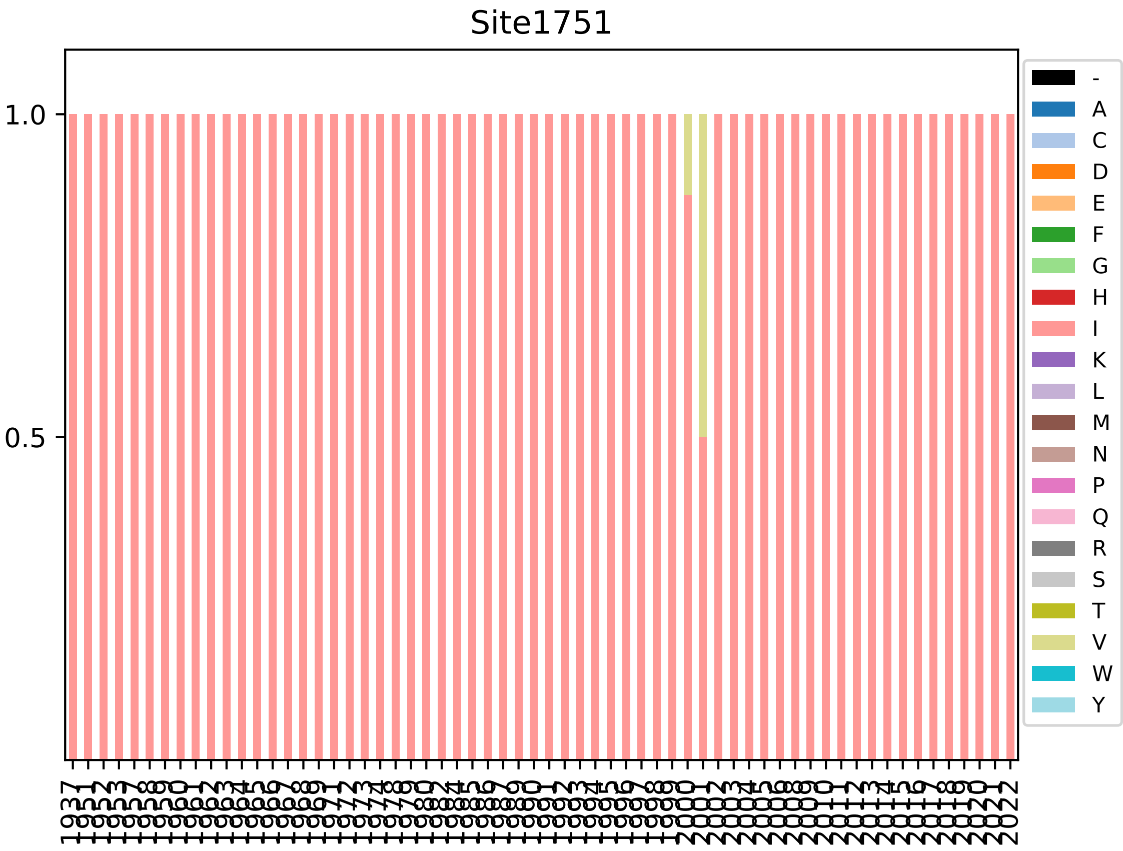

Site1751
1.0
0.5
-
A
C
D
E
F
G
H
I
K
L
M
N
P
Q
R
S
T
V
W
Y

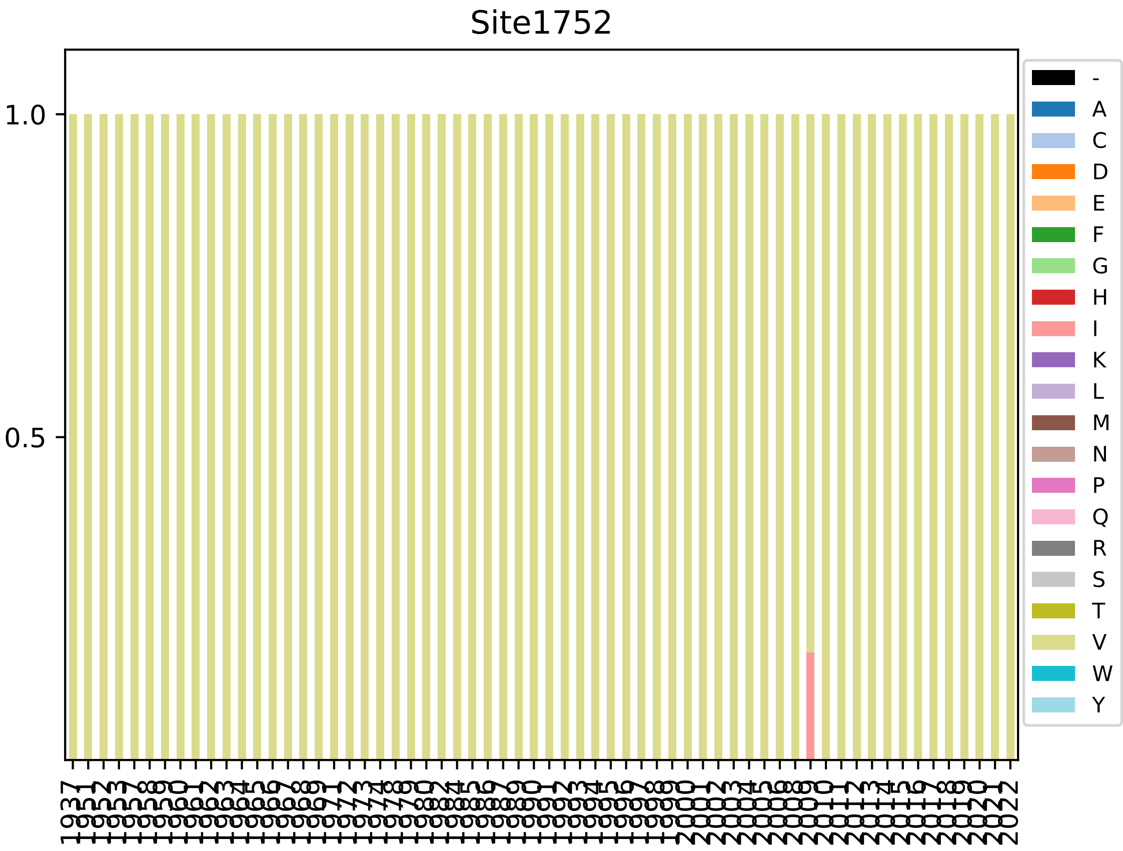
Site1752
1.0
0.5
-
A
C
D
E
F
G
H
I
K
L
M
N
P
Q
R
S
T
V
W
Y

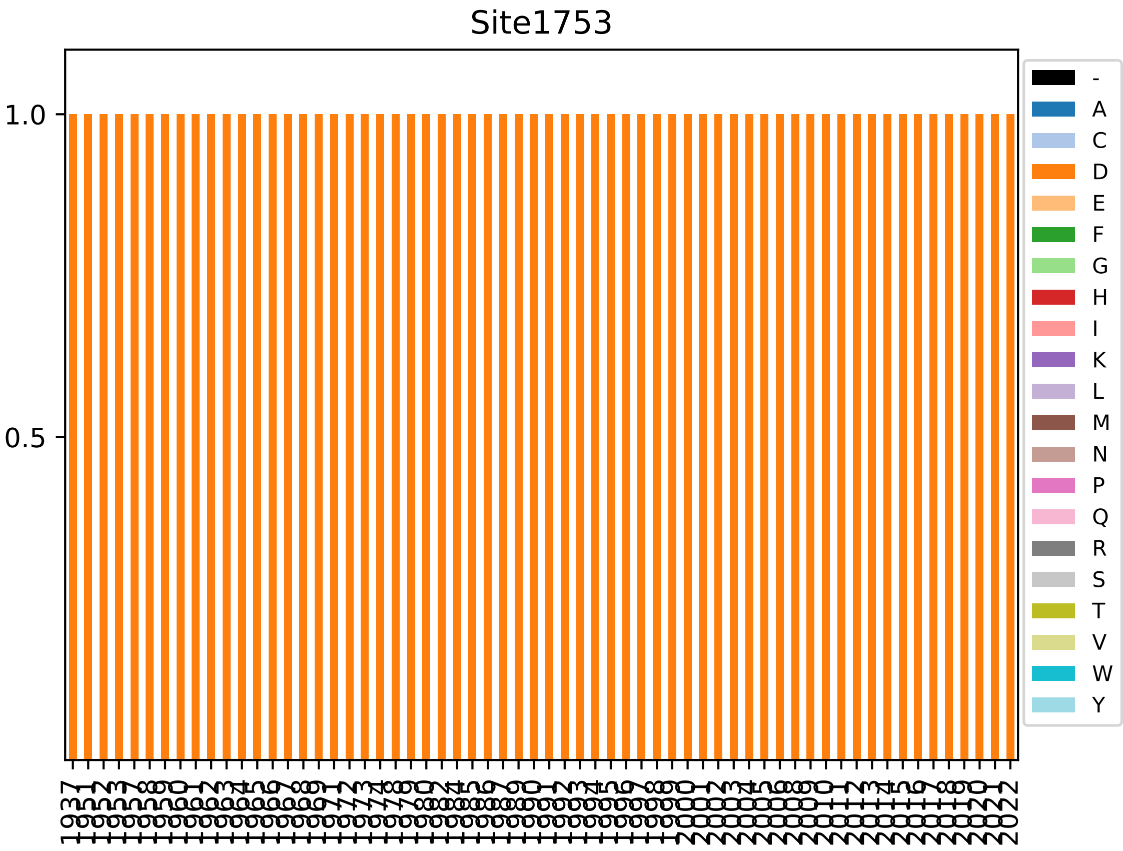
Site1753
1.0
0.5
-
A
C
D
E
F
G
H
I
K
L
M
N
P
Q
R
S
T
V
W
Y

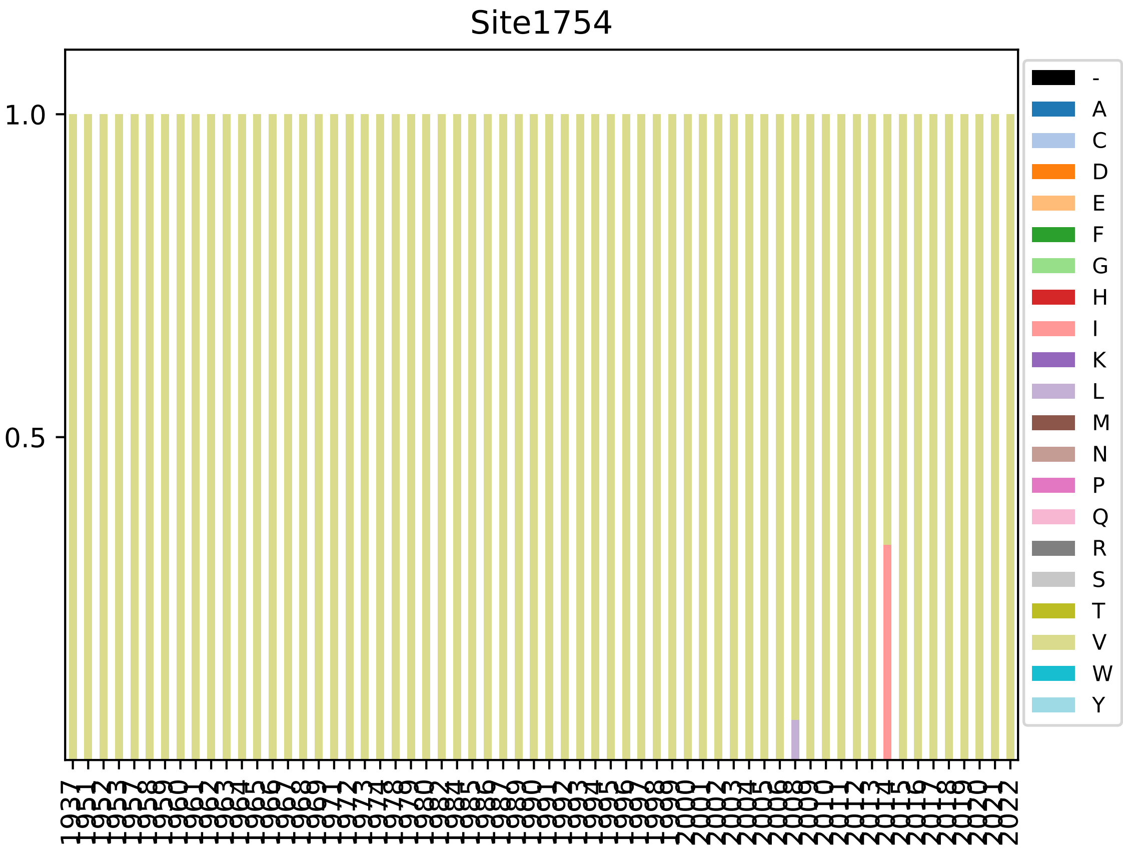
Site1754
1.0
0.5
-
A
C
D
E
F
G
H
I
K
L
M
N
P
Q
R
S
T
V
W
Y

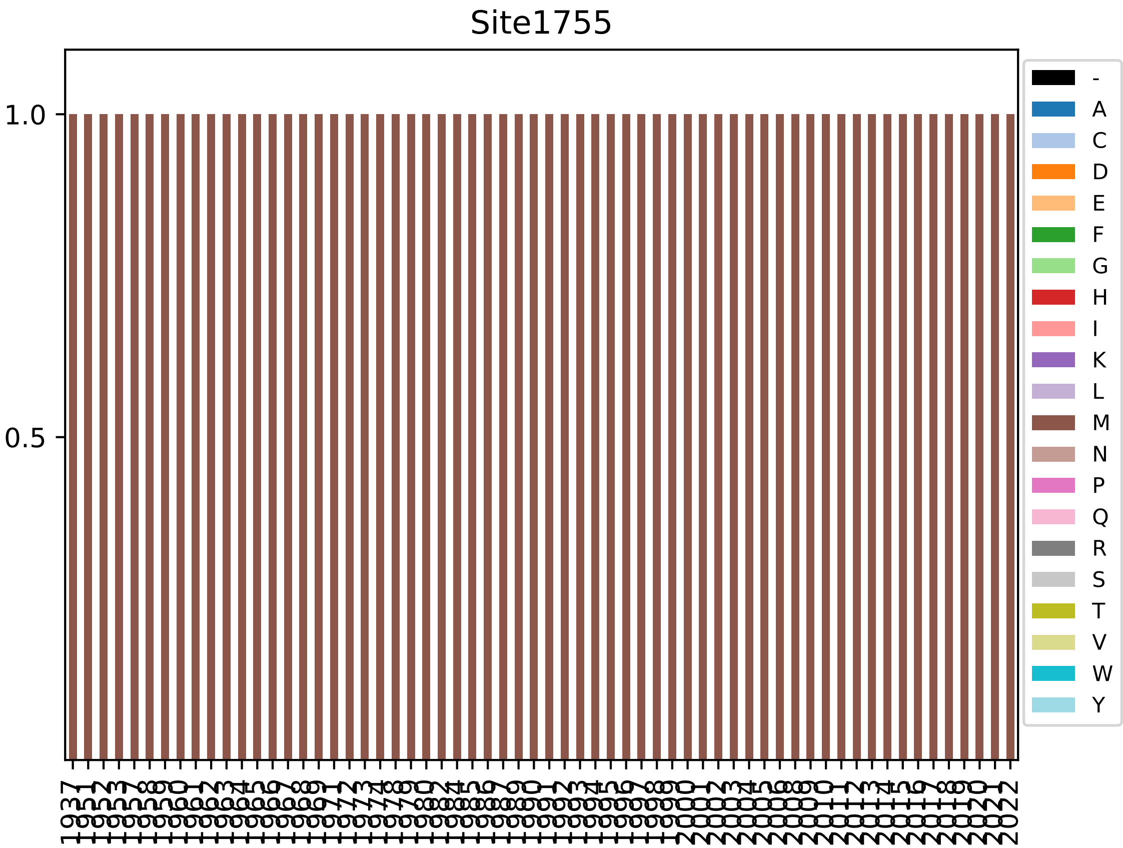
Site1755
1.0
0.5
-
A
C
D
E
F
G
H
I
K
L
M
N
P
Q
R
S
T
V
W
Y

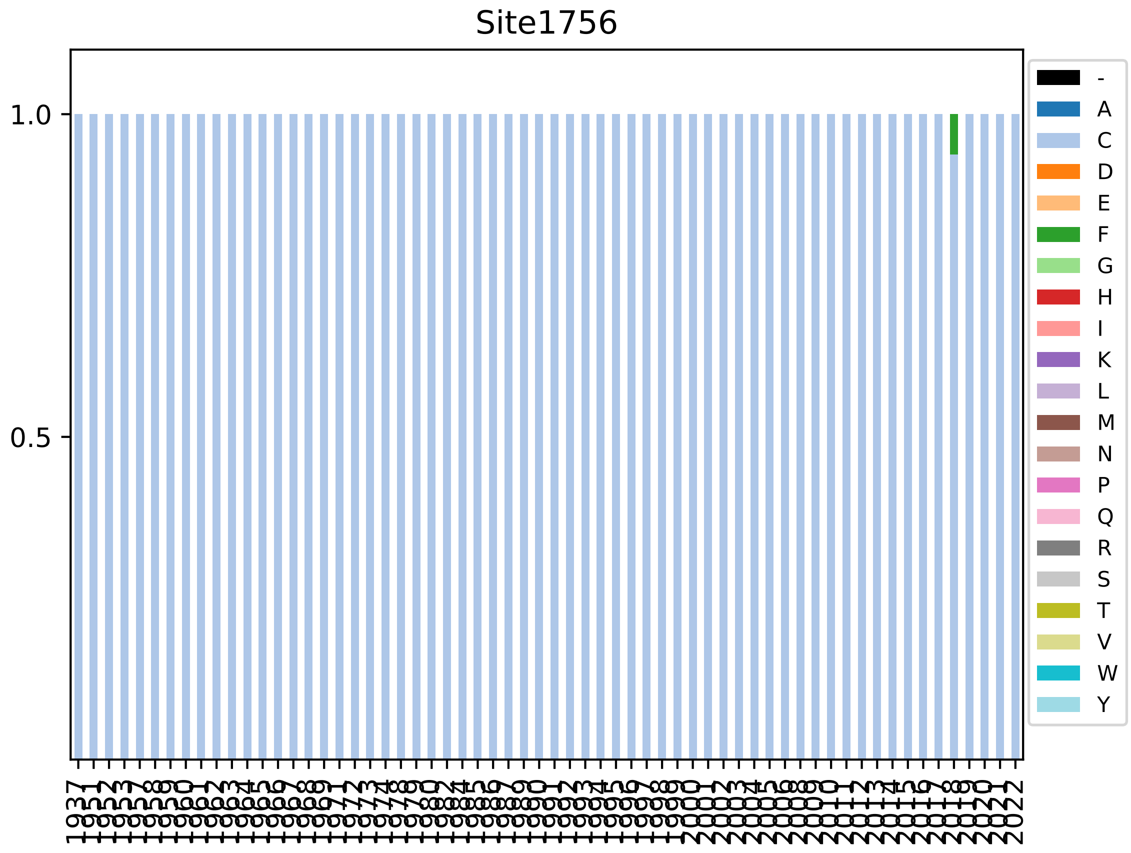
Site1756
1.0
0.5
-
A
C
D
E
F
G
H
I
K
L
M
N
P
Q
R
S
T
V
W
Y

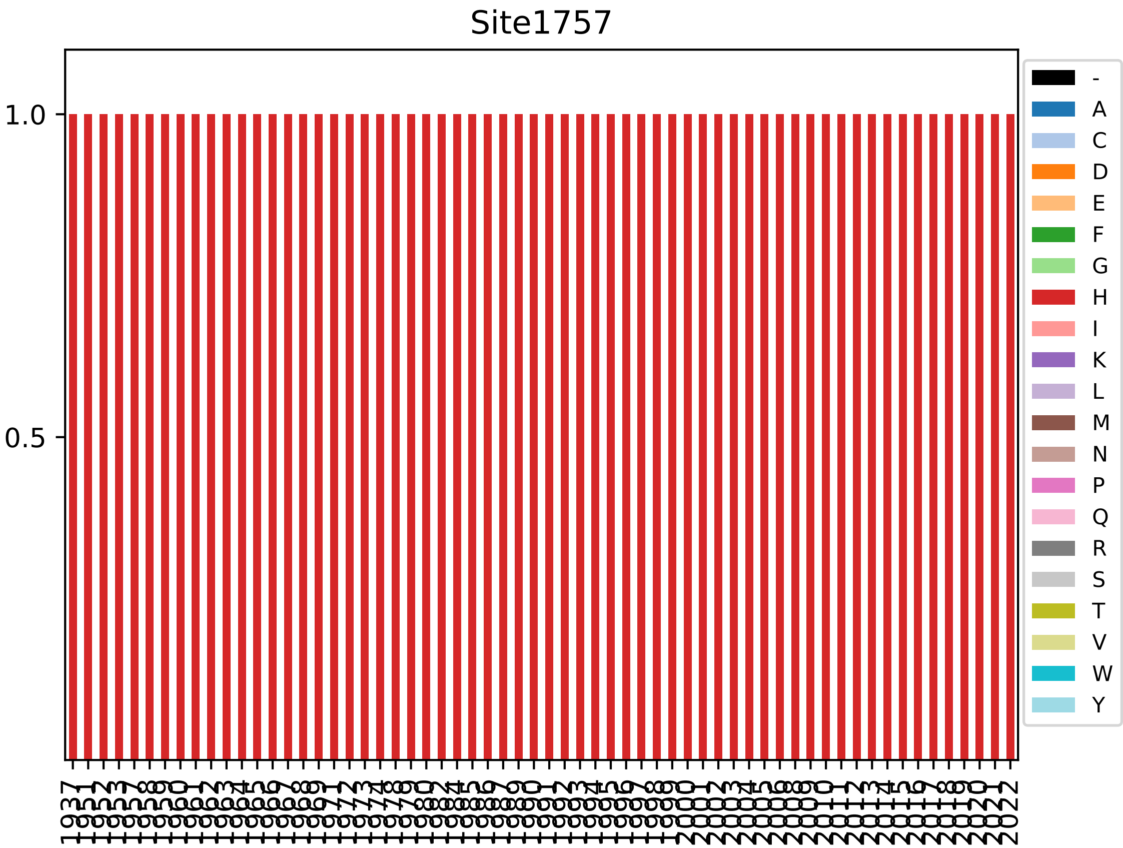
Site1757
1.0
0.5
-
A
C
D
E
F
G
H
I
K
L
M
N
P
Q
R
S
T
V
W
Y

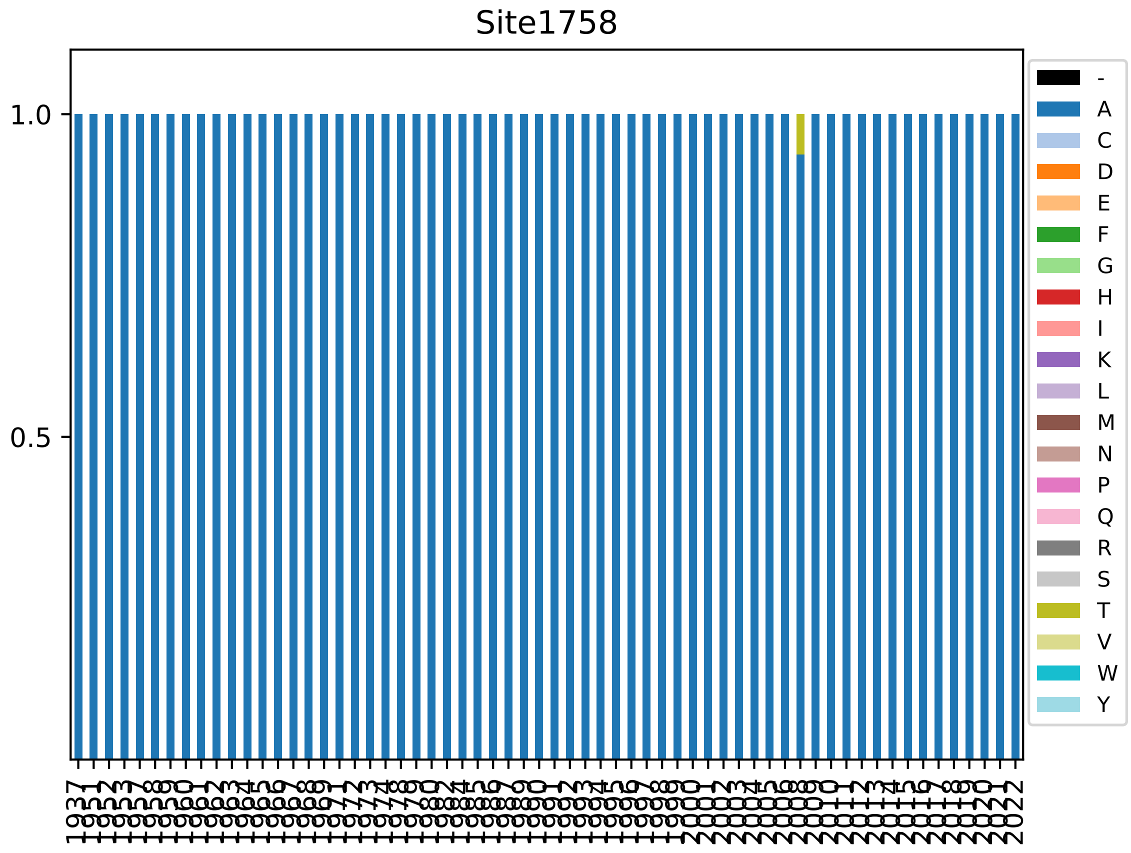

Site1758
1.0
0.5
-
A
C
D
E
F
G
H
I
K
L
M
N
P
Q
R
S
T
V
W
Y

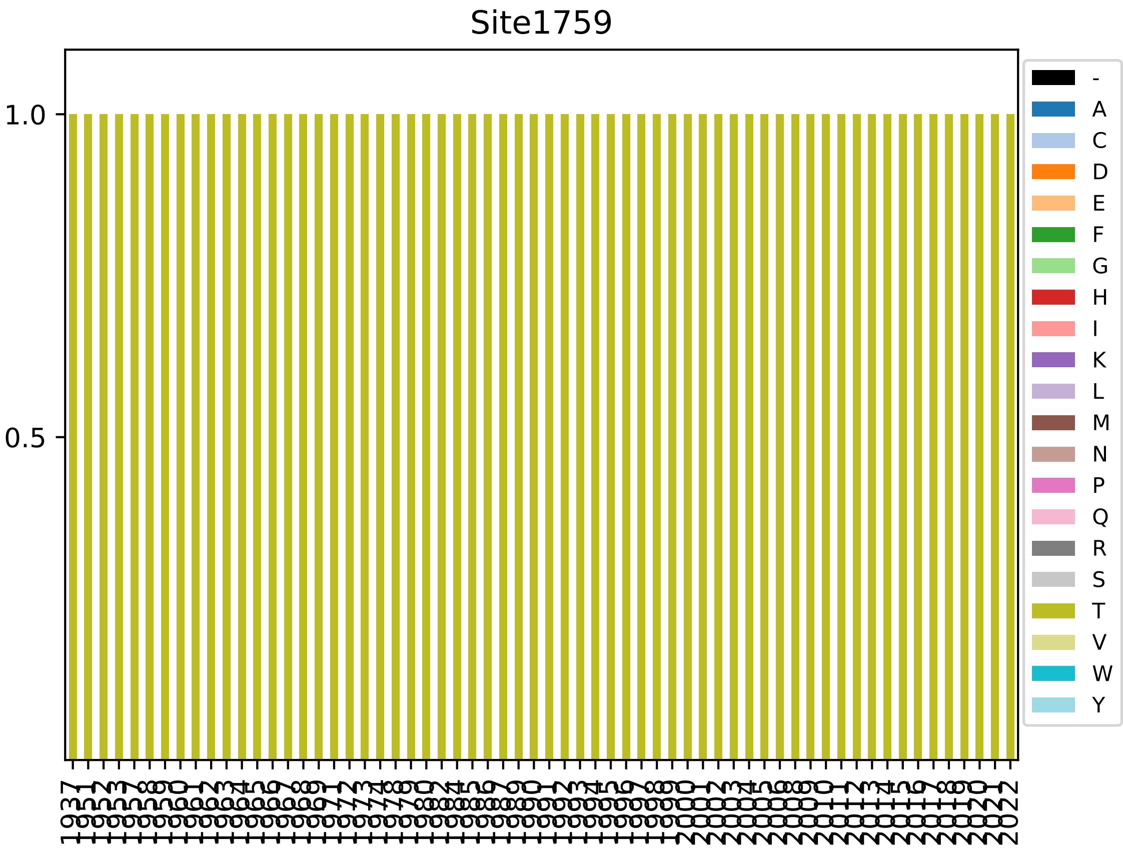
Site1759
1.0
0.5
-
A
C
D
E
F
G
H
I
K
L
M
N
P
Q
R
S
T
V
W
Y

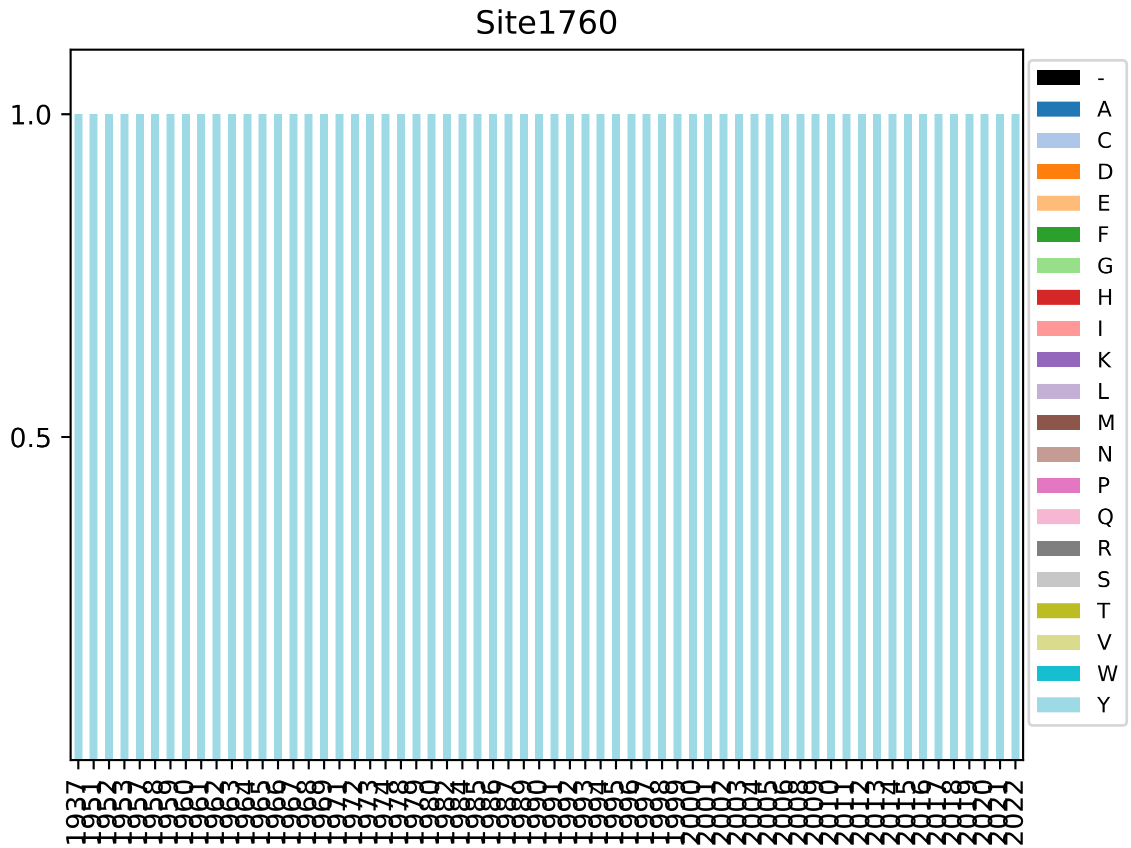
Site1760
1.0
0.5
-
A
C
D
E
F
G
H
I
K
L
M
N
P
Q
R
S
T
V
W
Y

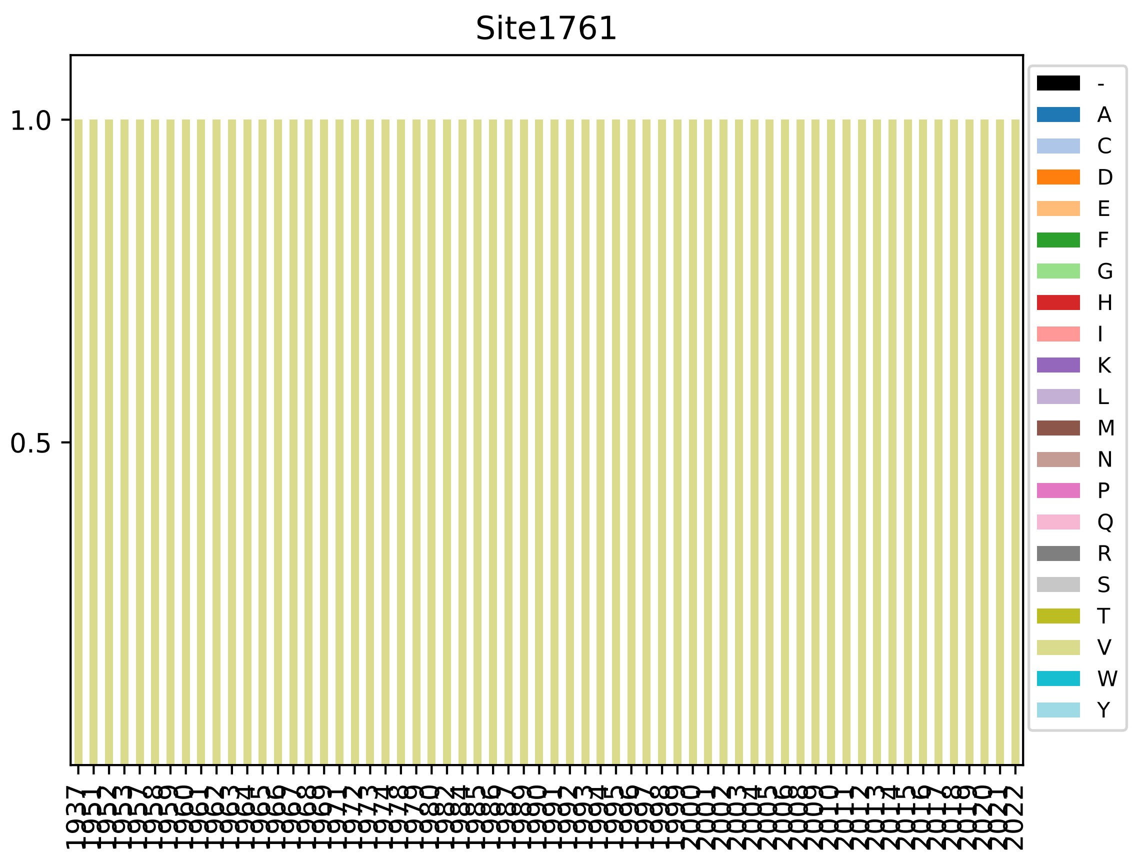

Site1761
1.0
0.5
-
A
C
D
E
F
G
H
I
K
L
M
N
P
Q
R
S
T
V
W
Y

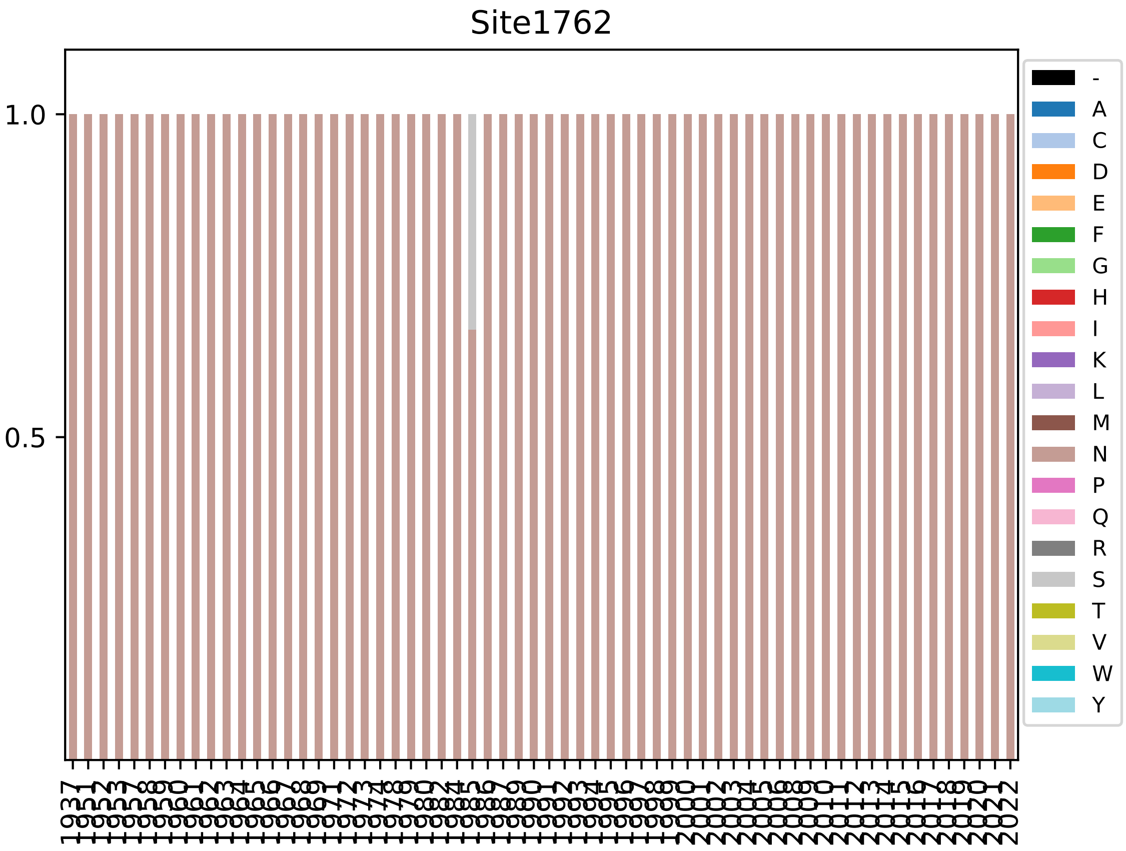
Site1762
1.0
0.5
-
A
C
D
E
F
G
H
I
K
L
M
N
P
Q
R
S
T
V
W
Y

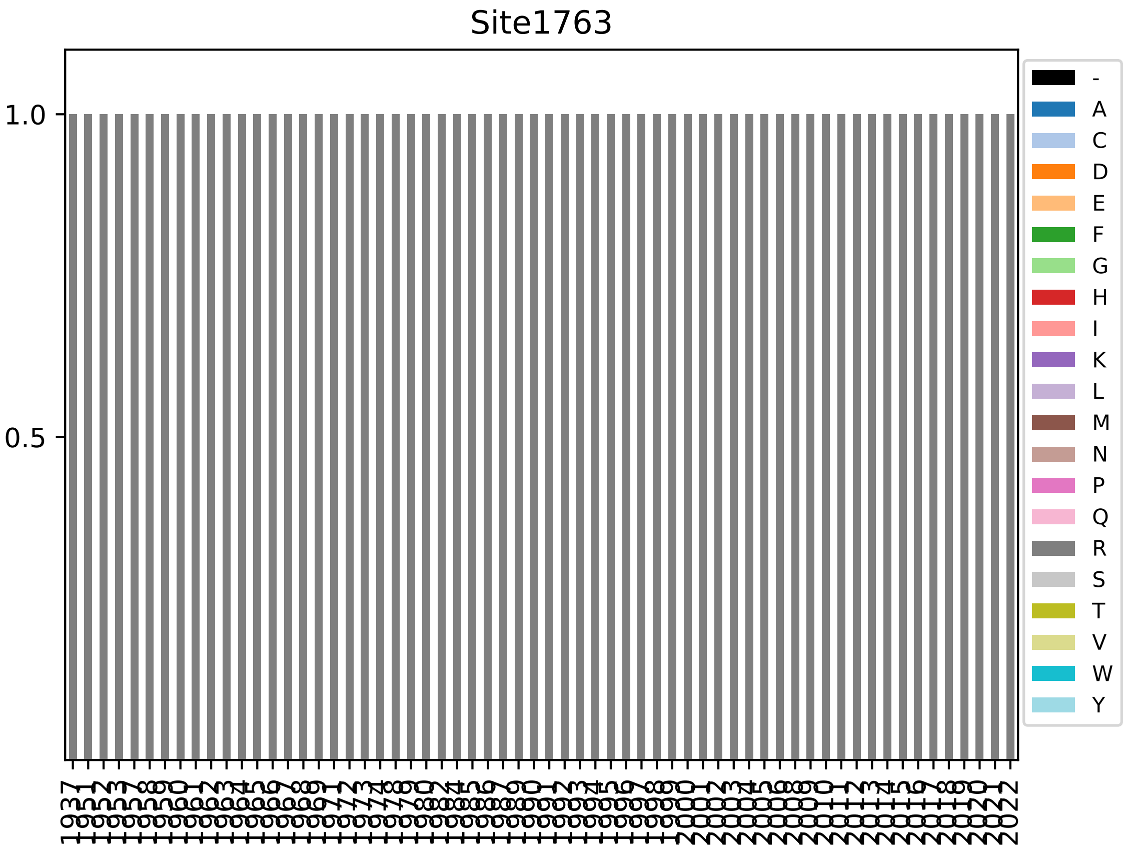
Site1763
1.0
0.5
-
A
C
D
E
F
G
H
I
K
L
M
N
P
Q
R
S
T
V
W
Y

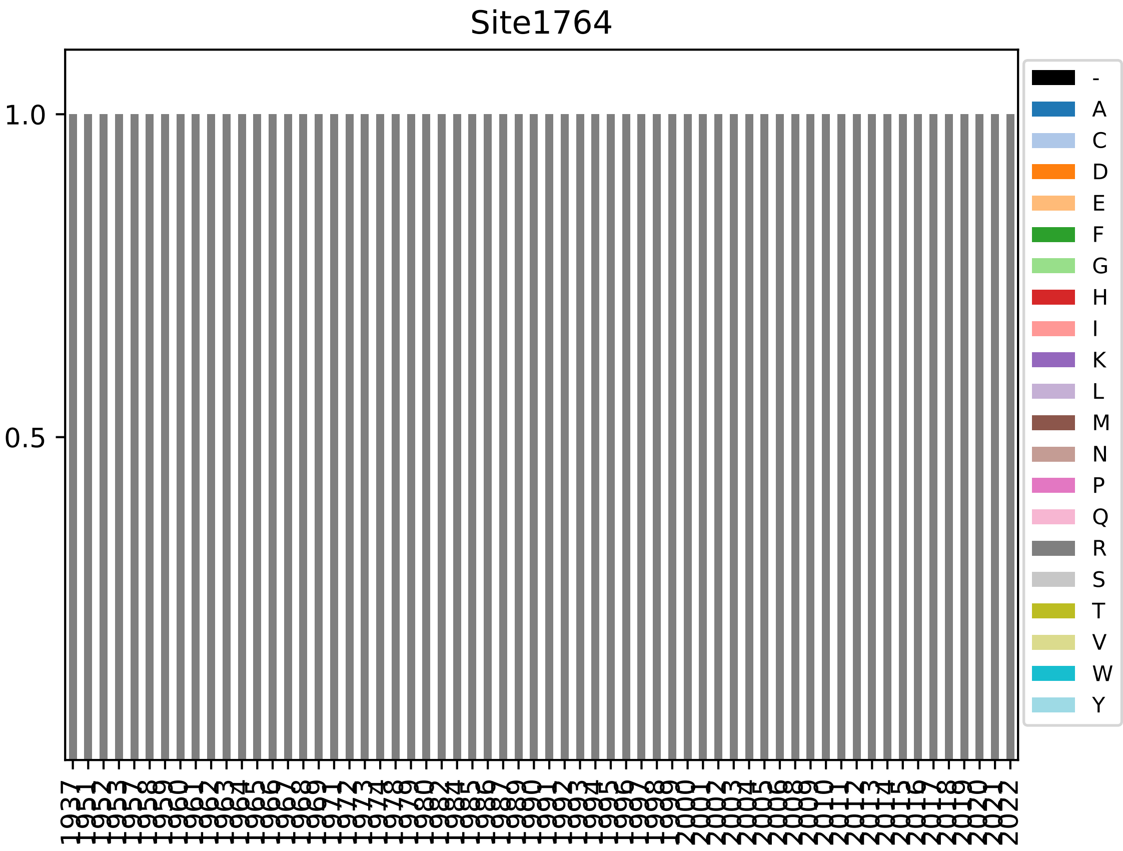
Site1764
1.0
0.5
-
A
C
D
E
F
G
H
I
K
L
M
N
P
Q
R
S
T
V
W
Y

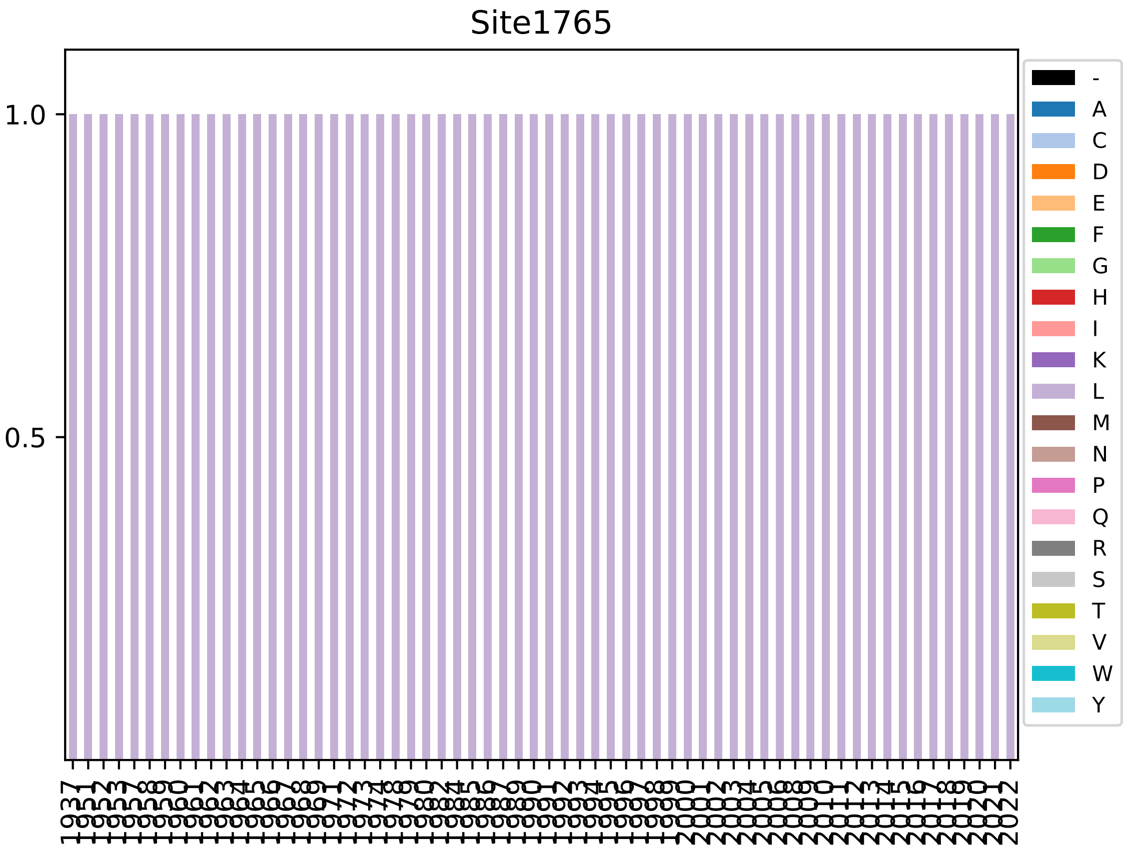
Site1765
1.0
0.5
-
A
C
D
E
F
G
H
I
K
L
M
N
P
Q
R
S
T
V
W
Y

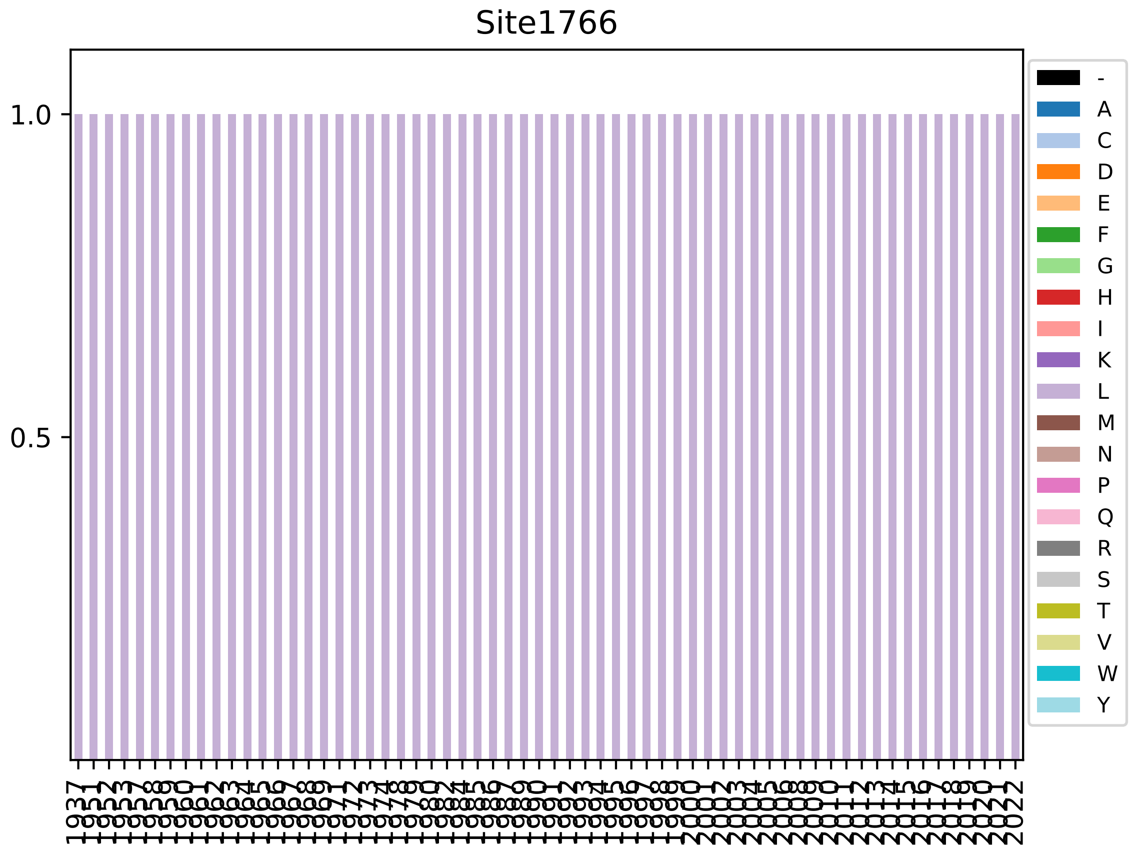
Site1766
1.0
0.5
-
A
C
D
E
F
G
H
I
K
L
M
N
P
Q
R
S
T
V
W
Y

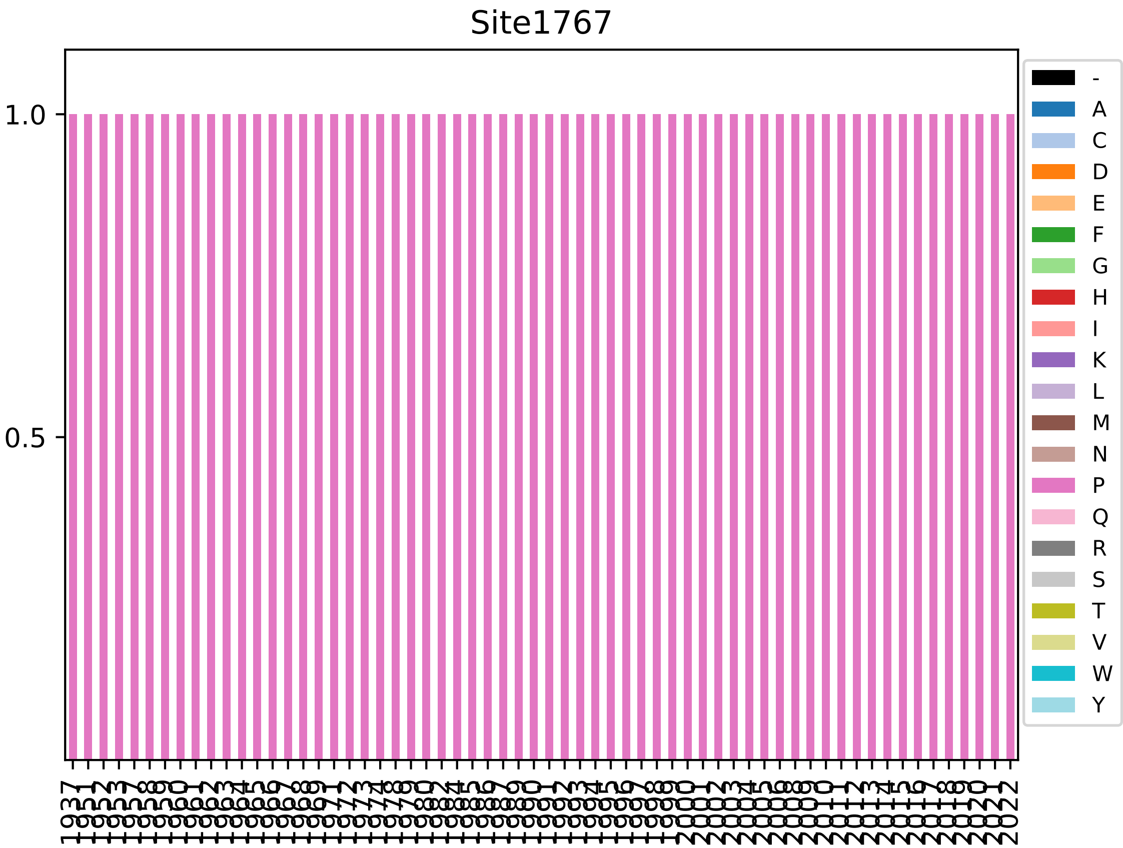
Site1767
1.0
0.5
-
A
C
D
E
F
G
H
I
K
L
M
N
P
Q
R
S
T
V
W
Y

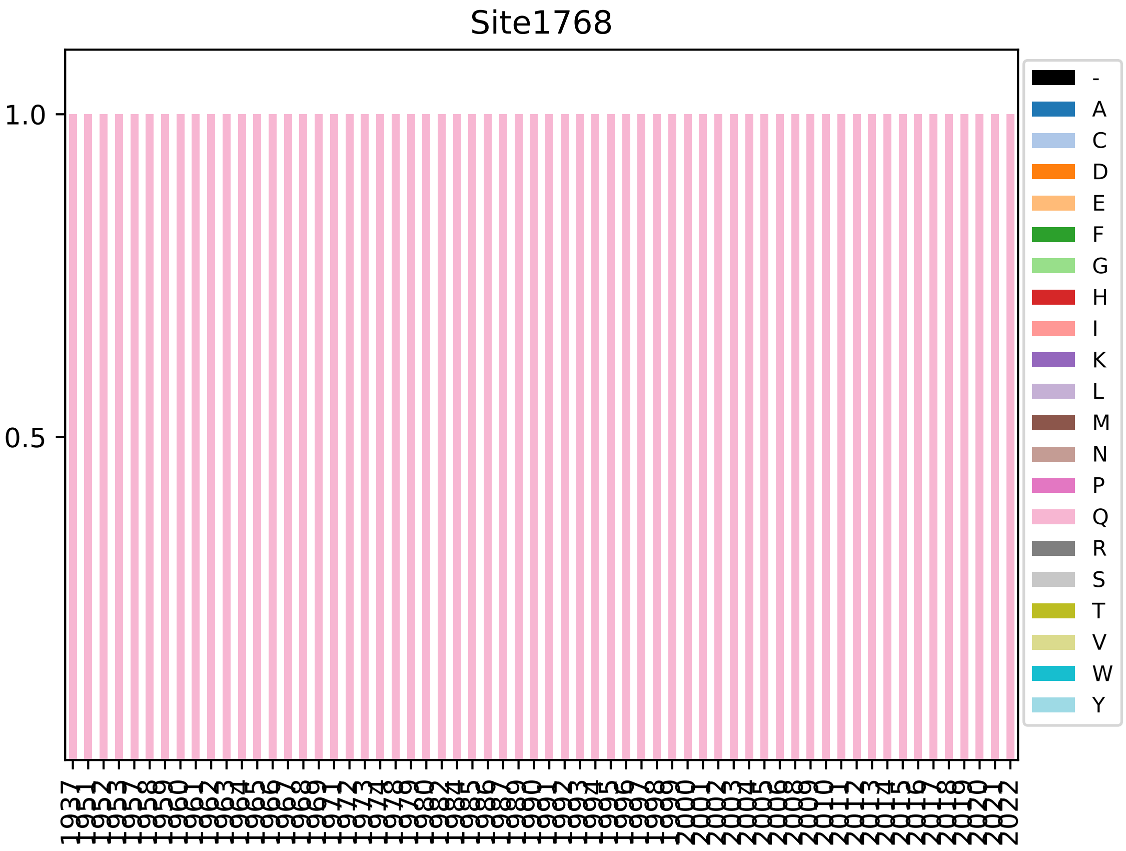

Site1768
1.0
0.5
-
A
C
D
E
F
G
H
I
K
L
M
N
P
Q
R
S
T
V
W
Y

Site1769

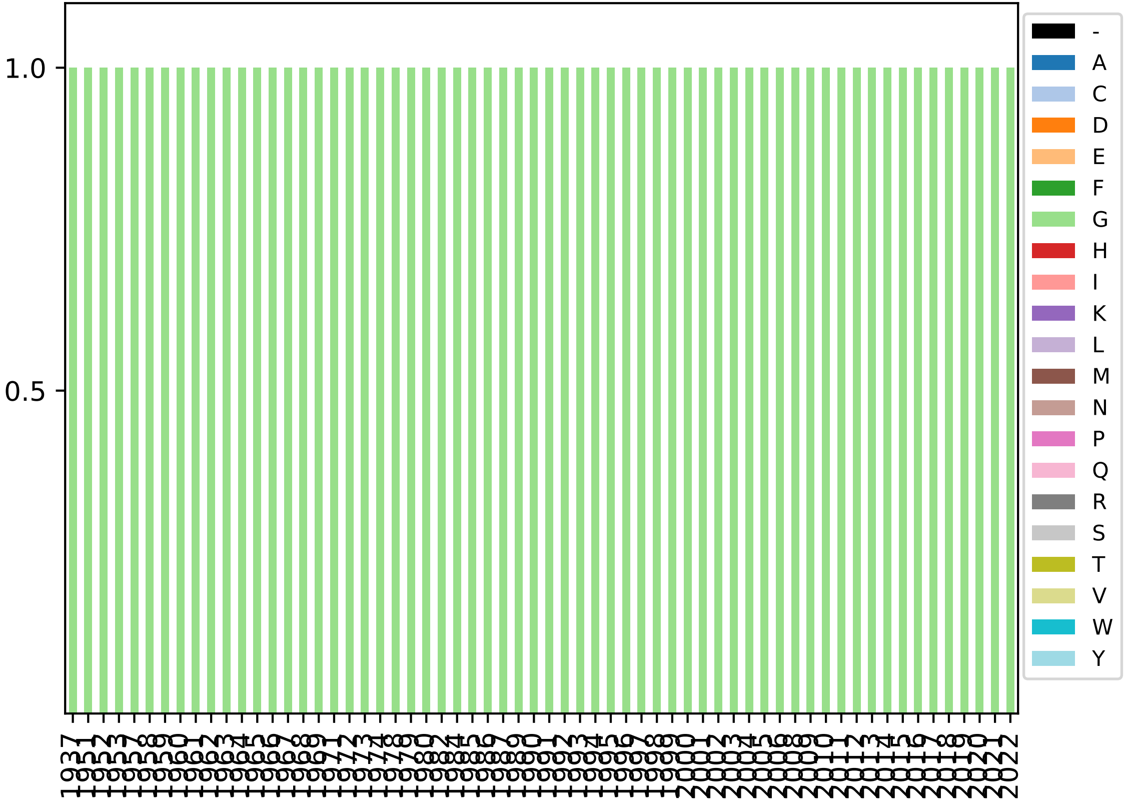

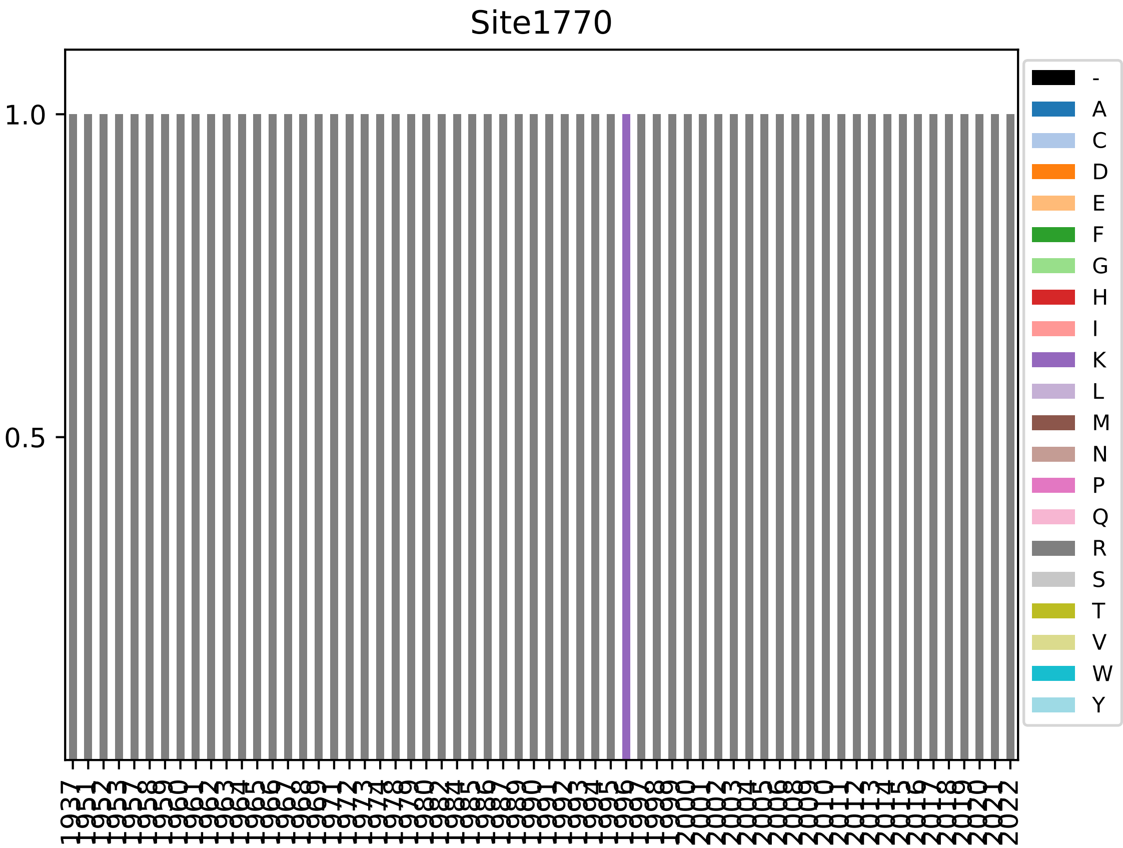
Site1770
1.0
0.5
-
A
C
D
E
F
G
H
I
K
L
M
N
P
Q
R
S
T
V
W
Y

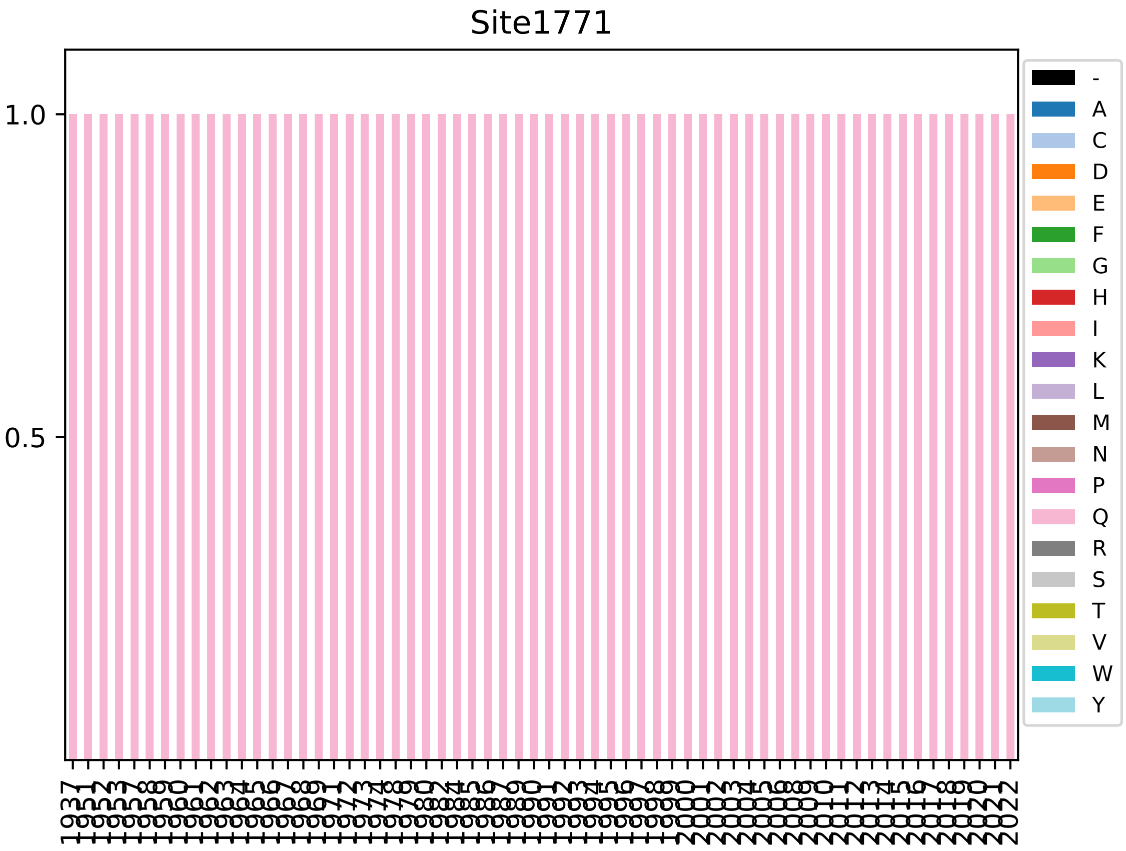
Site1771
1.0
0.5
-
A
C
D
E
F
G
H
I
K
L
M
N
P
Q
R
S
T
V
W
Y

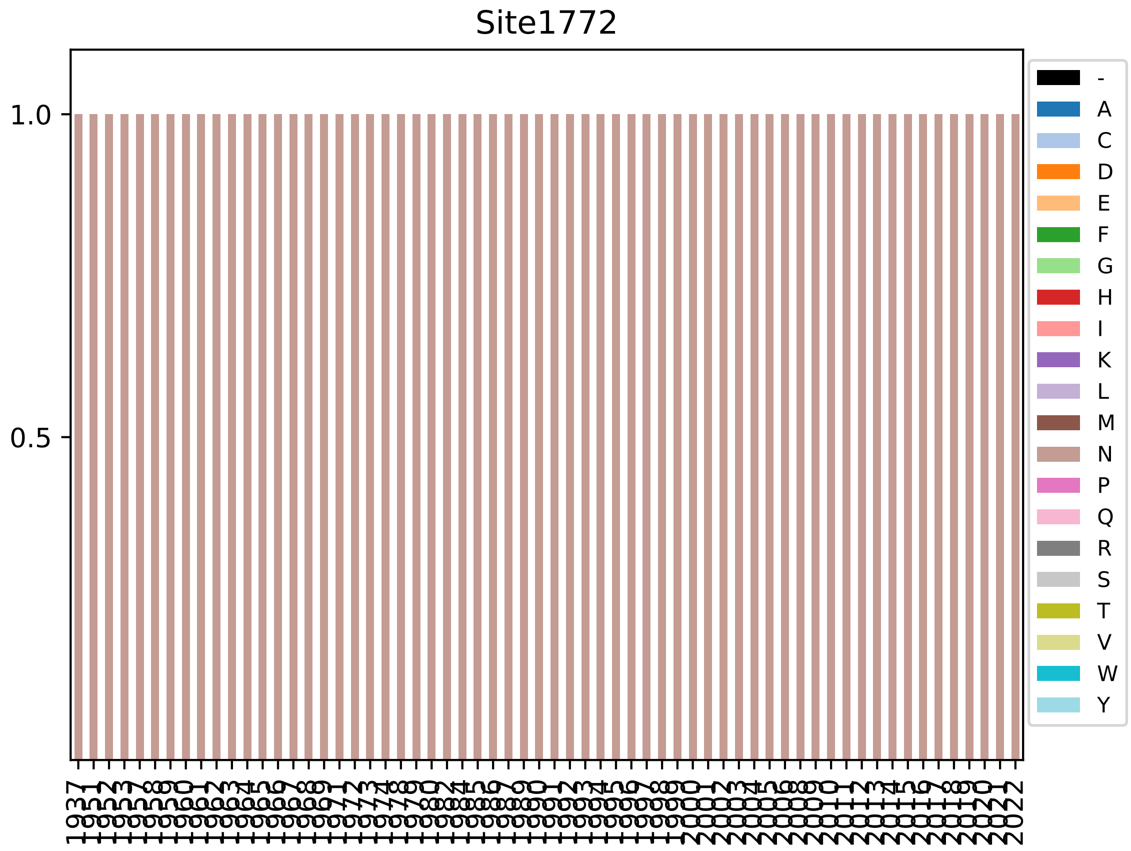
Site1772
1.0
0.5
-
A
C
D
E
F
G
H
I
K
L
M
N
P
Q
R
S
T
V
W
Y

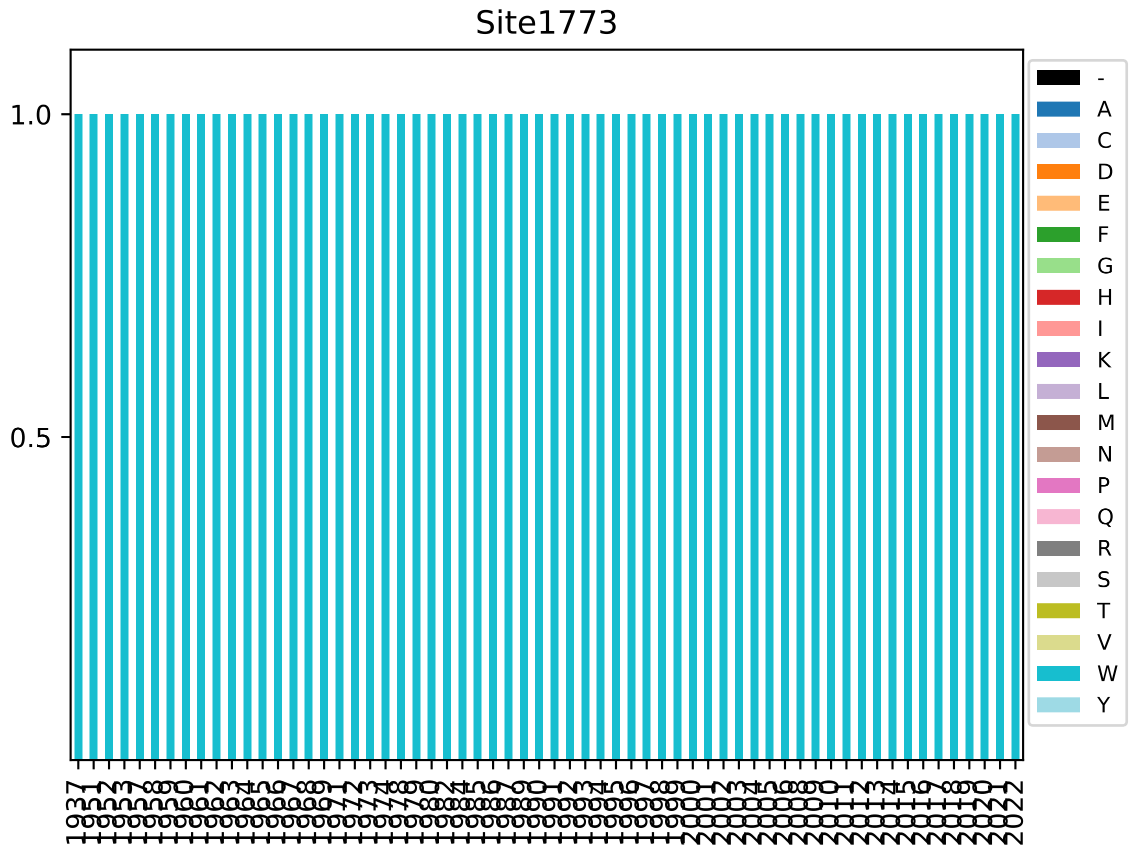
Site1773
1.0
0.5
-
A
C
D
E
F
G
H
I
K
L
M
N
P
Q
R
S
T
V
W
Y

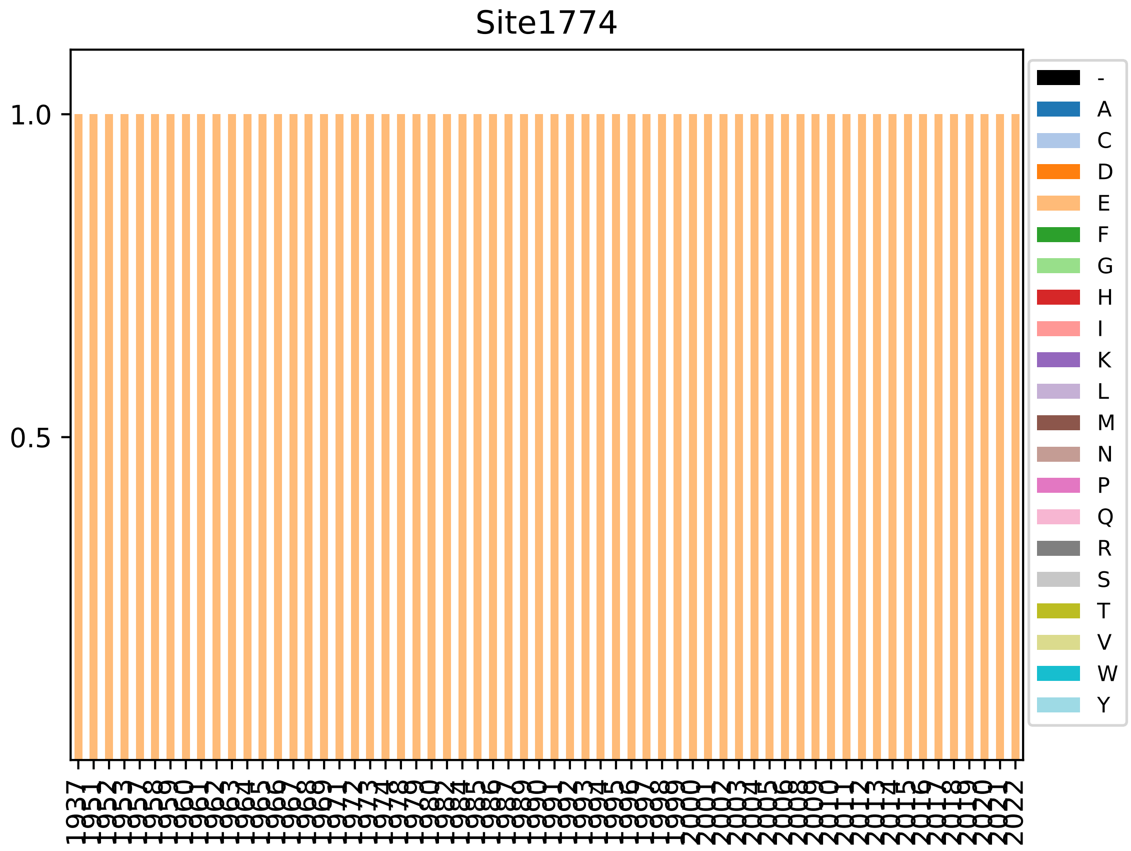
Site1774
1.0
0.5
-
A
C
D
E
F
G
H
I
K
L
M
N
P
Q
R
S
T
V
W
Y

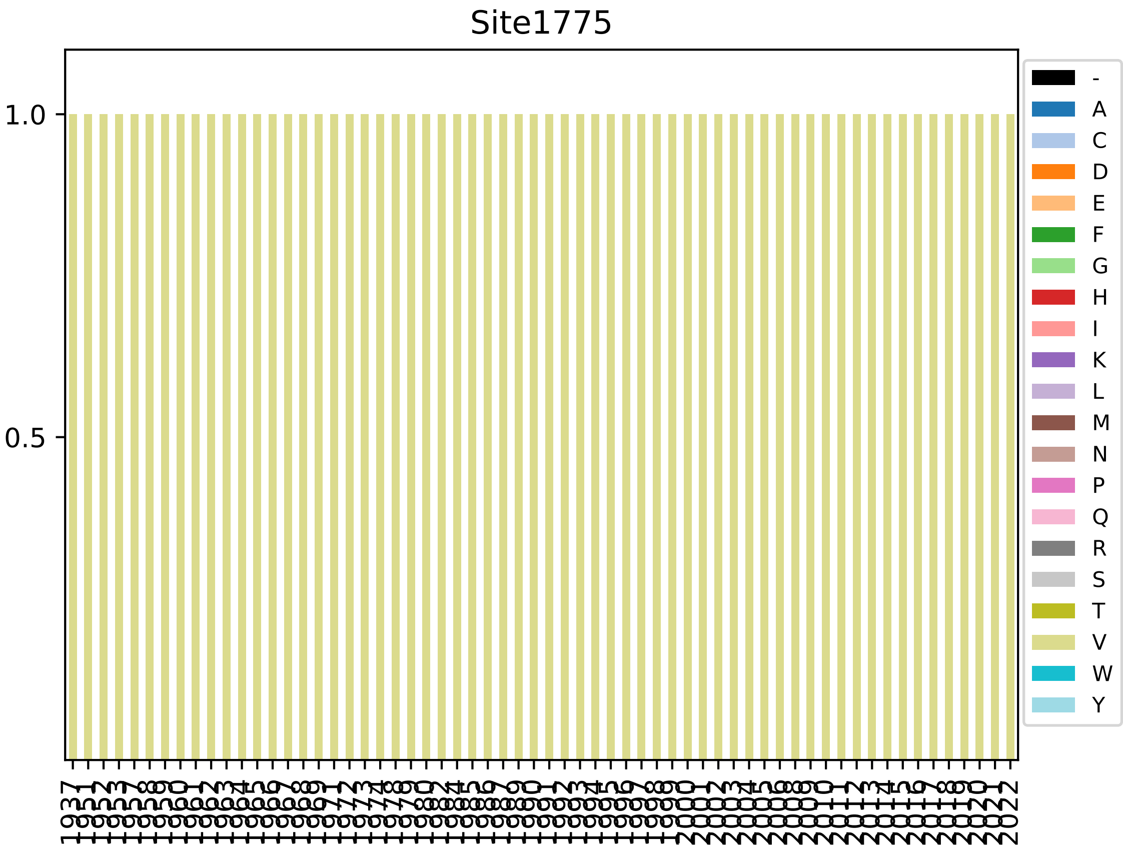
Site1775
1.0
0.5
-
A
C
D
E
F
G
H
I
K
L
M
N
P
Q
R
S
T
V
W
Y

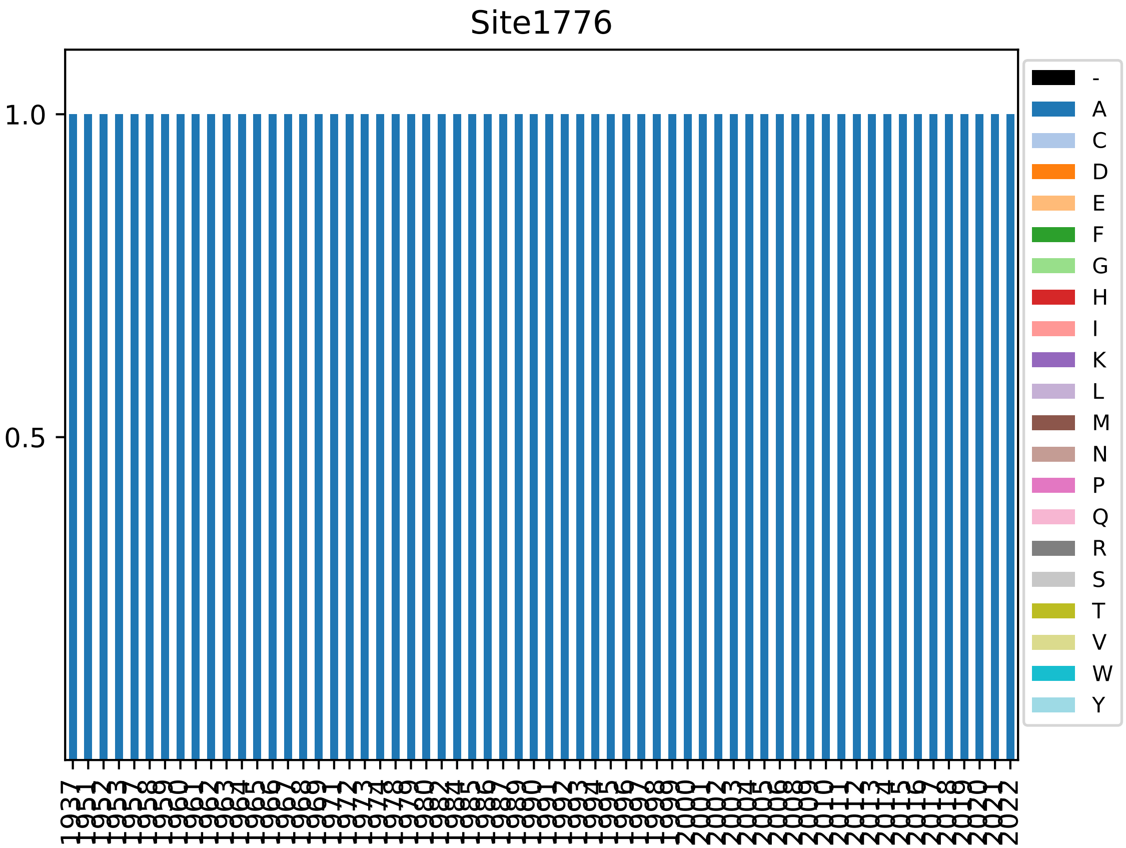
Site1776
1.0
0.5
-
A
C
D
E
F
G
H
I
K
L
M
N
P
Q
R
S
T
V
W
Y

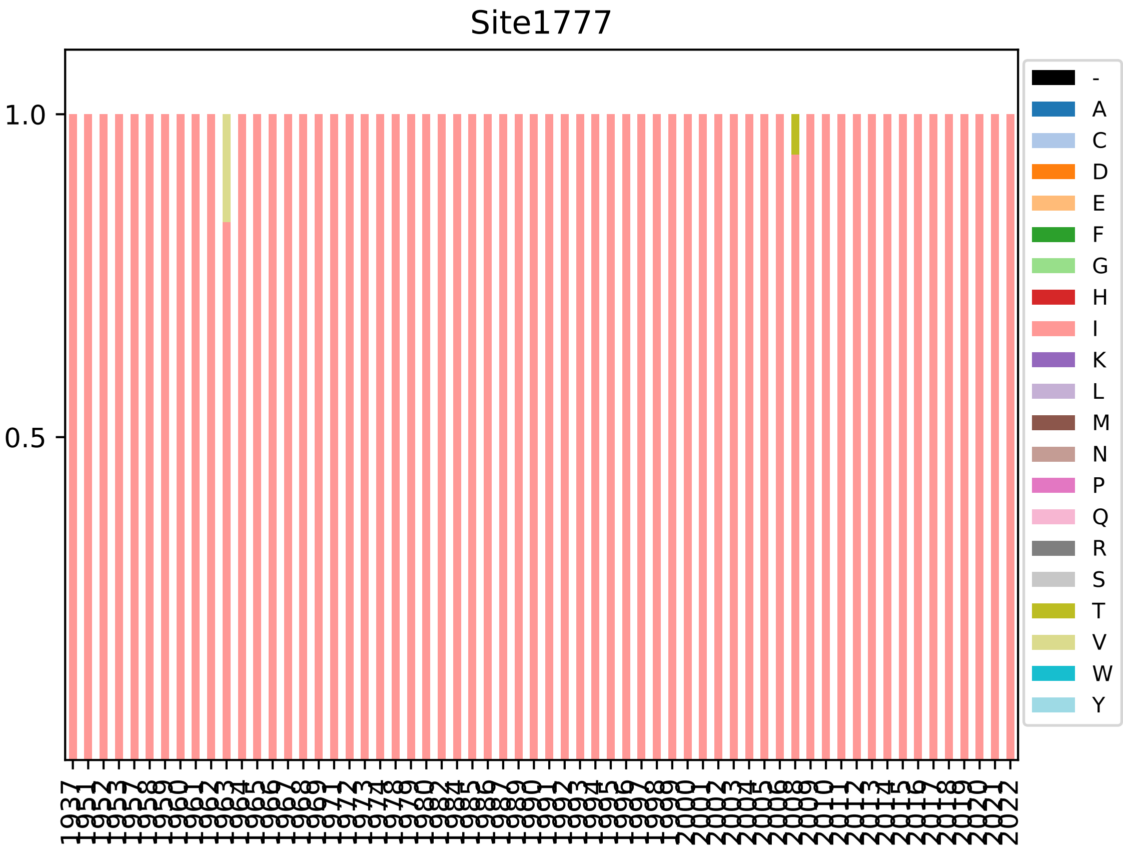

Site1777
1.0
0.5
-
A
C
D
E
F
G
H
I
K
L
M
N
P
Q
R
S
T
V
W
Y

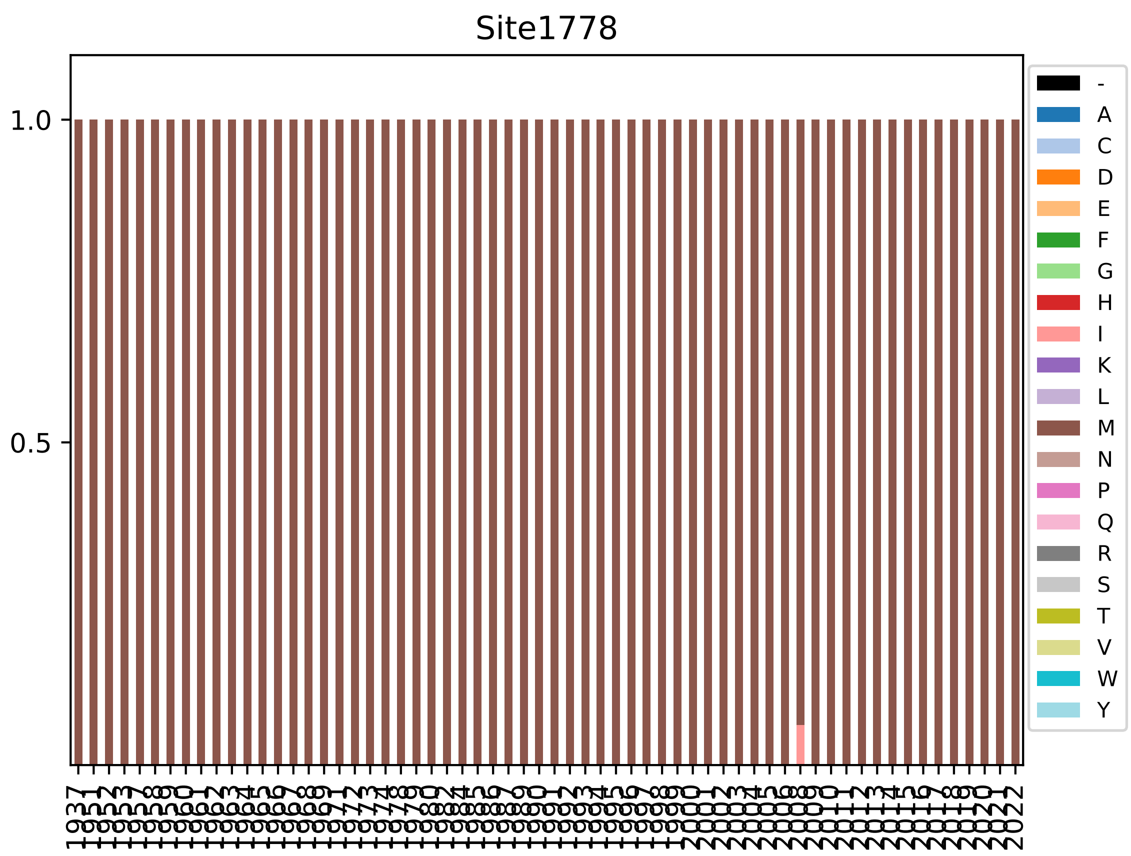

Site1778
1.0
0.5
-
A
C
D
E
F
G
H
I
K
L
M
N
P
Q
R
S
T
V
W
Y

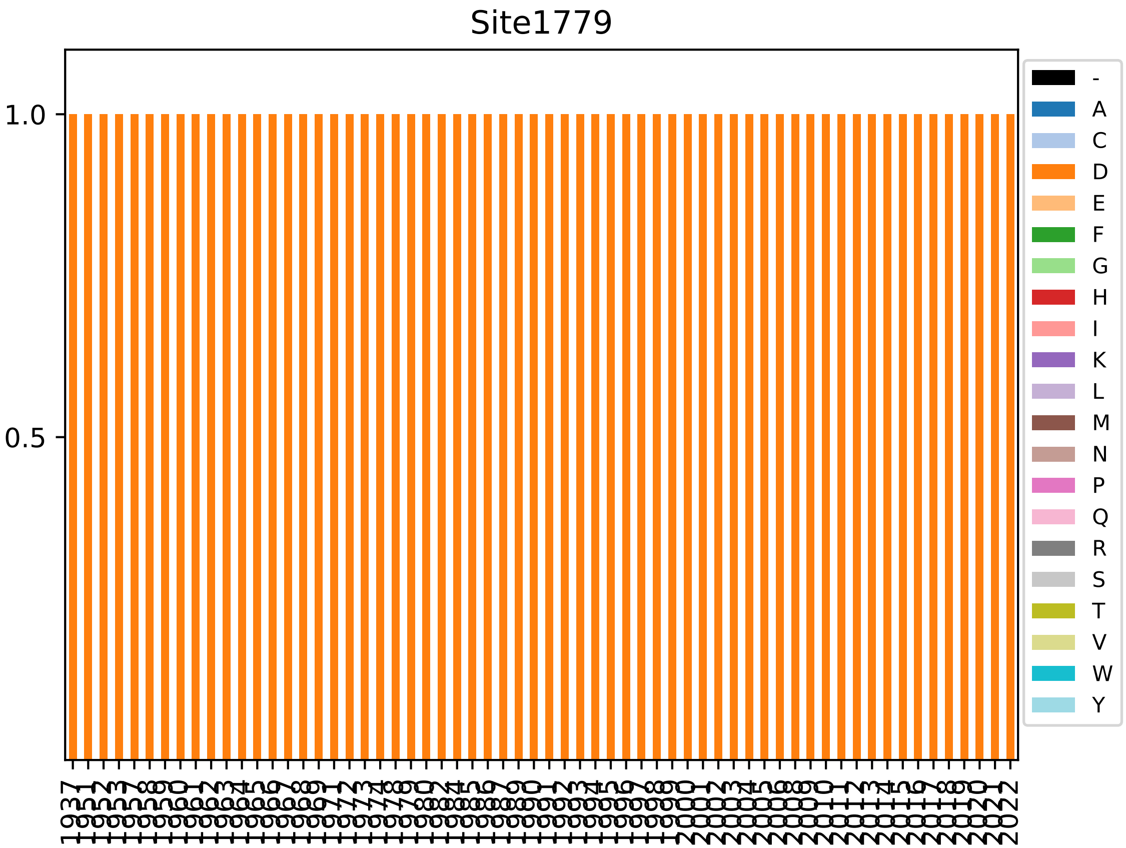
Site1779
1.0
0.5
-
A
C
D
E
F
G
H
I
K
L
M
N
P
Q
R
S
T
V
W
Y

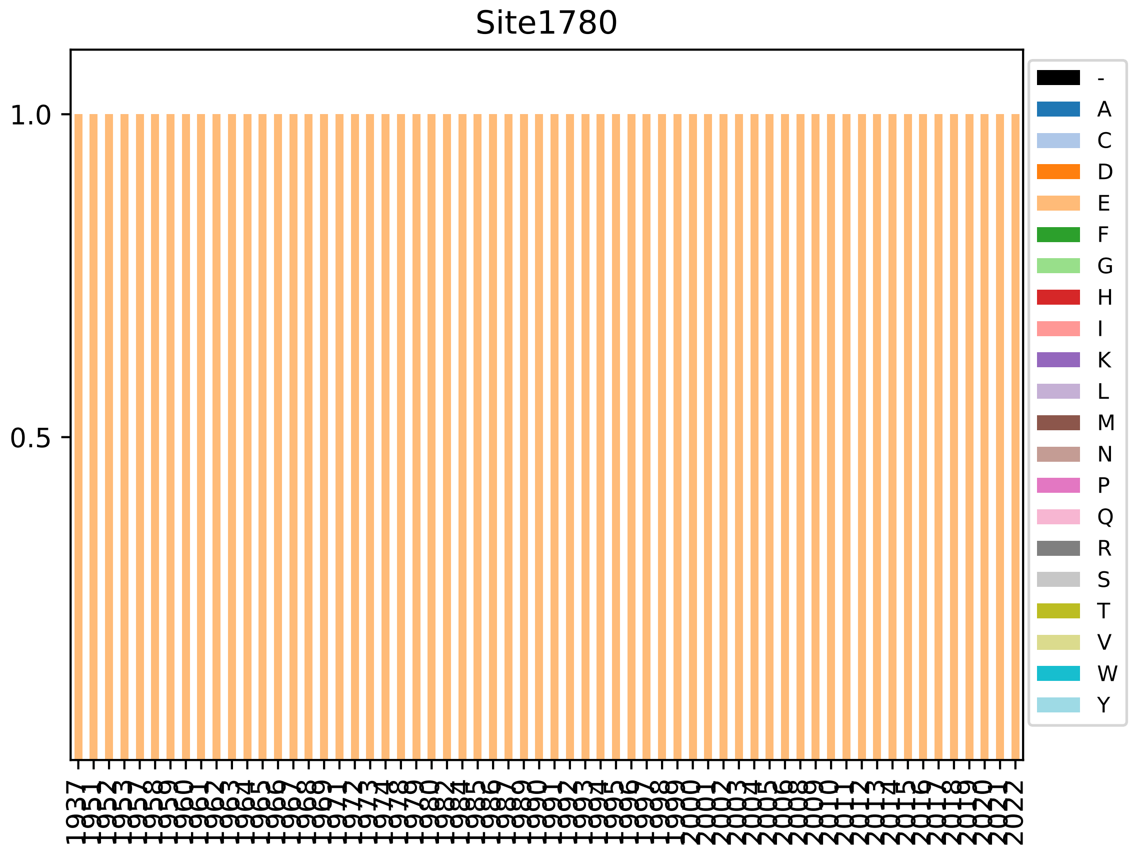
Site1780
1.0
0.5
-
A
C
D
E
F
G
H
I
K
L
M
N
P
Q
R
S
T
V
W
Y

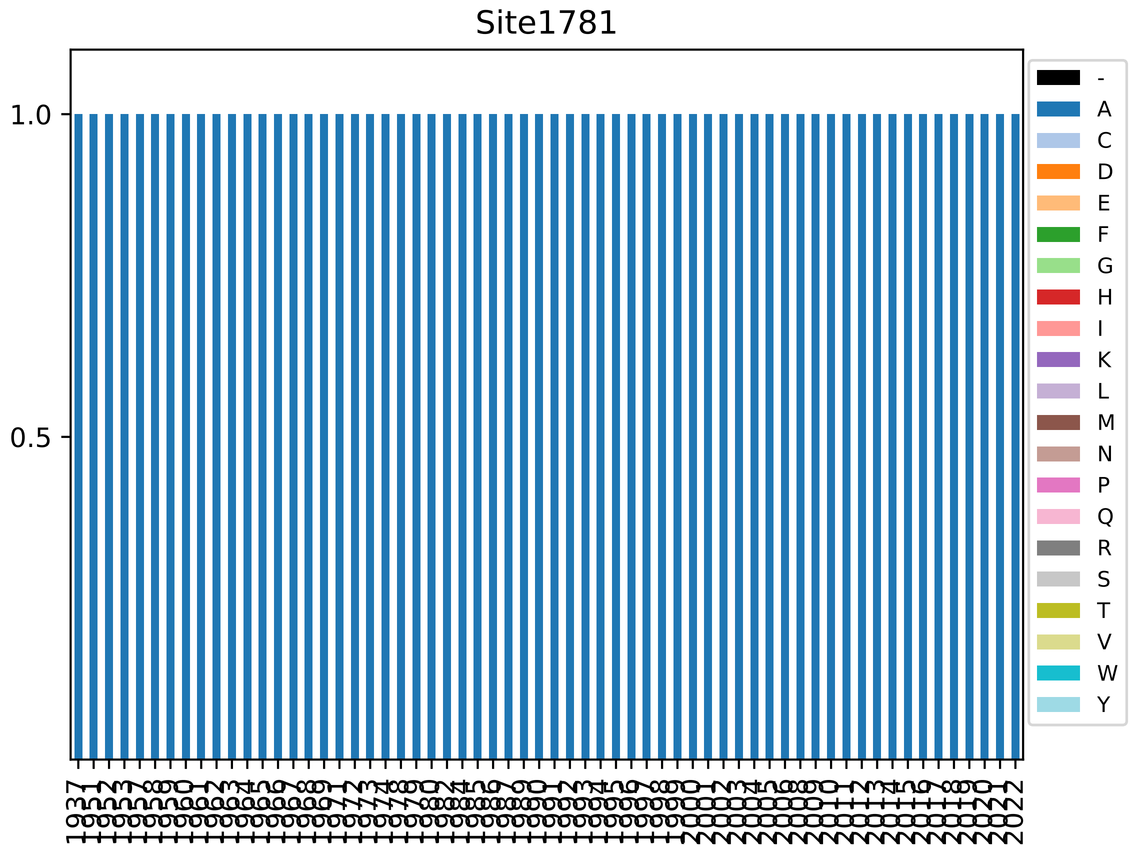
Site1781
1.0
0.5
-
A
C
D
E
F
G
H
I
K
L
M
N
P
Q
R
S
T
V
W
Y

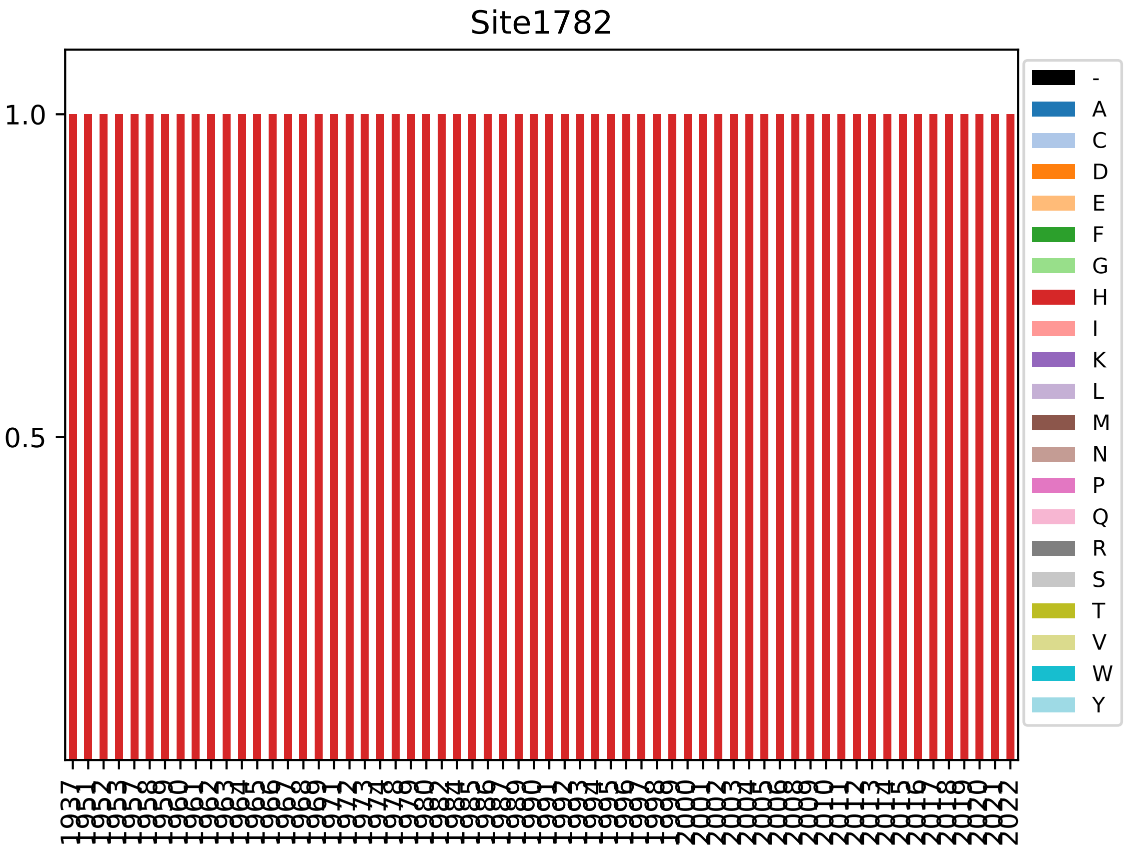
Site1782
1.0
0.5
-
A
C
D
E
F
G
H
I
K
L
M
N
P
Q
R
S
T
V
W
Y

Site1783

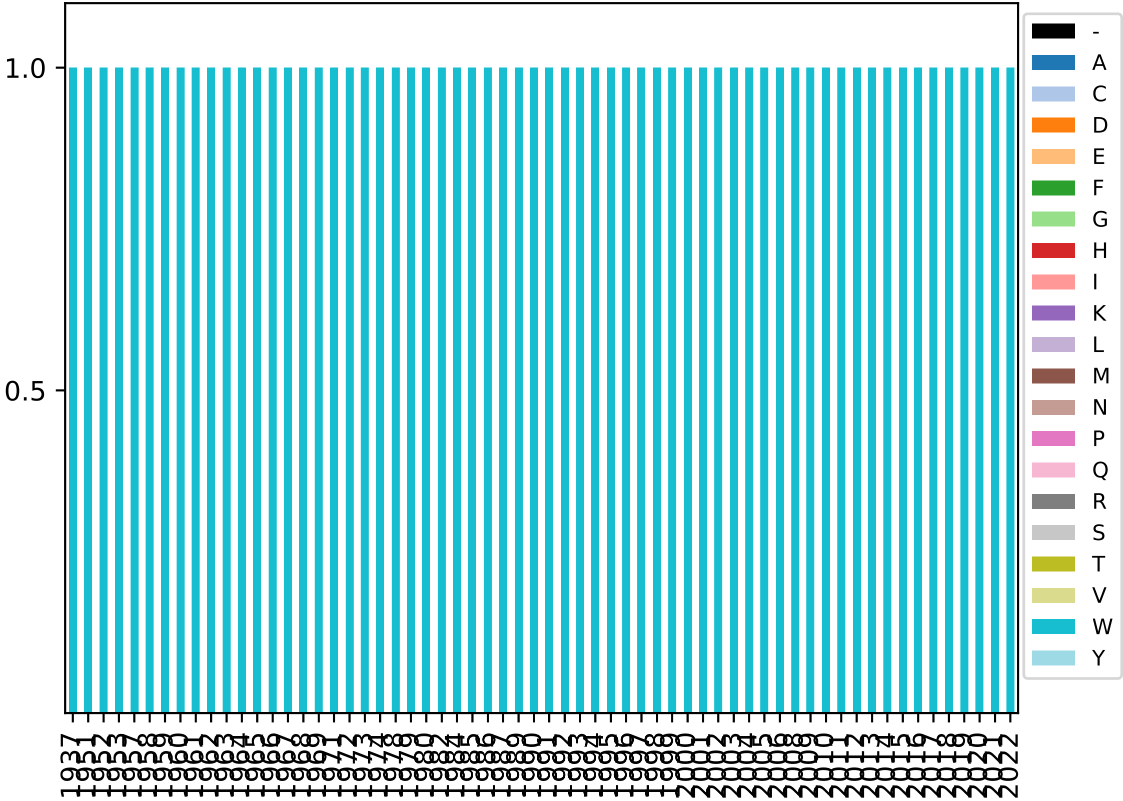

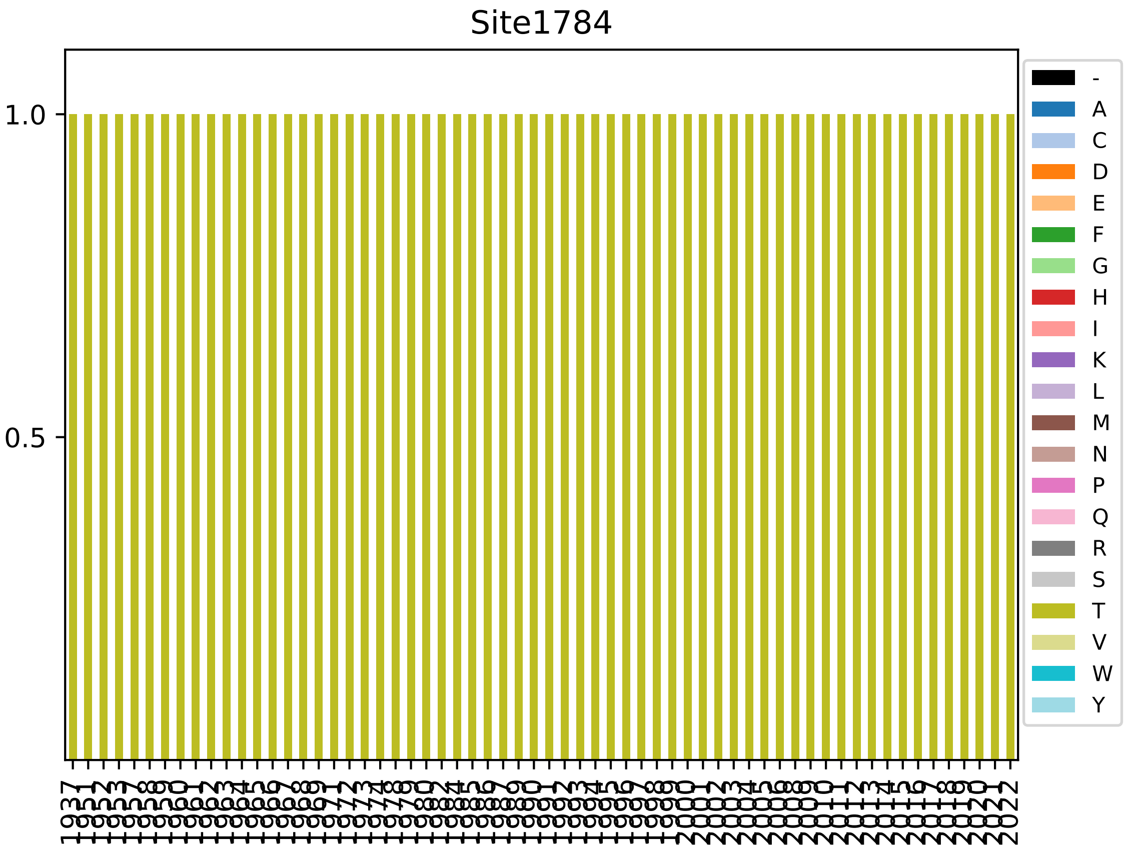
Site1784
1.0
0.5
-
A
C
D
E
F
G
H
I
K
L
M
N
P
Q
R
S
T
V
W
Y

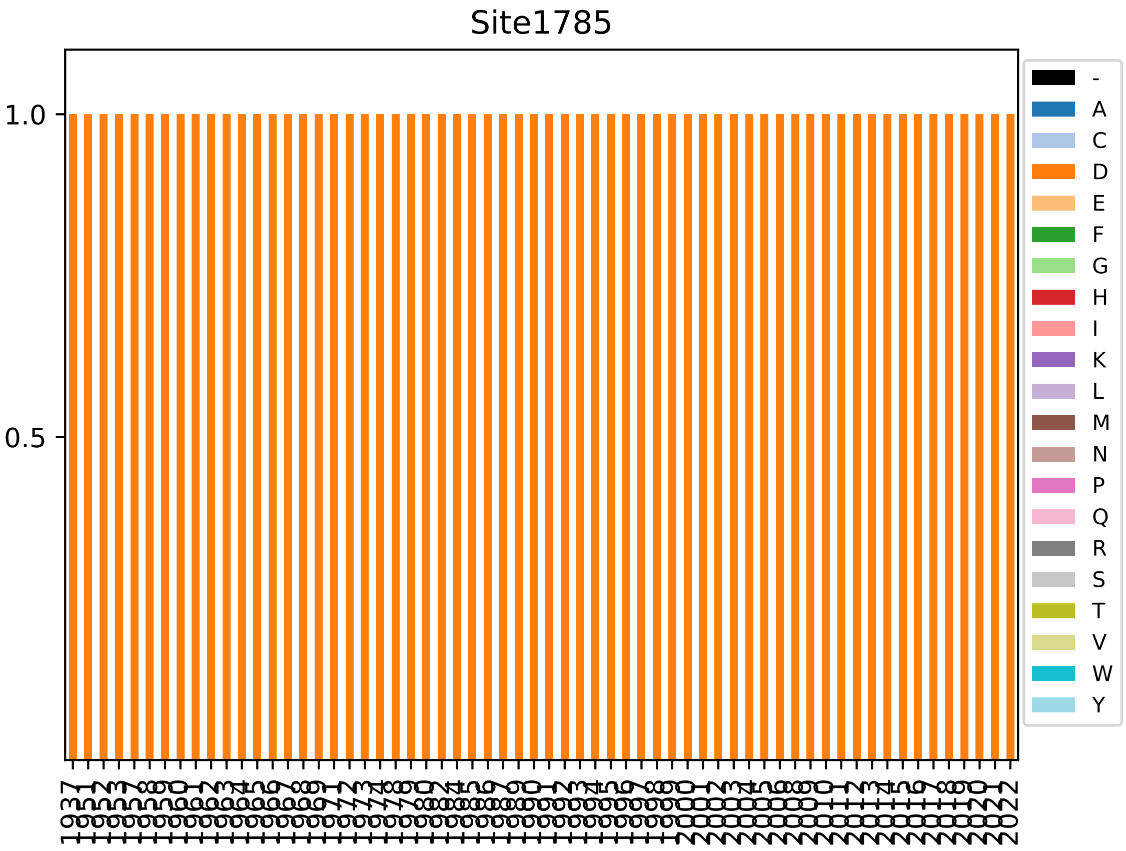
Site1785
1.0
0.5
-
A
C
D
E
F
G
H
I
K
L
M
N
P
Q
R
S
T
V
W
Y

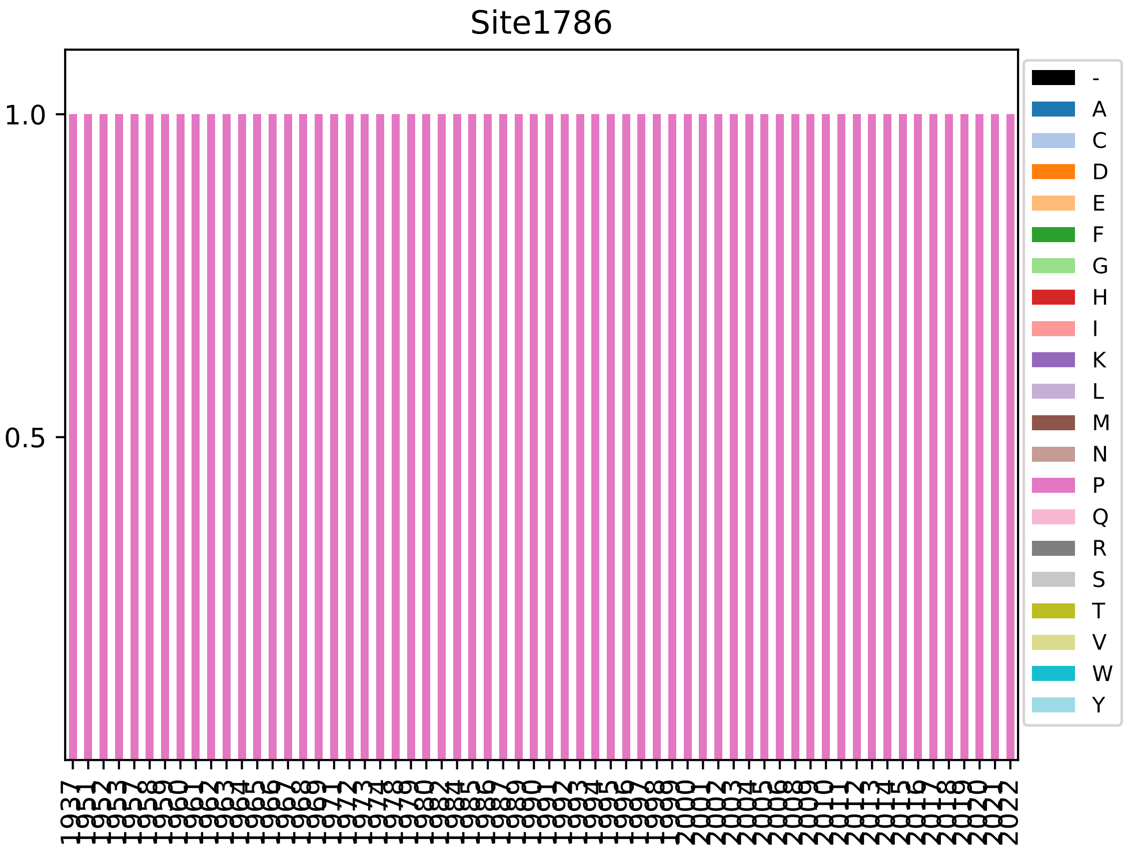
Site1786
1.0
0.5
-
A
C
D
E
F
G
H
I
K
L
M
N
P
Q
R
S
T
V
W
Y

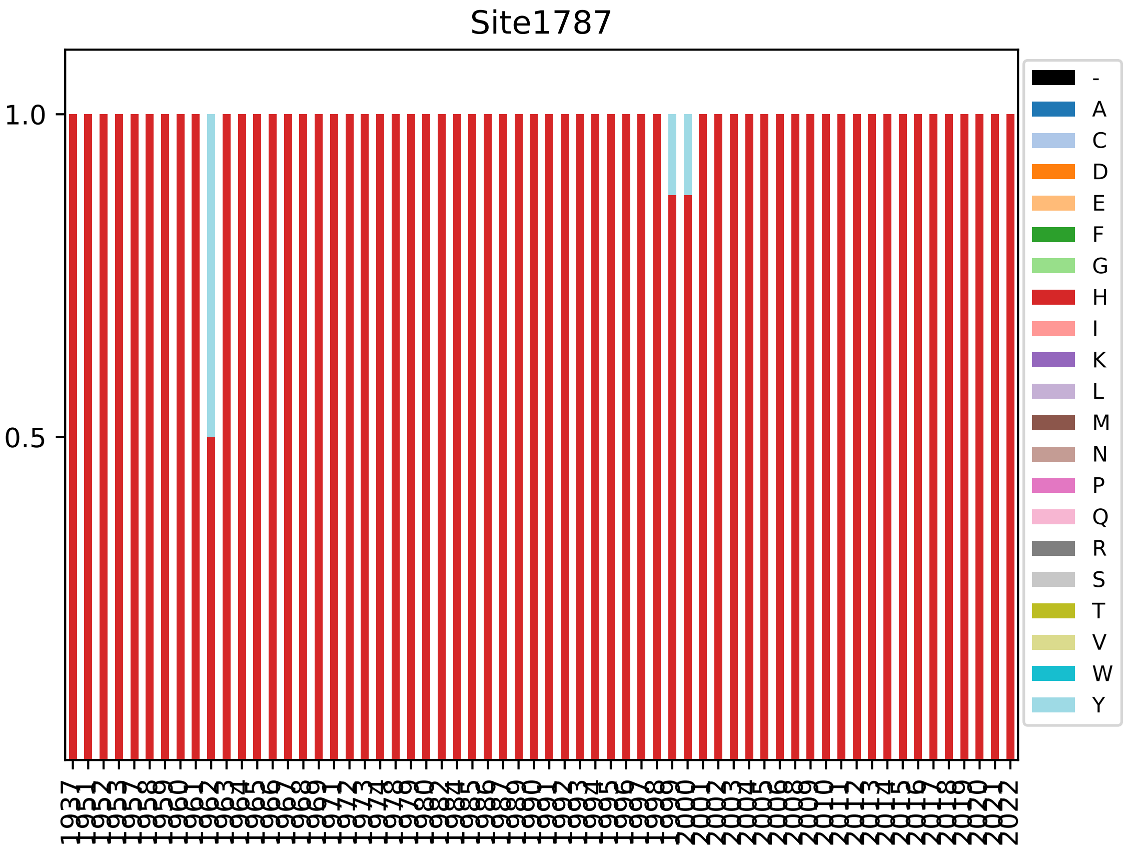

Site1787
1.0
0.5
-
A
C
D
E
F
G
H
I
K
L
M
N
P
Q
R
S
T
V
W
Y

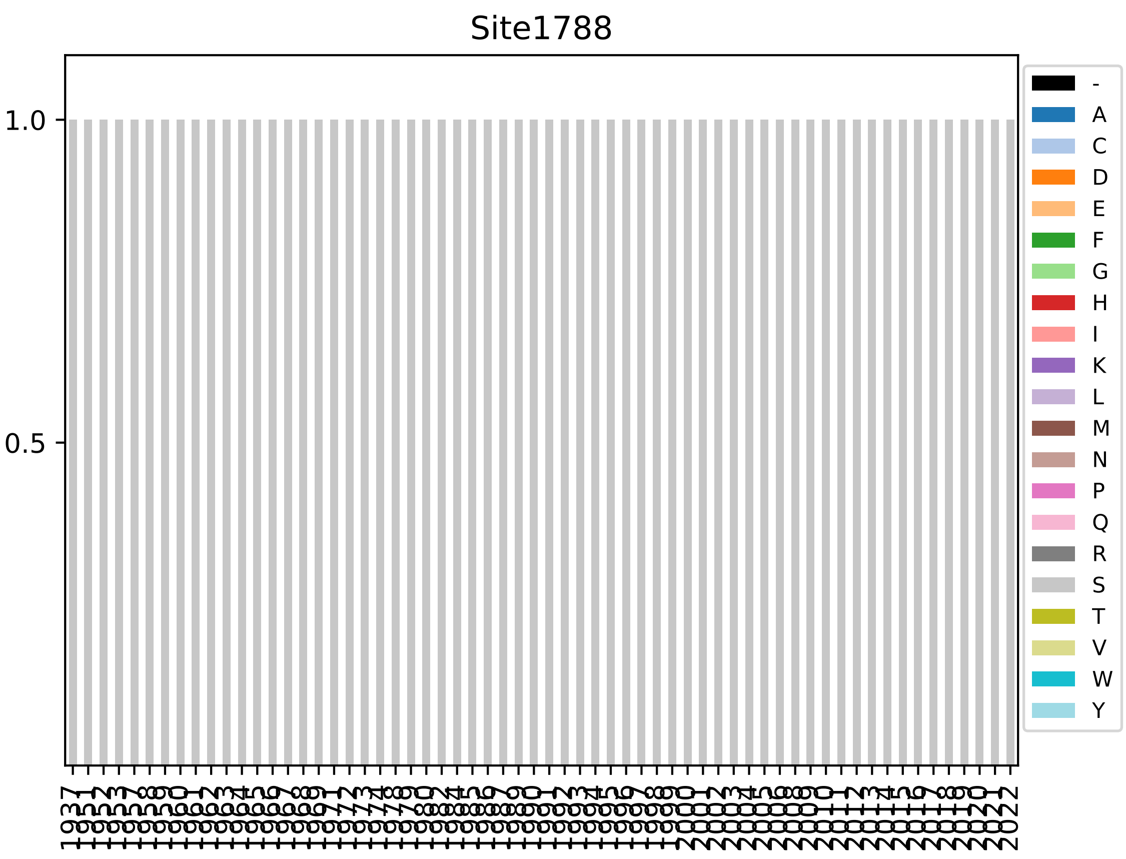

Site1788
1.0
0.5
-
A
C
D
E
F
G
H
I
K
L
M
N
P
Q
R
S
T
V
W
Y

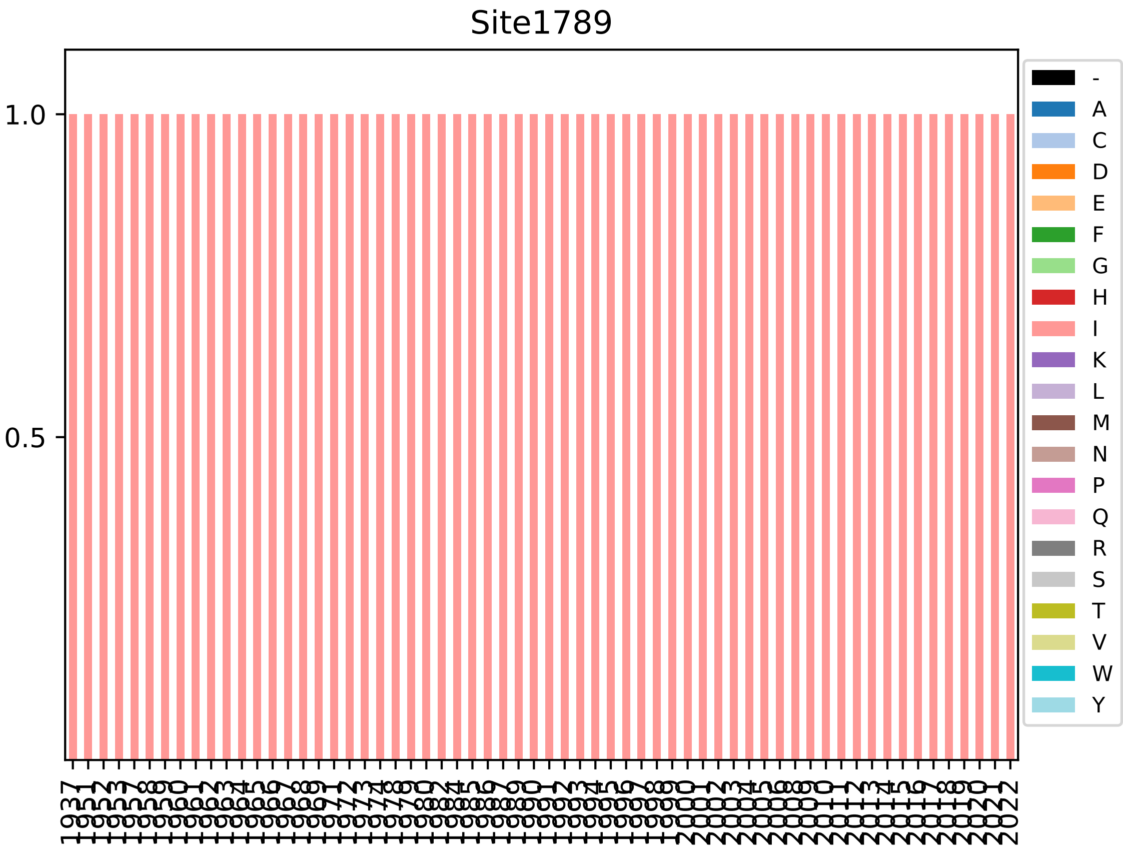
Site1789
1.0
0.5
-
A
C
D
E
F
G
H
I
K
L
M
N
P
Q
R
S
T
V
W
Y

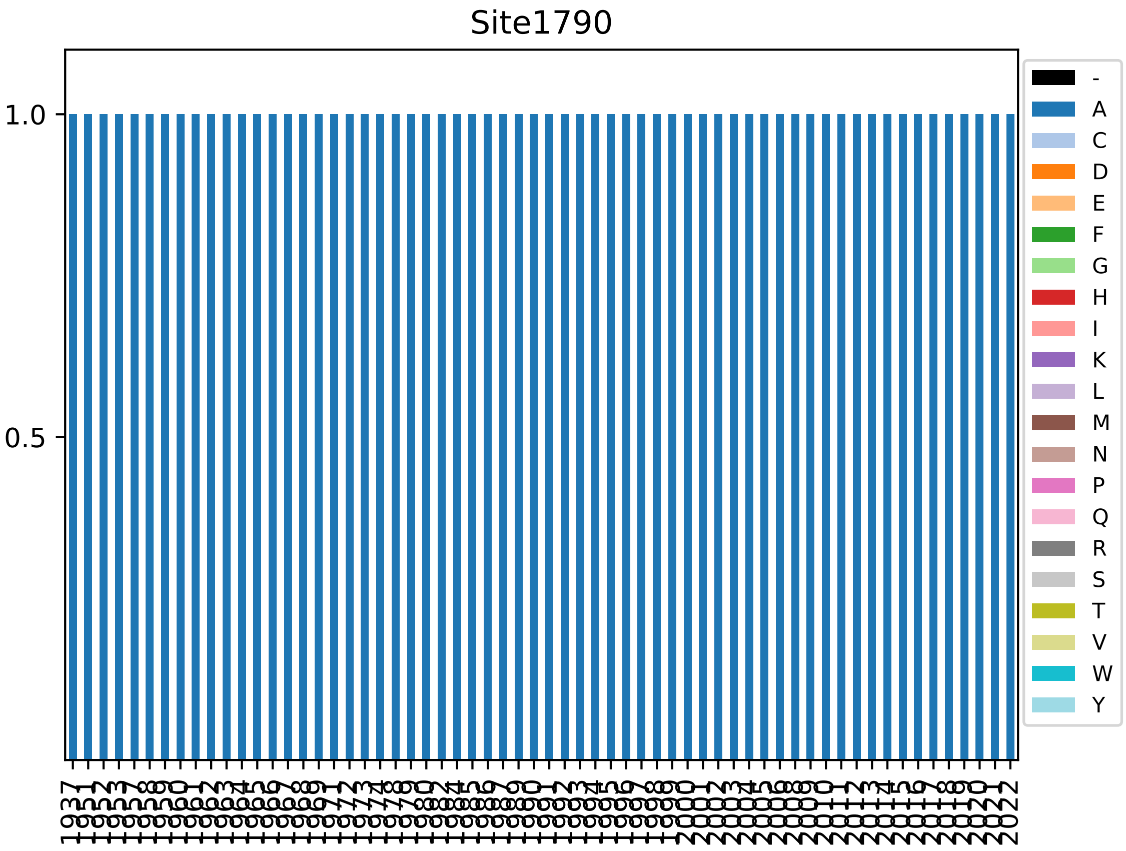
Site1790
1.0
0.5
-
A
C
D
E
F
G
H
I
K
L
M
N
P
Q
R
S
T
V
W
Y

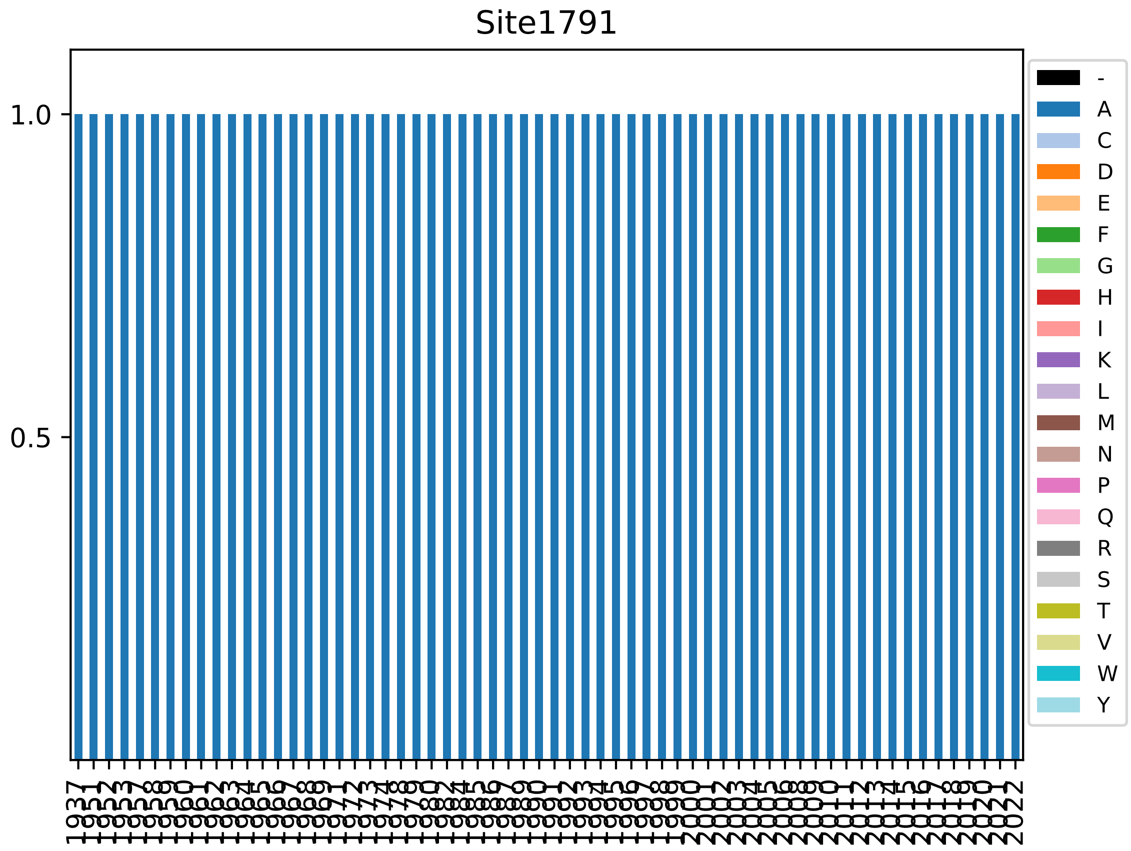
Site1791
1.0
0.5
-
A
C
D
E
F
G
H
I
K
L
M
N
P
Q
R
S
T
V
W
Y

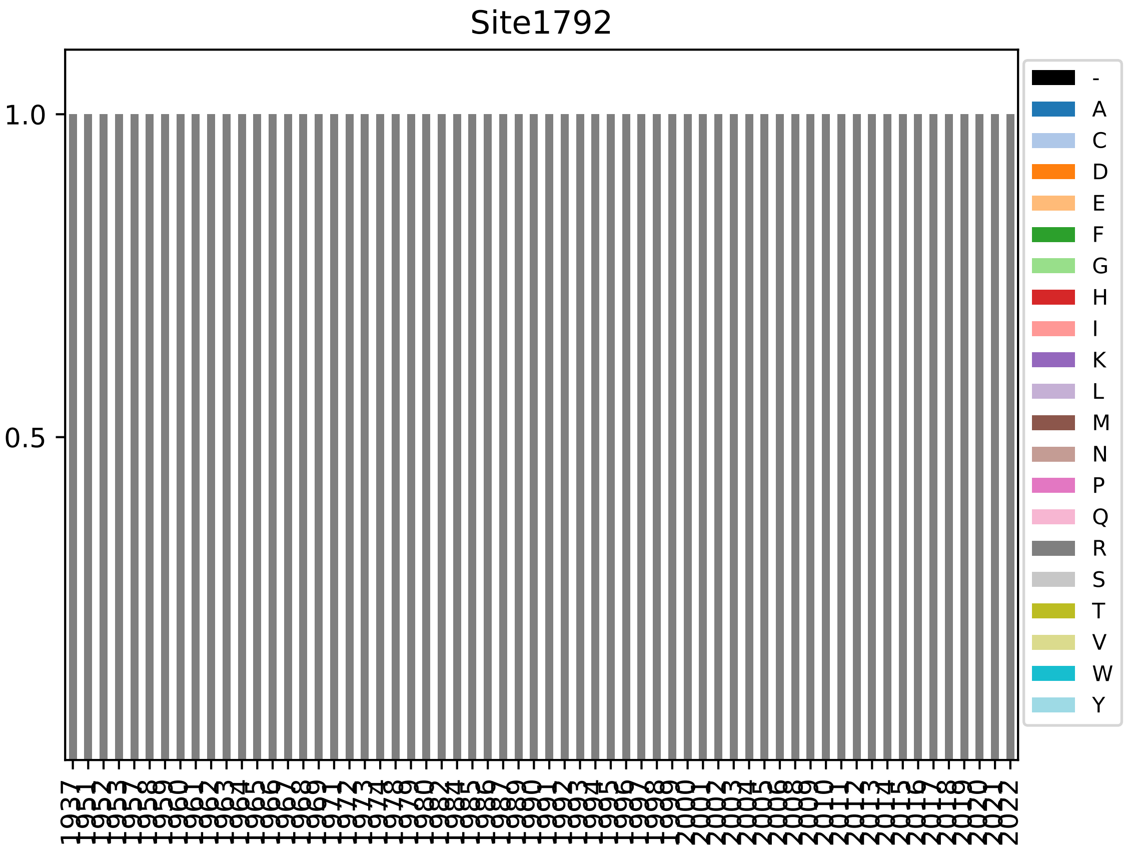
Site1792
1.0
0.5
-
A
C
D
E
F
G
H
I
K
L
M
N
P
Q
R
S
T
V
W
Y

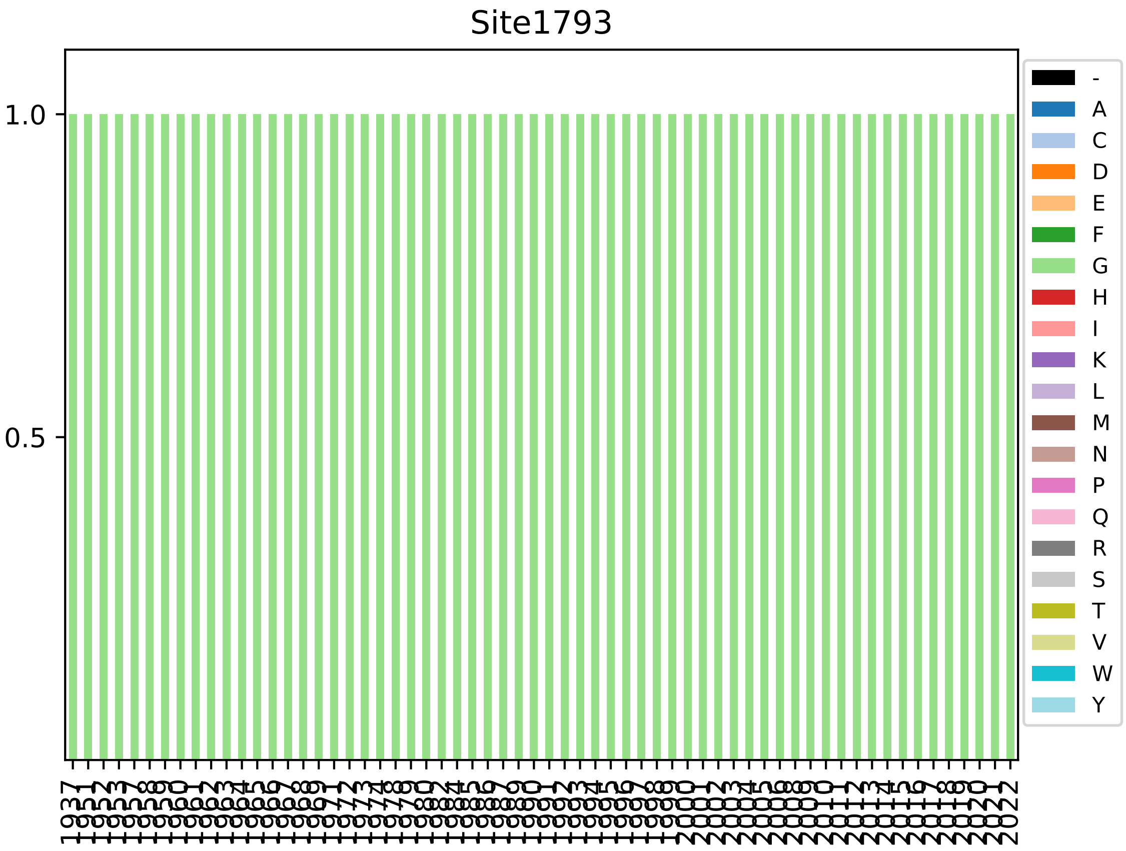
Site1793
1.0
0.5
-
A
C
D
E
F
G
H
I
K
L
M
N
P
Q
R
S
T
V
W
Y

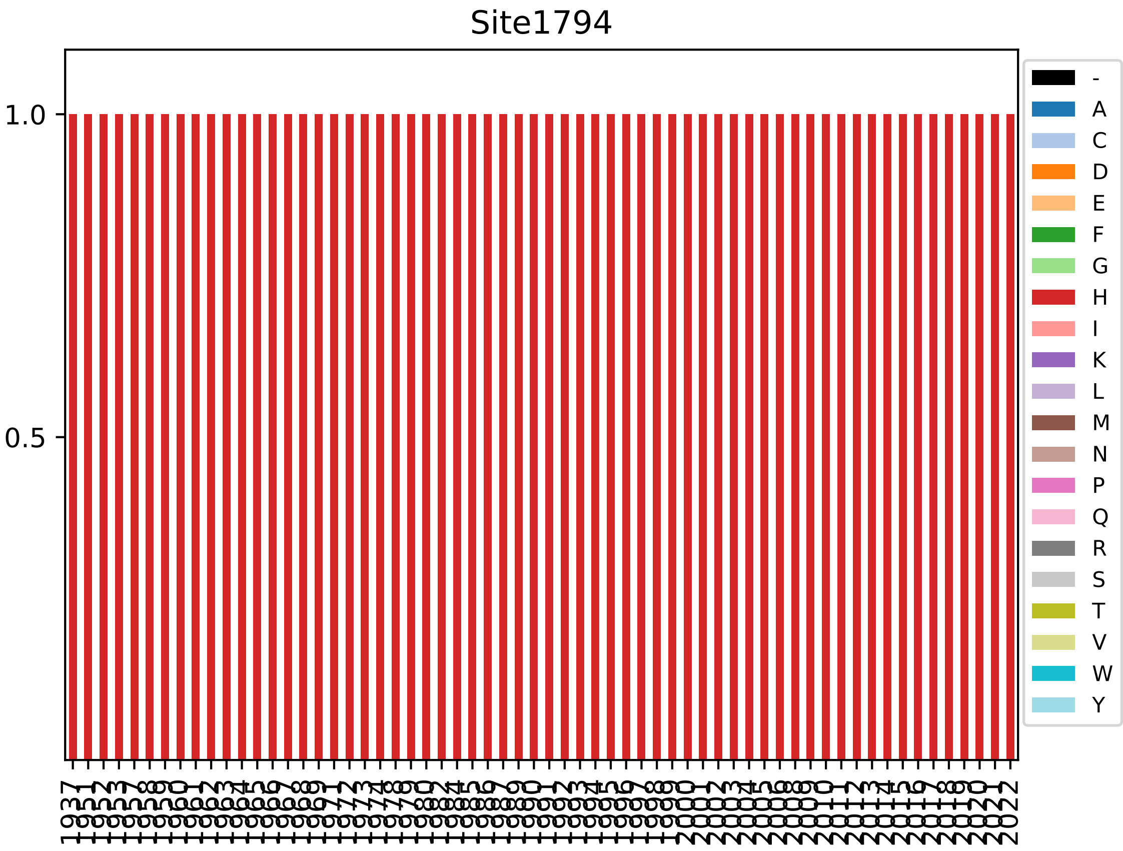
Site1794
1.0
0.5
-
A
C
D
E
F
G
H
I
K
L
M
N
P
Q
R
S
T
V
W
Y

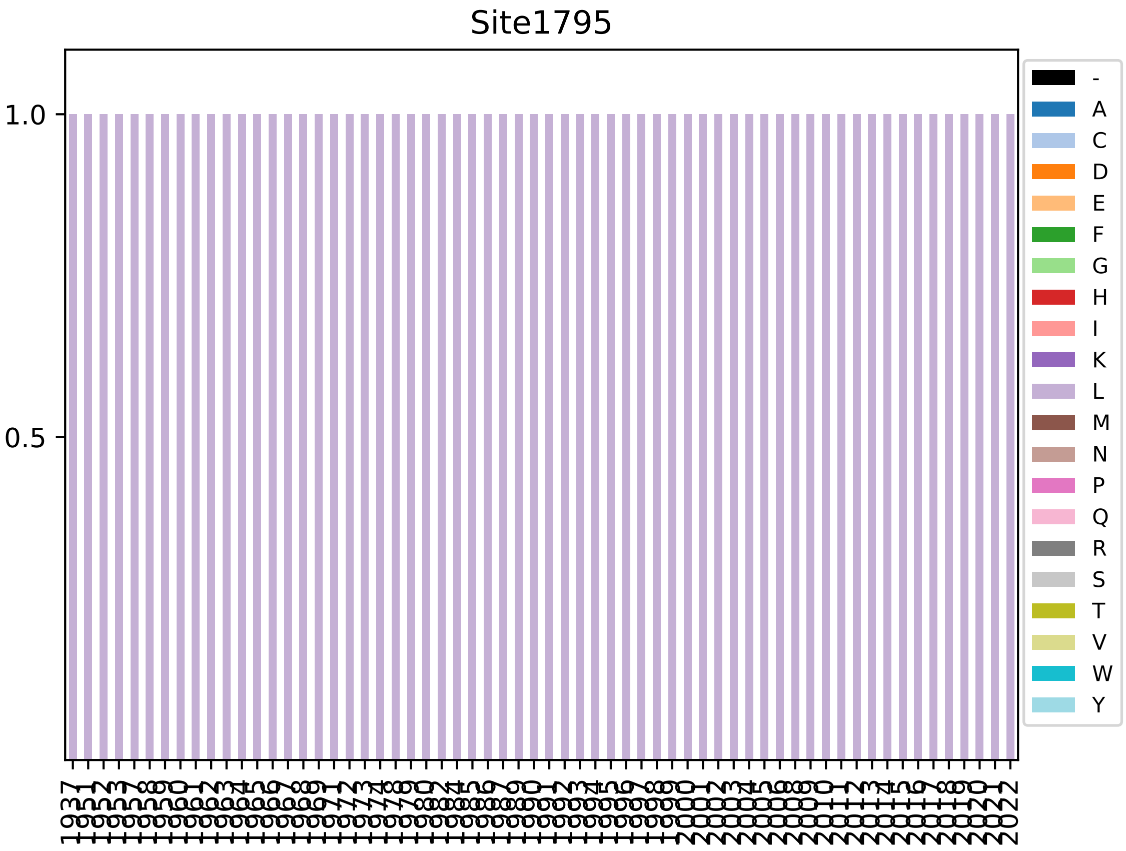
Site1795
1.0
0.5
-
A
C
D
E
F
G
H
I
K
L
M
N
P
Q
R
S
T
V
W
Y

Site1796

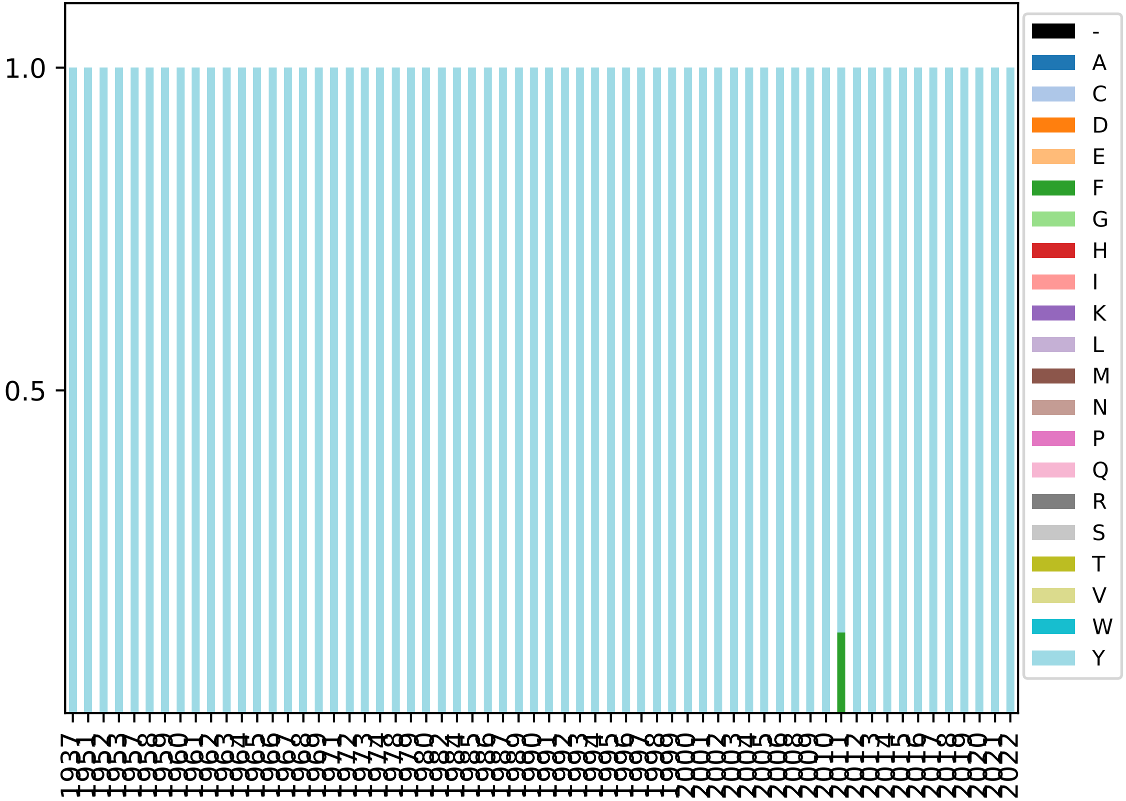

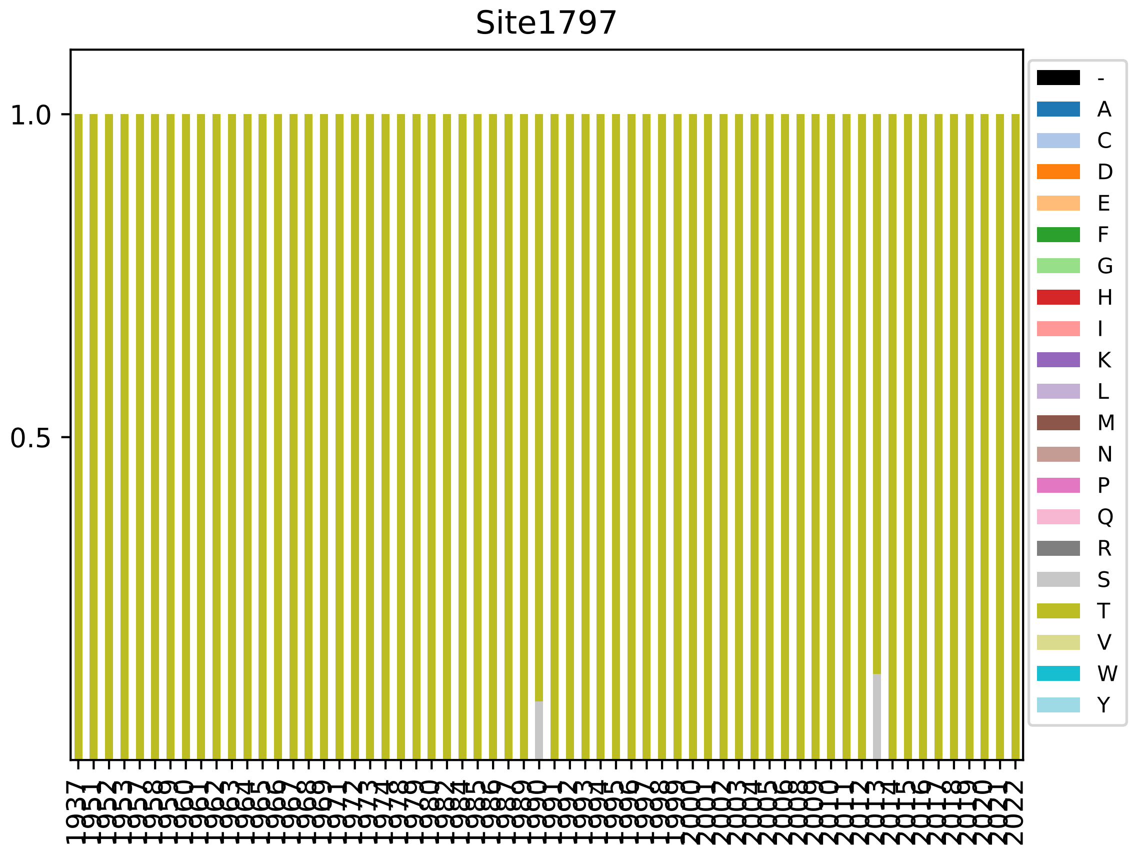
Site1797
1.0
0.5
-
A
C
D
E
F
G
H
I
K
L
M
N
P
Q
R
S
T
V
W
Y

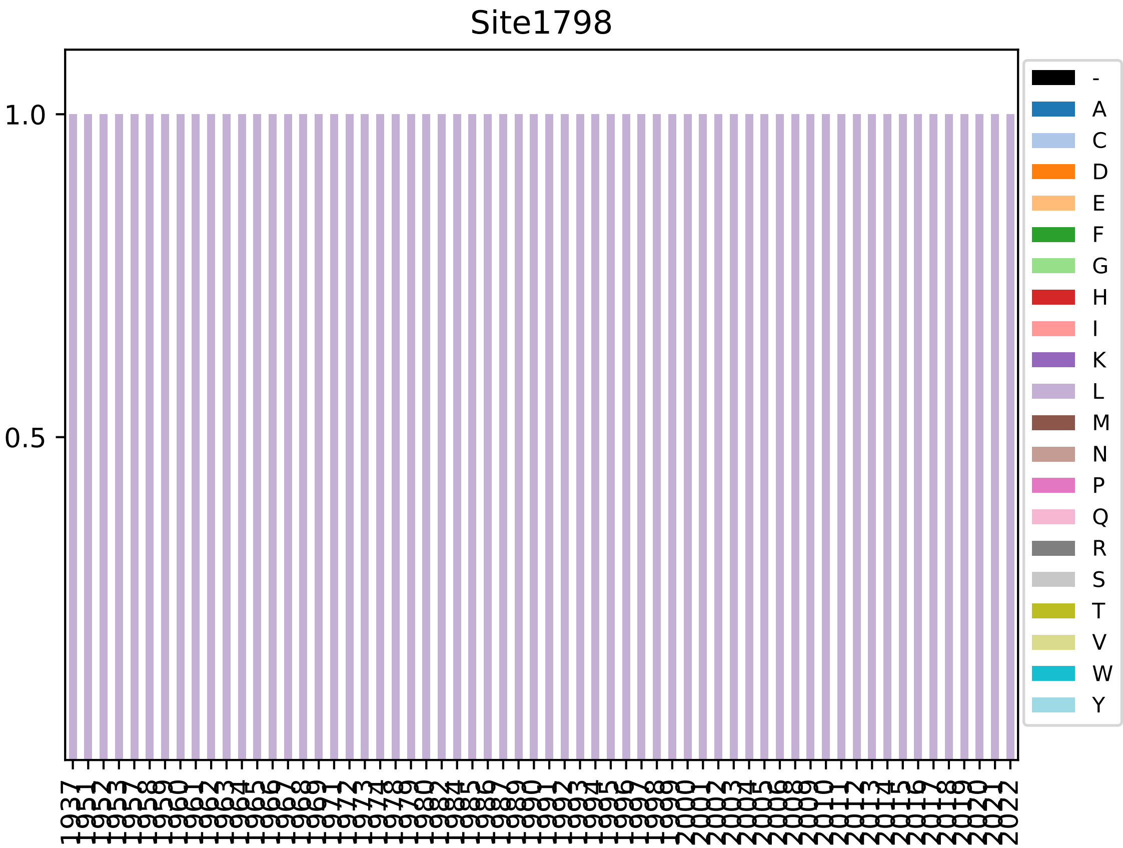
Site1798
1.0
0.5
-
A
C
D
E
F
G
H
I
K
L
M
N
P
Q
R
S
T
V
W
Y

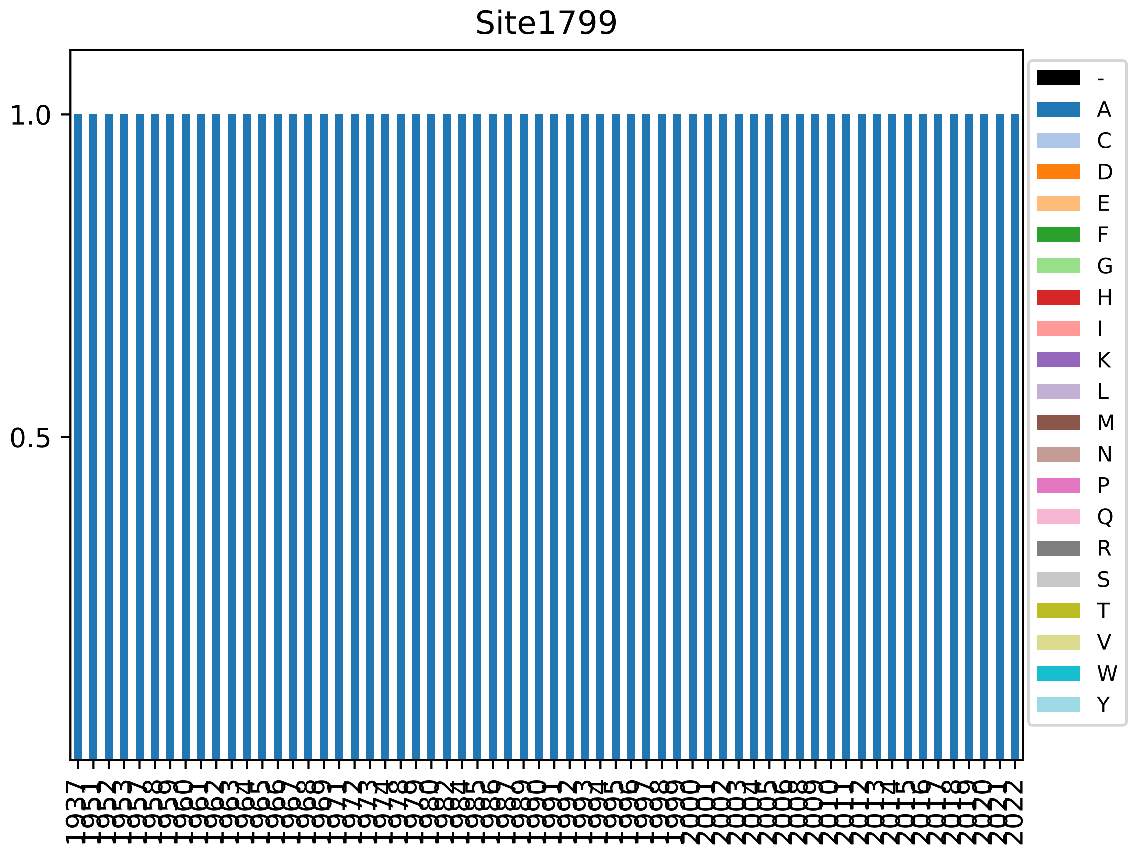
Site1799
1.0
0.5
-
A
C
D
E
F
G
H
I
K
L
M
N
P
Q
R
S
T
V
W
Y

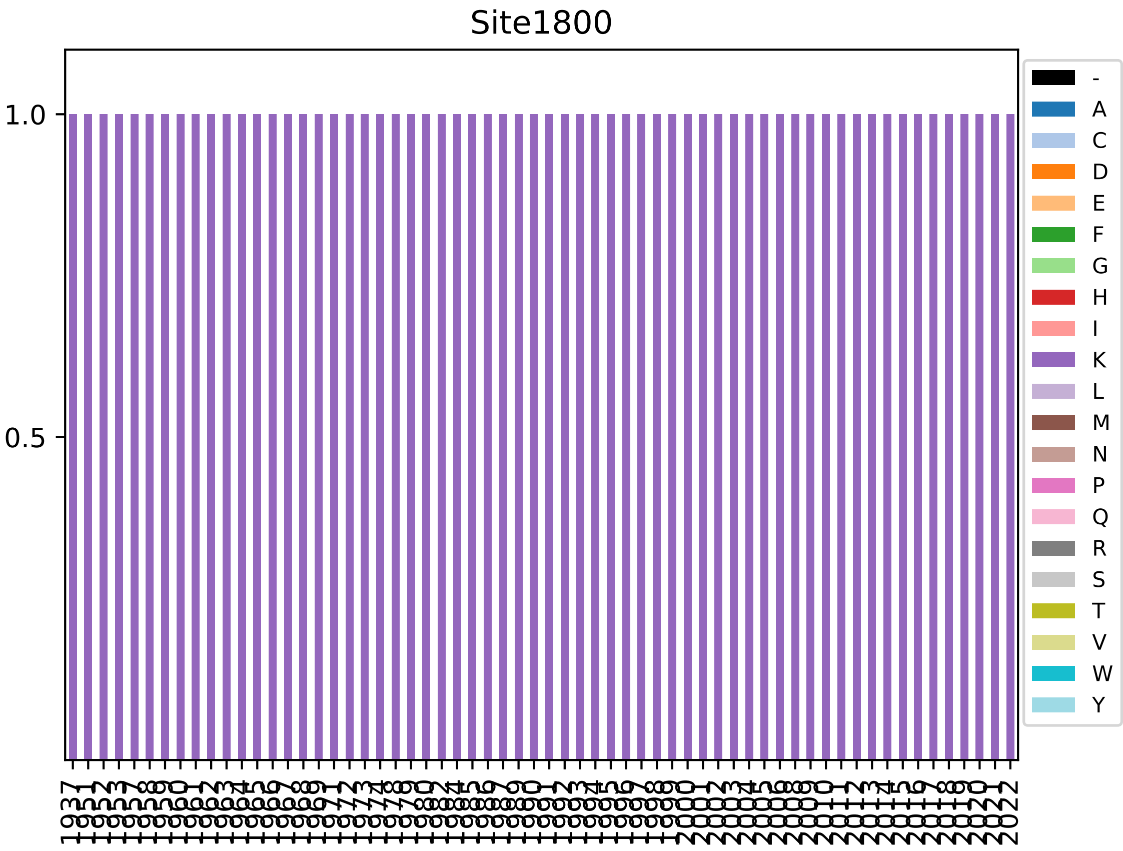
Site1800
1.0
0.5
-
A
C
D
E
F
G
H
I
K
L
M
N
P
Q
R
S
T
V
W
Y

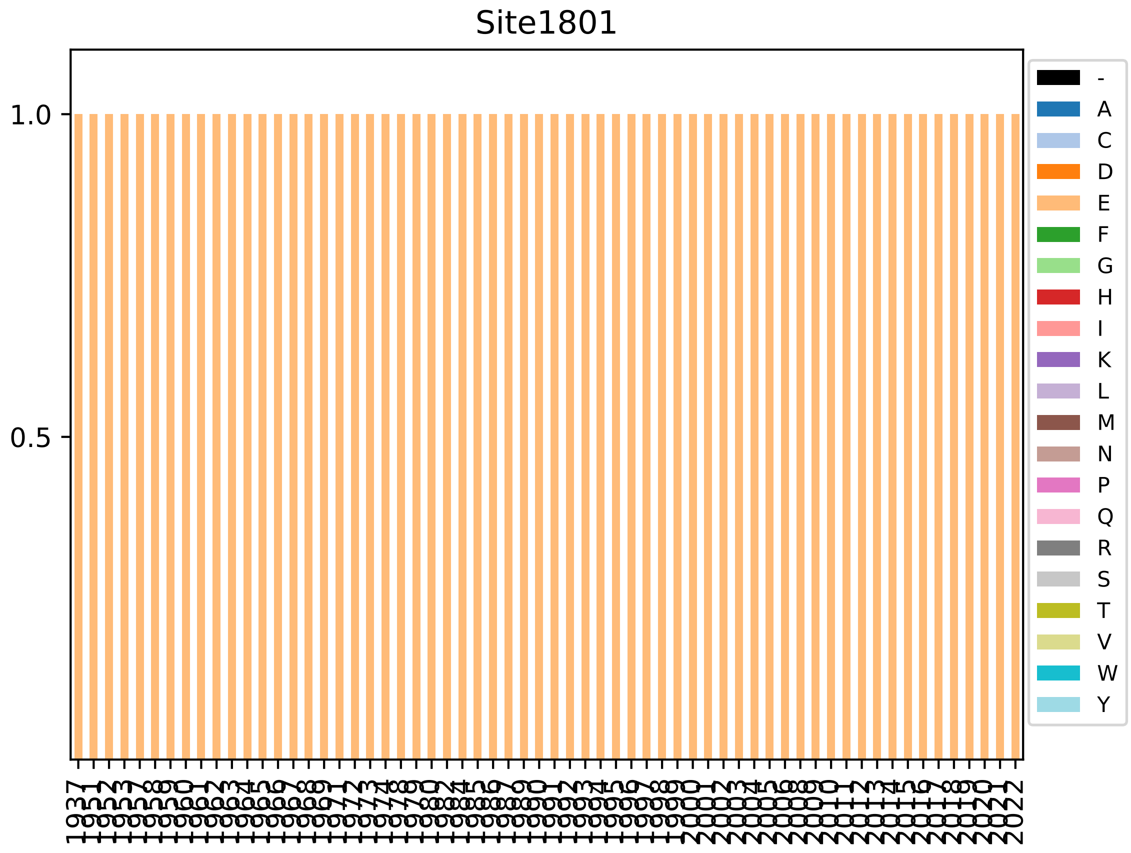
Site1801
1.0
0.5
-
A
C
D
E
F
G
H
I
K
L
M
N
P
Q
R
S
T
V
W
Y

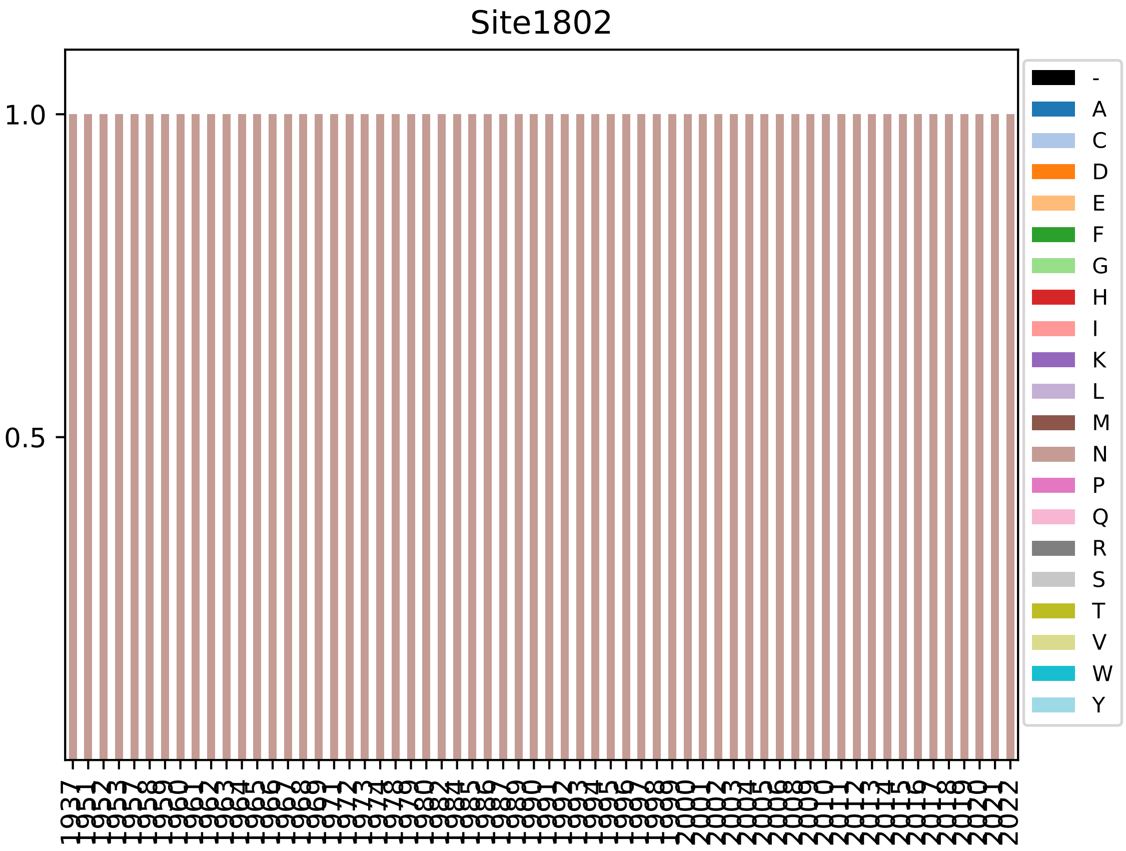
Site1802
1.0
0.5
-
A
C
D
E
F
G
H
I
K
L
M
N
P
Q
R
S
T
V
W
Y

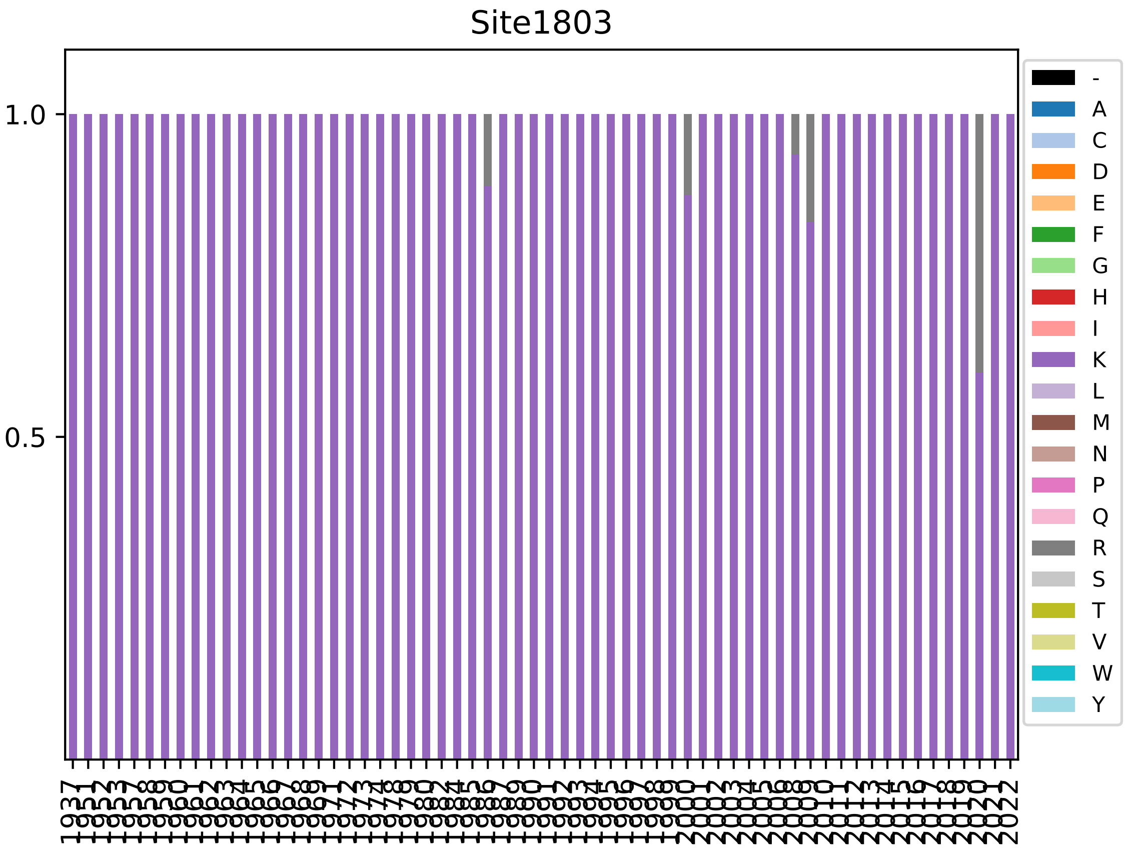
Site1803
1.0
0.5
-
A
C
D
E
F
G
H
I
K
L
M
N
P
Q
R
S
T
V
W
Y

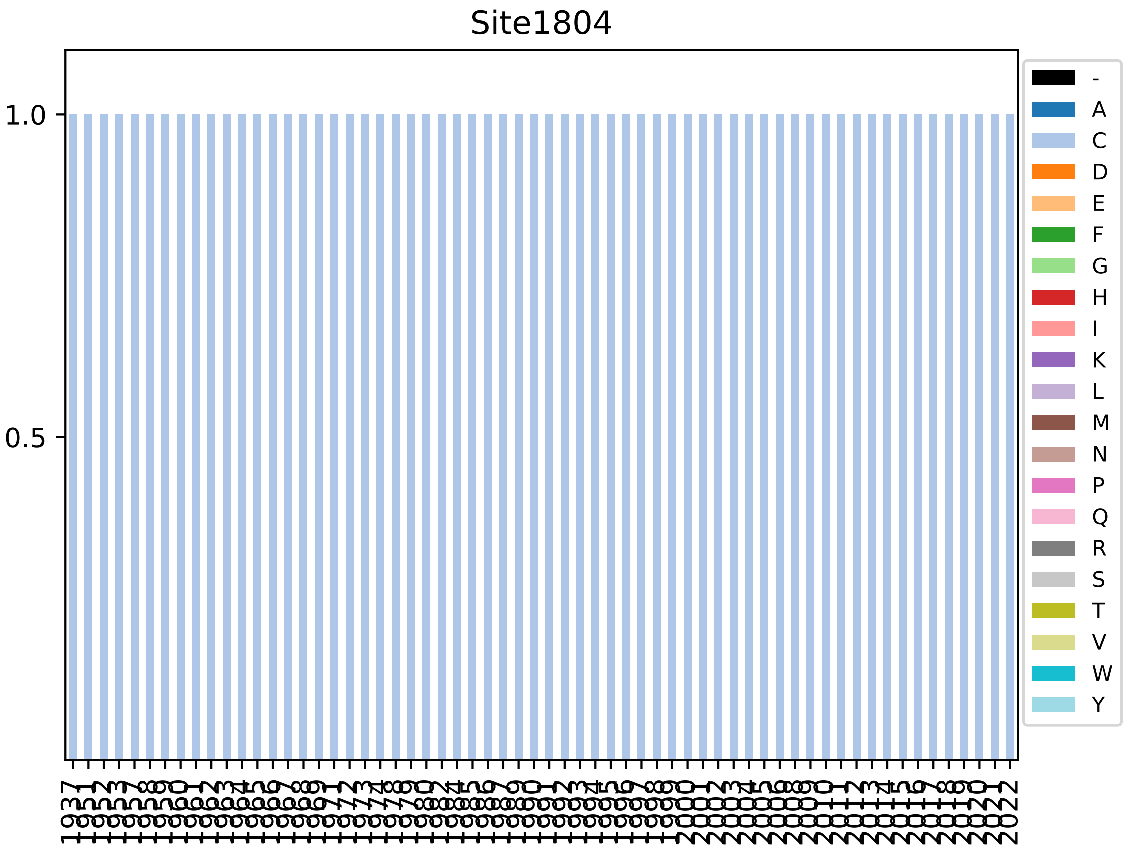
Site1804
1.0
0.5
-
A
C
D
E
F
G
H
I
K
L
M
N
P
Q
R
S
T
V
W
Y

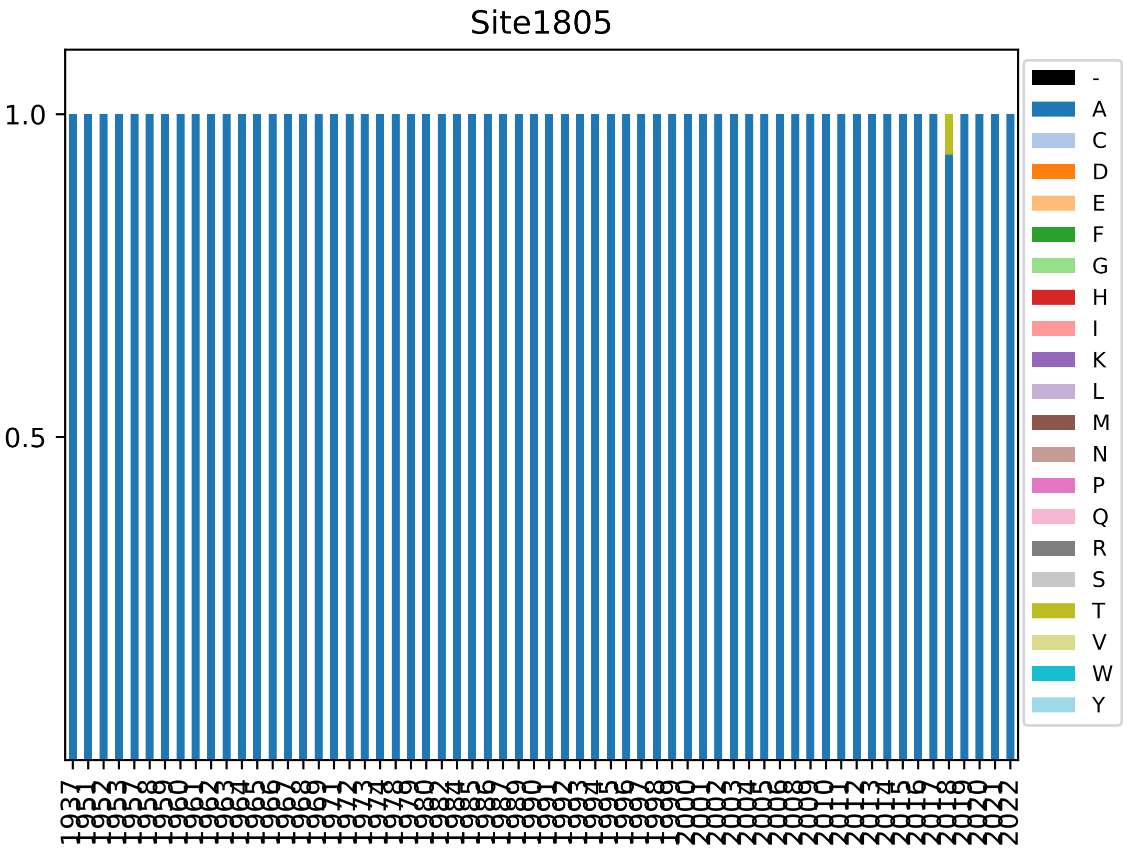
Site1805
1.0
0.5
-
A
C
D
E
F
G
H
I
K
L
M
N
P
Q
R
S
T
V
W
Y

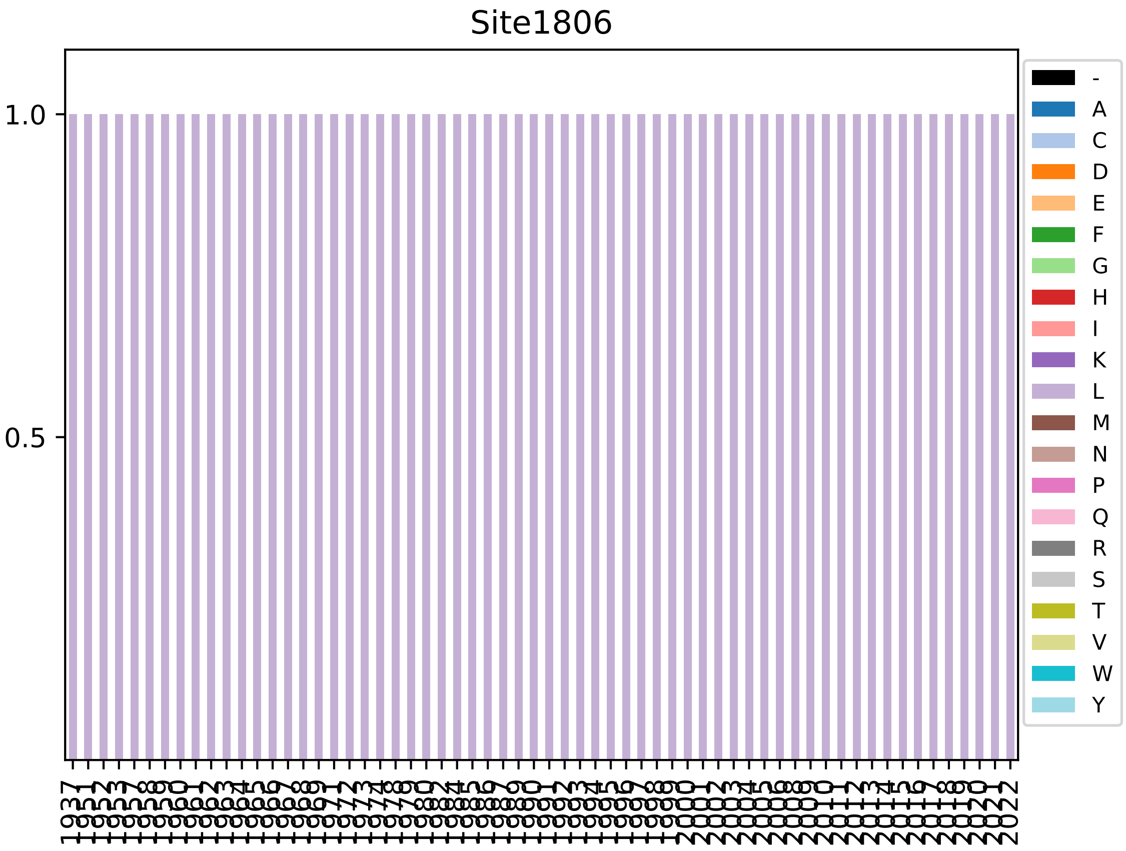
Site1806
1.0
0.5
-
A
C
D
E
F
G
H
I
K
L
M
N
P
Q
R
S
T
V
W
Y

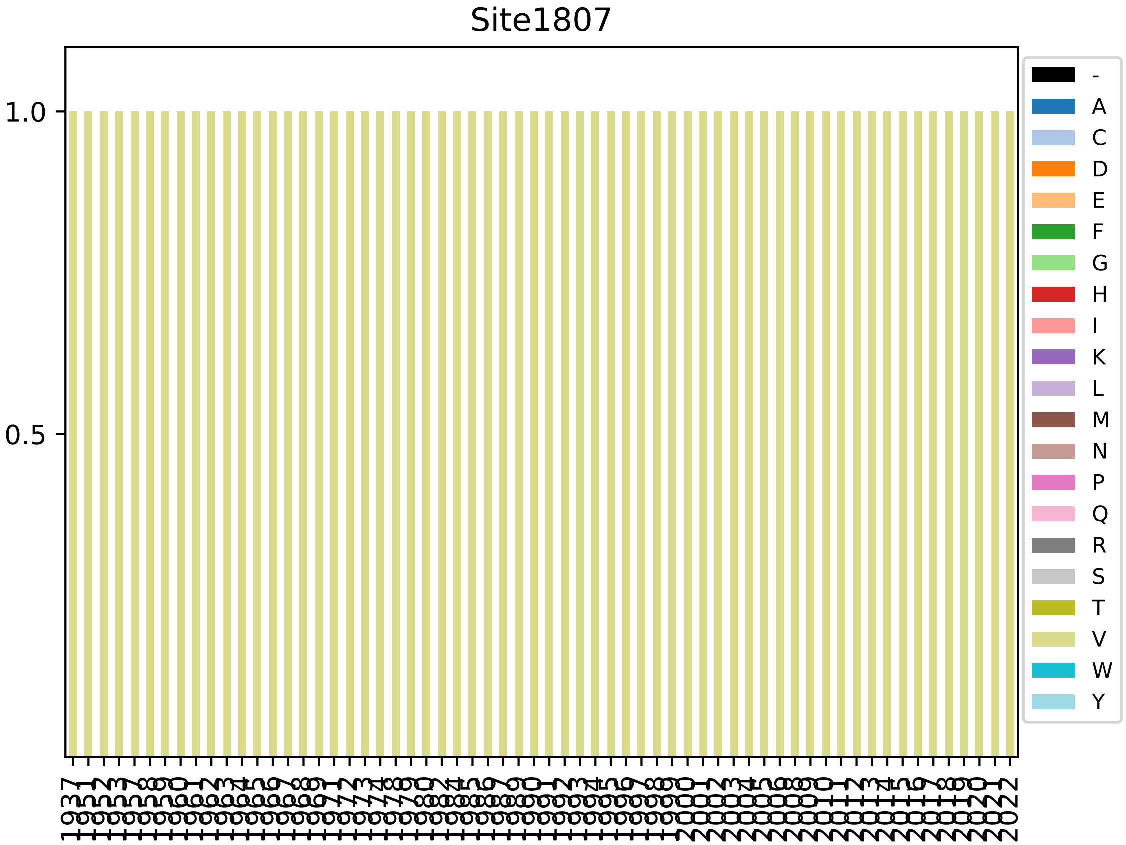
Site1807
1.0
0.5
-
A
C
D
E
F
G
H
I
K
L
M
N
P
Q
R
S
T
V
W
Y

Site1808

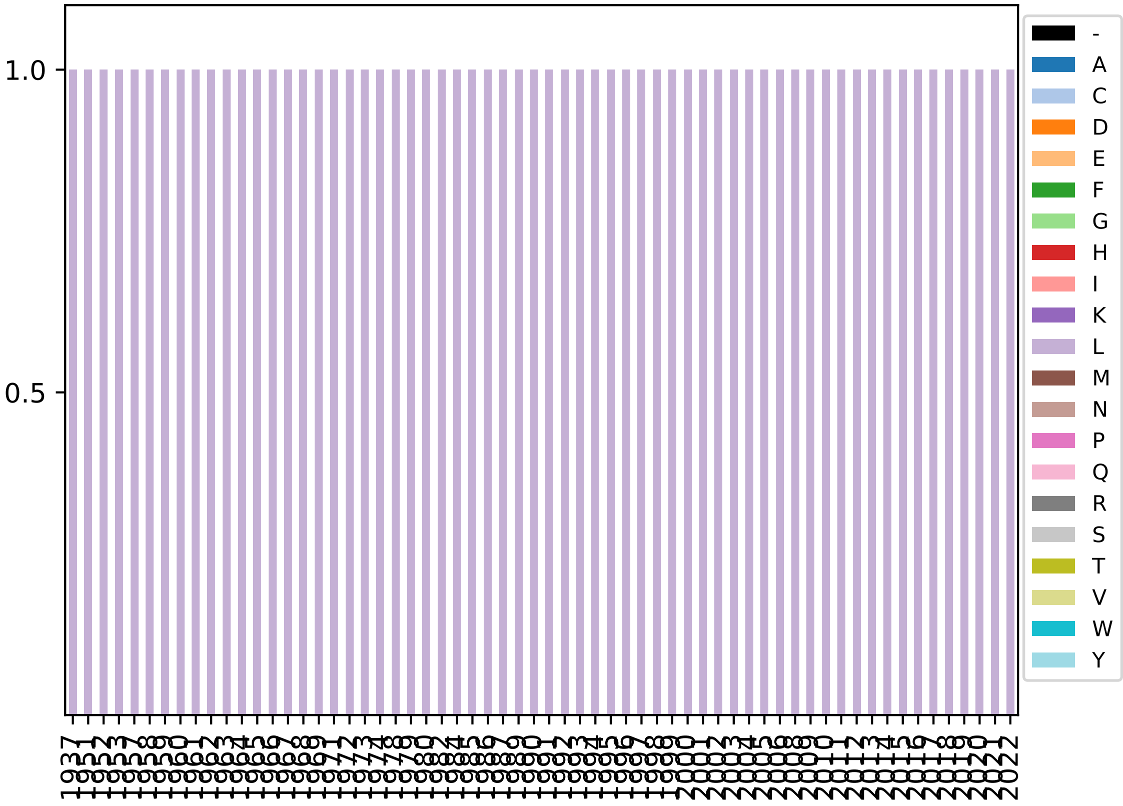

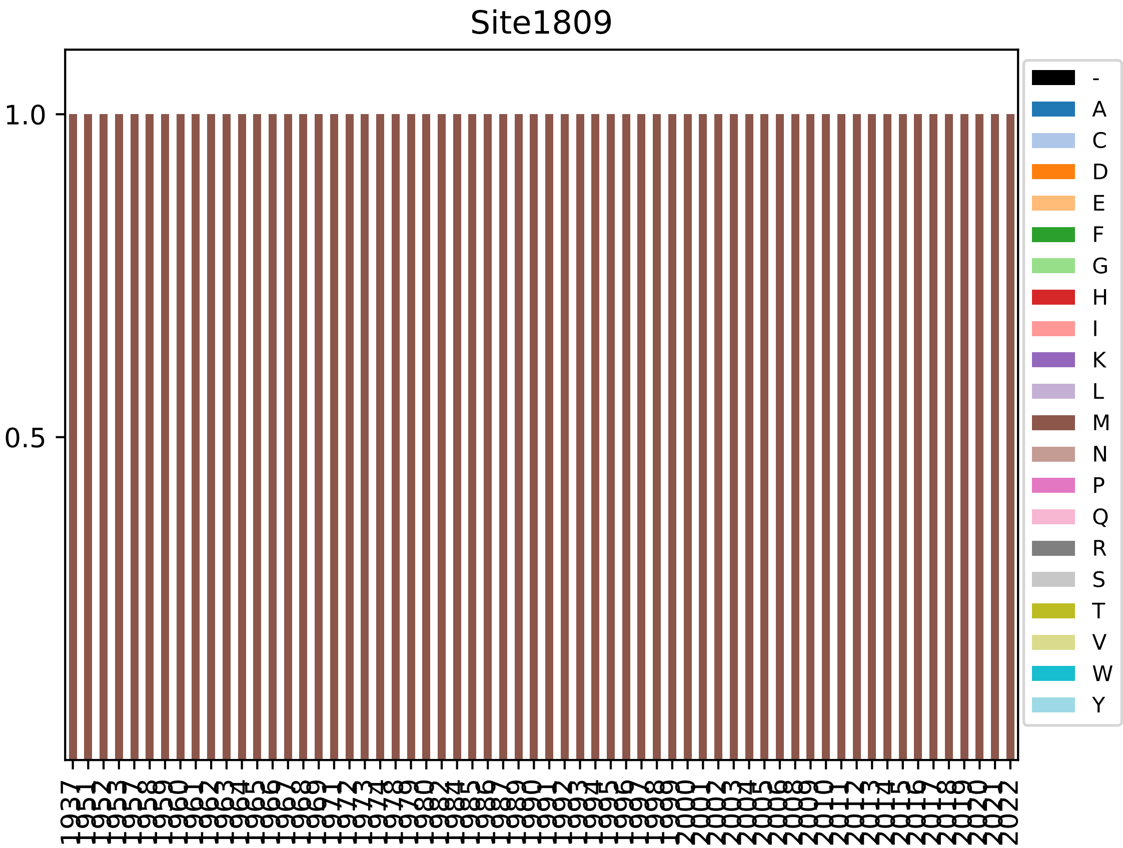
Site1809
1.0
0.5
-
A
C
D
E
F
G
H
I
K
L
M
N
P
Q
R
S
T
V
W
Y

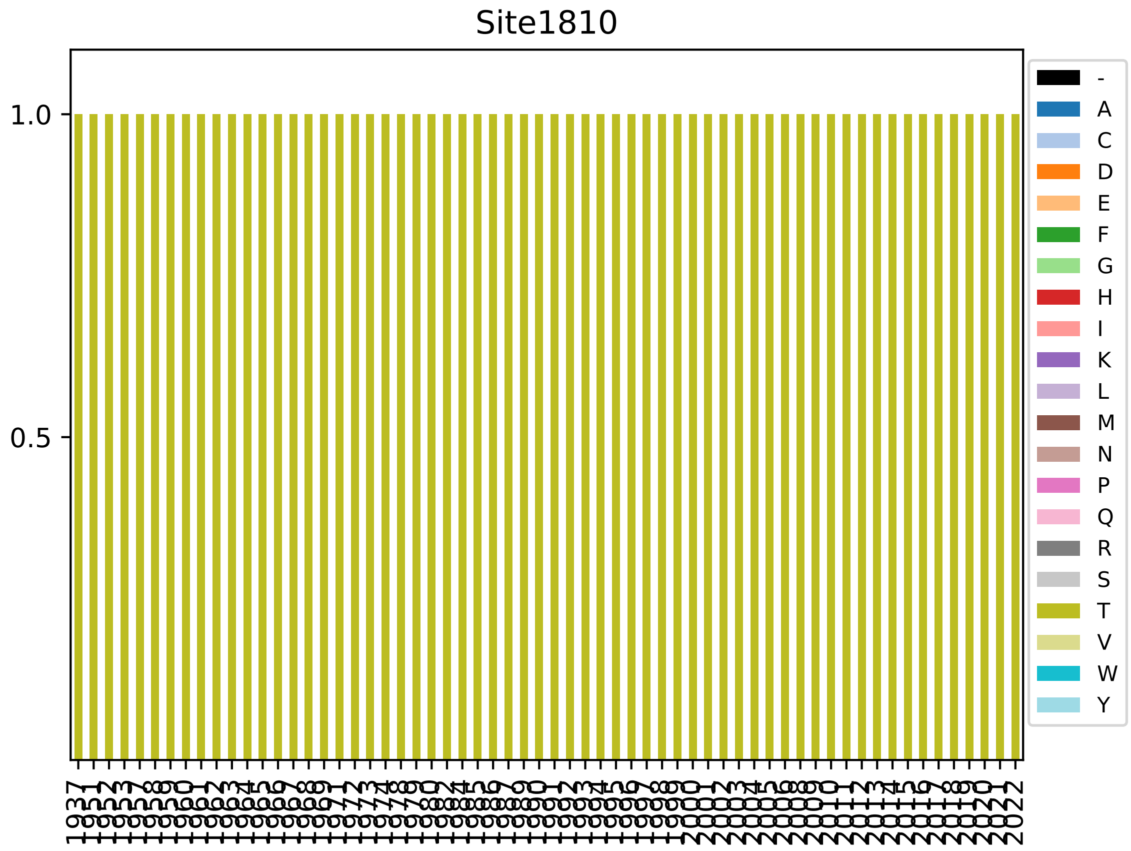
Site1810
1.0
0.5
-
A
C
D
E
F
G
H
I
K
L
M
N
P
Q
R
S
T
V
W
Y

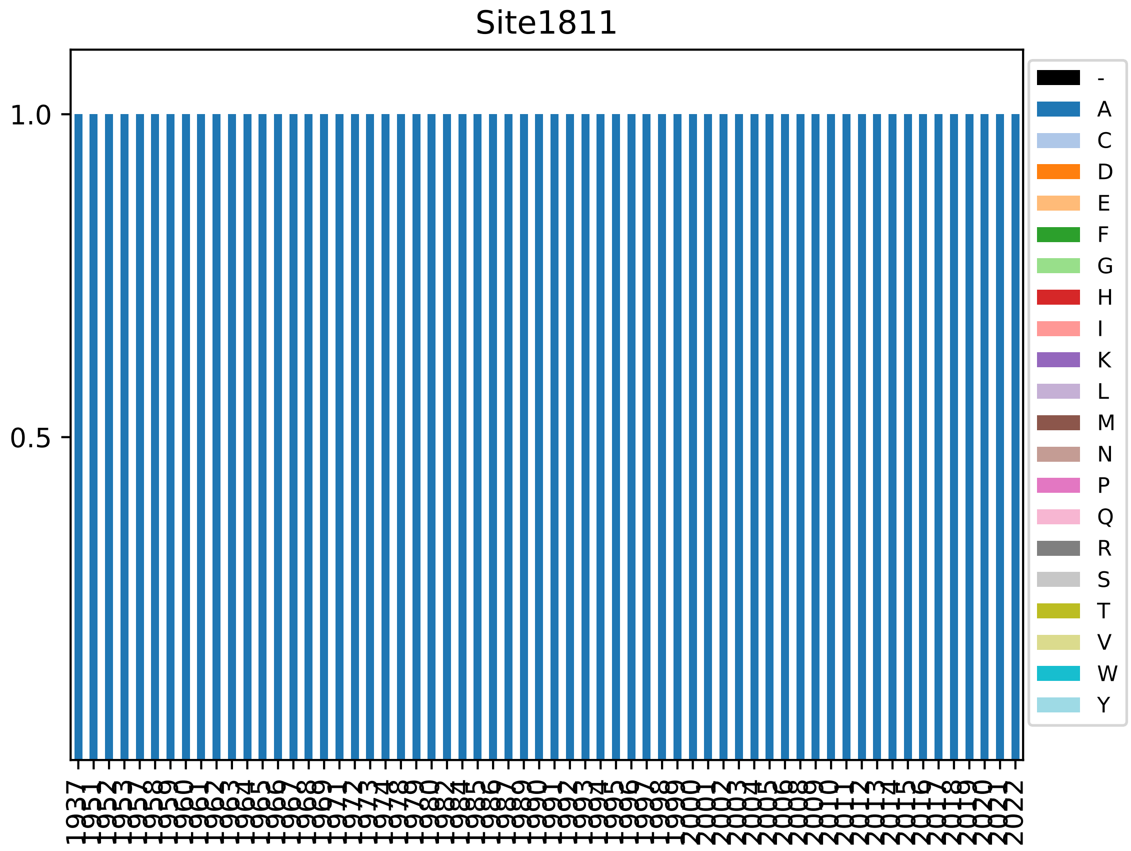
Site1811
1.0
0.5
-
A
C
D
E
F
G
H
I
K
L
M
N
P
Q
R
S
T
V
W
Y

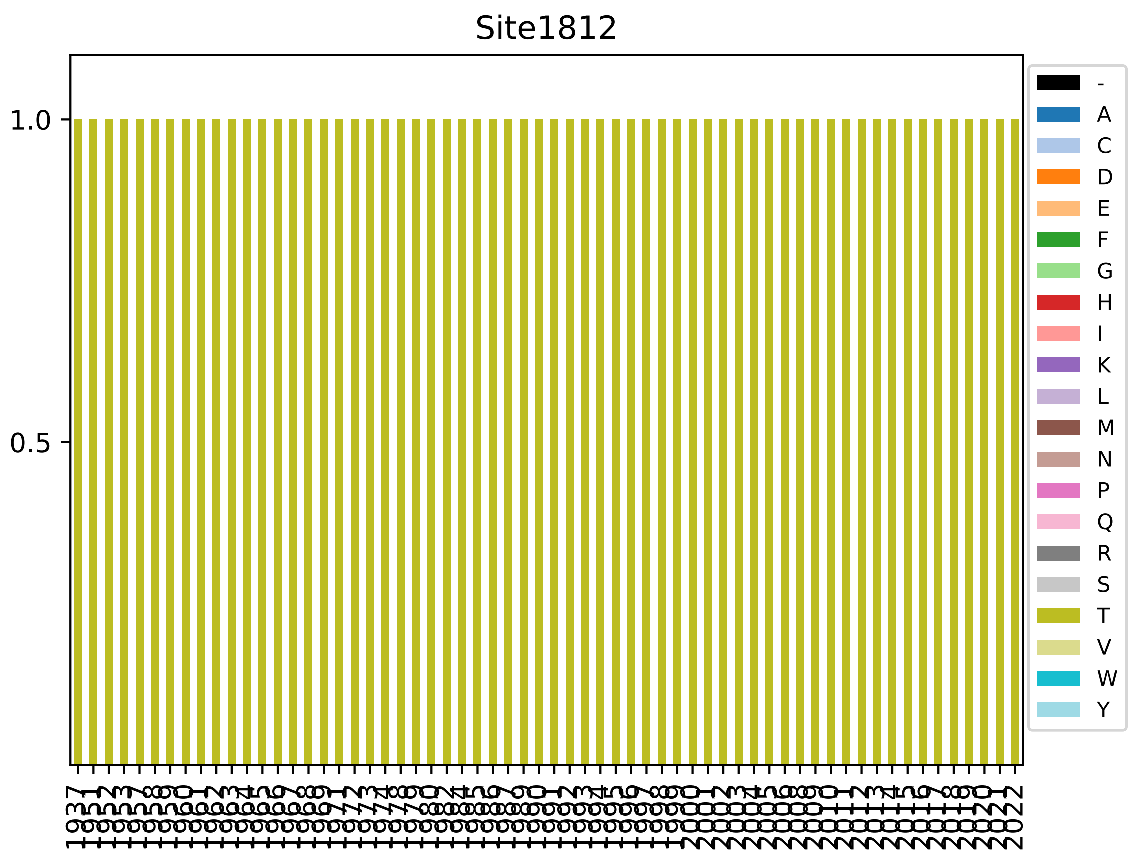
Site1812
1.0
0.5
-
A
C
D
E
F
G
H
I
K
L
M
N
P
Q
R
S
T
V
W
Y

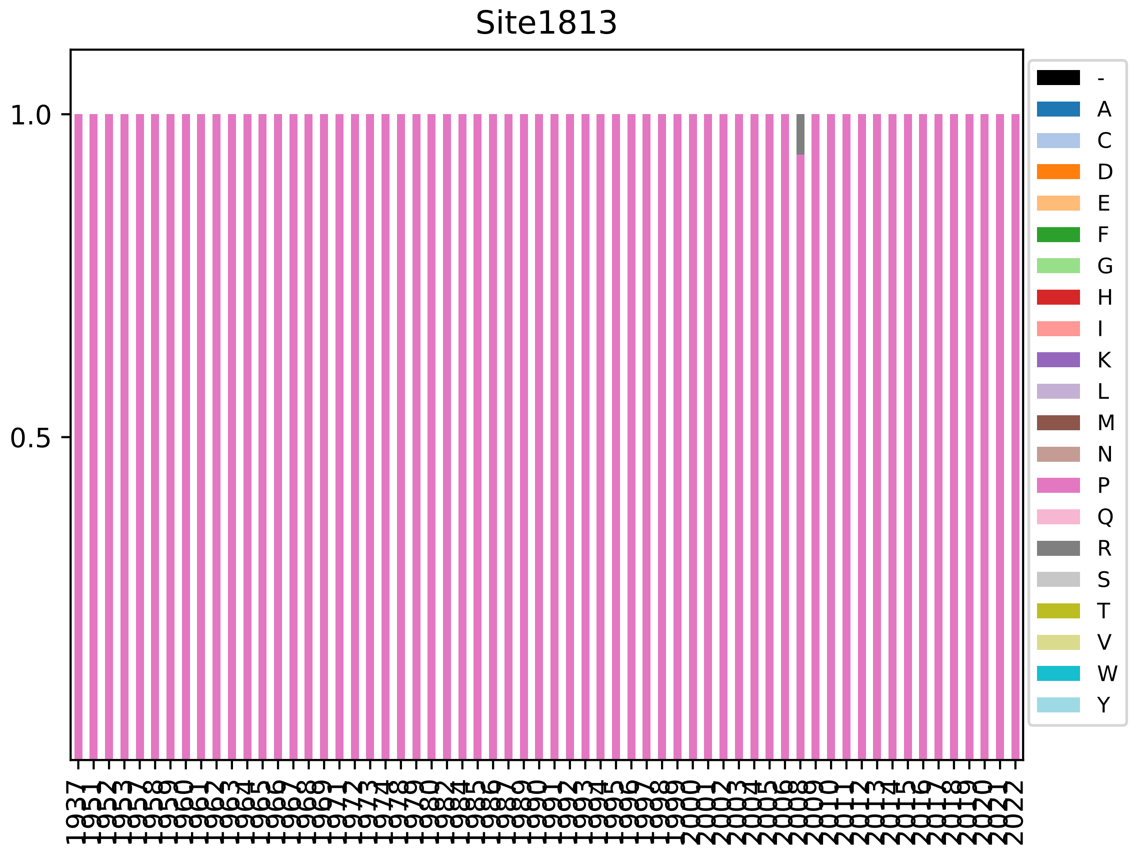
Site1813
1.0
0.5
-
A
C
D
E
F
G
H
I
K
L
M
N
P
Q
R
S
T
V
W
Y

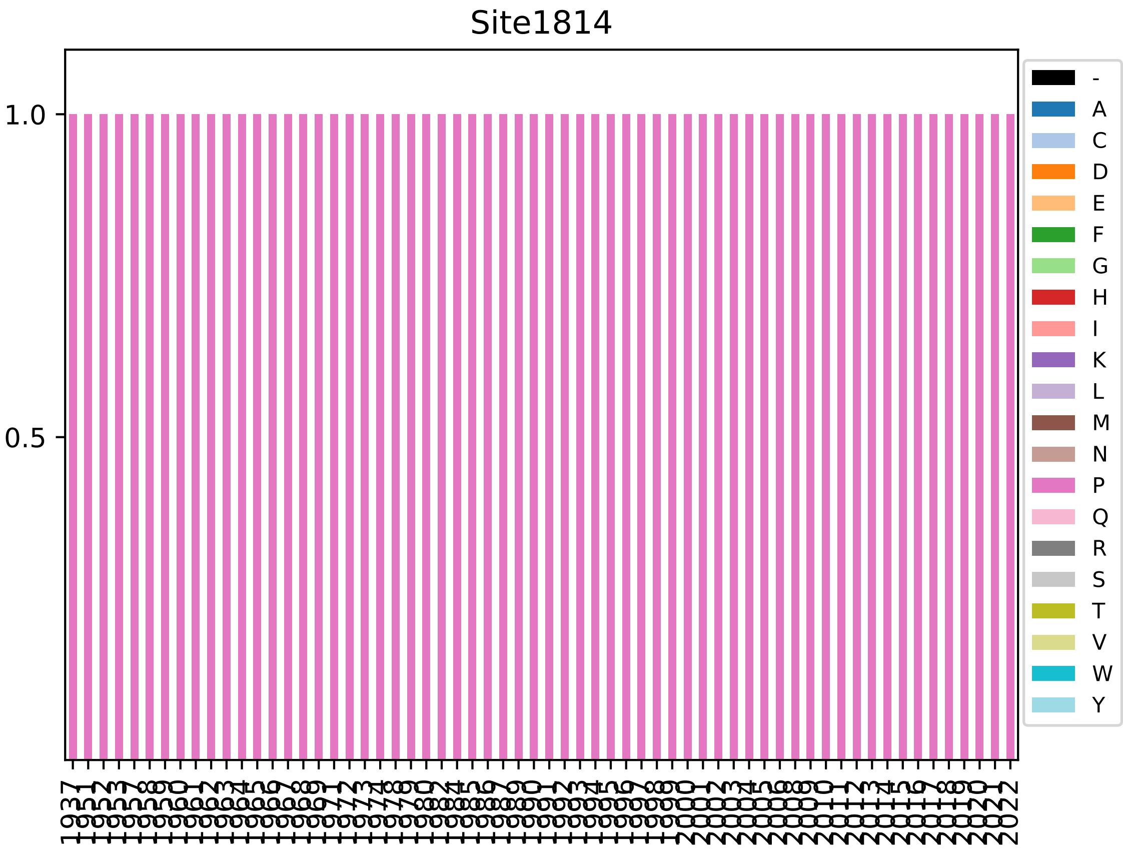
Site1814
1.0
0.5
-
A
C
D
E
F
G
H
I
K
L
M
N
P
Q
R
S
T
V
W
Y

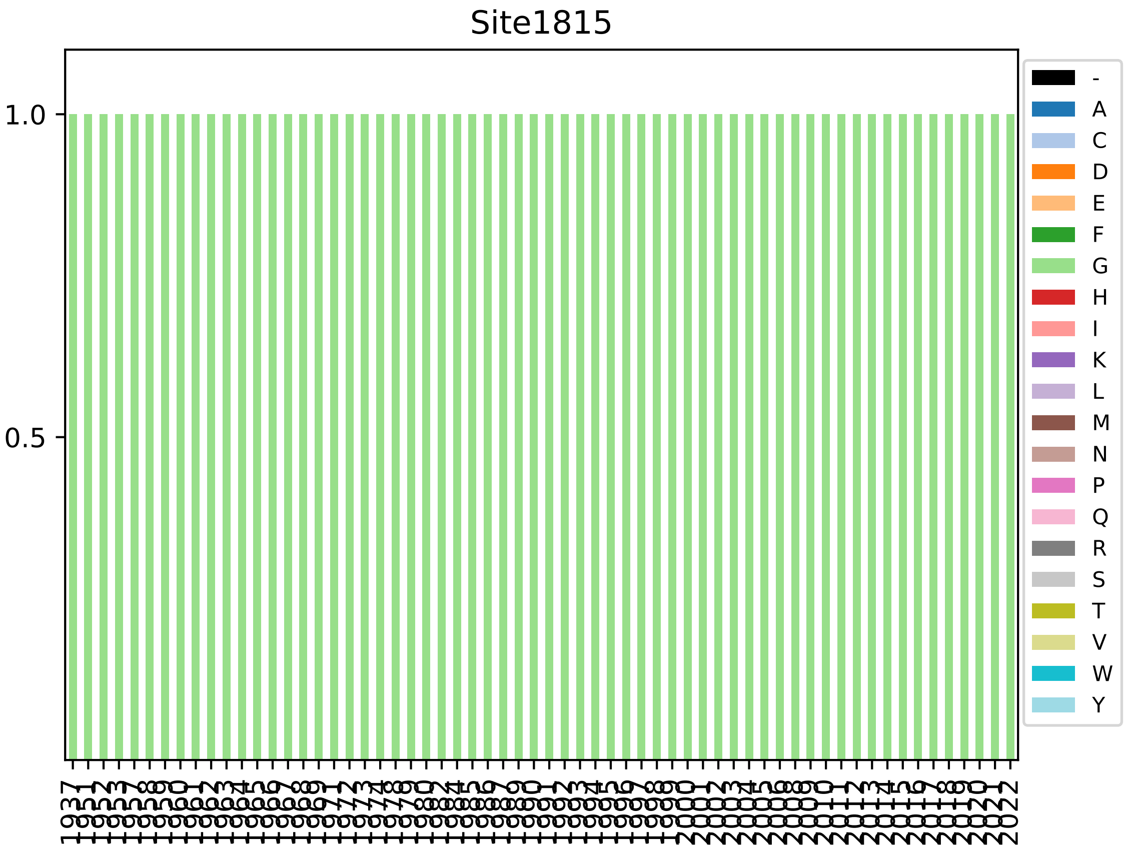
Site1815
1.0
0.5
-
A
C
D
E
F
G
H
I
K
L
M
N
P
Q
R
S
T
V
W
Y

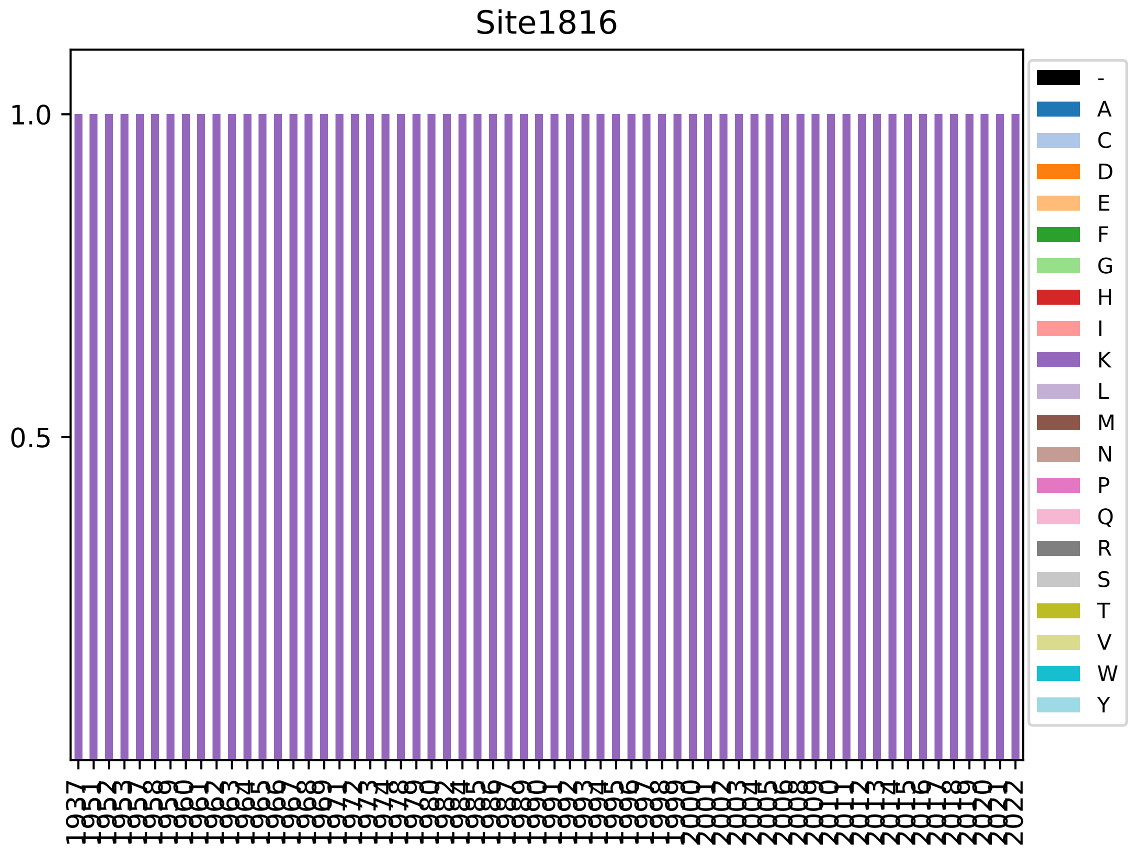
Site1816
1.0
0.5
-
A
C
D
E
F
G
H
I
K
L
M
N
P
Q
R
S
T
V
W
Y

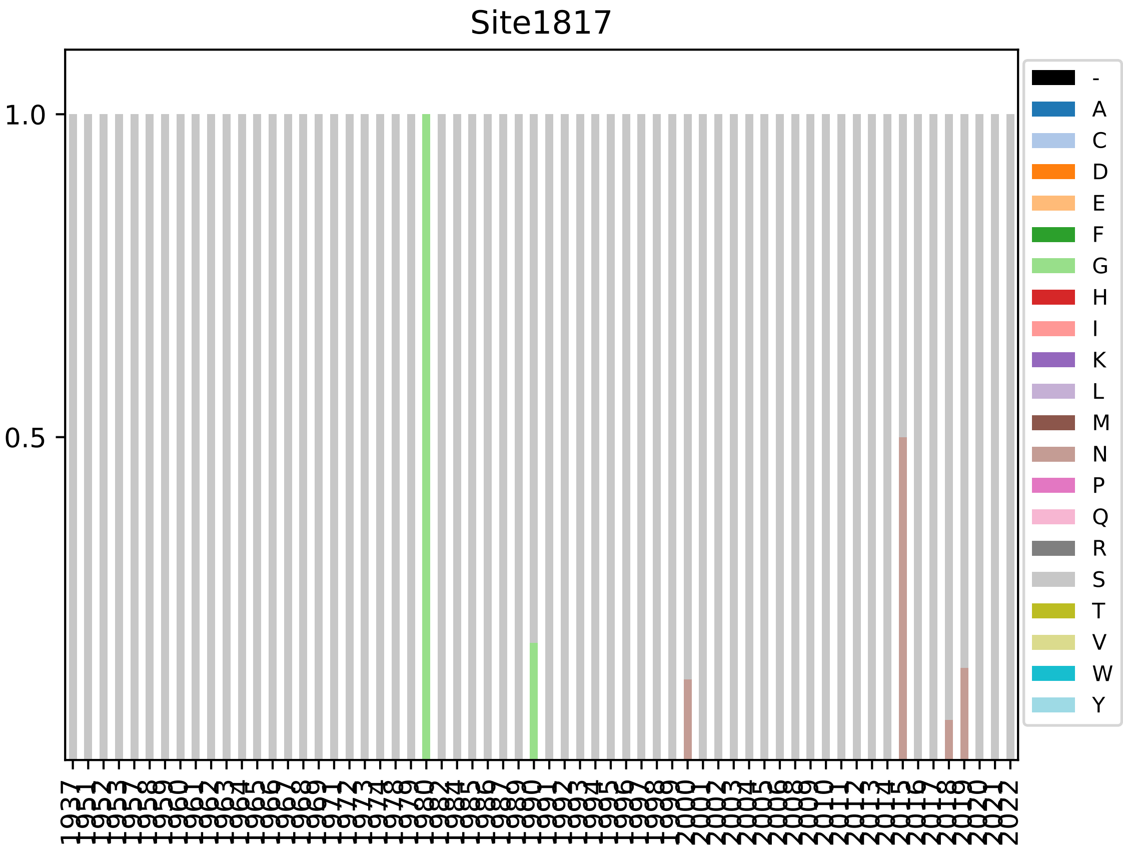

Site1817
1.0
0.5
-
A
C
D
E
F
G
H
I
K
L
M
N
P
Q
R
S
T
V
W
Y

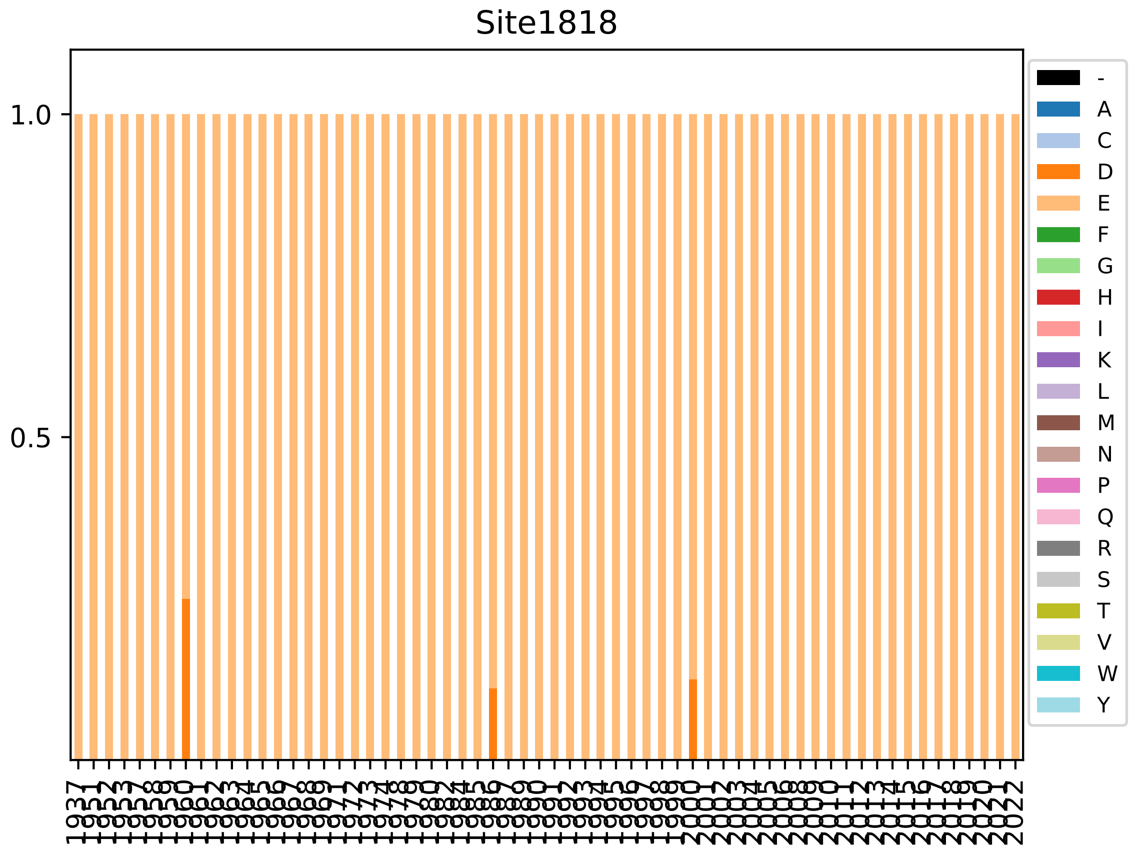
Site1818
1.0
0.5
-
A
C
D
E
F
G
H
I
K
L
M
N
P
Q
R
S
T
V
W
Y

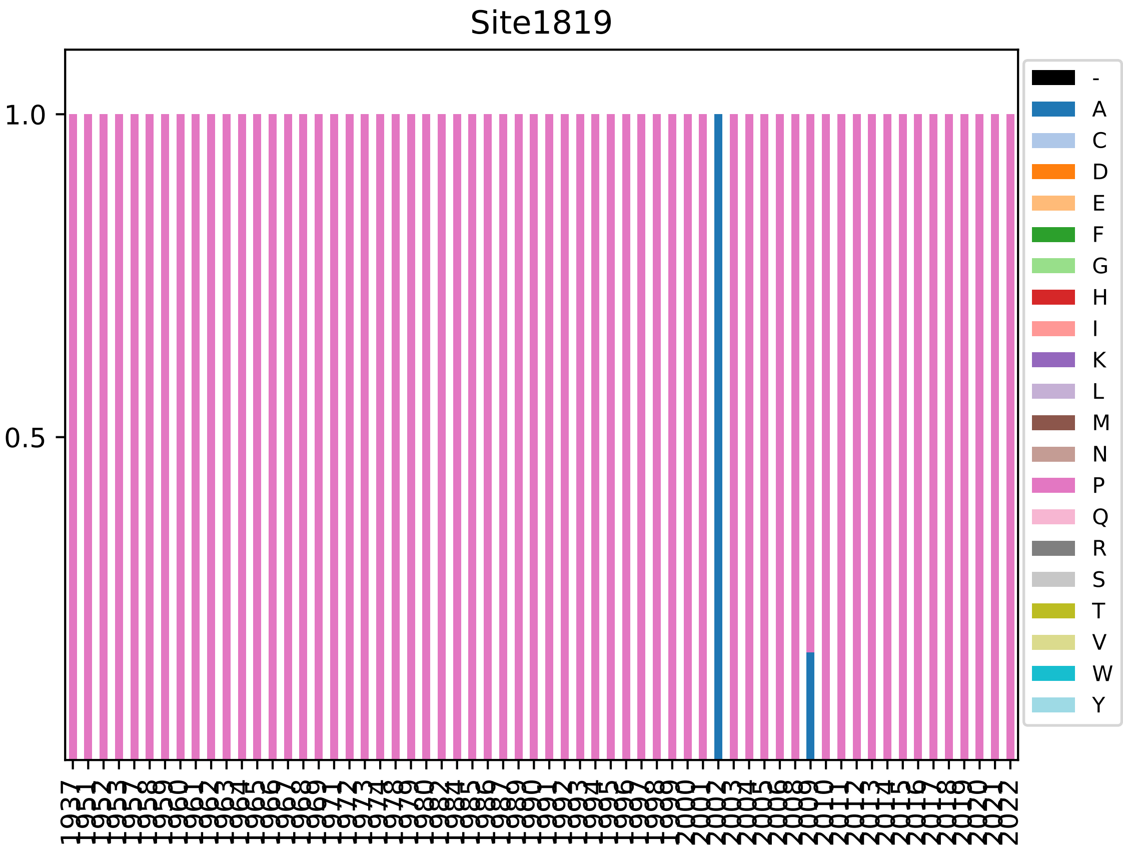

Site1819
1.0
0.5
-
A
C
D
E
F
G
H
I
K
L
M
N
P
Q
R
S
T
V
W
Y

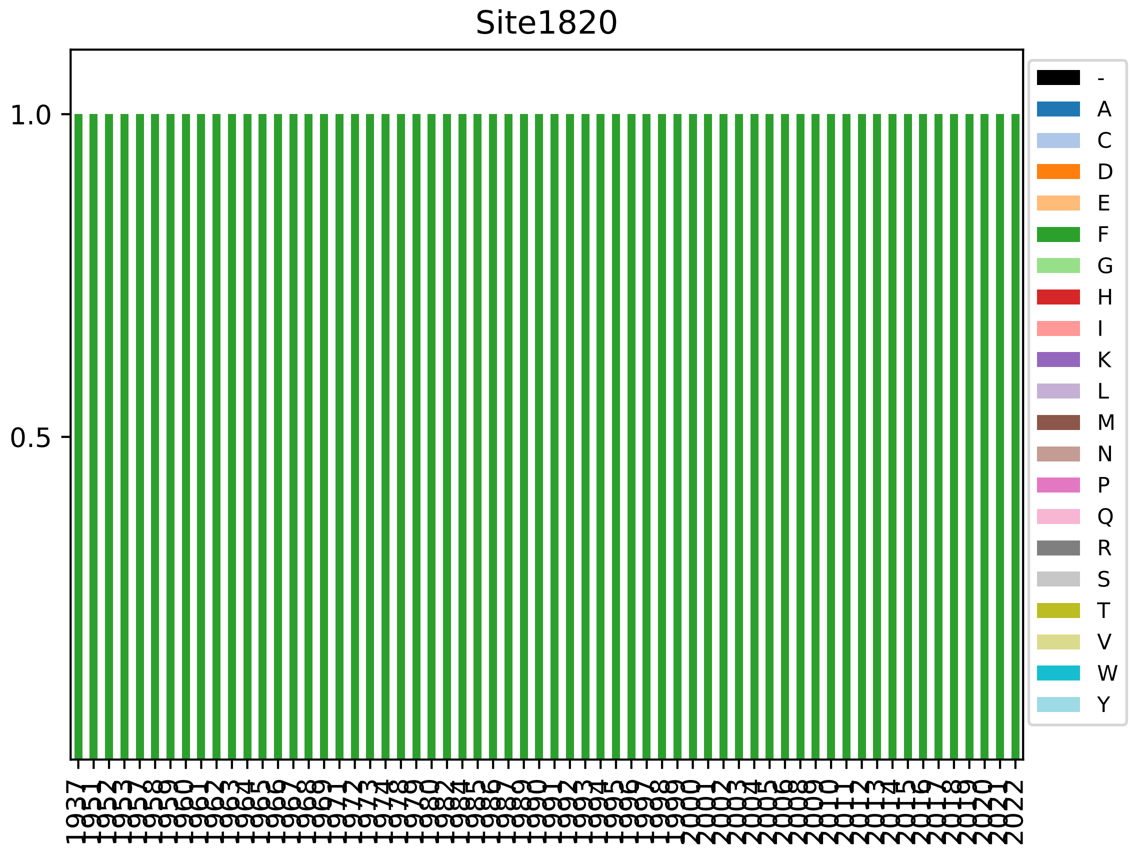
Site1820
1.0
0.5
-
A
C
D
E
F
G
H
I
K
L
M
N
P
Q
R
S
T
V
W
Y

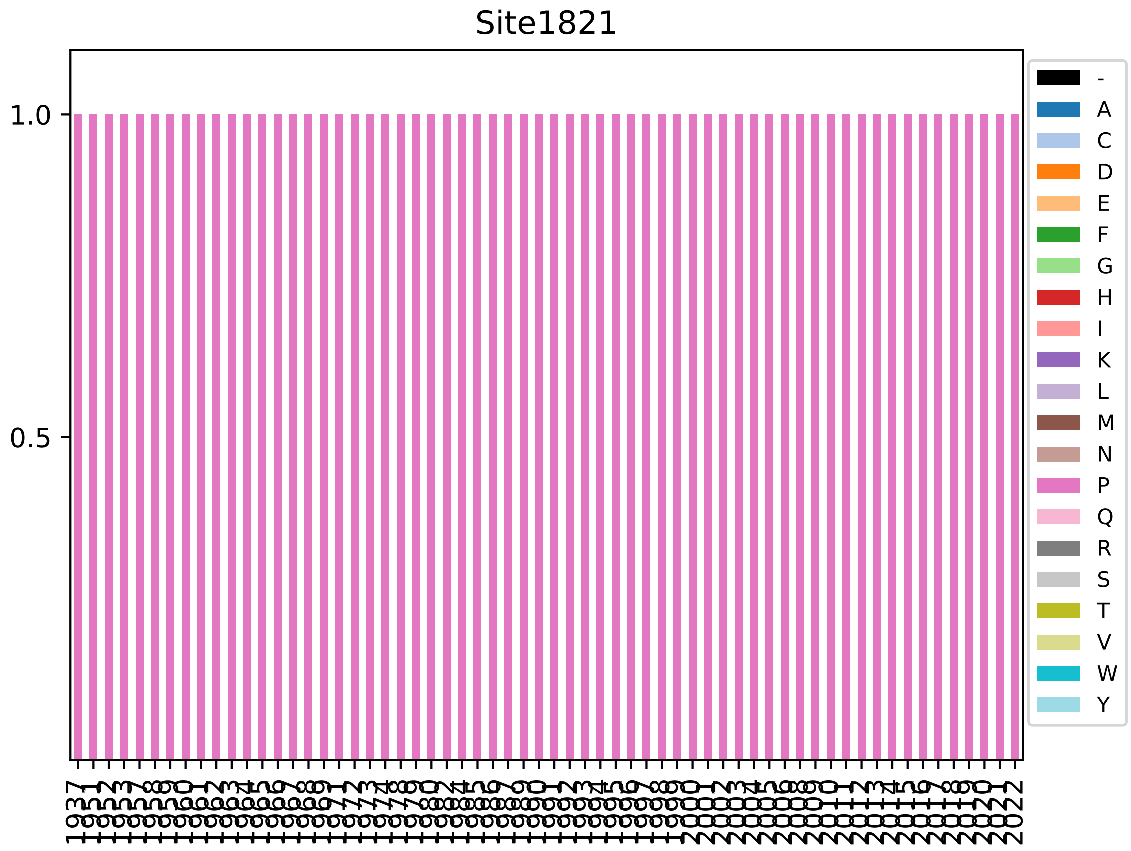
Site1821
1.0
0.5
-
A
C
D
E
F
G
H
I
K
L
M
N
P
Q
R
S
T
V
W
Y

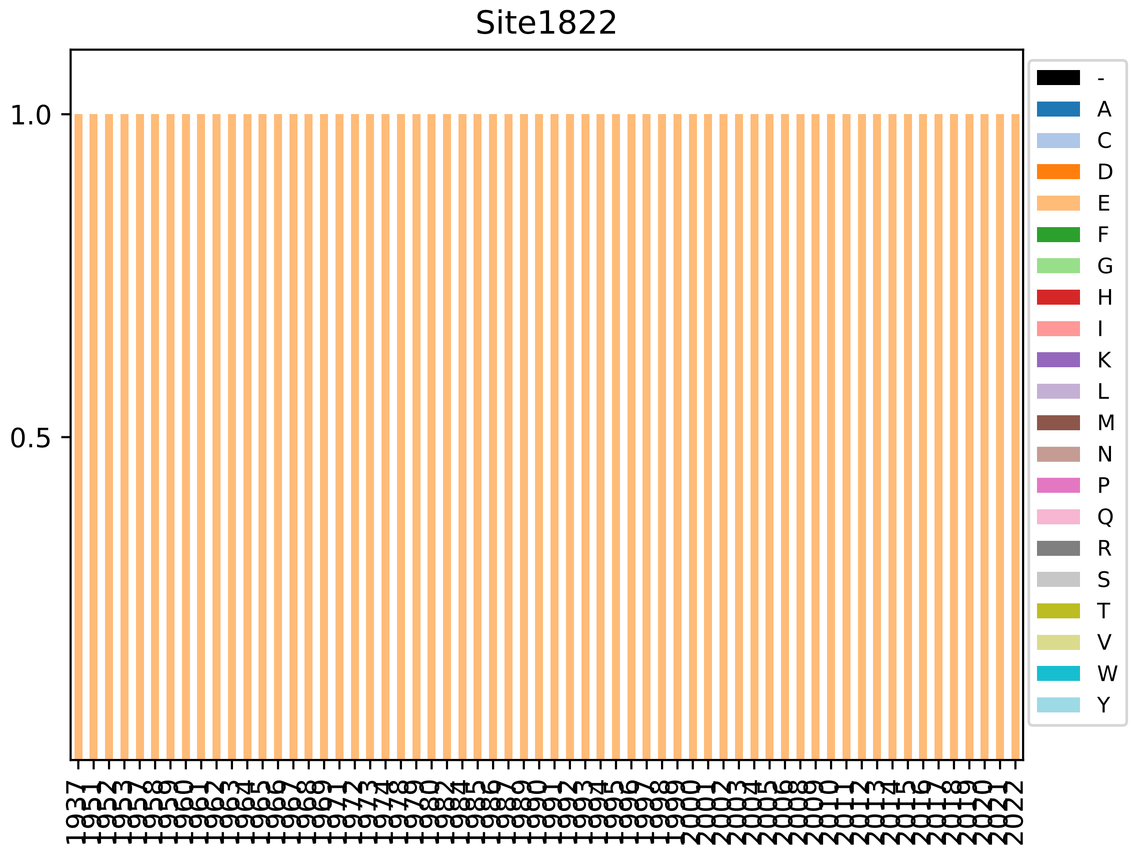
Site1822
1.0
0.5
-
A
C
D
E
F
G
H
I
K
L
M
N
P
Q
R
S
T
V
W
Y

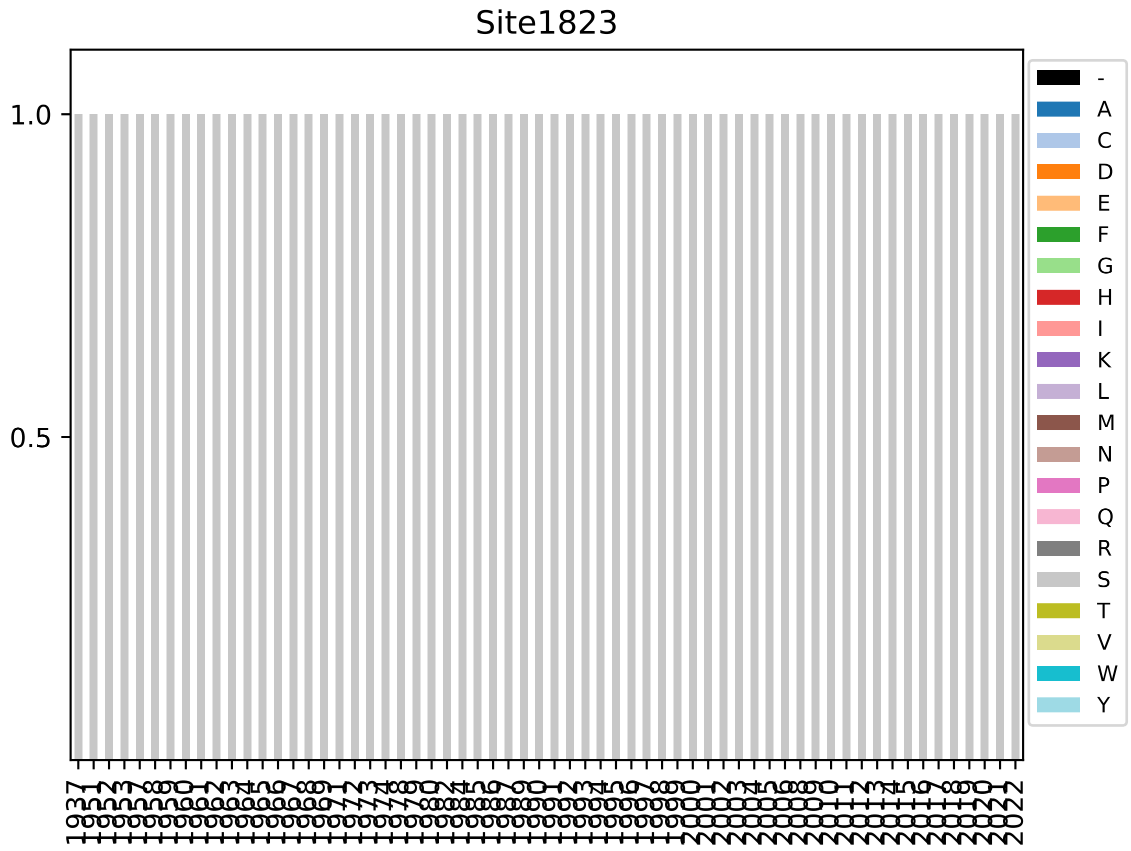
Site1823
1.0
0.5
-
A
C
D
E
F
G
H
I
K
L
M
N
P
Q
R
S
T
V
W
Y

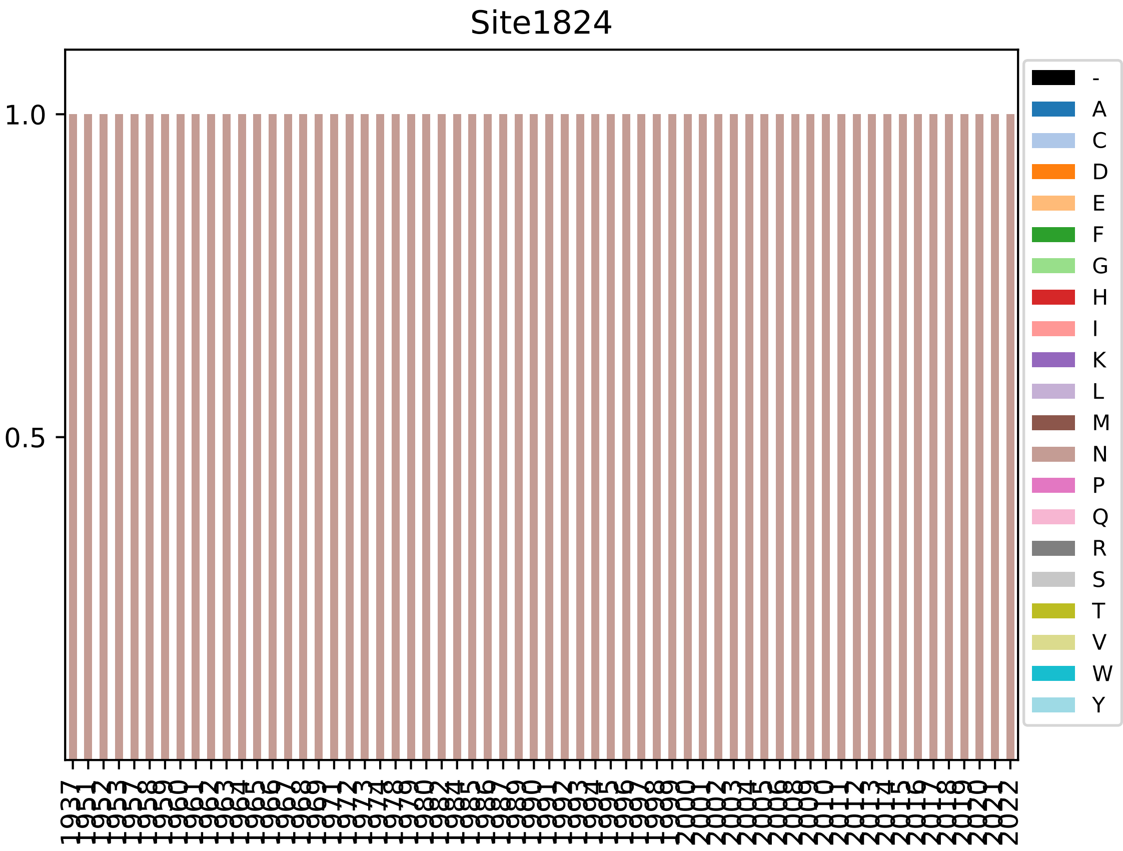
Site1824
1.0
0.5
-
A
C
D
E
F
G
H
I
K
L
M
N
P
Q
R
S
T
V
W
Y

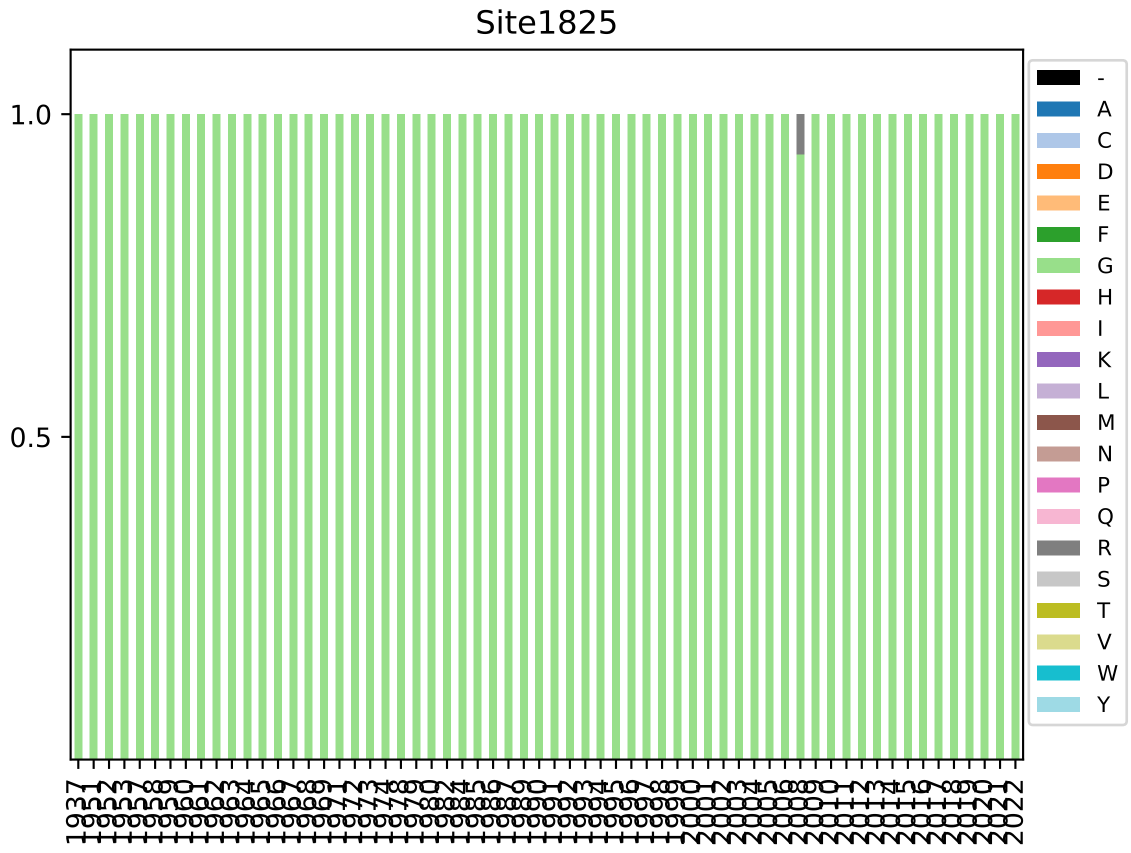
Site1825
1.0
0.5
-
A
C
D
E
F
G
H
I
K
L
M
N
P
Q
R
S
T
V
W
Y

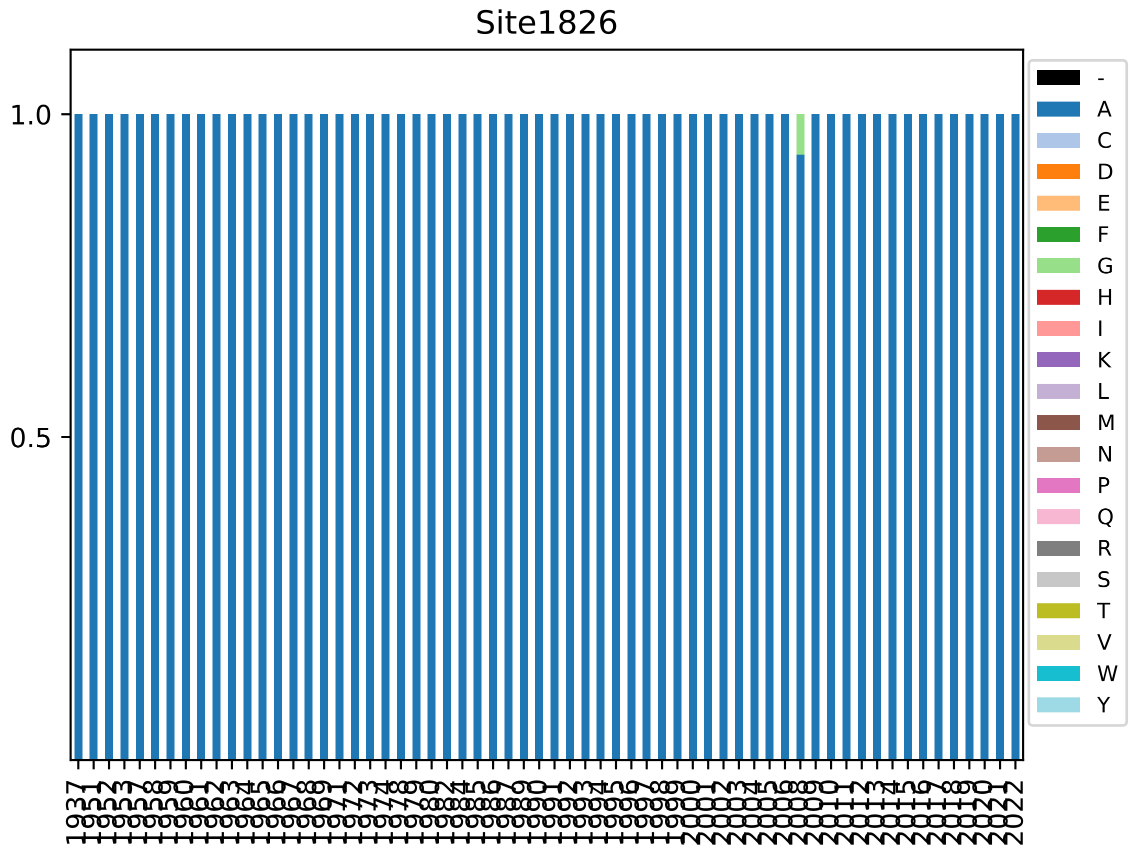

Site1826
1.0
0.5
-
A
C
D
E
F
G
H
I
K
L
M
N
P
Q
R
S
T
V
W
Y

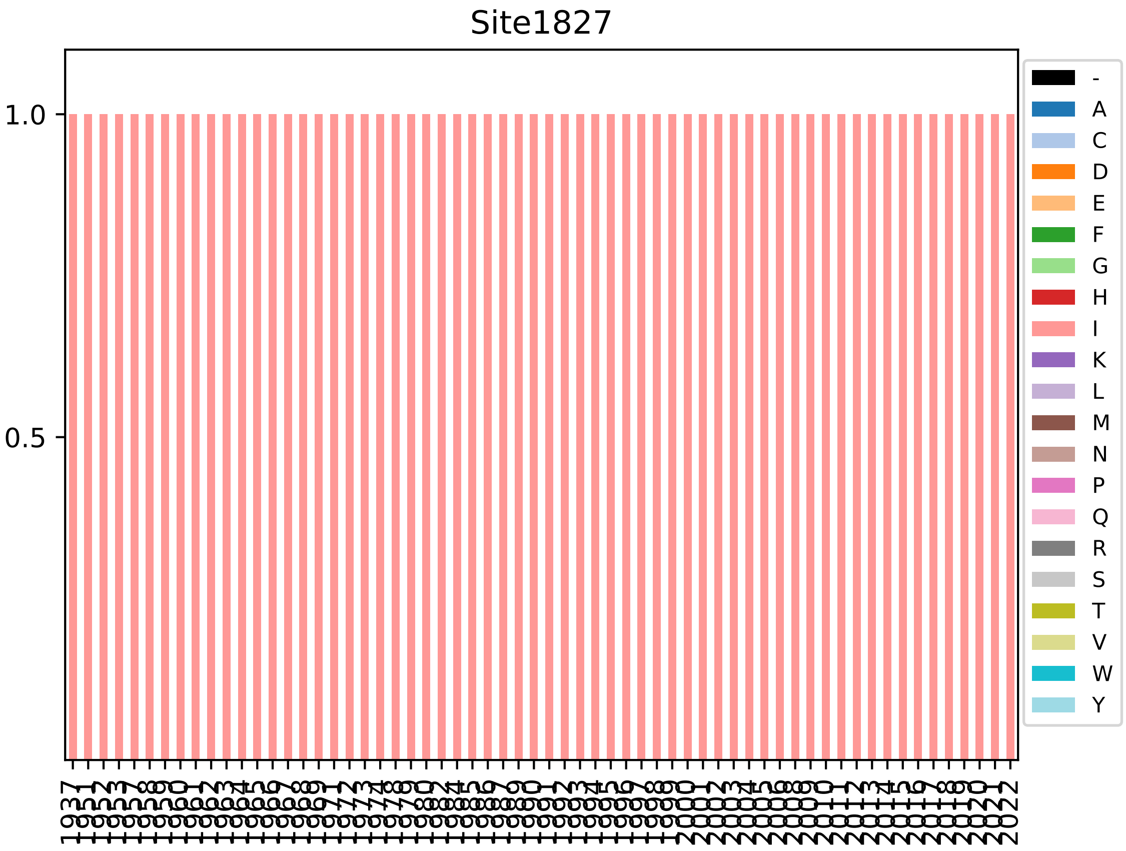
Site1827
1.0
0.5
-
A
C
D
E
F
G
H
I
K
L
M
N
P
Q
R
S
T
V
W
Y

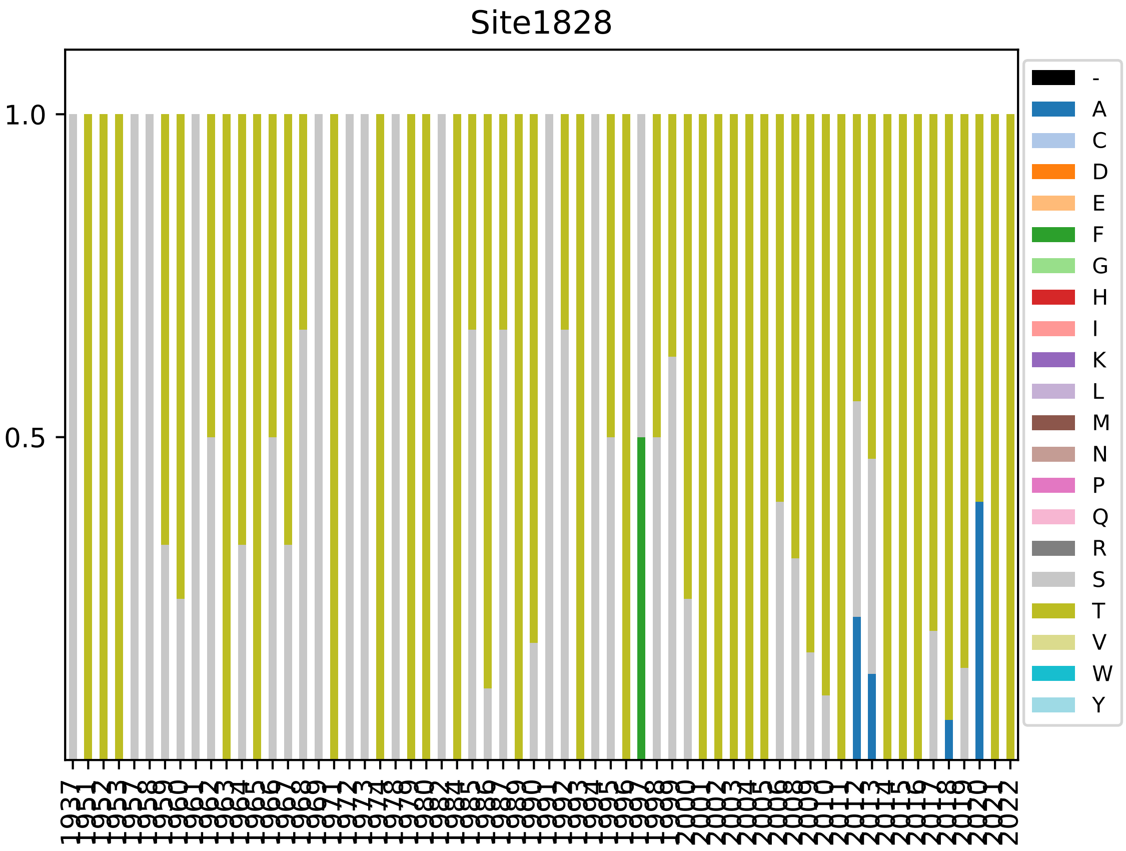
Site1828
1.0
0.5
-
A
C
D
E
F
G
H
I
K
L
M
N
P
Q
R
S
T
V
W
Y

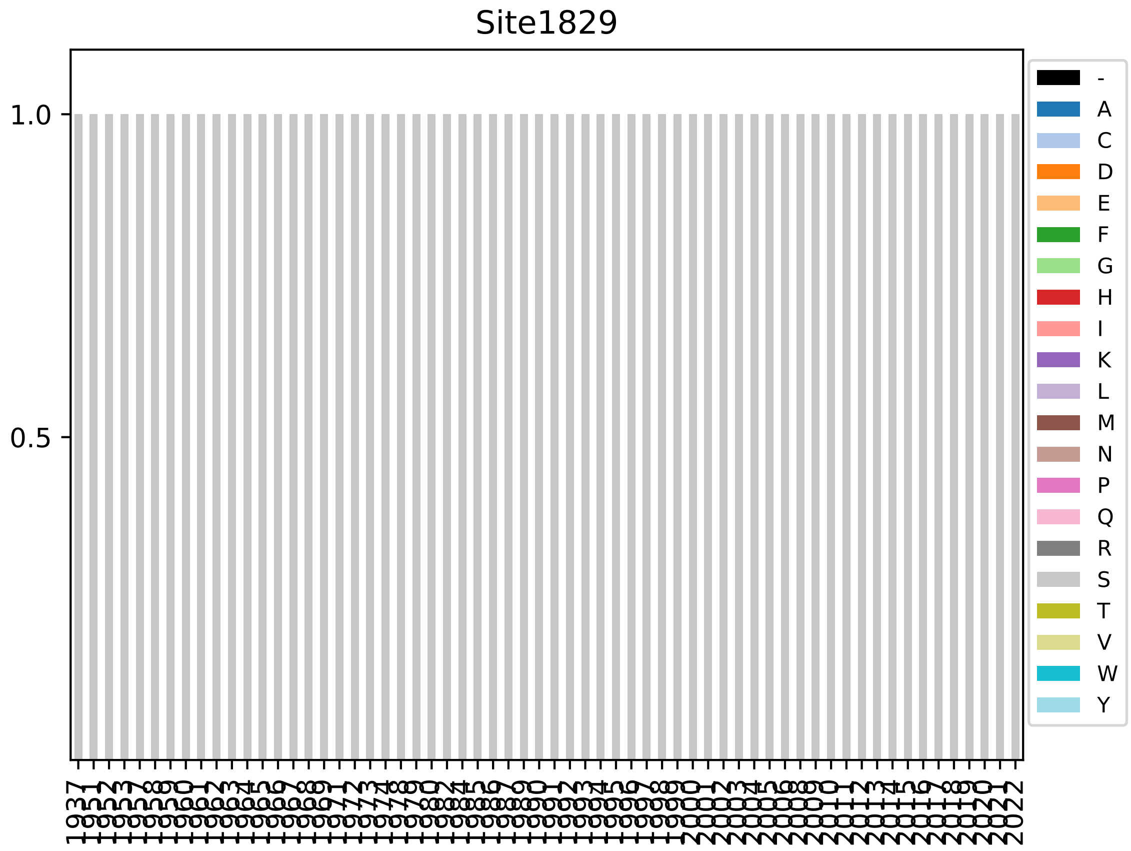
Site1829
1.0
0.5
-
A
C
D
E
F
G
H
I
K
L
M
N
P
Q
R
S
T
V
W
Y

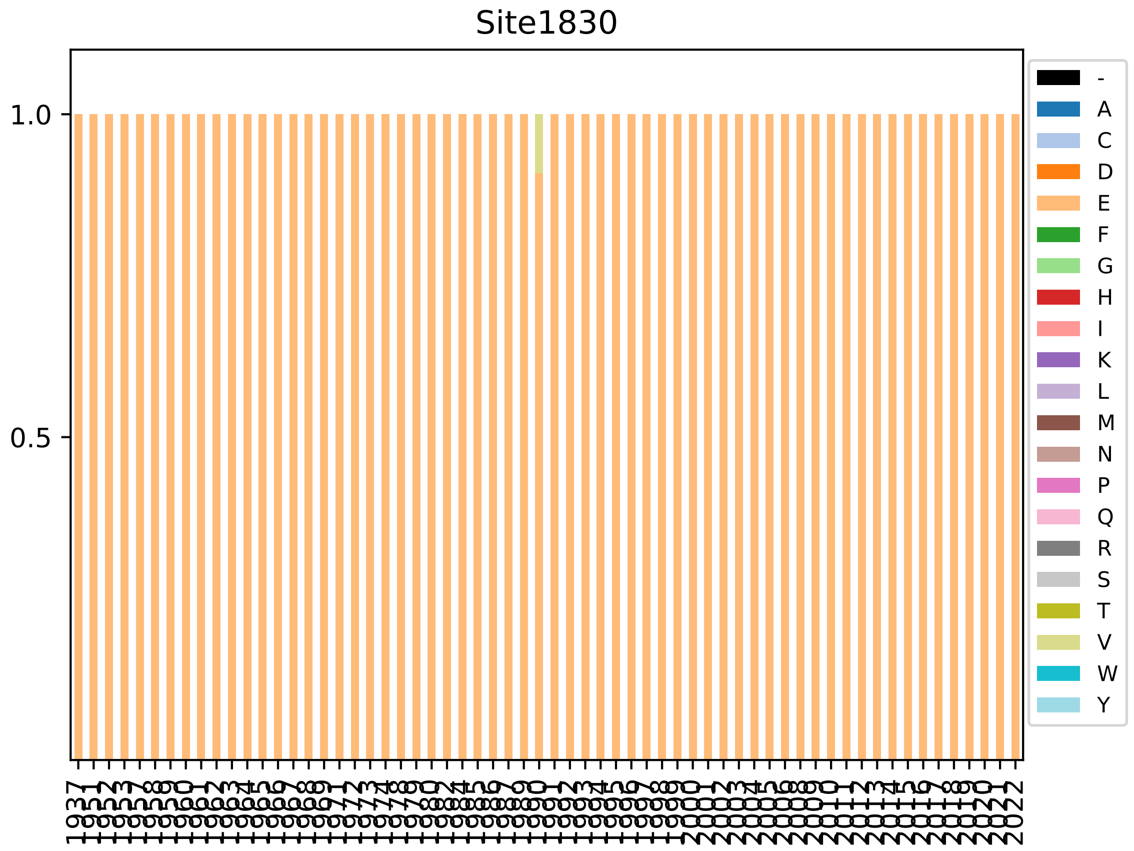
Site1830
1.0
0.5
-
A
C
D
E
F
G
H
I
K
L
M
N
P
Q
R
S
T
V
W
Y

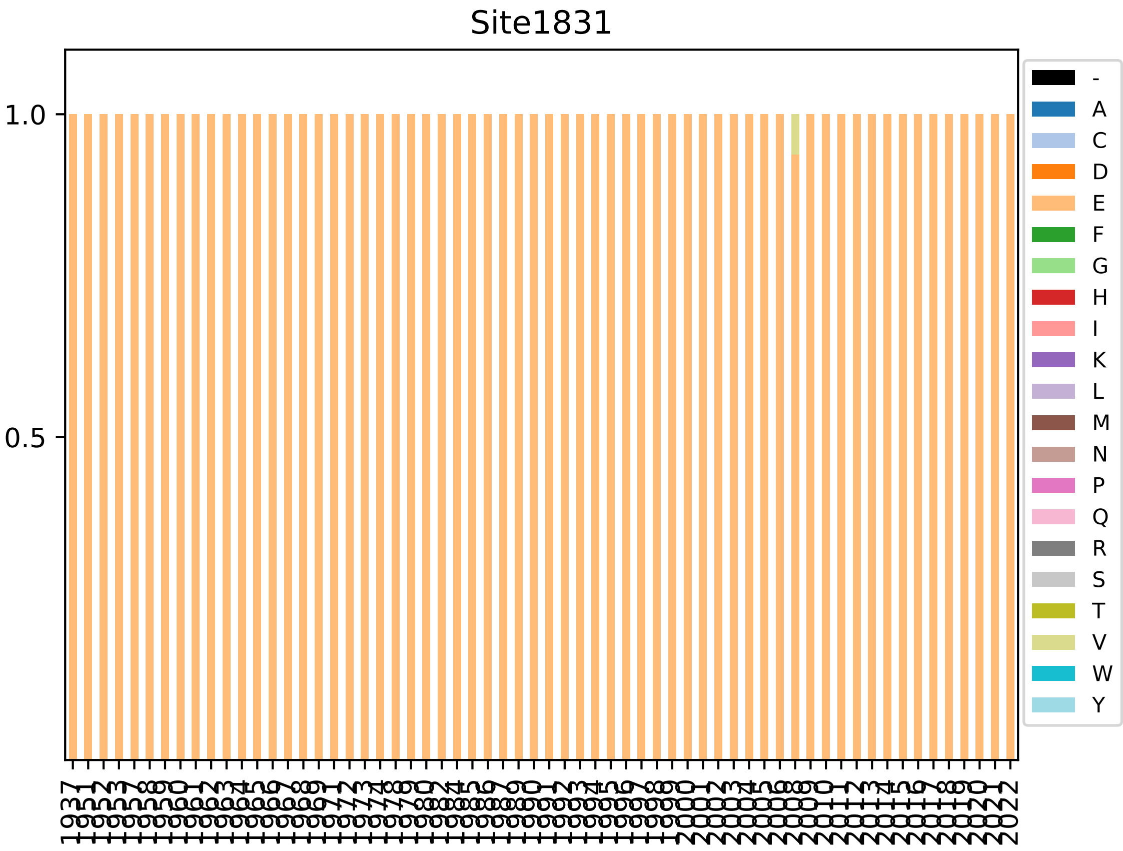
Site1831
1.0
0.5
-
A
C
D
E
F
G
H
I
K
L
M
N
P
Q
R
S
T
V
W
Y

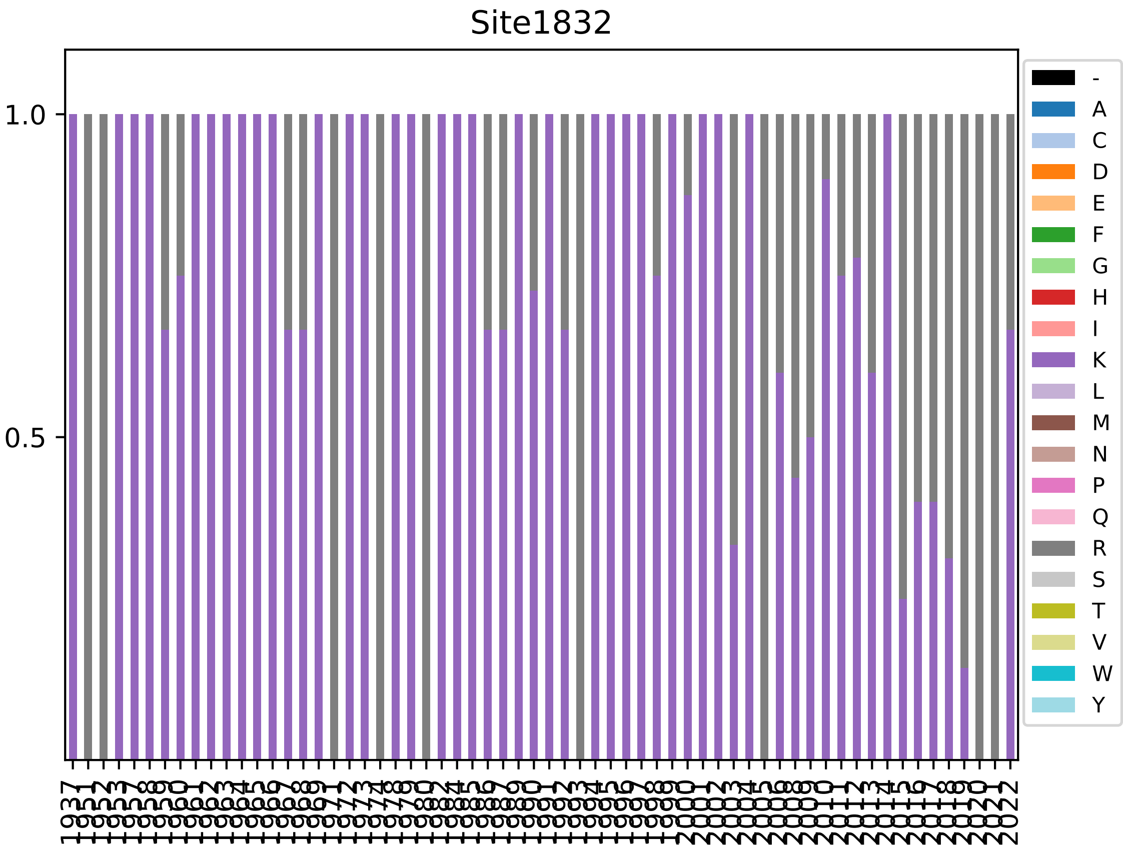
Site1832
1.0
0.5
-
A
C
D
E
F
G
H
I
K
L
M
N
P
Q
R
S
T
V
W
Y

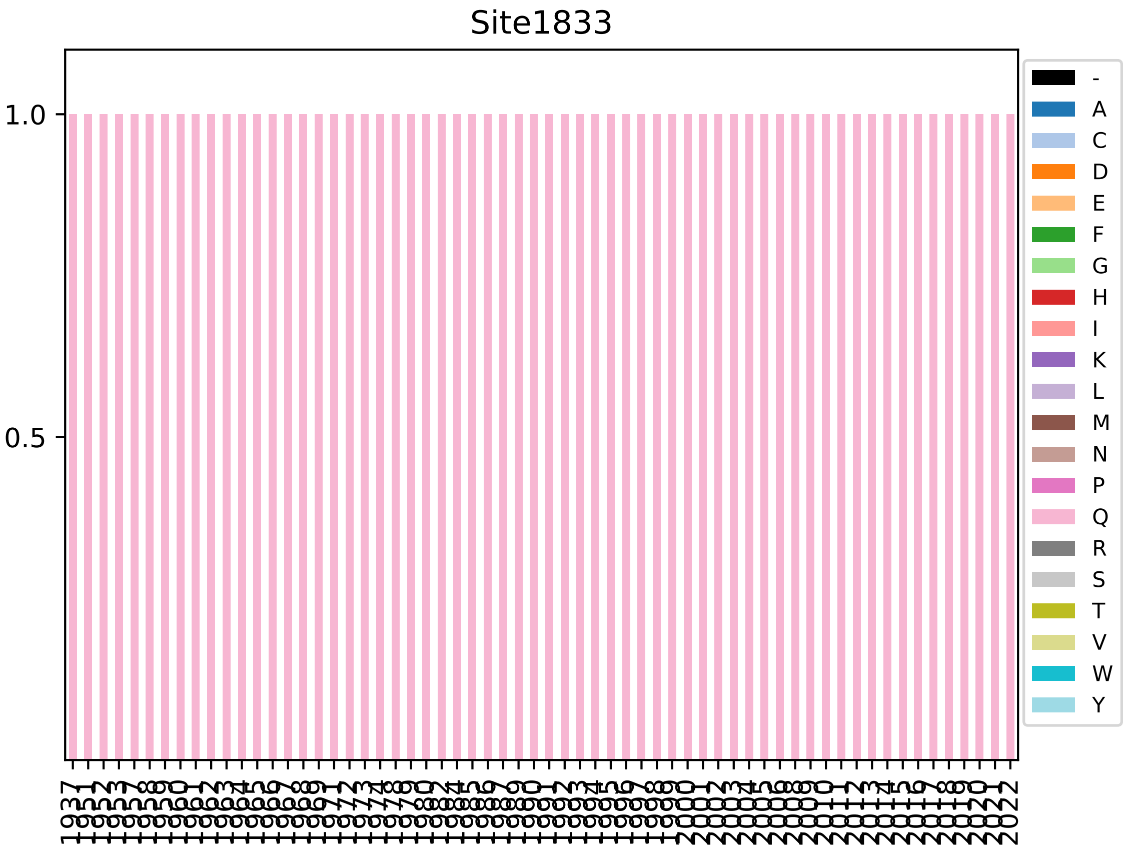
Site1833
1.0
0.5
-
A
C
D
E
F
G
H
I
K
L
M
N
P
Q
R
S
T
V
W
Y

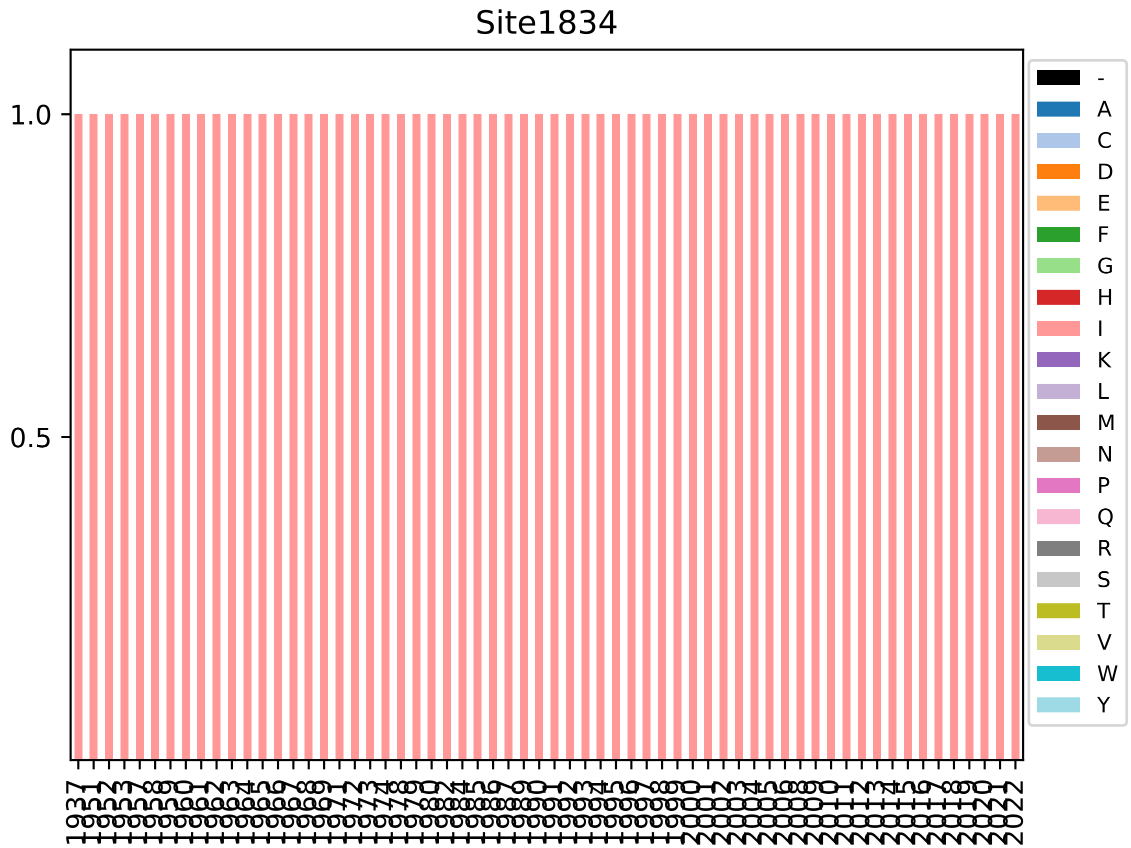
Site1834
1.0
0.5
-
A
C
D
E
F
G
H
I
K
L
M
N
P
Q
R
S
T
V
W
Y

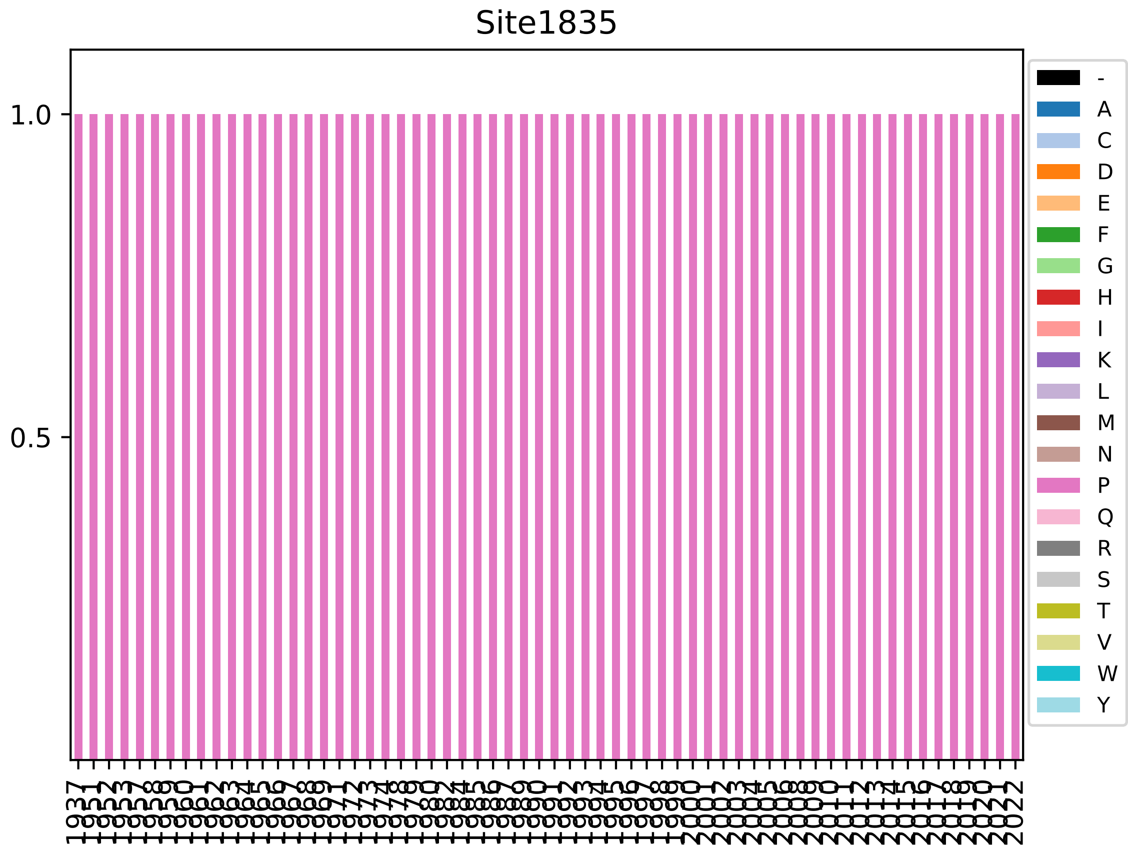
Site1835
1.0
0.5
-
A
C
D
E
F
G
H
I
K
L
M
N
P
Q
R
S
T
V
W
Y

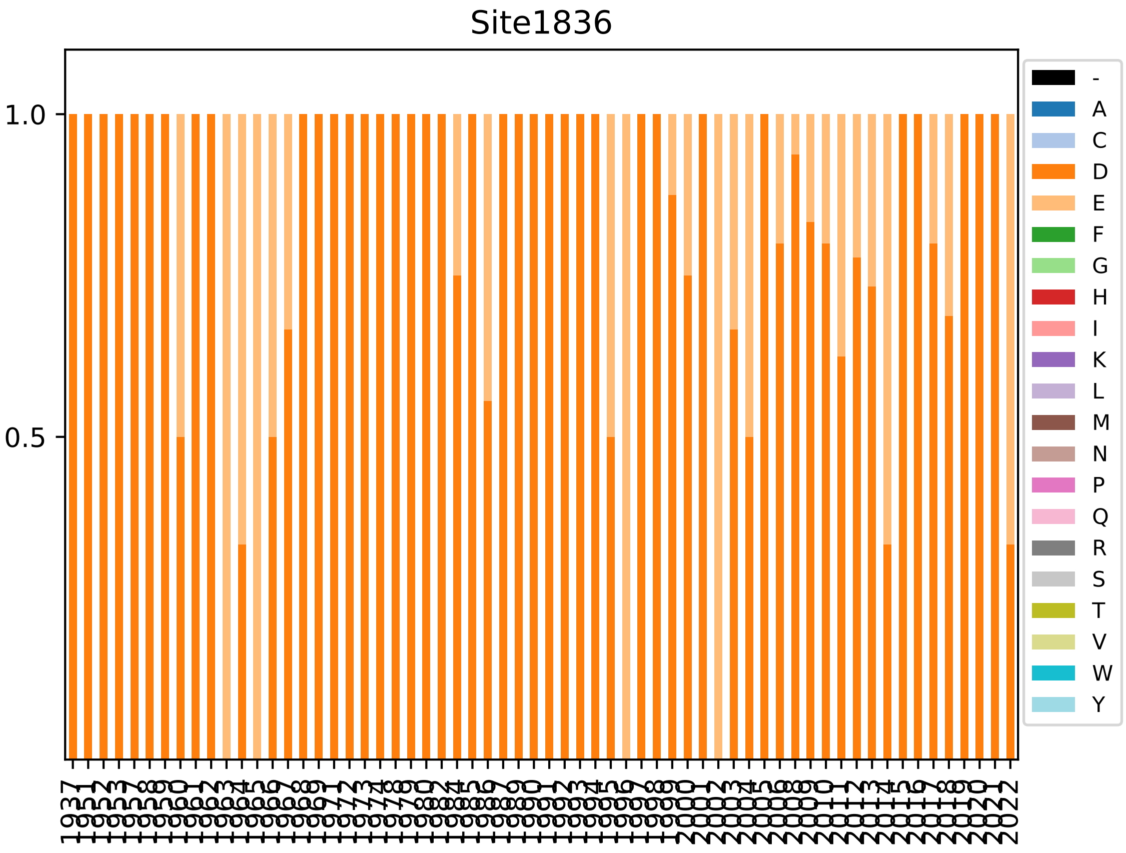
Site1836
1.0
0.5
-
A
C
D
E
F
G
H
I
K
L
M
N
P
Q
R
S
T
V
W
Y

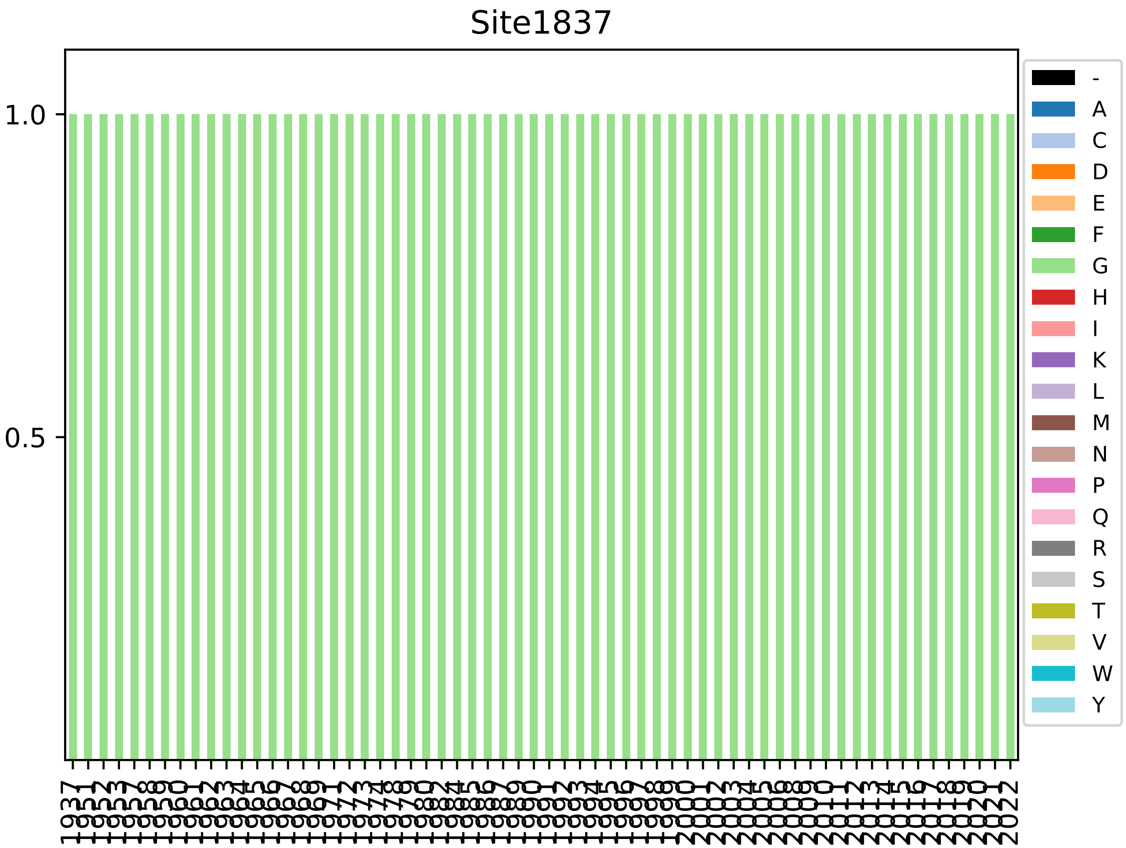
Site1837
1.0
0.5
-
A
C
D
E
F
G
H
I
K
L
M
N
P
Q
R
S
T
V
W
Y

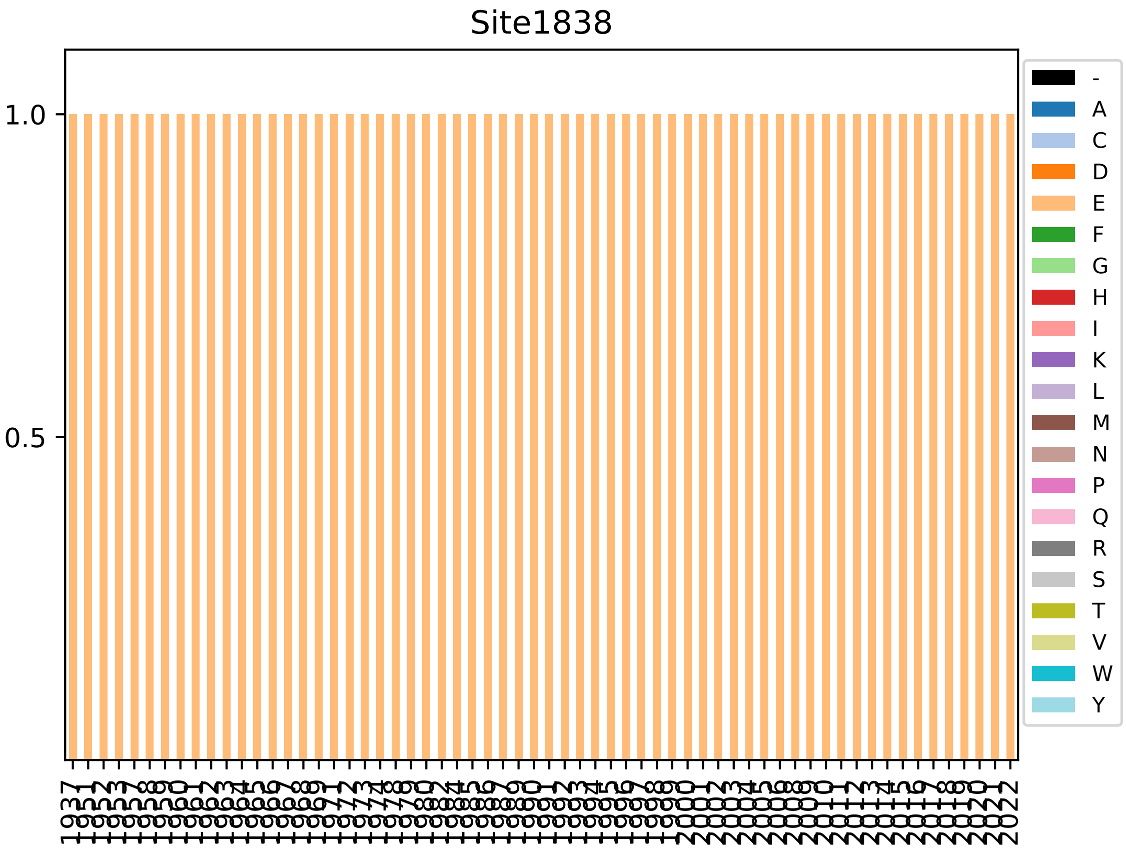

Site1838
1.0
0.5
-
A
C
D
E
F
G
H
I
K
L
M
N
P
Q
R
S
T
V
W
Y

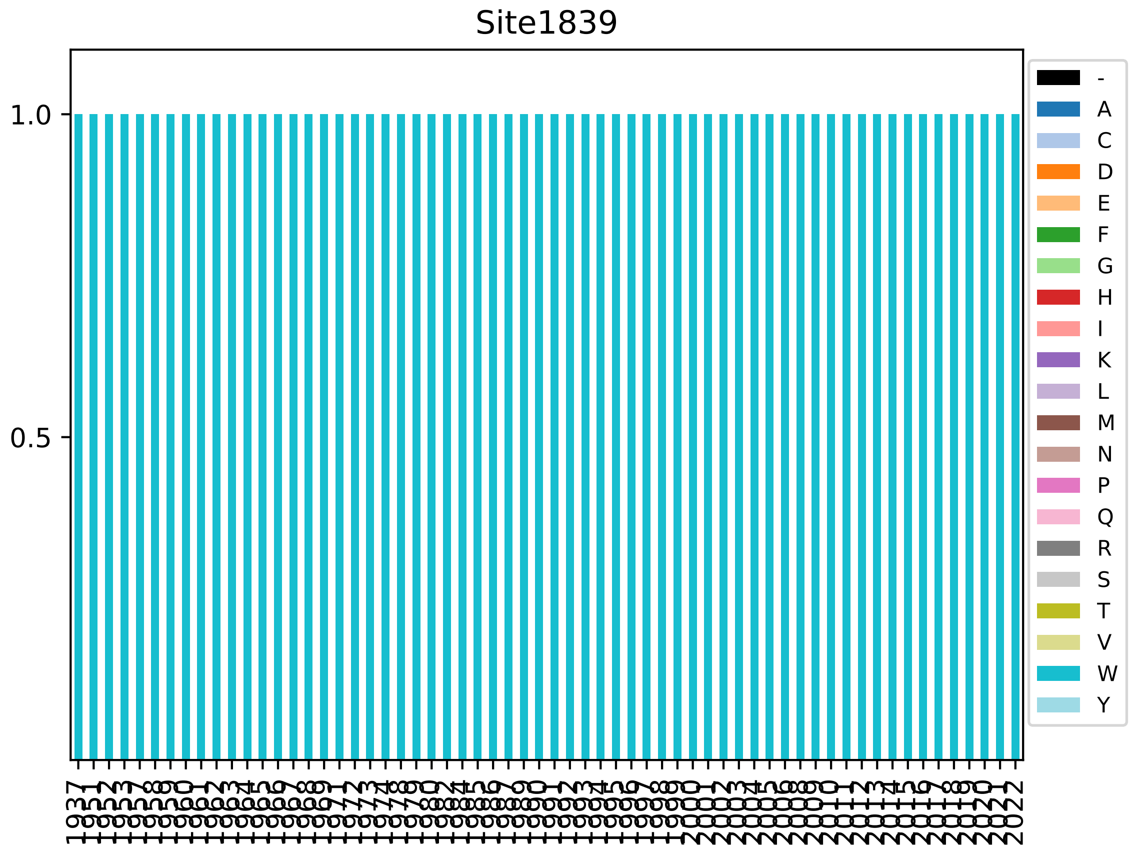

Site1839
1.0
0.5
-
A
C
D
E
F
G
H
I
K
L
M
N
P
Q
R
S
T
V
W
Y

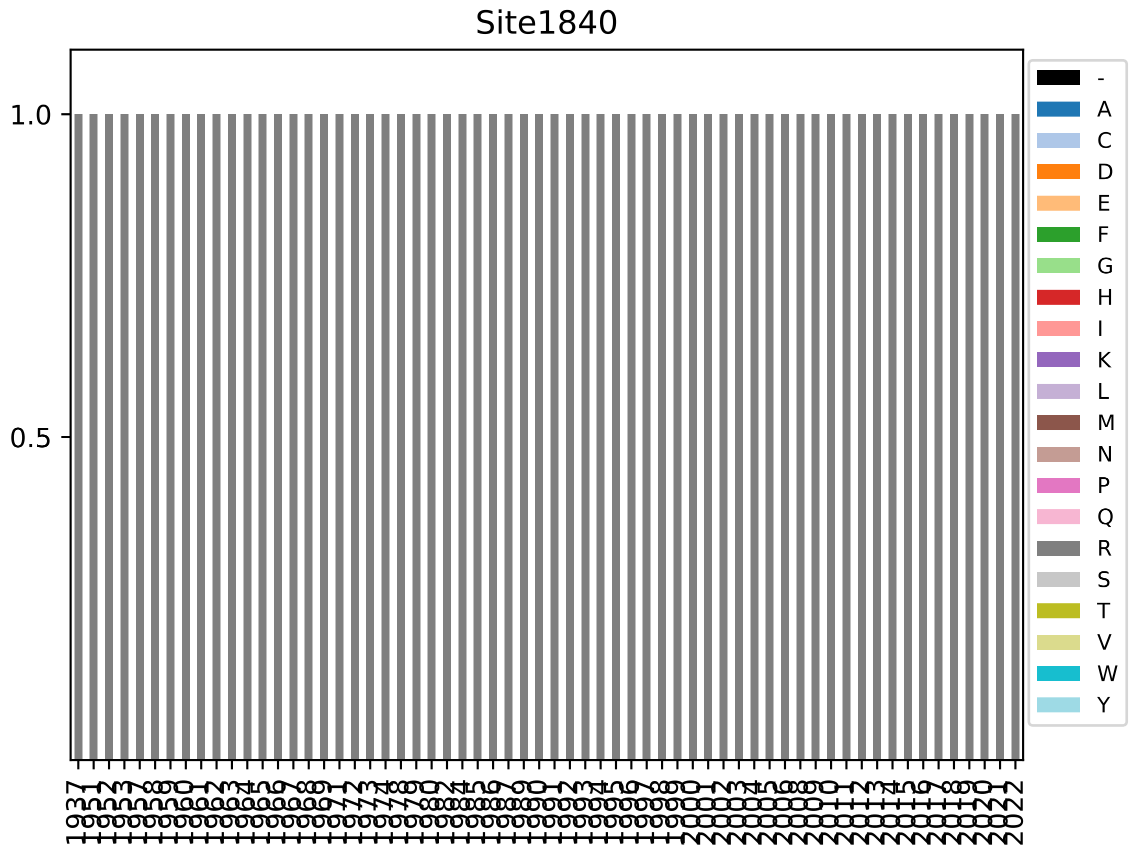
Site1840
1.0
0.5
-
A
C
D
E
F
G
H
I
K
L
M
N
P
Q
R
S
T
V
W
Y

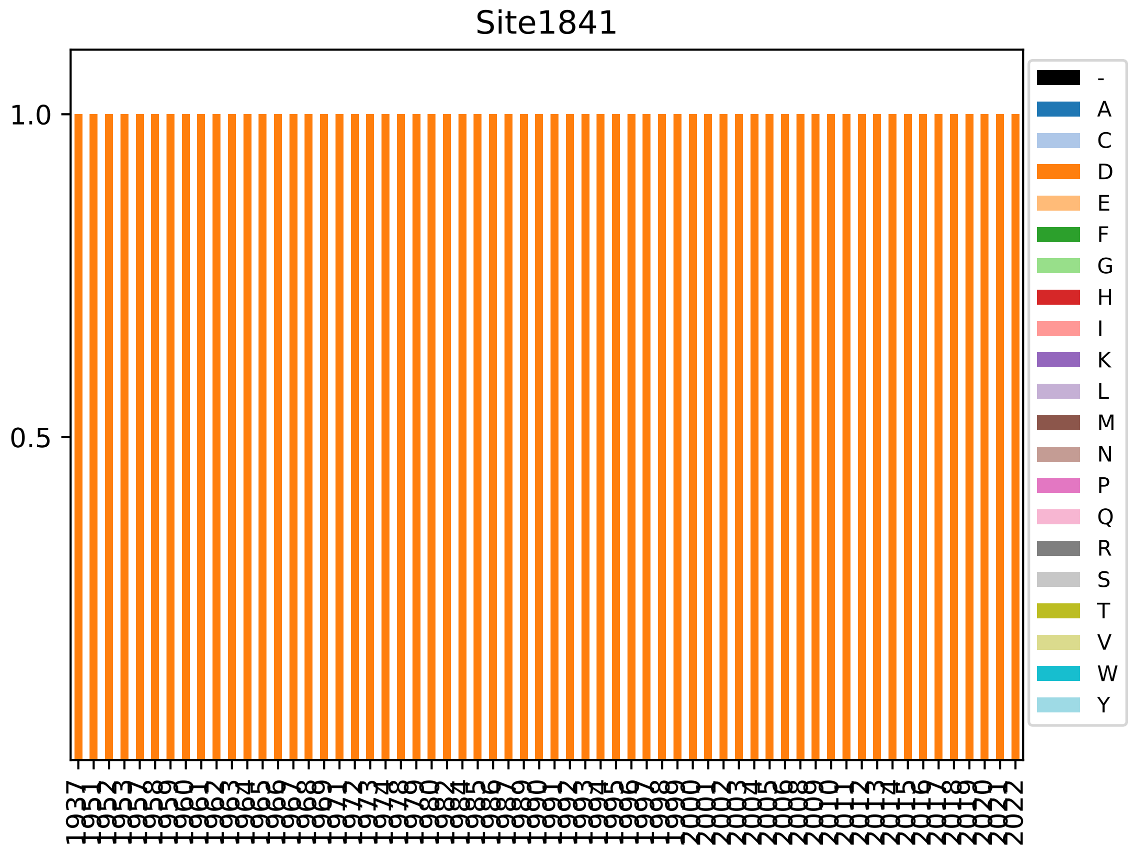
Site1841
1.0
0.5
-
A
C
D
E
F
G
H
I
K
L
M
N
P
Q
R
S
T
V
W
Y

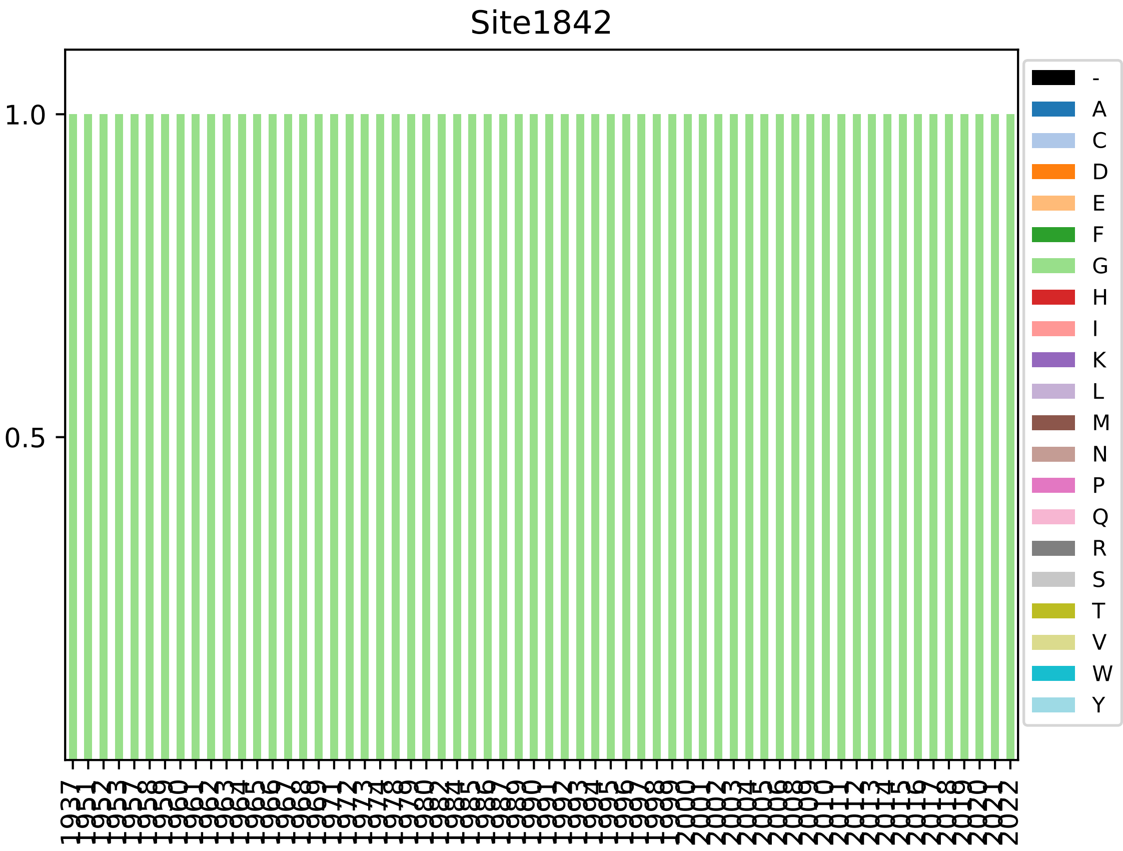
Site1842
1.0
0.5
-
A
C
D
E
F
G
H
I
K
L
M
N
P
Q
R
S
T
V
W
Y

Site1843

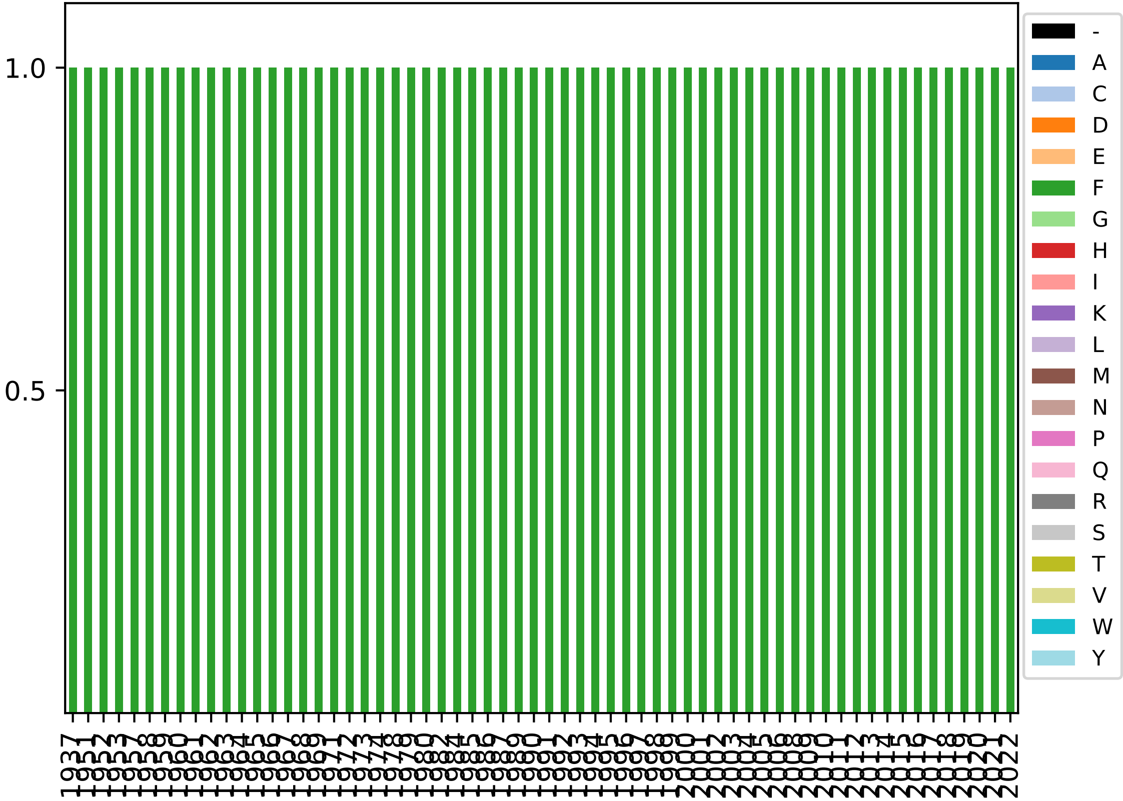

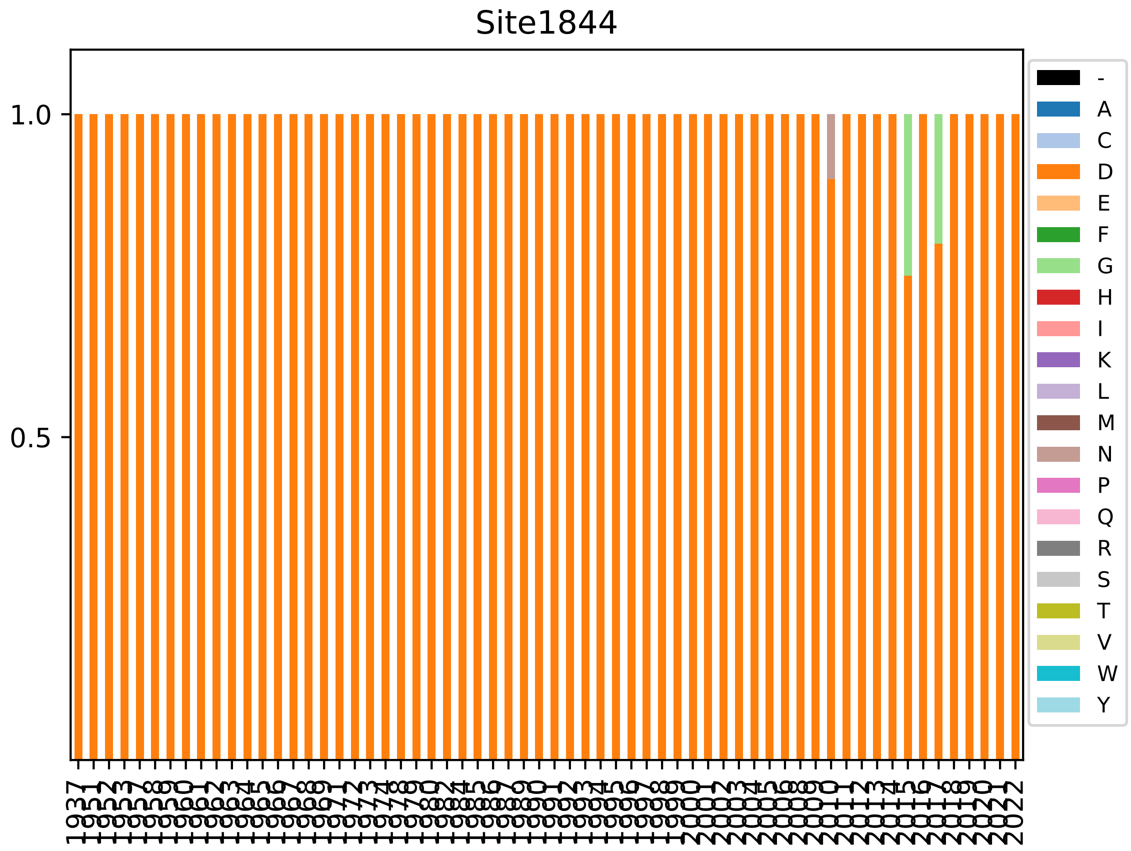
Site1844
1.0
0.5
-
A
C
D
E
F
G
H
I
K
L
M
N
P
Q
R
S
T
V
W
Y

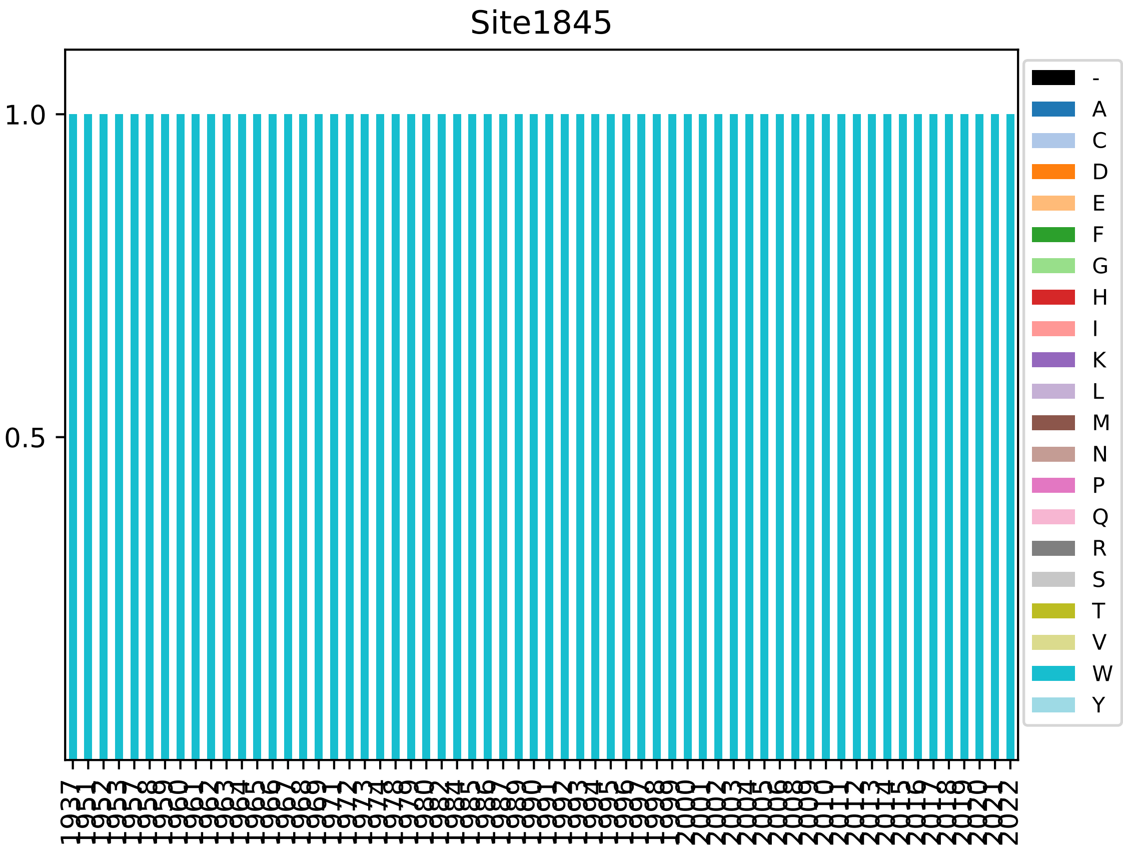
Site1845
1.0
0.5
-
A
C
D
E
F
G
H
I
K
L
M
N
P
Q
R
S
T
V
W
Y

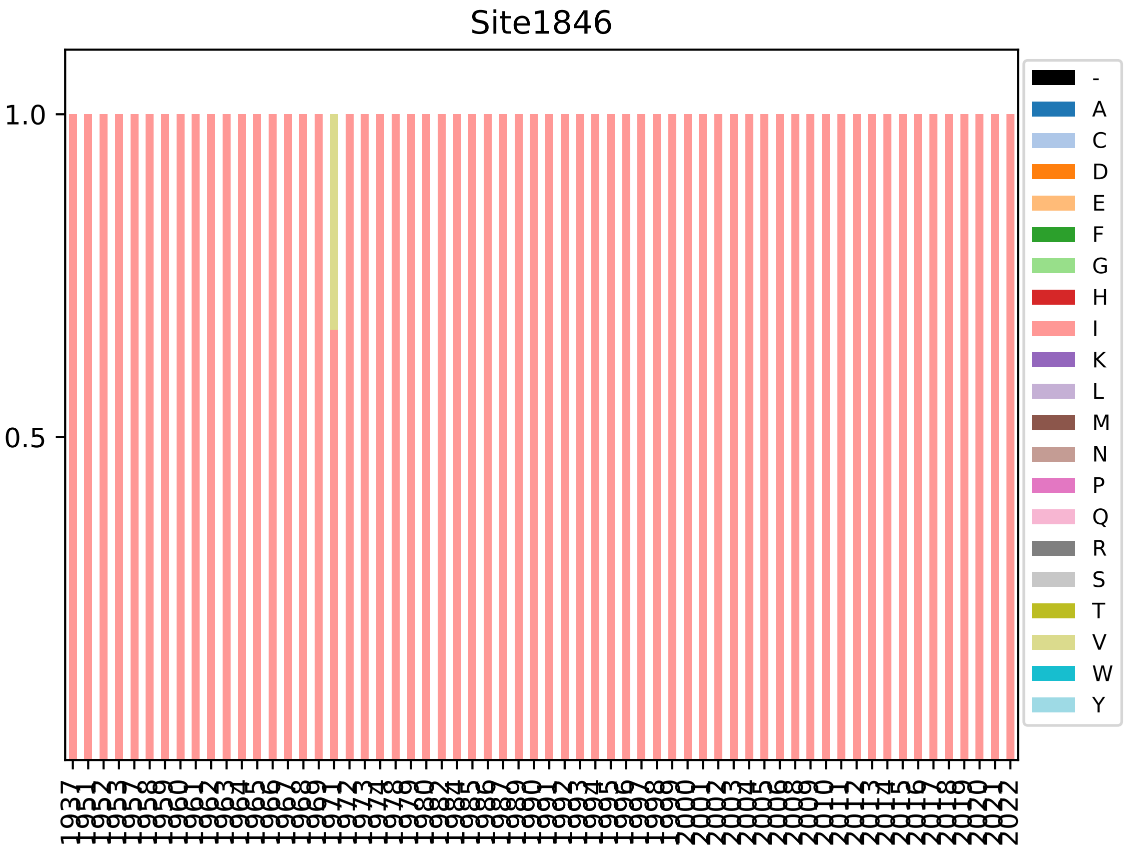
Site1846
1.0
0.5
-
A
C
D
E
F
G
H
I
K
L
M
N
P
Q
R
S
T
V
W
Y

Site1847

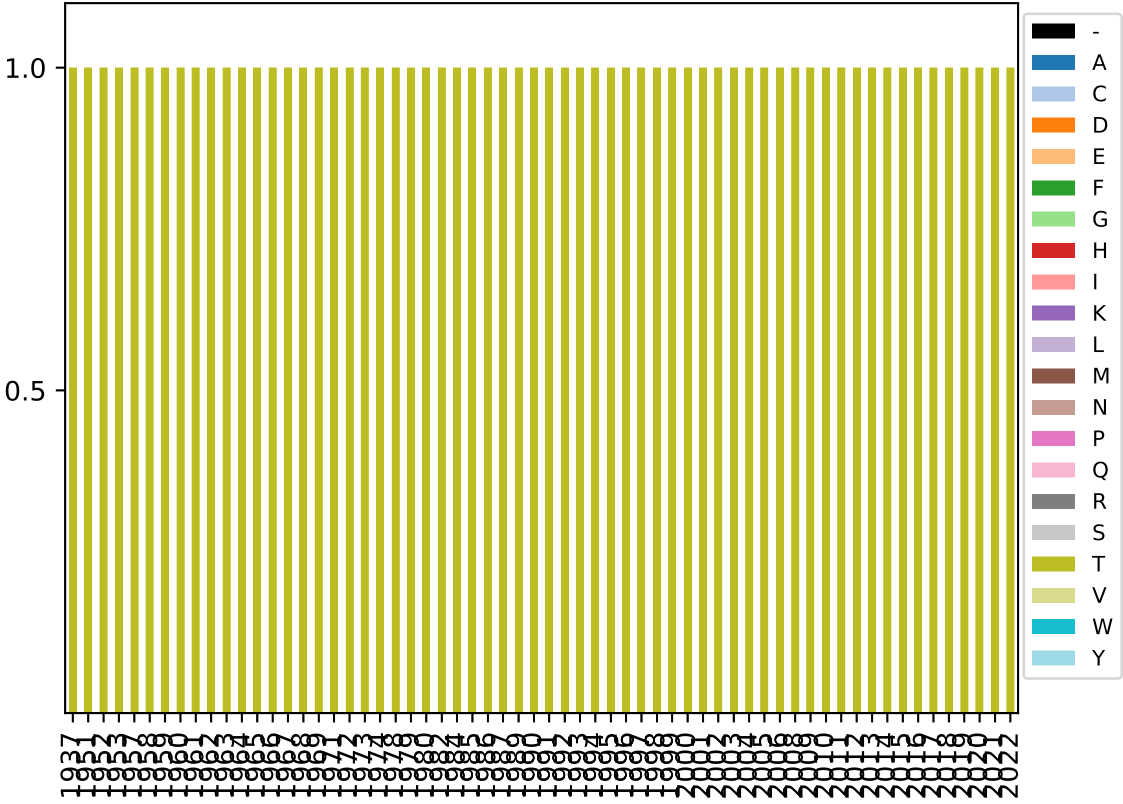

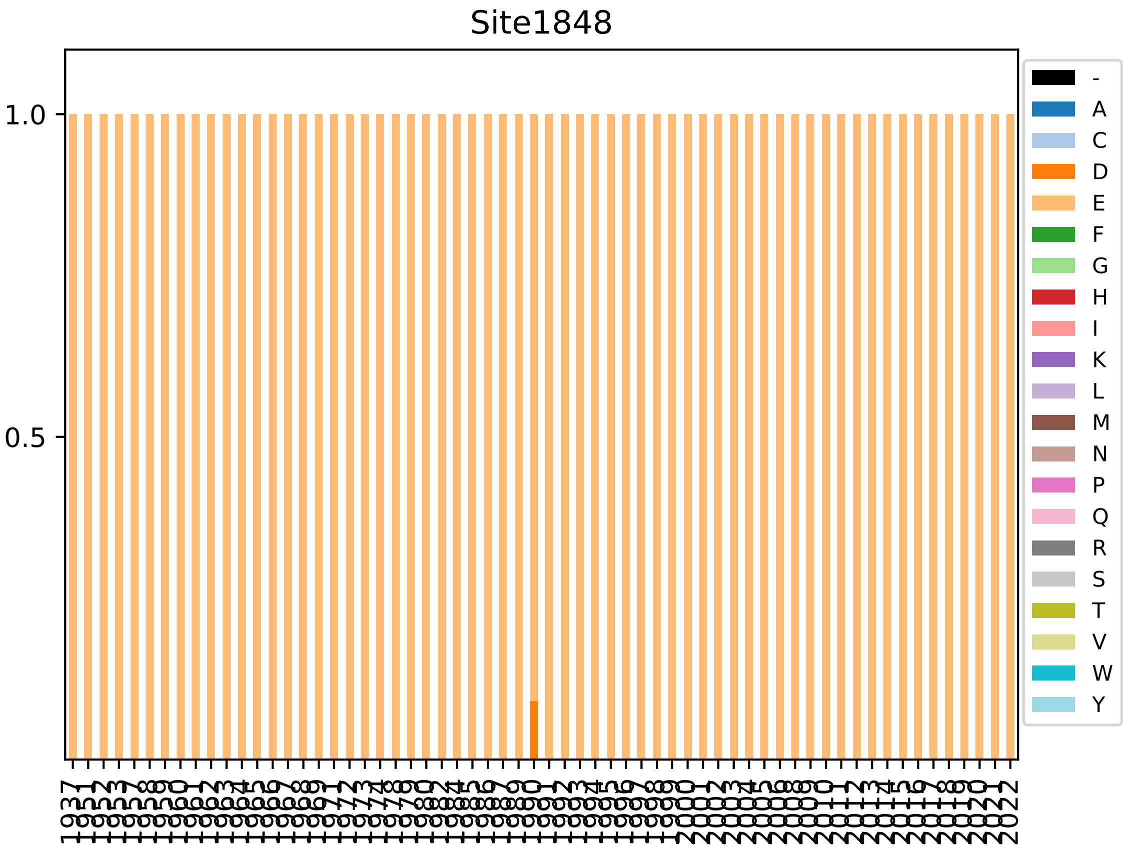
Site1848
1.0
0.5
-
A
C
D
E
F
G
H
I
K
L
M
N
P
Q
R
S
T
V
W
Y

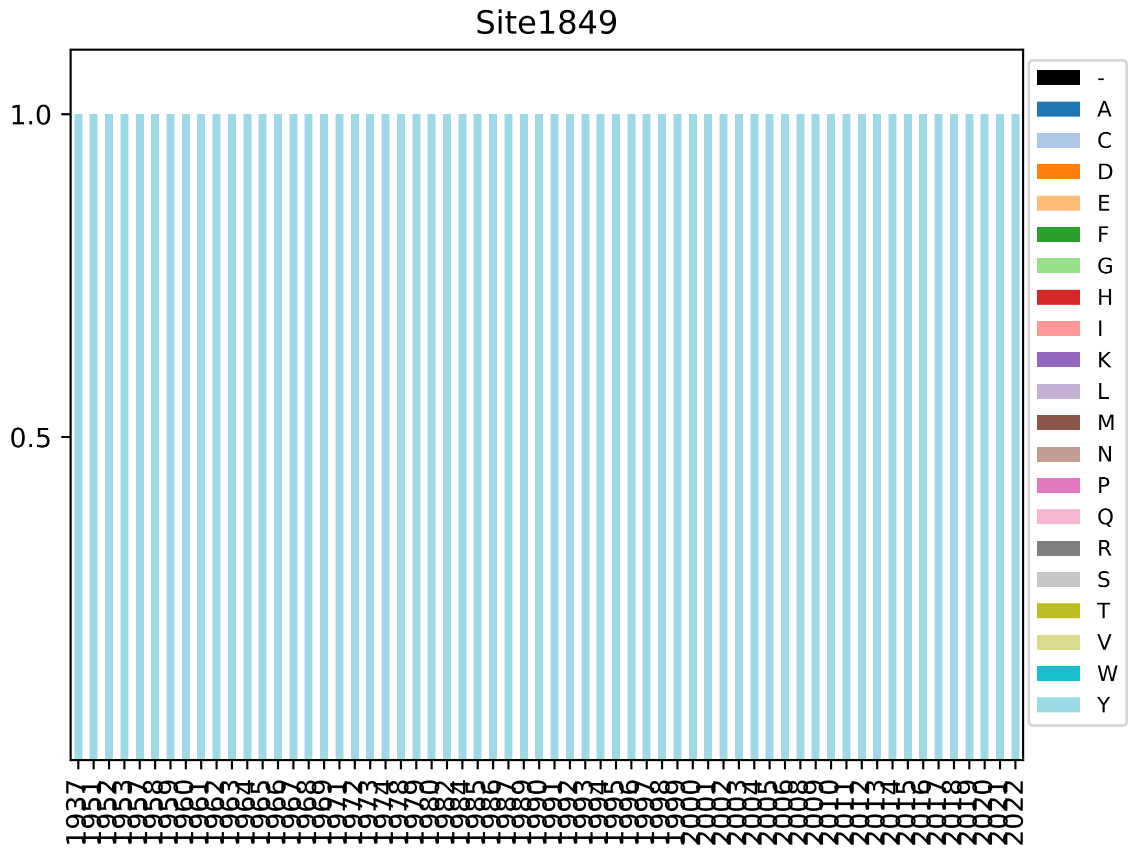
Site1849
1.0
0.5
-
A
C
D
E
F
G
H
I
K
L
M
N
P
Q
R
S
T
V
W
Y

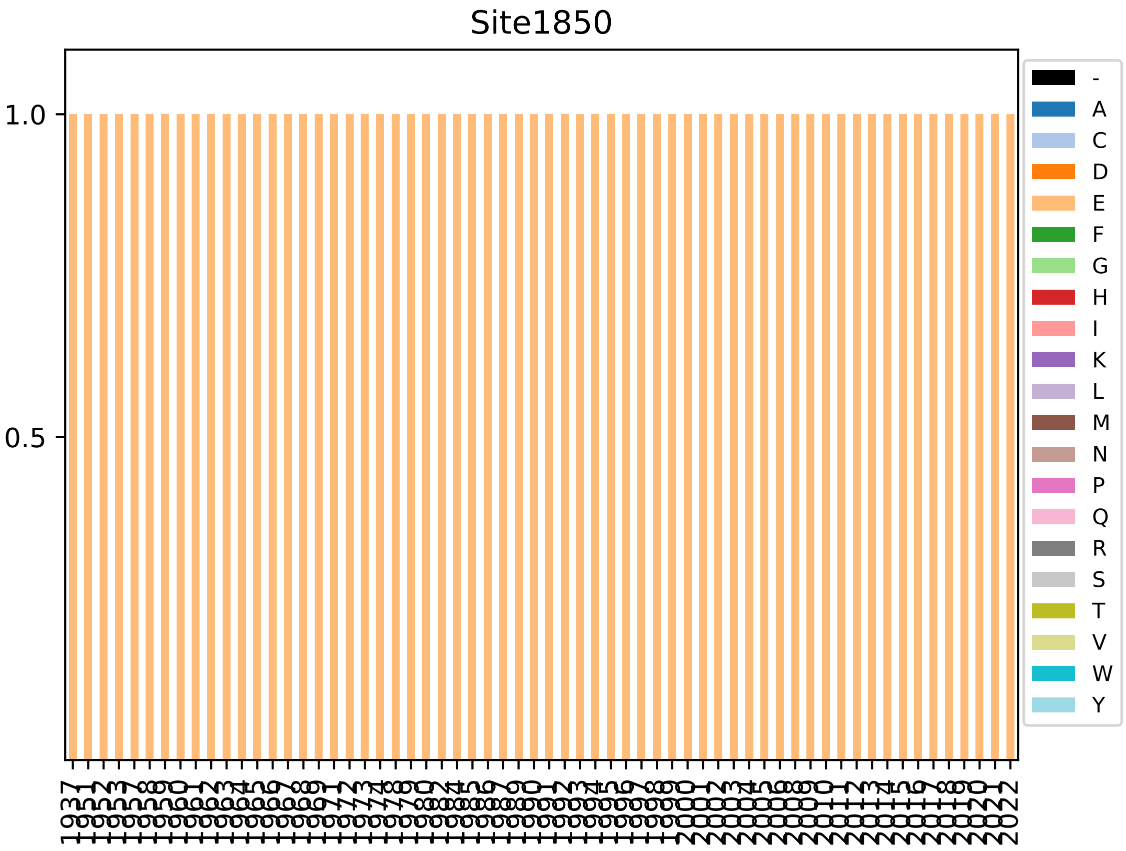
Site1850
1.0
0.5
-
A
C
D
E
F
G
H
I
K
L
M
N
P
Q
R
S
T
V
W
Y

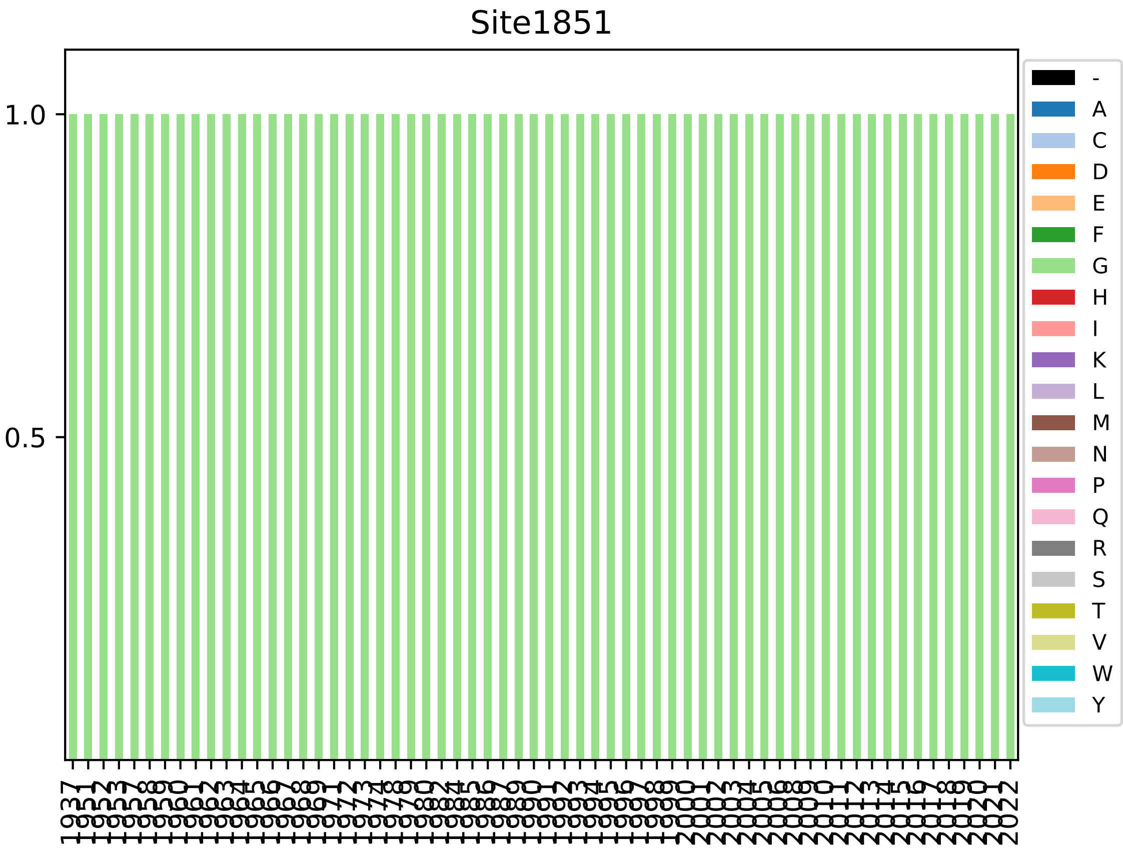
Site1851
1.0
0.5
-
A
C
D
E
F
G
H
I
K
L
M
N
P
Q
R
S
T
V
W
Y

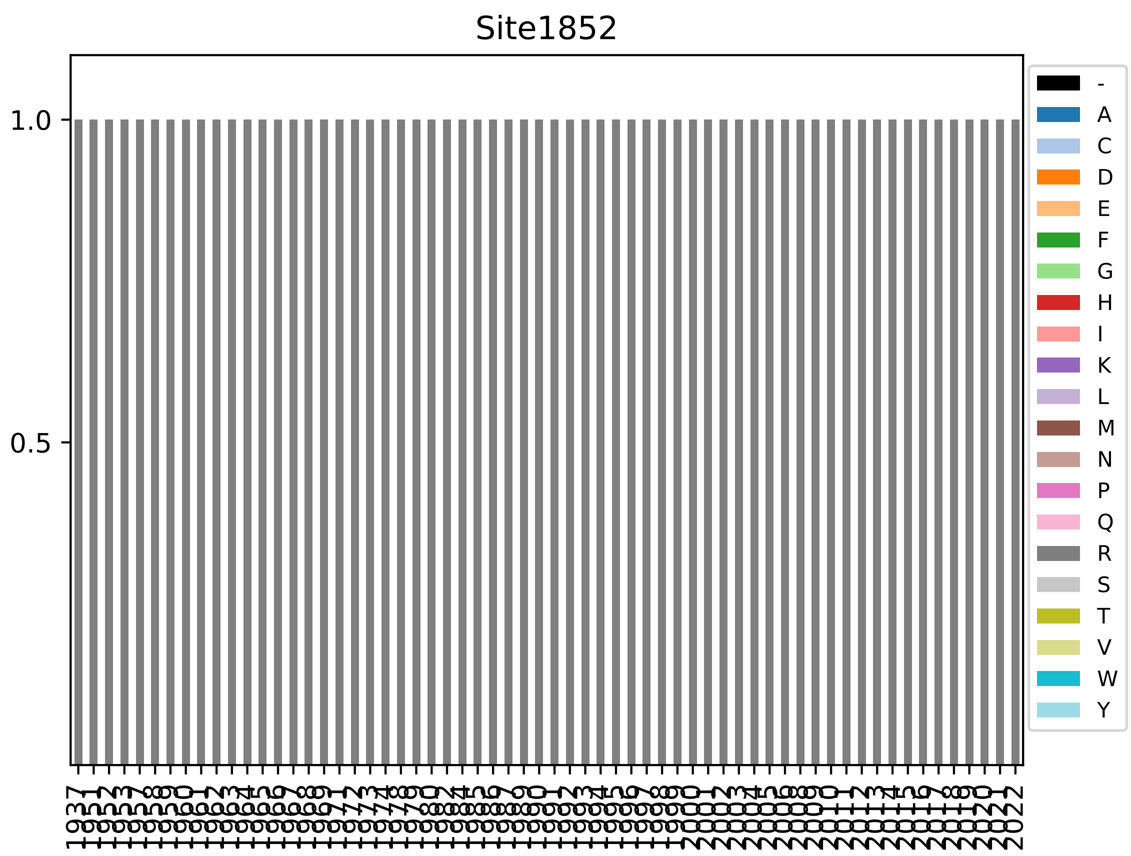

Site1852
1.0
0.5
-
A
C
D
E
F
G
H
I
K
L
M
N
P
Q
R
S
T
V
W
Y

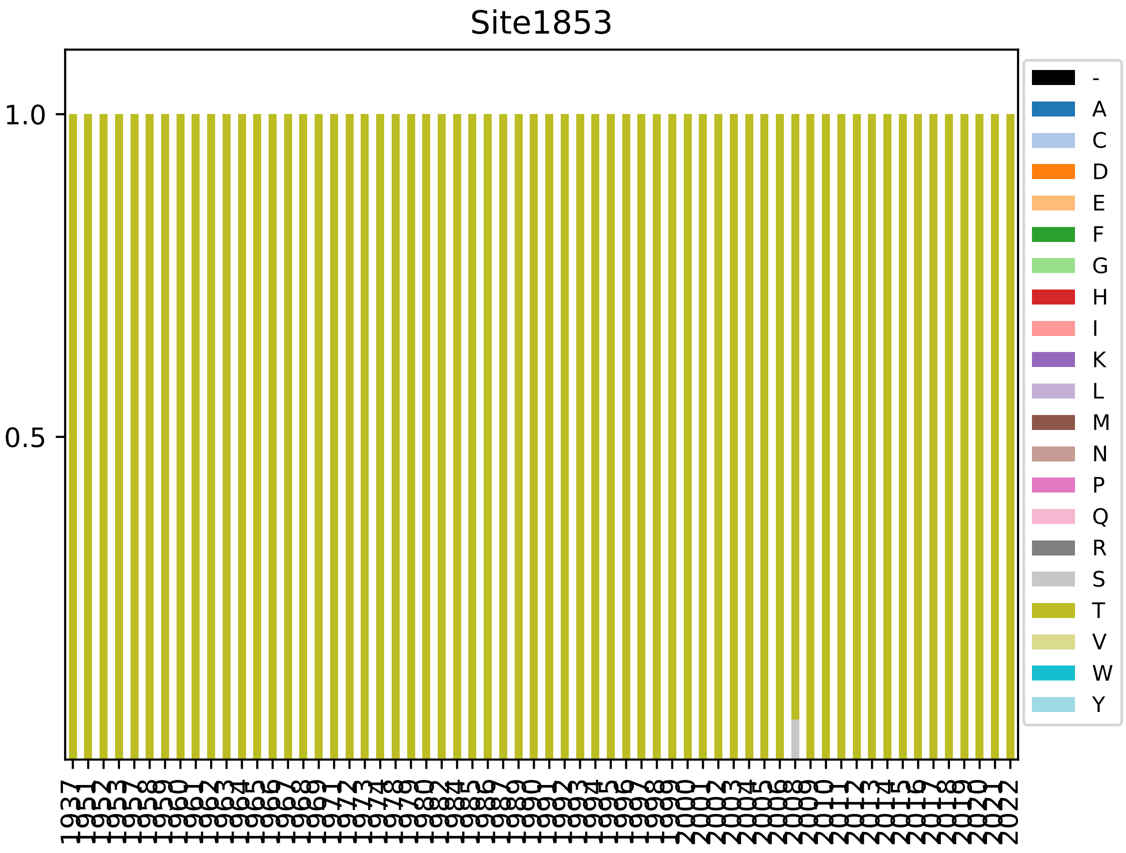

Site1853
1.0
0.5
-
A
C
D
E
F
G
H
I
K
L
M
N
P
Q
R
S
T
V
W
Y

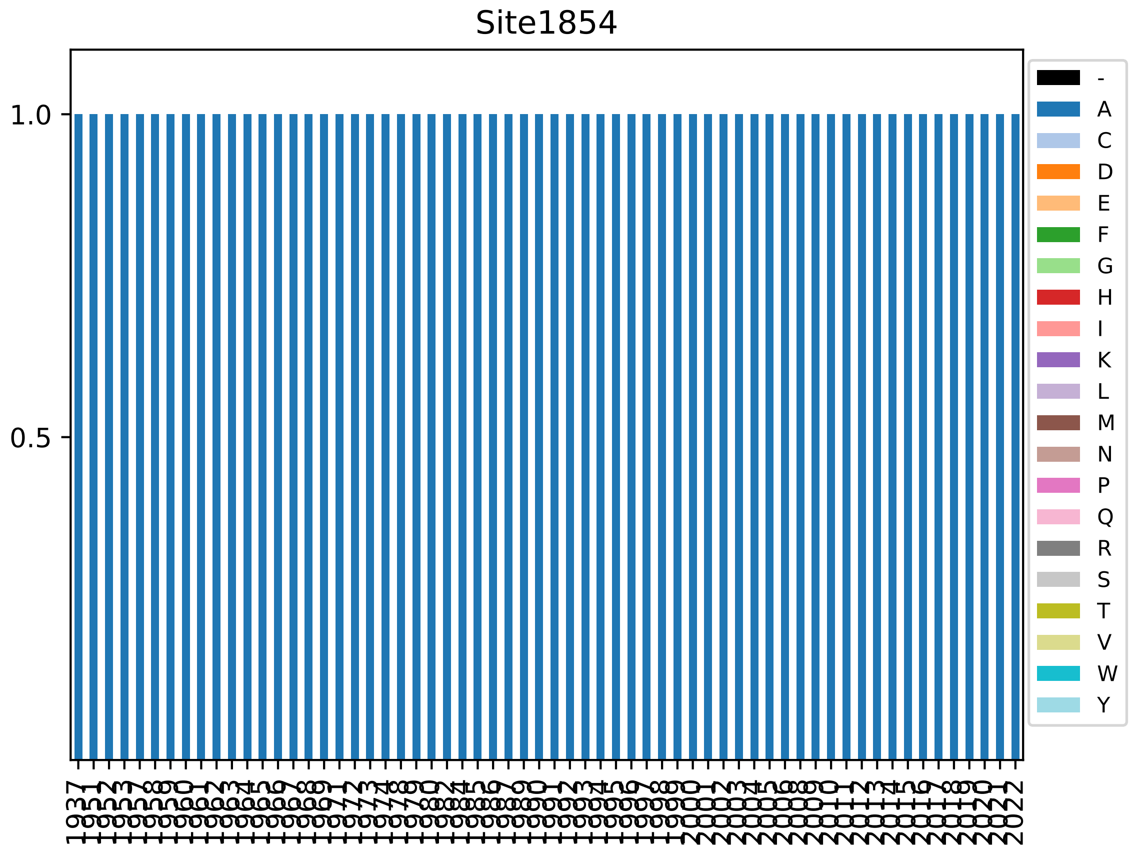
Site1854
1.0
0.5
-
A
C
D
E
F
G
H
I
K
L
M
N
P
Q
R
S
T
V
W
Y

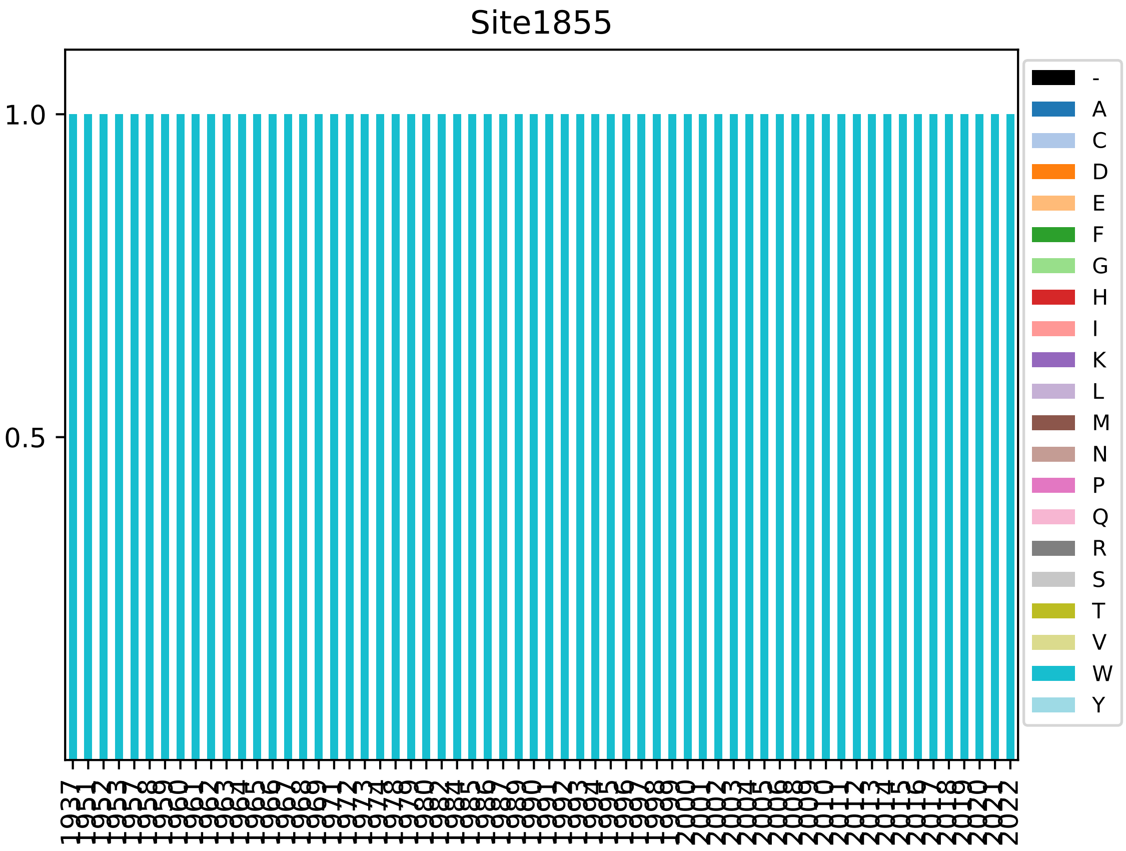
Site1855
1.0
0.5
-
A
C
D
E
F
G
H
I
K
L
M
N
P
Q
R
S
T
V
W
Y

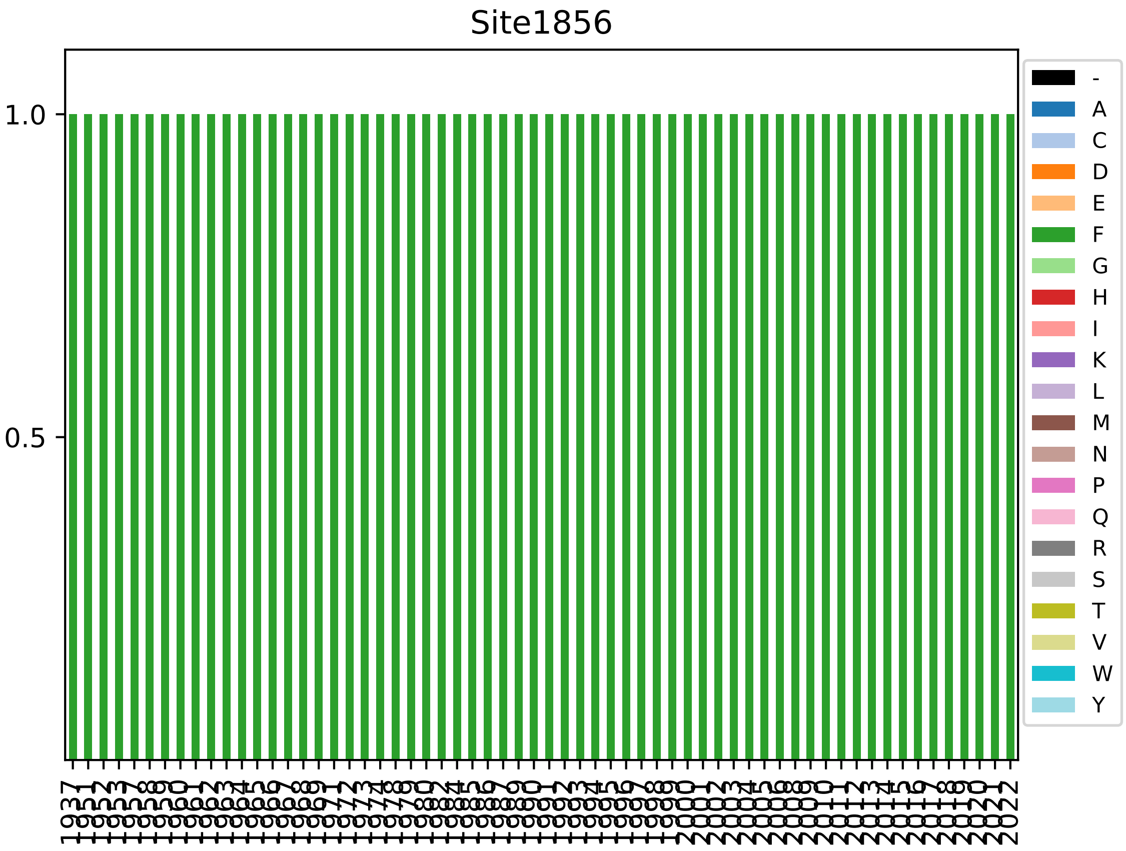
Site1856
1.0
0.5
-
A
C
D
E
F
G
H
I
K
L
M
N
P
Q
R
S
T
V
W
Y

Site1857

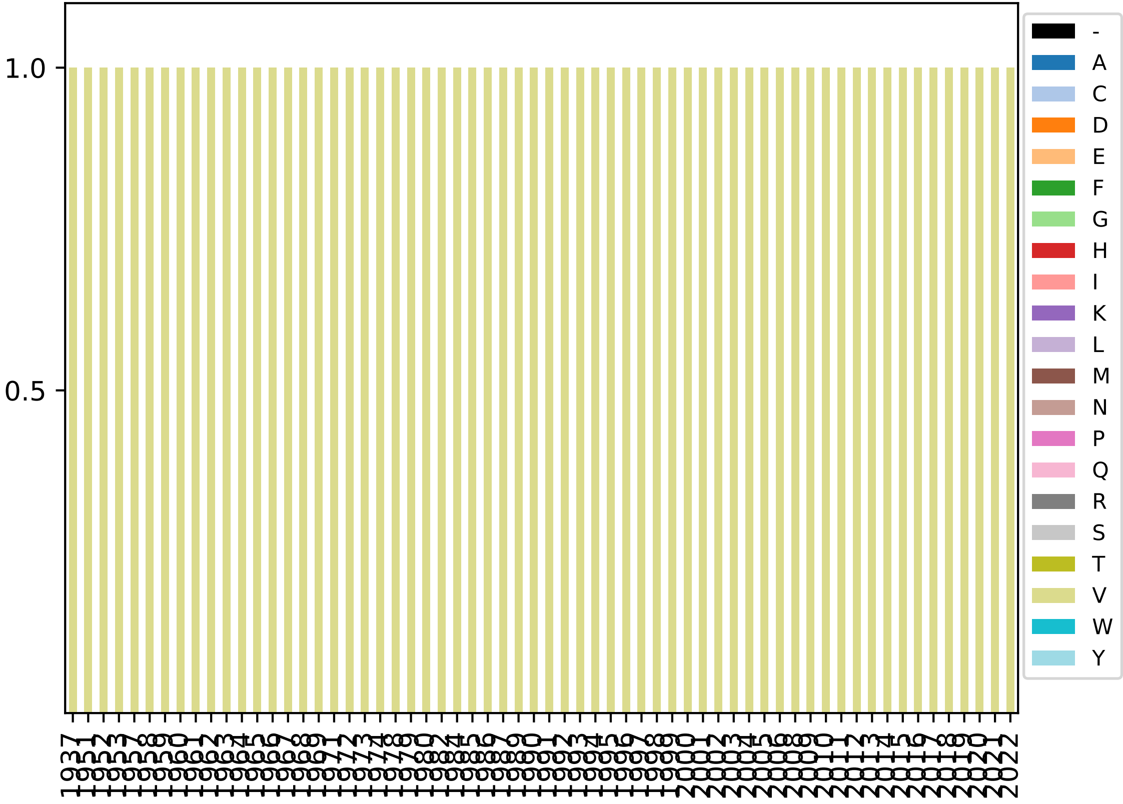

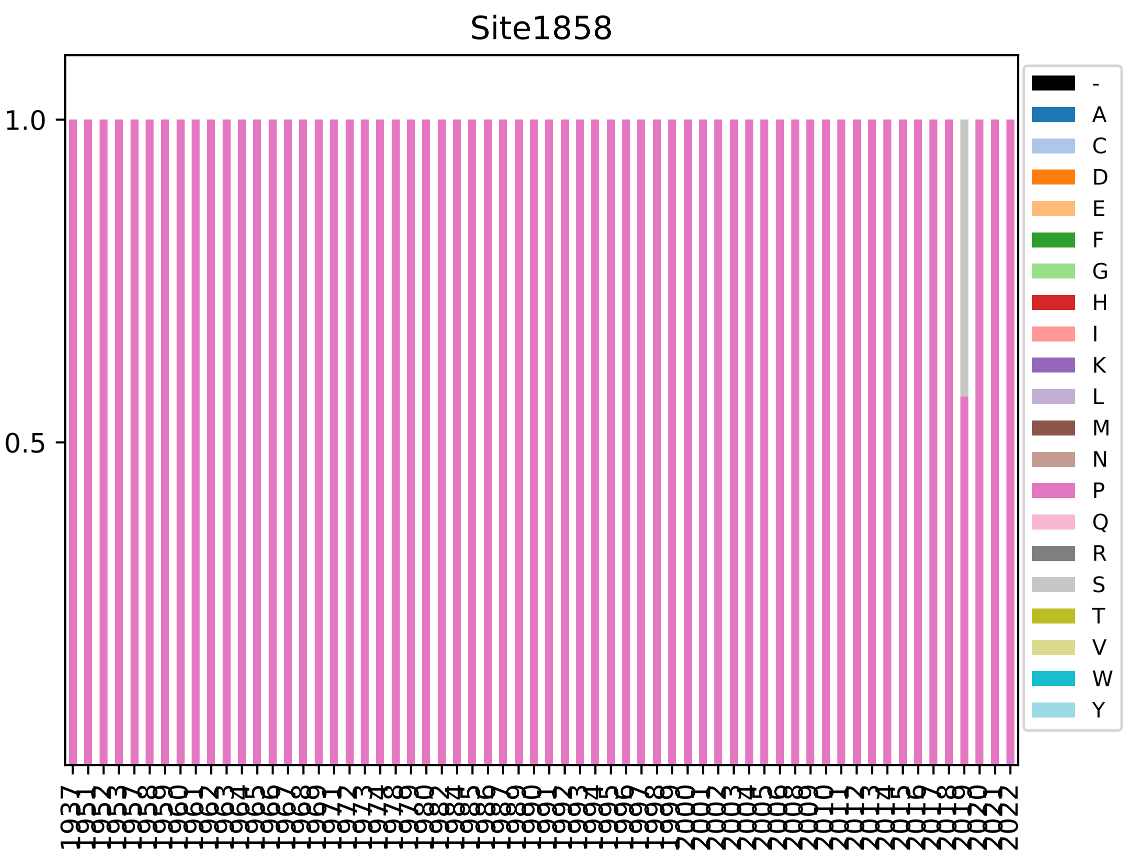
Site1858
1.0
0.5
-
A
C
D
E
F
G
H
I
K
L
M
N
P
Q
R
S
T
V
W
Y

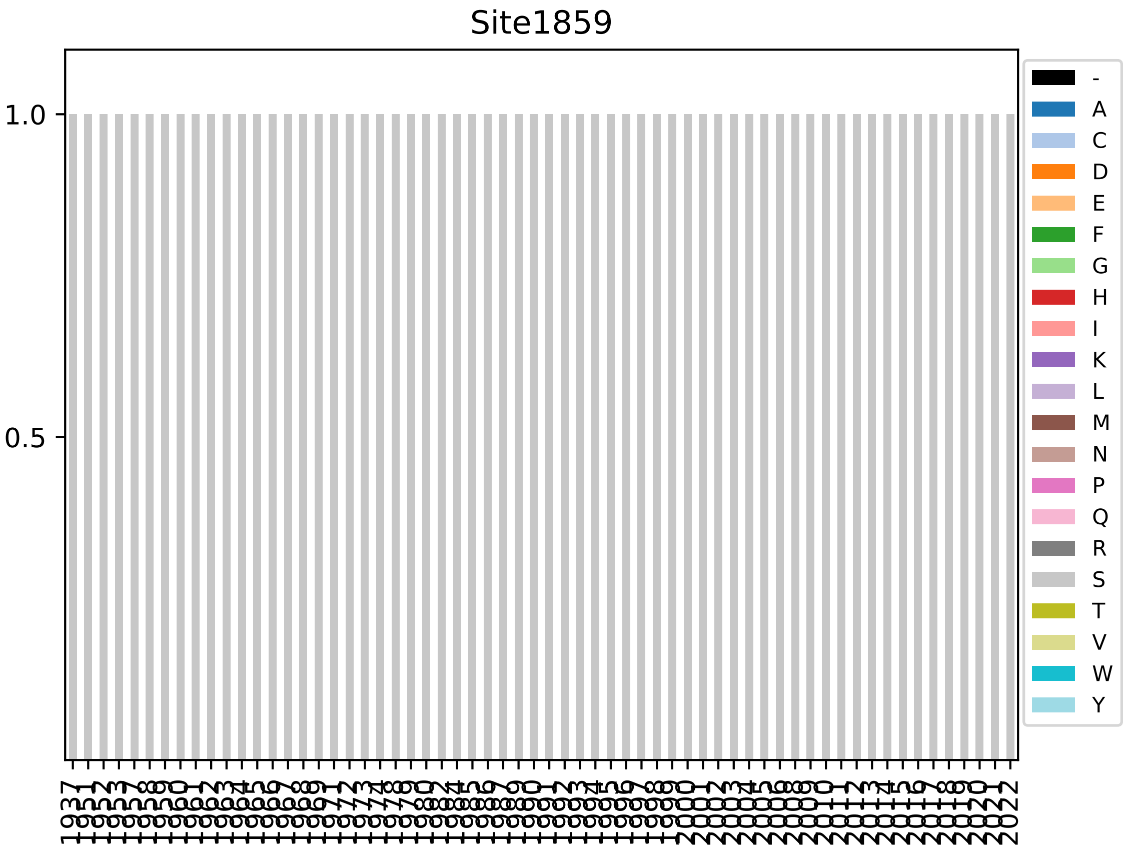
Site1859
1.0
0.5
-
A
C
D
E
F
G
H
I
K
L
M
N
P
Q
R
S
T
V
W
Y

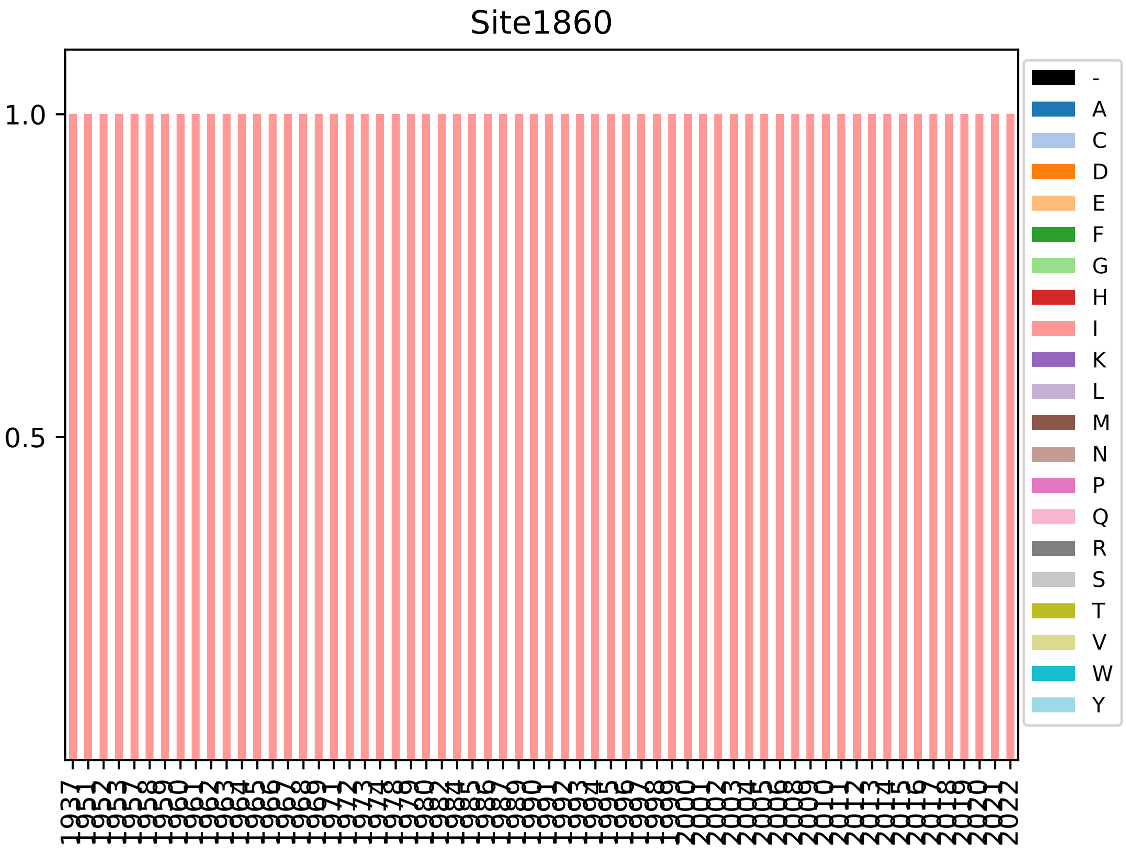
Site1860
1.0
0.5
-
A
C
D
E
F
G
H
I
K
L
M
N
P
Q
R
S
T
V
W
Y

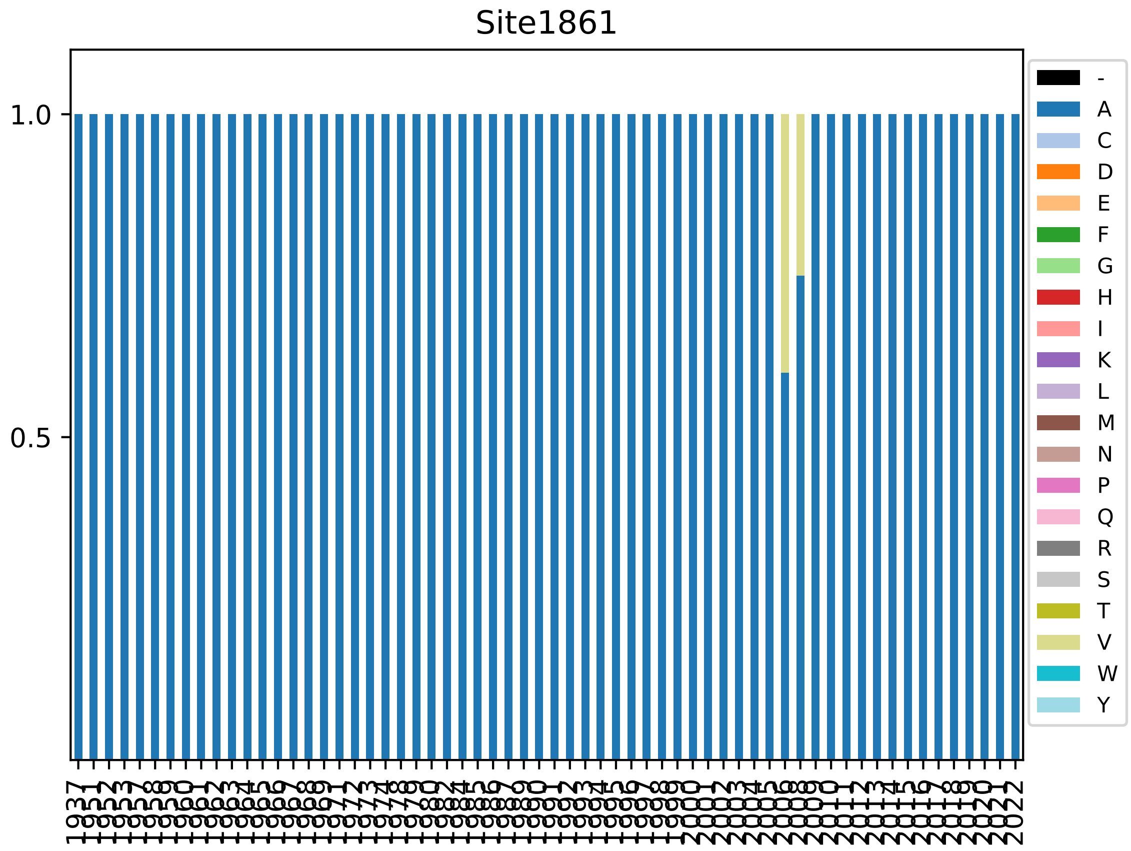
Site1861
1.0
0.5
-
A
C
D
E
F
G
H
I
K
L
M
N
P
Q
R
S
T
V
W
Y

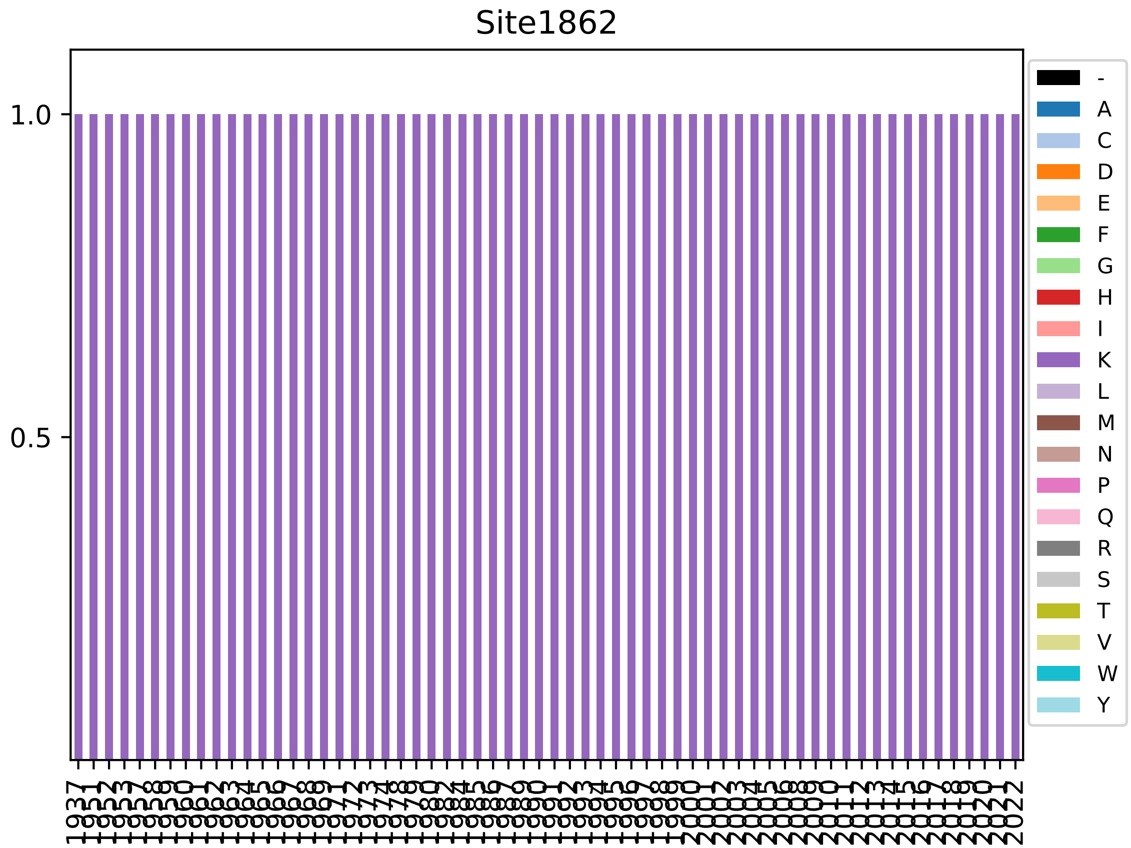
Site1862
1.0
0.5
-
A
C
D
E
F
G
H
I
K
L
M
N
P
Q
R
S
T
V
W
Y

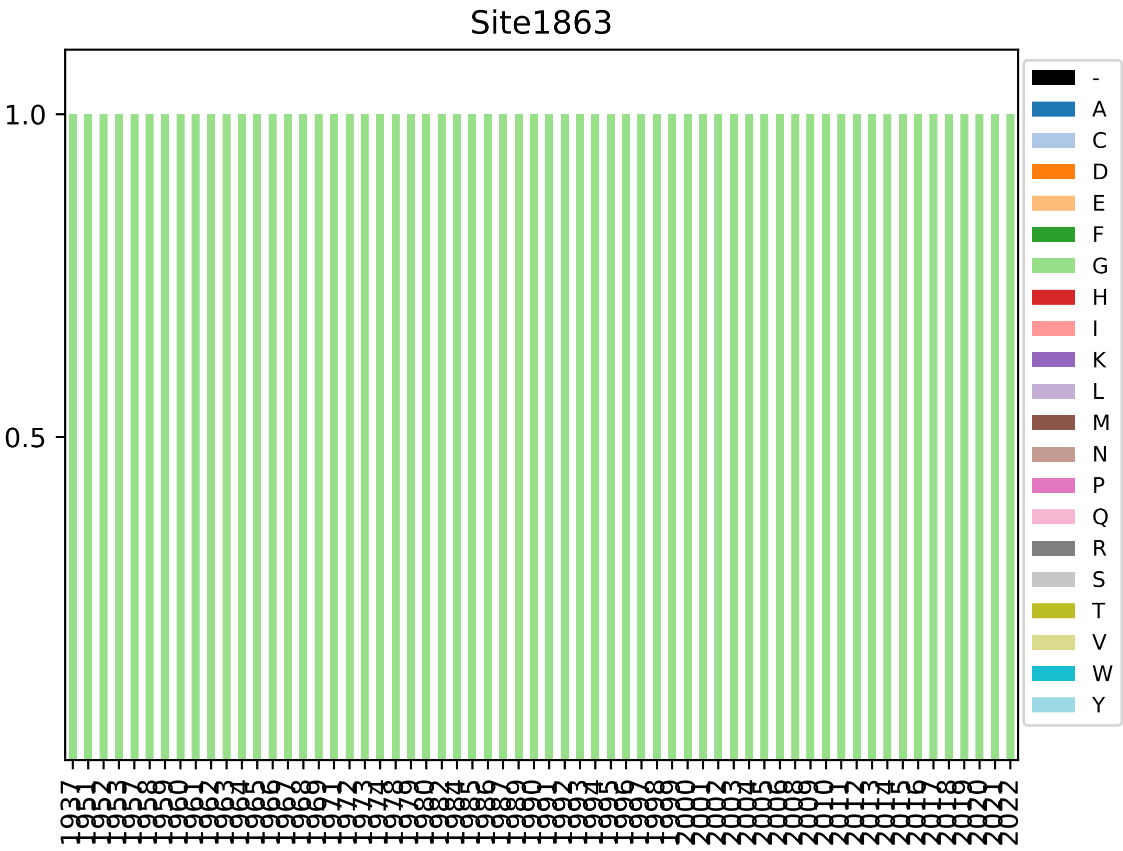
Site1863
1.0
0.5
-
A
C
D
E
F
G
H
I
K
L
M
N
P
Q
R
S
T
V
W
Y

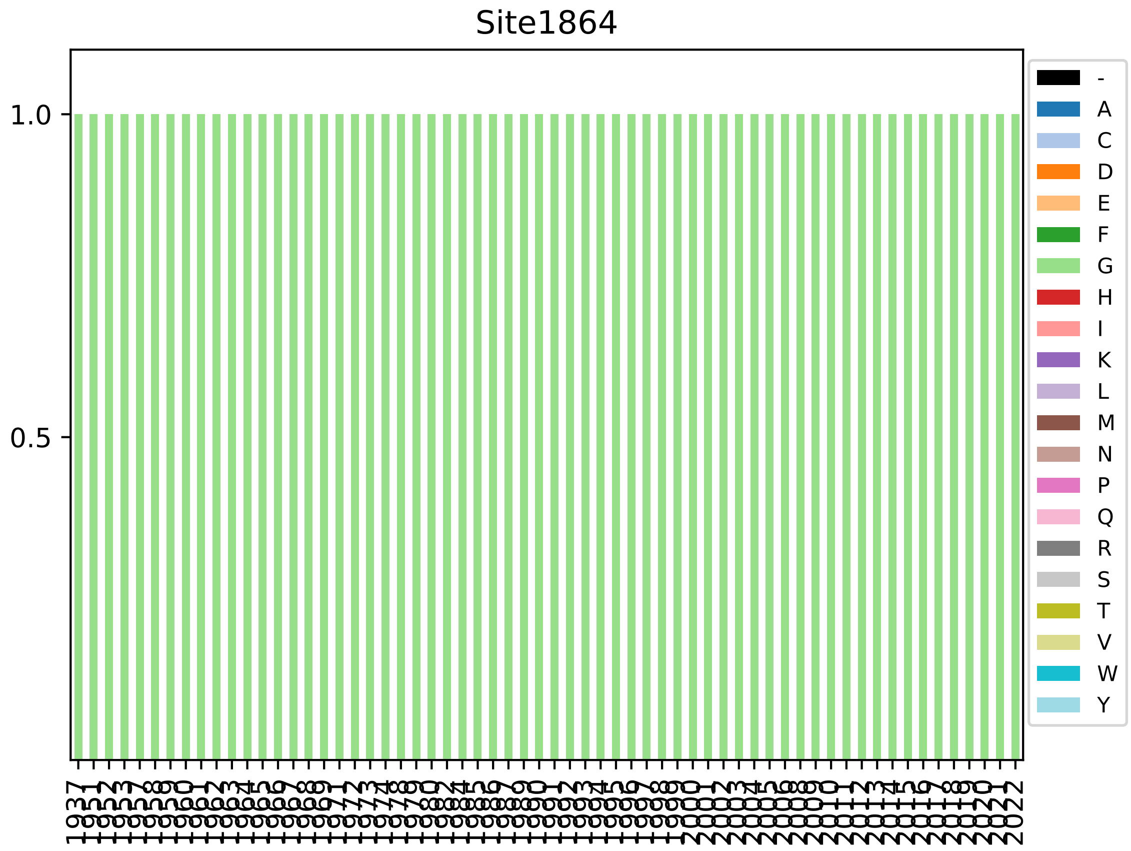
Site1864
1.0
0.5
-
A
C
D
E
F
G
H
I
K
L
M
N
P
Q
R
S
T
V
W
Y

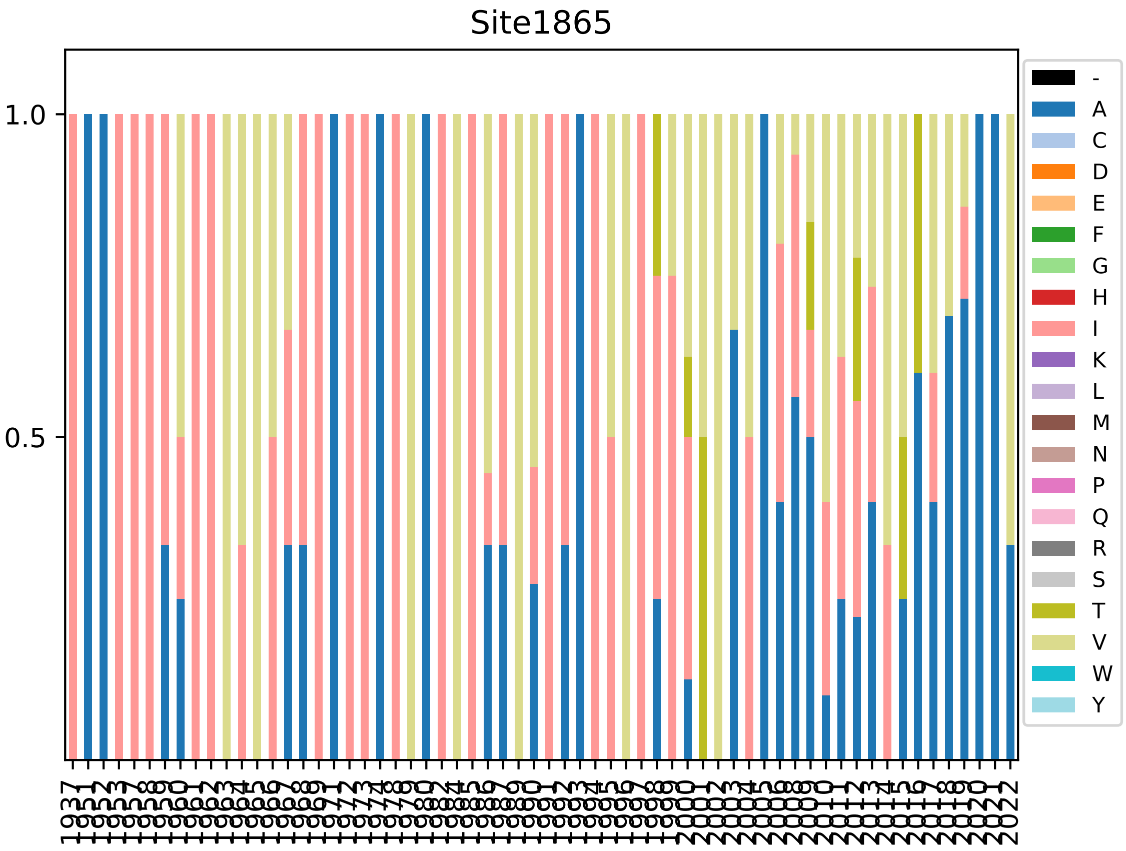

Site1865
1.0
0.5
-
A
C
D
E
F
G
H
I
K
L
M
N
P
Q
R
S
T
V
W
Y

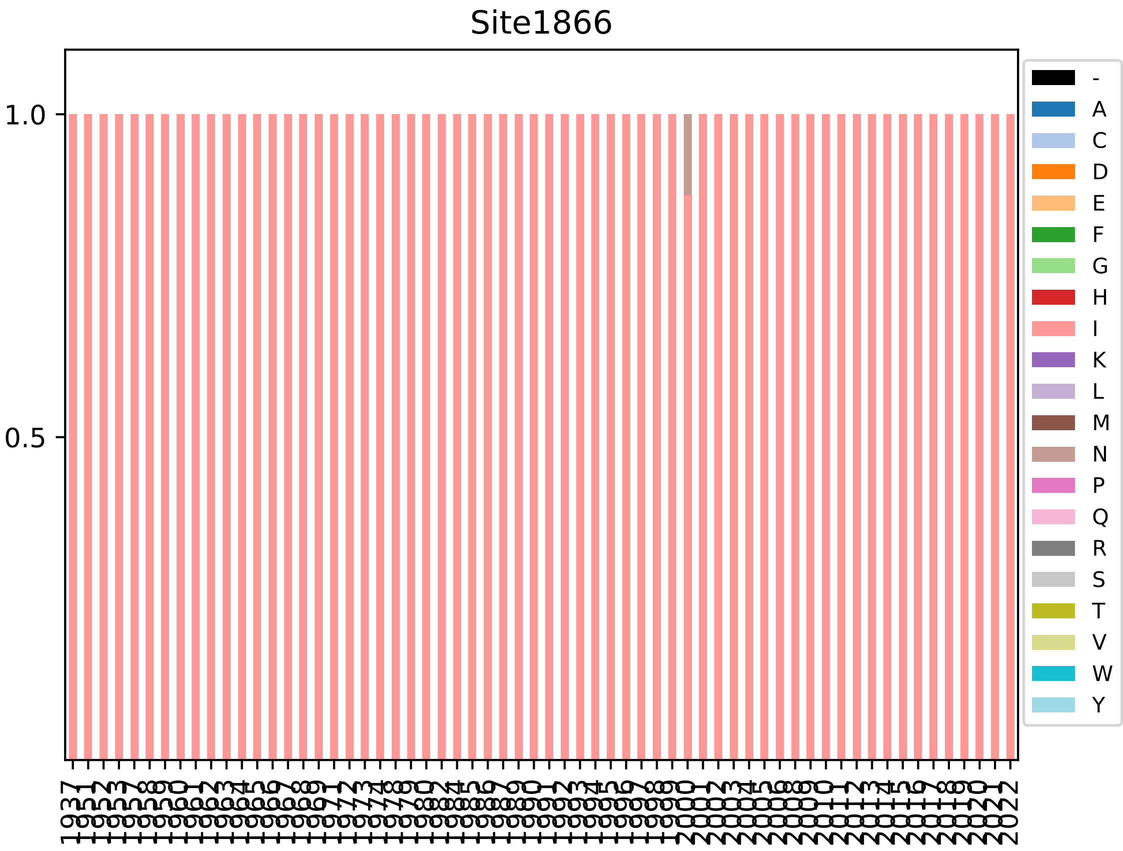
Site1866
1.0
0.5
-
A
C
D
E
F
G
H
I
K
L
M
N
P
Q
R
S
T
V
W
Y

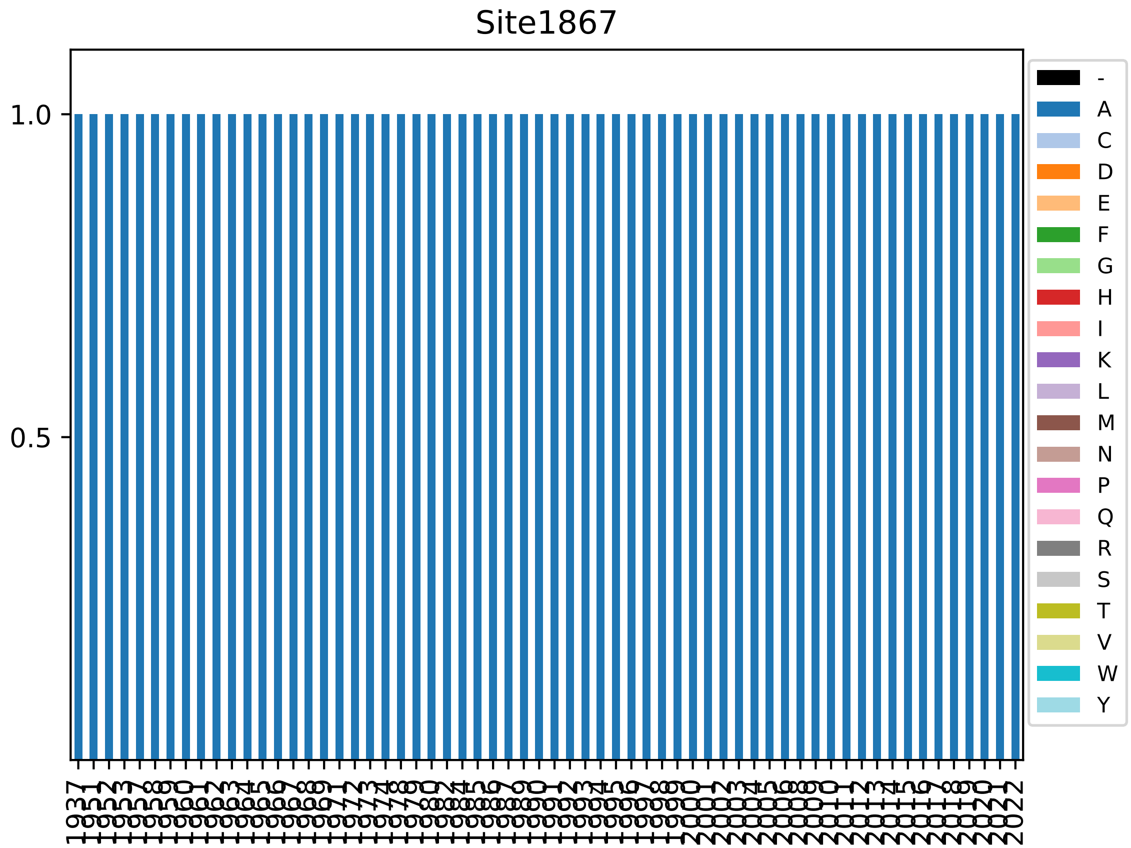
Site1867
1.0
0.5
-
A
C
D
E
F
G
H
I
K
L
M
N
P
Q
R
S
T
V
W
Y

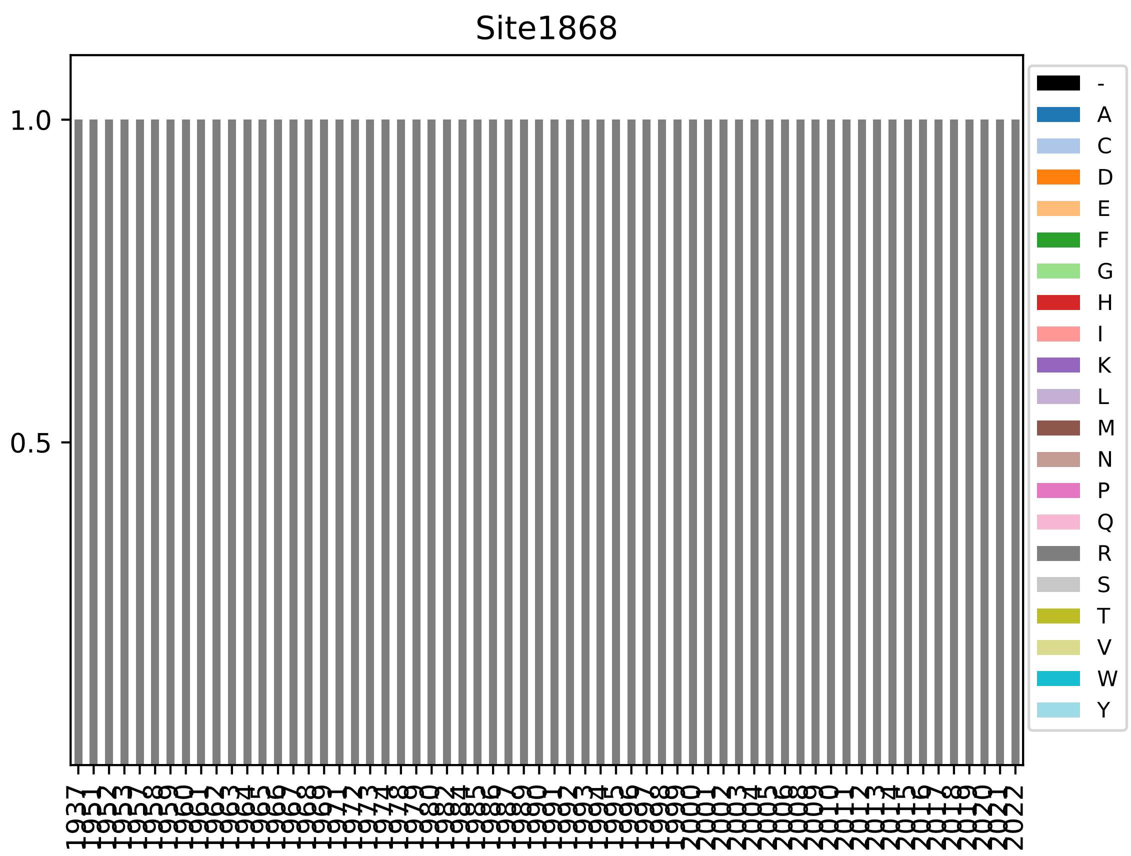
Site1868
1.0
0.5
-
A
C
D
E
F
G
H
I
K
L
M
N
P
Q
R
S
T
V
W
Y

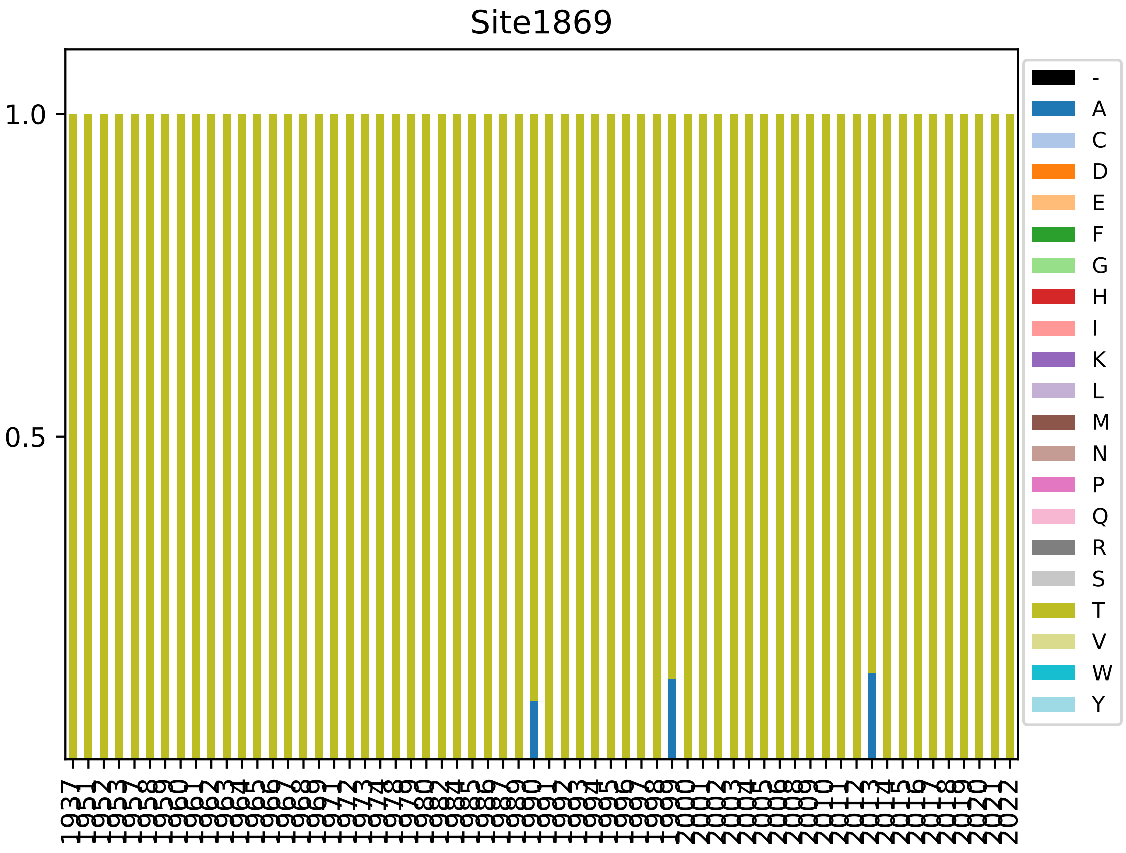
Site1869
1.0
0.5
-
A
C
D
E
F
G
H
I
K
L
M
N
P
Q
R
S
T
V
W
Y

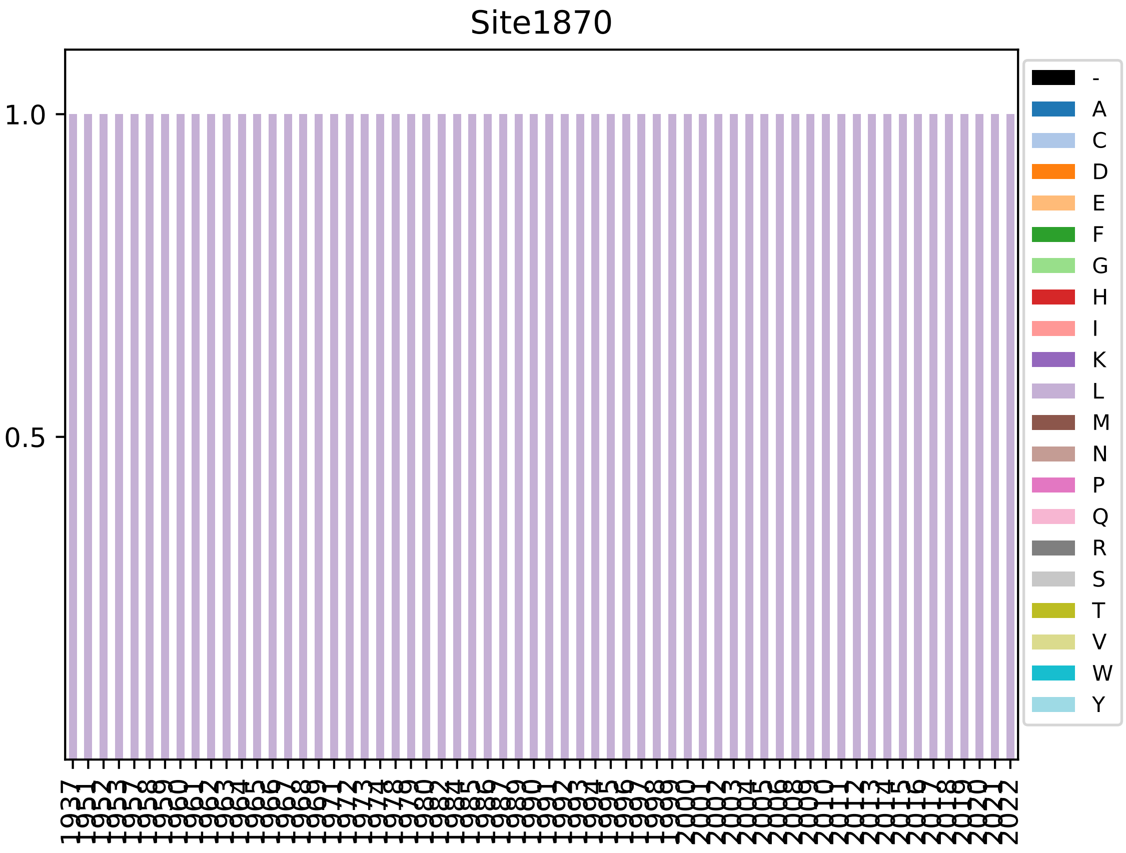
Site1870
1.0
0.5
-
A
C
D
E
F
G
H
I
K
L
M
N
P
Q
R
S
T
V
W
Y

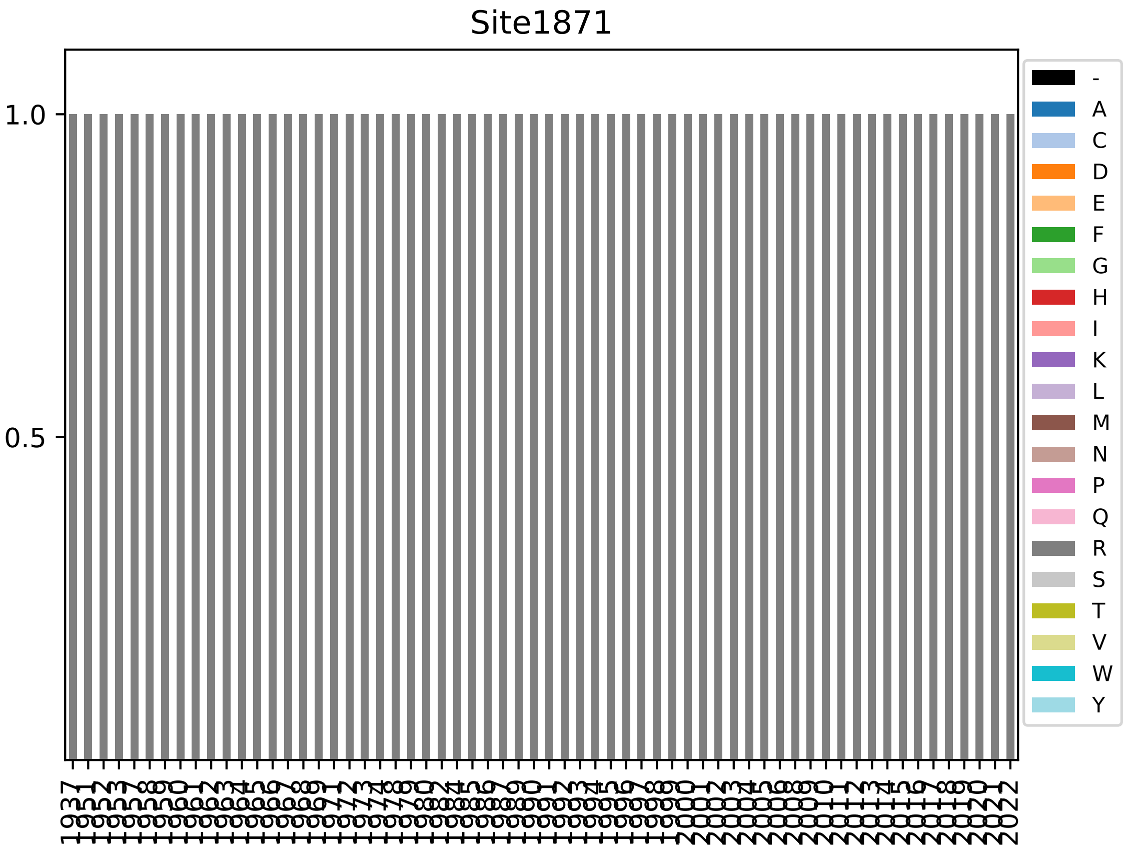

Site1871
1.0
0.5
-
A
C
D
E
F
G
H
I
K
L
M
N
P
Q
R
S
T
V
W
Y

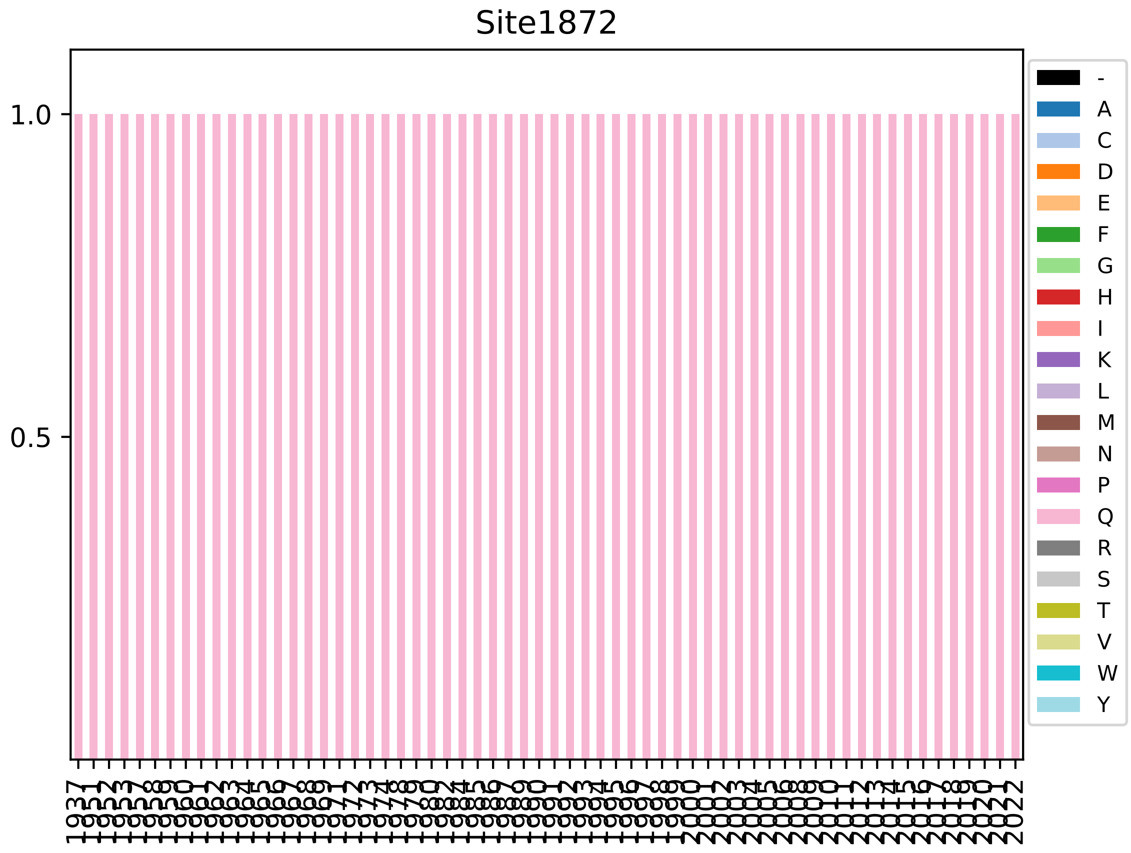
Site1872
1.0
0.5
-
A
C
D
E
F
G
H
I
K
L
M
N
P
Q
R
S
T
V
W
Y

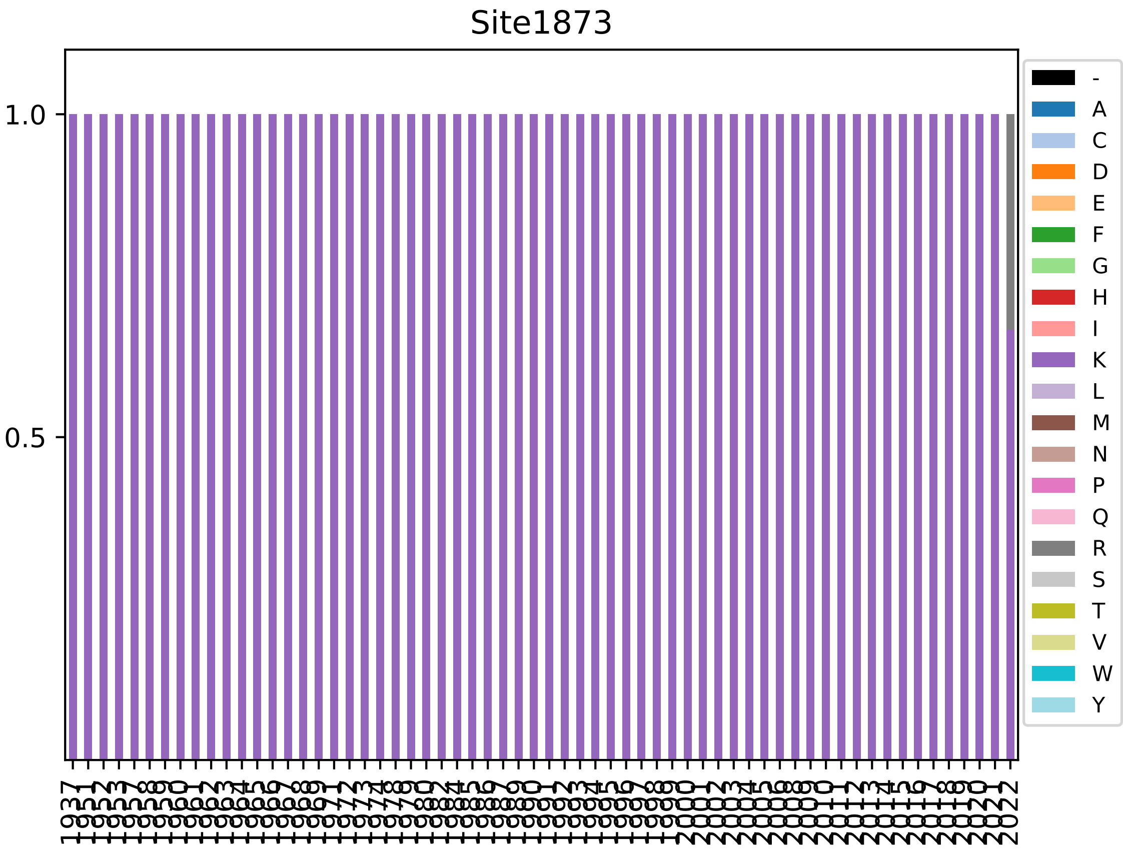

Site1873
1.0
0.5
-
A
C
D
E
F
G
H
I
K
L
M
N
P
Q
R
S
T
V
W
Y

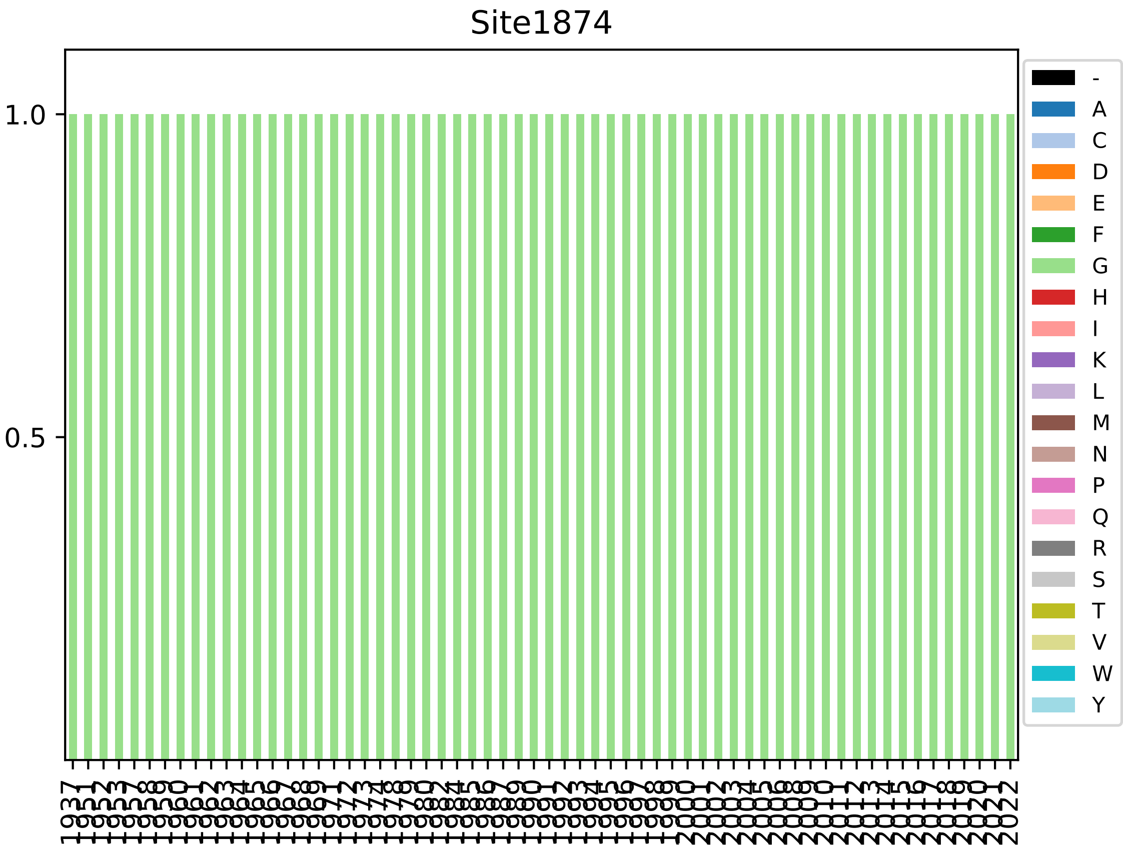
Site1874
1.0
0.5
-
A
C
D
E
F
G
H
I
K
L
M
N
P
Q
R
S
T
V
W
Y

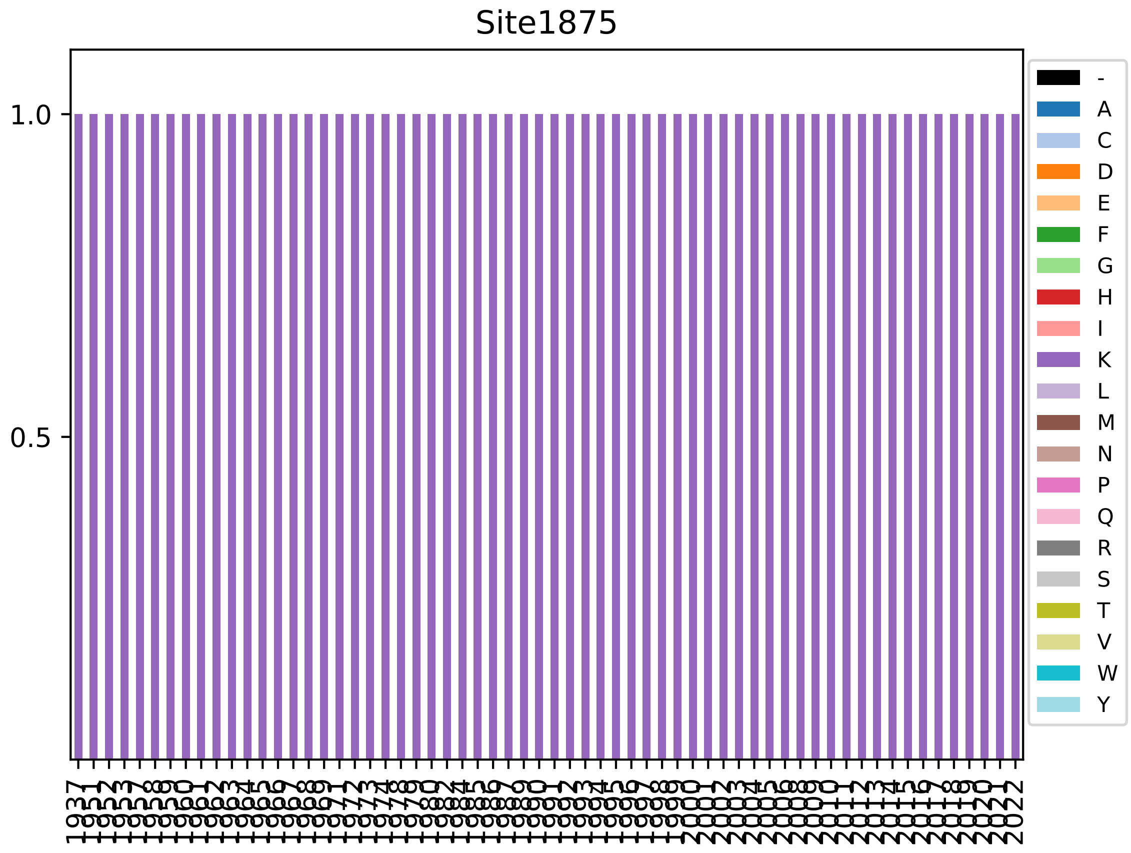
Site1875
1.0
0.5
-
A
C
D
E
F
G
H
I
K
L
M
N
P
Q
R
S
T
V
W
Y

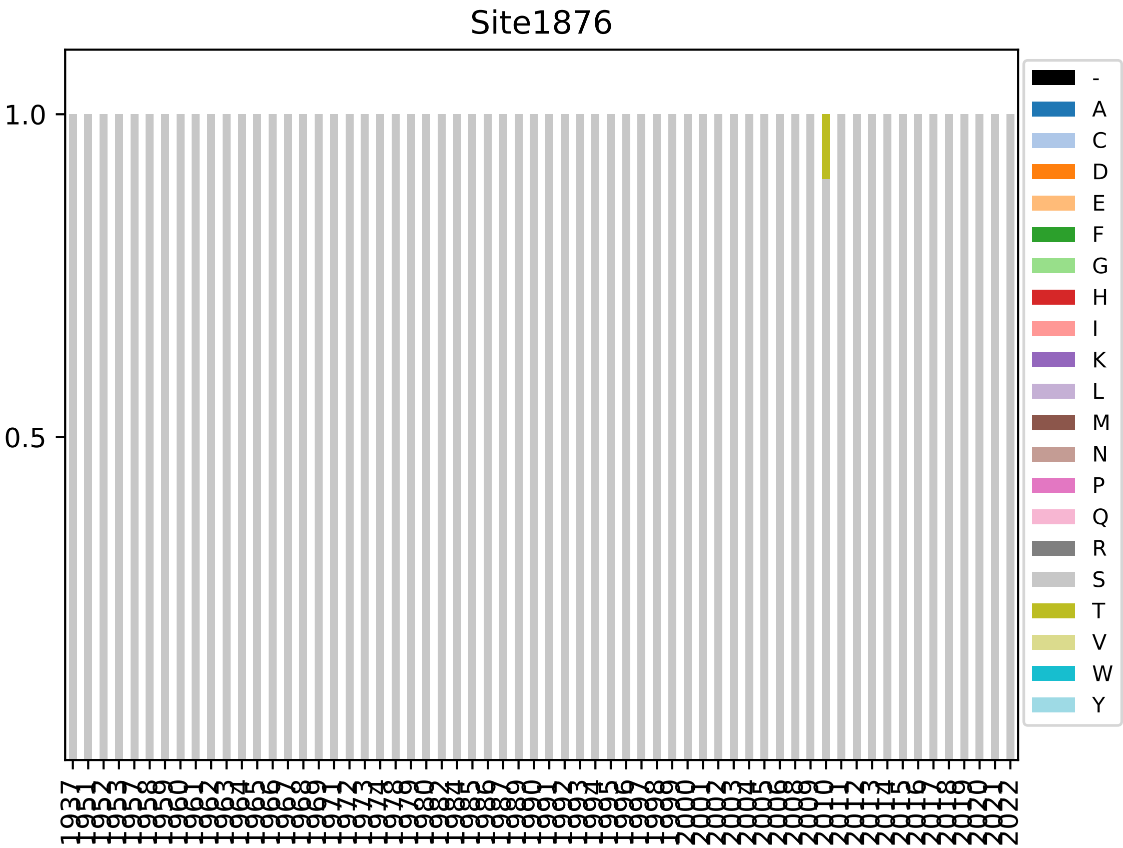
Site1876
1.0
0.5
-
A
C
D
E
F
G
H
I
K
L
M
N
P
Q
R
S
T
V
W
Y

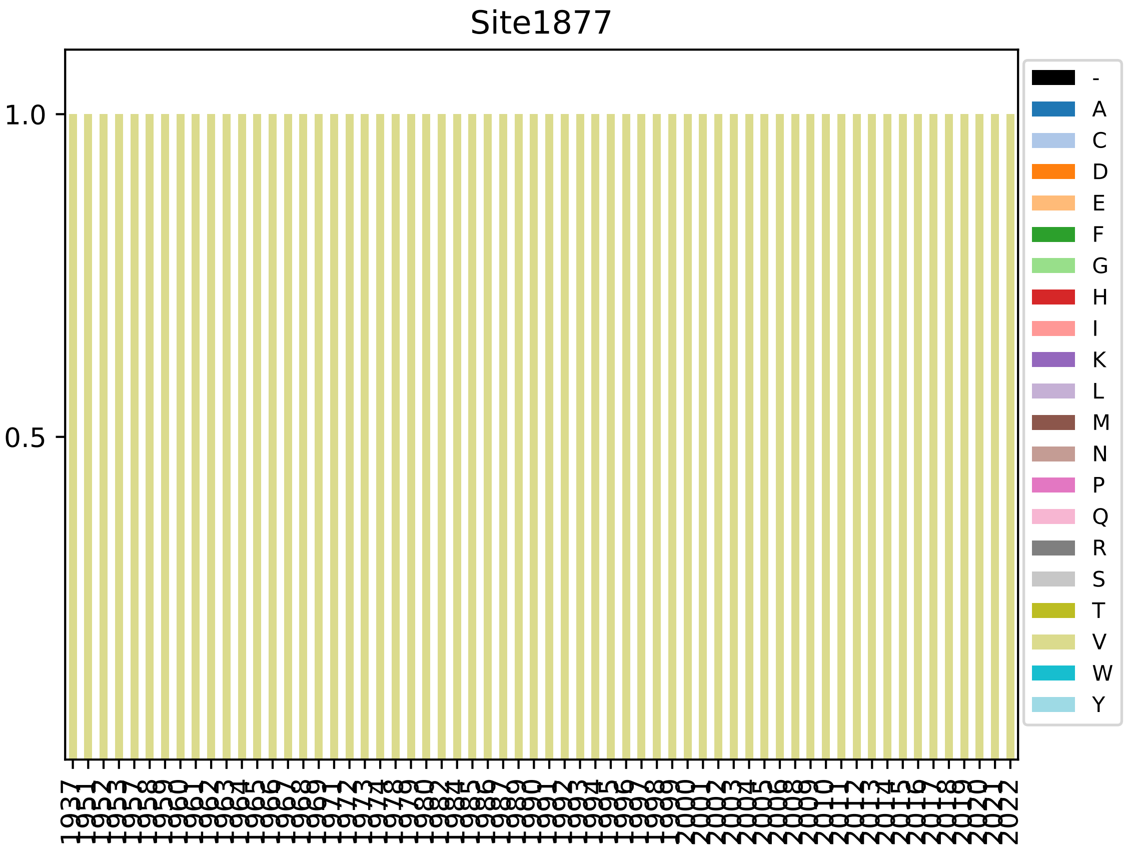
Site1877
1.0
0.5
-
A
C
D
E
F
G
H
I
K
L
M
N
P
Q
R
S
T
V
W
Y

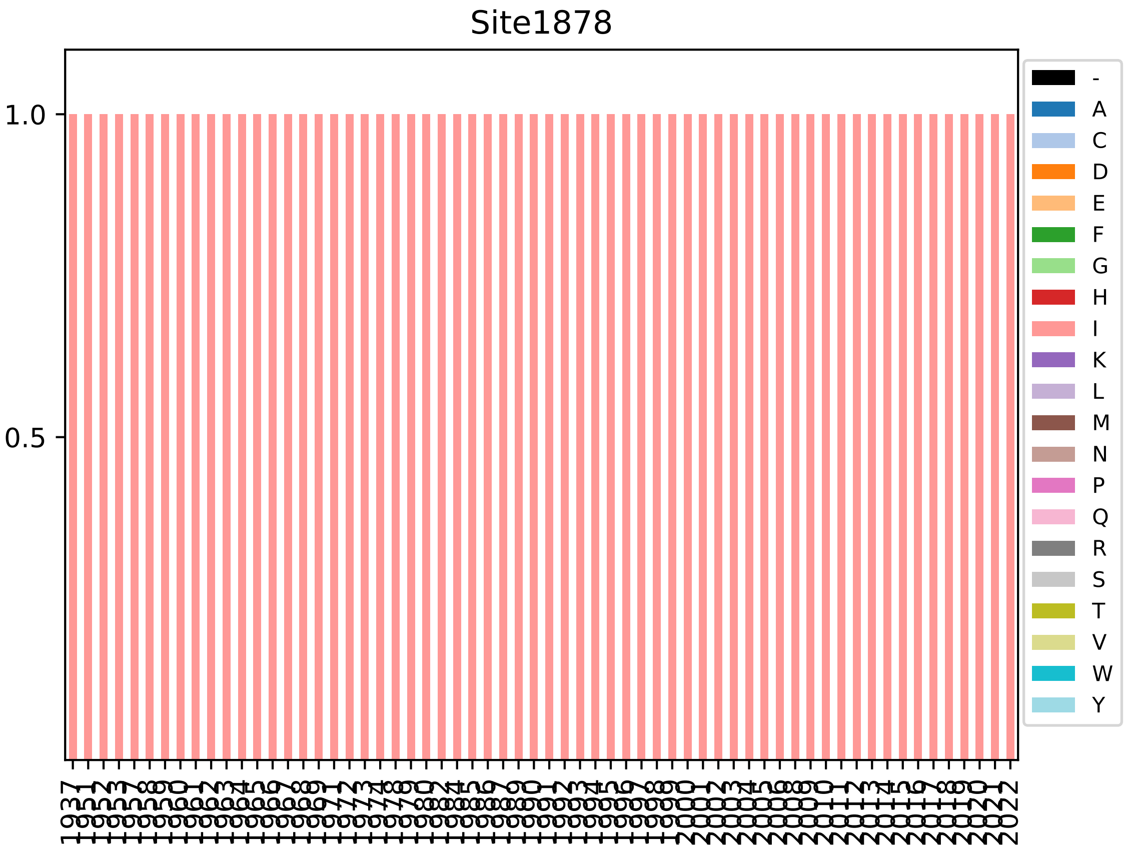
Site1878
1.0
0.5
-
A
C
D
E
F
G
H
I
K
L
M
N
P
Q
R
S
T
V
W
Y

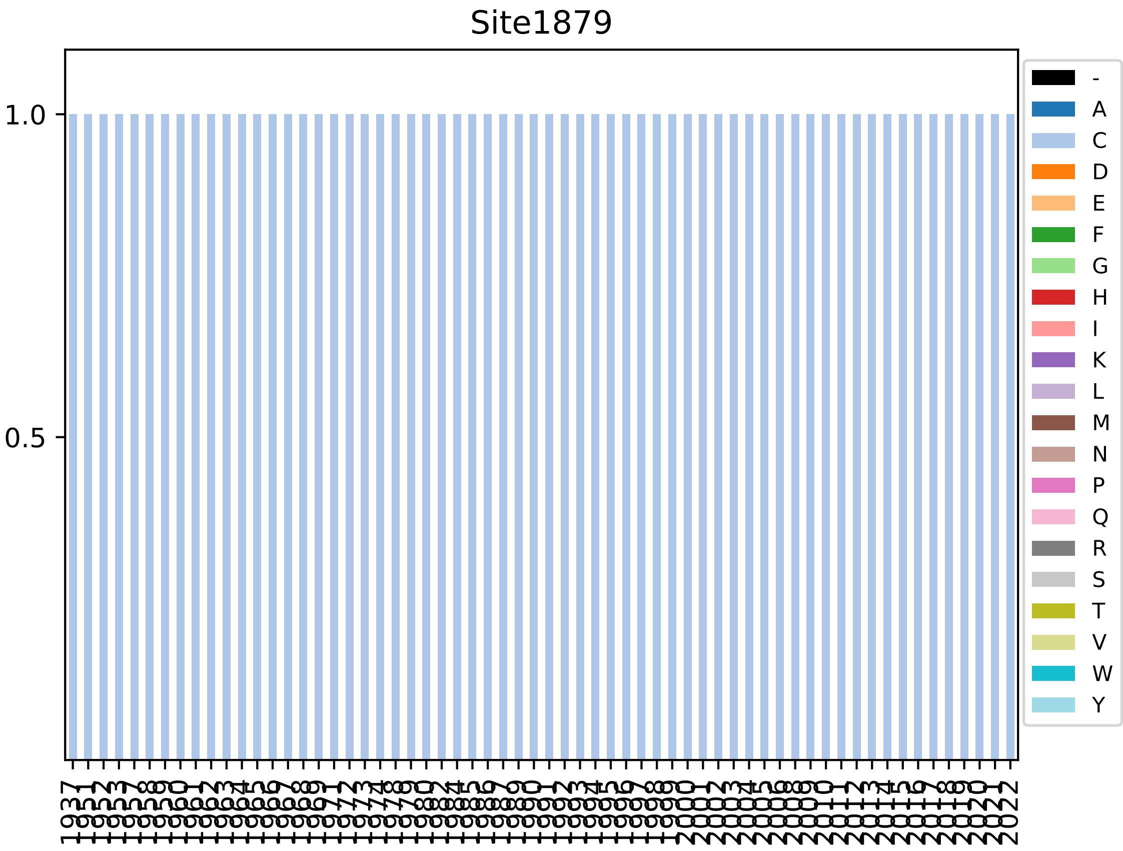
Site1879
1.0
0.5
-
A
C
D
E
F
G
H
I
K
L
M
N
P
Q
R
S
T
V
W
Y

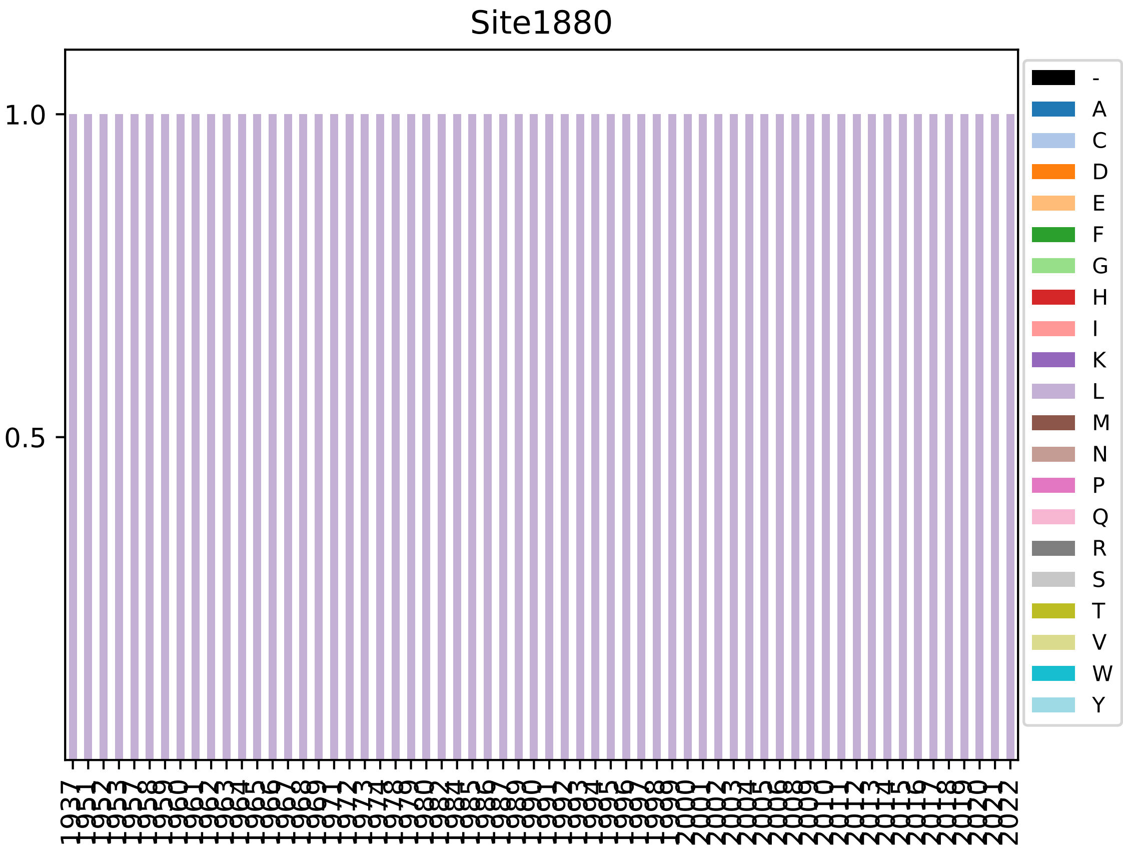
Site1880
1.0
0.5
-
A
C
D
E
F
G
H
I
K
L
M
N
P
Q
R
S
T
V
W
Y

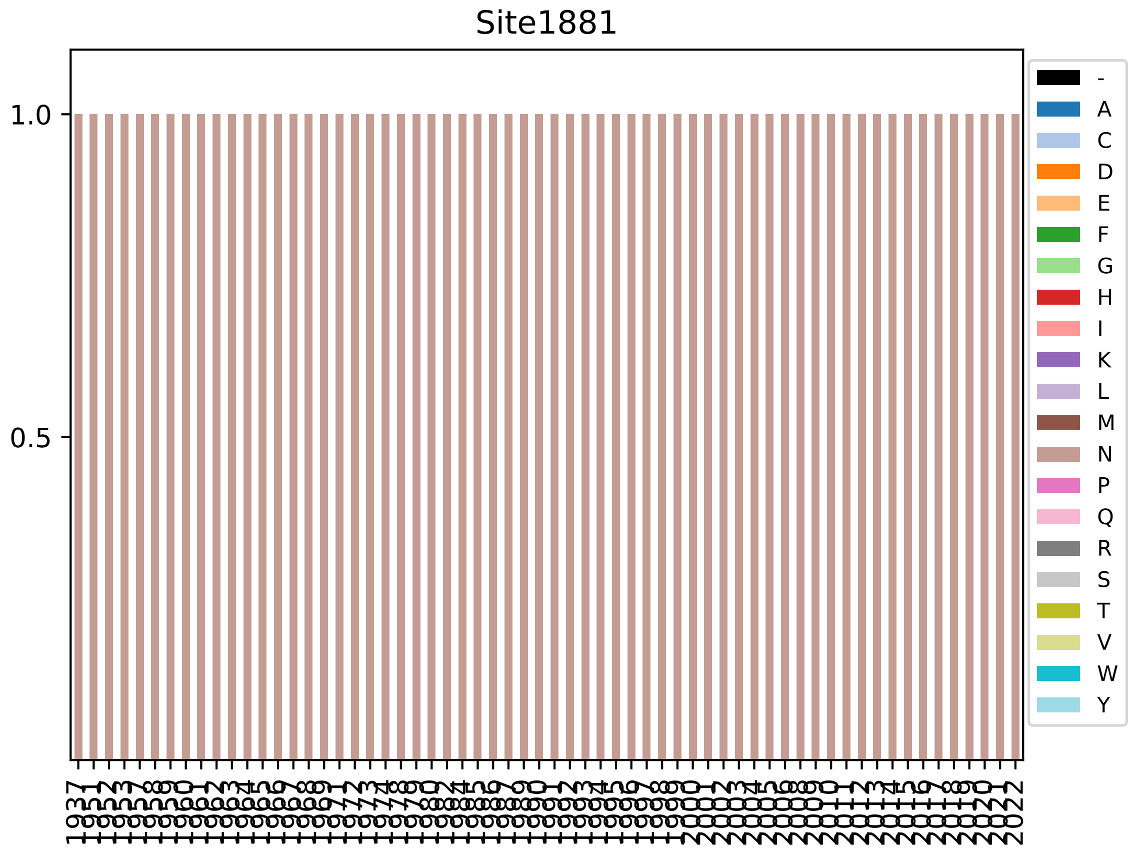
Site1881
1.0
0.5
-
A
C
D
E
F
G
H
I
K
L
M
N
P
Q
R
S
T
V
W
Y

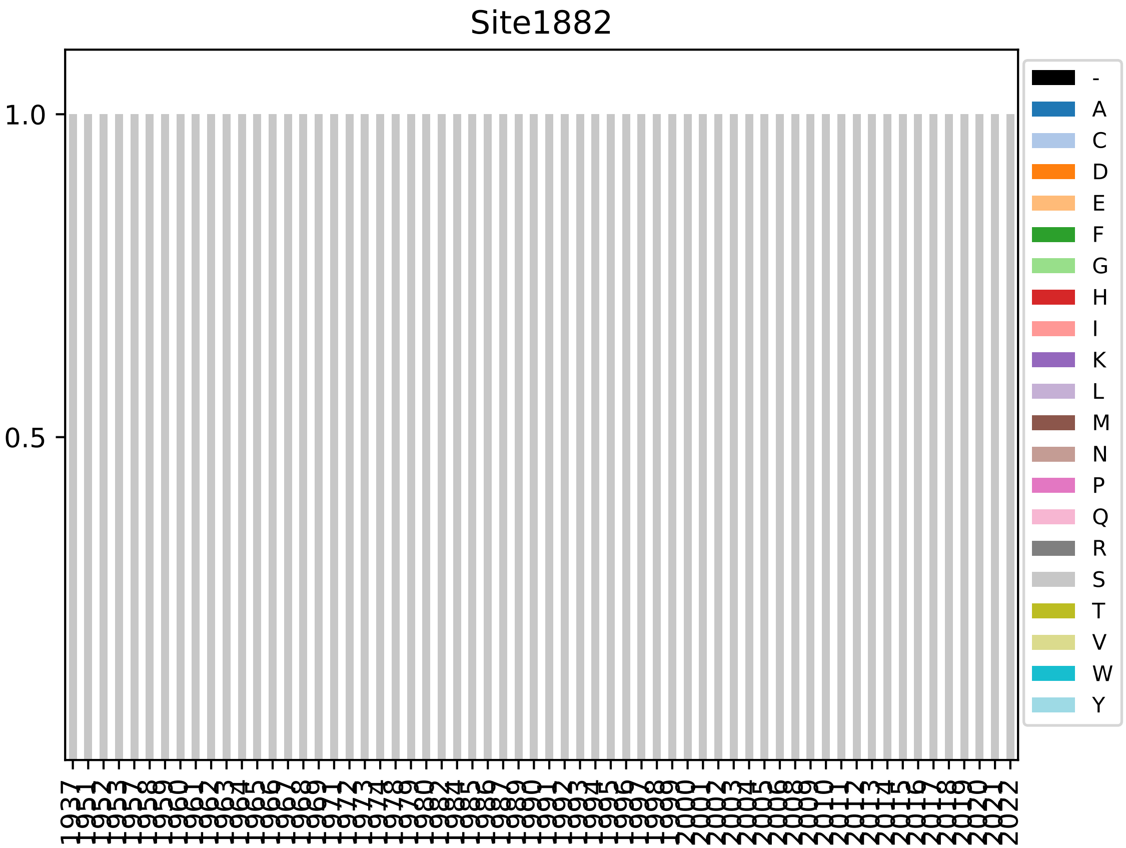
Site1882
1.0
0.5
-
A
C
D
E
F
G
H
I
K
L
M
N
P
Q
R
S
T
V
W
Y

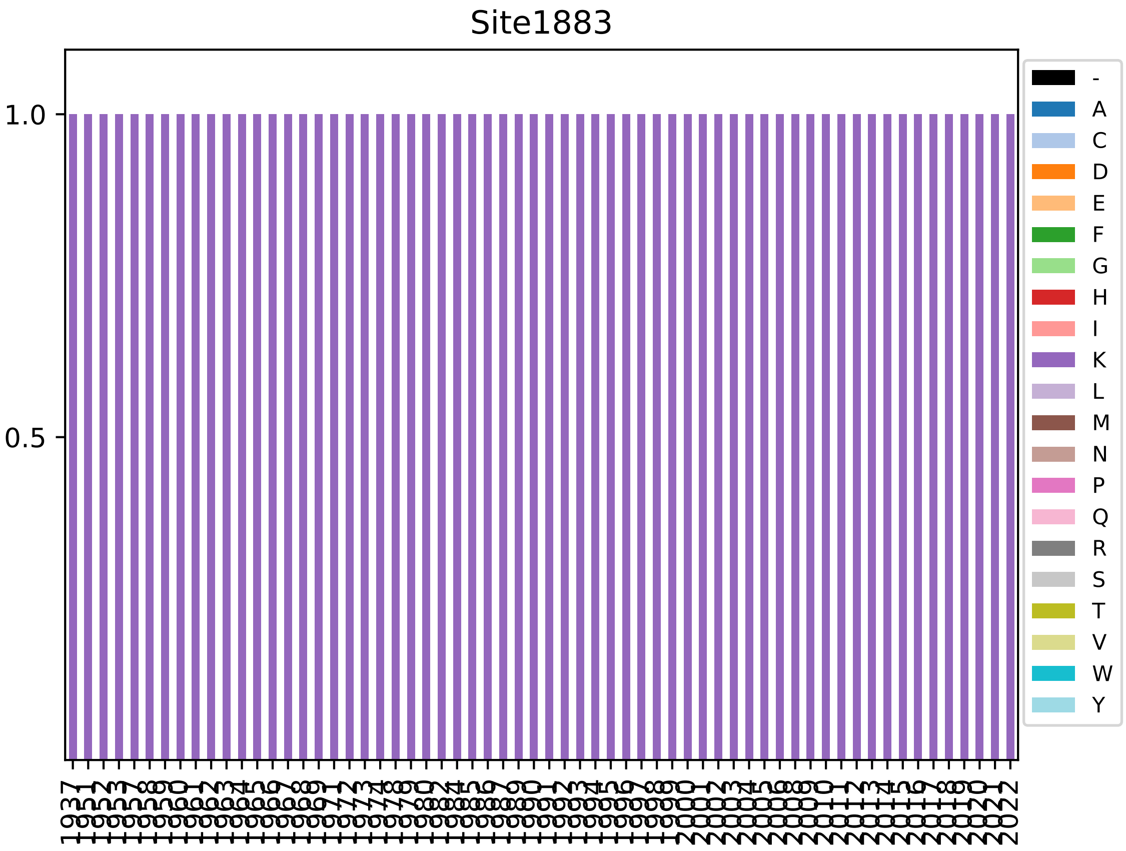
Site1883
1.0
0.5
-
A
C
D
E
F
G
H
I
K
L
M
N
P
Q
R
S
T
V
W
Y

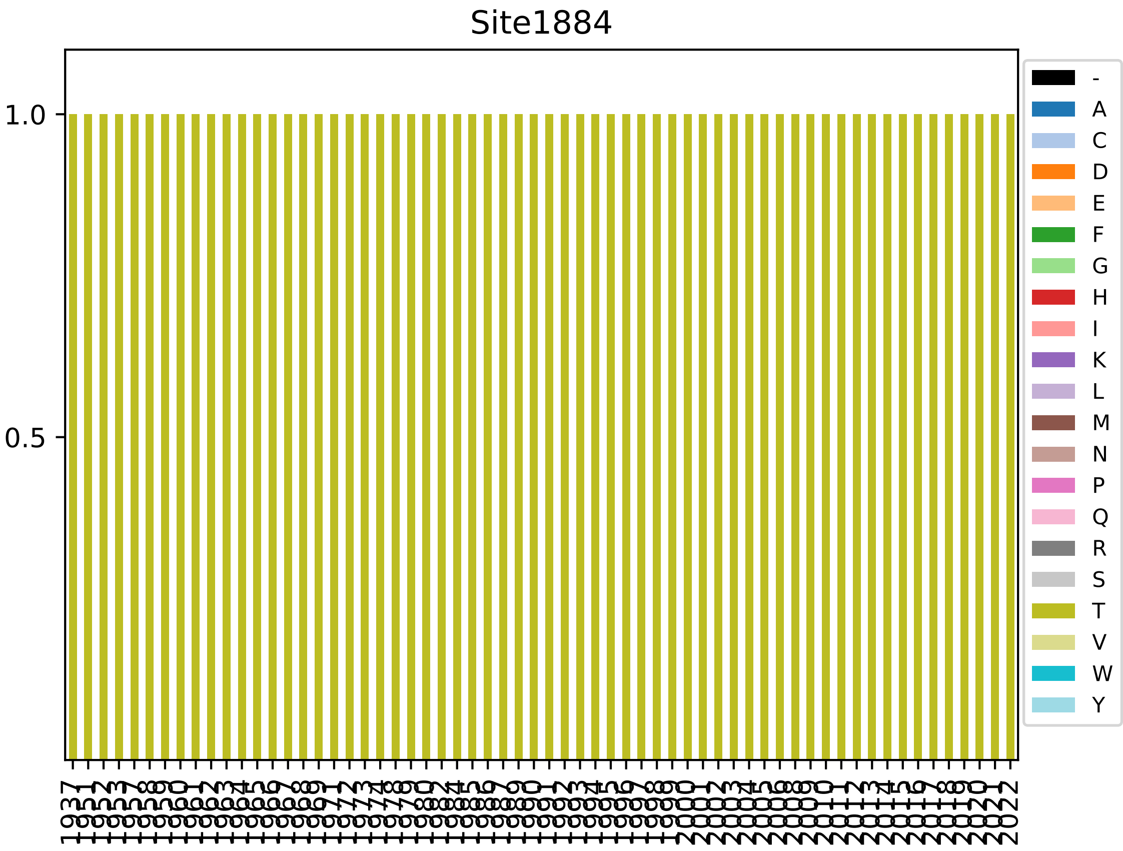
Site1884
1.0
0.5
-
A
C
D
E
F
G
H
I
K
L
M
N
P
Q
R
S
T
V
W
Y

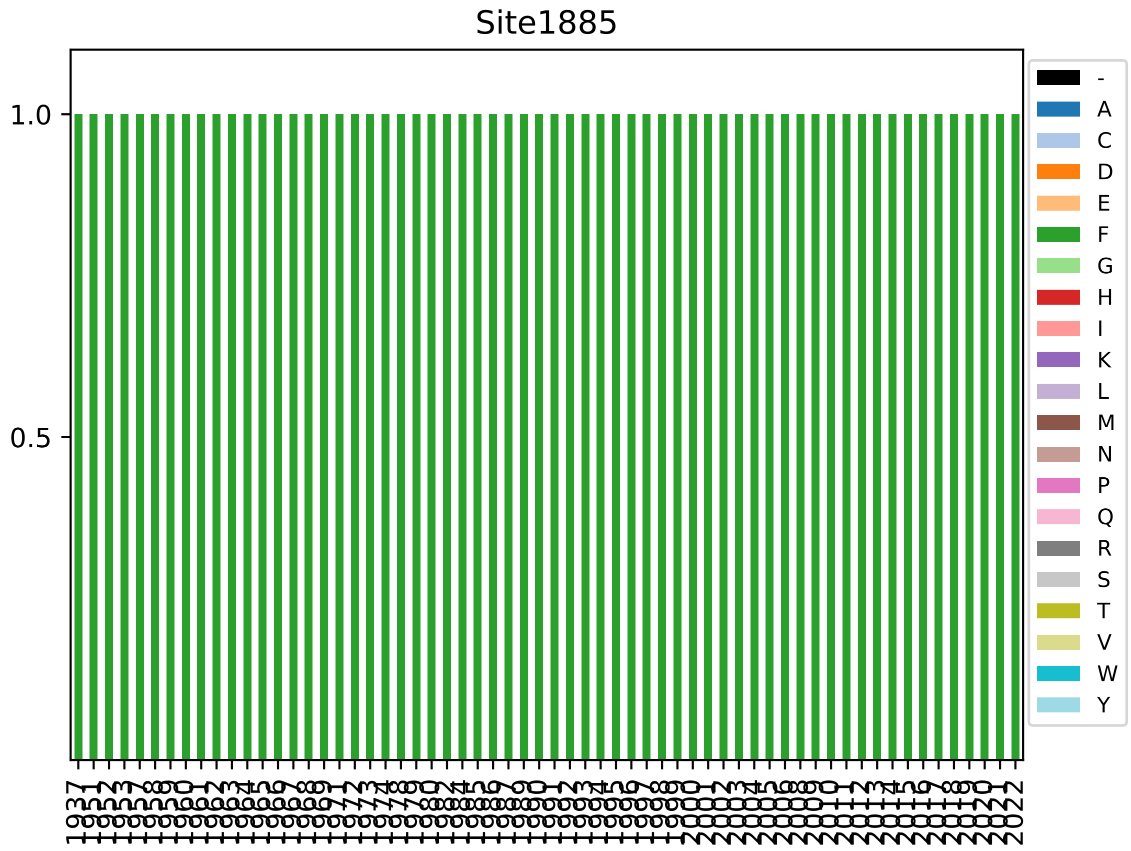
Site1885
1.0
0.5
-
A
C
D
E
F
G
H
I
K
L
M
N
P
Q
R
S
T
V
W
Y

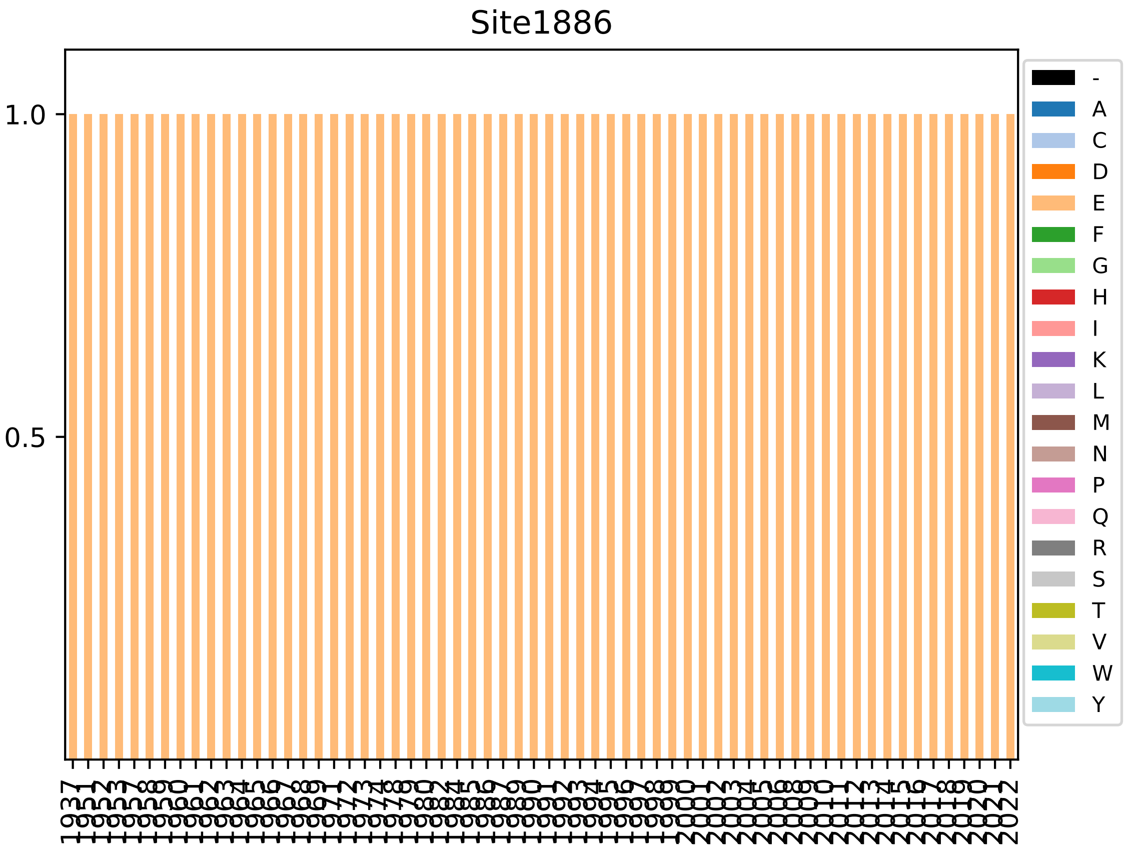
Site1886
1.0
0.5
-
A
C
D
E
F
G
H
I
K
L
M
N
P
Q
R
S
T
V
W
Y

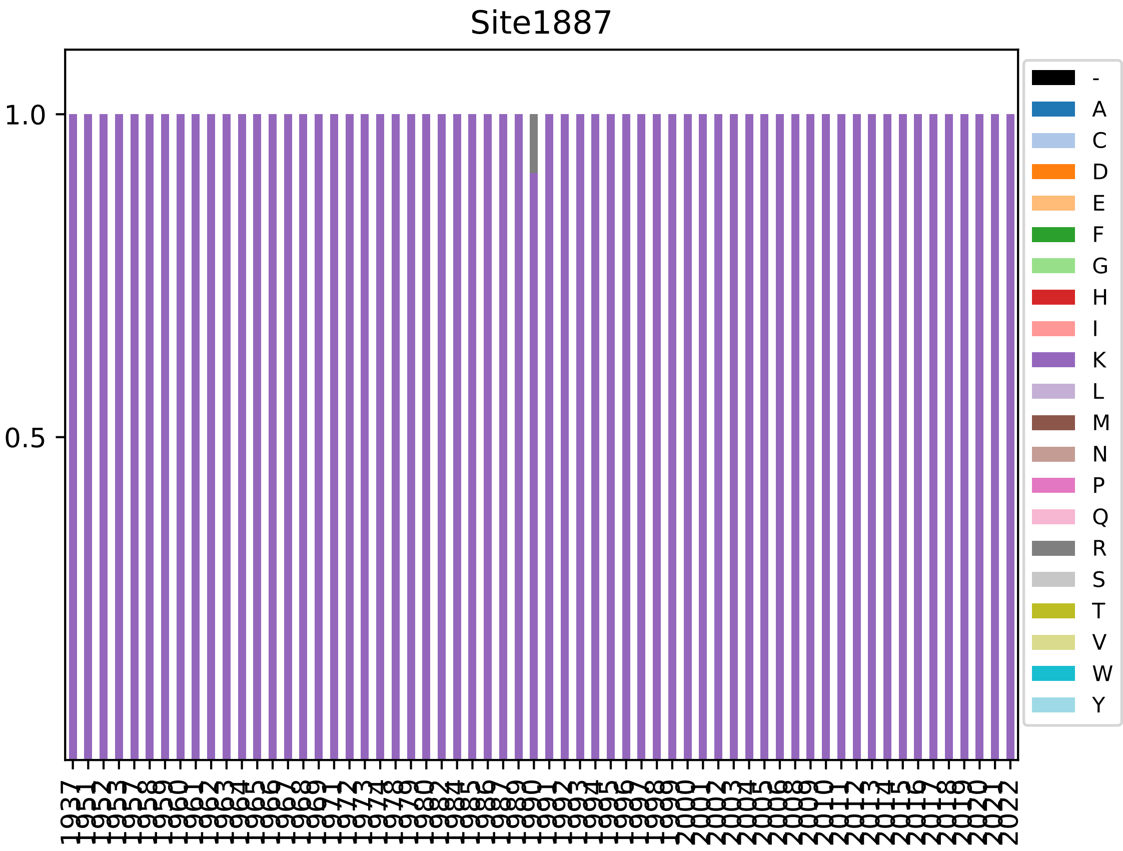

Site1887
1.0
0.5
-
A
C
D
E
F
G
H
I
K
L
M
N
P
Q
R
S
T
V
W
Y

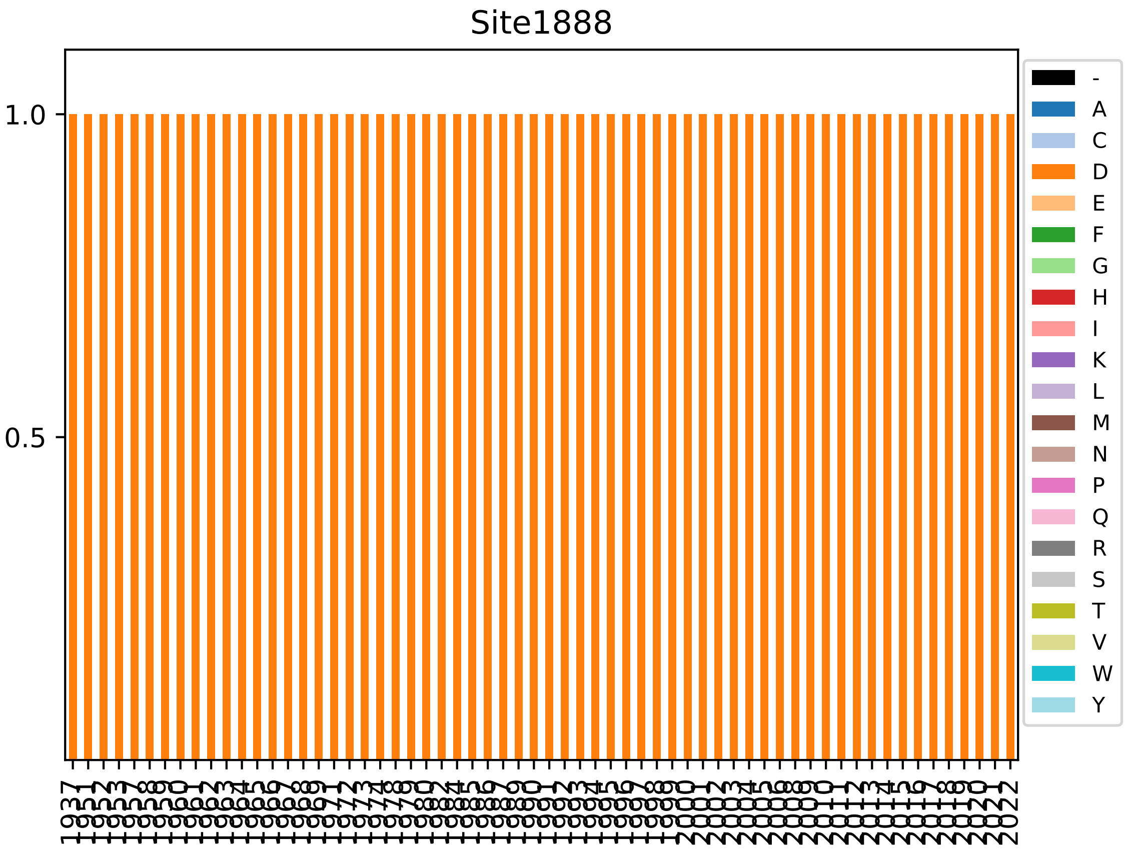
Site1888
1.0
0.5
-
A
C
D
E
F
G
H
I
K
L
M
N
P
Q
R
S
T
V
W
Y

Site1889

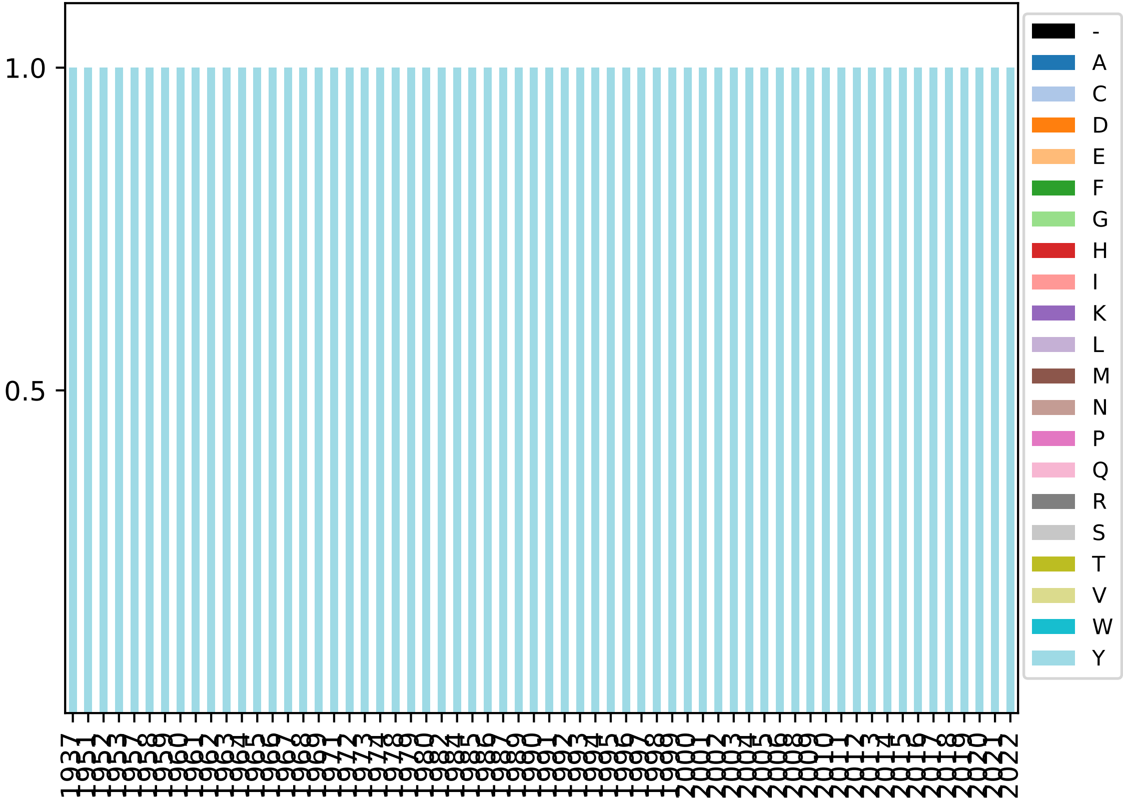

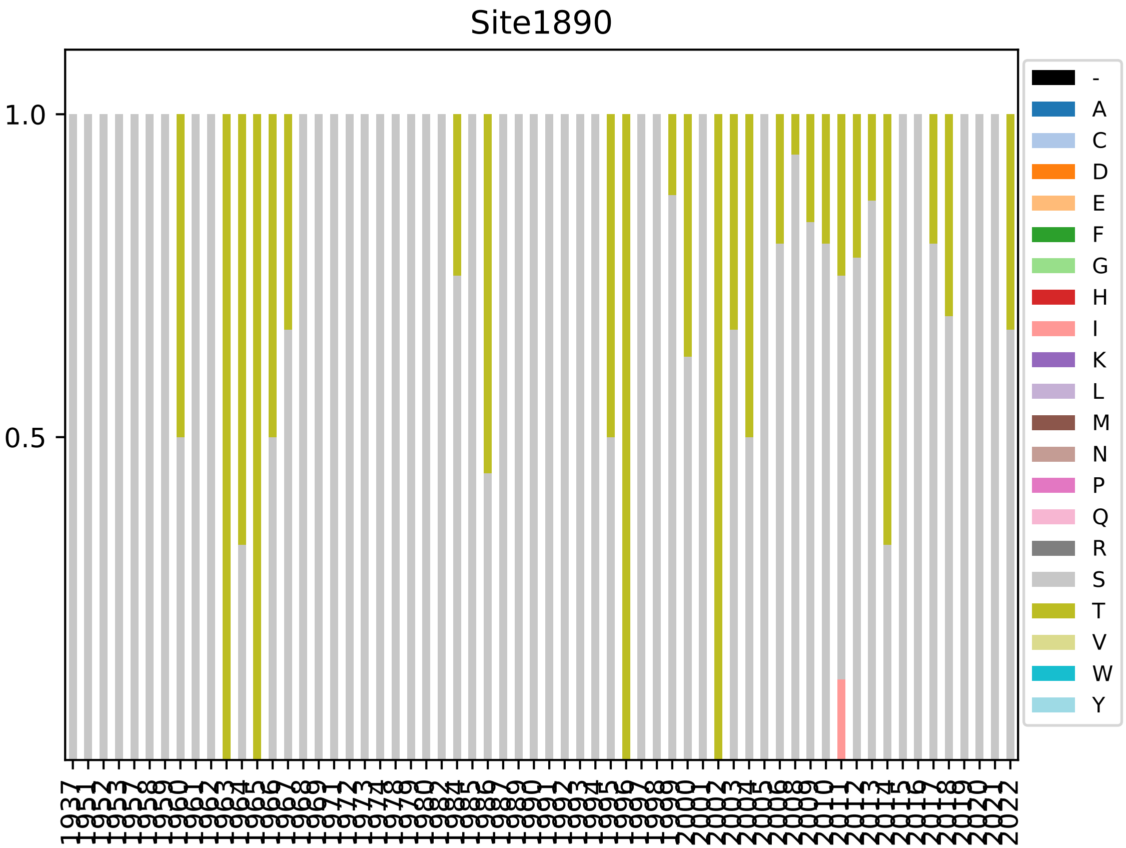
Site1890
1.0
0.5
-
A
C
D
E
F
G
H
I
K
L
M
N
P
Q
R
S
T
V
W
Y

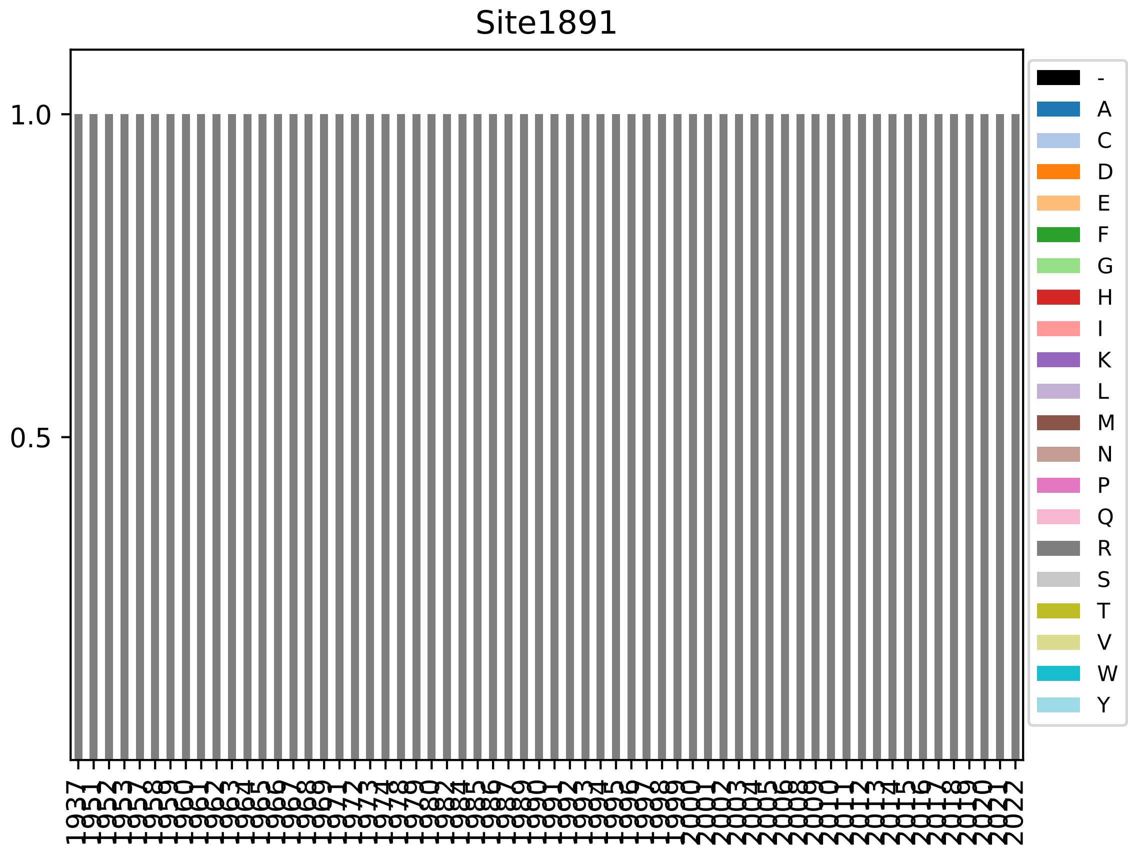
Site1891
1.0
0.5
-
A
C
D
E
F
G
H
I
K
L
M
N
P
Q
R
S
T
V
W
Y

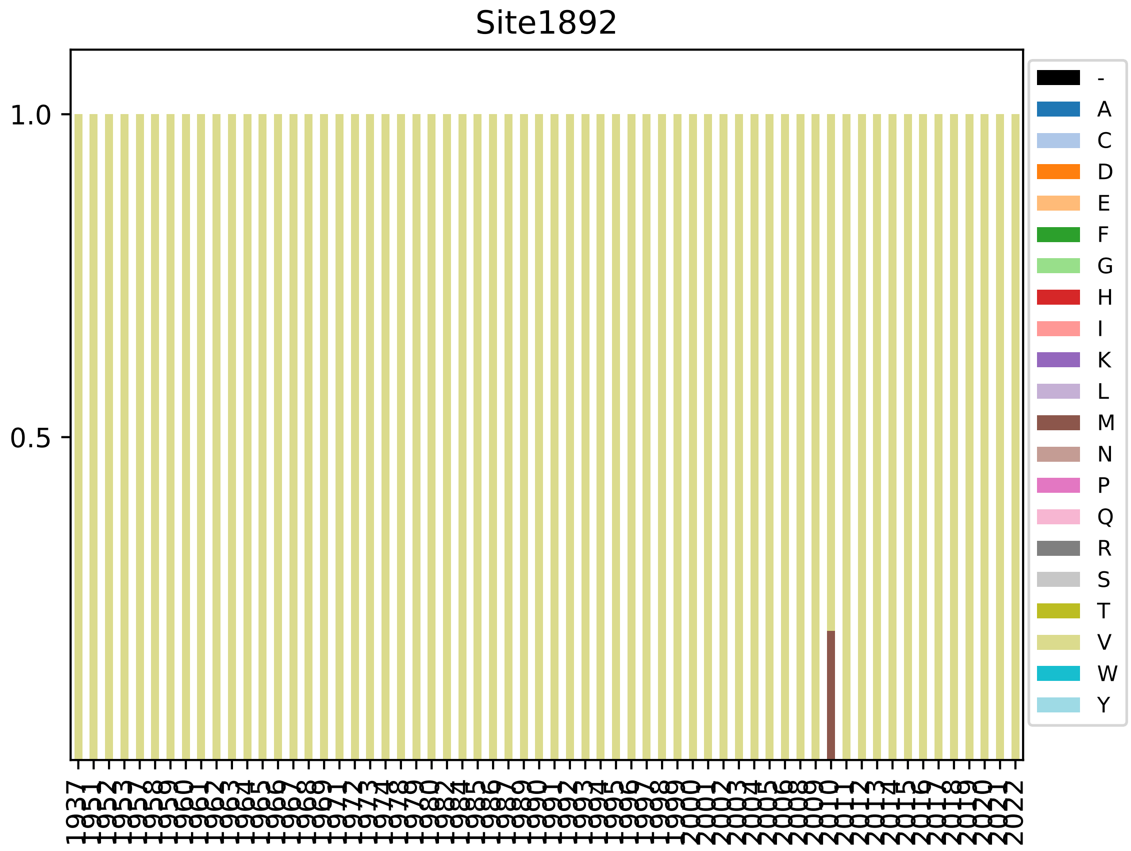
Site1892
1.0
0.5
-
A
C
D
E
F
G
H
I
K
L
M
N
P
Q
R
S
T
V
W
Y

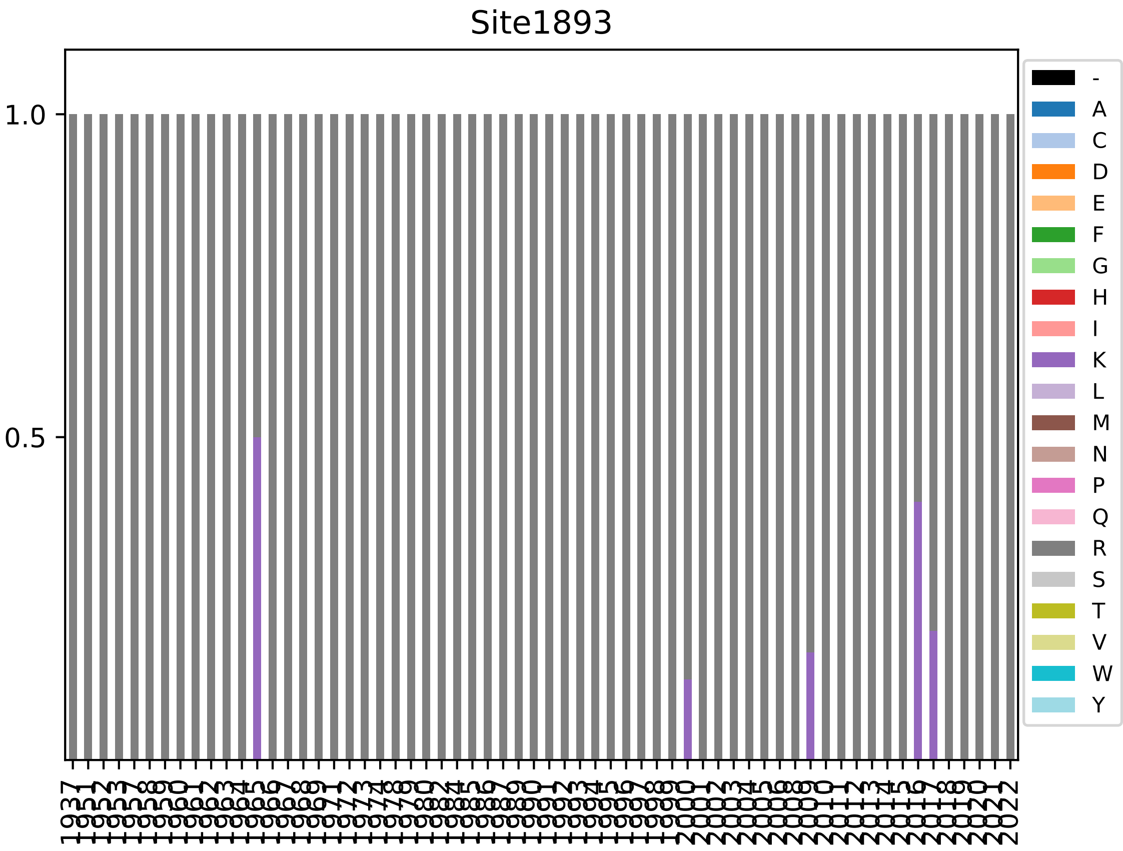
Site1893
1.0
0.5
-
A
C
D
E
F
G
H
I
K
L
M
N
P
Q
R
S
T
V
W
Y

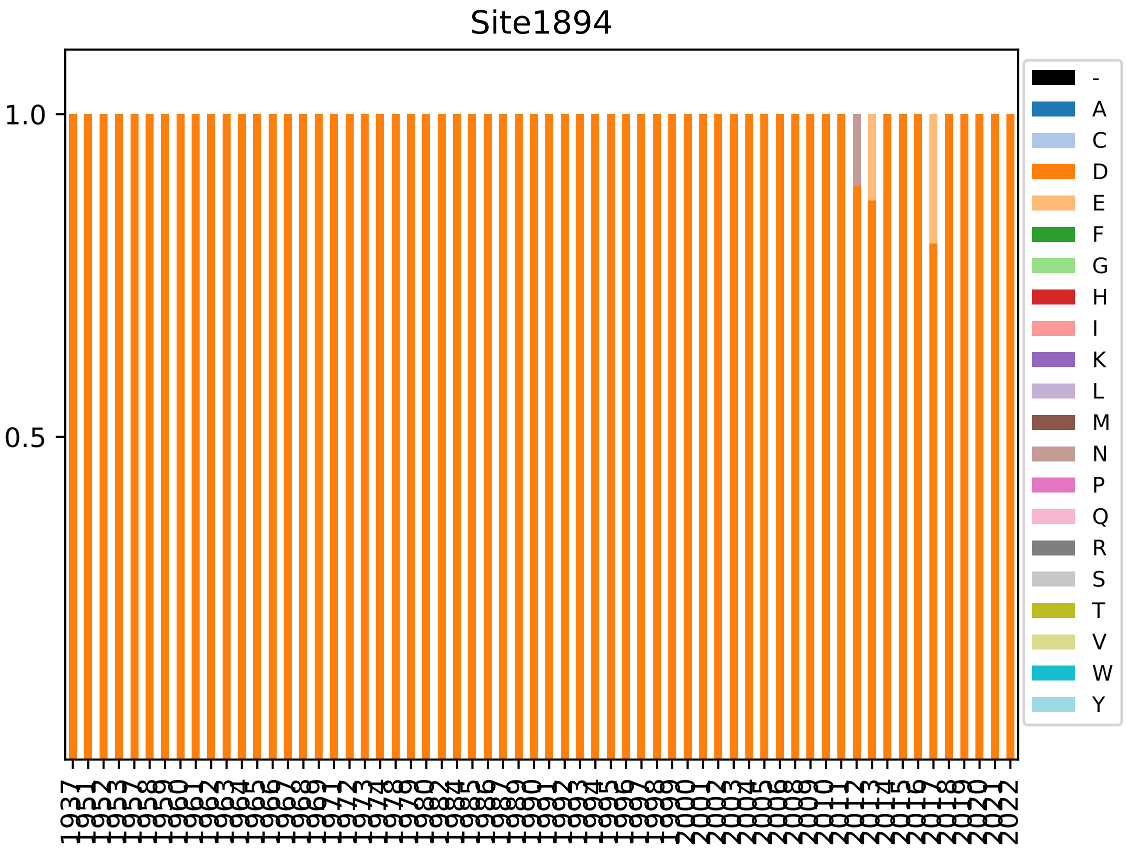
Site1894
1.0
0.5
-
A
C
D
E
F
G
H
I
K
L
M
N
P
Q
R
S
T
V
W
Y

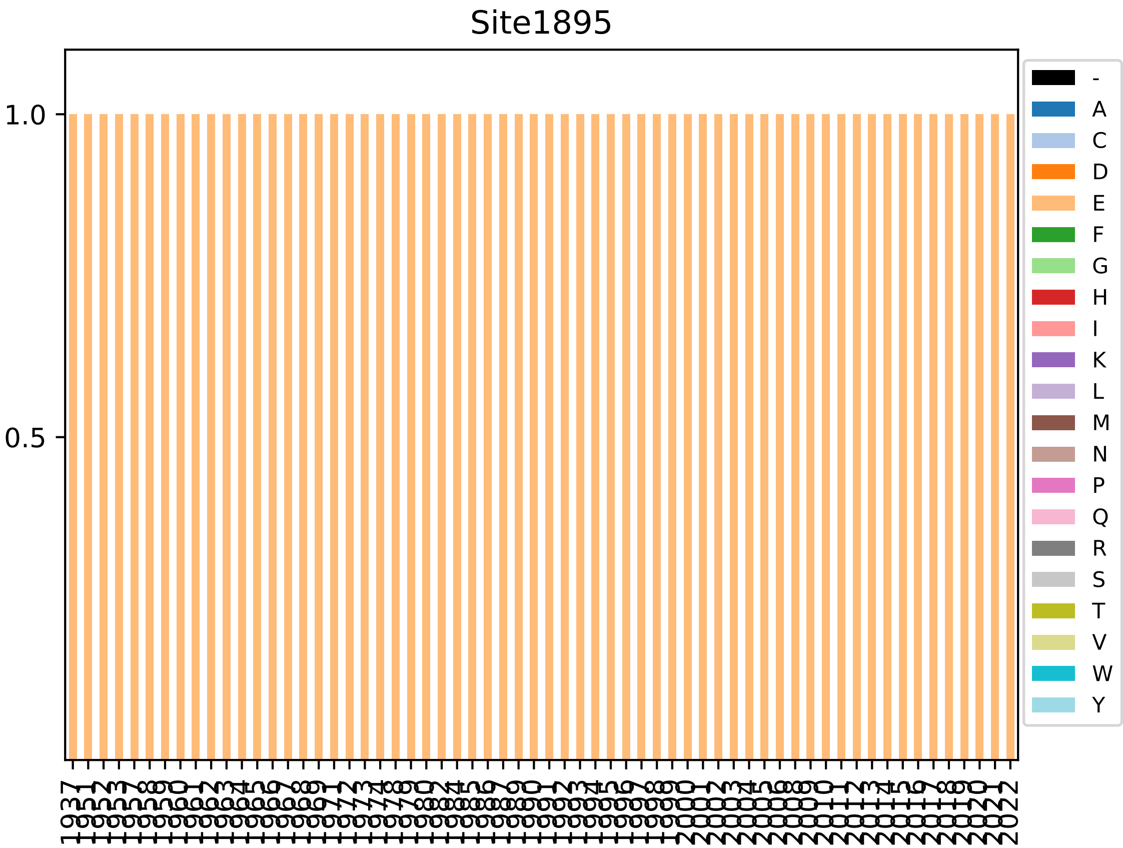
Site1895
1.0
0.5
-
A
C
D
E
F
G
H
I
K
L
M
N
P
Q
R
S
T
V
W
Y

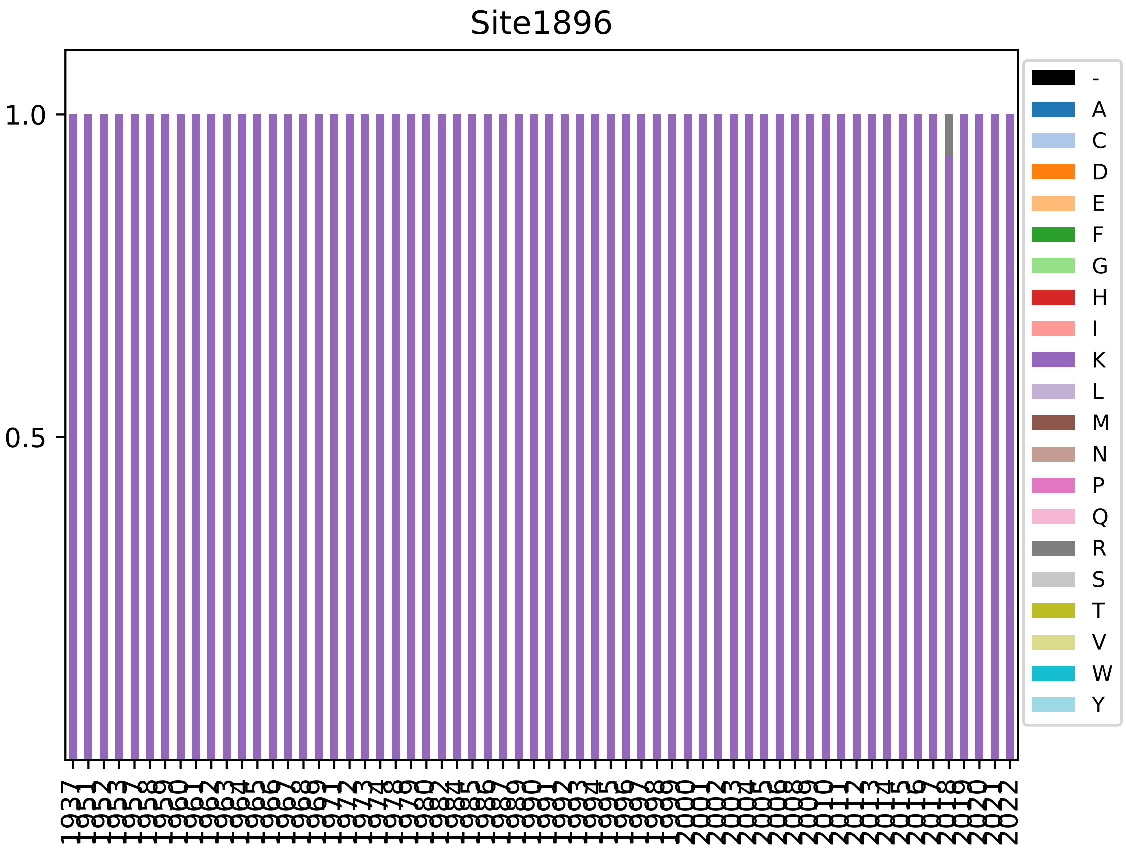
Site1896
1.0
0.5
-
A
C
D
E
F
G
H
I
K
L
M
N
P
Q
R
S
T
V
W
Y

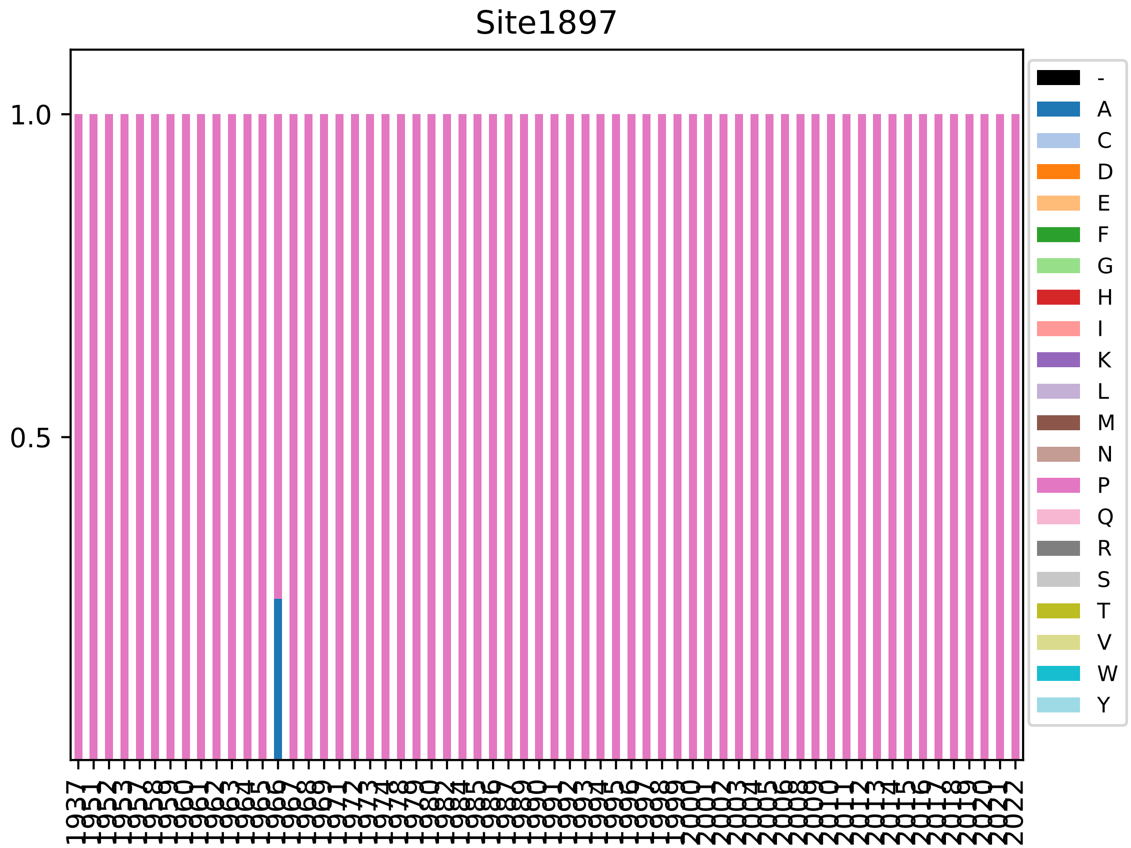
Site1897
1.0
0.5
-
A
C
D
E
F
G
H
I
K
L
M
N
P
Q
R
S
T
V
W
Y

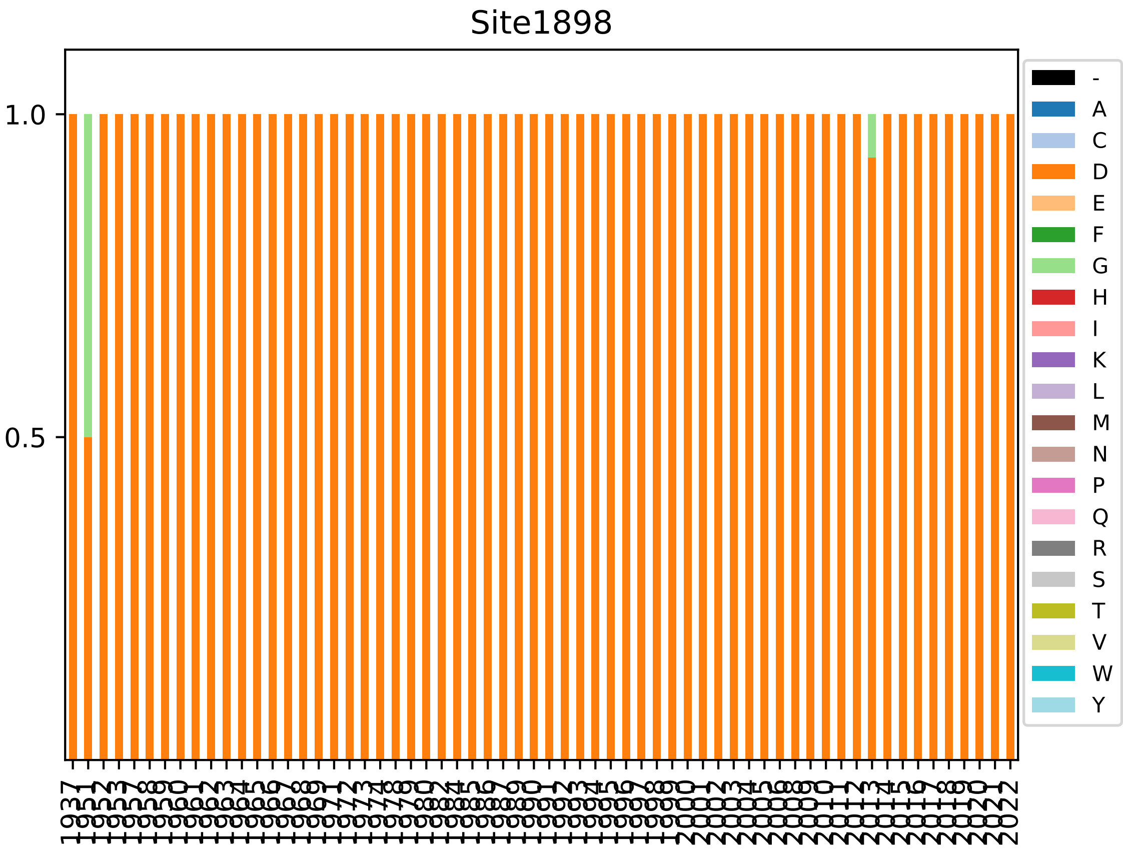

Site1898
1.0
0.5
-
A
C
D
E
F
G
H
I
K
L
M
N
P
Q
R
S
T
V
W
Y

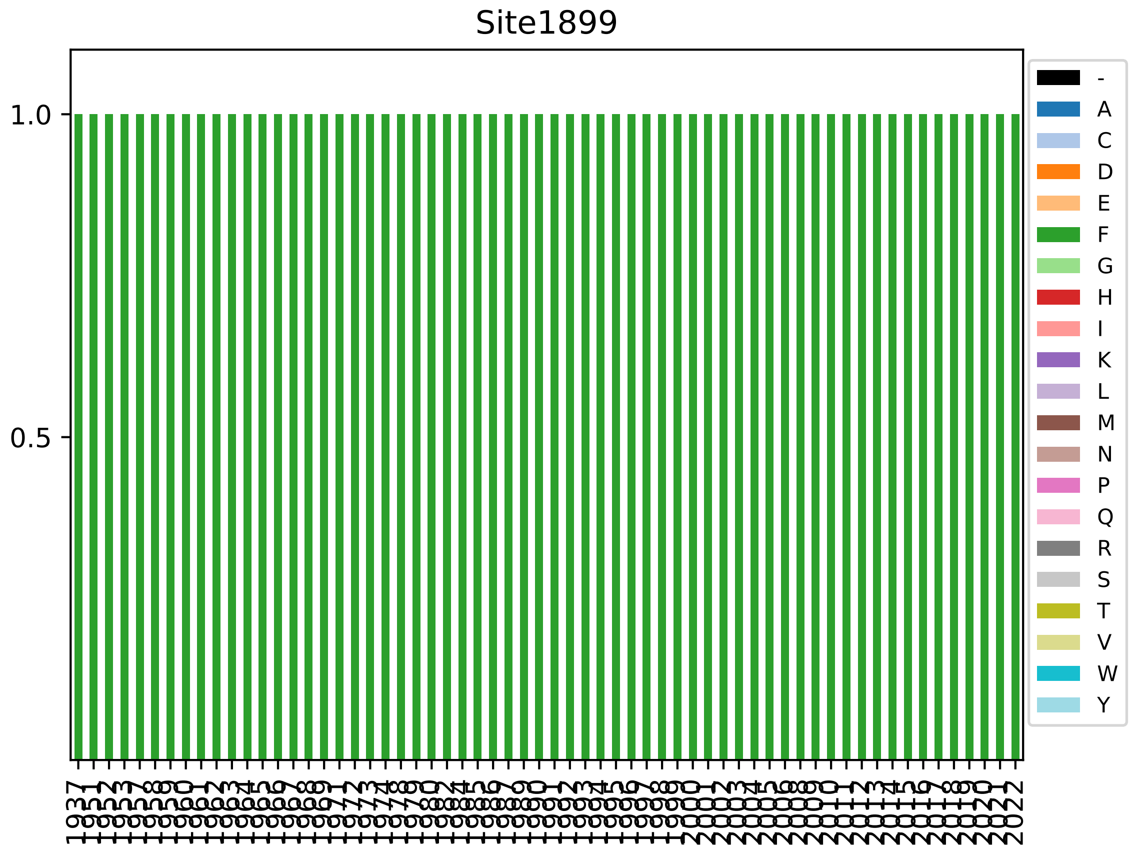
Site1899
1.0
0.5
-
A
C
D
E
F
G
H
I
K
L
M
N
P
Q
R
S
T
V
W
Y

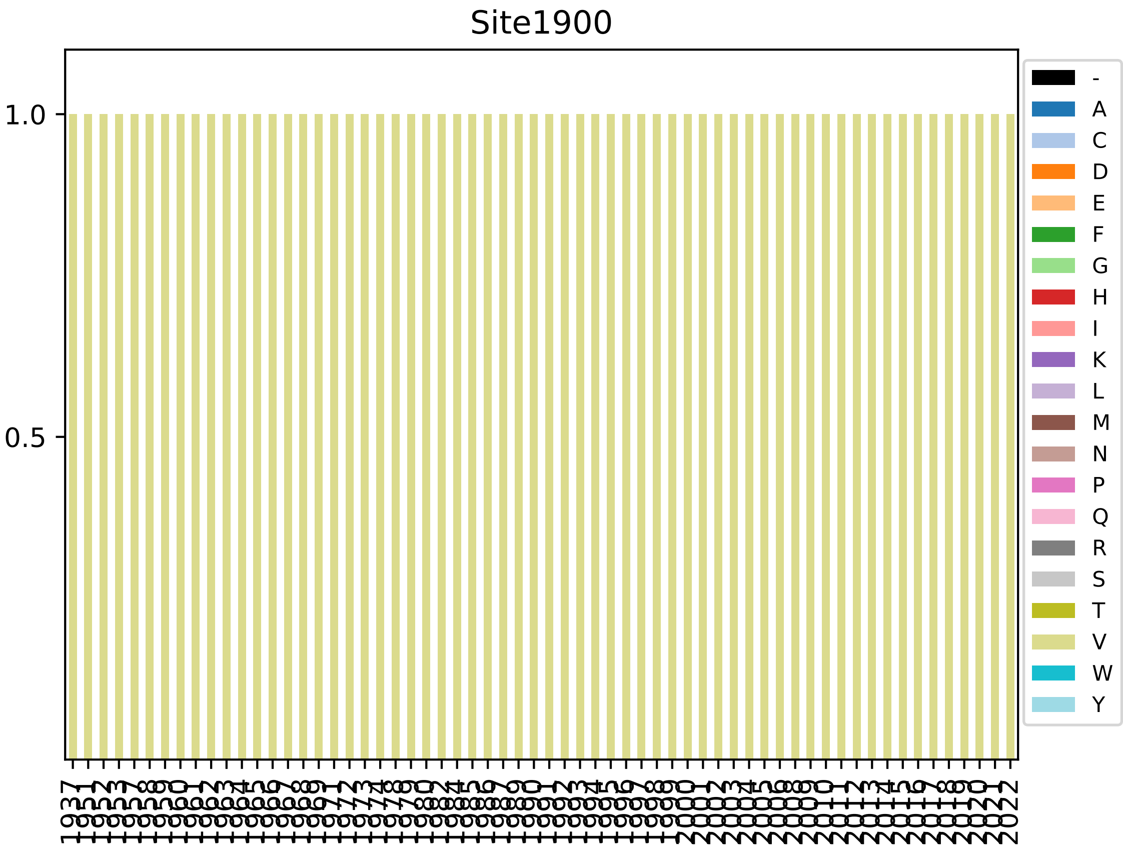
Site1900
1.0
0.5
-
A
C
D
E
F
G
H
I
K
L
M
N
P
Q
R
S
T
V
W
Y

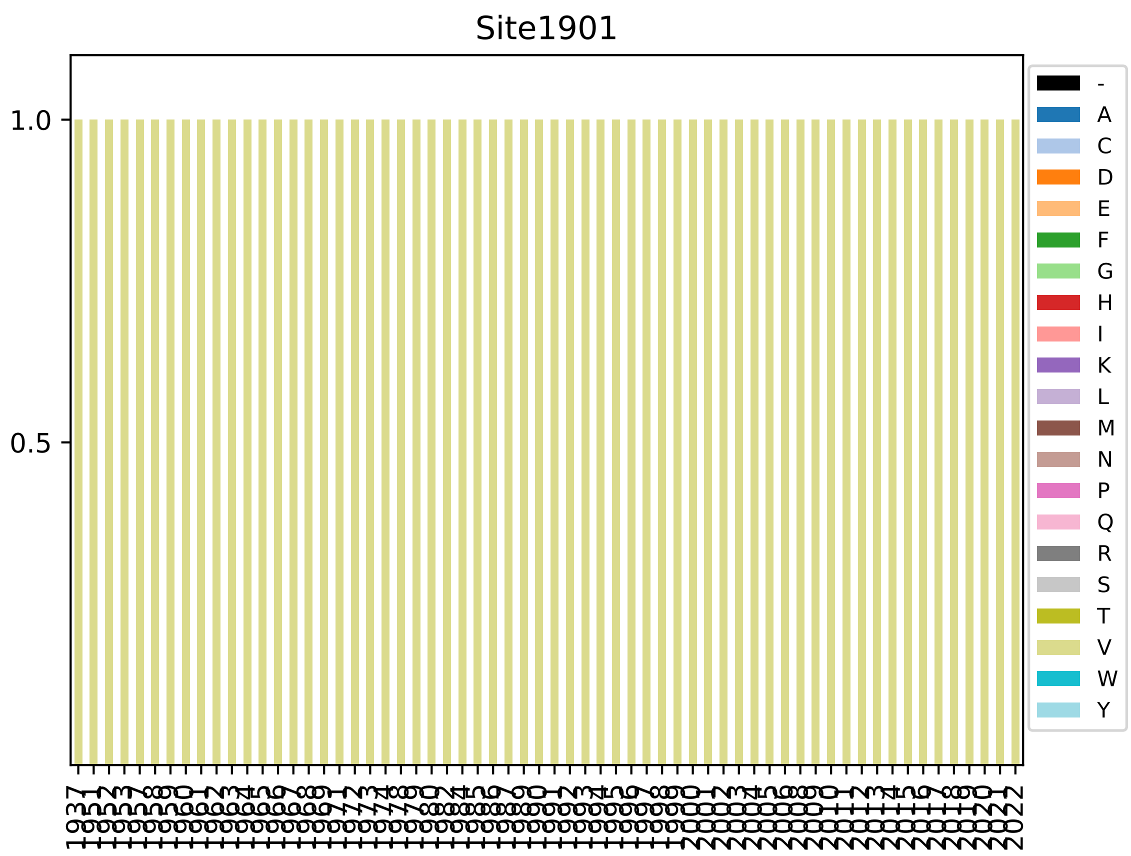
Site1901
1.0
0.5
-
A
C
D
E
F
G
H
I
K
L
M
N
P
Q
R
S
T
V
W
Y

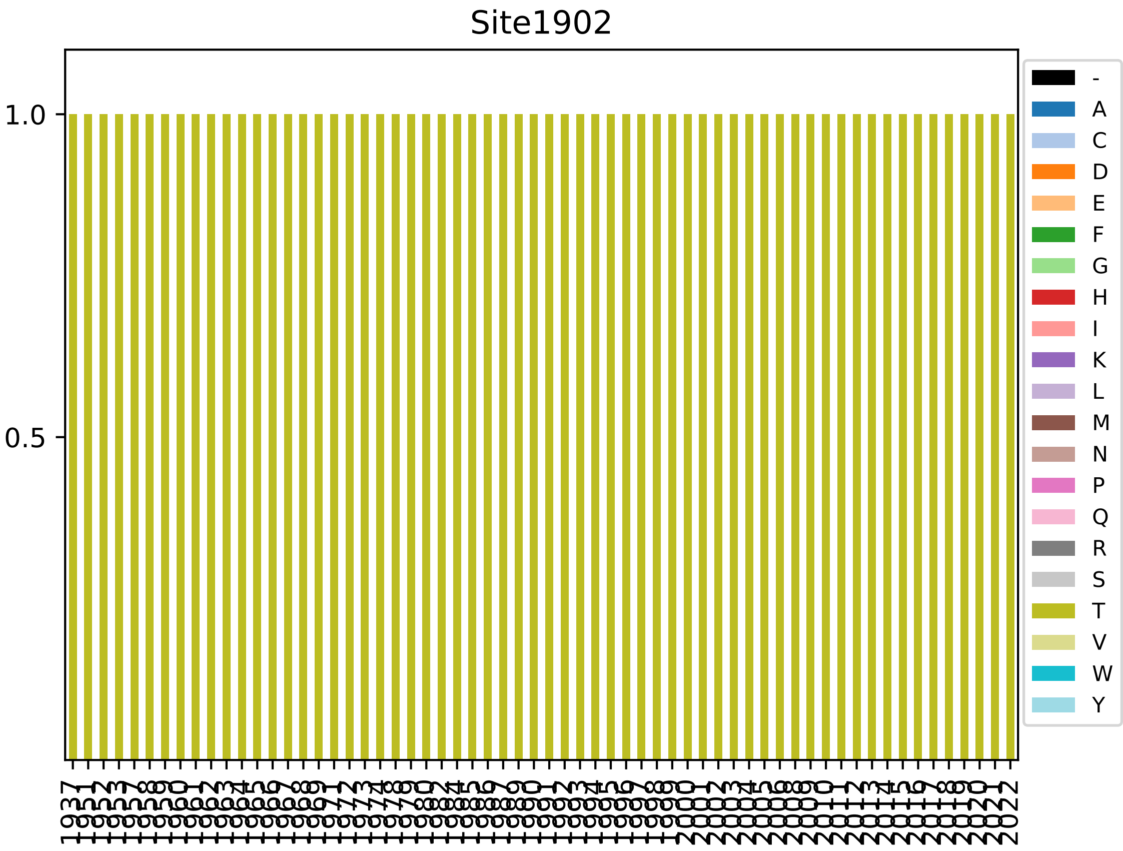
Site1902
1.0
0.5
-
A
C
D
E
F
G
H
I
K
L
M
N
P
Q
R
S
T
V
W
Y

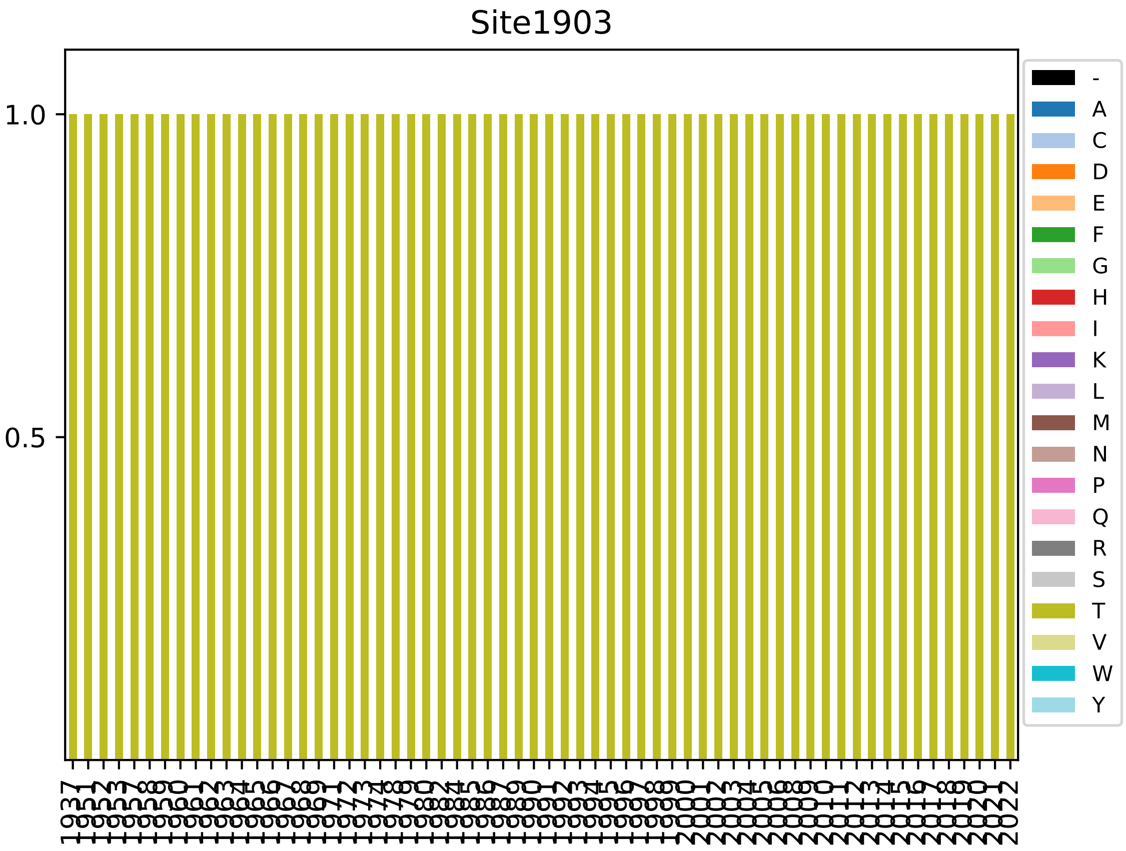
Site1903
1.0
0.5
-
A
C
D
E
F
G
H
I
K
L
M
N
P
Q
R
S
T
V
W
Y

Site1904

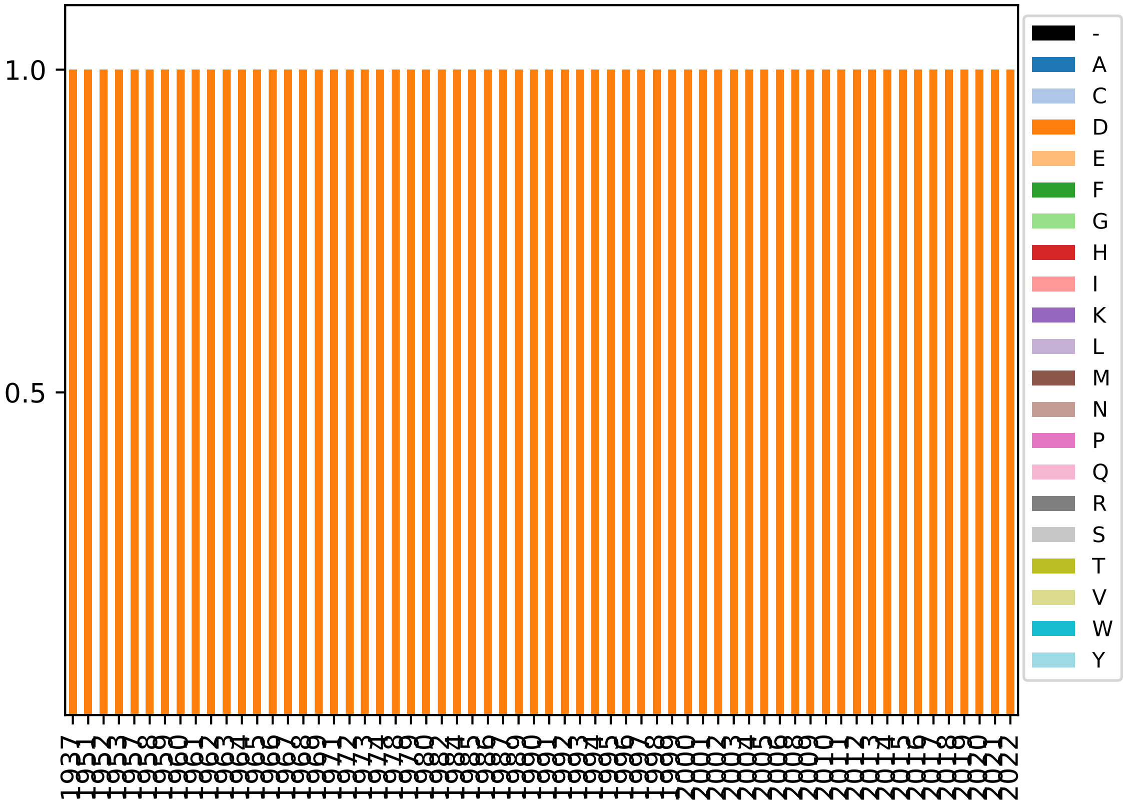

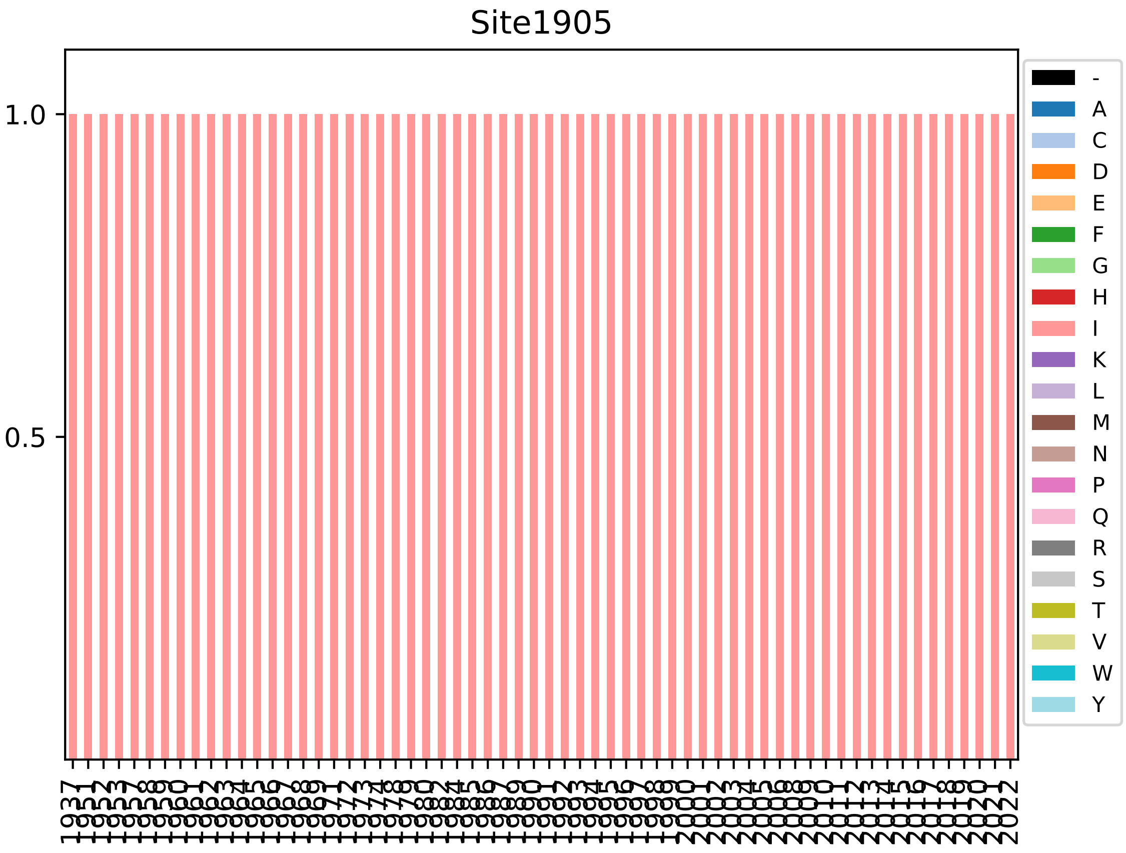

Site1905
1.0
0.5
-
A
C
D
E
F
G
H
I
K
L
M
N
P
Q
R
S
T
V
W
Y

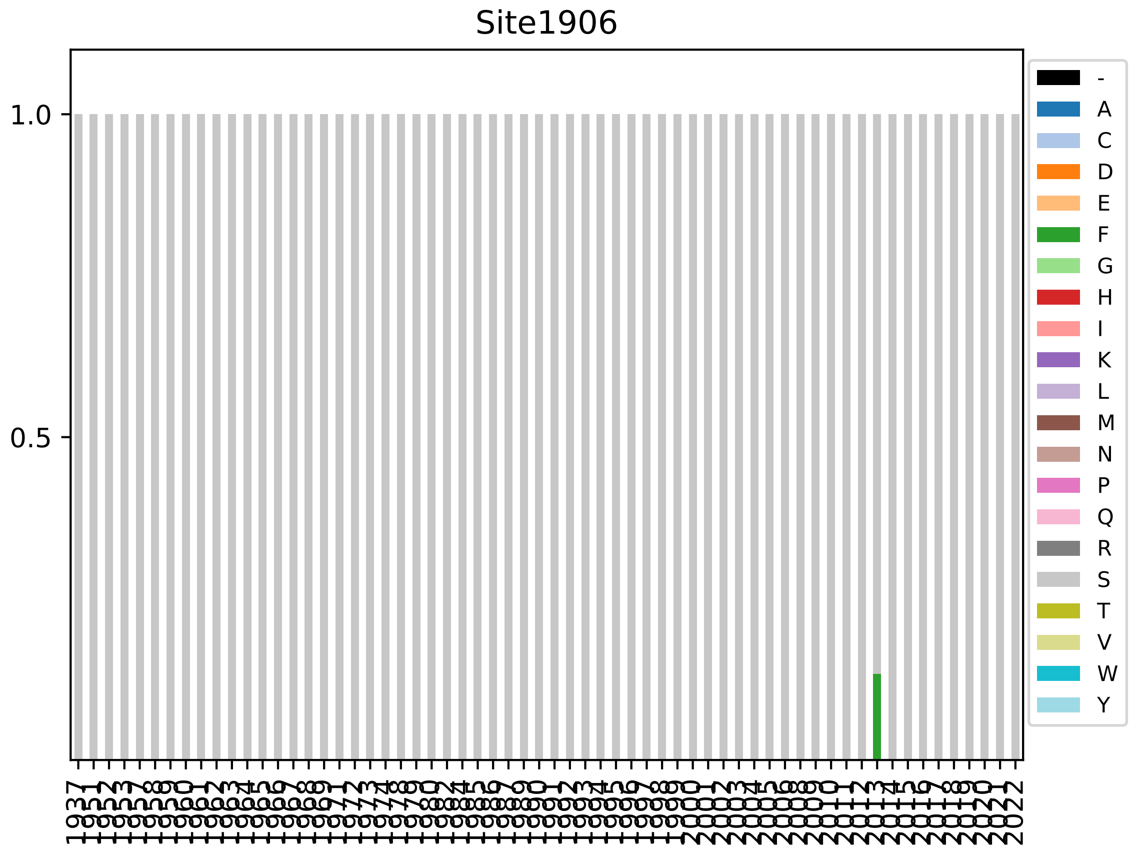
Site1906
1.0
0.5
-
A
C
D
E
F
G
H
I
K
L
M
N
P
Q
R
S
T
V
W
Y

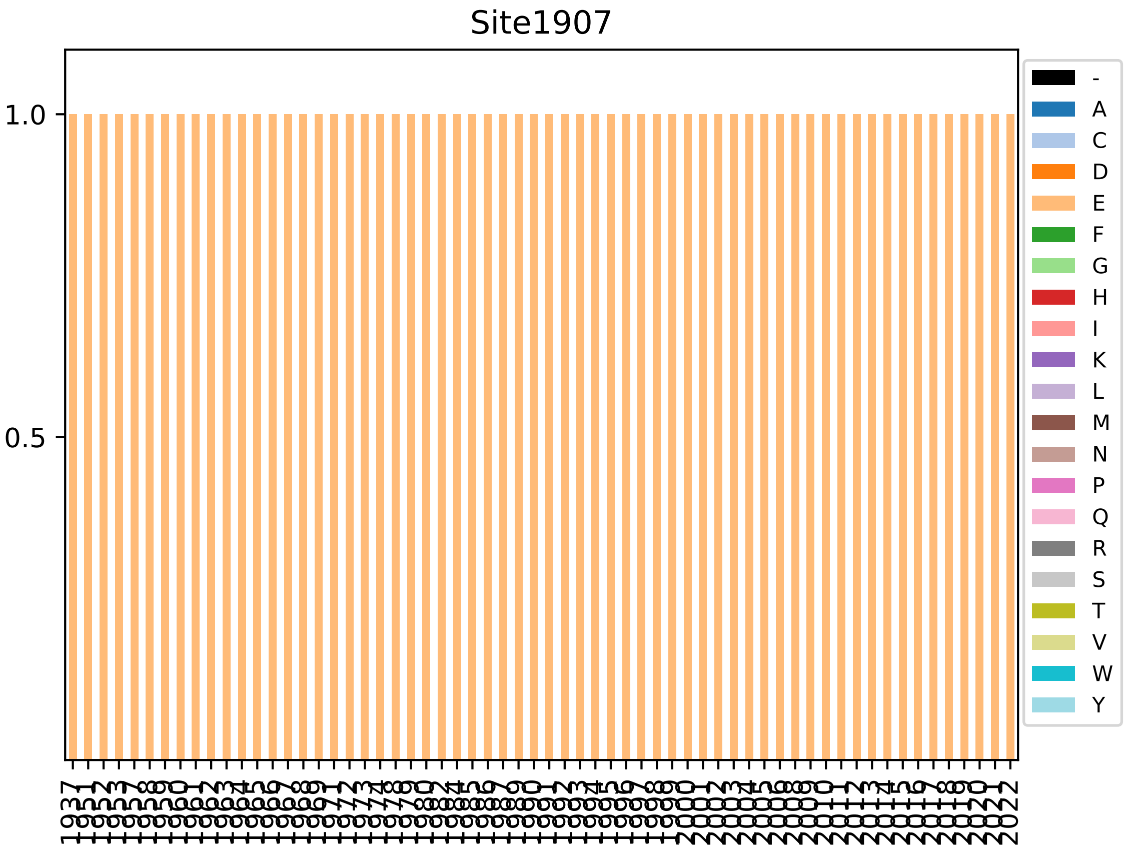
Site1907
1.0
0.5
-
A
C
D
E
F
G
H
I
K
L
M
N
P
Q
R
S
T
V
W
Y

Site1908

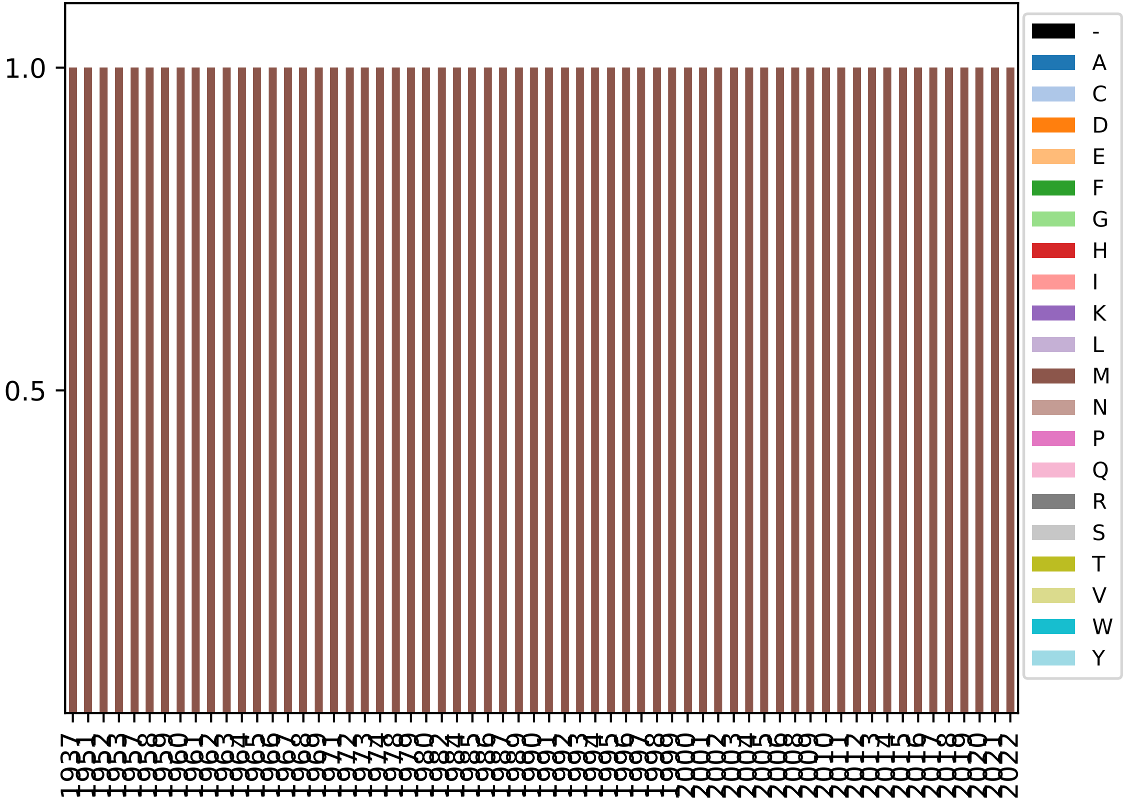

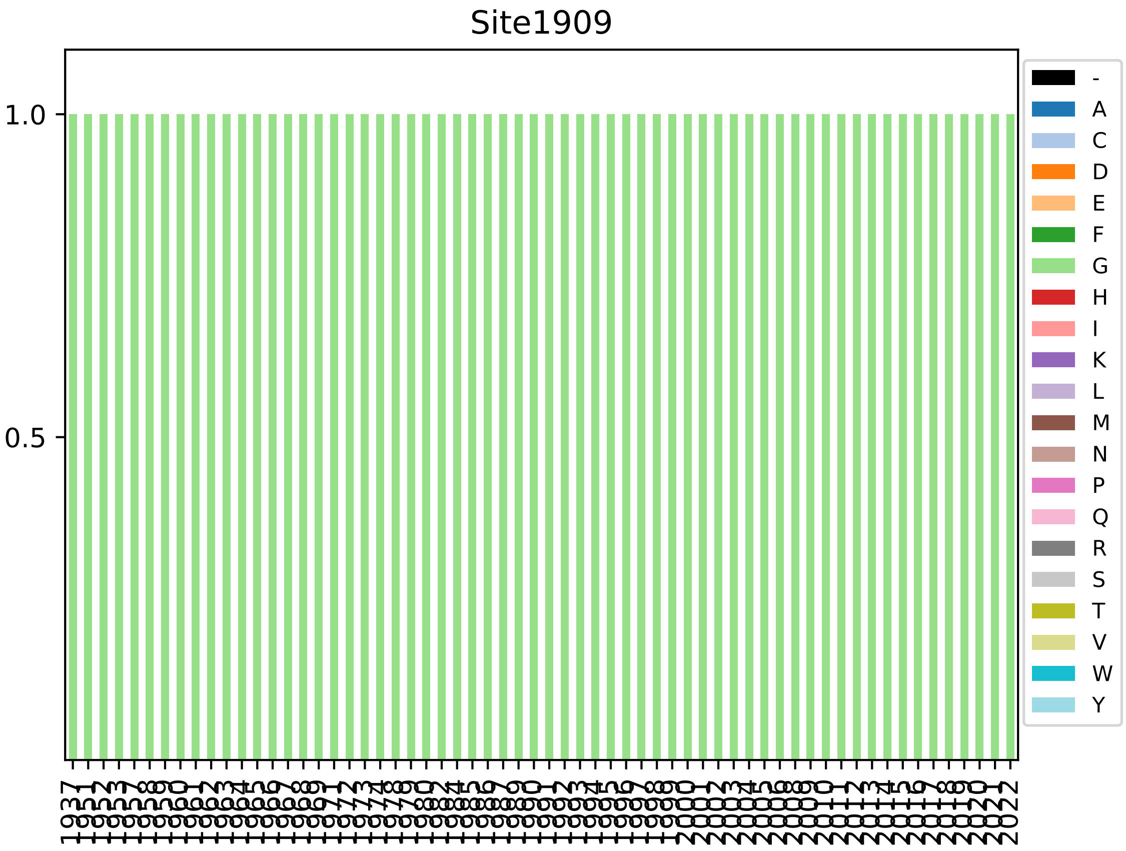
Site1909
1.0
0.5
-
A
C
D
E
F
G
H
I
K
L
M
N
P
Q
R
S
T
V
W
Y

Site1910

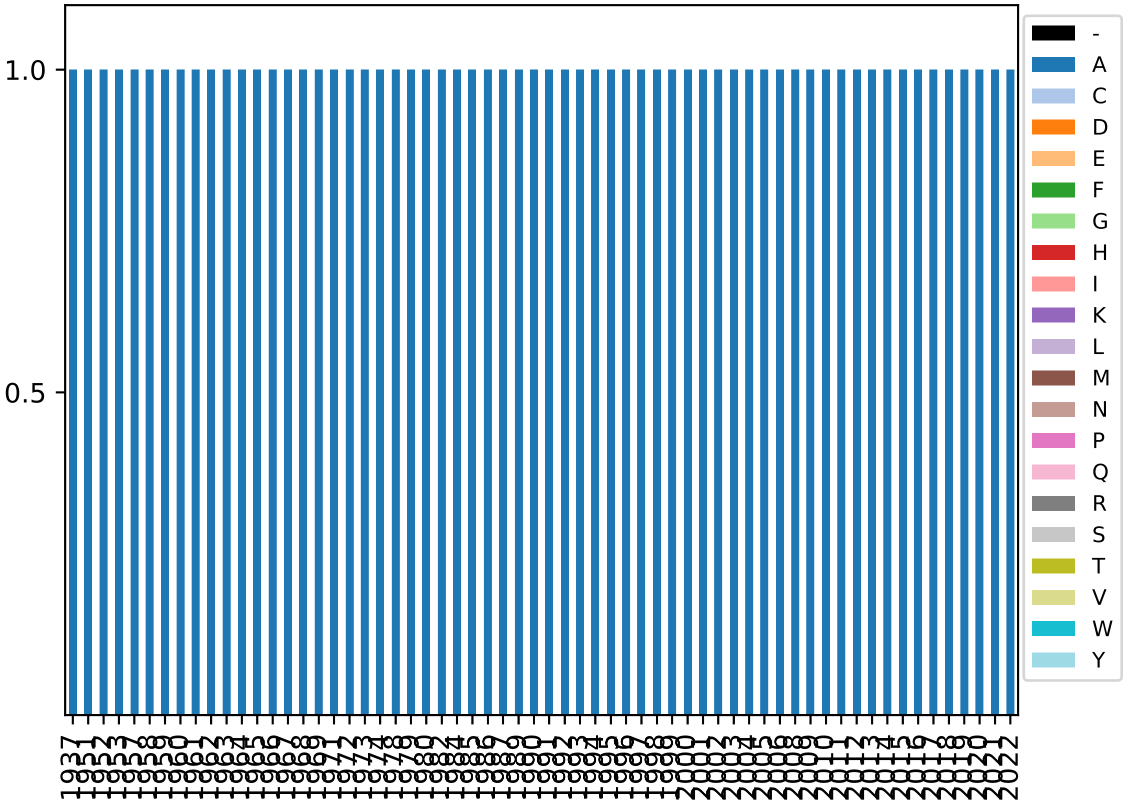

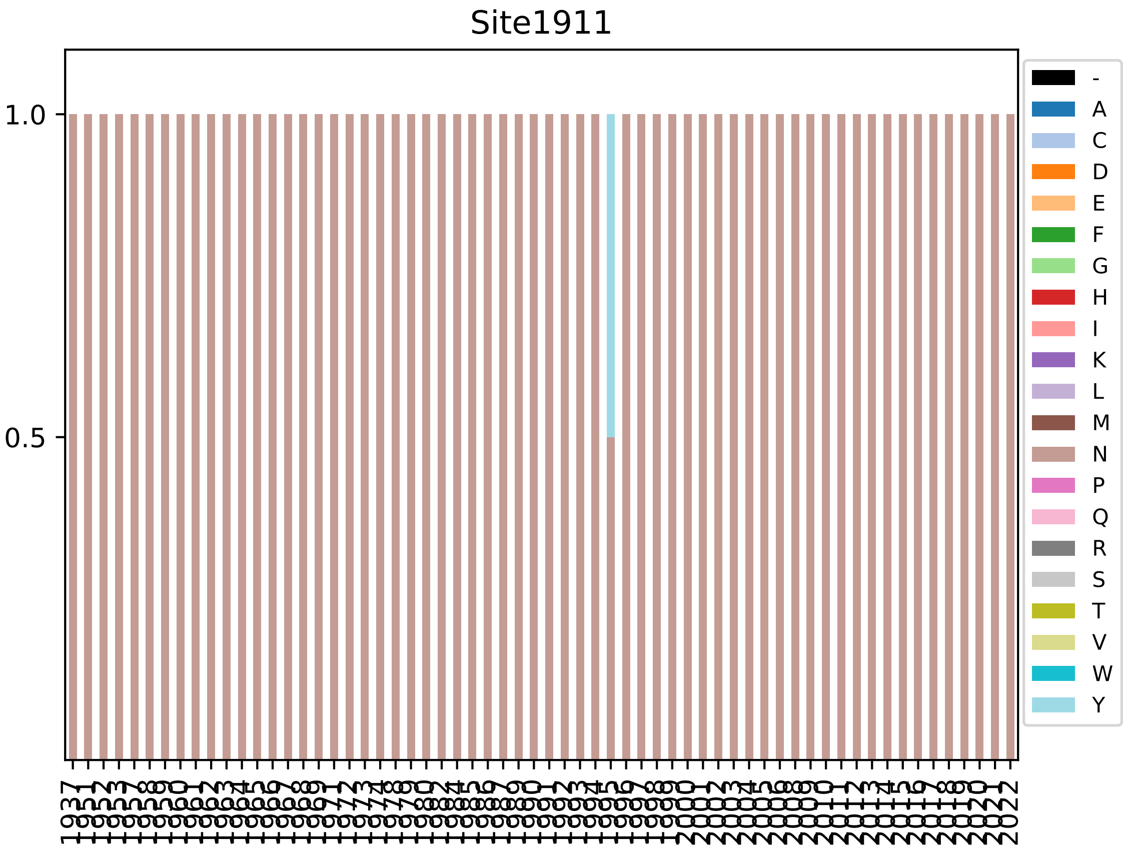
Site1911
1.0
0.5
-
A
C
D
E
F
G
H
I
K
L
M
N
P
Q
R
S
T
V
W
Y

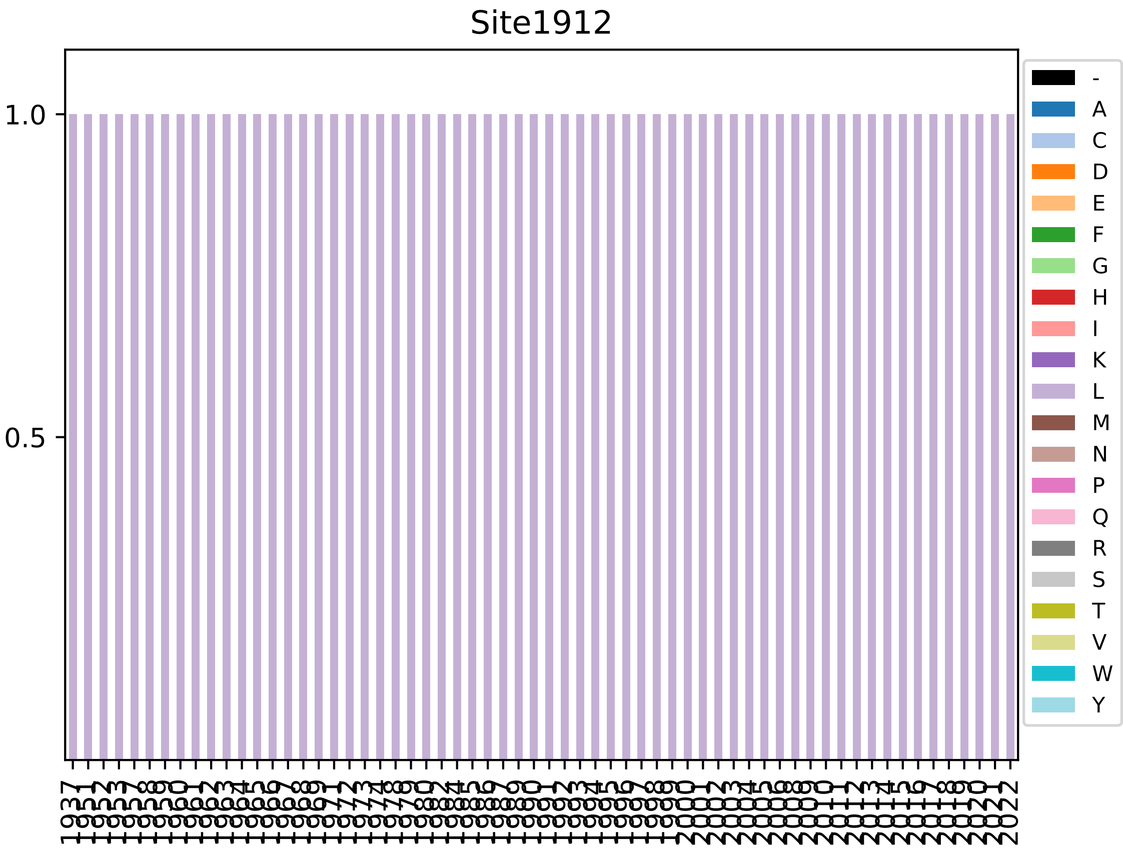
Site1912
1.0
0.5
-
A
C
D
E
F
G
H
I
K
L
M
N
P
Q
R
S
T
V
W
Y

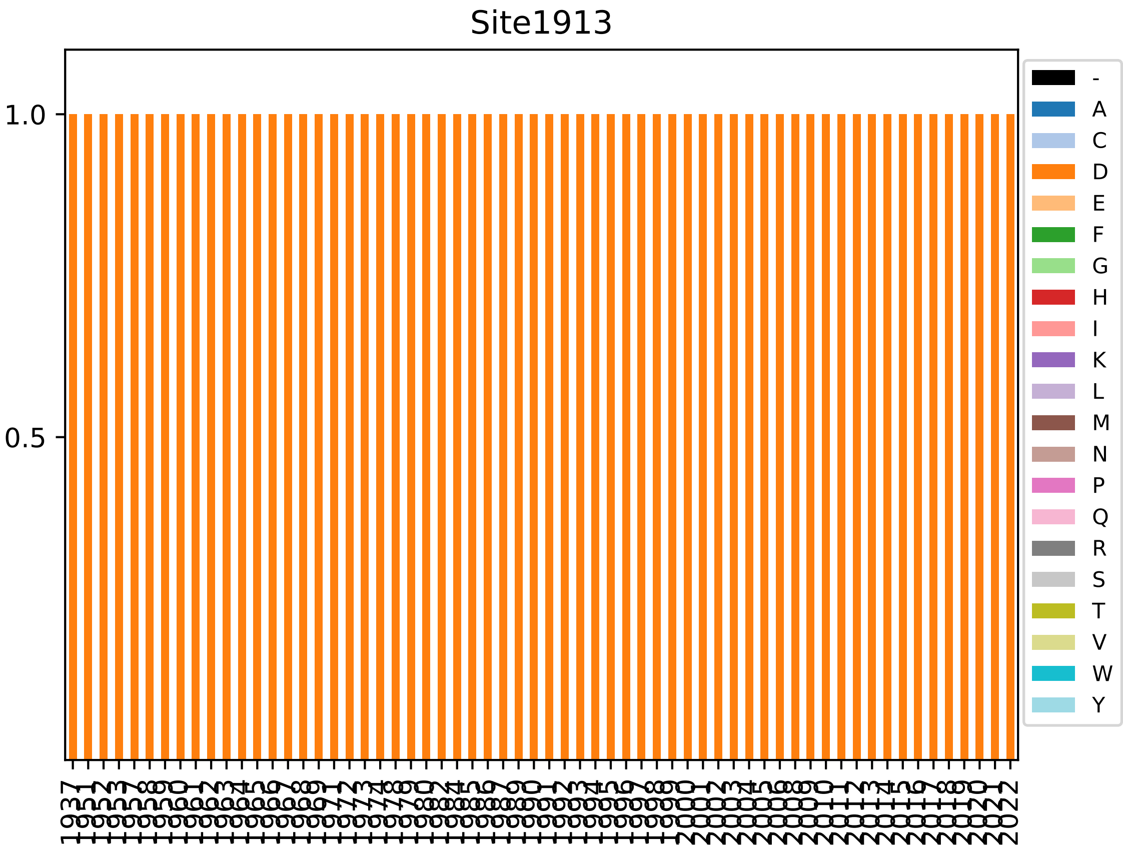
Site1913
1.0
0.5
-
A
C
D
E
F
G
H
I
K
L
M
N
P
Q
R
S
T
V
W
Y

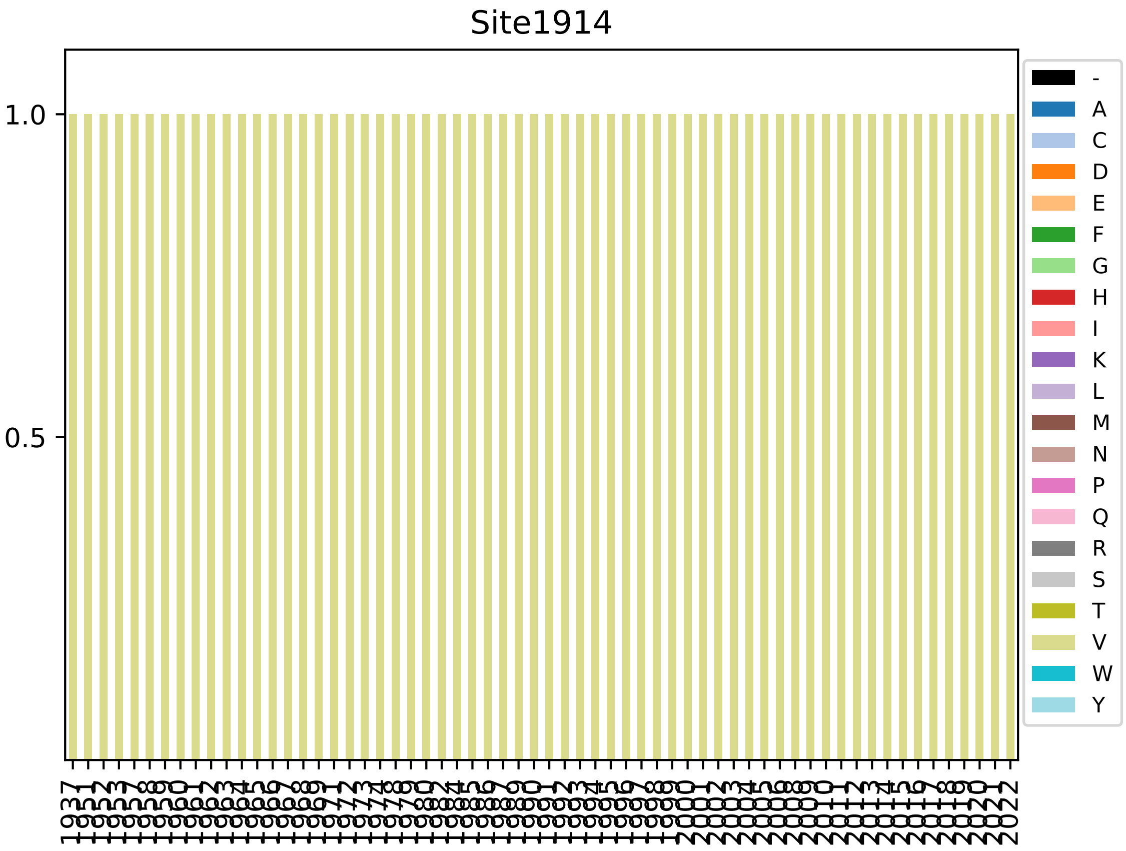
Site1914
1.0
0.5
-
A
C
D
E
F
G
H
I
K
L
M
N
P
Q
R
S
T
V
W
Y

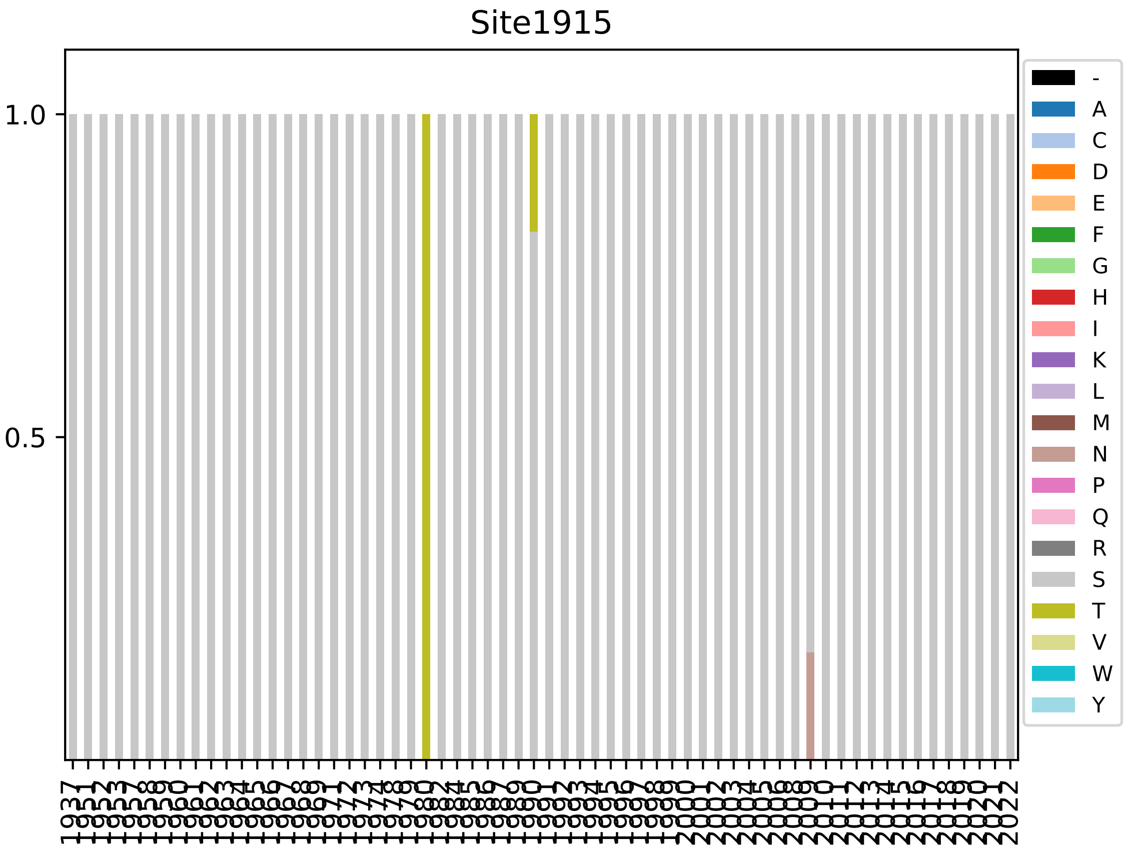
Site1915
1.0
0.5
-
A
C
D
E
F
G
H
I
K
L
M
N
P
Q
R
S
T
V
W
Y

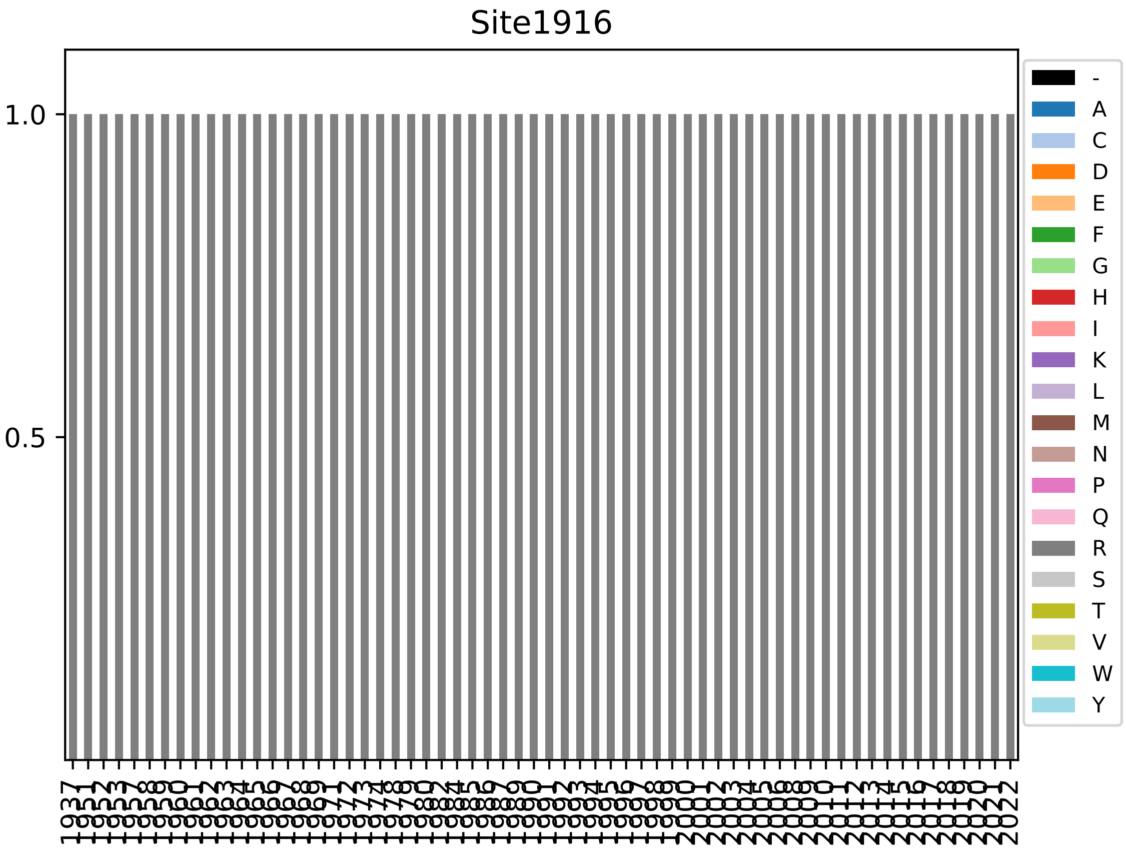
Site1916
1.0
0.5
-
A
C
D
E
F
G
H
I
K
L
M
N
P
Q
R
S
T
V
W
Y

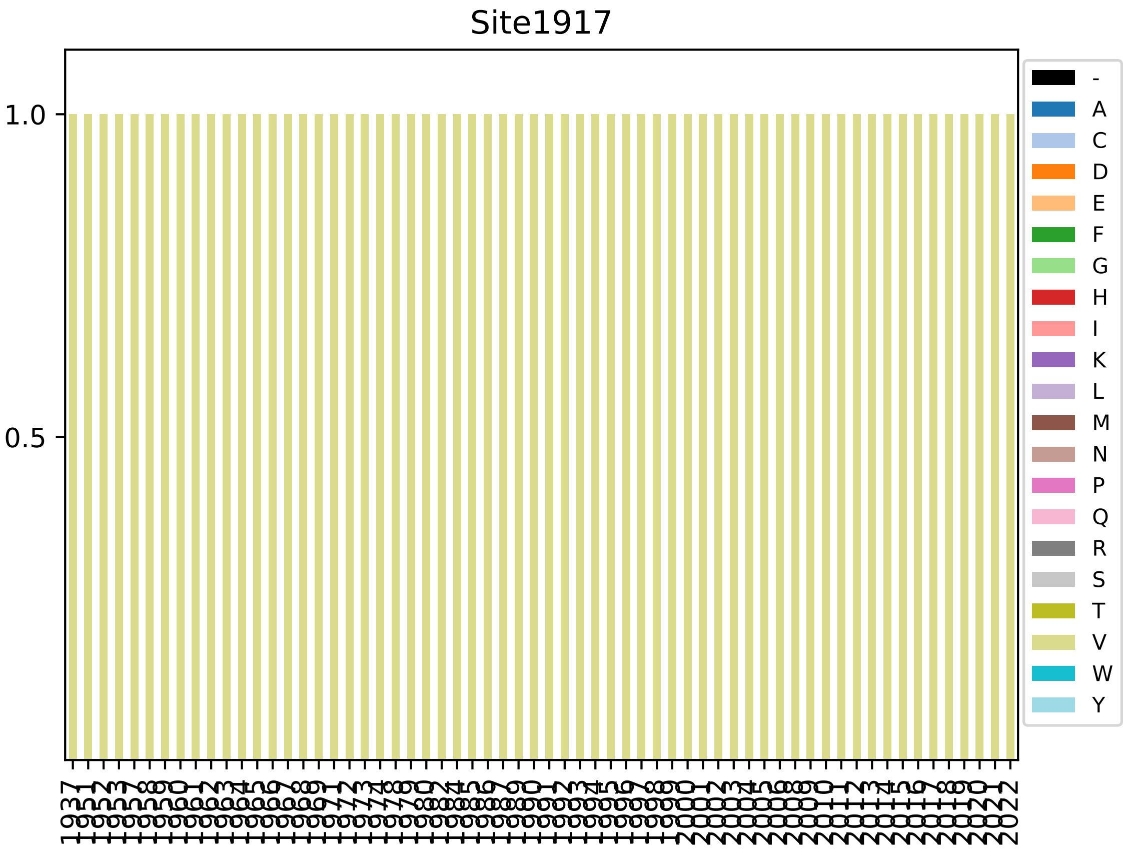
Site1917
1.0
0.5
-
A
C
D
E
F
G
H
I
K
L
M
N
P
Q
R
S
T
V
W
Y

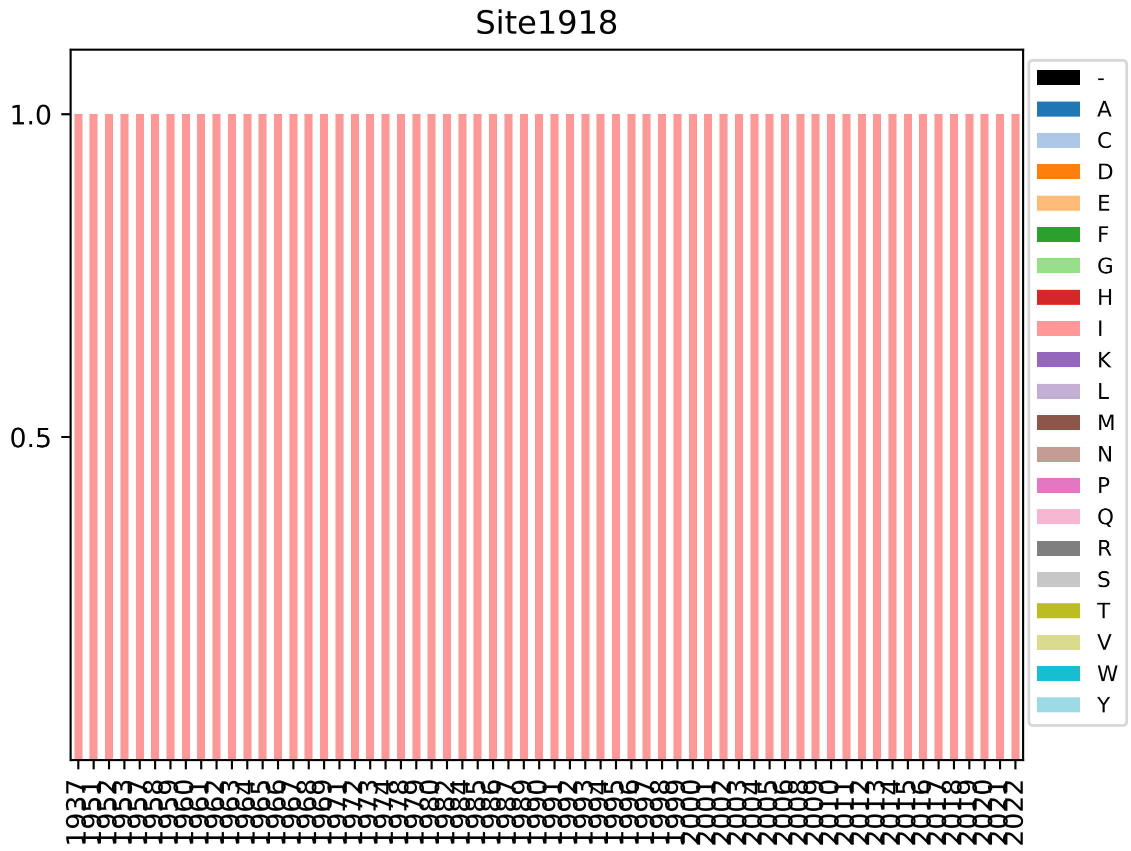
Site1918
1.0
0.5
-
A
C
D
E
F
G
H
I
K
L
M
N
P
Q
R
S
T
V
W
Y

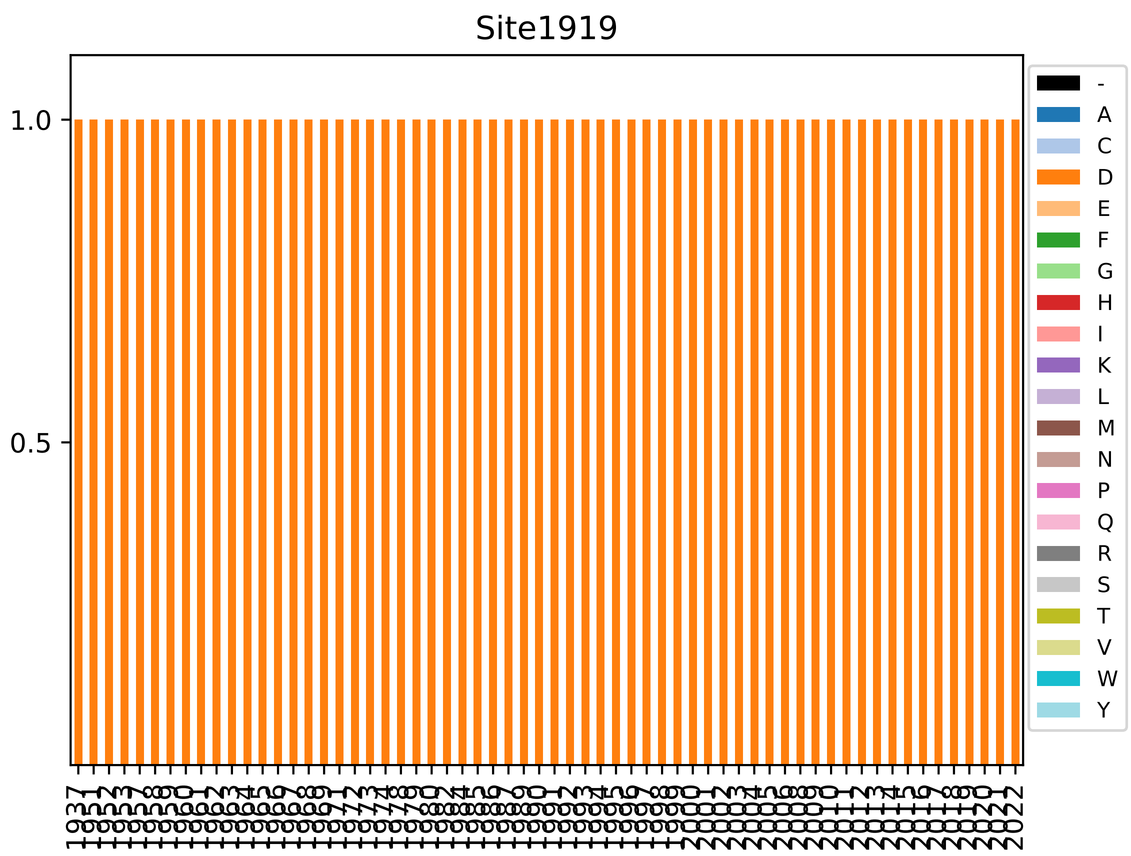
Site1919
1.0
0.5
-
A
C
D
E
F
G
H
I
K
L
M
N
P
Q
R
S
T
V
W
Y

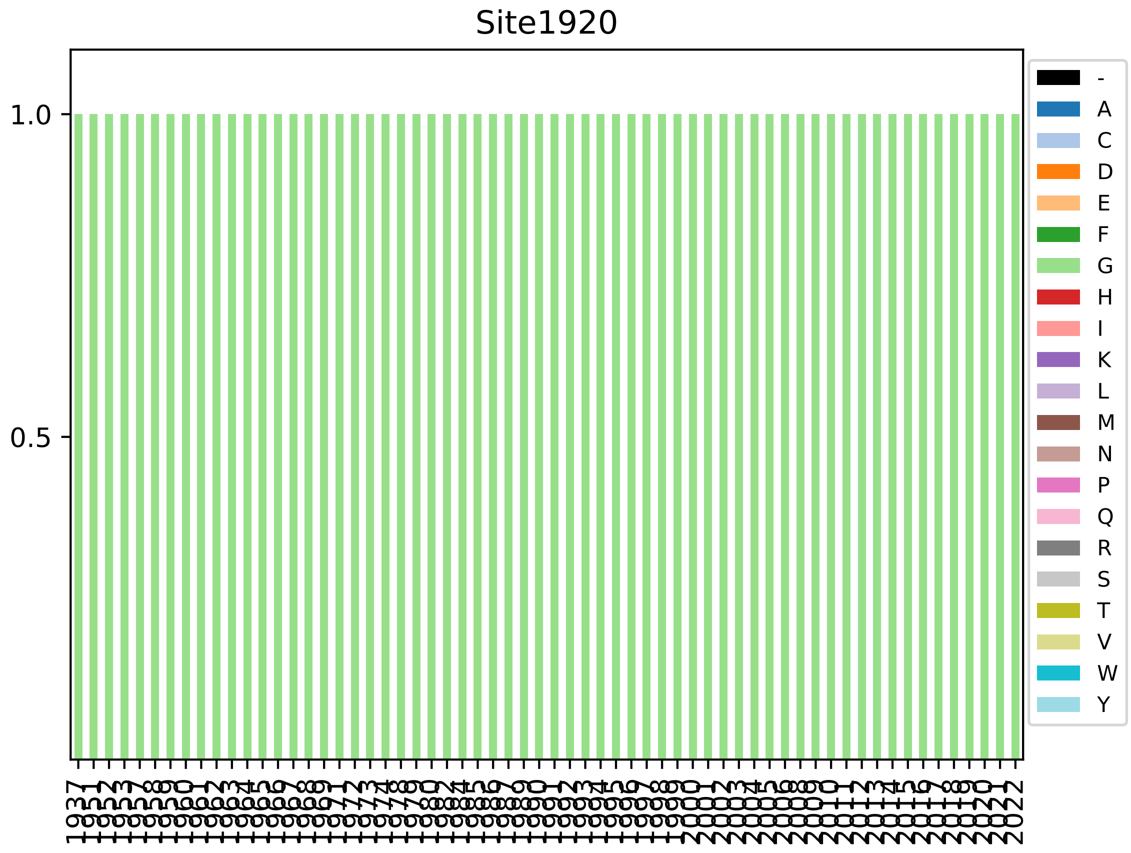
Site1920
1.0
0.5
-
A
C
D
E
F
G
H
I
K
L
M
N
P
Q
R
S
T
V
W
Y

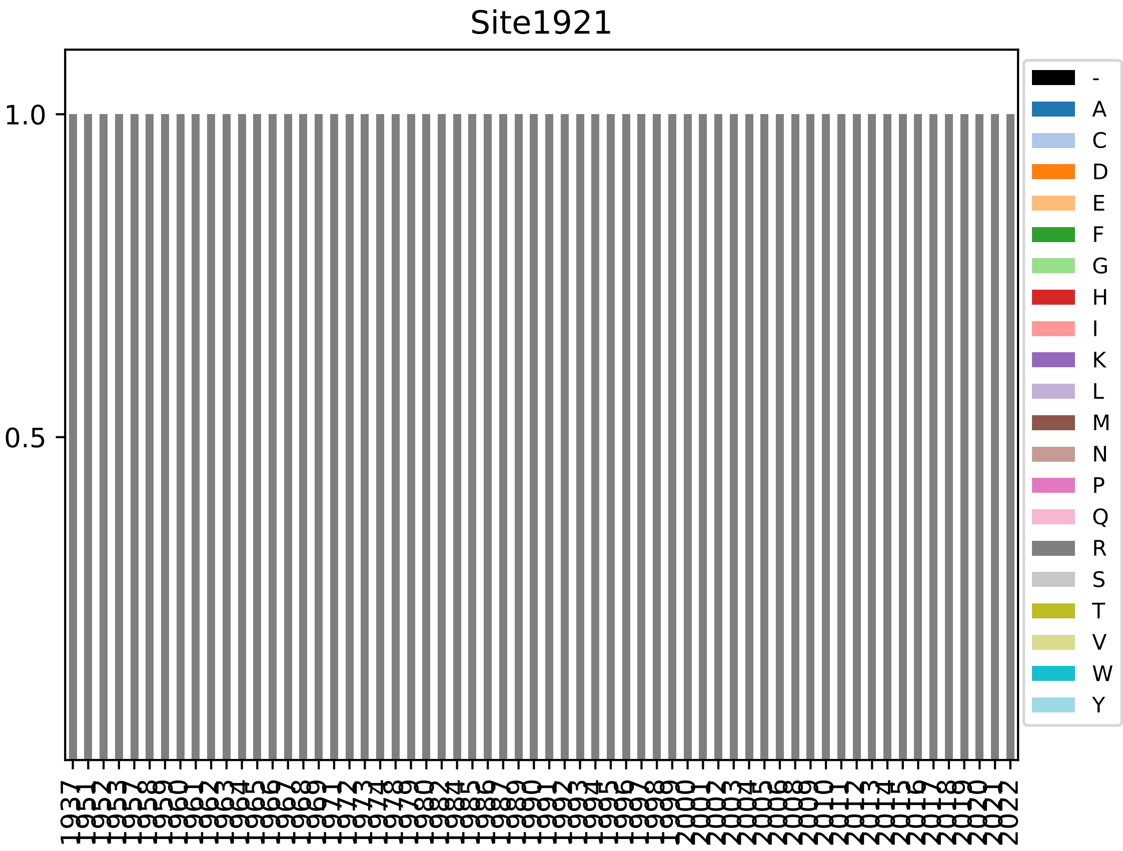
Site1921
1.0
0.5
-
A
C
D
E
F
G
H
I
K
L
M
N
P
Q
R
S
T
V
W
Y

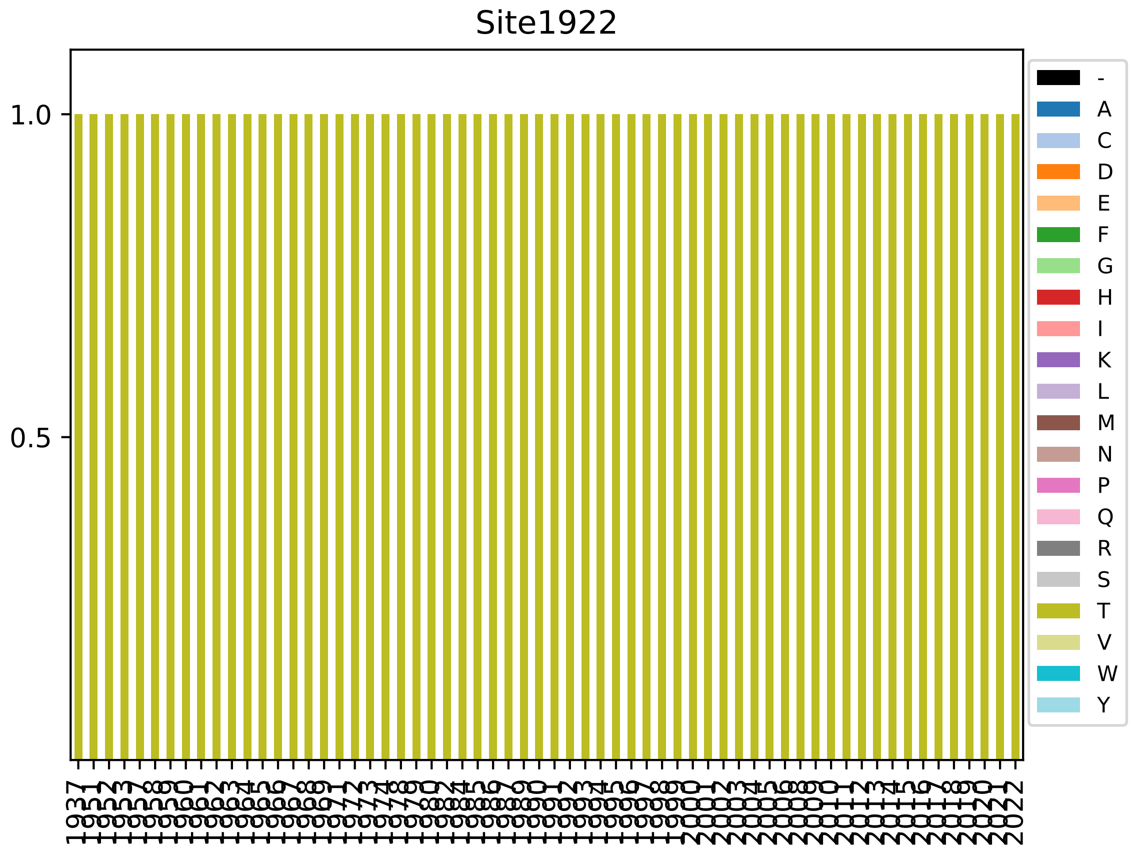
Site1922
1.0
0.5
-
A
C
D
E
F
G
H
I
K
L
M
N
P
Q
R
S
T
V
W
Y

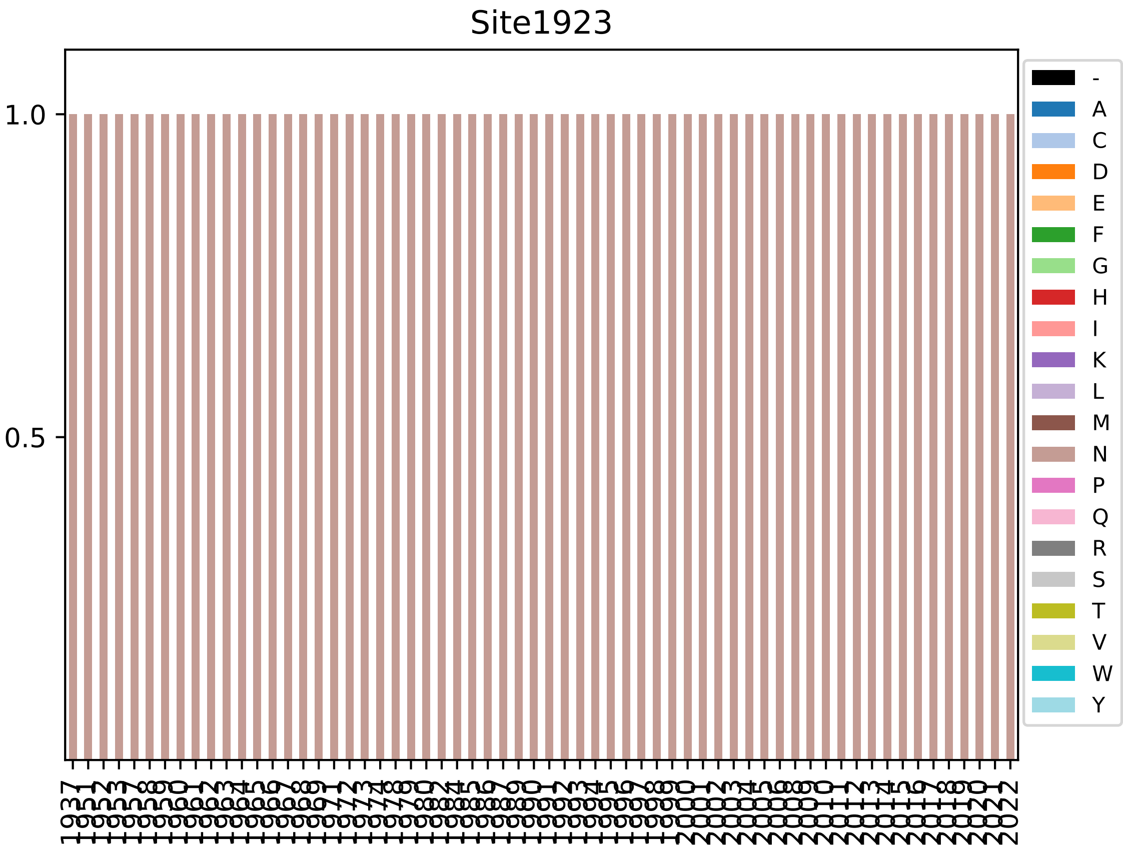
Site1923
1.0
0.5
-
A
C
D
E
F
G
H
I
K
L
M
N
P
Q
R
S
T
V
W
Y

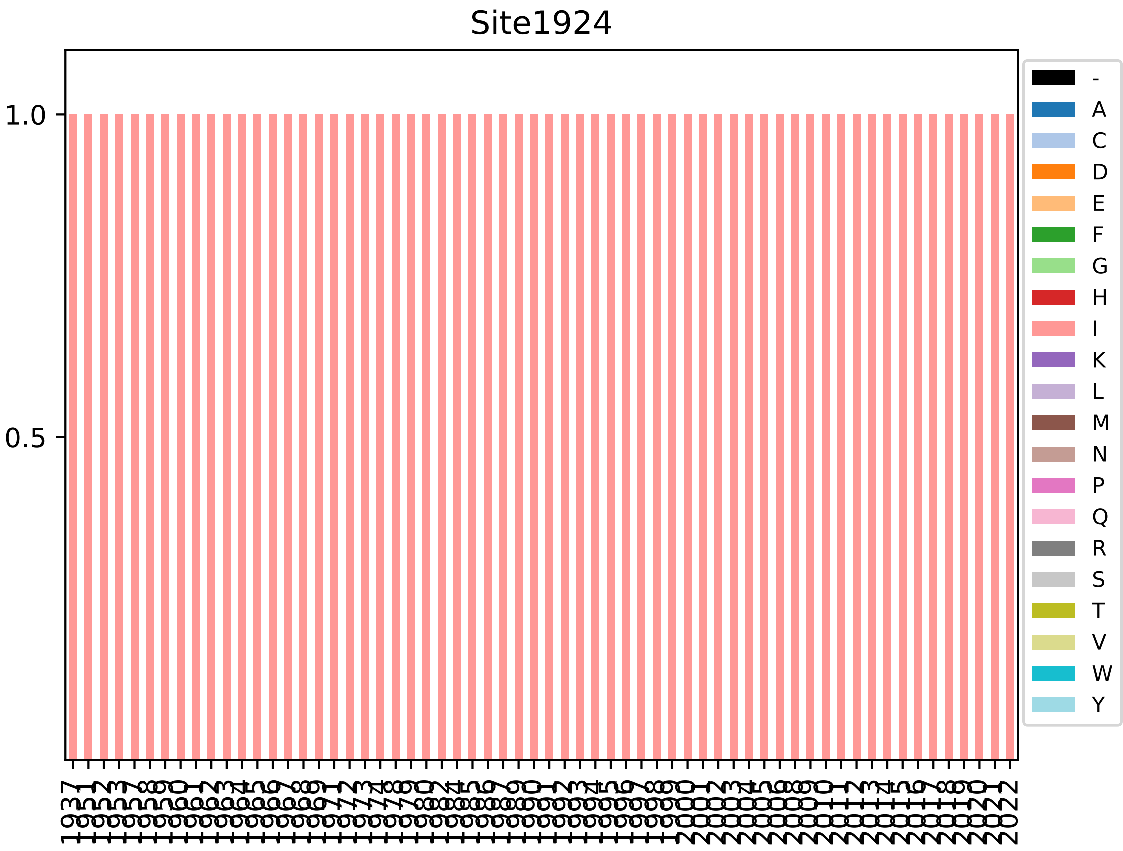

Site1924
1.0
0.5
-
A
C
D
E
F
G
H
I
K
L
M
N
P
Q
R
S
T
V
W
Y

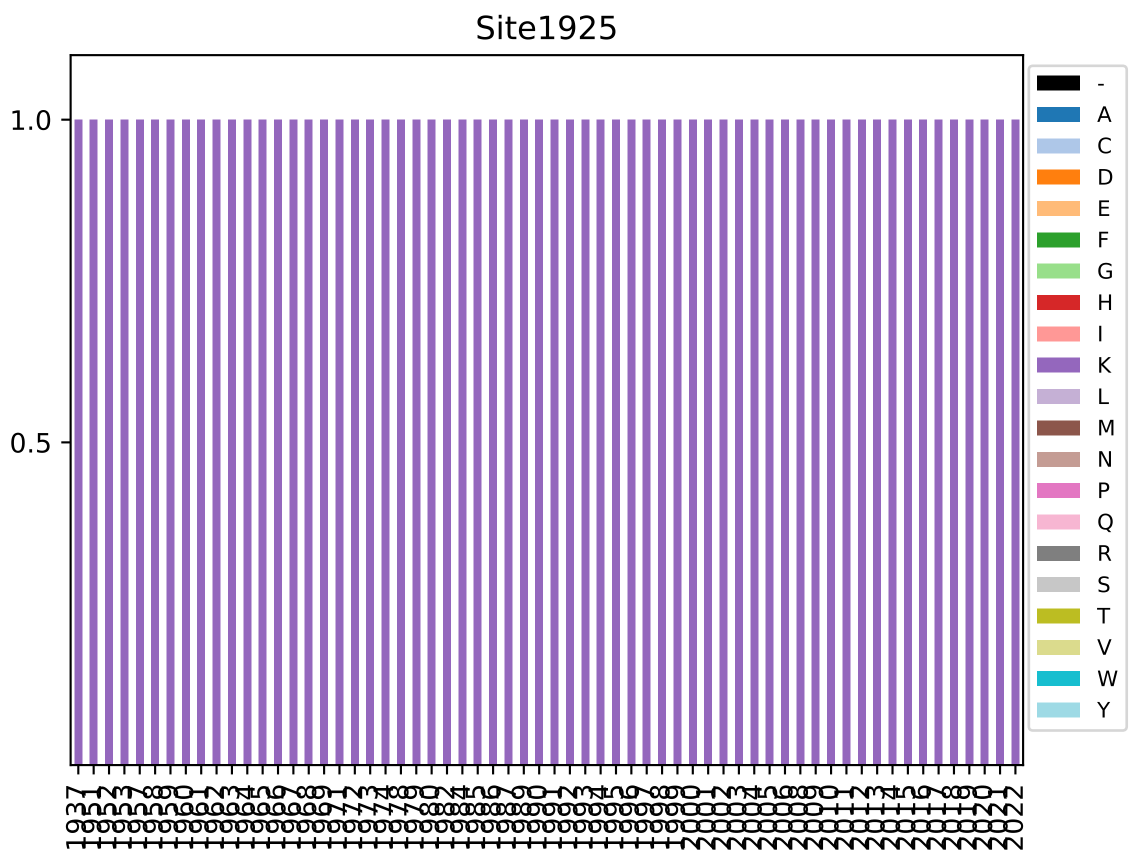
Site1925
1.0
0.5
-
A
C
D
E
F
G
H
I
K
L
M
N
P
Q
R
S
T
V
W
Y

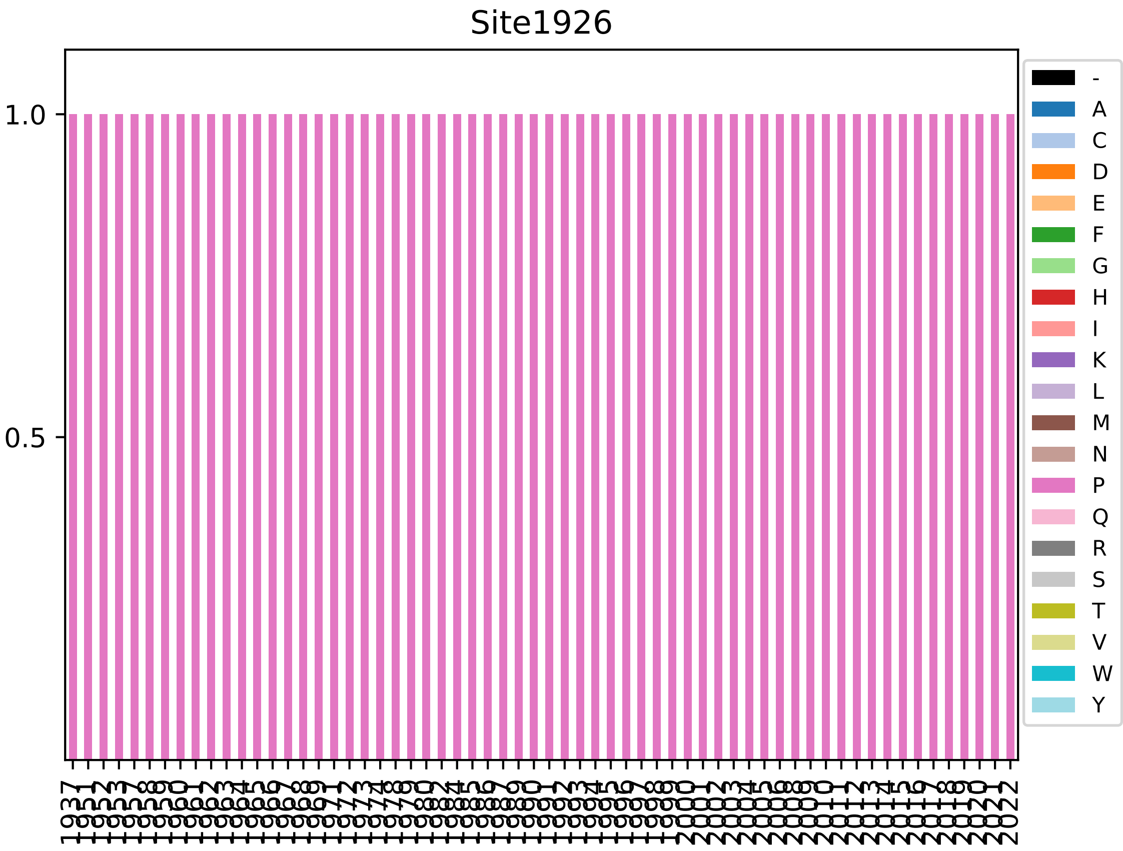
Site1926
1.0
0.5
-
A
C
D
E
F
G
H
I
K
L
M
N
P
Q
R
S
T
V
W
Y

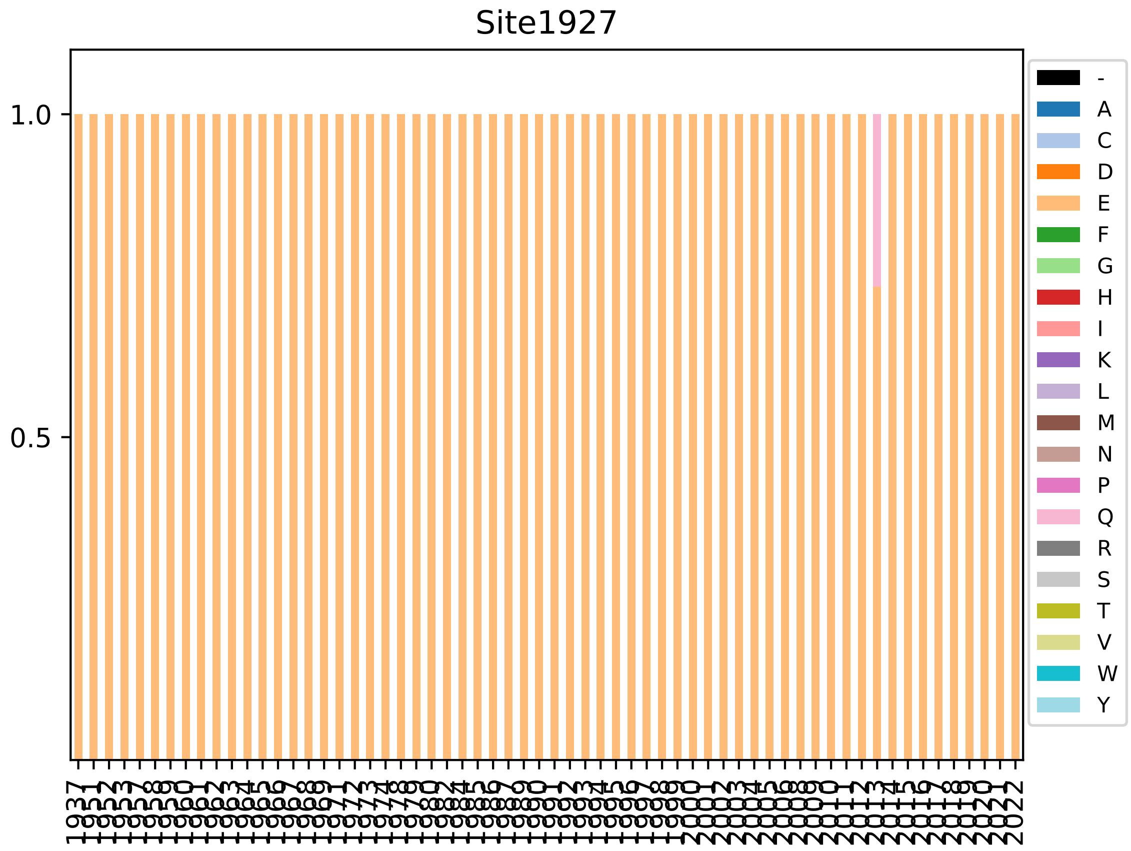
Site1927
1.0
0.5
-
A
C
D
E
F
G
H
I
K
L
M
N
P
Q
R
S
T
V
W
Y

Site1928

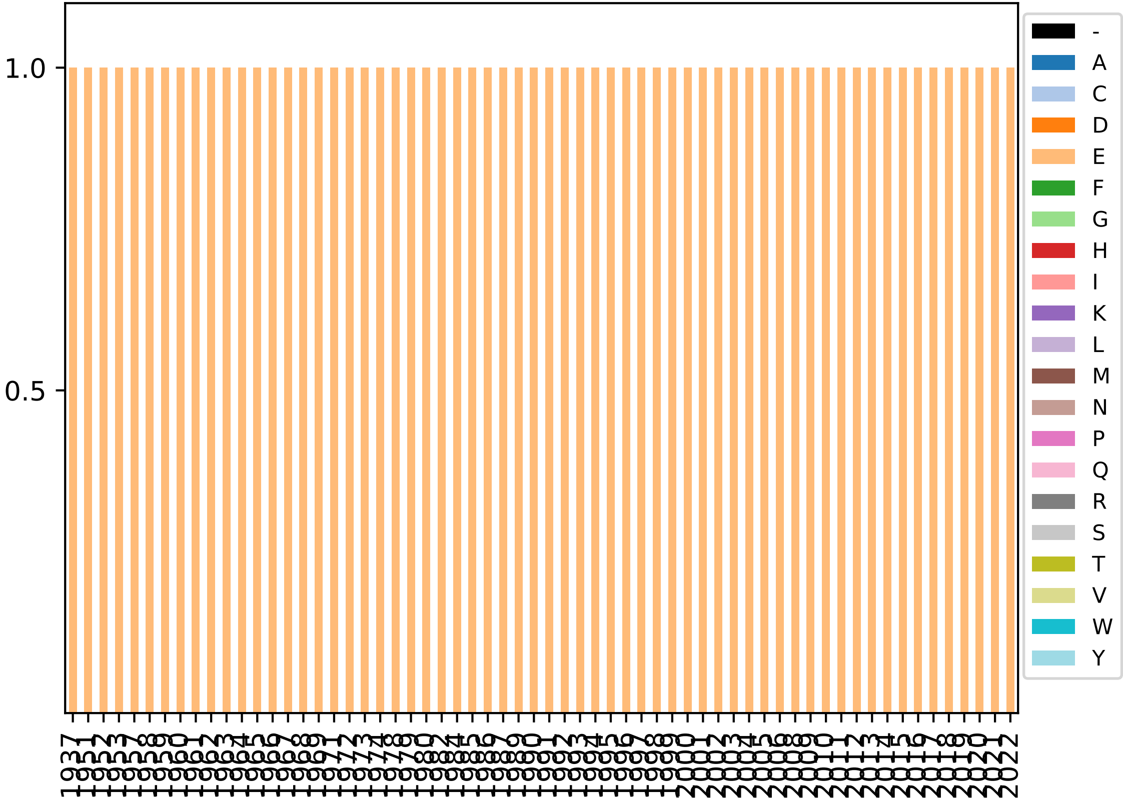

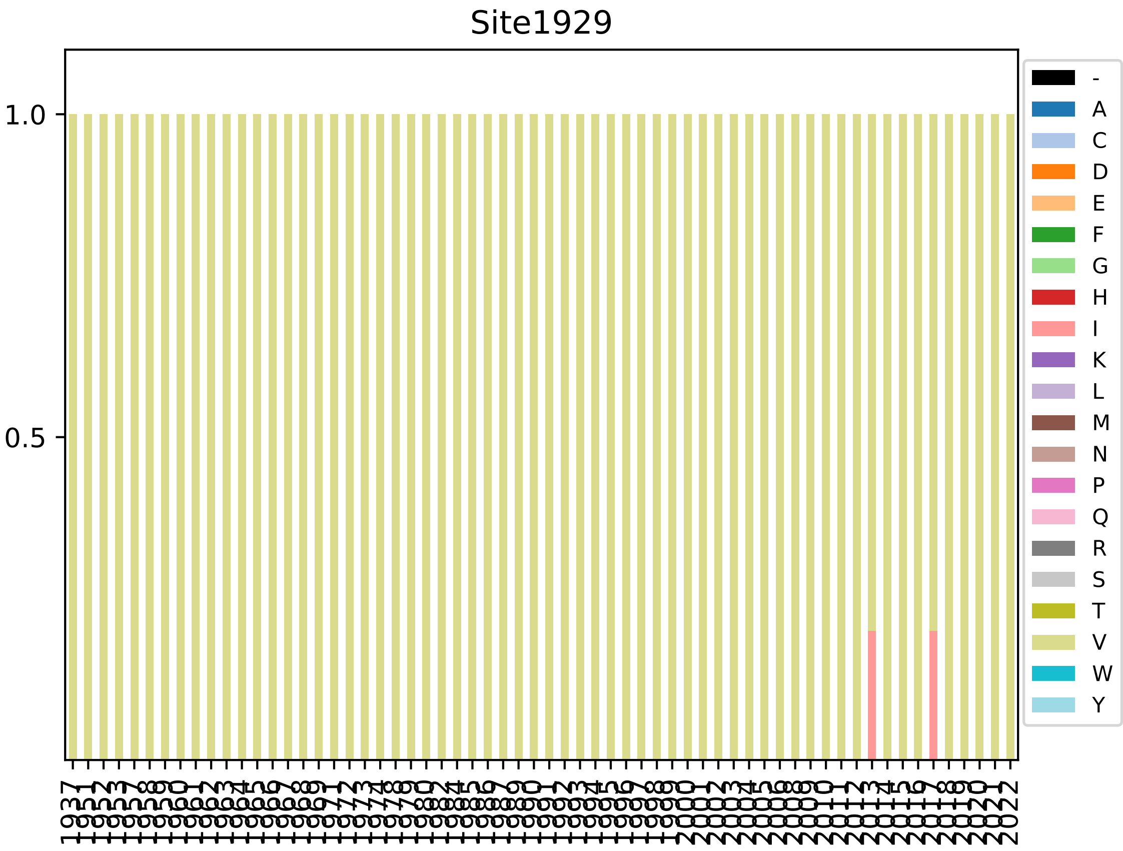
Site1929
1.0
0.5
-
A
C
D
E
F
G
H
I
K
L
M
N
P
Q
R
S
T
V
W
Y

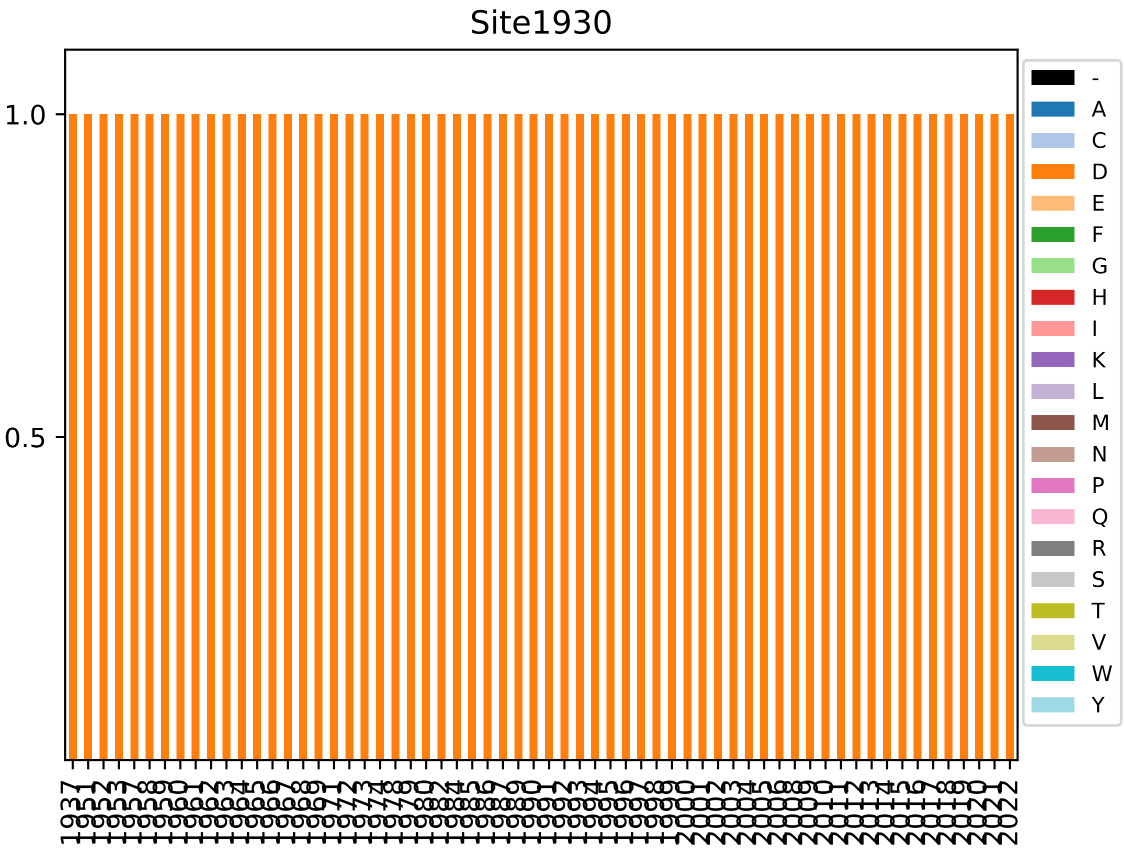
Site1930
1.0
0.5
-
A
C
D
E
F
G
H
I
K
L
M
N
P
Q
R
S
T
V
W
Y

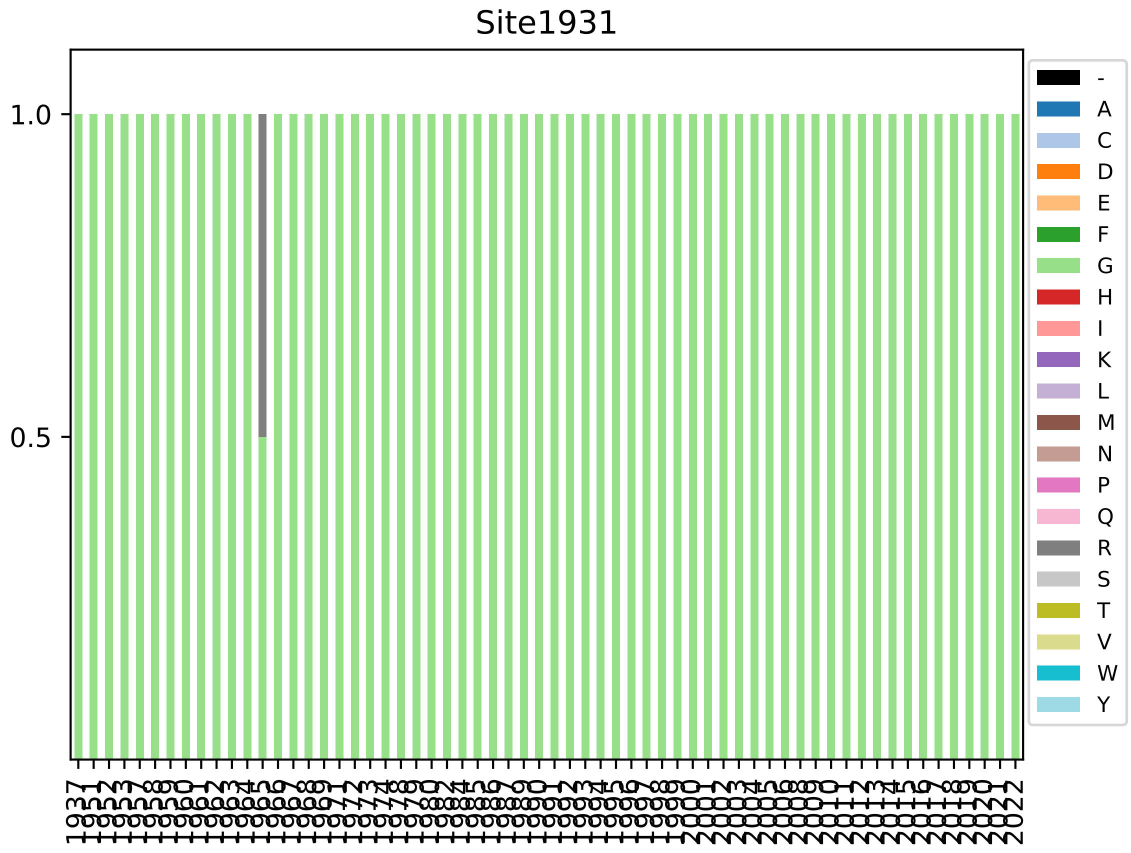
Site1931
1.0
0.5
-
A
C
D
E
F
G
H
I
K
L
M
N
P
Q
R
S
T
V
W
Y

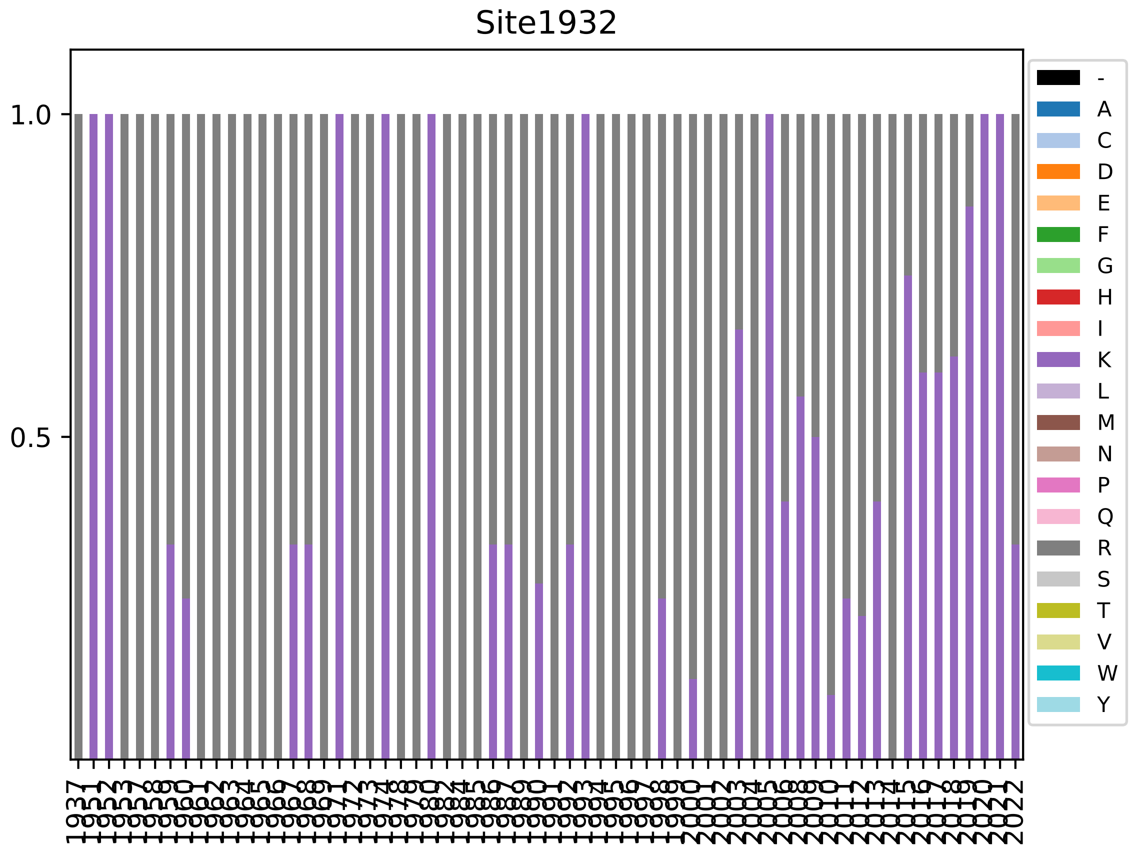
Site1932
1.0
0.5
-
A
C
D
E
F
G
H
I
K
L
M
N
P
Q
R
S
T
V
W
Y

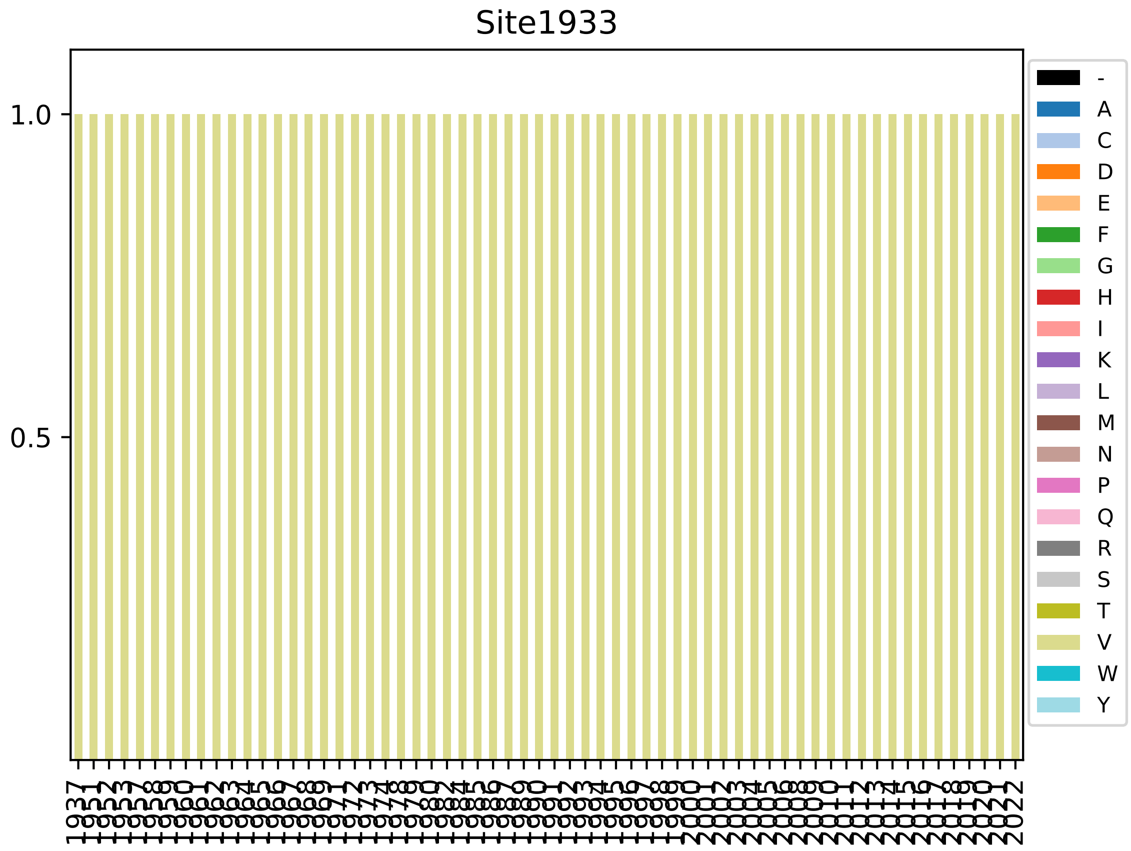
Site1933
1.0
0.5
-
A
C
D
E
F
G
H
I
K
L
M
N
P
Q
R
S
T
V
W
Y

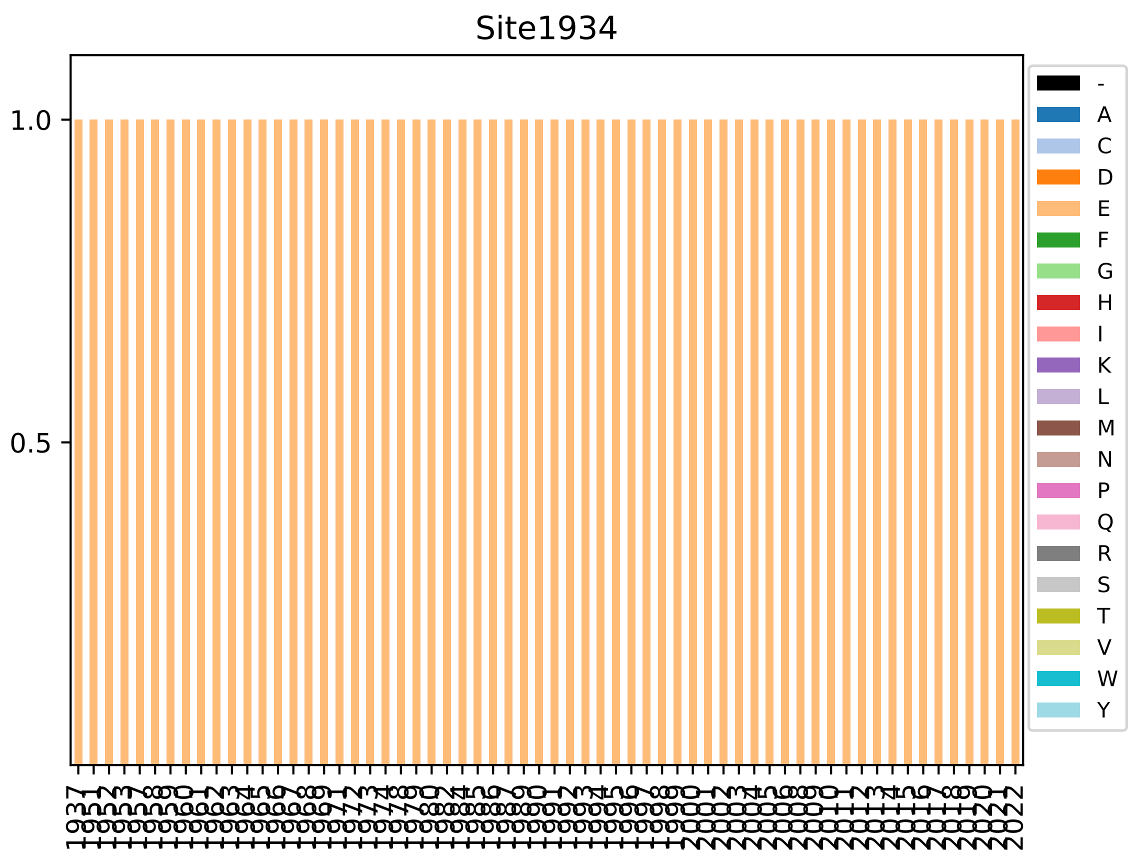
Site1934
1.0
0.5
-
A
C
D
E
F
G
H
I
K
L
M
N
P
Q
R
S
T
V
W
Y

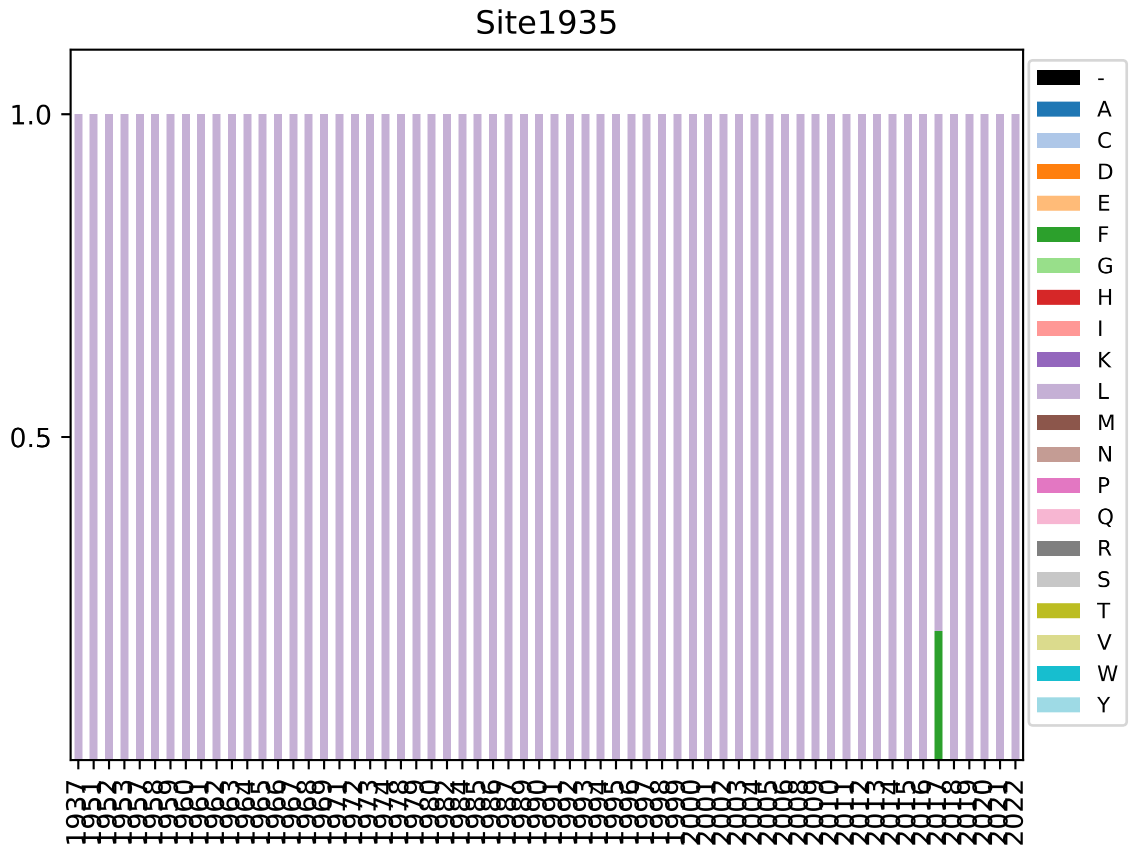
Site1935
1.0
0.5
-
A
C
D
E
F
G
H
I
K
L
M
N
P
Q
R
S
T
V
W
Y

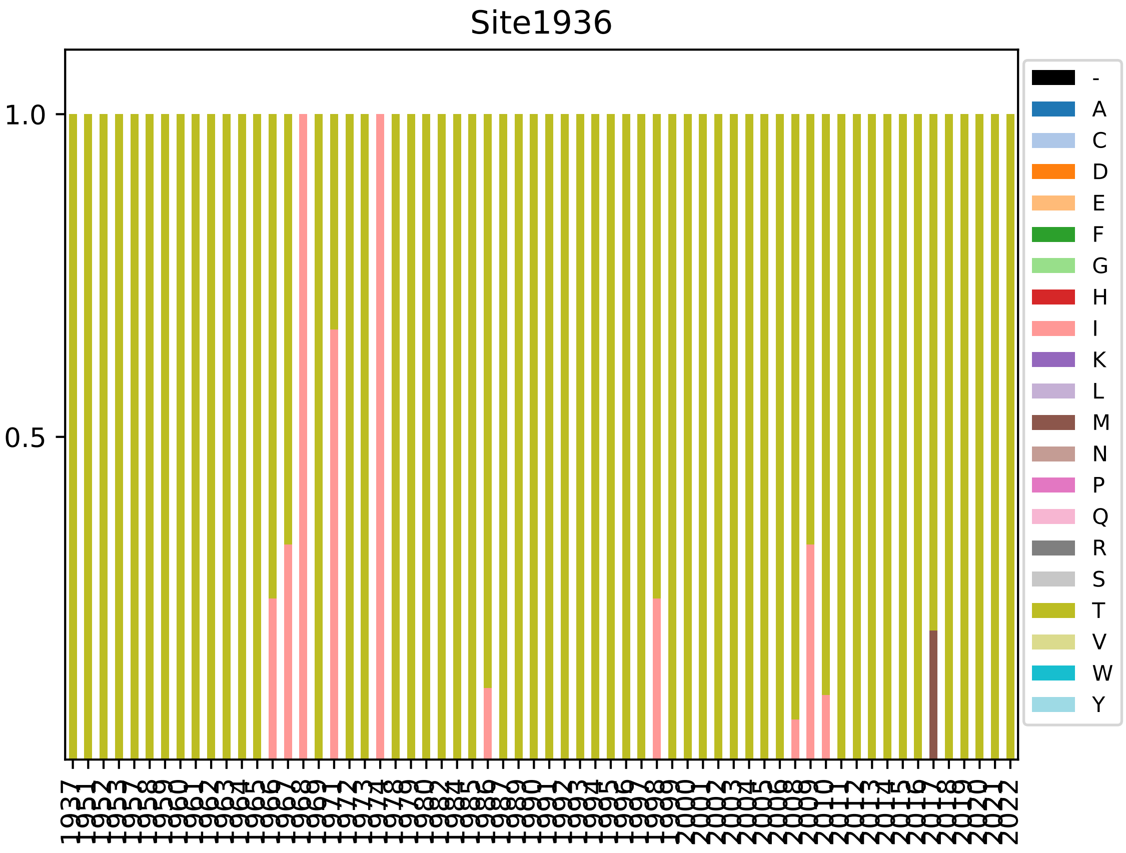
Site1936
1.0
0.5
-
A
C
D
E
F
G
H
I
K
L
M
N
P
Q
R
S
T
V
W
Y

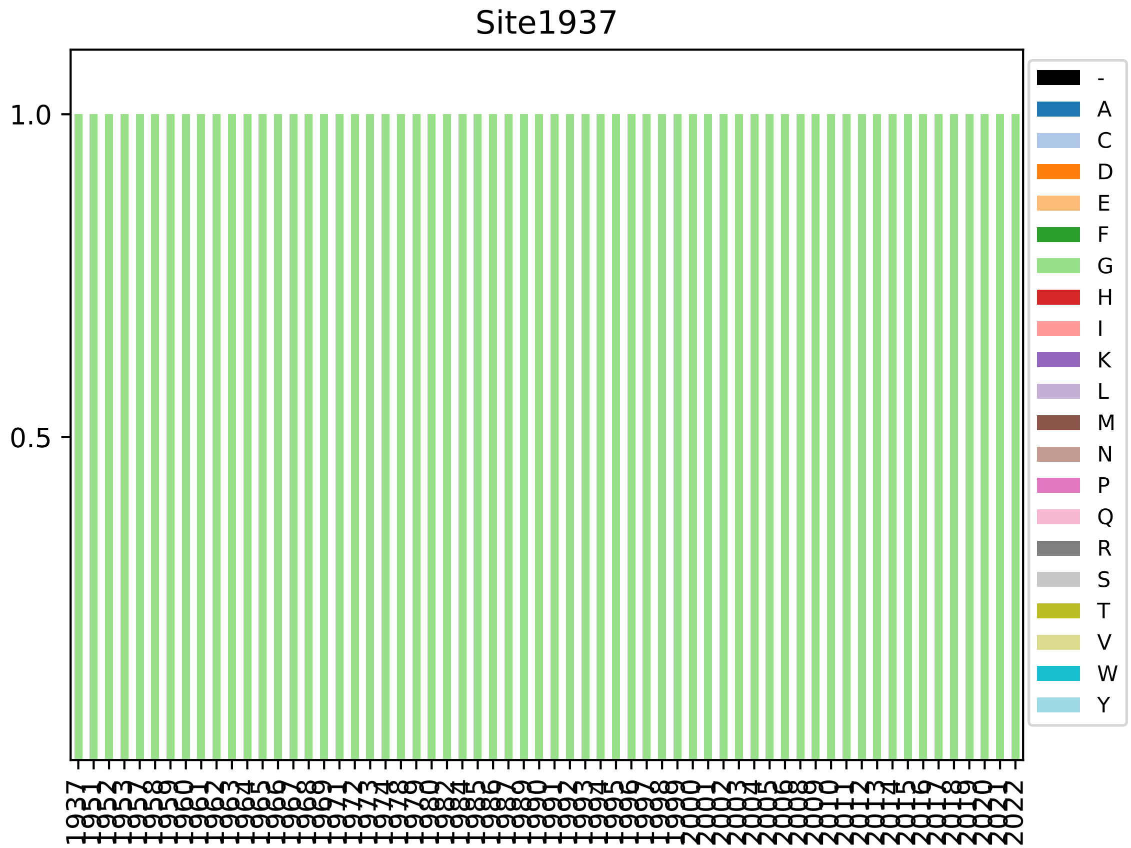
Site1937
1.0
0.5
-
A
C
D
E
F
G
H
I
K
L
M
N
P
Q
R
S
T
V
W
Y

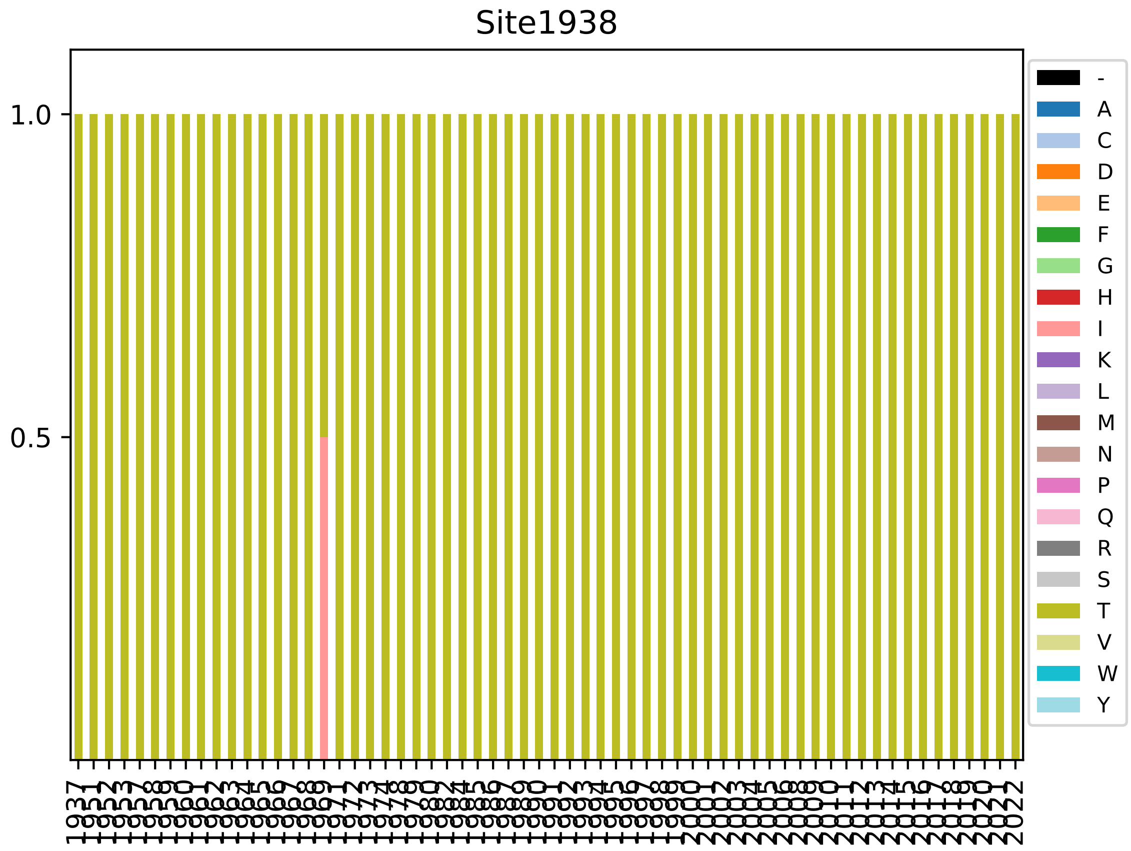
Site1938
1.0
0.5
-
A
C
D
E
F
G
H
I
K
L
M
N
P
Q
R
S
T
V
W
Y

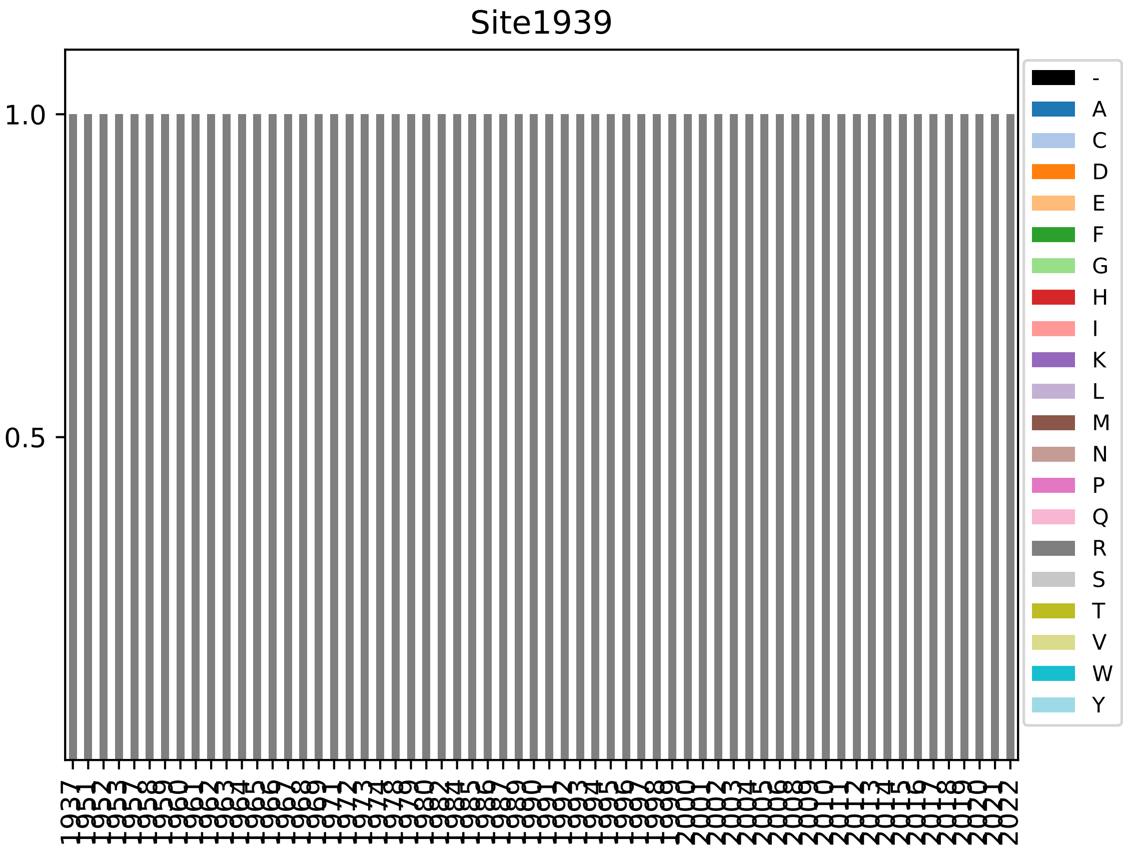

Site1939
1.0
0.5
-
A
C
D
E
F
G
H
I
K
L
M
N
P
Q
R
S
T
V
W
Y

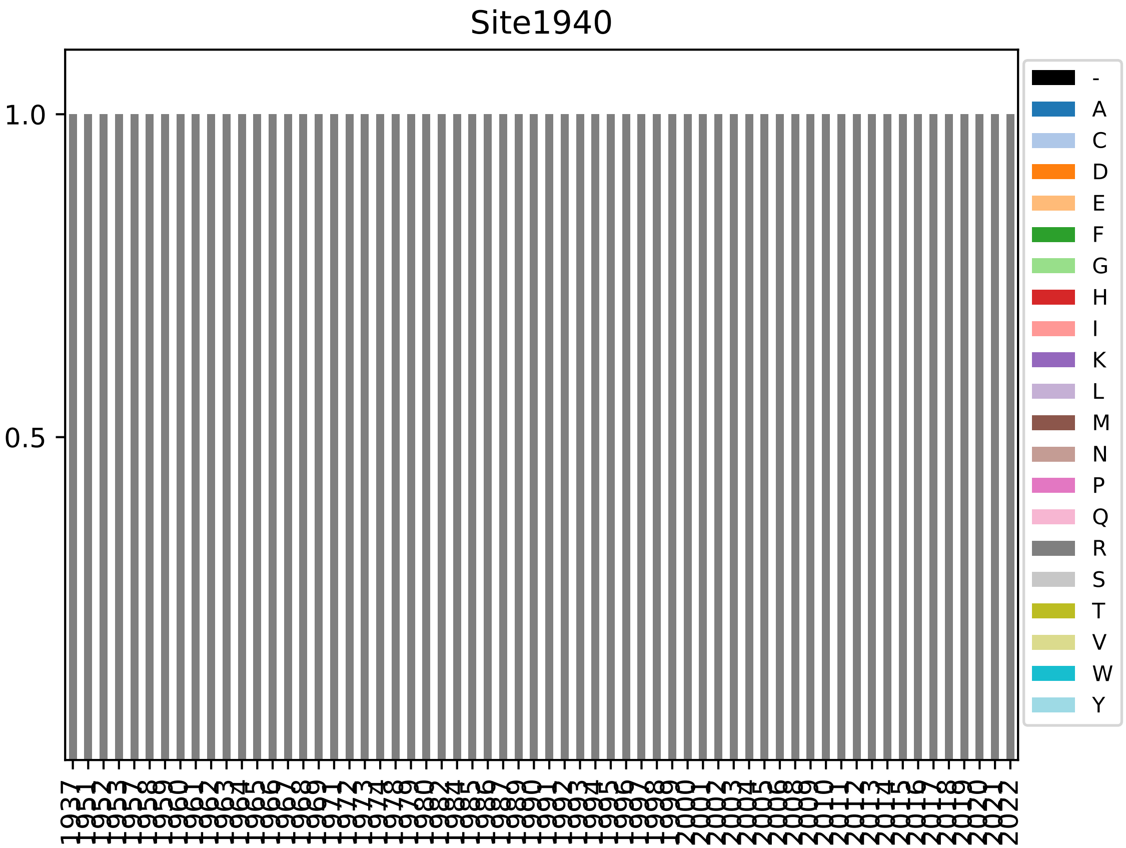
Site1940
1.0
0.5
-
A
C
D
E
F
G
H
I
K
L
M
N
P
Q
R
S
T
V
W
Y

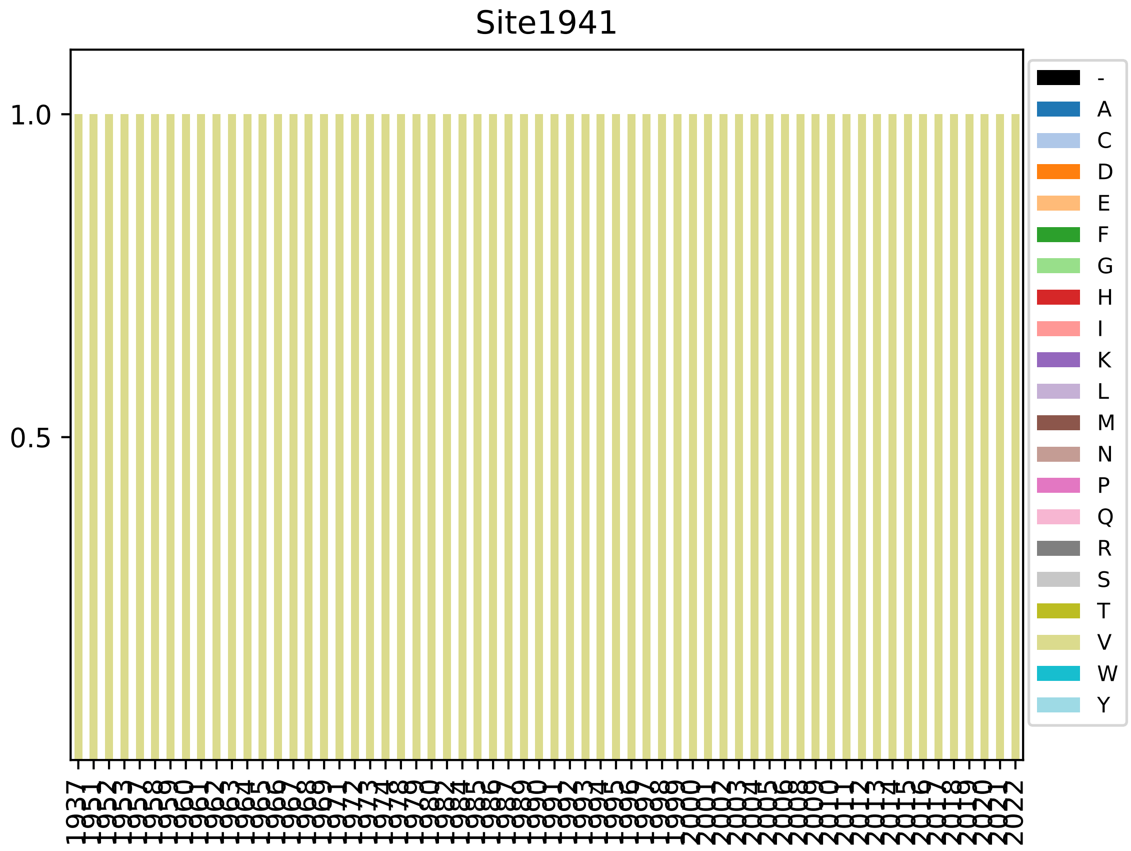
Site1941
1.0
0.5
-
A
C
D
E
F
G
H
I
K
L
M
N
P
Q
R
S
T
V
W
Y

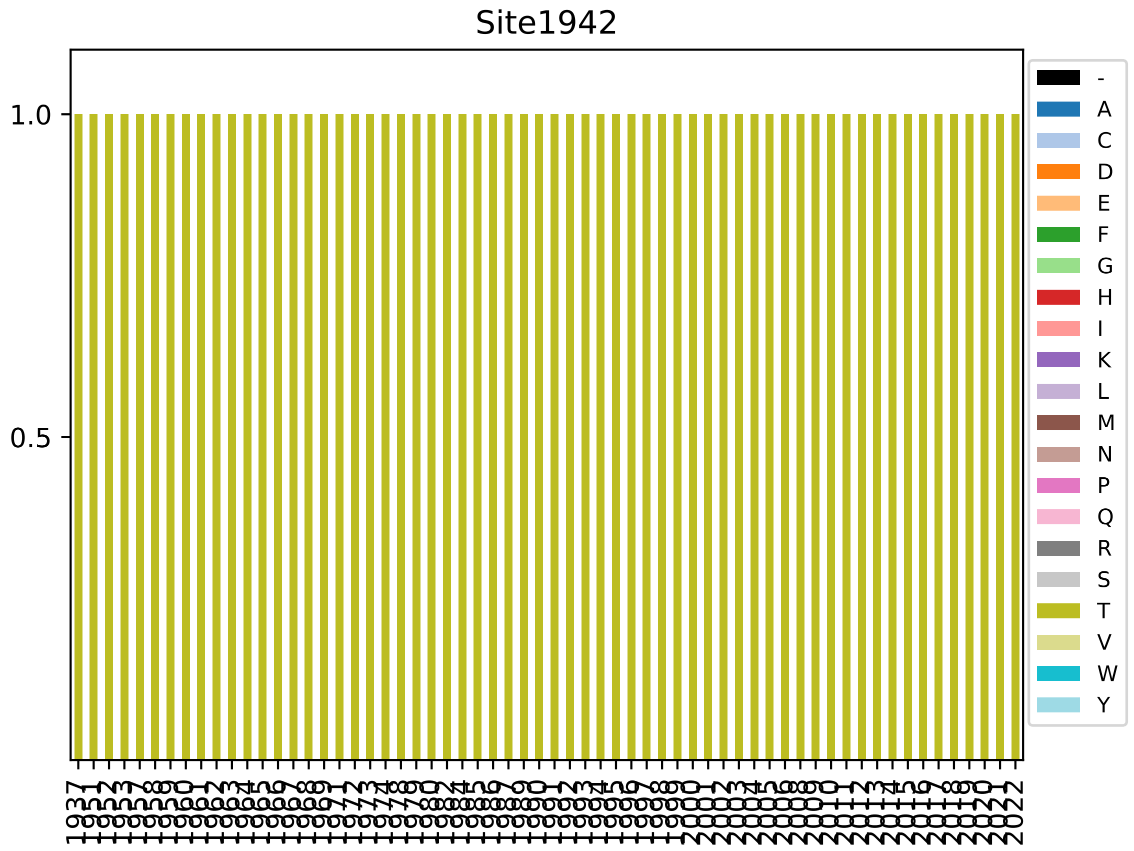

Site1942
1.0
0.5
-
A
C
D
E
F
G
H
I
K
L
M
N
P
Q
R
S
T
V
W
Y

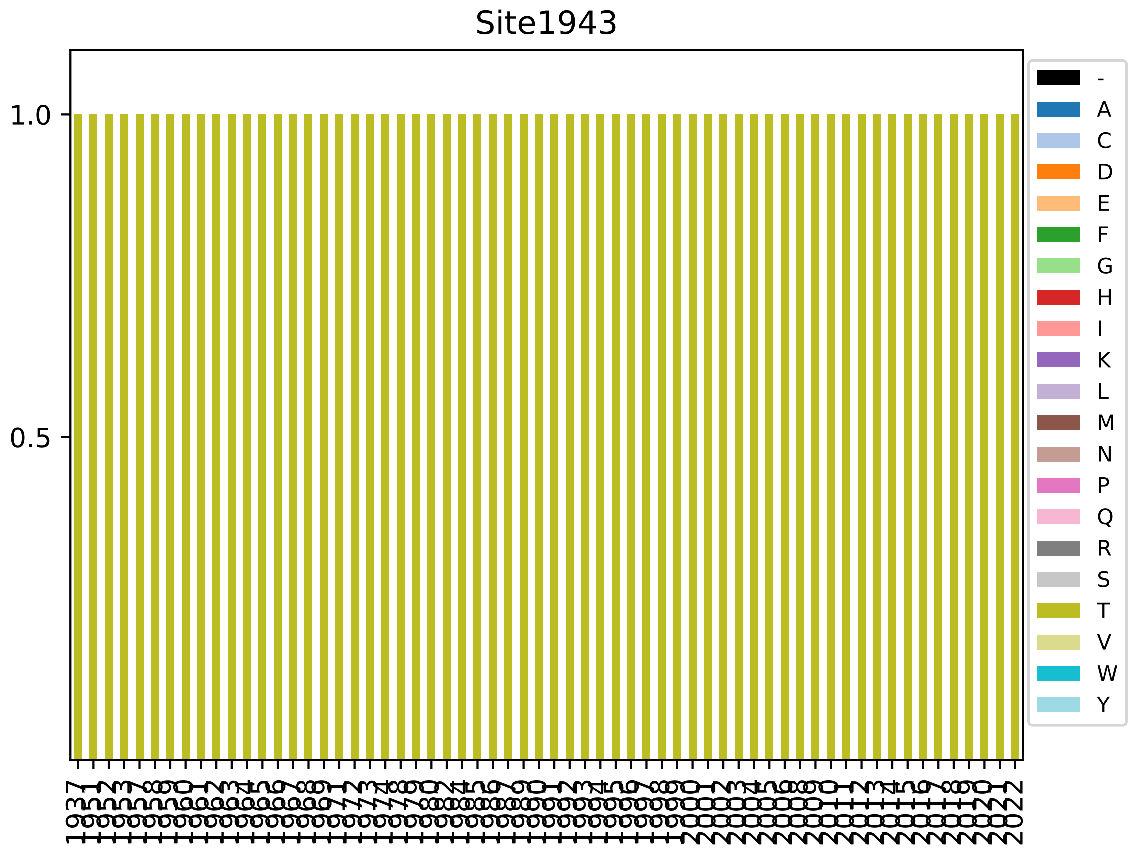
Site1943
1.0
0.5
-
A
C
D
E
F
G
H
I
K
L
M
N
P
Q
R
S
T
V
W
Y

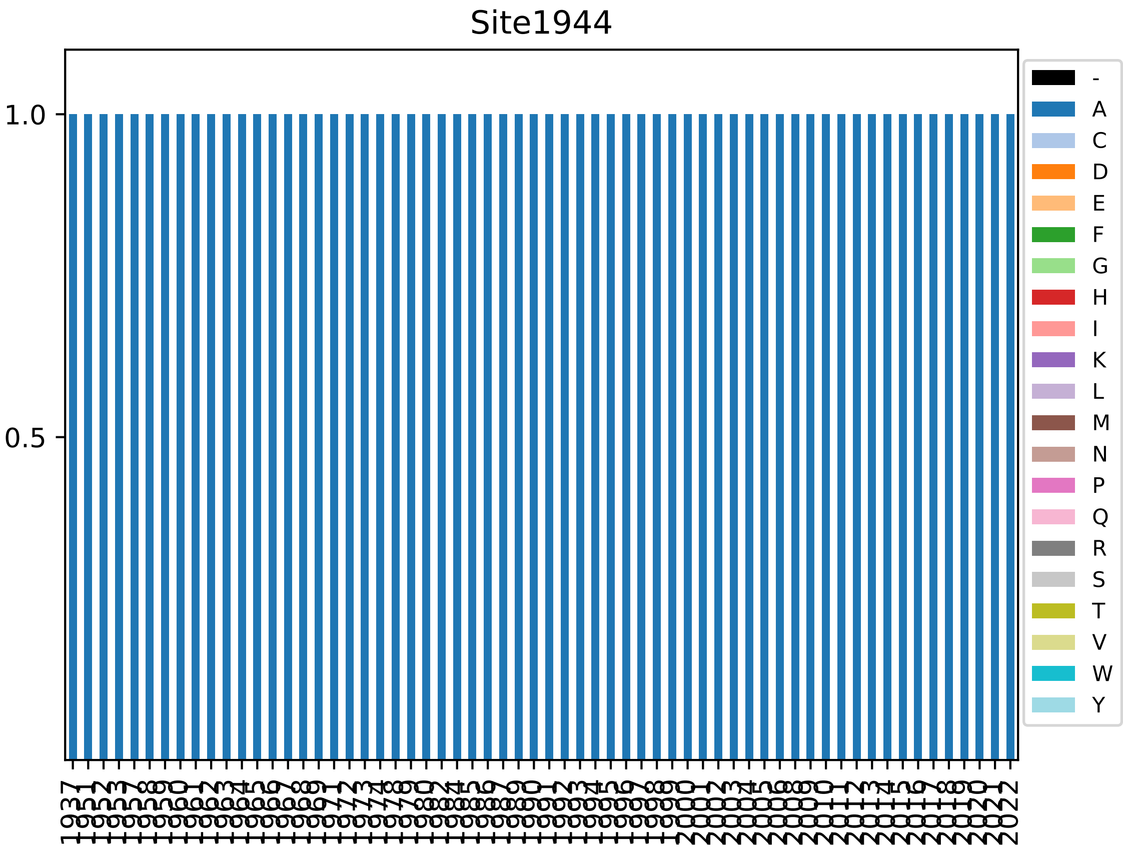
Site1944
1.0
0.5
-
A
C
D
E
F
G
H
I
K
L
M
N
P
Q
R
S
T
V
W
Y

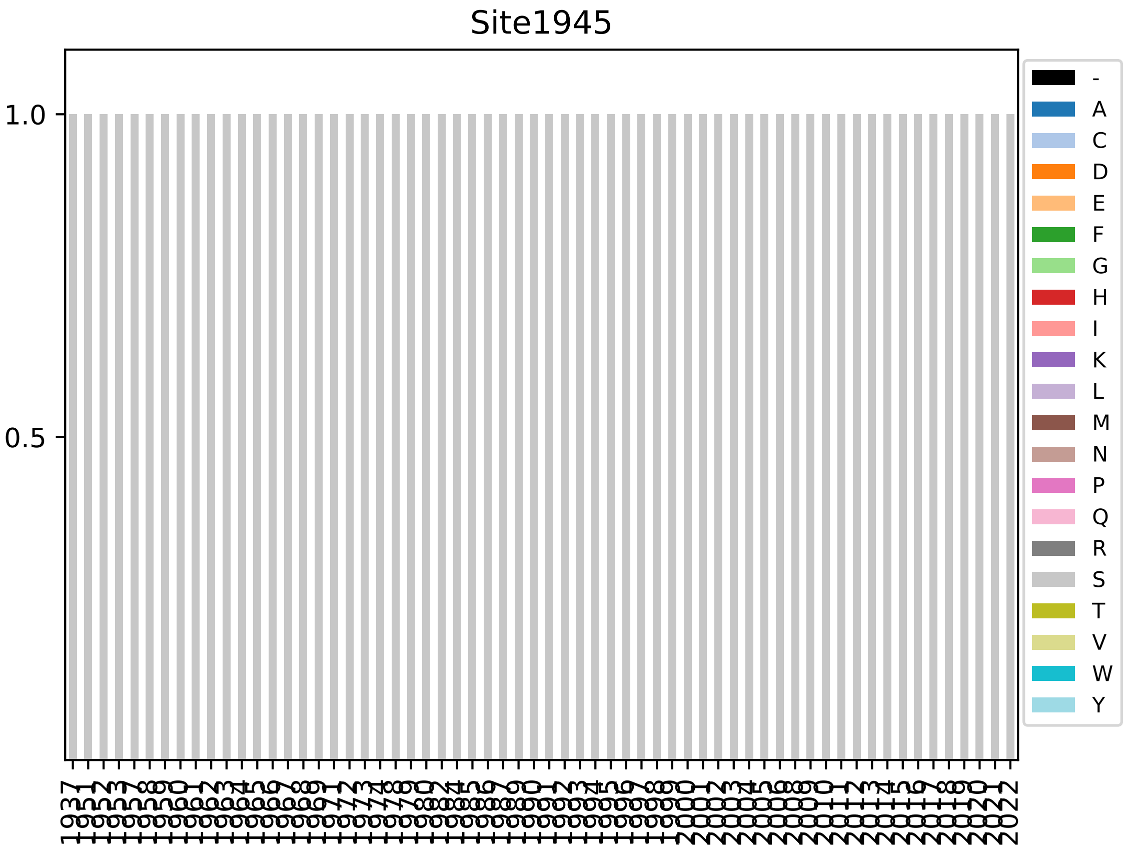
Site1945
1.0
0.5
-
A
C
D
E
F
G
H
I
K
L
M
N
P
Q
R
S
T
V
W
Y

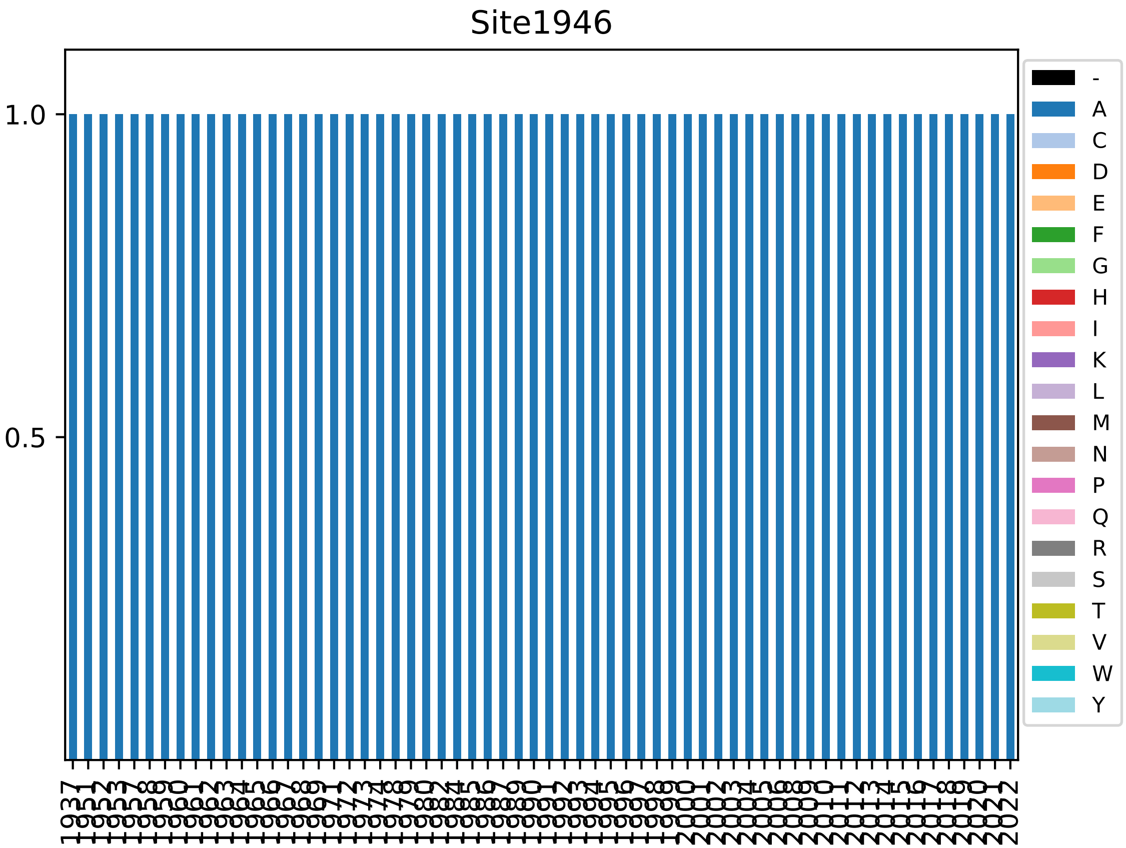
Site1946
1.0
0.5
-
A
C
D
E
F
G
H
I
K
L
M
N
P
Q
R
S
T
V
W
Y

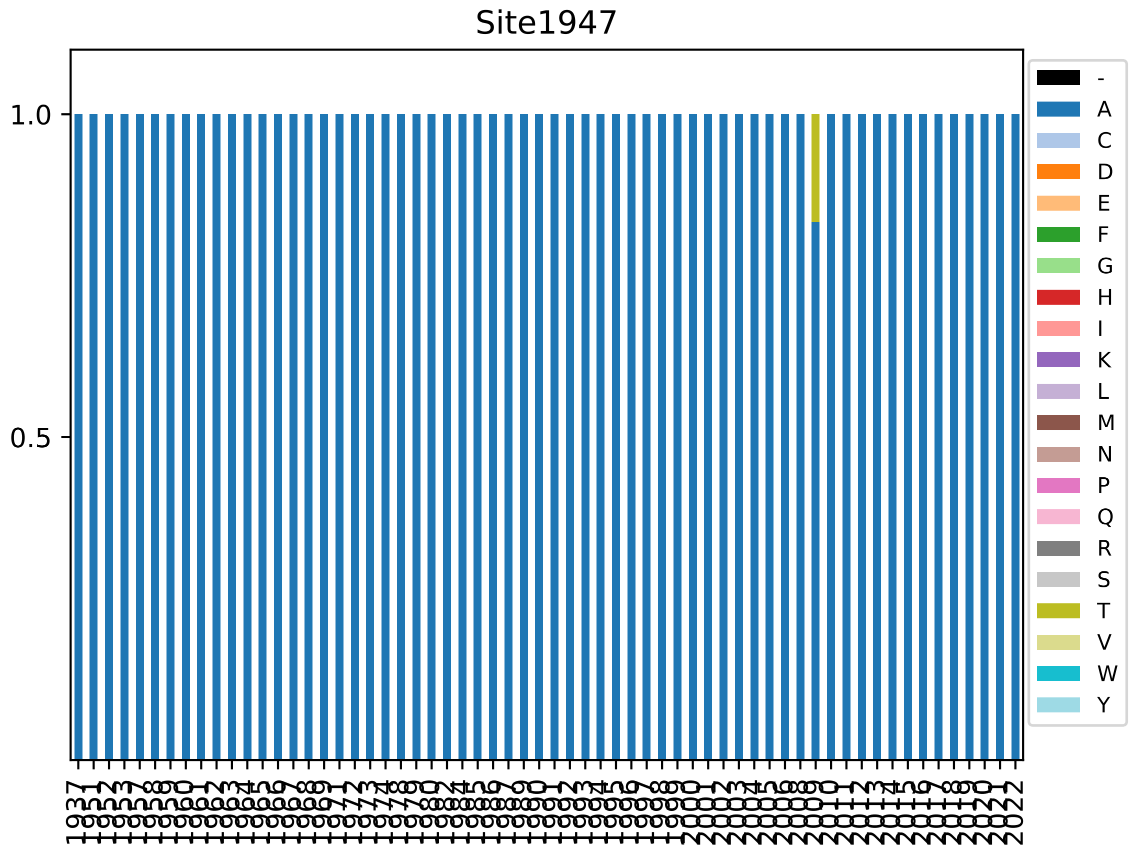
Site1947
1.0
0.5
-
A
C
D
E
F
G
H
I
K
L
M
N
P
Q
R
S
T
V
W
Y

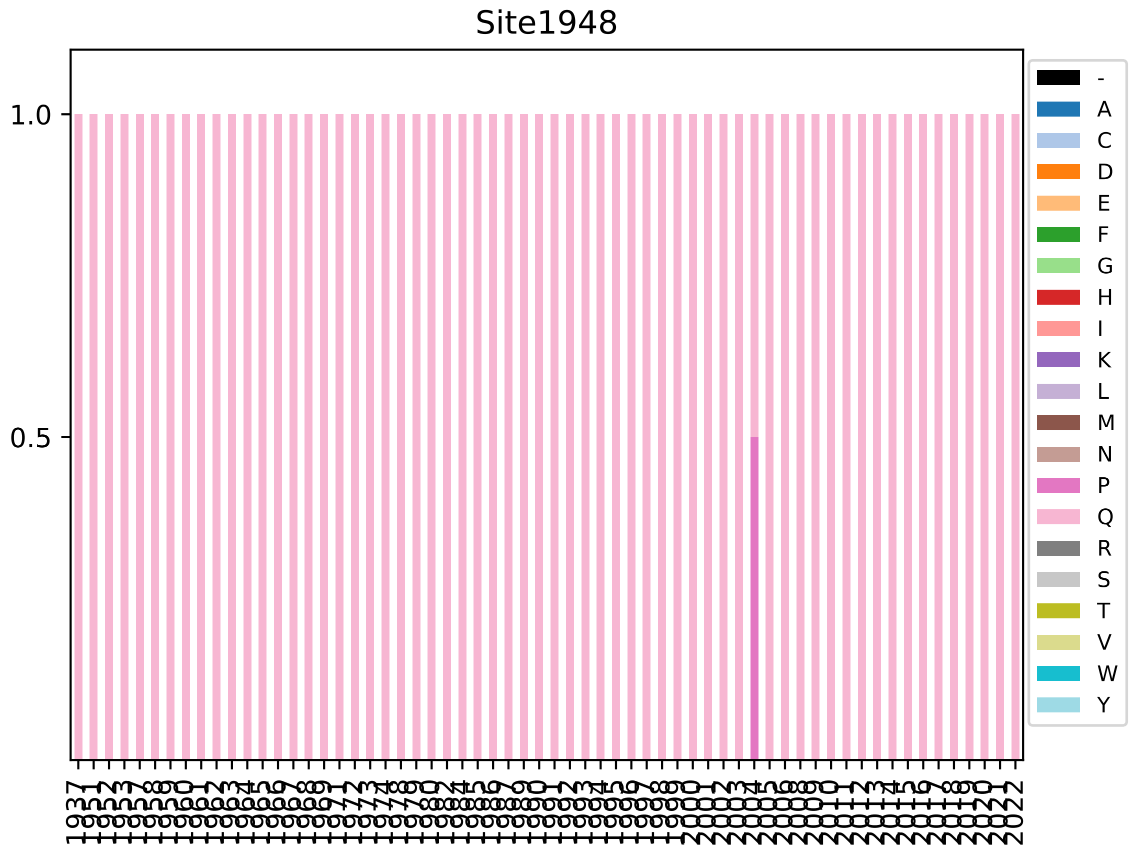
Site1948
1.0
0.5
-
A
C
D
E
F
G
H
I
K
L
M
N
P
Q
R
S
T
V
W
Y

Site1949

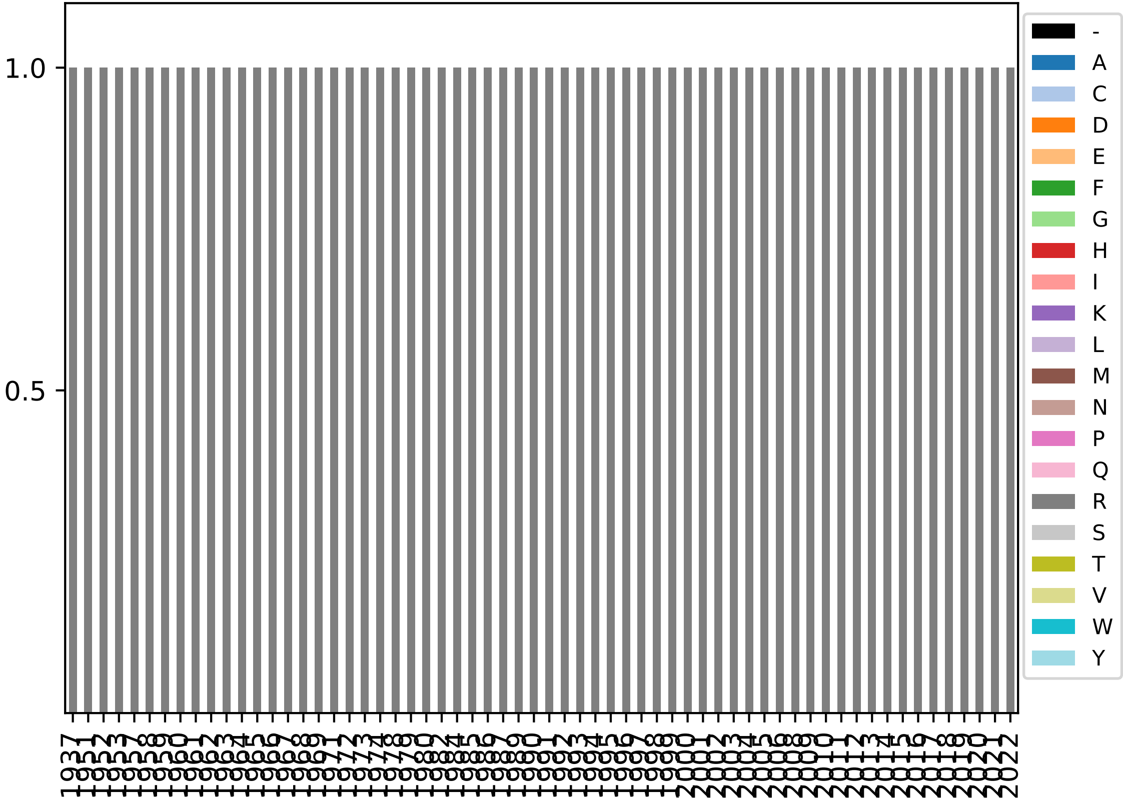

Site1950

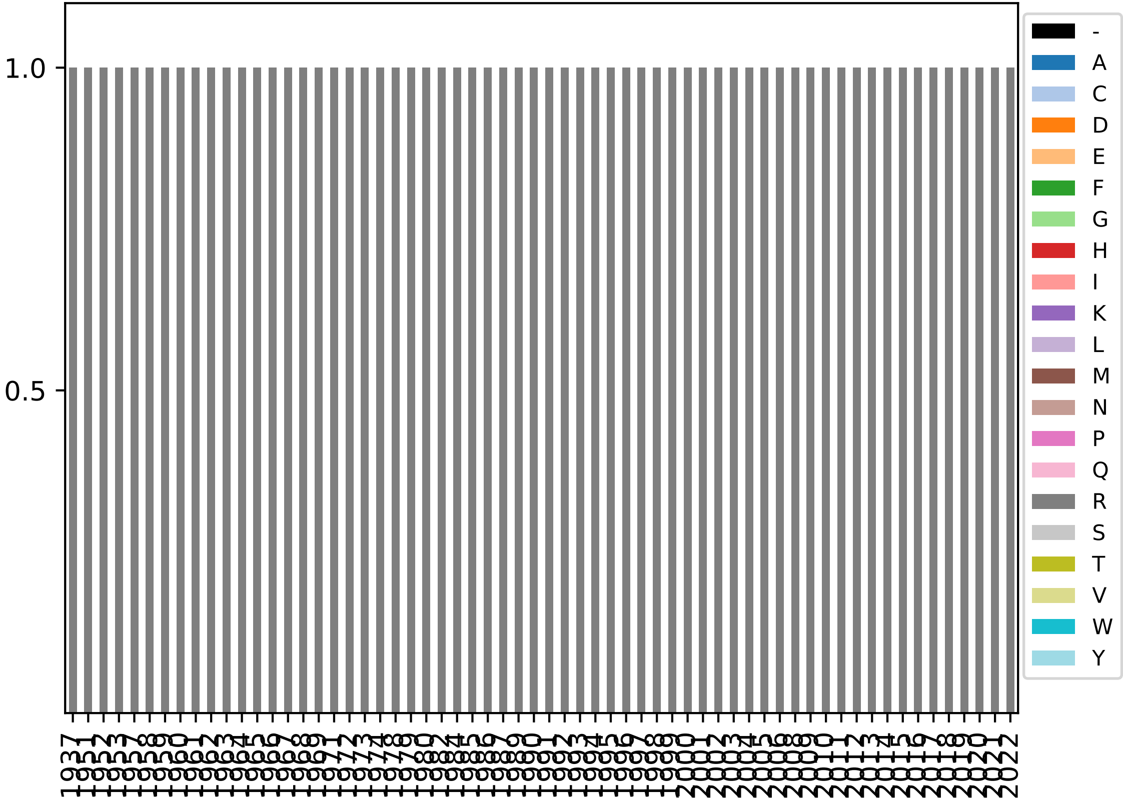

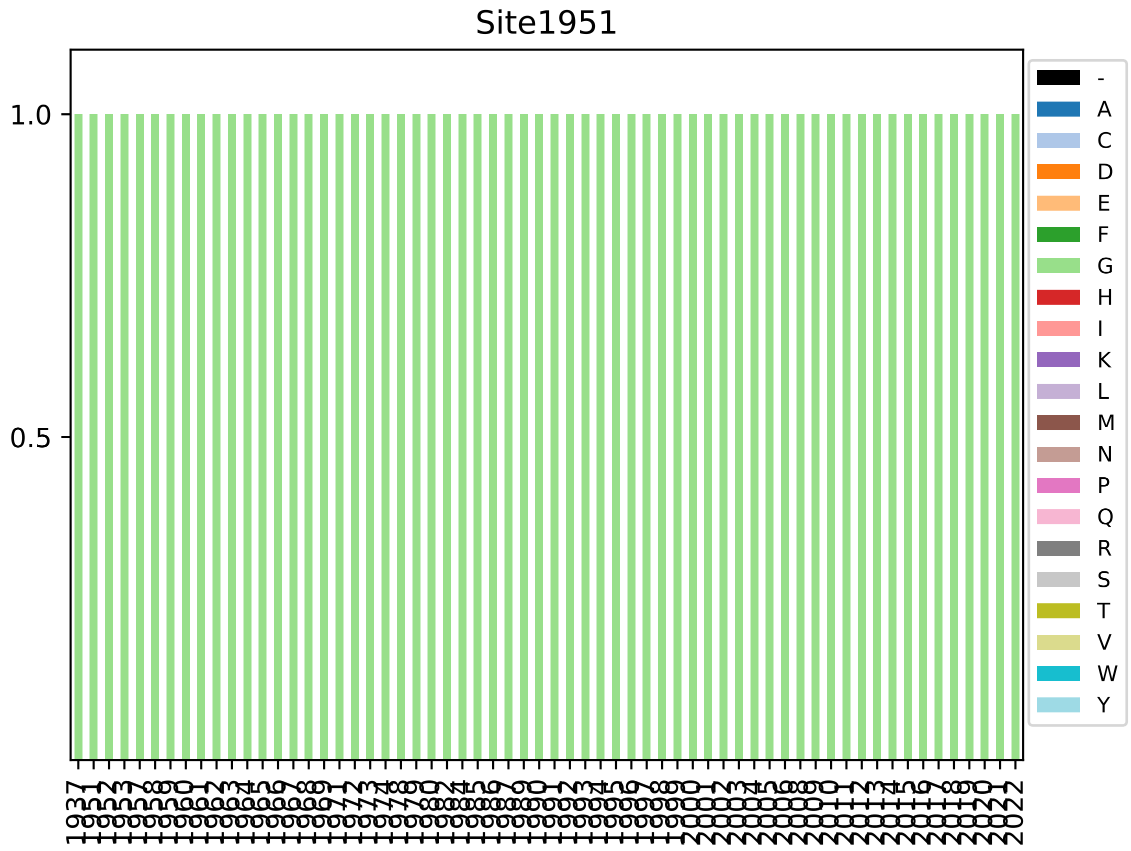
Site1951
1.0
0.5
-
A
C
D
E
F
G
H
I
K
L
M
N
P
Q
R
S
T
V
W
Y

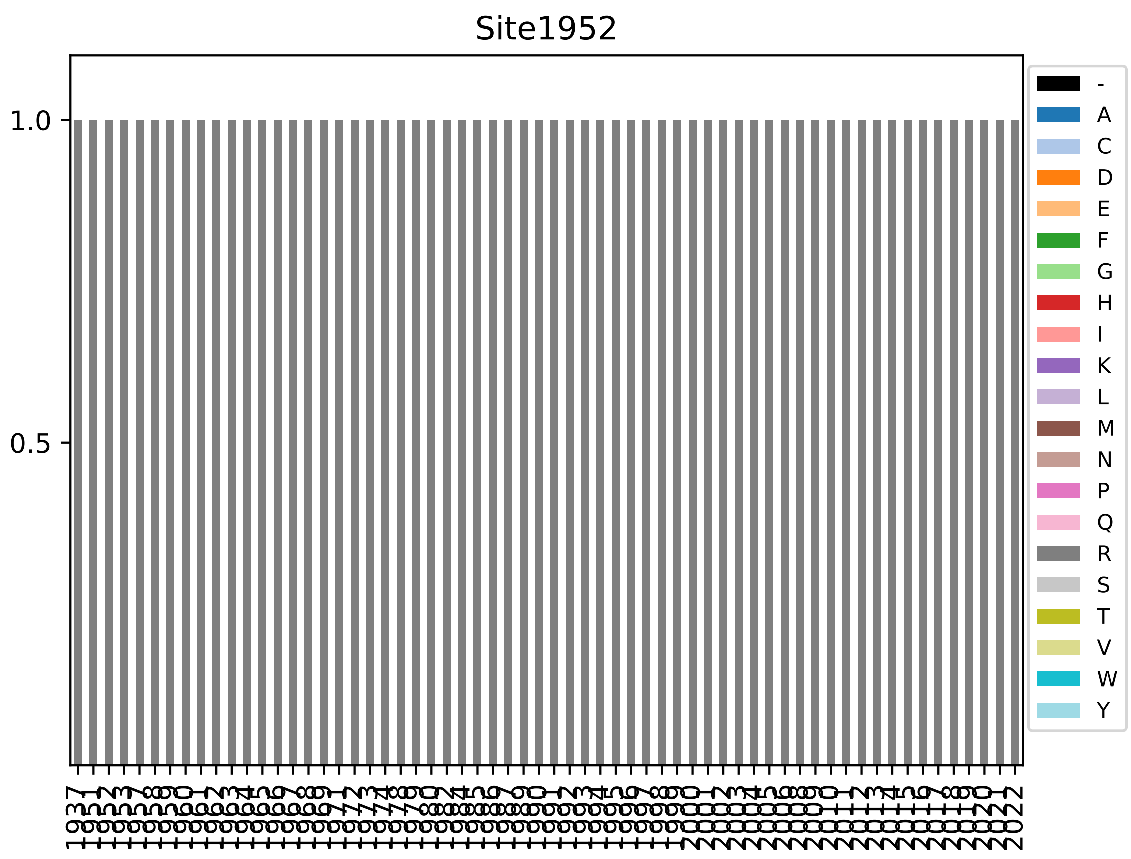

Site1952
1.0
0.5
-
A
C
D
E
F
G
H
I
K
L
M
N
P
Q
R
S
T
V
W
Y

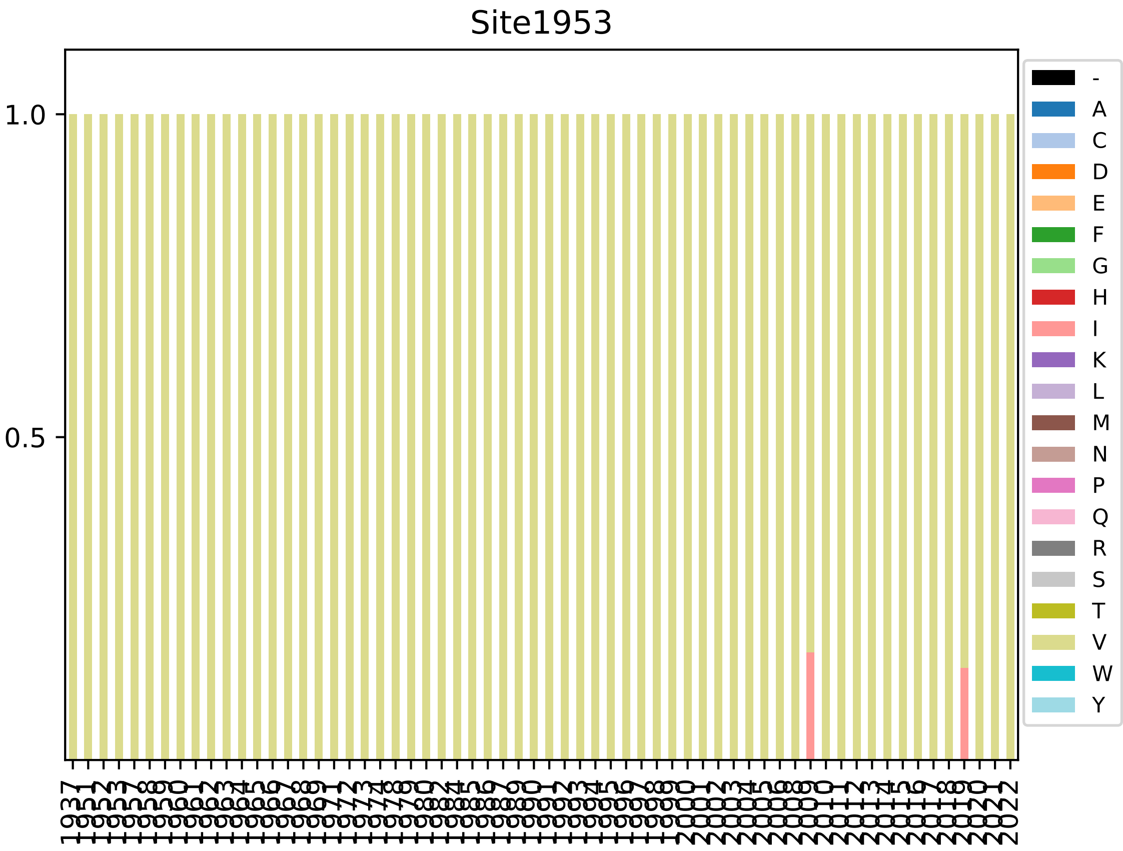
Site1953
1.0
0.5
-
A
C
D
E
F
G
H
I
K
L
M
N
P
Q
R
S
T
V
W
Y

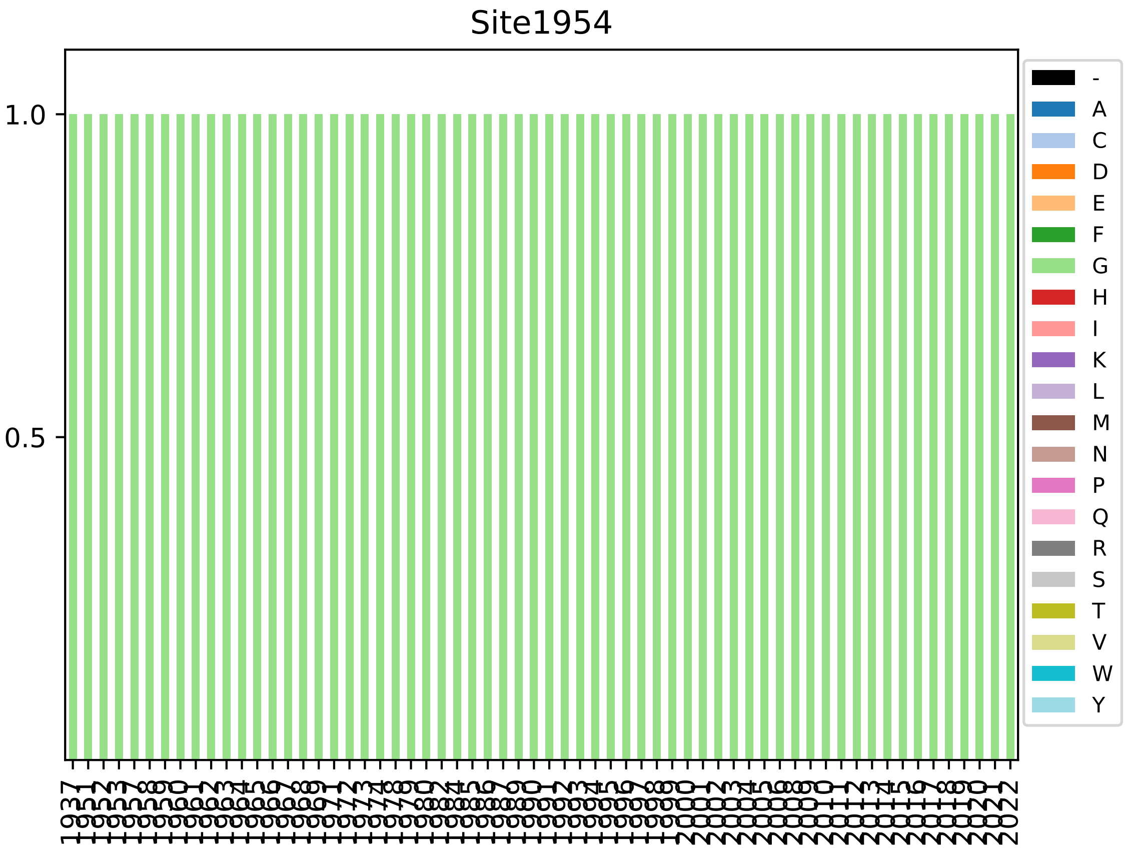
Site1954
1.0
0.5
-
A
C
D
E
F
G
H
I
K
L
M
N
P
Q
R
S
T
V
W
Y

Site1955

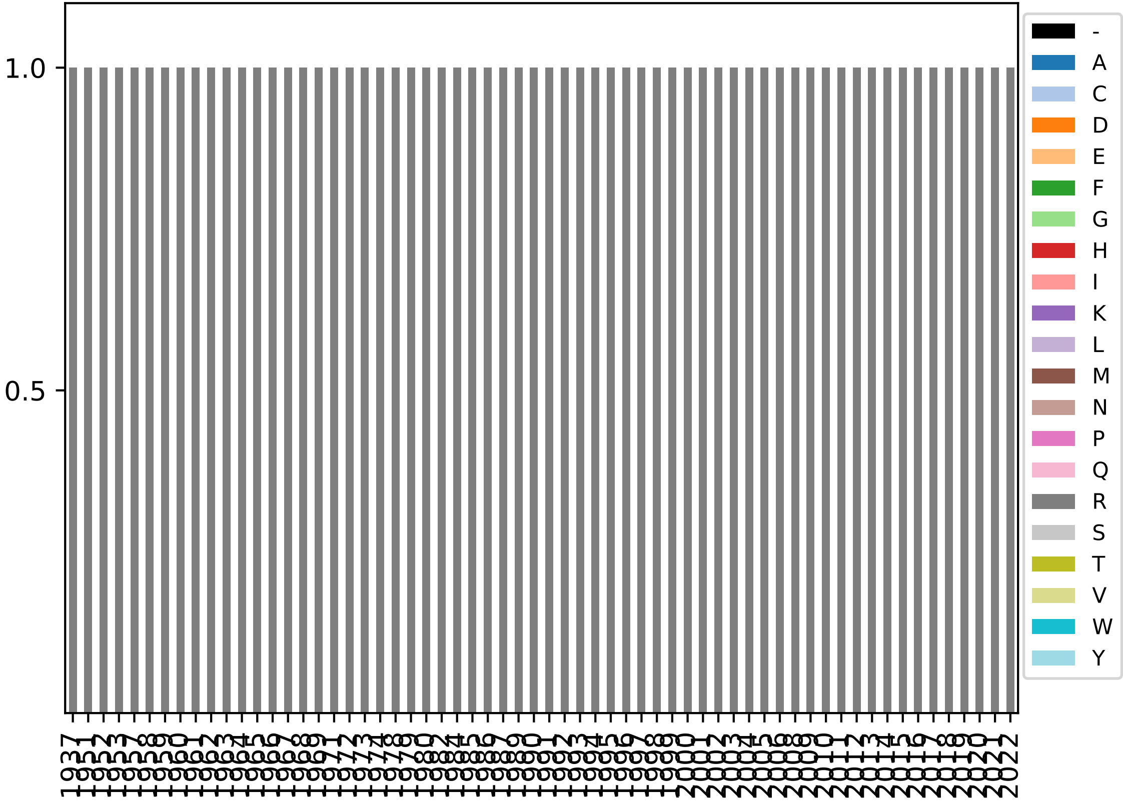

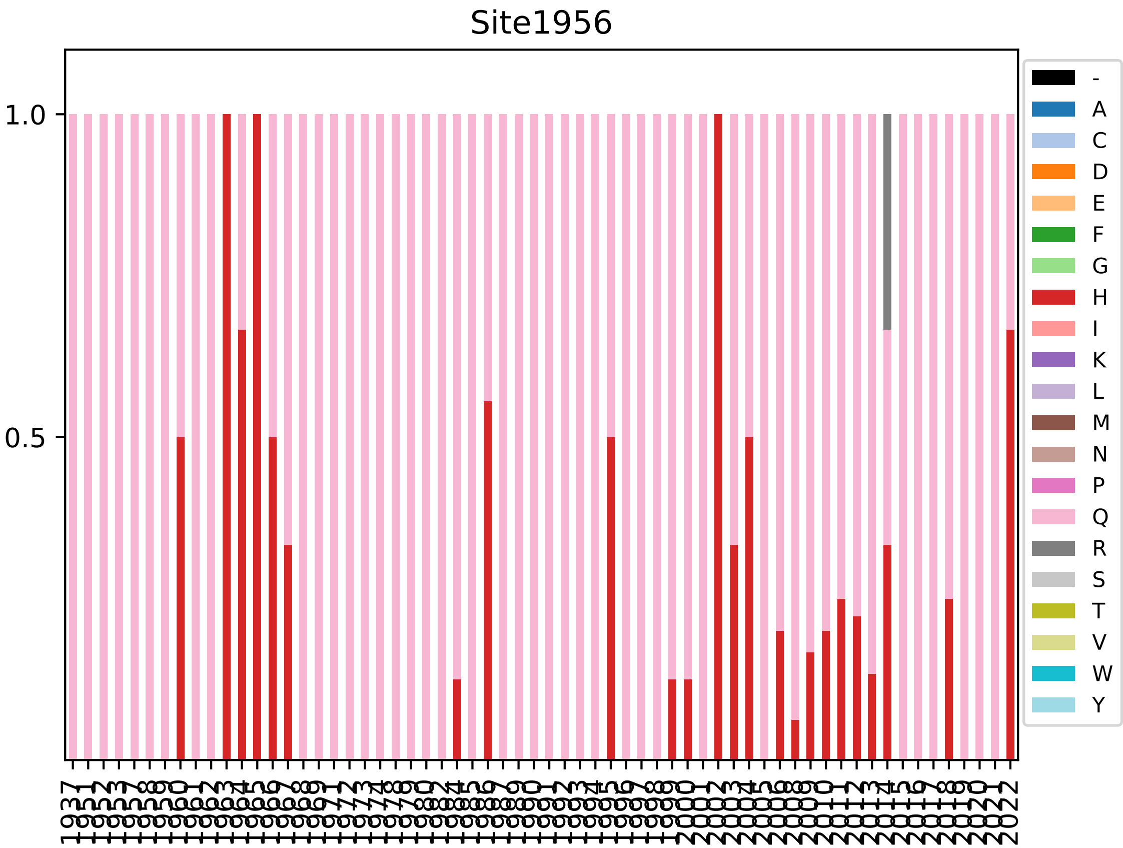
Site1956
1.0
0.5
-
A
C
D
E
F
G
H
I
K
L
M
N
P
Q
R
S
T
V
W
Y

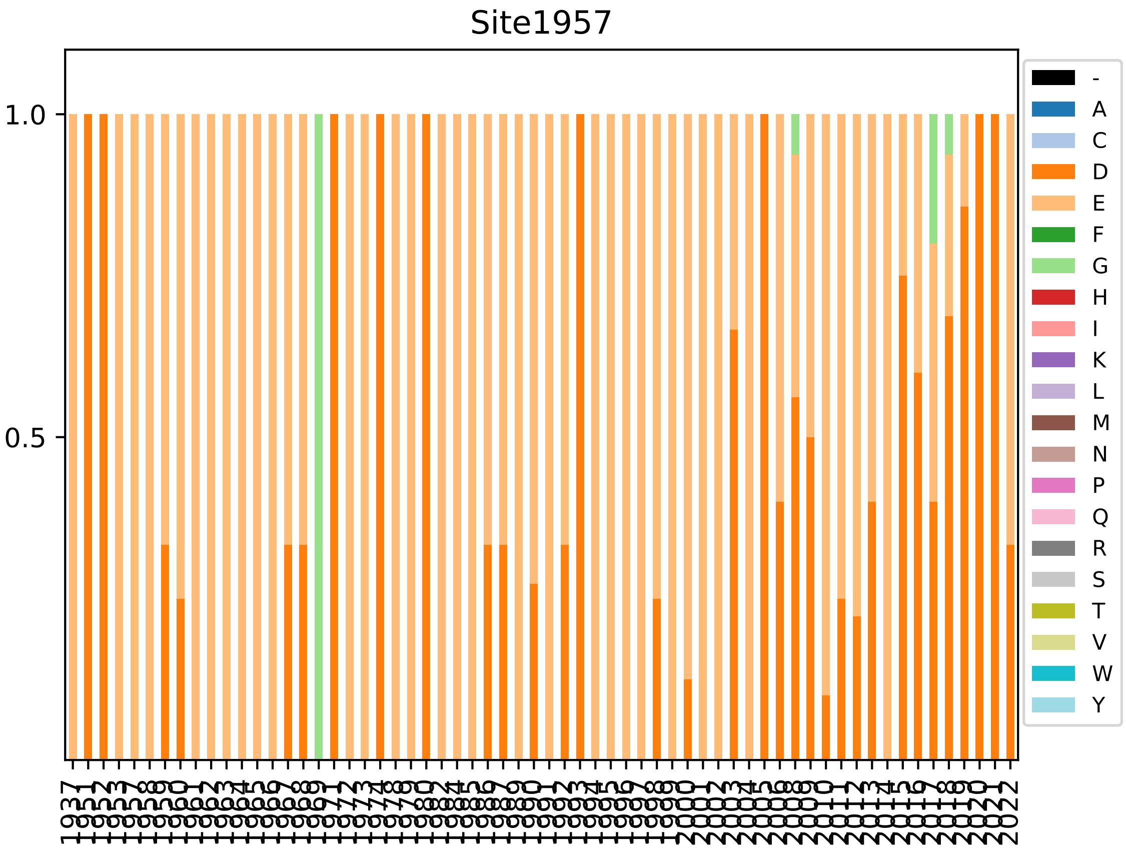
Site1957
1.0
0.5
-
A
C
D
E
F
G
H
I
K
L
M
N
P
Q
R
S
T
V
W
Y

Site1958

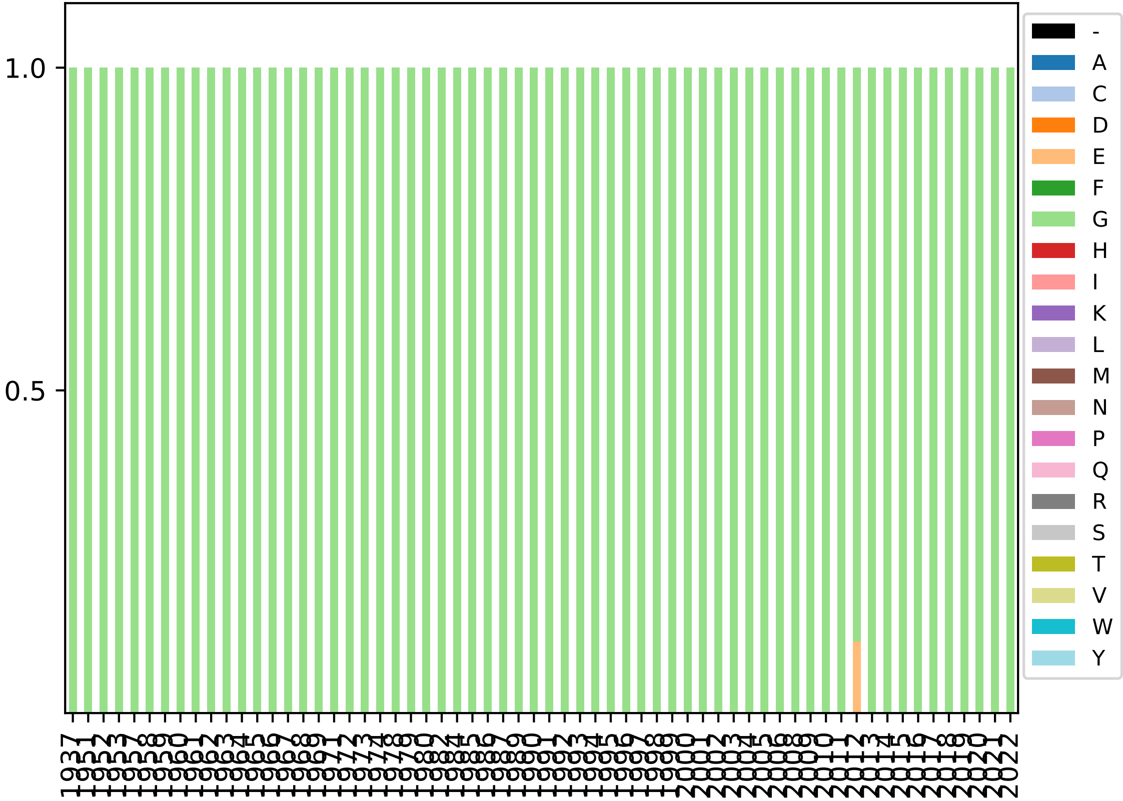

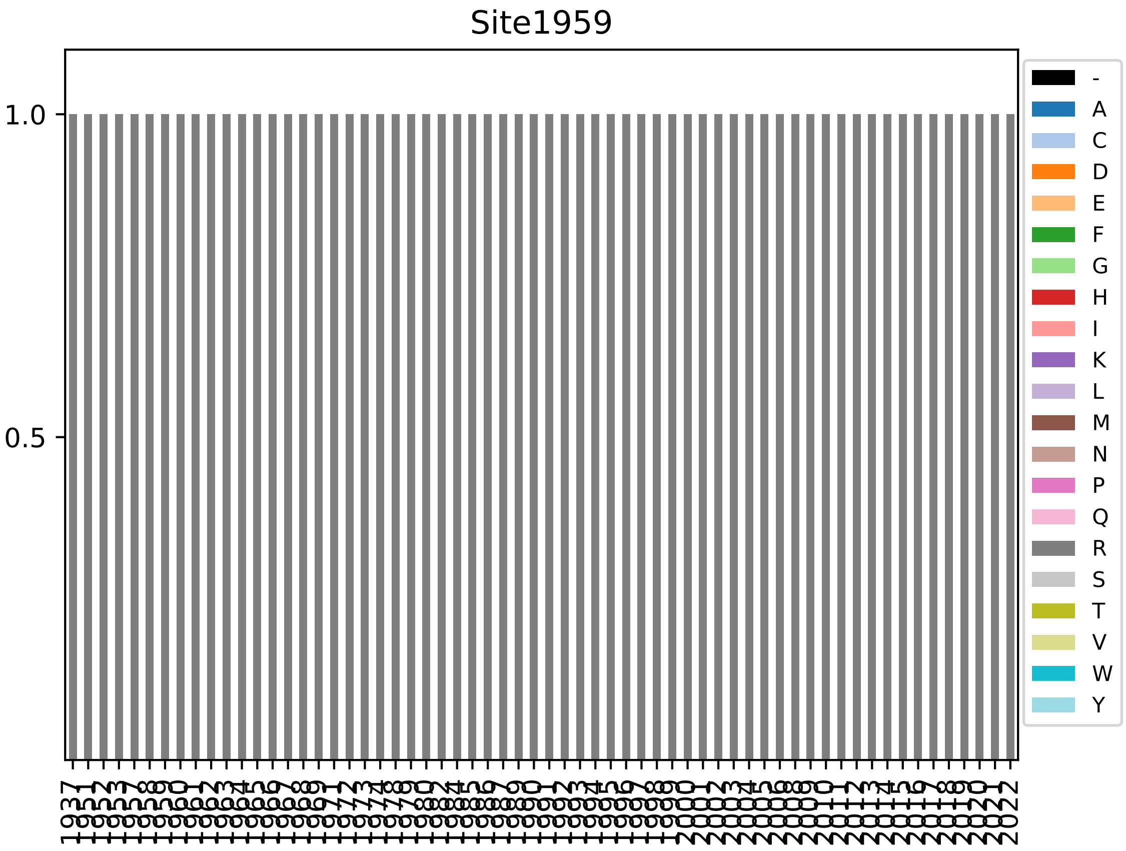

Site1959
1.0
0.5
-
A
C
D
E
F
G
H
I
K
L
M
N
P
Q
R
S
T
V
W
Y

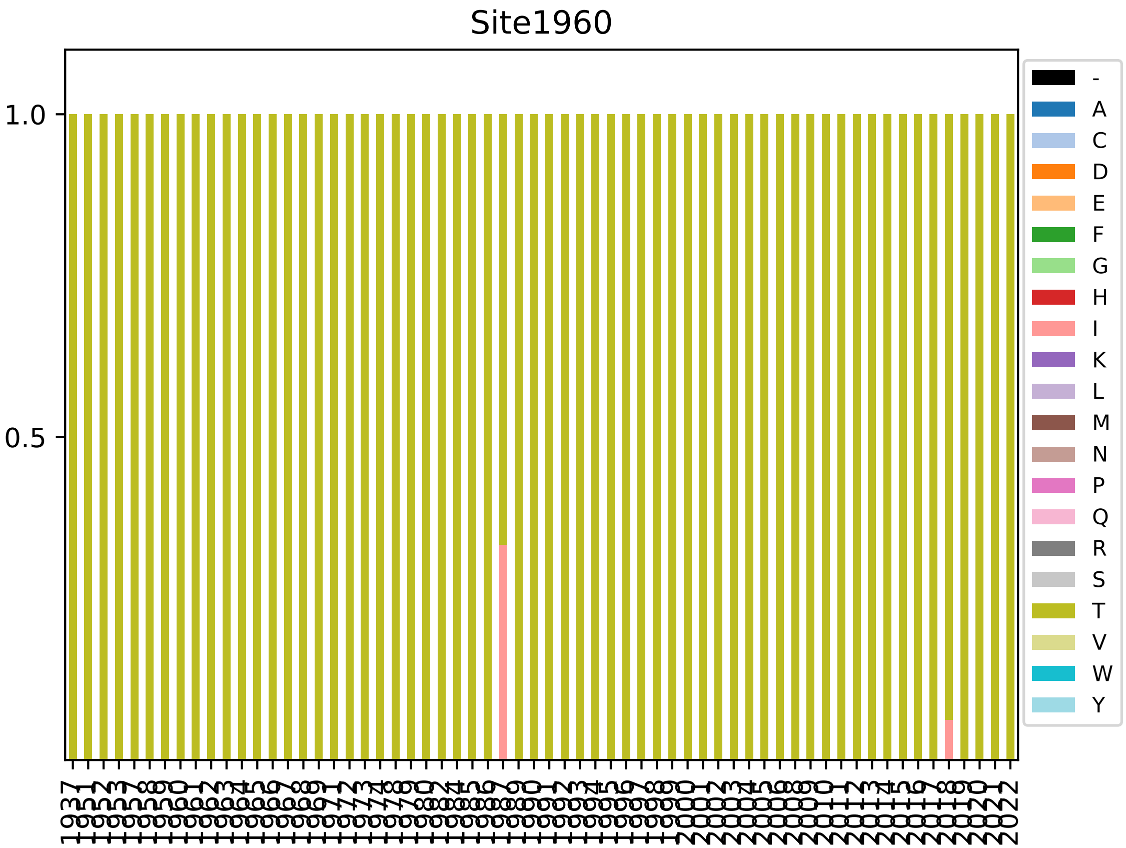
Site1960
1.0
0.5
-
A
C
D
E
F
G
H
I
K
L
M
N
P
Q
R
S
T
V
W
Y

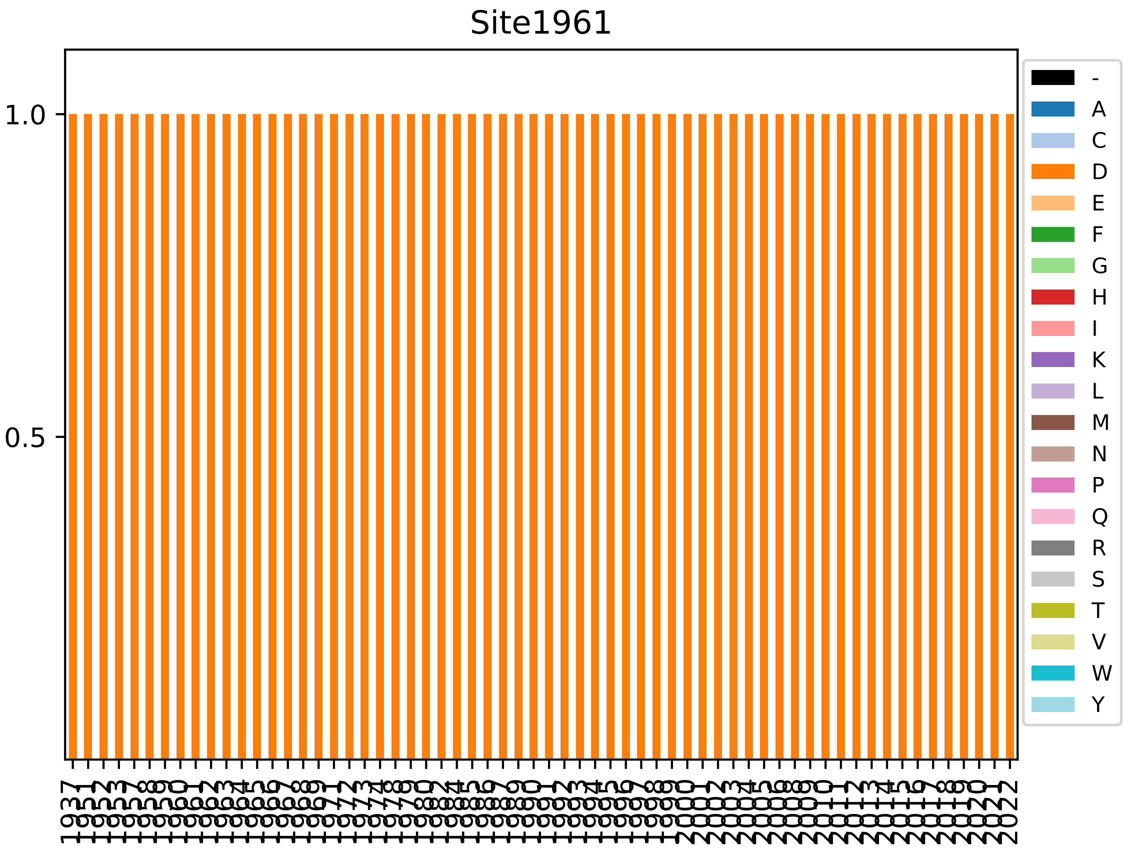
Site1961
1.0
0.5
-
A
C
D
E
F
G
H
I
K
L
M
N
P
Q
R
S
T
V
W
Y

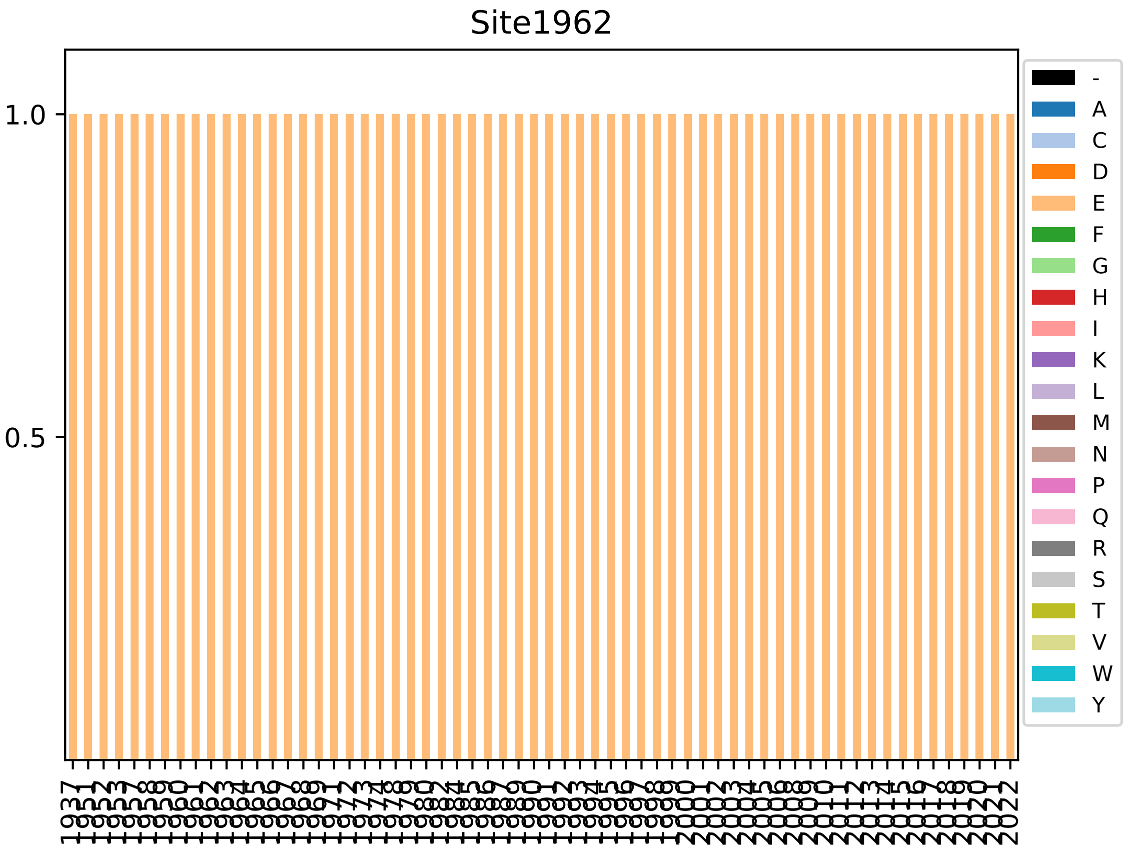
Site1962
1.0
0.5
-
A
C
D
E
F
G
H
I
K
L
M
N
P
Q
R
S
T
V
W
Y

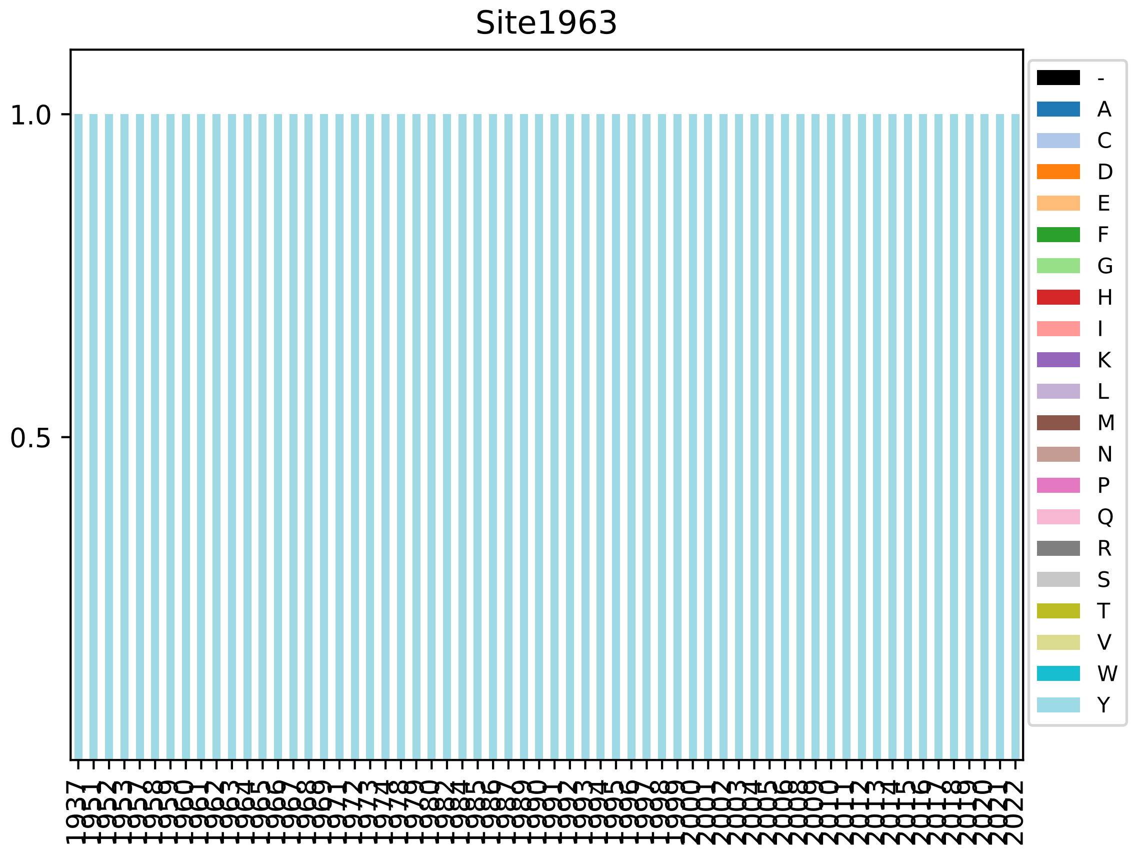
Site1963
1.0
0.5
-
A
C
D
E
F
G
H
I
K
L
M
N
P
Q
R
S
T
V
W
Y

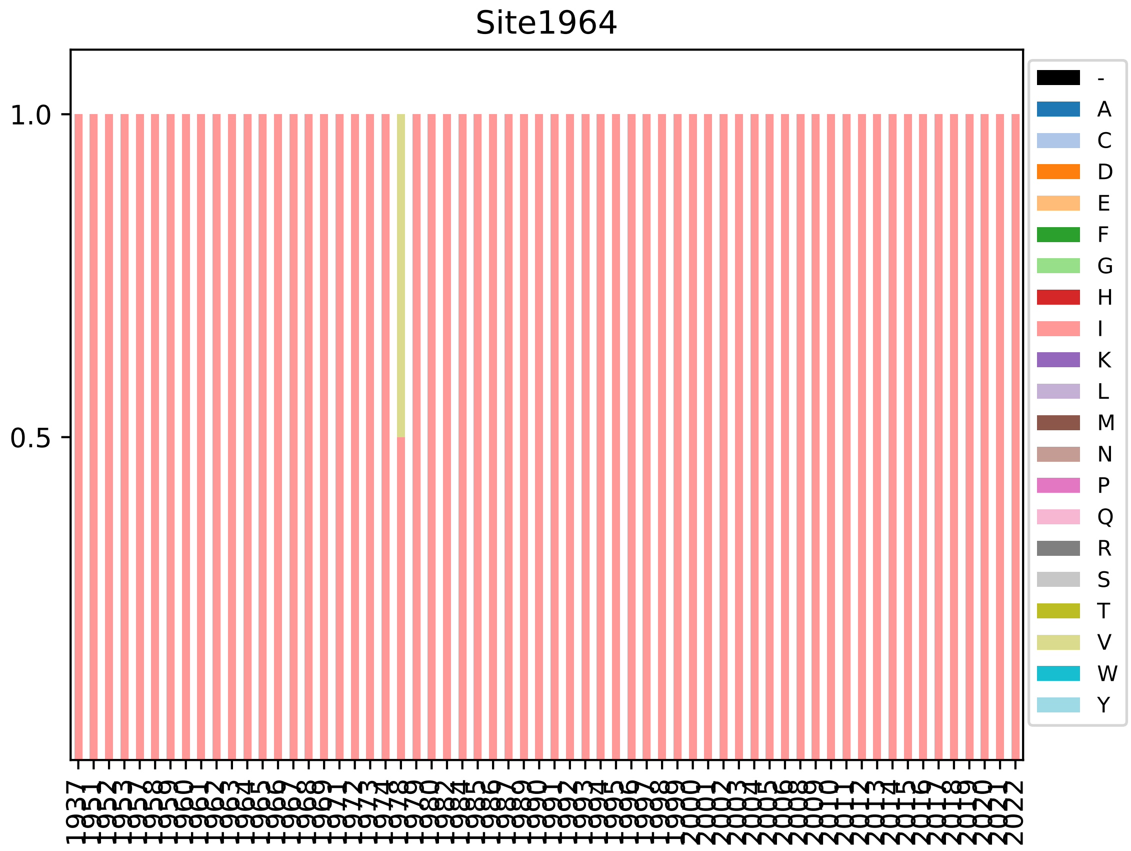
Site1964
1.0
0.5
-
A
C
D
E
F
G
H
I
K
L
M
N
P
Q
R
S
T
V
W
Y

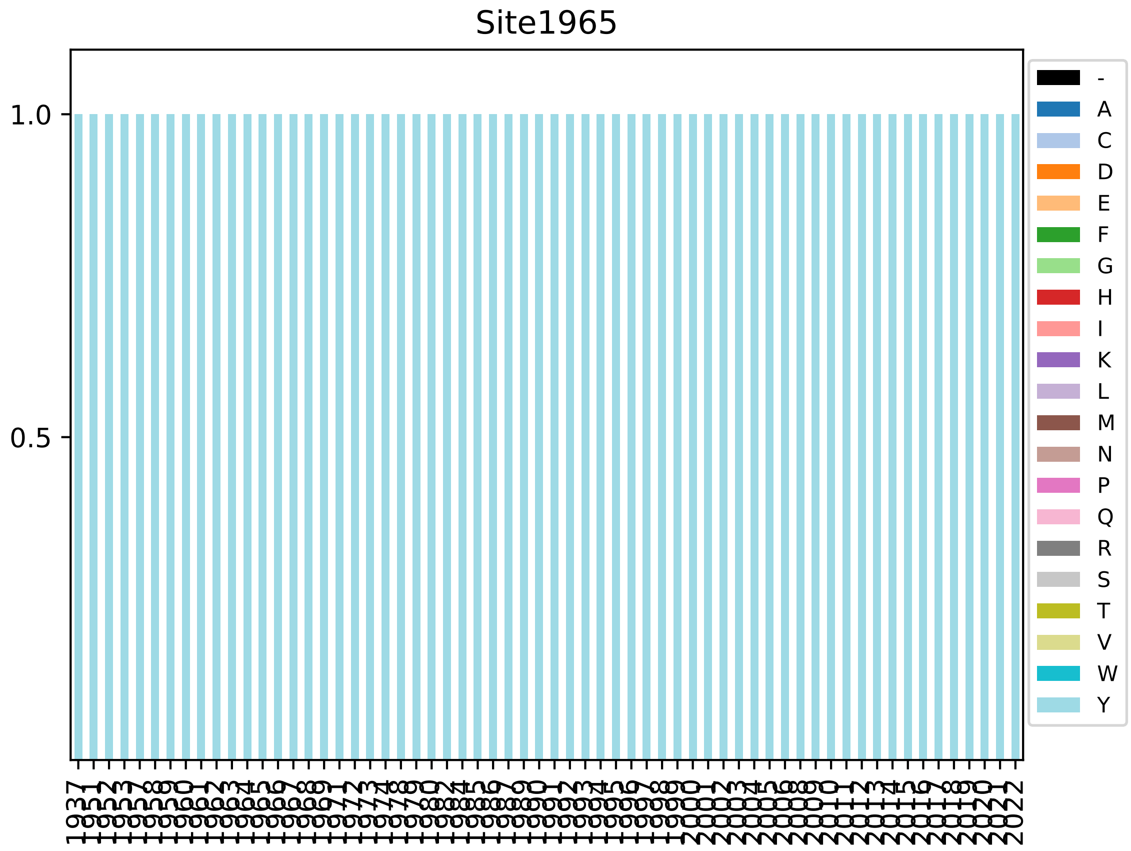
Site1965
1.0
0.5
-
A
C
D
E
F
G
H
I
K
L
M
N
P
Q
R
S
T
V
W
Y

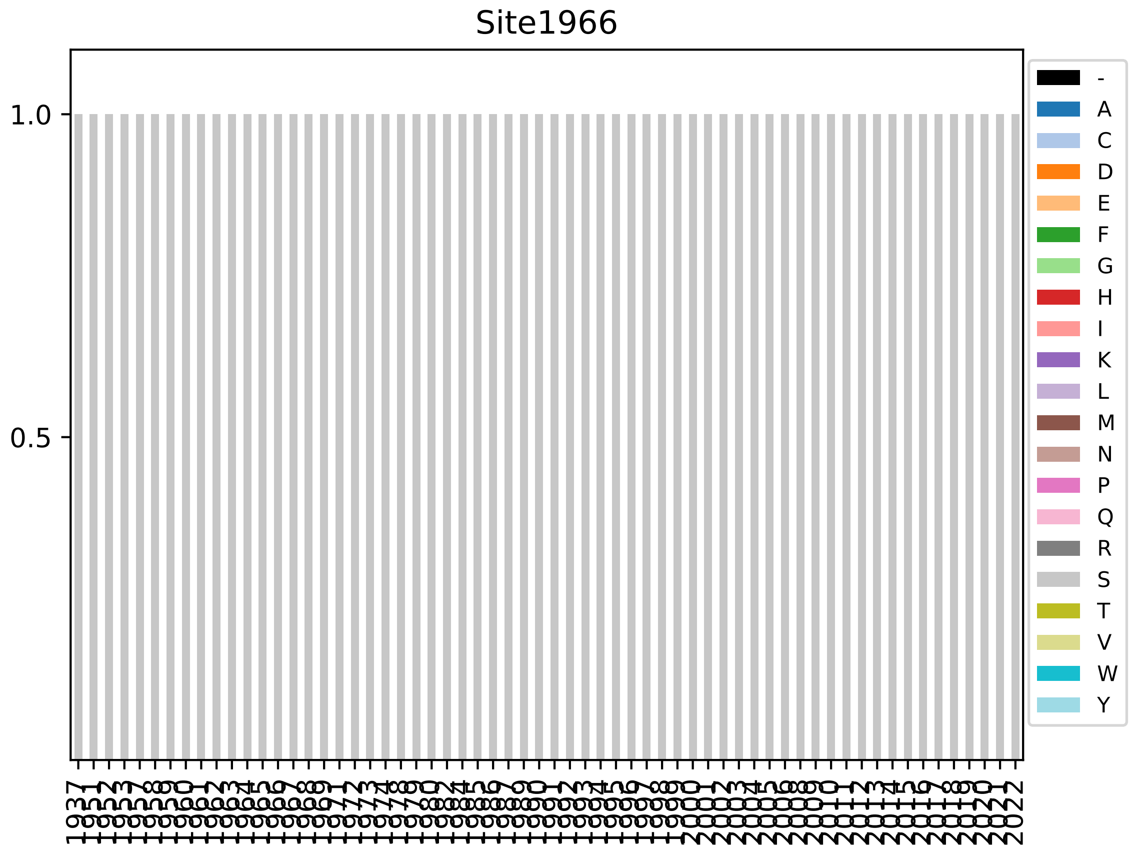
Site1966
1.0
0.5
-
A
C
D
E
F
G
H
I
K
L
M
N
P
Q
R
S
T
V
W
Y

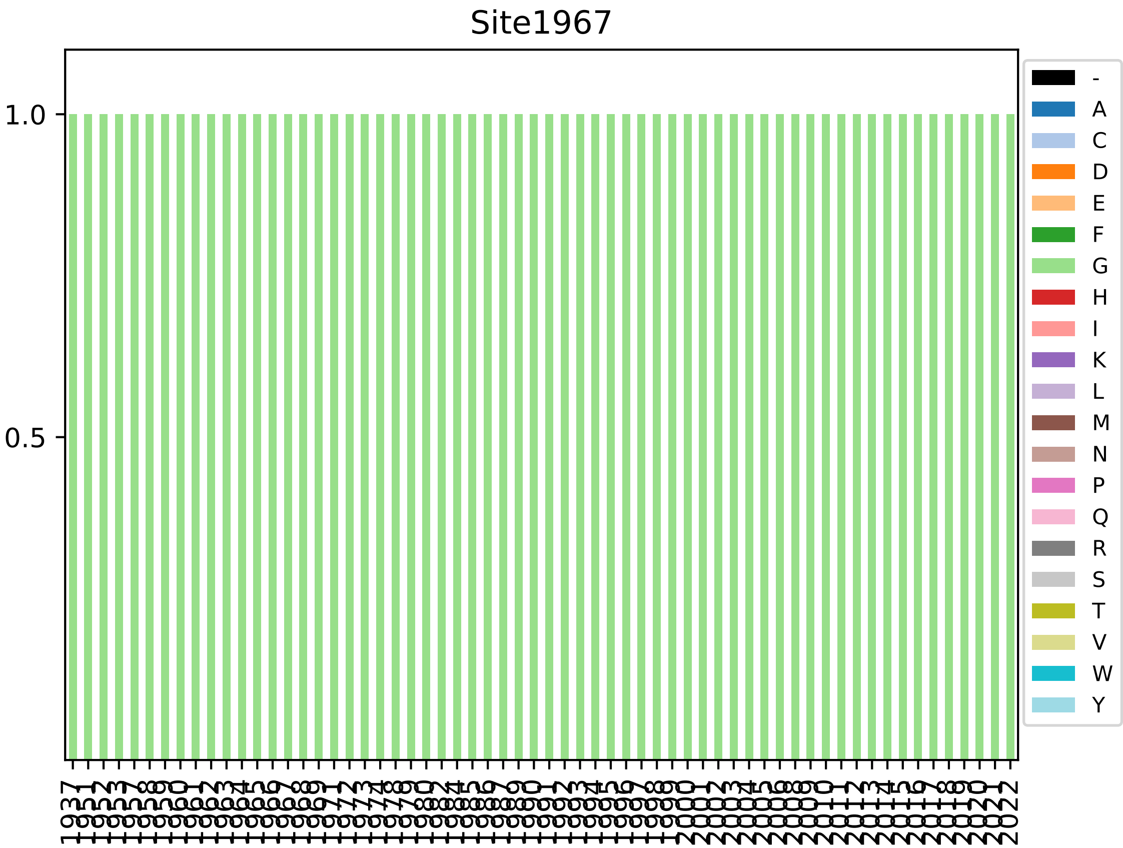

Site1967
1.0
0.5
-
A
C
D
E
F
G
H
I
K
L
M
N
P
Q
R
S
T
V
W
Y

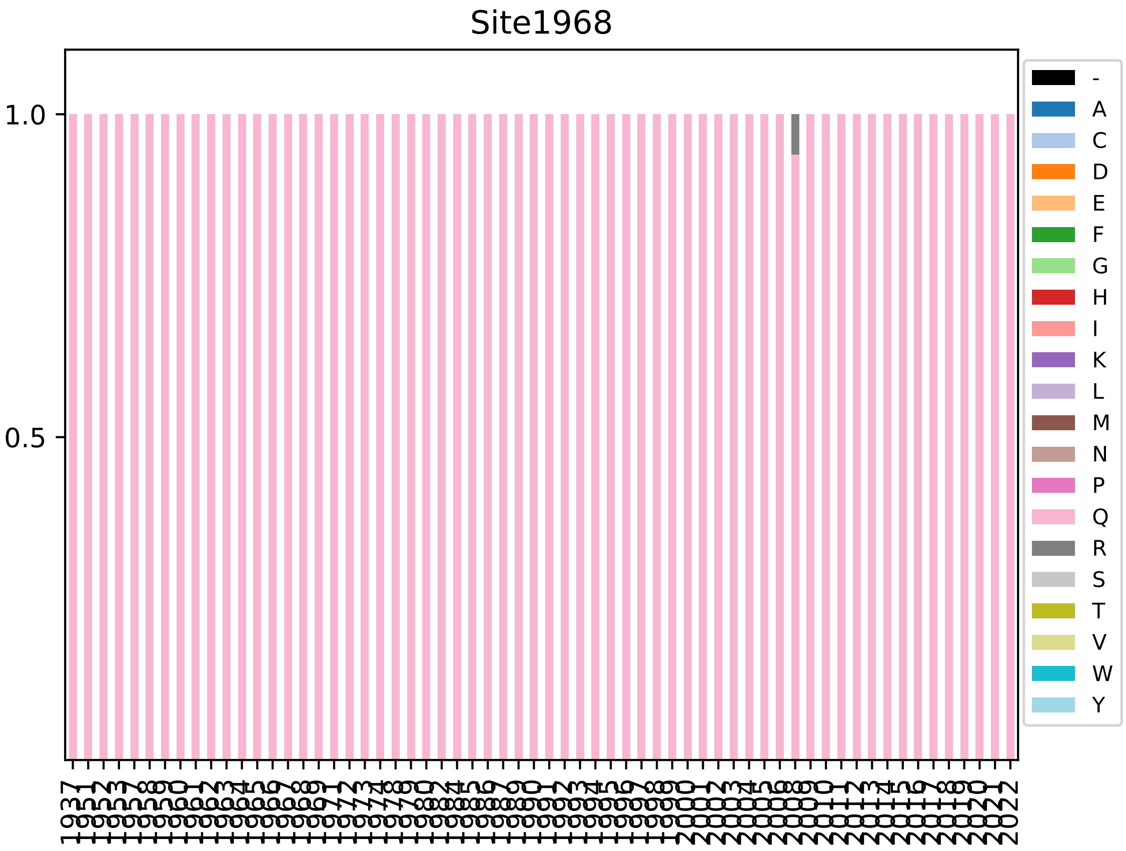
Site1968
1.0
0.5
-
A
C
D
E
F
G
H
I
K
L
M
N
P
Q
R
S
T
V
W
Y

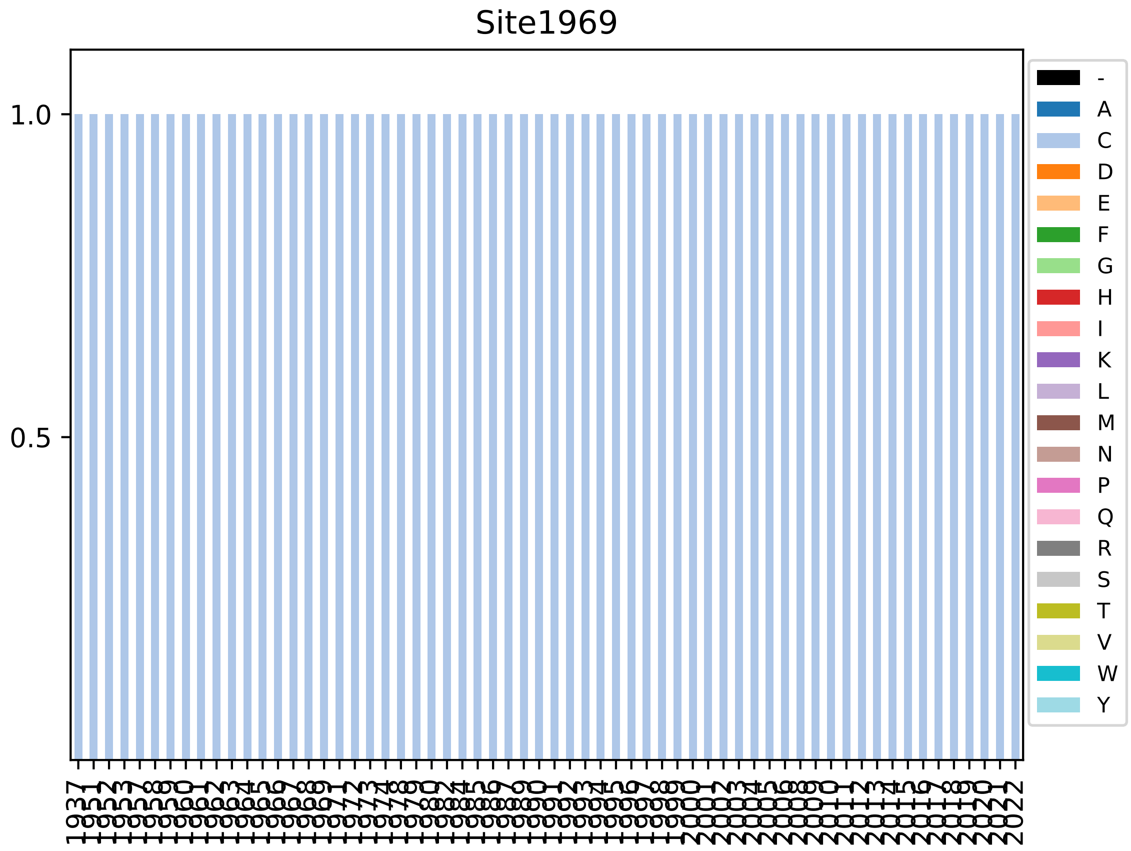
Site1969
1.0
0.5
-
A
C
D
E
F
G
H
I
K
L
M
N
P
Q
R
S
T
V
W
Y

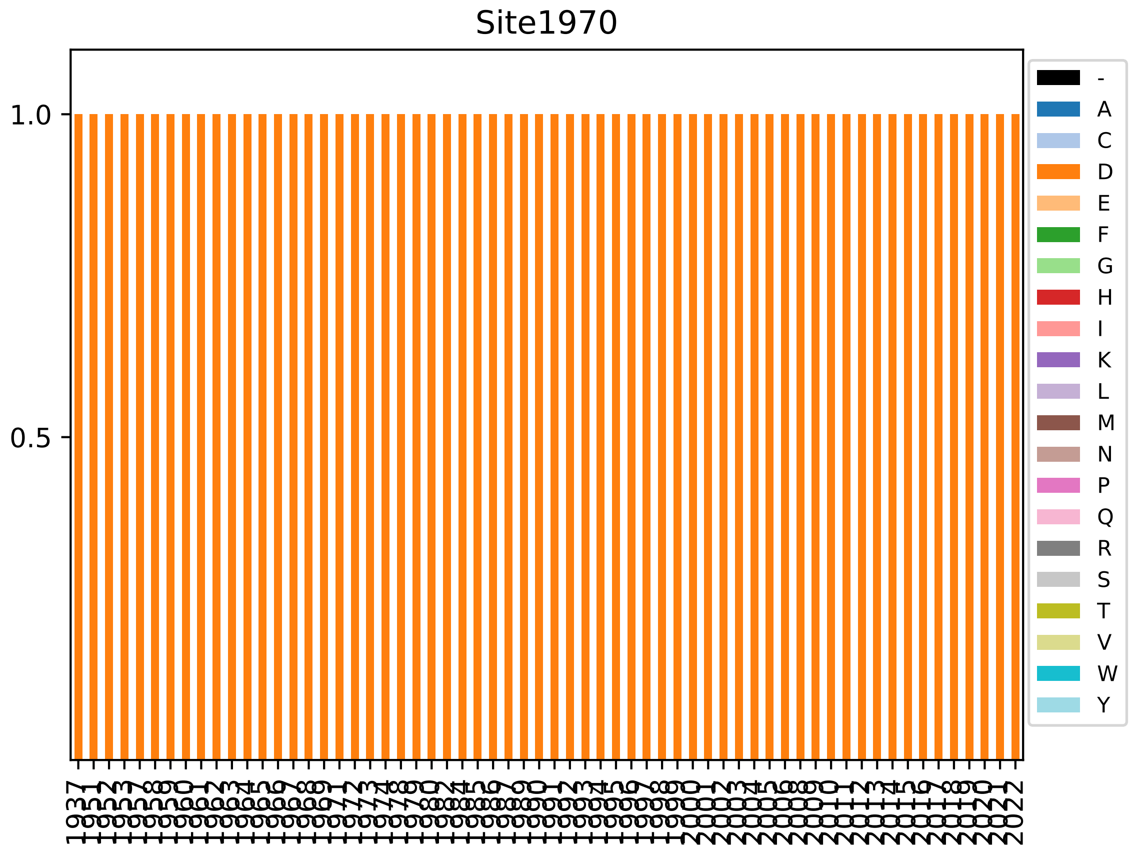
Site1970
1.0
0.5
-
A
C
D
E
F
G
H
I
K
L
M
N
P
Q
R
S
T
V
W
Y

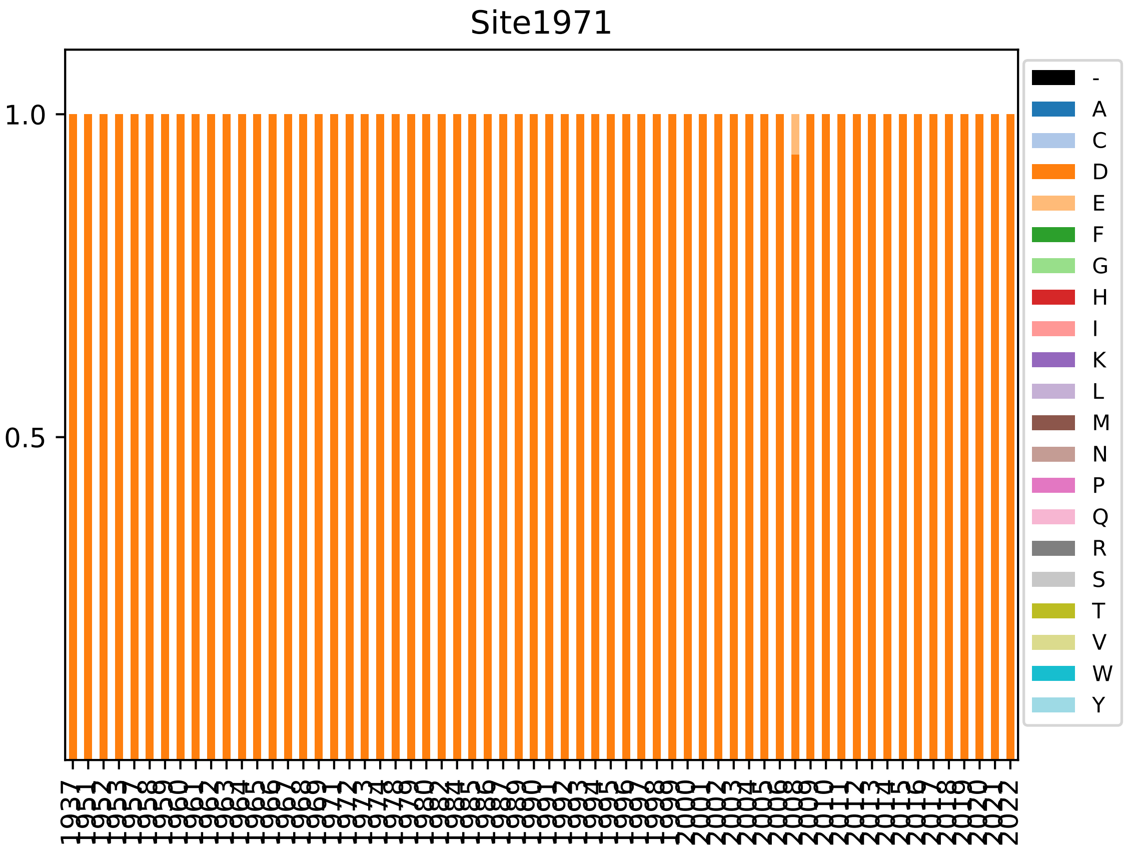
Site1971
1.0
0.5
-
A
C
D
E
F
G
H
I
K
L
M
N
P
Q
R
S
T
V
W
Y

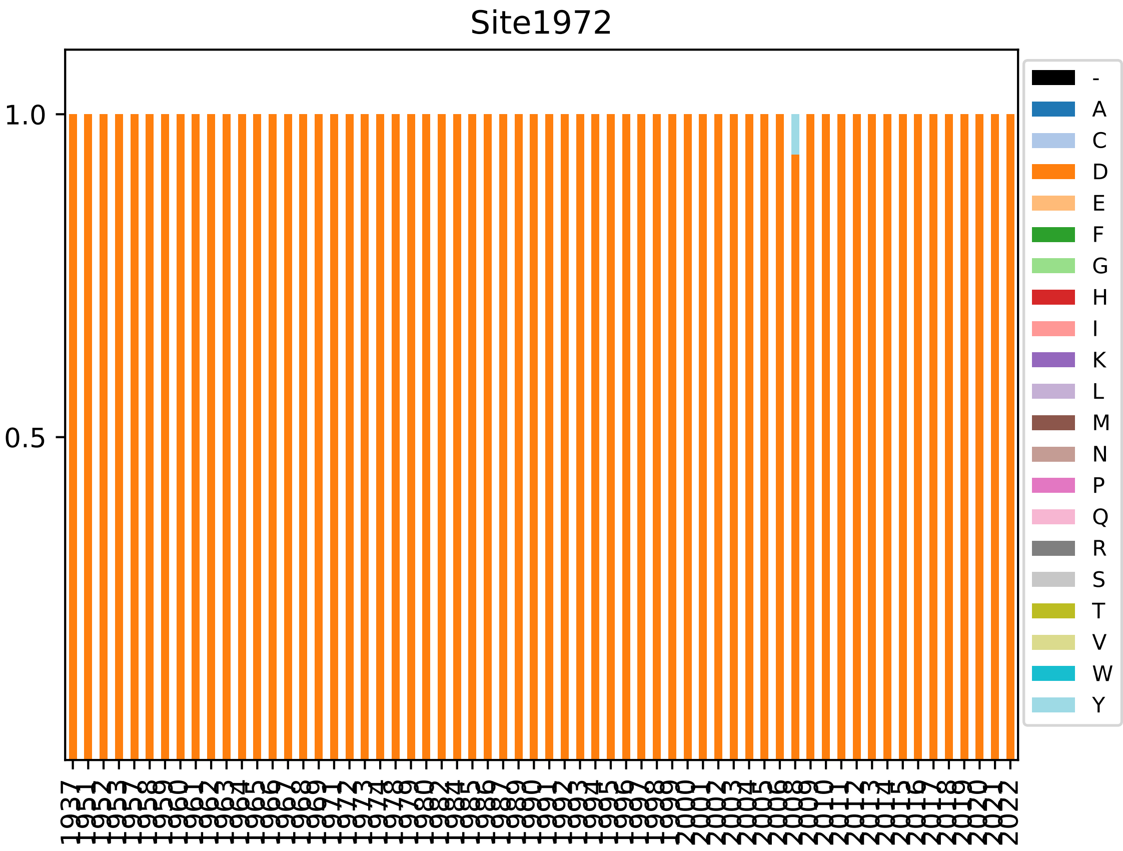
Site1972
1.0
0.5
-
A
C
D
E
F
G
H
I
K
L
M
N
P
Q
R
S
T
V
W
Y

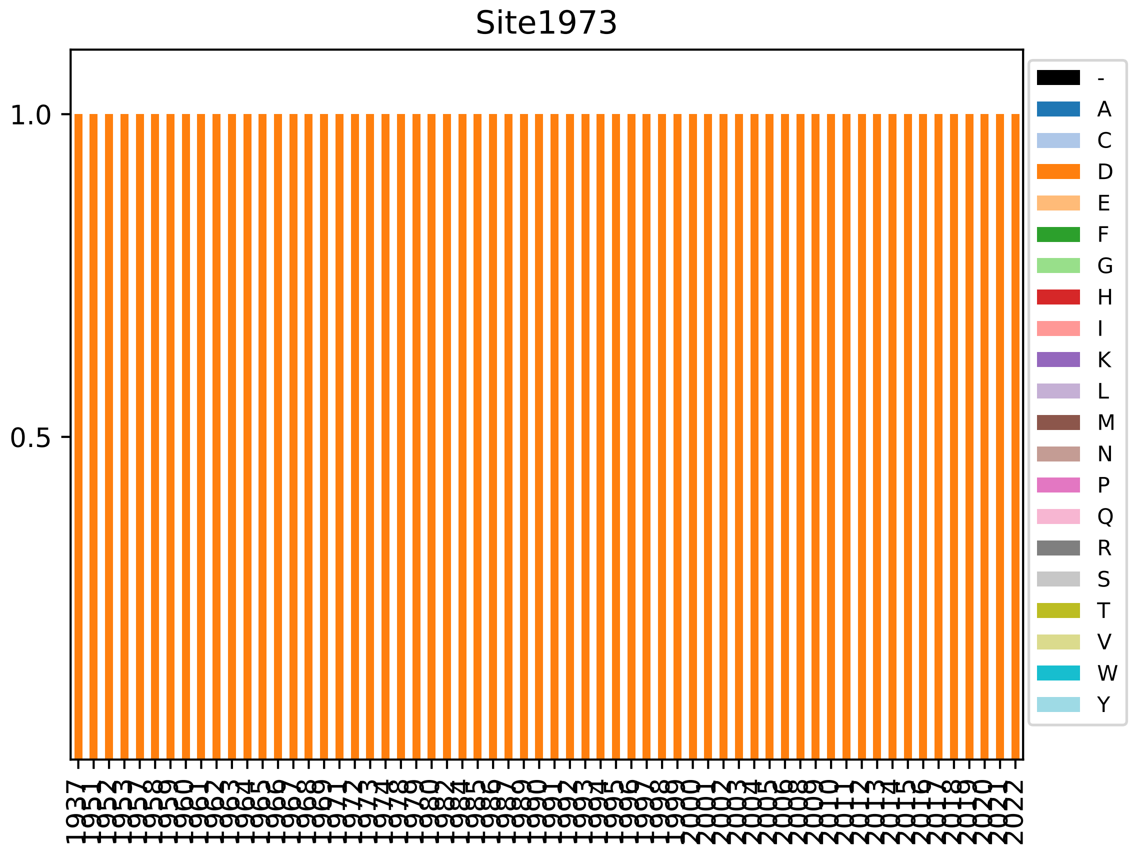
Site1973
1.0
0.5
-
A
C
D
E
F
G
H
I
K
L
M
N
P
Q
R
S
T
V
W
Y

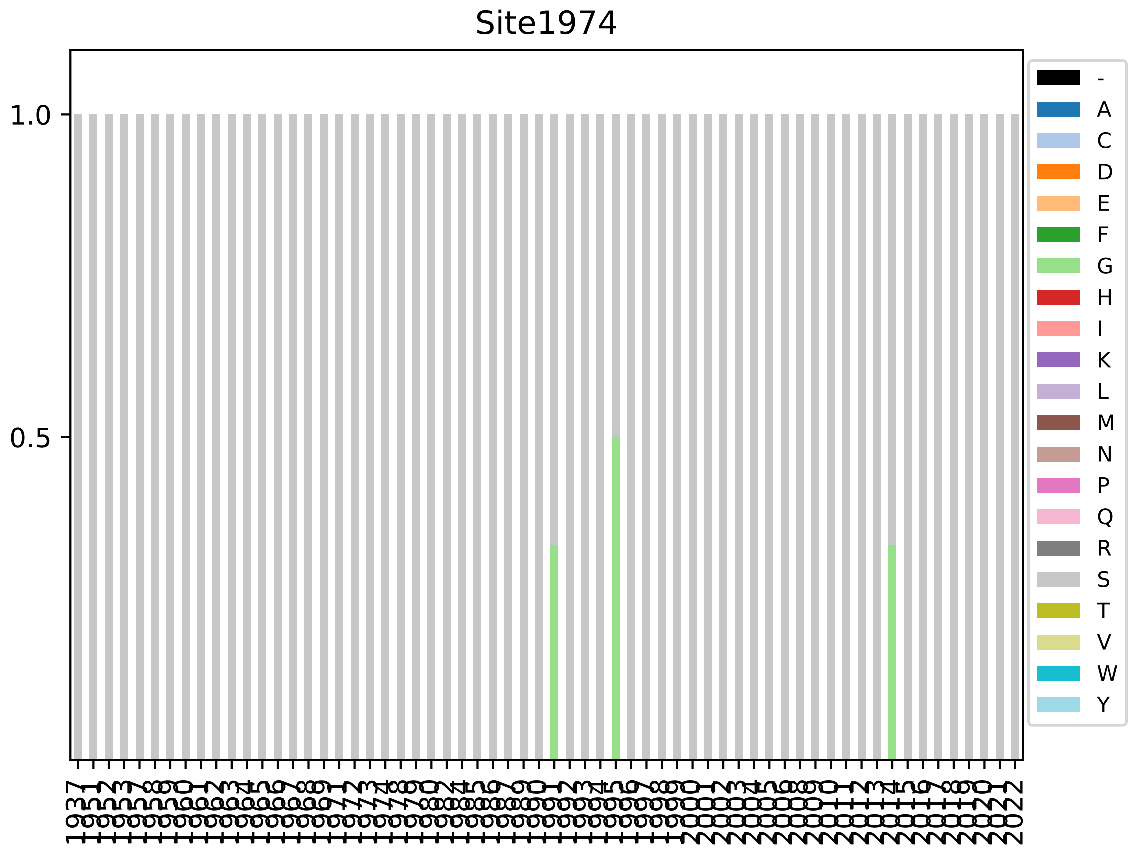
Site1974
1.0
0.5
-
A
C
D
E
F
G
H
I
K
L
M
N
P
Q
R
S
T
V
W
Y

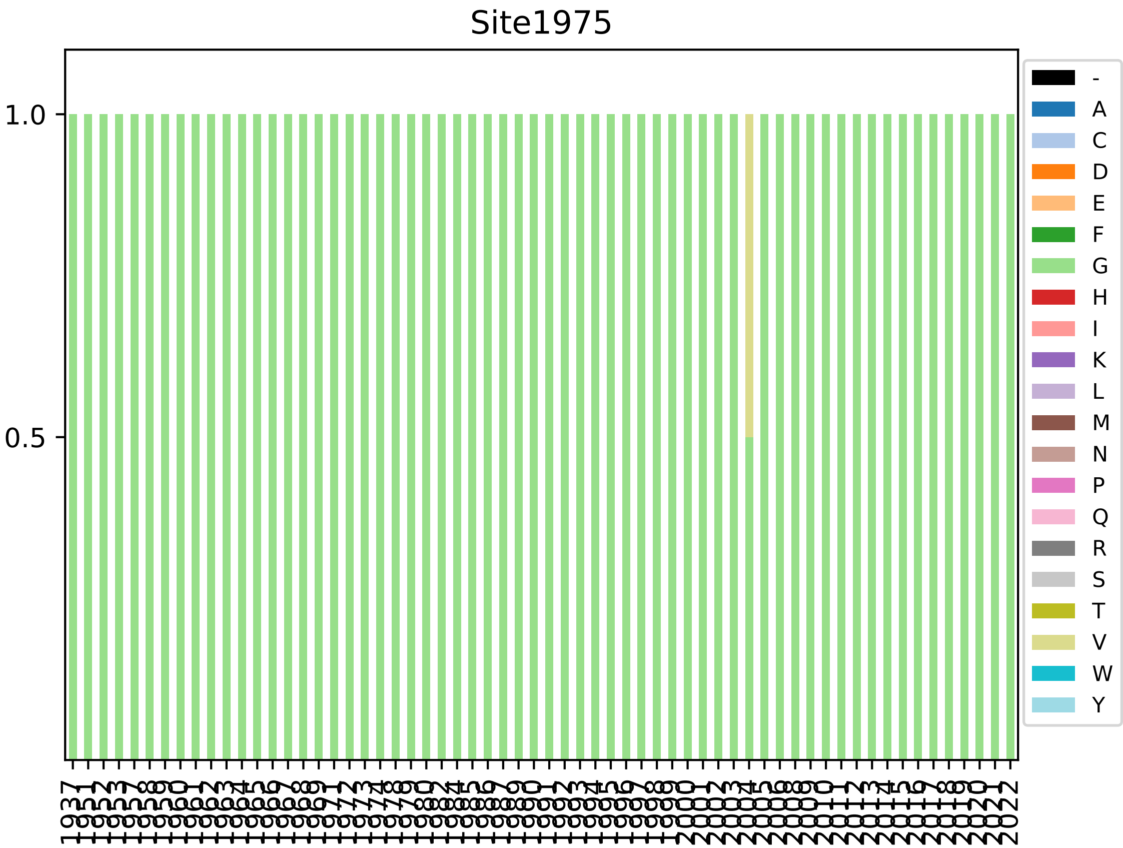
Site1975
1.0
0.5
-
A
C
D
E
F
G
H
I
K
L
M
N
P
Q
R
S
T
V
W
Y

Site1976

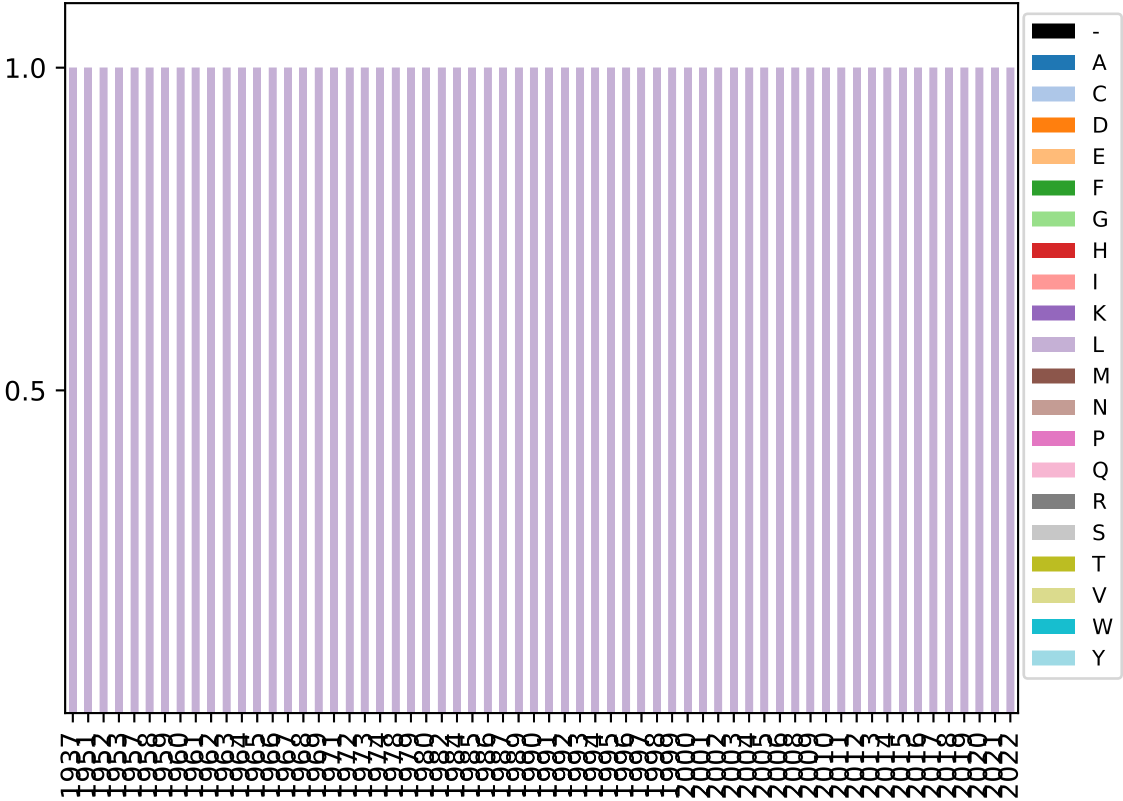

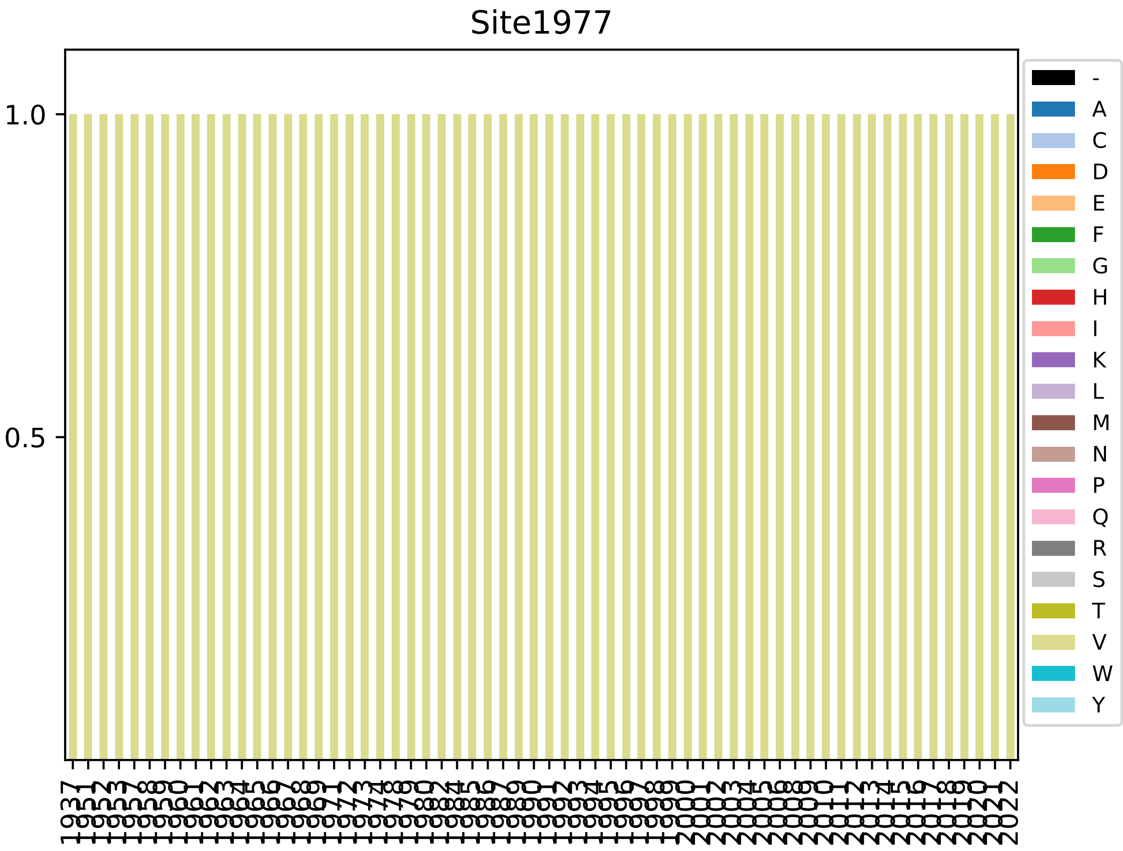

Site1977
1.0
0.5
-
A
C
D
E
F
G
H
I
K
L
M
N
P
Q
R
S
T
V
W
Y

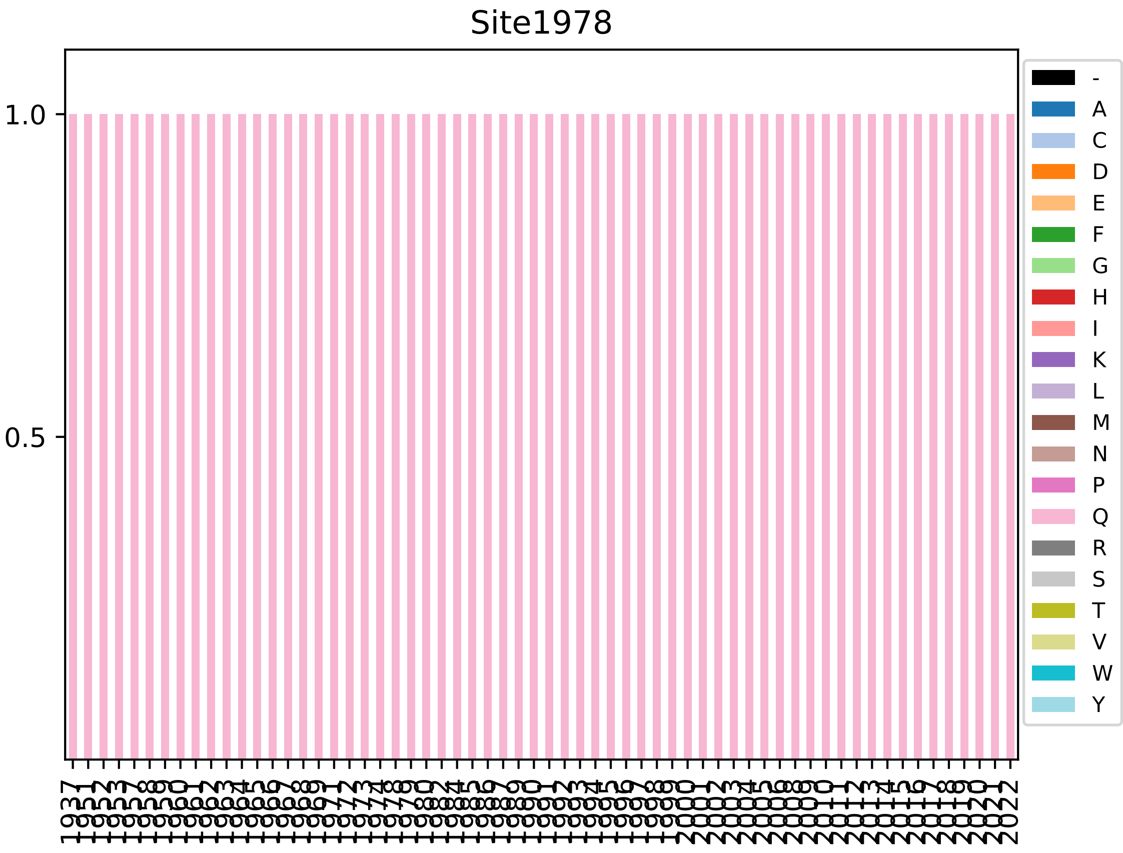

Site1978
1.0
0.5
-
A
C
D
E
F
G
H
I
K
L
M
N
P
Q
R
S
T
V
W
Y

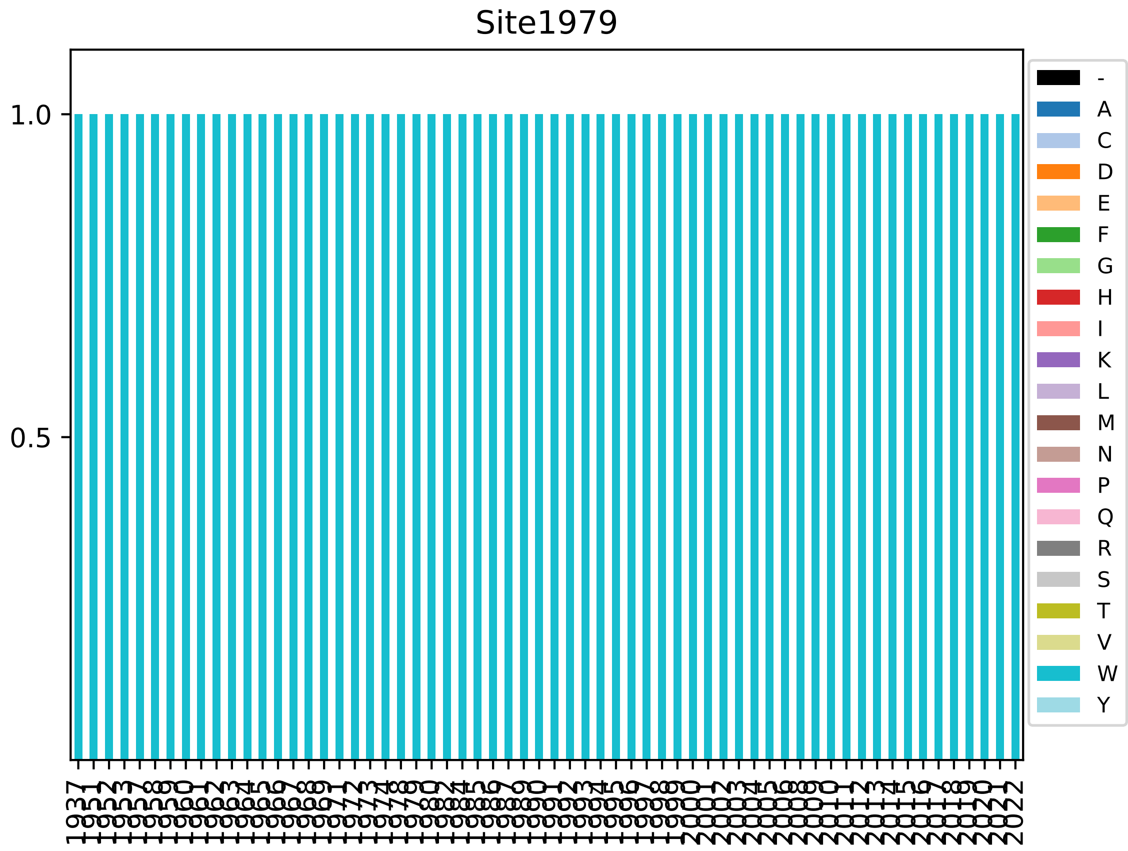
Site1979
1.0
0.5
-
A
C
D
E
F
G
H
I
K
L
M
N
P
Q
R
S
T
V
W
Y

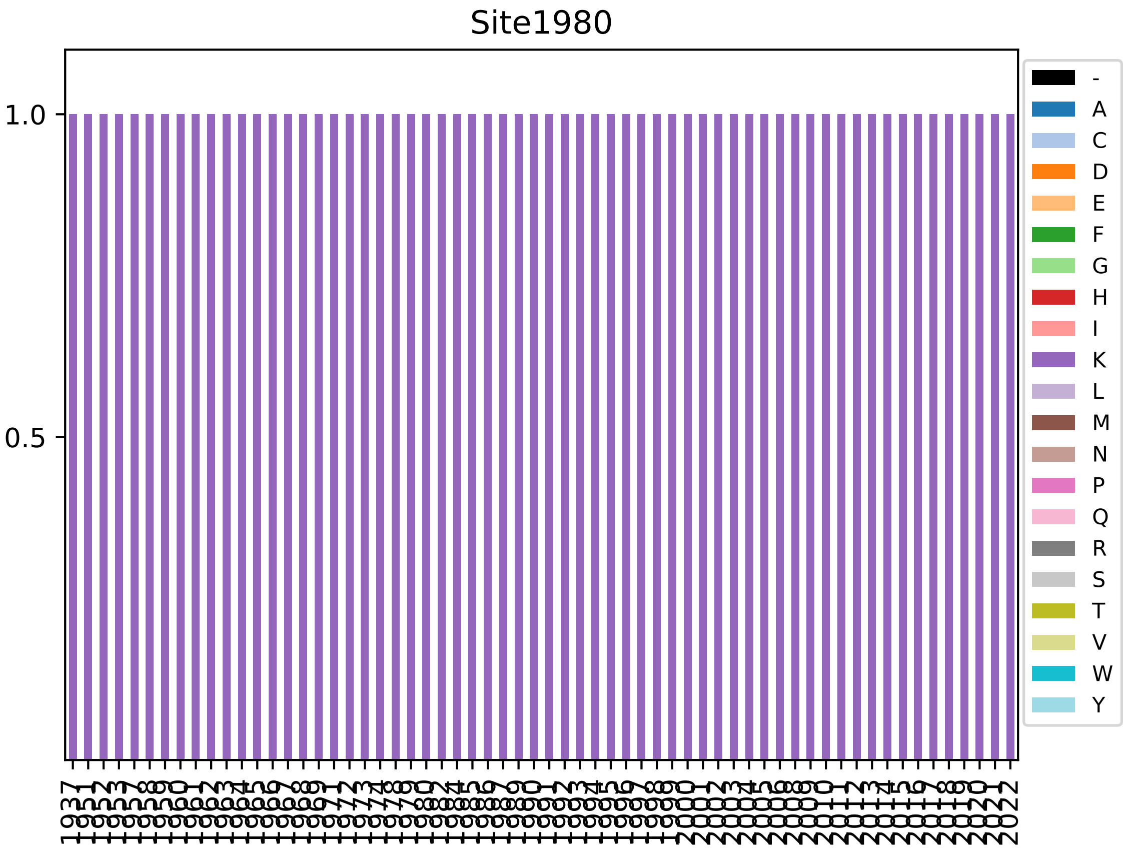

Site1980
1.0
0.5
-
A
C
D
E
F
G
H
I
K
L
M
N
P
Q
R
S
T
V
W
Y

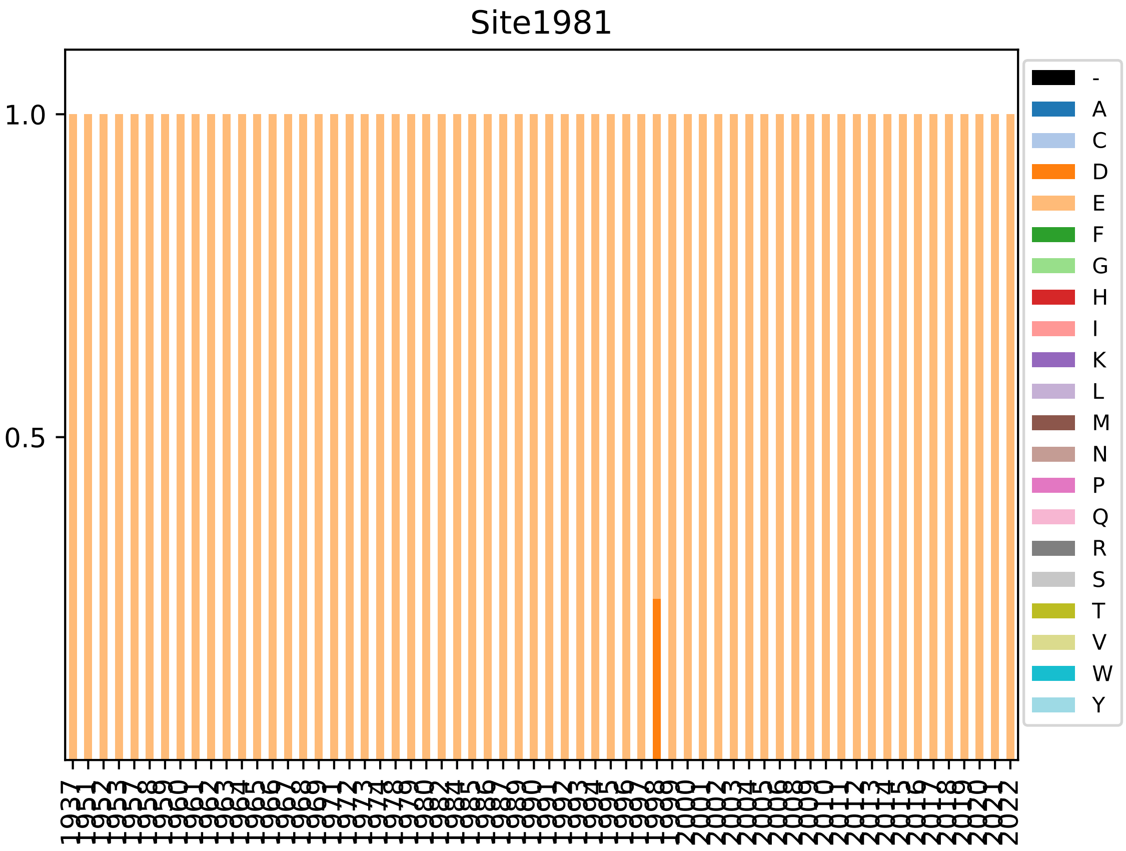

Site1981
1.0
0.5
-
A
C
D
E
F
G
H
I
K
L
M
N
P
Q
R
S
T
V
W
Y

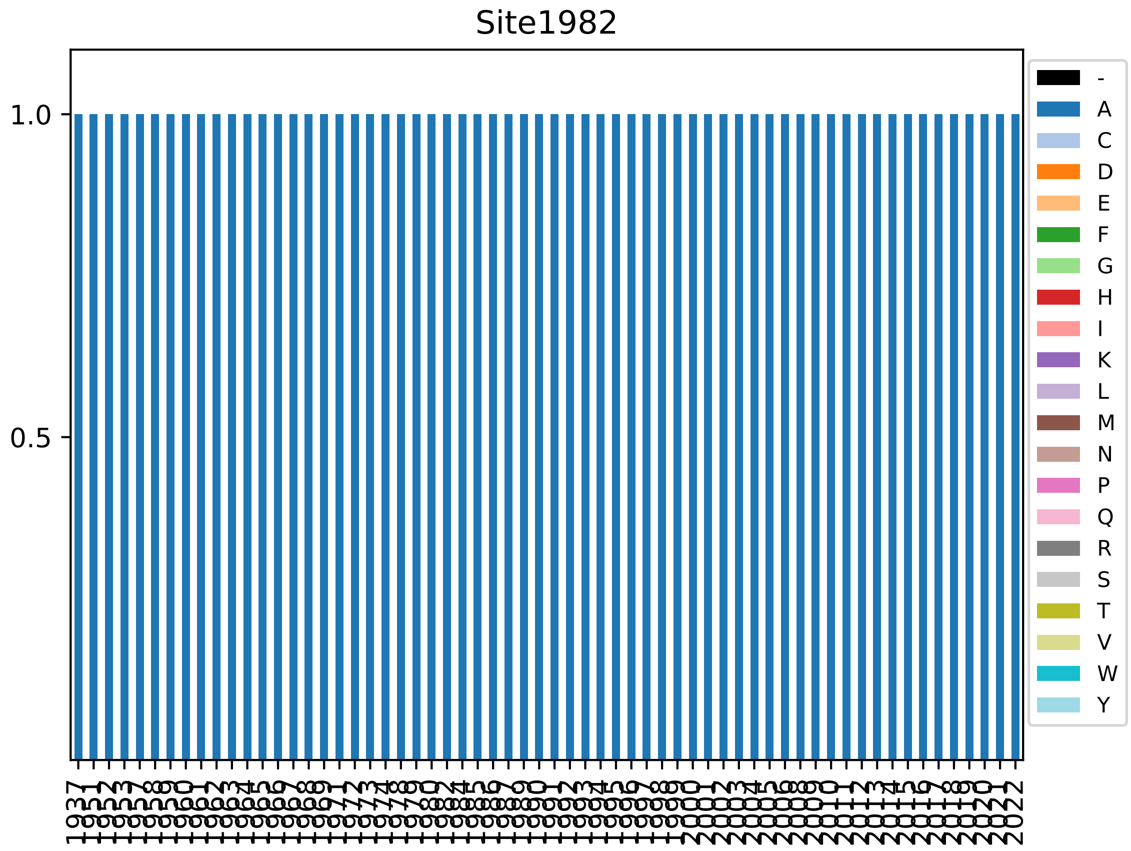
Site1982
1.0
0.5
-
A
C
D
E
F
G
H
I
K
L
M
N
P
Q
R
S
T
V
W
Y

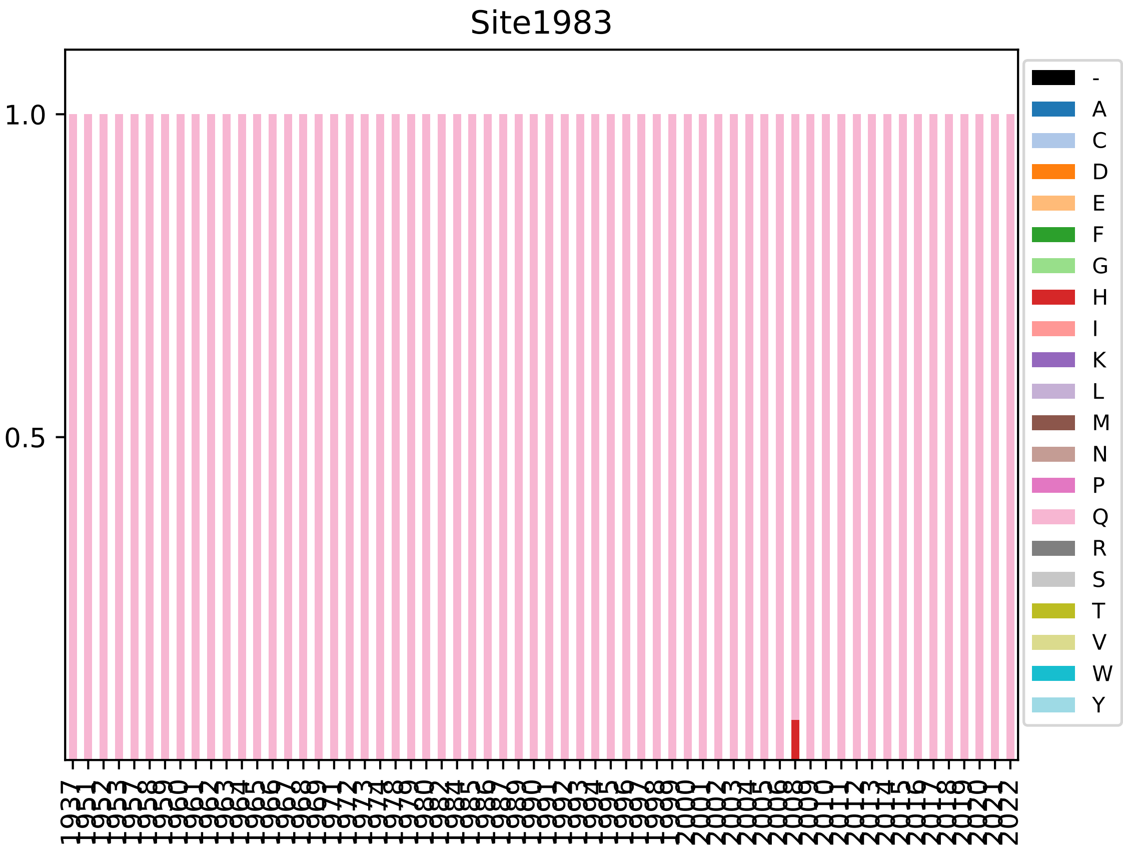
Site1983
1.0
0.5
-
A
C
D
E
F
G
H
I
K
L
M
N
P
Q
R
S
T
V
W
Y

Site1984

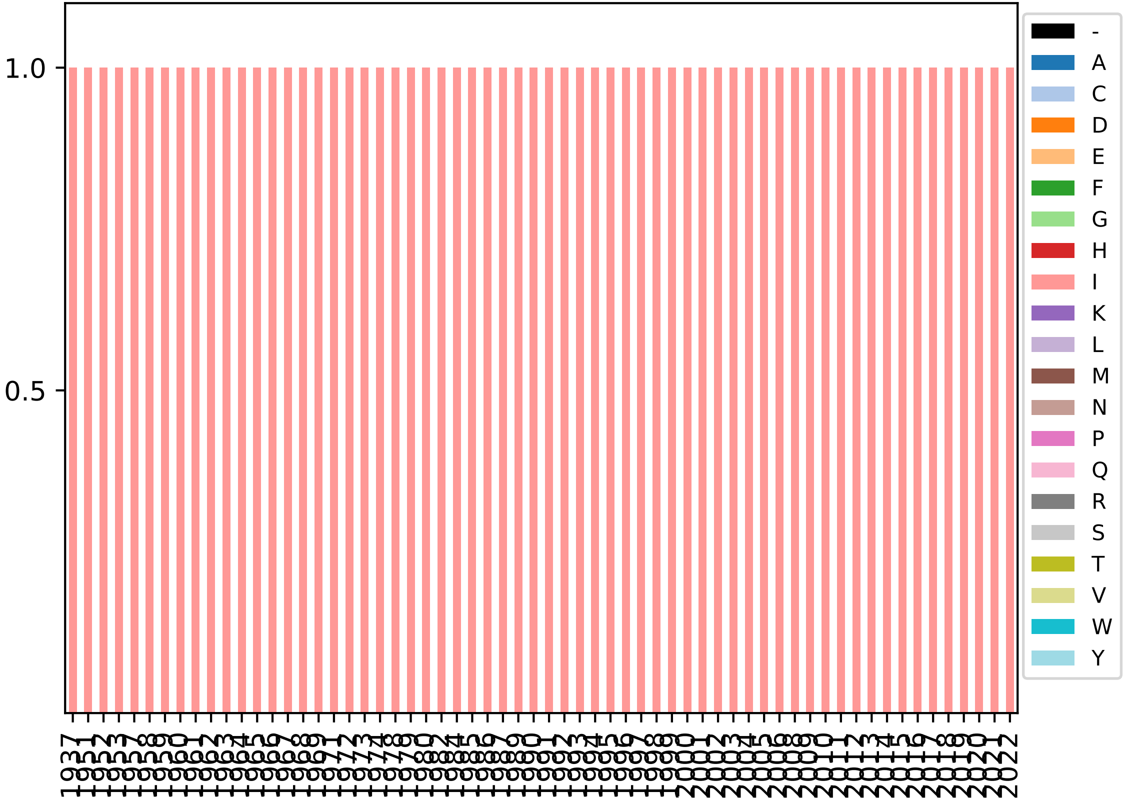

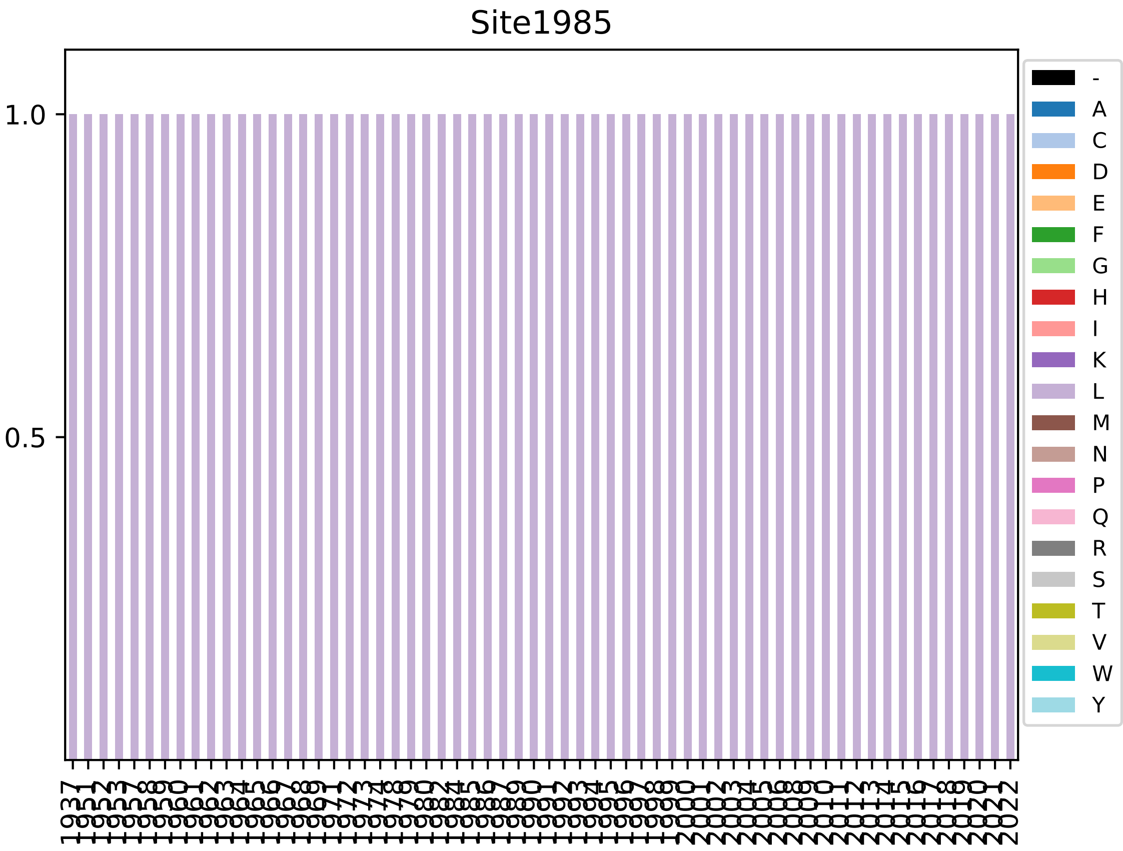

Site1985
1.0
0.5
-
A
C
D
E
F
G
H
I
K
L
M
N
P
Q
R
S
T
V
W
Y

Site1986

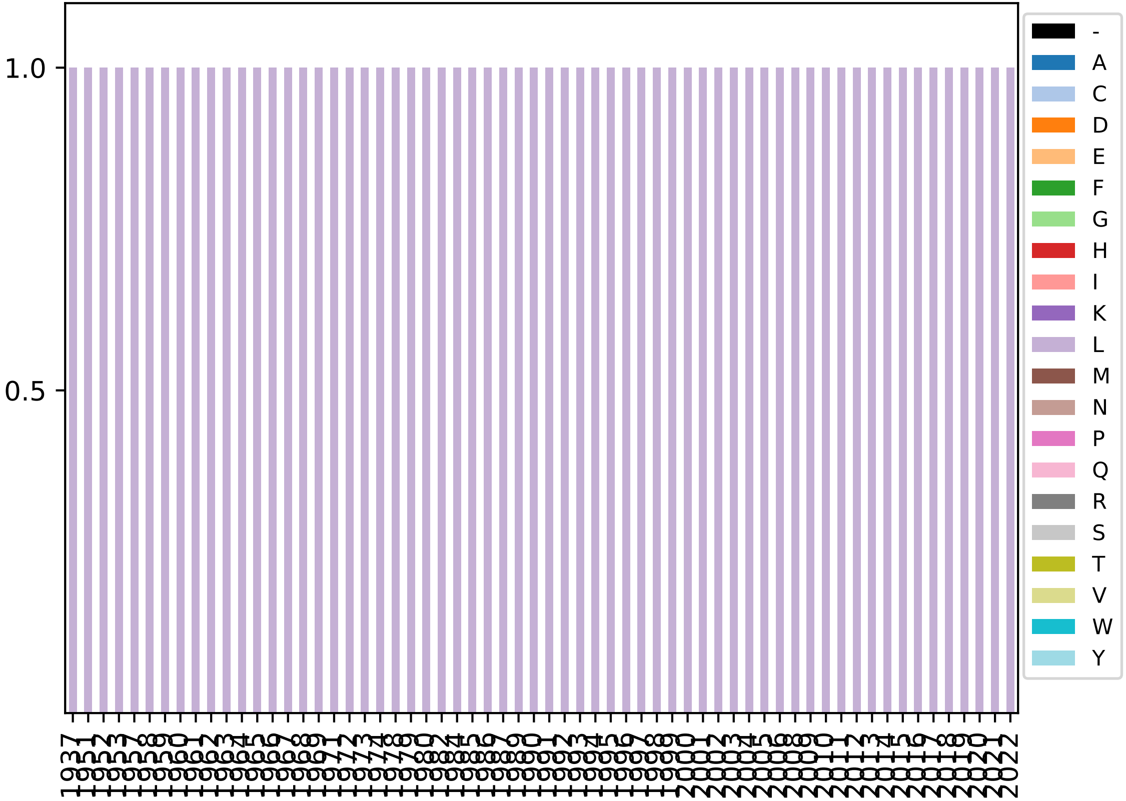

Site1987

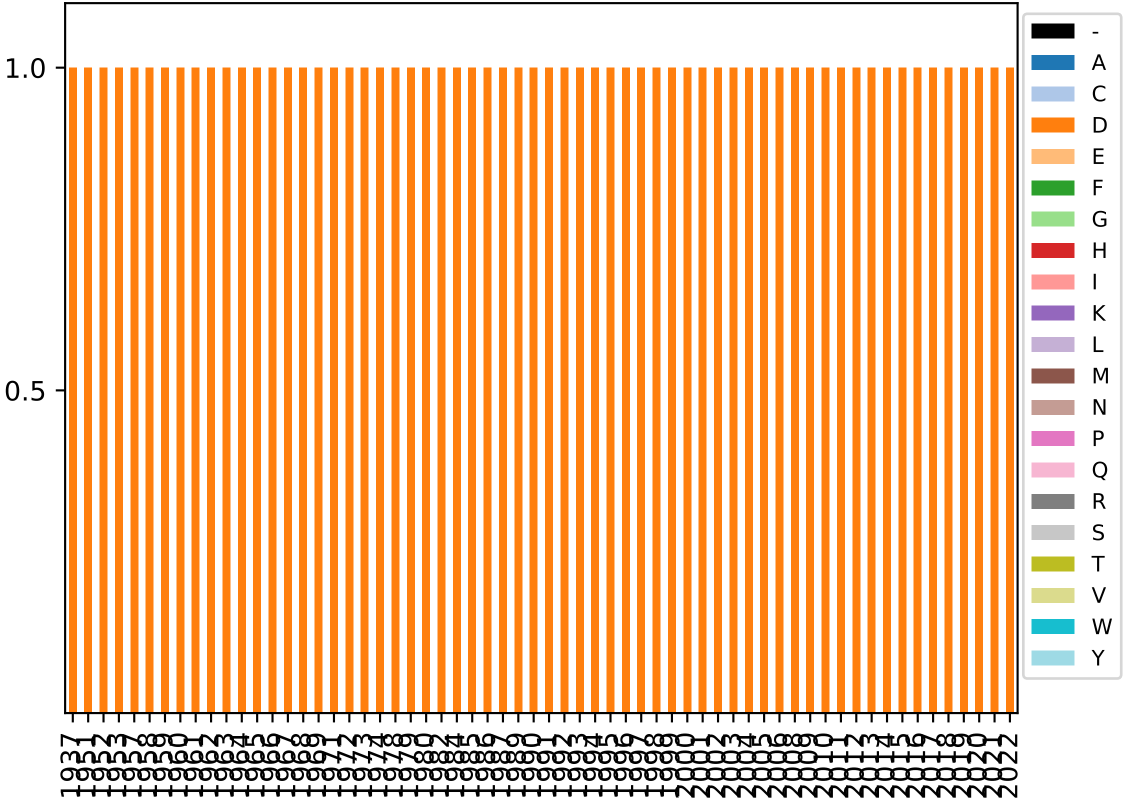

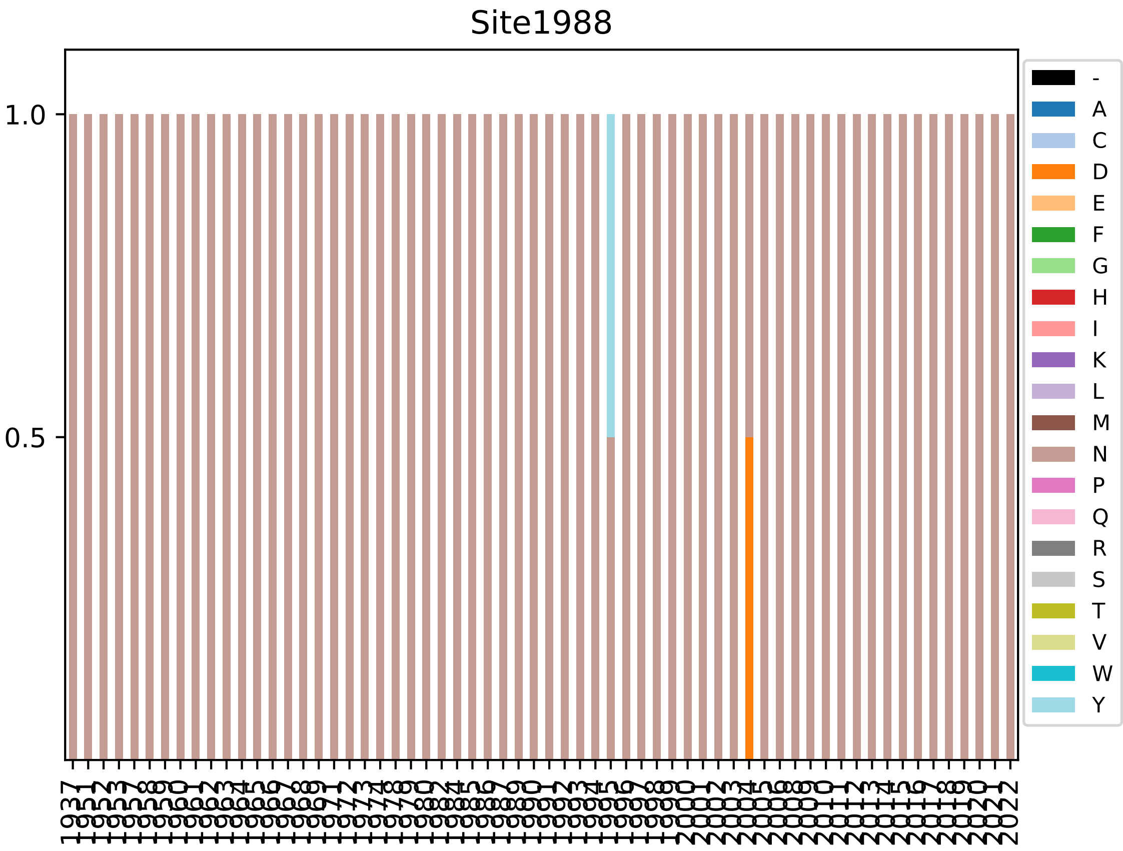

Site1988
1.0
0.5
-
A
C
D
E
F
G
H
I
K
L
M
N
P
Q
R
S
T
V
W
Y

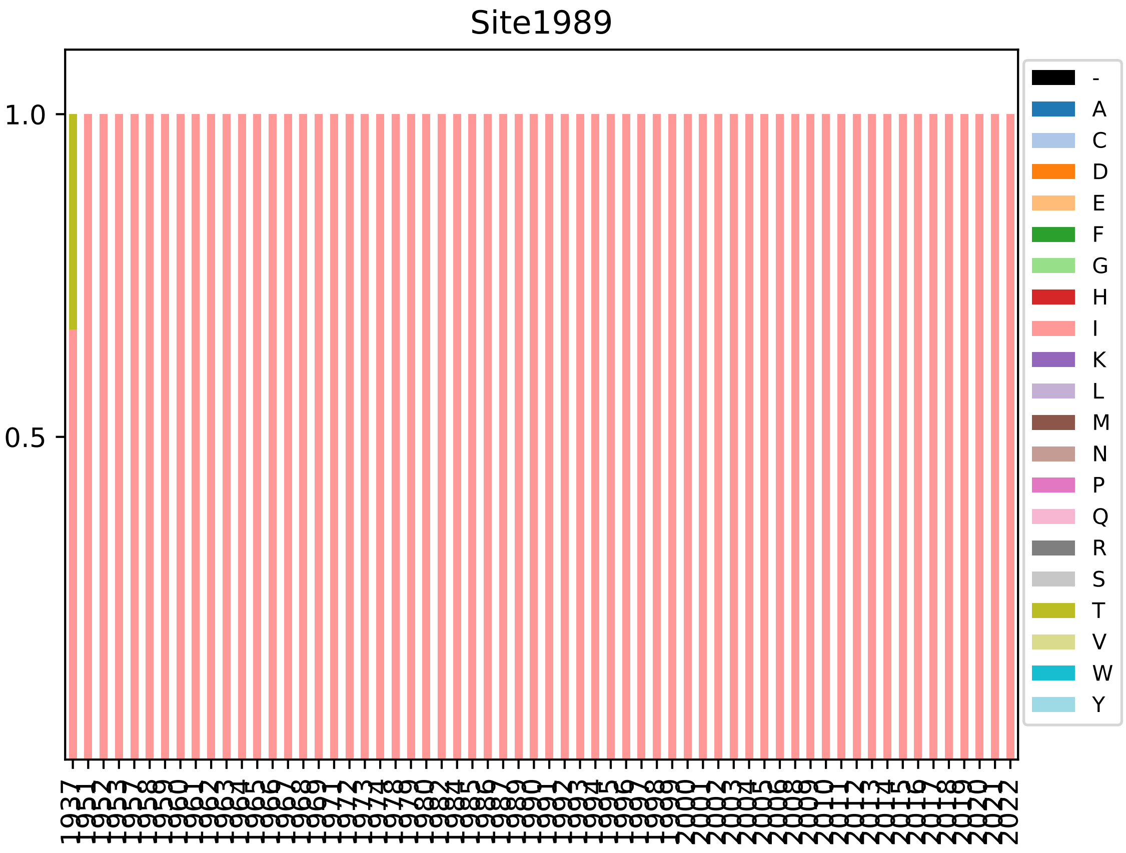

Site1989
1.0
0.5
-
A
C
D
E
F
G
H
I
K
L
M
N
P
Q
R
S
T
V
W
Y

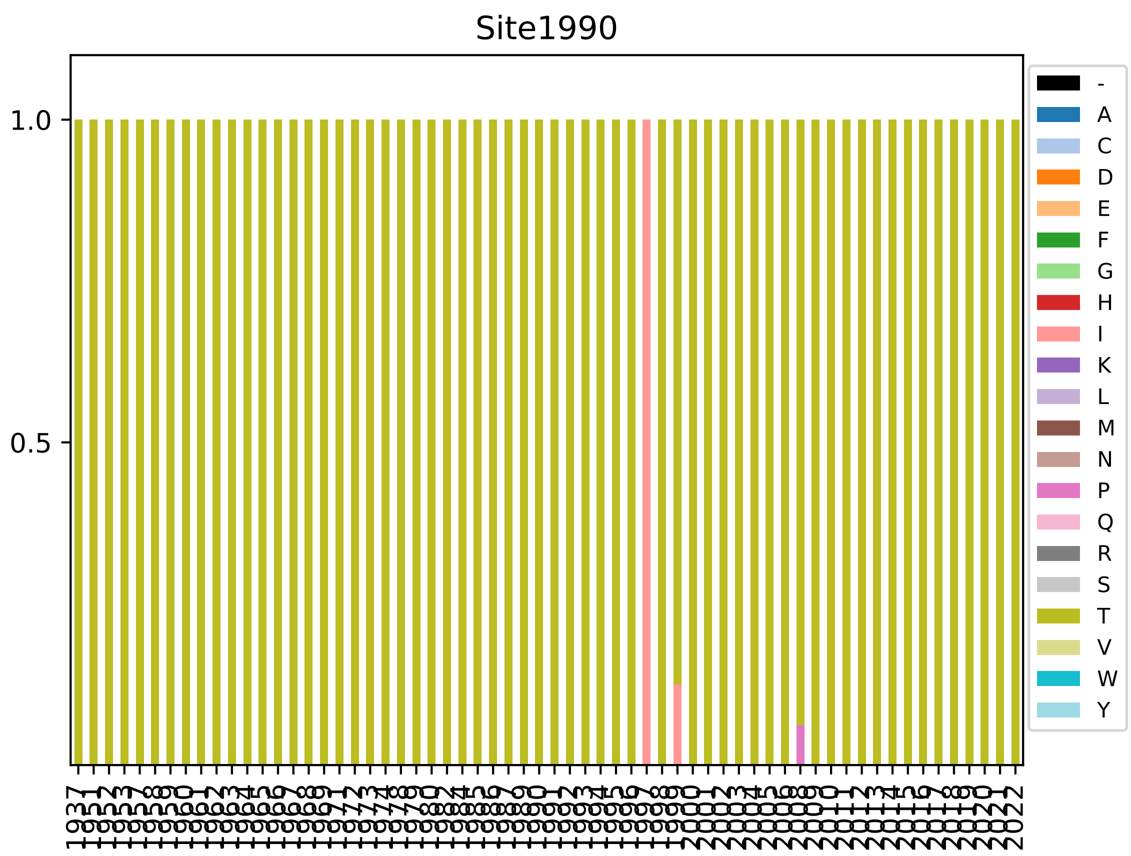

Site1990
1.0
0.5
-
A
C
D
E
F
G
H
I
K
L
M
N
P
Q
R
S
T
V
W
Y

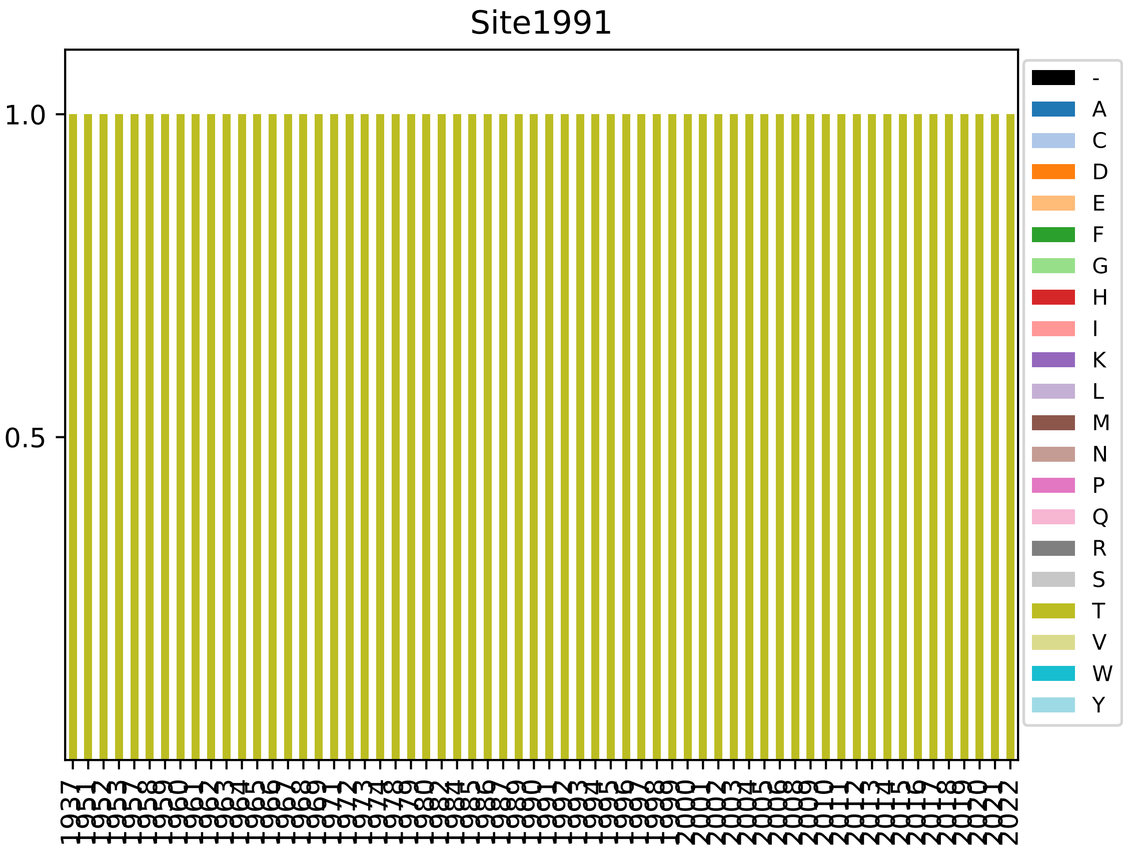
Site1991
1.0
0.5
-
A
C
D
E
F
G
H
I
K
L
M
N
P
Q
R
S
T
V
W
Y

Site1992

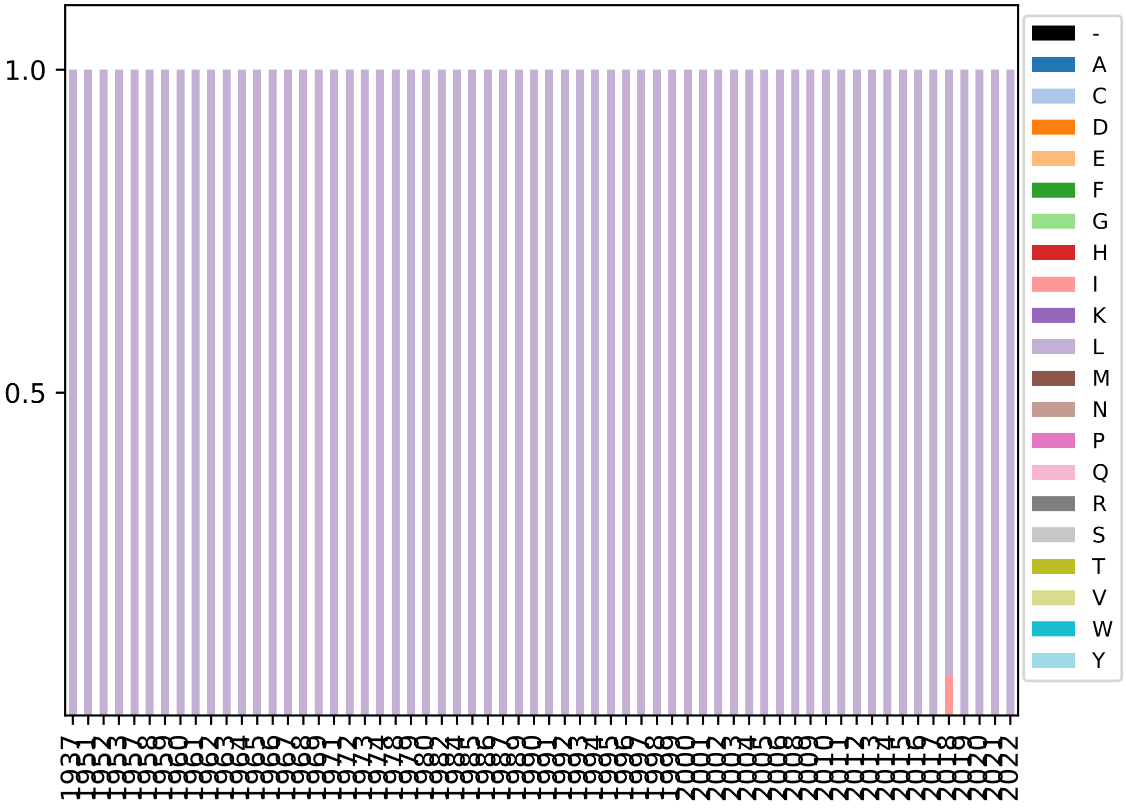

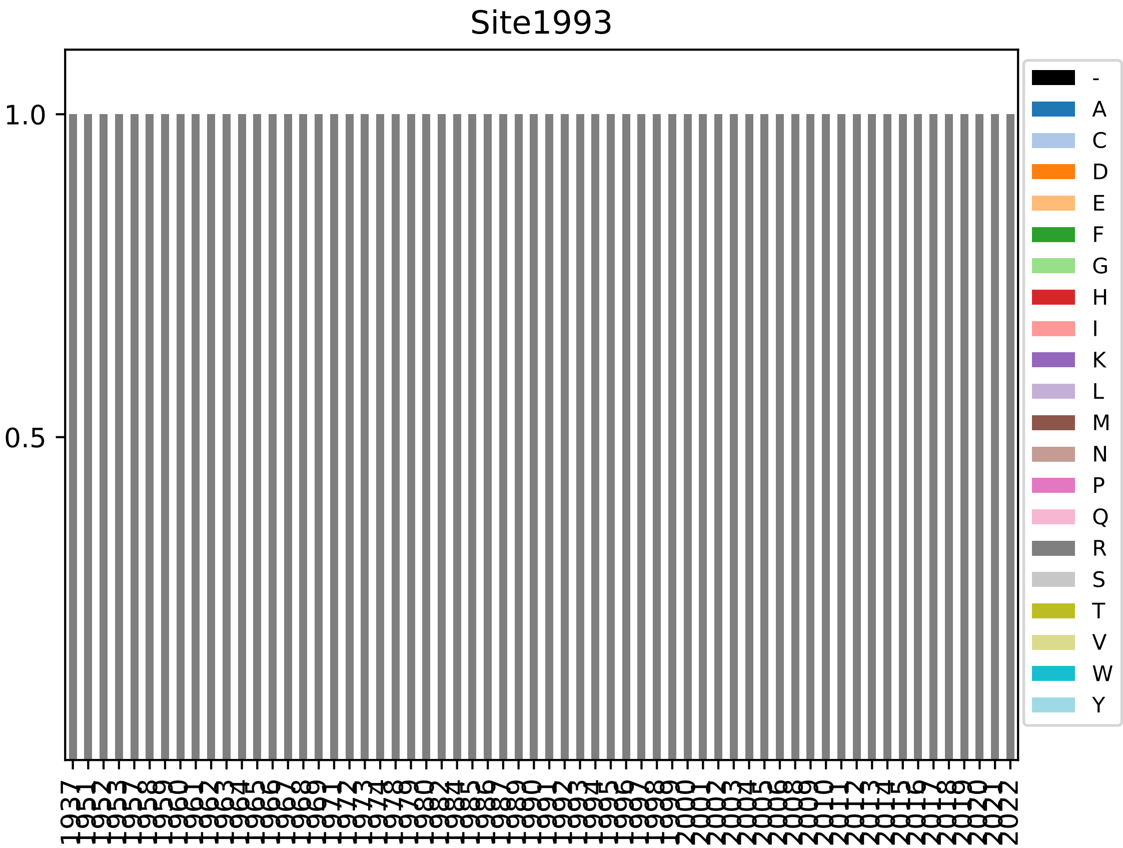
Site1993
1.0
0.5
-
A
C
D
E
F
G
H
I
K
L
M
N
P
Q
R
S
T
V
W
Y

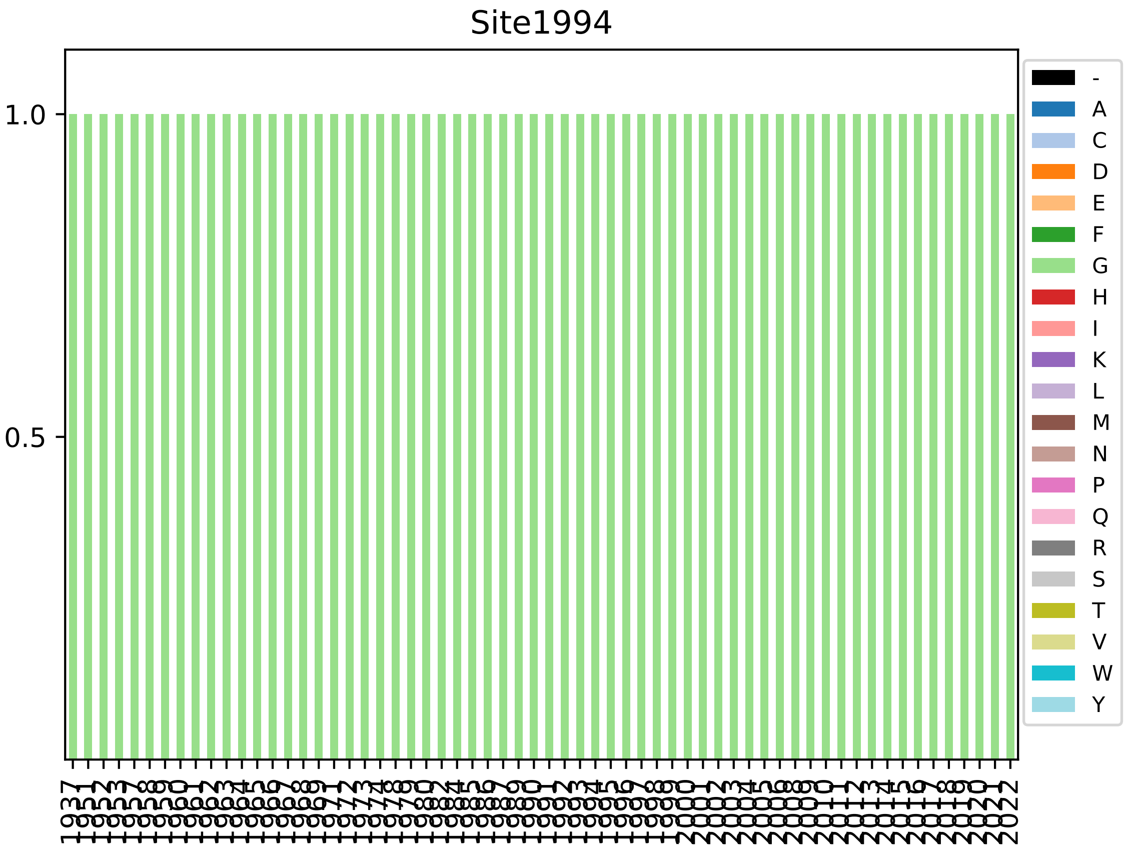
Site1994
1.0
0.5
-
A
C
D
E
F
G
H
I
K
L
M
N
P
Q
R
S
T
V
W
Y

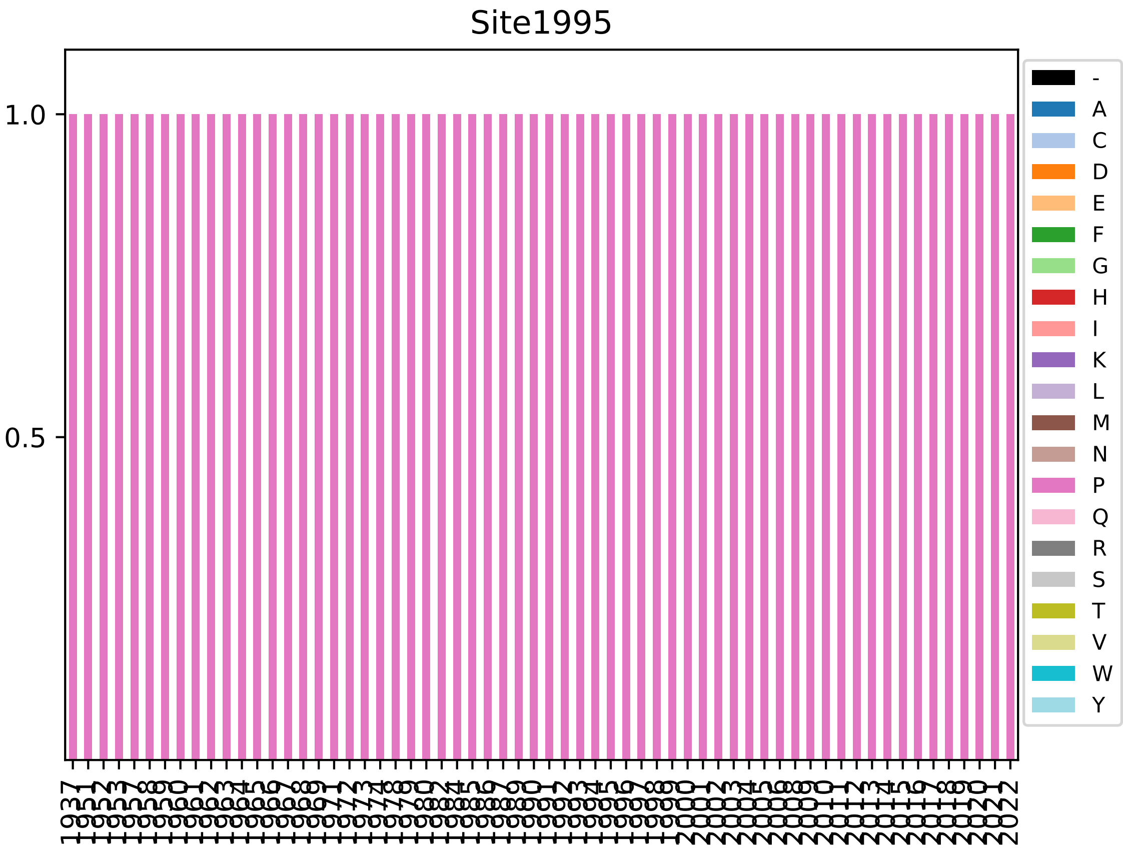
Site1995
1.0
0.5
-
A
C
D
E
F
G
H
I
K
L
M
N
P
Q
R
S
T
V
W
Y

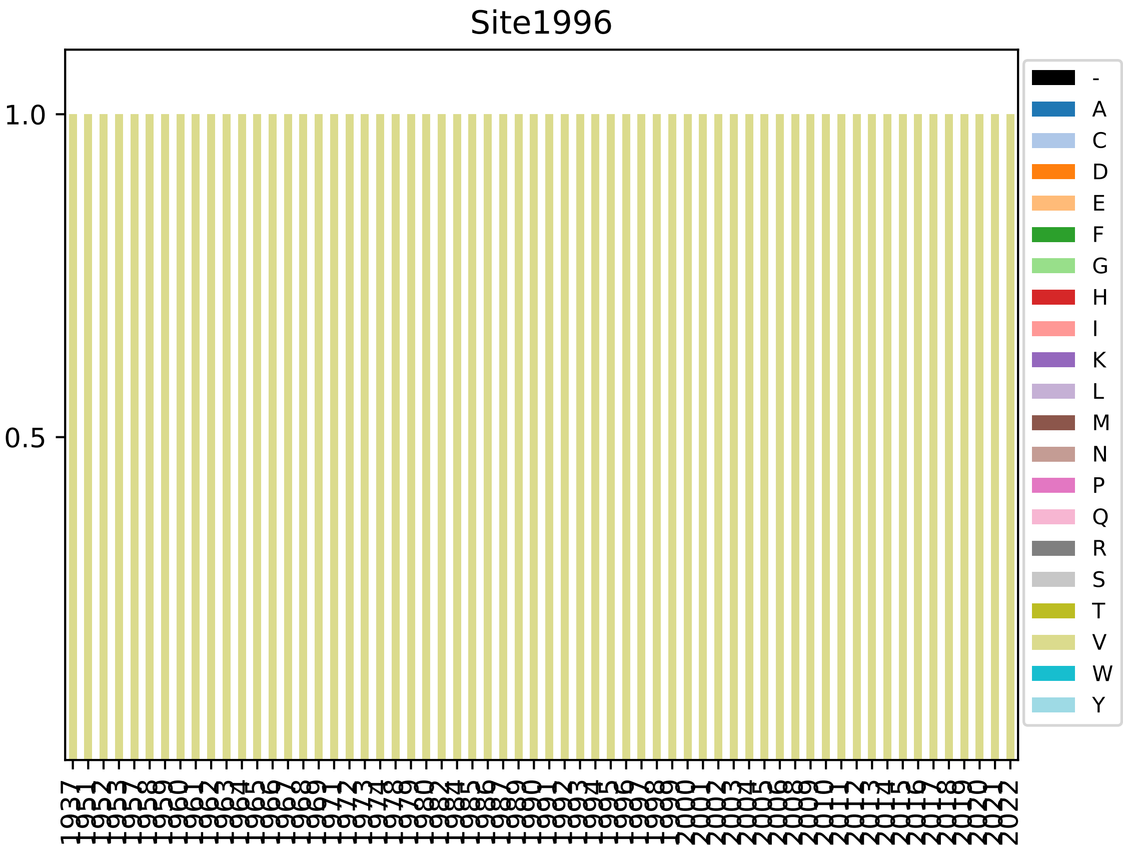
Site1996
1.0
0.5
-
A
C
D
E
F
G
H
I
K
L
M
N
P
Q
R
S
T
V
W
Y

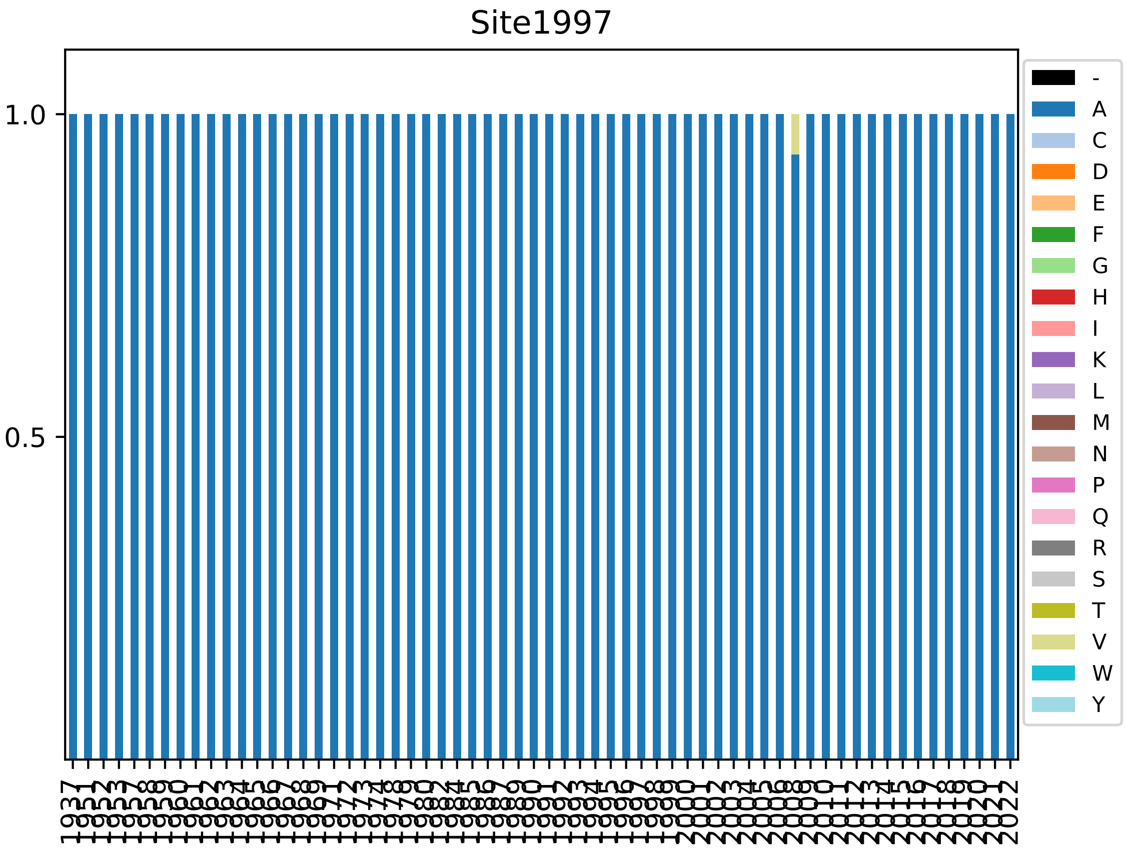

Site1997
1.0
0.5
-
A
C
D
E
F
G
H
I
K
L
M
N
P
Q
R
S
T
V
W
Y

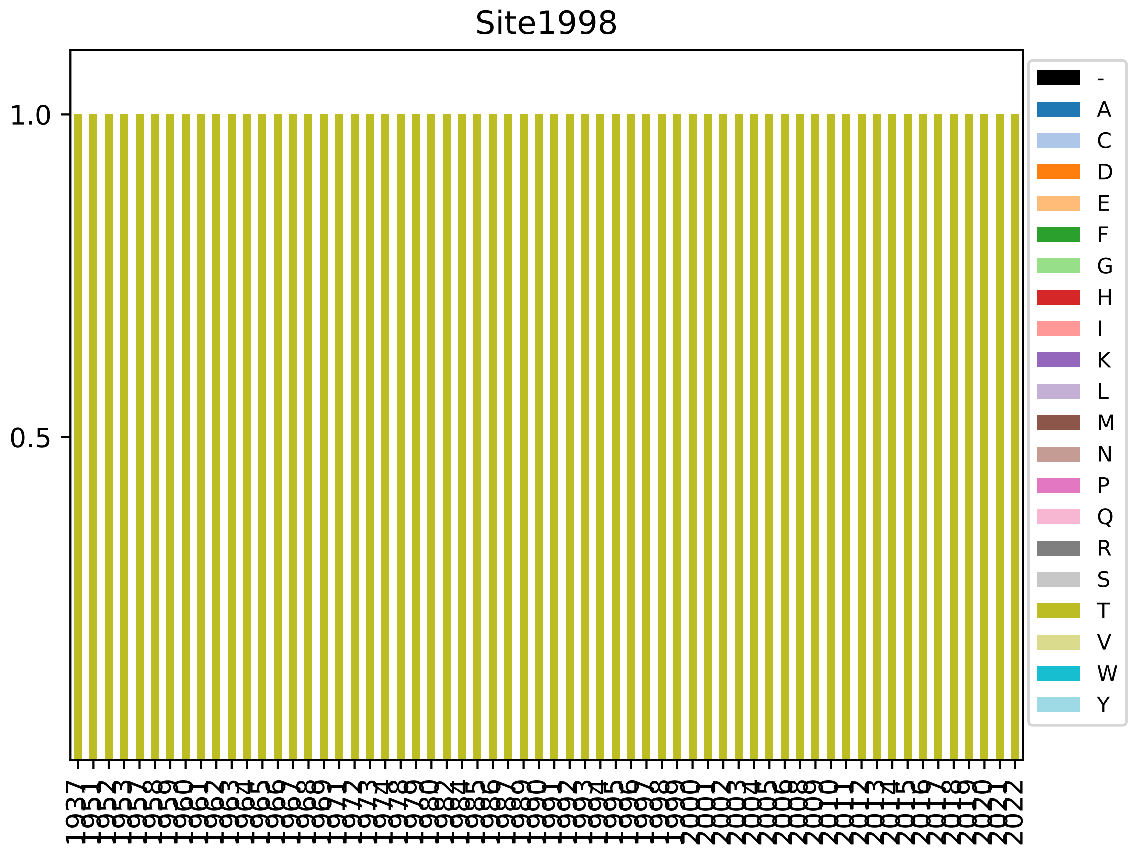
Site1998
1.0
0.5
-
A
C
D
E
F
G
H
I
K
L
M
N
P
Q
R
S
T
V
W
Y

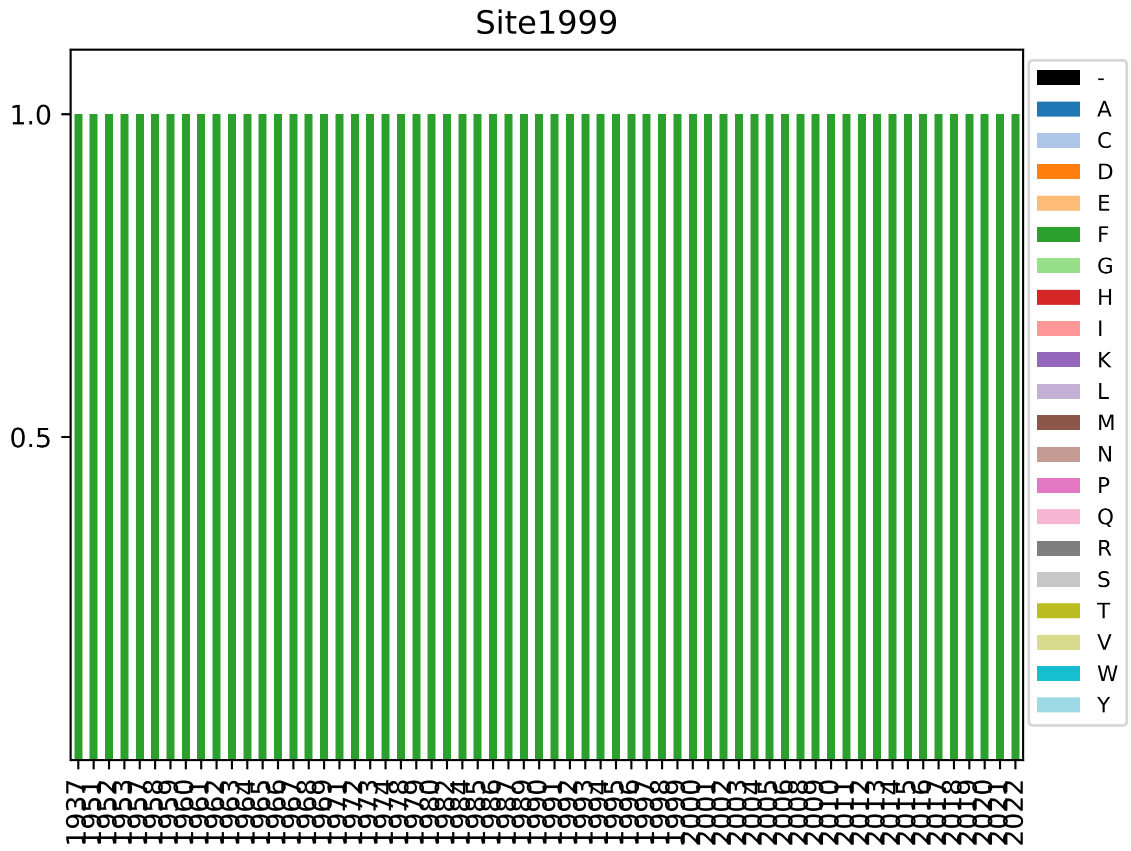
Site1999
1.0
0.5
-
A
C
D
E
F
G
H
I
K
L
M
N
P
Q
R
S
T
V
W
Y

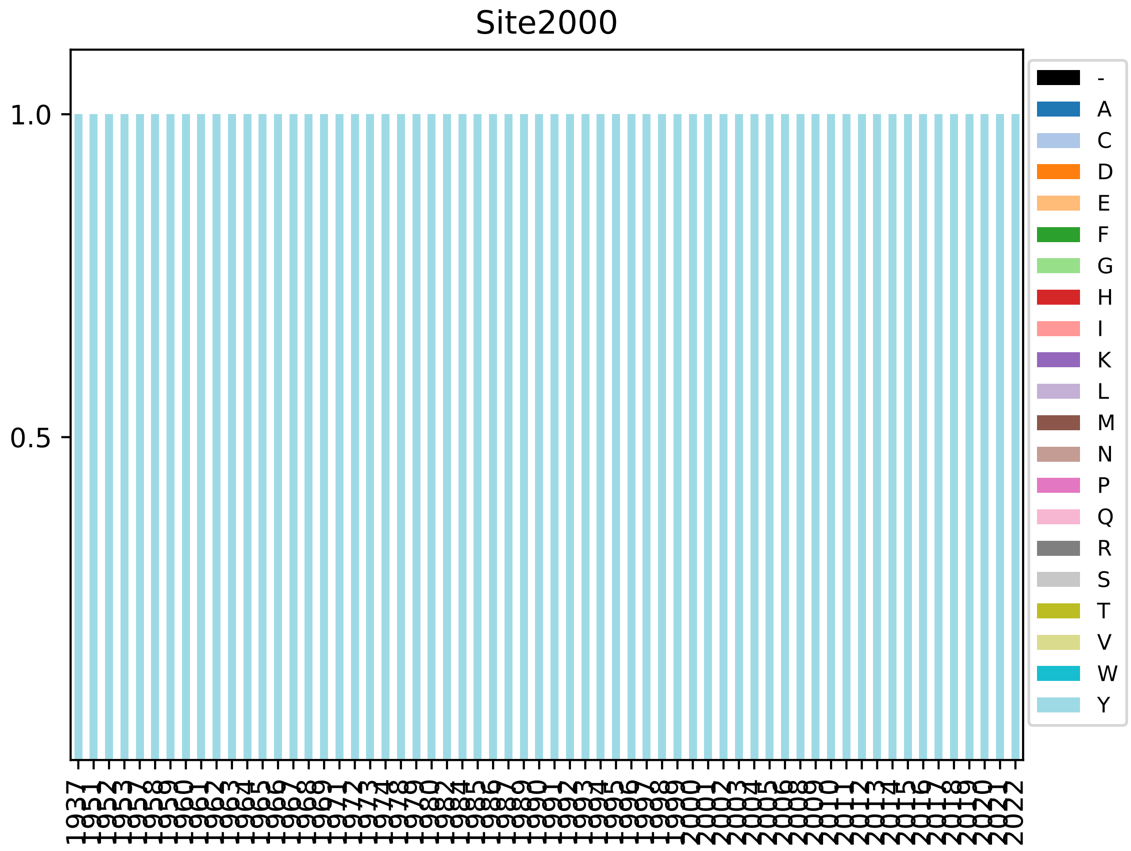
Site2000
1.0
0.5
-
A
C
D
E
F
G
H
I
K
L
M
N
P
Q
R
S
T
V
W
Y

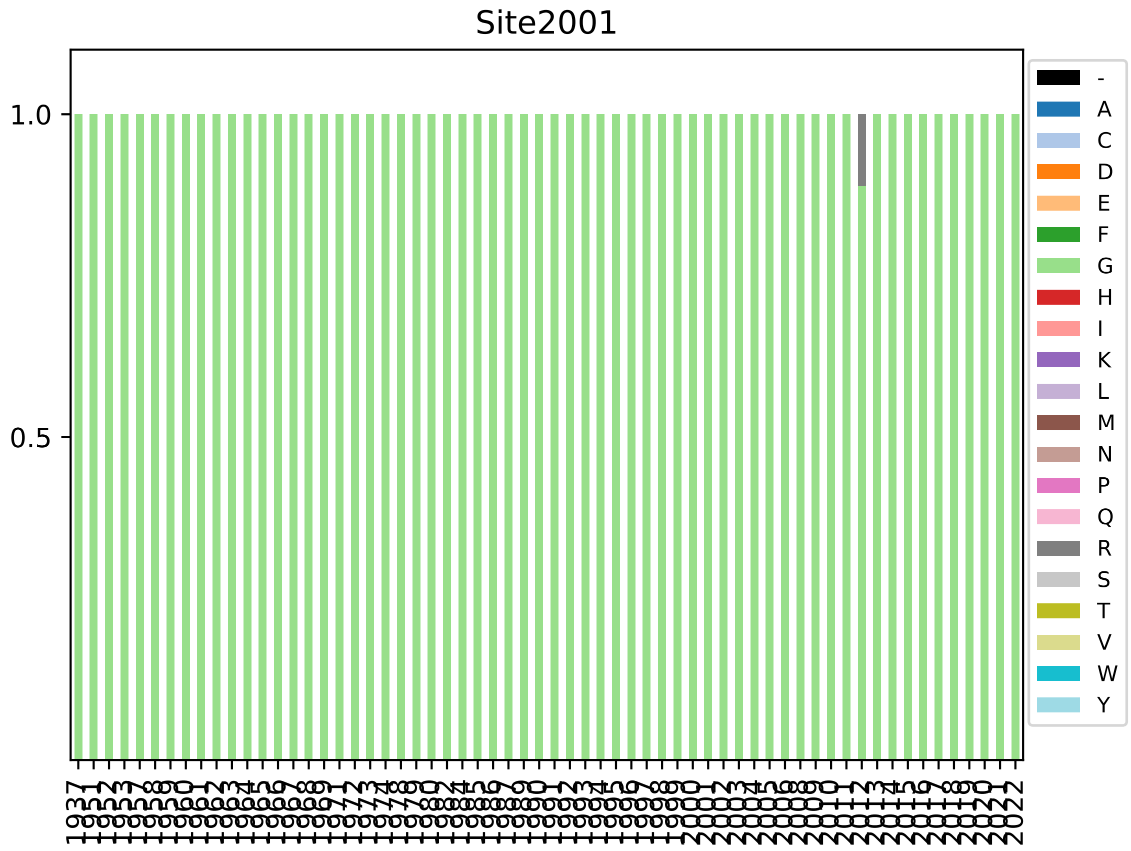
Site2001
1.0
0.5
-
A
C
D
E
F
G
H
I
K
L
M
N
P
Q
R
S
T
V
W
Y

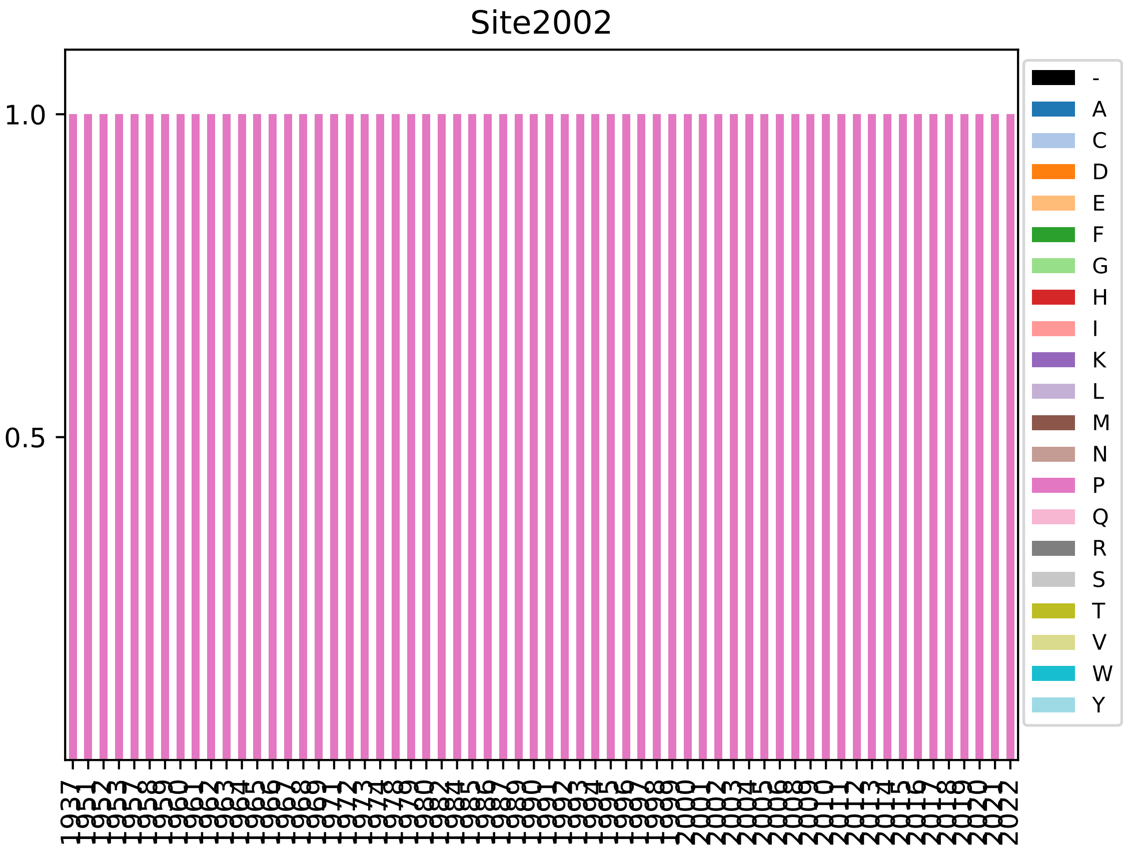
Site2002
1.0
0.5
-
A
C
D
E
F
G
H
I
K
L
M
N
P
Q
R
S
T
V
W
Y

Site2003

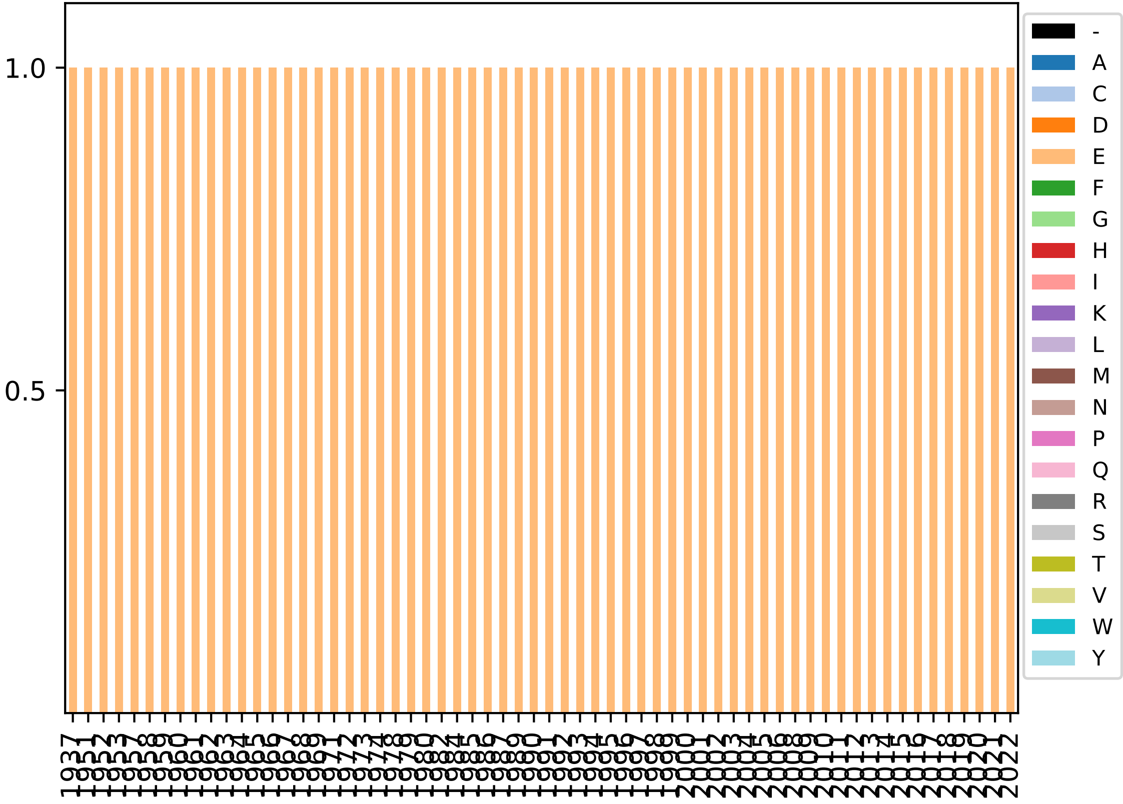

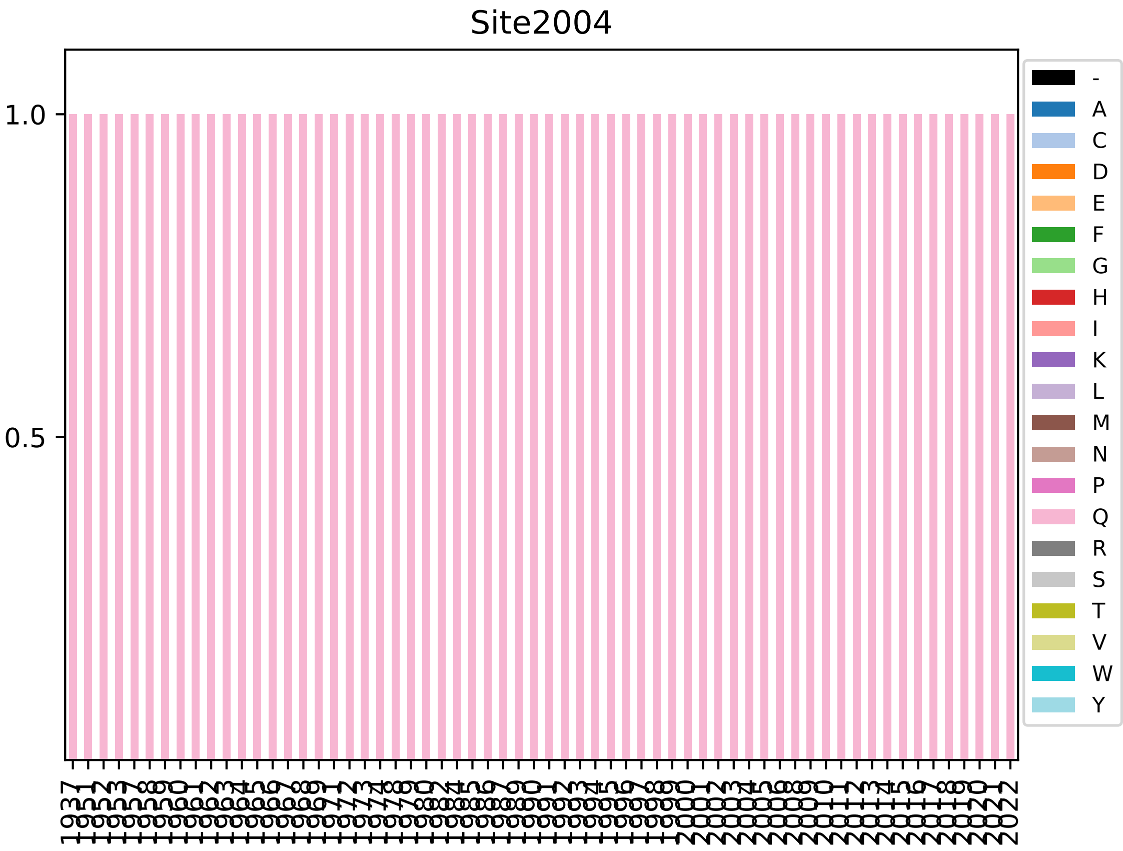
Site2004
1.0
0.5
-
A
C
D
E
F
G
H
I
K
L
M
N
P
Q
R
S
T
V
W
Y

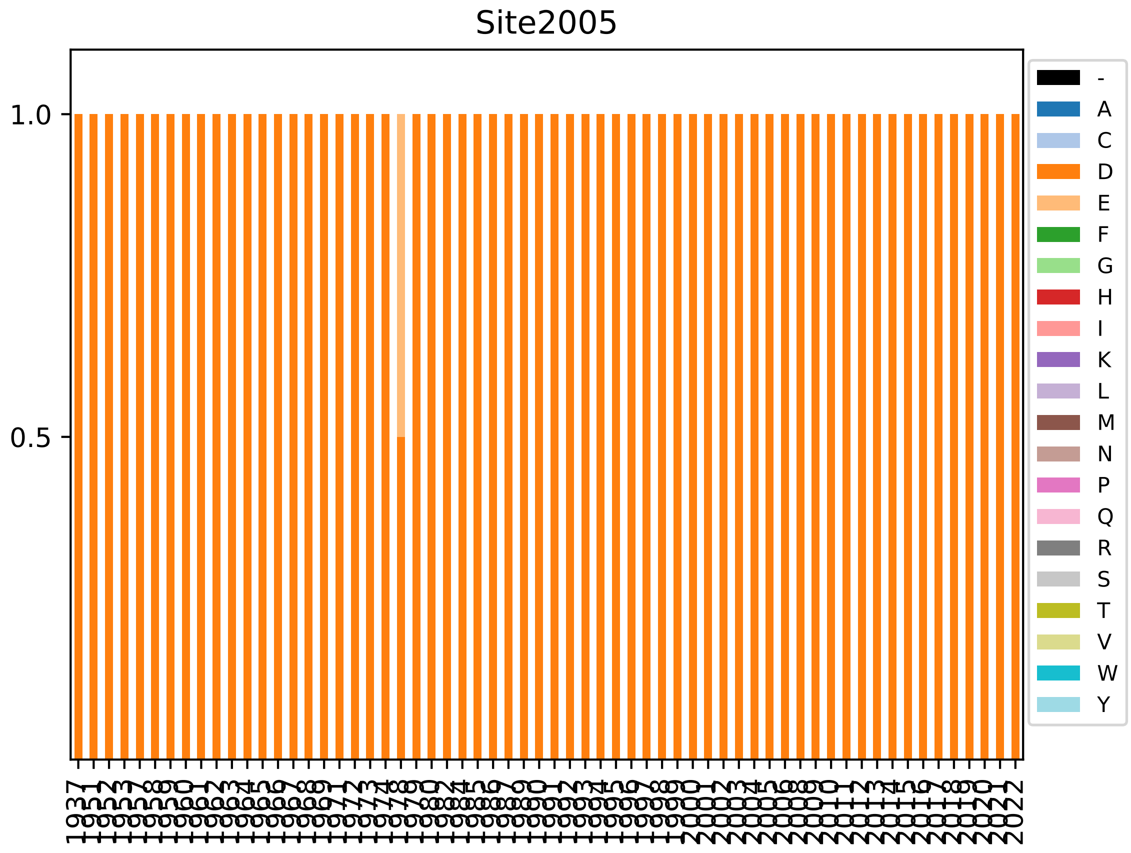
Site2005
1.0
0.5
-
A
C
D
E
F
G
H
I
K
L
M
N
P
Q
R
S
T
V
W
Y

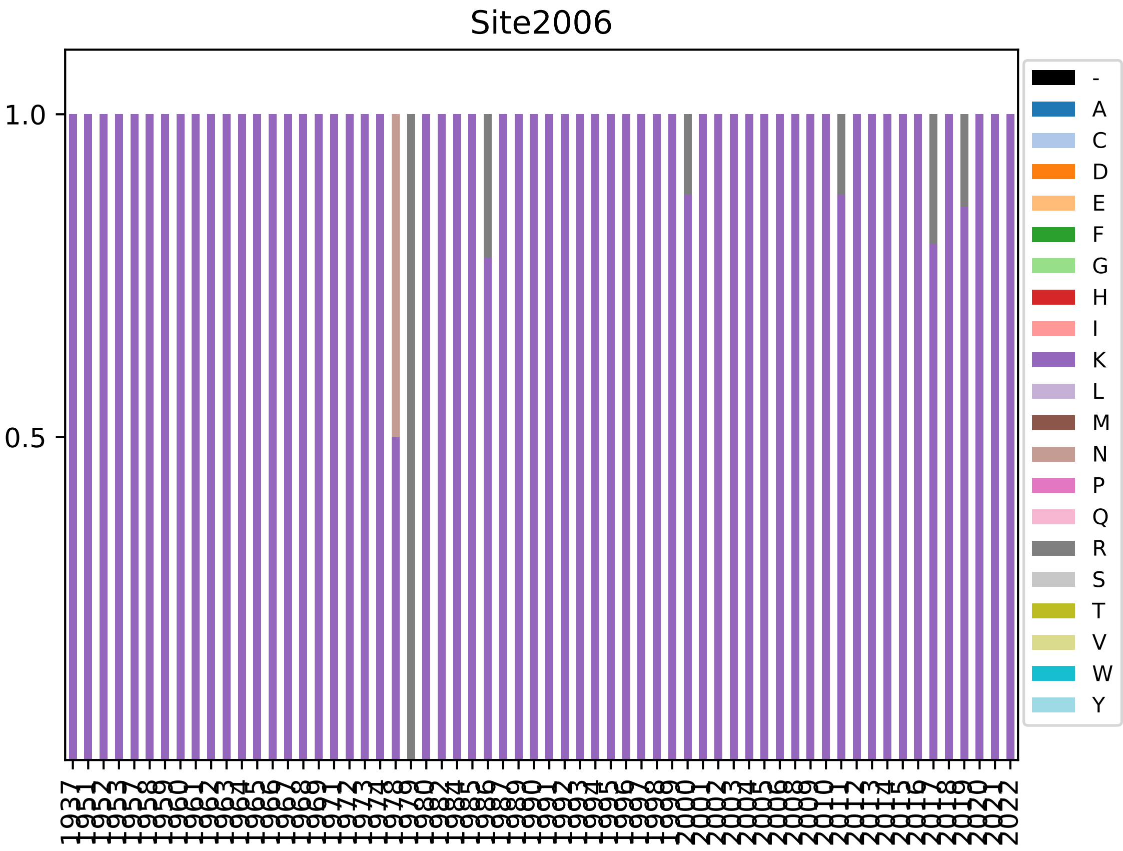
Site2006
1.0
0.5
-
A
C
D
E
F
G
H
I
K
L
M
N
P
Q
R
S
T
V
W
Y

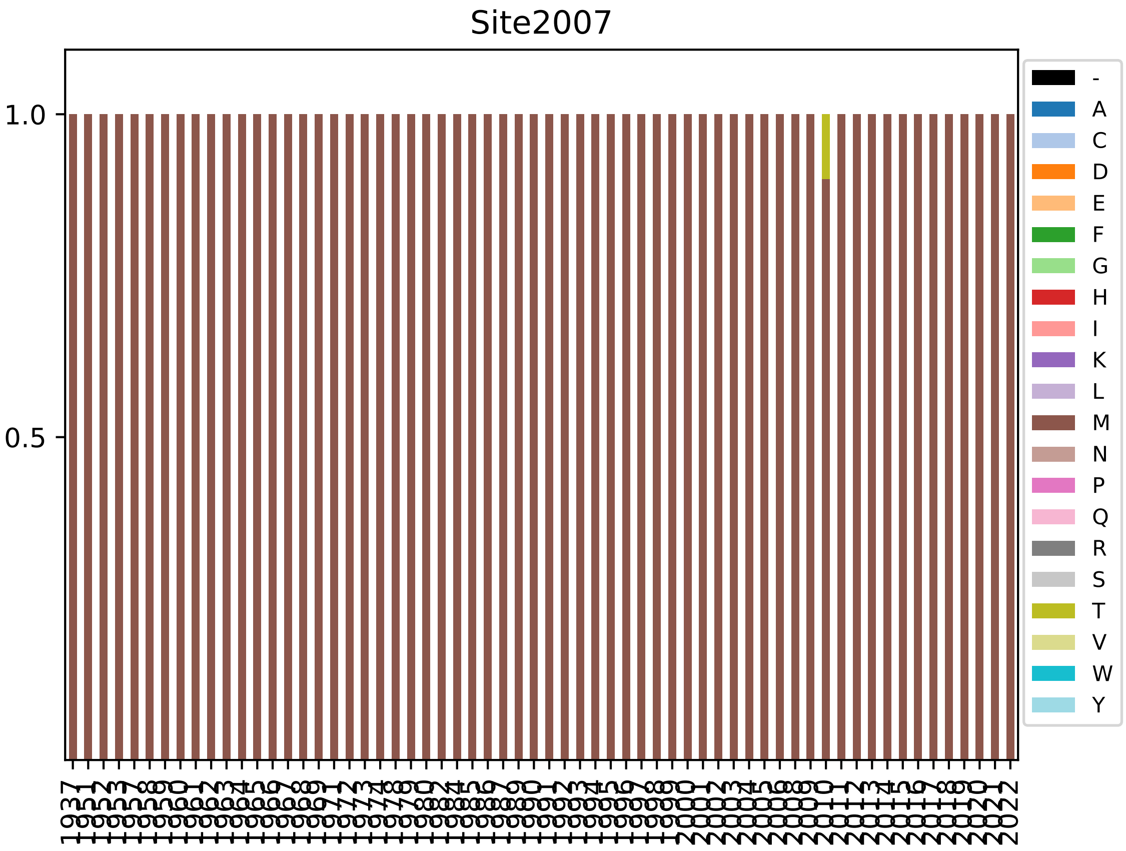
Site2007
1.0
0.5
-
A
C
D
E
F
G
H
I
K
L
M
N
P
Q
R
S
T
V
W
Y

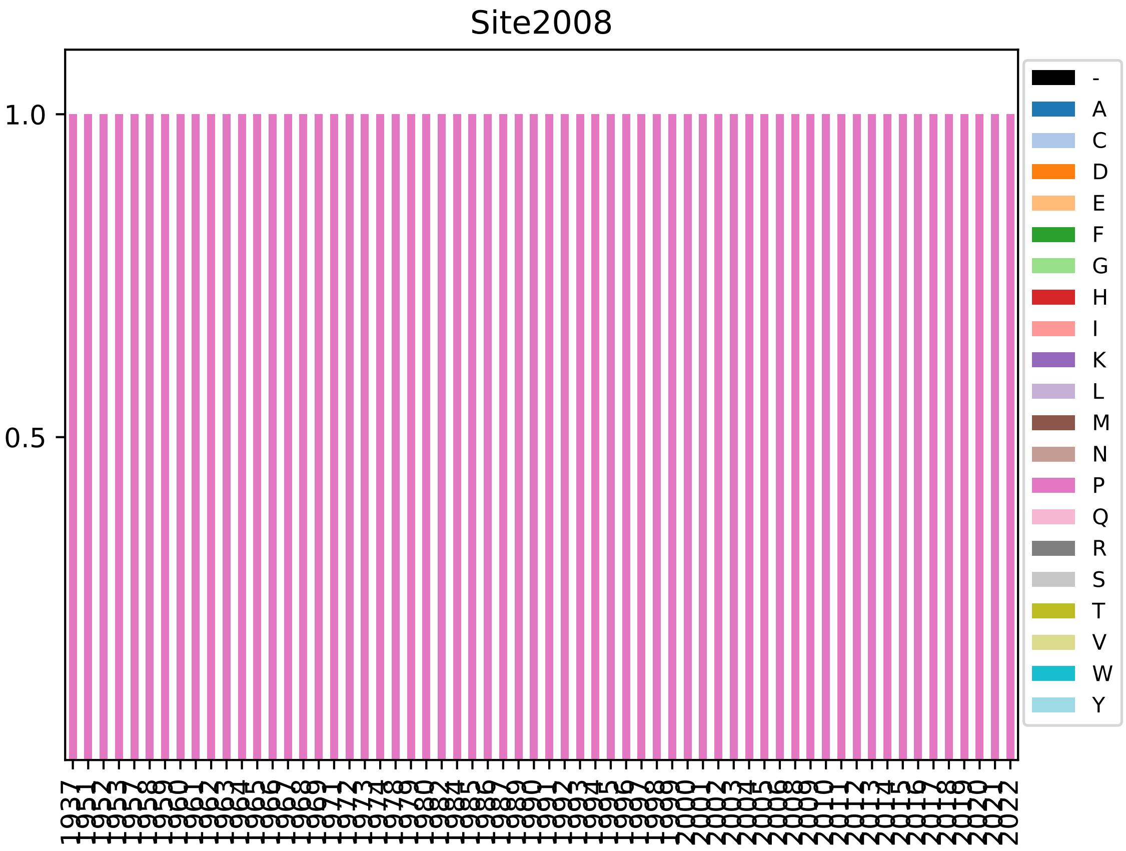
Site2008
1.0
0.5
-
A
C
D
E
F
G
H
I
K
L
M
N
P
Q
R
S
T
V
W
Y

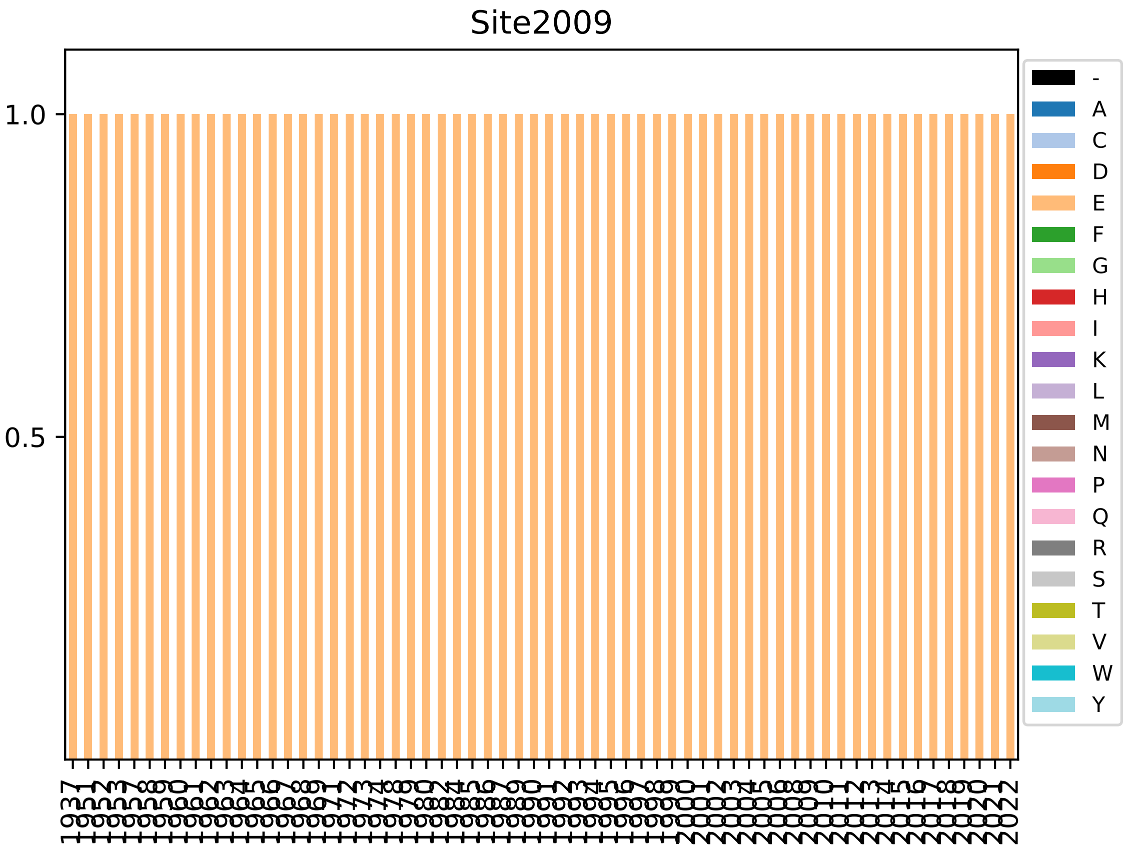
Site2009
1.0
0.5
-
A
C
D
E
F
G
H
I
K
L
M
N
P
Q
R
S
T
V
W
Y

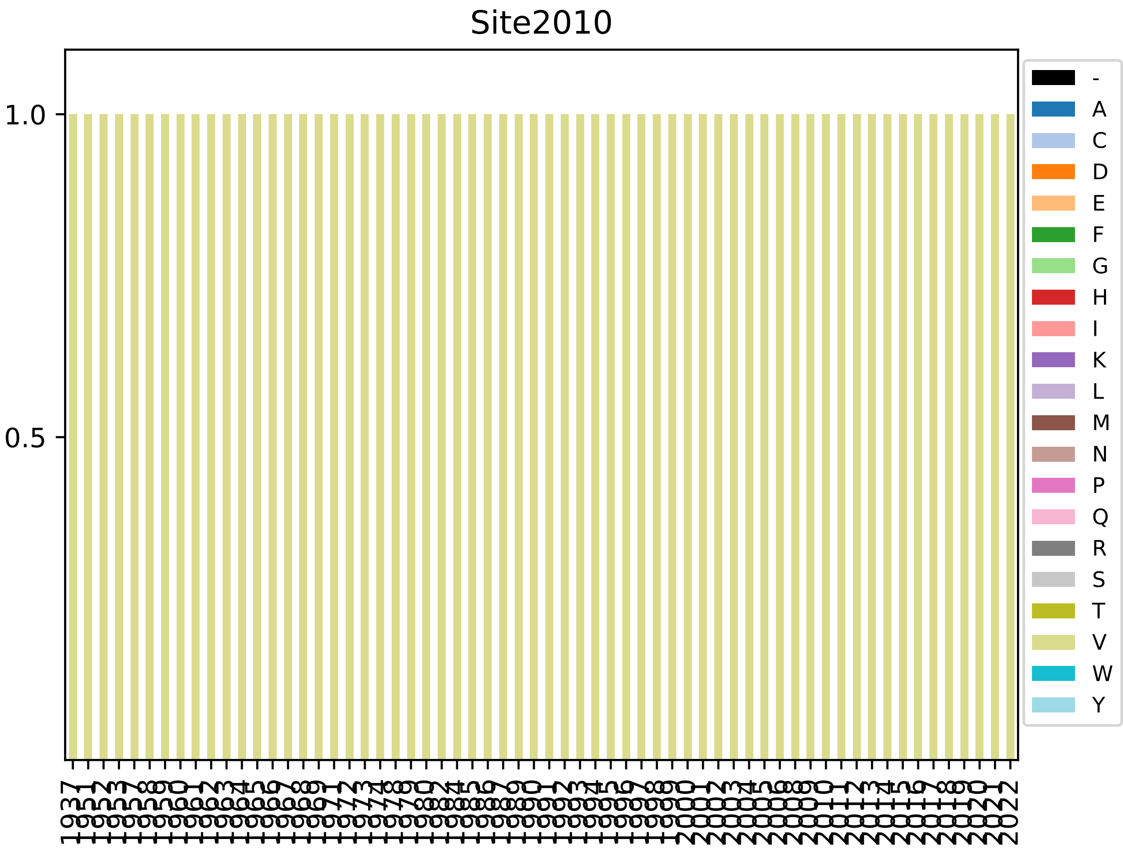
Site2010
1.0
0.5
-
A
C
D
E
F
G
H
I
K
L
M
N
P
Q
R
S
T
V
W
Y

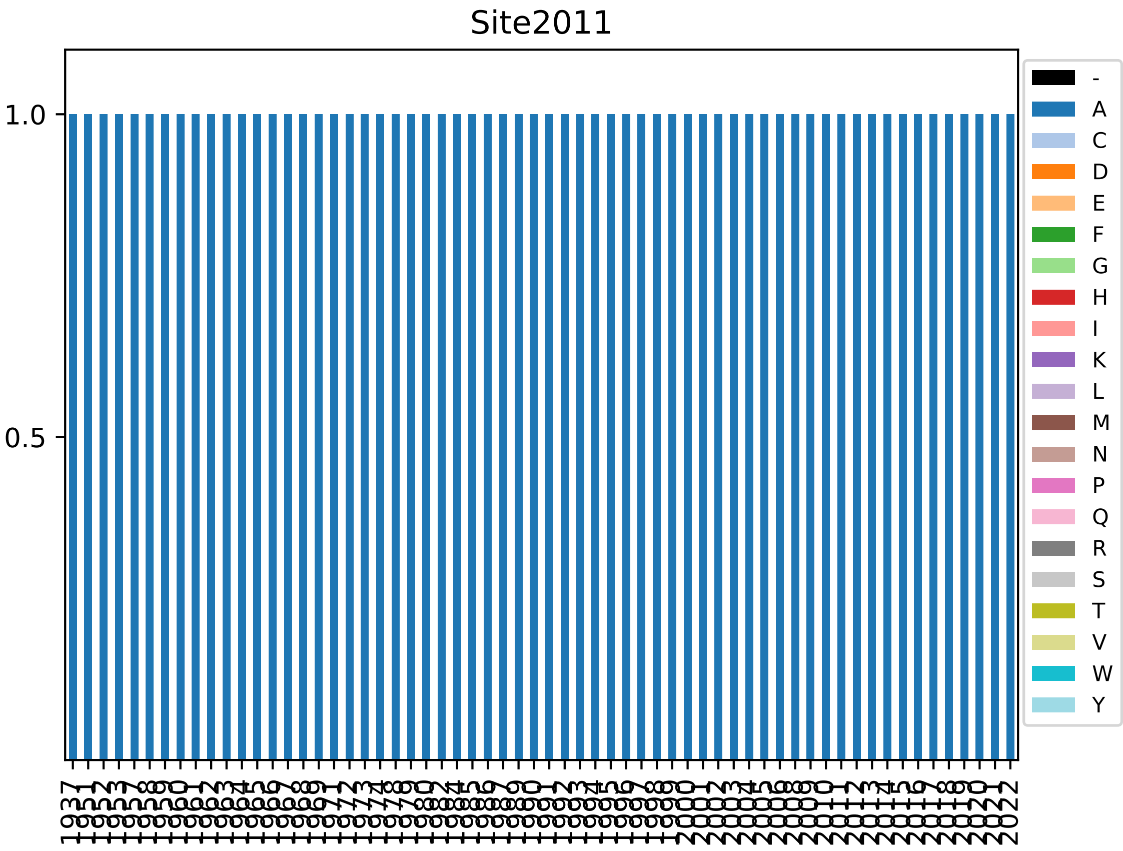
Site2011
1.0
0.5
-
A
C
D
E
F
G
H
I
K
L
M
N
P
Q
R
S
T
V
W
Y

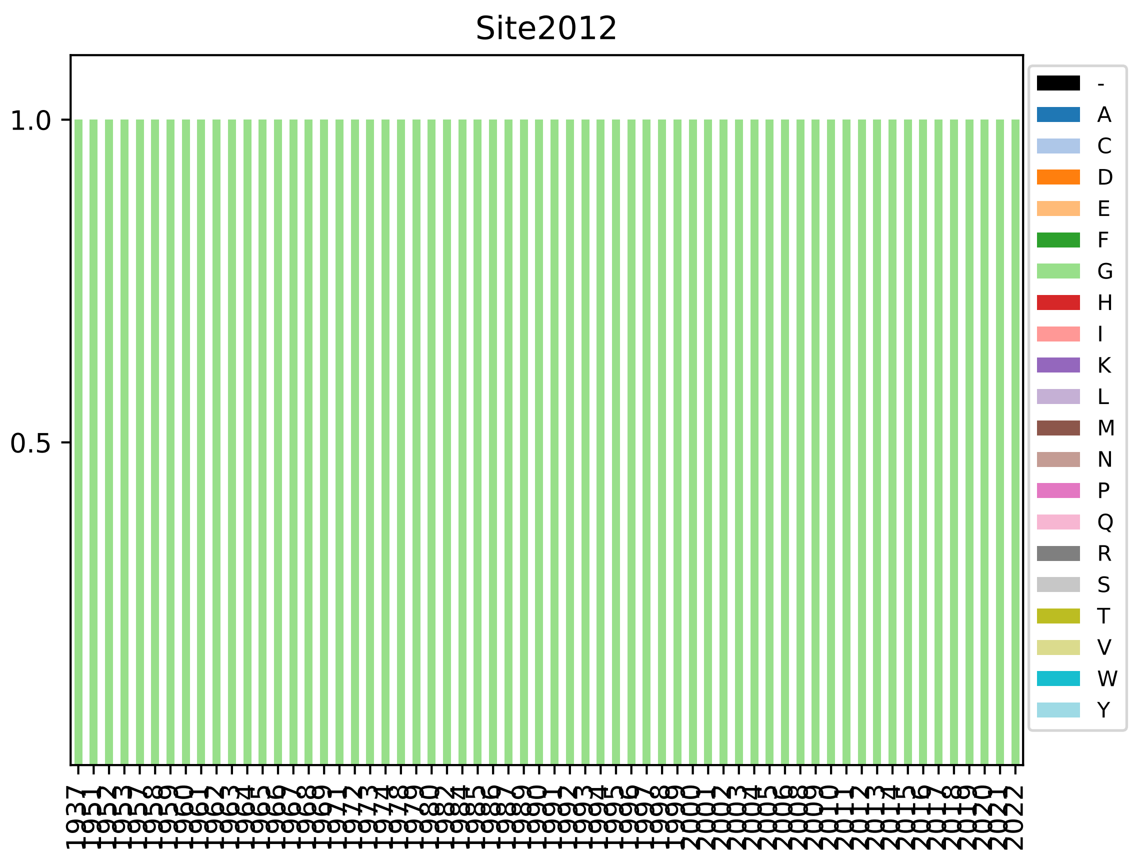
Site2012
1.0
0.5
-
A
C
D
E
F
G
H
I
K
L
M
N
P
Q
R
S
T
V
W
Y

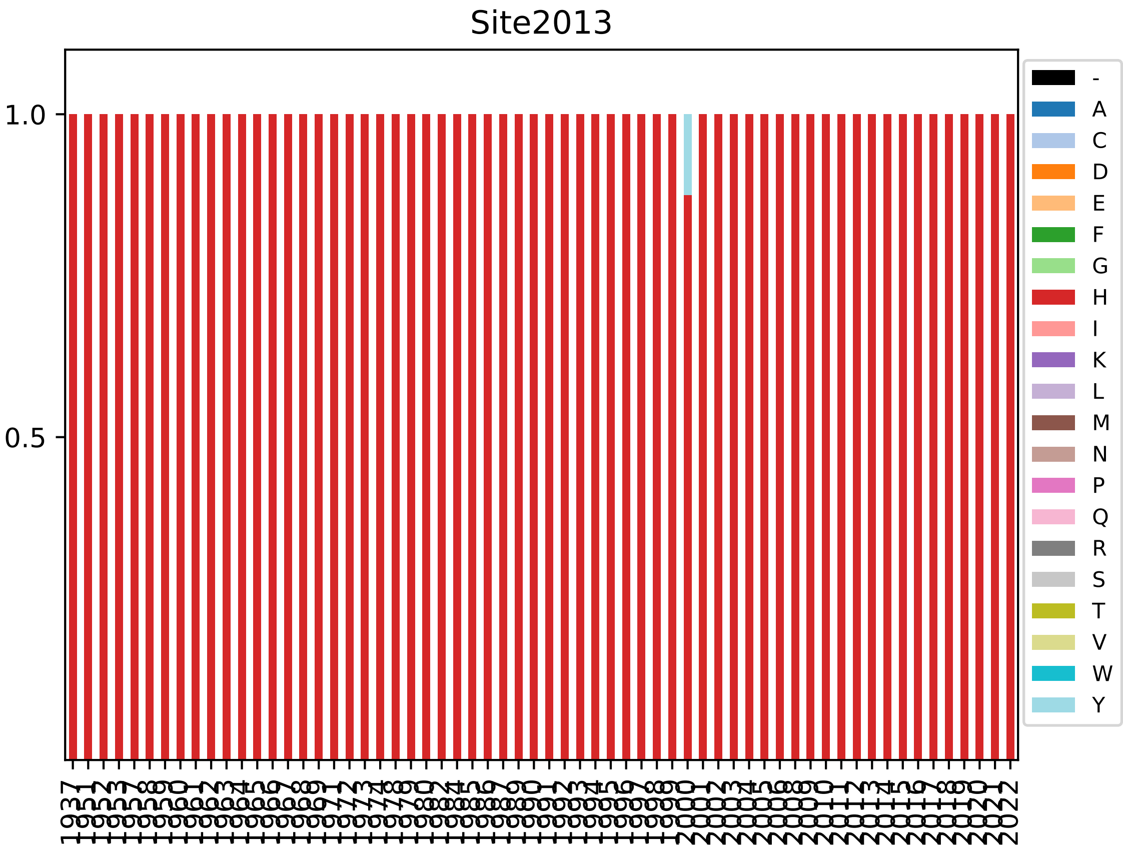
Site2013
1.0
0.5
-
A
C
D
E
F
G
H
I
K
L
M
N
P
Q
R
S
T
V
W
Y

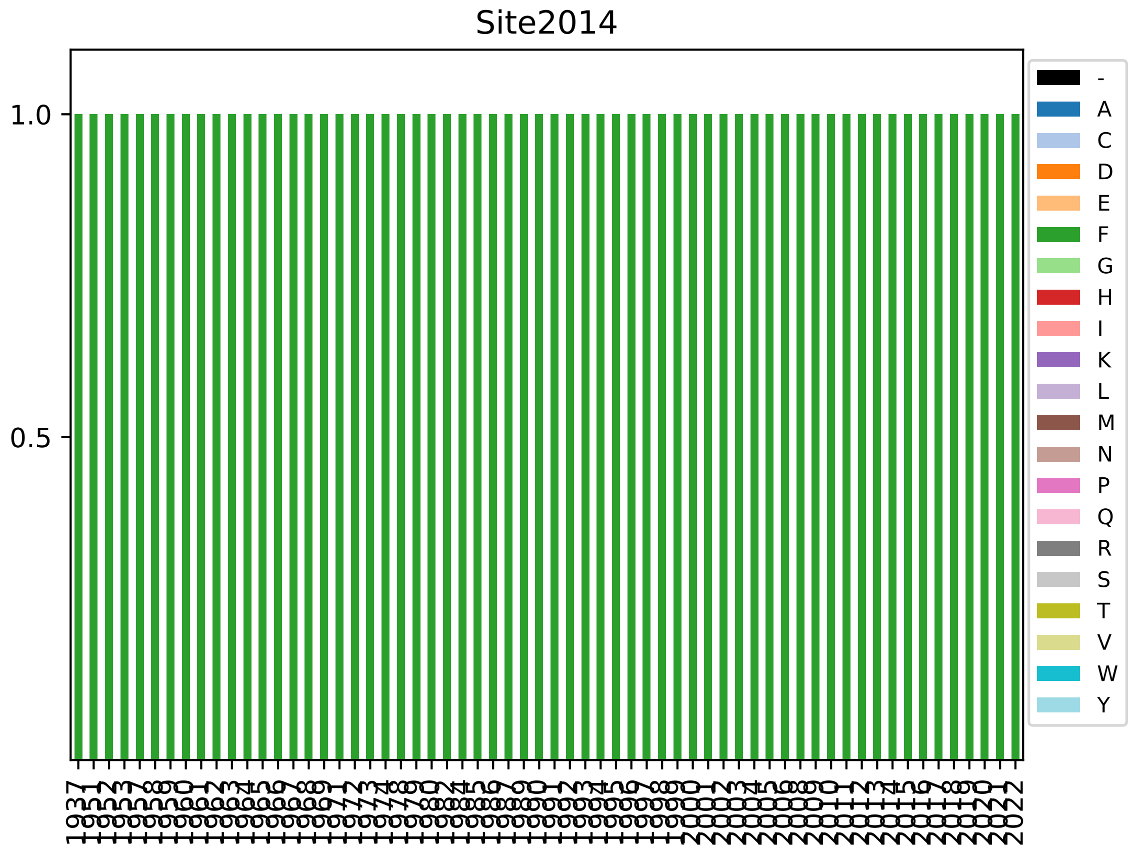
Site2014
1.0
0.5
-
A
C
D
E
F
G
H
I
K
L
M
N
P
Q
R
S
T
V
W
Y

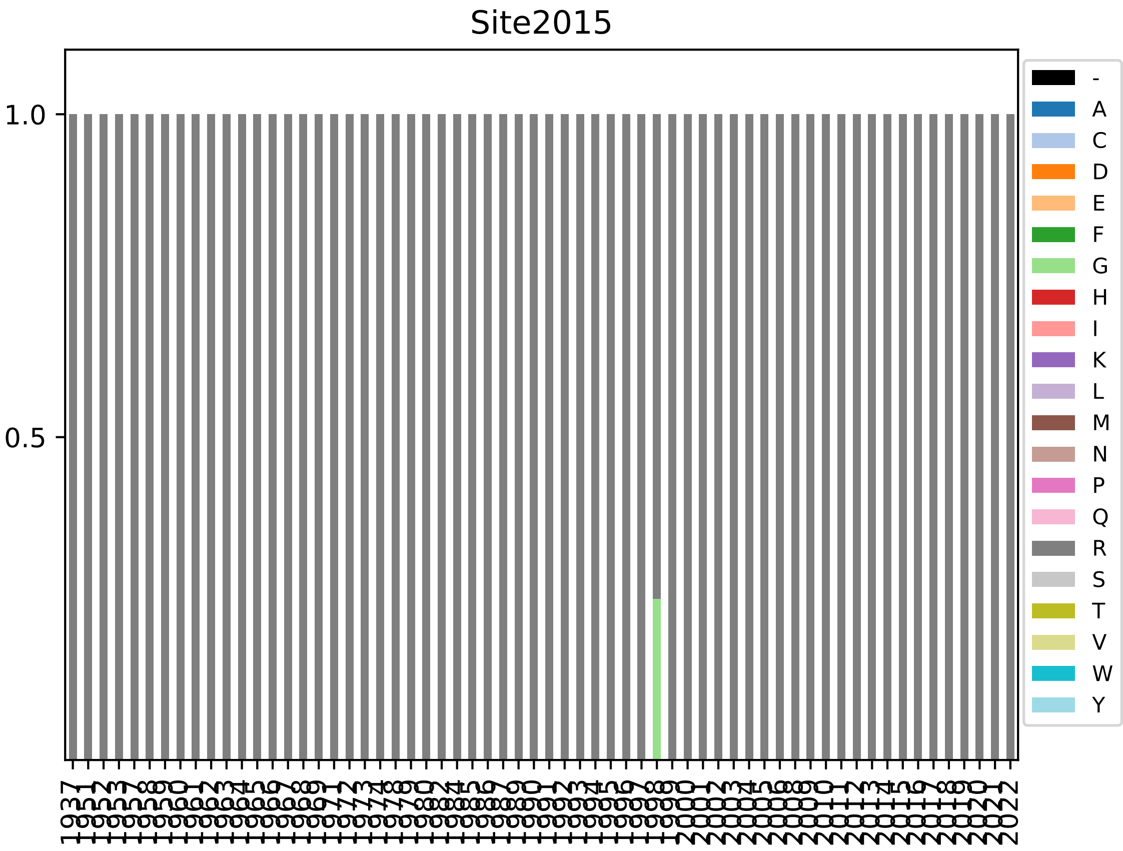
Site2015
1.0
0.5
-
A
C
D
E
F
G
H
I
K
L
M
N
P
Q
R
S
T
V
W
Y

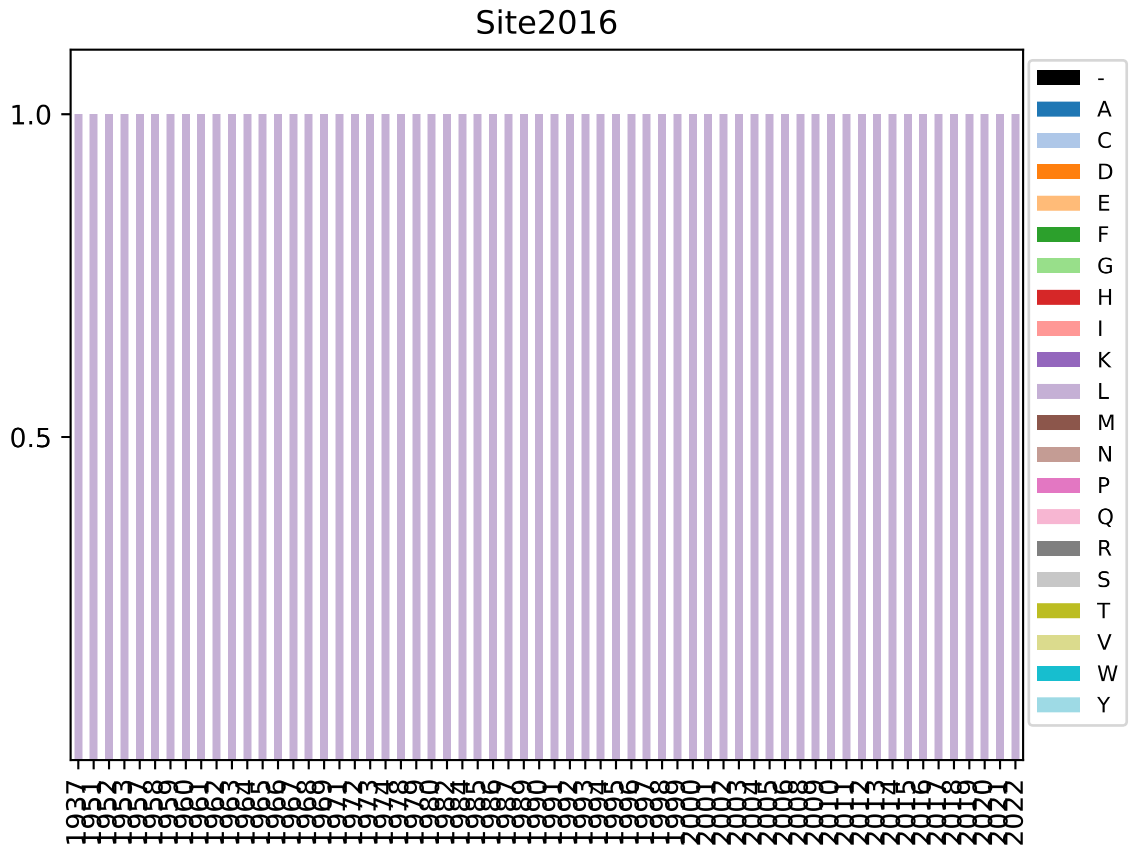
Site2016
1.0
0.5
-
A
C
D
E
F
G
H
I
K
L
M
N
P
Q
R
S
T
V
W
Y

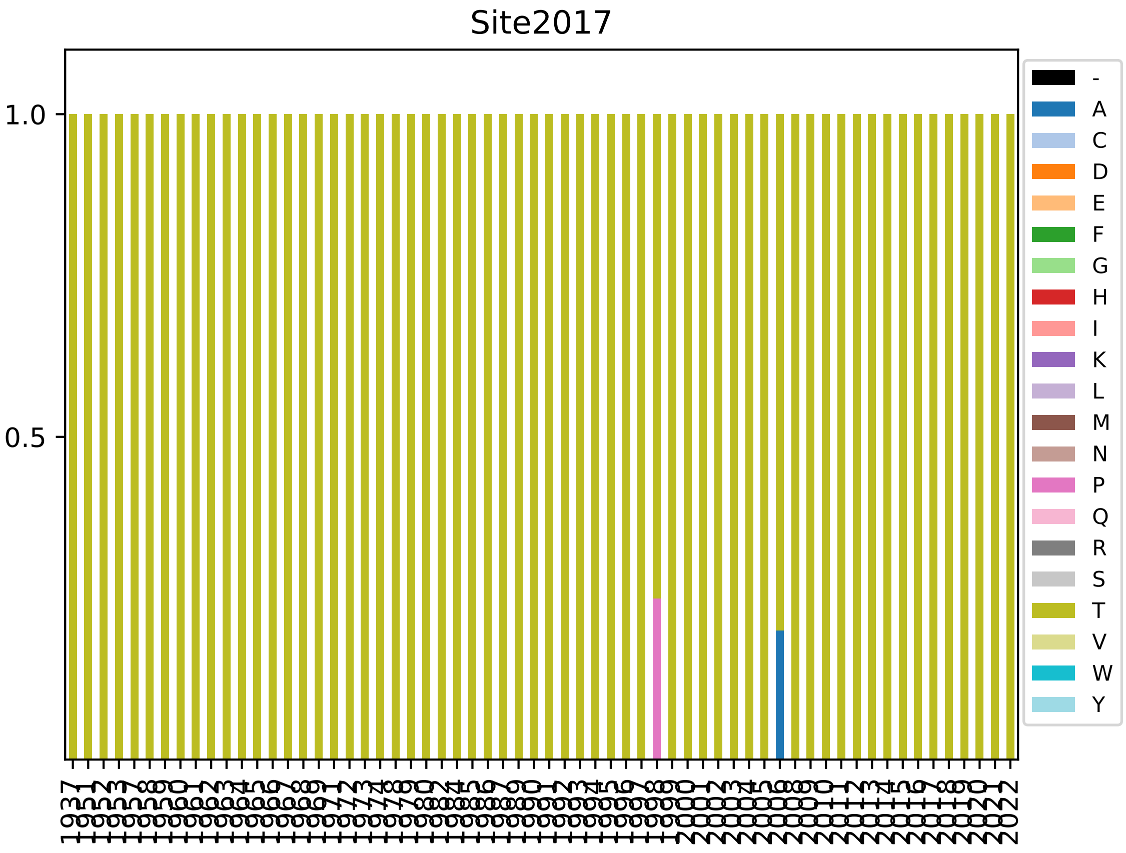
Site2017
1.0
0.5
-
A
C
D
E
F
G
H
I
K
L
M
N
P
Q
R
S
T
V
W
Y

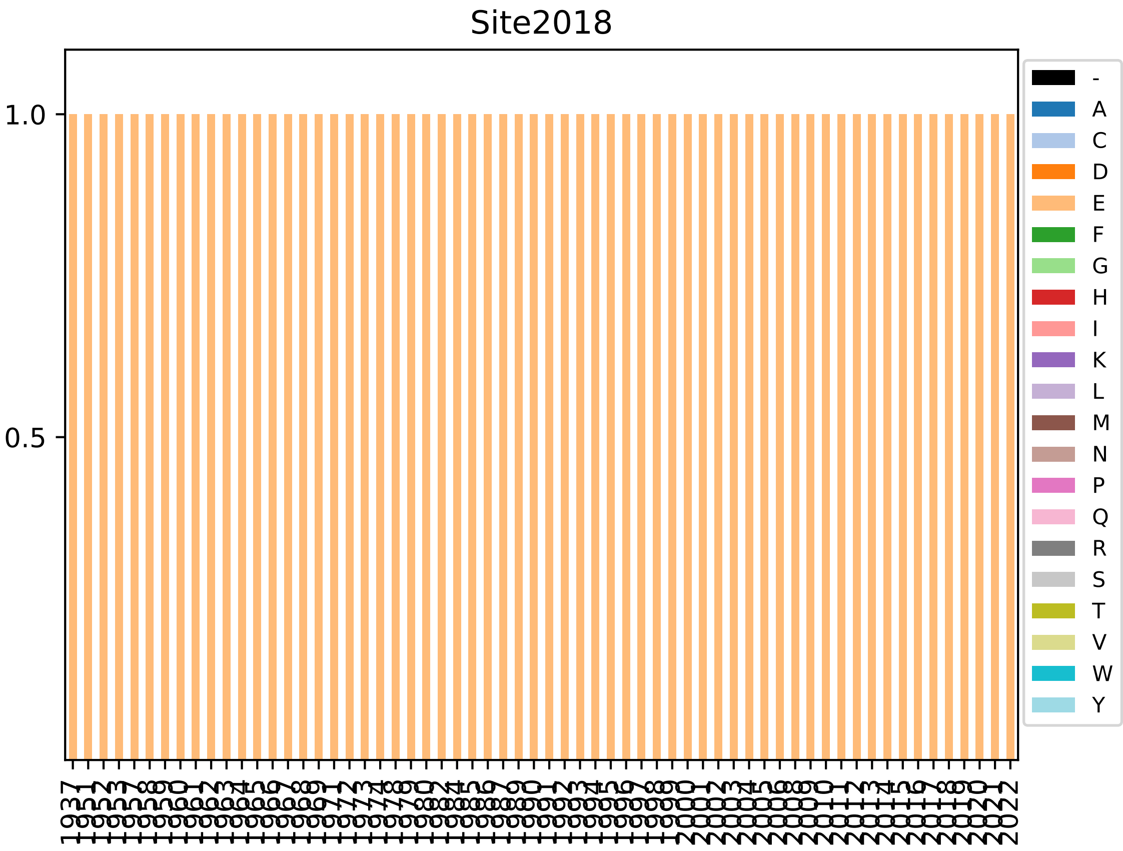
Site2018
1.0
0.5
-
A
C
D
E
F
G
H
I
K
L
M
N
P
Q
R
S
T
V
W
Y

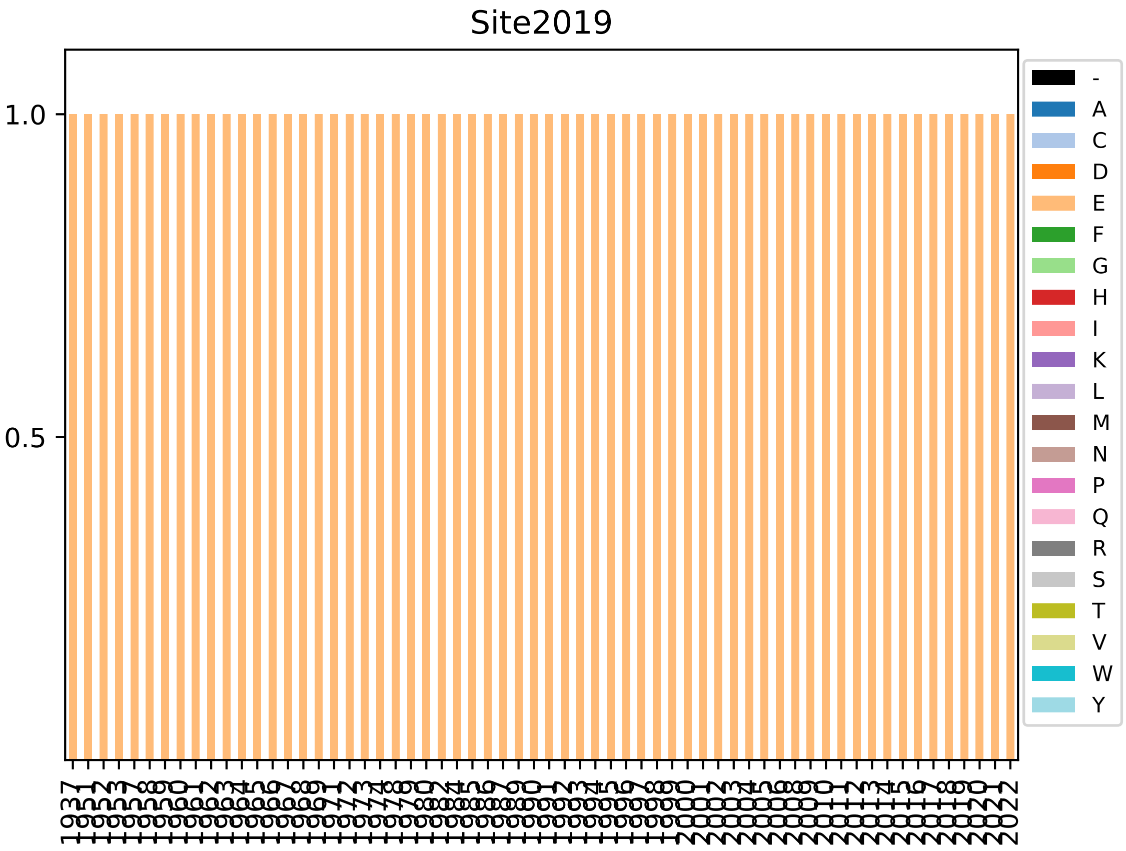
Site2019
1.0
0.5
-
A
C
D
E
F
G
H
I
K
L
M
N
P
Q
R
S
T
V
W
Y

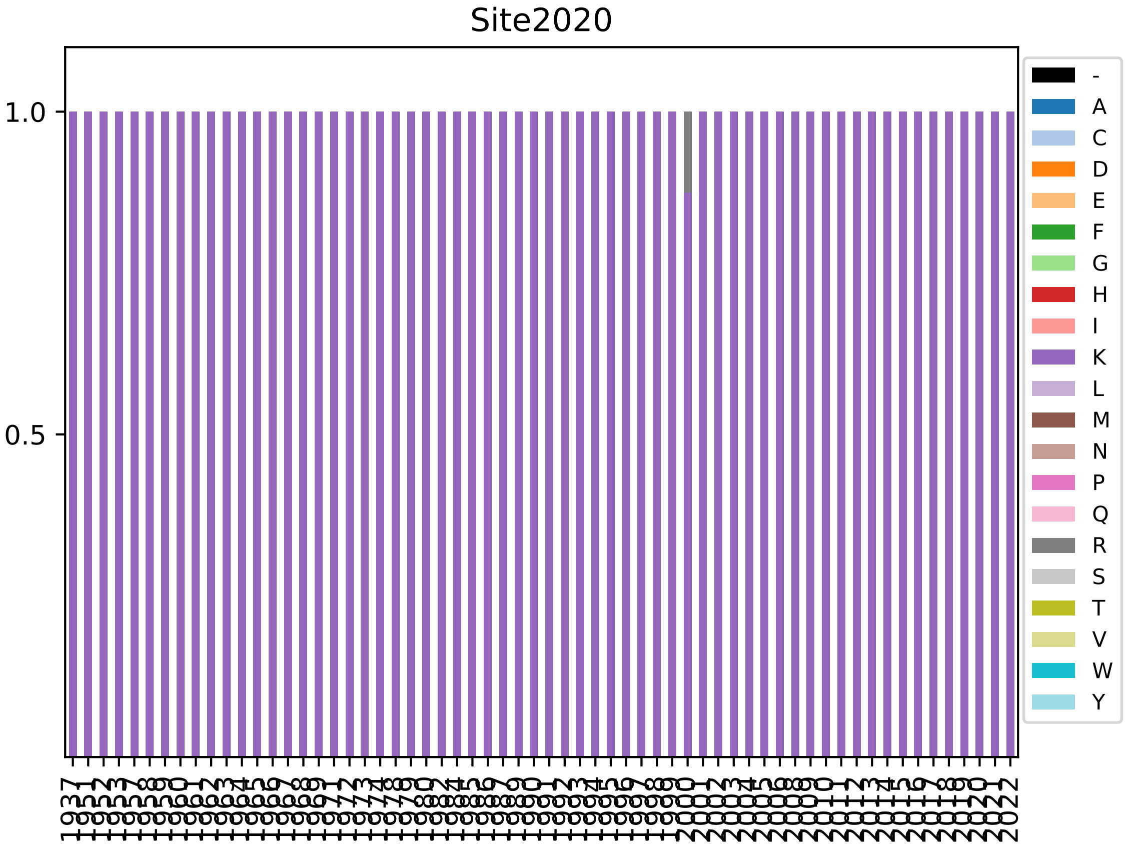
Site2020
1.0
0.5
-
A
C
D
E
F
G
H
I
K
L
M
N
P
Q
R
S
T
V
W
Y

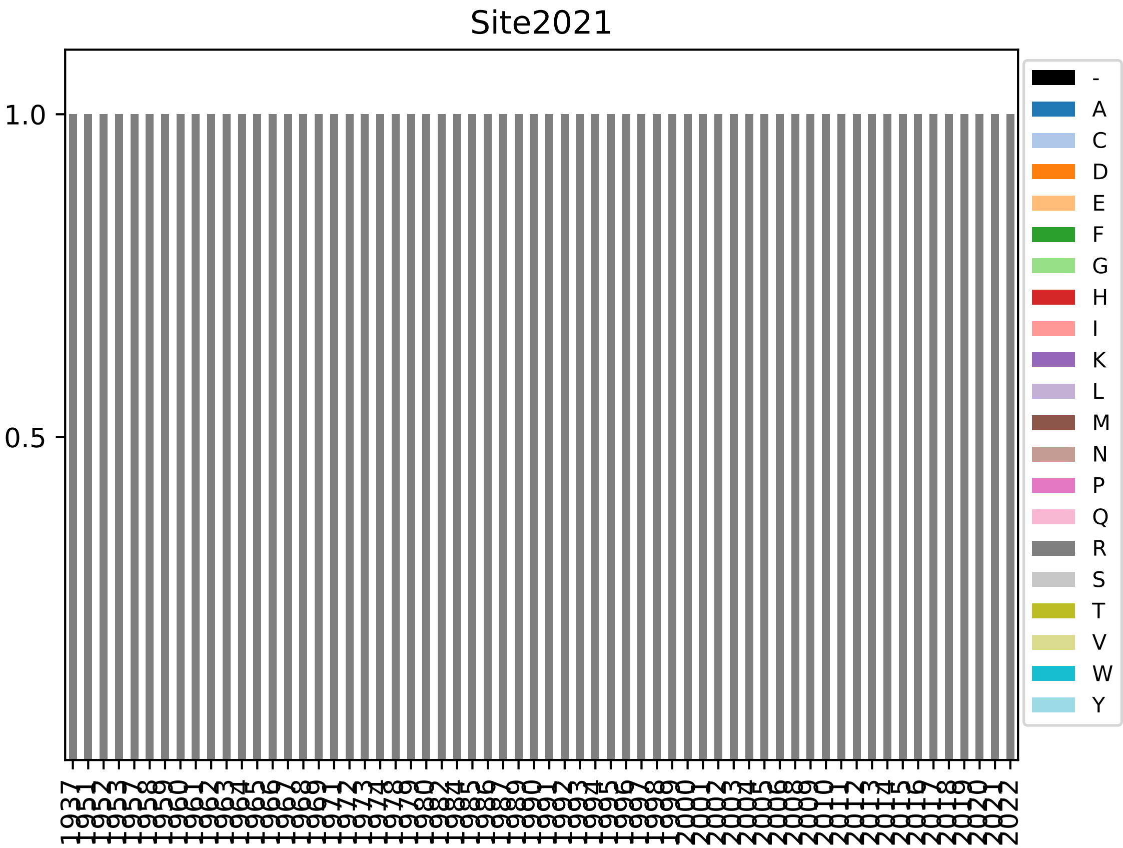
Site2021
1.0
0.5
-
A
C
D
E
F
G
H
I
K
L
M
N
P
Q
R
S
T
V
W
Y

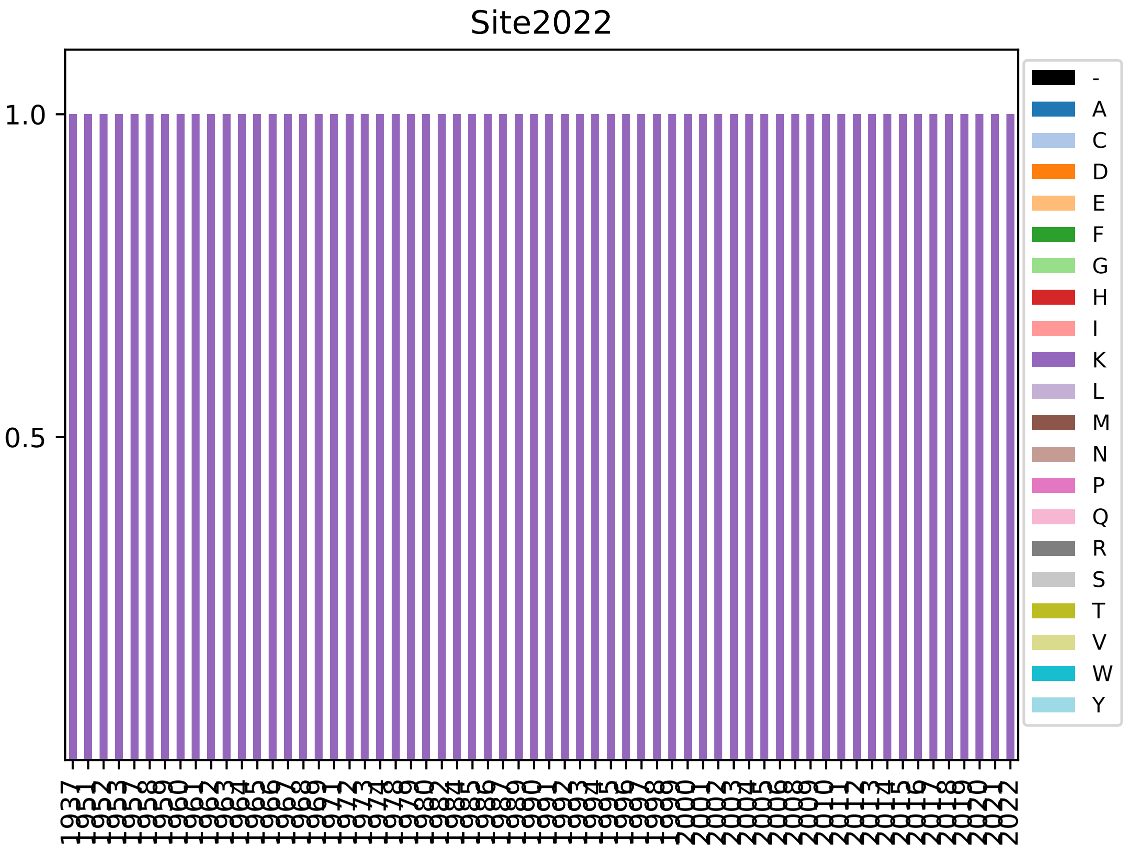

Site2022
1.0
0.5
-
A
C
D
E
F
G
H
I
K
L
M
N
P
Q
R
S
T
V
W
Y

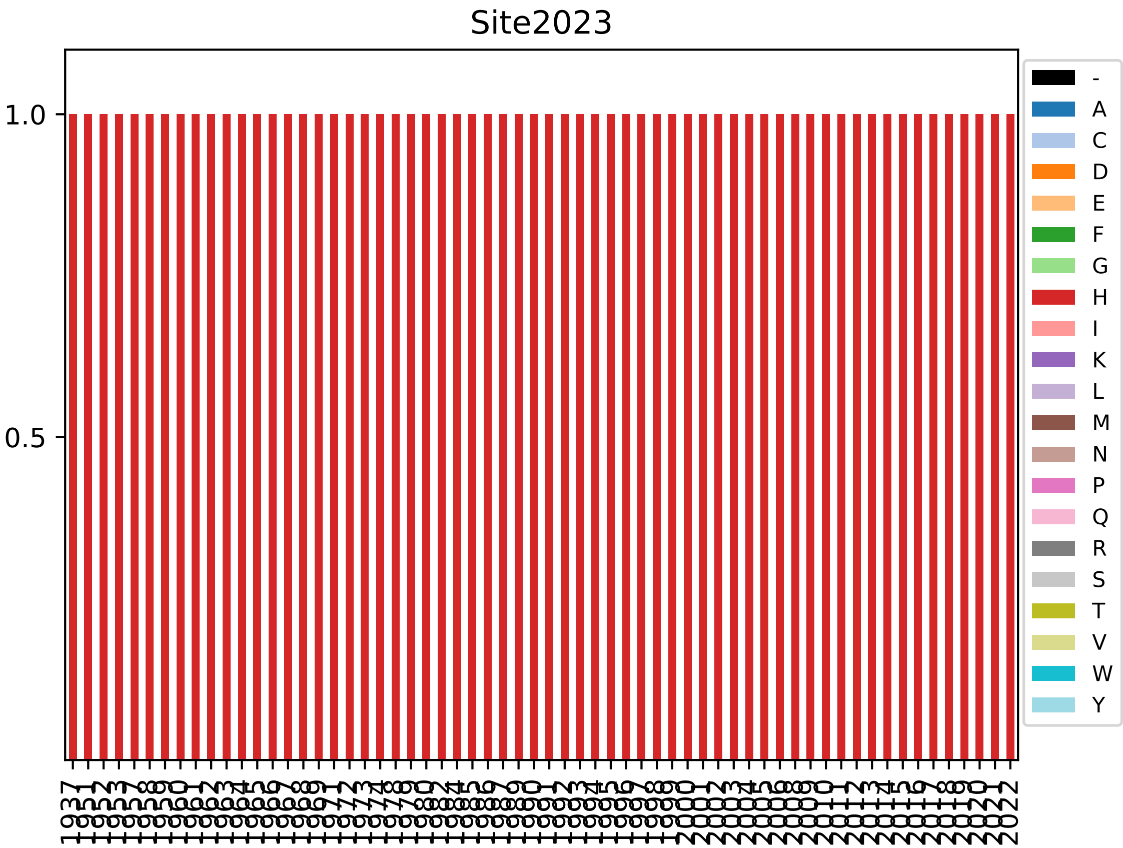
Site2023
1.0
0.5
-
A
C
D
E
F
G
H
I
K
L
M
N
P
Q
R
S
T
V
W
Y

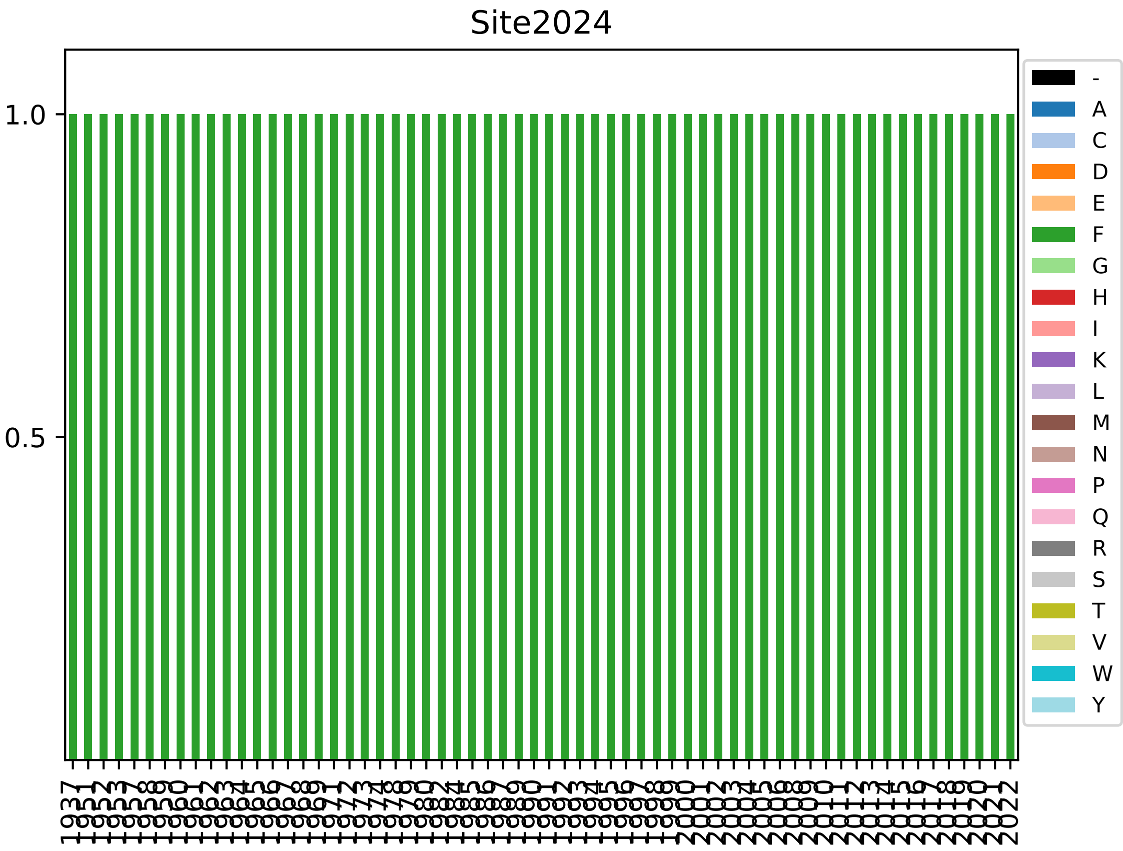
Site2024
1.0
0.5
-
A
C
D
E
F
G
H
I
K
L
M
N
P
Q
R
S
T
V
W
Y

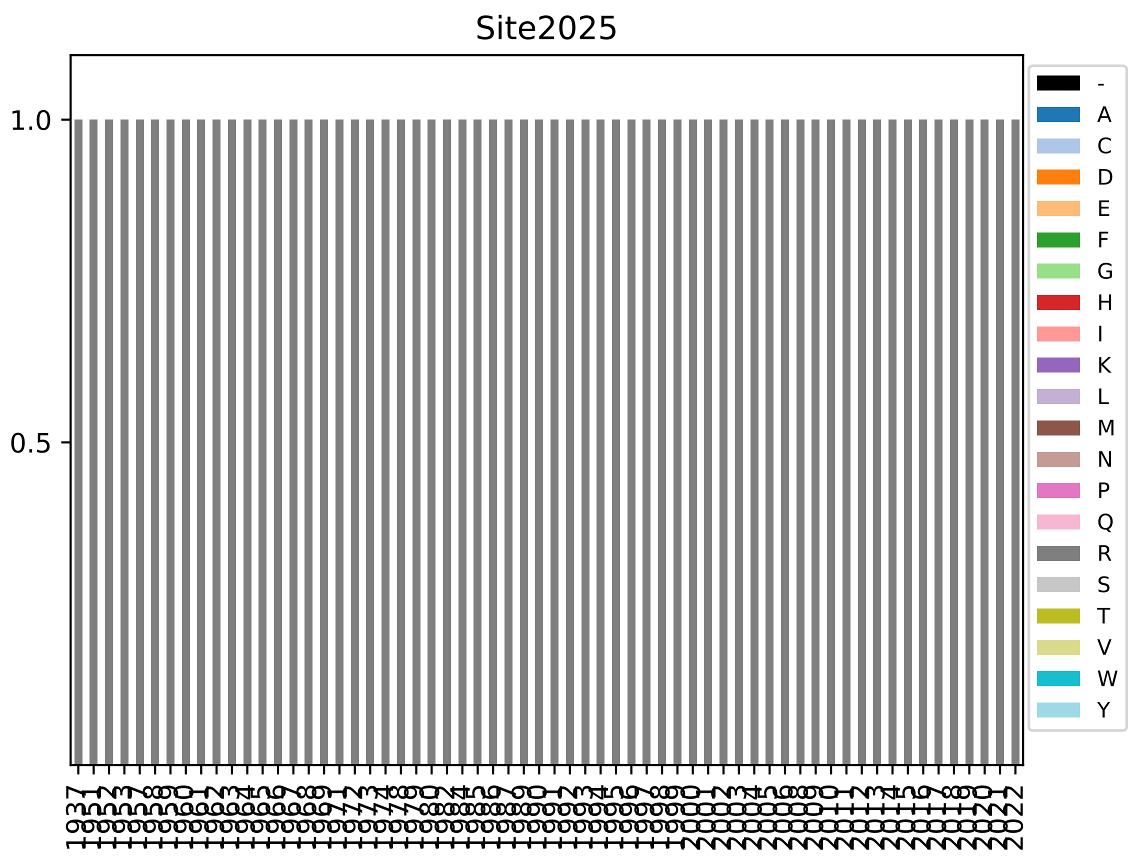

Site2025
1.0
0.5
-
A
C
D
E
F
G
H
I
K
L
M
N
P
Q
R
S
T
V
W
Y

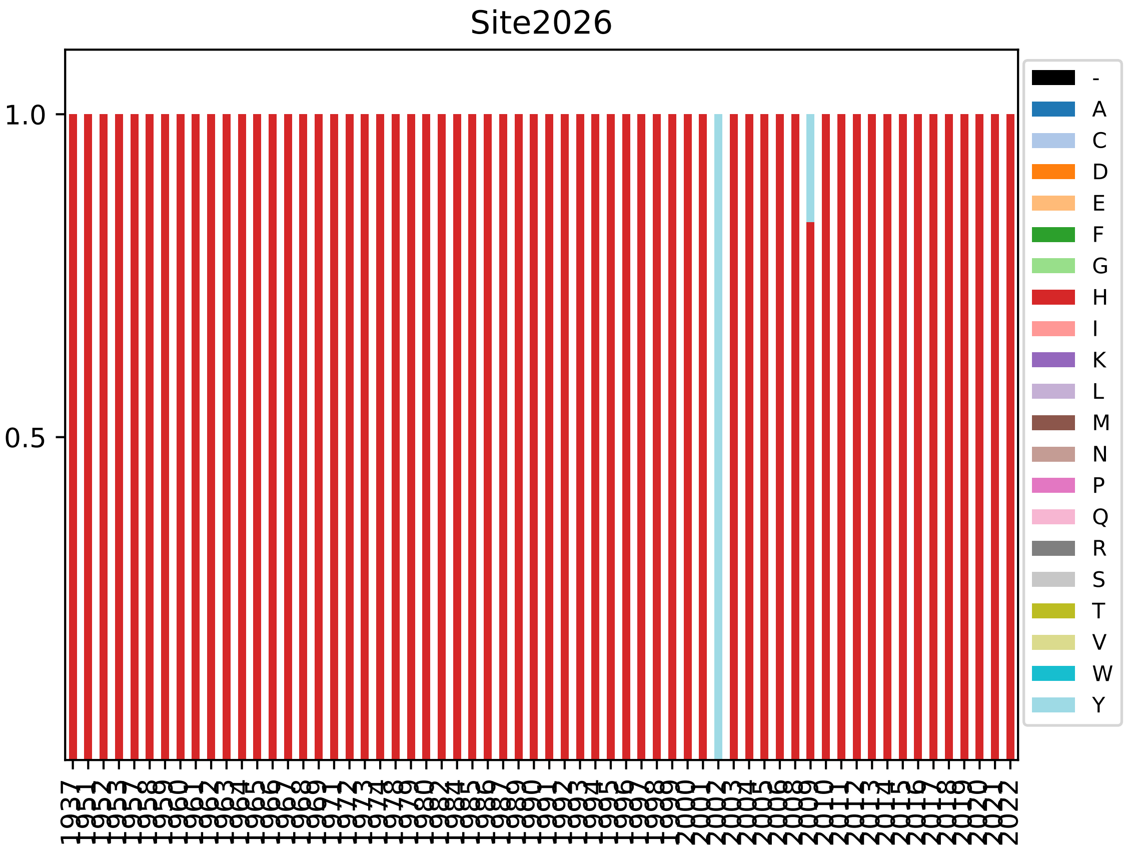
Site2026
1.0
0.5
-
A
C
D
E
F
G
H
I
K
L
M
N
P
Q
R
S
T
V
W
Y

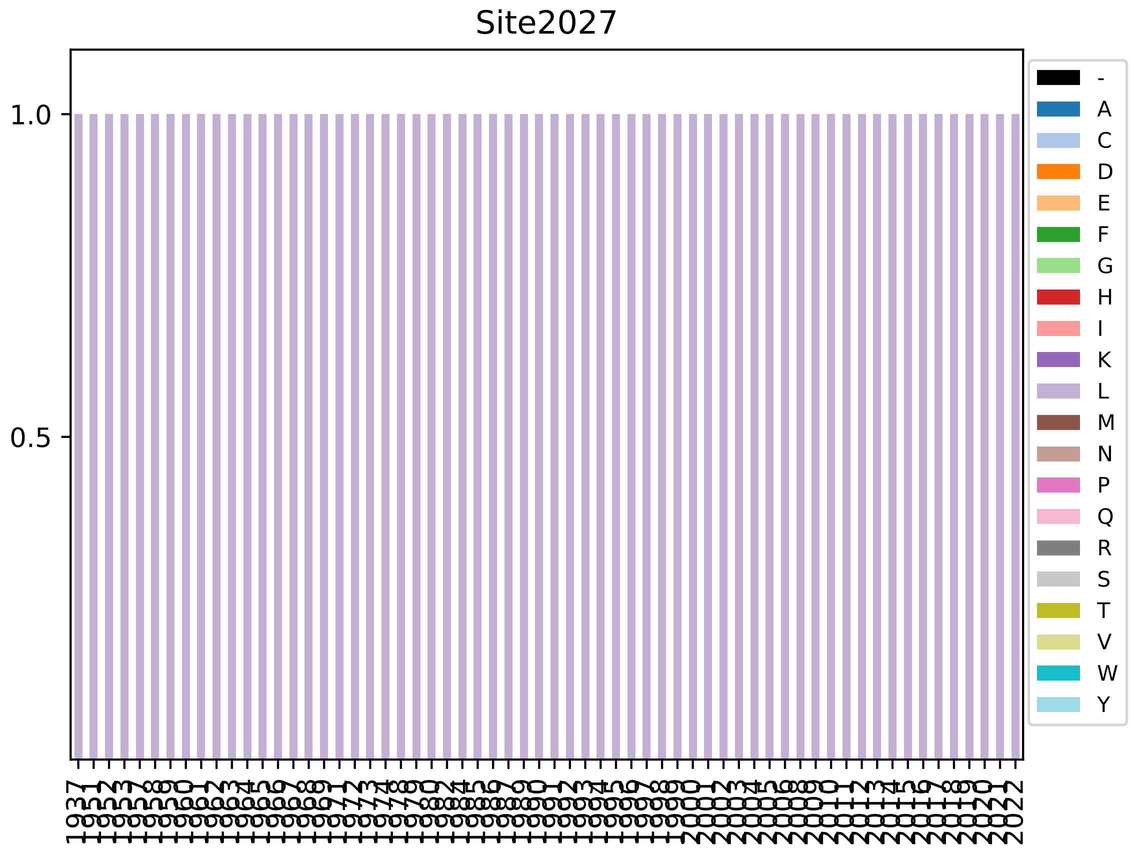
Site2027
1.0
0.5
-
A
C
D
E
F
G
H
I
K
L
M
N
P
Q
R
S
T
V
W
Y

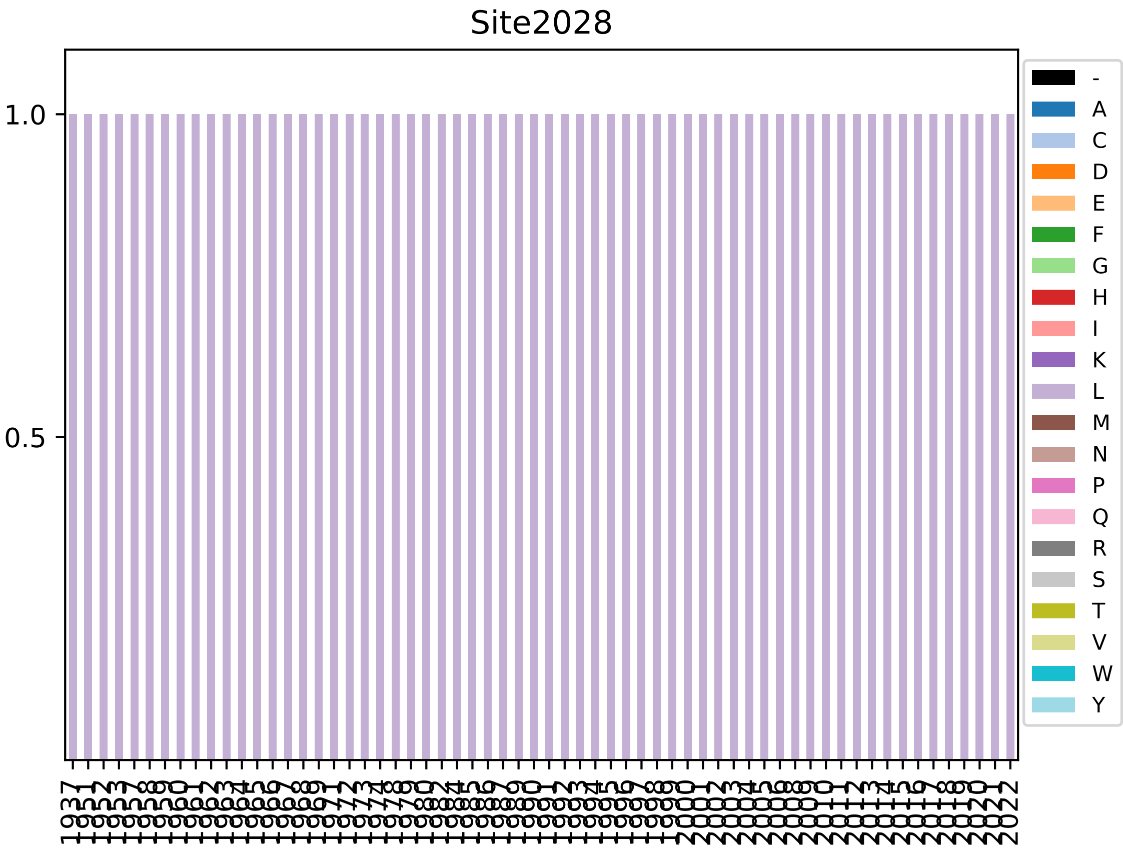
Site2028
1.0
0.5
-
A
C
D
E
F
G
H
I
K
L
M
N
P
Q
R
S
T
V
W
Y

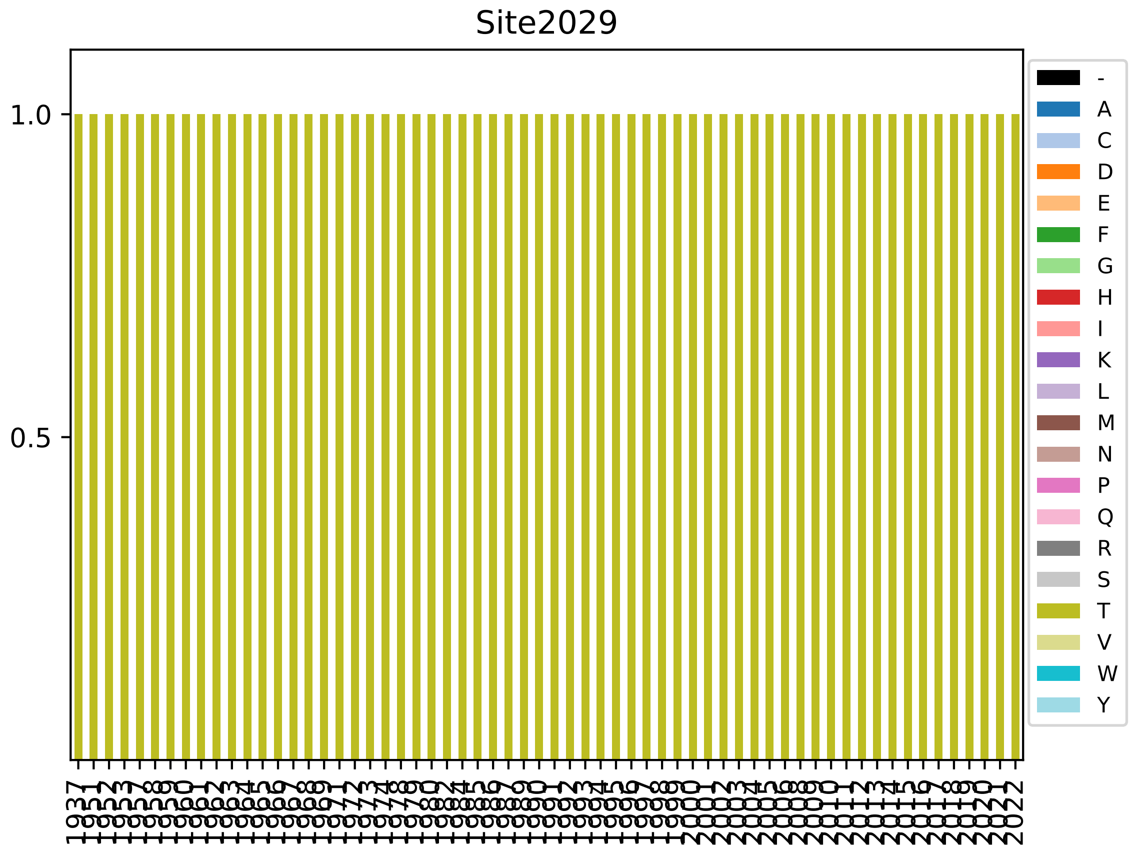
Site2029
1.0
0.5
-
A
C
D
E
F
G
H
I
K
L
M
N
P
Q
R
S
T
V
W
Y

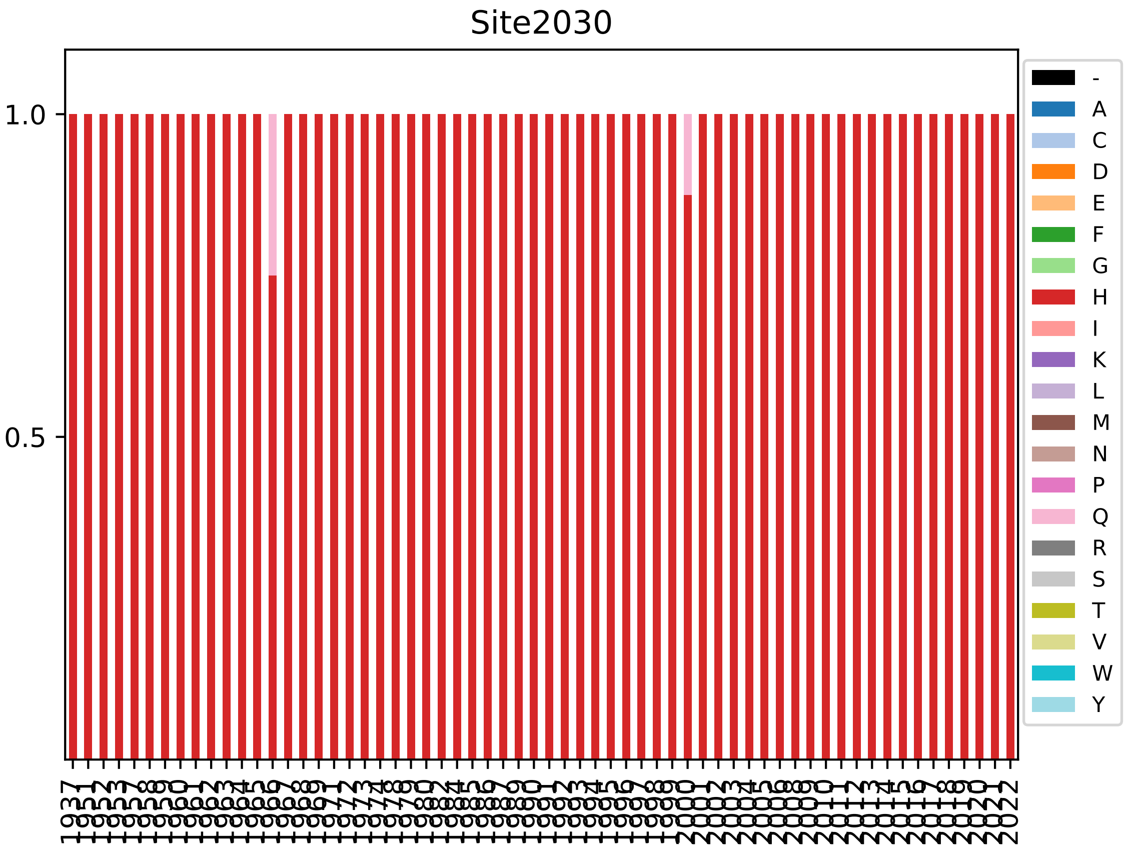
Site2030
1.0
0.5
-
A
C
D
E
F
G
H
I
K
L
M
N
P
Q
R
S
T
V
W
Y

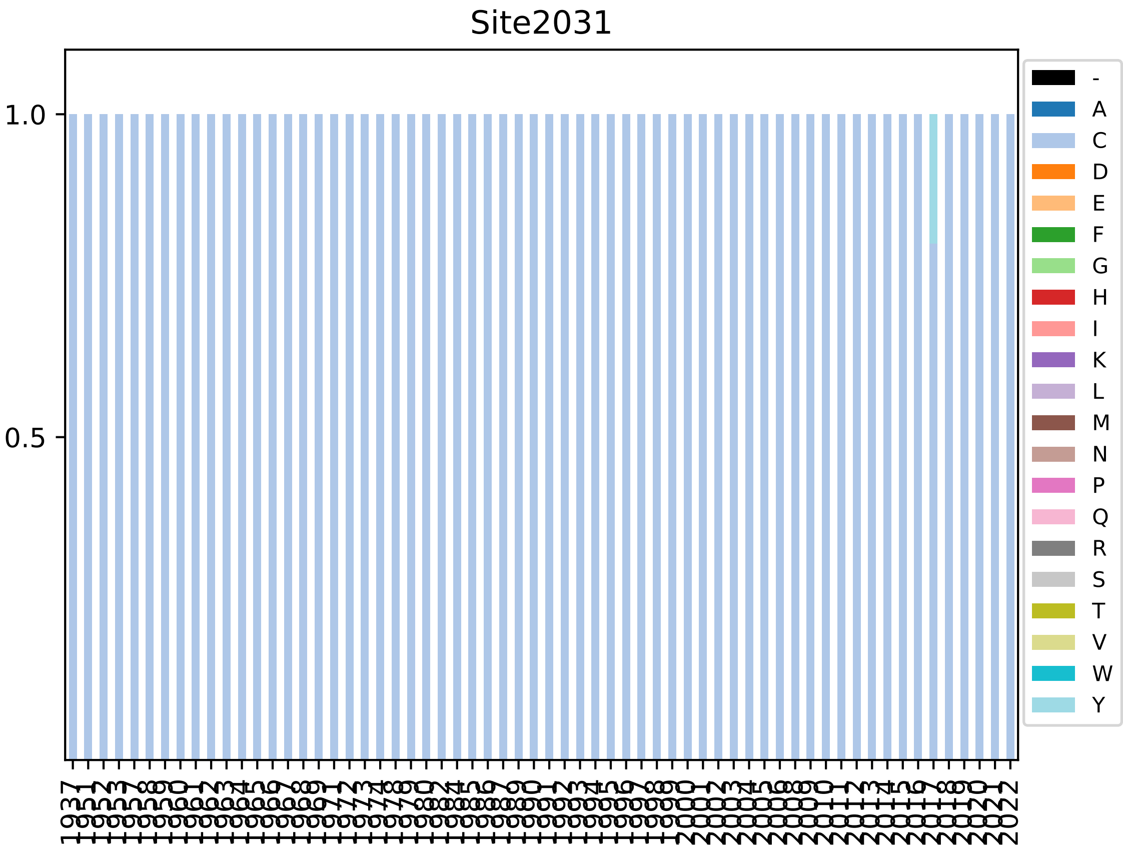
Site2031
1.0
0.5
-
A
C
D
E
F
G
H
I
K
L
M
N
P
Q
R
S
T
V
W
Y

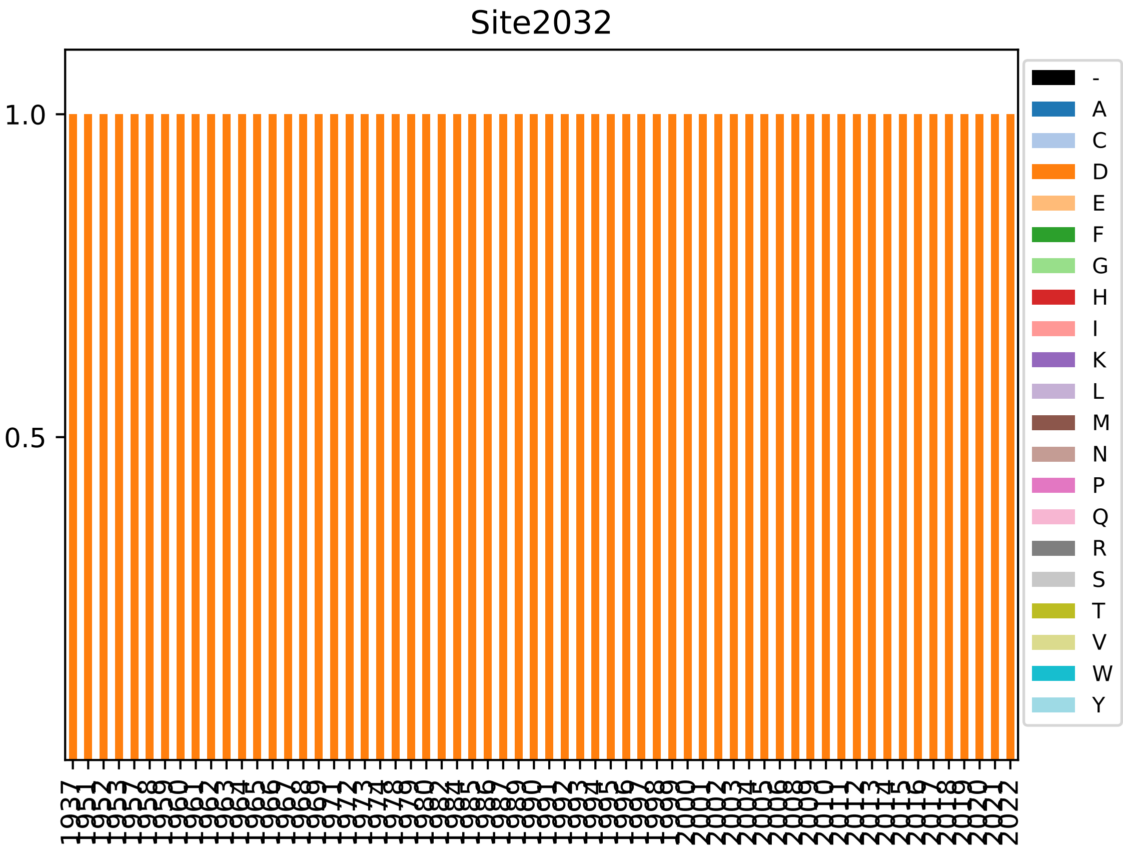

Site2032
1.0
0.5
-
A
C
D
E
F
G
H
I
K
L
M
N
P
Q
R
S
T
V
W
Y

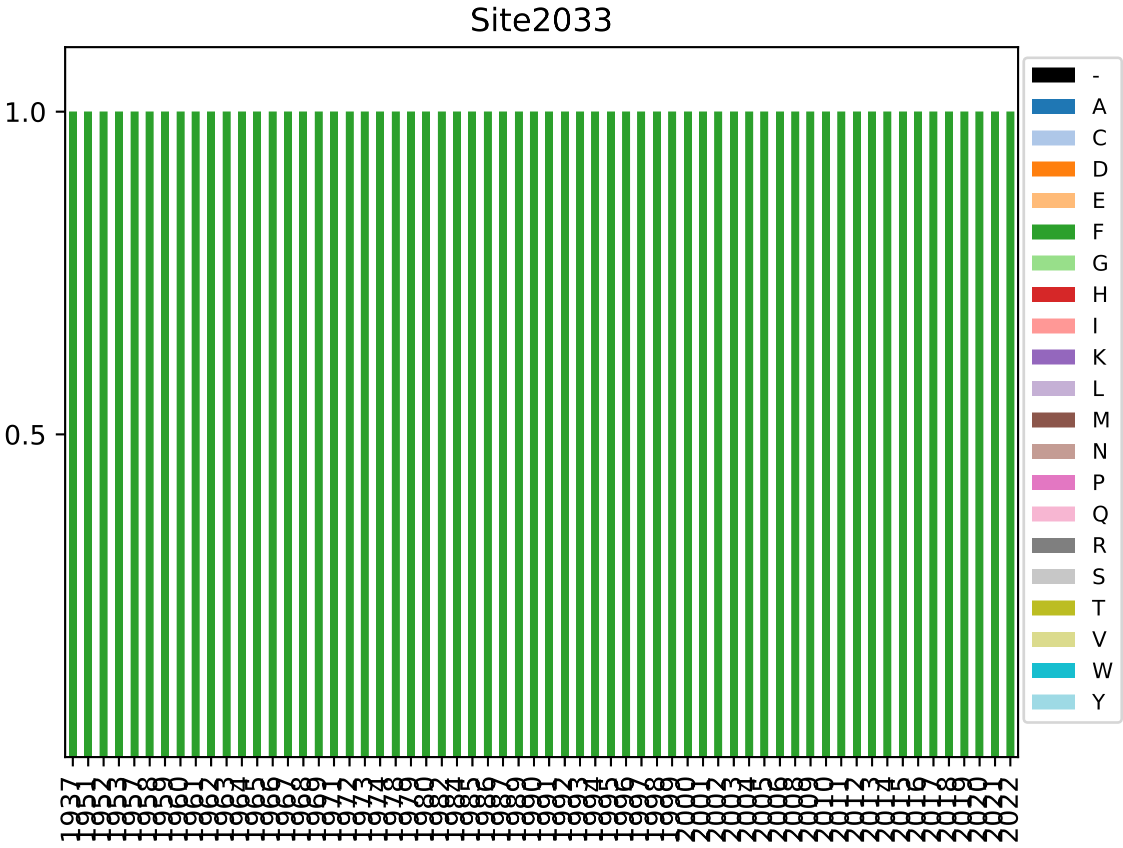
Site2033
1.0
0.5
-
A
C
D
E
F
G
H
I
K
L
M
N
P
Q
R
S
T
V
W
Y

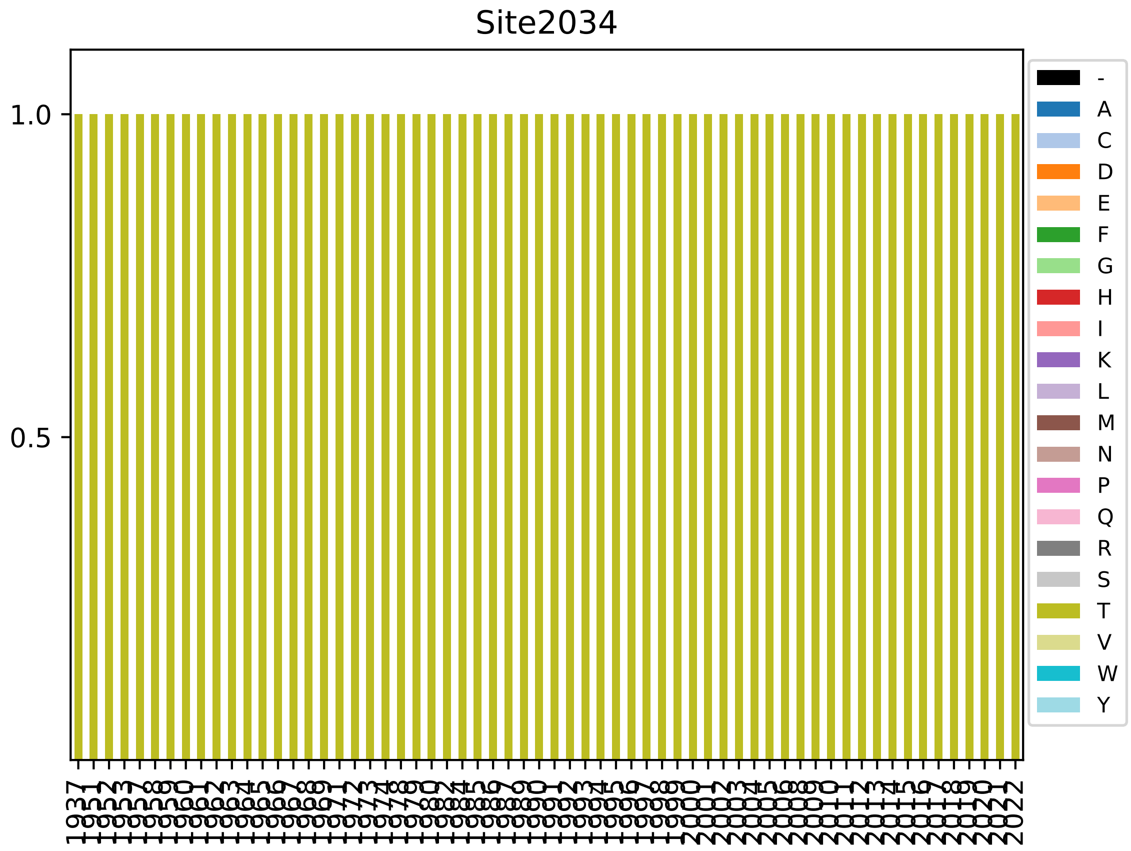
Site2034
1.0
0.5
-
A
C
D
E
F
G
H
I
K
L
M
N
P
Q
R
S
T
V
W
Y

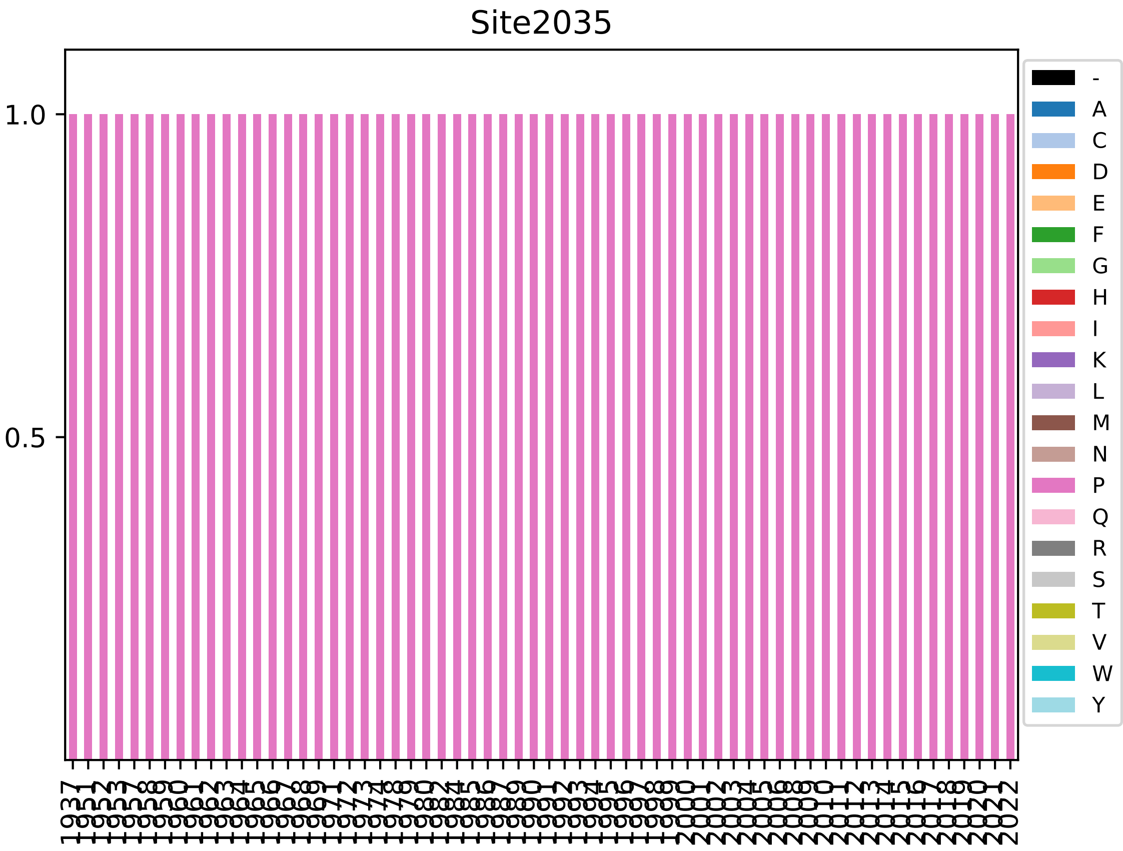
Site2035
1.0
0.5
-
A
C
D
E
F
G
H
I
K
L
M
N
P
Q
R
S
T
V
W
Y

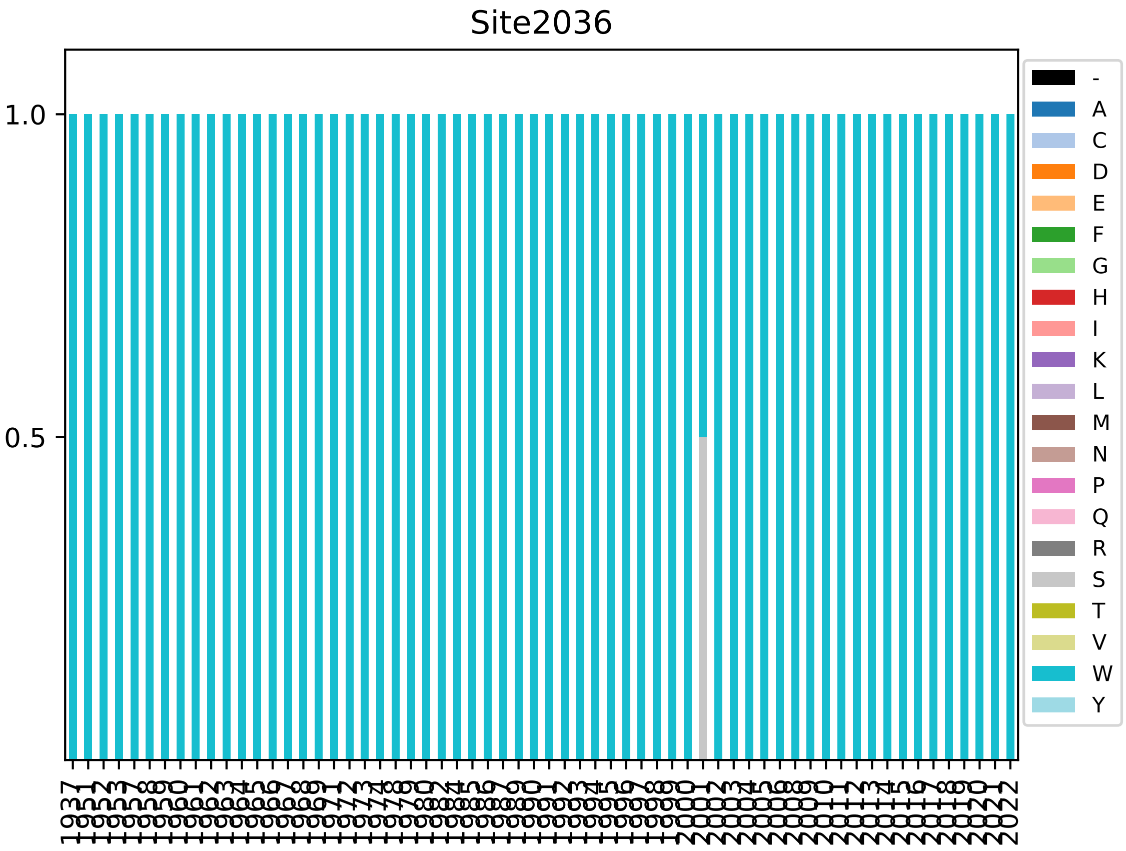
Site2036
1.0
0.5
-
A
C
D
E
F
G
H
I
K
L
M
N
P
Q
R
S
T
V
W
Y

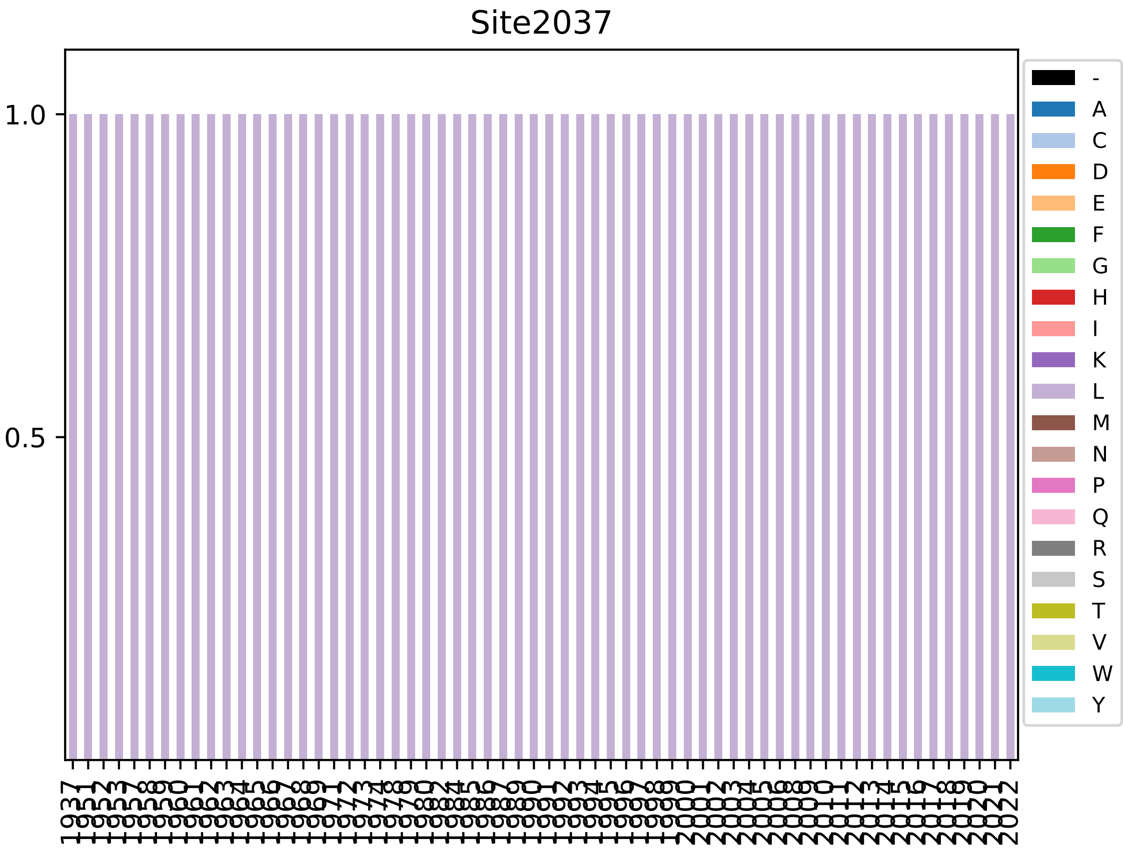
Site2037
1.0
0.5
-
A
C
D
E
F
G
H
I
K
L
M
N
P
Q
R
S
T
V
W
Y

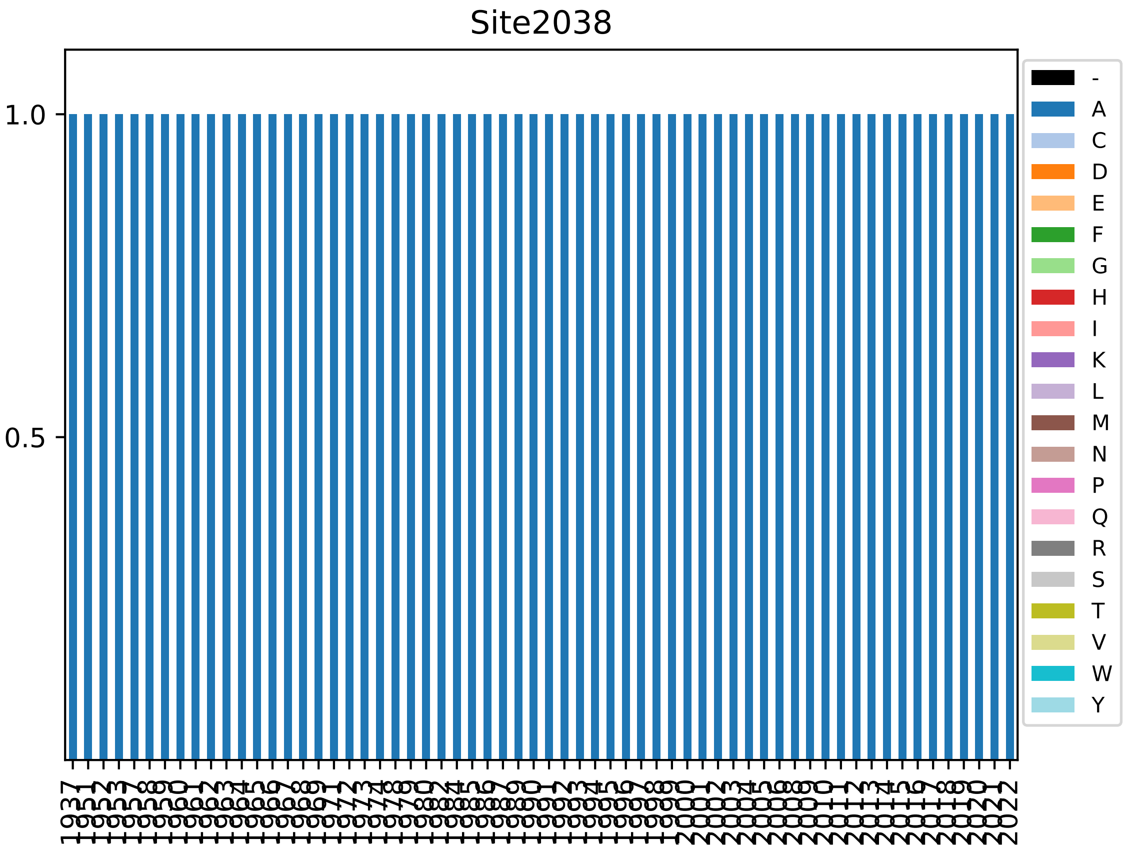
Site2038
1.0
0.5
-
A
C
D
E
F
G
H
I
K
L
M
N
P
Q
R
S
T
V
W
Y

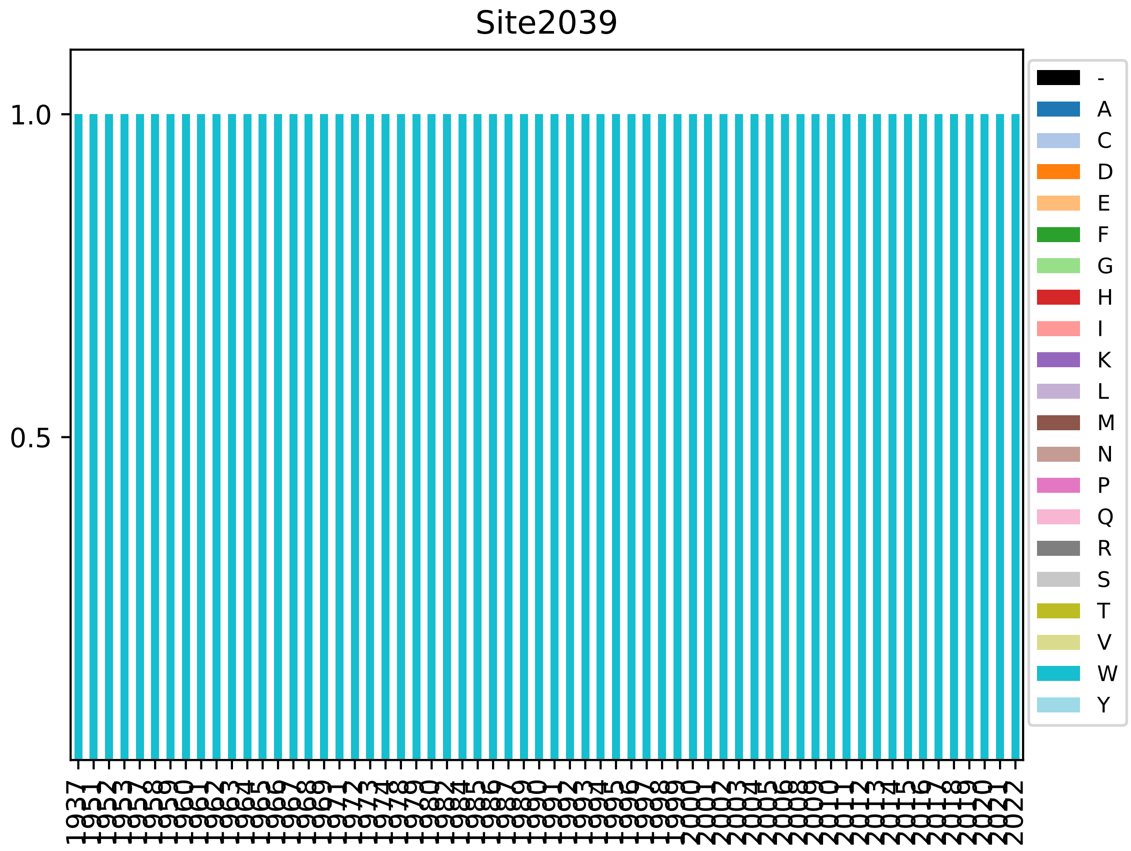
Site2039
1.0
0.5
-
A
C
D
E
F
G
H
I
K
L
M
N
P
Q
R
S
T
V
W
Y

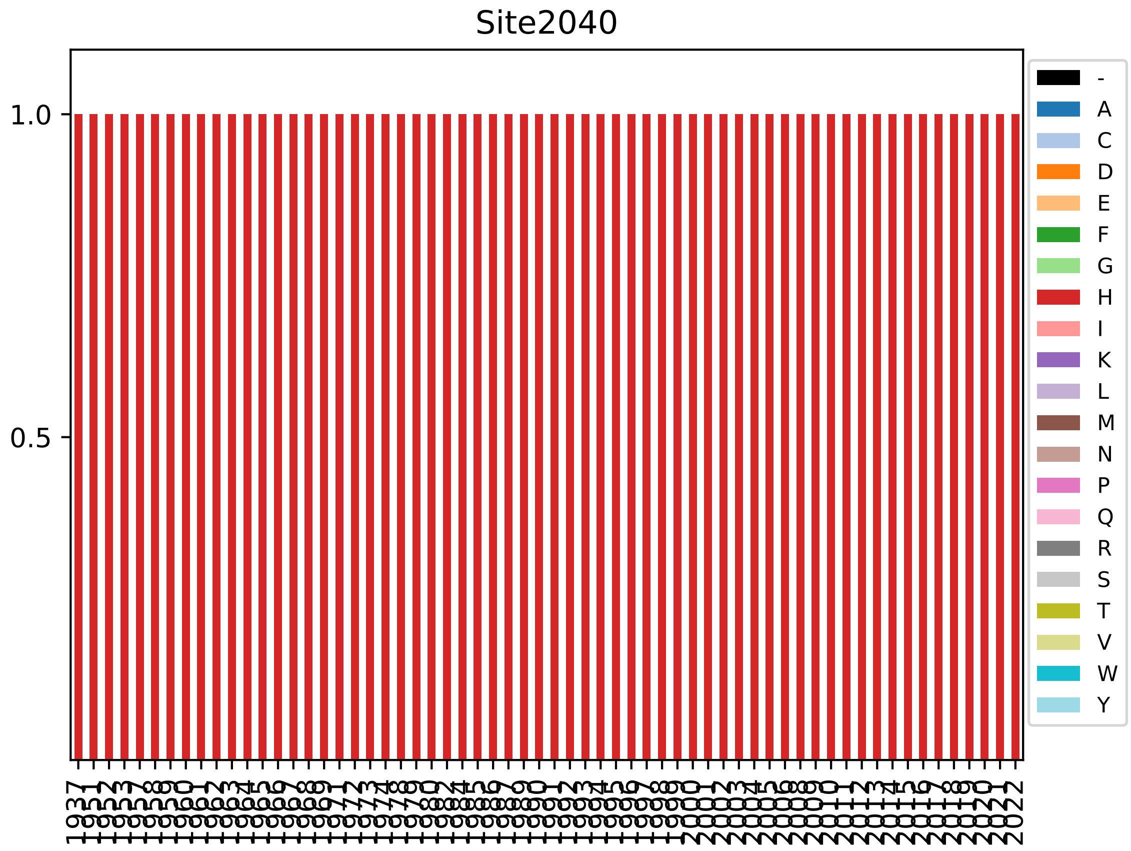

Site2040
1.0
0.5
-
A
C
D
E
F
G
H
I
K
L
M
N
P
Q
R
S
T
V
W
Y

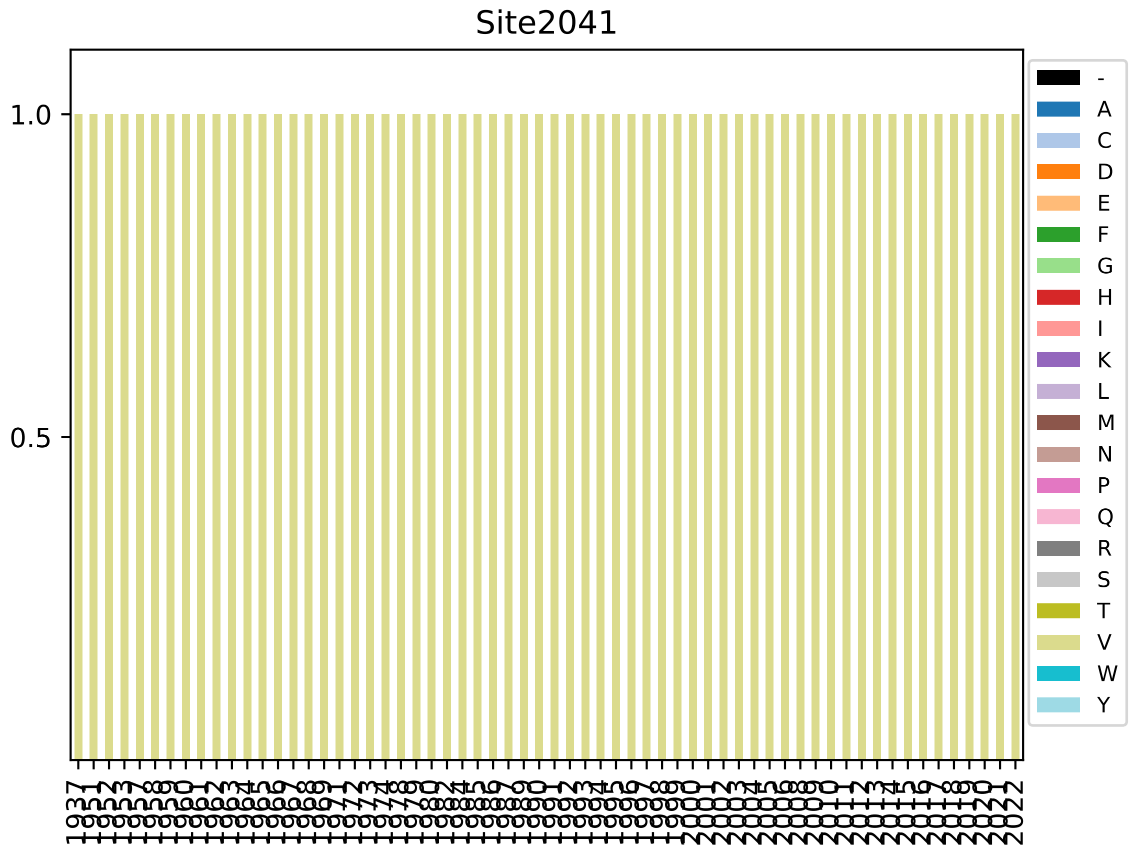
Site2041
1.0
0.5
-
A
C
D
E
F
G
H
I
K
L
M
N
P
Q
R
S
T
V
W
Y

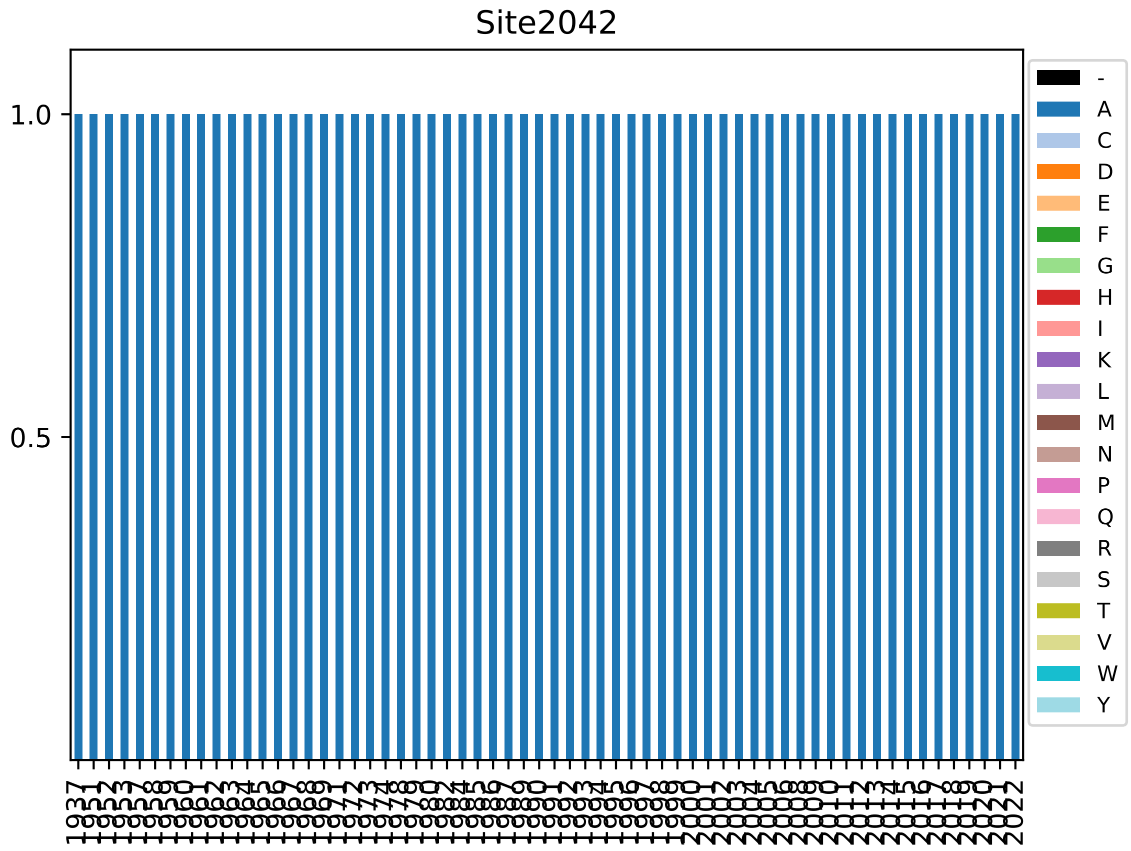
Site2042
1.0
0.5
-
A
C
D
E
F
G
H
I
K
L
M
N
P
Q
R
S
T
V
W
Y

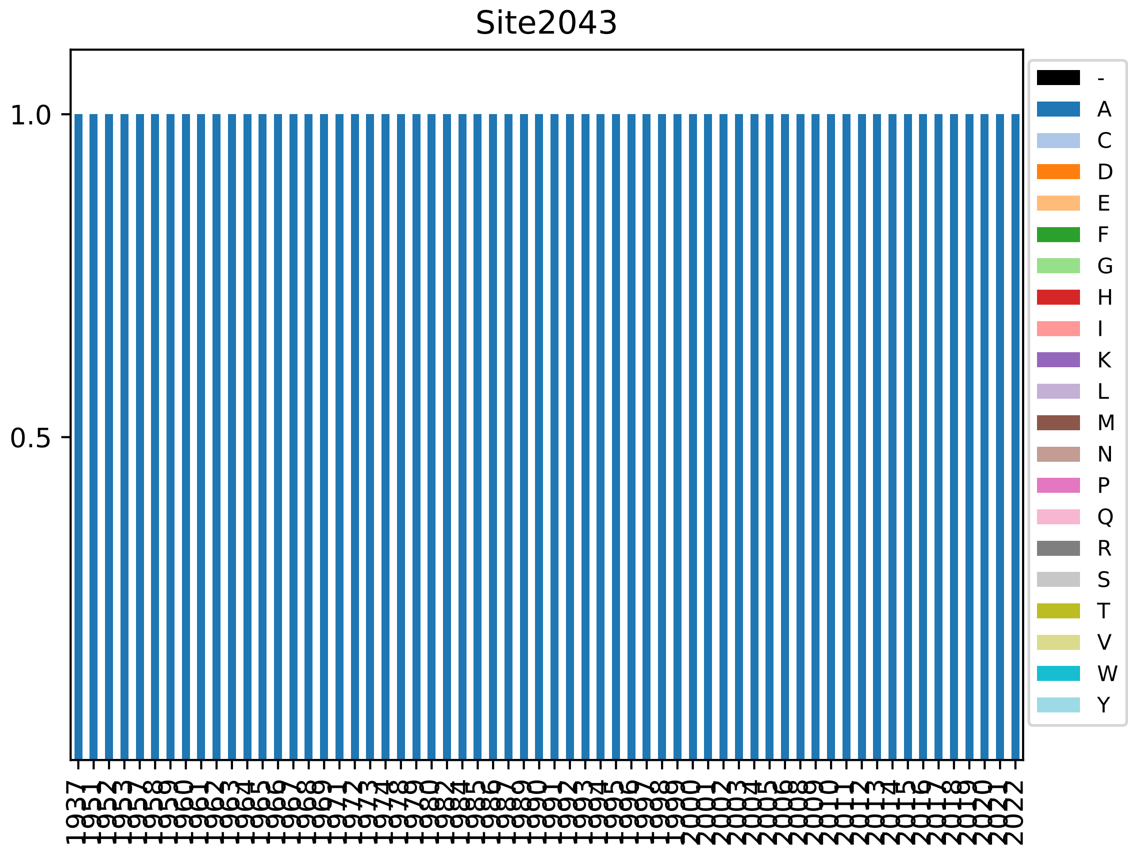
Site2043
1.0
0.5
-
A
C
D
E
F
G
H
I
K
L
M
N
P
Q
R
S
T
V
W
Y

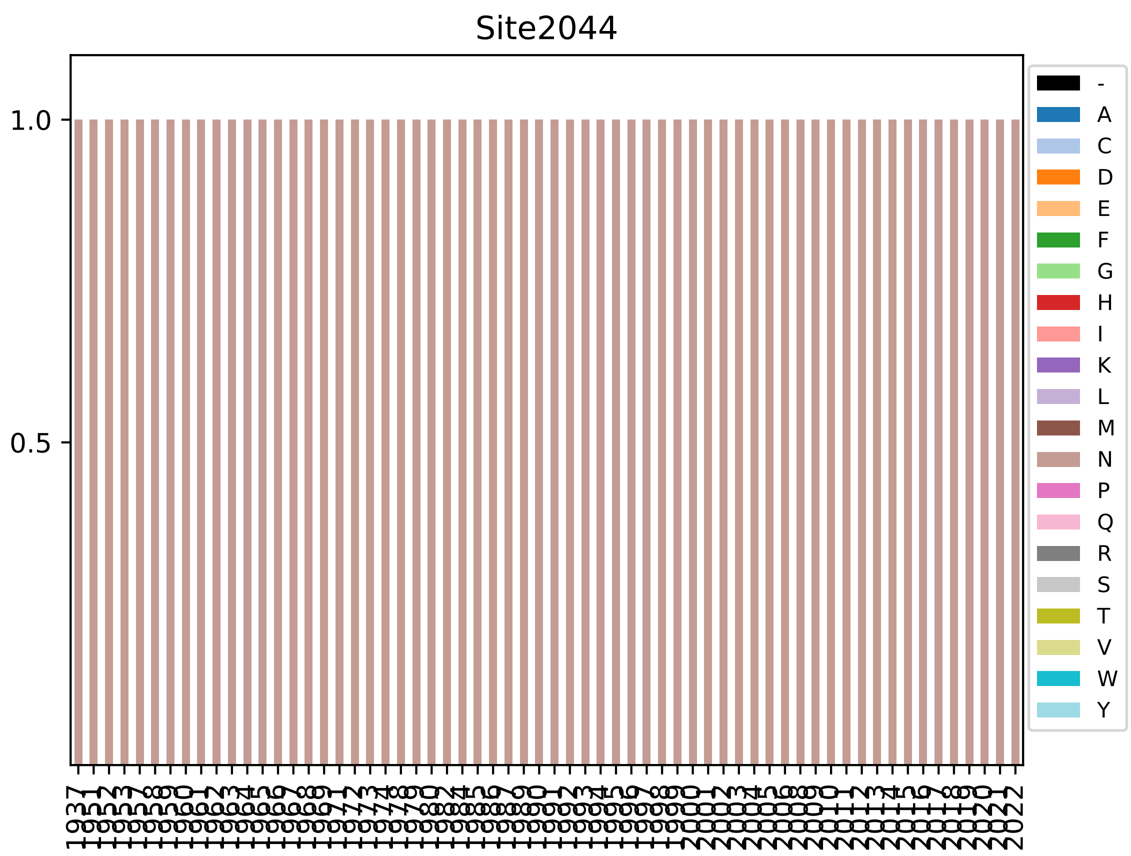
Site2044
1.0
0.5
-
A
C
D
E
F
G
H
I
K
L
M
N
P
Q
R
S
T
V
W
Y

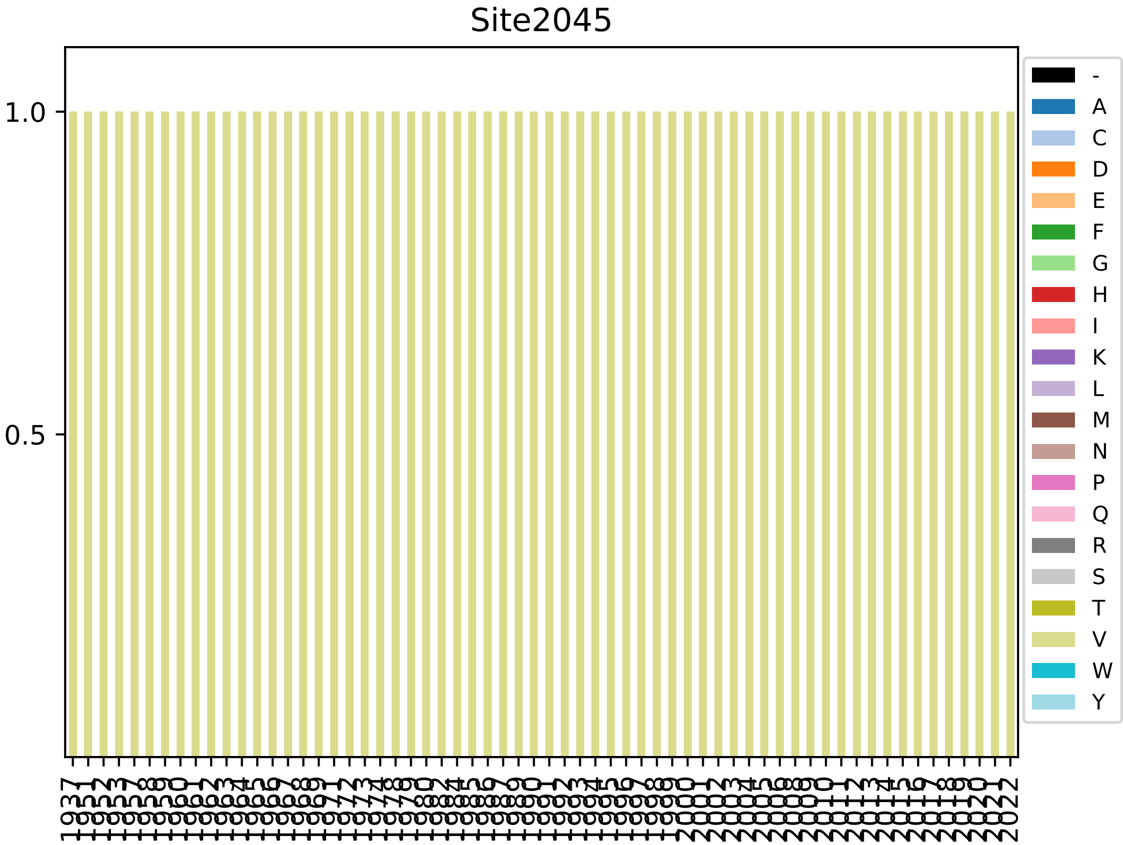
Site2045
1.0
0.5
-
A
C
D
E
F
G
H
I
K
L
M
N
P
Q
R
S
T
V
W
Y

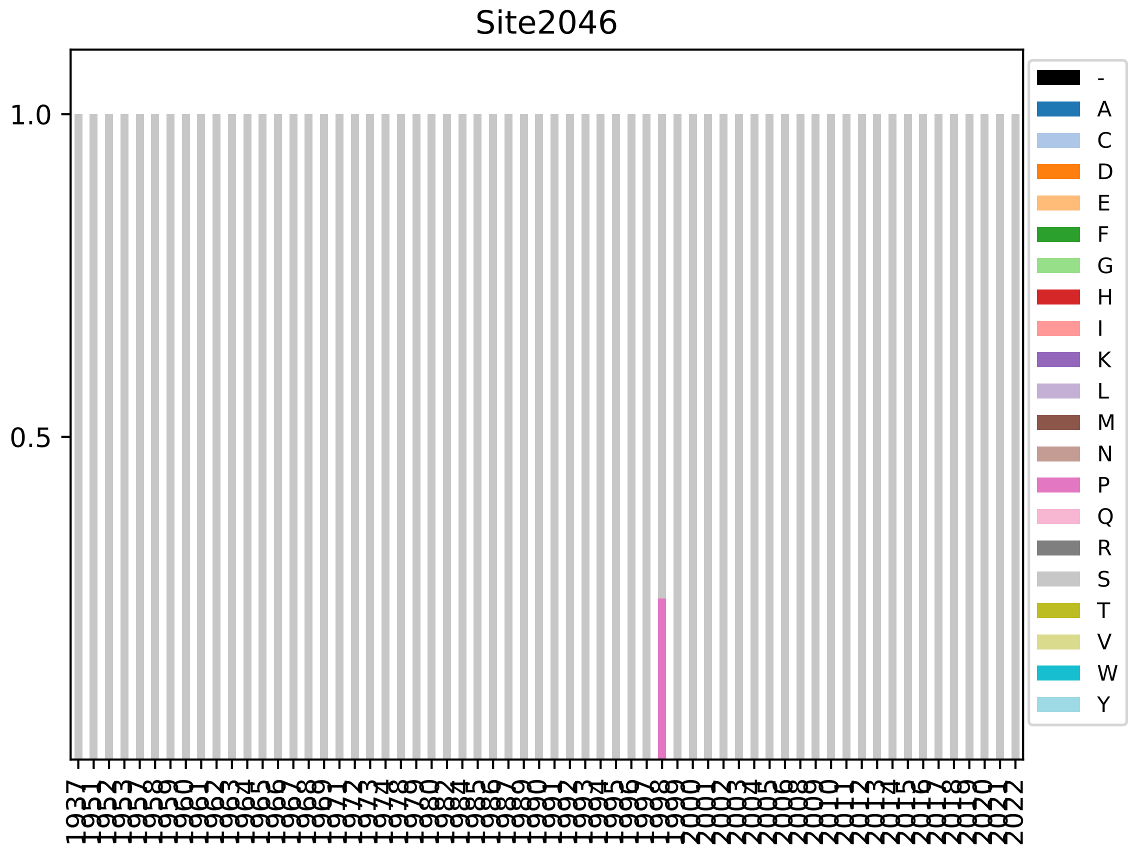
Site2046
1.0
0.5
-
A
C
D
E
F
G
H
I
K
L
M
N
P
Q
R
S
T
V
W
Y

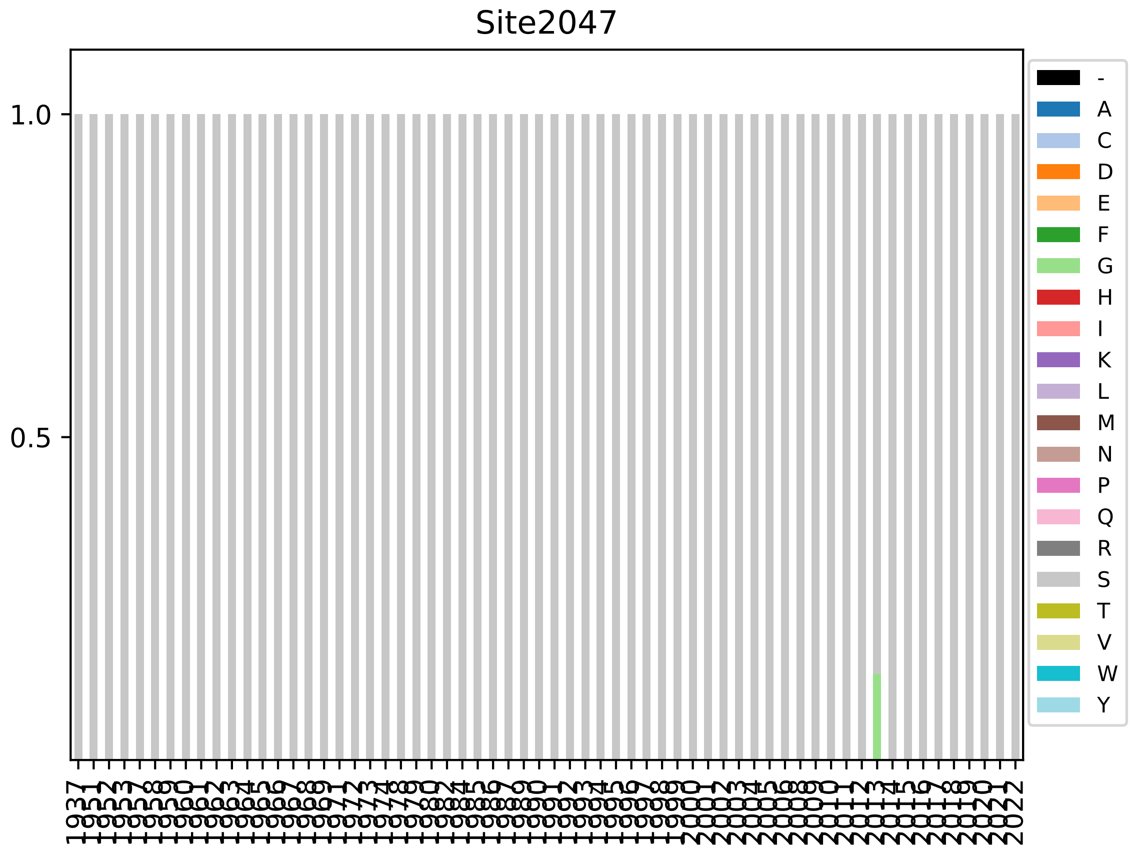
Site2047
1.0
0.5
-
A
C
D
E
F
G
H
I
K
L
M
N
P
Q
R
S
T
V
W
Y

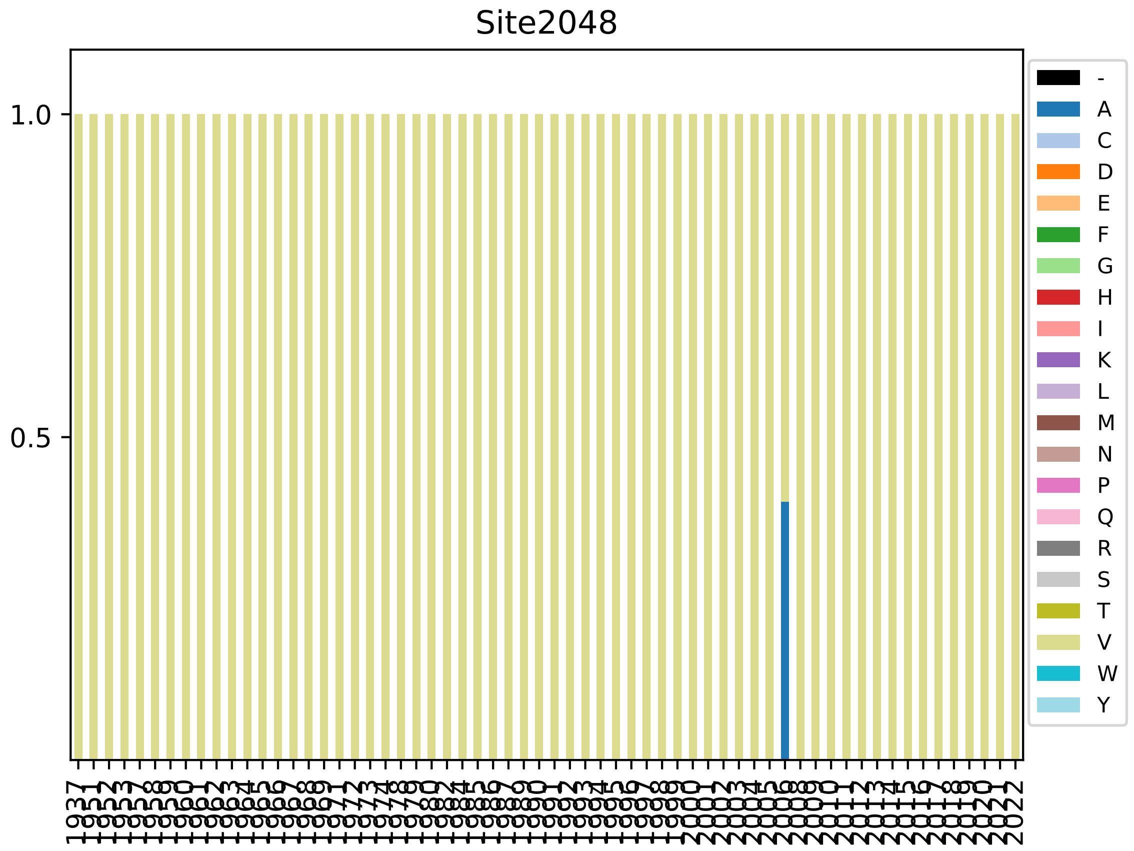
Site2048
1.0
0.5
-
A
C
D
E
F
G
H
I
K
L
M
N
P
Q
R
S
T
V
W
Y

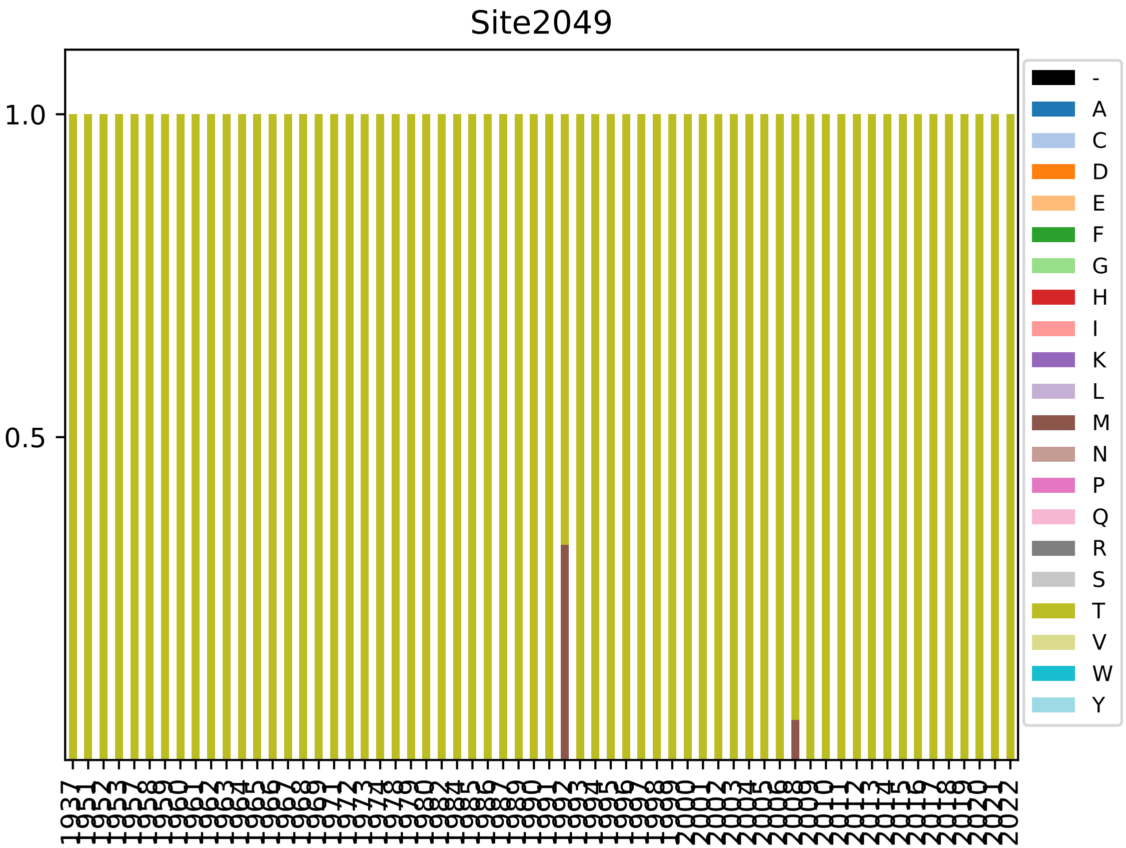
Site2049
1.0
0.5
-
A
C
D
E
F
G
H
I
K
L
M
N
P
Q
R
S
T
V
W
Y

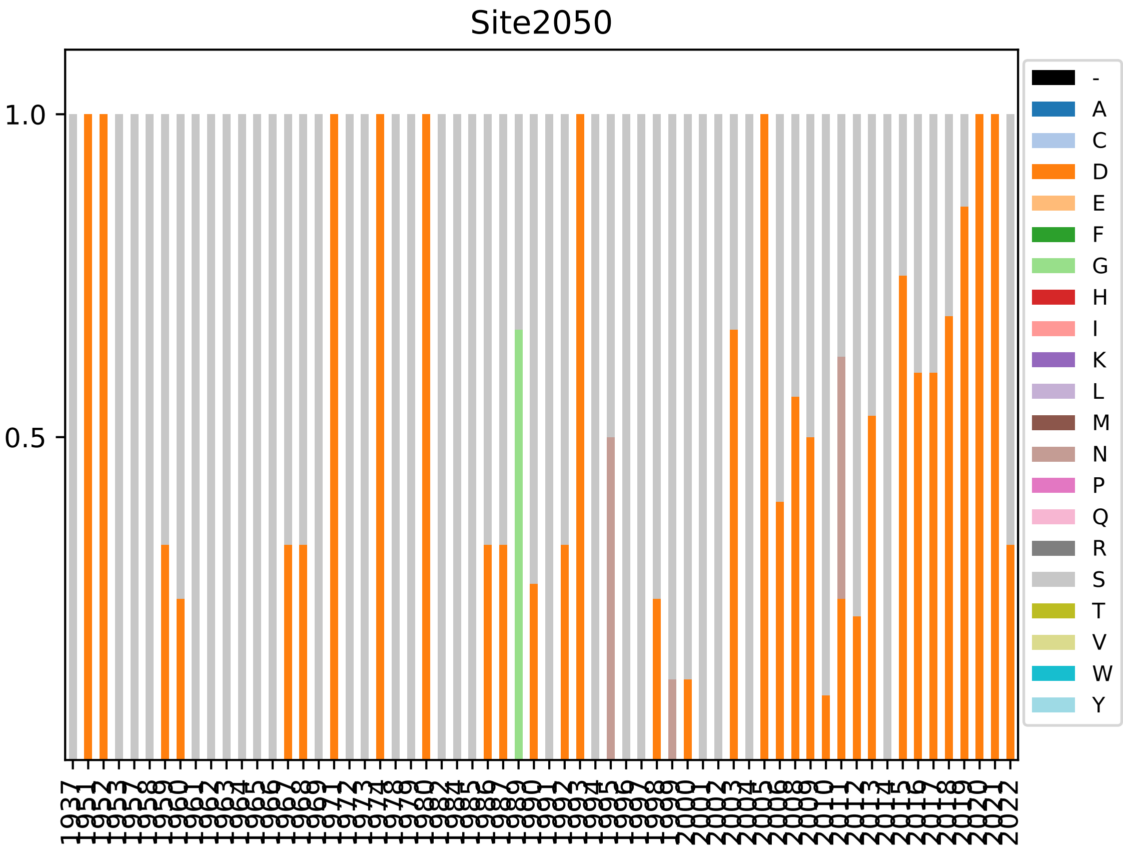
Site2050
1.0
0.5
-
A
C
D
E
F
G
H
I
K
L
M
N
P
Q
R
S
T
V
W
Y

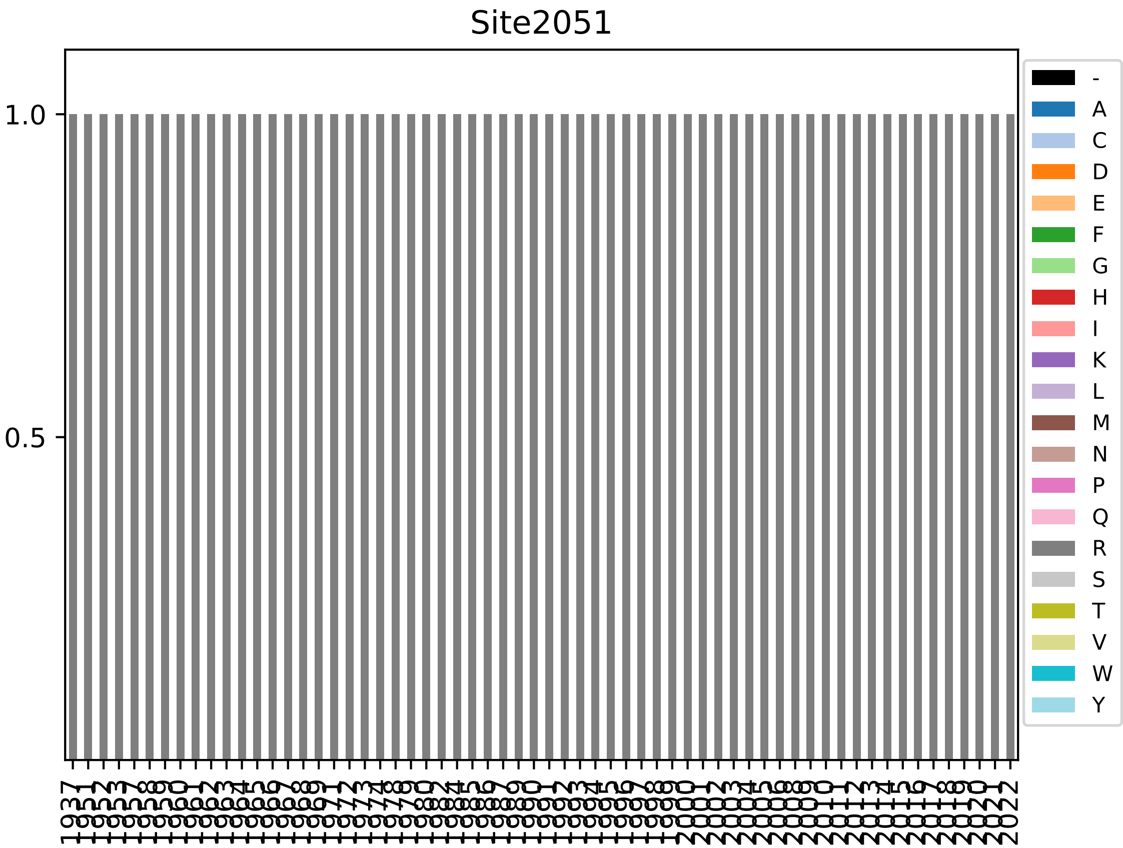
Site2051
1.0
0.5
-
A
C
D
E
F
G
H
I
K
L
M
N
P
Q
R
S
T
V
W
Y

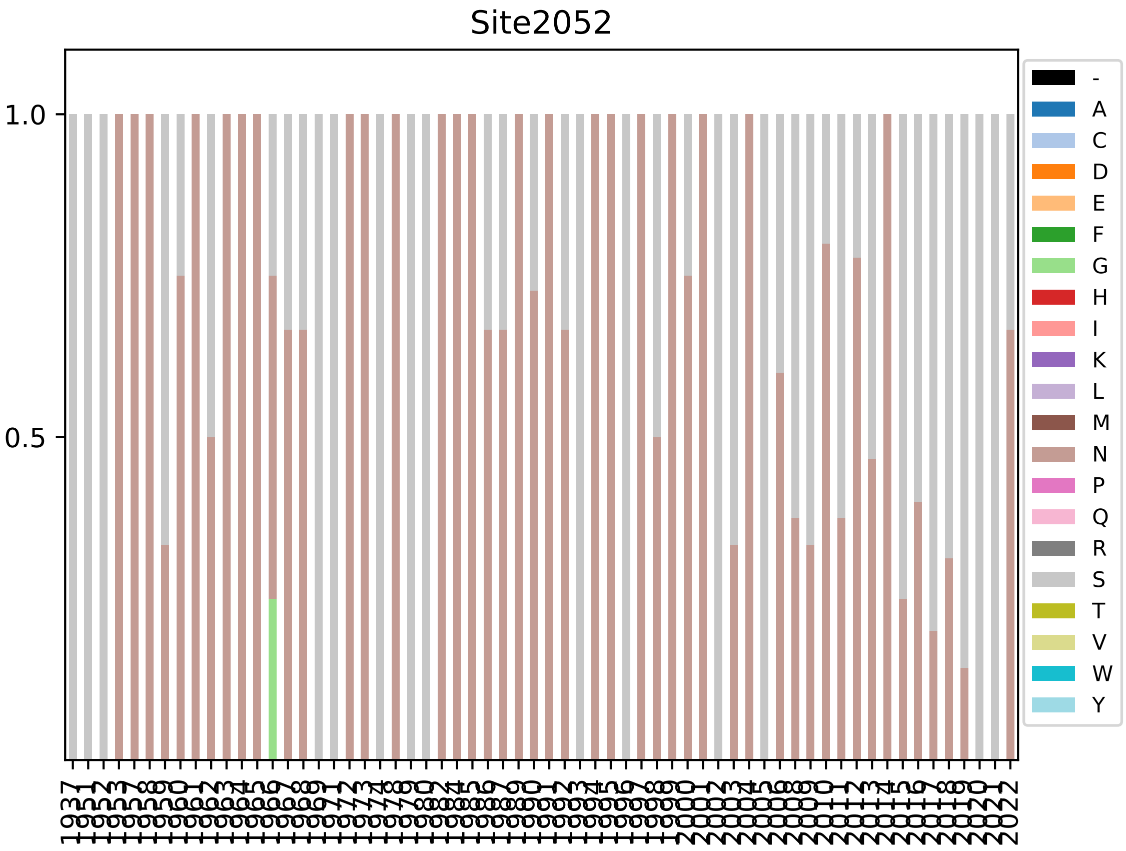
Site2052
1.0
0.5
-
A
C
D
E
F
G
H
I
K
L
M
N
P
Q
R
S
T
V
W
Y

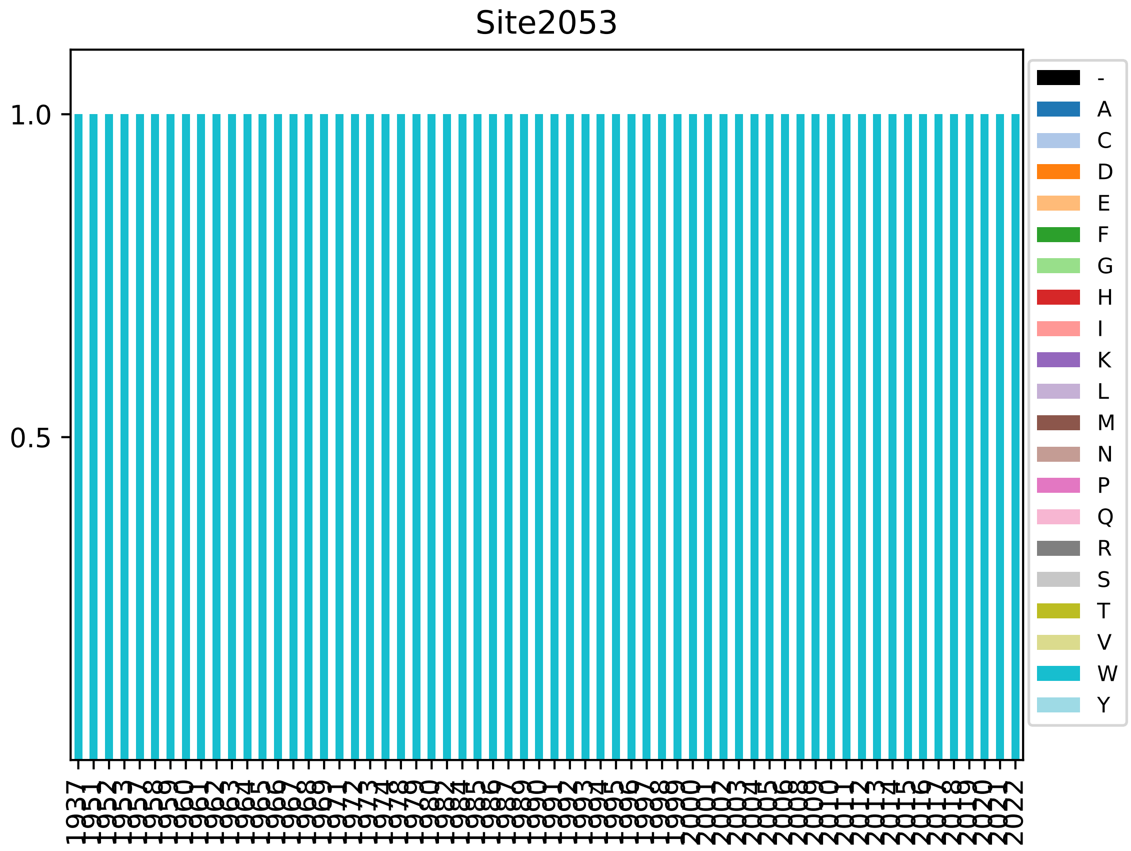
Site2053
1.0
0.5
-
A
C
D
E
F
G
H
I
K
L
M
N
P
Q
R
S
T
V
W
Y

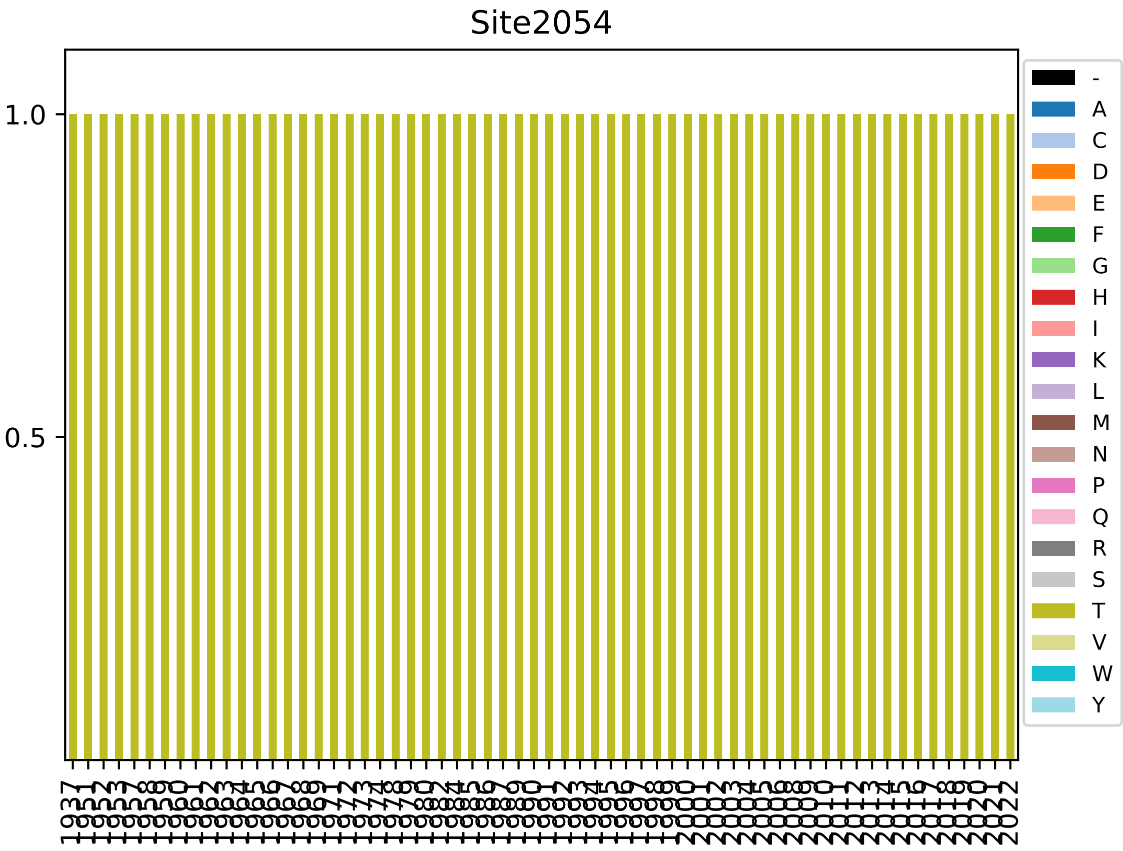
Site2054
1.0
0.5
-
A
C
D
E
F
G
H
I
K
L
M
N
P
Q
R
S
T
V
W
Y

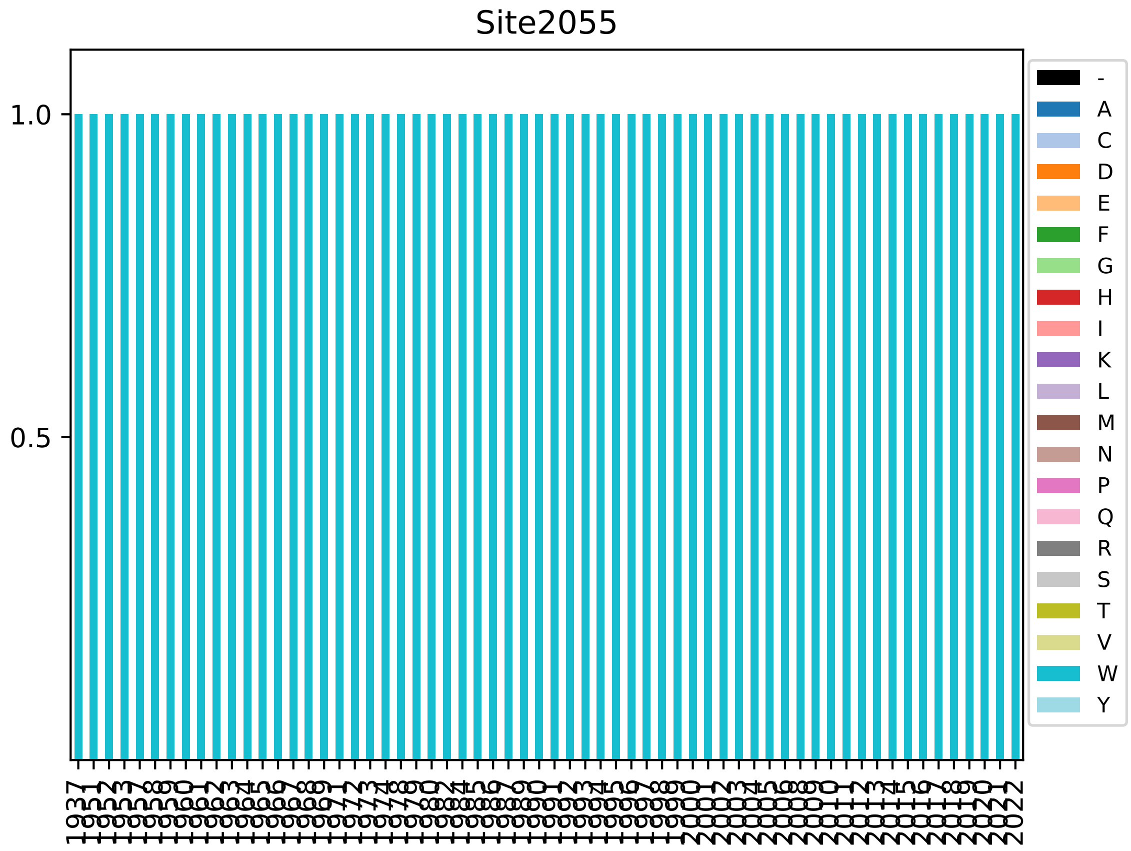
Site2055
1.0
0.5
-
A
C
D
E
F
G
H
I
K
L
M
N
P
Q
R
S
T
V
W
Y

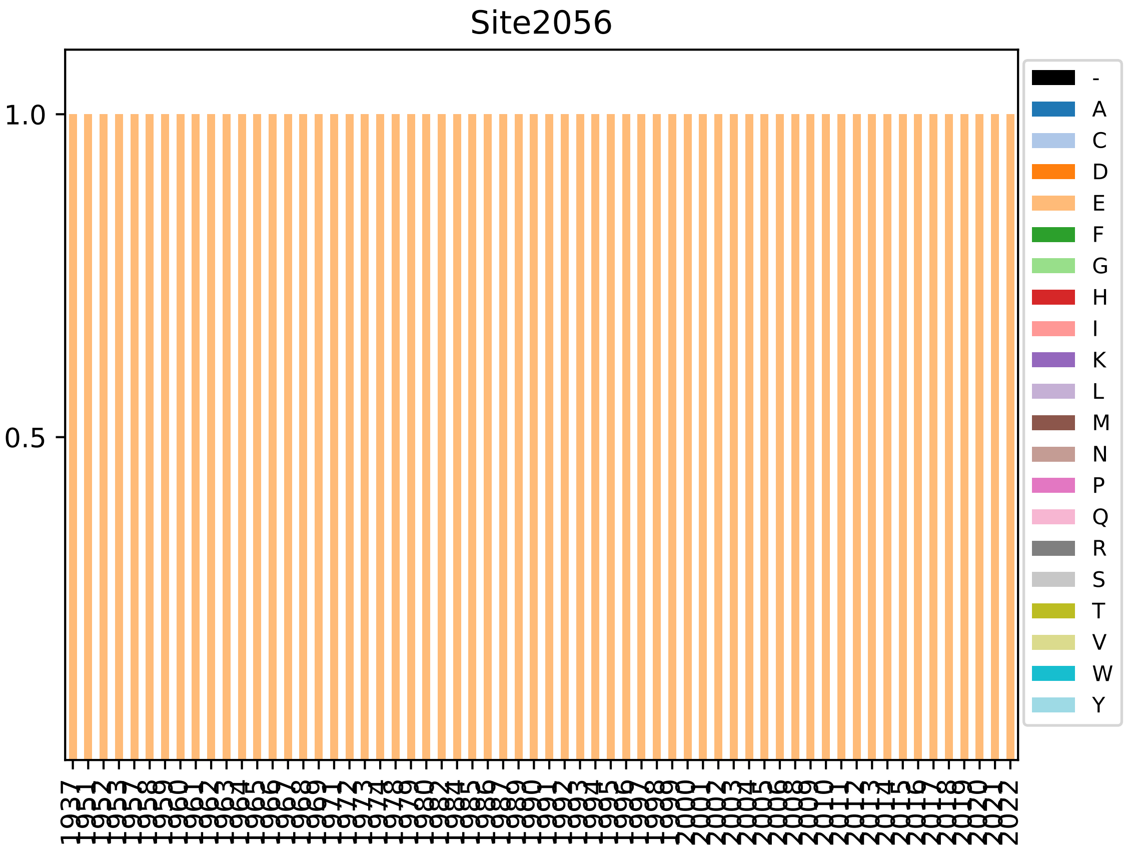

Site2056
1.0
0.5
-
A
C
D
E
F
G
H
I
K
L
M
N
P
Q
R
S
T
V
W
Y

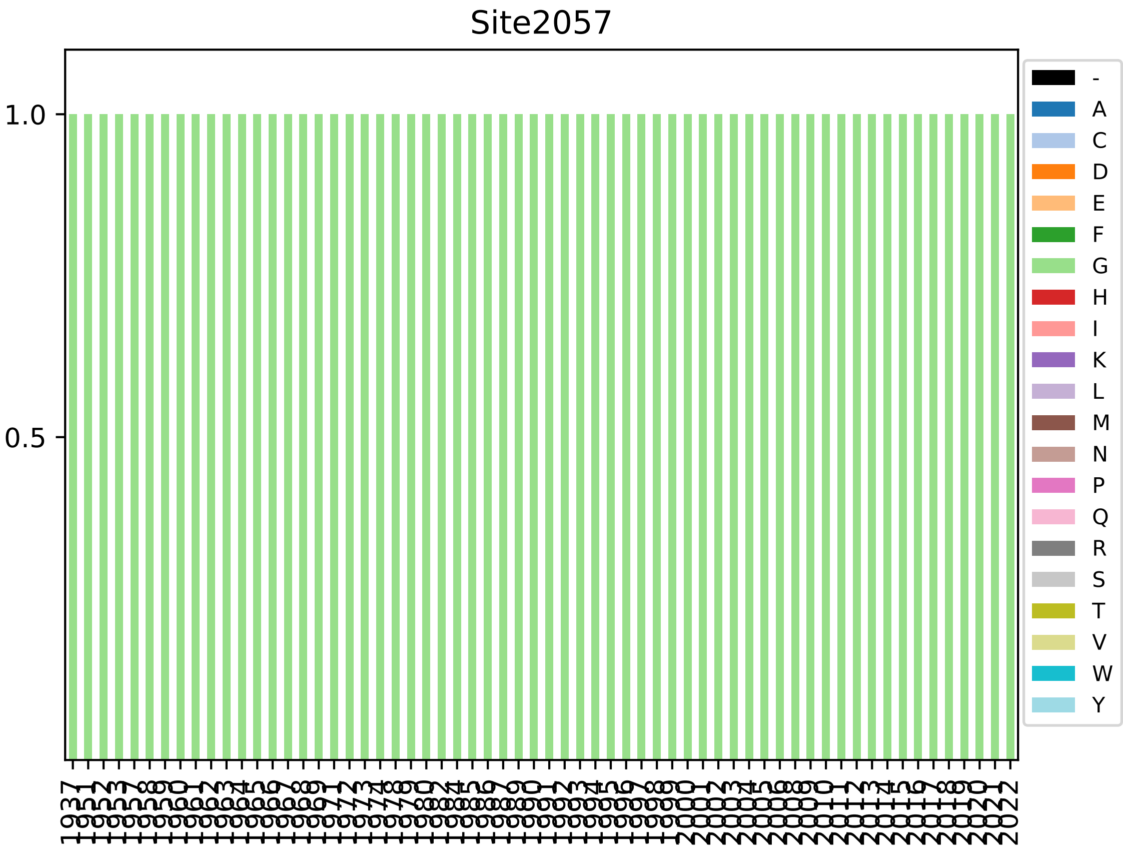
Site2057
1.0
0.5
-
A
C
D
E
F
G
H
I
K
L
M
N
P
Q
R
S
T
V
W
Y

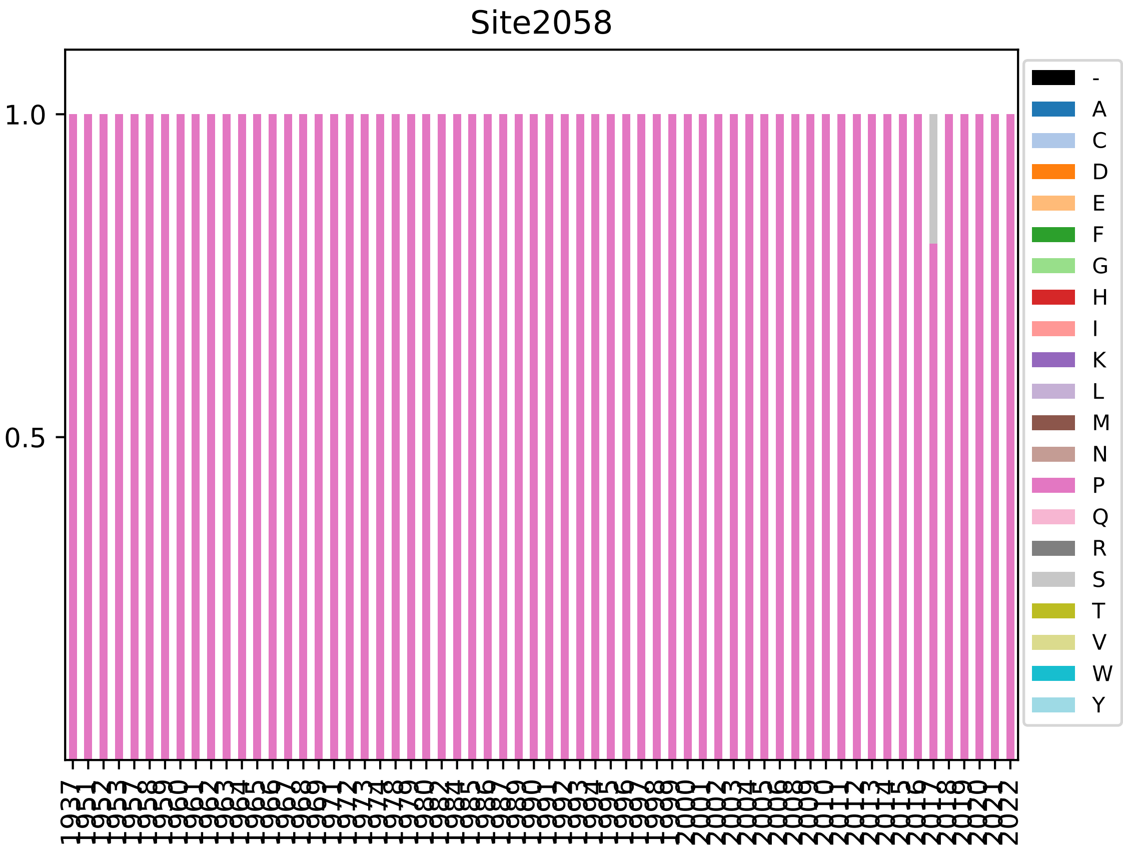
Site2058
1.0
0.5
-
A
C
D
E
F
G
H
I
K
L
M
N
P
Q
R
S
T
V
W
Y

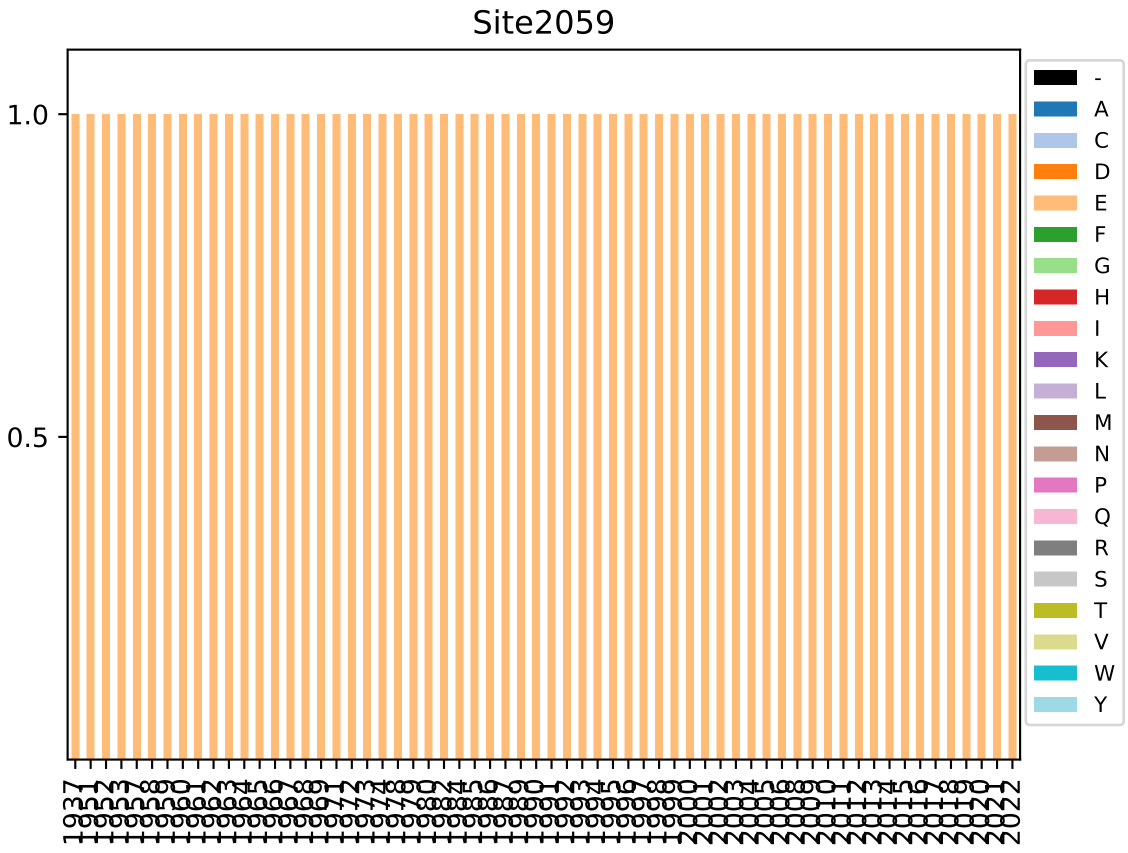
Site2059
1.0
0.5
-
A
C
D
E
F
G
H
I
K
L
M
N
P
Q
R
S
T
V
W
Y

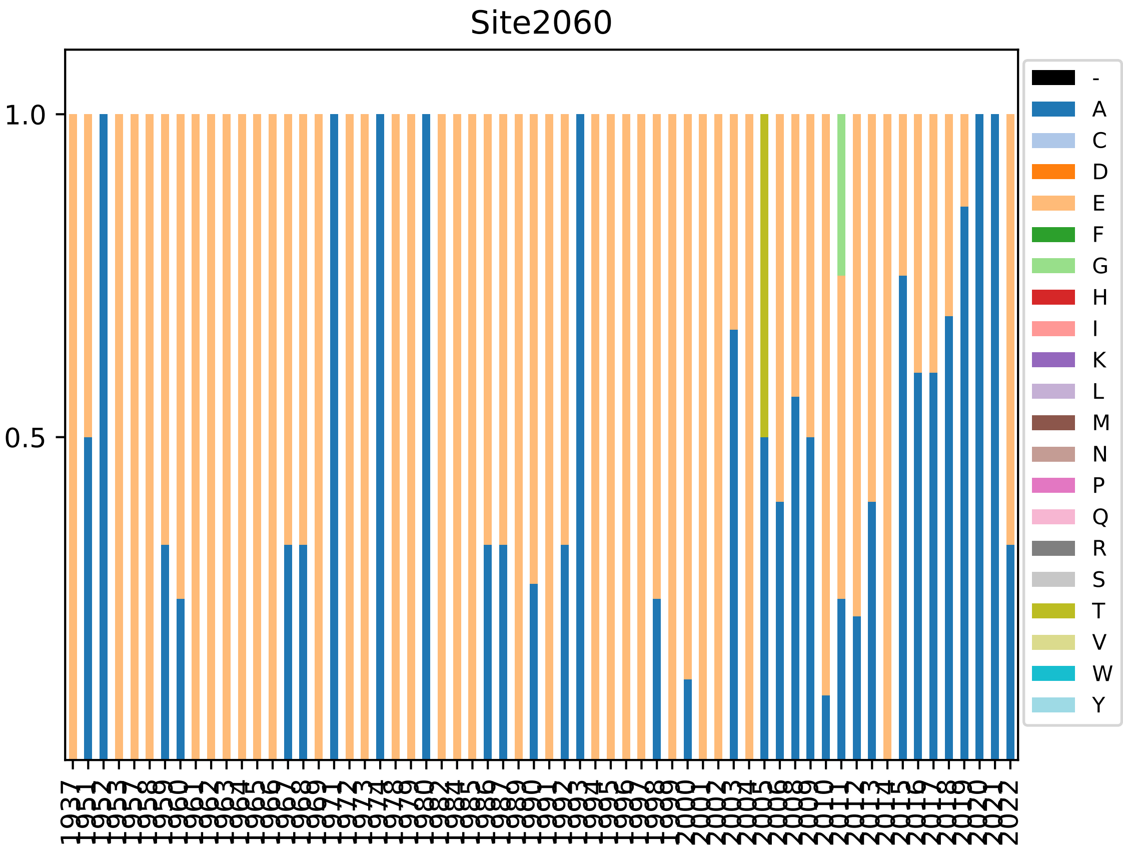
Site2060
1.0
0.5
-
A
C
D
E
F
G
H
I
K
L
M
N
P
Q
R
S
T
V
W
Y

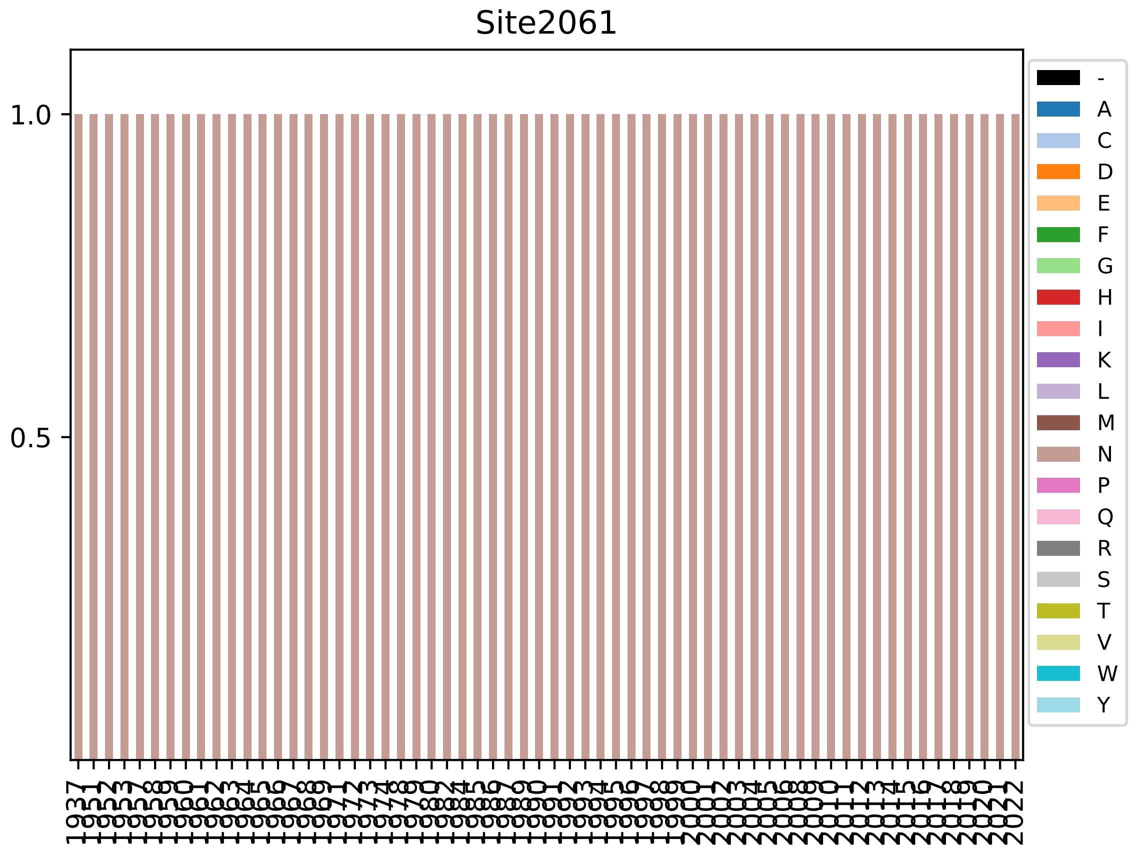
Site2061
1.0
0.5
-
A
C
D
E
F
G
H
I
K
L
M
N
P
Q
R
S
T
V
W
Y

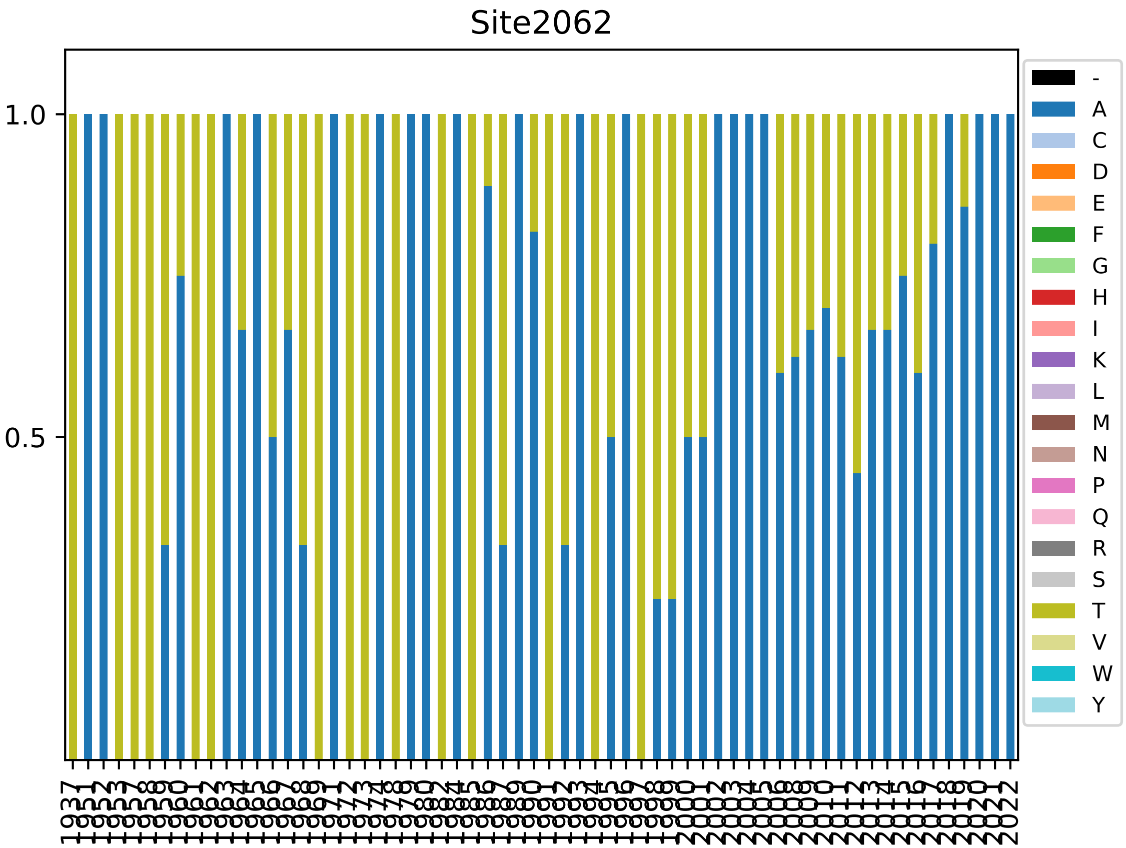

Site2062
1.0
0.5
-
A
C
D
E
F
G
H
I
K
L
M
N
P
Q
R
S
T
V
W
Y

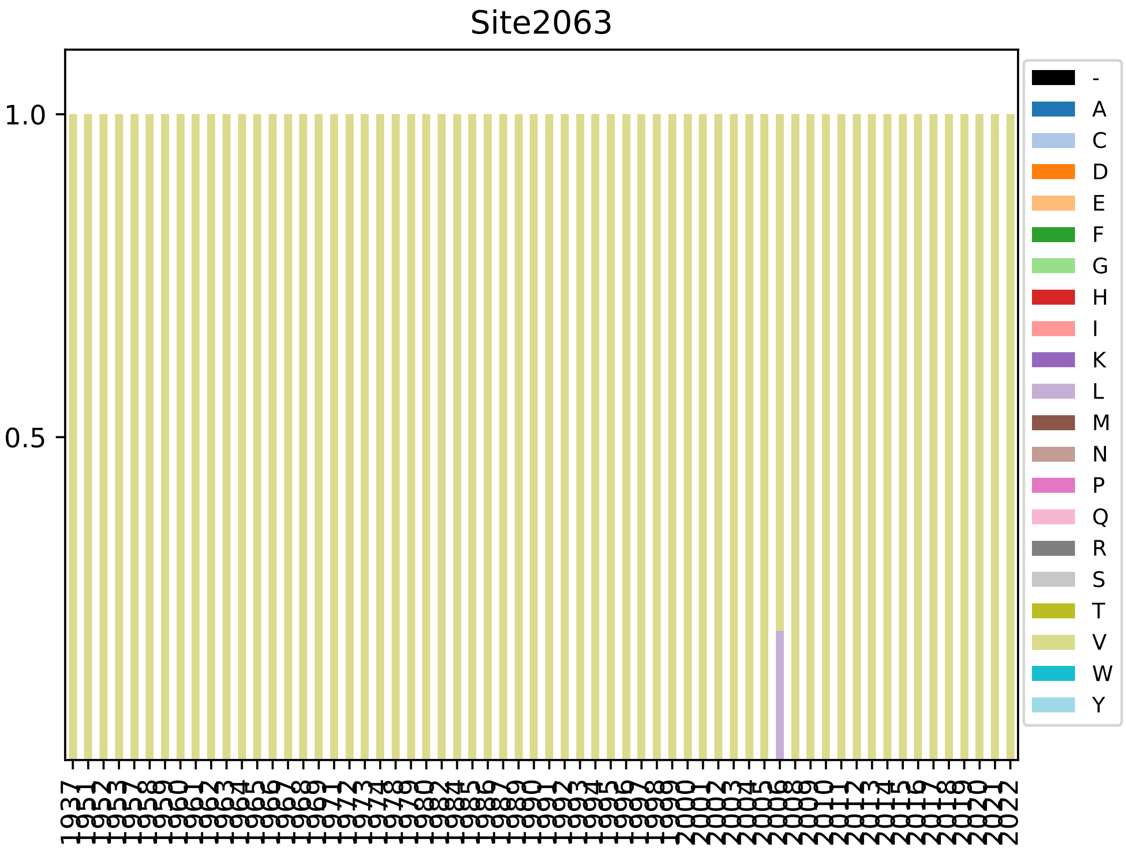
Site2063
1.0
0.5
-
A
C
D
E
F
G
H
I
K
L
M
N
P
Q
R
S
T
V
W
Y

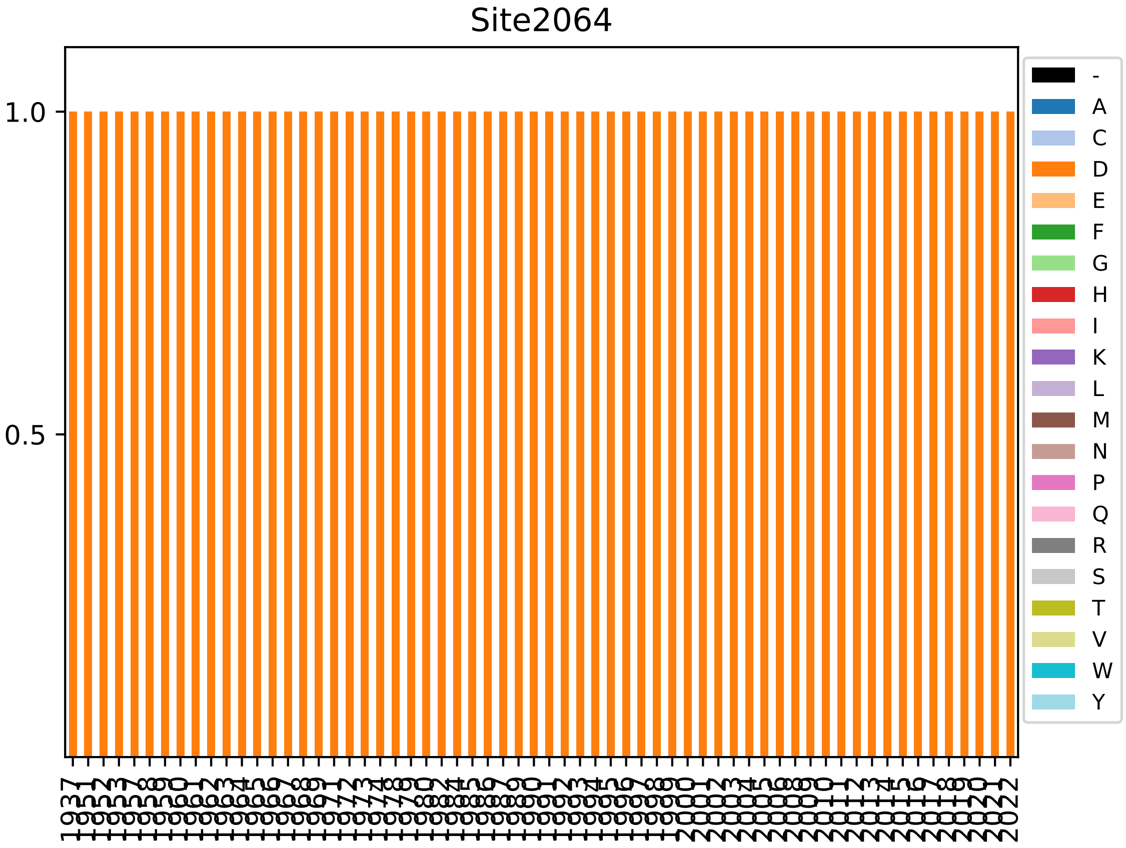
Site2064
1.0
0.5
-
A
C
D
E
F
G
H
I
K
L
M
N
P
Q
R
S
T
V
W
Y

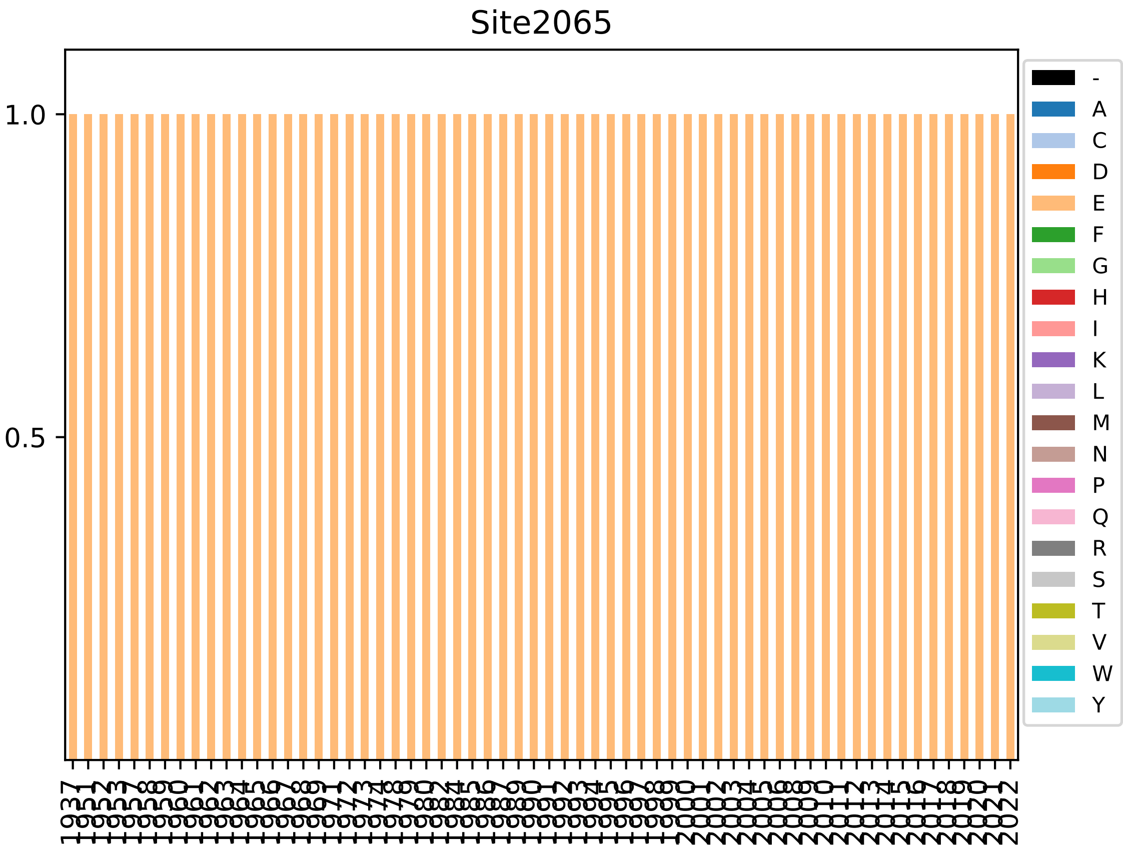

Site2065
1.0
0.5
-
A
C
D
E
F
G
H
I
K
L
M
N
P
Q
R
S
T
V
W
Y

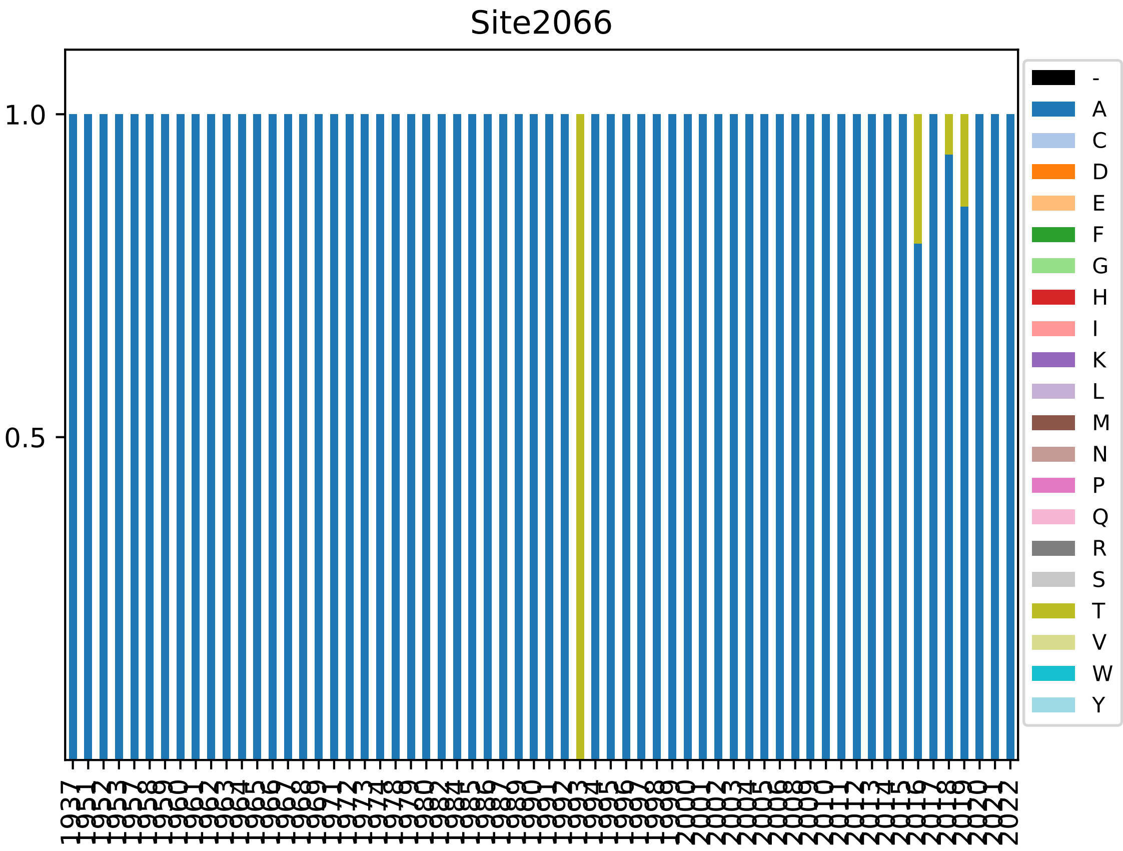
Site2066
1.0
0.5
-
A
C
D
E
F
G
H
I
K
L
M
N
P
Q
R
S
T
V
W
Y

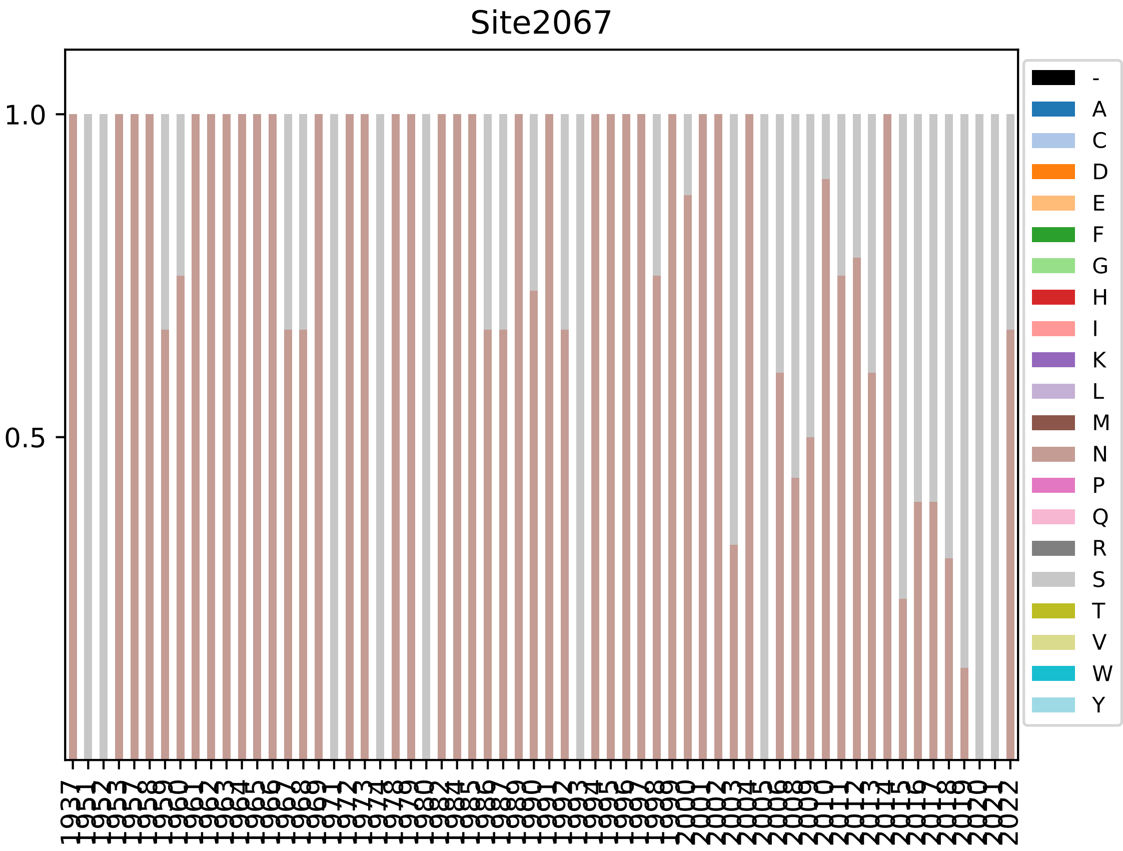
Site2067
1.0
0.5
-
A
C
D
E
F
G
H
I
K
L
M
N
P
Q
R
S
T
V
W
Y

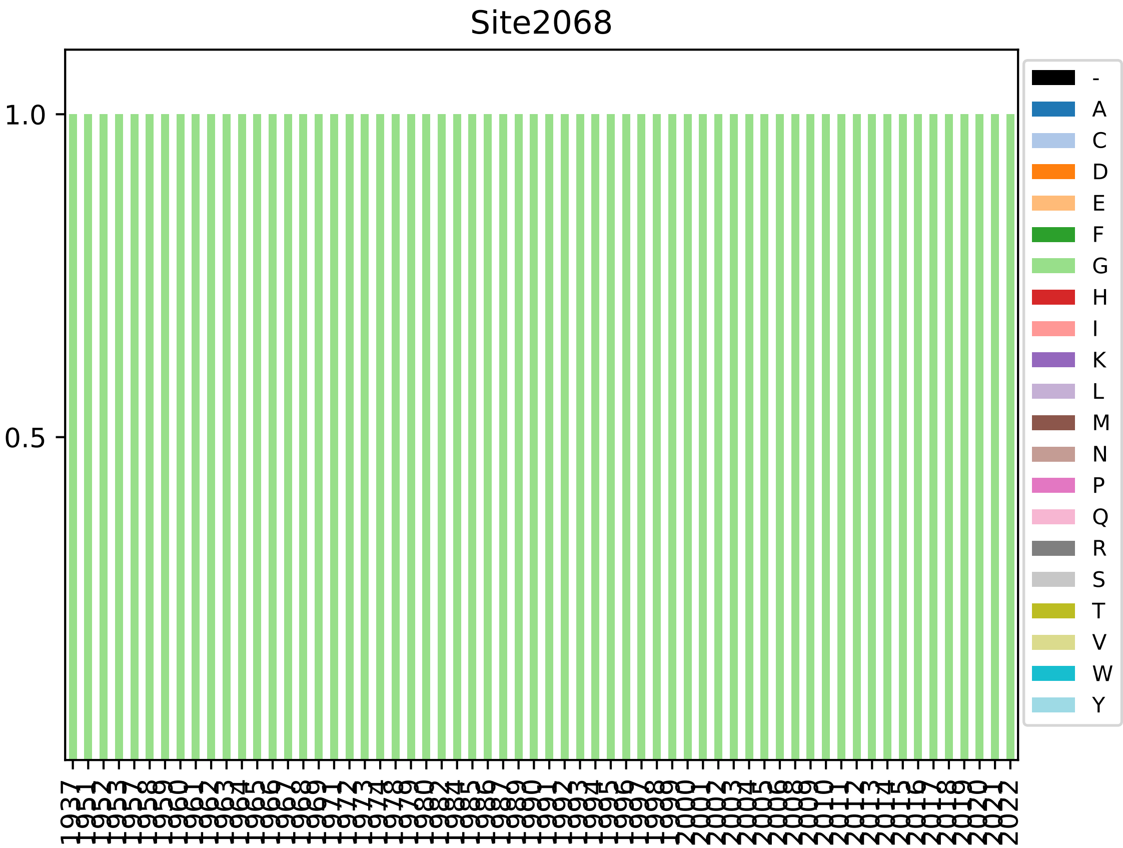
Site2068
1.0
0.5
-
A
C
D
E
F
G
H
I
K
L
M
N
P
Q
R
S
T
V
W
Y

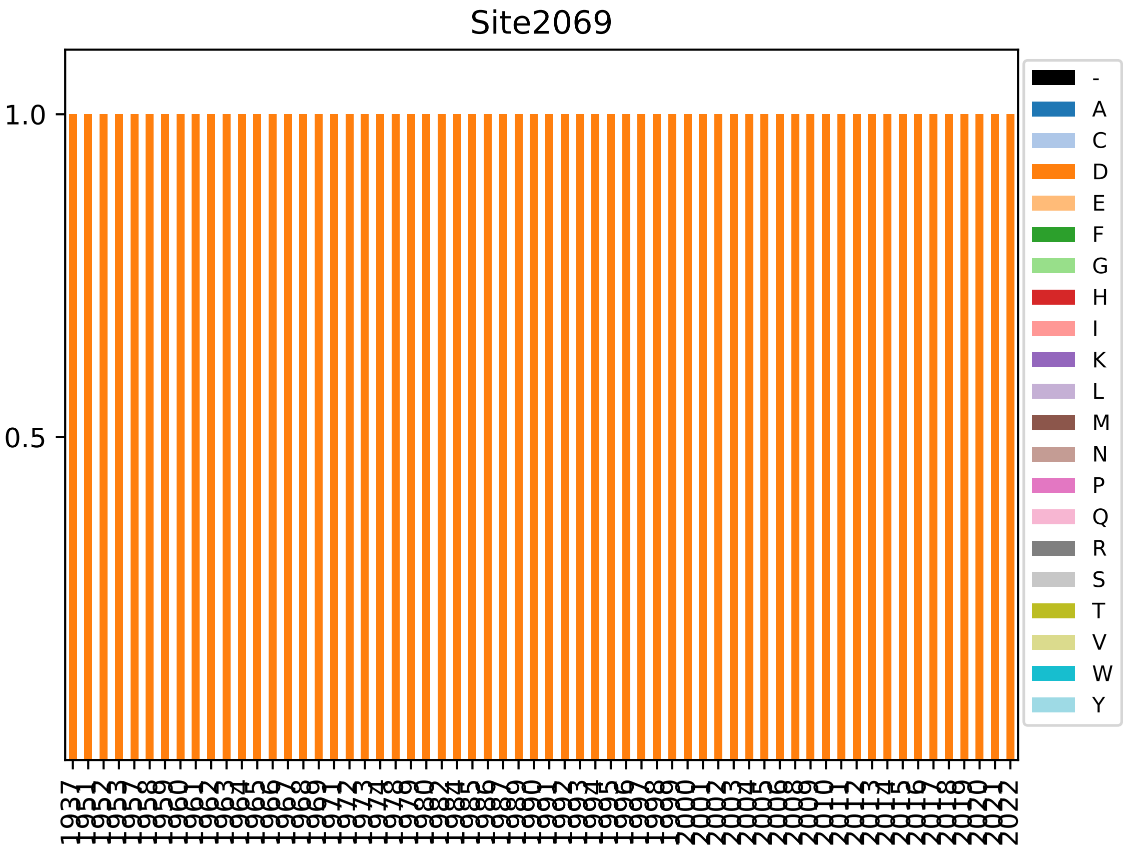
Site2069
1.0
0.5
-
A
C
D
E
F
G
H
I
K
L
M
N
P
Q
R
S
T
V
W
Y

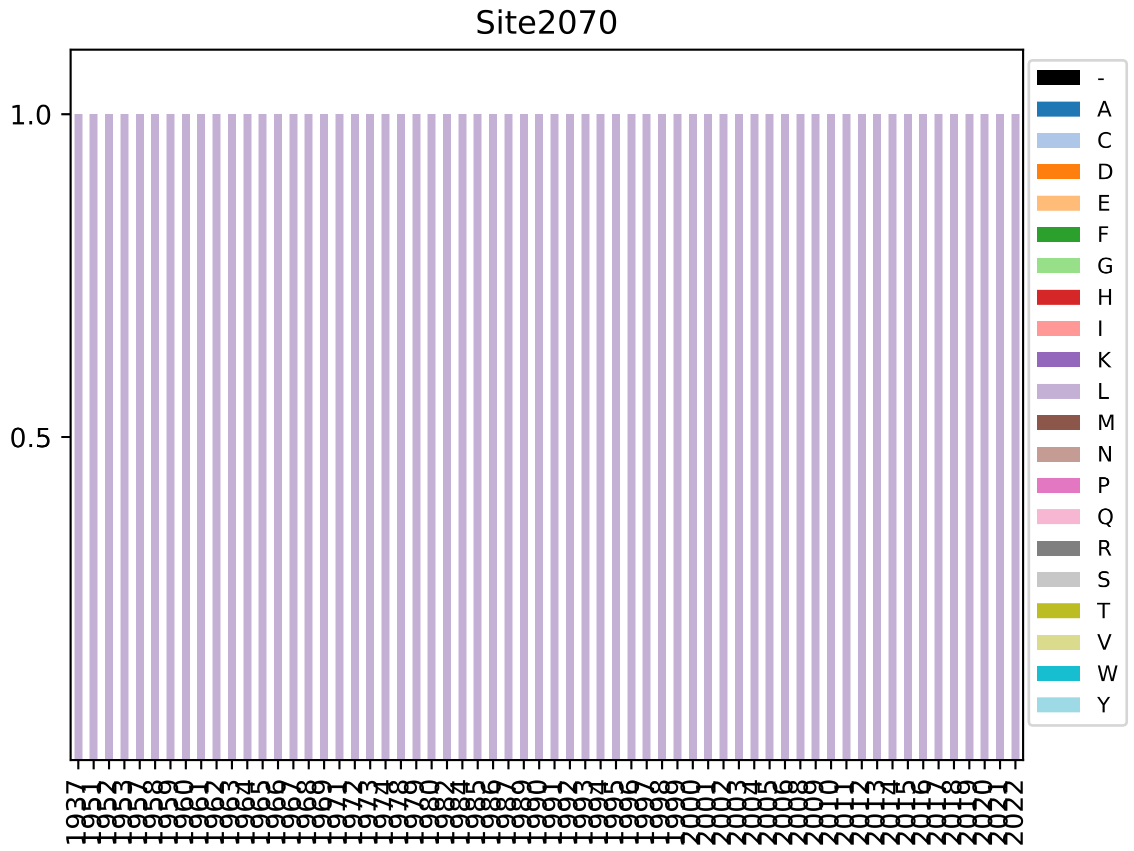

Site2070
1.0
0.5
-
A
C
D
E
F
G
H
I
K
L
M
N
P
Q
R
S
T
V
W
Y

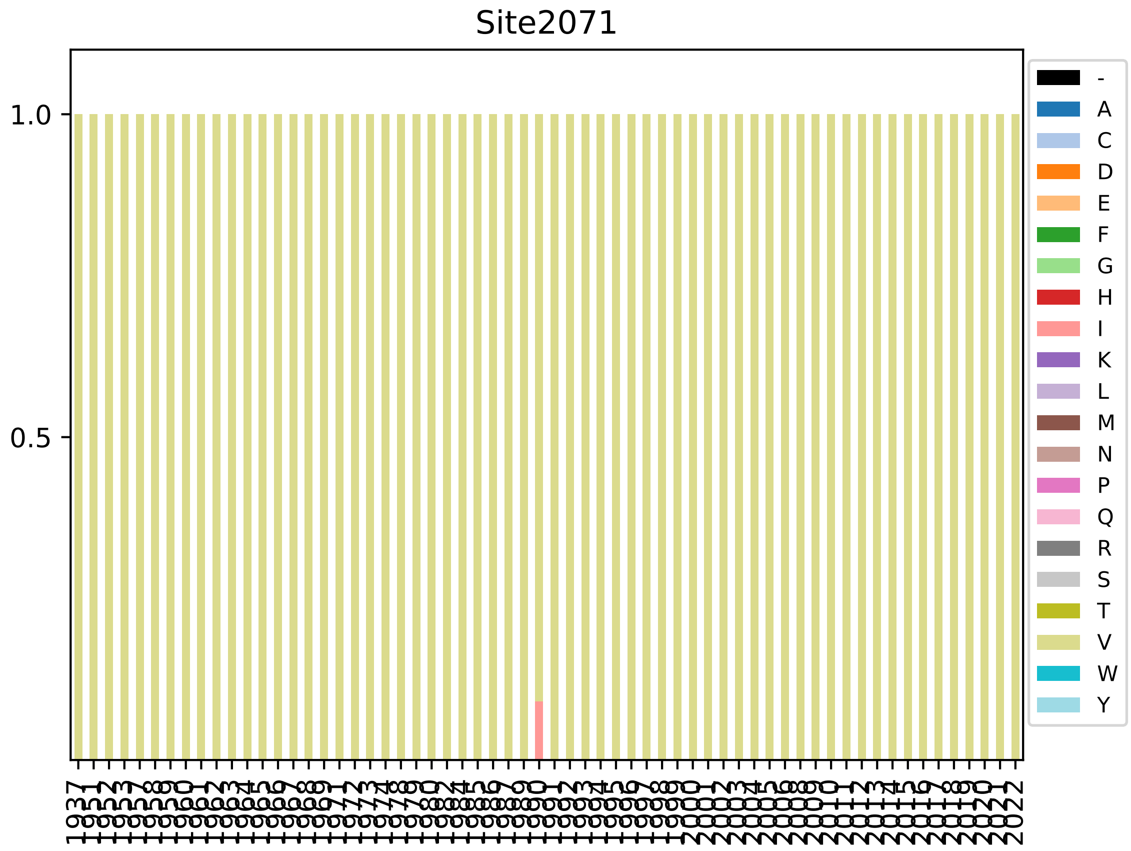
Site2071
1.0
0.5
-
A
C
D
E
F
G
H
I
K
L
M
N
P
Q
R
S
T
V
W
Y

Site2072

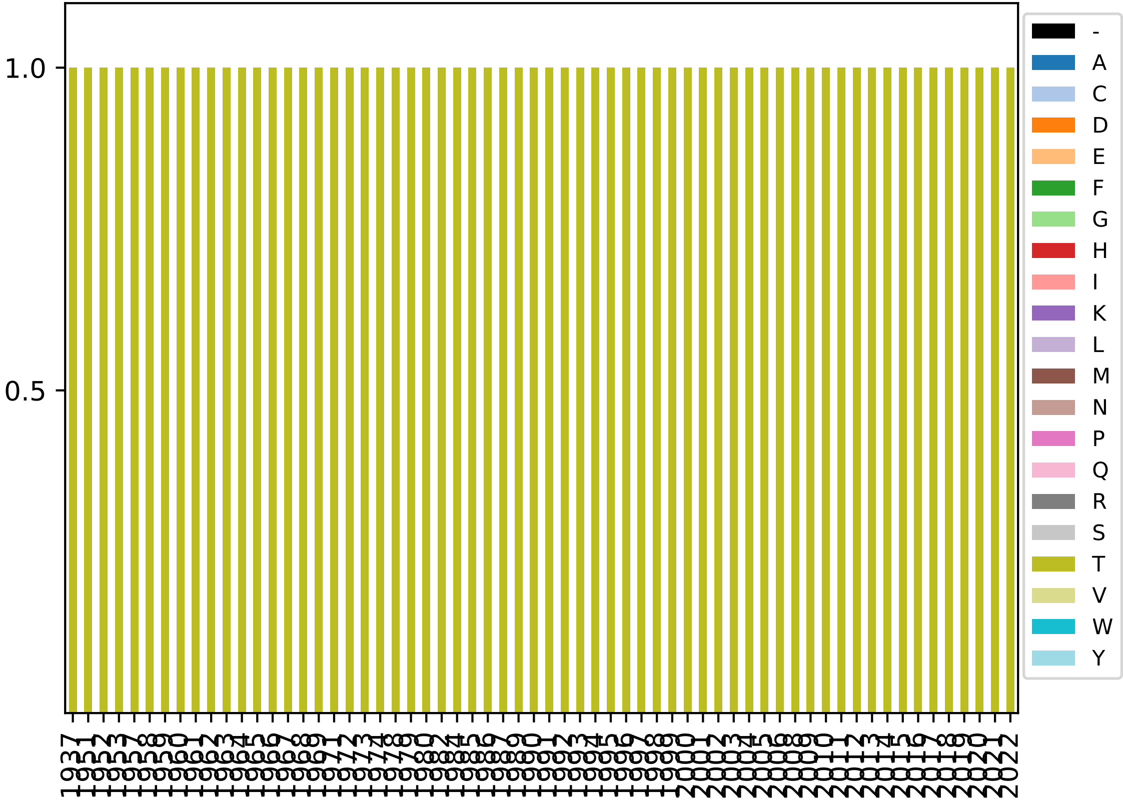

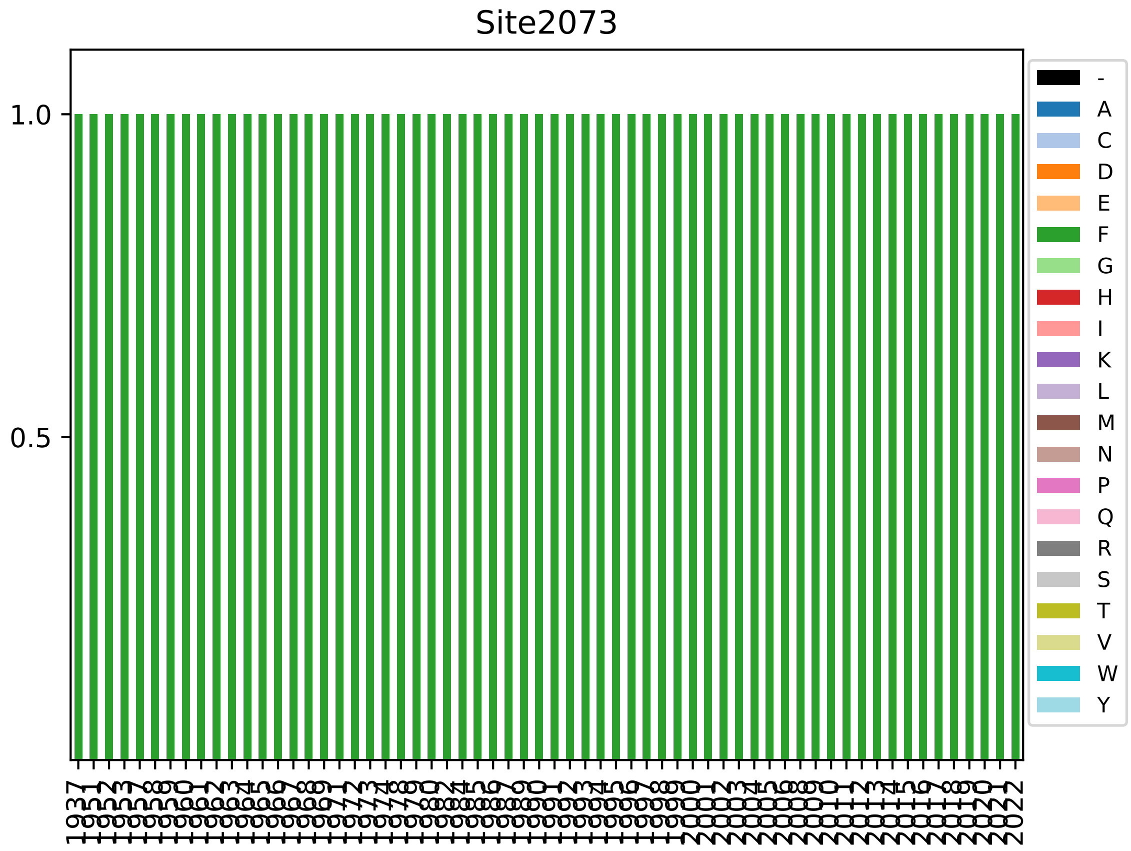
Site2073
1.0
0.5
-
A
C
D
E
F
G
H
I
K
L
M
N
P
Q
R
S
T
V
W
Y

Site2074

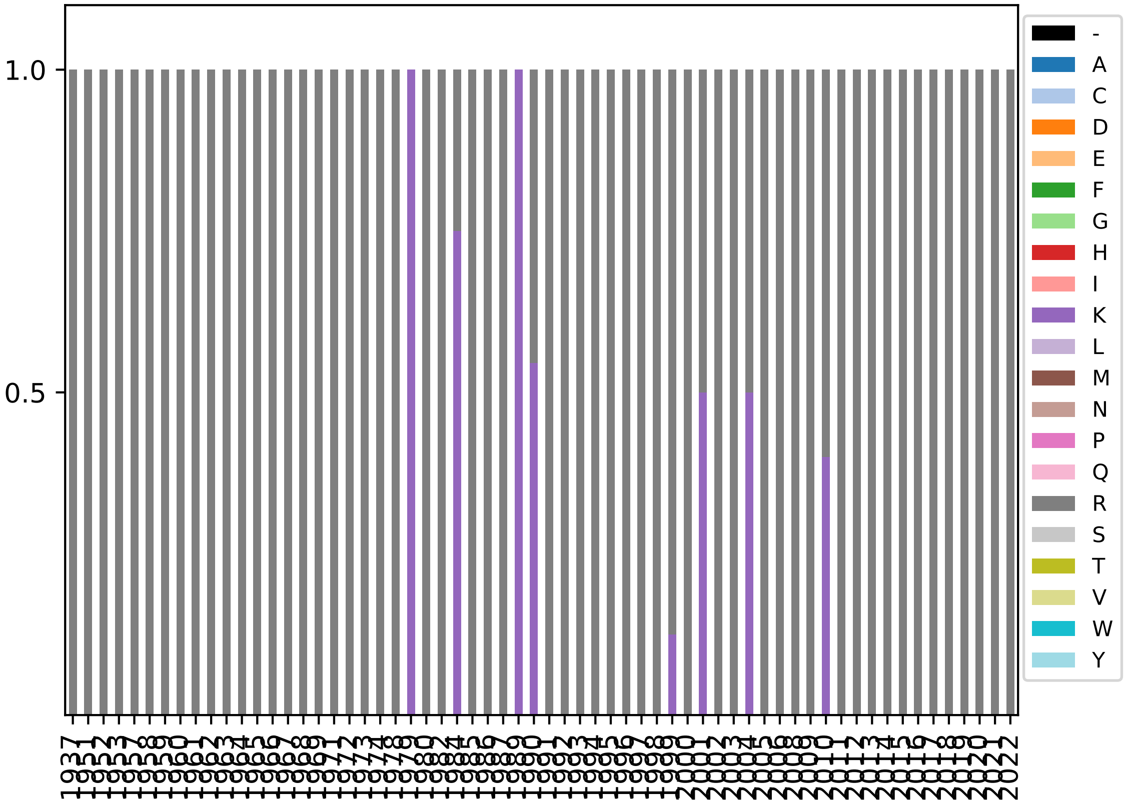

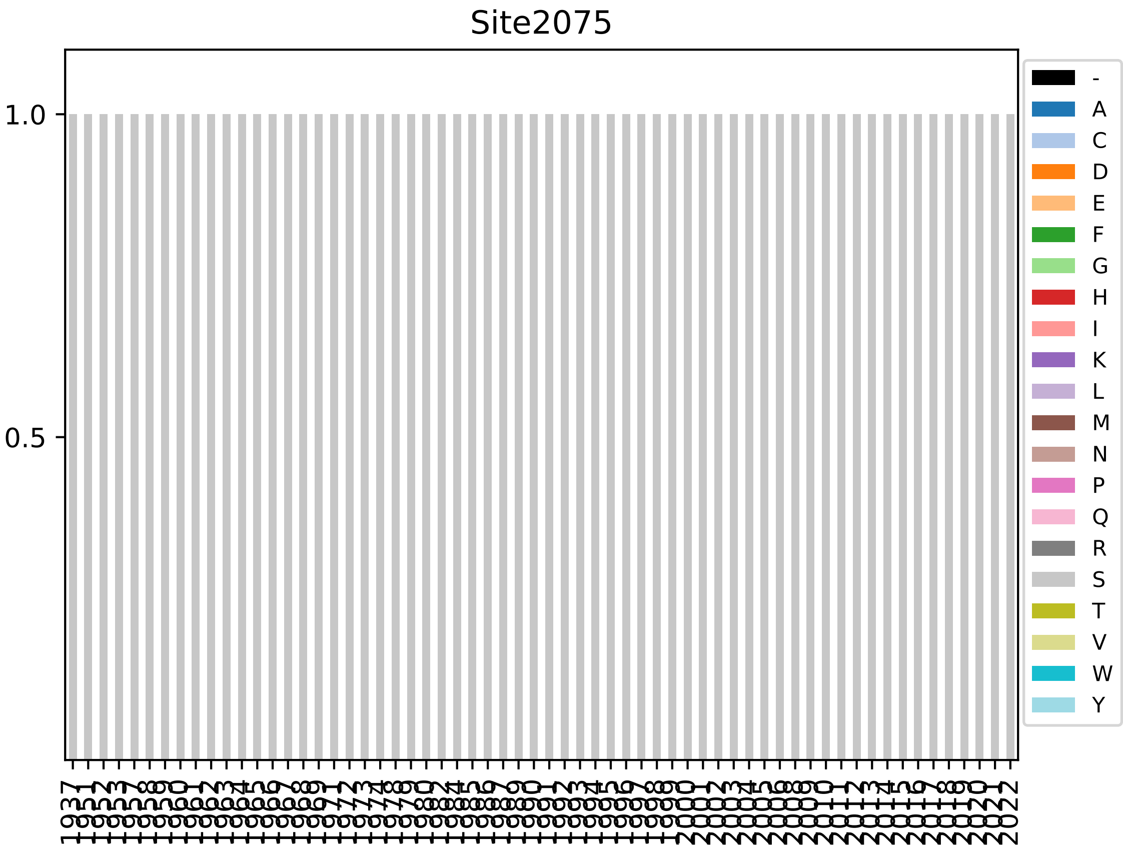
Site2075
1.0
0.5
-
A
C
D
E
F
G
H
I
K
L
M
N
P
Q
R
S
T
V
W
Y

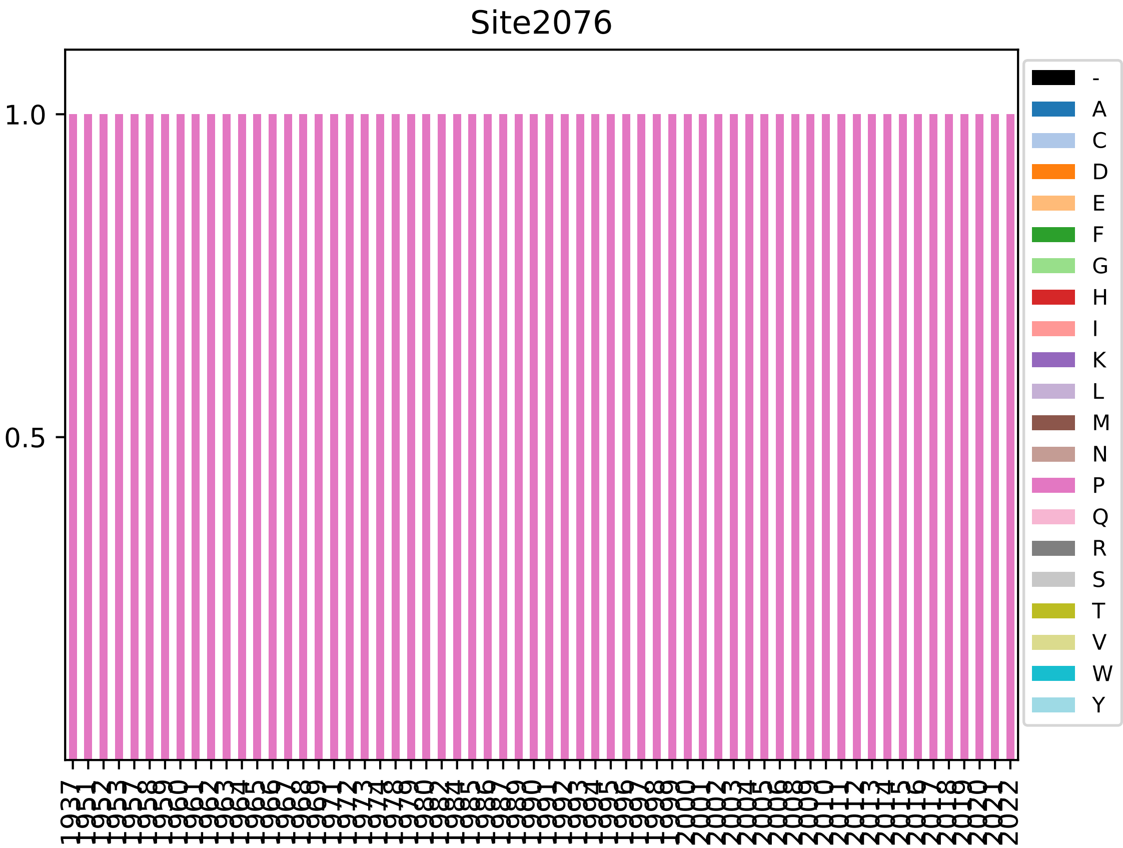
Site2076
1.0
0.5
-
A
C
D
E
F
G
H
I
K
L
M
N
P
Q
R
S
T
V
W
Y

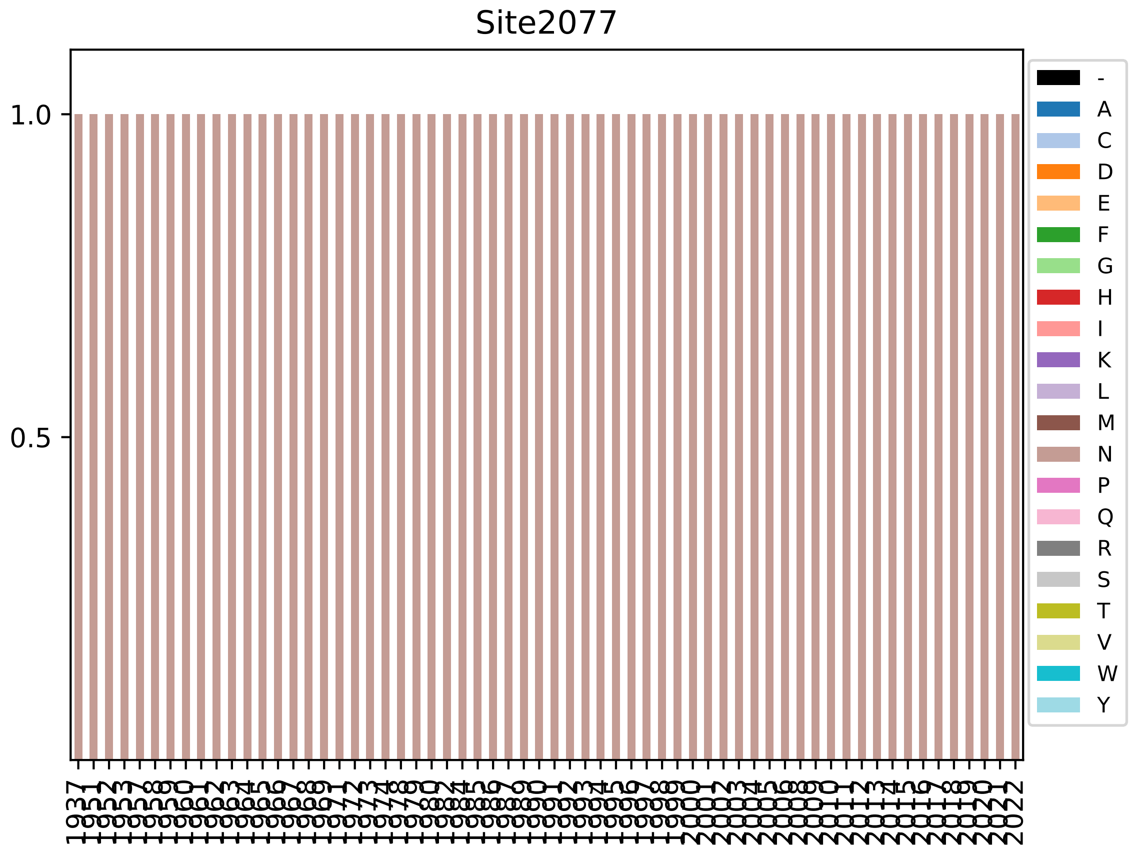
Site2077
1.0
0.5
-
A
C
D
E
F
G
H
I
K
L
M
N
P
Q
R
S
T
V
W
Y

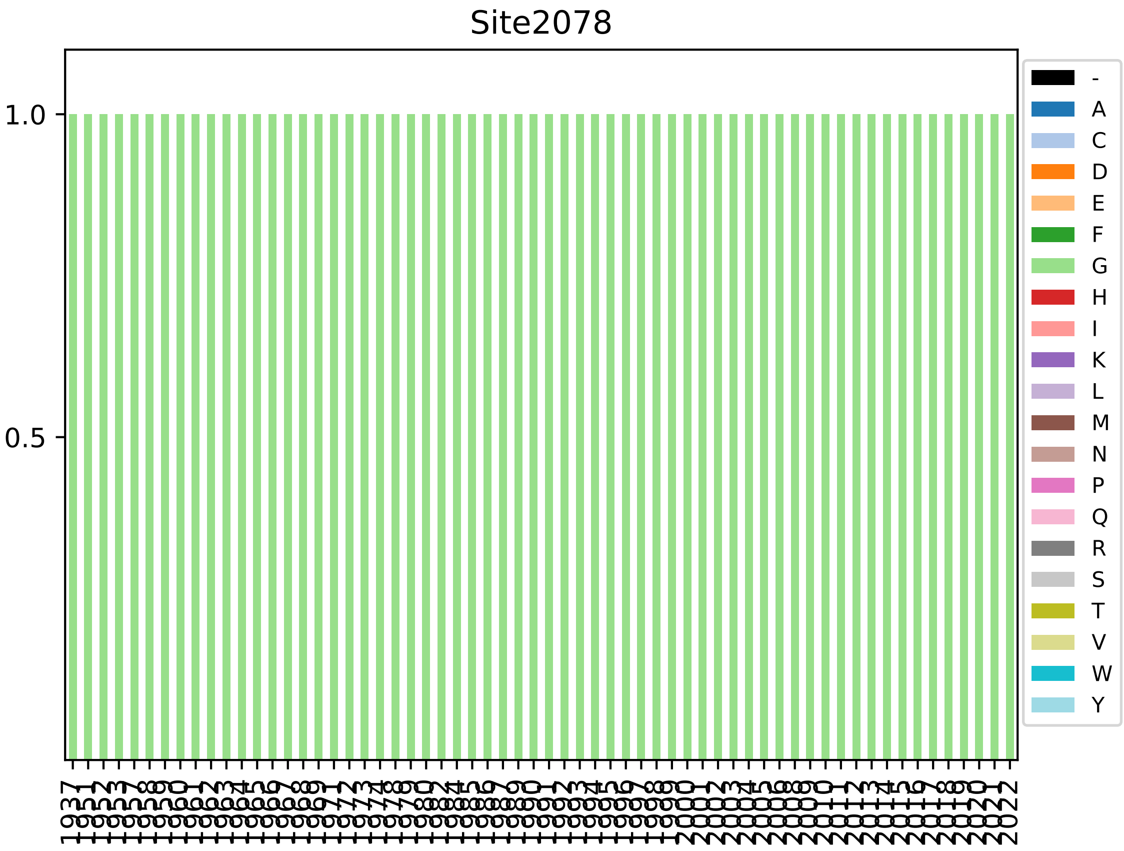
Site2078
1.0
0.5
-
A
C
D
E
F
G
H
I
K
L
M
N
P
Q
R
S
T
V
W
Y

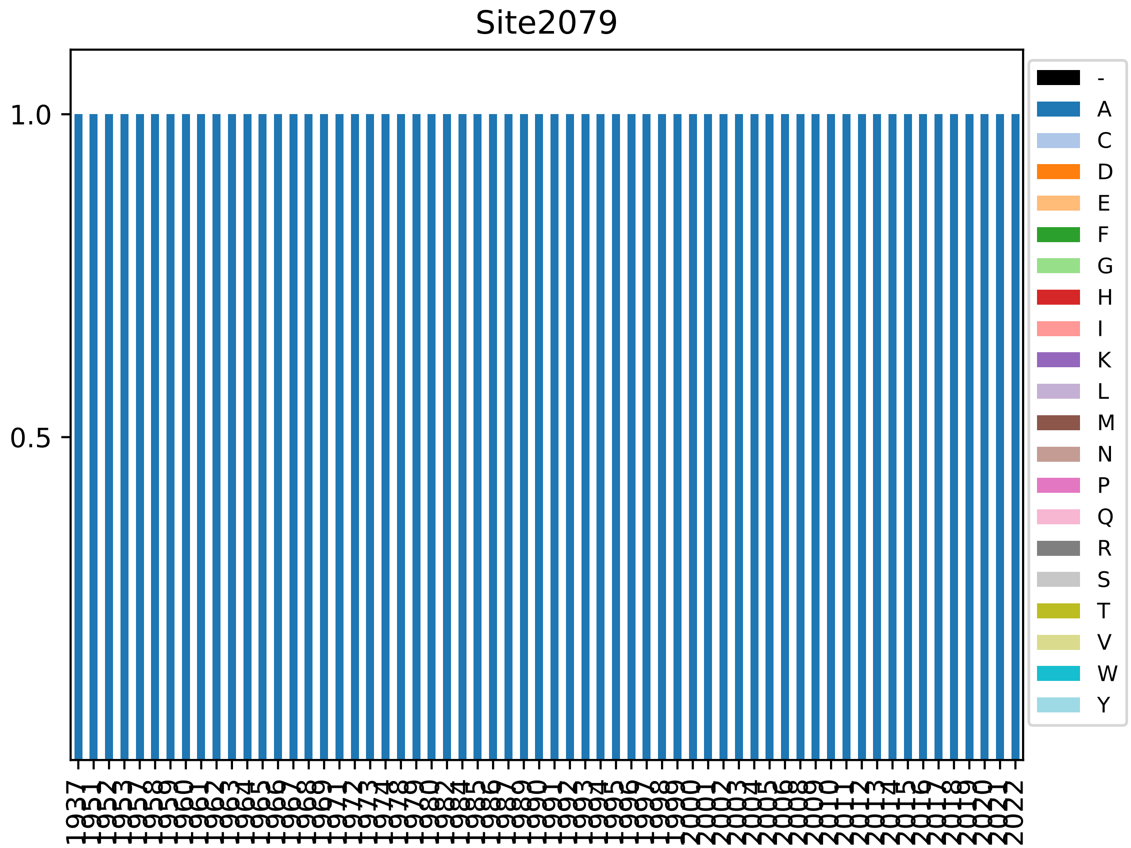
Site2079
1.0
0.5
-
A
C
D
E
F
G
H
I
K
L
M
N
P
Q
R
S
T
V
W
Y

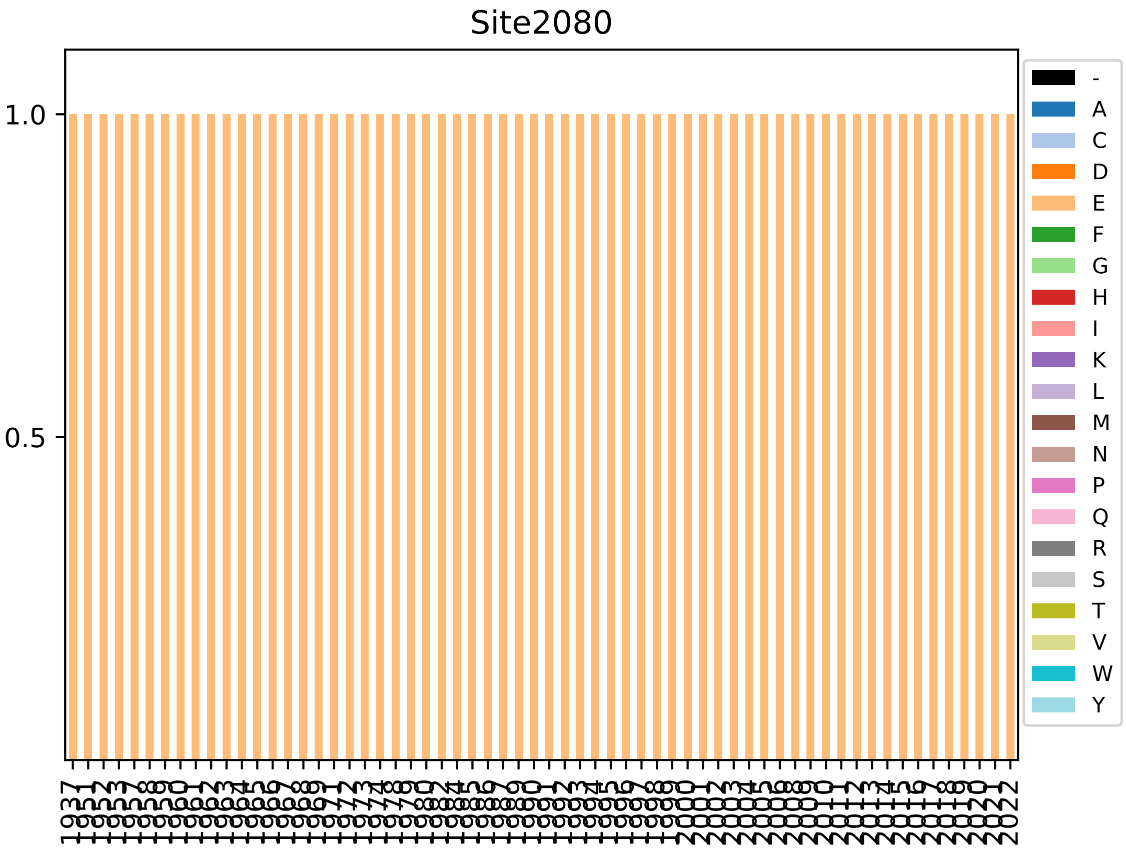
Site2080
1.0
0.5
-
A
C
D
E
F
G
H
I
K
L
M
N
P
Q
R
S
T
V
W
Y

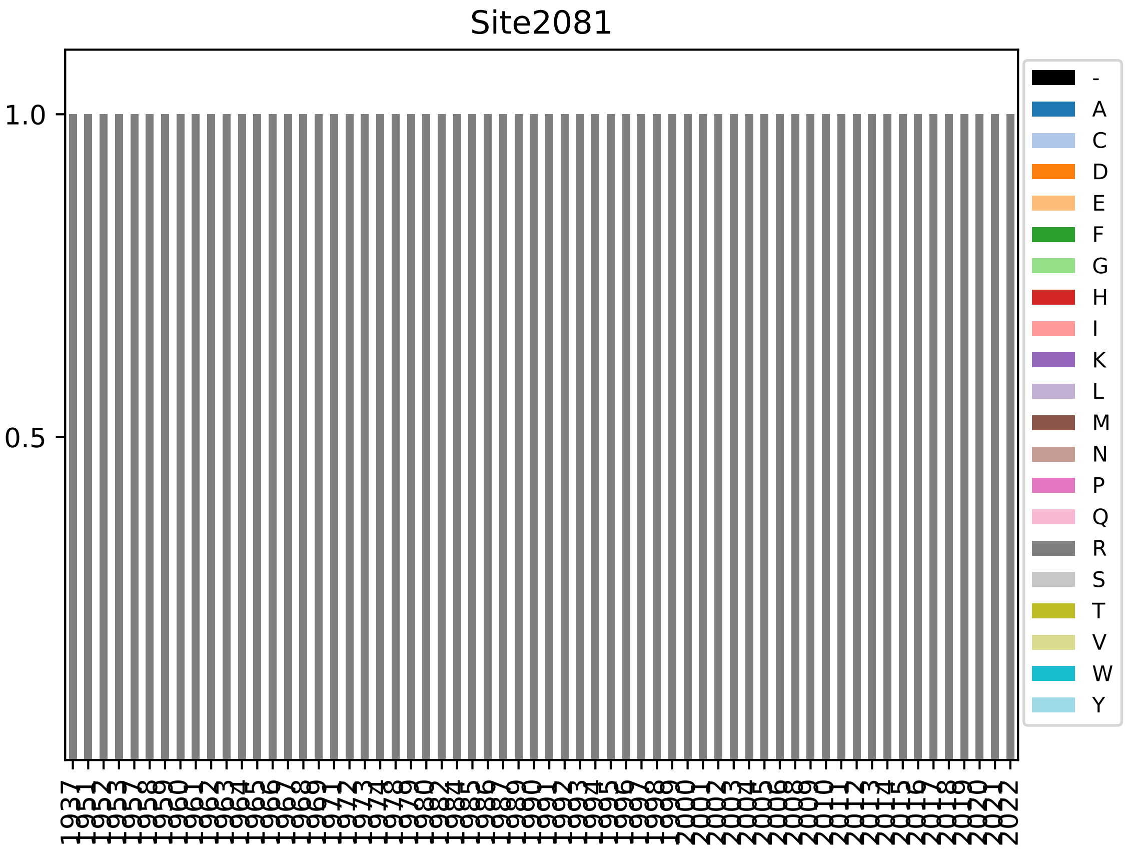
Site2081
1.0
0.5
-
A
C
D
E
F
G
H
I
K
L
M
N
P
Q
R
S
T
V
W
Y

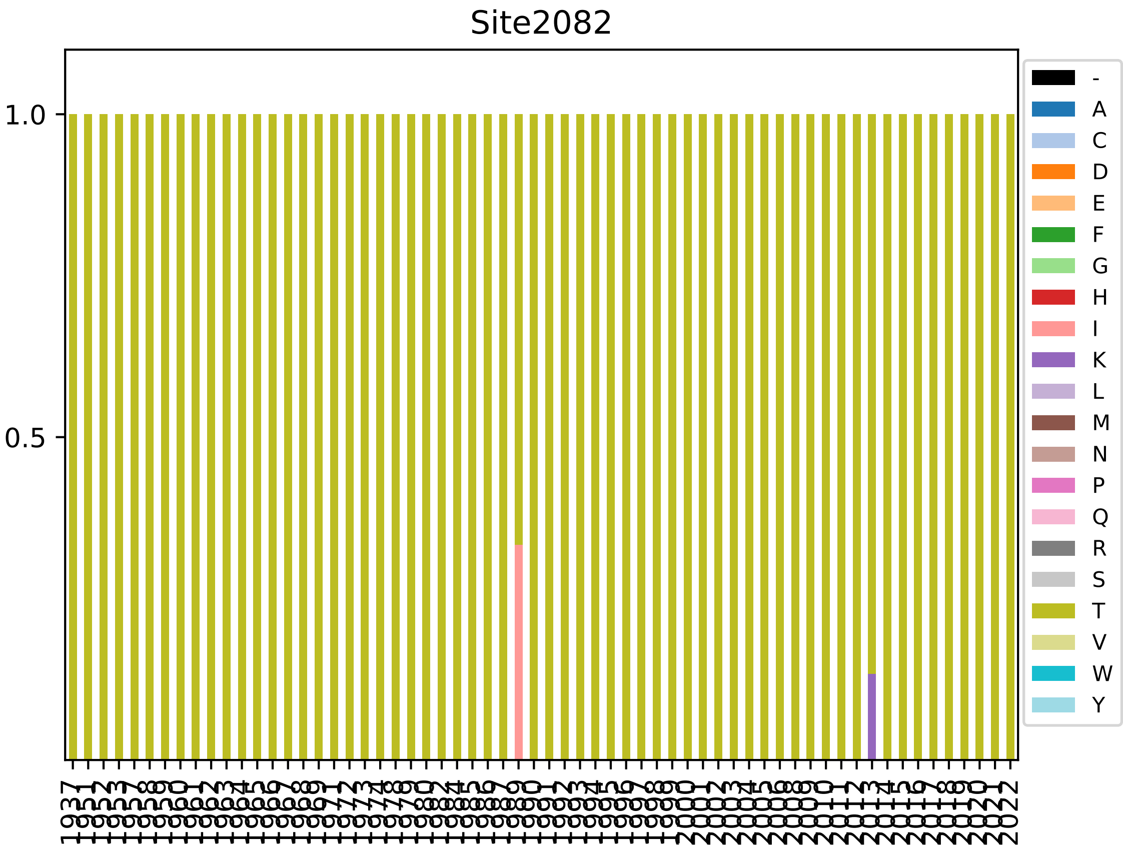

Site2082
1.0
0.5
-
A
C
D
E
F
G
H
I
K
L
M
N
P
Q
R
S
T
V
W
Y

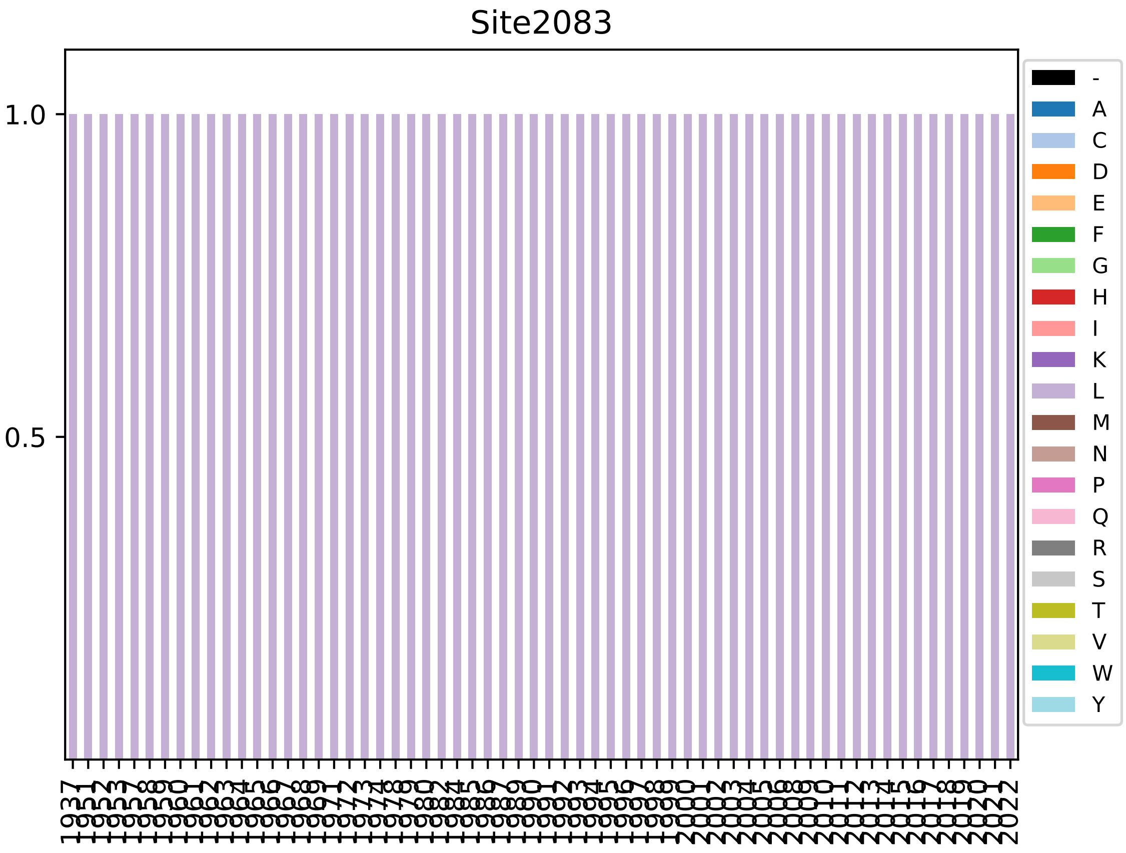

Site2083
1.0
0.5
-
A
C
D
E
F
G
H
I
K
L
M
N
P
Q
R
S
T
V
W
Y

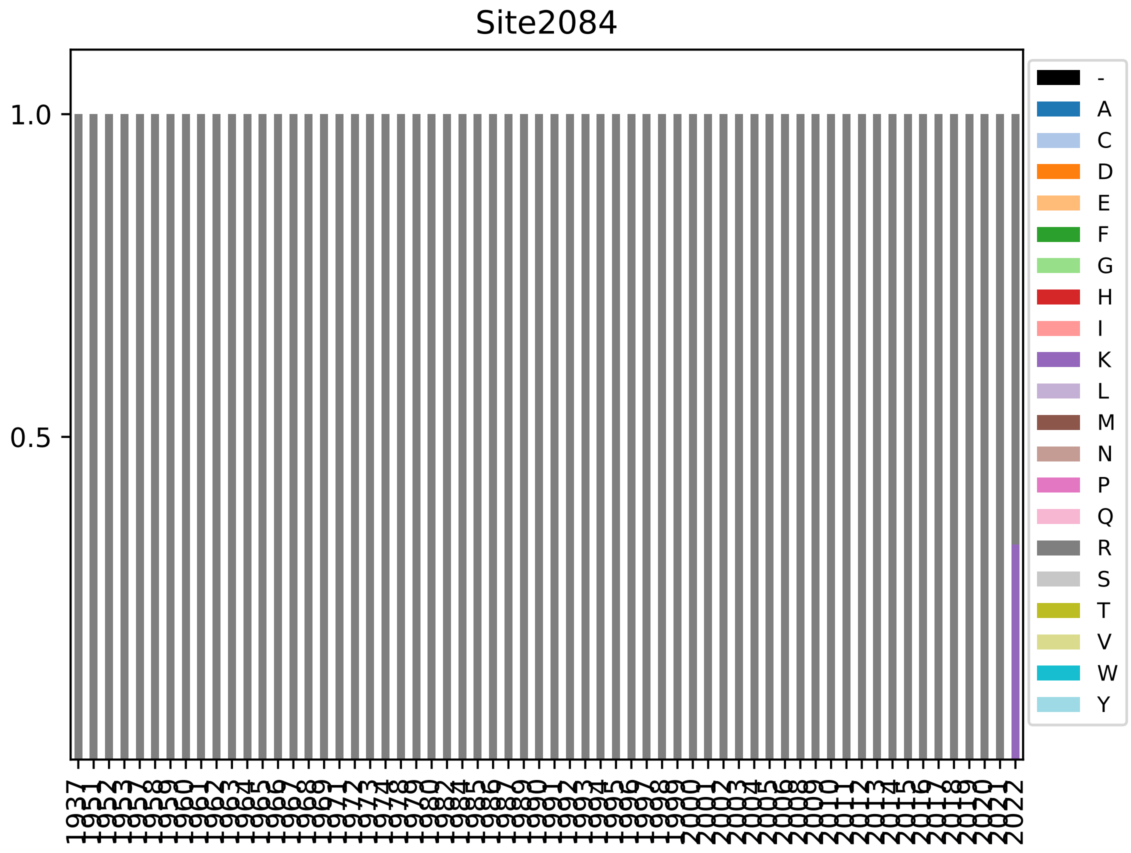

Site2084
1.0
0.5
-
A
C
D
E
F
G
H
I
K
L
M
N
P
Q
R
S
T
V
W
Y

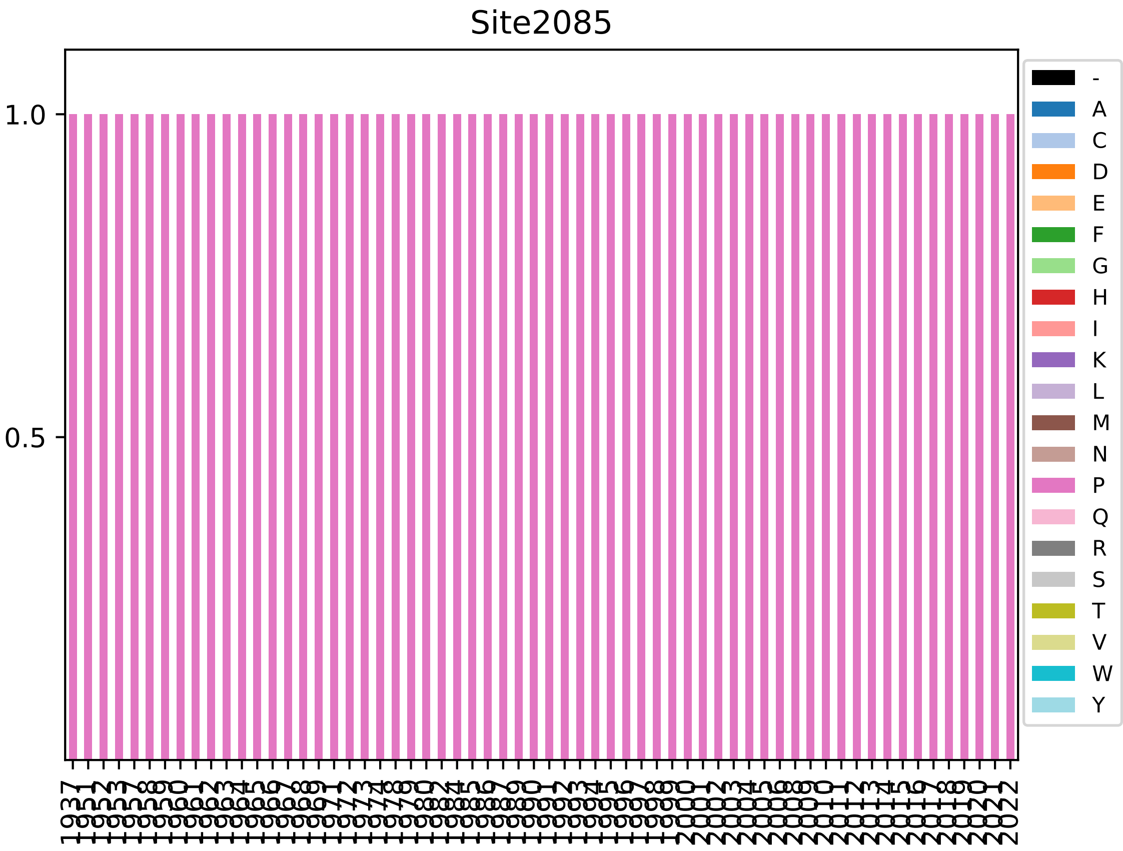
Site2085
1.0
0.5
-
A
C
D
E
F
G
H
I
K
L
M
N
P
Q
R
S
T
V
W
Y

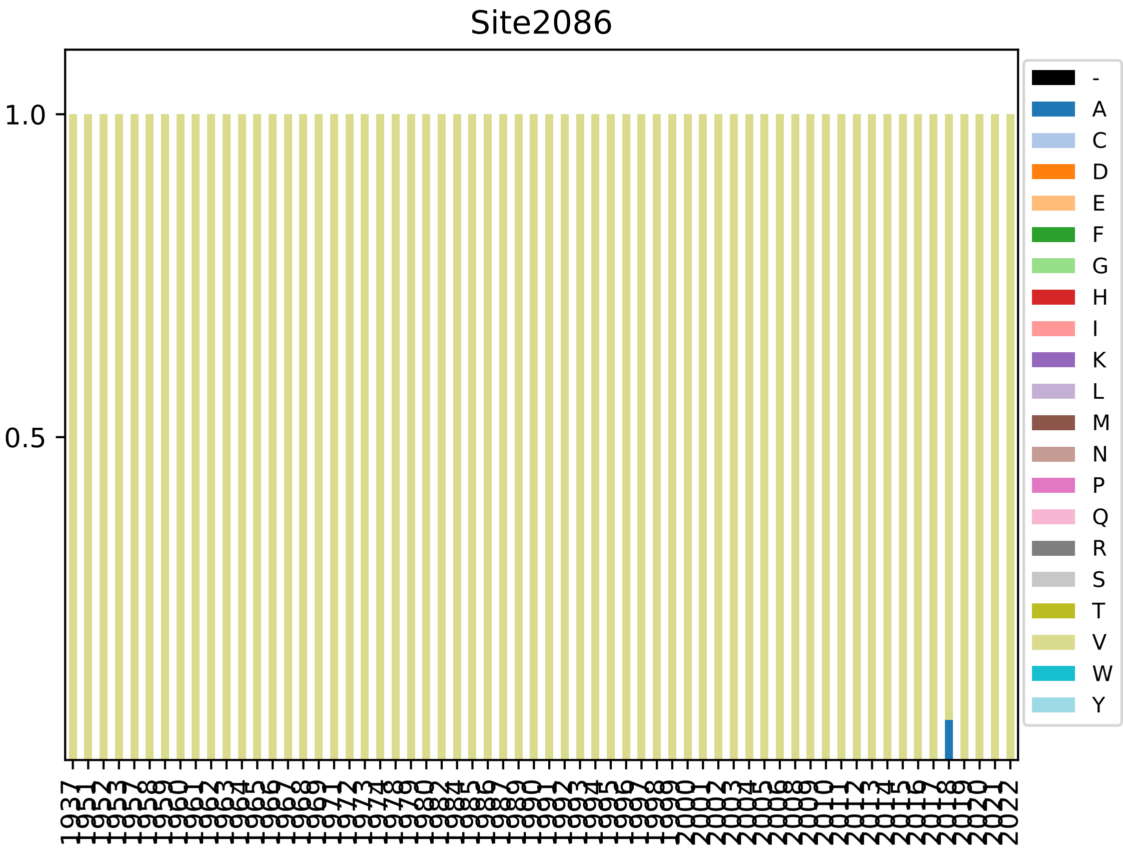
Site2086
1.0
0.5
-
A
C
D
E
F
G
H
I
K
L
M
N
P
Q
R
S
T
V
W
Y

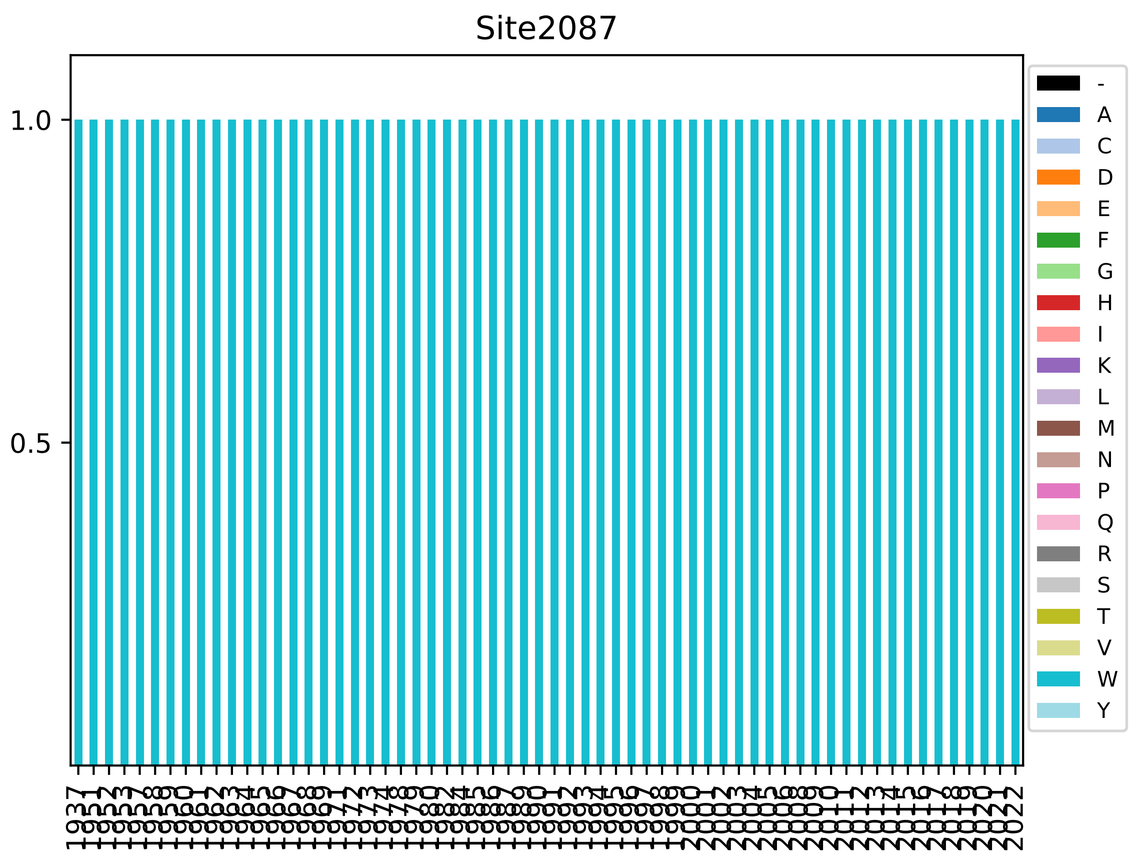
Site2087
1.0
0.5
-
A
C
D
E
F
G
H
I
K
L
M
N
P
Q
R
S
T
V
W
Y

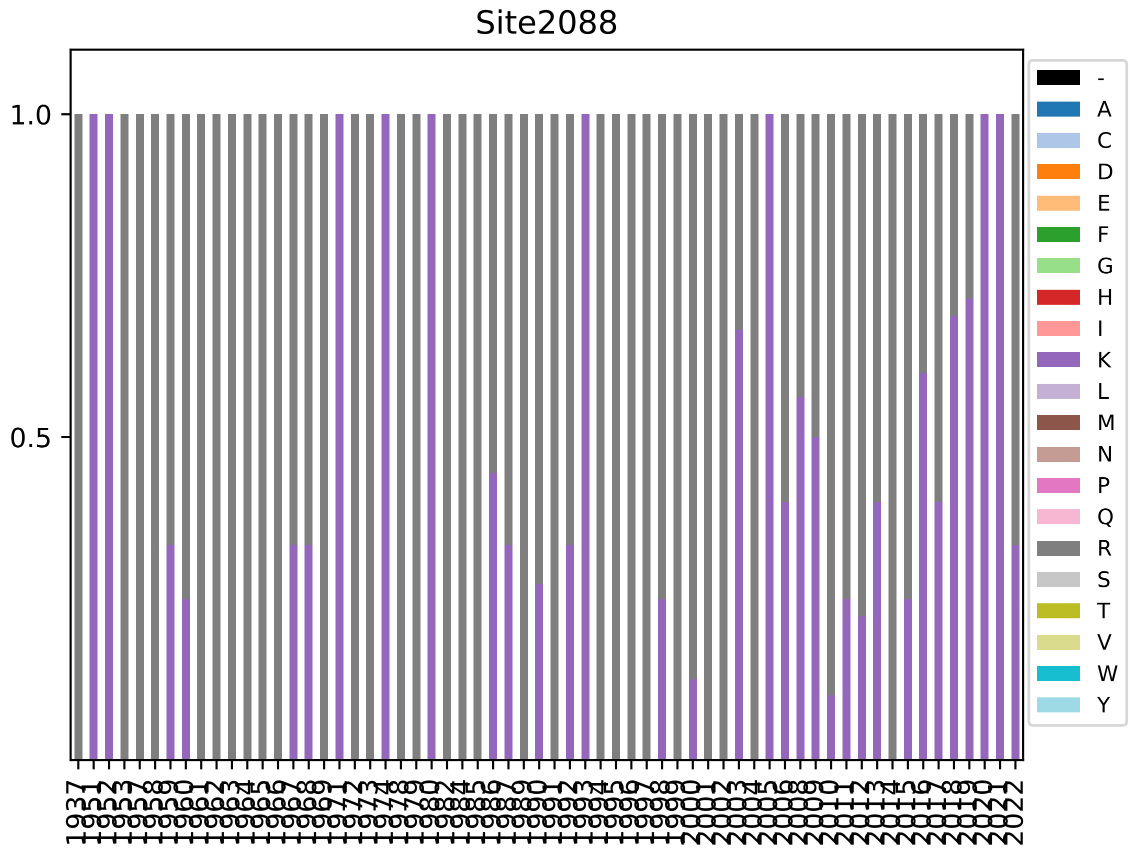
Site2088
1.0
0.5
-
A
C
D
E
F
G
H
I
K
L
M
N
P
Q
R
S
T
V
W
Y

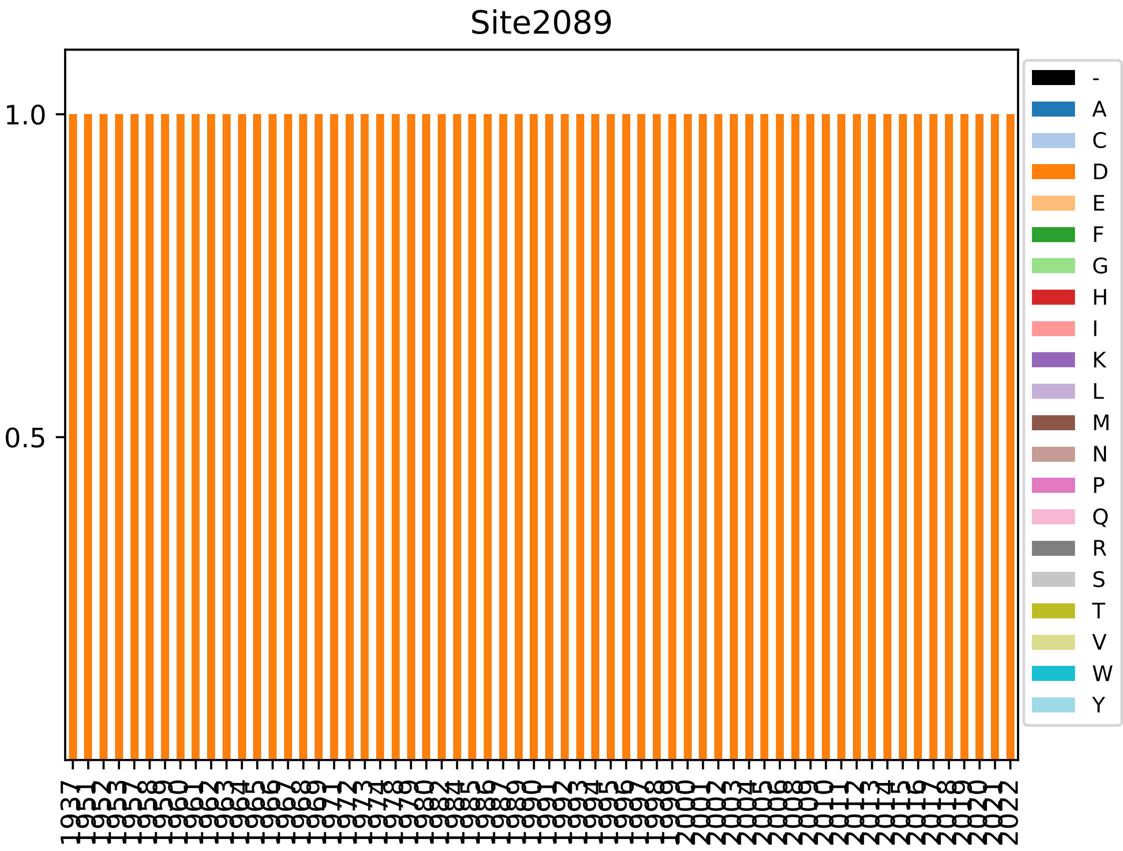
Site2089
1.0
0.5
-
A
C
D
E
F
G
H
I
K
L
M
N
P
Q
R
S
T
V
W
Y

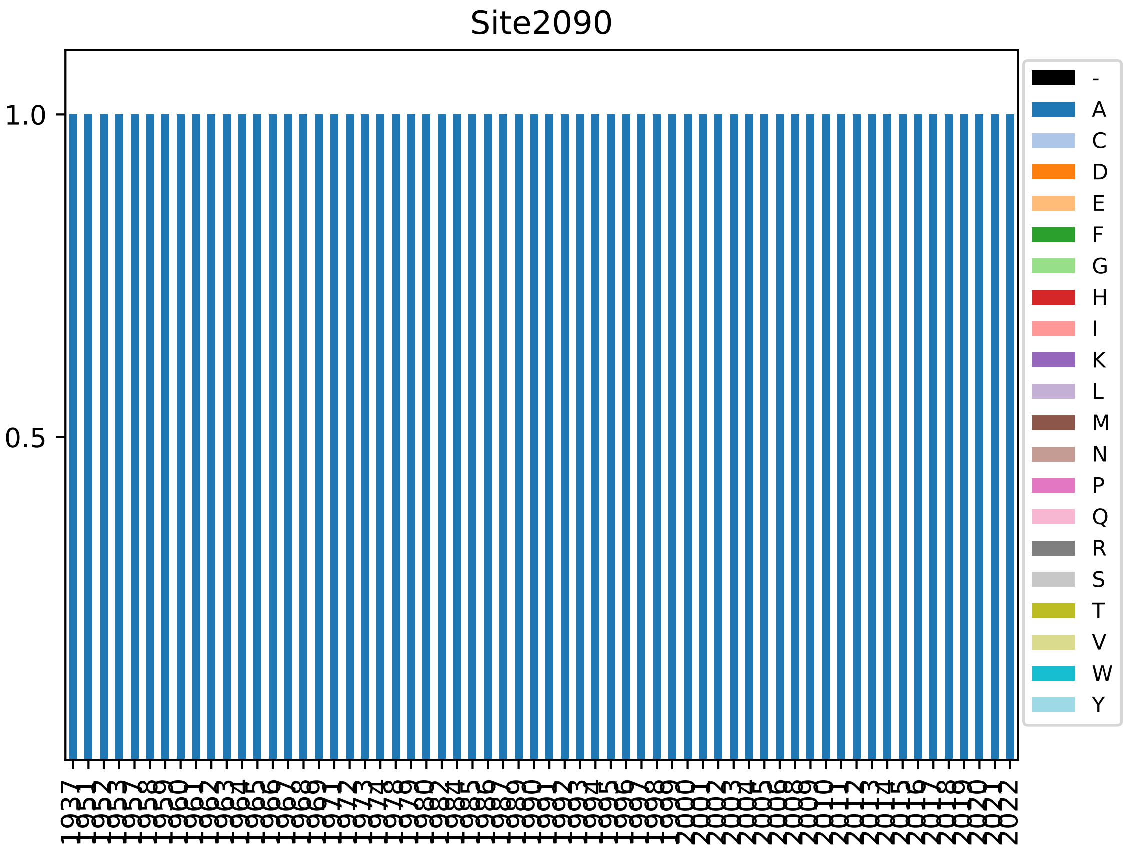
Site2090
1.0
0.5
-
A
C
D
E
F
G
H
I
K
L
M
N
P
Q
R
S
T
V
W
Y

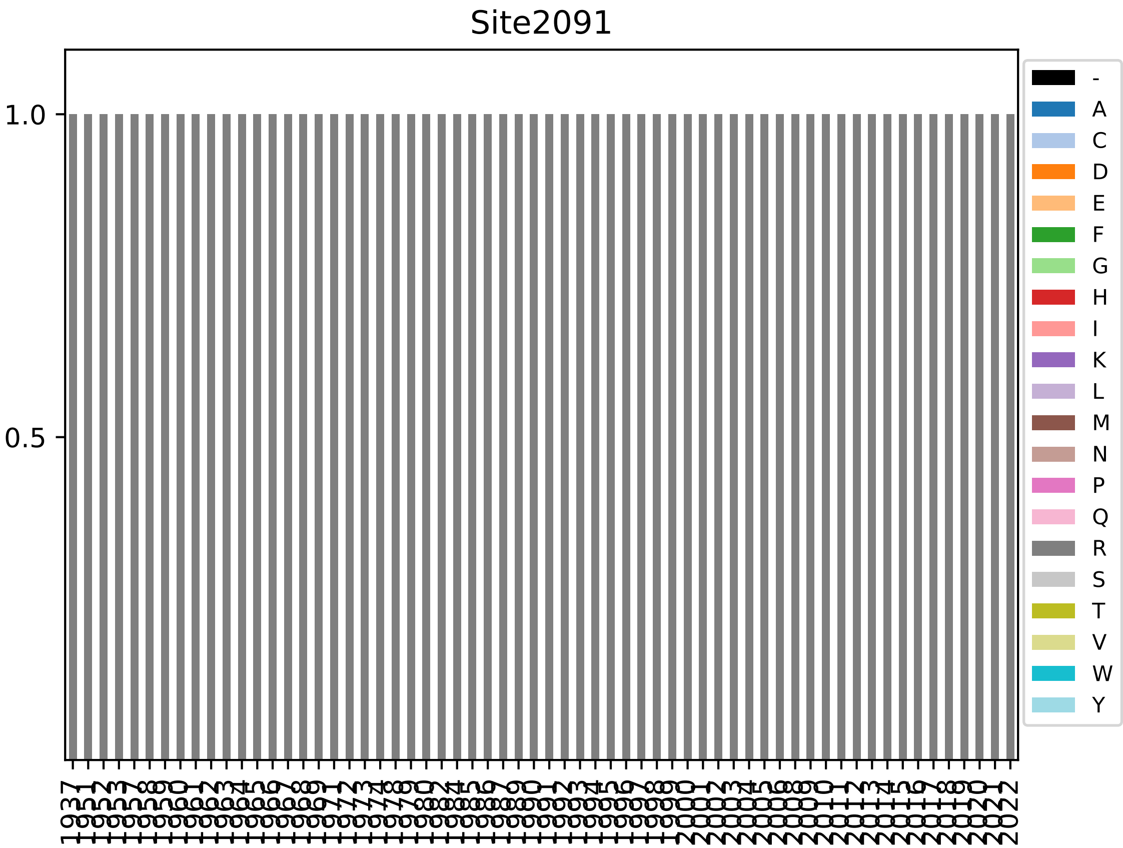
Site2091
1.0
0.5
-
A
C
D
E
F
G
H
I
K
L
M
N
P
Q
R
S
T
V
W
Y

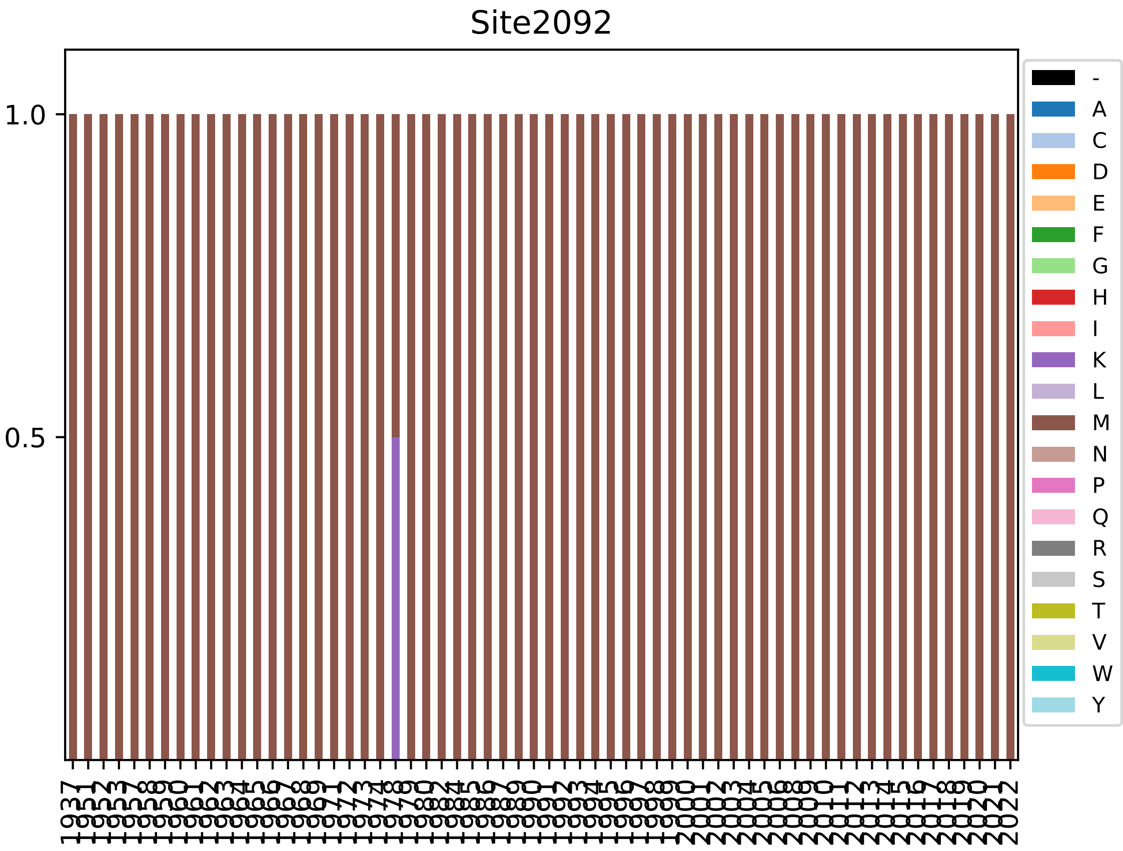

Site2092
1.0
0.5
-
A
C
D
E
F
G
H
I
K
L
M
N
P
Q
R
S
T
V
W
Y

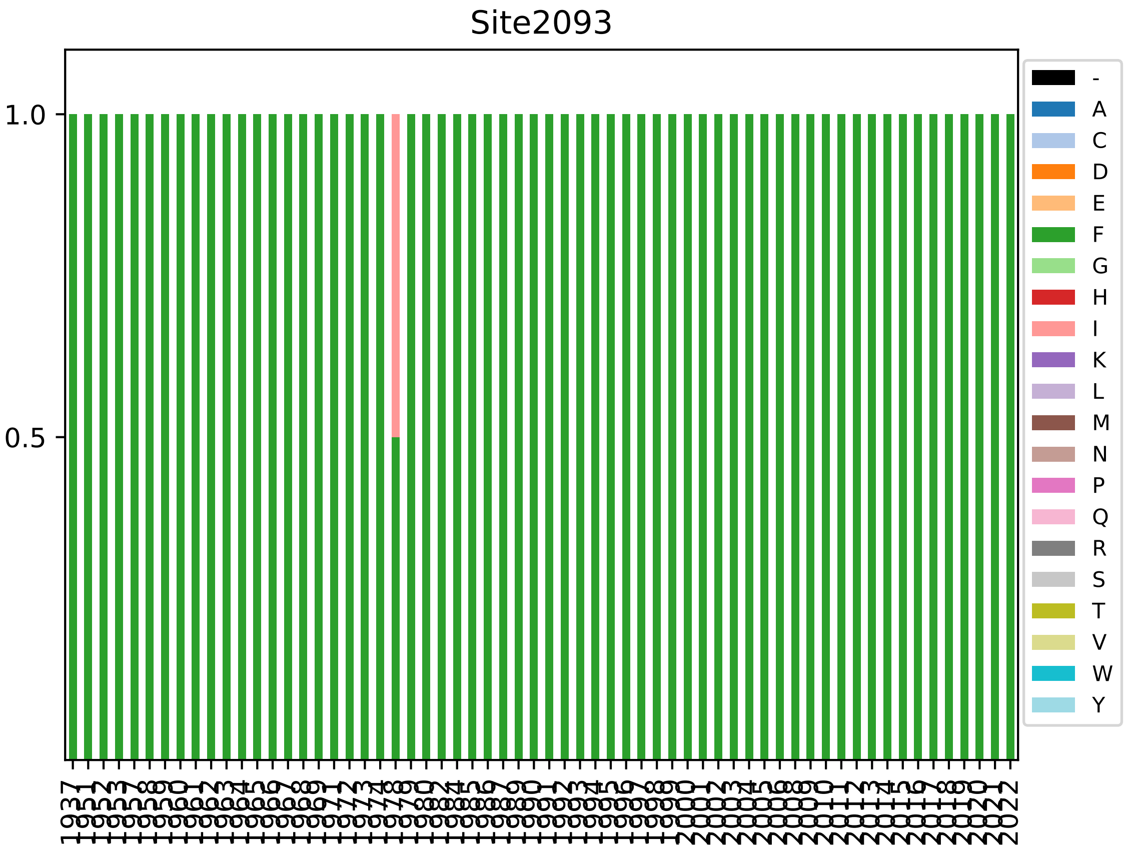
Site2093
1.0
0.5
-
A
C
D
E
F
G
H
I
K
L
M
N
P
Q
R
S
T
V
W
Y

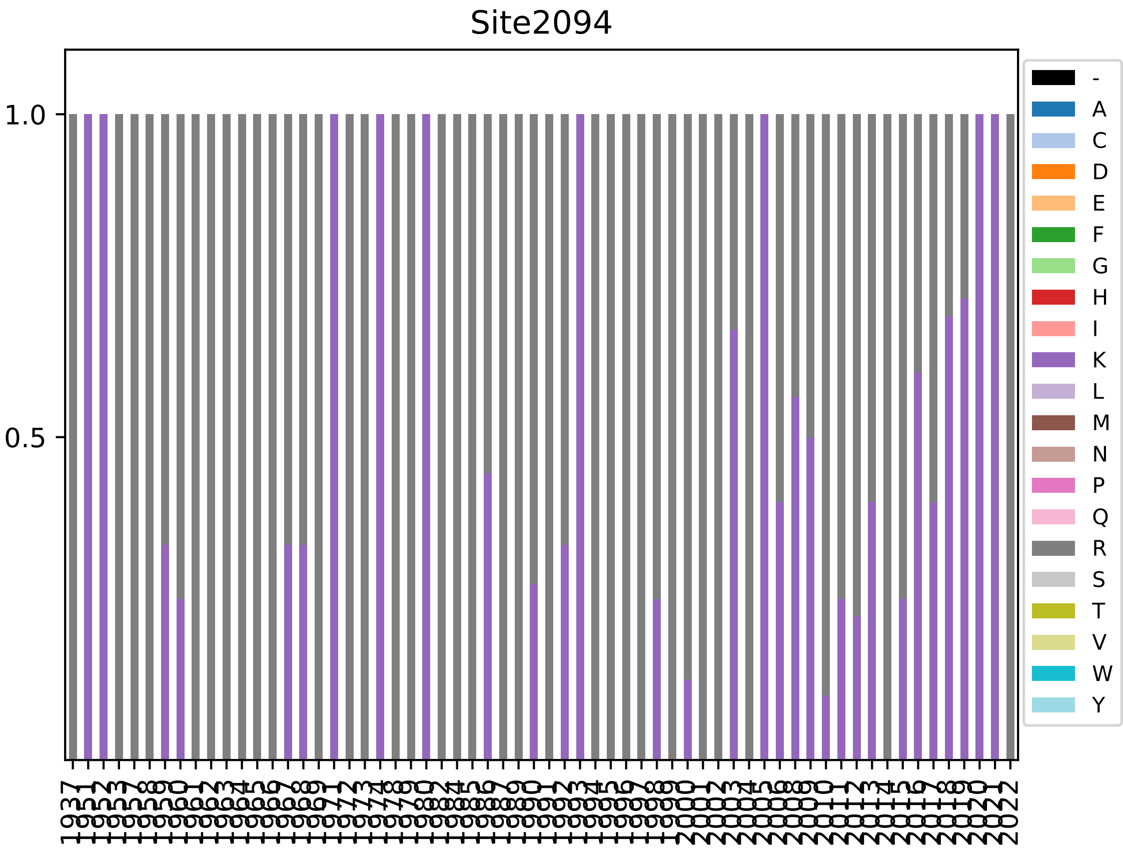
Site2094
1.0
0.5
-
A
C
D
E
F
G
H
I
K
L
M
N
P
Q
R
S
T
V
W
Y

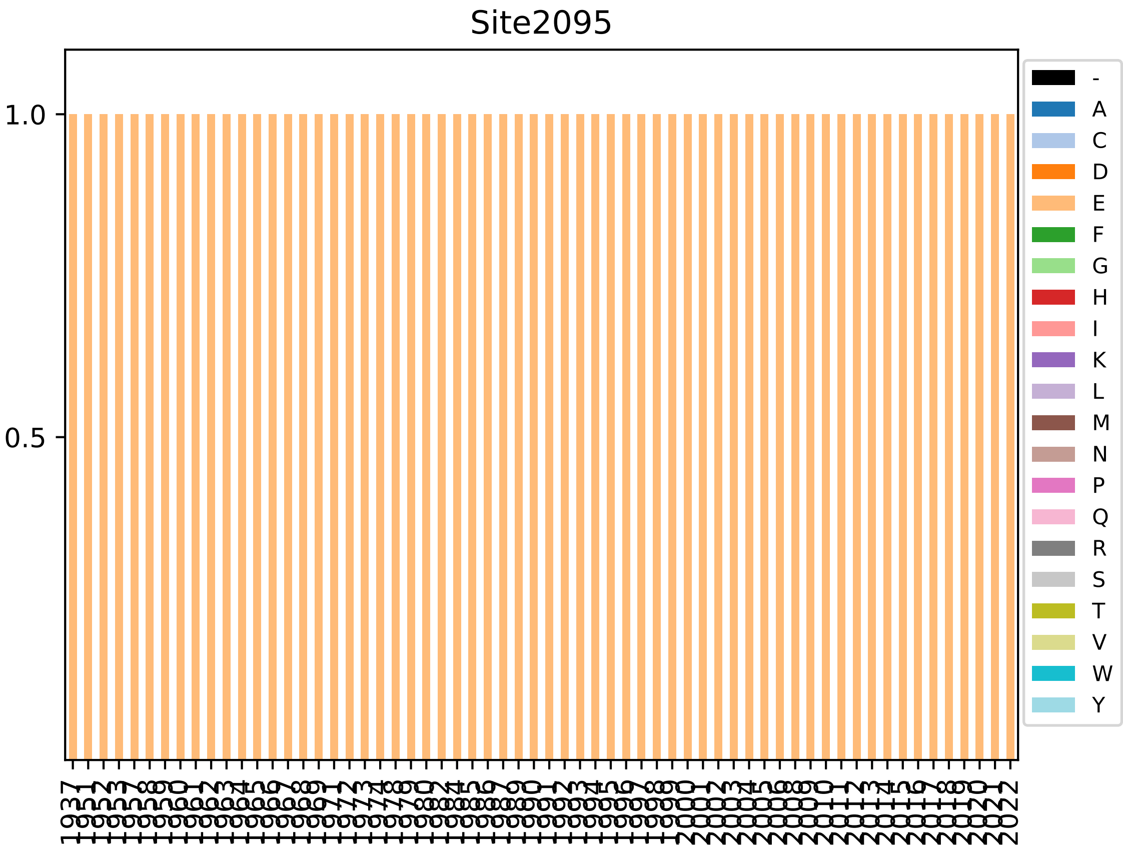
Site2095
1.0
0.5
-
A
C
D
E
F
G
H
I
K
L
M
N
P
Q
R
S
T
V
W
Y

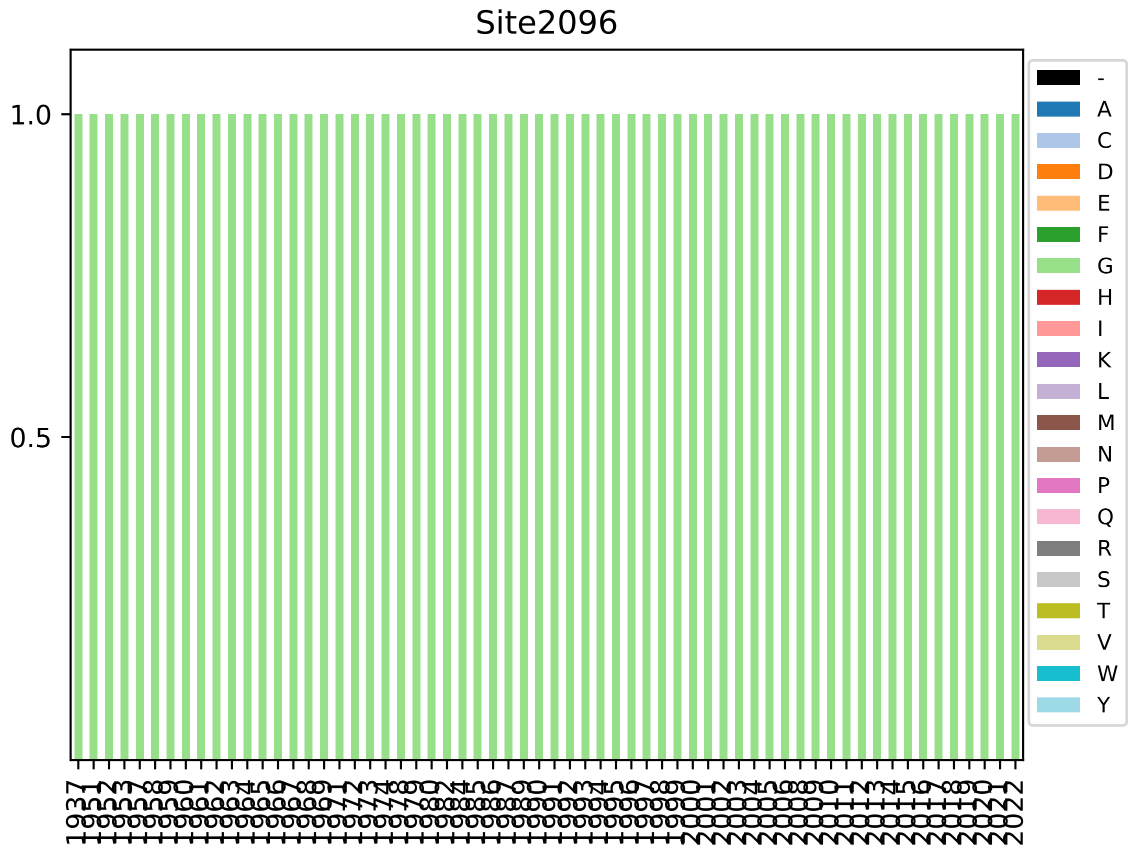

Site2096
1.0
0.5
-
A
C
D
E
F
G
H
I
K
L
M
N
P
Q
R
S
T
V
W
Y

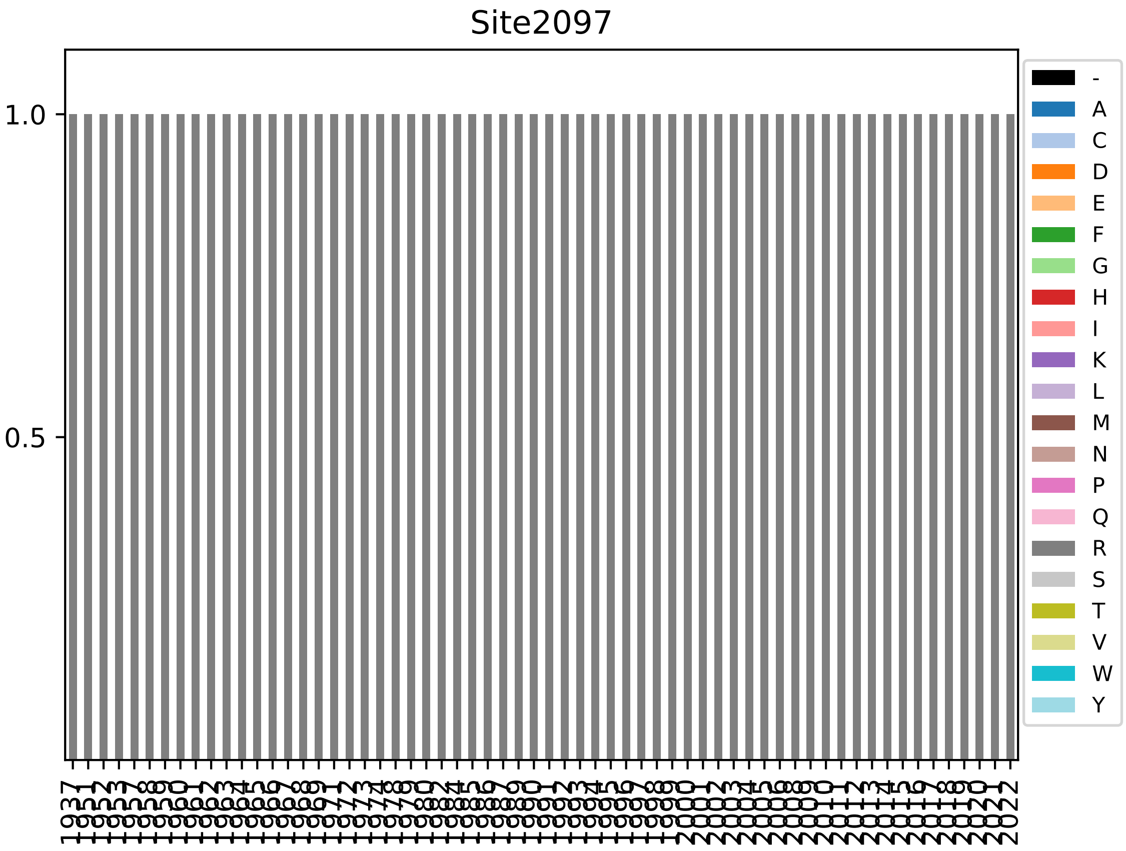
Site2097
1.0
0.5
-
A
C
D
E
F
G
H
I
K
L
M
N
P
Q
R
S
T
V
W
Y

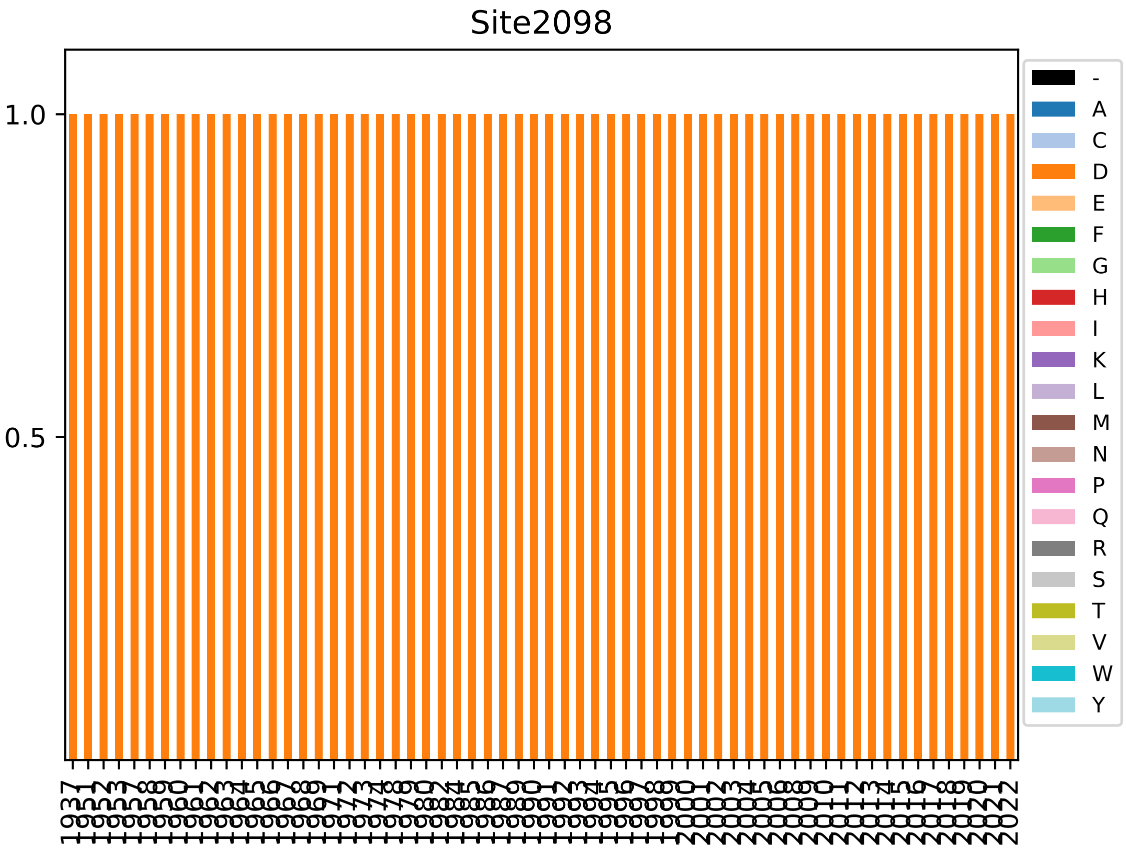
Site2098
1.0
0.5
-
A
C
D
E
F
G
H
I
K
L
M
N
P
Q
R
S
T
V
W
Y

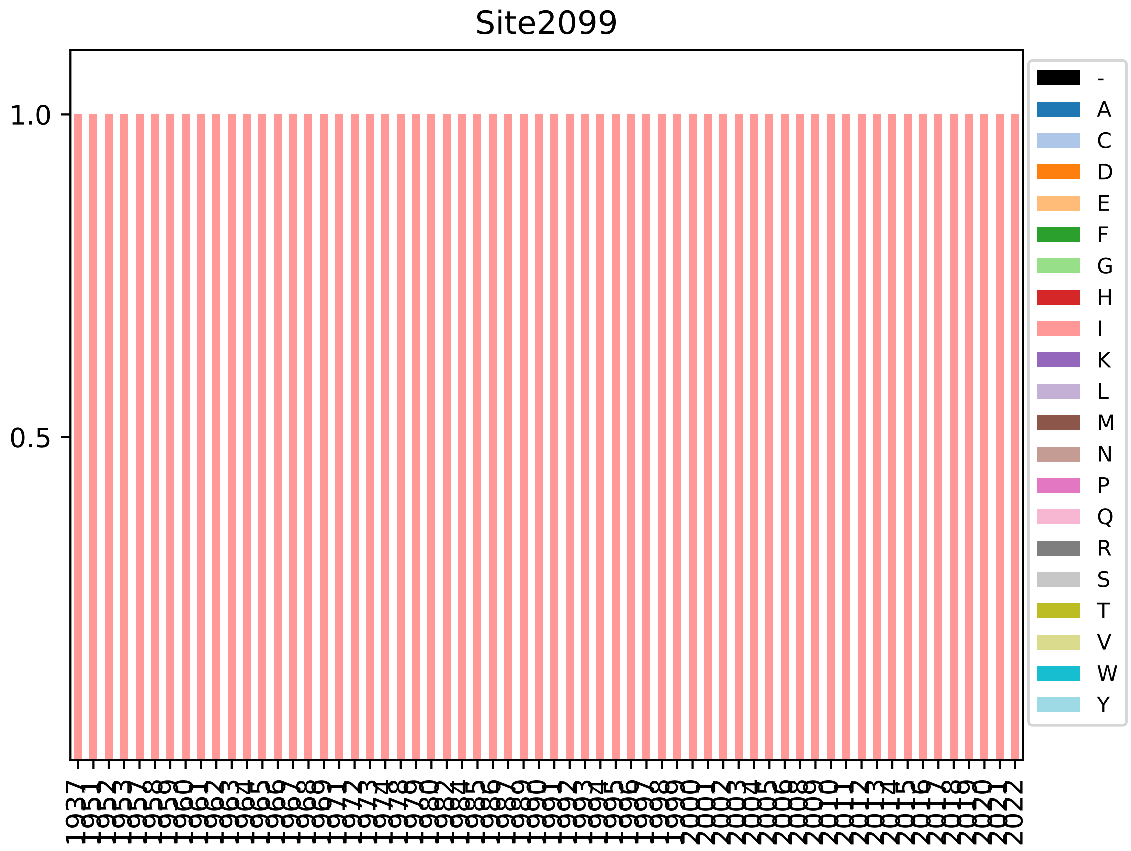
Site2099
1.0
0.5
-
A
C
D
E
F
G
H
I
K
L
M
N
P
Q
R
S
T
V
W
Y

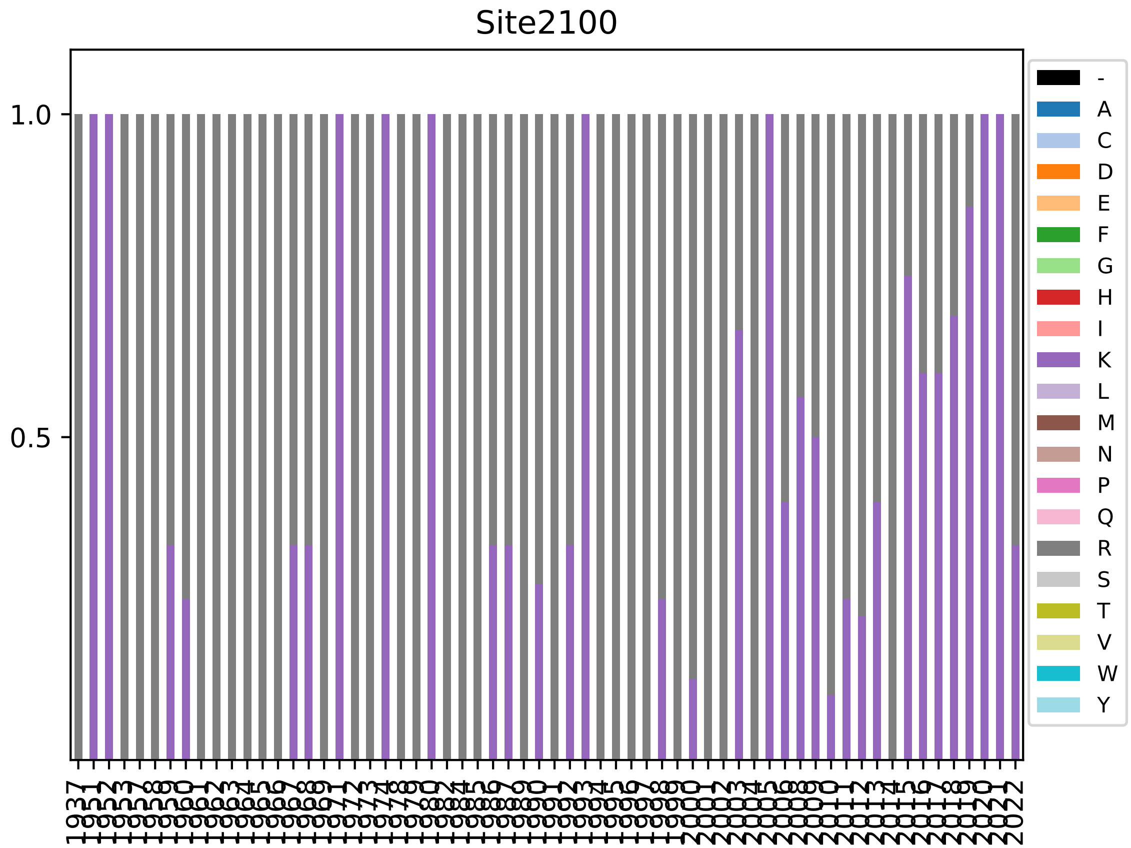
Site2100
1.0
0.5
-
A
C
D
E
F
G
H
I
K
L
M
N
P
Q
R
S
T
V
W
Y

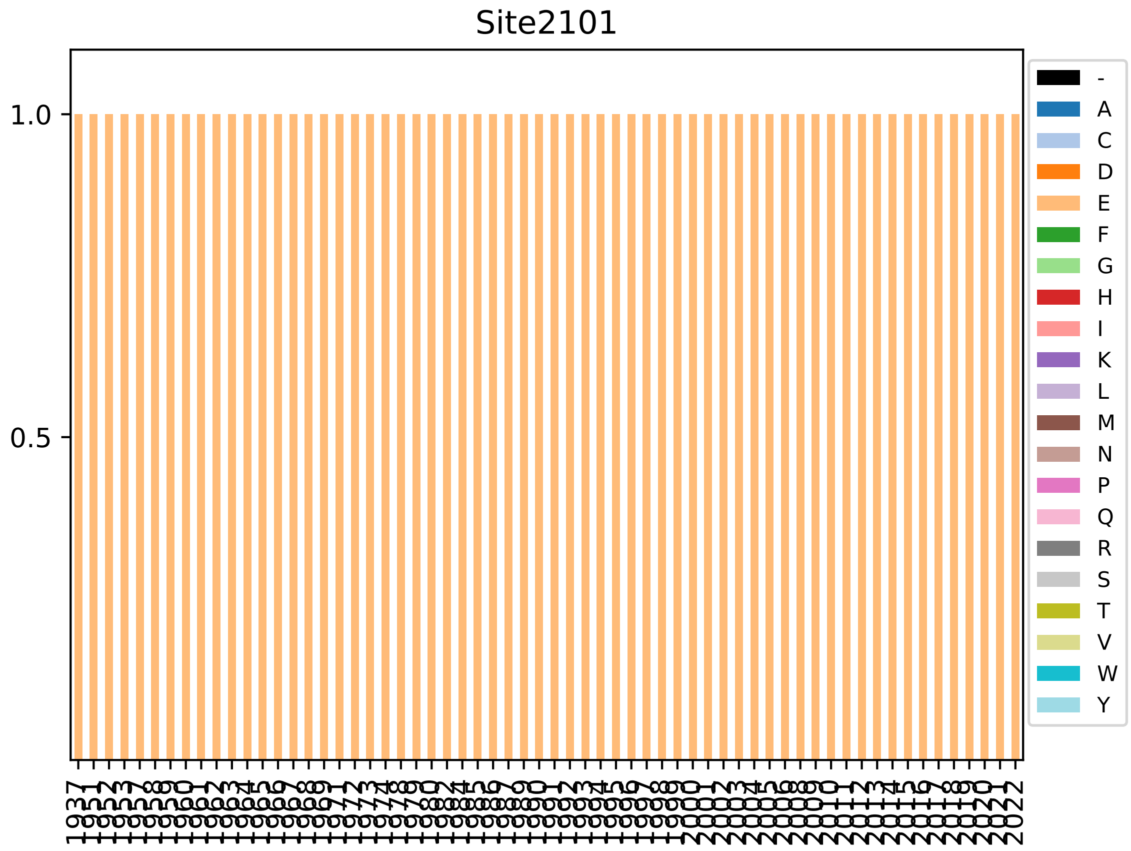
Site2101
1.0
0.5
-
A
C
D
E
F
G
H
I
K
L
M
N
P
Q
R
S
T
V
W
Y

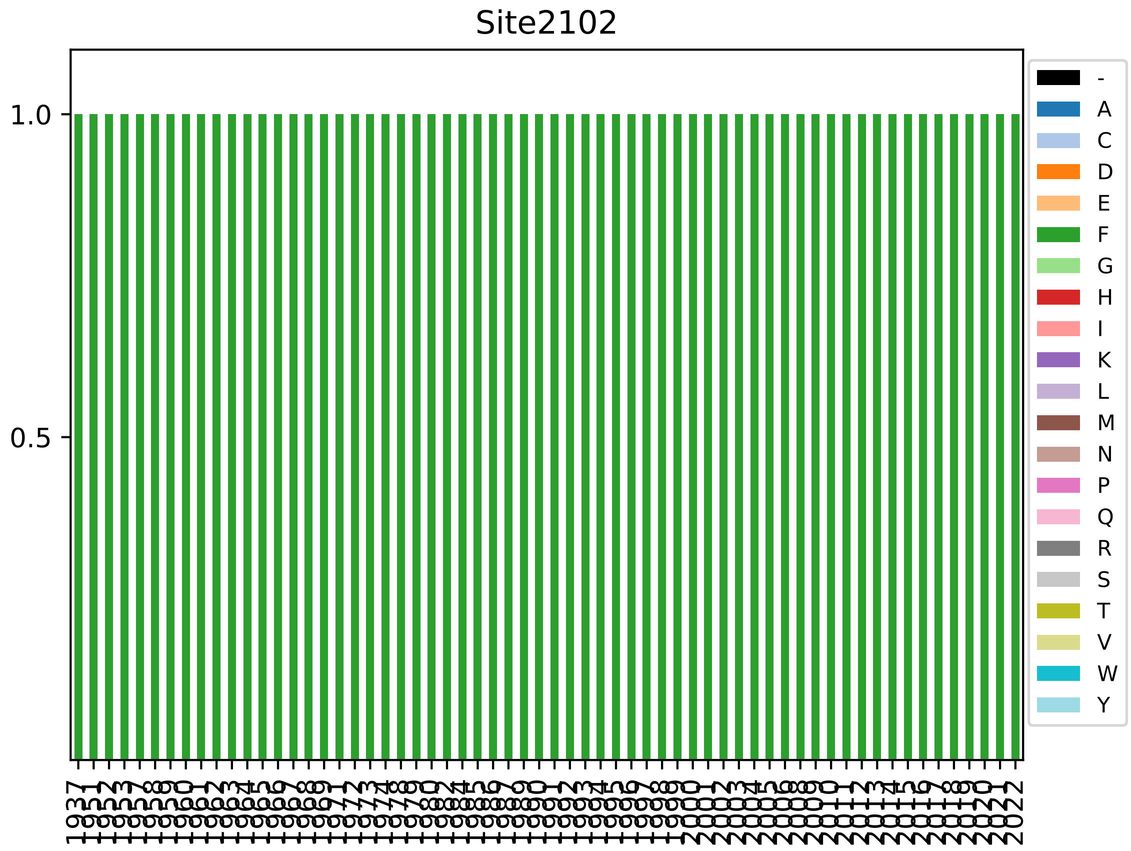
Site2102
1.0
0.5
-
A
C
D
E
F
G
H
I
K
L
M
N
P
Q
R
S
T
V
W
Y

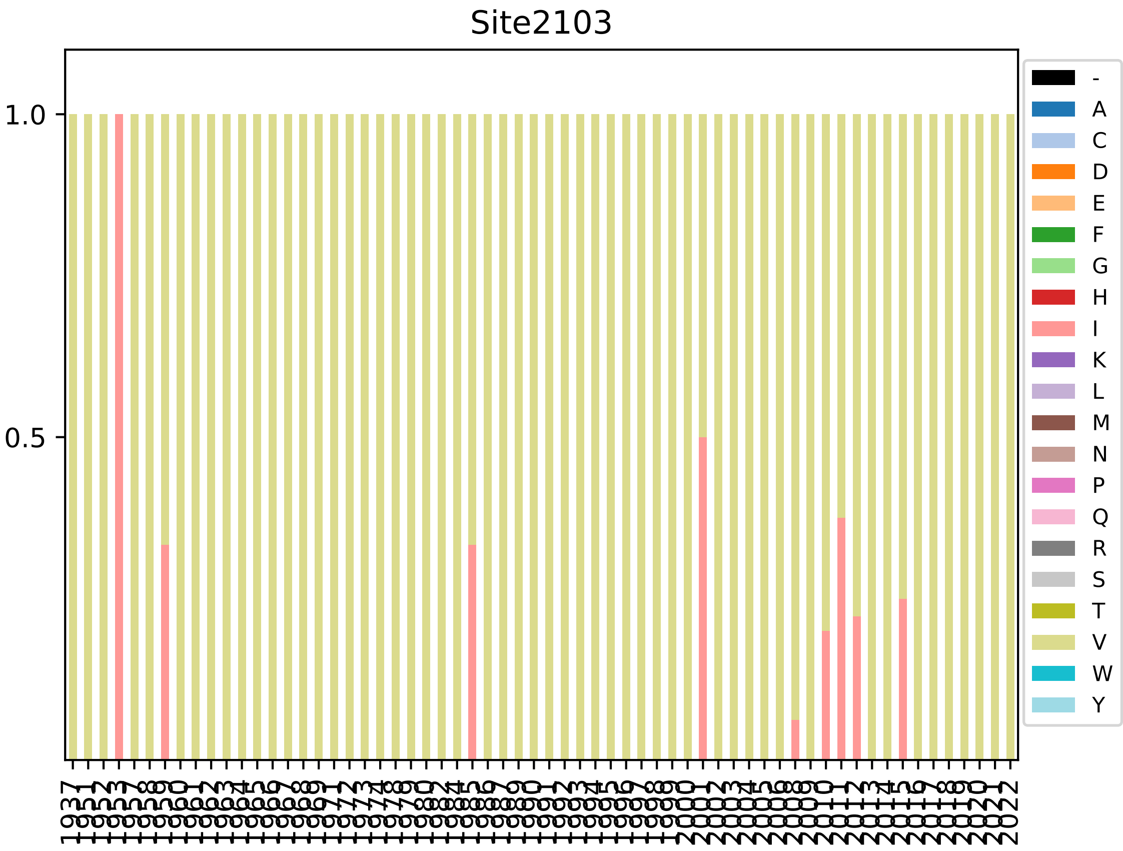
Site2103
1.0
0.5
-
A
C
D
E
F
G
H
I
K
L
M
N
P
Q
R
S
T
V
W
Y

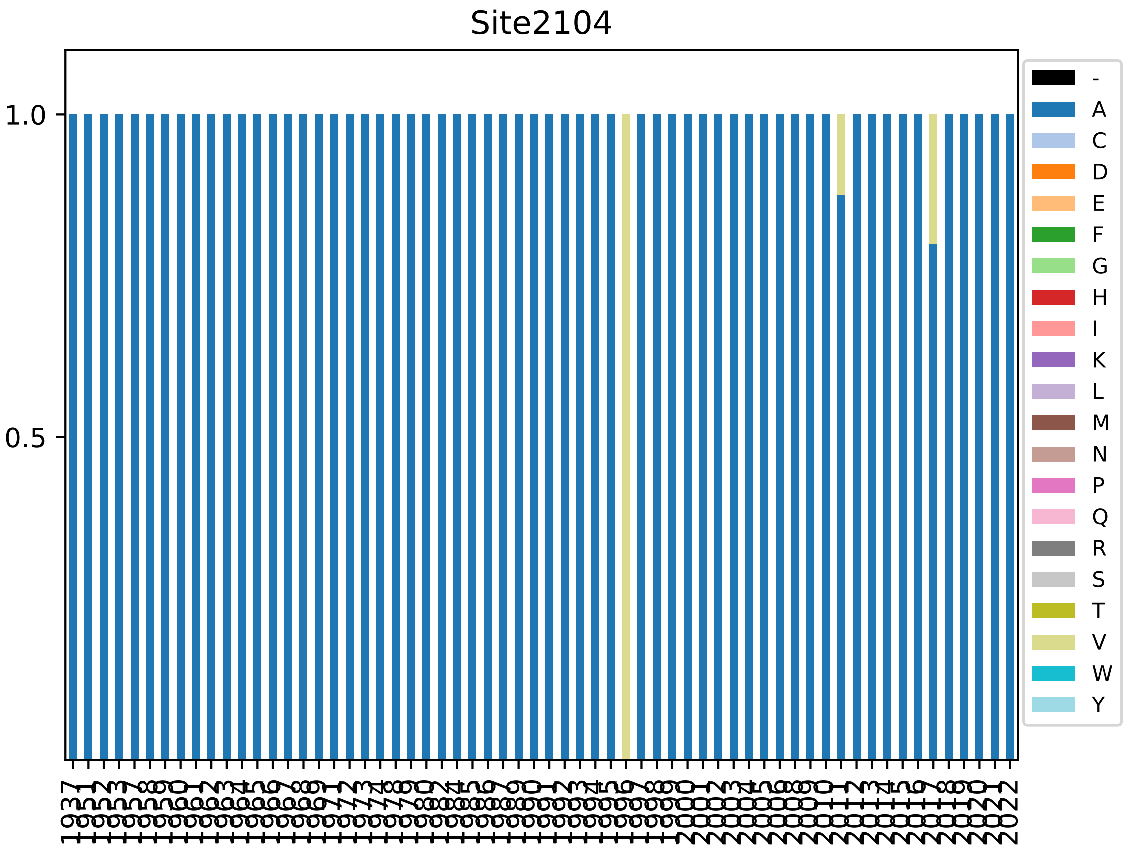
Site2104
1.0
0.5
-
A
C
D
E
F
G
H
I
K
L
M
N
P
Q
R
S
T
V
W
Y

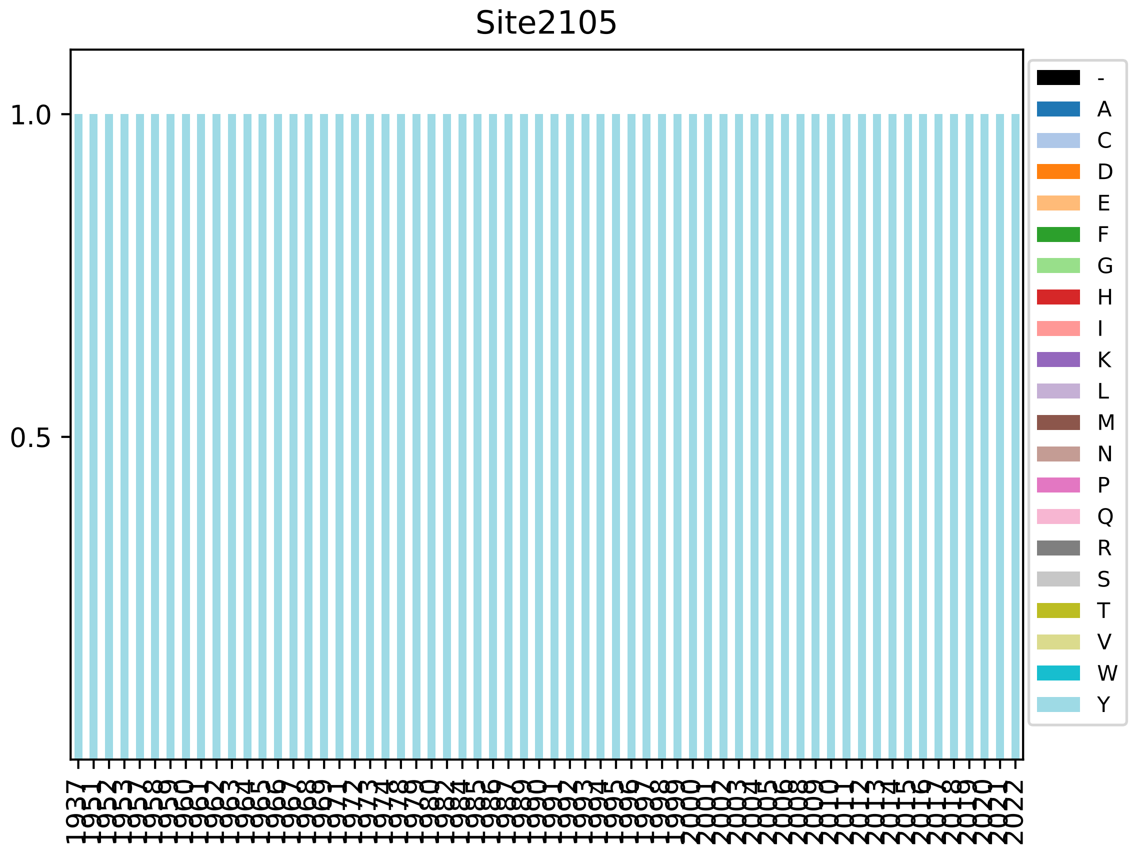
Site2105
1.0
0.5
-
A
C
D
E
F
G
H
I
K
L
M
N
P
Q
R
S
T
V
W
Y

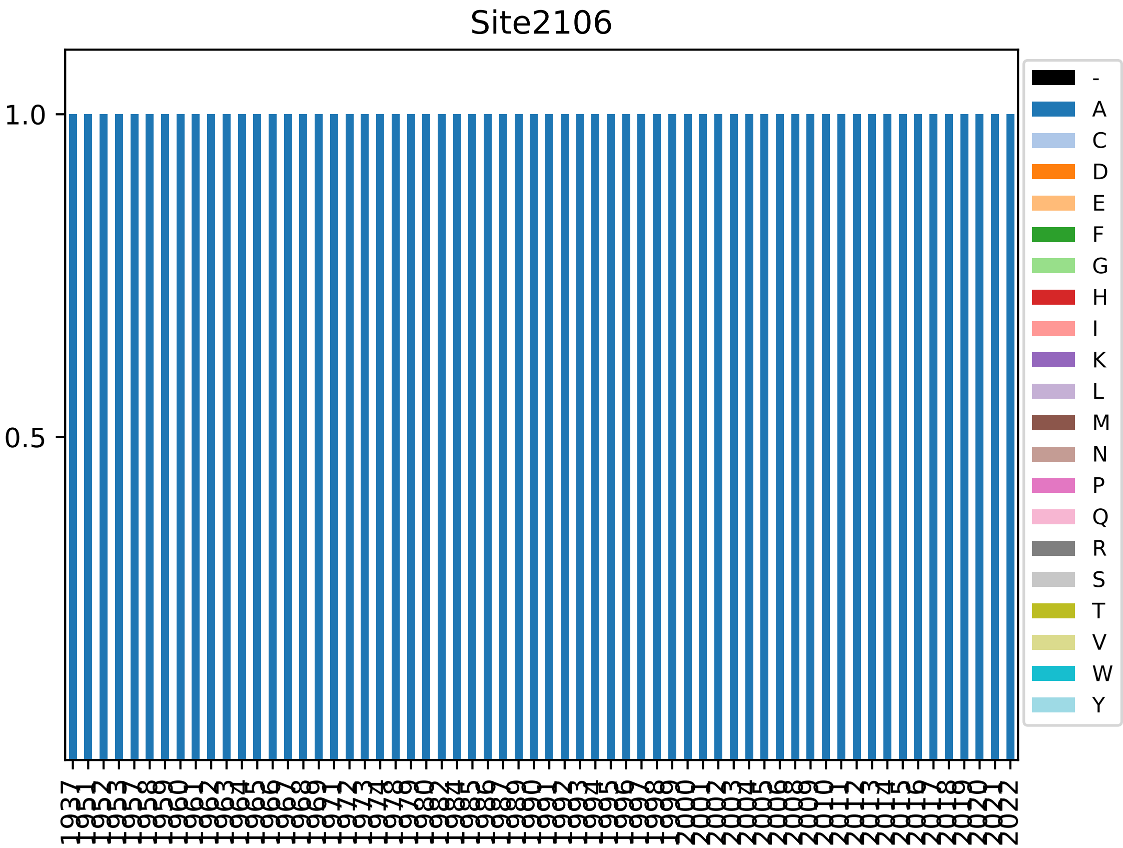

Site2106
1.0
0.5
-
A
C
D
E
F
G
H
I
K
L
M
N
P
Q
R
S
T
V
W
Y

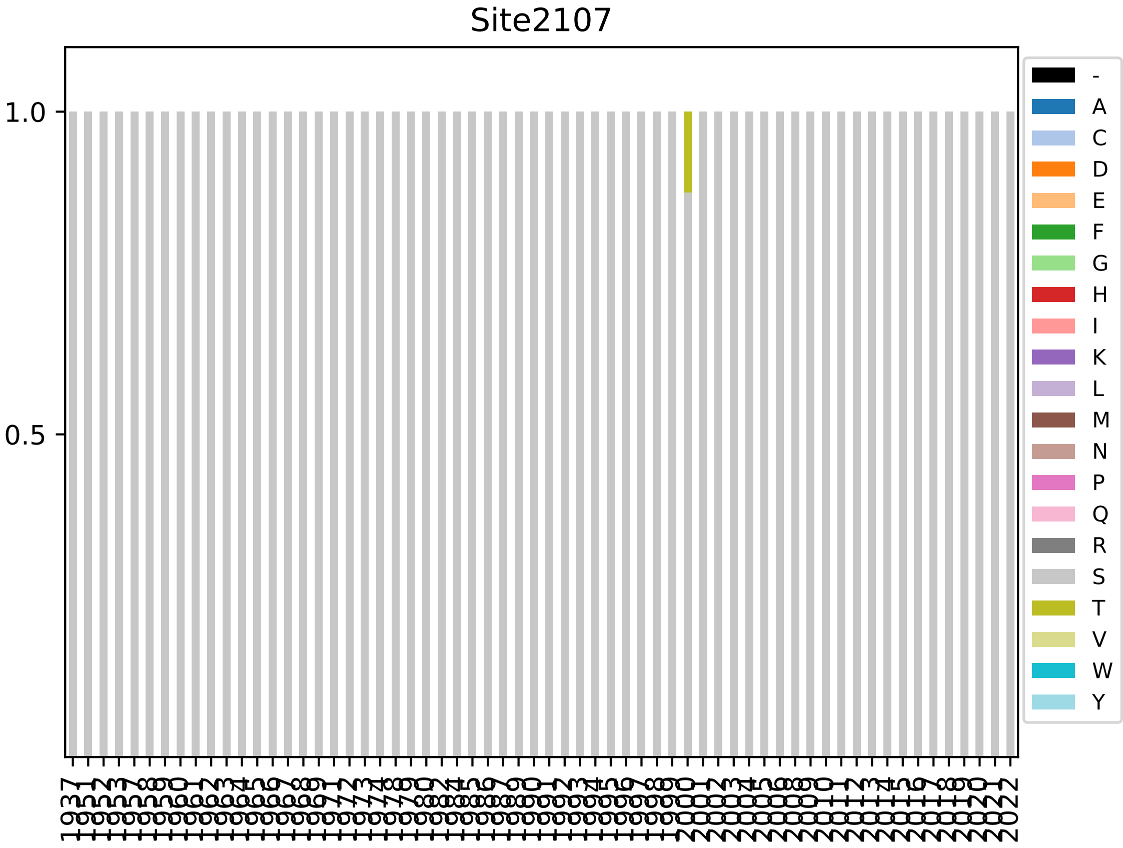
Site2107
1.0
0.5
-
A
C
D
E
F
G
H
I
K
L
M
N
P
Q
R
S
T
V
W
Y

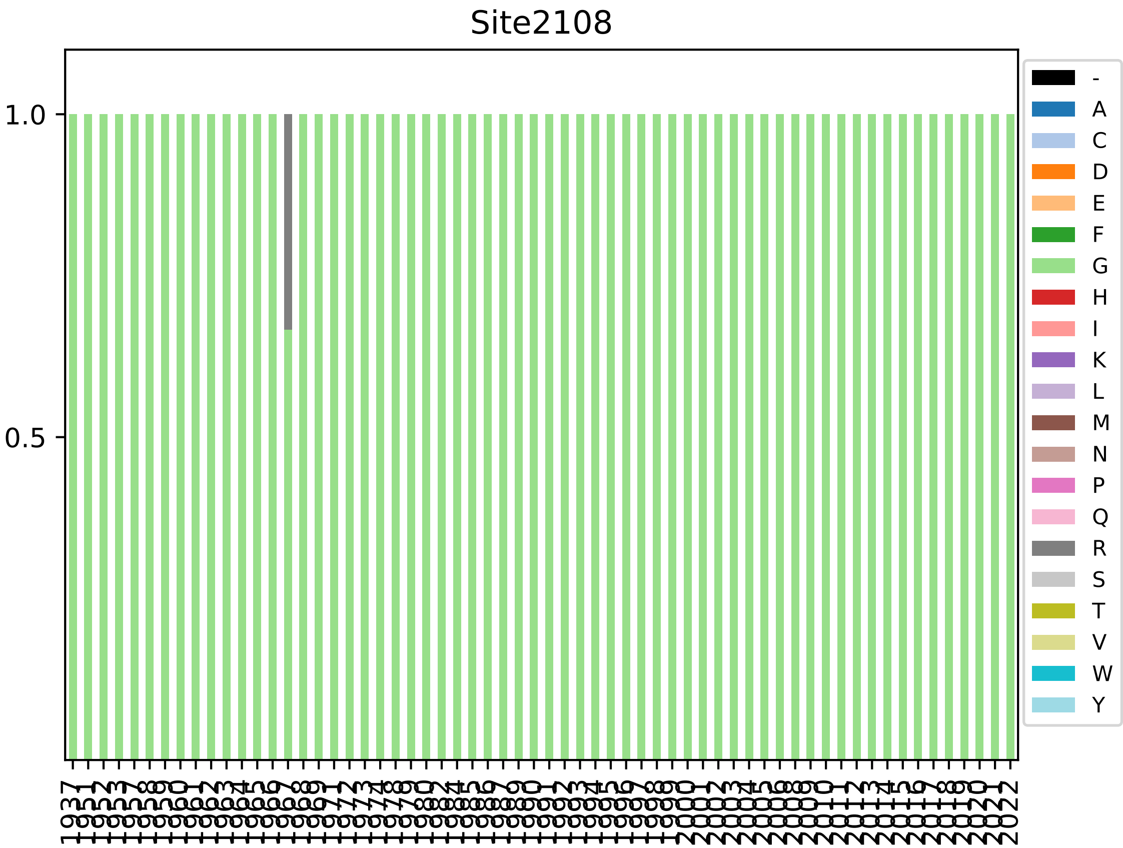
Site2108
1.0
0.5
-
A
C
D
E
F
G
H
I
K
L
M
N
P
Q
R
S
T
V
W
Y

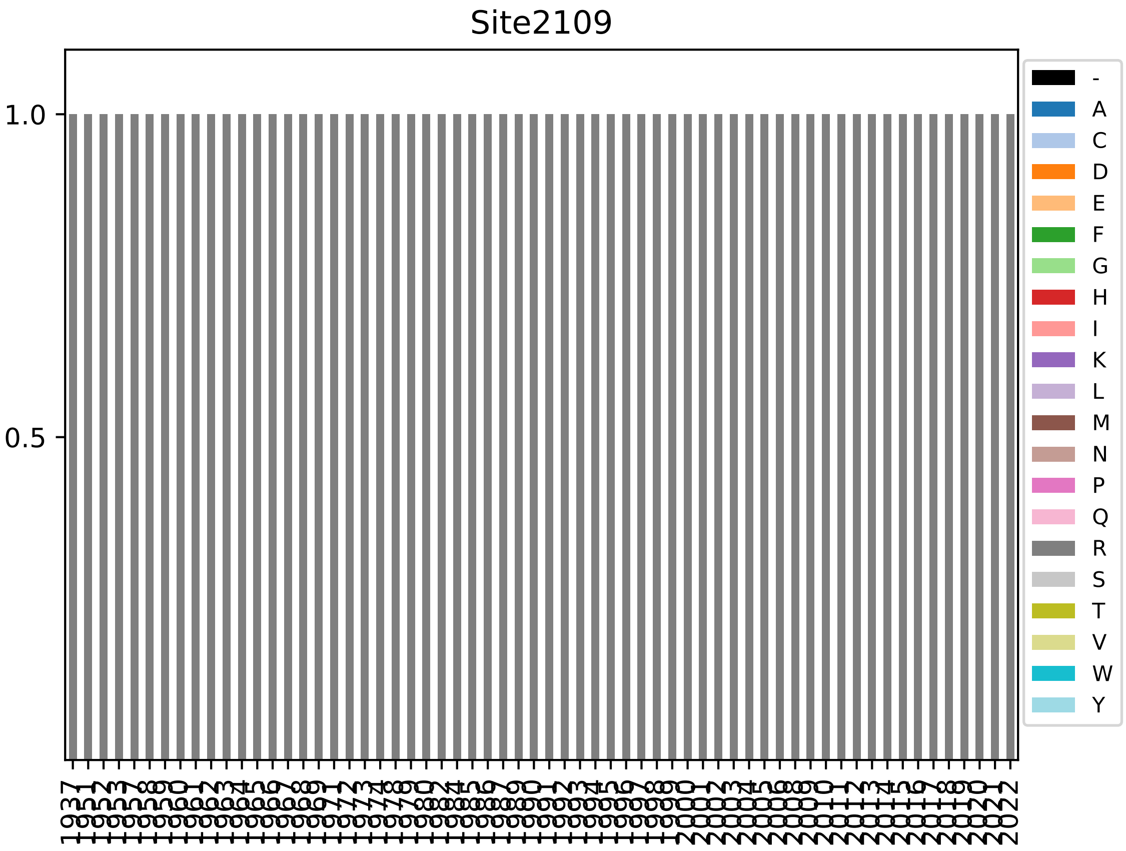
Site2109
1.0
0.5
-
A
C
D
E
F
G
H
I
K
L
M
N
P
Q
R
S
T
V
W
Y

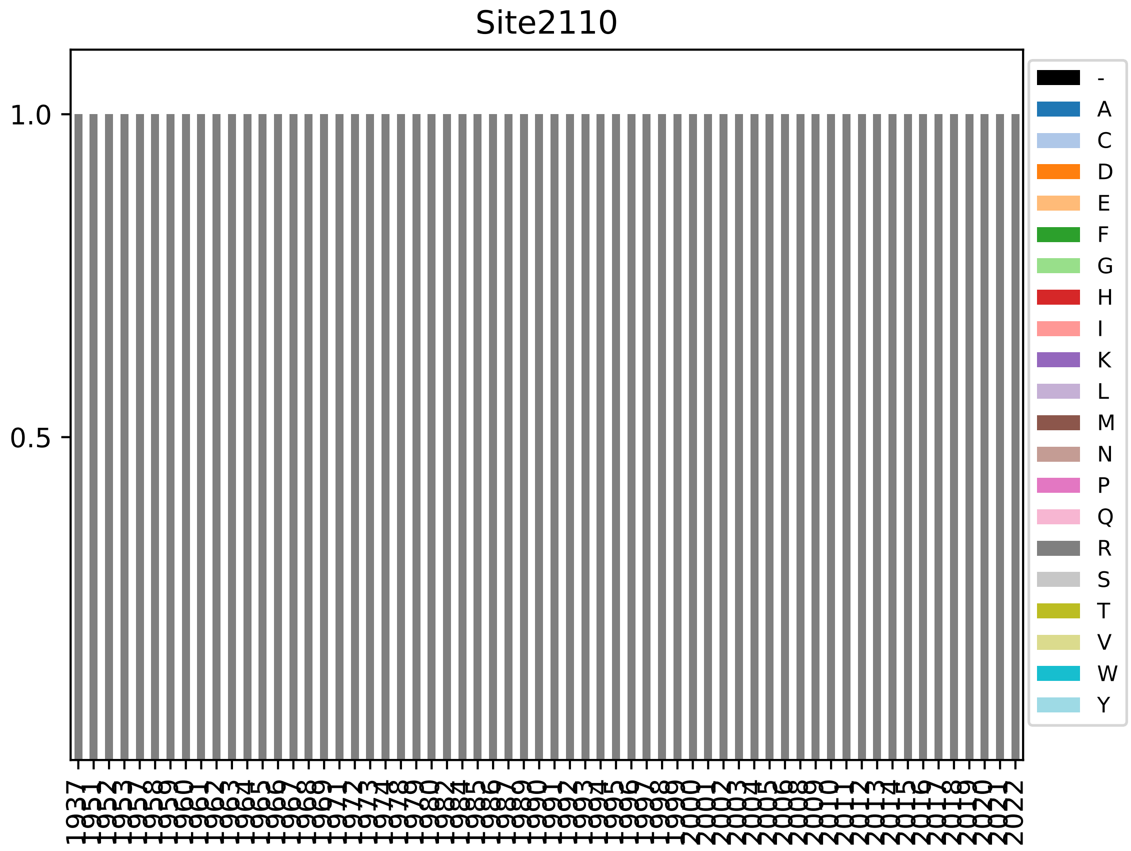
Site2110
1.0
0.5
-
A
C
D
E
F
G
H
I
K
L
M
N
P
Q
R
S
T
V
W
Y

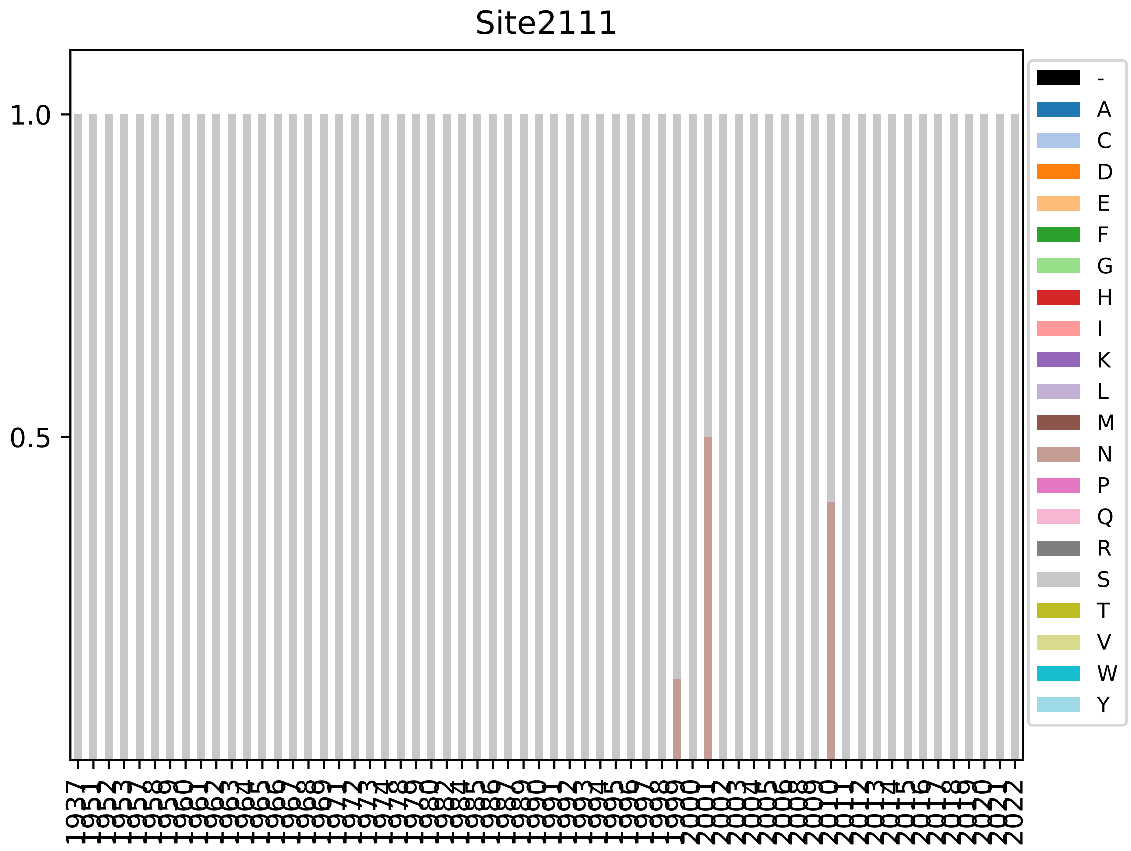

Site2111
1.0
0.5
-
A
C
D
E
F
G
H
I
K
L
M
N
P
Q
R
S
T
V
W
Y

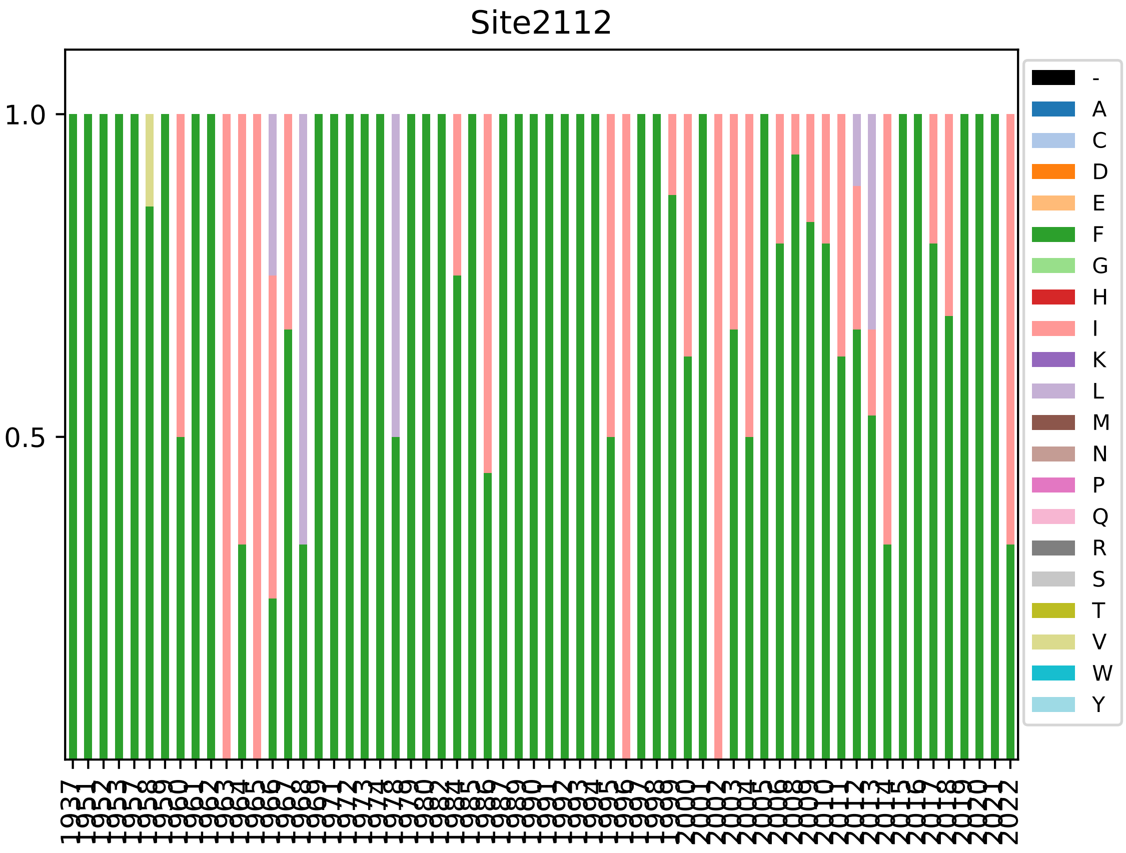
Site2112
1.0
0.5
-
A
C
D
E
F
G
H
I
K
L
M
N
P
Q
R
S
T
V
W
Y

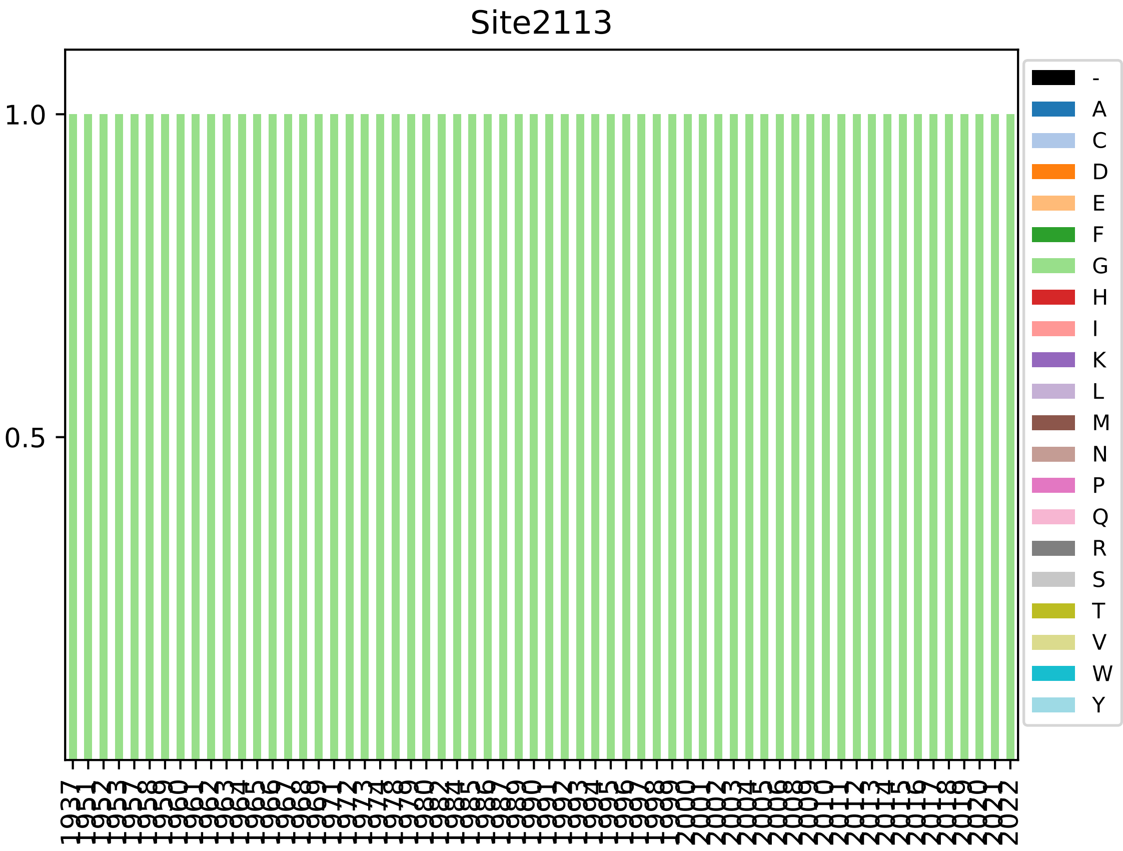
Site2113
1.0
0.5
-
A
C
D
E
F
G
H
I
K
L
M
N
P
Q
R
S
T
V
W
Y

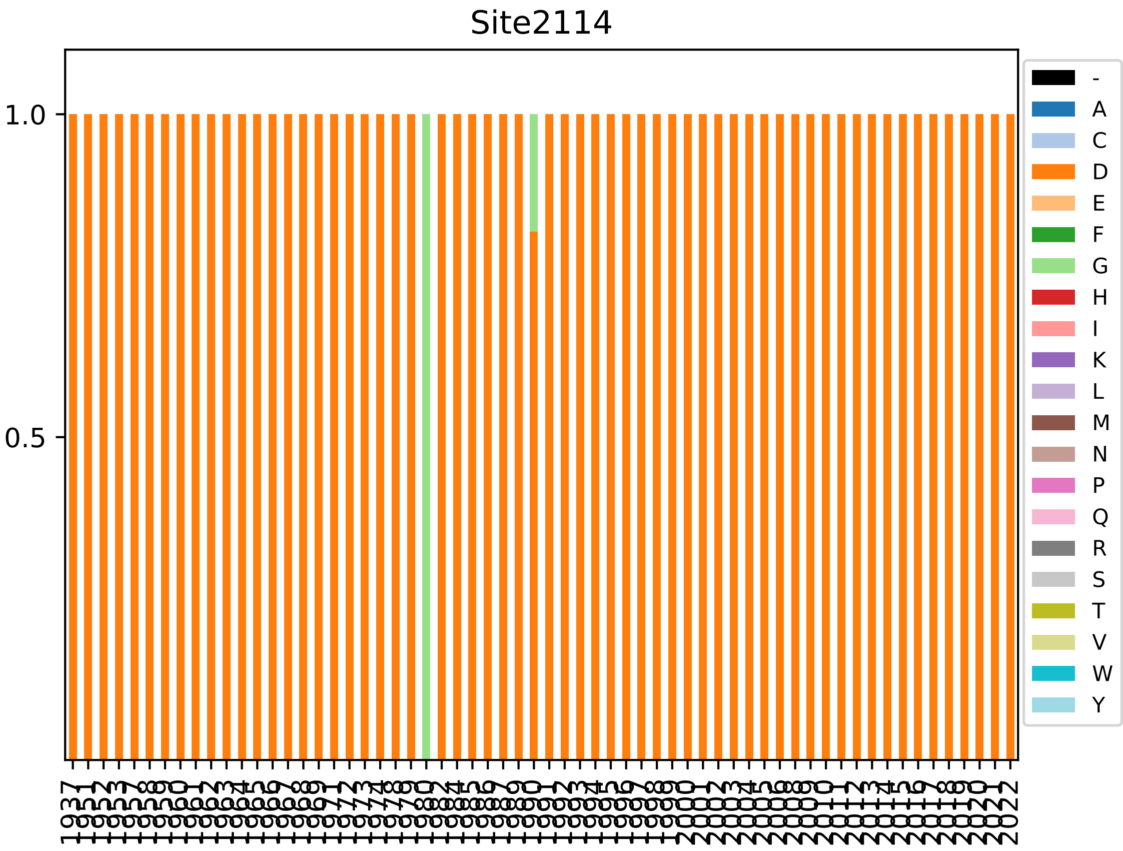
Site2114
1.0
0.5
-
A
C
D
E
F
G
H
I
K
L
M
N
P
Q
R
S
T
V
W
Y

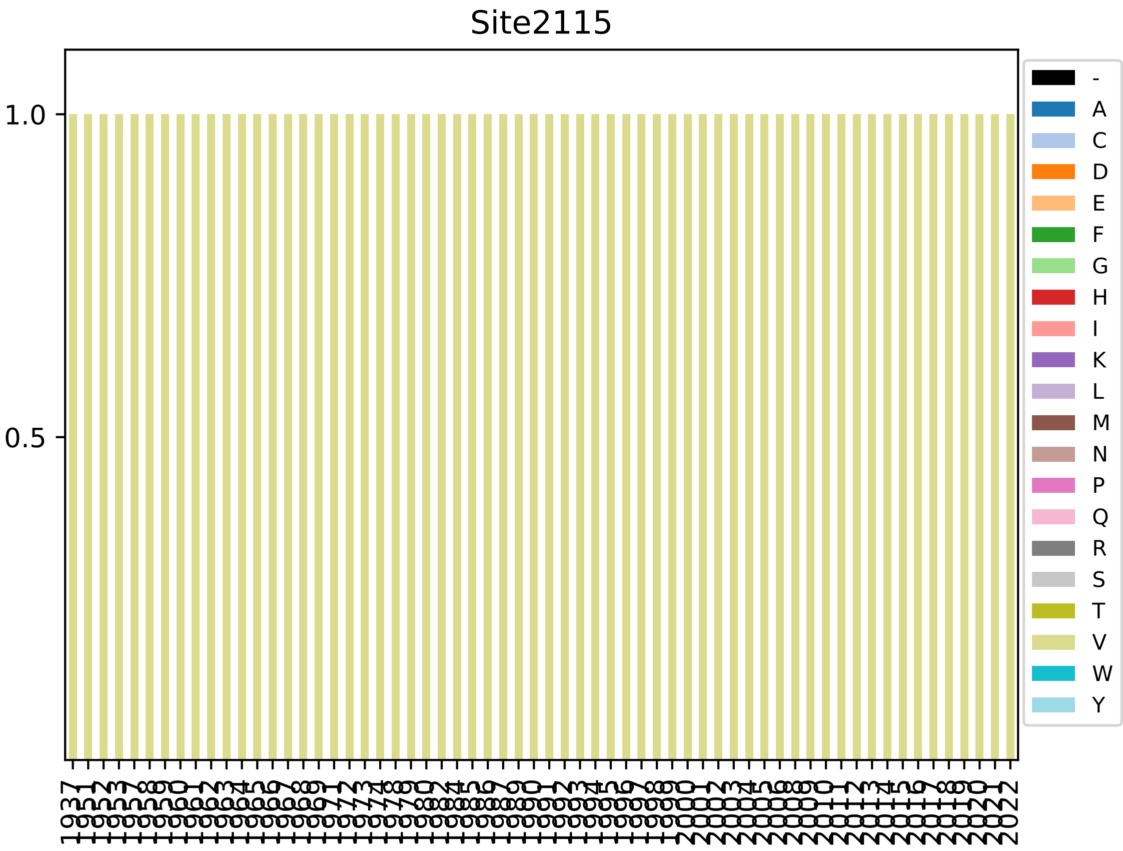
Site2115
1.0
0.5
-
A
C
D
E
F
G
H
I
K
L
M
N
P
Q
R
S
T
V
W
Y

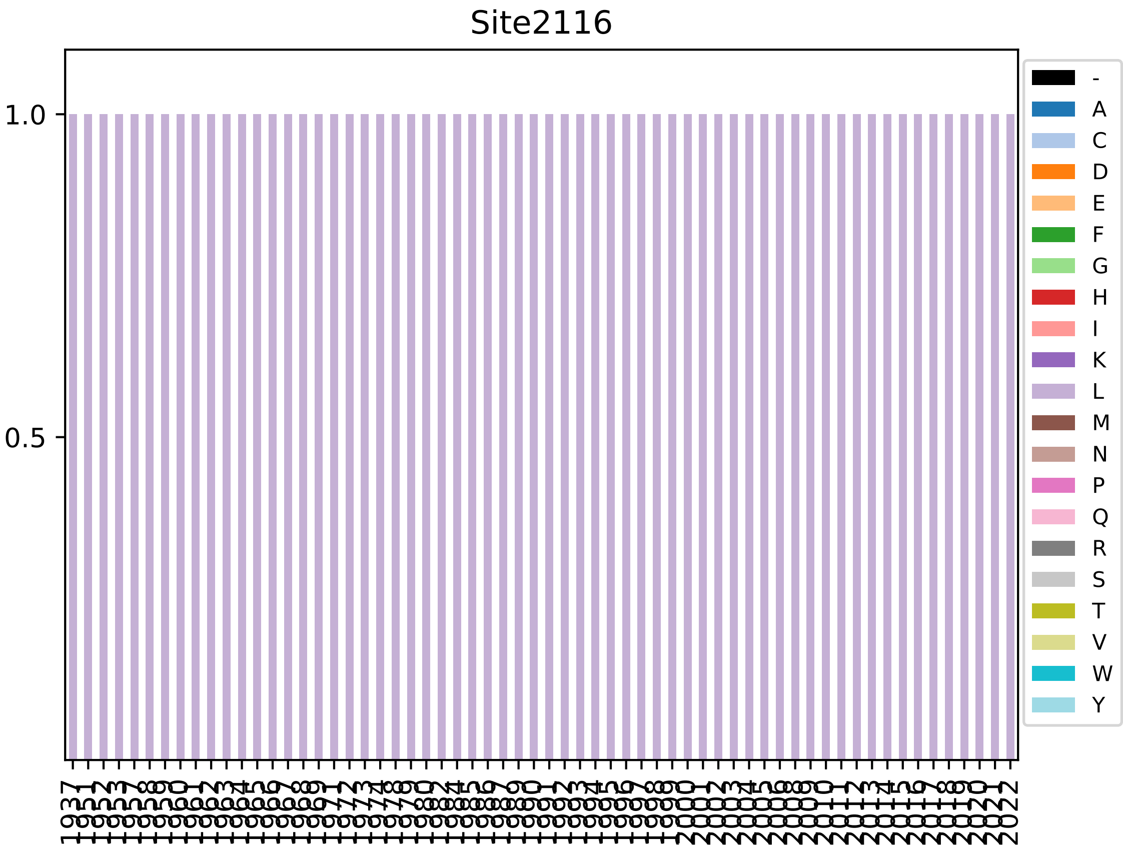
Site2116
1.0
0.5
-
A
C
D
E
F
G
H
I
K
L
M
N
P
Q
R
S
T
V
W
Y

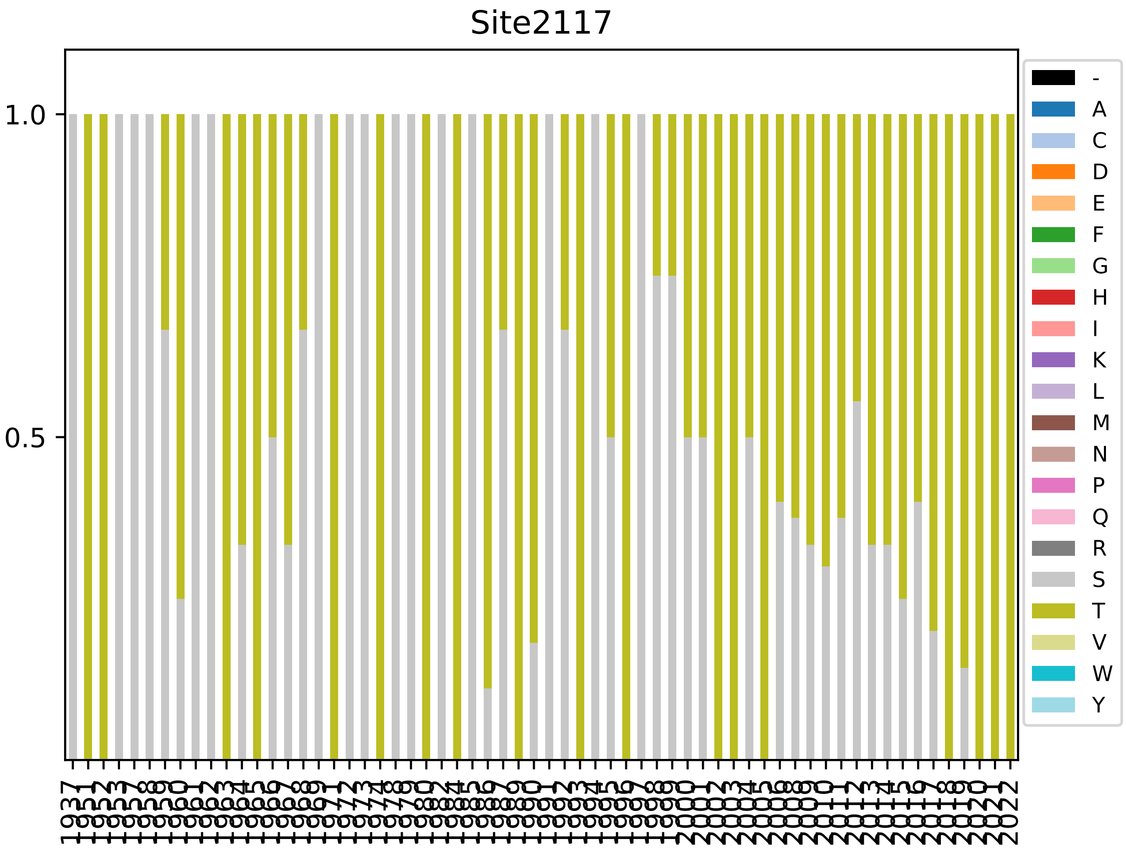
Site2117
1.0
0.5
-
A
C
D
E
F
G
H
I
K
L
M
N
P
Q
R
S
T
V
W
Y

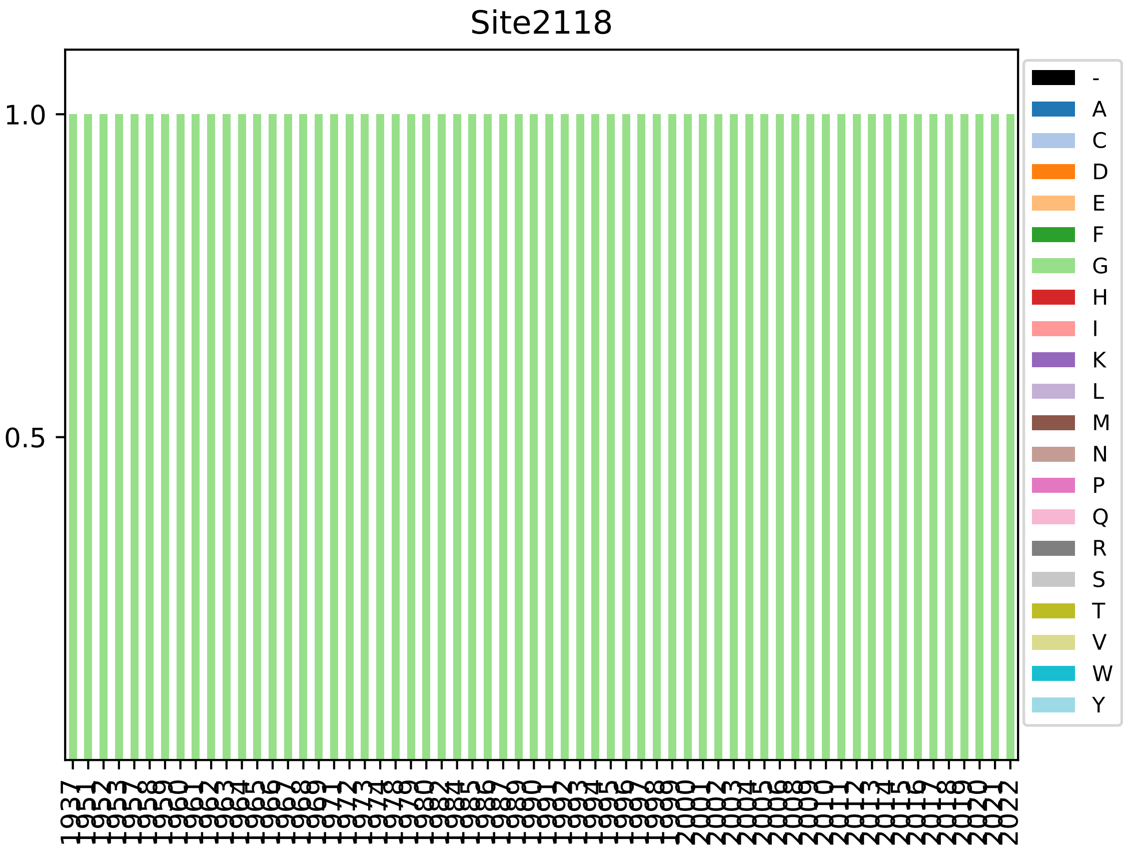
Site2118
1.0
0.5
-
A
C
D
E
F
G
H
I
K
L
M
N
P
Q
R
S
T
V
W
Y

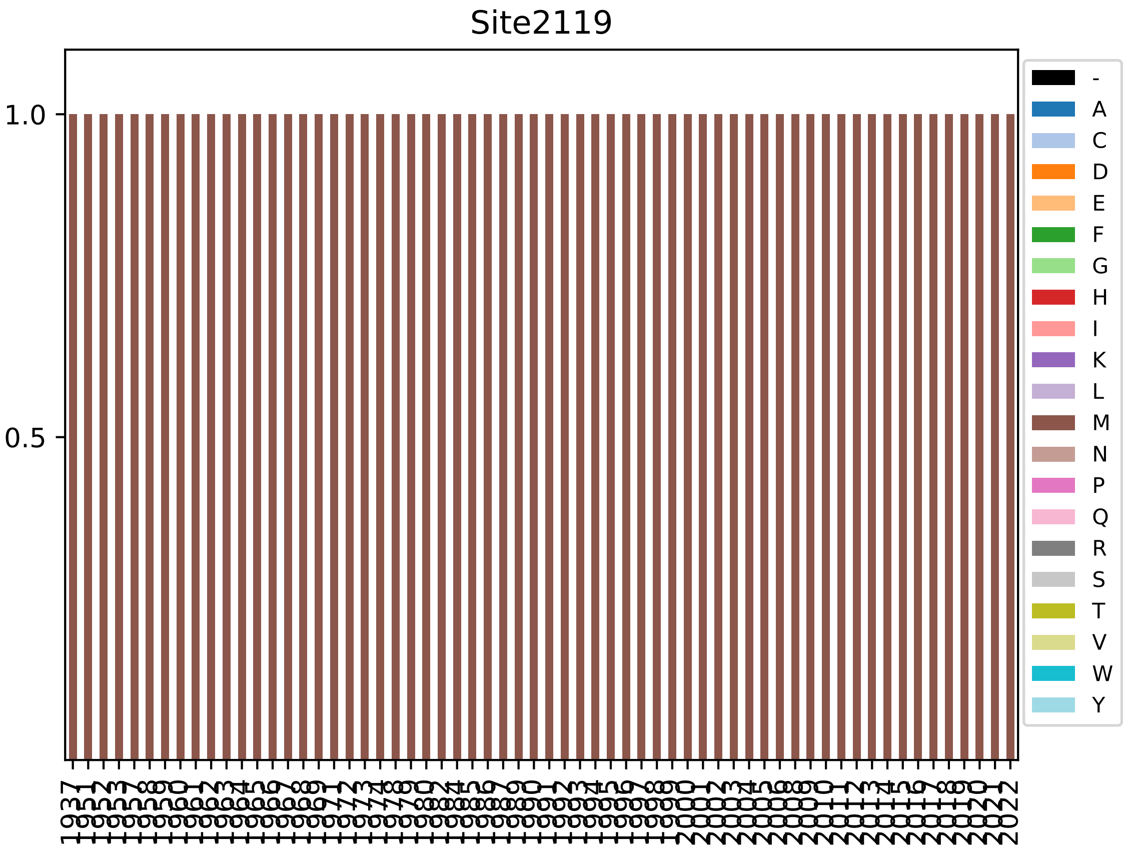
Site2119
1.0
0.5
-
A
C
D
E
F
G
H
I
K
L
M
N
P
Q
R
S
T
V
W
Y

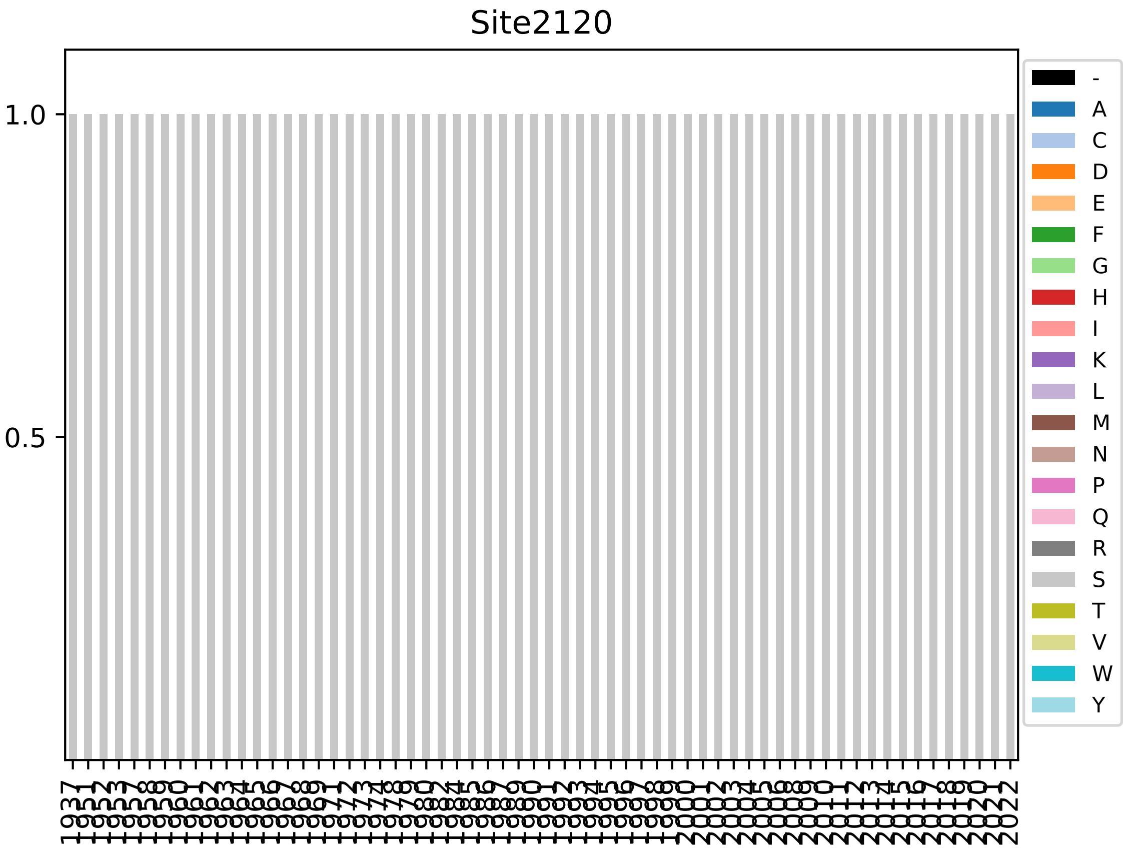
Site2120
1.0
0.5
-
A
C
D
E
F
G
H
I
K
L
M
N
P
Q
R
S
T
V
W
Y

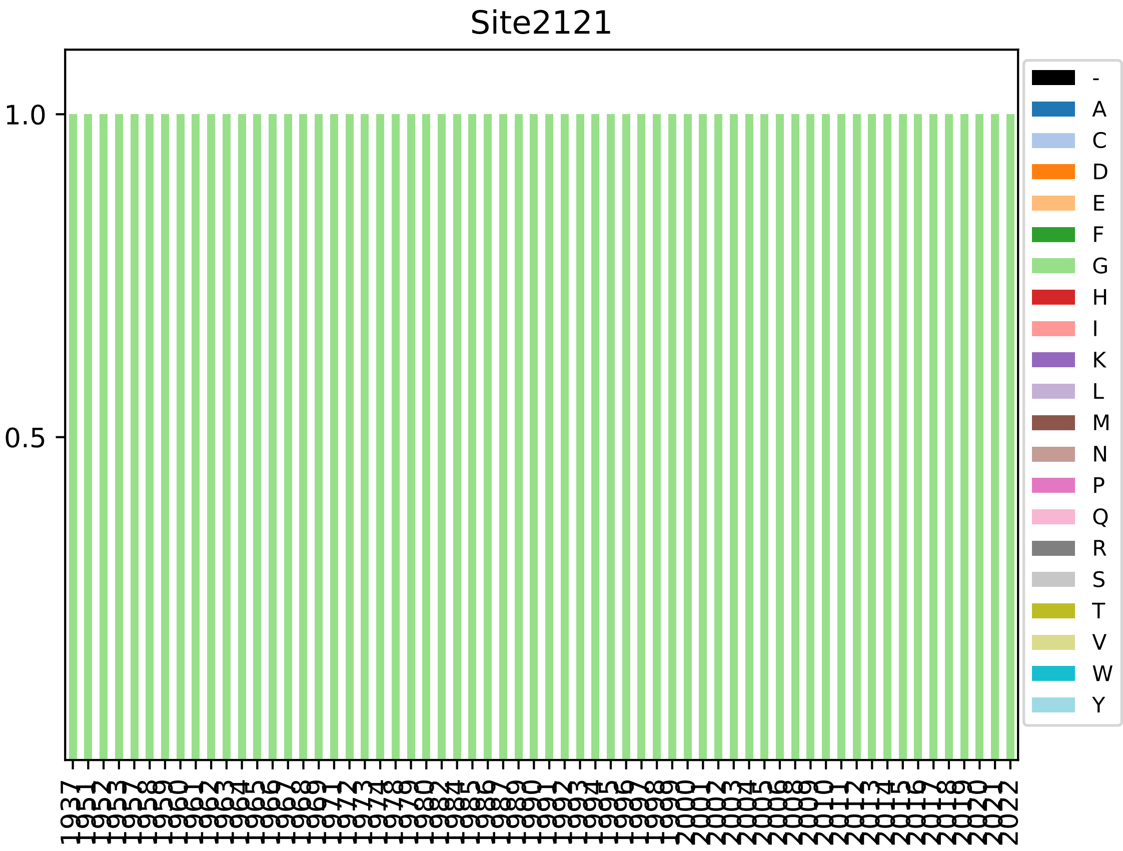

Site2121
1.0
0.5
-
A
C
D
E
F
G
H
I
K
L
M
N
P
Q
R
S
T
V
W
Y

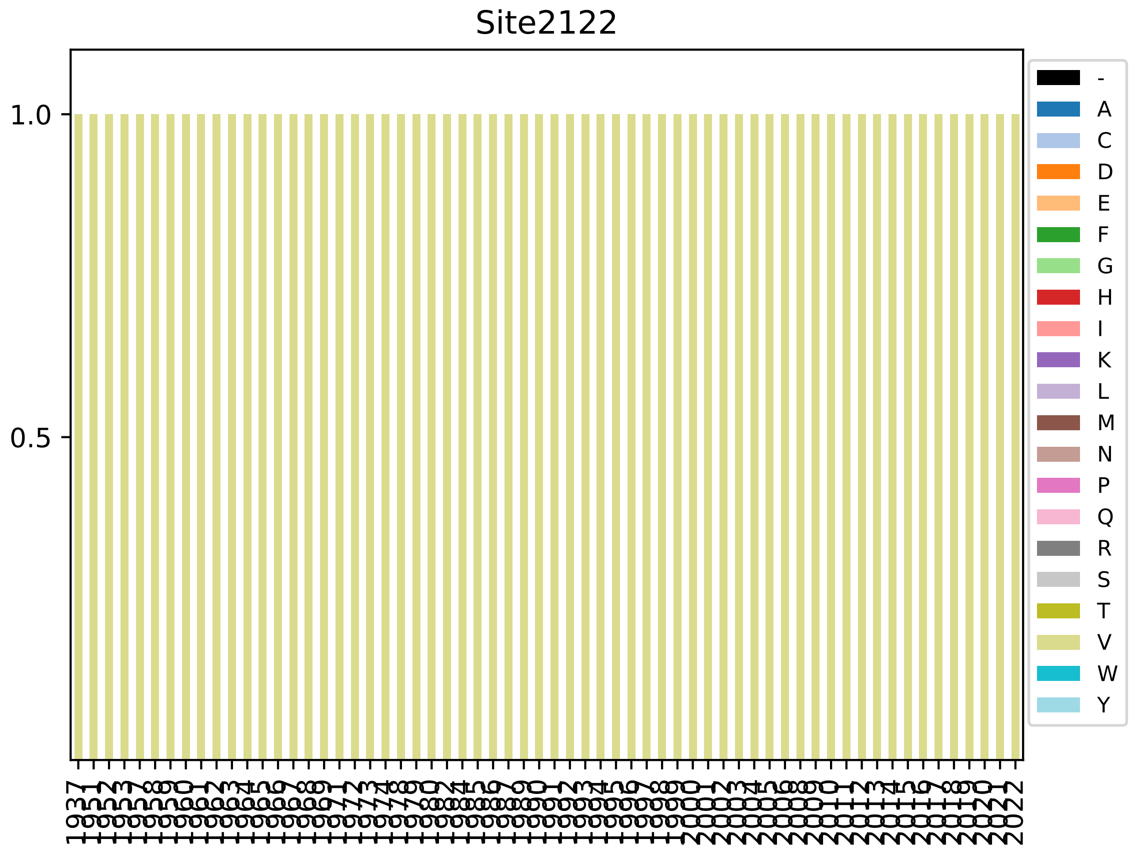
Site2122
1.0
0.5
-
A
C
D
E
F
G
H
I
K
L
M
N
P
Q
R
S
T
V
W
Y

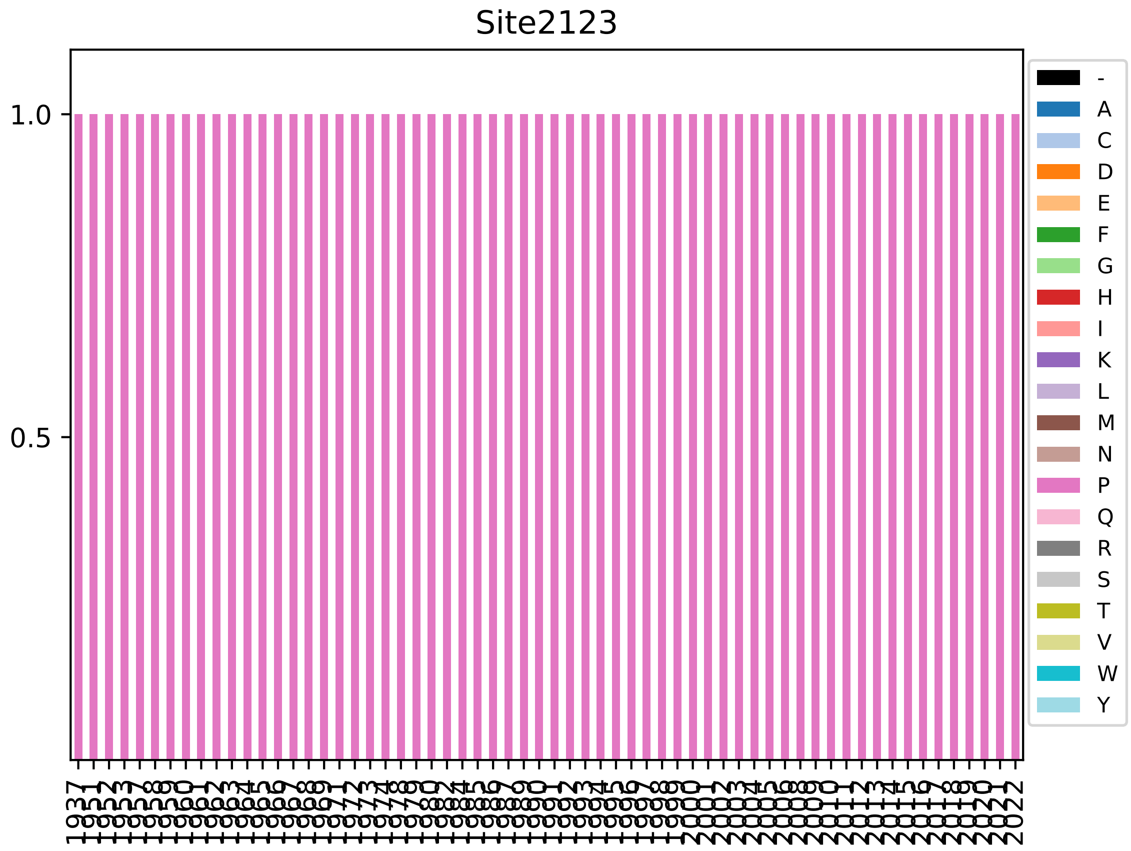
Site2123
1.0
0.5
-
A
C
D
E
F
G
H
I
K
L
M
N
P
Q
R
S
T
V
W
Y

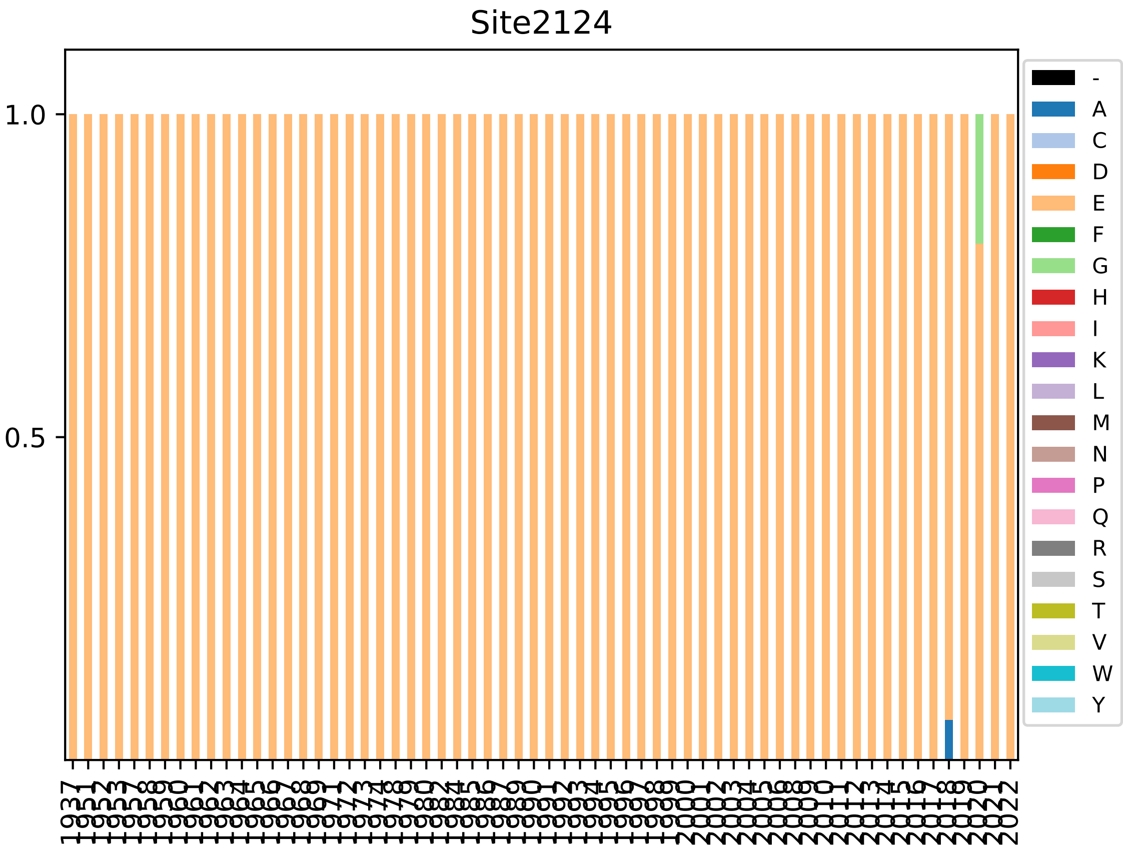
Site2124
1.0
0.5
-
A
C
D
E
F
G
H
I
K
L
M
N
P
Q
R
S
T
V
W
Y

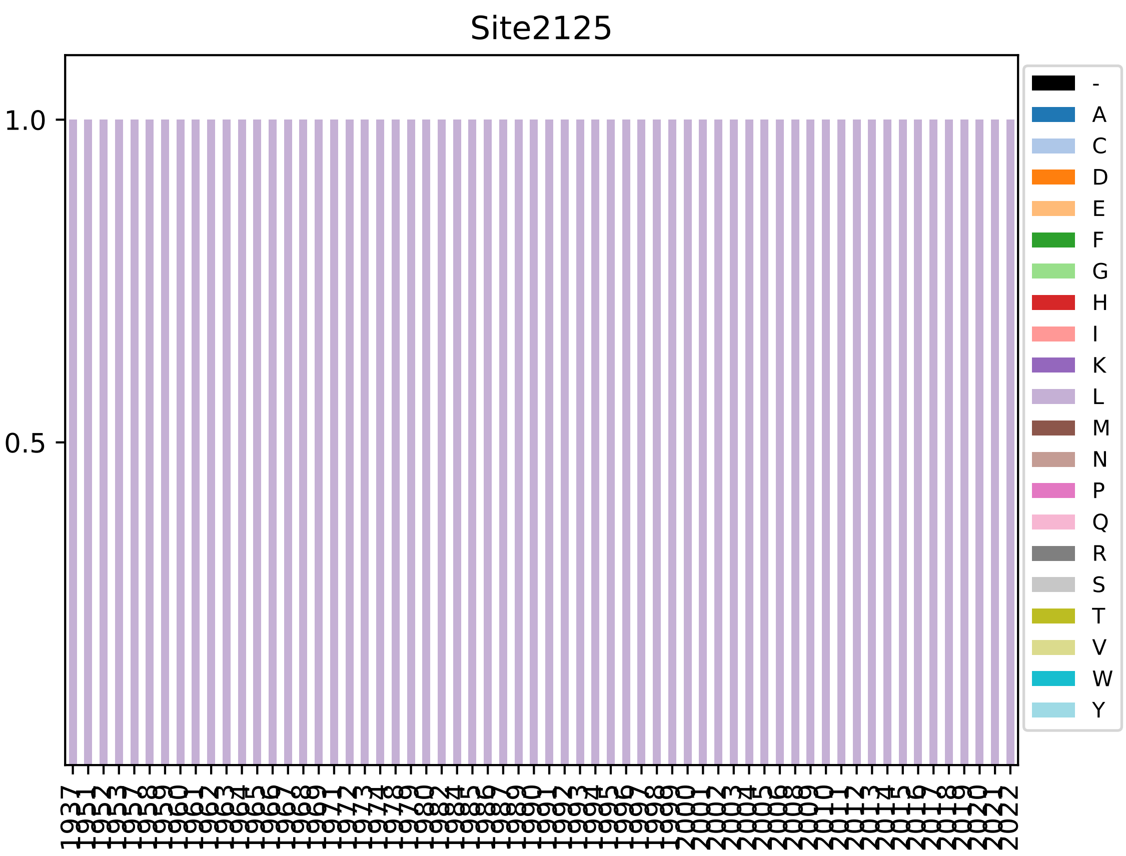
Site2125
1.0
0.5
-
A
C
D
E
F
G
H
I
K
L
M
N
P
Q
R
S
T
V
W
Y

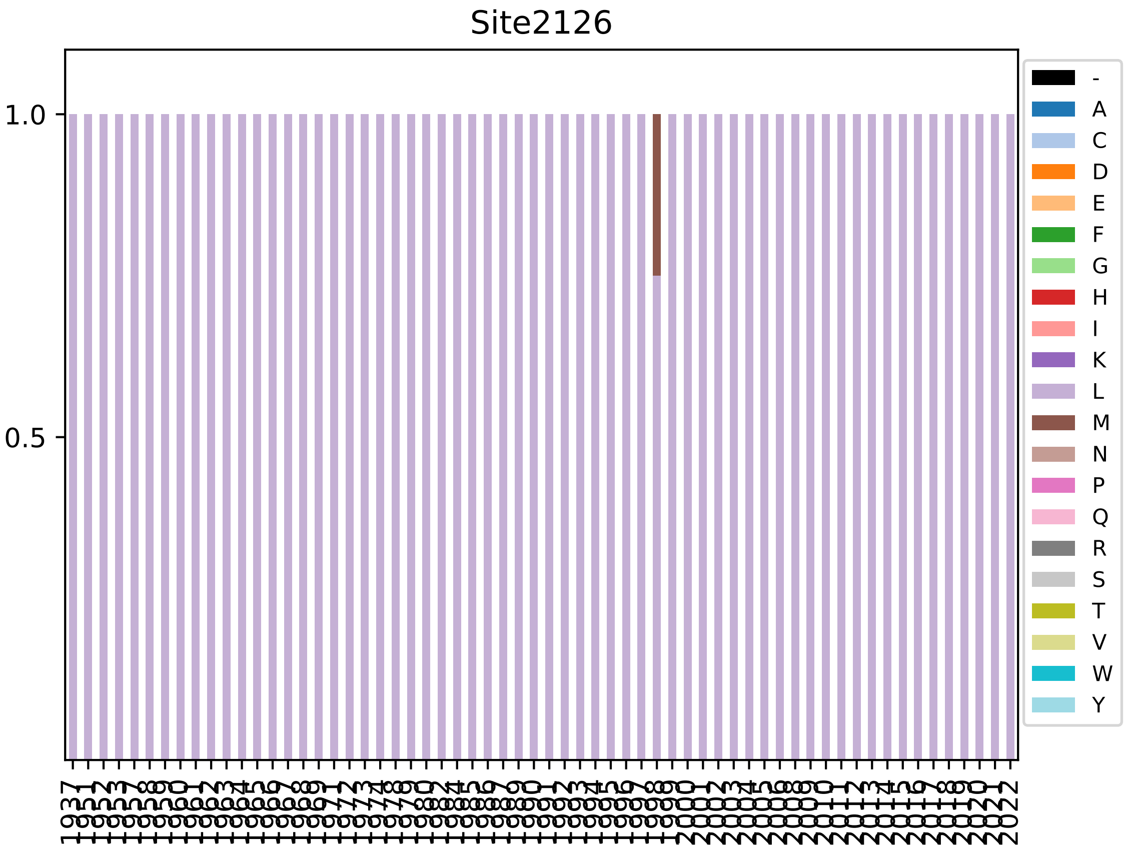
Site2126
1.0
0.5
-
A
C
D
E
F
G
H
I
K
L
M
N
P
Q
R
S
T
V
W
Y

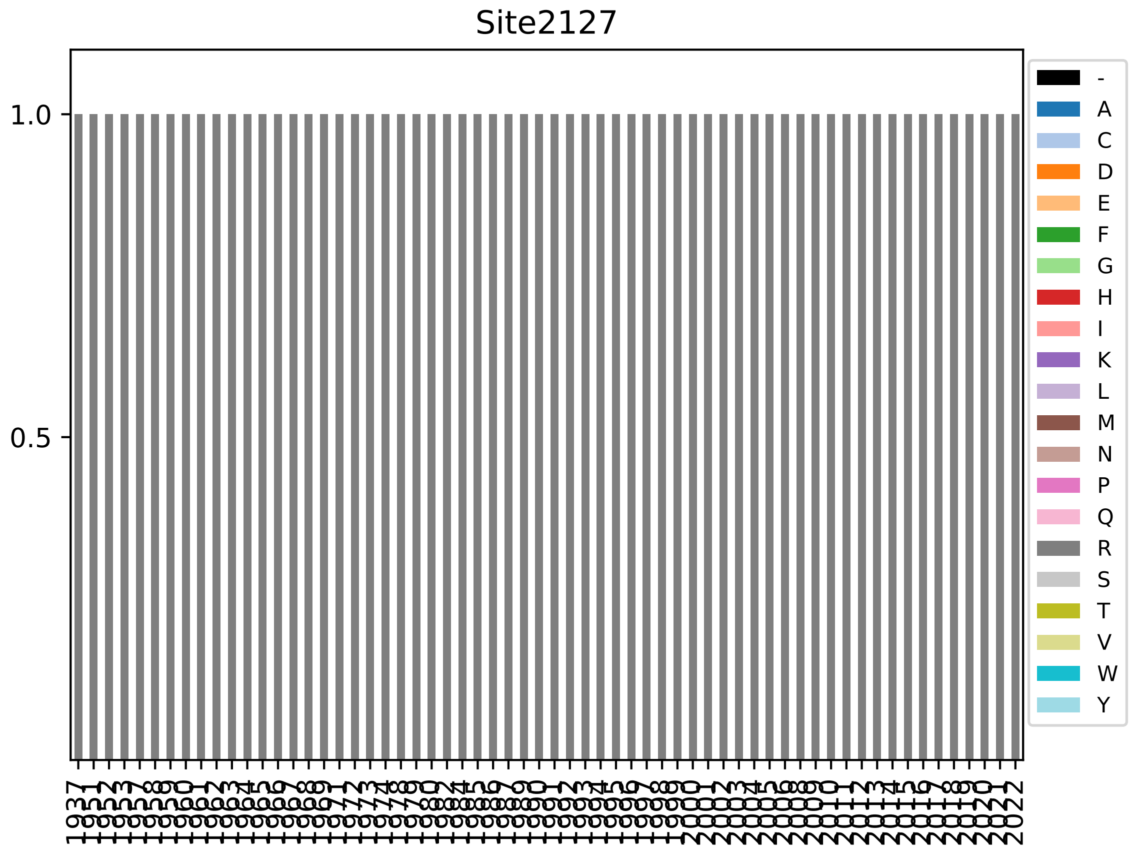
Site2127
1.0
0.5
-
A
C
D
E
F
G
H
I
K
L
M
N
P
Q
R
S
T
V
W
Y

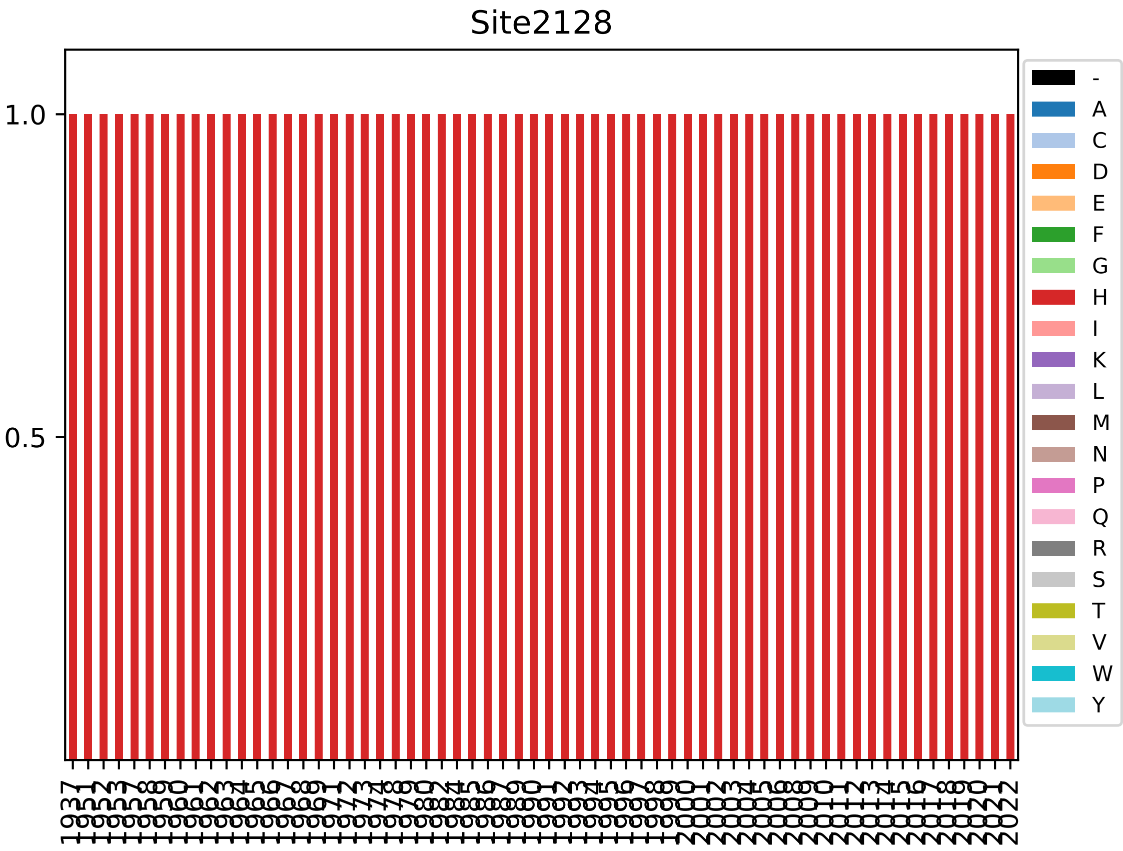
Site2128
1.0
0.5
-
A
C
D
E
F
G
H
I
K
L
M
N
P
Q
R
S
T
V
W
Y

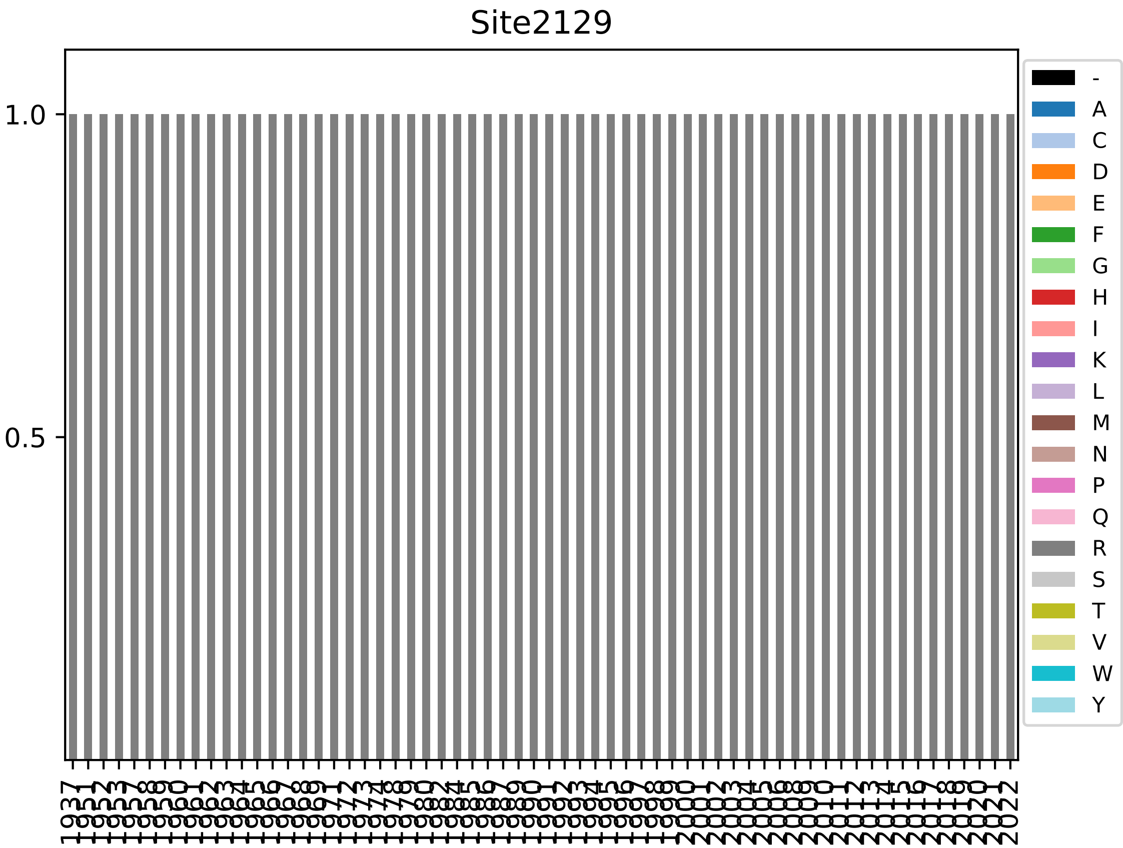
Site2129
1.0
0.5
-
A
C
D
E
F
G
H
I
K
L
M
N
P
Q
R
S
T
V
W
Y

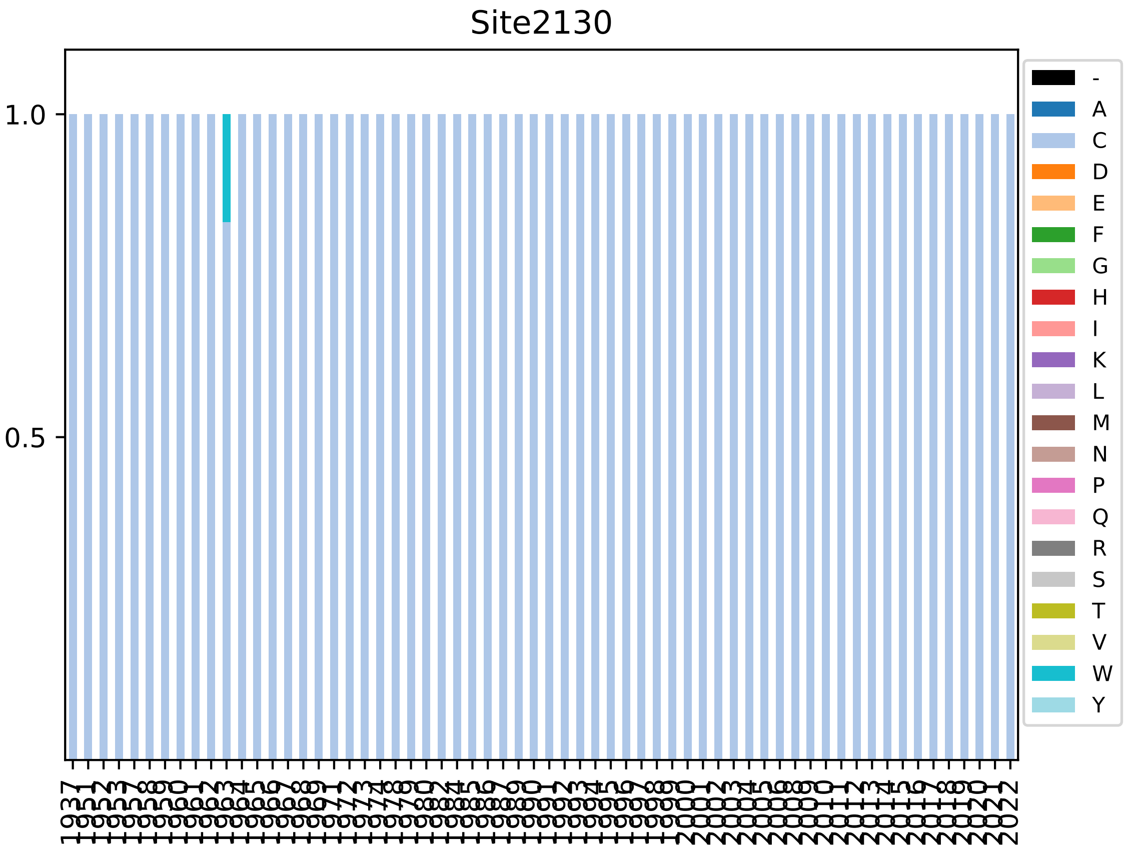
Site2130
1.0
0.5
-
A
C
D
E
F
G
H
I
K
L
M
N
P
Q
R
S
T
V
W
Y

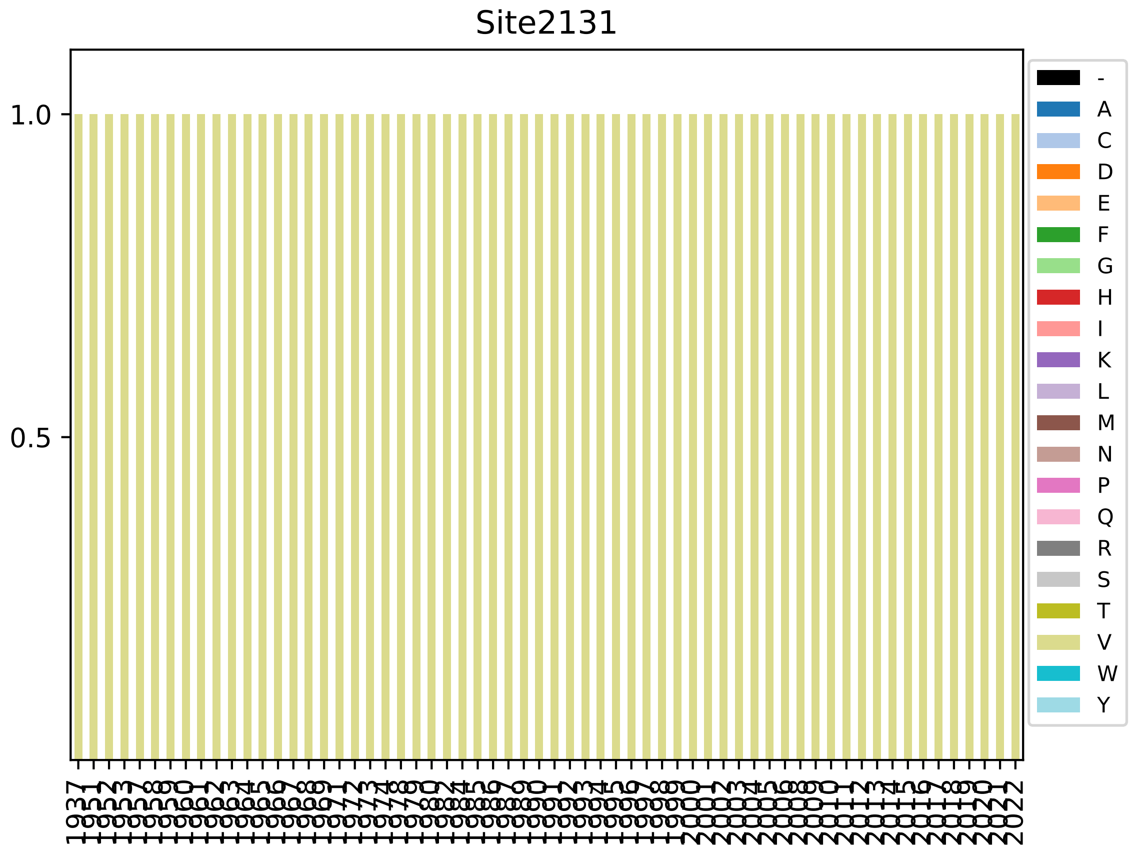
Site2131
1.0
0.5
-
A
C
D
E
F
G
H
I
K
L
M
N
P
Q
R
S
T
V
W
Y

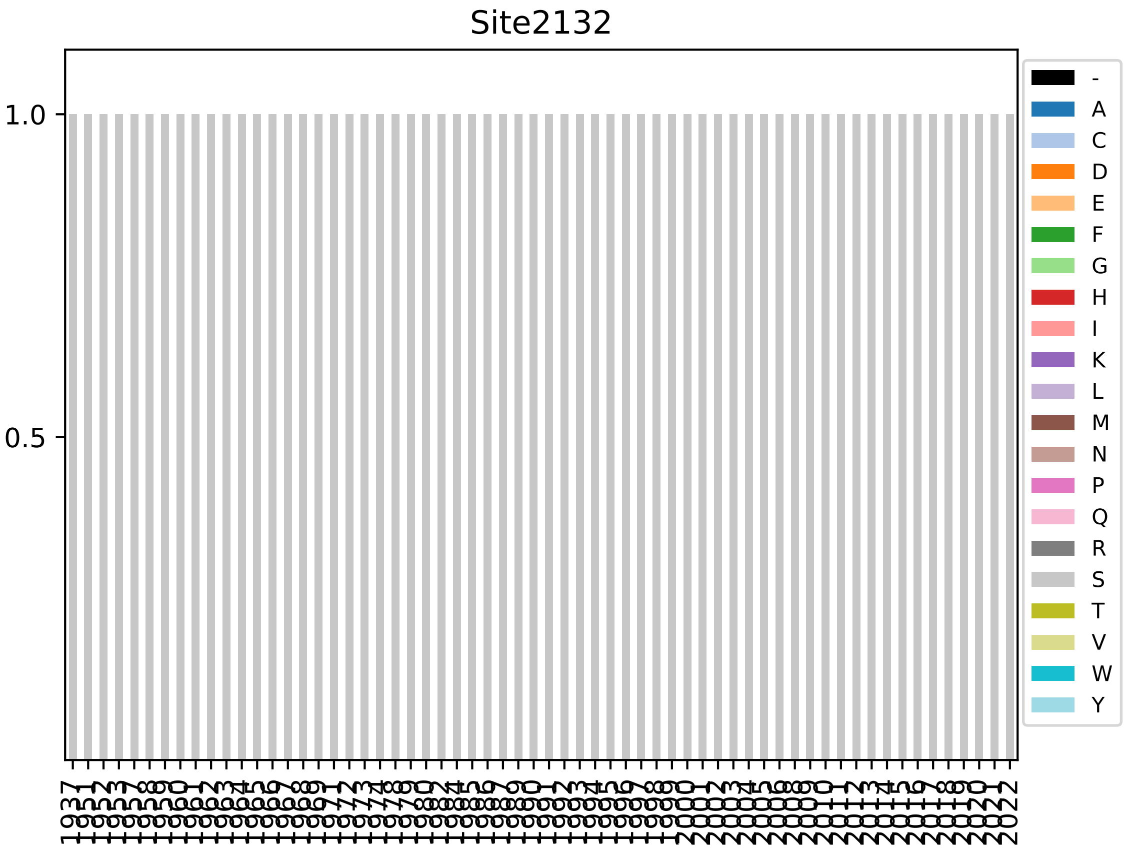
Site2132
1.0
0.5
-
A
C
D
E
F
G
H
I
K
L
M
N
P
Q
R
S
T
V
W
Y

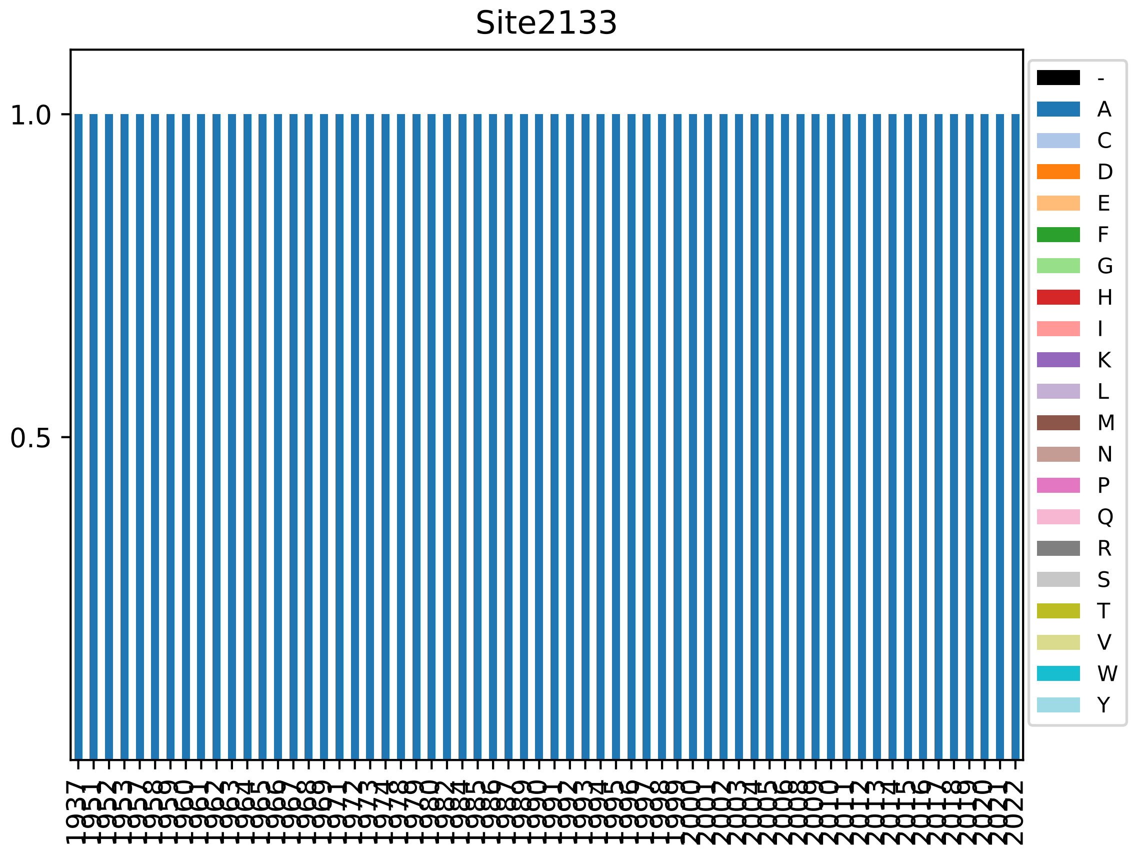
Site2133
1.0
0.5
-
A
C
D
E
F
G
H
I
K
L
M
N
P
Q
R
S
T
V
W
Y

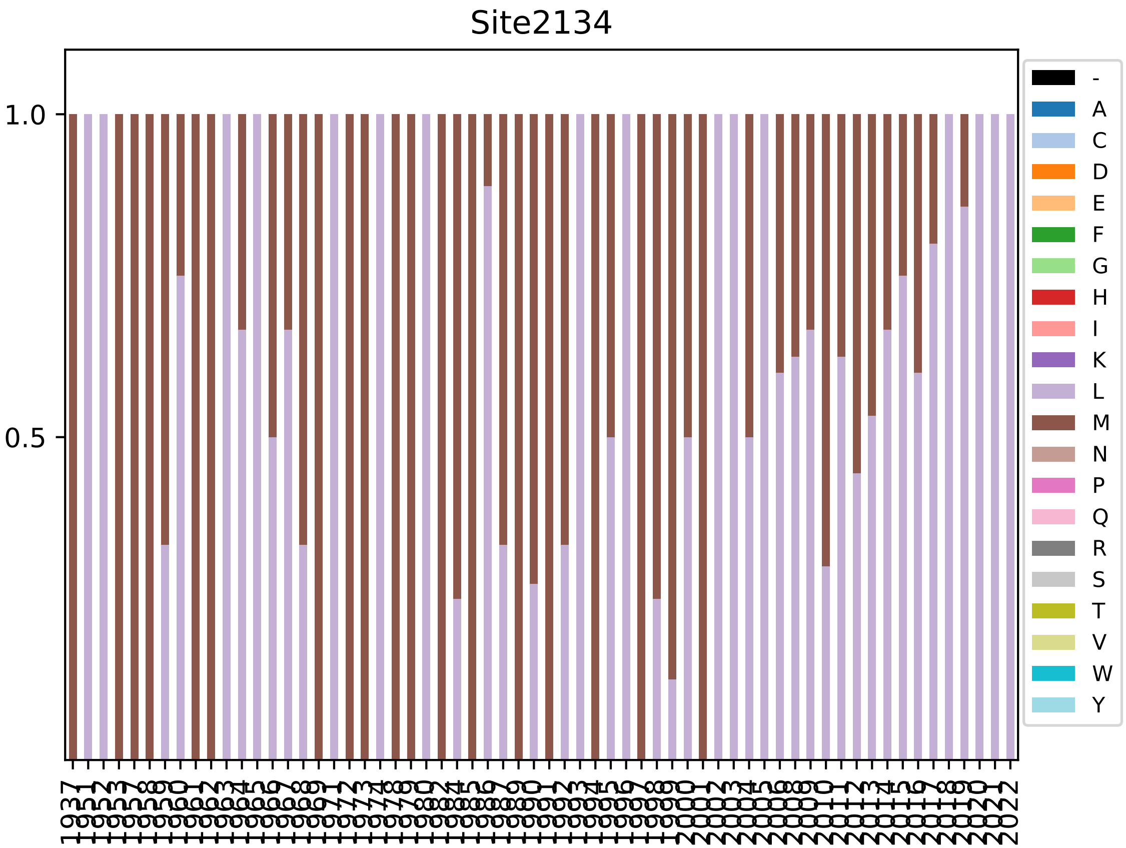
Site2134
1.0
0.5
-
A
C
D
E
F
G
H
I
K
L
M
N
P
Q
R
S
T
V
W
Y

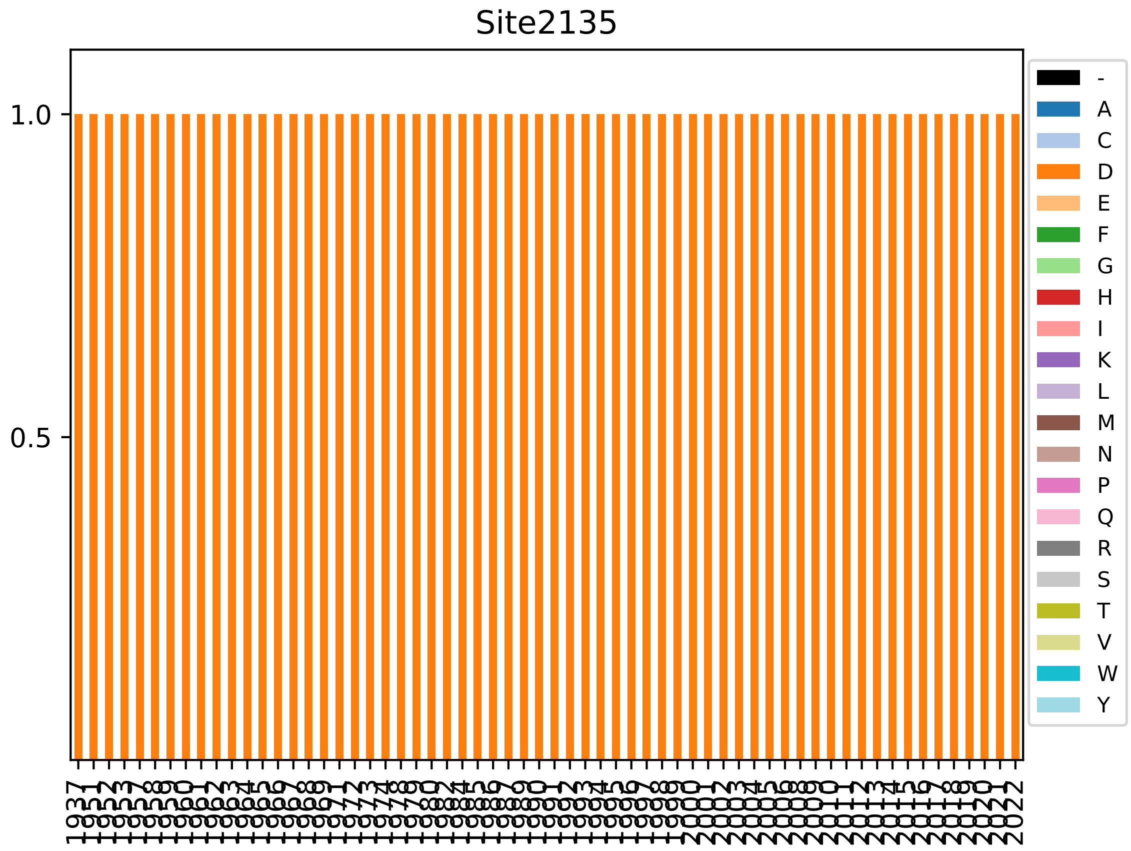
Site2135
1.0
0.5
-
A
C
D
E
F
G
H
I
K
L
M
N
P
Q
R
S
T
V
W
Y

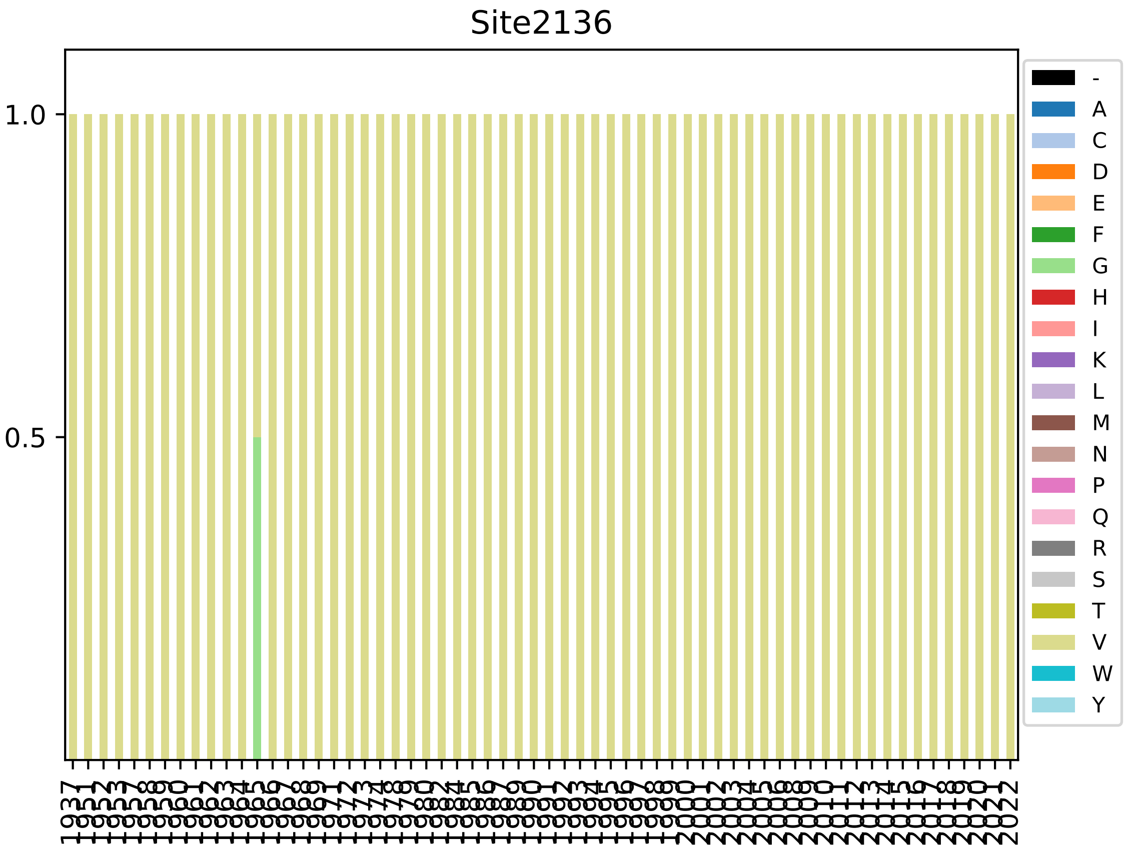
Site2136
1.0
0.5
-
A
C
D
E
F
G
H
I
K
L
M
N
P
Q
R
S
T
V
W
Y

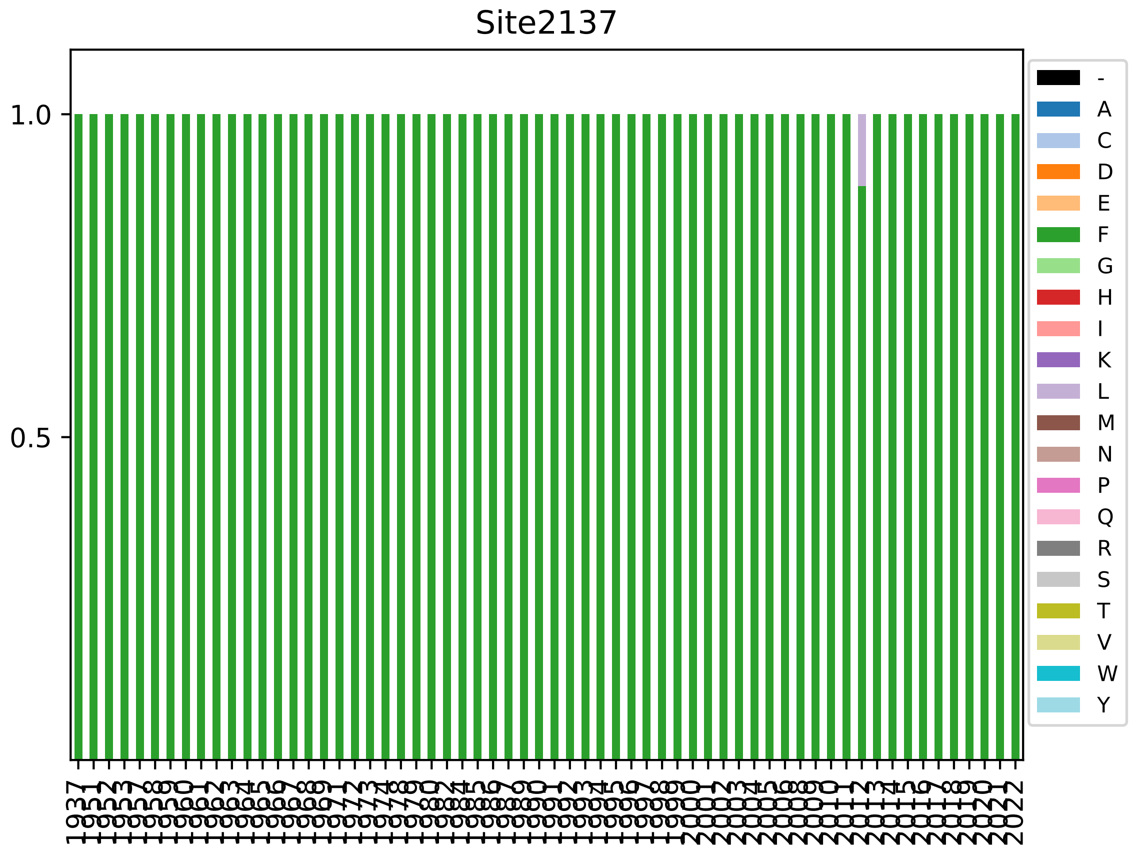
Site2137
1.0
0.5
-
A
C
D
E
F
G
H
I
K
L
M
N
P
Q
R
S
T
V
W
Y

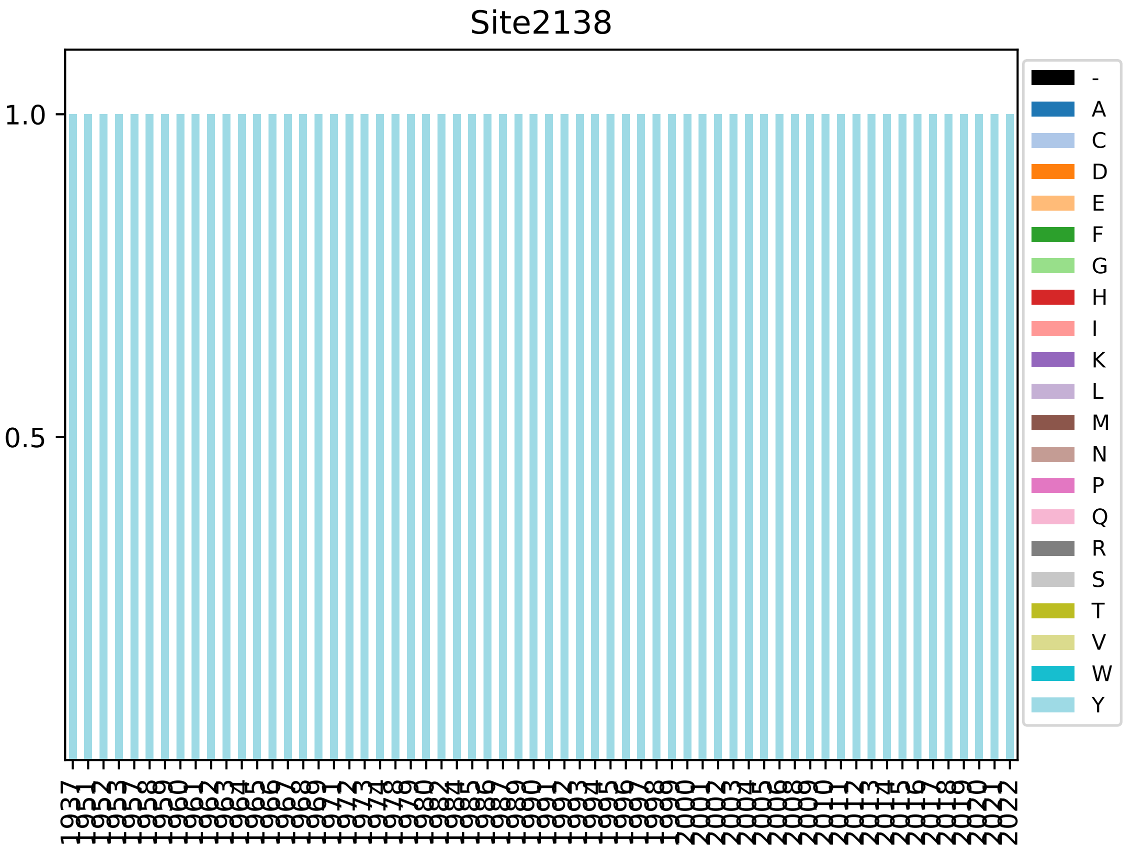
Site2138
1.0
0.5
-
A
C
D
E
F
G
H
I
K
L
M
N
P
Q
R
S
T
V
W
Y

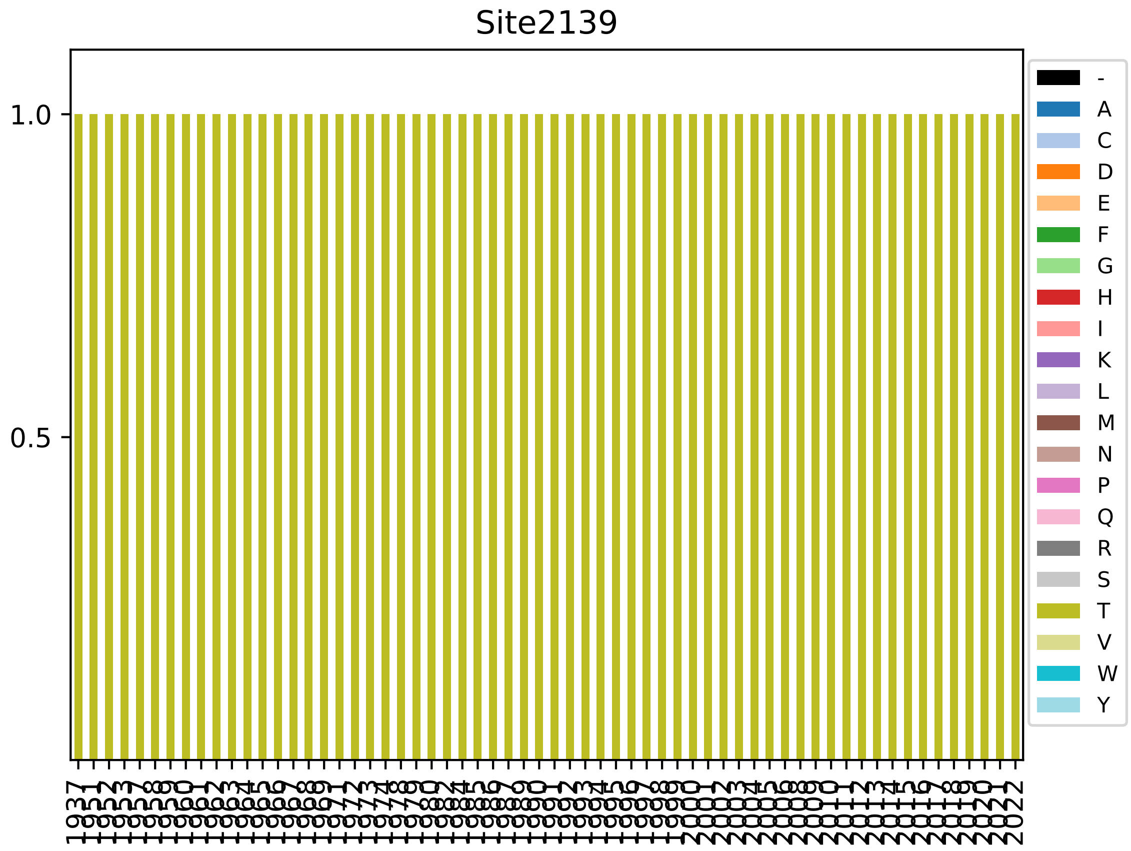
Site2139
1.0
0.5
-
A
C
D
E
F
G
H
I
K
L
M
N
P
Q
R
S
T
V
W
Y

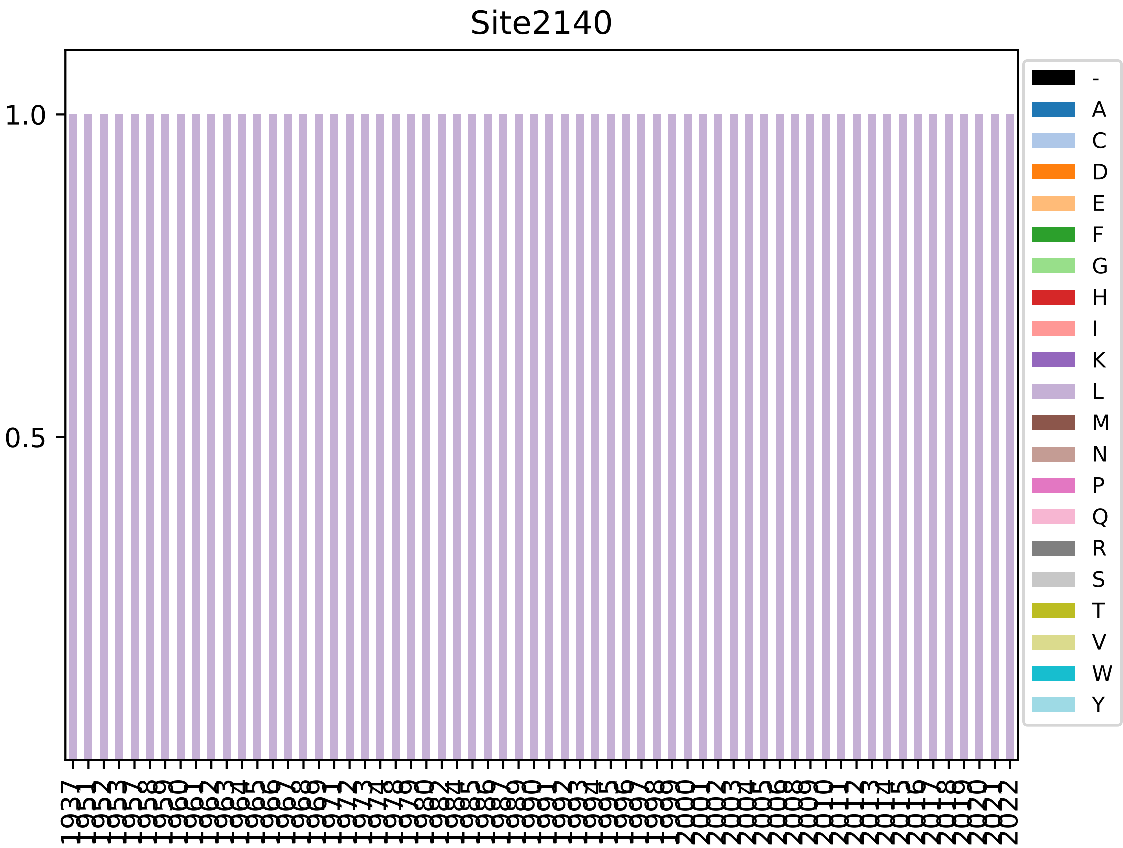
Site2140
1.0
0.5
-
A
C
D
E
F
G
H
I
K
L
M
N
P
Q
R
S
T
V
W
Y

Site2141

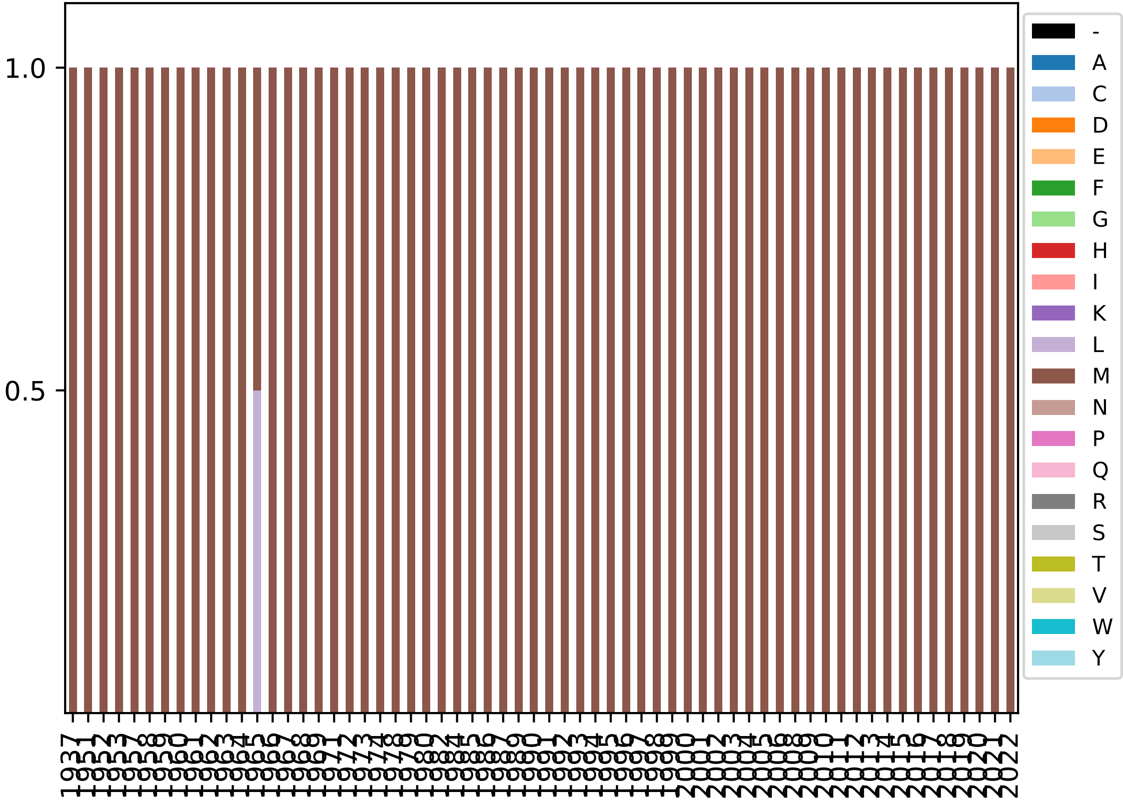

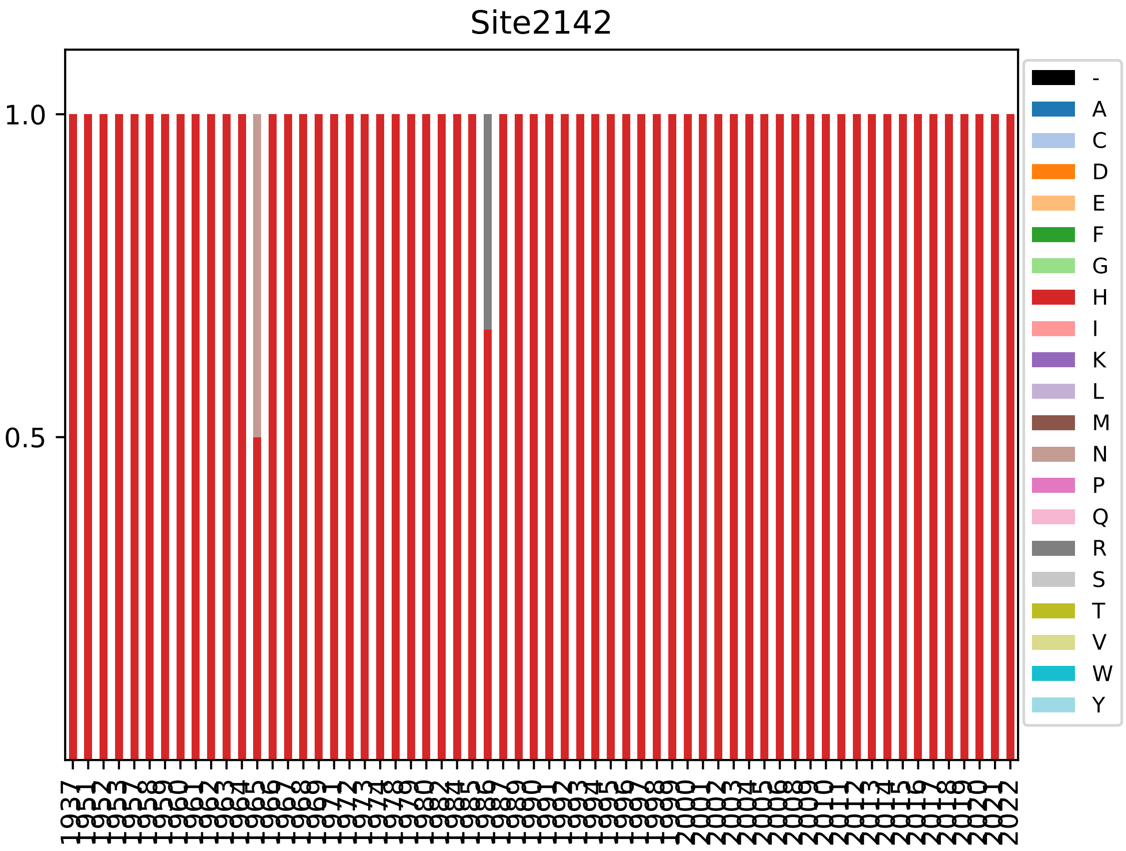
Site2142
1.0
0.5
-
A
C
D
E
F
G
H
I
K
L
M
N
P
Q
R
S
T
V
W
Y

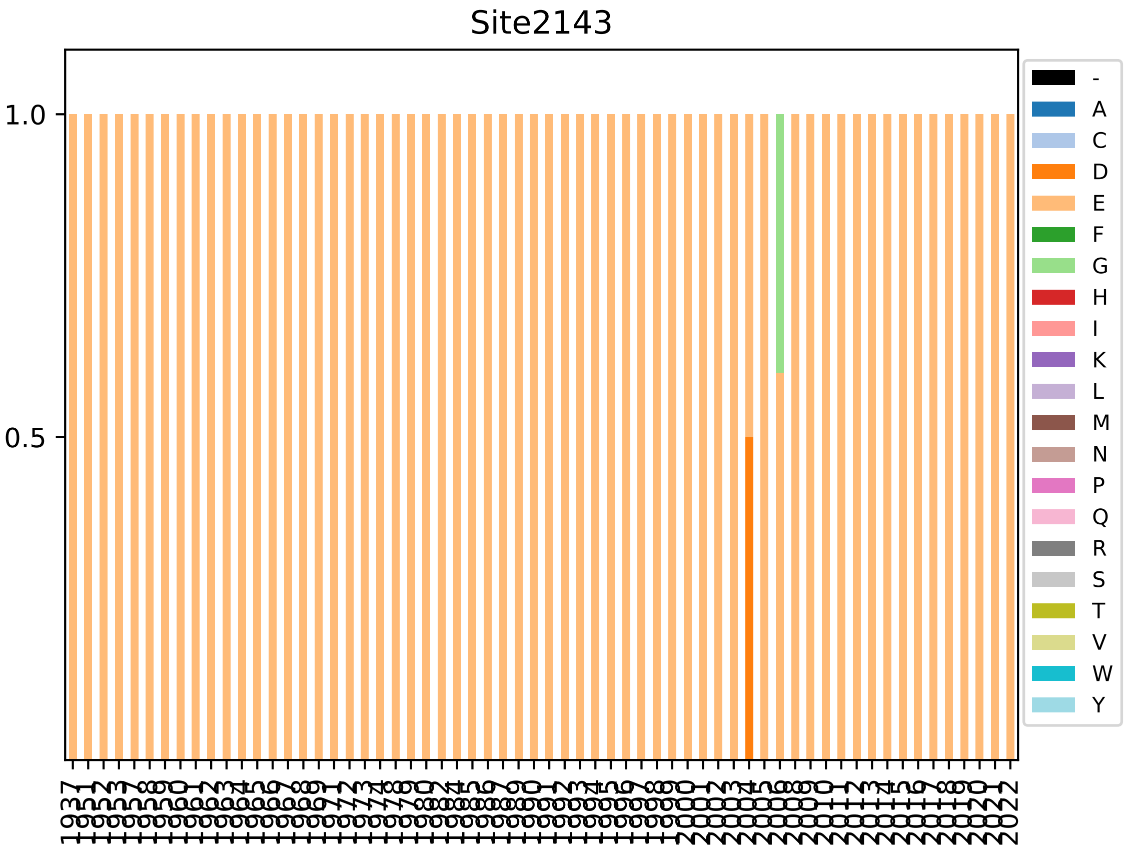
Site2143
1.0
0.5
-
A
C
D
E
F
G
H
I
K
L
M
N
P
Q
R
S
T
V
W
Y

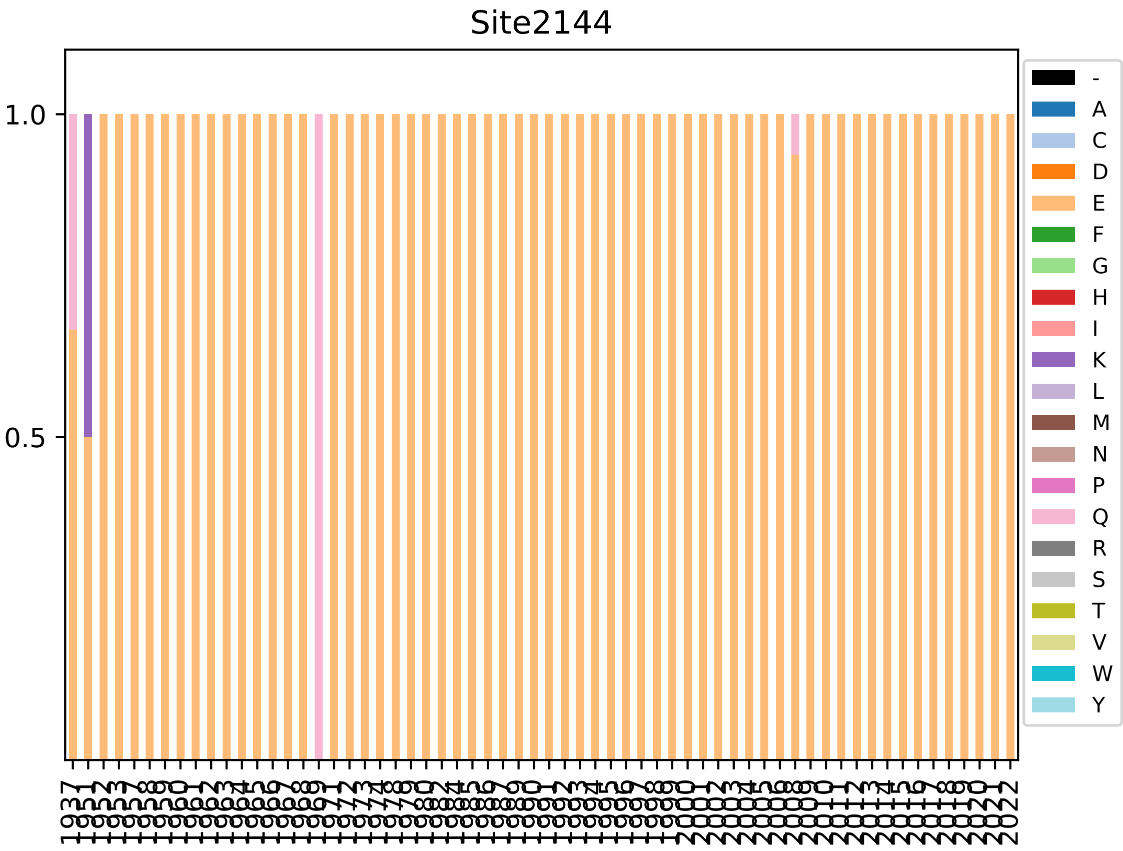

Site2144
1.0
0.5
-
A
C
D
E
F
G
H
I
K
L
M
N
P
Q
R
S
T
V
W
Y

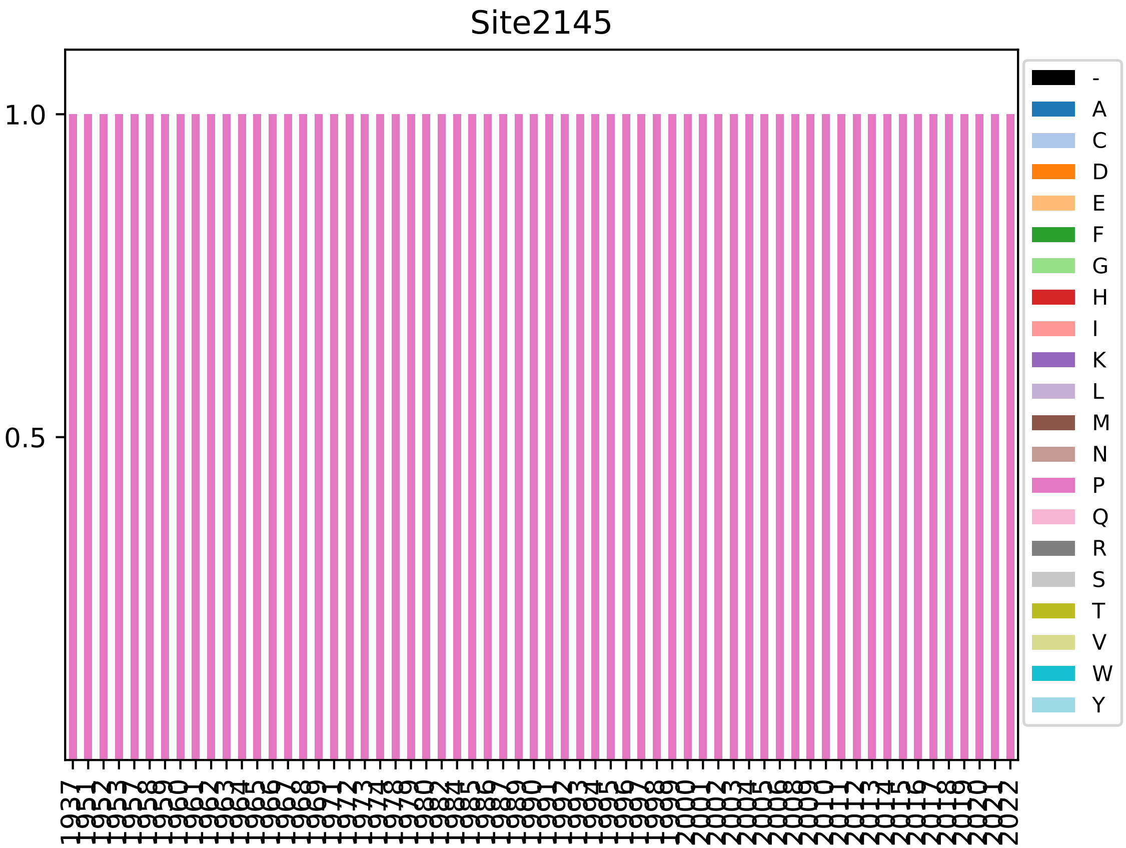
Site2145
1.0
0.5
-
A
C
D
E
F
G
H
I
K
L
M
N
P
Q
R
S
T
V
W
Y

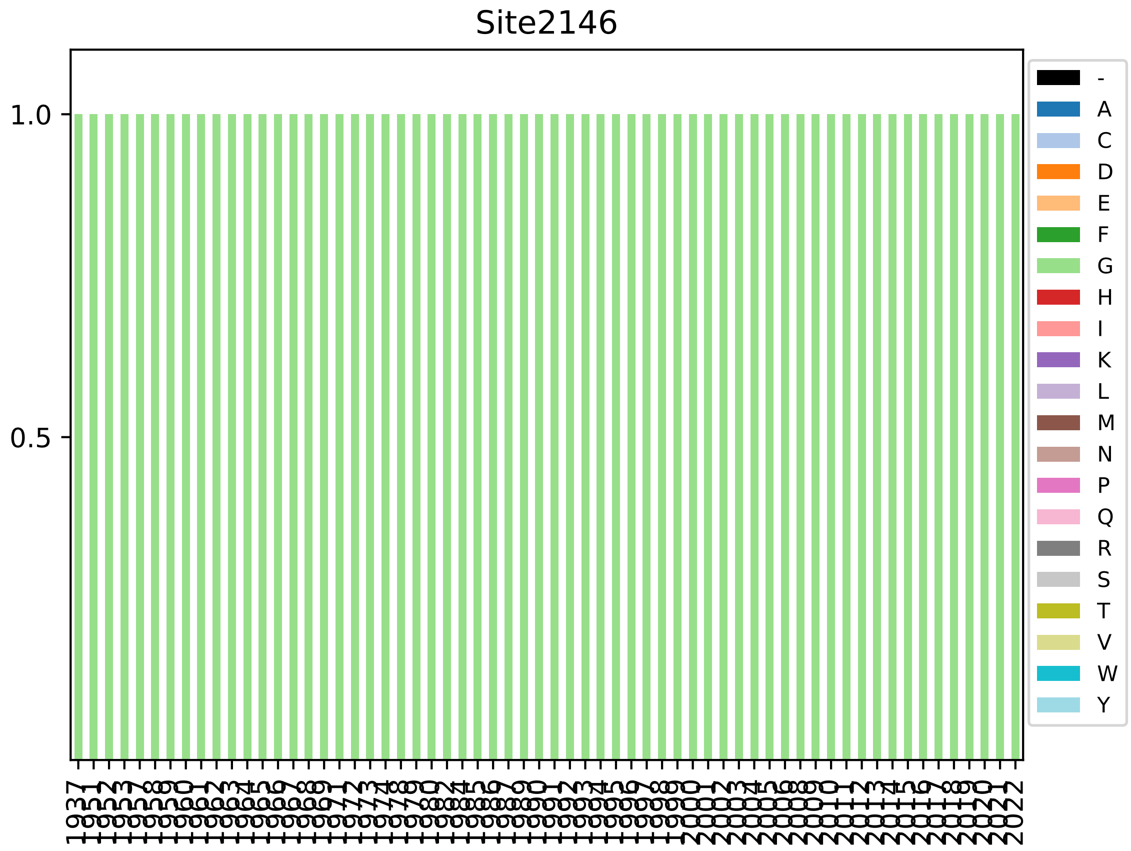
Site2146
1.0
0.5
-
A
C
D
E
F
G
H
I
K
L
M
N
P
Q
R
S
T
V
W
Y

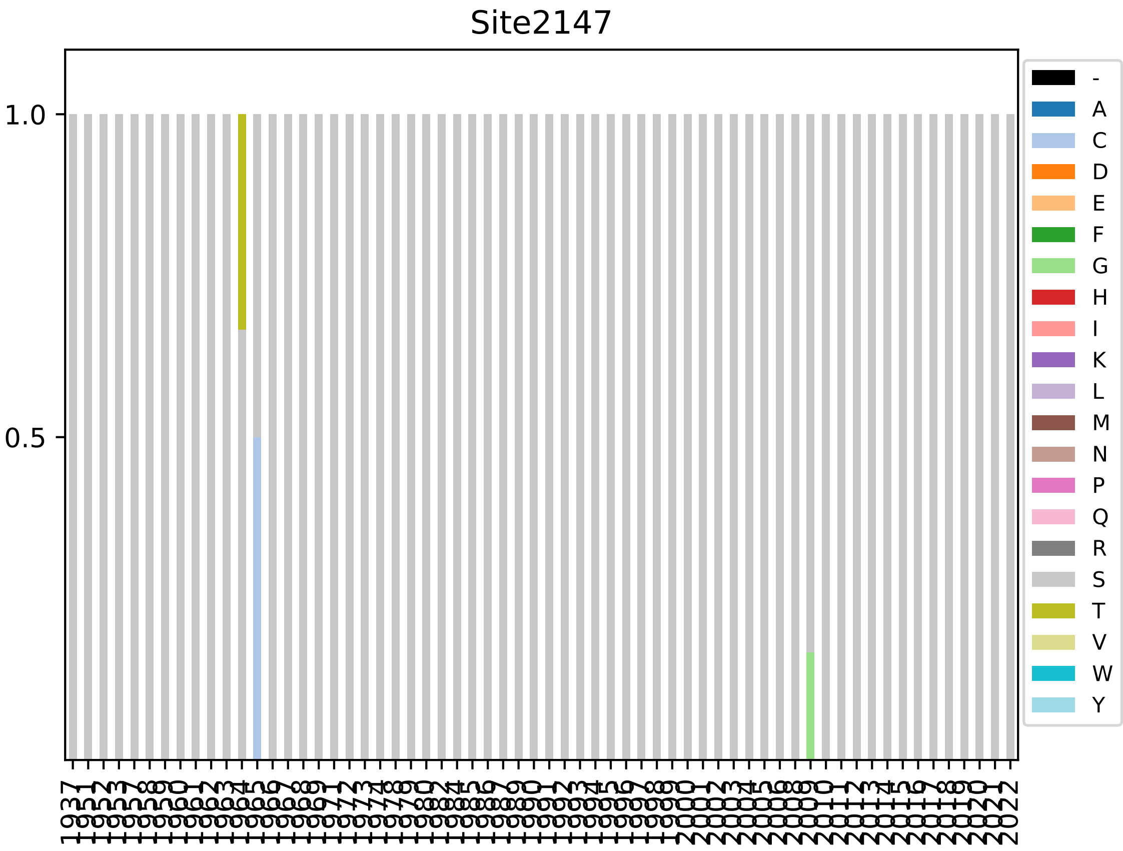
Site2147
1.0
0.5
-
A
C
D
E
F
G
H
I
K
L
M
N
P
Q
R
S
T
V
W
Y

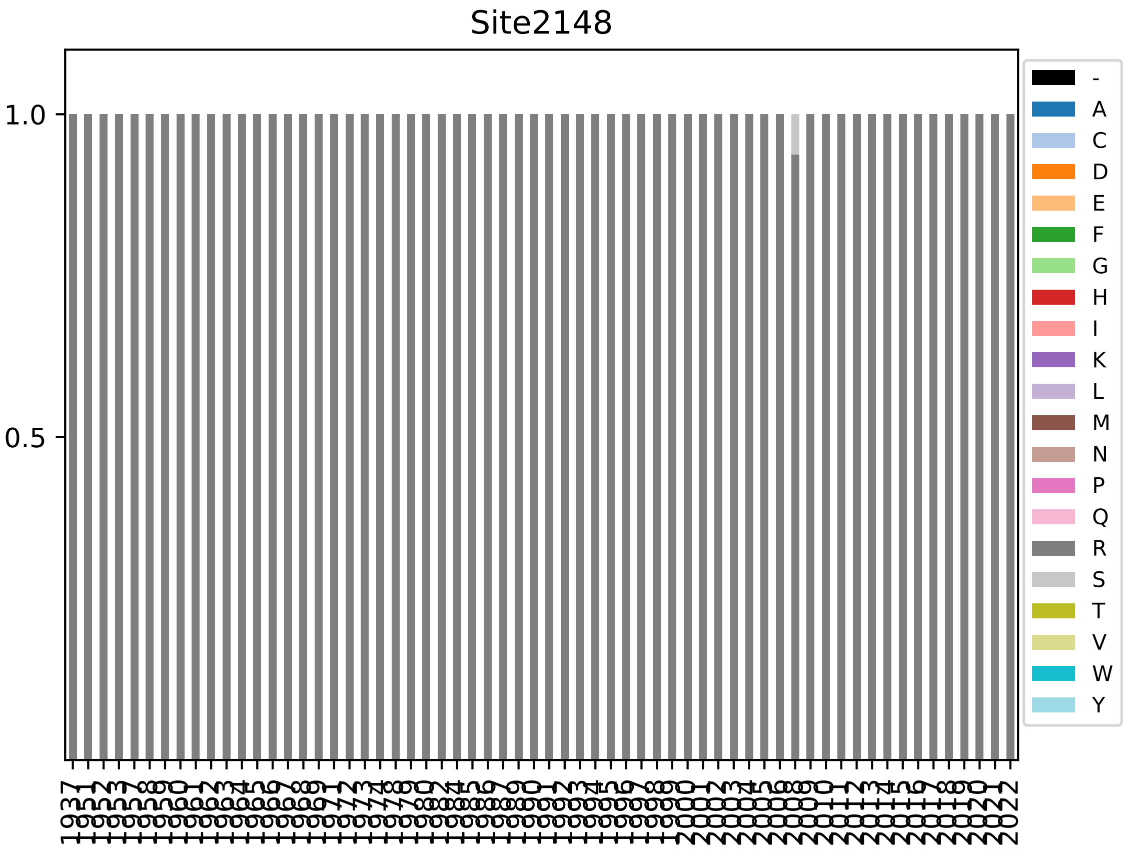
Site2148
1.0
0.5
-
A
C
D
E
F
G
H
I
K
L
M
N
P
Q
R
S
T
V
W
Y

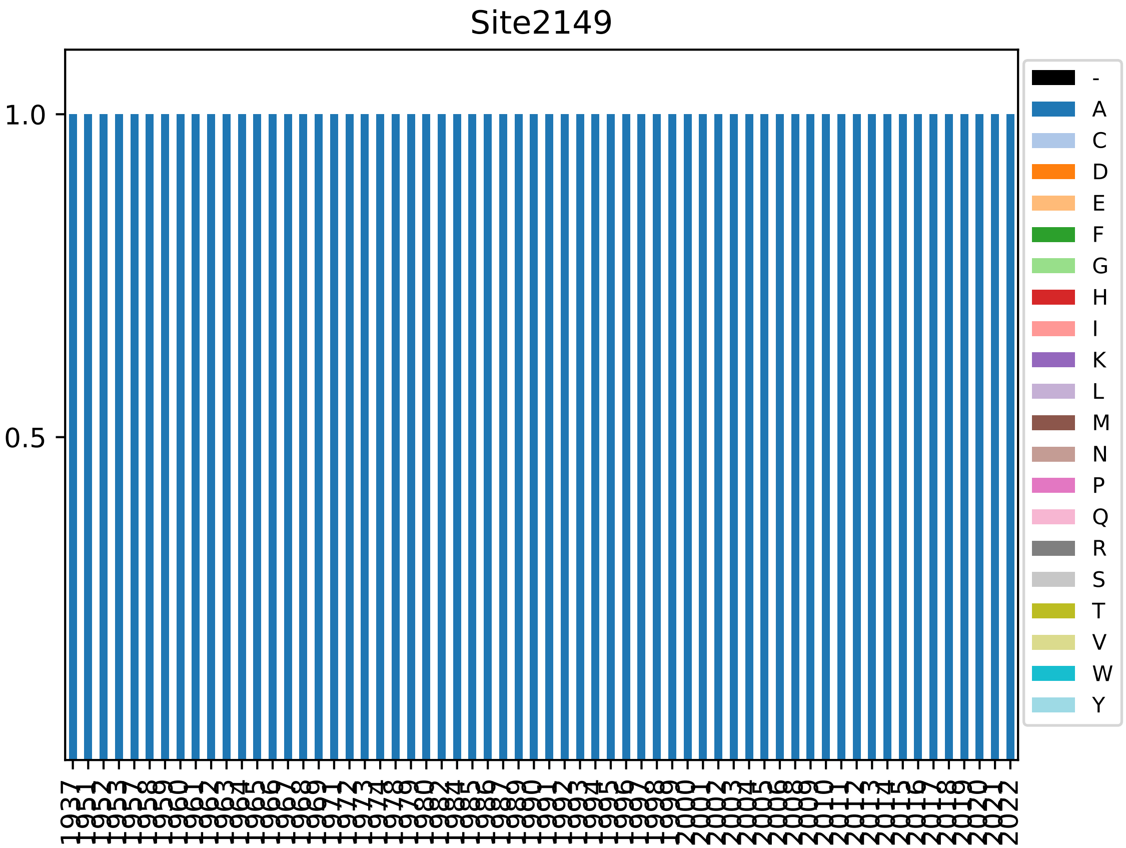

Site2149
1.0
0.5
-
A
C
D
E
F
G
H
I
K
L
M
N
P
Q
R
S
T
V
W
Y

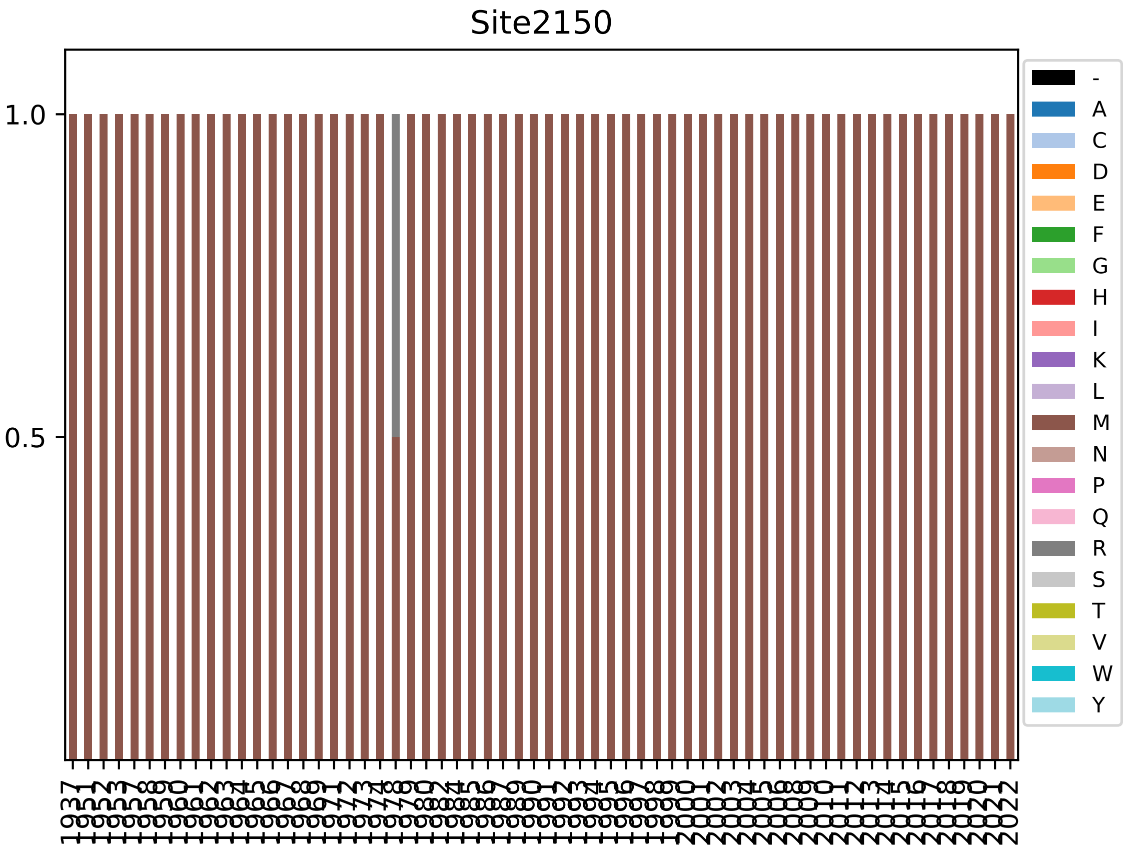
Site2150
1.0
0.5
-
A
C
D
E
F
G
H
I
K
L
M
N
P
Q
R
S
T
V
W
Y

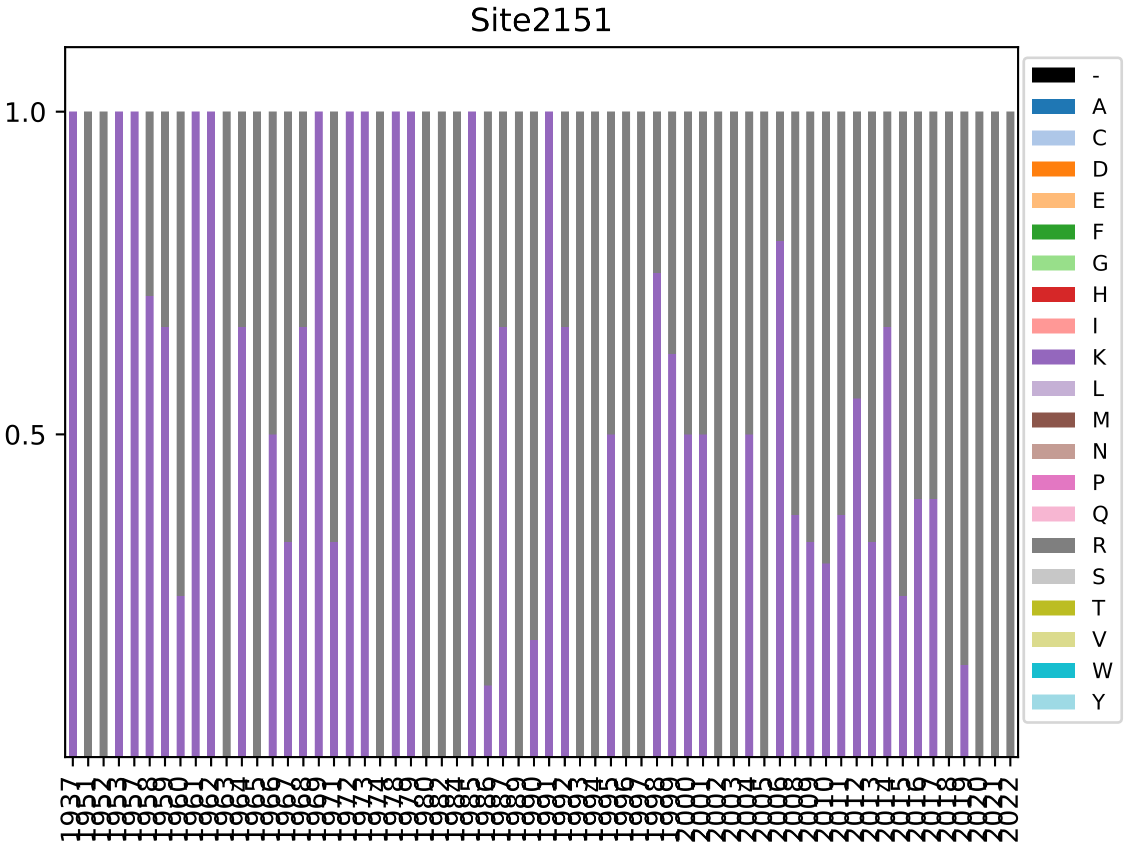
Site2151
1.0
0.5
-
A
C
D
E
F
G
H
I
K
L
M
N
P
Q
R
S
T
V
W
Y

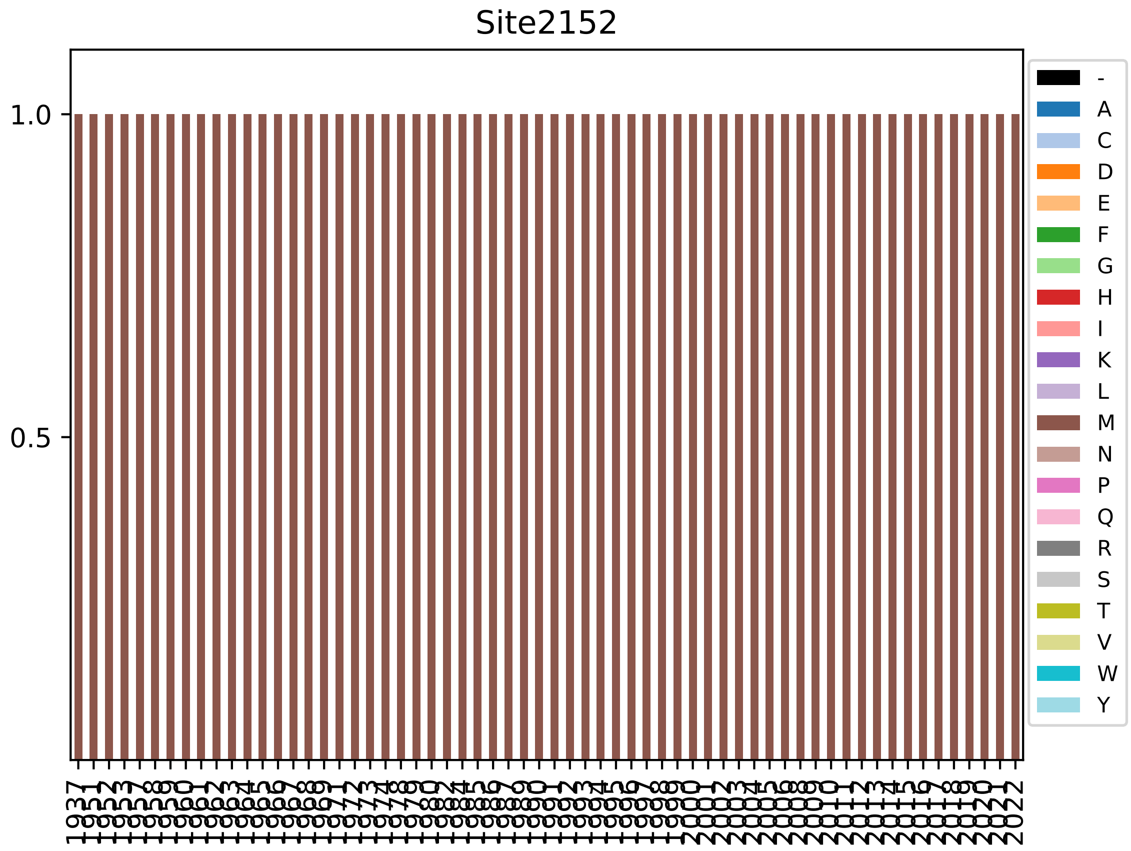
Site2152
1.0
0.5
-
A
C
D
E
F
G
H
I
K
L
M
N
P
Q
R
S
T
V
W
Y

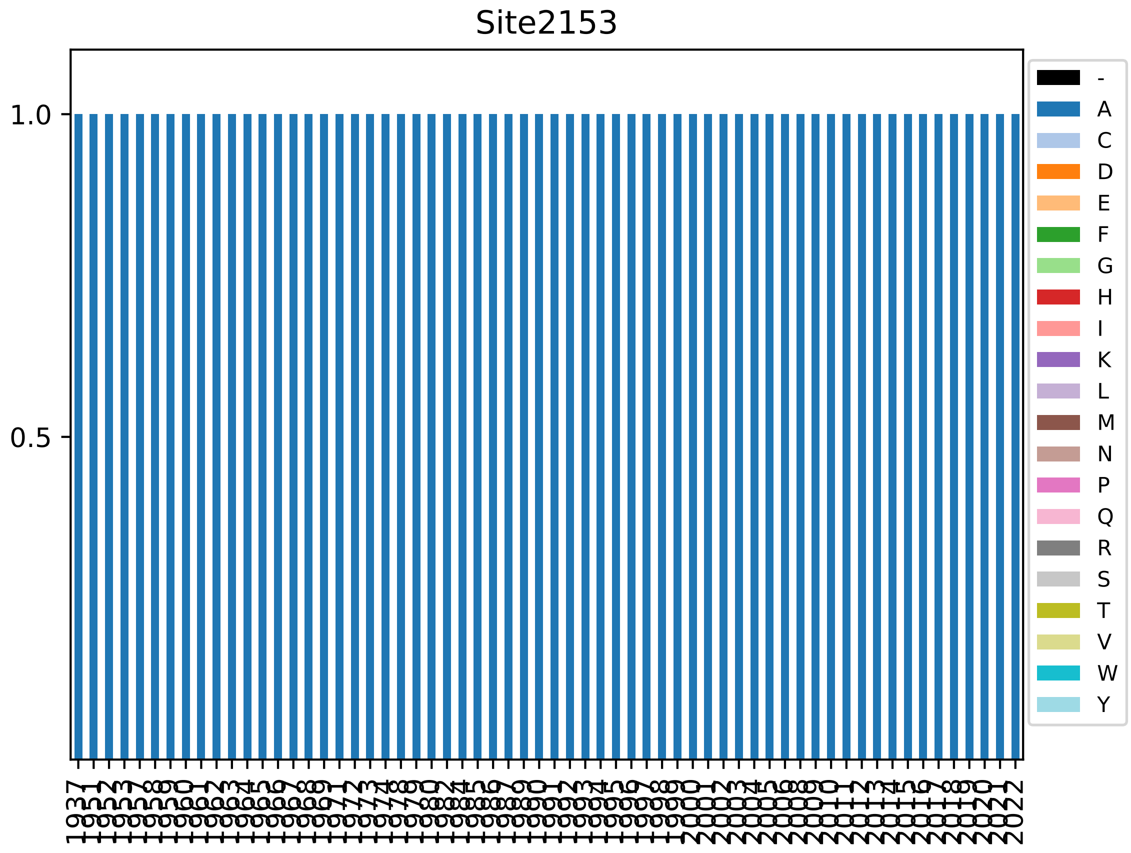
Site2153
1.0
0.5
-
A
C
D
E
F
G
H
I
K
L
M
N
P
Q
R
S
T
V
W
Y

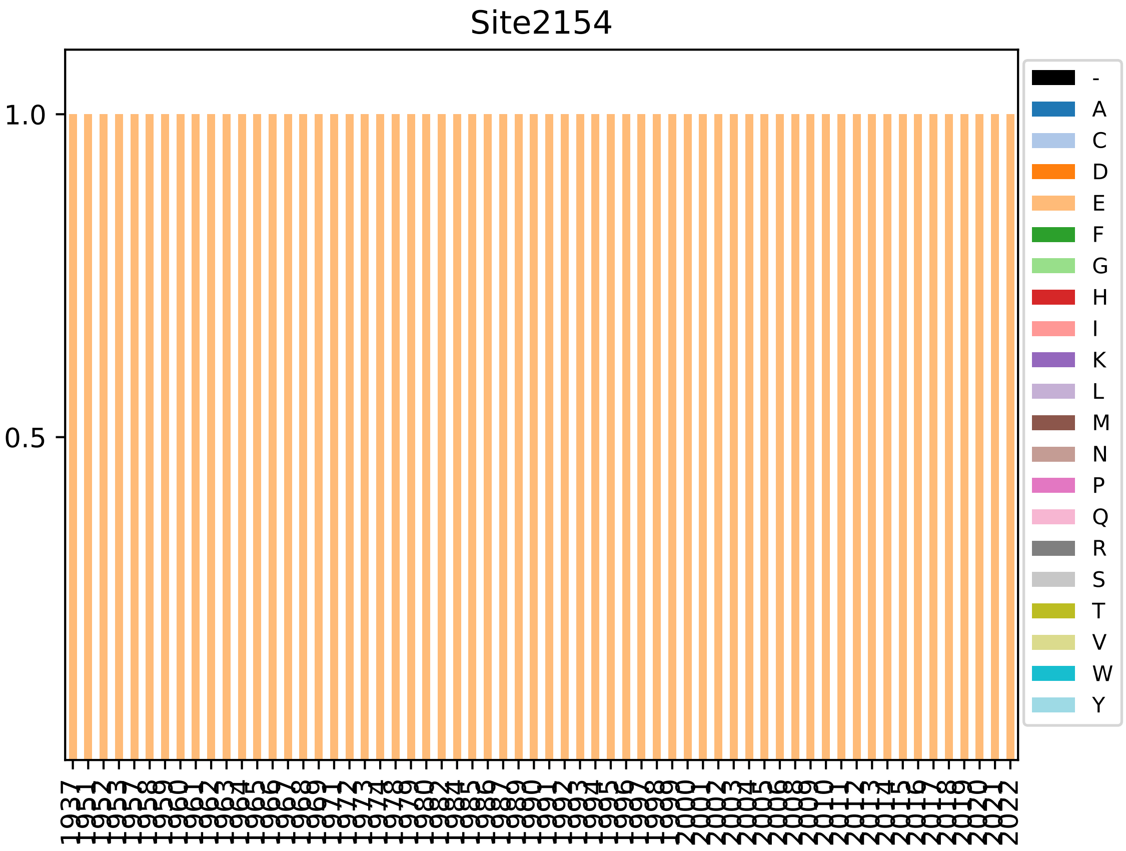
Site2154
1.0
0.5
-
A
C
D
E
F
G
H
I
K
L
M
N
P
Q
R
S
T
V
W
Y

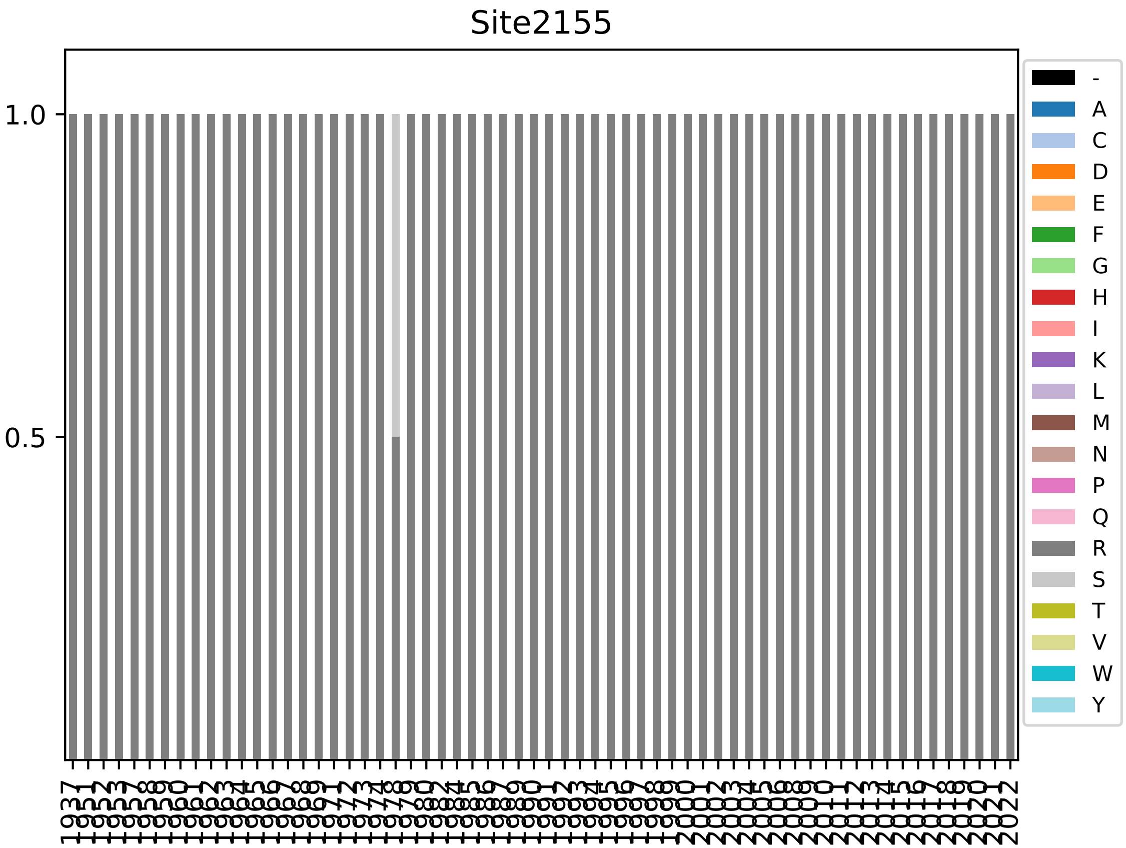
Site2155
1.0
0.5
-
A
C
D
E
F
G
H
I
K
L
M
N
P
Q
R
S
T
V
W
Y

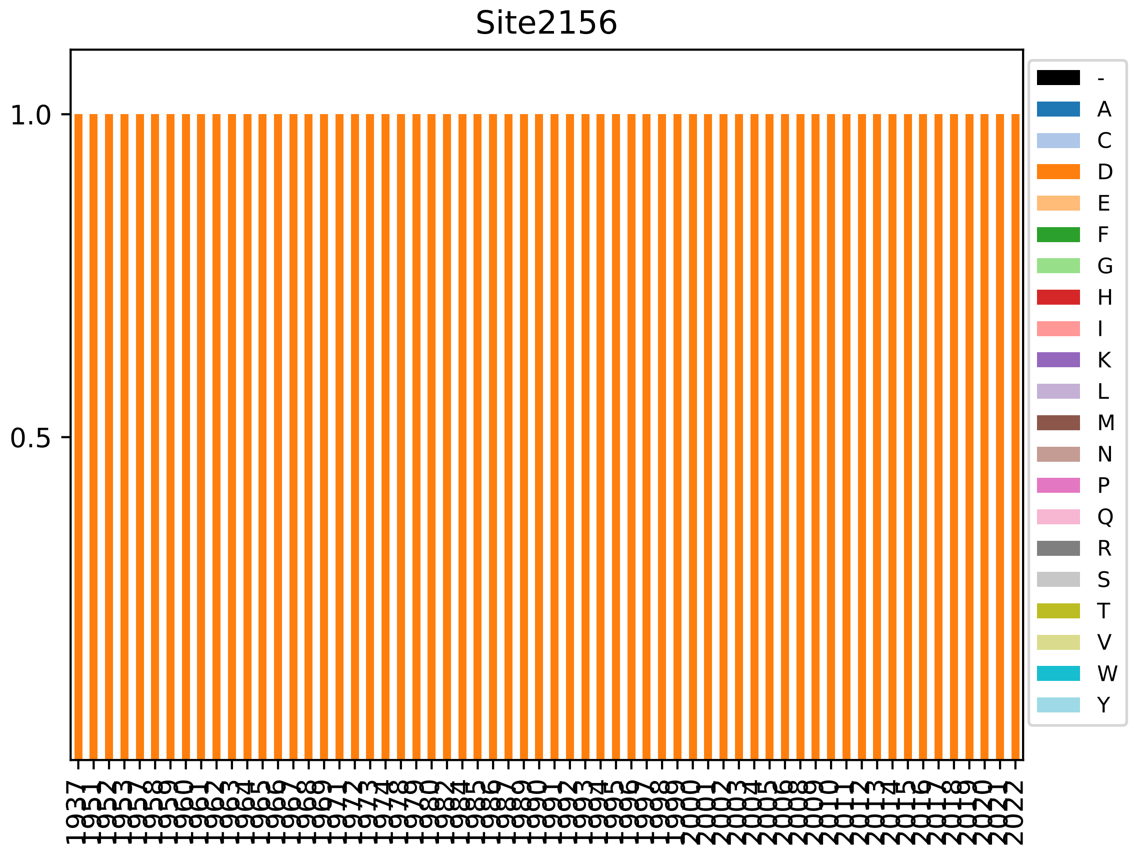
Site2156
1.0
0.5
-
A
C
D
E
F
G
H
I
K
L
M
N
P
Q
R
S
T
V
W
Y

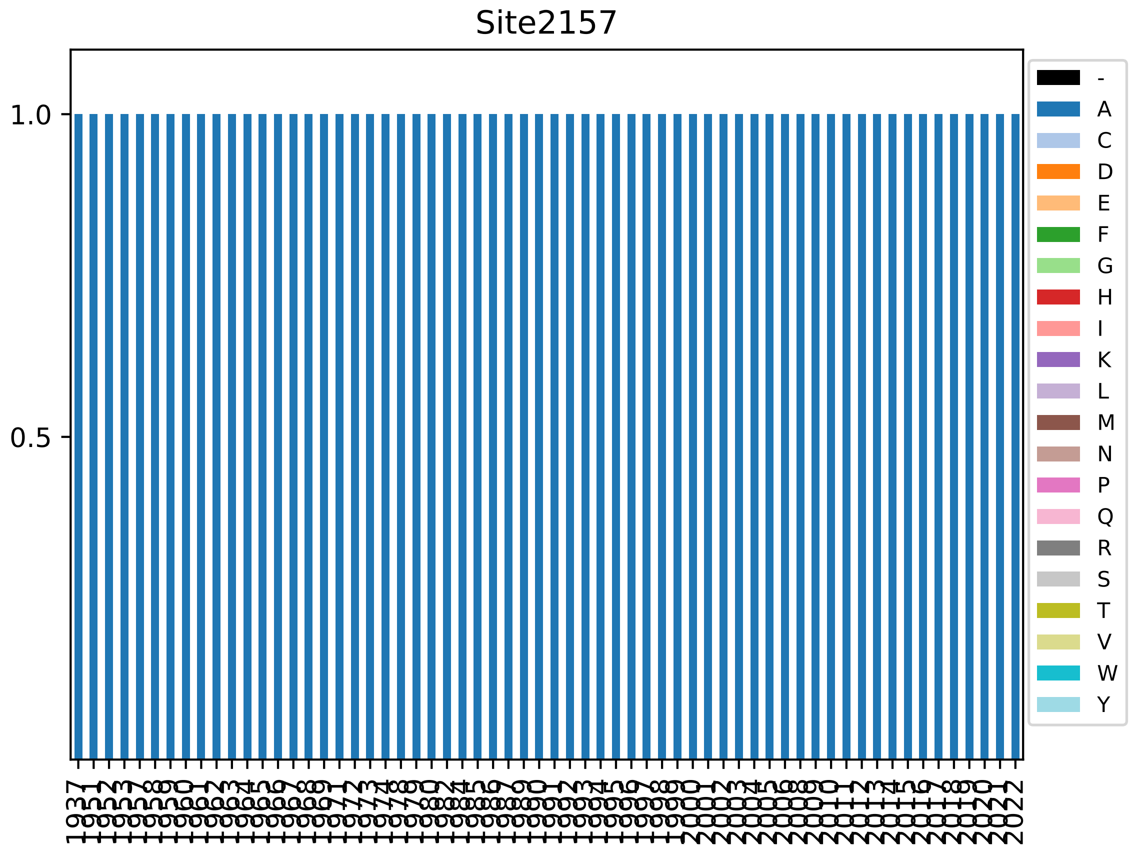
Site2157
1.0
0.5
-
A
C
D
E
F
G
H
I
K
L
M
N
P
Q
R
S
T
V
W
Y

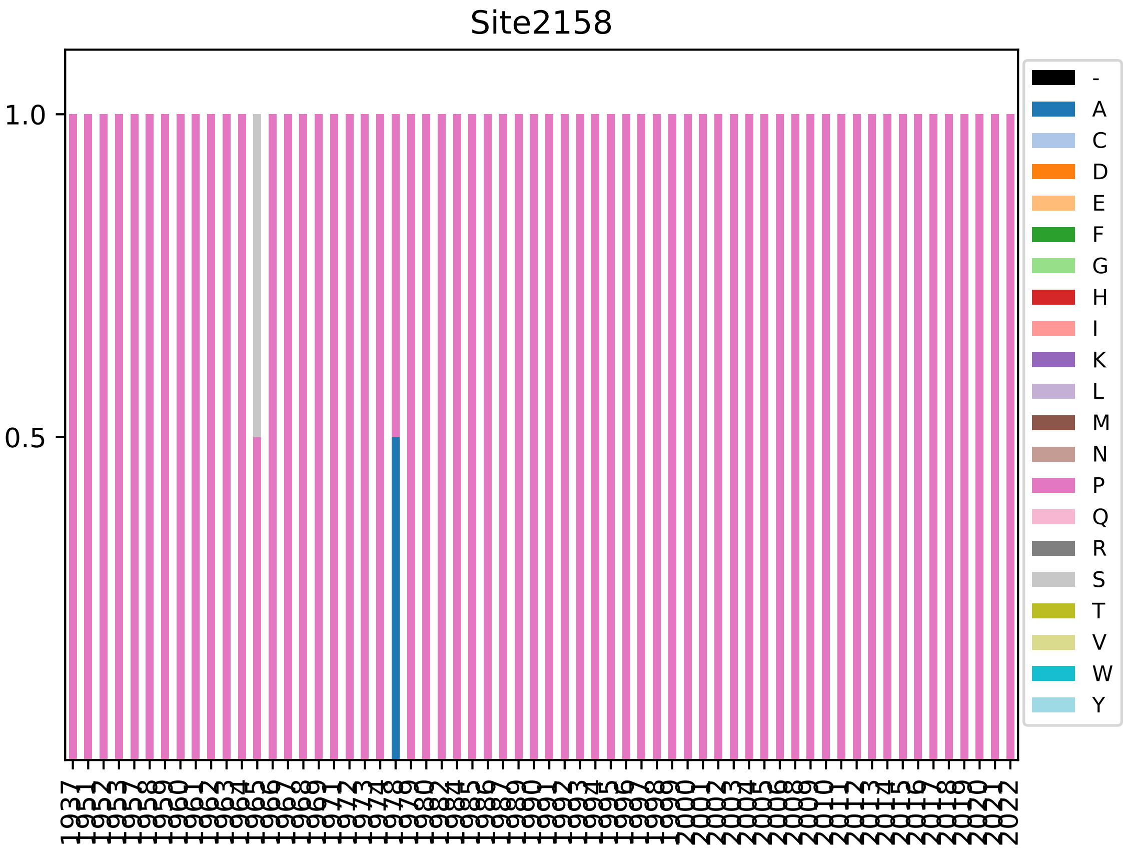
Site2158
1.0
0.5
-
A
C
D
E
F
G
H
I
K
L
M
N
P
Q
R
S
T
V
W
Y

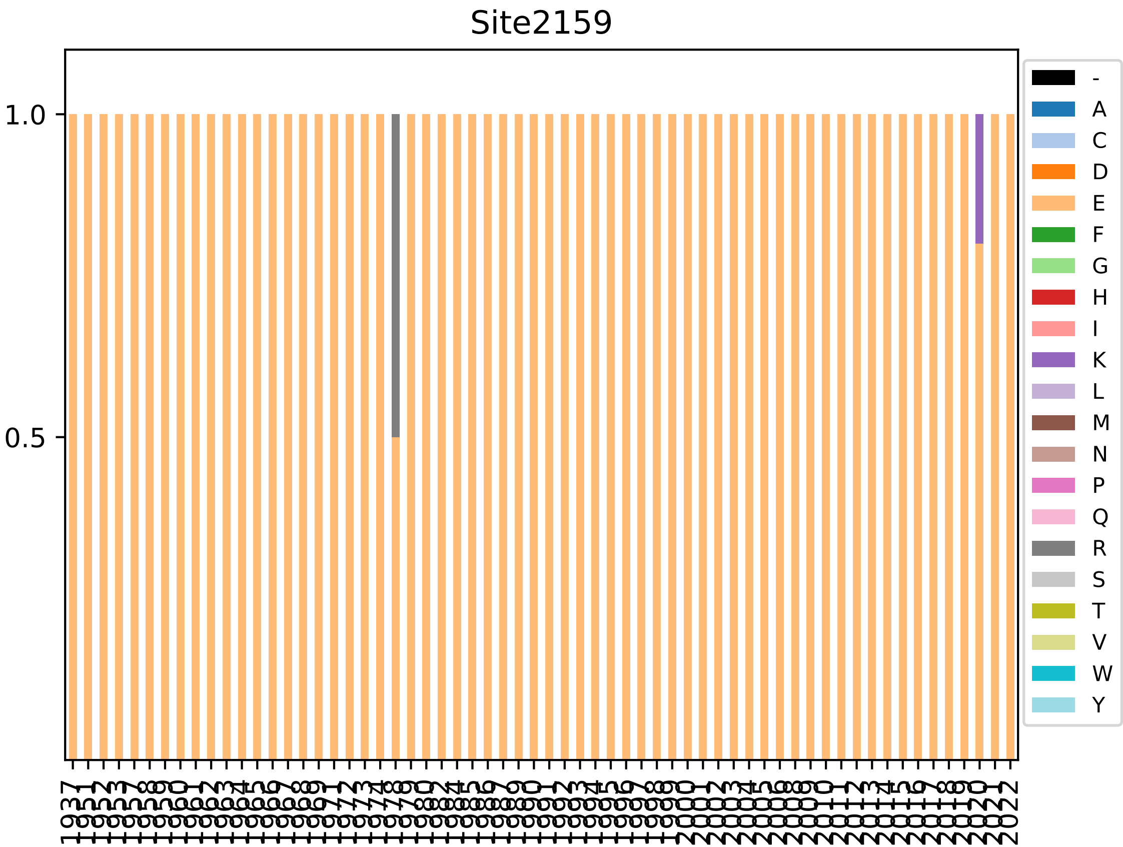
Site2159
1.0
0.5
-
A
C
D
E
F
G
H
I
K
L
M
N
P
Q
R
S
T
V
W
Y

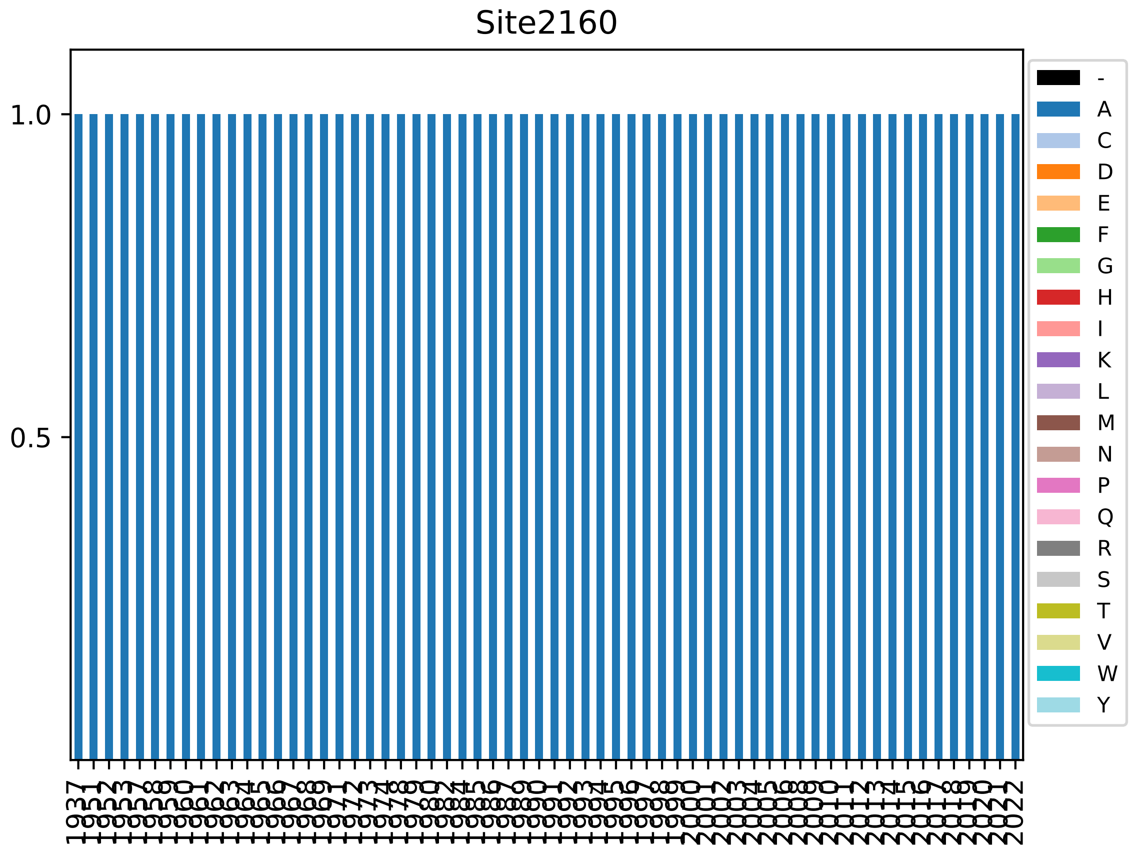
Site2160
1.0
0.5
-
A
C
D
E
F
G
H
I
K
L
M
N
P
Q
R
S
T
V
W
Y

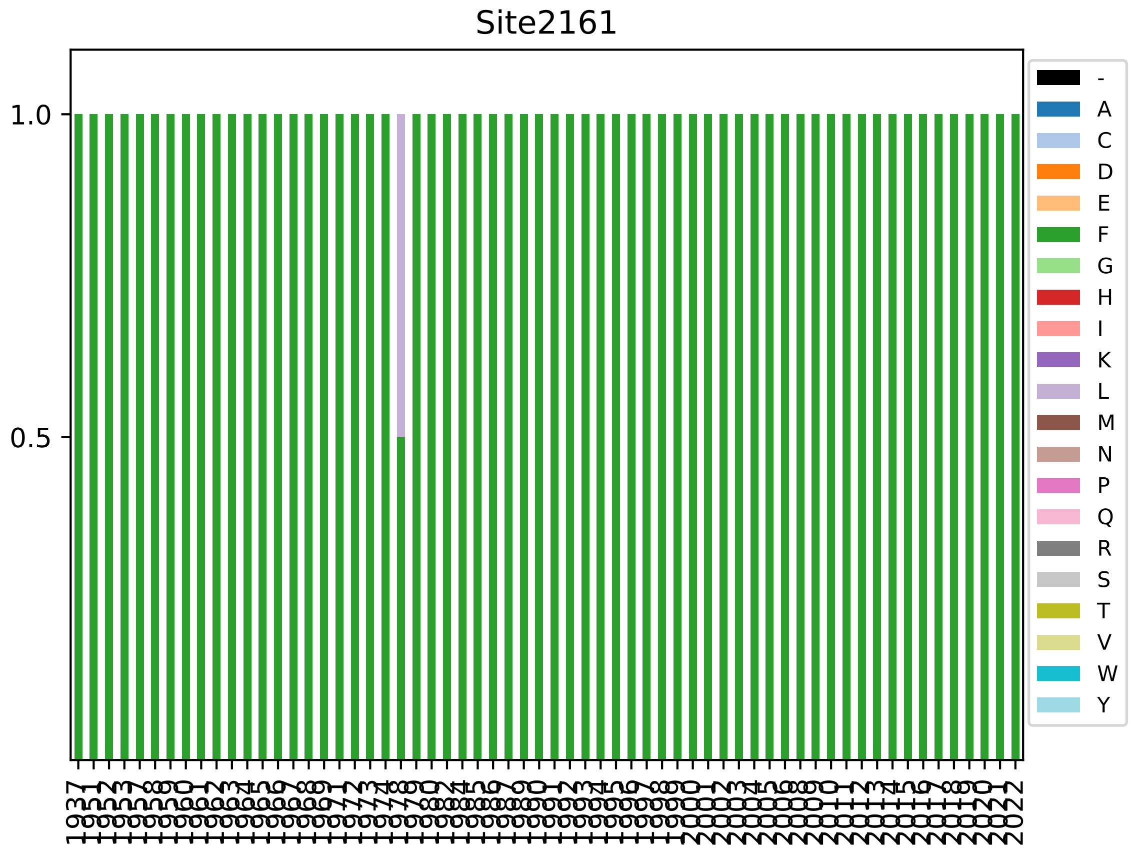
Site2161
1.0
0.5
-
A
C
D
E
F
G
H
I
K
L
M
N
P
Q
R
S
T
V
W
Y

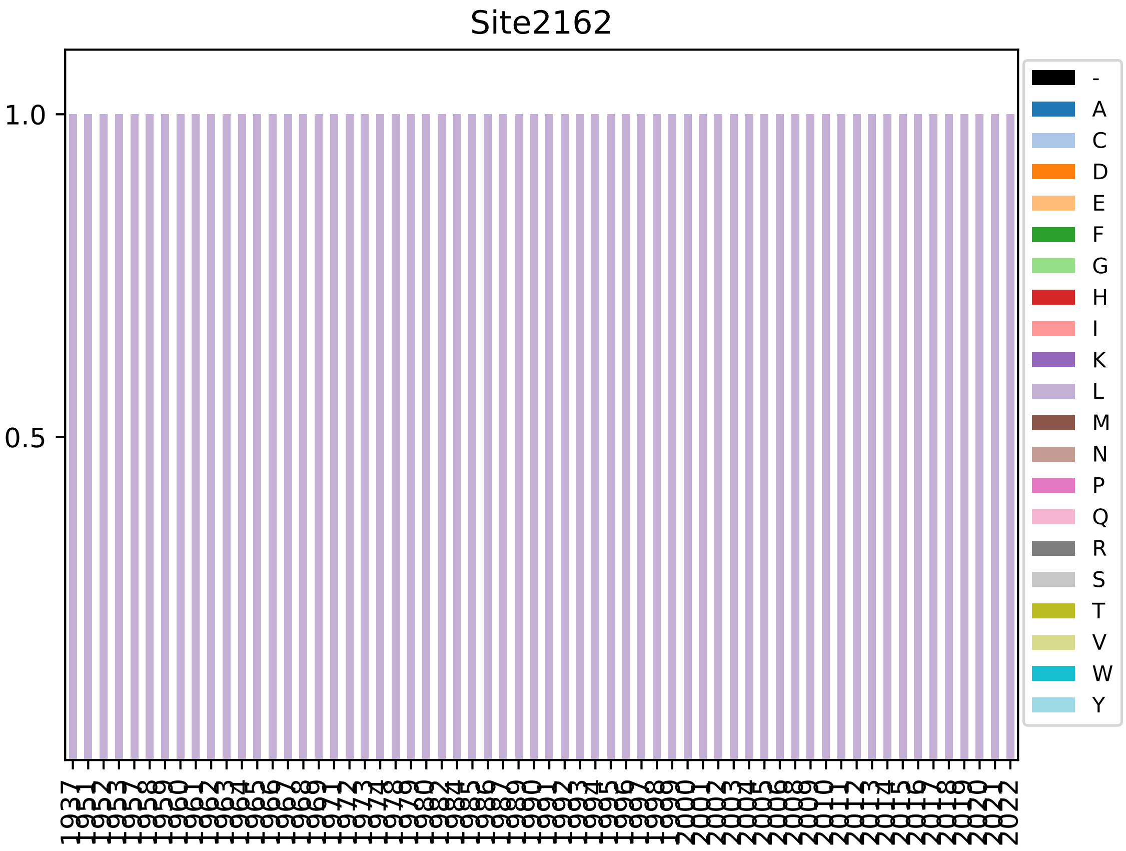
Site2162
1.0
0.5
-
A
C
D
E
F
G
H
I
K
L
M
N
P
Q
R
S
T
V
W
Y

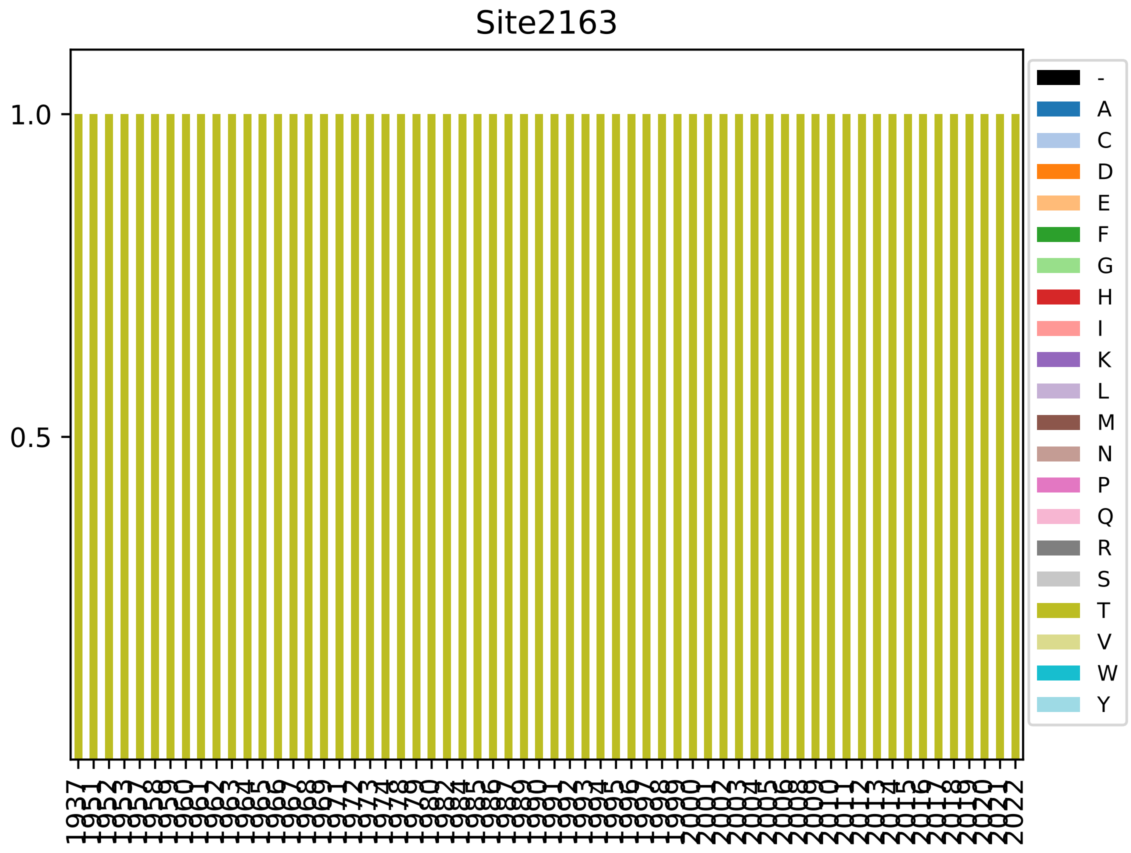
Site2163
1.0
0.5
-
A
C
D
E
F
G
H
I
K
L
M
N
P
Q
R
S
T
V
W
Y

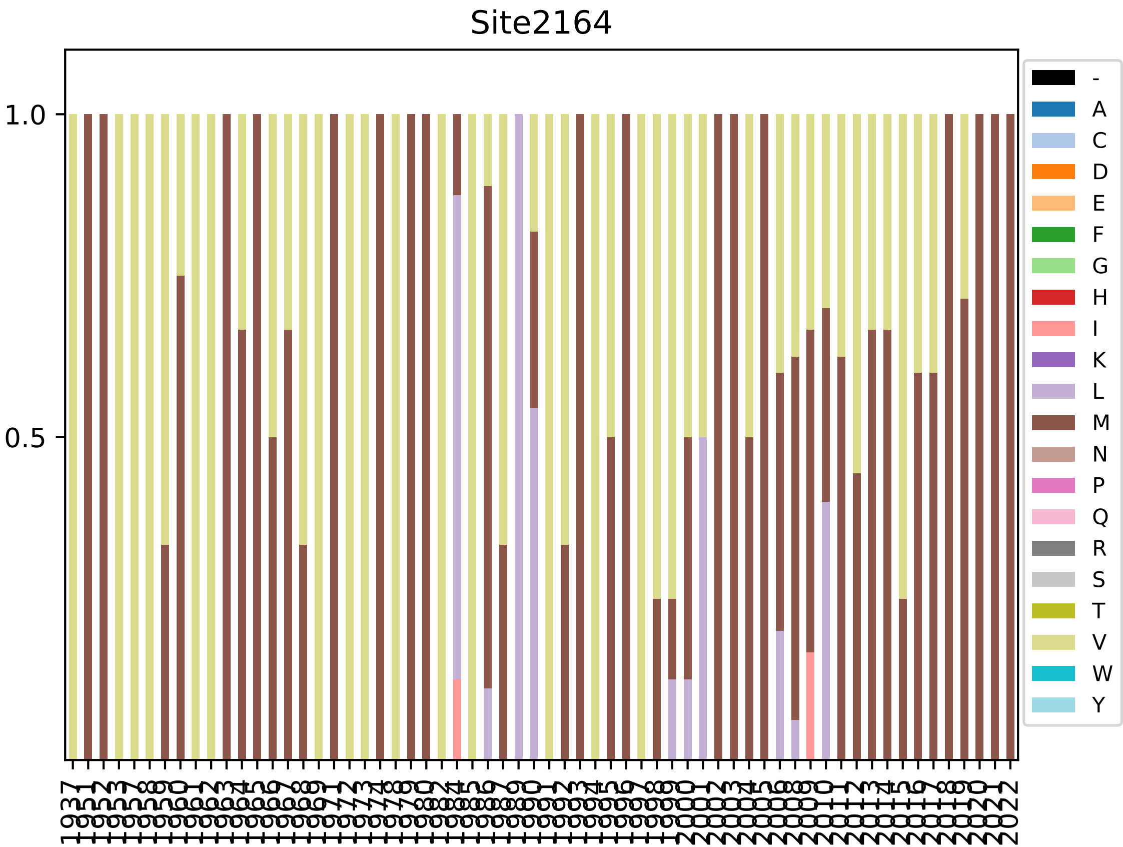
Site2164
1.0
0.5
-
A
C
D
E
F
G
H
I
K
L
M
N
P
Q
R
S
T
V
W
Y

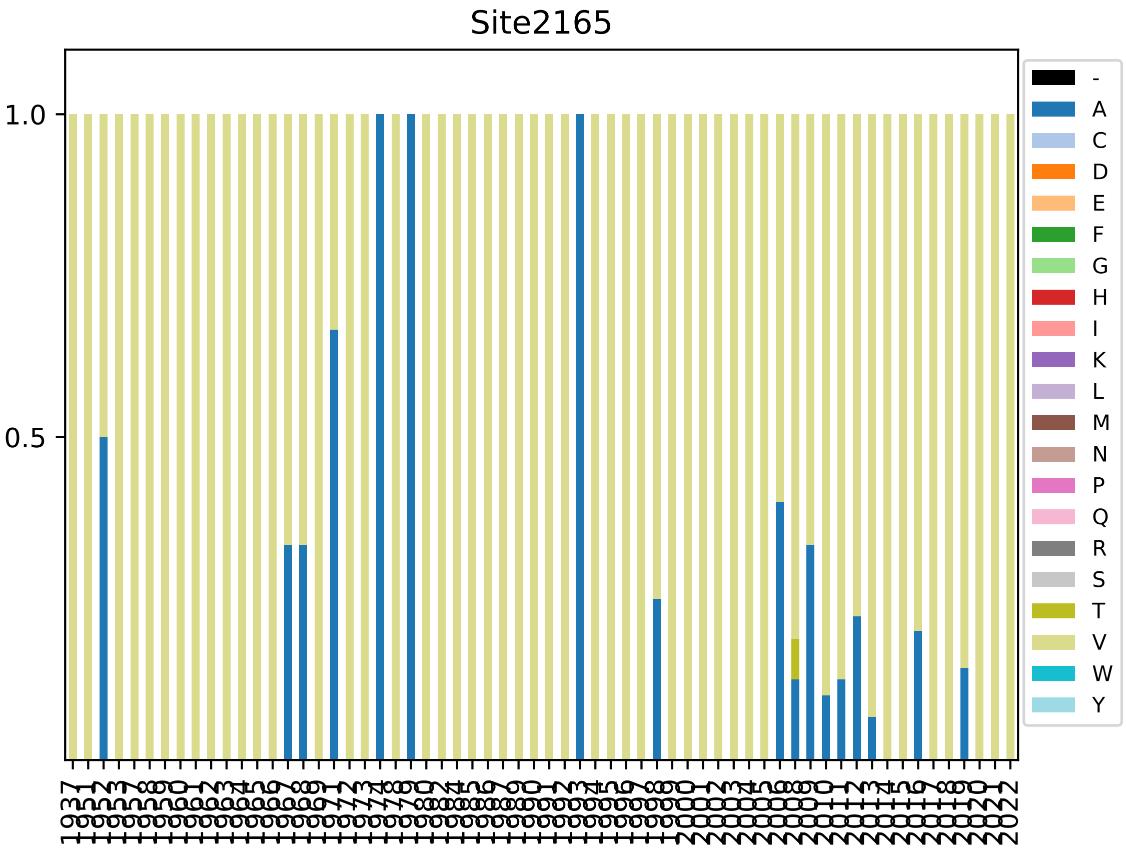

Site2165
1.0
0.5
-
A
C
D
E
F
G
H
I
K
L
M
N
P
Q
R
S
T
V
W
Y

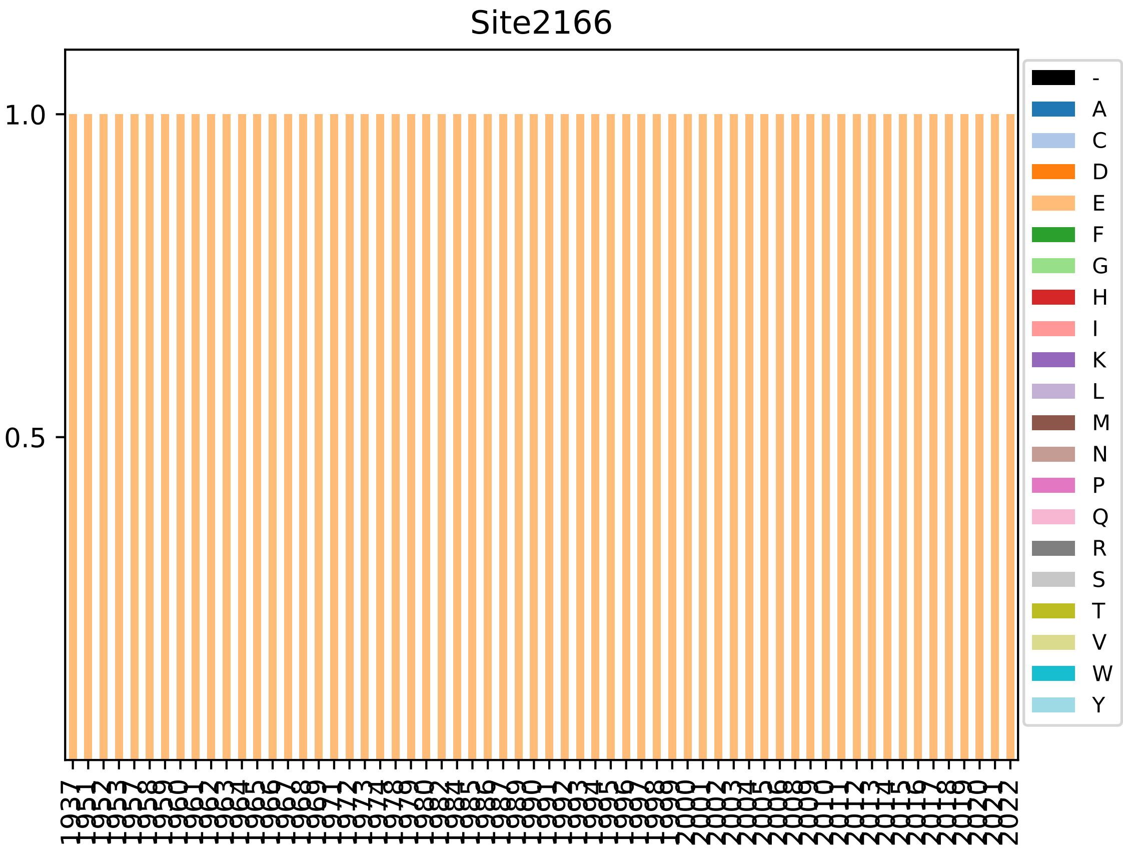
Site2166
1.0
0.5
-
A
C
D
E
F
G
H
I
K
L
M
N
P
Q
R
S
T
V
W
Y

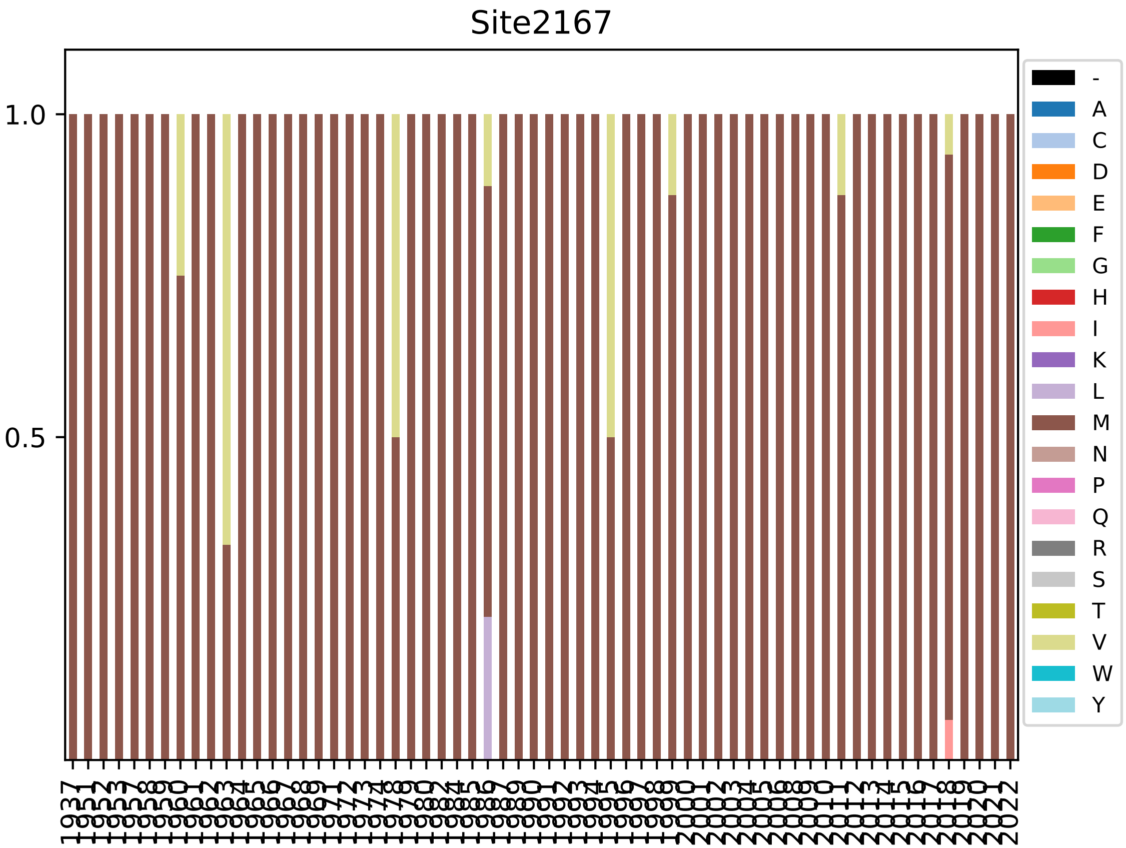
Site2167
1.0
0.5
-
A
C
D
E
F
G
H
I
K
L
M
N
P
Q
R
S
T
V
W
Y

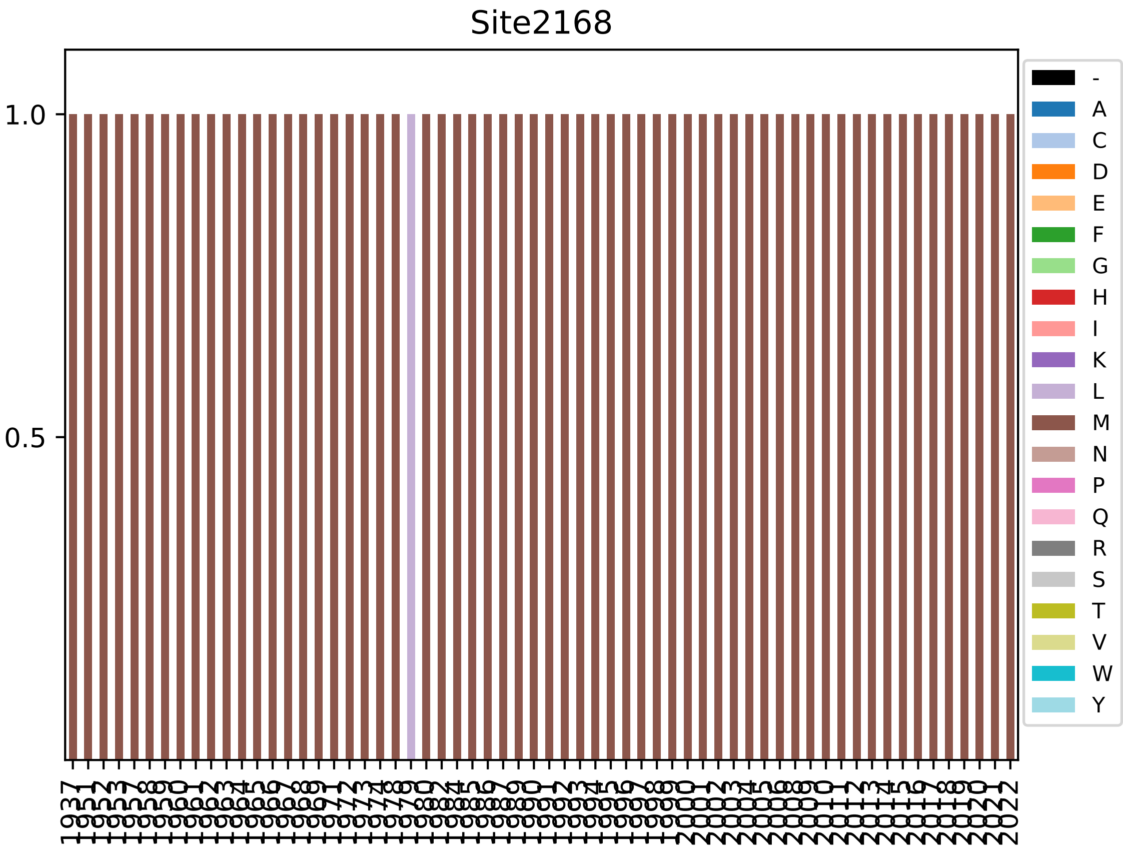
Site2168
1.0
0.5
-
A
C
D
E
F
G
H
I
K
L
M
N
P
Q
R
S
T
V
W
Y

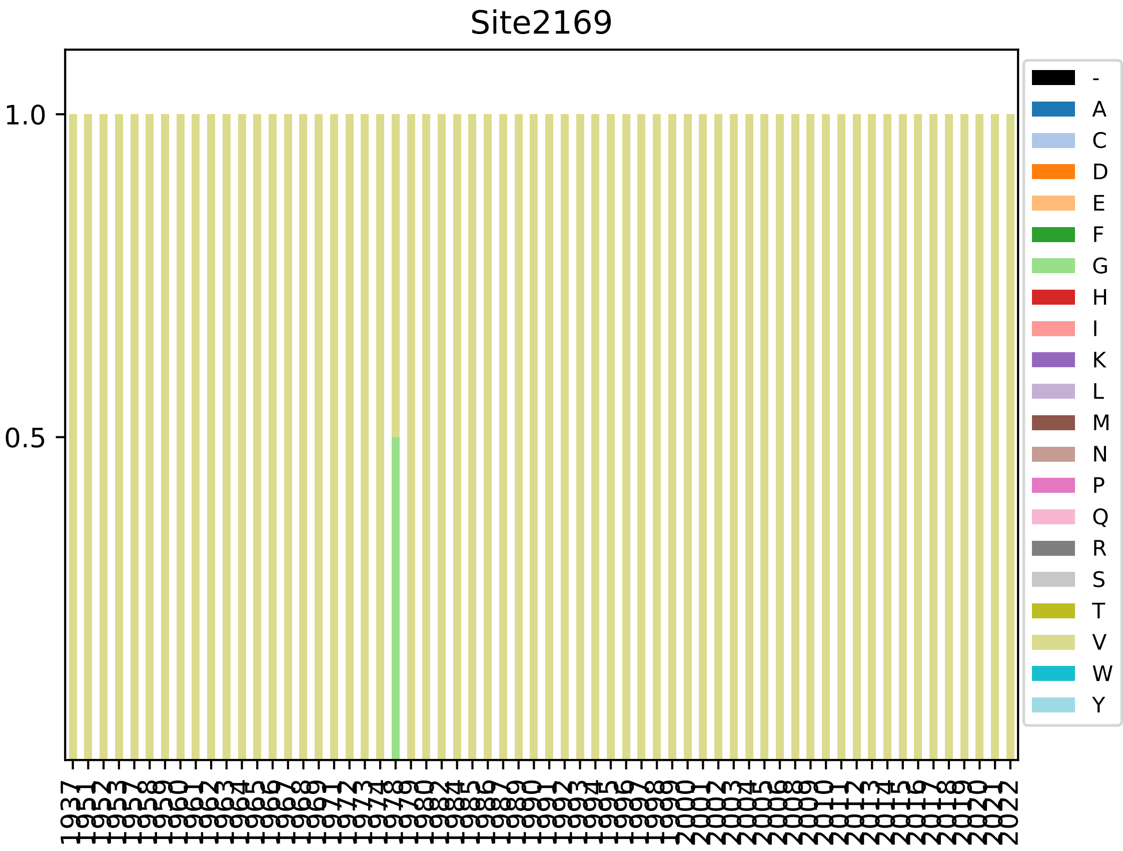
Site2169
1.0
0.5
-
A
C
D
E
F
G
H
I
K
L
M
N
P
Q
R
S
T
V
W
Y

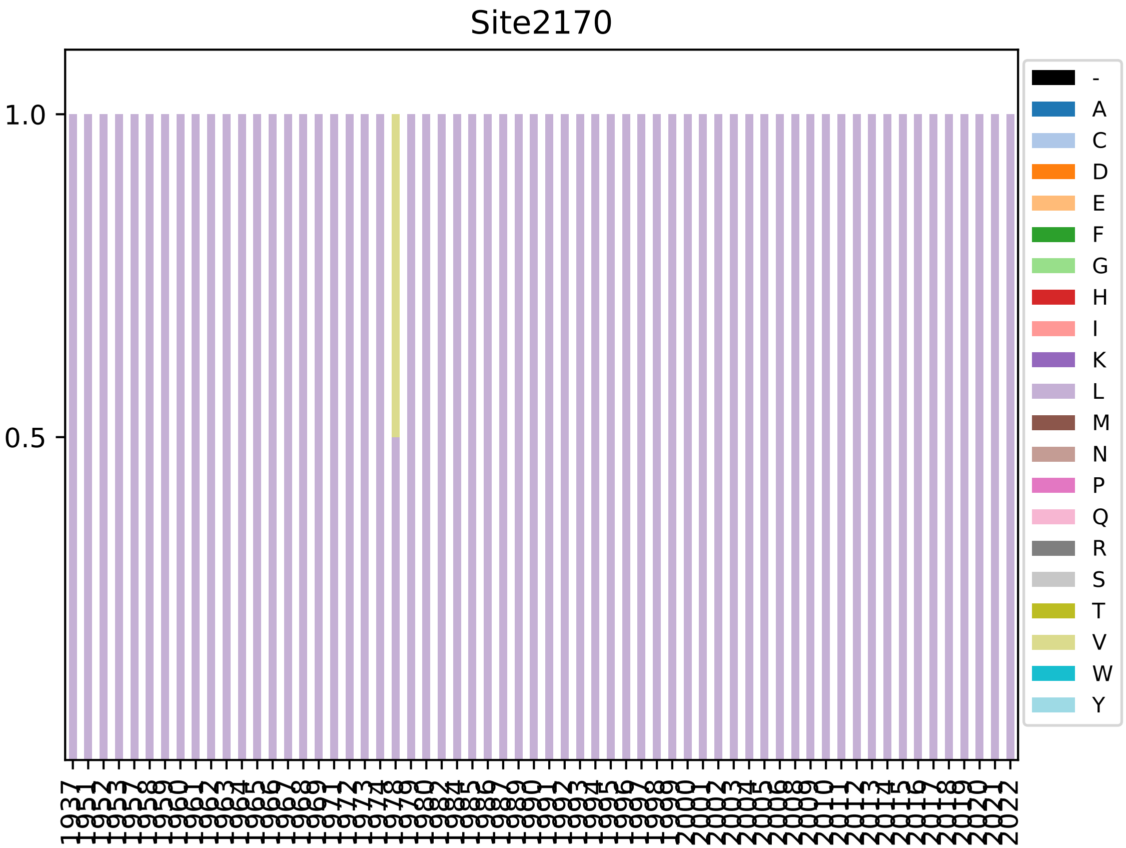
Site2170
1.0
0.5
-
A
C
D
E
F
G
H
I
K
L
M
N
P
Q
R
S
T
V
W
Y

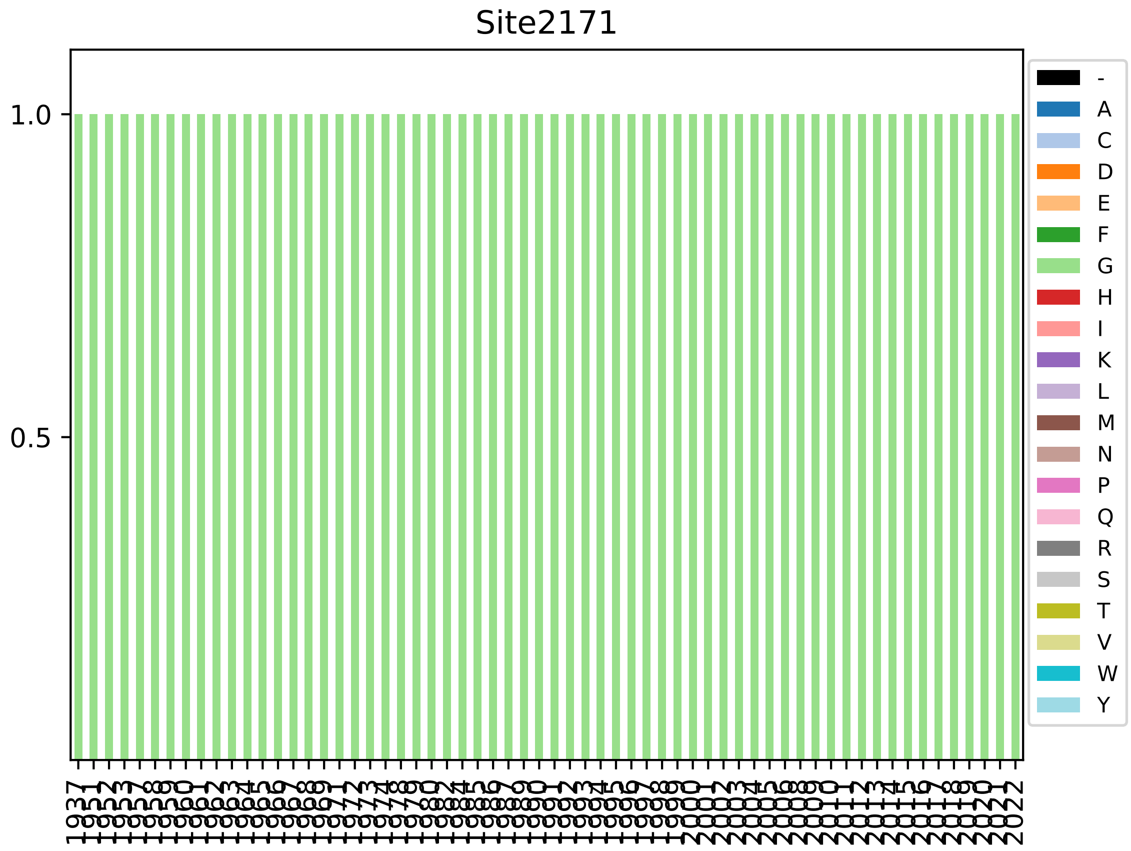
Site2171
1.0
0.5
-
A
C
D
E
F
G
H
I
K
L
M
N
P
Q
R
S
T
V
W
Y

Site2172

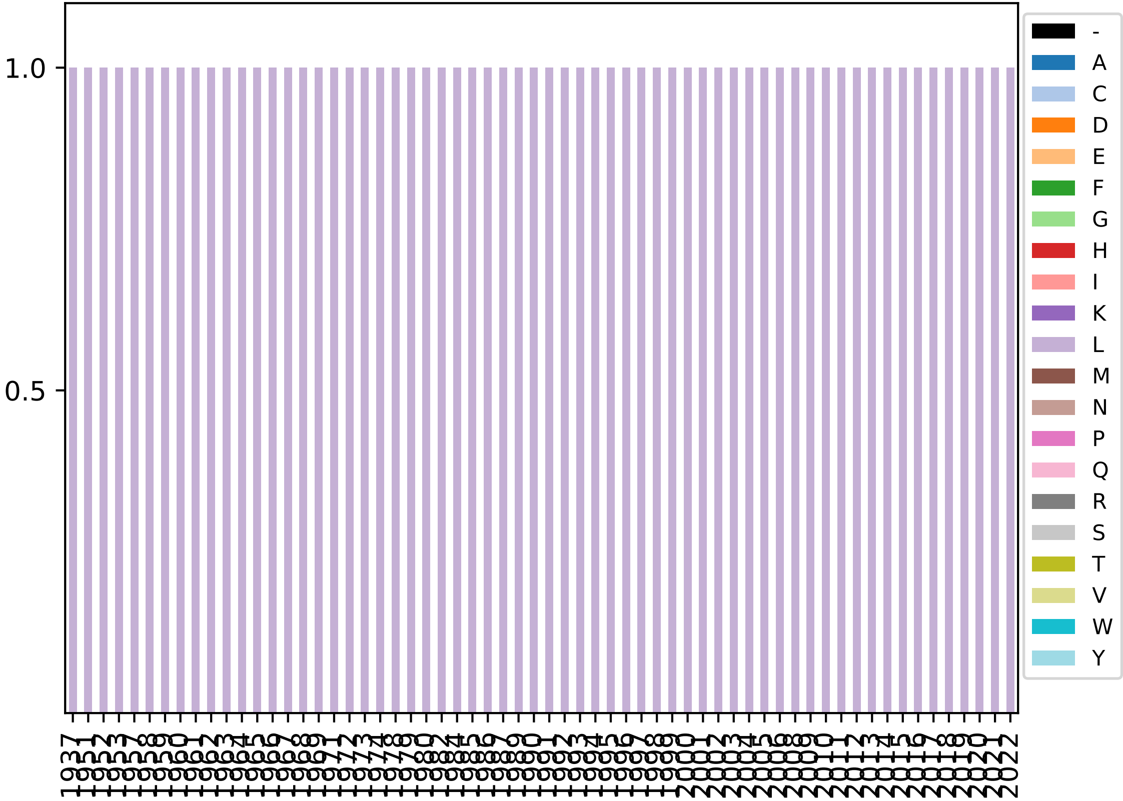

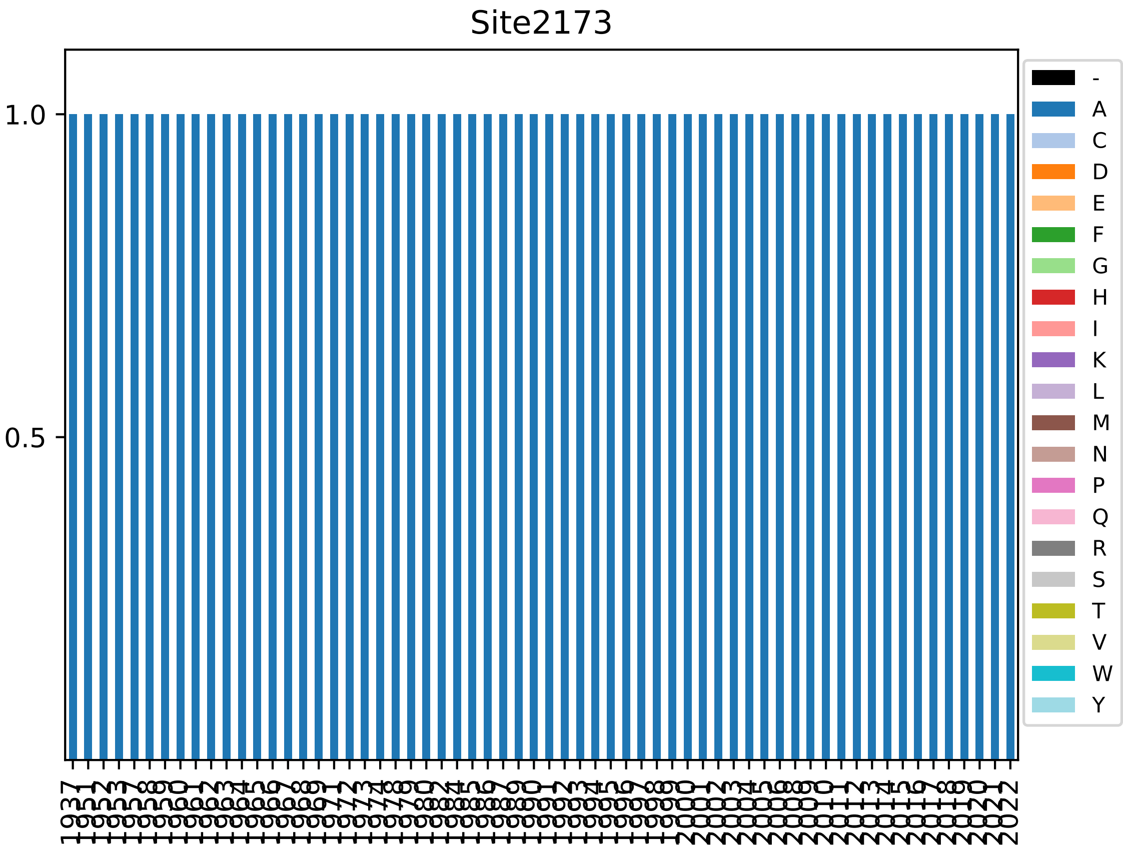
Site2173
1.0
0.5
-
A
C
D
E
F
G
H
I
K
L
M
N
P
Q
R
S
T
V
W
Y

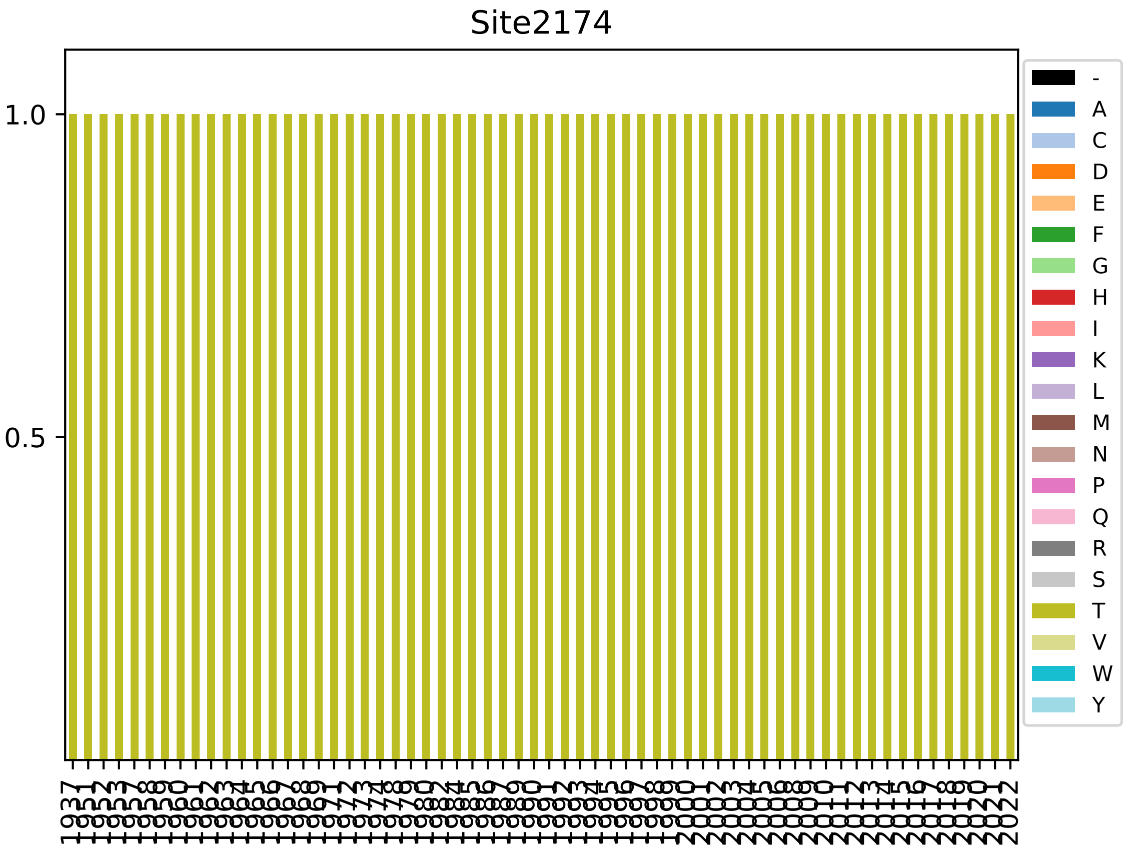
Site2174
1.0
0.5
-
A
C
D
E
F
G
H
I
K
L
M
N
P
Q
R
S
T
V
W
Y

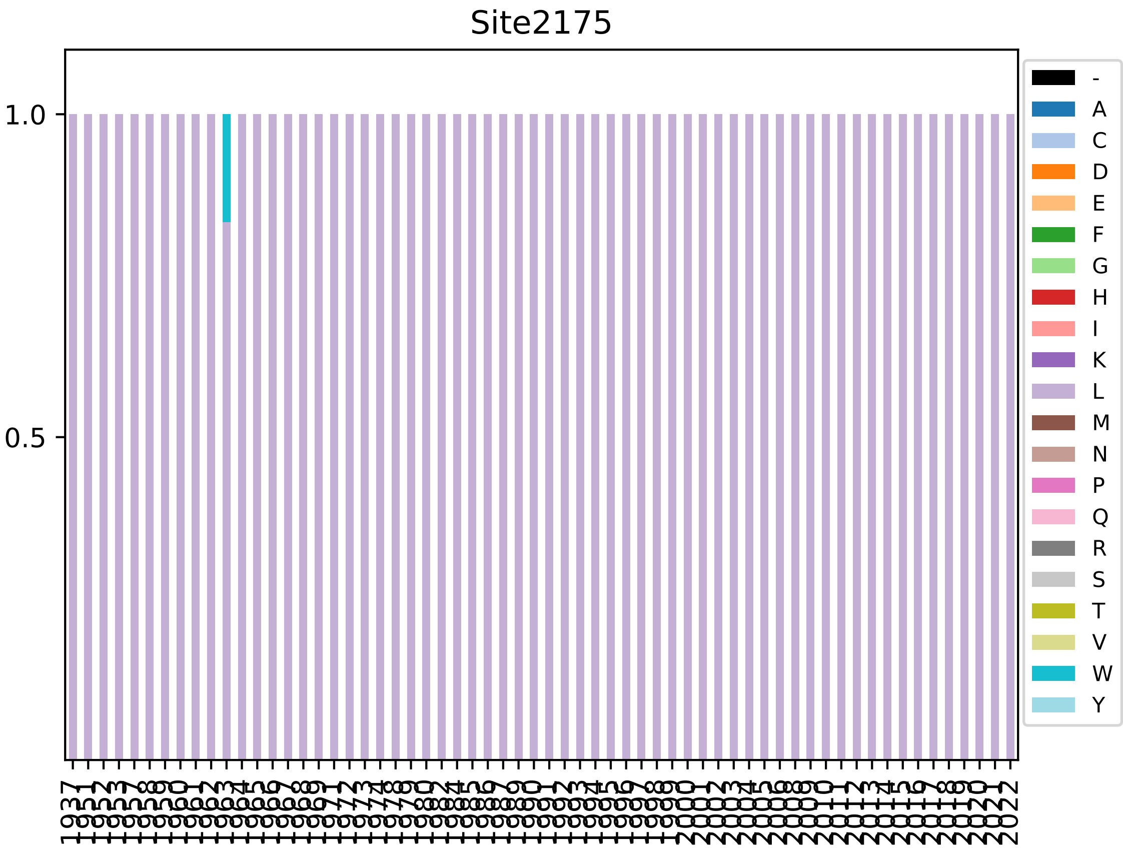
Site2175
1.0
0.5
-
A
C
D
E
F
G
H
I
K
L
M
N
P
Q
R
S
T
V
W
Y

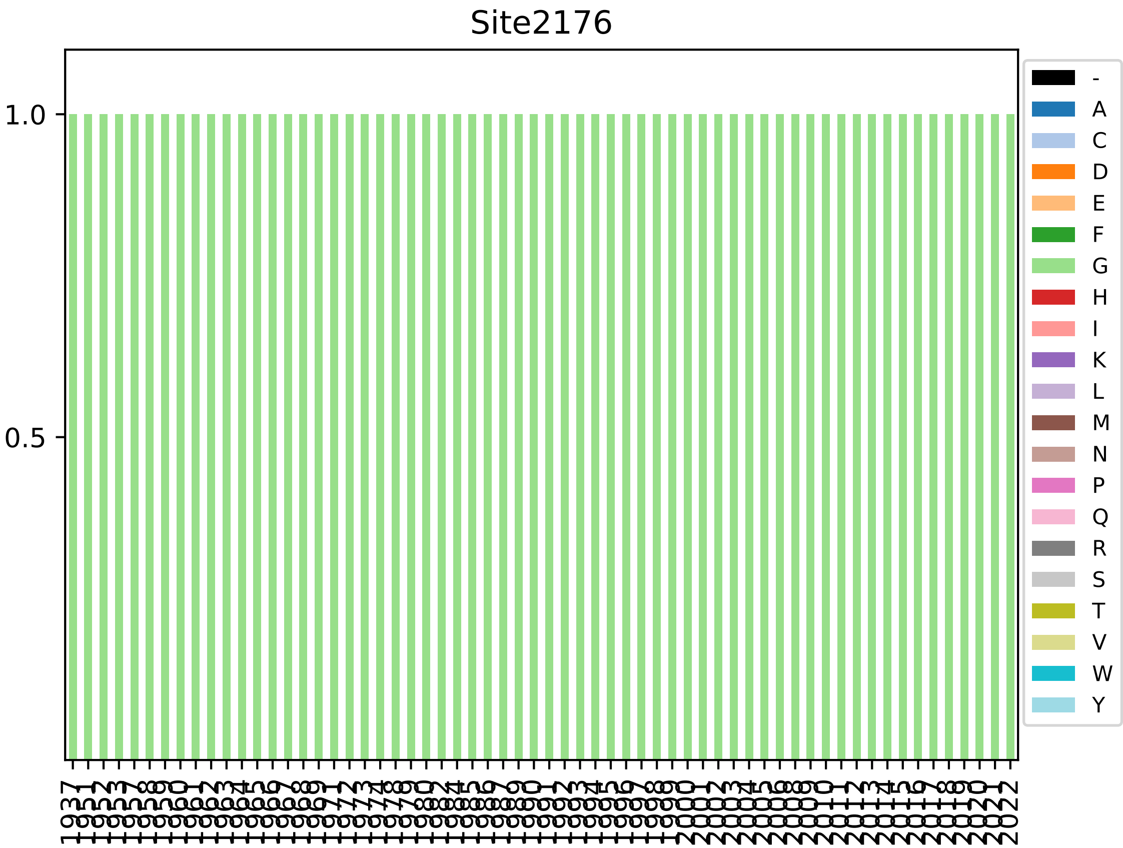
Site2176
1.0
0.5
-
A
C
D
E
F
G
H
I
K
L
M
N
P
Q
R
S
T
V
W
Y

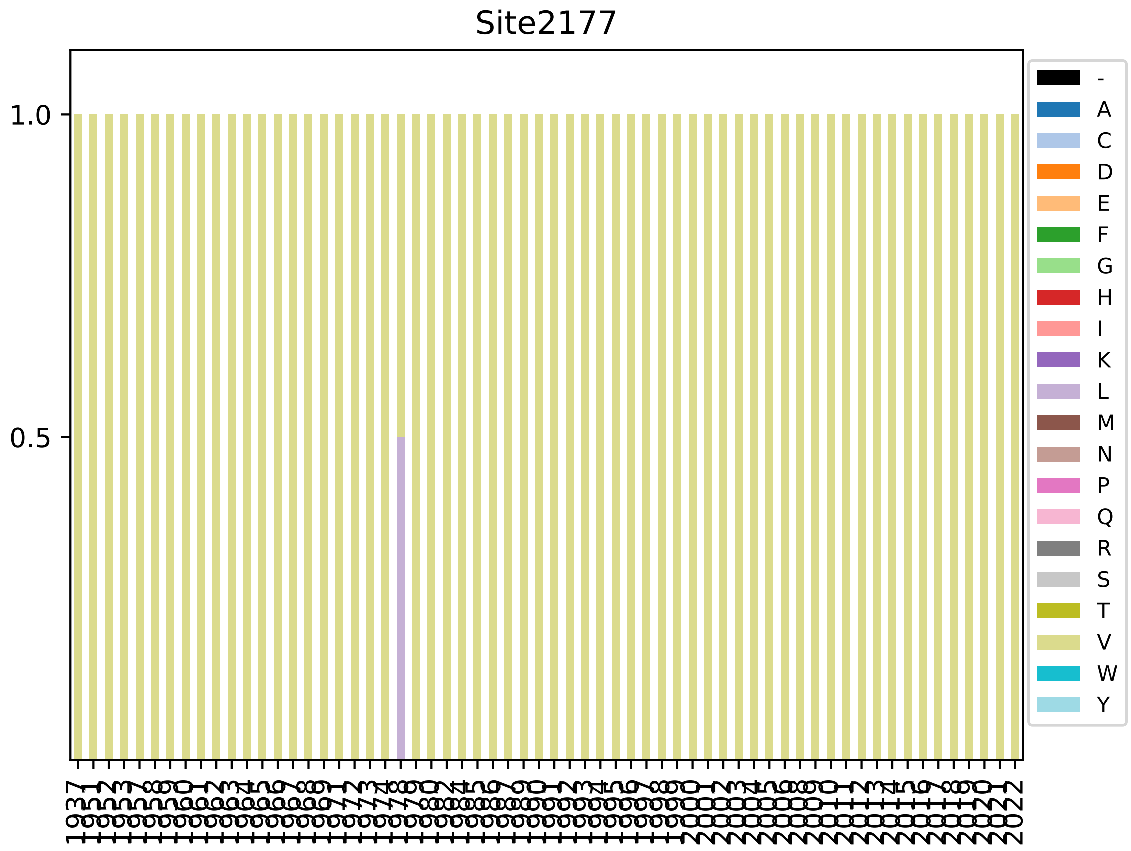
Site2177
1.0
0.5
-
A
C
D
E
F
G
H
I
K
L
M
N
P
Q
R
S
T
V
W
Y

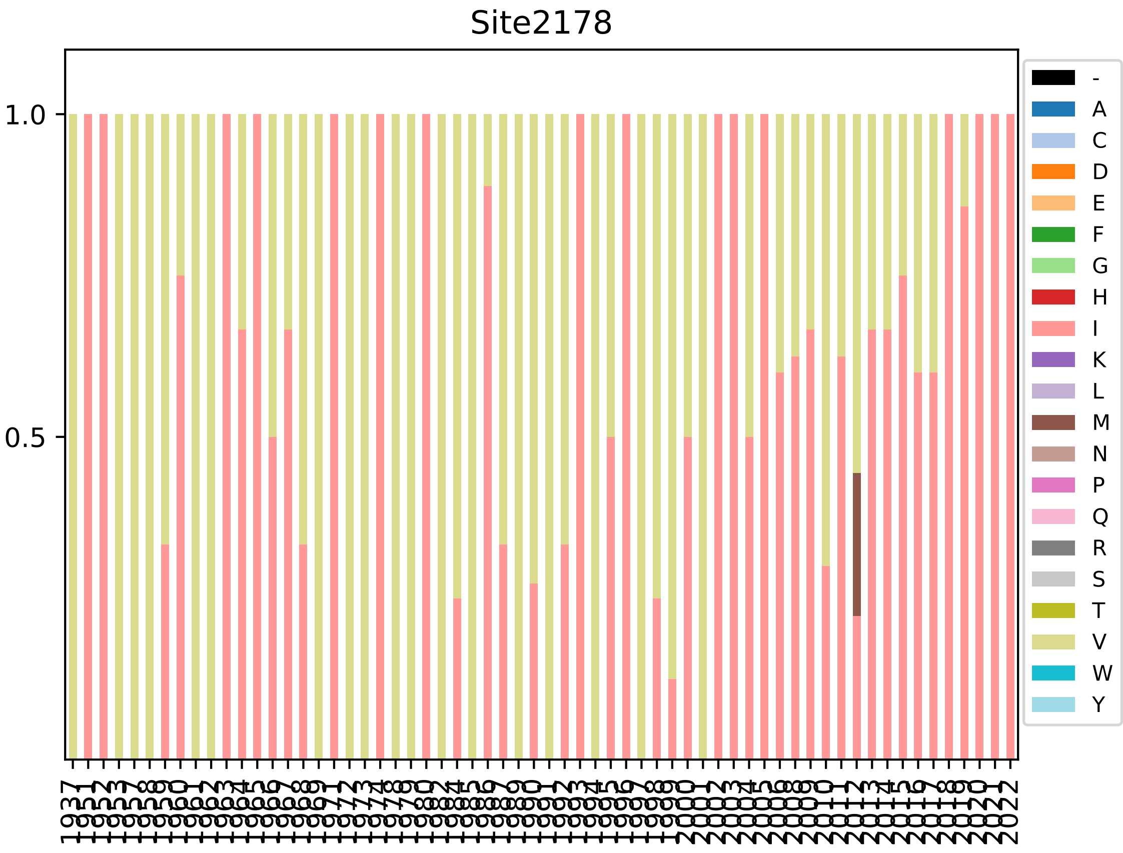
Site2178
1.0
0.5
-
A
C
D
E
F
G
H
I
K
L
M
N
P
Q
R
S
T
V
W
Y

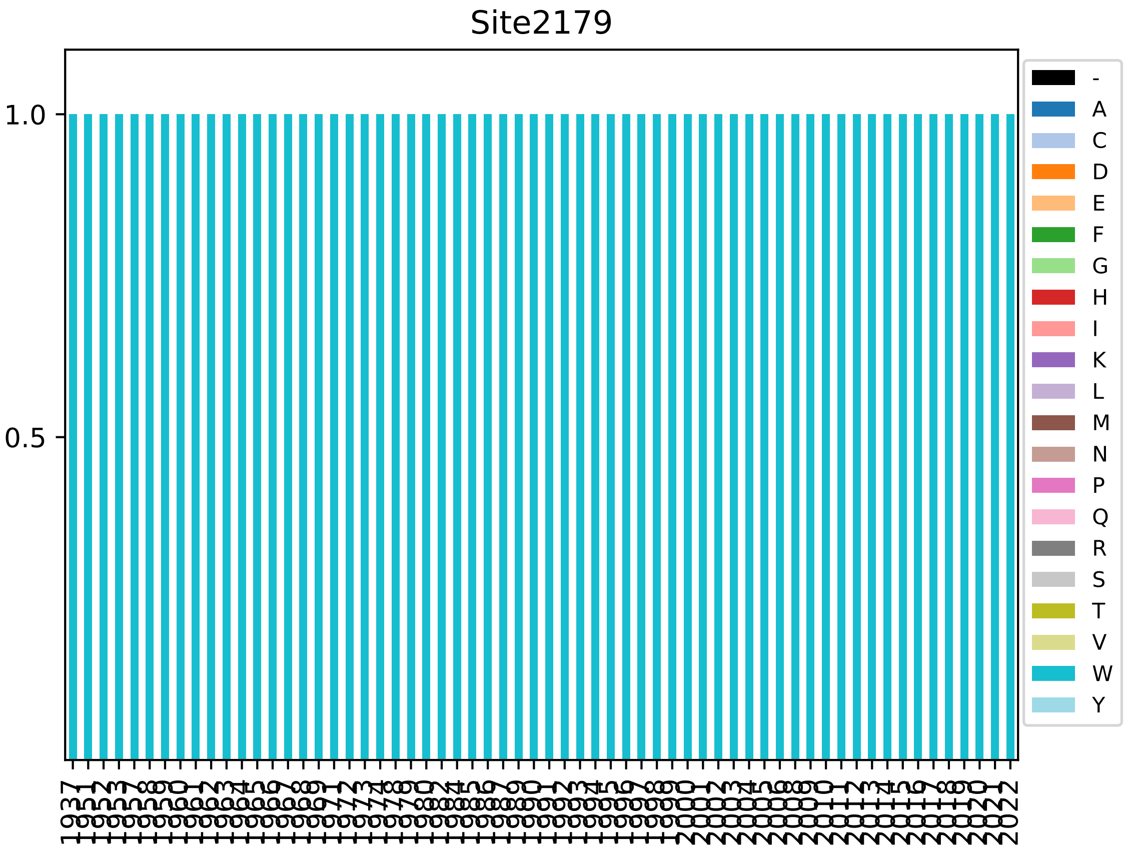

Site2179
1.0
0.5
-
A
C
D
E
F
G
H
I
K
L
M
N
P
Q
R
S
T
V
W
Y

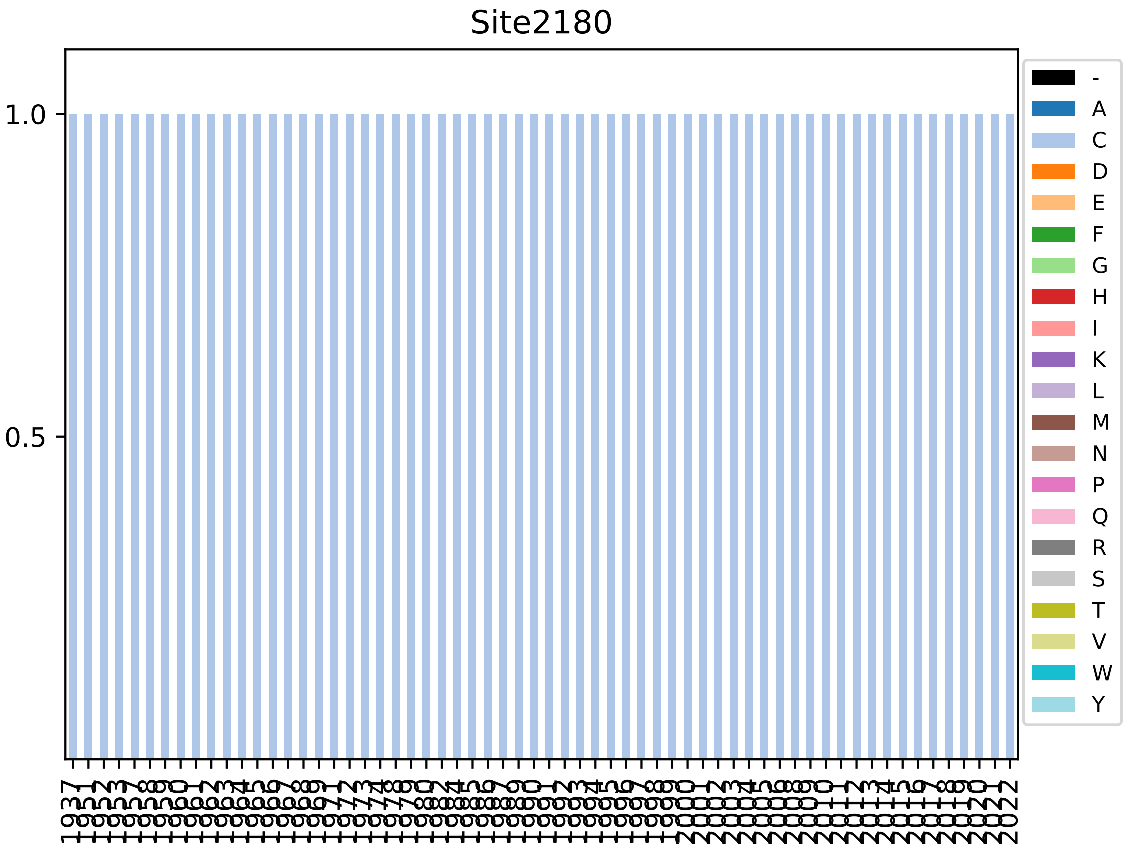
Site2180
1.0
0.5
-
A
C
D
E
F
G
H
I
K
L
M
N
P
Q
R
S
T
V
W
Y

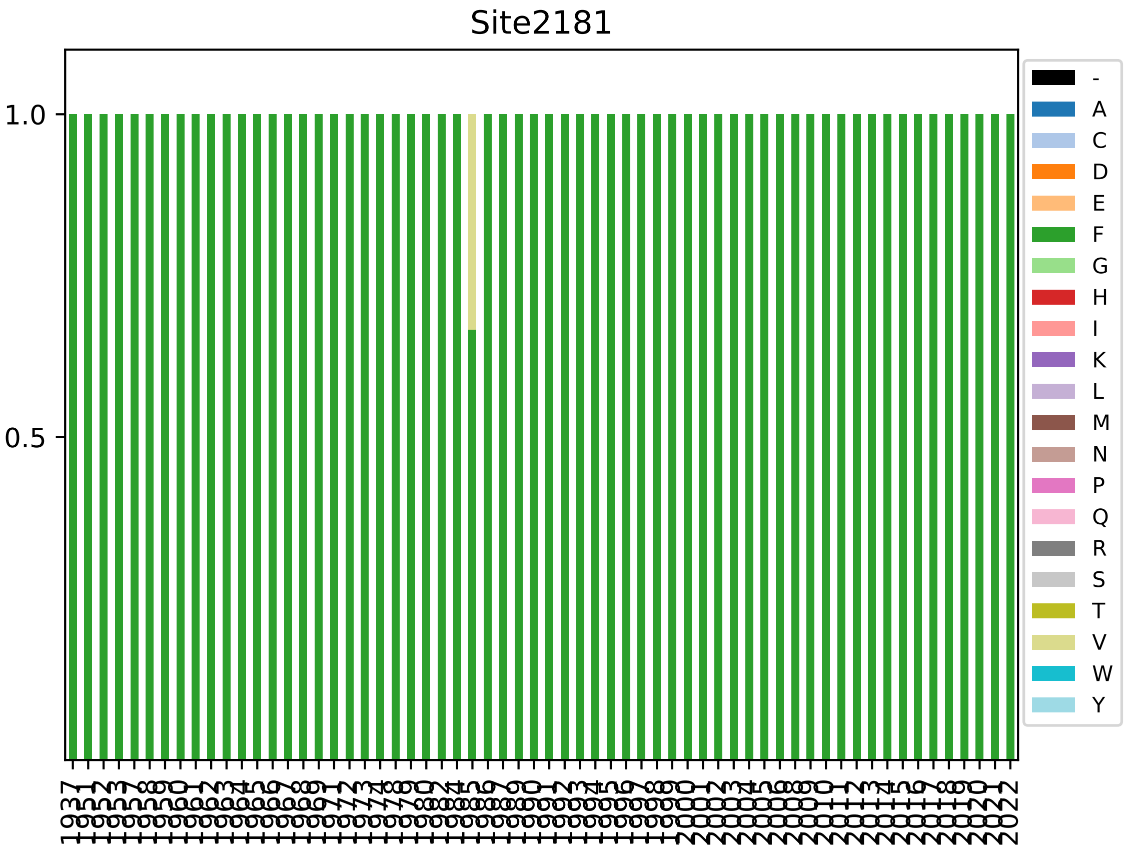
Site2181
1.0
0.5
-
A
C
D
E
F
G
H
I
K
L
M
N
P
Q
R
S
T
V
W
Y

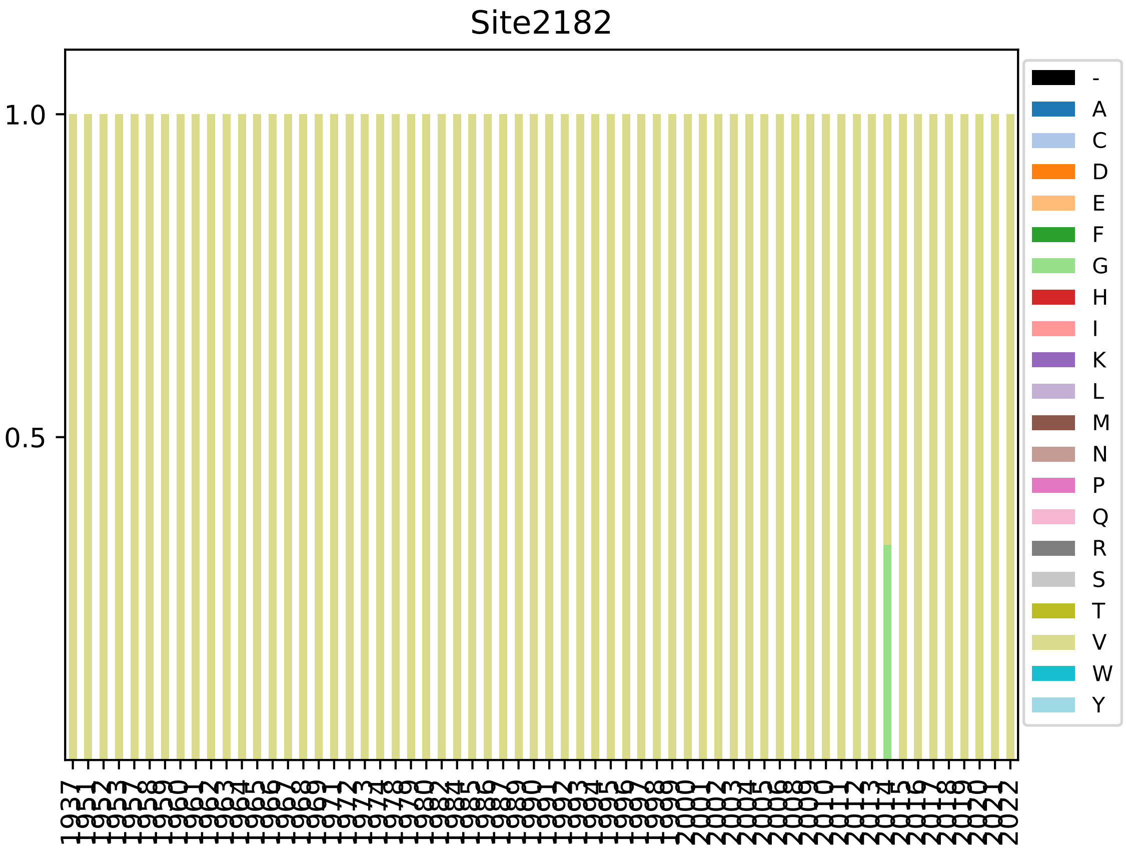

Site2182
1.0
0.5
-
A
C
D
E
F
G
H
I
K
L
M
N
P
Q
R
S
T
V
W
Y

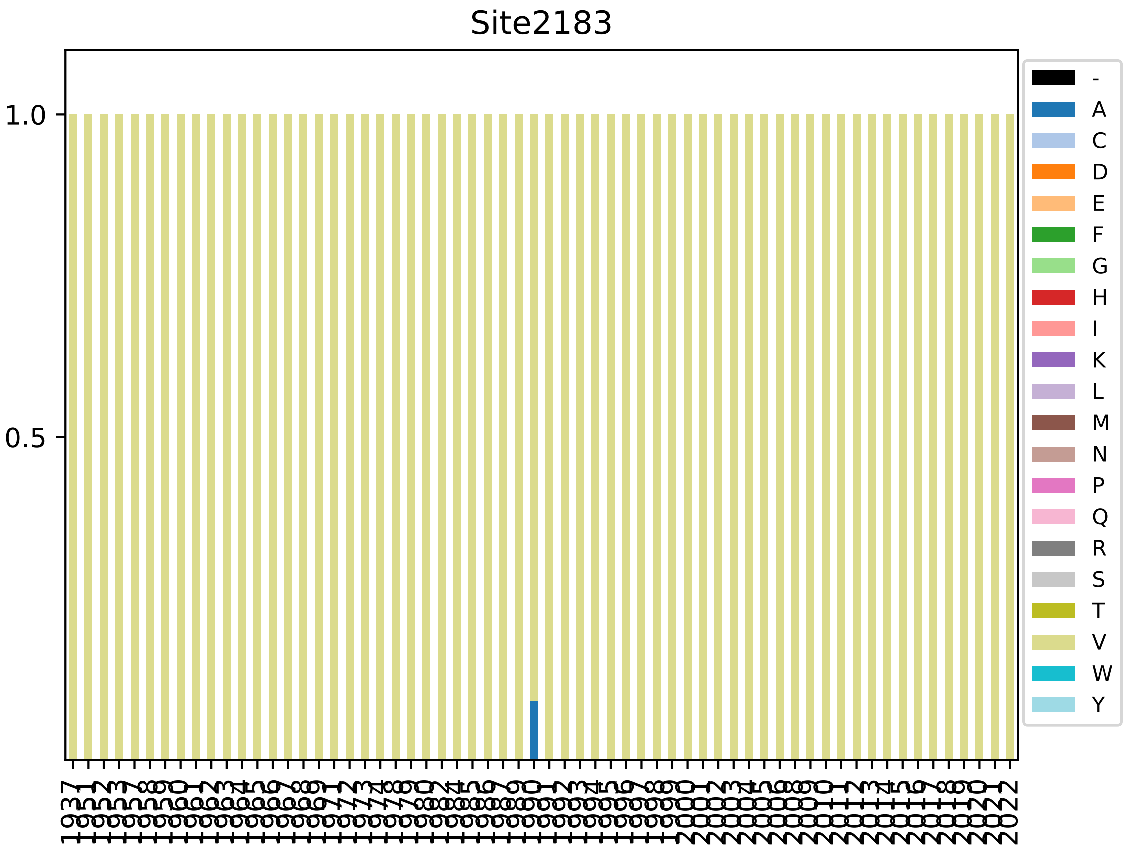
Site2183
1.0
0.5
-
A
C
D
E
F
G
H
I
K
L
M
N
P
Q
R
S
T
V
W
Y

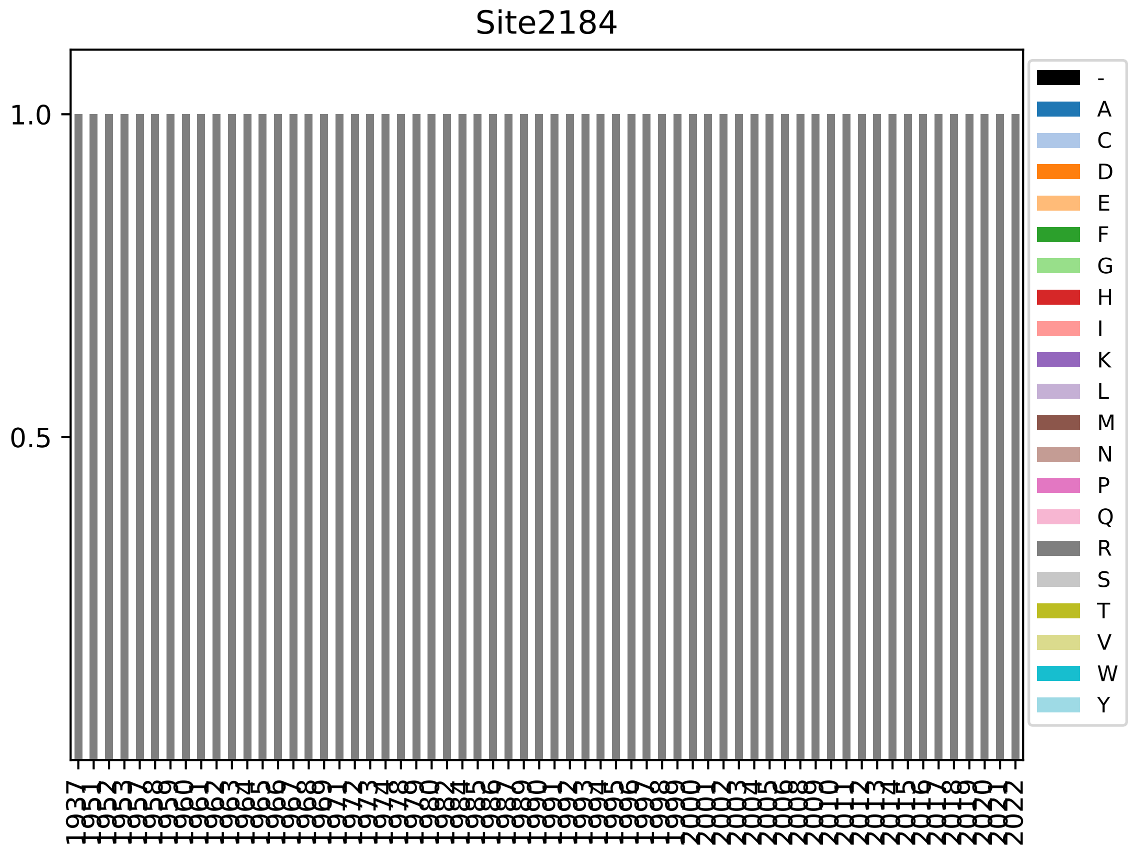
Site2184
1.0
0.5
-
A
C
D
E
F
G
H
I
K
L
M
N
P
Q
R
S
T
V
W
Y

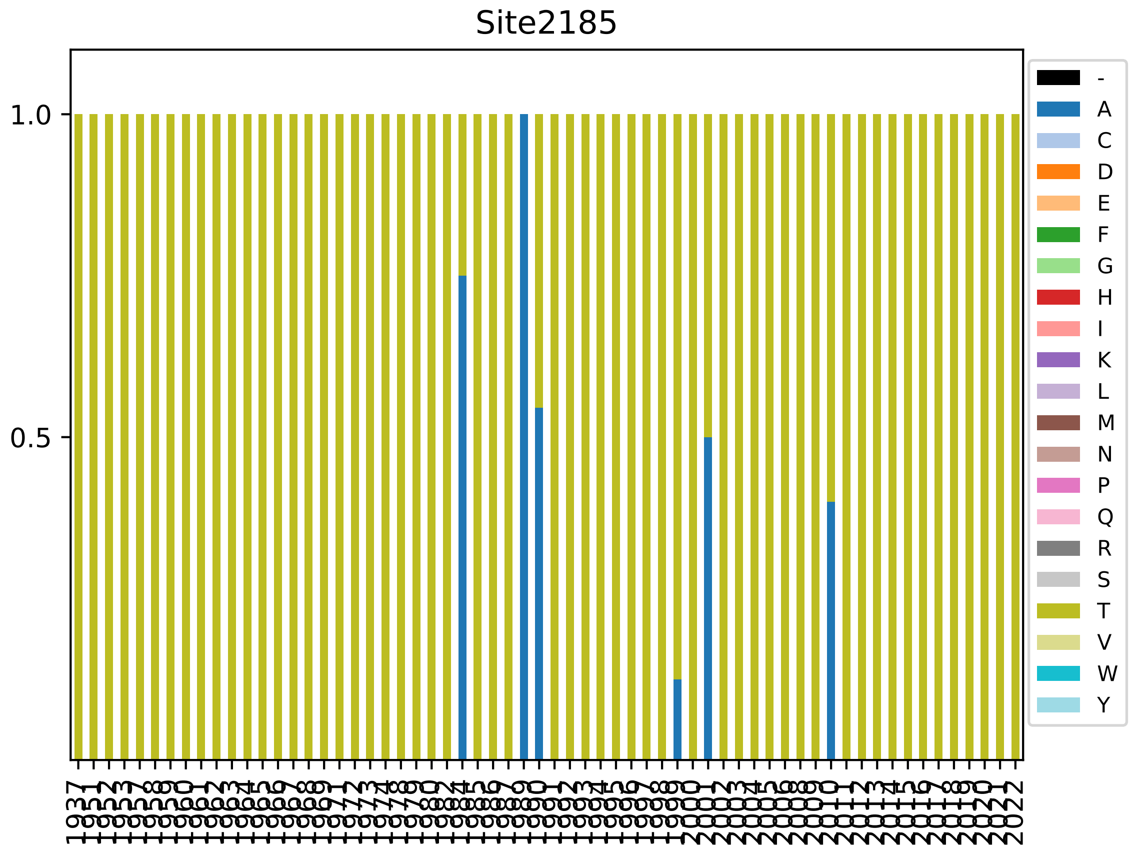

Site2185
1.0
0.5
-
A
C
D
E
F
G
H
I
K
L
M
N
P
Q
R
S
T
V
W
Y

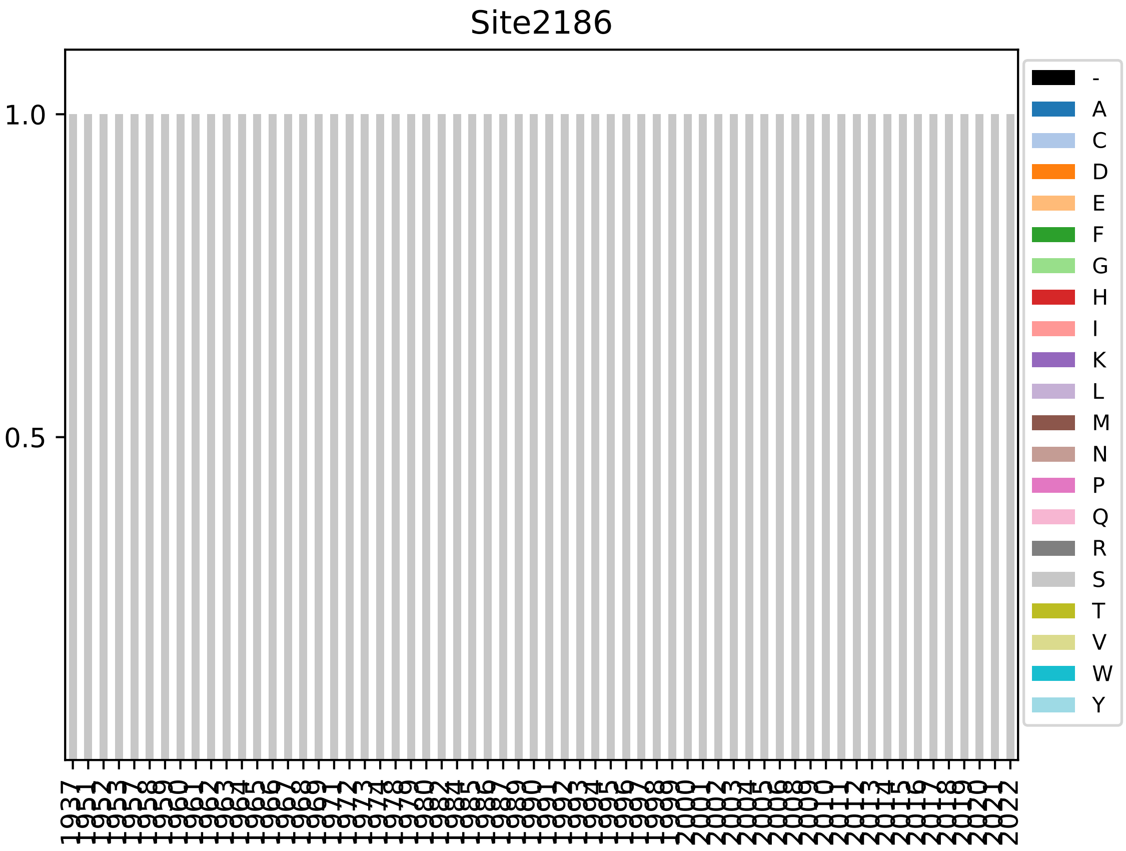
Site2186
1.0
0.5
-
A
C
D
E
F
G
H
I
K
L
M
N
P
Q
R
S
T
V
W
Y

Site2187

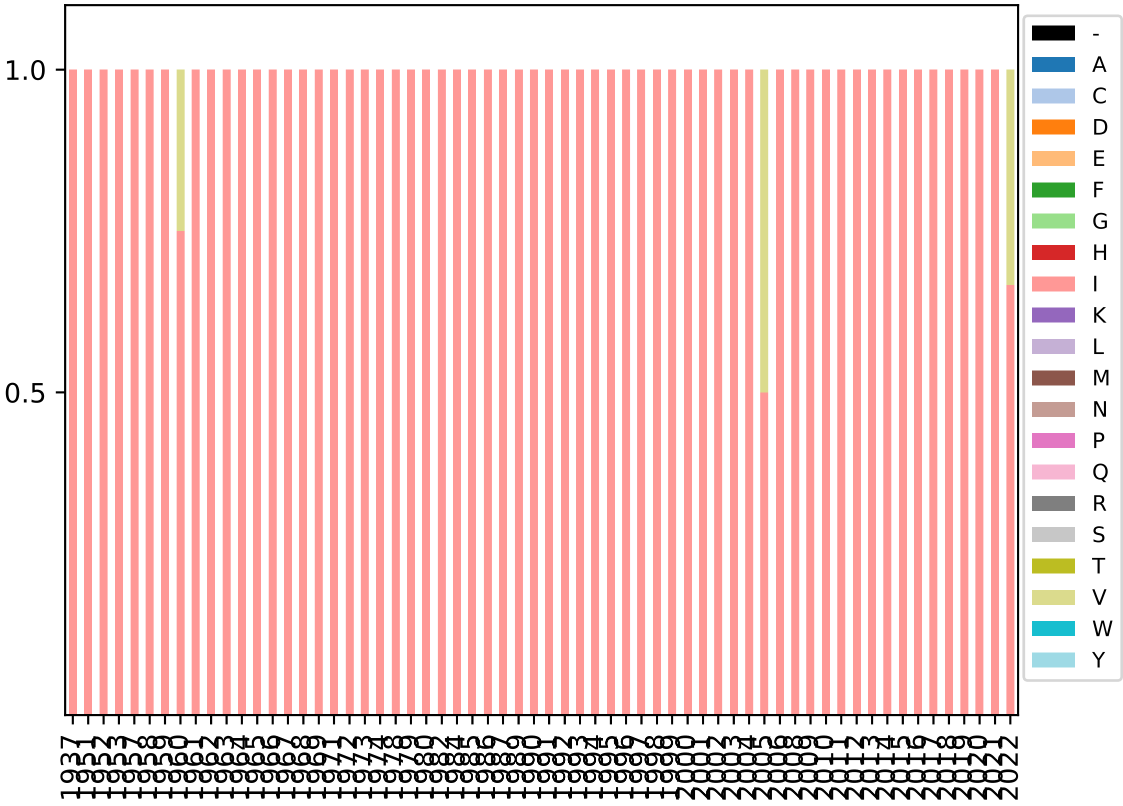

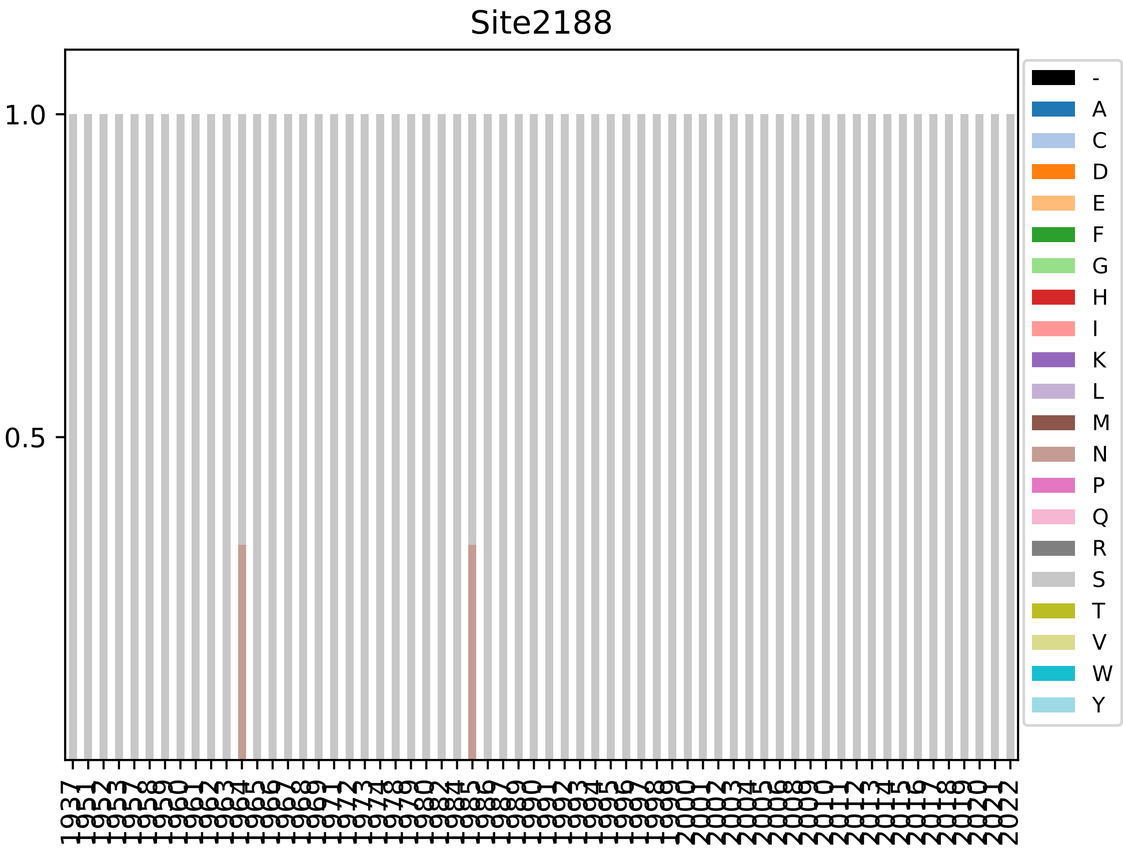
Site2188
1.0
0.5
-
A
C
D
E
F
G
H
I
K
L
M
N
P
Q
R
S
T
V
W
Y

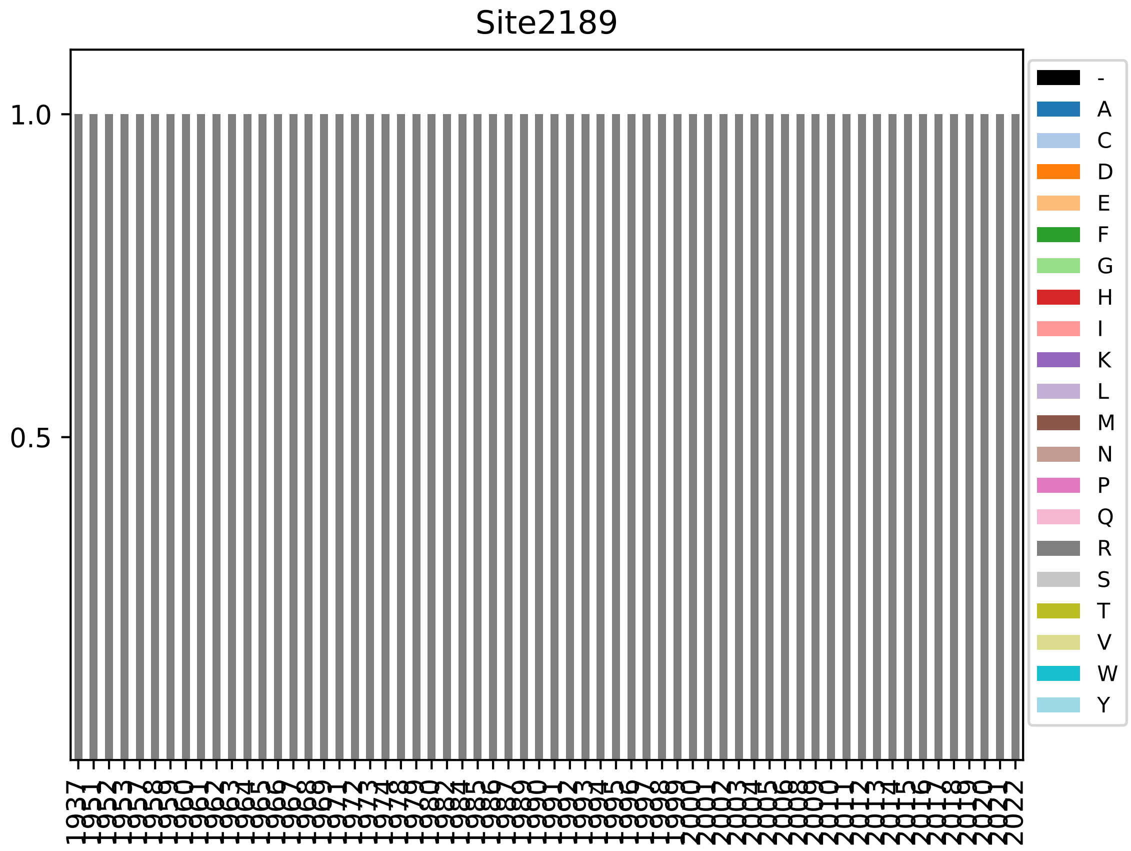
Site2189
1.0
0.5
-
A
C
D
E
F
G
H
I
K
L
M
N
P
Q
R
S
T
V
W
Y

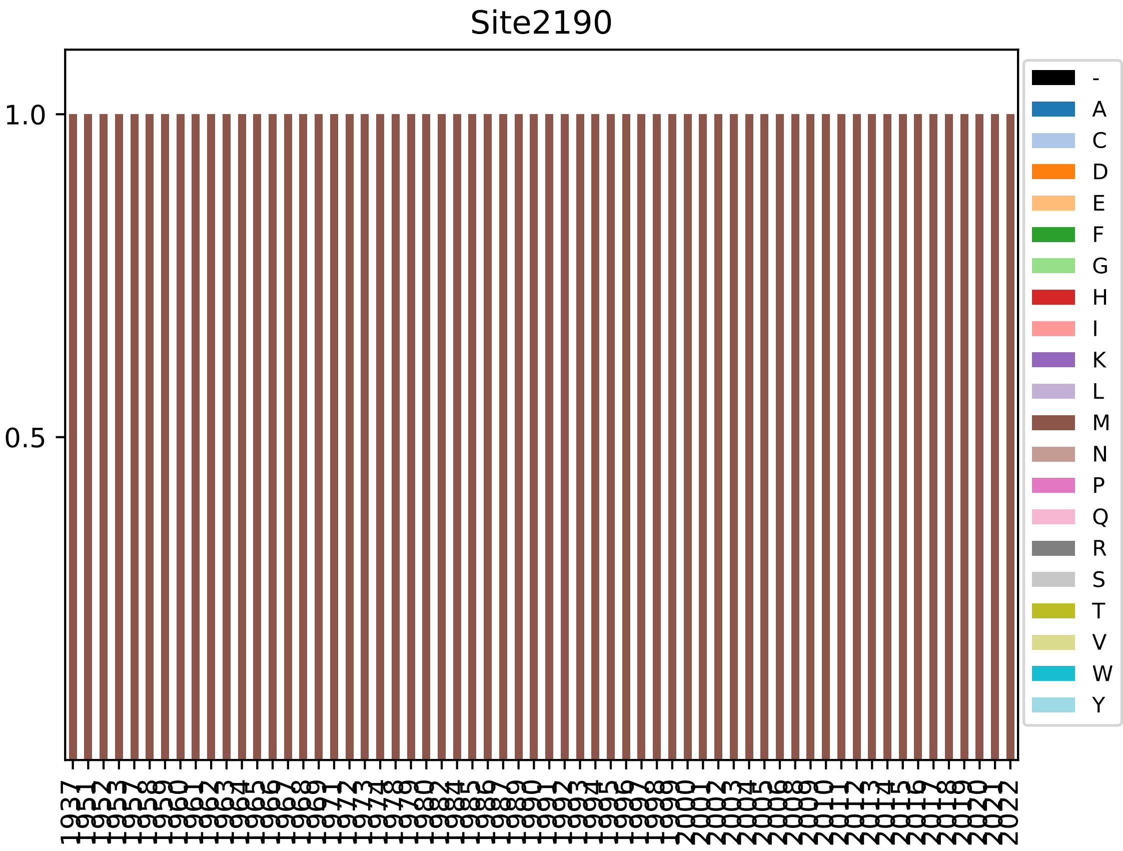
Site2190
1.0
0.5
-
A
C
D
E
F
G
H
I
K
L
M
N
P
Q
R
S
T
V
W
Y

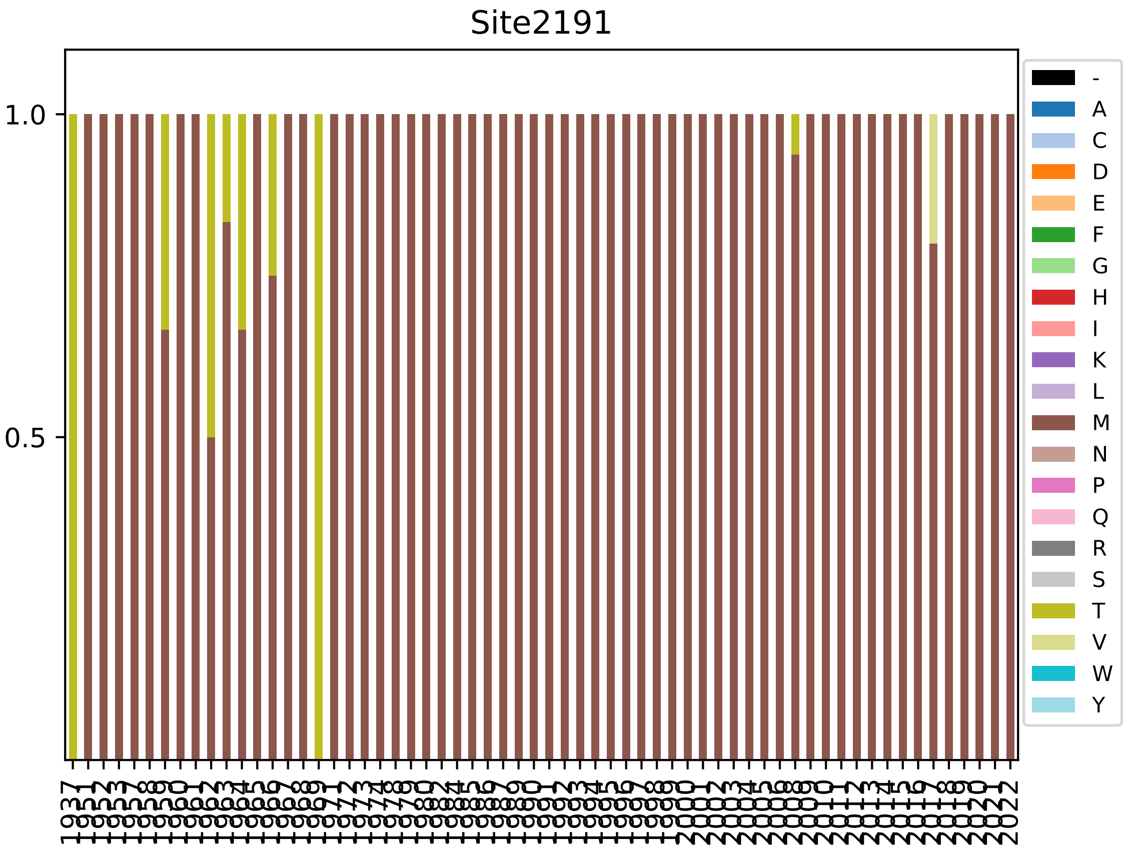

Site2191
1.0
0.5
-
A
C
D
E
F
G
H
I
K
L
M
N
P
Q
R
S
T
V
W
Y

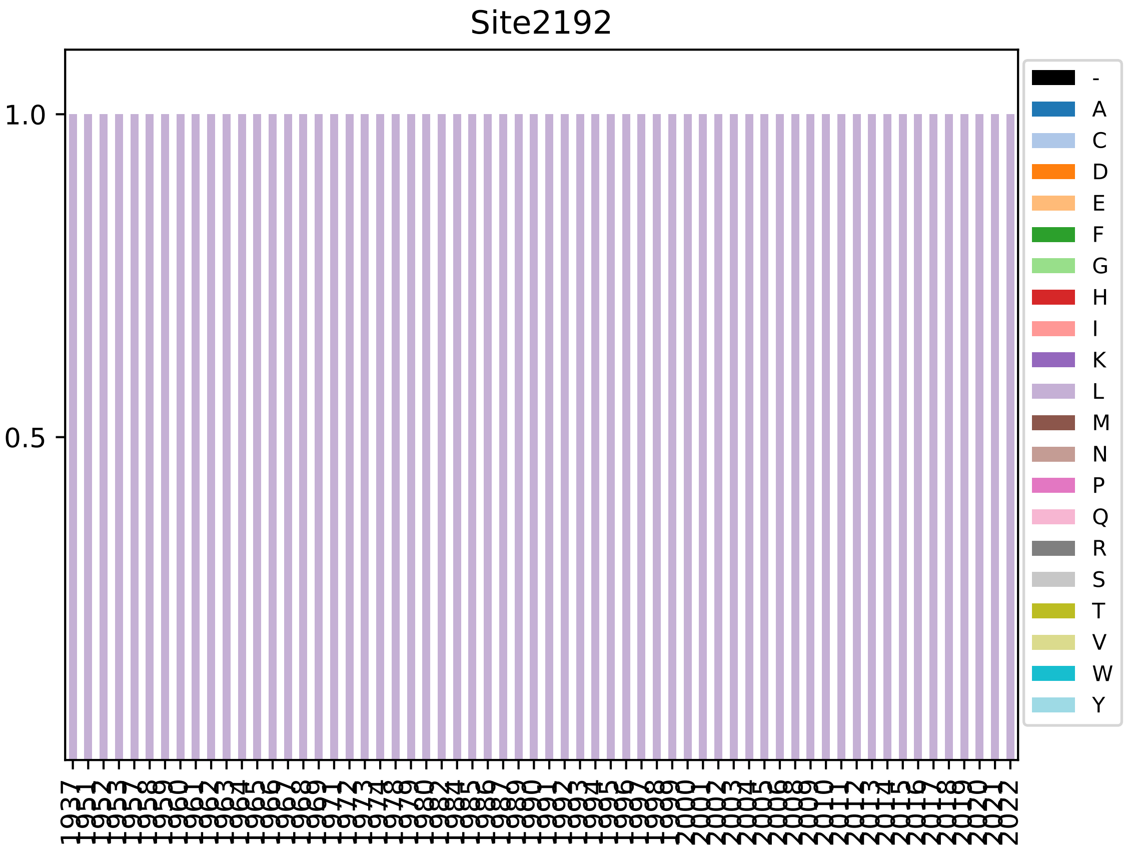
Site2192
1.0
0.5
-
A
C
D
E
F
G
H
I
K
L
M
N
P
Q
R
S
T
V
W
Y

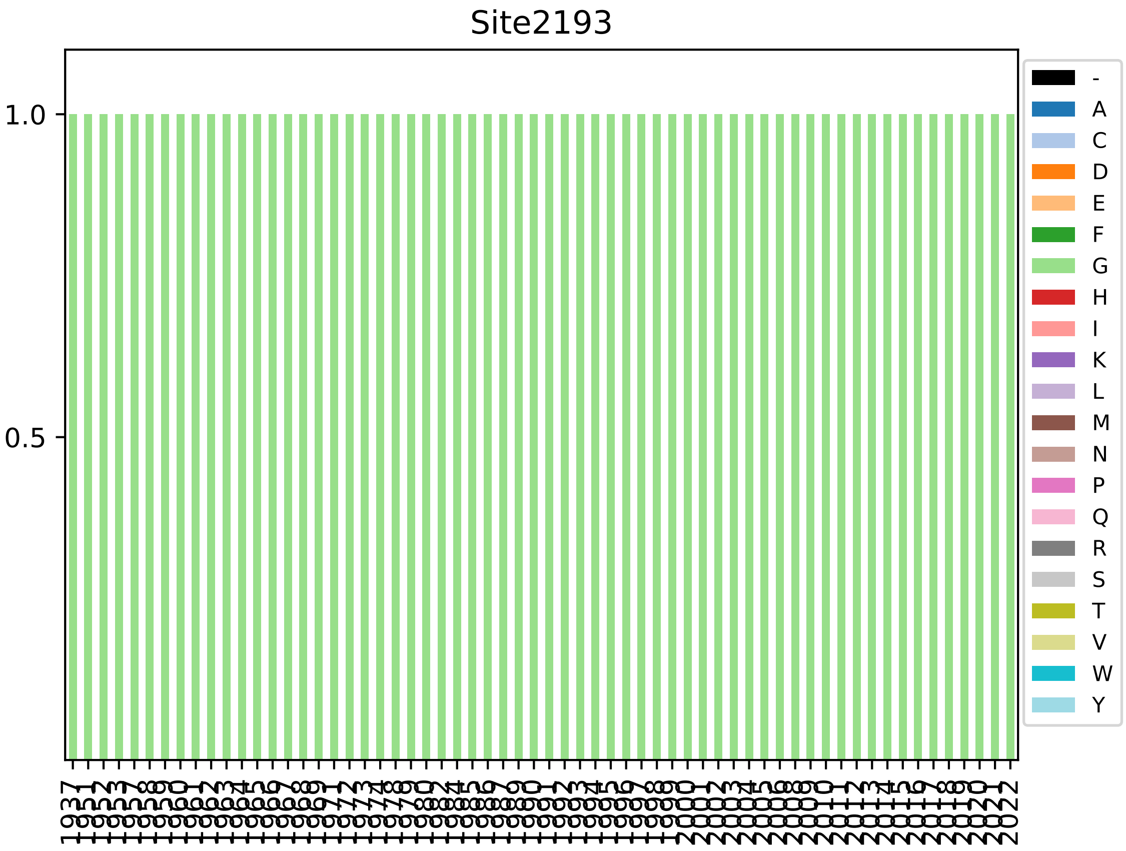
Site2193
1.0
0.5
-
A
C
D
E
F
G
H
I
K
L
M
N
P
Q
R
S
T
V
W
Y

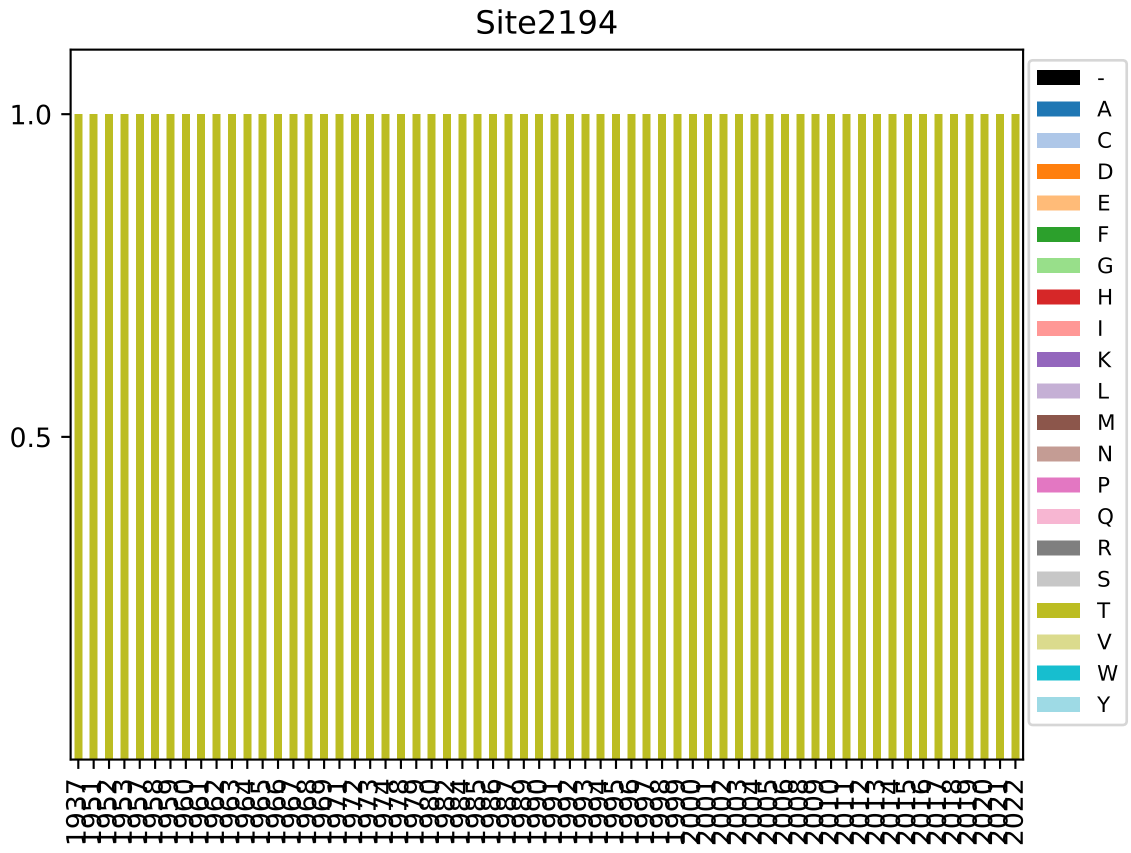
Site2194
1.0
0.5
-
A
C
D
E
F
G
H
I
K
L
M
N
P
Q
R
S
T
V
W
Y

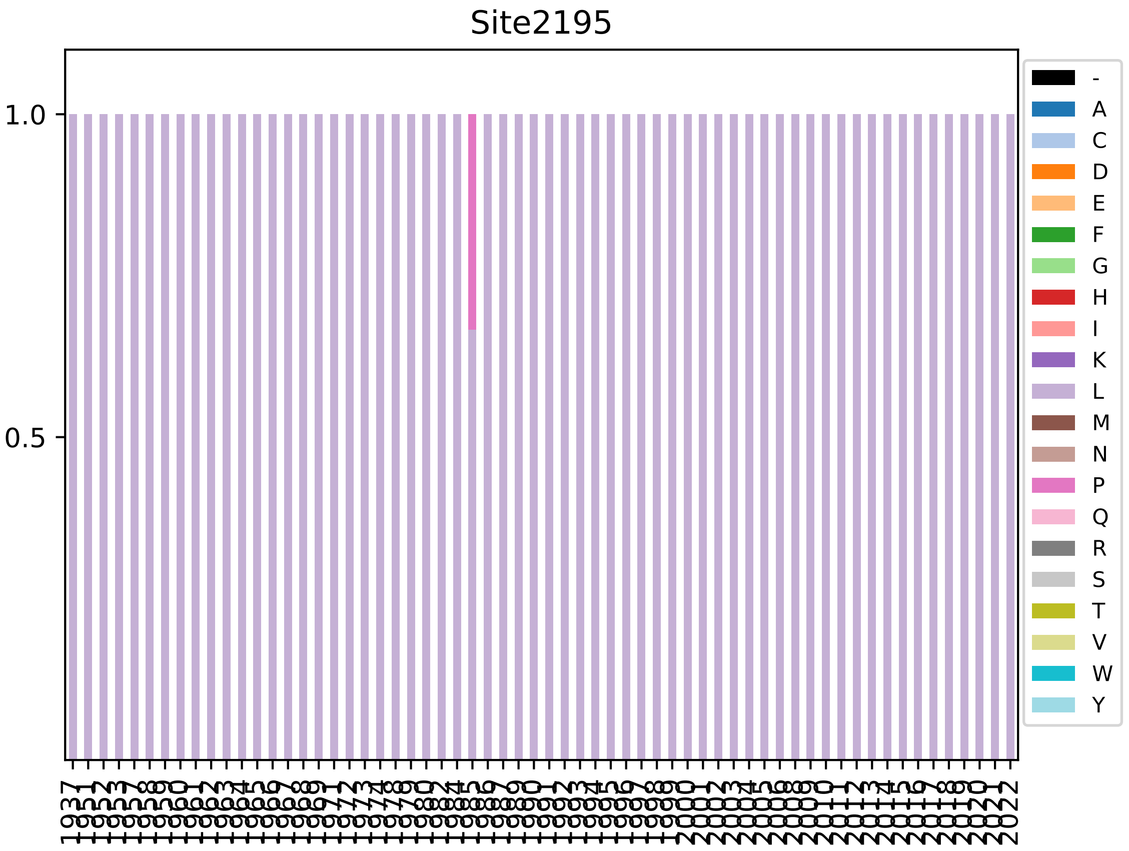

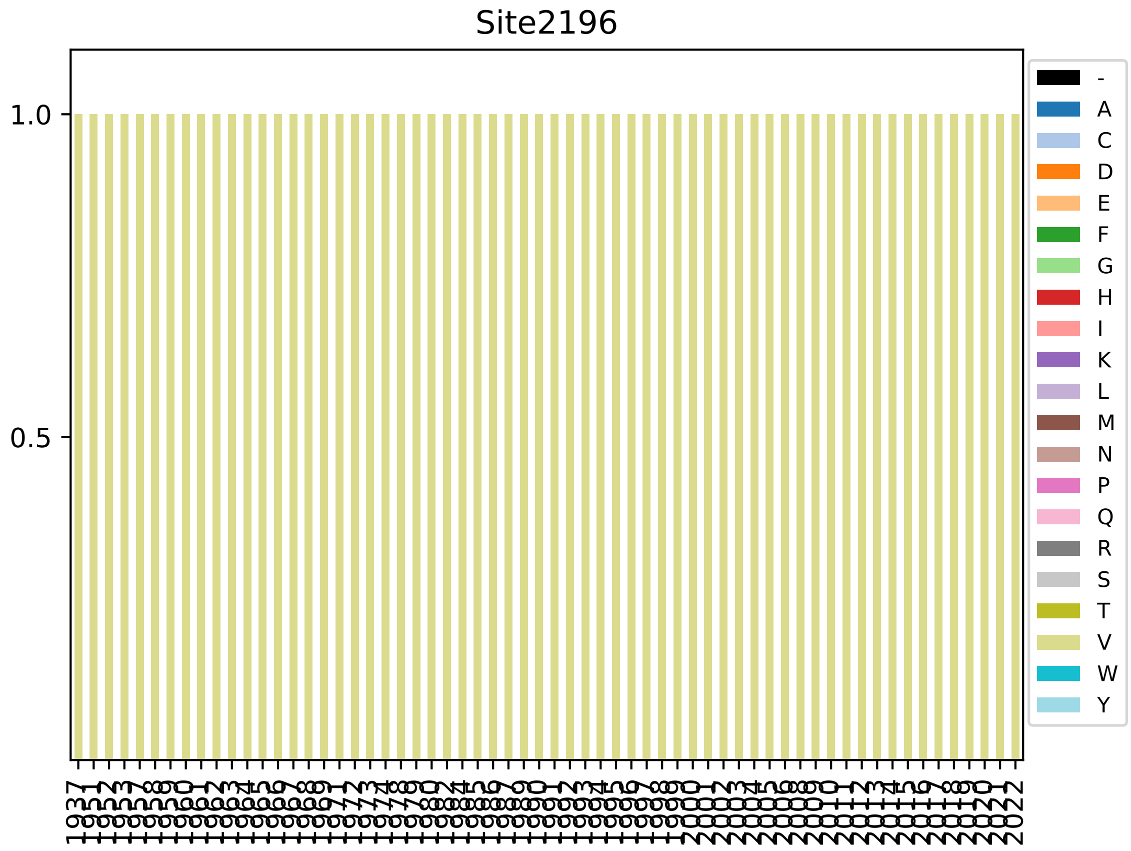
Site2196
1.0
0.5
-
A
C
D
E
F
G
H
I
K
L
M
N
P
Q
R
S
T
V
W
Y

Site2197

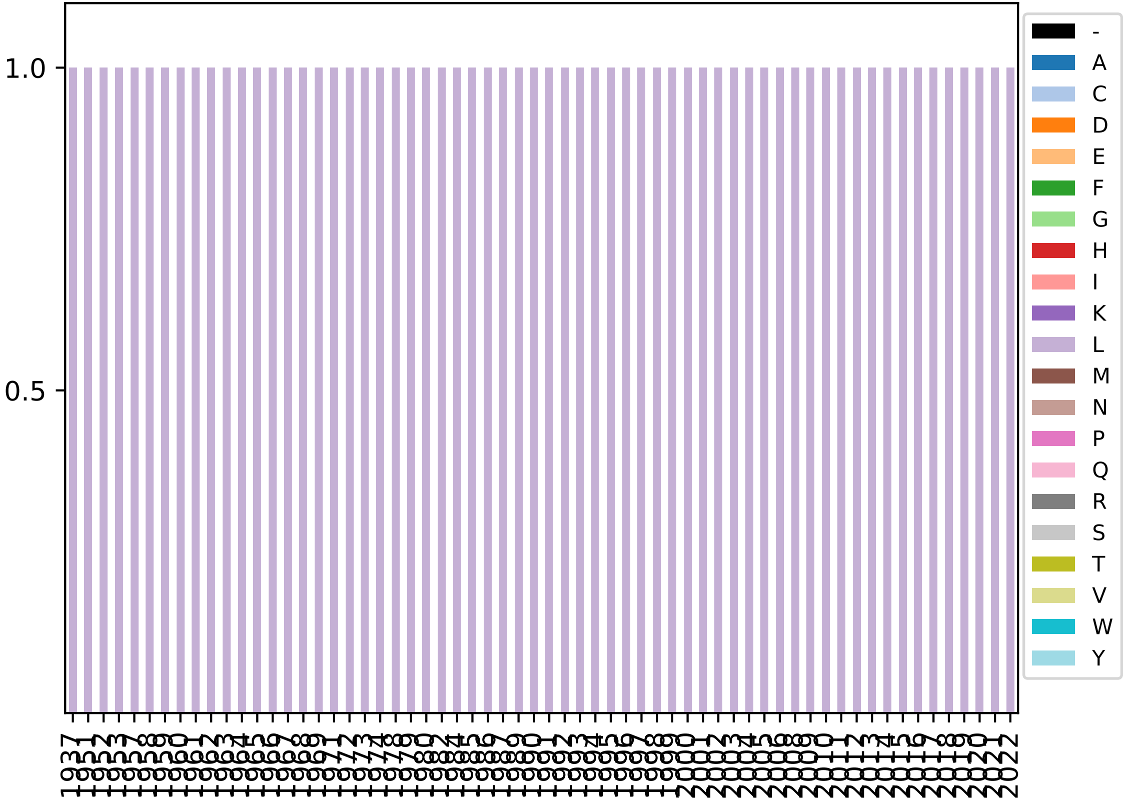

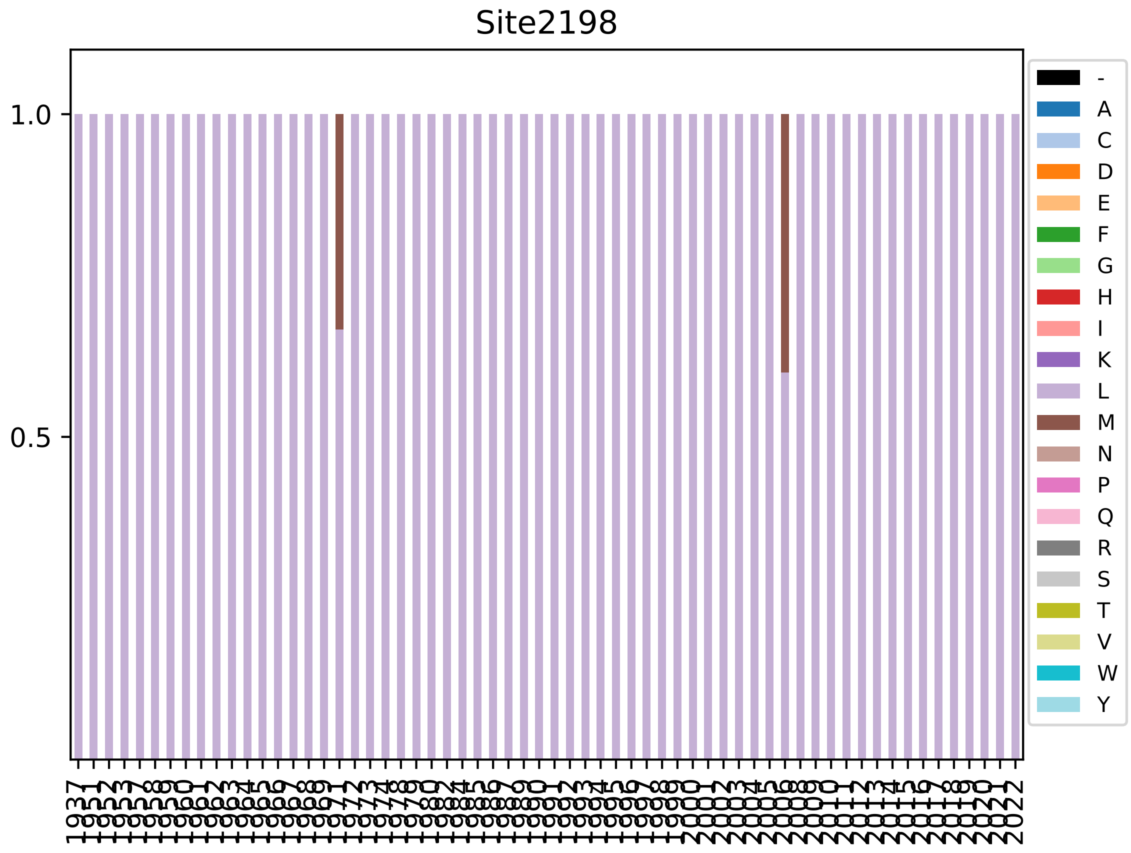

Site2198
1.0
0.5
-
A
C
D
E
F
G
H
I
K
L
M
N
P
Q
R
S
T
V
W
Y

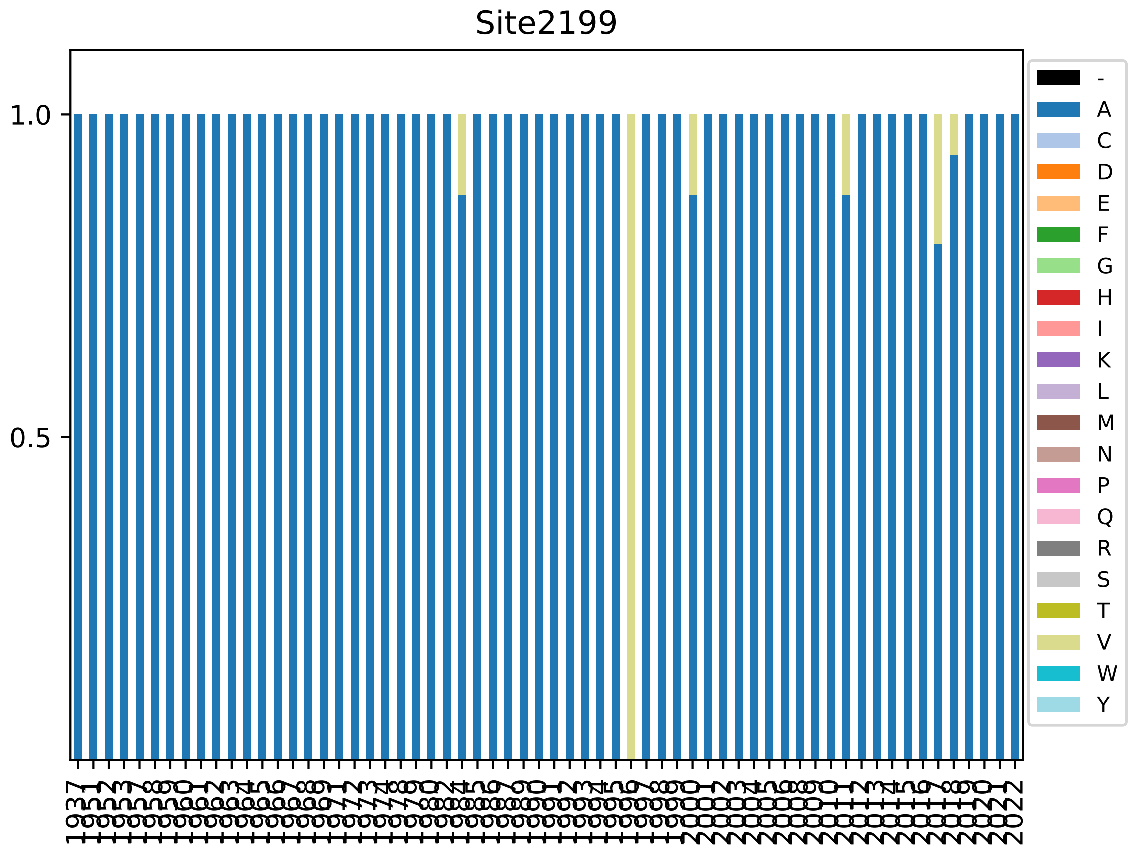
Site2199
1.0
0.5
-
A
C
D
E
F
G
H
I
K
L
M
N
P
Q
R
S
T
V
W
Y

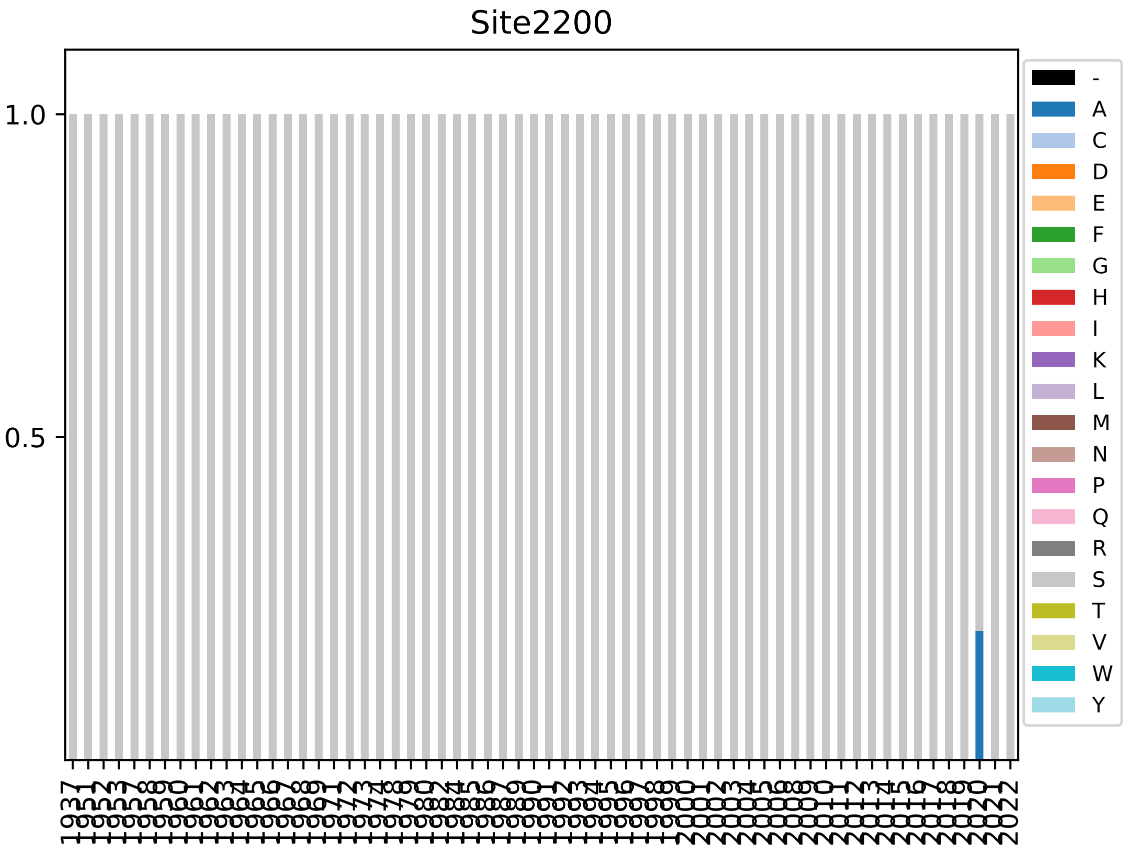
Site2200
1.0
0.5
-
A
C
D
E
F
G
H
I
K
L
M
N
P
Q
R
S
T
V
W
Y

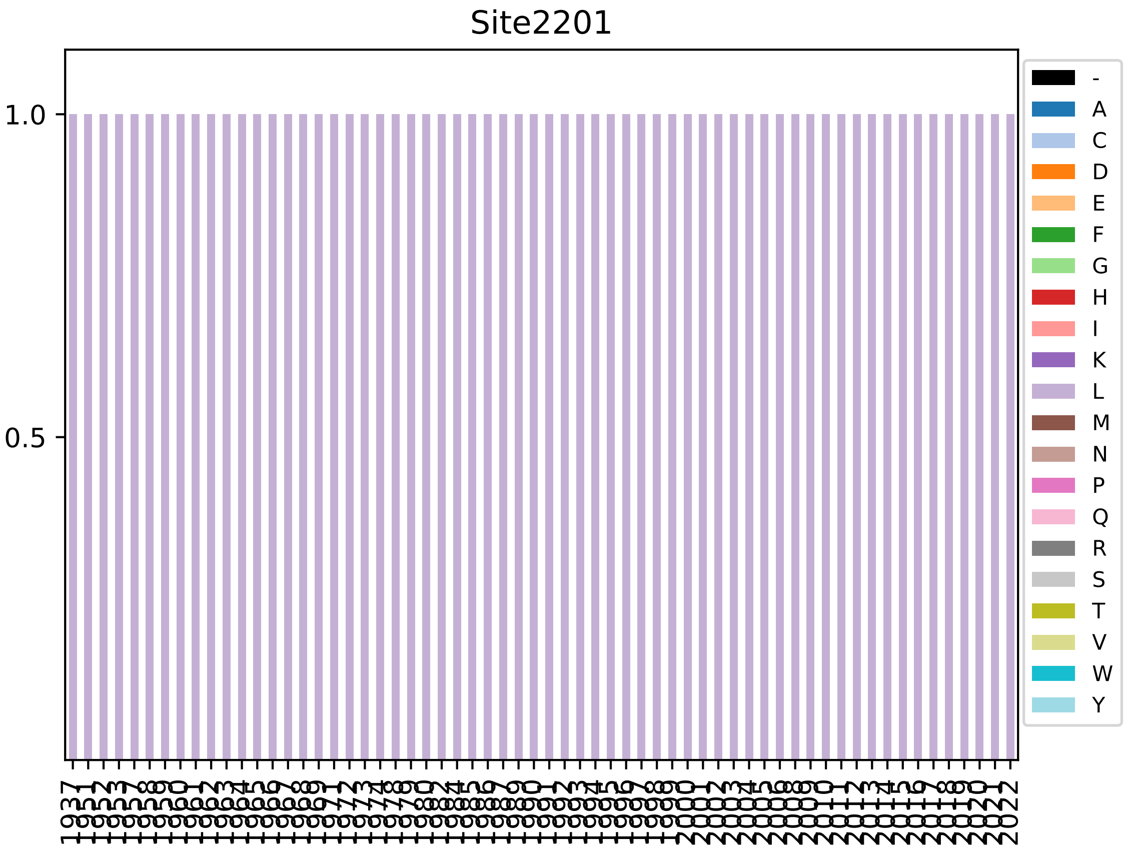
Site2201
1.0
0.5
-
A
C
D
E
F
G
H
I
K
L
M
N
P
Q
R
S
T
V
W
Y

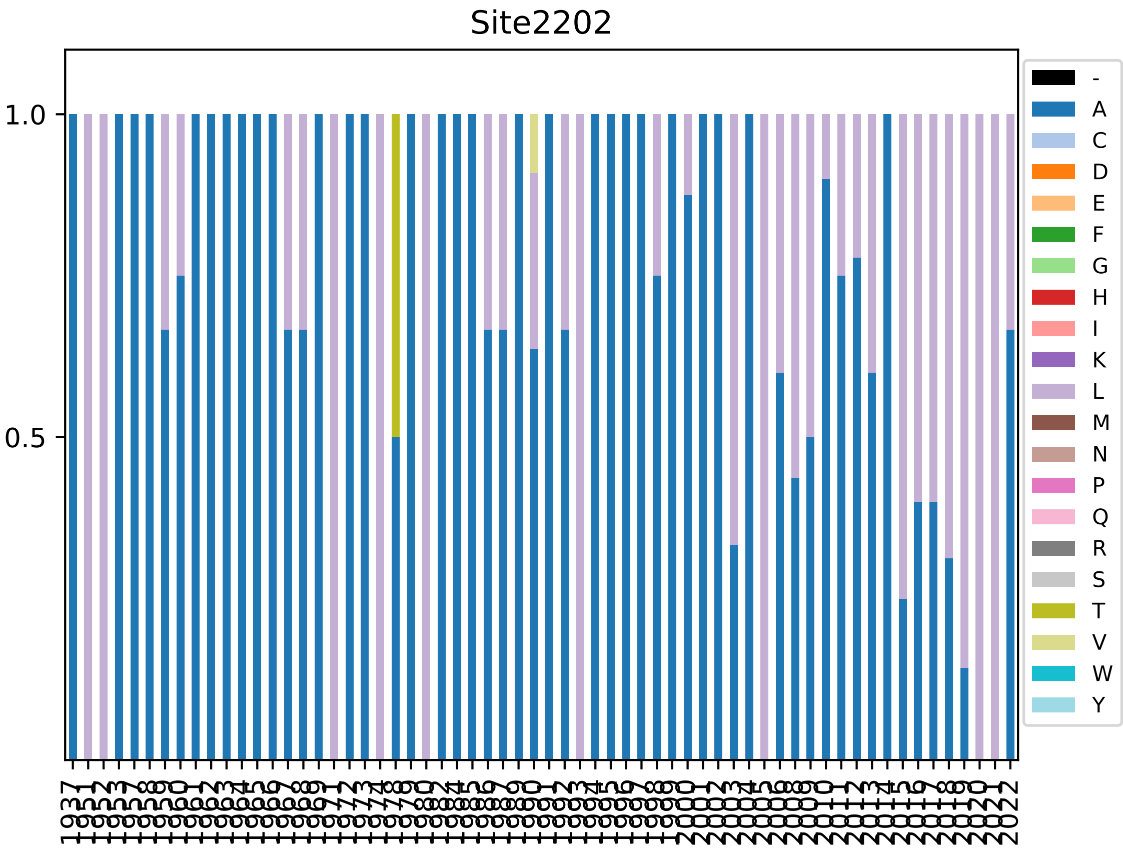

Site2202
1.0
0.5
-
A
C
D
E
F
G
H
I
K
L
M
N
P
Q
R
S
T
V
W
Y

Site2203

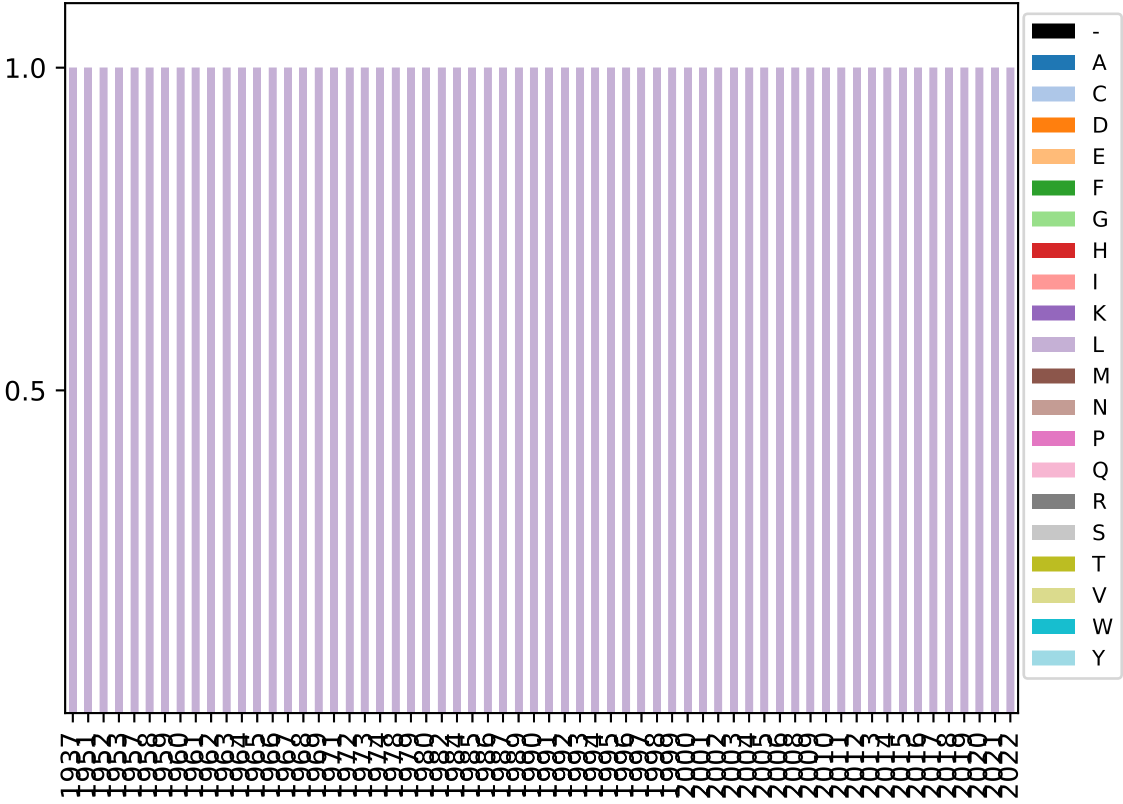

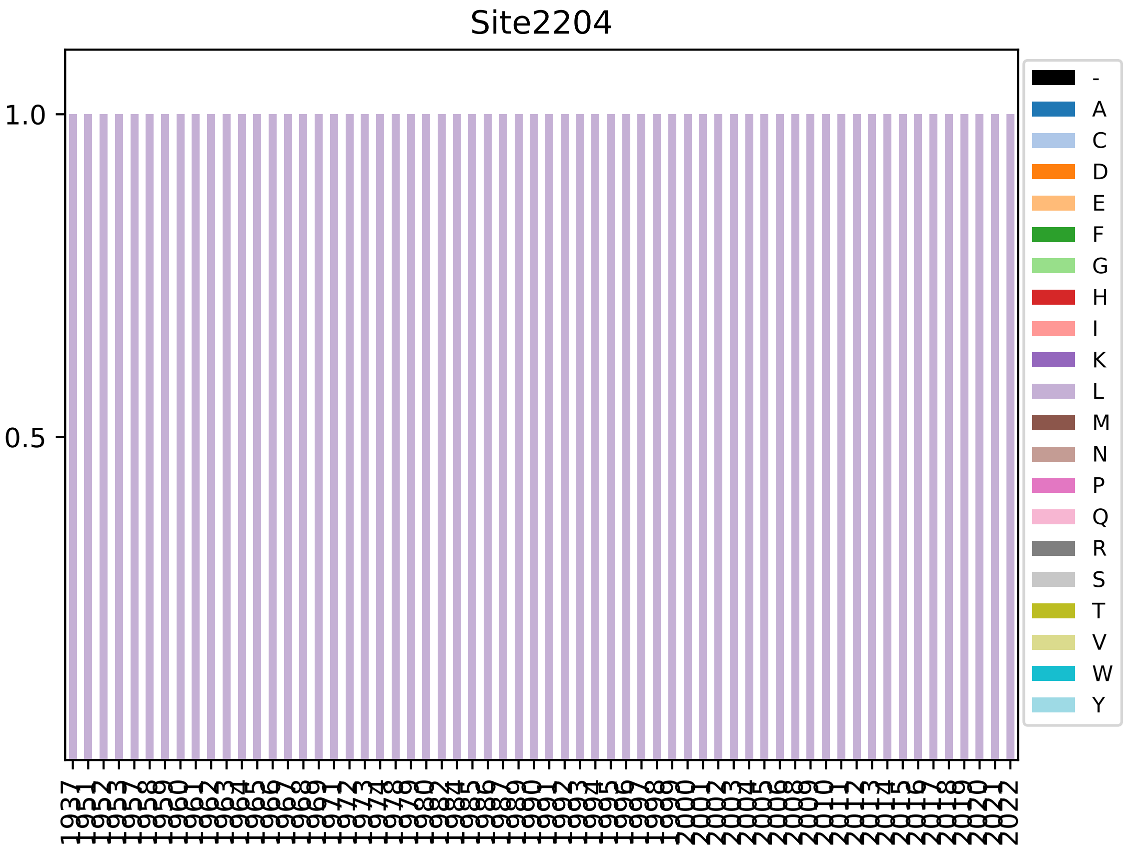

Site2204
1.0
0.5
-
A
C
D
E
F
G
H
I
K
L
M
N
P
Q
R
S
T
V
W
Y

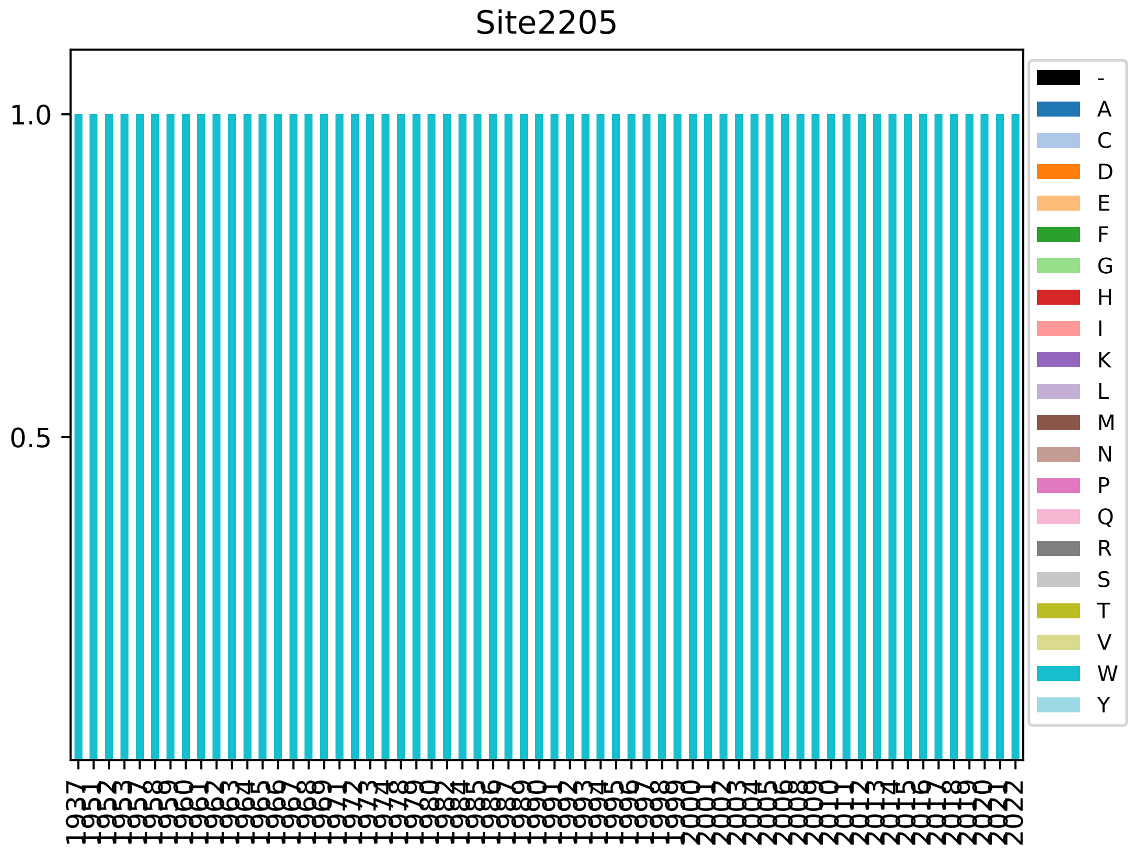

Site2205
1.0
0.5
-
A
C
D
E
F
G
H
I
K
L
M
N
P
Q
R
S
T
V
W
Y

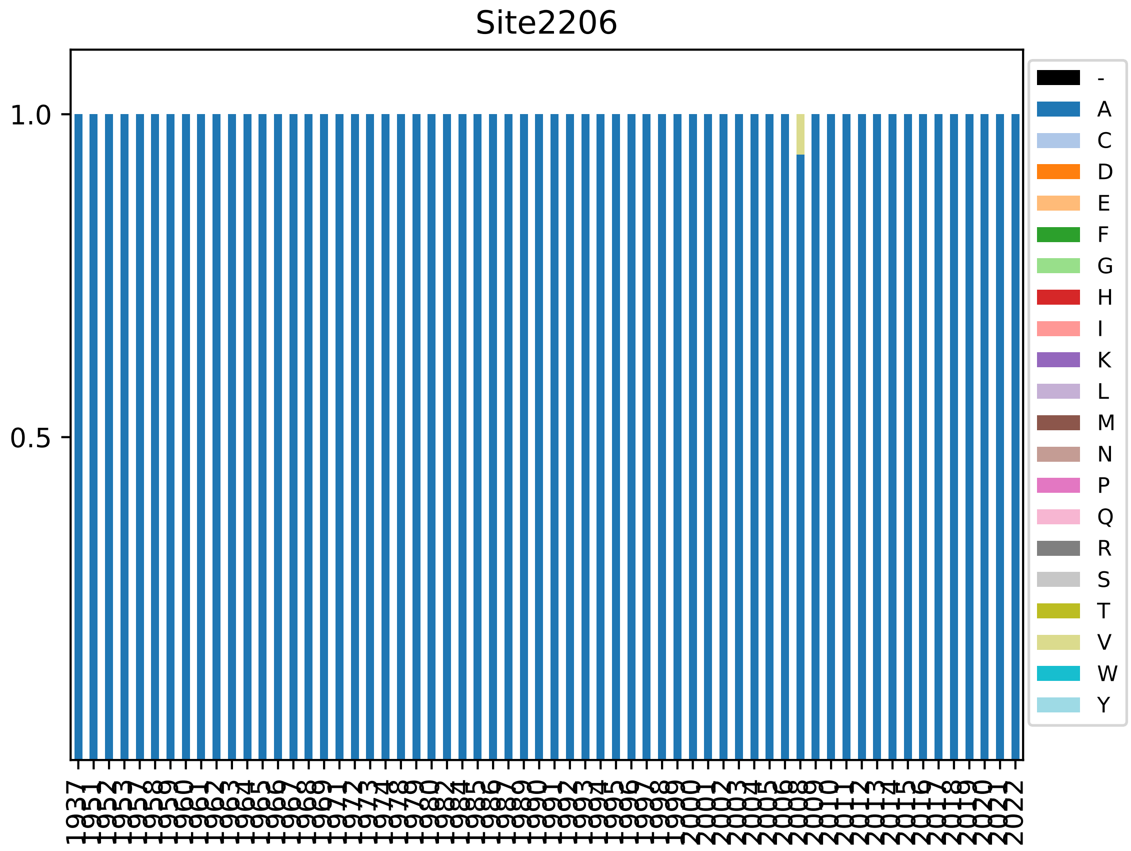
Site2206
1.0
0.5
-
A
C
D
E
F
G
H
I
K
L
M
N
P
Q
R
S
T
V
W
Y

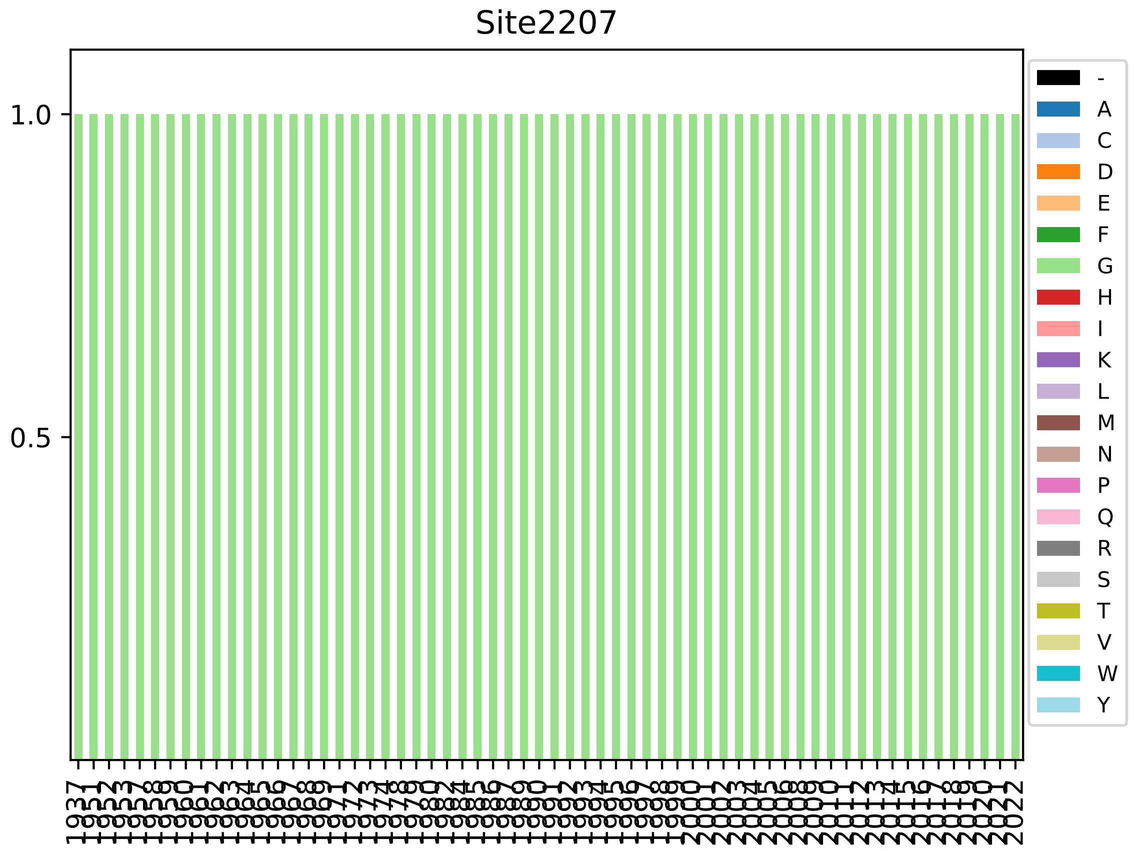
Site2207
1.0
0.5
-
A
C
D
E
F
G
H
I
K
L
M
N
P
Q
R
S
T
V
W
Y

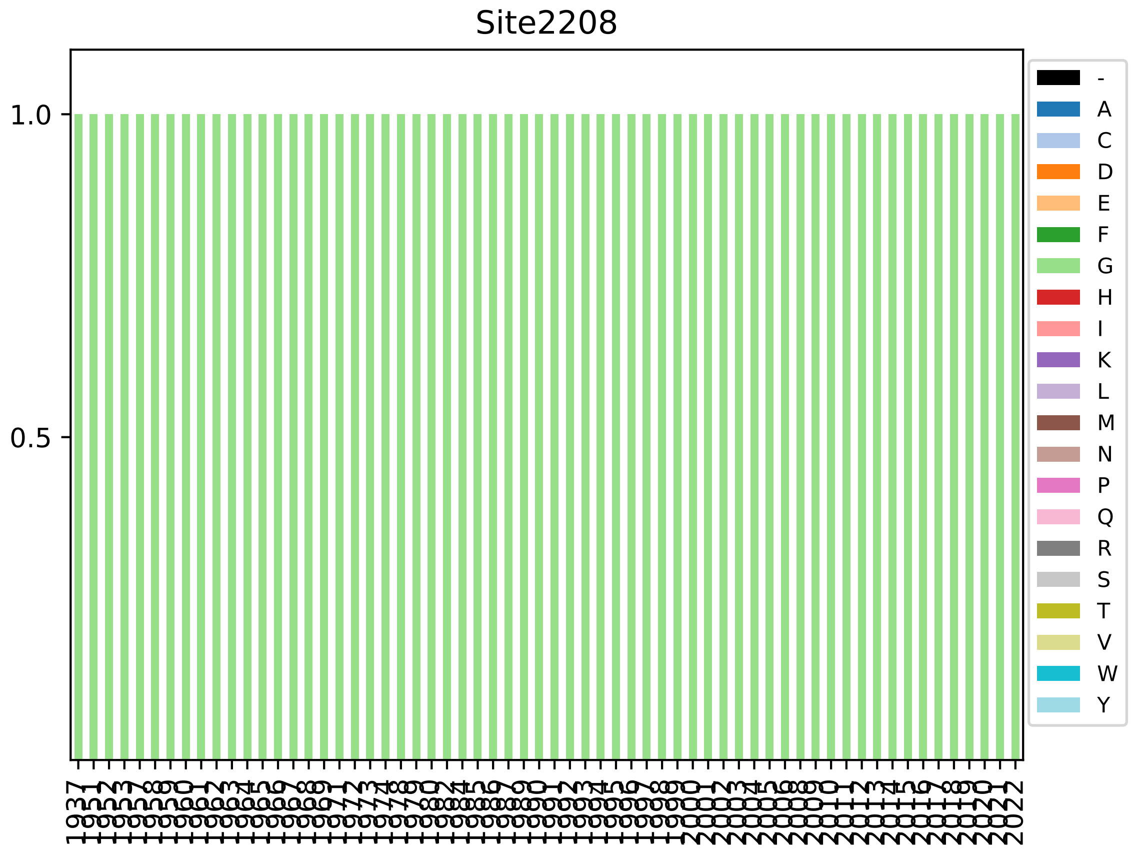
Site2208
1.0
0.5
-
A
C
D
E
F
G
H
I
K
L
M
N
P
Q
R
S
T
V
W
Y

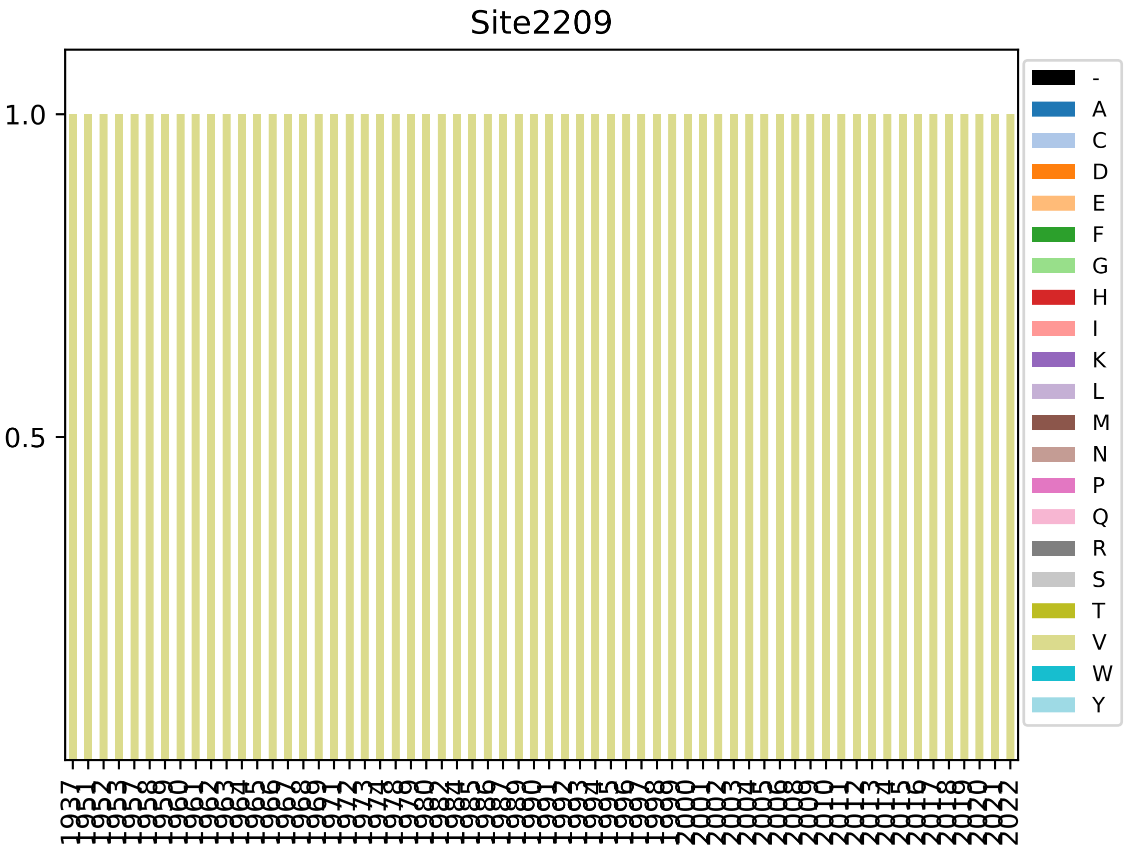
Site2209
1.0
0.5
-
A
C
D
E
F
G
H
I
K
L
M
N
P
Q
R
S
T
V
W
Y

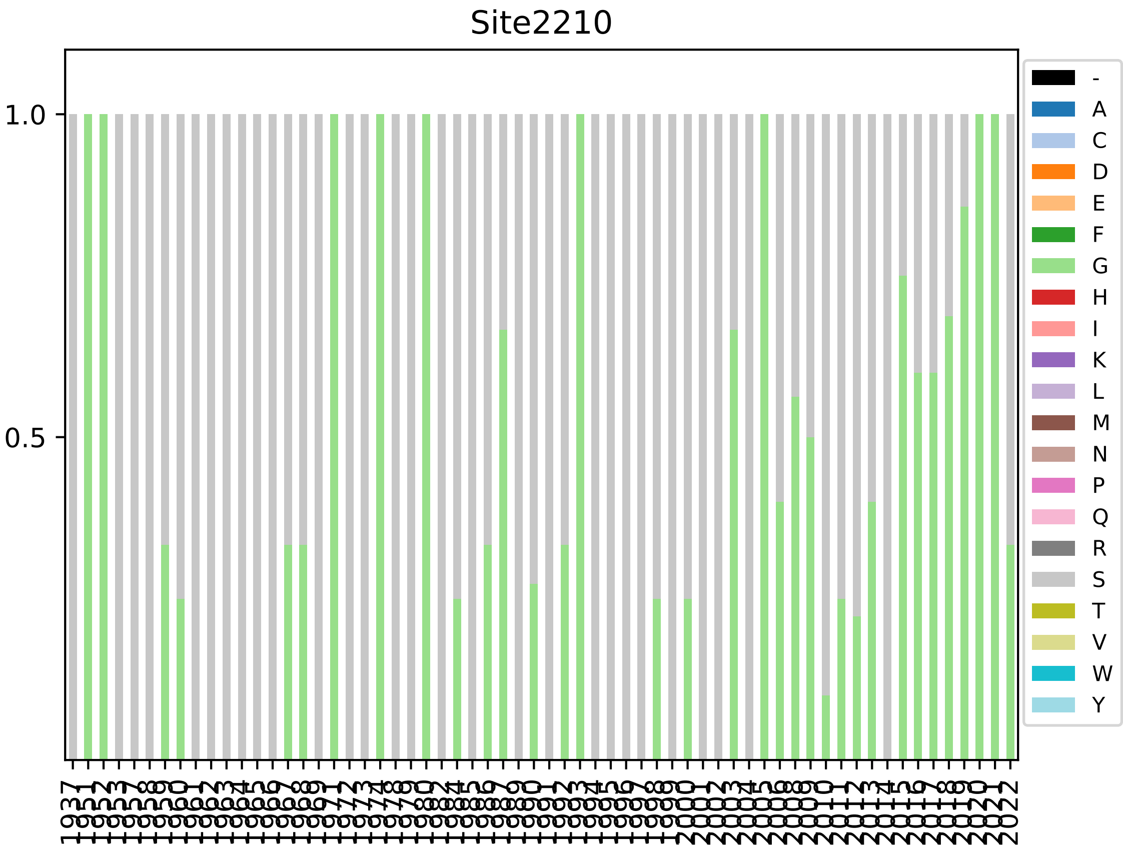

Site2210
1.0
0.5
-
A
C
D
E
F
G
H
I
K
L
M
N
P
Q
R
S
T
V
W
Y

Site2211

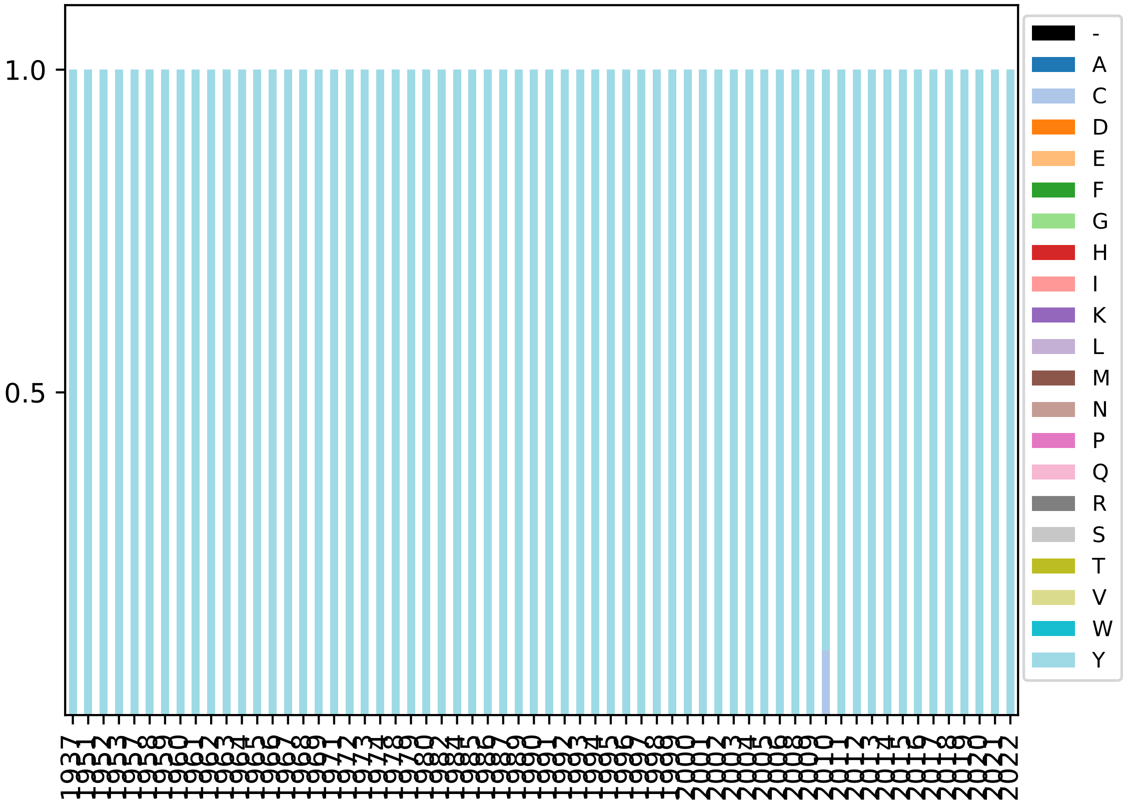

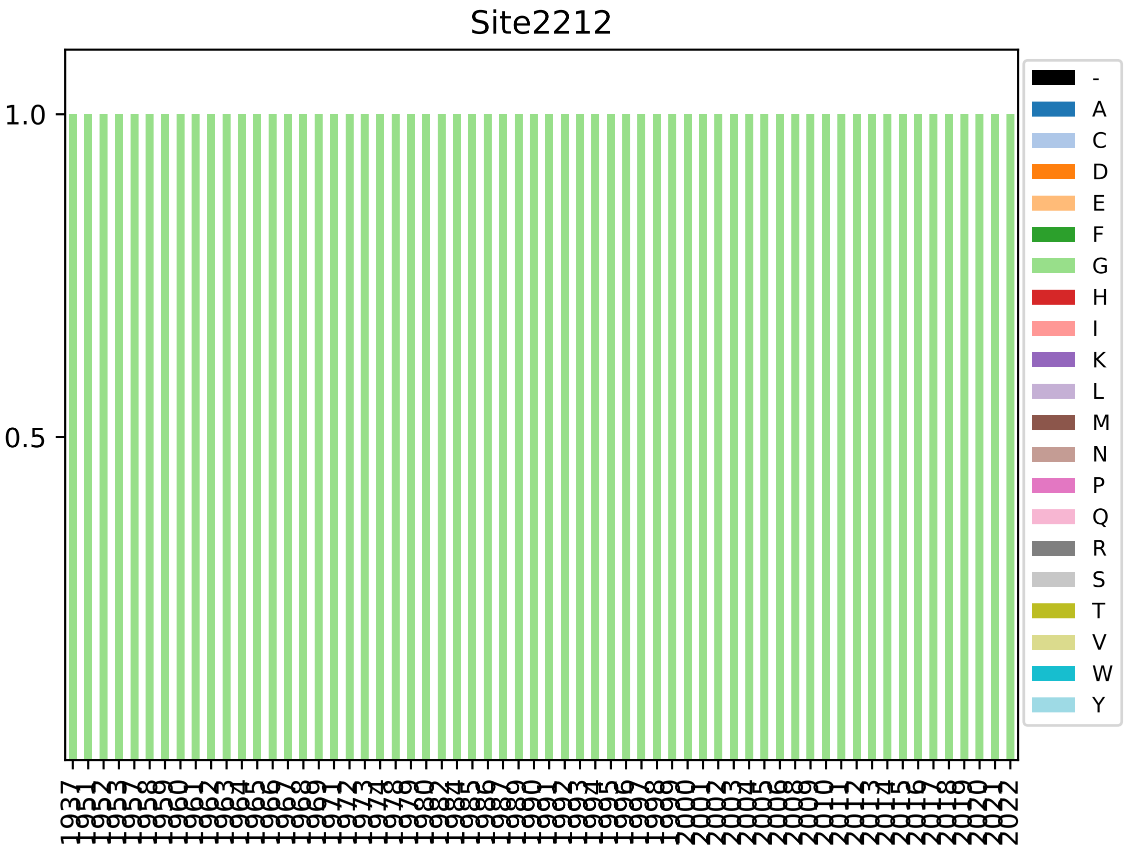
Site2212
1.0
0.5
-
A
C
D
E
F
G
H
I
K
L
M
N
P
Q
R
S
T
V
W
Y

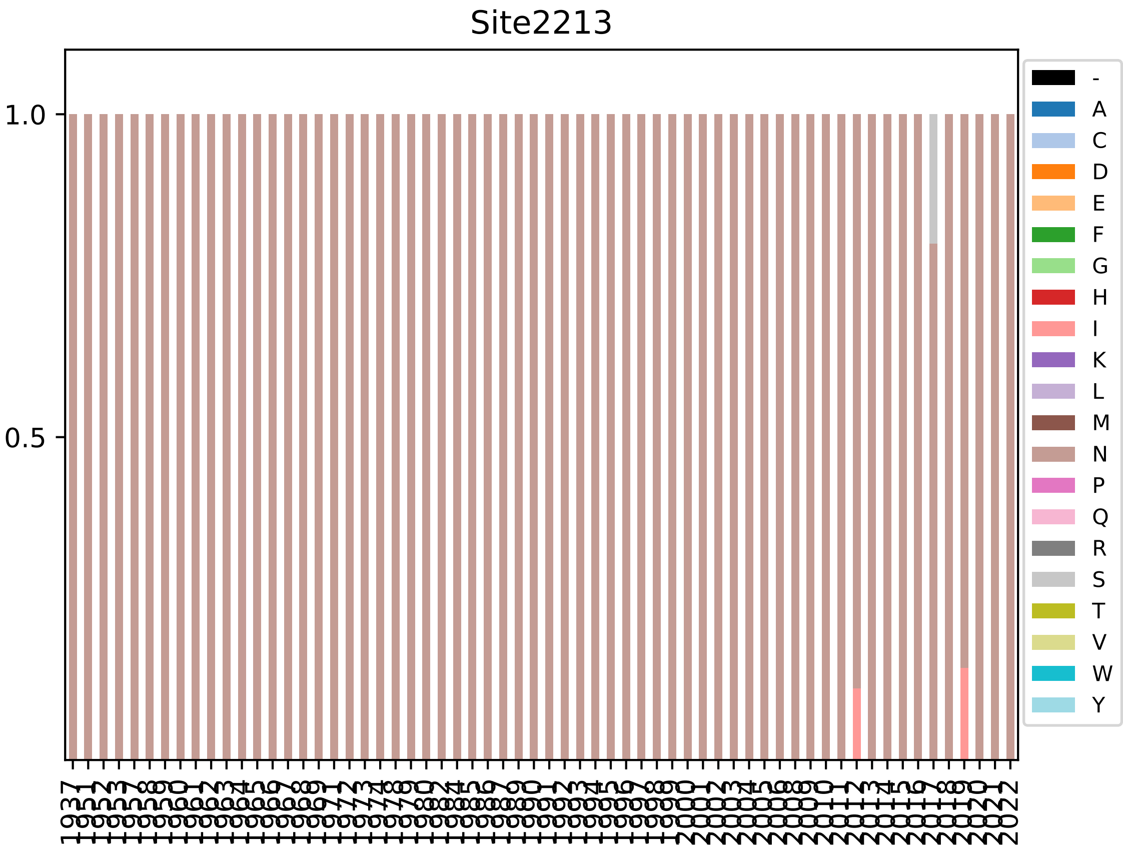
Site2213
1.0
0.5
-
A
C
D
E
F
G
H
I
K
L
M
N
P
Q
R
S
T
V
W
Y

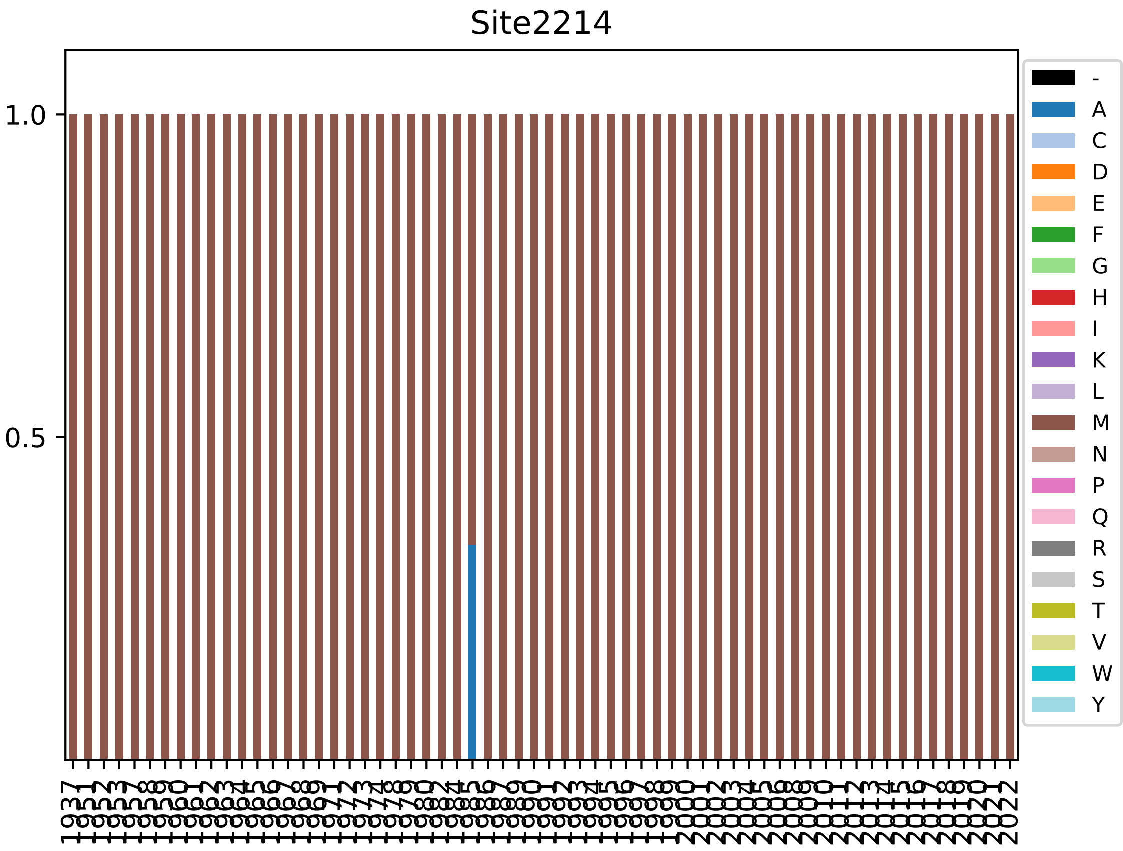
Site2214
1.0
0.5
-
A
C
D
E
F
G
H
I
K
L
M
N
P
Q
R
S
T
V
W
Y

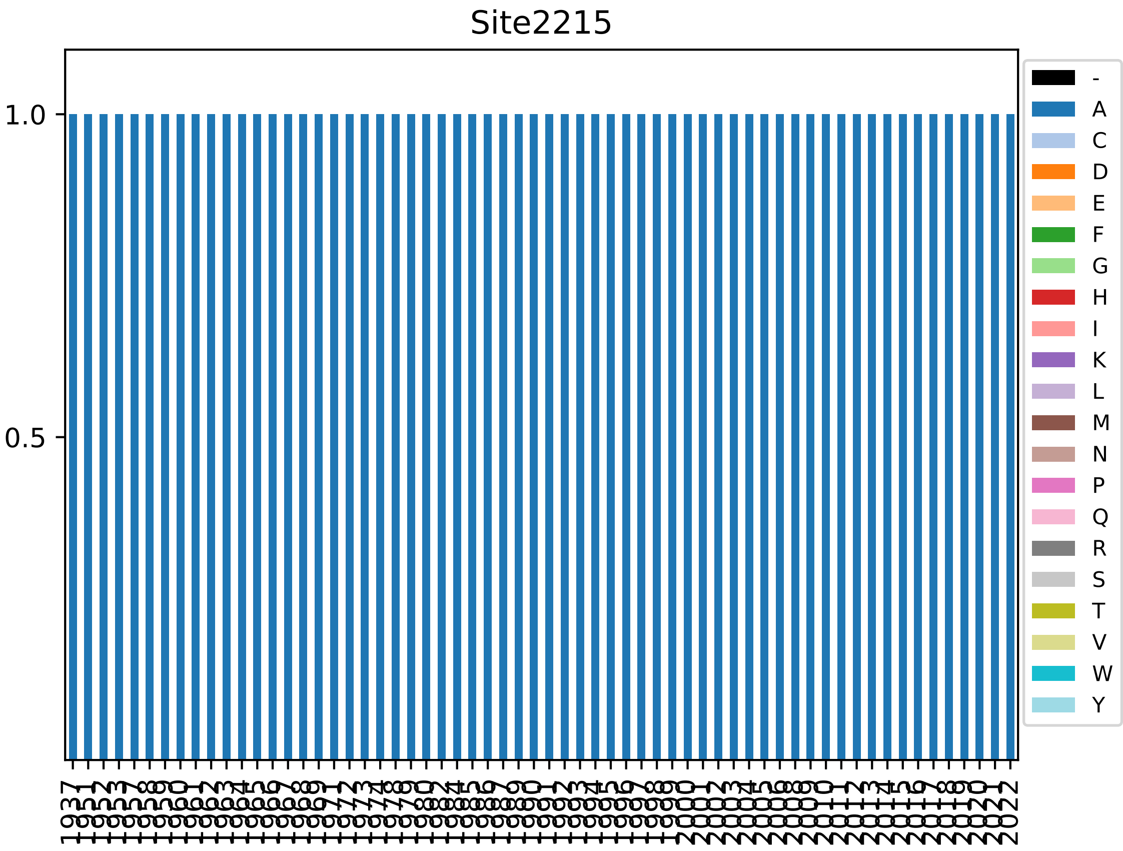
Site2215
1.0
0.5
-
A
C
D
E
F
G
H
I
K
L
M
N
P
Q
R
S
T
V
W
Y

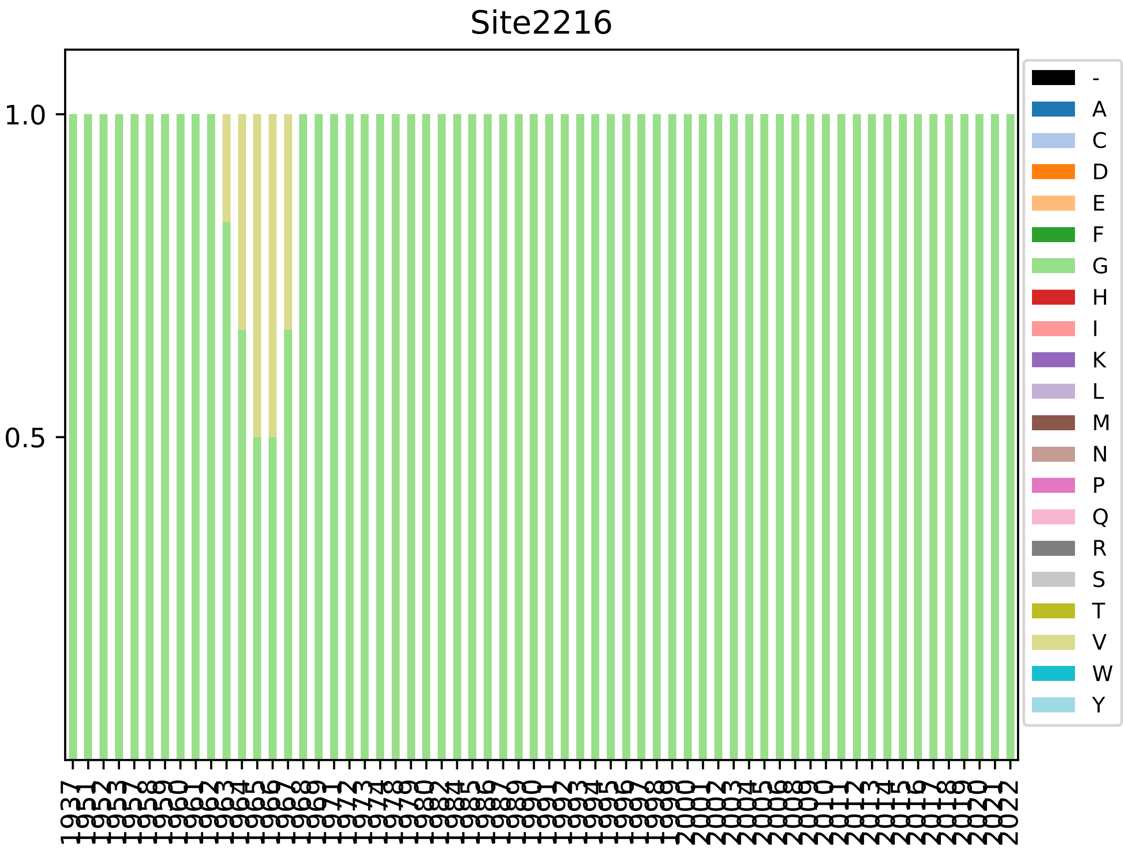
Site2216
1.0
0.5
-
A
C
D
E
F
G
H
I
K
L
M
N
P
Q
R
S
T
V
W
Y

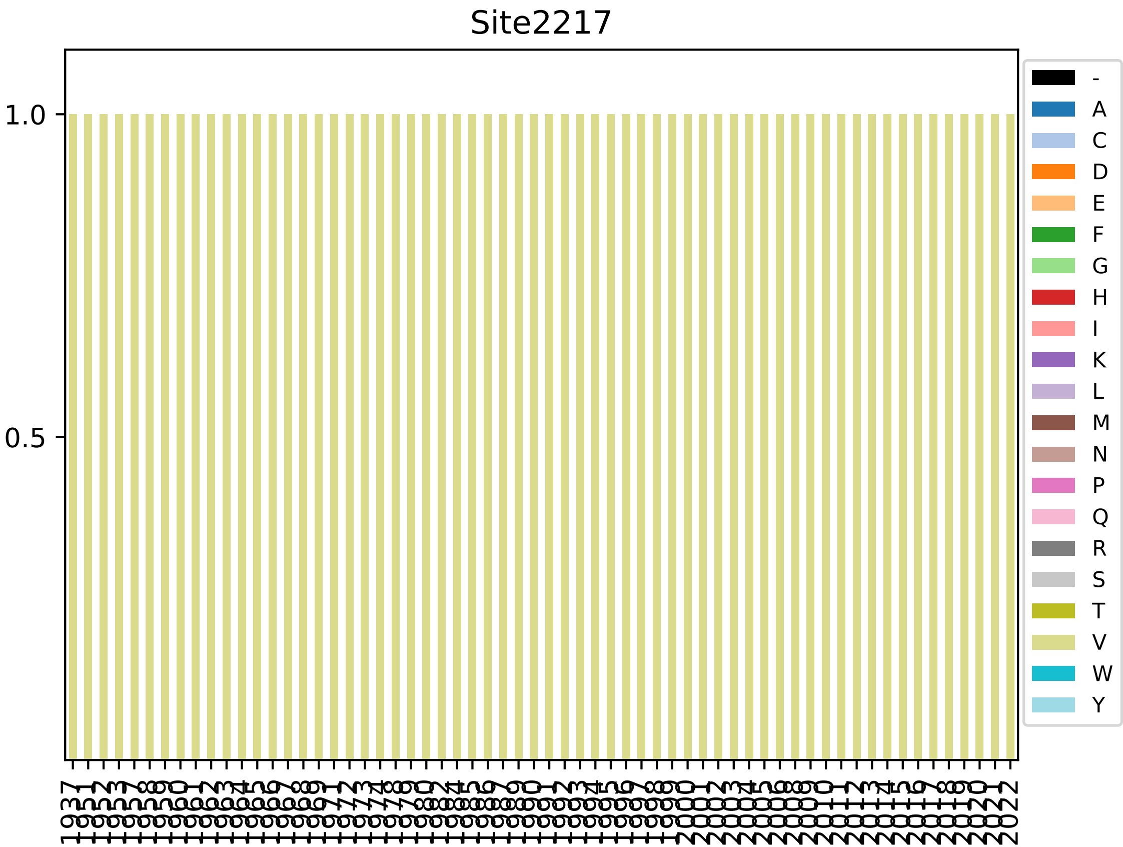
Site2217
1.0
0.5
-
A
C
D
E
F
G
H
I
K
L
M
N
P
Q
R
S
T
V
W
Y

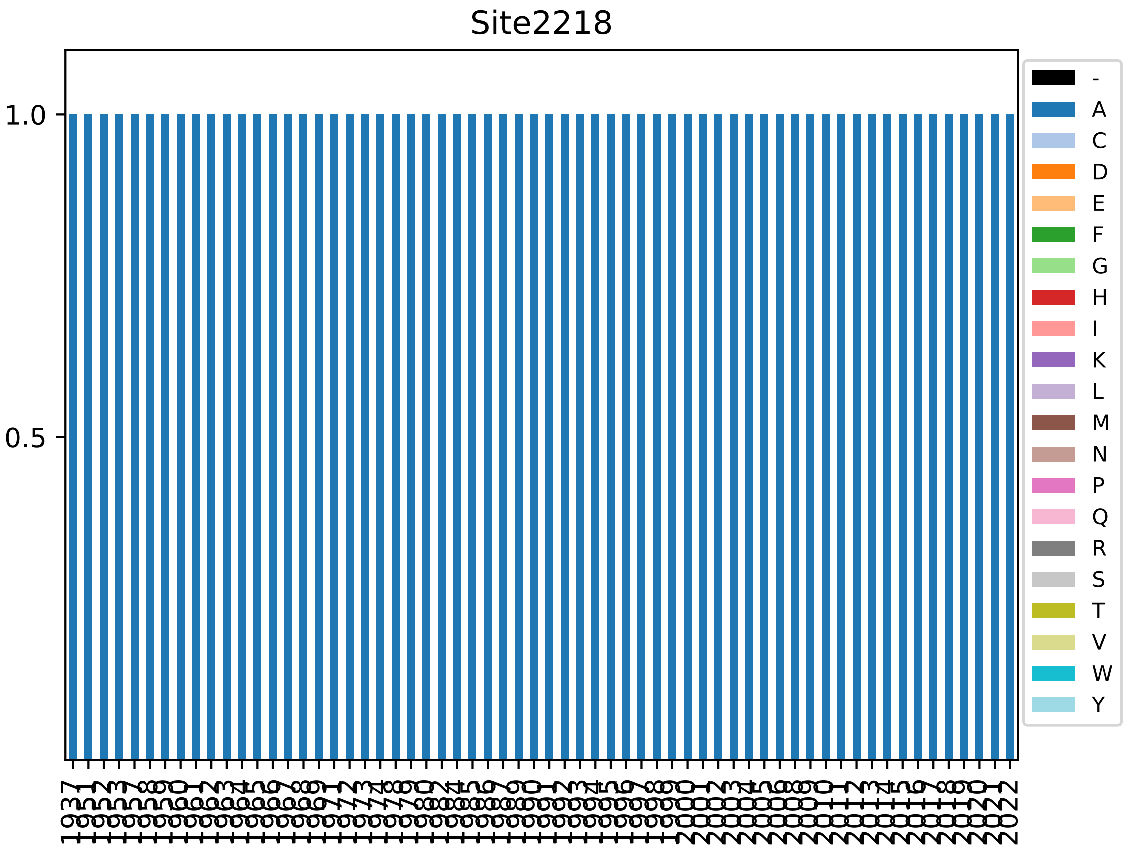

Site2218
1.0
0.5
-
A
C
D
E
F
G
H
I
K
L
M
N
P
Q
R
S
T
V
W
Y

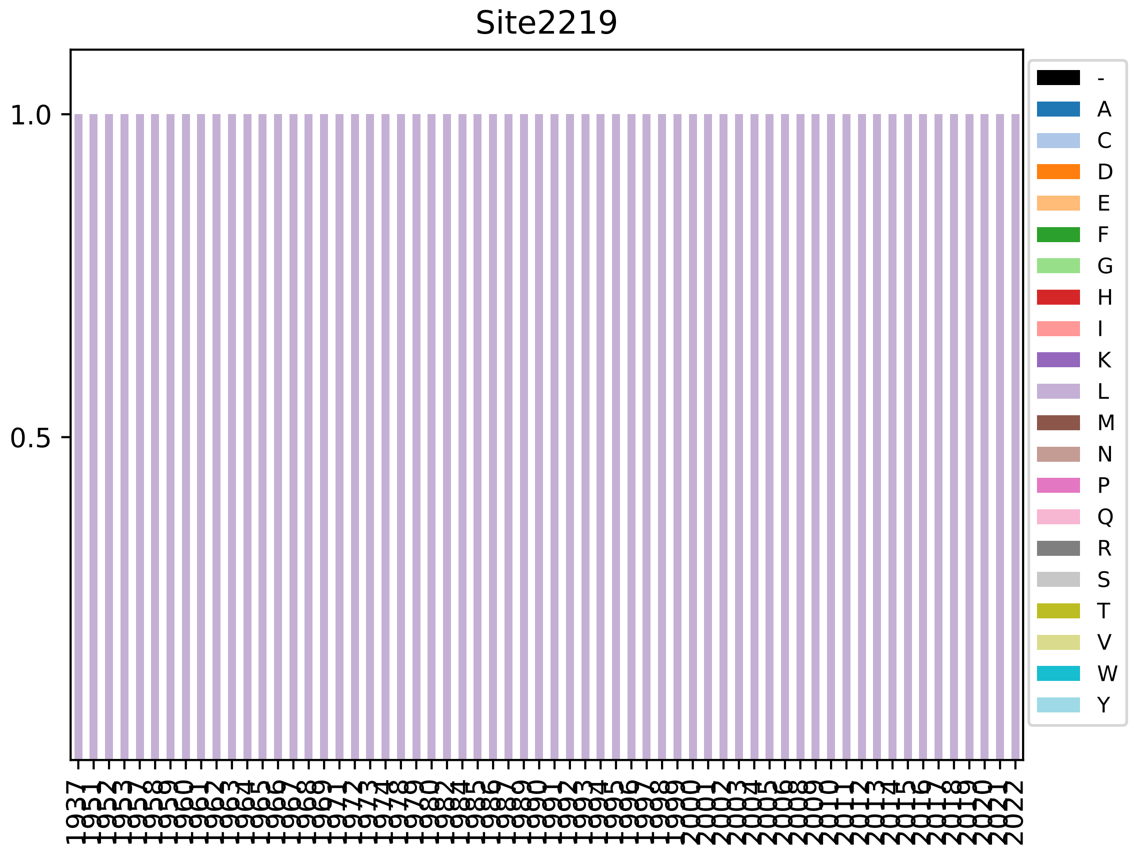
Site2219
1.0
0.5
-
A
C
D
E
F
G
H
I
K
L
M
N
P
Q
R
S
T
V
W
Y

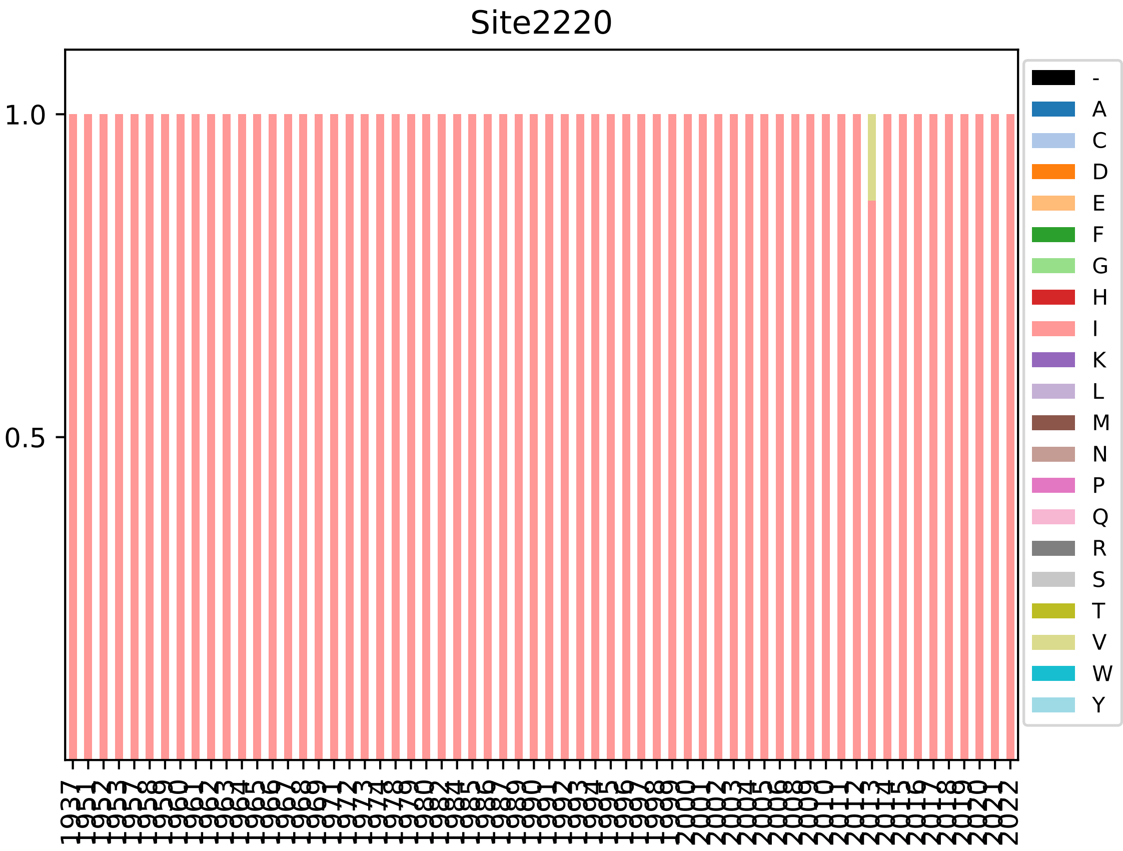

Site2220
1.0
0.5
-
A
C
D
E
F
G
H
I
K
L
M
N
P
Q
R
S
T
V
W
Y

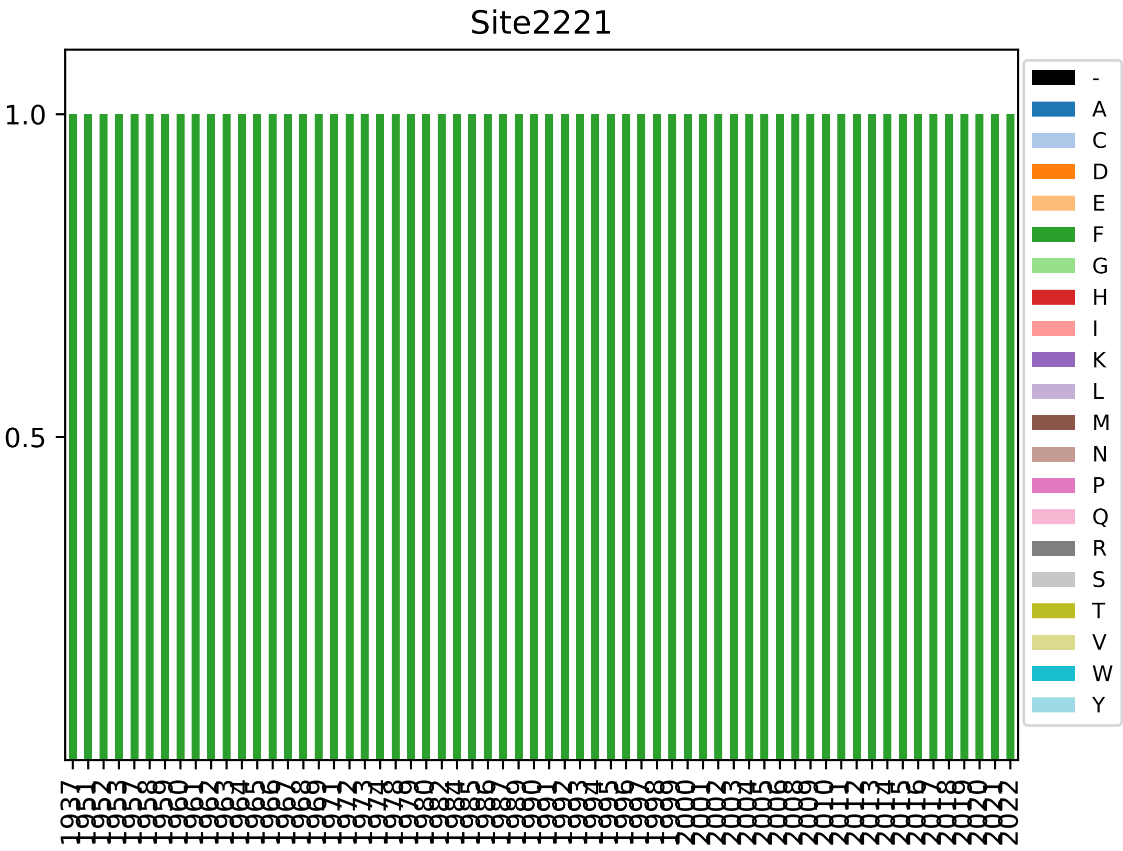
Site2221
1.0
0.5
-
A
C
D
E
F
G
H
I
K
L
M
N
P
Q
R
S
T
V
W
Y

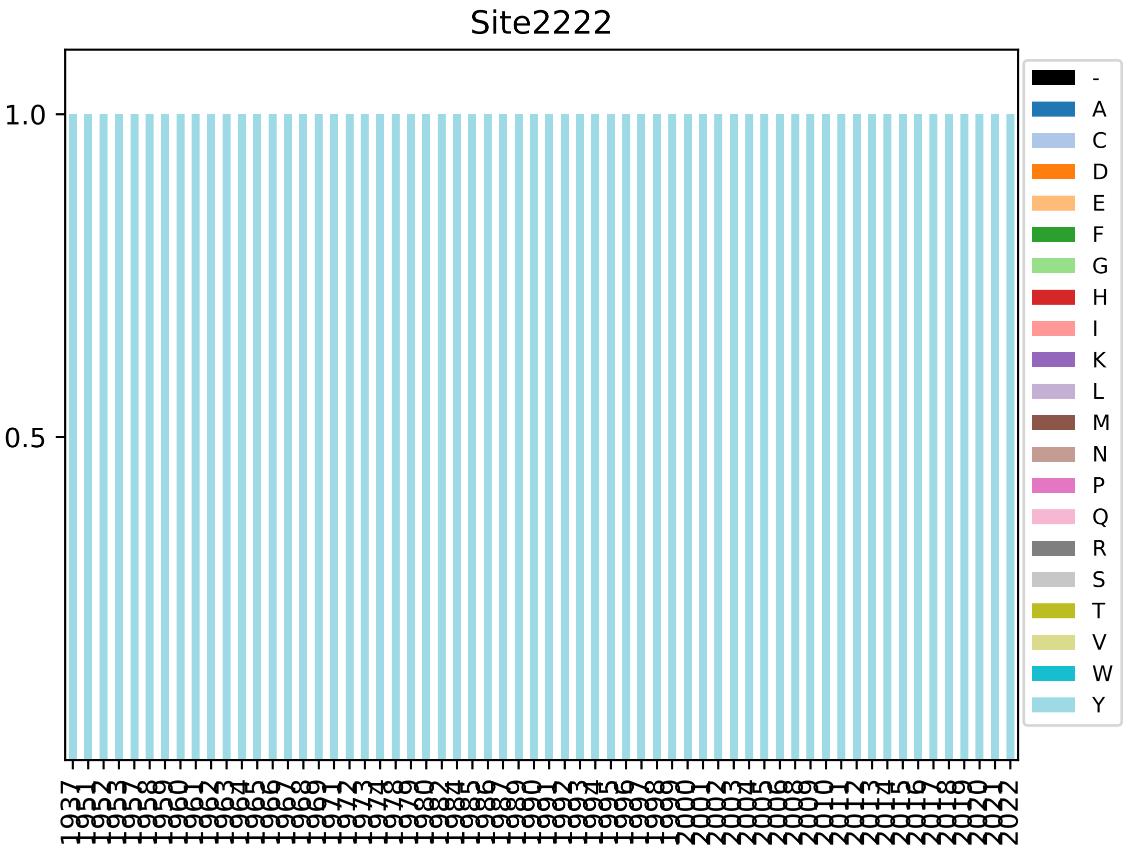
Site2222
1.0
0.5
-
A
C
D
E
F
G
H
I
K
L
M
N
P
Q
R
S
T
V
W
Y

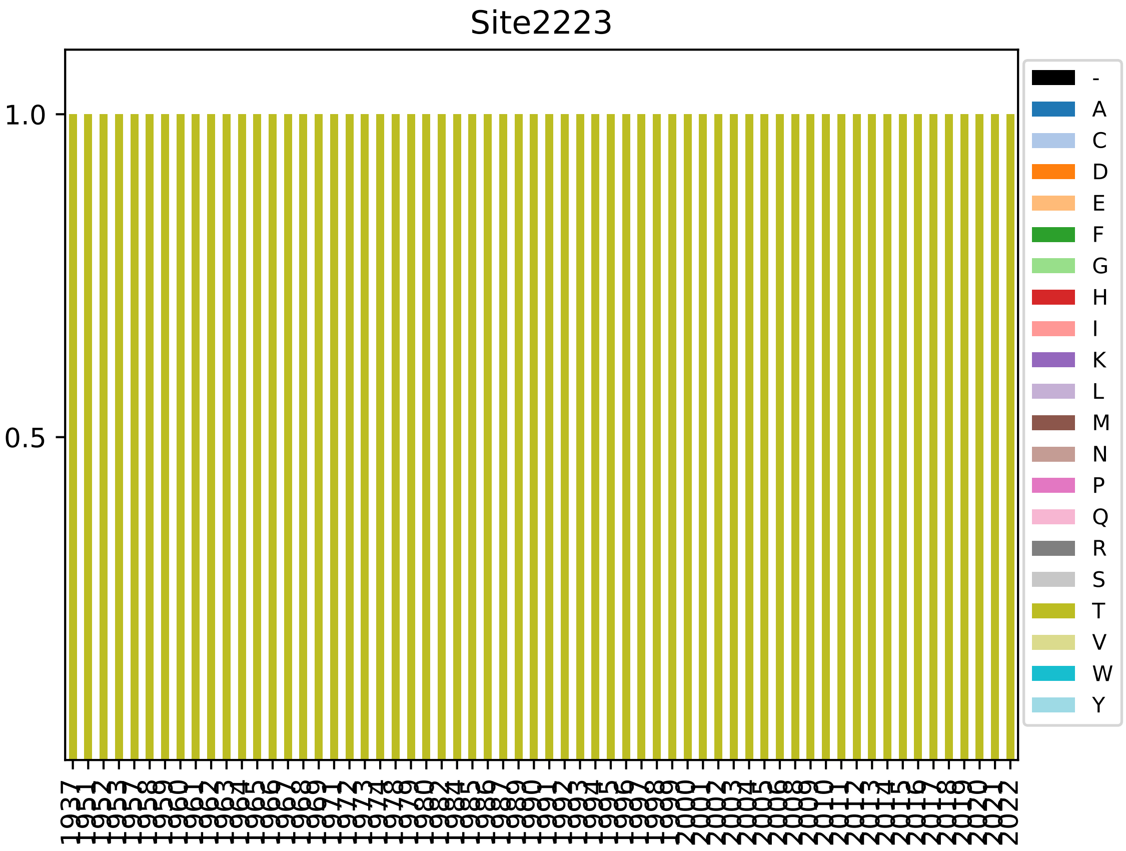
Site2223
1.0
0.5
-
A
C
D
E
F
G
H
I
K
L
M
N
P
Q
R
S
T
V
W
Y

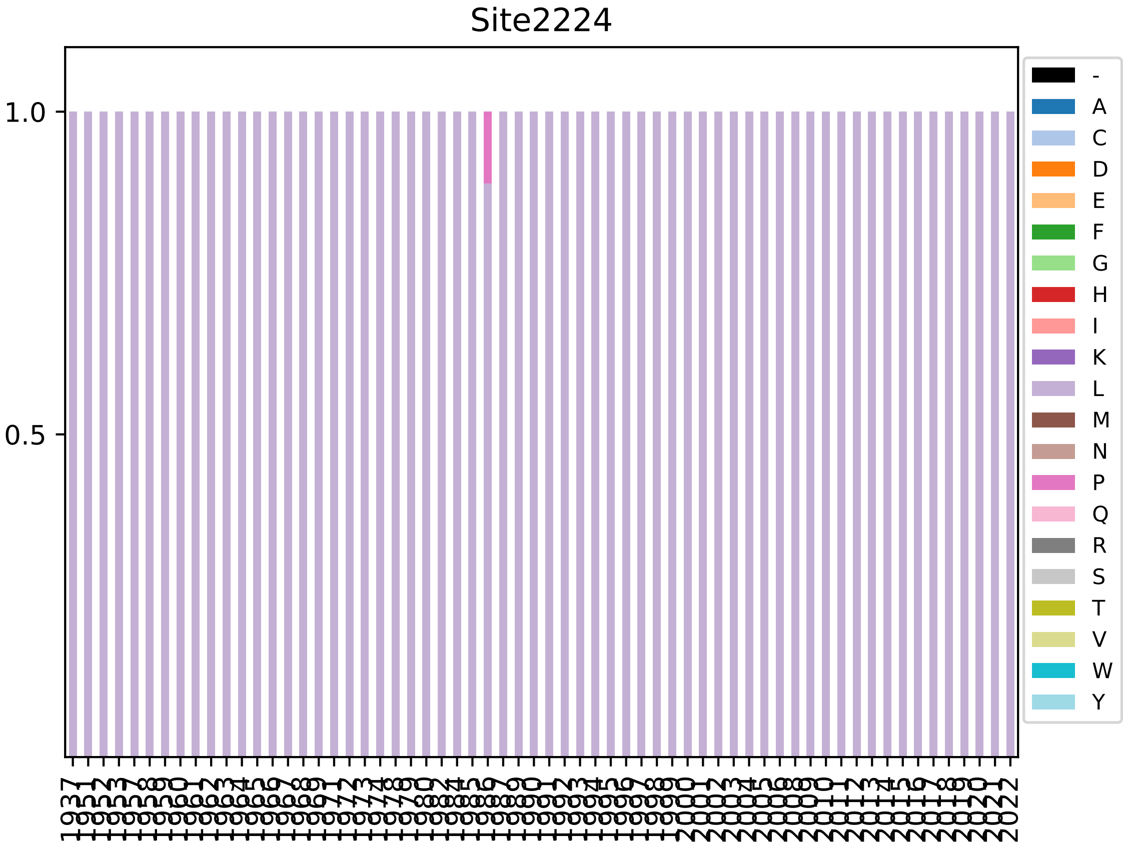
Site2224
1.0
0.5
-
A
C
D
E
F
G
H
I
K
L
M
N
P
Q
R
S
T
V
W
Y

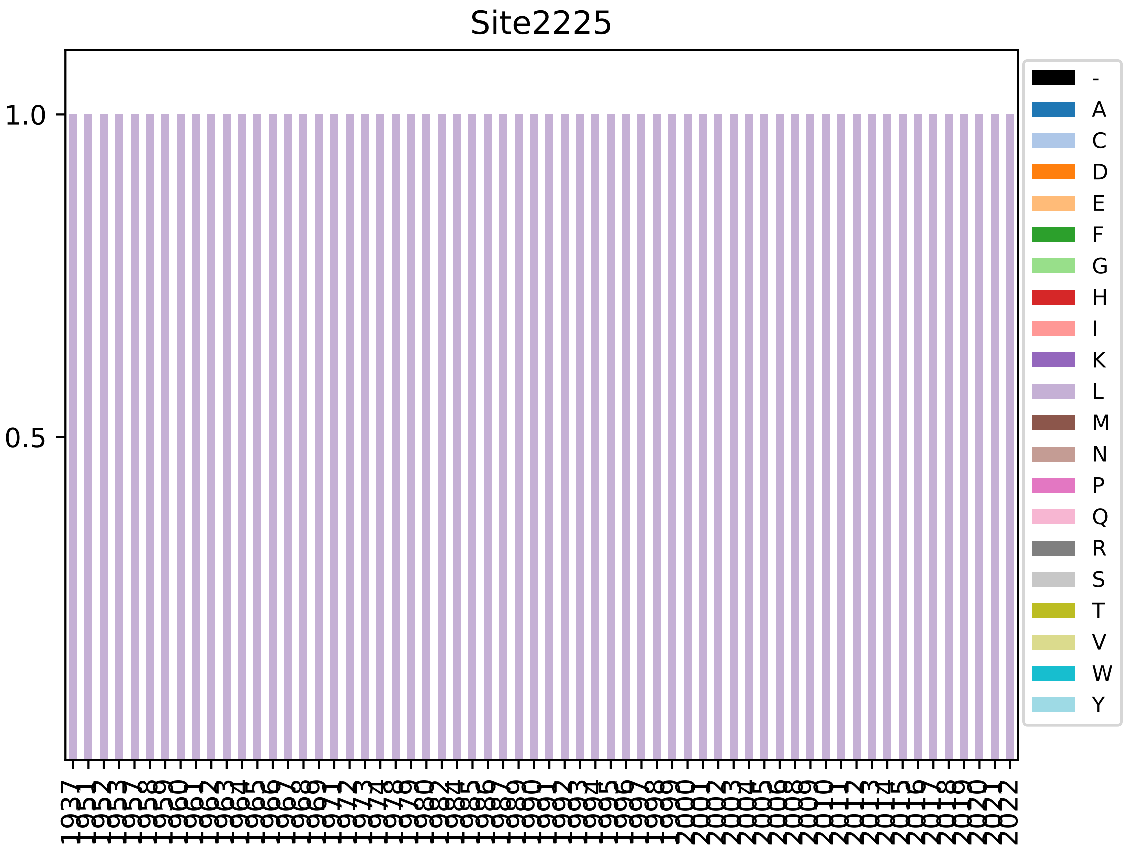
Site2225
1.0
0.5
-
A
C
D
E
F
G
H
I
K
L
M
N
P
Q
R
S
T
V
W
Y

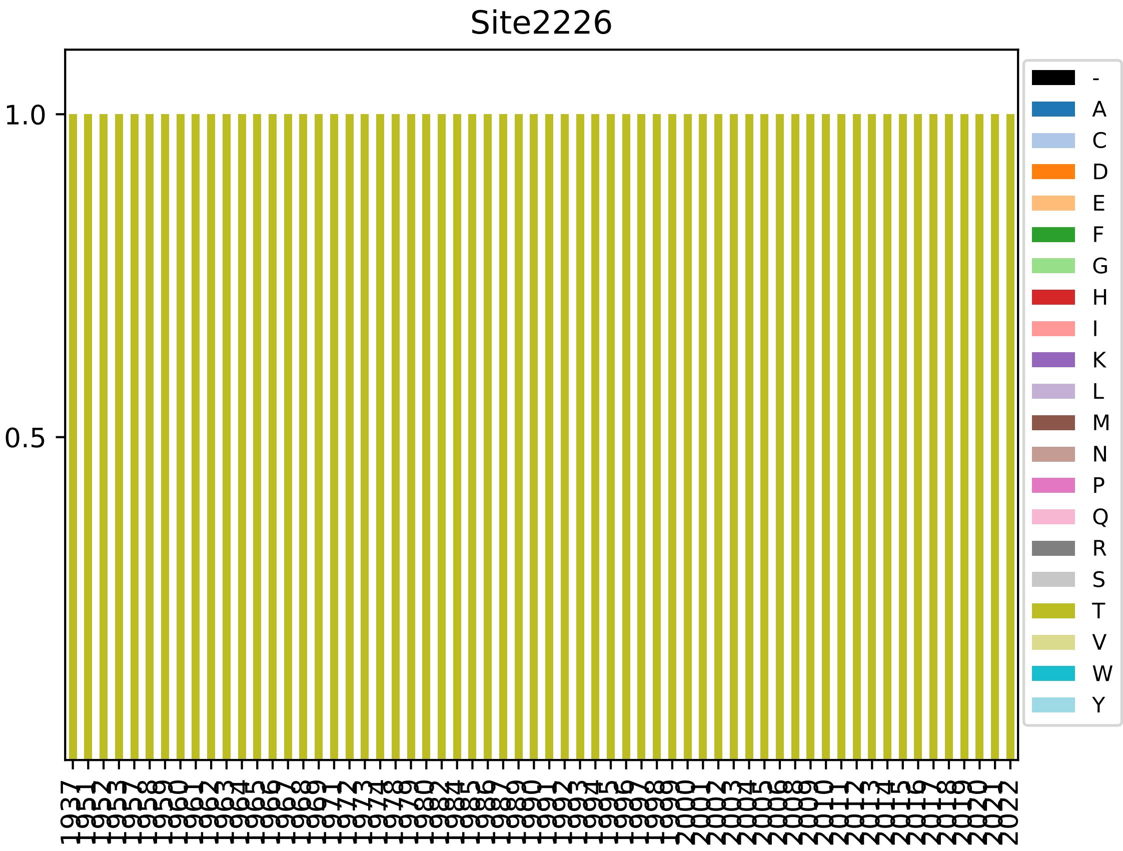
Site2226
1.0
0.5
-
A
C
D
E
F
G
H
I
K
L
M
N
P
Q
R
S
T
V
W
Y

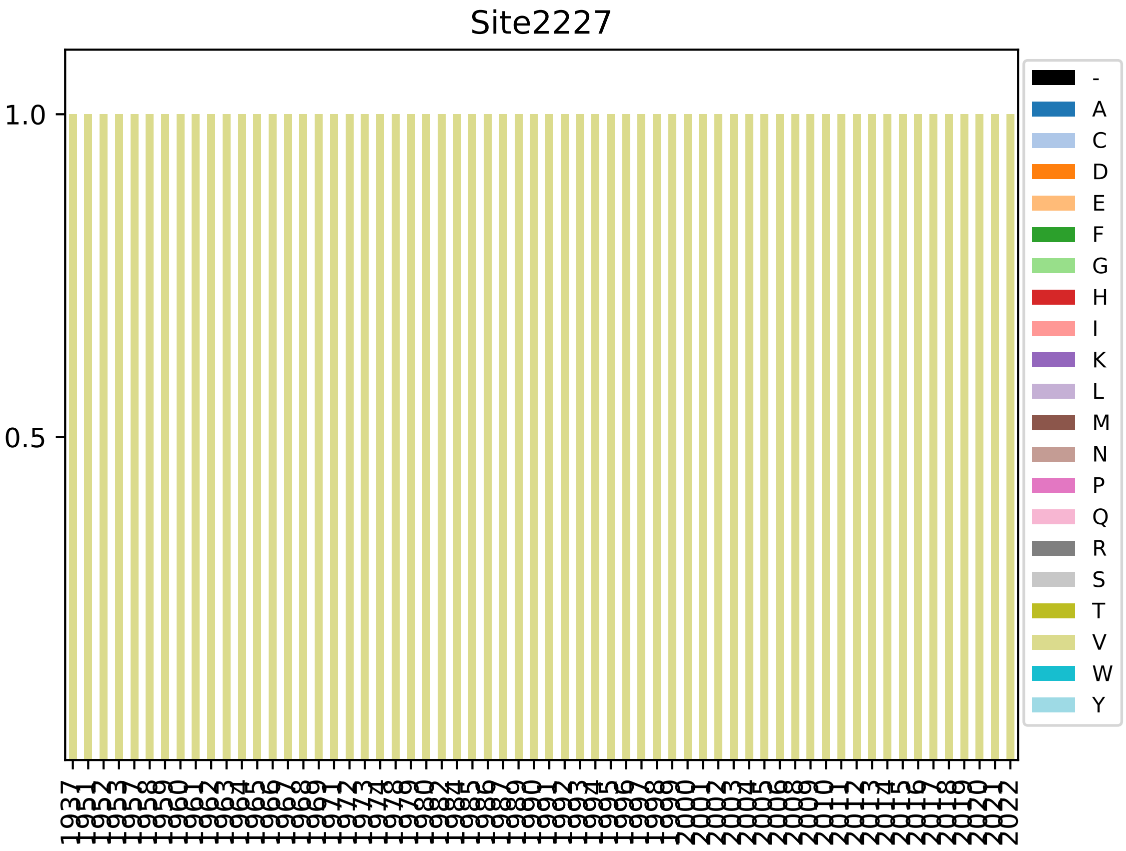
Site2227
1.0
0.5
-
A
C
D
E
F
G
H
I
K
L
M
N
P
Q
R
S
T
V
W
Y

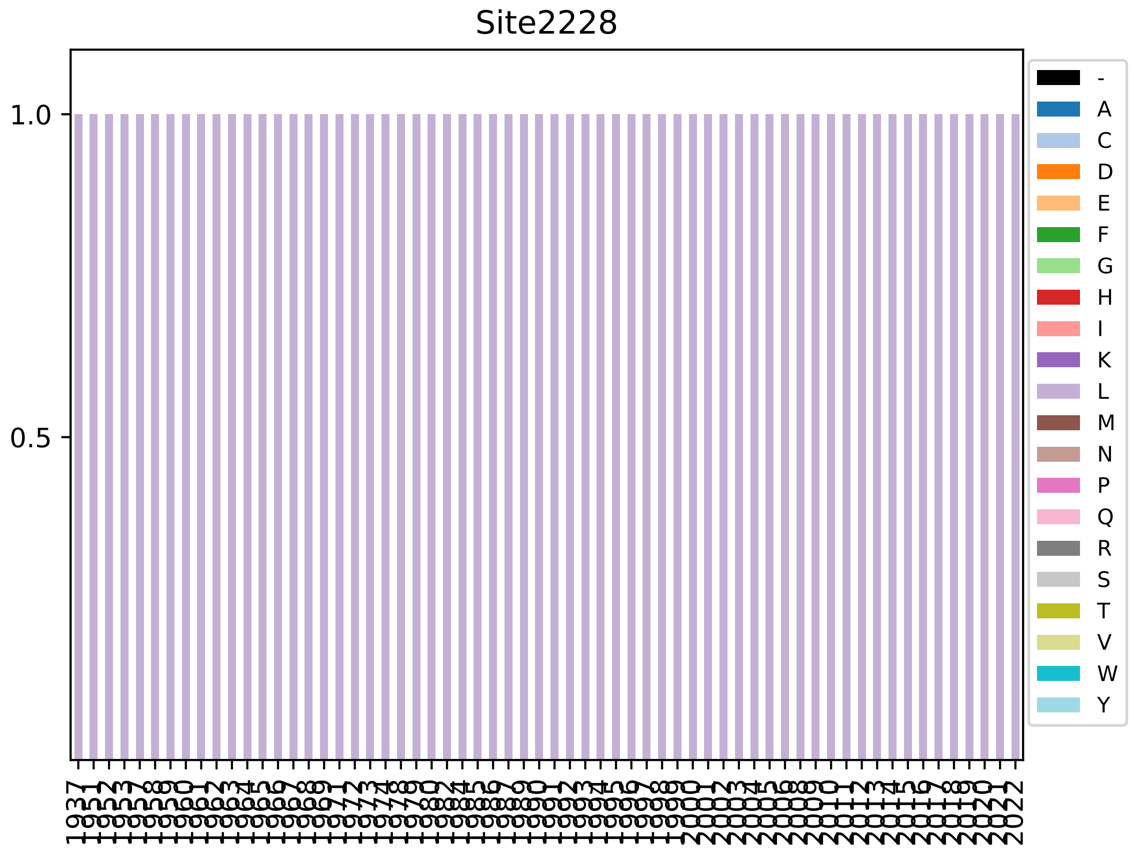
Site2228
1.0
0.5
-
A
C
D
E
F
G
H
I
K
L
M
N
P
Q
R
S
T
V
W
Y

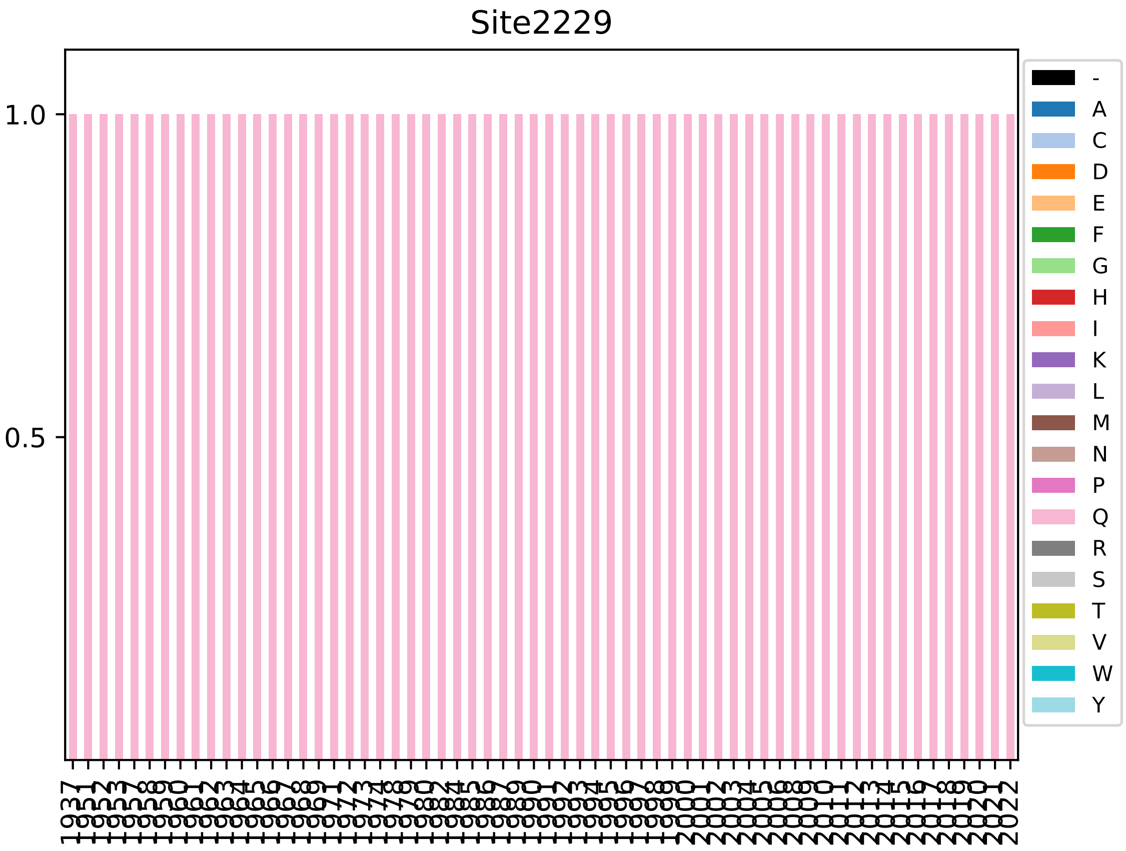
Site2229
1.0
0.5
-
A
C
D
E
F
G
H
I
K
L
M
N
P
Q
R
S
T
V
W
Y

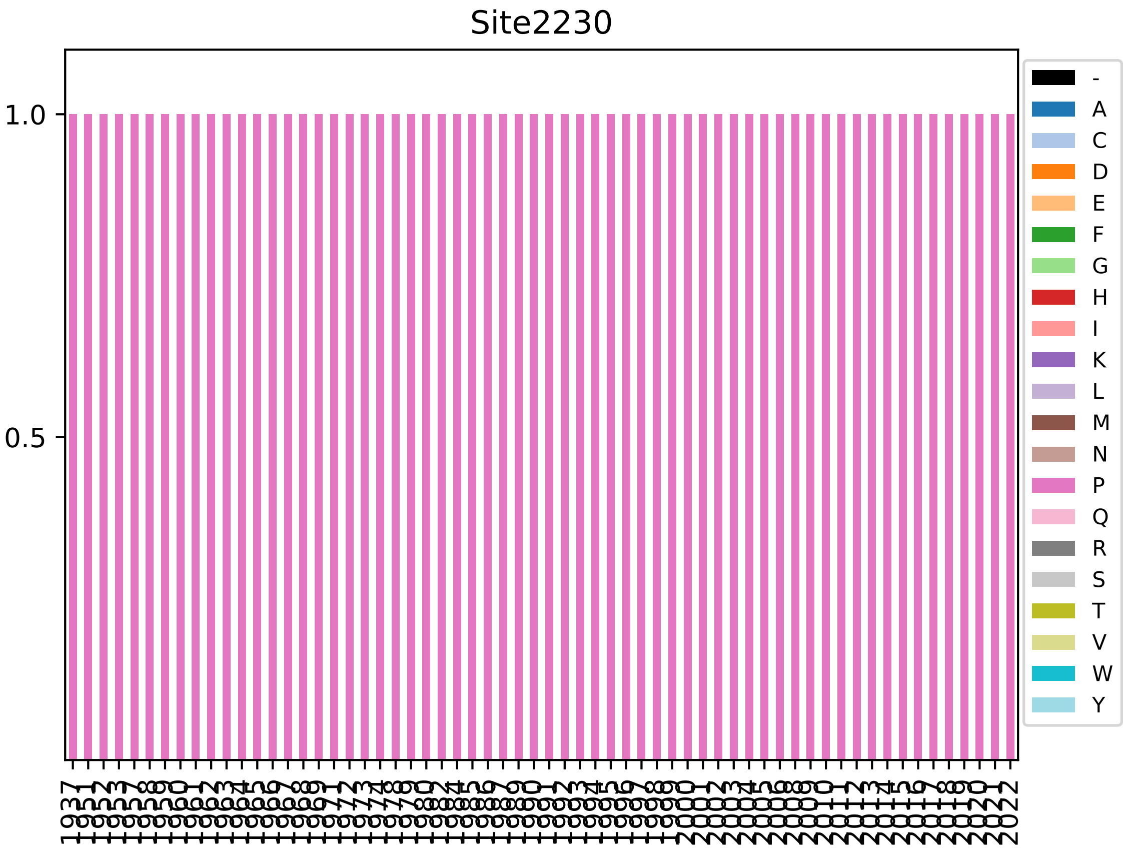
Site2230
1.0
0.5
-
A
C
D
E
F
G
H
I
K
L
M
N
P
Q
R
S
T
V
W
Y

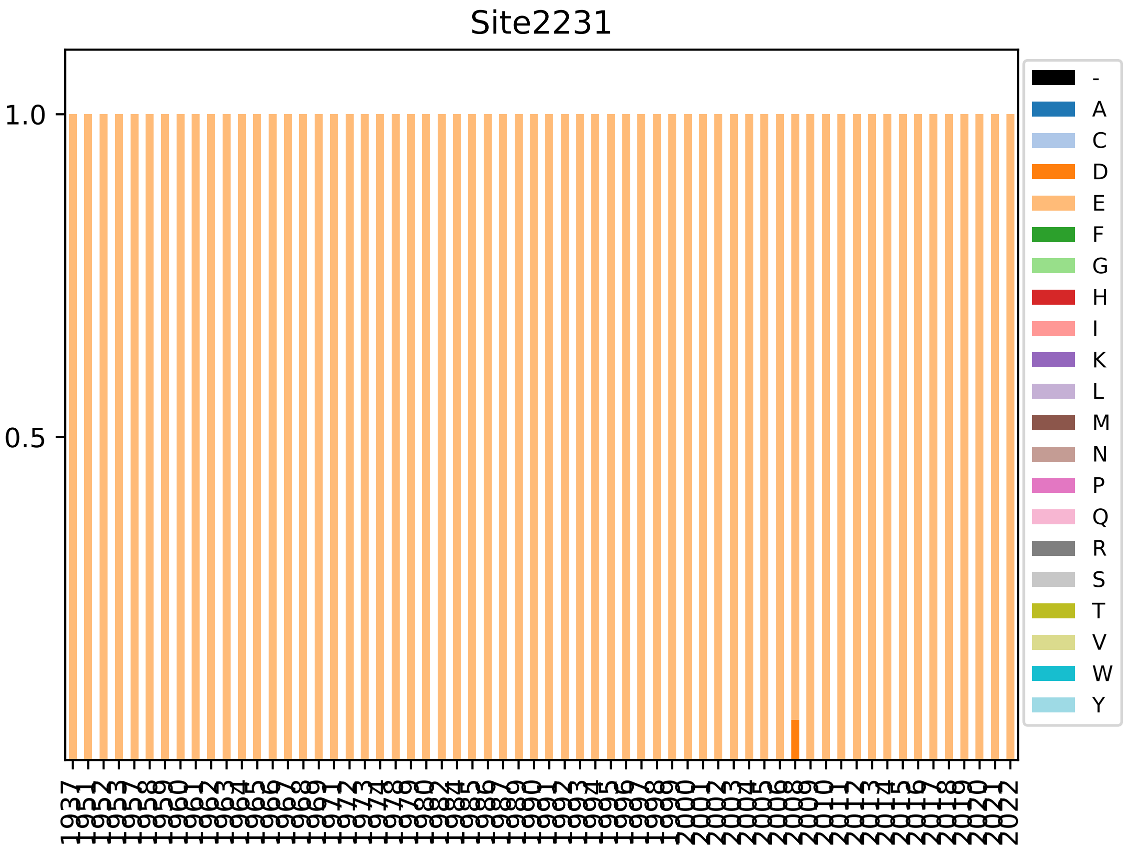
Site2231
1.0
0.5
-
A
C
D
E
F
G
H
I
K
L
M
N
P
Q
R
S
T
V
W
Y

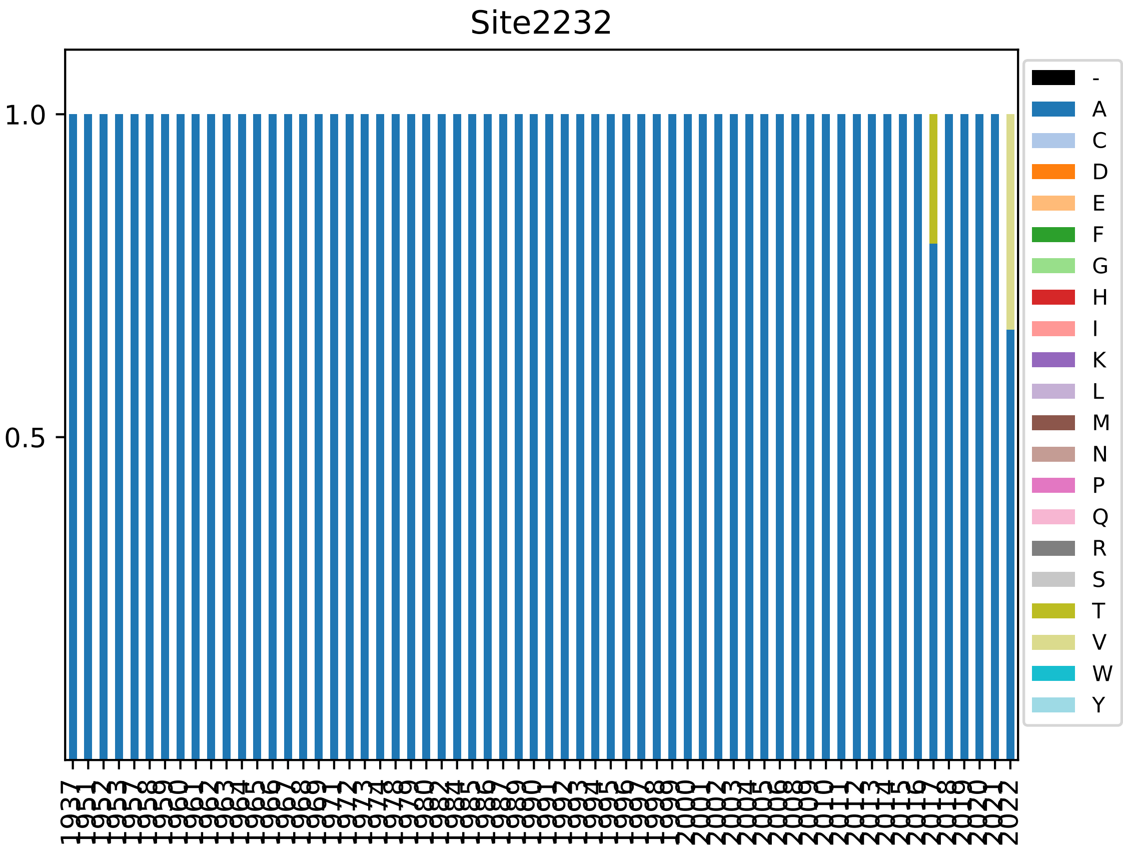
Site2232
1.0
0.5
-
A
C
D
E
F
G
H
I
K
L
M
N
P
Q
R
S
T
V
W
Y

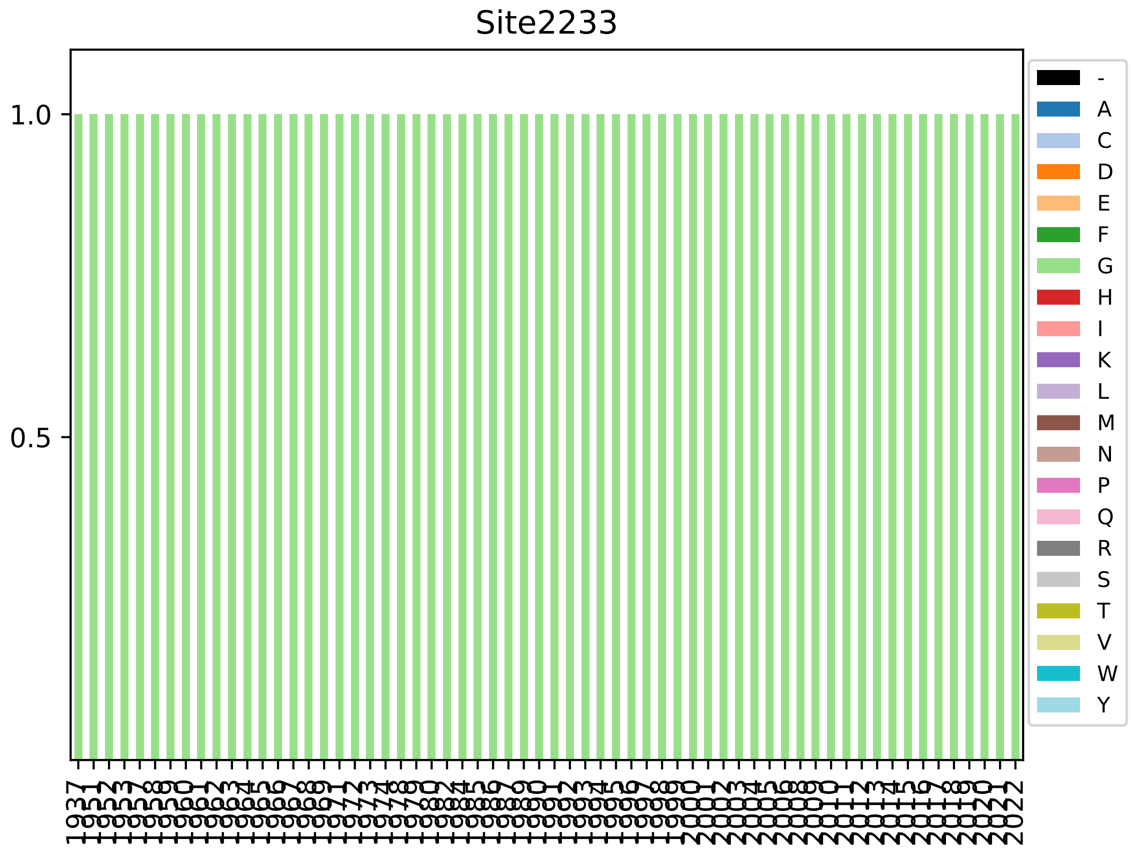
Site2233
1.0
0.5
-
A
C
D
E
F
G
H
I
K
L
M
N
P
Q
R
S
T
V
W
Y

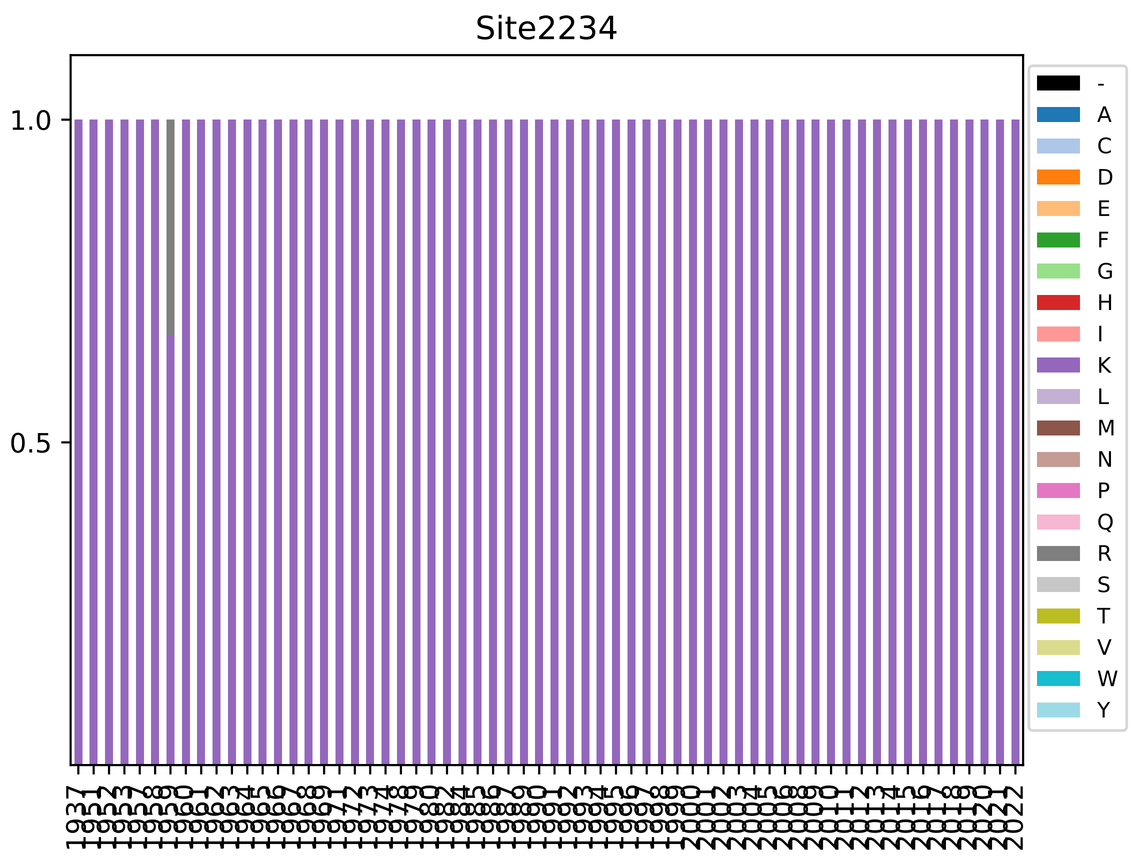
Site2234
1.0
0.5
-
A
C
D
E
F
G
H
I
K
L
M
N
P
Q
R
S
T
V
W
Y

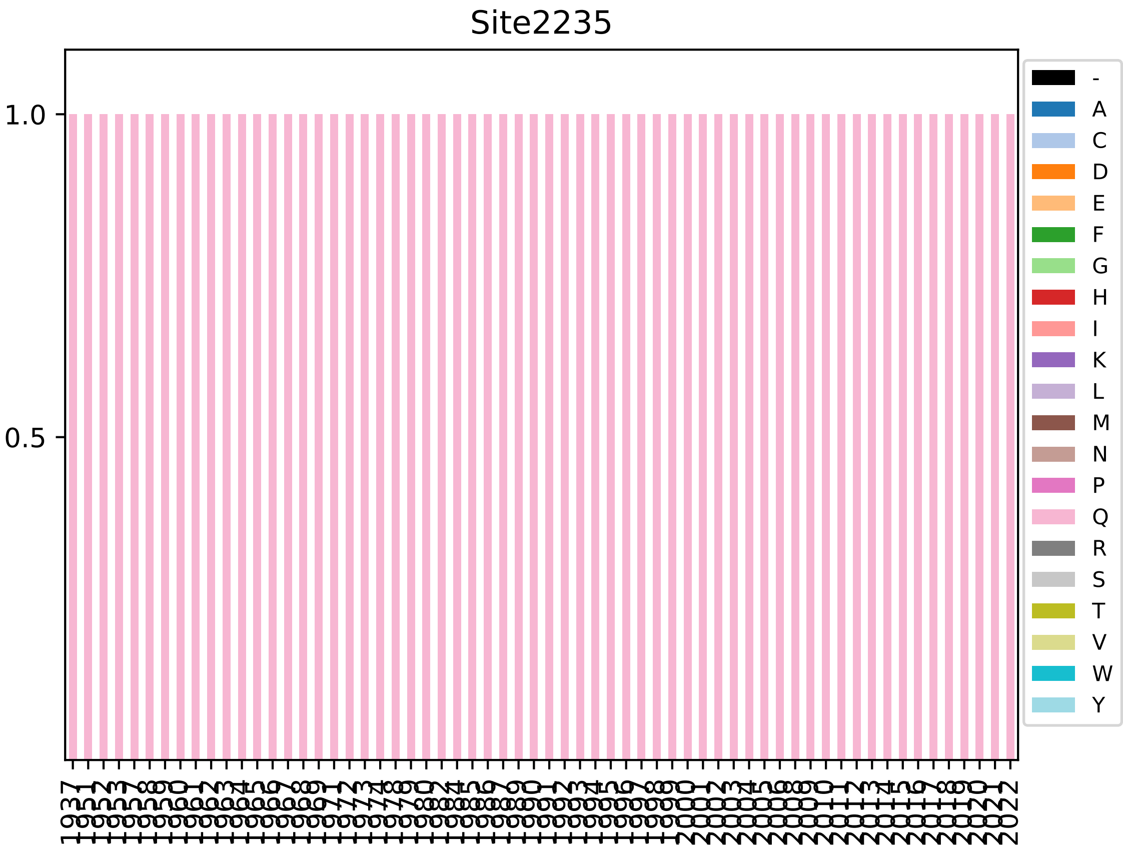
Site2235
1.0
0.5
-
A
C
D
E
F
G
H
I
K
L
M
N
P
Q
R
S
T
V
W
Y

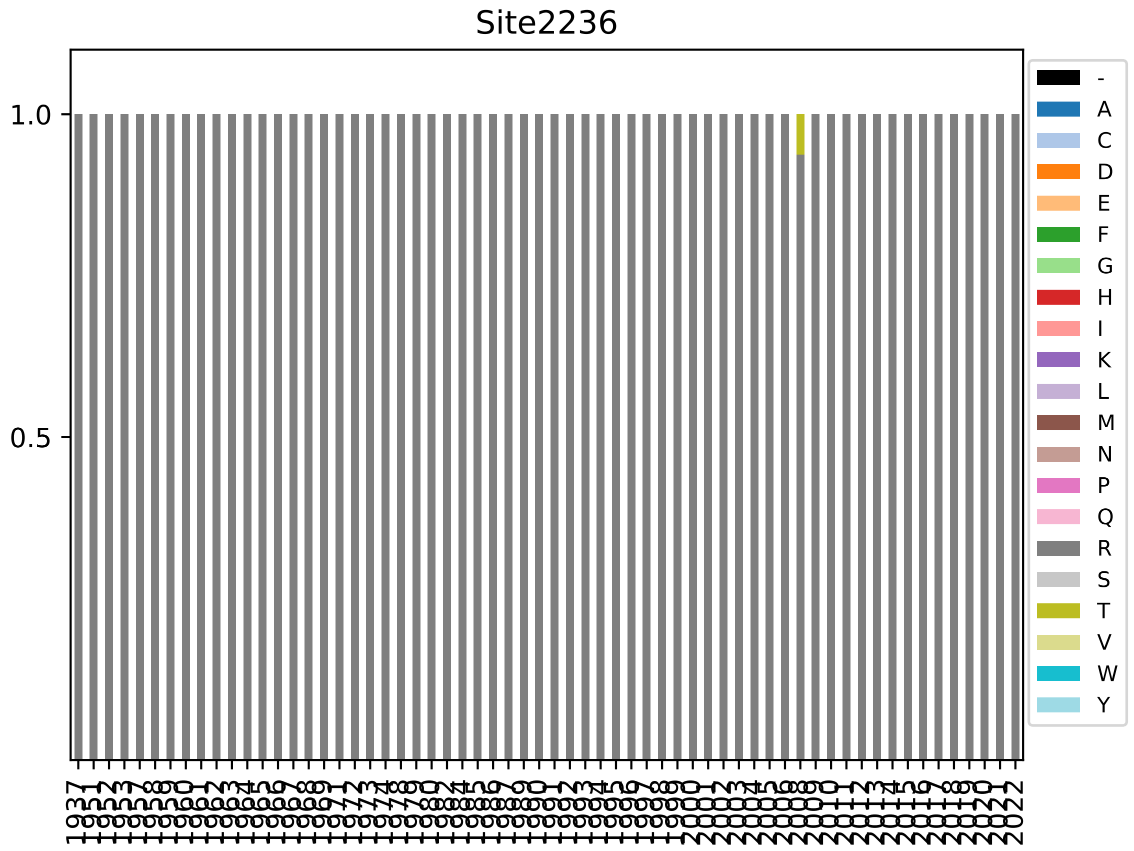
Site2236
1.0
0.5
-
A
C
D
E
F
G
H
I
K
L
M
N
P
Q
R
S
T
V
W
Y

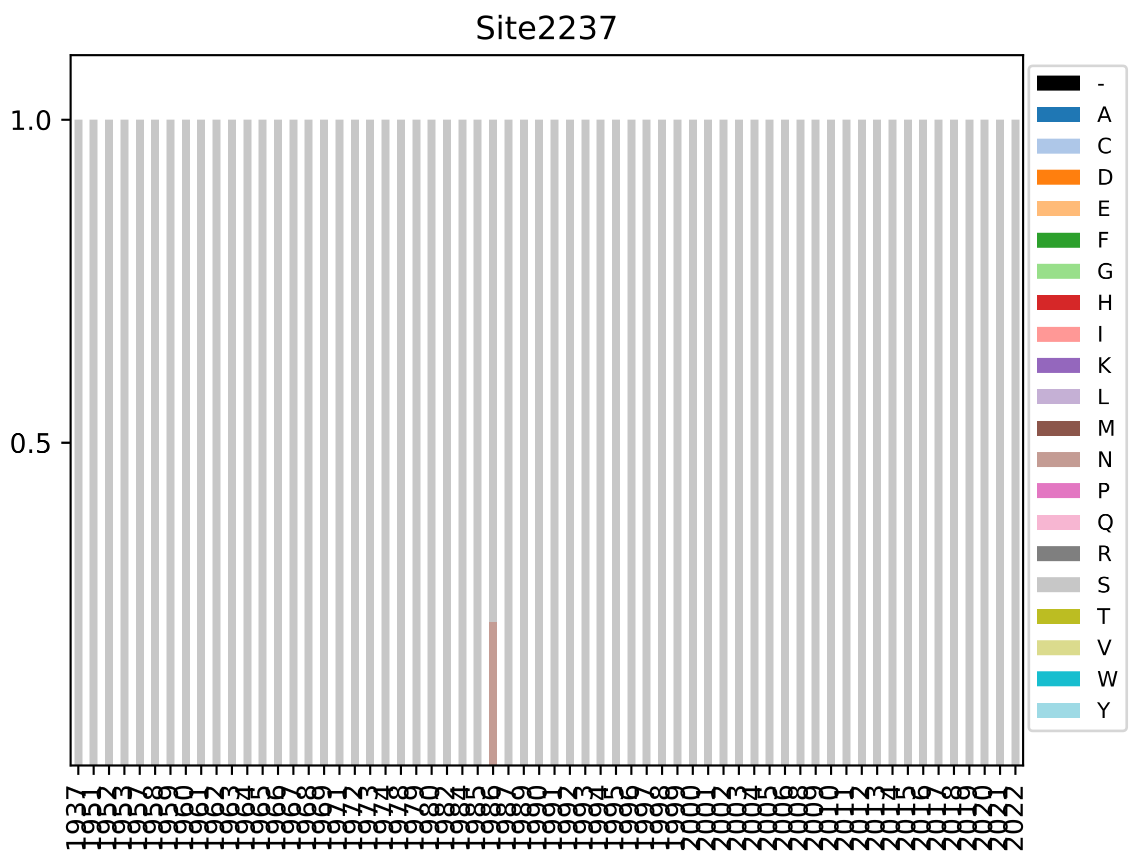
Site2237
1.0
0.5
-
A
C
D
E
F
G
H
I
K
L
M
N
P
Q
R
S
T
V
W
Y

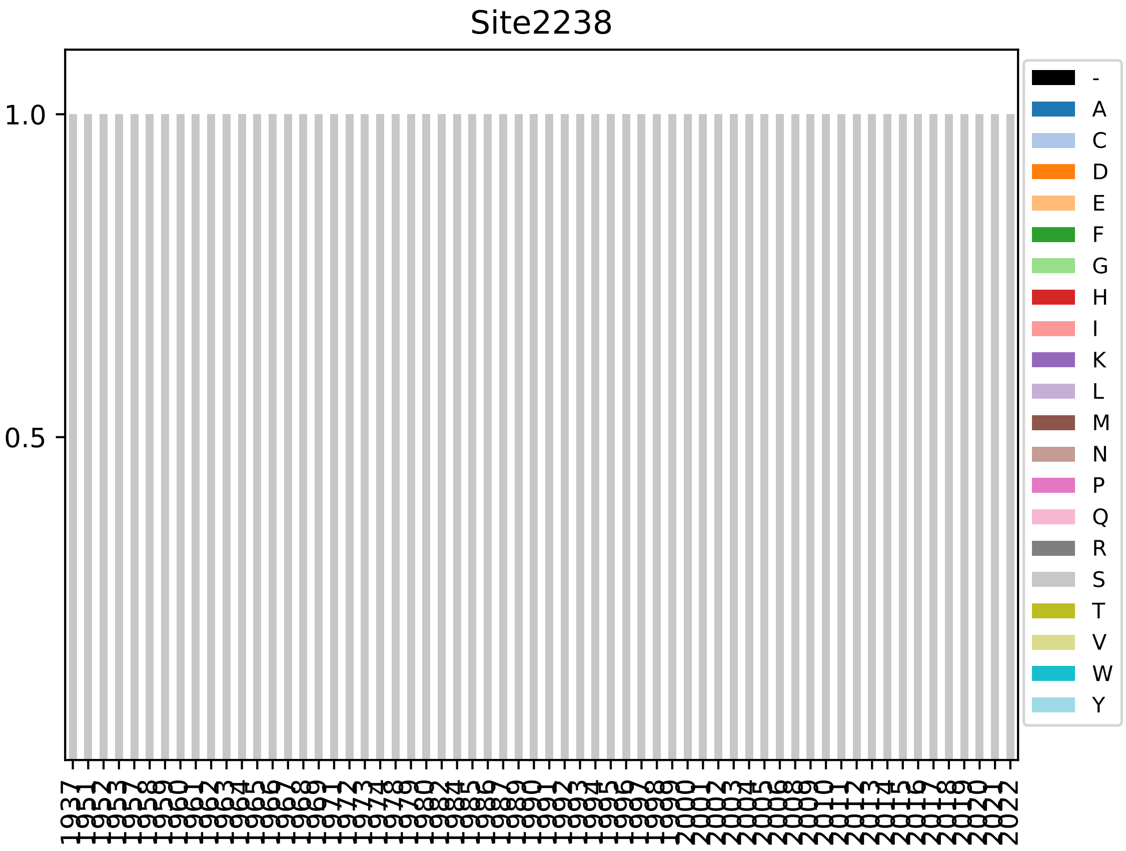
Site2238
1.0
0.5
-
A
C
D
E
F
G
H
I
K
L
M
N
P
Q
R
S
T
V
W
Y

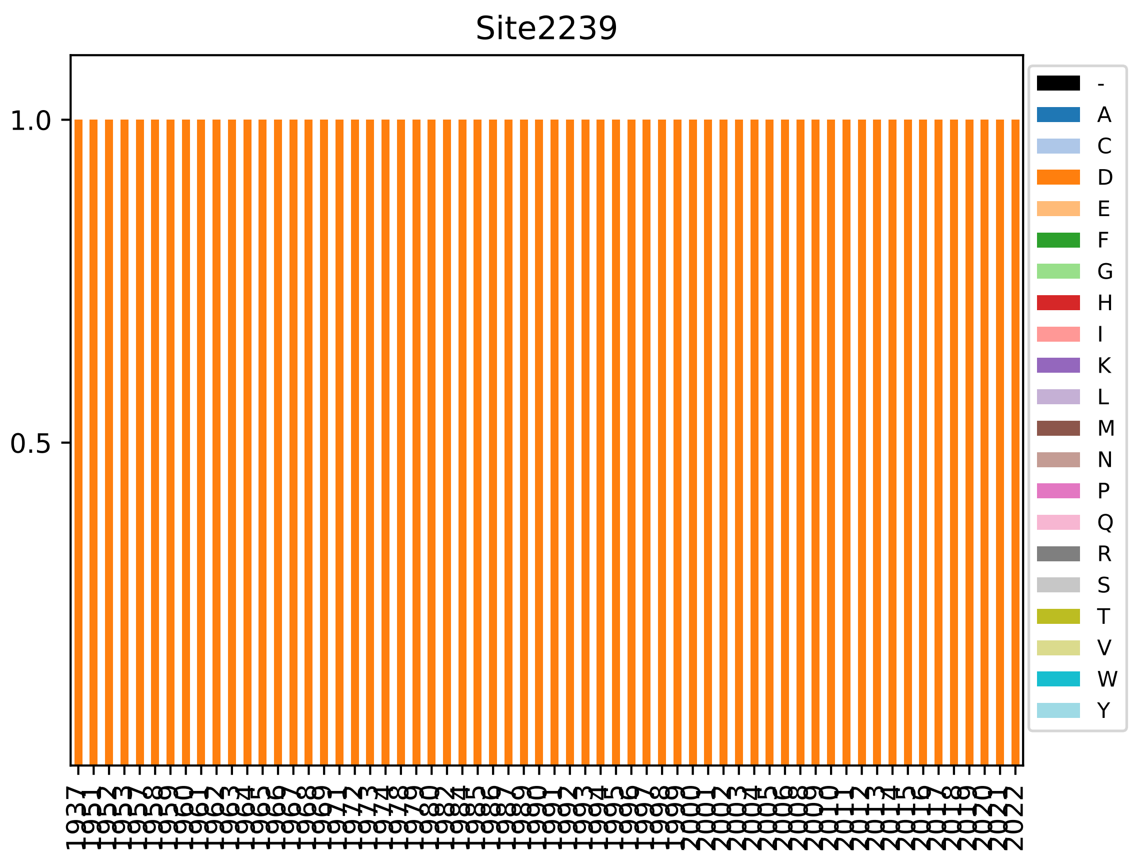
Site2239
1.0
0.5
-
A
C
D
E
F
G
H
I
K
L
M
N
P
Q
R
S
T
V
W
Y

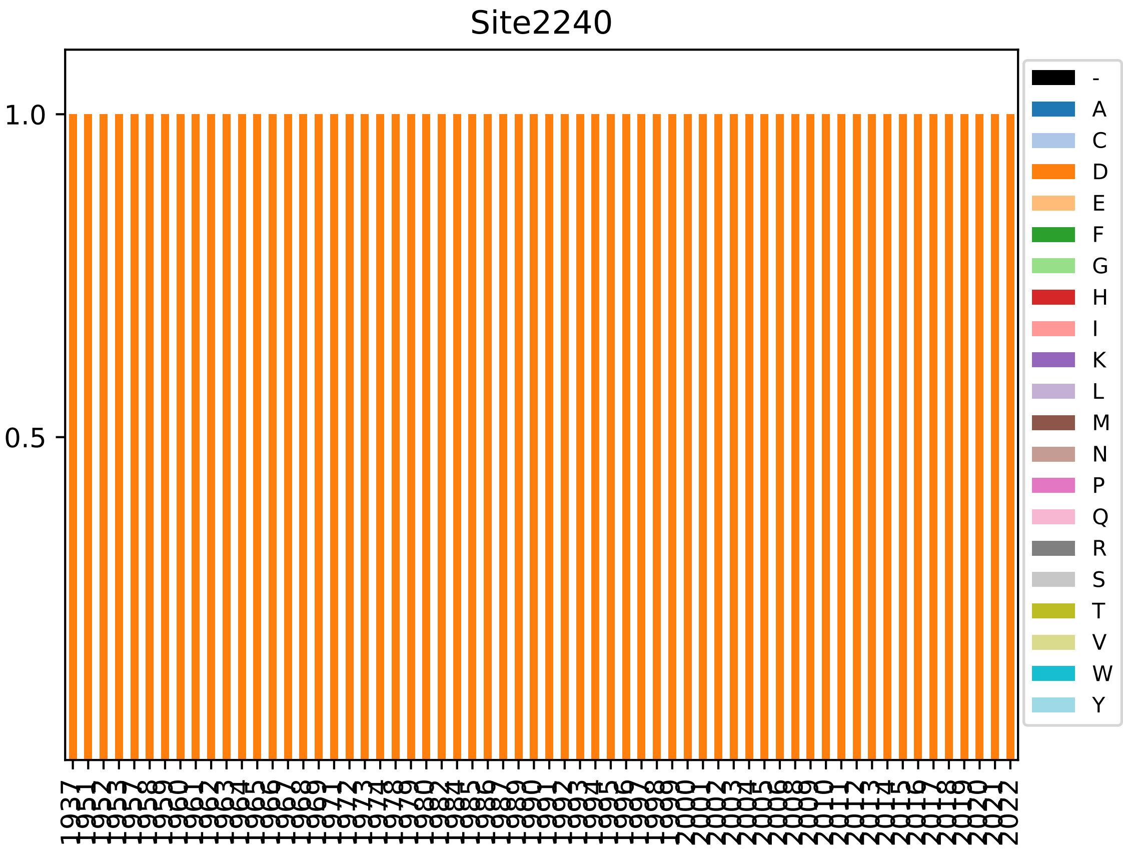
Site2240
1.0
0.5
-
A
C
D
E
F
G
H
I
K
L
M
N
P
Q
R
S
T
V
W
Y

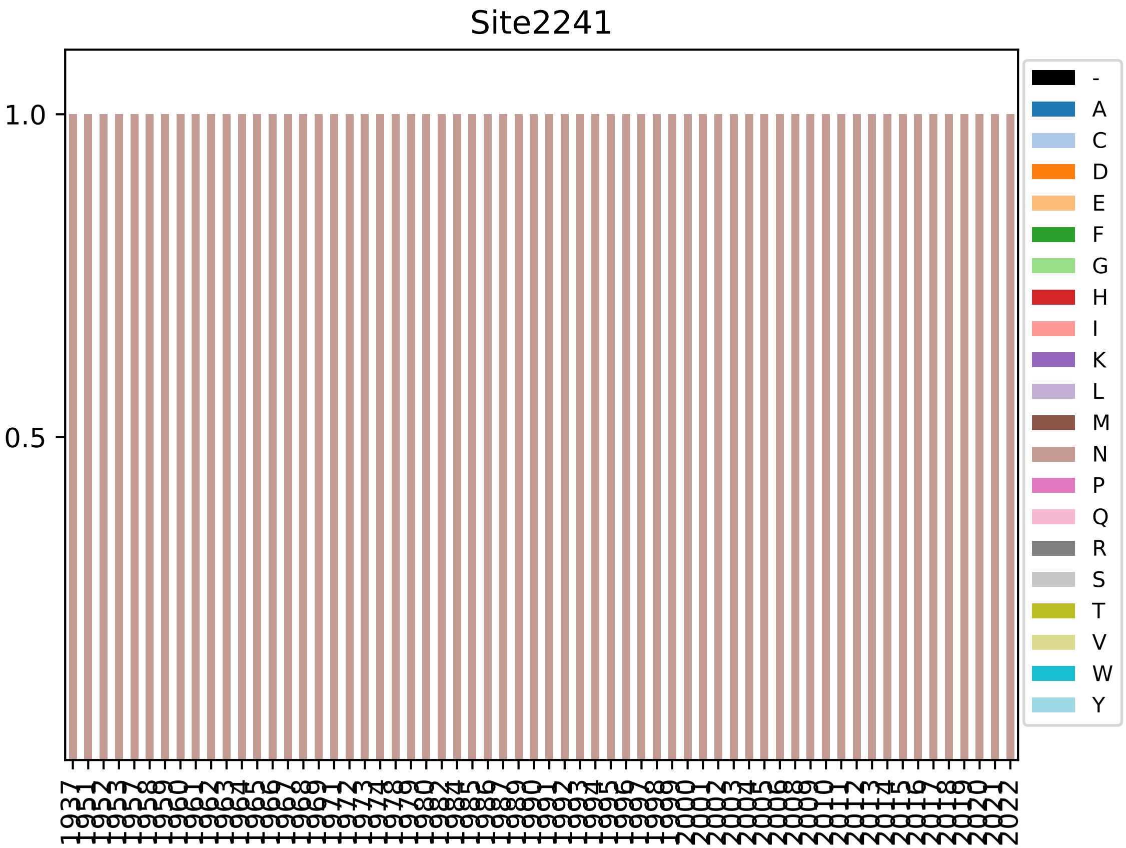

Site2241
1.0
0.5
-
A
C
D
E
F
G
H
I
K
L
M
N
P
Q
R
S
T
V
W
Y

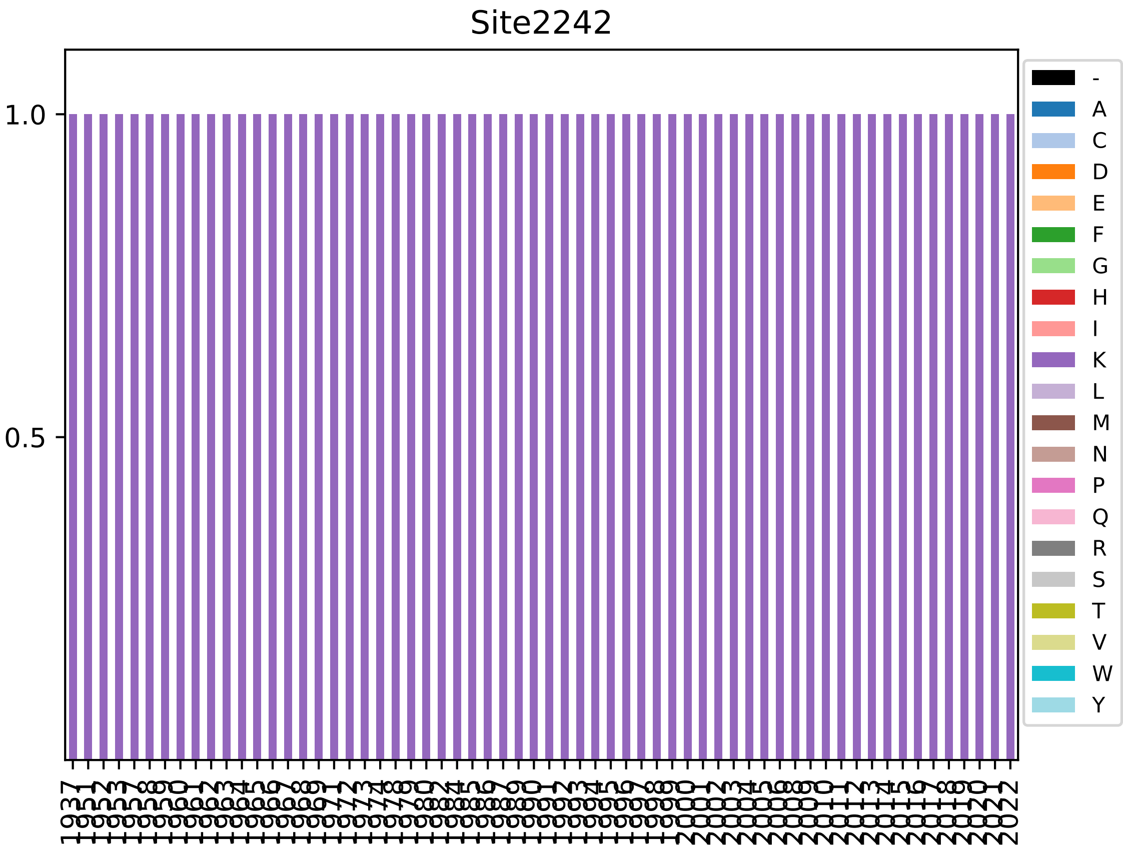
Site2242
1.0
0.5
-
A
C
D
E
F
G
H
I
K
L
M
N
P
Q
R
S
T
V
W
Y

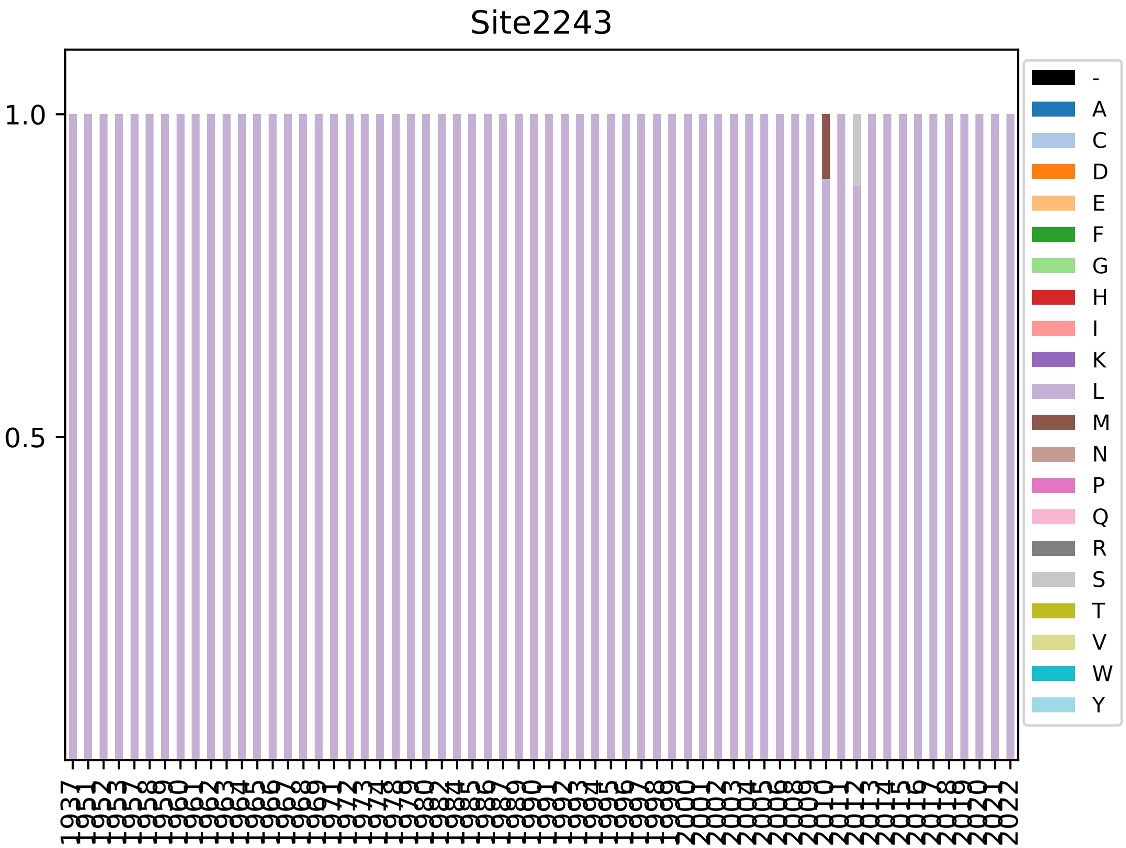
Site2243
1.0
0.5
-
A
C
D
E
F
G
H
I
K
L
M
N
P
Q
R
S
T
V
W
Y

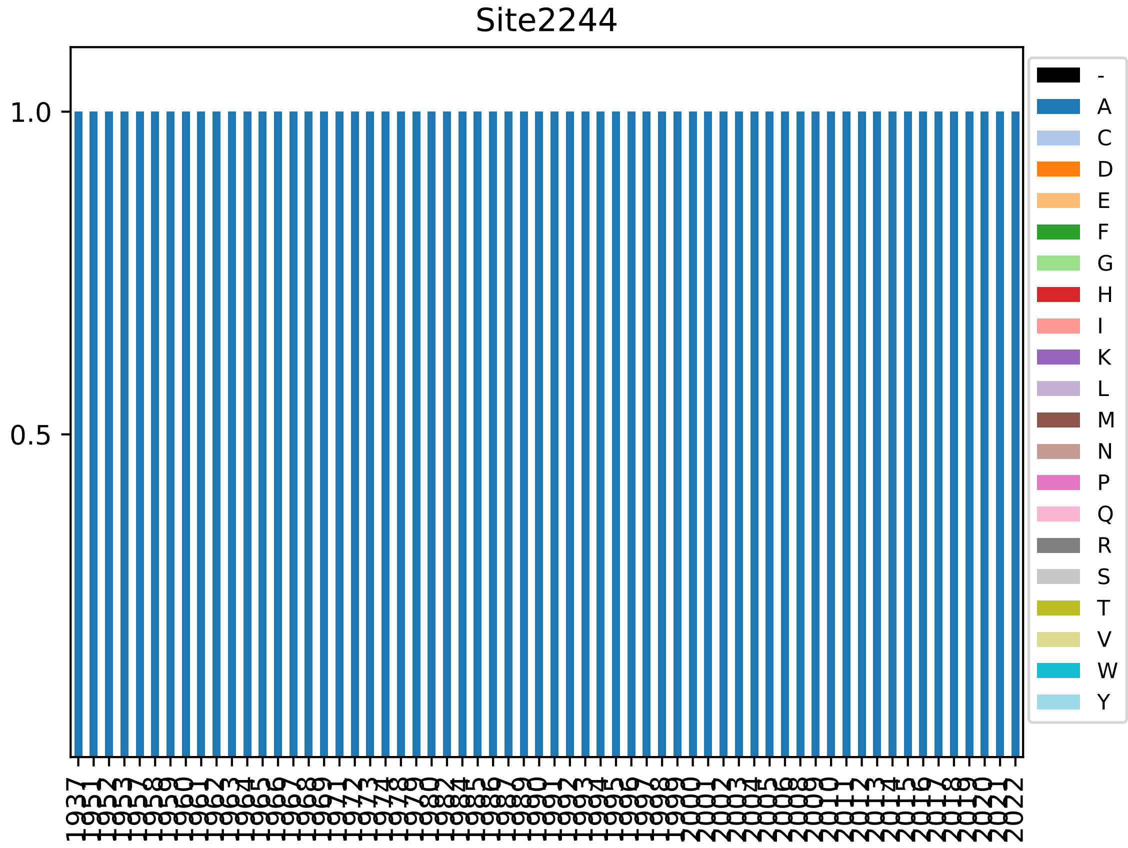
Site2244
1.0
0.5
-
A
C
D
E
F
G
H
I
K
L
M
N
P
Q
R
S
T
V
W
Y

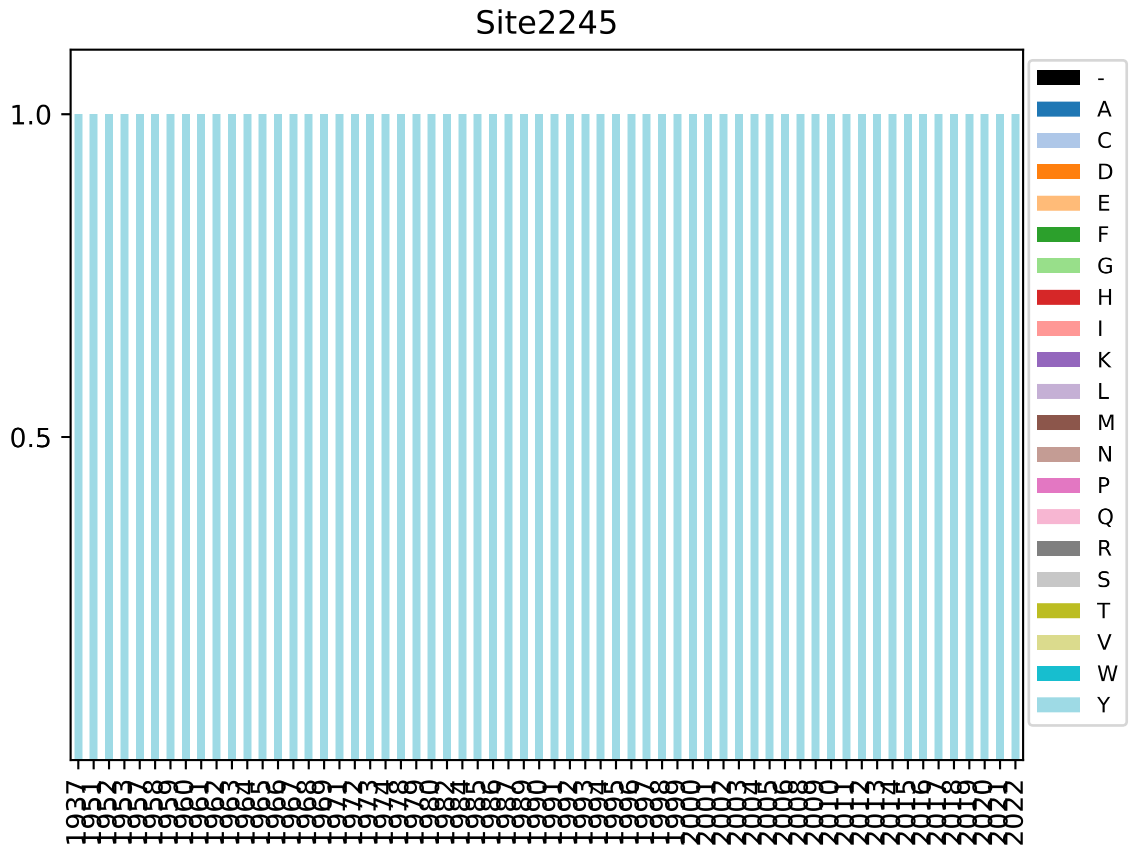
Site2245
1.0
0.5
-
A
C
D
E
F
G
H
I
K
L
M
N
P
Q
R
S
T
V
W
Y

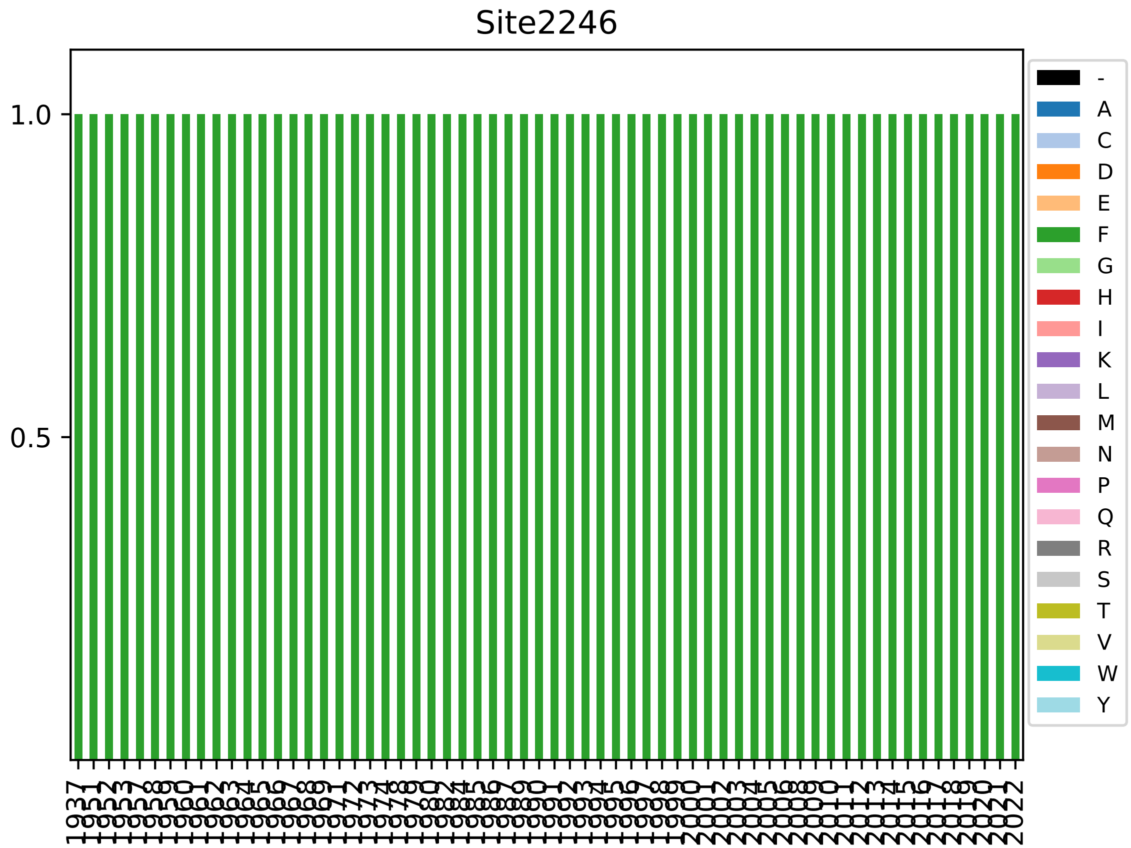
Site2246
1.0
0.5
-
A
C
D
E
F
G
H
I
K
L
M
N
P
Q
R
S
T
V
W
Y

Site2247

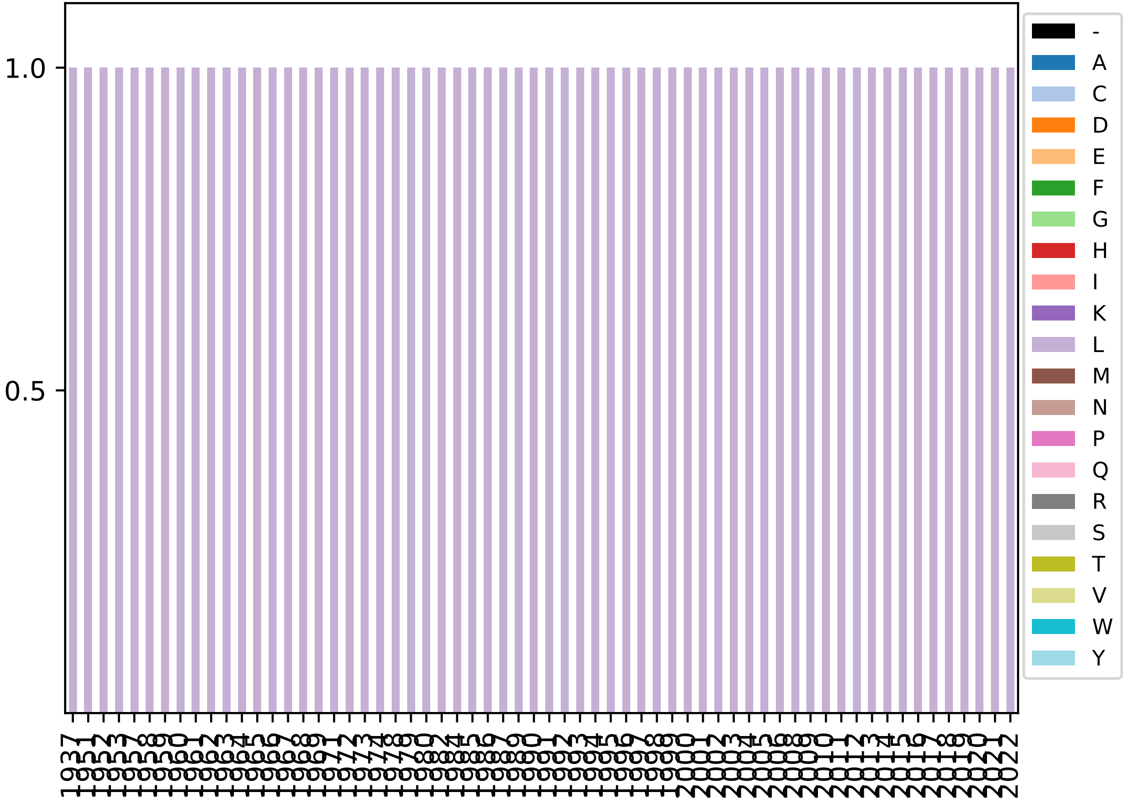

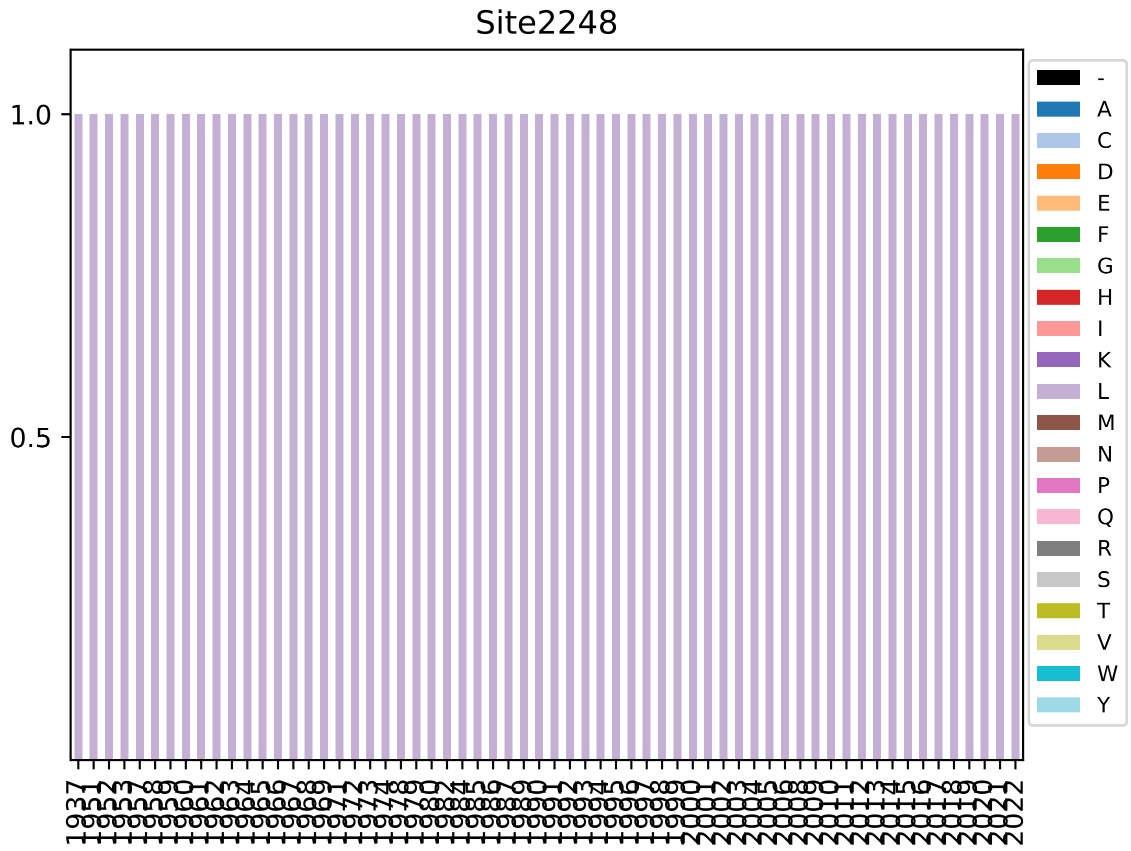
Site2248
1.0
0.5
-
A
C
D
E
F
G
H
I
K
L
M
N
P
Q
R
S
T
V
W
Y

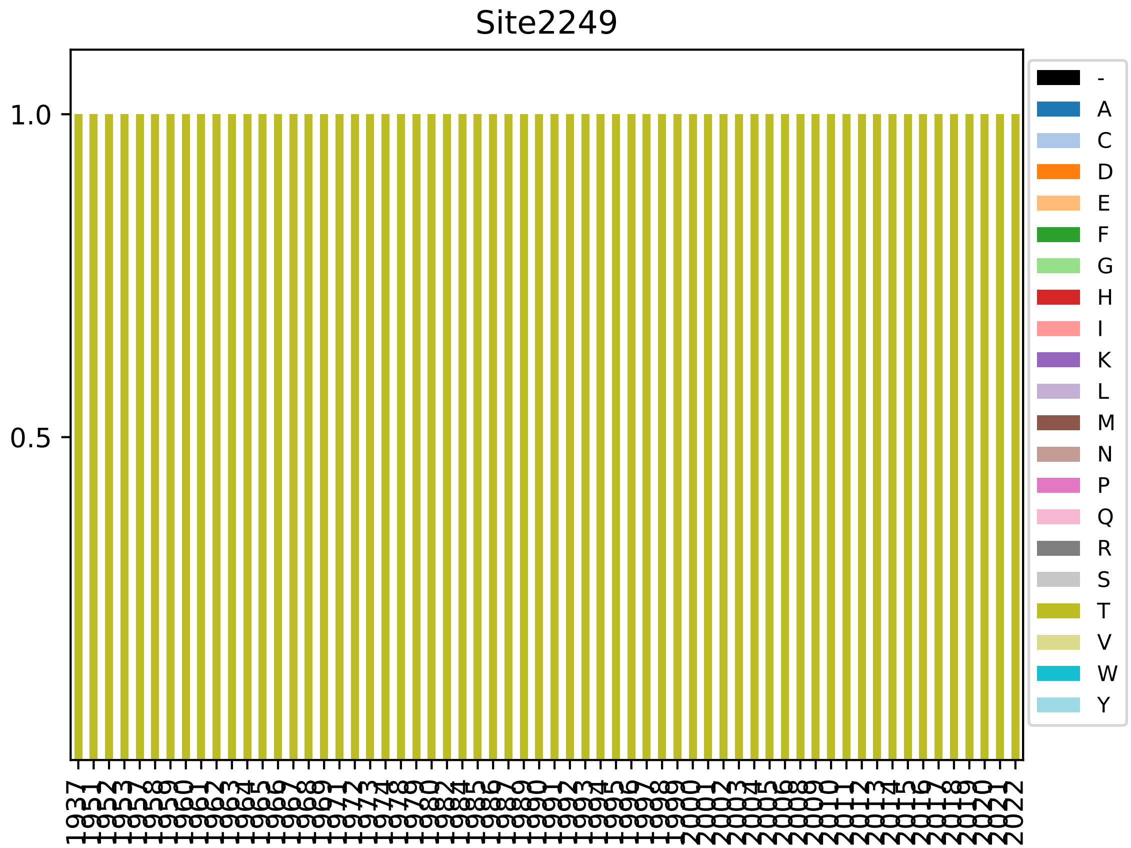
Site2249
1.0
0.5
-
A
C
D
E
F
G
H
I
K
L
M
N
P
Q
R
S
T
V
W
Y

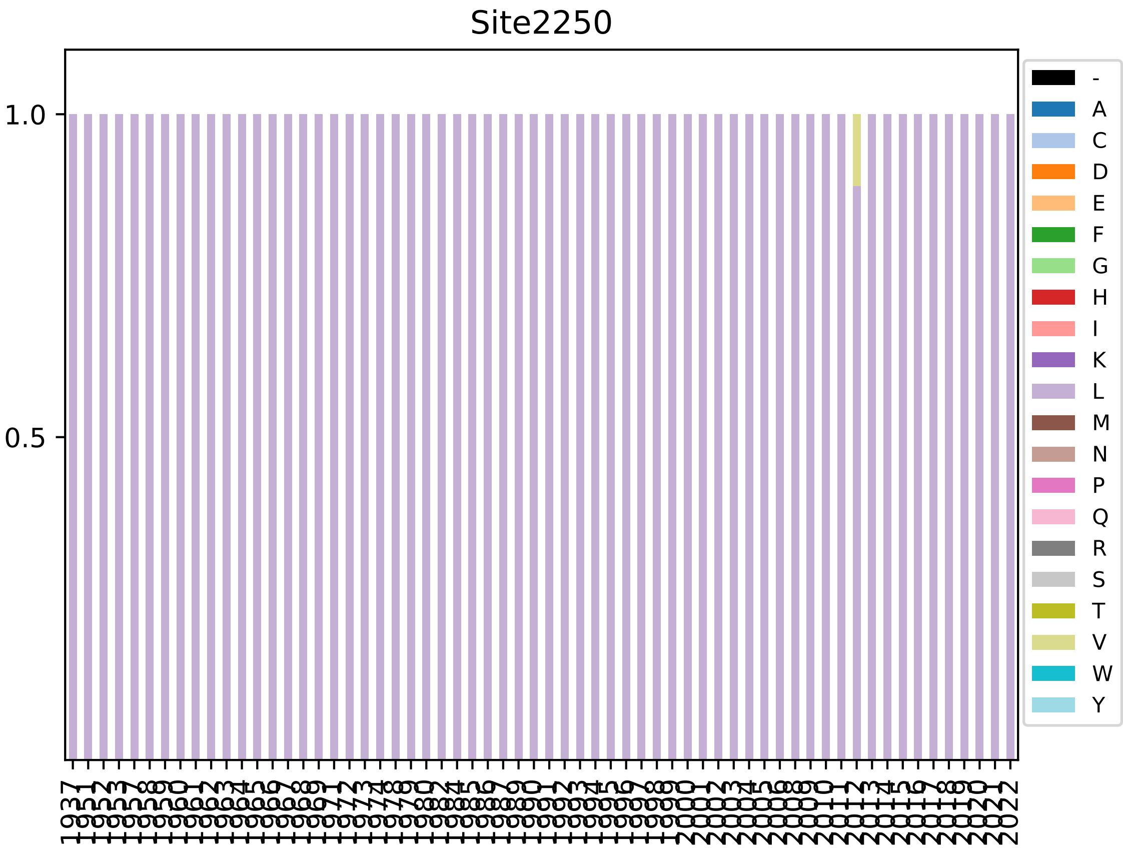
Site2250
1.0
0.5
-
A
C
D
E
F
G
H
I
K
L
M
N
P
Q
R
S
T
V
W
Y

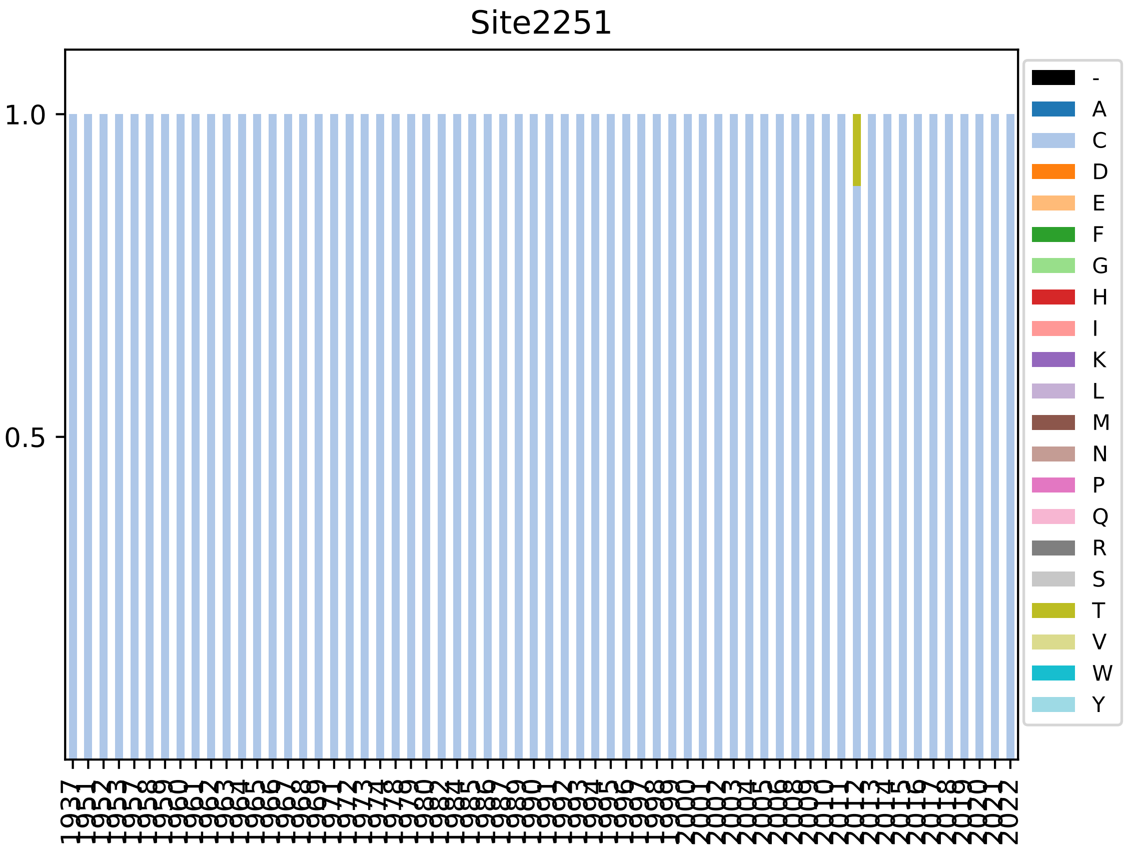
Site2251
1.0
0.5
-
A
C
D
E
F
G
H
I
K
L
M
N
P
Q
R
S
T
V
W
Y

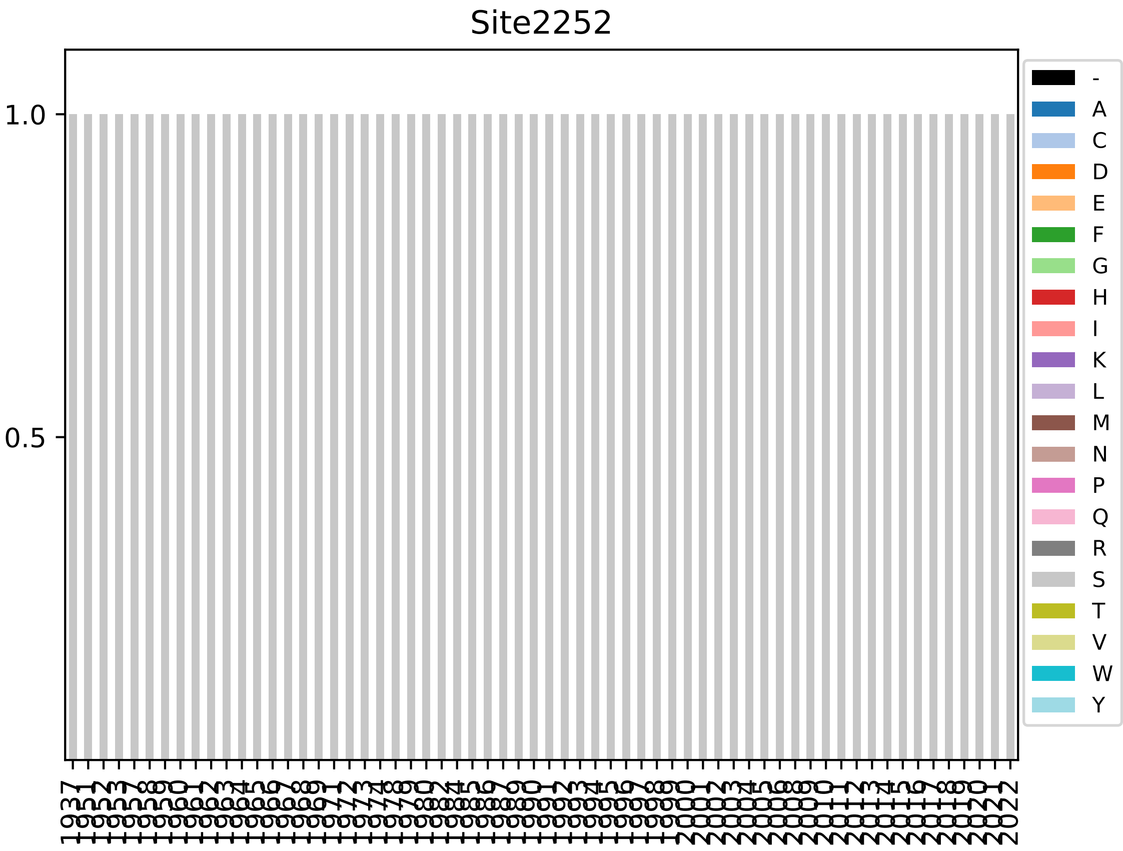
Site2252
1.0
0.5
-
A
C
D
E
F
G
H
I
K
L
M
N
P
Q
R
S
T
V
W
Y

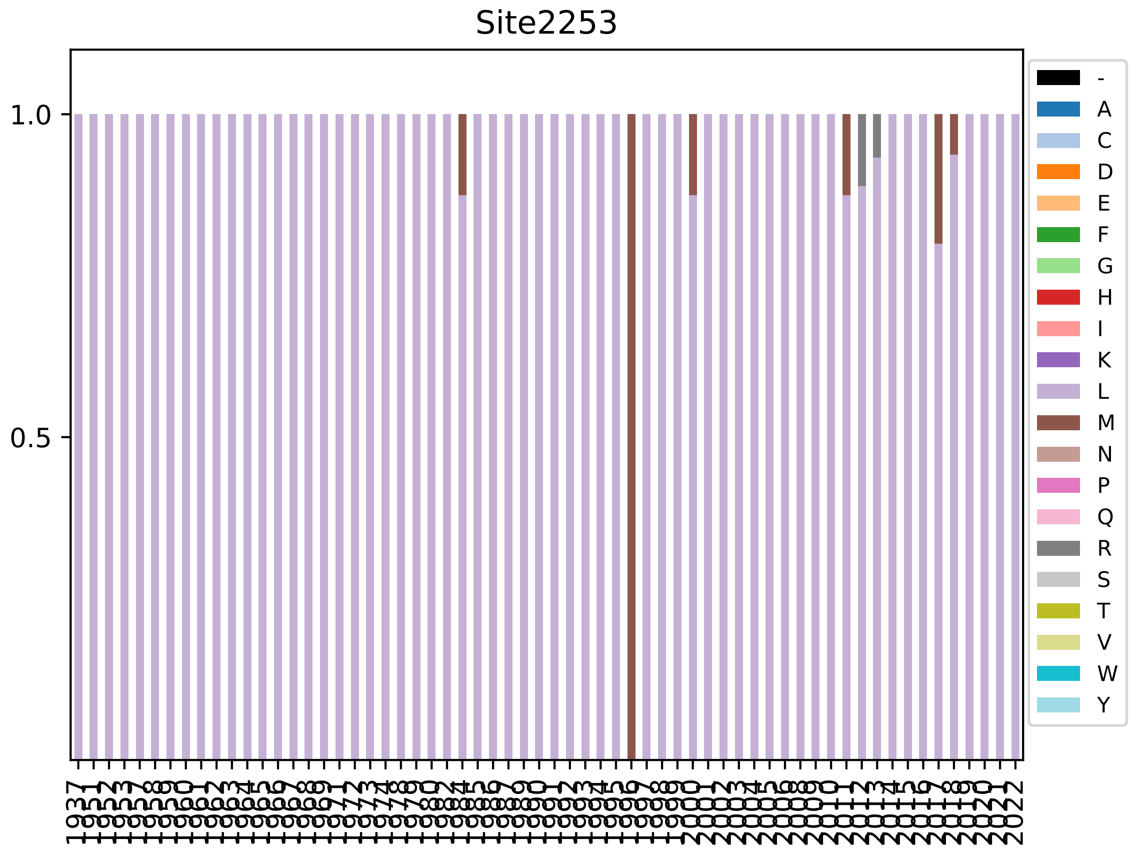
Site2253
1.0
0.5
-
A
C
D
E
F
G
H
I
K
L
M
N
P
Q
R
S
T
V
W
Y

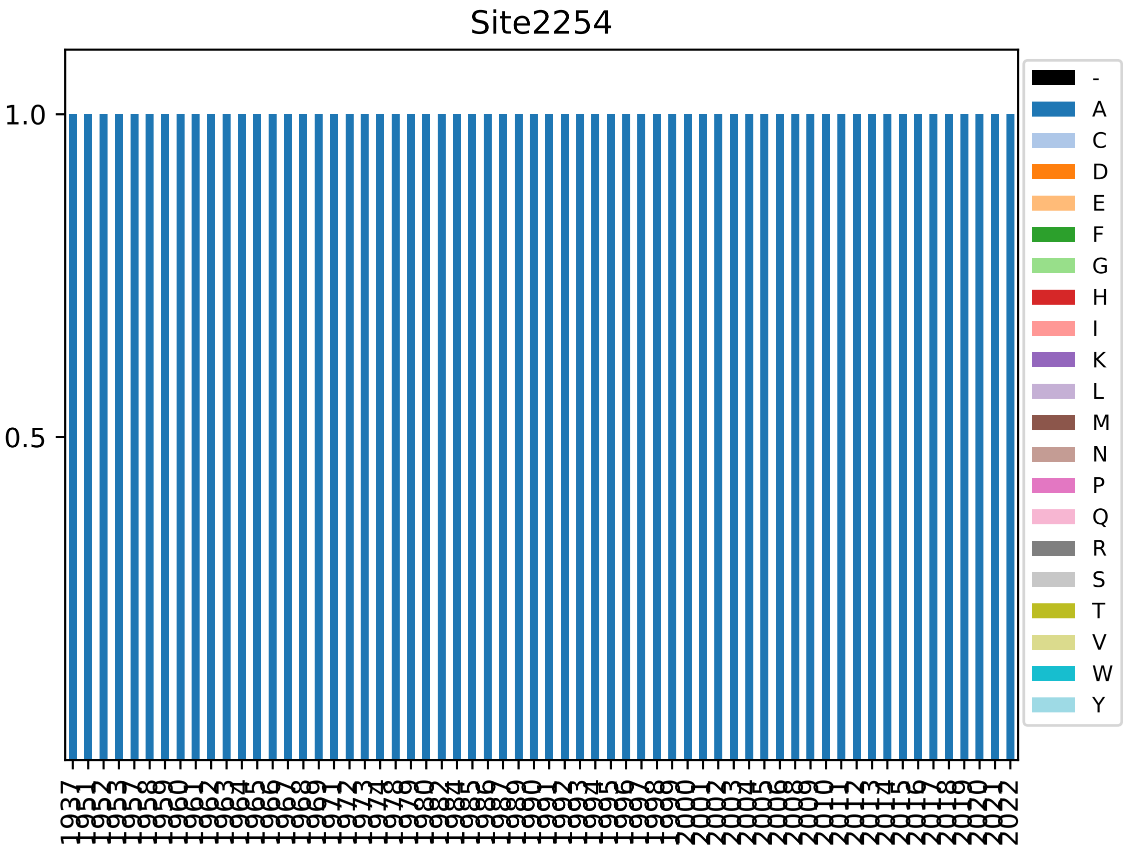
Site2254
1.0
0.5
-
A
C
D
E
F
G
H
I
K
L
M
N
P
Q
R
S
T
V
W
Y

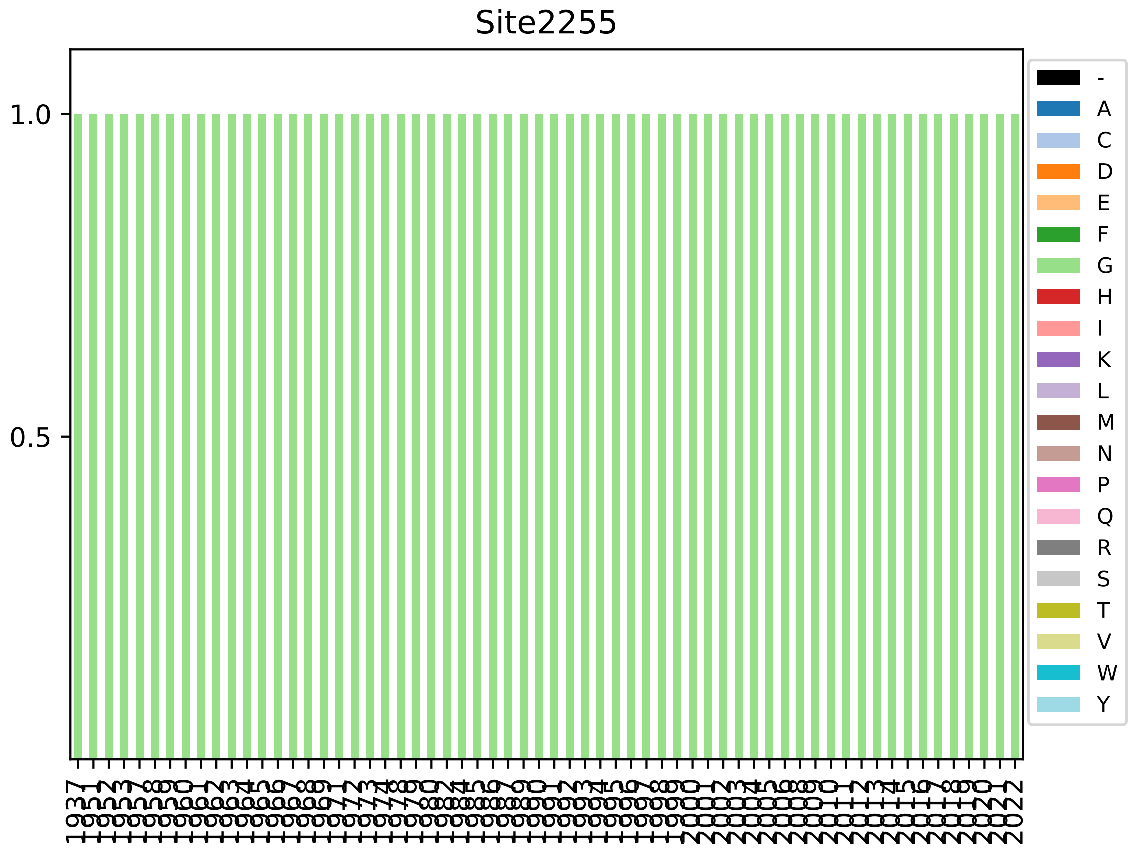
Site2255
1.0
0.5
-
A
C
D
E
F
G
H
I
K
L
M
N
P
Q
R
S
T
V
W
Y

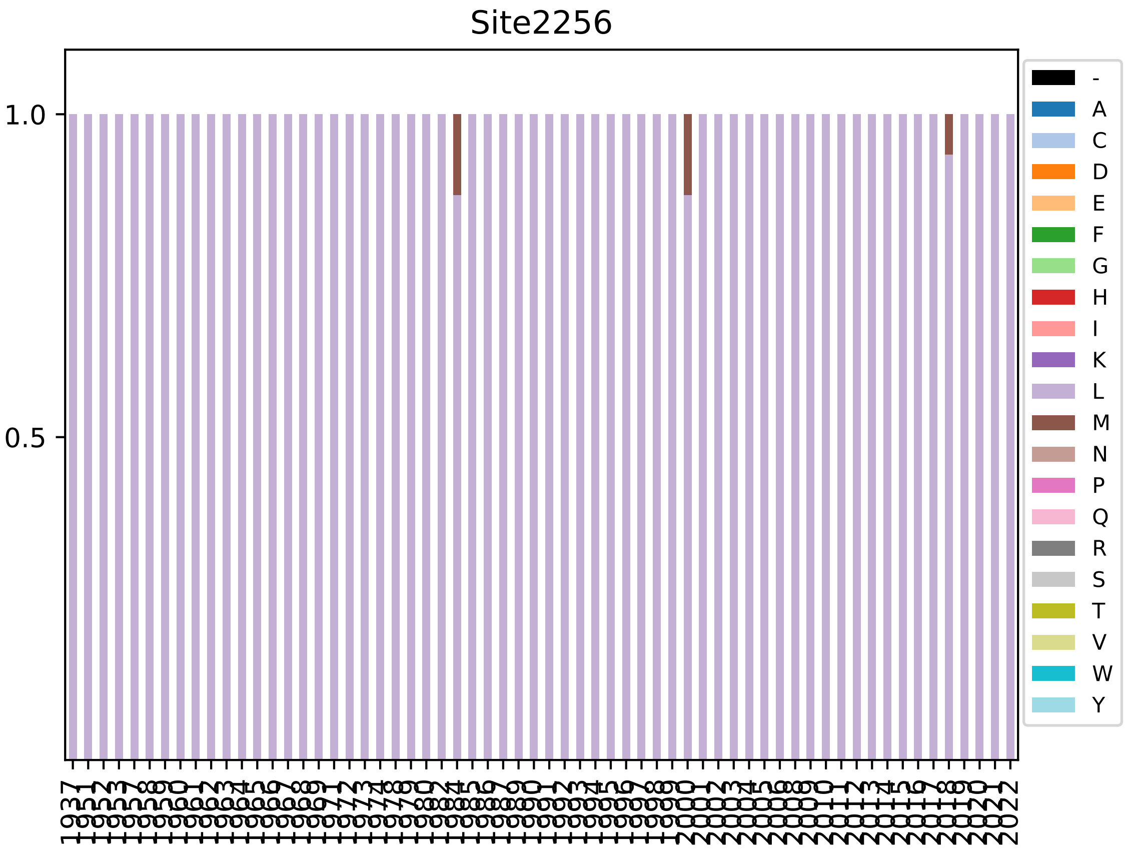
Site2256
1.0
0.5
-
A
C
D
E
F
G
H
I
K
L
M
N
P
Q
R
S
T
V
W
Y

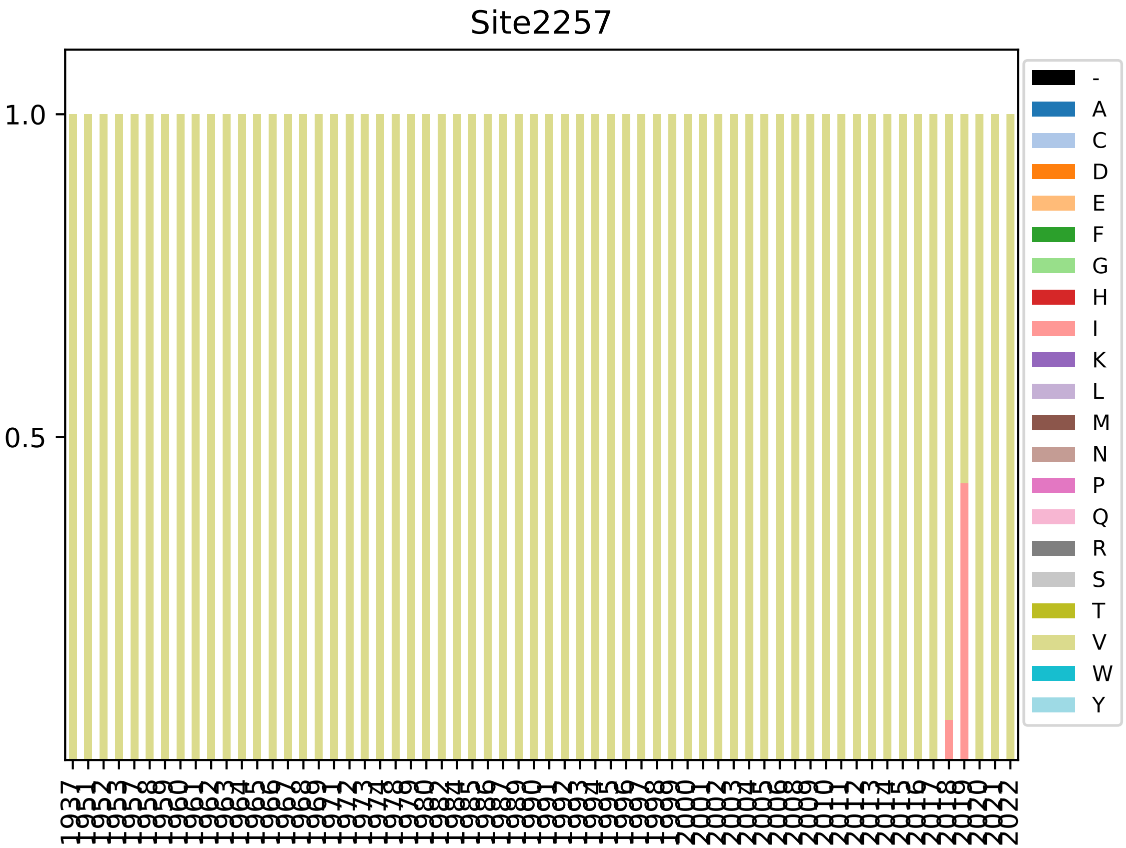
Site2257
1.0
0.5
-
A
C
D
E
F
G
H
I
K
L
M
N
P
Q
R
S
T
V
W
Y

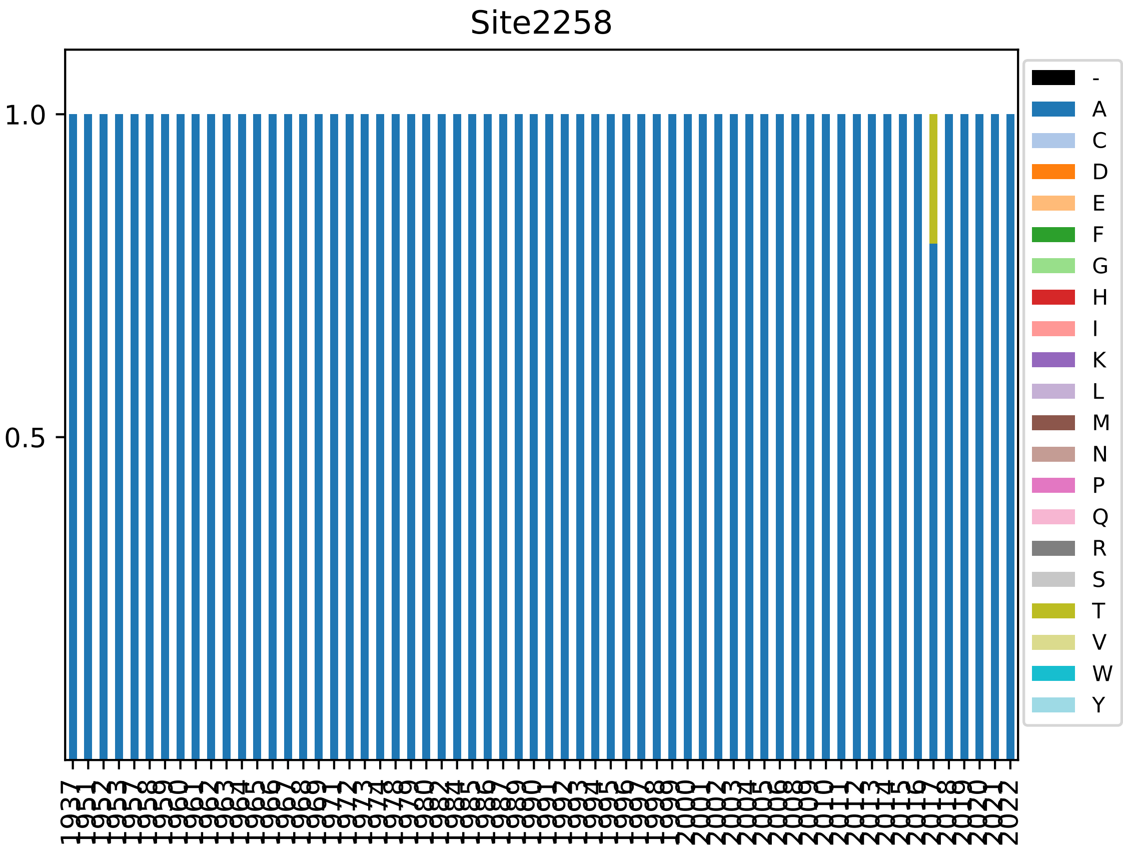
Site2258
1.0
0.5
-
A
C
D
E
F
G
H
I
K
L
M
N
P
Q
R
S
T
V
W
Y

Site2259

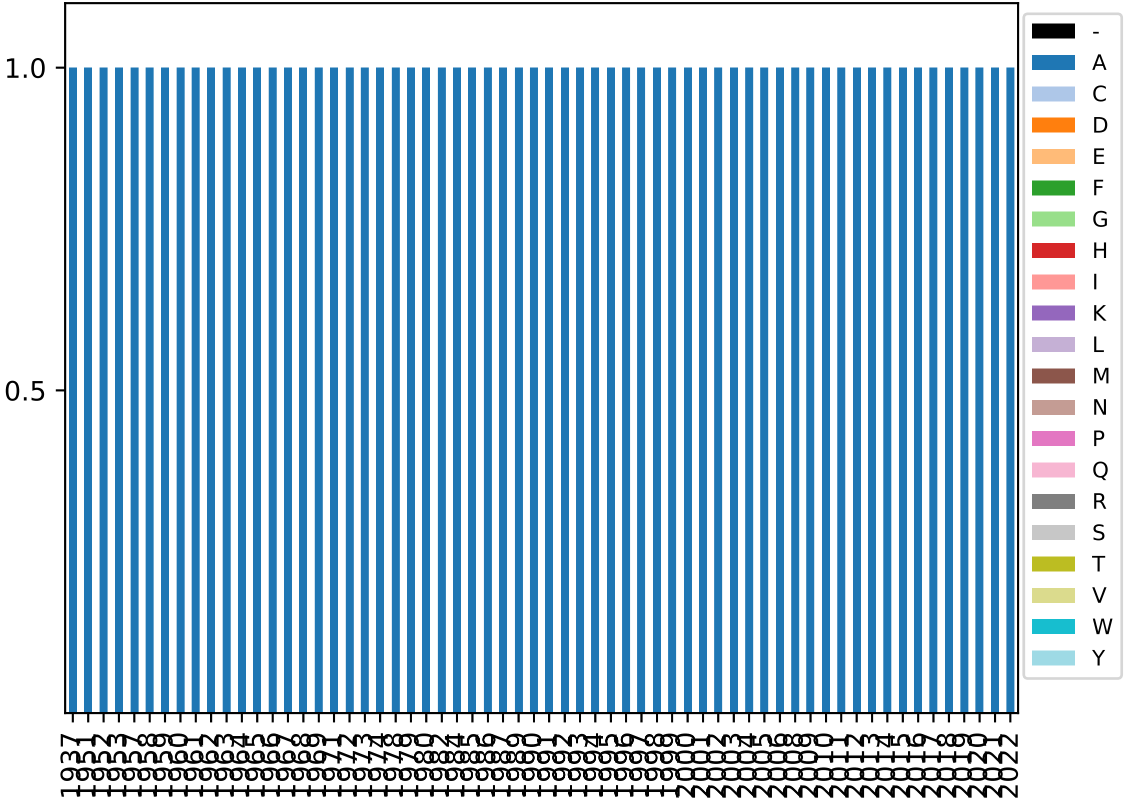

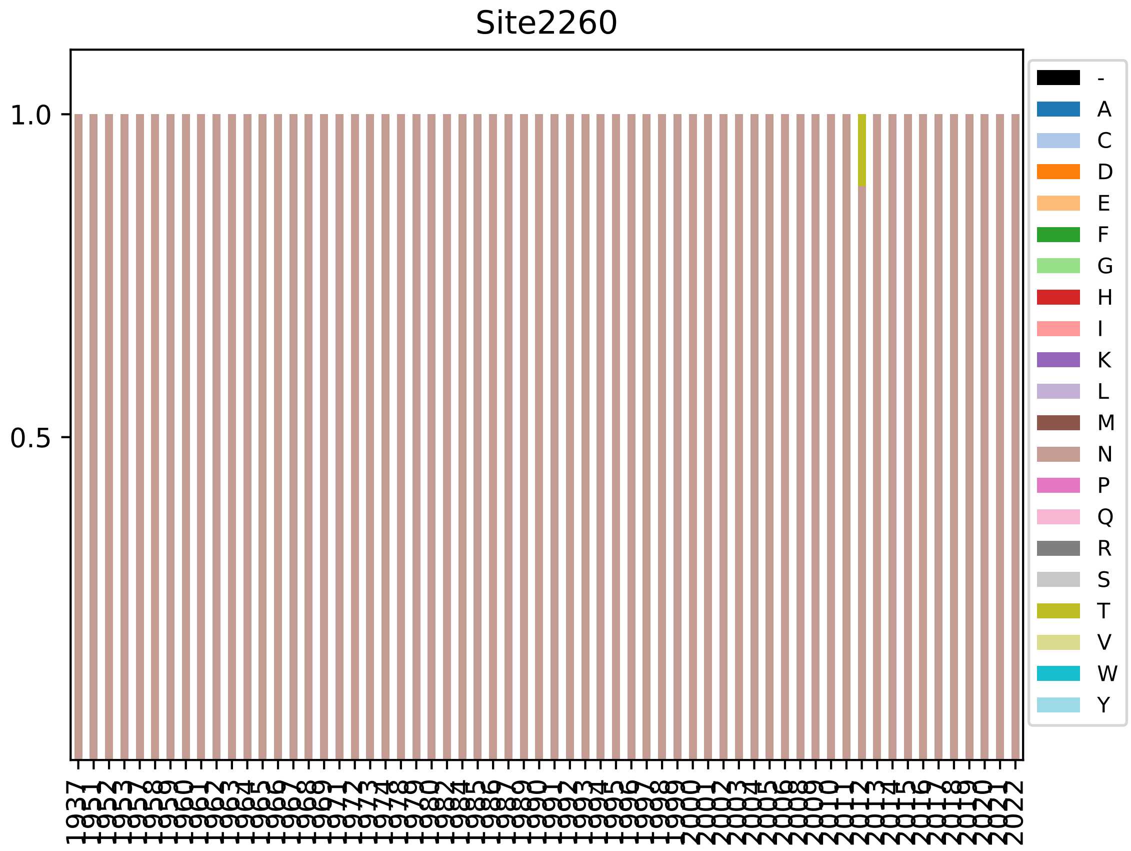
Site2260
1.0
0.5
-
A
C
D
E
F
G
H
I
K
L
M
N
P
Q
R
S
T
V
W
Y

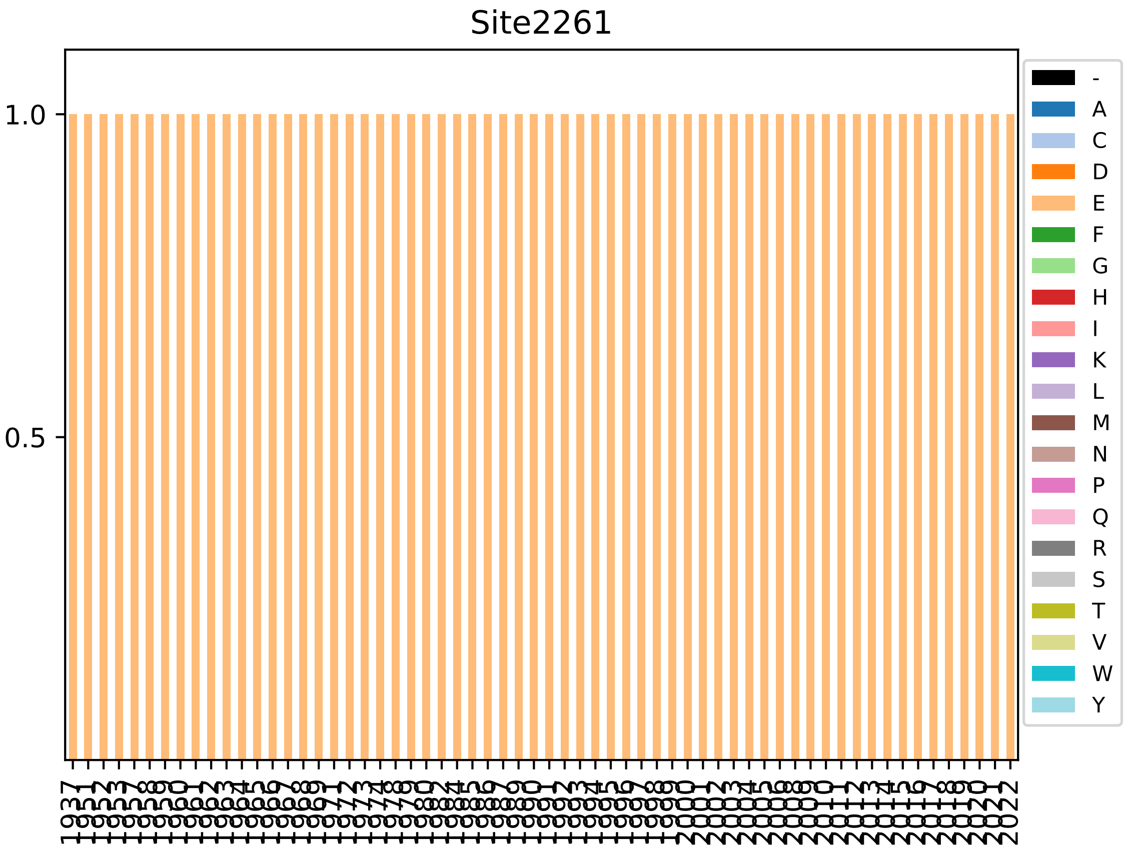
Site2261
1.0
0.5
-
A
C
D
E
F
G
H
I
K
L
M
N
P
Q
R
S
T
V
W
Y

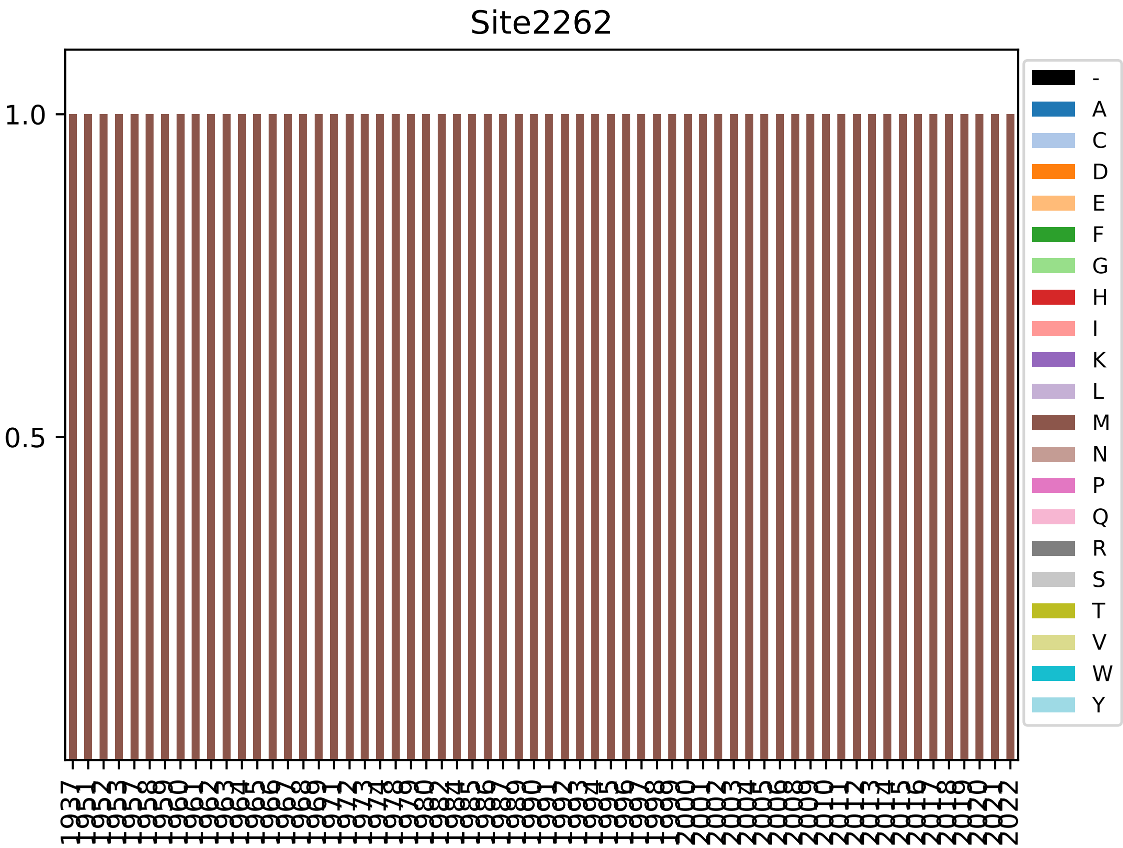
Site2262
1.0
0.5
-
A
C
D
E
F
G
H
I
K
L
M
N
P
Q
R
S
T
V
W
Y

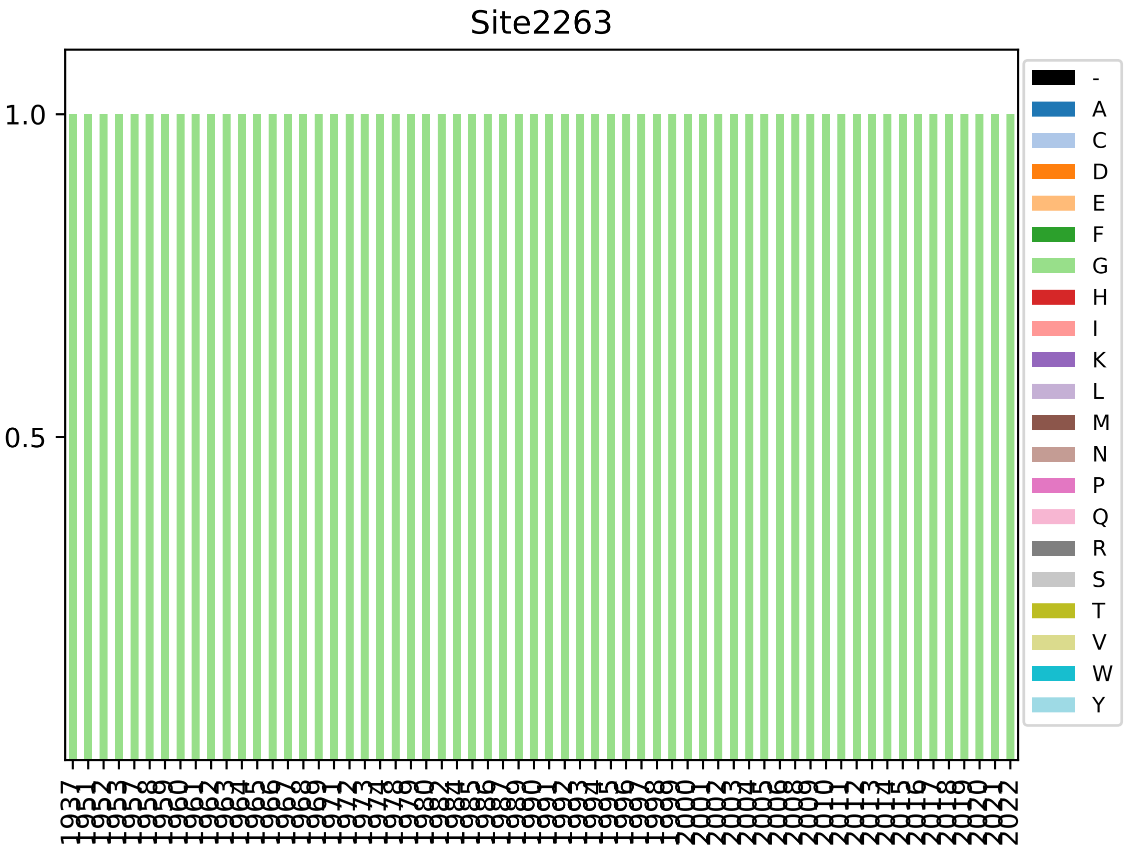

Site2263
1.0
0.5
-
A
C
D
E
F
G
H
I
K
L
M
N
P
Q
R
S
T
V
W
Y

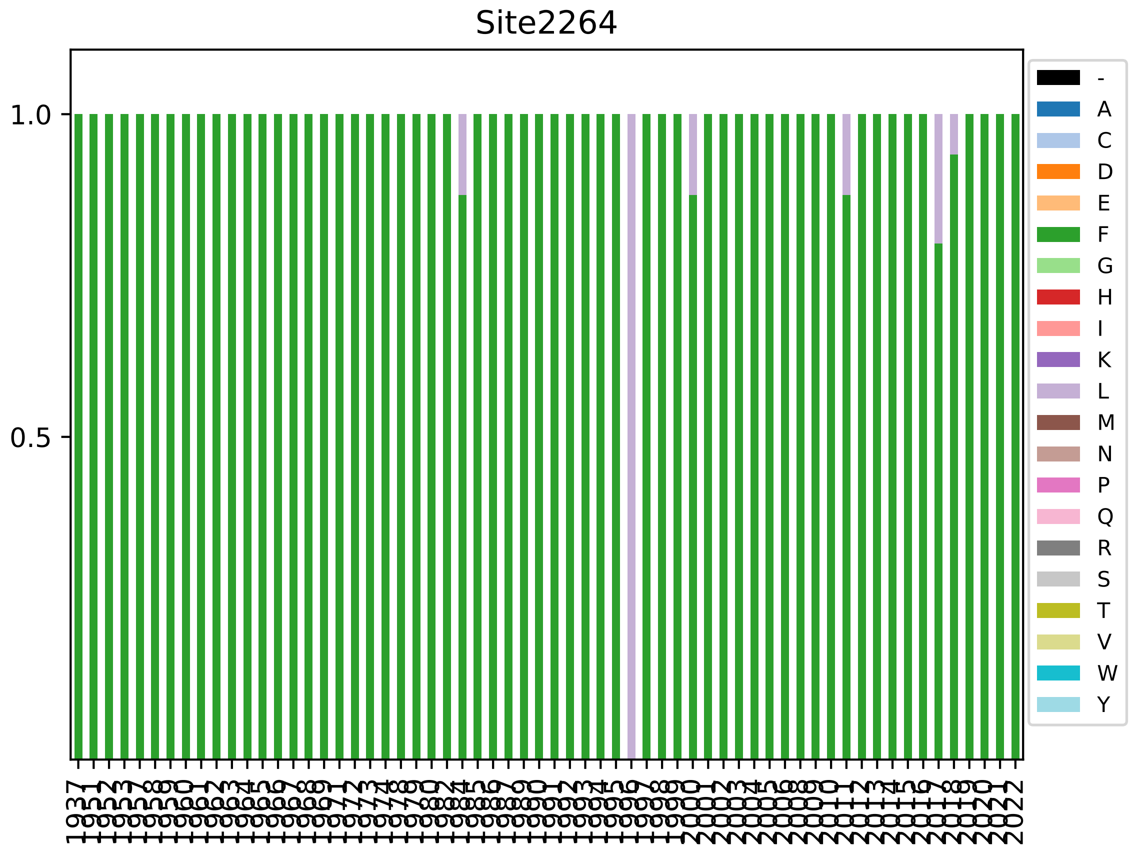
Site2264
1.0
0.5
-
A
C
D
E
F
G
H
I
K
L
M
N
P
Q
R
S
T
V
W
Y

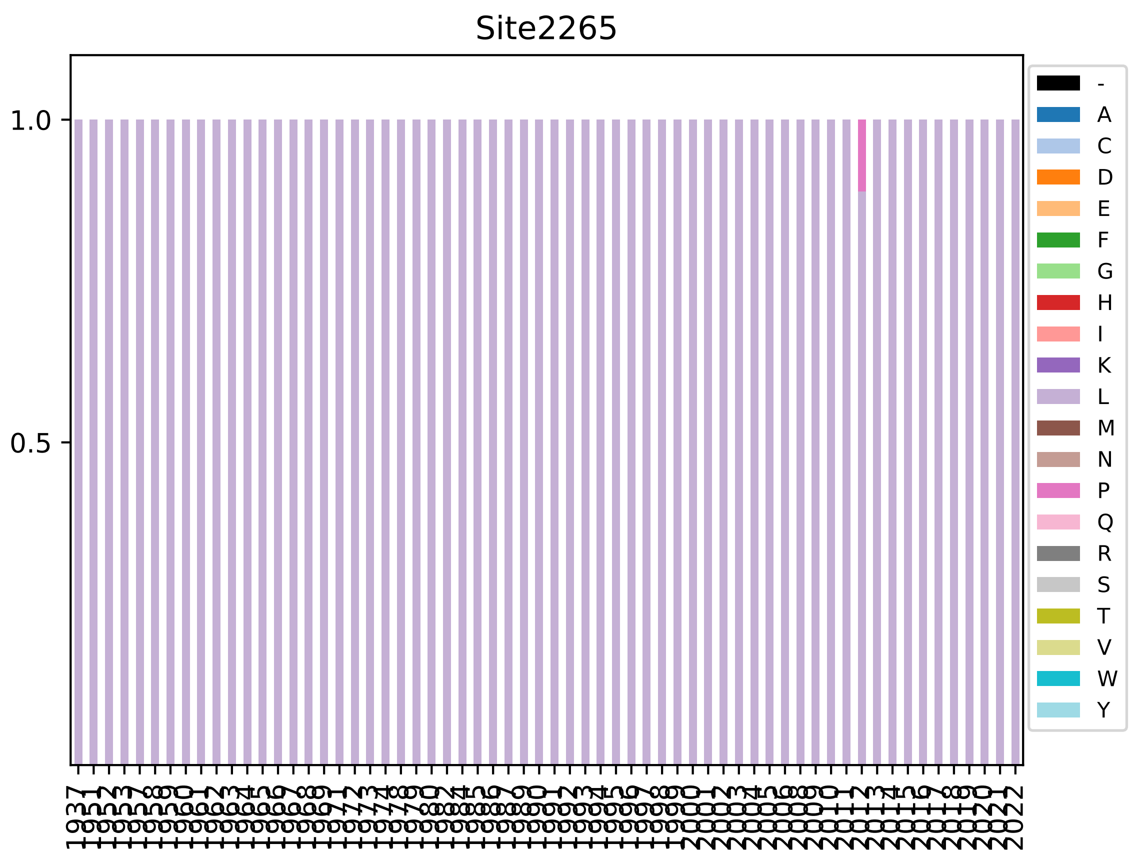
Site2265
1.0
0.5
-
A
C
D
E
F
G
H
I
K
L
M
N
P
Q
R
S
T
V
W
Y

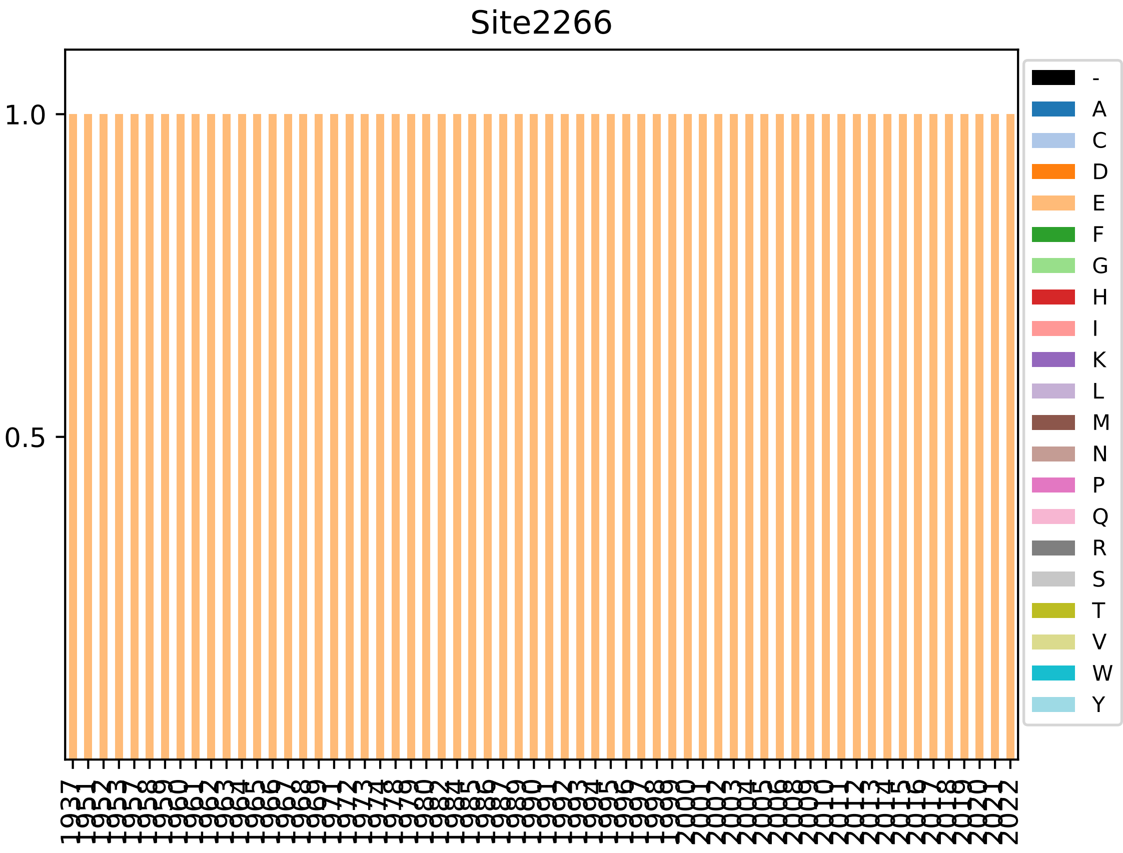
Site2266
1.0
0.5
-
A
C
D
E
F
G
H
I
K
L
M
N
P
Q
R
S
T
V
W
Y

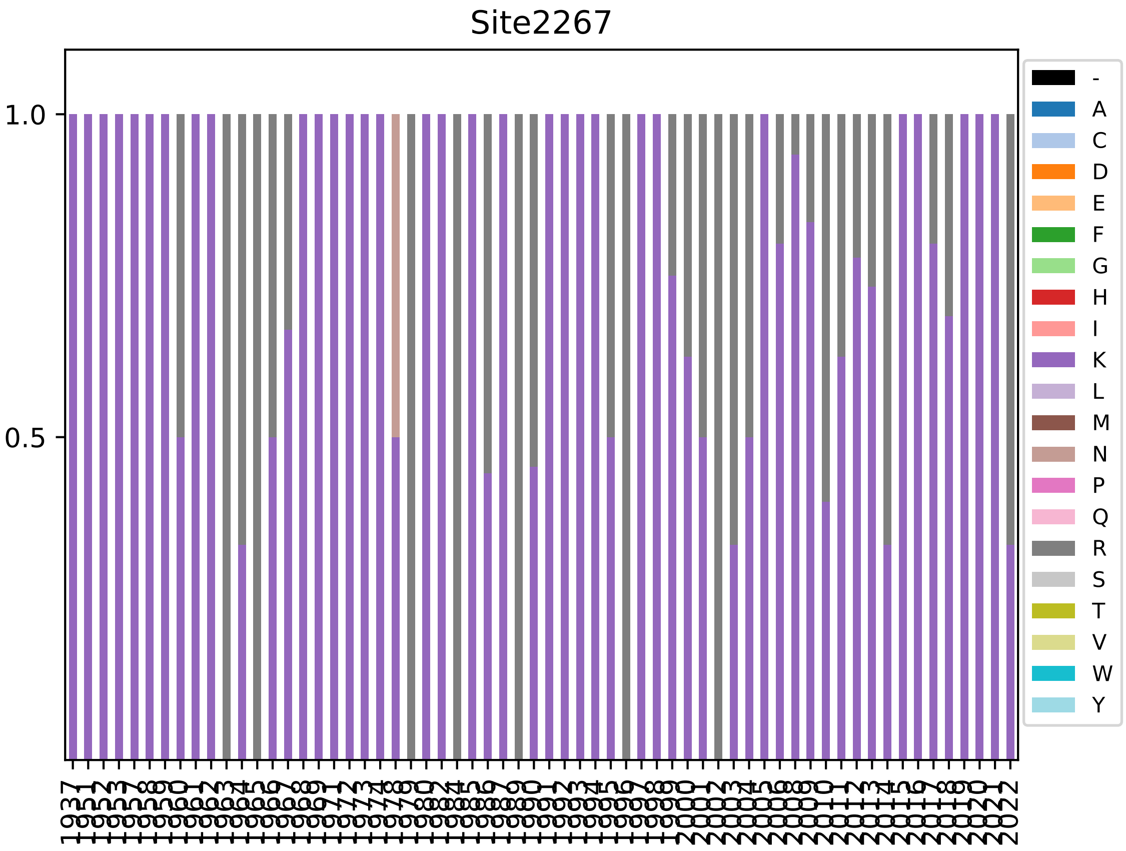
Site2267
1.0
0.5
-
A
C
D
E
F
G
H
I
K
L
M
N
P
Q
R
S
T
V
W
Y

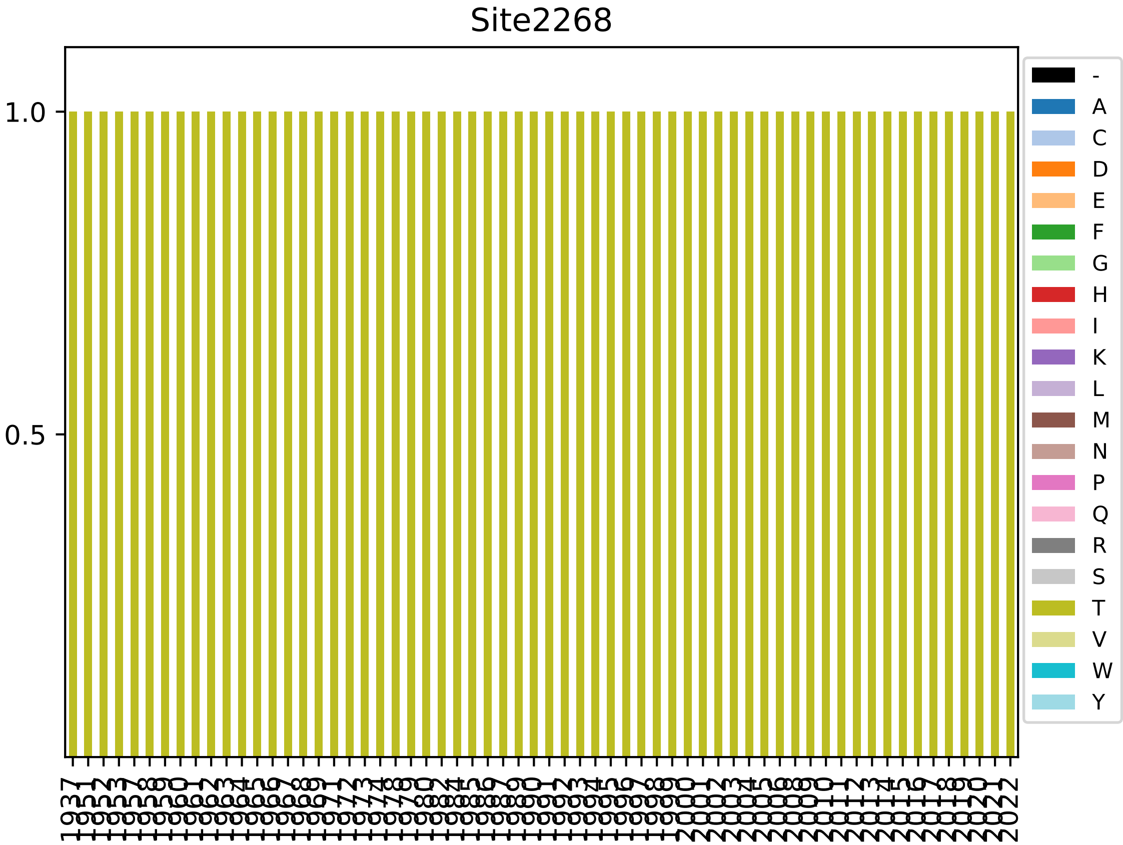
Site2268
1.0
0.5
-
A
C
D
E
F
G
H
I
K
L
M
N
P
Q
R
S
T
V
W
Y

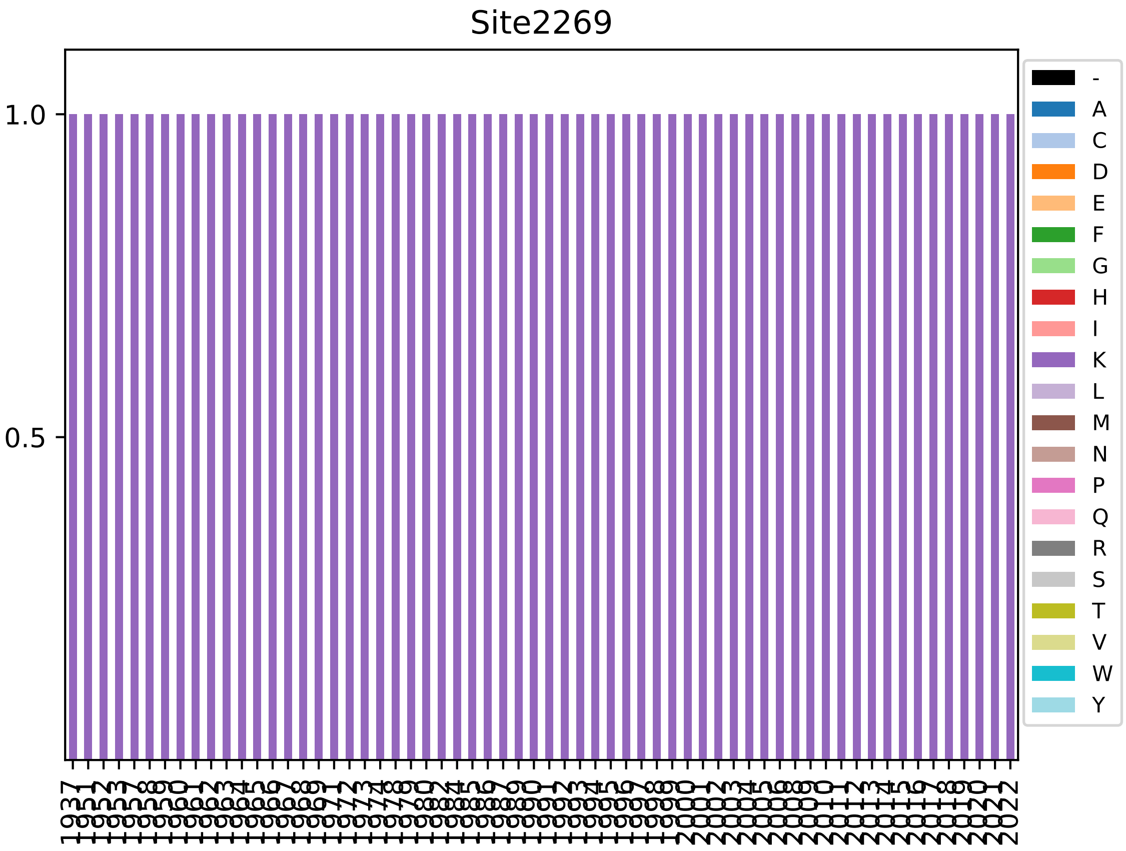
Site2269
1.0
0.5
-
A
C
D
E
F
G
H
I
K
L
M
N
P
Q
R
S
T
V
W
Y

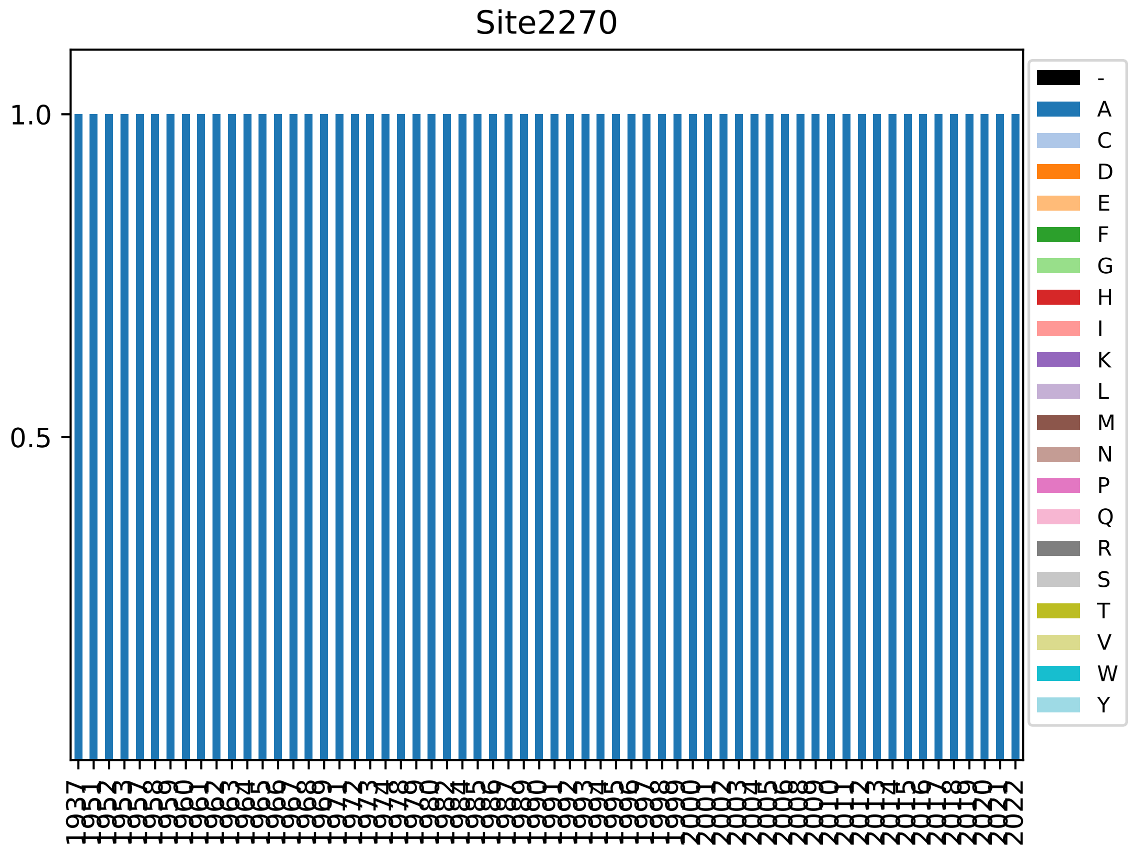
Site2270
1.0
0.5
-
A
C
D
E
F
G
H
I
K
L
M
N
P
Q
R
S
T
V
W
Y

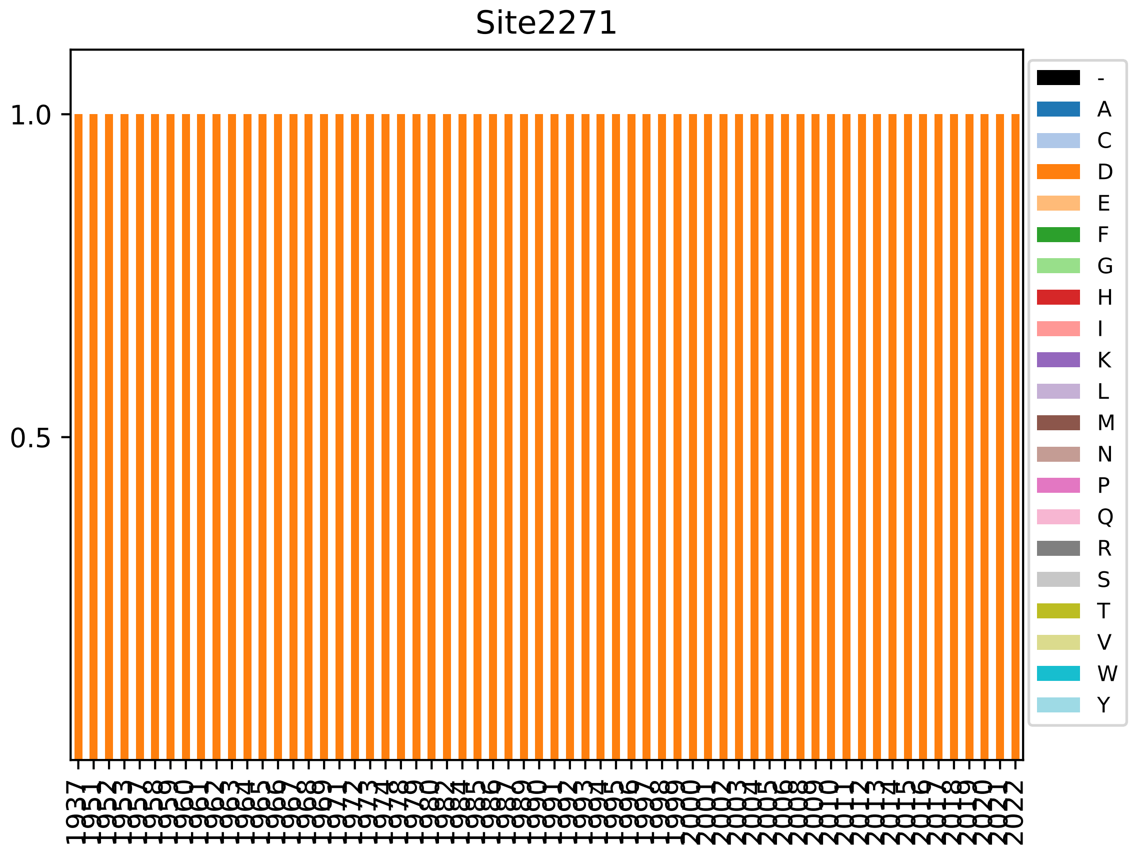
Site2271
1.0
0.5
-
A
C
D
E
F
G
H
I
K
L
M
N
P
Q
R
S
T
V
W
Y

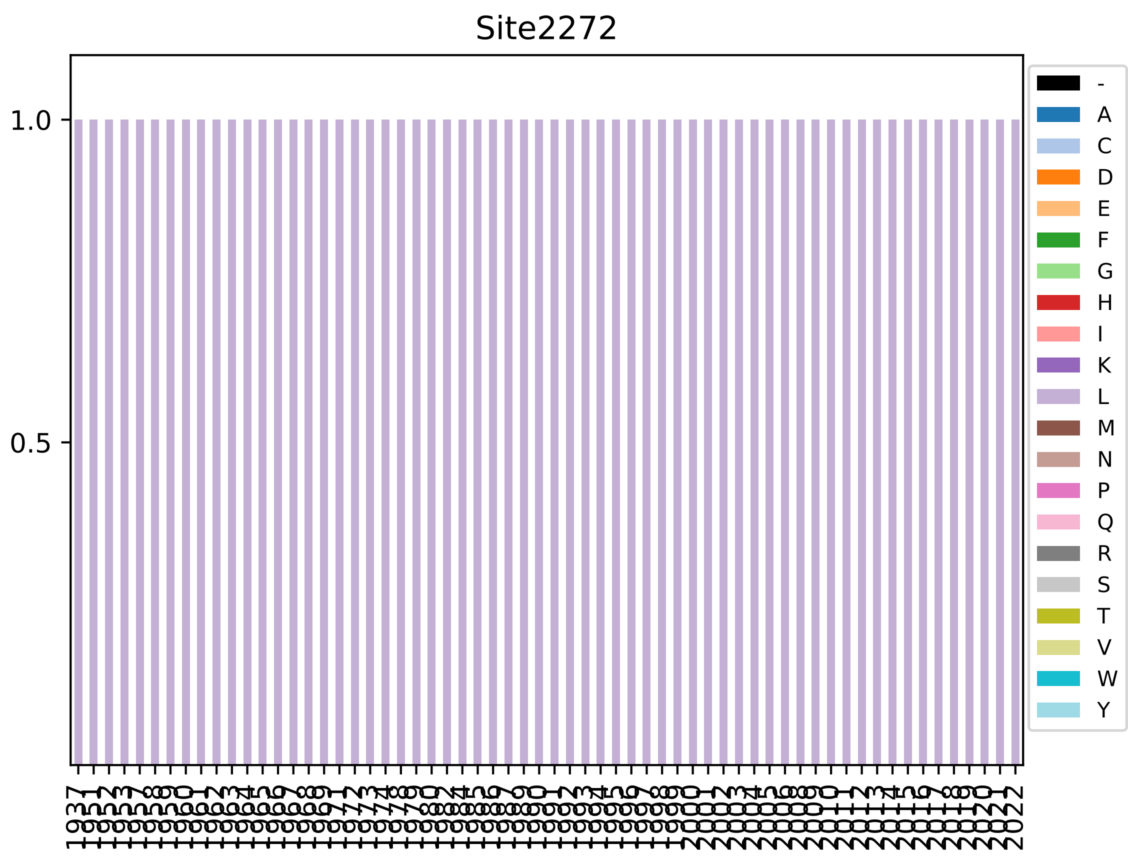
Site2272
1.0
0.5
-
A
C
D
E
F
G
H
I
K
L
M
N
P
Q
R
S
T
V
W
Y

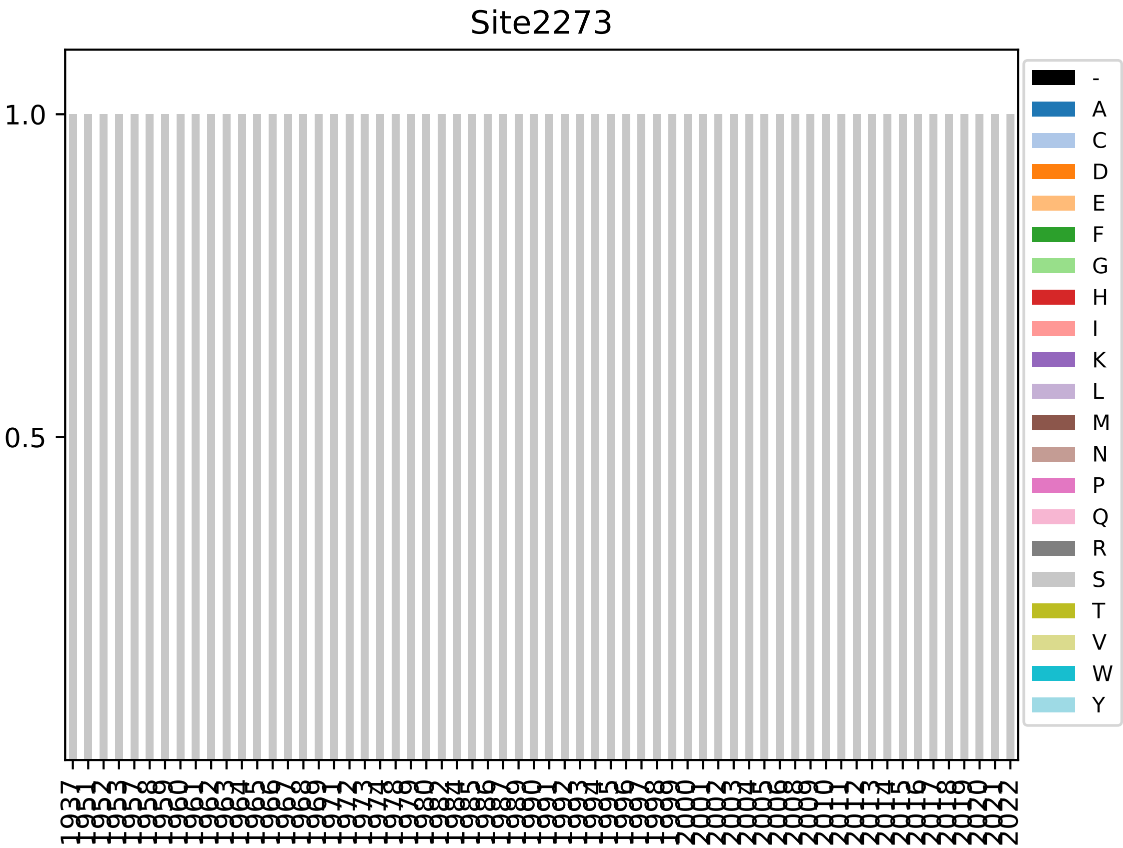
Site2273
1.0
0.5
-
A
C
D
E
F
G
H
I
K
L
M
N
P
Q
R
S
T
V
W
Y

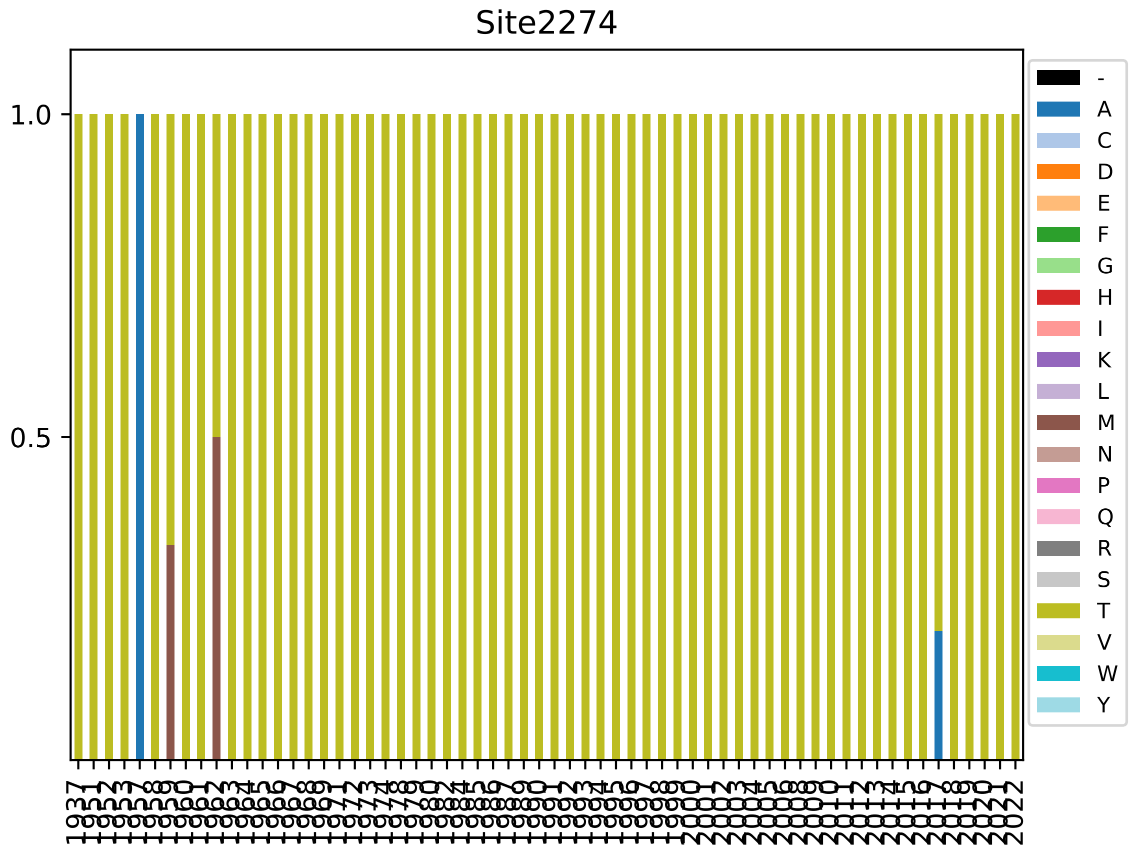
Site2274
1.0
0.5
-
A
C
D
E
F
G
H
I
K
L
M
N
P
Q
R
S
T
V
W
Y

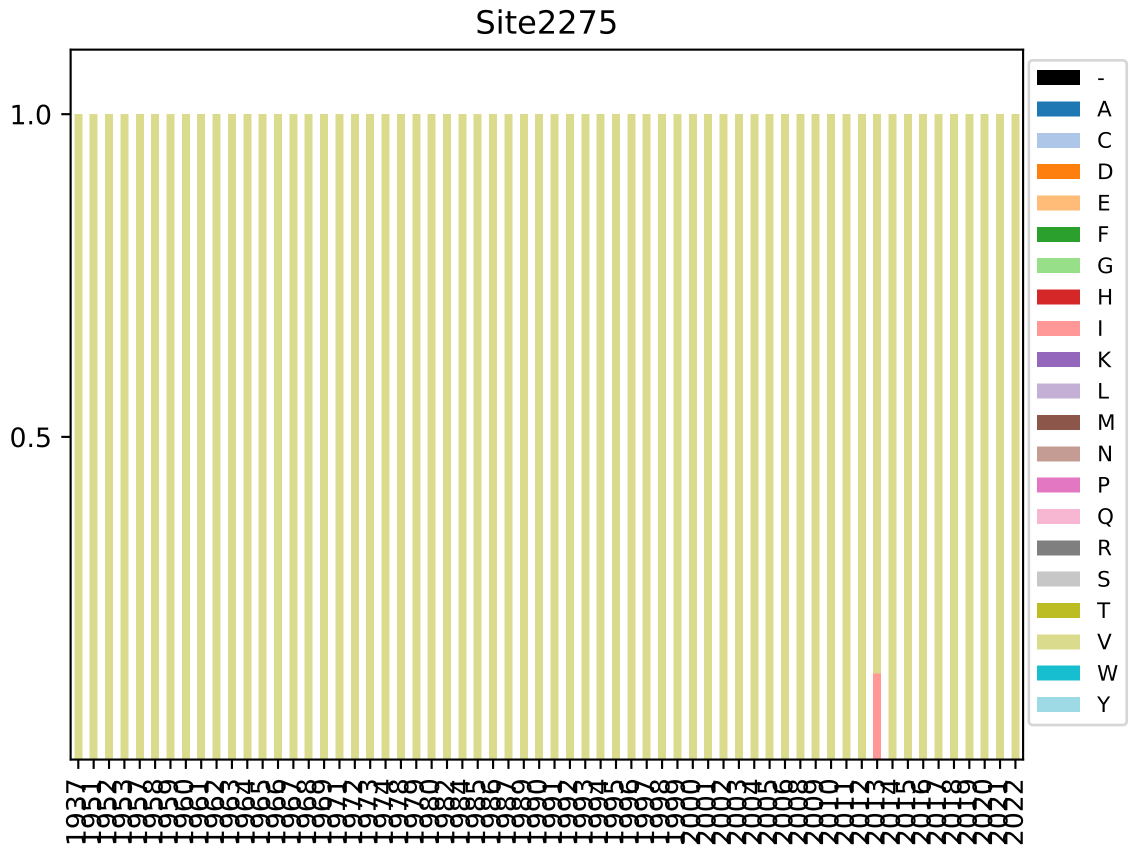
Site2275
1.0
0.5
-
A
C
D
E
F
G
H
I
K
L
M
N
P
Q
R
S
T
V
W
Y

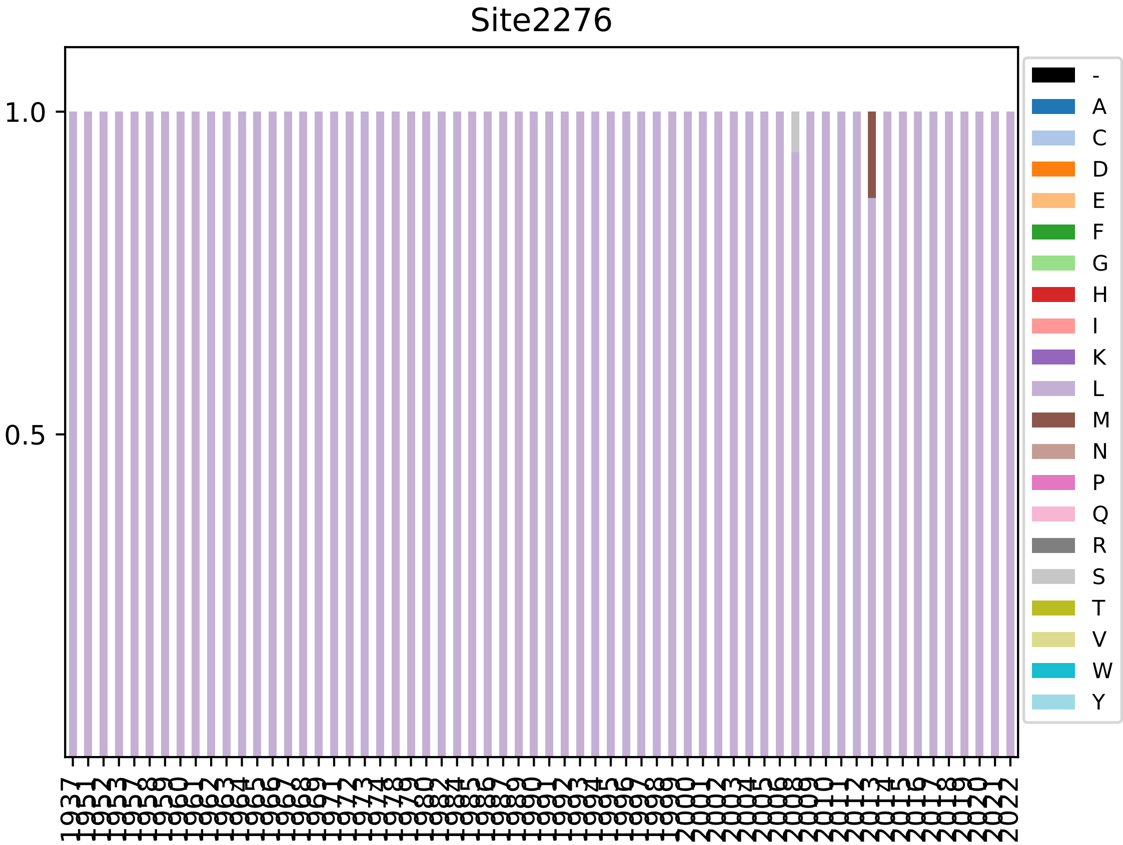
Site2276
1.0
0.5
-
A
C
D
E
F
G
H
I
K
L
M
N
P
Q
R
S
T
V
W
Y

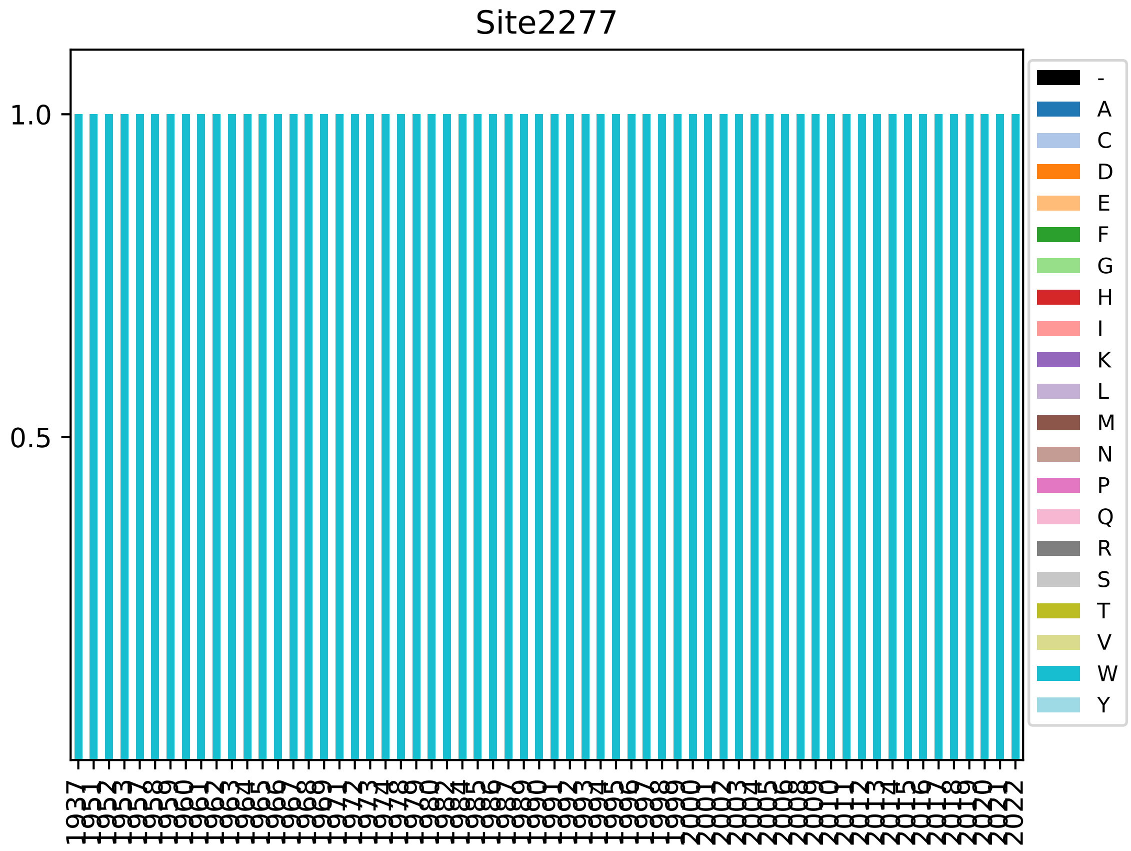
Site2277
1.0
0.5
-
A
C
D
E
F
G
H
I
K
L
M
N
P
Q
R
S
T
V
W
Y

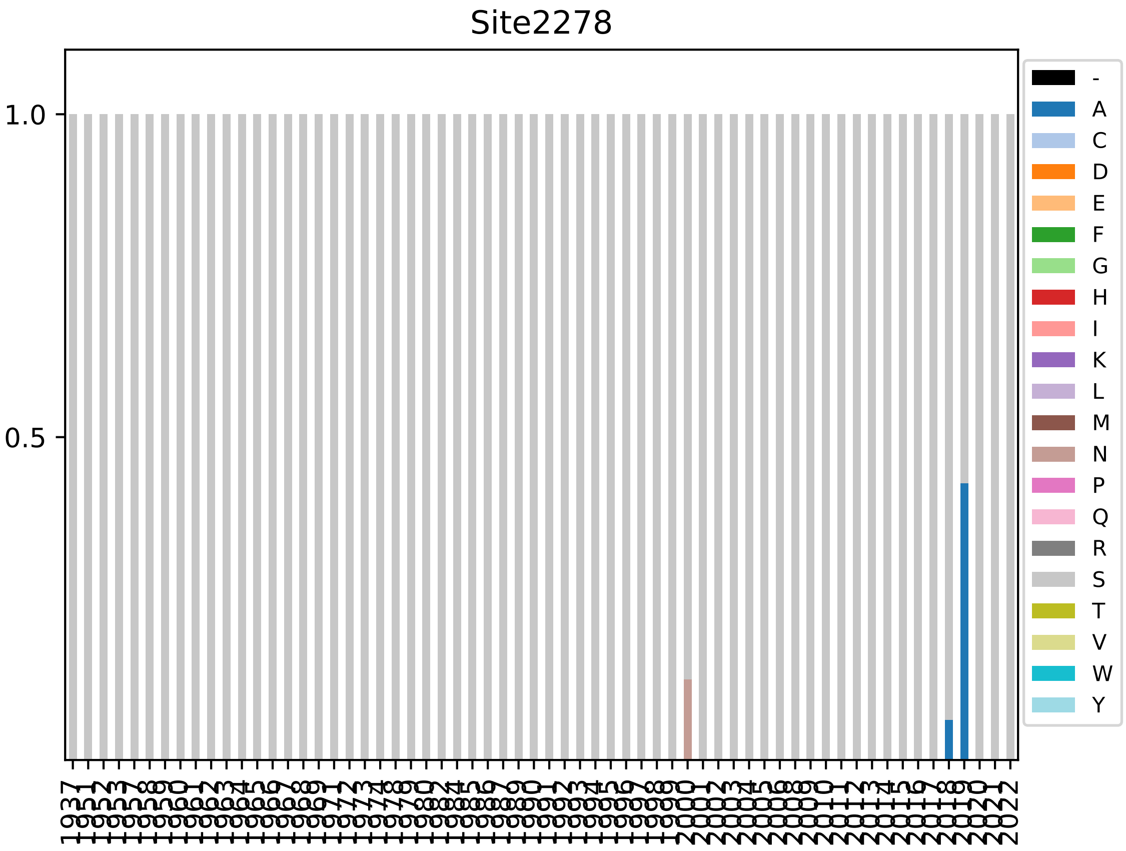
Site2278
1.0
0.5
-
A
C
D
E
F
G
H
I
K
L
M
N
P
Q
R
S
T
V
W
Y

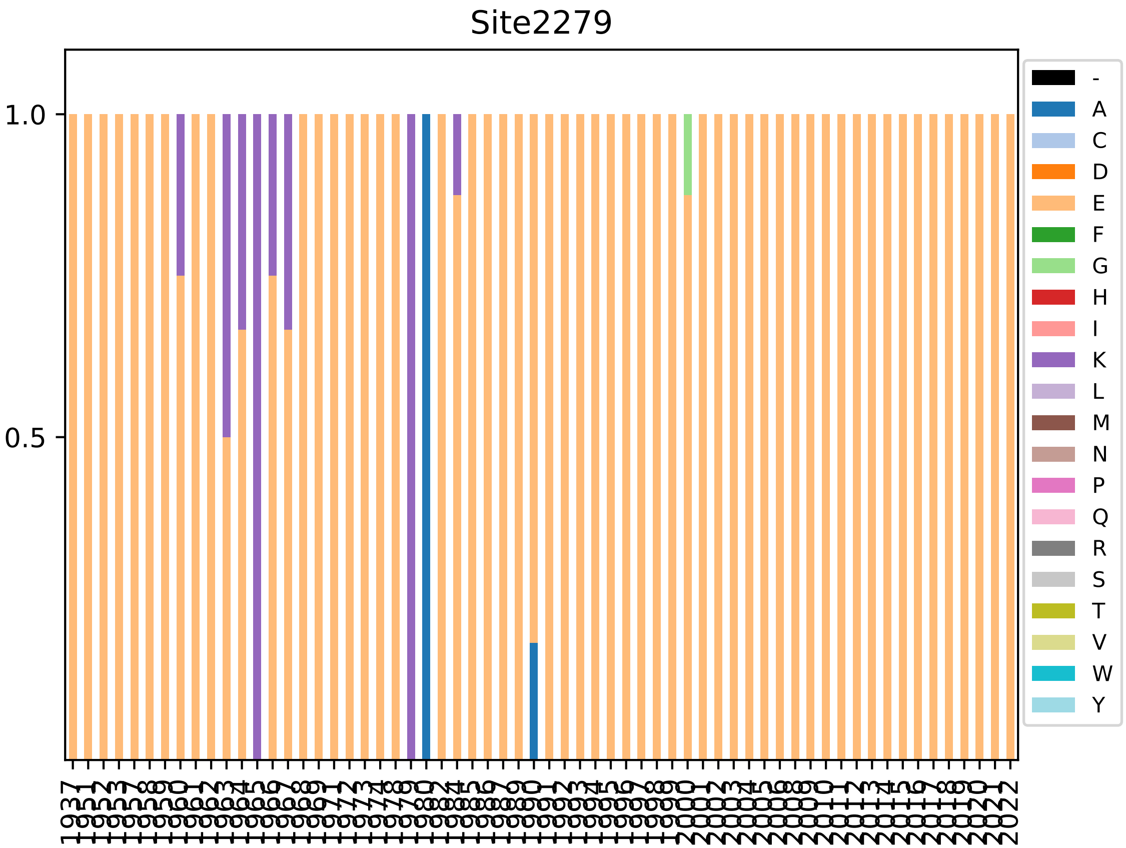
Site2279
1.0
0.5
-
A
C
D
E
F
G
H
I
K
L
M
N
P
Q
R
S
T
V
W
Y

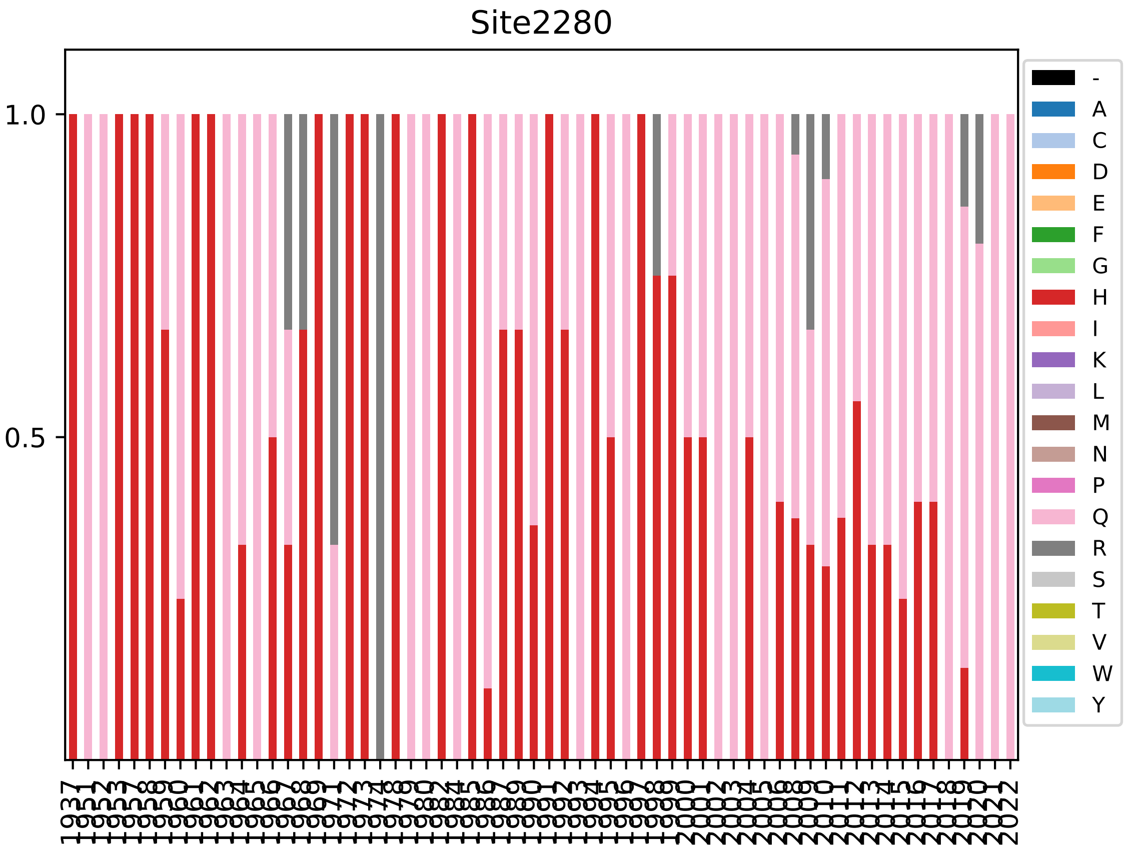
Site2280
1.0
0.5
-
A
C
D
E
F
G
H
I
K
L
M
N
P
Q
R
S
T
V
W
Y

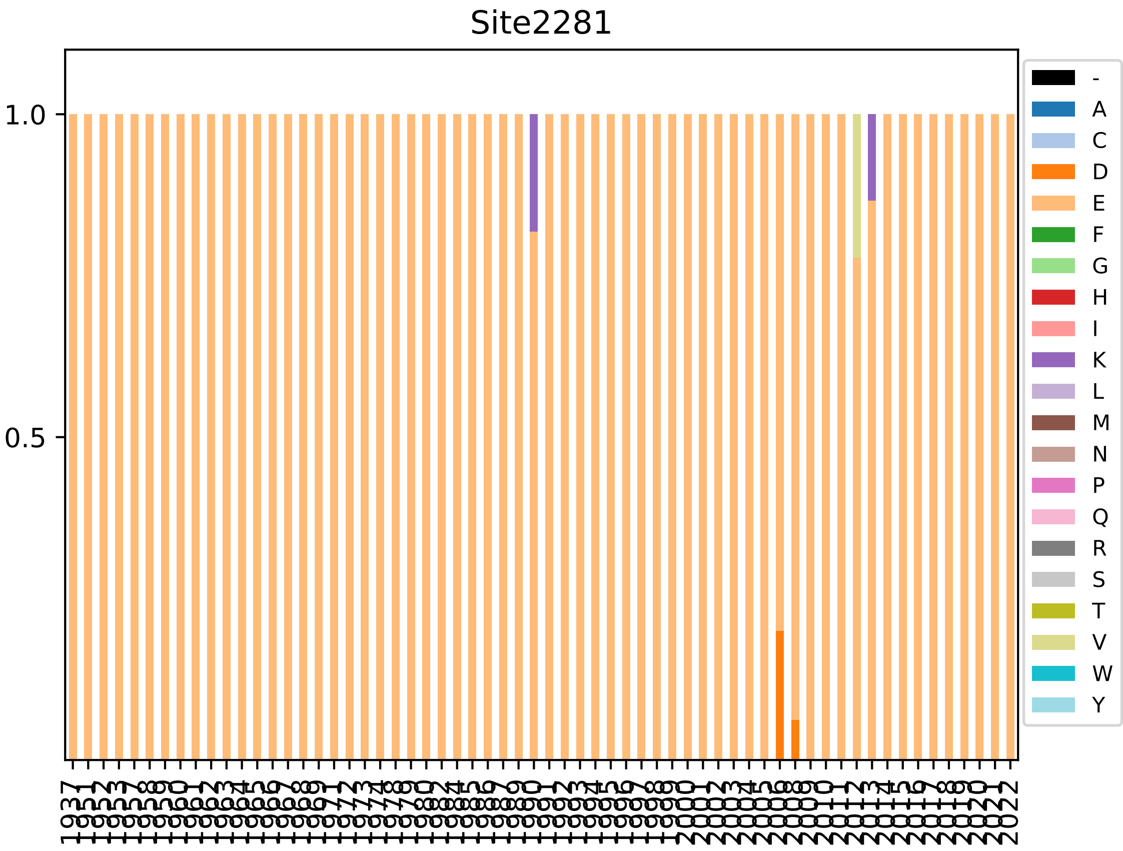
Site2281
1.0
0.5
-
A
C
D
E
F
G
H
I
K
L
M
N
P
Q
R
S
T
V
W
Y

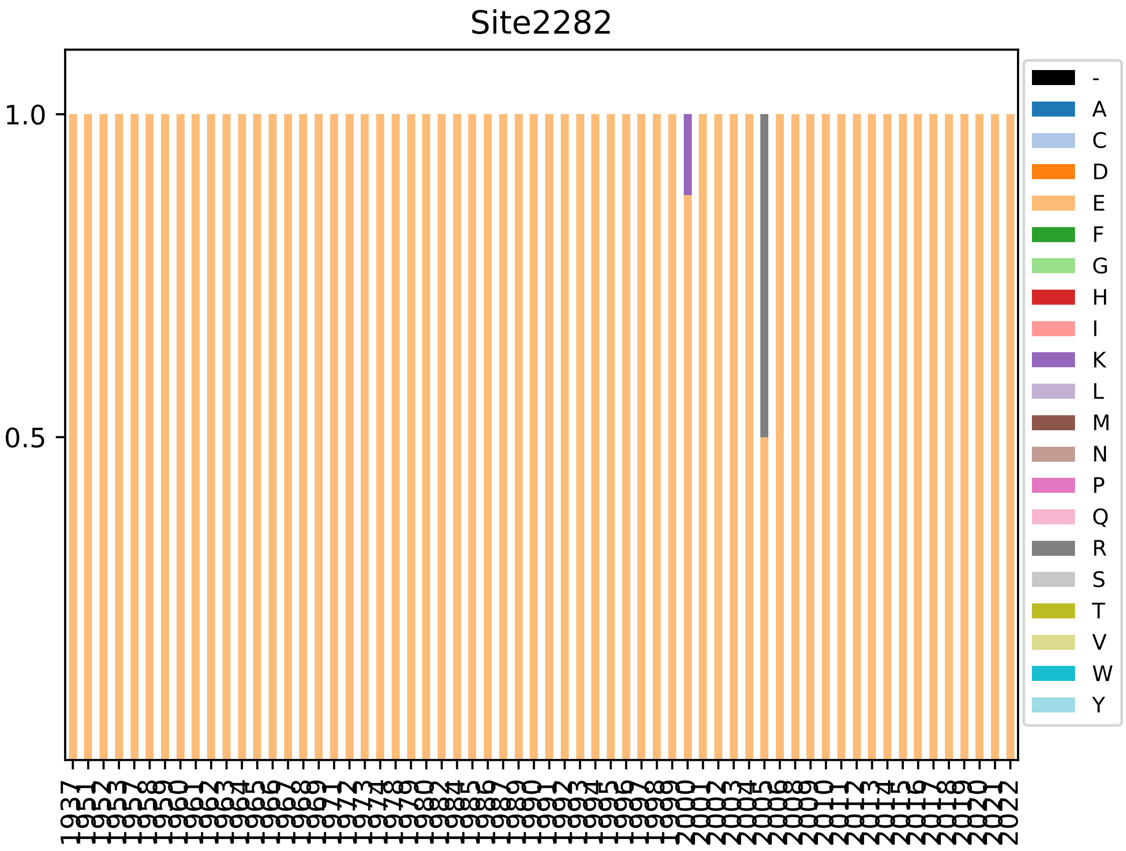
Site2282
1.0
0.5
-
A
C
D
E
F
G
H
I
K
L
M
N
P
Q
R
S
T
V
W
Y

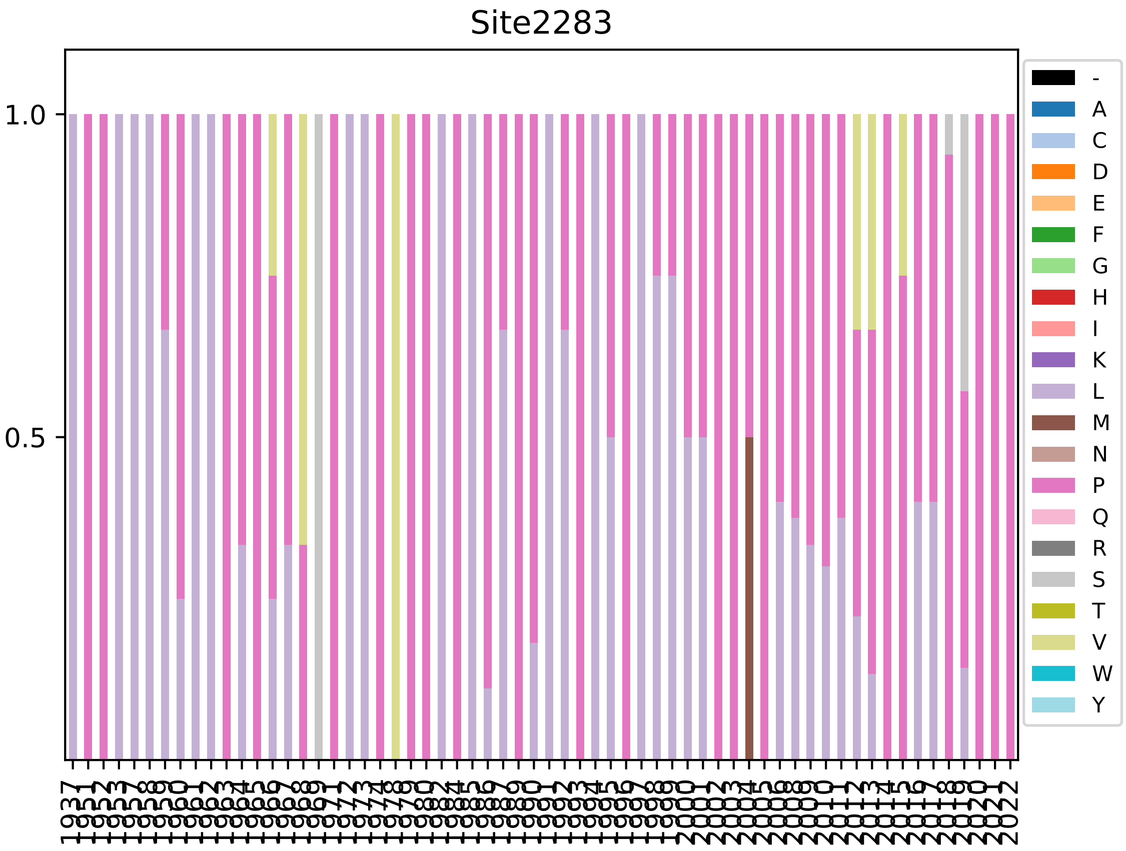
Site2283
1.0
0.5
-
A
C
D
E
F
G
H
I
K
L
M
N
P
Q
R
S
T
V
W
Y

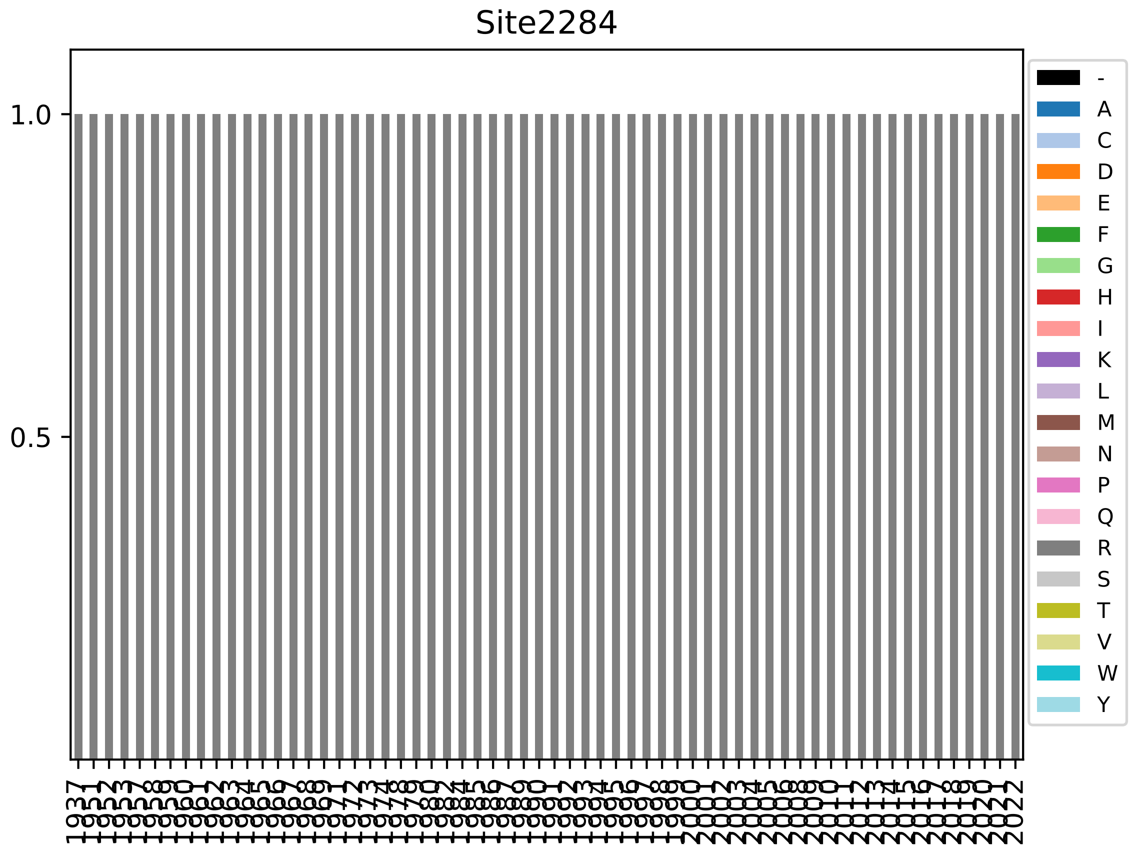
Site2284
1.0
0.5
-
A
C
D
E
F
G
H
I
K
L
M
N
P
Q
R
S
T
V
W
Y

Site2285

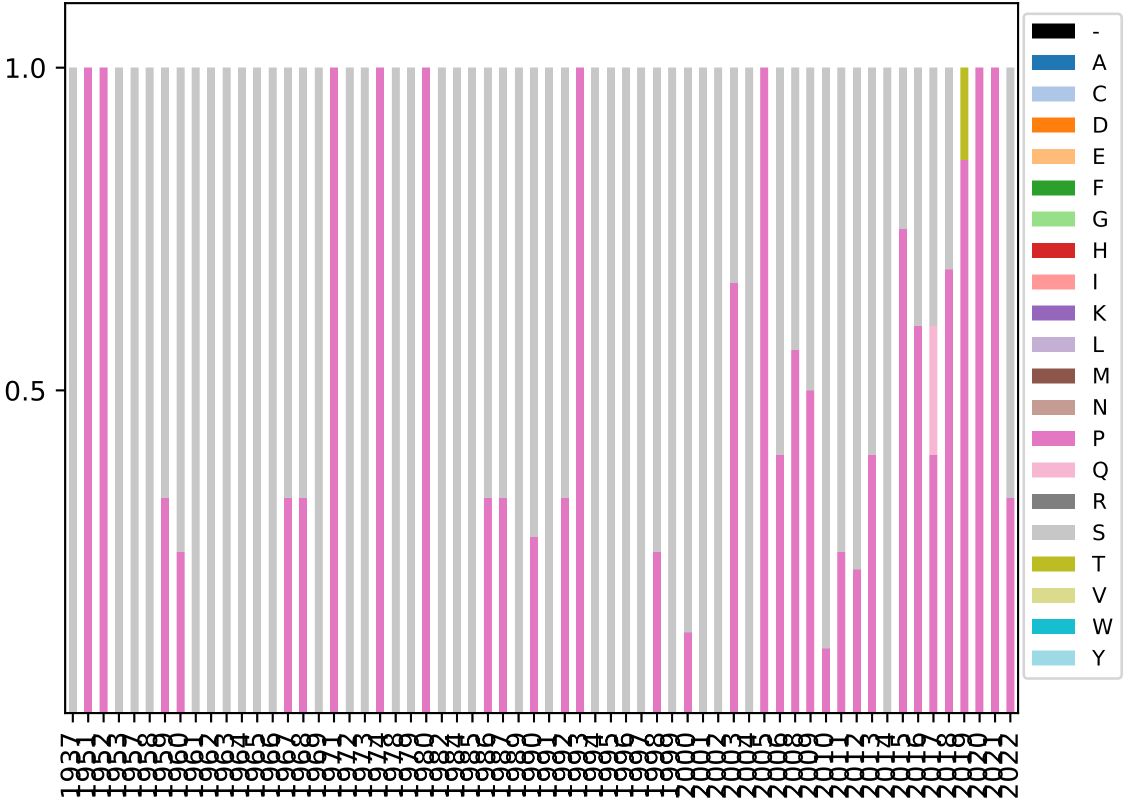

Site2286

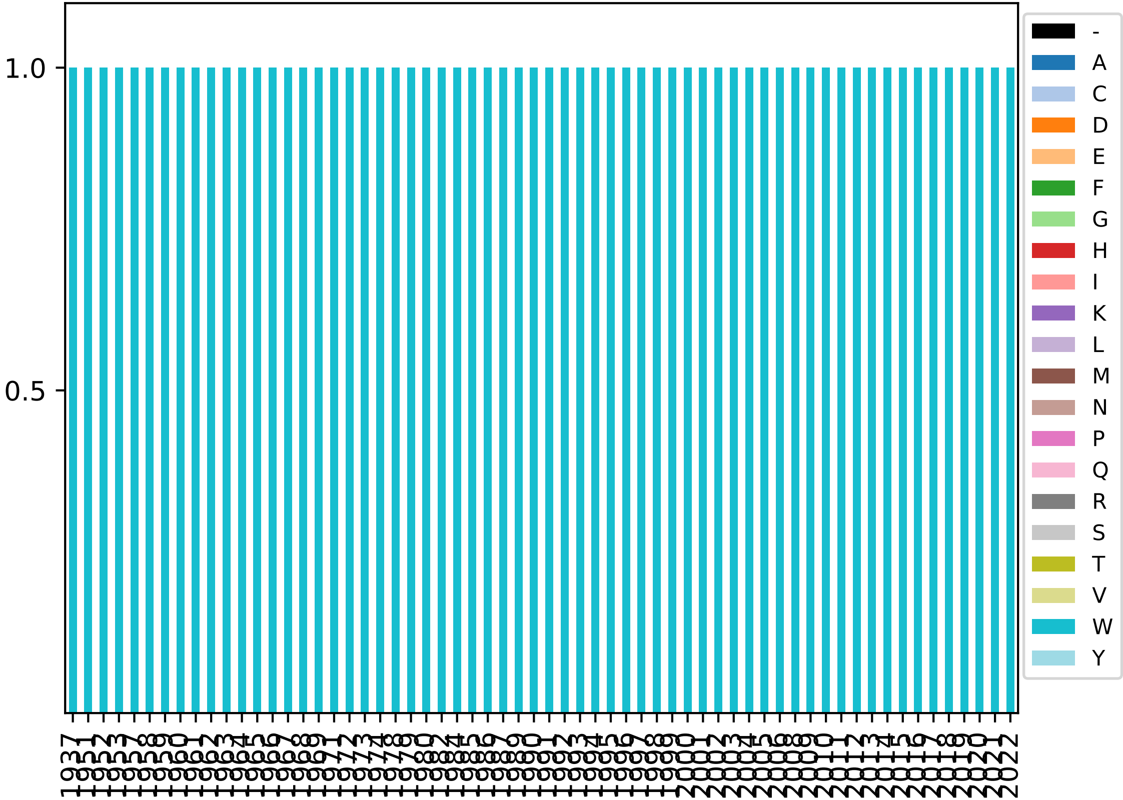

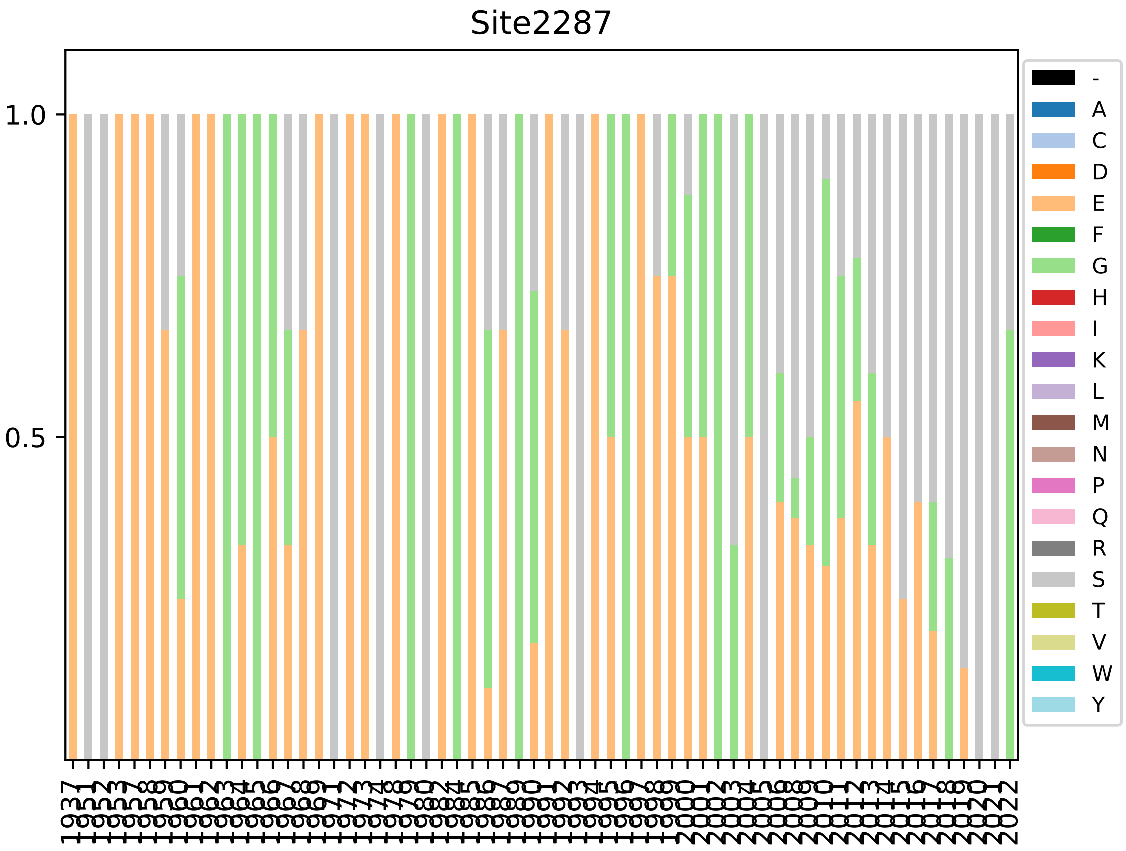
Site2287
1.0
0.5
-
A
C
D
E
F
G
H
I
K
L
M
N
P
Q
R
S
T
V
W
Y

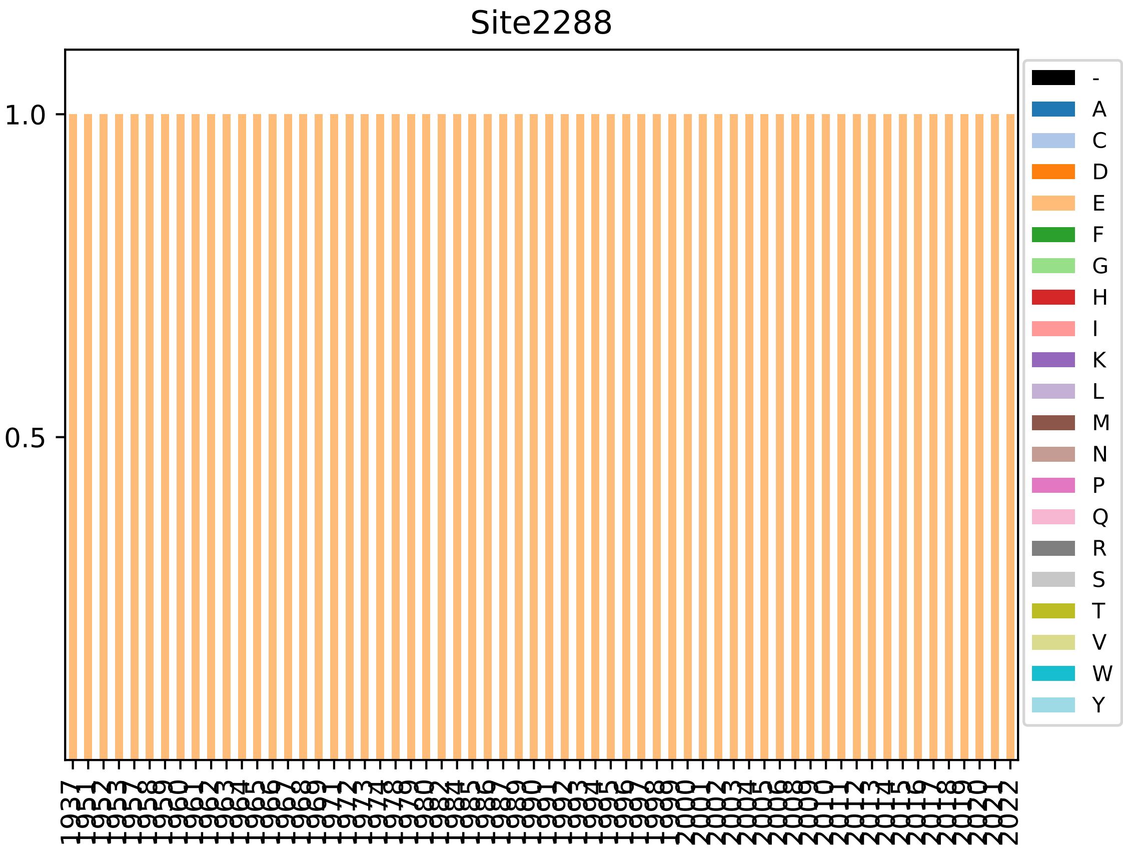
Site2288
1.0
0.5
-
A
C
D
E
F
G
H
I
K
L
M
N
P
Q
R
S
T
V
W
Y

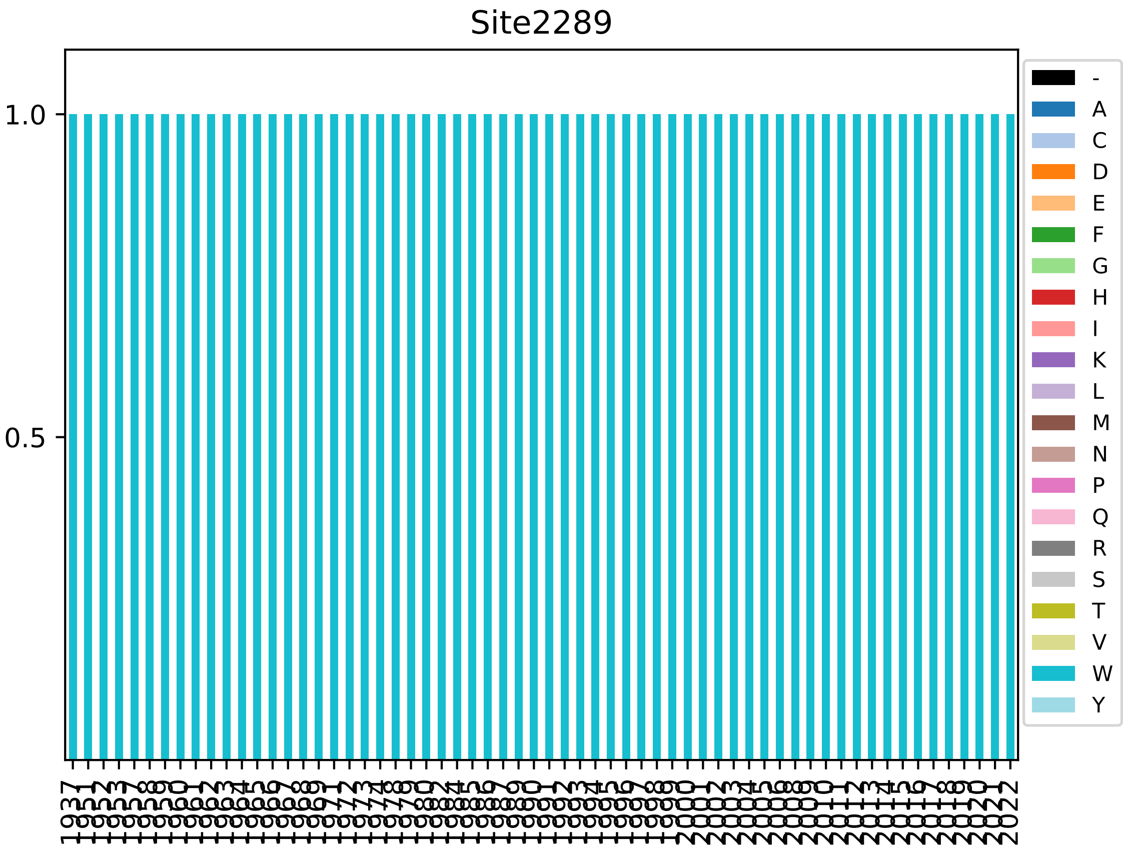
Site2289
1.0
0.5
-
A
C
D
E
F
G
H
I
K
L
M
N
P
Q
R
S
T
V
W
Y

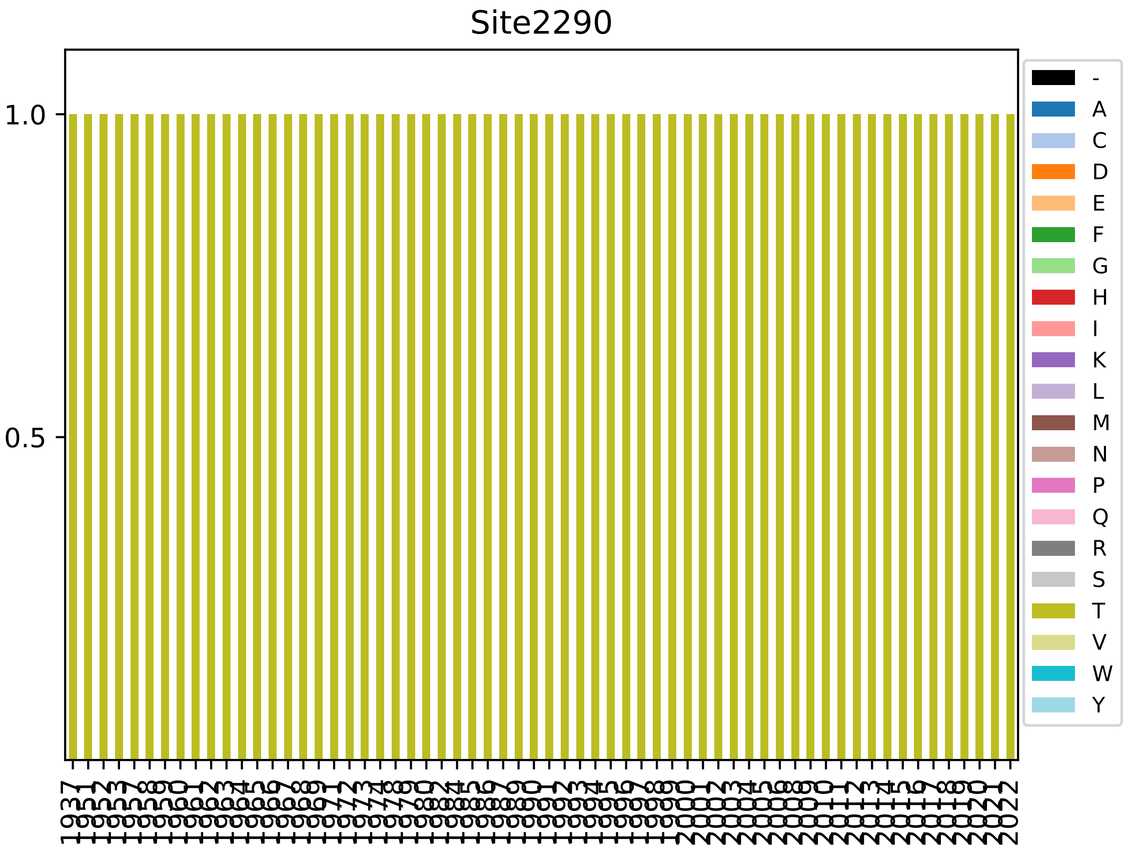

Site2290
1.0
0.5
-
A
C
D
E
F
G
H
I
K
L
M
N
P
Q
R
S
T
V
W
Y

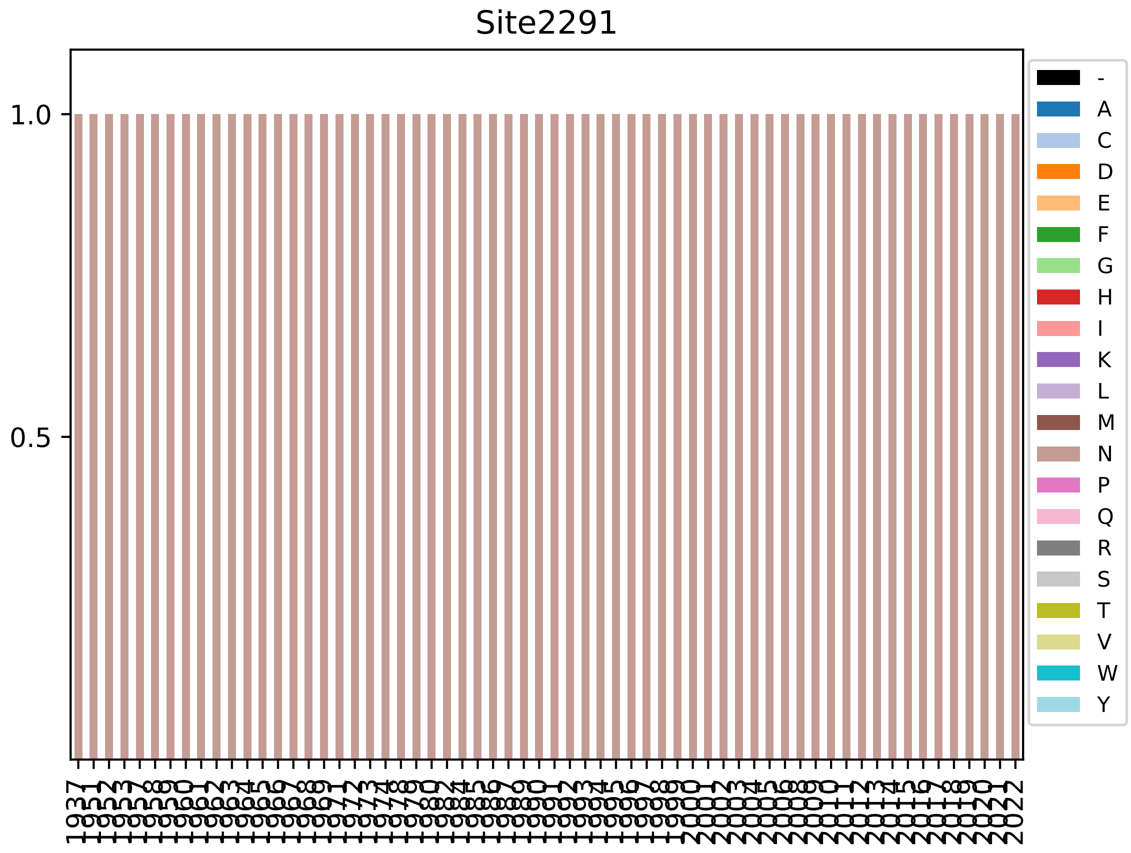
Site2291
1.0
0.5
-
A
C
D
E
F
G
H
I
K
L
M
N
P
Q
R
S
T
V
W
Y

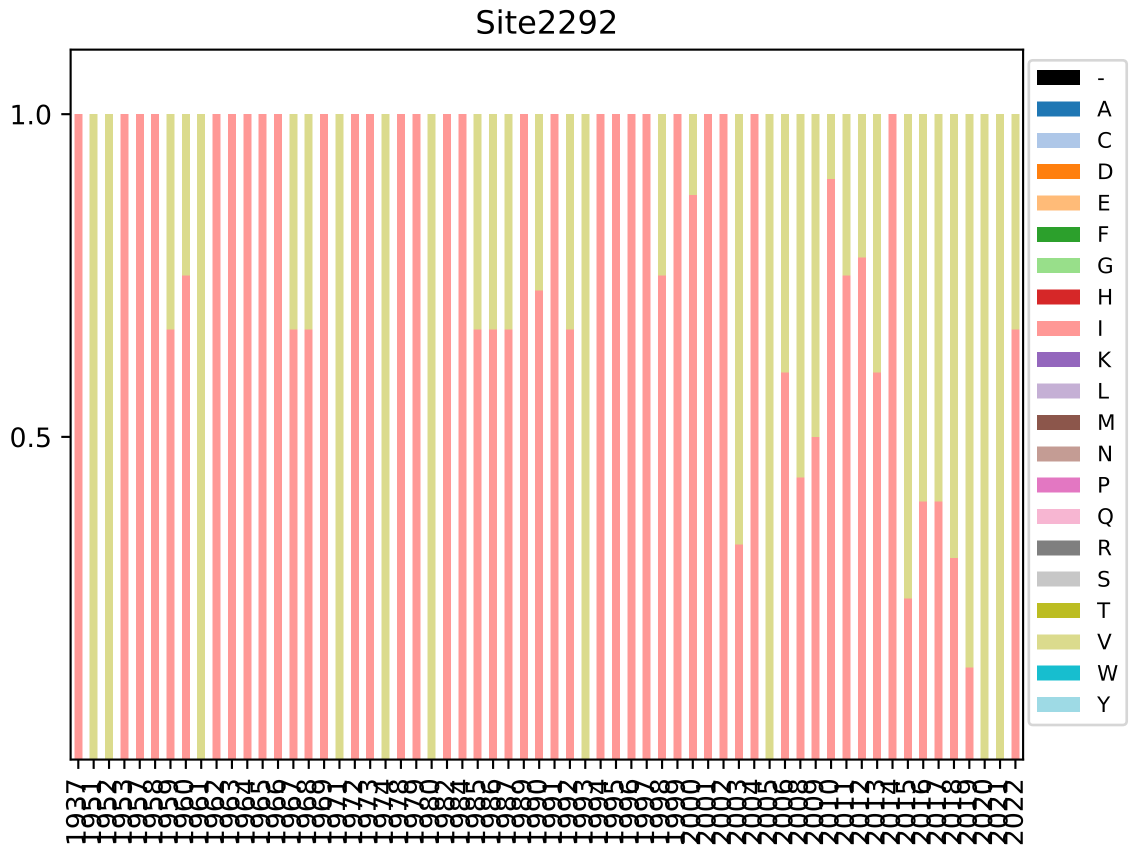
Site2292
1.0
0.5
-
A
C
D
E
F
G
H
I
K
L
M
N
P
Q
R
S
T
V
W
Y

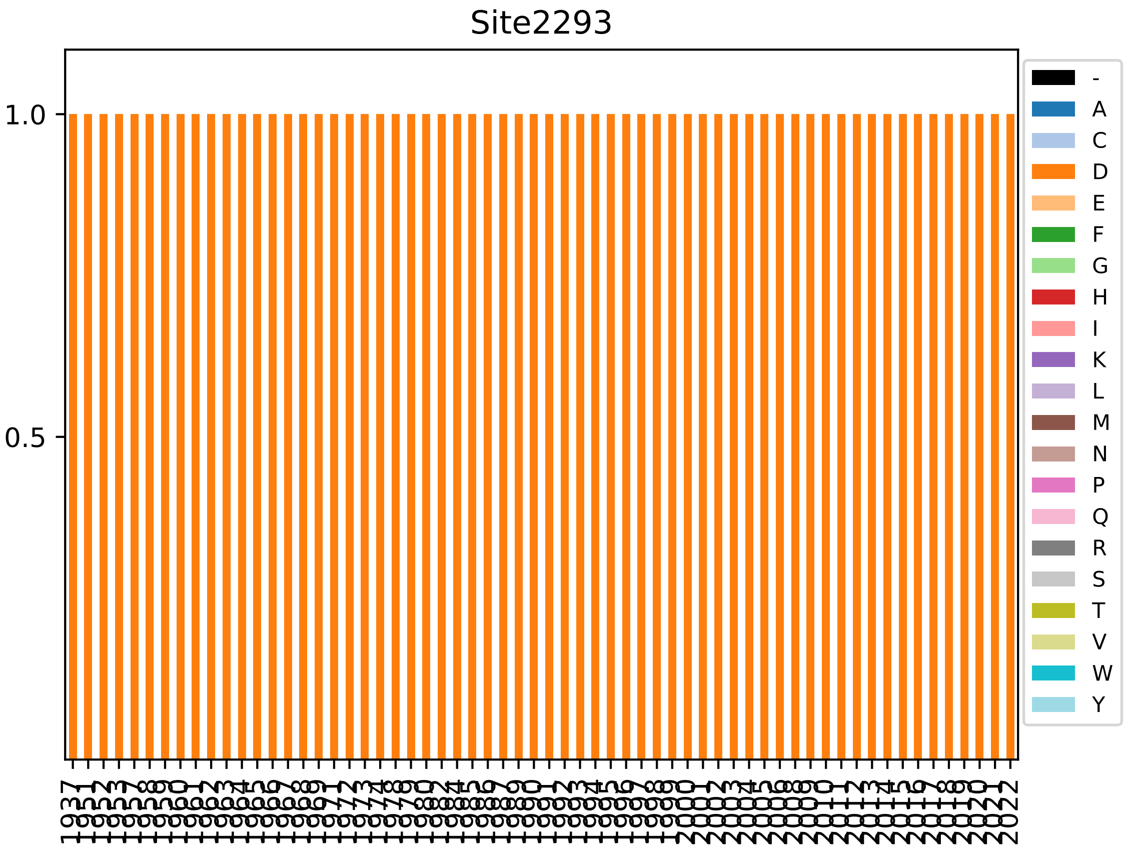
Site2293
1.0
0.5
-
A
C
D
E
F
G
H
I
K
L
M
N
P
Q
R
S
T
V
W
Y

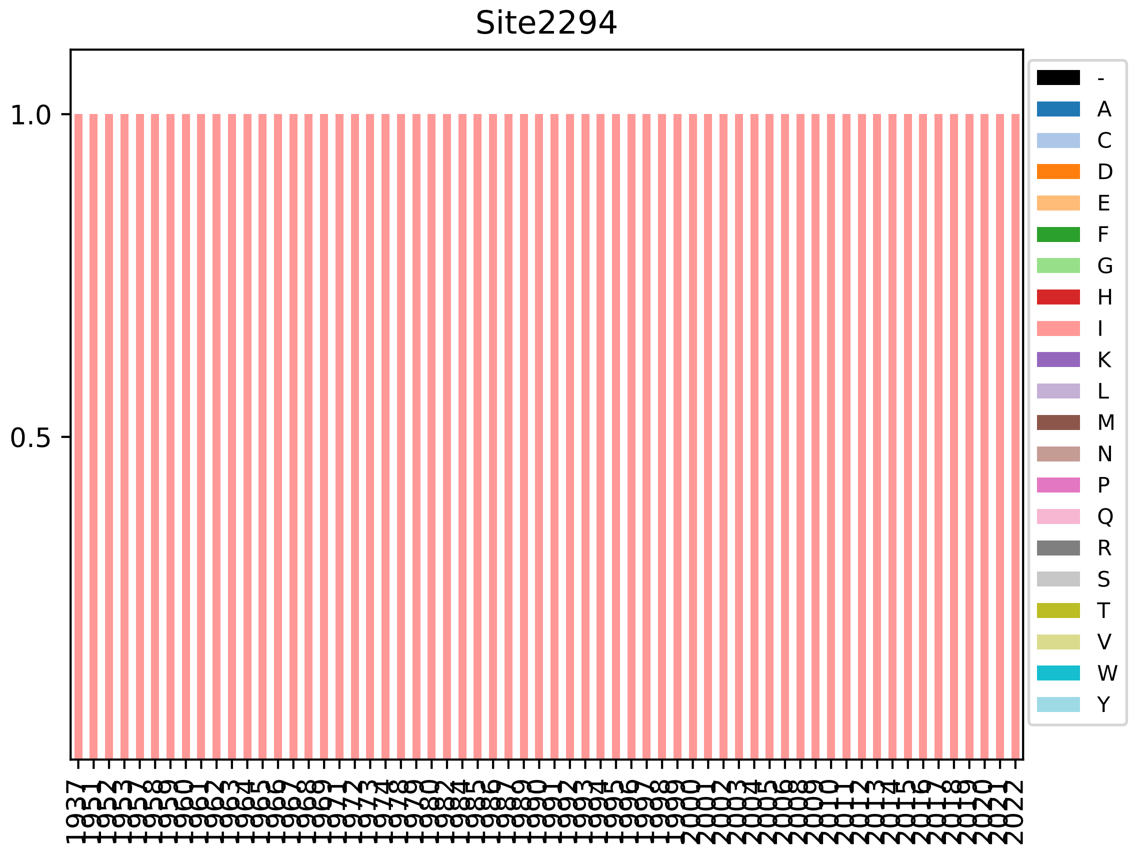
Site2294
1.0
0.5
-
A
C
D
E
F
G
H
I
K
L
M
N
P
Q
R
S
T
V
W
Y

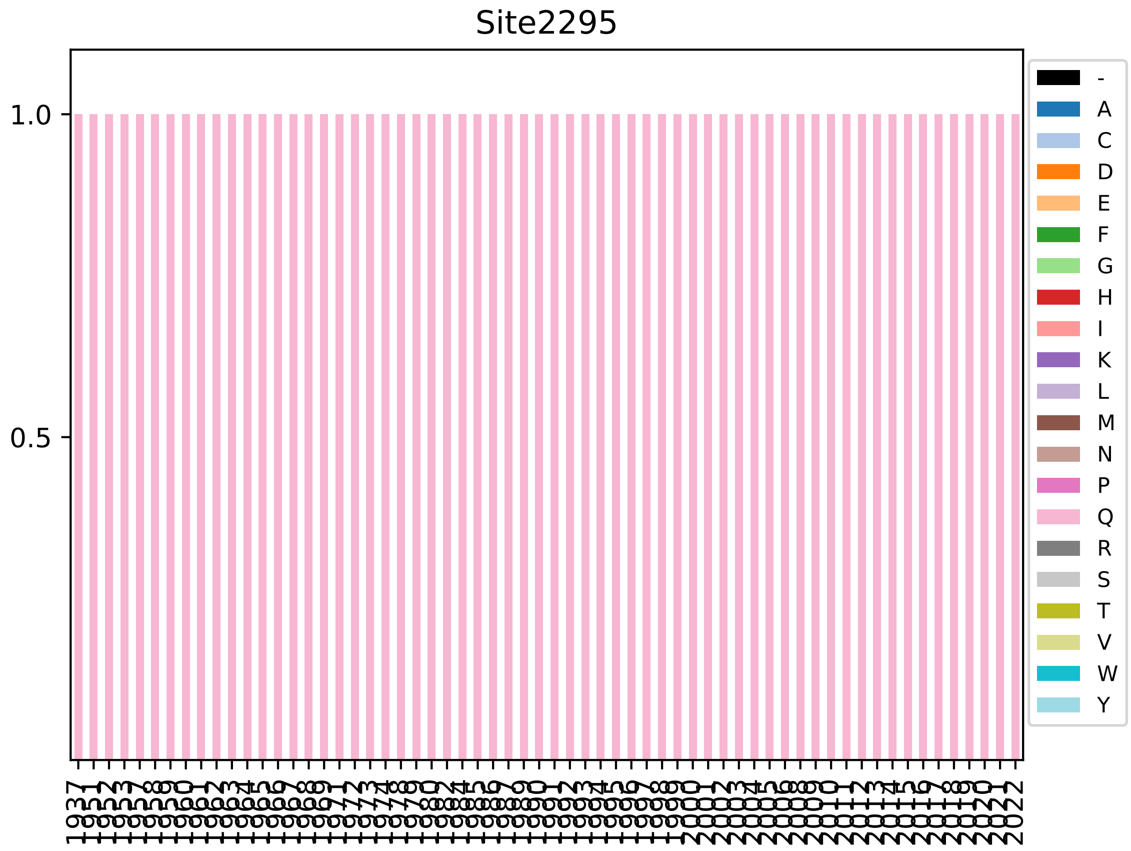
Site2295
1.0
0.5
-
A
C
D
E
F
G
H
I
K
L
M
N
P
Q
R
S
T
V
W
Y

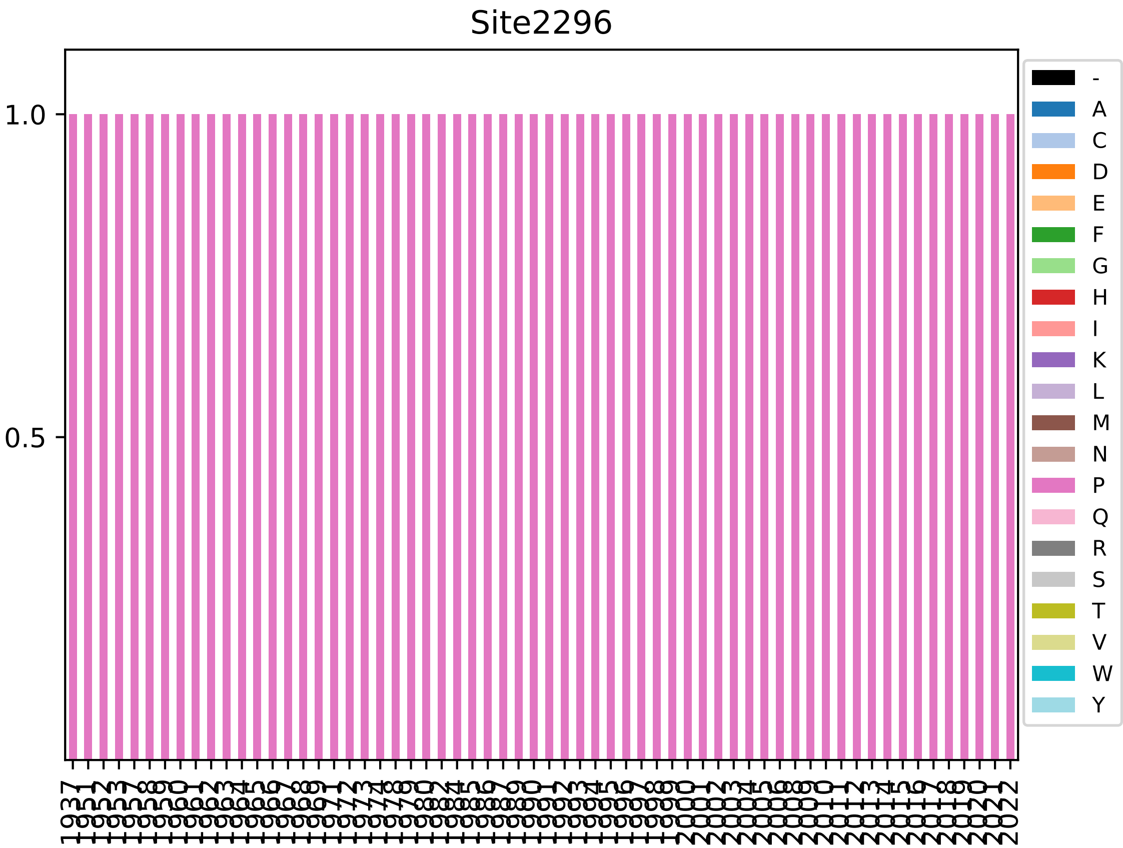
Site2296
1.0
0.5
-
A
C
D
E
F
G
H
I
K
L
M
N
P
Q
R
S
T
V
W
Y

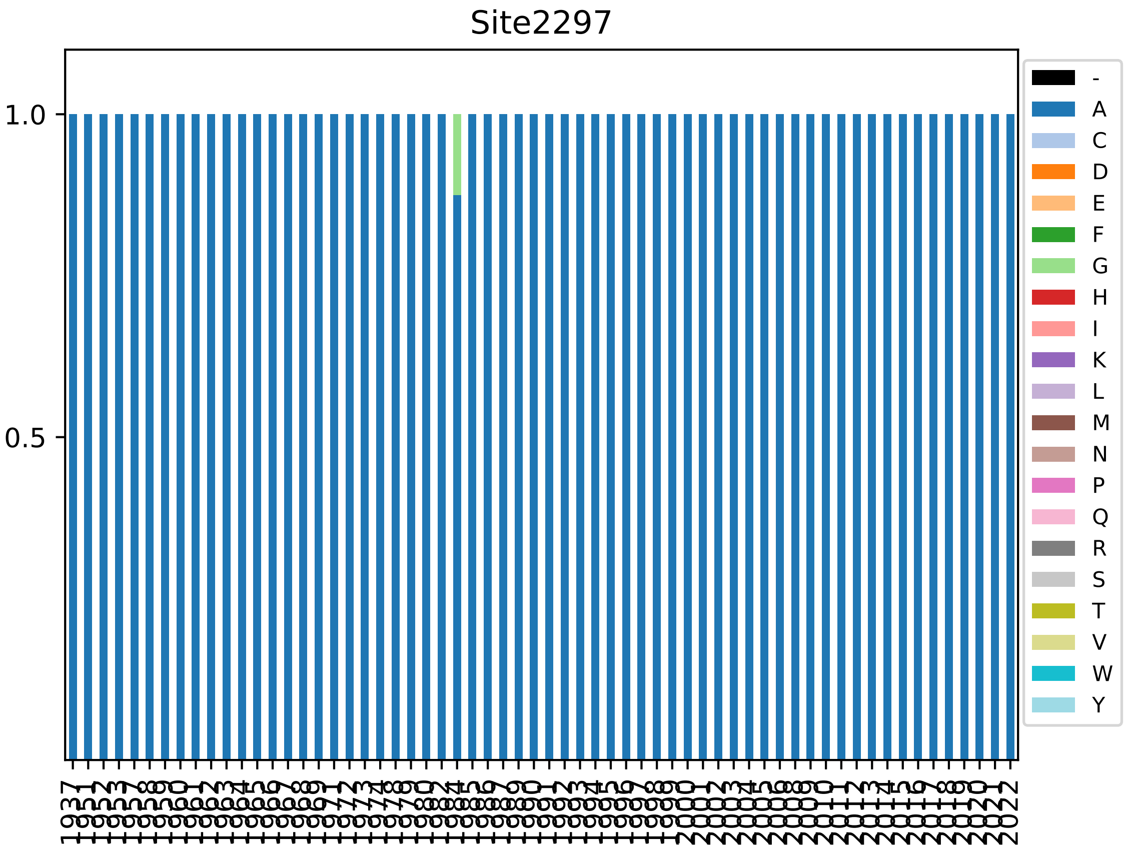
Site2297
1.0
0.5
-
A
C
D
E
F
G
H
I
K
L
M
N
P
Q
R
S
T
V
W
Y

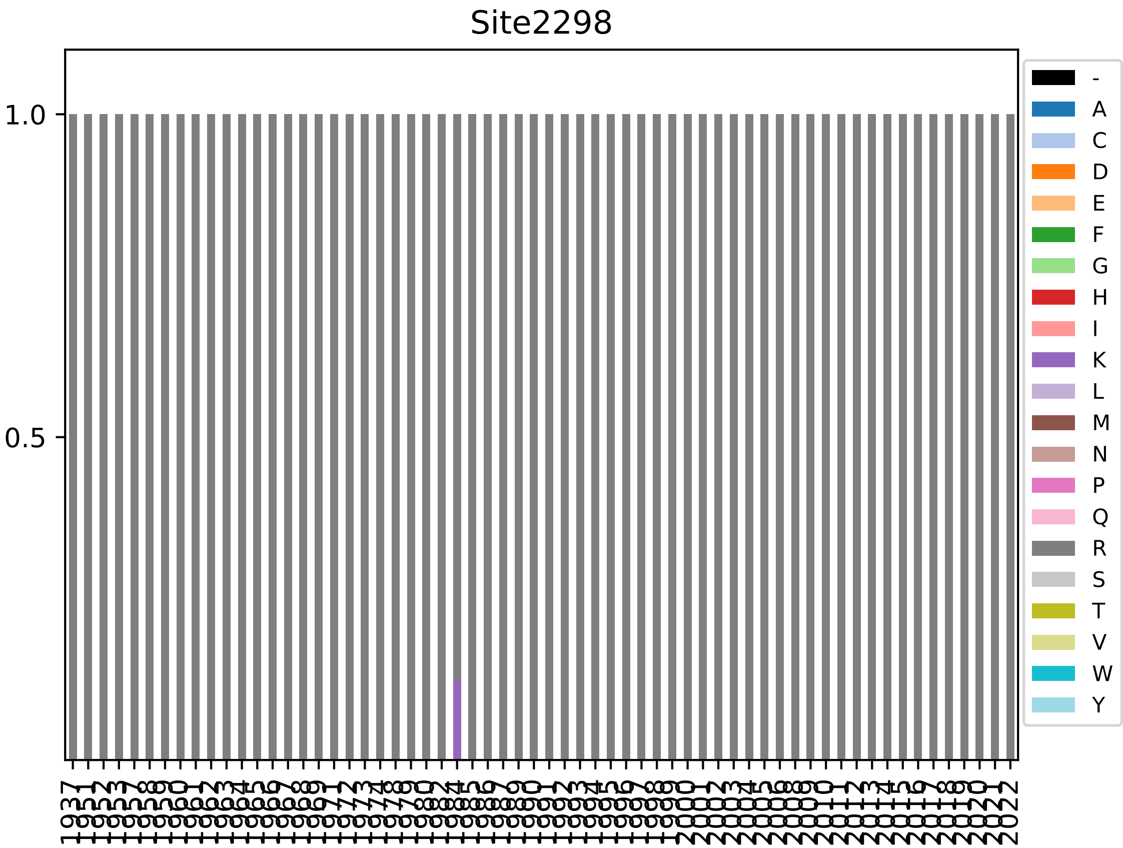
Site2298
1.0
0.5
-
A
C
D
E
F
G
H
I
K
L
M
N
P
Q
R
S
T
V
W
Y

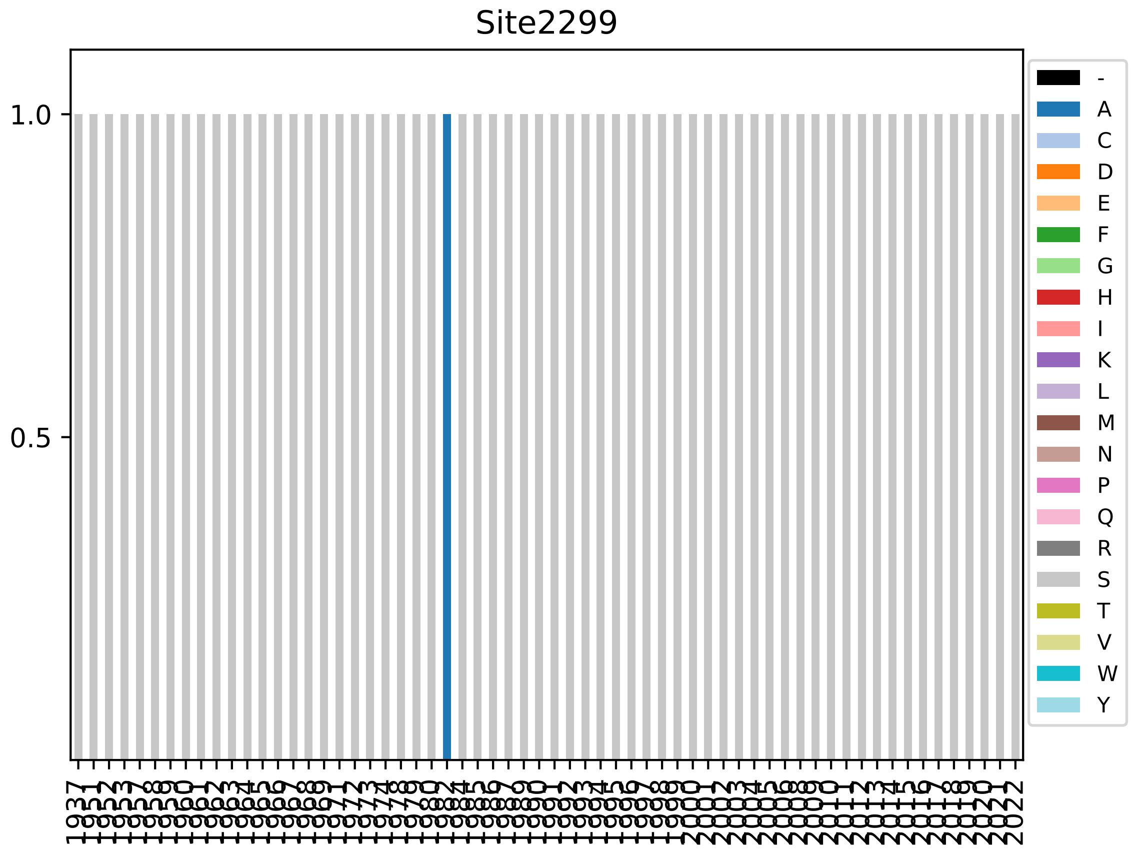
Site2299
1.0
0.5
-
A
C
D
E
F
G
H
I
K
L
M
N
P
Q
R
S
T
V
W
Y

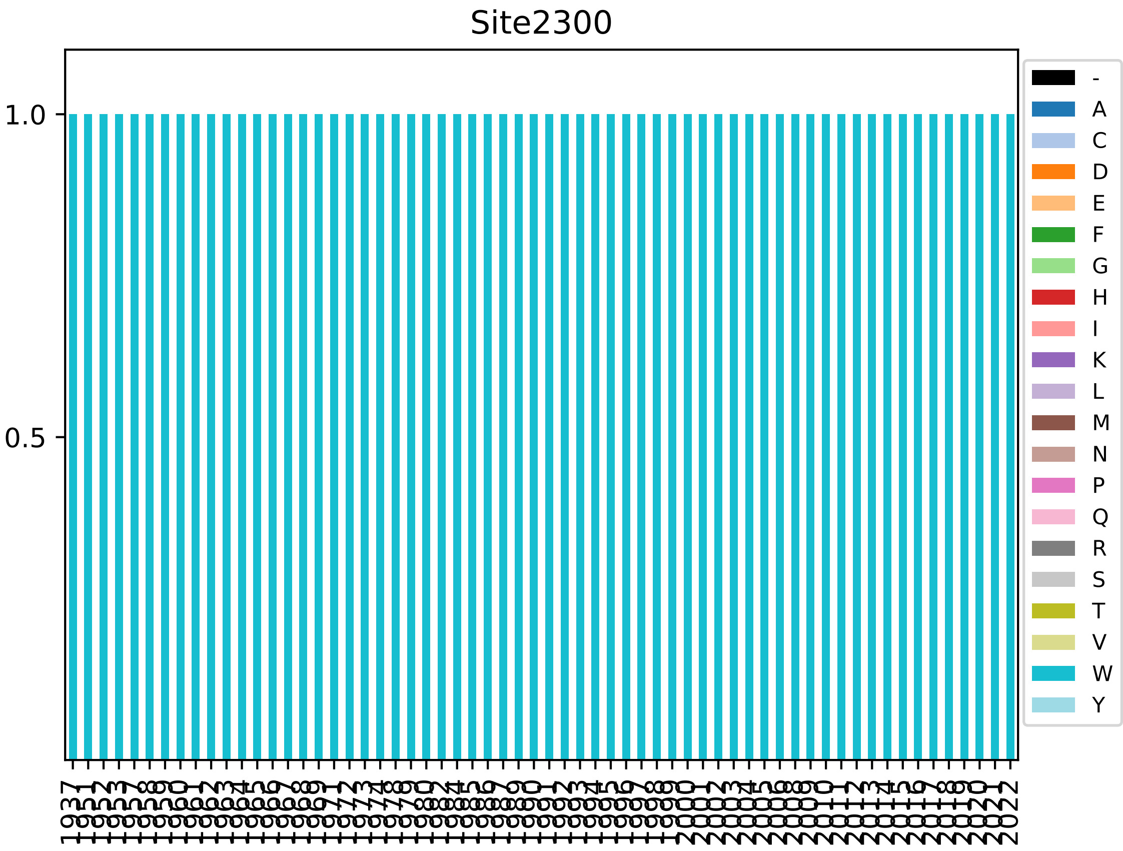
Site2300
1.0
0.5
-
A
C
D
E
F
G
H
I
K
L
M
N
P
Q
R
S
T
V
W
Y

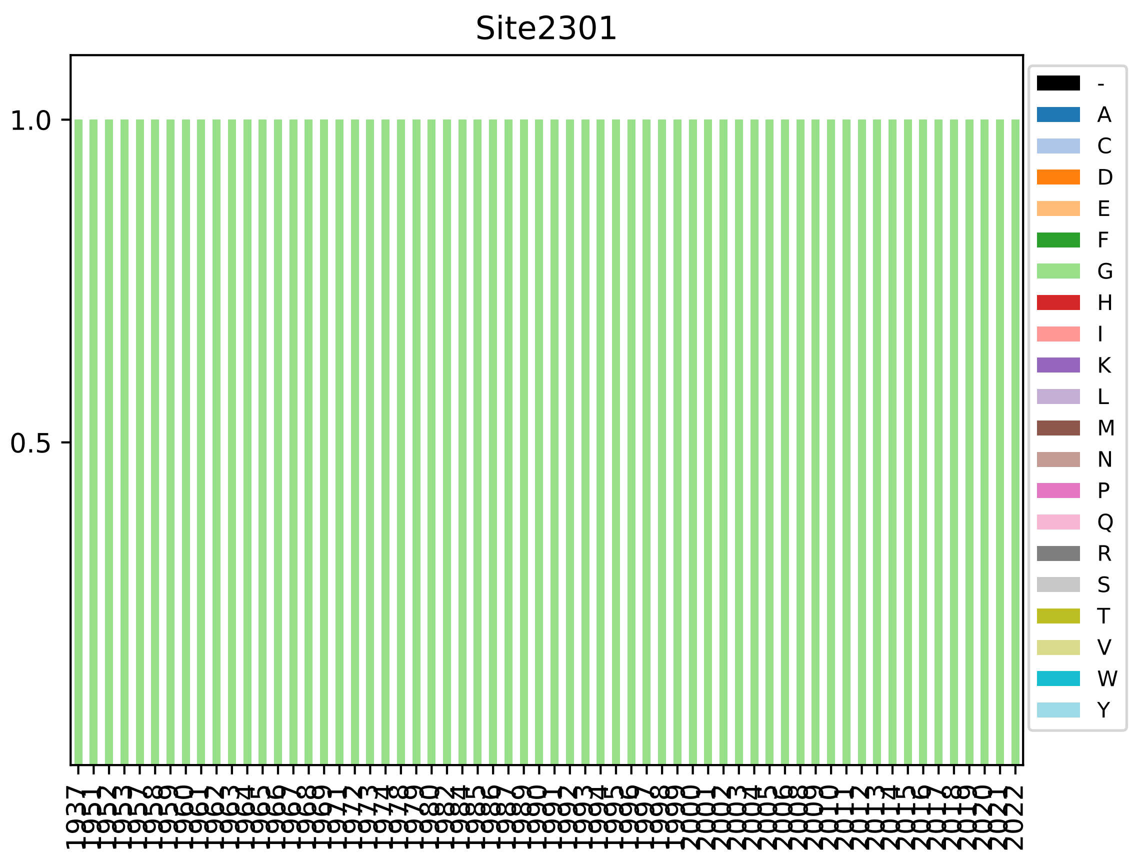

Site2301
1.0
0.5
-
A
C
D
E
F
G
H
I
K
L
M
N
P
Q
R
S
T
V
W
Y

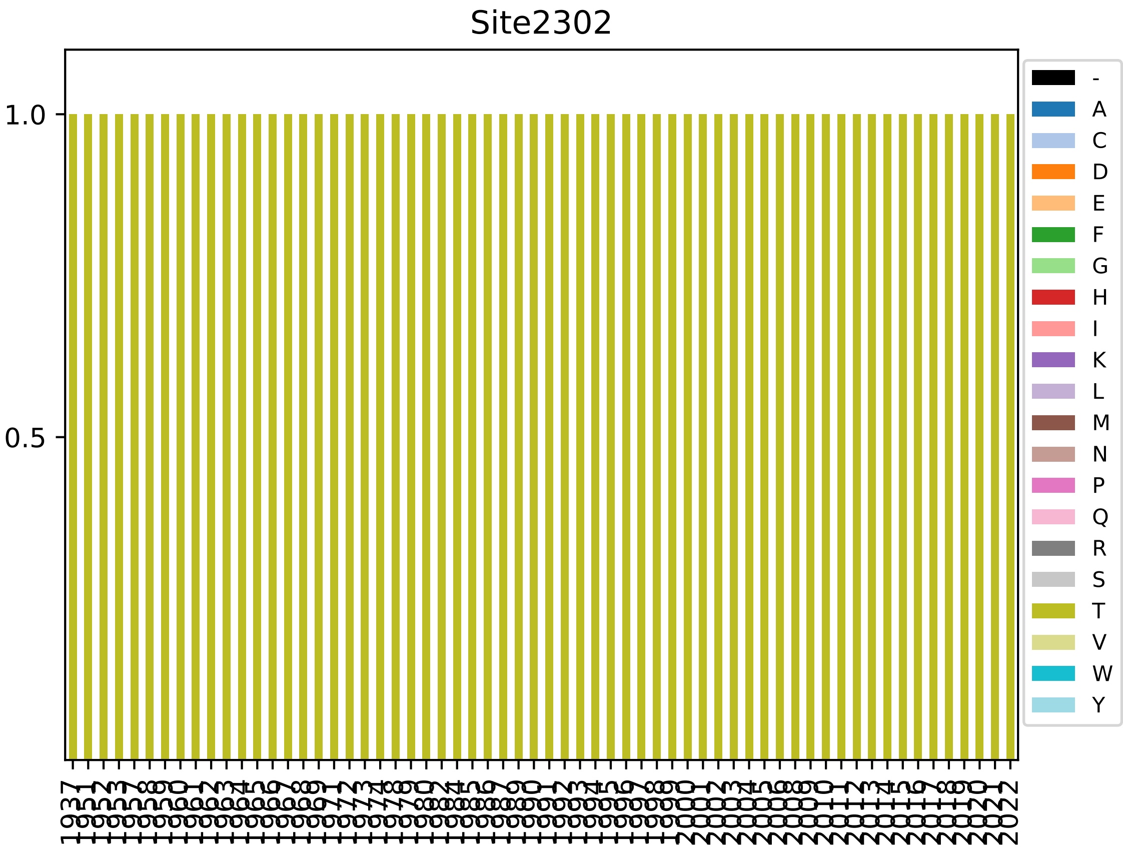
Site2302
1.0
0.5
-
A
C
D
E
F
G
H
I
K
L
M
N
P
Q
R
S
T
V
W
Y

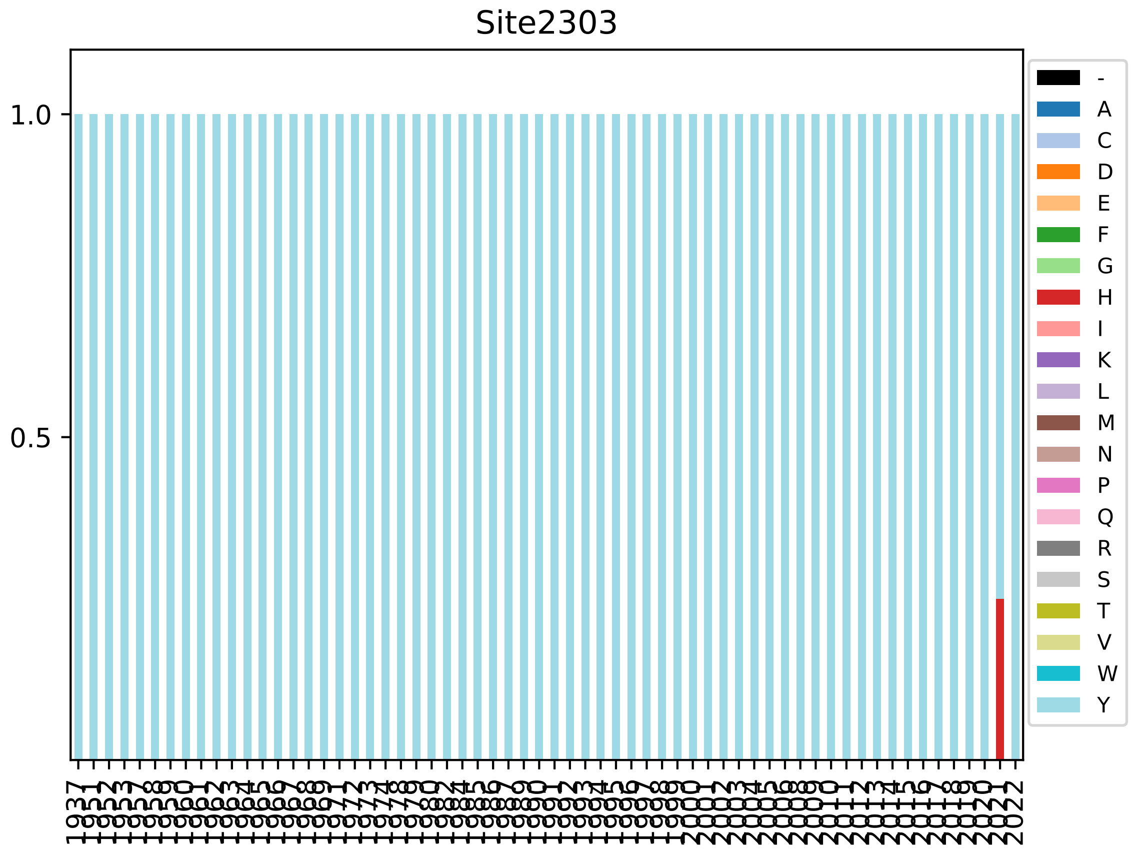
Site2303
1.0
0.5
-
A
C
D
E
F
G
H
I
K
L
M
N
P
Q
R
S
T
V
W
Y

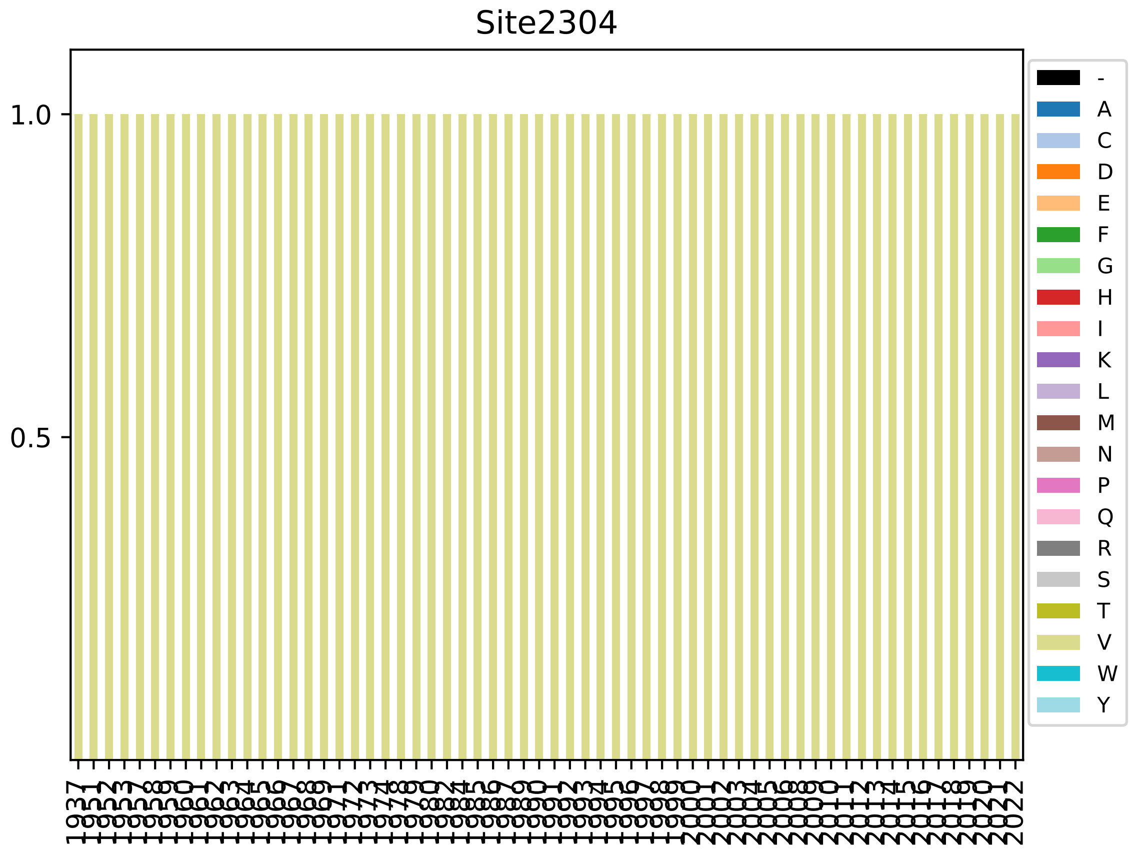
Site2304
1.0
0.5
-
A
C
D
E
F
G
H
I
K
L
M
N
P
Q
R
S
T
V
W
Y

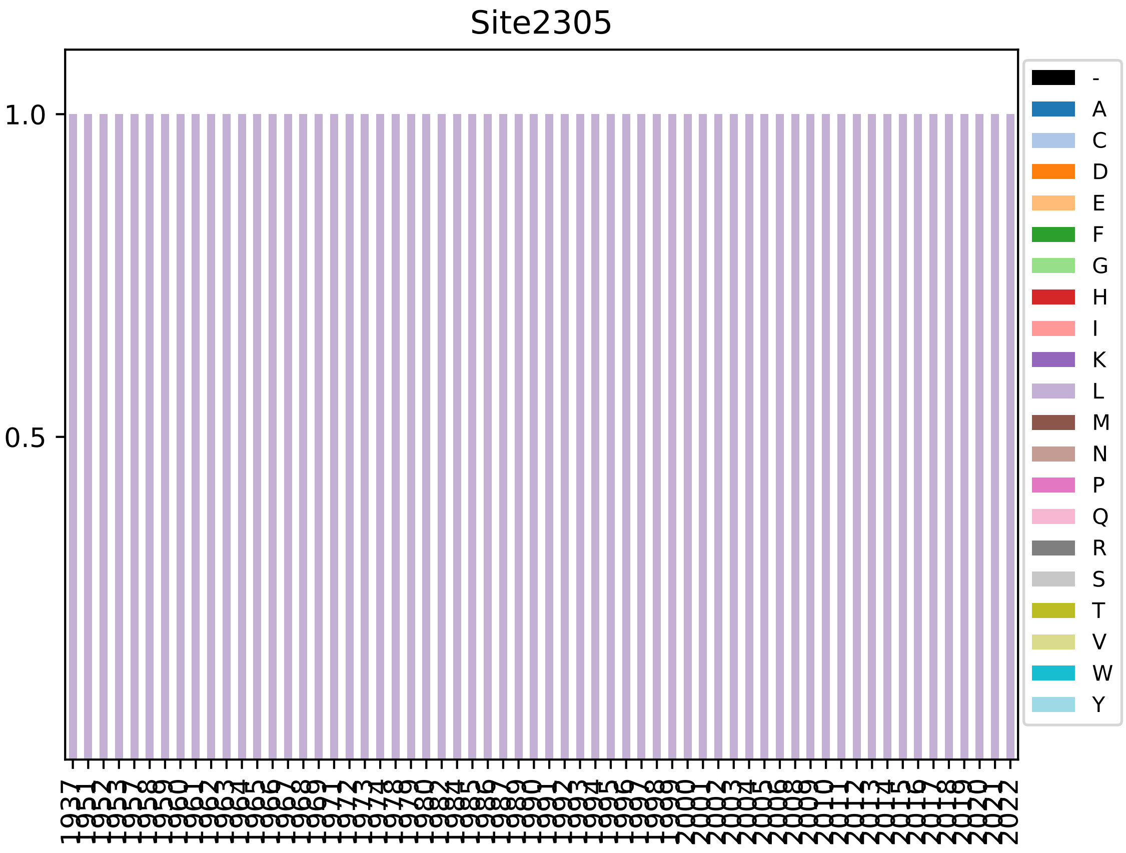
Site2305
1.0
0.5
-
A
C
D
E
F
G
H
I
K
L
M
N
P
Q
R
S
T
V
W
Y

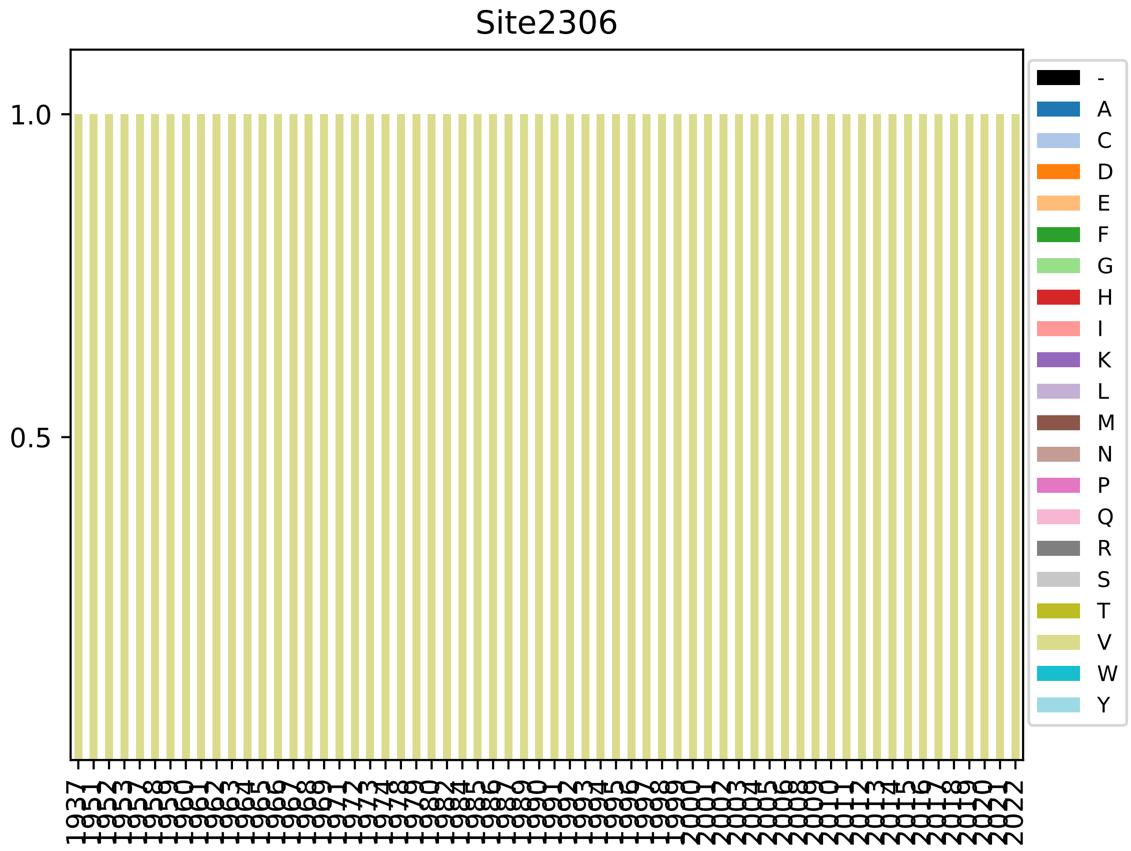
Site2306
1.0
0.5
-
A
C
D
E
F
G
H
I
K
L
M
N
P
Q
R
S
T
V
W
Y

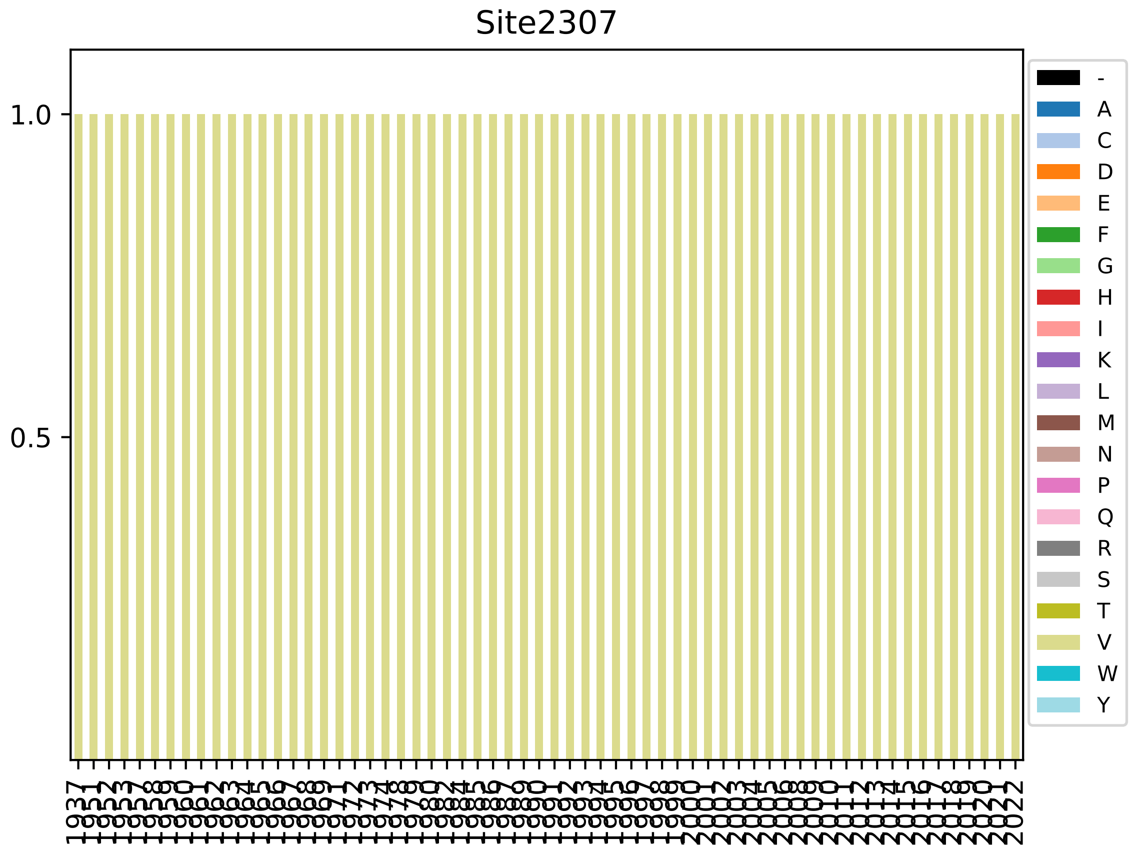
Site2307
1.0
0.5
-
A
C
D
E
F
G
H
I
K
L
M
N
P
Q
R
S
T
V
W
Y

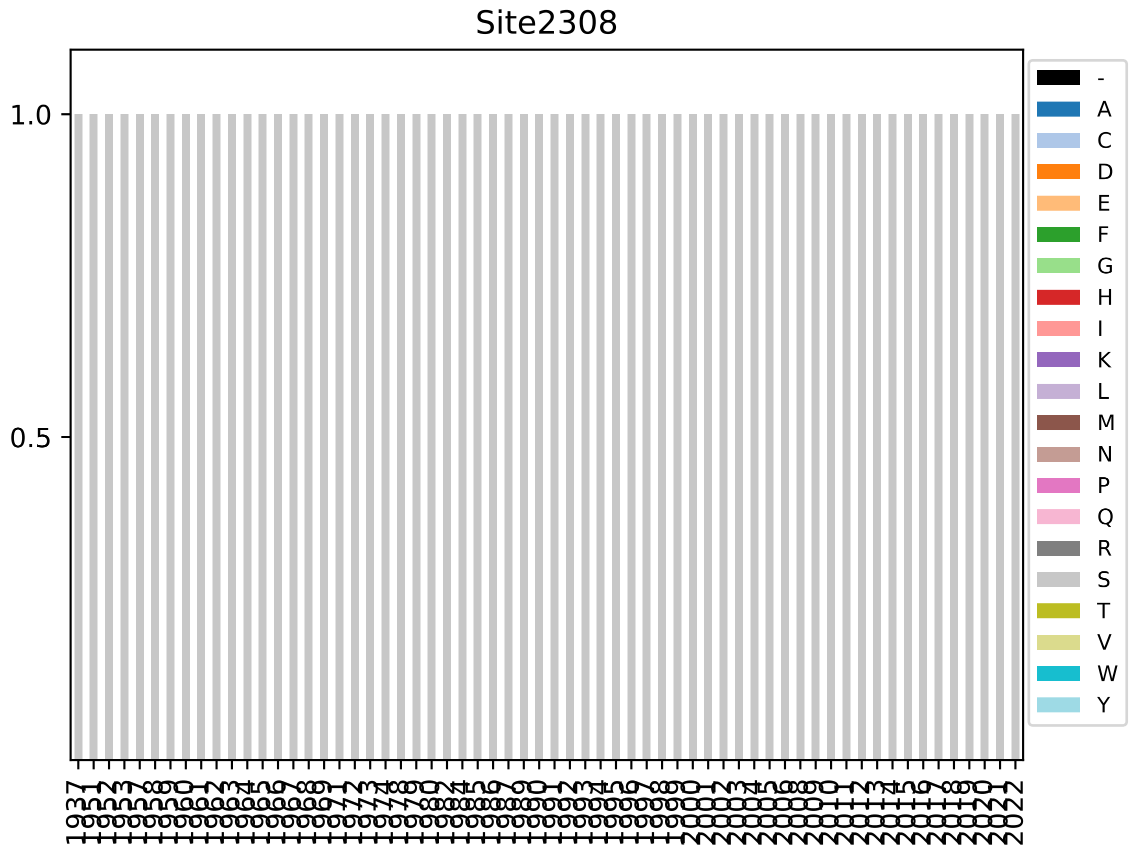

Site2308
1.0
0.5
-
A
C
D
E
F
G
H
I
K
L
M
N
P
Q
R
S
T
V
W
Y

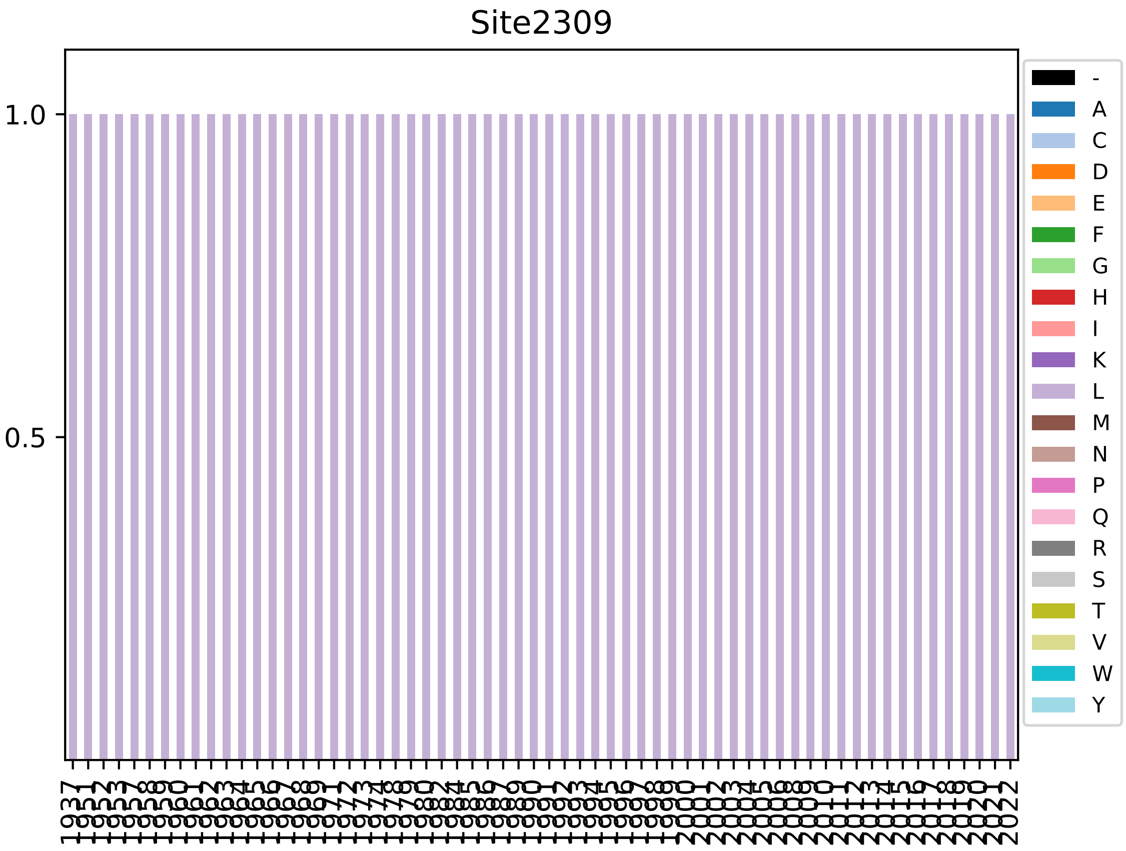
Site2309
1.0
0.5
-
A
C
D
E
F
G
H
I
K
L
M
N
P
Q
R
S
T
V
W
Y

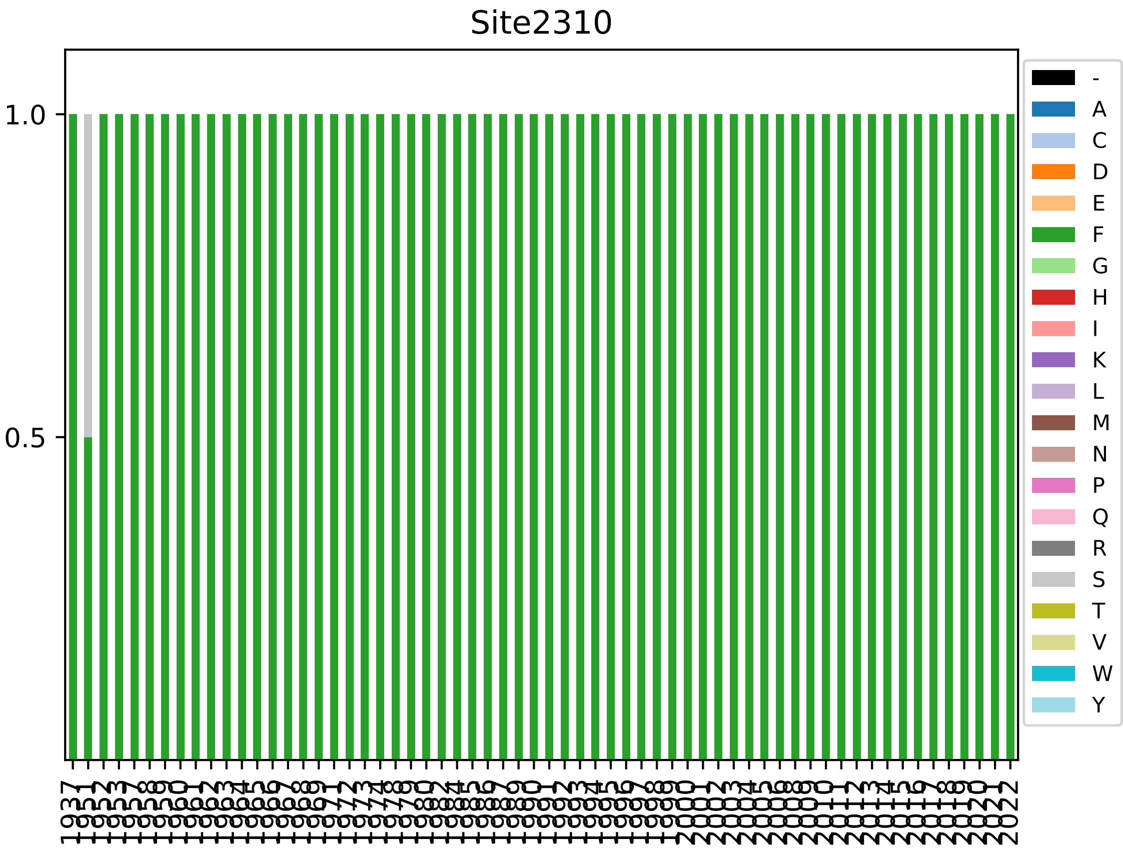
Site2310
1.0
0.5
-
A
C
D
E
F
G
H
I
K
L
M
N
P
Q
R
S
T
V
W
Y

Site2311

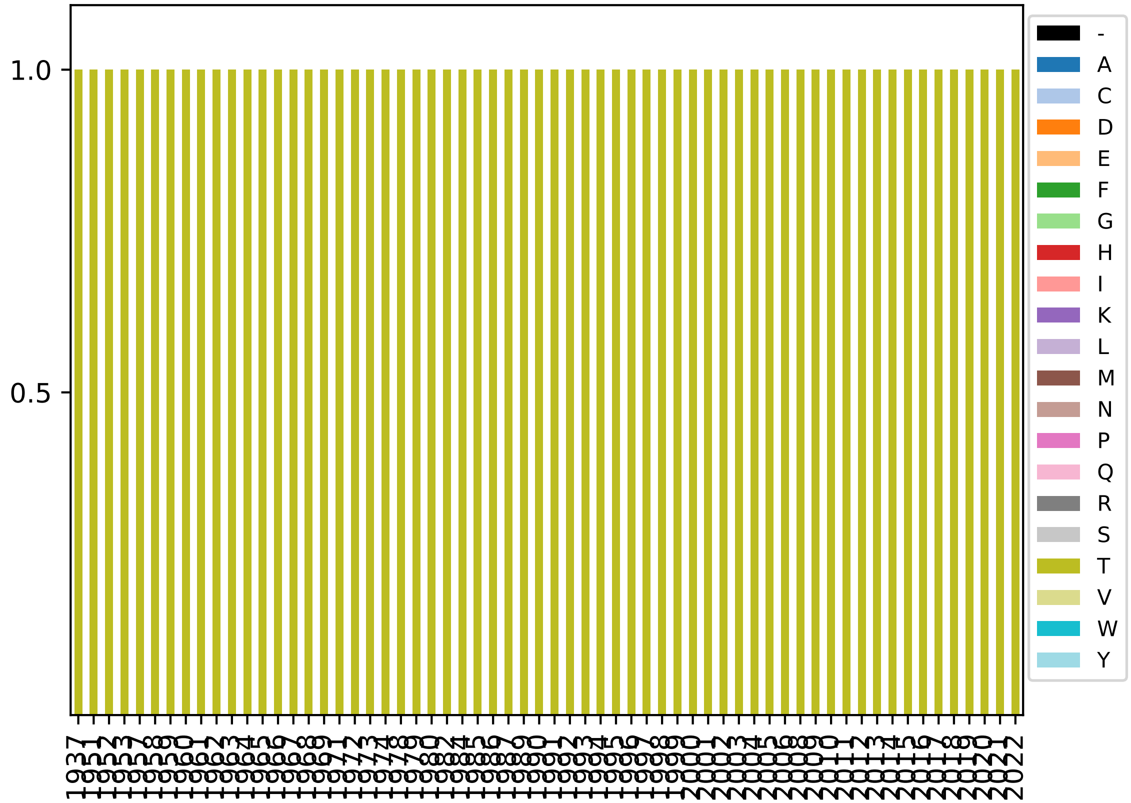

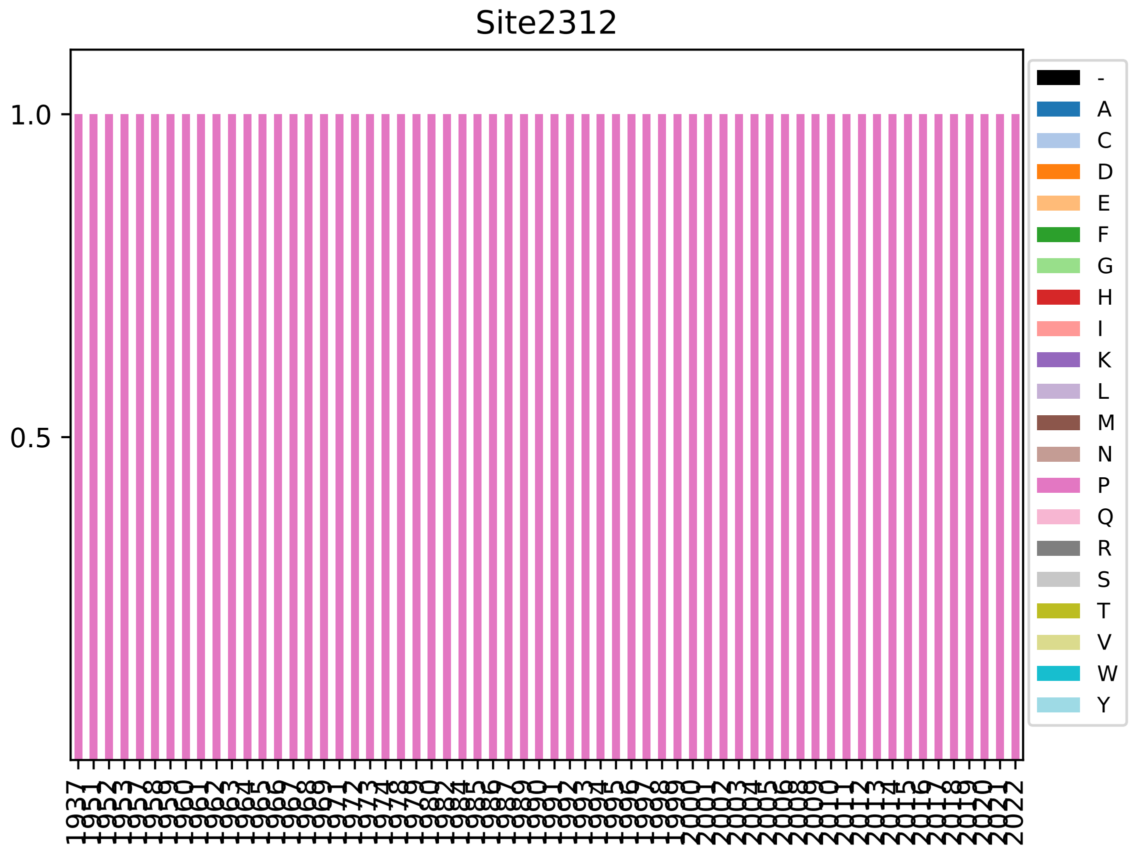

Site2312
1.0
0.5
-
A
C
D
E
F
G
H
I
K
L
M
N
P
Q
R
S
T
V
W
Y

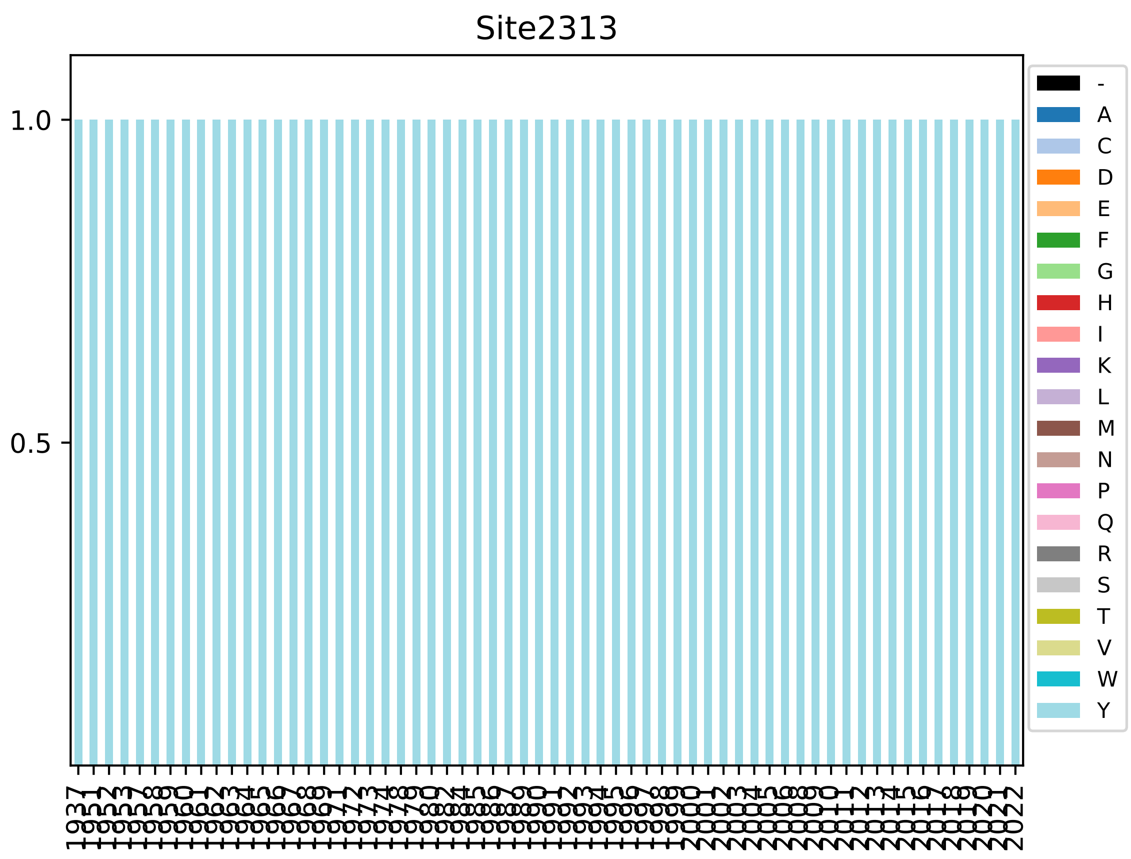
Site2313
1.0
0.5
-
A
C
D
E
F
G
H
I
K
L
M
N
P
Q
R
S
T
V
W
Y

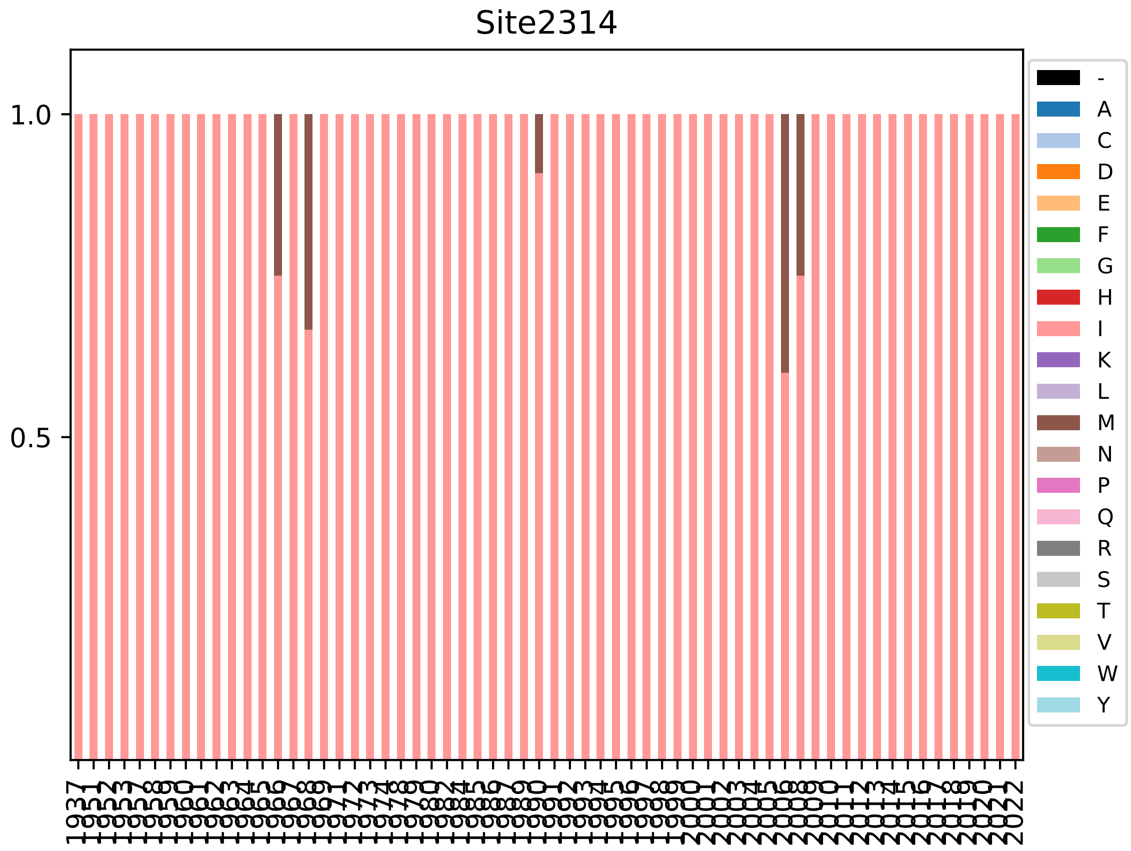
Site2314
1.0
0.5
-
A
C
D
E
F
G
H
I
K
L
M
N
P
Q
R
S
T
V
W
Y

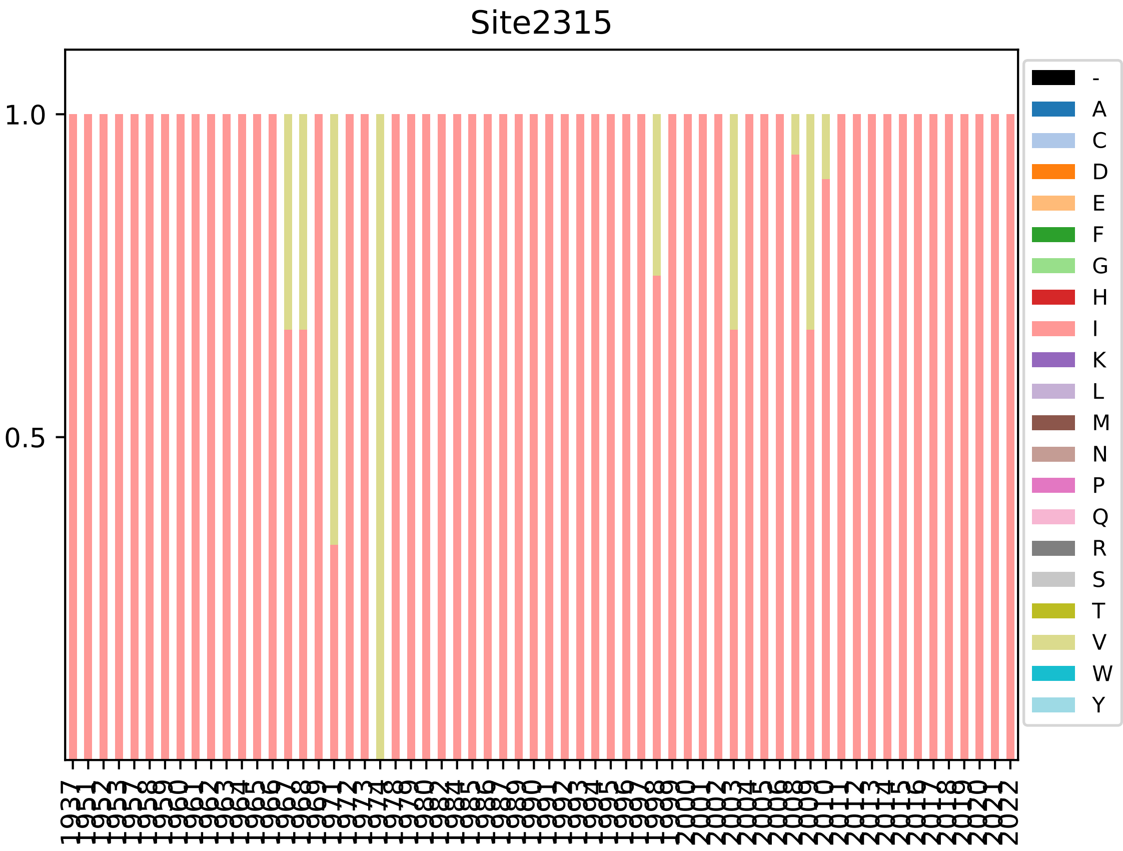
Site2315
1.0
0.5
-
A
C
D
E
F
G
H
I
K
L
M
N
P
Q
R
S
T
V
W
Y

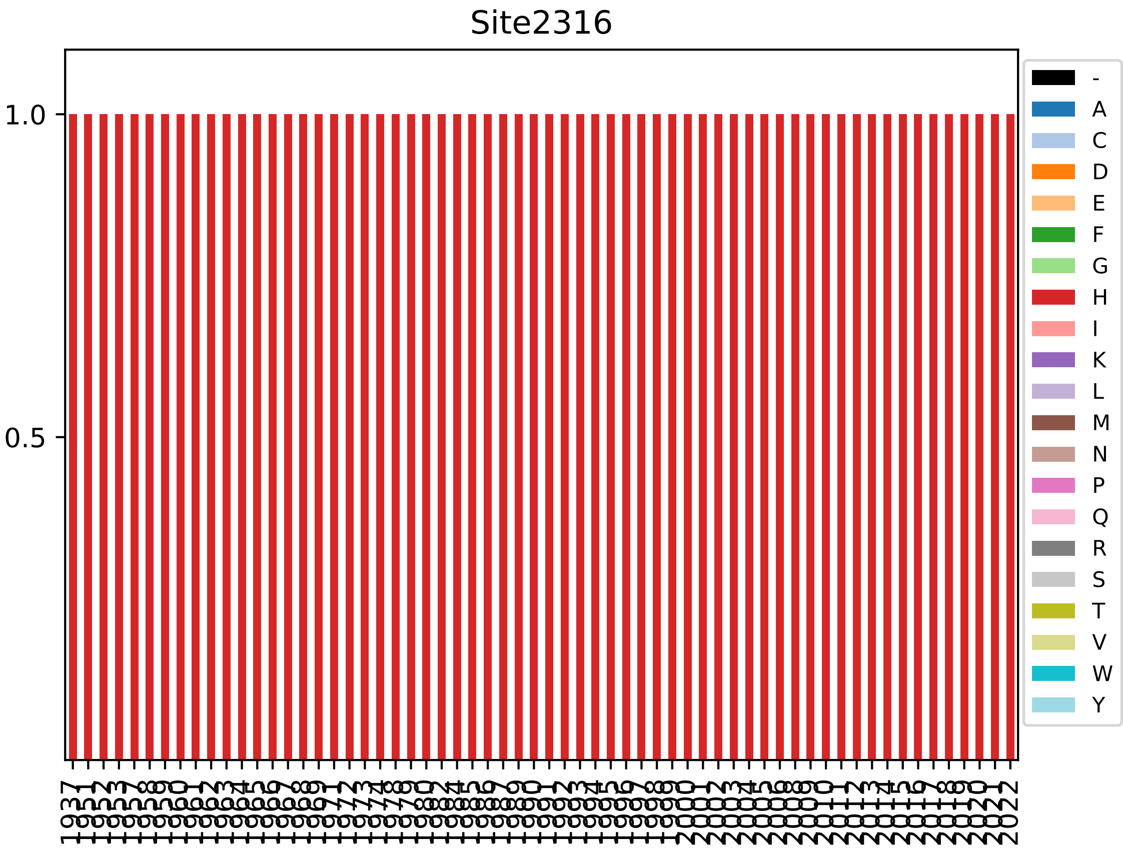
Site2316
1.0
0.5
-
A
C
D
E
F
G
H
I
K
L
M
N
P
Q
R
S
T
V
W
Y

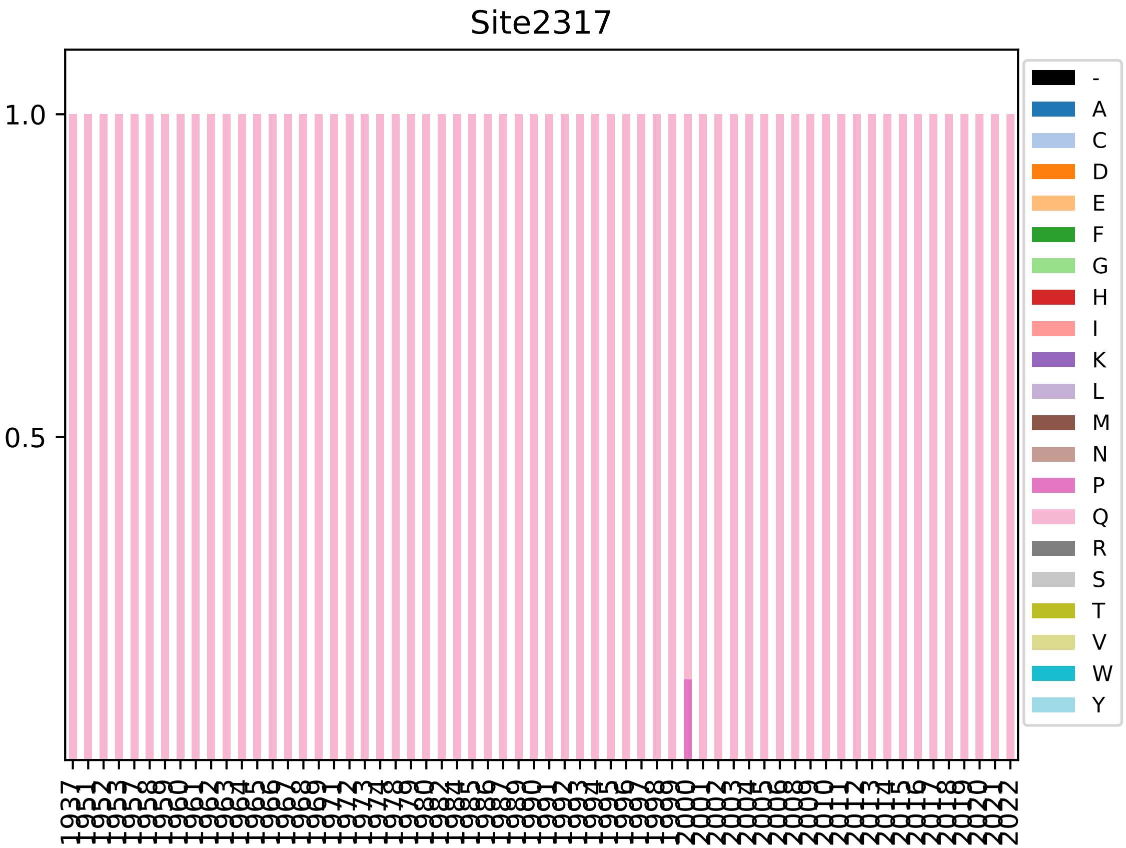

Site2317
1.0
0.5
-
A
C
D
E
F
G
H
I
K
L
M
N
P
Q
R
S
T
V
W
Y

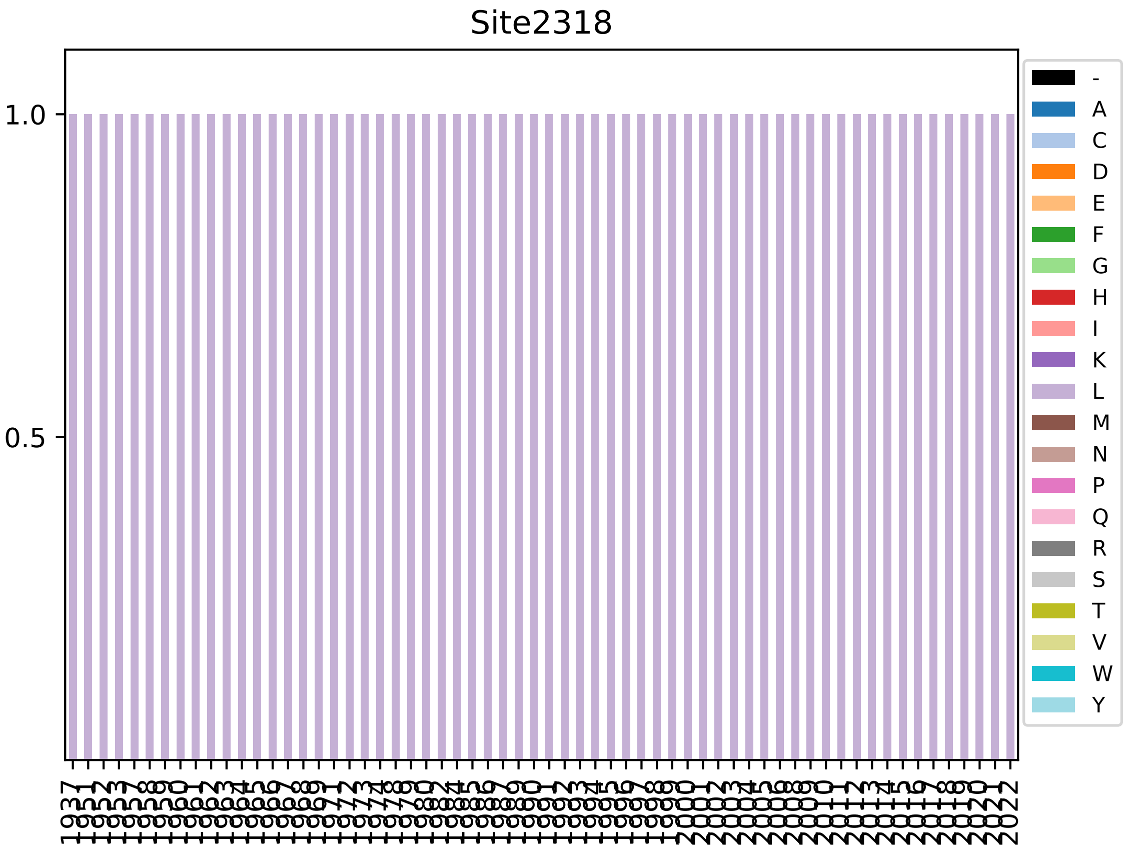
Site2318
1.0
0.5
-
A
C
D
E
F
G
H
I
K
L
M
N
P
Q
R
S
T
V
W
Y

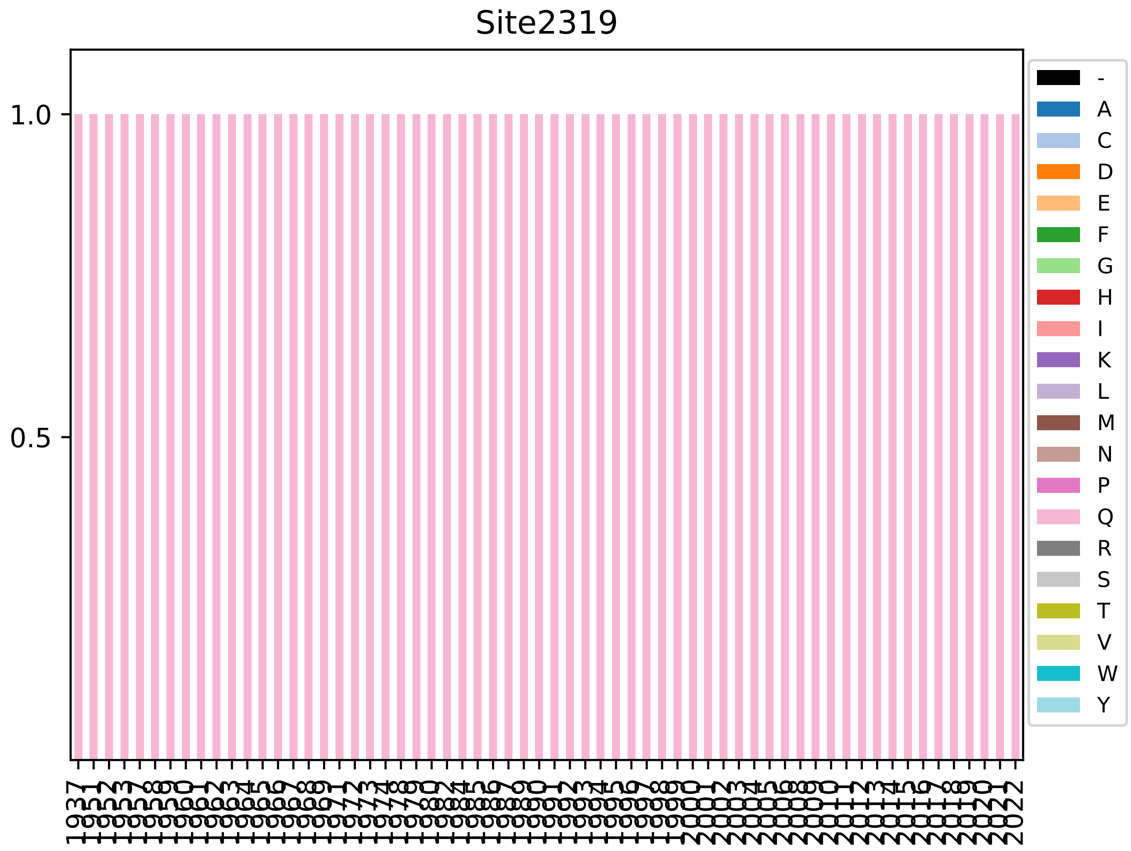

Site2319
1.0
0.5
-
A
C
D
E
F
G
H
I
K
L
M
N
P
Q
R
S
T
V
W
Y

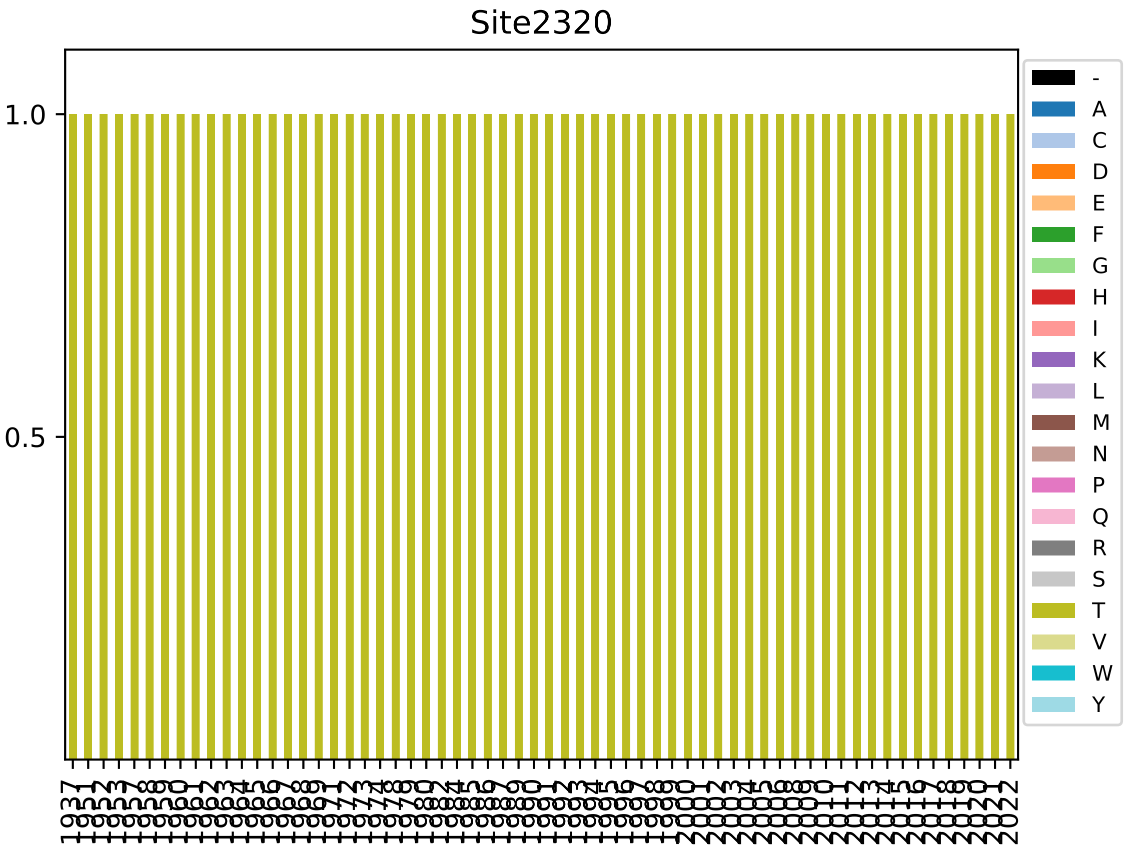
Site2320
1.0
0.5
-
A
C
D
E
F
G
H
I
K
L
M
N
P
Q
R
S
T
V
W
Y

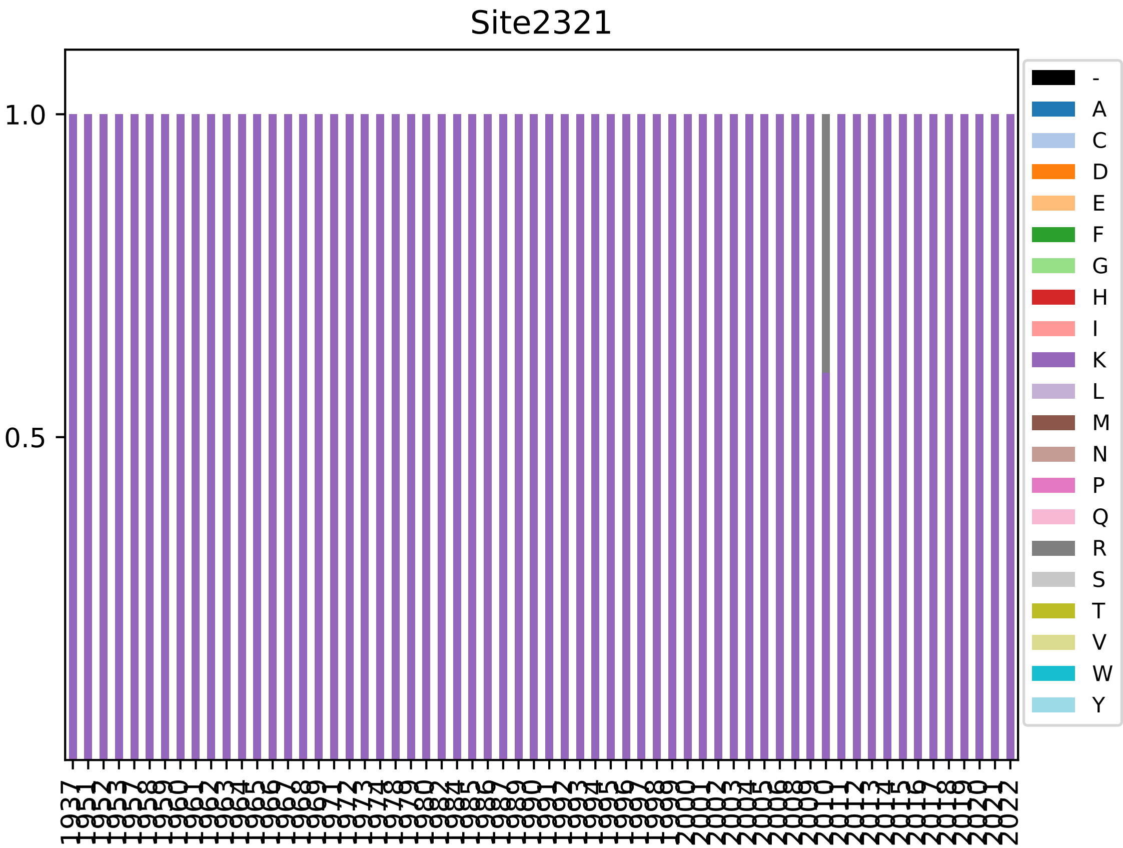

Site2321
1.0
0.5
-
A
C
D
E
F
G
H
I
K
L
M
N
P
Q
R
S
T
V
W
Y

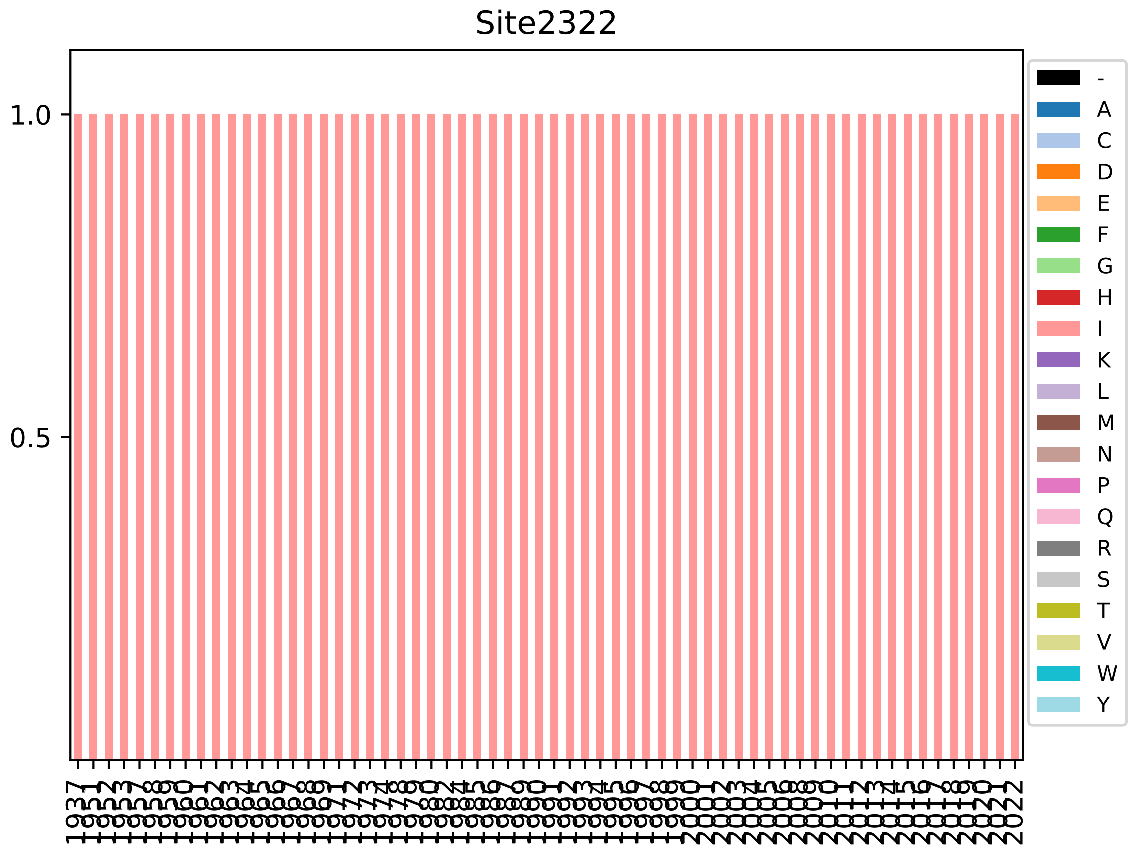
Site2322
1.0
0.5
-
A
C
D
E
F
G
H
I
K
L
M
N
P
Q
R
S
T
V
W
Y

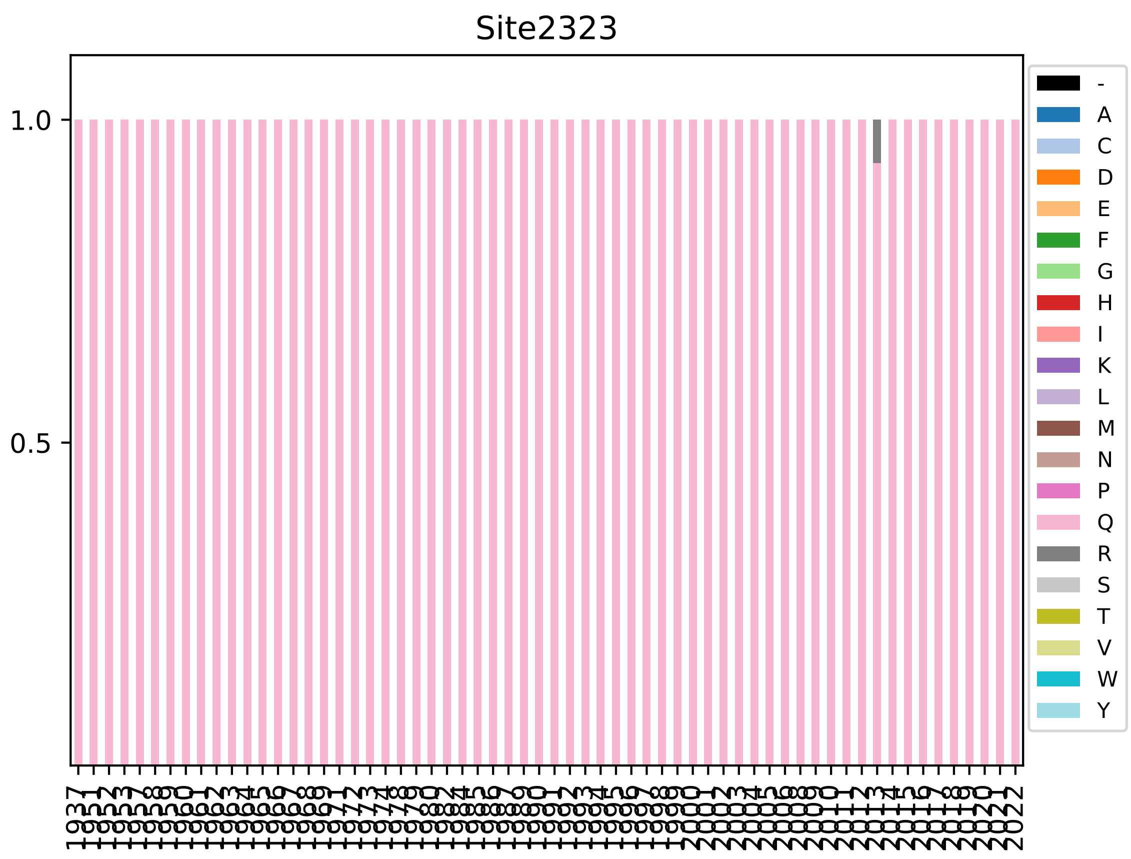
Site2323
1.0
0.5
-
A
C
D
E
F
G
H
I
K
L
M
N
P
Q
R
S
T
V
W
Y

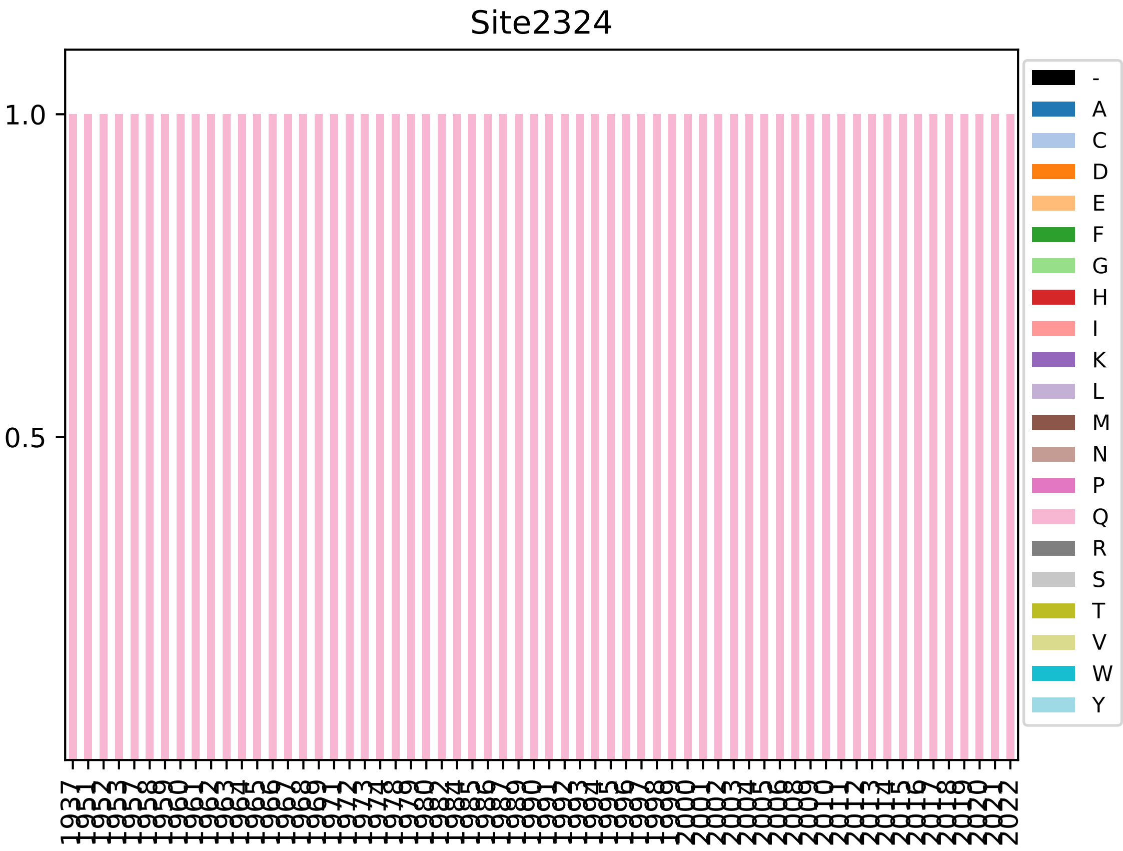

Site2324
1.0
0.5
-
A
C
D
E
F
G
H
I
K
L
M
N
P
Q
R
S
T
V
W
Y

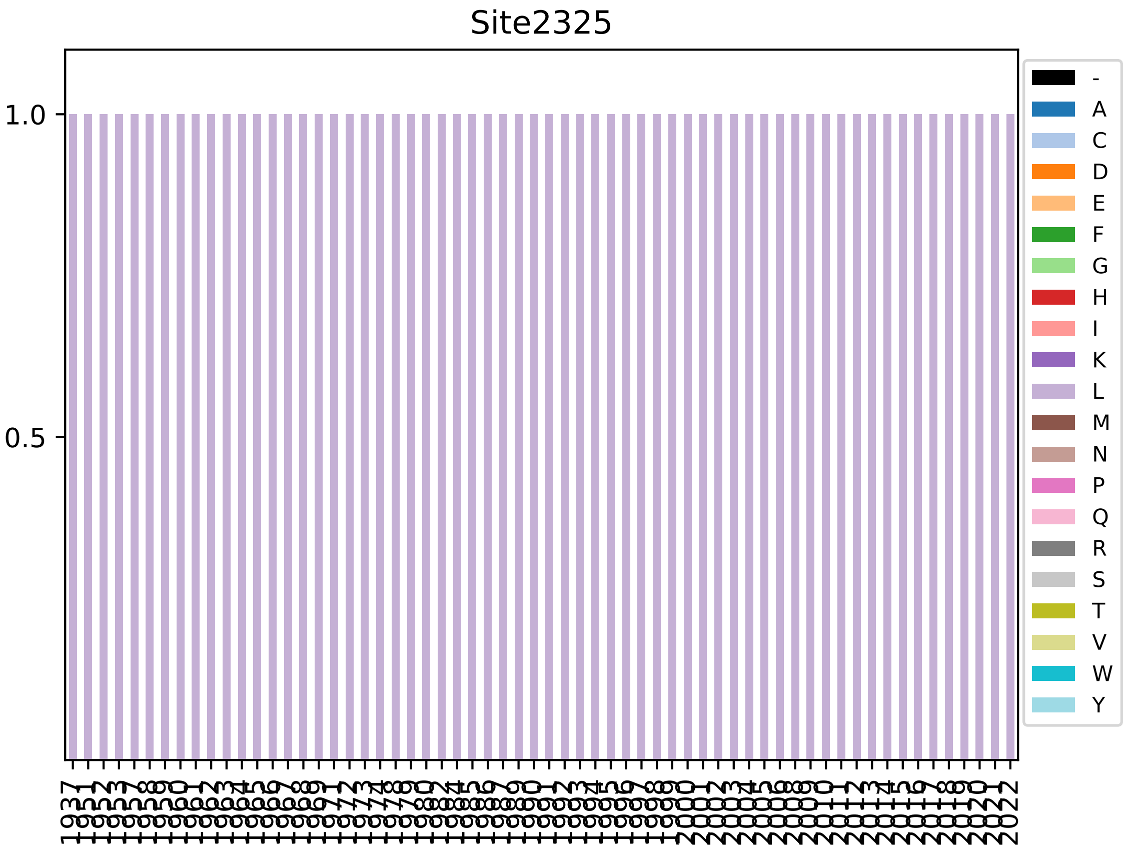
Site2325
1.0
0.5
-
A
C
D
E
F
G
H
I
K
L
M
N
P
Q
R
S
T
V
W
Y

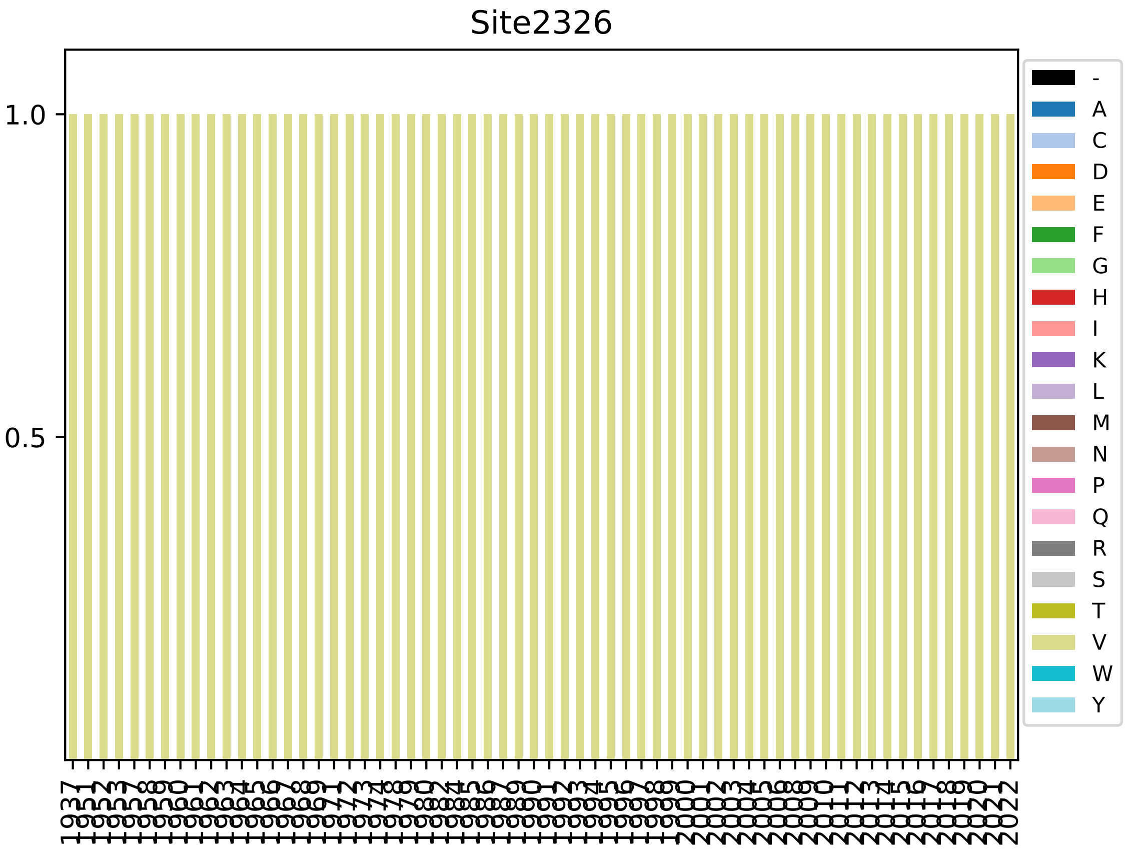
Site2326
1.0
0.5
-
A
C
D
E
F
G
H
I
K
L
M
N
P
Q
R
S
T
V
W
Y

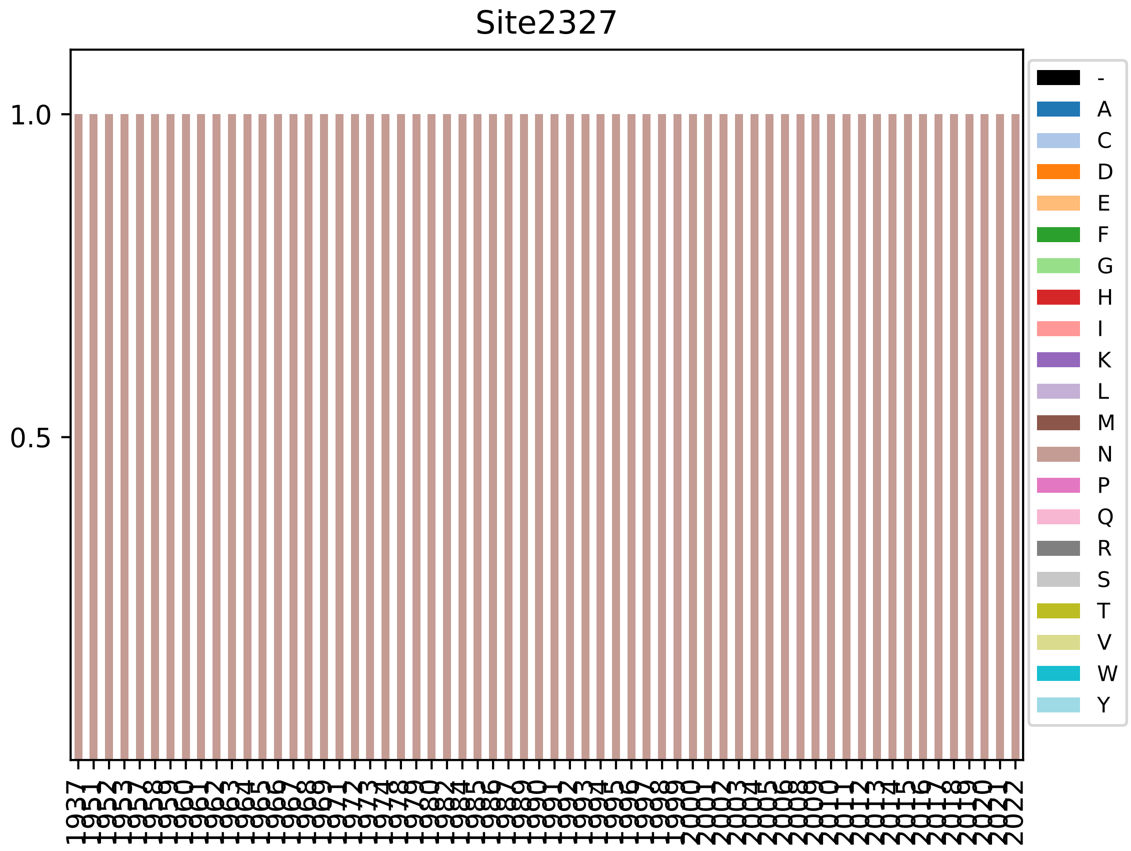
Site2327
1.0
0.5
-
A
C
D
E
F
G
H
I
K
L
M
N
P
Q
R
S
T
V
W
Y

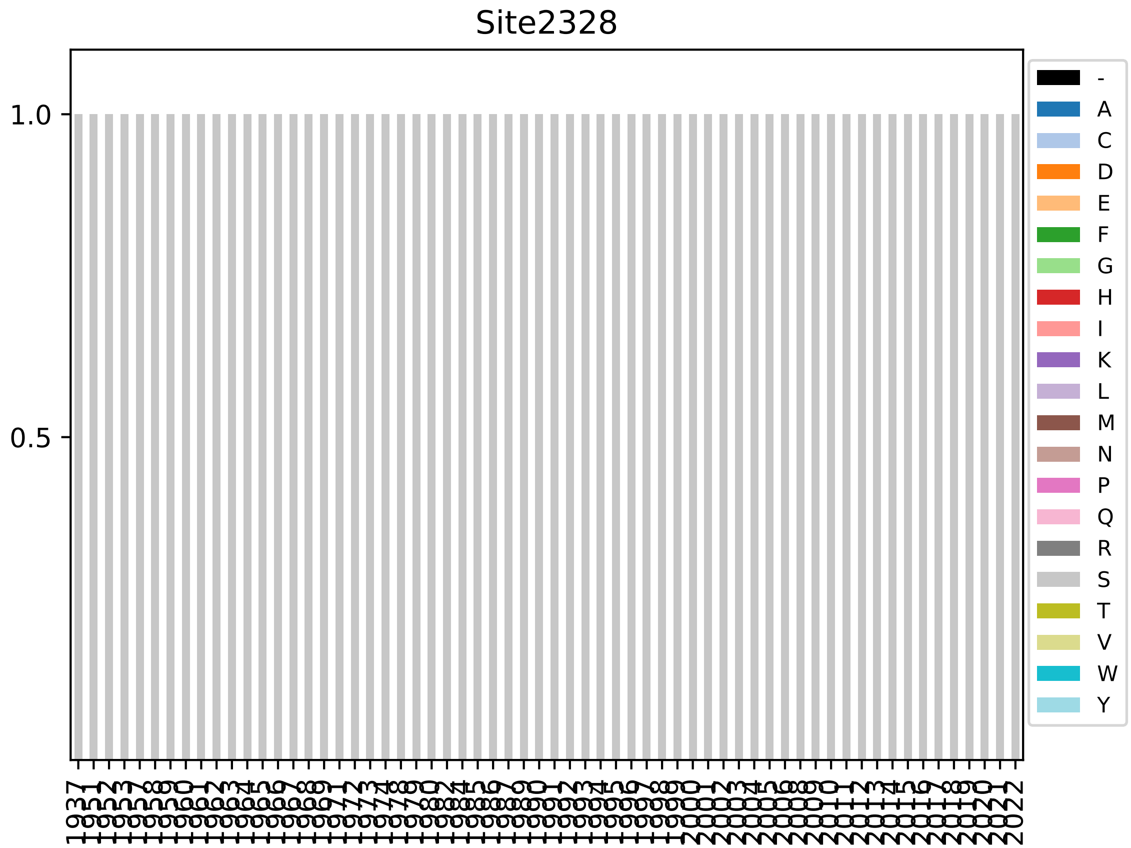
Site2328
1.0
0.5
-
A
C
D
E
F
G
H
I
K
L
M
N
P
Q
R
S
T
V
W
Y

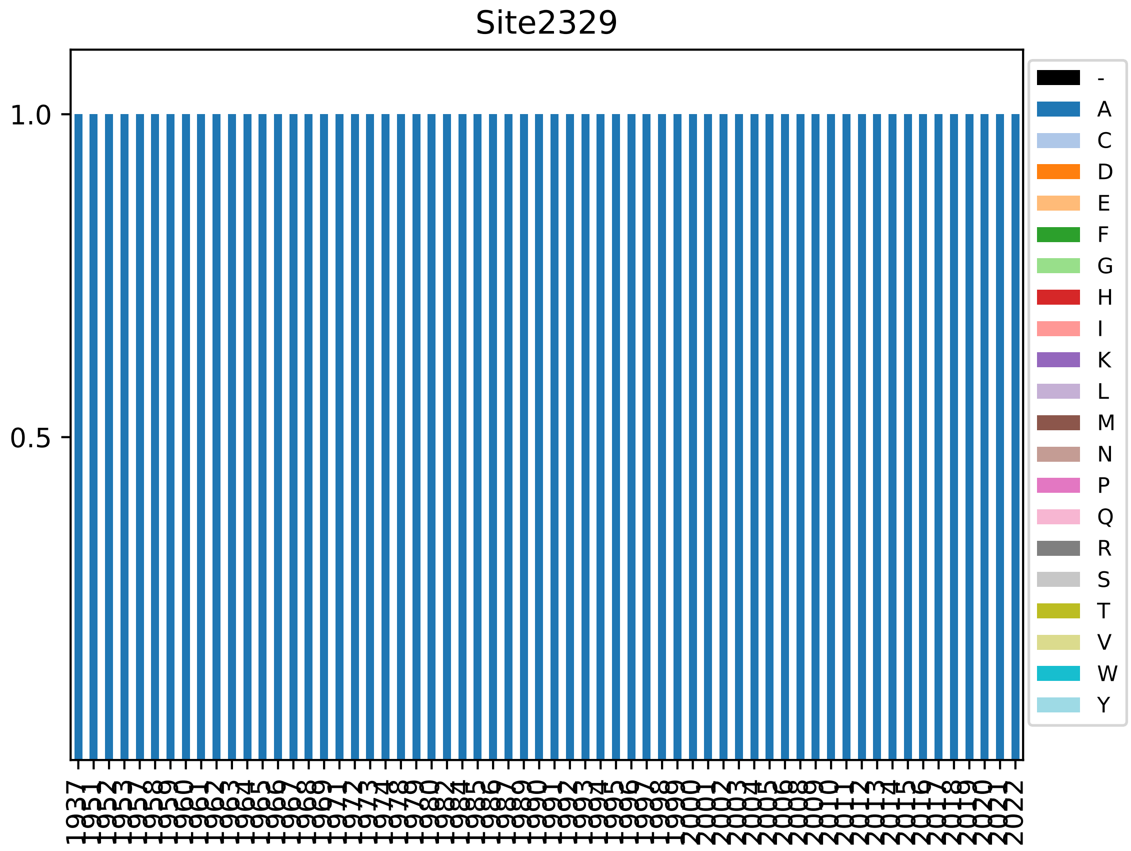

Site2329
1.0
0.5
-
A
C
D
E
F
G
H
I
K
L
M
N
P
Q
R
S
T
V
W
Y

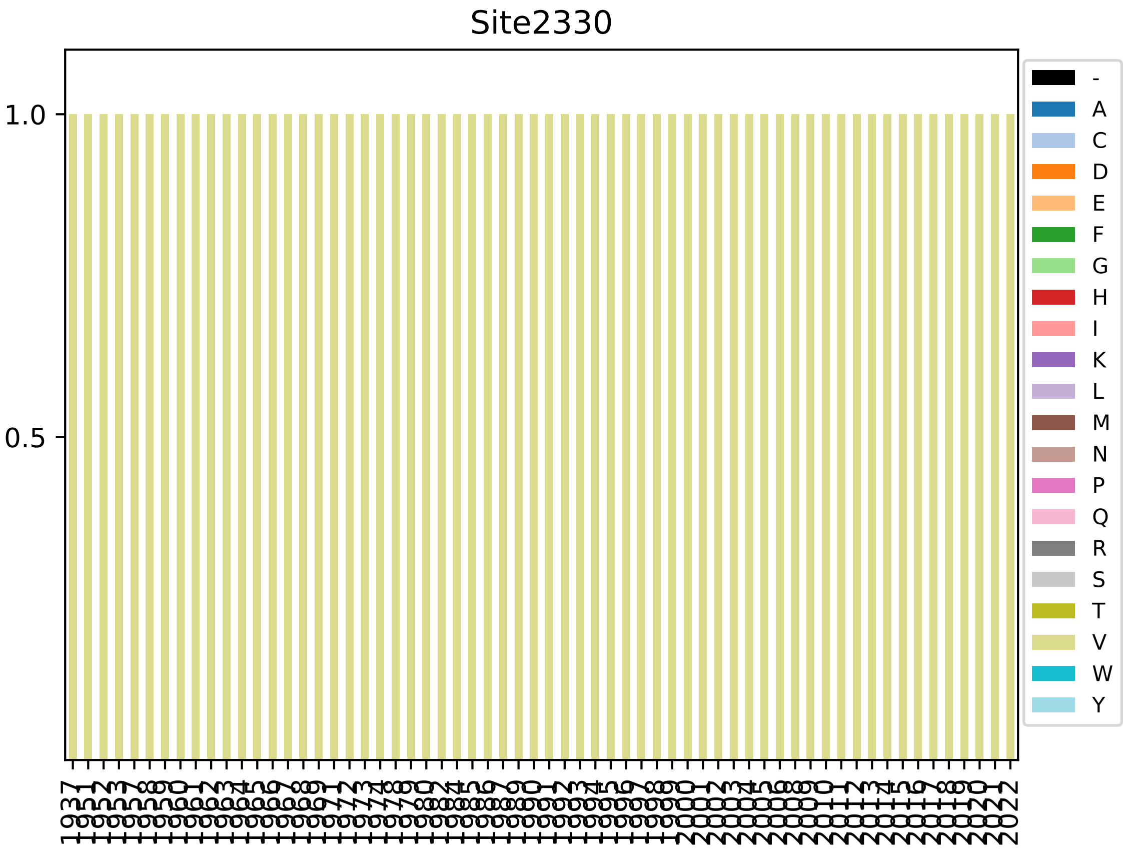
Site2330
1.0
0.5
-
A
C
D
E
F
G
H
I
K
L
M
N
P
Q
R
S
T
V
W
Y

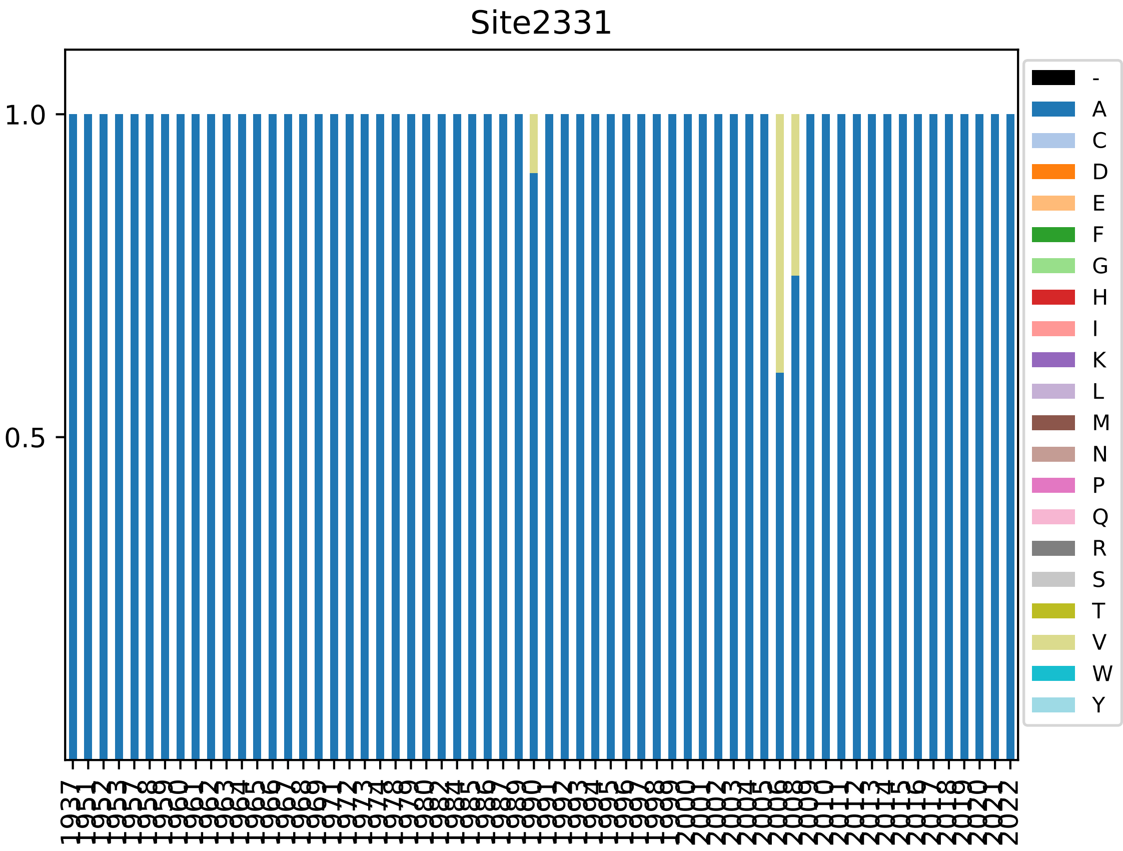
Site2331
1.0
0.5
-
A
C
D
E
F
G
H
I
K
L
M
N
P
Q
R
S
T
V
W
Y

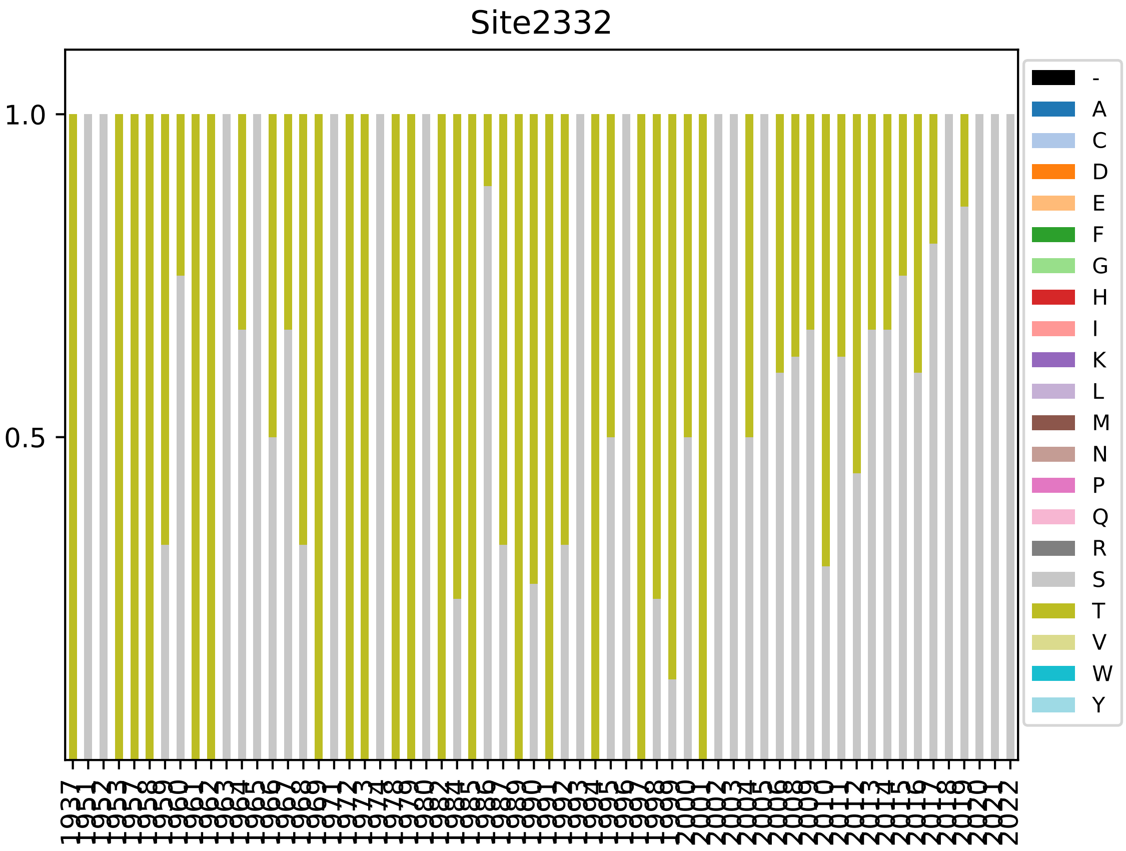
Site2332
1.0
0.5
-
A
C
D
E
F
G
H
I
K
L
M
N
P
Q
R
S
T
V
W
Y

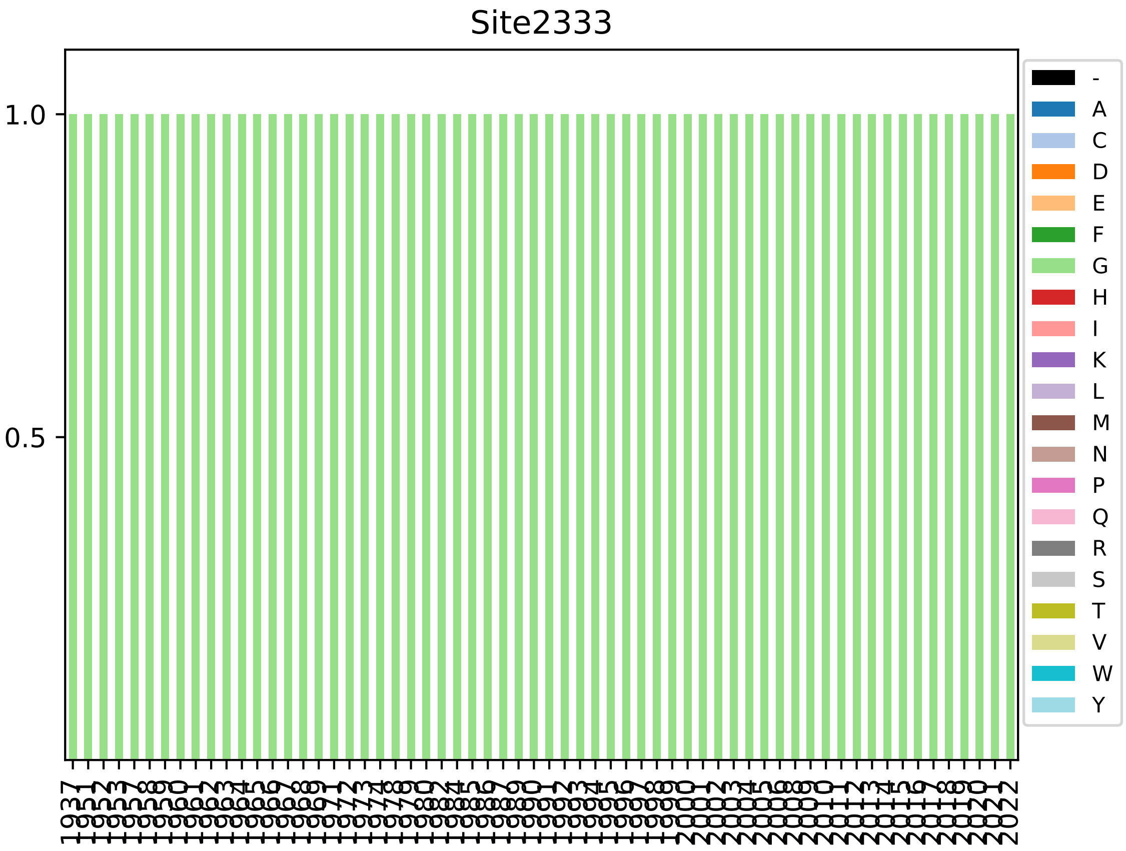
Site2333
1.0
0.5
-
A
C
D
E
F
G
H
I
K
L
M
N
P
Q
R
S
T
V
W
Y

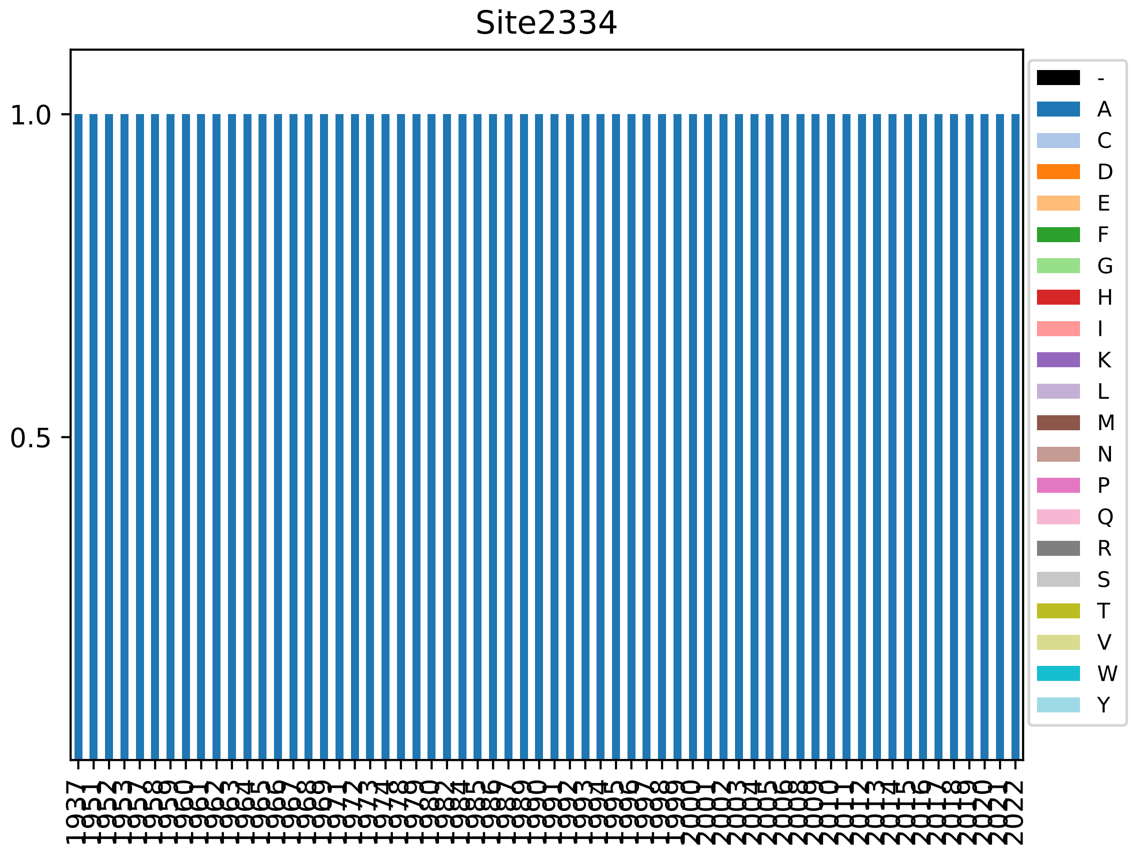
Site2334
1.0
0.5
-
A
C
D
E
F
G
H
I
K
L
M
N
P
Q
R
S
T
V
W
Y

Site2335

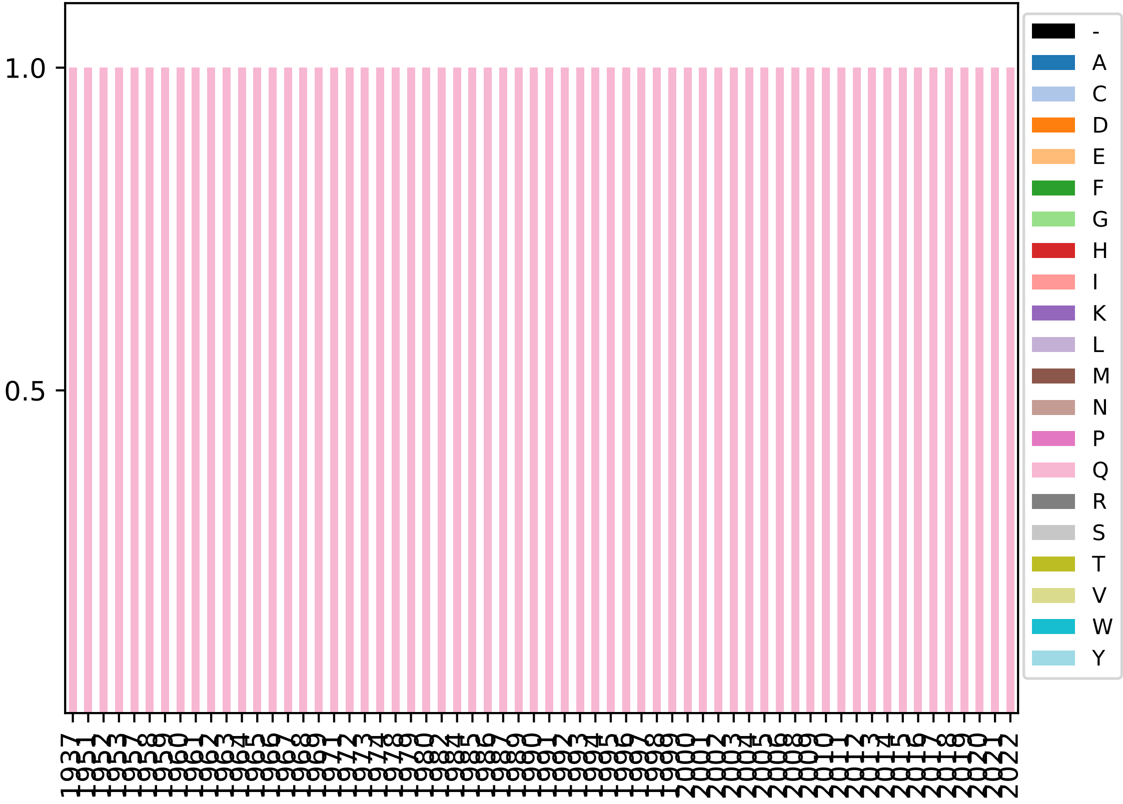

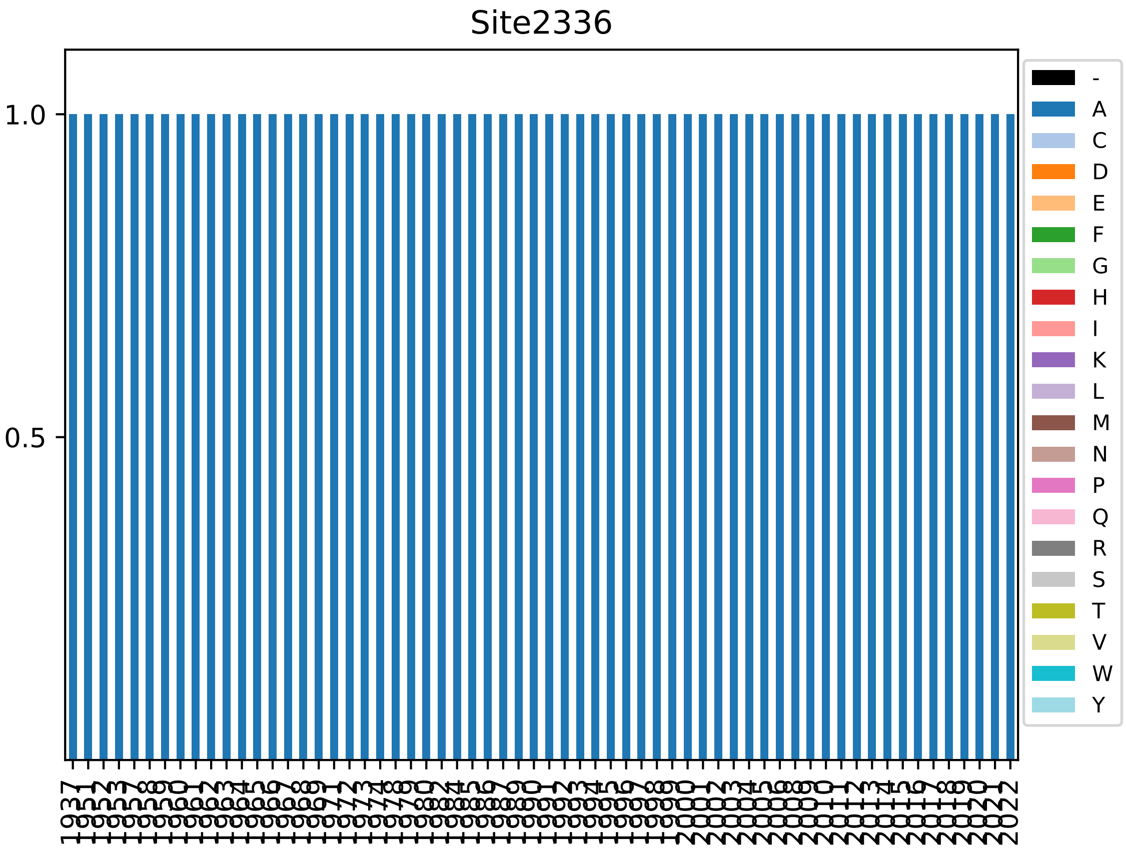
Site2336
1.0
0.5
-
A
C
D
E
F
G
H
I
K
L
M
N
P
Q
R
S
T
V
W
Y

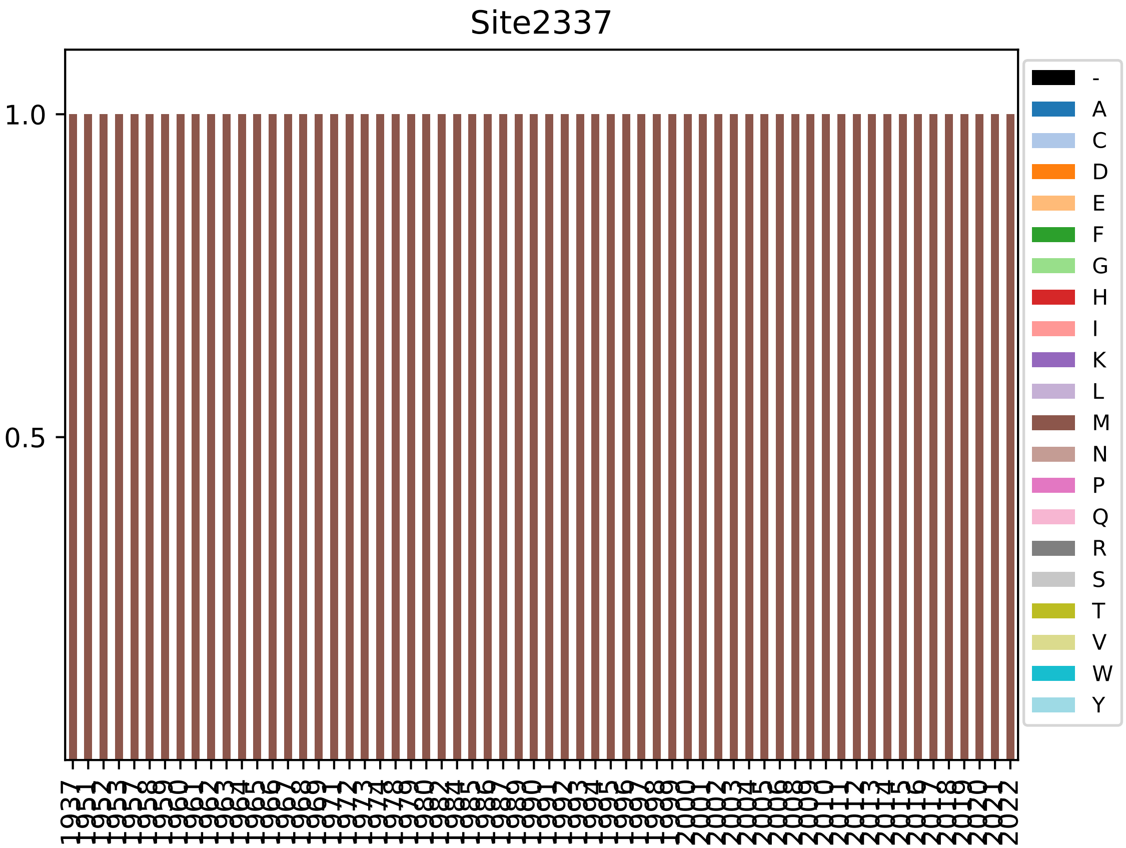

Site2337
1.0
0.5
-
A
C
D
E
F
G
H
I
K
L
M
N
P
Q
R
S
T
V
W
Y

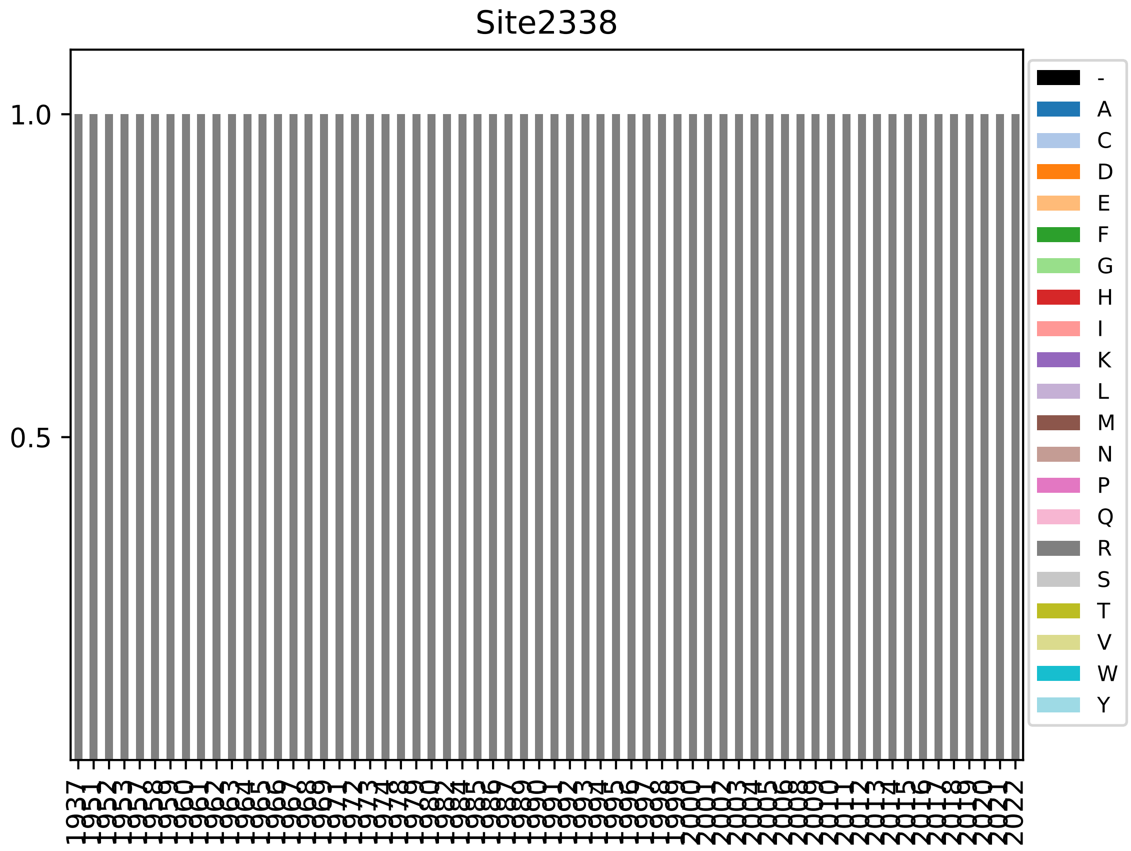
Site2338
1.0
0.5
-
A
C
D
E
F
G
H
I
K
L
M
N
P
Q
R
S
T
V
W
Y

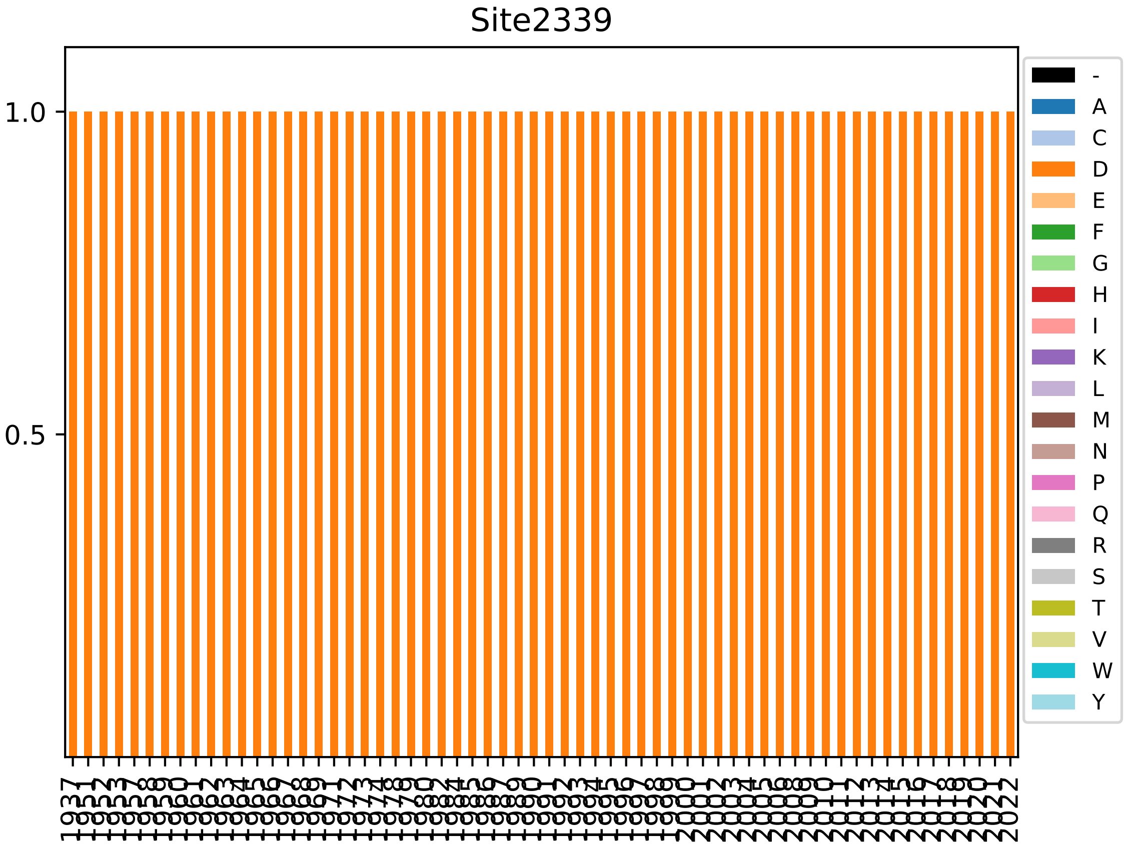
Site2339
1.0
0.5
-
A
C
D
E
F
G
H
I
K
L
M
N
P
Q
R
S
T
V
W
Y

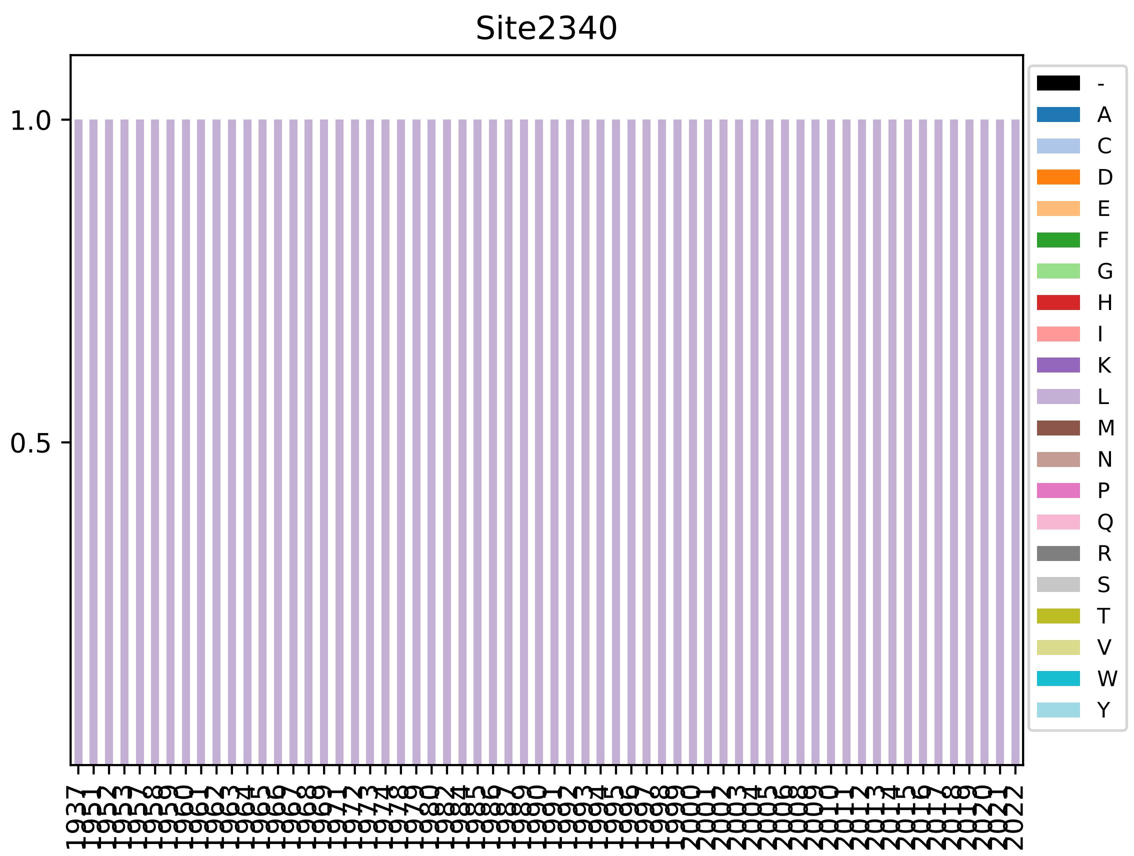
Site2340
1.0
0.5
-
A
C
D
E
F
G
H
I
K
L
M
N
P
Q
R
S
T
V
W
Y

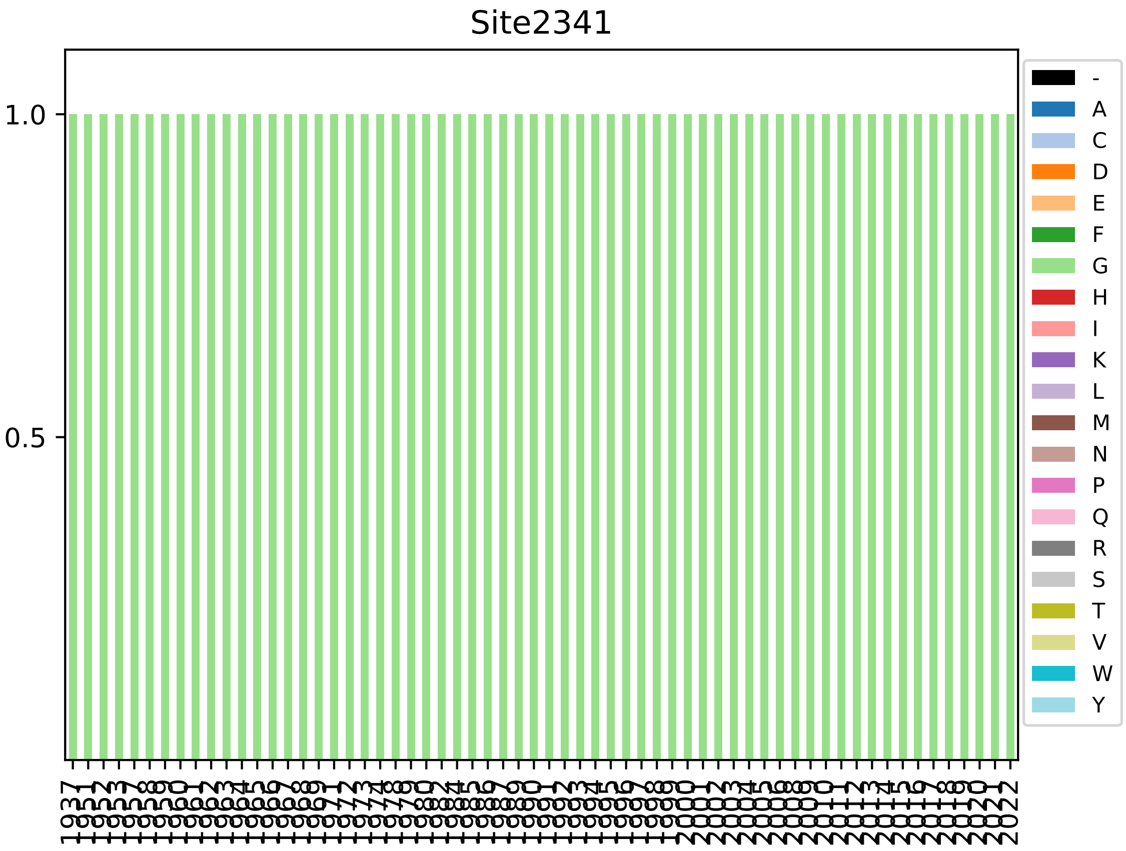
Site2341
1.0
0.5
-
A
C
D
E
F
G
H
I
K
L
M
N
P
Q
R
S
T
V
W
Y

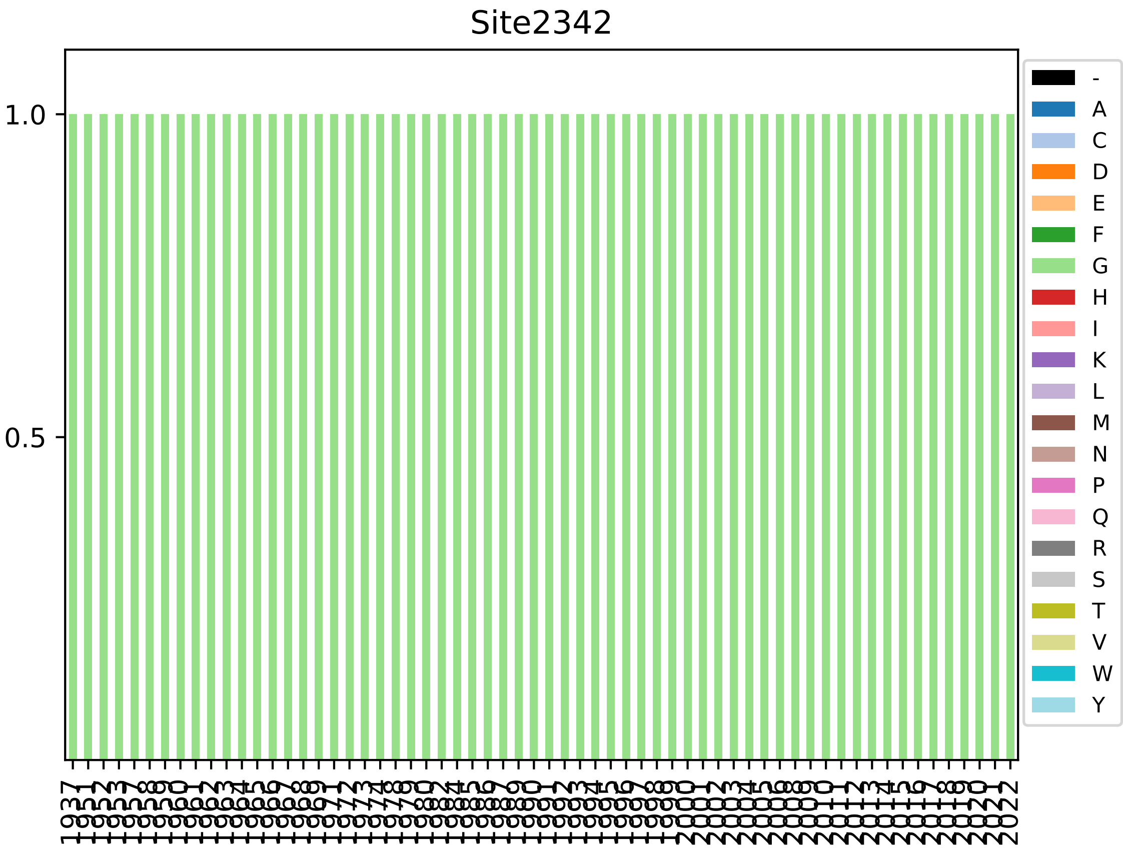
Site2342
1.0
0.5
-
A
C
D
E
F
G
H
I
K
L
M
N
P
Q
R
S
T
V
W
Y

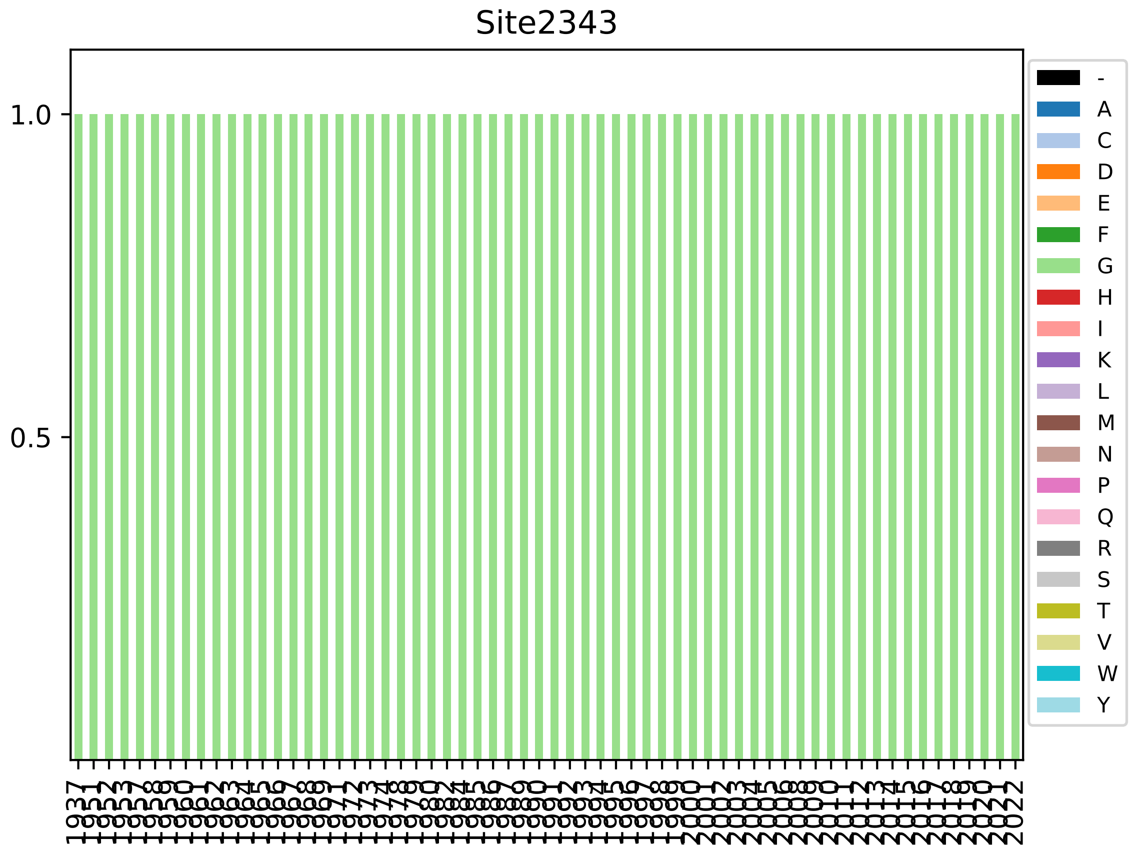
Site2343
1.0
0.5
-
A
C
D
E
F
G
H
I
K
L
M
N
P
Q
R
S
T
V
W
Y

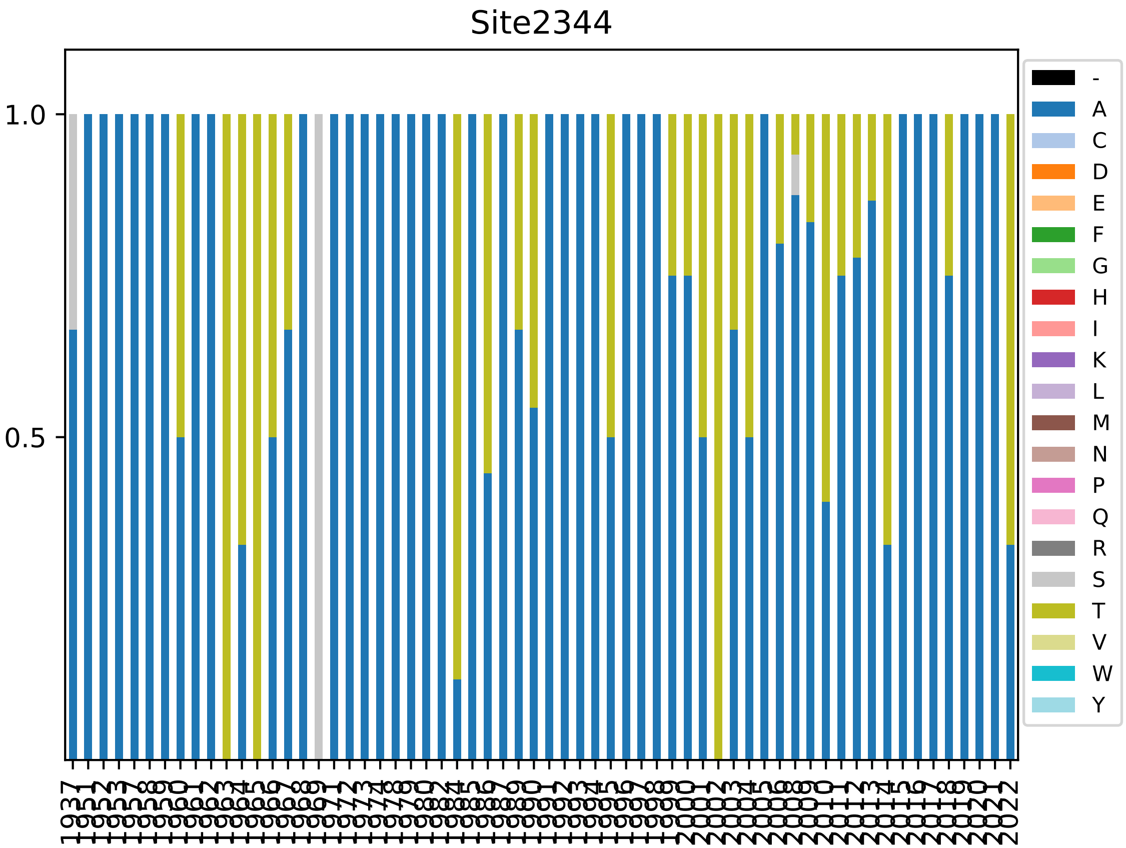
Site2344
1.0
0.5
-
A
C
D
E
F
G
H
I
K
L
M
N
P
Q
R
S
T
V
W
Y

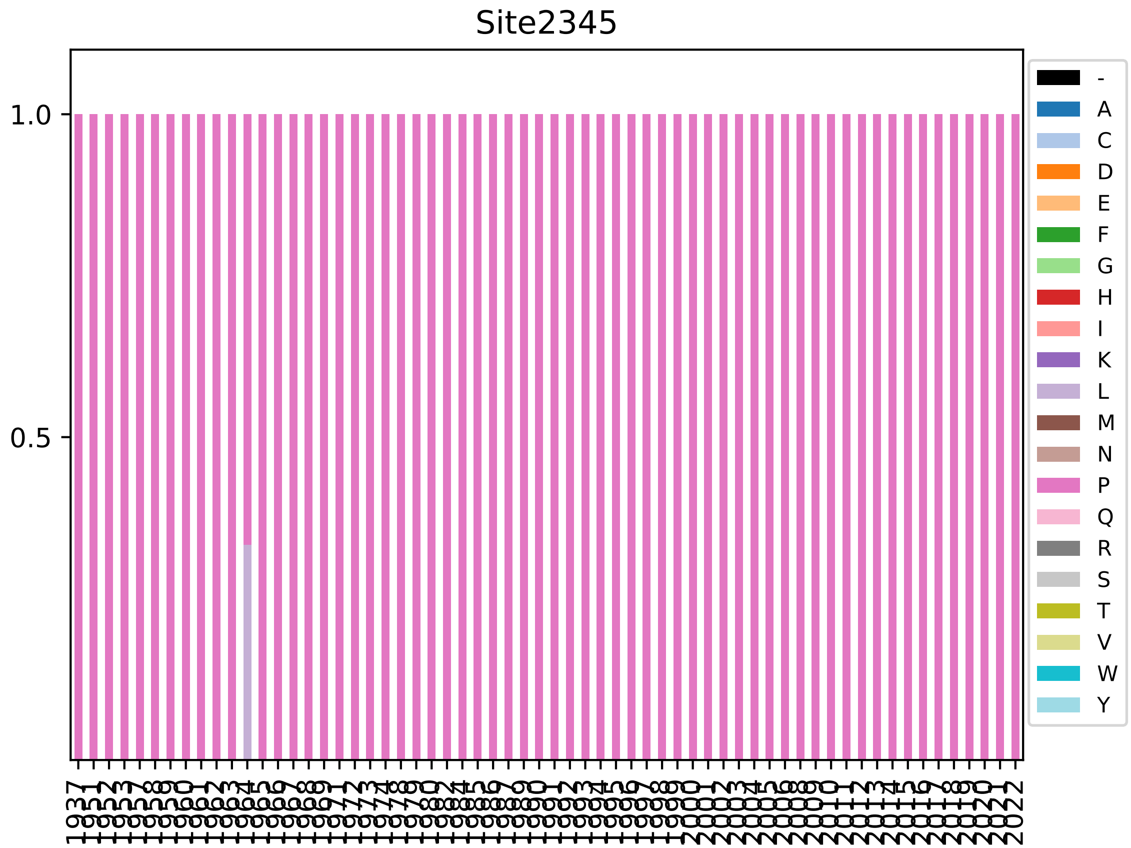
Site2345
1.0
0.5
-
A
C
D
E
F
G
H
I
K
L
M
N
P
Q
R
S
T
V
W
Y

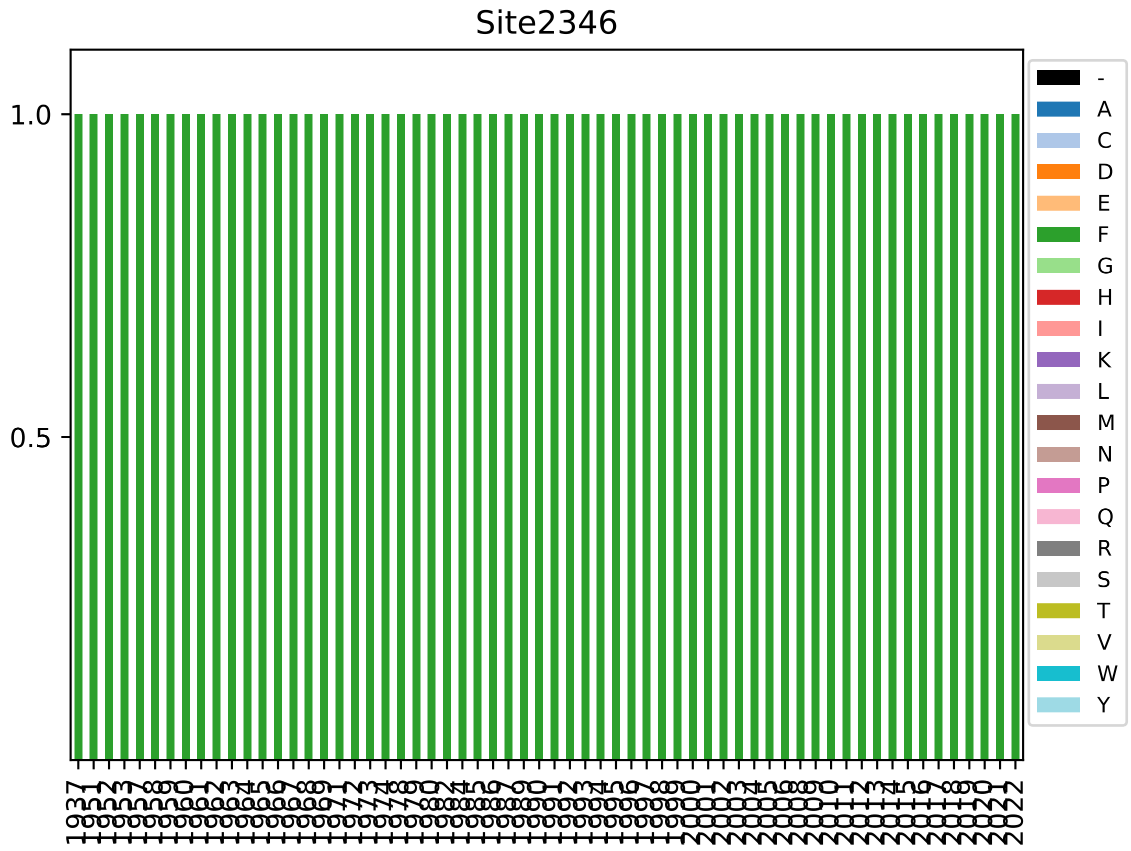
Site2346
1.0
0.5
-
A
C
D
E
F
G
H
I
K
L
M
N
P
Q
R
S
T
V
W
Y

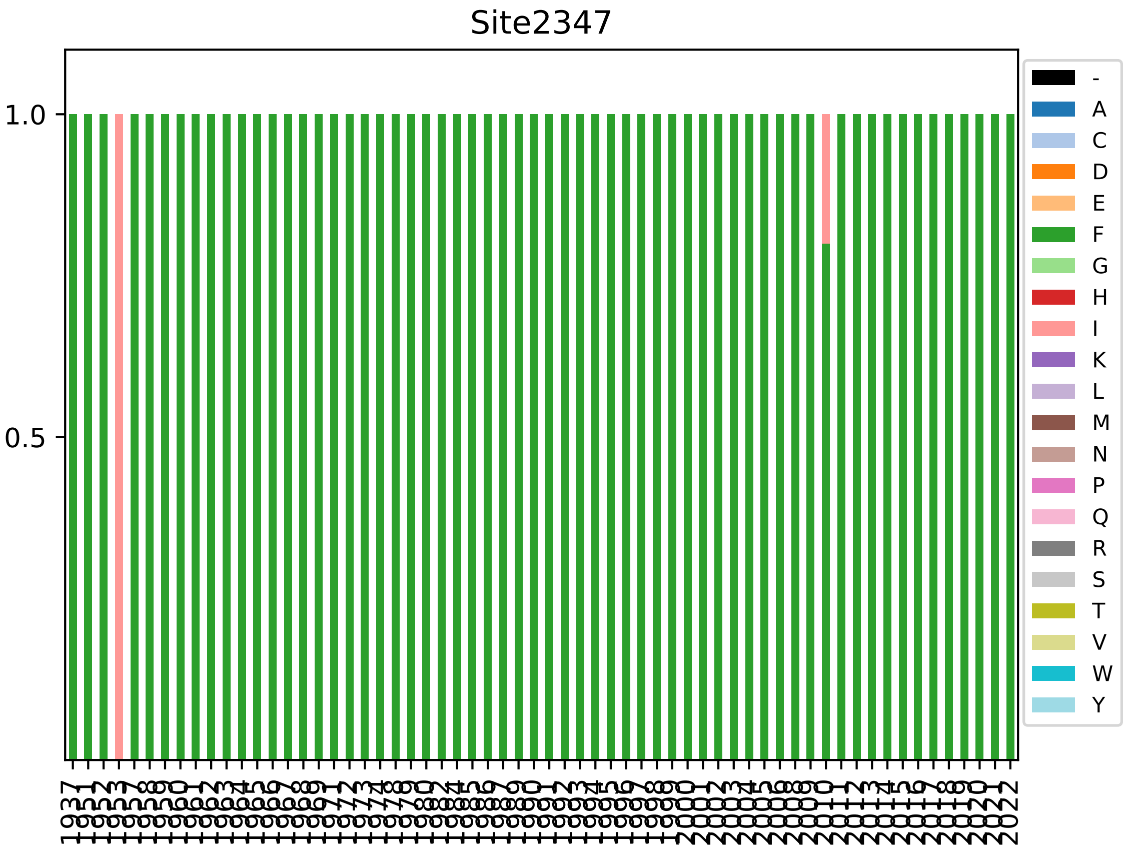
Site2347
1.0
0.5
-
A
C
D
E
F
G
H
I
K
L
M
N
P
Q
R
S
T
V
W
Y

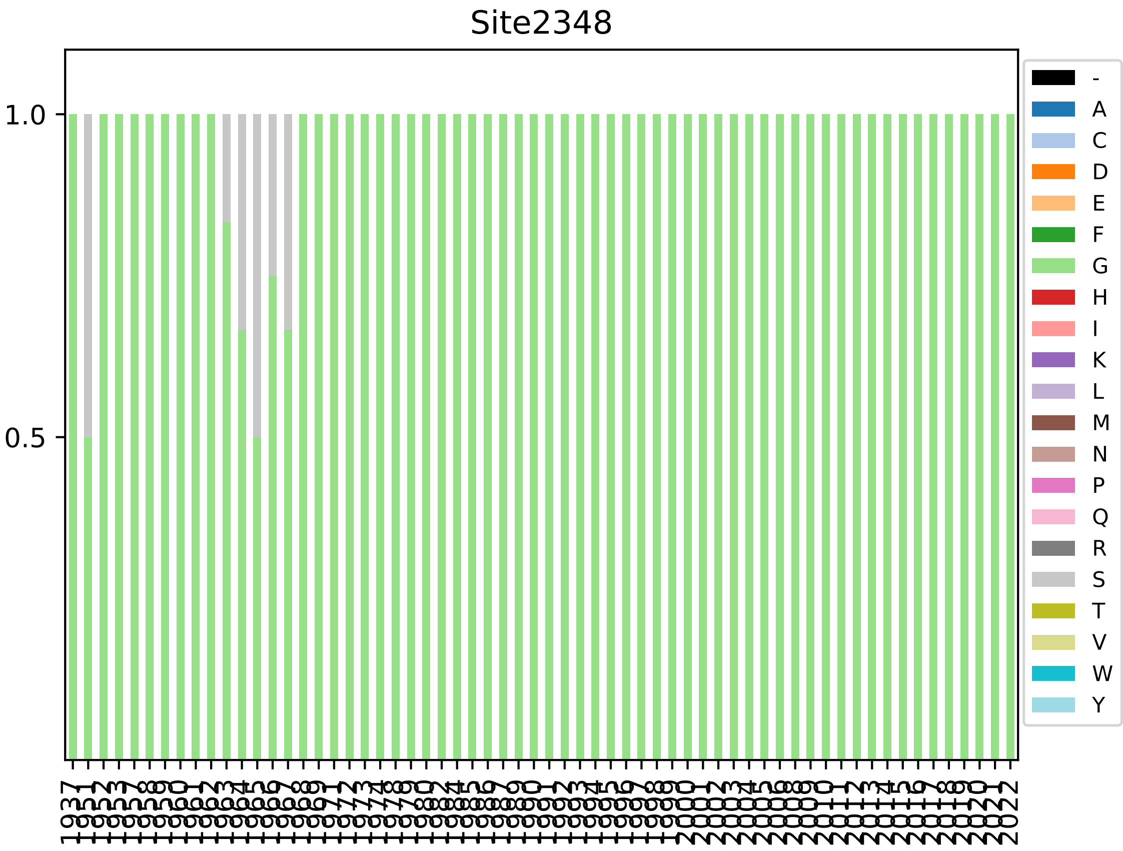
Site2348
1.0
0.5
-
A
C
D
E
F
G
H
I
K
L
M
N
P
Q
R
S
T
V
W
Y

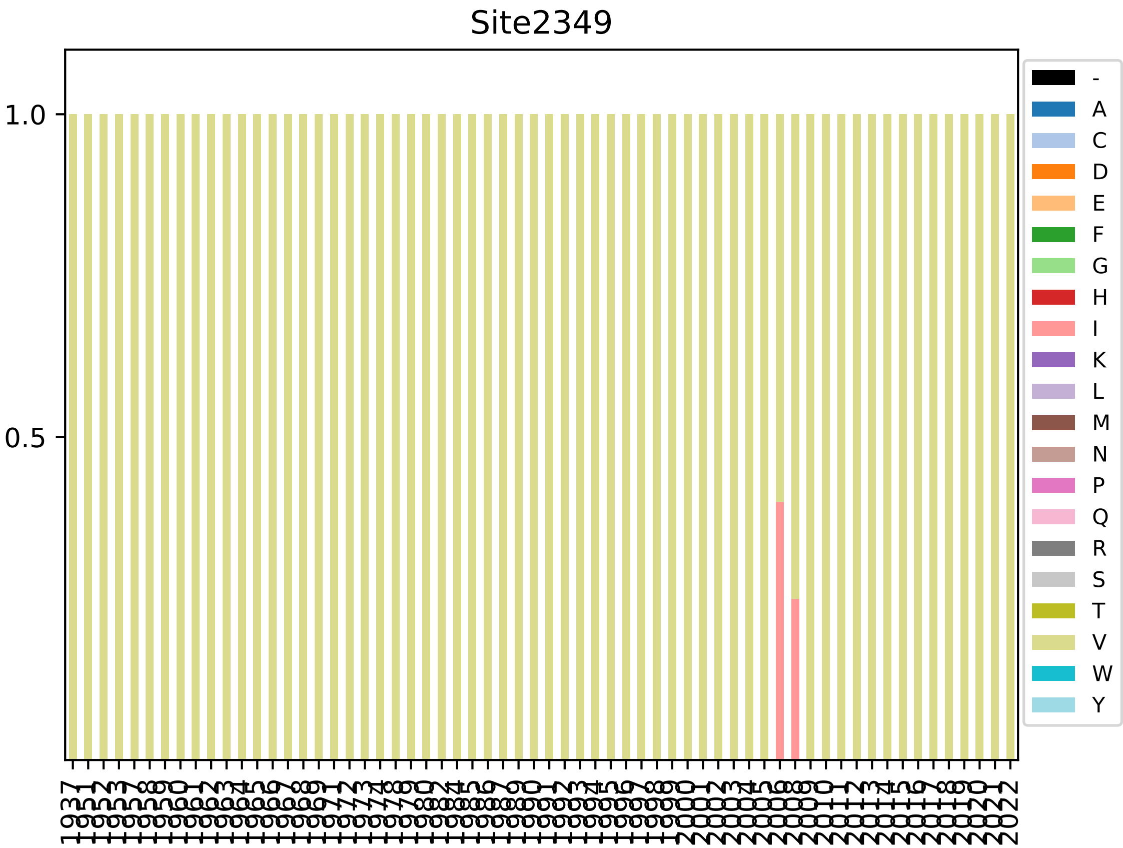
Site2349
1.0
0.5
-
A
C
D
E
F
G
H
I
K
L
M
N
P
Q
R
S
T
V
W
Y

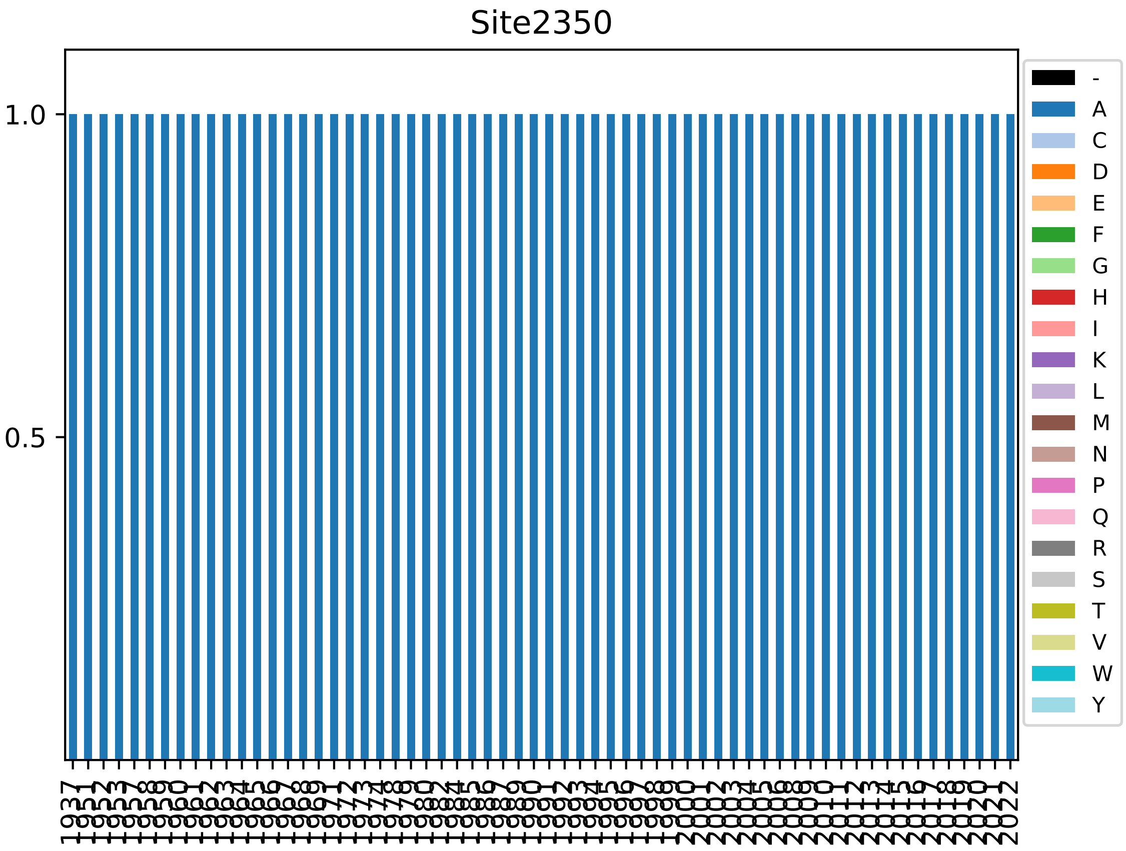
Site2350
1.0
0.5
-
A
C
D
E
F
G
H
I
K
L
M
N
P
Q
R
S
T
V
W
Y

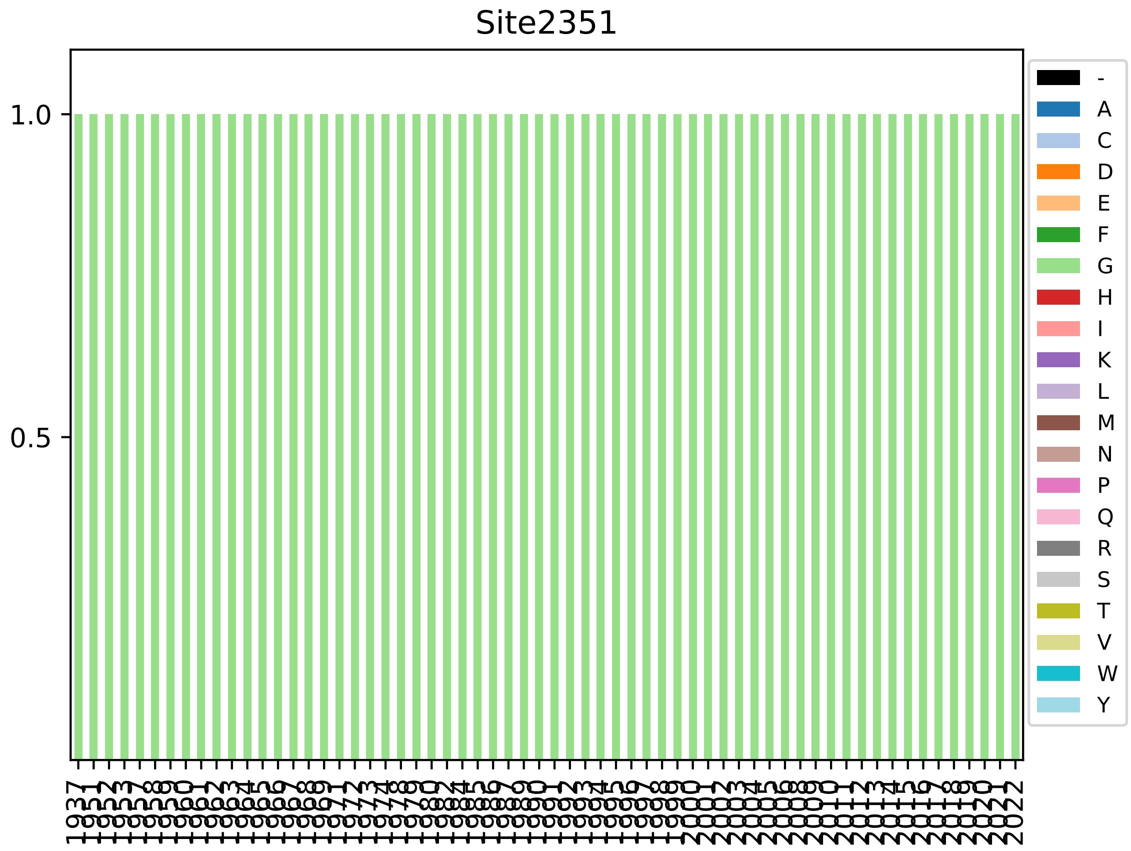
Site2351
1.0
0.5
-
A
C
D
E
F
G
H
I
K
L
M
N
P
Q
R
S
T
V
W
Y

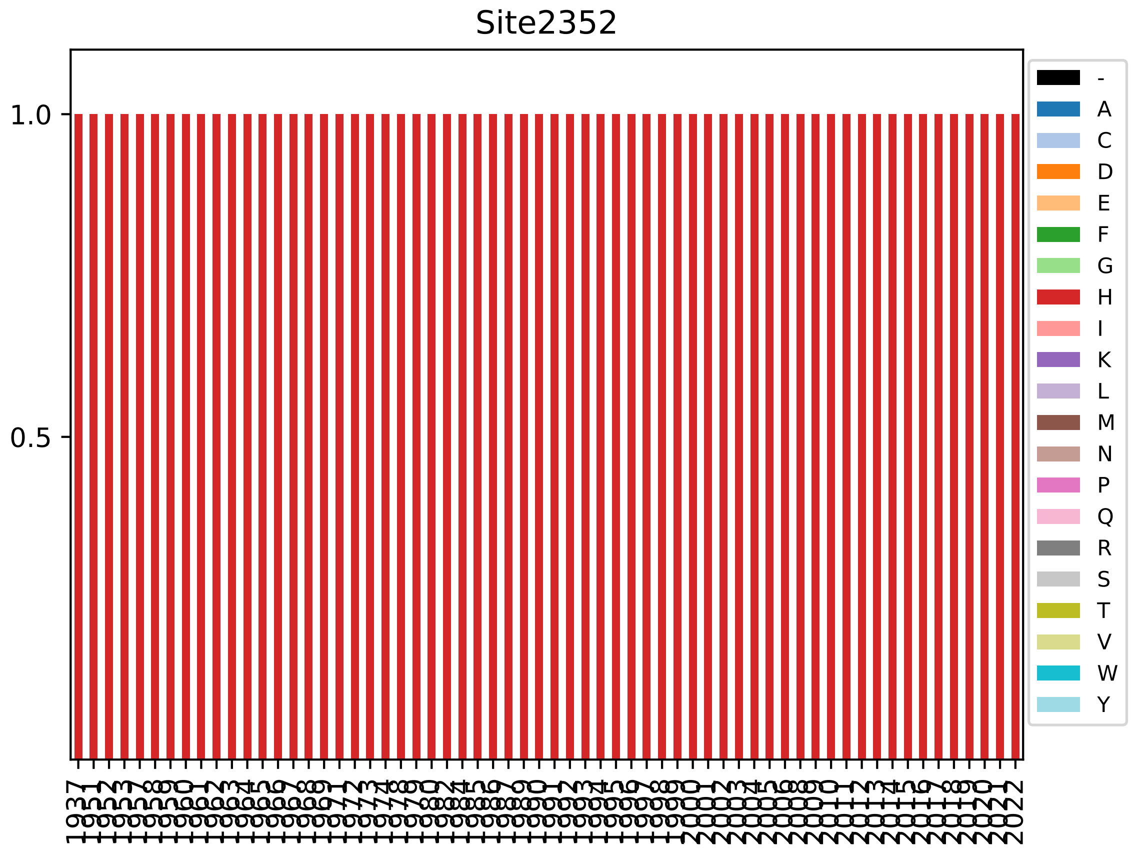
Site2352
1.0
0.5
-
A
C
D
E
F
G
H
I
K
L
M
N
P
Q
R
S
T
V
W
Y

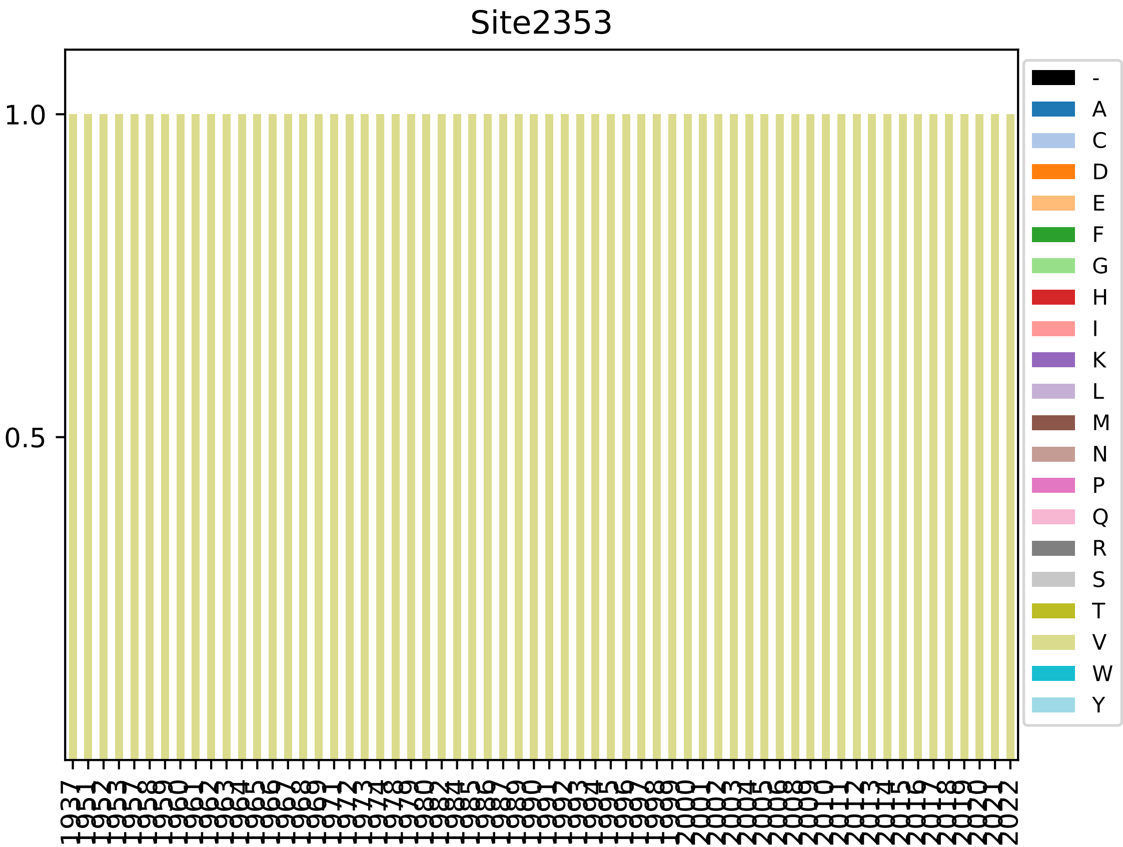

Site2353
1.0
0.5
-
A
C
D
E
F
G
H
I
K
L
M
N
P
Q
R
S
T
V
W
Y

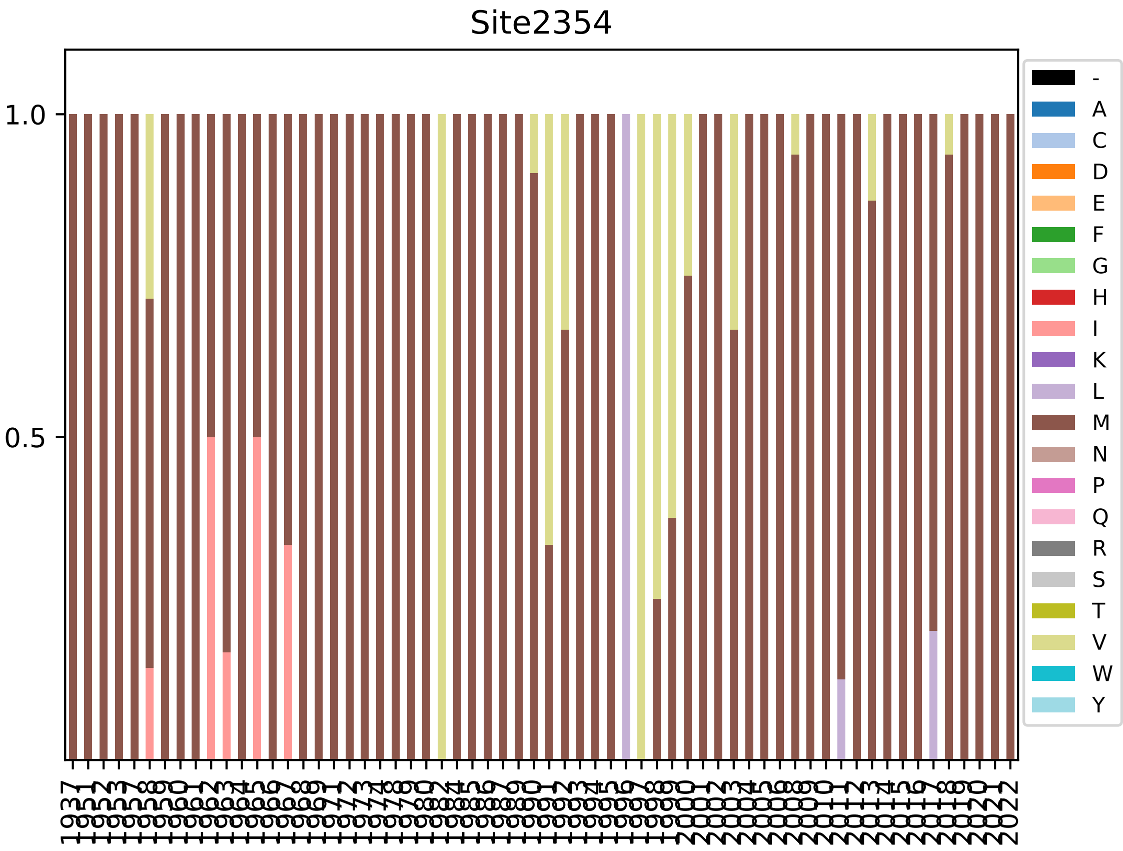
Site2354
1.0
0.5
-
A
C
D
E
F
G
H
I
K
L
M
N
P
Q
R
S
T
V
W
Y

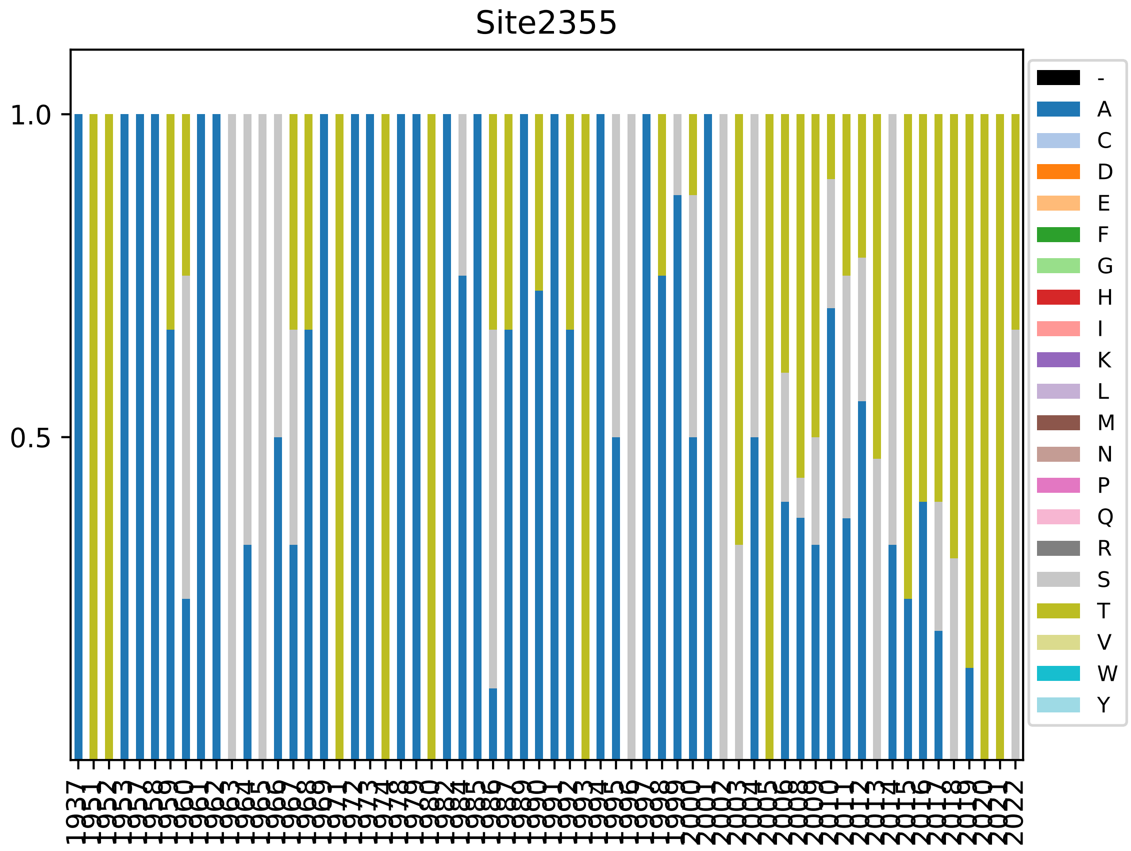

Site2355
1.0
0.5
-
A
C
D
E
F
G
H
I
K
L
M
N
P
Q
R
S
T
V
W
Y

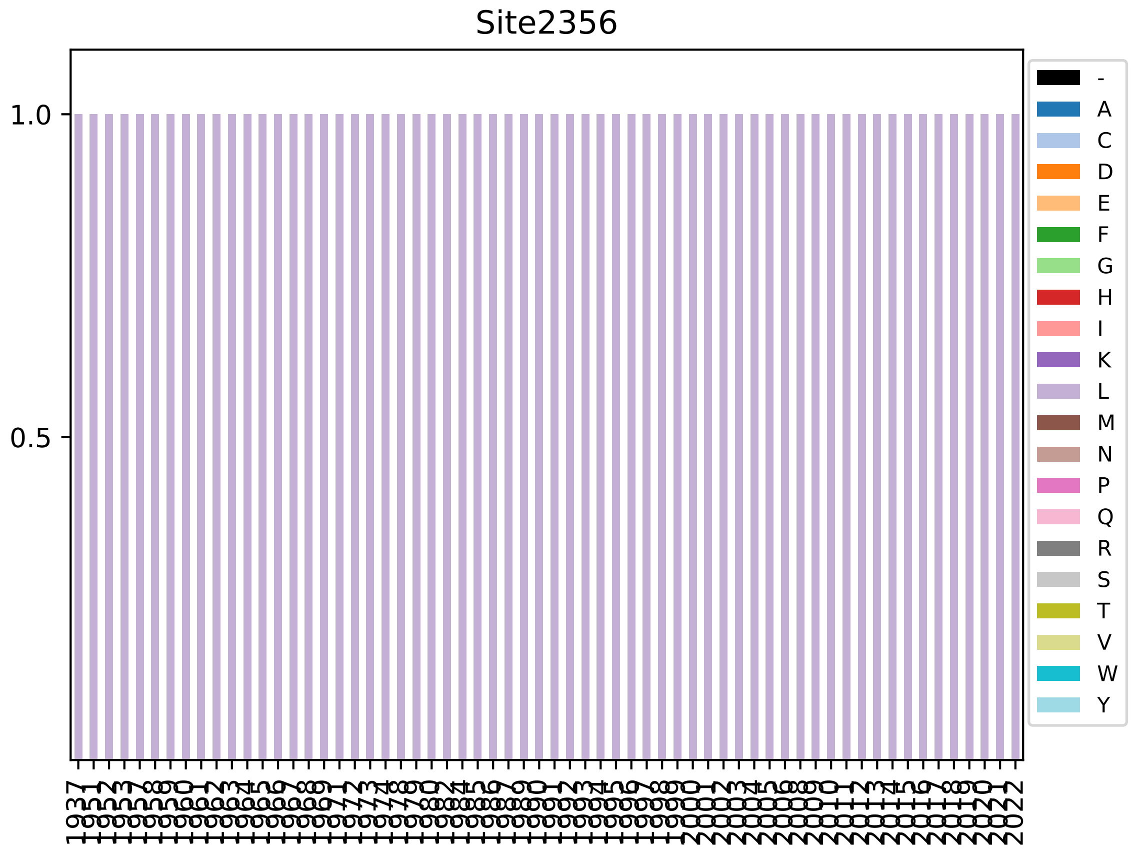
Site2356
1.0
0.5
-
A
C
D
E
F
G
H
I
K
L
M
N
P
Q
R
S
T
V
W
Y

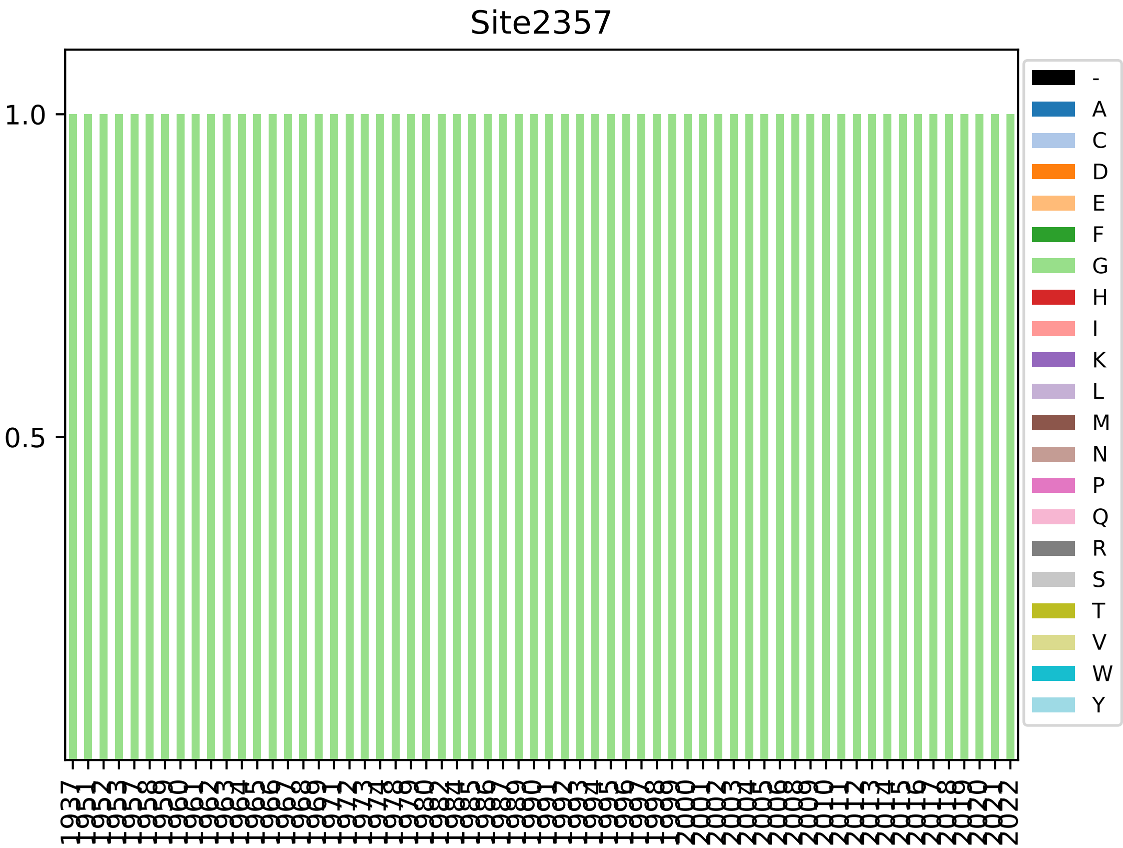
Site2357
1.0
0.5
-
A
C
D
E
F
G
H
I
K
L
M
N
P
Q
R
S
T
V
W
Y

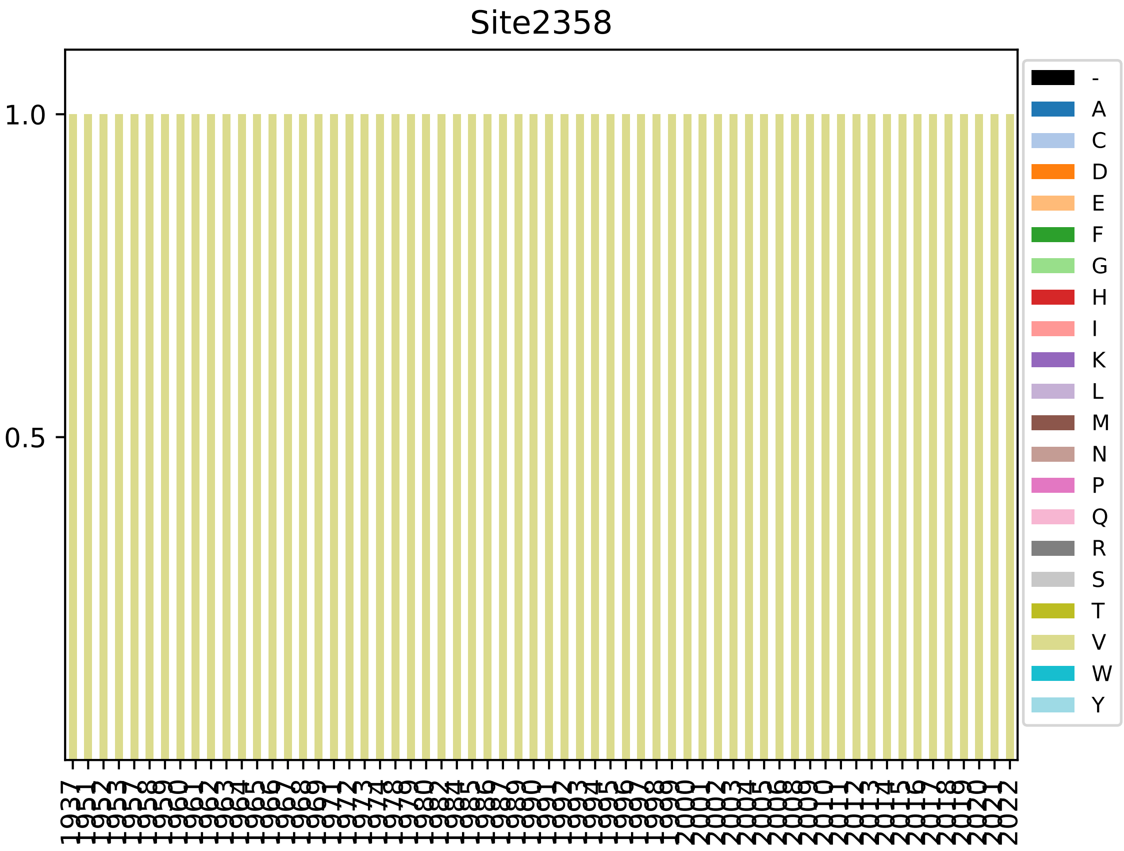
Site2358
1.0
0.5
-
A
C
D
E
F
G
H
I
K
L
M
N
P
Q
R
S
T
V
W
Y

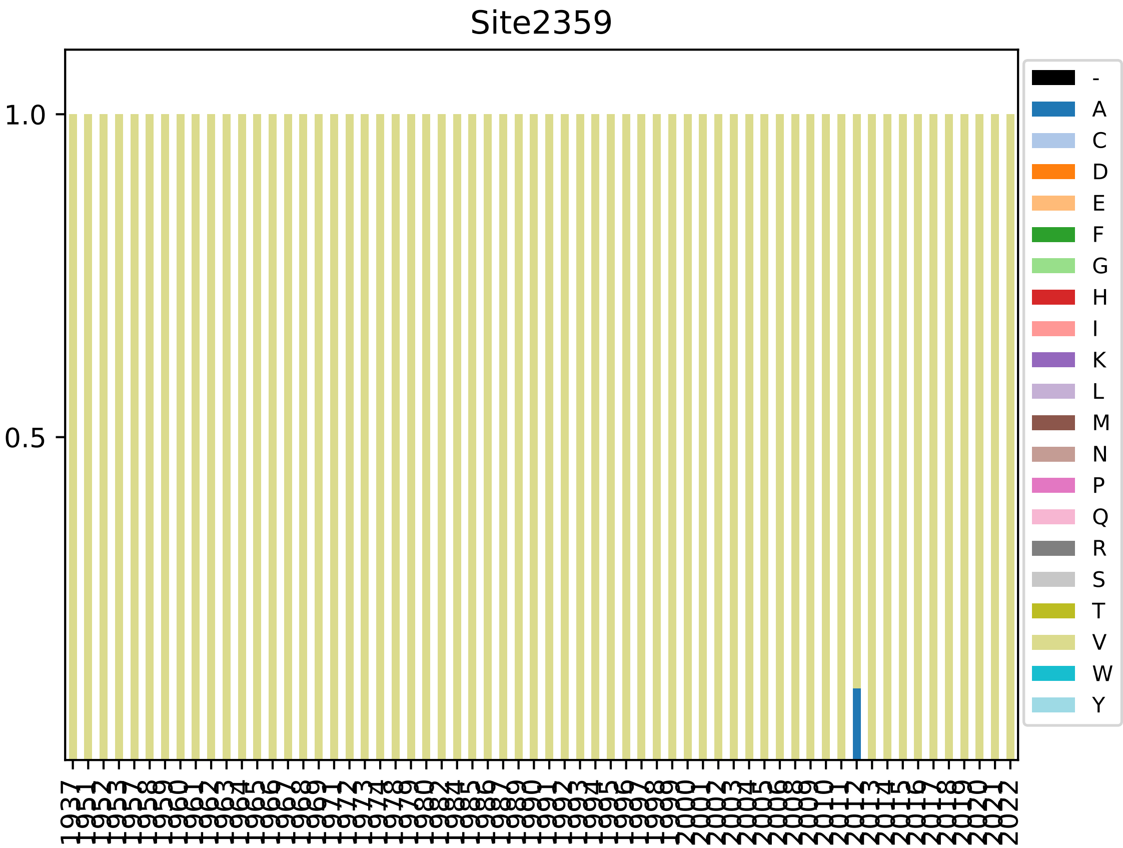
Site2359
1.0
0.5
-
A
C
D
E
F
G
H
I
K
L
M
N
P
Q
R
S
T
V
W
Y

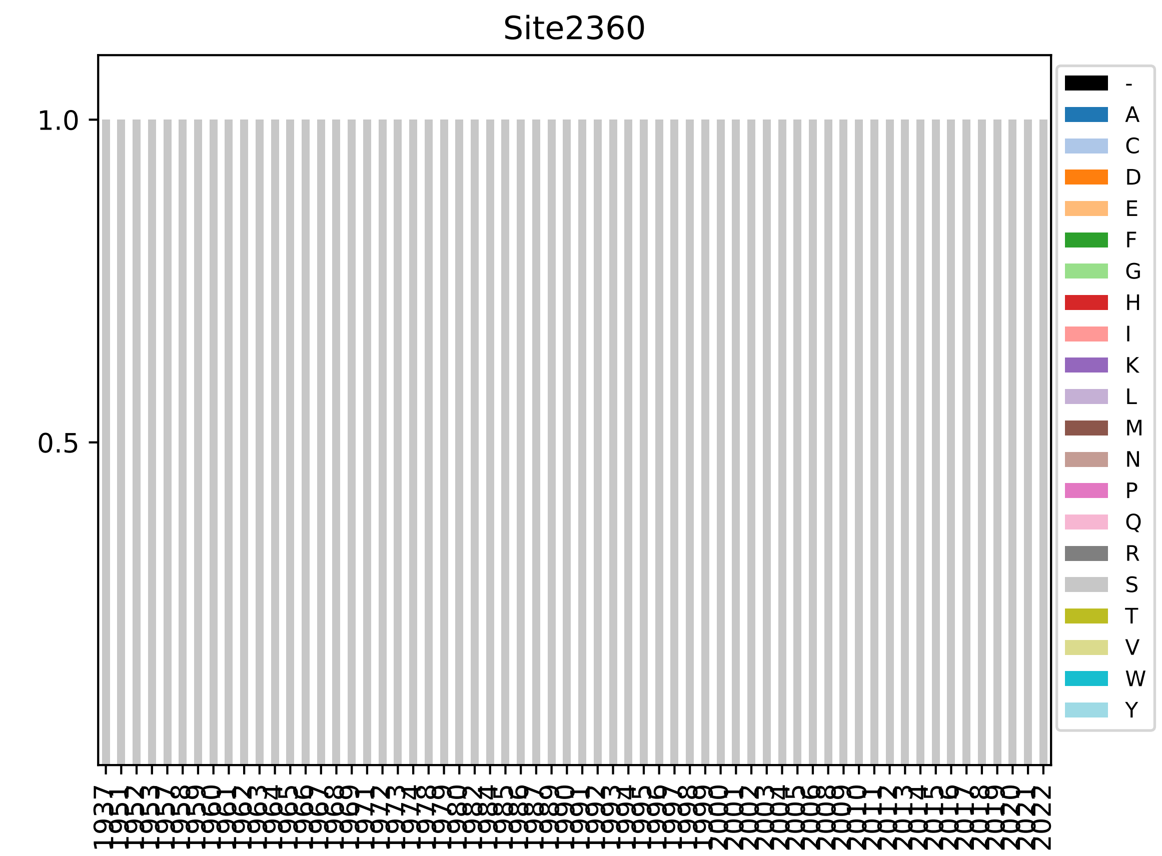

Site2360
1.0
0.5
-
A
C
D
E
F
G
H
I
K
L
M
N
P
Q
R
S
T
V
W
Y

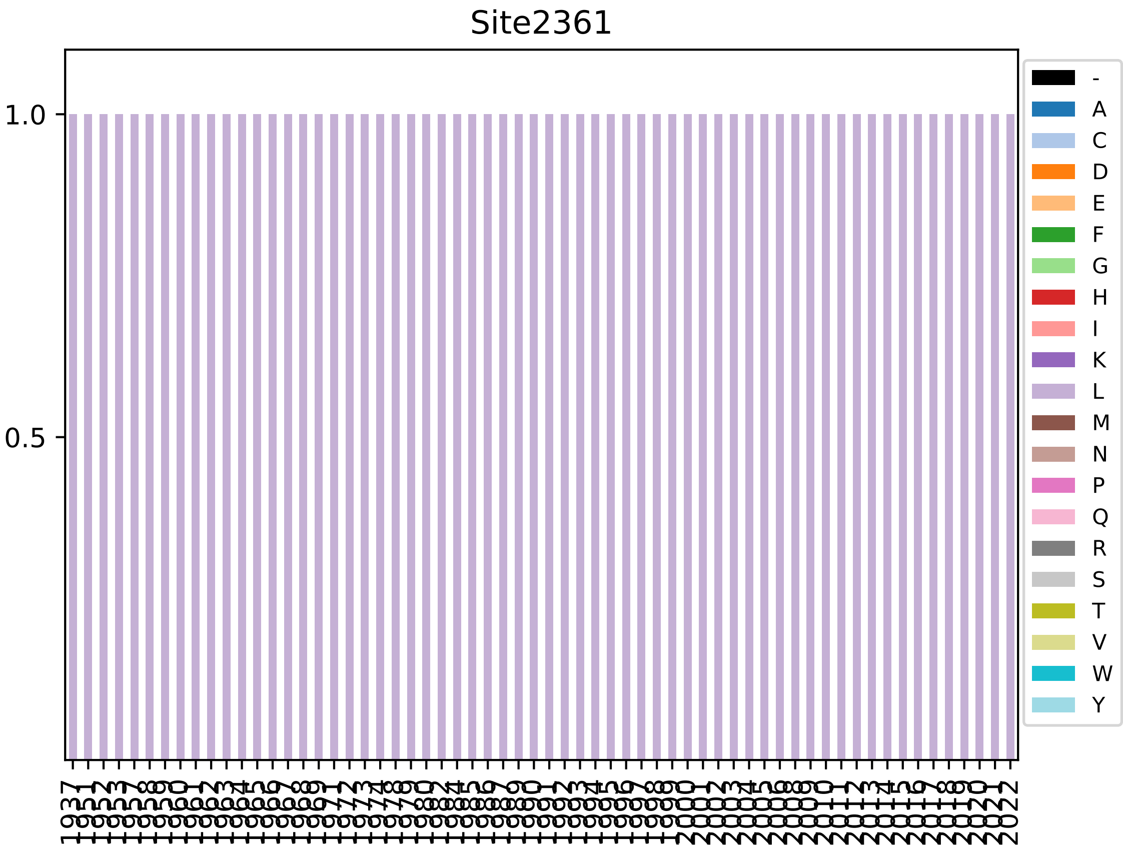
Site2361
1.0
0.5
-
A
C
D
E
F
G
H
I
K
L
M
N
P
Q
R
S
T
V
W
Y

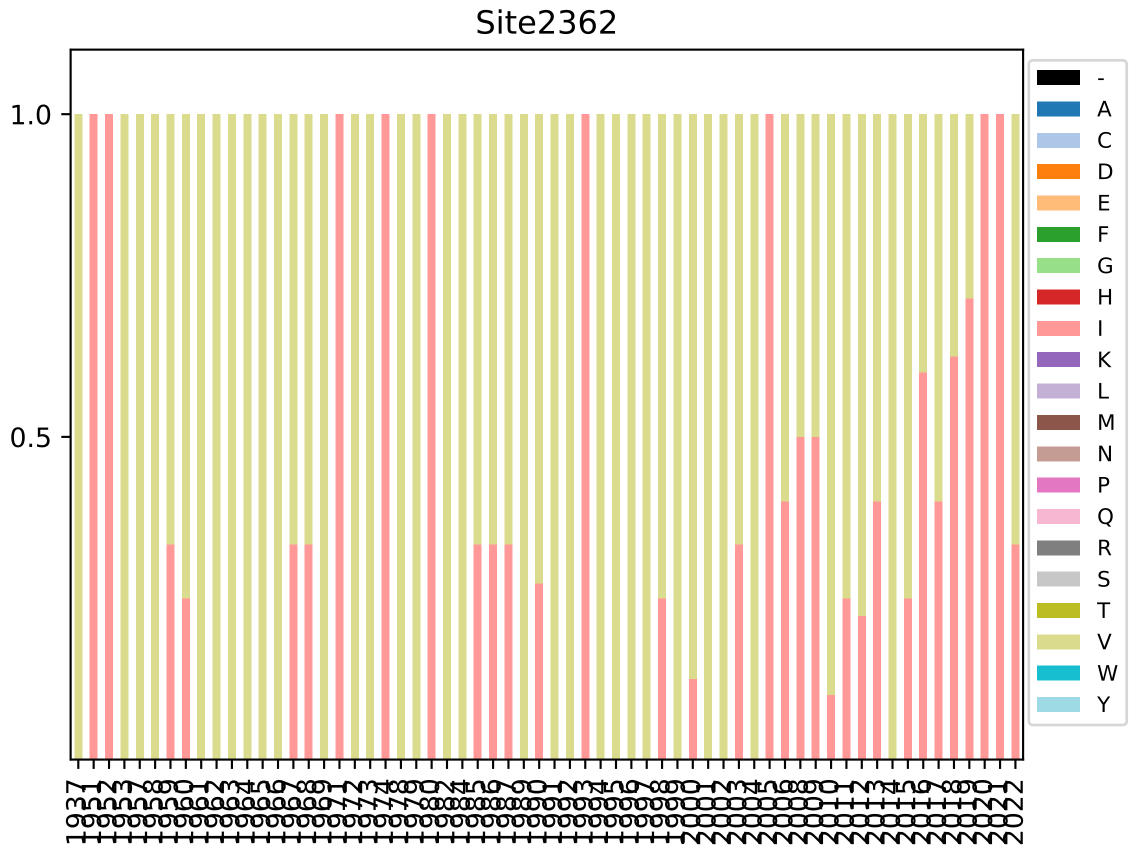
Site2362
1.0
0.5
-
A
C
D
E
F
G
H
I
K
L
M
N
P
Q
R
S
T
V
W
Y

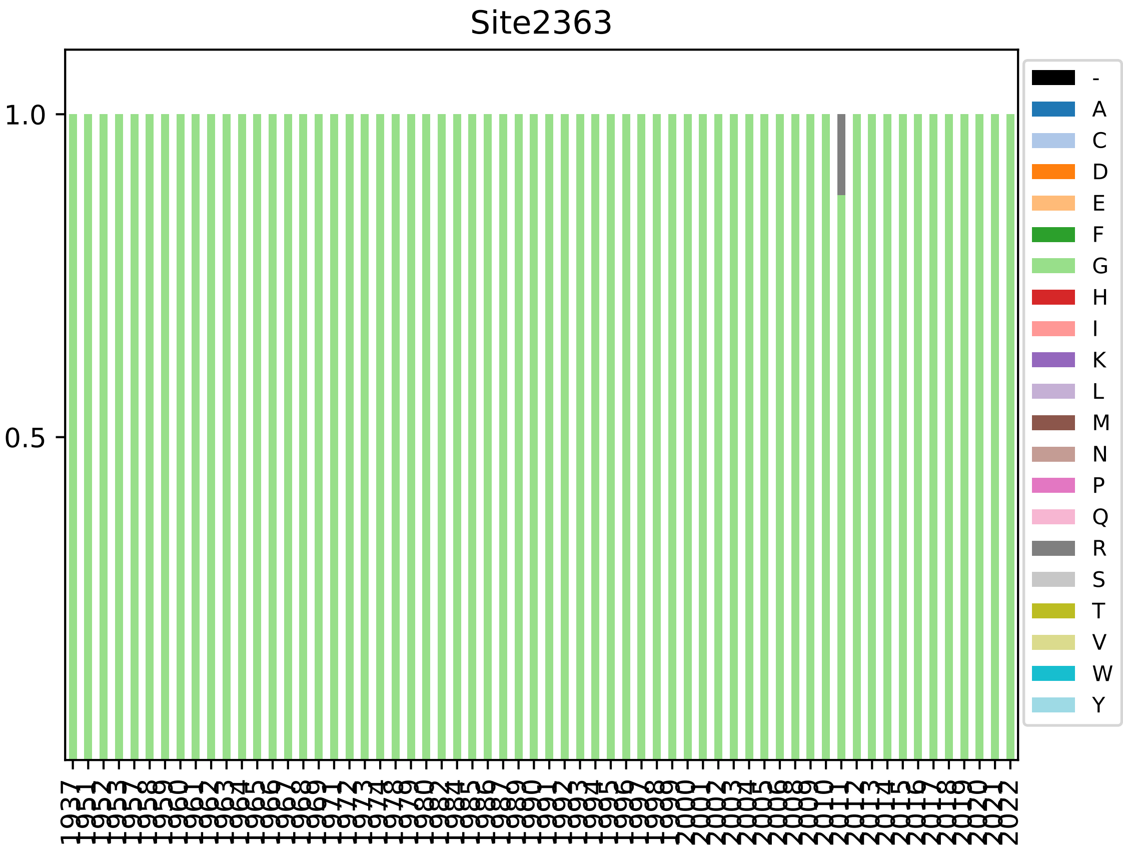
Site2363
1.0
0.5
-
A
C
D
E
F
G
H
I
K
L
M
N
P
Q
R
S
T
V
W
Y

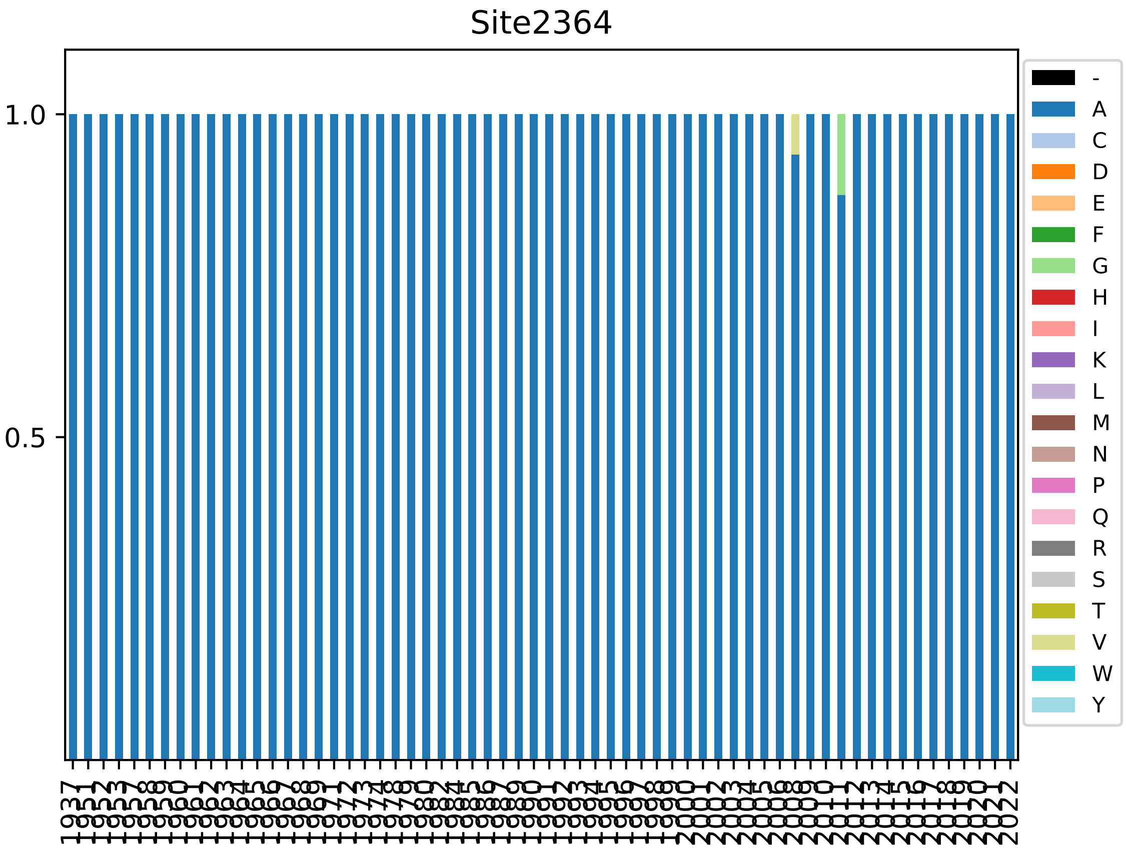
Site2364
1.0
0.5
-
A
C
D
E
F
G
H
I
K
L
M
N
P
Q
R
S
T
V
W
Y

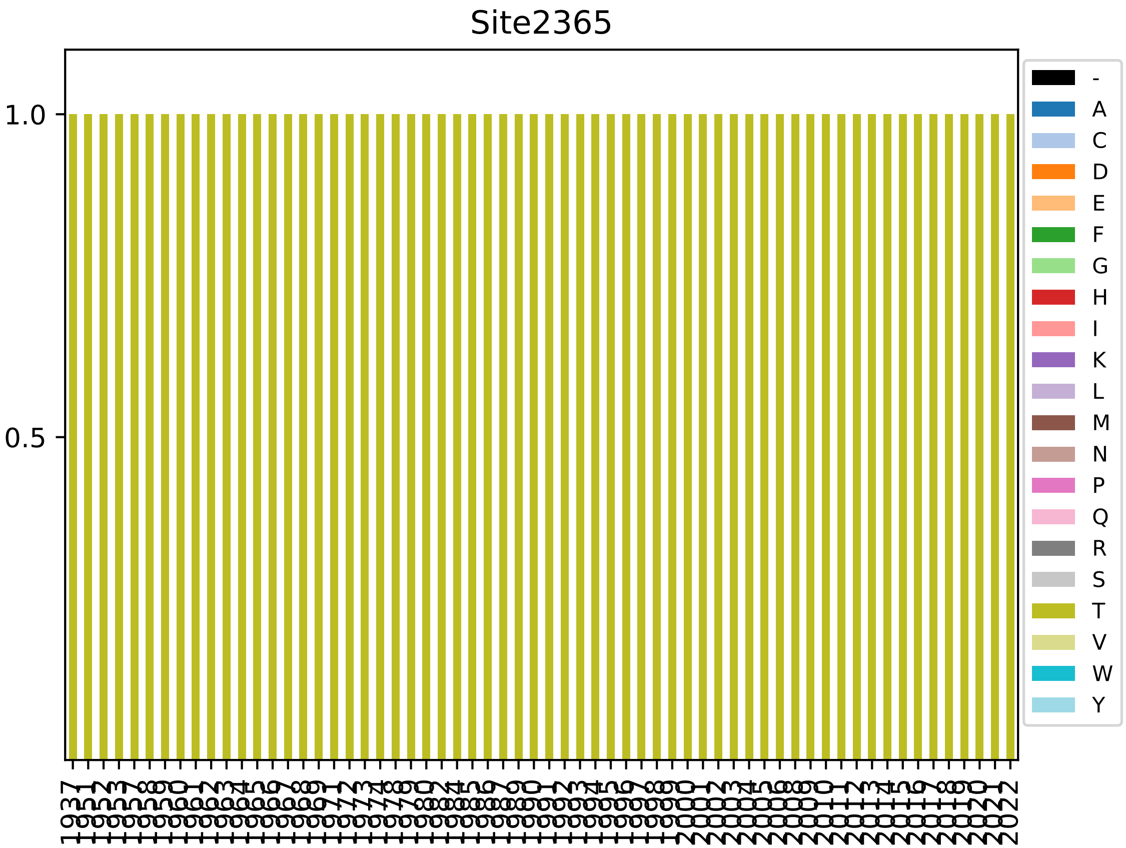
Site2365
1.0
0.5
-
A
C
D
E
F
G
H
I
K
L
M
N
P
Q
R
S
T
V
W
Y

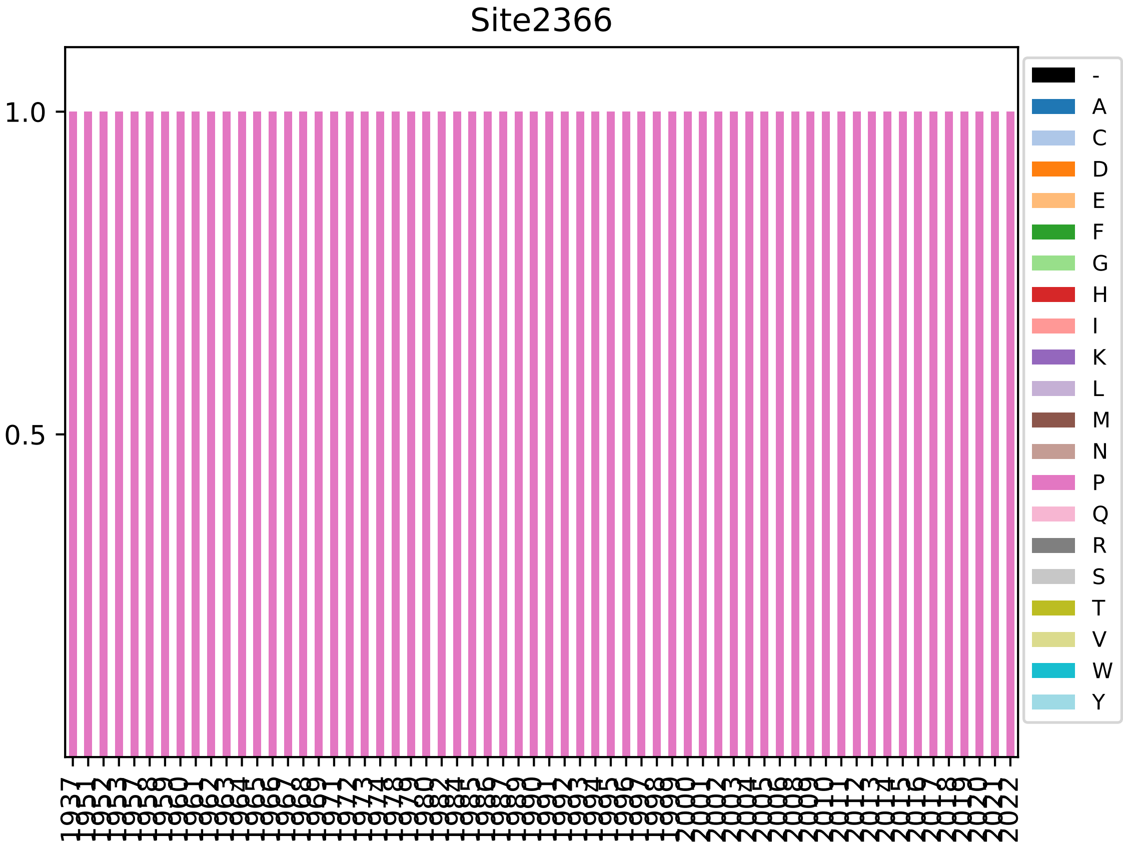
Site2366
1.0
0.5
-
A
C
D
E
F
G
H
I
K
L
M
N
P
Q
R
S
T
V
W
Y

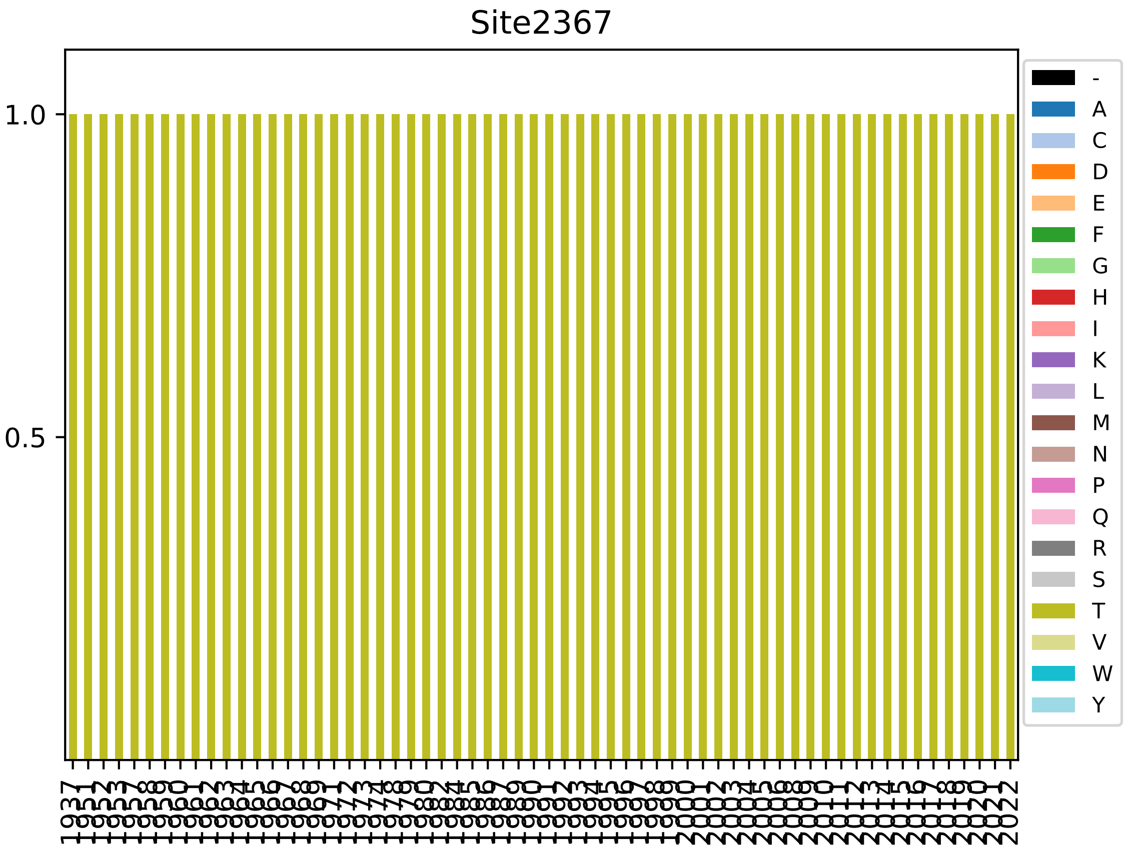
Site2367
1.0
0.5
-
A
C
D
E
F
G
H
I
K
L
M
N
P
Q
R
S
T
V
W
Y

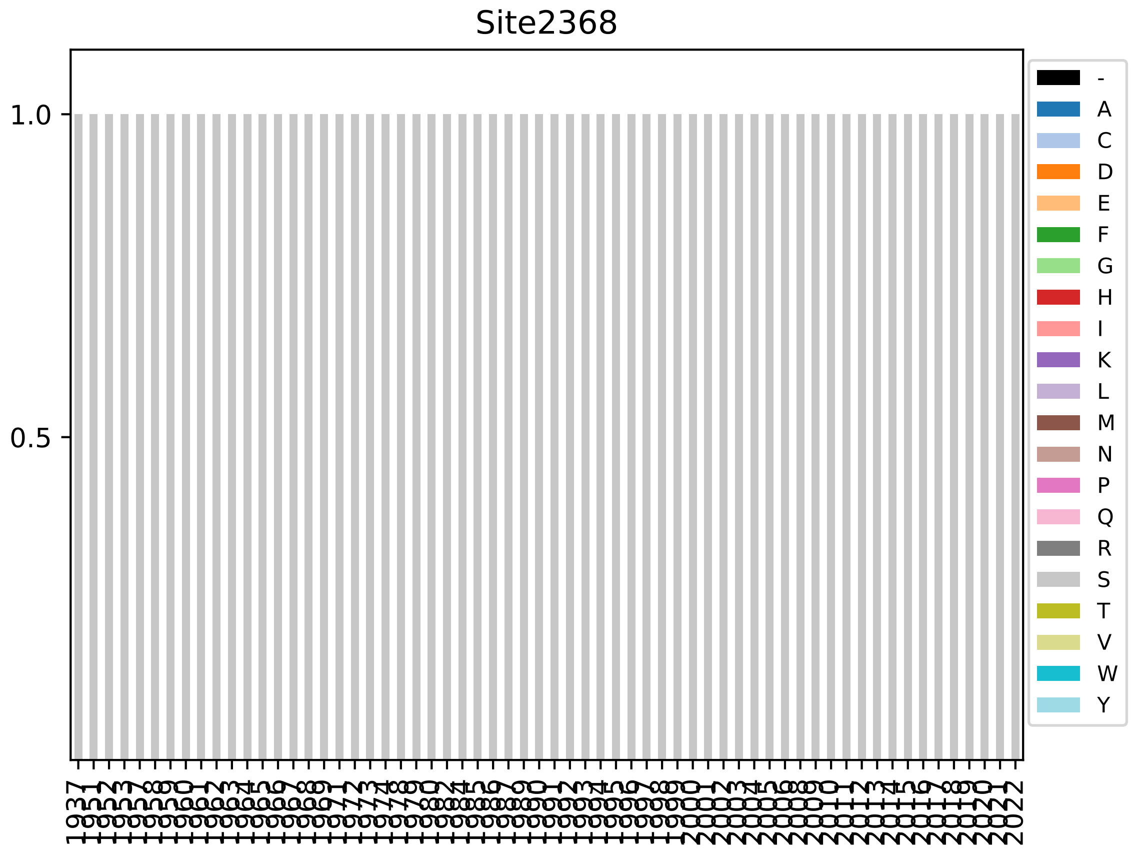
Site2368
1.0
0.5
-
A
C
D
E
F
G
H
I
K
L
M
N
P
Q
R
S
T
V
W
Y

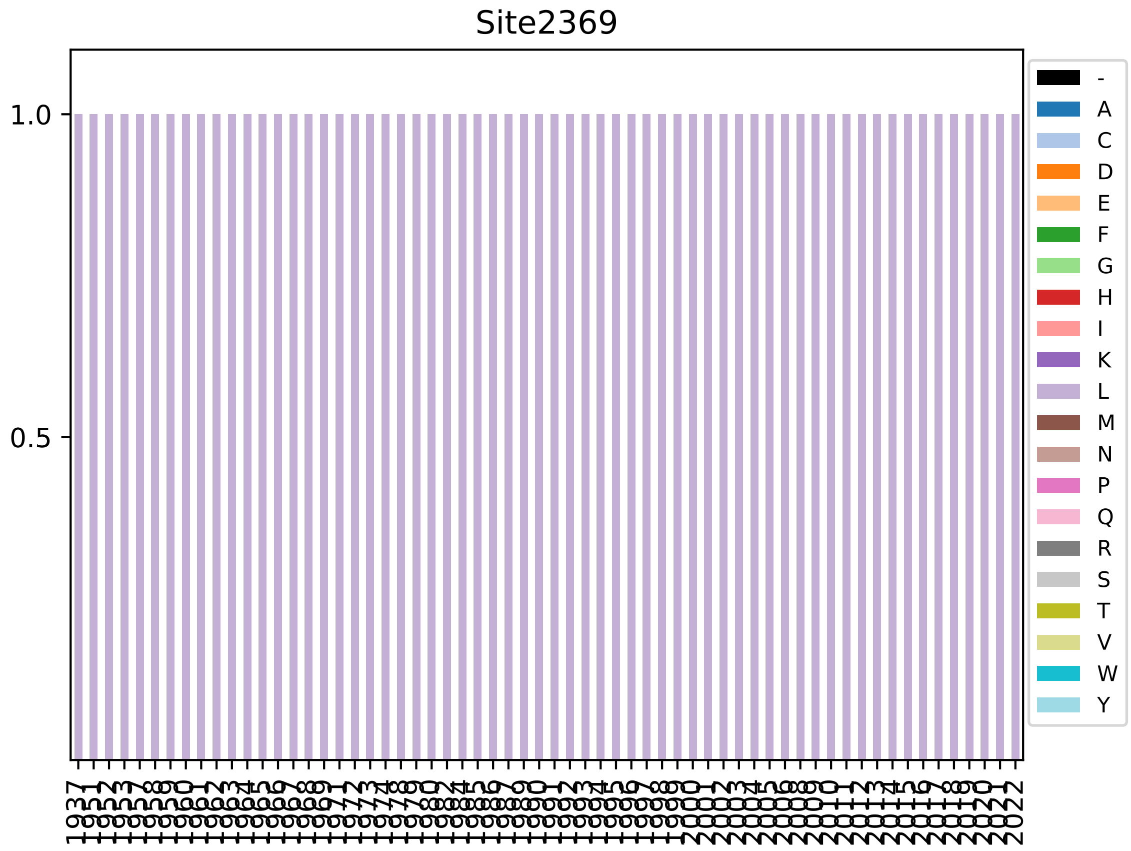
Site2369
1.0
0.5
-
A
C
D
E
F
G
H
I
K
L
M
N
P
Q
R
S
T
V
W
Y

Site2370

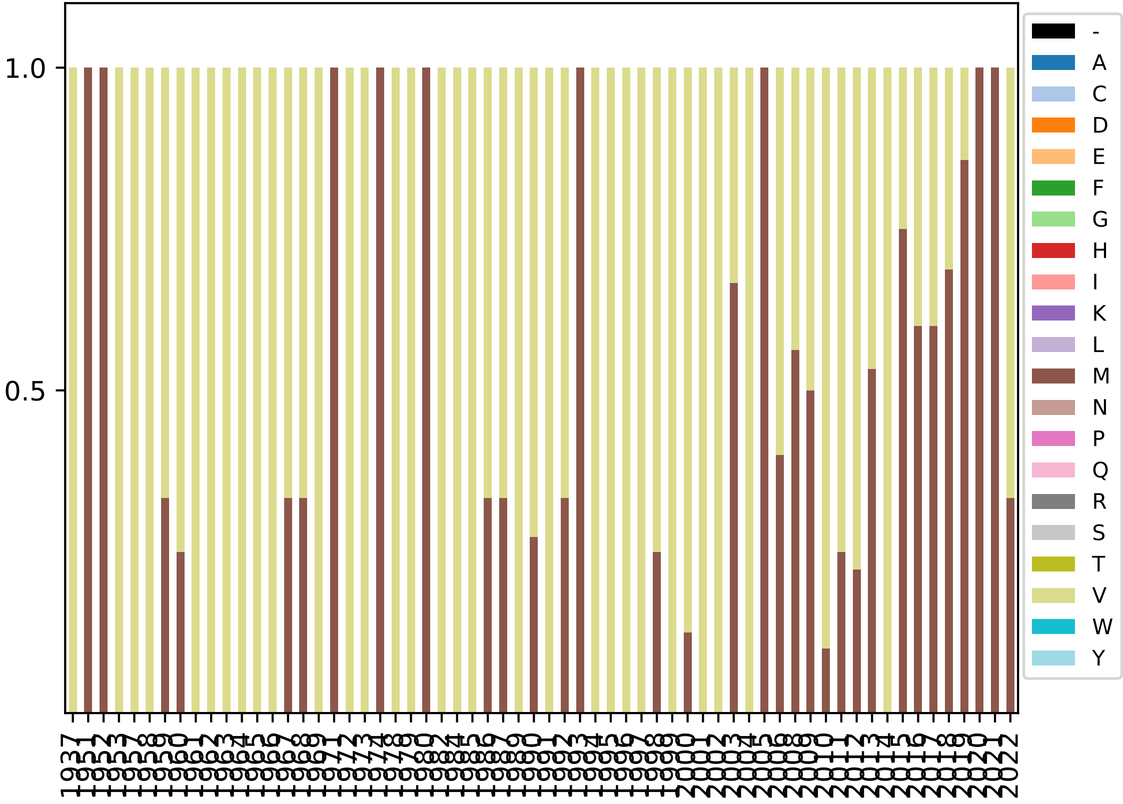

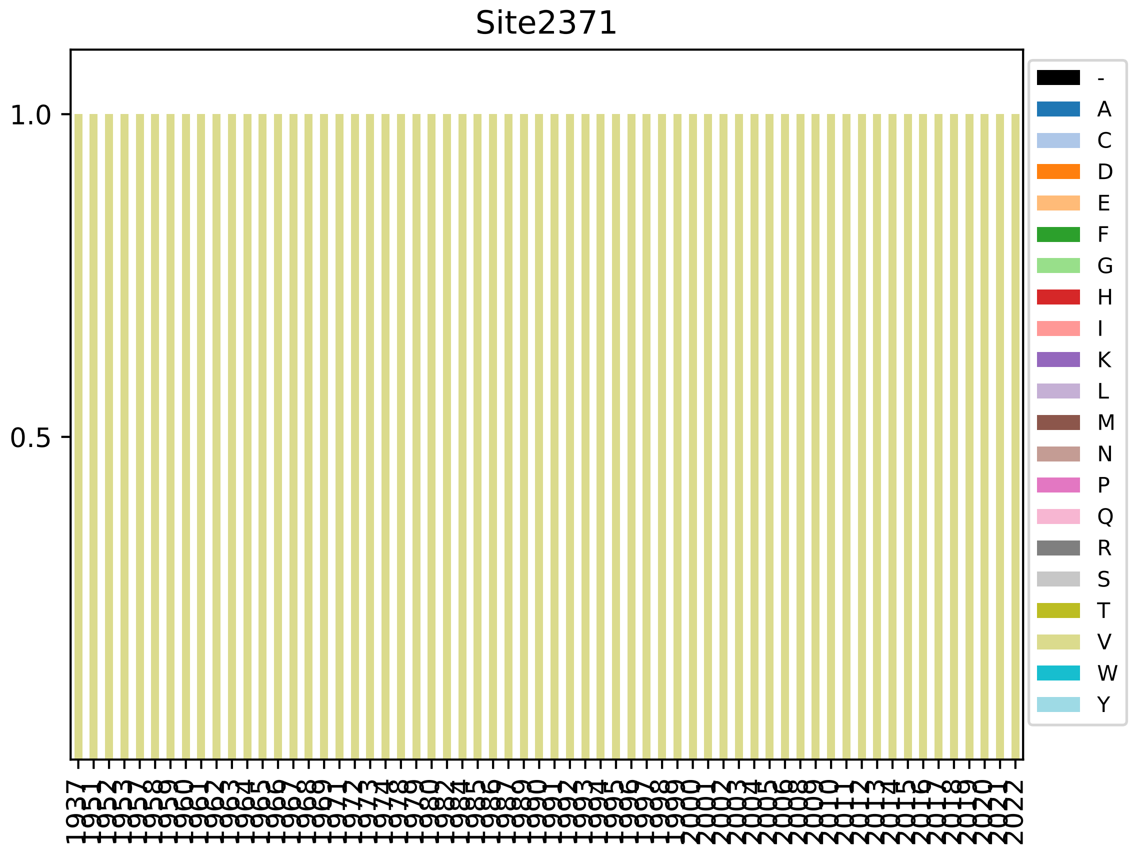
Site2371
1.0
0.5
-
A
C
D
E
F
G
H
I
K
L
M
N
P
Q
R
S
T
V
W
Y

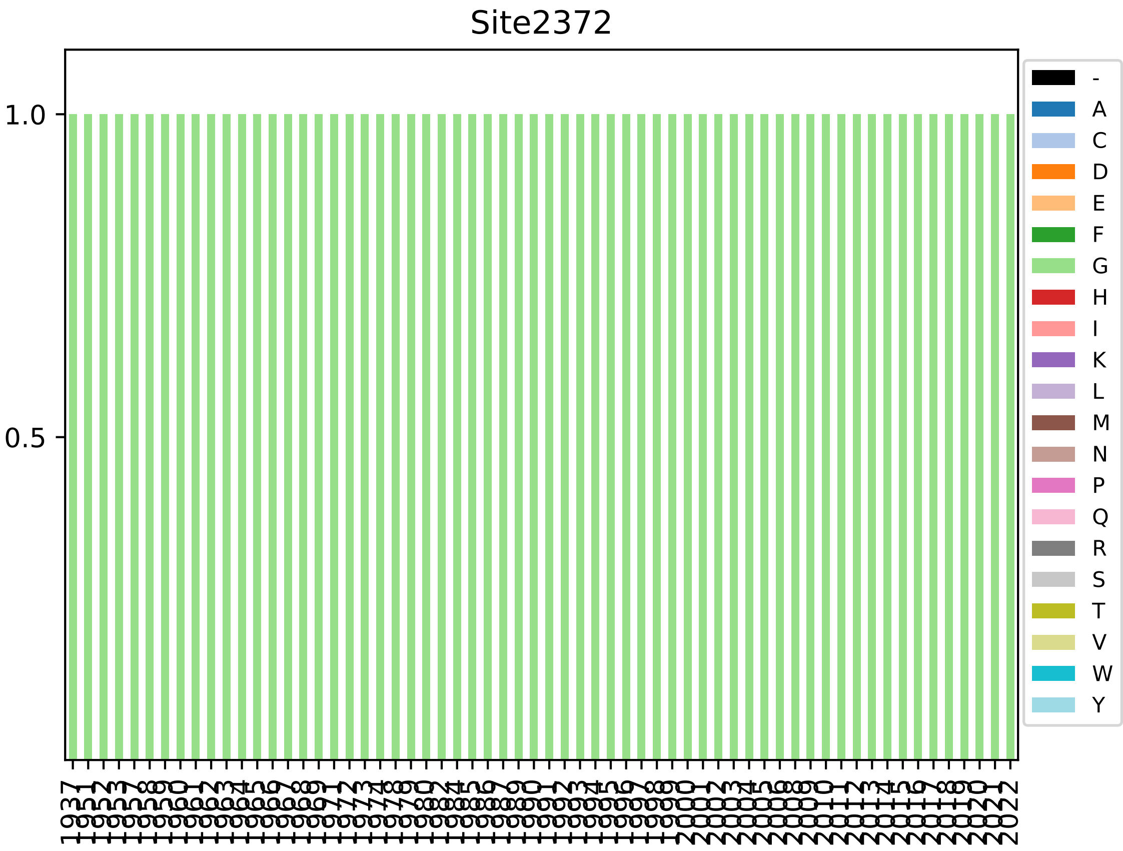
Site2372
1.0
0.5
-
A
C
D
E
F
G
H
I
K
L
M
N
P
Q
R
S
T
V
W
Y

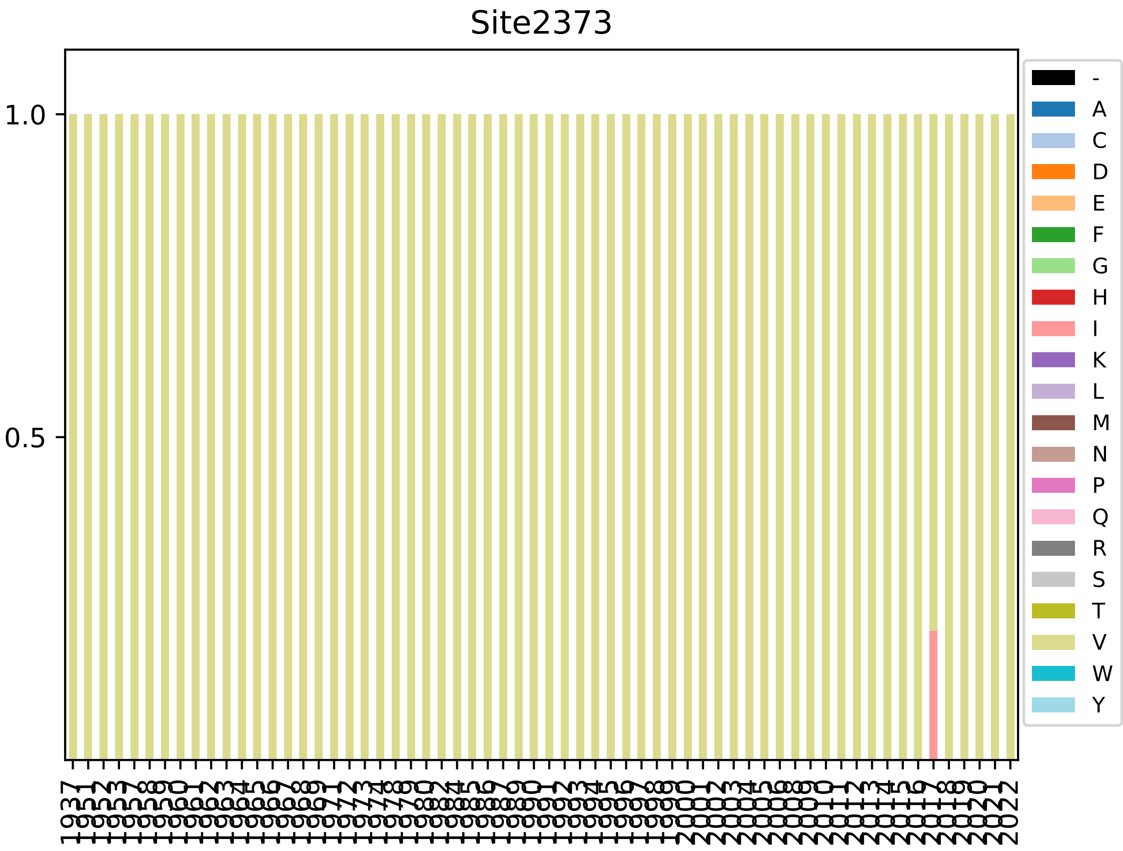
Site2373
1.0
0.5
-
A
C
D
E
F
G
H
I
K
L
M
N
P
Q
R
S
T
V
W
Y

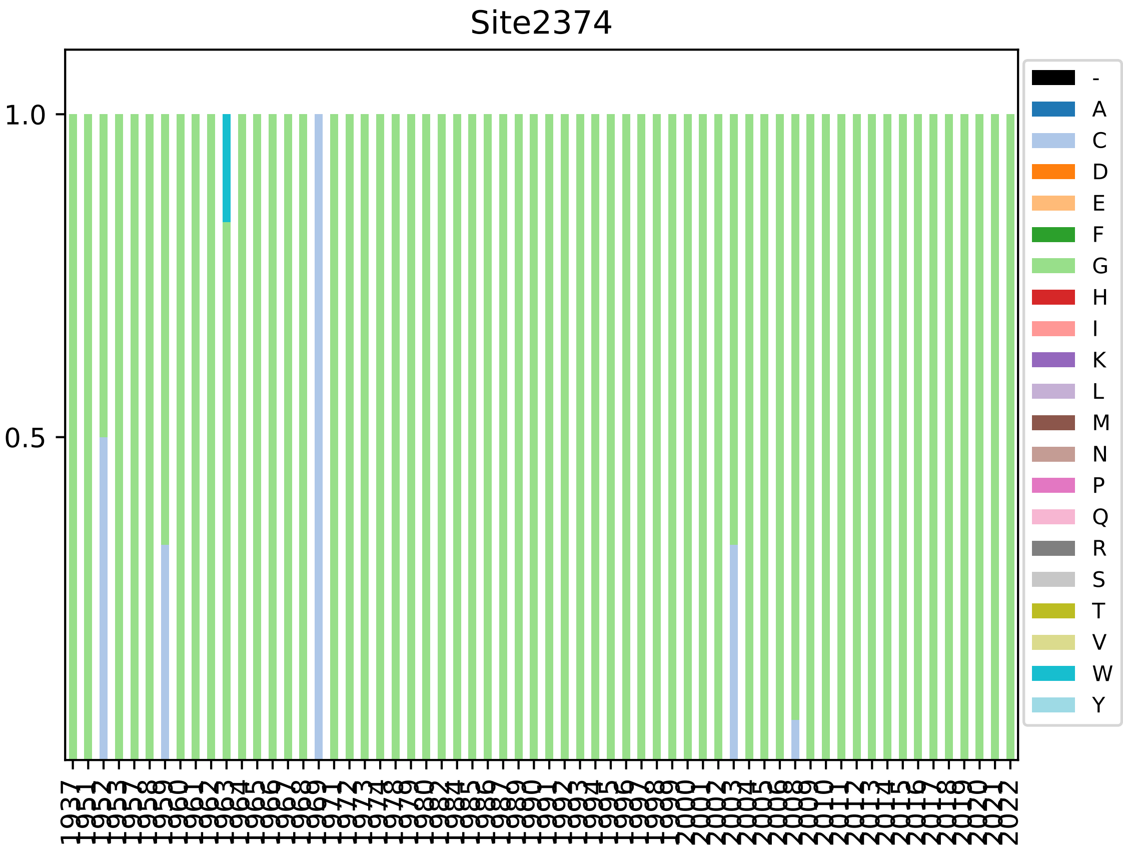
Site2374
1.0
0.5
-
A
C
D
E
F
G
H
I
K
L
M
N
P
Q
R
S
T
V
W
Y

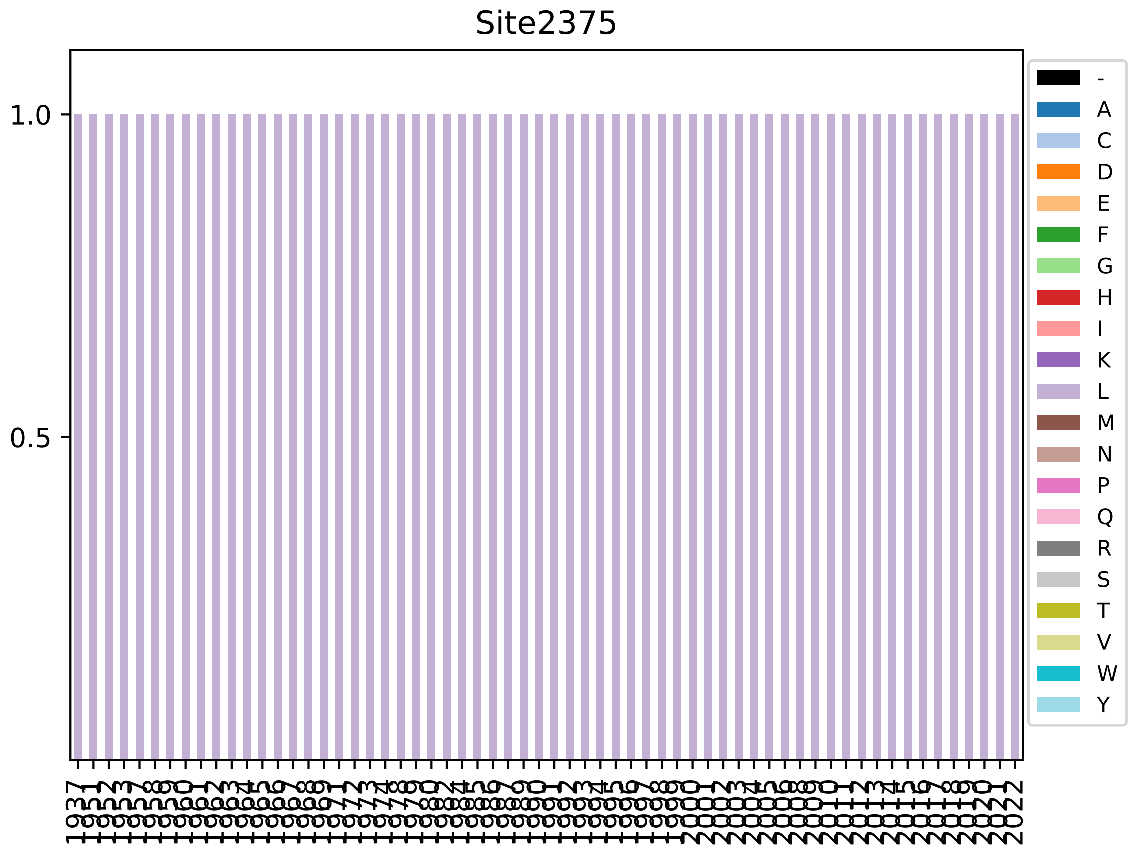
Site2375
1.0
0.5
-
A
C
D
E
F
G
H
I
K
L
M
N
P
Q
R
S
T
V
W
Y

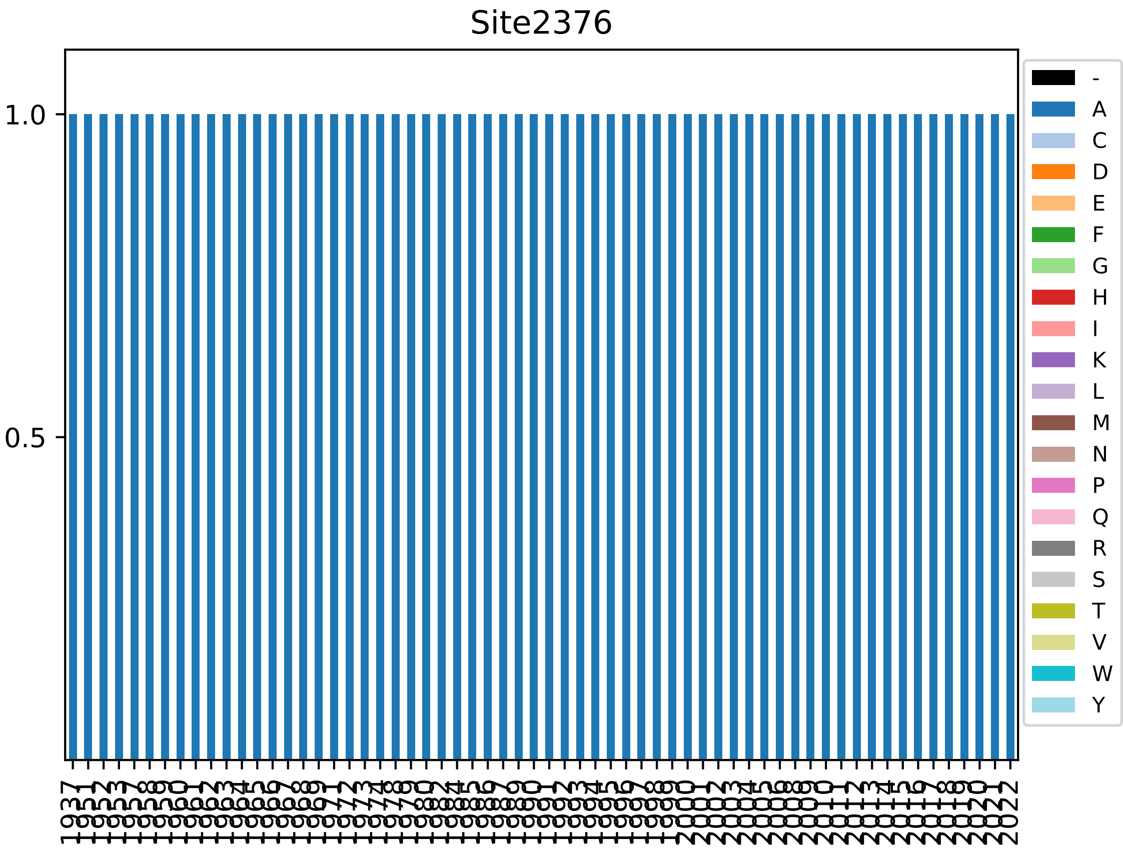

Site2376
1.0
0.5
-
A
C
D
E
F
G
H
I
K
L
M
N
P
Q
R
S
T
V
W
Y

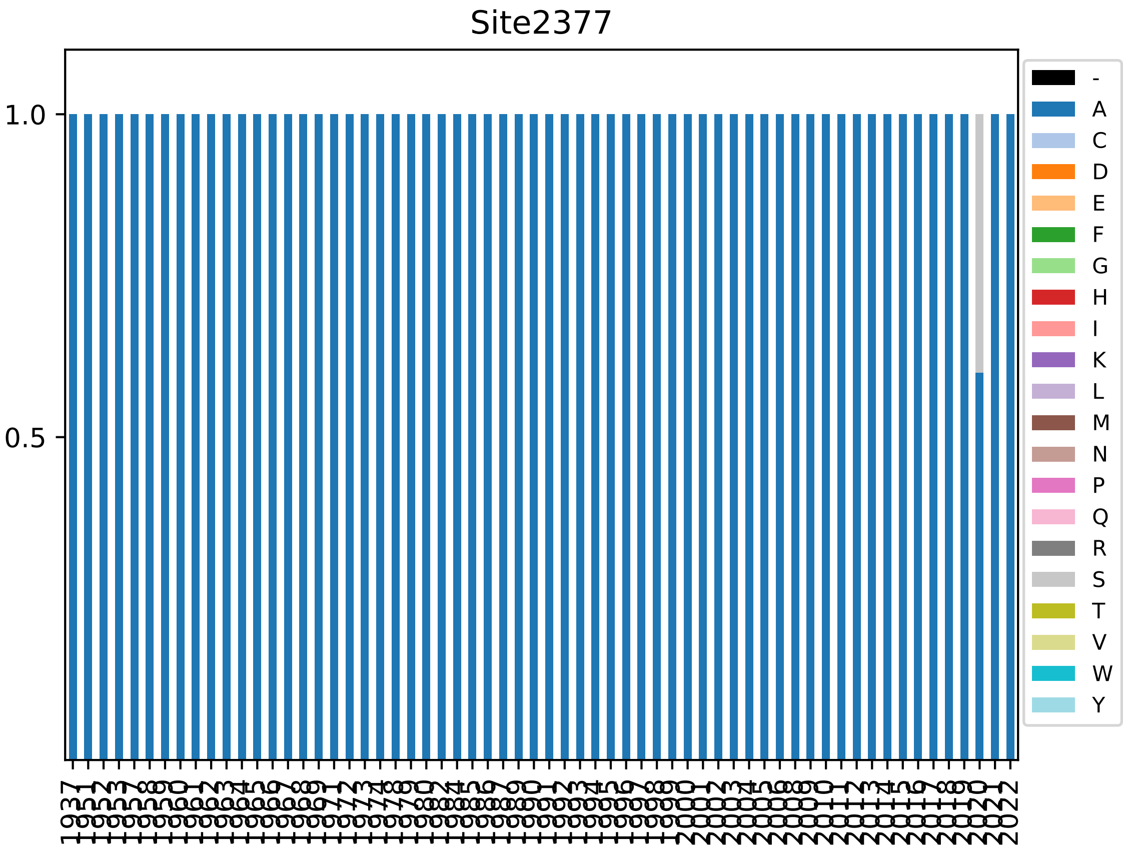
Site2377
1.0
0.5
-
A
C
D
E
F
G
H
I
K
L
M
N
P
Q
R
S
T
V
W
Y

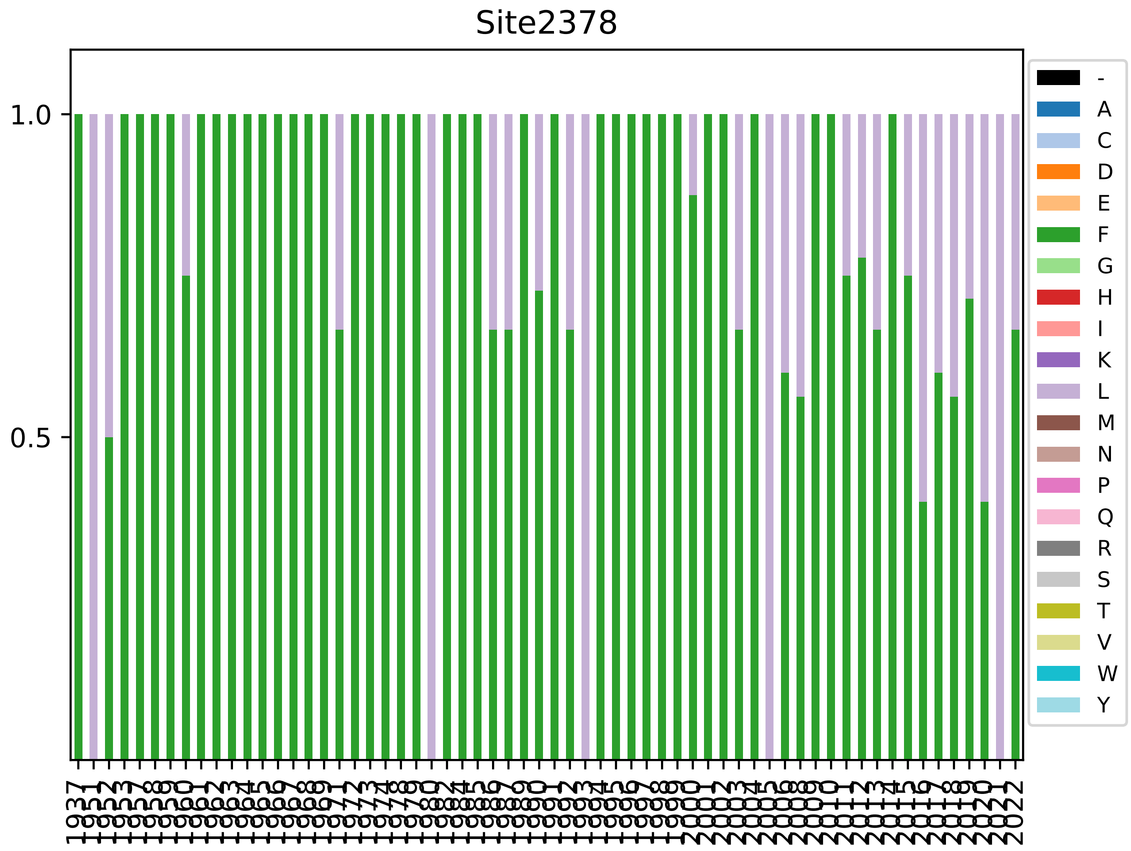
Site2378
1.0
0.5
-
A
C
D
E
F
G
H
I
K
L
M
N
P
Q
R
S
T
V
W
Y

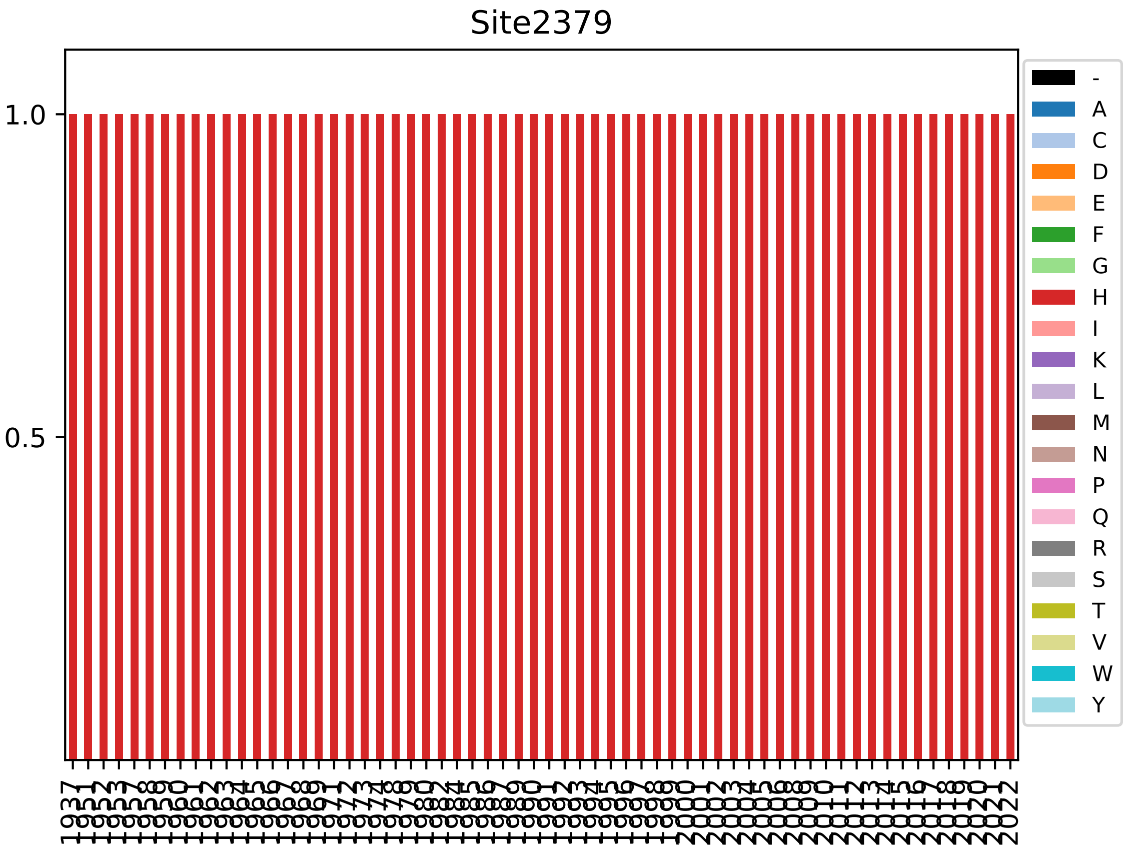
Site2379
1.0
0.5
-
A
C
D
E
F
G
H
I
K
L
M
N
P
Q
R
S
T
V
W
Y

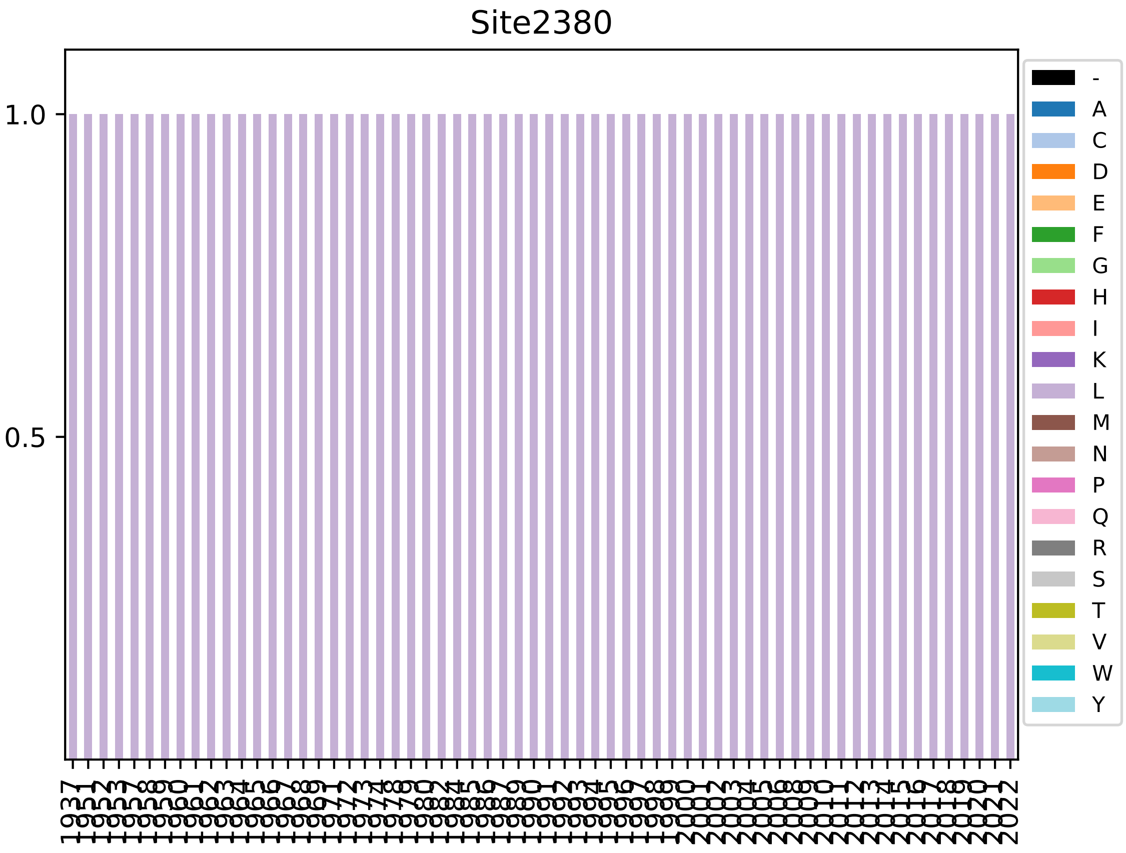
Site2380
1.0
0.5
-
A
C
D
E
F
G
H
I
K
L
M
N
P
Q
R
S
T
V
W
Y

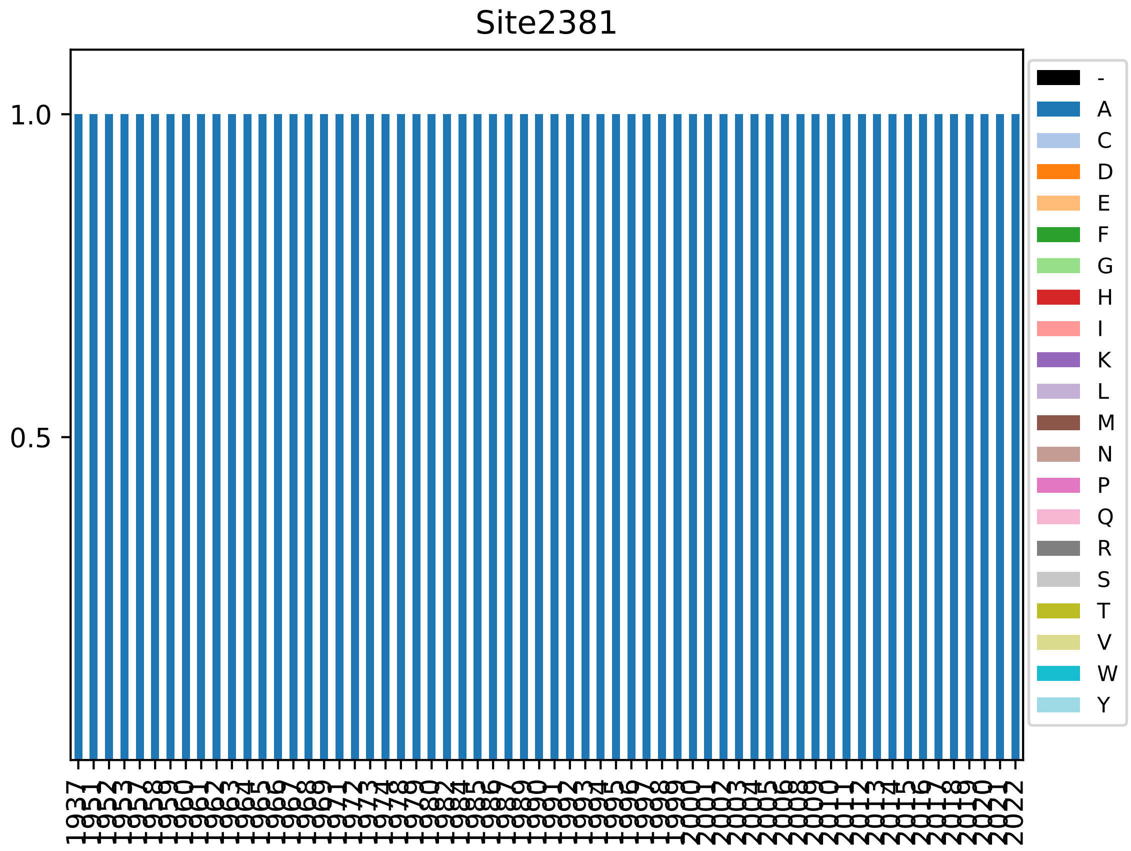
Site2381
1.0
0.5
-
A
C
D
E
F
G
H
I
K
L
M
N
P
Q
R
S
T
V
W
Y

Site2382

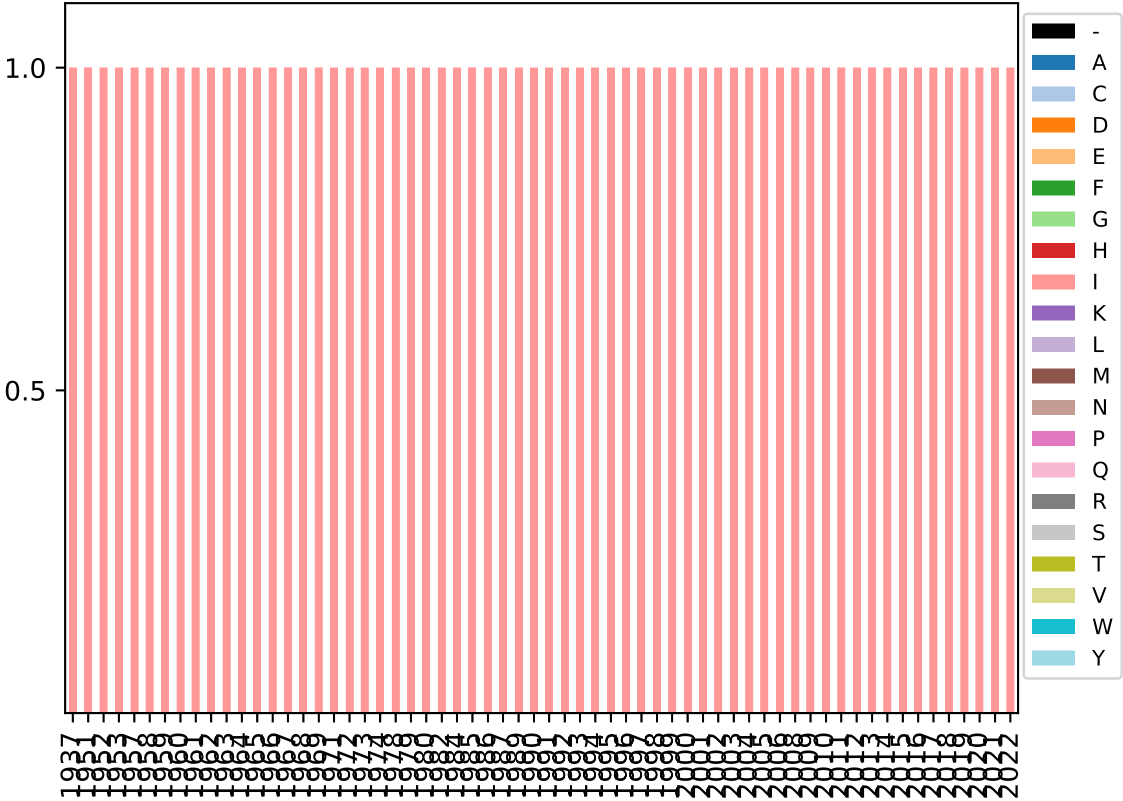

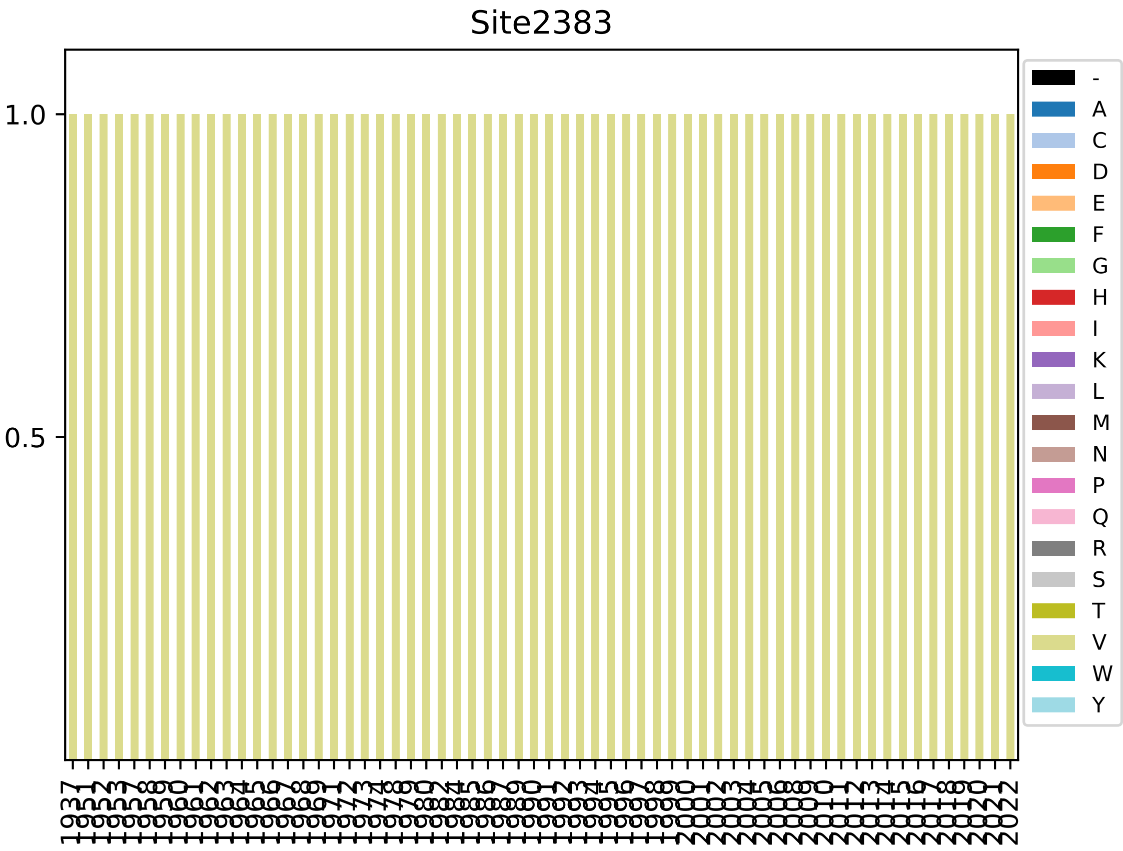

Site2383
1.0
0.5
-
A
C
D
E
F
G
H
I
K
L
M
N
P
Q
R
S
T
V
W
Y

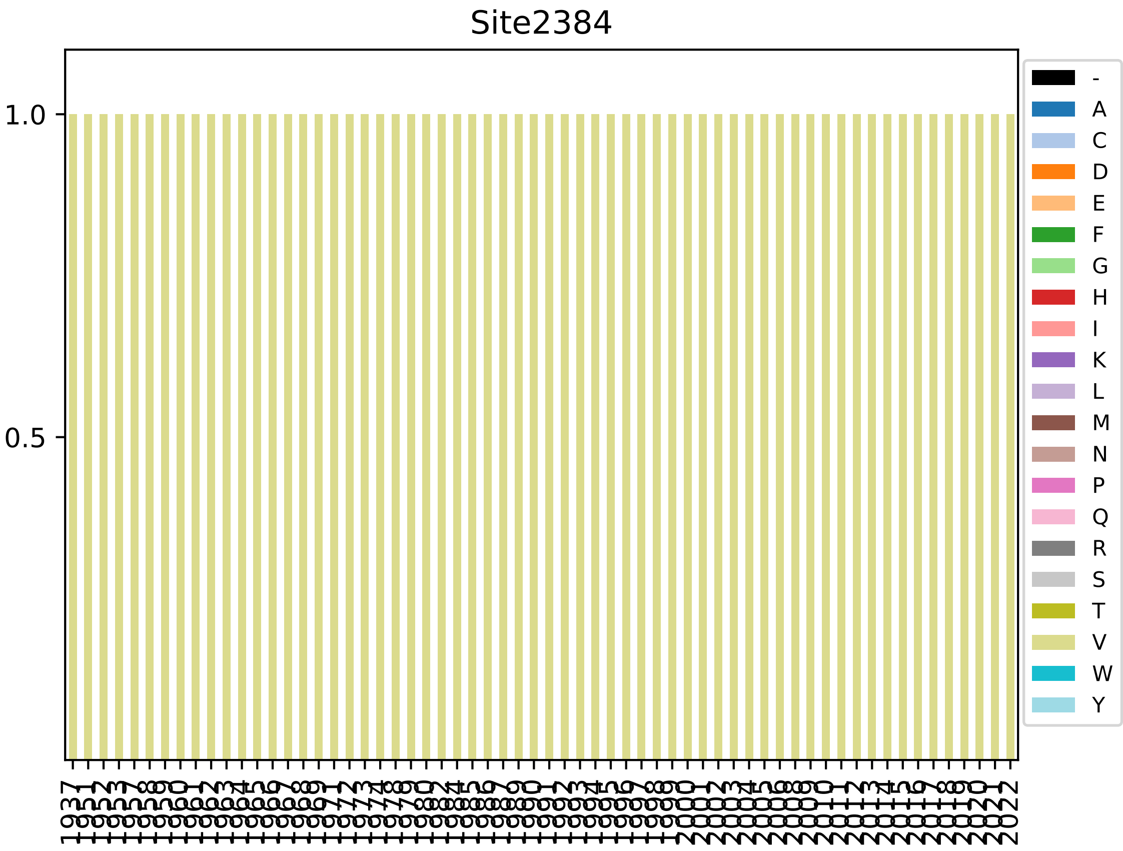
Site2384
1.0
0.5
-
A
C
D
E
F
G
H
I
K
L
M
N
P
Q
R
S
T
V
W
Y

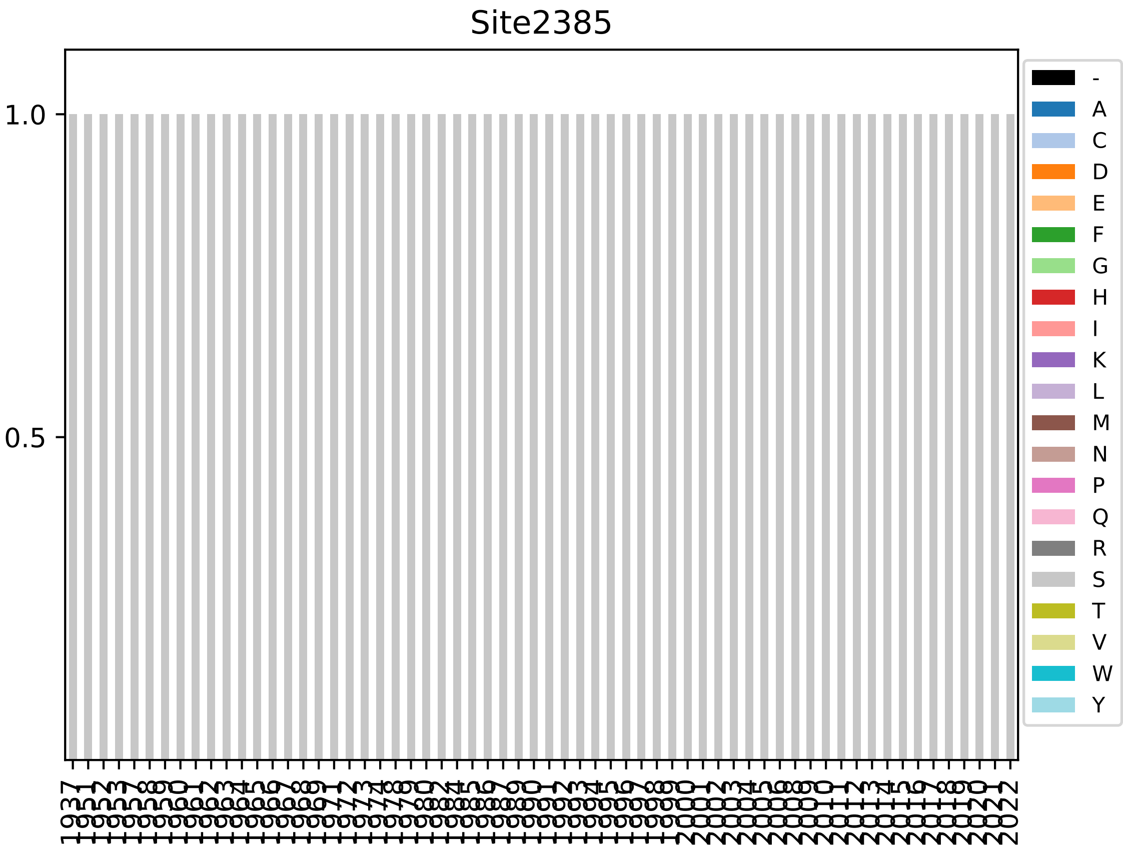
Site2385
1.0
0.5
-
A
C
D
E
F
G
H
I
K
L
M
N
P
Q
R
S
T
V
W
Y

Site2386

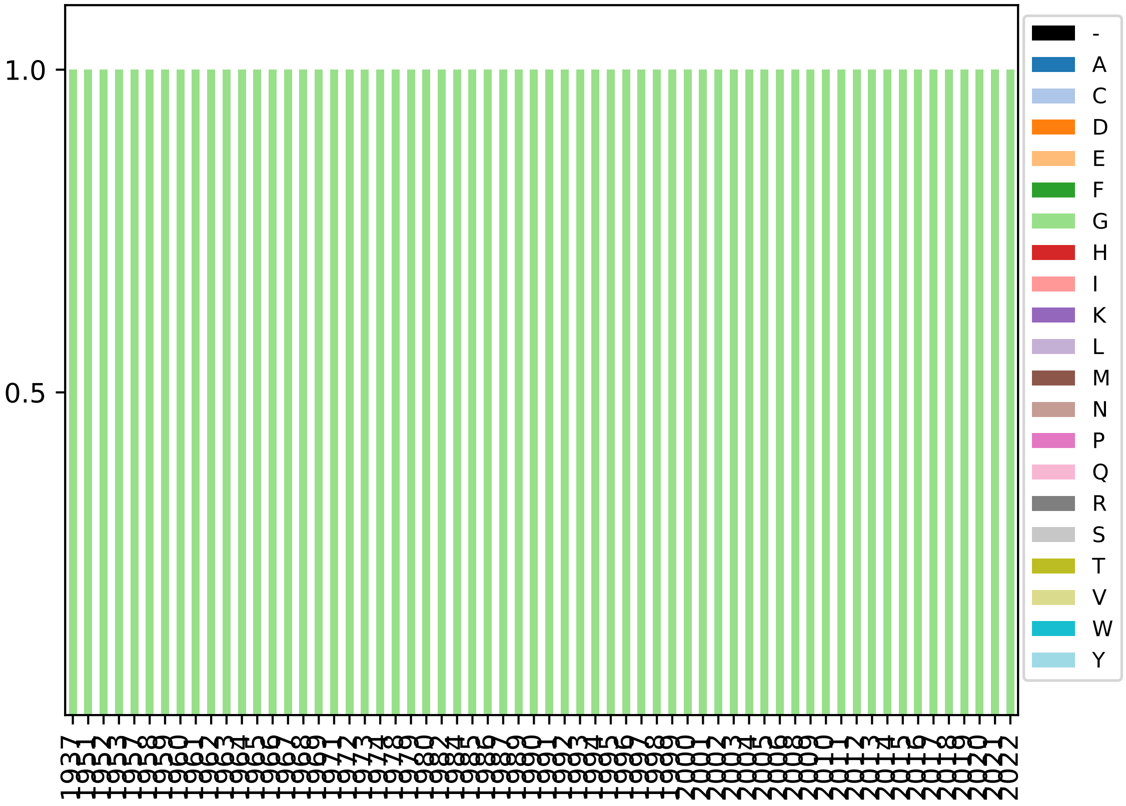

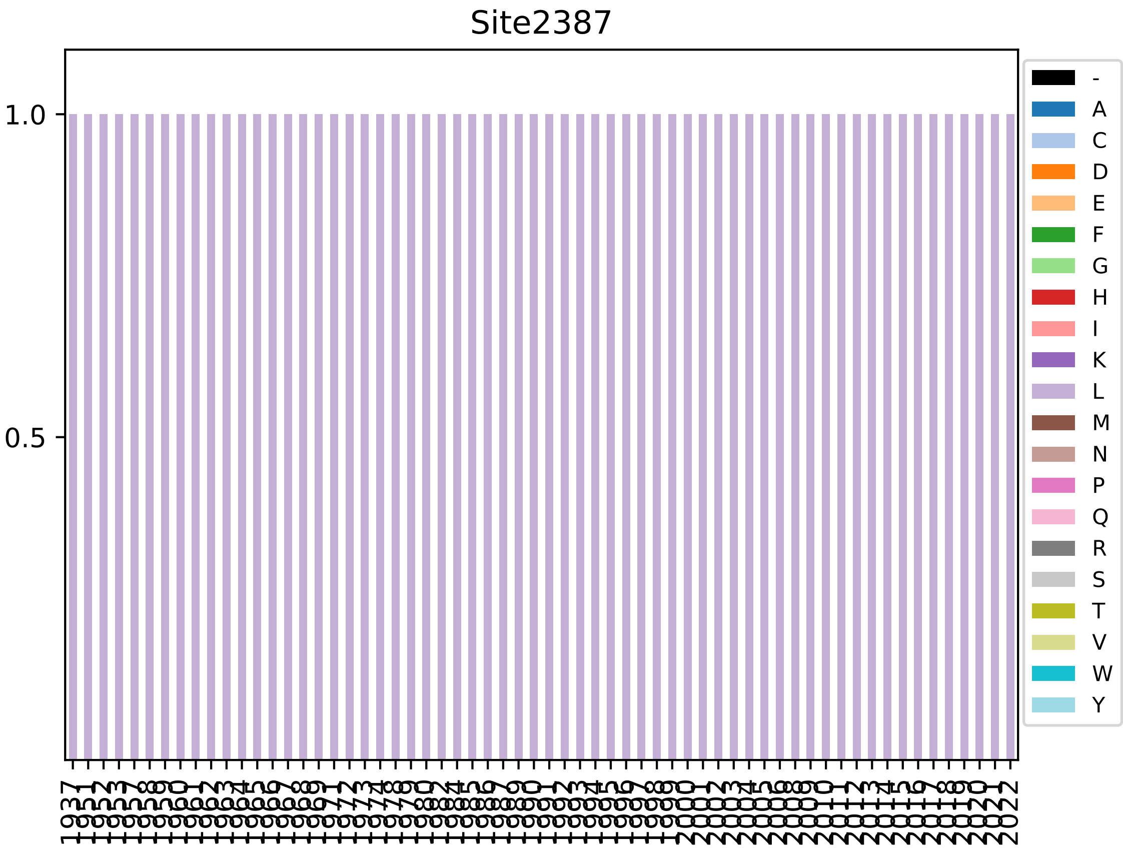
Site2387
1.0
0.5
-
A
C
D
E
F
G
H
I
K
L
M
N
P
Q
R
S
T
V
W
Y

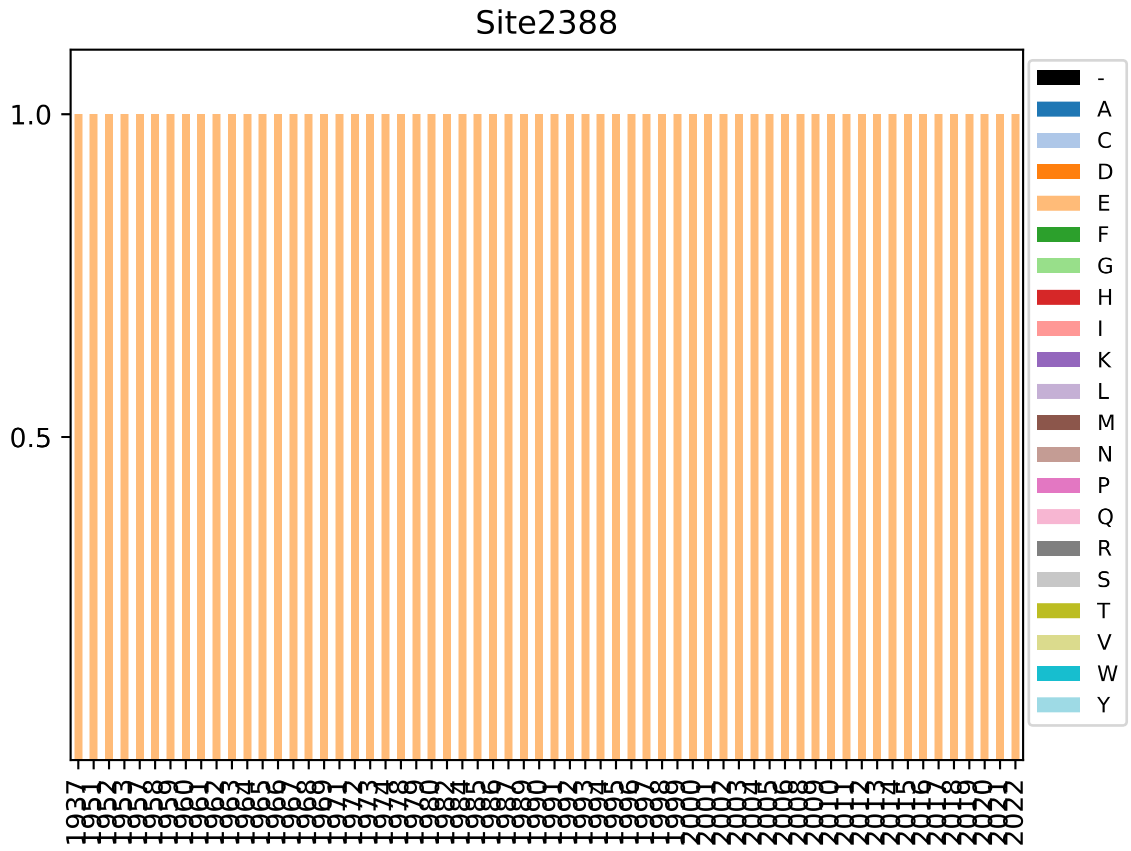
Site2388
1.0
0.5
-
A
C
D
E
F
G
H
I
K
L
M
N
P
Q
R
S
T
V
W
Y

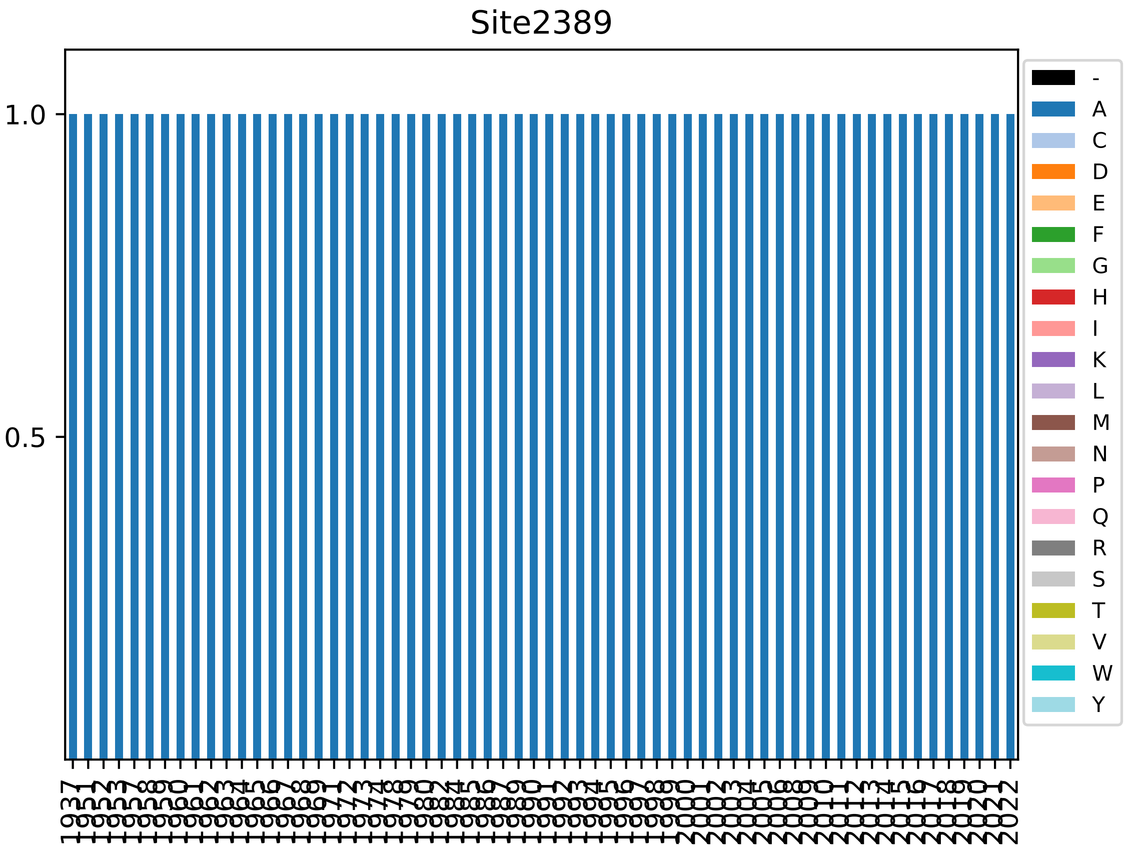
Site2389
1.0
0.5
-
A
C
D
E
F
G
H
I
K
L
M
N
P
Q
R
S
T
V
W
Y

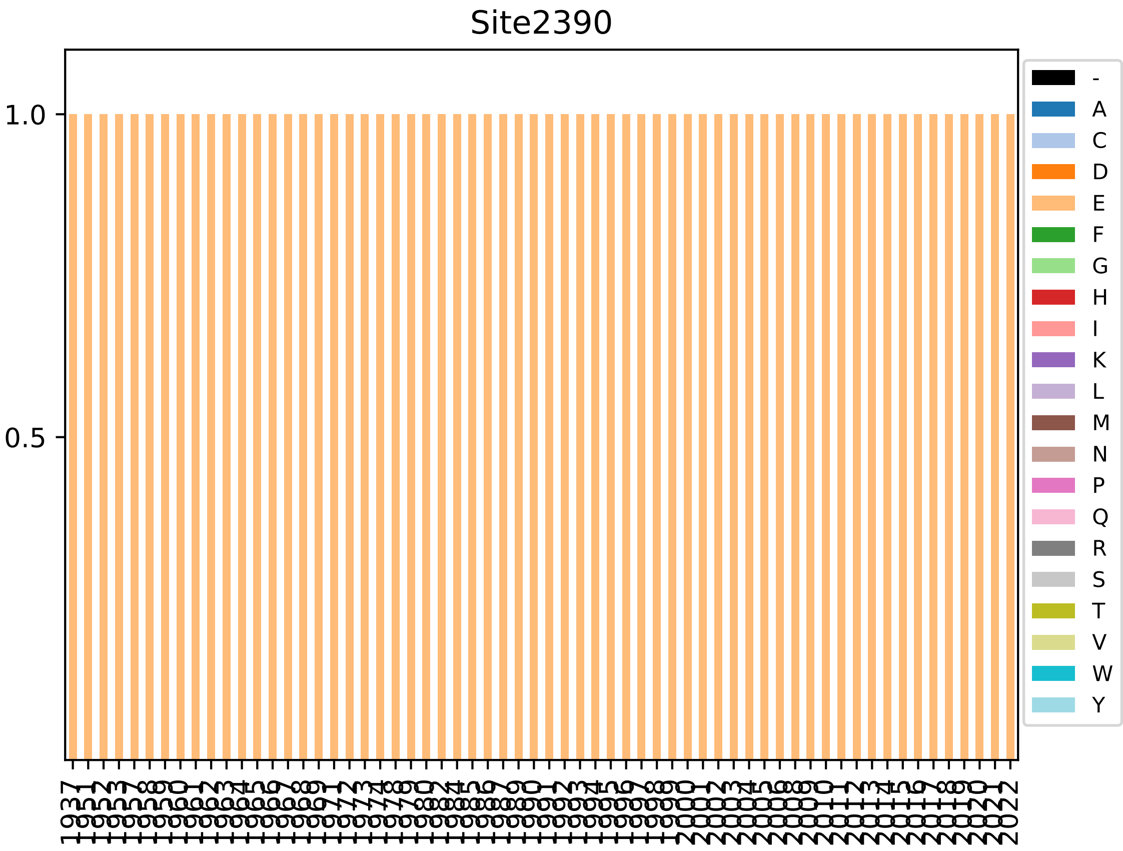

Site2390
1.0
0.5
-
A
C
D
E
F
G
H
I
K
L
M
N
P
Q
R
S
T
V
W
Y

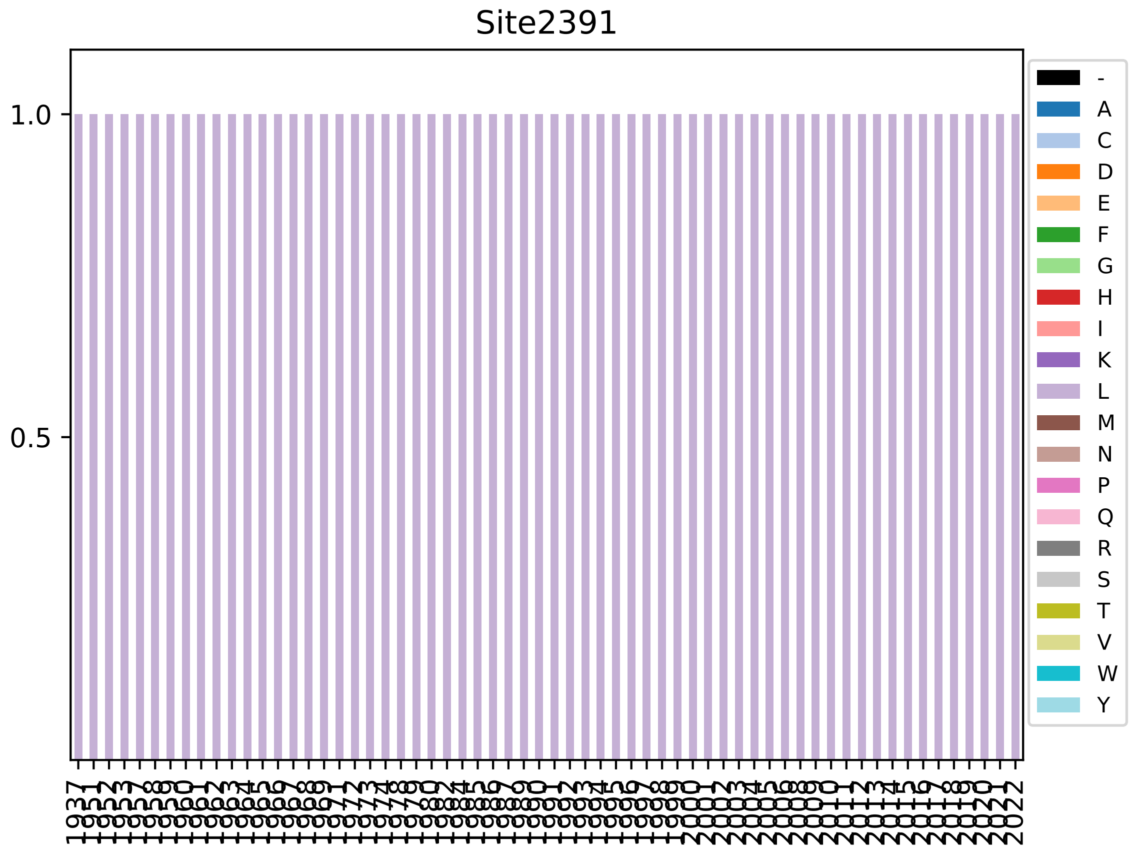
Site2391
1.0
0.5
-
A
C
D
E
F
G
H
I
K
L
M
N
P
Q
R
S
T
V
W
Y

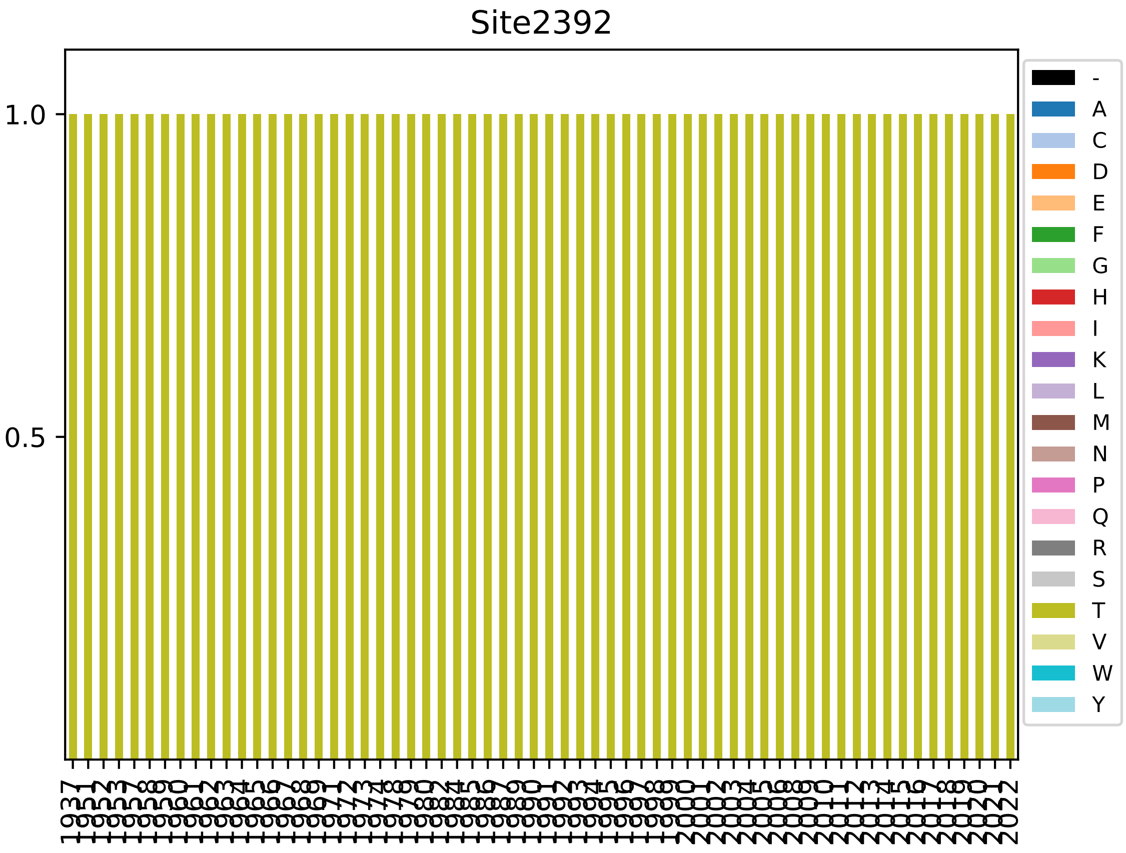
Site2392
1.0
0.5
-
A
C
D
E
F
G
H
I
K
L
M
N
P
Q
R
S
T
V
W
Y

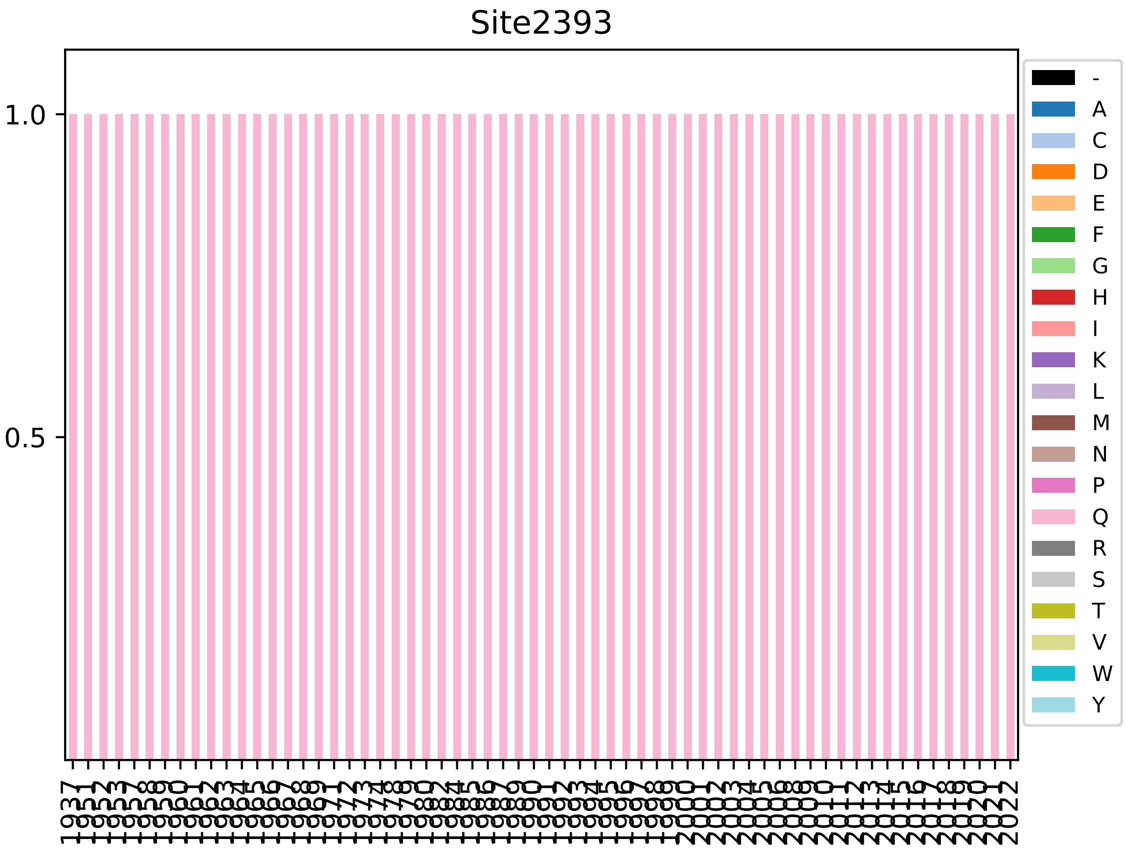
Site2393
1.0
0.5
-
A
C
D
E
F
G
H
I
K
L
M
N
P
Q
R
S
T
V
W
Y

Site2394

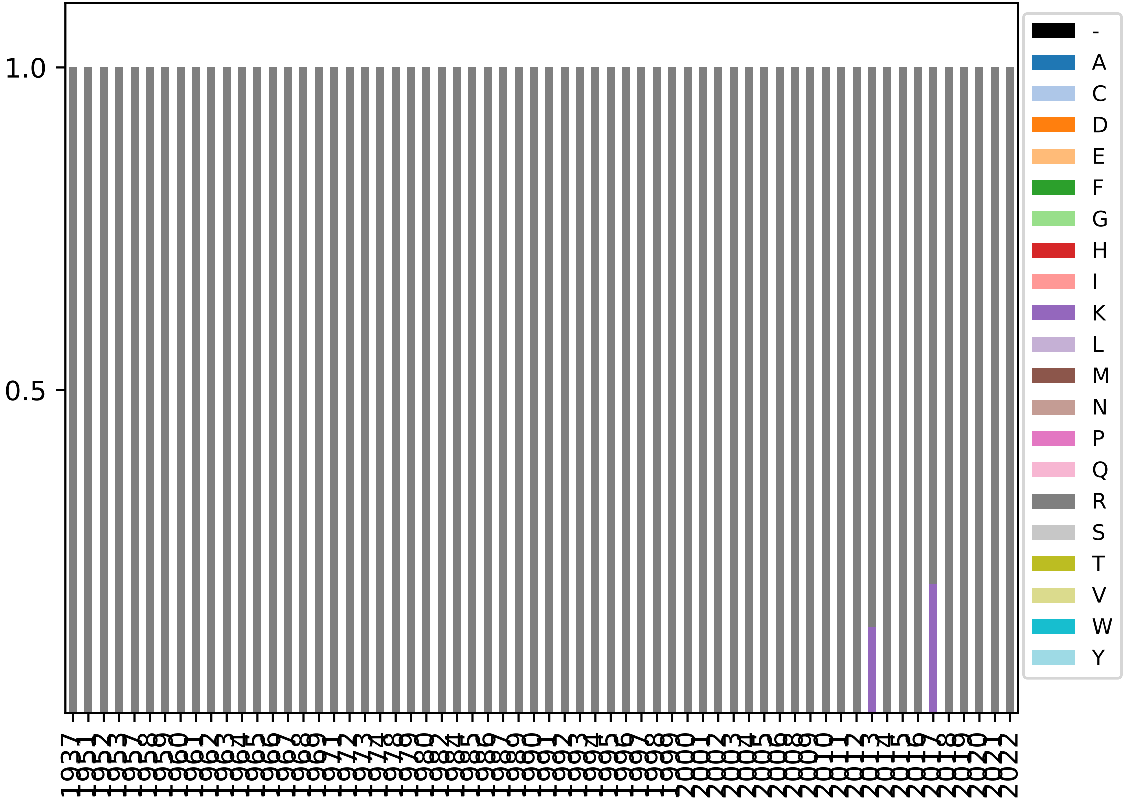

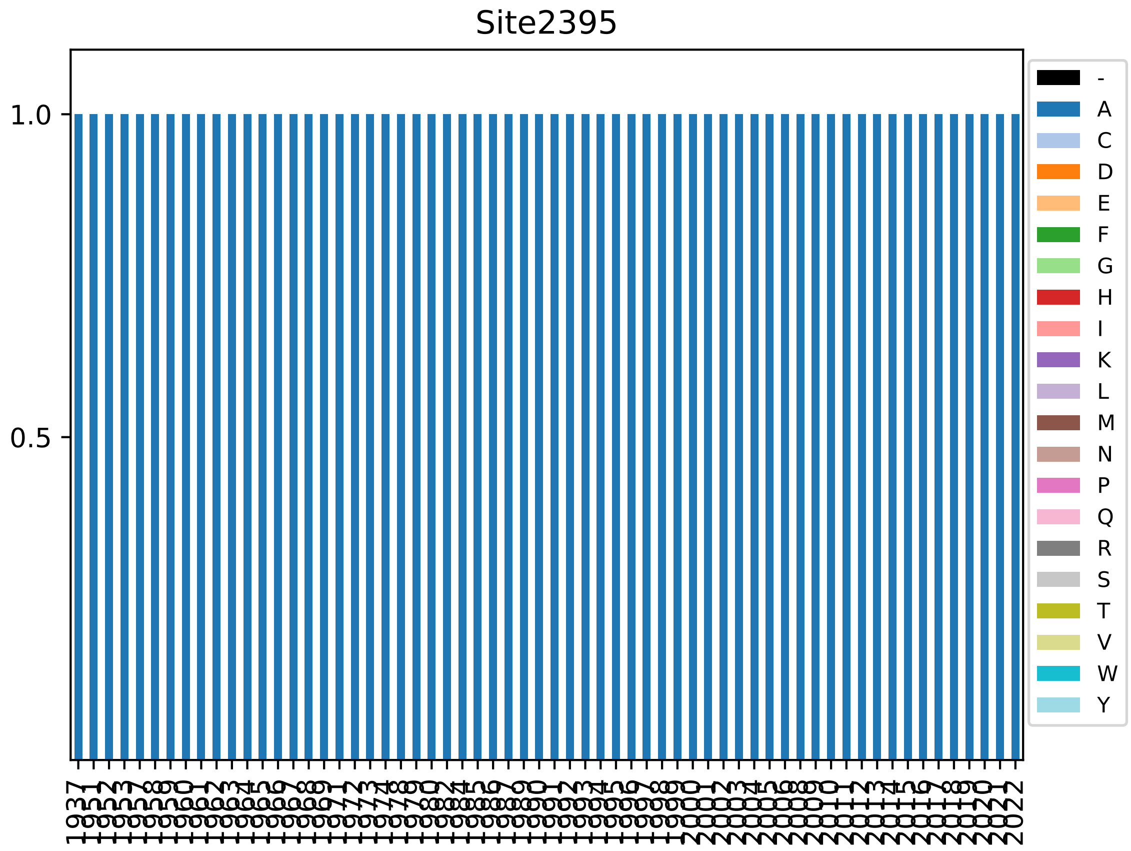
Site2395
1.0
0.5
-
A
C
D
E
F
G
H
I
K
L
M
N
P
Q
R
S
T
V
W
Y

Site2396

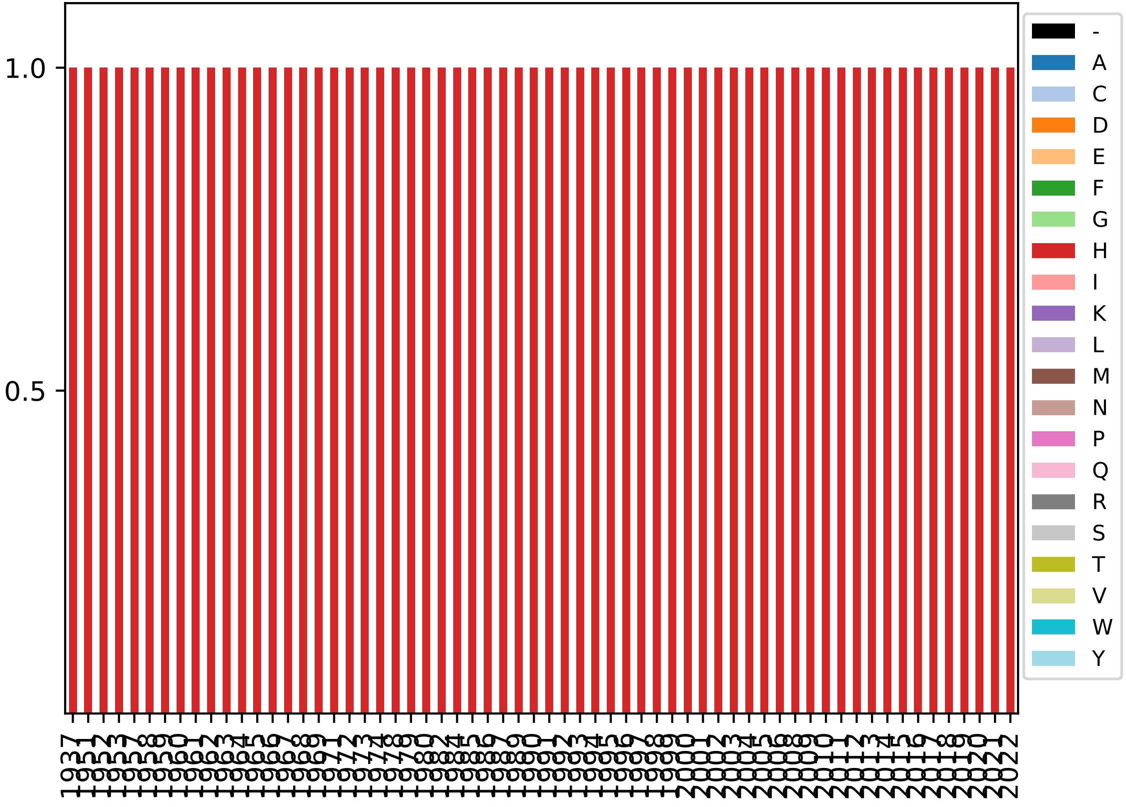

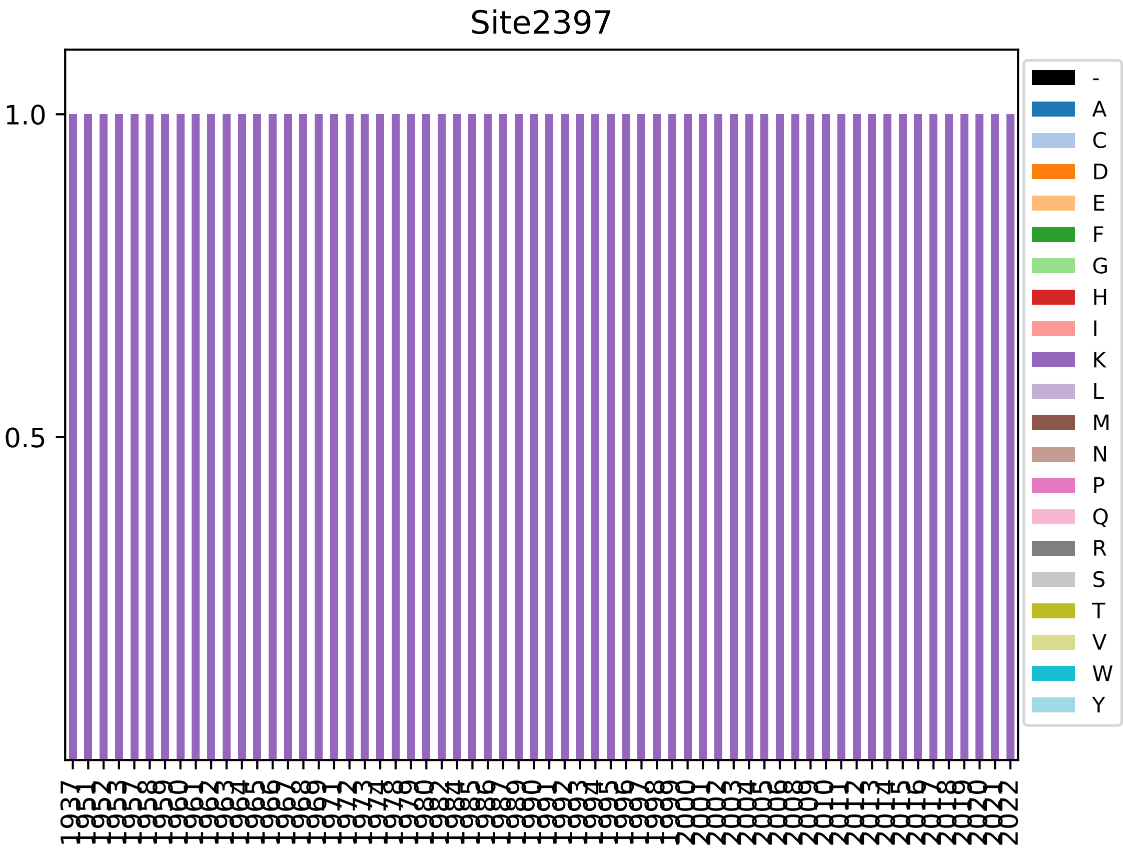
Site2397
1.0
0.5
-
A
C
D
E
F
G
H
I
K
L
M
N
P
Q
R
S
T
V
W
Y

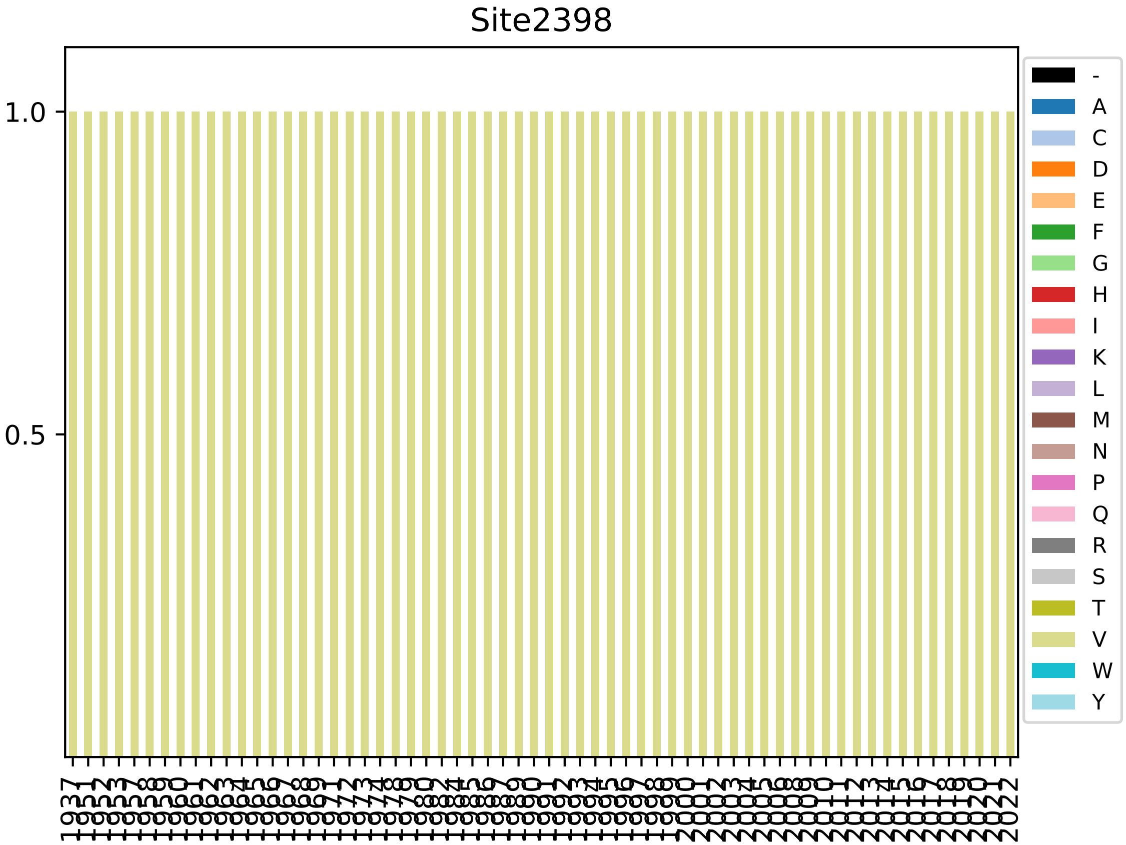
Site2398
1.0
0.5
-
A
C
D
E
F
G
H
I
K
L
M
N
P
Q
R
S
T
V
W
Y

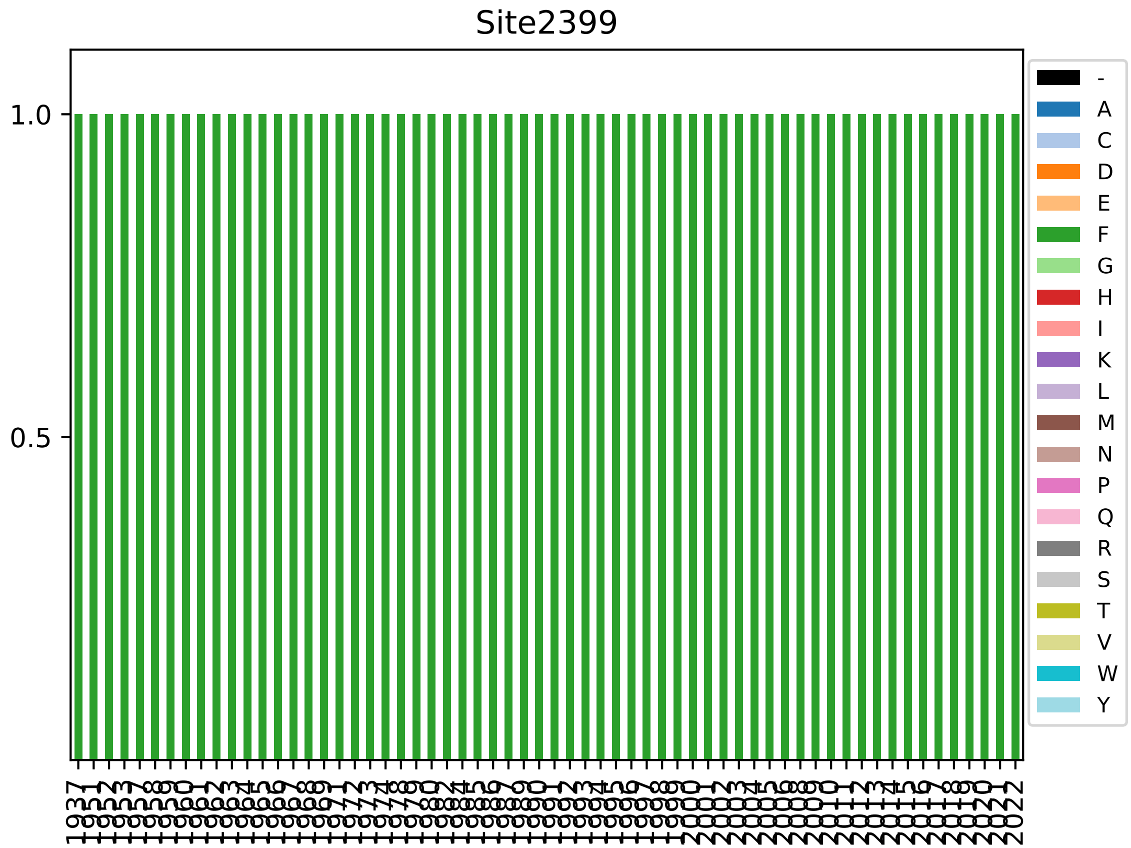
Site2399
1.0
0.5
-
A
C
D
E
F
G
H
I
K
L
M
N
P
Q
R
S
T
V
W
Y

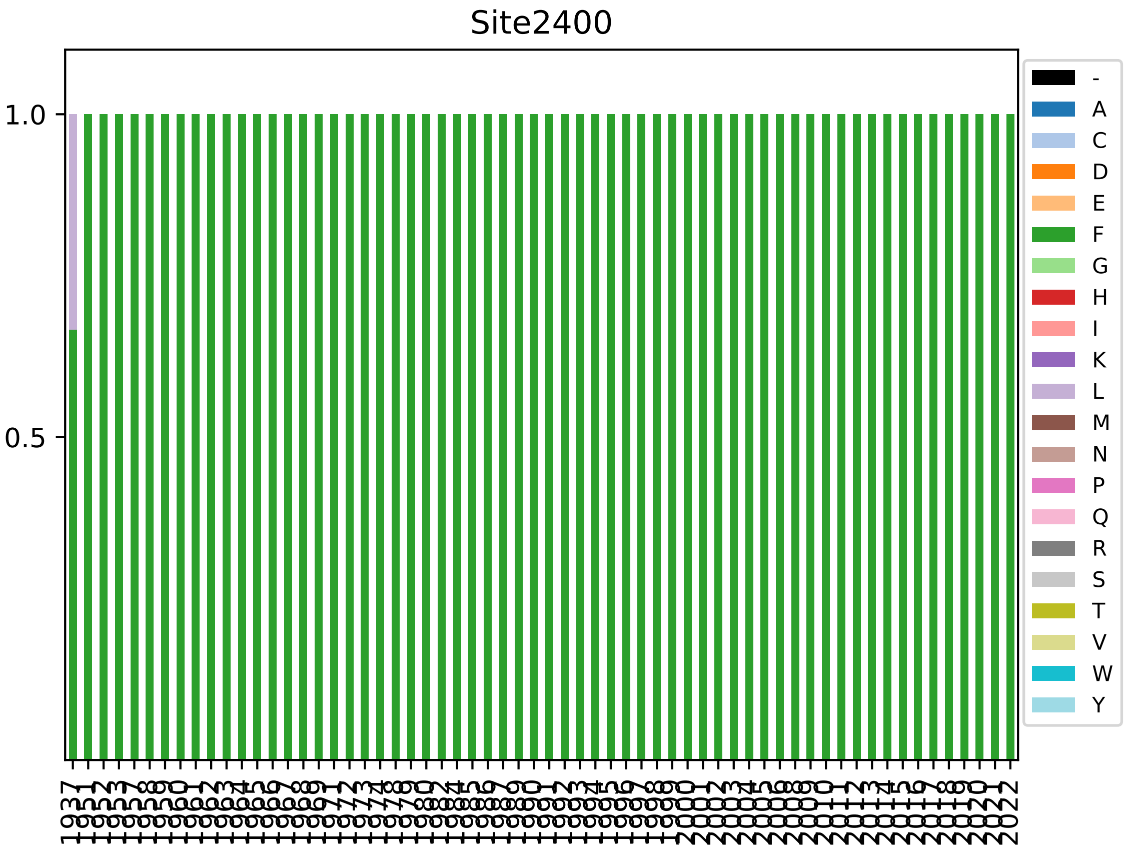
Site2400
1.0
0.5
-
A
C
D
E
F
G
H
I
K
L
M
N
P
Q
R
S
T
V
W
Y

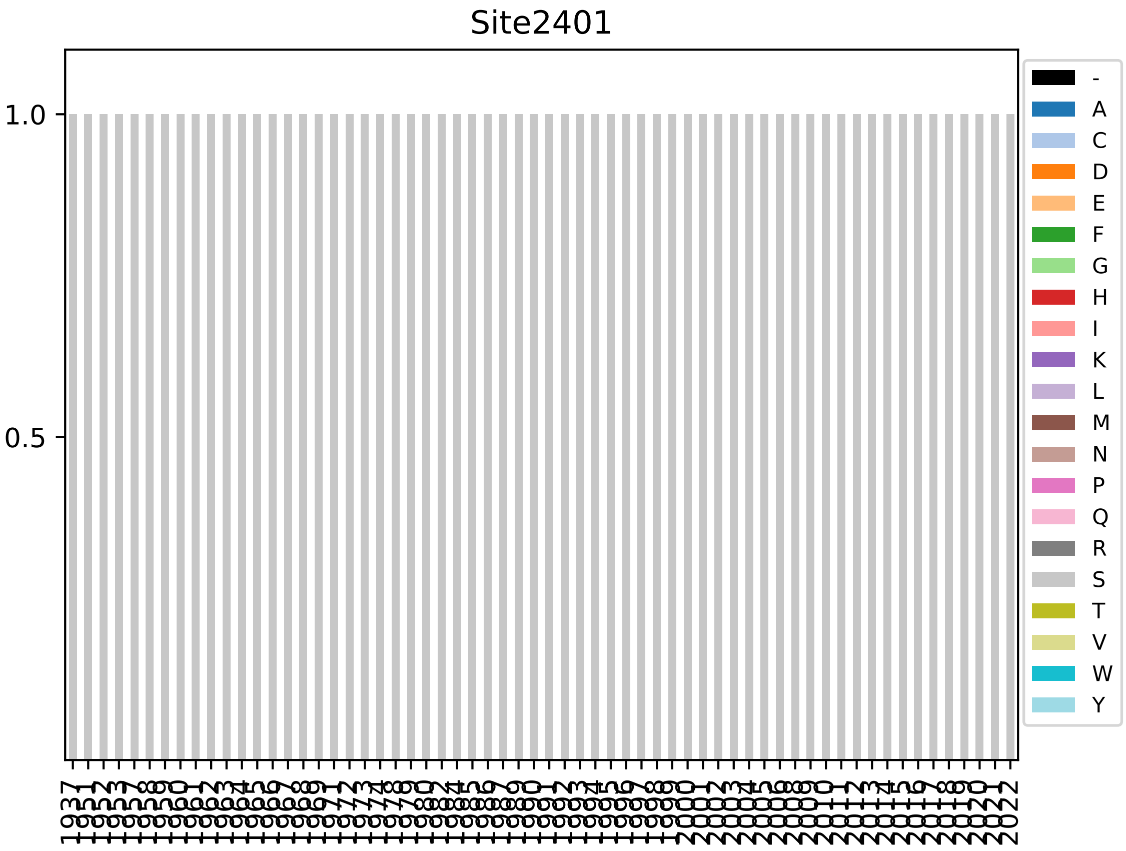
Site2401
1.0
0.5
-
A
C
D
E
F
G
H
I
K
L
M
N
P
Q
R
S
T
V
W
Y

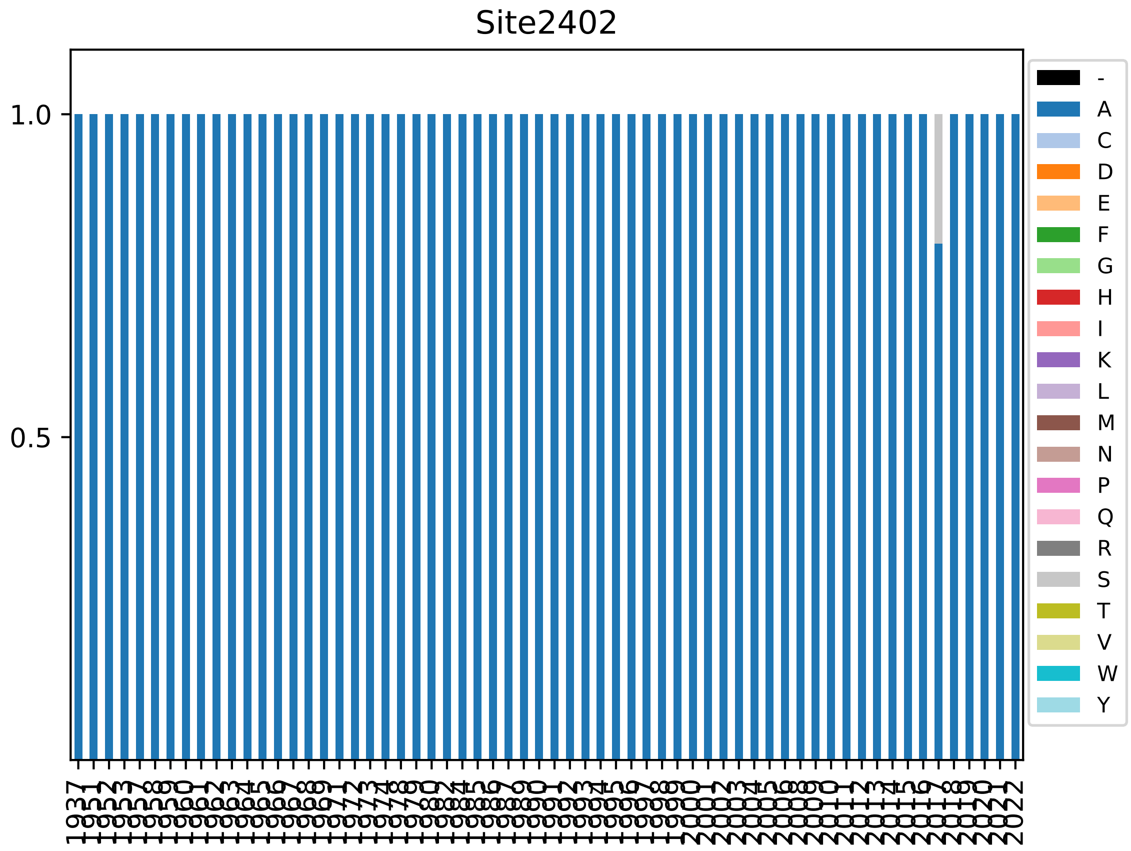
Site2402
1.0
0.5
-
A
C
D
E
F
G
H
I
K
L
M
N
P
Q
R
S
T
V
W
Y

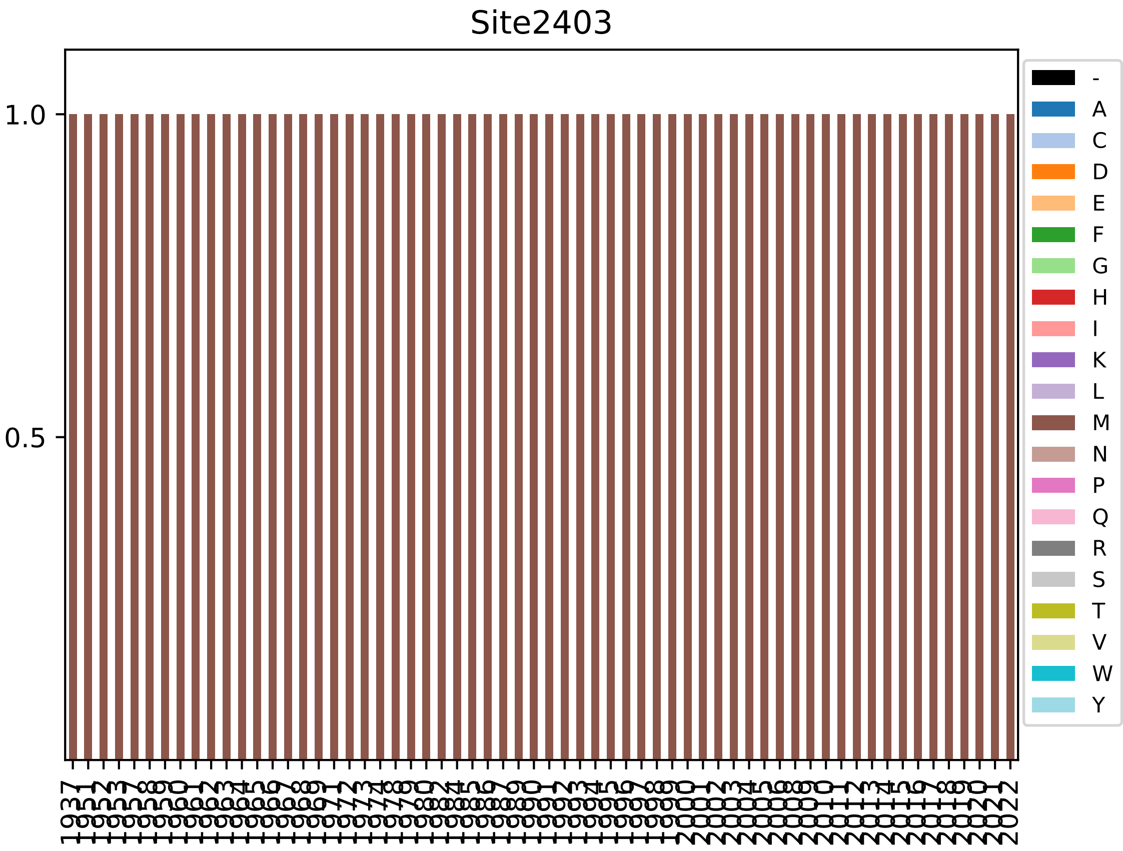
Site2403
1.0
0.5
-
A
C
D
E
F
G
H
I
K
L
M
N
P
Q
R
S
T
V
W
Y

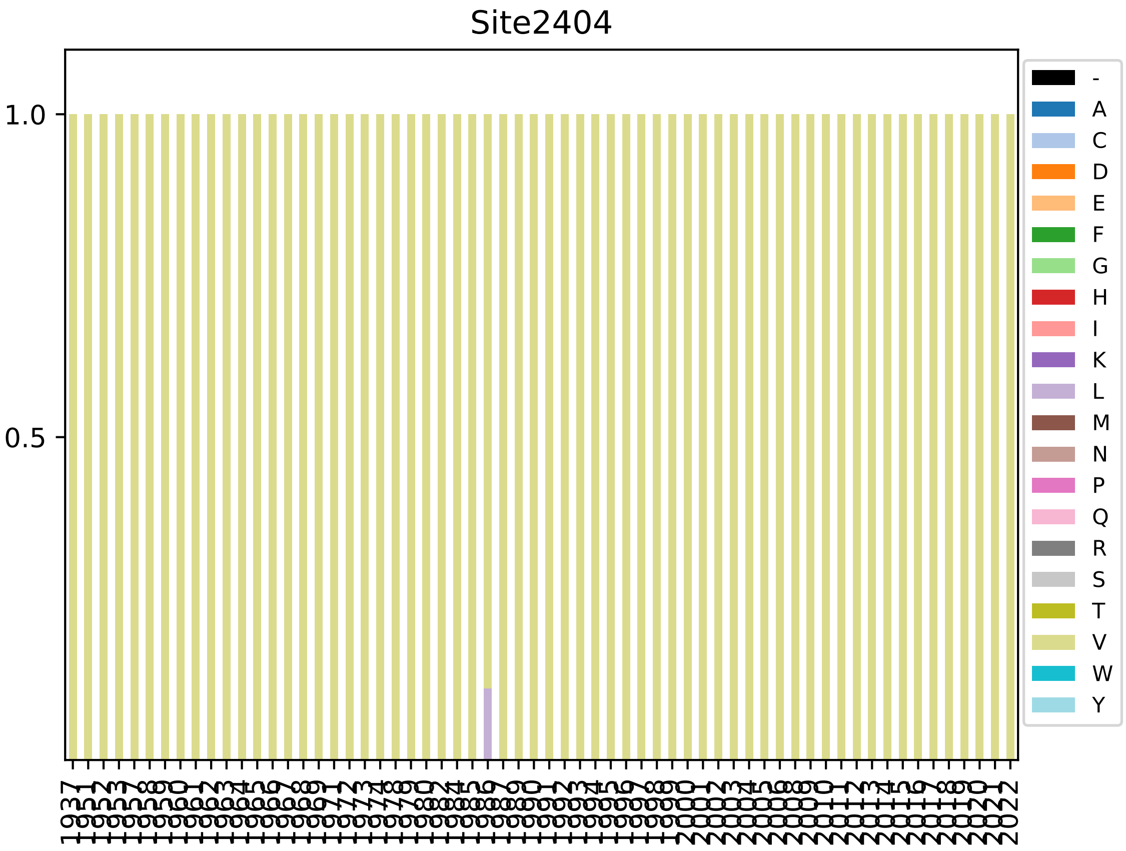
Site2404
1.0
0.5
-
A
C
D
E
F
G
H
I
K
L
M
N
P
Q
R
S
T
V
W
Y

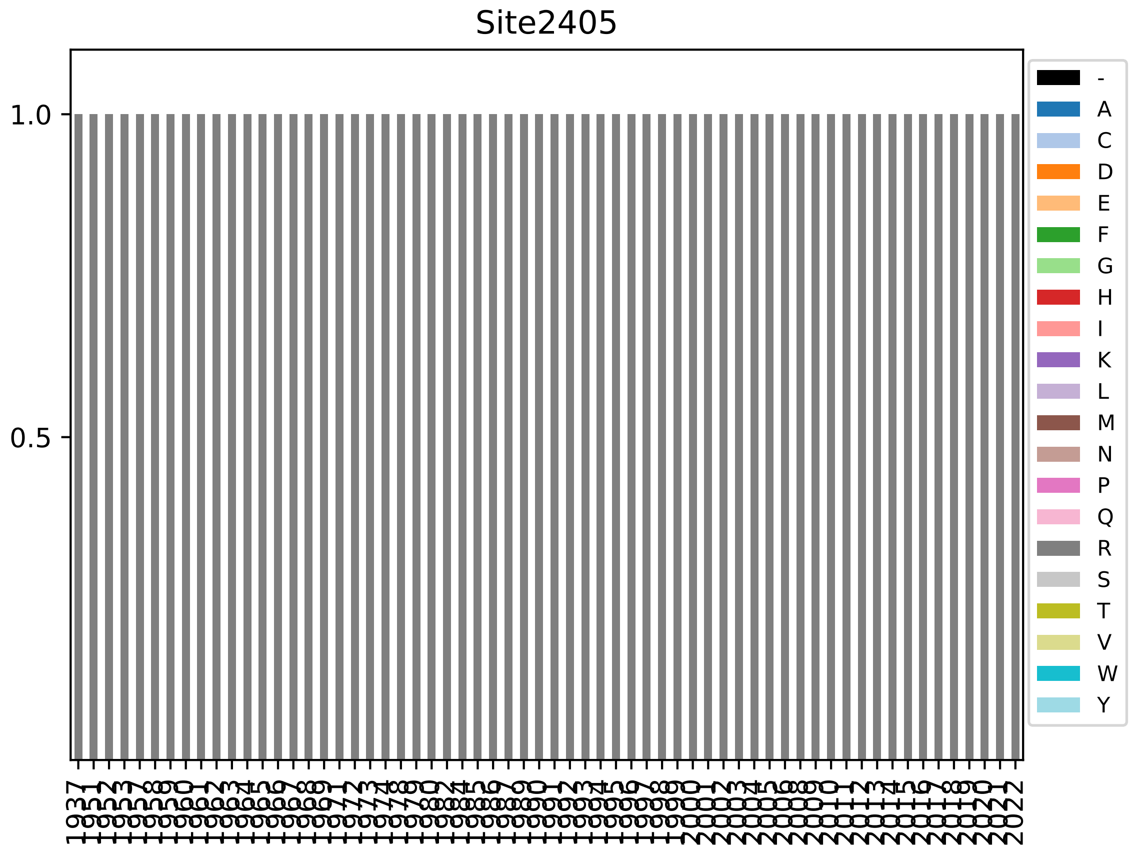
Site2405
1.0
0.5
-
A
C
D
E
F
G
H
I
K
L
M
N
P
Q
R
S
T
V
W
Y

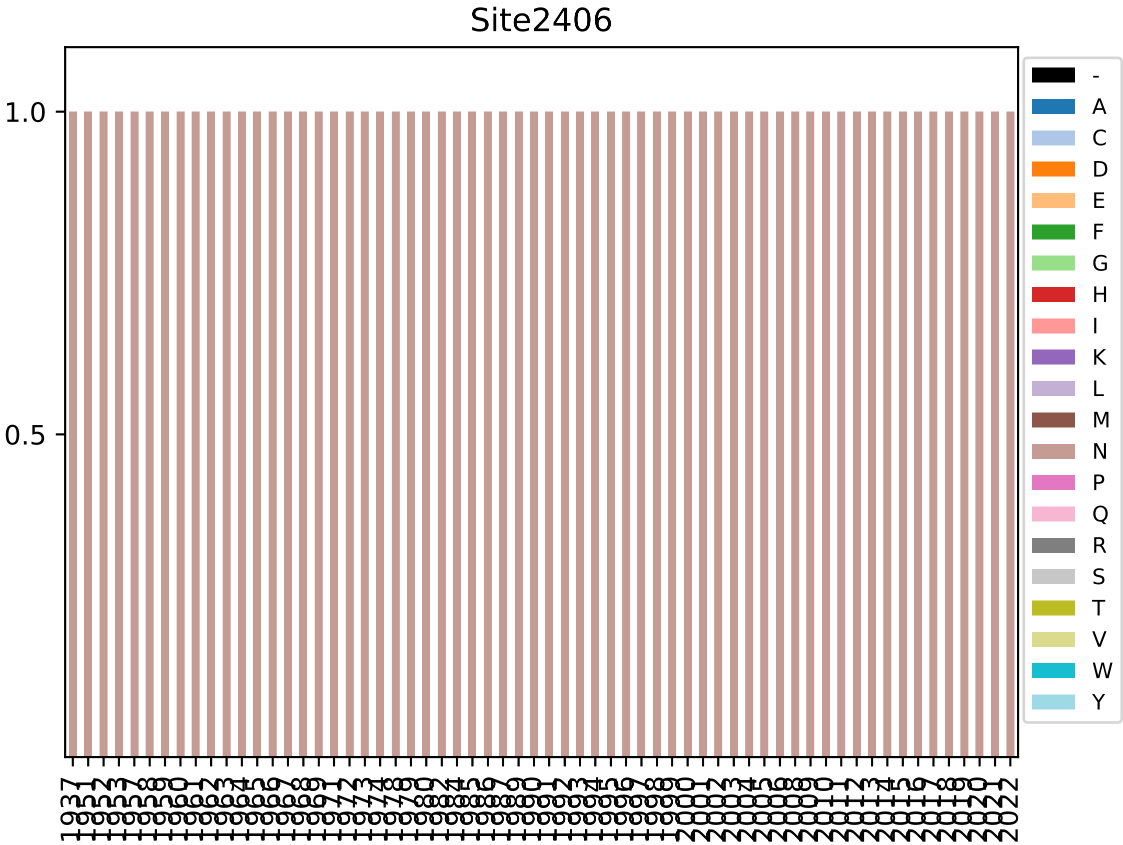
Site2406
1.0
0.5
-
A
C
D
E
F
G
H
I
K
L
M
N
P
Q
R
S
T
V
W
Y

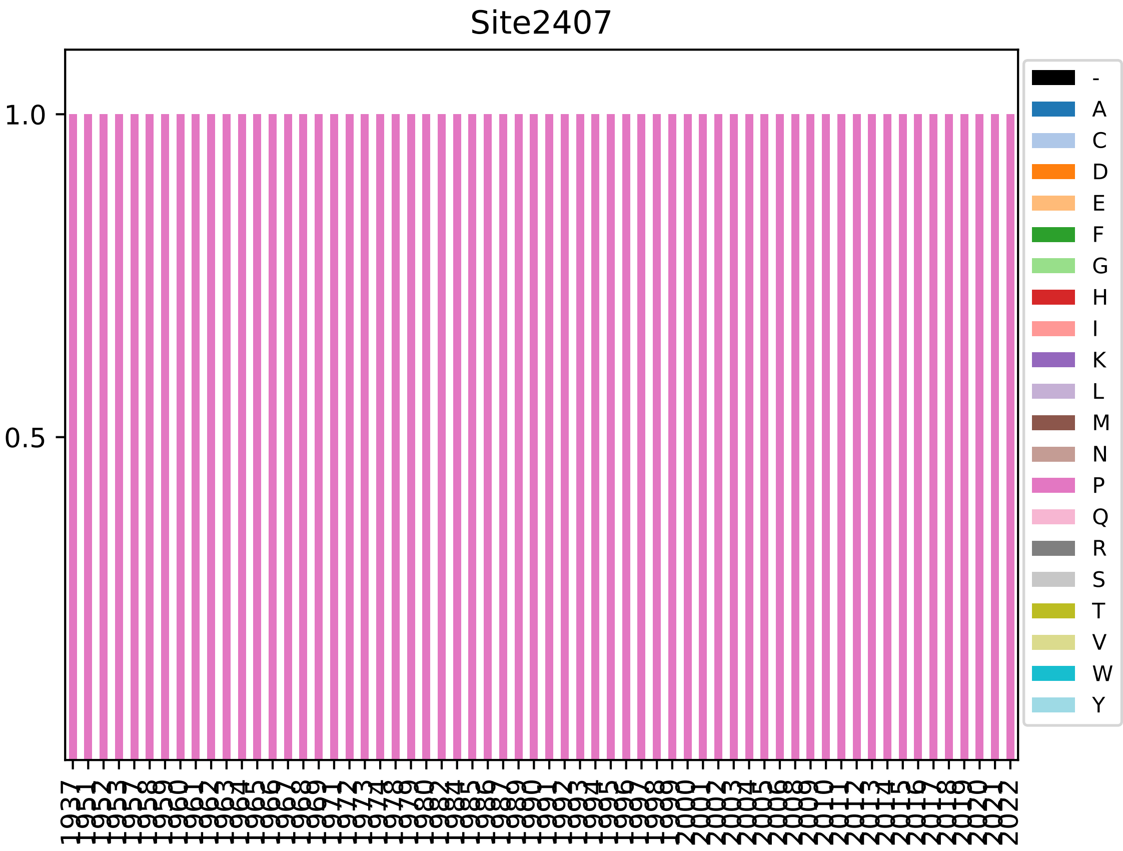
Site2407
1.0
0.5
-
A
C
D
E
F
G
H
I
K
L
M
N
P
Q
R
S
T
V
W
Y

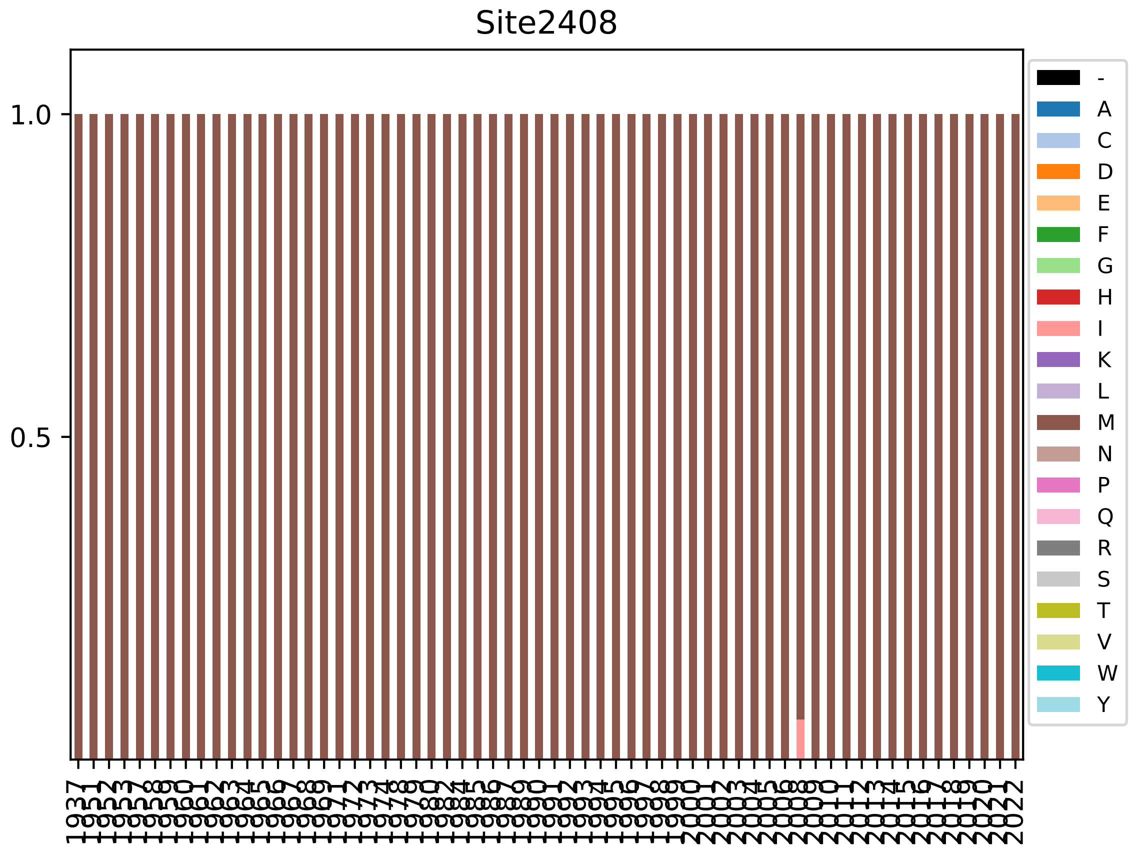
Site2408
1.0
0.5
-
A
C
D
E
F
G
H
I
K
L
M
N
P
Q
R
S
T
V
W
Y

Site2409

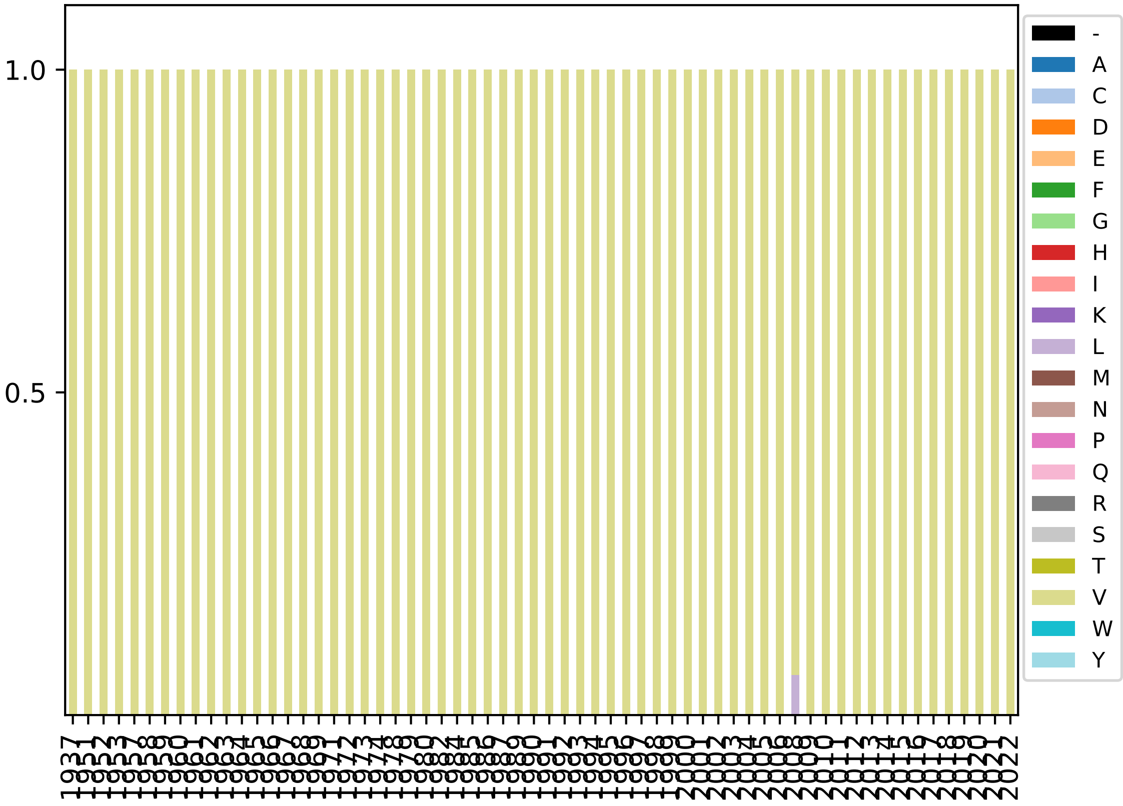

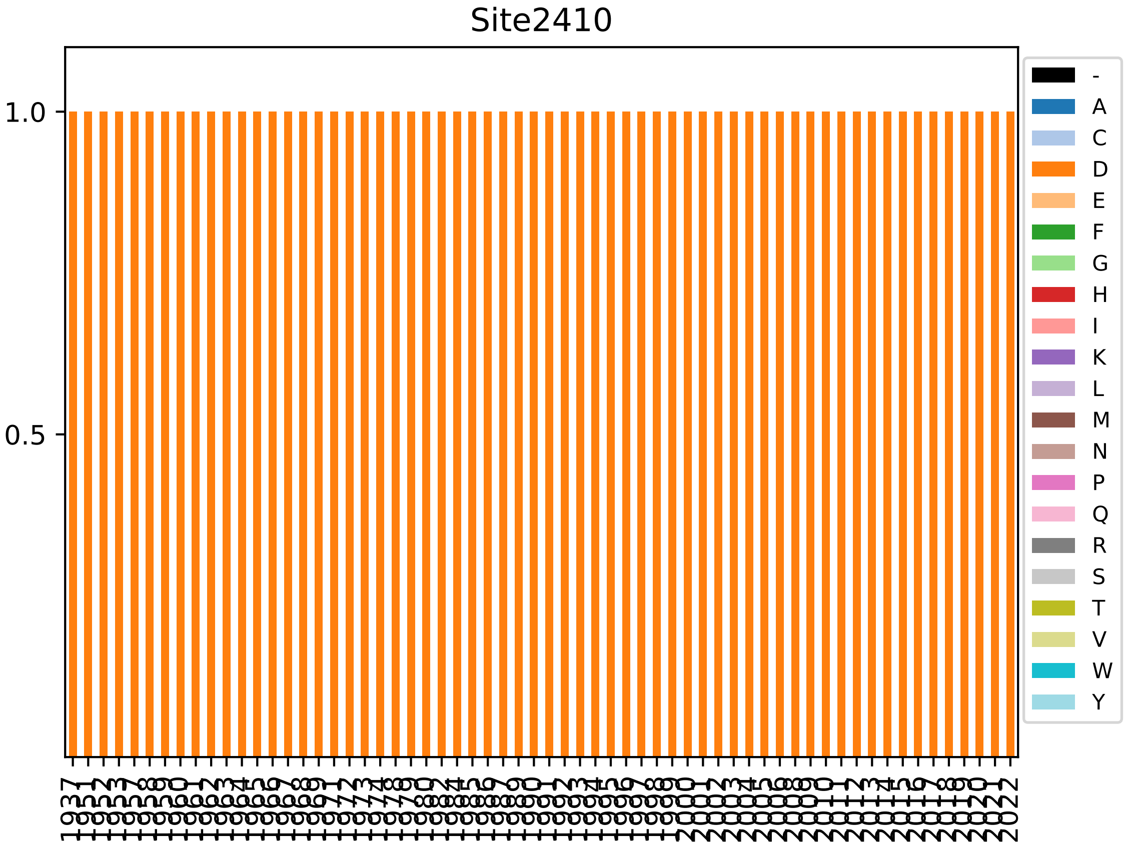

Site2410
1.0
0.5
-
A
C
D
E
F
G
H
I
K
L
M
N
P
Q
R
S
T
V
W
Y

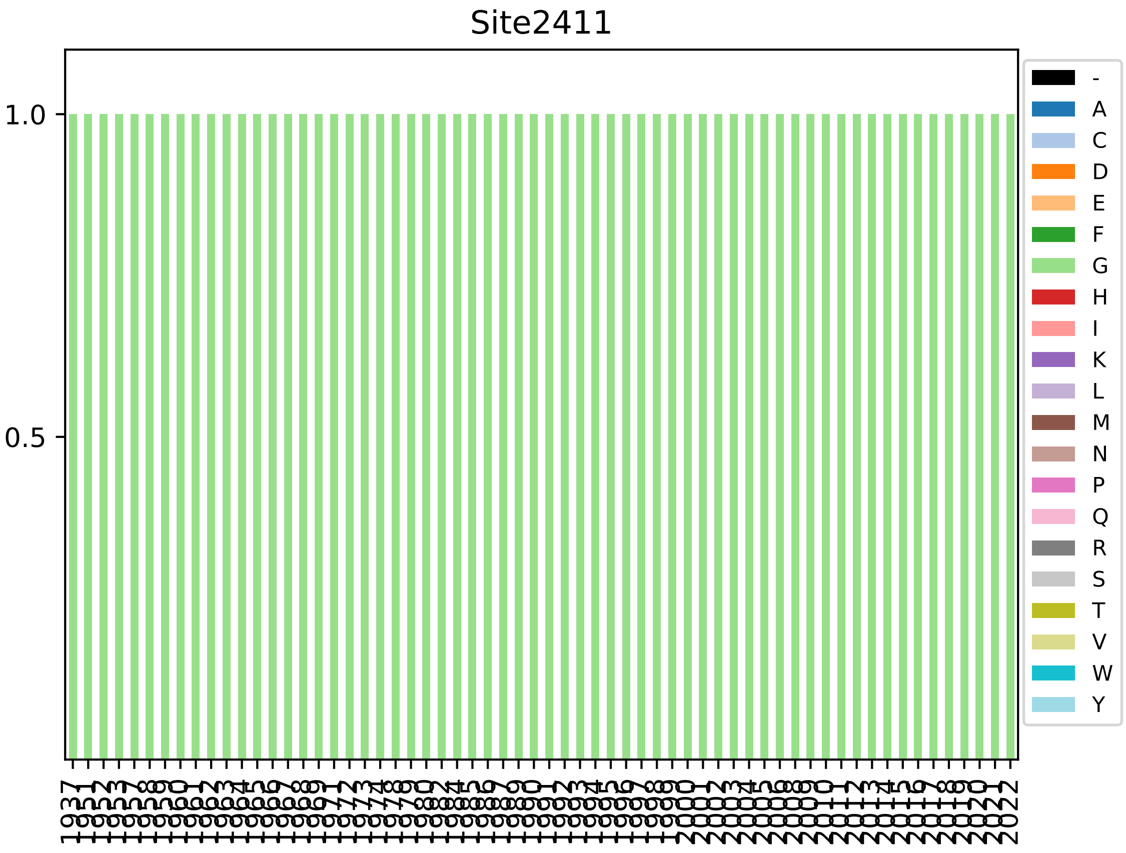
Site2411
1.0
0.5
-
A
C
D
E
F
G
H
I
K
L
M
N
P
Q
R
S
T
V
W
Y

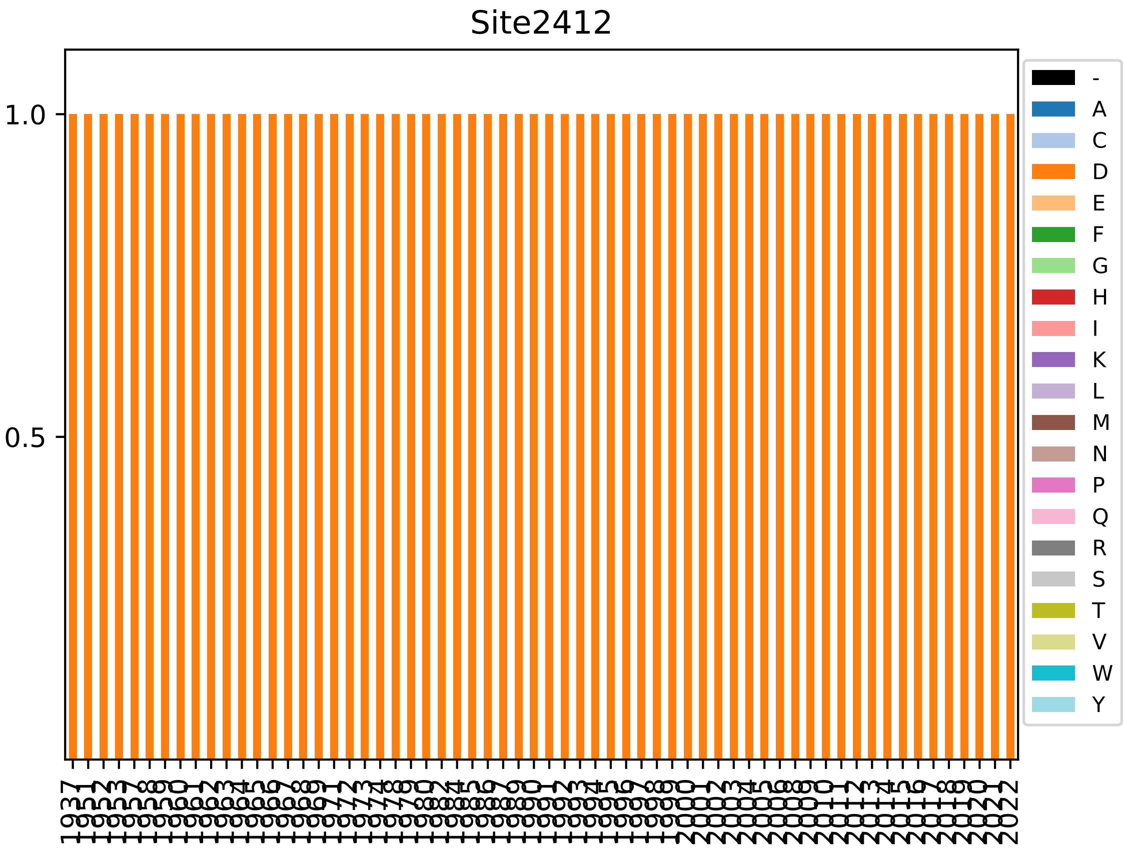
Site2412
1.0
0.5
-
A
C
D
E
F
G
H
I
K
L
M
N
P
Q
R
S
T
V
W
Y

Site2413

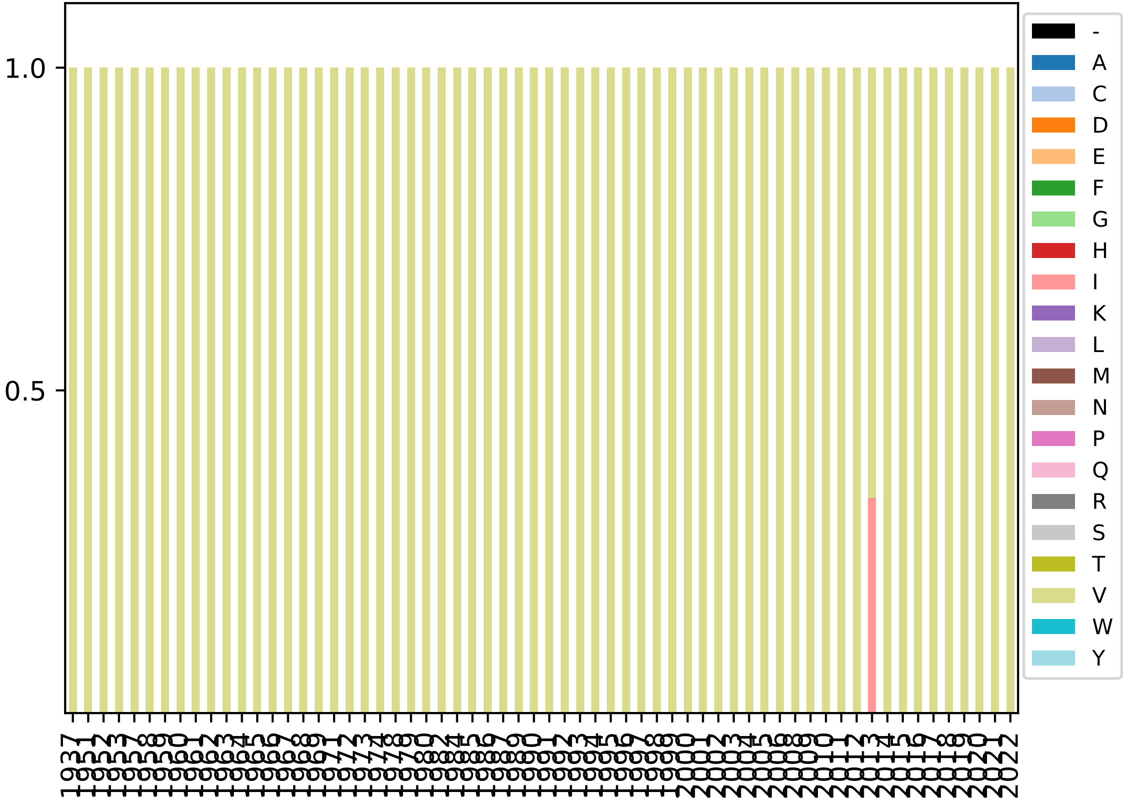

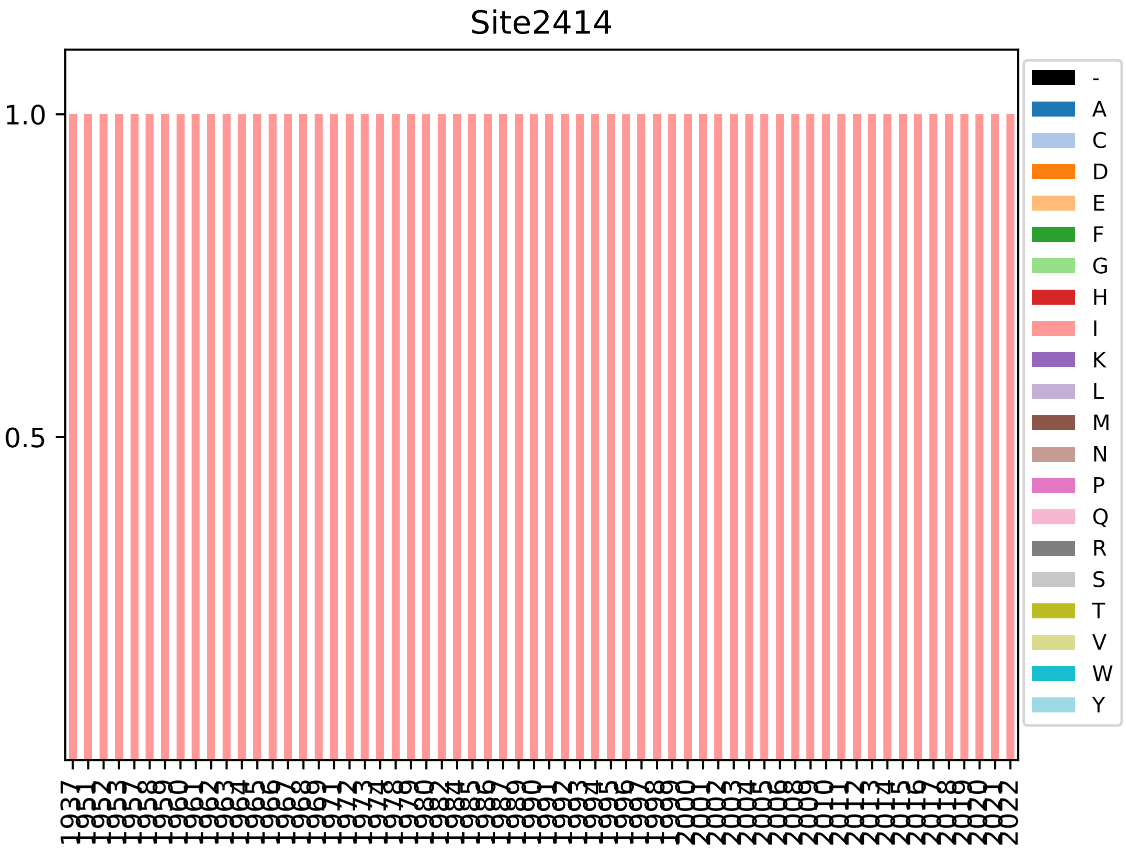
Site2414
1.0
0.5
-
A
C
D
E
F
G
H
I
K
L
M
N
P
Q
R
S
T
V
W
Y

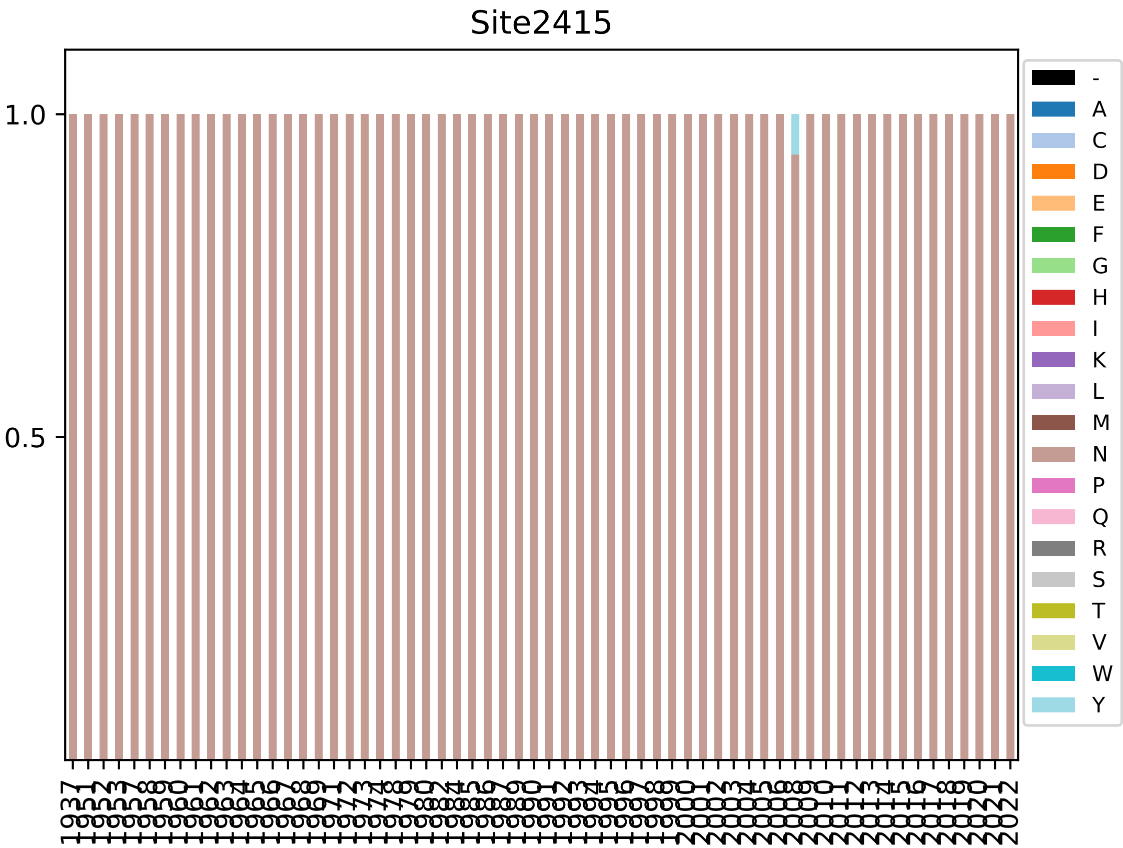
Site2415
1.0
0.5
-
A
C
D
E
F
G
H
I
K
L
M
N
P
Q
R
S
T
V
W
Y

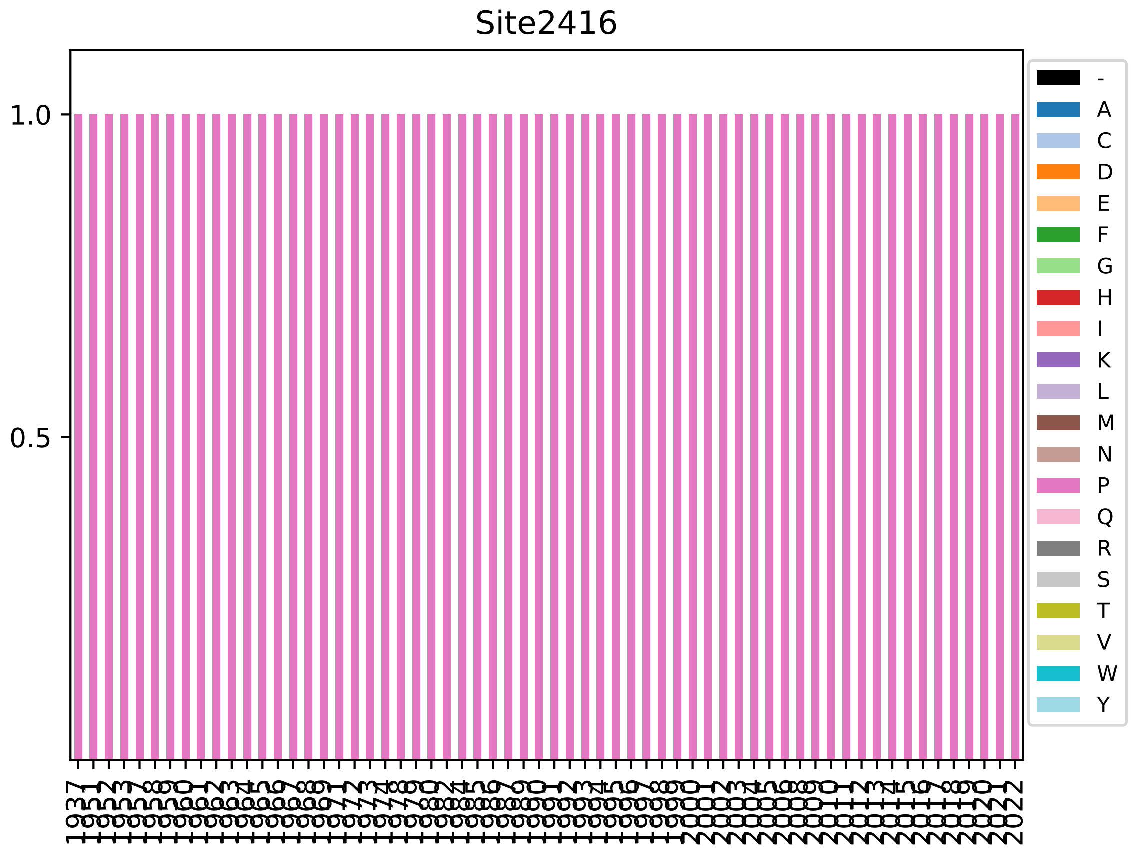
Site2416
1.0
0.5
-
A
C
D
E
F
G
H
I
K
L
M
N
P
Q
R
S
T
V
W
Y

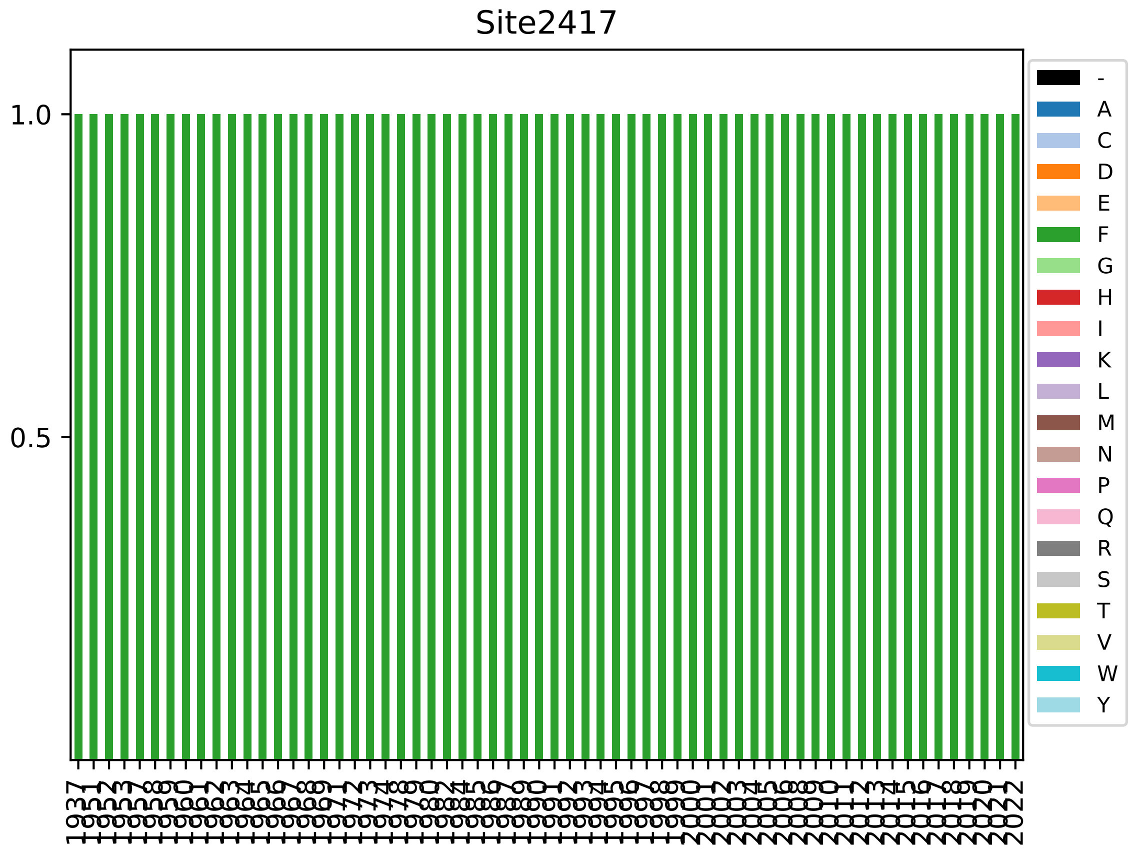
Site2417
1.0
0.5
-
A
C
D
E
F
G
H
I
K
L
M
N
P
Q
R
S
T
V
W
Y

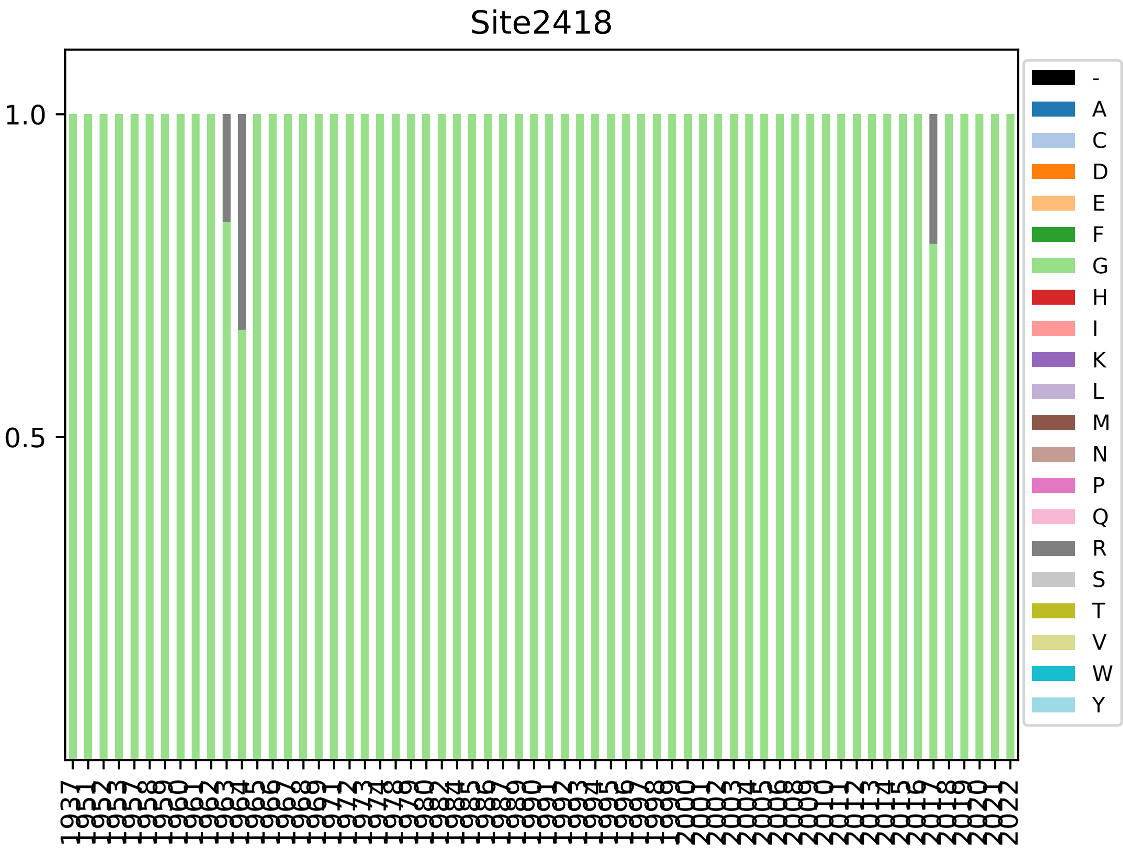
Site2418
1.0
0.5
-
A
C
D
E
F
G
H
I
K
L
M
N
P
Q
R
S
T
V
W
Y

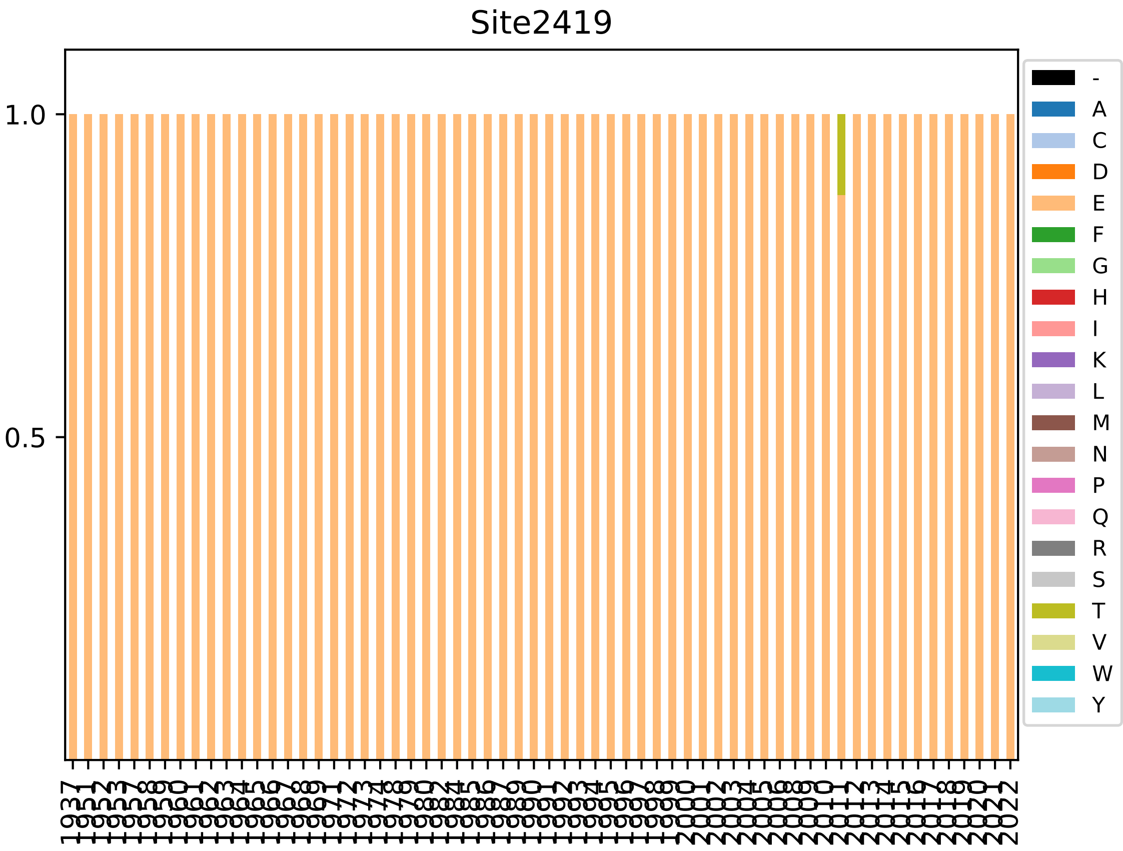
Site2419
1.0
0.5
-
A
C
D
E
F
G
H
I
K
L
M
N
P
Q
R
S
T
V
W
Y

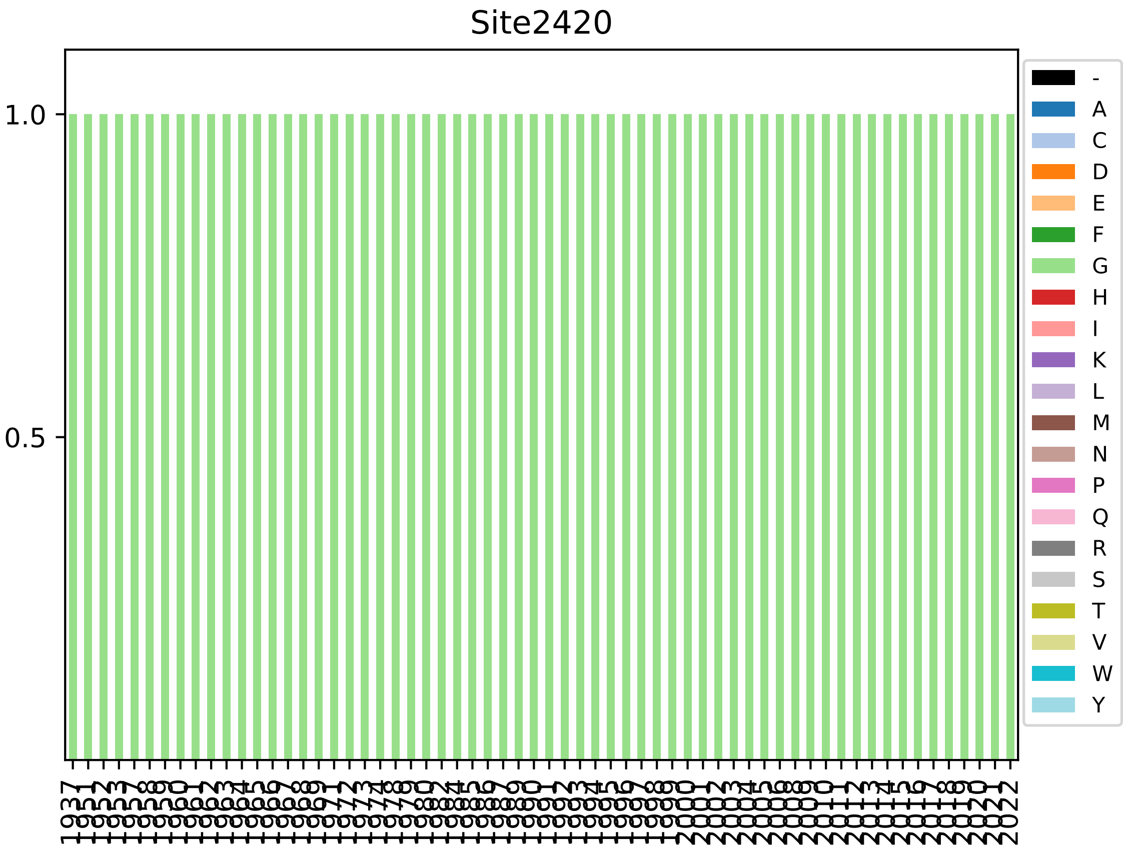

Site2420
1.0
0.5
-
A
C
D
E
F
G
H
I
K
L
M
N
P
Q
R
S
T
V
W
Y

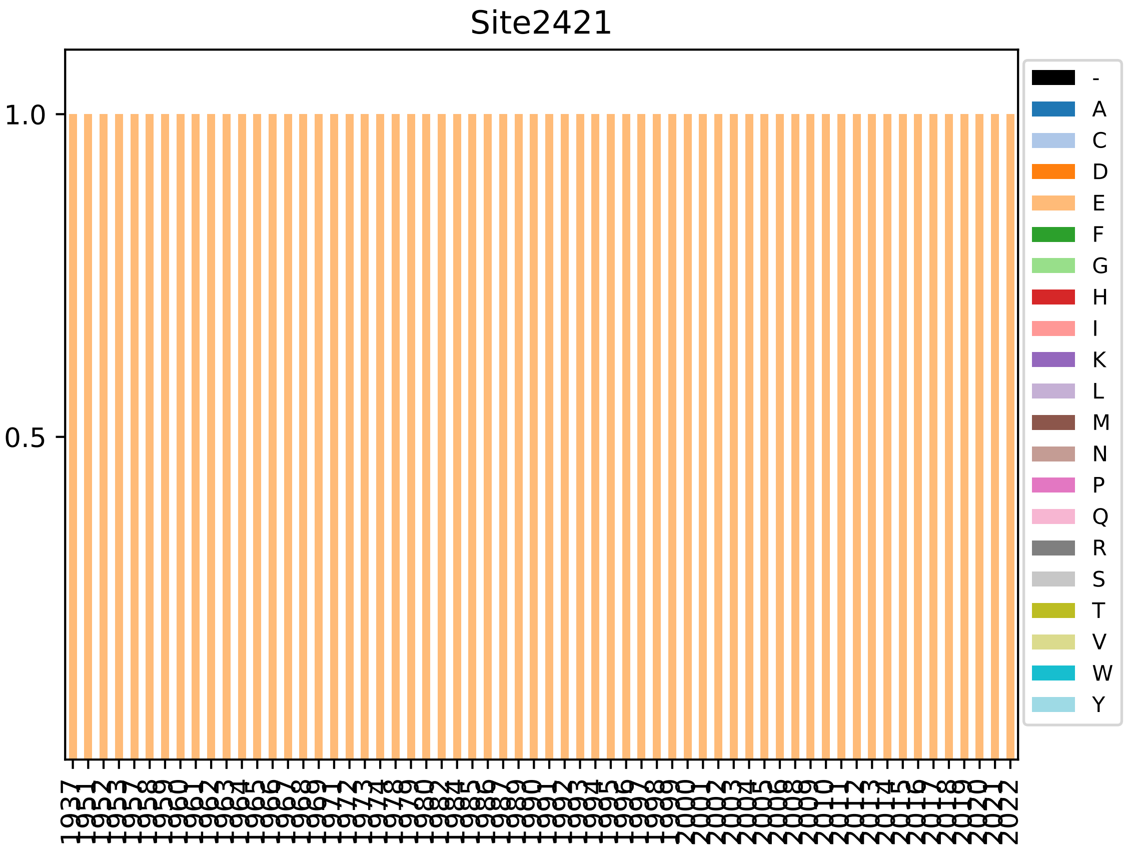
Site2421
1.0
0.5
-
A
C
D
E
F
G
H
I
K
L
M
N
P
Q
R
S
T
V
W
Y

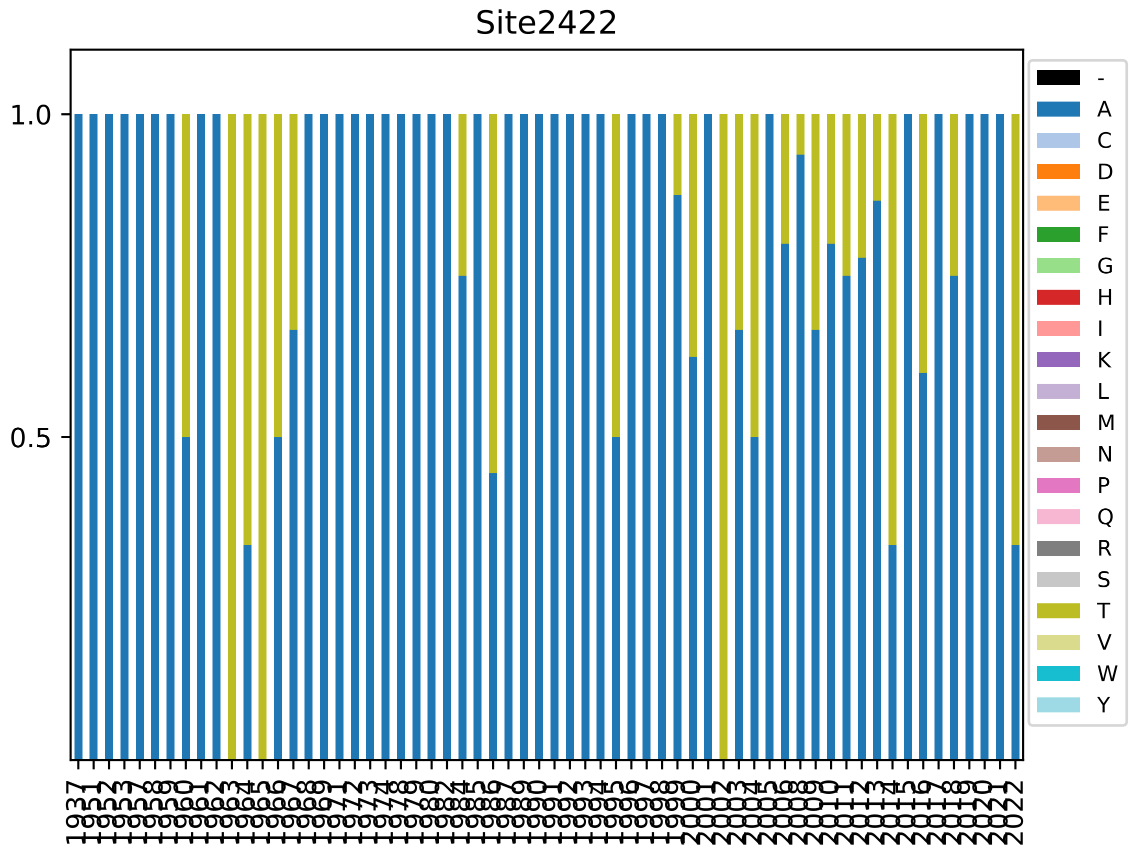
Site2422
1.0
0.5
-
A
C
D
E
F
G
H
I
K
L
M
N
P
Q
R
S
T
V
W
Y

Site2423

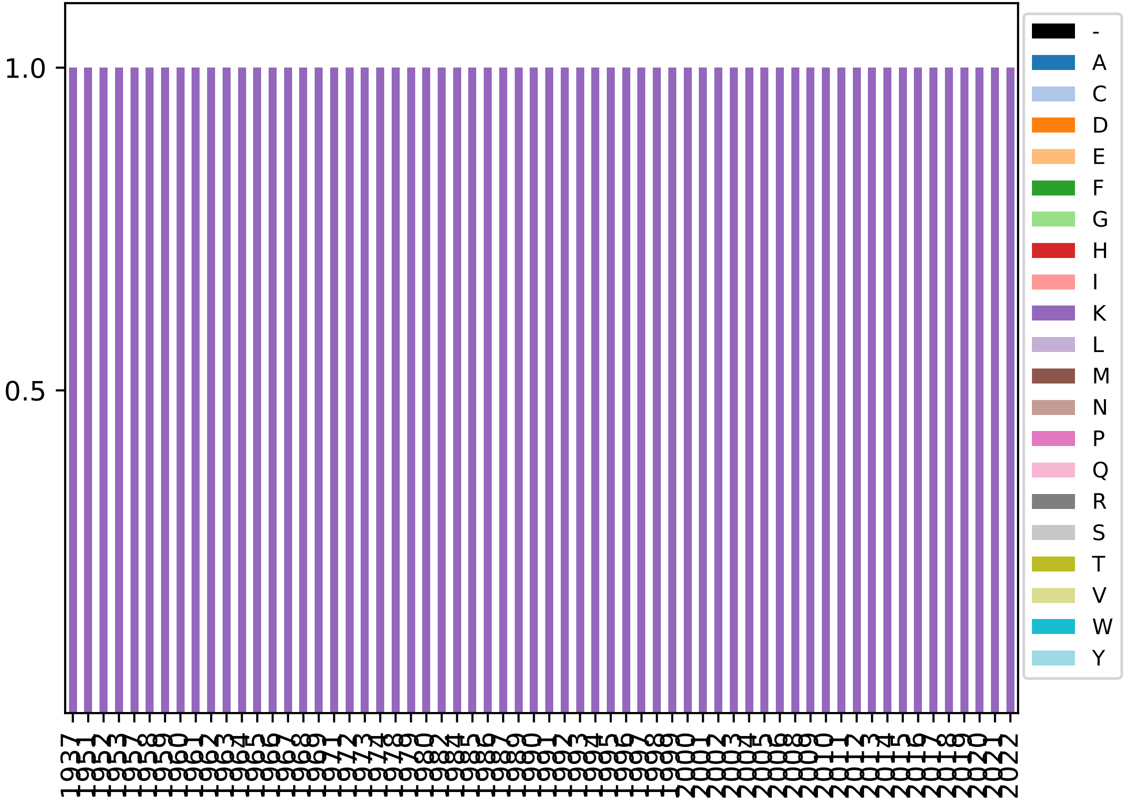

Site2424

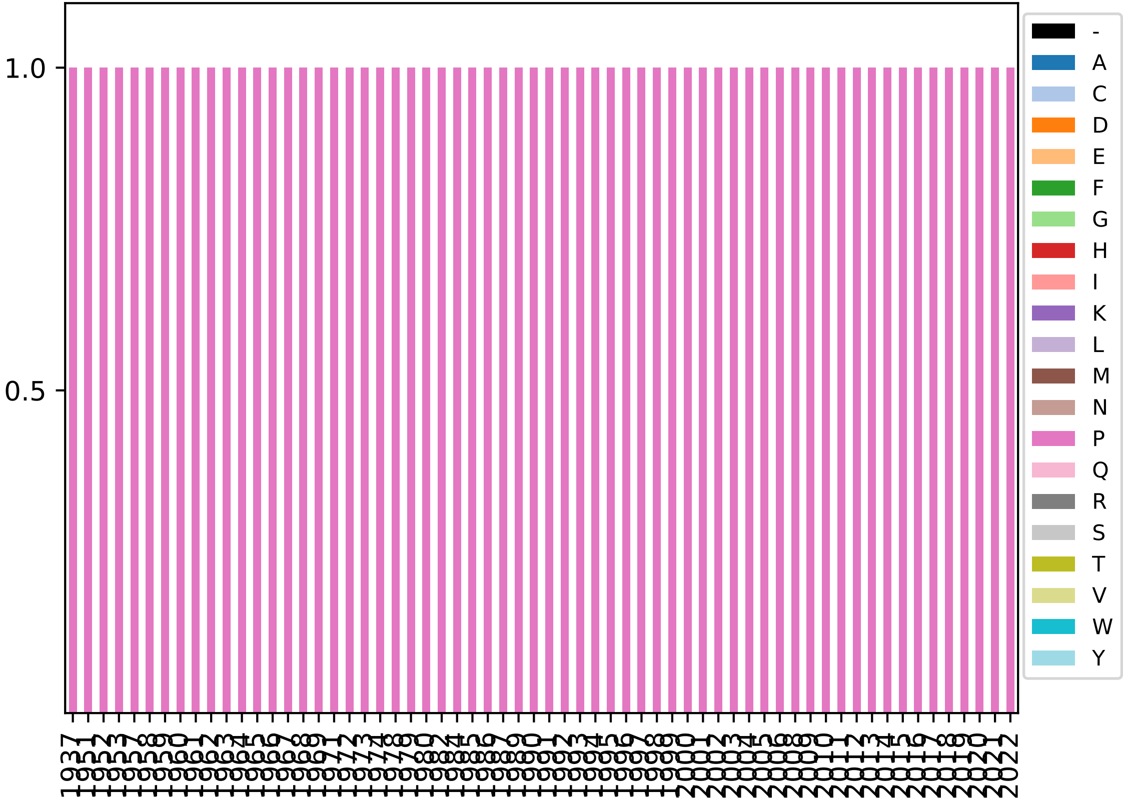

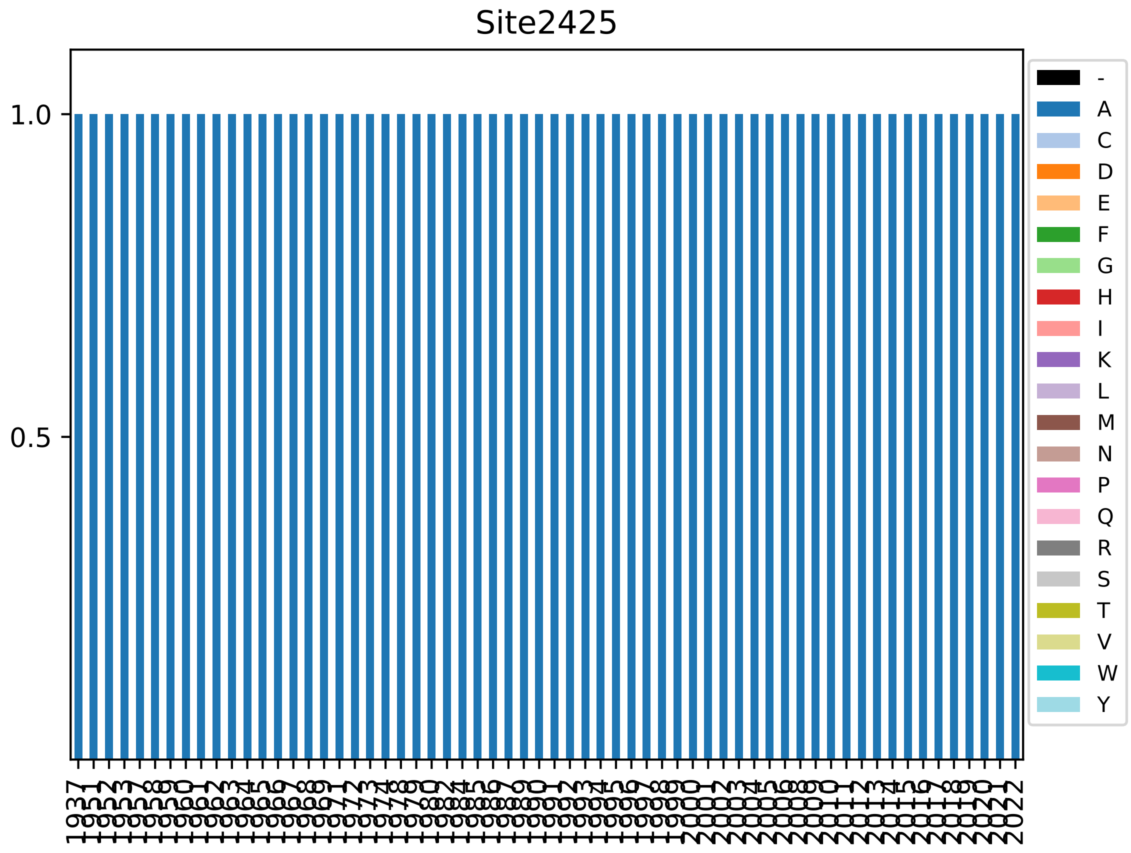
Site2425
1.0
0.5
-
A
C
D
E
F
G
H
I
K
L
M
N
P
Q
R
S
T
V
W
Y

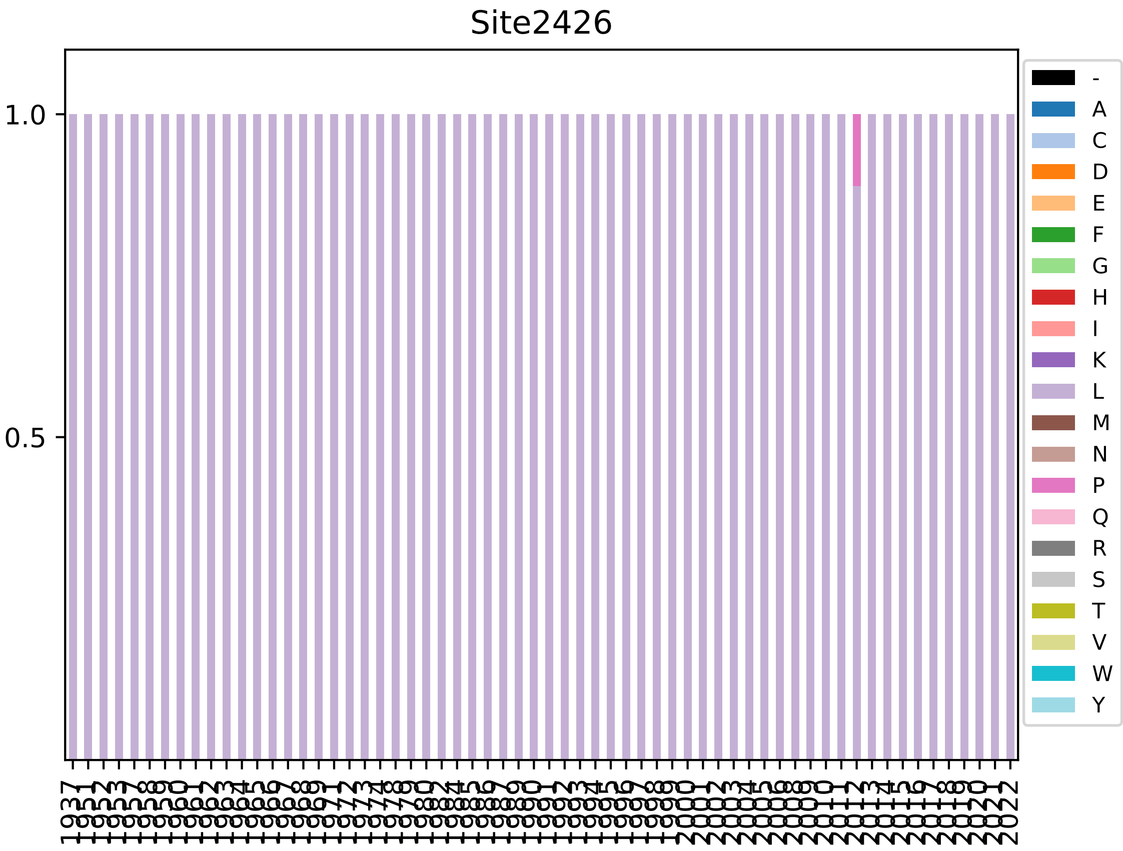
Site2426
1.0
0.5
-
A
C
D
E
F
G
H
I
K
L
M
N
P
Q
R
S
T
V
W
Y

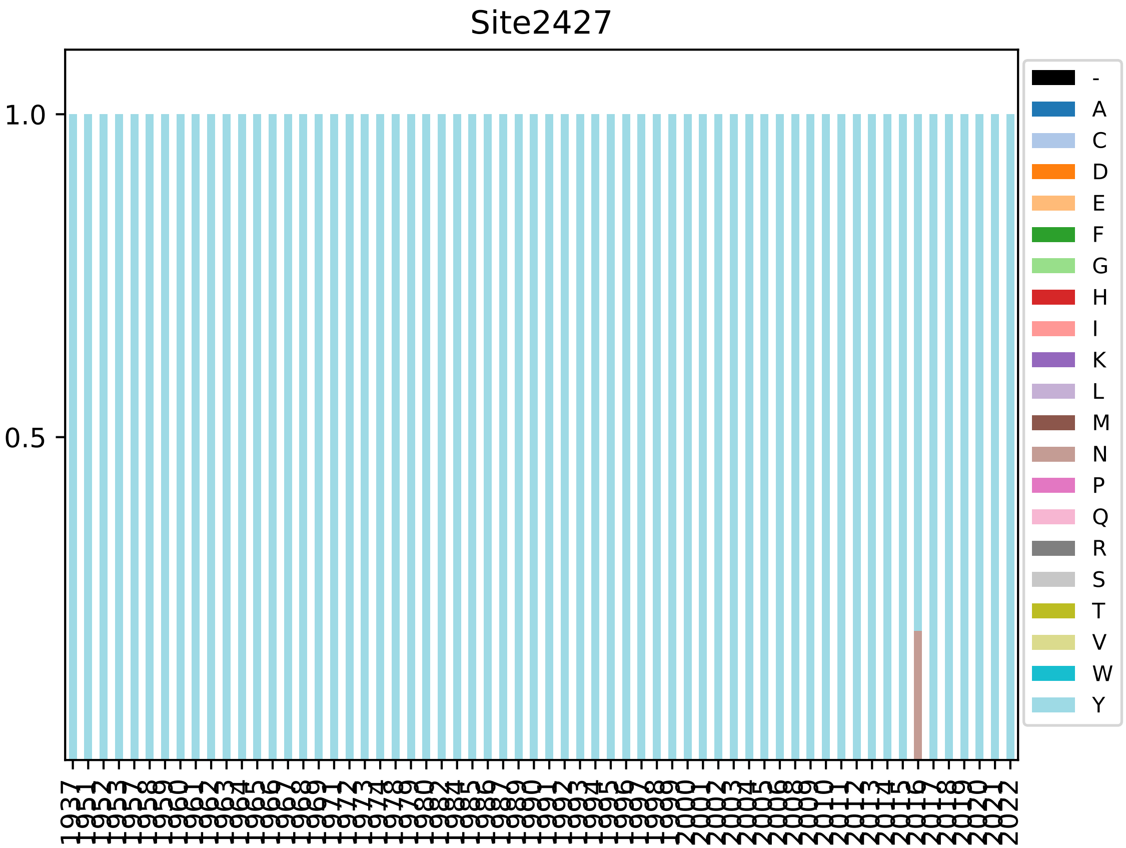
Site2427
1.0
0.5
-
A
C
D
E
F
G
H
I
K
L
M
N
P
Q
R
S
T
V
W
Y

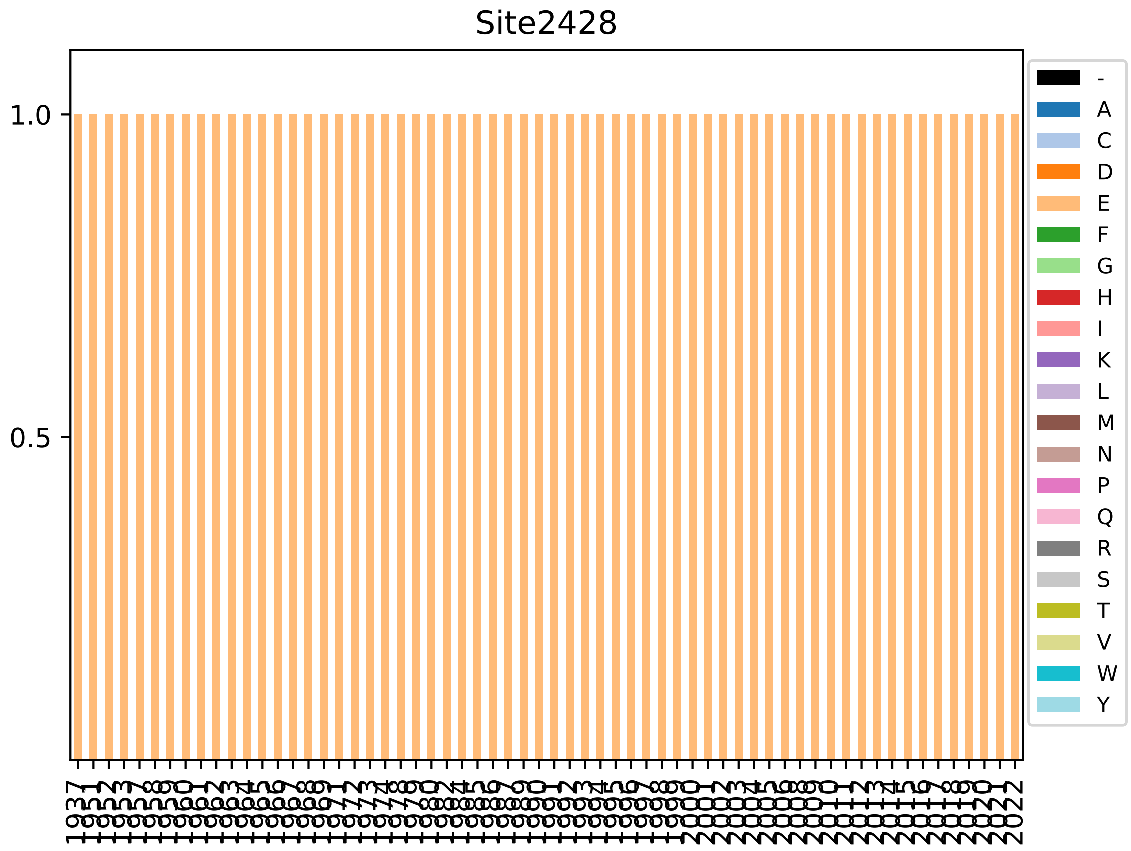

Site2428
1.0
0.5
-
A
C
D
E
F
G
H
I
K
L
M
N
P
Q
R
S
T
V
W
Y

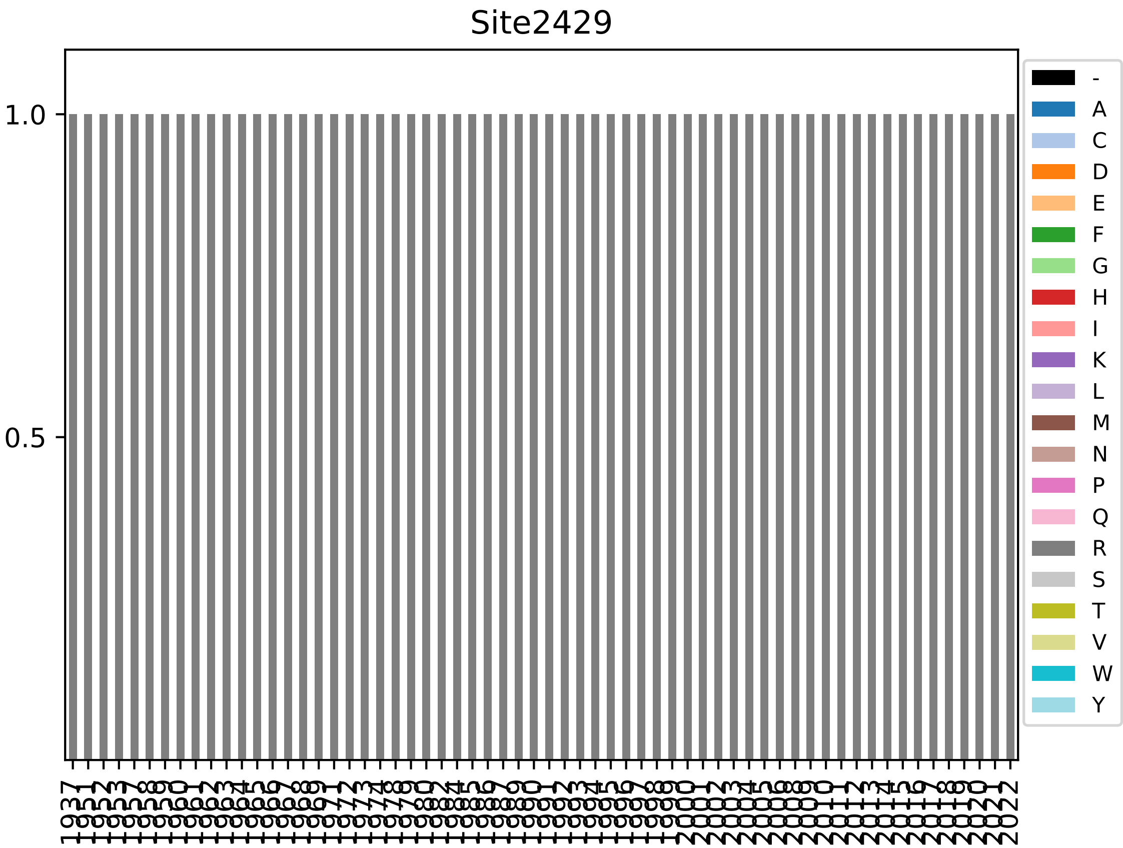
Site2429
1.0
0.5
-
A
C
D
E
F
G
H
I
K
L
M
N
P
Q
R
S
T
V
W
Y

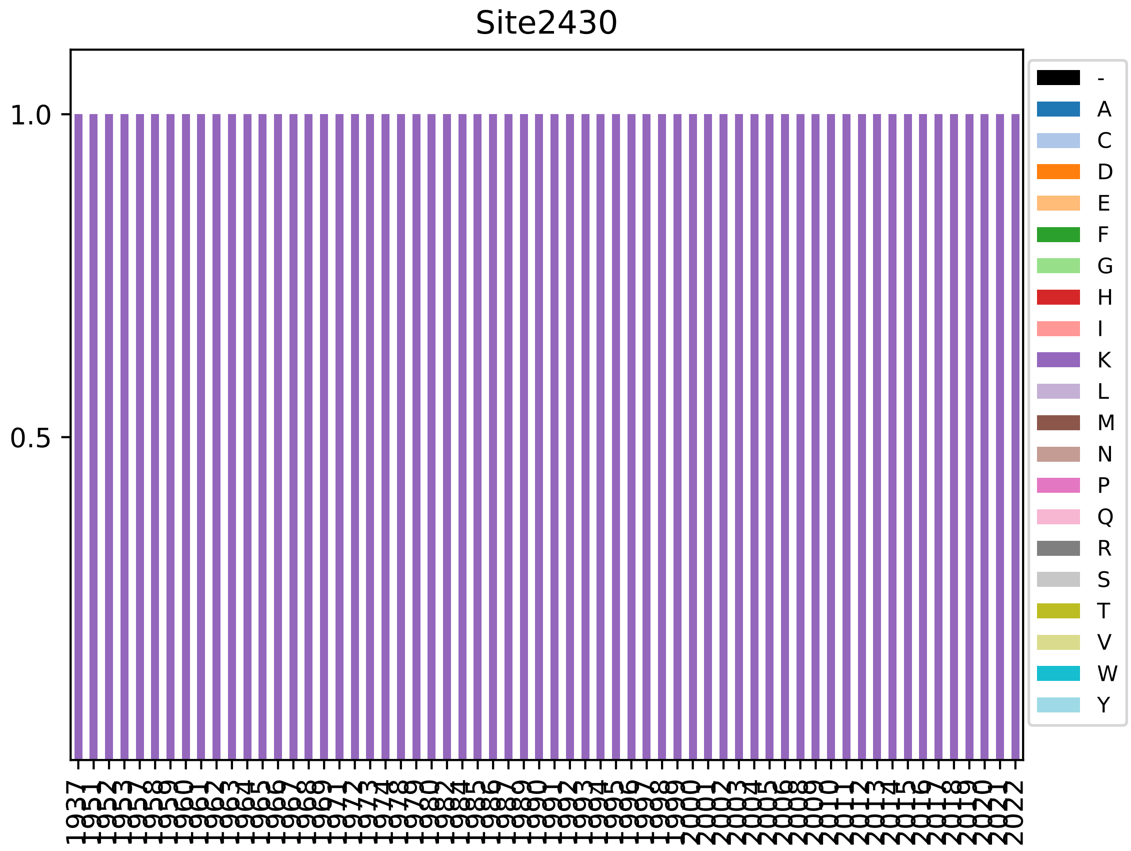
Site2430
1.0
0.5
-
A
C
D
E
F
G
H
I
K
L
M
N
P
Q
R
S
T
V
W
Y

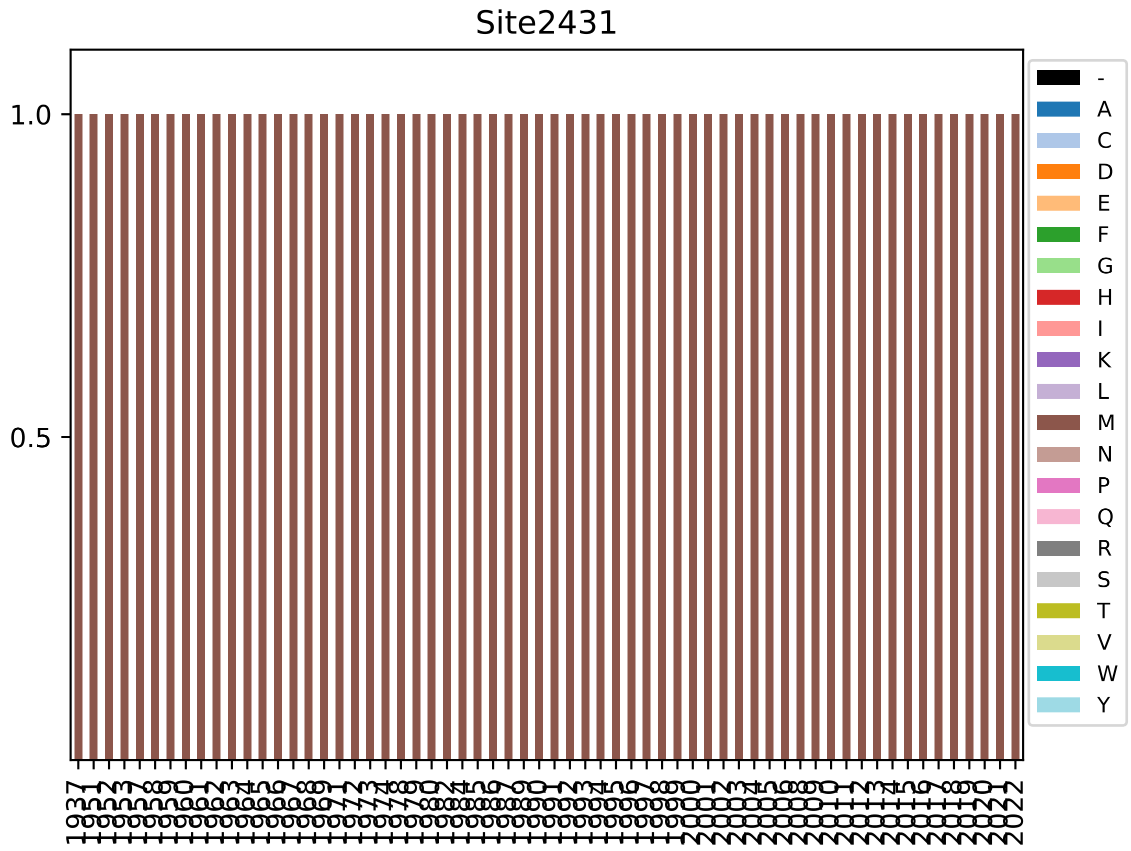
Site2431
1.0
0.5
-
A
C
D
E
F
G
H
I
K
L
M
N
P
Q
R
S
T
V
W
Y

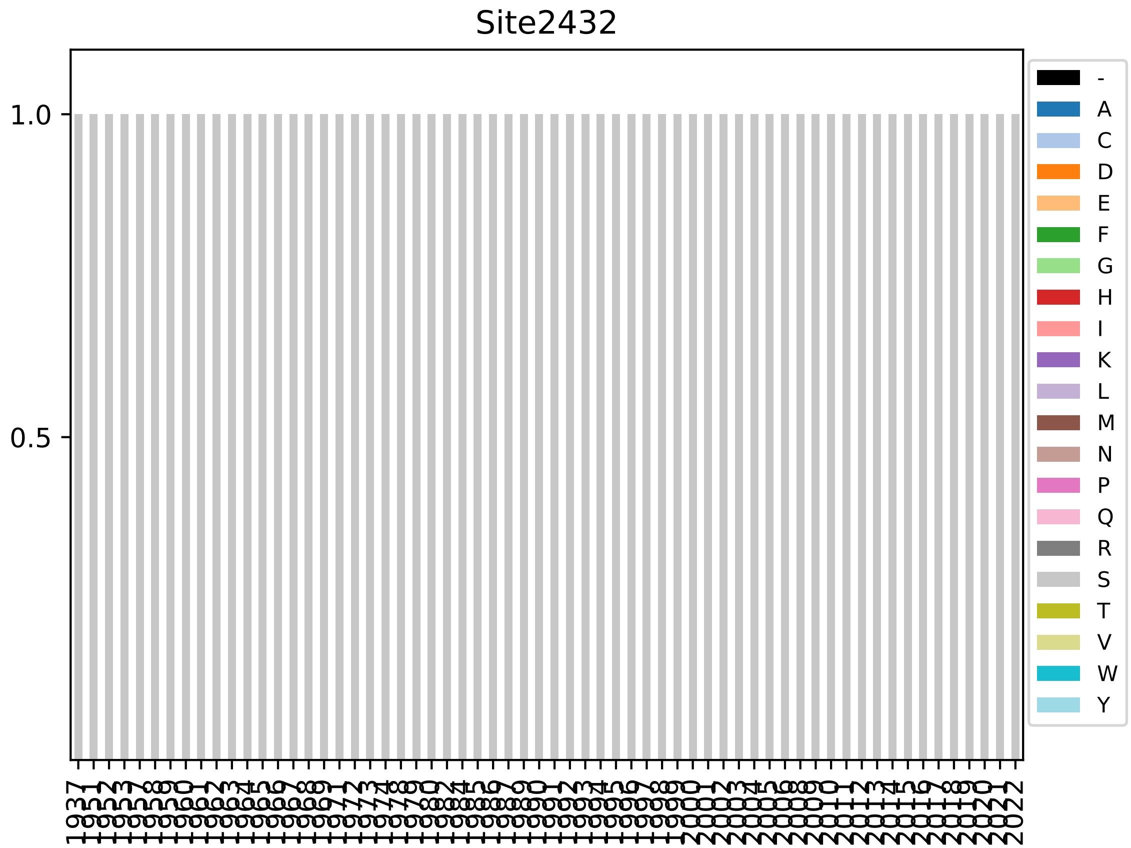
Site2432
1.0
0.5
-
A
C
D
E
F
G
H
I
K
L
M
N
P
Q
R
S
T
V
W
Y

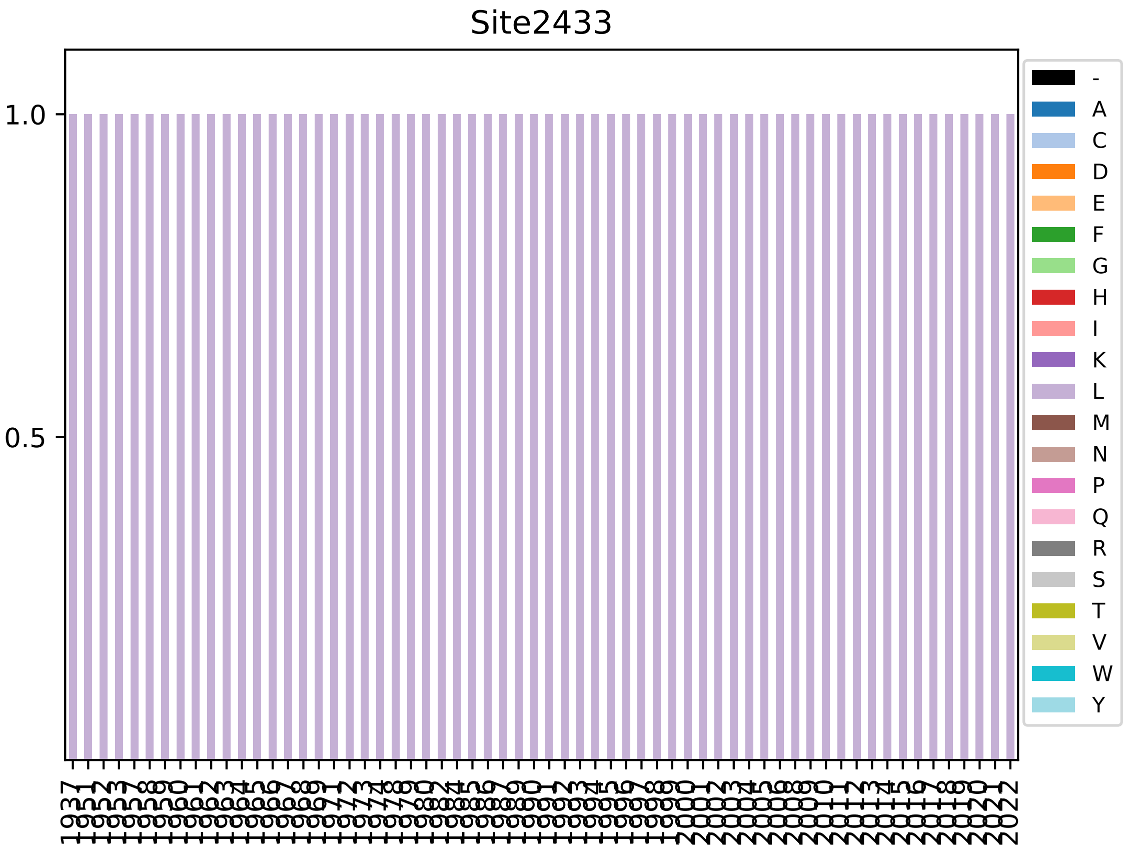
Site2433
1.0
0.5
-
A
C
D
E
F
G
H
I
K
L
M
N
P
Q
R
S
T
V
W
Y

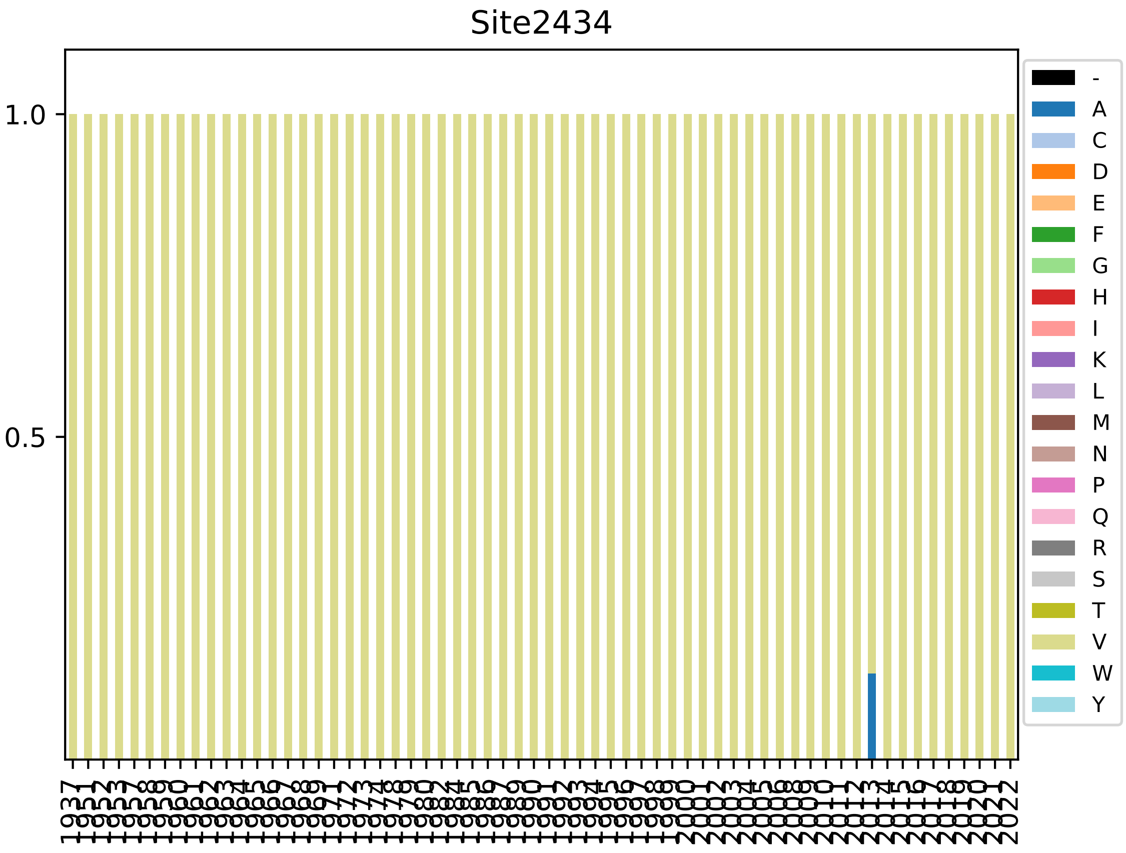
Site2434
1.0
0.5
-
A
C
D
E
F
G
H
I
K
L
M
N
P
Q
R
S
T
V
W
Y

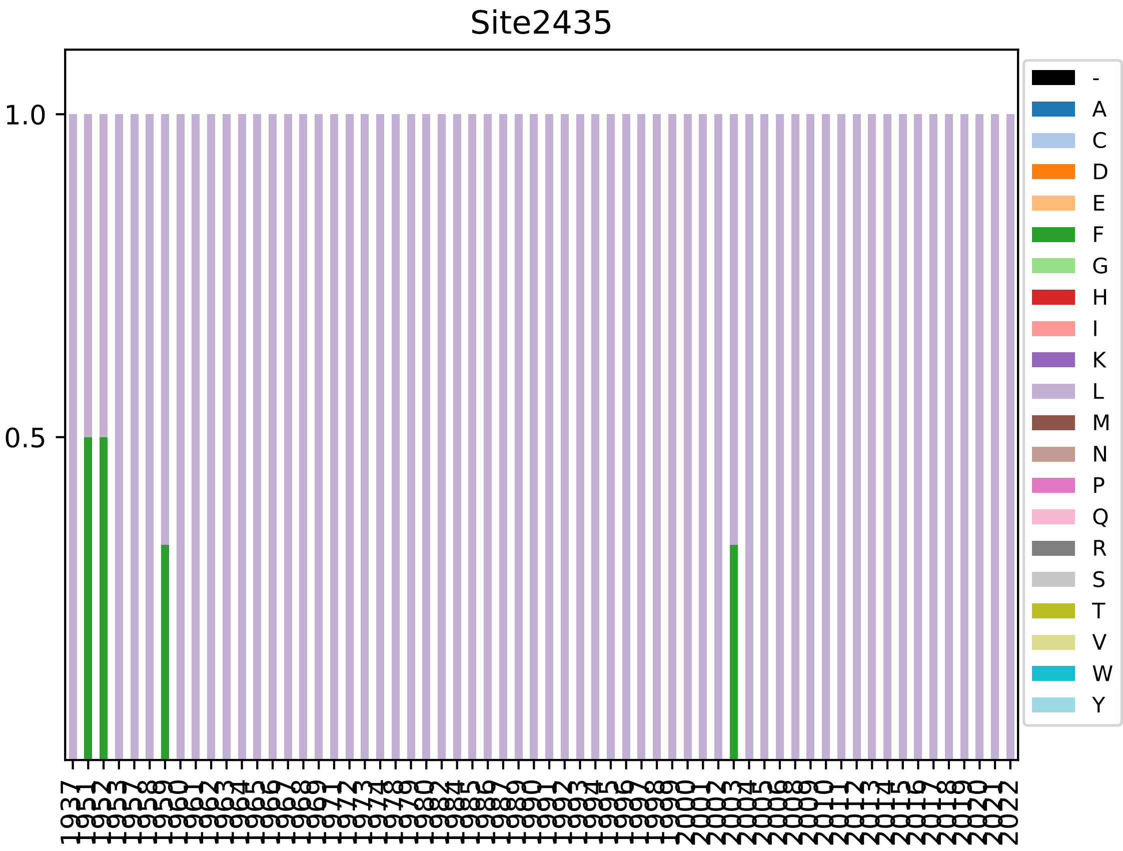
Site2435
1.0
0.5
-
A
C
D
E
F
G
H
I
K
L
M
N
P
Q
R
S
T
V
W
Y

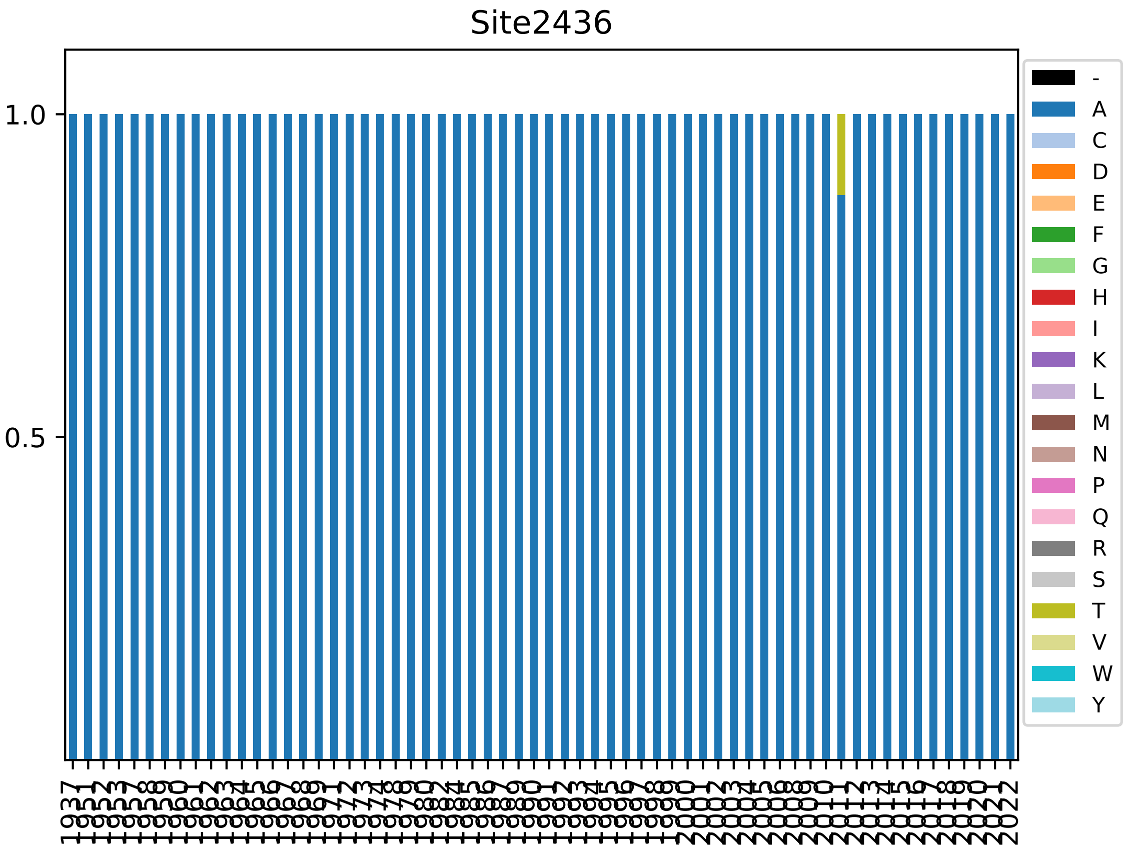
Site2436
1.0
0.5
-
A
C
D
E
F
G
H
I
K
L
M
N
P
Q
R
S
T
V
W
Y

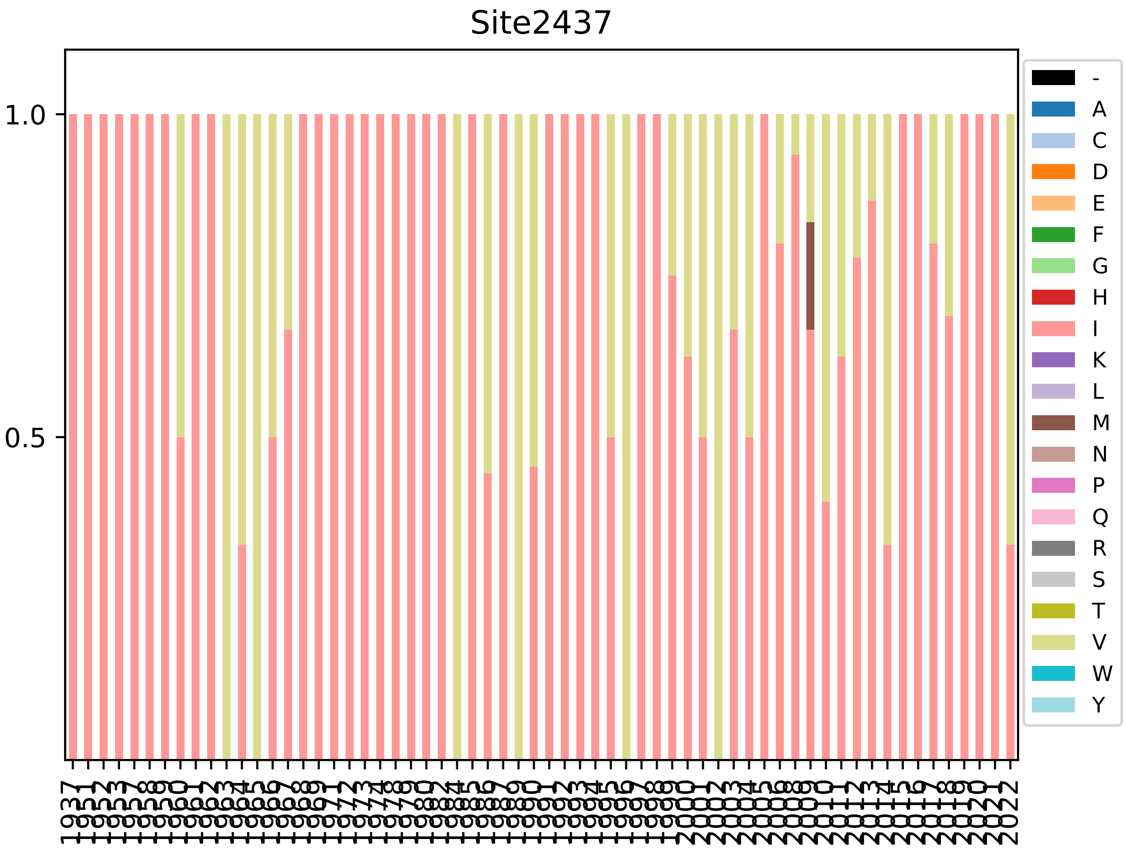
Site2437
1.0
0.5
-
A
C
D
E
F
G
H
I
K
L
M
N
P
Q
R
S
T
V
W
Y

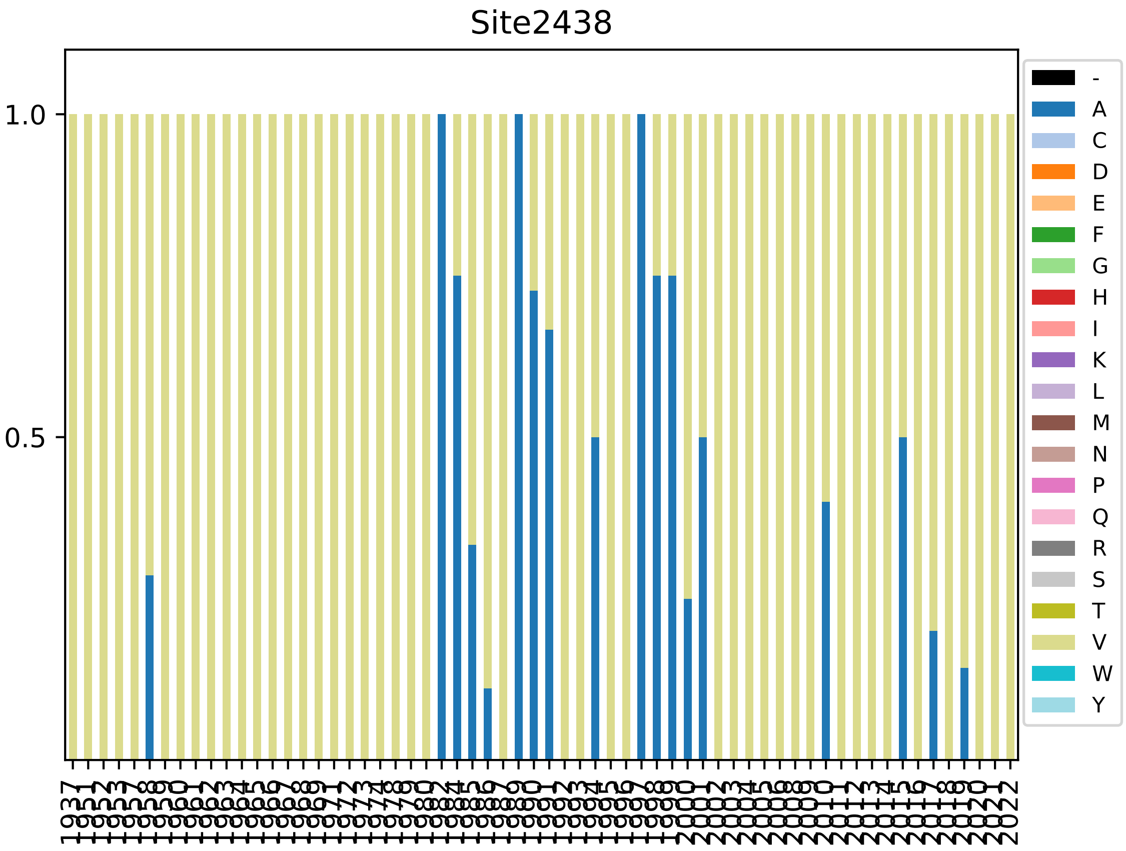
Site2438
1.0
0.5
-
A
C
D
E
F
G
H
I
K
L
M
N
P
Q
R
S
T
V
W
Y

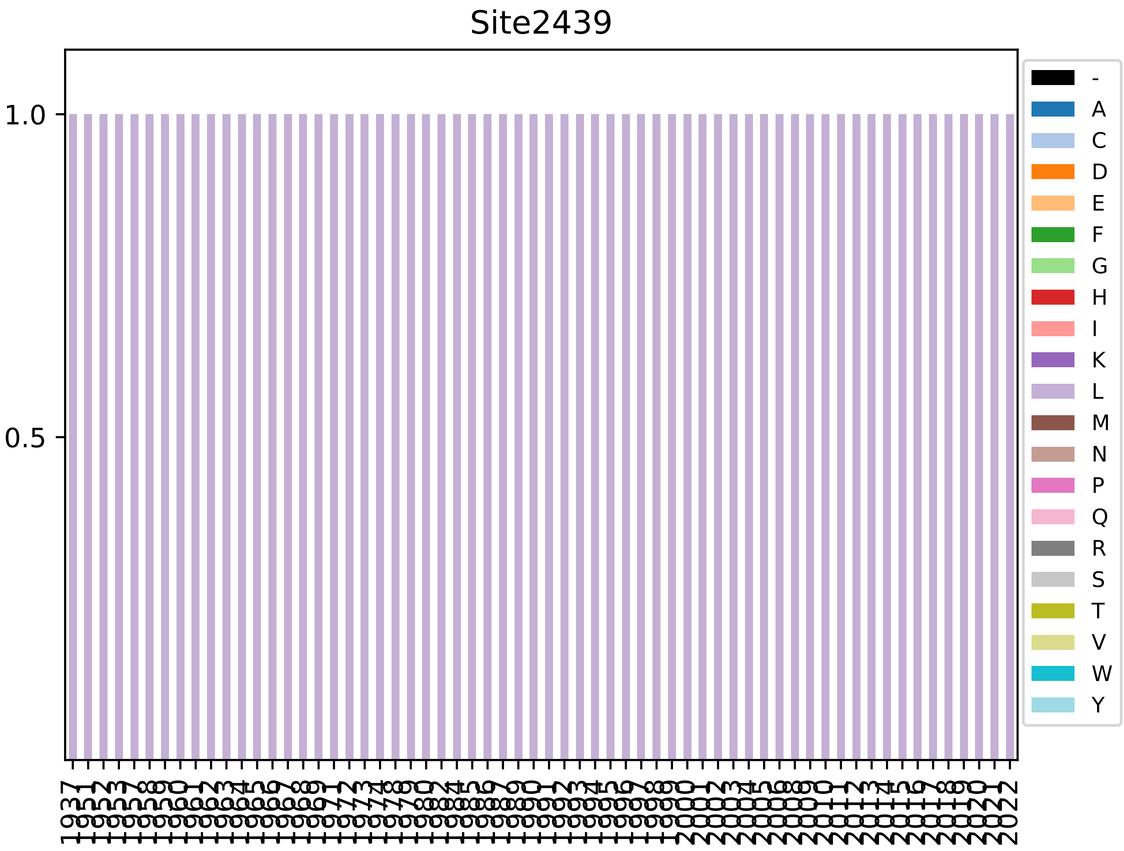
Site2439
1.0
0.5
-
A
C
D
E
F
G
H
I
K
L
M
N
P
Q
R
S
T
V
W
Y

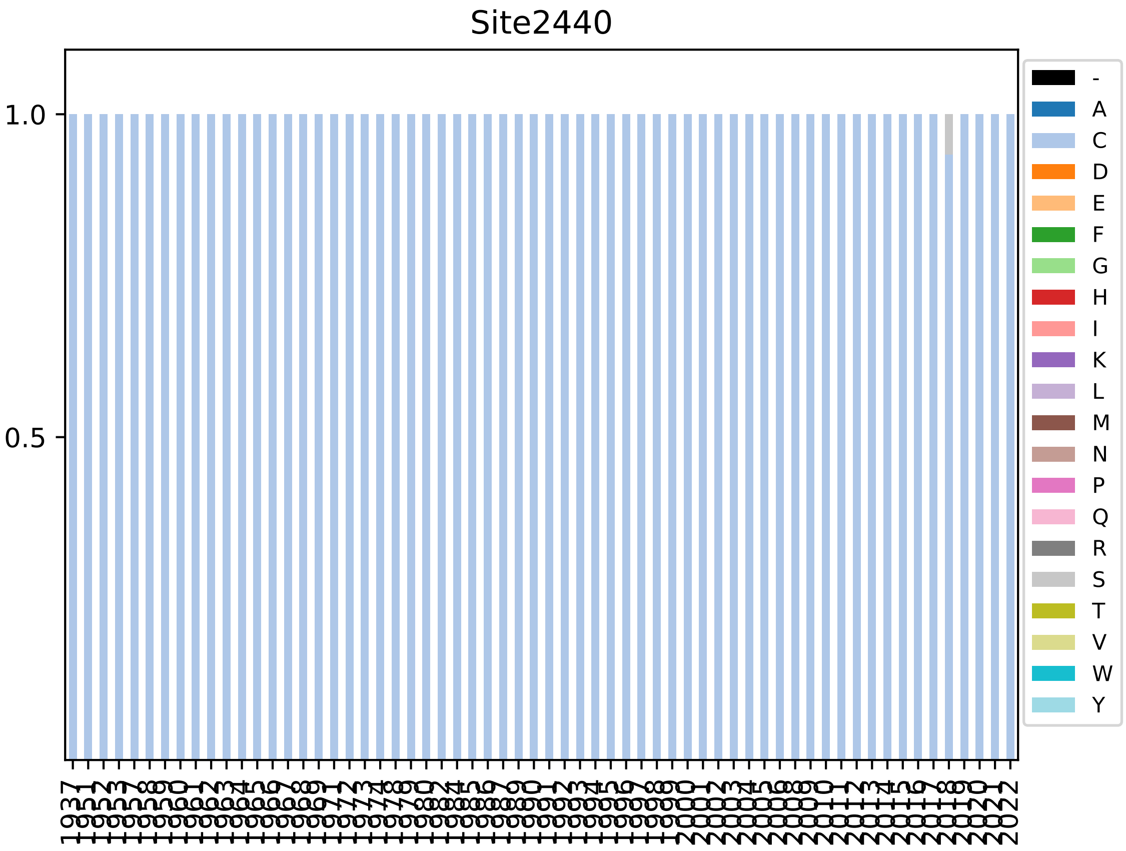
Site2440
1.0
0.5
-
A
C
D
E
F
G
H
I
K
L
M
N
P
Q
R
S
T
V
W
Y

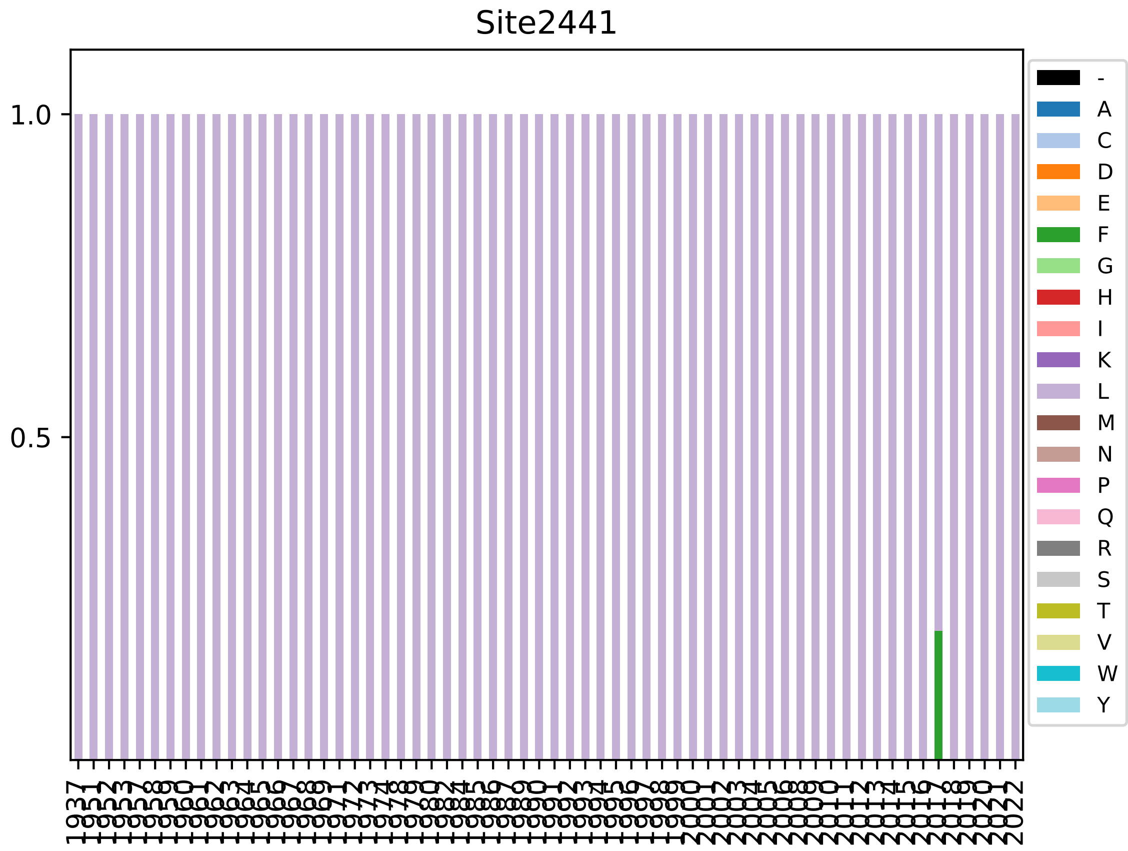
Site2441
1.0
0.5
-
A
C
D
E
F
G
H
I
K
L
M
N
P
Q
R
S
T
V
W
Y

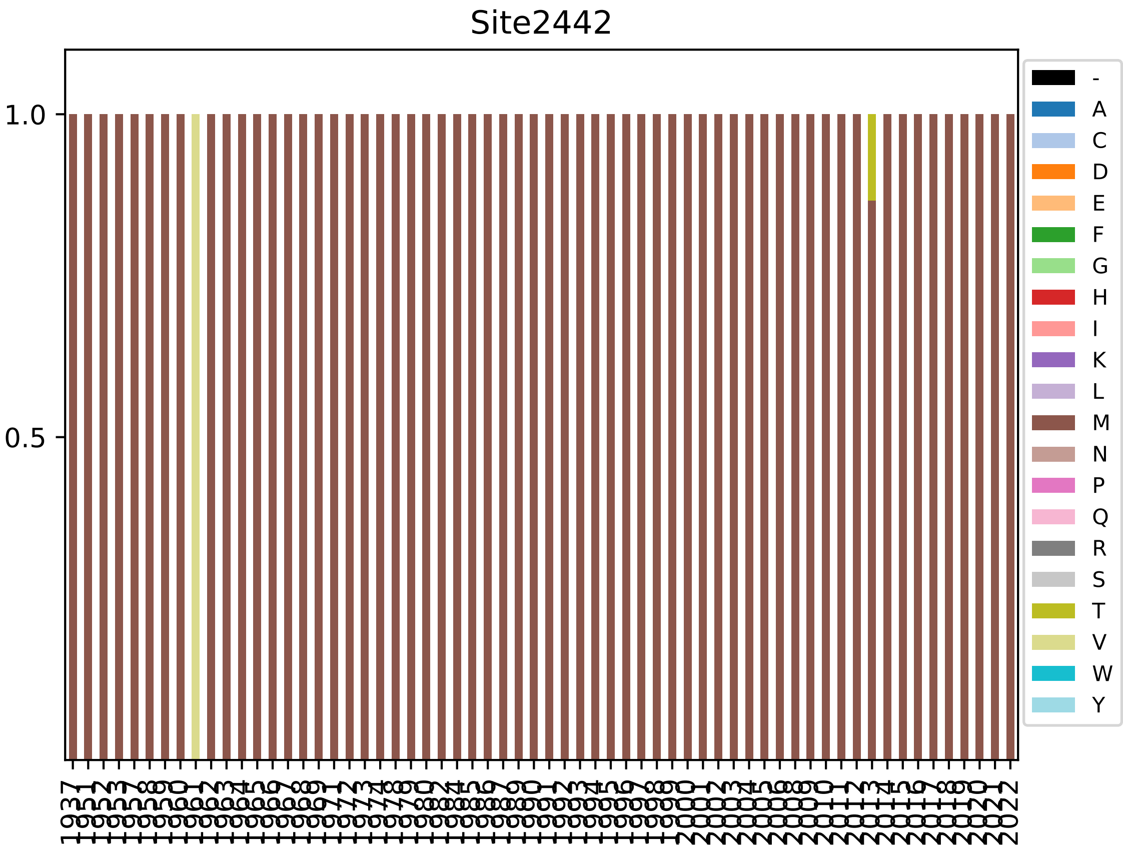
Site2442
1.0
0.5
-
A
C
D
E
F
G
H
I
K
L
M
N
P
Q
R
S
T
V
W
Y

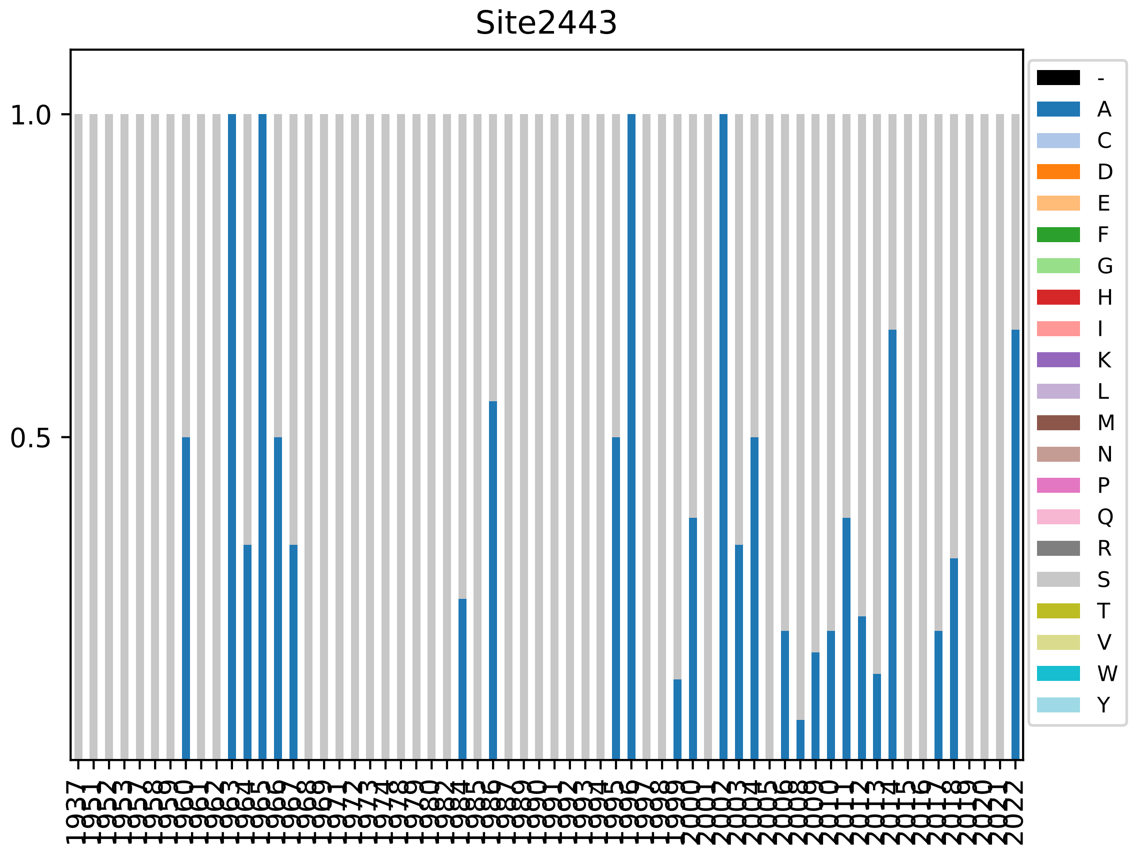
Site2443
1.0
0.5
-
A
C
D
E
F
G
H
I
K
L
M
N
P
Q
R
S
T
V
W
Y

Site2444

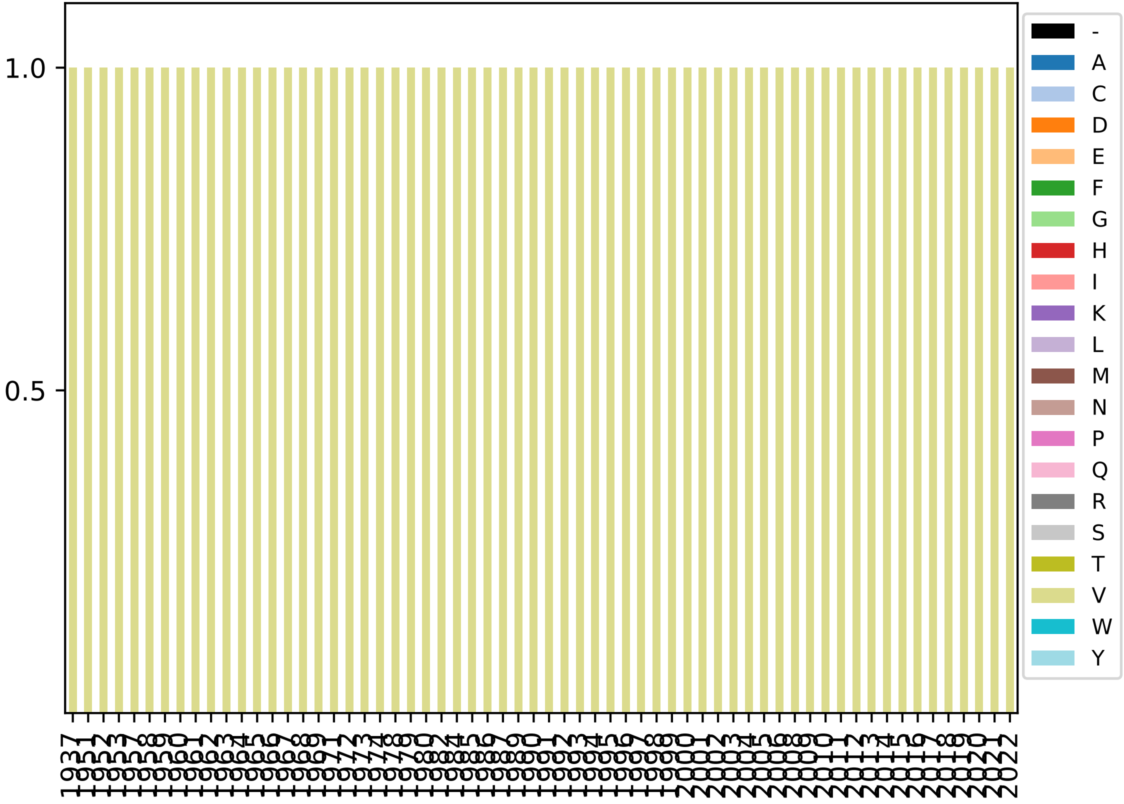

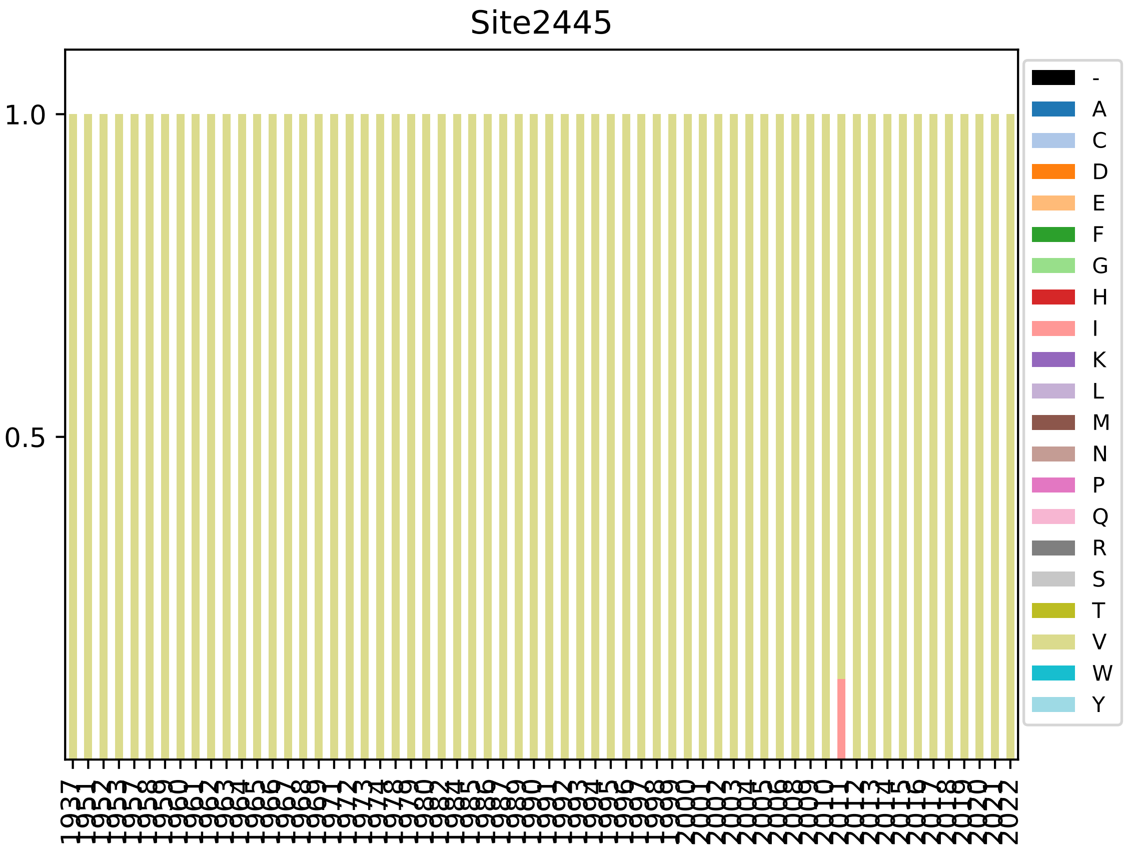
Site2445
1.0
0.5
-
A
C
D
E
F
G
H
I
K
L
M
N
P
Q
R
S
T
V
W
Y

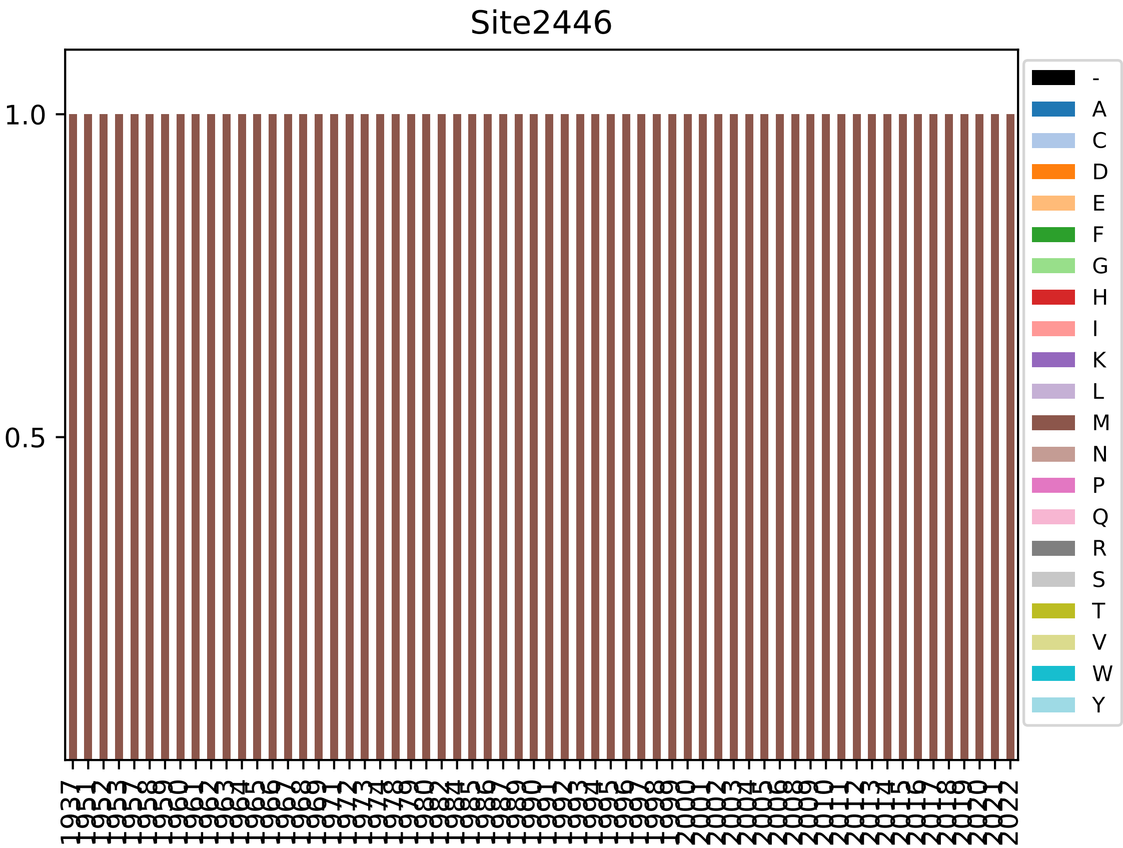

Site2446
1.0
0.5
-
A
C
D
E
F
G
H
I
K
L
M
N
P
Q
R
S
T
V
W
Y

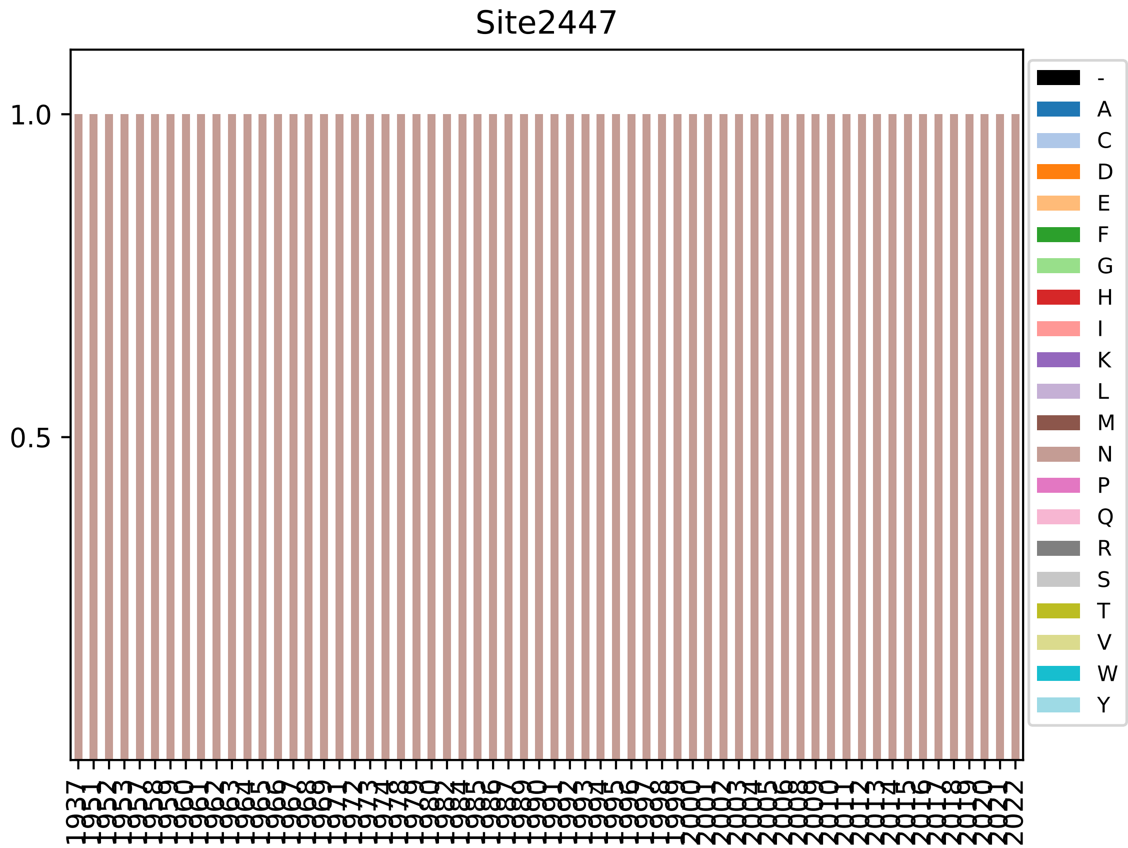
Site2447
1.0
0.5
-
A
C
D
E
F
G
H
I
K
L
M
N
P
Q
R
S
T
V
W
Y

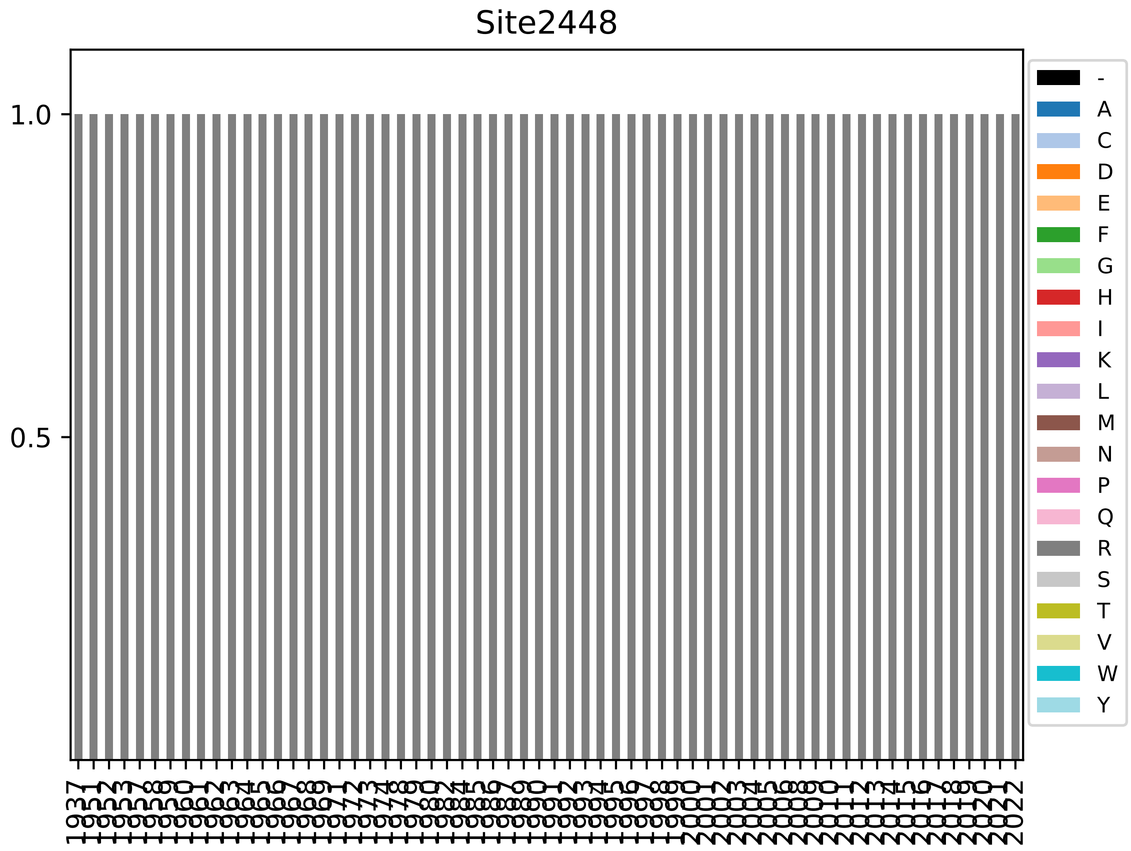
Site2448
1.0
0.5
-
A
C
D
E
F
G
H
I
K
L
M
N
P
Q
R
S
T
V
W
Y

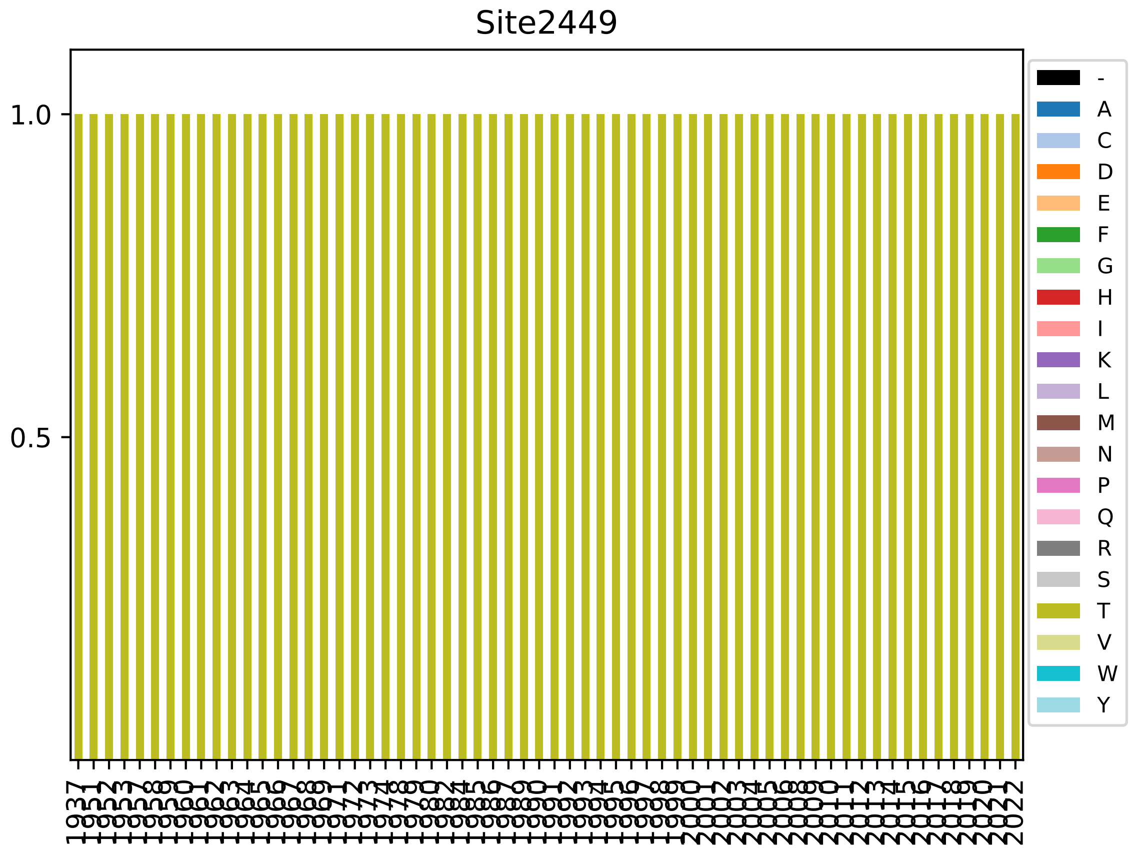
Site2449
1.0
0.5
-
A
C
D
E
F
G
H
I
K
L
M
N
P
Q
R
S
T
V
W
Y

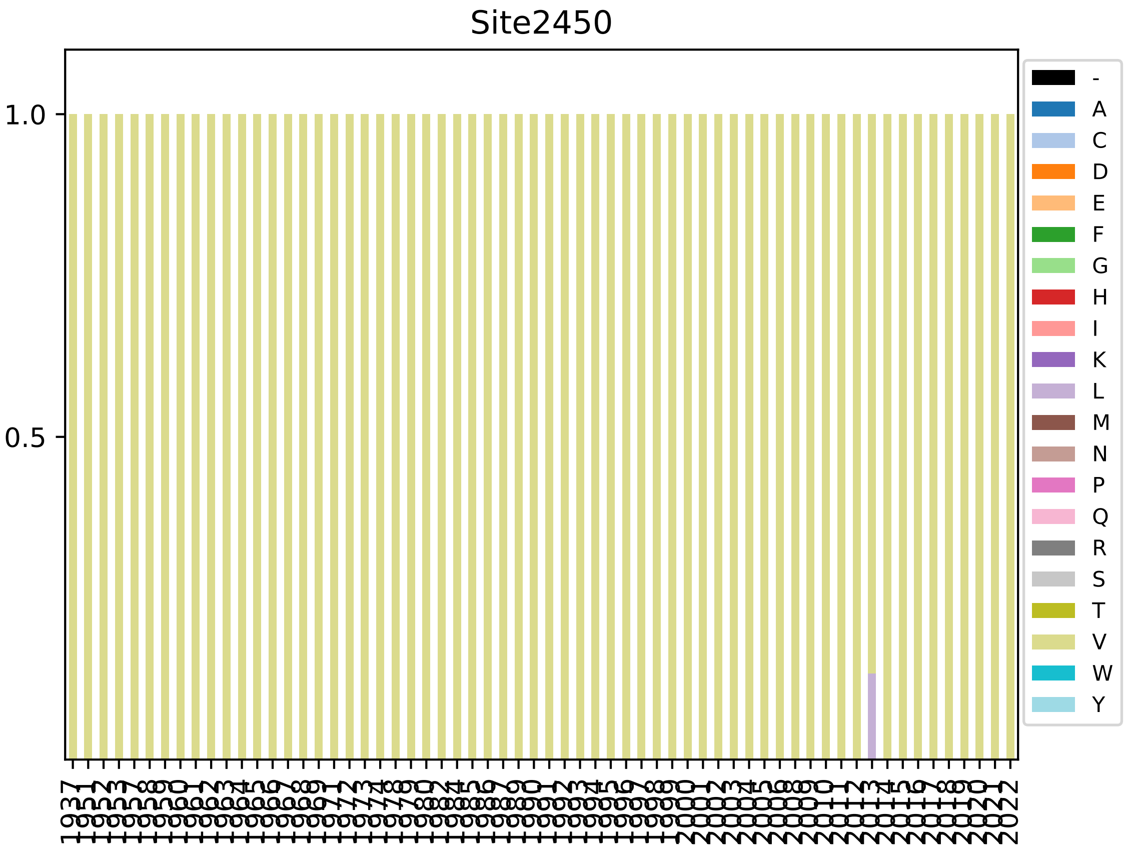
Site2450
1.0
0.5
-
A
C
D
E
F
G
H
I
K
L
M
N
P
Q
R
S
T
V
W
Y

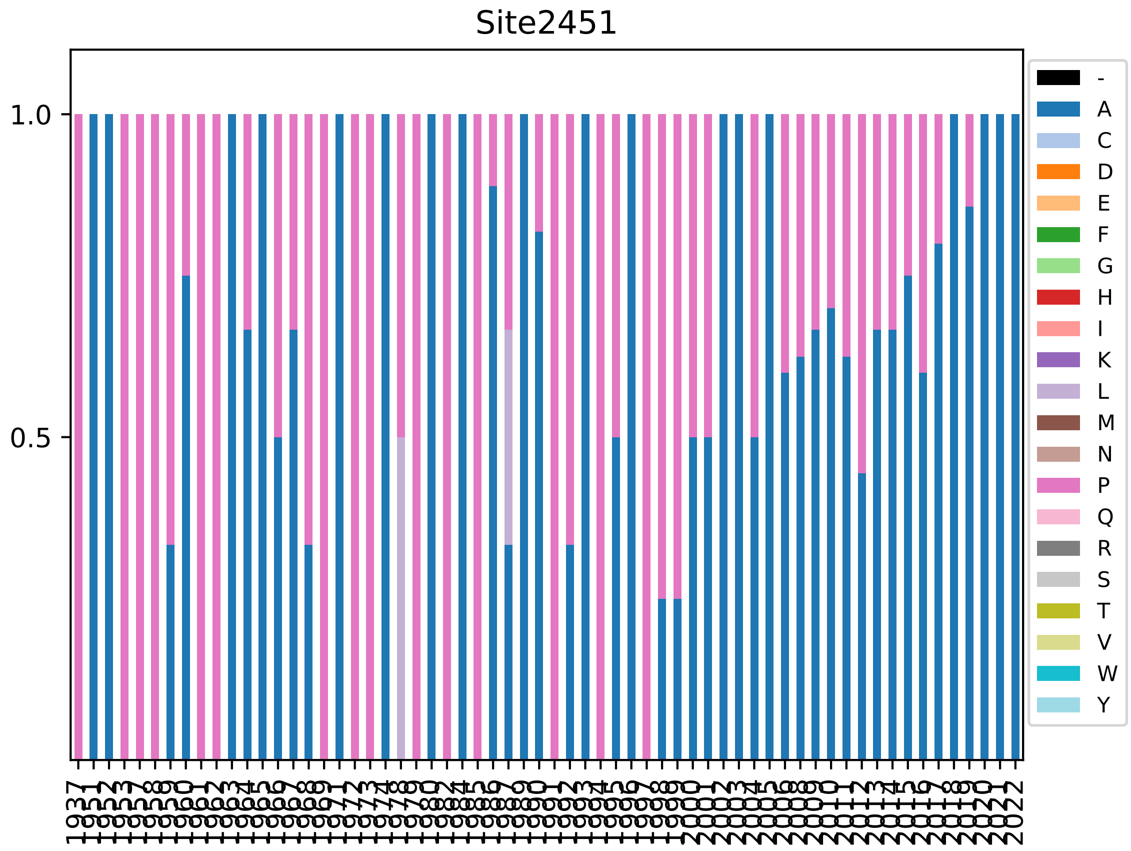
Site2451
1.0
0.5
-
A
C
D
E
F
G
H
I
K
L
M
N
P
Q
R
S
T
V
W
Y

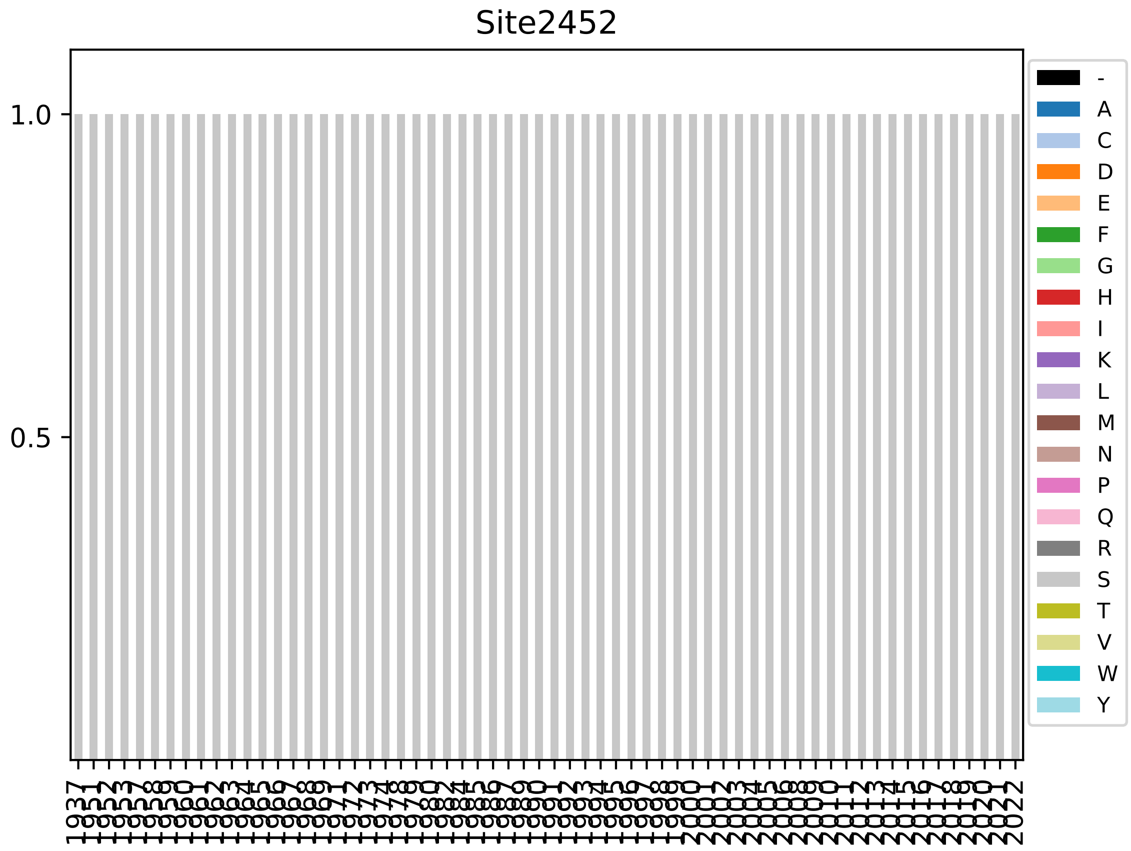
Site2452
1.0
0.5
-
A
C
D
E
F
G
H
I
K
L
M
N
P
Q
R
S
T
V
W
Y

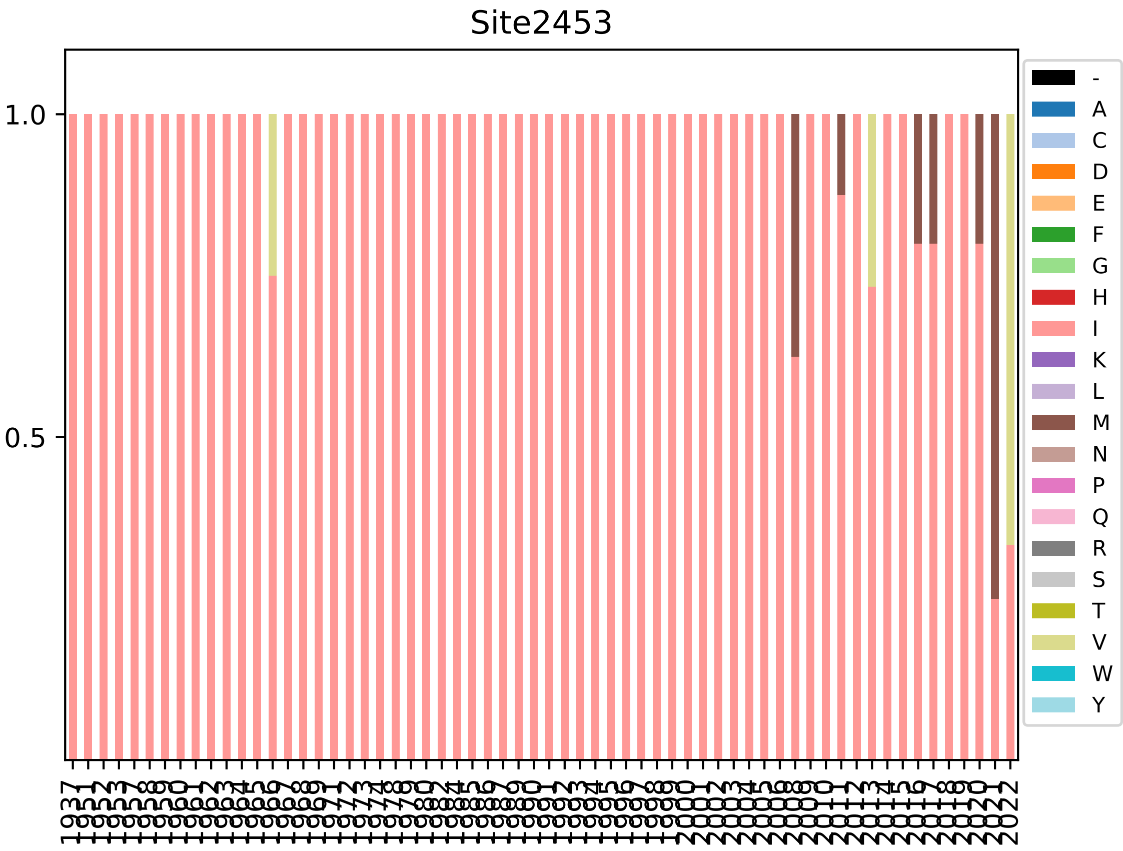
Site2453
1.0
0.5
-
A
C
D
E
F
G
H
I
K
L
M
N
P
Q
R
S
T
V
W
Y

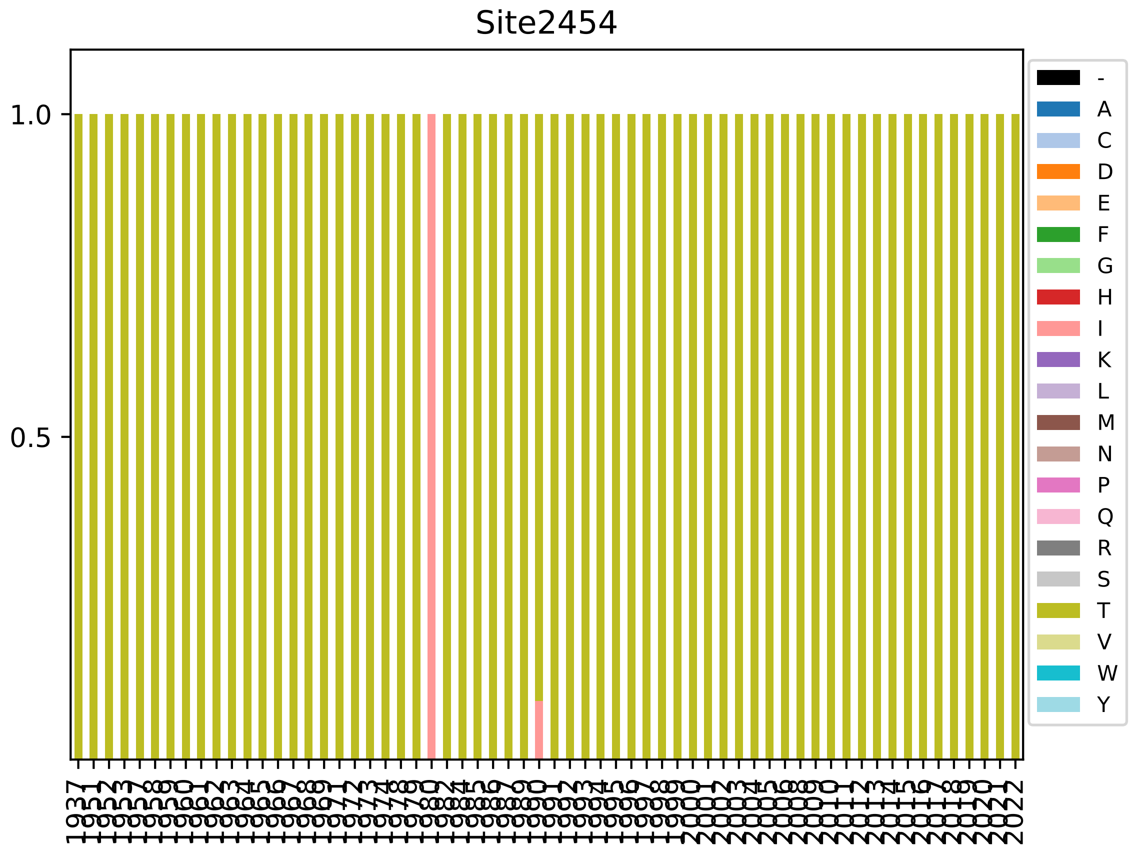
Site2454
1.0
0.5
-
A
C
D
E
F
G
H
I
K
L
M
N
P
Q
R
S
T
V
W
Y

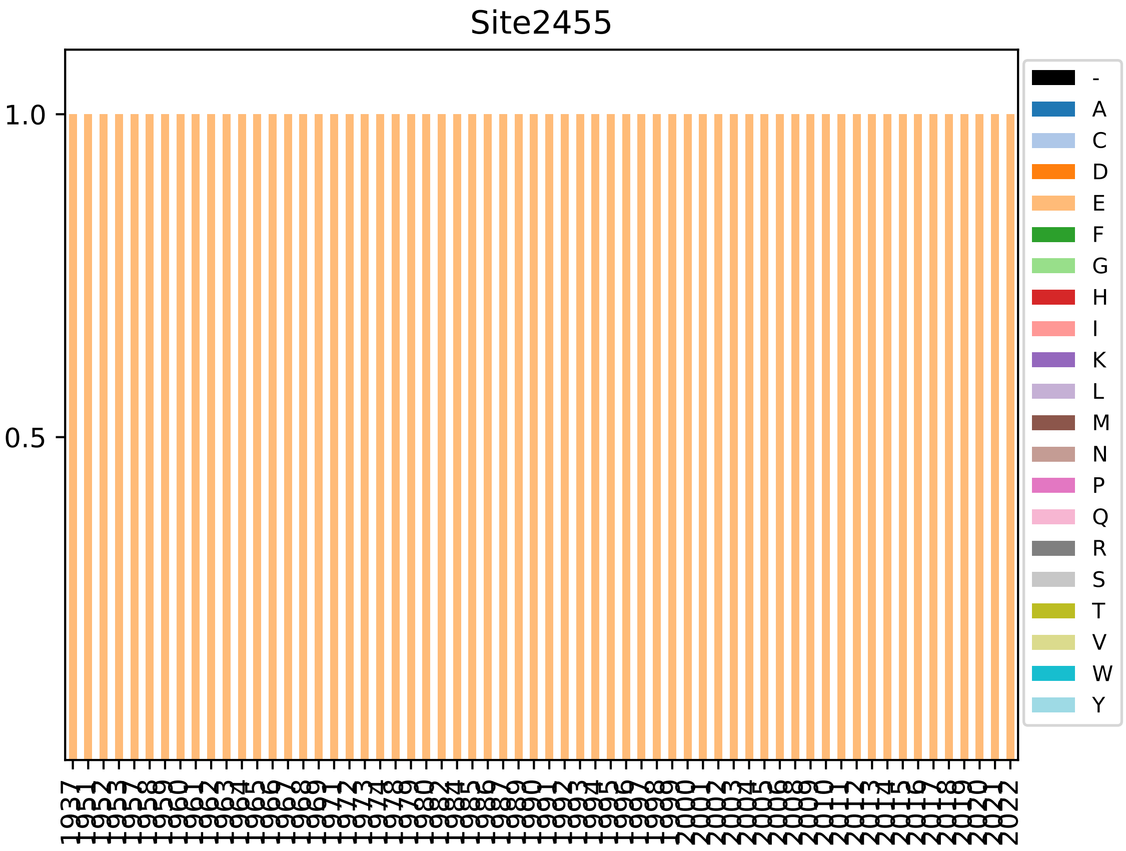
Site2455
1.0
0.5
-
A
C
D
E
F
G
H
I
K
L
M
N
P
Q
R
S
T
V
W
Y

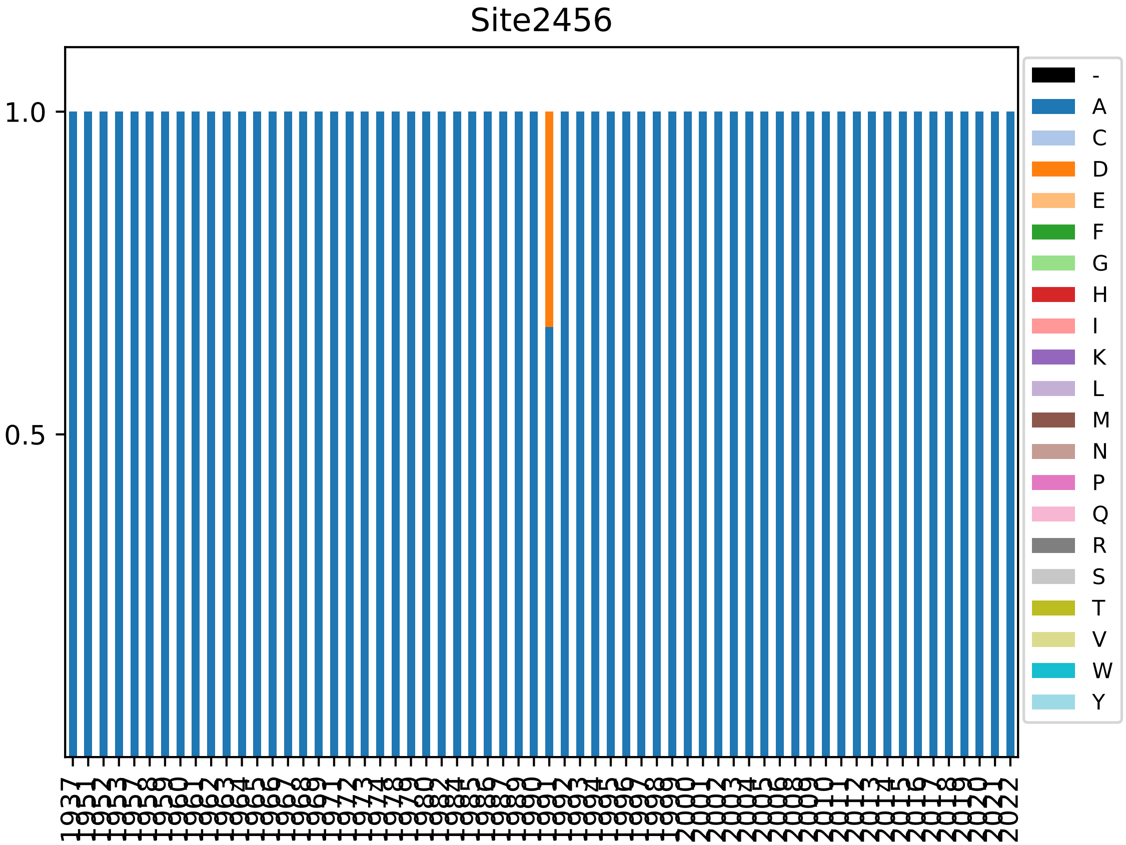
Site2456
1.0
0.5
-
A
C
D
E
F
G
H
I
K
L
M
N
P
Q
R
S
T
V
W
Y

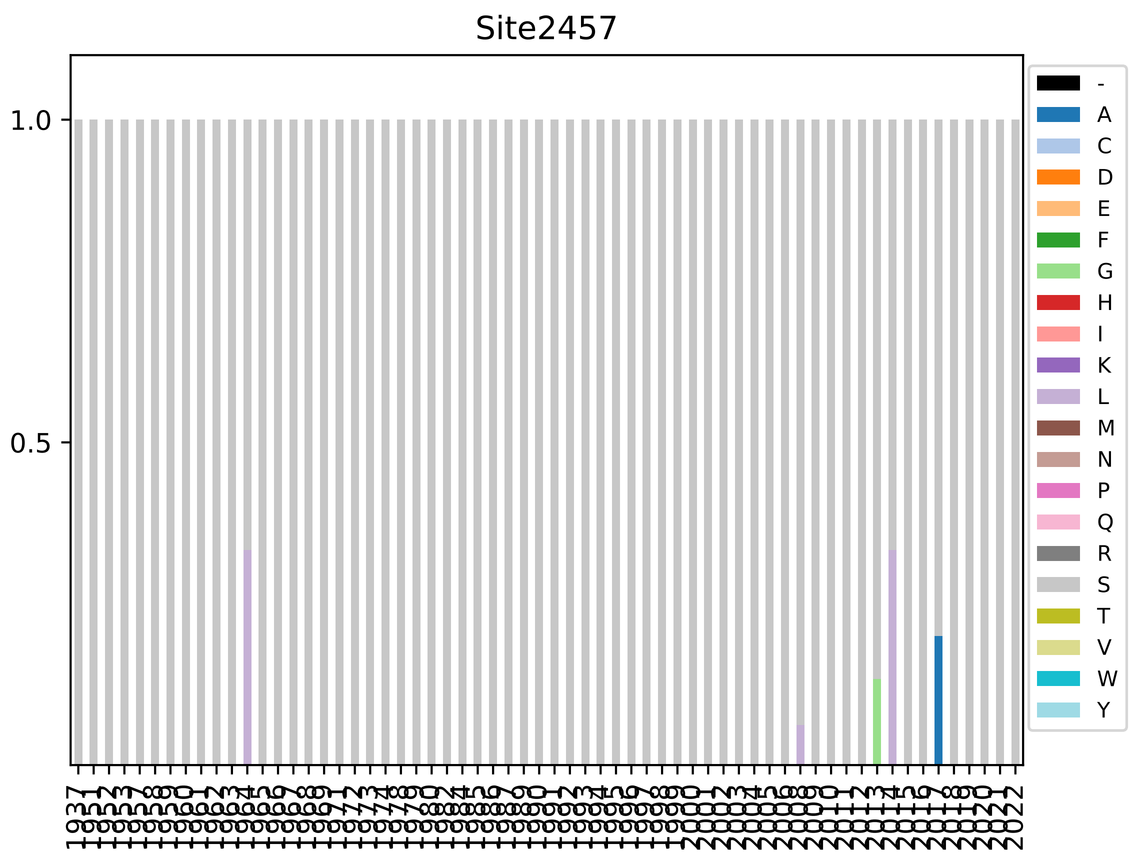
Site2457
1.0
0.5
-
A
C
D
E
F
G
H
I
K
L
M
N
P
Q
R
S
T
V
W
Y

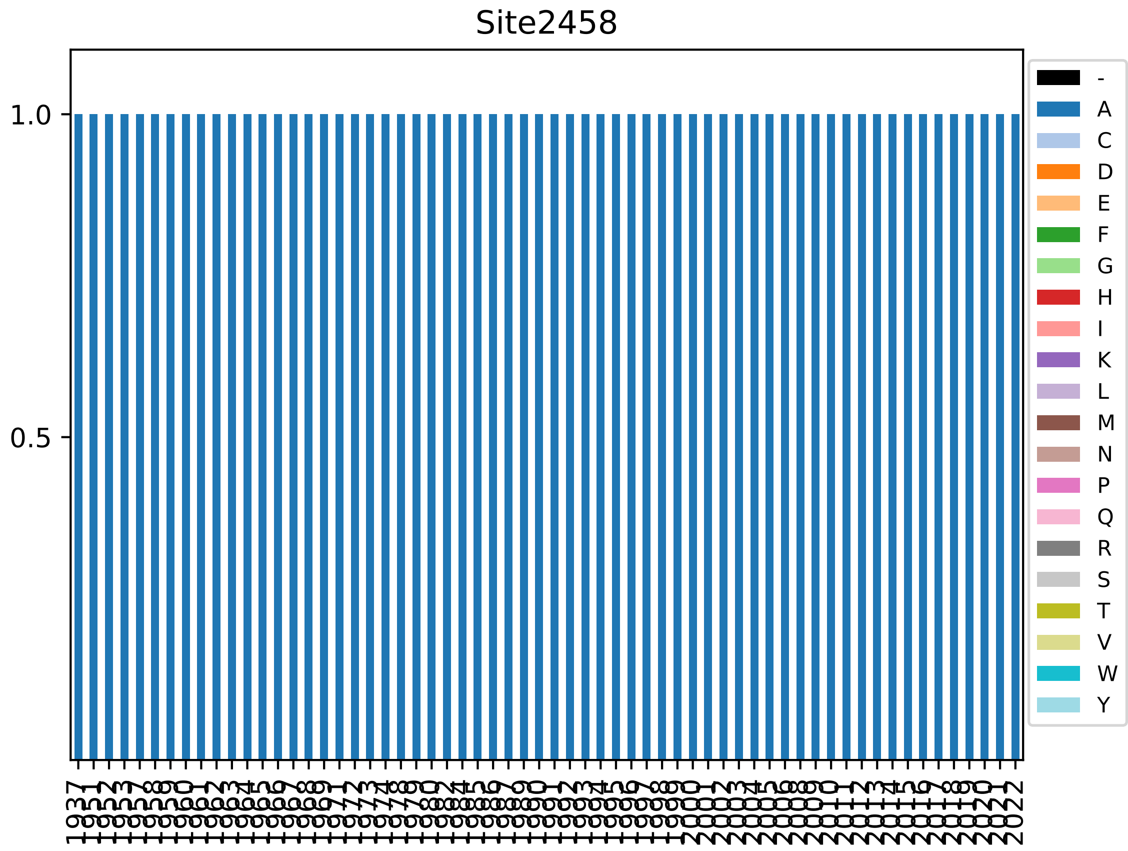
Site2458
1.0
0.5
-
A
C
D
E
F
G
H
I
K
L
M
N
P
Q
R
S
T
V
W
Y

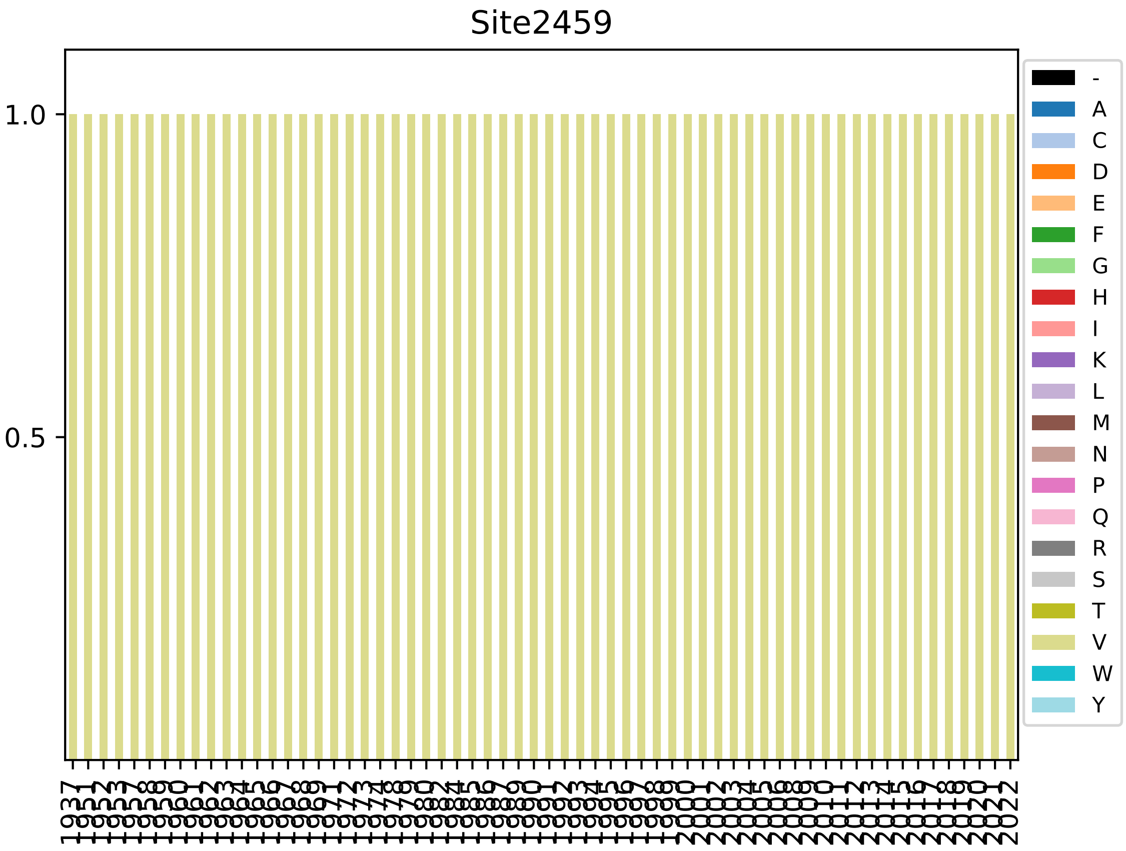
Site2459
1.0
0.5
-
A
C
D
E
F
G
H
I
K
L
M
N
P
Q
R
S
T
V
W
Y

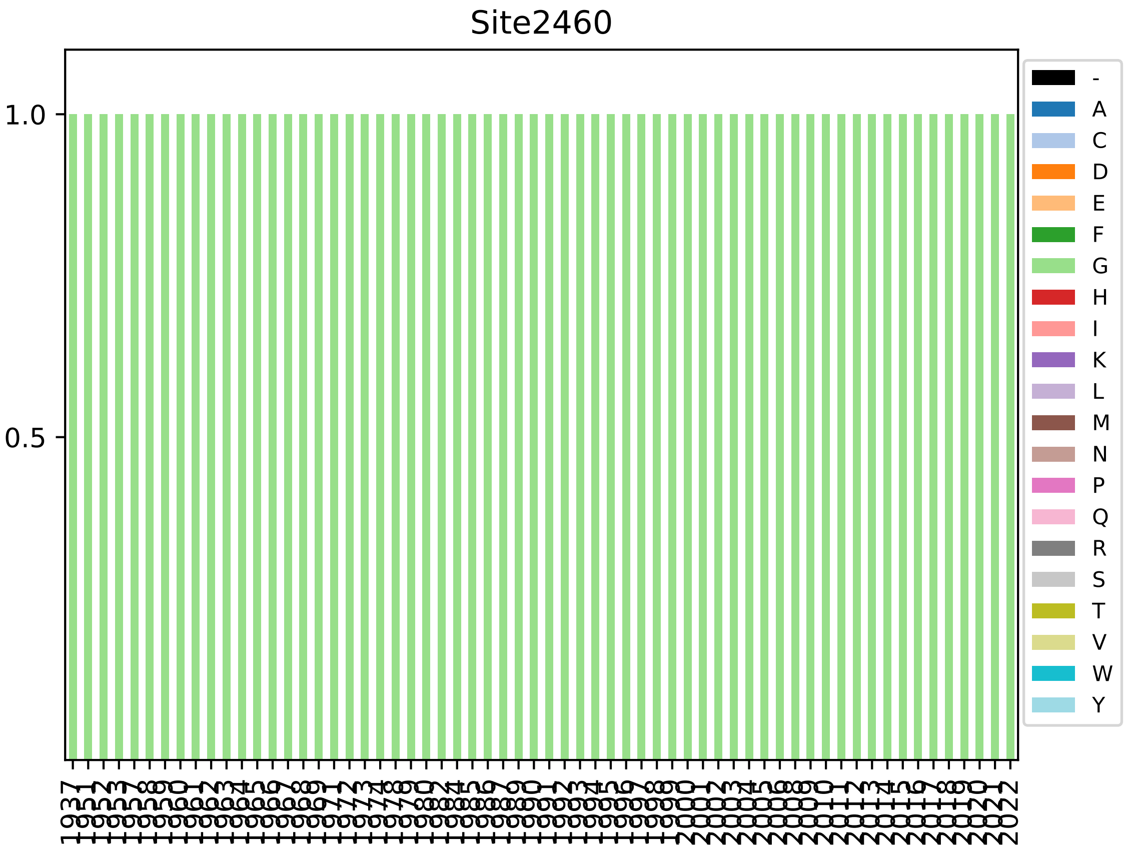
Site2460
1.0
0.5
-
A
C
D
E
F
G
H
I
K
L
M
N
P
Q
R
S
T
V
W
Y

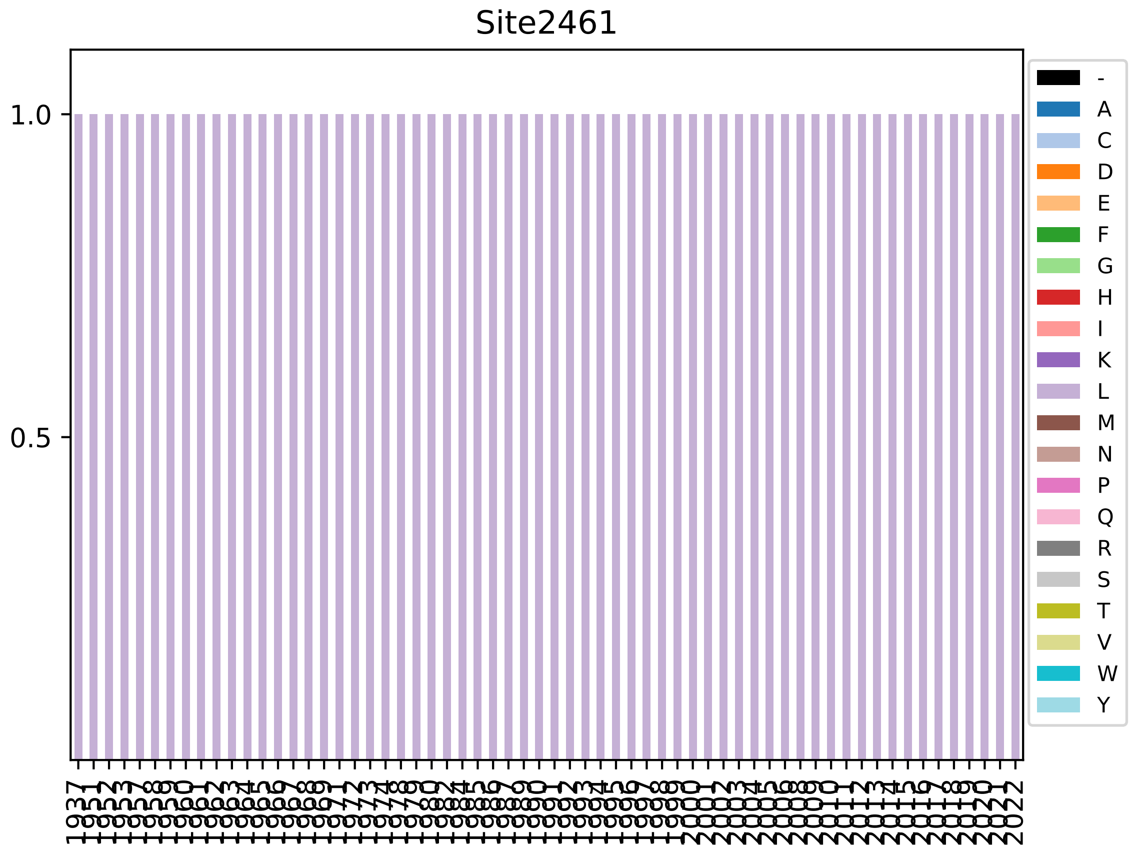
Site2461
1.0
0.5
-
A
C
D
E
F
G
H
I
K
L
M
N
P
Q
R
S
T
V
W
Y

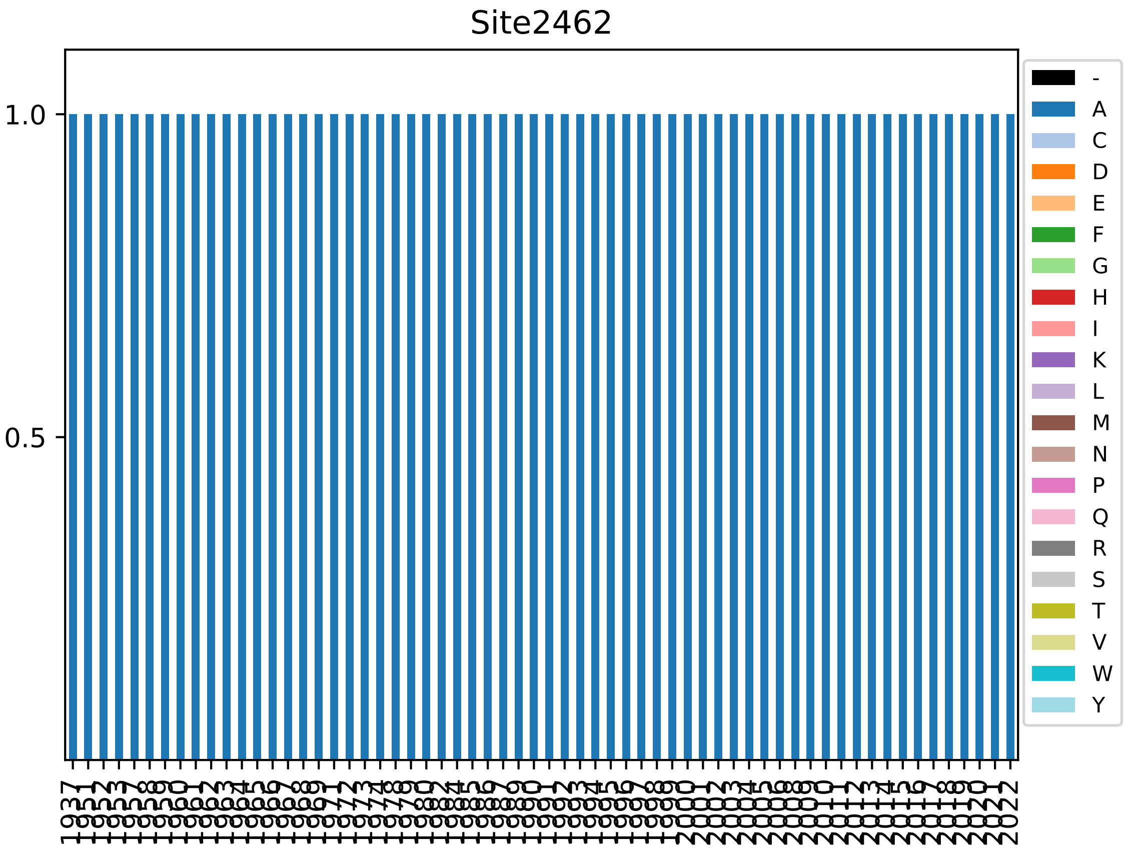
Site2462
1.0
0.5
-
A
C
D
E
F
G
H
I
K
L
M
N
P
Q
R
S
T
V
W
Y

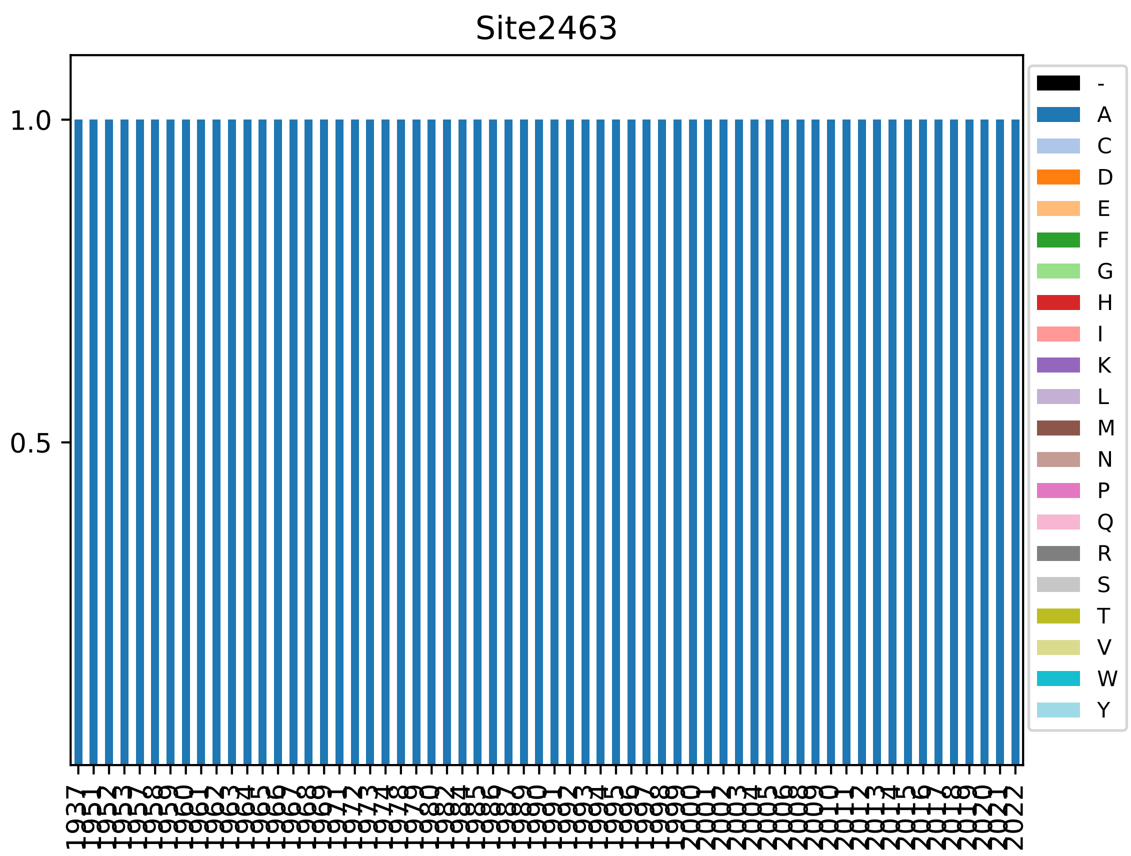
Site2463
1.0
0.5
-
A
C
D
E
F
G
H
I
K
L
M
N
P
Q
R
S
T
V
W
Y

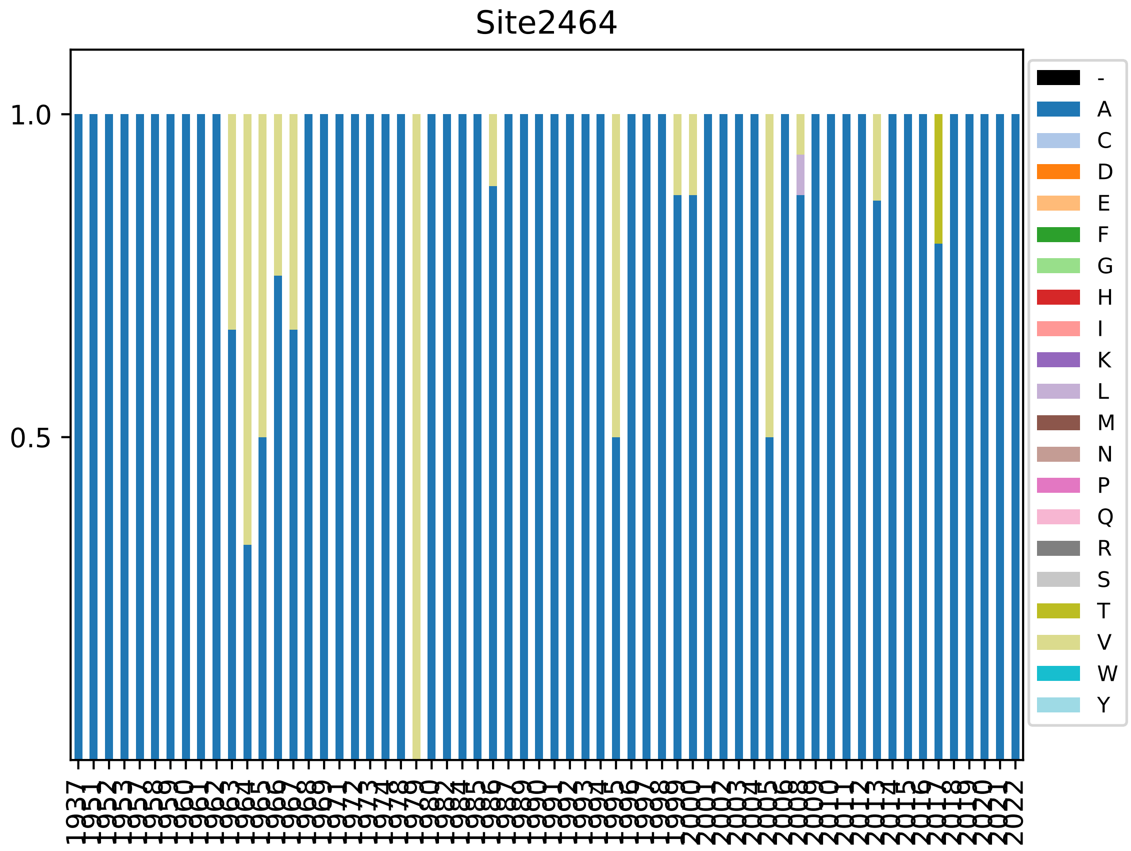
Site2464
1.0
0.5
-
A
C
D
E
F
G
H
I
K
L
M
N
P
Q
R
S
T
V
W
Y

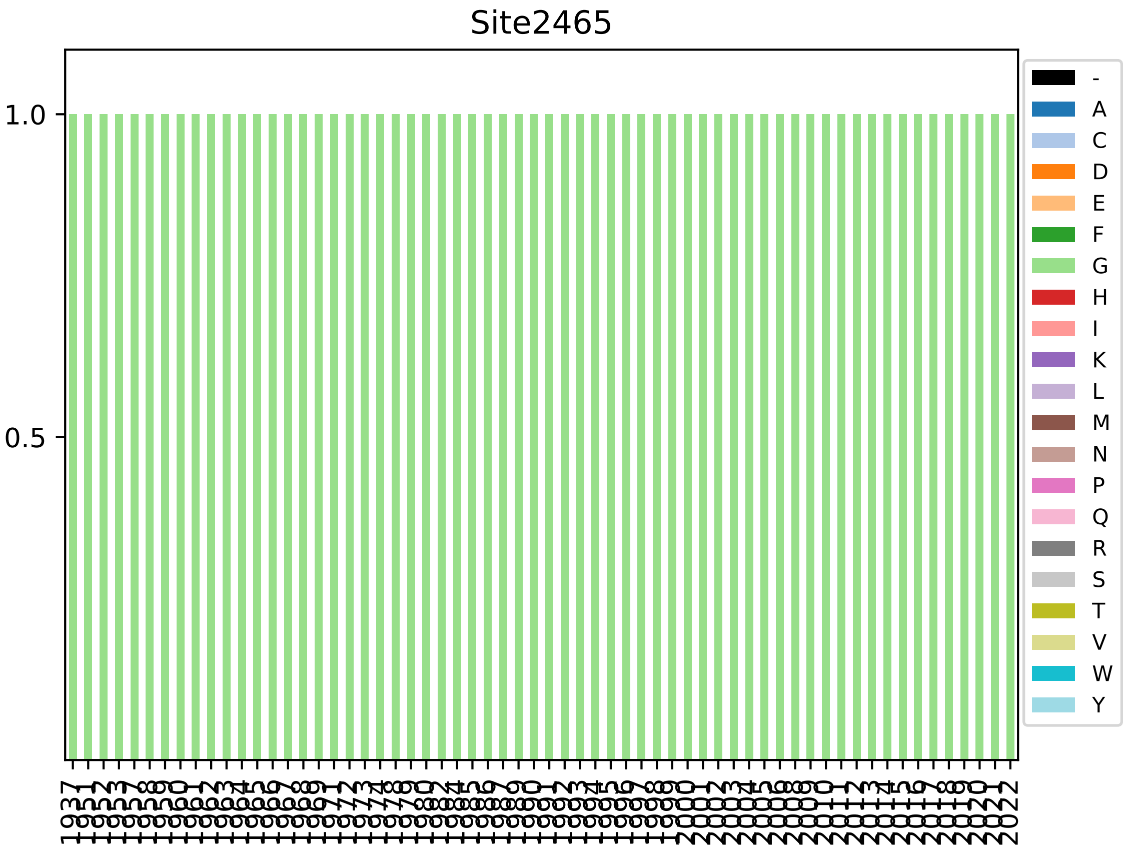
Site2465
1.0
0.5
-
A
C
D
E
F
G
H
I
K
L
M
N
P
Q
R
S
T
V
W
Y

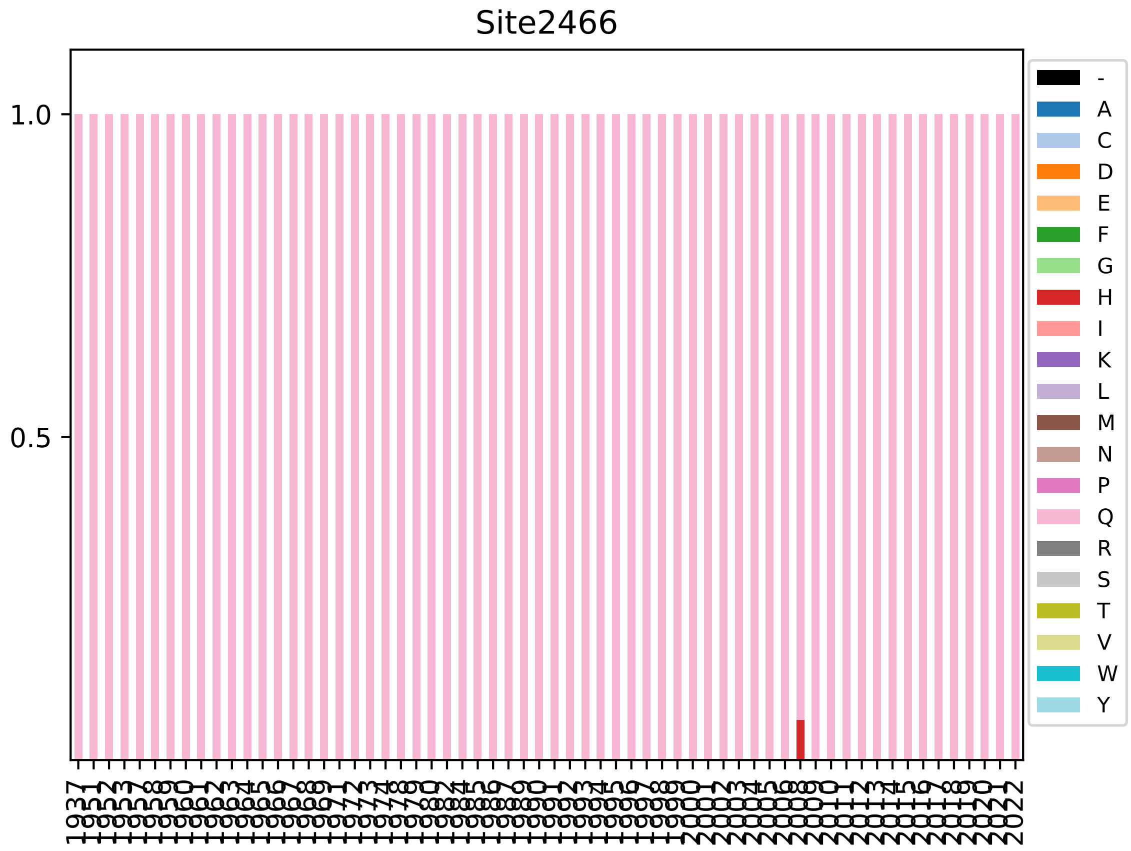
Site2466
1.0
0.5
-
A
C
D
E
F
G
H
I
K
L
M
N
P
Q
R
S
T
V
W
Y

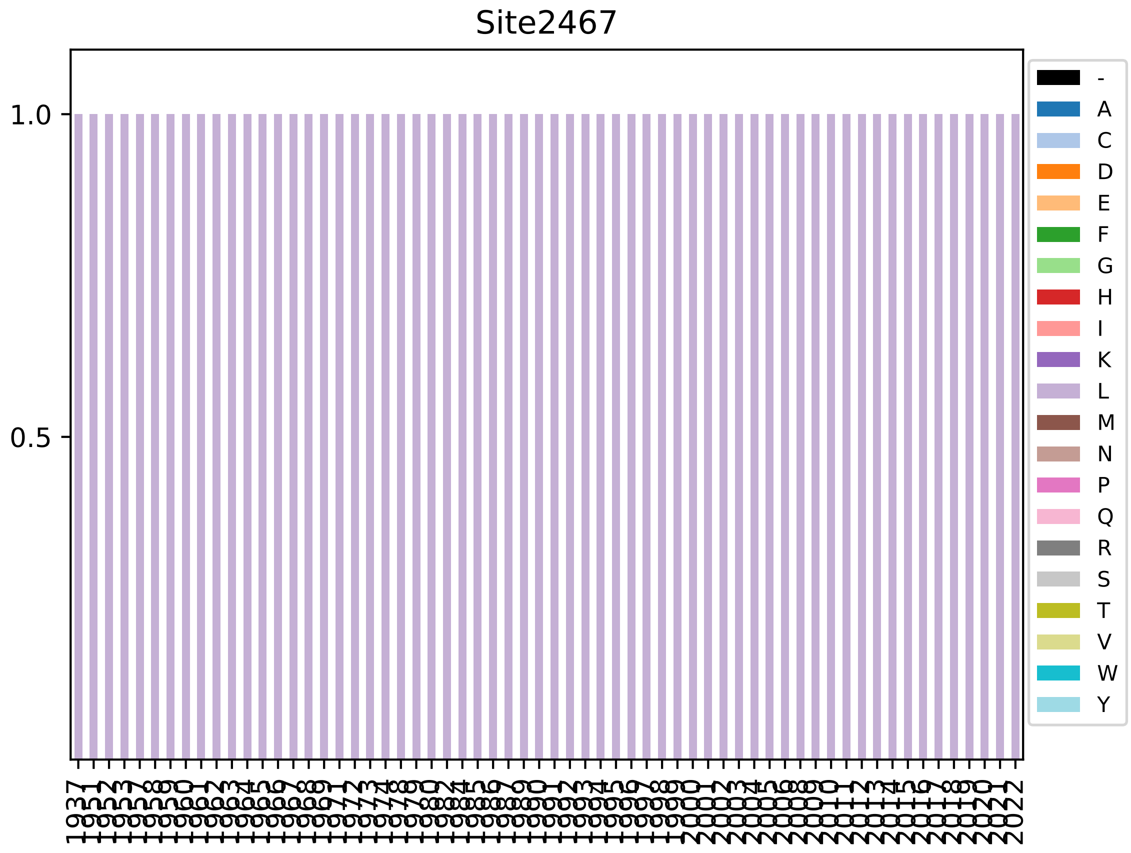
Site2467
1.0
0.5
-
A
C
D
E
F
G
H
I
K
L
M
N
P
Q
R
S
T
V
W
Y

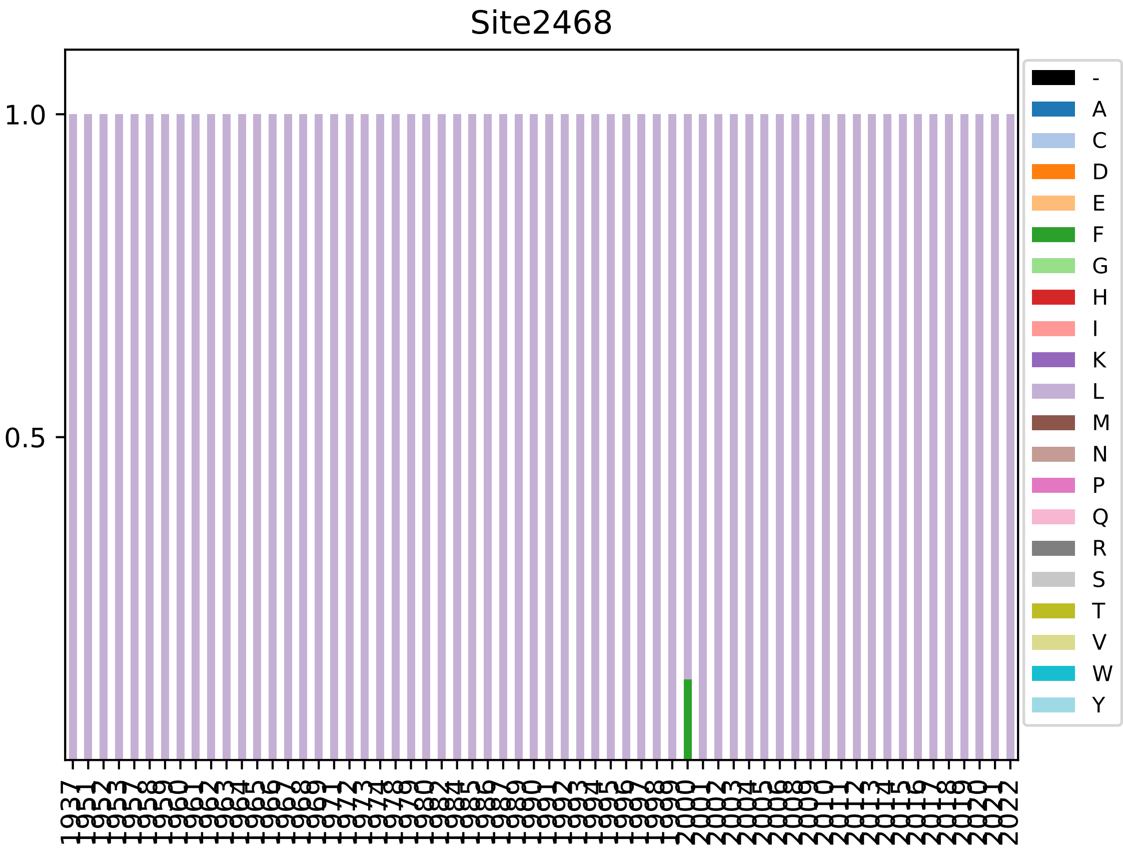
Site2468
1.0
0.5
-
A
C
D
E
F
G
H
I
K
L
M
N
P
Q
R
S
T
V
W
Y

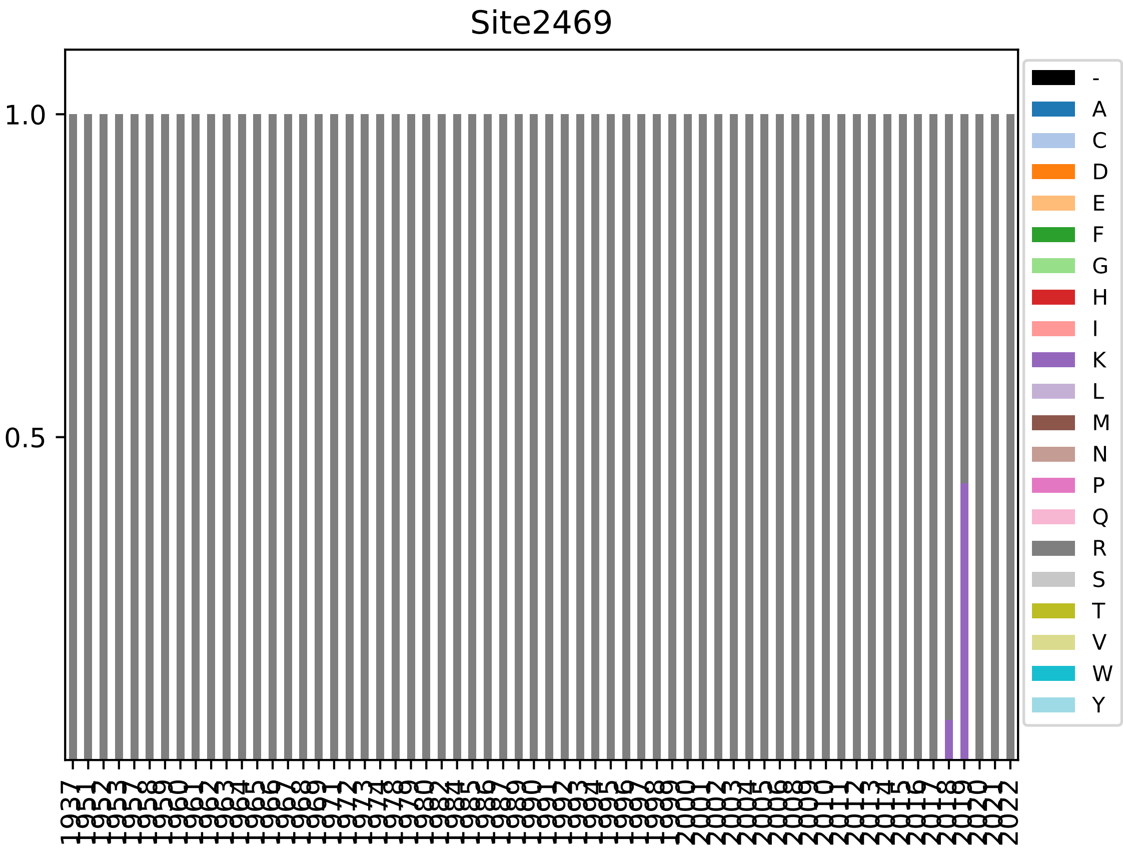

Site2469
1.0
0.5
-
A
C
D
E
F
G
H
I
K
L
M
N
P
Q
R
S
T
V
W
Y

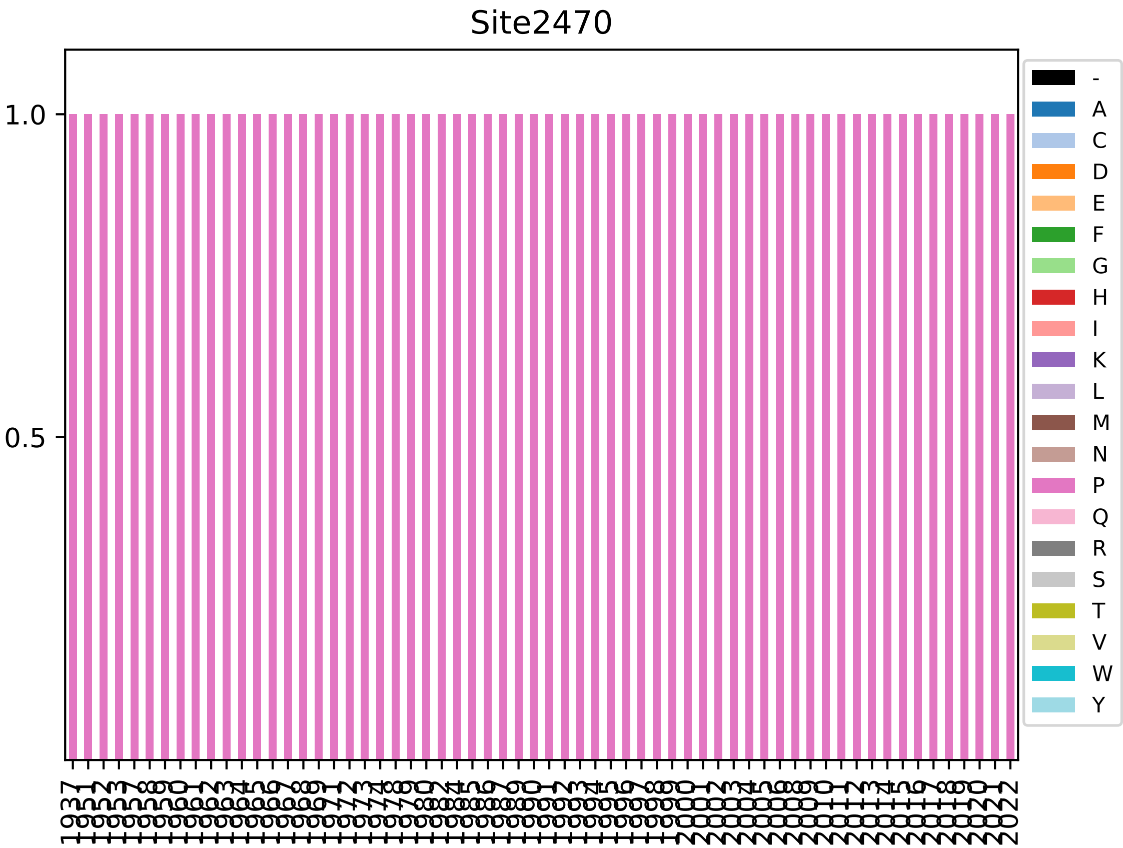
Site2470
1.0
0.5
-
A
C
D
E
F
G
H
I
K
L
M
N
P
Q
R
S
T
V
W
Y

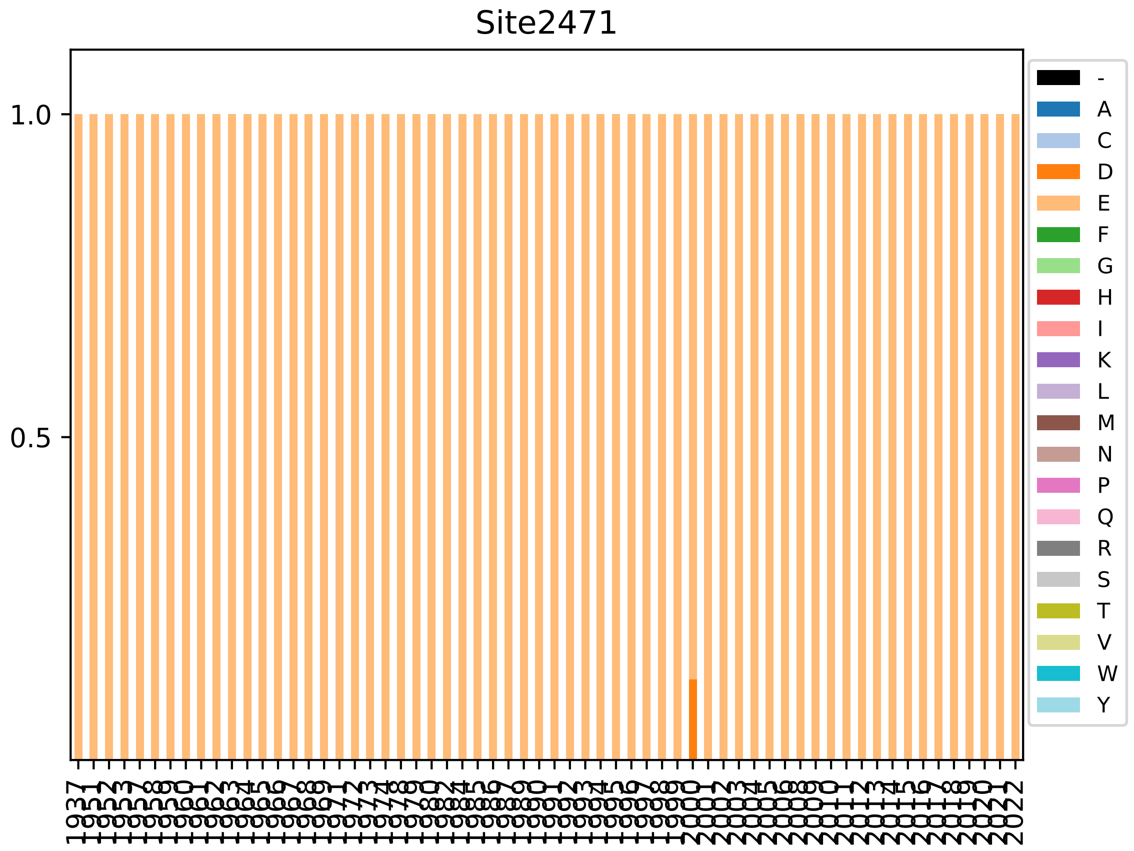
Site2471
1.0
0.5
-
A
C
D
E
F
G
H
I
K
L
M
N
P
Q
R
S
T
V
W
Y

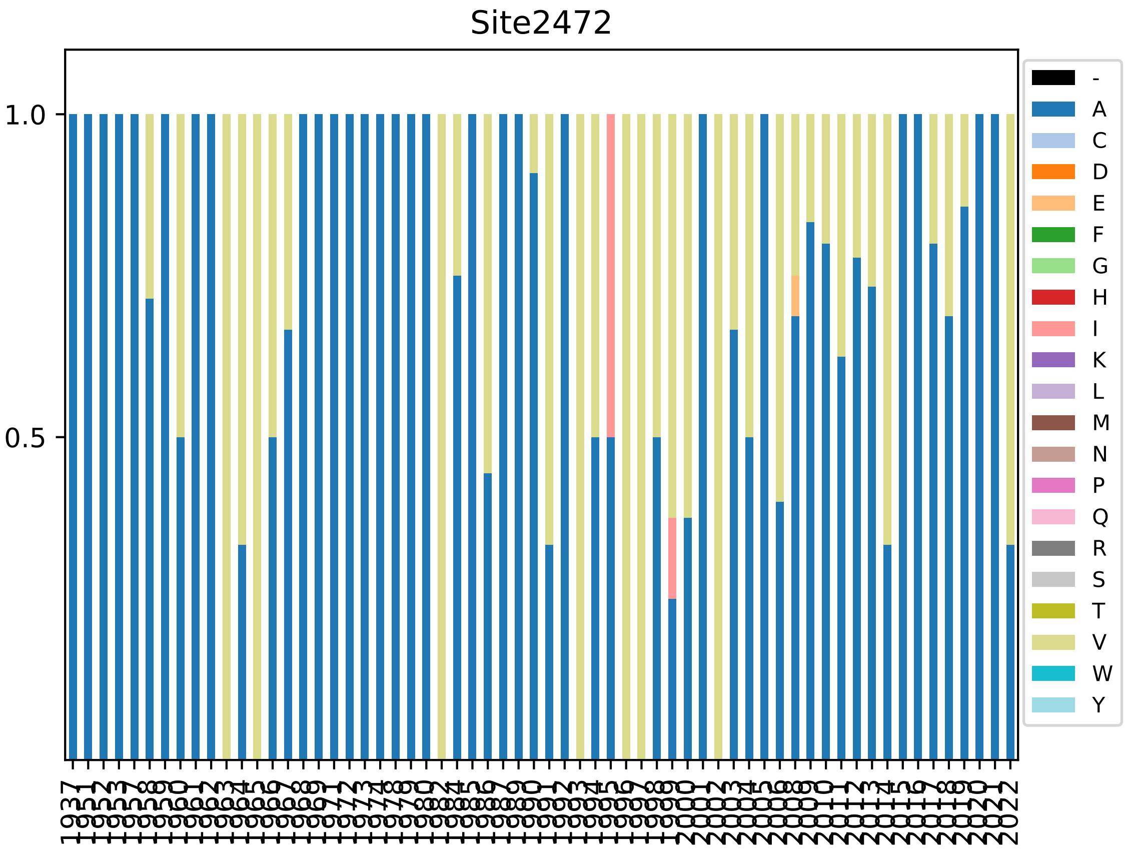
Site2472
1.0
0.5
-
A
C
D
E
F
G
H
I
K
L
M
N
P
Q
R
S
T
V
W
Y

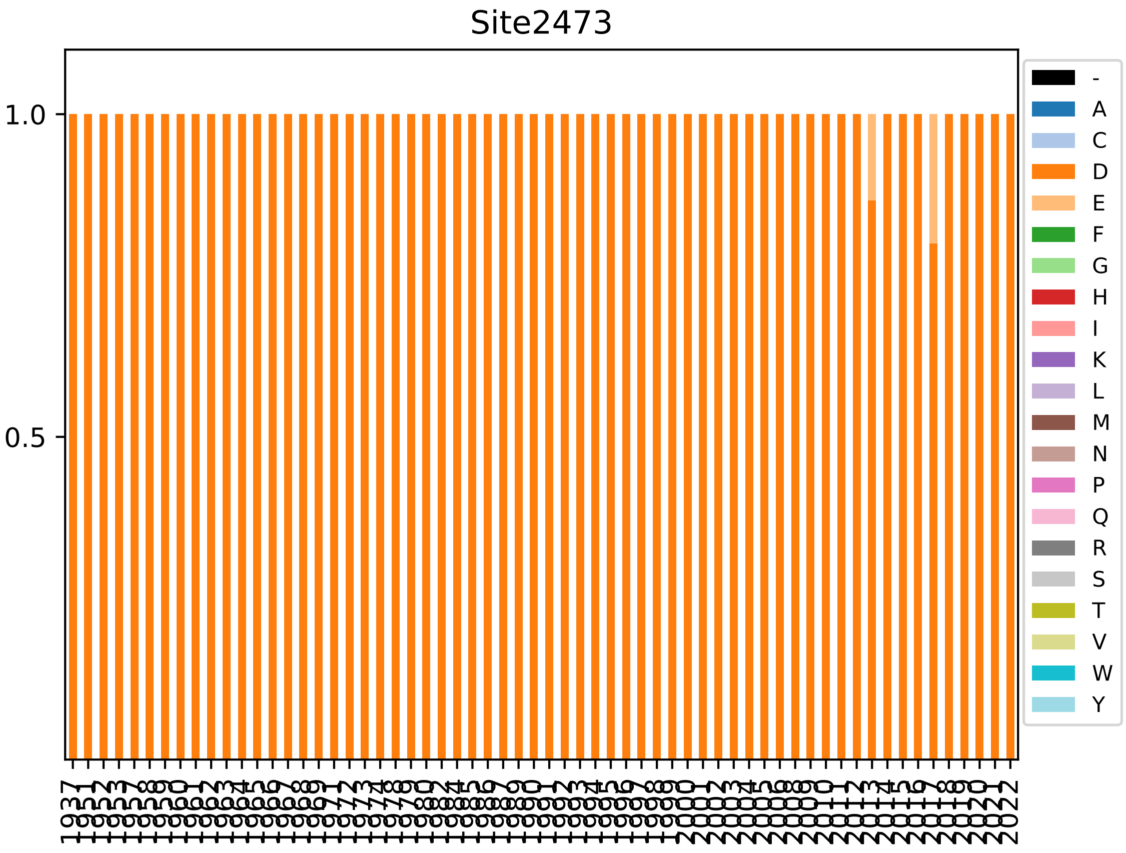
Site2473
1.0
0.5
-
A
C
D
E
F
G
H
I
K
L
M
N
P
Q
R
S
T
V
W
Y

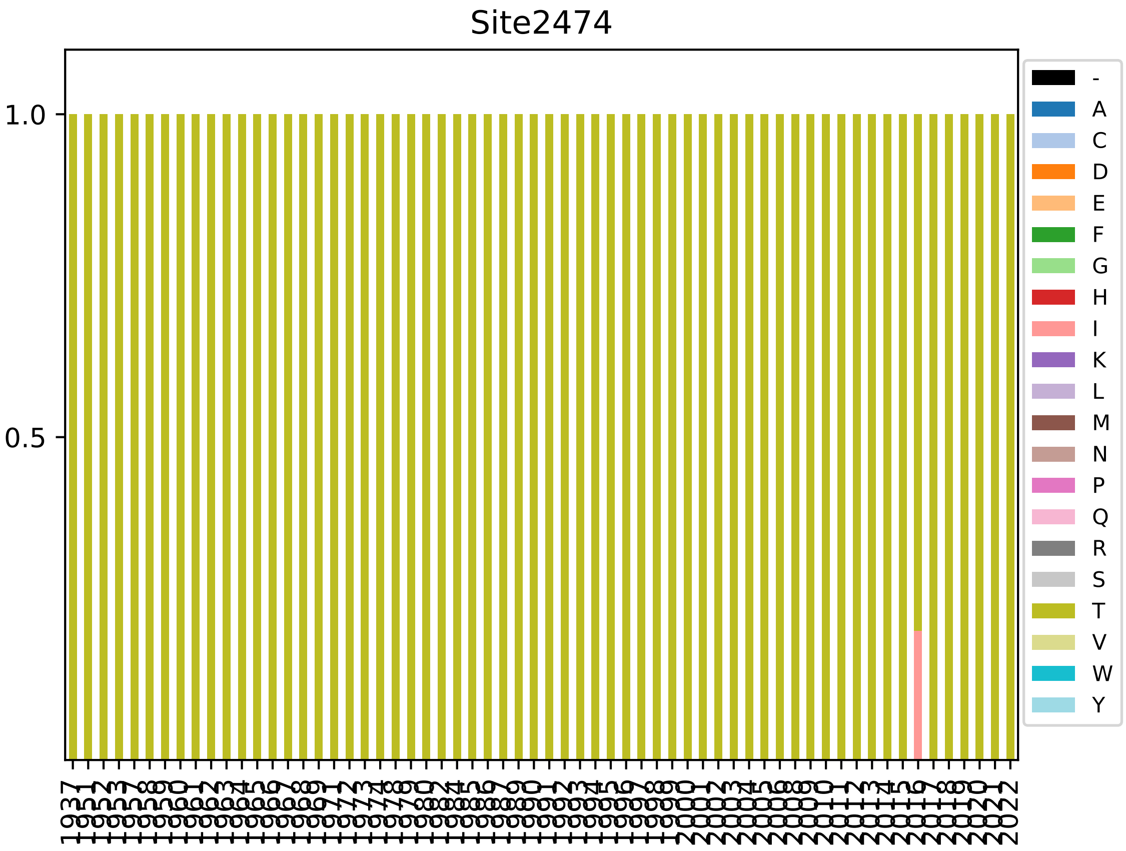
Site2474
1.0
0.5
-
A
C
D
E
F
G
H
I
K
L
M
N
P
Q
R
S
T
V
W
Y

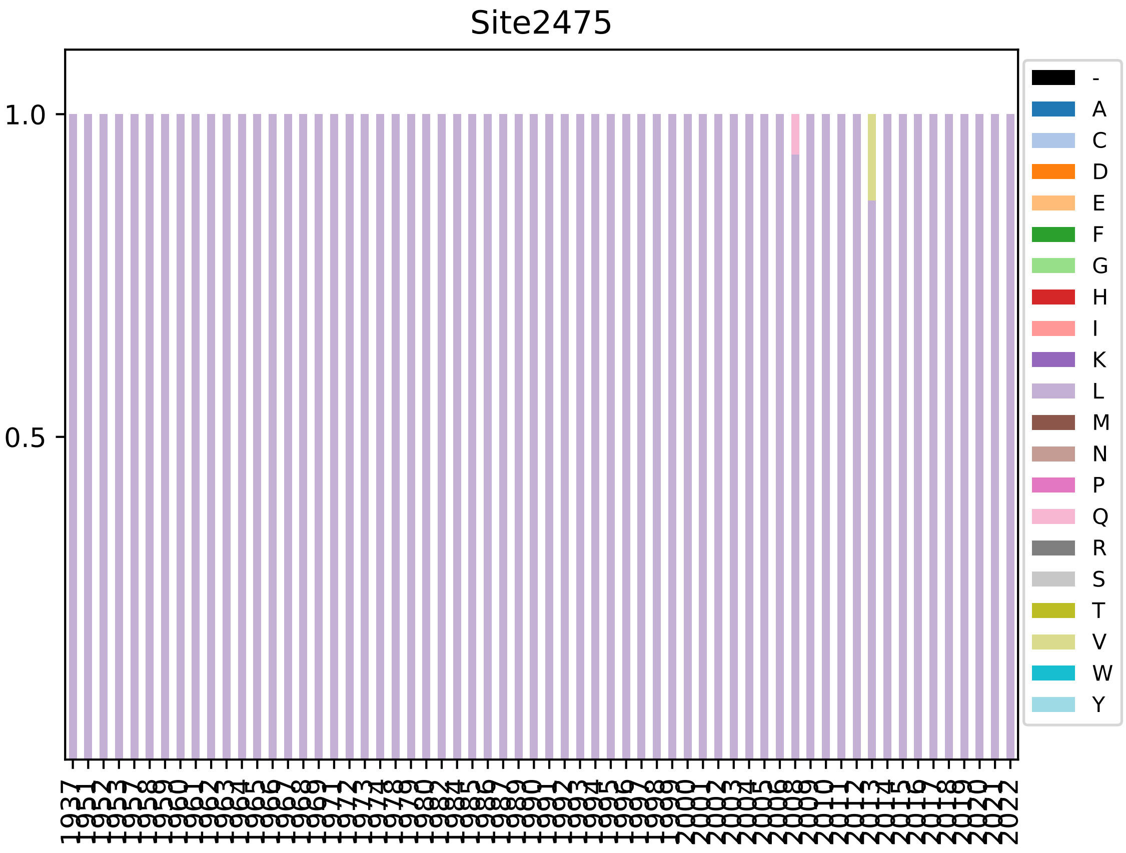
Site2475
1.0
0.5
-
A
C
D
E
F
G
H
I
K
L
M
N
P
Q
R
S
T
V
W
Y

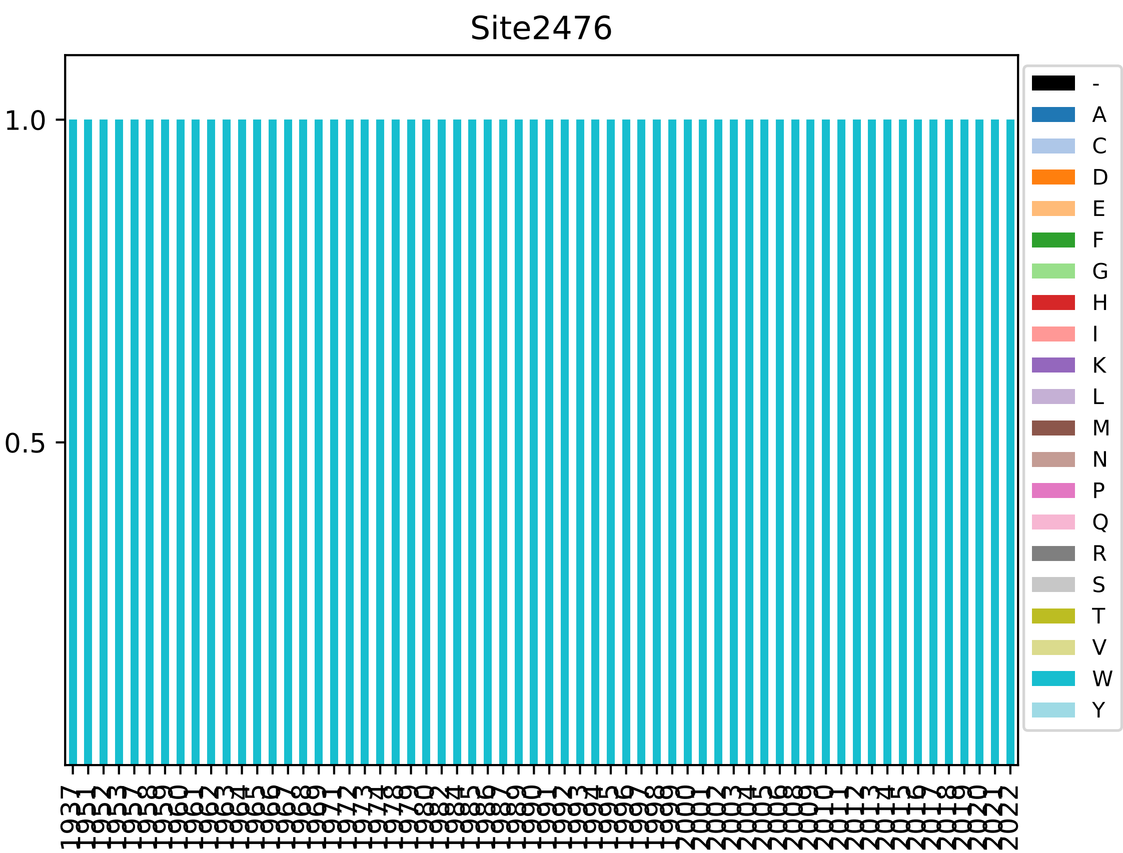
Site2476
1.0
0.5
-
A
C
D
E
F
G
H
I
K
L
M
N
P
Q
R
S
T
V
W
Y

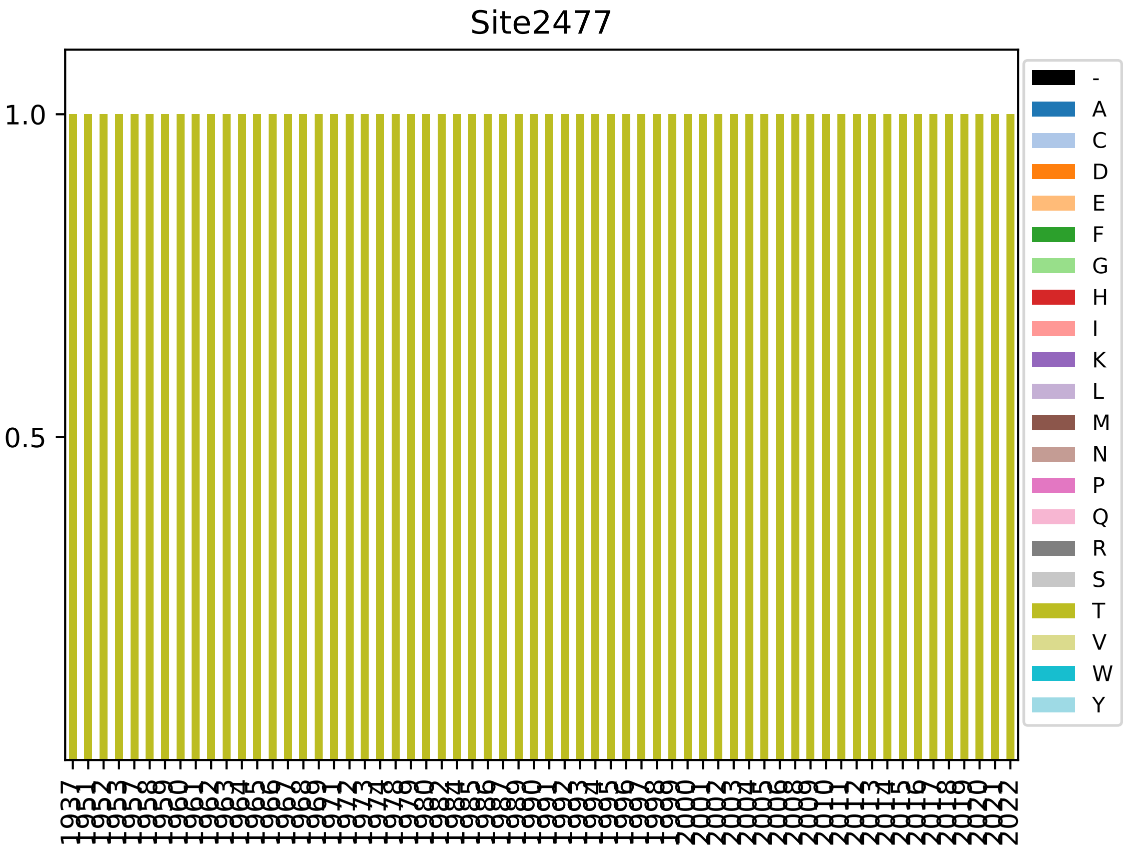
Site2477
1.0
0.5
-
A
C
D
E
F
G
H
I
K
L
M
N
P
Q
R
S
T
V
W
Y

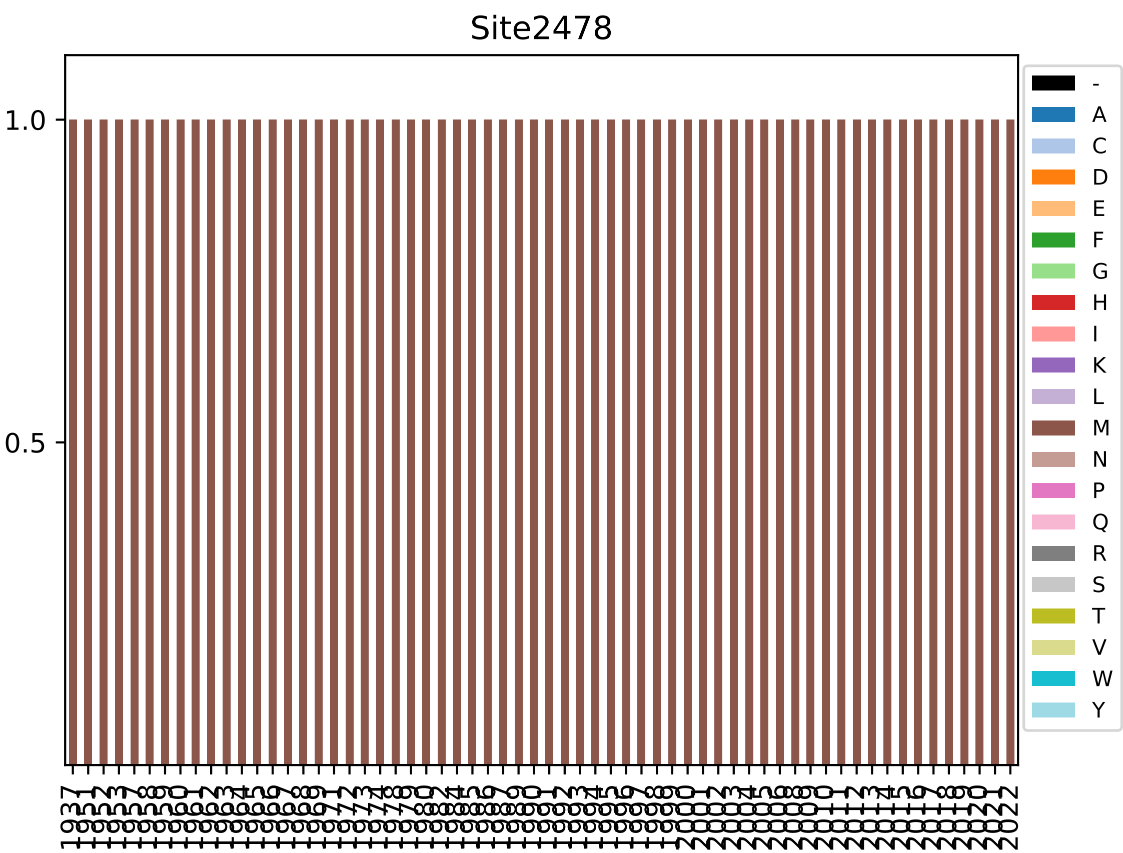
Site2478
1.0
0.5
-
A
C
D
E
F
G
H
I
K
L
M
N
P
Q
R
S
T
V
W
Y

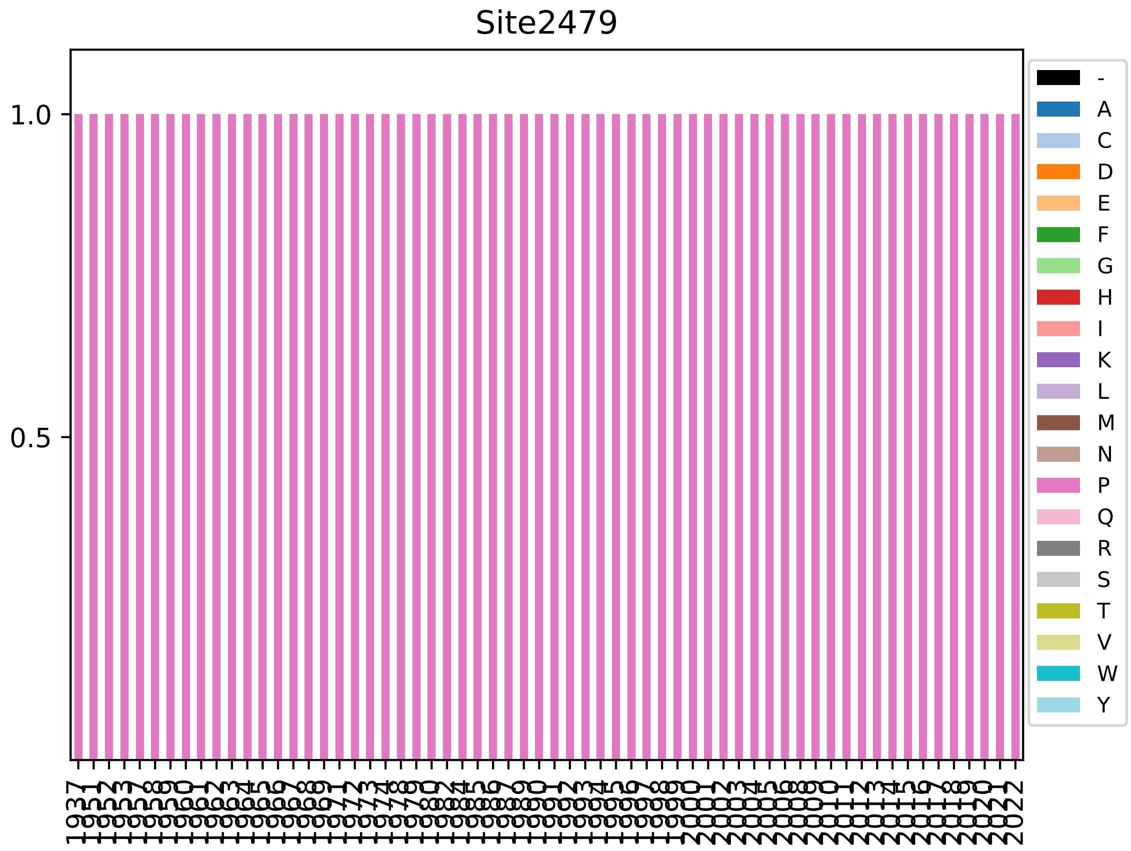
Site2479
1.0
0.5
-
A
C
D
E
F
G
H
I
K
L
M
N
P
Q
R
S
T
V
W
Y

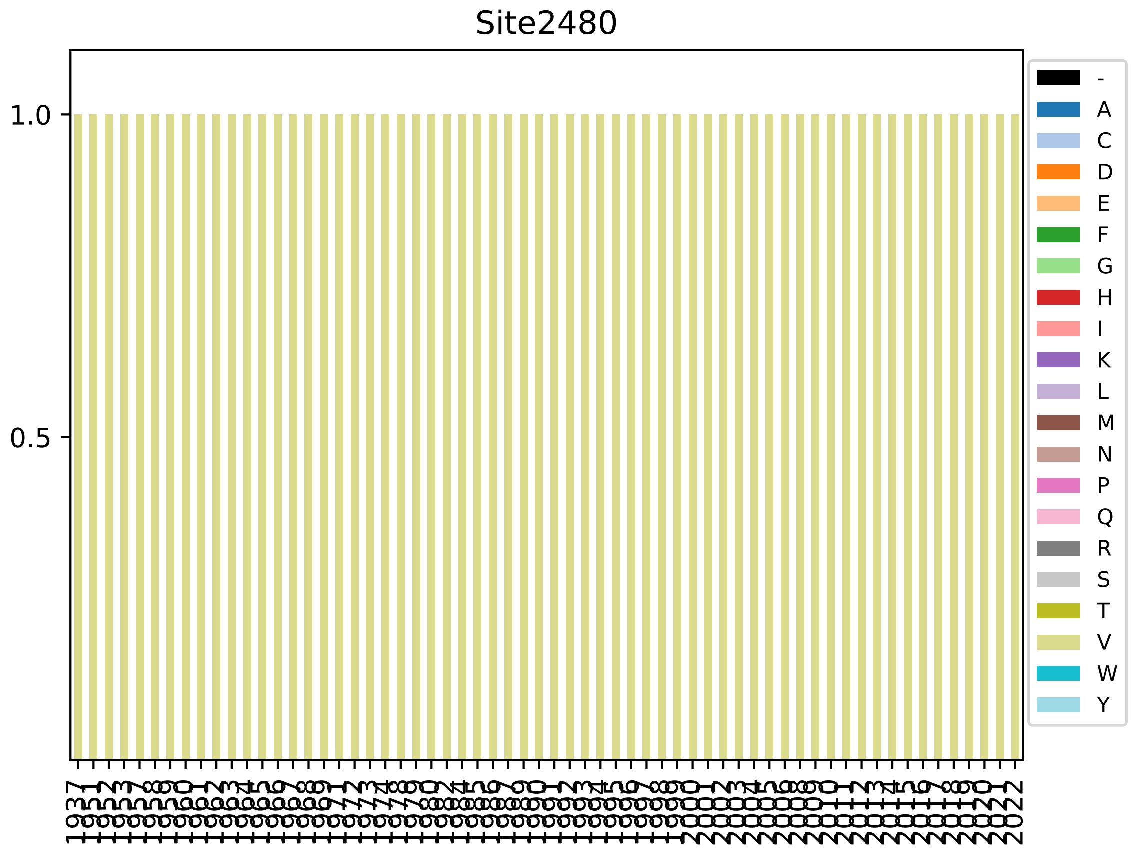
Site2480
1.0
0.5
-
A
C
D
E
F
G
H
I
K
L
M
N
P
Q
R
S
T
V
W
Y

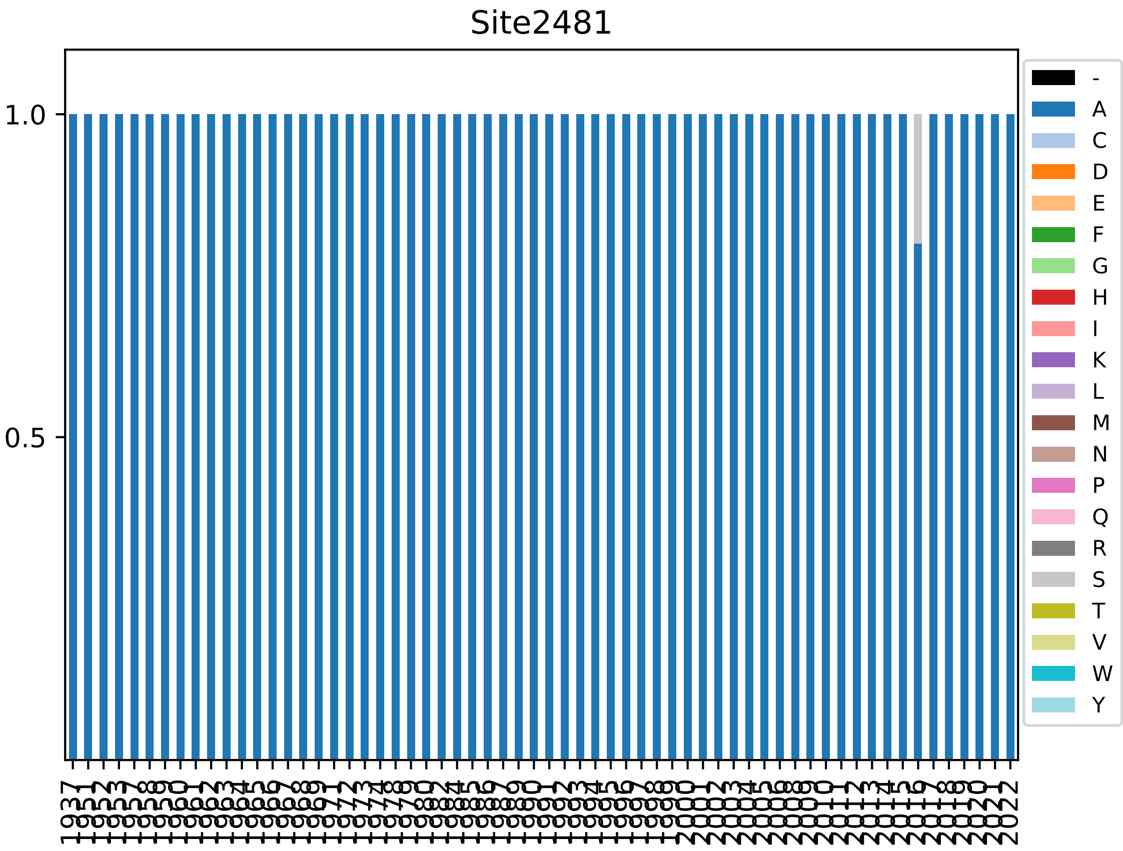
Site2481
1.0
0.5
-
A
C
D
E
F
G
H
I
K
L
M
N
P
Q
R
S
T
V
W
Y

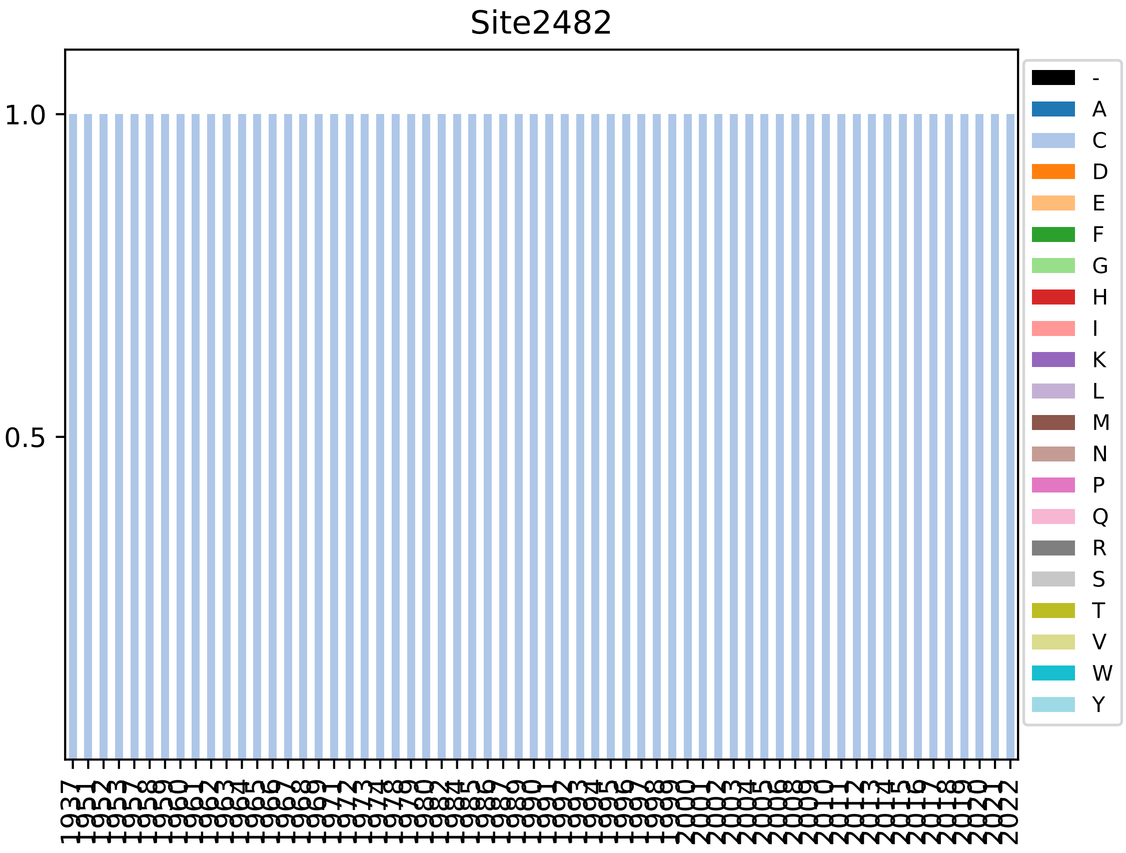
Site2482
1.0
0.5
-
A
C
D
E
F
G
H
I
K
L
M
N
P
Q
R
S
T
V
W
Y

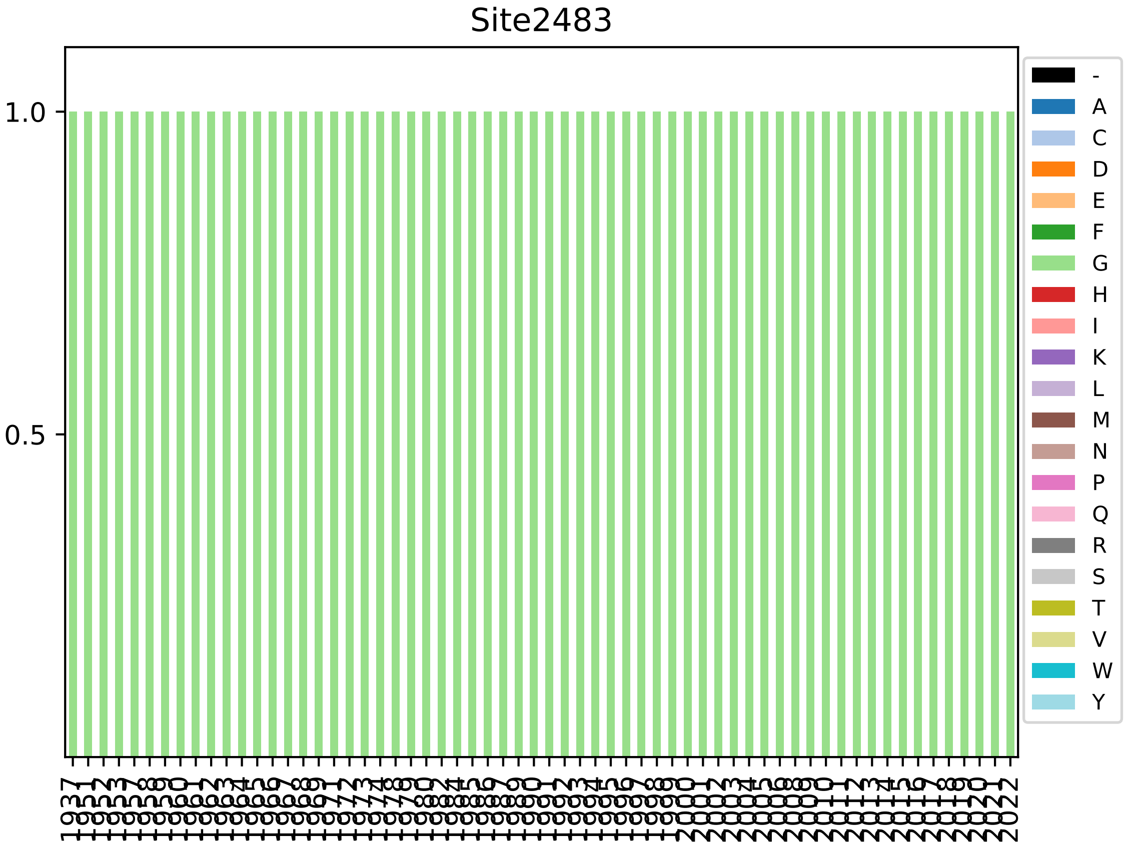
Site2483
1.0
0.5
-
A
C
D
E
F
G
H
I
K
L
M
N
P
Q
R
S
T
V
W
Y

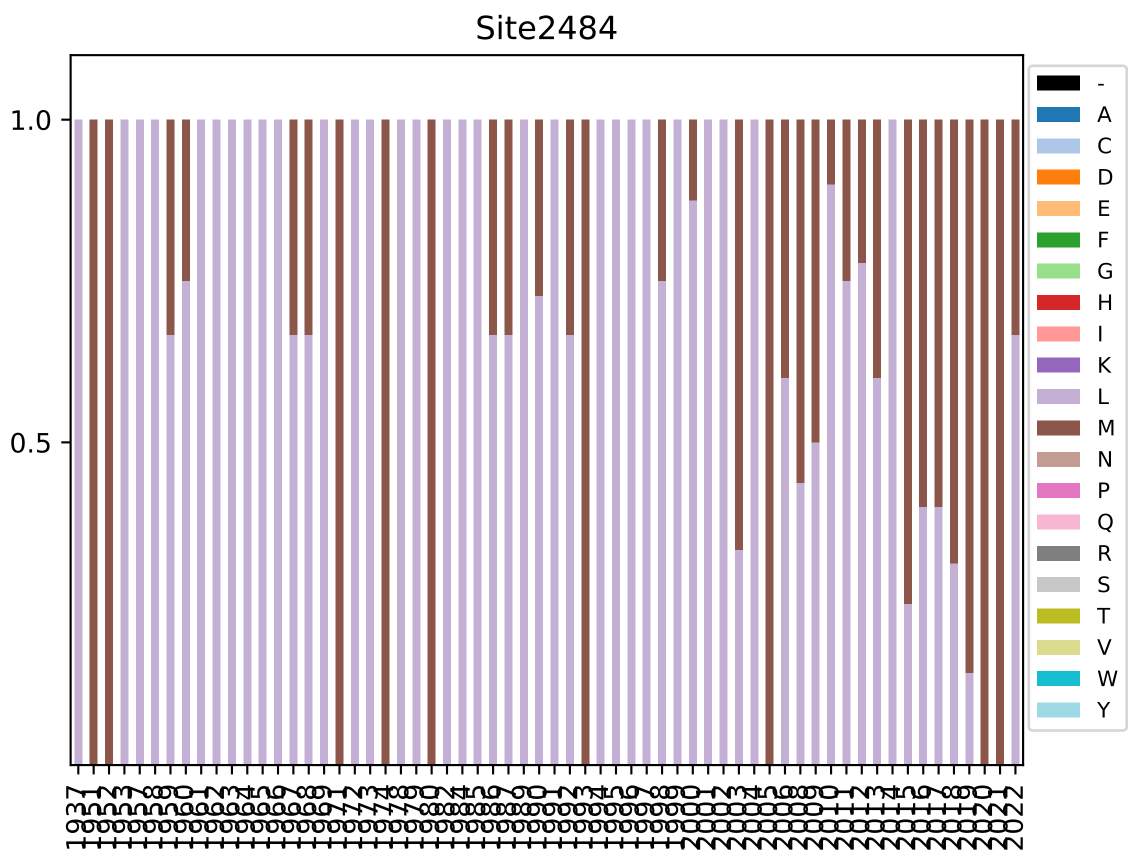
Site2484
1.0
0.5
-
A
C
D
E
F
G
H
I
K
L
M
N
P
Q
R
S
T
V
W
Y

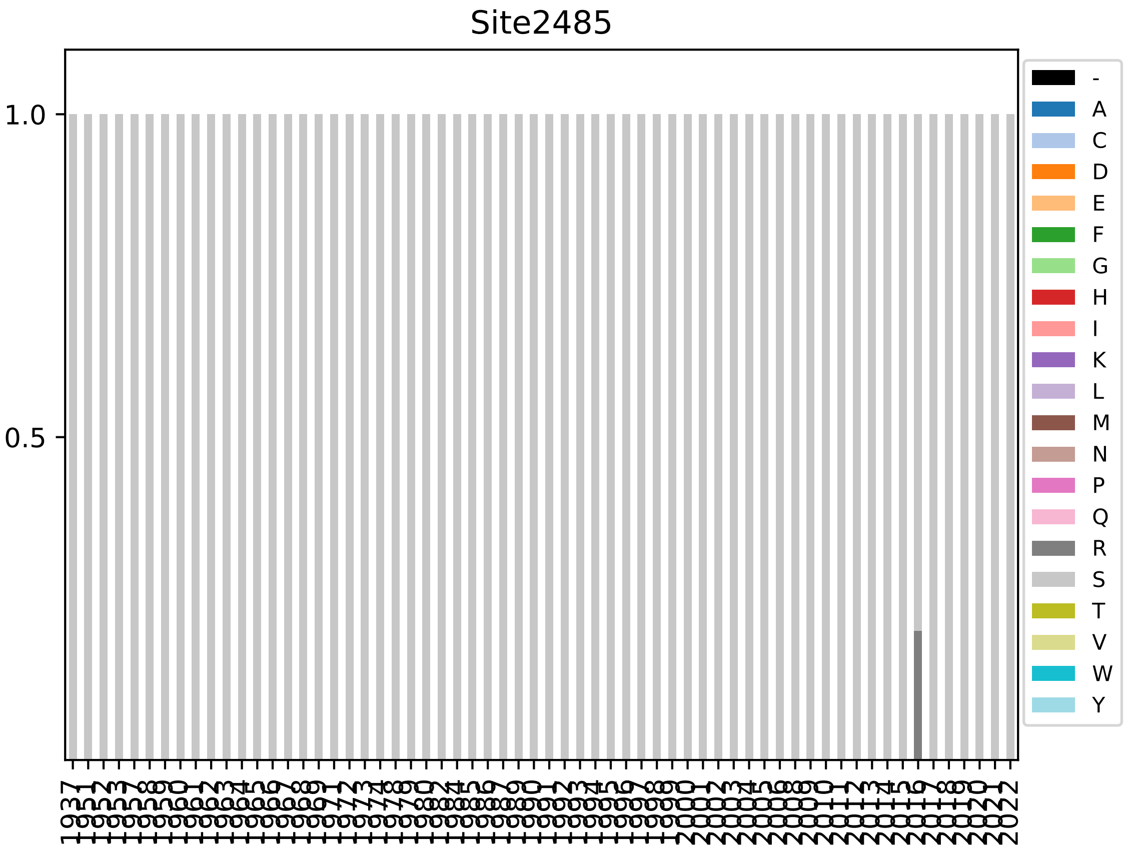
Site2485
1.0
0.5
-
A
C
D
E
F
G
H
I
K
L
M
N
P
Q
R
S
T
V
W
Y

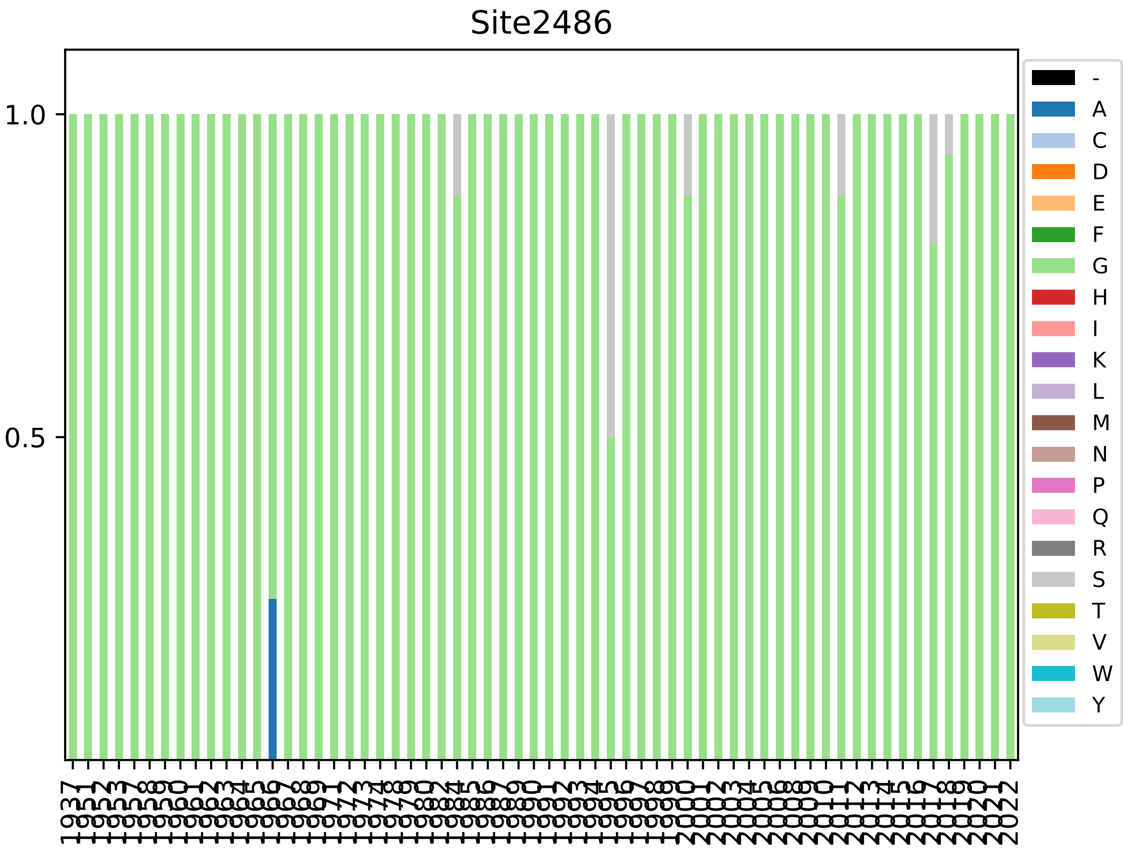
Site2486
1.0
0.5
-
A
C
D
E
F
G
H
I
K
L
M
N
P
Q
R
S
T
V
W
Y

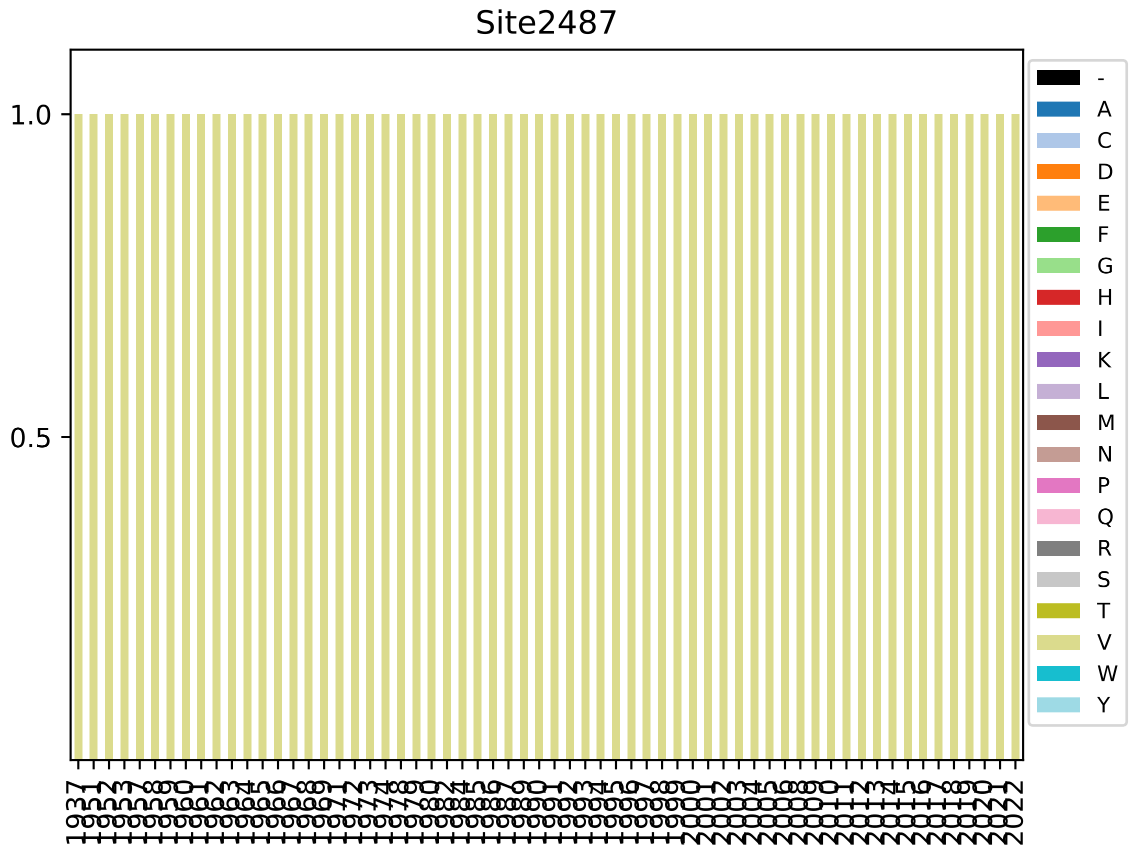
Site2487
1.0
0.5
-
A
C
D
E
F
G
H
I
K
L
M
N
P
Q
R
S
T
V
W
Y

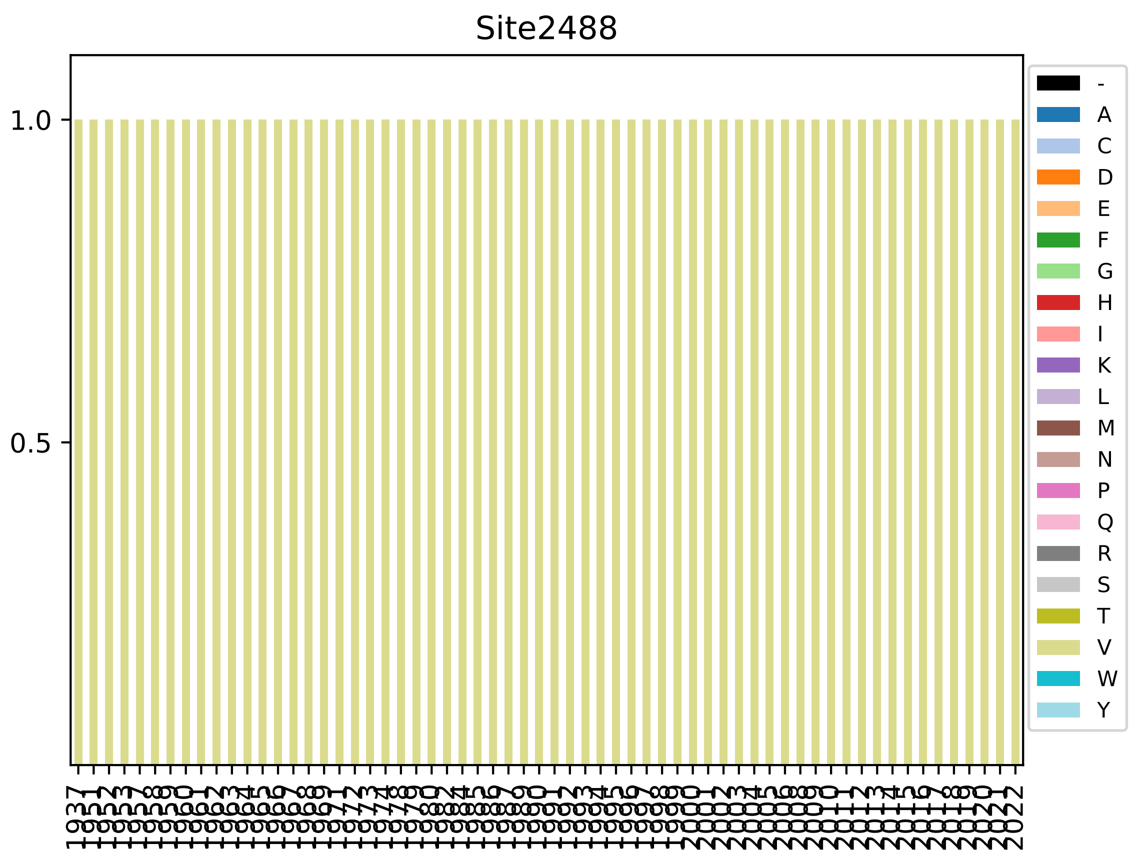
Site2488
1.0
0.5
-
A
C
D
E
F
G
H
I
K
L
M
N
P
Q
R
S
T
V
W
Y

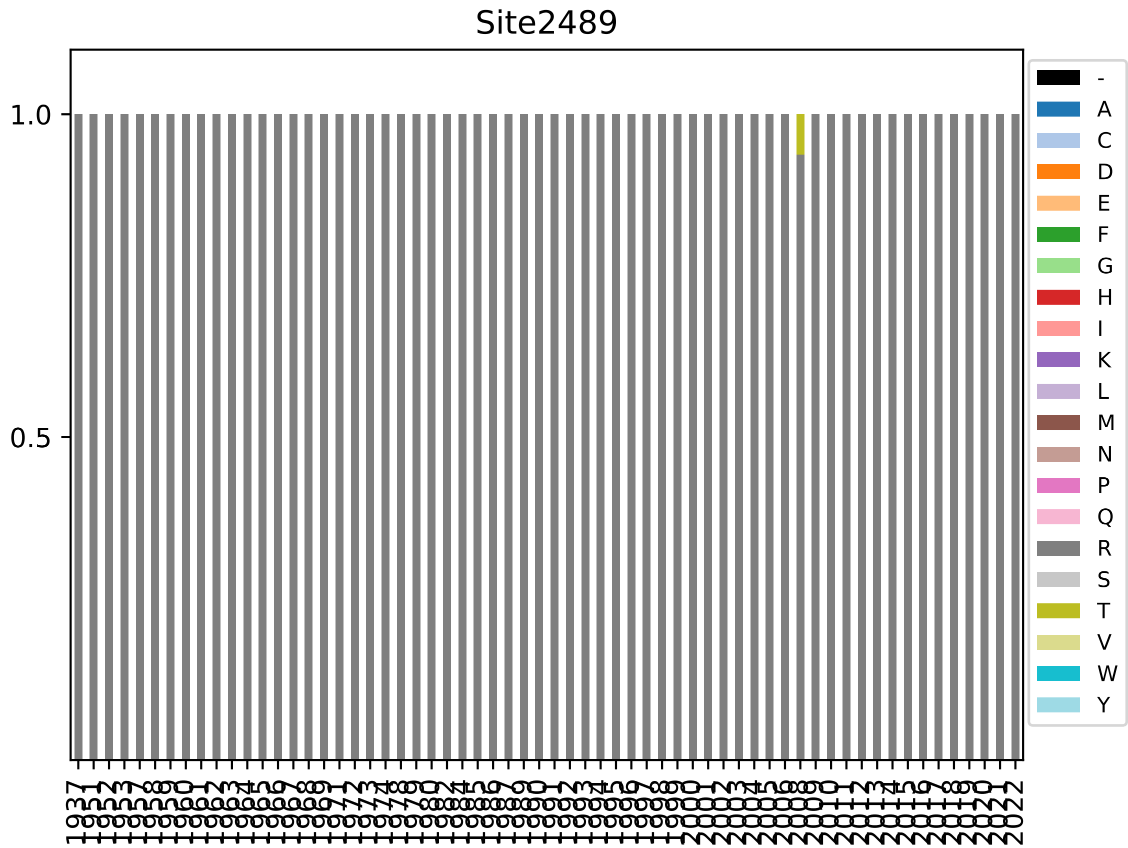
Site2489
1.0
0.5
-
A
C
D
E
F
G
H
I
K
L
M
N
P
Q
R
S
T
V
W
Y

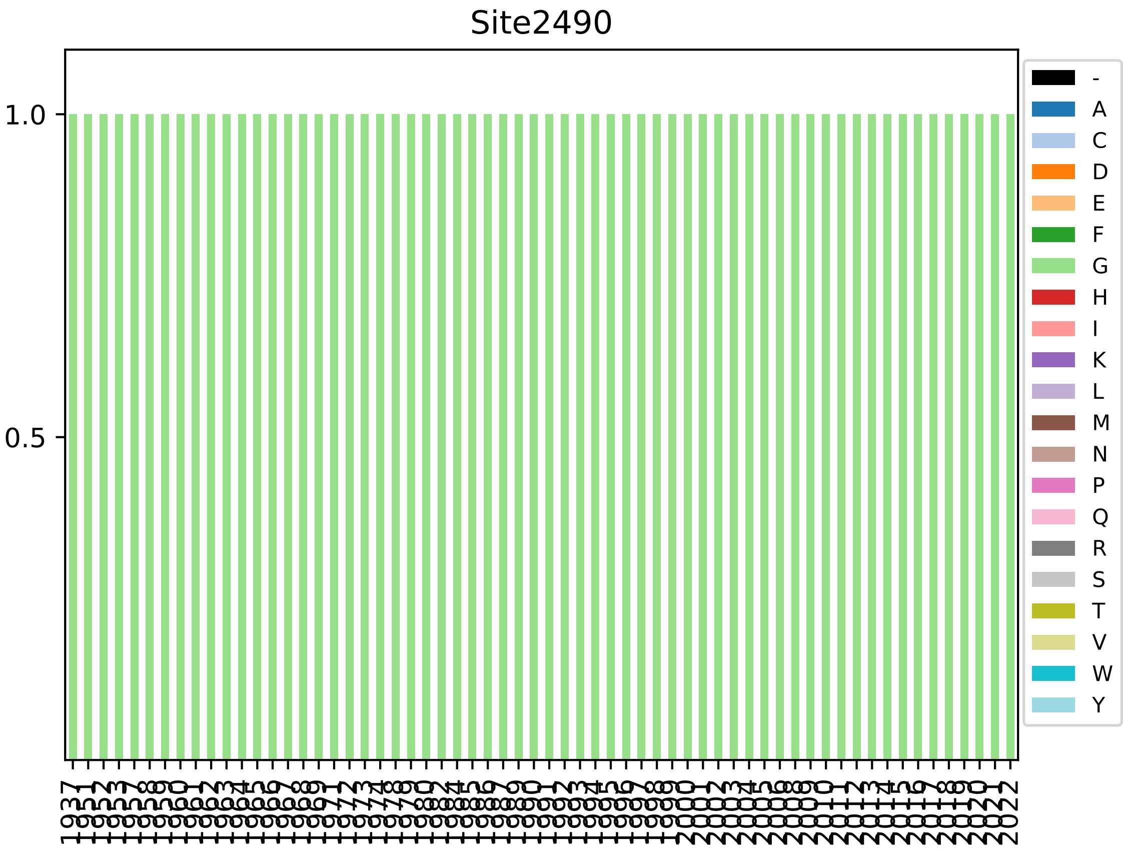
Site2490
1.0
0.5
-
A
C
D
E
F
G
H
I
K
L
M
N
P
Q
R
S
T
V
W
Y

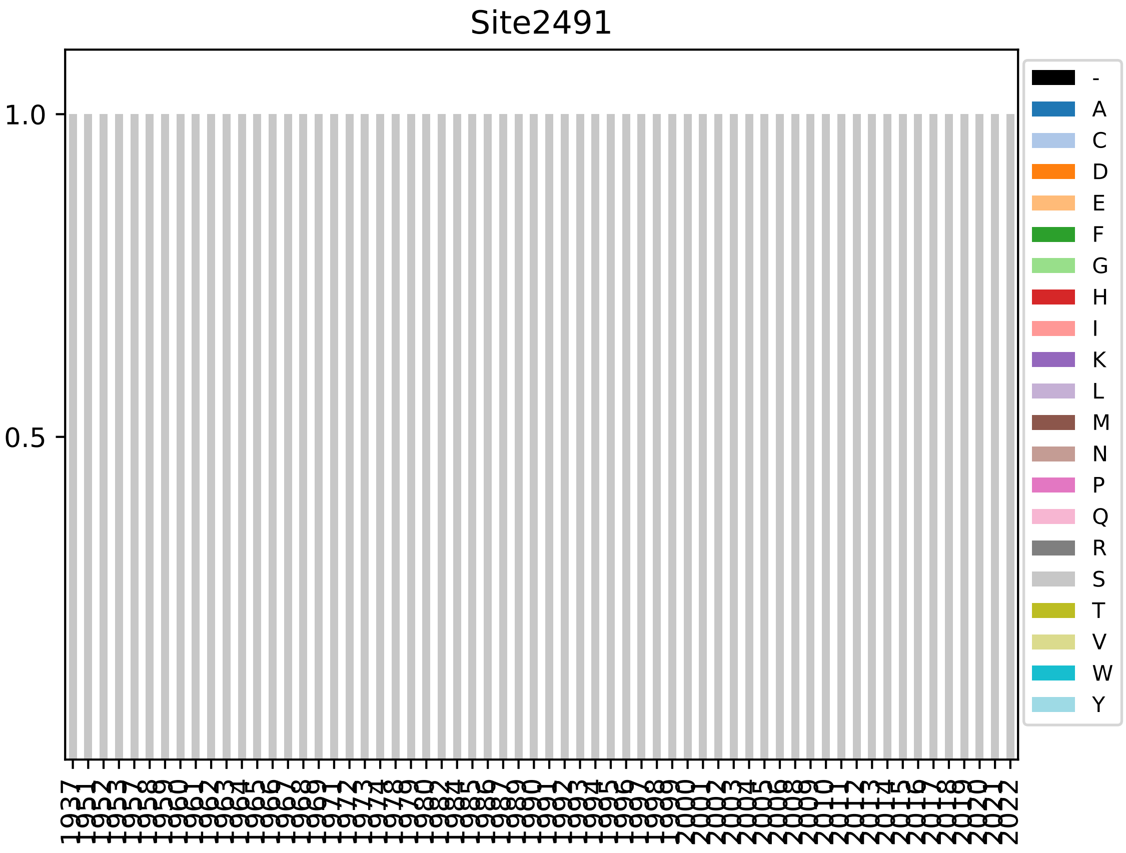
Site2491
1.0
0.5
-
A
C
D
E
F
G
H
I
K
L
M
N
P
Q
R
S
T
V
W
Y

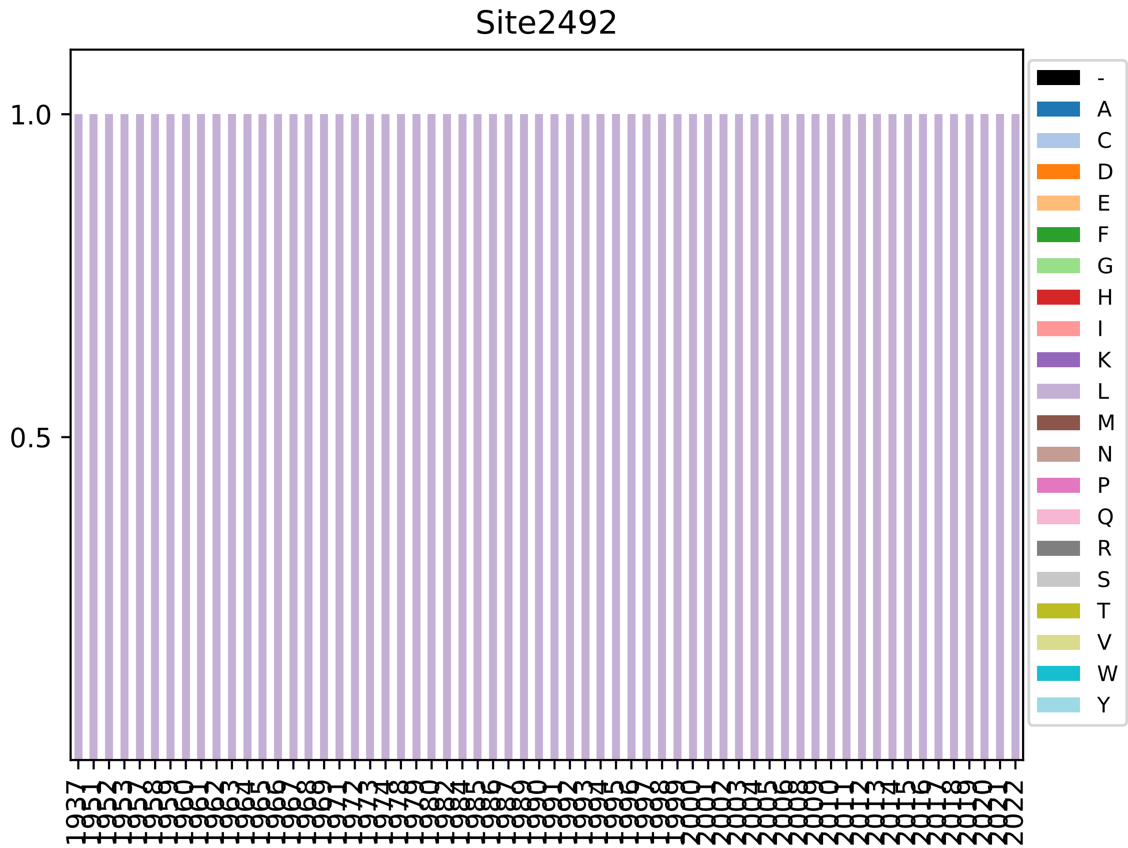
Site2492
1.0
0.5
-
A
C
D
E
F
G
H
I
K
L
M
N
P
Q
R
S
T
V
W
Y

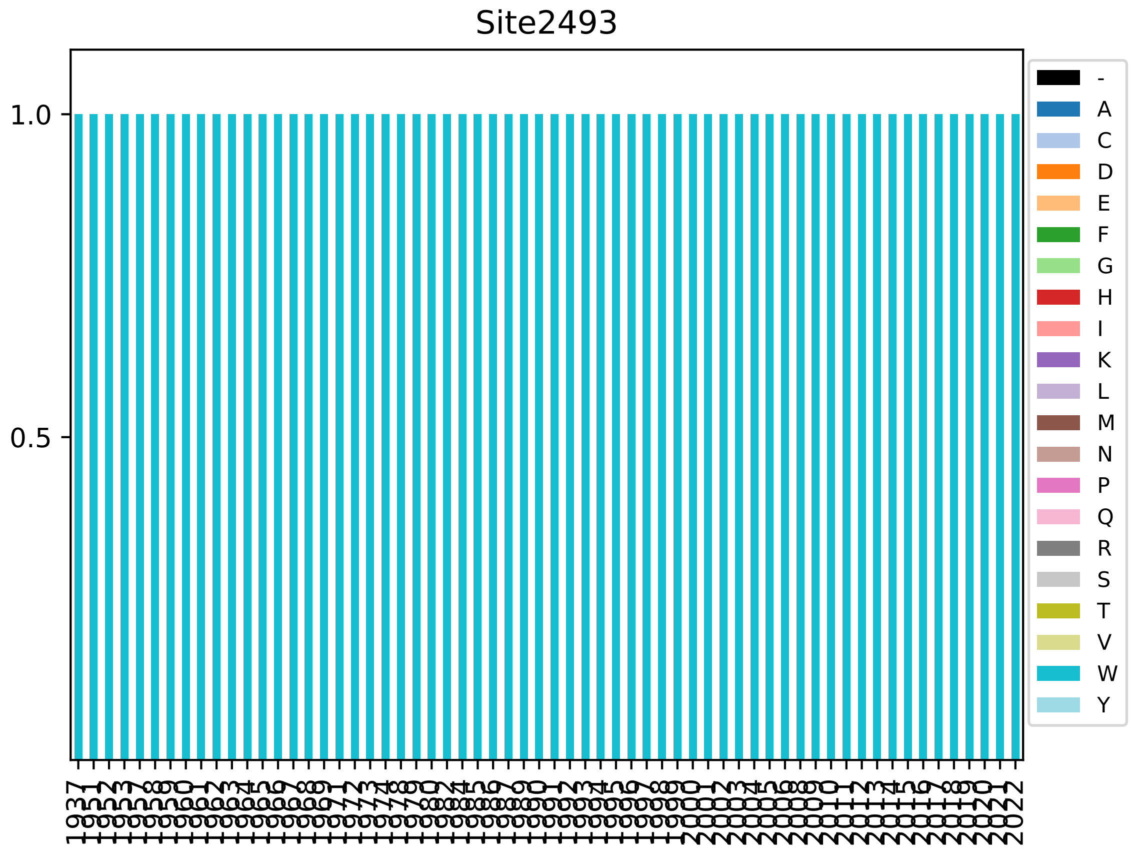
Site2493
1.0
0.5
-
A
C
D
E
F
G
H
I
K
L
M
N
P
Q
R
S
T
V
W
Y

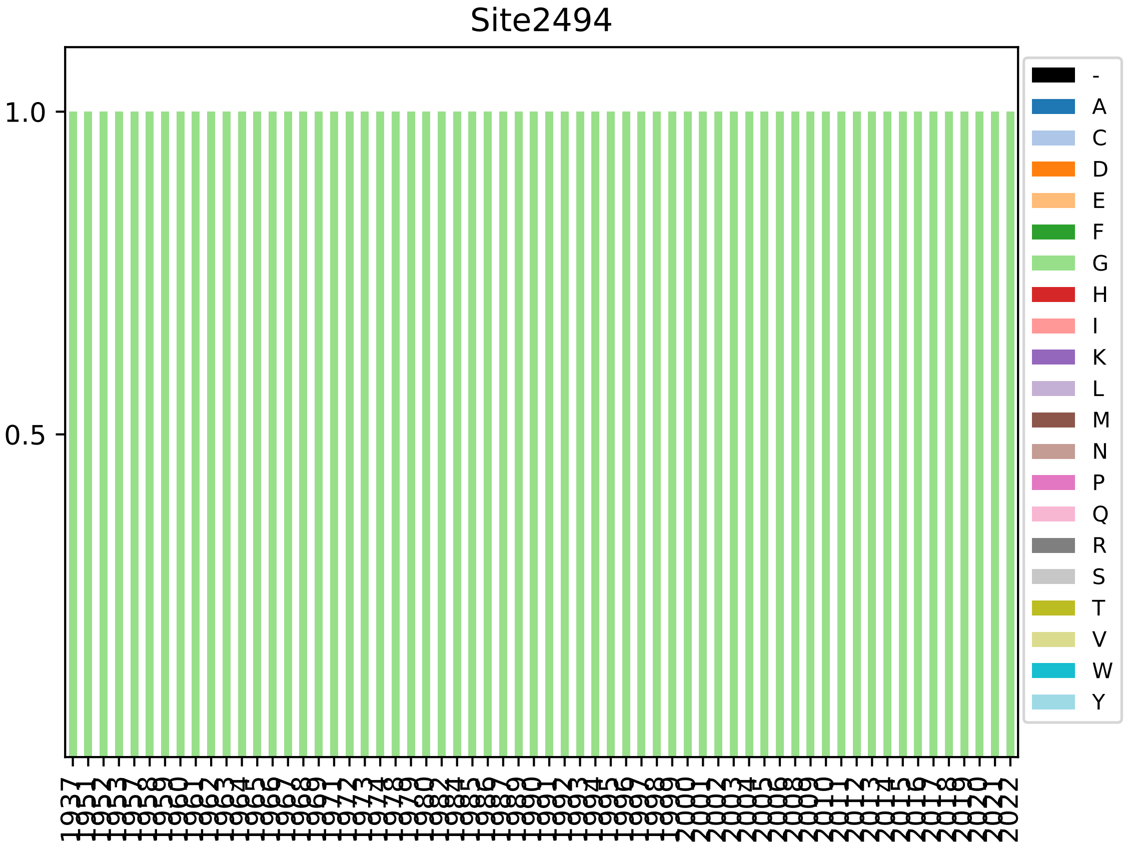
Site2494
1.0
0.5
-
A
C
D
E
F
G
H
I
K
L
M
N
P
Q
R
S
T
V
W
Y

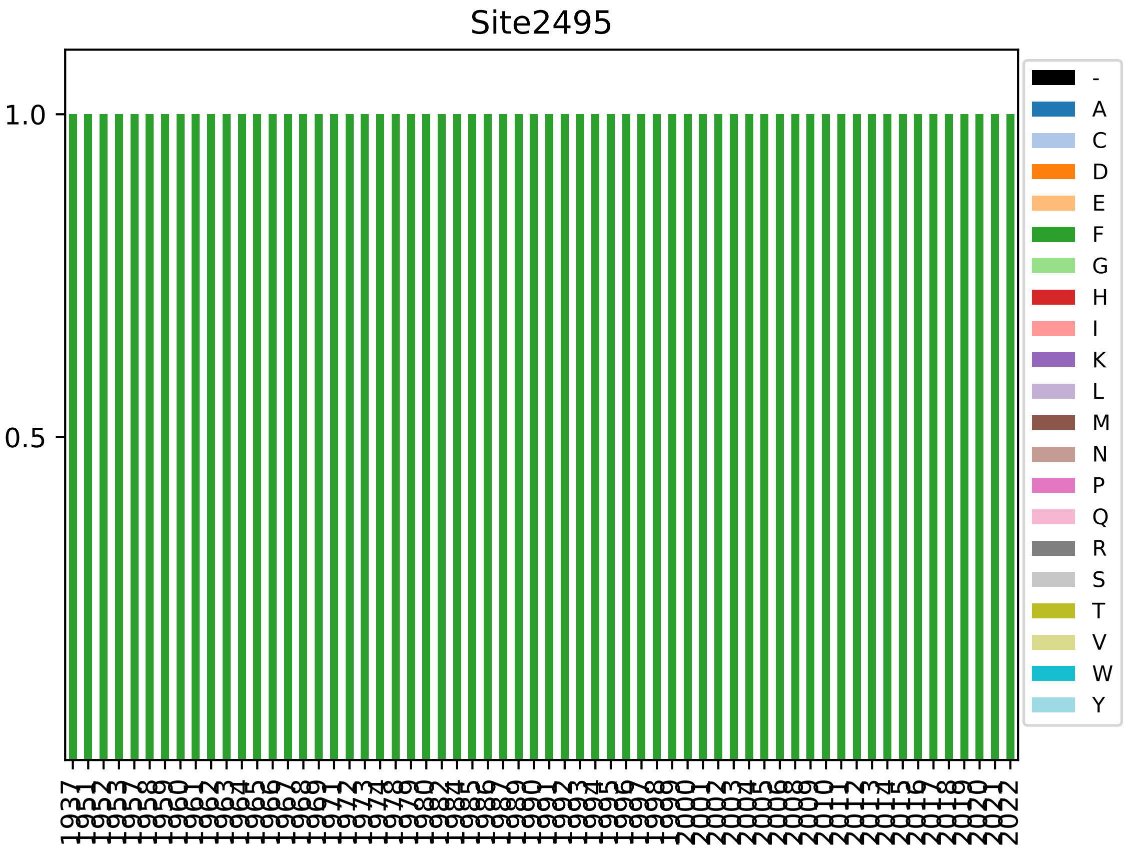
Site2495
1.0
0.5
-
A
C
D
E
F
G
H
I
K
L
M
N
P
Q
R
S
T
V
W
Y

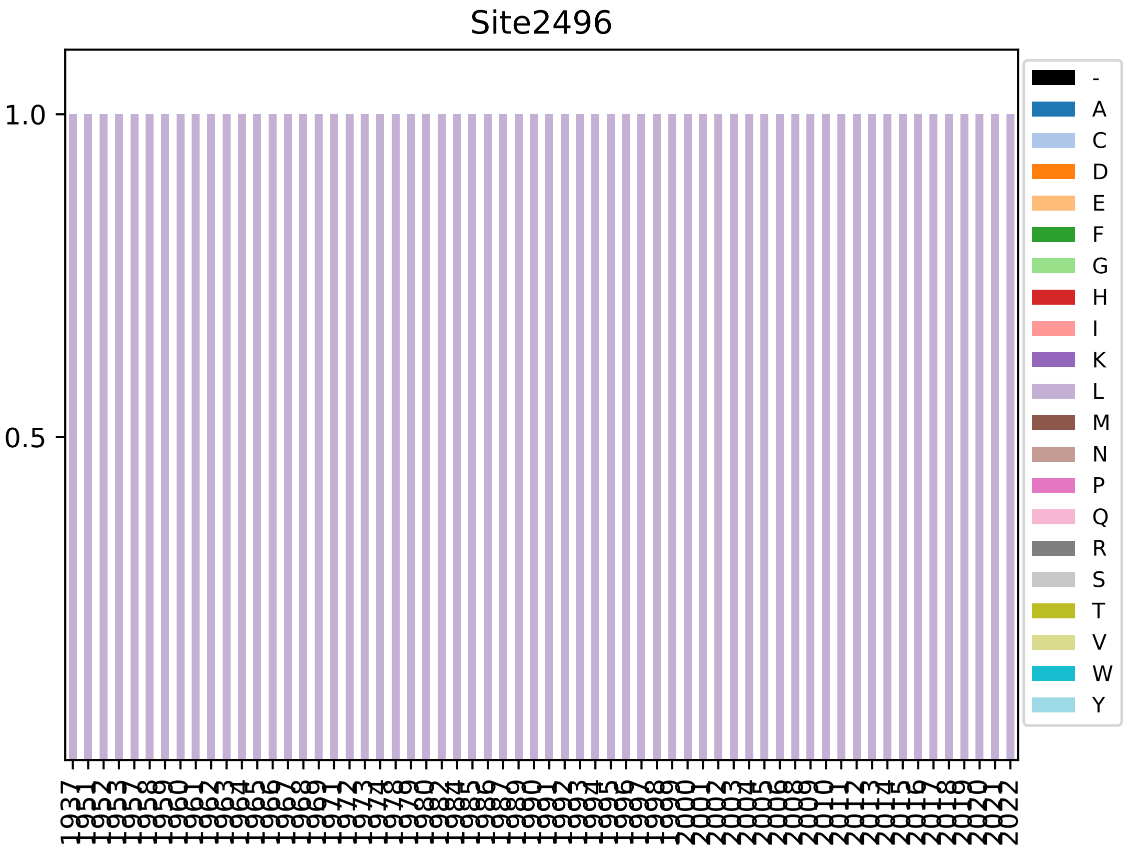
Site2496
1.0
0.5
-
A
C
D
E
F
G
H
I
K
L
M
N
P
Q
R
S
T
V
W
Y

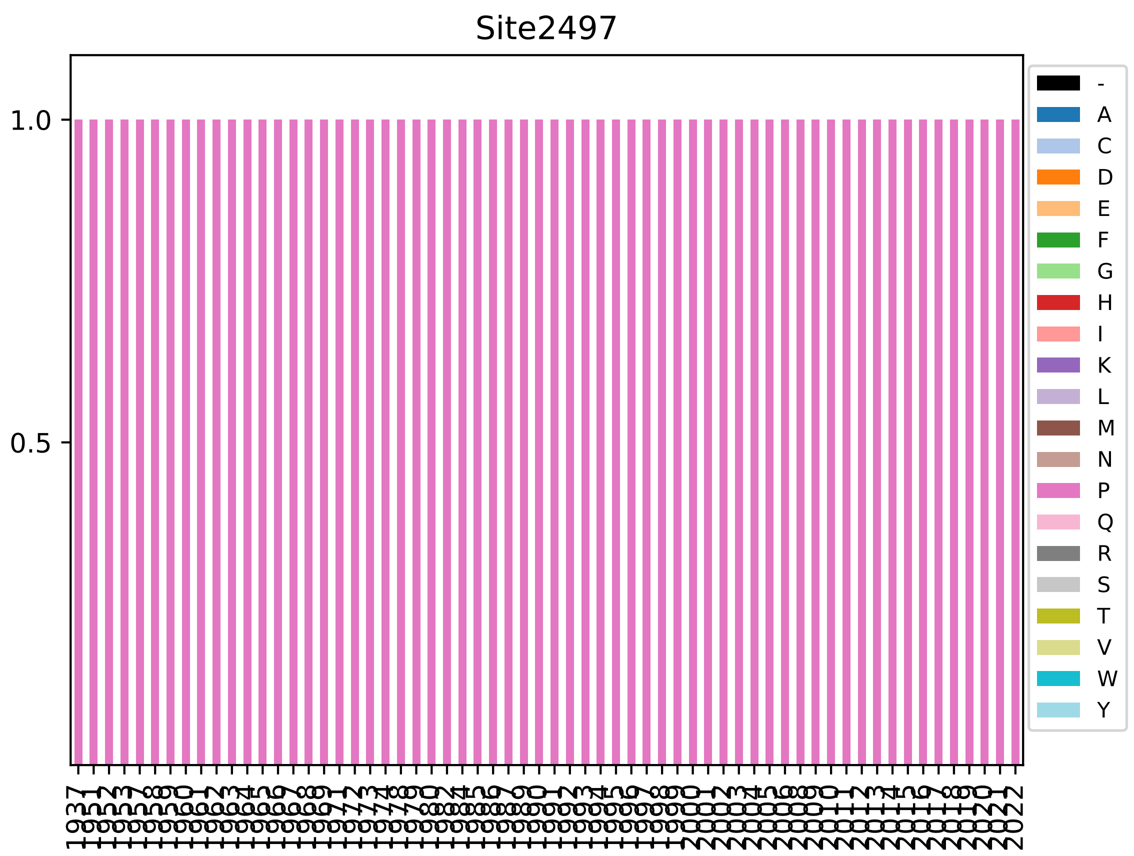

Site2497
1.0
0.5
-
A
C
D
E
F
G
H
I
K
L
M
N
P
Q
R
S
T
V
W
Y

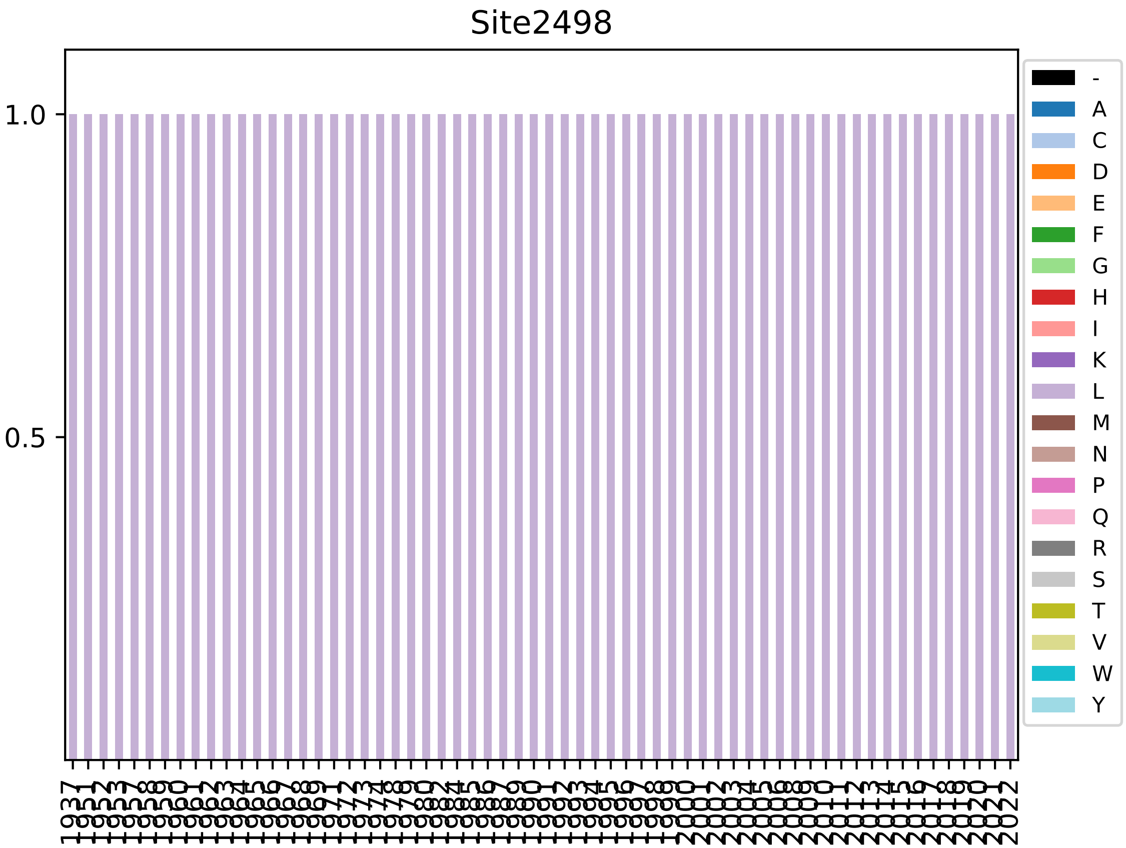

Site2498
1.0
0.5
-
A
C
D
E
F
G
H
I
K
L
M
N
P
Q
R
S
T
V
W
Y

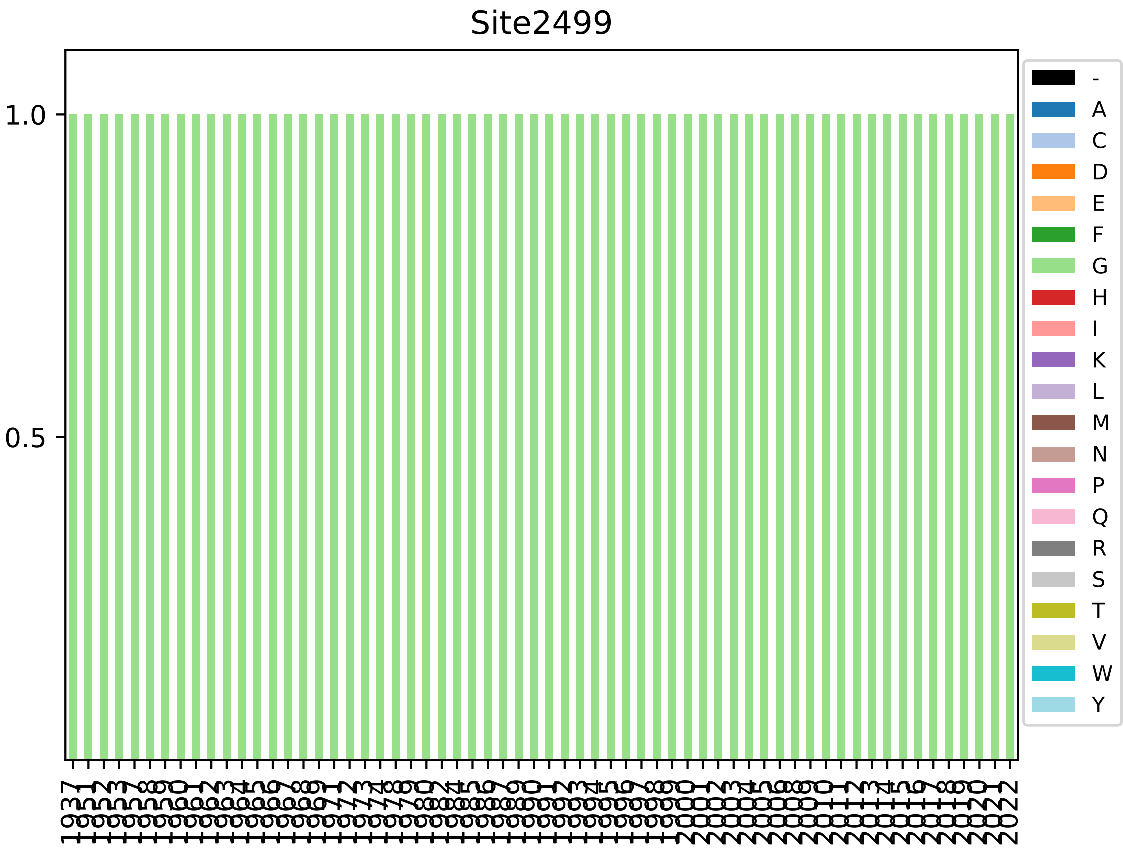

Site2499
1.0
0.5
-
A
C
D
E
F
G
H
I
K
L
M
N
P
Q
R
S
T
V
W
Y

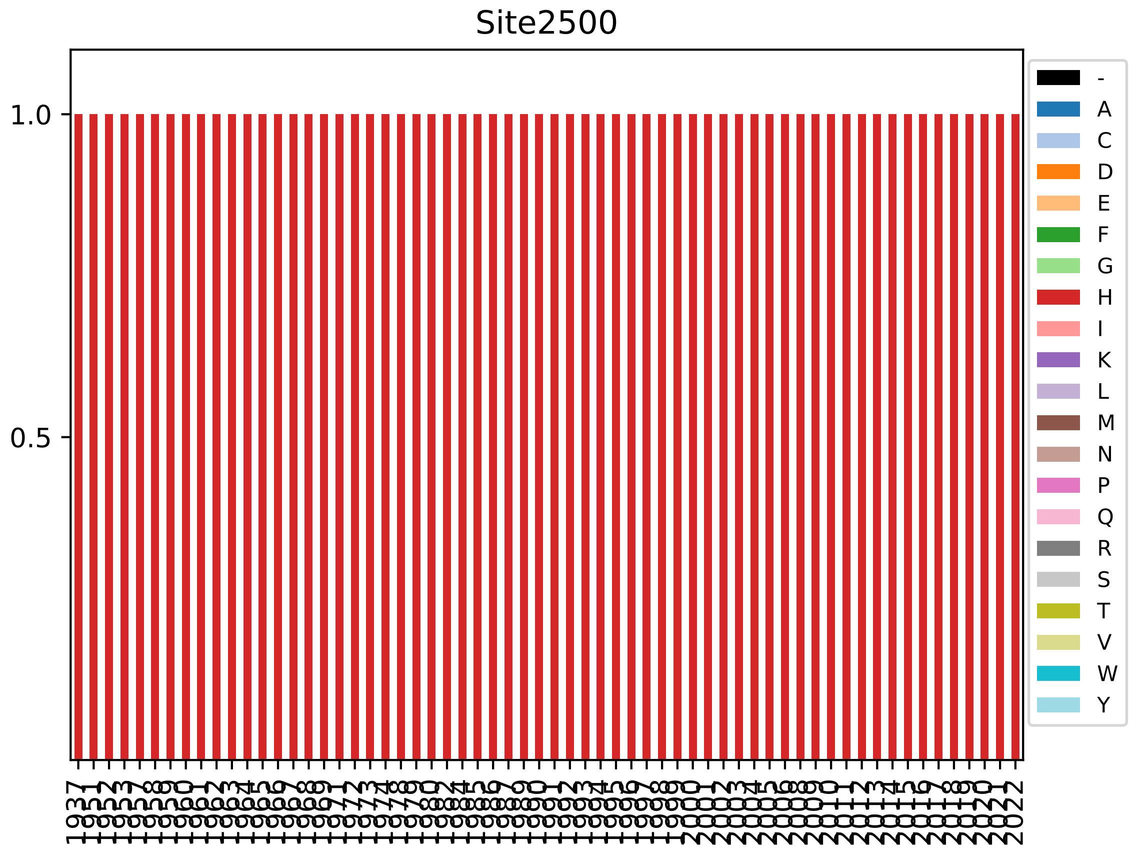
Site2500
1.0
0.5
-
A
C
D
E
F
G
H
I
K
L
M
N
P
Q
R
S
T
V
W
Y

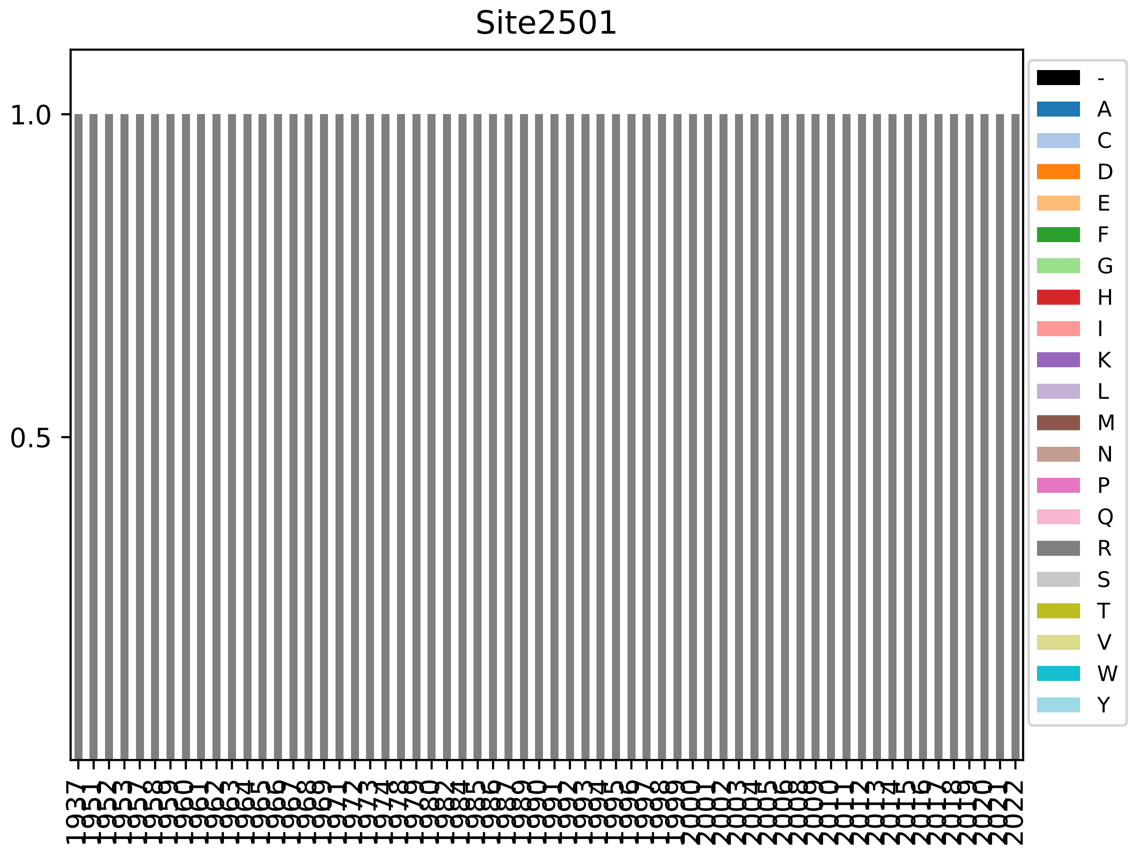
Site2501
1.0
0.5
-
A
C
D
E
F
G
H
I
K
L
M
N
P
Q
R
S
T
V
W
Y

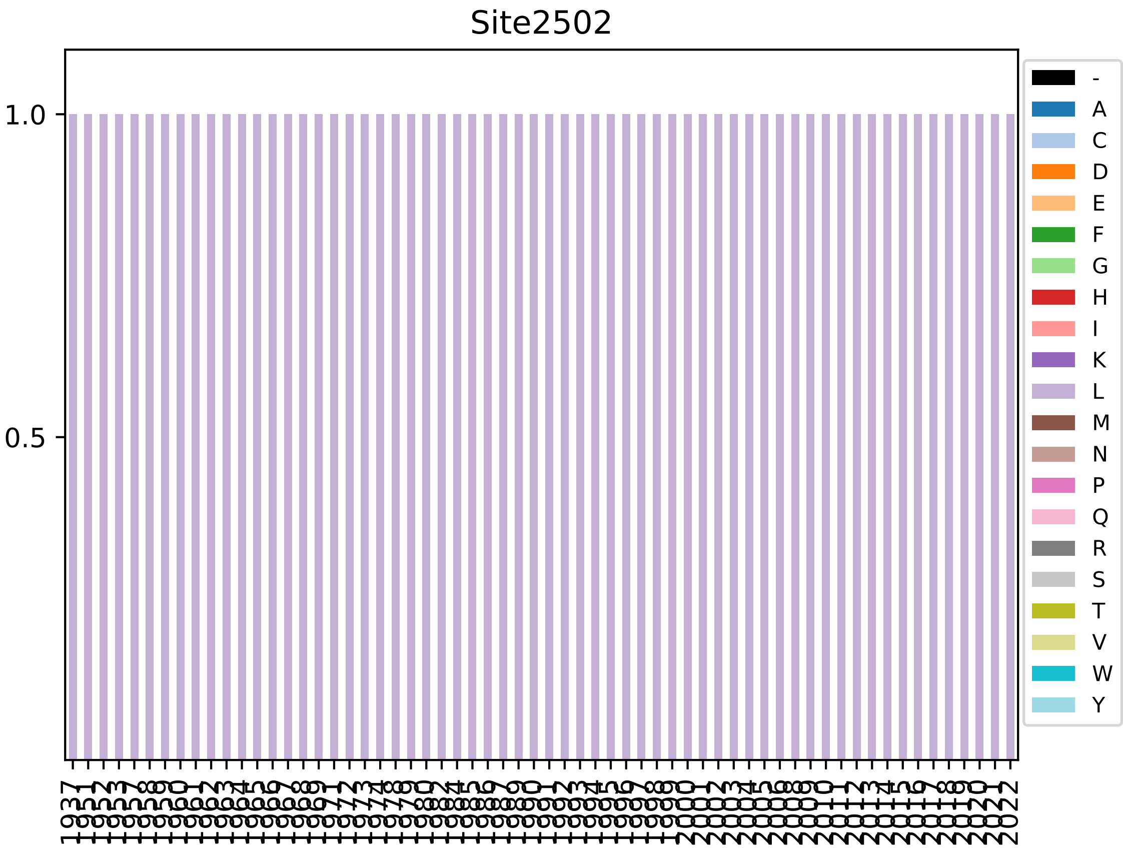
Site2502
1.0
0.5
-
A
C
D
E
F
G
H
I
K
L
M
N
P
Q
R
S
T
V
W
Y

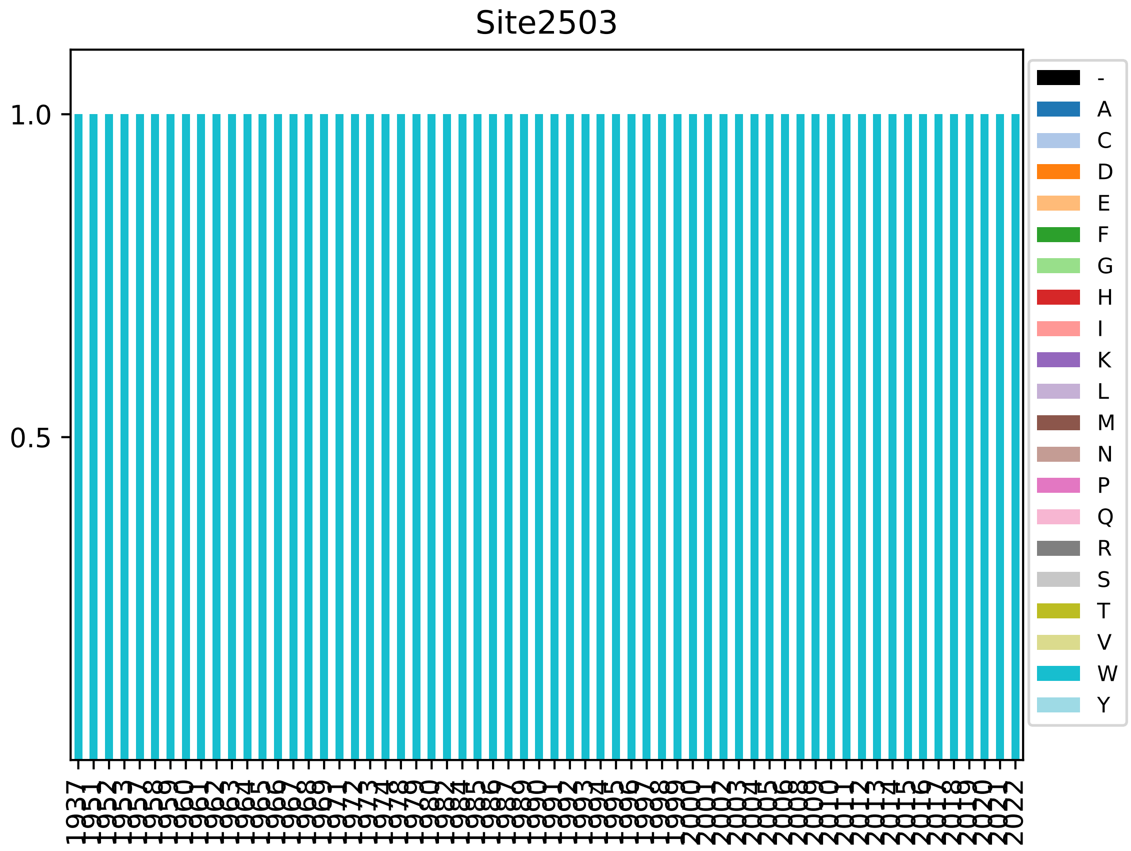
Site2503
1.0
0.5
-
A
C
D
E
F
G
H
I
K
L
M
N
P
Q
R
S
T
V
W
Y

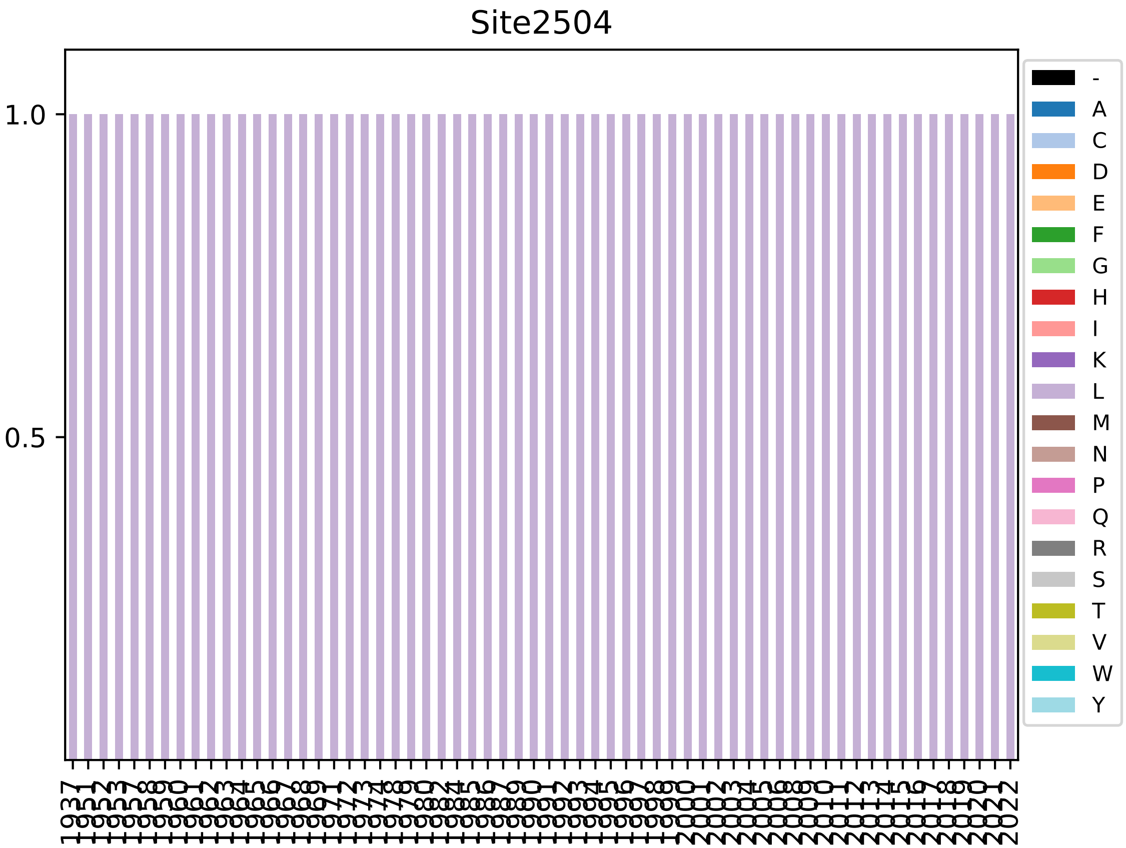
Site2504
1.0
0.5
-
A
C
D
E
F
G
H
I
K
L
M
N
P
Q
R
S
T
V
W
Y

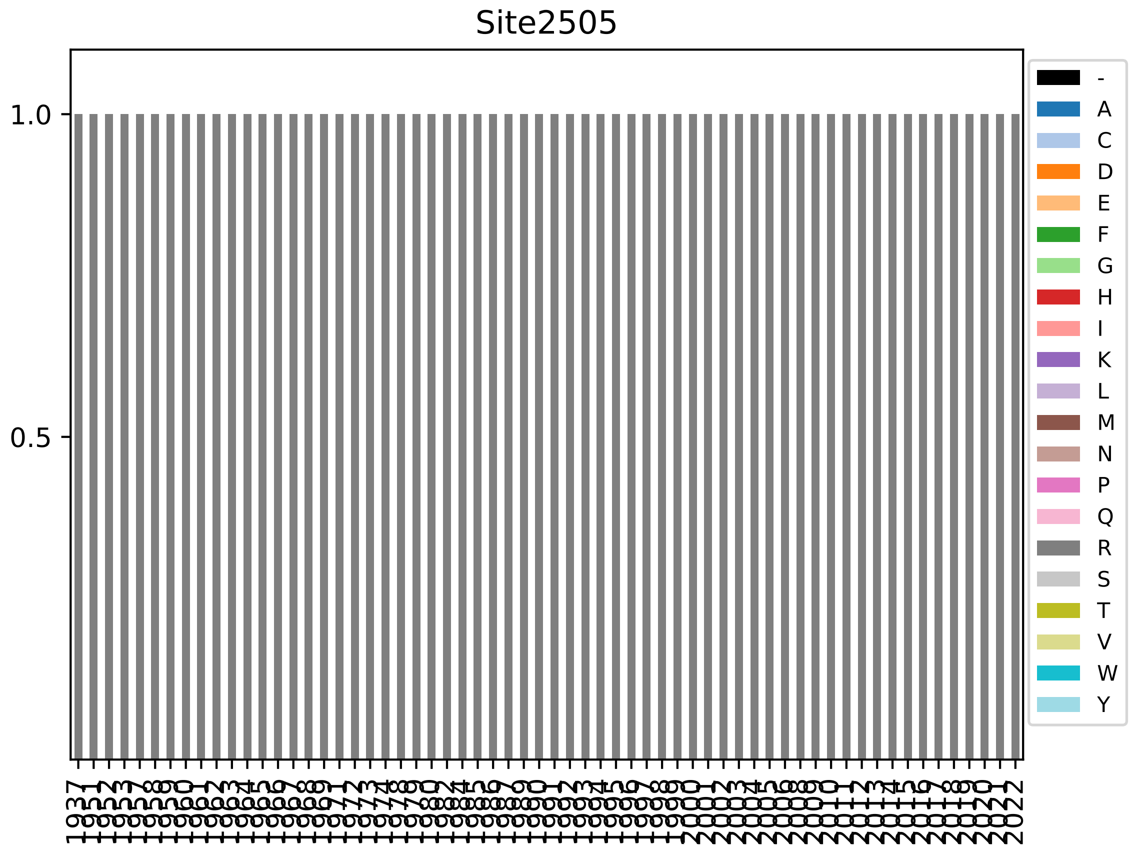

Site2505
1.0
0.5
-
A
C
D
E
F
G
H
I
K
L
M
N
P
Q
R
S
T
V
W
Y

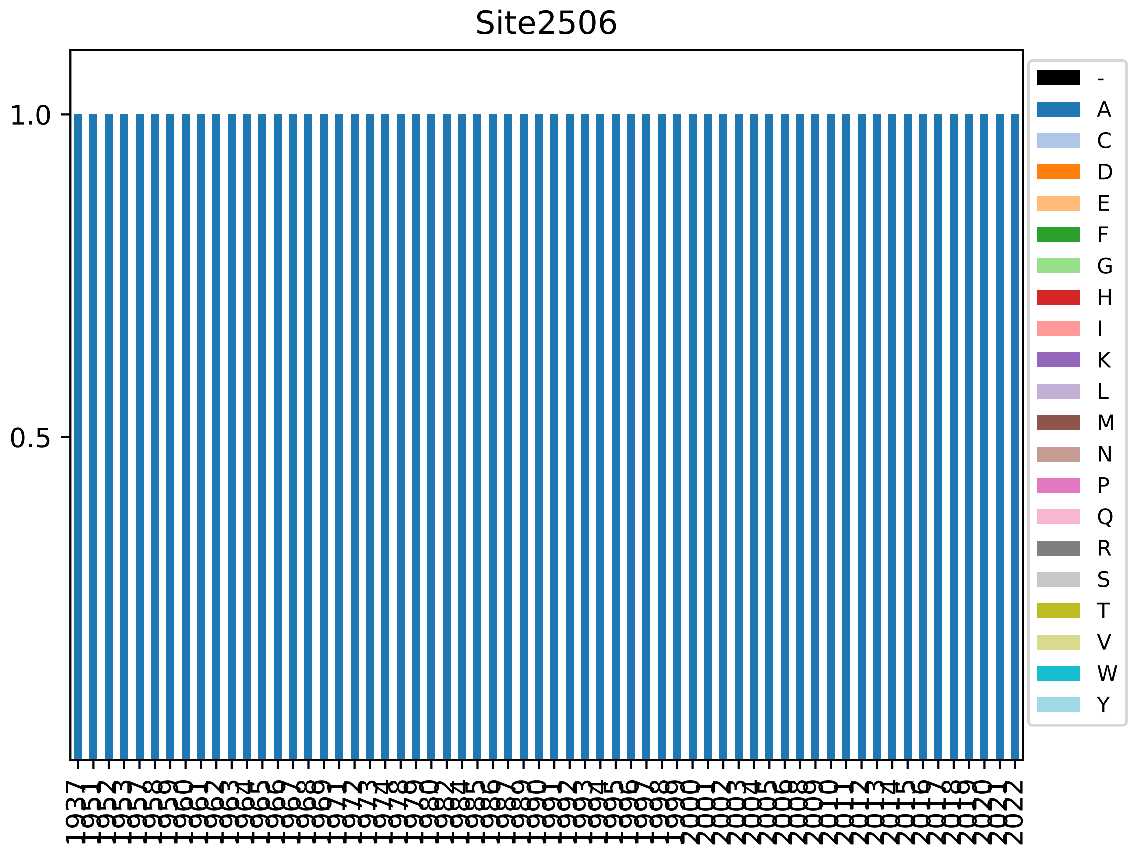
Site2506
1.0
0.5
-
A
C
D
E
F
G
H
I
K
L
M
N
P
Q
R
S
T
V
W
Y

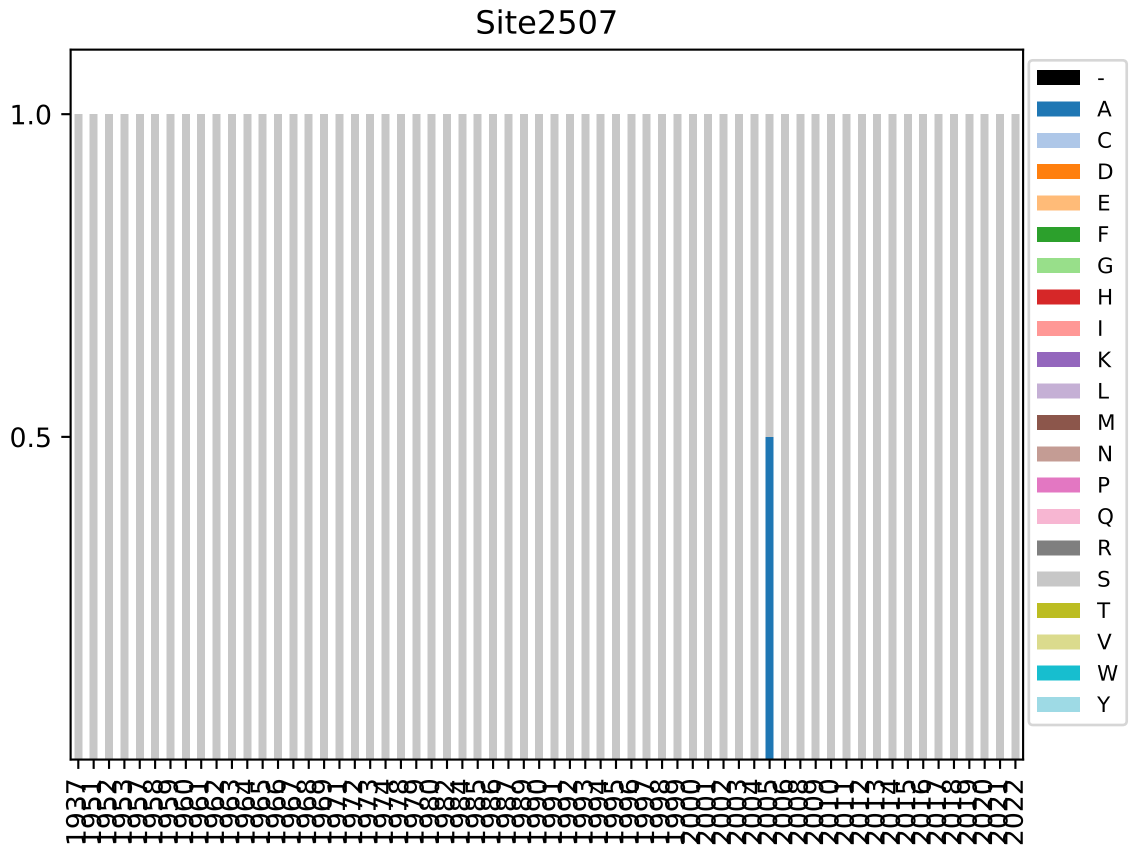

Site2507
1.0
0.5
-
A
C
D
E
F
G
H
I
K
L
M
N
P
Q
R
S
T
V
W
Y

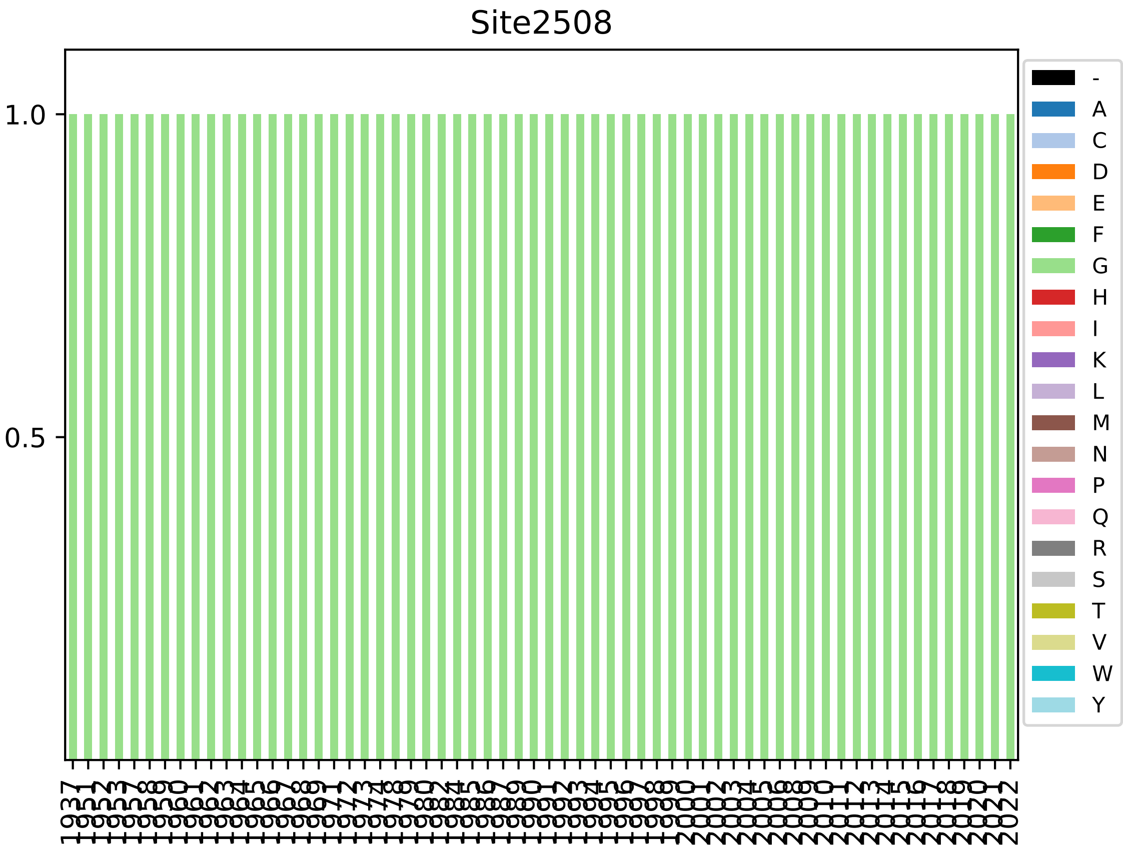
Site2508
1.0
0.5
-
A
C
D
E
F
G
H
I
K
L
M
N
P
Q
R
S
T
V
W
Y

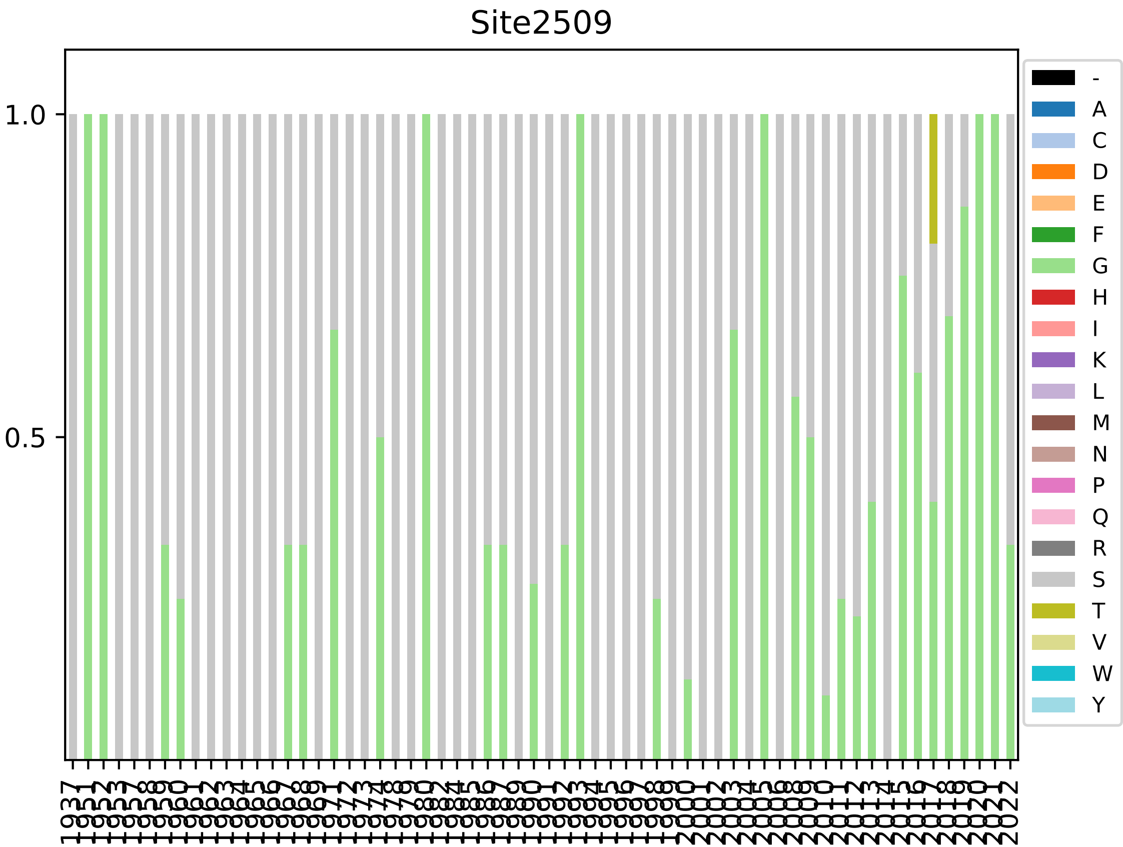
Site2509
1.0
0.5
-
A
C
D
E
F
G
H
I
K
L
M
N
P
Q
R
S
T
V
W
Y

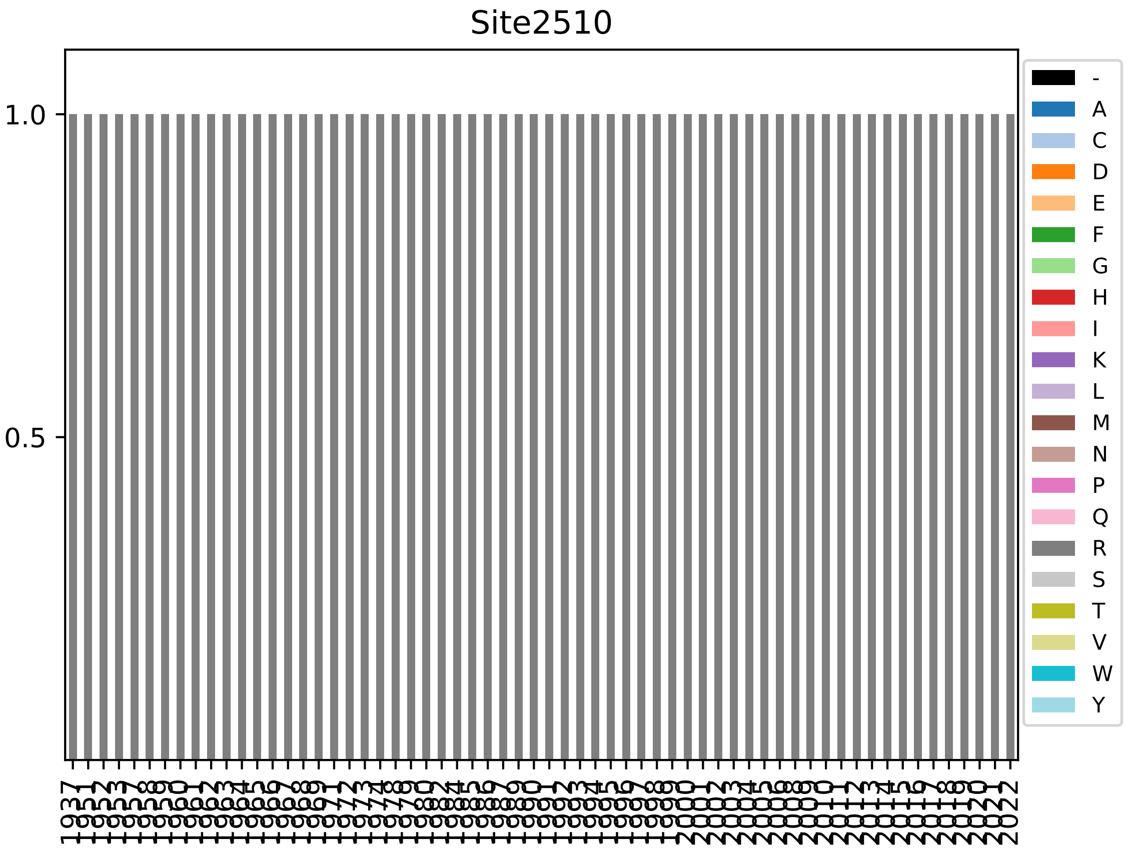
Site2510
1.0
0.5
-
A
C
D
E
F
G
H
I
K
L
M
N
P
Q
R
S
T
V
W
Y

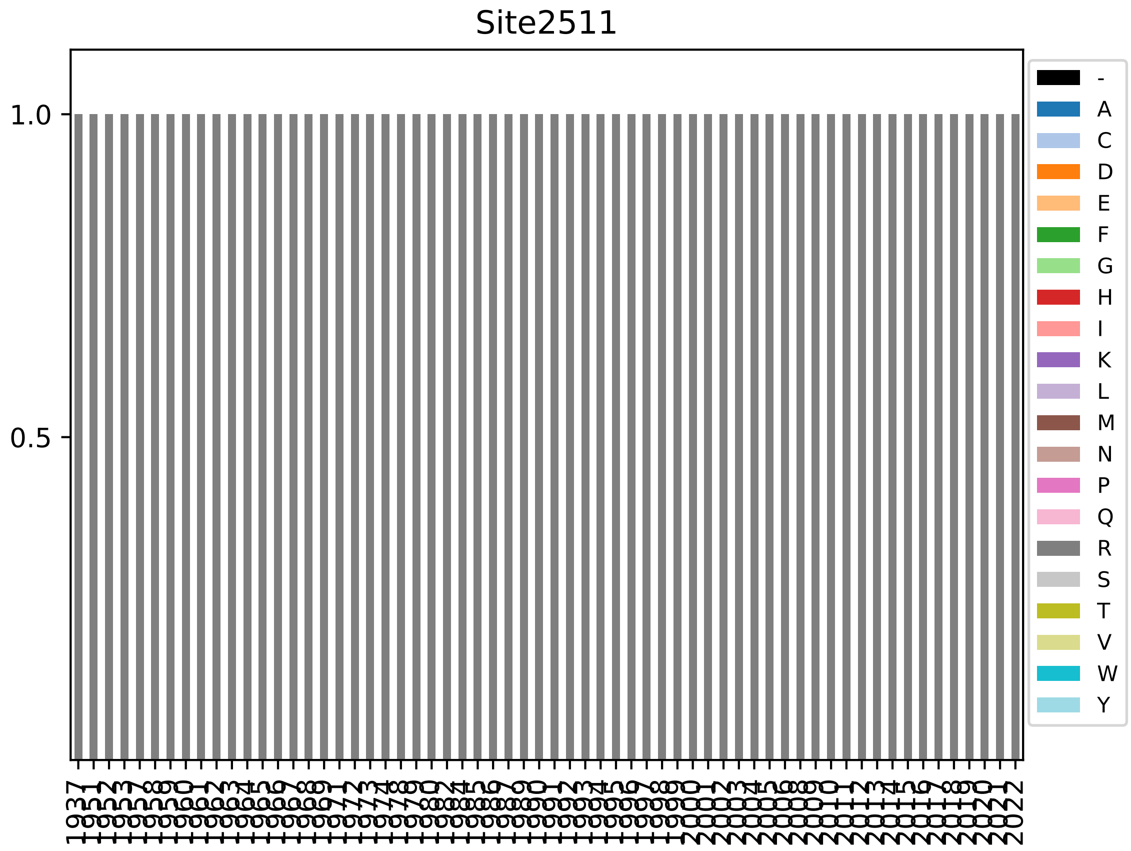
Site2511
1.0
0.5
-
A
C
D
E
F
G
H
I
K
L
M
N
P
Q
R
S
T
V
W
Y

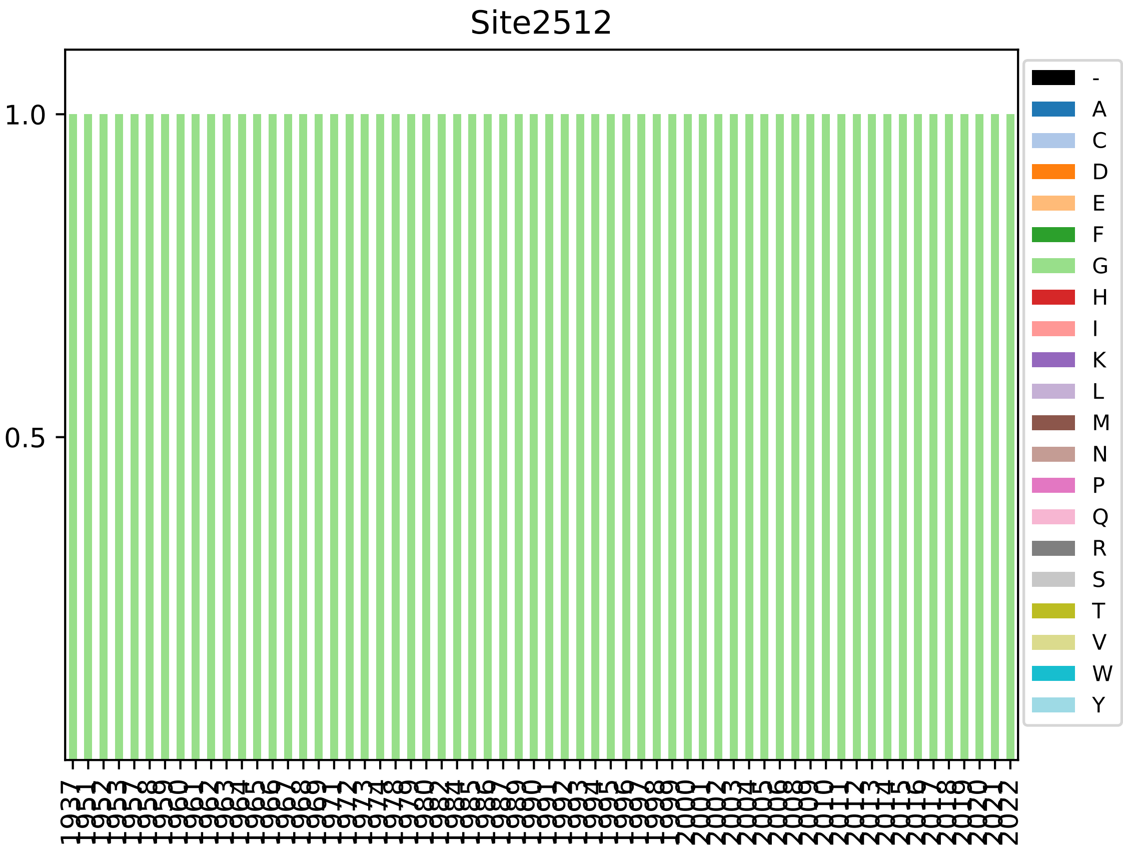
Site2512
1.0
0.5
-
A
C
D
E
F
G
H
I
K
L
M
N
P
Q
R
S
T
V
W
Y

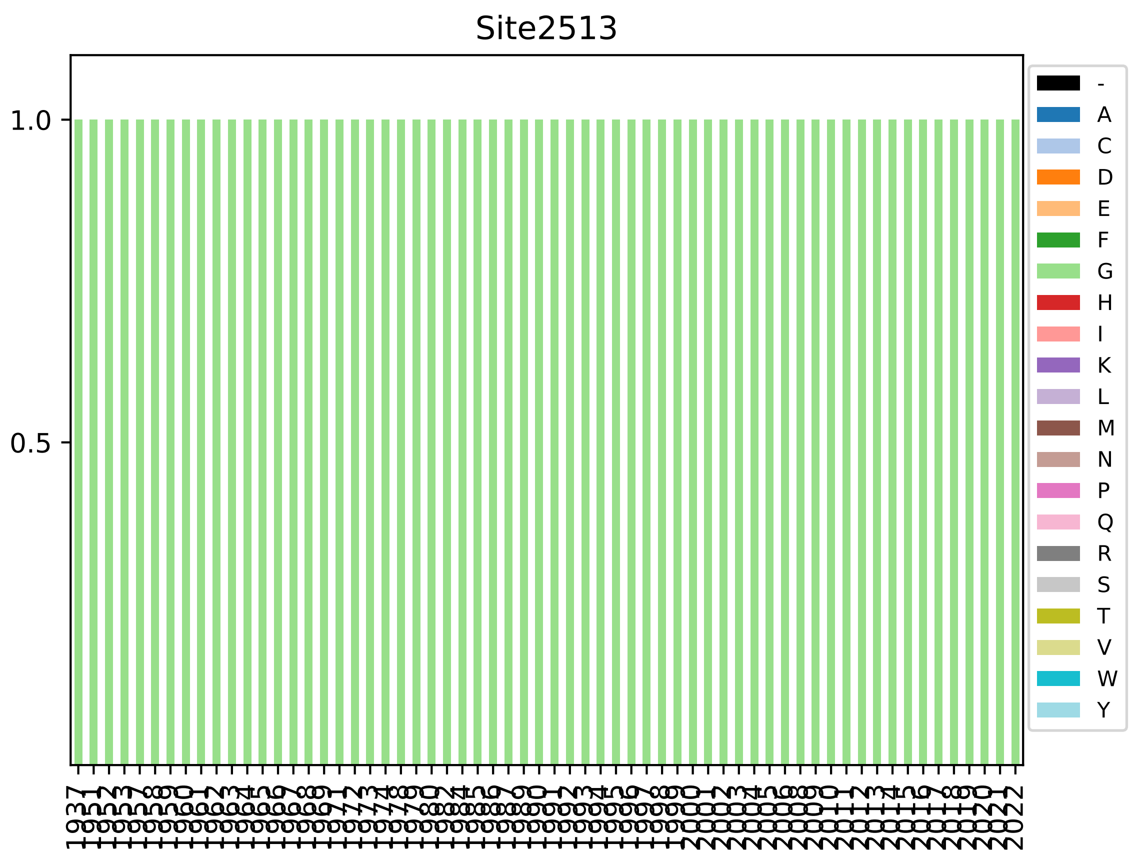
Site2513
1.0
0.5
-
A
C
D
E
F
G
H
I
K
L
M
N
P
Q
R
S
T
V
W
Y

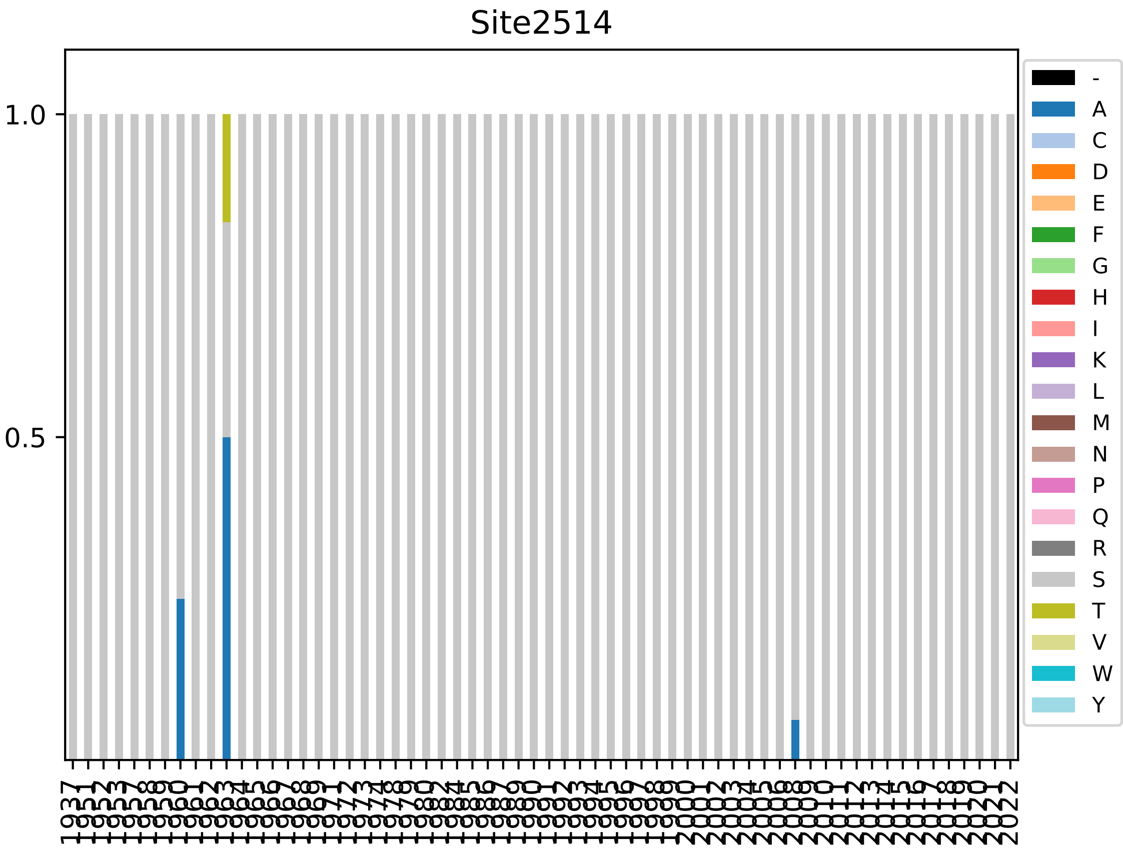

Site2514
1.0
0.5
-
A
C
D
E
F
G
H
I
K
L
M
N
P
Q
R
S
T
V
W
Y

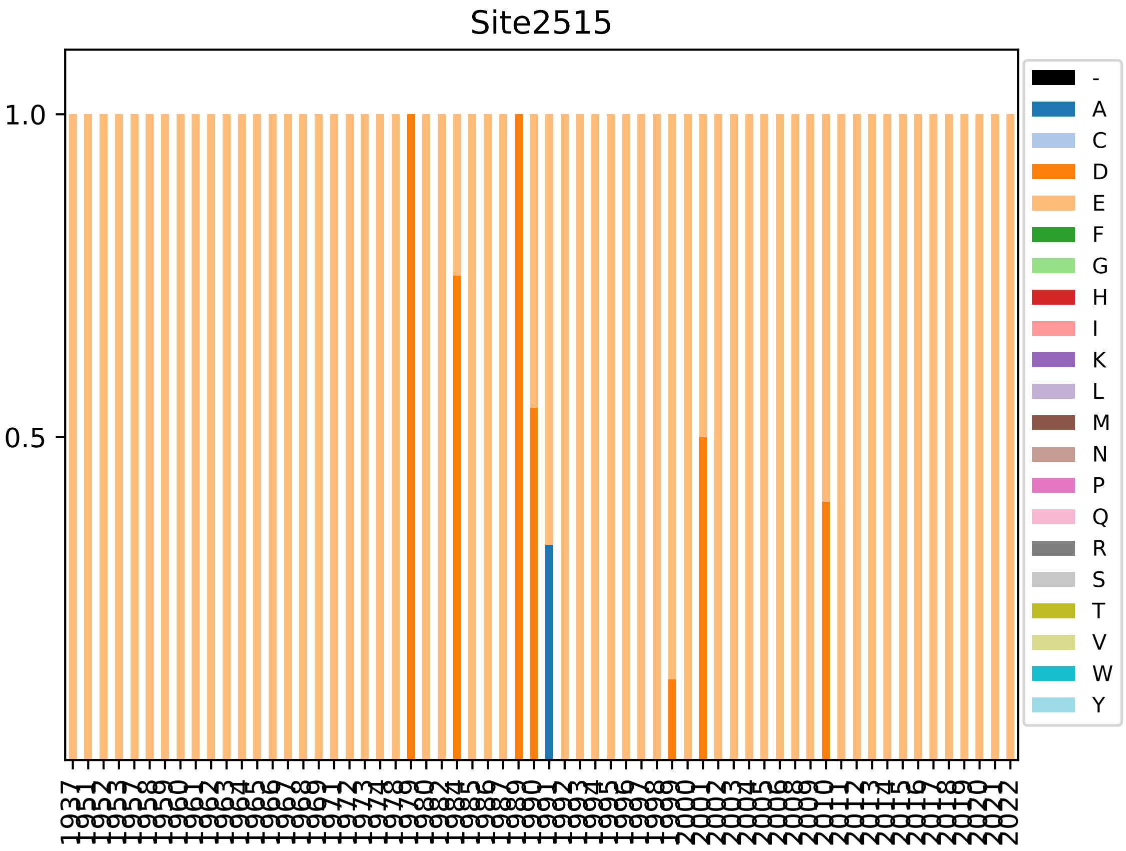

Site2515
1.0
0.5
-
A
C
D
E
F
G
H
I
K
L
M
N
P
Q
R
S
T
V
W
Y

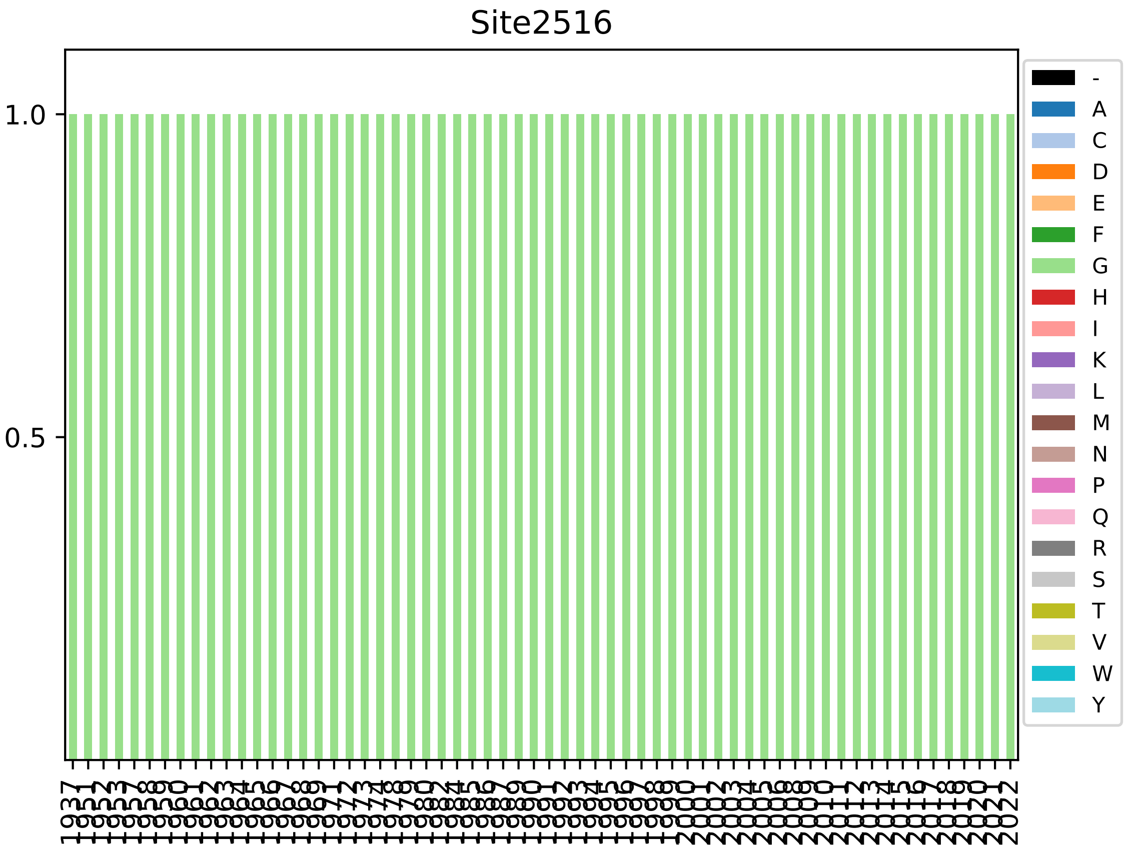
Site2516
1.0
0.5
-
A
C
D
E
F
G
H
I
K
L
M
N
P
Q
R
S
T
V
W
Y

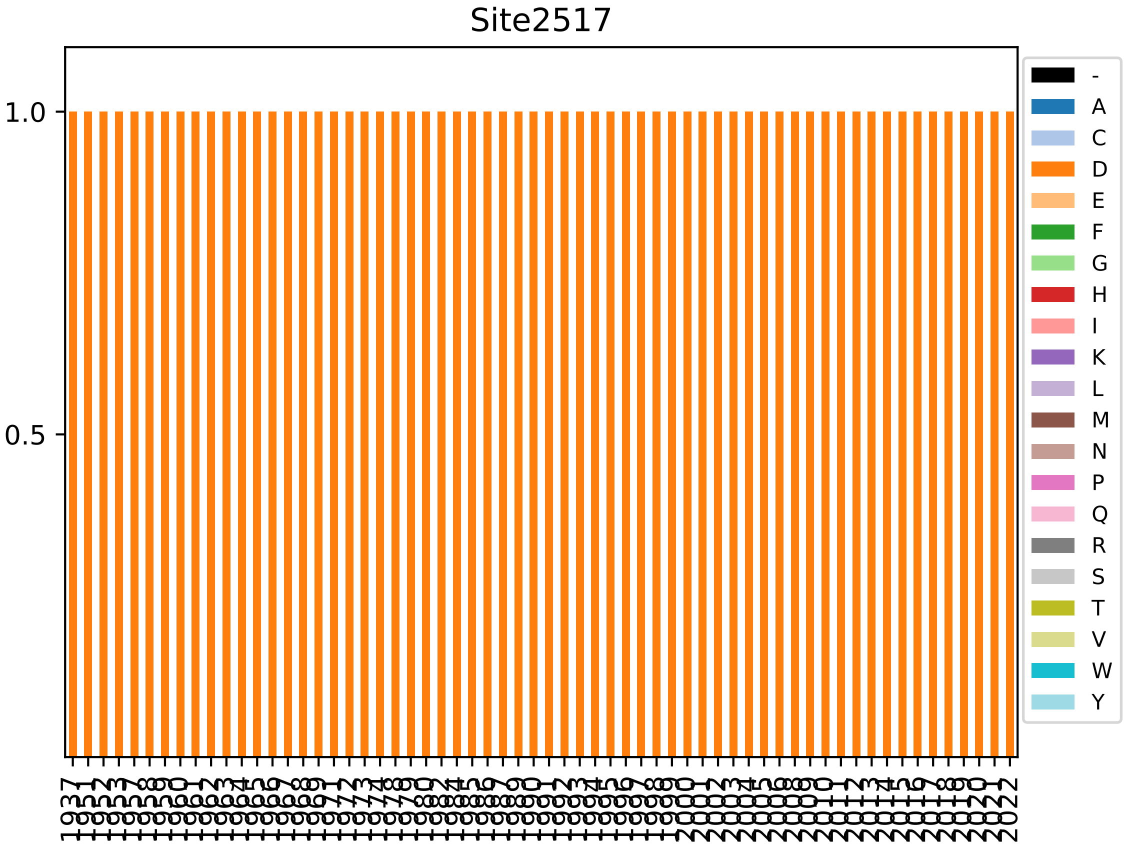
Site2517
1.0
0.5
-
A
C
D
E
F
G
H
I
K
L
M
N
P
Q
R
S
T
V
W
Y

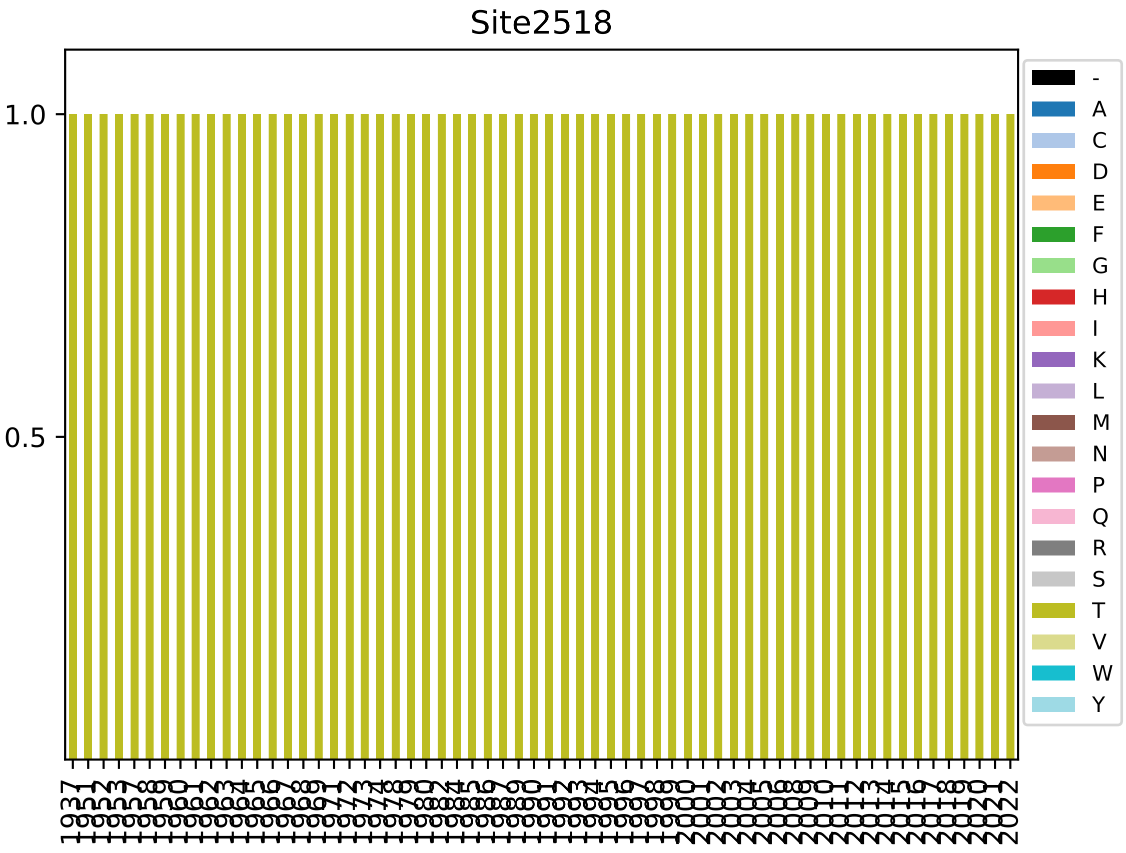
Site2518
1.0
0.5
-
A
C
D
E
F
G
H
I
K
L
M
N
P
Q
R
S
T
V
W
Y

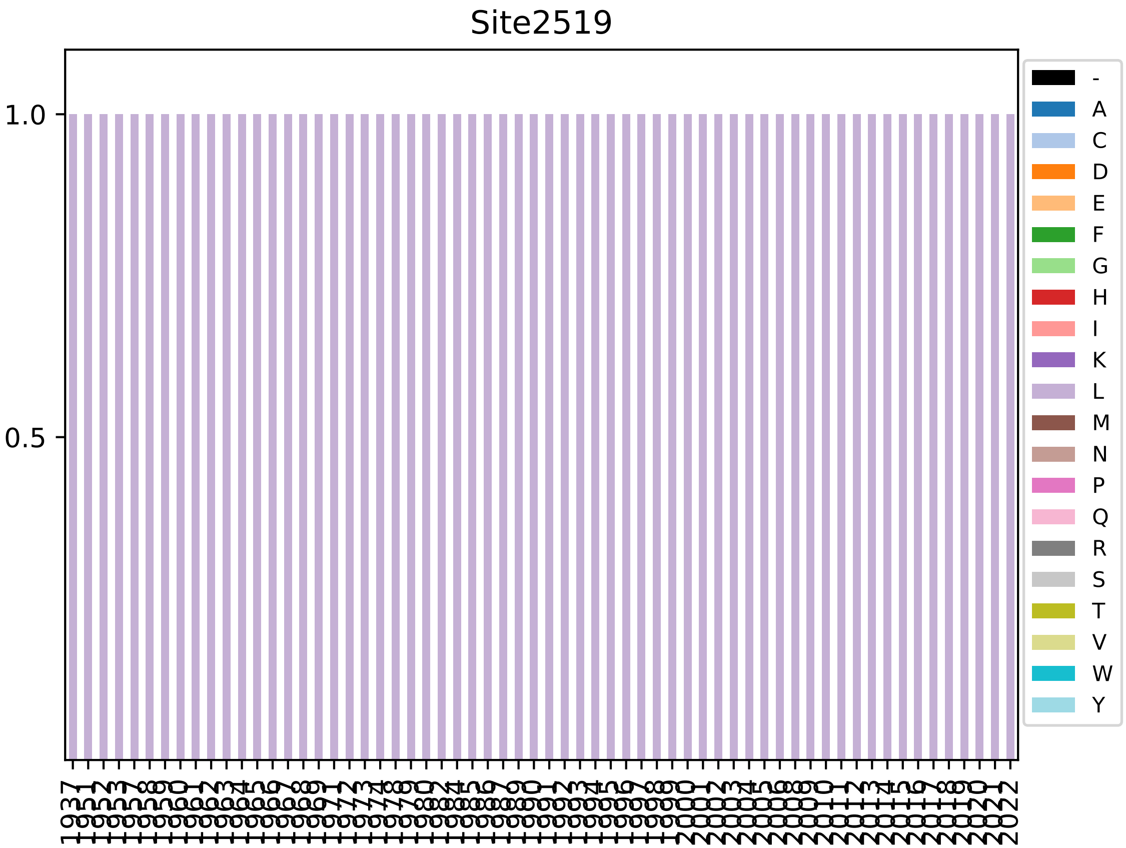
Site2519
1.0
0.5
-
A
C
D
E
F
G
H
I
K
L
M
N
P
Q
R
S
T
V
W
Y

Site2520

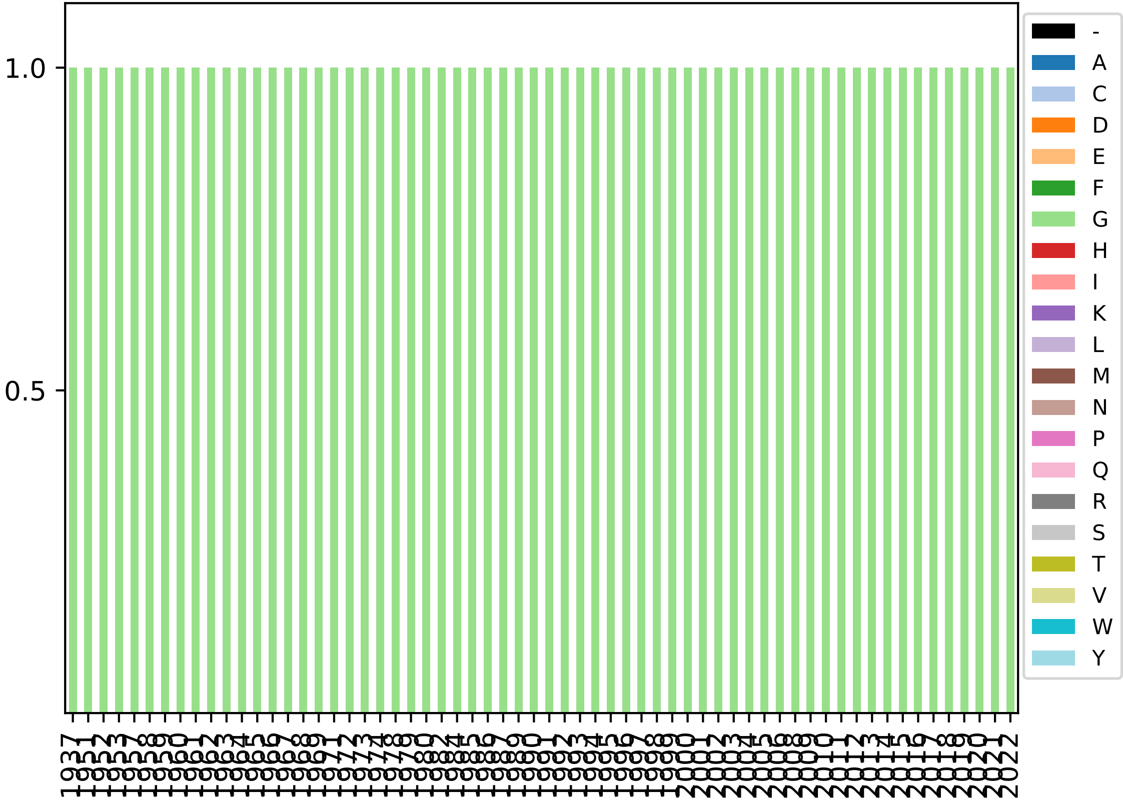

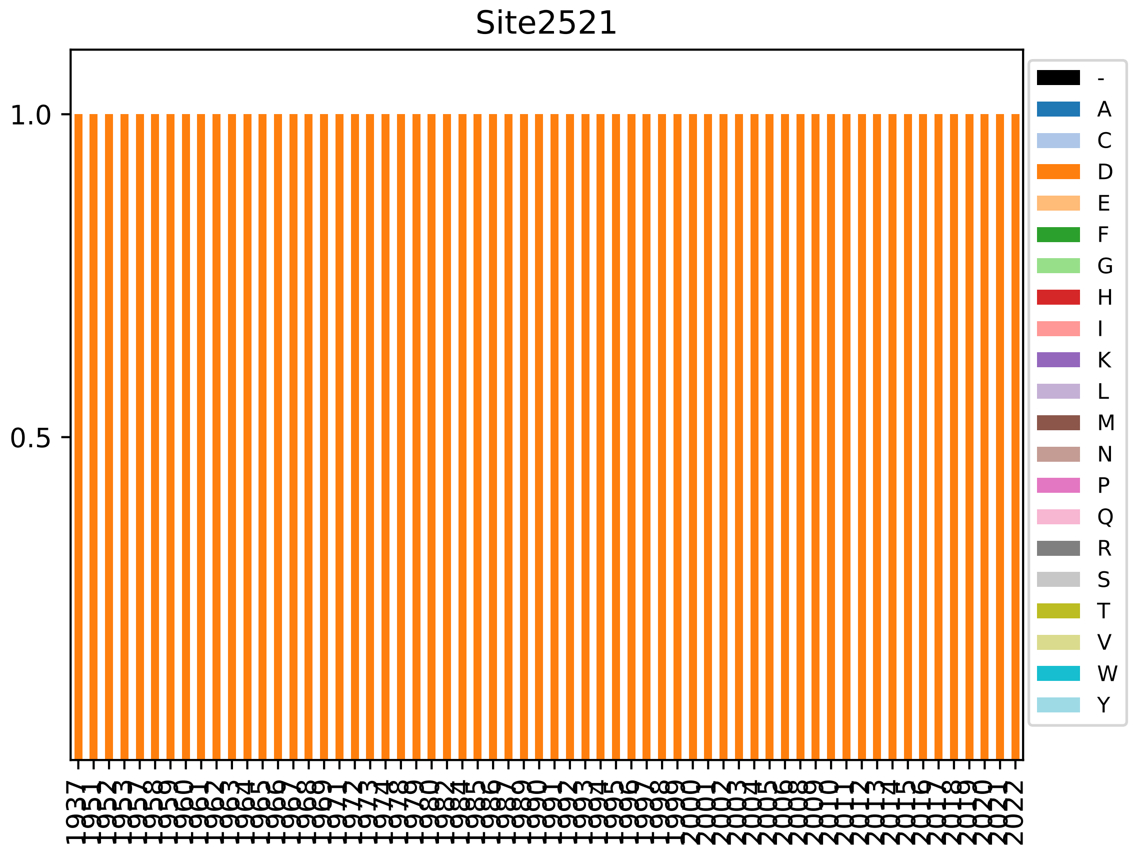
Site2521
1.0
0.5
-
A
C
D
E
F
G
H
I
K
L
M
N
P
Q
R
S
T
V
W
Y

Site2522

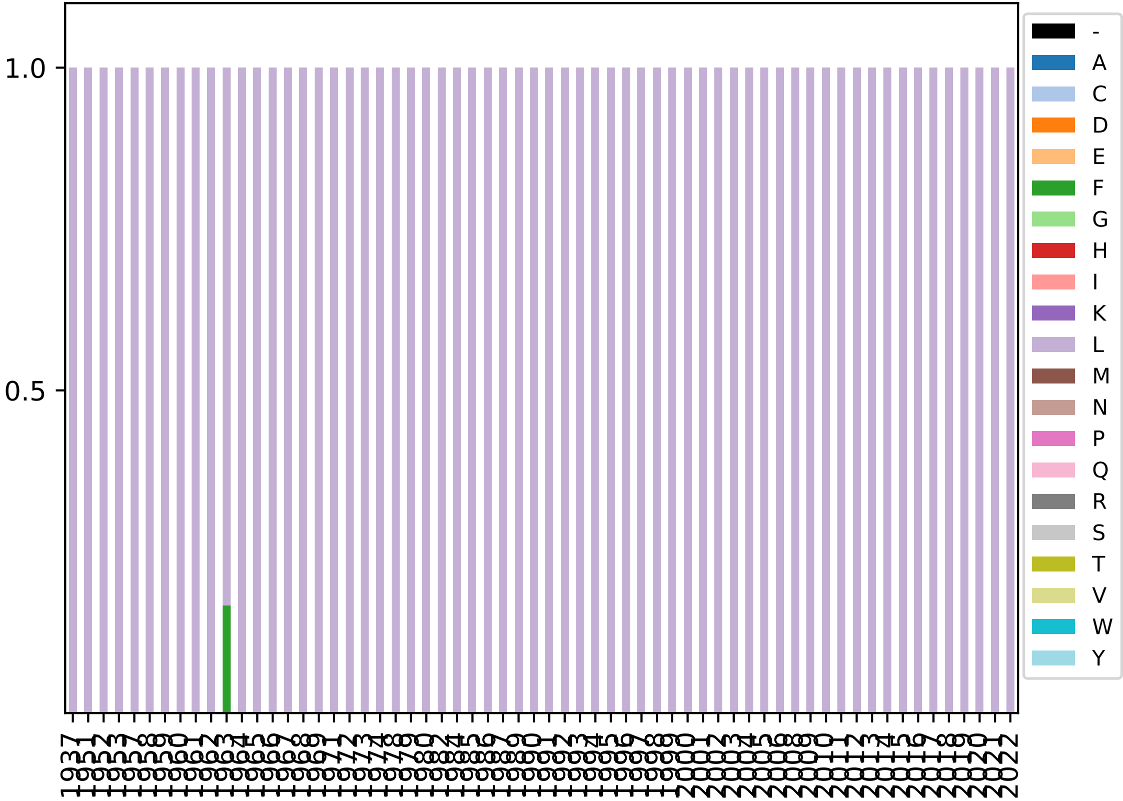

Site2523

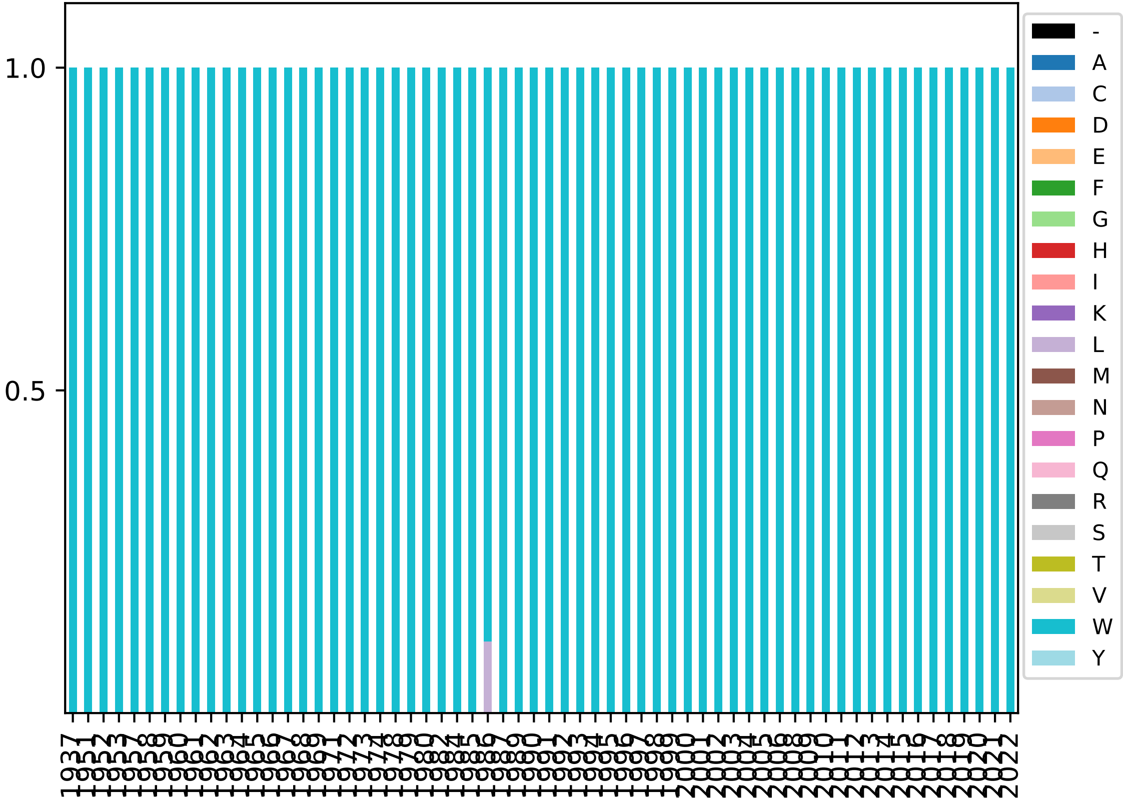

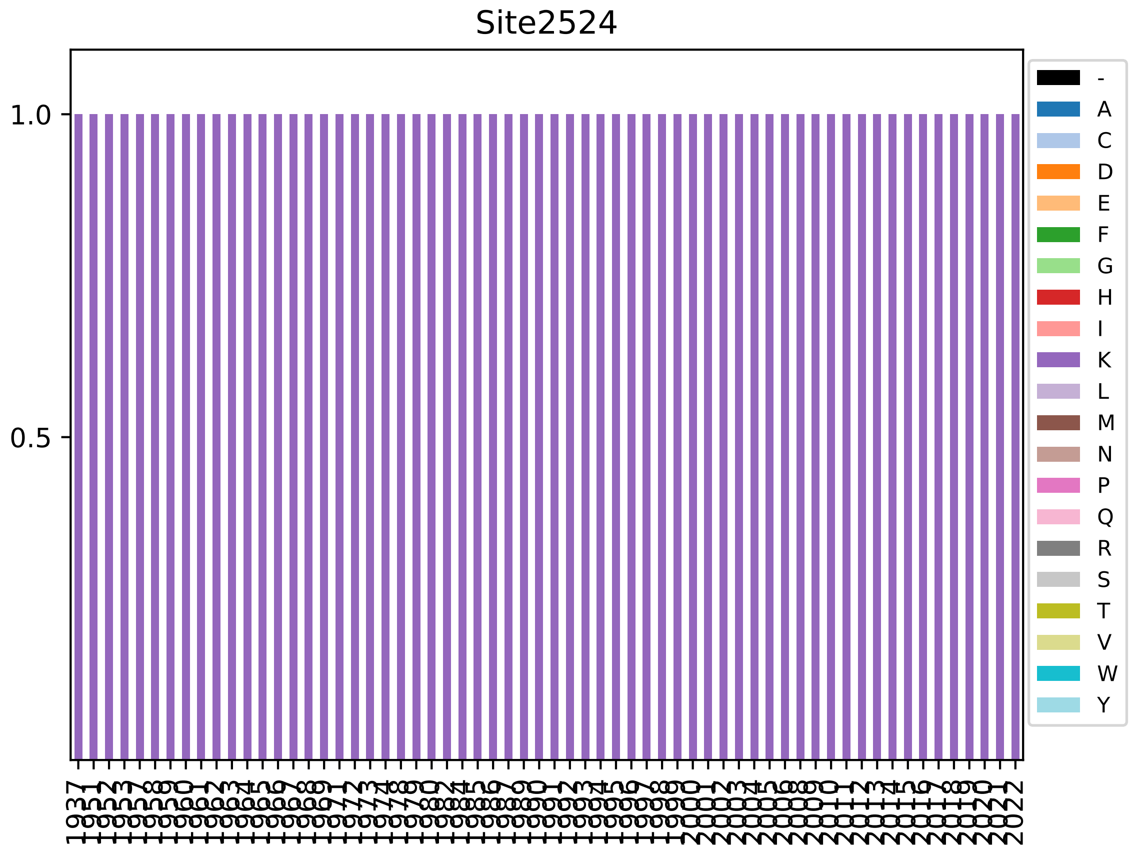
Site2524
1.0
0.5
-
A
C
D
E
F
G
H
I
K
L
M
N
P
Q
R
S
T
V
W
Y

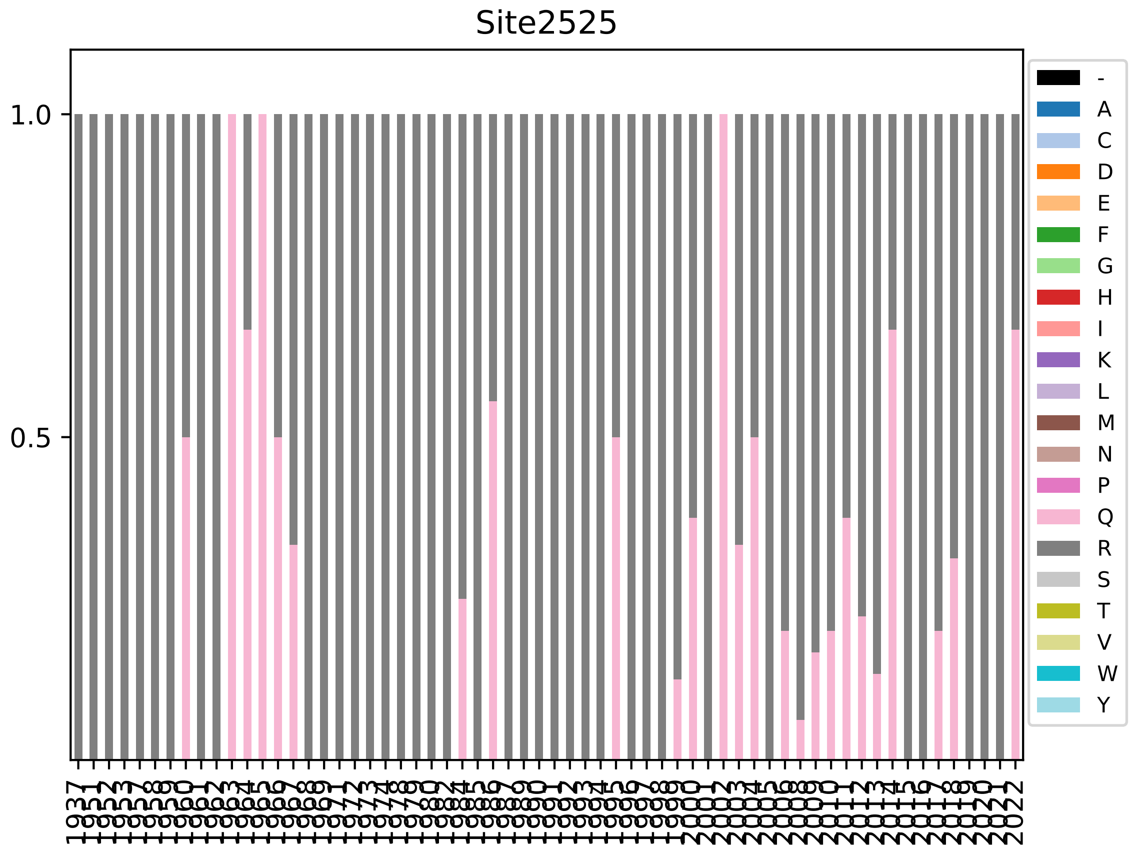
Site2525
1.0
0.5
-
A
C
D
E
F
G
H
I
K
L
M
N
P
Q
R
S
T
V
W
Y

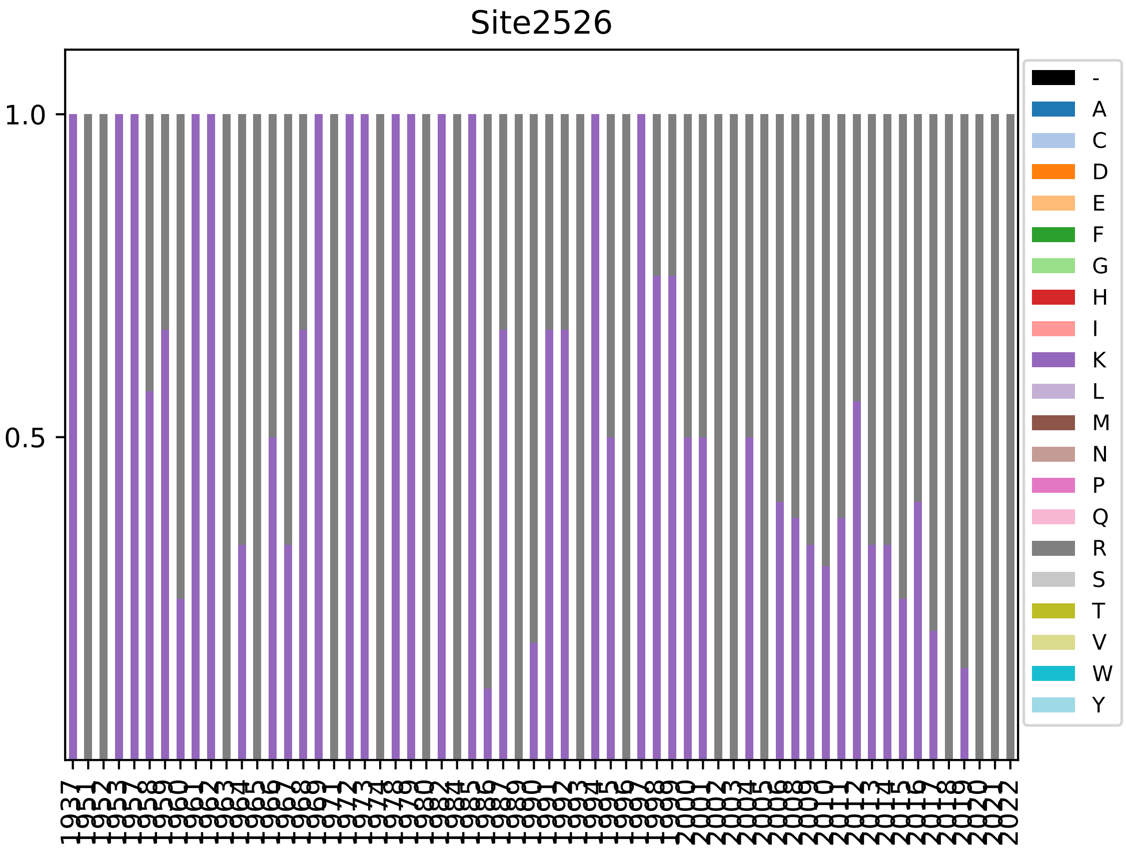

Site2526
1.0
0.5
-
A
C
D
E
F
G
H
I
K
L
M
N
P
Q
R
S
T
V
W
Y

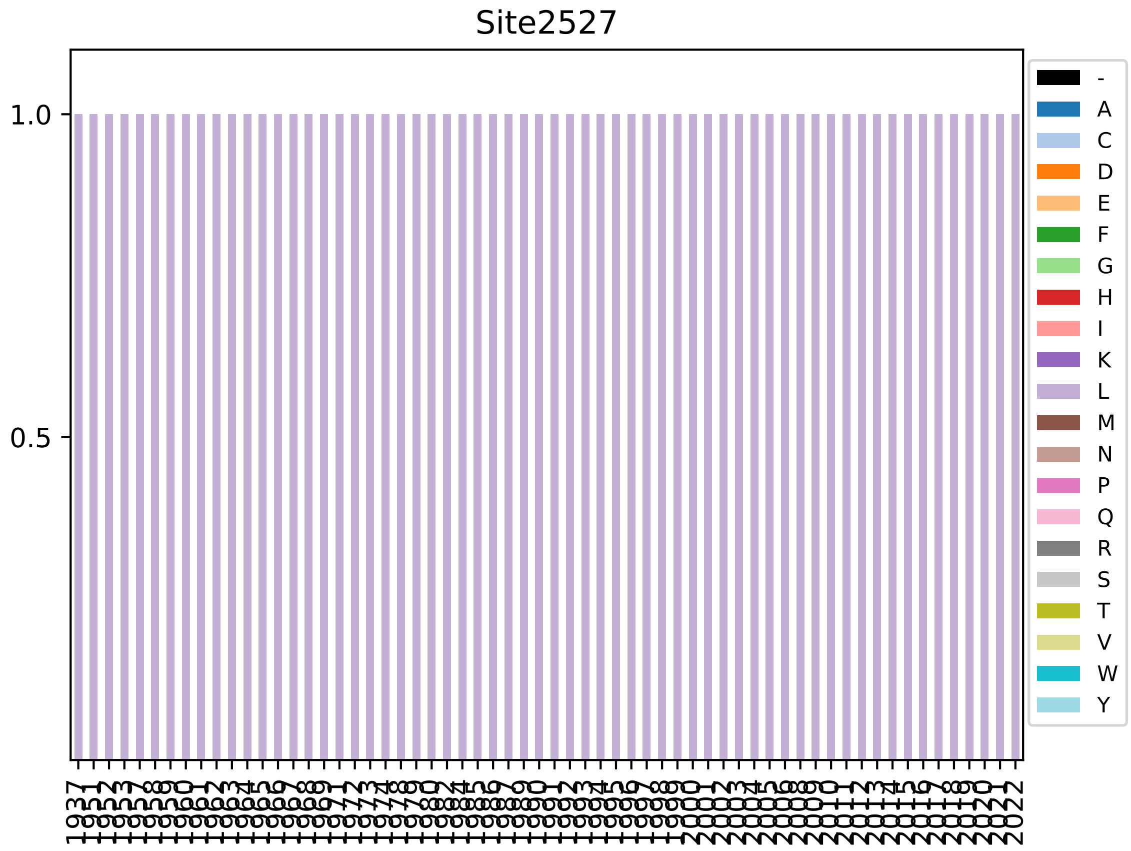
Site2527
1.0
0.5
-
A
C
D
E
F
G
H
I
K
L
M
N
P
Q
R
S
T
V
W
Y

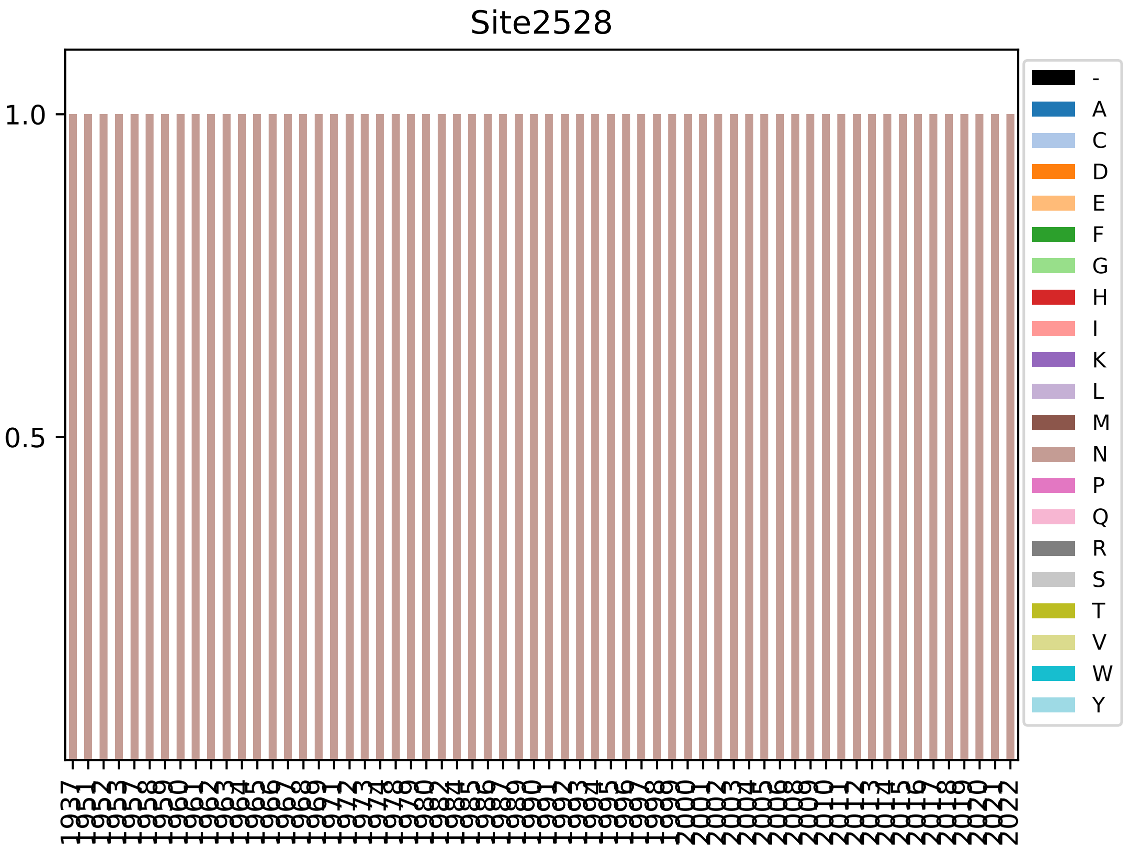
Site2528
1.0
0.5
-
A
C
D
E
F
G
H
I
K
L
M
N
P
Q
R
S
T
V
W
Y

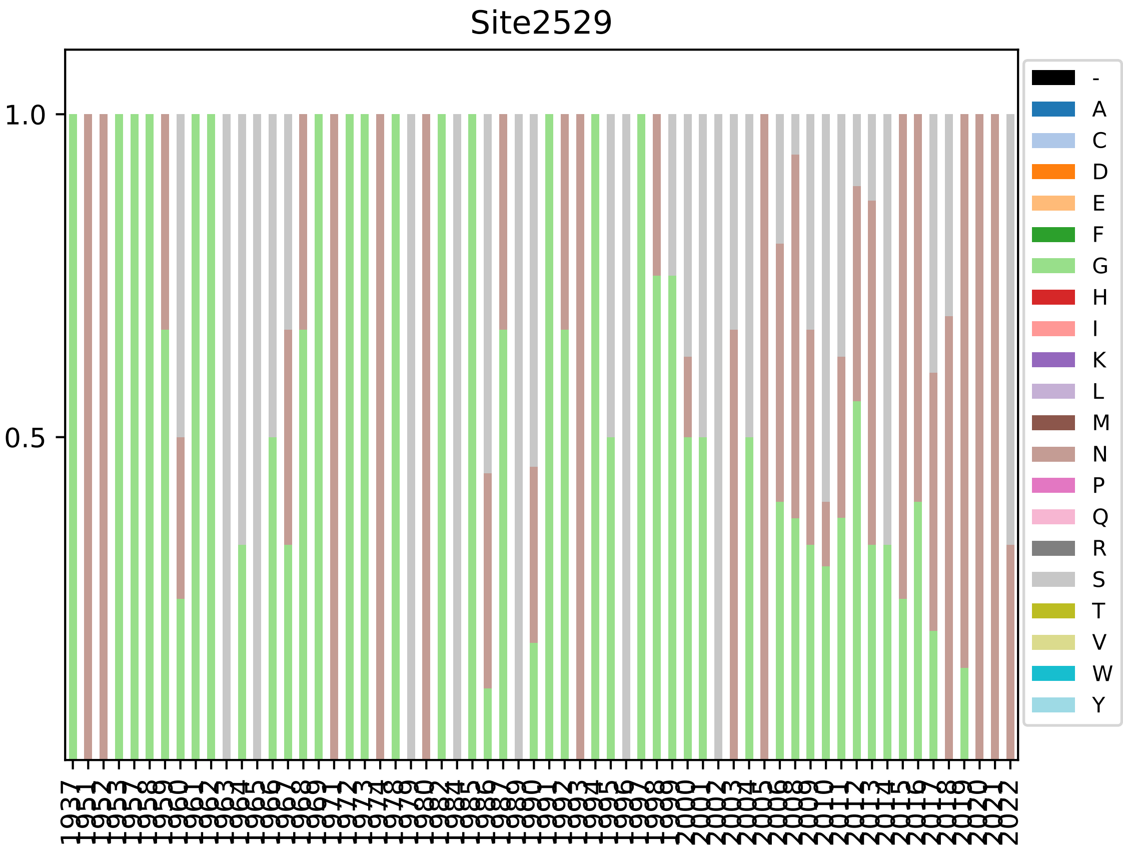
Site2529
1.0
0.5
-
A
C
D
E
F
G
H
I
K
L
M
N
P
Q
R
S
T
V
W
Y

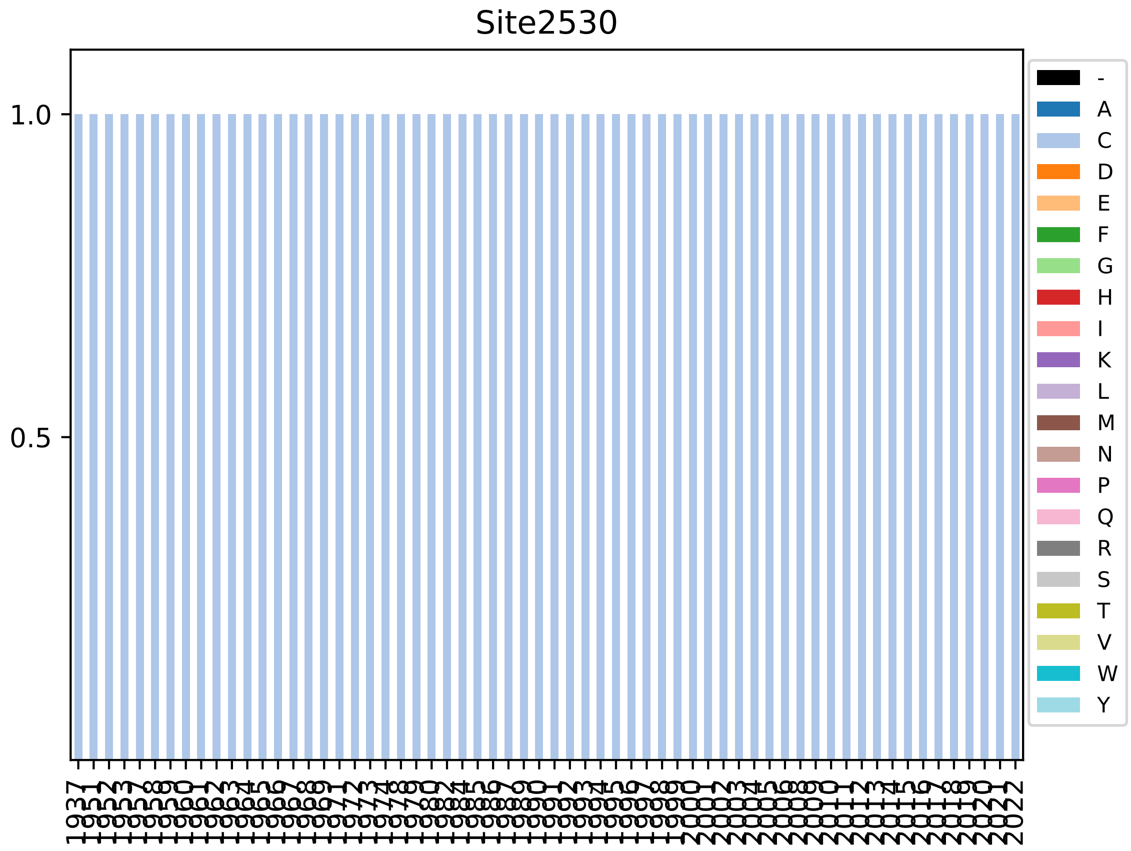

Site2530
1.0
0.5
-
A
C
D
E
F
G
H
I
K
L
M
N
P
Q
R
S
T
V
W
Y

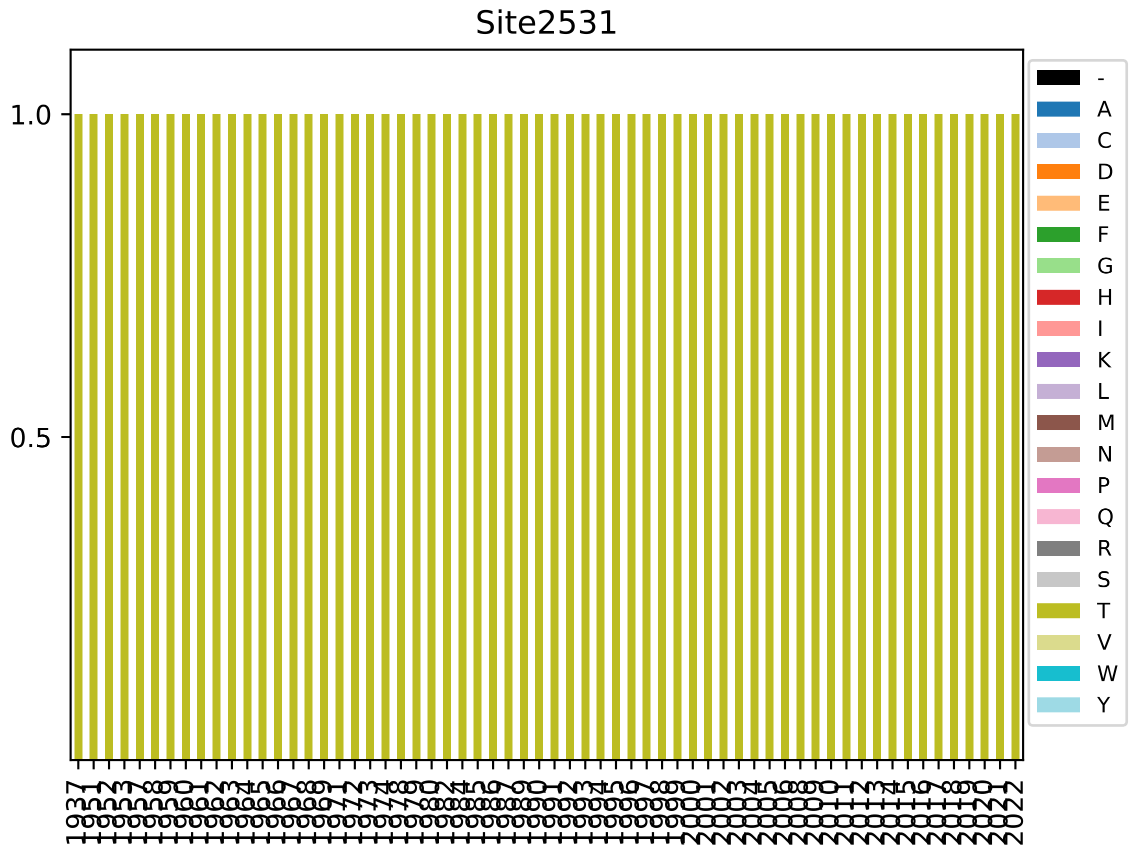
Site2531
1.0
0.5
-
A
C
D
E
F
G
H
I
K
L
M
N
P
Q
R
S
T
V
W
Y

Site2532

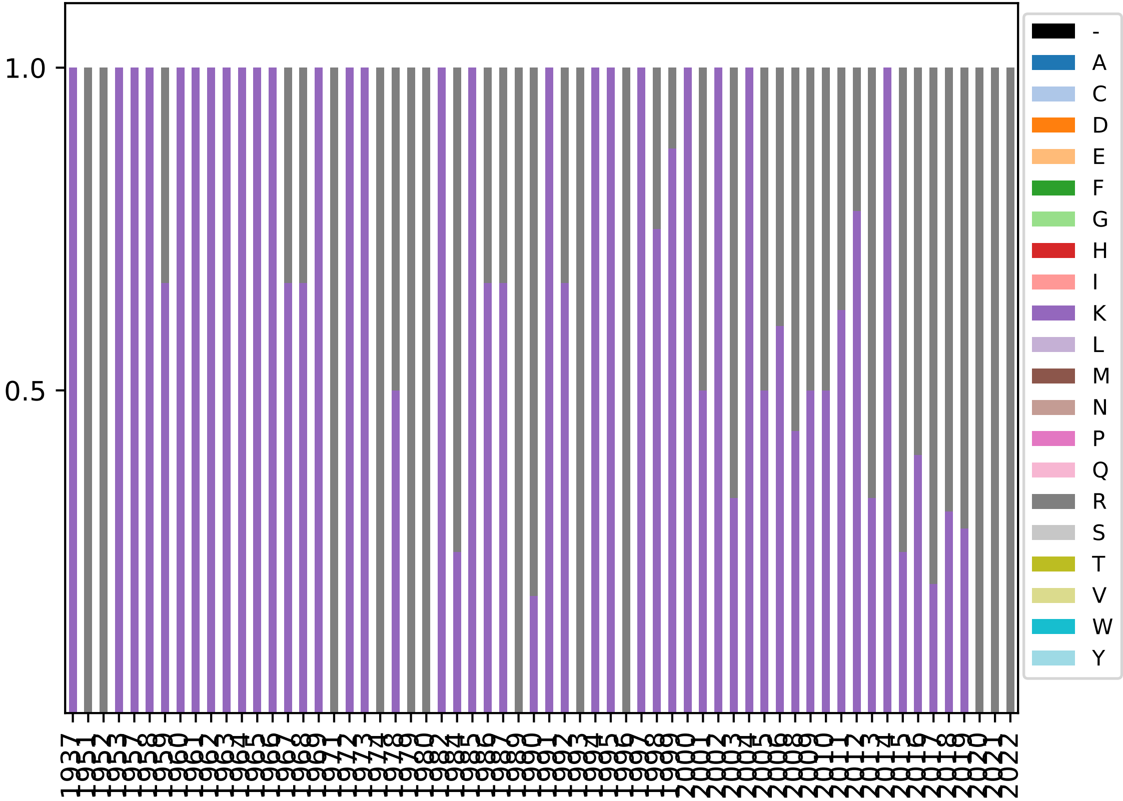

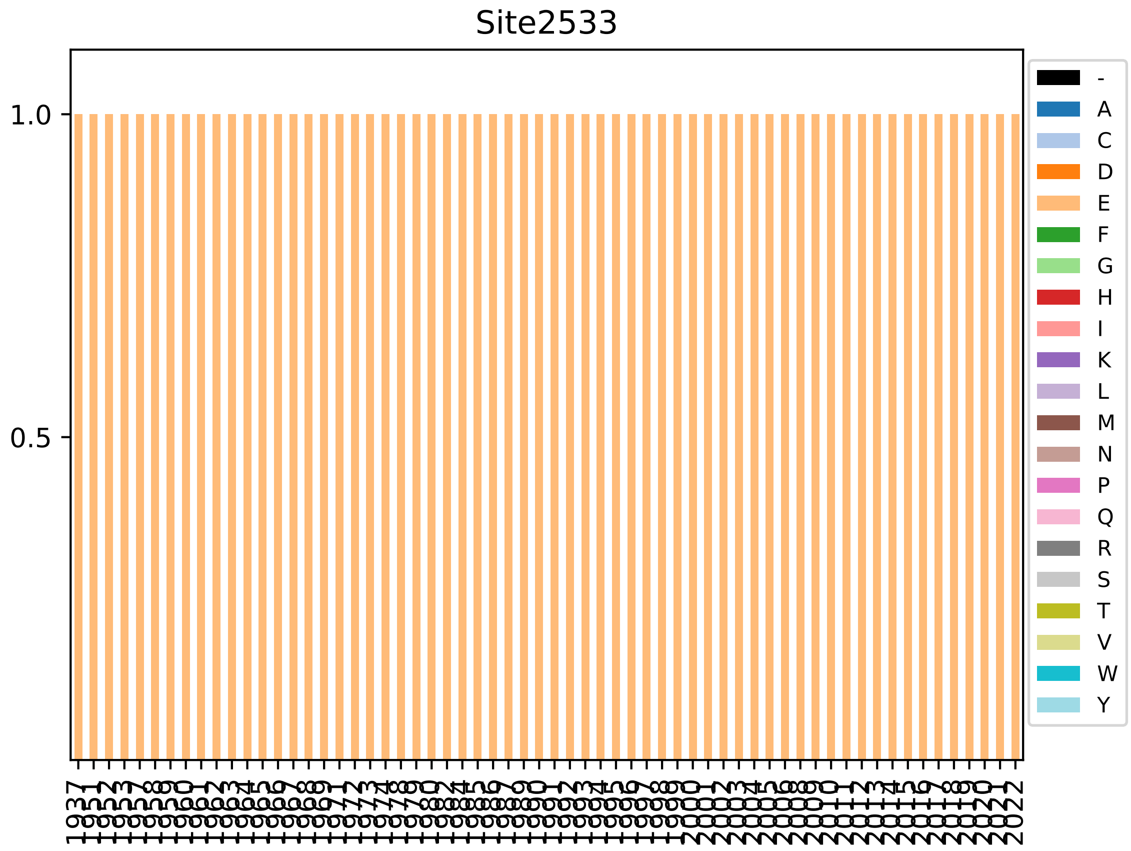
Site2533
1.0
0.5
-
A
C
D
E
F
G
H
I
K
L
M
N
P
Q
R
S
T
V
W
Y

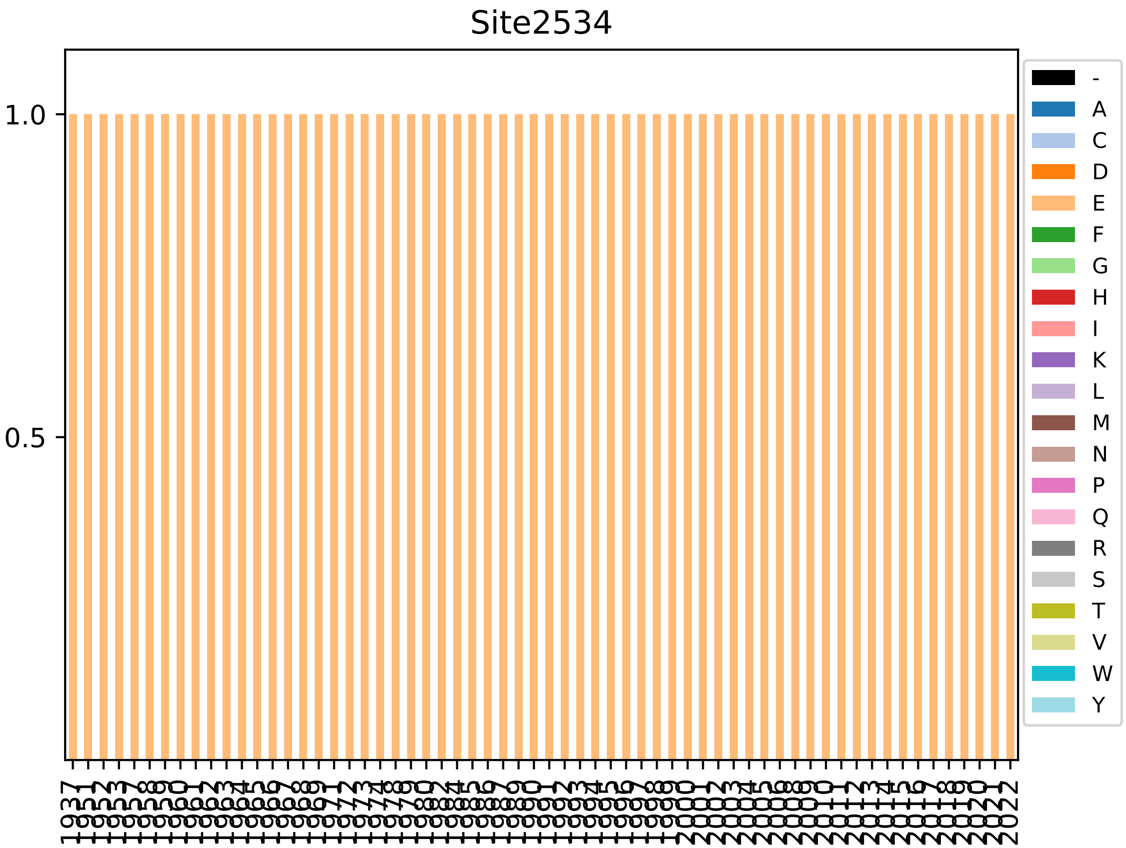
Site2534
1.0
0.5
-
A
C
D
E
F
G
H
I
K
L
M
N
P
Q
R
S
T
V
W
Y

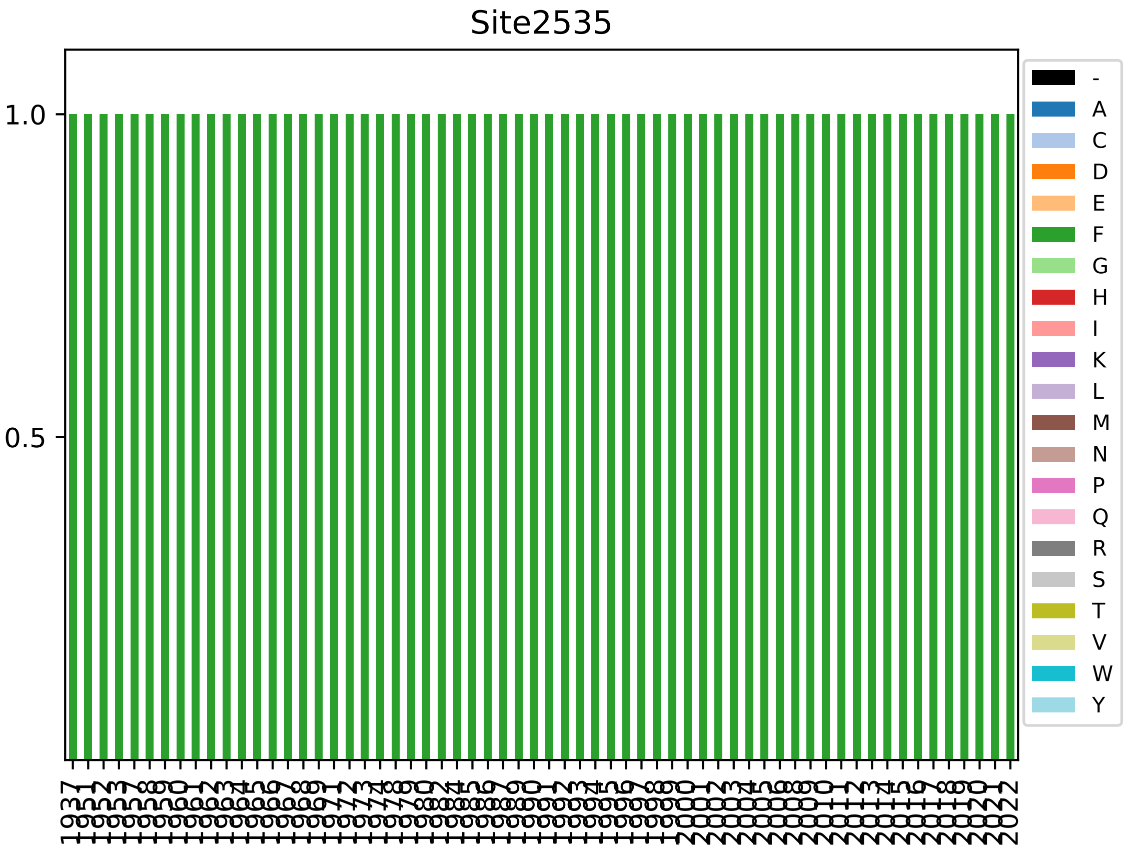
Site2535
1.0
0.5
-
A
C
D
E
F
G
H
I
K
L
M
N
P
Q
R
S
T
V
W
Y

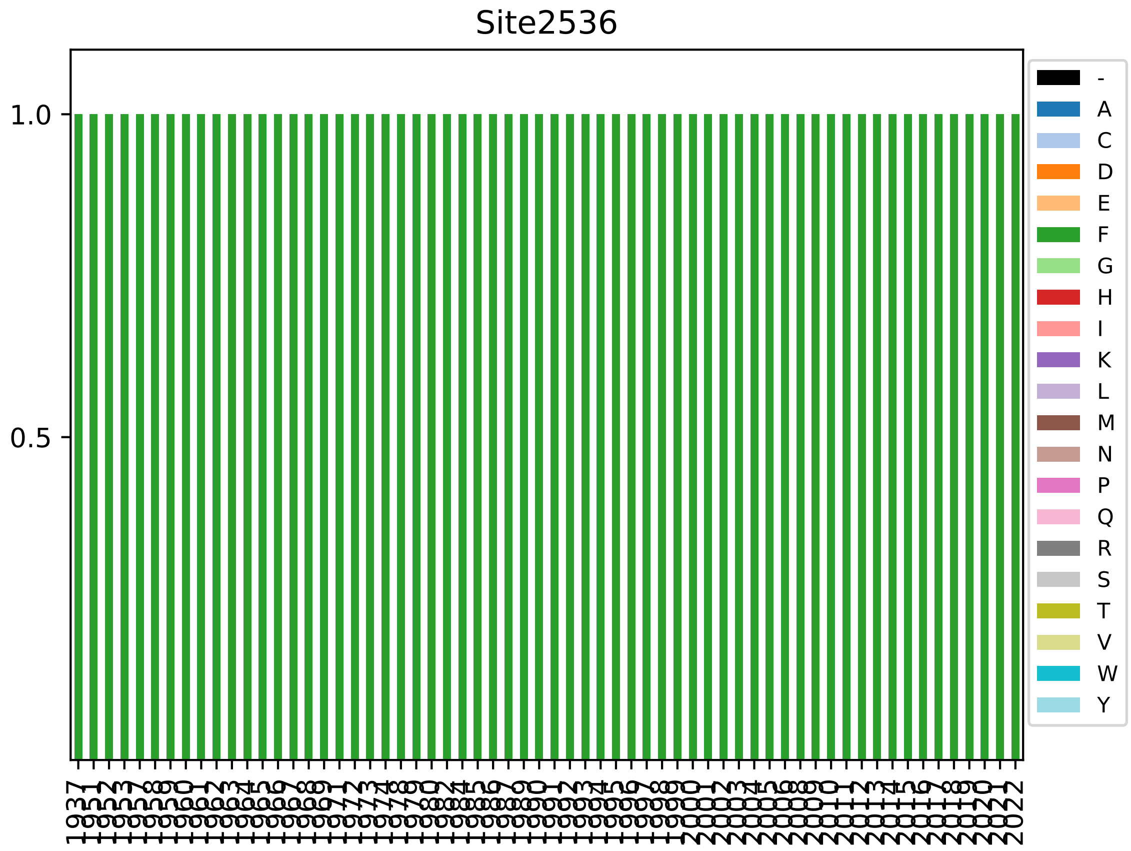
Site2536
1.0
0.5
-
A
C
D
E
F
G
H
I
K
L
M
N
P
Q
R
S
T
V
W
Y

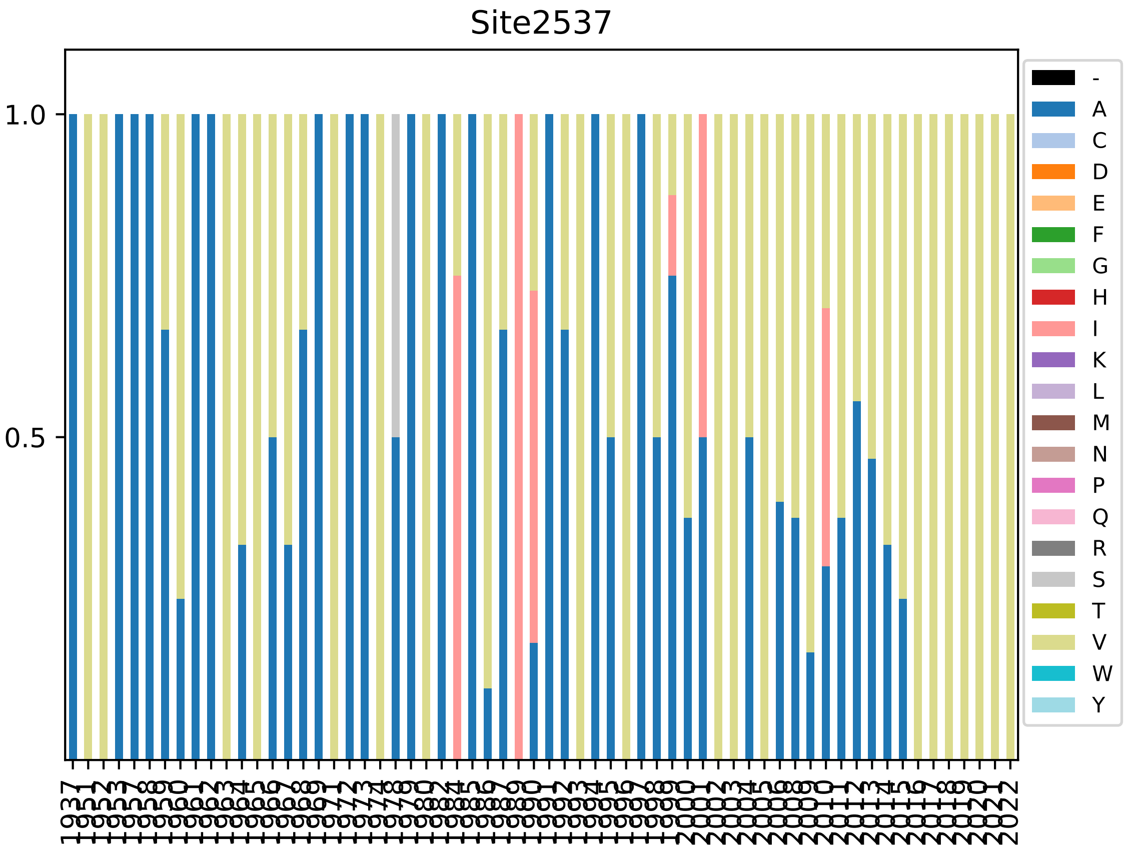

Site2537
1.0
0.5
-
A
C
D
E
F
G
H
I
K
L
M
N
P
Q
R
S
T
V
W
Y

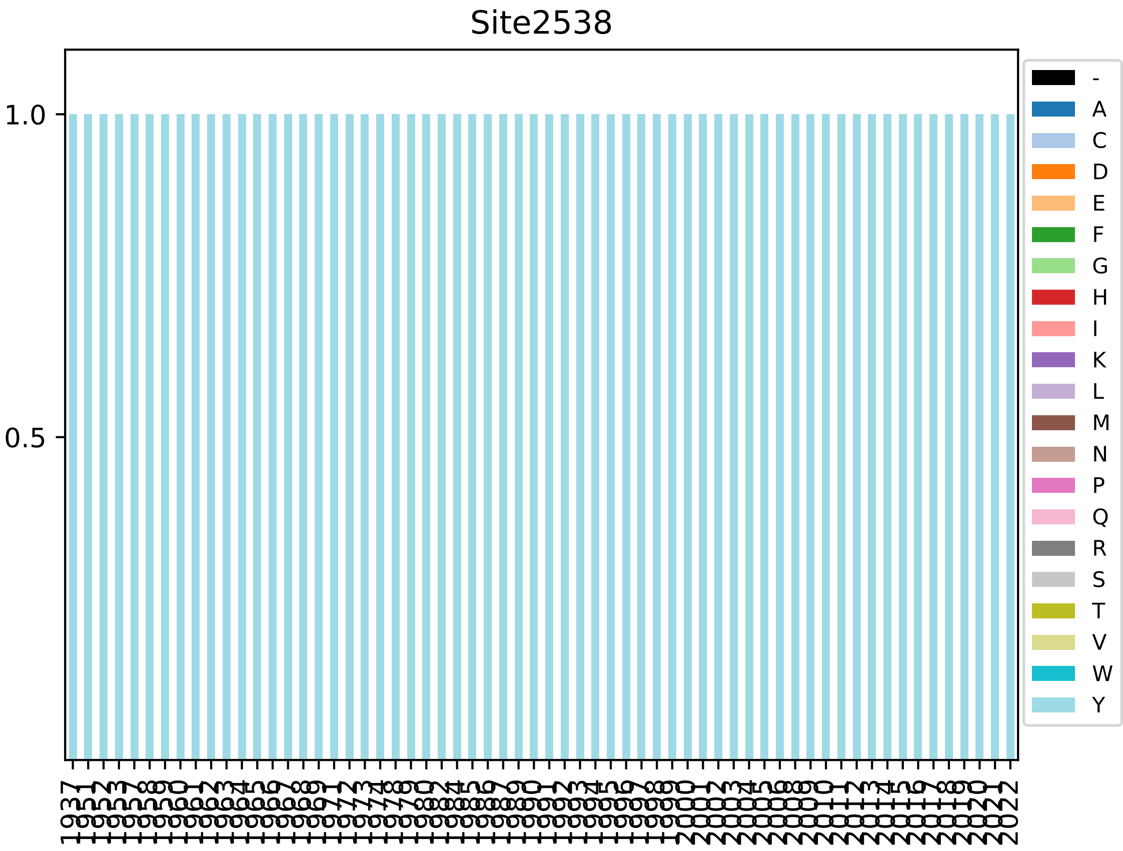
Site2538
1.0
0.5
-
A
C
D
E
F
G
H
I
K
L
M
N
P
Q
R
S
T
V
W
Y

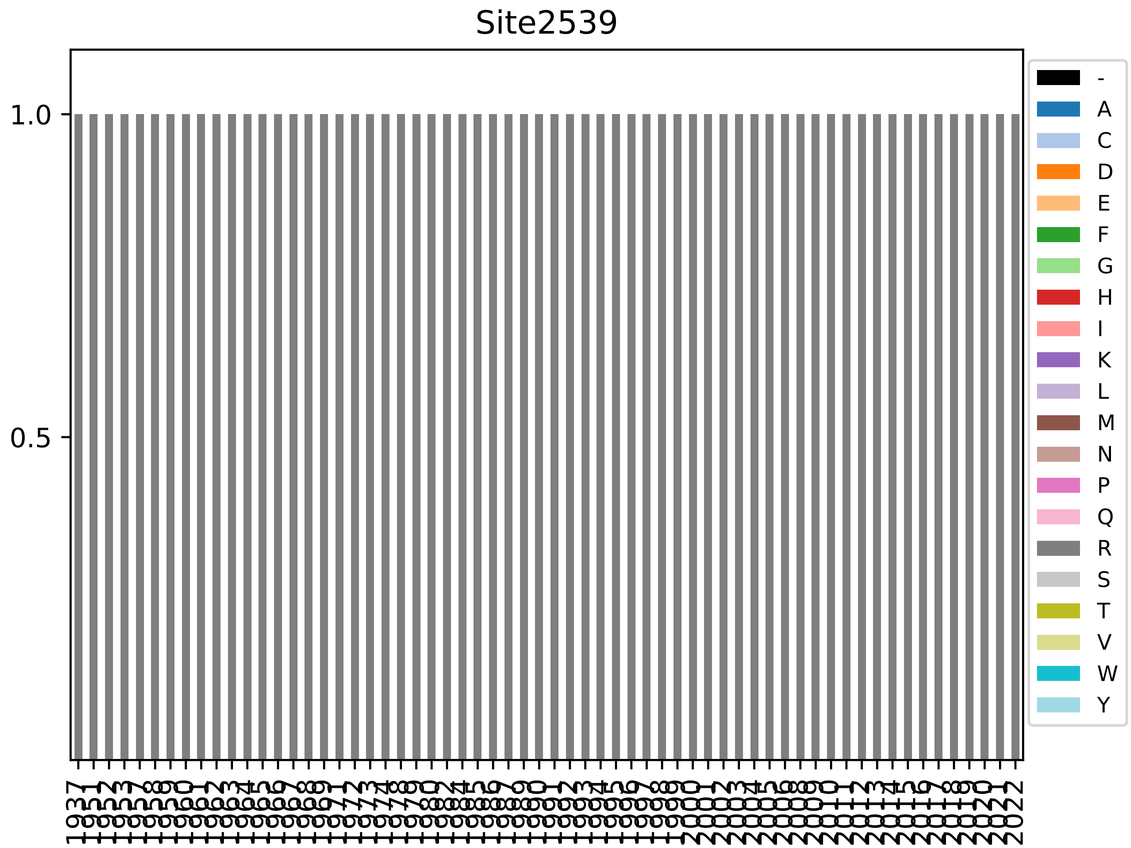
Site2539
1.0
0.5
-
A
C
D
E
F
G
H
I
K
L
M
N
P
Q
R
S
T
V
W
Y

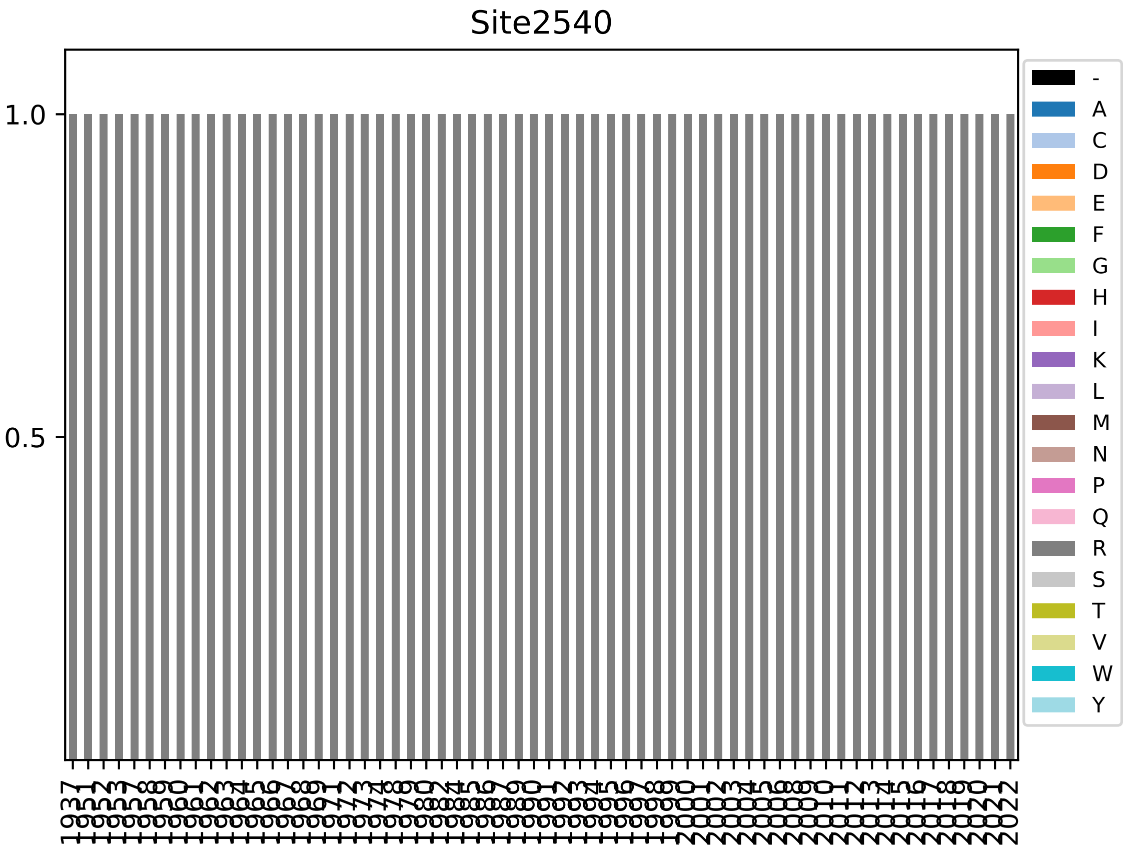
Site2540
1.0
0.5
-
A
C
D
E
F
G
H
I
K
L
M
N
P
Q
R
S
T
V
W
Y

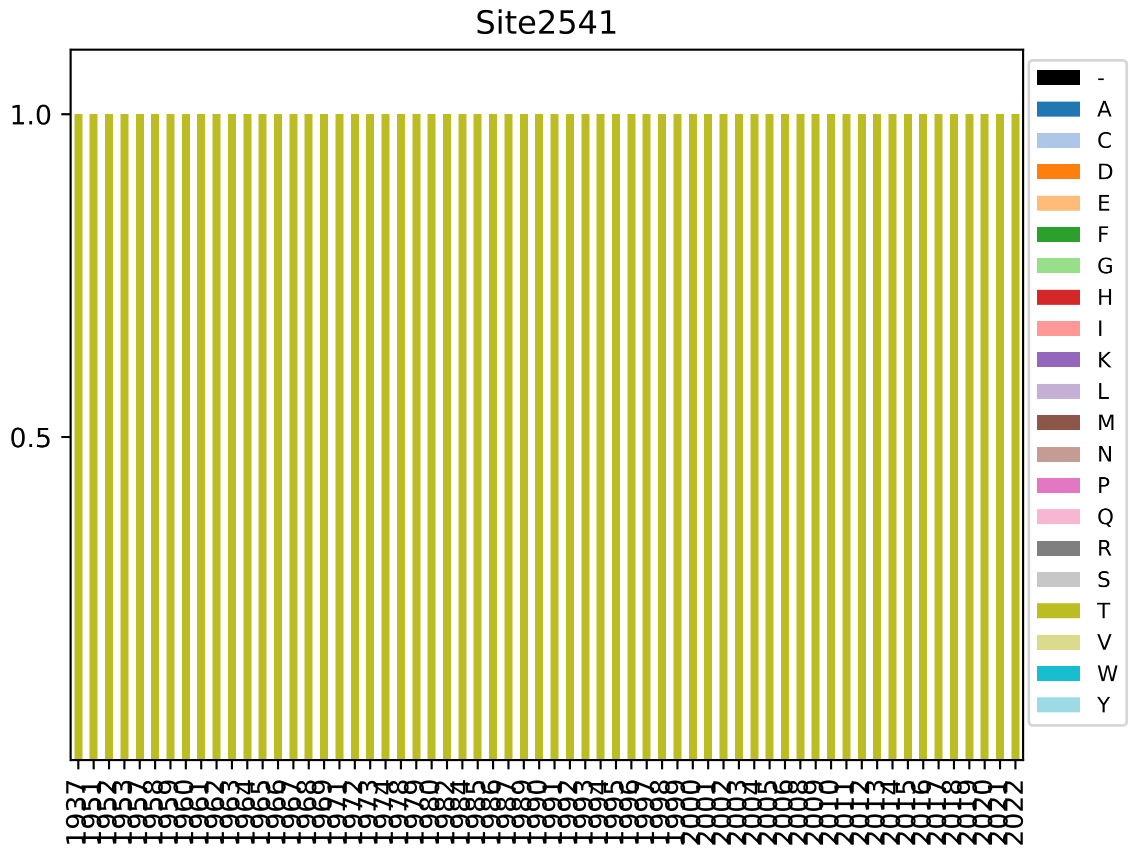
Site2541
1.0
0.5
-
A
C
D
E
F
G
H
I
K
L
M
N
P
Q
R
S
T
V
W
Y

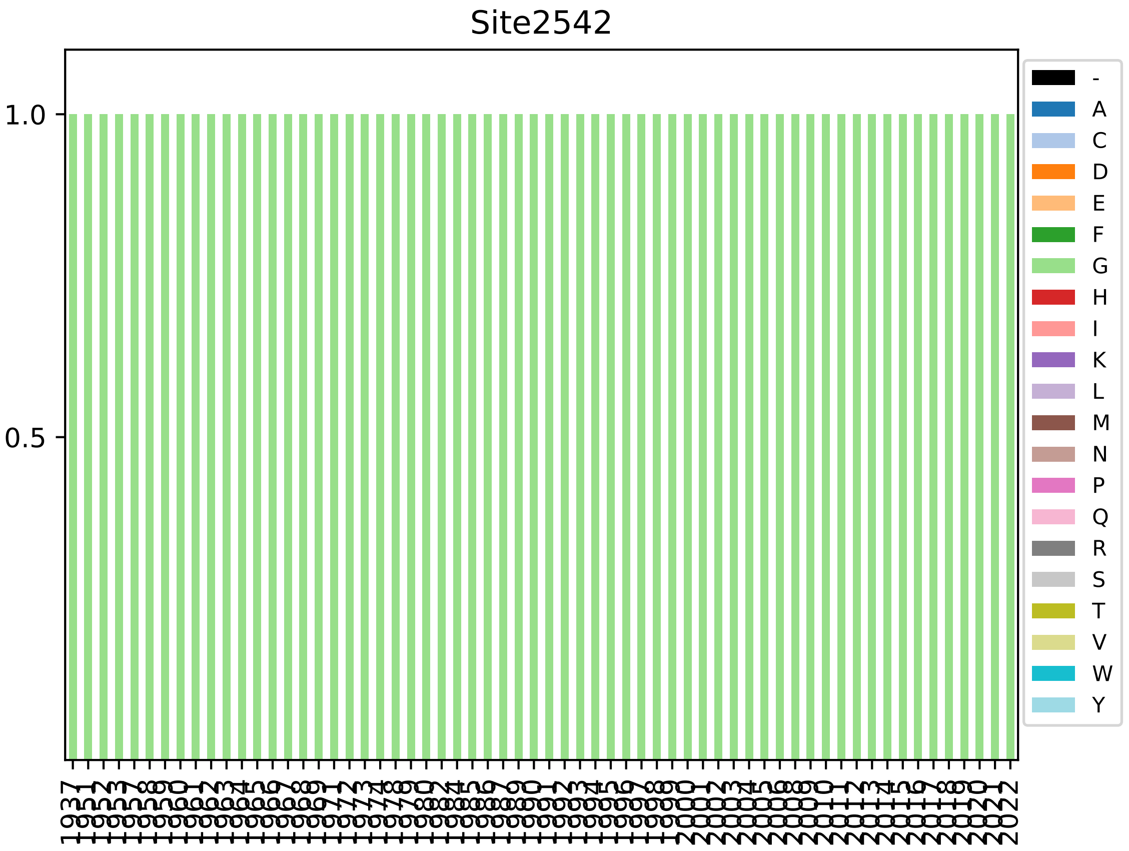
Site2542
1.0
0.5
-
A
C
D
E
F
G
H
I
K
L
M
N
P
Q
R
S
T
V
W
Y

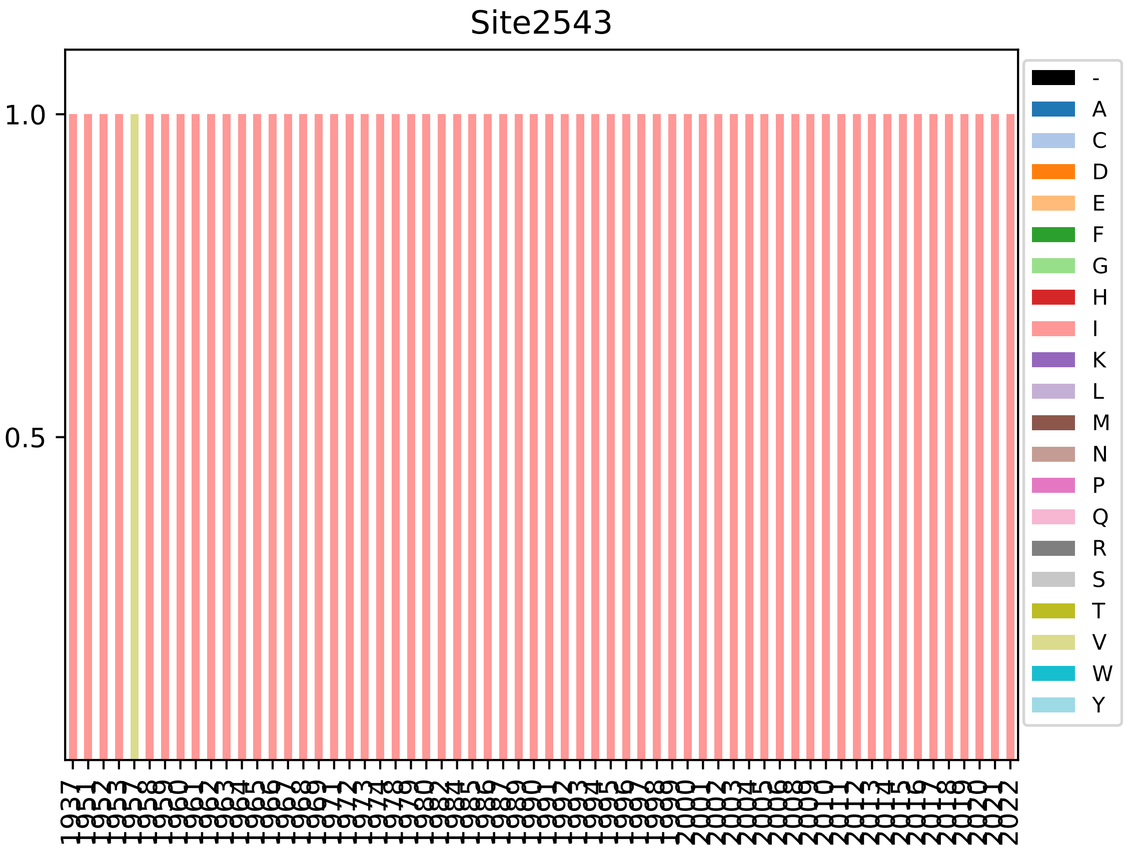

Site2543
1.0
0.5
-
A
C
D
E
F
G
H
I
K
L
M
N
P
Q
R
S
T
V
W
Y

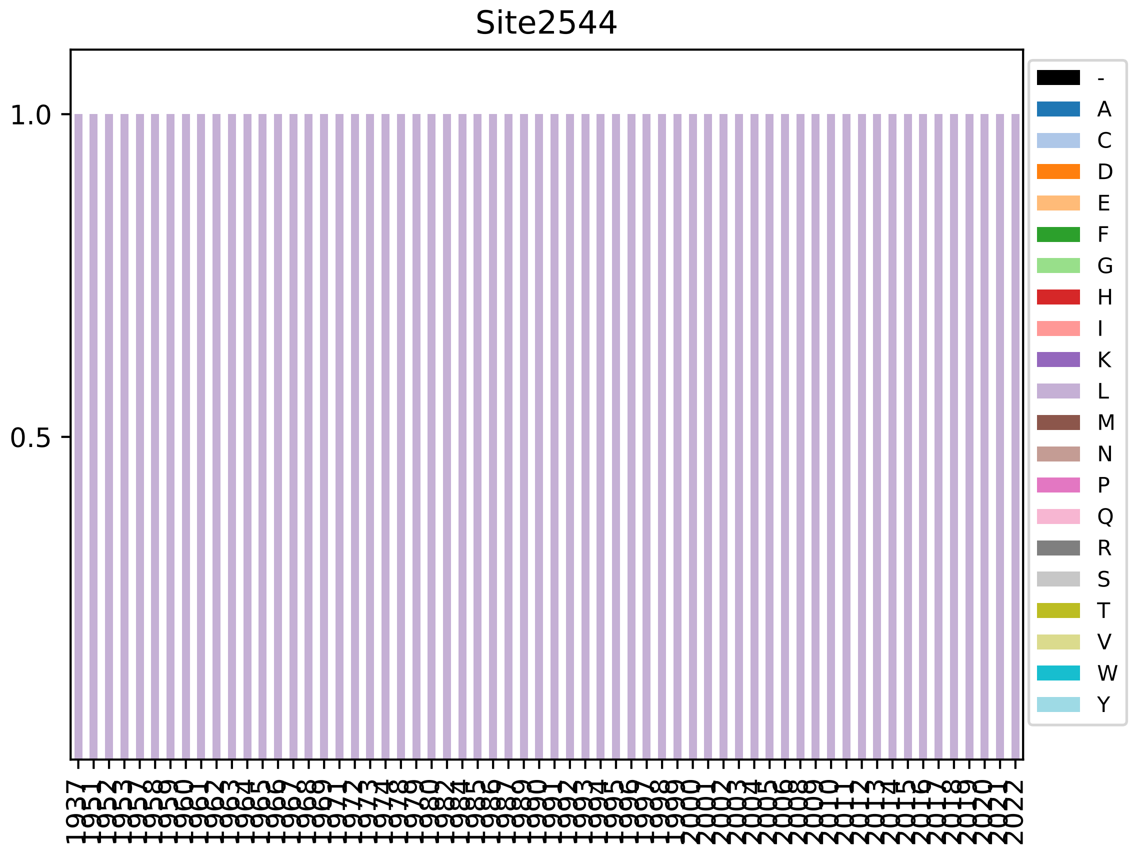
Site2544
1.0
0.5
-
A
C
D
E
F
G
H
I
K
L
M
N
P
Q
R
S
T
V
W
Y

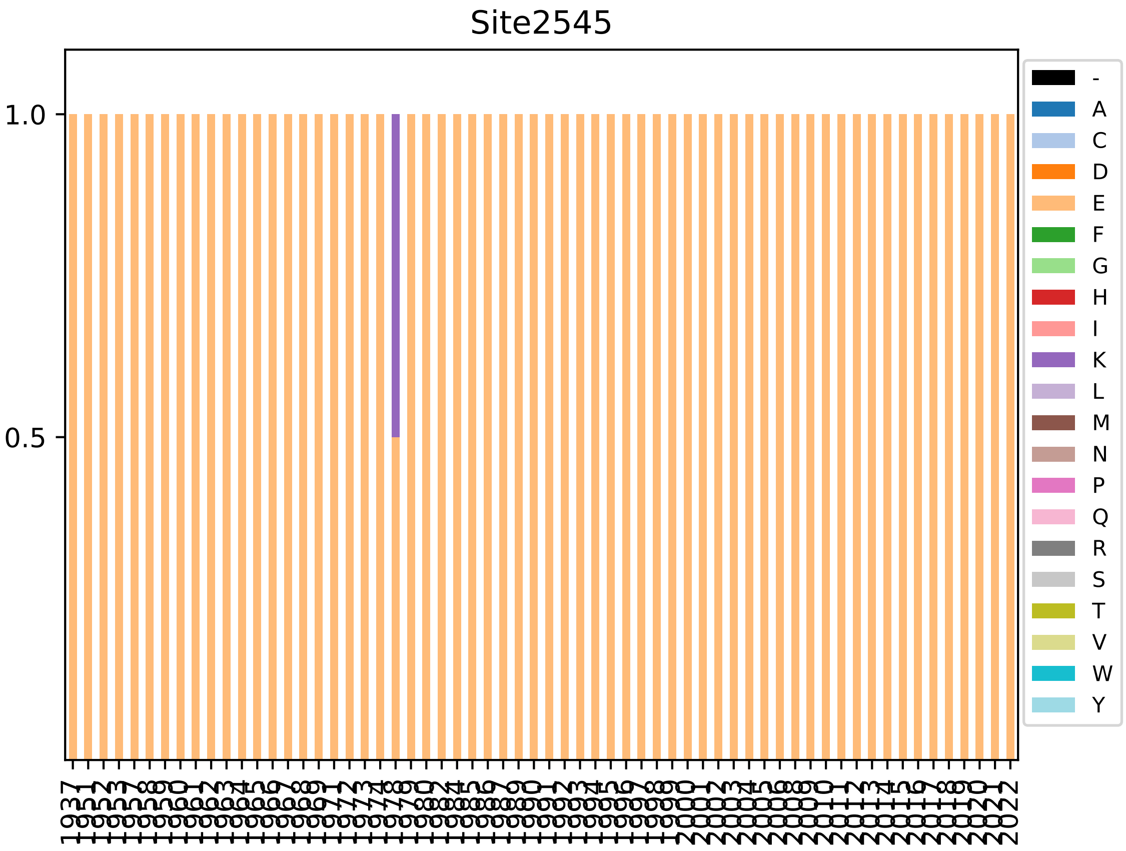
Site2545
1.0
0.5
-
A
C
D
E
F
G
H
I
K
L
M
N
P
Q
R
S
T
V
W
Y

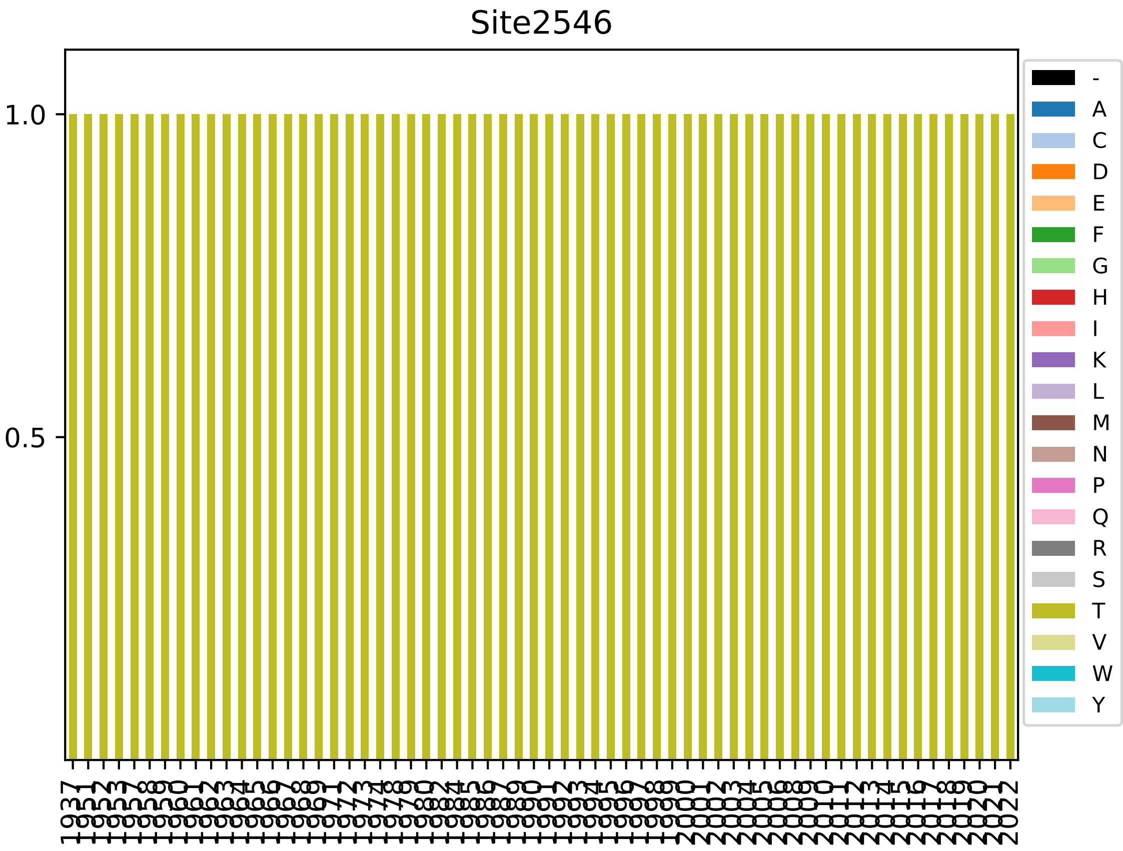
Site2546
1.0
0.5
-
A
C
D
E
F
G
H
I
K
L
M
N
P
Q
R
S
T
V
W
Y

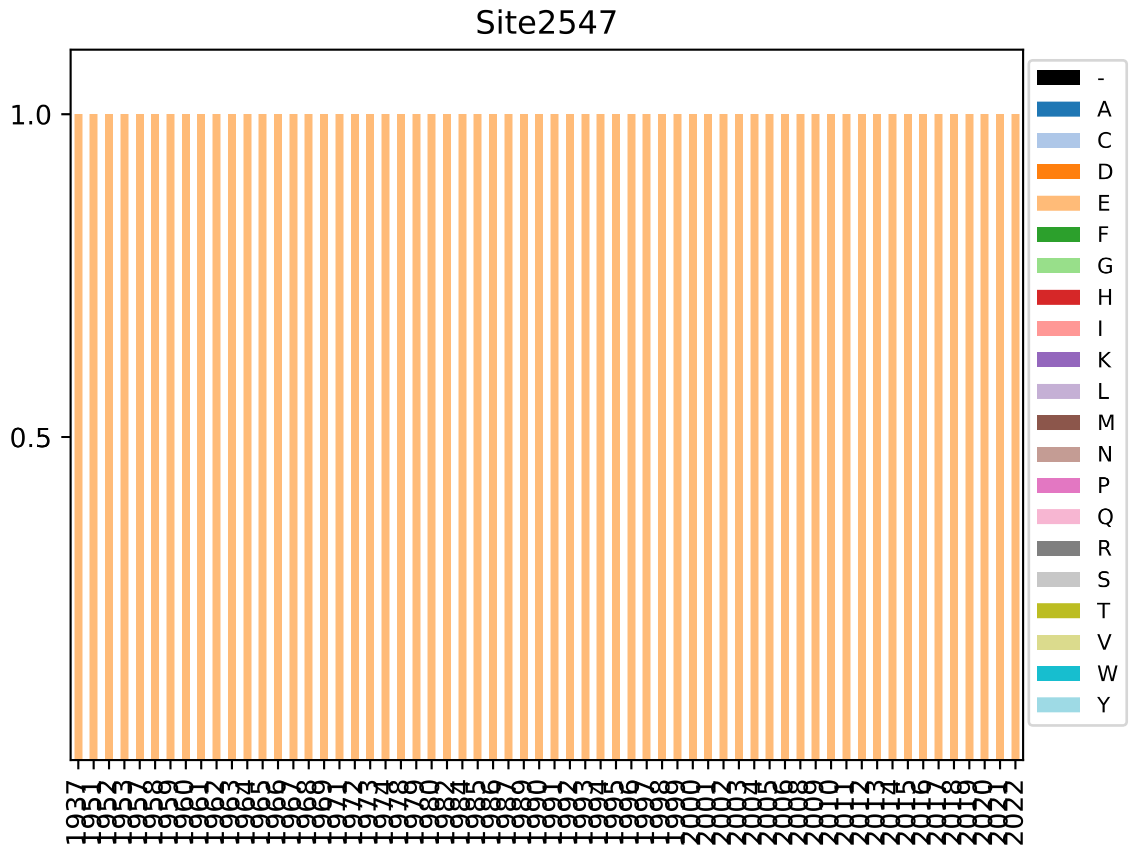
Site2547
1.0
0.5
-
A
C
D
E
F
G
H
I
K
L
M
N
P
Q
R
S
T
V
W
Y

Site2548

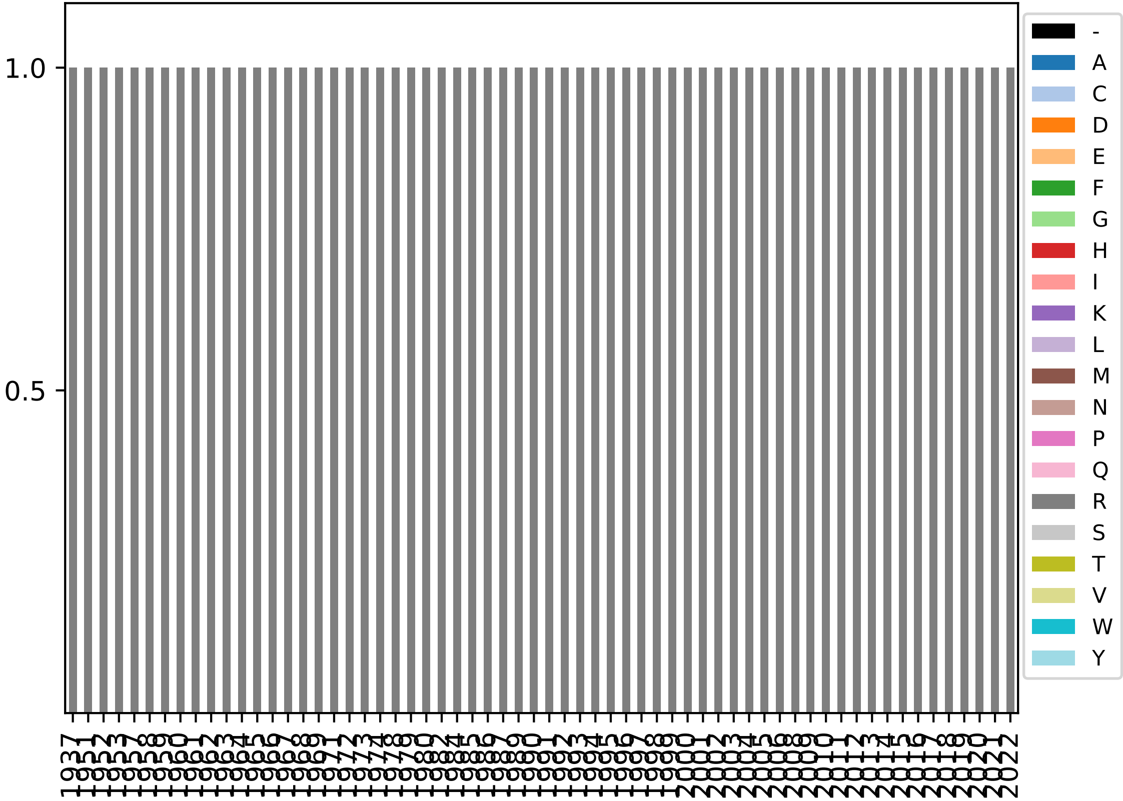

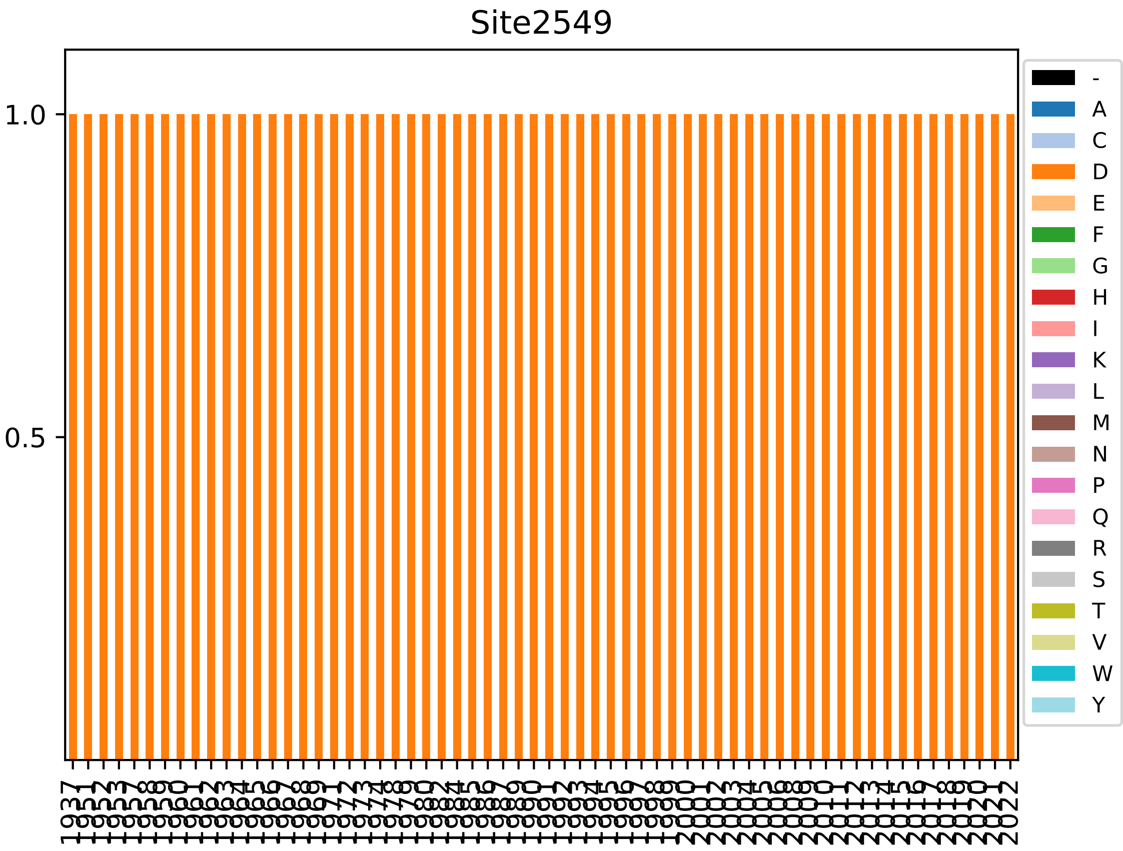
Site2549
1.0
0.5
-
A
C
D
E
F
G
H
I
K
L
M
N
P
Q
R
S
T
V
W
Y

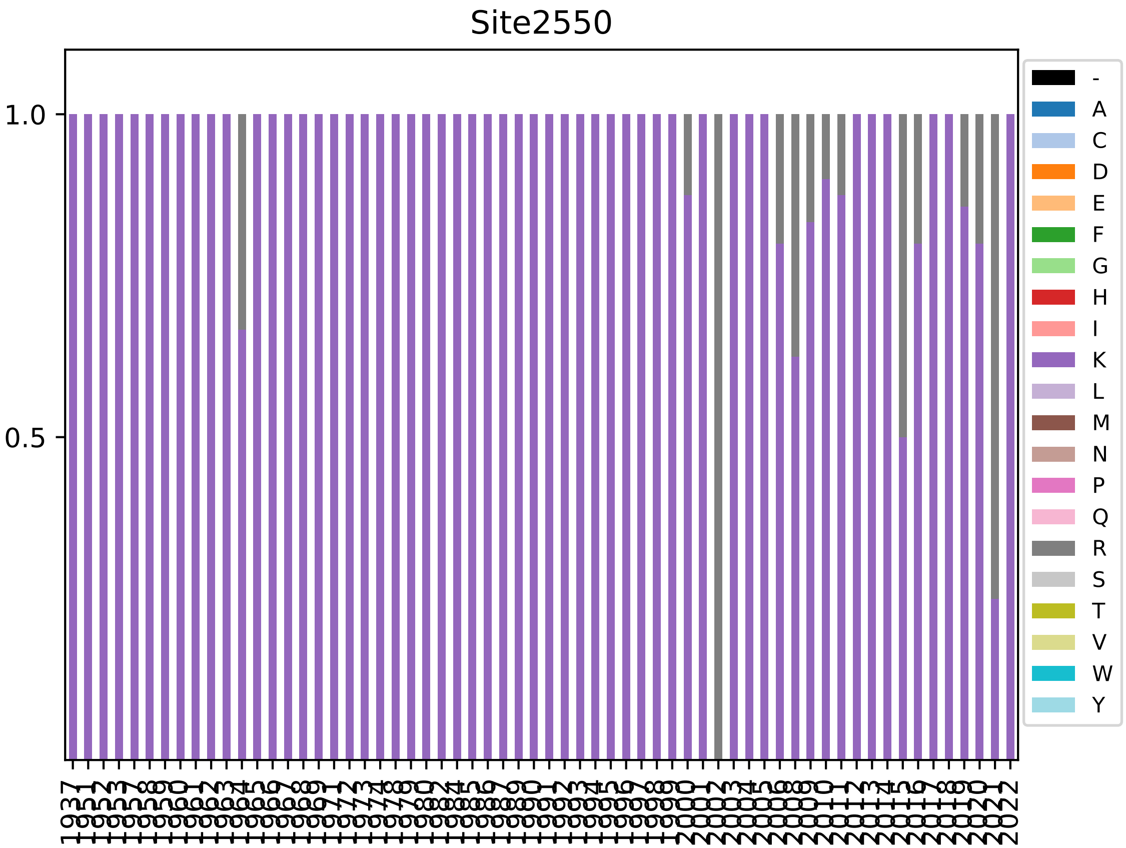

Site2550
1.0
0.5
-
A
C
D
E
F
G
H
I
K
L
M
N
P
Q
R
S
T
V
W
Y

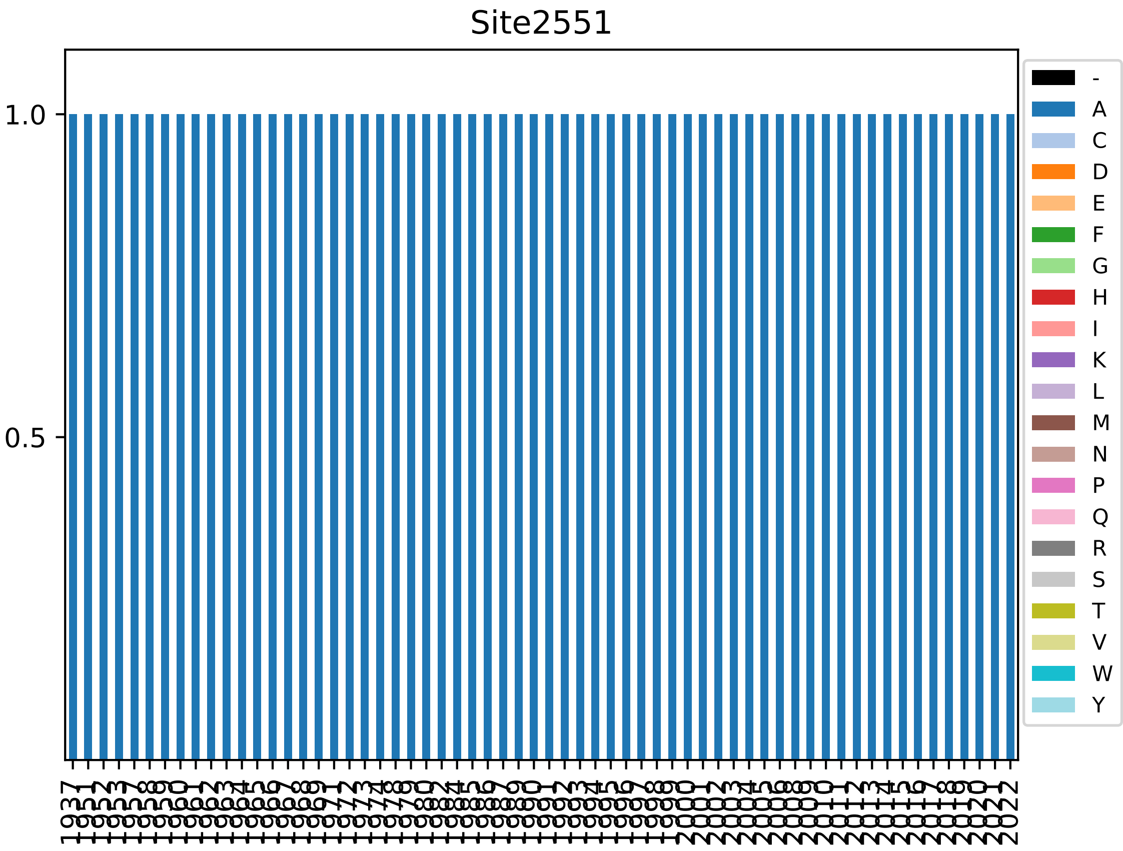
Site2551
1.0
0.5
-
A
C
D
E
F
G
H
I
K
L
M
N
P
Q
R
S
T
V
W
Y

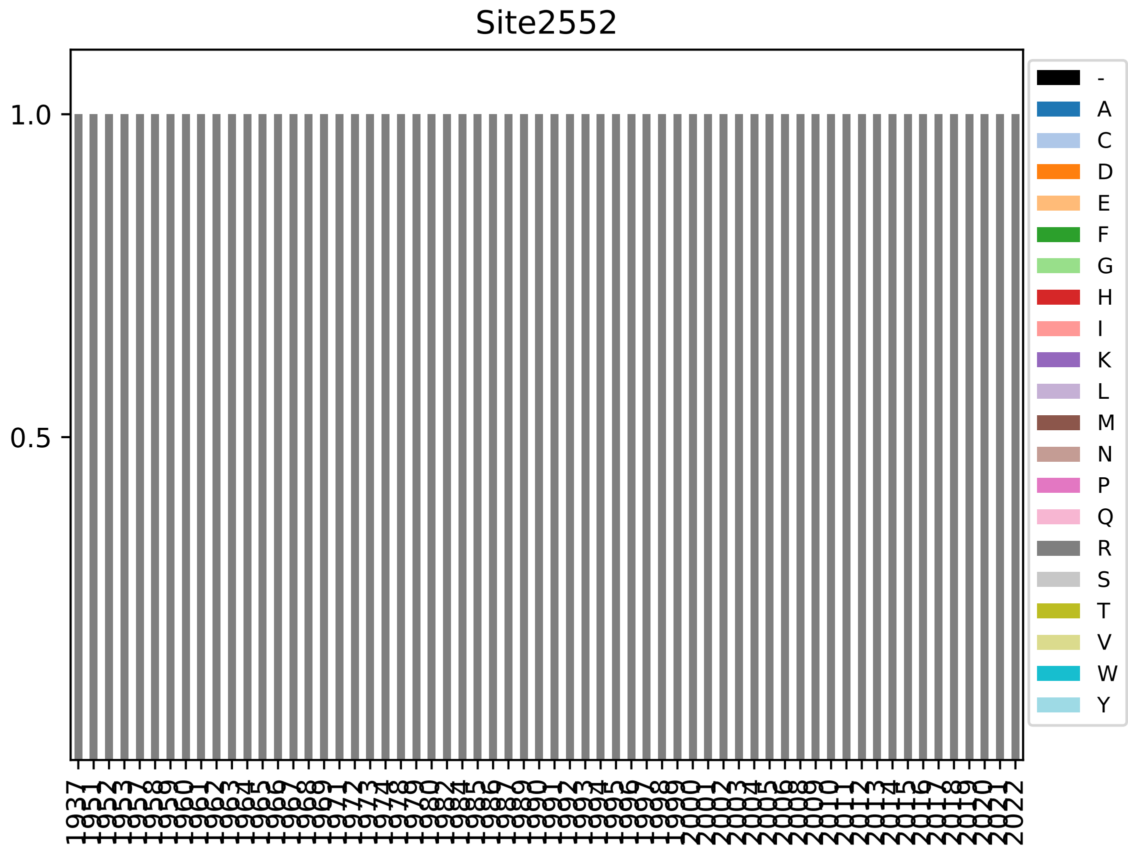
Site2552
1.0
0.5
-
A
C
D
E
F
G
H
I
K
L
M
N
P
Q
R
S
T
V
W
Y

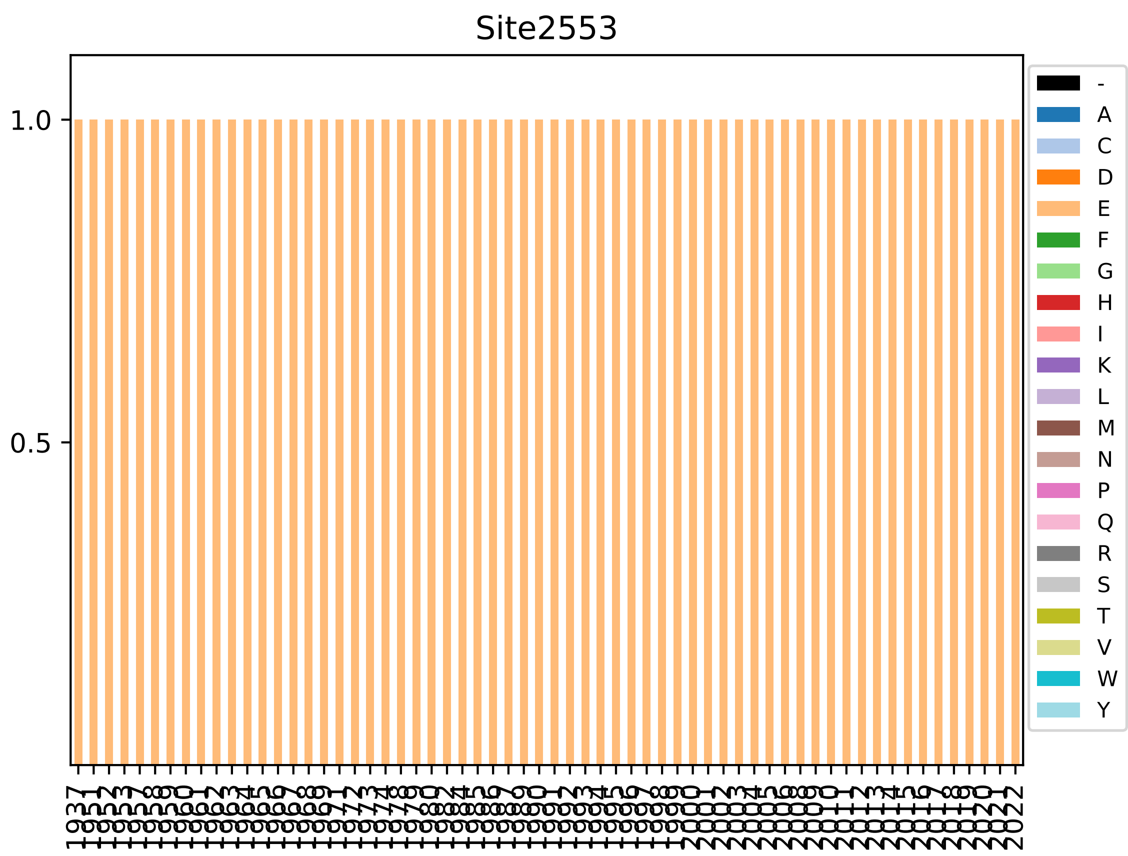
Site2553
1.0
0.5
-
A
C
D
E
F
G
H
I
K
L
M
N
P
Q
R
S
T
V
W
Y

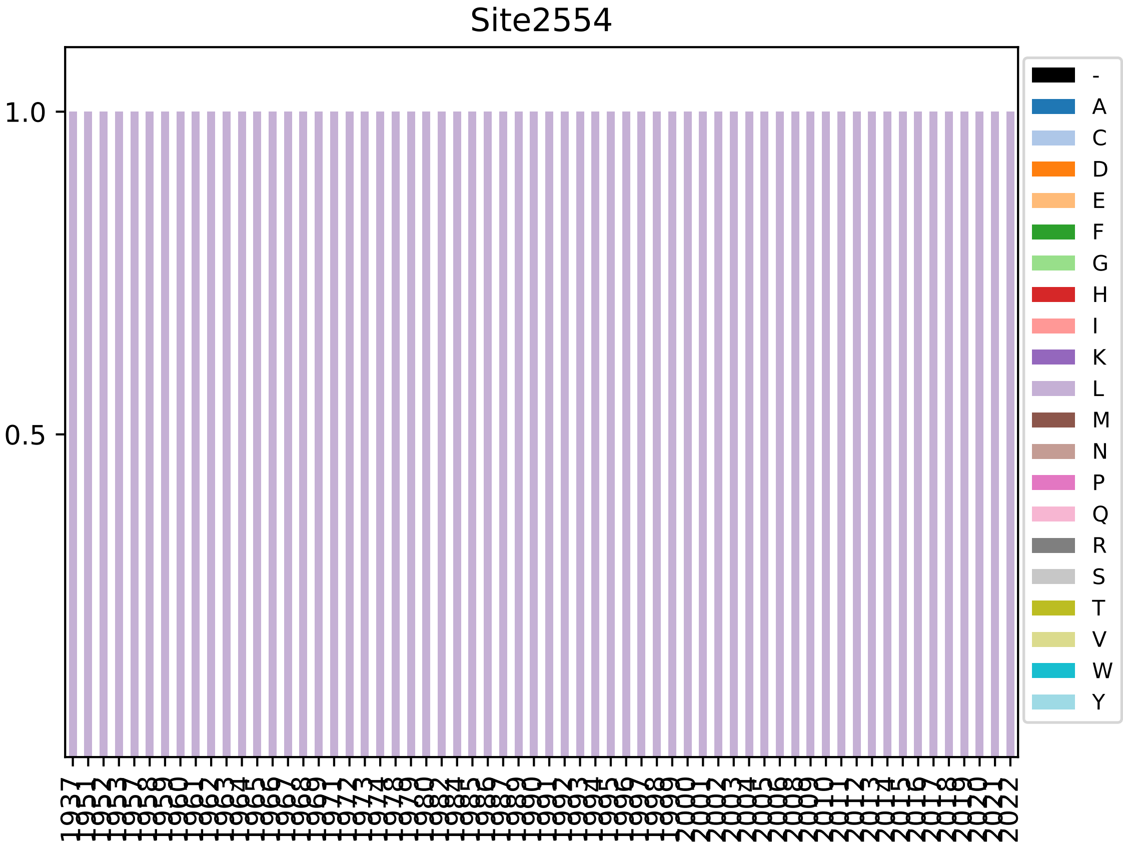
Site2554
1.0
0.5
-
A
C
D
E
F
G
H
I
K
L
M
N
P
Q
R
S
T
V
W
Y

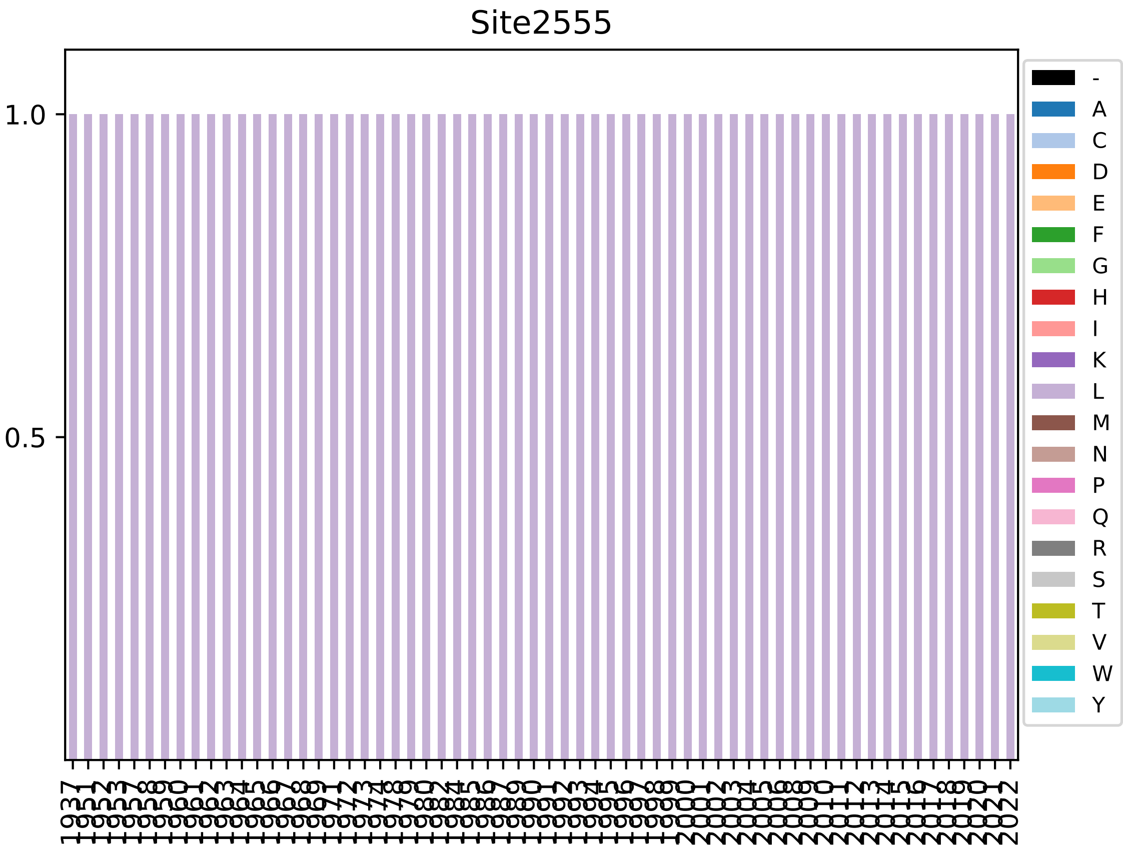
Site2555
1.0
0.5
-
A
C
D
E
F
G
H
I
K
L
M
N
P
Q
R
S
T
V
W
Y

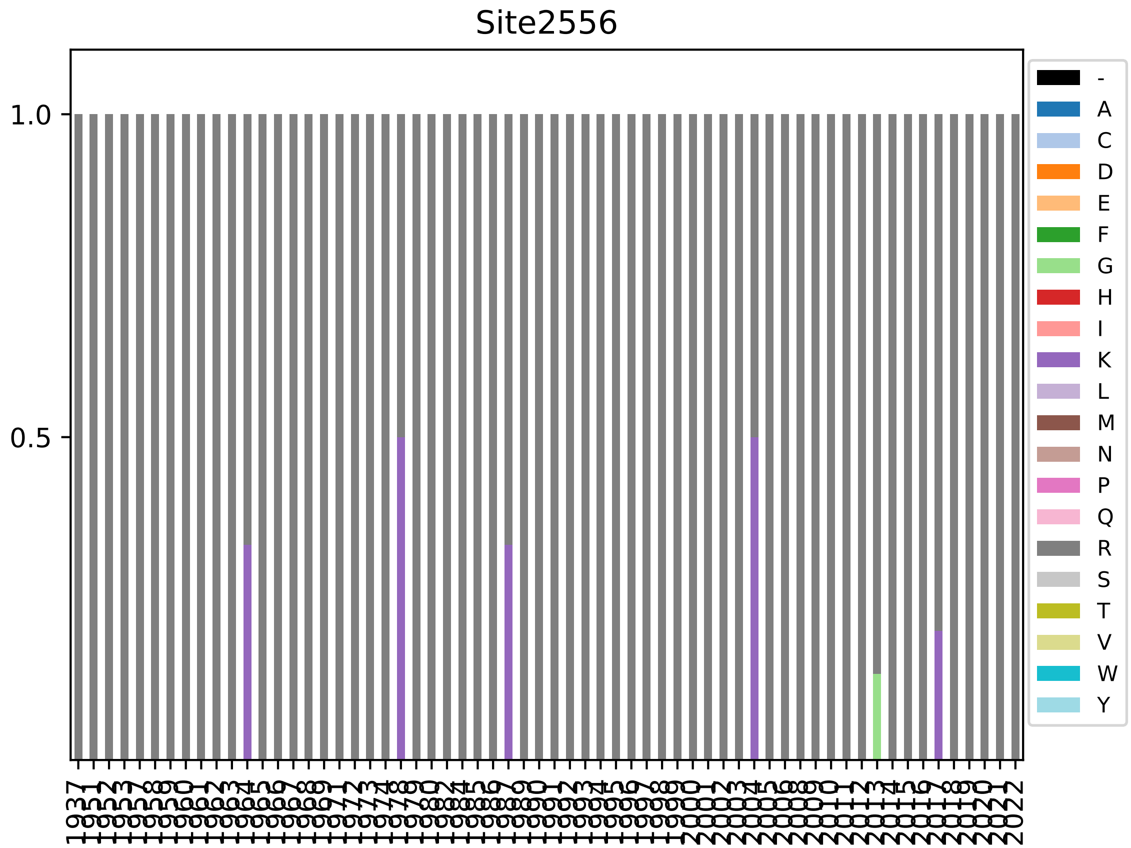
Site2556
1.0
0.5
-
A
C
D
E
F
G
H
I
K
L
M
N
P
Q
R
S
T
V
W
Y

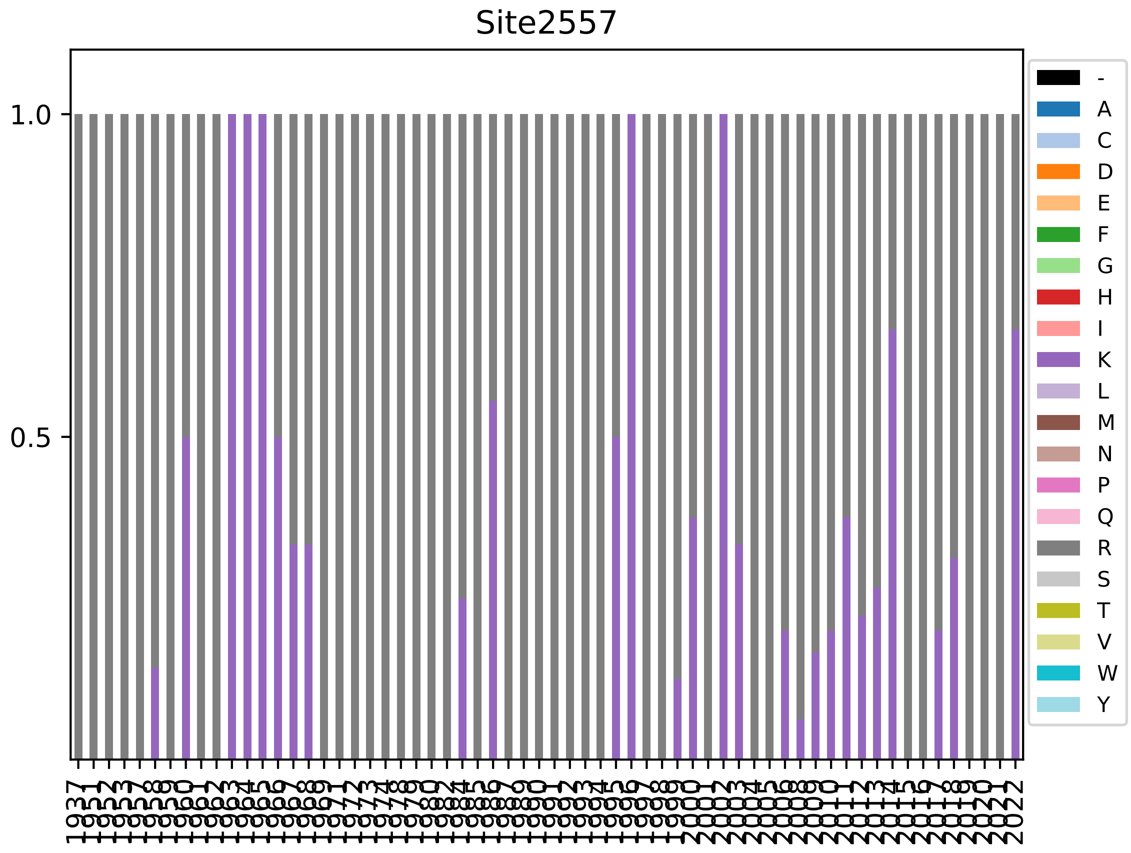
Site2557
1.0
0.5
-
A
C
D
E
F
G
H
I
K
L
M
N
P
Q
R
S
T
V
W
Y

Site2558

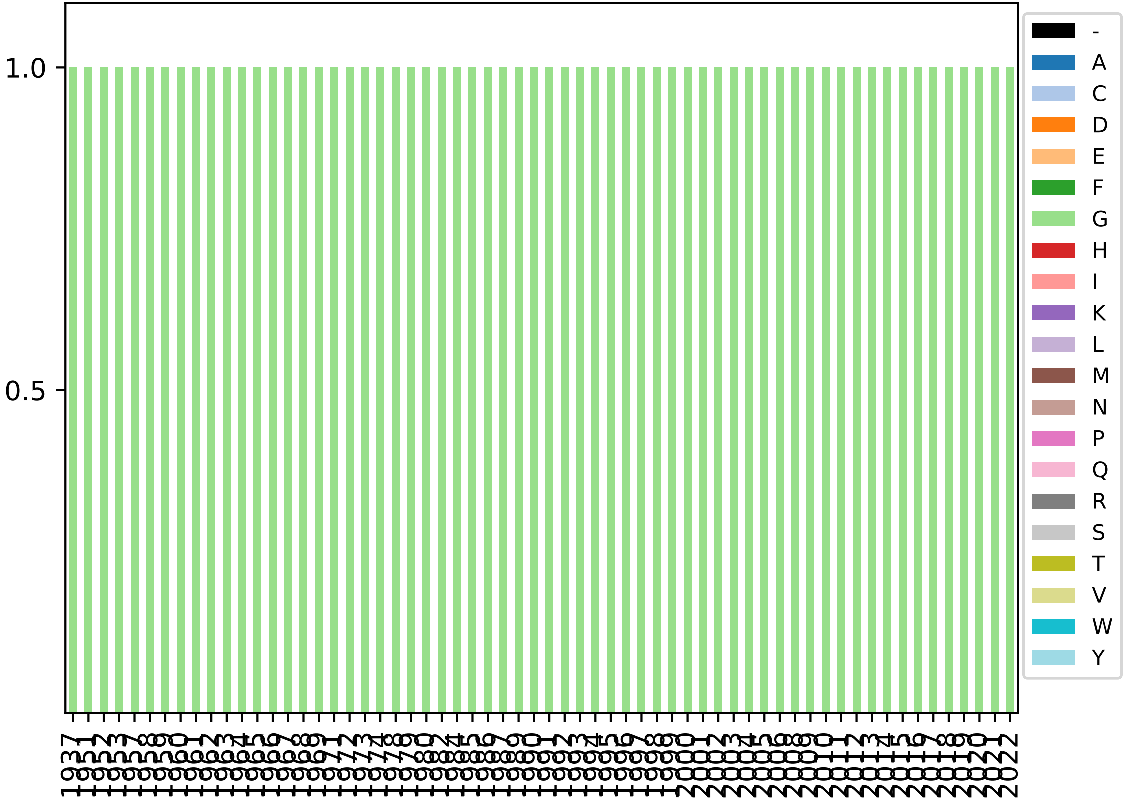

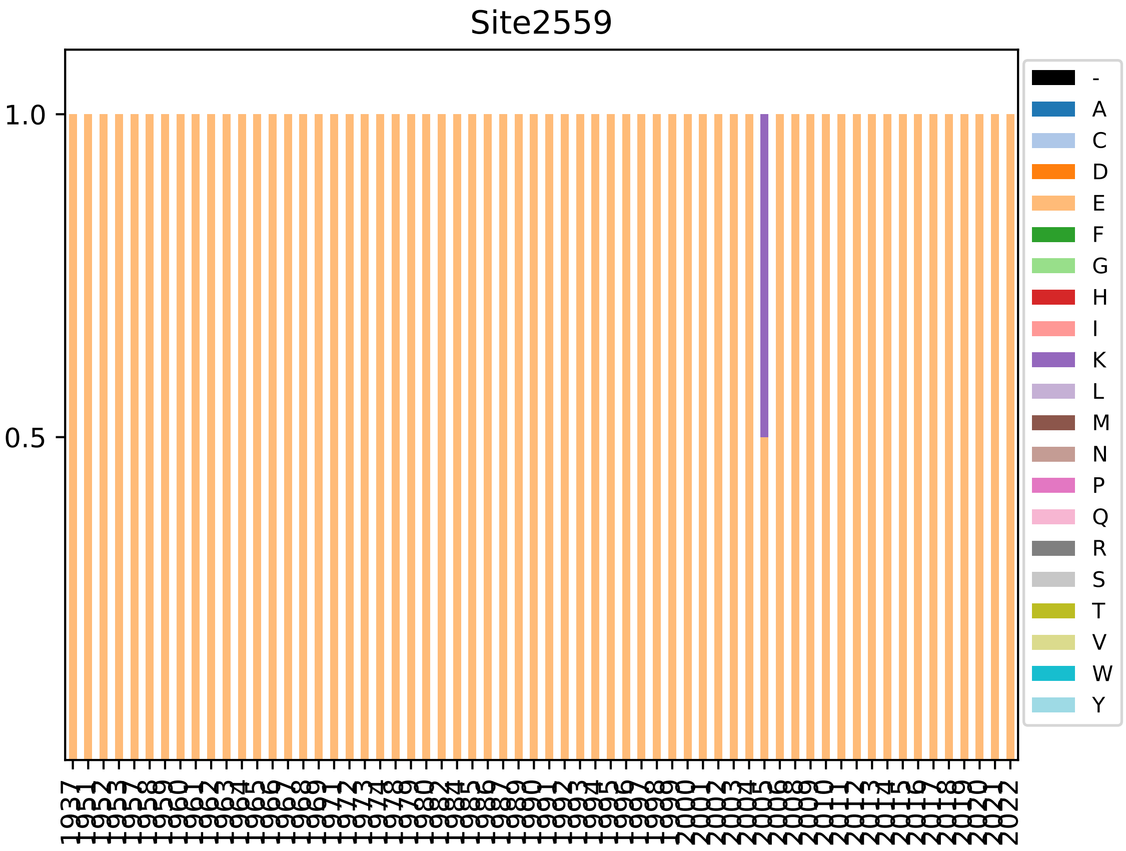
Site2559
1.0
0.5
-
A
C
D
E
F
G
H
I
K
L
M
N
P
Q
R
S
T
V
W
Y

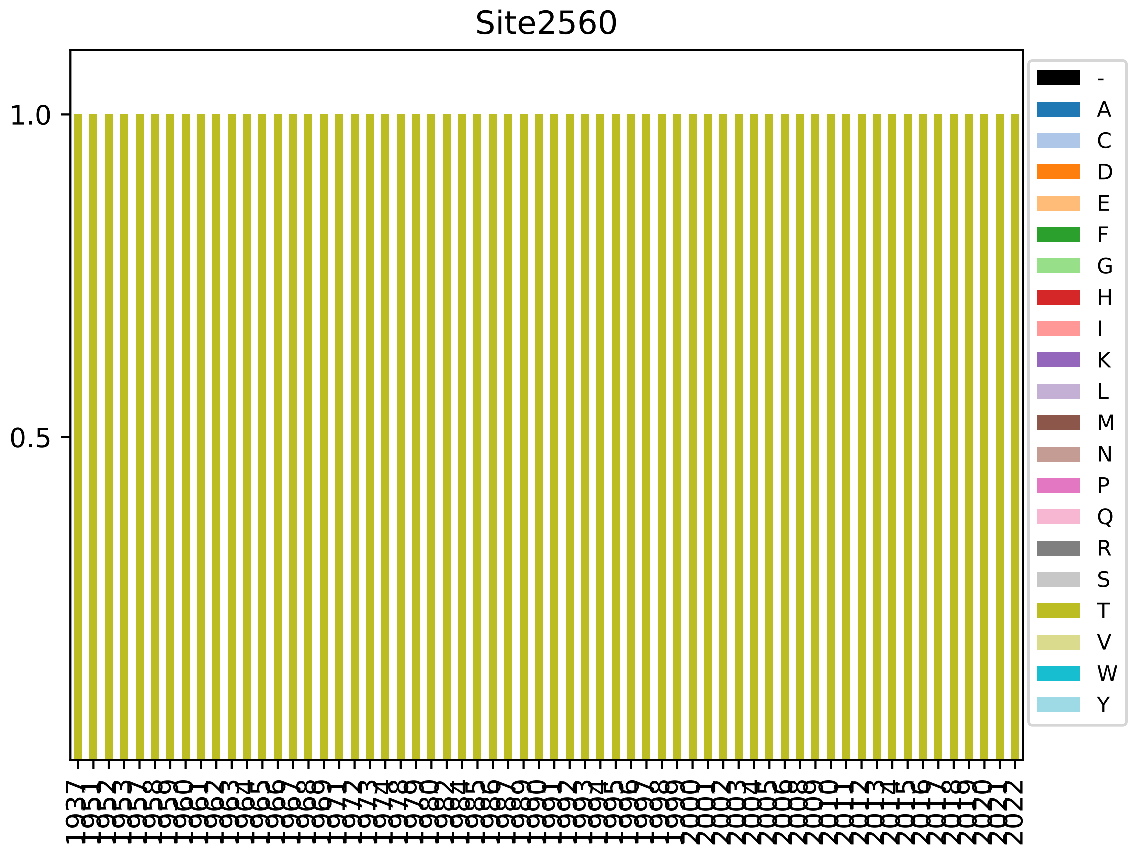
Site2560
1.0
0.5
-
A
C
D
E
F
G
H
I
K
L
M
N
P
Q
R
S
T
V
W
Y

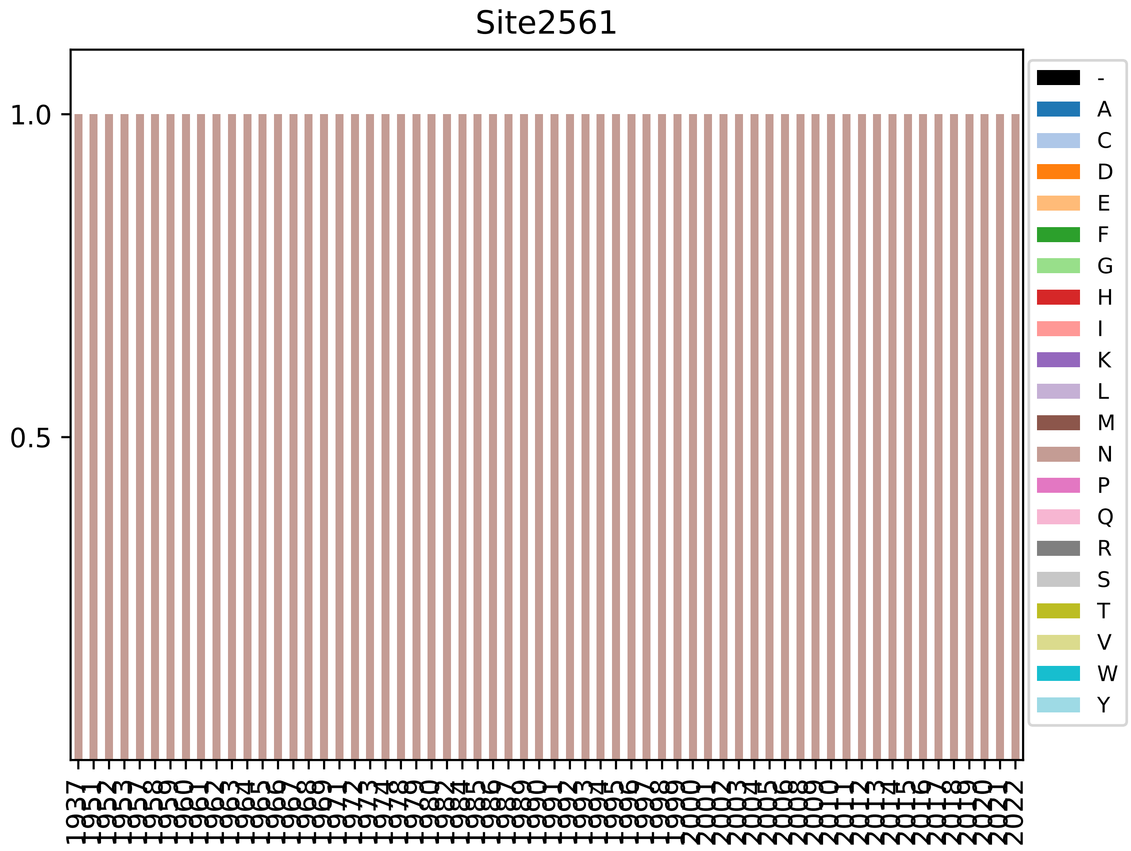
Site2561
1.0
0.5
-
A
C
D
E
F
G
H
I
K
L
M
N
P
Q
R
S
T
V
W
Y

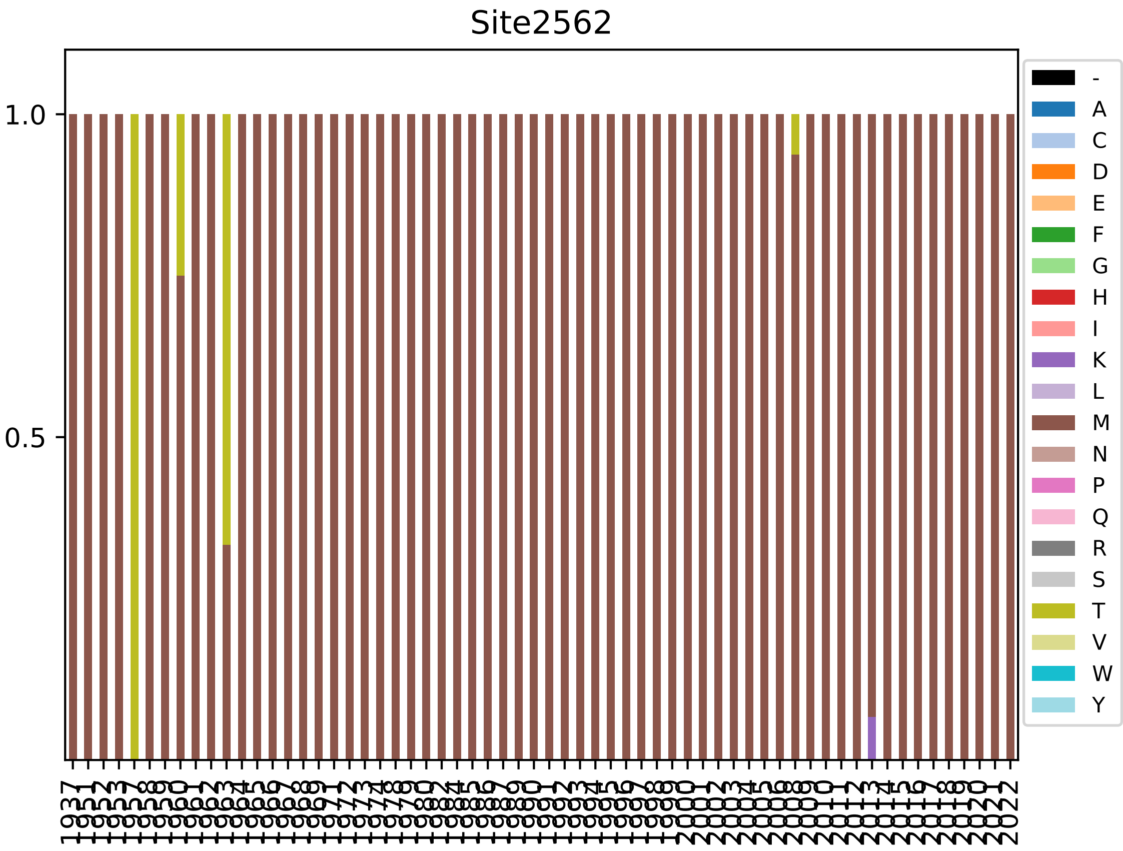
Site2562
1.0
0.5
-
A
C
D
E
F
G
H
I
K
L
M
N
P
Q
R
S
T
V
W
Y

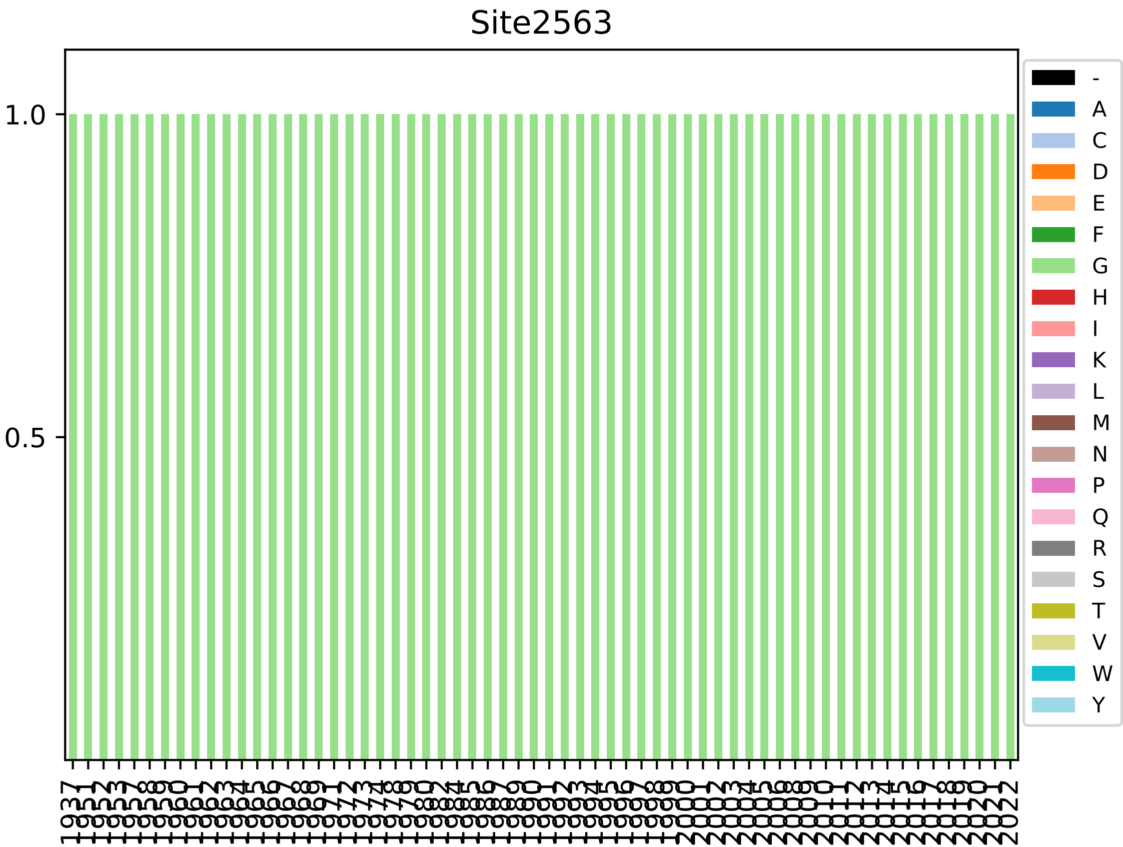
Site2563
1.0
0.5
-
A
C
D
E
F
G
H
I
K
L
M
N
P
Q
R
S
T
V
W
Y

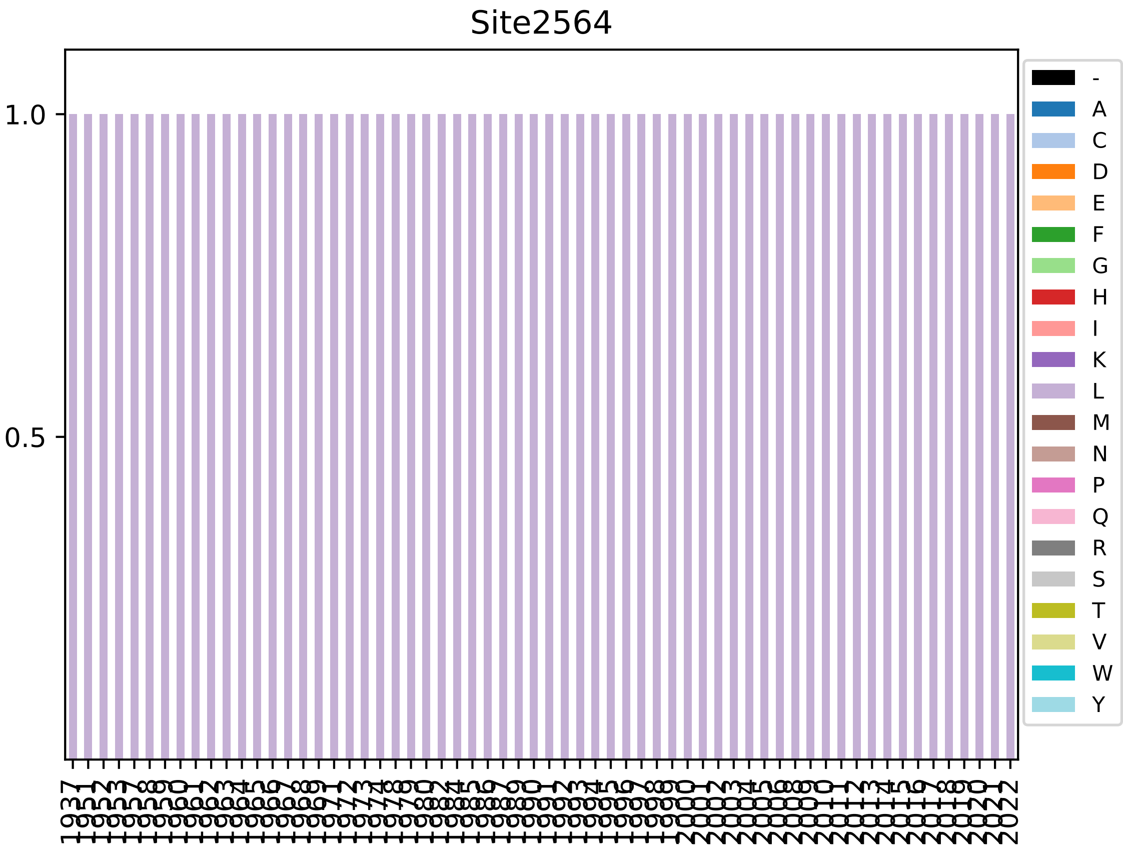
Site2564
1.0
0.5
-
A
C
D
E
F
G
H
I
K
L
M
N
P
Q
R
S
T
V
W
Y

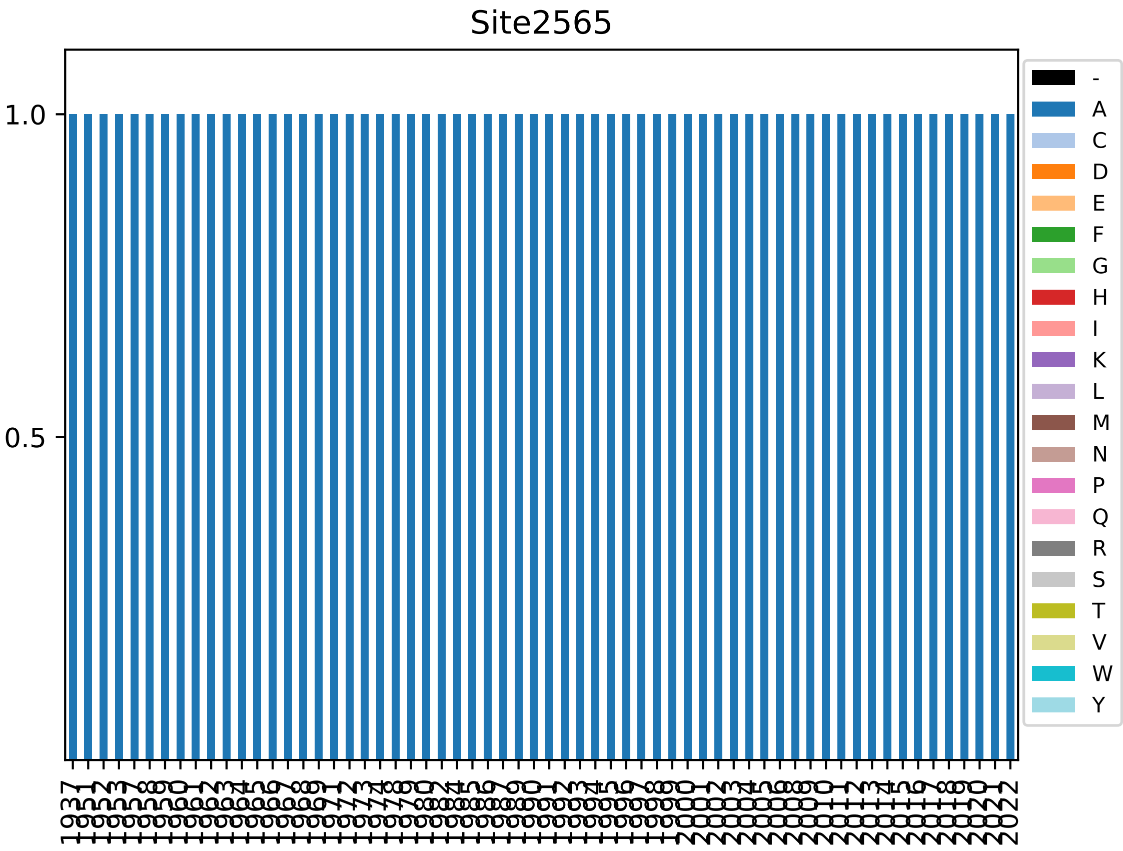
Site2565
1.0
0.5
-
A
C
D
E
F
G
H
I
K
L
M
N
P
Q
R
S
T
V
W
Y

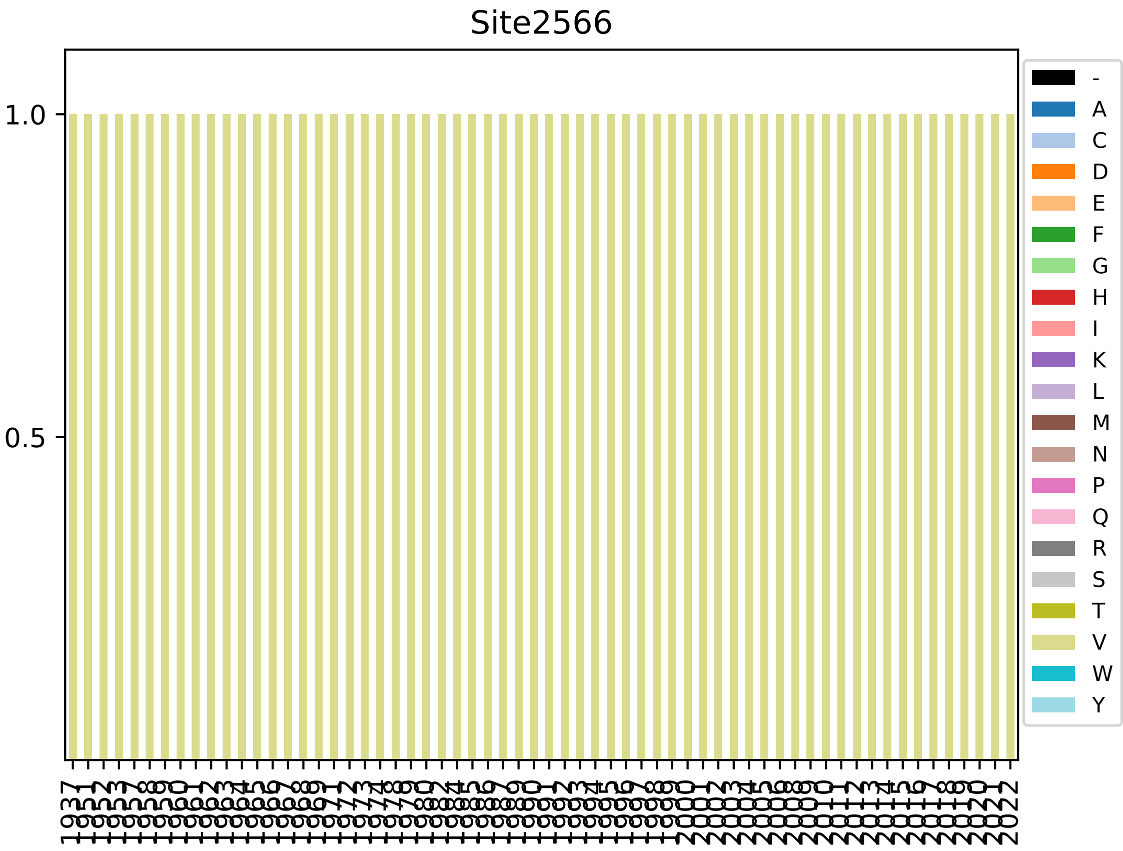
Site2566
1.0
0.5
-
A
C
D
E
F
G
H
I
K
L
M
N
P
Q
R
S
T
V
W
Y

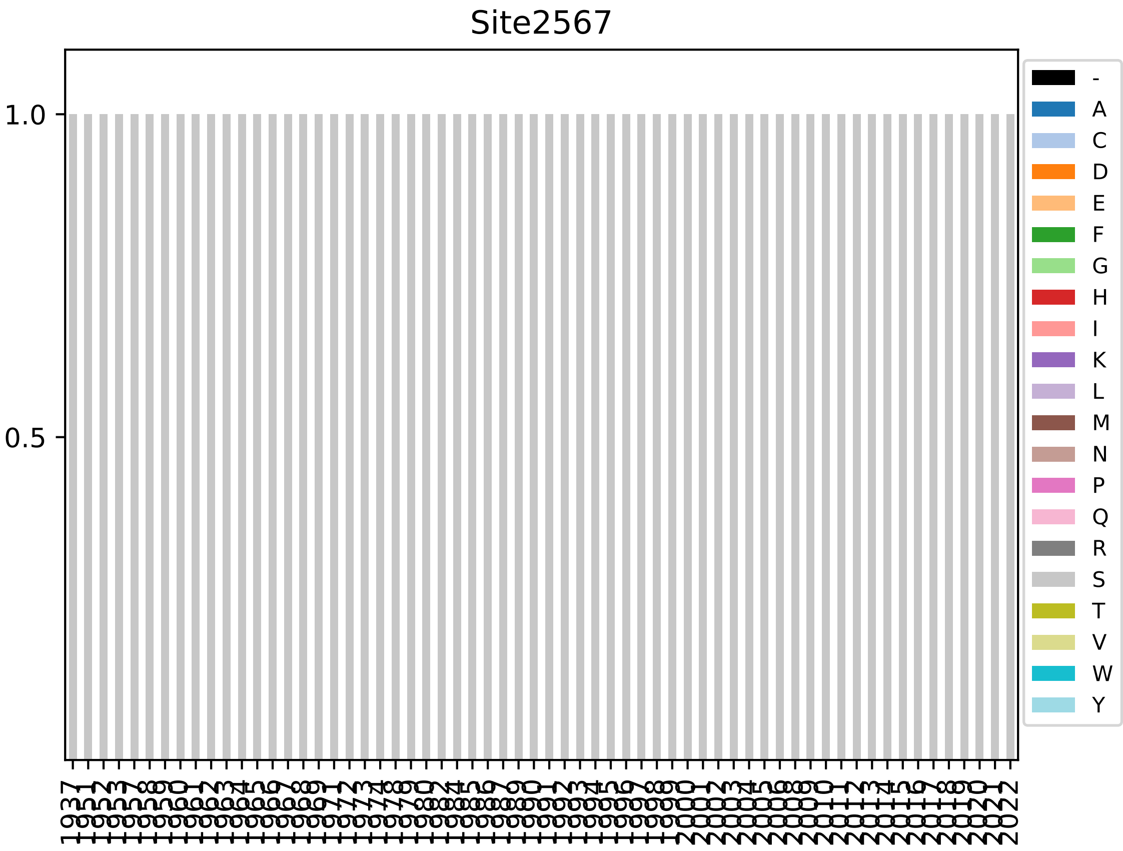
Site2567
1.0
0.5
-
A
C
D
E
F
G
H
I
K
L
M
N
P
Q
R
S
T
V
W
Y

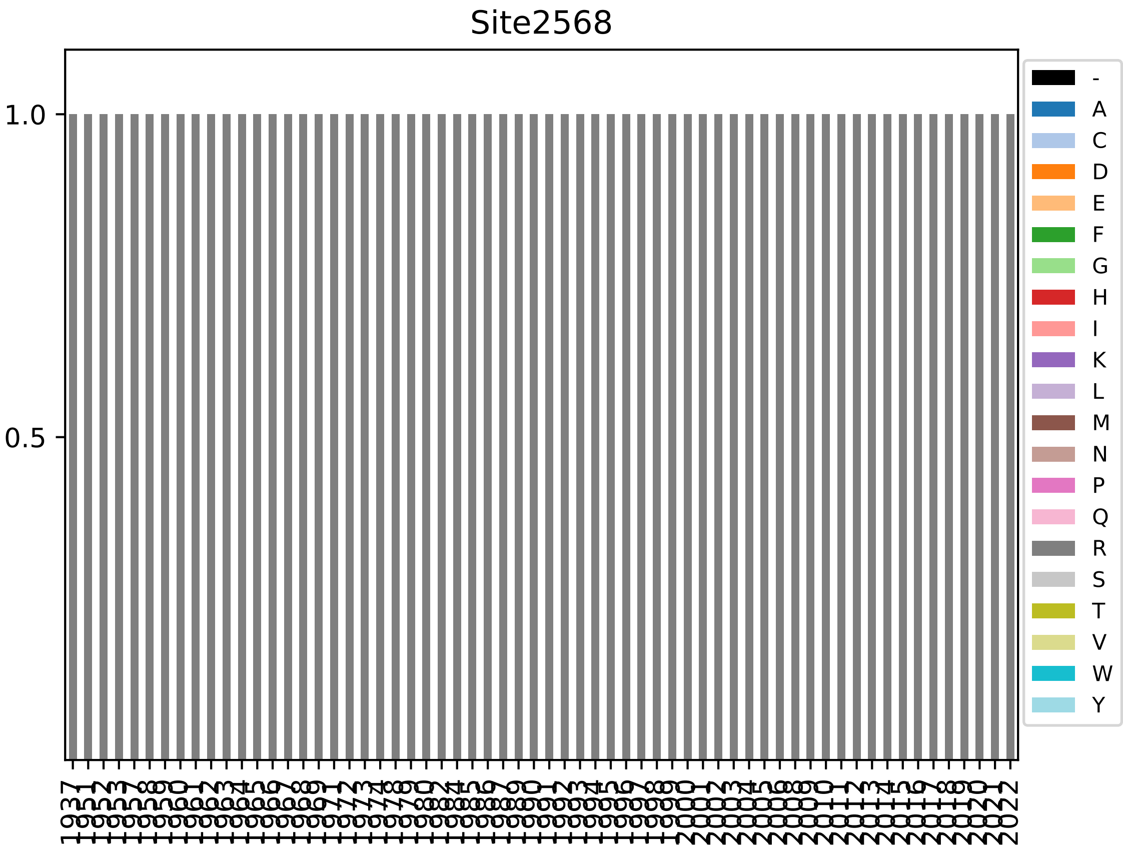
Site2568
1.0
0.5
-
A
C
D
E
F
G
H
I
K
L
M
N
P
Q
R
S
T
V
W
Y

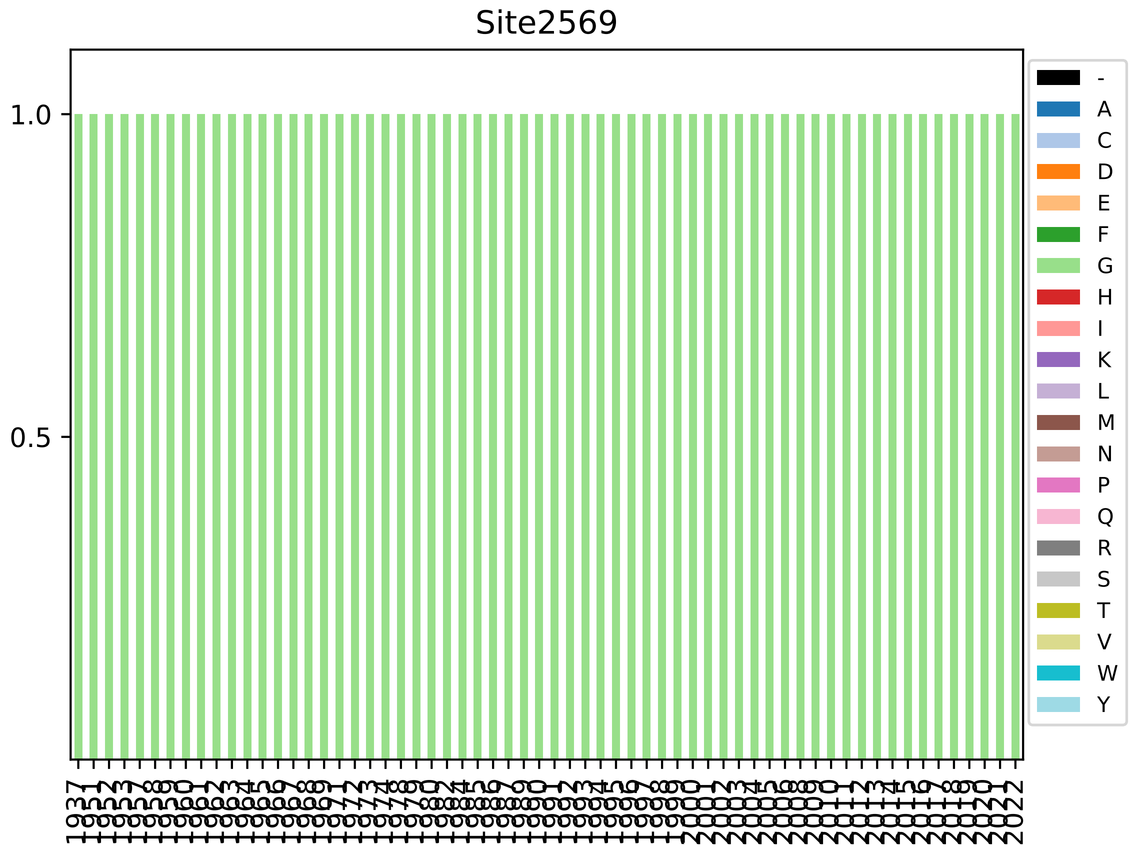
Site2569
1.0
0.5
-
A
C
D
E
F
G
H
I
K
L
M
N
P
Q
R
S
T
V
W
Y

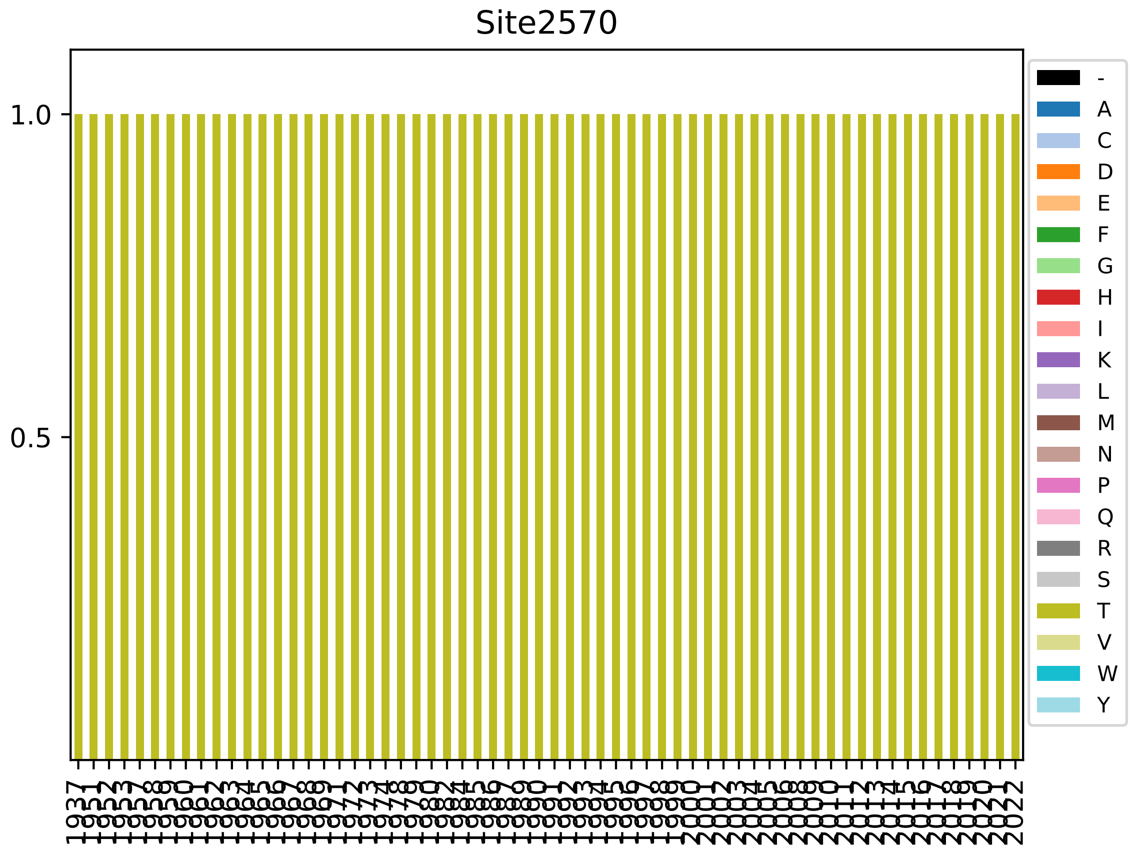
Site2570
1.0
0.5
-
A
C
D
E
F
G
H
I
K
L
M
N
P
Q
R
S
T
V
W
Y

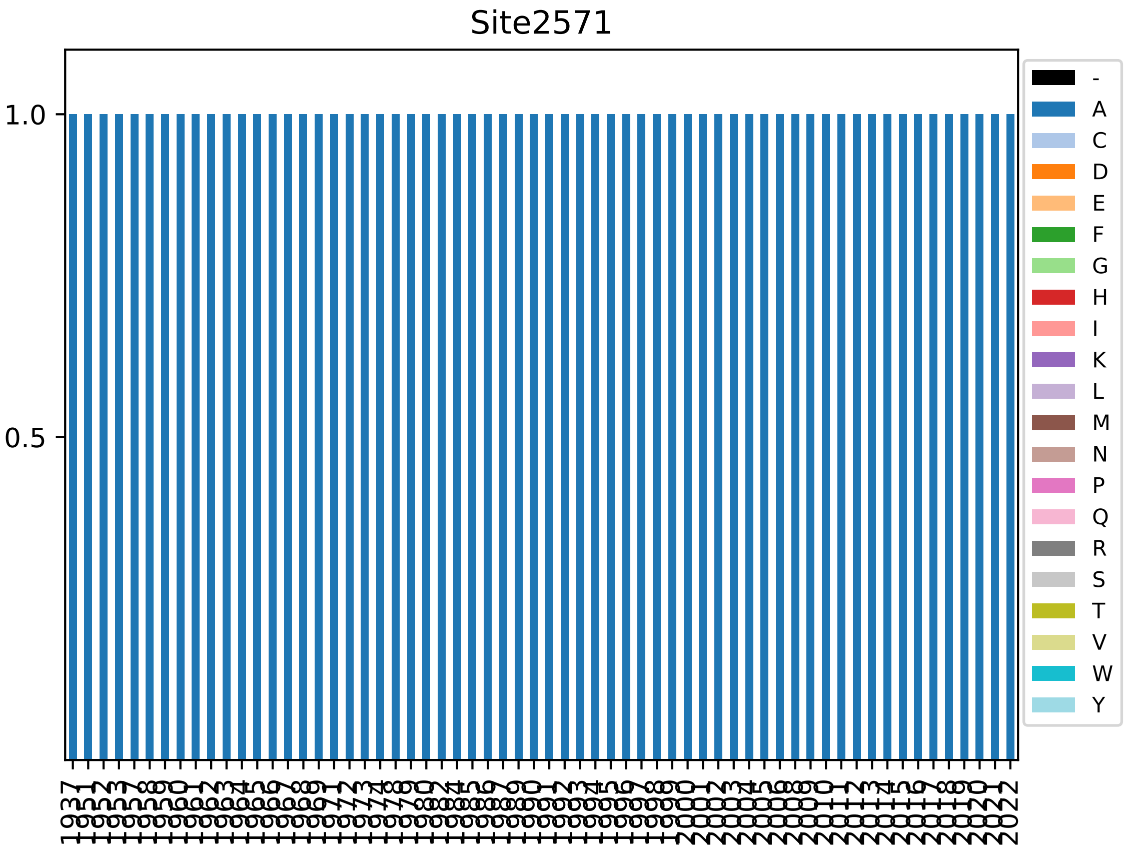
Site2571
1.0
0.5
-
A
C
D
E
F
G
H
I
K
L
M
N
P
Q
R
S
T
V
W
Y

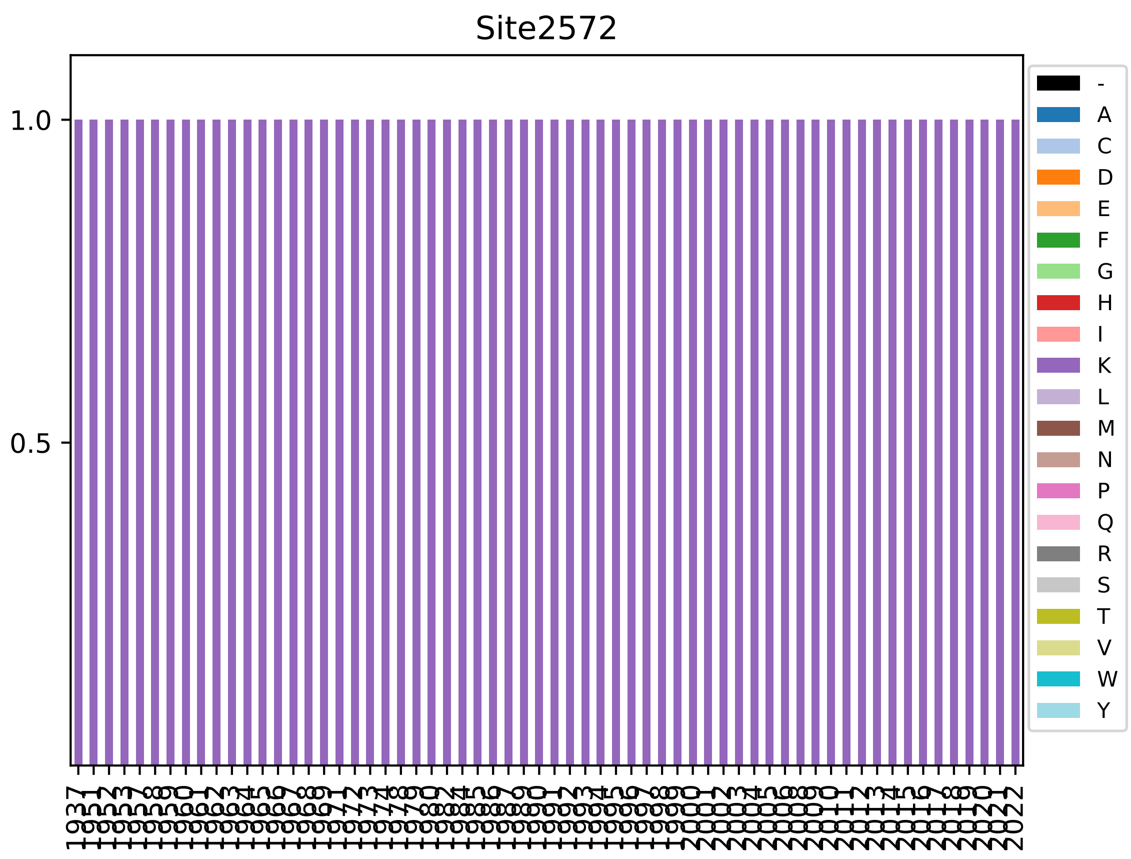
Site2572
1.0
0.5
-
A
C
D
E
F
G
H
I
K
L
M
N
P
Q
R
S
T
V
W
Y

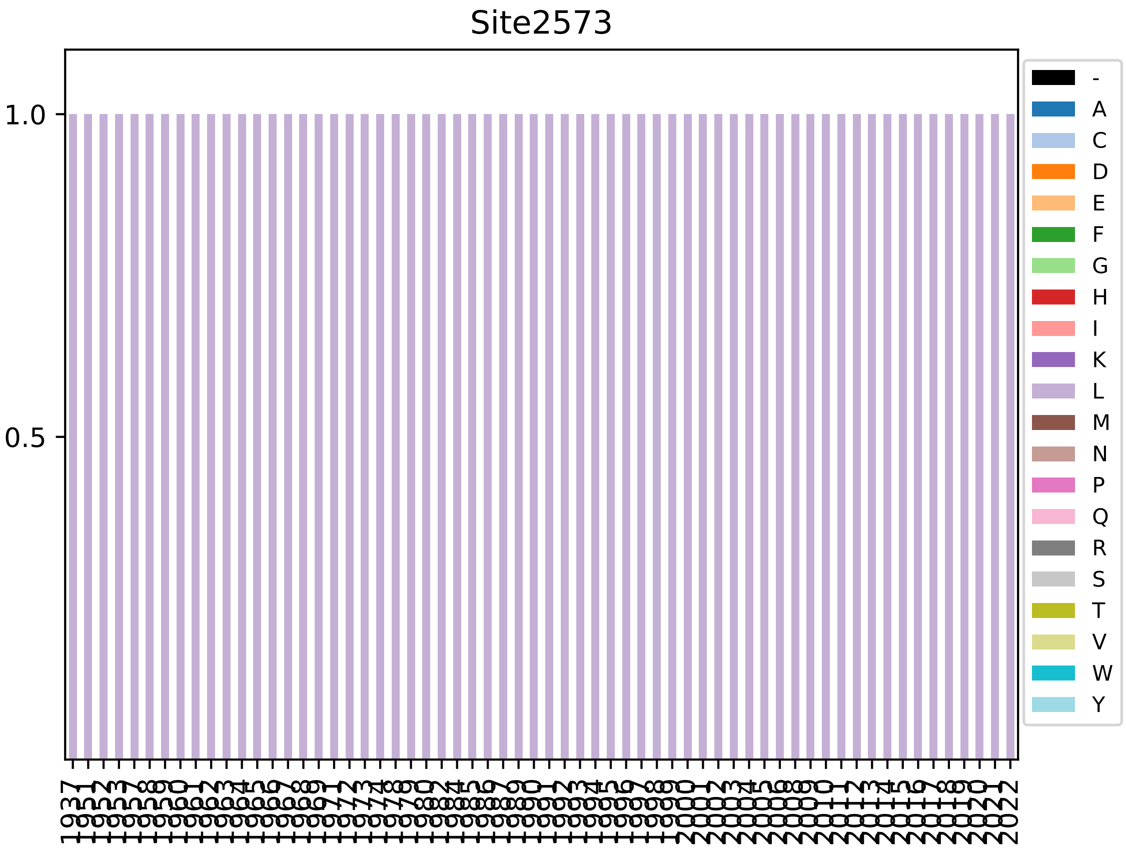
Site2573
1.0
0.5
-
A
C
D
E
F
G
H
I
K
L
M
N
P
Q
R
S
T
V
W
Y

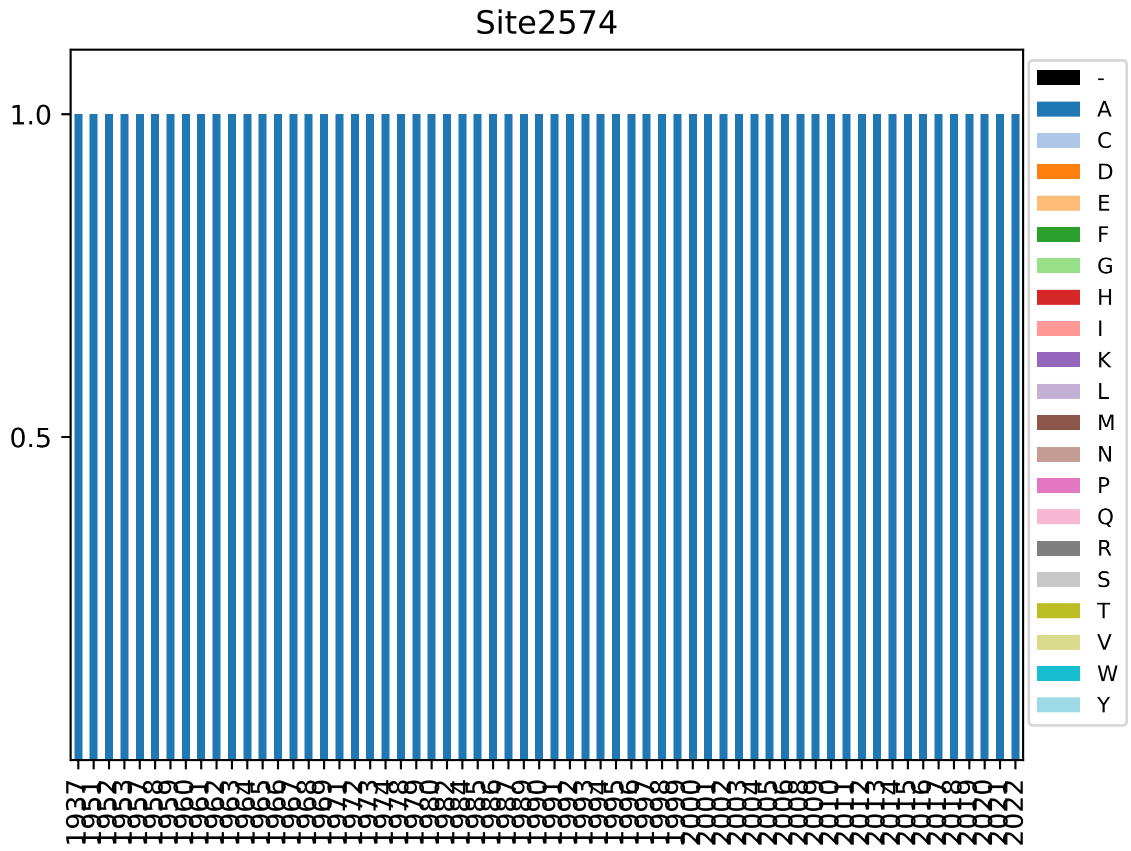
Site2574
1.0
0.5
-
A
C
D
E
F
G
H
I
K
L
M
N
P
Q
R
S
T
V
W
Y

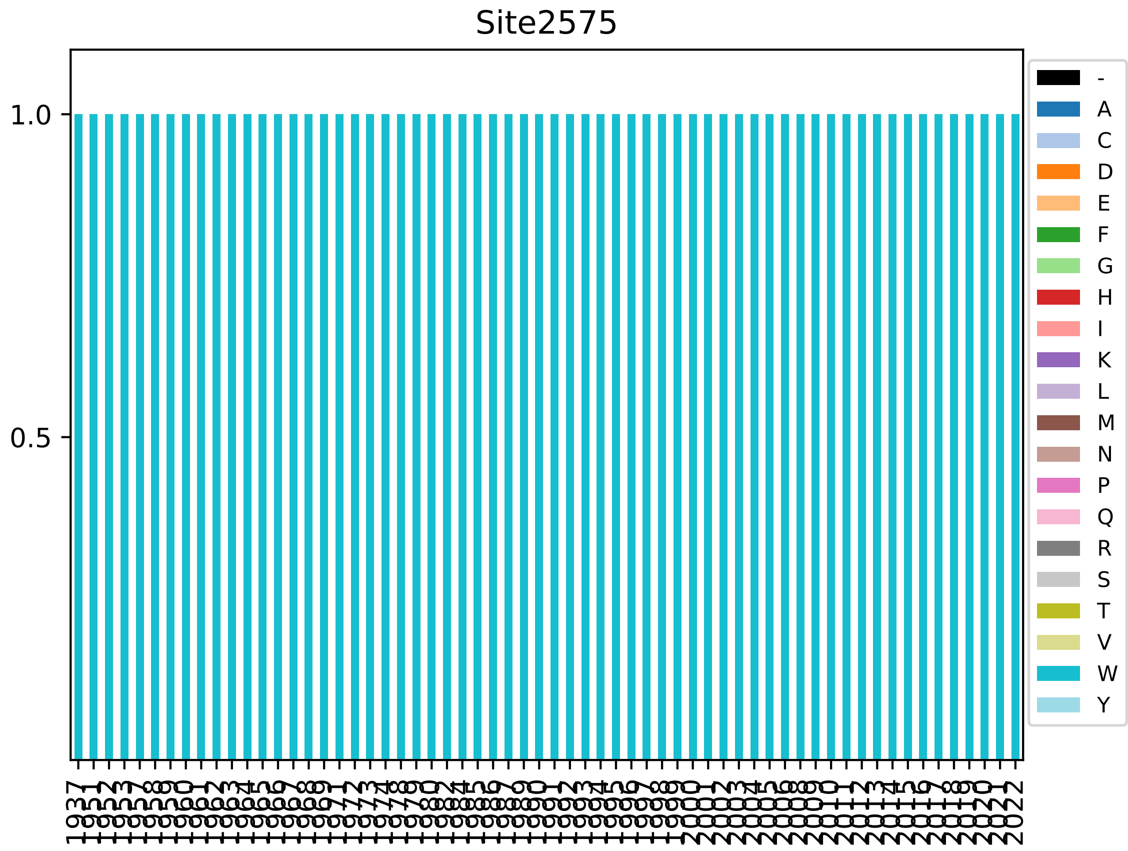
Site2575
1.0
0.5
-
A
C
D
E
F
G
H
I
K
L
M
N
P
Q
R
S
T
V
W
Y

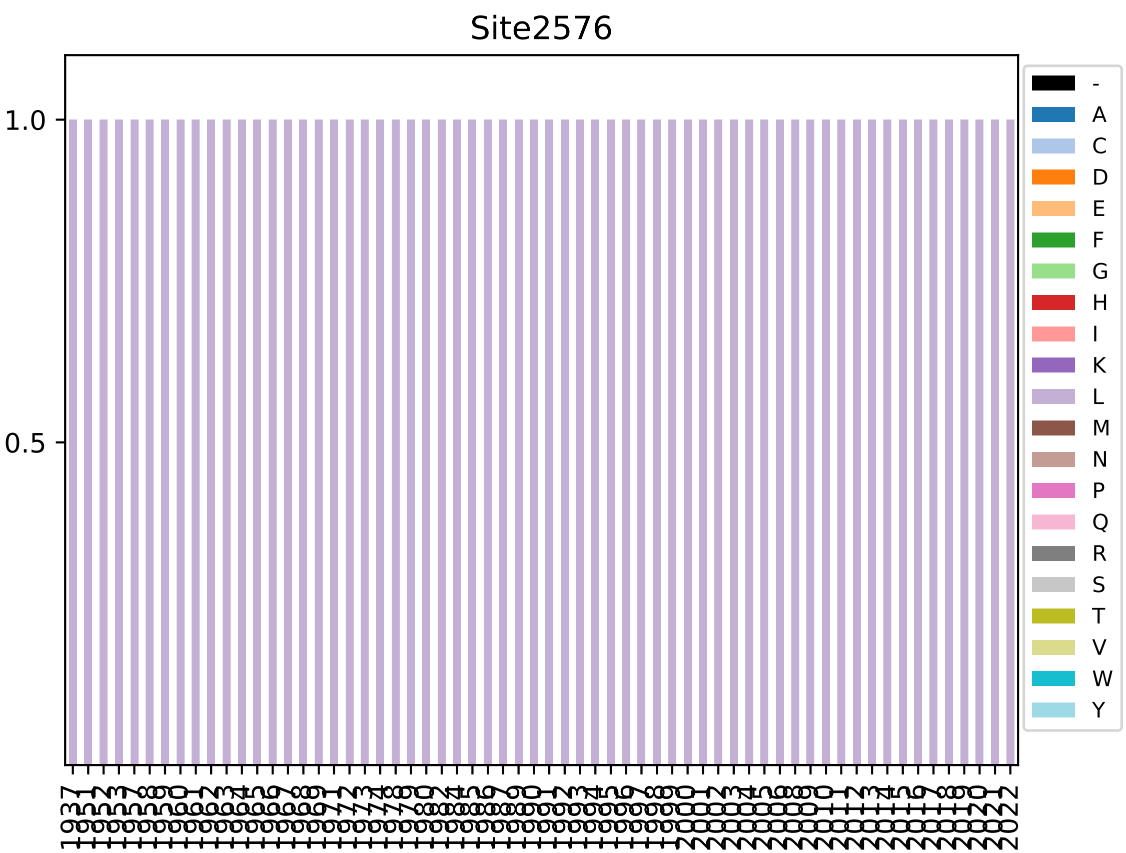
Site2576
1.0
0.5
-
A
C
D
E
F
G
H
I
K
L
M
N
P
Q
R
S
T
V
W
Y

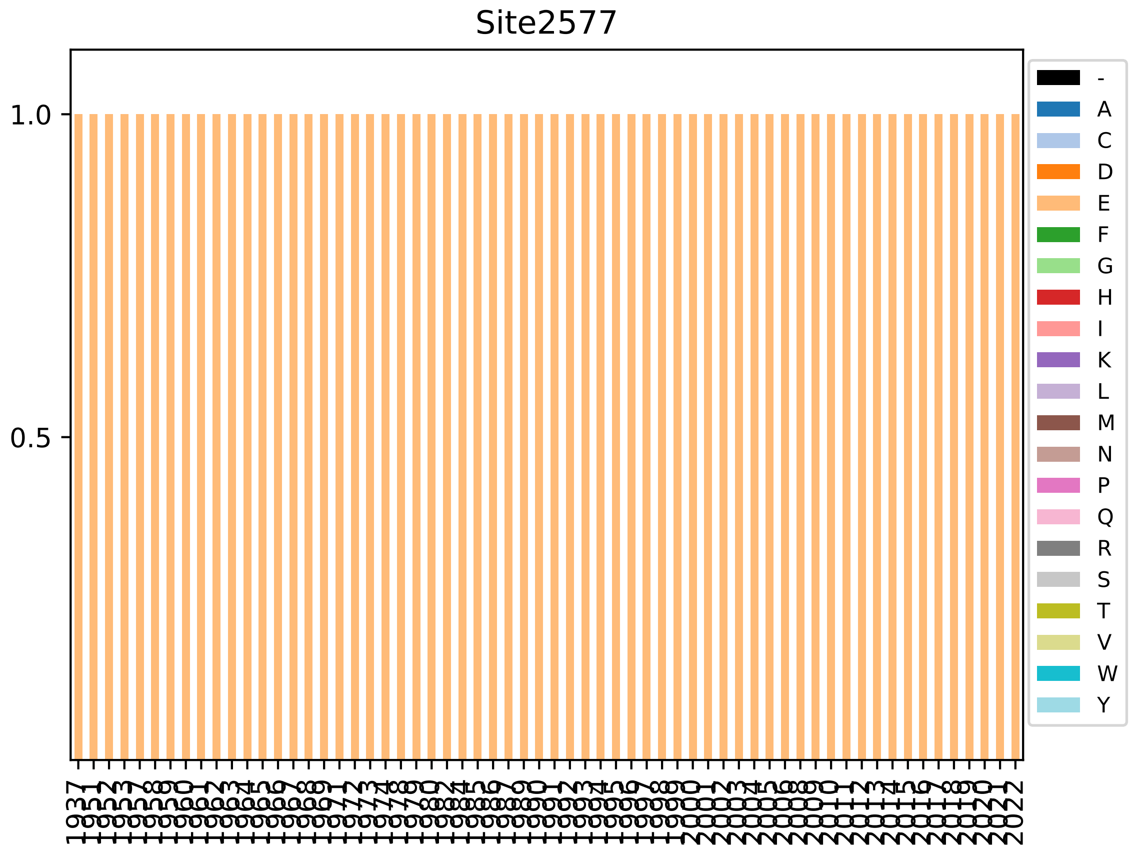
Site2577
1.0
0.5
-
A
C
D
E
F
G
H
I
K
L
M
N
P
Q
R
S
T
V
W
Y

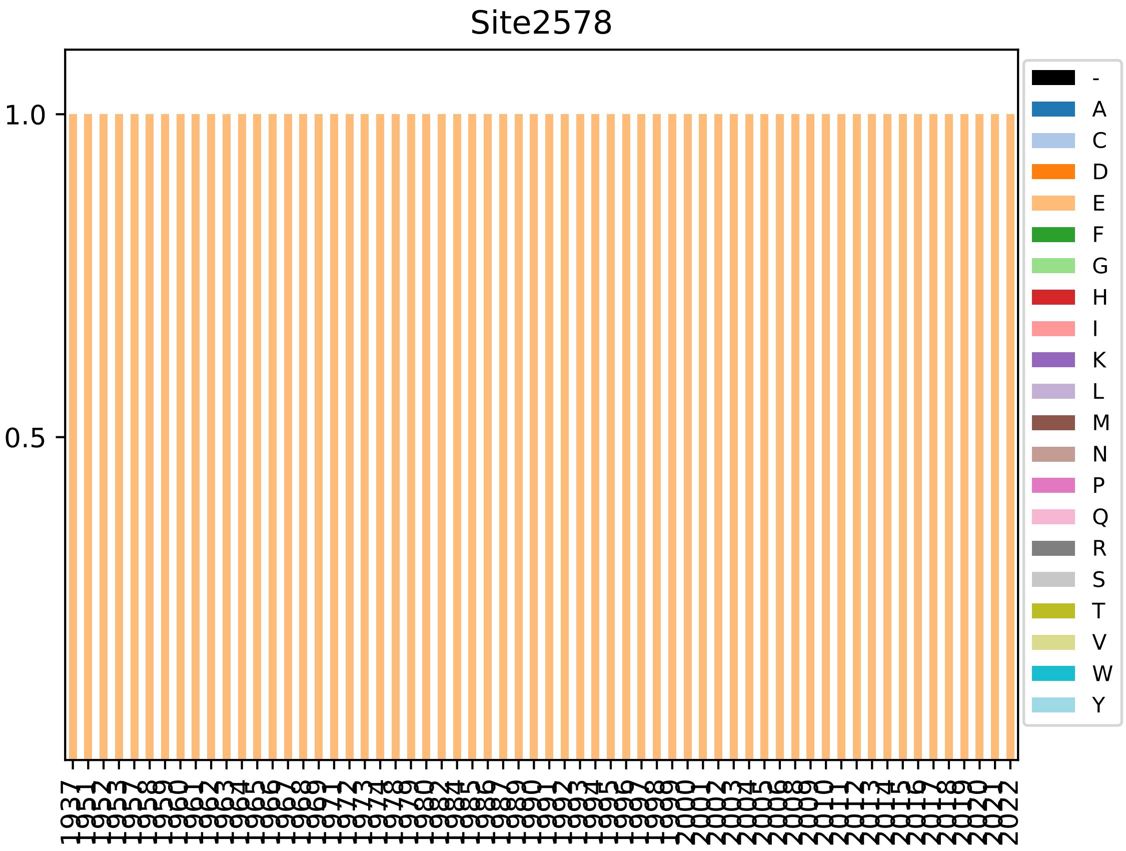

Site2578
1.0
0.5
-
A
C
D
E
F
G
H
I
K
L
M
N
P
Q
R
S
T
V
W
Y

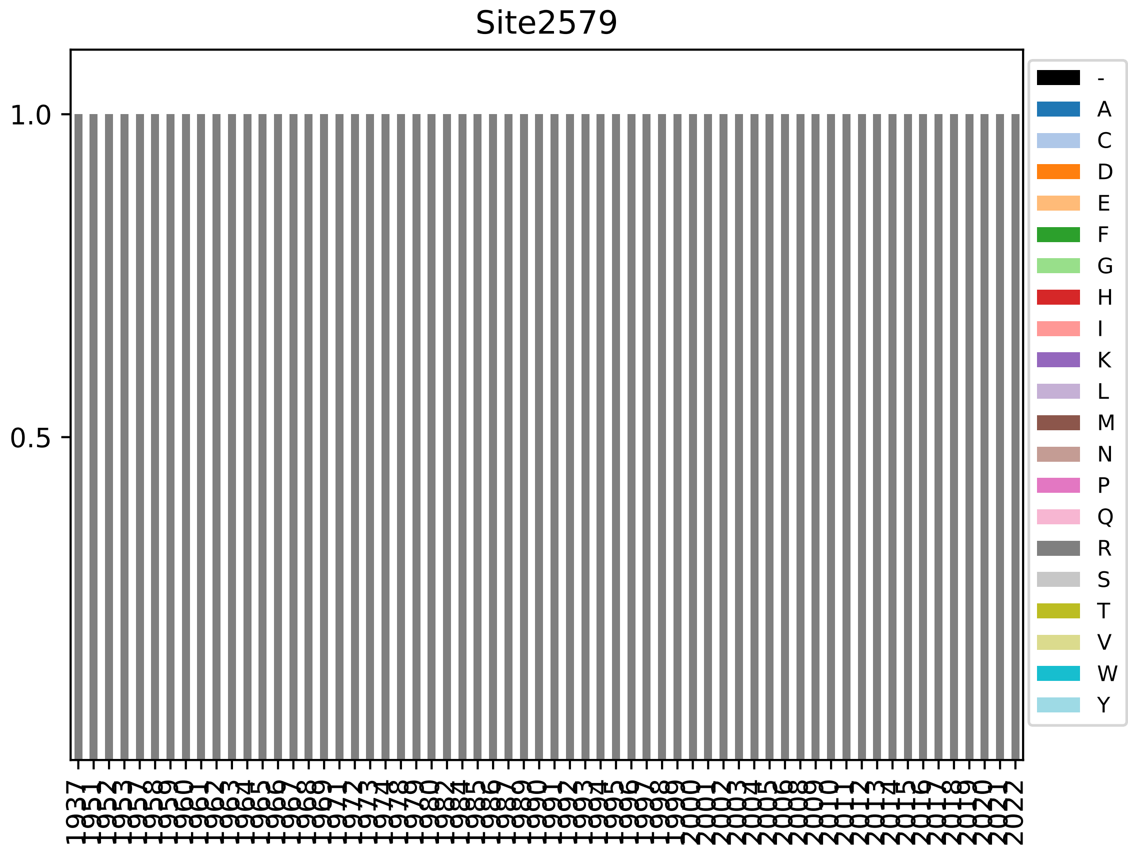
Site2579
1.0
0.5
-
A
C
D
E
F
G
H
I
K
L
M
N
P
Q
R
S
T
V
W
Y

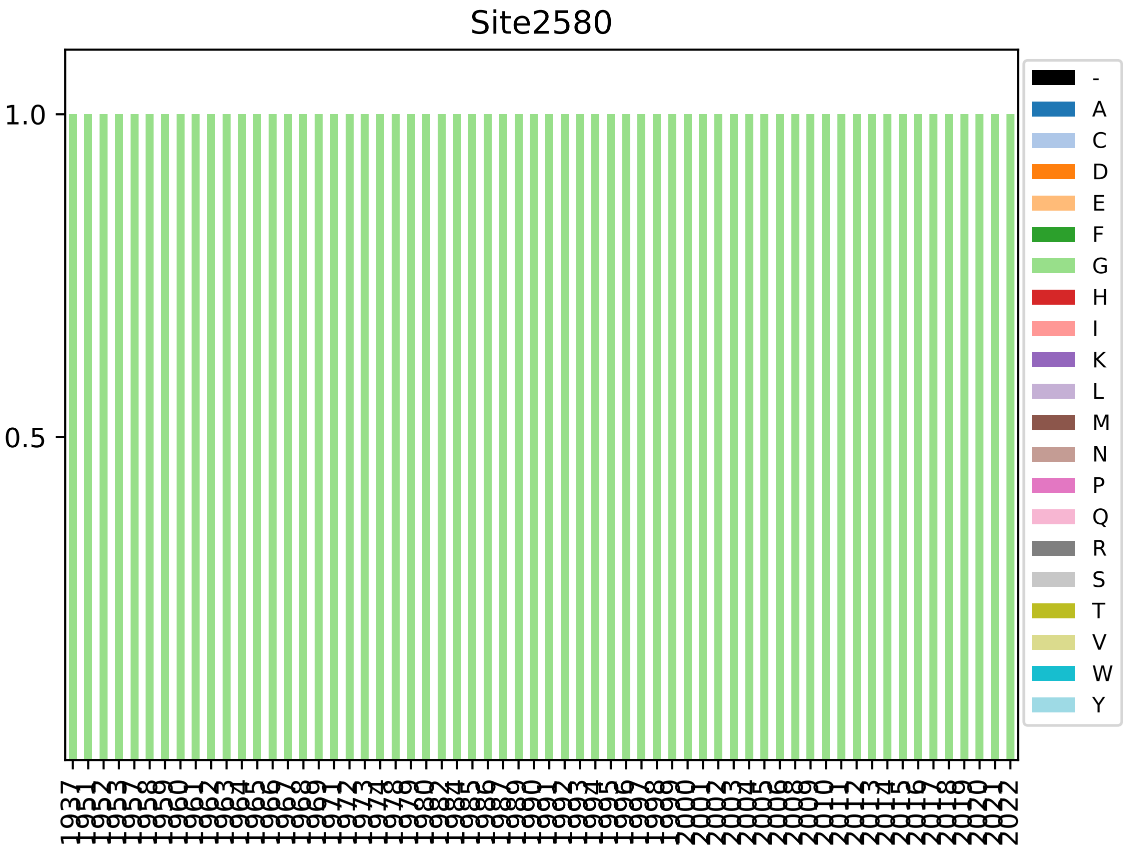
Site2580
1.0
0.5
-
A
C
D
E
F
G
H
I
K
L
M
N
P
Q
R
S
T
V
W
Y

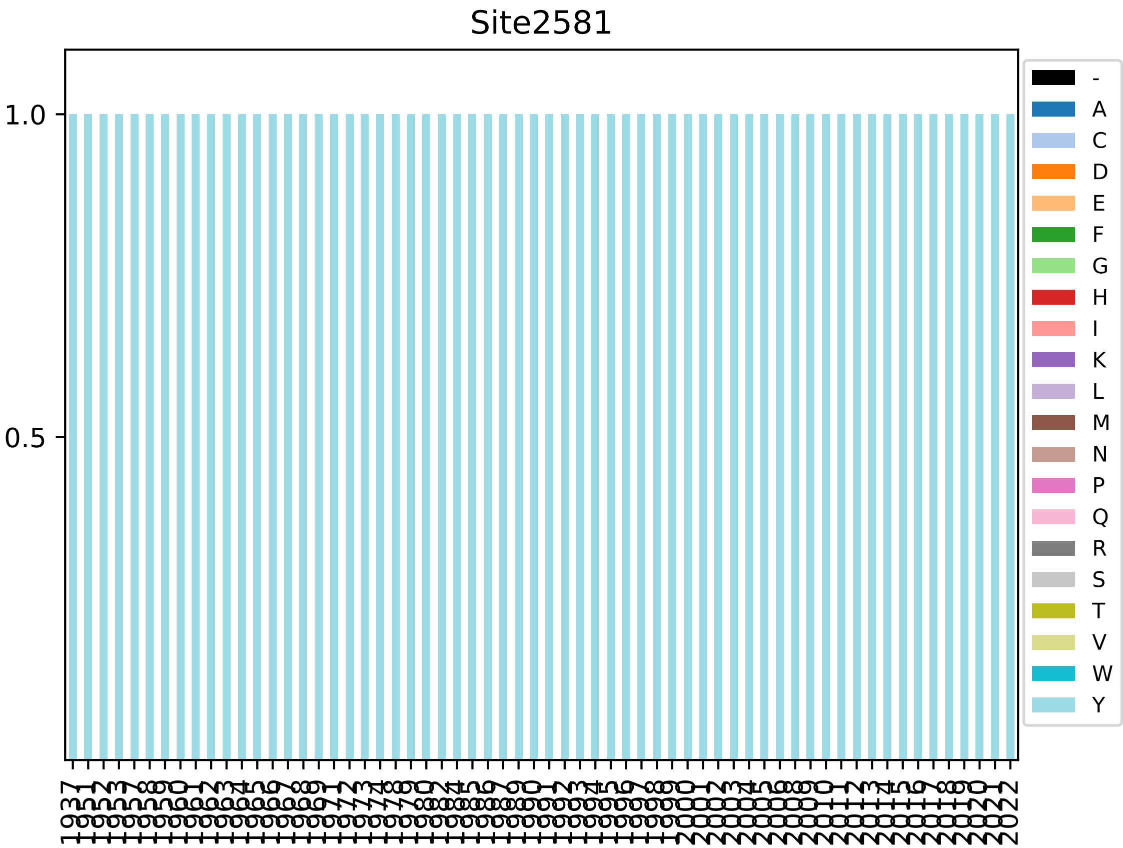
Site2581
1.0
0.5
-
A
C
D
E
F
G
H
I
K
L
M
N
P
Q
R
S
T
V
W
Y

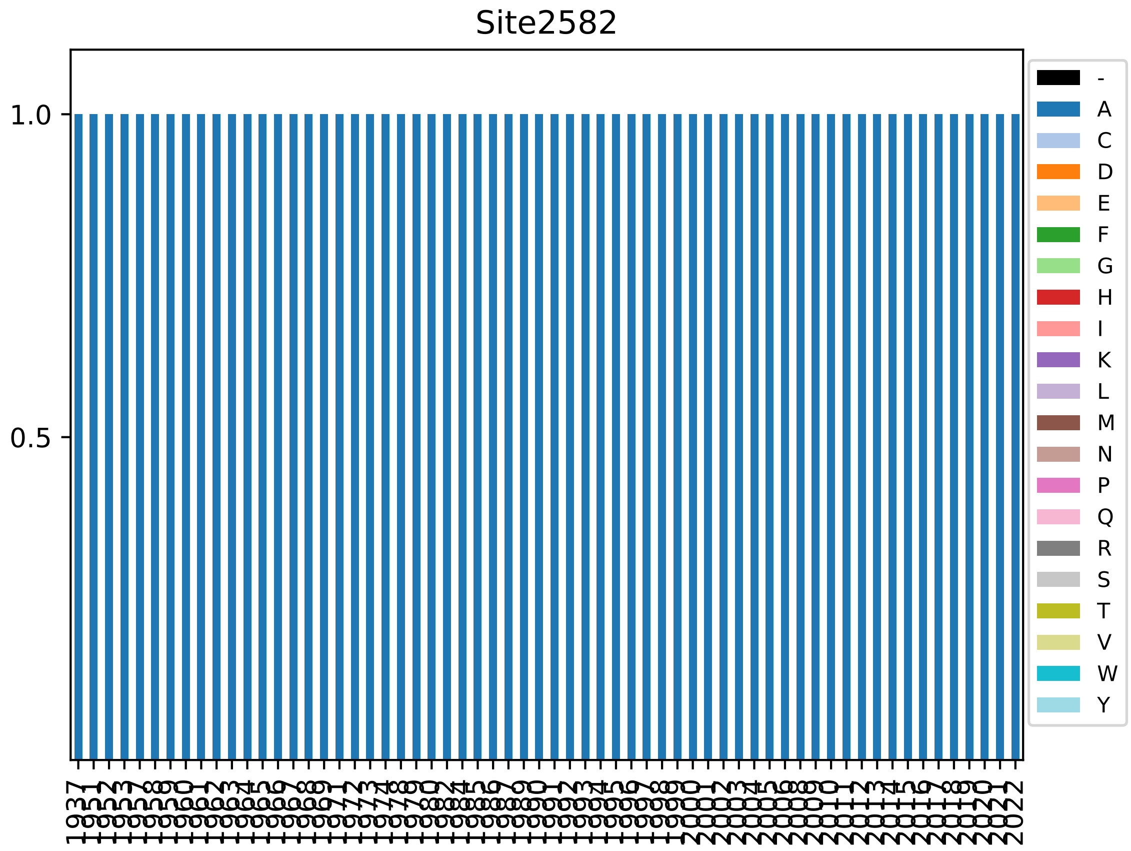
Site2582
1.0
0.5
-
A
C
D
E
F
G
H
I
K
L
M
N
P
Q
R
S
T
V
W
Y

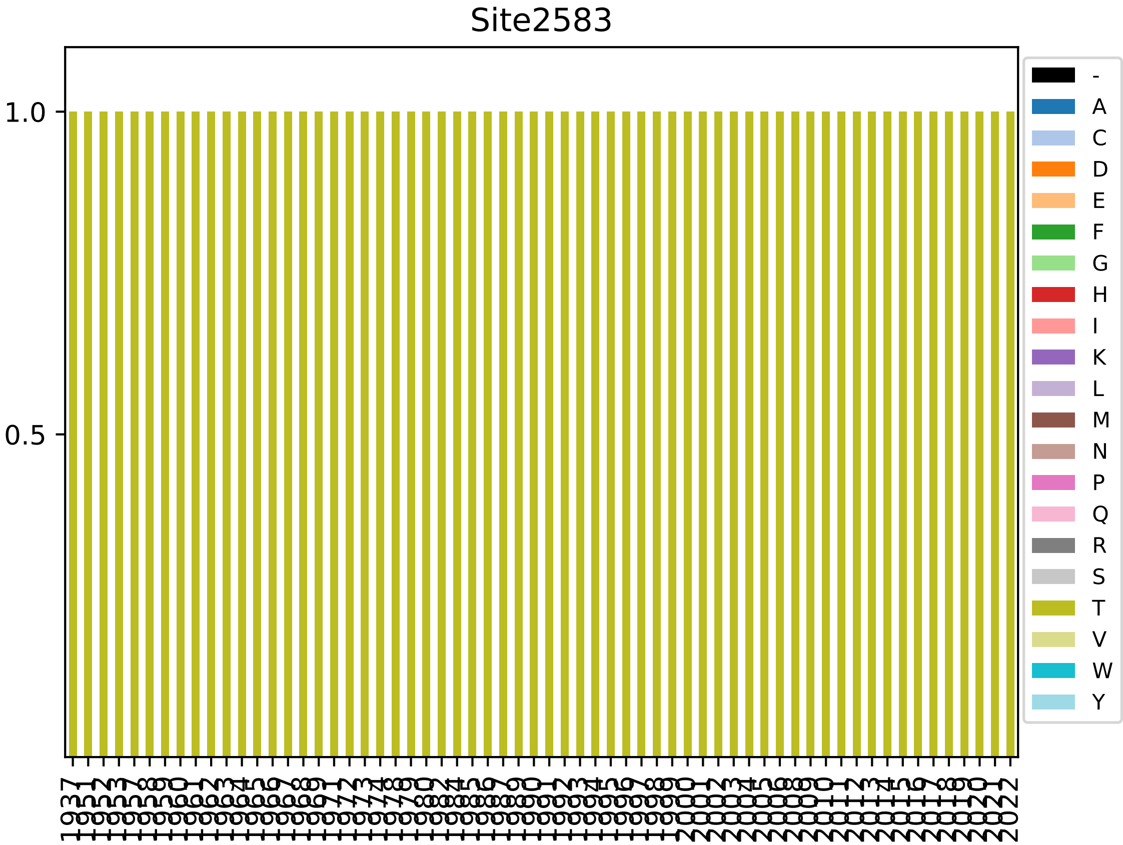
Site2583
1.0
0.5
-
A
C
D
E
F
G
H
I
K
L
M
N
P
Q
R
S
T
V
W
Y

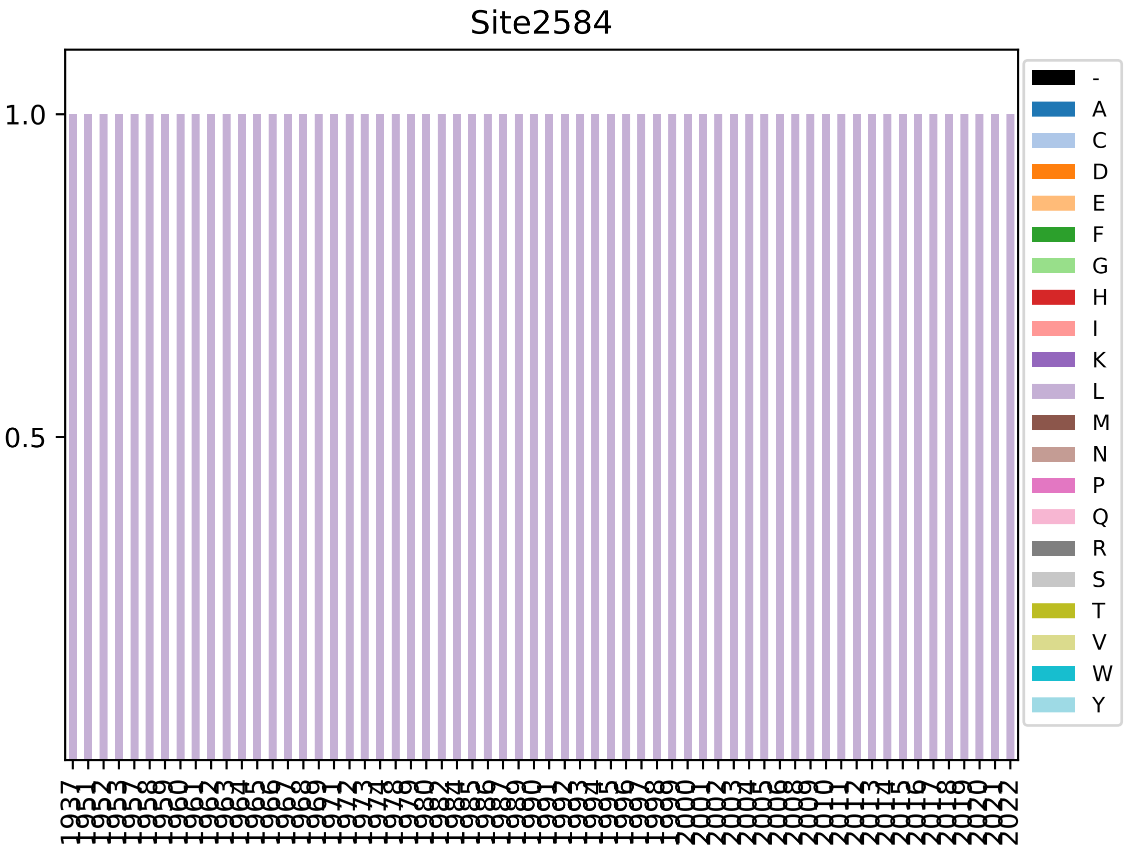

Site2584
1.0
0.5
-
A
C
D
E
F
G
H
I
K
L
M
N
P
Q
R
S
T
V
W
Y

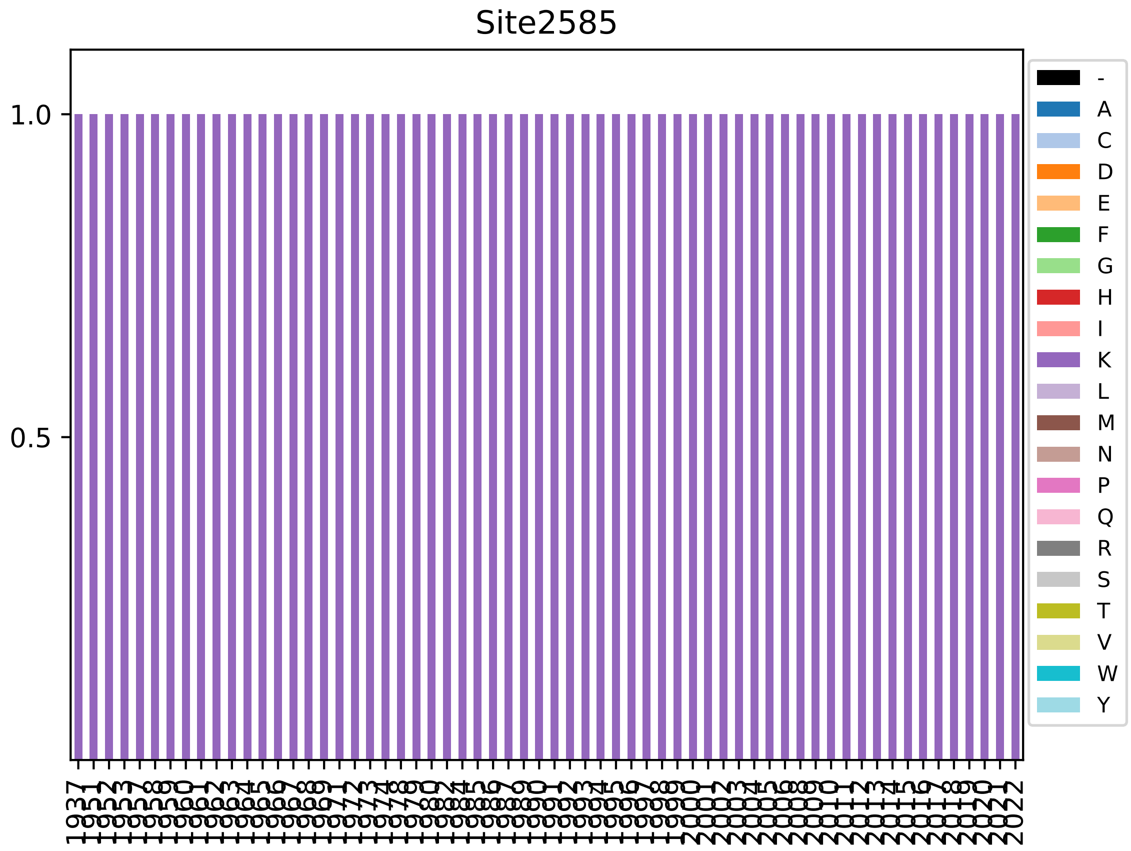
Site2585
1.0
0.5
-
A
C
D
E
F
G
H
I
K
L
M
N
P
Q
R
S
T
V
W
Y

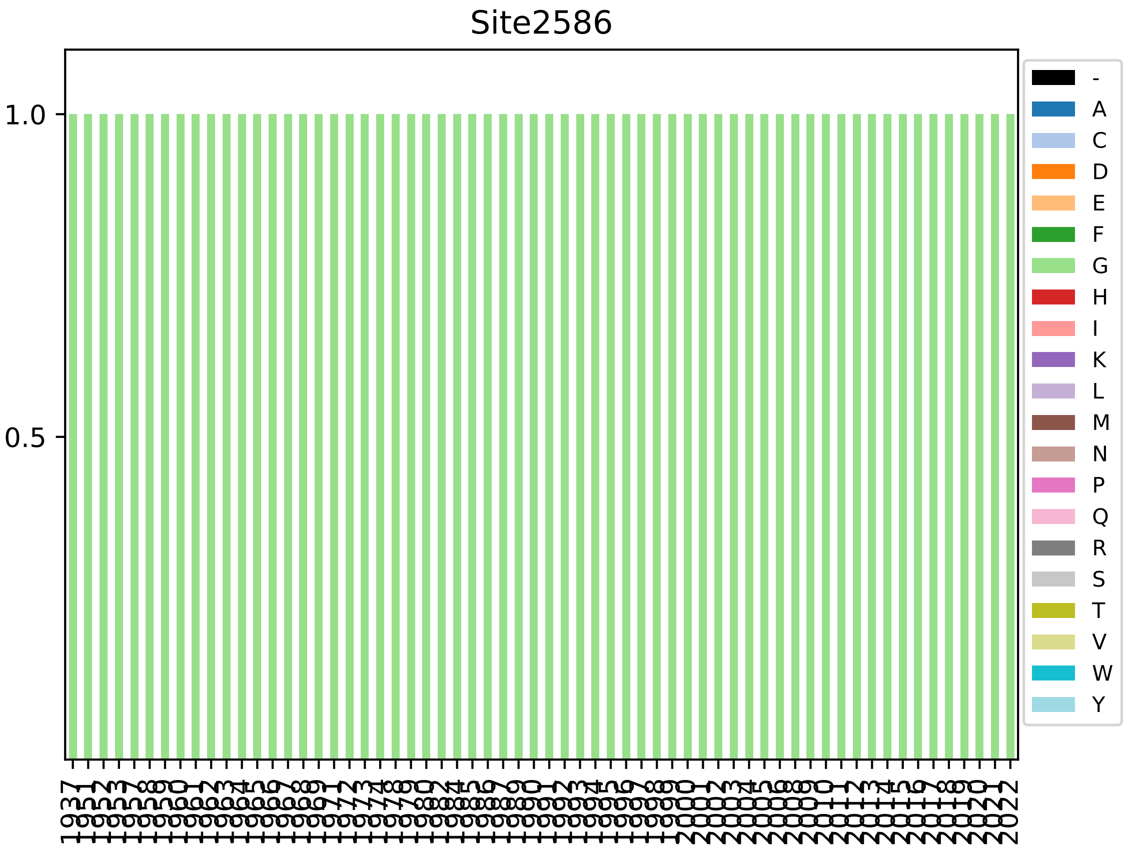
Site2586
1.0
0.5
-
A
C
D
E
F
G
H
I
K
L
M
N
P
Q
R
S
T
V
W
Y

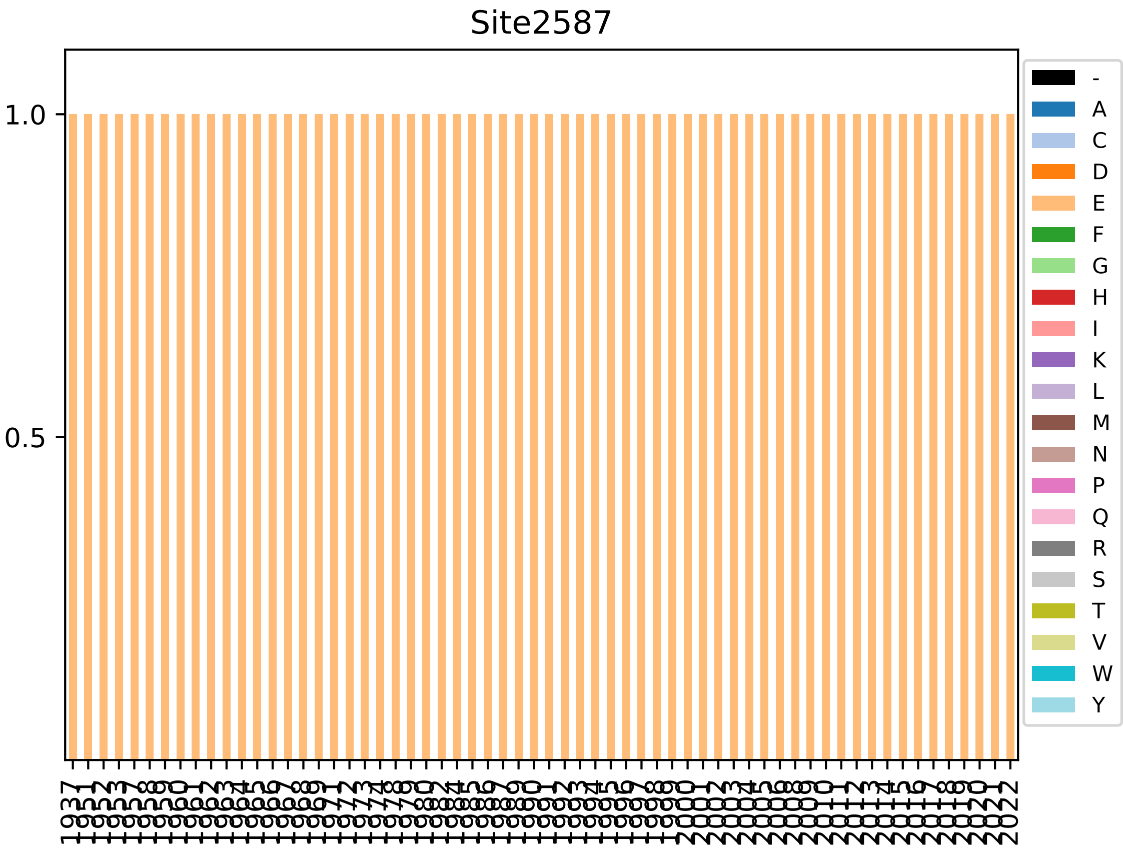
Site2587
1.0
0.5
-
A
C
D
E
F
G
H
I
K
L
M
N
P
Q
R
S
T
V
W
Y

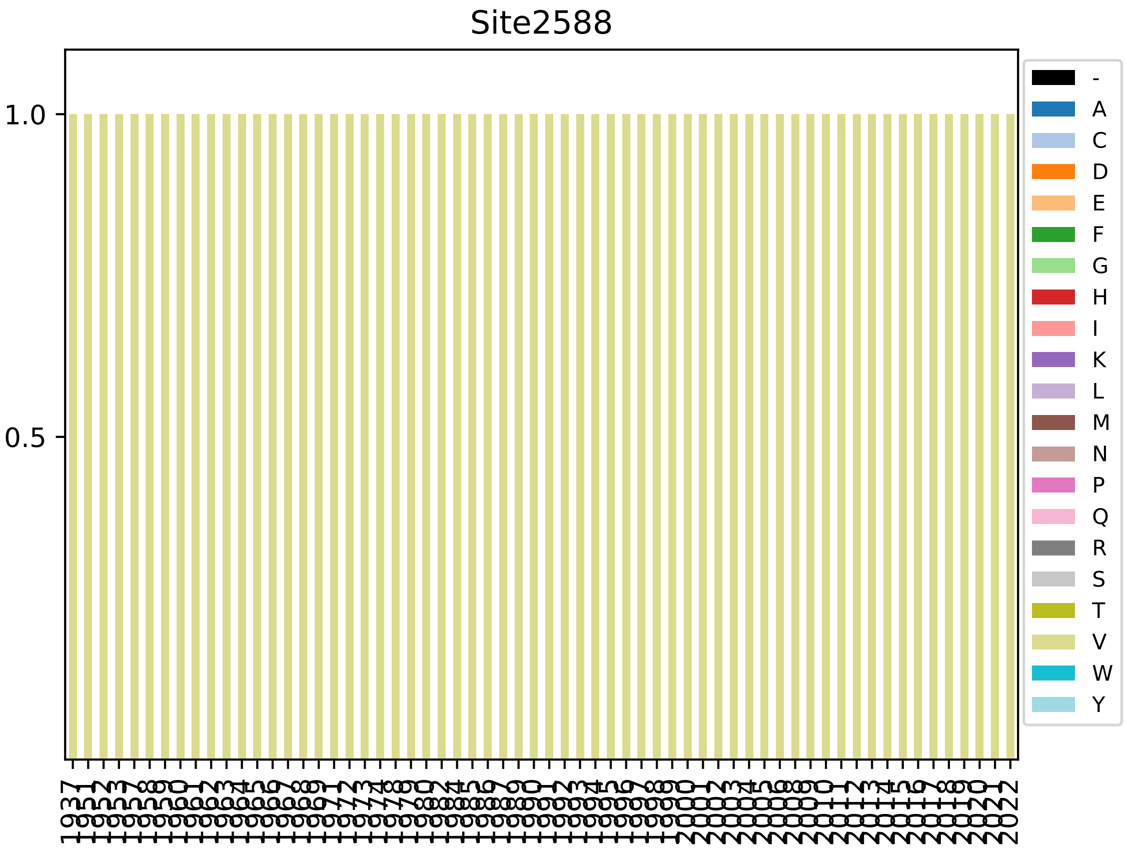
Site2588
1.0
0.5
-
A
C
D
E
F
G
H
I
K
L
M
N
P
Q
R
S
T
V
W
Y

Site2589

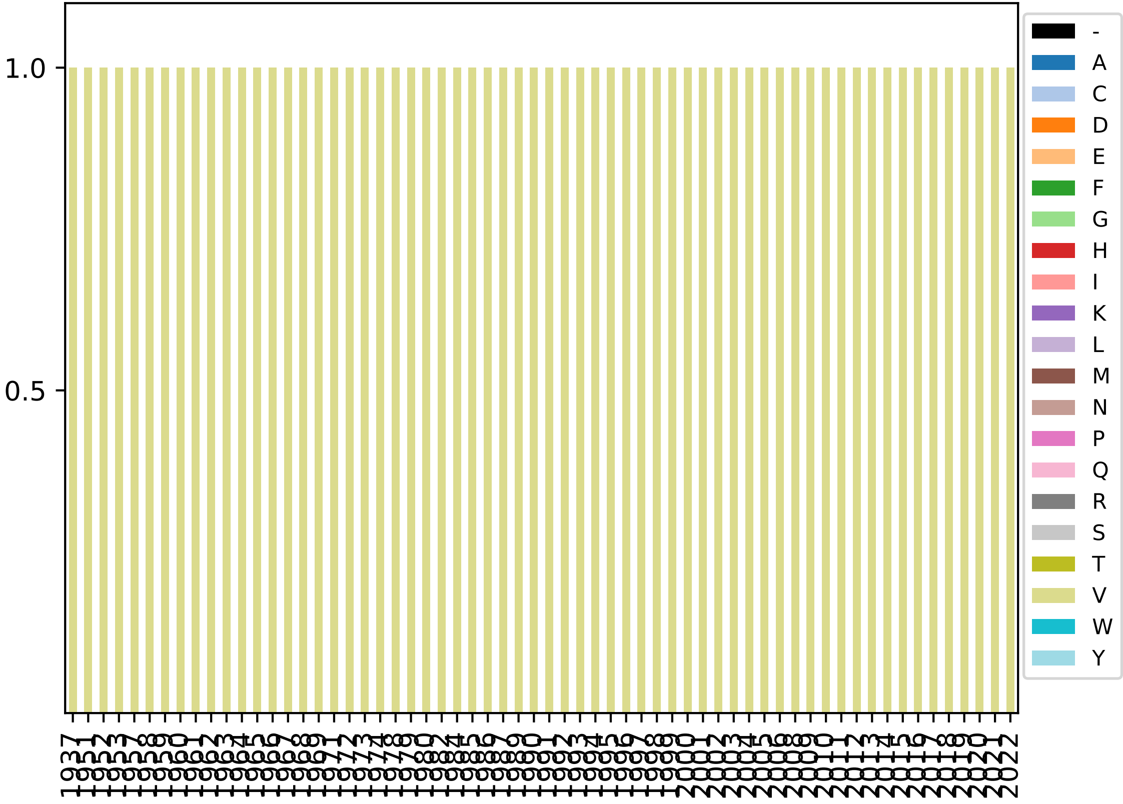

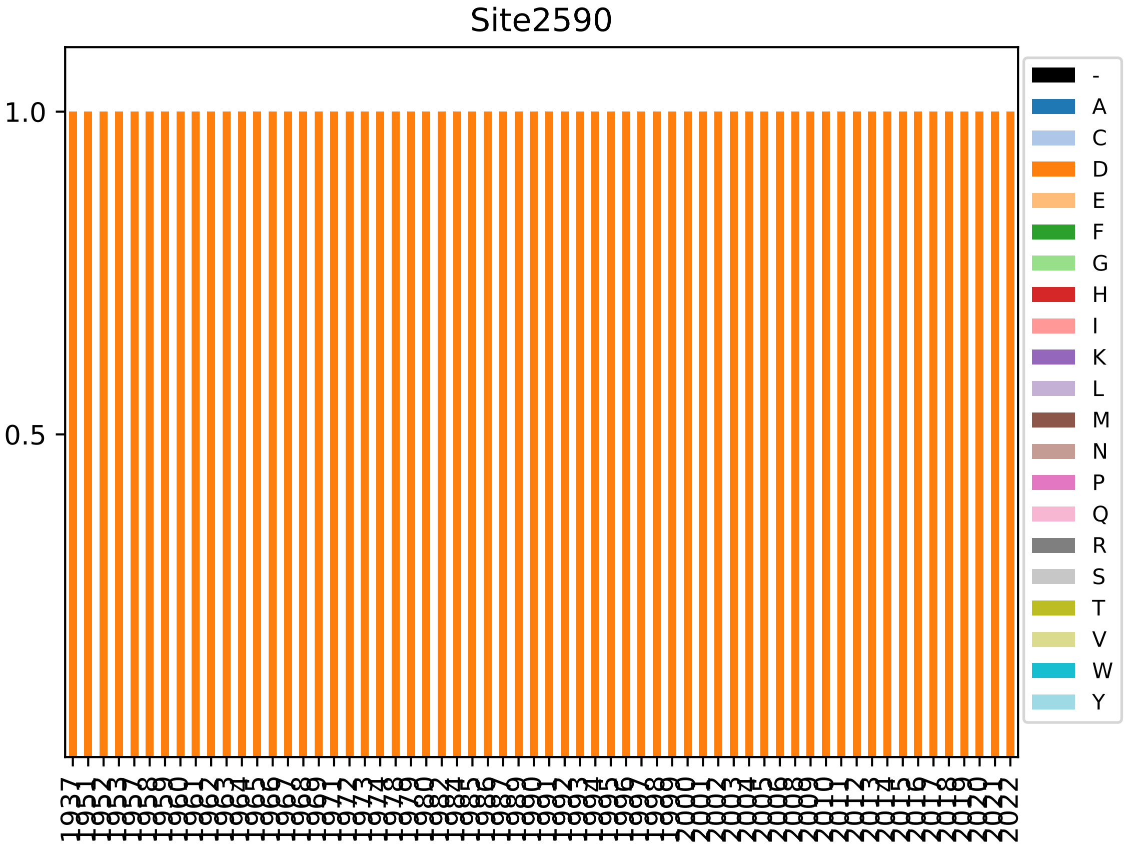
Site2590
1.0
0.5
-
A
C
D
E
F
G
H
I
K
L
M
N
P
Q
R
S
T
V
W
Y

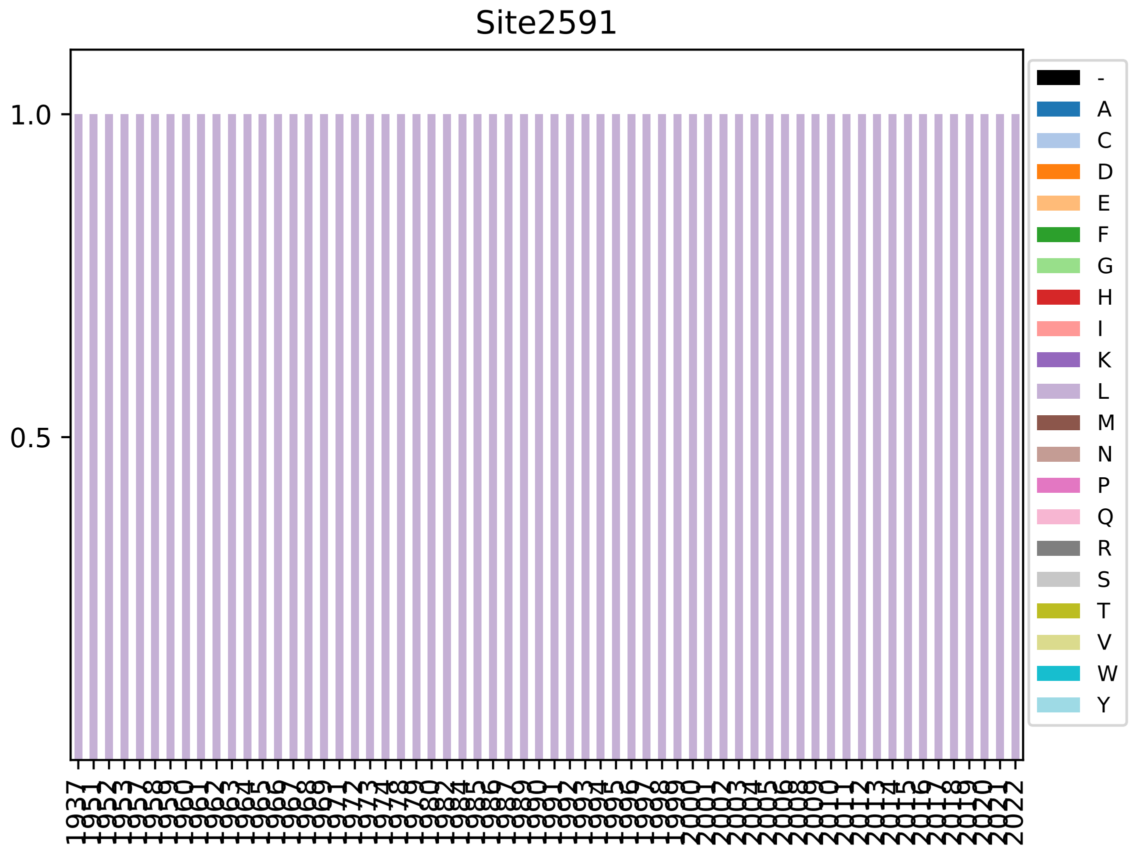
Site2591
1.0
0.5
-
A
C
D
E
F
G
H
I
K
L
M
N
P
Q
R
S
T
V
W
Y

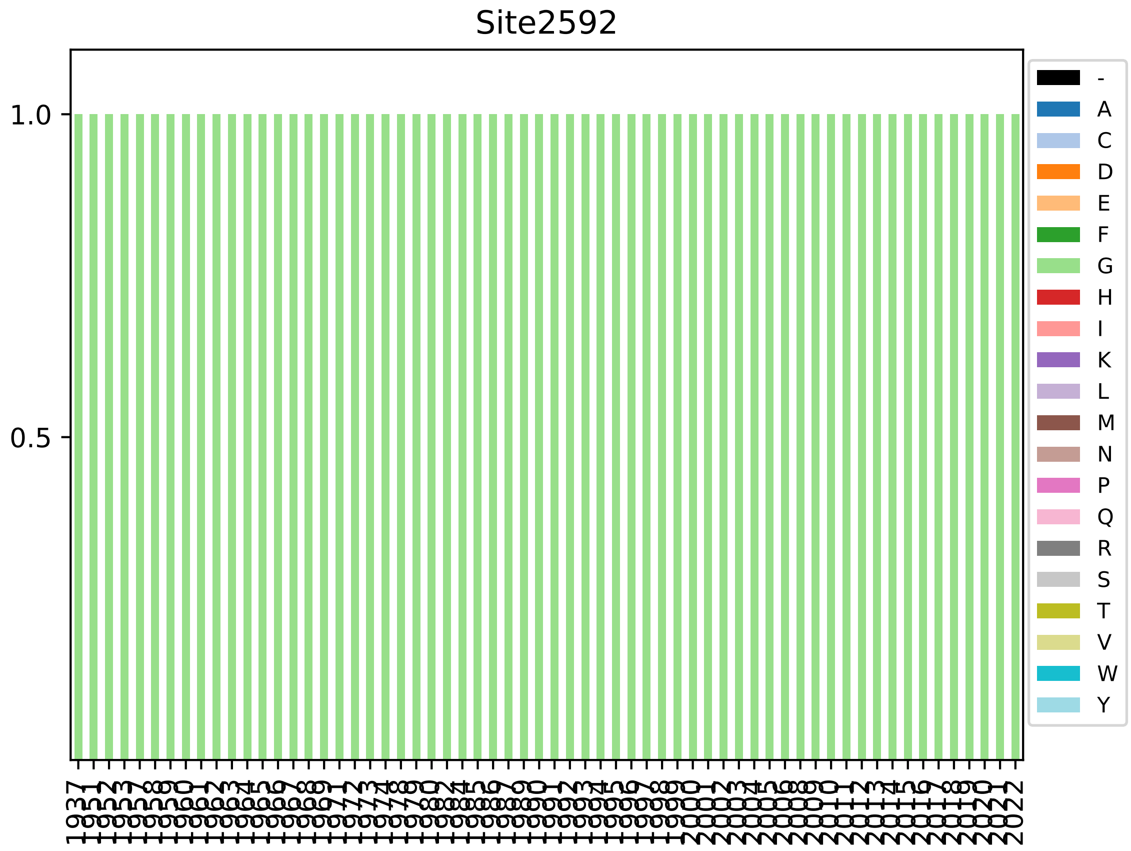
Site2592
1.0
0.5
-
A
C
D
E
F
G
H
I
K
L
M
N
P
Q
R
S
T
V
W
Y

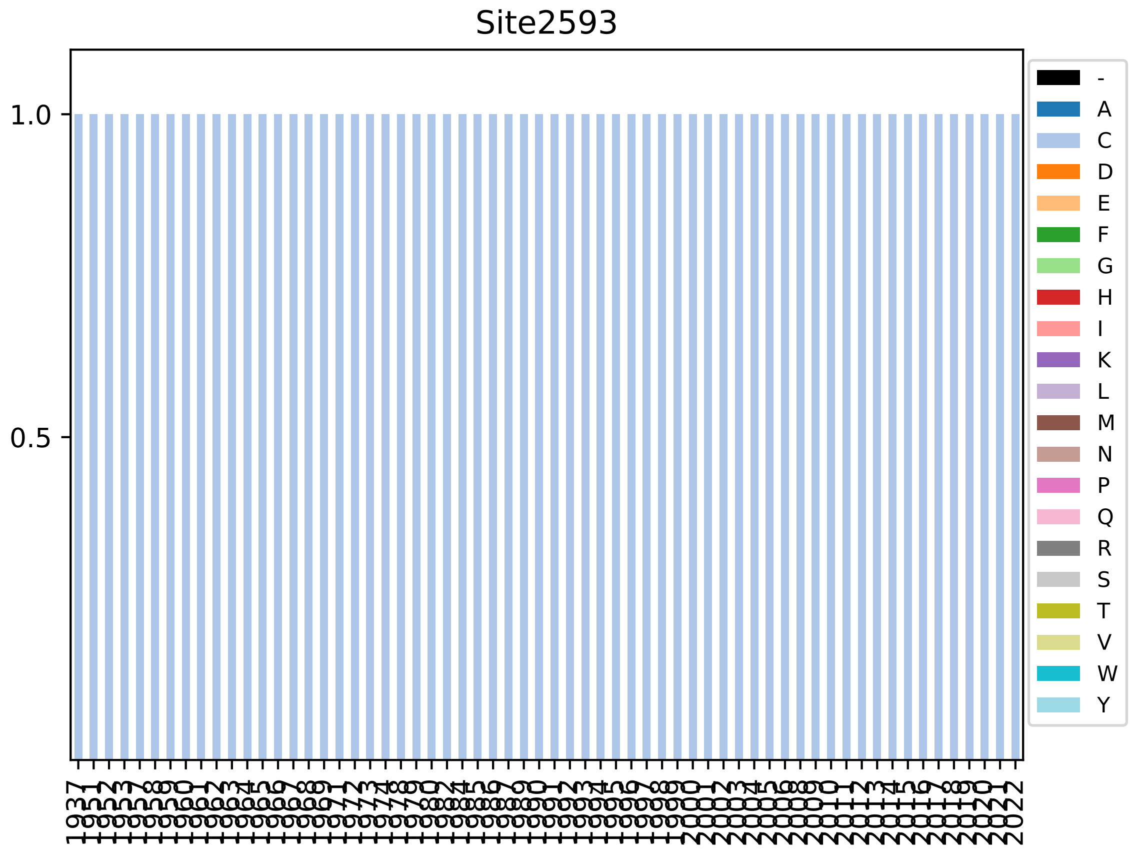
Site2593
1.0
0.5
-
A
C
D
E
F
G
H
I
K
L
M
N
P
Q
R
S
T
V
W
Y

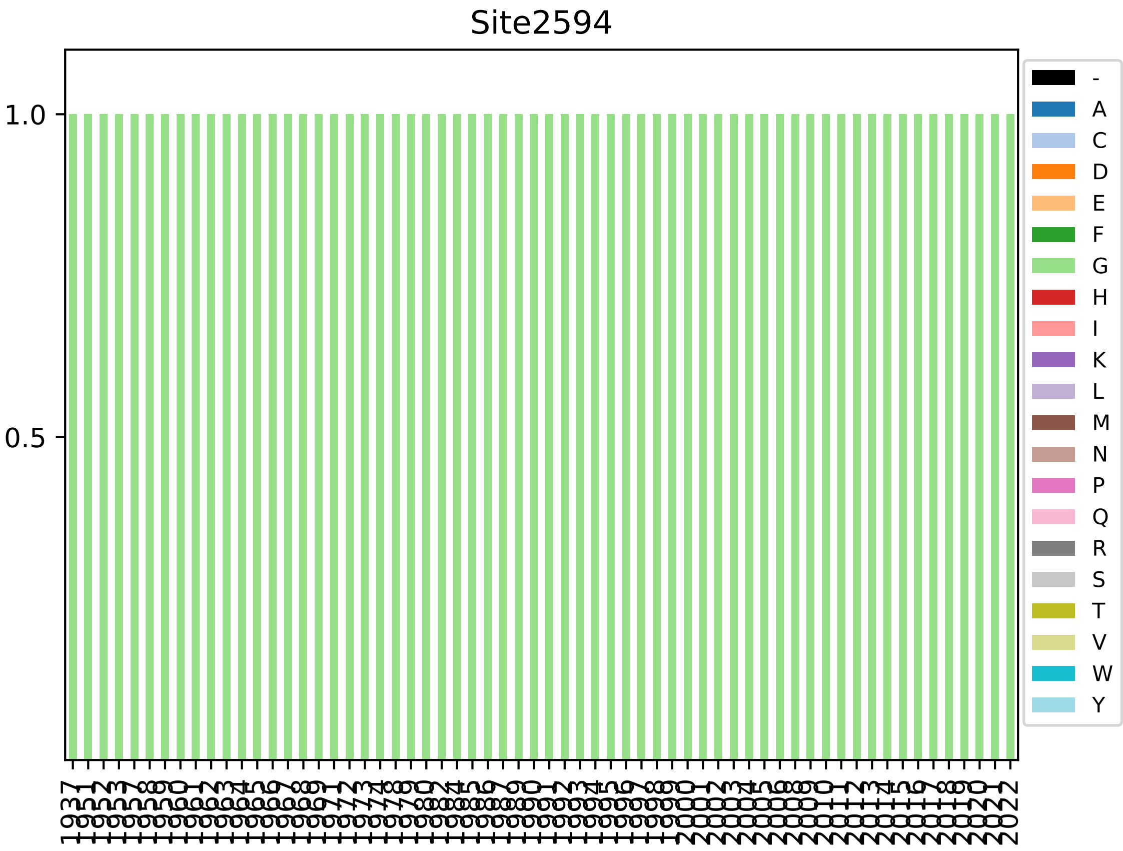

Site2594
1.0
0.5
-
A
C
D
E
F
G
H
I
K
L
M
N
P
Q
R
S
T
V
W
Y

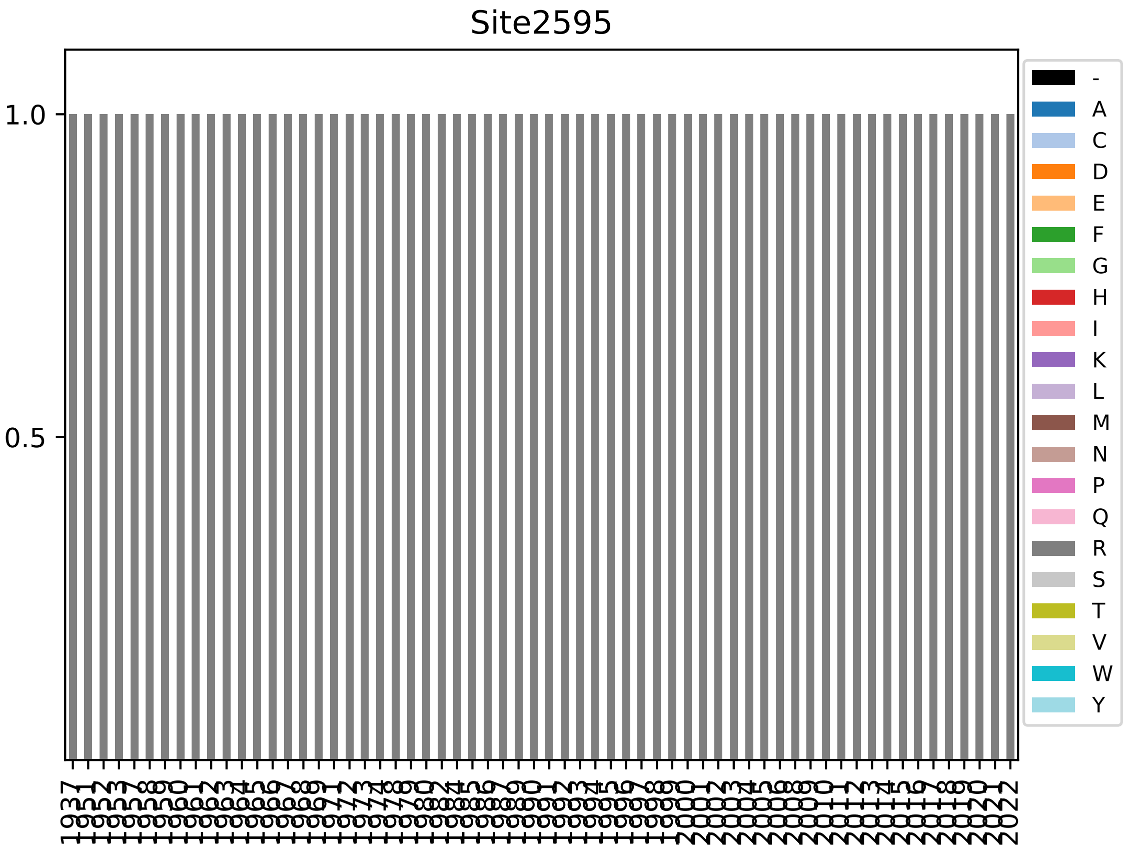
Site2595
1.0
0.5
-
A
C
D
E
F
G
H
I
K
L
M
N
P
Q
R
S
T
V
W
Y

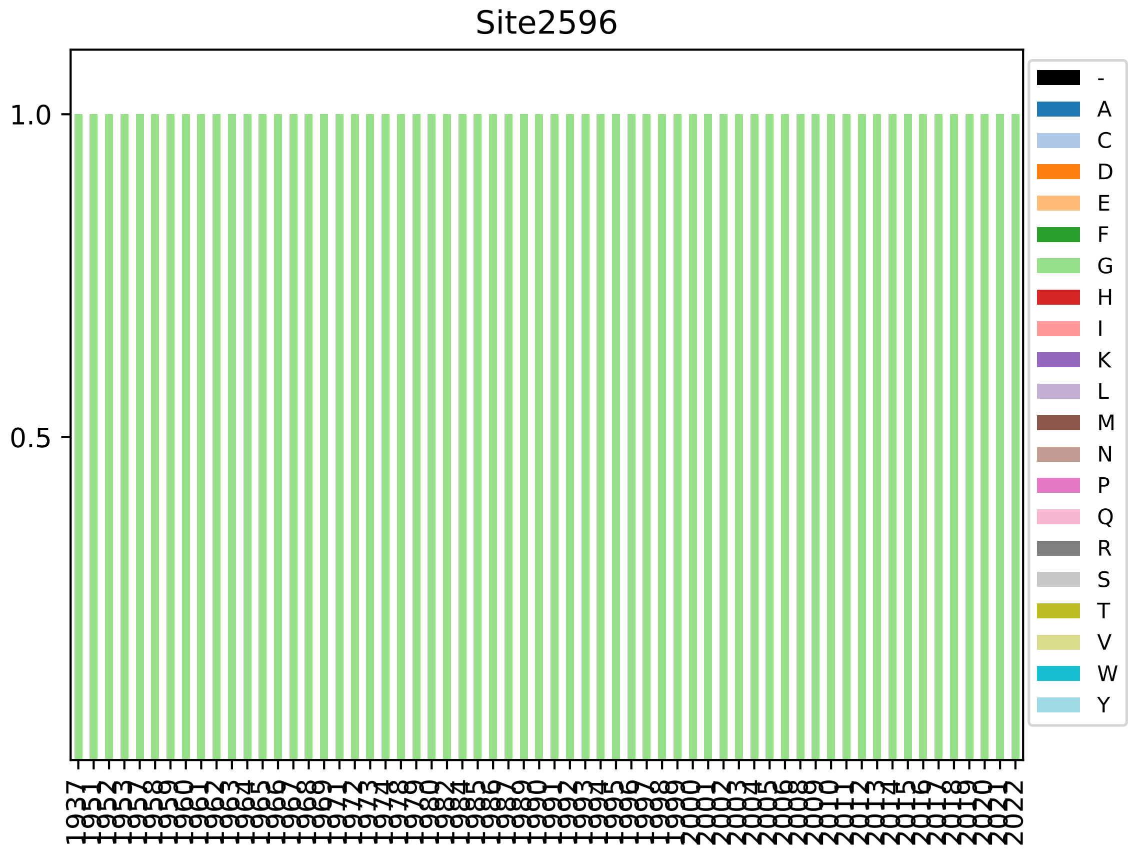
Site2596
1.0
0.5
-
A
C
D
E
F
G
H
I
K
L
M
N
P
Q
R
S
T
V
W
Y

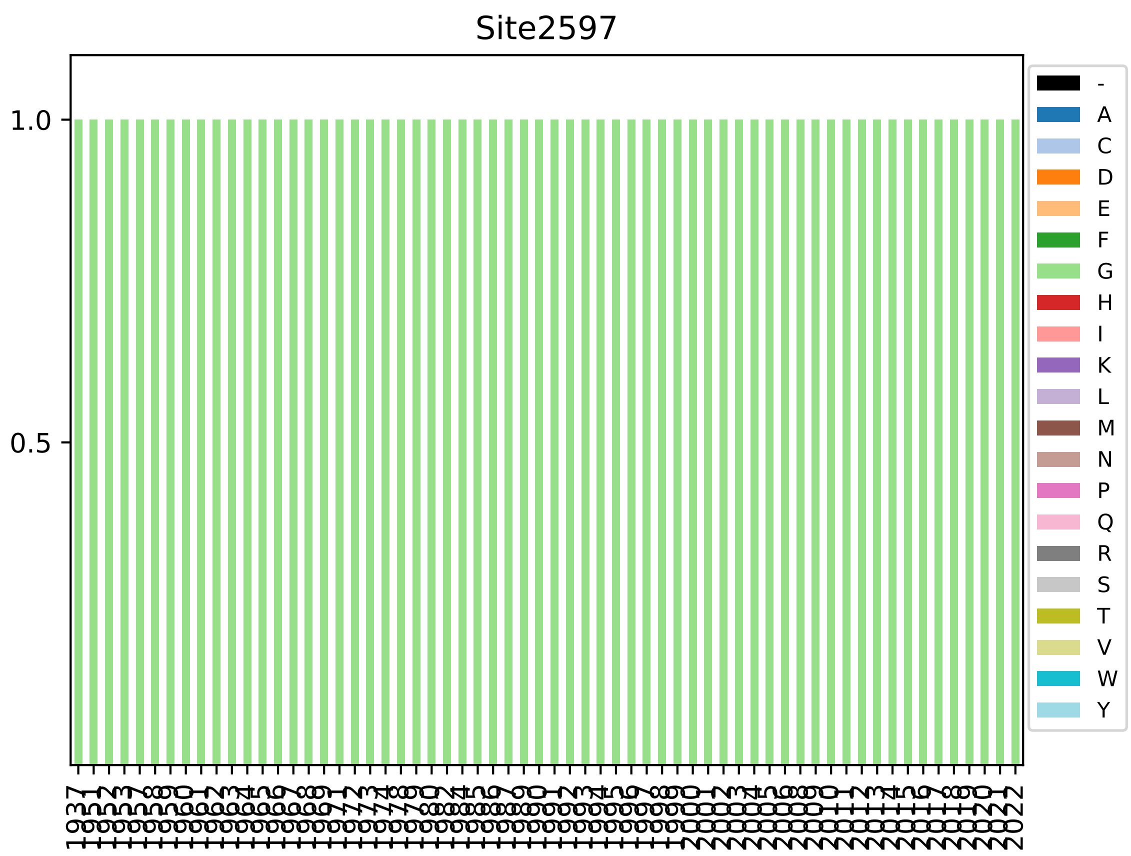
Site2597
1.0
0.5
-
A
C
D
E
F
G
H
I
K
L
M
N
P
Q
R
S
T
V
W
Y

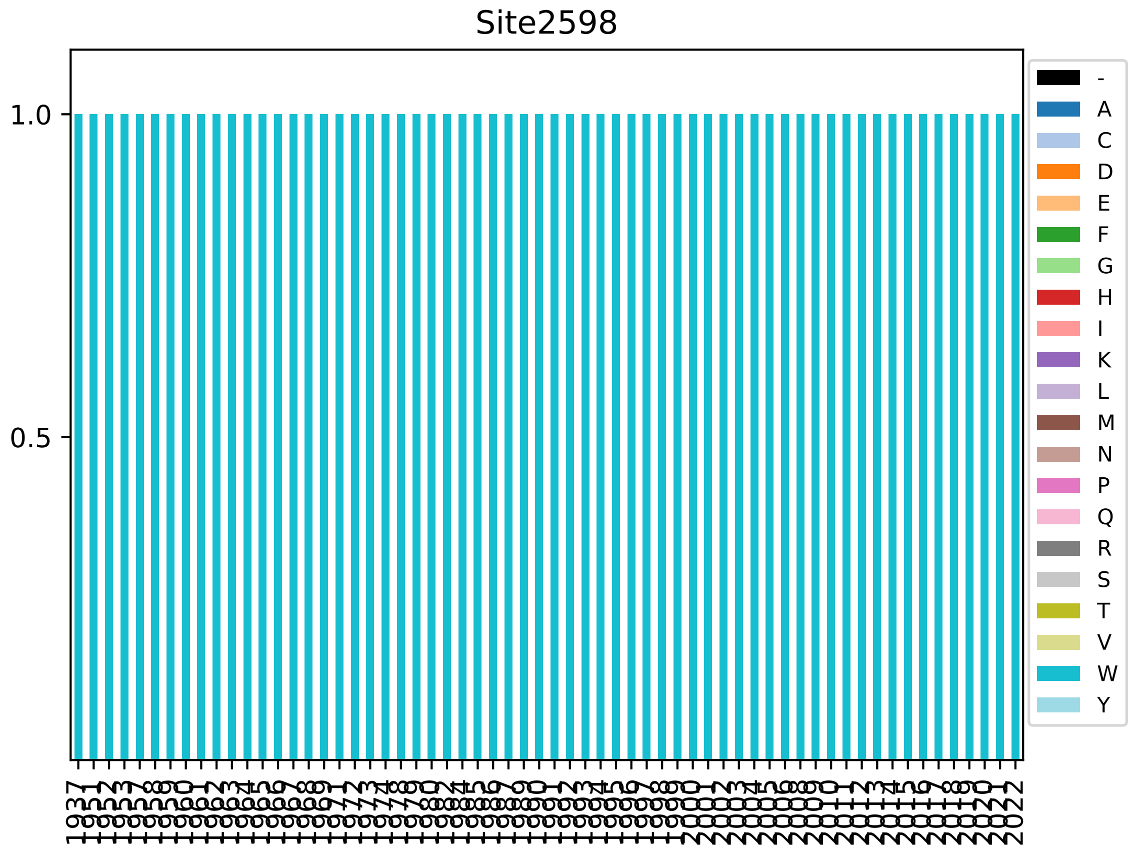
Site2598
1.0
0.5
-
A
C
D
E
F
G
H
I
K
L
M
N
P
Q
R
S
T
V
W
Y

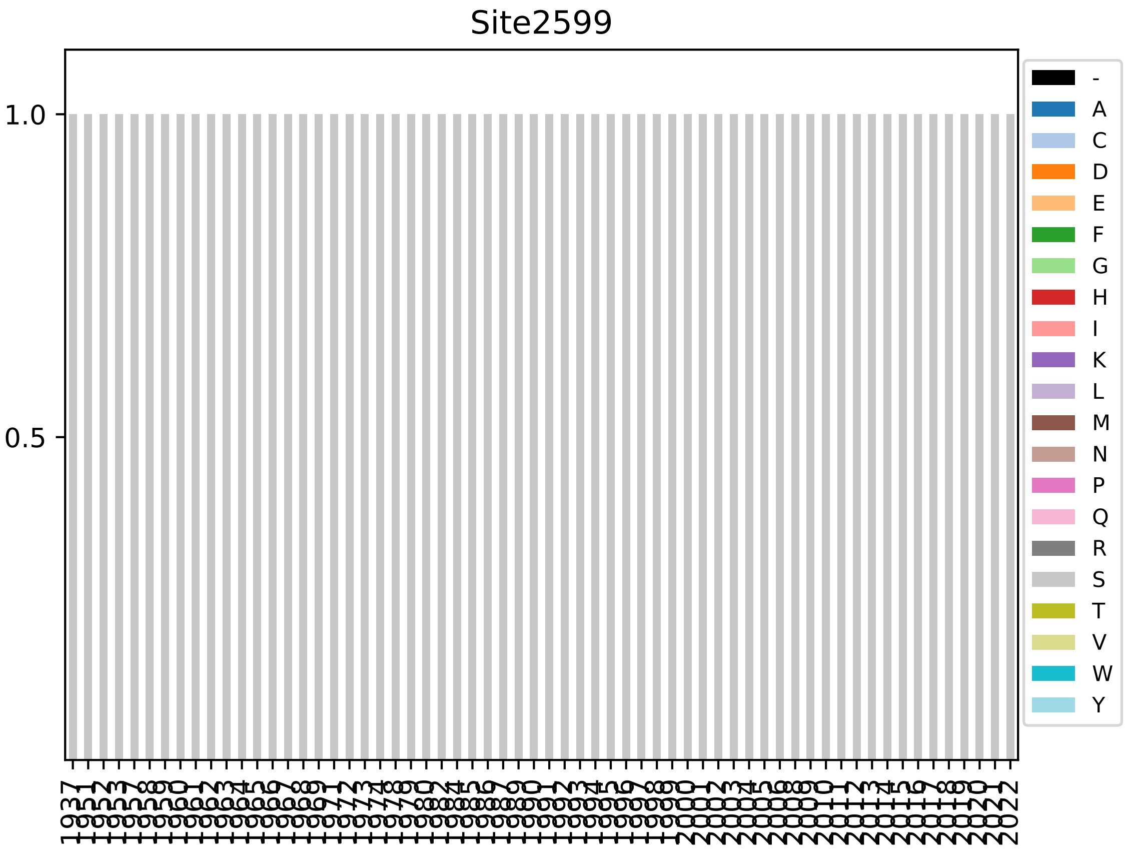
Site2599
1.0
0.5
-
A
C
D
E
F
G
H
I
K
L
M
N
P
Q
R
S
T
V
W
Y

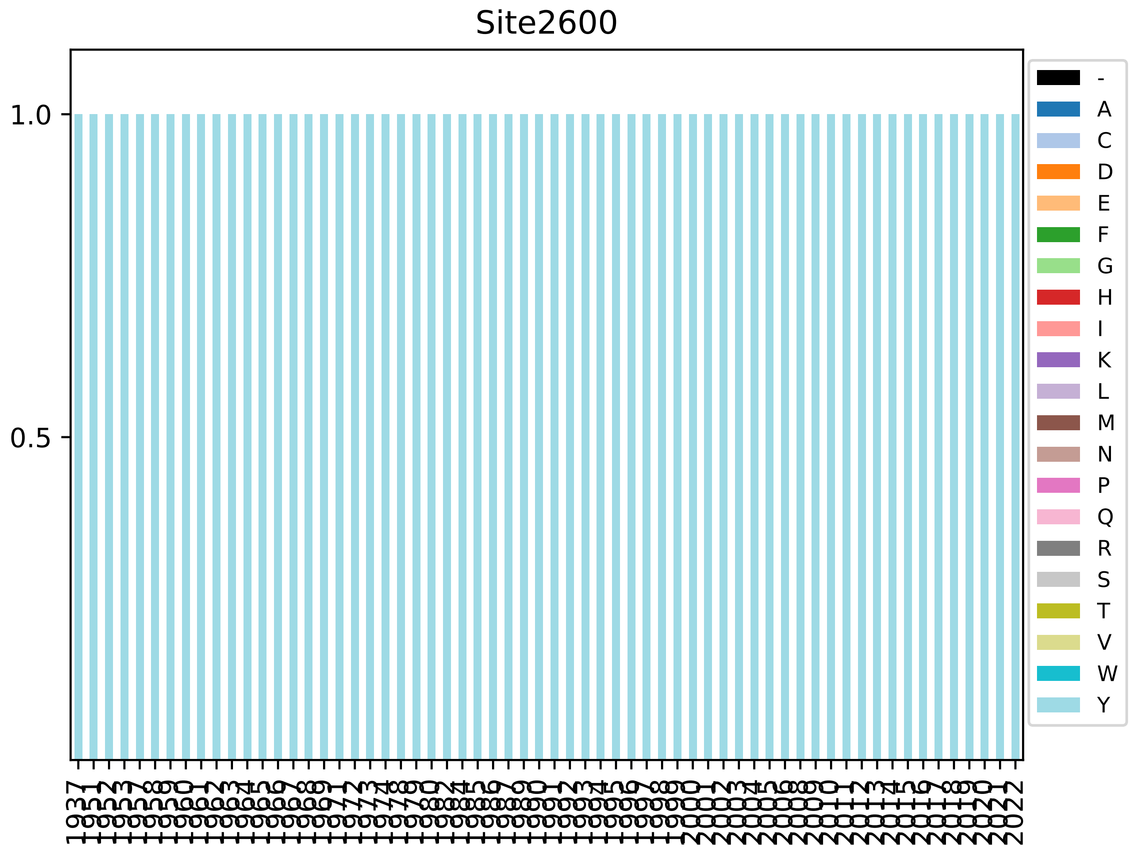

Site2600
1.0
0.5
-
A
C
D
E
F
G
H
I
K
L
M
N
P
Q
R
S
T
V
W
Y

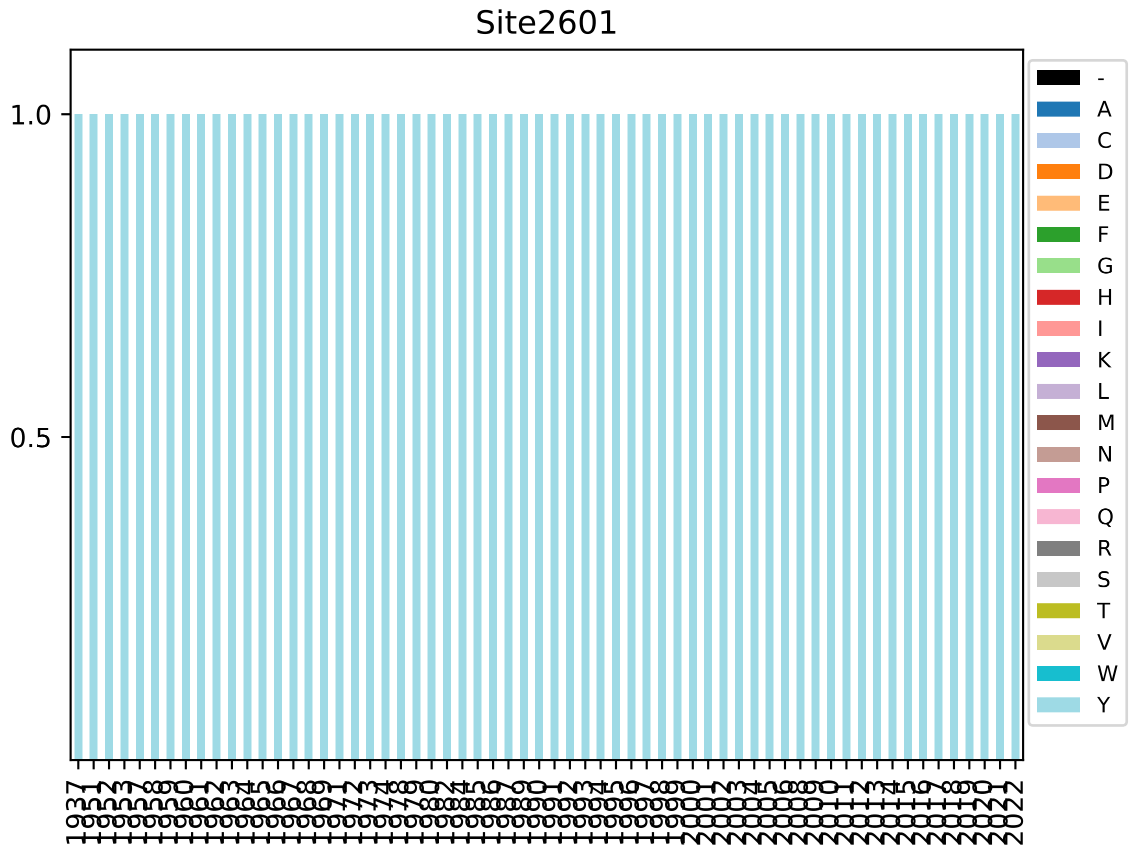
Site2601
1.0
0.5
-
A
C
D
E
F
G
H
I
K
L
M
N
P
Q
R
S
T
V
W
Y

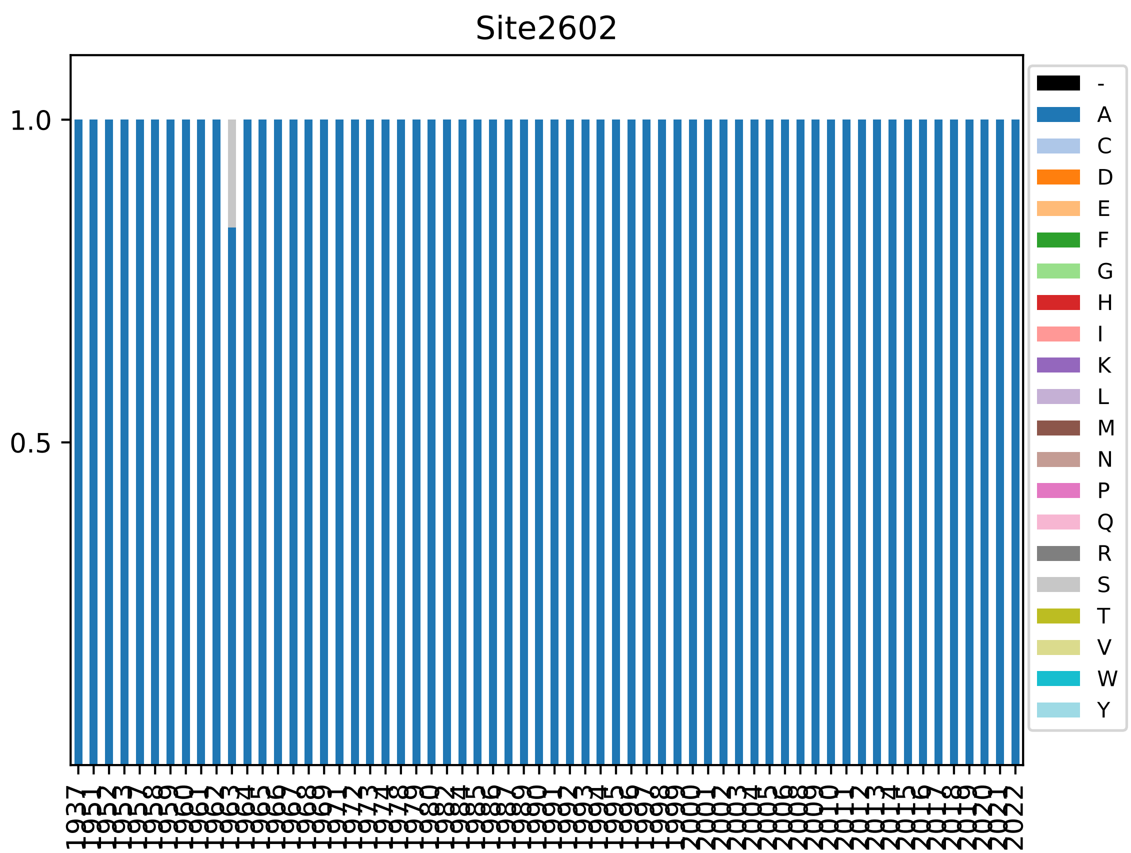
Site2602
1.0
0.5
-
A
C
D
E
F
G
H
I
K
L
M
N
P
Q
R
S
T
V
W
Y

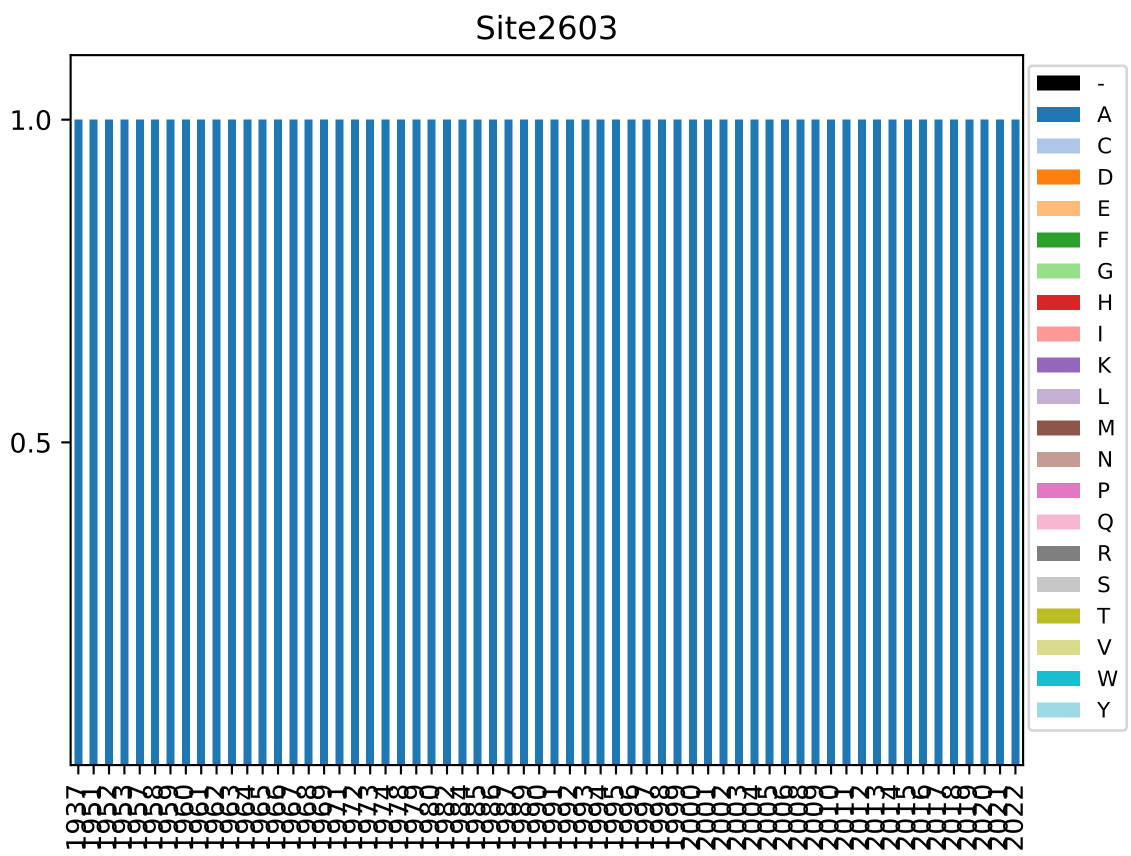
Site2603
1.0
0.5
-
A
C
D
E
F
G
H
I
K
L
M
N
P
Q
R
S
T
V
W
Y

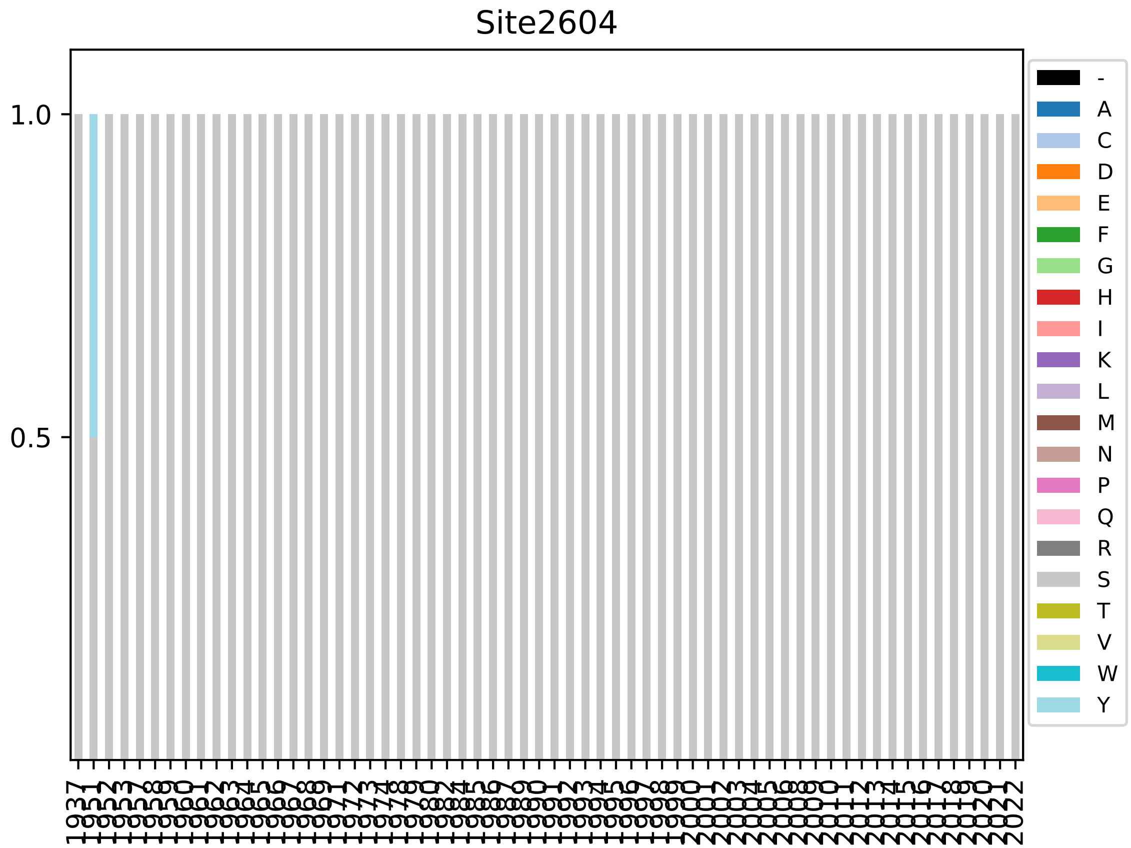
Site2604
1.0
0.5
-
A
C
D
E
F
G
H
I
K
L
M
N
P
Q
R
S
T
V
W
Y

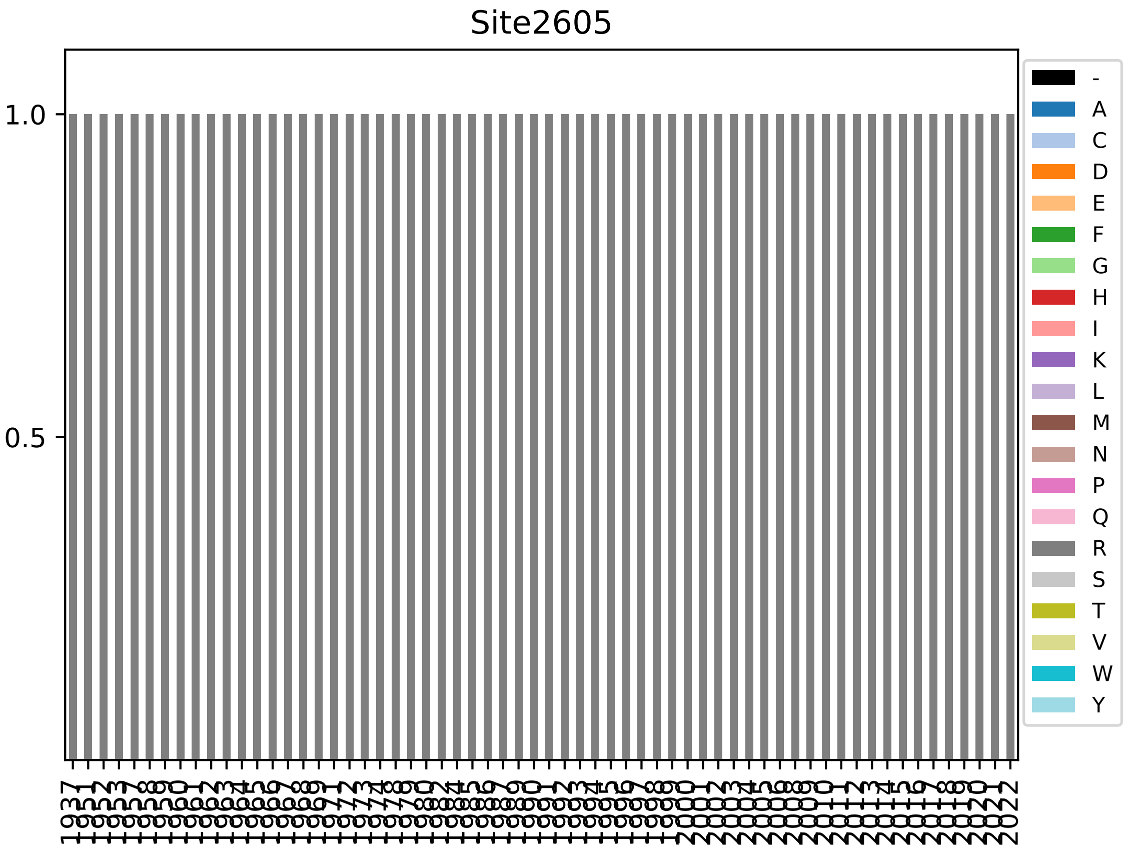
Site2605
1.0
0.5
-
A
C
D
E
F
G
H
I
K
L
M
N
P
Q
R
S
T
V
W
Y

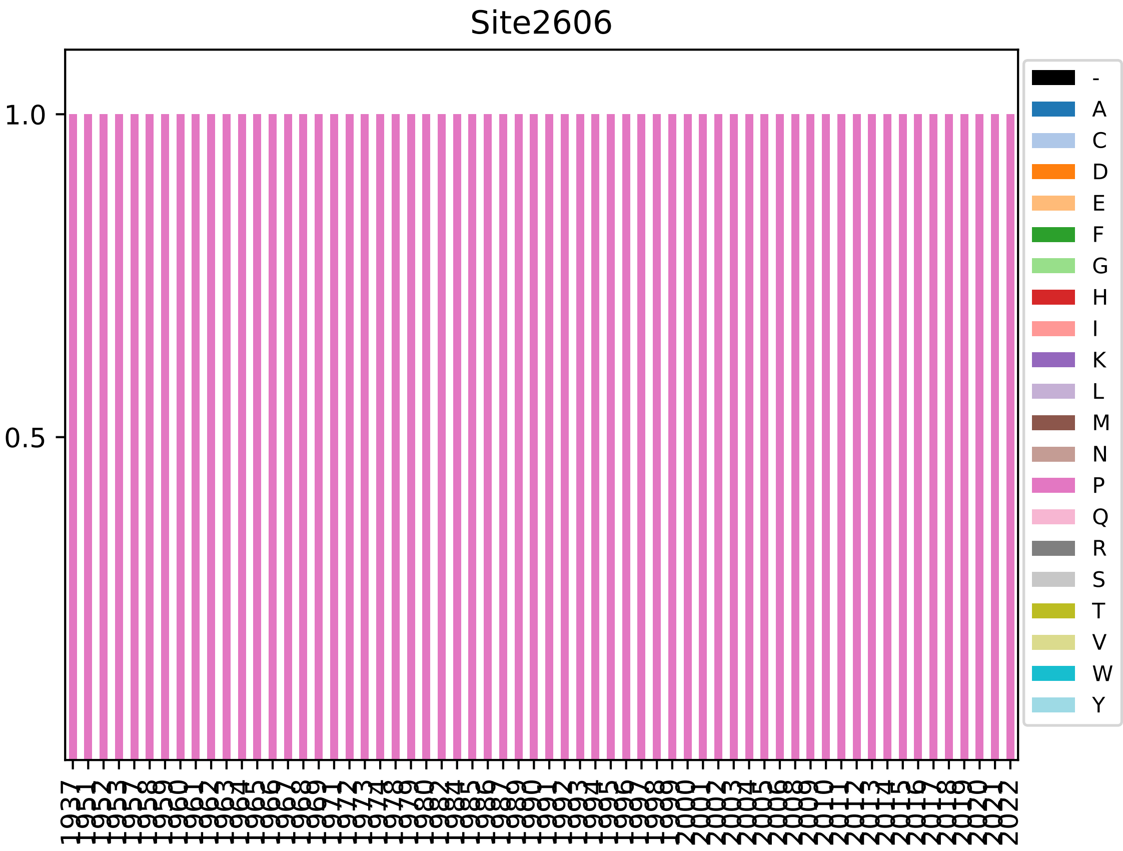
Site2606
1.0
0.5
-
A
C
D
E
F
G
H
I
K
L
M
N
P
Q
R
S
T
V
W
Y

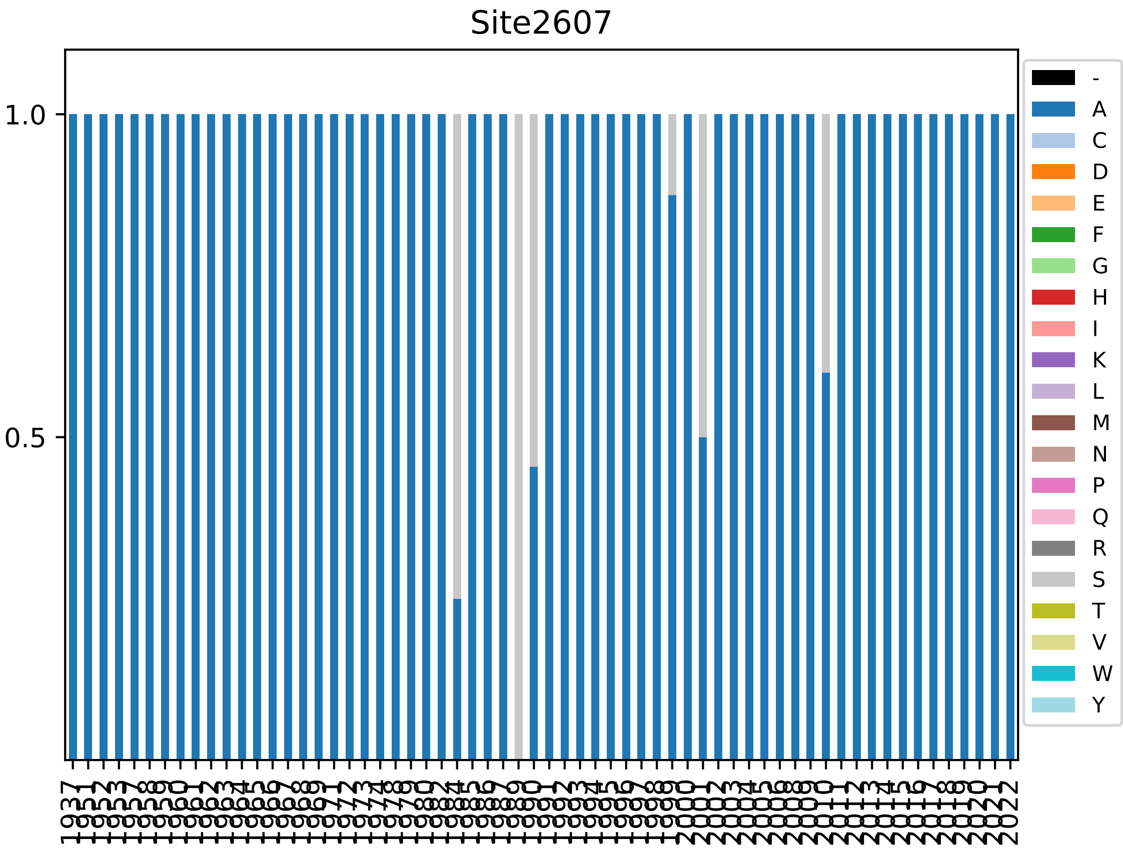
Site2607
1.0
0.5
-
A
C
D
E
F
G
H
I
K
L
M
N
P
Q
R
S
T
V
W
Y

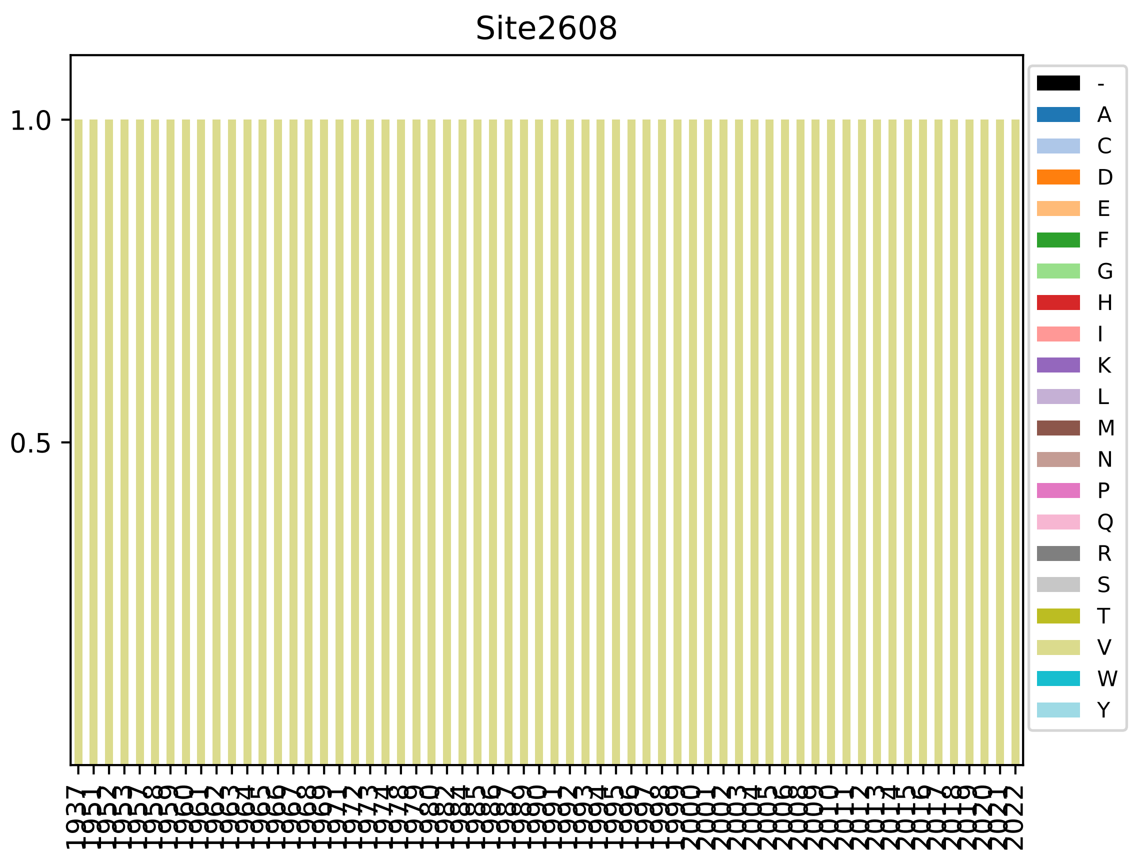
Site2608
1.0
0.5
-
A
C
D
E
F
G
H
I
K
L
M
N
P
Q
R
S
T
V
W
Y

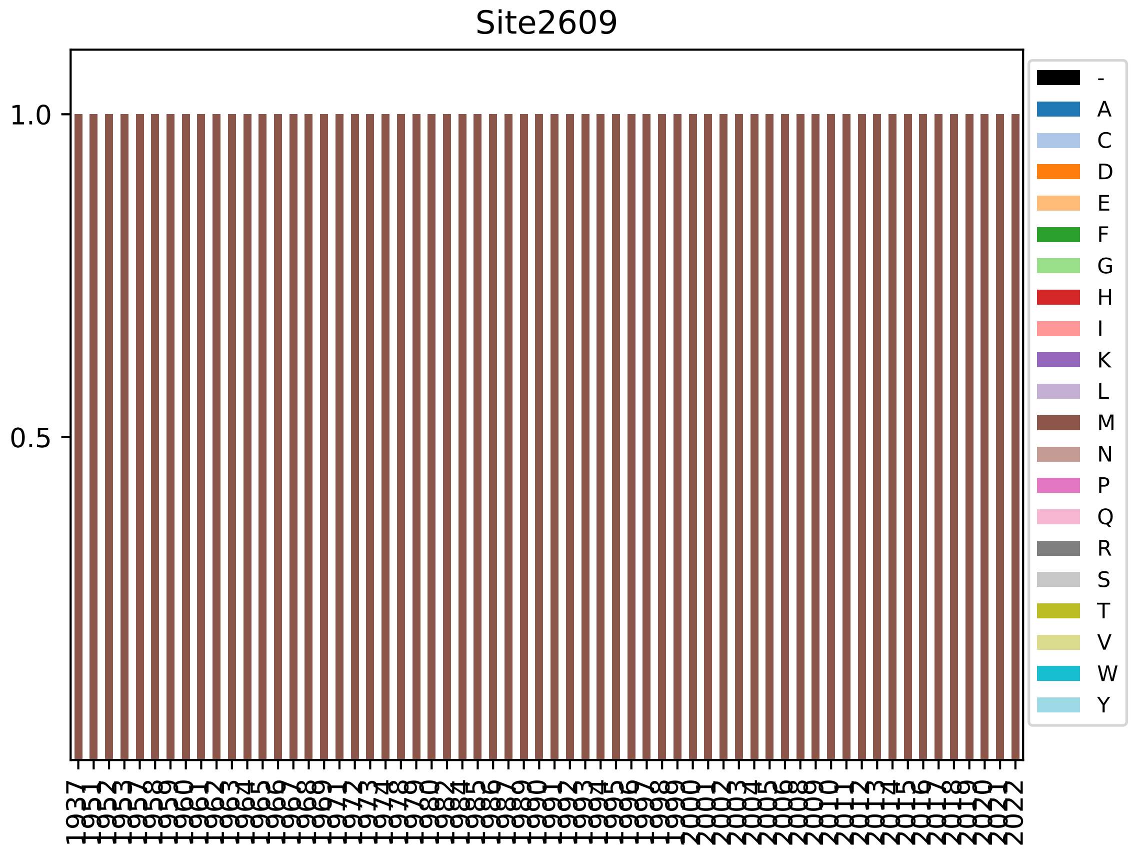

Site2609
1.0
0.5
-
A
C
D
E
F
G
H
I
K
L
M
N
P
Q
R
S
T
V
W
Y
1937
1951
1952
1953
1957
1958
1959
1960
1961
1962
1963
1964
1965
1966
1967
1968
1969
1971
1972
1973
1974
1978
1980
1982
1984
1985
1986
1987
1989
1990
1991
1992
1993
1994
1995
1996
1997
1998
1999
2000
2001
2002
2003
2004
2005
2006
2007
2008
2009
2010
2011
2012
2013
2014
2015
2016
2017
2018
2019
2020
2021
2022

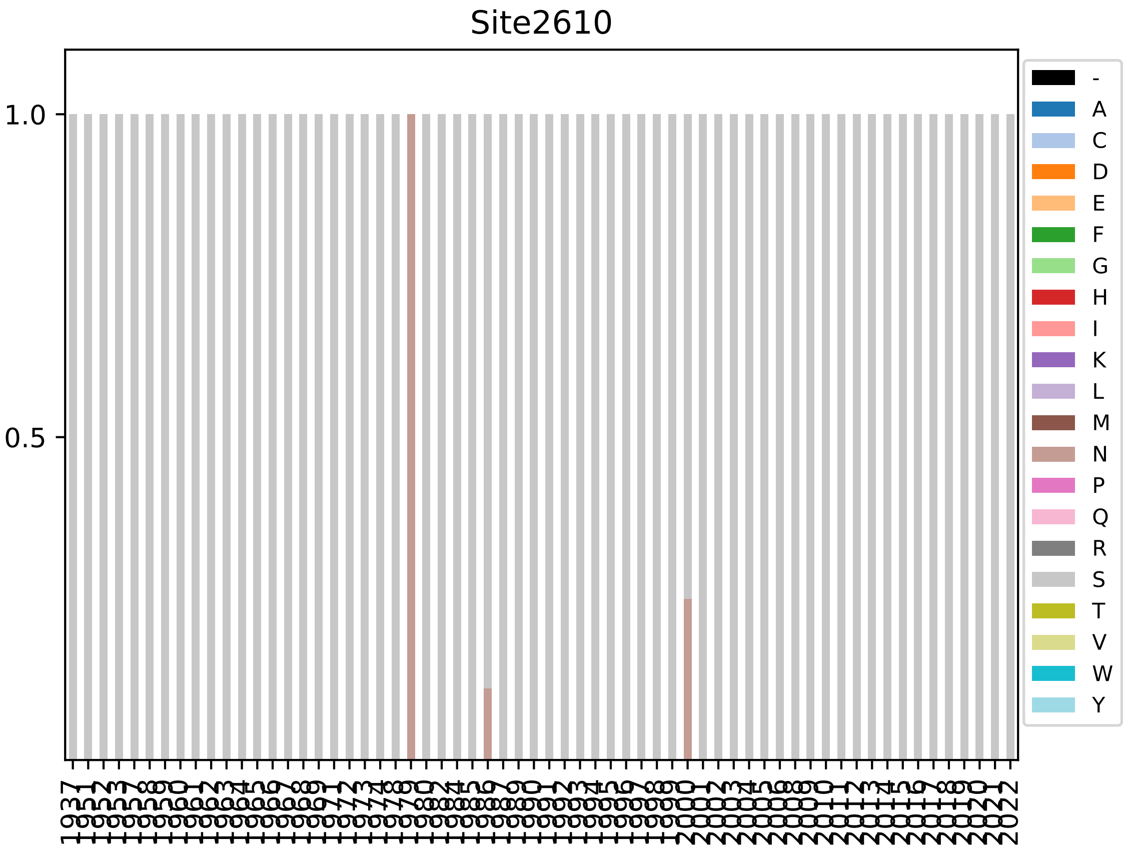
Site2610
1.0
0.5
-
A
C
D
E
F
G
H
I
K
L
M
N
P
Q
R
S
T
V
W
Y

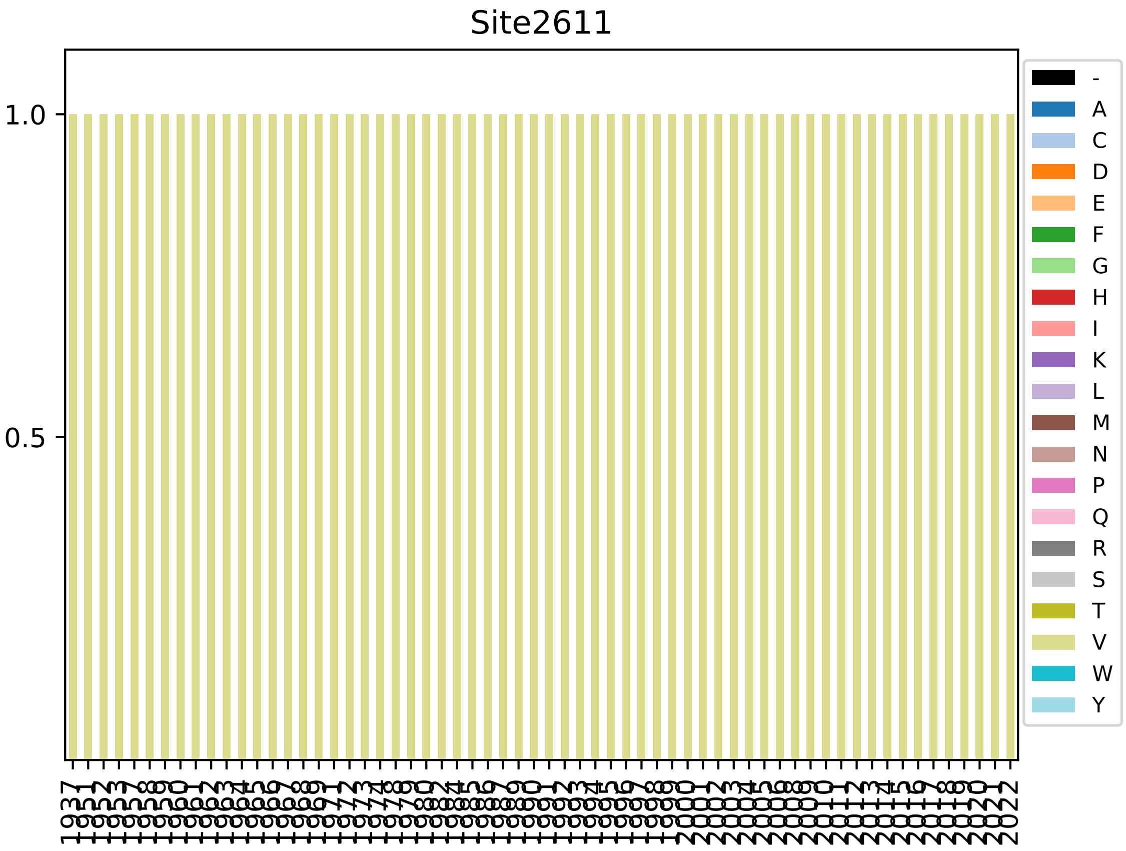
Site2611
1.0
0.5
-
A
C
D
E
F
G
H
I
K
L
M
N
P
Q
R
S
T
V
W
Y

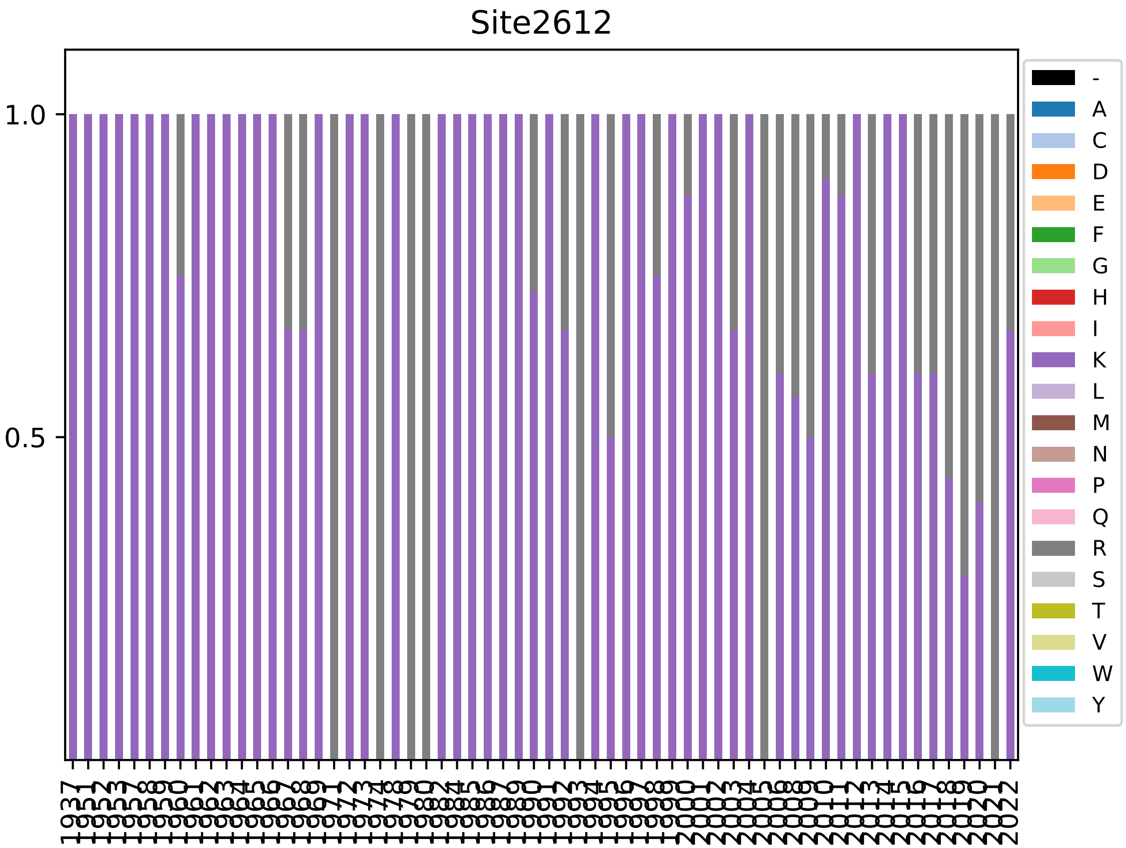
Site2612
1.0
0.5
-
A
C
D
E
F
G
H
I
K
L
M
N
P
Q
R
S
T
V
W
Y

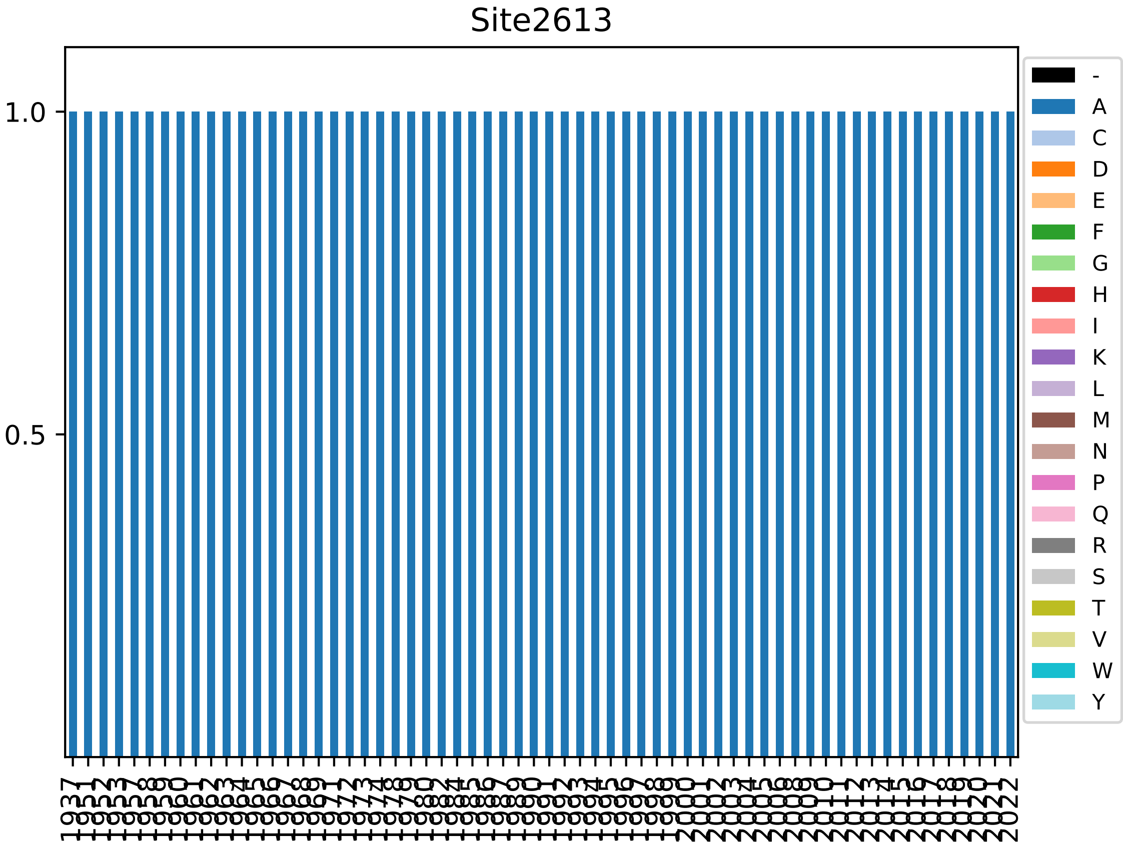
Site2613
1.0
0.5
-
A
C
D
E
F
G
H
I
K
L
M
N
P
Q
R
S
T
V
W
Y

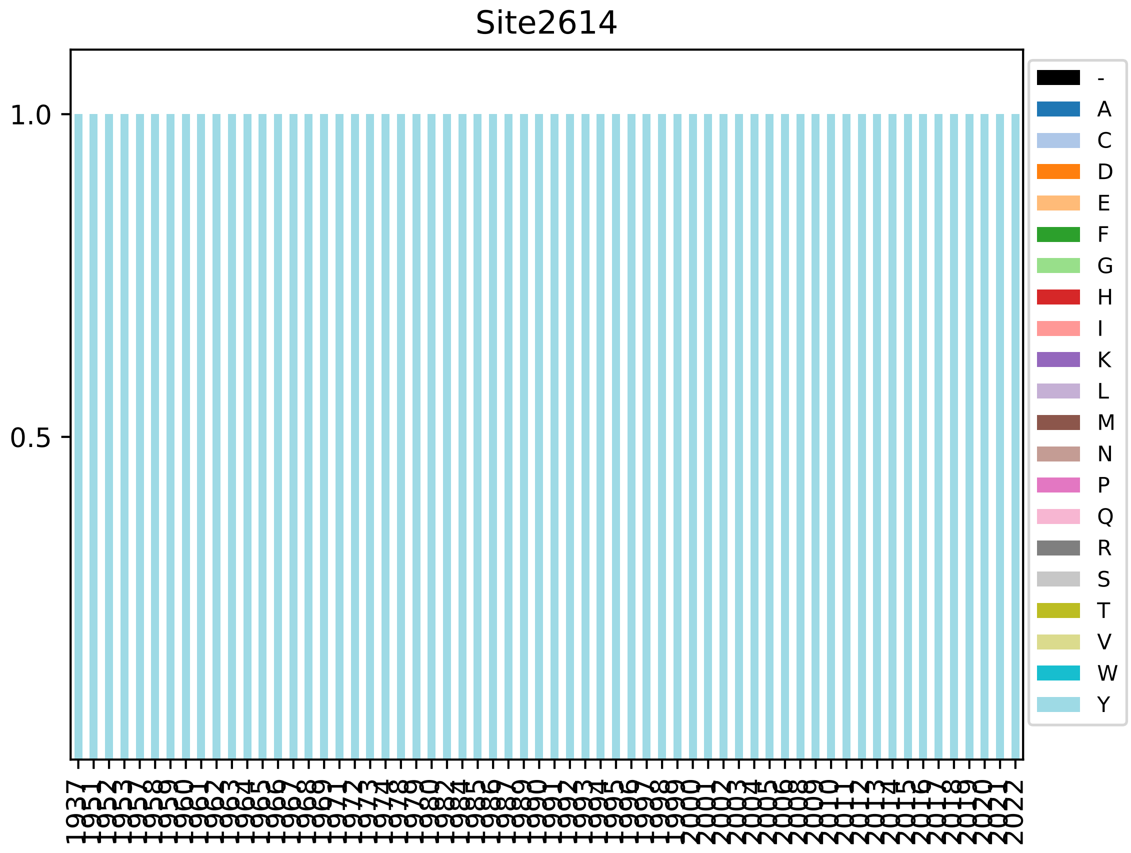
Site2614
1.0
0.5
-
A
C
D
E
F
G
H
I
K
L
M
N
P
Q
R
S
T
V
W
Y

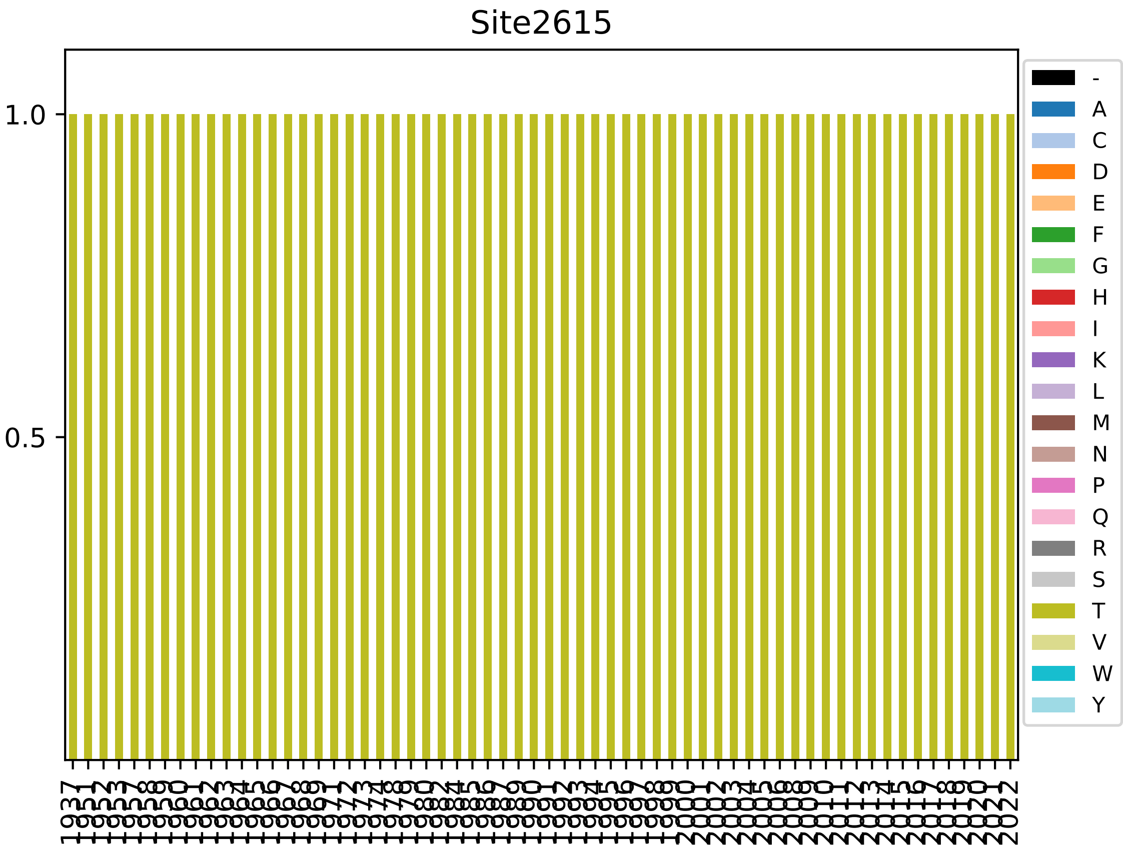
Site2615
1.0
0.5
-
A
C
D
E
F
G
H
I
K
L
M
N
P
Q
R
S
T
V
W
Y

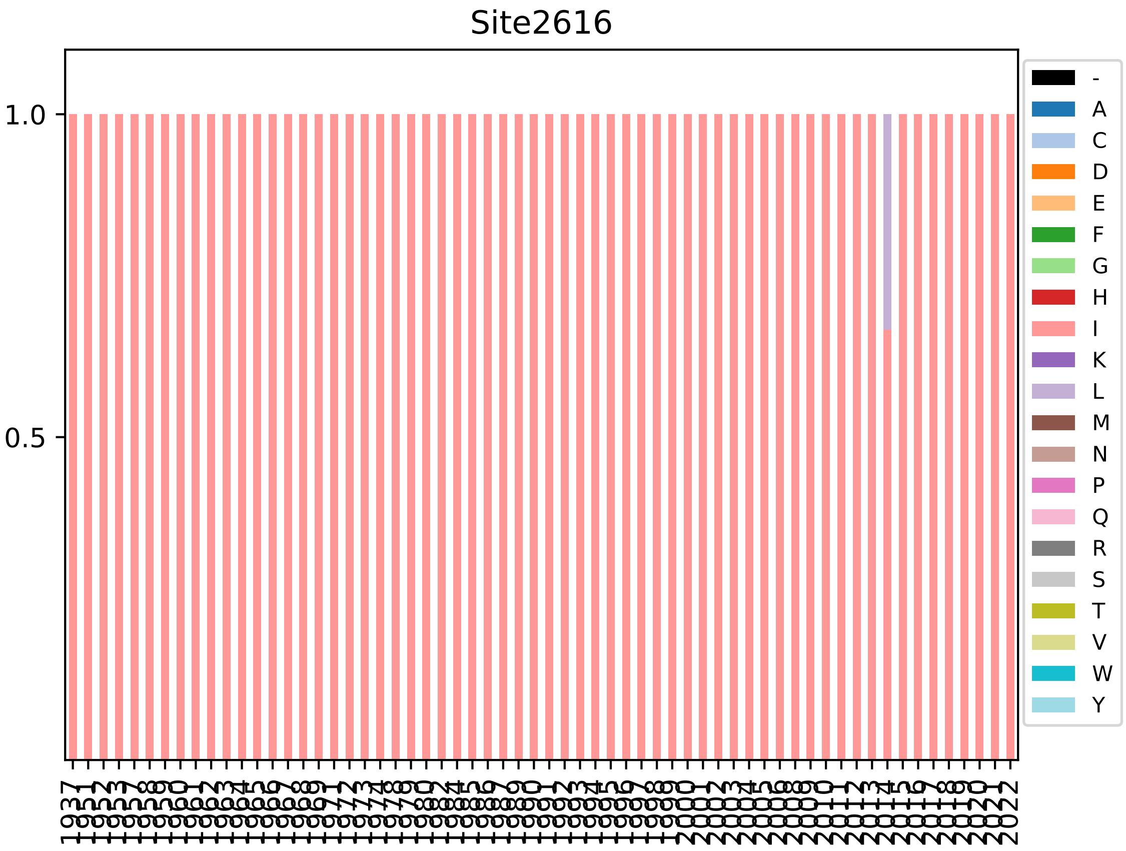
Site2616
1.0
0.5
-
A
C
D
E
F
G
H
I
K
L
M
N
P
Q
R
S
T
V
W
Y

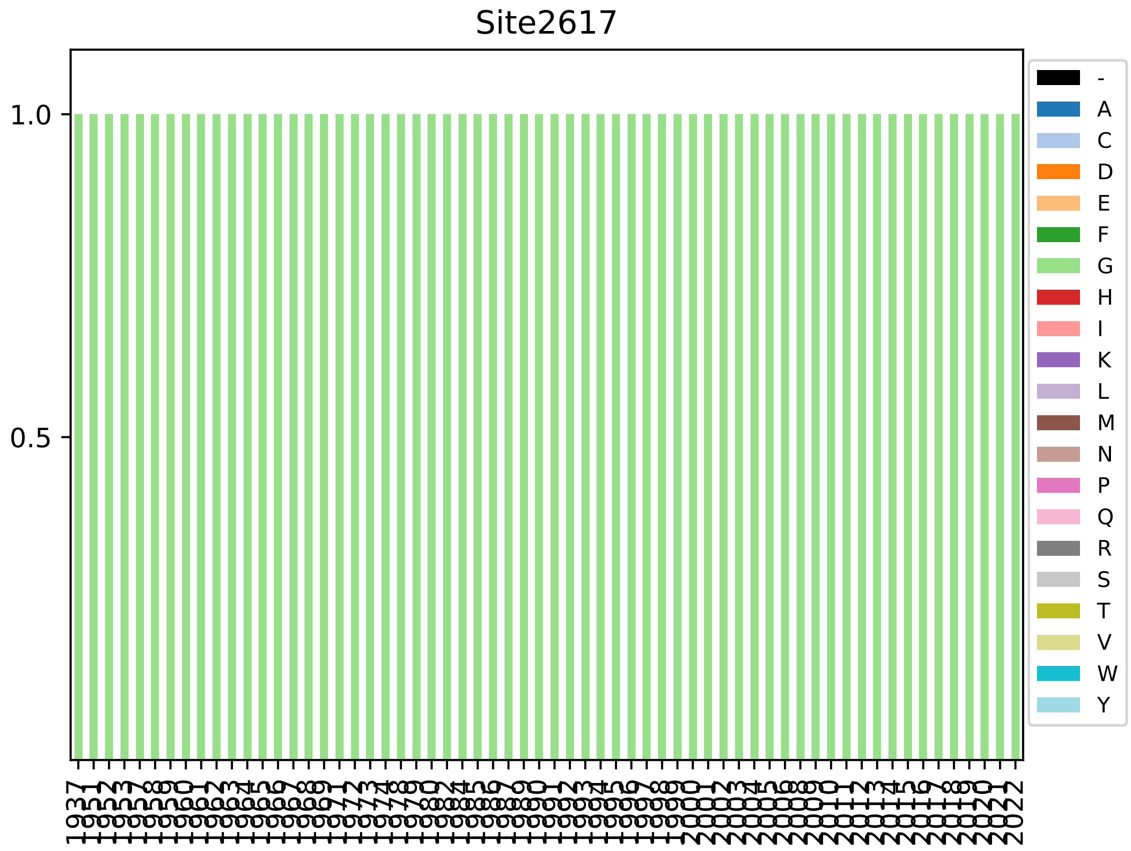
Site2617
1.0
0.5
-
A
C
D
E
F
G
H
I
K
L
M
N
P
Q
R
S
T
V
W
Y

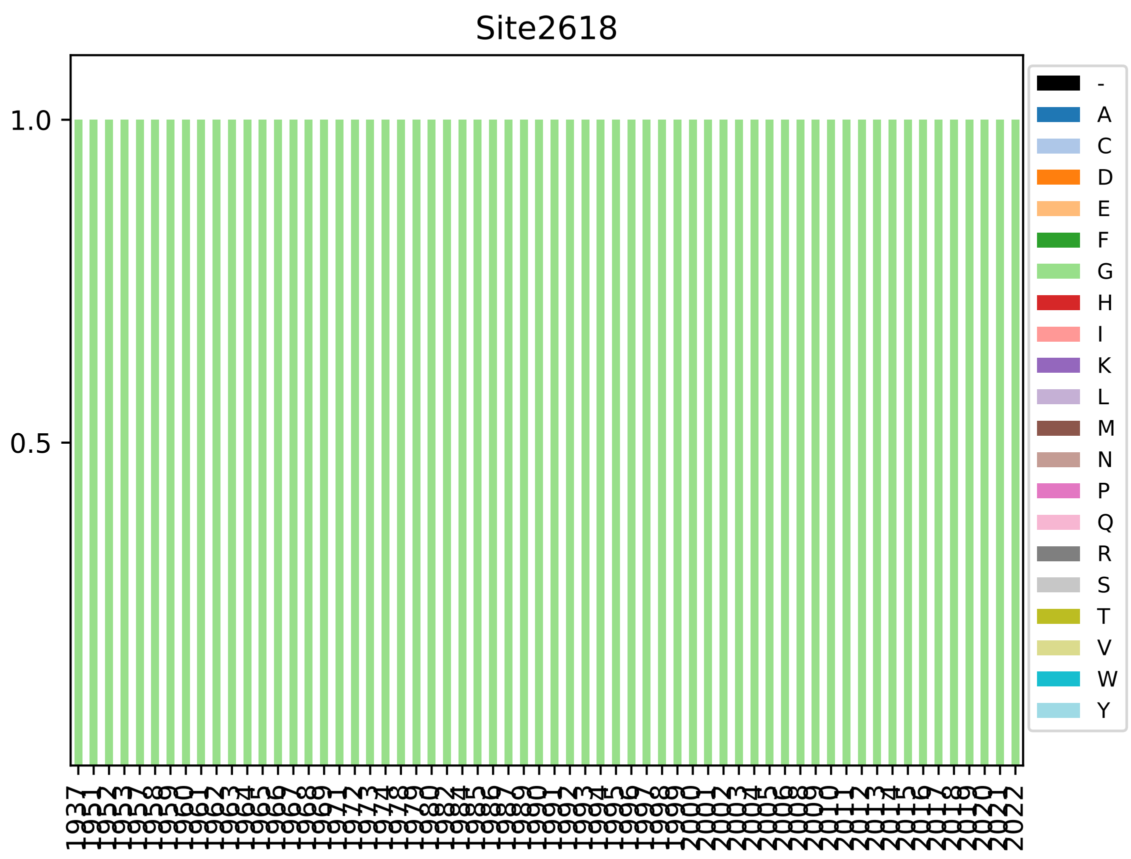

Site2618
1.0
0.5
-
A
C
D
E
F
G
H
I
K
L
M
N
P
Q
R
S
T
V
W
Y

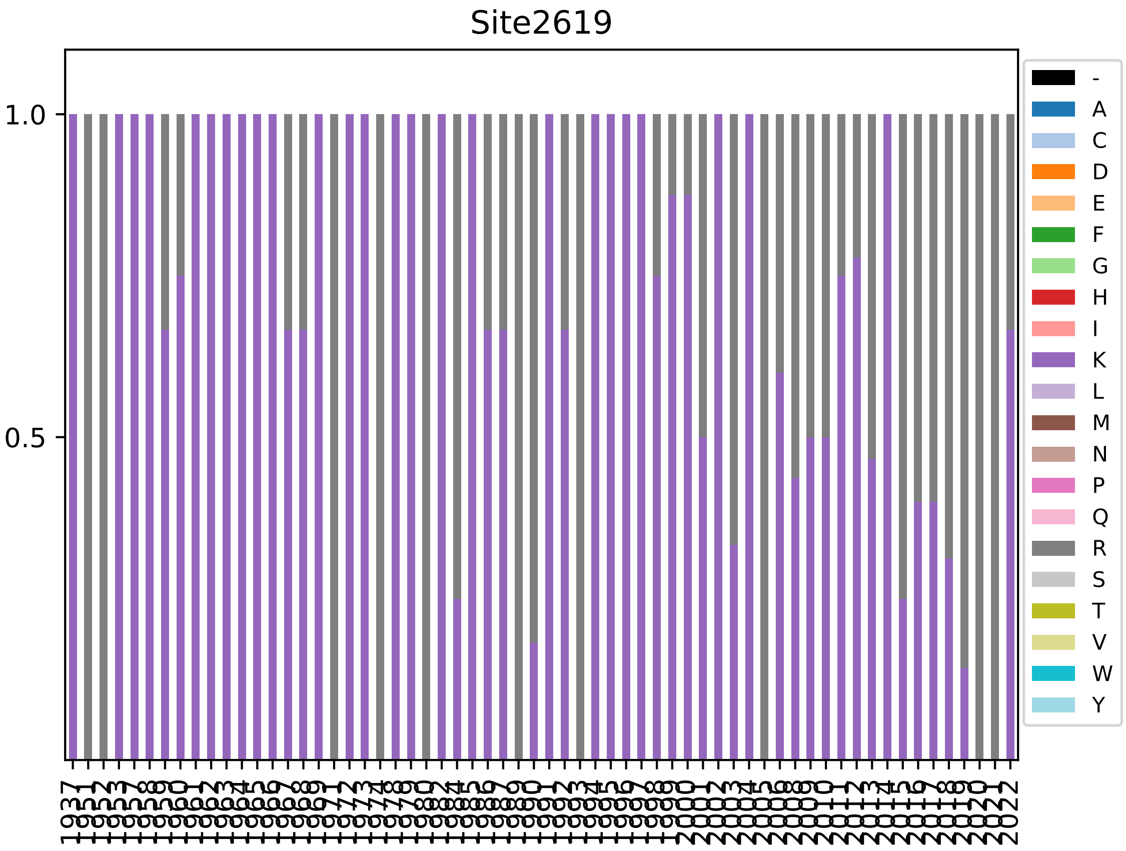
Site2619
1.0
0.5
-
A
C
D
E
F
G
H
I
K
L
M
N
P
Q
R
S
T
V
W
Y

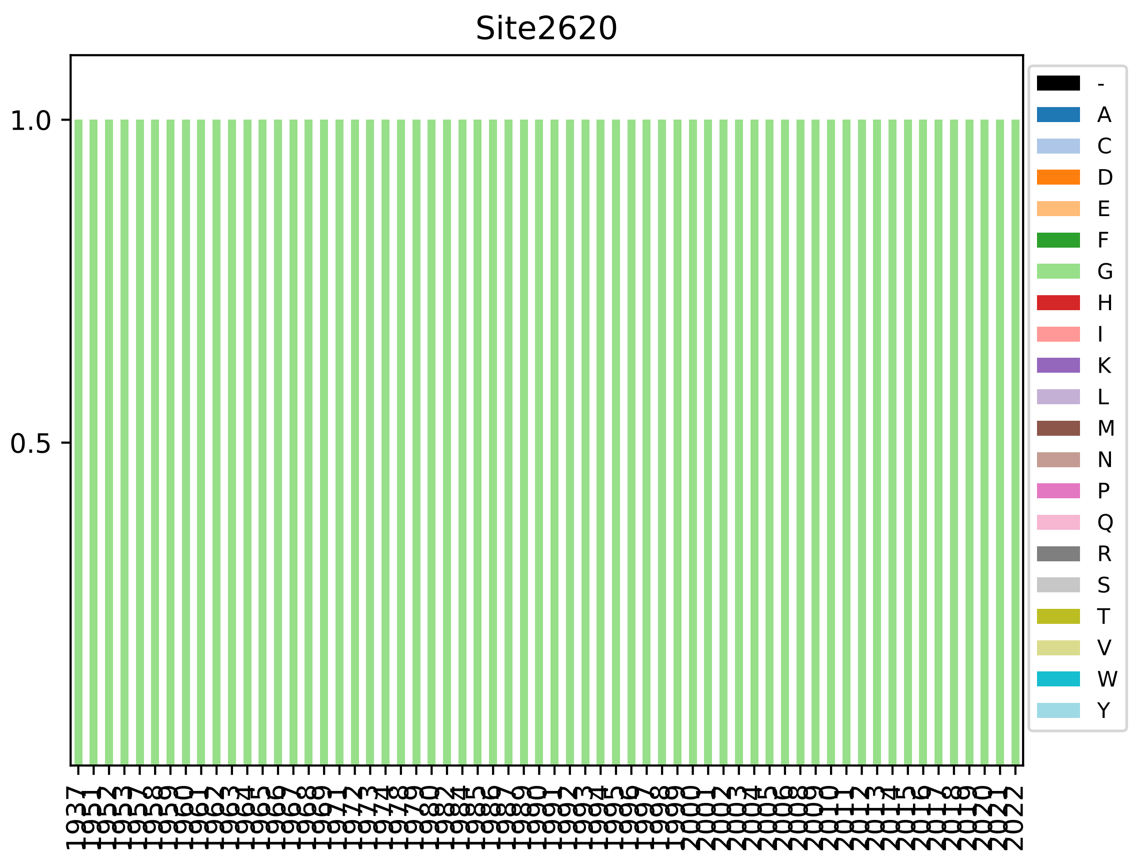
Site2620
1.0
0.5
-
A
C
D
E
F
G
H
I
K
L
M
N
P
Q
R
S
T
V
W
Y

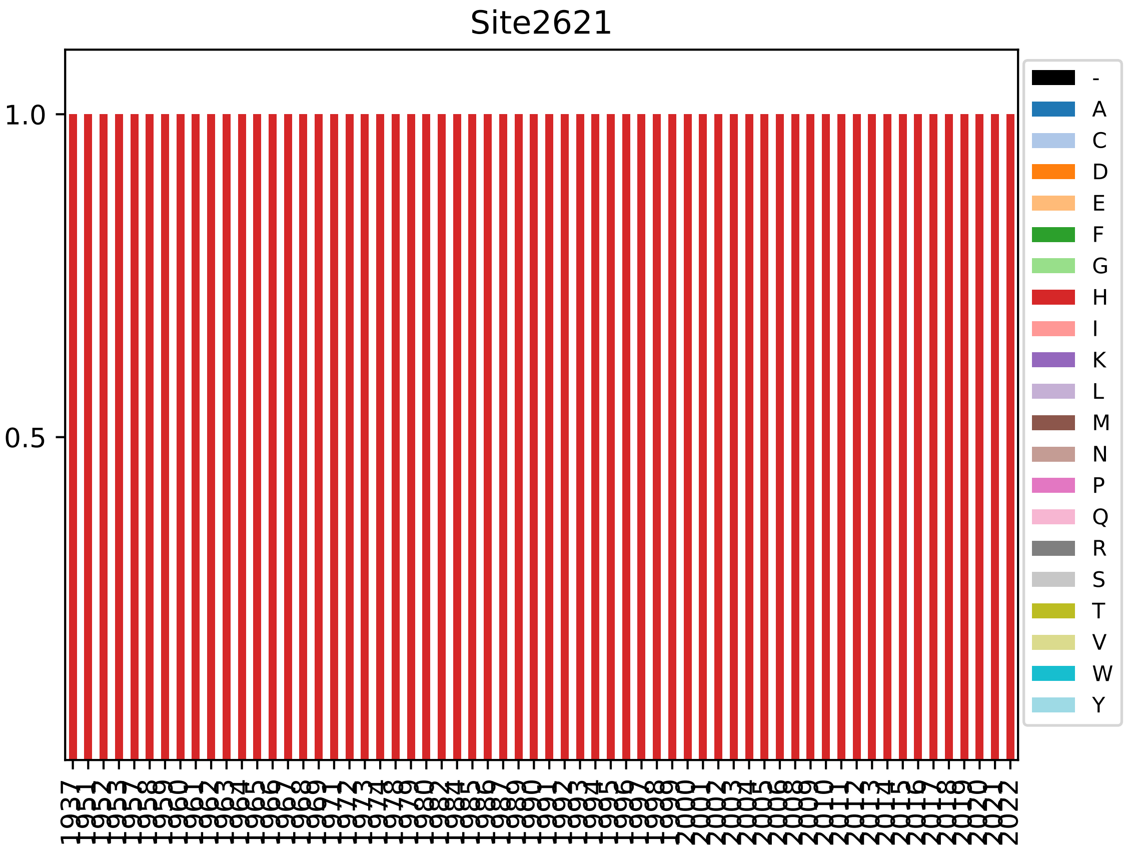
Site2621
1.0
0.5
-
A
C
D
E
F
G
H
I
K
L
M
N
P
Q
R
S
T
V
W
Y

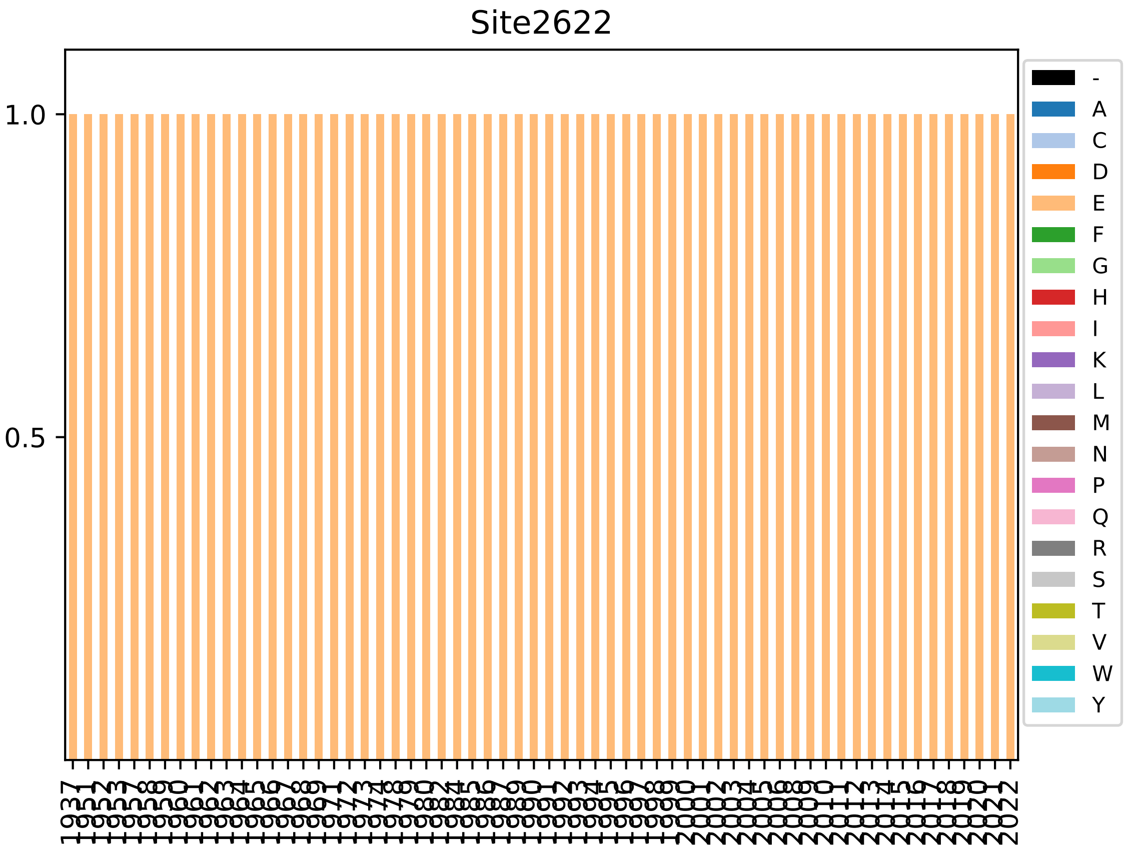
Site2622
1.0
0.5
-
A
C
D
E
F
G
H
I
K
L
M
N
P
Q
R
S
T
V
W
Y

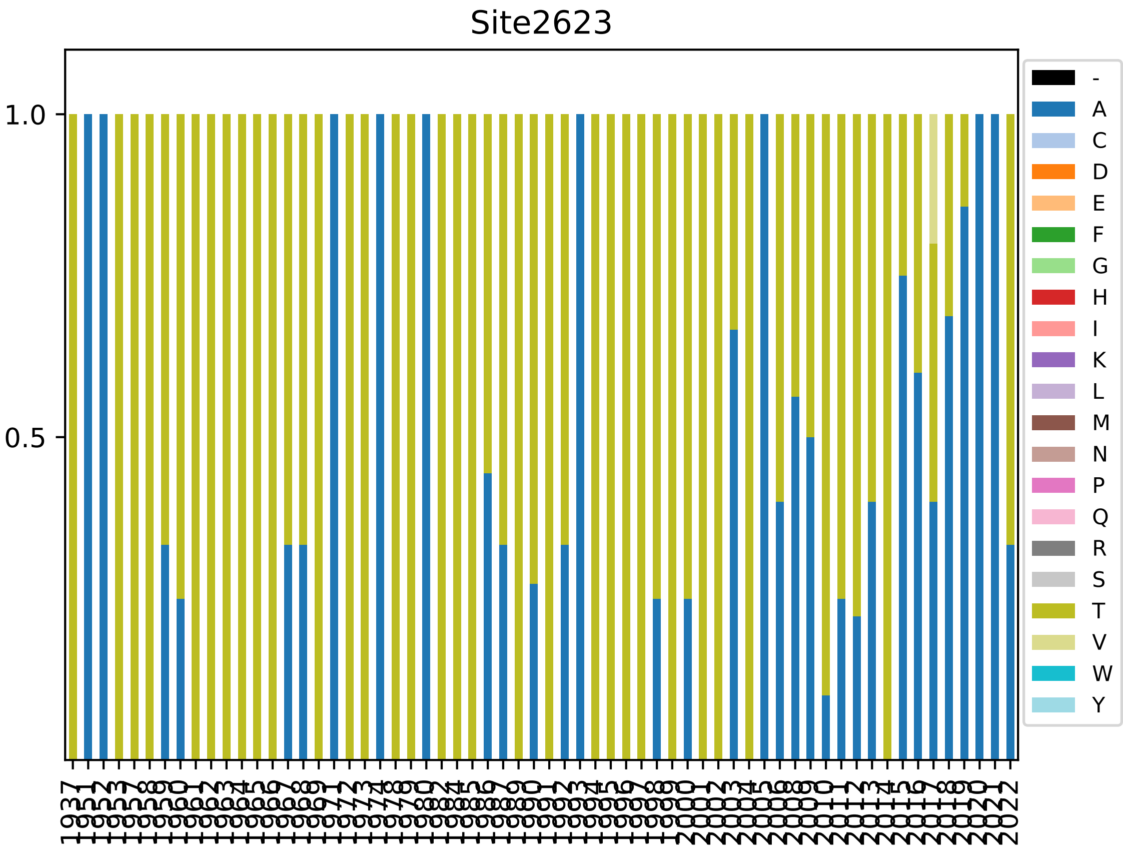
Site2623
1.0
0.5
-
A
C
D
E
F
G
H
I
K
L
M
N
P
Q
R
S
T
V
W
Y

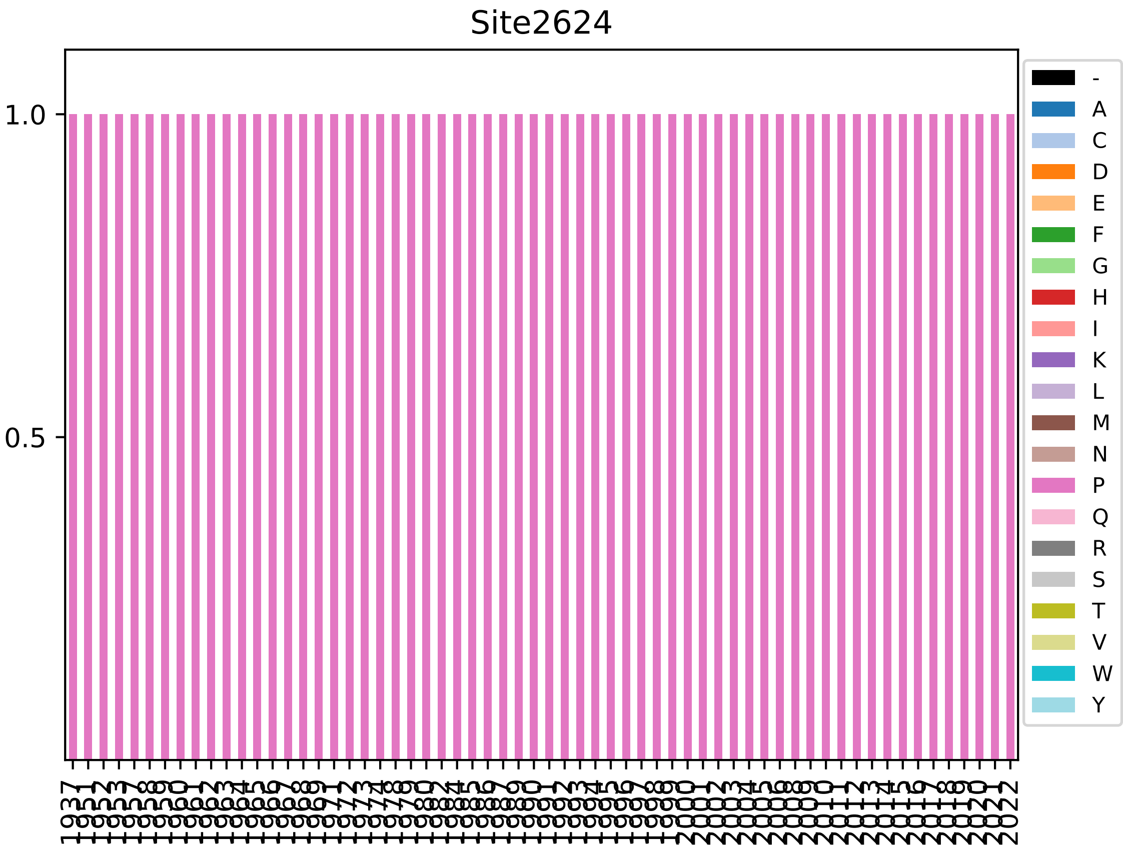
Site2624
1.0
0.5
-
A
C
D
E
F
G
H
I
K
L
M
N
P
Q
R
S
T
V
W
Y

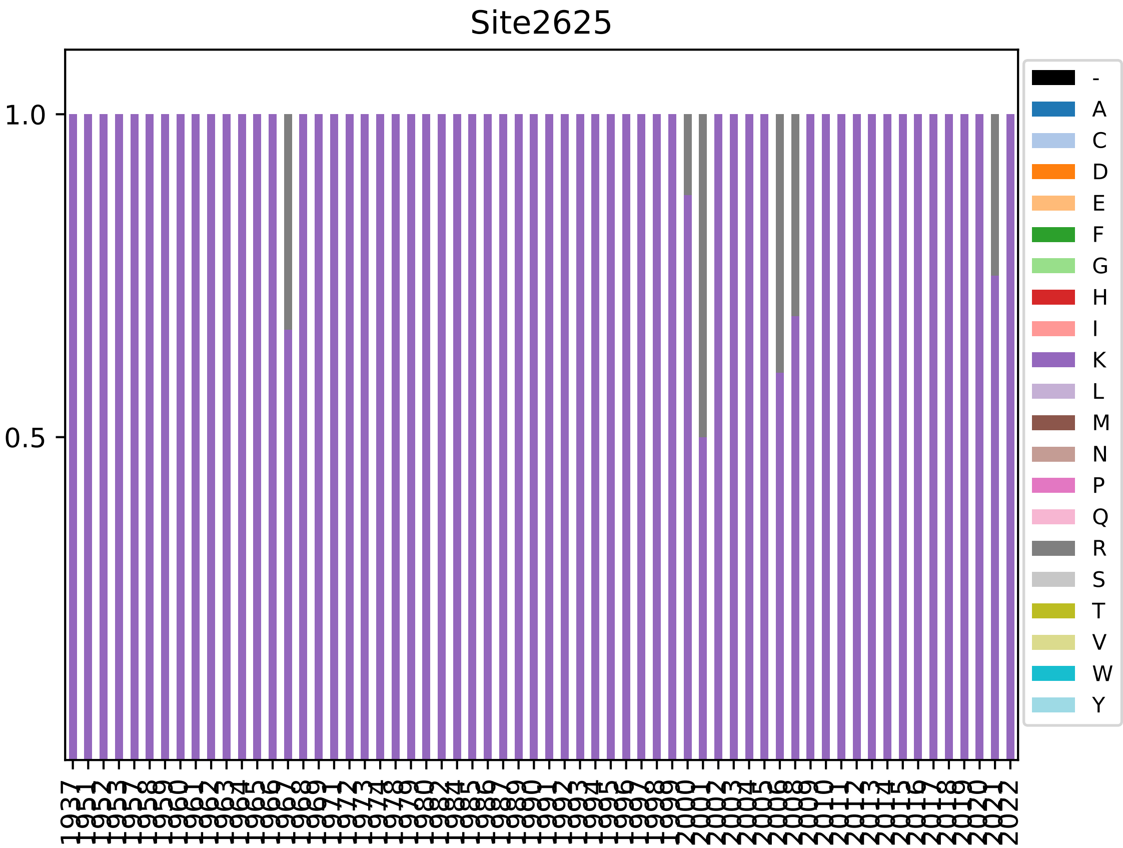
Site2625
1.0
0.5
-
A
C
D
E
F
G
H
I
K
L
M
N
P
Q
R
S
T
V
W
Y

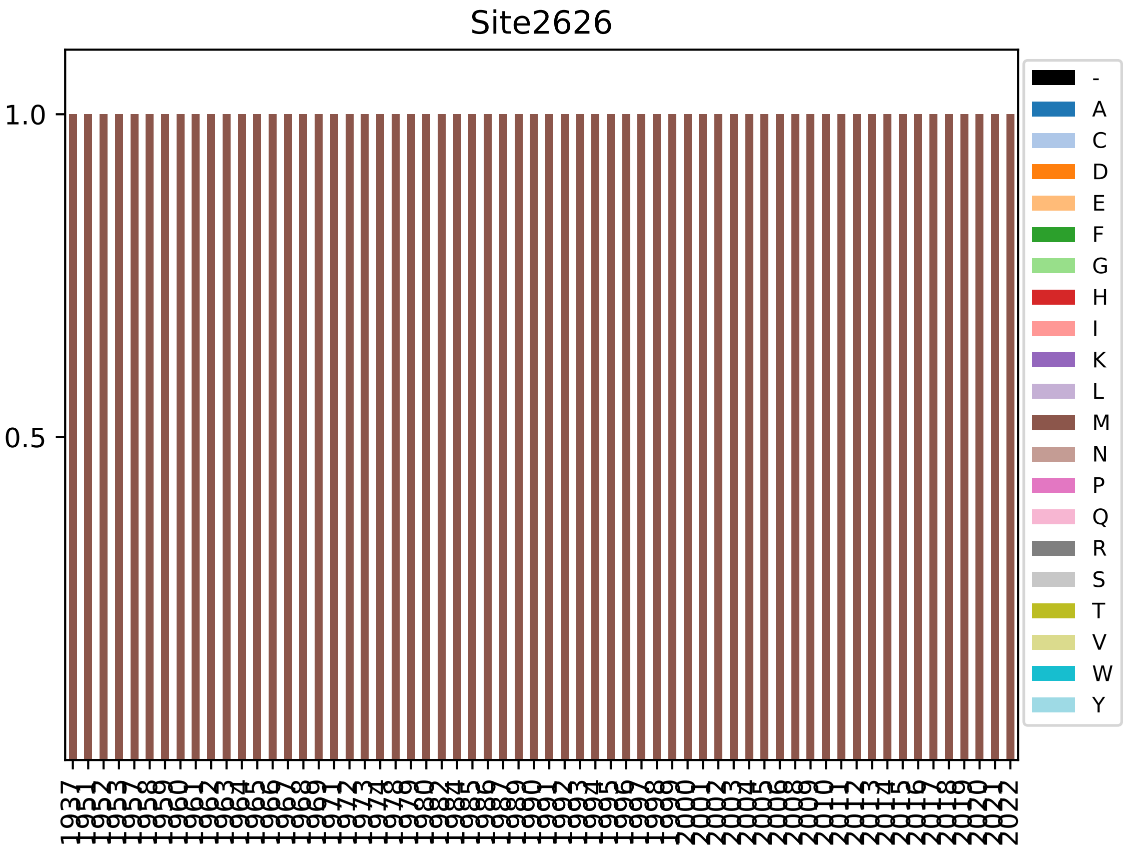
Site2626
1.0
0.5
-
A
C
D
E
F
G
H
I
K
L
M
N
P
Q
R
S
T
V
W
Y

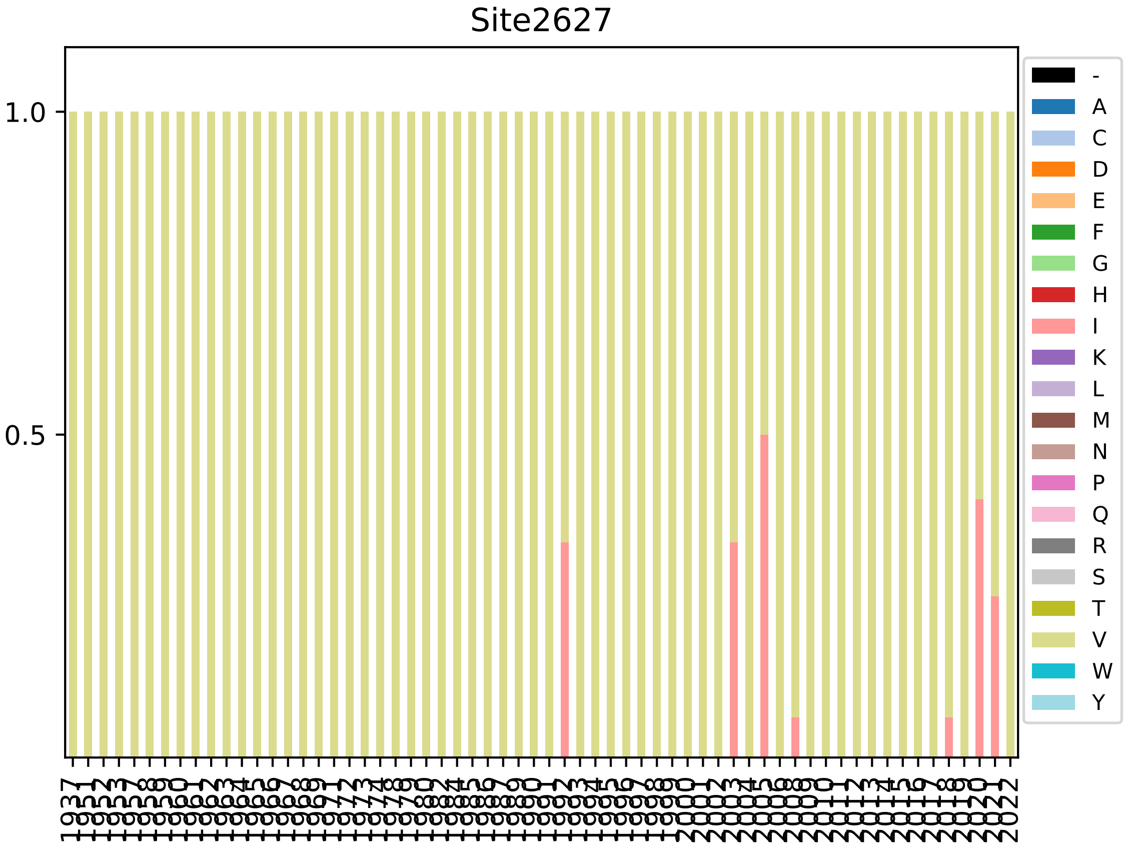
Site2627
1.0
0.5
-
A
C
D
E
F
G
H
I
K
L
M
N
P
Q
R
S
T
V
W
Y

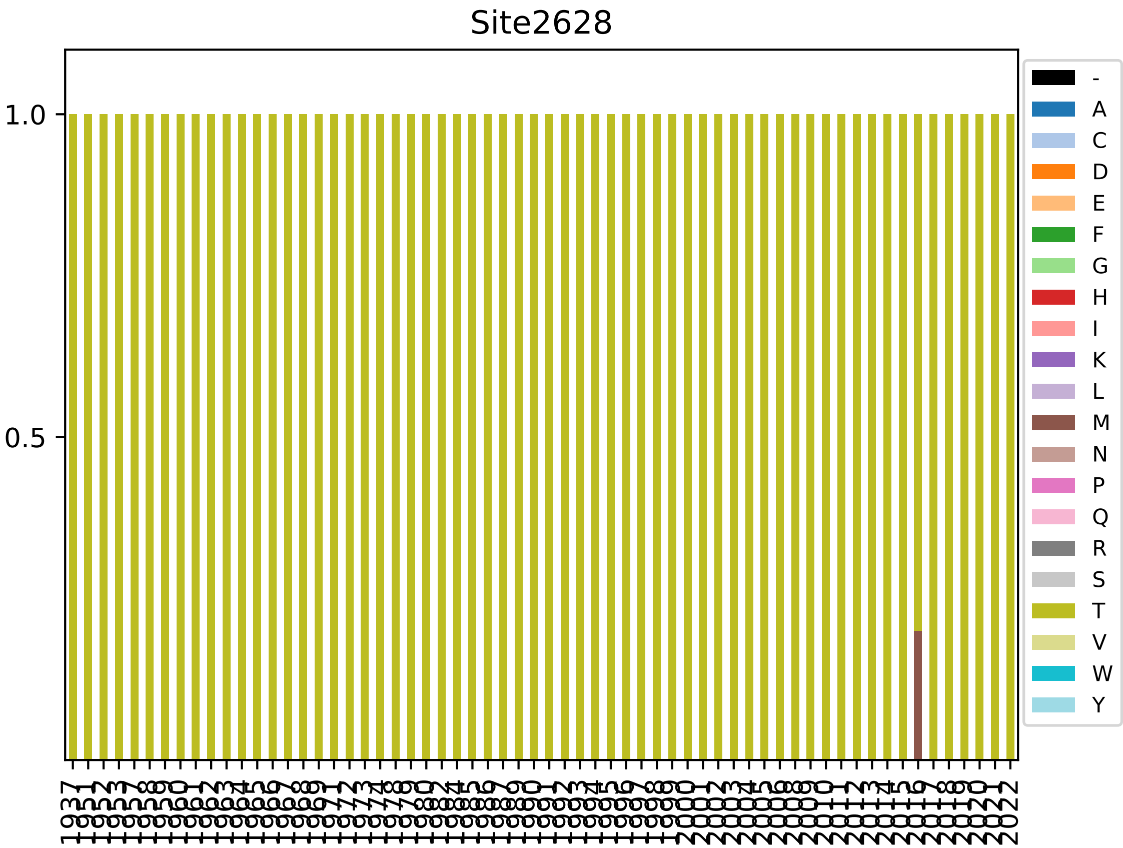
Site2628
1.0
0.5
-
A
C
D
E
F
G
H
I
K
L
M
N
P
Q
R
S
T
V
W
Y

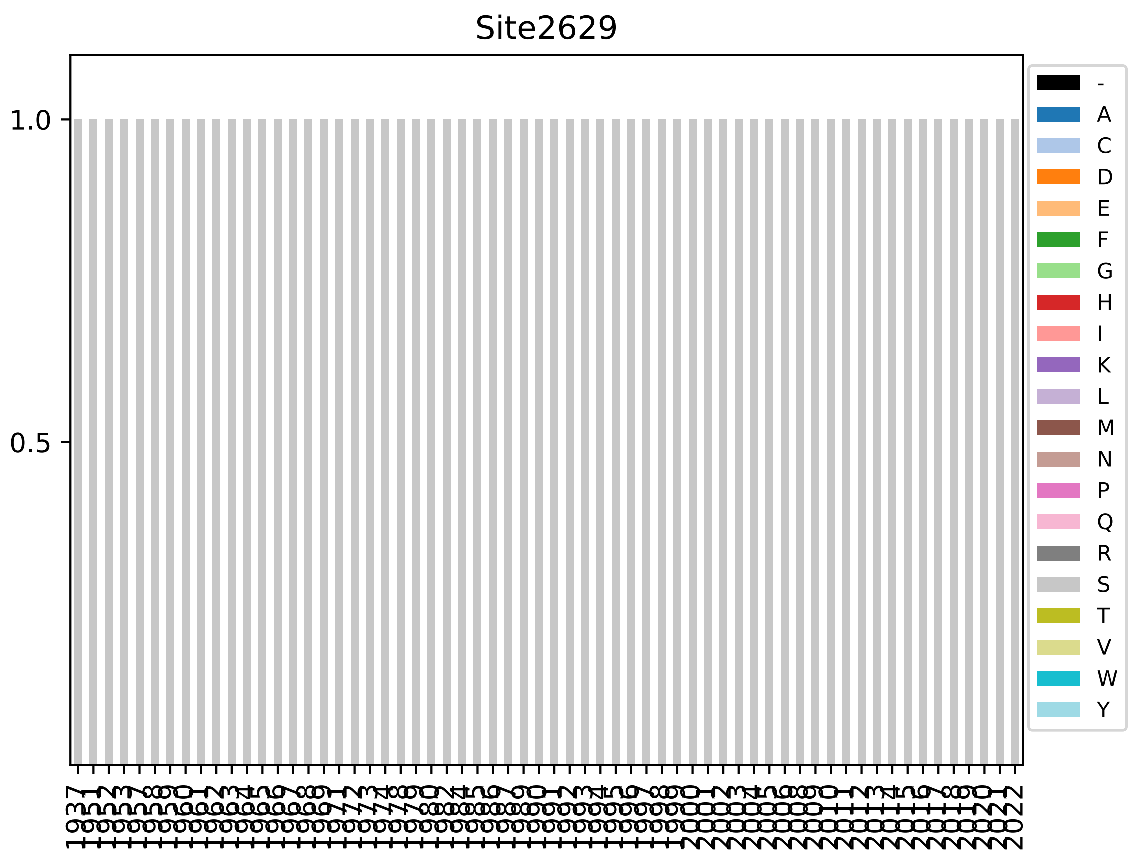
Site2629
1.0
0.5
-
A
C
D
E
F
G
H
I
K
L
M
N
P
Q
R
S
T
V
W
Y

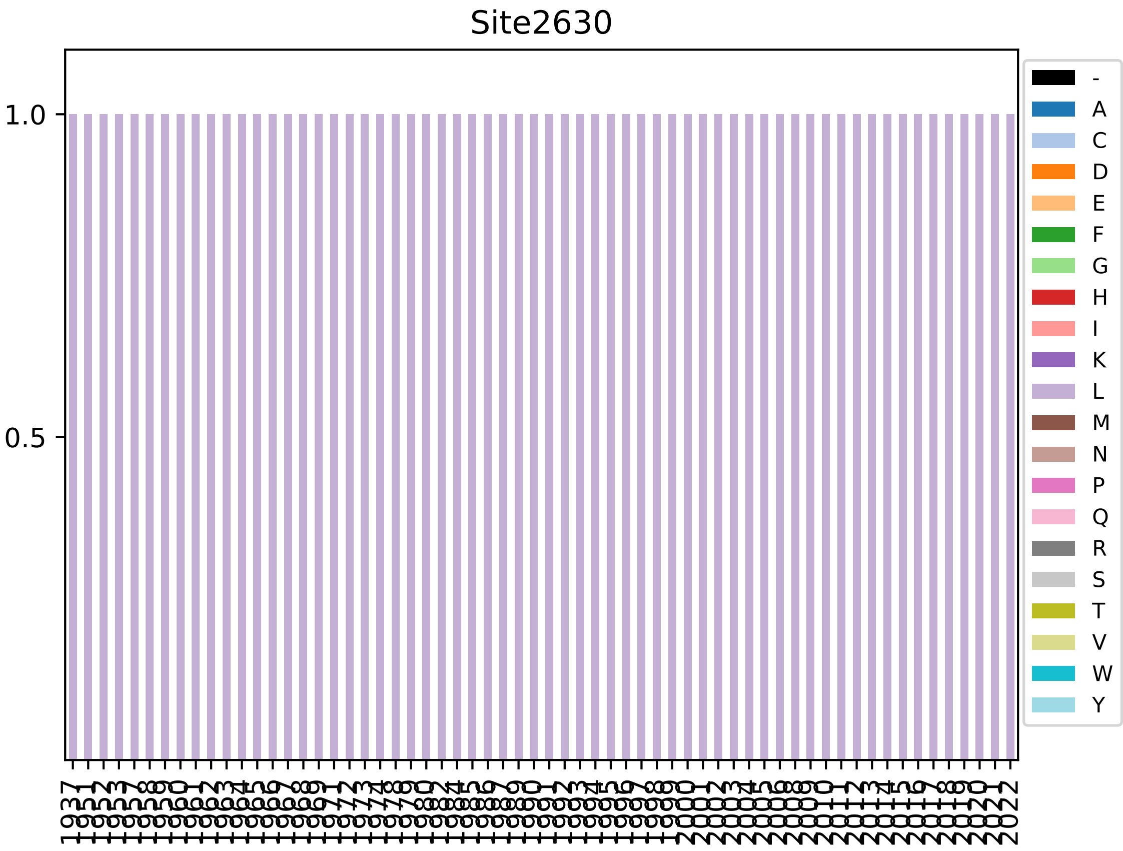
Site2630
1.0
0.5
-
A
C
D
E
F
G
H
I
K
L
M
N
P
Q
R
S
T
V
W
Y

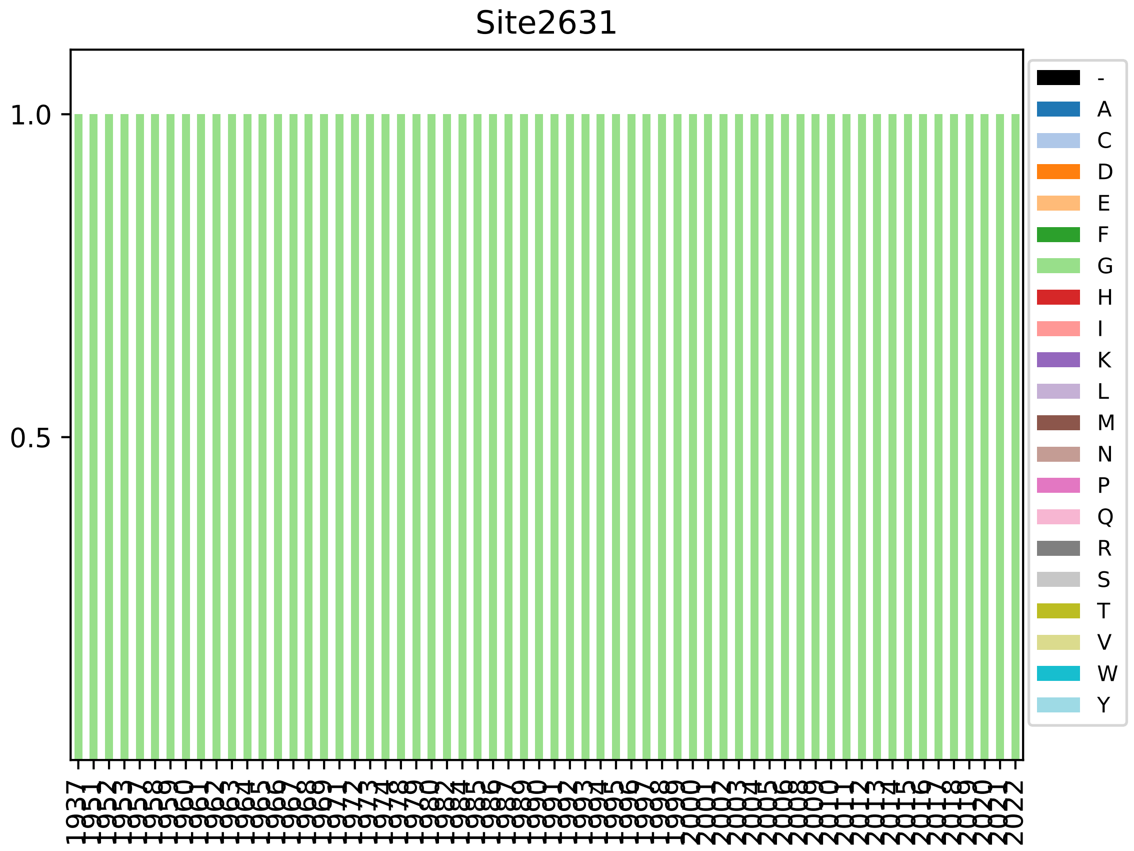
Site2631
1.0
0.5
-
A
C
D
E
F
G
H
I
K
L
M
N
P
Q
R
S
T
V
W
Y

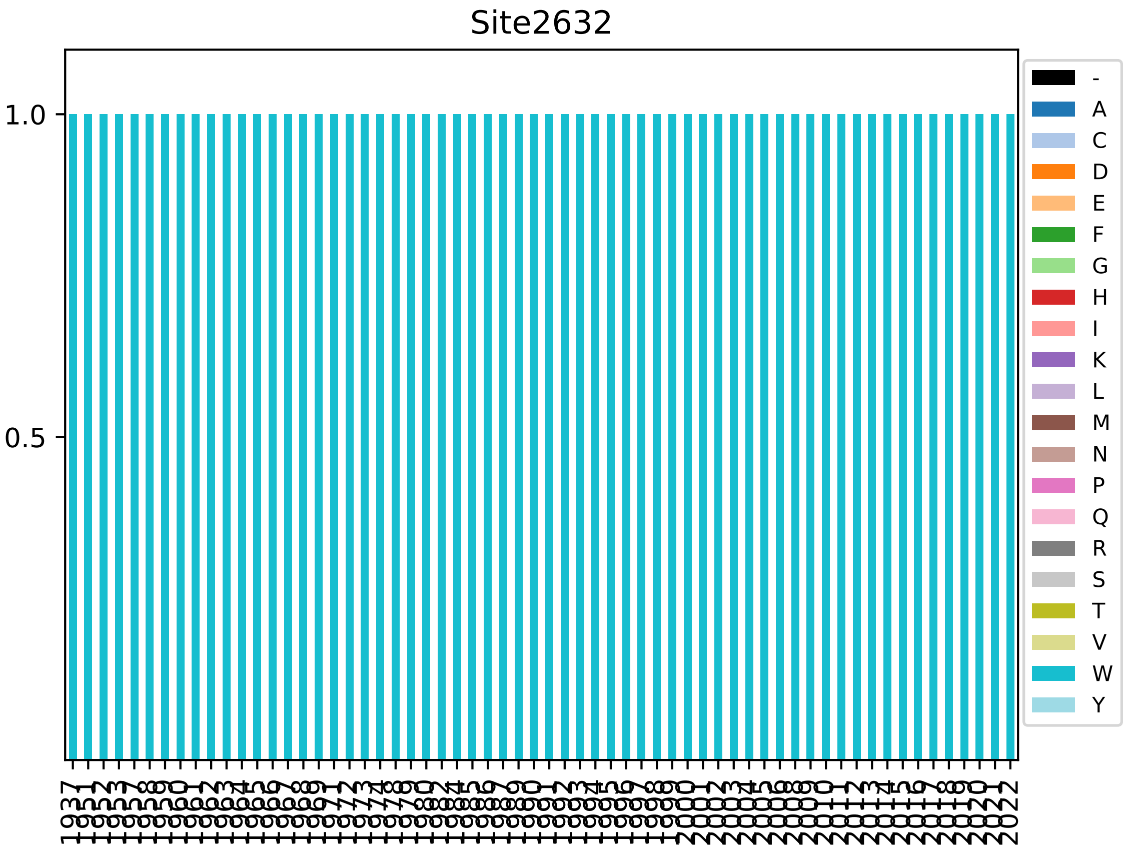
Site2632
1.0
0.5
-
A
C
D
E
F
G
H
I
K
L
M
N
P
Q
R
S
T
V
W
Y

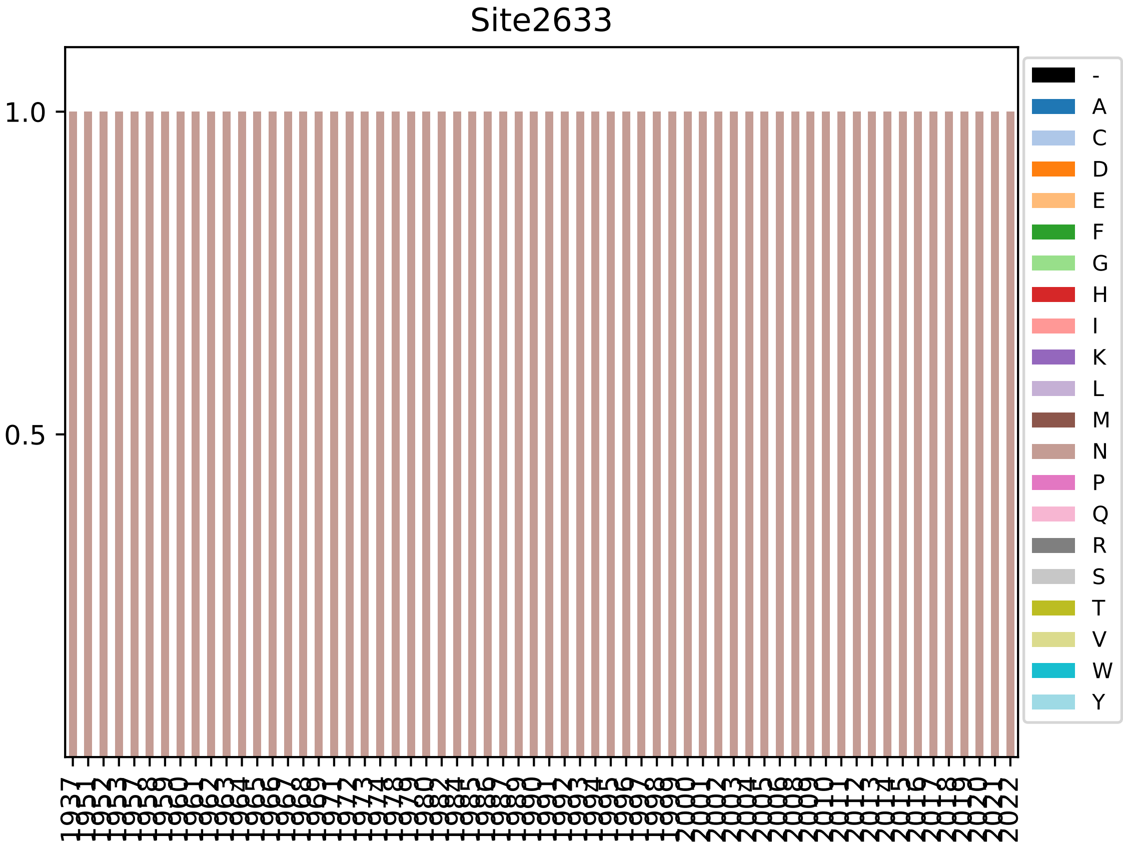
Site2633
1.0
0.5
-
A
C
D
E
F
G
H
I
K
L
M
N
P
Q
R
S
T
V
W
Y

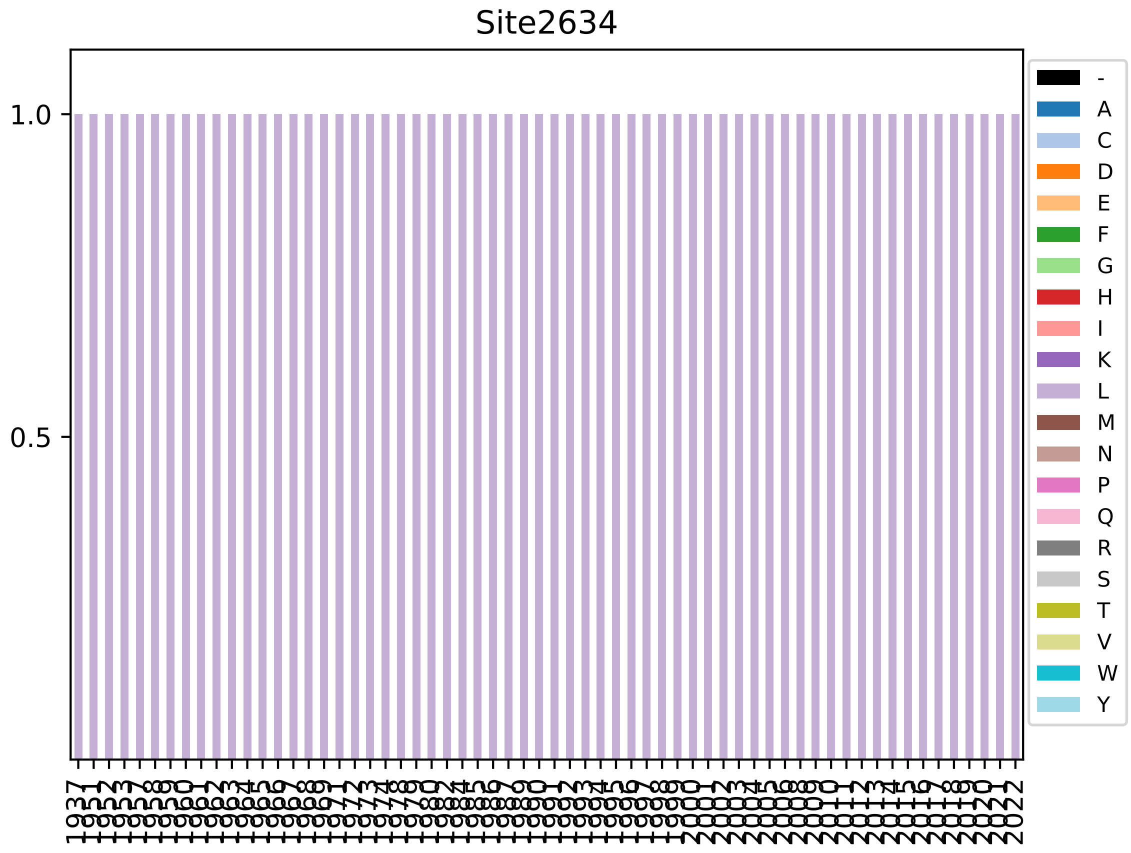

Site2634
1.0
0.5
-
A
C
D
E
F
G
H
I
K
L
M
N
P
Q
R
S
T
V
W
Y

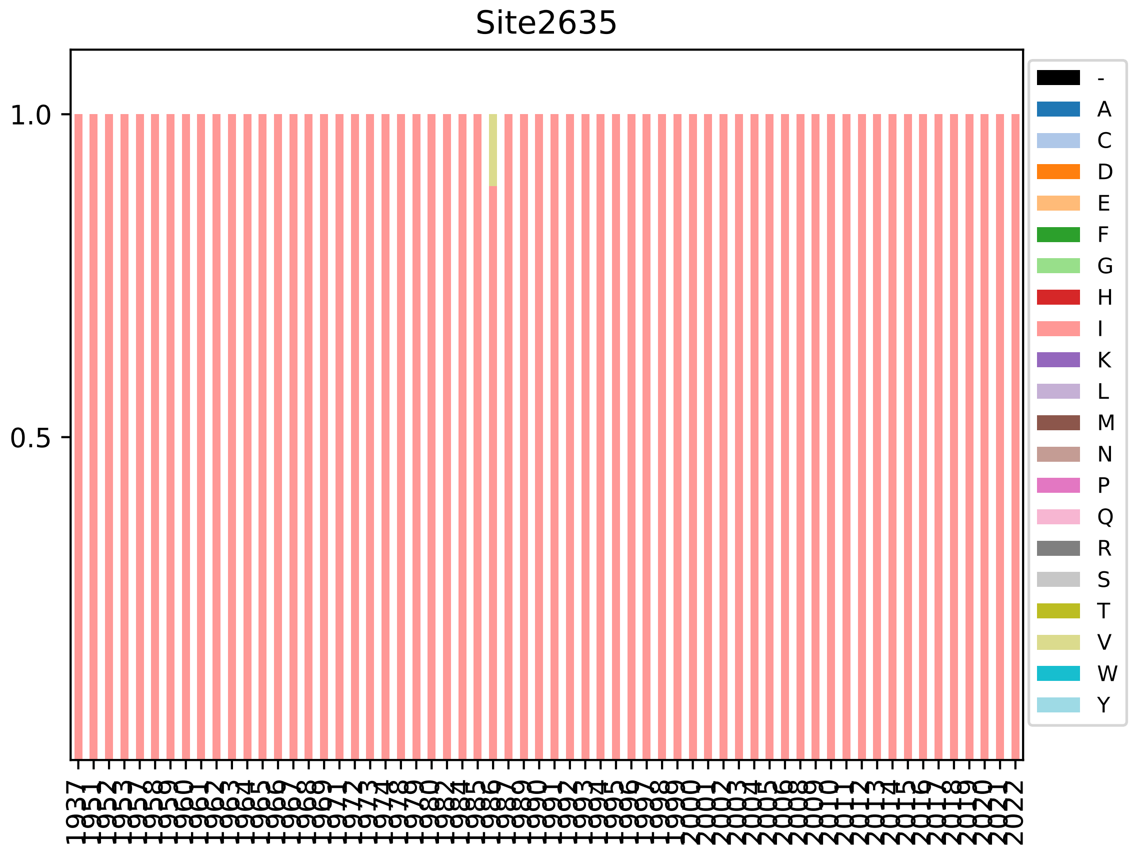
Site2635
1.0
0.5
-
A
C
D
E
F
G
H
I
K
L
M
N
P
Q
R
S
T
V
W
Y

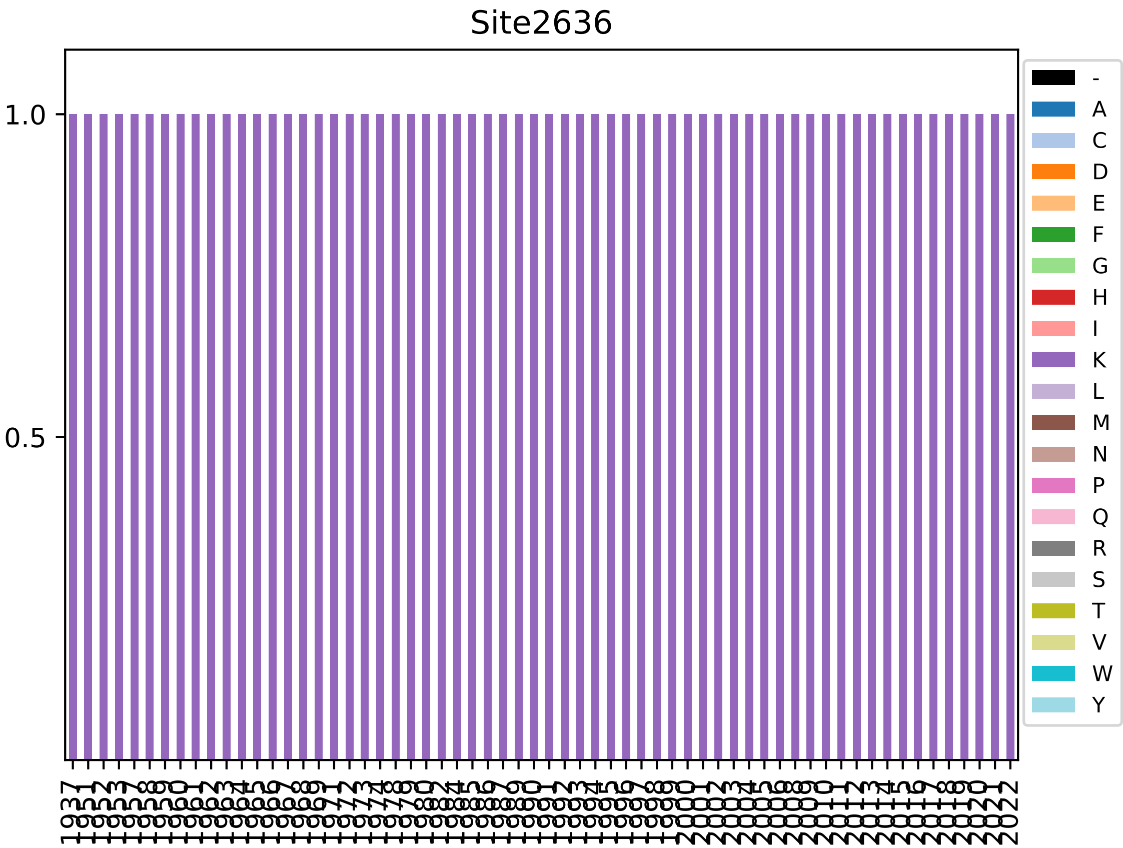
Site2636
1.0
0.5
-
A
C
D
E
F
G
H
I
K
L
M
N
P
Q
R
S
T
V
W
Y

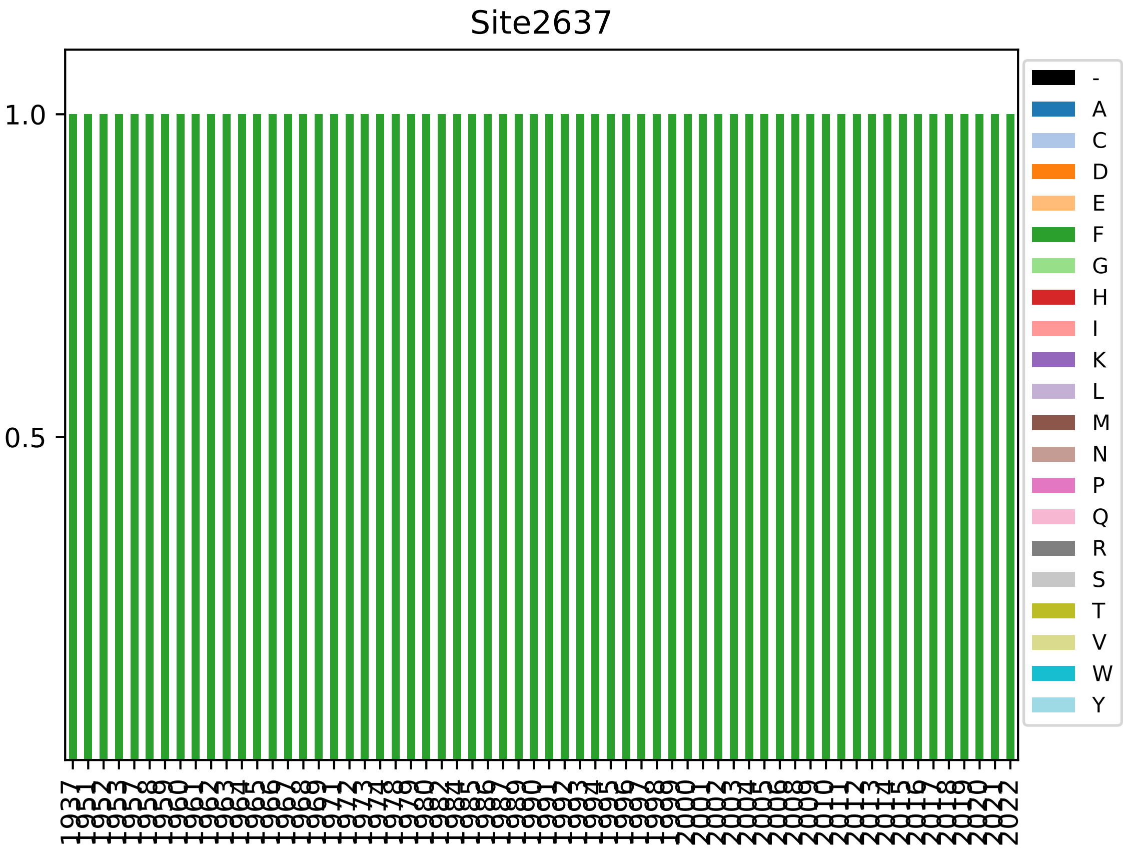
Site2637
1.0
0.5
-
A
C
D
E
F
G
H
I
K
L
M
N
P
Q
R
S
T
V
W
Y

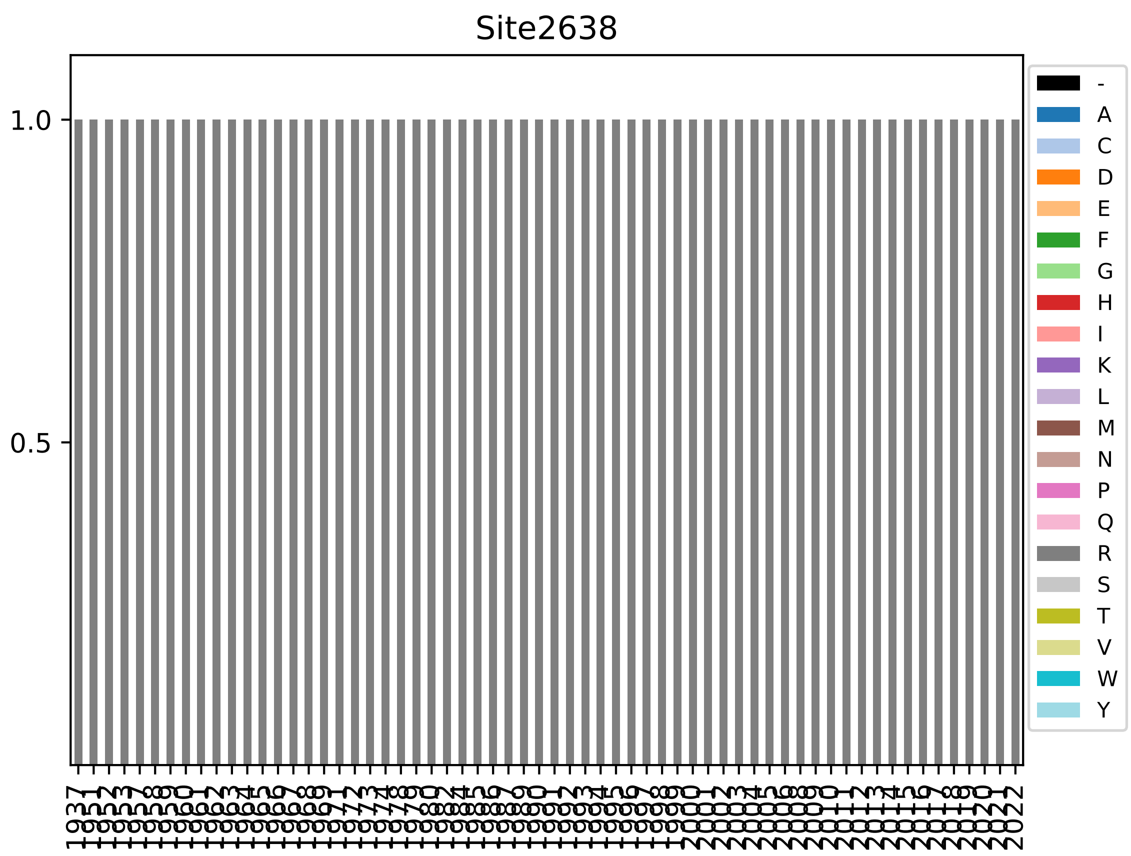
Site2638
1.0
0.5
-
A
C
D
E
F
G
H
I
K
L
M
N
P
Q
R
S
T
V
W
Y

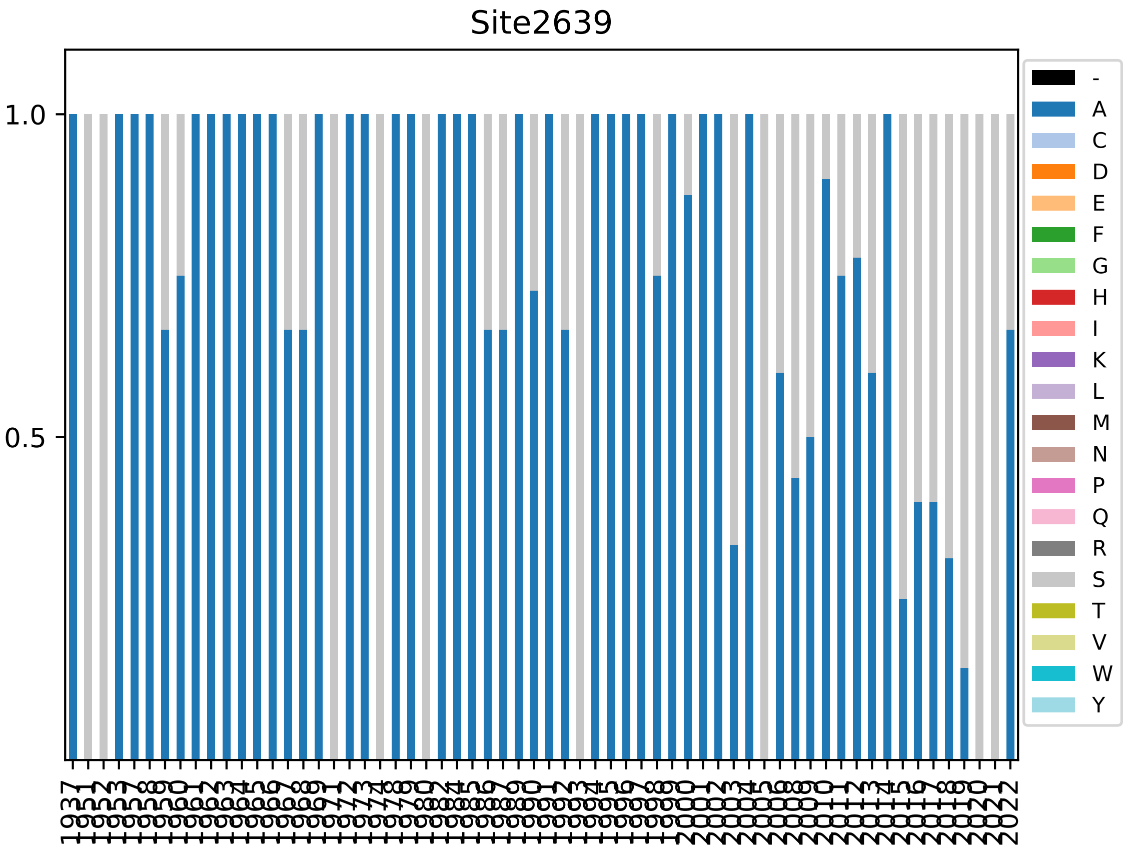
Site2639
1.0
0.5
-
A
C
D
E
F
G
H
I
K
L
M
N
P
Q
R
S
T
V
W
Y

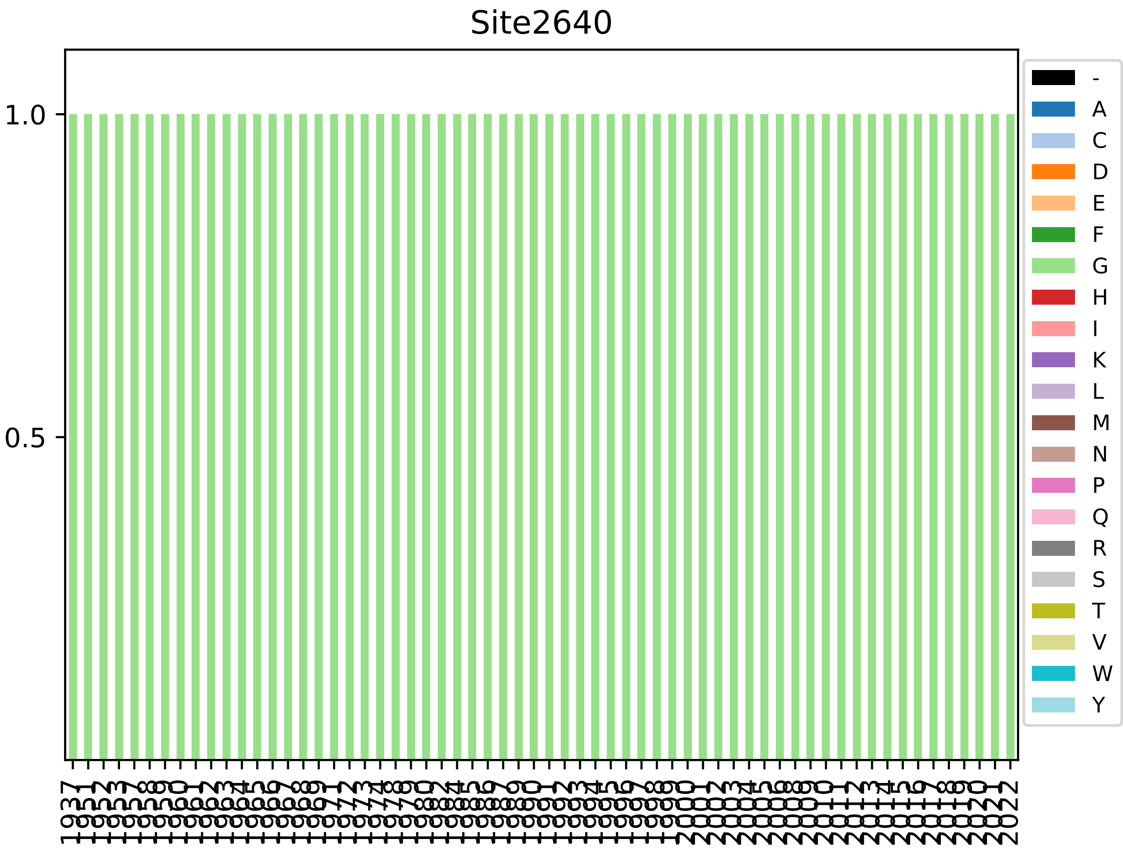

Site2640
1.0
0.5
-
A
C
D
E
F
G
H
I
K
L
M
N
P
Q
R
S
T
V
W
Y

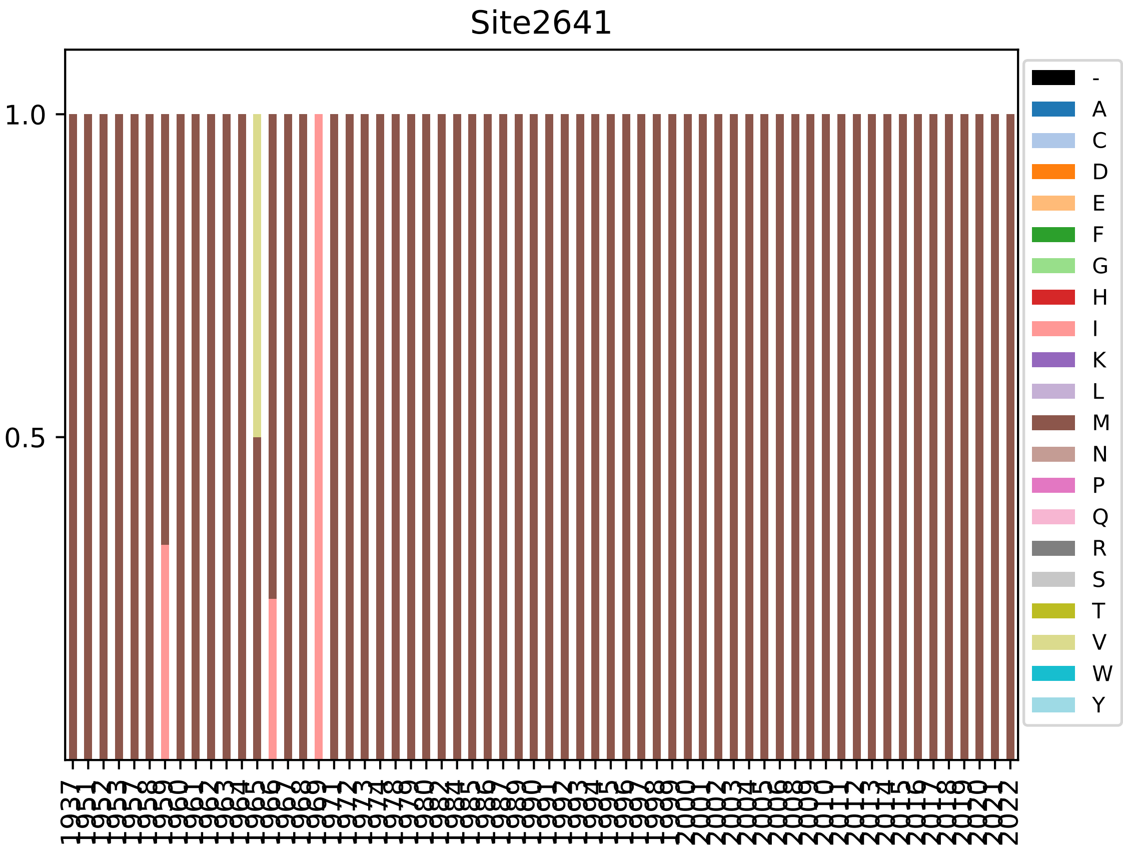
Site2641
1.0
0.5
-
A
C
D
E
F
G
H
I
K
L
M
N
P
Q
R
S
T
V
W
Y

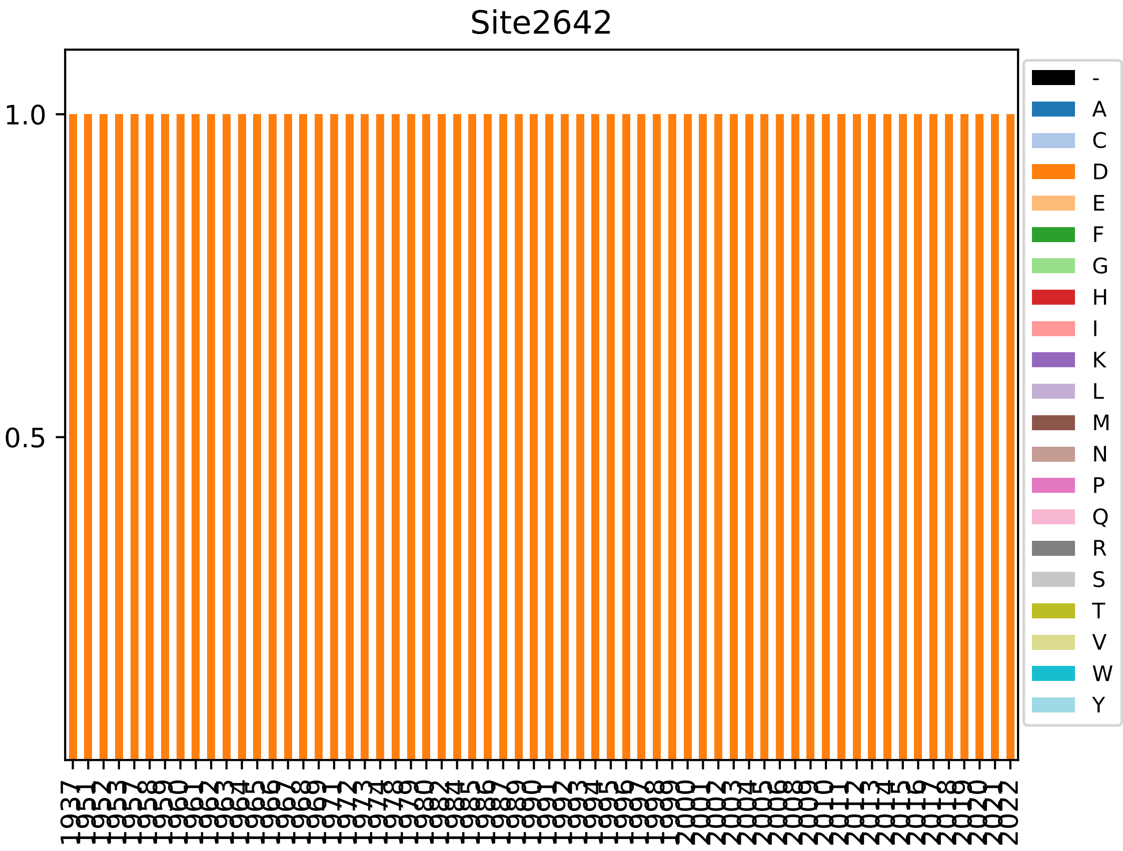
Site2642
1.0
0.5
-
A
C
D
E
F
G
H
I
K
L
M
N
P
Q
R
S
T
V
W
Y

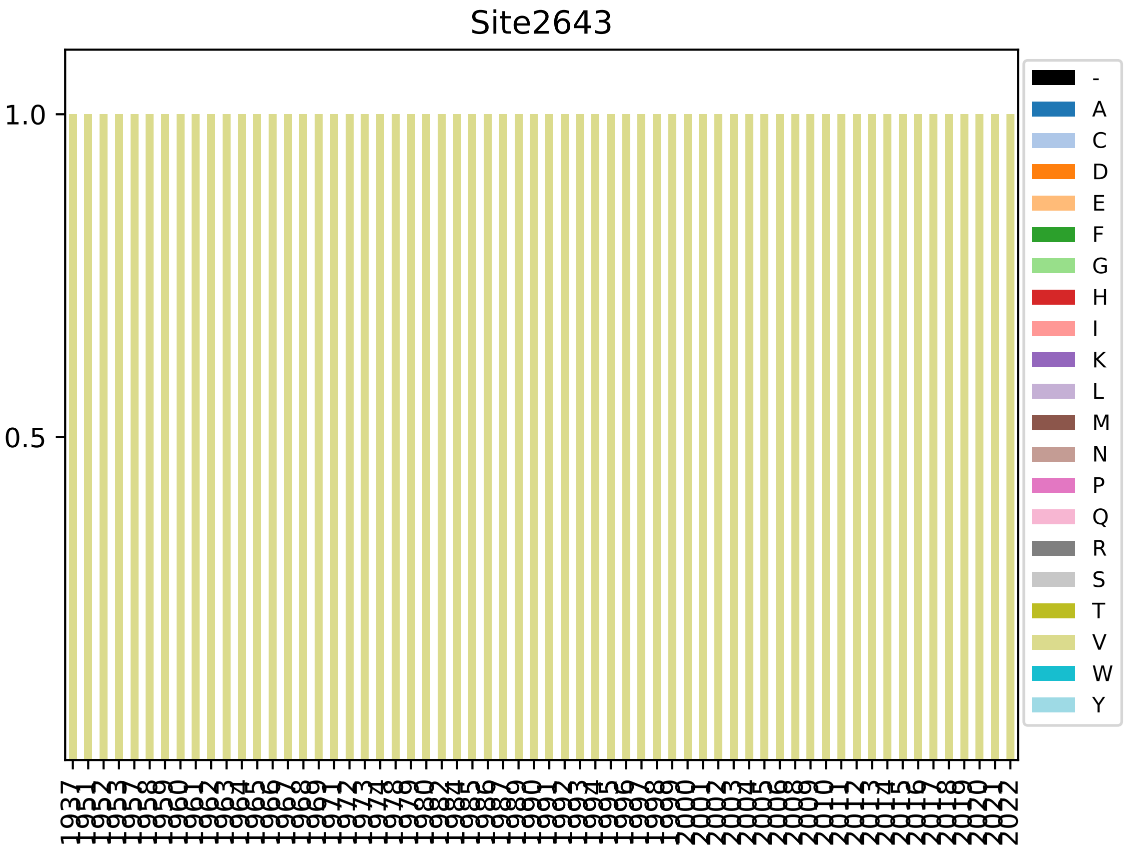
Site2643
1.0
0.5
-
A
C
D
E
F
G
H
I
K
L
M
N
P
Q
R
S
T
V
W
Y

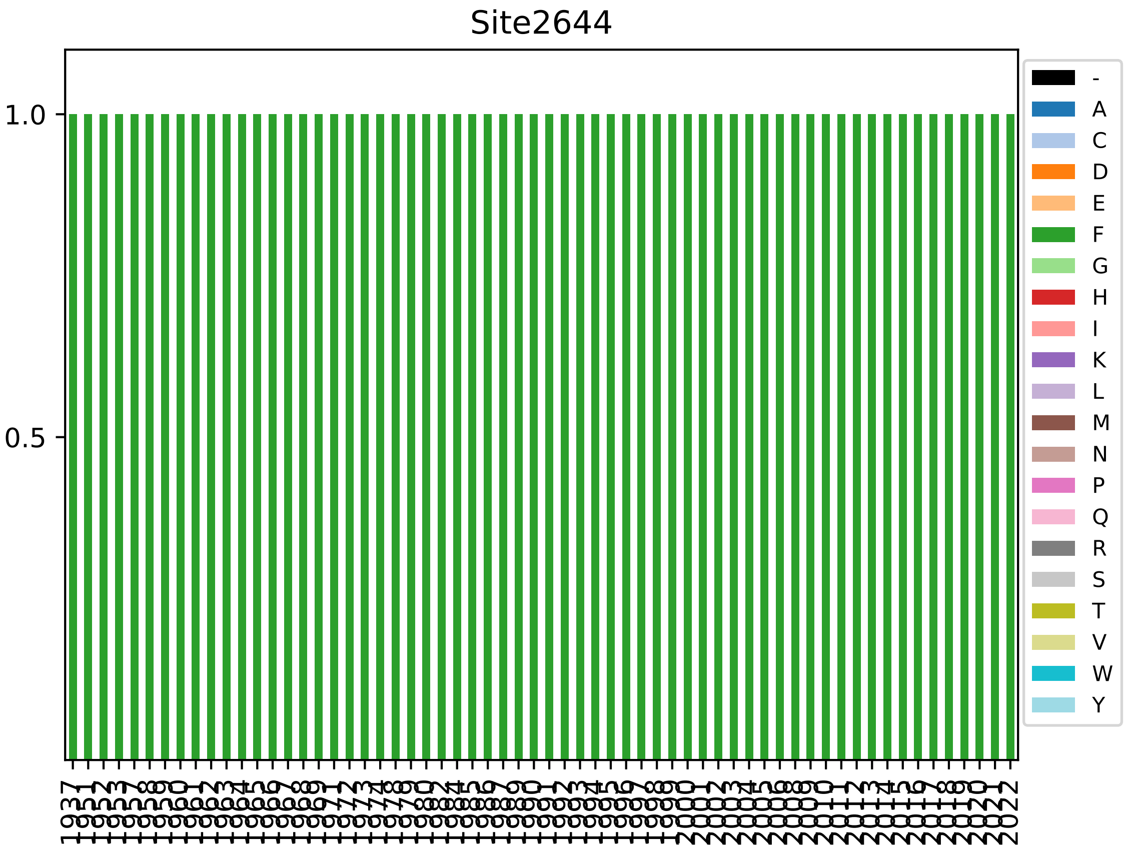

Site2644
1.0
0.5
-
A
C
D
E
F
G
H
I
K
L
M
N
P
Q
R
S
T
V
W
Y

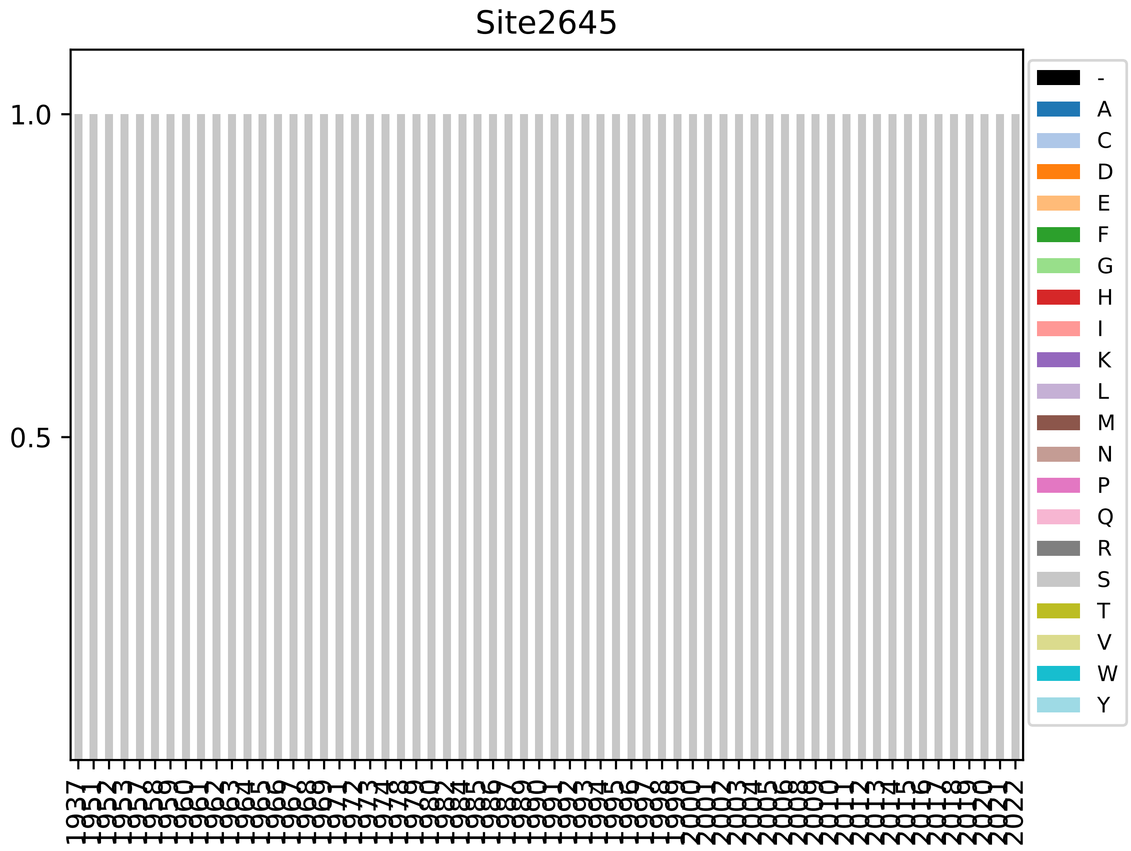
Site2645
1.0
0.5
-
A
C
D
E
F
G
H
I
K
L
M
N
P
Q
R
S
T
V
W
Y

Site2646

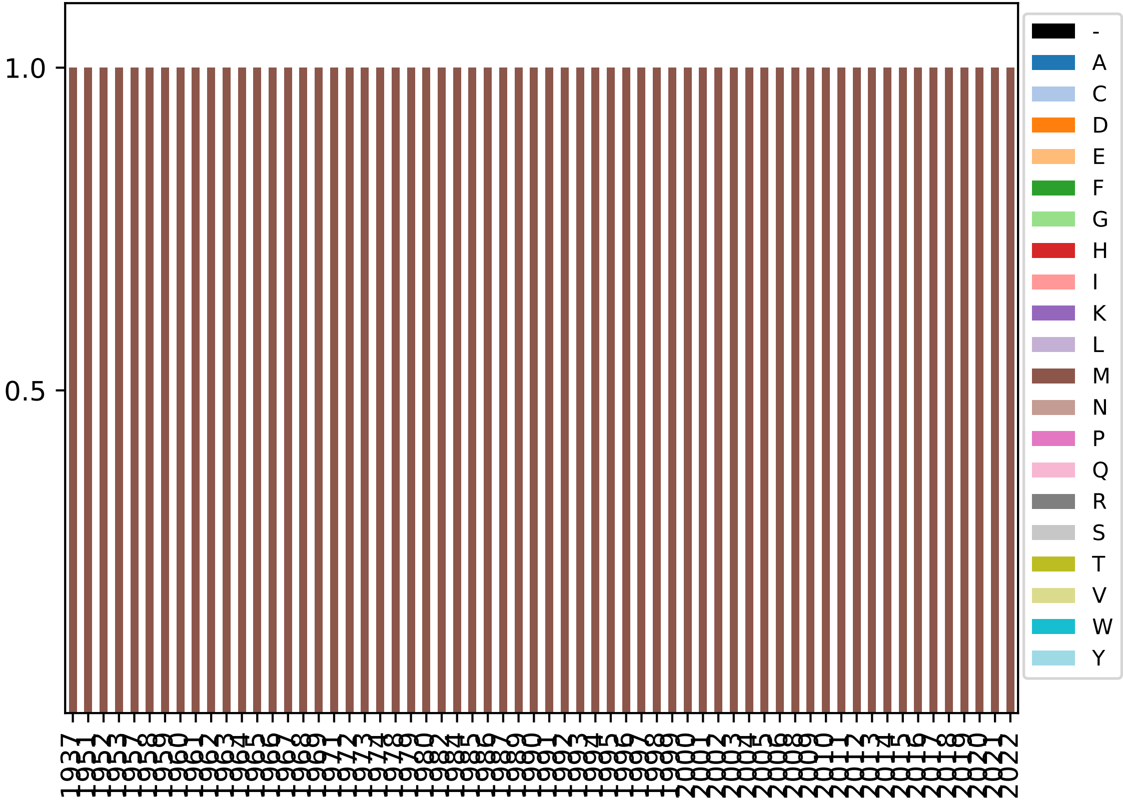

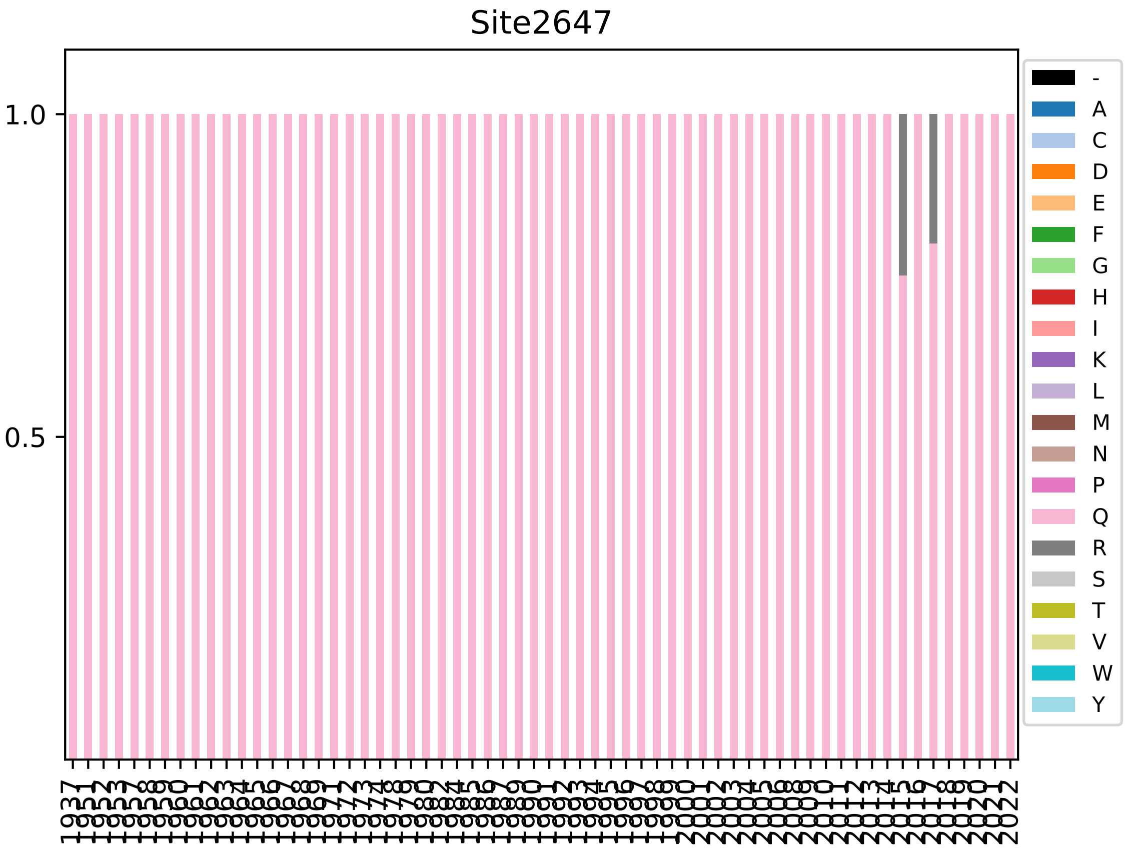
Site2647
1.0
0.5
-
A
C
D
E
F
G
H
I
K
L
M
N
P
Q
R
S
T
V
W
Y

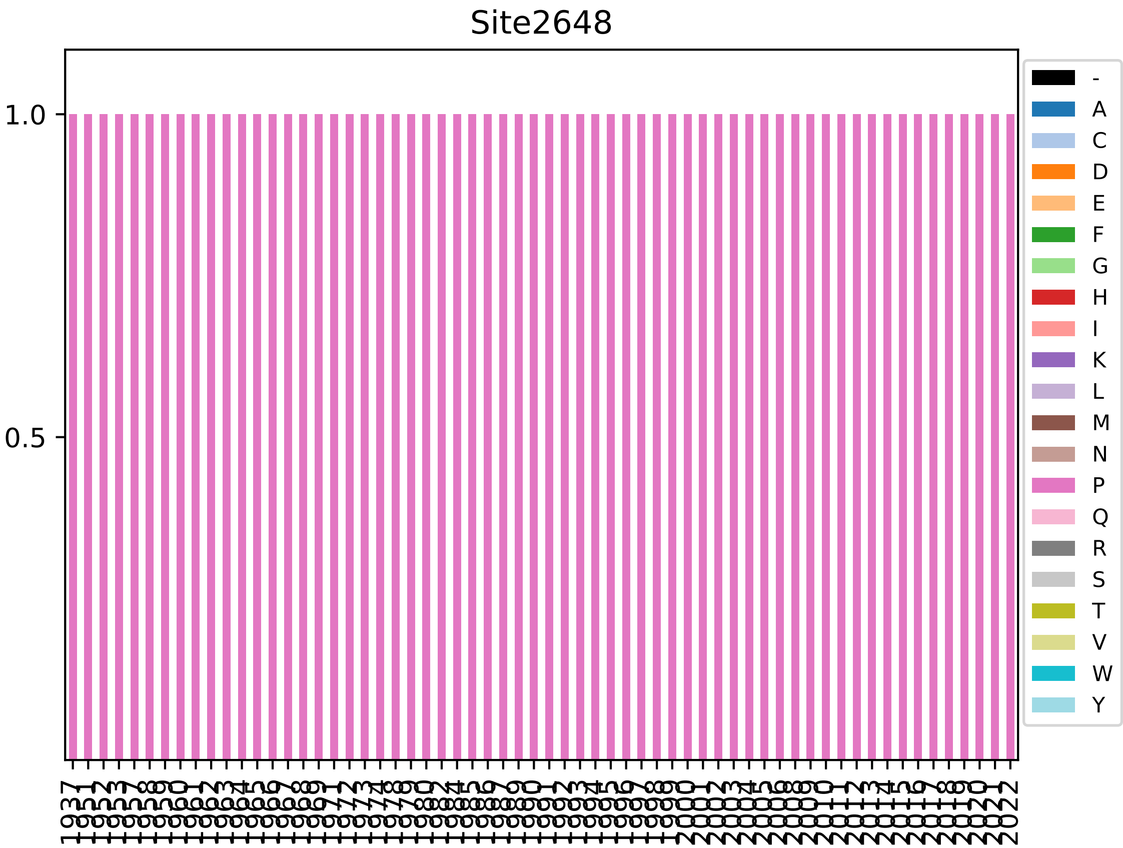
Site2648
1.0
0.5
-
A
C
D
E
F
G
H
I
K
L
M
N
P
Q
R
S
T
V
W
Y

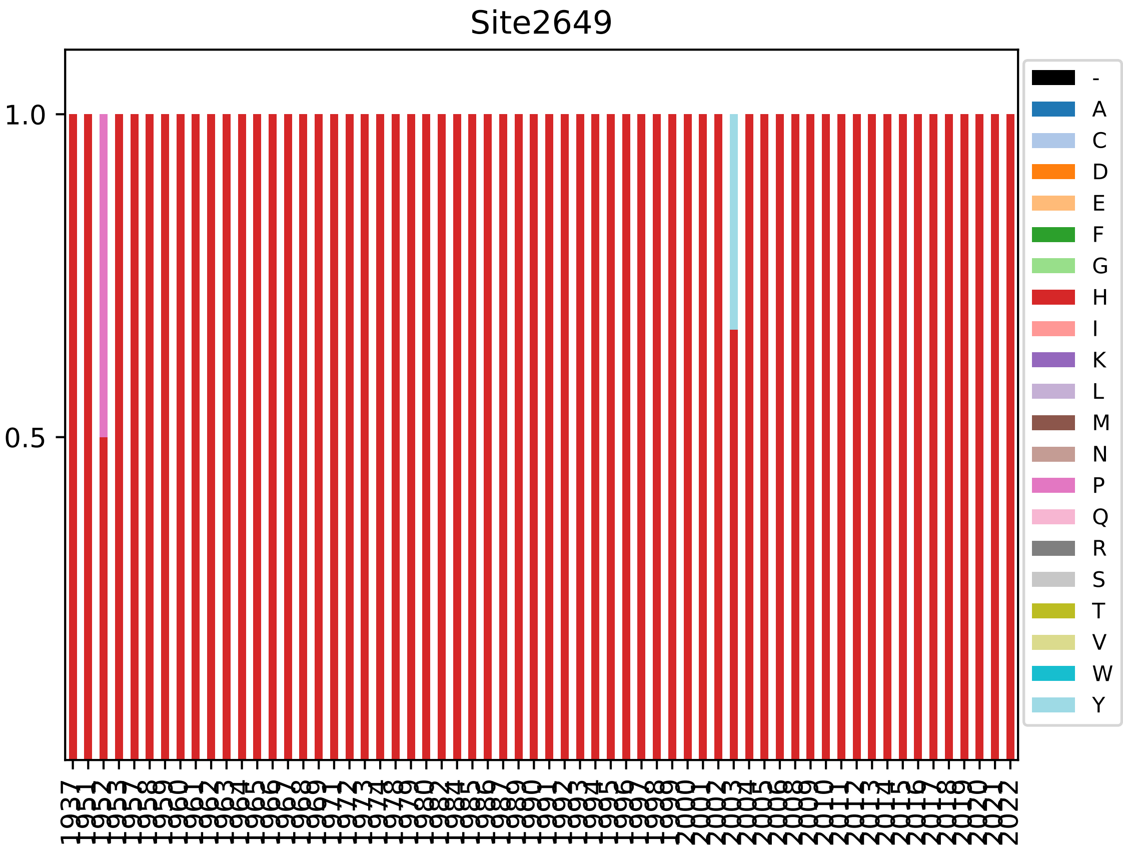
Site2649
1.0
0.5
-
A
C
D
E
F
G
H
I
K
L
M
N
P
Q
R
S
T
V
W
Y

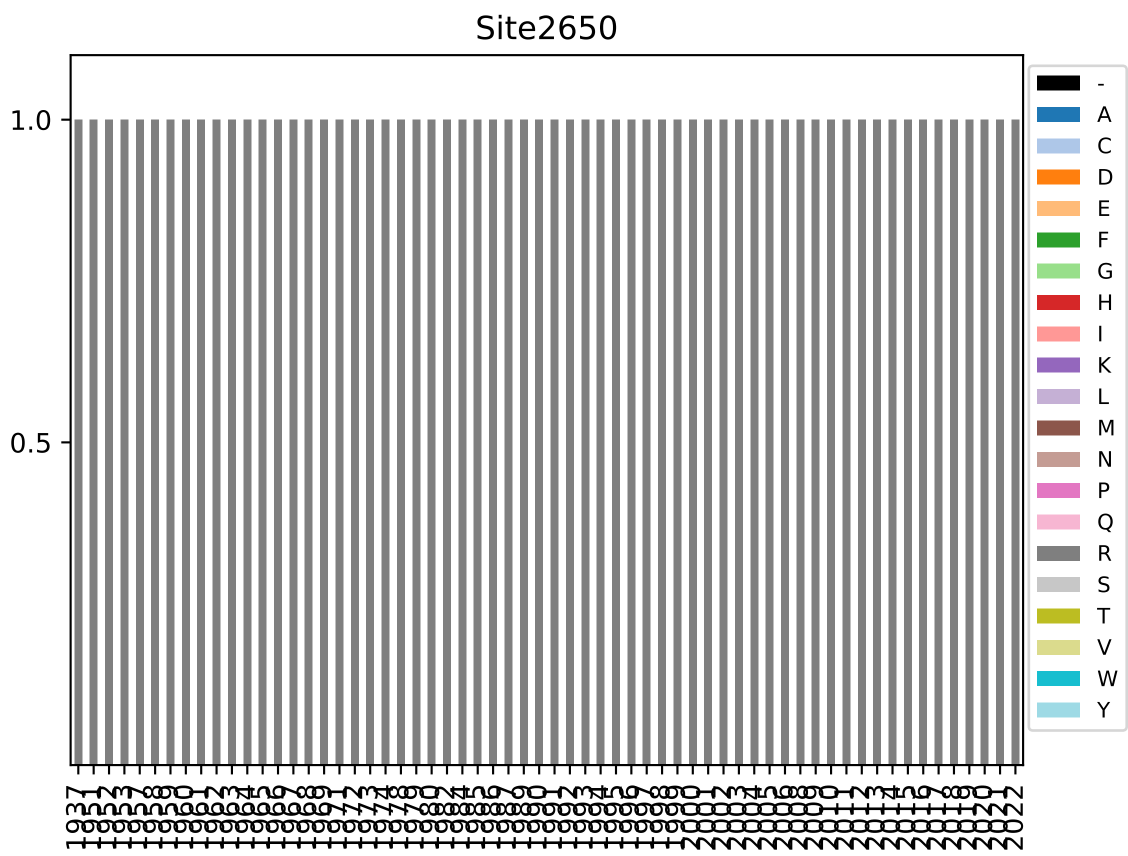

Site2650
1.0
0.5
-
A
C
D
E
F
G
H
I
K
L
M
N
P
Q
R
S
T
V
W
Y

Site2651

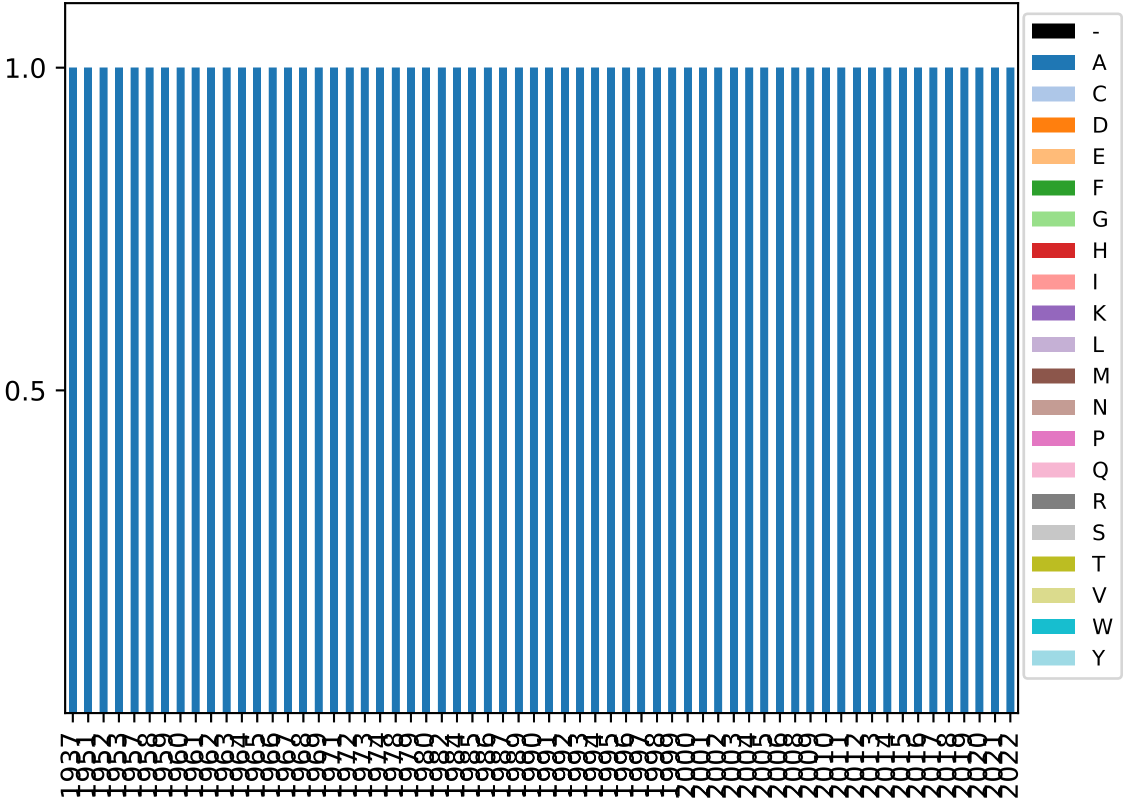

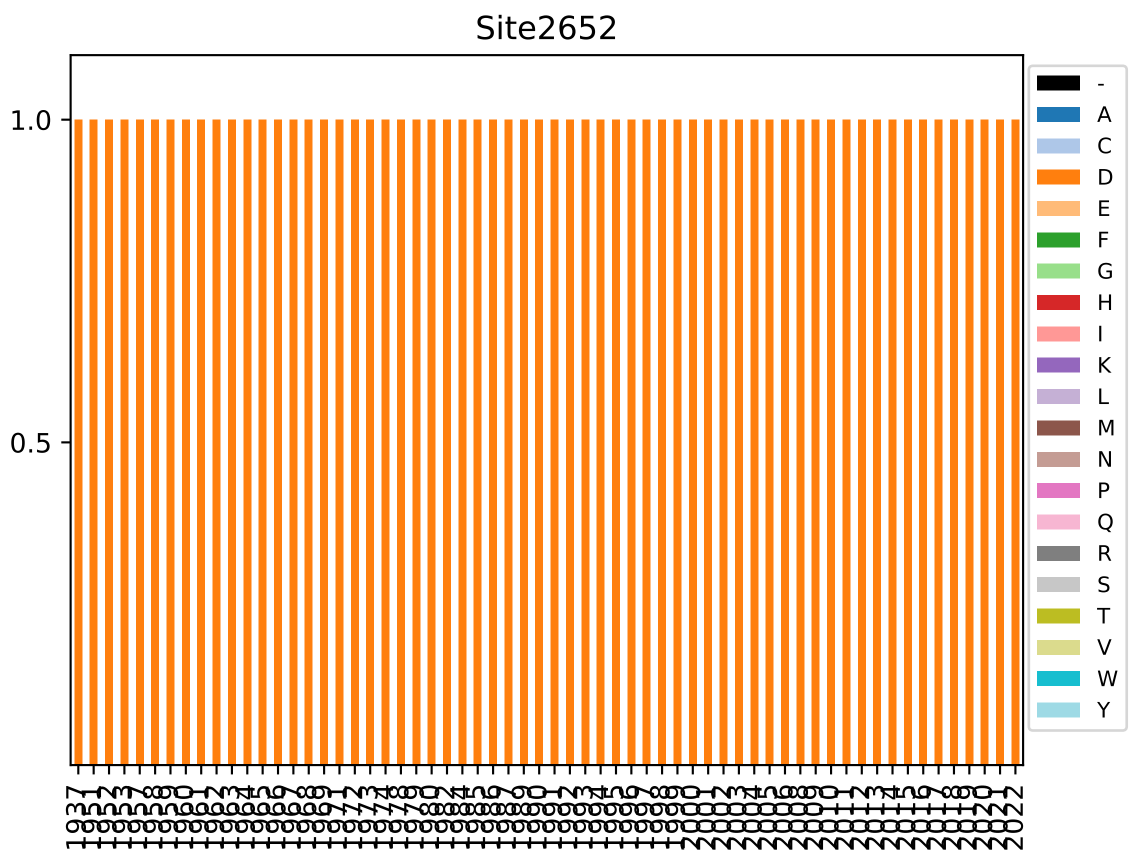
Site2652
1.0
0.5
-
A
C
D
E
F
G
H
I
K
L
M
N
P
Q
R
S
T
V
W
Y

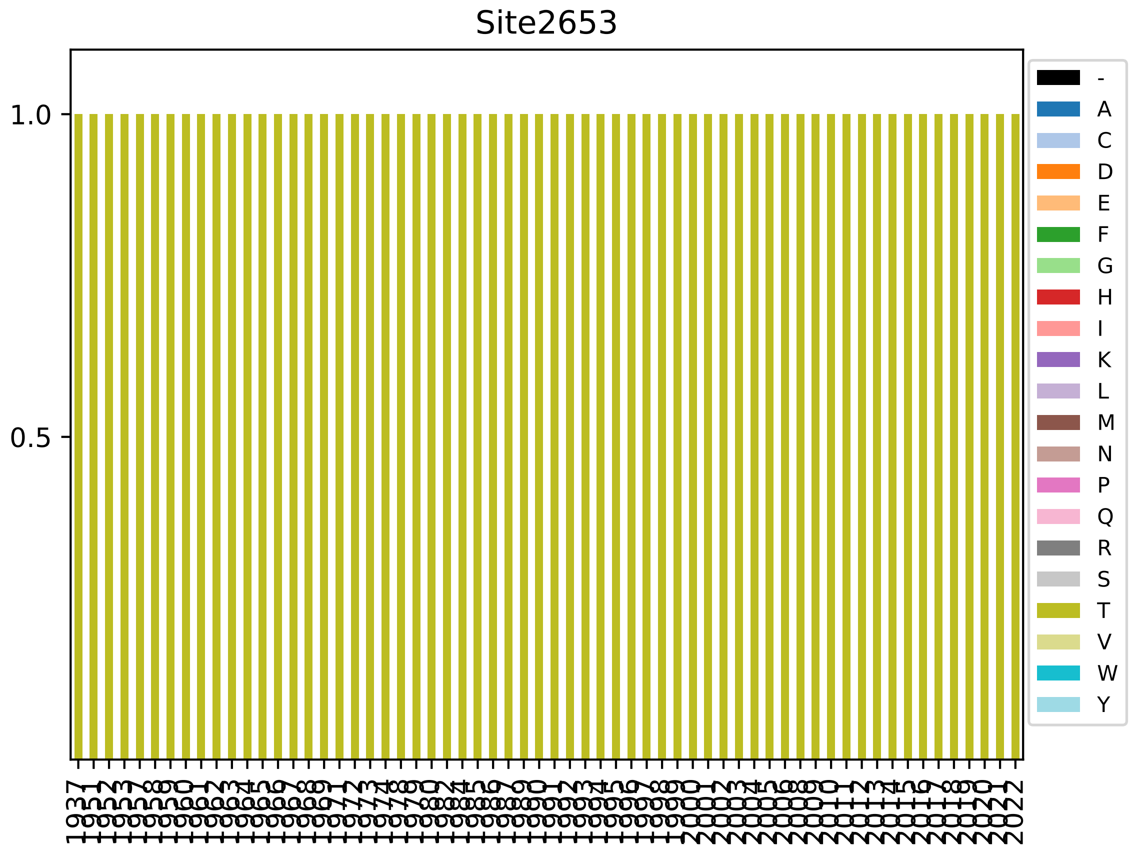
Site2653
1.0
0.5
-
A
C
D
E
F
G
H
I
K
L
M
N
P
Q
R
S
T
V
W
Y

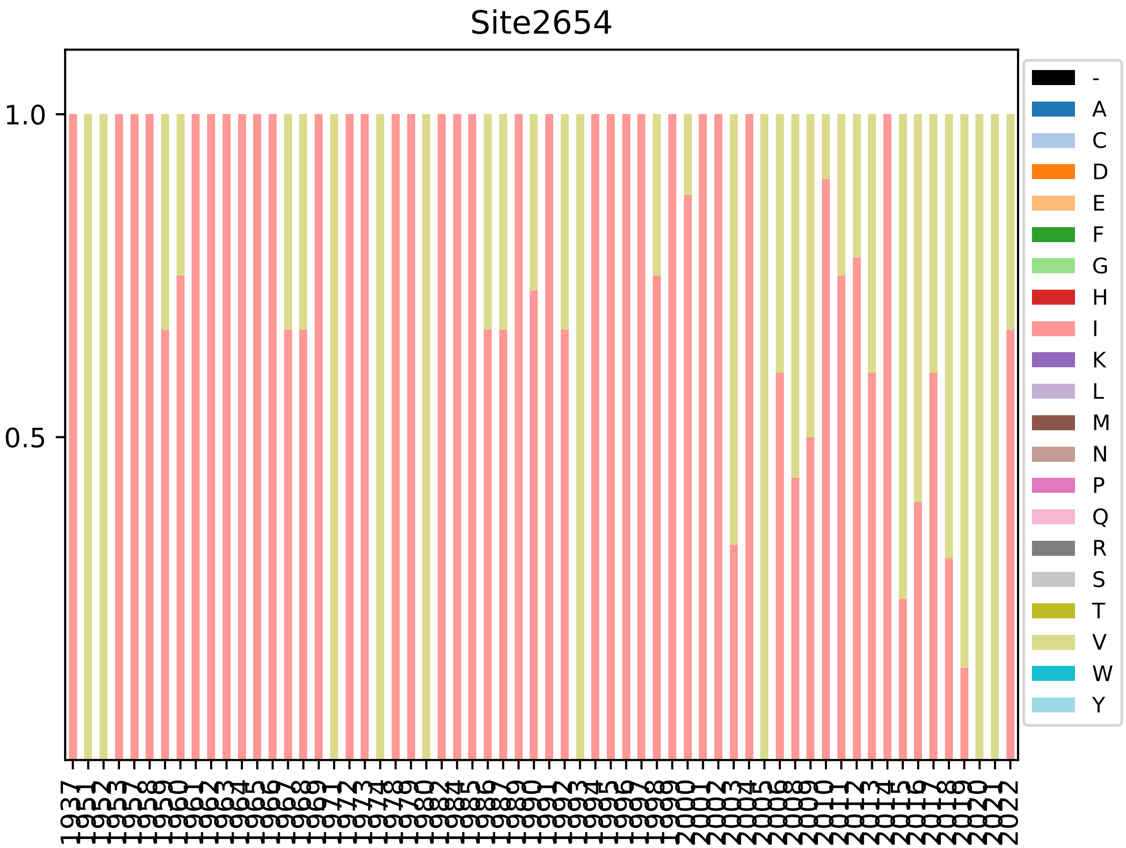

Site2654
1.0
0.5
-
A
C
D
E
F
G
H
I
K
L
M
N
P
Q
R
S
T
V
W
Y

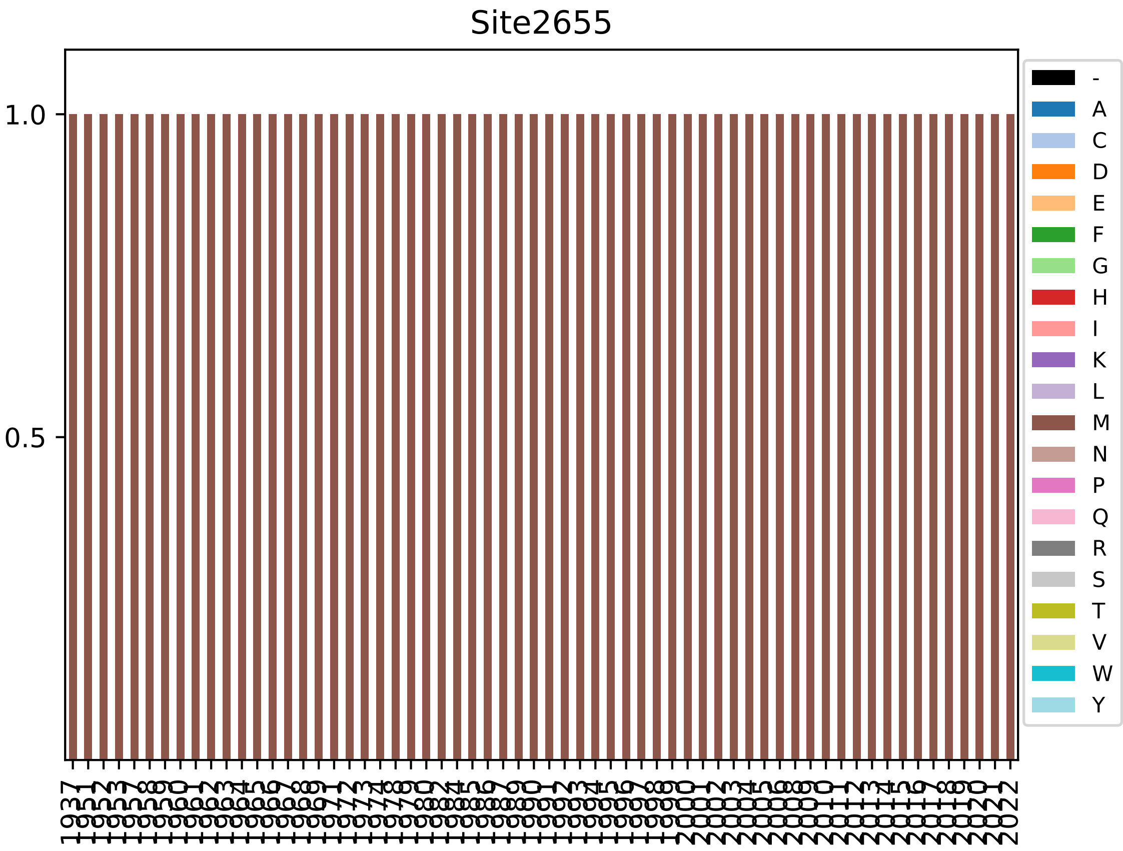

Site2655
1.0
0.5
-
A
C
D
E
F
G
H
I
K
L
M
N
P
Q
R
S
T
V
W
Y

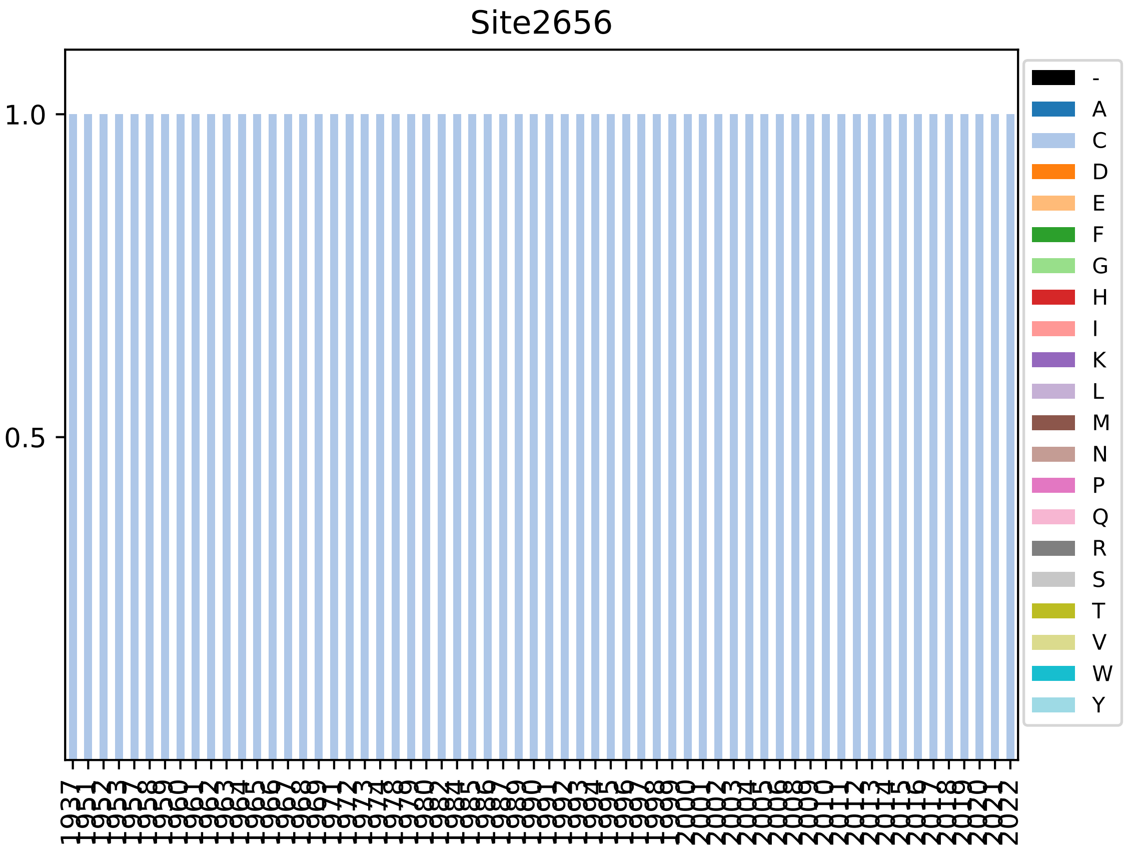
Site2656
1.0
0.5
-
A
C
D
E
F
G
H
I
K
L
M
N
P
Q
R
S
T
V
W
Y

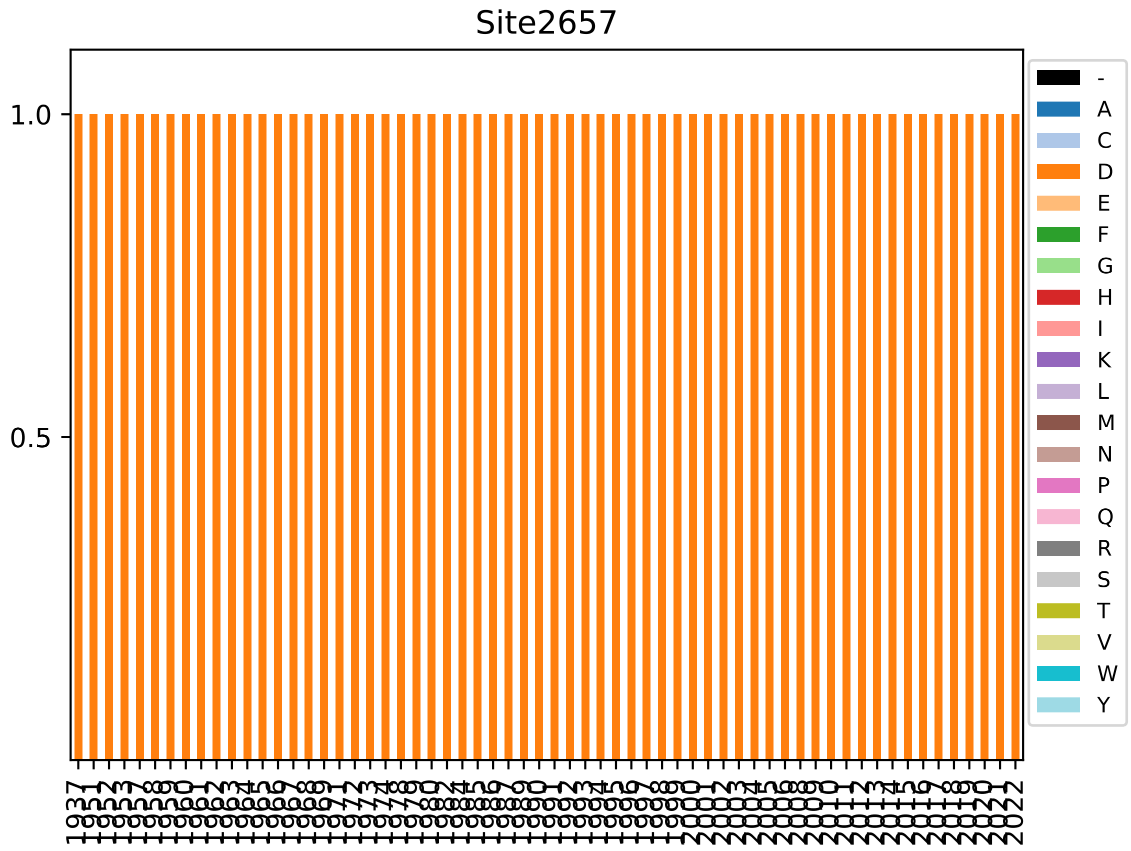
Site2657
1.0
0.5
-
A
C
D
E
F
G
H
I
K
L
M
N
P
Q
R
S
T
V
W
Y

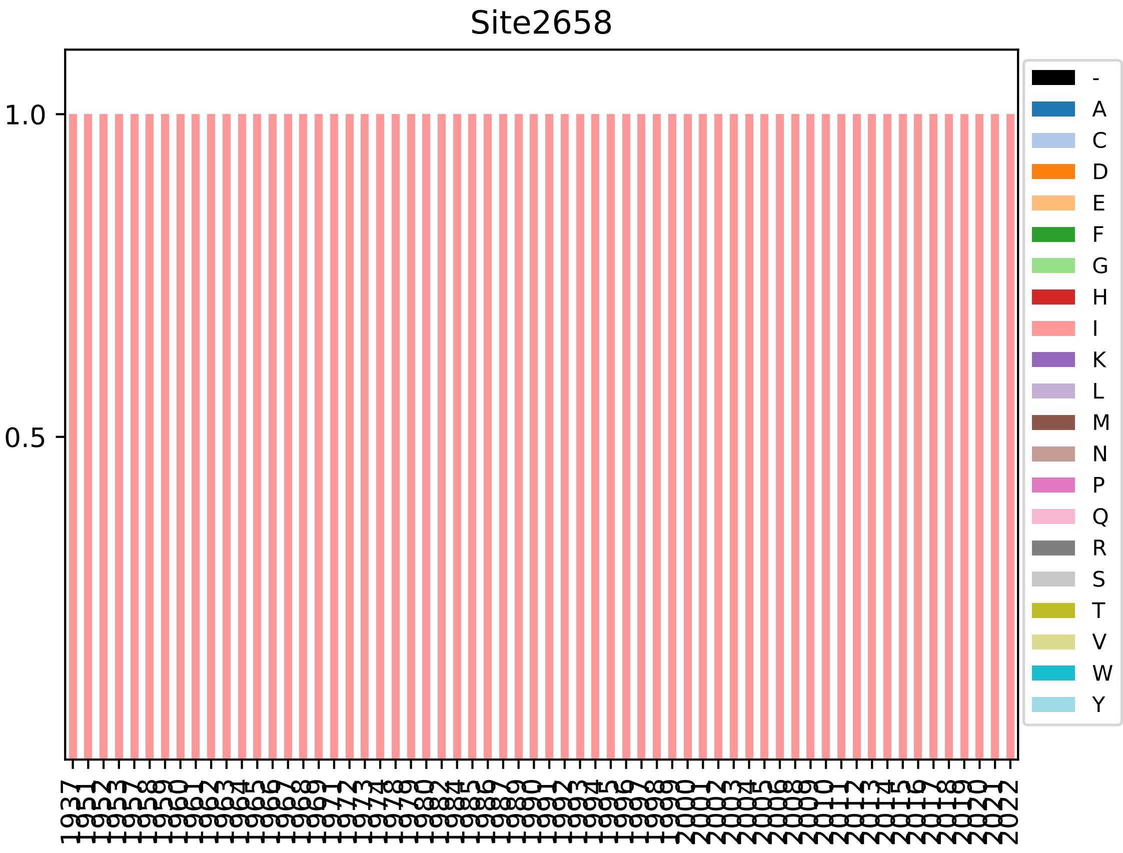
Site2658
1.0
0.5
-
A
C
D
E
F
G
H
I
K
L
M
N
P
Q
R
S
T
V
W
Y

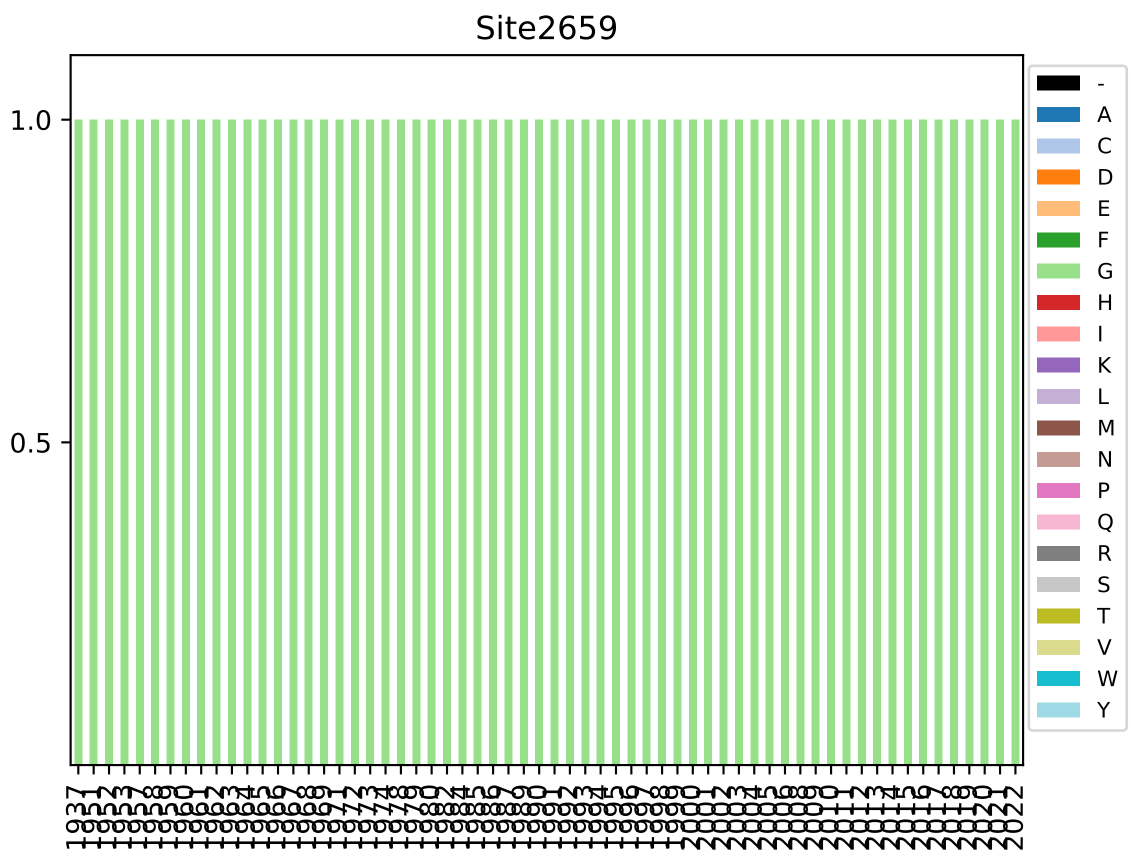
Site2659
1.0
0.5
-
A
C
D
E
F
G
H
I
K
L
M
N
P
Q
R
S
T
V
W
Y

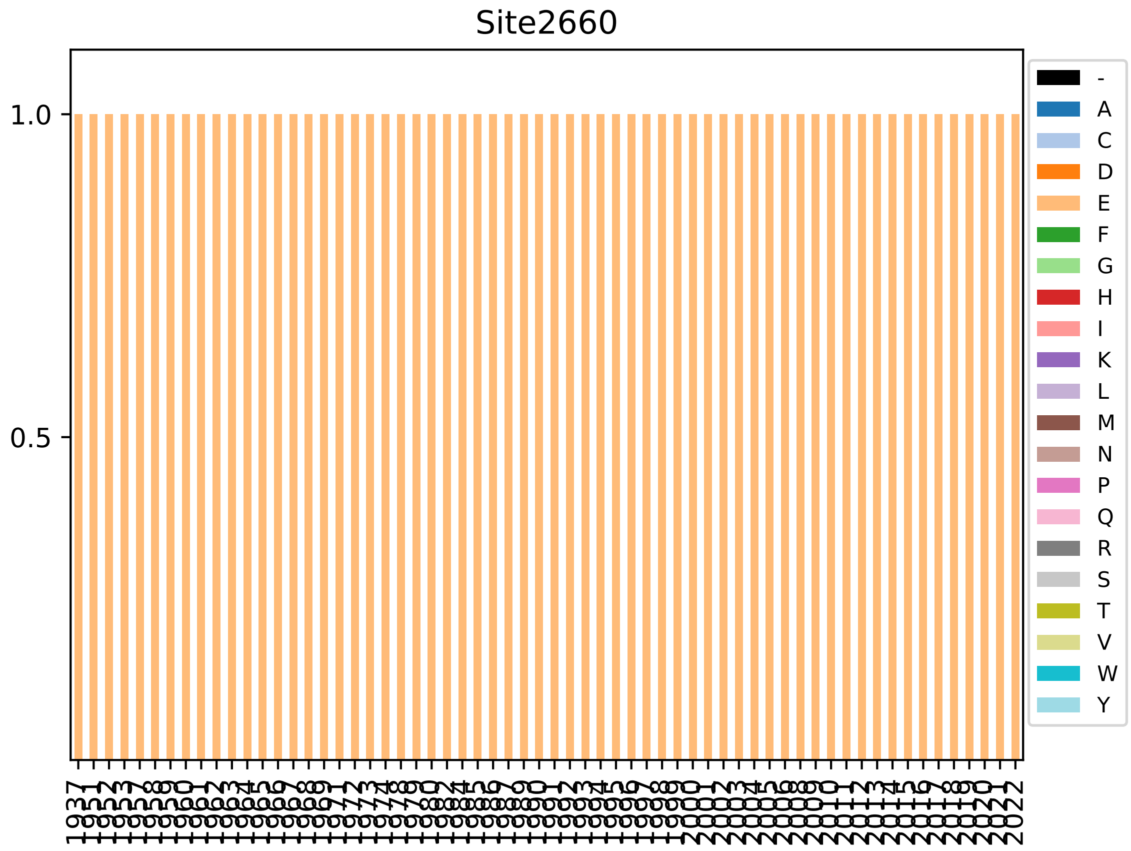
Site2660
1.0
0.5
-
A
C
D
E
F
G
H
I
K
L
M
N
P
Q
R
S
T
V
W
Y

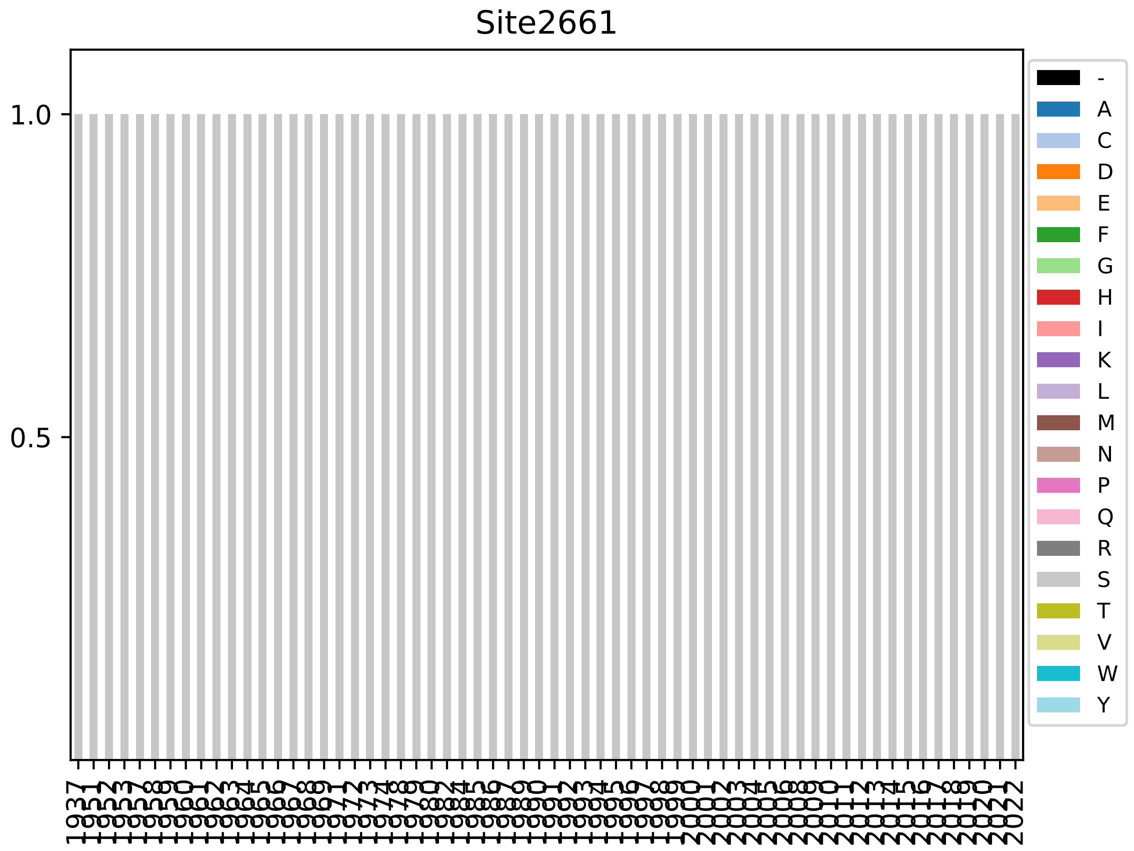
Site2661
1.0
0.5
-
A
C
D
E
F
G
H
I
K
L
M
N
P
Q
R
S
T
V
W
Y

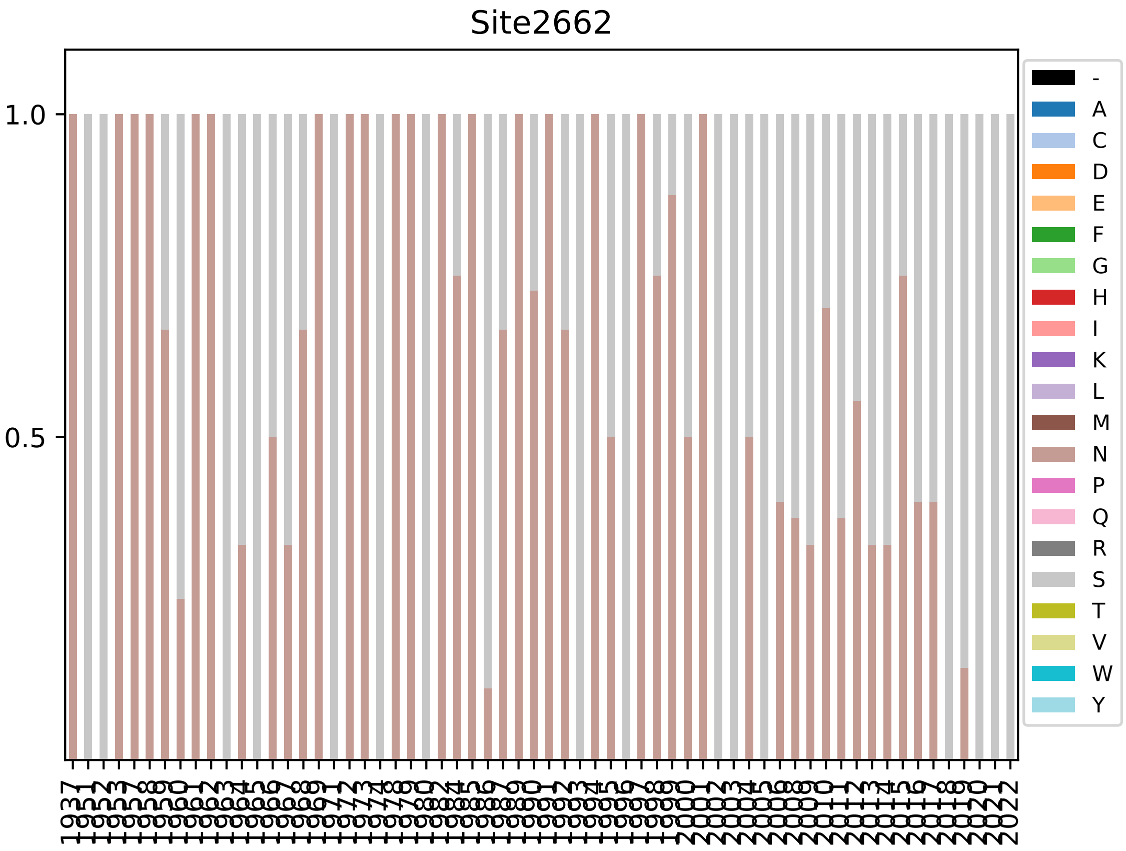
Site2662
1.0
0.5
-
A
C
D
E
F
G
H
I
K
L
M
N
P
Q
R
S
T
V
W
Y

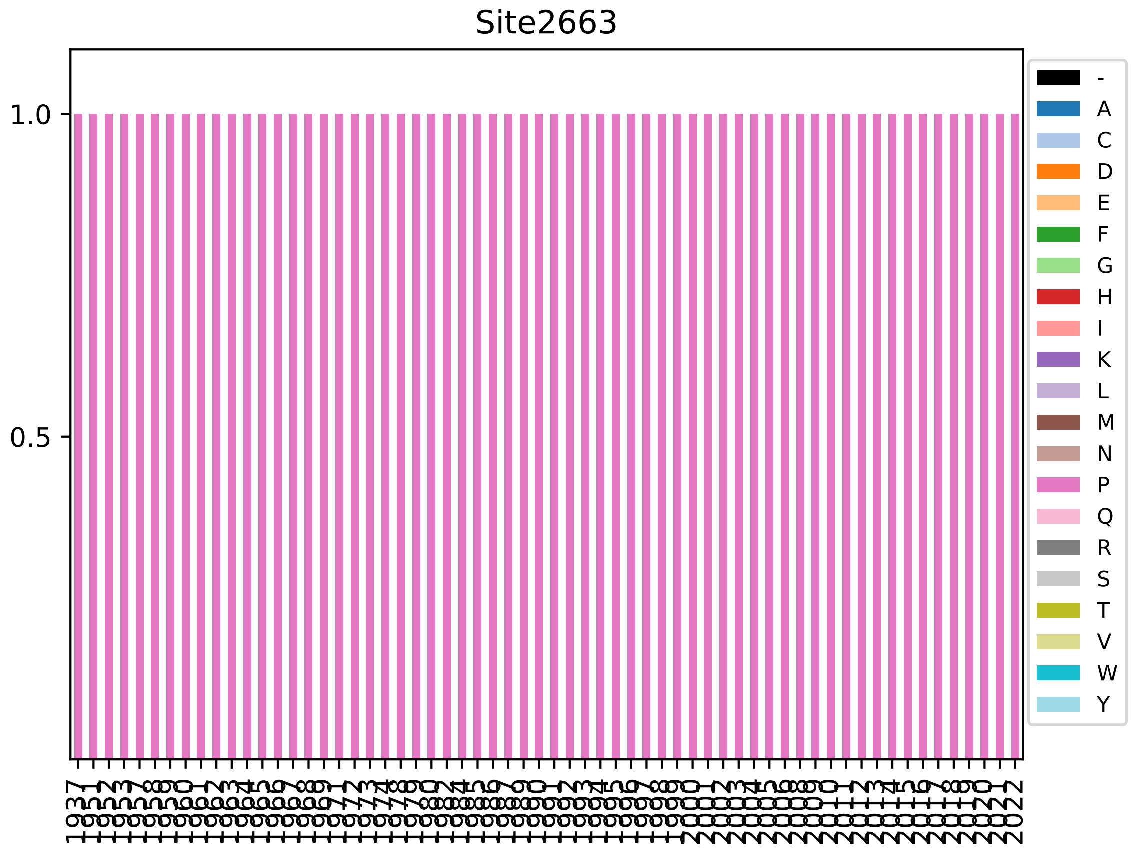
Site2663
1.0
0.5
-
A
C
D
E
F
G
H
I
K
L
M
N
P
Q
R
S
T
V
W
Y

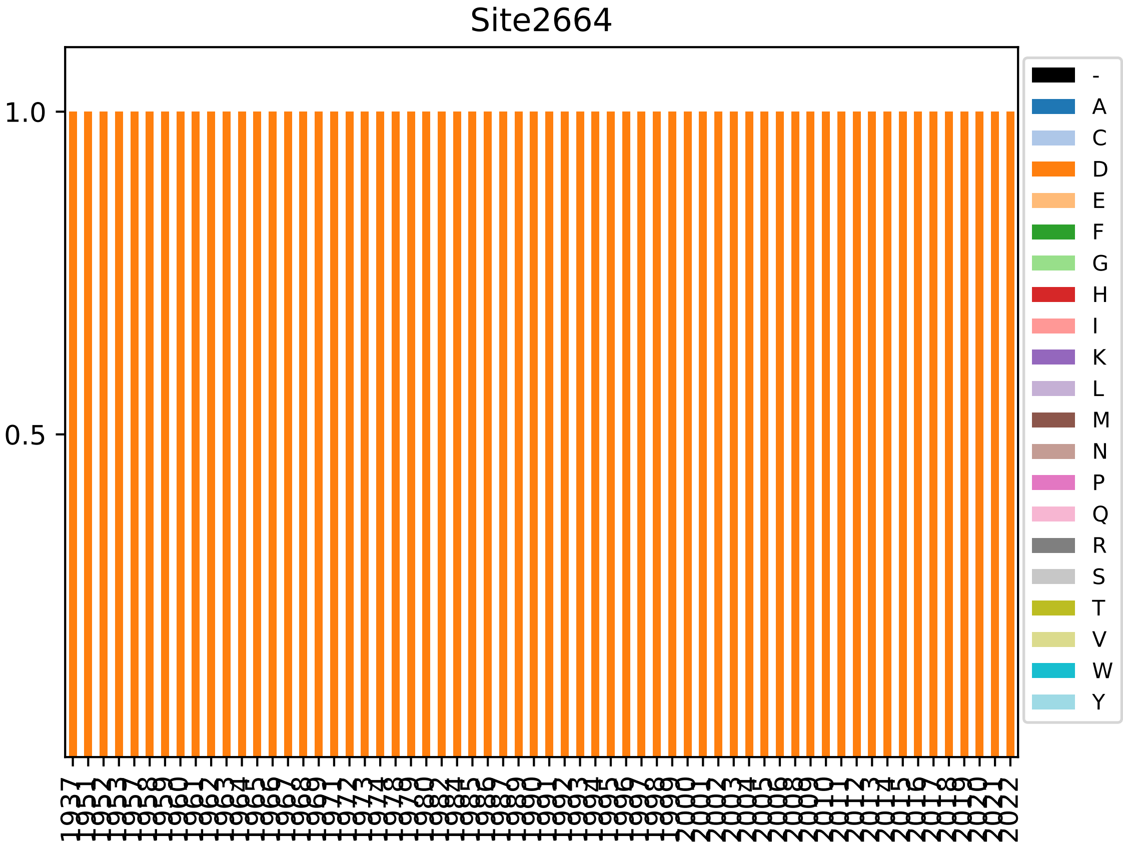
Site2664
1.0
0.5
-
A
C
D
E
F
G
H
I
K
L
M
N
P
Q
R
S
T
V
W
Y

Site2665

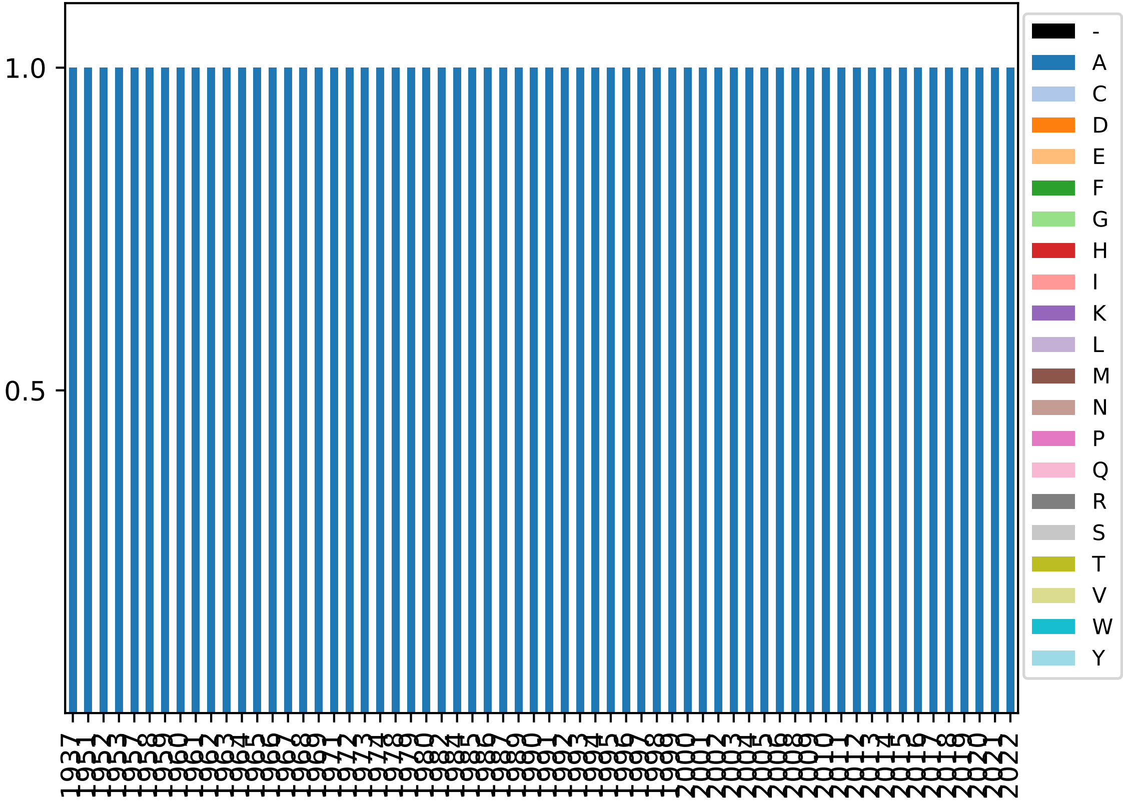

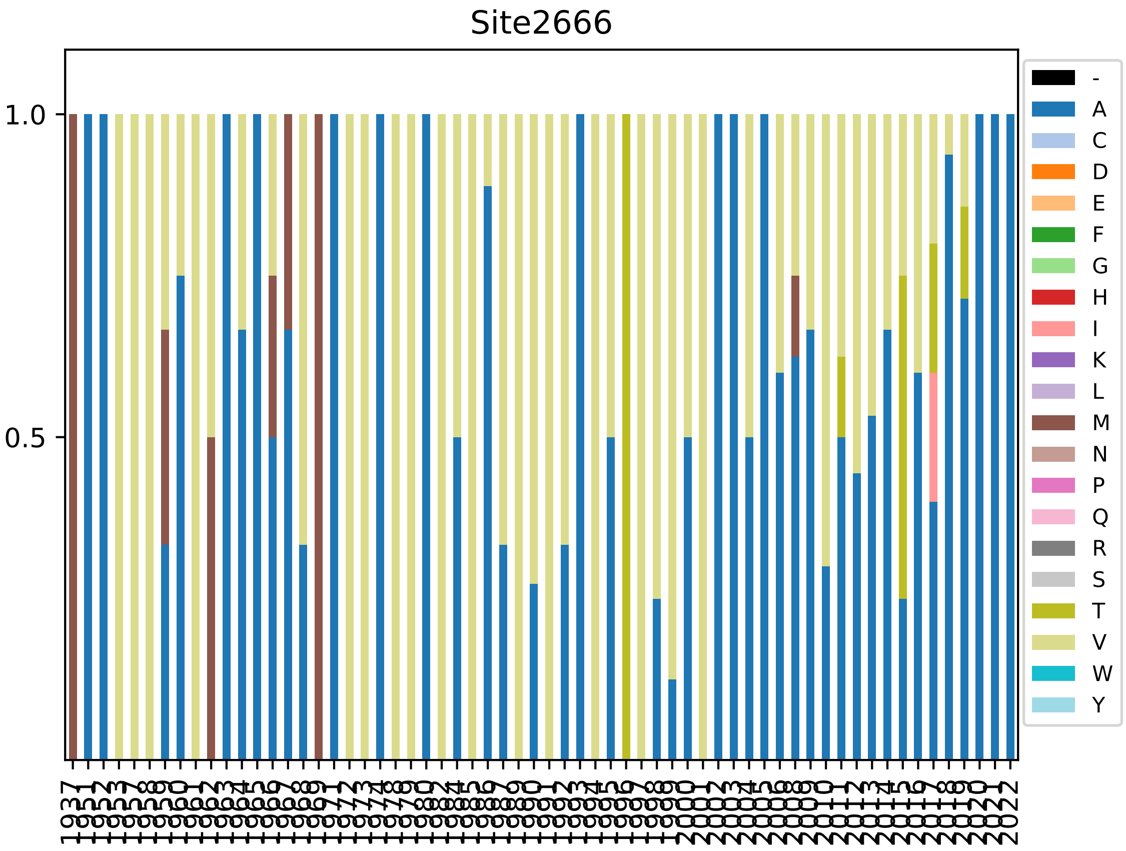
Site2666
1.0
0.5
-
A
C
D
E
F
G
H
I
K
L
M
N
P
Q
R
S
T
V
W
Y

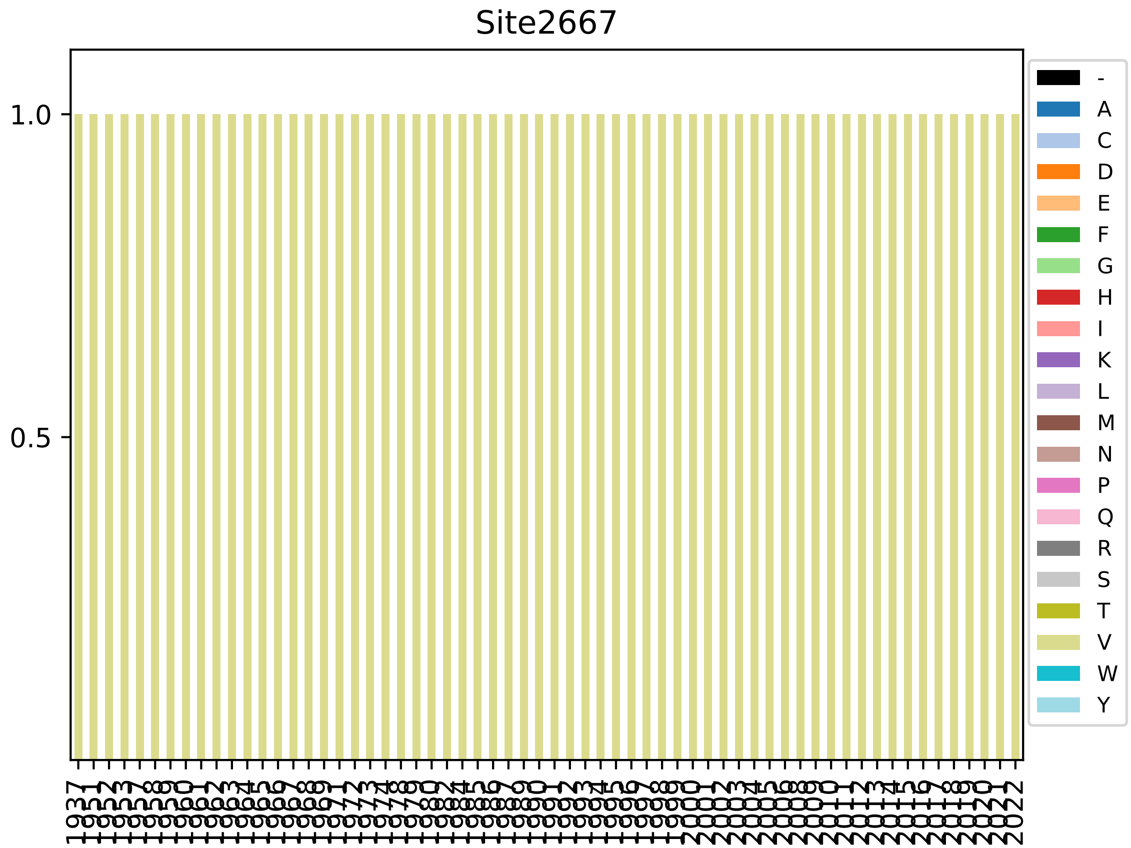
Site2667
1.0
0.5
-
A
C
D
E
F
G
H
I
K
L
M
N
P
Q
R
S
T
V
W
Y

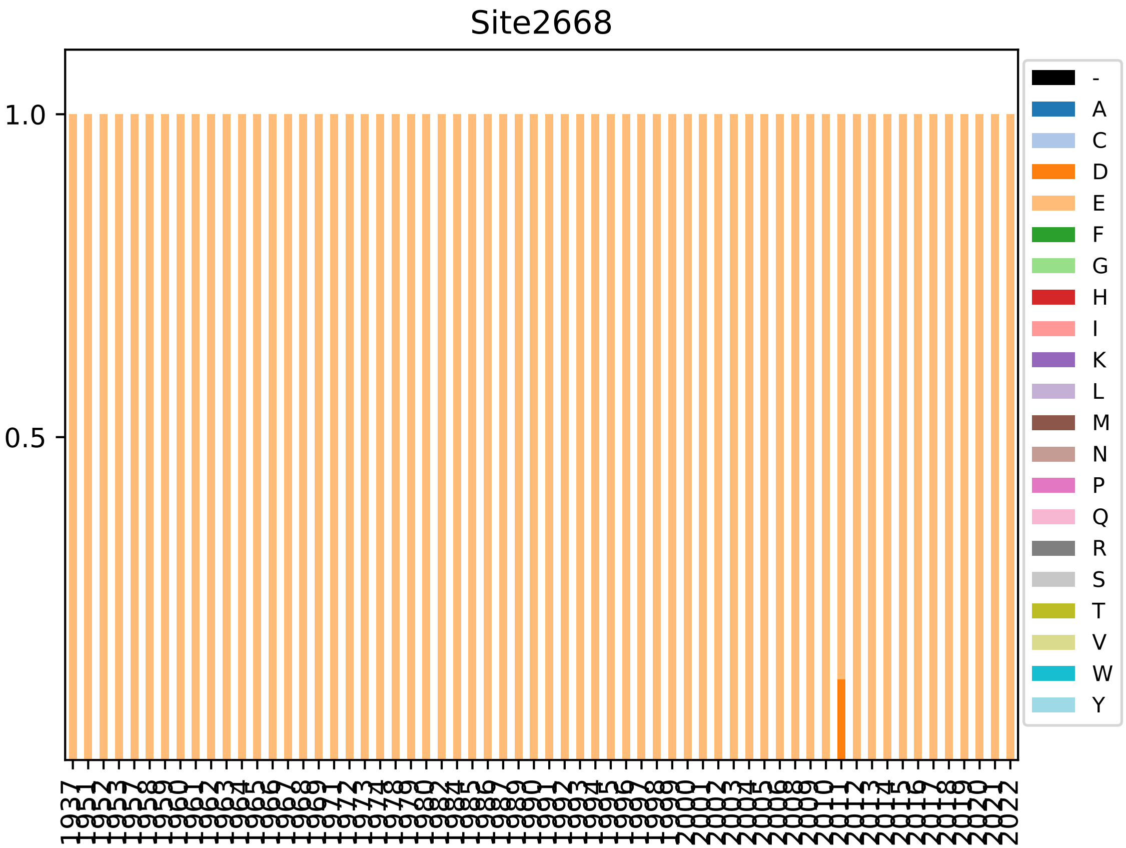

Site2668
1.0
0.5
-
A
C
D
E
F
G
H
I
K
L
M
N
P
Q
R
S
T
V
W
Y

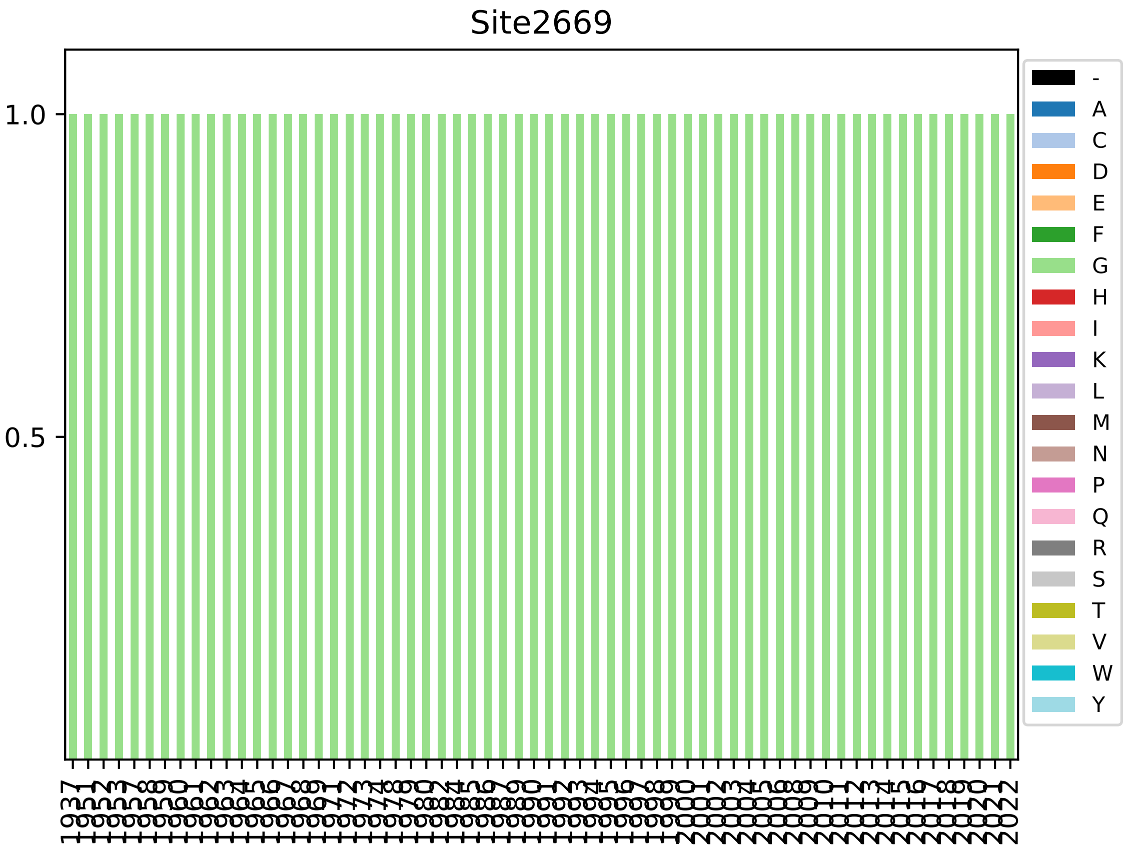
Site2669
1.0
0.5
-
A
C
D
E
F
G
H
I
K
L
M
N
P
Q
R
S
T
V
W
Y

Site2670

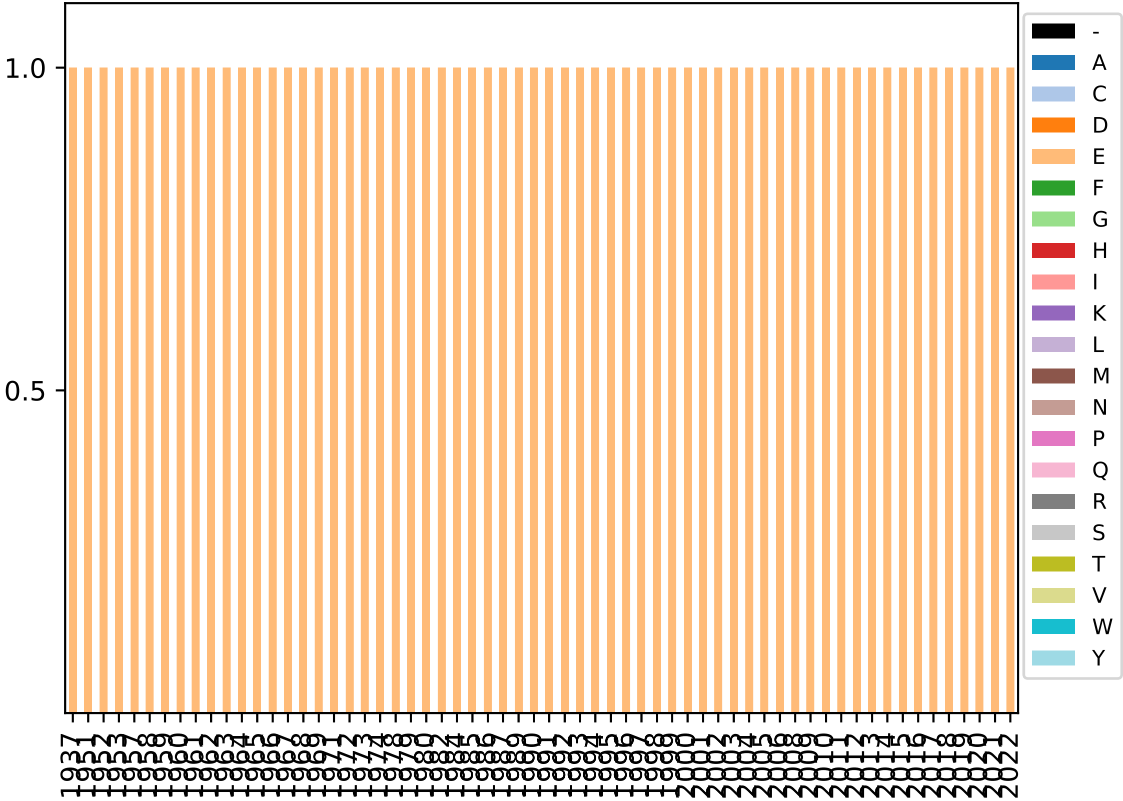

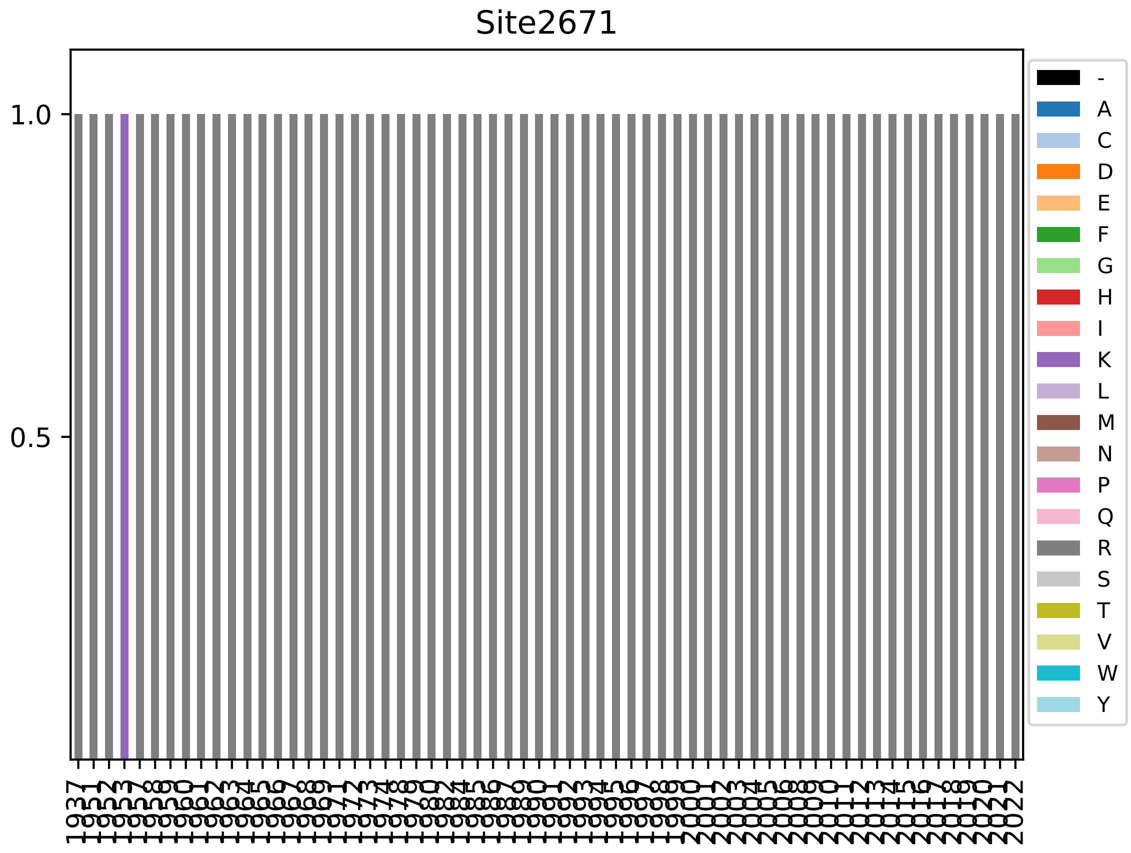
Site2671
1.0
0.5
-
A
C
D
E
F
G
H
I
K
L
M
N
P
Q
R
S
T
V
W
Y

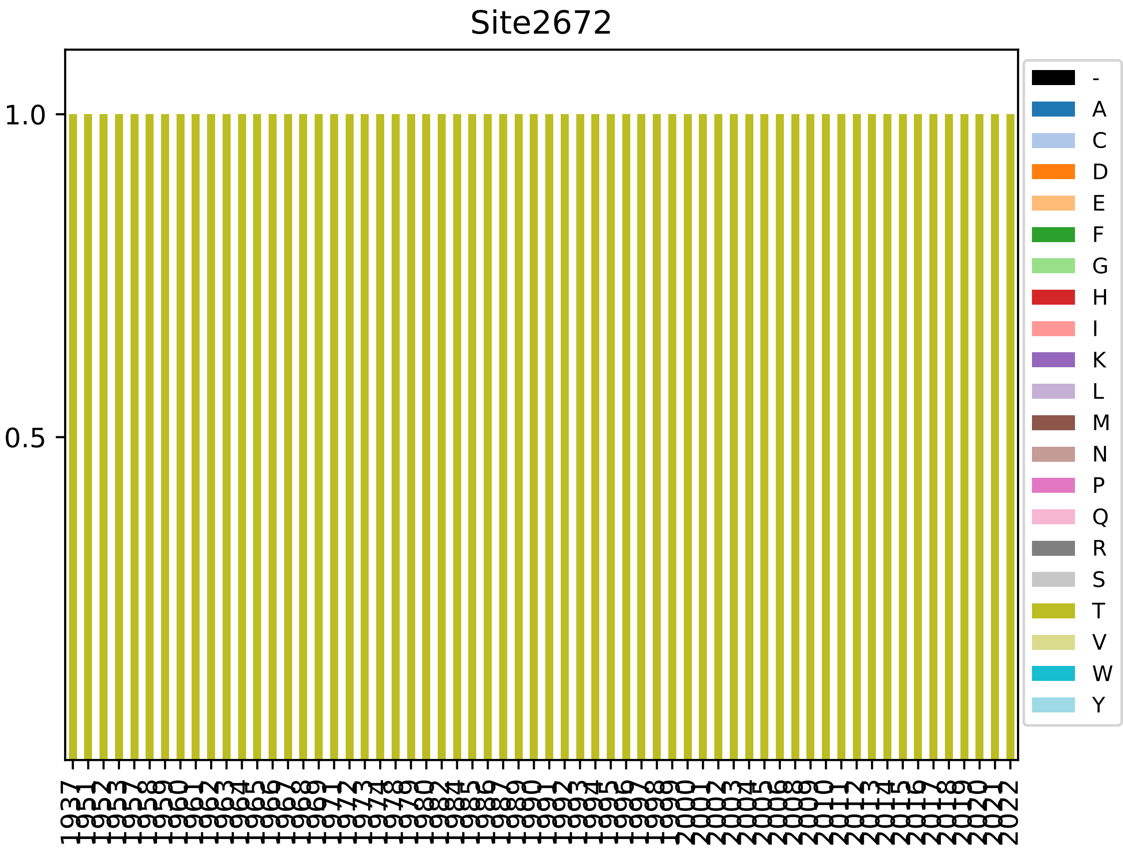
Site2672
1.0
0.5
-
A
C
D
E
F
G
H
I
K
L
M
N
P
Q
R
S
T
V
W
Y

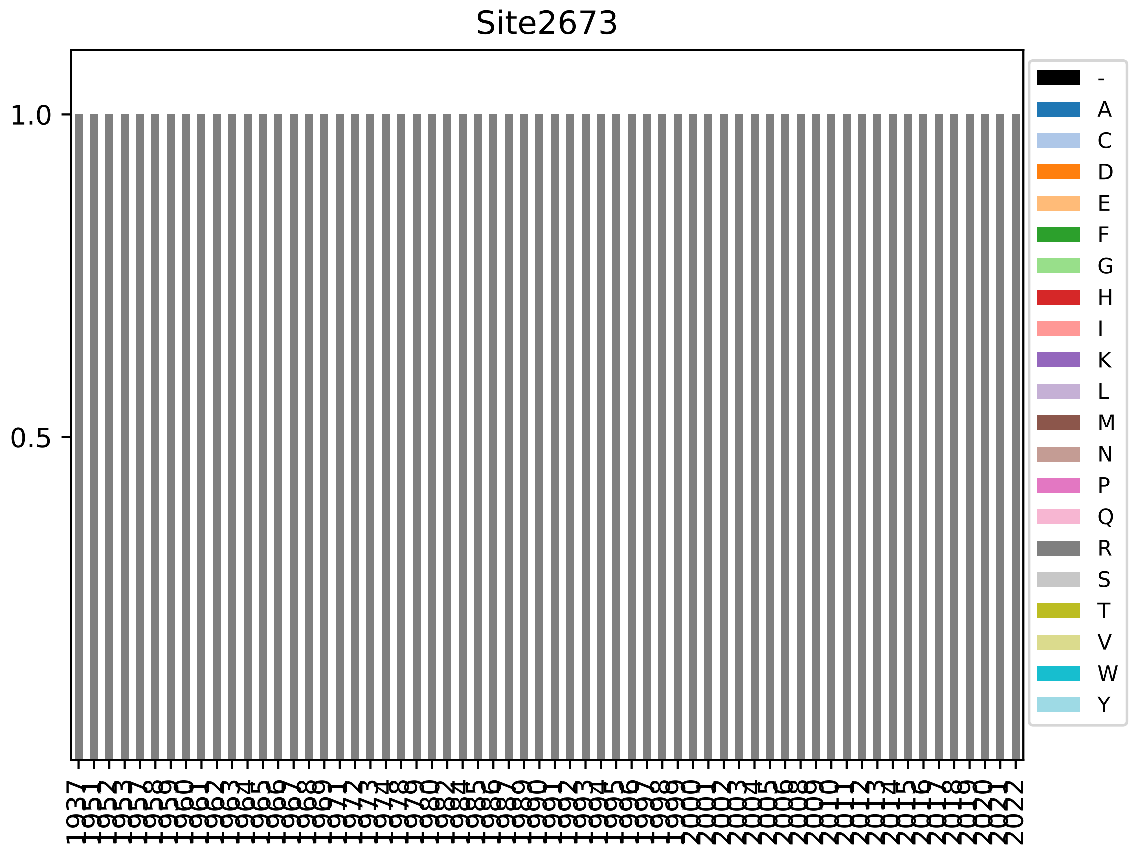
Site2673
1.0
0.5
-
A
C
D
E
F
G
H
I
K
L
M
N
P
Q
R
S
T
V
W
Y

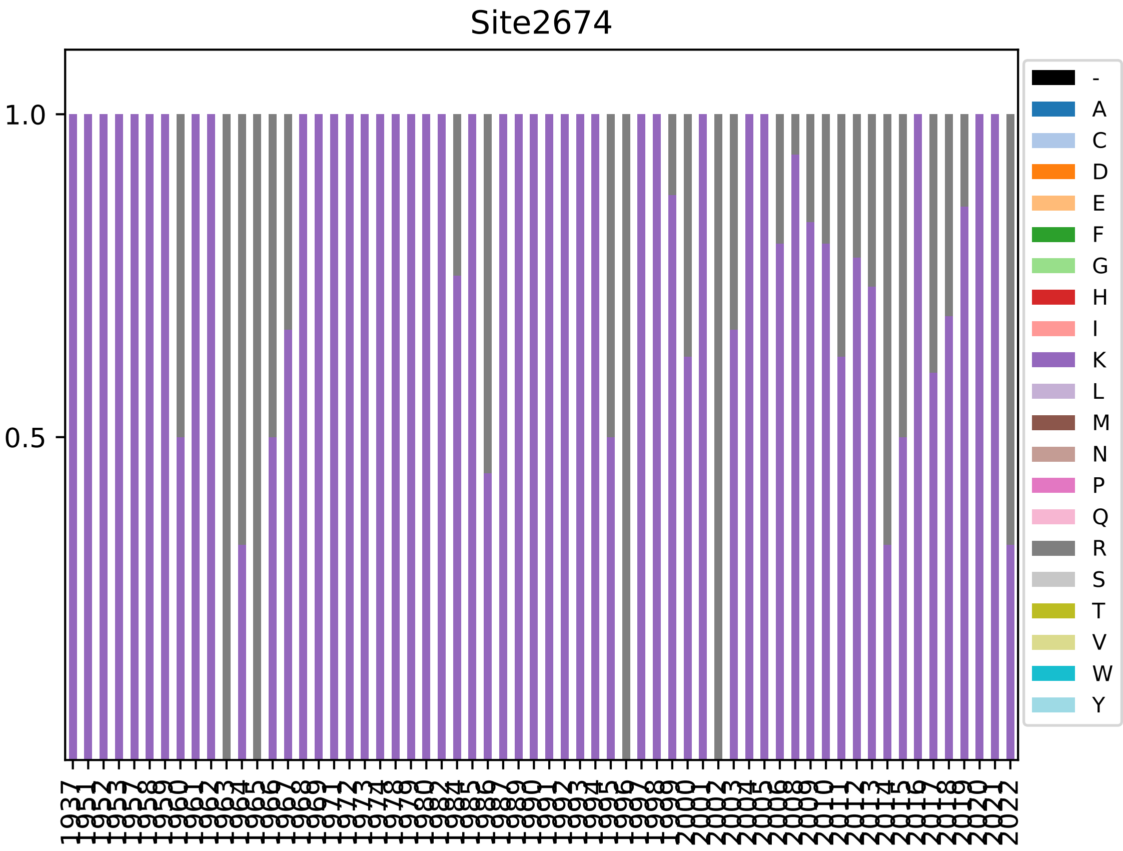
Site2674
1.0
0.5
-
A
C
D
E
F
G
H
I
K
L
M
N
P
Q
R
S
T
V
W
Y

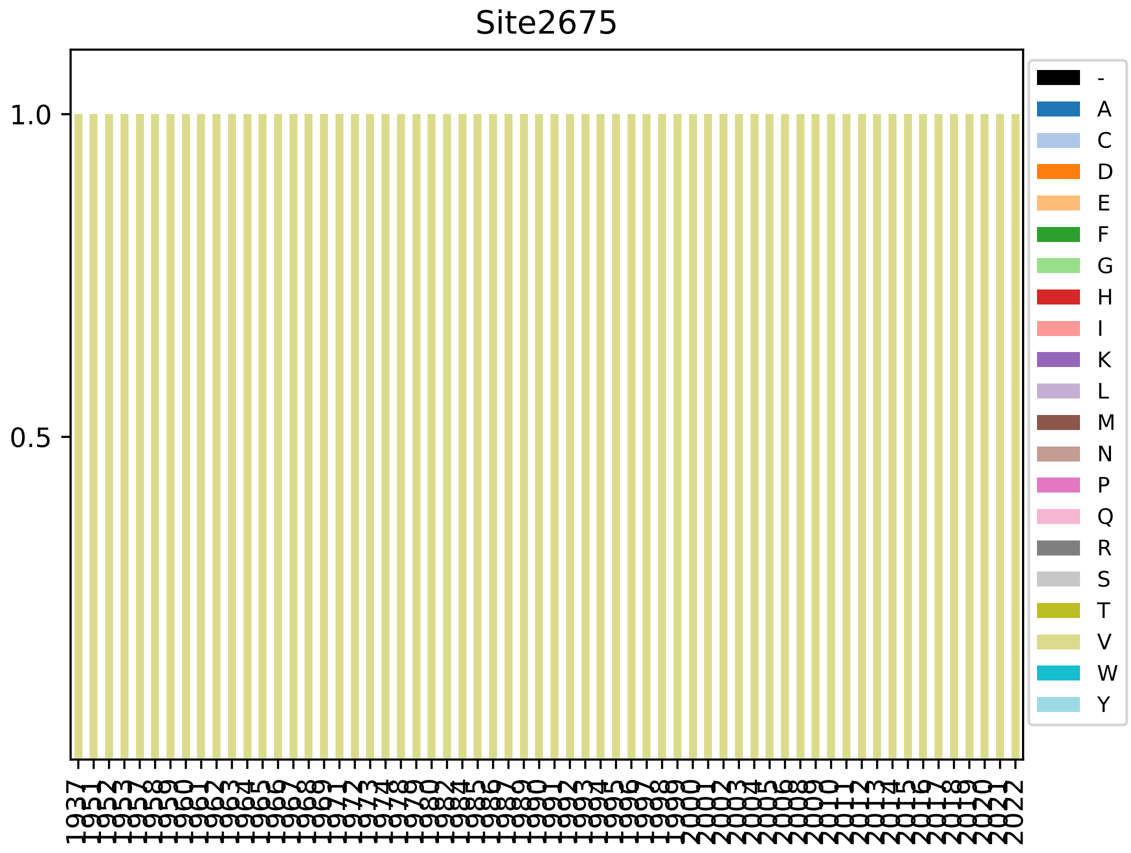
Site2675
1.0
0.5
-
A
C
D
E
F
G
H
I
K
L
M
N
P
Q
R
S
T
V
W
Y

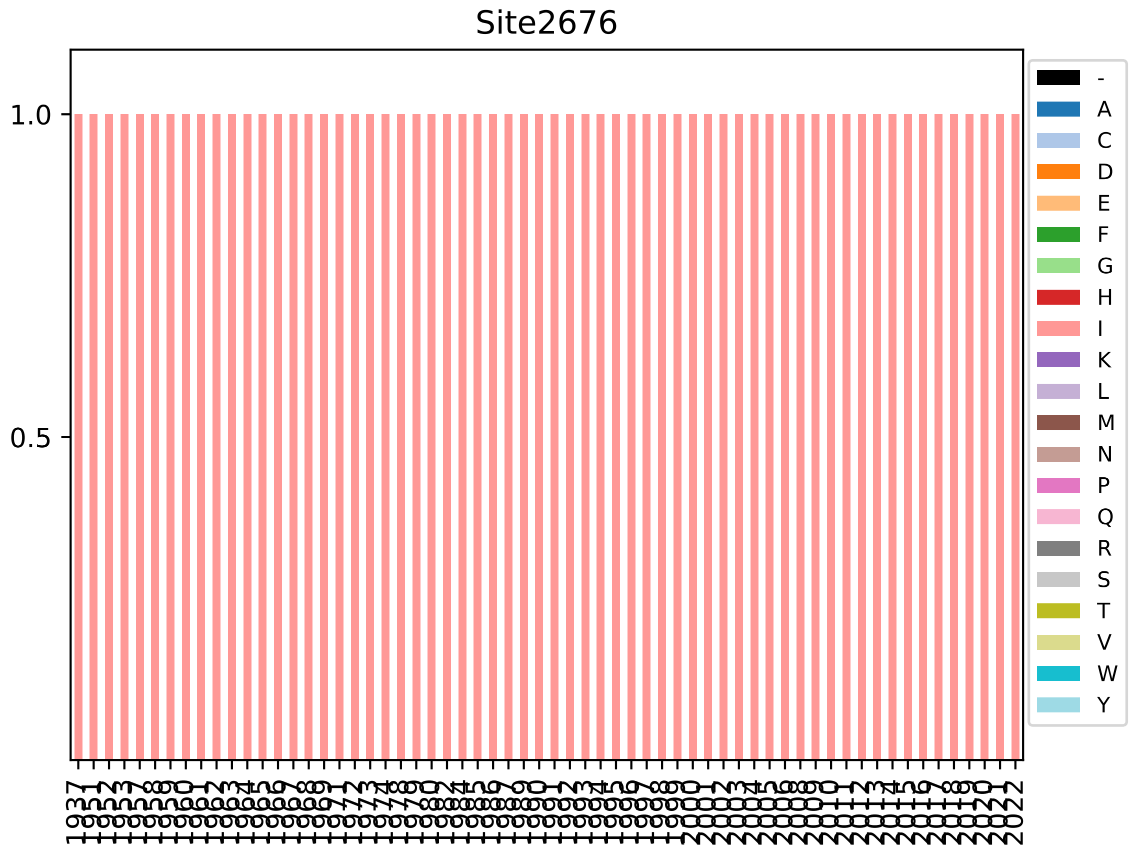
Site2676
1.0
0.5
-
A
C
D
E
F
G
H
I
K
L
M
N
P
Q
R
S
T
V
W
Y

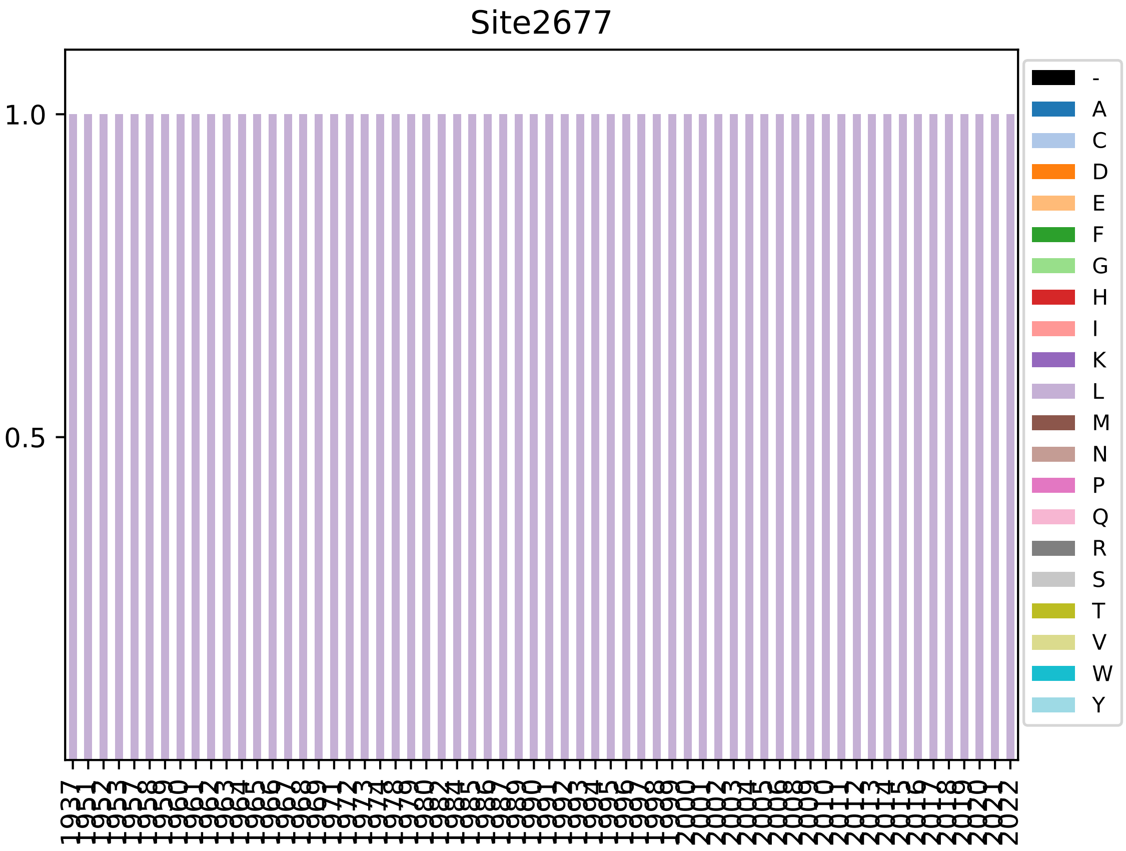
Site2677
1.0
0.5
-
A
C
D
E
F
G
H
I
K
L
M
N
P
Q
R
S
T
V
W
Y

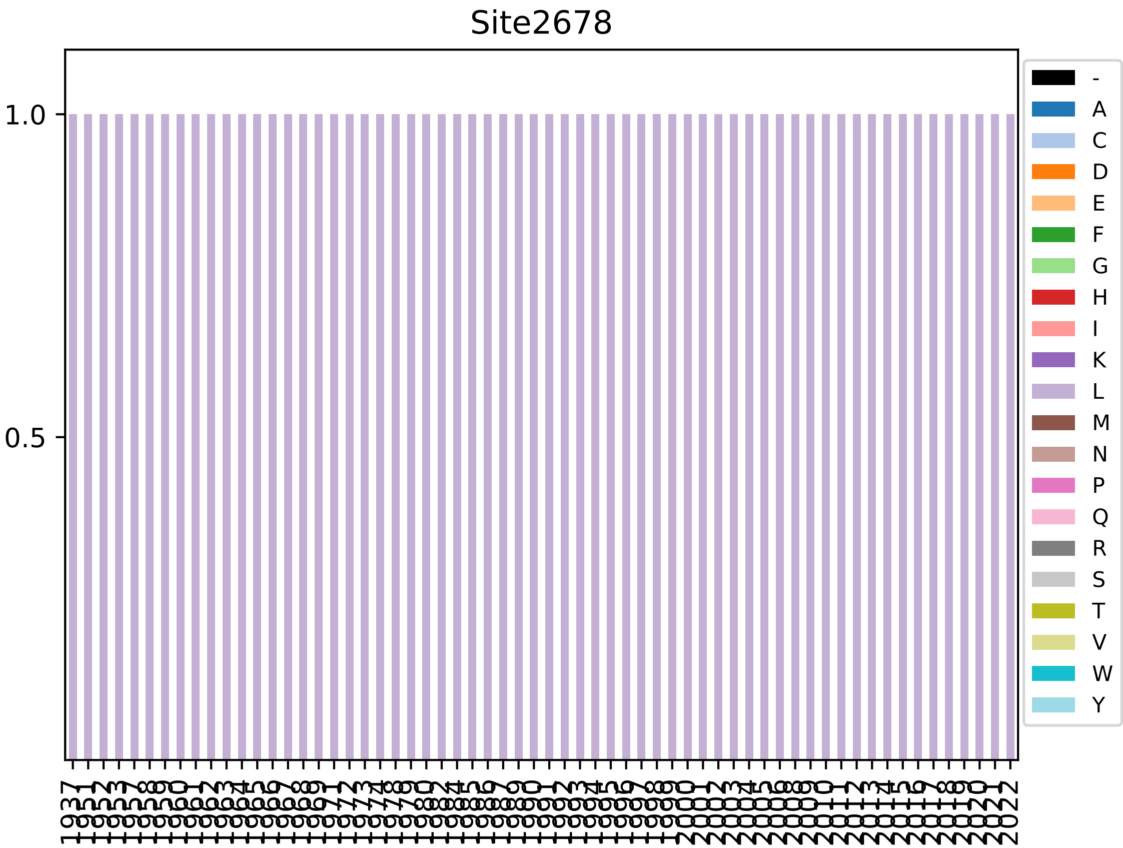
Site2678
1.0
0.5
-
A
C
D
E
F
G
H
I
K
L
M
N
P
Q
R
S
T
V
W
Y

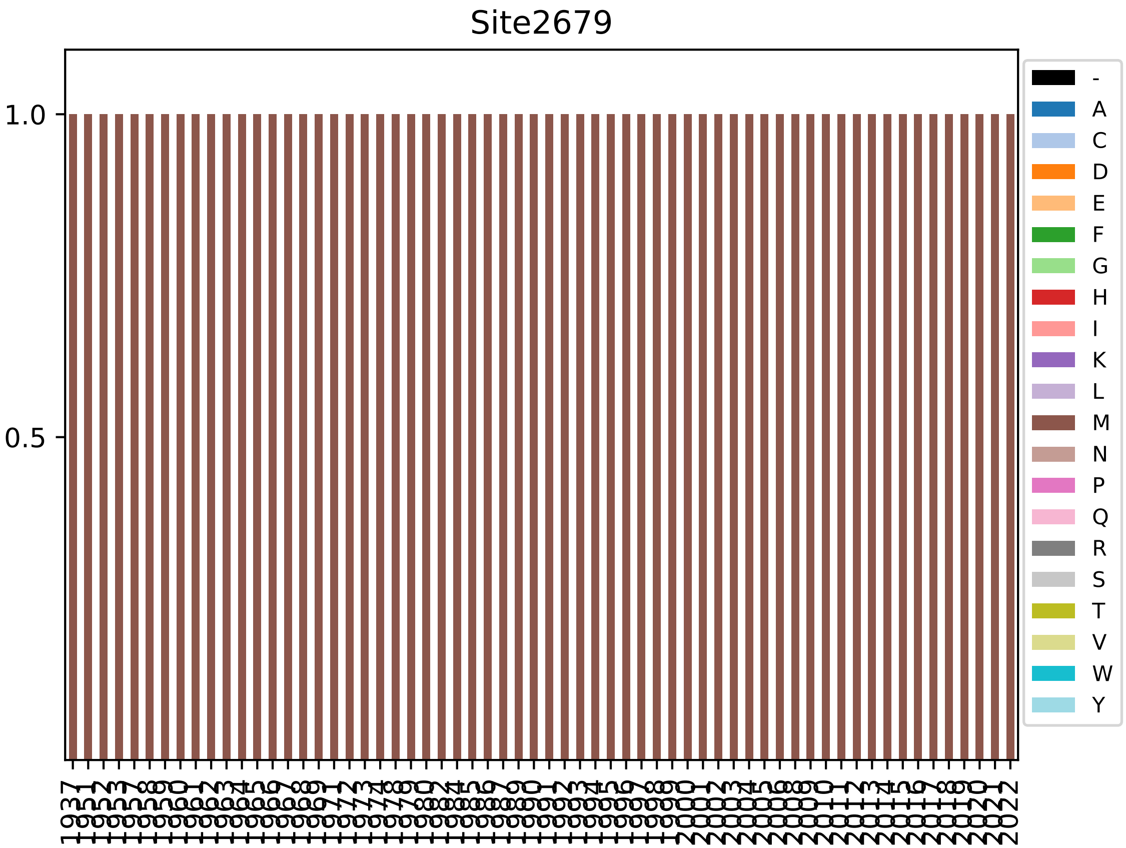
Site2679
1.0
0.5
-
A
C
D
E
F
G
H
I
K
L
M
N
P
Q
R
S
T
V
W
Y

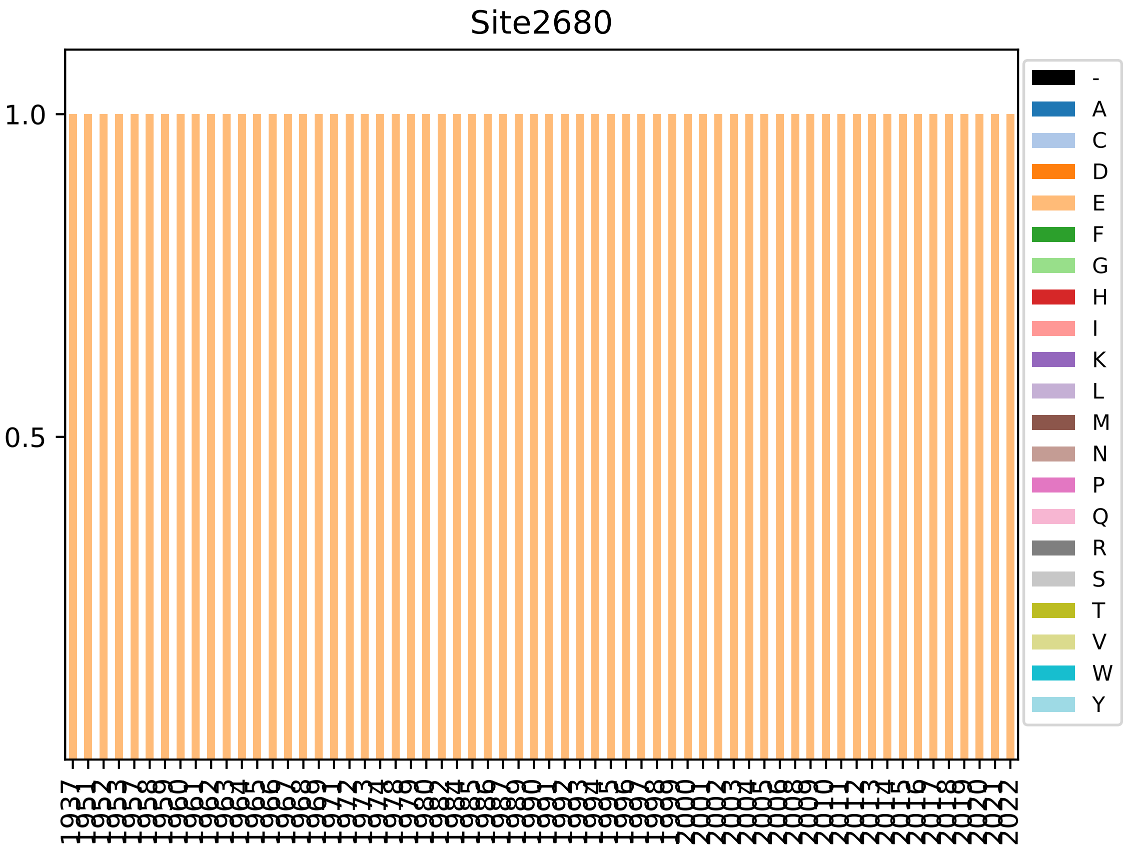
Site2680
1.0
0.5
-
A
C
D
E
F
G
H
I
K
L
M
N
P
Q
R
S
T
V
W
Y

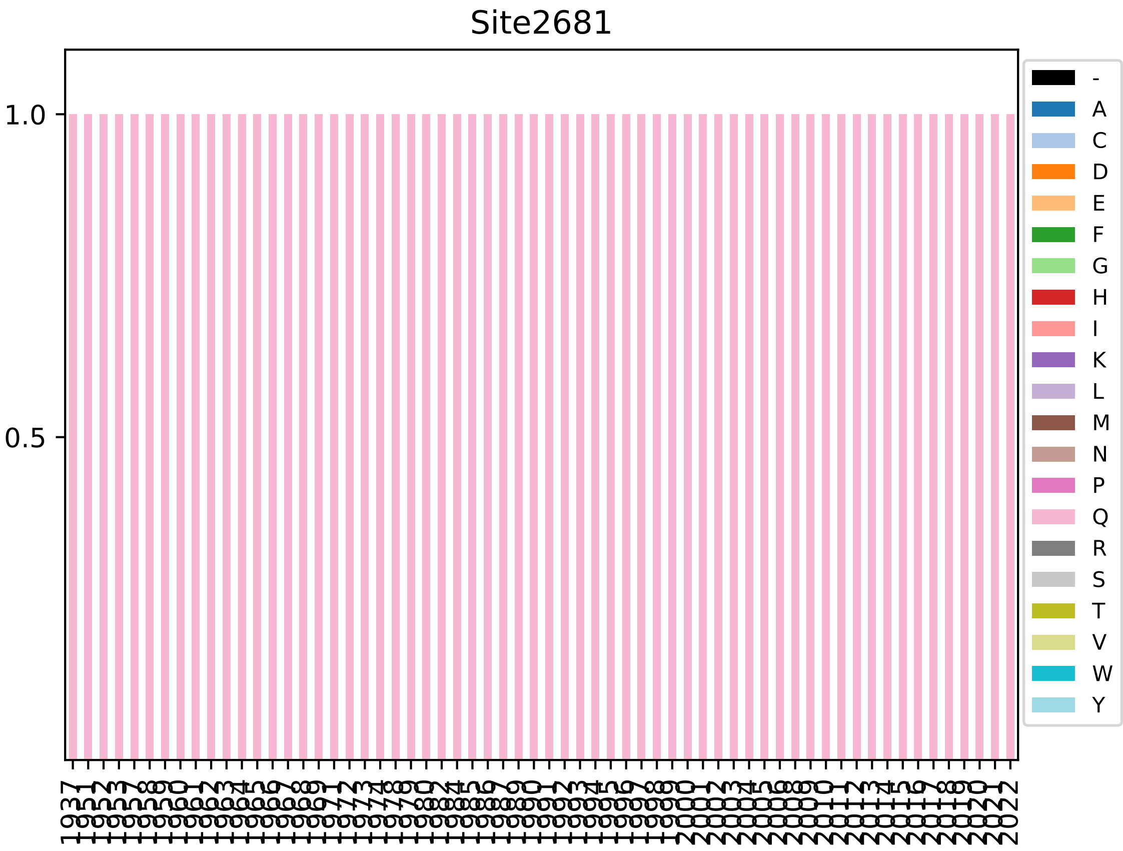
Site2681
1.0
0.5
-
A
C
D
E
F
G
H
I
K
L
M
N
P
Q
R
S
T
V
W
Y

Site2682

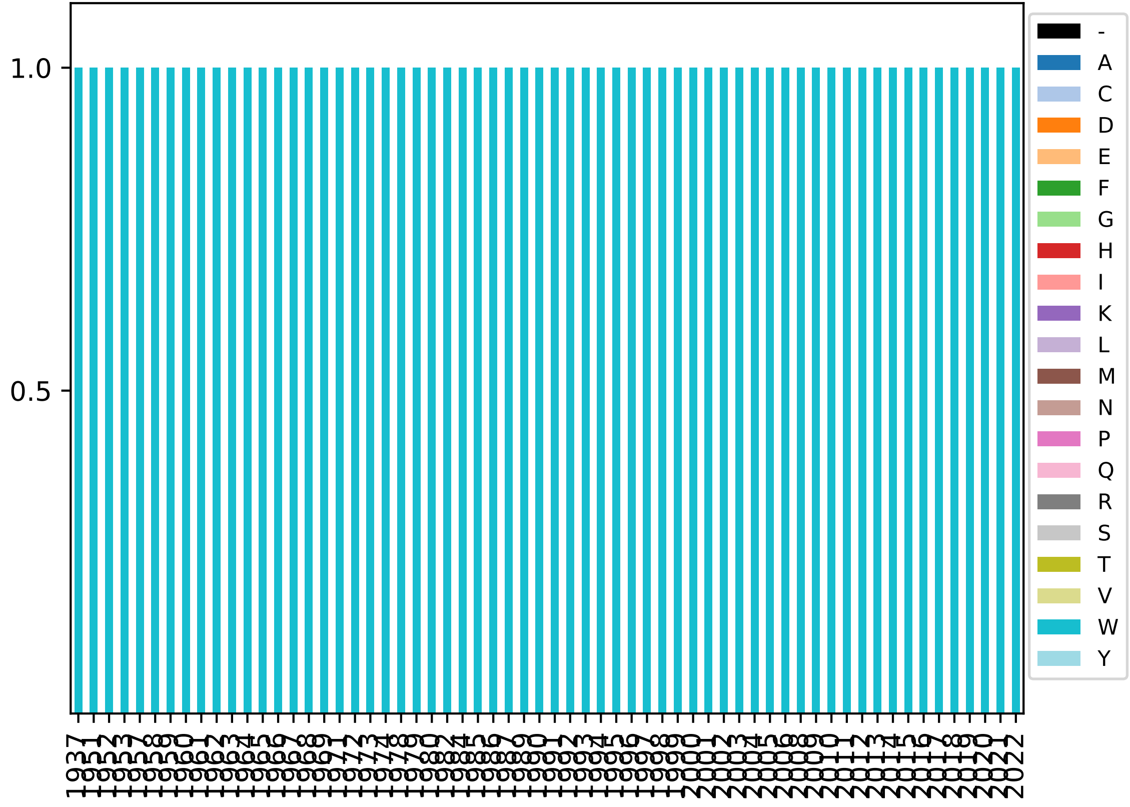

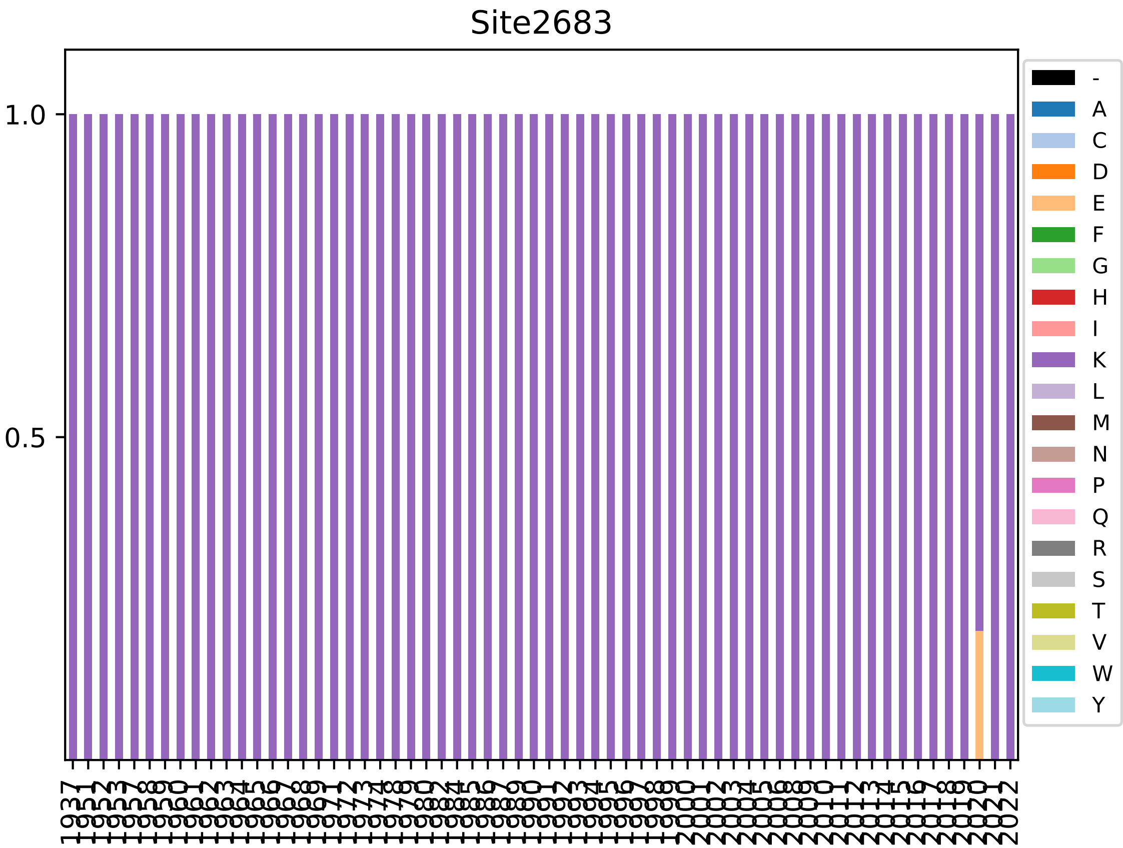
Site2683
1.0
0.5
-
A
C
D
E
F
G
H
I
K
L
M
N
P
Q
R
S
T
V
W
Y

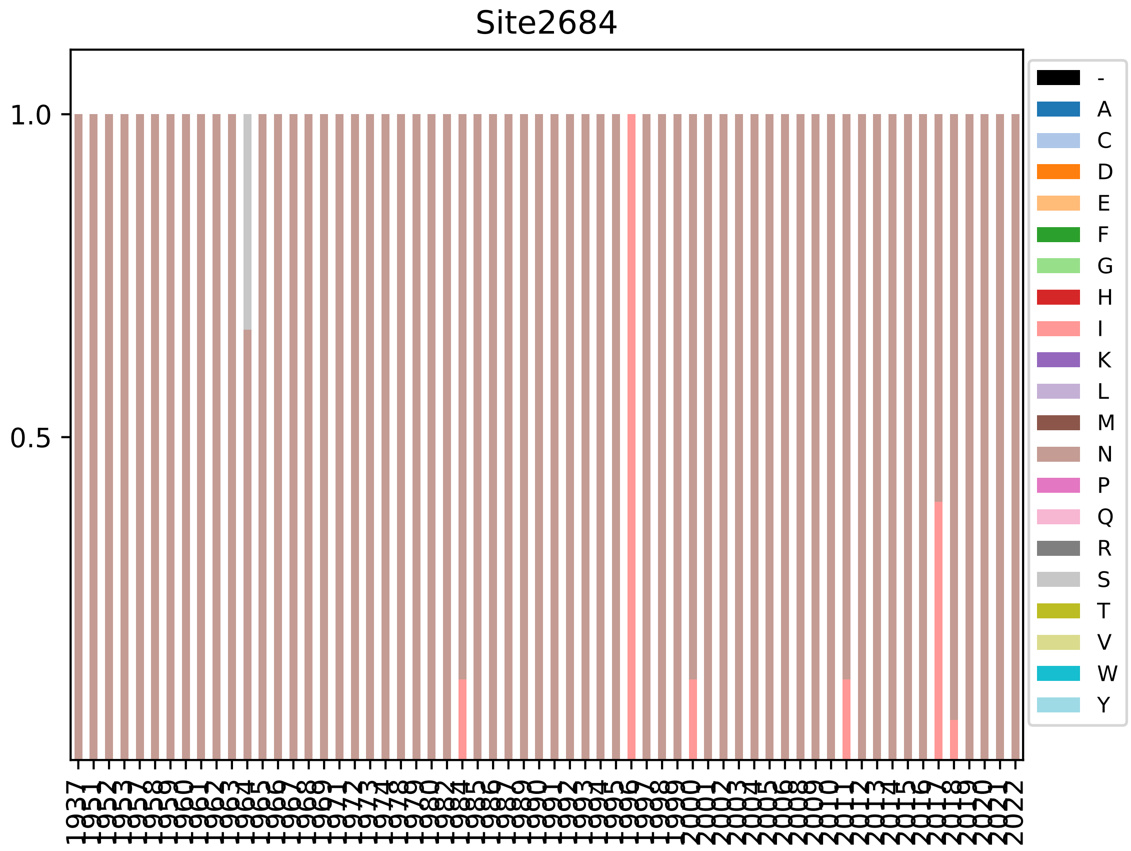
Site2684
1.0
0.5
-
A
C
D
E
F
G
H
I
K
L
M
N
P
Q
R
S
T
V
W
Y

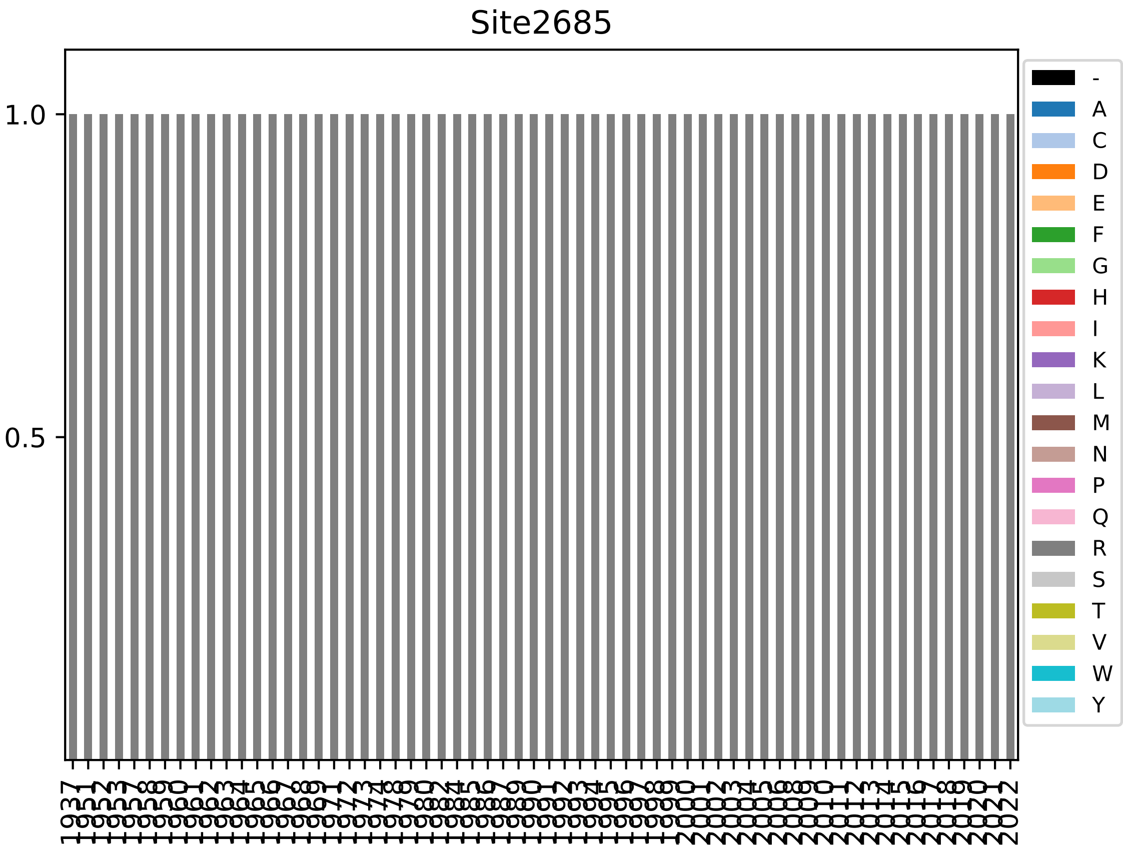
Site2685
1.0
0.5
-
A
C
D
E
F
G
H
I
K
L
M
N
P
Q
R
S
T
V
W
Y

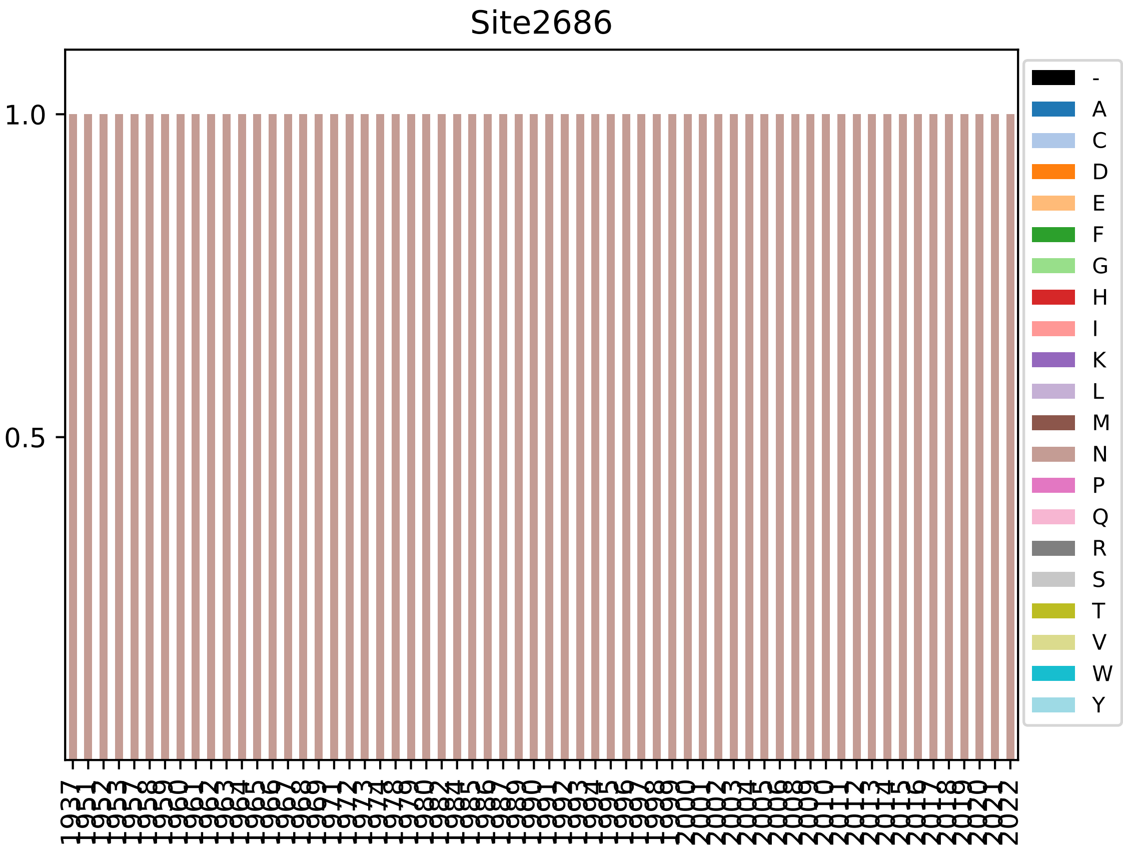

Site2686
1.0
0.5
-
A
C
D
E
F
G
H
I
K
L
M
N
P
Q
R
S
T
V
W
Y

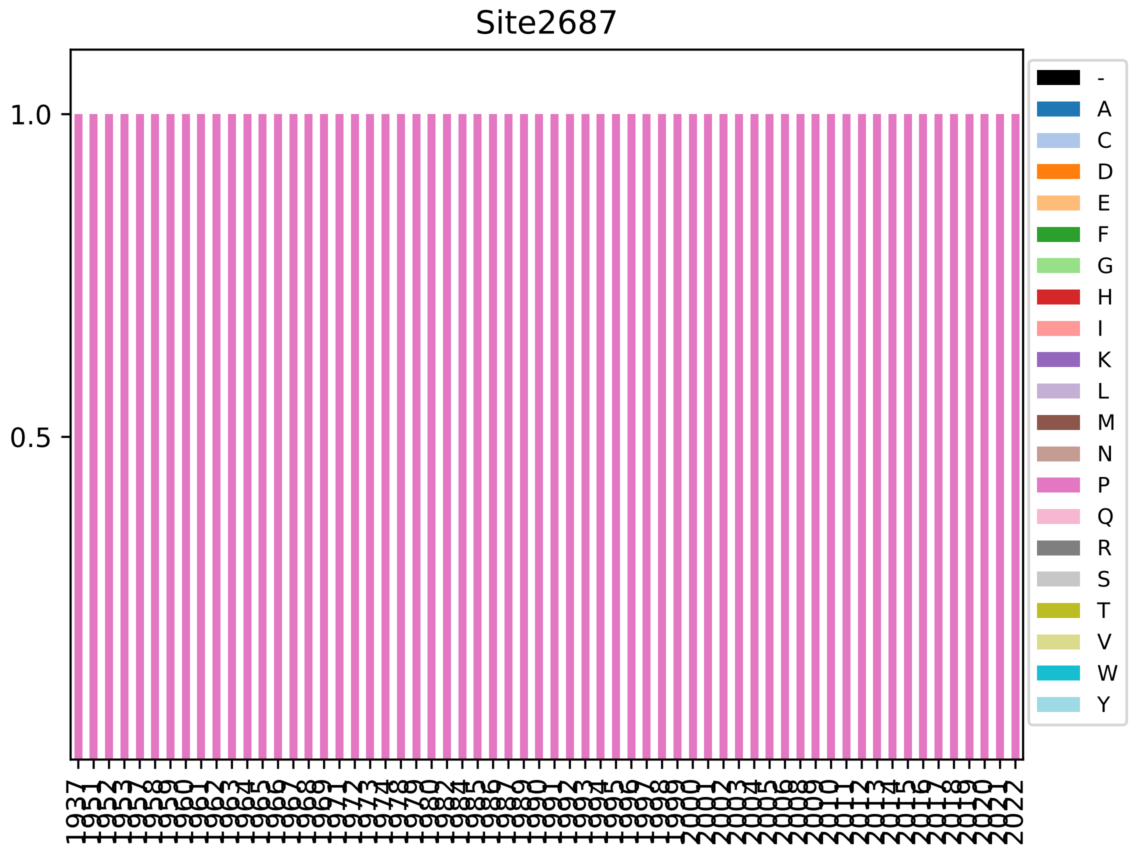
Site2687
1.0
0.5
-
A
C
D
E
F
G
H
I
K
L
M
N
P
Q
R
S
T
V
W
Y

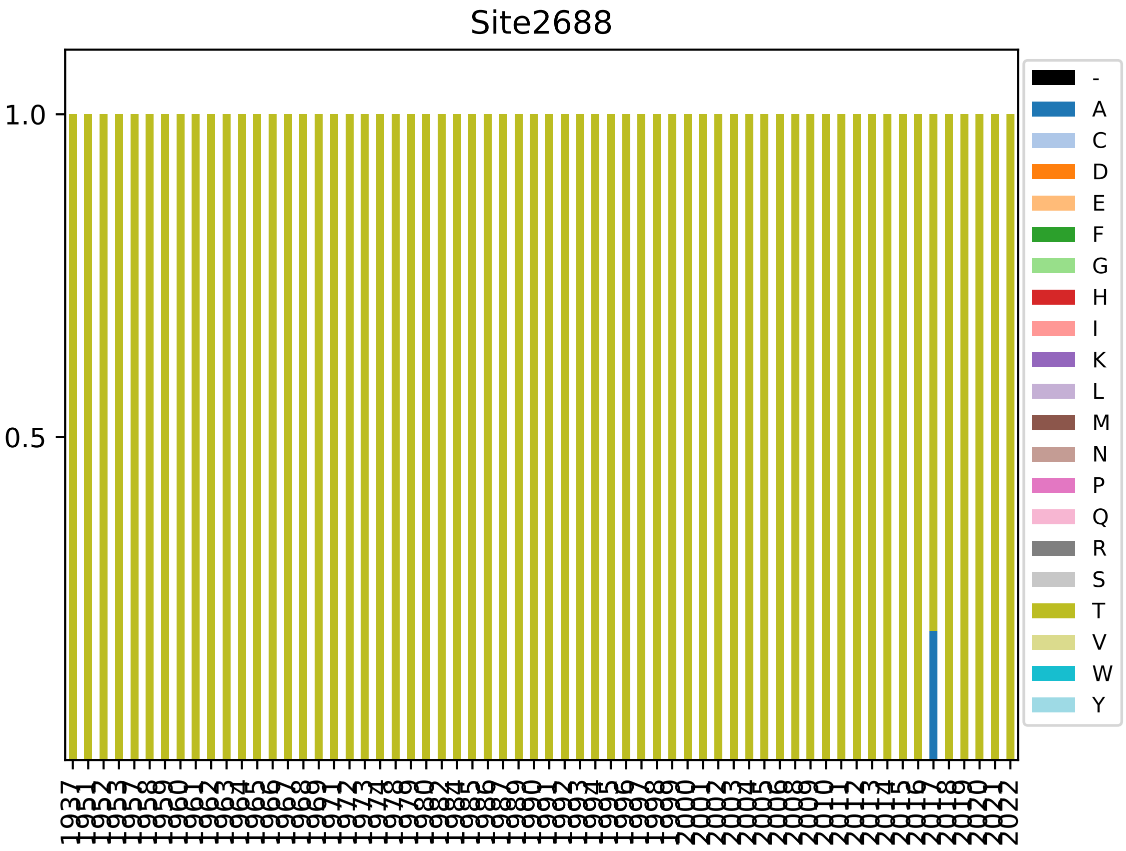
Site2688
1.0
0.5
-
A
C
D
E
F
G
H
I
K
L
M
N
P
Q
R
S
T
V
W
Y

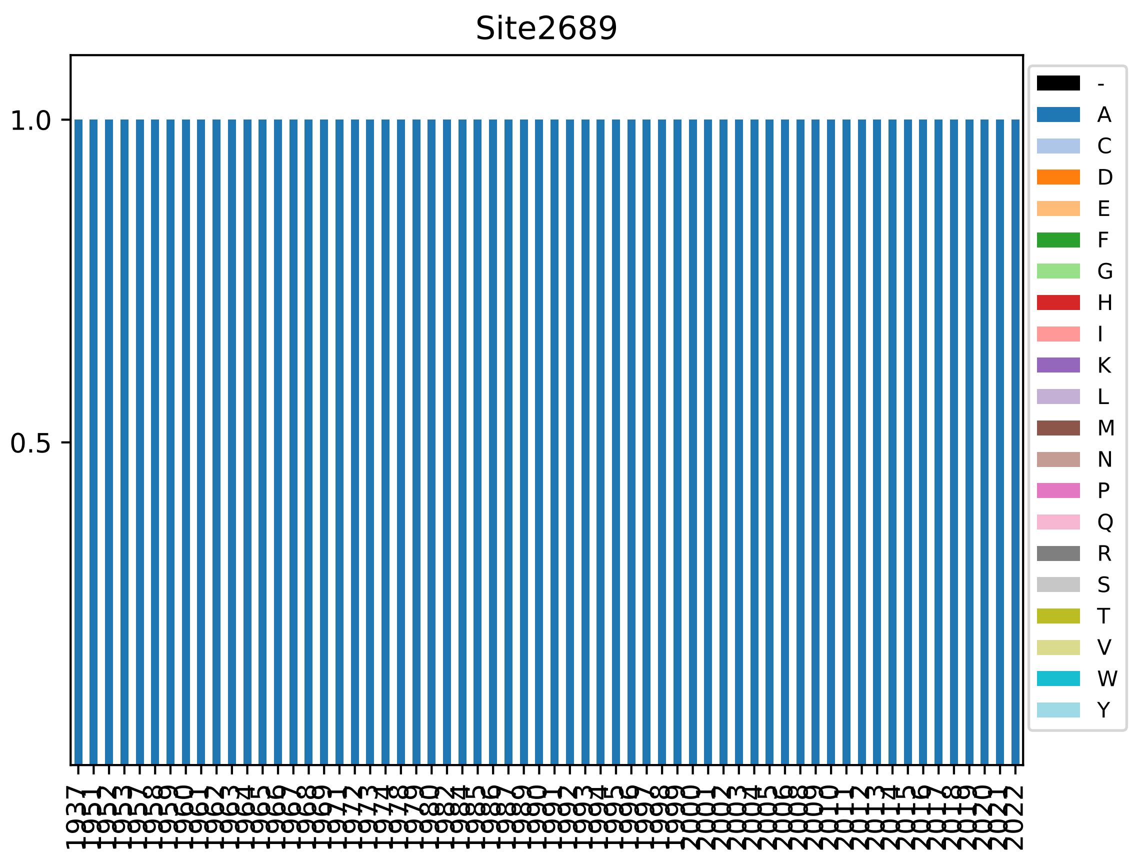
Site2689
1.0
0.5
-
A
C
D
E
F
G
H
I
K
L
M
N
P
Q
R
S
T
V
W
Y

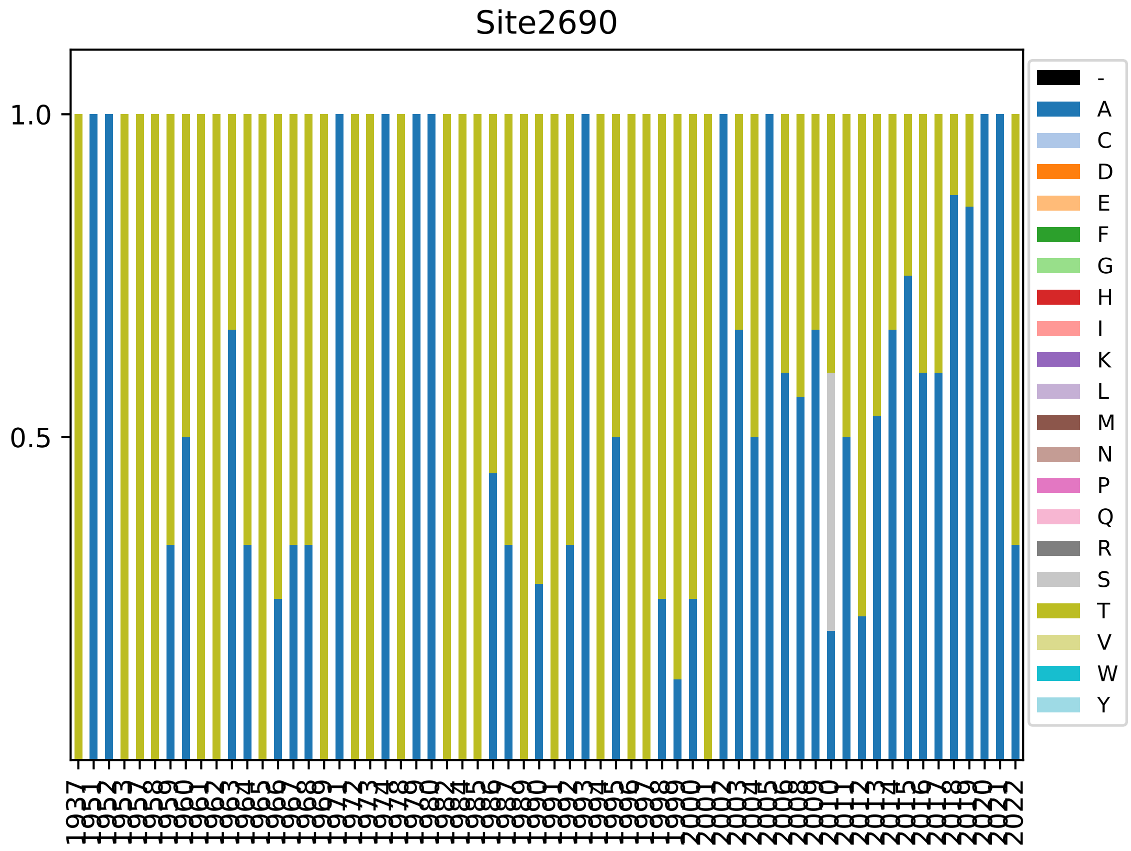
Site2690
1.0
0.5
-
A
C
D
E
F
G
H
I
K
L
M
N
P
Q
R
S
T
V
W
Y

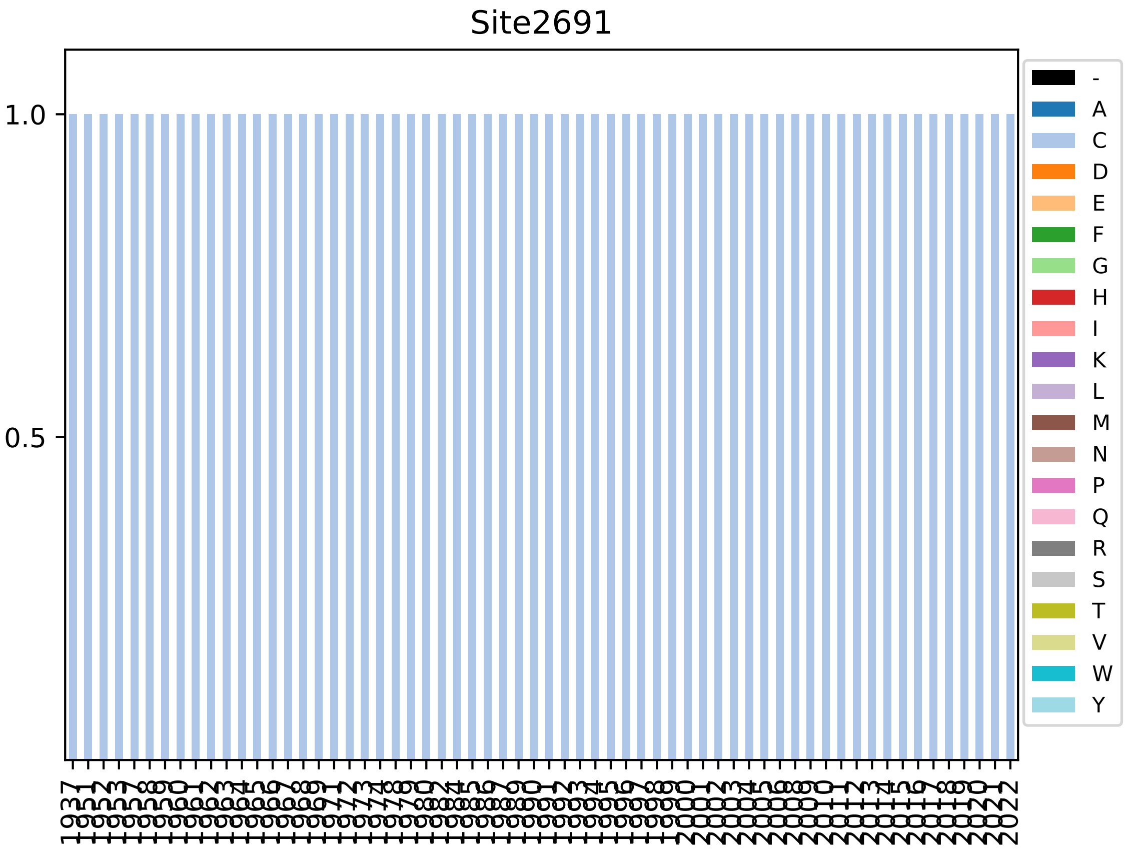
Site2691
1.0
0.5
-
A
C
D
E
F
G
H
I
K
L
M
N
P
Q
R
S
T
V
W
Y

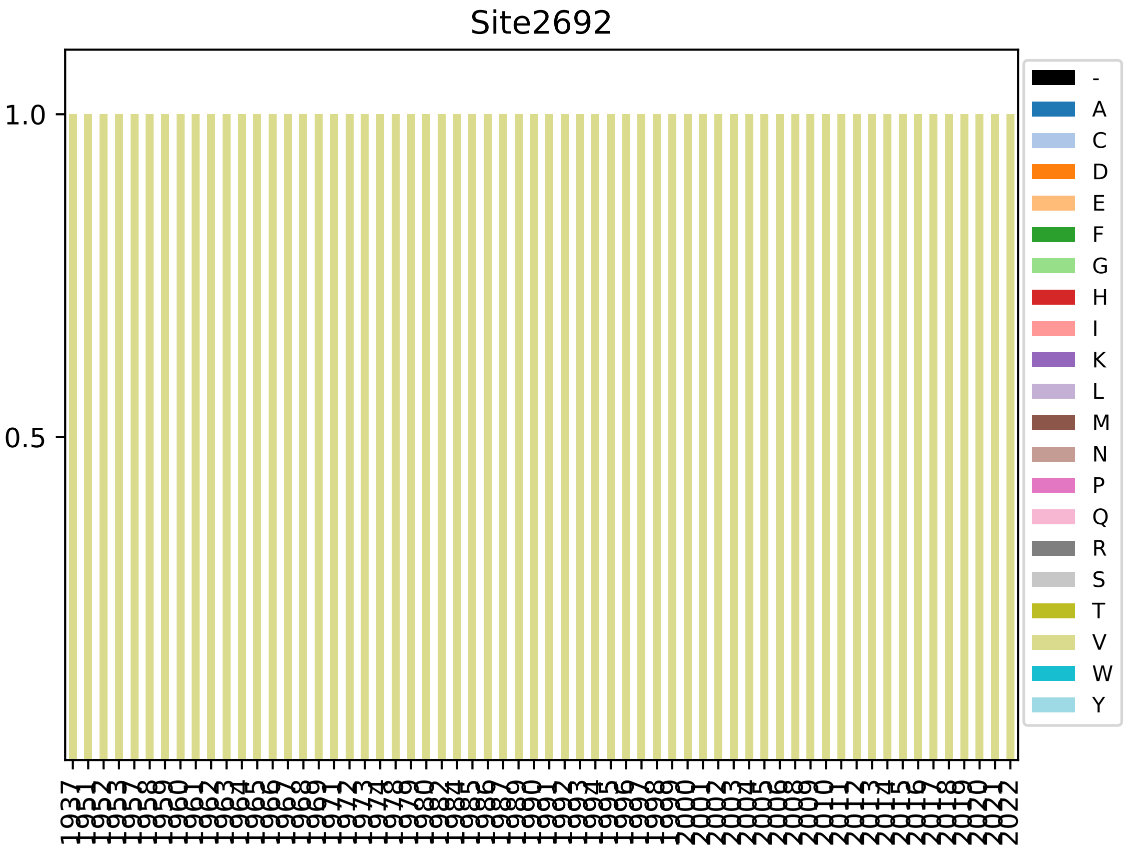
Site2692
1.0
0.5
-
A
C
D
E
F
G
H
I
K
L
M
N
P
Q
R
S
T
V
W
Y

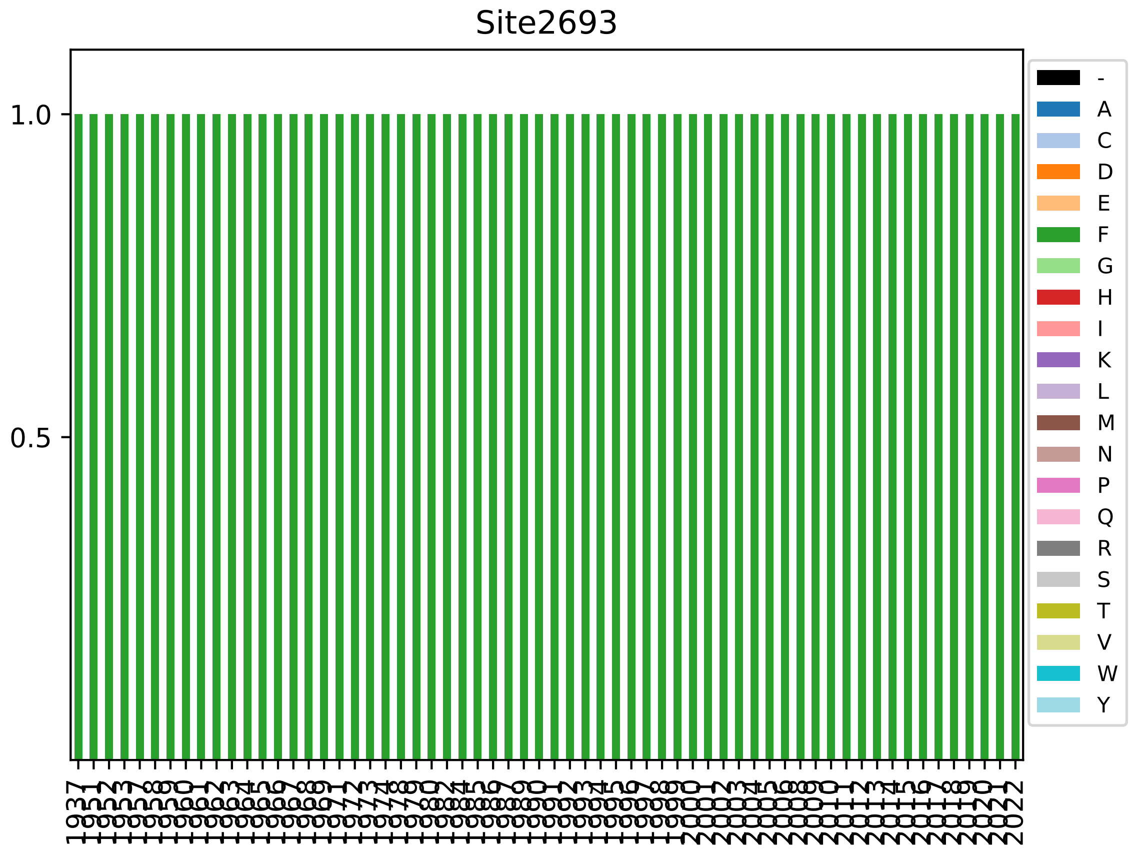

Site2693
1.0
0.5
-
A
C
D
E
F
G
H
I
K
L
M
N
P
Q
R
S
T
V
W
Y

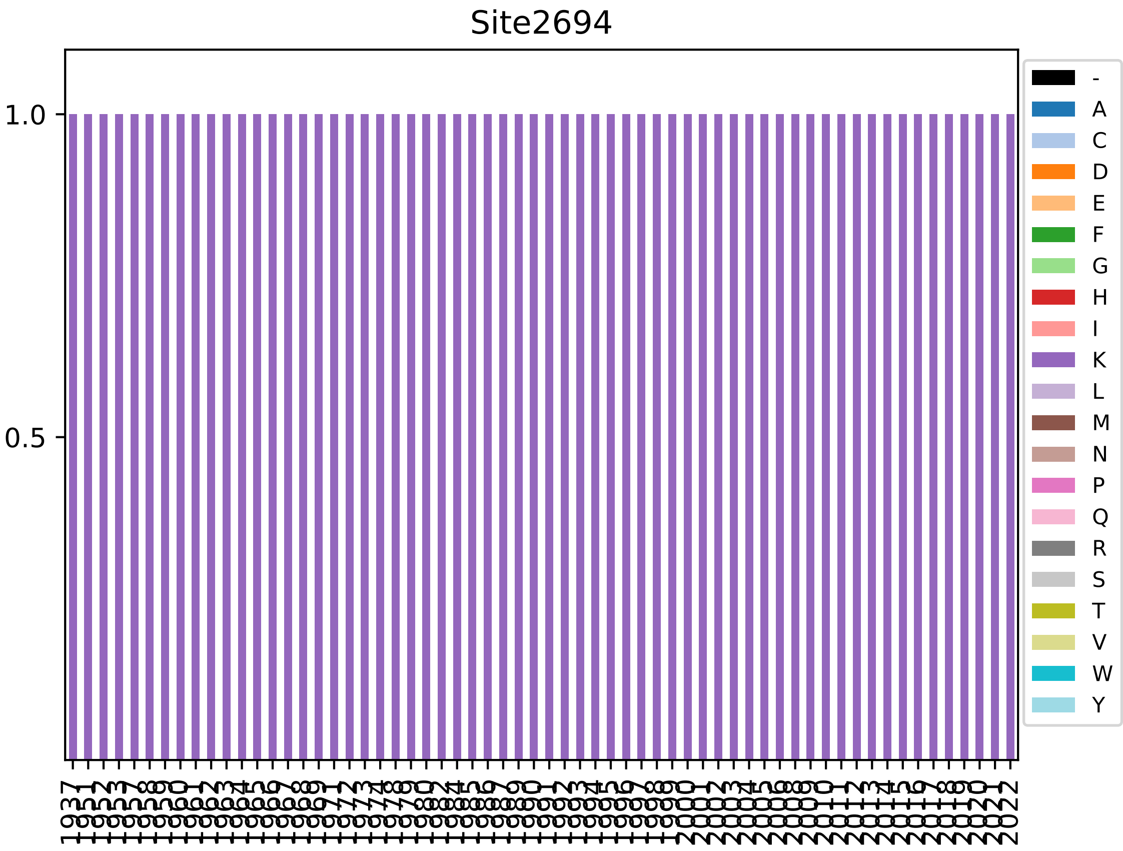

Site2694
1.0
0.5
-
A
C
D
E
F
G
H
I
K
L
M
N
P
Q
R
S
T
V
W
Y

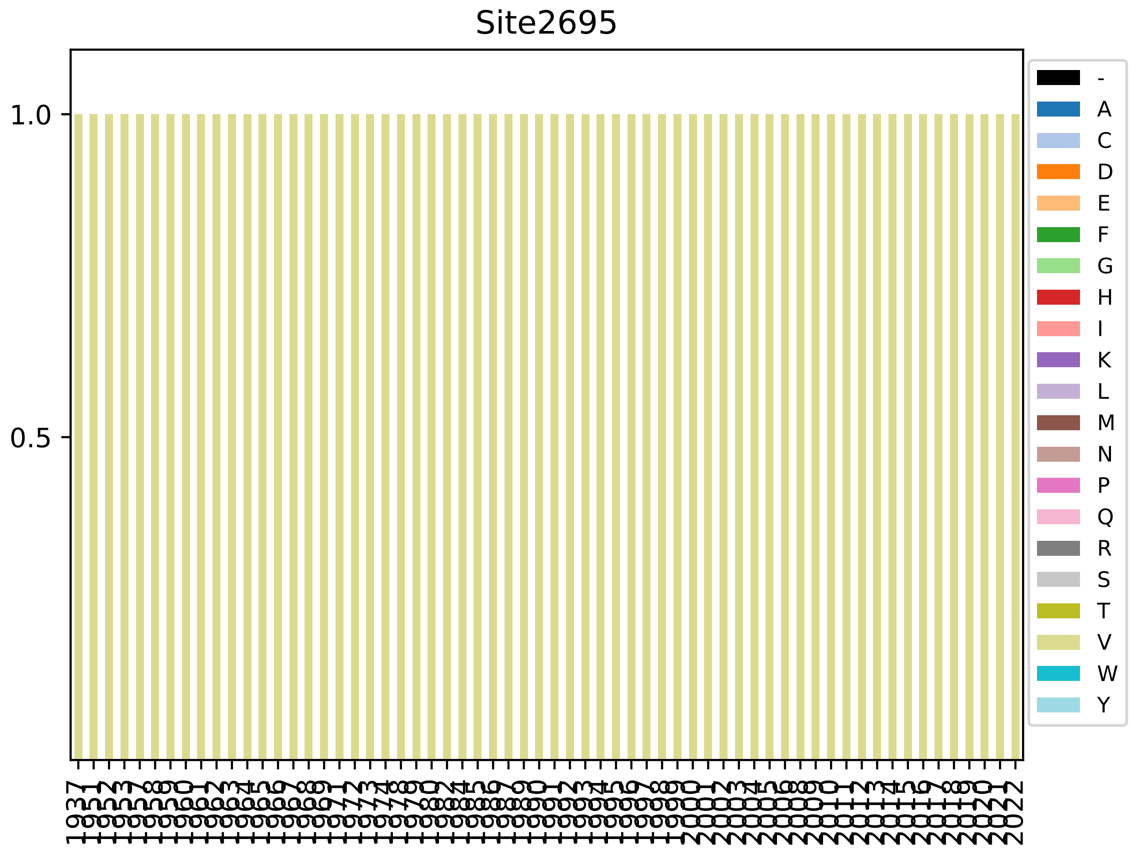
Site2695
1.0
0.5
-
A
C
D
E
F
G
H
I
K
L
M
N
P
Q
R
S
T
V
W
Y

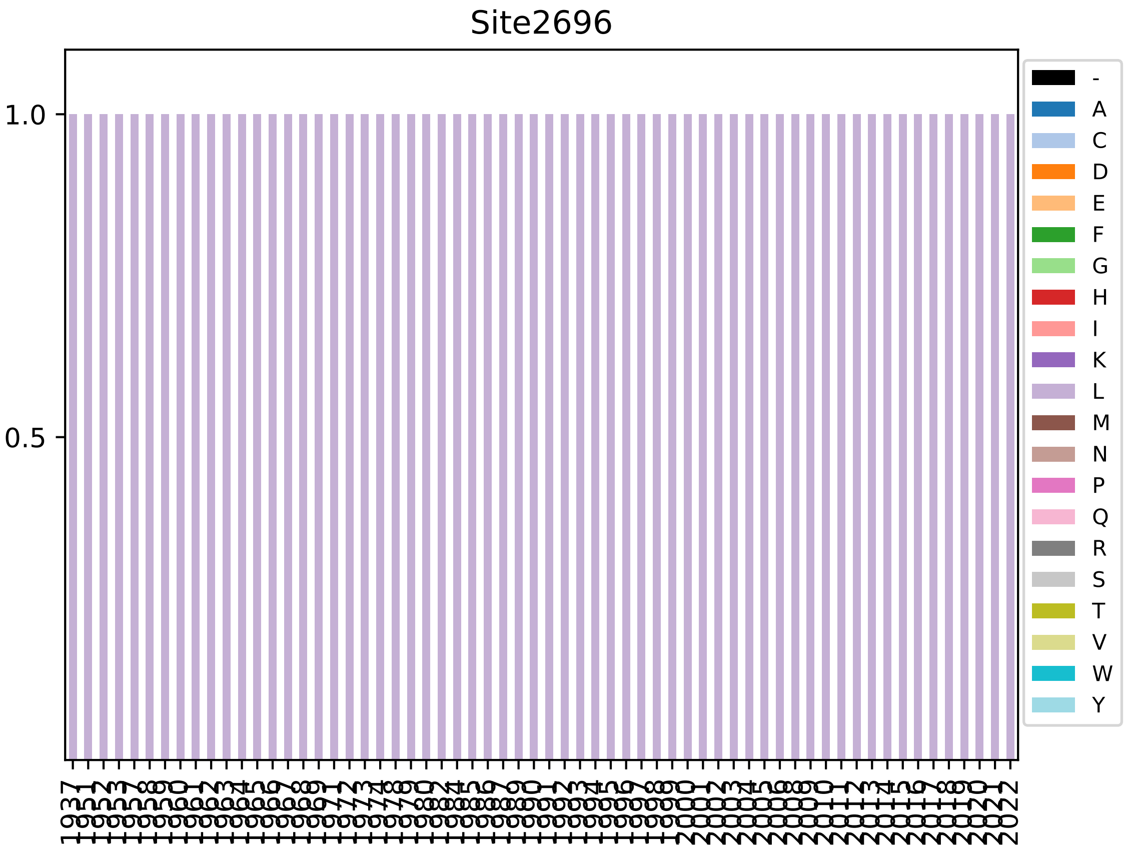
Site2696
1.0
0.5
-
A
C
D
E
F
G
H
I
K
L
M
N
P
Q
R
S
T
V
W
Y

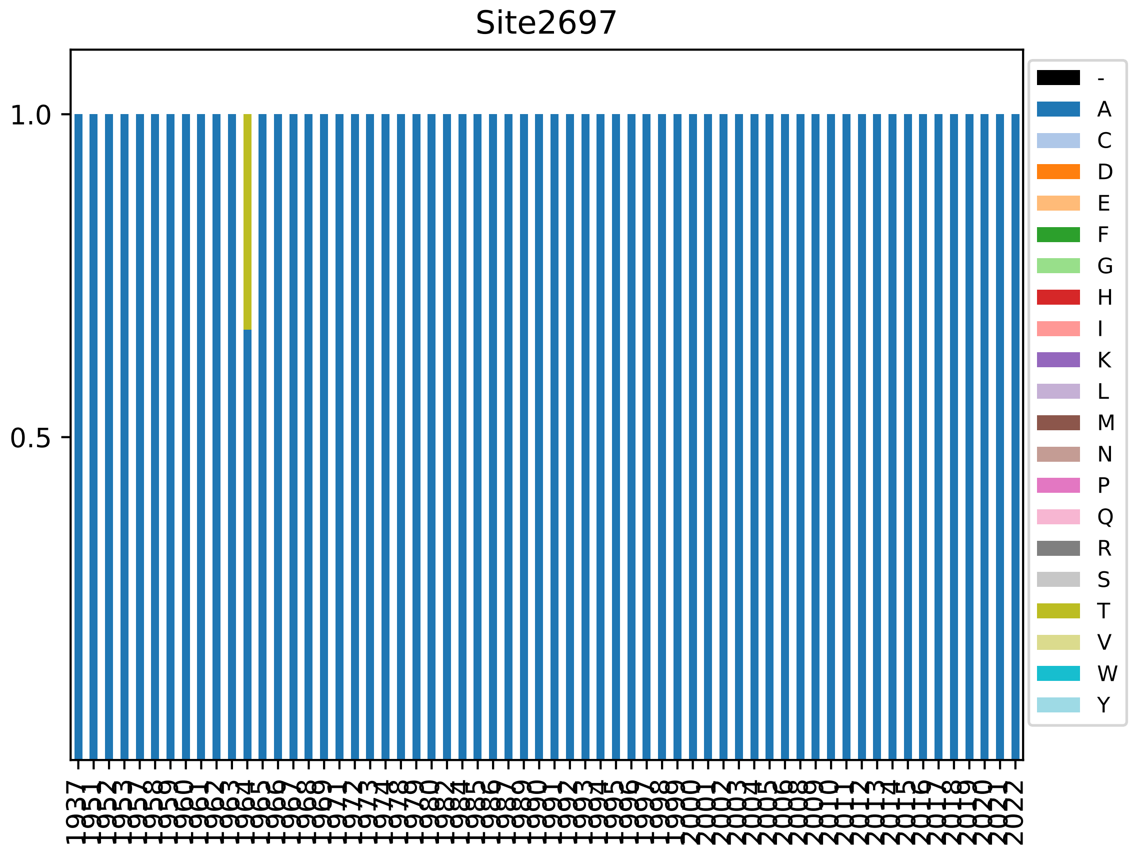
Site2697
1.0
0.5
-
A
C
D
E
F
G
H
I
K
L
M
N
P
Q
R
S
T
V
W
Y

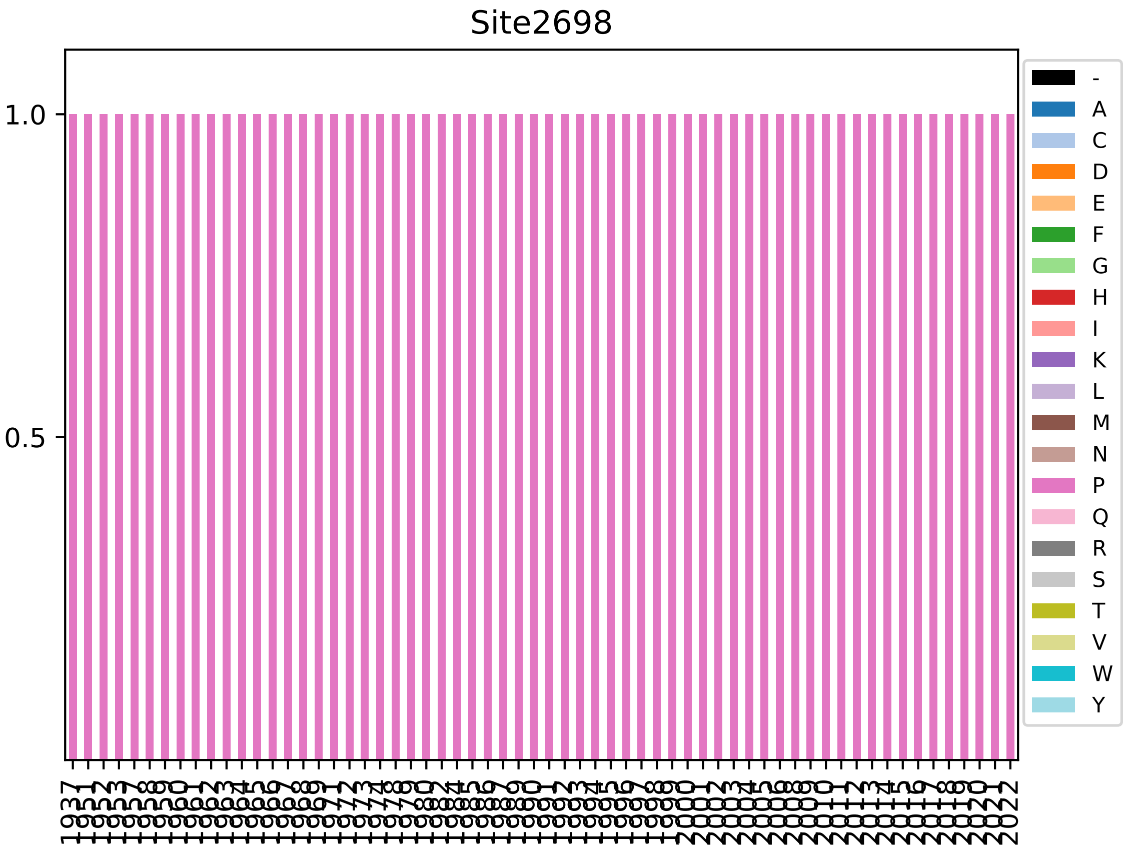
Site2698
1.0
0.5
-
A
C
D
E
F
G
H
I
K
L
M
N
P
Q
R
S
T
V
W
Y

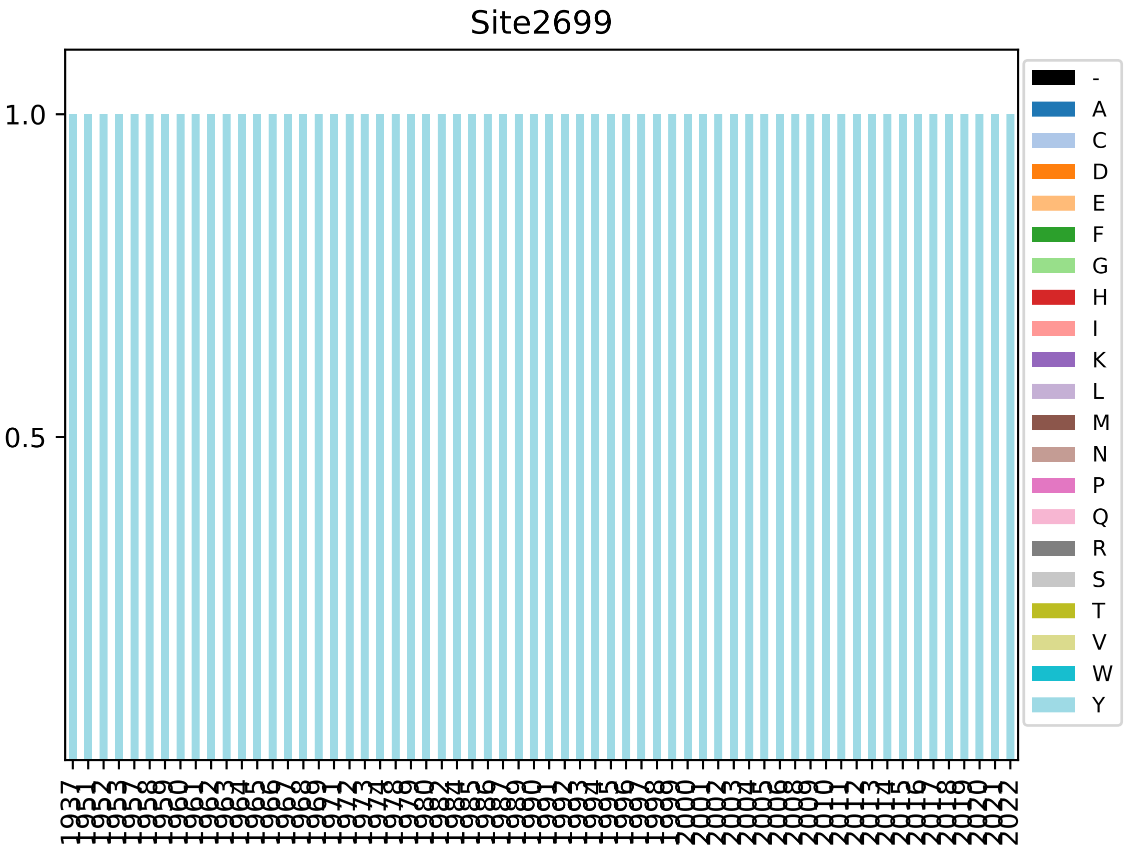
Site2699
1.0
0.5
-
A
C
D
E
F
G
H
I
K
L
M
N
P
Q
R
S
T
V
W
Y

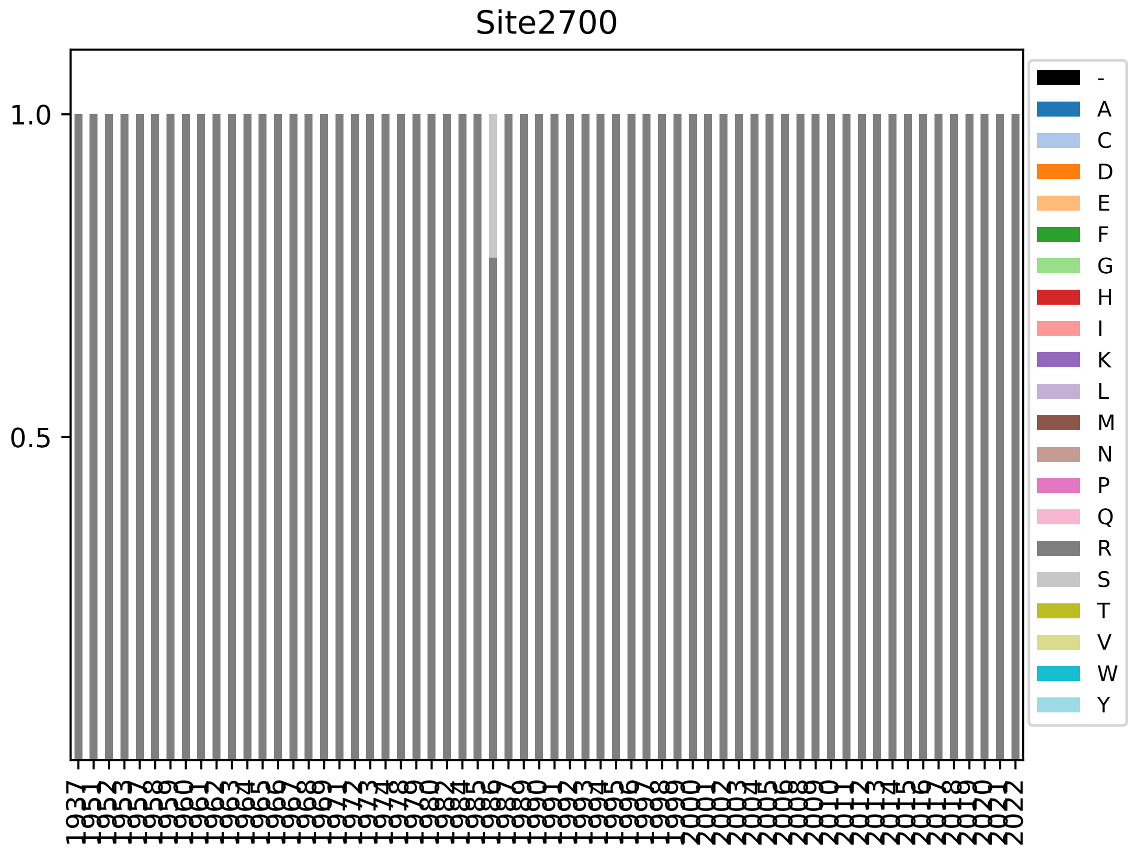
Site2700
1.0
0.5
-
A
C
D
E
F
G
H
I
K
L
M
N
P
Q
R
S
T
V
W
Y

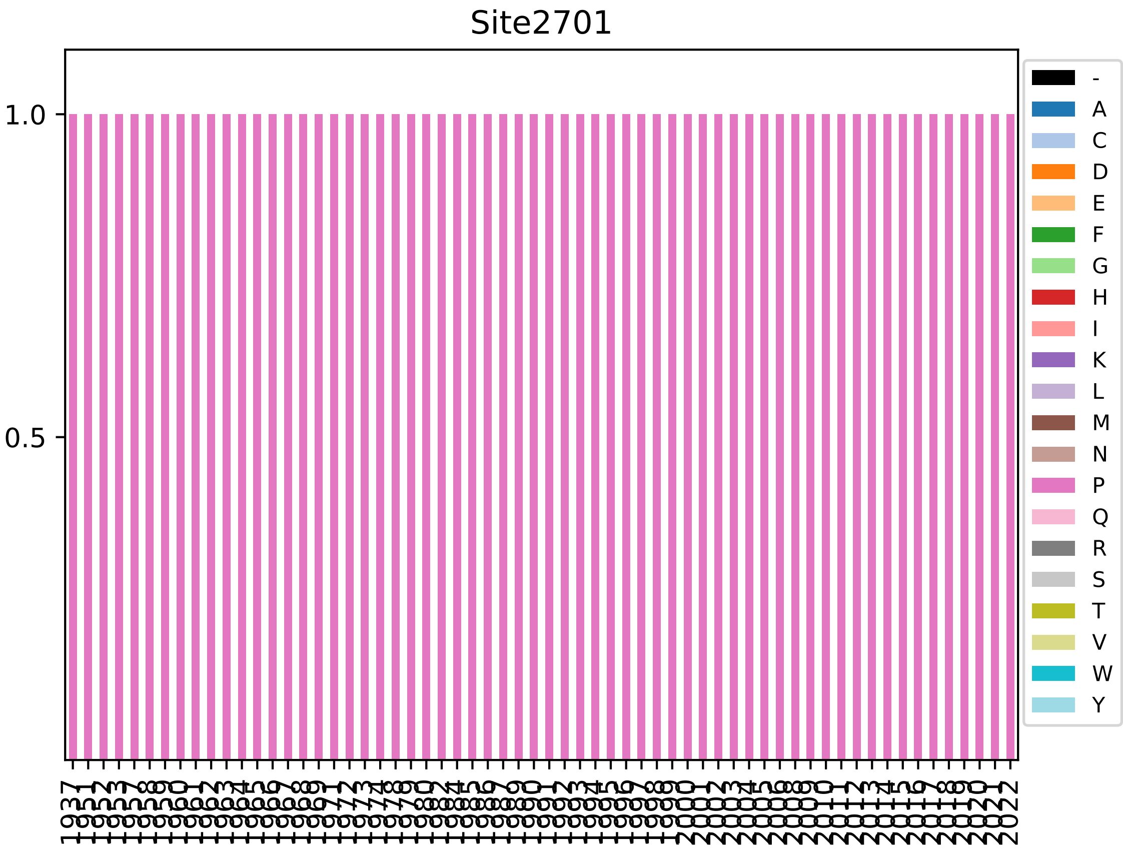

Site2701
1.0
0.5
-
A
C
D
E
F
G
H
I
K
L
M
N
P
Q
R
S
T
V
W
Y

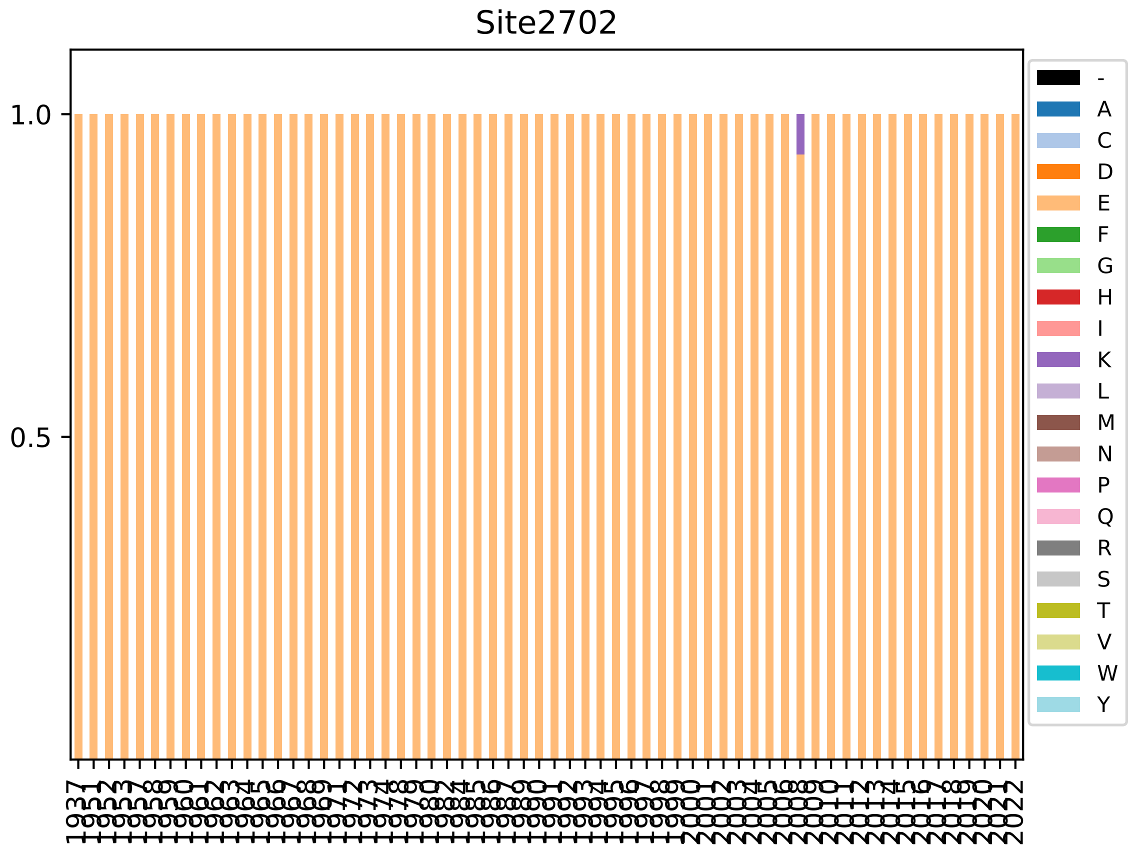
Site2702
1.0
0.5
-
A
C
D
E
F
G
H
I
K
L
M
N
P
Q
R
S
T
V
W
Y

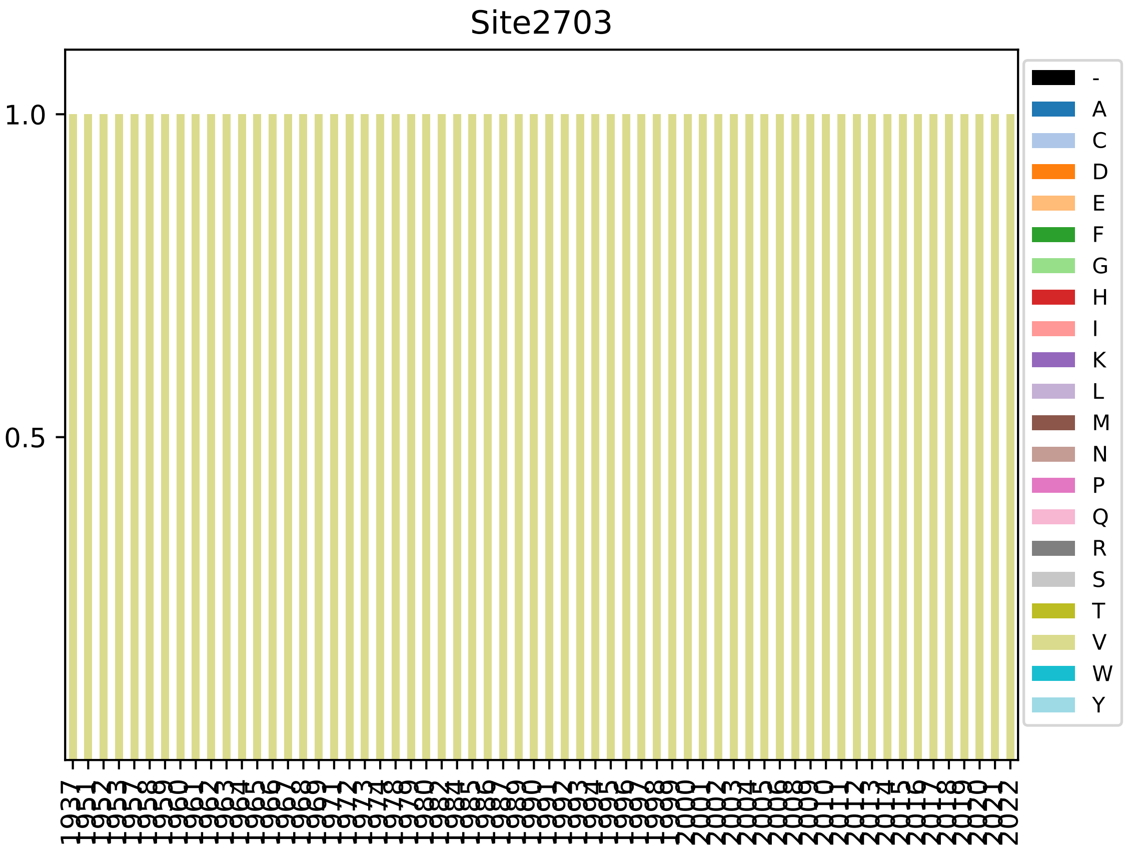
Site2703
1.0
0.5
-
A
C
D
E
F
G
H
I
K
L
M
N
P
Q
R
S
T
V
W
Y

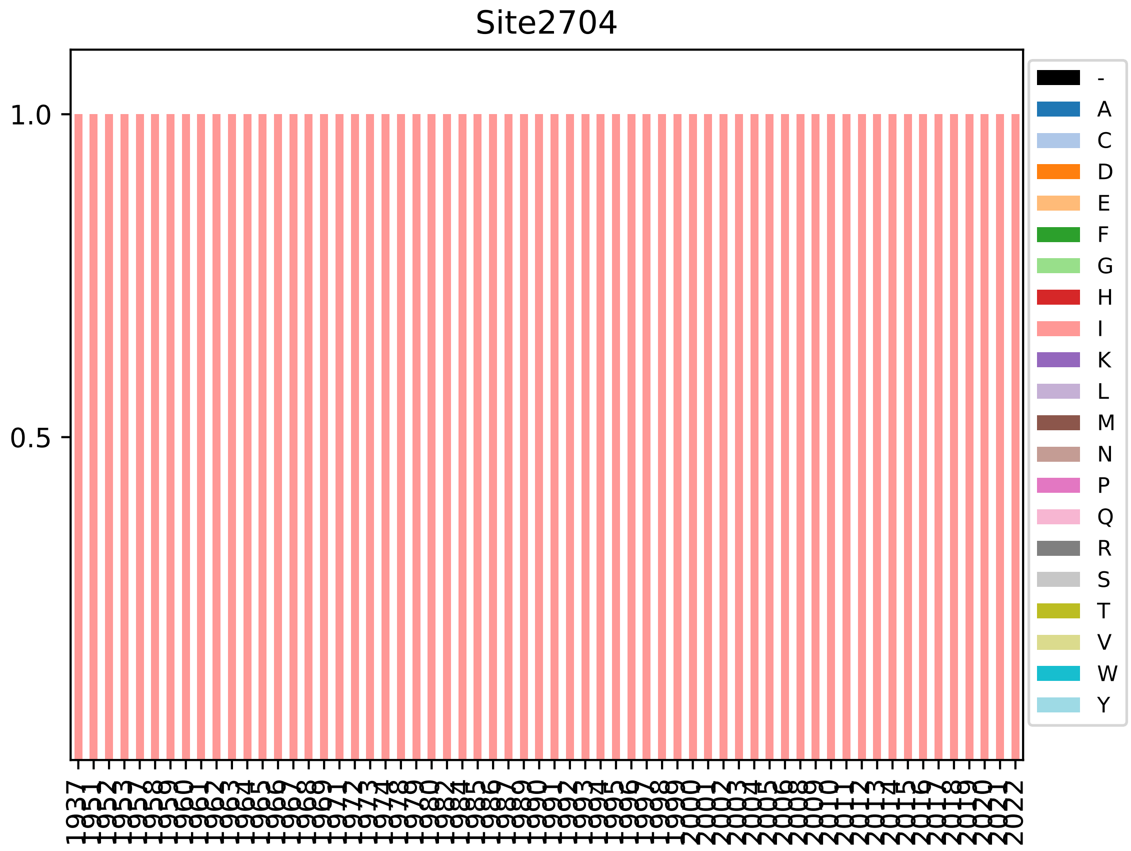
Site2704
1.0
0.5
-
A
C
D
E
F
G
H
I
K
L
M
N
P
Q
R
S
T
V
W
Y

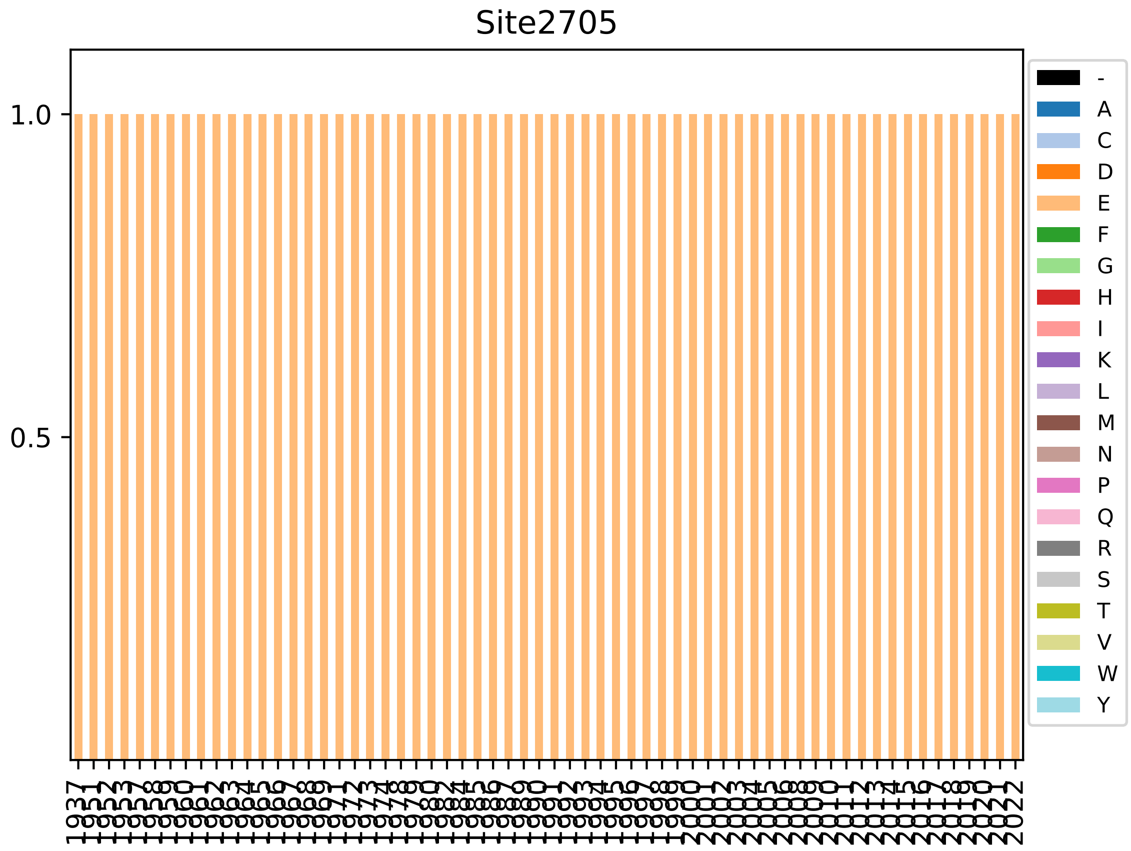
Site2705
1.0
0.5
-
A
C
D
E
F
G
H
I
K
L
M
N
P
Q
R
S
T
V
W
Y

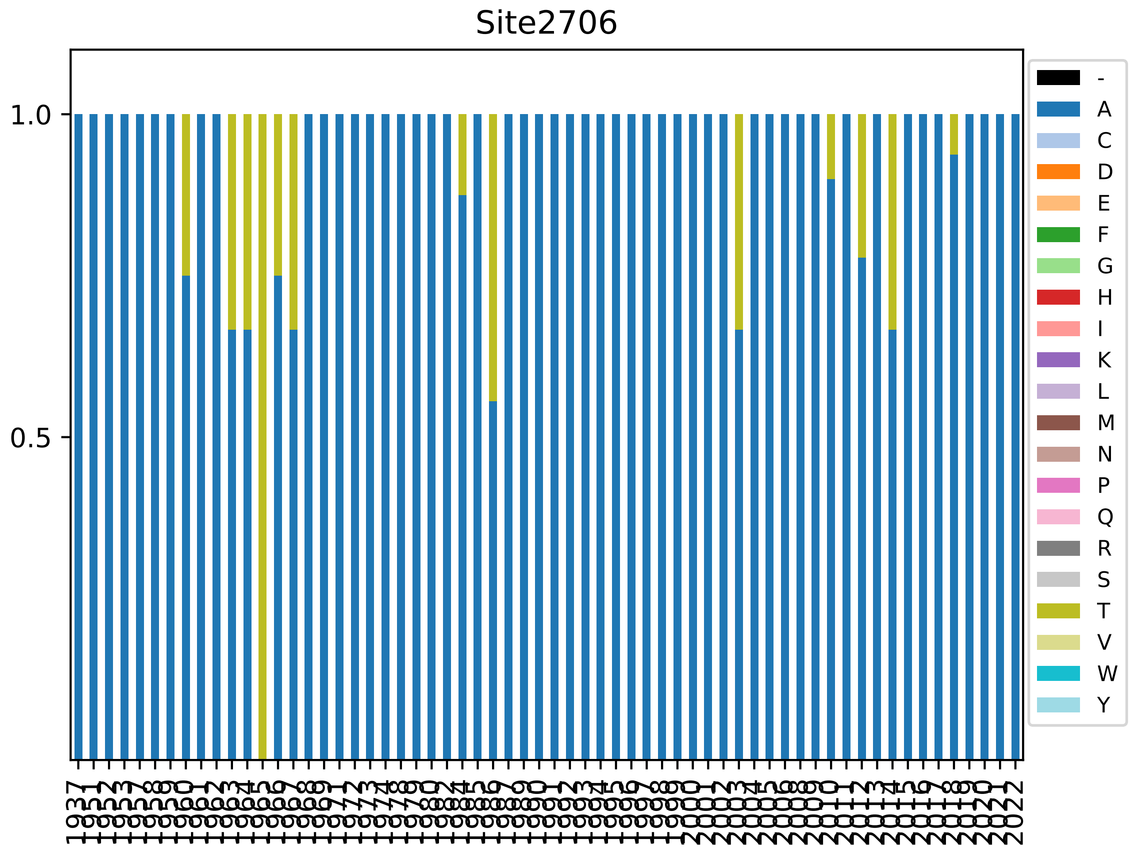
Site2706
1.0
0.5
-
A
C
D
E
F
G
H
I
K
L
M
N
P
Q
R
S
T
V
W
Y

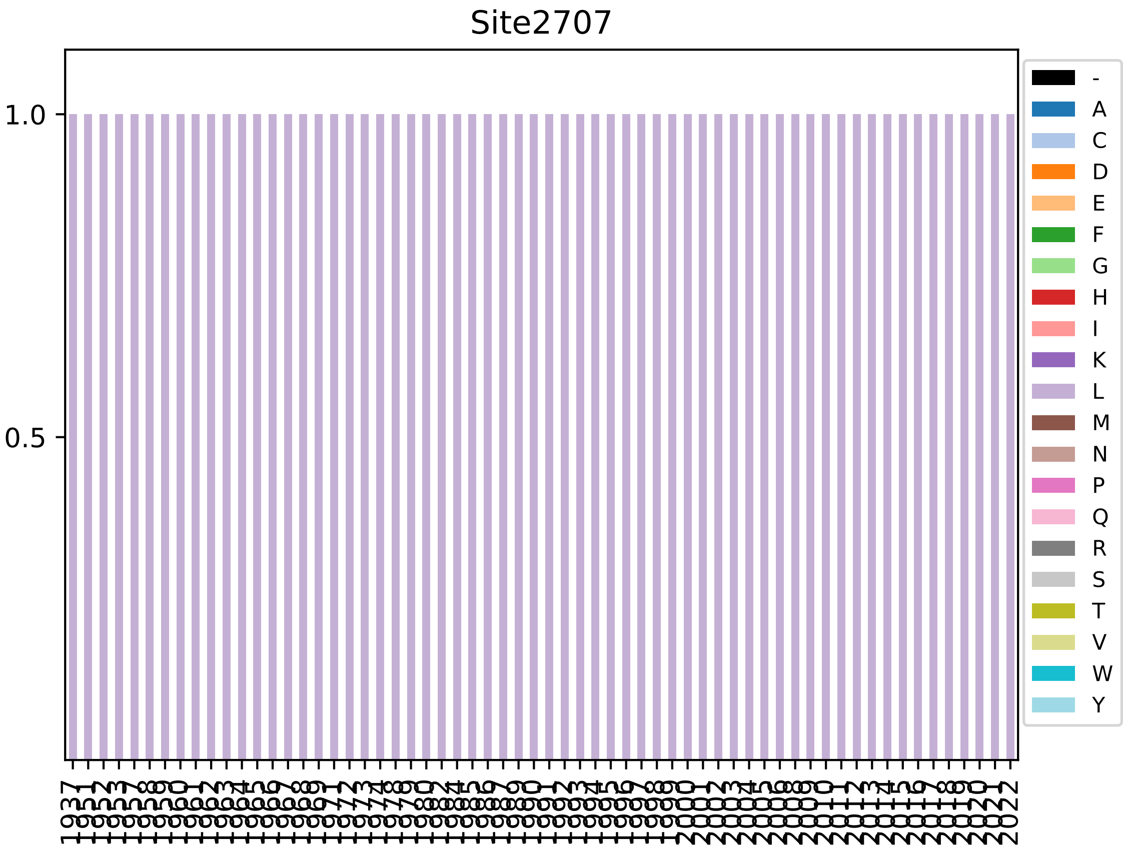
Site2707
1.0
0.5
-
A
C
D
E
F
G
H
I
K
L
M
N
P
Q
R
S
T
V
W
Y

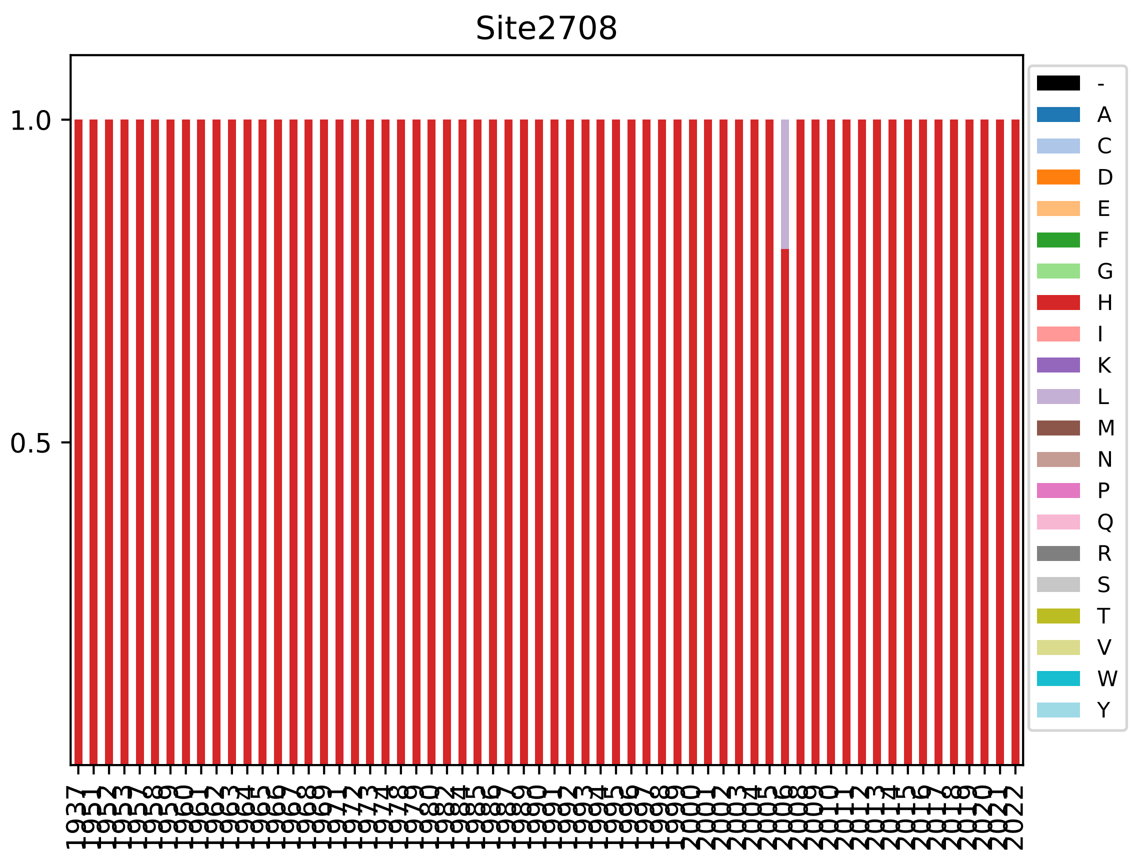
Site2708
1.0
0.5
-
A
C
D
E
F
G
H
I
K
L
M
N
P
Q
R
S
T
V
W
Y

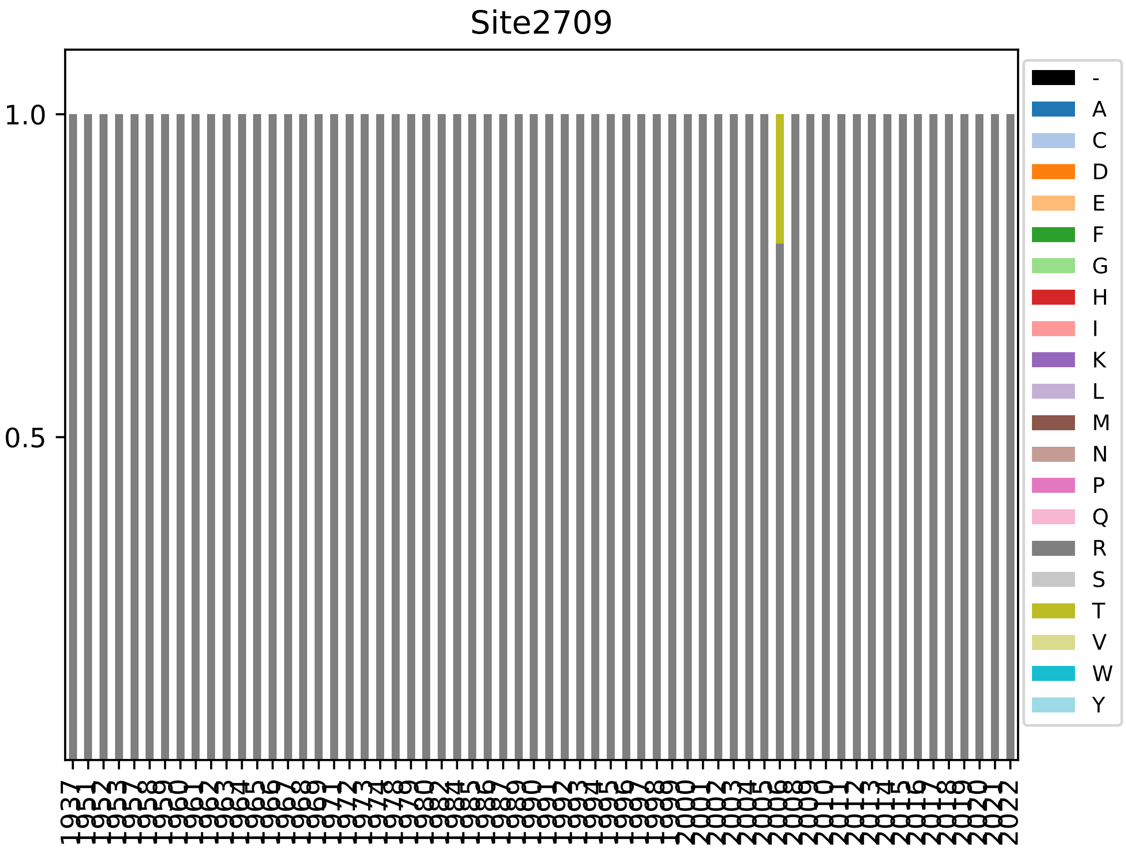

Site2709
1.0
0.5
-
A
C
D
E
F
G
H
I
K
L
M
N
P
Q
R
S
T
V
W
Y

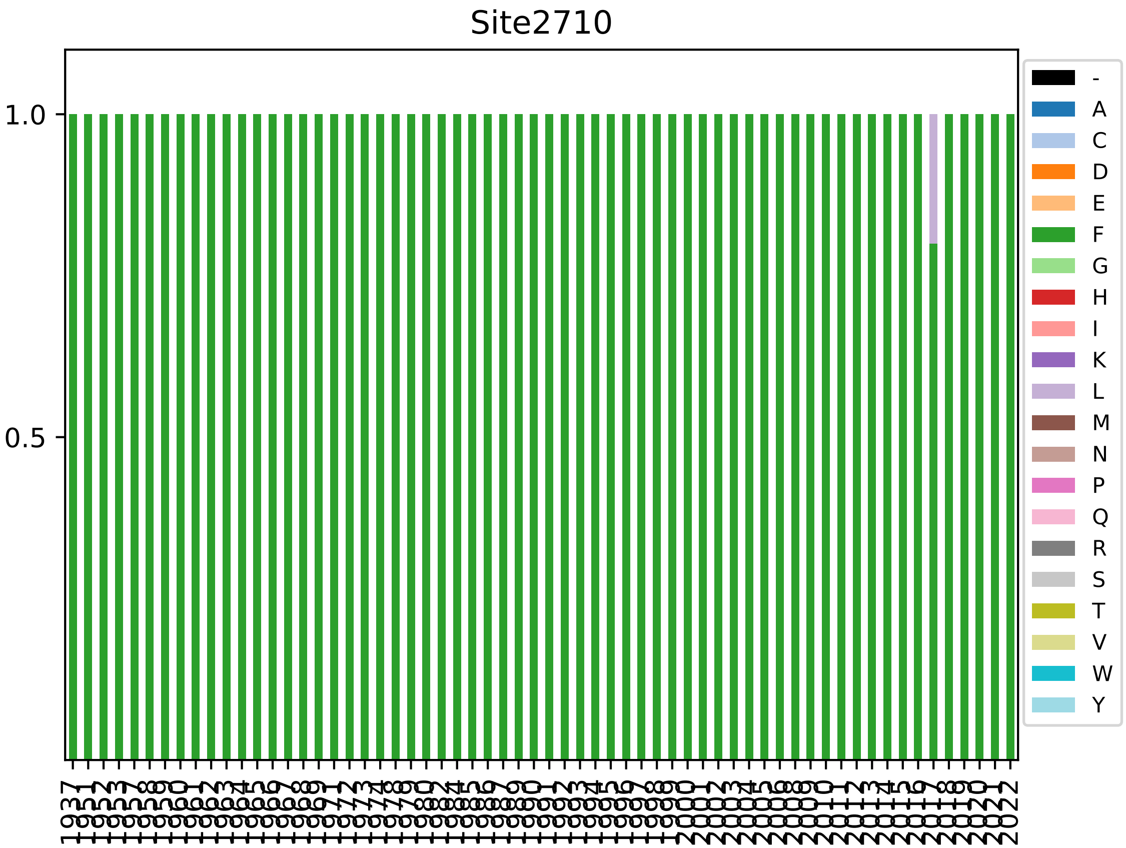
Site2710
1.0
0.5
-
A
C
D
E
F
G
H
I
K
L
M
N
P
Q
R
S
T
V
W
Y

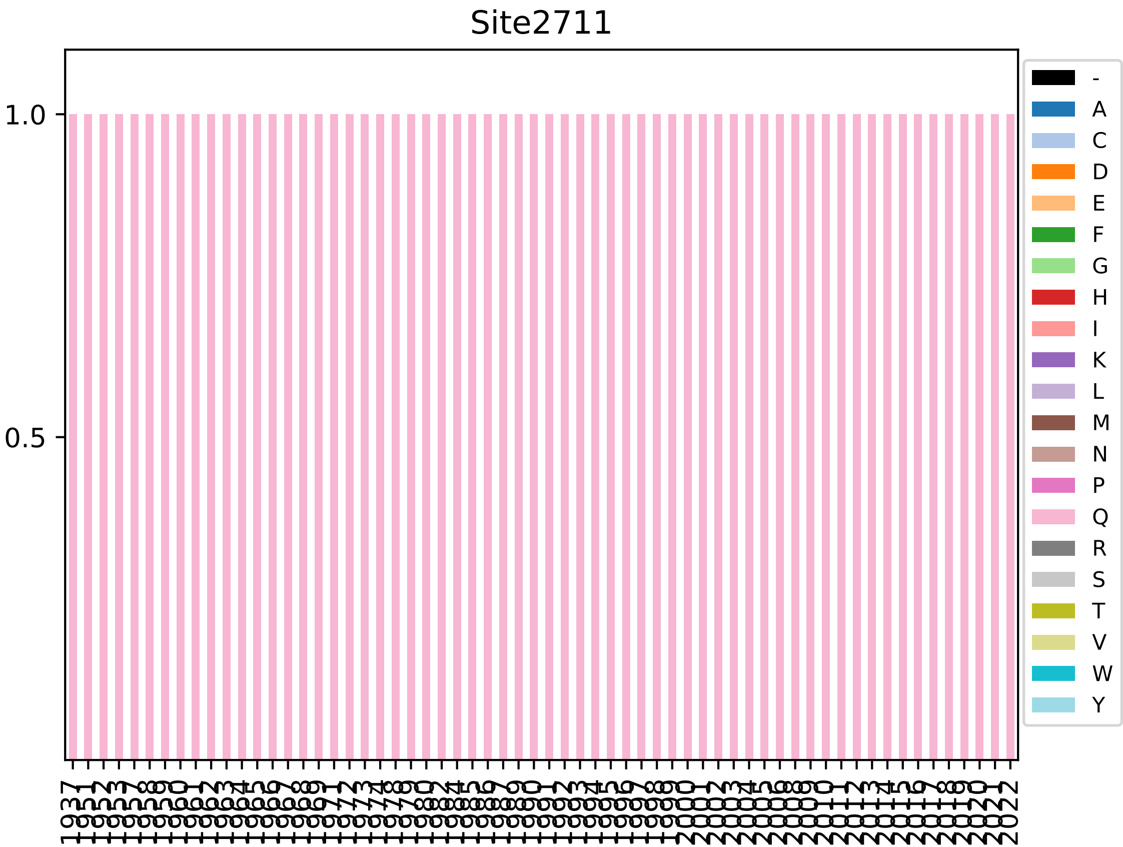
Site2711
1.0
0.5
-
A
C
D
E
F
G
H
I
K
L
M
N
P
Q
R
S
T
V
W
Y

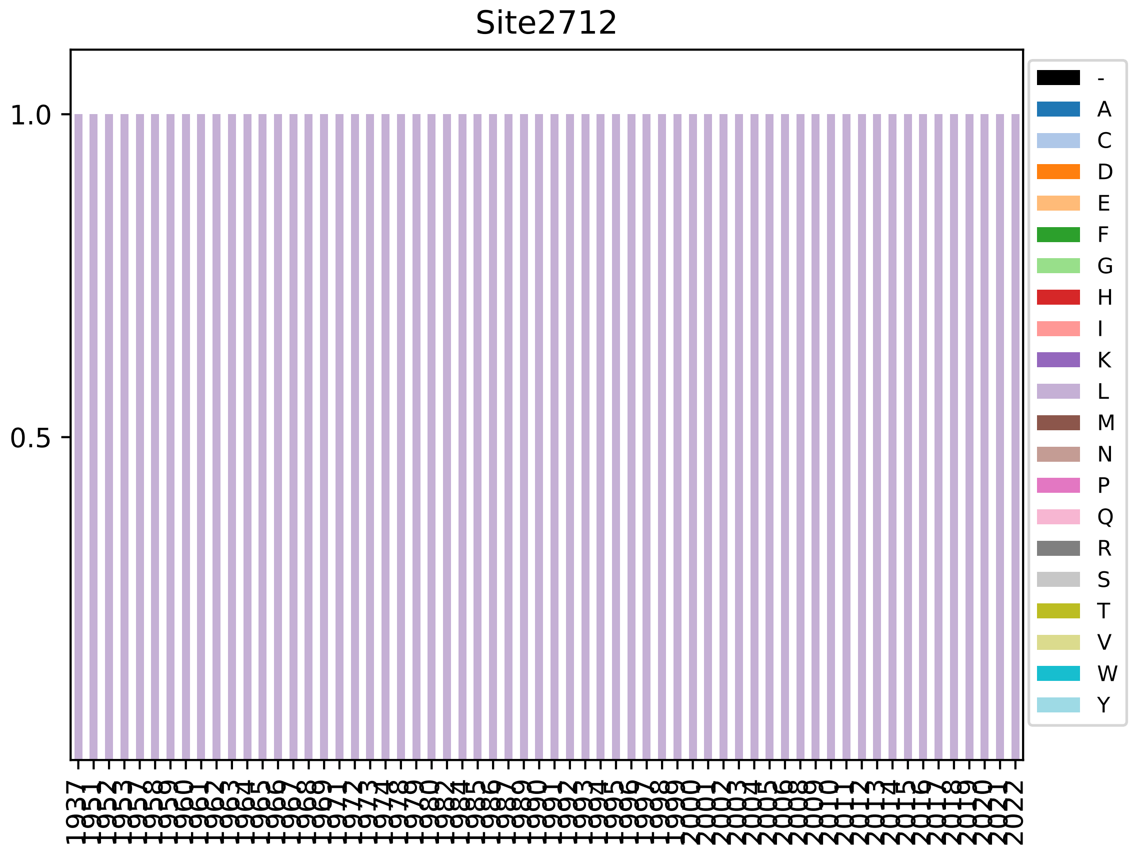
Site2712
1.0
0.5
-
A
C
D
E
F
G
H
I
K
L
M
N
P
Q
R
S
T
V
W
Y

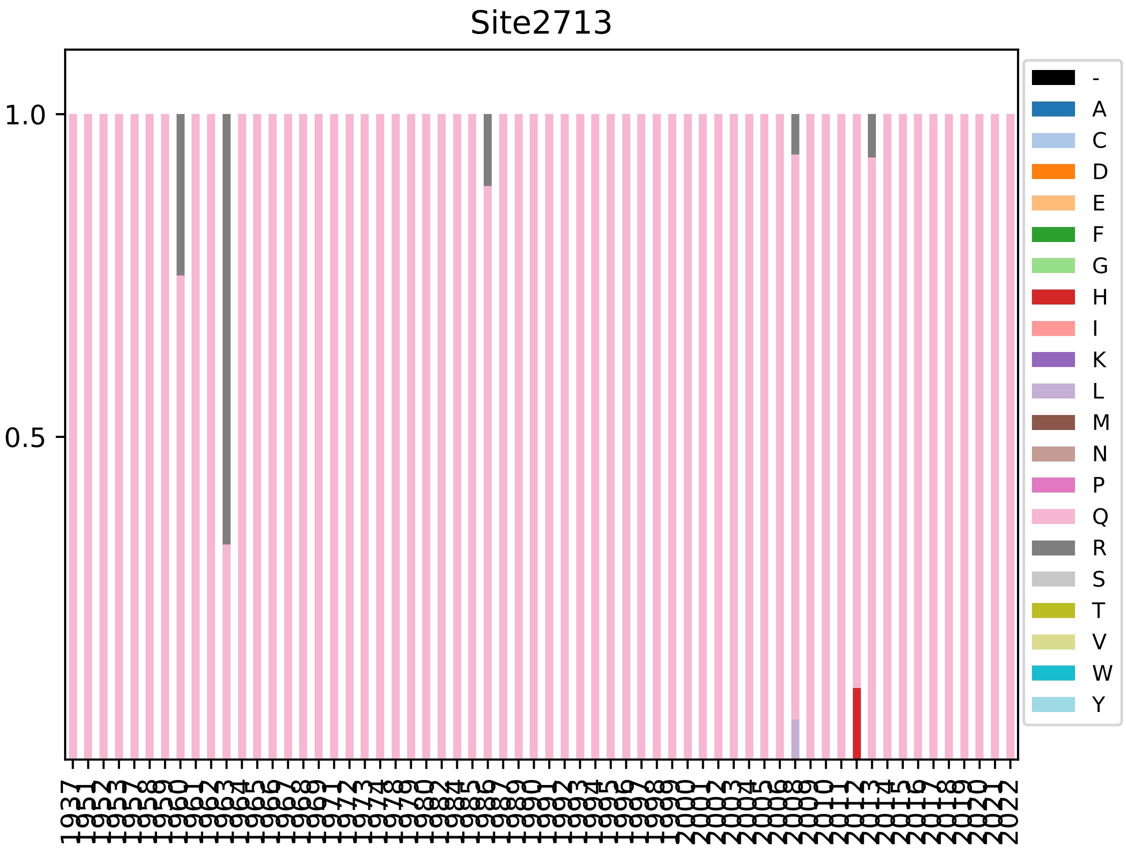

Site2713
1.0
0.5
-
A
C
D
E
F
G
H
I
K
L
M
N
P
Q
R
S
T
V
W
Y

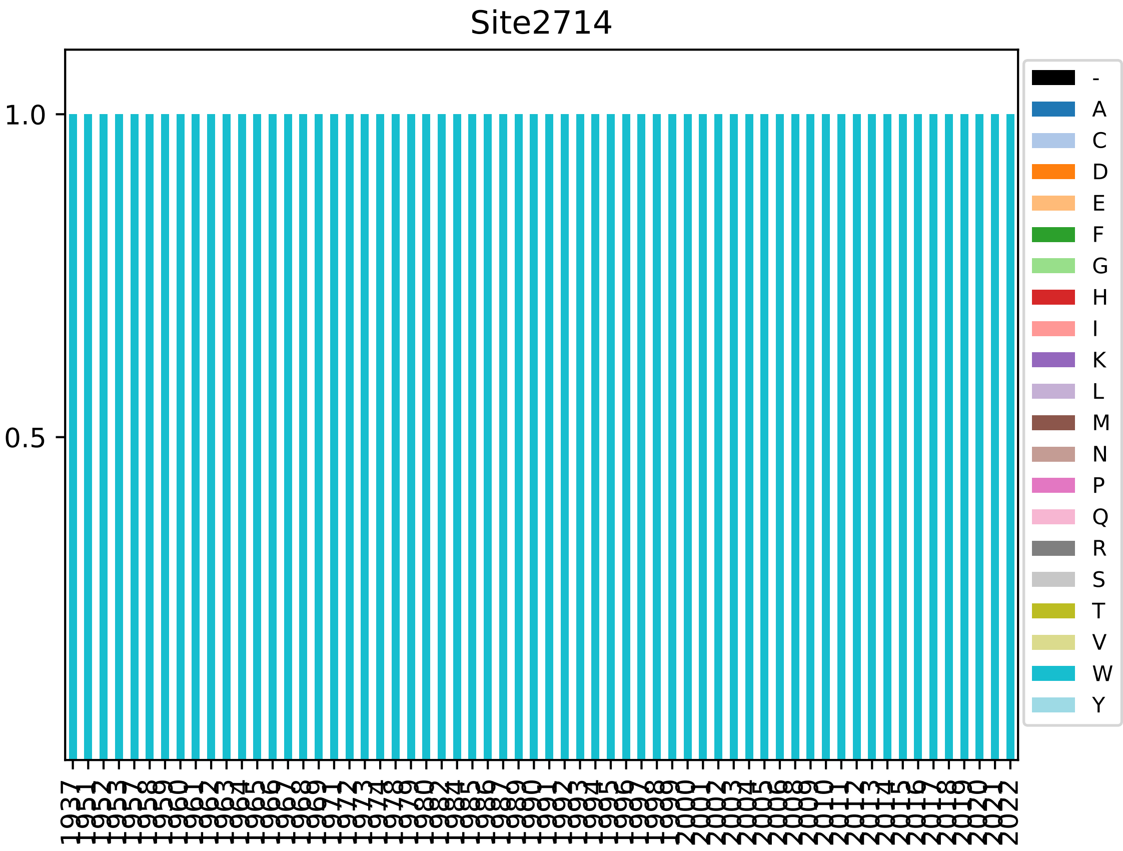
Site2714
1.0
0.5
-
A
C
D
E
F
G
H
I
K
L
M
N
P
Q
R
S
T
V
W
Y

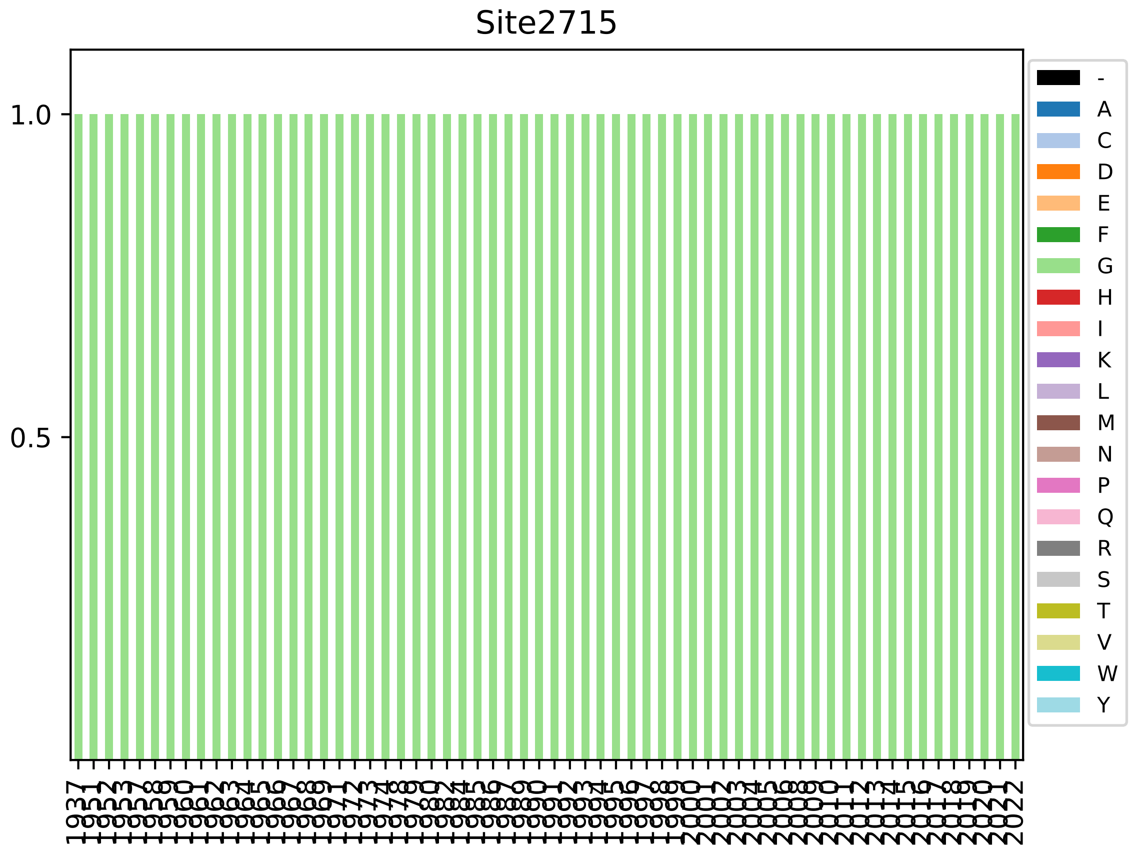
Site2715
1.0
0.5
-
A
C
D
E
F
G
H
I
K
L
M
N
P
Q
R
S
T
V
W
Y

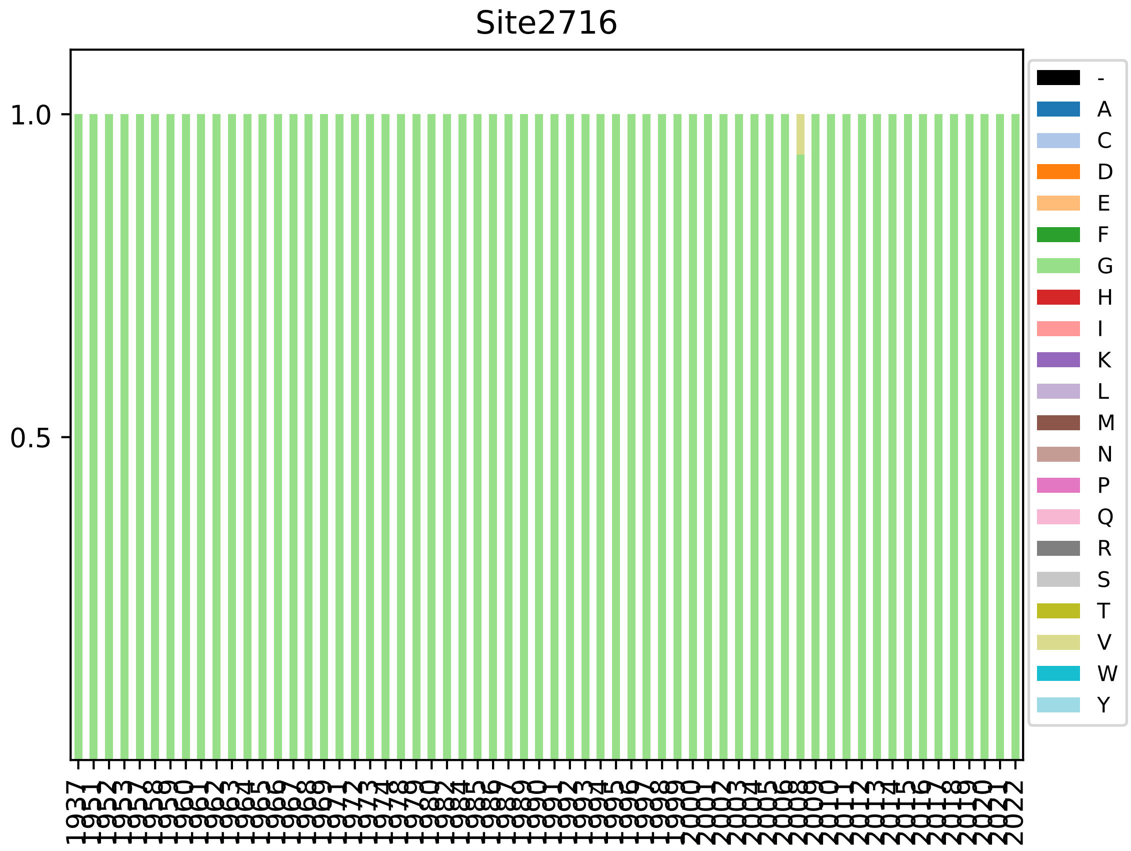
Site2716
1.0
0.5
-
A
C
D
E
F
G
H
I
K
L
M
N
P
Q
R
S
T
V
W
Y

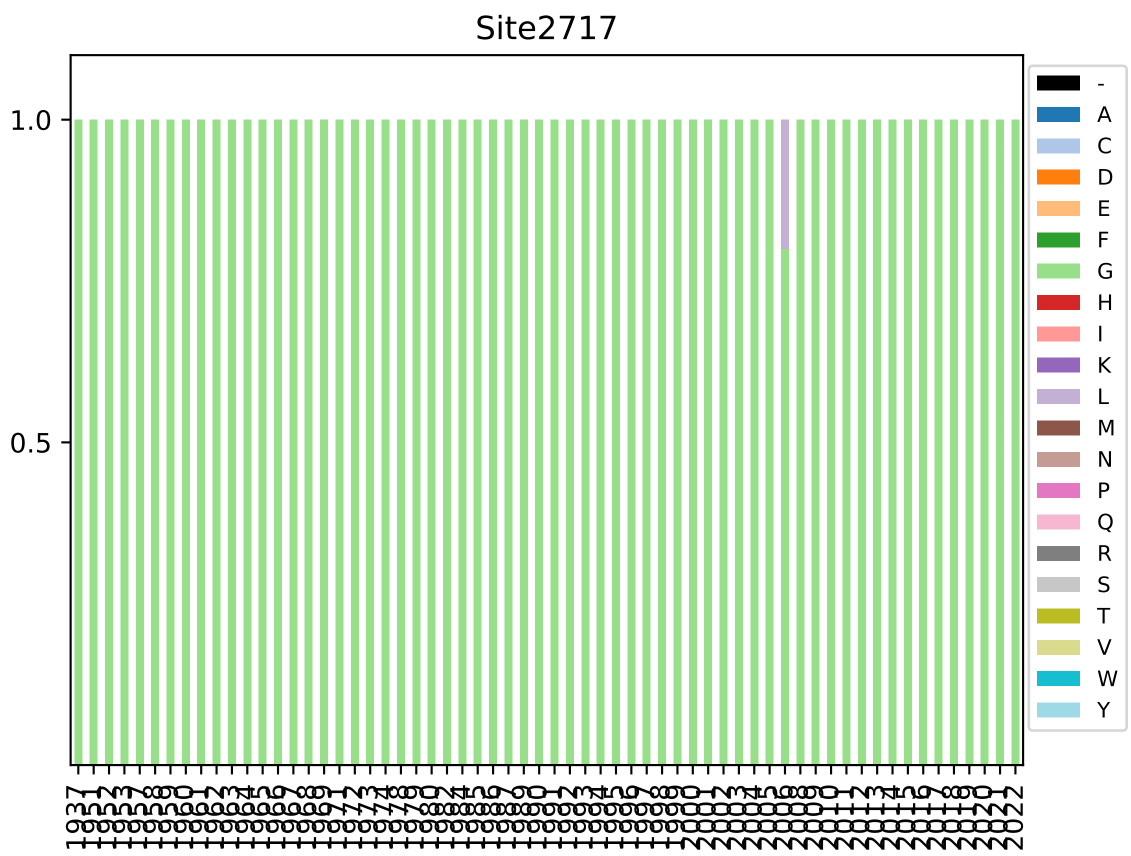
Site2717
1.0
0.5
-
A
C
D
E
F
G
H
I
K
L
M
N
P
Q
R
S
T
V
W
Y

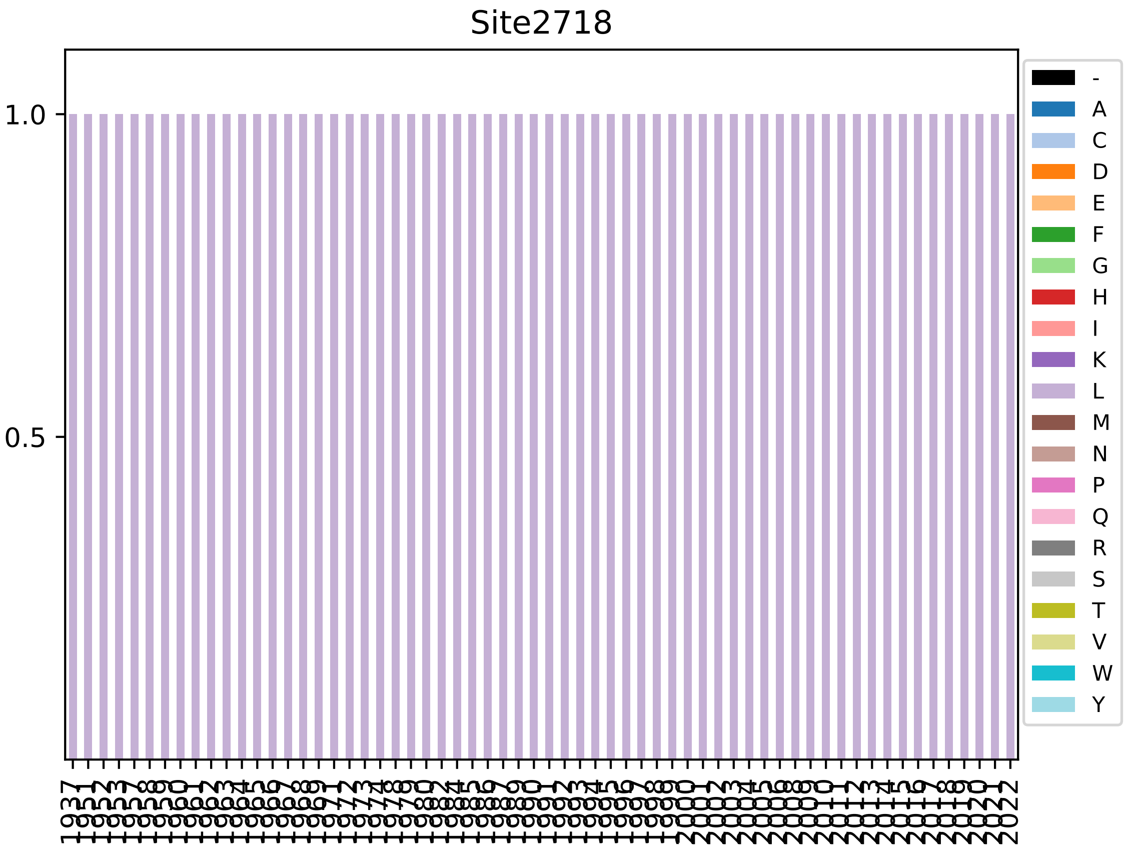

Site2718
1.0
0.5
-
A
C
D
E
F
G
H
I
K
L
M
N
P
Q
R
S
T
V
W
Y

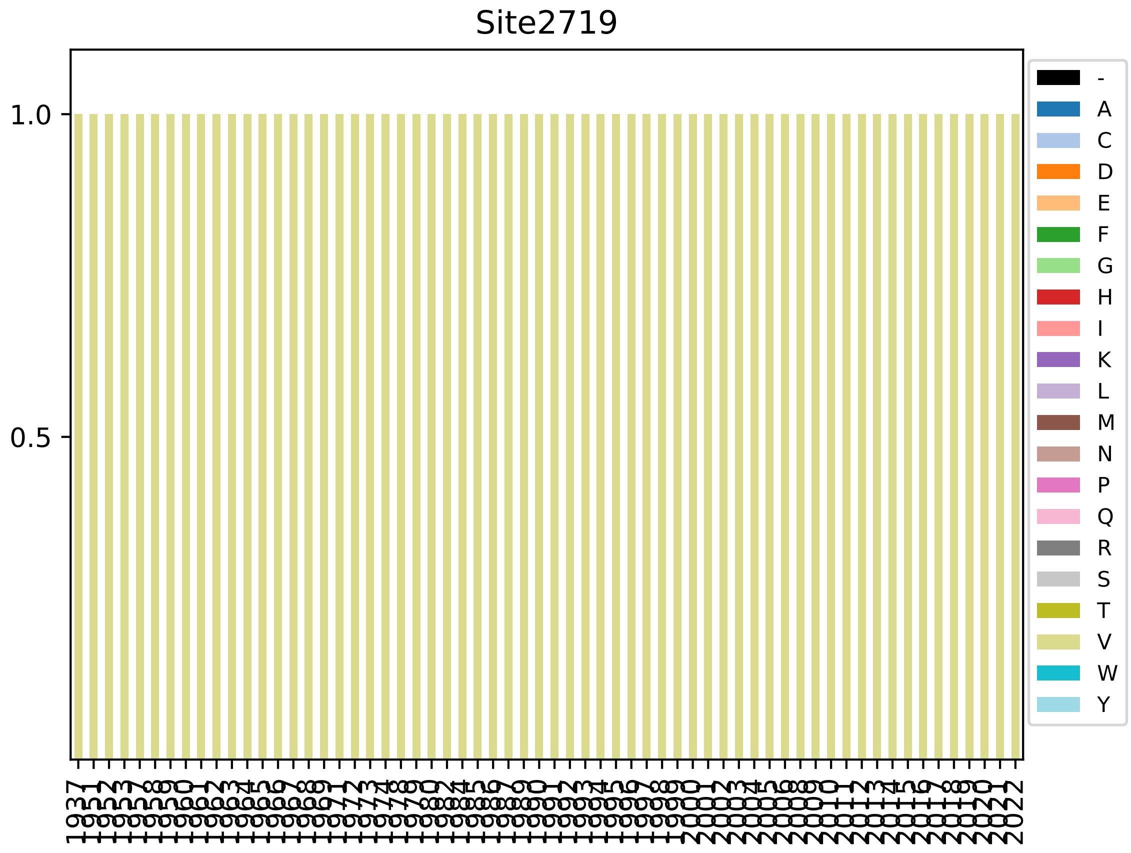
Site2719
1.0
0.5
-
A
C
D
E
F
G
H
I
K
L
M
N
P
Q
R
S
T
V
W
Y

Site2720

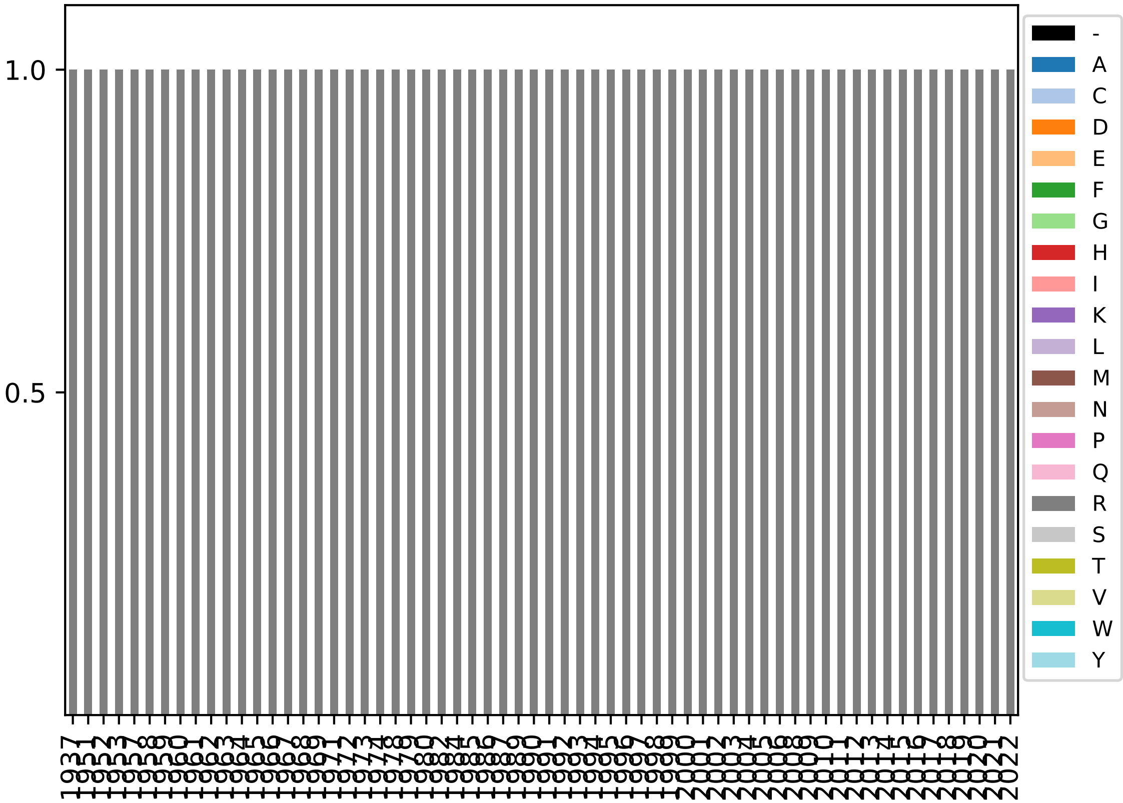

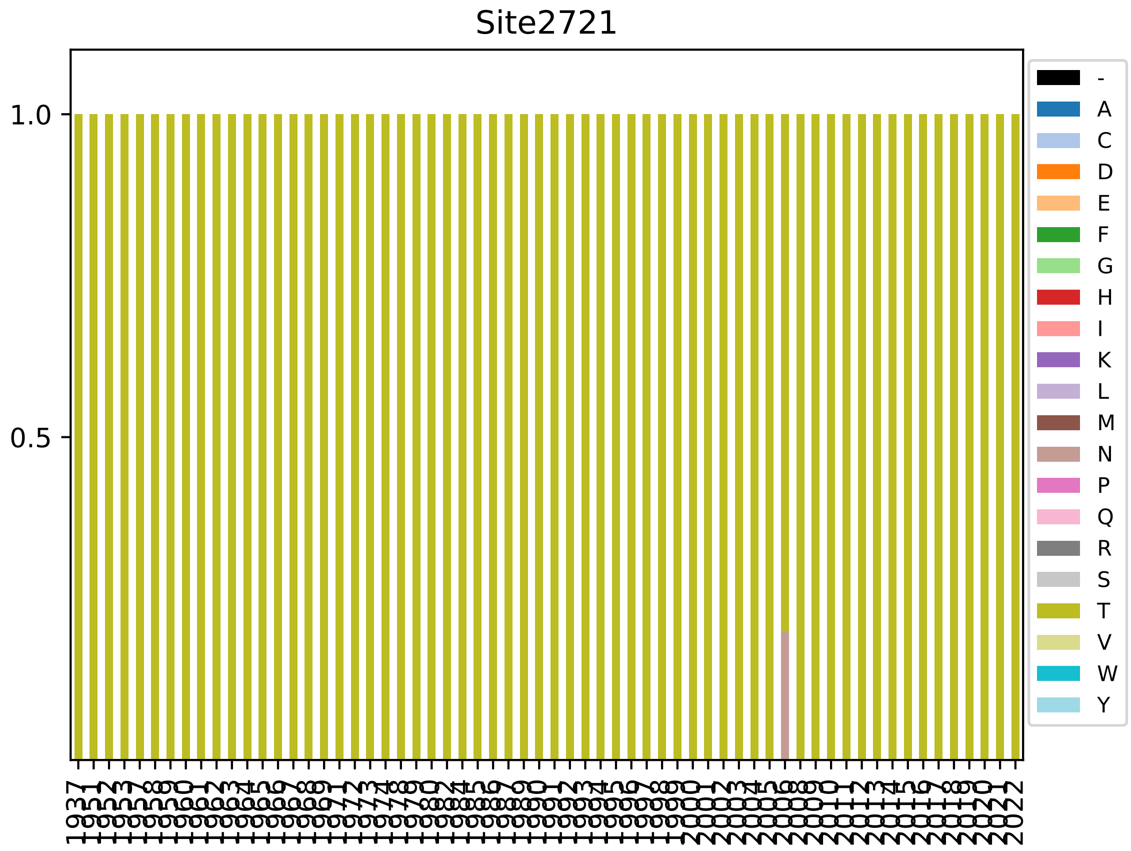
Site2721
1.0
0.5
-
A
C
D
E
F
G
H
I
K
L
M
N
P
Q
R
S
T
V
W
Y

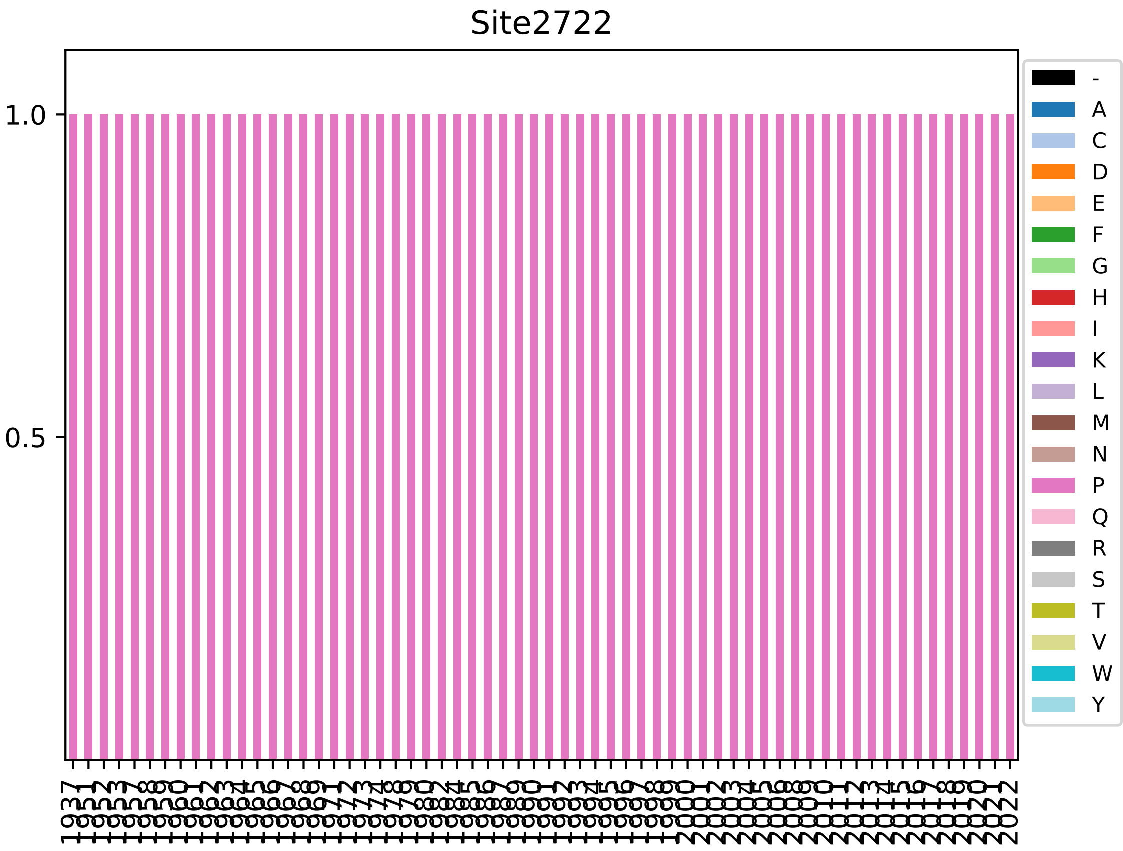
Site2722
1.0
0.5
-
A
C
D
E
F
G
H
I
K
L
M
N
P
Q
R
S
T
V
W
Y

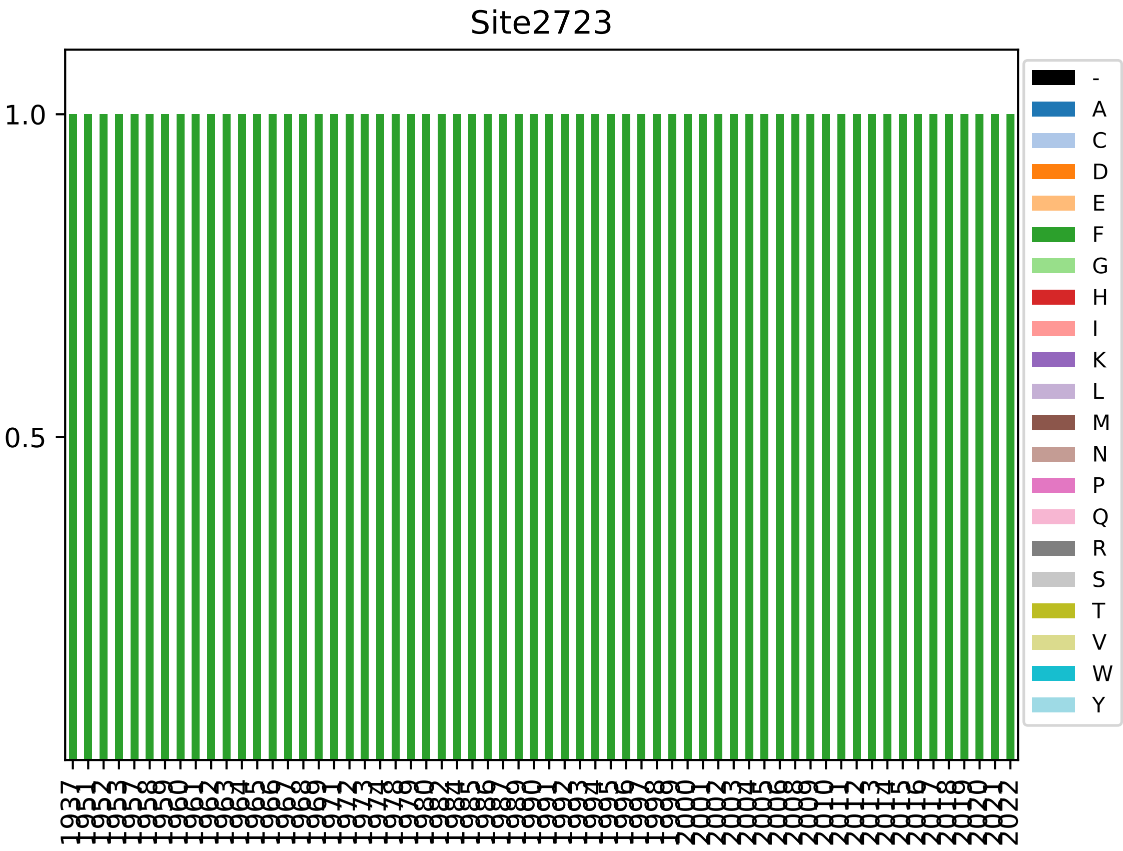
Site2723
1.0
0.5
-
A
C
D
E
F
G
H
I
K
L
M
N
P
Q
R
S
T
V
W
Y

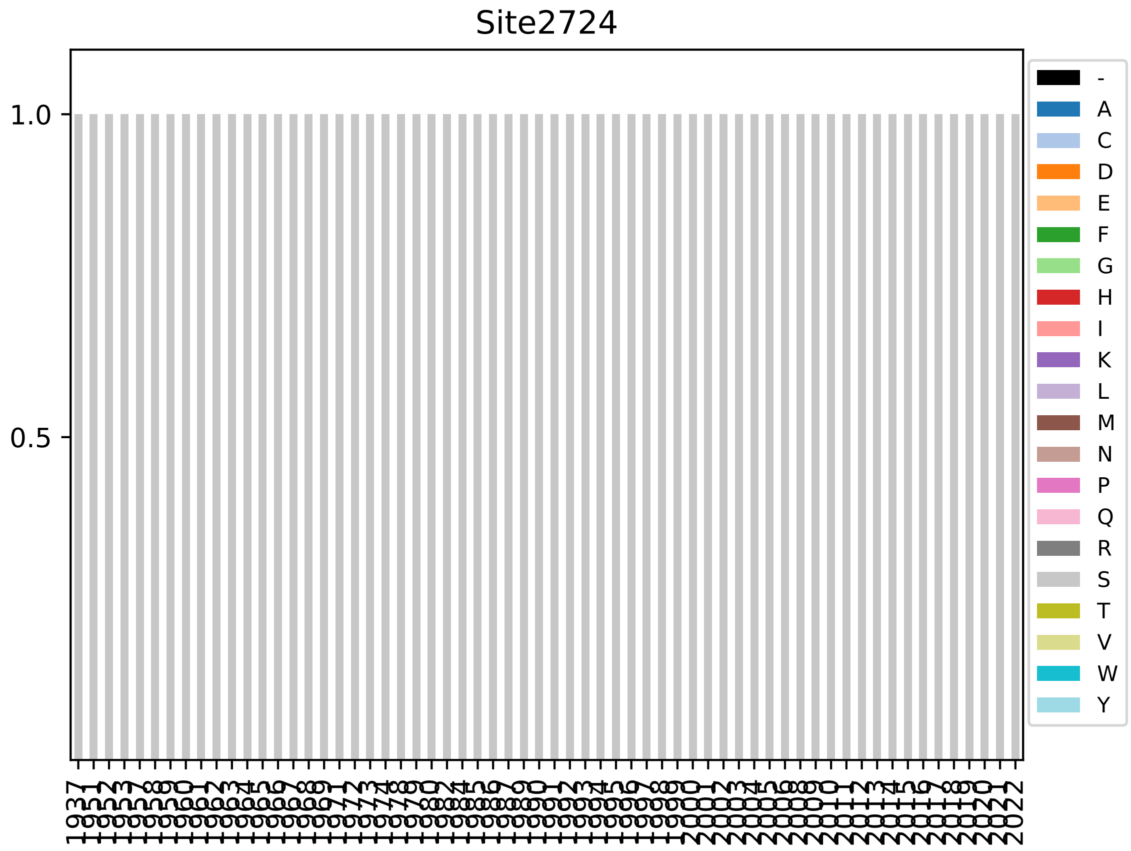

Site2724
1.0
0.5
-
A
C
D
E
F
G
H
I
K
L
M
N
P
Q
R
S
T
V
W
Y

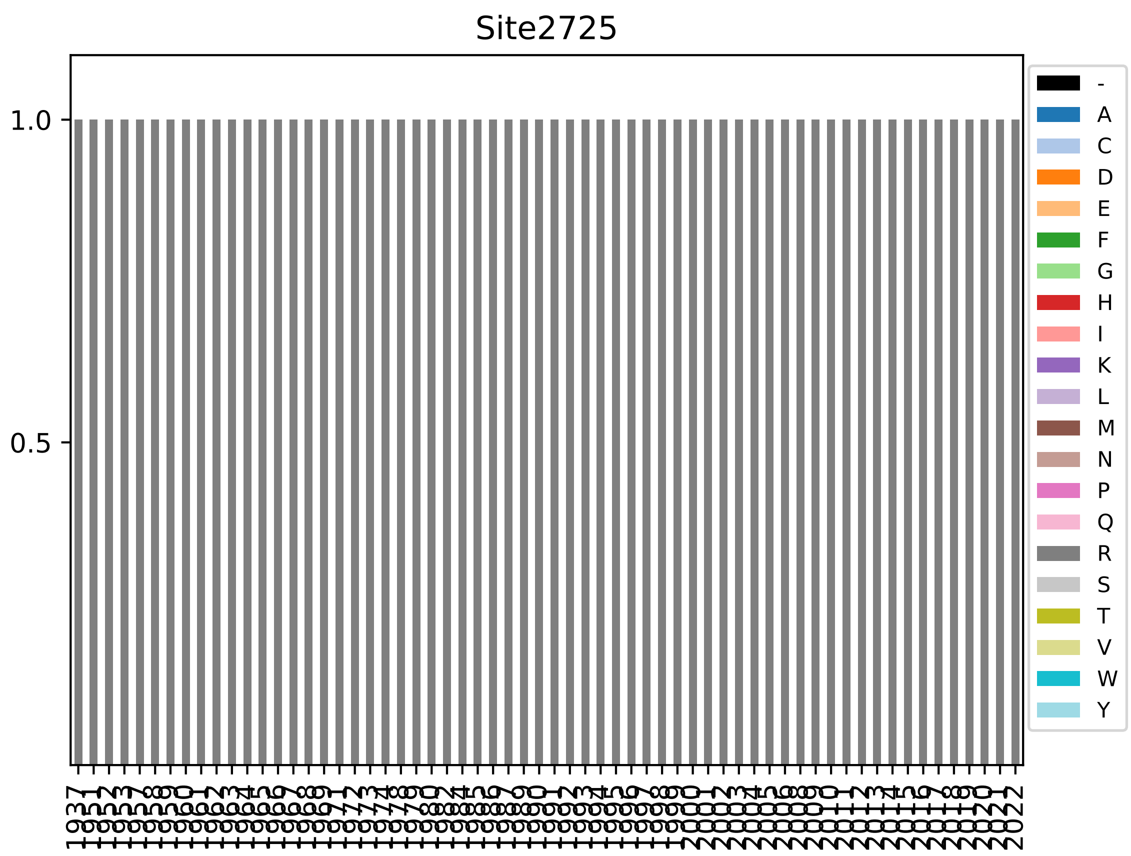

Site2725
1.0
0.5
-
A
C
D
E
F
G
H
I
K
L
M
N
P
Q
R
S
T
V
W
Y

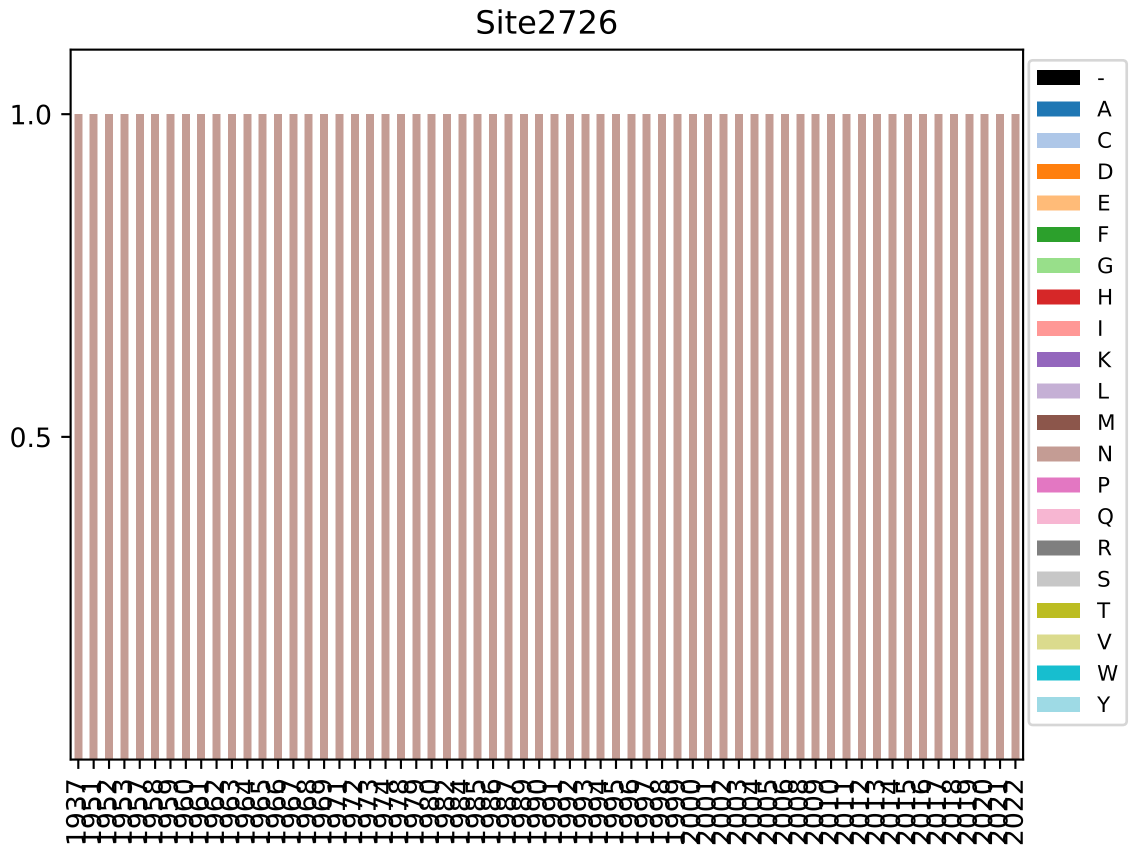
Site2726
1.0
0.5
-
A
C
D
E
F
G
H
I
K
L
M
N
P
Q
R
S
T
V
W
Y

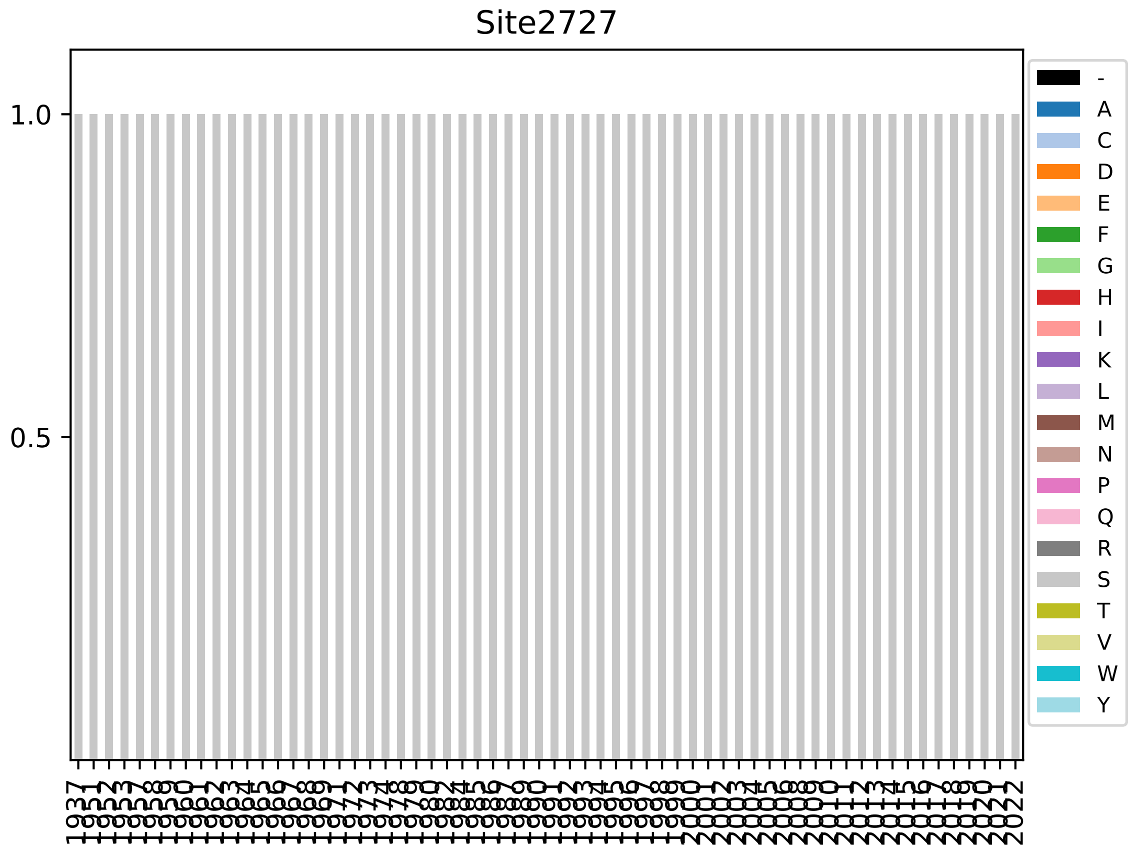
Site2727
1.0
0.5
-
A
C
D
E
F
G
H
I
K
L
M
N
P
Q
R
S
T
V
W
Y

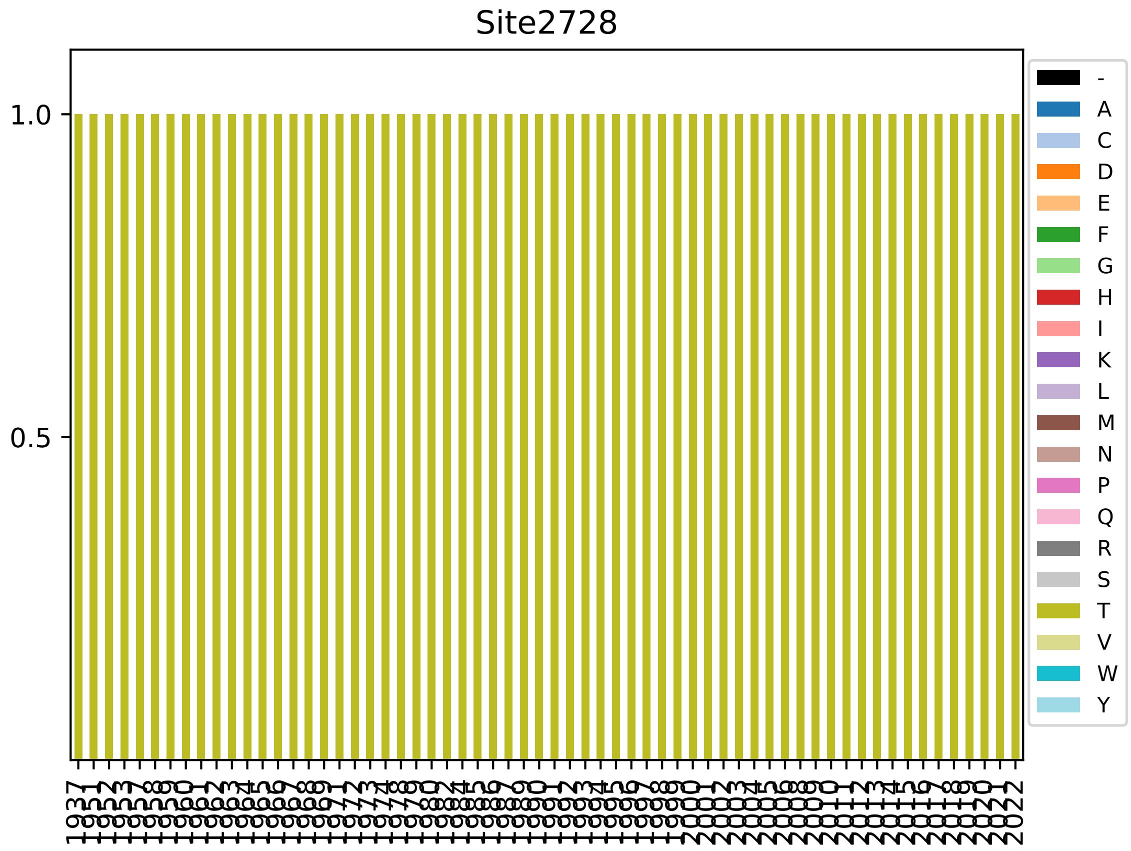
Site2728
1.0
0.5
-
A
C
D
E
F
G
H
I
K
L
M
N
P
Q
R
S
T
V
W
Y

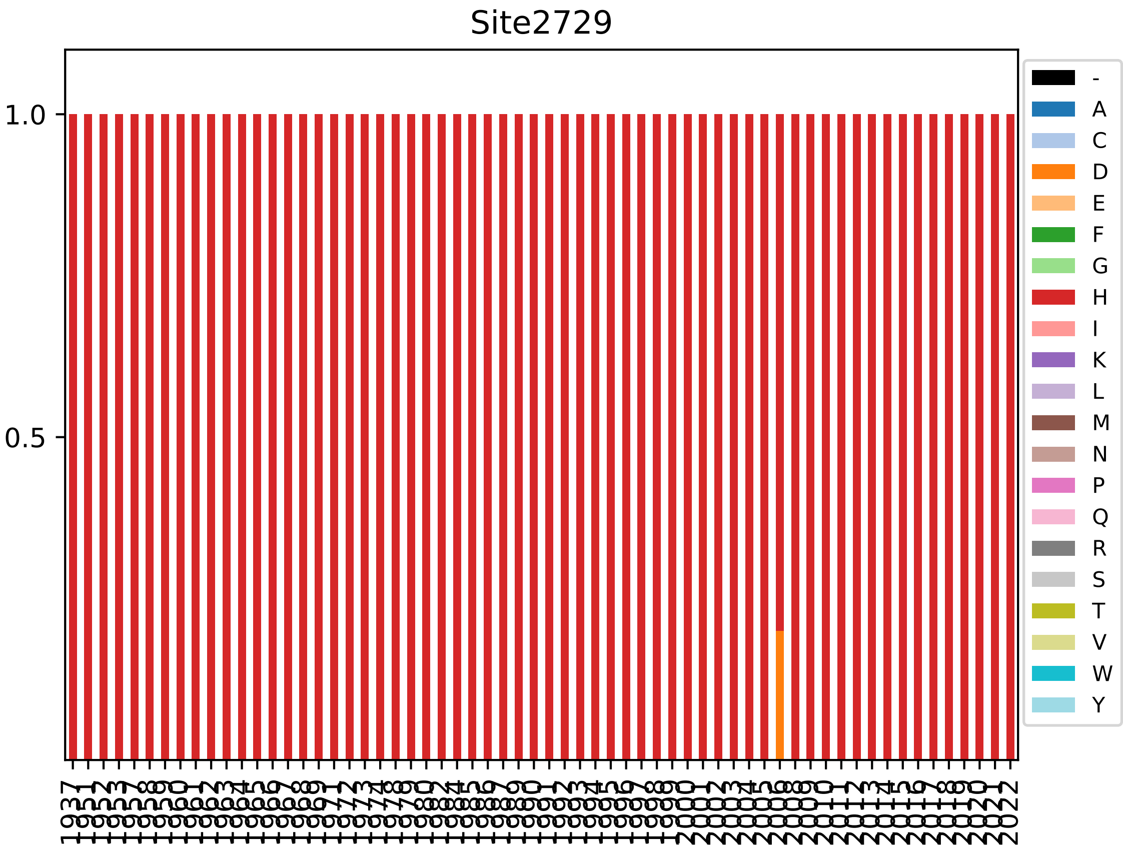
Site2729
1.0
0.5
-
A
C
D
E
F
G
H
I
K
L
M
N
P
Q
R
S
T
V
W
Y

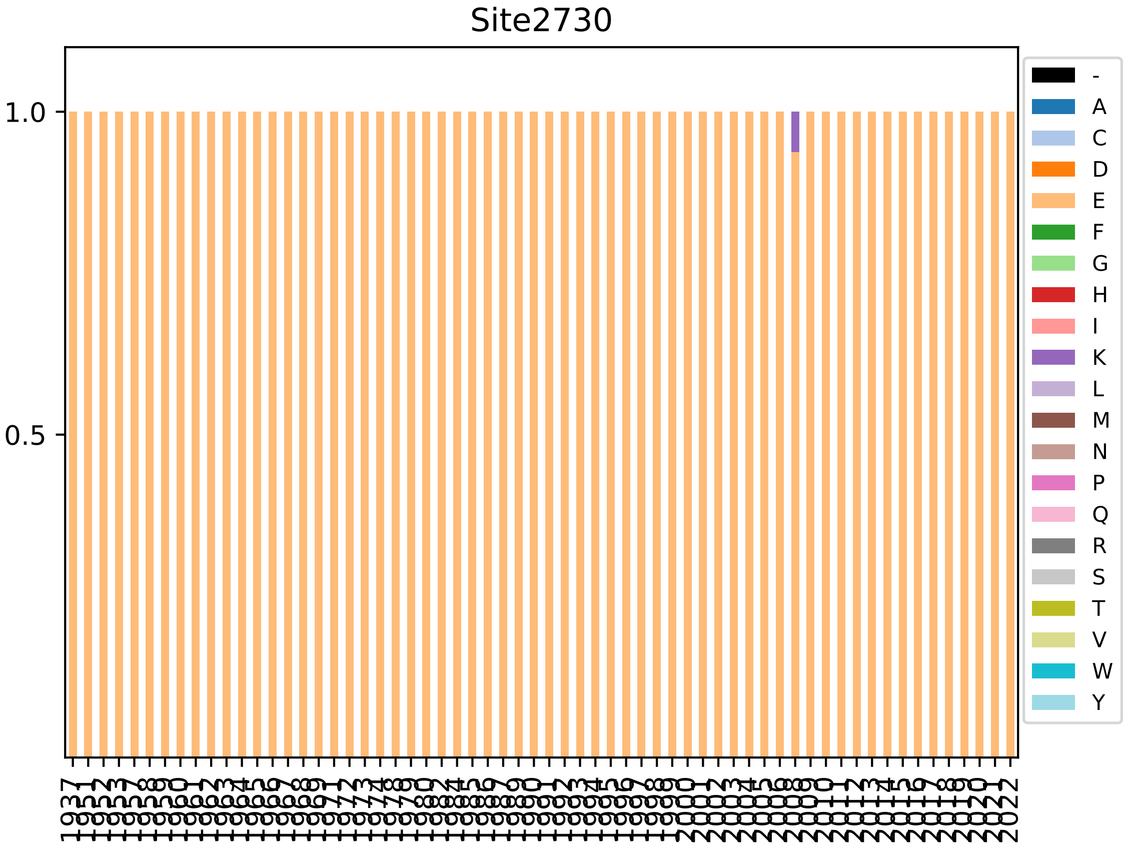
Site2730
1.0
0.5
-
A
C
D
E
F
G
H
I
K
L
M
N
P
Q
R
S
T
V
W
Y

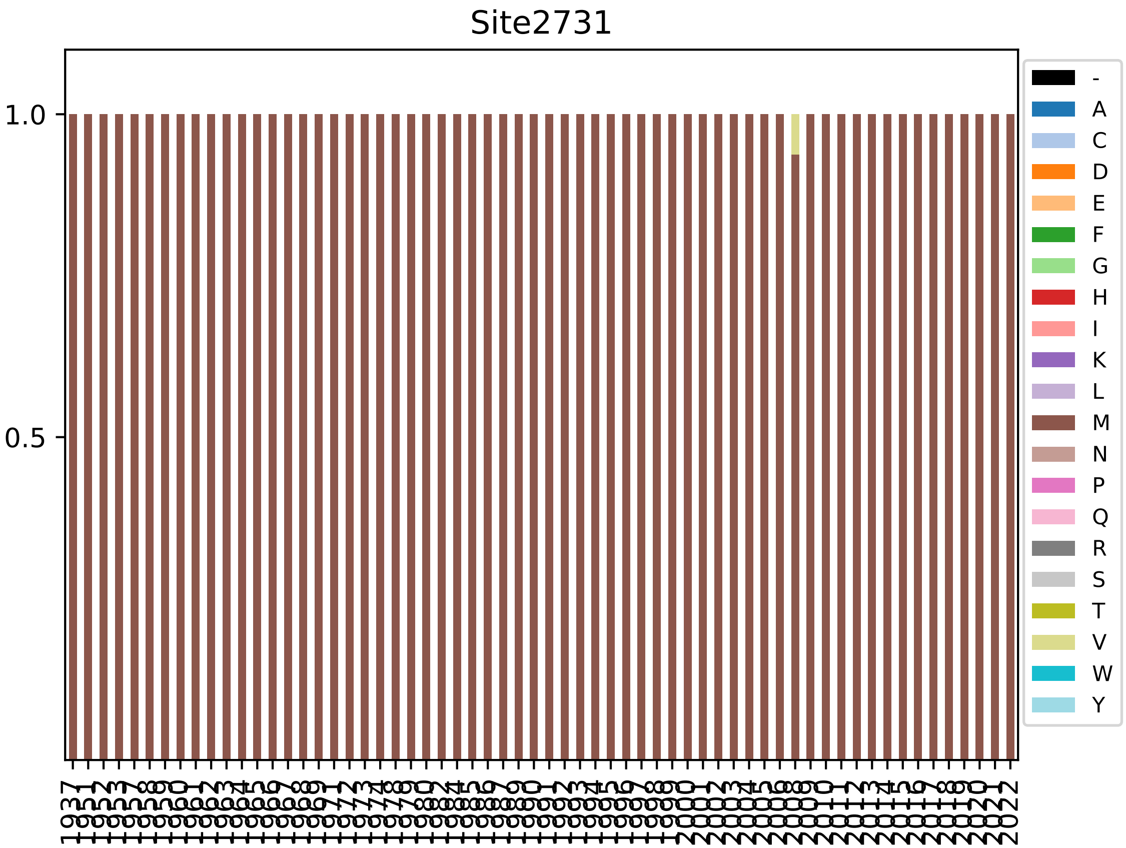
Site2731
1.0
0.5
-
A
C
D
E
F
G
H
I
K
L
M
N
P
Q
R
S
T
V
W
Y

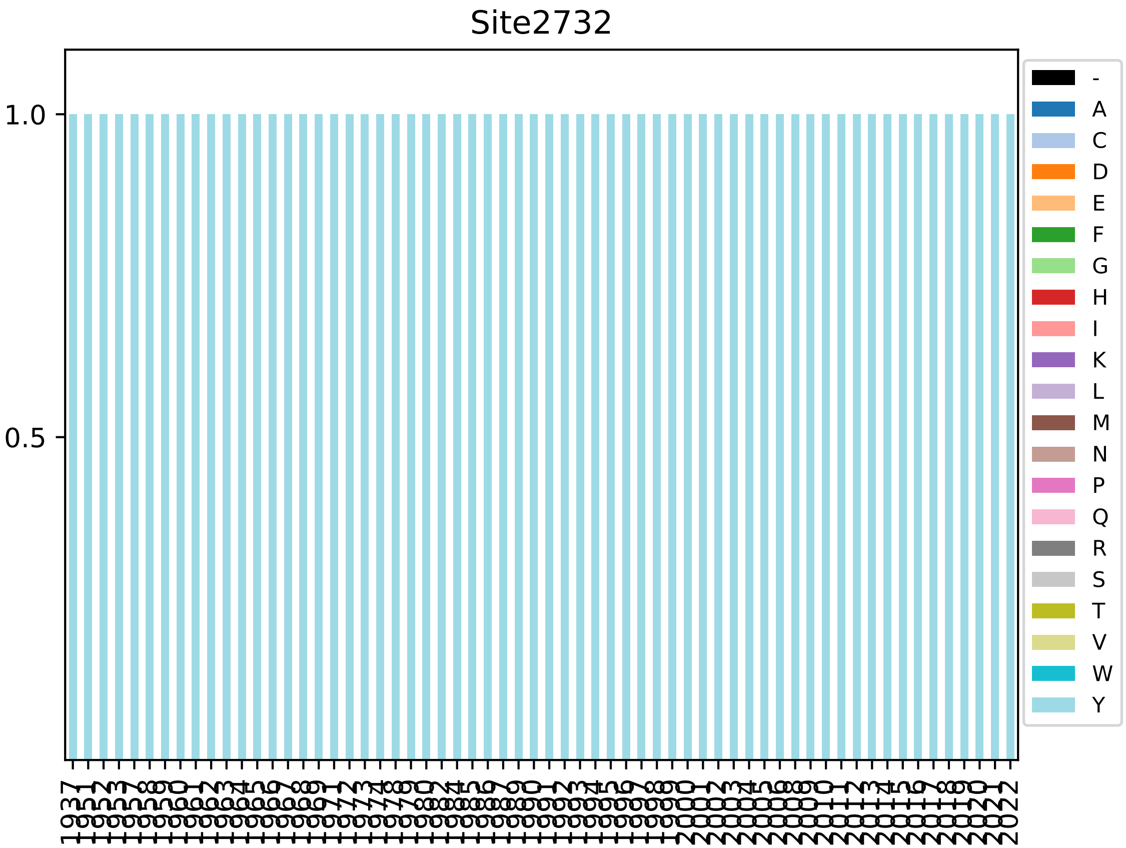
Site2732
1.0
0.5
-
A
C
D
E
F
G
H
I
K
L
M
N
P
Q
R
S
T
V
W
Y

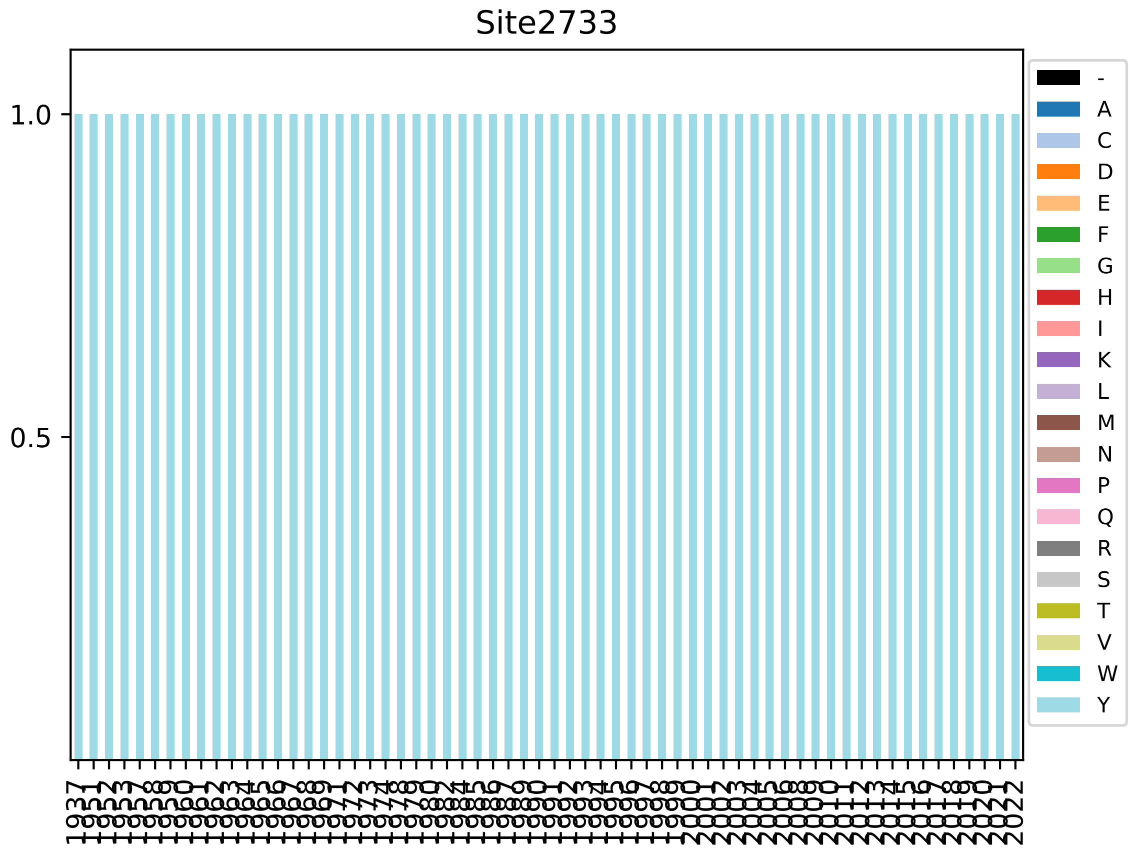

Site2733
1.0
0.5
-
A
C
D
E
F
G
H
I
K
L
M
N
P
Q
R
S
T
V
W
Y

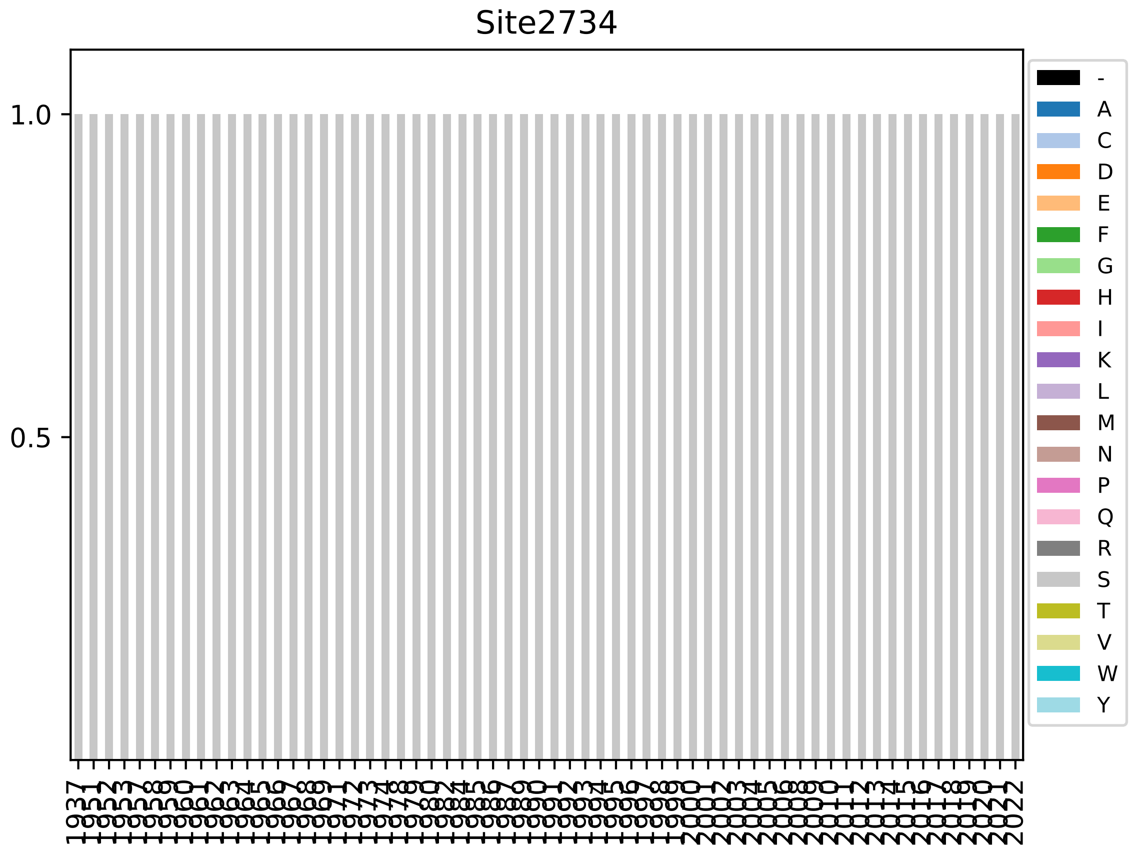

Site2734
1.0
0.5
-
A
C
D
E
F
G
H
I
K
L
M
N
P
Q
R
S
T
V
W
Y

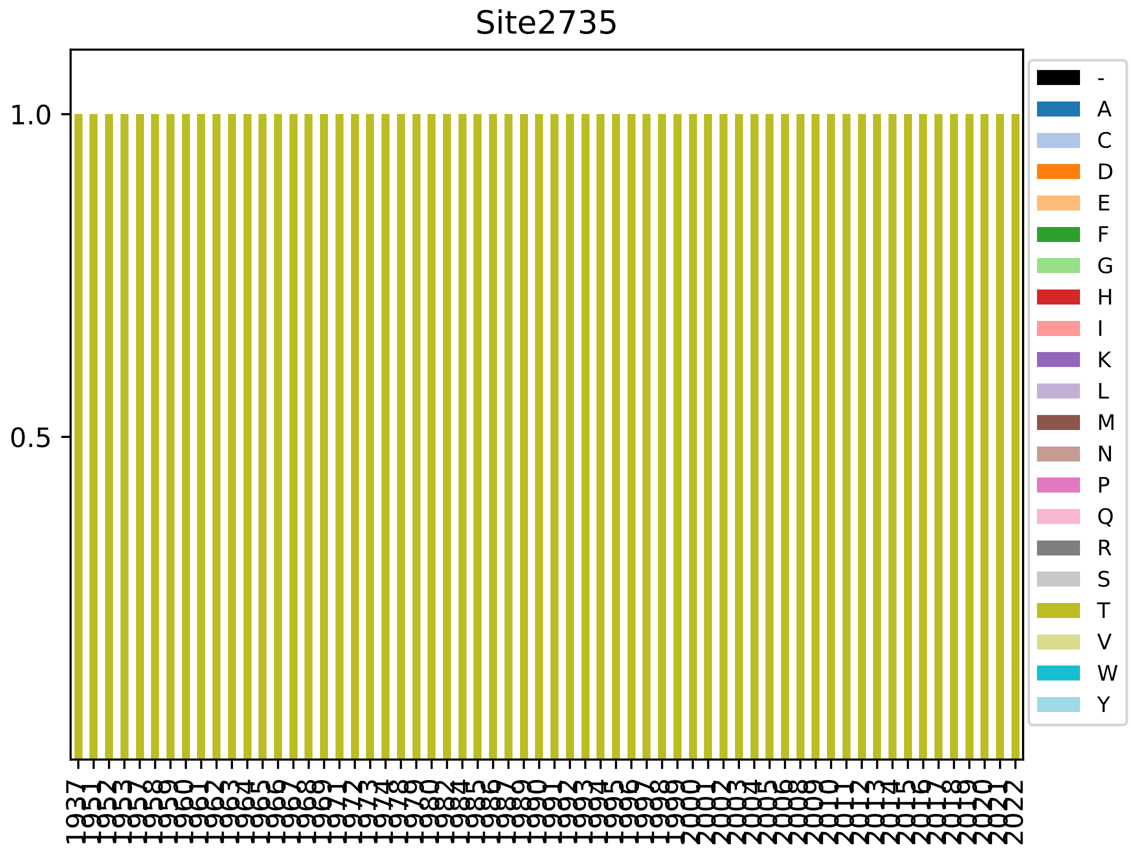
Site2735
1.0
0.5
-
A
C
D
E
F
G
H
I
K
L
M
N
P
Q
R
S
T
V
W
Y

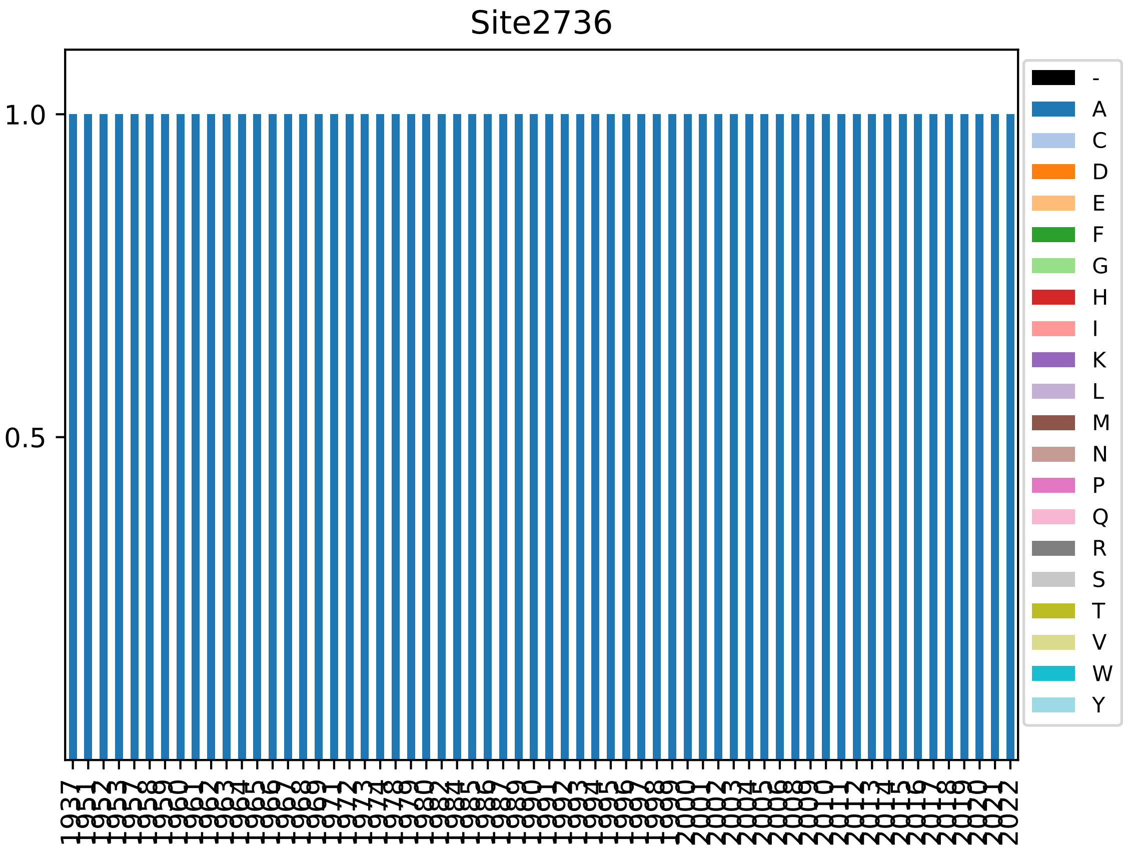
Site2736
1.0
0.5
-
A
C
D
E
F
G
H
I
K
L
M
N
P
Q
R
S
T
V
W
Y

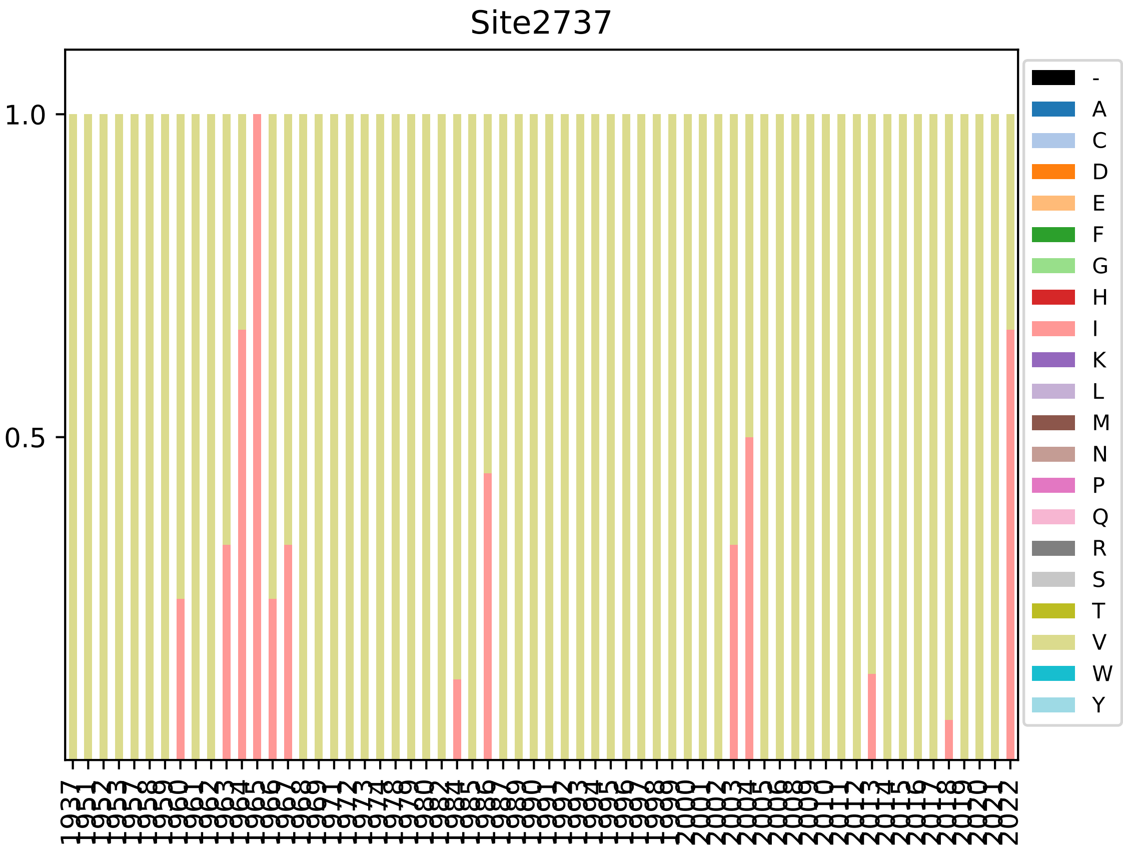
Site2737
1.0
0.5
-
A
C
D
E
F
G
H
I
K
L
M
N
P
Q
R
S
T
V
W
Y

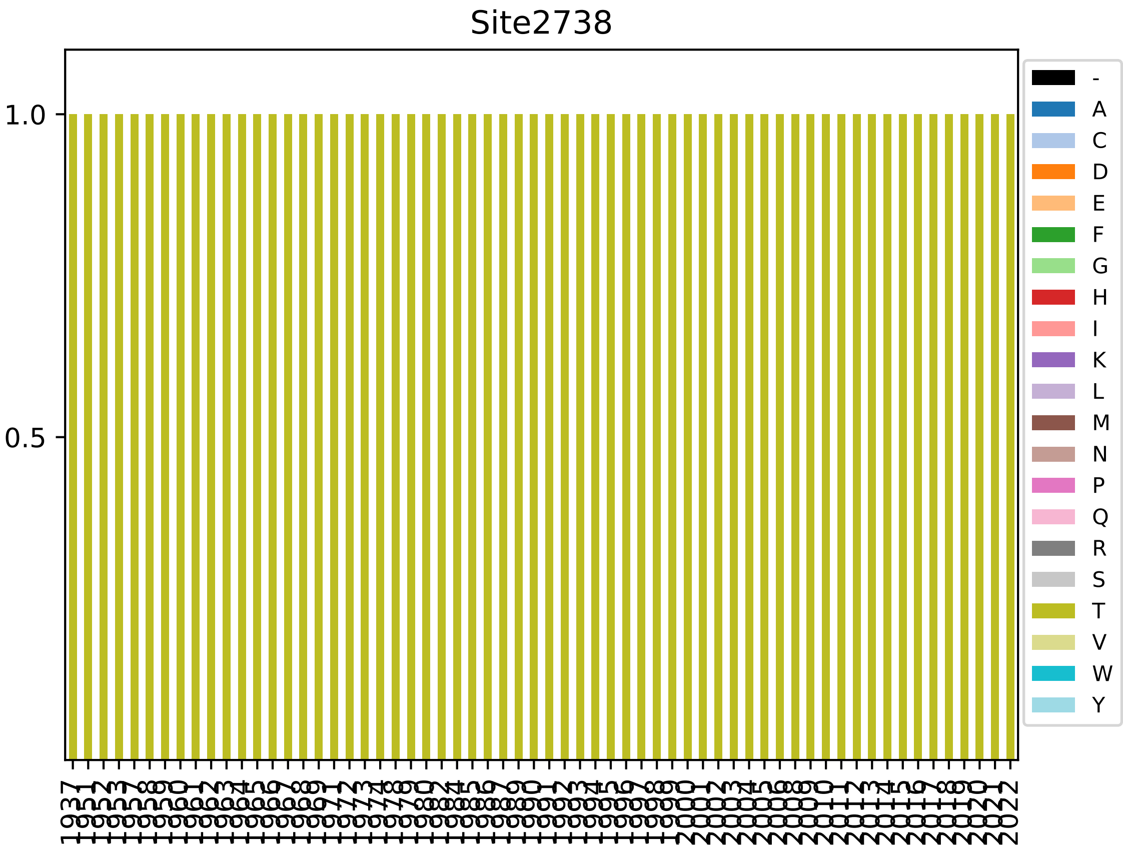
Site2738
1.0
0.5
-
A
C
D
E
F
G
H
I
K
L
M
N
P
Q
R
S
T
V
W
Y

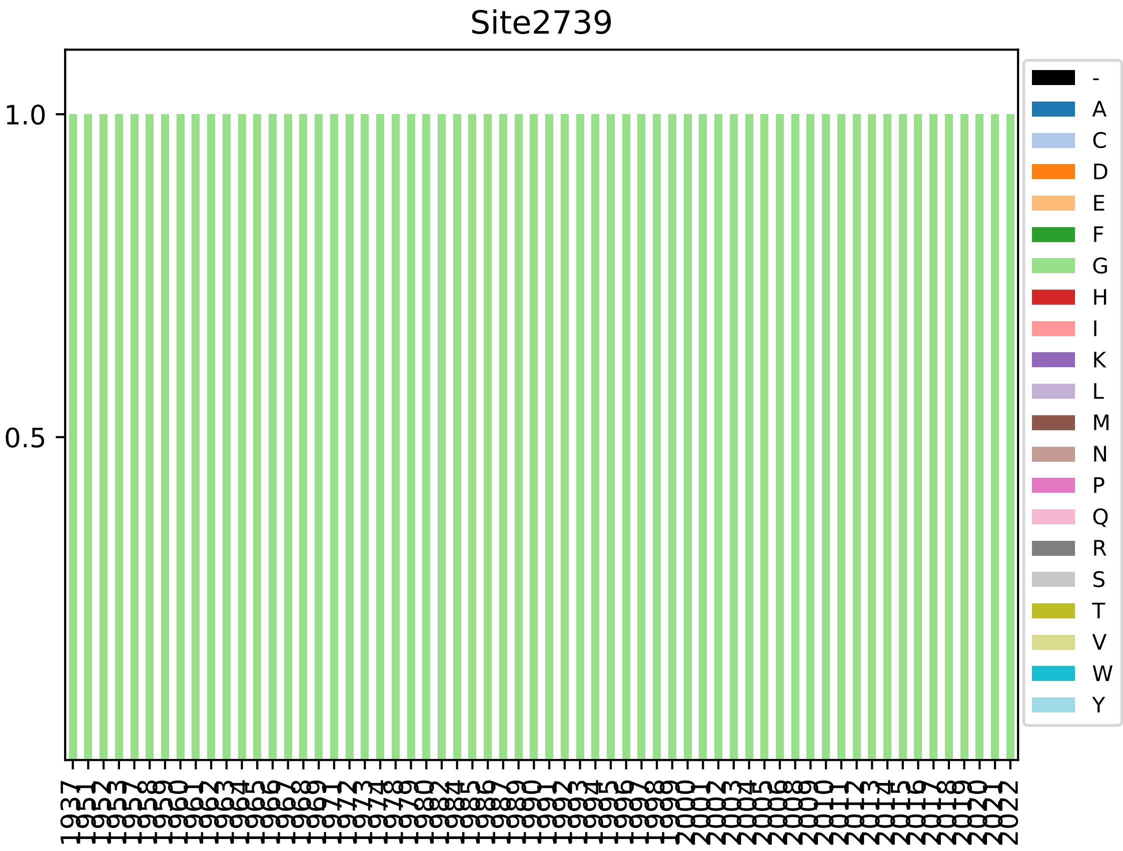
Site2739
1.0
0.5
-
A
C
D
E
F
G
H
I
K
L
M
N
P
Q
R
S
T
V
W
Y

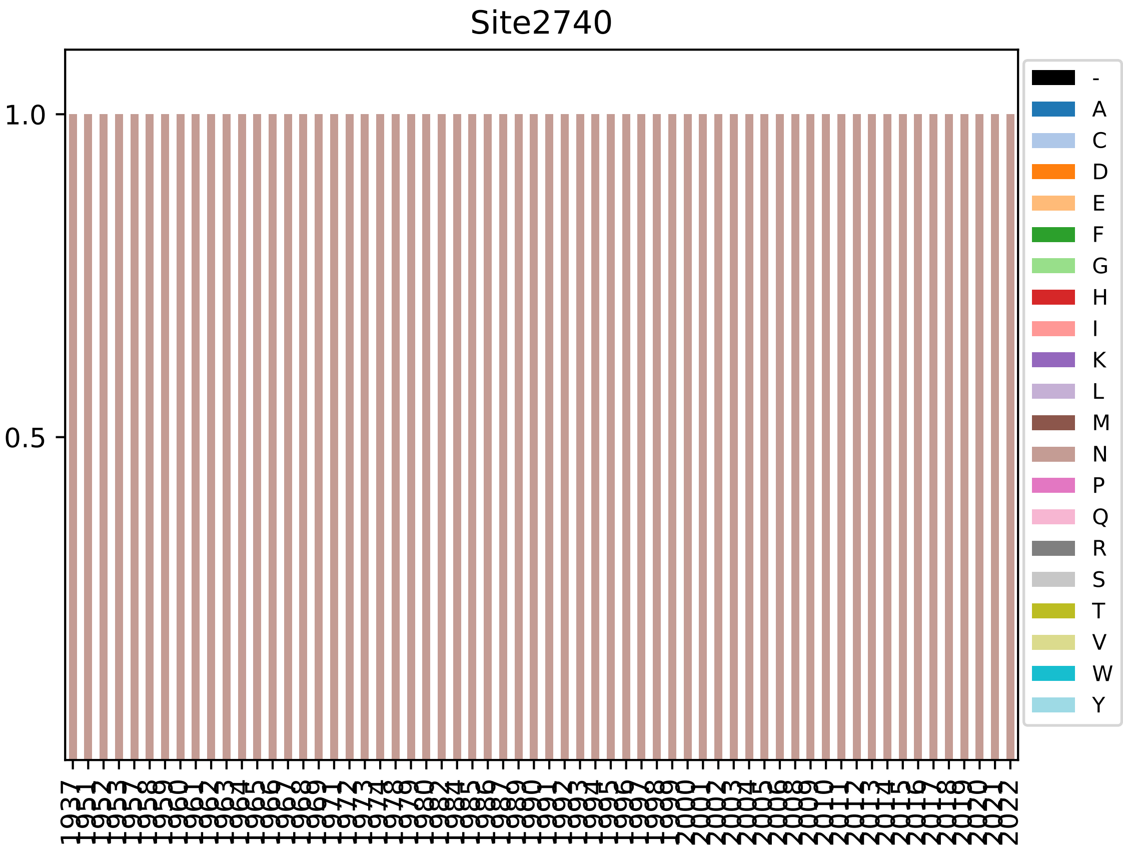
Site2740
1.0
0.5
-
A
C
D
E
F
G
H
I
K
L
M
N
P
Q
R
S
T
V
W
Y

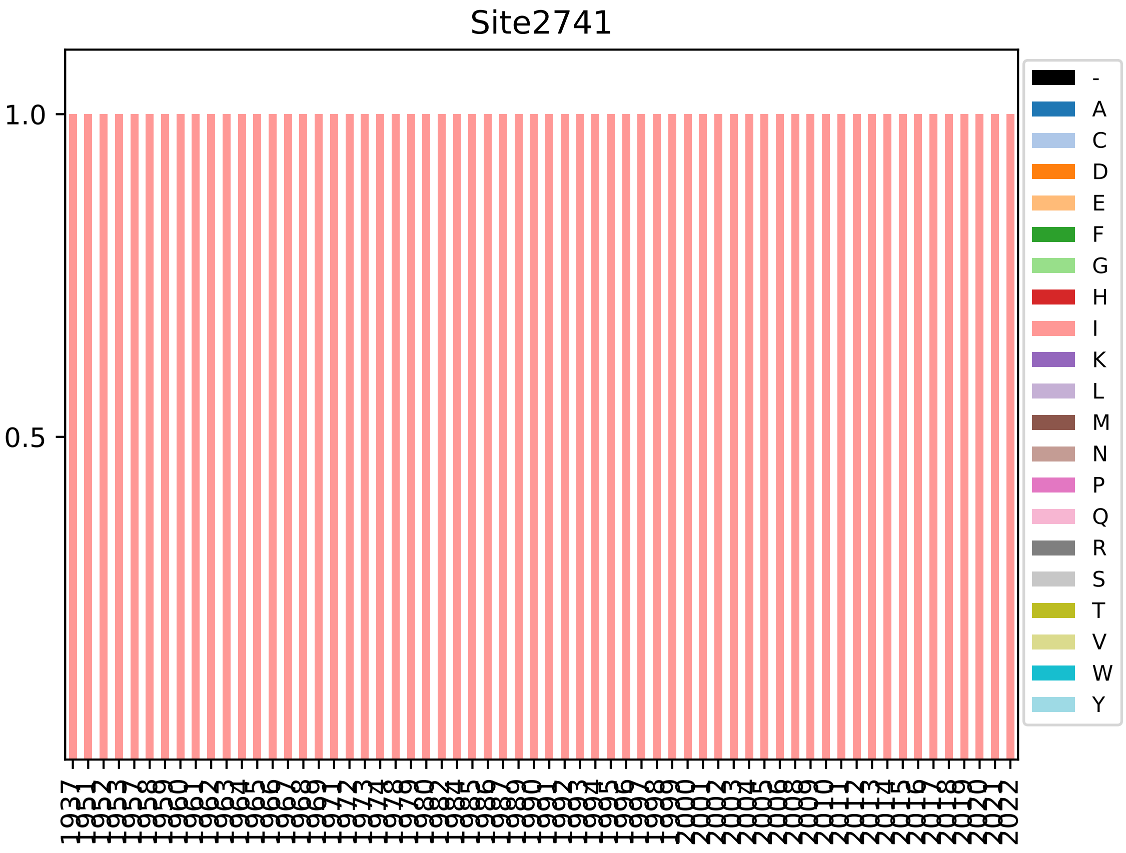

Site2741
1.0
0.5
-
A
C
D
E
F
G
H
I
K
L
M
N
P
Q
R
S
T
V
W
Y

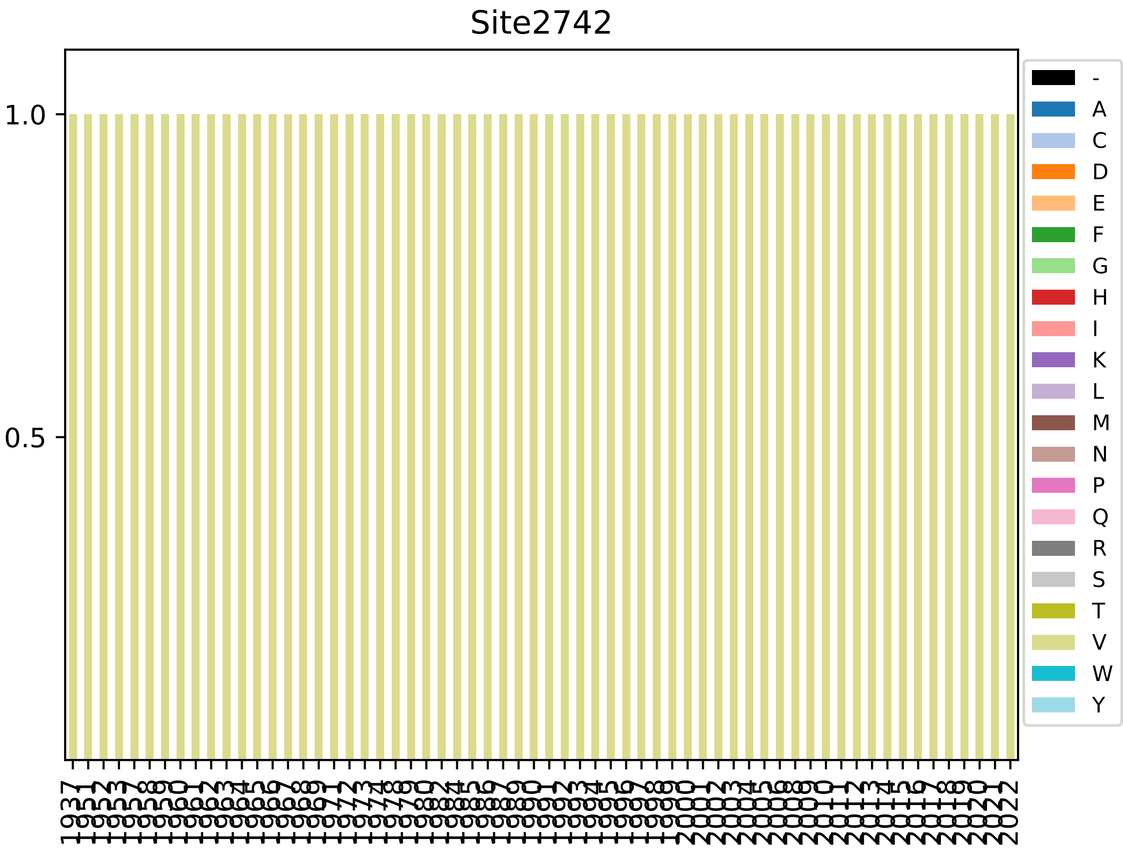
Site2742
1.0
0.5
-
A
C
D
E
F
G
H
I
K
L
M
N
P
Q
R
S
T
V
W
Y

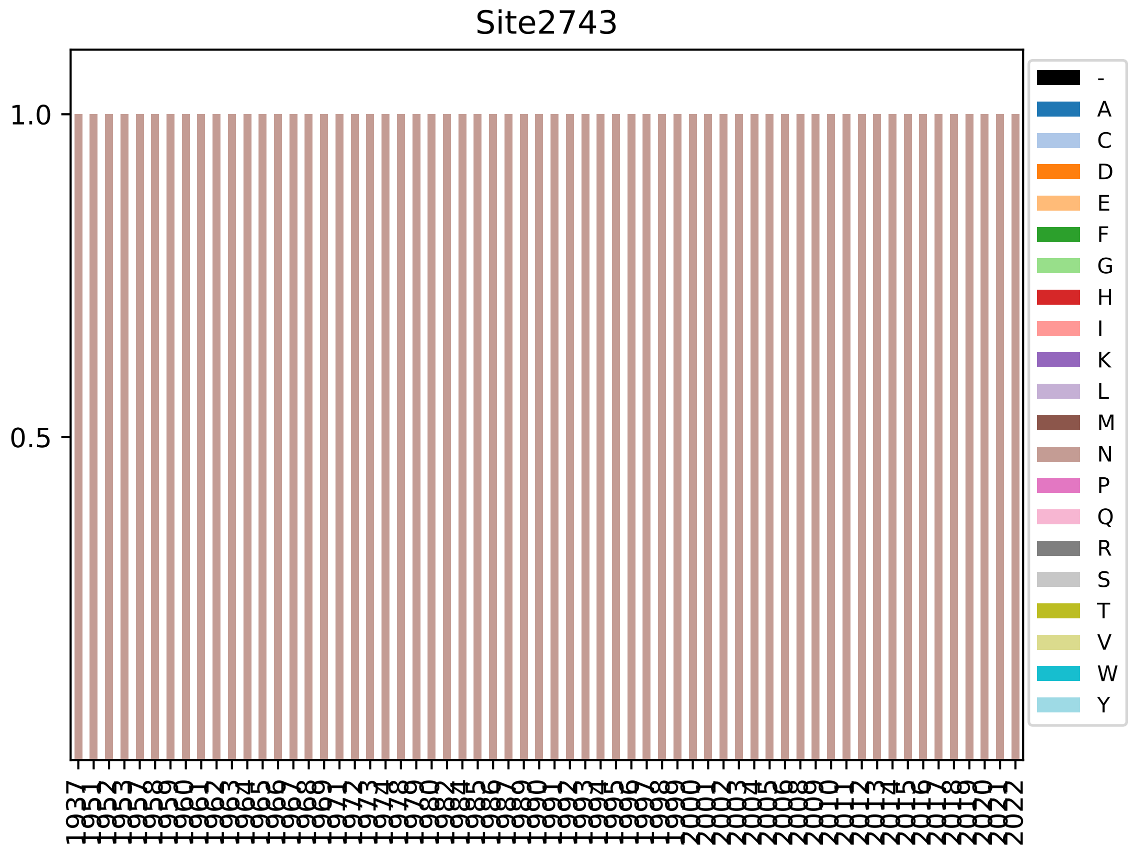
Site2743
1.0
0.5
-
A
C
D
E
F
G
H
I
K
L
M
N
P
Q
R
S
T
V
W
Y

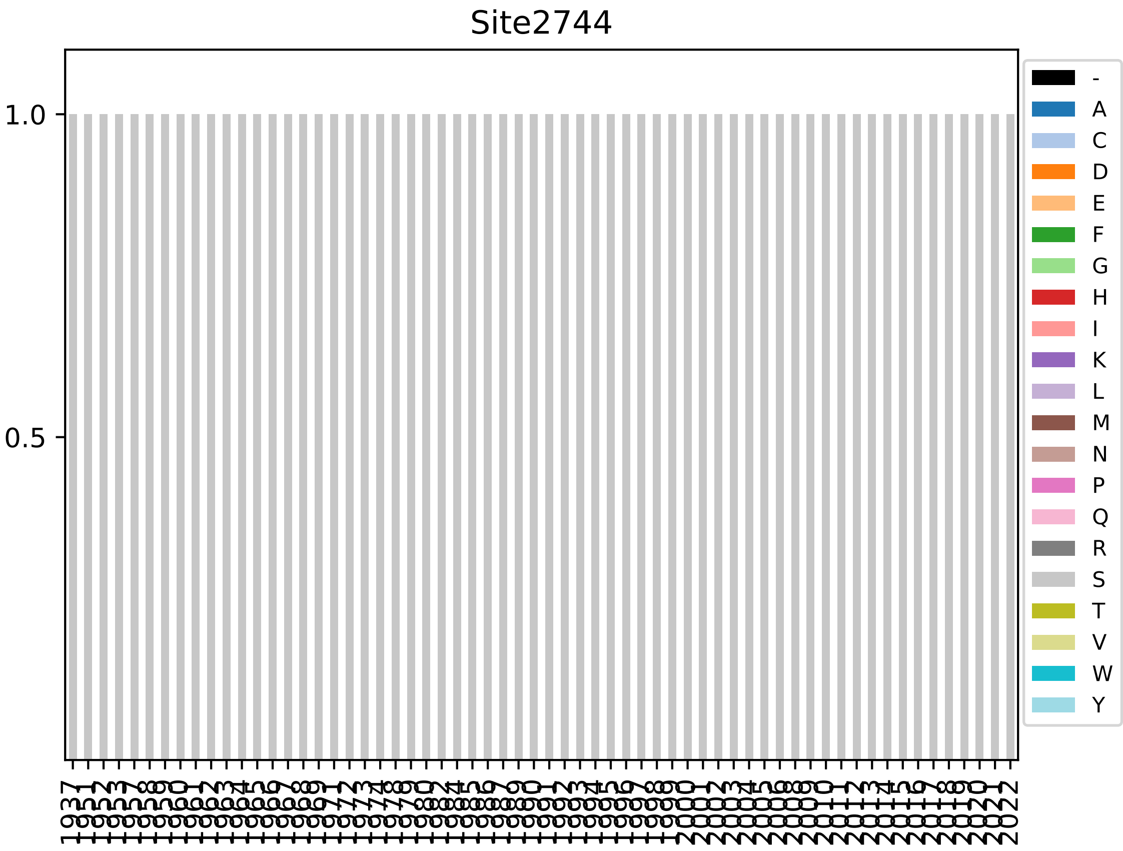
Site2744
1.0
0.5
-
A
C
D
E
F
G
H
I
K
L
M
N
P
Q
R
S
T
V
W
Y

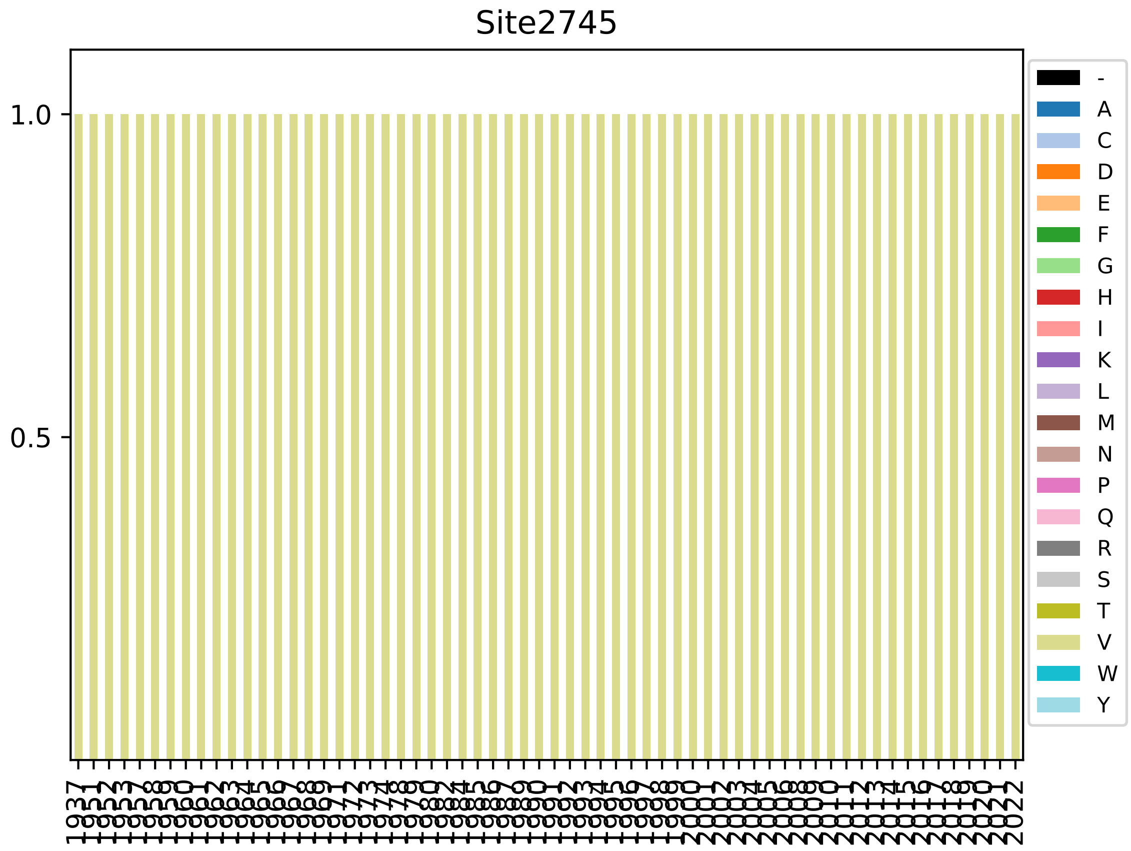

Site2745
1.0
0.5
-
A
C
D
E
F
G
H
I
K
L
M
N
P
Q
R
S
T
V
W
Y

Site2746

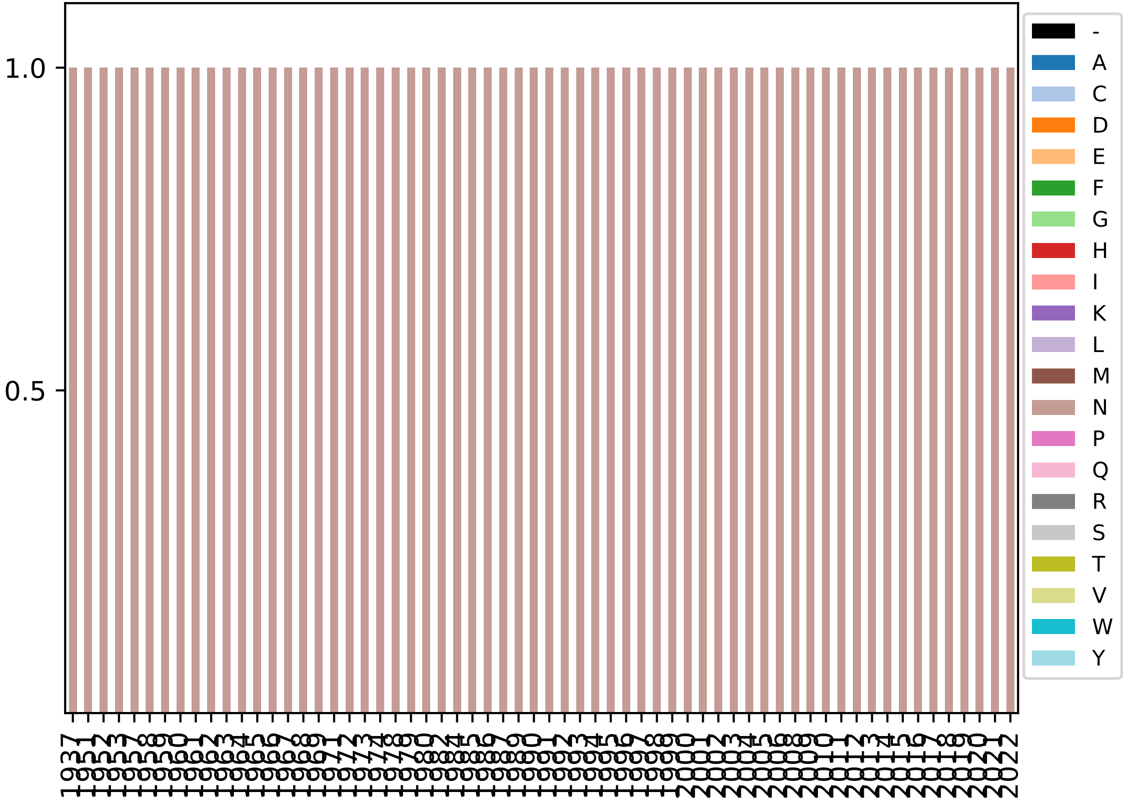

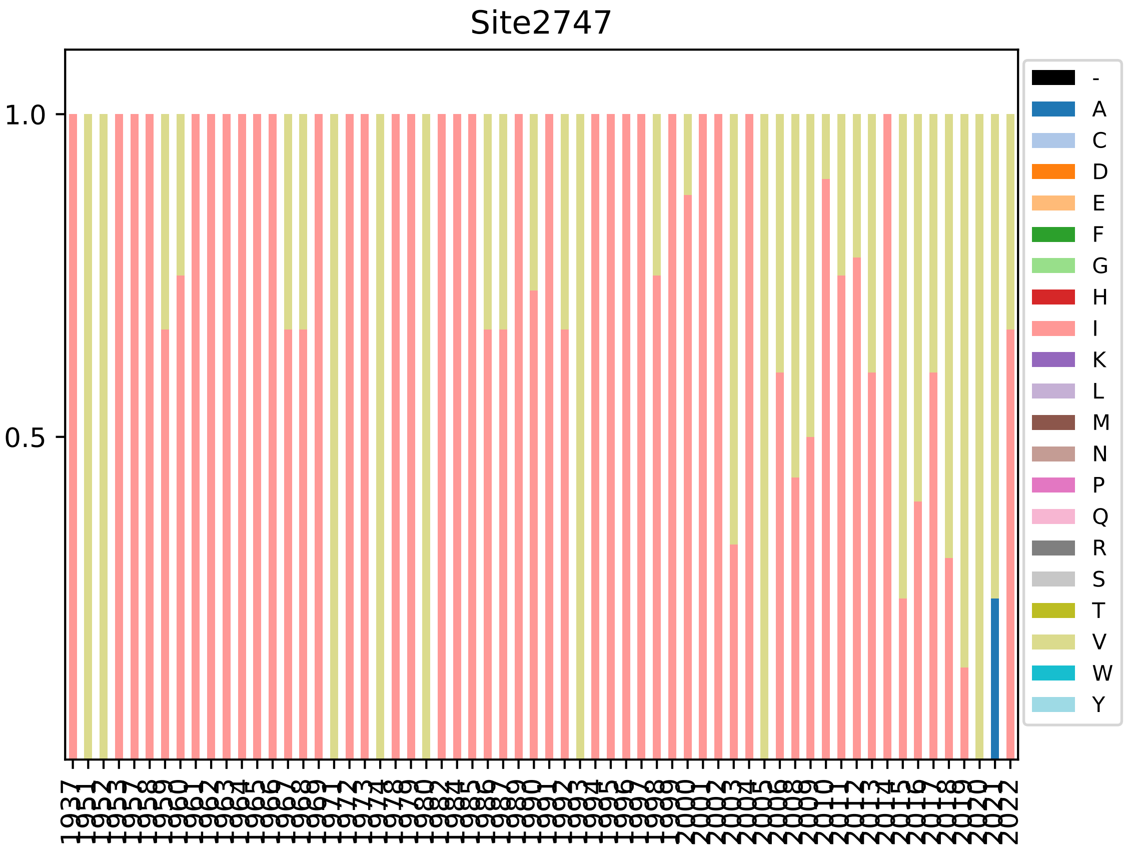
Site2747
1.0
0.5
-
A
C
D
E
F
G
H
I
K
L
M
N
P
Q
R
S
T
V
W
Y

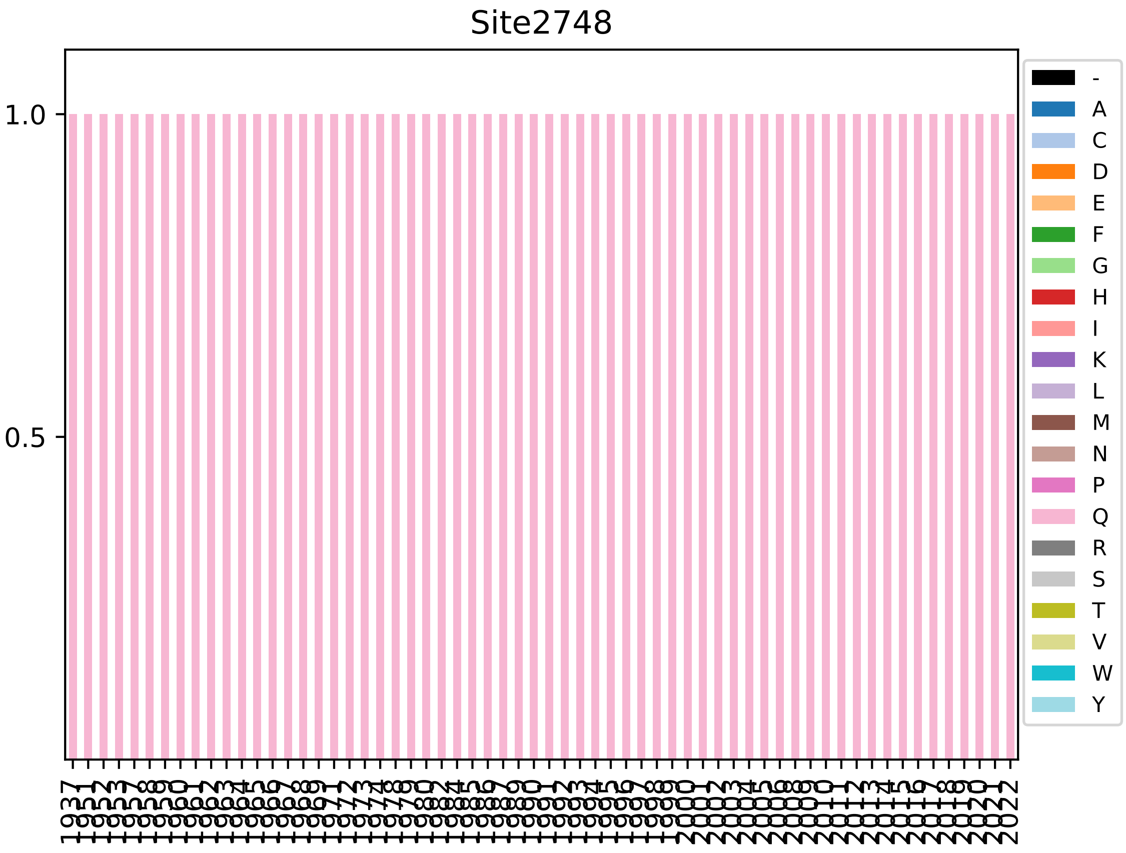
Site2748
1.0
0.5
-
A
C
D
E
F
G
H
I
K
L
M
N
P
Q
R
S
T
V
W
Y

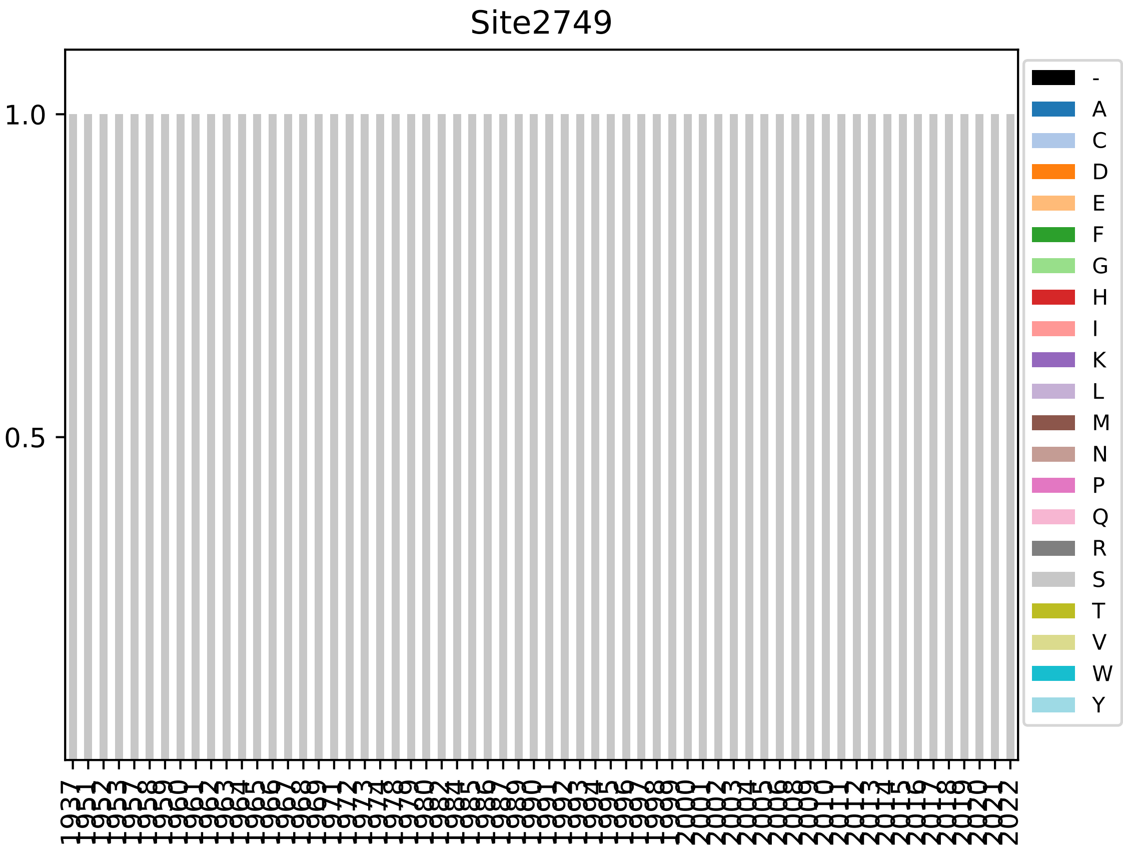
Site2749
1.0
0.5
-
A
C
D
E
F
G
H
I
K
L
M
N
P
Q
R
S
T
V
W
Y

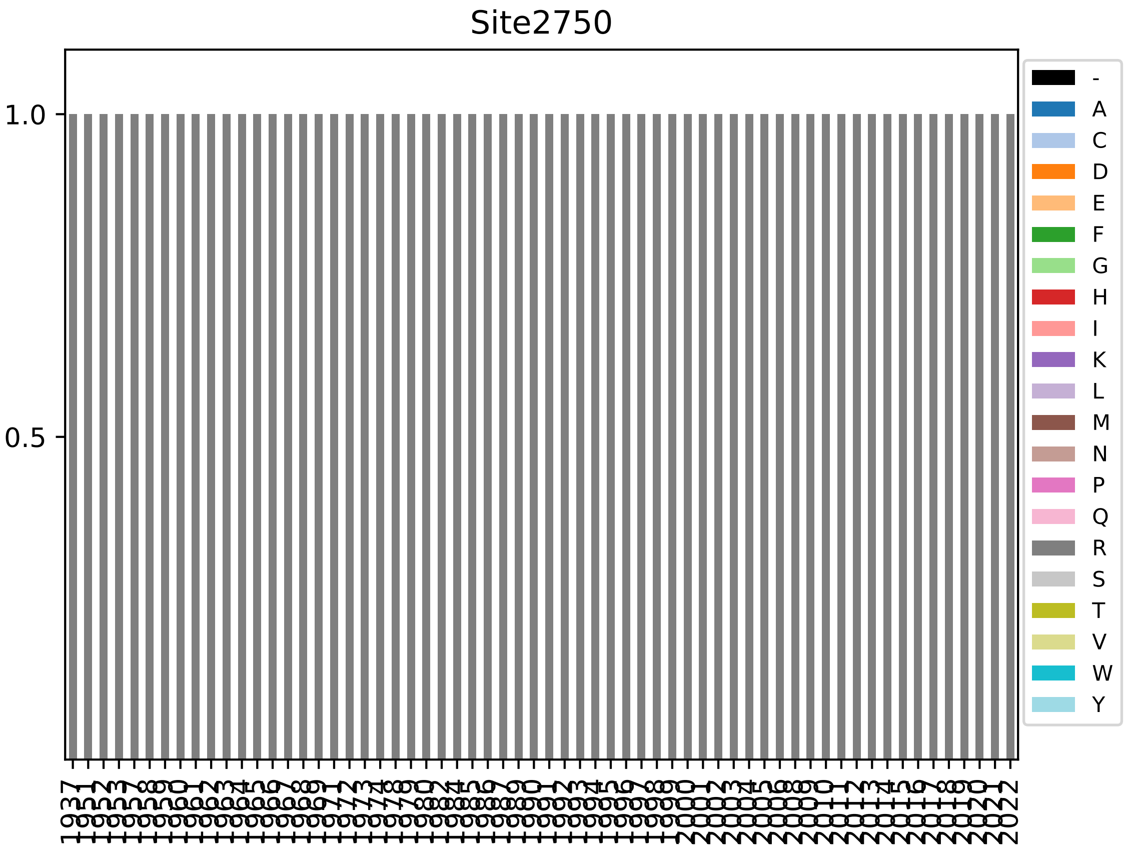
Site2750
1.0
0.5
-
A
C
D
E
F
G
H
I
K
L
M
N
P
Q
R
S
T
V
W
Y

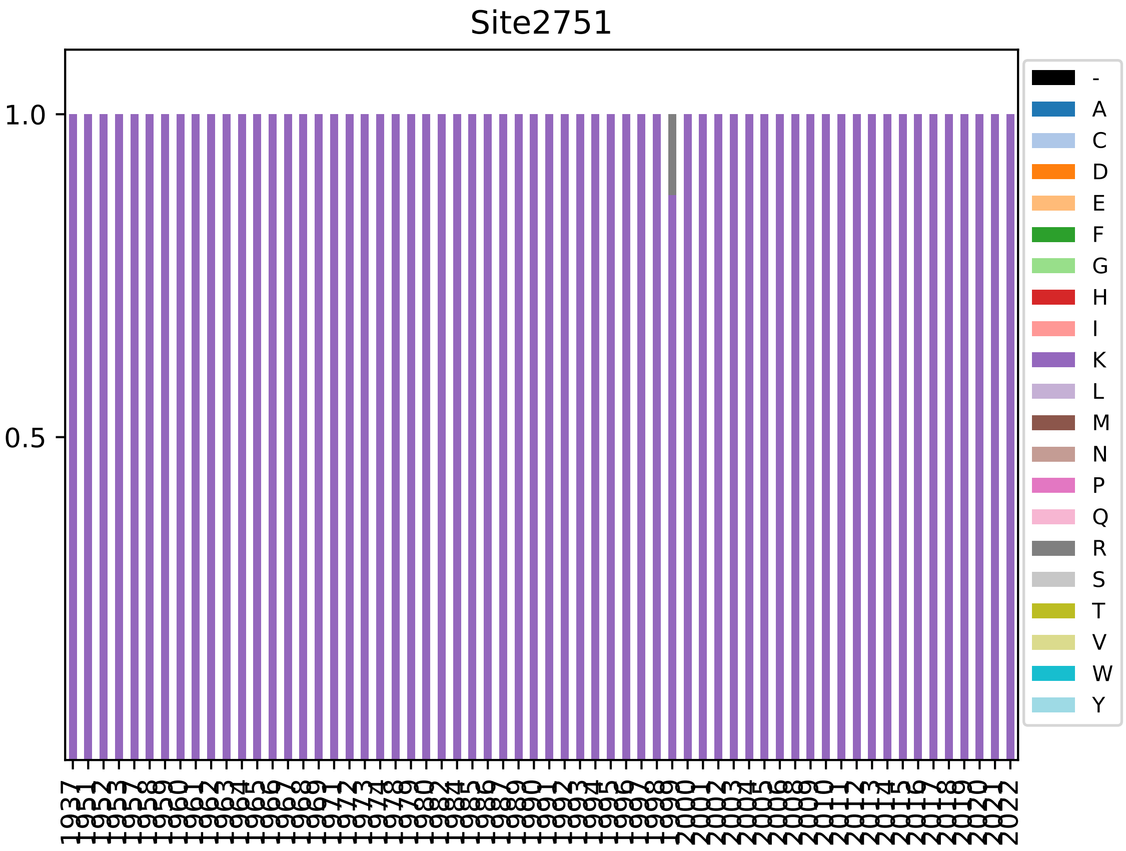

Site2751
1.0
0.5
-
A
C
D
E
F
G
H
I
K
L
M
N
P
Q
R
S
T
V
W
Y

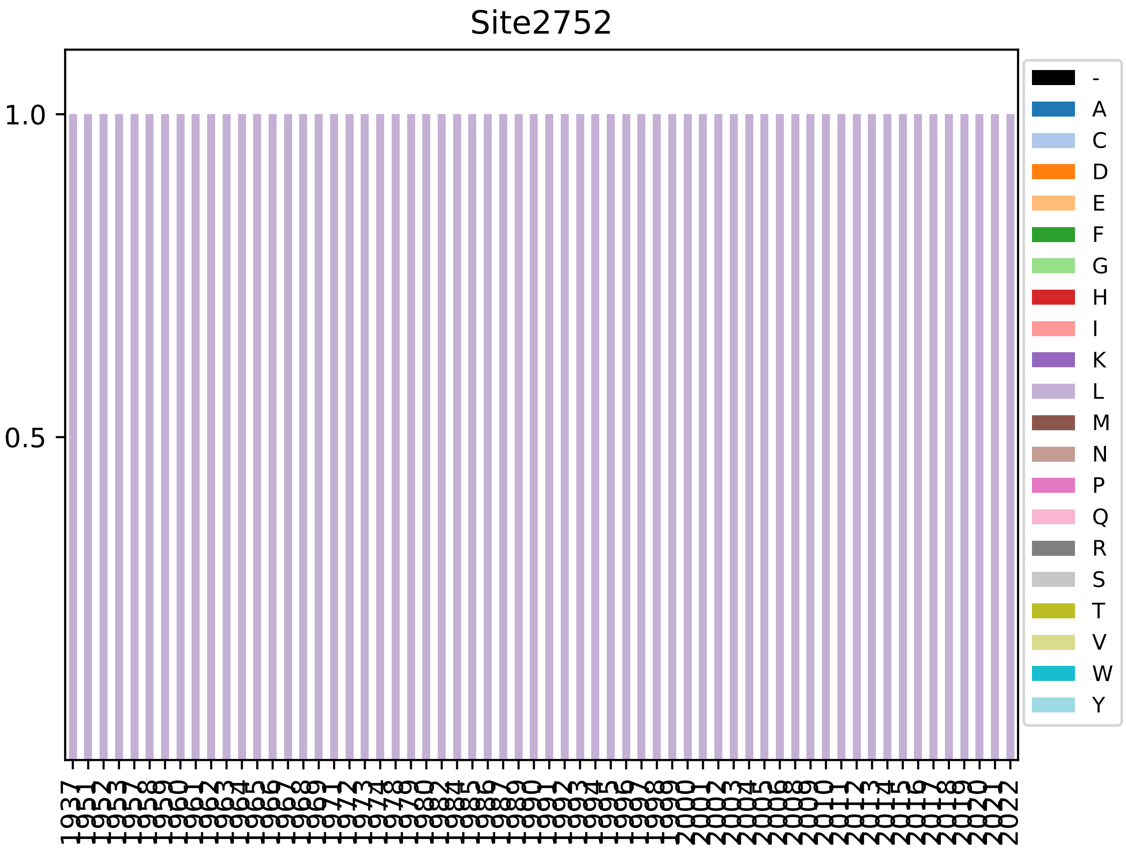
Site2752
1.0
0.5
-
A
C
D
E
F
G
H
I
K
L
M
N
P
Q
R
S
T
V
W
Y

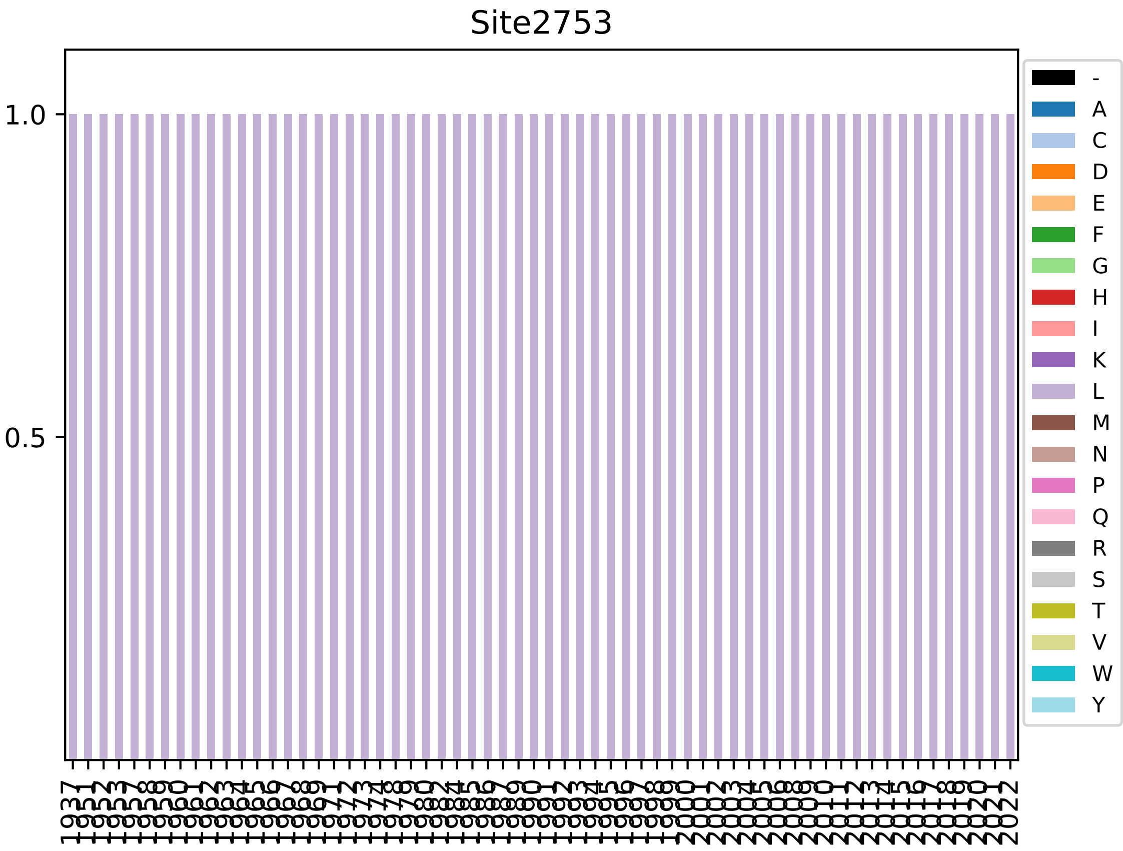
Site2753
1.0
0.5
-
A
C
D
E
F
G
H
I
K
L
M
N
P
Q
R
S
T
V
W
Y

Site2754

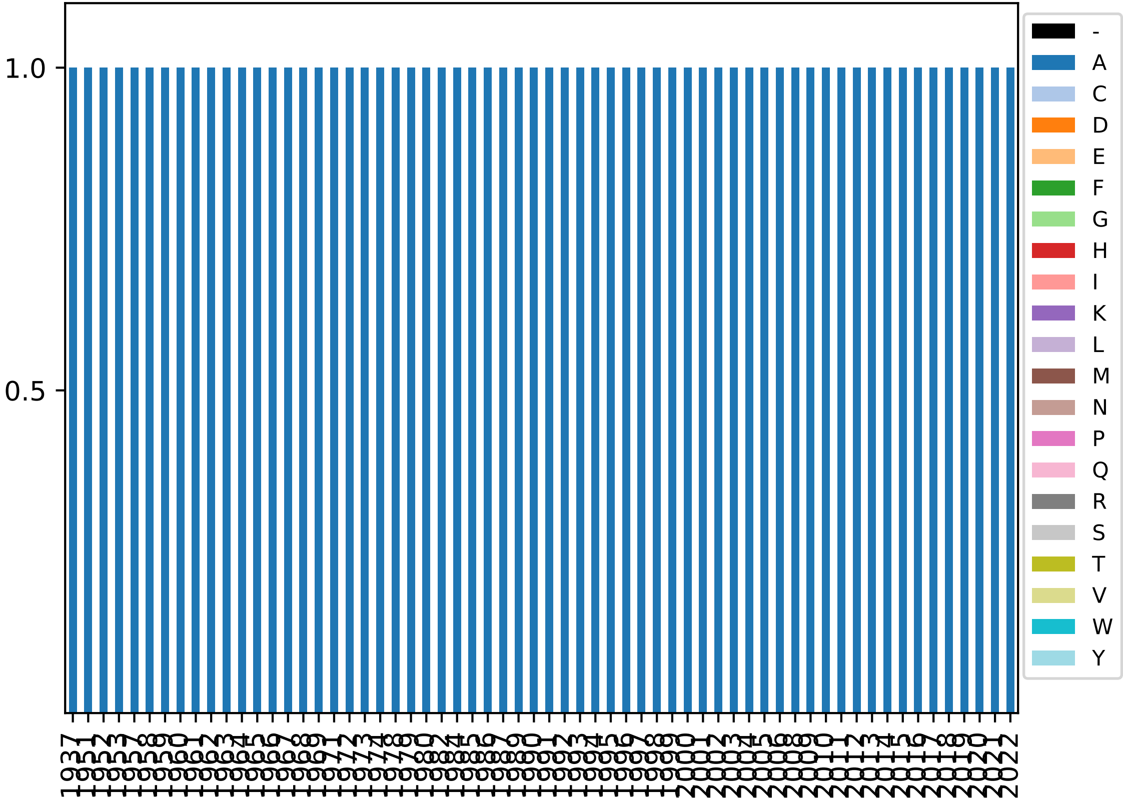

Site2755

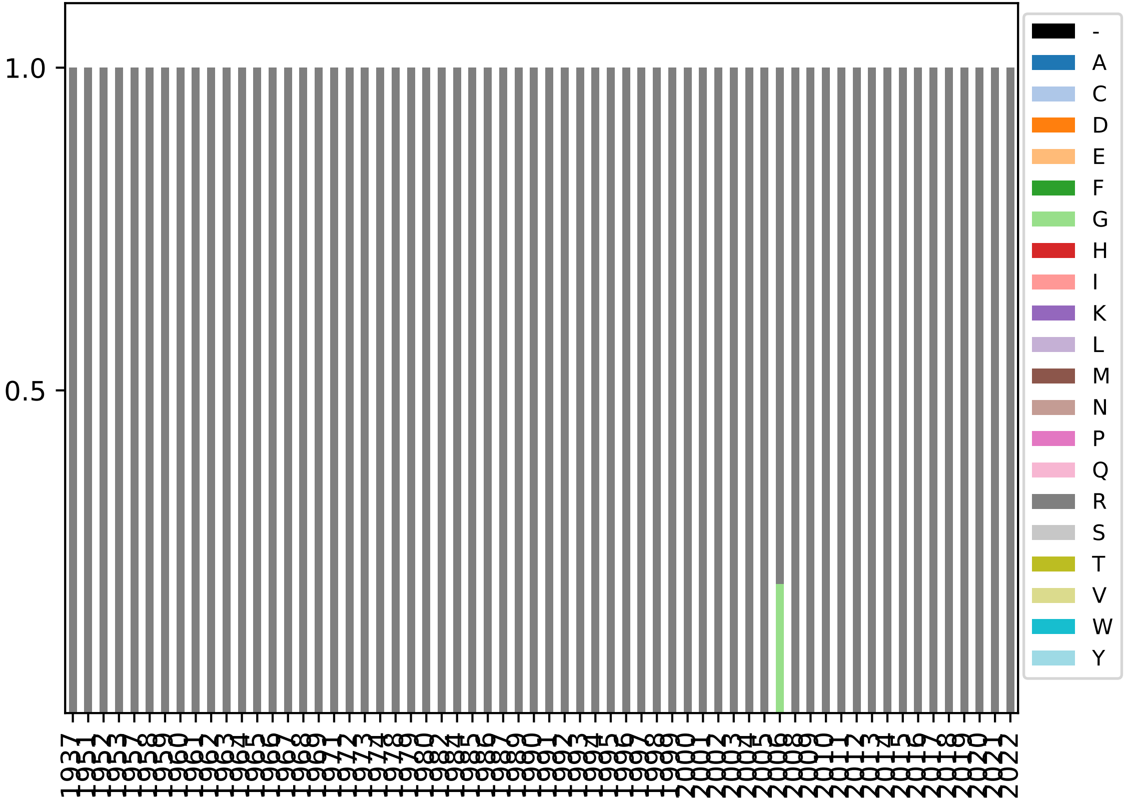

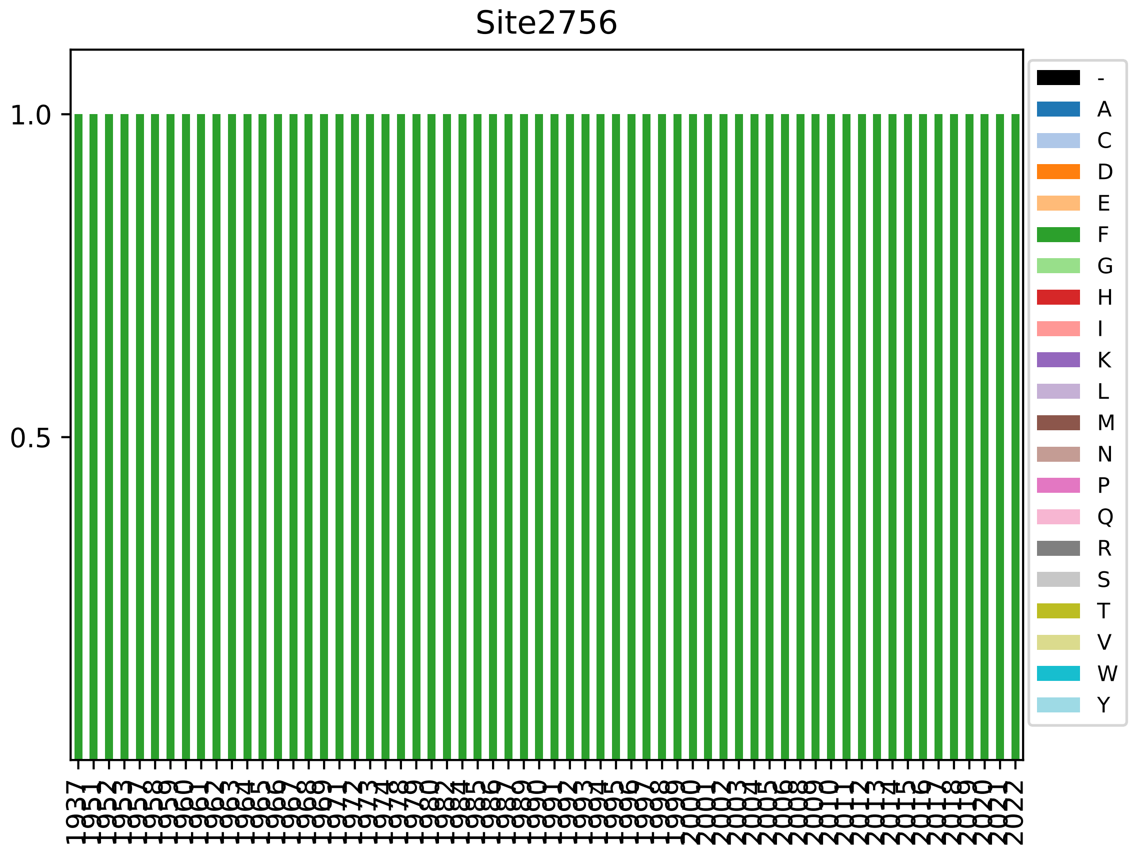
Site2756
1.0
0.5
-
A
C
D
E
F
G
H
I
K
L
M
N
P
Q
R
S
T
V
W
Y

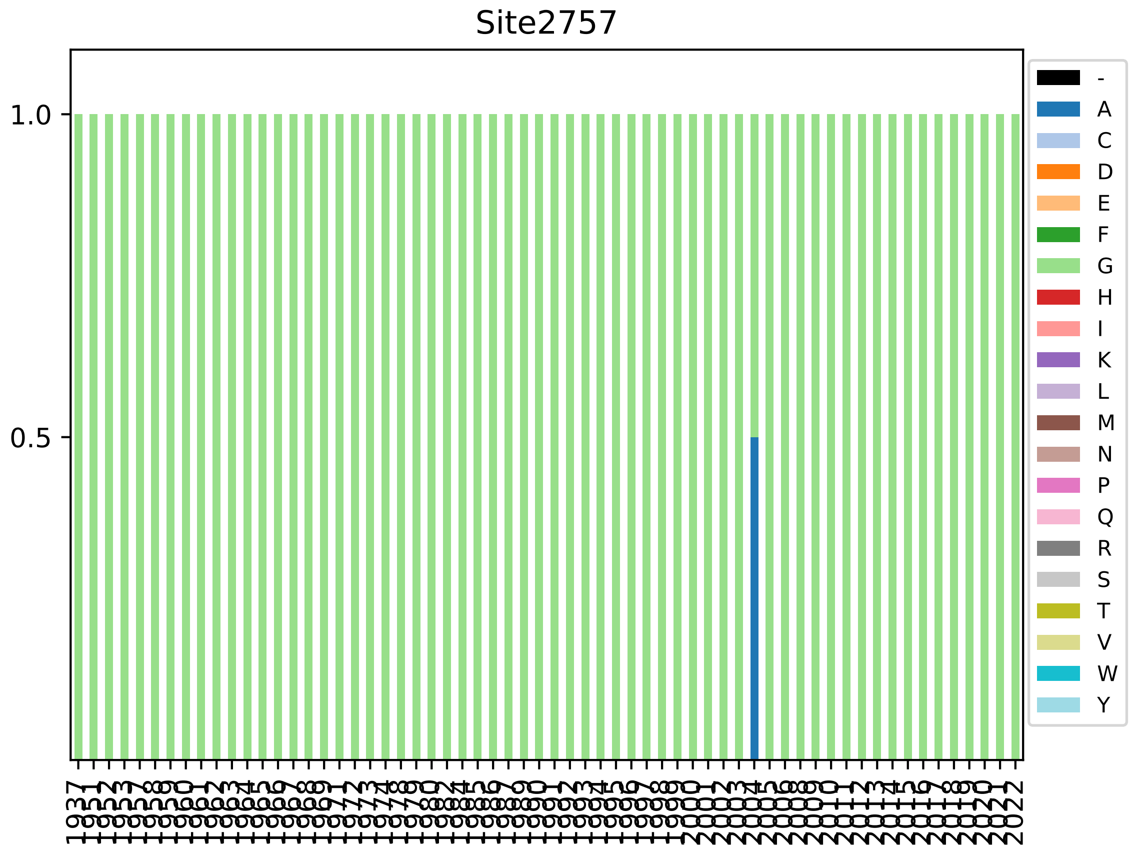
Site2757
1.0
0.5
-
A
C
D
E
F
G
H
I
K
L
M
N
P
Q
R
S
T
V
W
Y

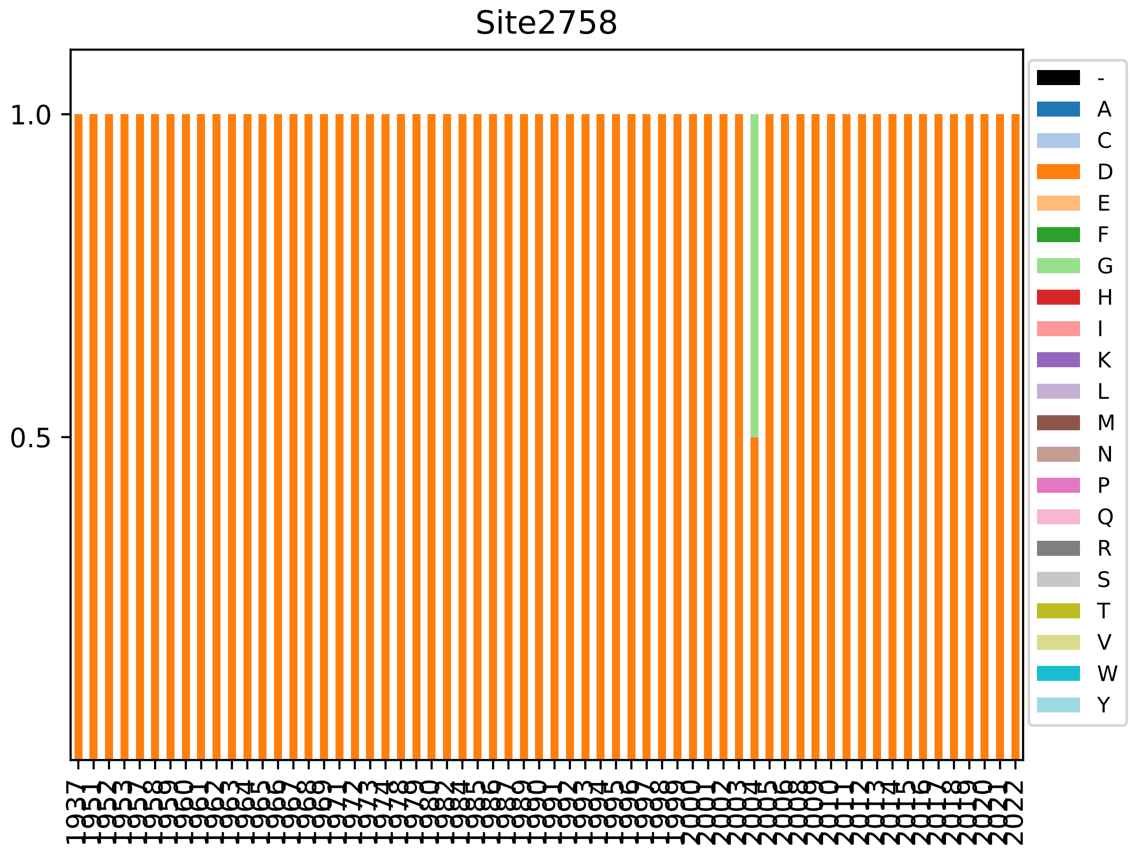
Site2758
1.0
0.5
-
A
C
D
E
F
G
H
I
K
L
M
N
P
Q
R
S
T
V
W
Y

Site2759

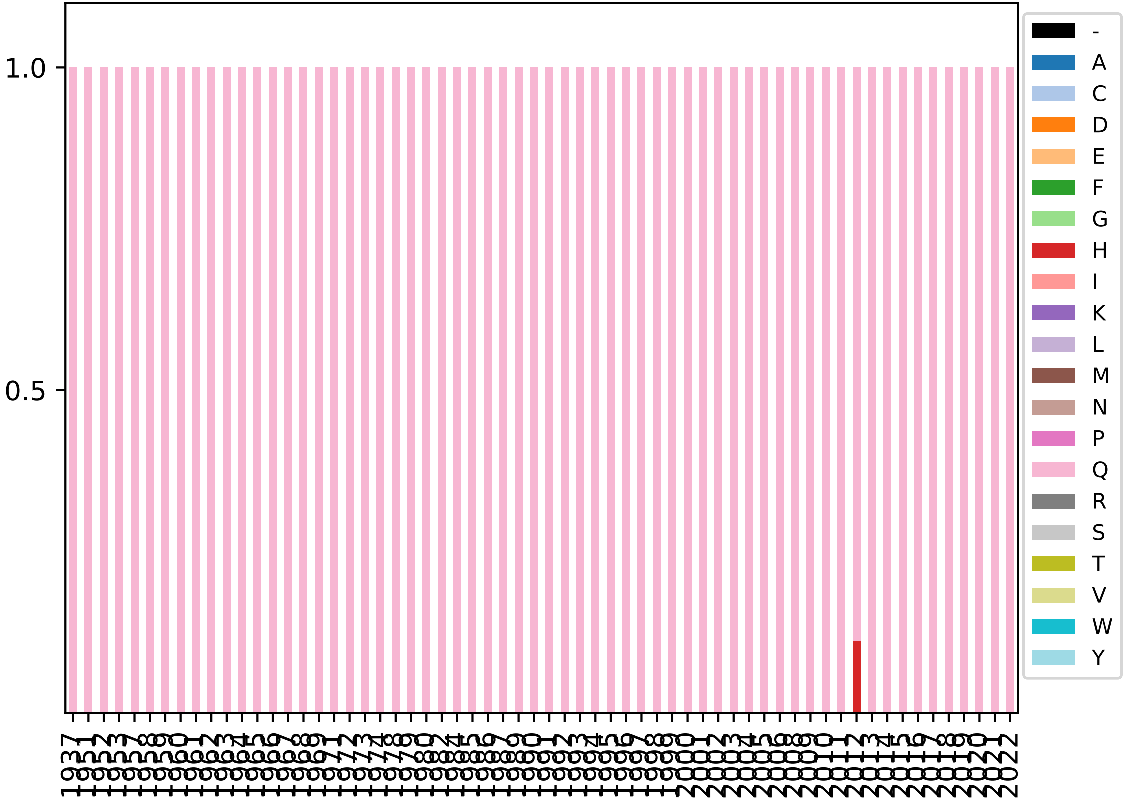

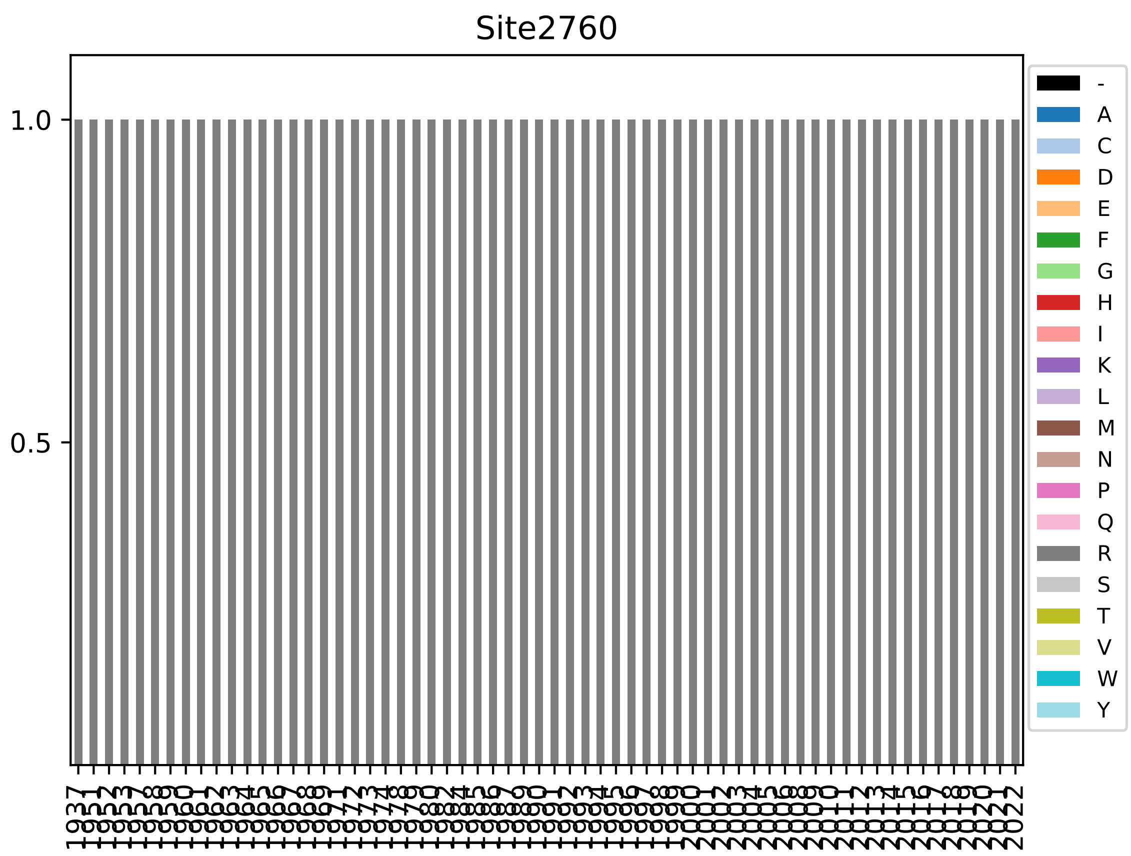
Site2760
1.0
0.5
-
A
C
D
E
F
G
H
I
K
L
M
N
P
Q
R
S
T
V
W
Y

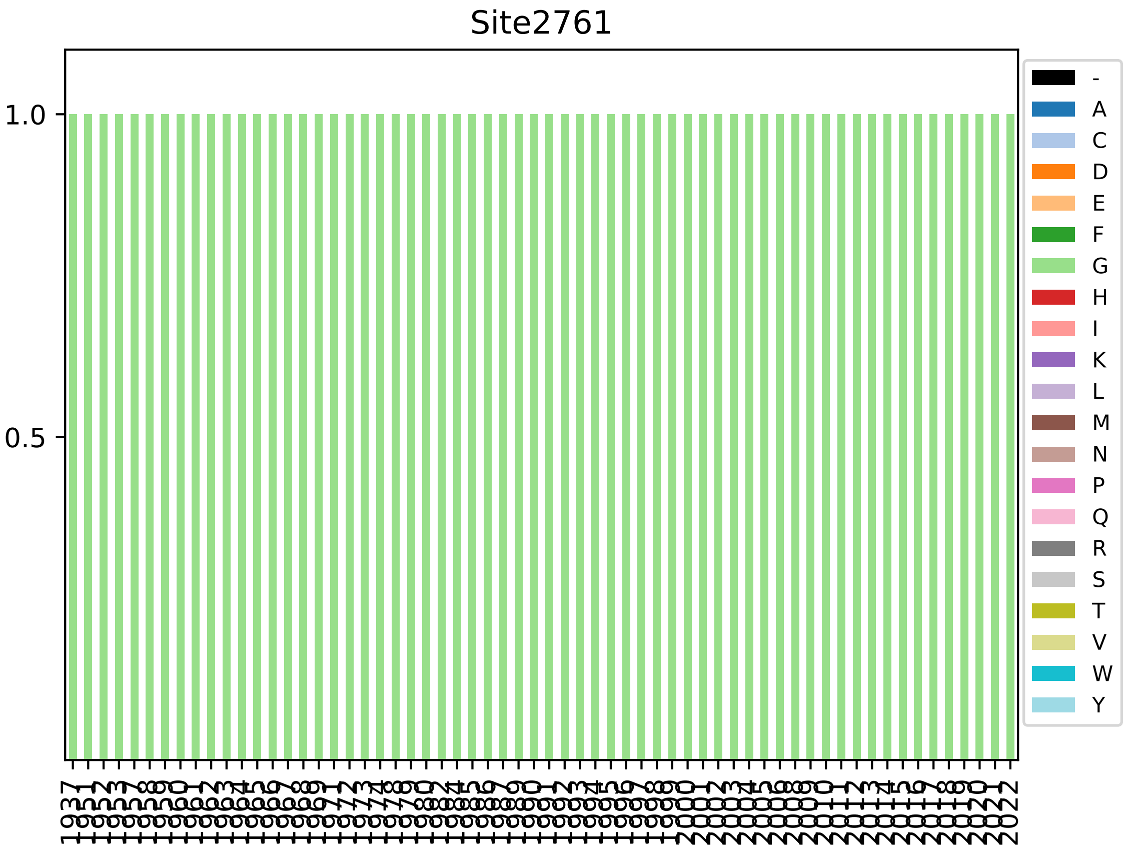

Site2761
1.0
0.5
-
A
C
D
E
F
G
H
I
K
L
M
N
P
Q
R
S
T
V
W
Y

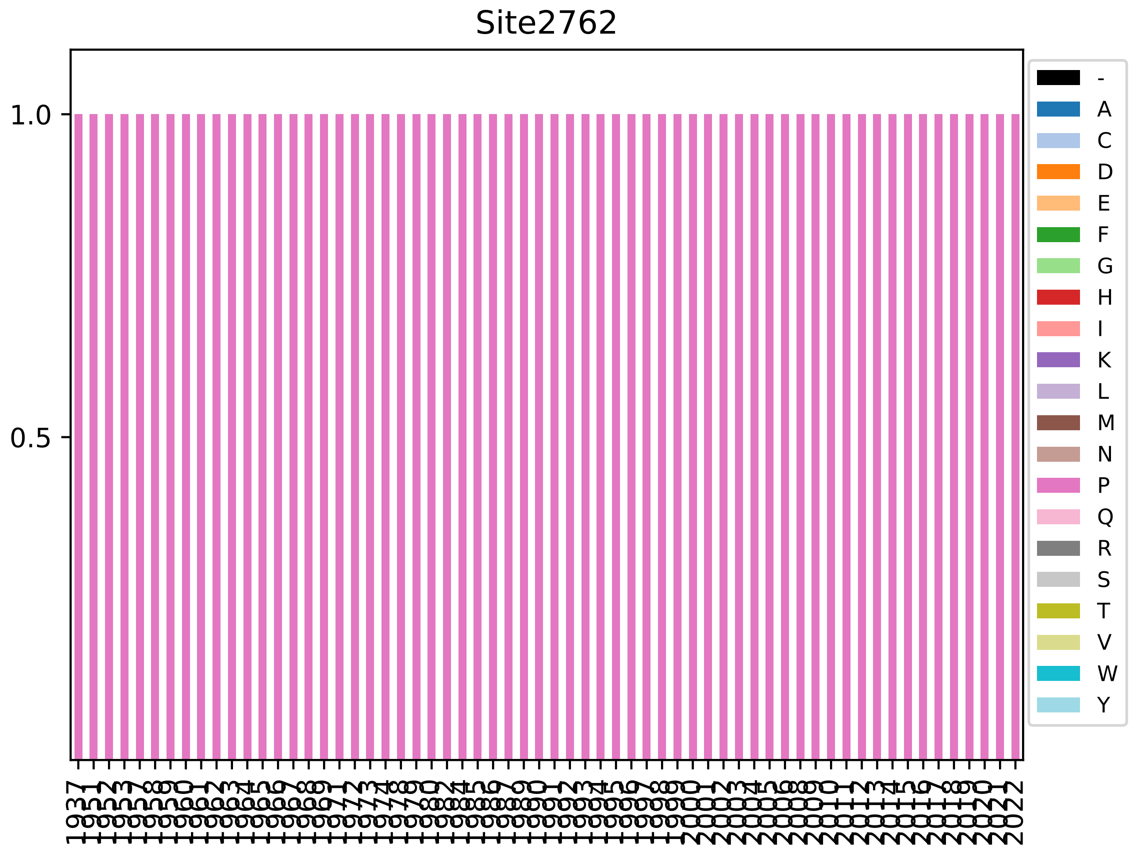
Site2762
1.0
0.5
-
A
C
D
E
F
G
H
I
K
L
M
N
P
Q
R
S
T
V
W
Y

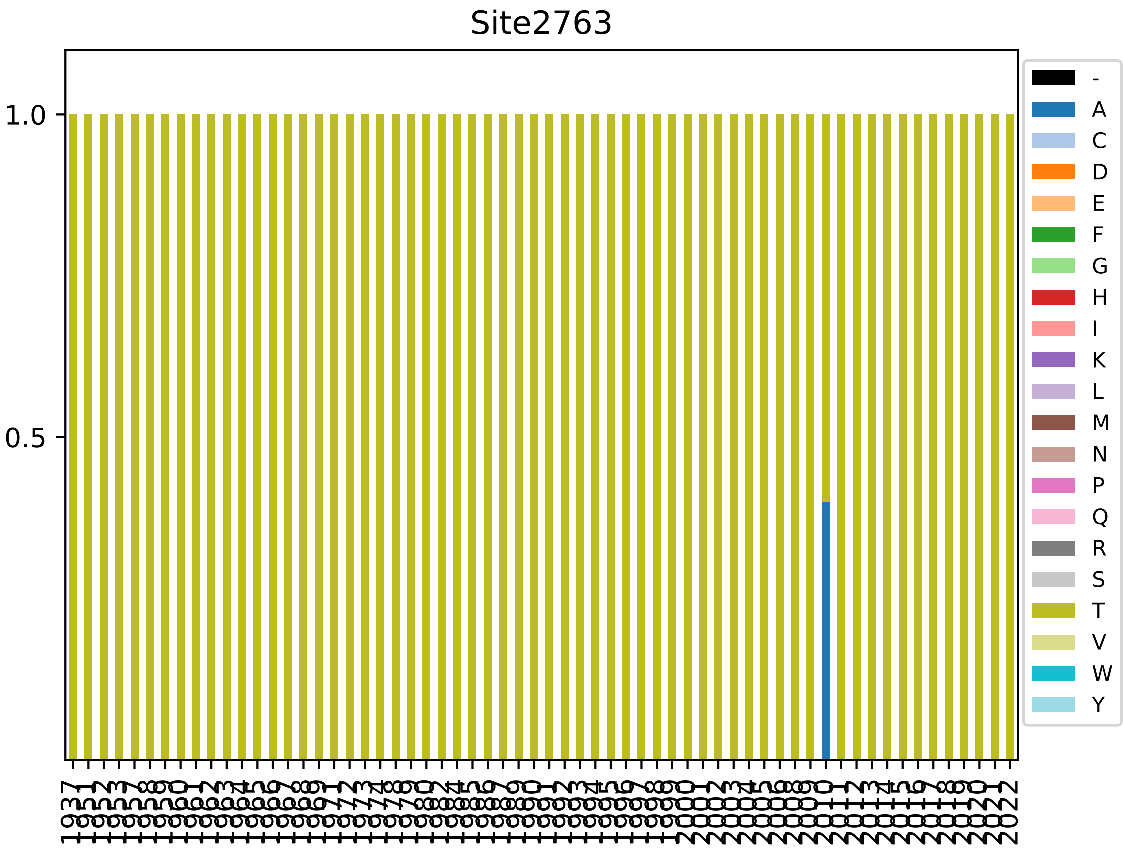
Site2763
1.0
0.5
-
A
C
D
E
F
G
H
I
K
L
M
N
P
Q
R
S
T
V
W
Y

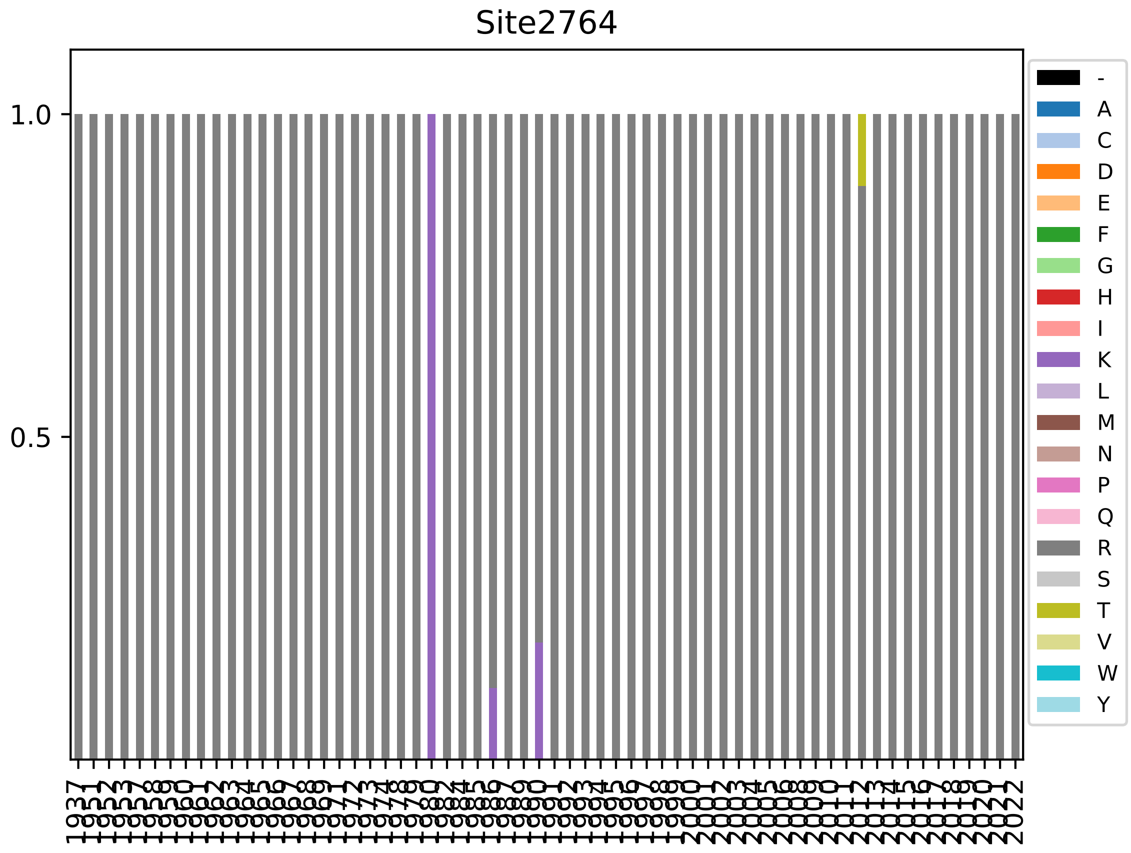
Site2764
1.0
0.5
-
A
C
D
E
F
G
H
I
K
L
M
N
P
Q
R
S
T
V
W
Y

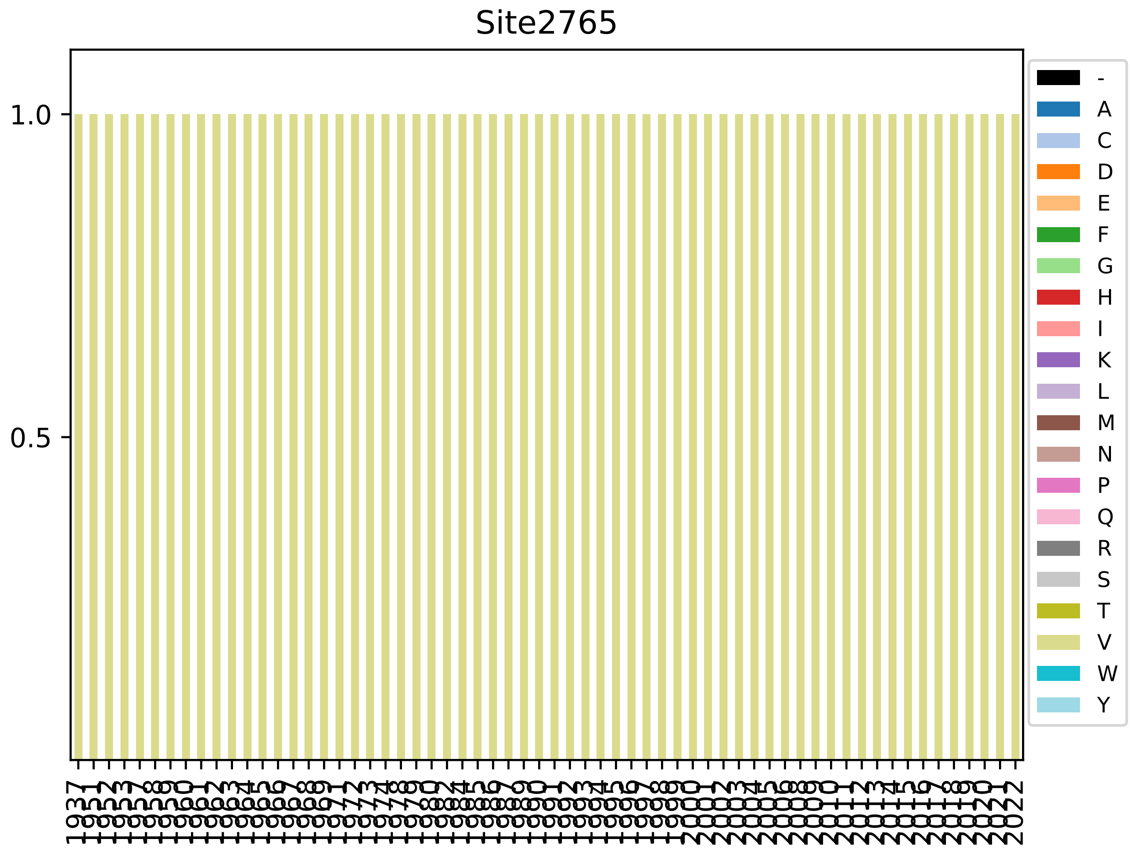
Site2765
1.0
0.5
-
A
C
D
E
F
G
H
I
K
L
M
N
P
Q
R
S
T
V
W
Y

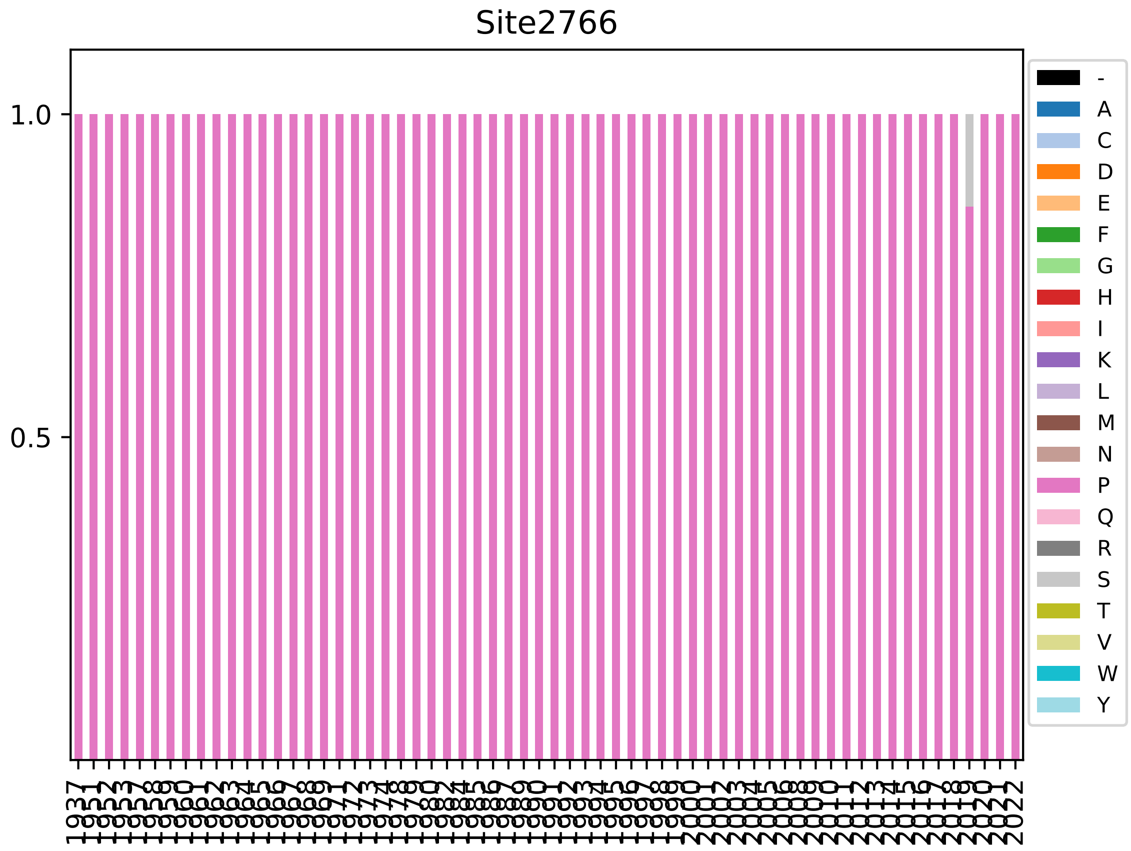
Site2766
1.0
0.5
-
A
C
D
E
F
G
H
I
K
L
M
N
P
Q
R
S
T
V
W
Y

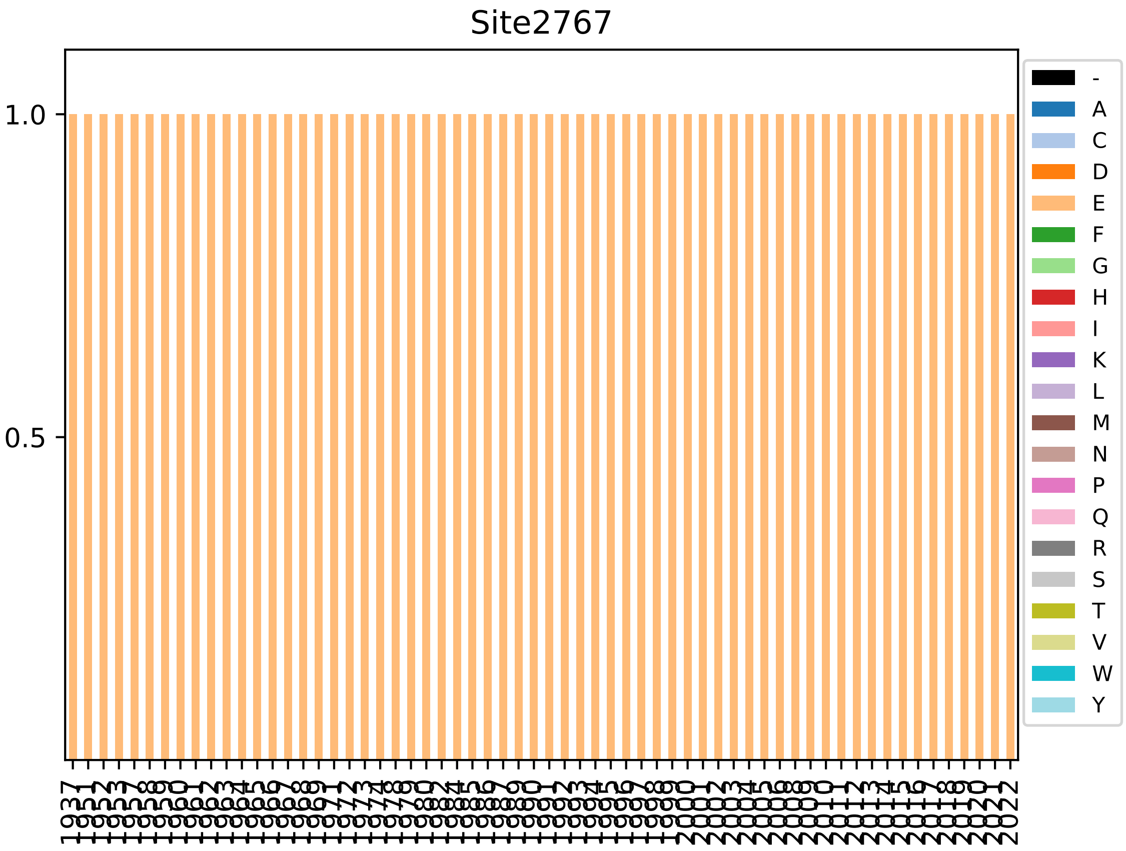
Site2767
1.0
0.5
-
A
C
D
E
F
G
H
I
K
L
M
N
P
Q
R
S
T
V
W
Y

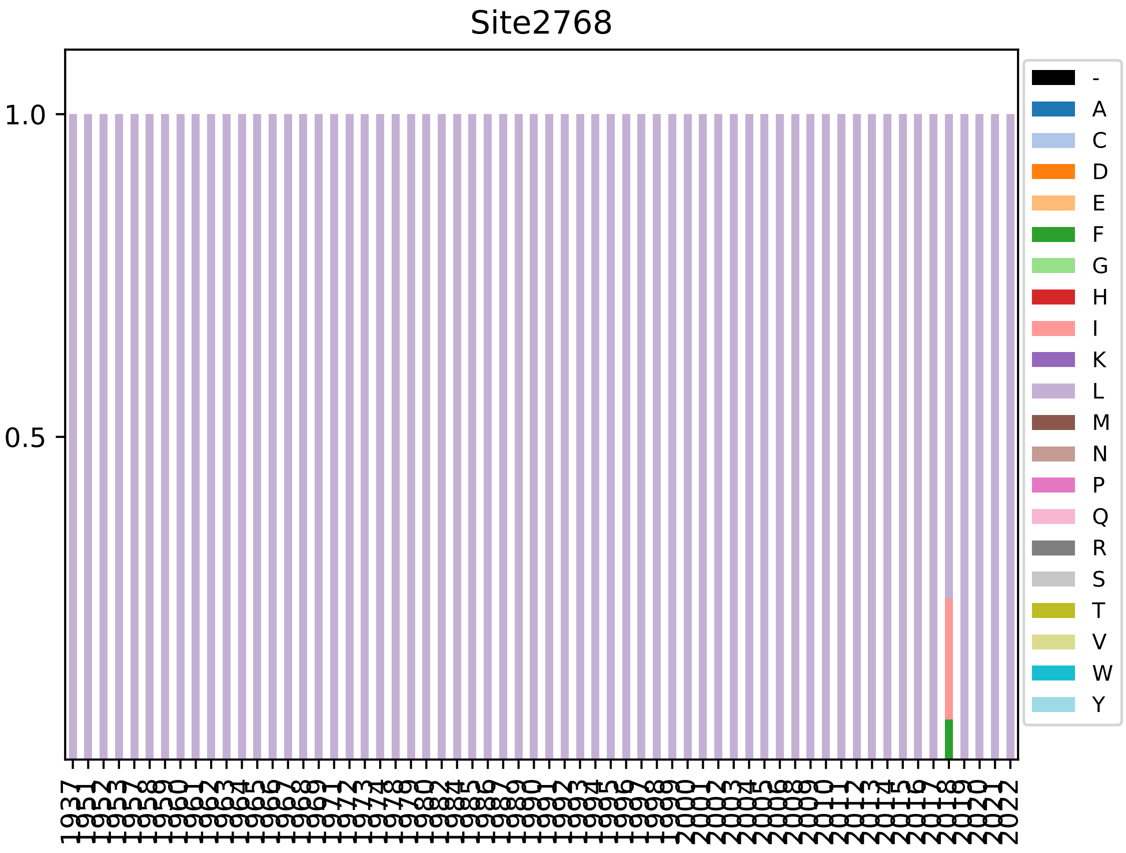
Site2768
1.0
0.5
-
A
C
D
E
F
G
H
I
K
L
M
N
P
Q
R
S
T
V
W
Y

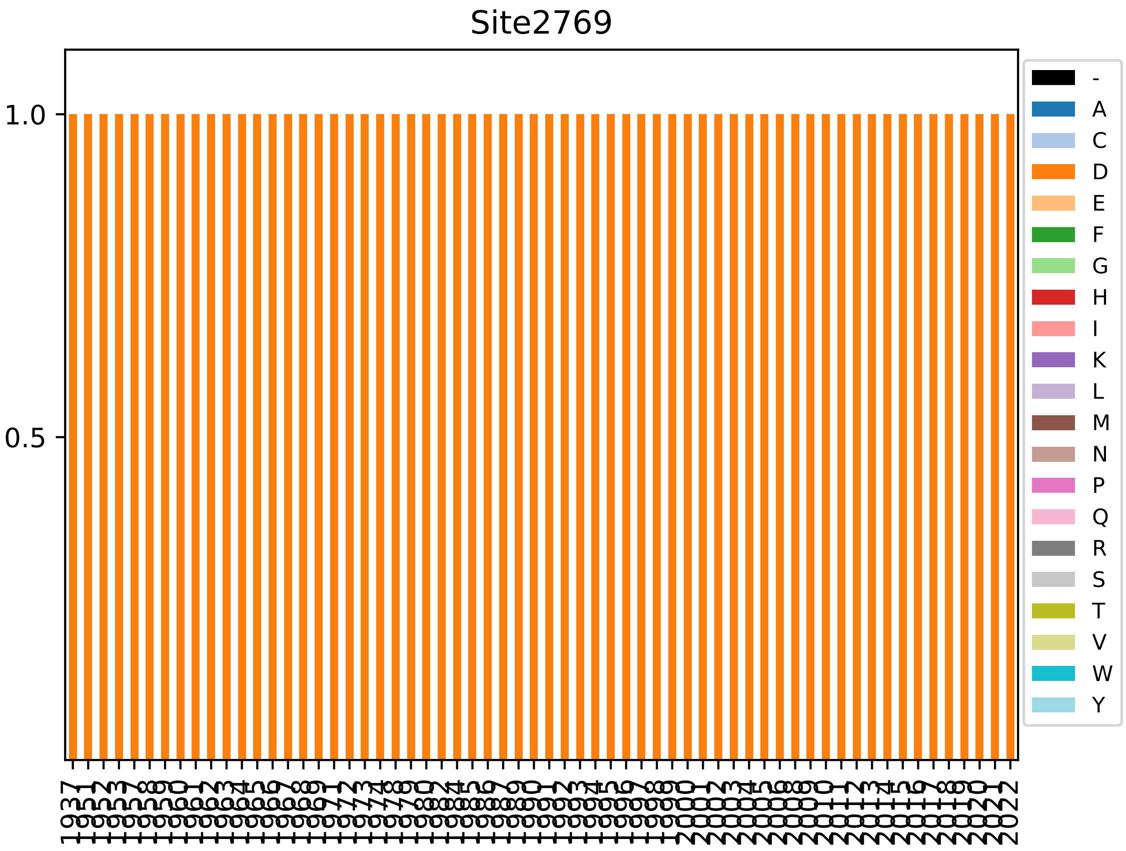

Site2769
1.0
0.5
-
A
C
D
E
F
G
H
I
K
L
M
N
P
Q
R
S
T
V
W
Y

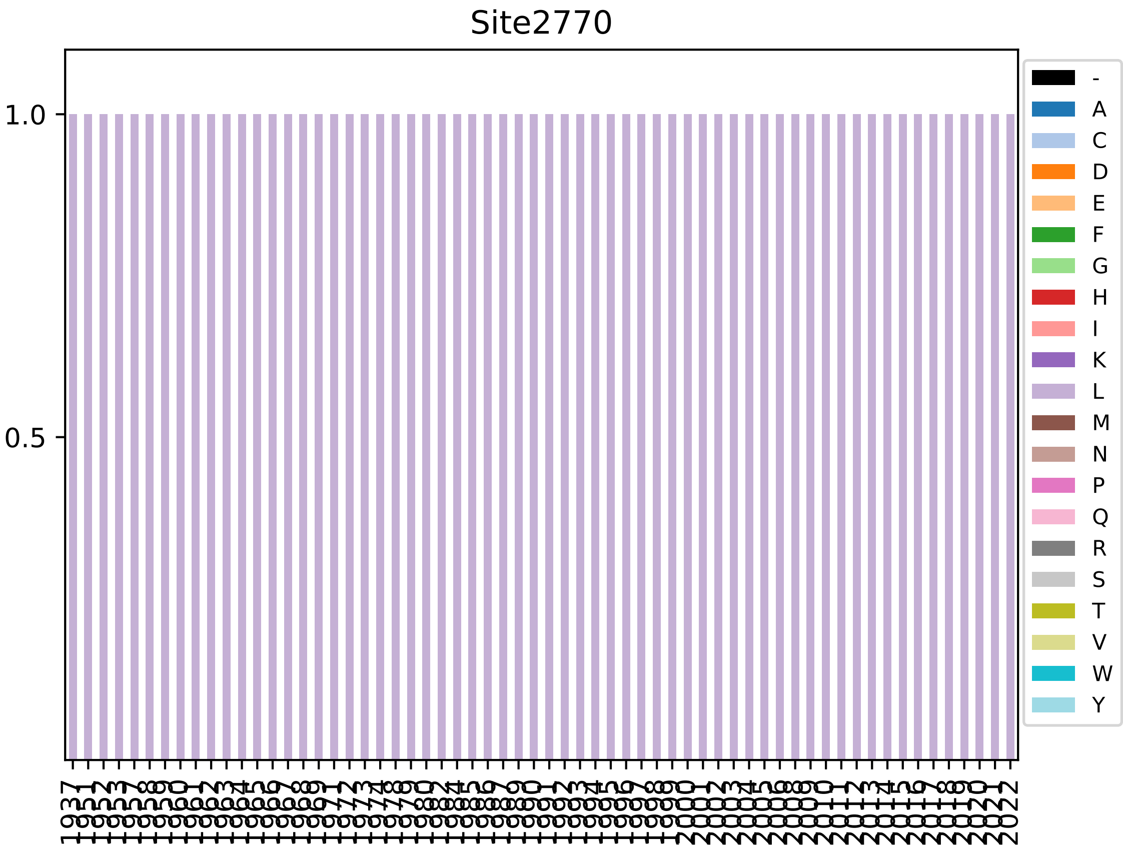
Site2770
1.0
0.5
1937
2022
-
A
C
D
E
F
G
H
I
K
L
M
N
P
Q
R
S
T
V
W
Y

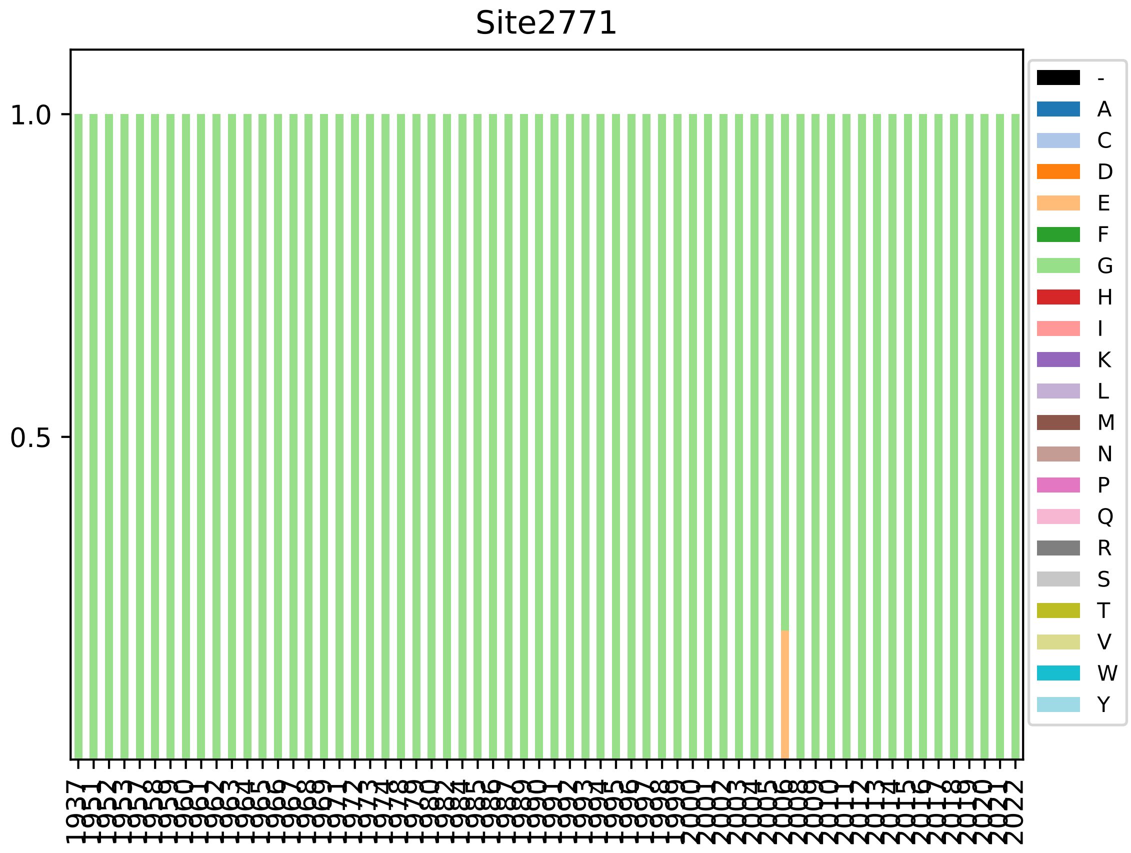
Site2771
1.0
0.5
-
A
C
D
E
F
G
H
I
K
L
M
N
P
Q
R
S
T
V
W
Y

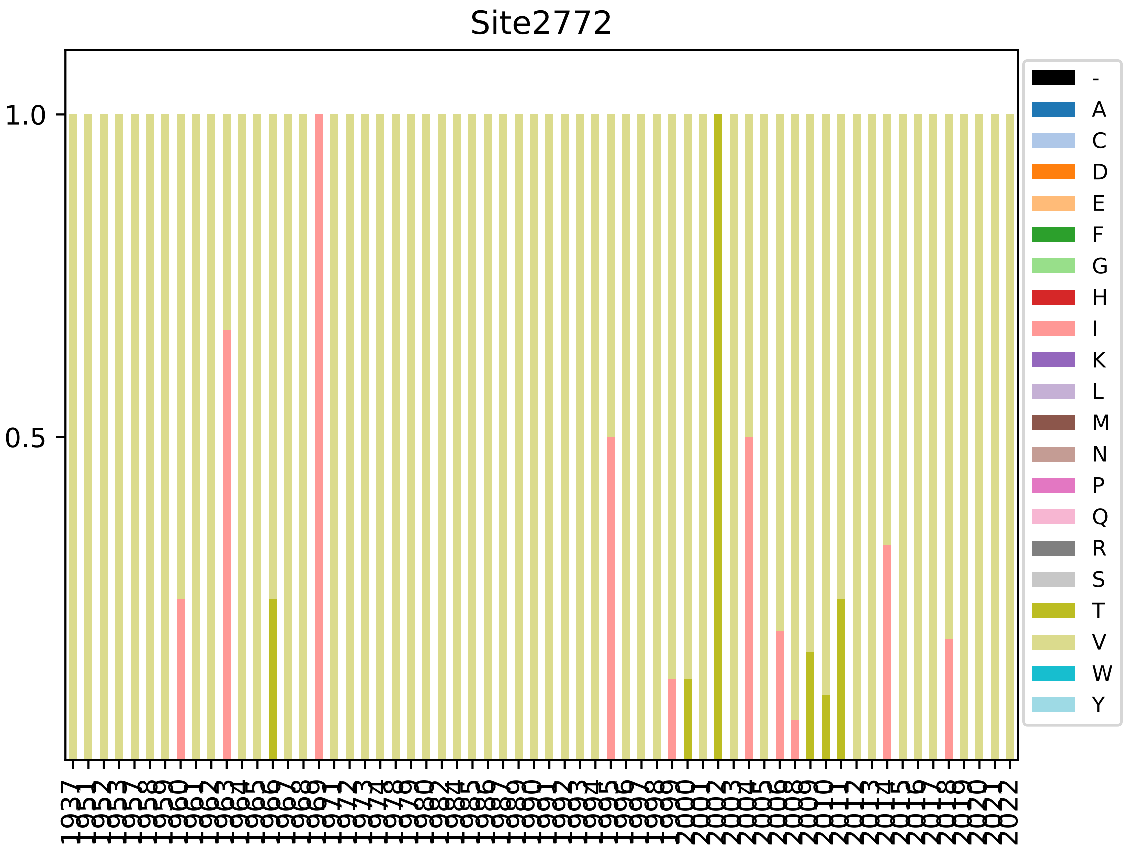
Site2772
1.0
0.5
-
A
C
D
E
F
G
H
I
K
L
M
N
P
Q
R
S
T
V
W
Y

Site2773

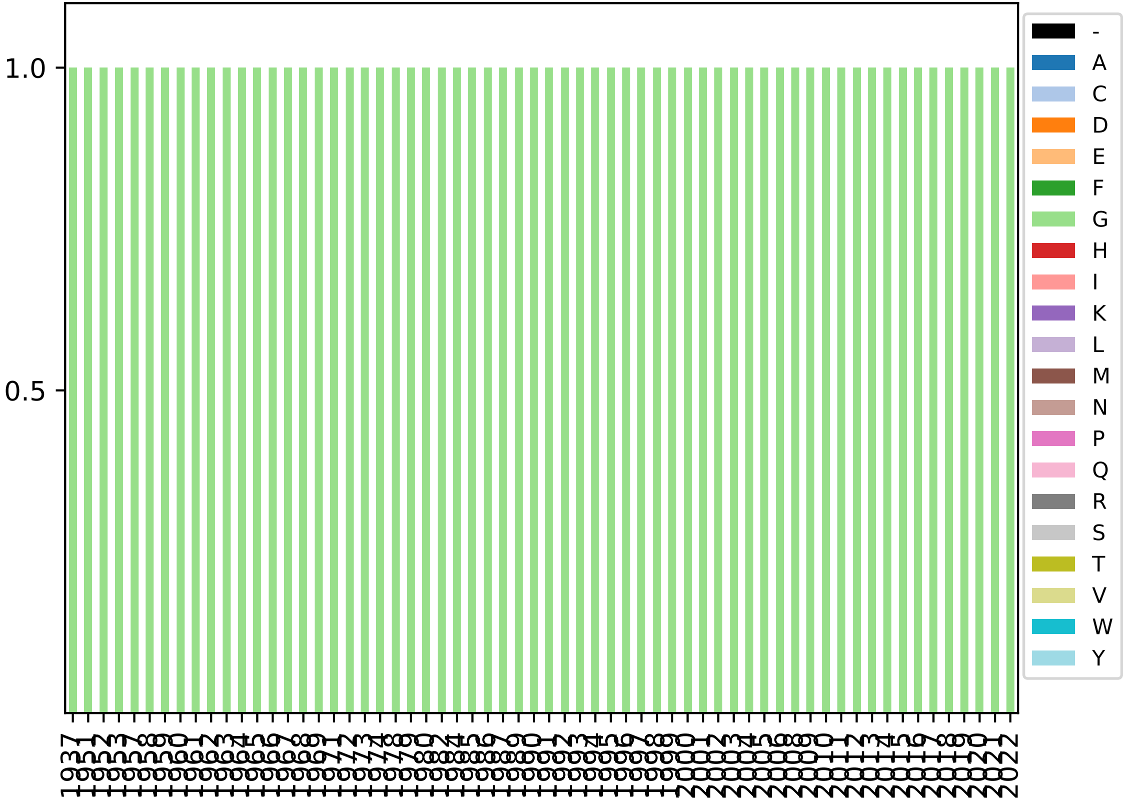

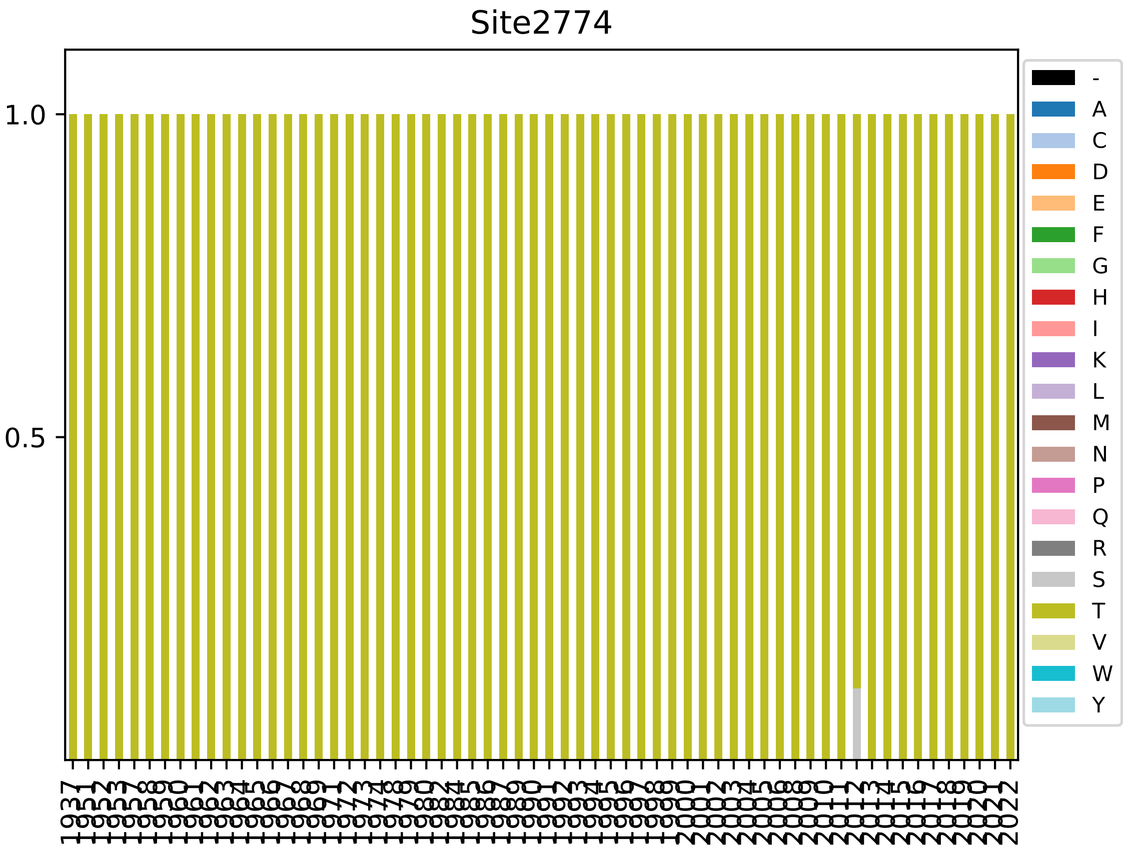
Site2774
1.0
0.5
-
A
C
D
E
F
G
H
I
K
L
M
N
P
Q
R
S
T
V
W
Y

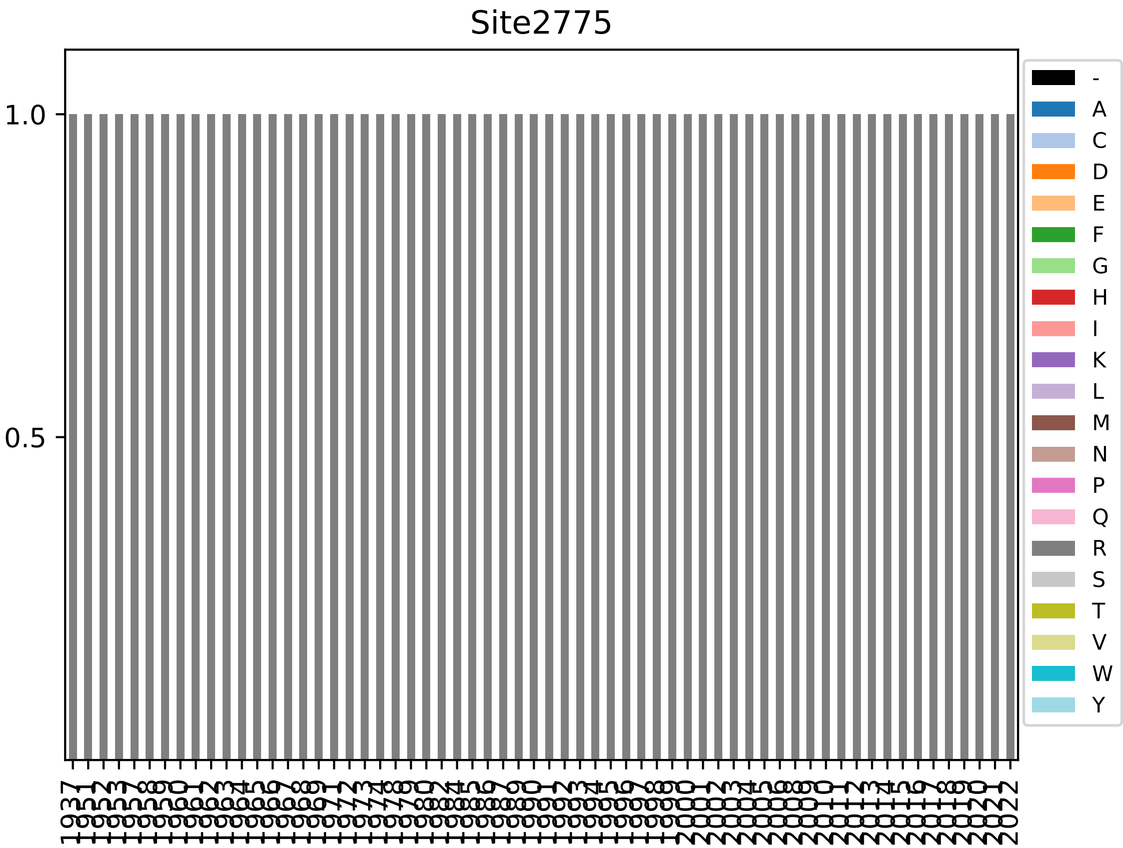
Site2775
1.0
0.5
-
A
C
D
E
F
G
H
I
K
L
M
N
P
Q
R
S
T
V
W
Y

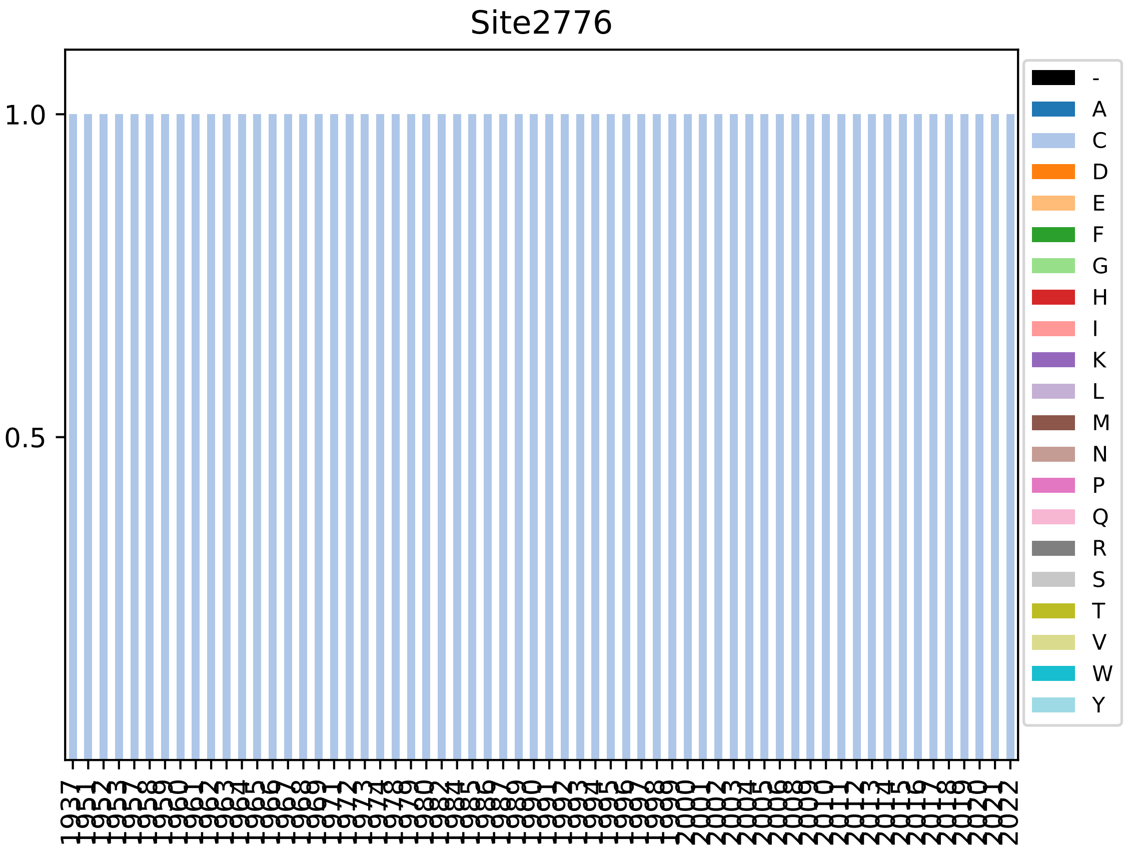
Site2776
1.0
0.5
-
A
C
D
E
F
G
H
I
K
L
M
N
P
Q
R
S
T
V
W
Y

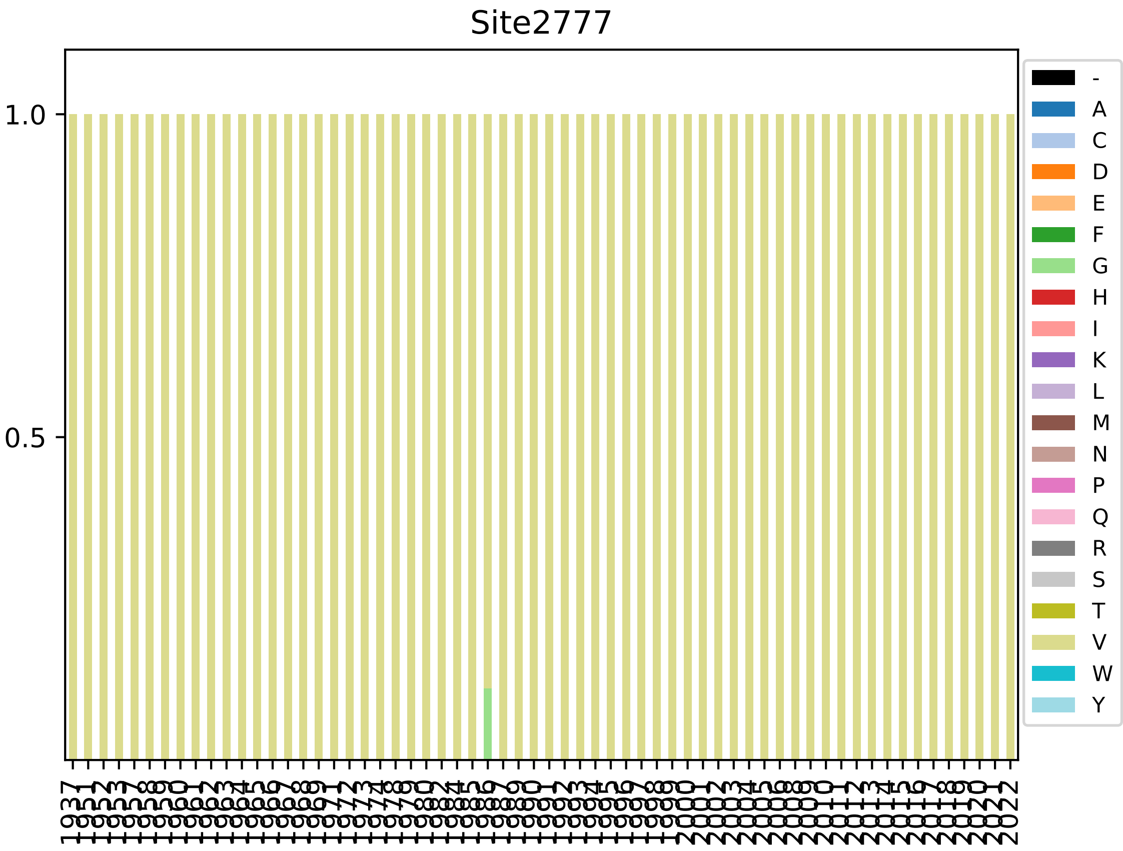
Site2777
1.0
0.5
-
A
C
D
E
F
G
H
I
K
L
M
N
P
Q
R
S
T
V
W
Y

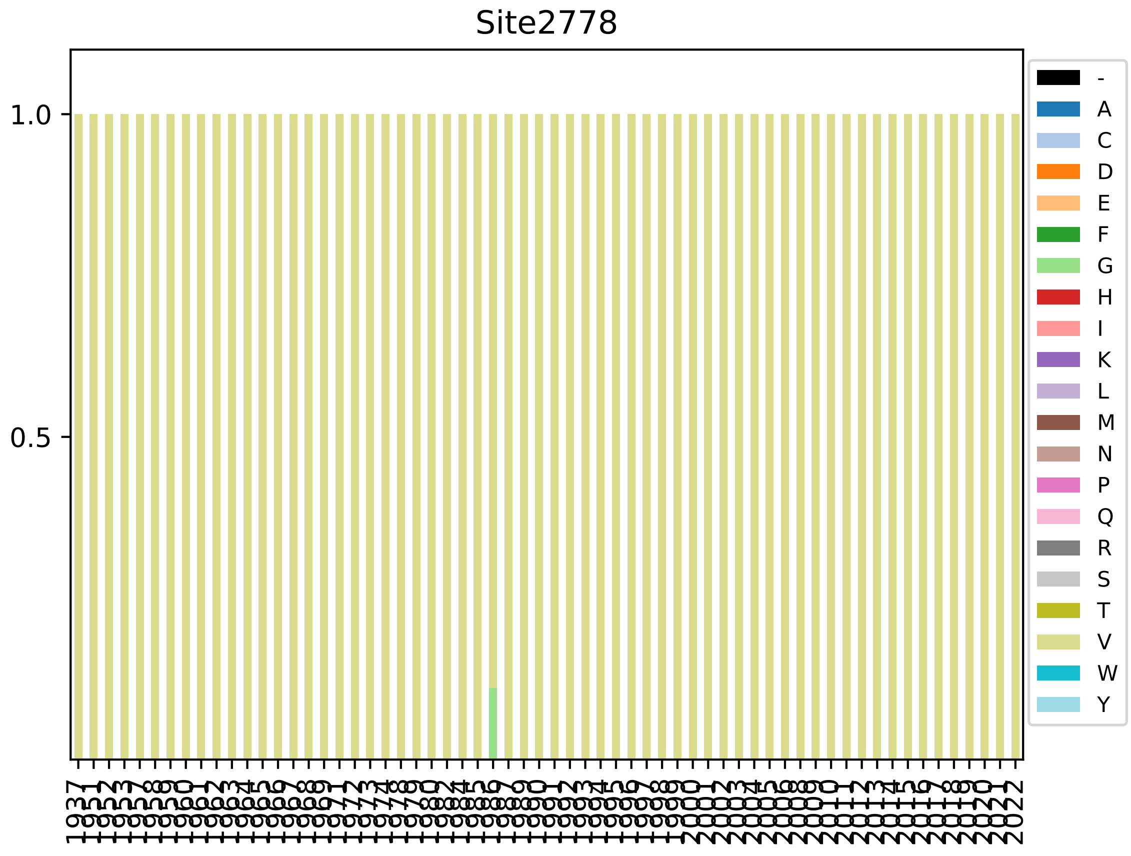
Site2778
1.0
0.5
-
A
C
D
E
F
G
H
I
K
L
M
N
P
Q
R
S
T
V
W
Y

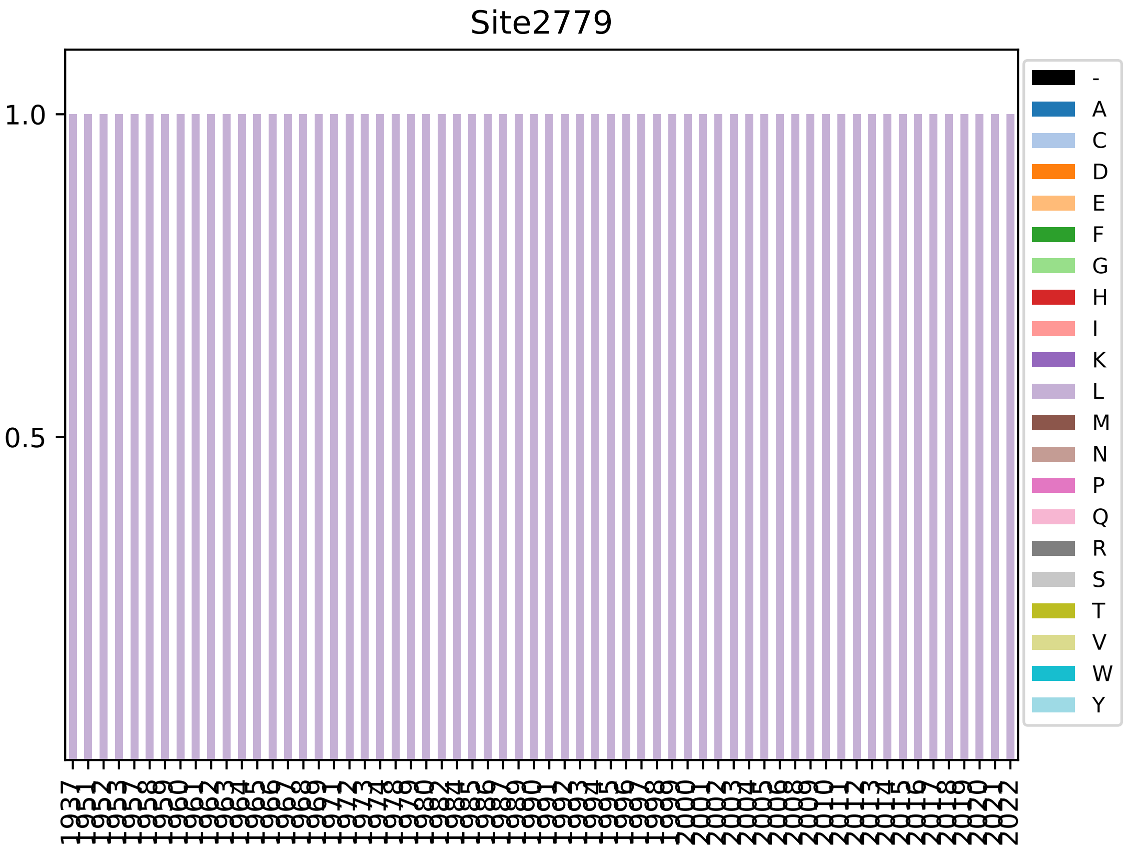
Site2779
1.0
0.5
-
A
C
D
E
F
G
H
I
K
L
M
N
P
Q
R
S
T
V
W
Y

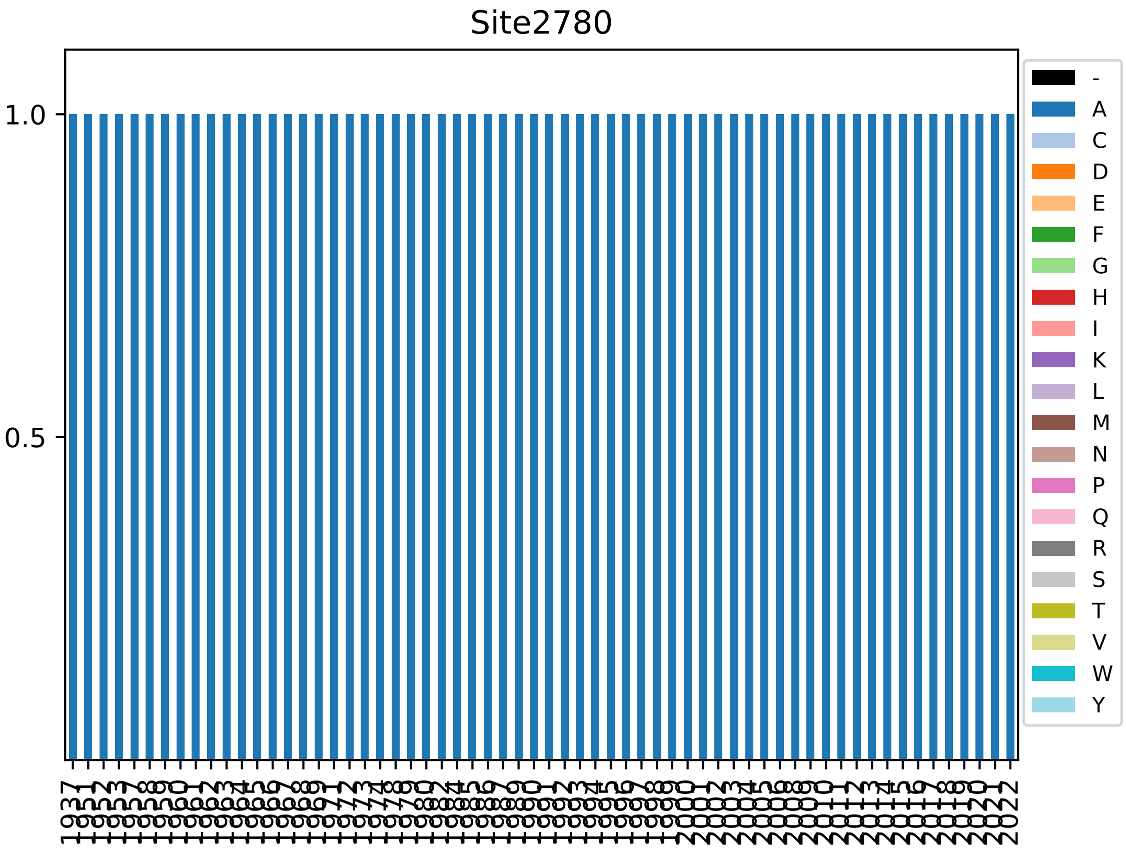
Site2780
1.0
0.5
-
A
C
D
E
F
G
H
I
K
L
M
N
P
Q
R
S
T
V
W
Y

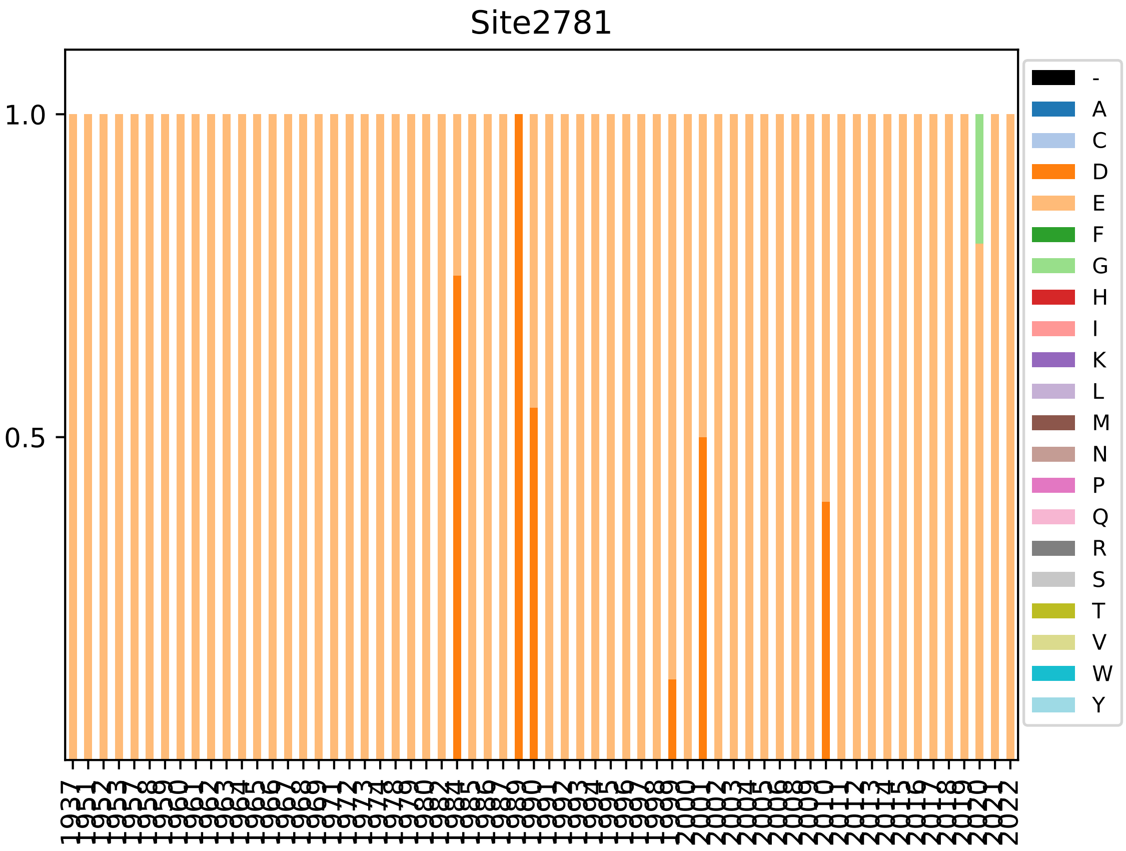
Site2781
1.0
0.5
-
A
C
D
E
F
G
H
I
K
L
M
N
P
Q
R
S
T
V
W
Y

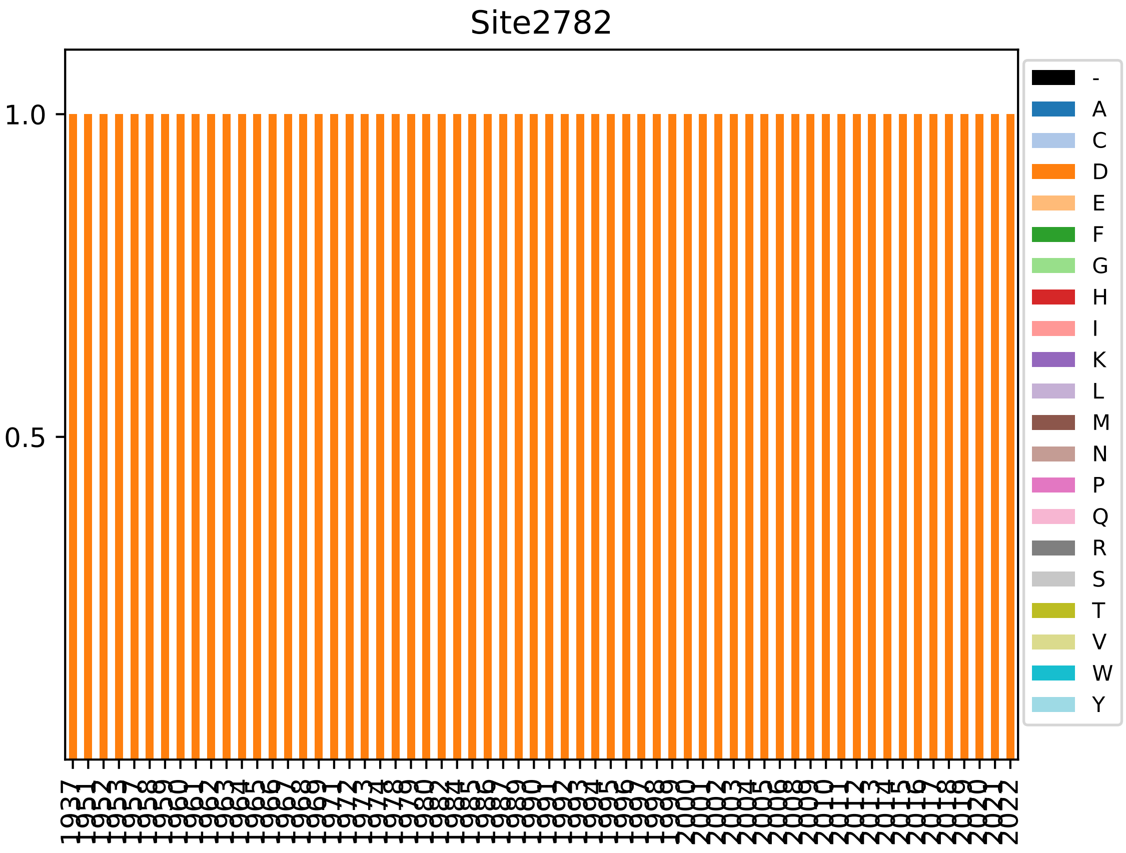

Site2782
1.0
0.5
-
A
C
D
E
F
G
H
I
K
L
M
N
P
Q
R
S
T
V
W
Y

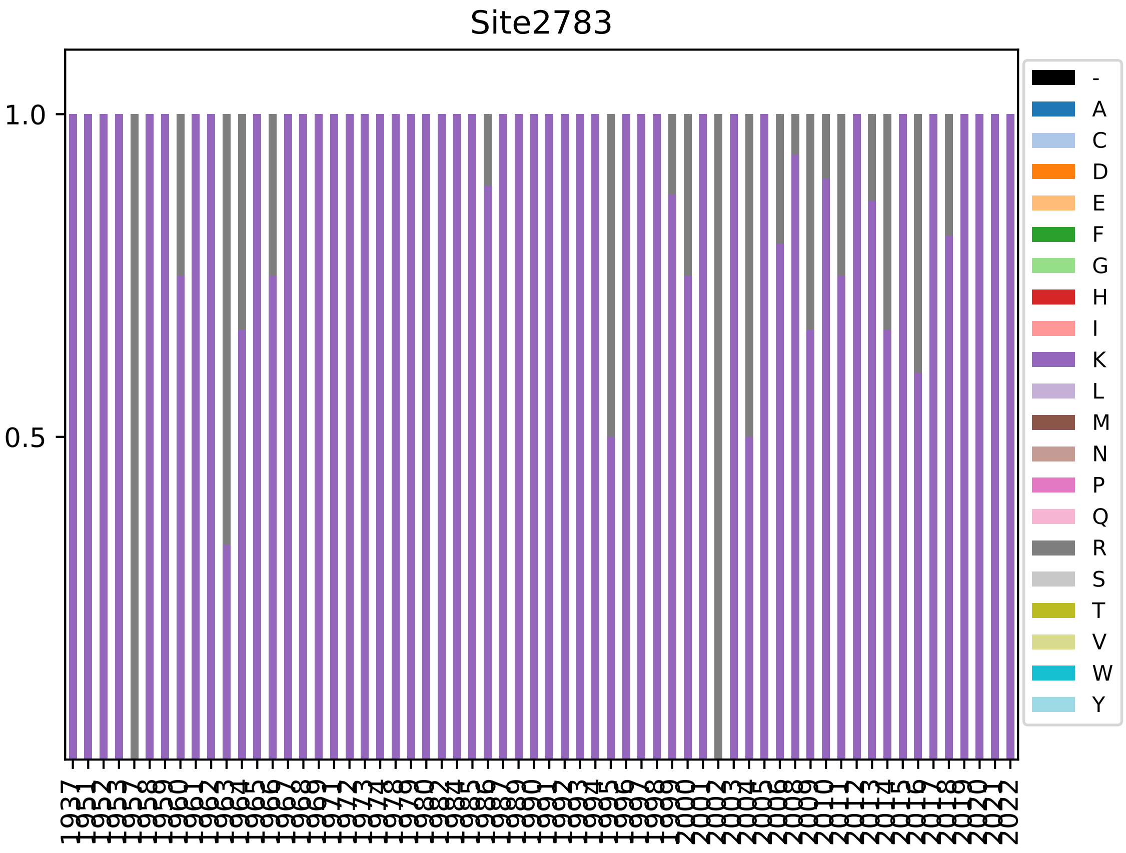
Site2783
1.0
0.5
-
A
C
D
E
F
G
H
I
K
L
M
N
P
Q
R
S
T
V
W
Y

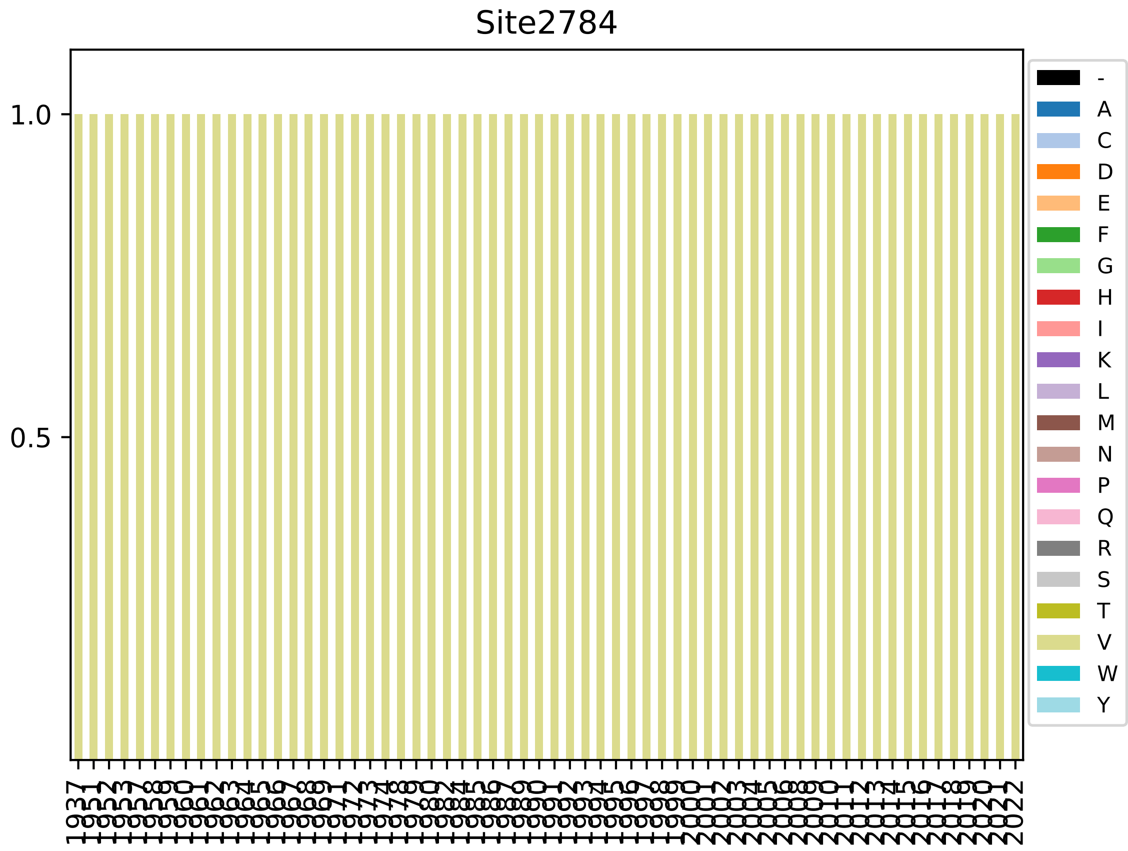
Site2784
1.0
0.5
-
A
C
D
E
F
G
H
I
K
L
M
N
P
Q
R
S
T
V
W
Y

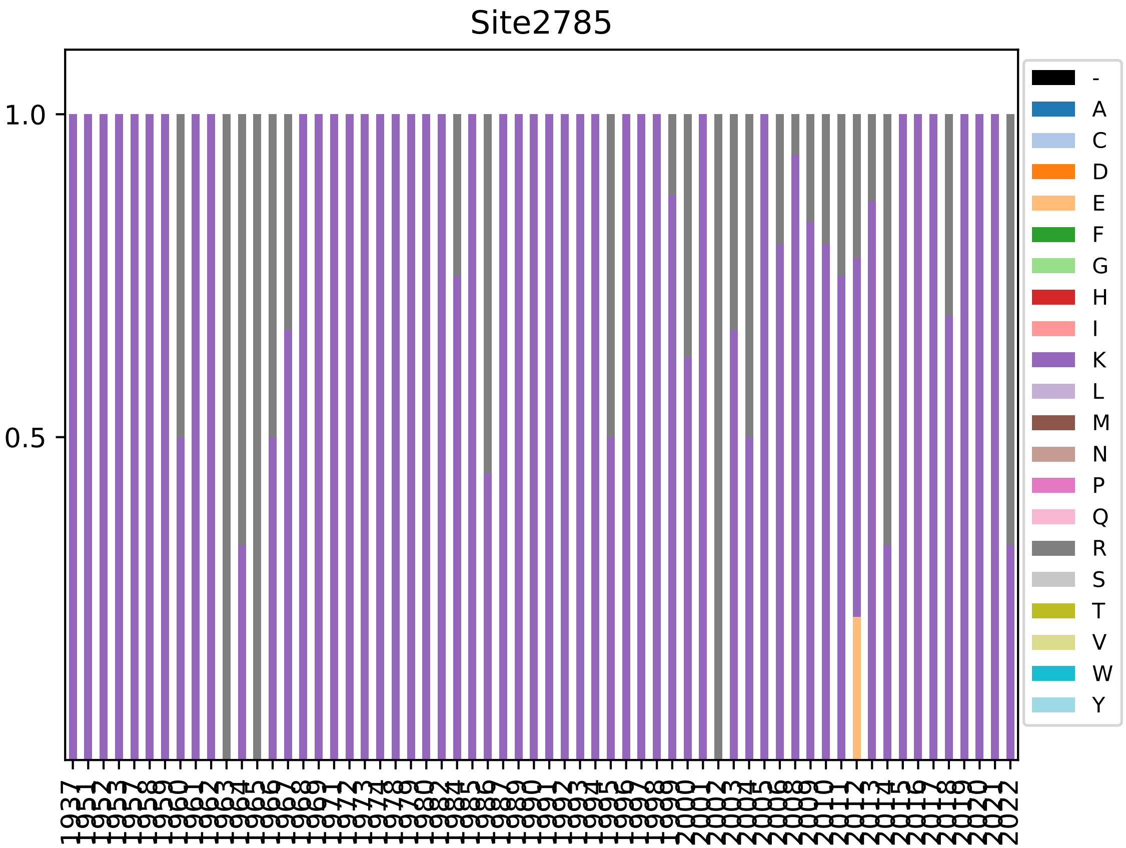
Site2785
1.0
0.5
-
A
C
D
E
F
G
H
I
K
L
M
N
P
Q
R
S
T
V
W
Y

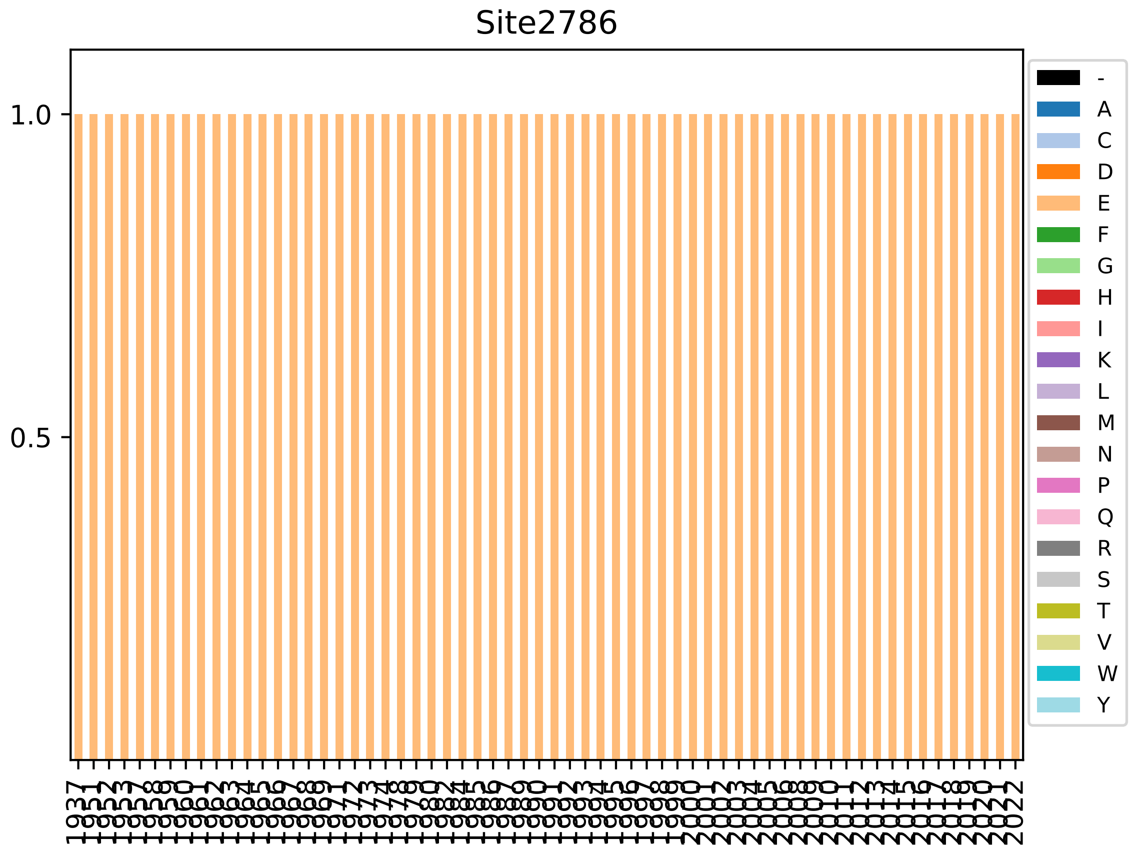
Site2786
1.0
0.5
-
A
C
D
E
F
G
H
I
K
L
M
N
P
Q
R
S
T
V
W
Y

Site2787

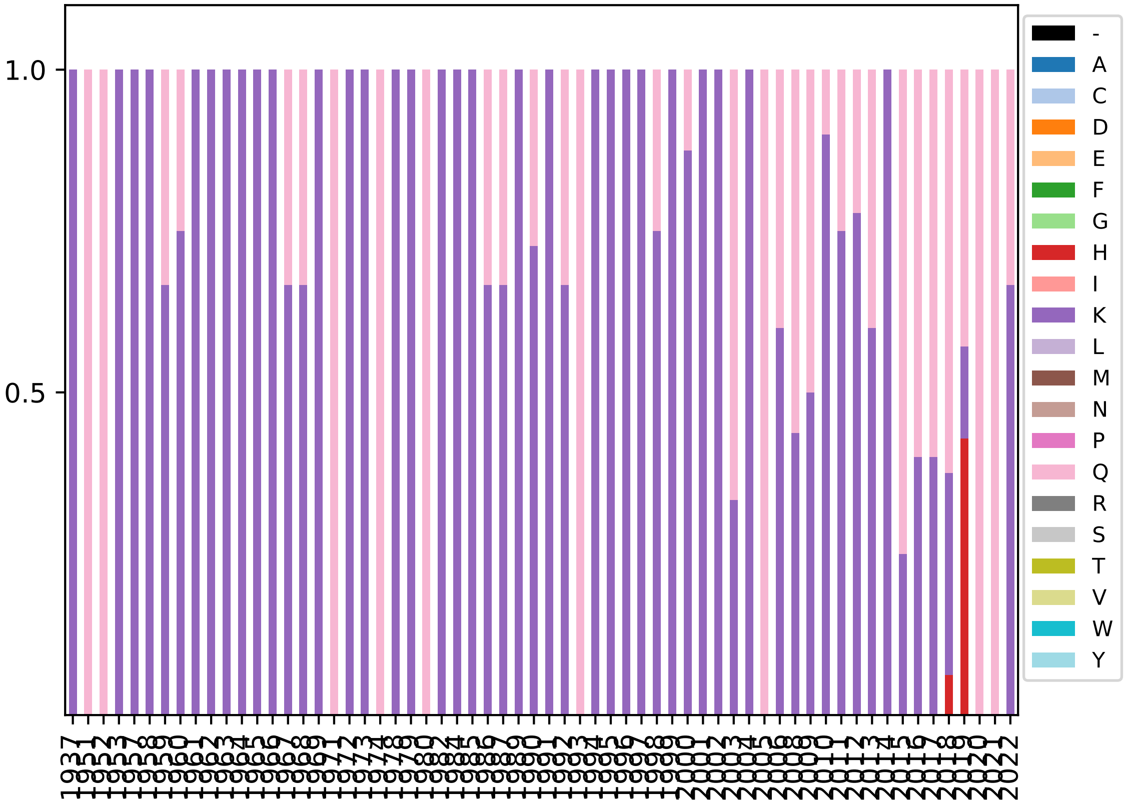

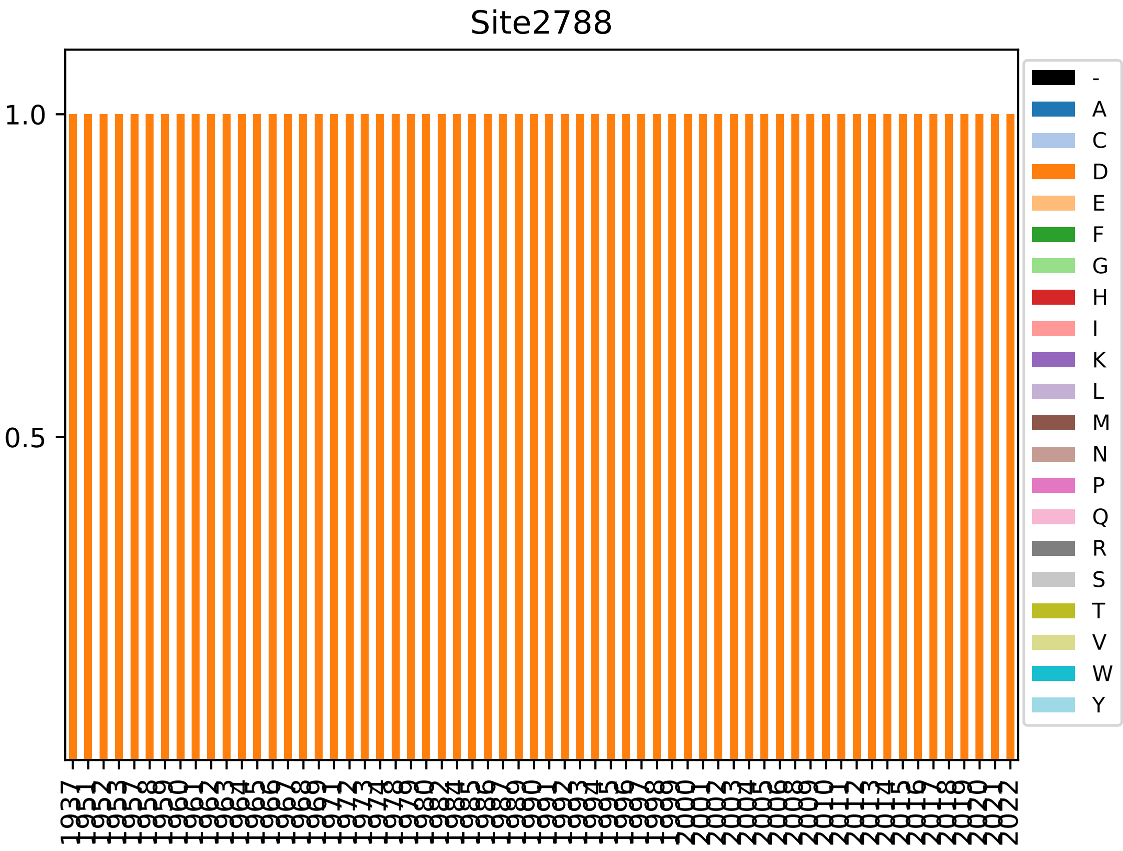
Site2788
1.0
0.5
-
A
C
D
E
F
G
H
I
K
L
M
N
P
Q
R
S
T
V
W
Y

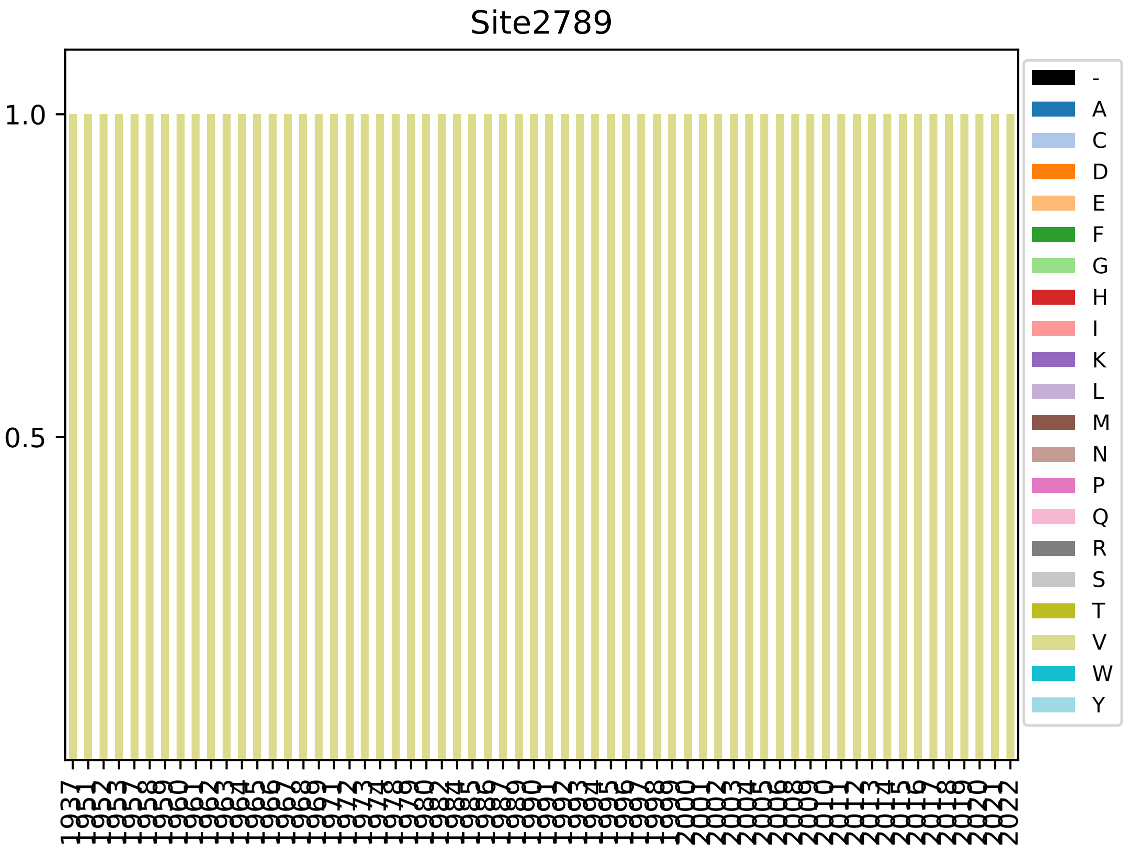
Site2789
1.0
0.5
-
A
C
D
E
F
G
H
I
K
L
M
N
P
Q
R
S
T
V
W
Y

Site2790

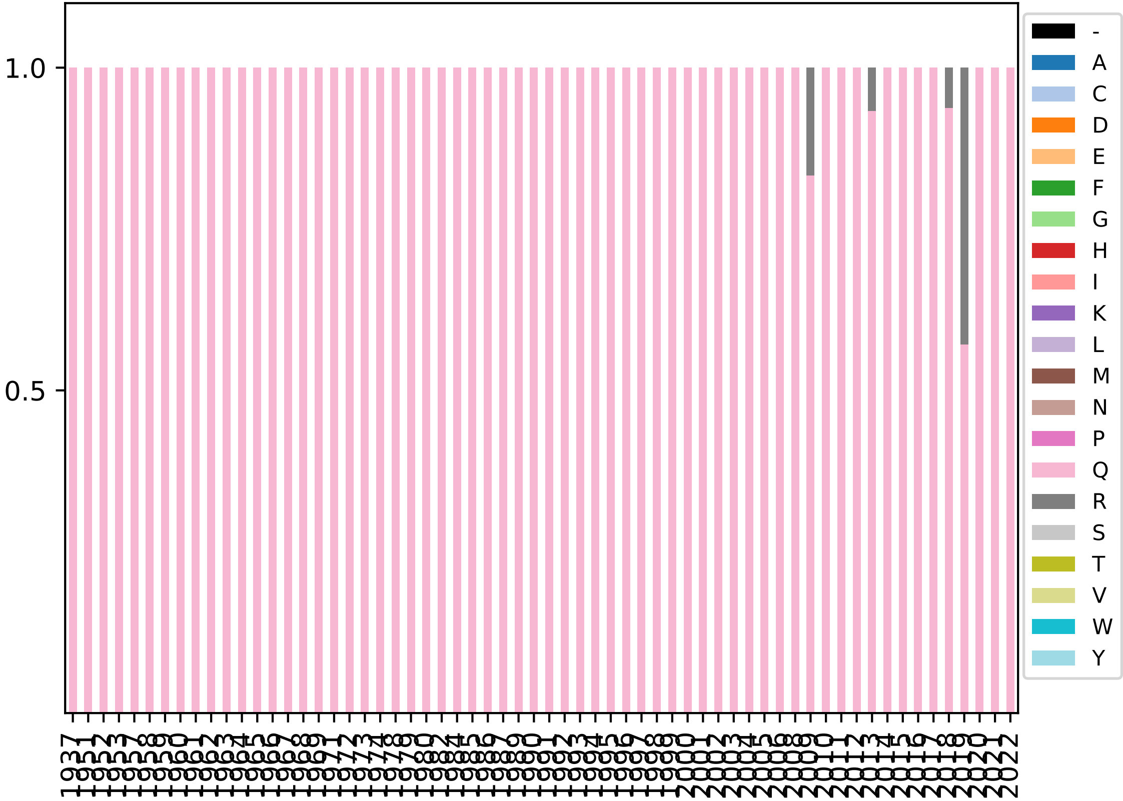

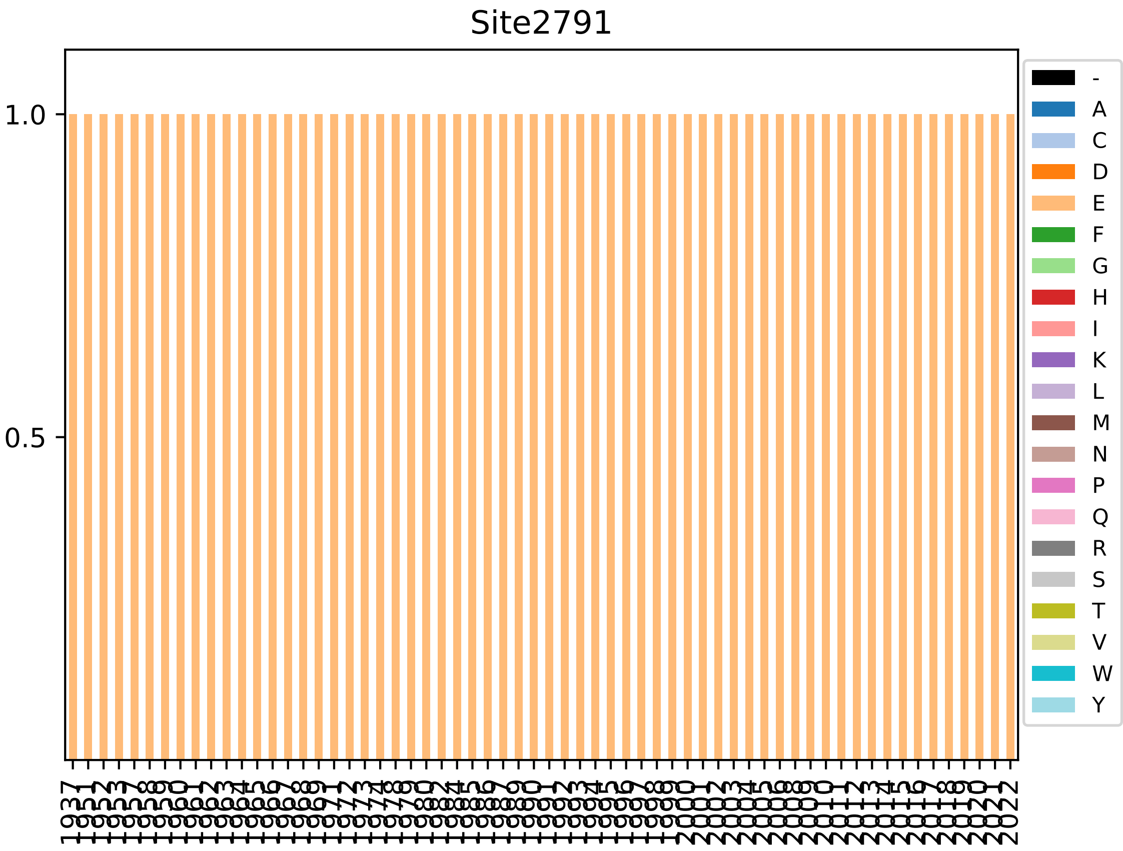
Site2791
1.0
0.5
-
A
C
D
E
F
G
H
I
K
L
M
N
P
Q
R
S
T
V
W
Y

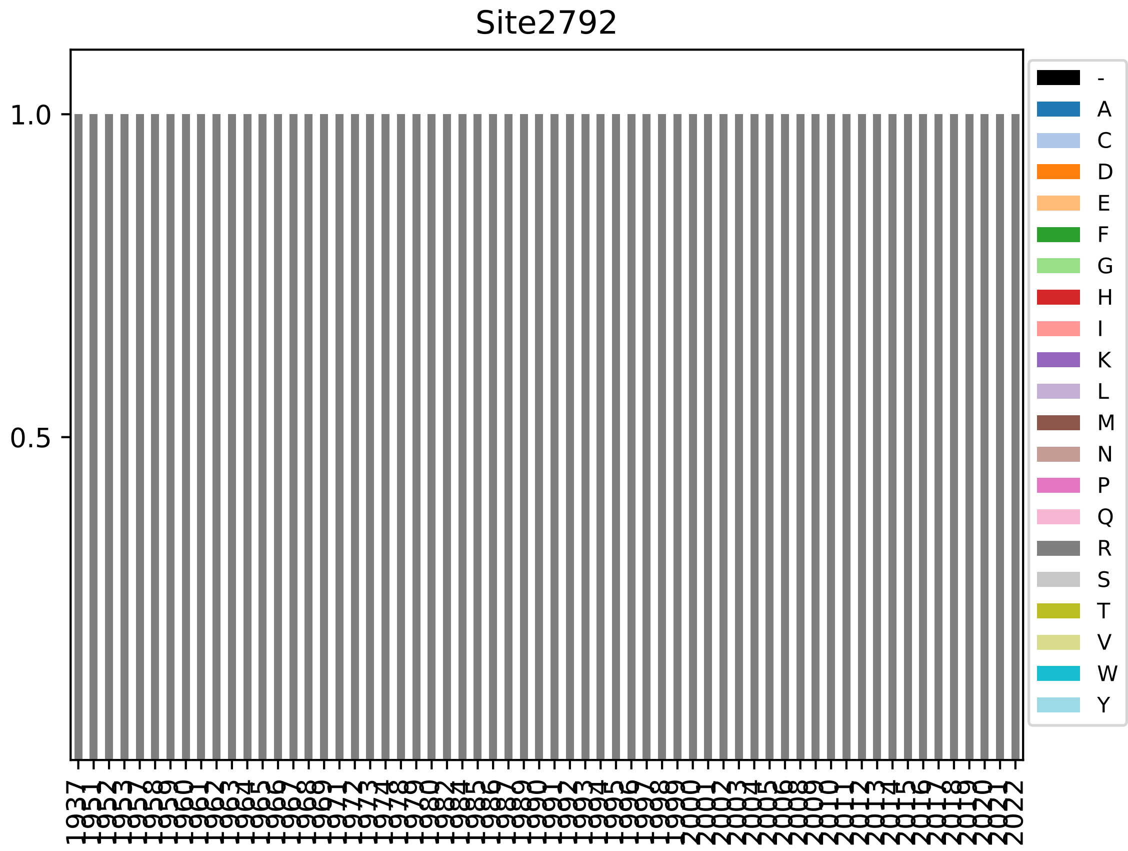

Site2792
1.0
0.5
-
A
C
D
E
F
G
H
I
K
L
M
N
P
Q
R
S
T
V
W
Y

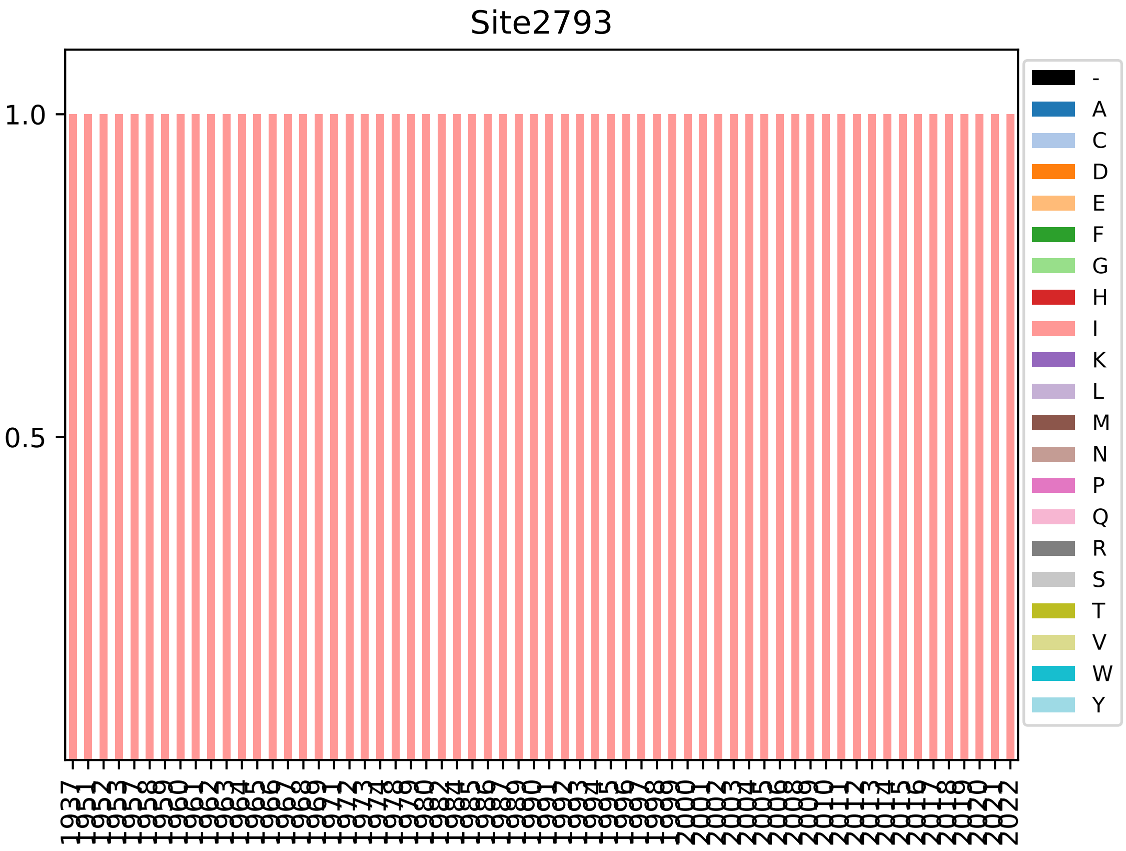
Site2793
1.0
0.5
-
A
C
D
E
F
G
H
I
K
L
M
N
P
Q
R
S
T
V
W
Y

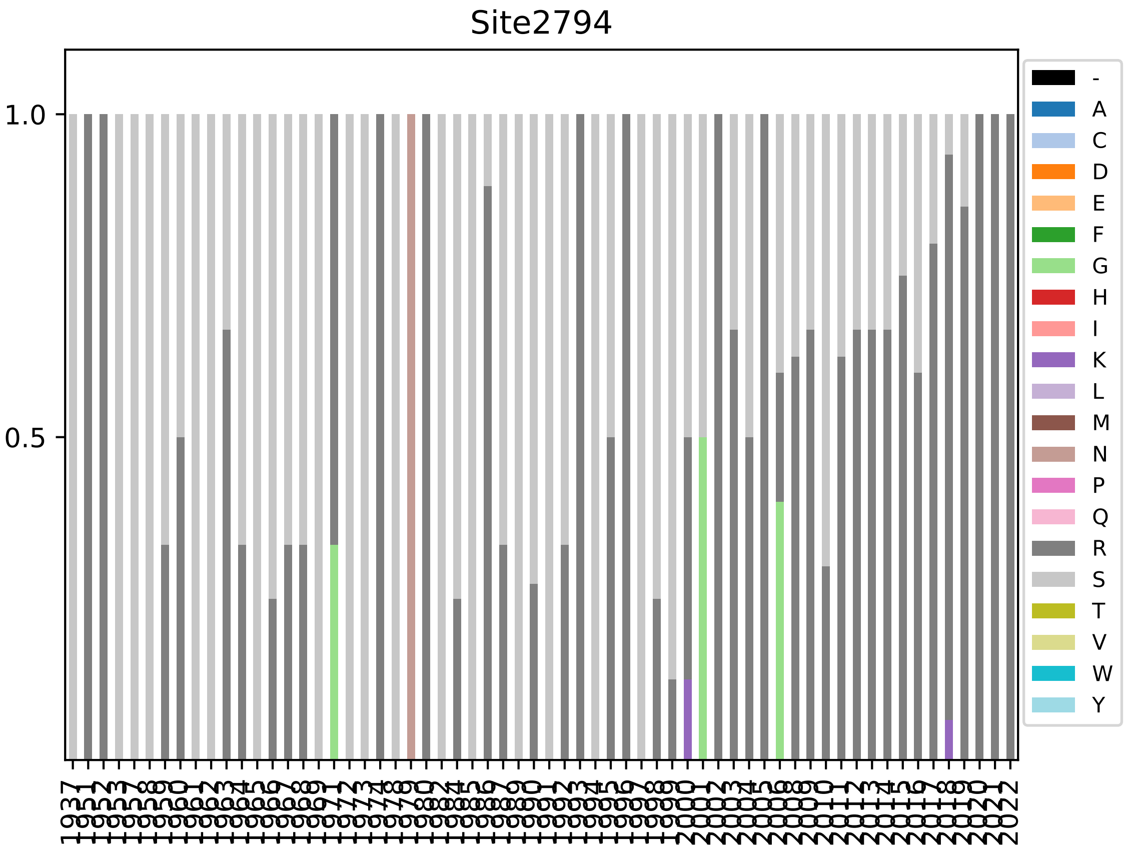

Site2794
1.0
0.5
-
A
C
D
E
F
G
H
I
K
L
M
N
P
Q
R
S
T
V
W
Y

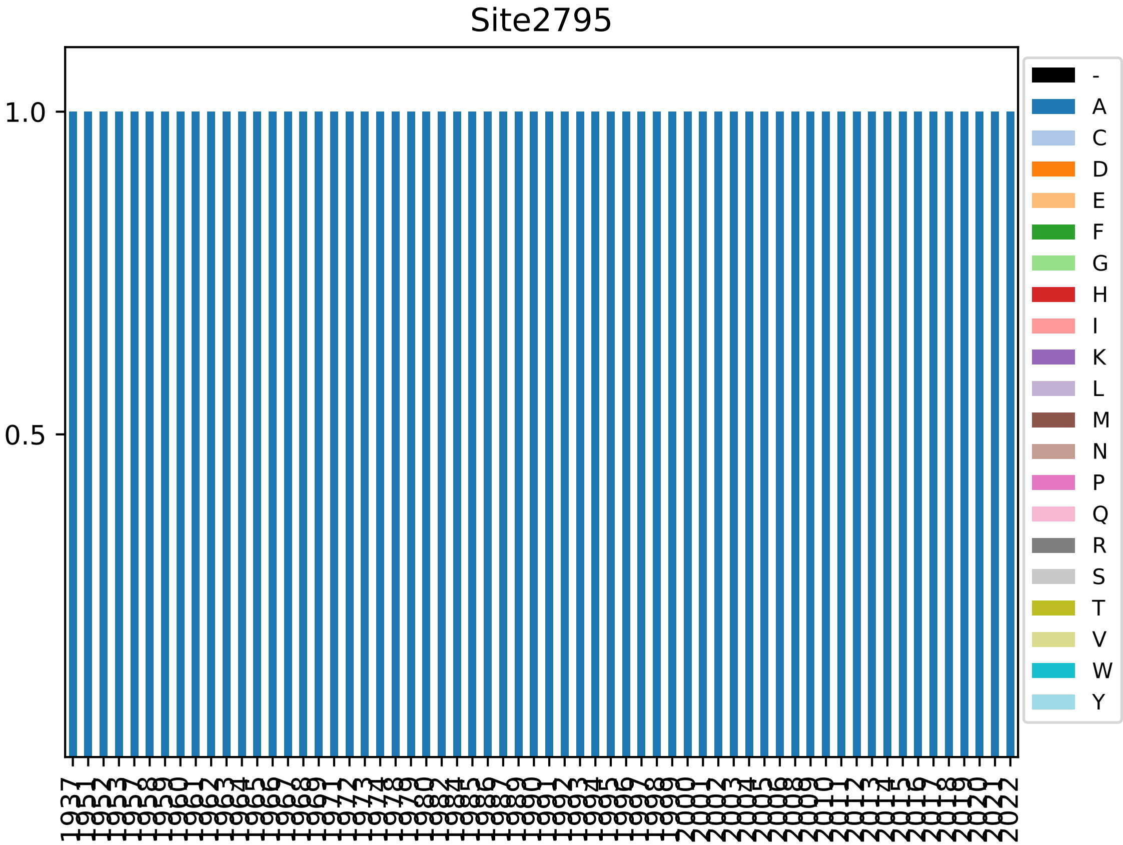
Site2795
1.0
0.5
-
A
C
D
E
F
G
H
I
K
L
M
N
P
Q
R
S
T
V
W
Y

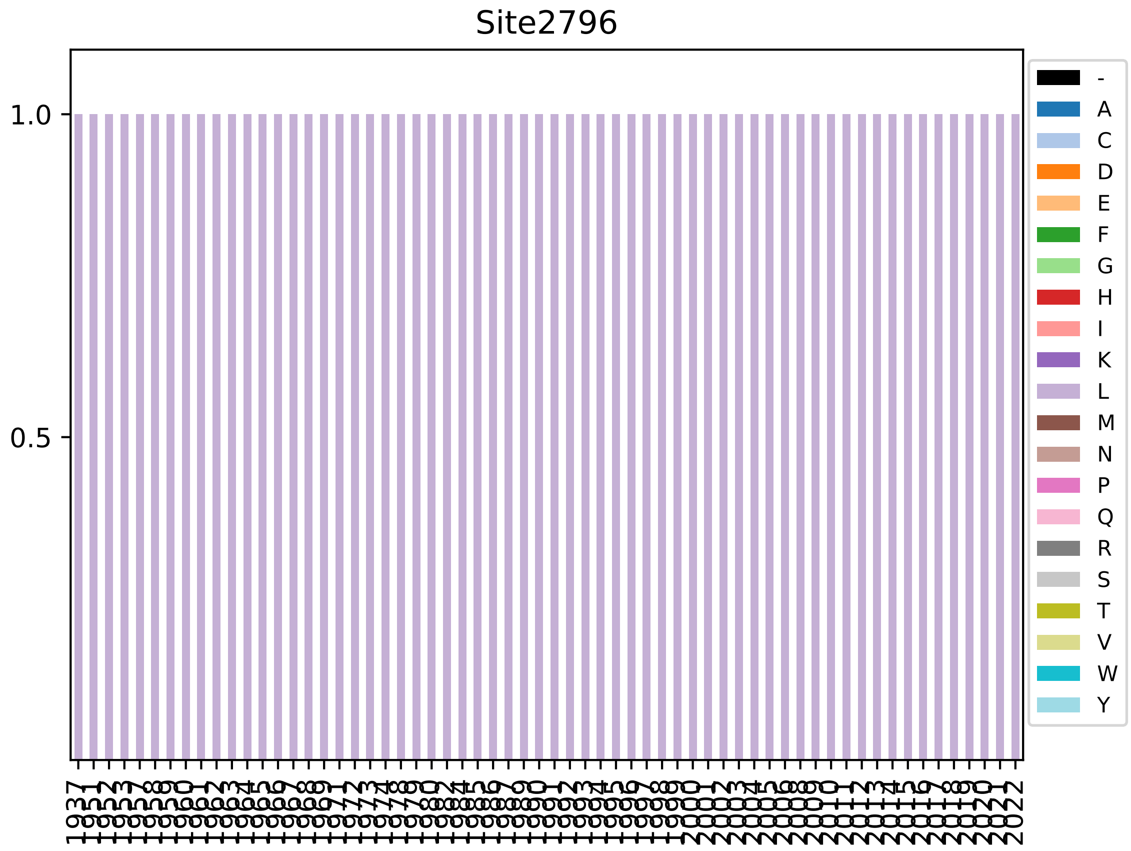
Site2796
1.0
0.5
-
A
C
D
E
F
G
H
I
K
L
M
N
P
Q
R
S
T
V
W
Y

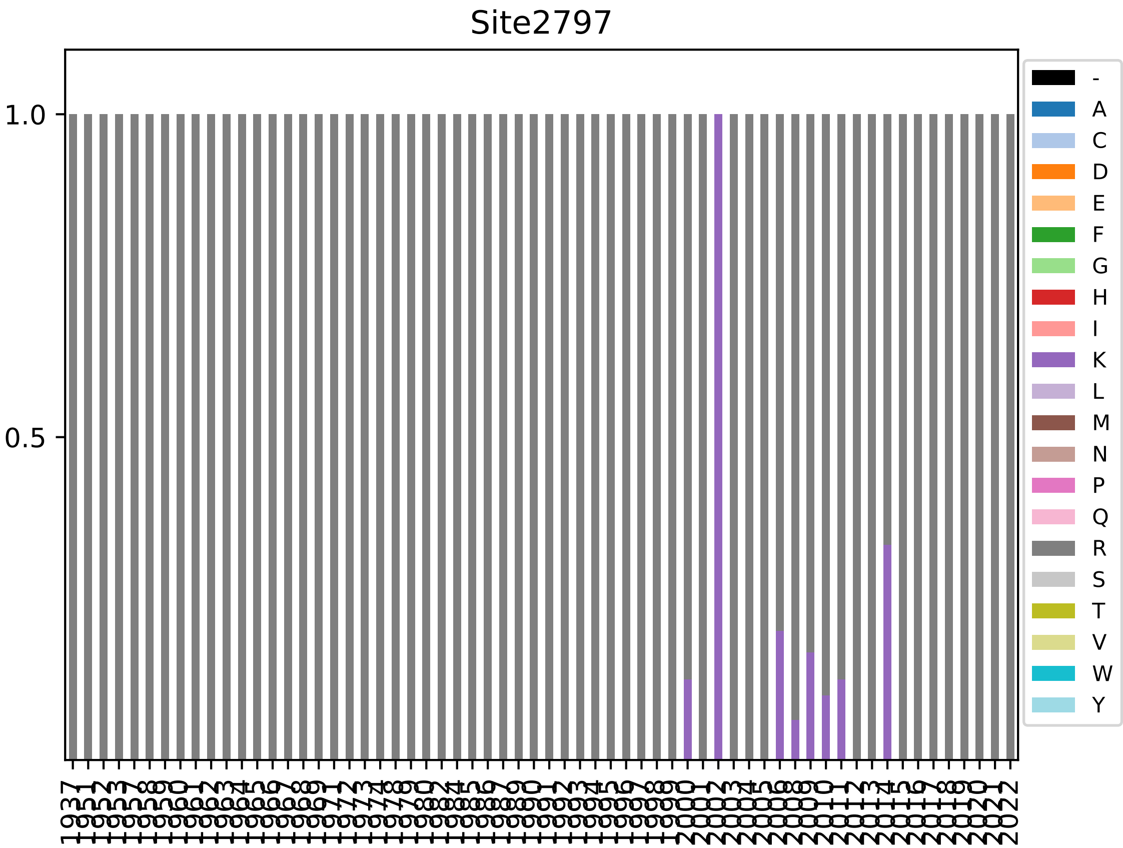
Site2797
1.0
0.5
-
A
C
D
E
F
G
H
I
K
L
M
N
P
Q
R
S
T
V
W
Y

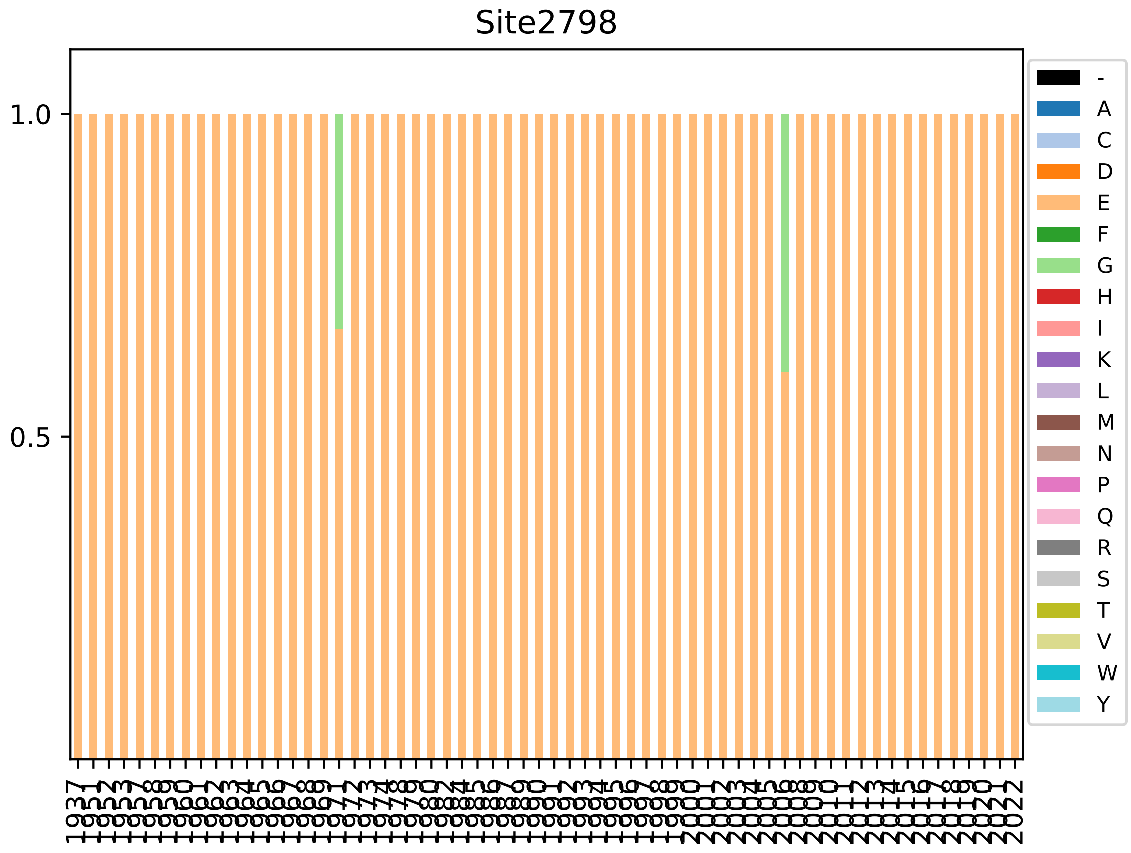
Site2798
1.0
0.5
-
A
C
D
E
F
G
H
I
K
L
M
N
P
Q
R
S
T
V
W
Y

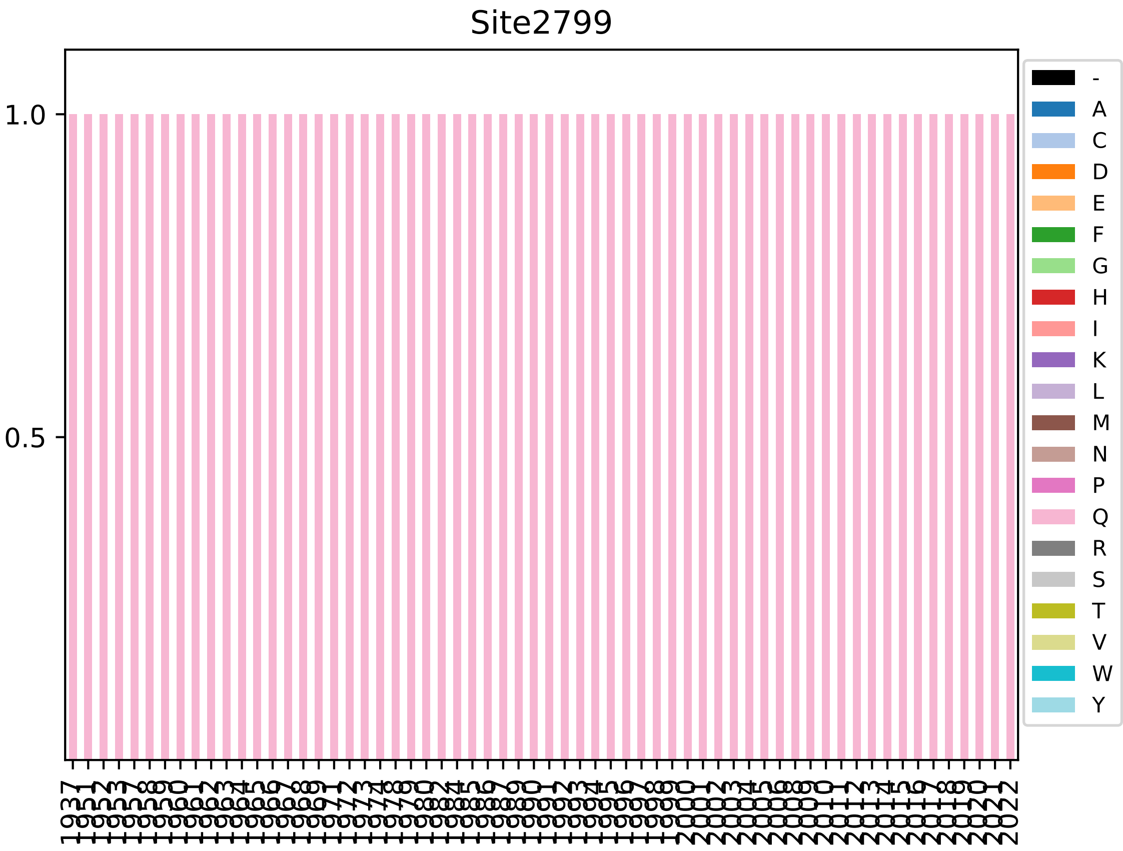
Site2799
1.0
0.5
-
A
C
D
E
F
G
H
I
K
L
M
N
P
Q
R
S
T
V
W
Y

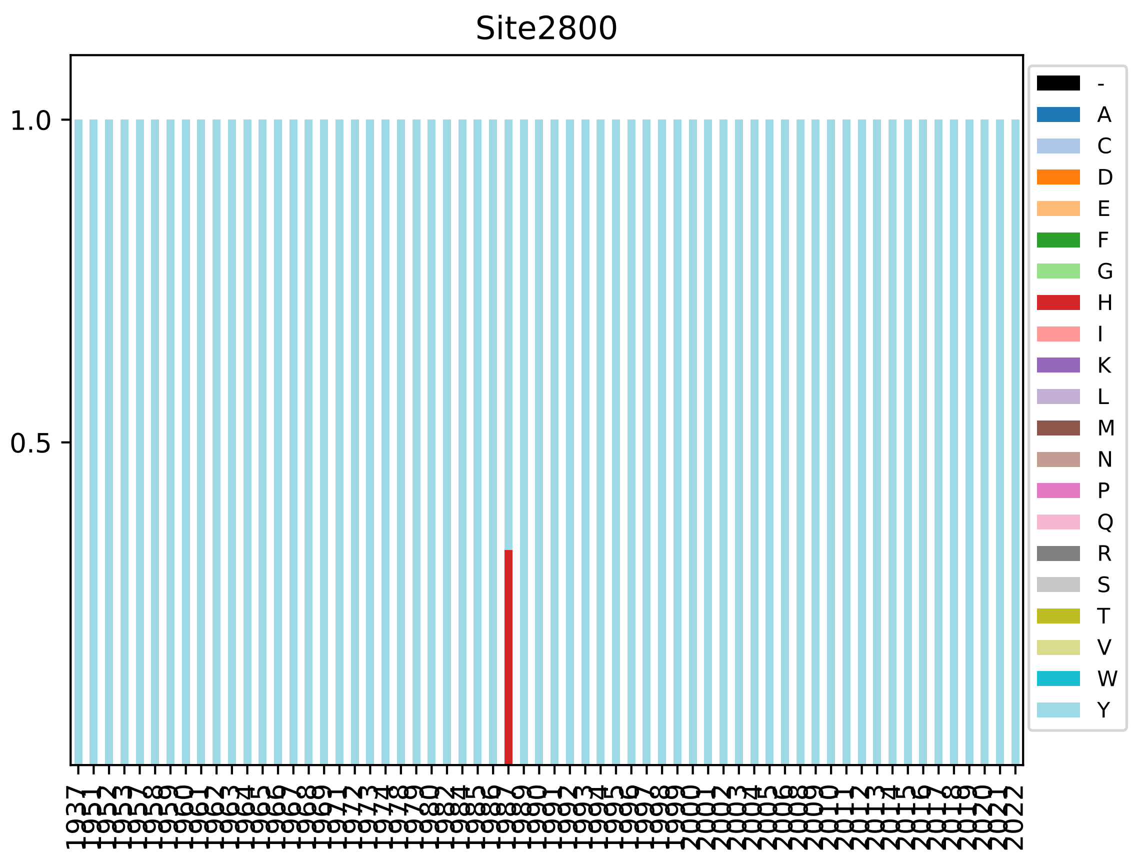
Site2800
1.0
0.5
-
A
C
D
E
F
G
H
I
K
L
M
N
P
Q
R
S
T
V
W
Y

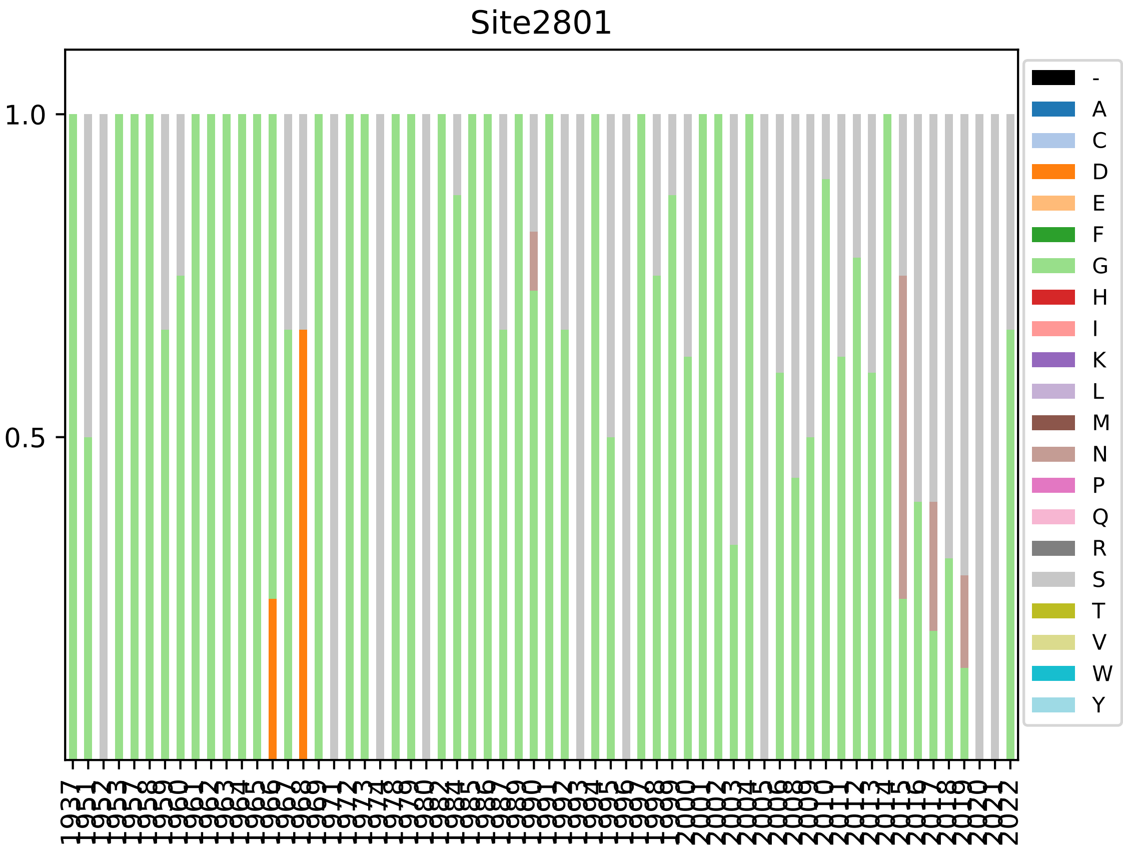
Site2801
1.0
0.5
-
A
C
D
E
F
G
H
I
K
L
M
N
P
Q
R
S
T
V
W
Y

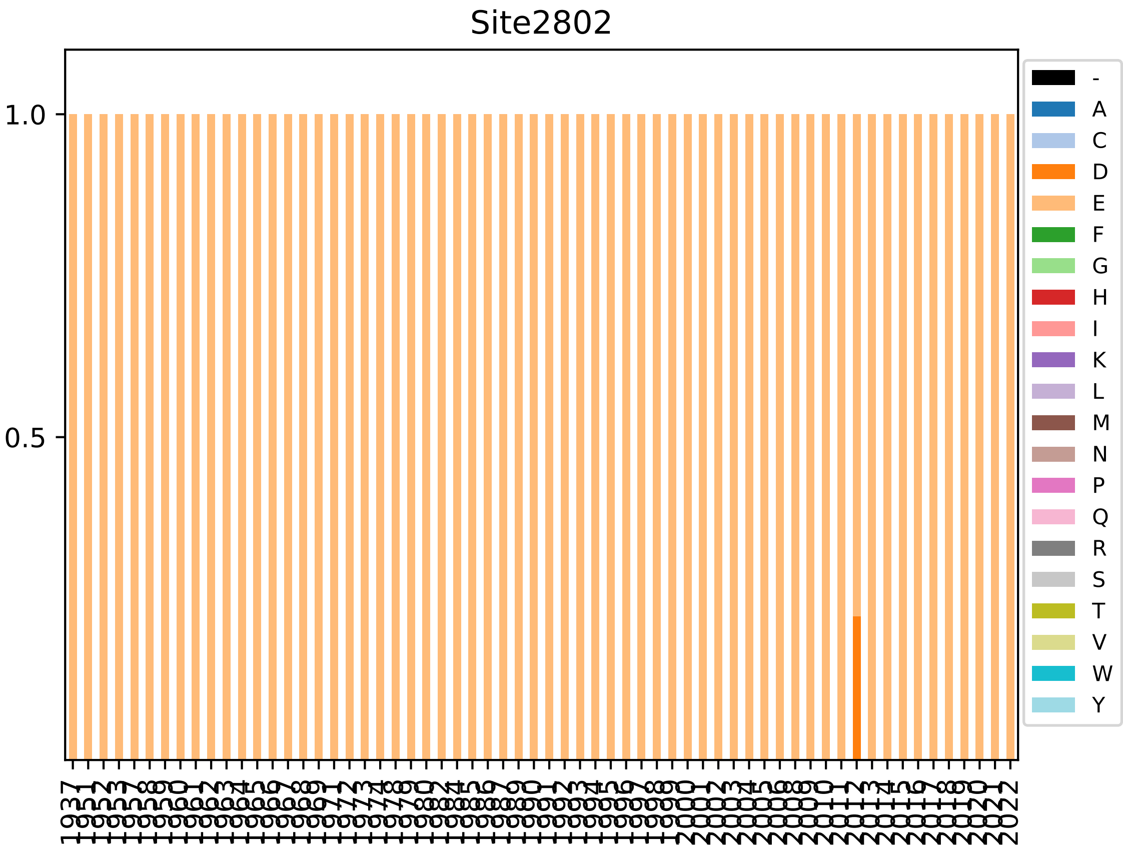
Site2802
1.0
0.5
-
A
C
D
E
F
G
H
I
K
L
M
N
P
Q
R
S
T
V
W
Y

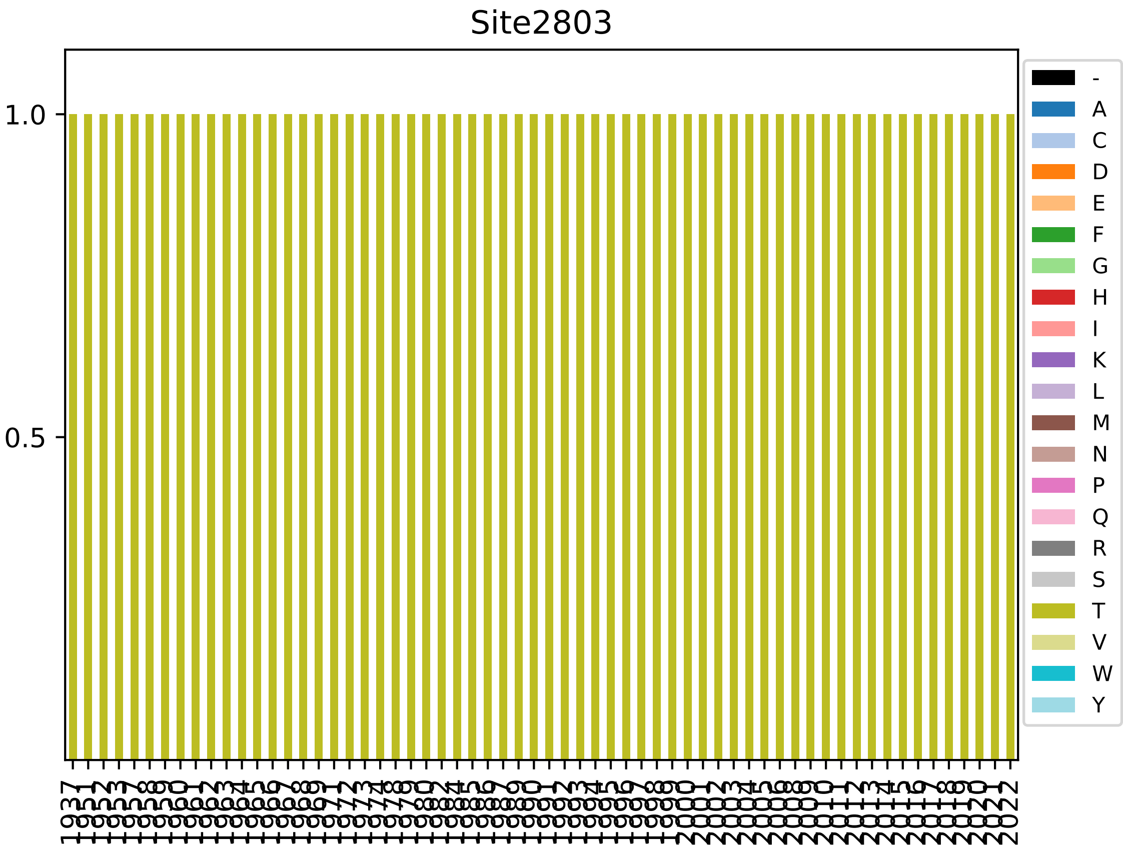
Site2803
1.0
0.5
-
A
C
D
E
F
G
H
I
K
L
M
N
P
Q
R
S
T
V
W
Y

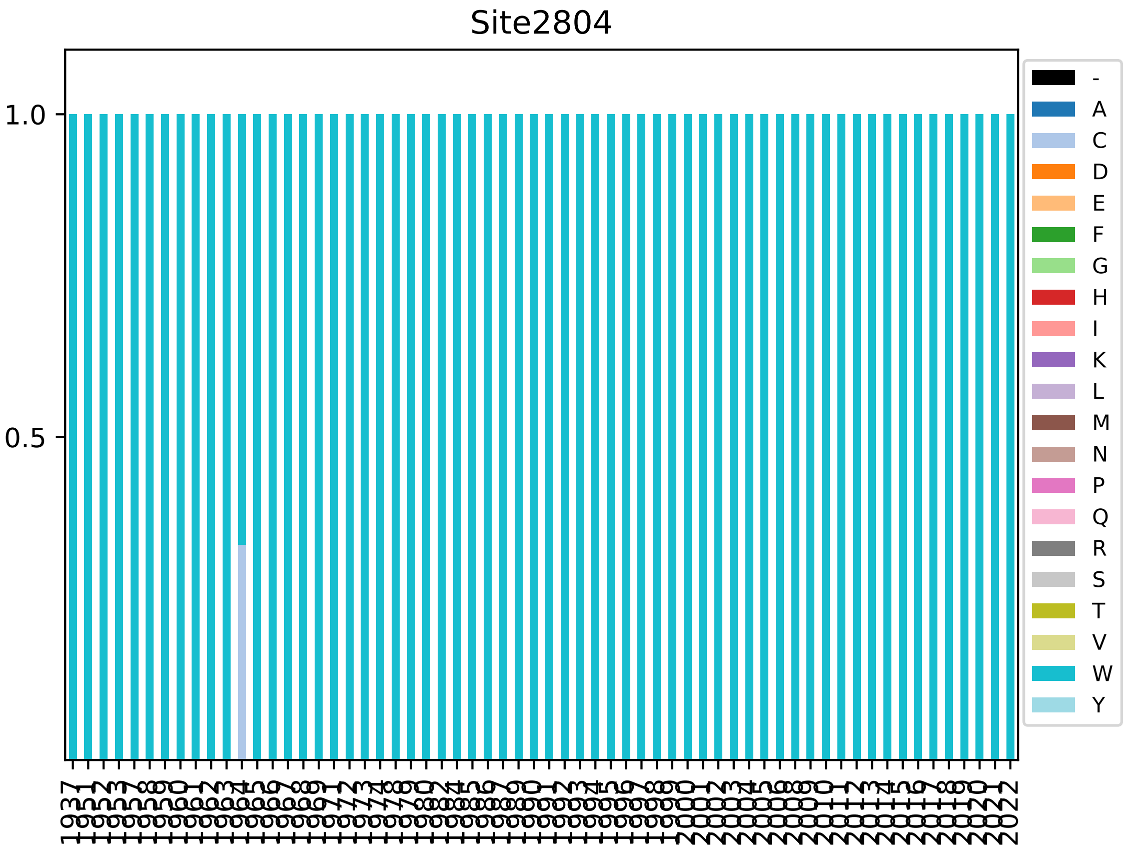

Site2804
1.0
0.5
-
A
C
D
E
F
G
H
I
K
L
M
N
P
Q
R
S
T
V
W
Y

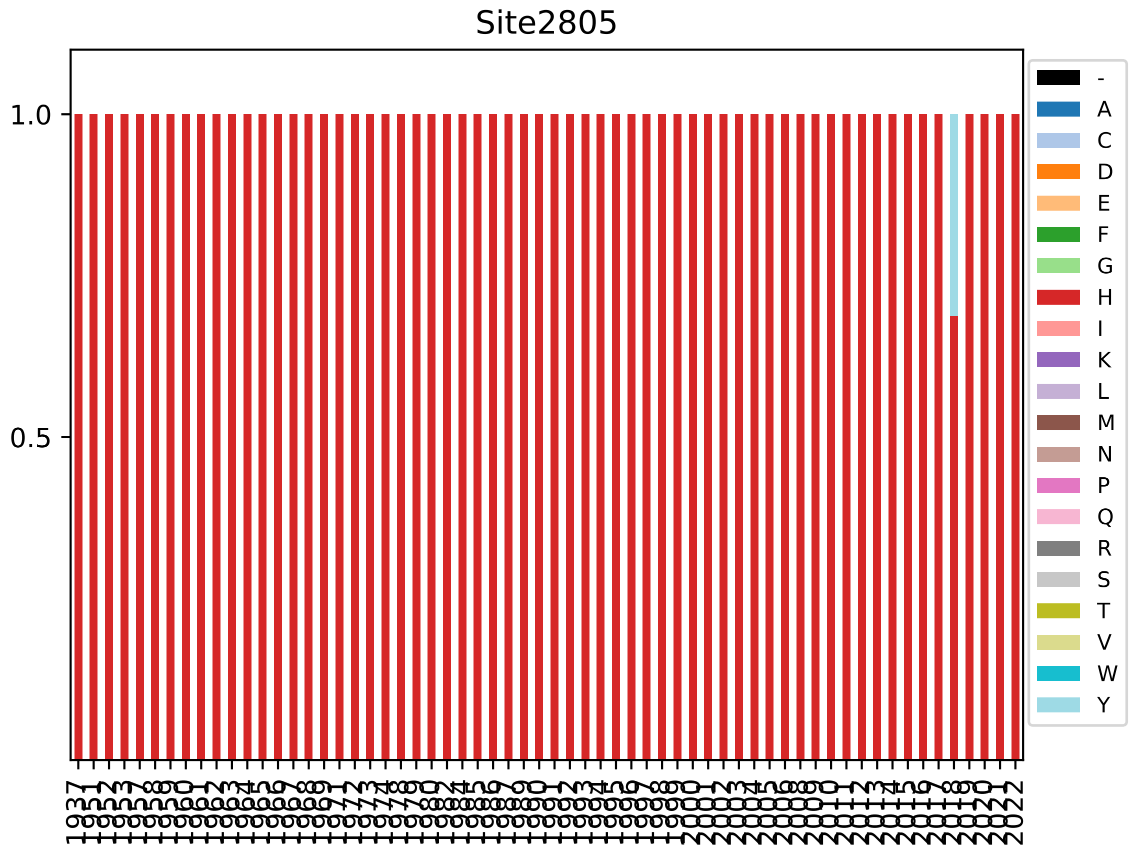
Site2805
1.0
0.5
-
A
C
D
E
F
G
H
I
K
L
M
N
P
Q
R
S
T
V
W
Y

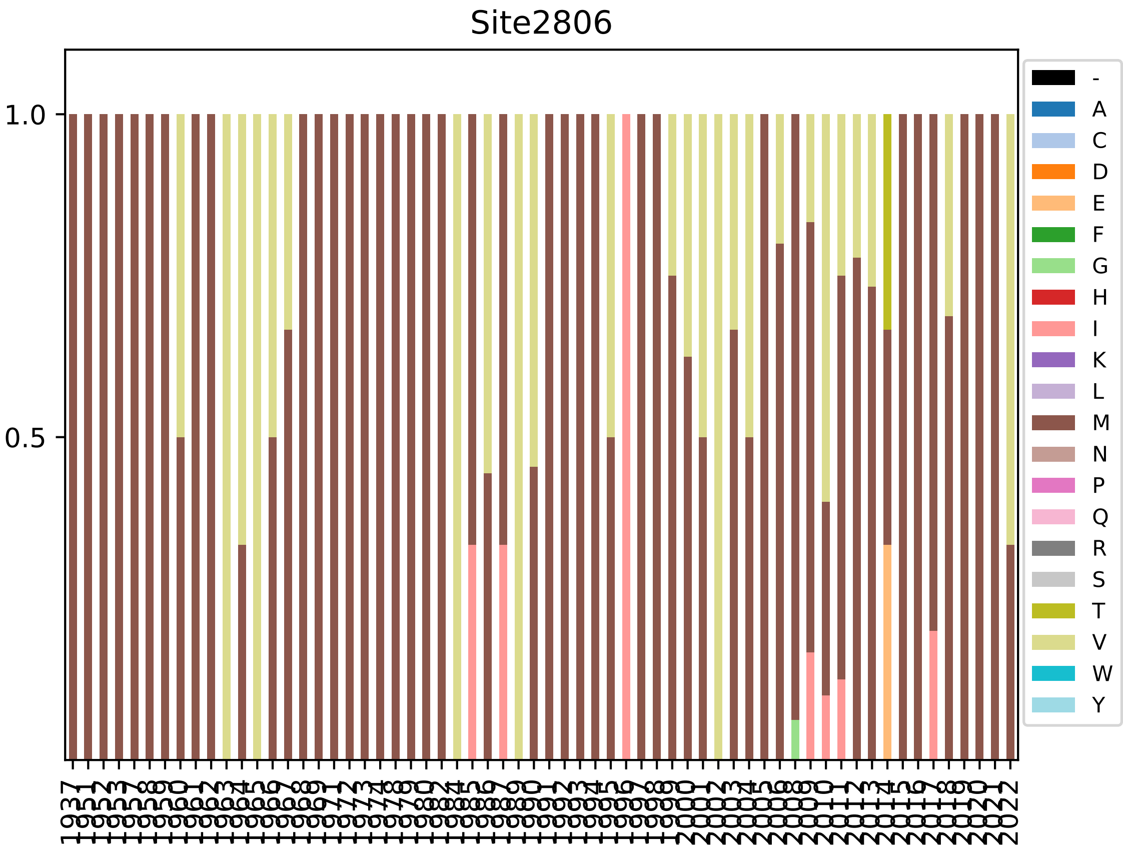

Site2806
1.0
0.5
-
A
C
D
E
F
G
H
I
K
L
M
N
P
Q
R
S
T
V
W
Y

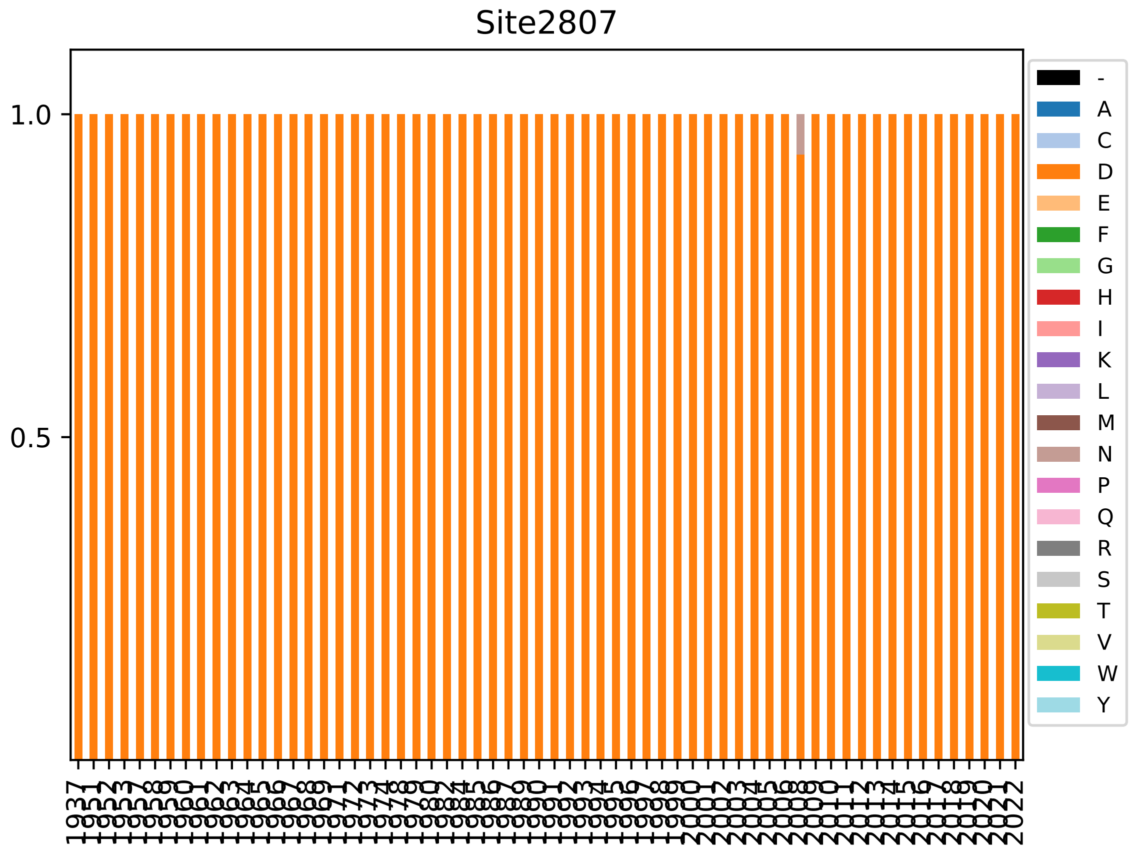
Site2807
1.0
0.5
-
A
C
D
E
F
G
H
I
K
L
M
N
P
Q
R
S
T
V
W
Y

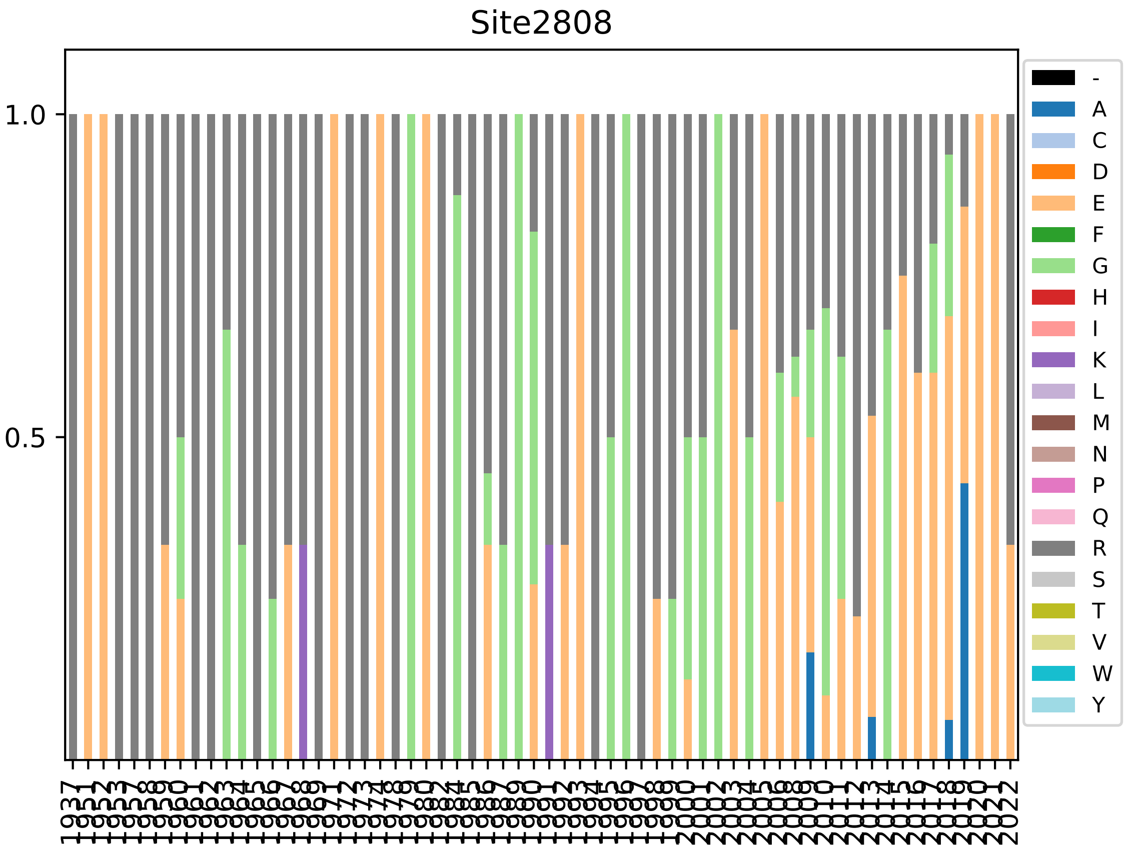
Site2808
1.0
0.5
-
A
C
D
E
F
G
H
I
K
L
M
N
P
Q
R
S
T
V
W
Y

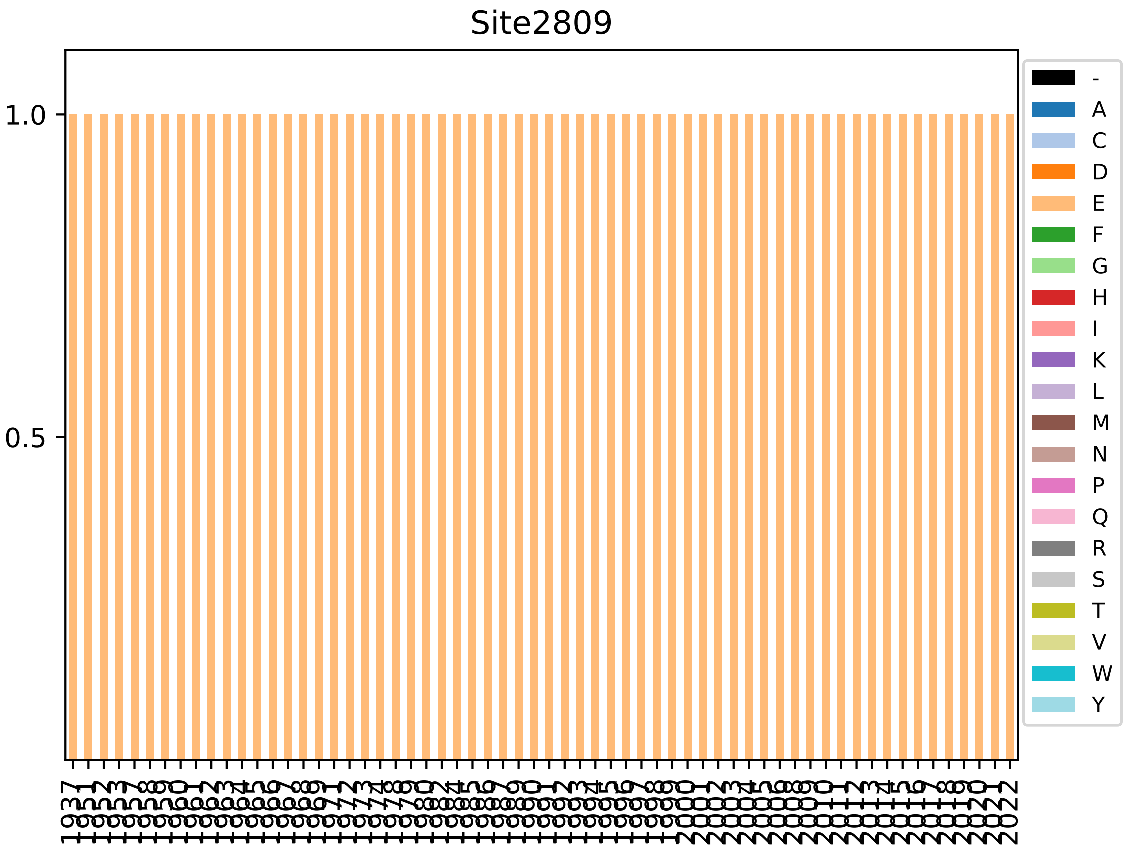
Site2809
1.0
0.5
-
A
C
D
E
F
G
H
I
K
L
M
N
P
Q
R
S
T
V
W
Y

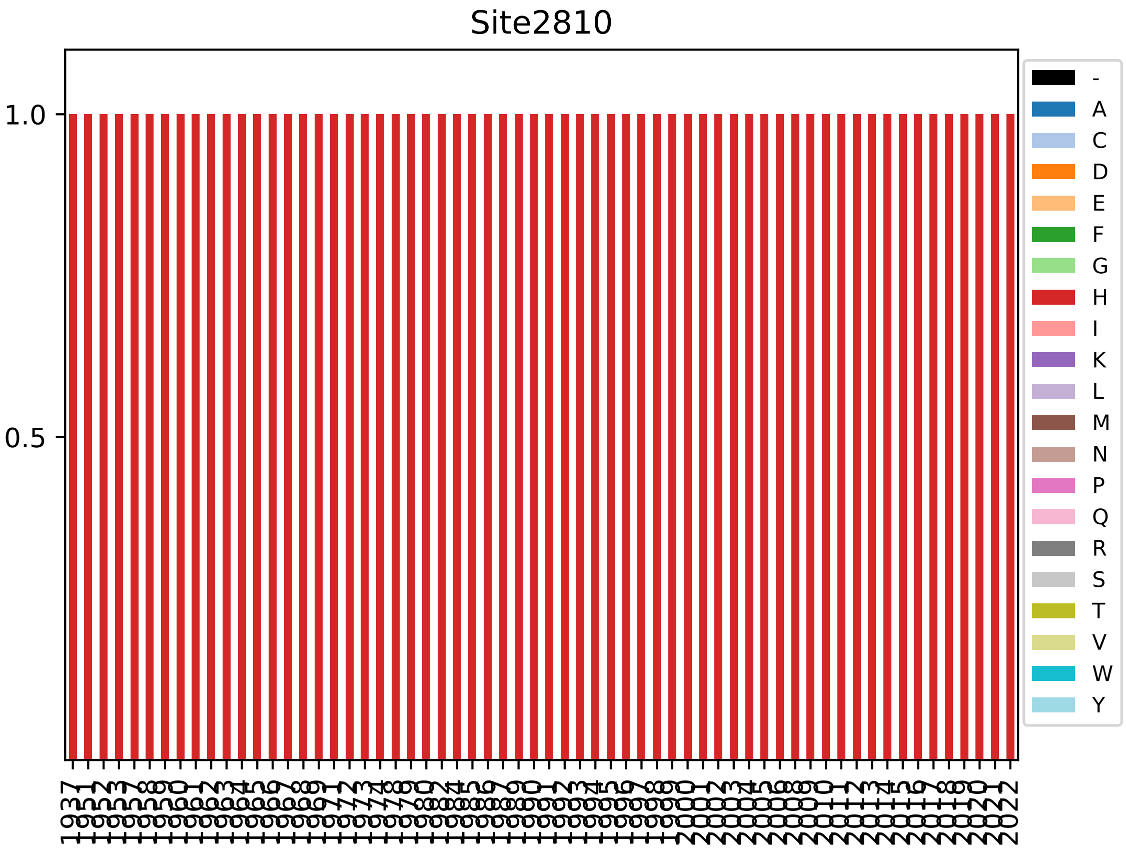
Site2810
1.0
0.5
-
A
C
D
E
F
G
H
I
K
L
M
N
P
Q
R
S
T
V
W
Y

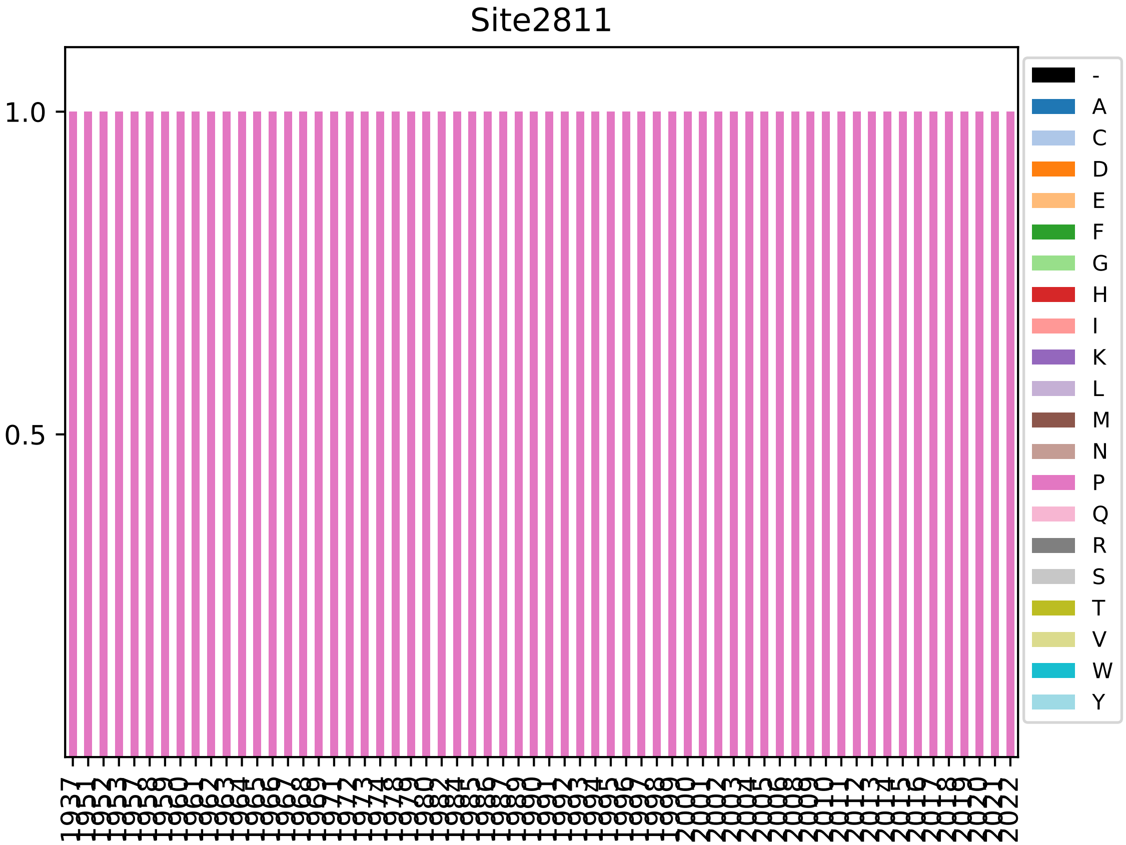
Site2811
1.0
0.5
-
A
C
D
E
F
G
H
I
K
L
M
N
P
Q
R
S
T
V
W
Y

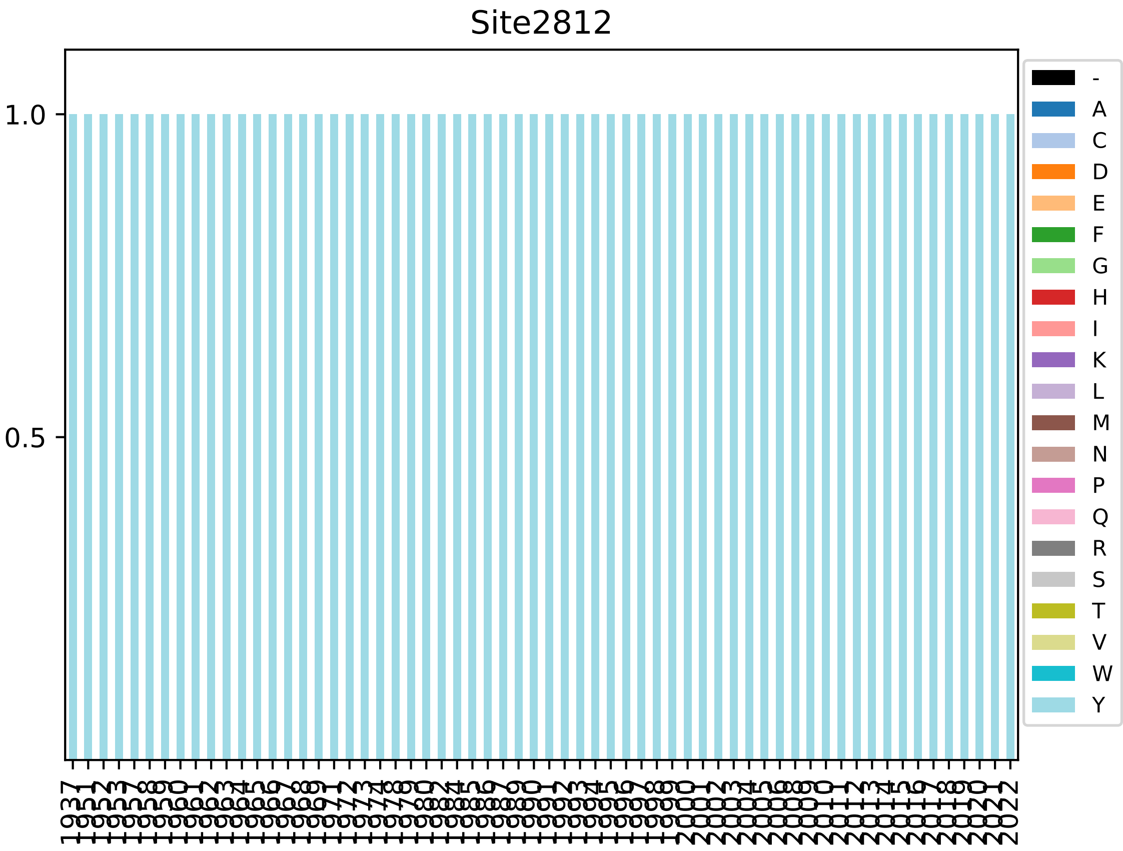

Site2812
1.0
0.5
-
A
C
D
E
F
G
H
I
K
L
M
N
P
Q
R
S
T
V
W
Y

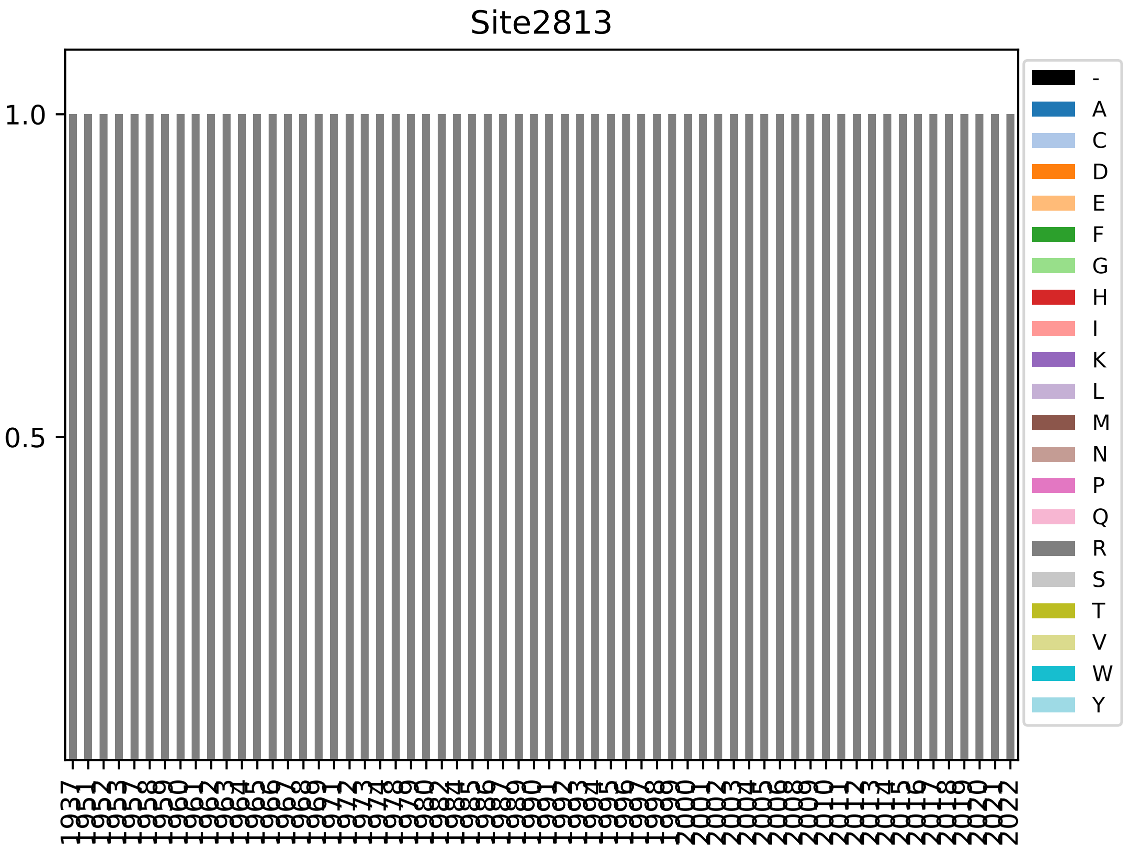
Site2813
1.0
0.5
-
A
C
D
E
F
G
H
I
K
L
M
N
P
Q
R
S
T
V
W
Y

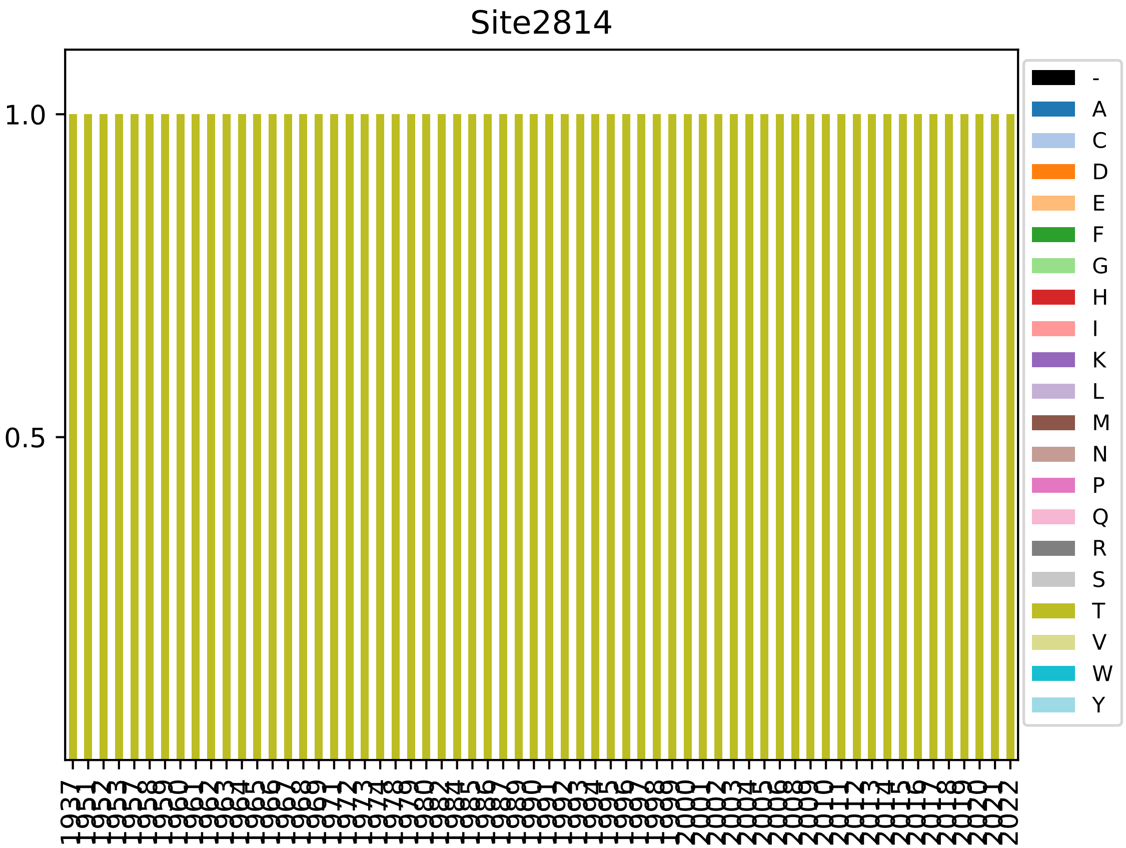
Site2814
1.0
0.5
-
A
C
D
E
F
G
H
I
K
L
M
N
P
Q
R
S
T
V
W
Y

Site2815

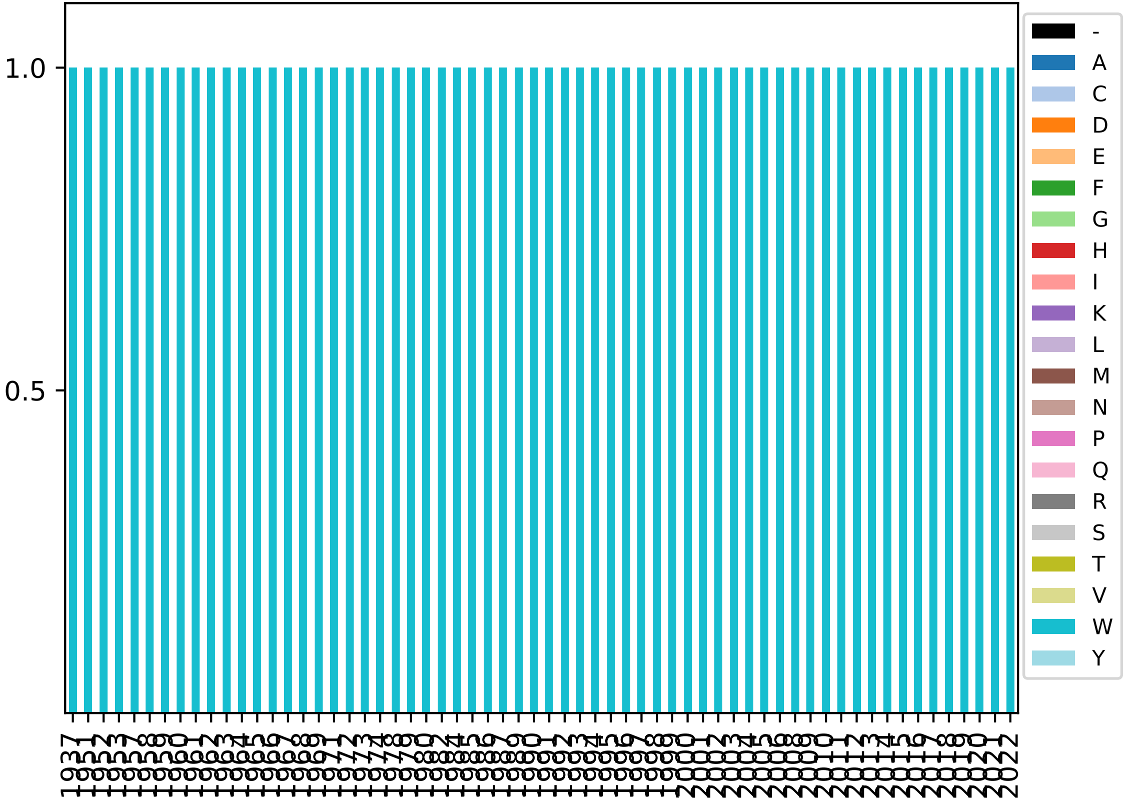

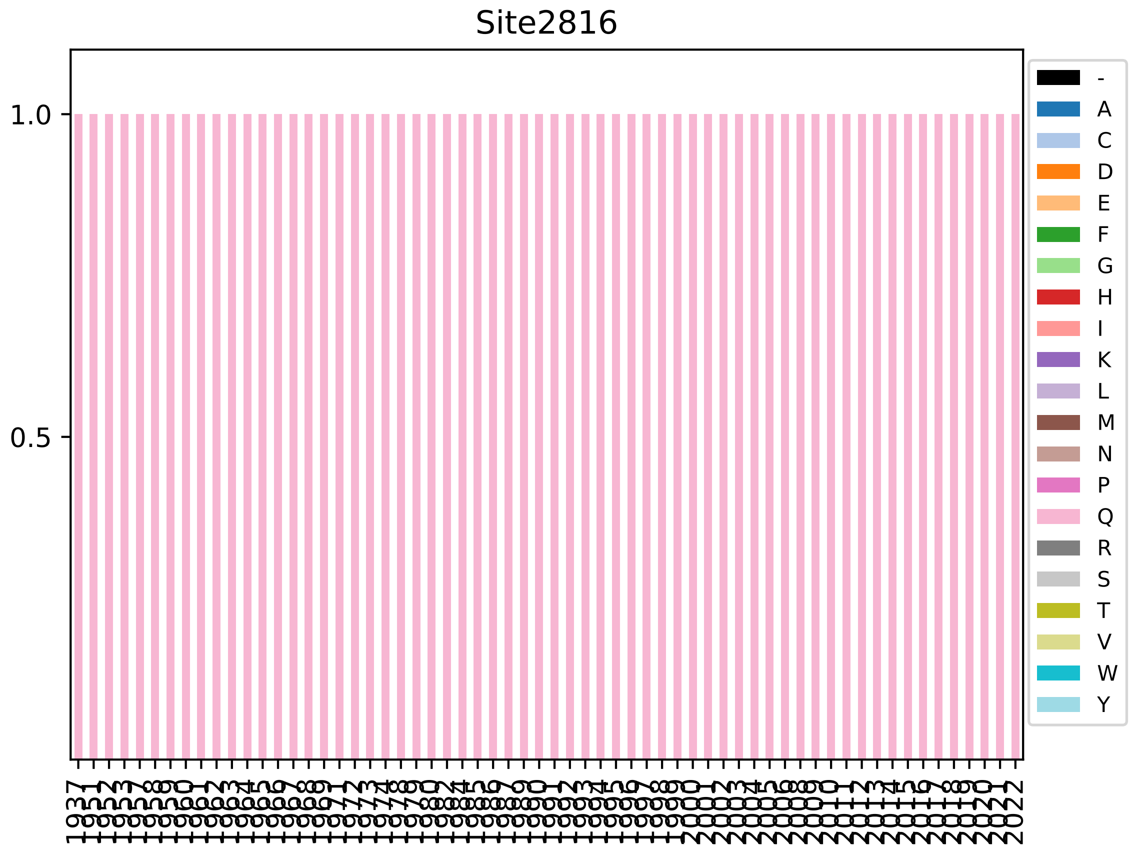

Site2816
1.0
0.5
-
A
C
D
E
F
G
H
I
K
L
M
N
P
Q
R
S
T
V
W
Y

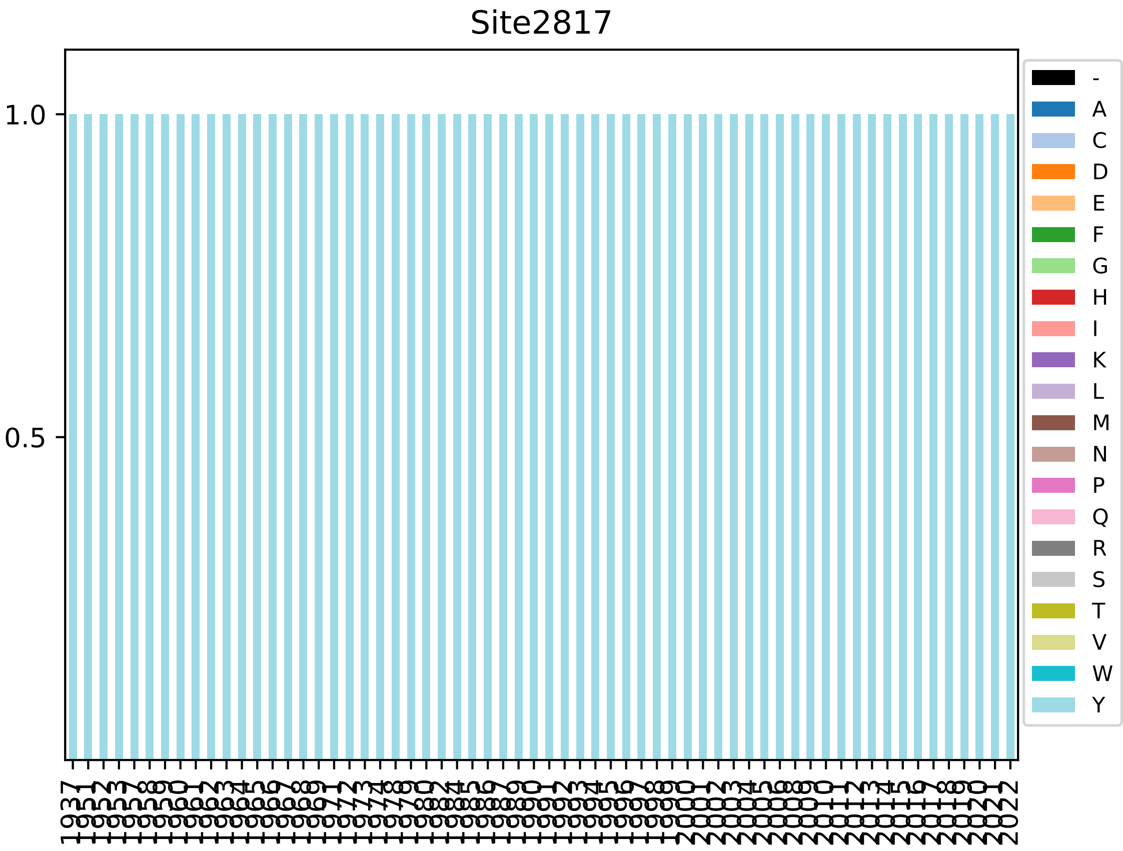
Site2817
1.0
0.5
-
A
C
D
E
F
G
H
I
K
L
M
N
P
Q
R
S
T
V
W
Y

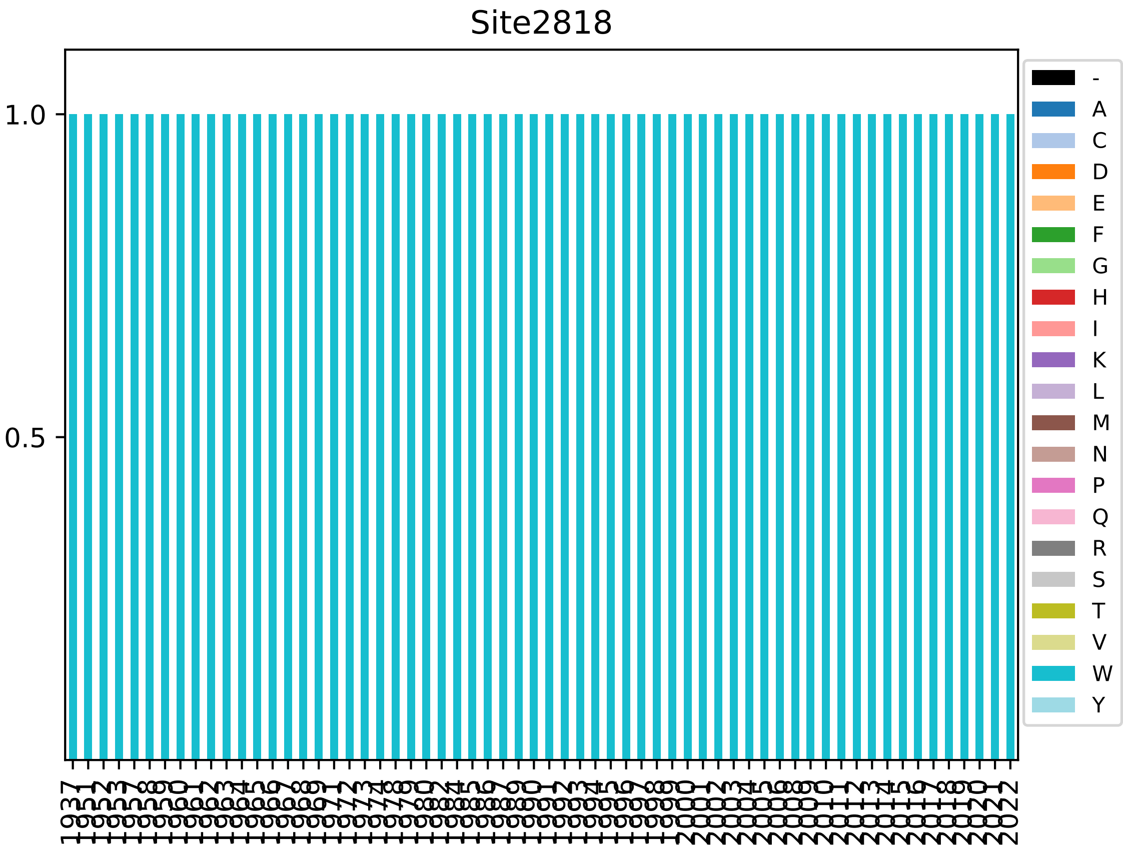
Site2818
1.0
0.5
-
A
C
D
E
F
G
H
I
K
L
M
N
P
Q
R
S
T
V
W
Y

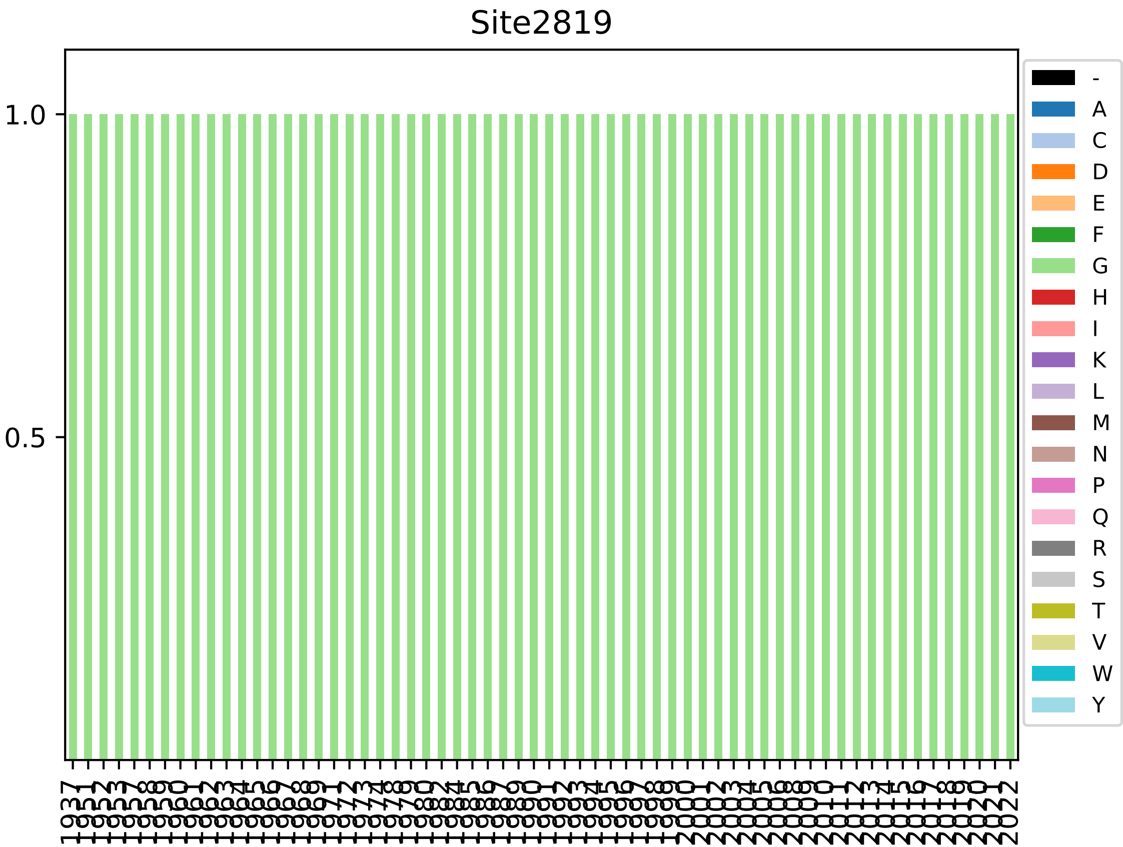
Site2819
1.0
0.5
-
A
C
D
E
F
G
H
I
K
L
M
N
P
Q
R
S
T
V
W
Y

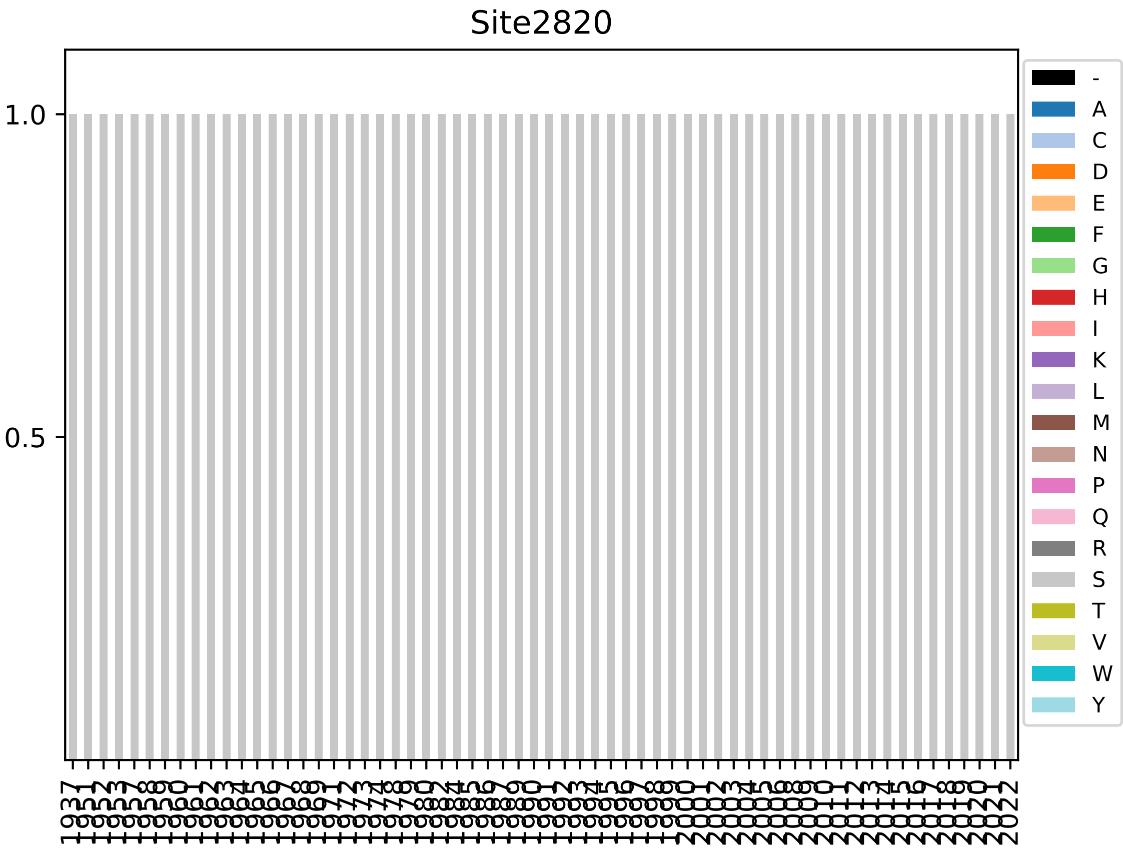
Site2820
1.0
0.5
-
A
C
D
E
F
G
H
I
K
L
M
N
P
Q
R
S
T
V
W
Y

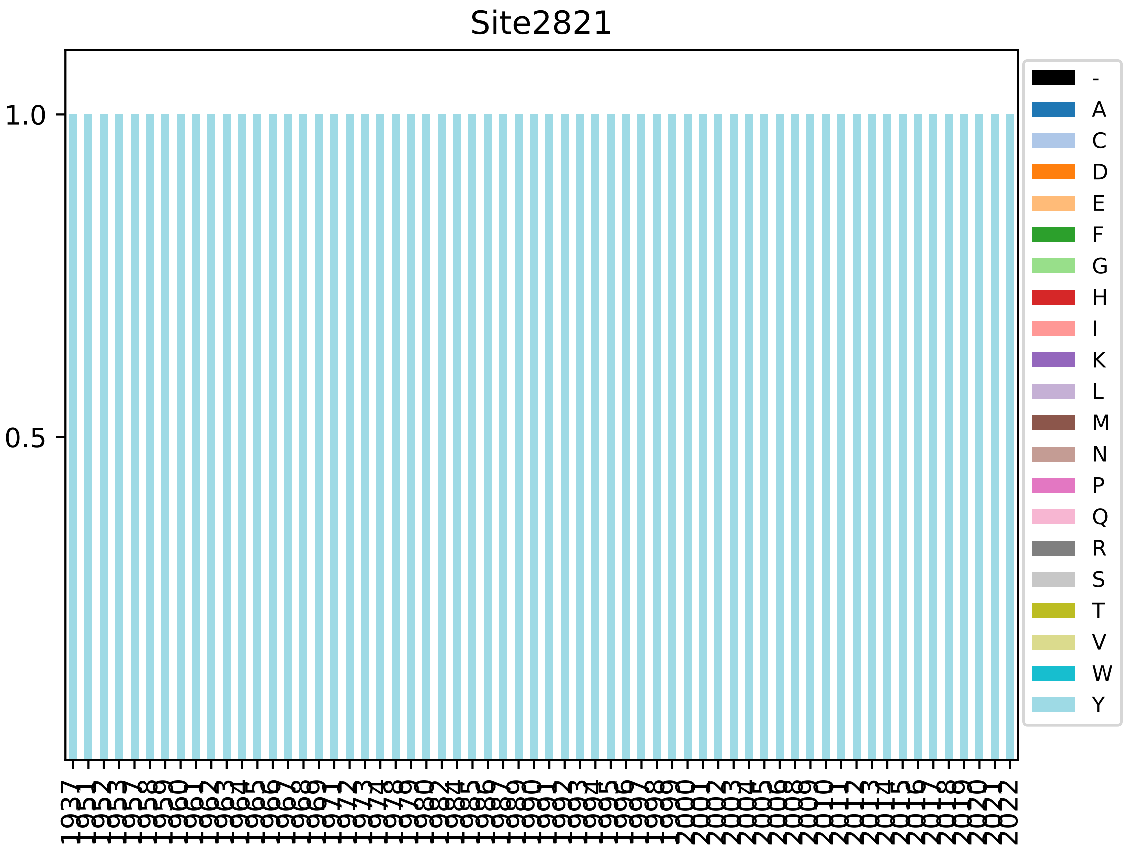
Site2821
1.0
0.5
-
A
C
D
E
F
G
H
I
K
L
M
N
P
Q
R
S
T
V
W
Y

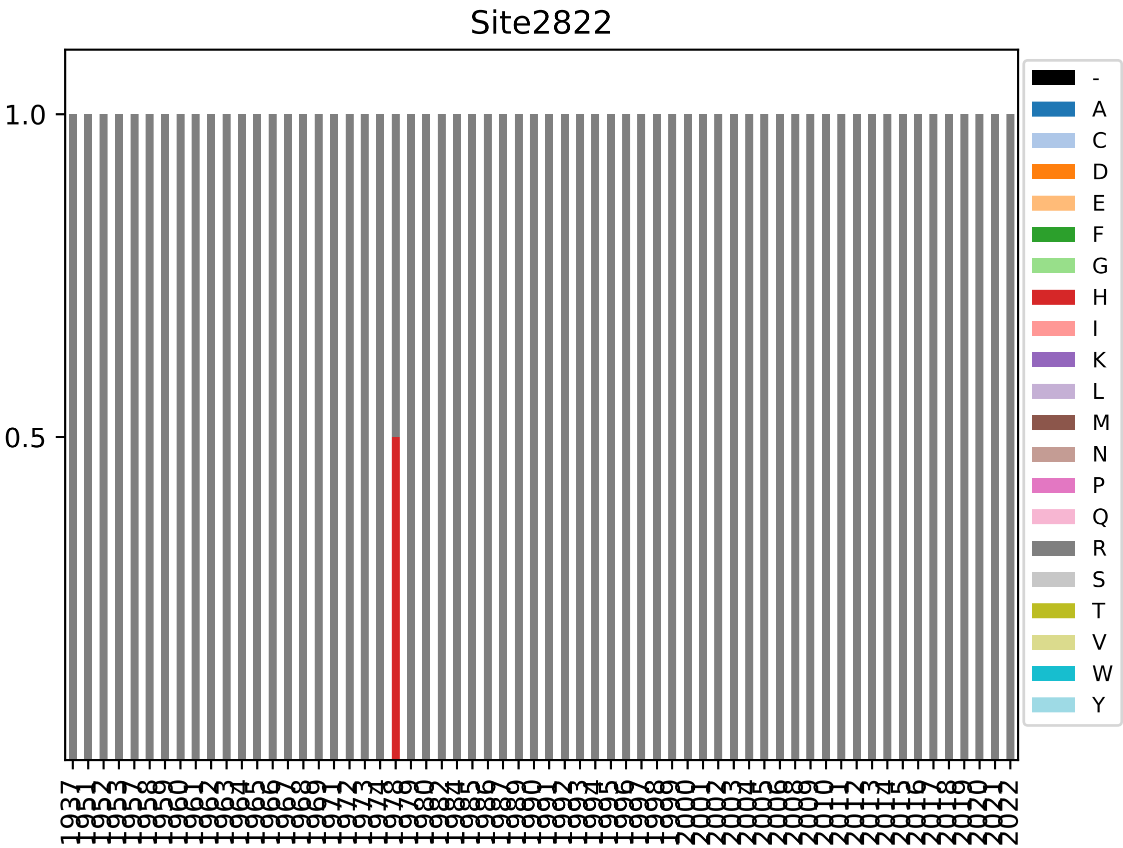
Site2822
1.0
0.5
-
A
C
D
E
F
G
H
I
K
L
M
N
P
Q
R
S
T
V
W
Y

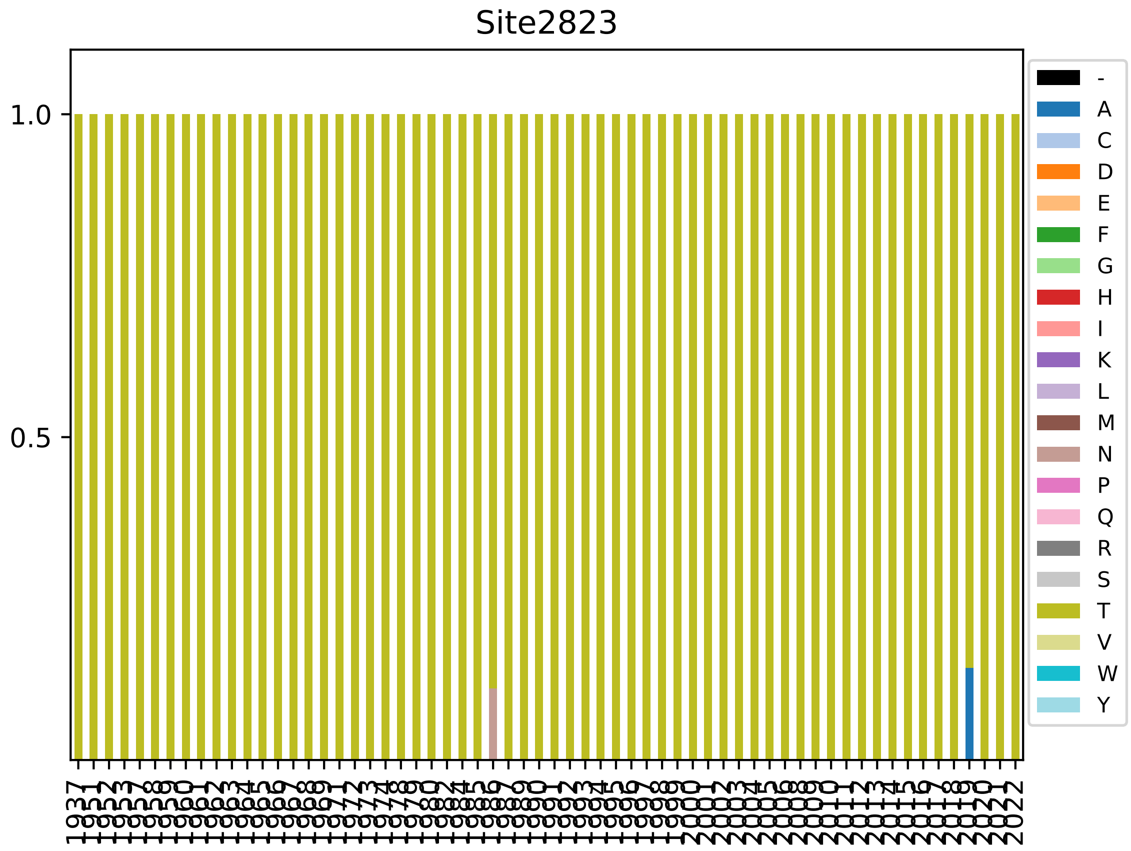
Site2823
1.0
0.5
-
A
C
D
E
F
G
H
I
K
L
M
N
P
Q
R
S
T
V
W
Y

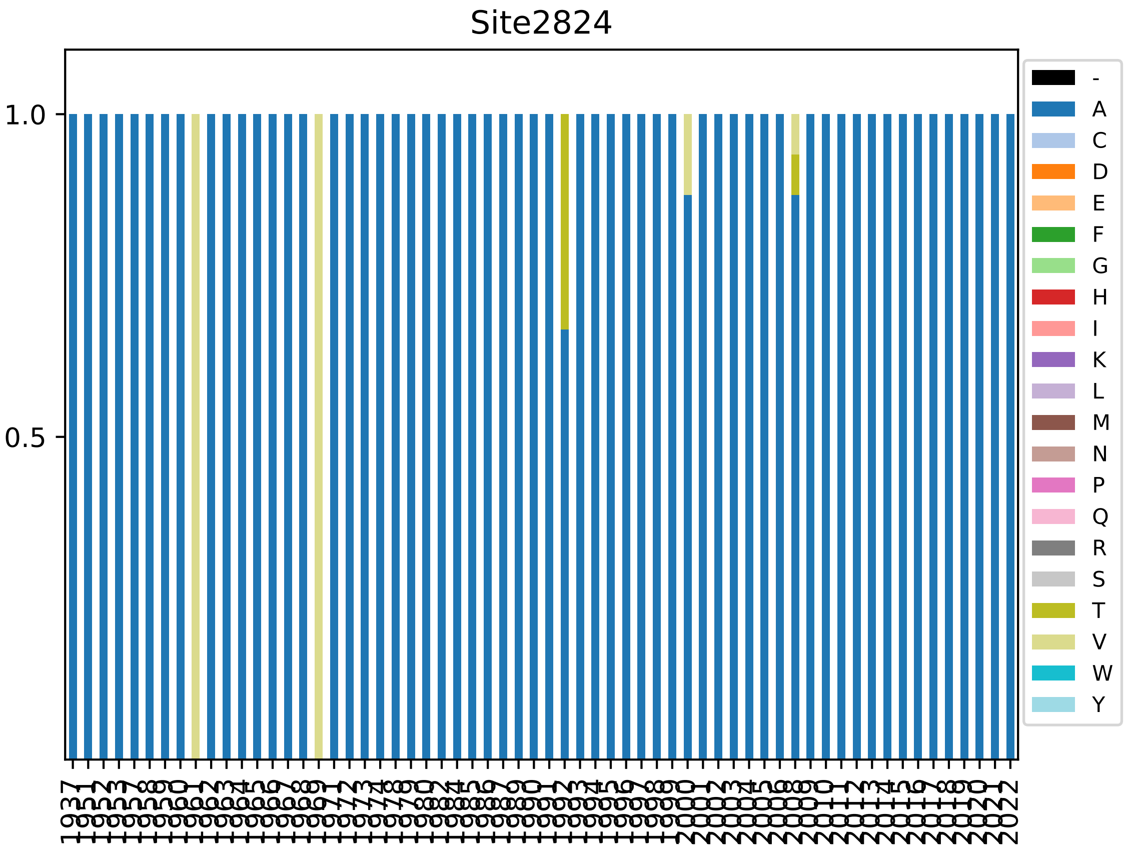
Site2824
1.0
0.5
-
A
C
D
E
F
G
H
I
K
L
M
N
P
Q
R
S
T
V
W
Y

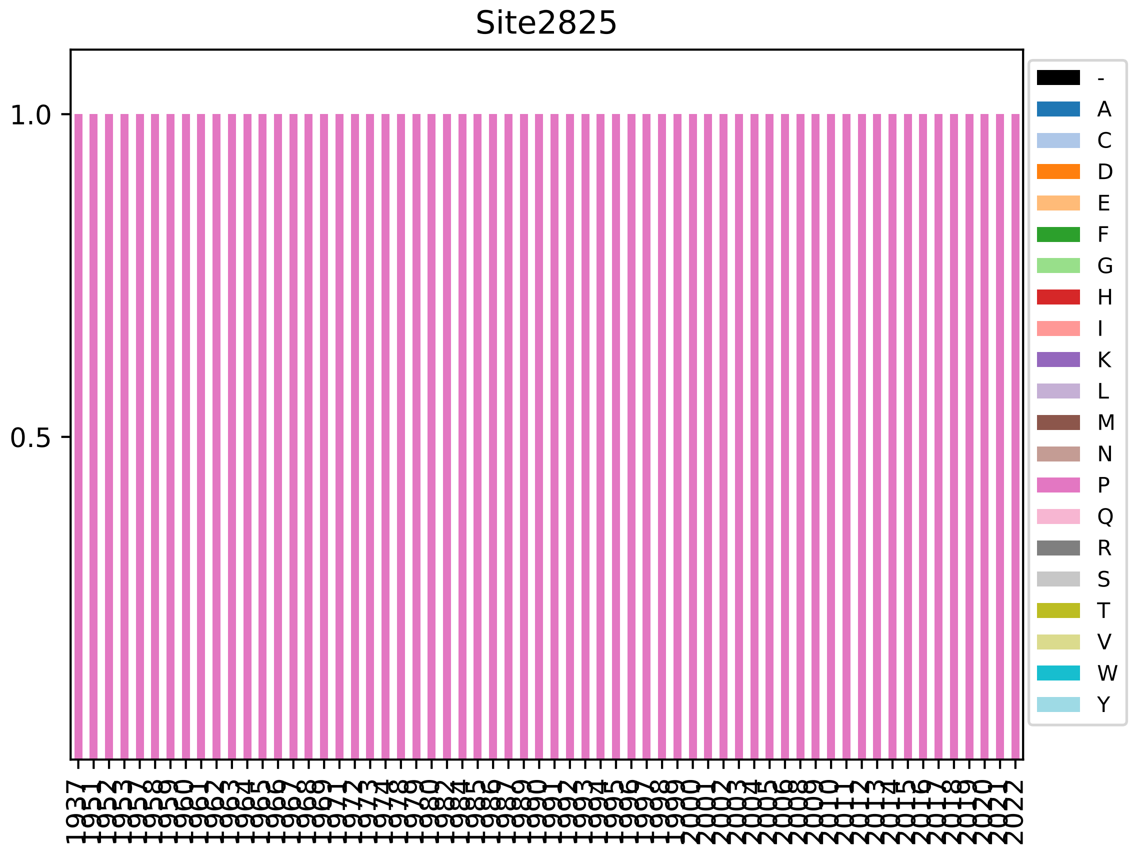

Site2825
1.0
0.5
-
A
C
D
E
F
G
H
I
K
L
M
N
P
Q
R
S
T
V
W
Y

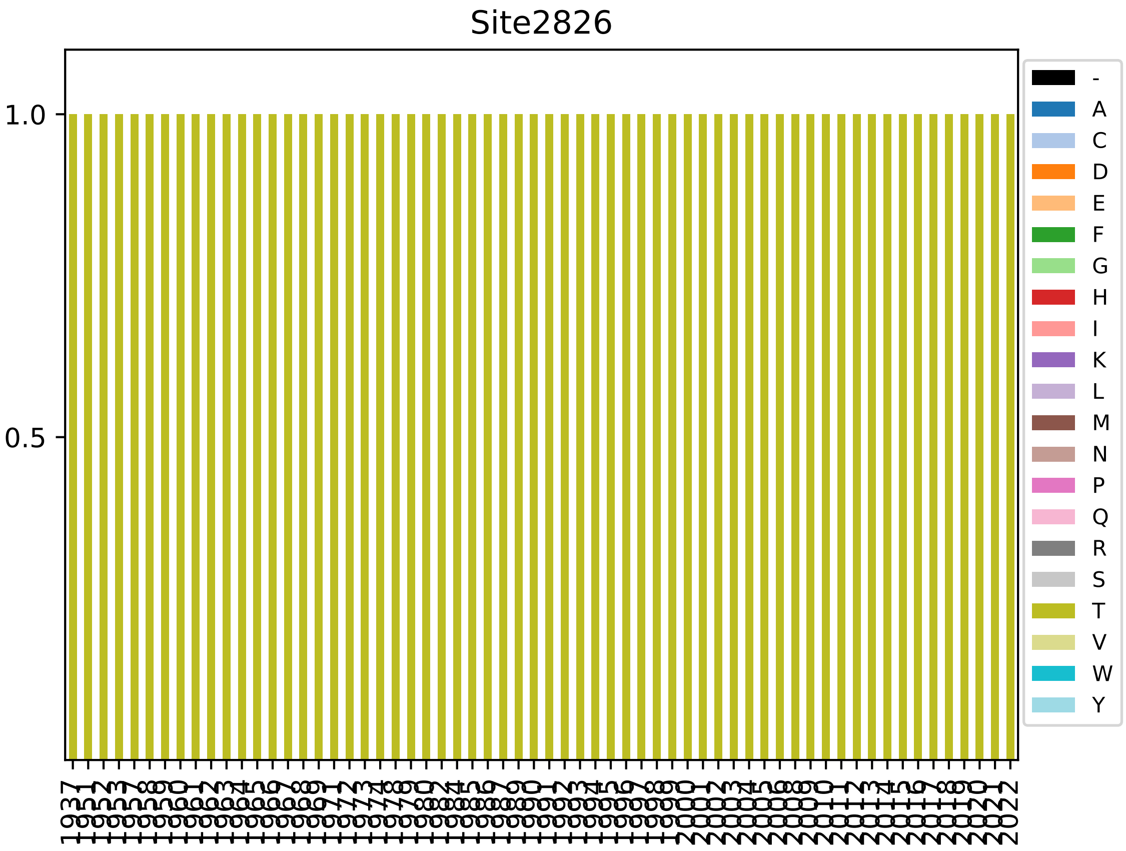
Site2826
1.0
0.5
-
A
C
D
E
F
G
H
I
K
L
M
N
P
Q
R
S
T
V
W
Y

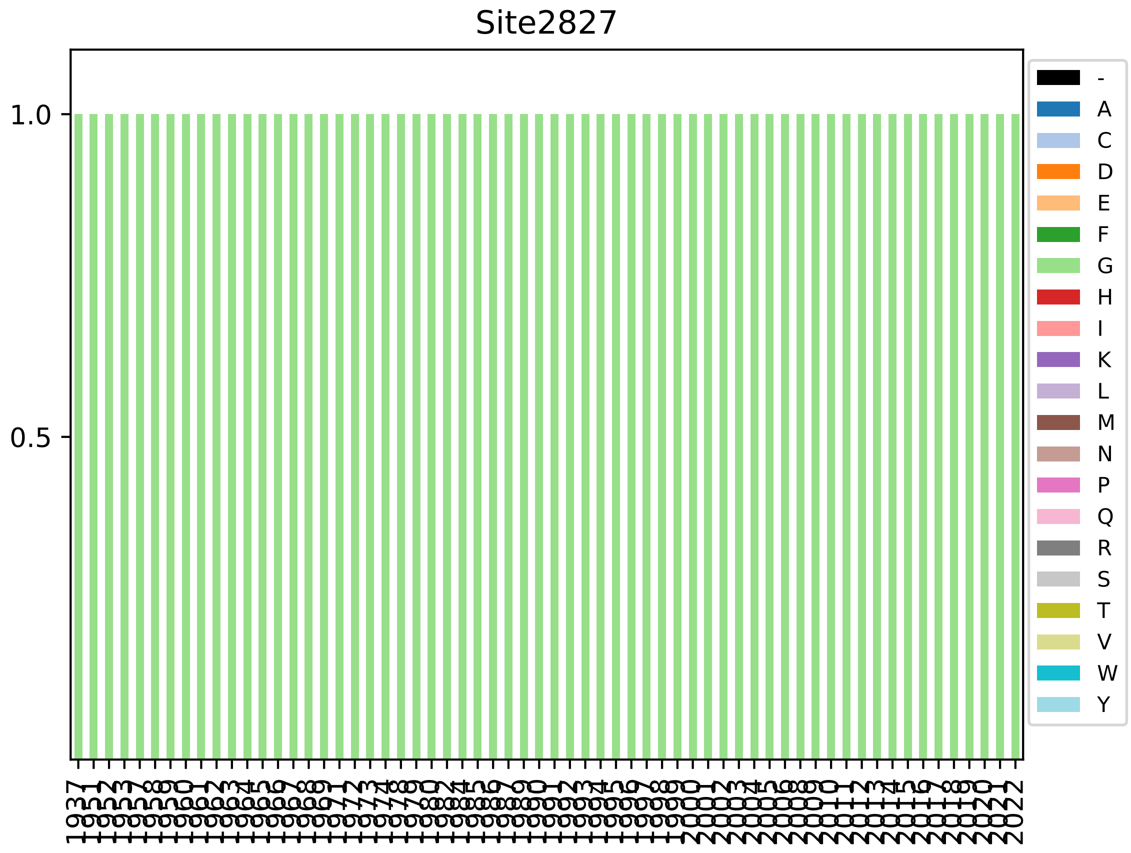
Site2827
1.0
0.5
-
A
C
D
E
F
G
H
I
K
L
M
N
P
Q
R
S
T
V
W
Y

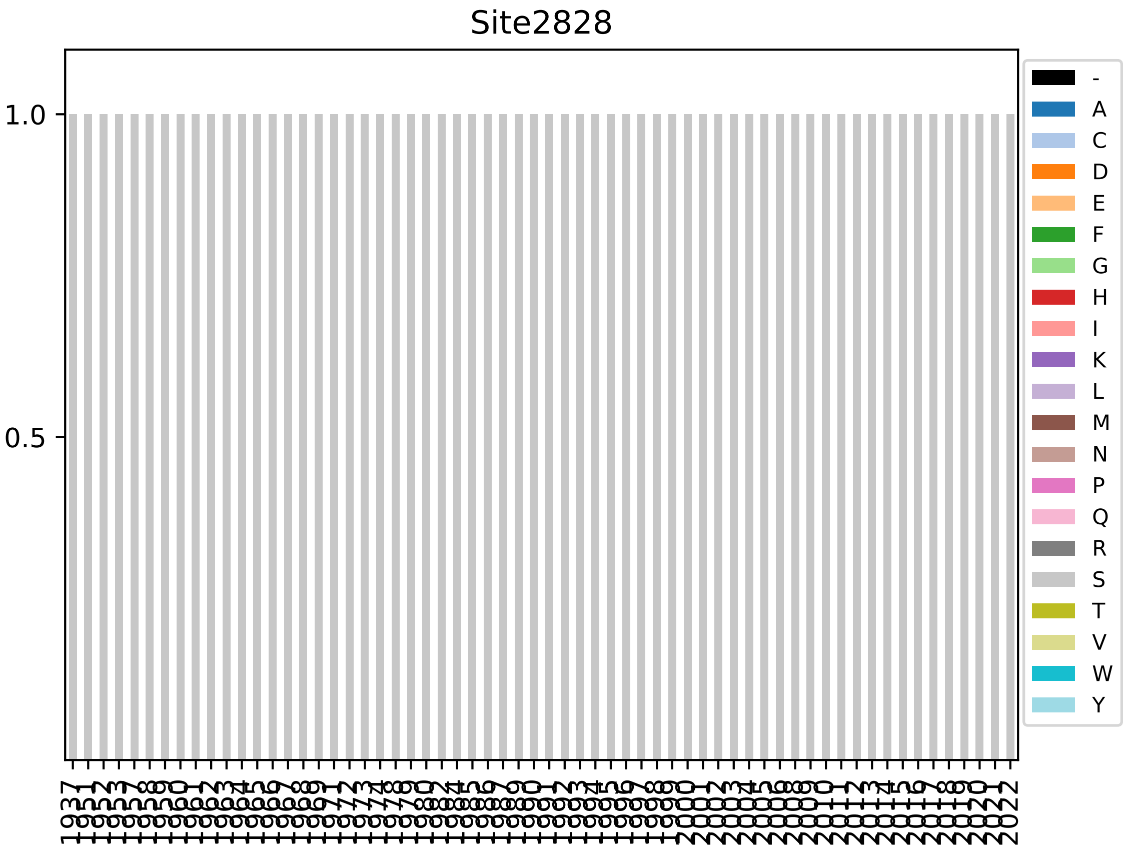
Site2828
1.0
0.5
-
A
C
D
E
F
G
H
I
K
L
M
N
P
Q
R
S
T
V
W
Y

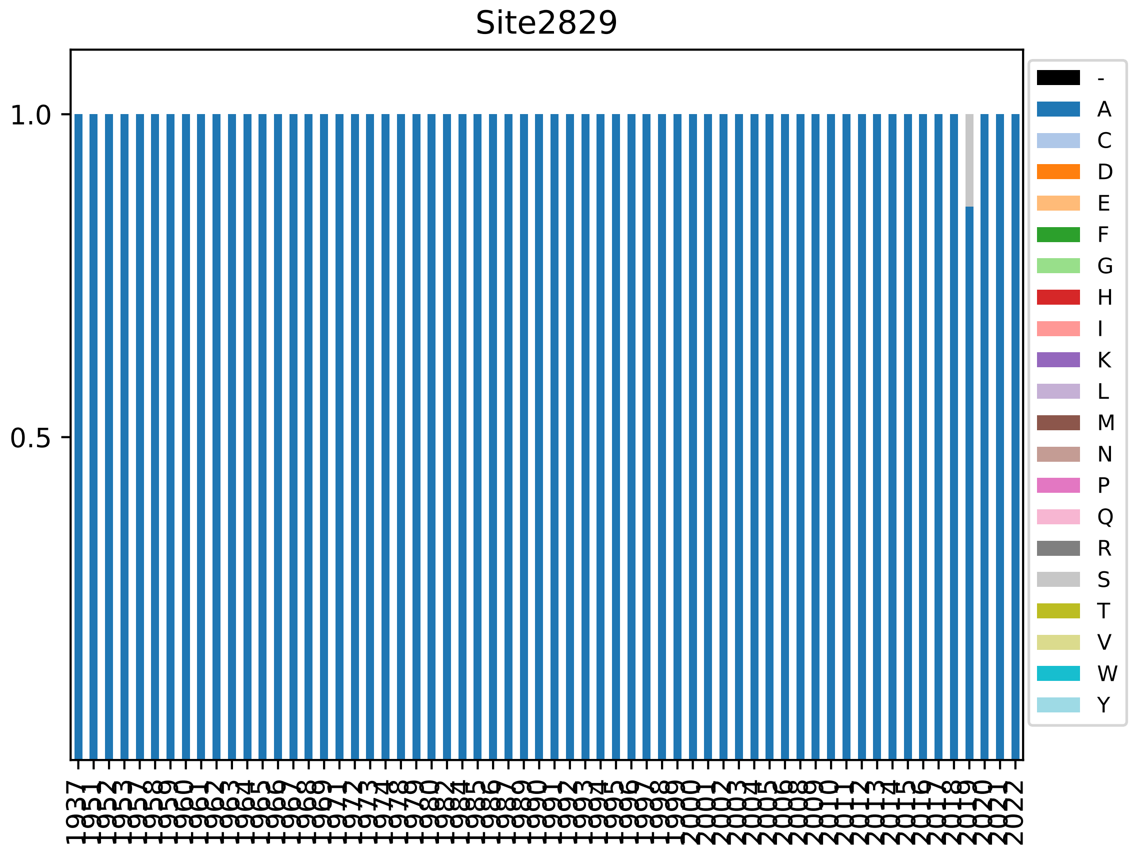
Site2829
1.0
0.5
-
A
C
D
E
F
G
H
I
K
L
M
N
P
Q
R
S
T
V
W
Y

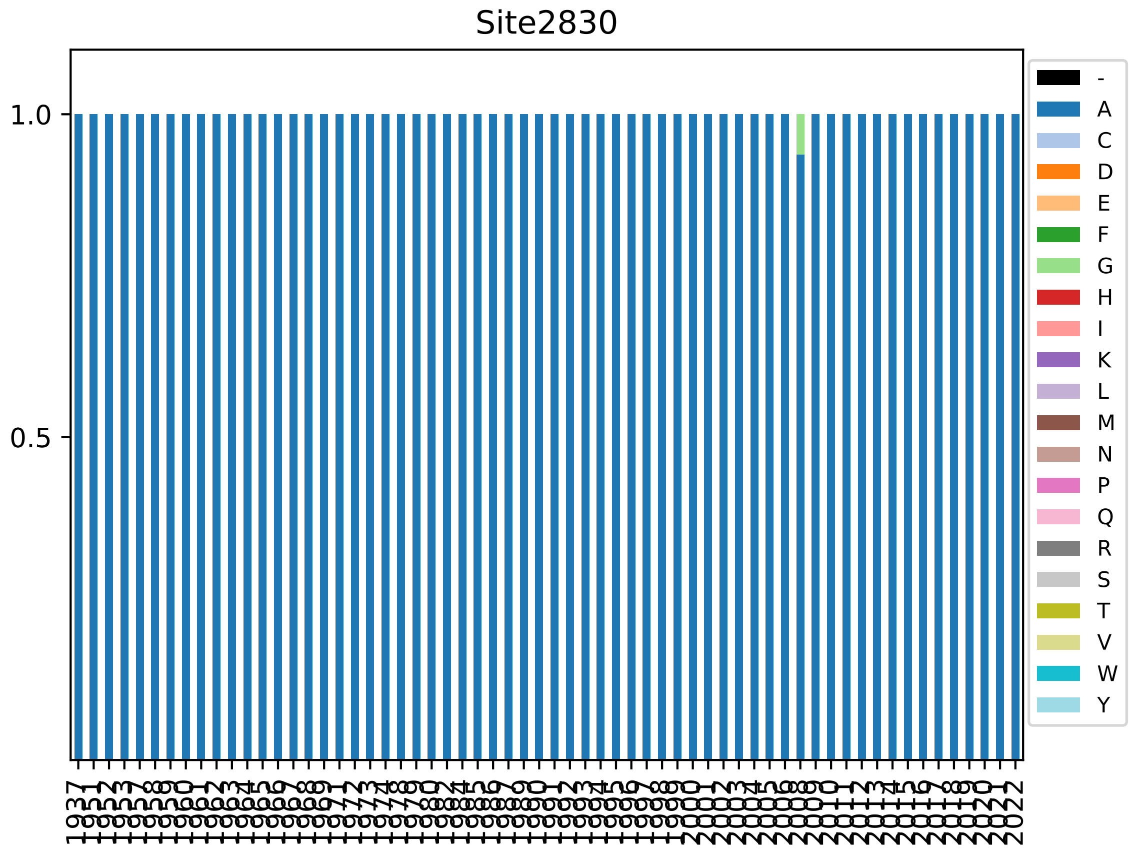
Site2830
1.0
0.5
-
A
C
D
E
F
G
H
I
K
L
M
N
P
Q
R
S
T
V
W
Y

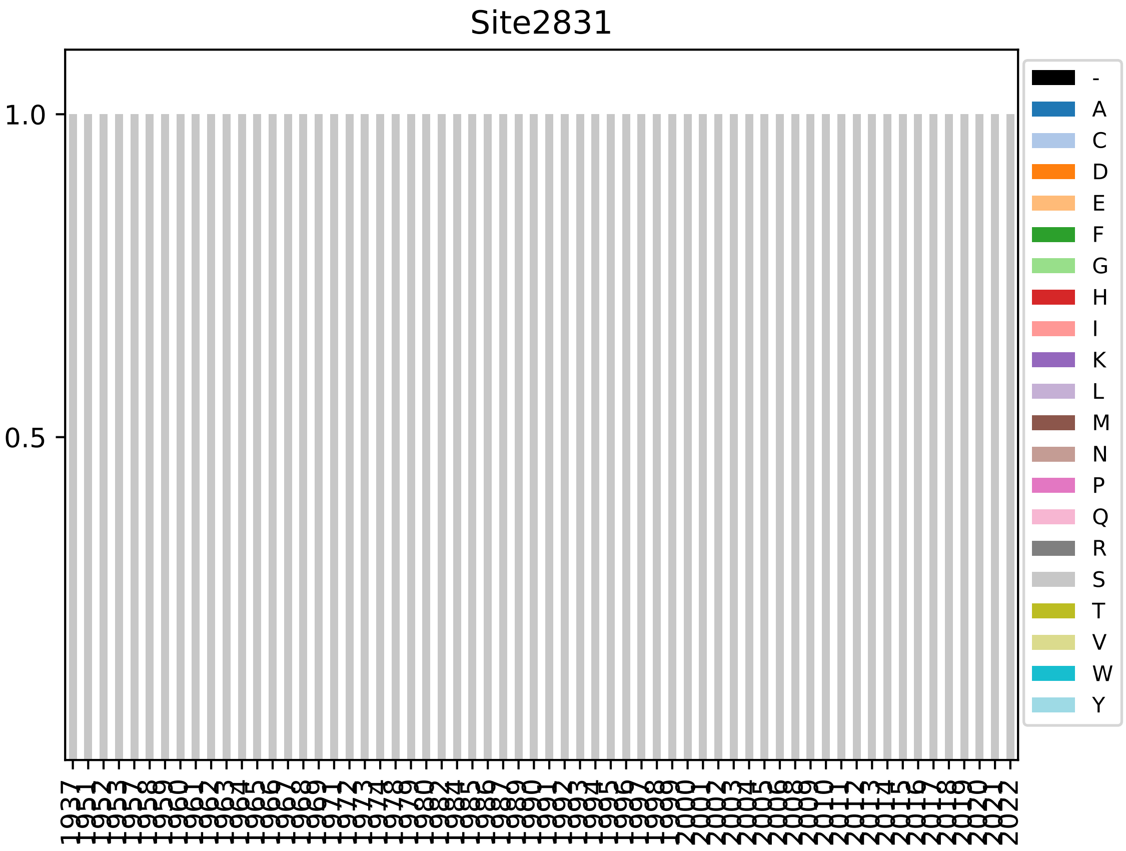

Site2831
1.0
0.5
-
A
C
D
E
F
G
H
I
K
L
M
N
P
Q
R
S
T
V
W
Y

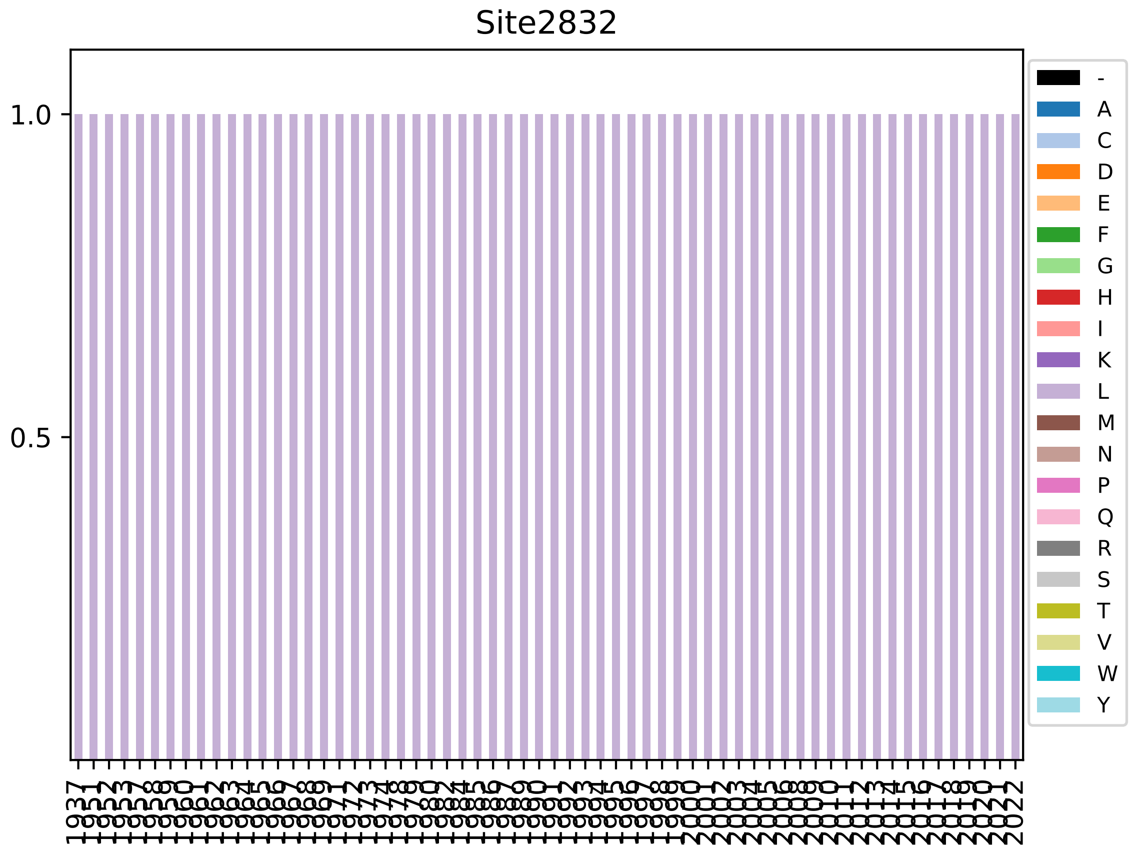
Site2832
1.0
0.5
-
A
C
D
E
F
G
H
I
K
L
M
N
P
Q
R
S
T
V
W
Y

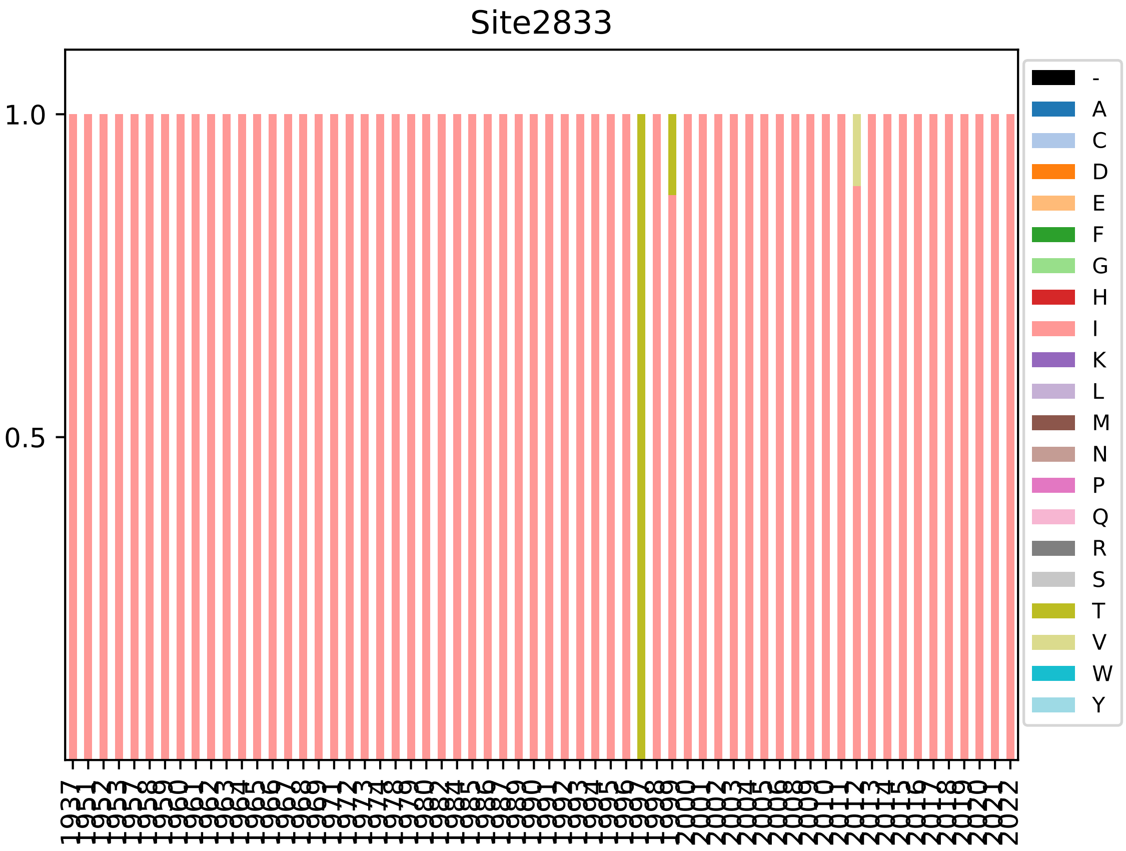
Site2833
1.0
0.5
-
A
C
D
E
F
G
H
I
K
L
M
N
P
Q
R
S
T
V
W
Y

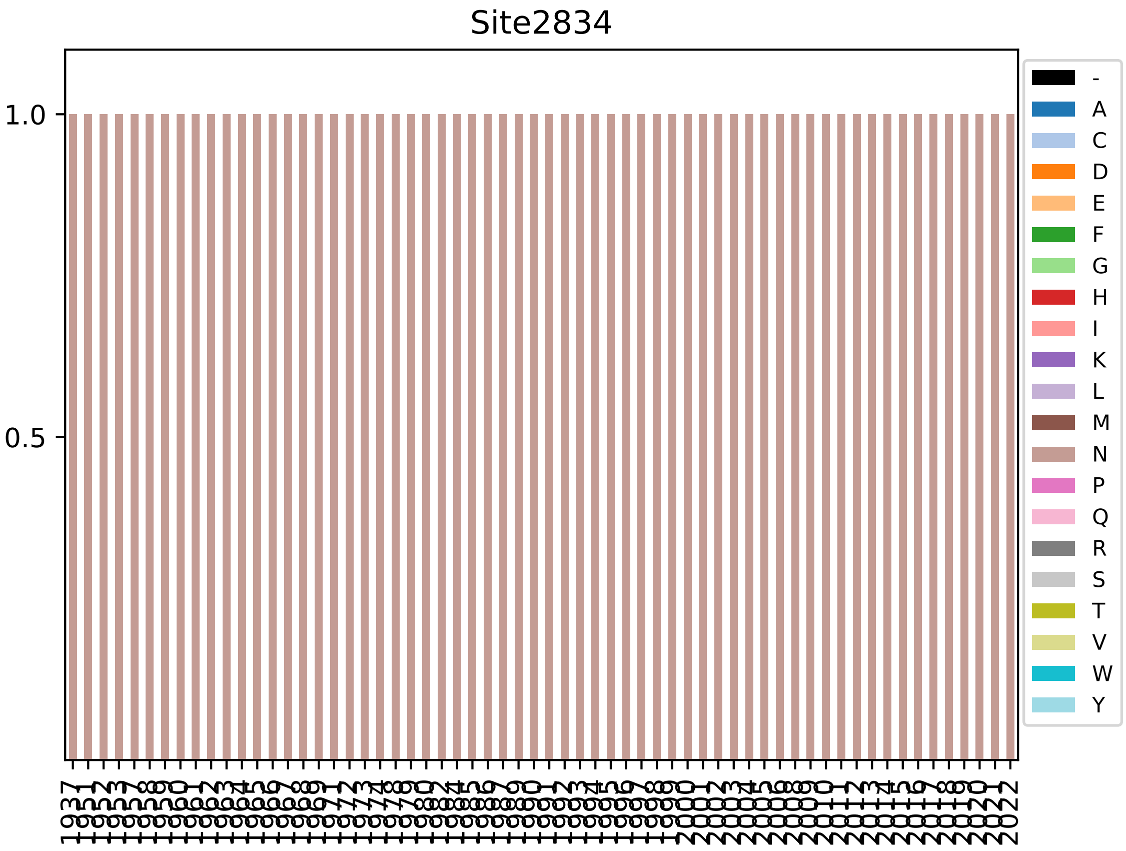
Site2834
1.0
0.5
-
A
C
D
E
F
G
H
I
K
L
M
N
P
Q
R
S
T
V
W
Y

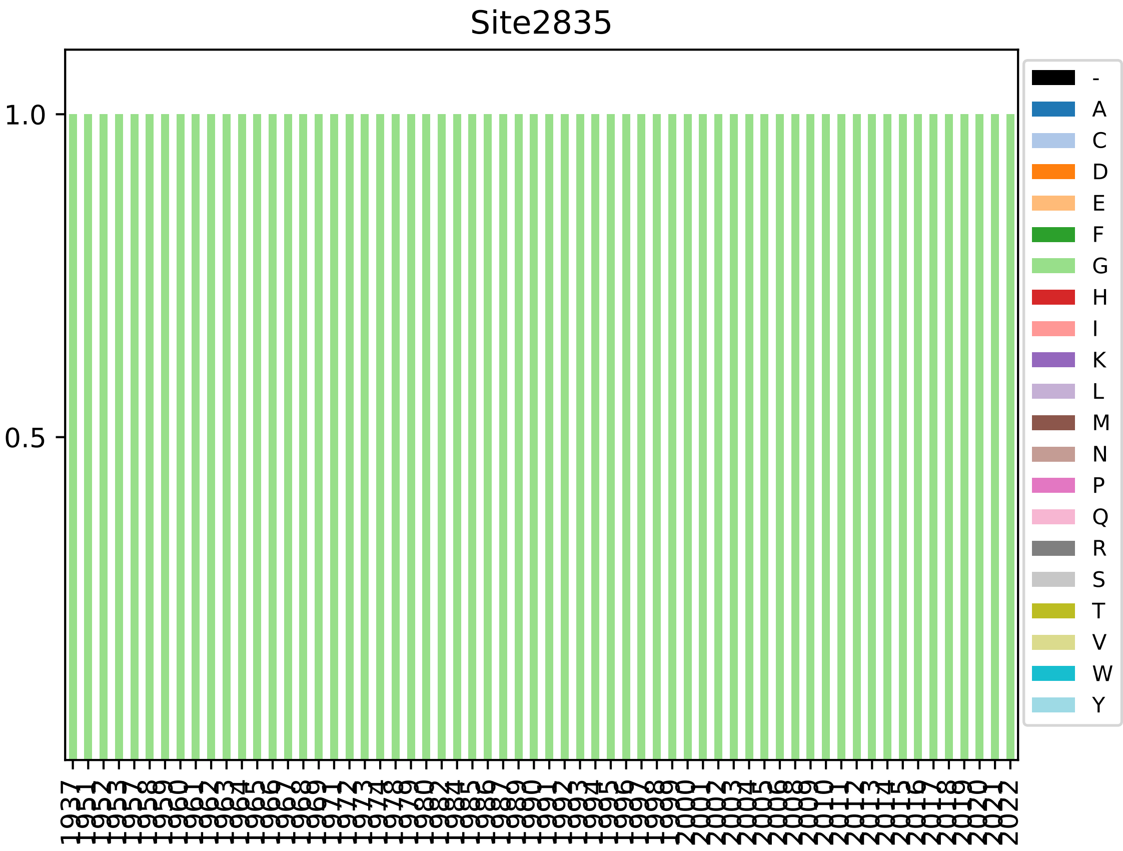
Site2835
1.0
0.5
-
A
C
D
E
F
G
H
I
K
L
M
N
P
Q
R
S
T
V
W
Y

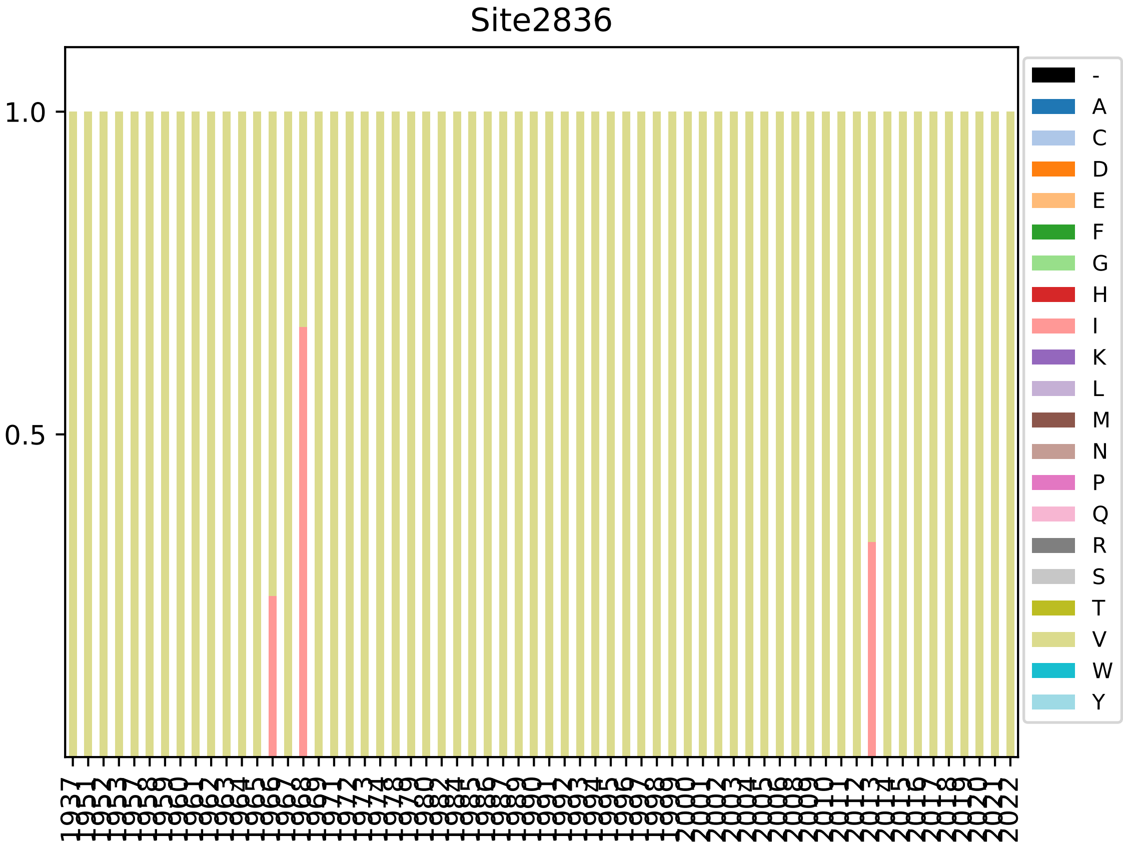

Site2836
1.0
0.5
-
A
C
D
E
F
G
H
I
K
L
M
N
P
Q
R
S
T
V
W
Y

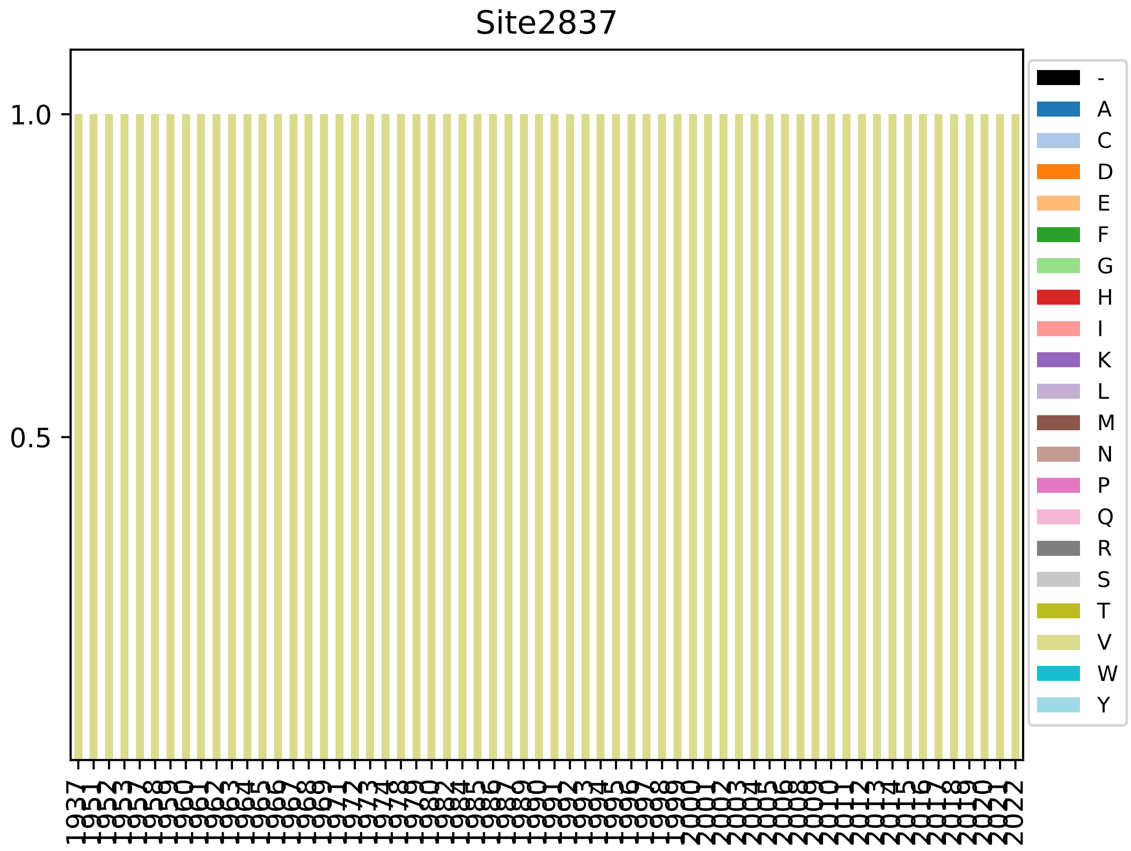
Site2837
1.0
0.5
-
A
C
D
E
F
G
H
I
K
L
M
N
P
Q
R
S
T
V
W
Y

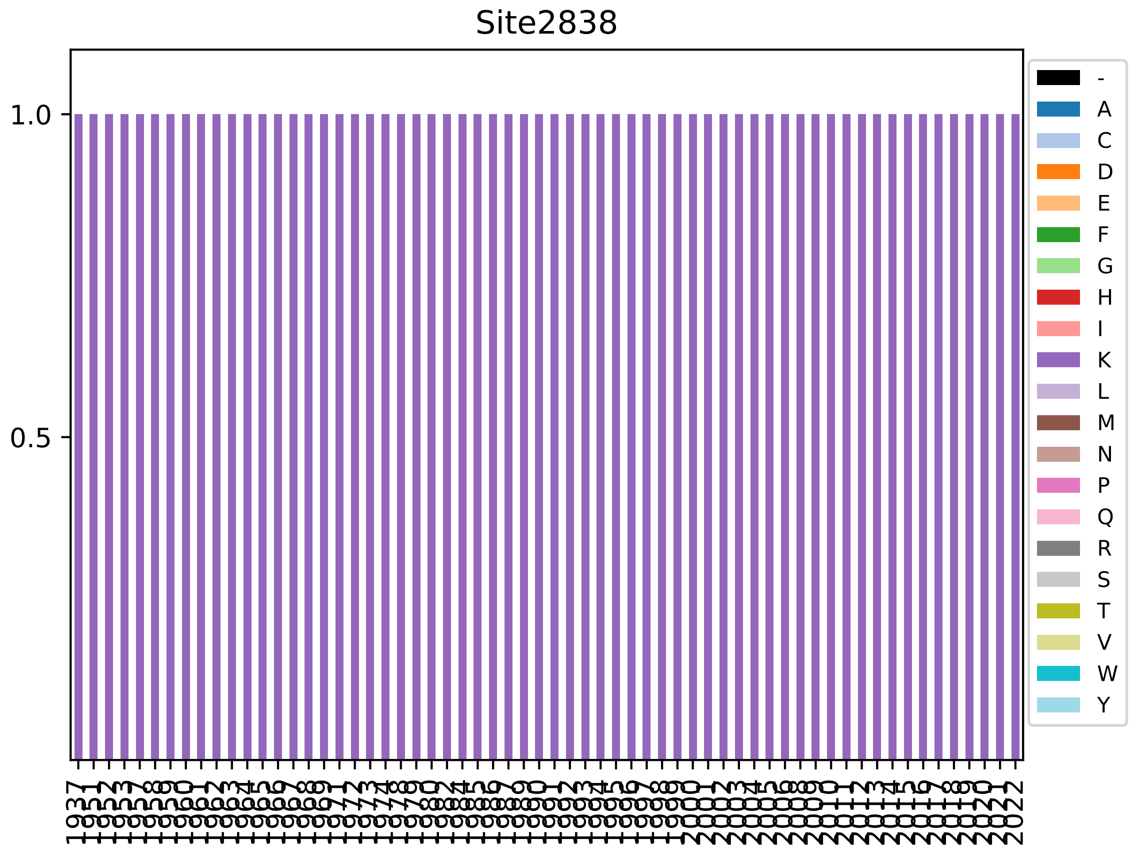
Site2838
1.0
0.5
-
A
C
D
E
F
G
H
I
K
L
M
N
P
Q
R
S
T
V
W
Y

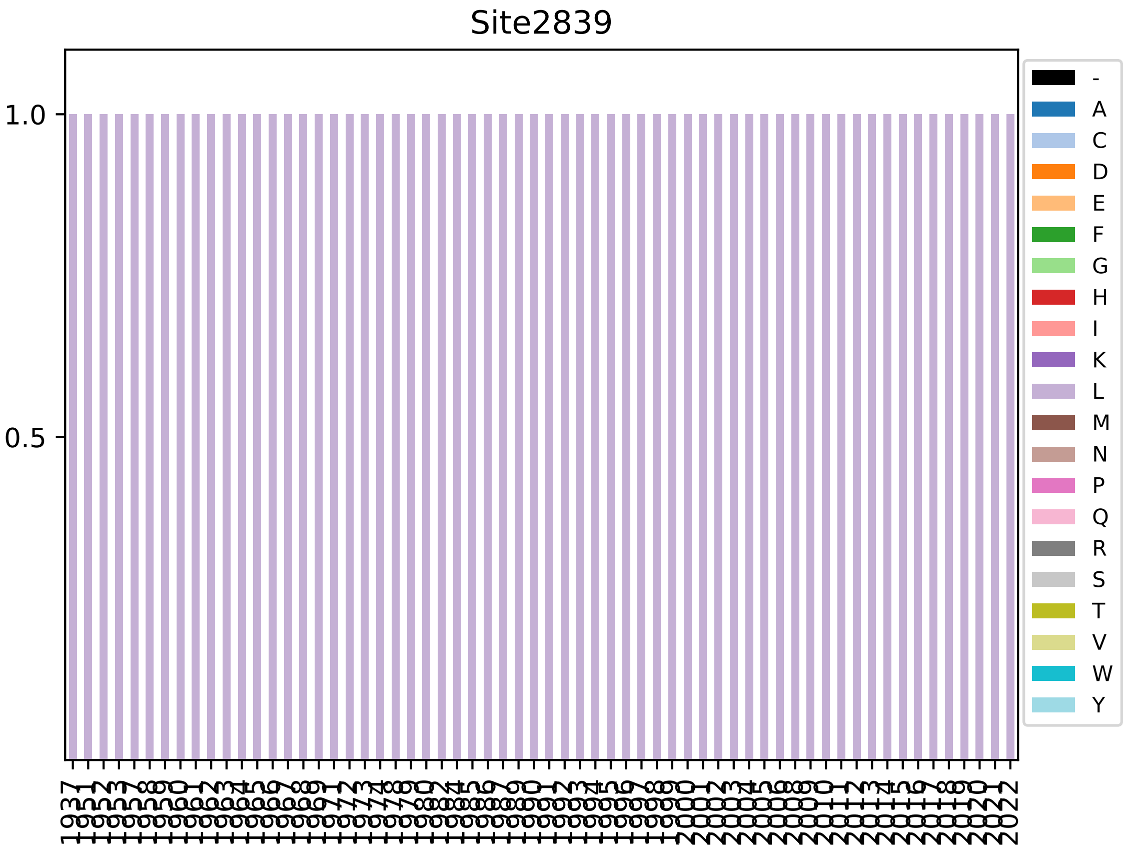
Site2839
1.0
0.5
-
A
C
D
E
F
G
H
I
K
L
M
N
P
Q
R
S
T
V
W
Y

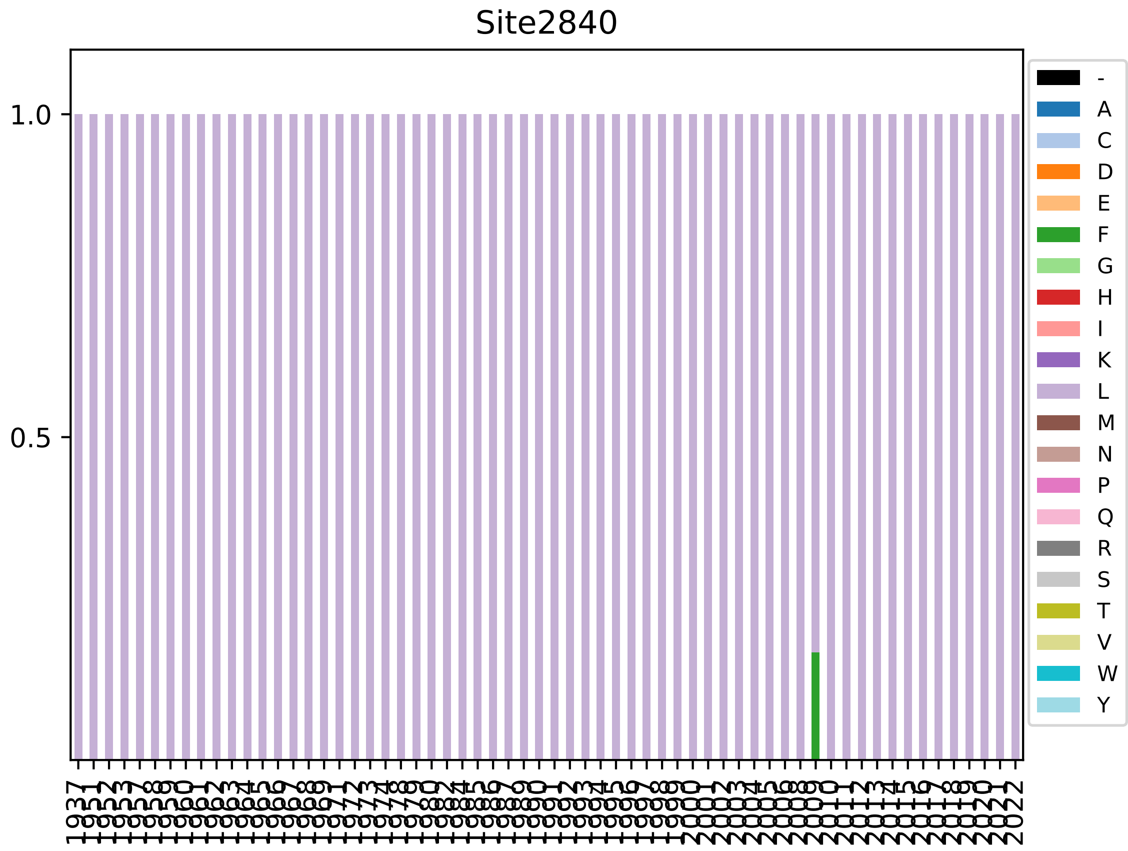
Site2840
1.0
0.5
-
A
C
D
E
F
G
H
I
K
L
M
N
P
Q
R
S
T
V
W
Y

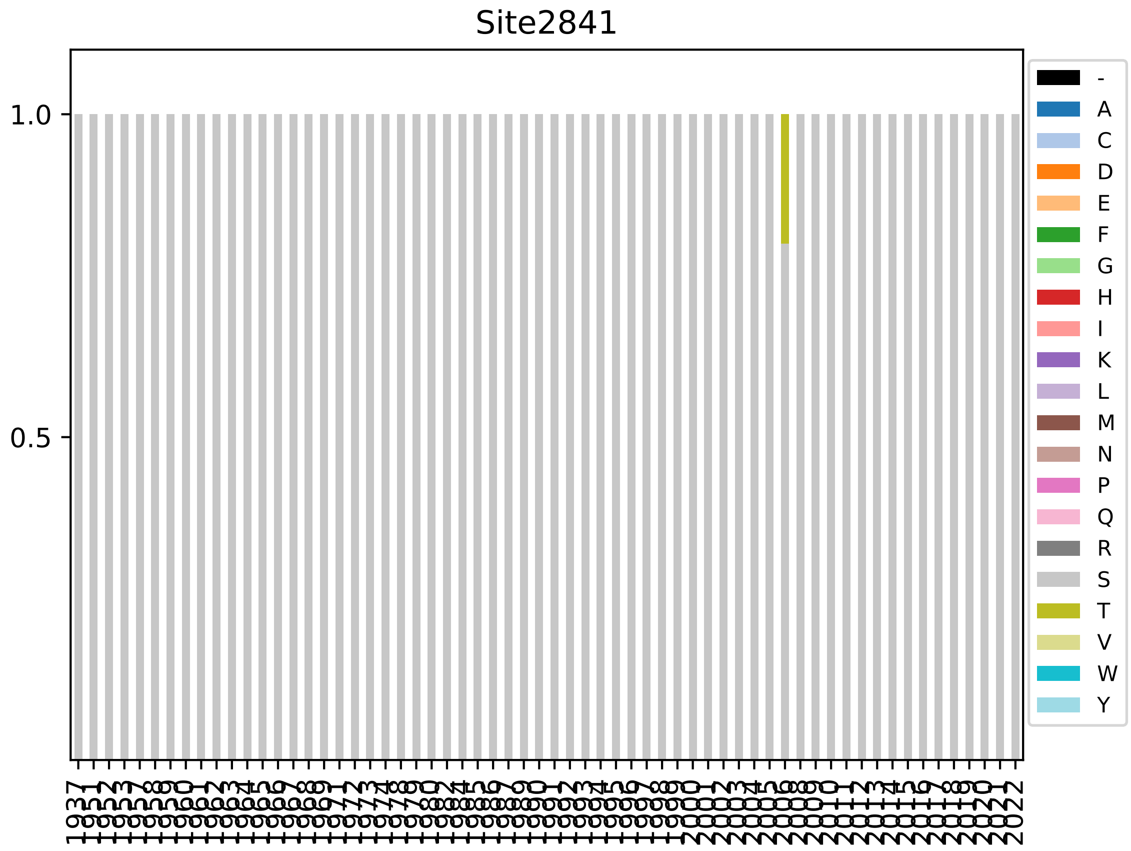
Site2841
1.0
0.5
-
A
C
D
E
F
G
H
I
K
L
M
N
P
Q
R
S
T
V
W
Y

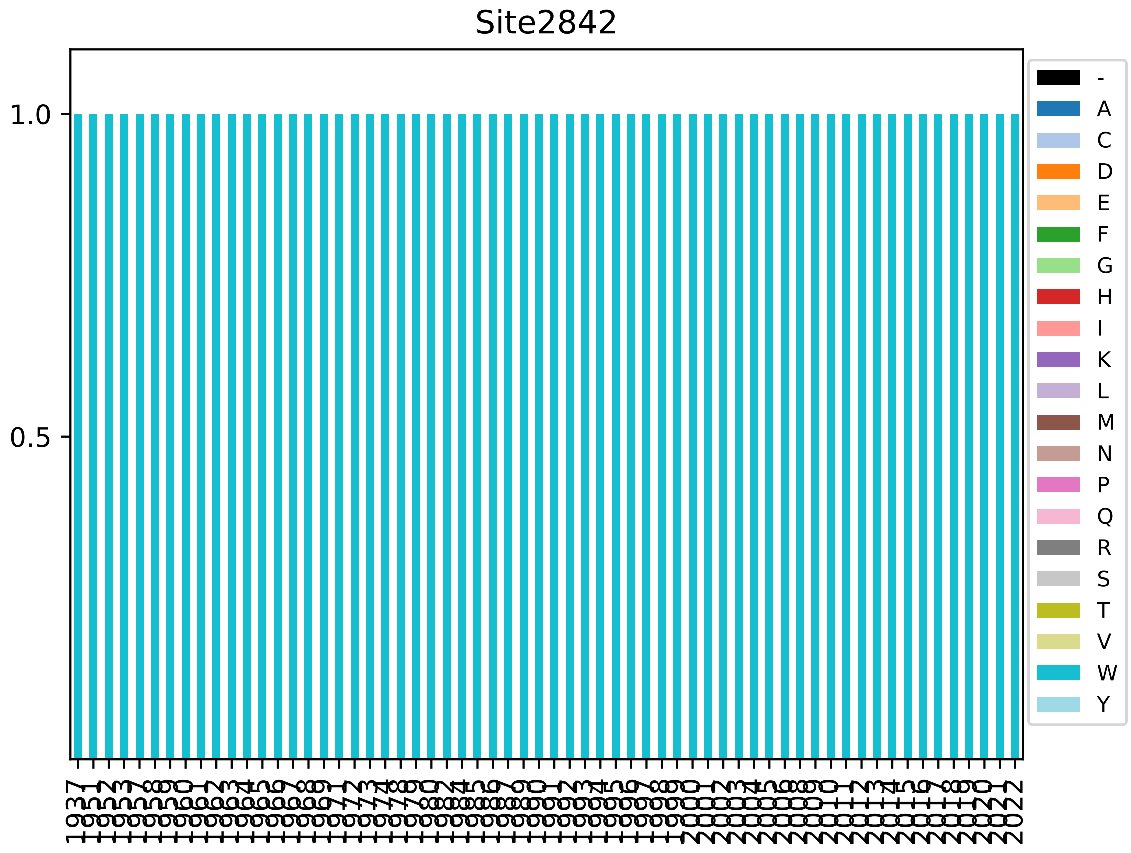
Site2842
1.0
0.5
-
A
C
D
E
F
G
H
I
K
L
M
N
P
Q
R
S
T
V
W
Y

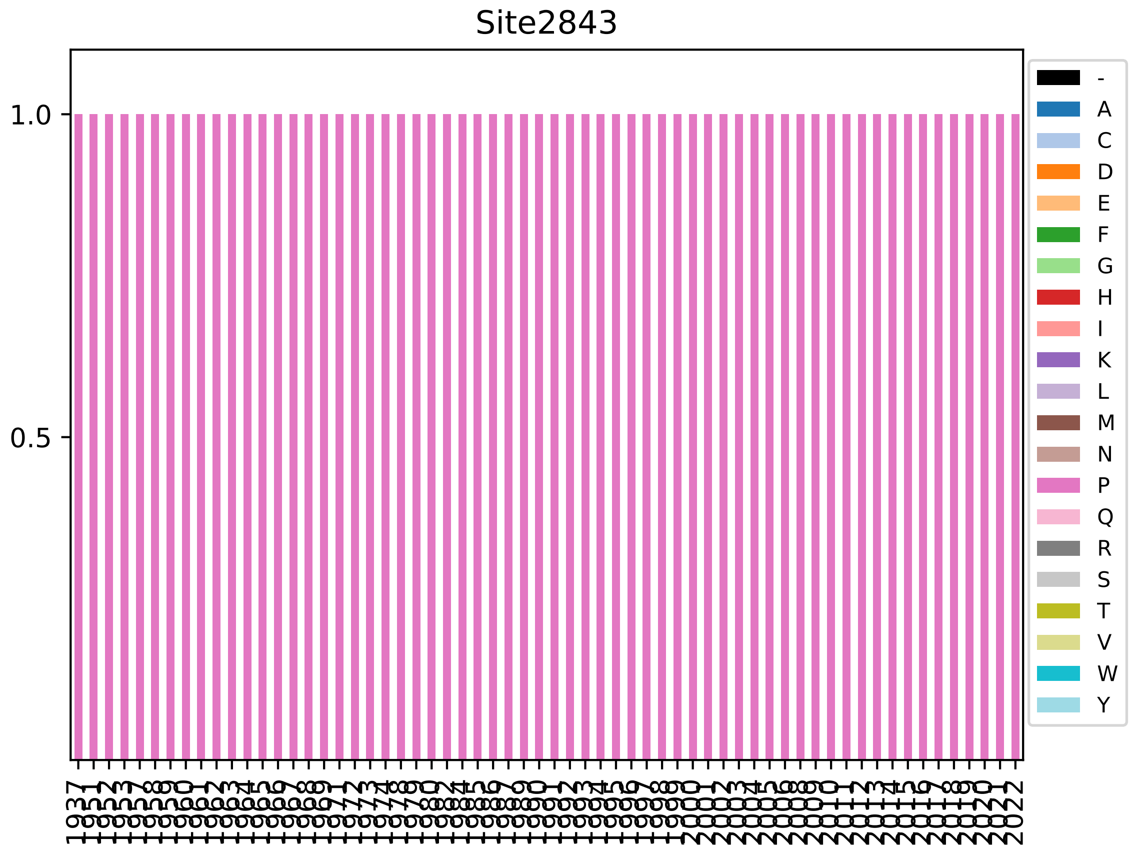
Site2843
1.0
0.5
-
A
C
D
E
F
G
H
I
K
L
M
N
P
Q
R
S
T
V
W
Y

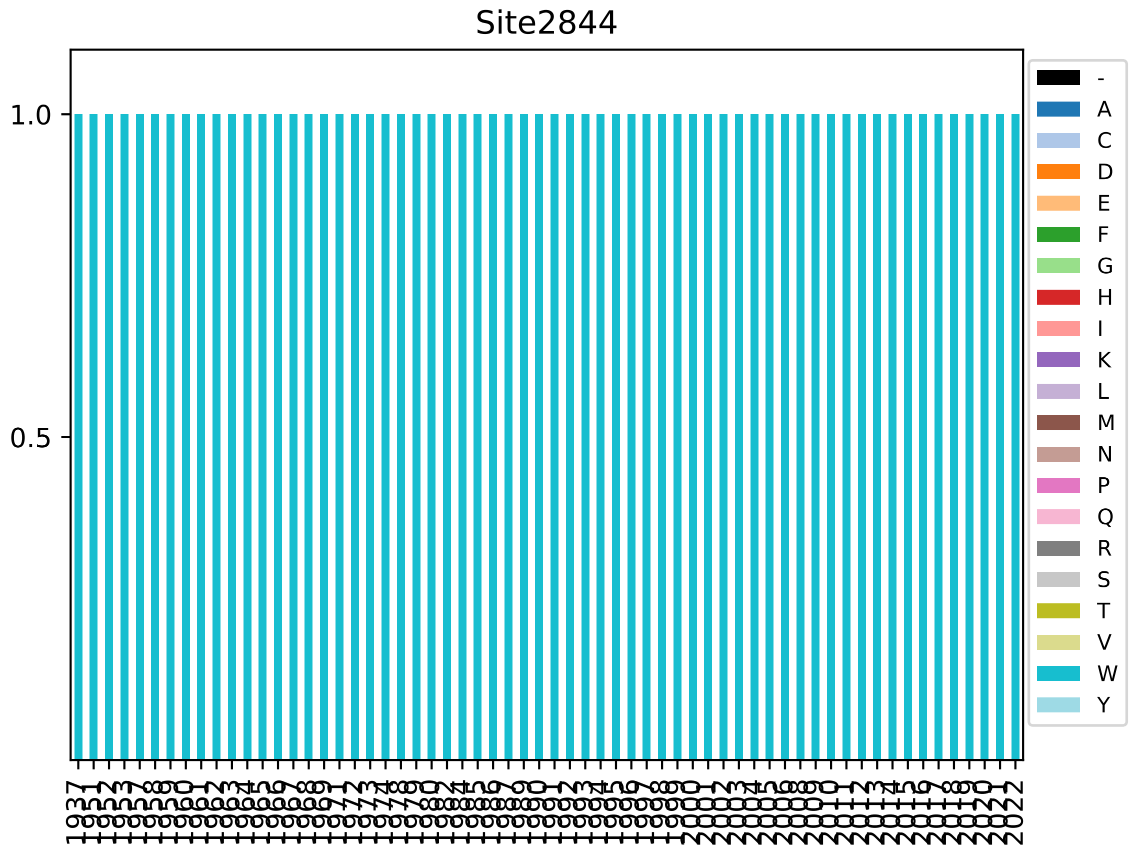

Site2844
1.0
0.5
-
A
C
D
E
F
G
H
I
K
L
M
N
P
Q
R
S
T
V
W
Y

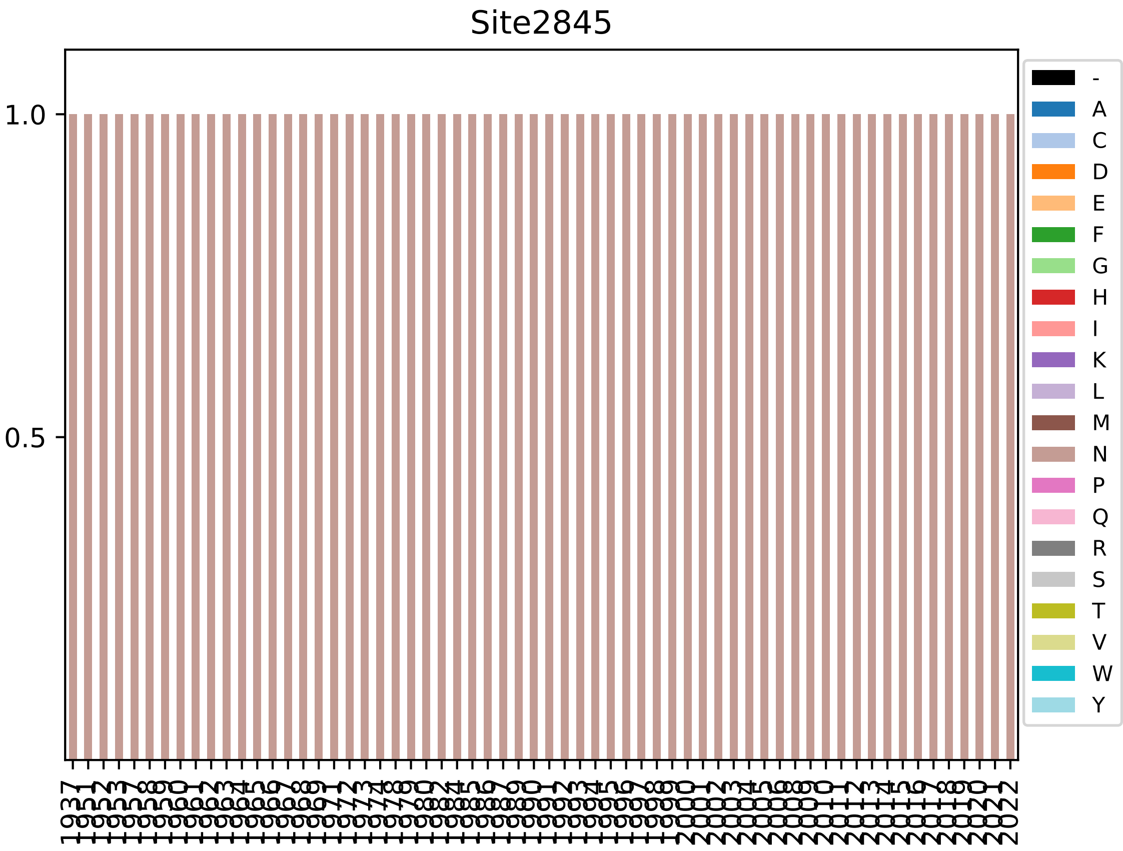
Site2845
1.0
0.5
-
A
C
D
E
F
G
H
I
K
L
M
N
P
Q
R
S
T
V
W
Y

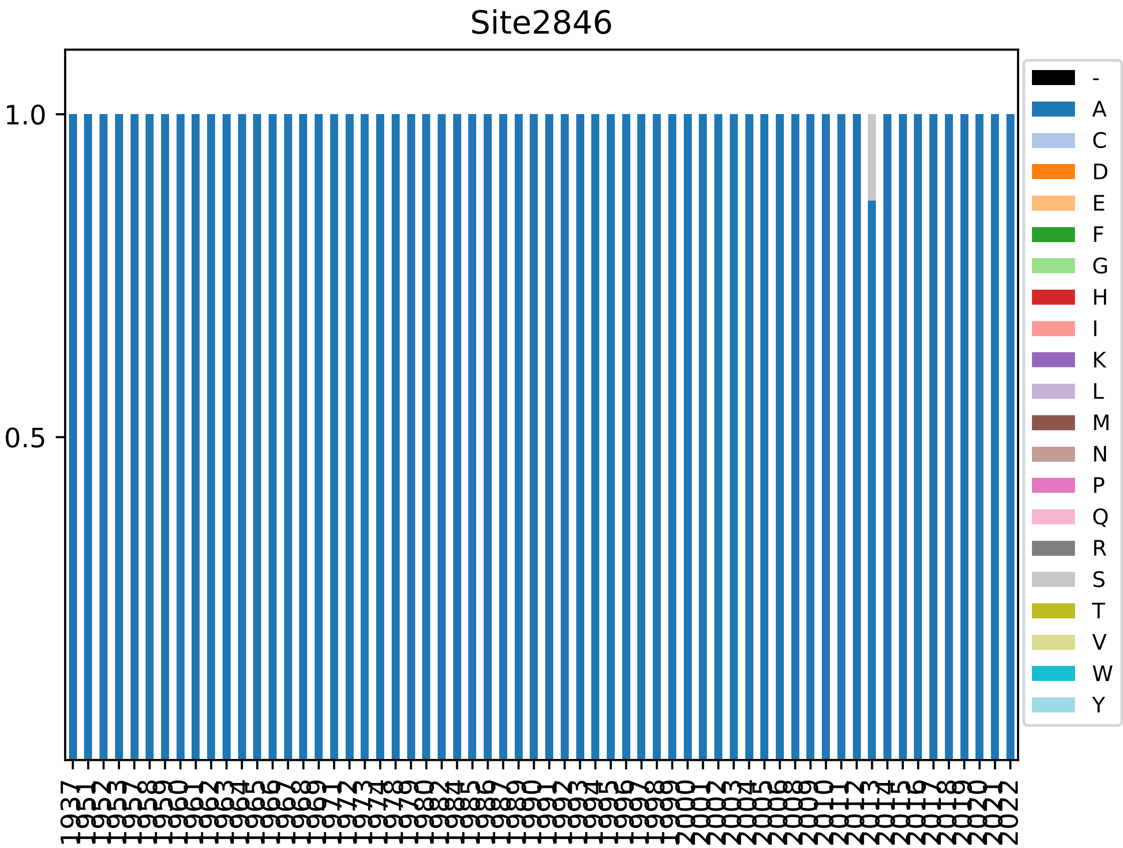
Site2846
1.0
0.5
-
A
C
D
E
F
G
H
I
K
L
M
N
P
Q
R
S
T
V
W
Y

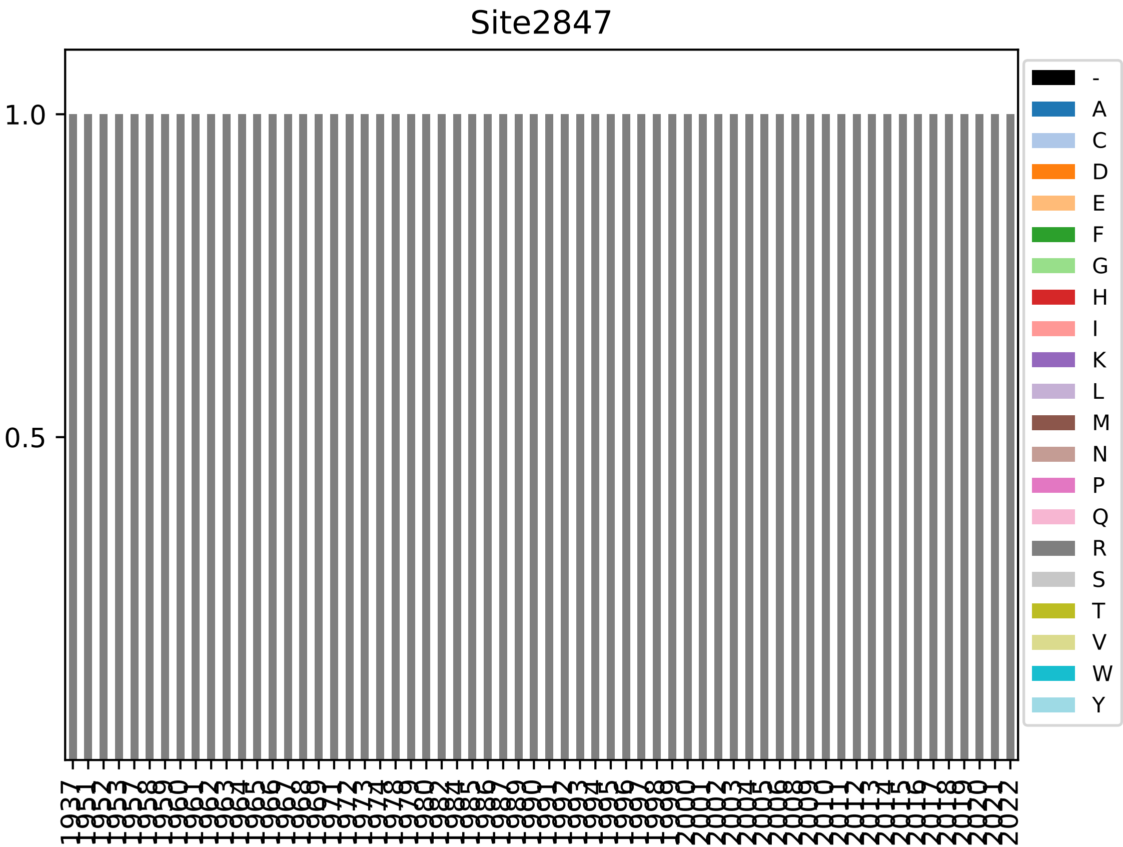
Site2847
1.0
0.5
-
A
C
D
E
F
G
H
I
K
L
M
N
P
Q
R
S
T
V
W
Y

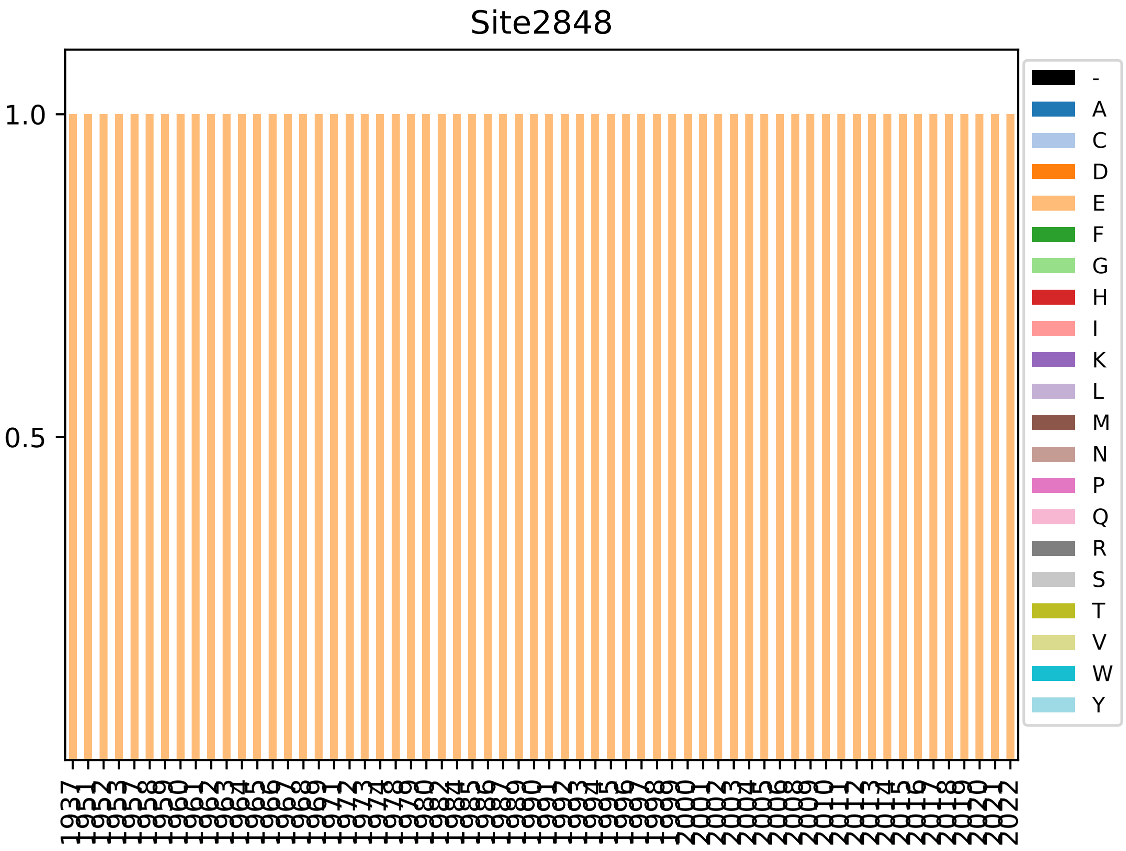
Site2848
1.0
0.5
-
A
C
D
E
F
G
H
I
K
L
M
N
P
Q
R
S
T
V
W
Y

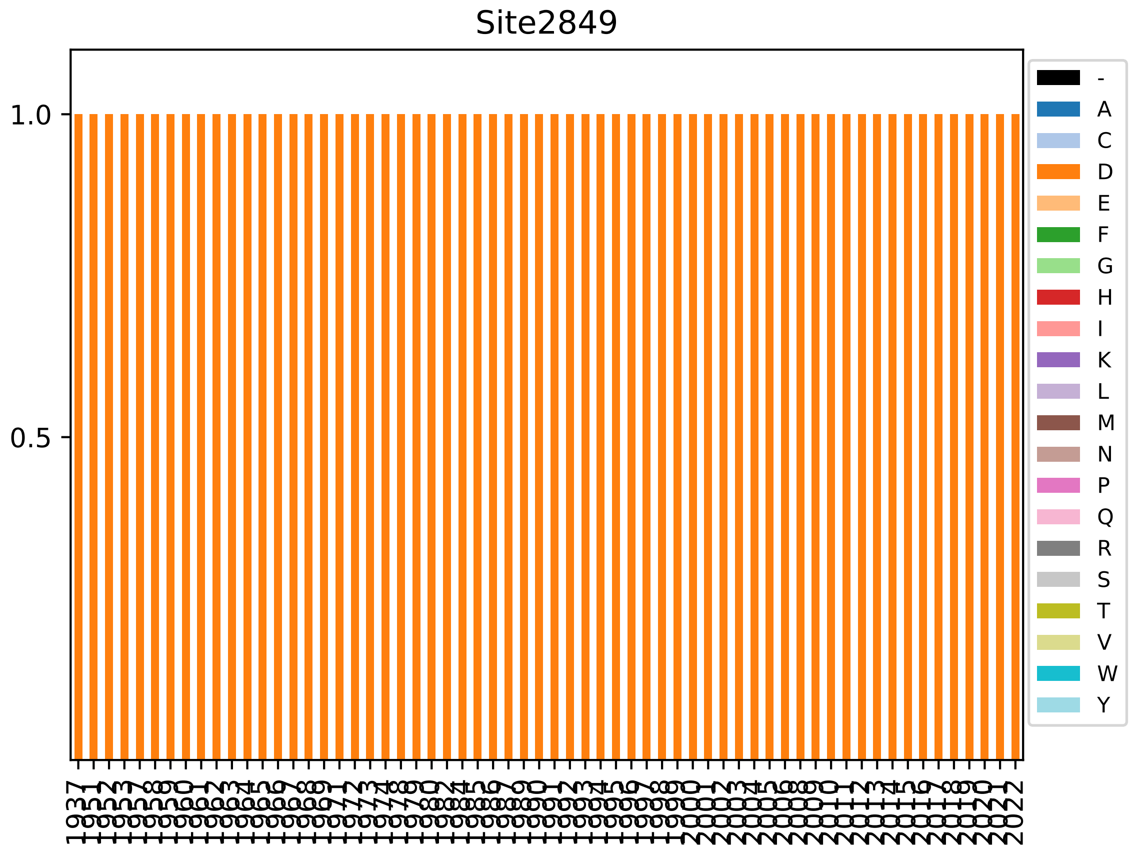
Site2849
1.0
0.5
-
A
C
D
E
F
G
H
I
K
L
M
N
P
Q
R
S
T
V
W
Y

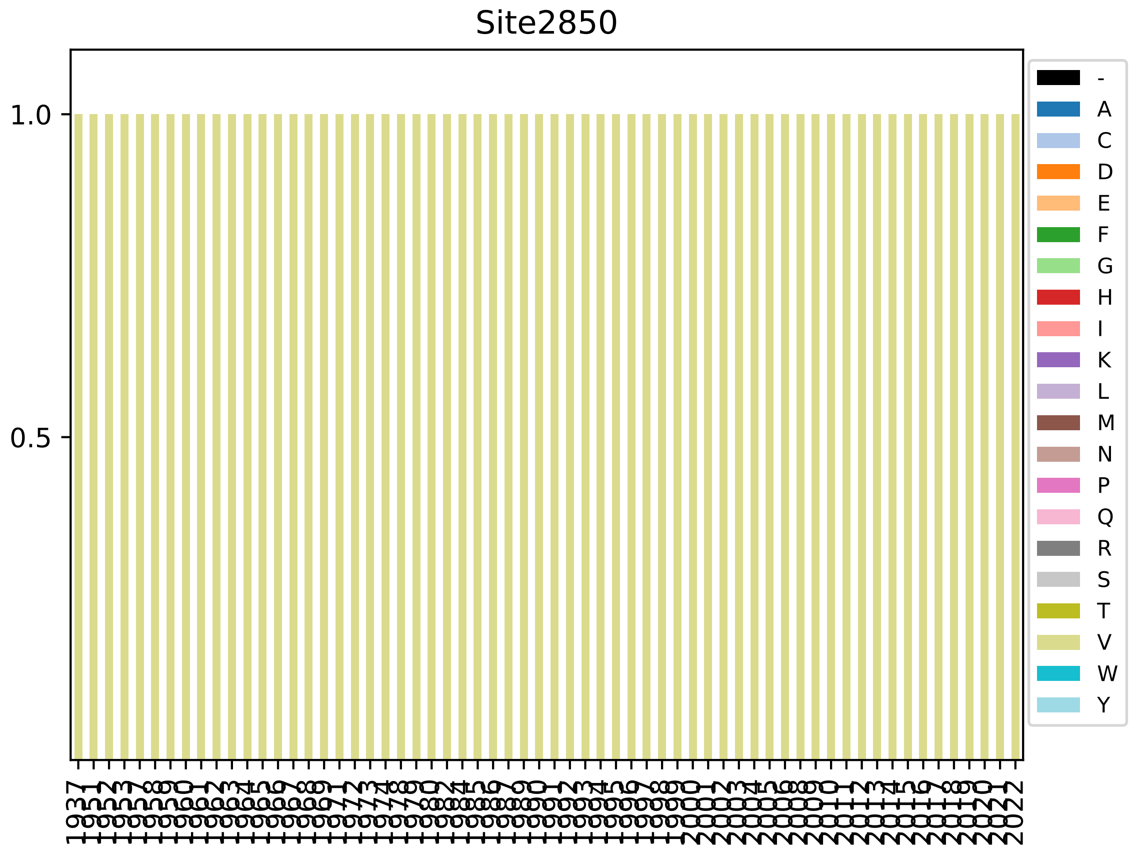
Site2850
1.0
0.5
-
A
C
D
E
F
G
H
I
K
L
M
N
P
Q
R
S
T
V
W
Y

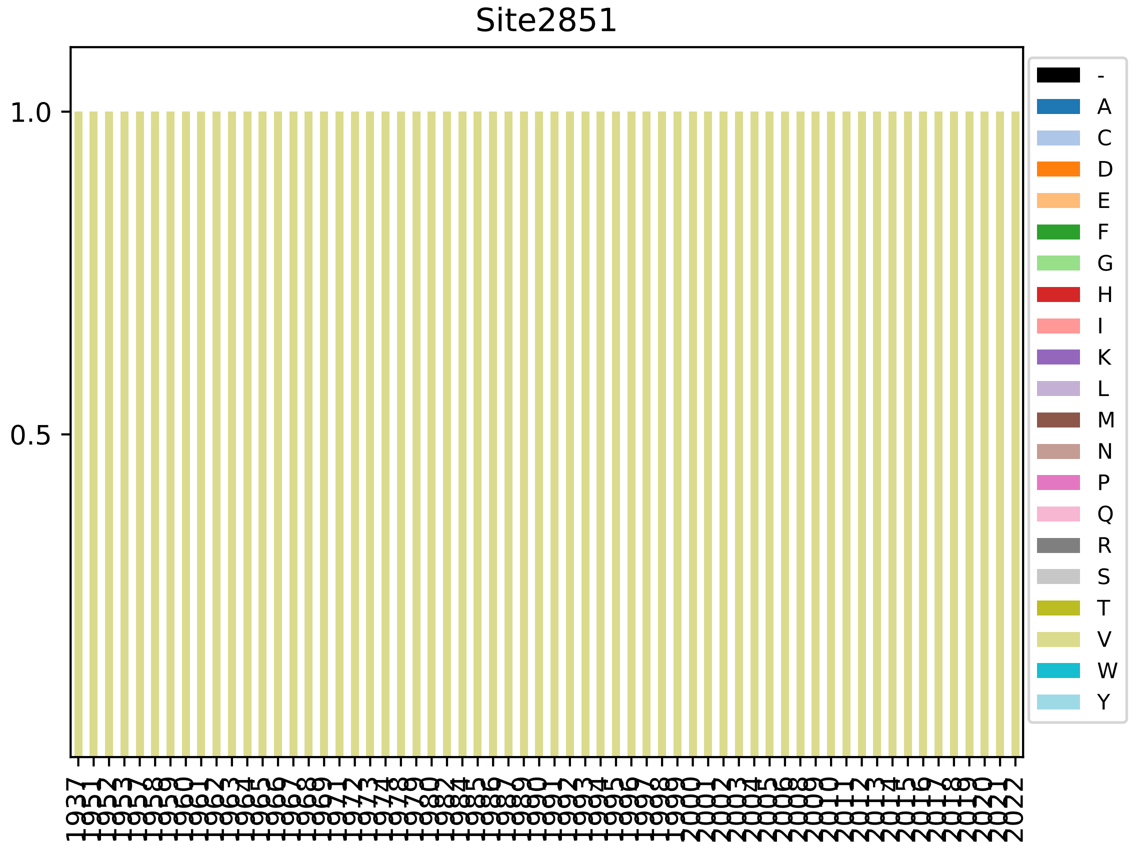
Site2851
1.0
0.5
-
A
C
D
E
F
G
H
I
K
L
M
N
P
Q
R
S
T
V
W
Y

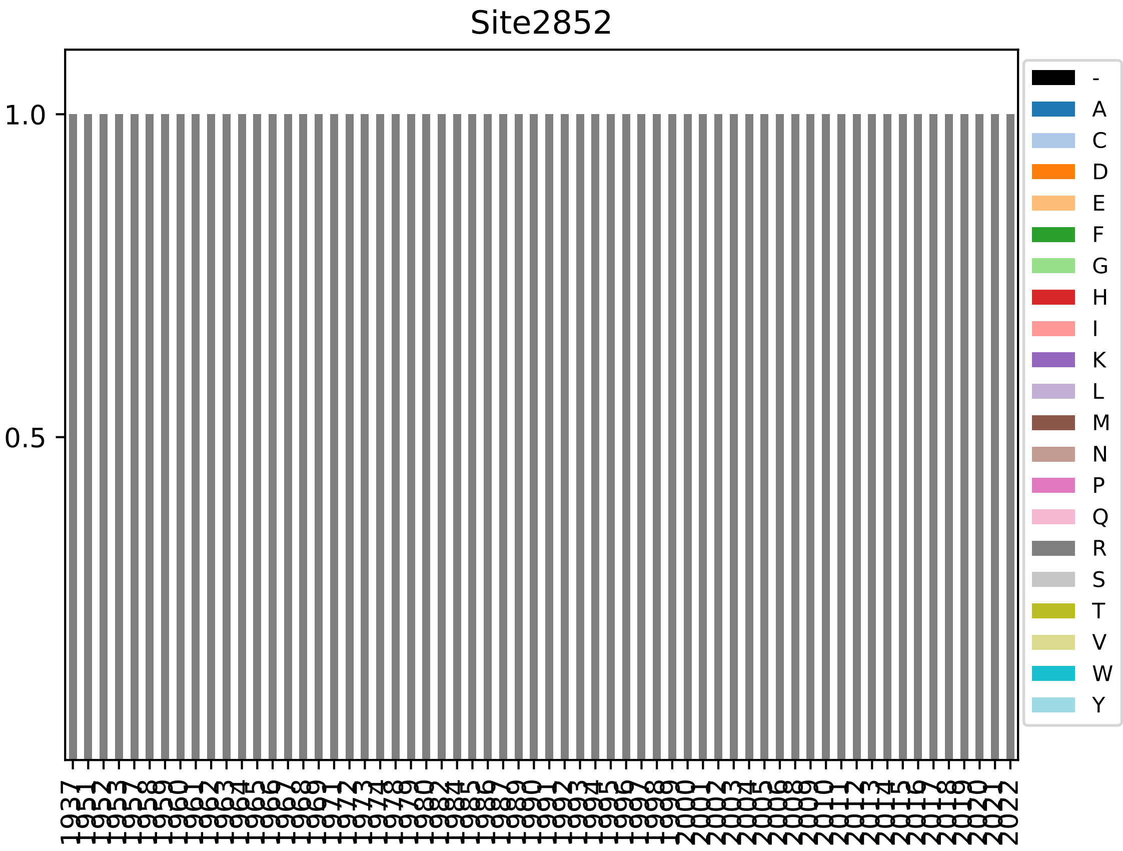
Site2852
1.0
0.5
-
A
C
D
E
F
G
H
I
K
L
M
N
P
Q
R
S
T
V
W
Y

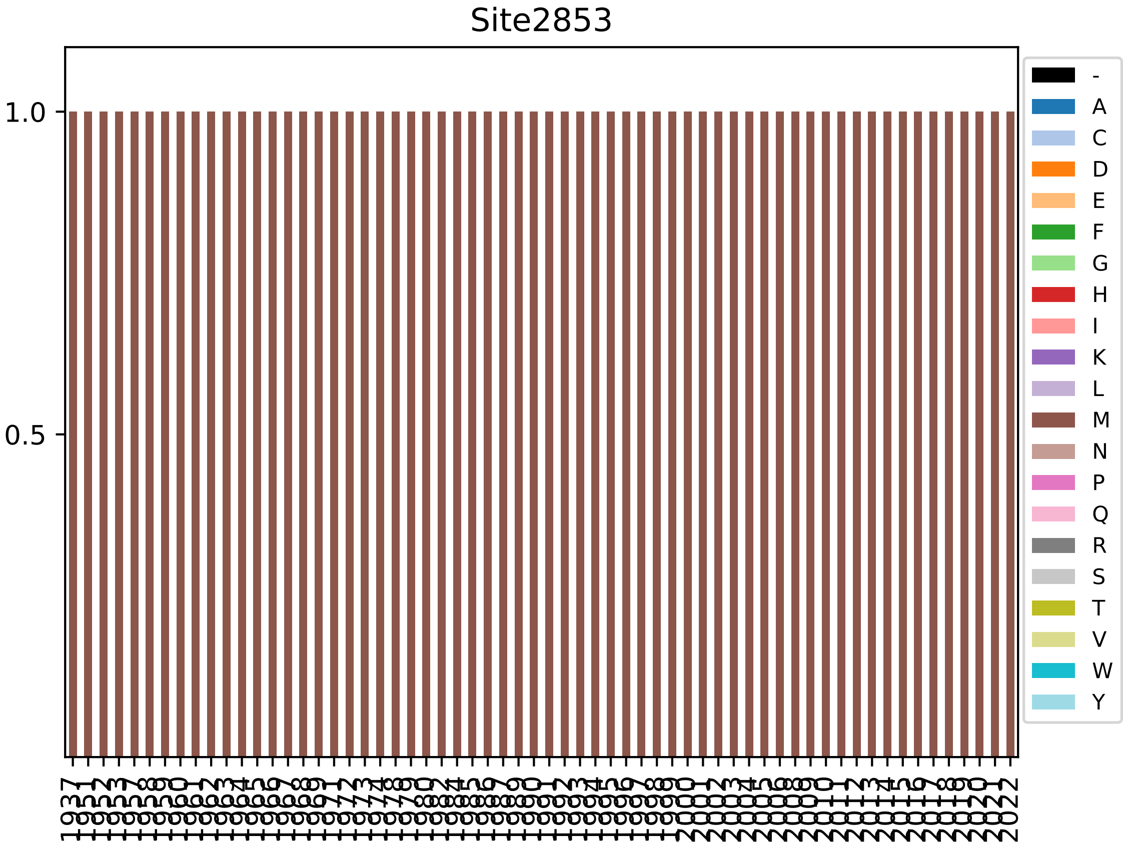
Site2853
1.0
0.5
-
A
C
D
E
F
G
H
I
K
L
M
N
P
Q
R
S
T
V
W
Y

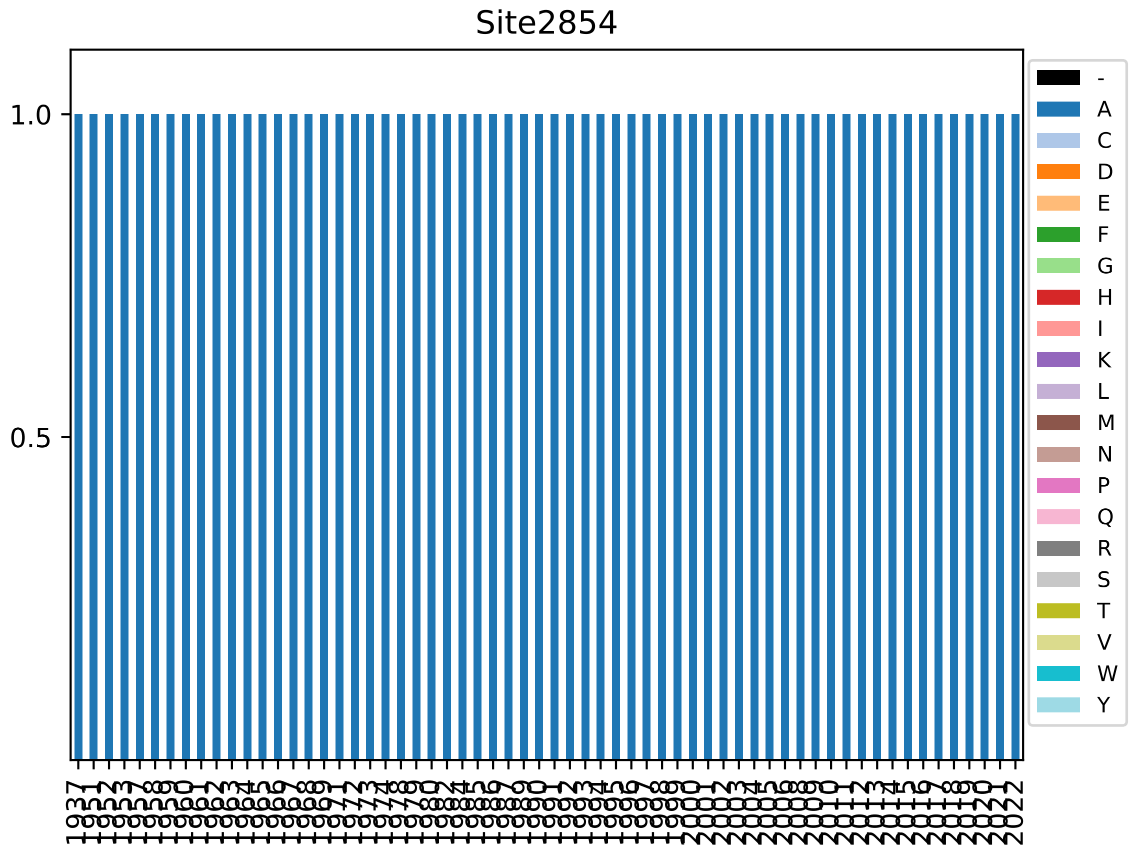
Site2854
1.0
0.5
-
A
C
D
E
F
G
H
I
K
L
M
N
P
Q
R
S
T
V
W
Y

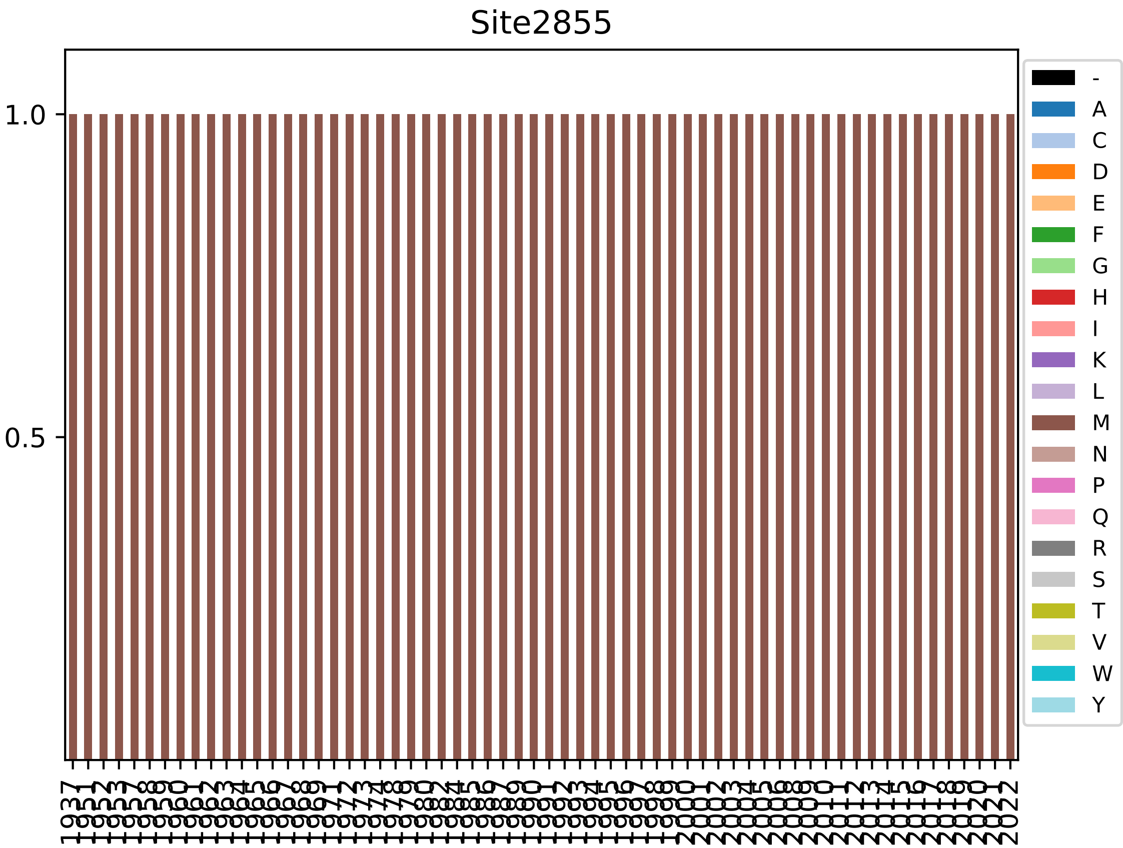

Site2855
1.0
0.5
-
A
C
D
E
F
G
H
I
K
L
M
N
P
Q
R
S
T
V
W
Y

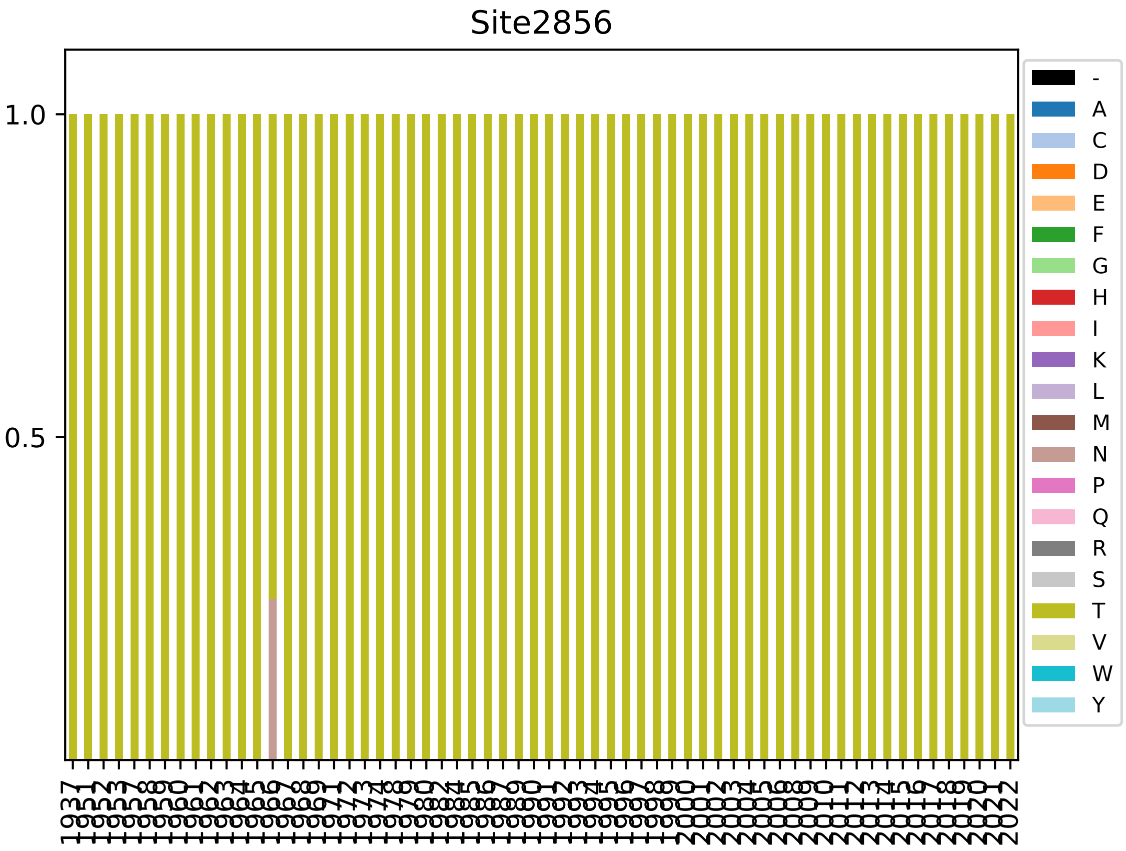

Site2856
1.0
0.5
-
A
C
D
E
F
G
H
I
K
L
M
N
P
Q
R
S
T
V
W
Y

Site2857

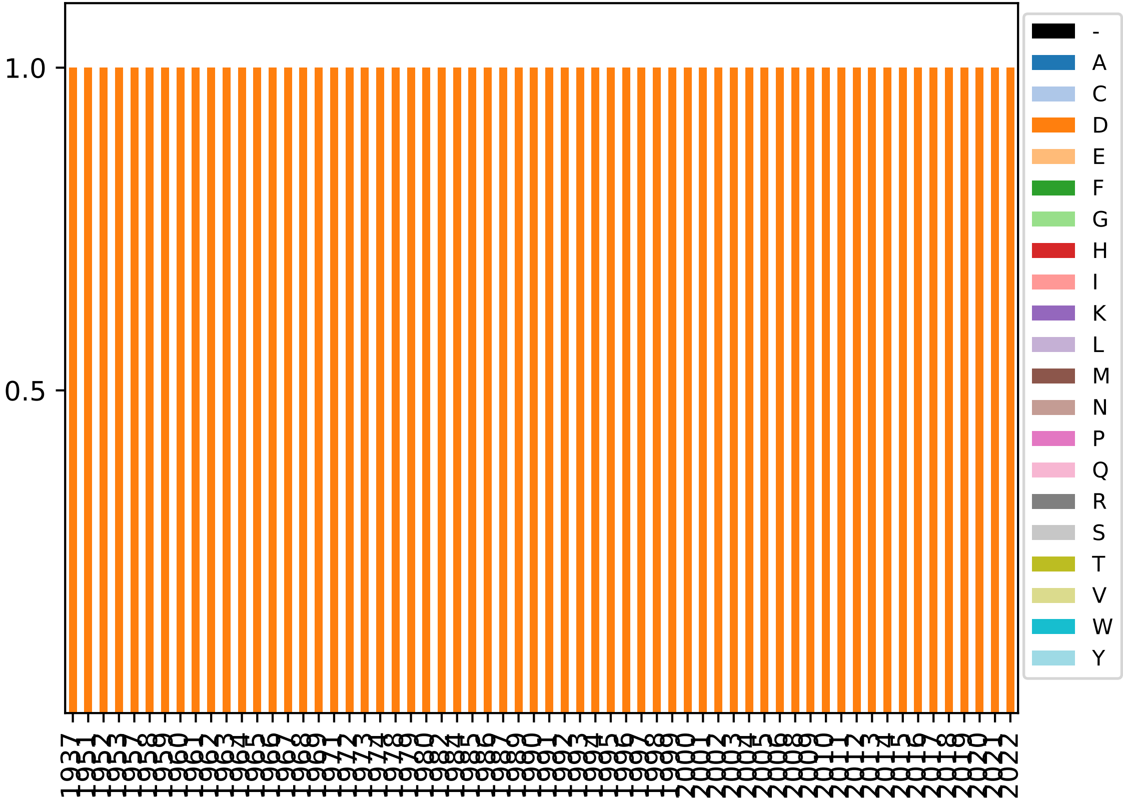

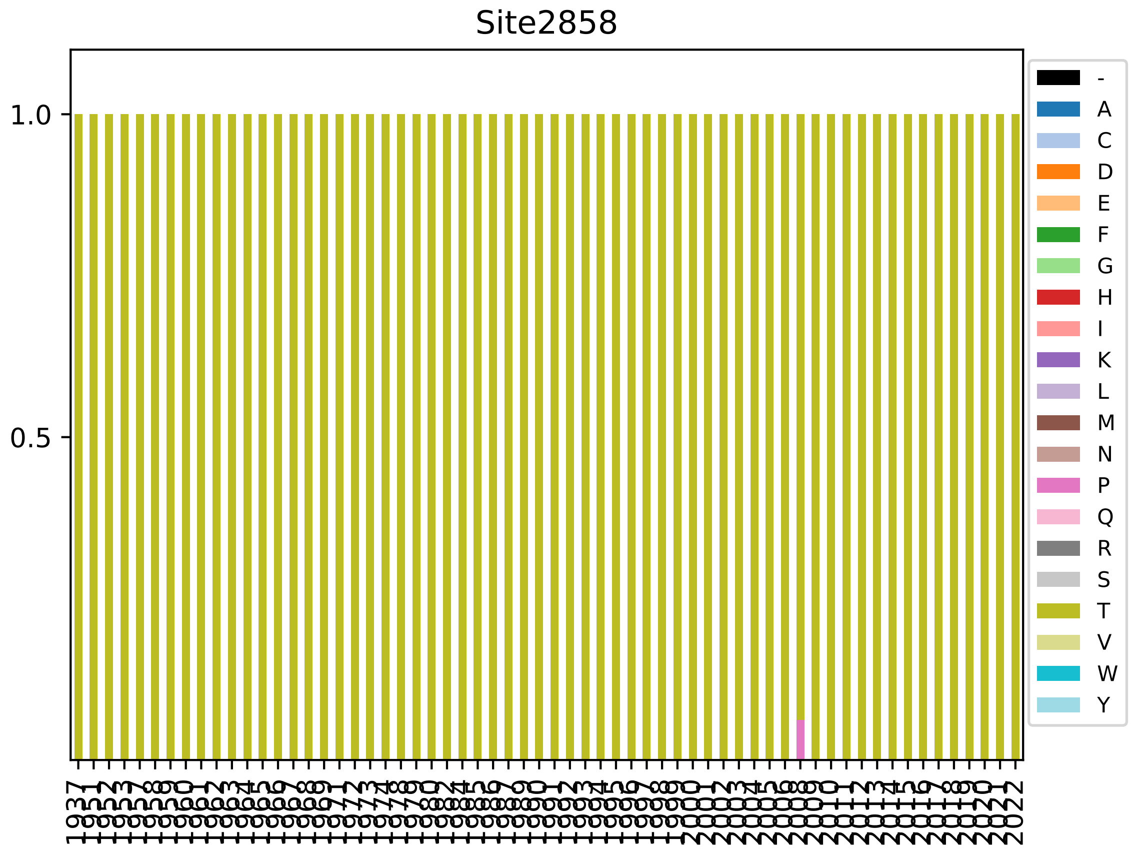
Site2858
1.0
0.5
-
A
C
D
E
F
G
H
I
K
L
M
N
P
Q
R
S
T
V
W
Y

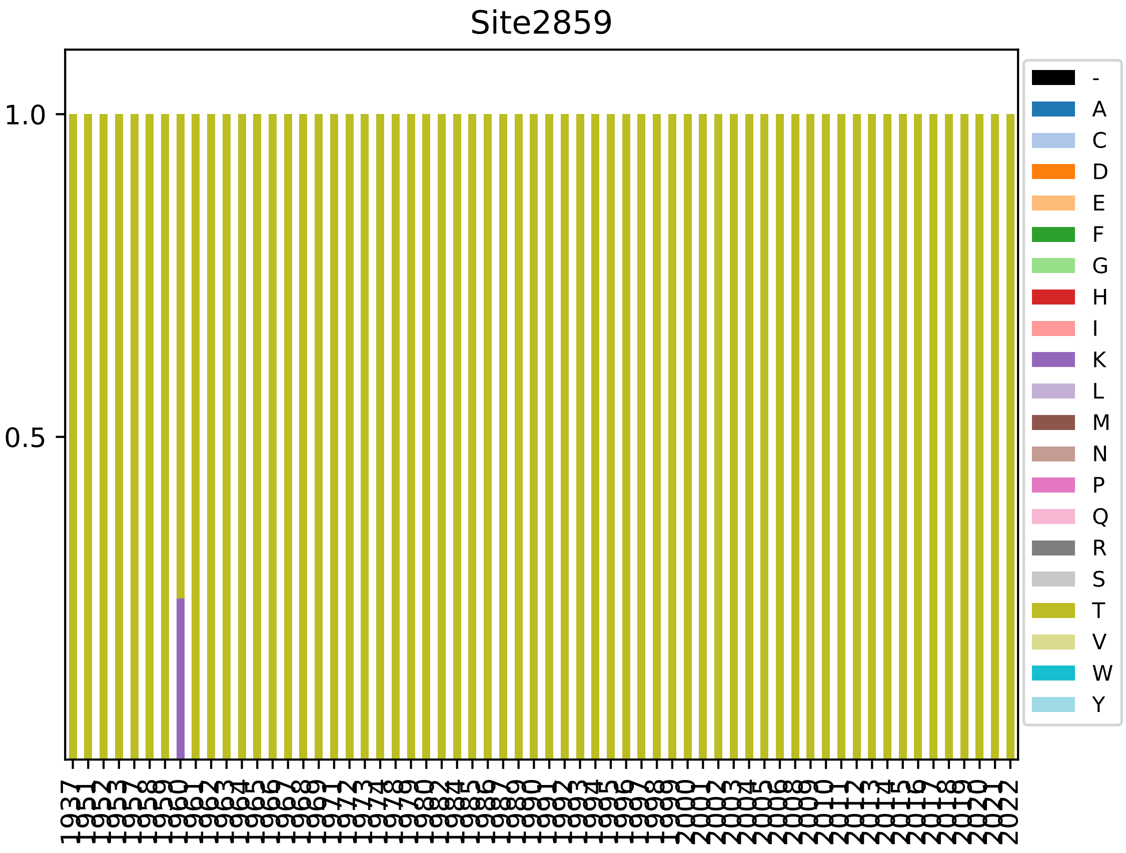
Site2859
1.0
0.5
-
A
C
D
E
F
G
H
I
K
L
M
N
P
Q
R
S
T
V
W
Y

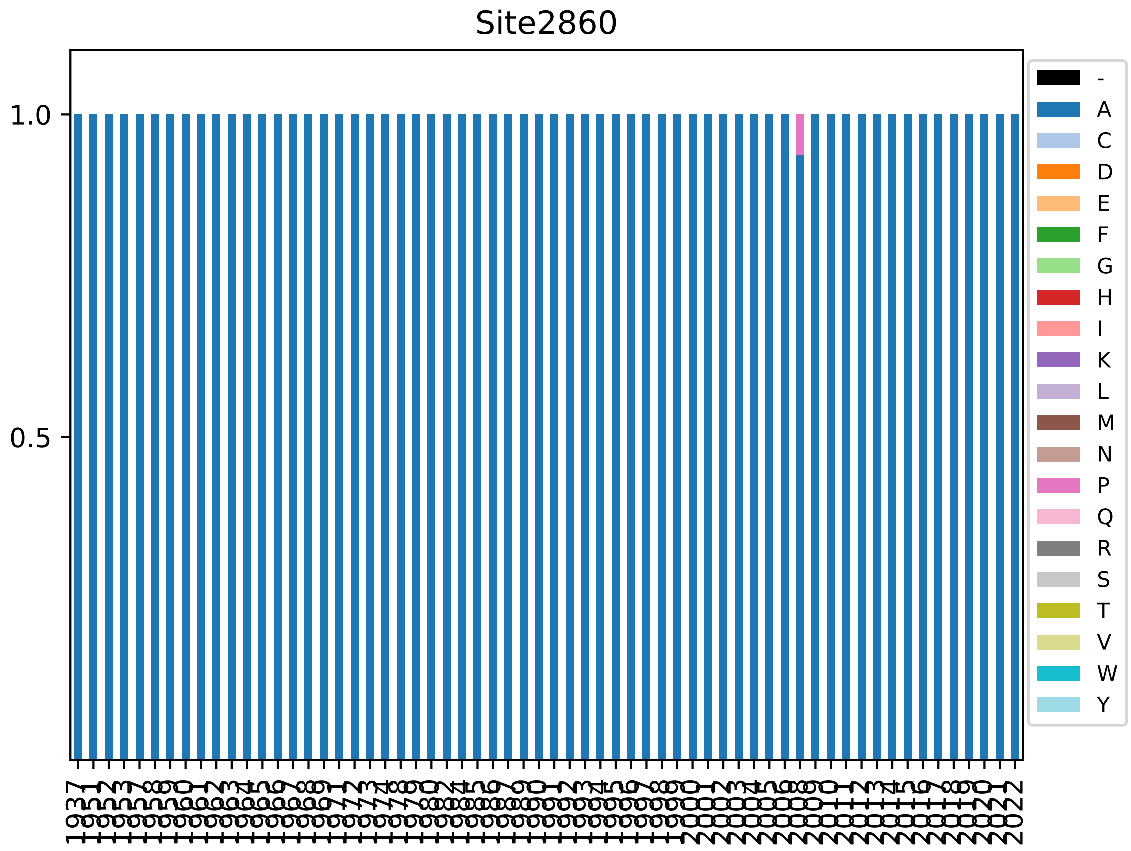
Site2860
1.0
0.5
-
A
C
D
E
F
G
H
I
K
L
M
N
P
Q
R
S
T
V
W
Y

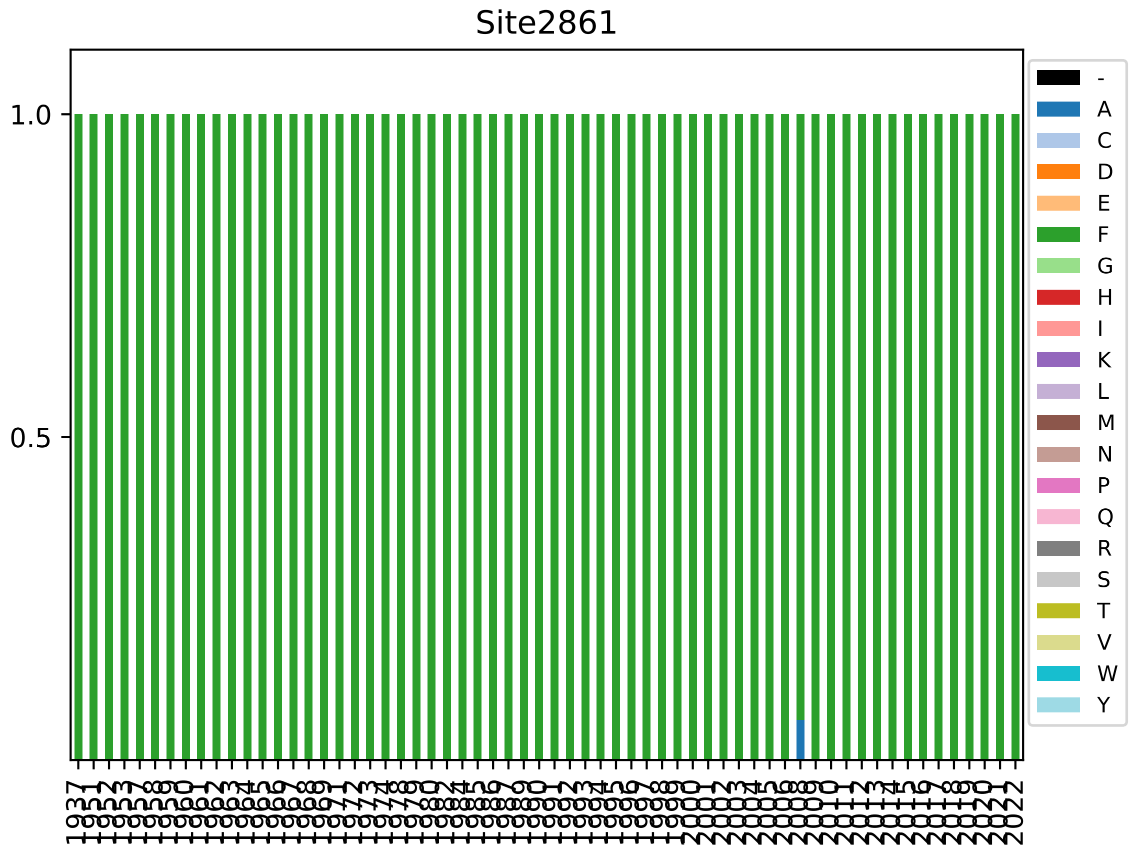
Site2861
1.0
0.5
-
A
C
D
E
F
G
H
I
K
L
M
N
P
Q
R
S
T
V
W
Y

Site2862

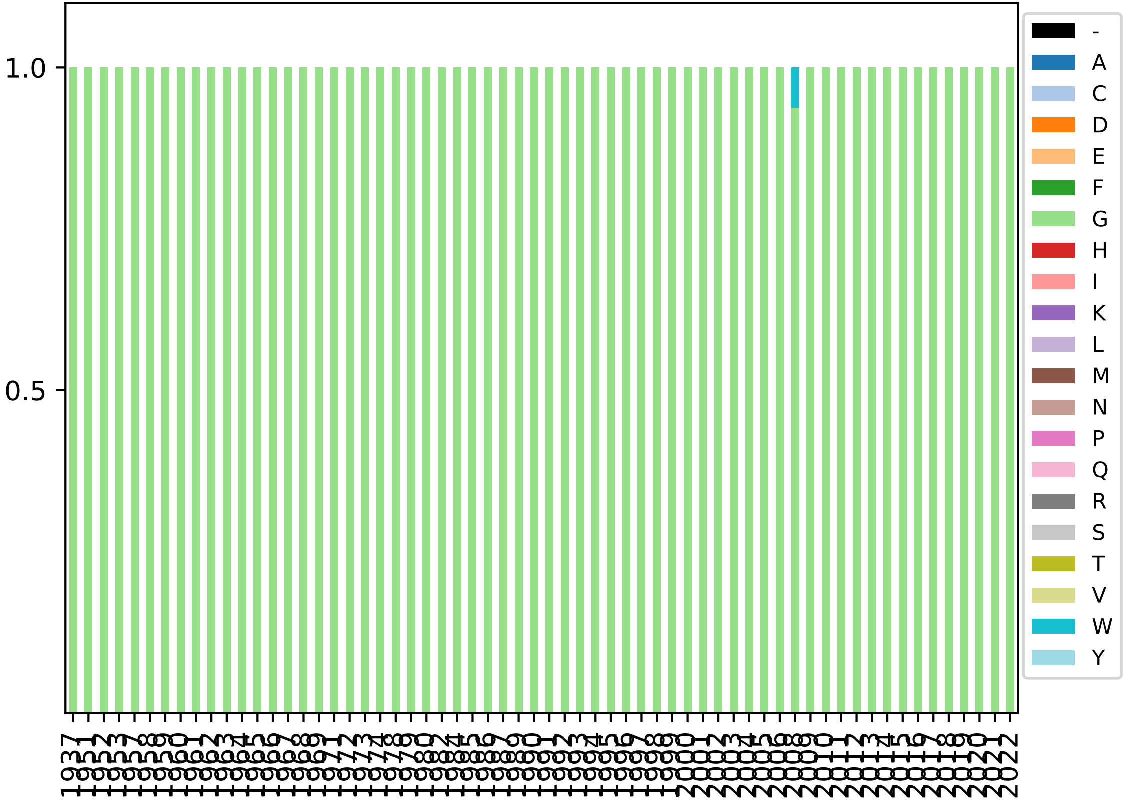

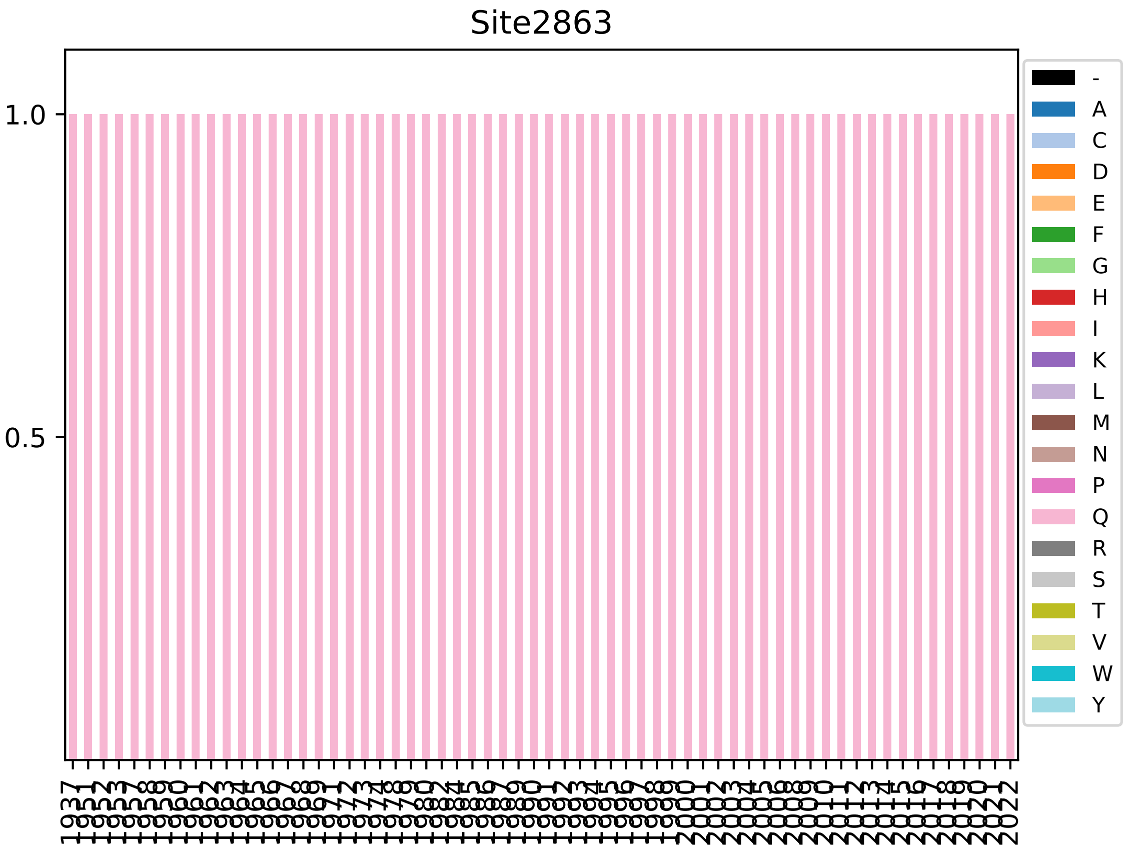
Site2863
1.0
0.5
-
A
C
D
E
F
G
H
I
K
L
M
N
P
Q
R
S
T
V
W
Y

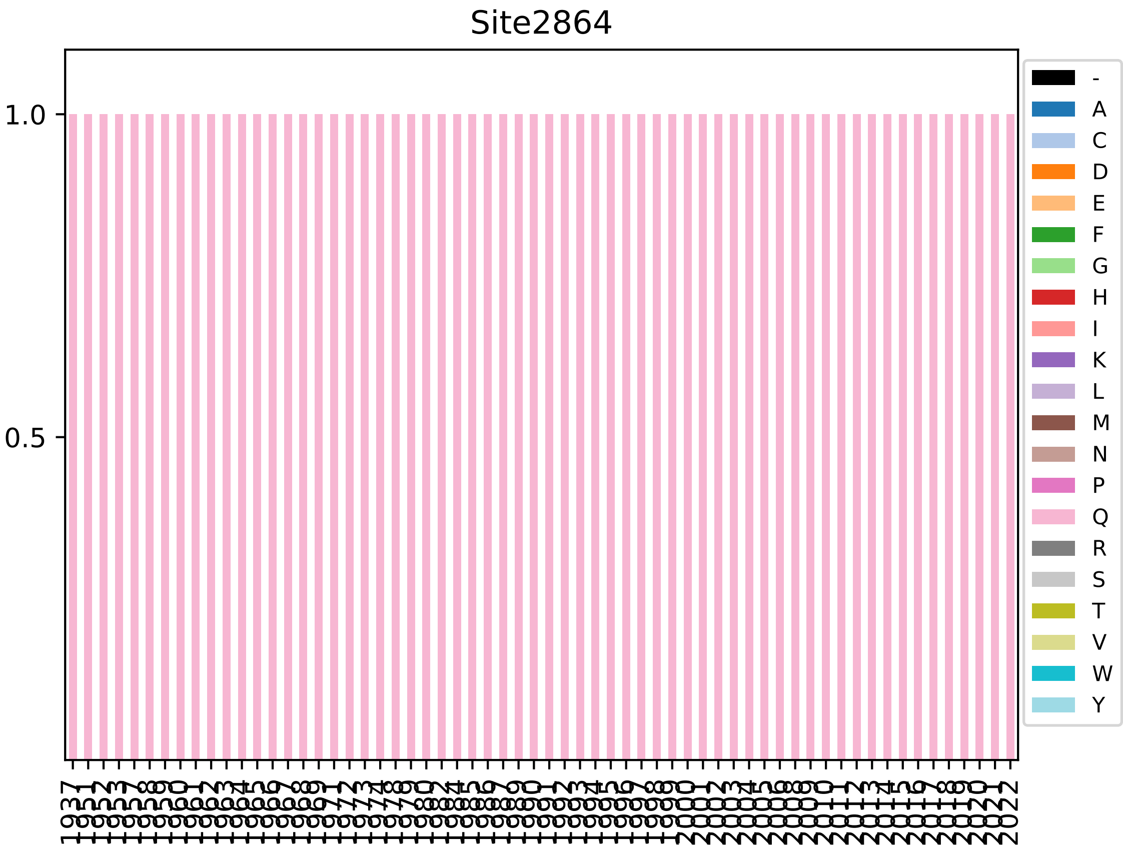
Site2864
1.0
0.5
-
A
C
D
E
F
G
H
I
K
L
M
N
P
Q
R
S
T
V
W
Y

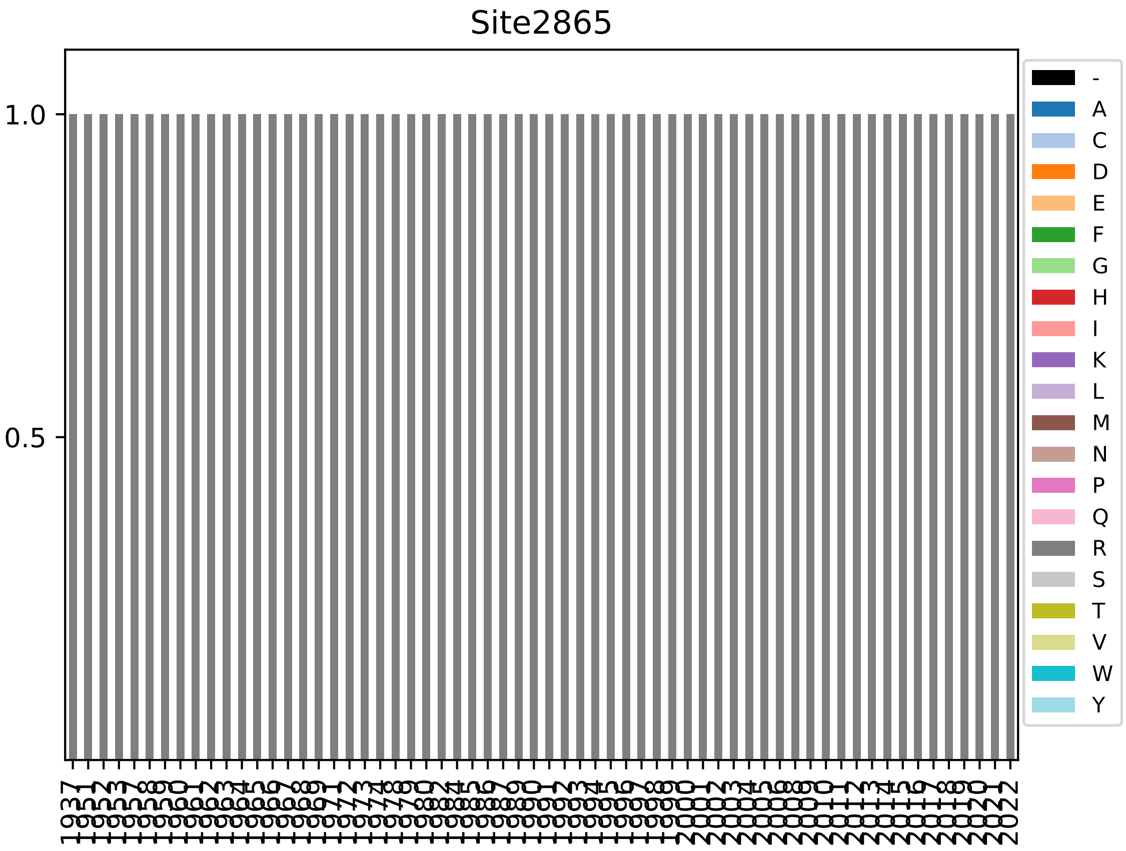
Site2865
1.0
0.5
-
A
C
D
E
F
G
H
I
K
L
M
N
P
Q
R
S
T
V
W
Y

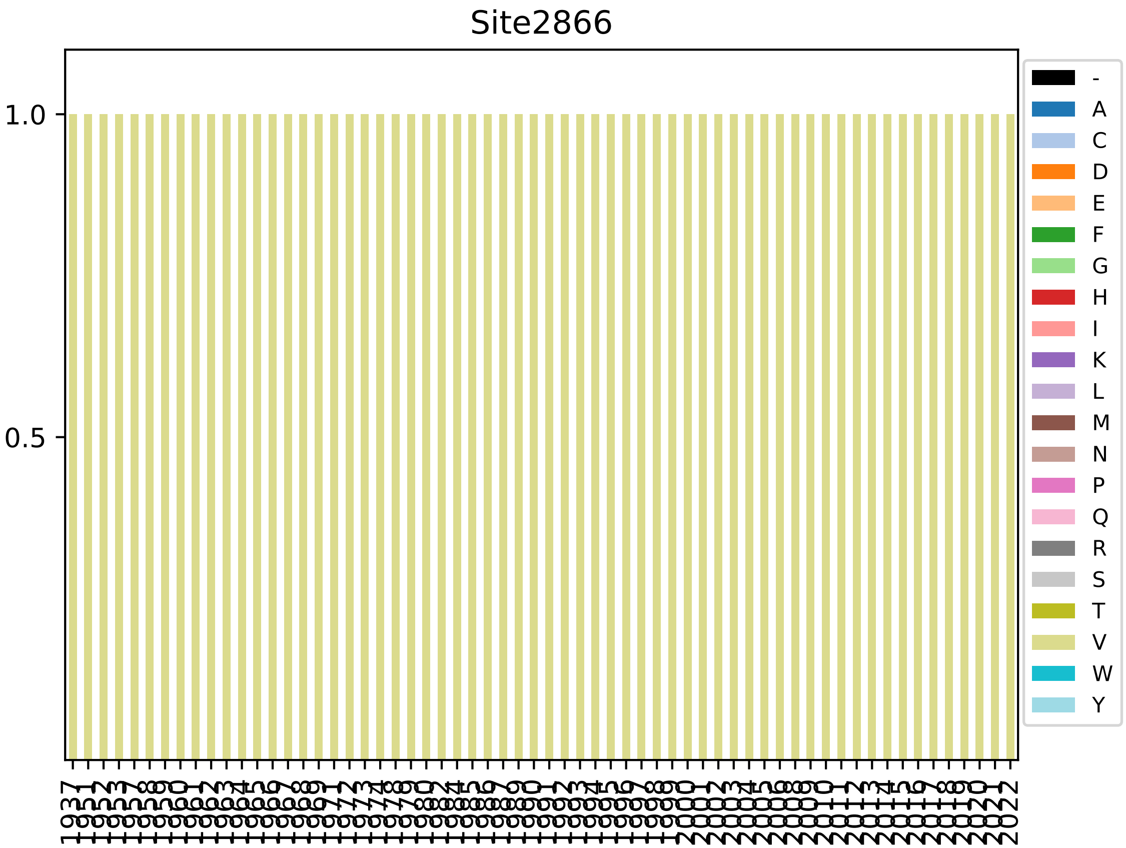
Site2866
1.0
0.5
-
A
C
D
E
F
G
H
I
K
L
M
N
P
Q
R
S
T
V
W
Y

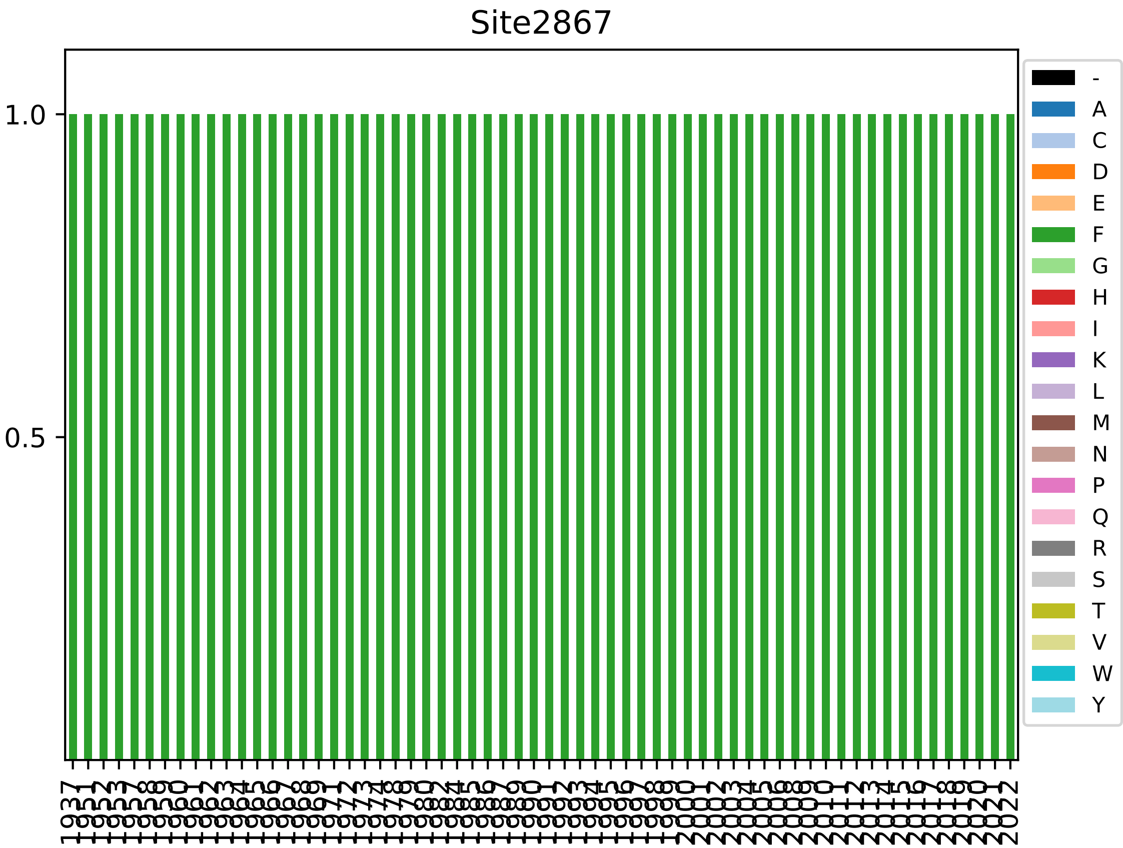
Site2867
1.0
0.5
-
A
C
D
E
F
G
H
I
K
L
M
N
P
Q
R
S
T
V
W
Y

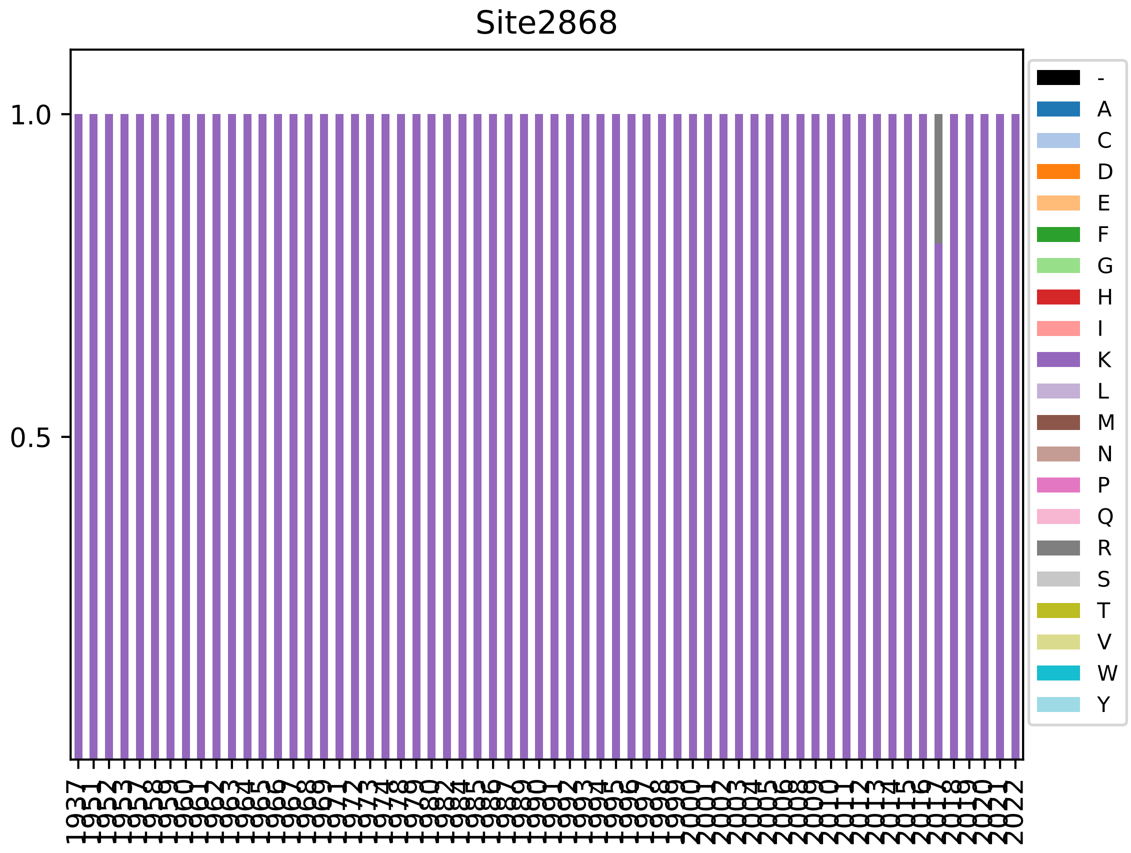

Site2868
1.0
0.5
-
A
C
D
E
F
G
H
I
K
L
M
N
P
Q
R
S
T
V
W
Y

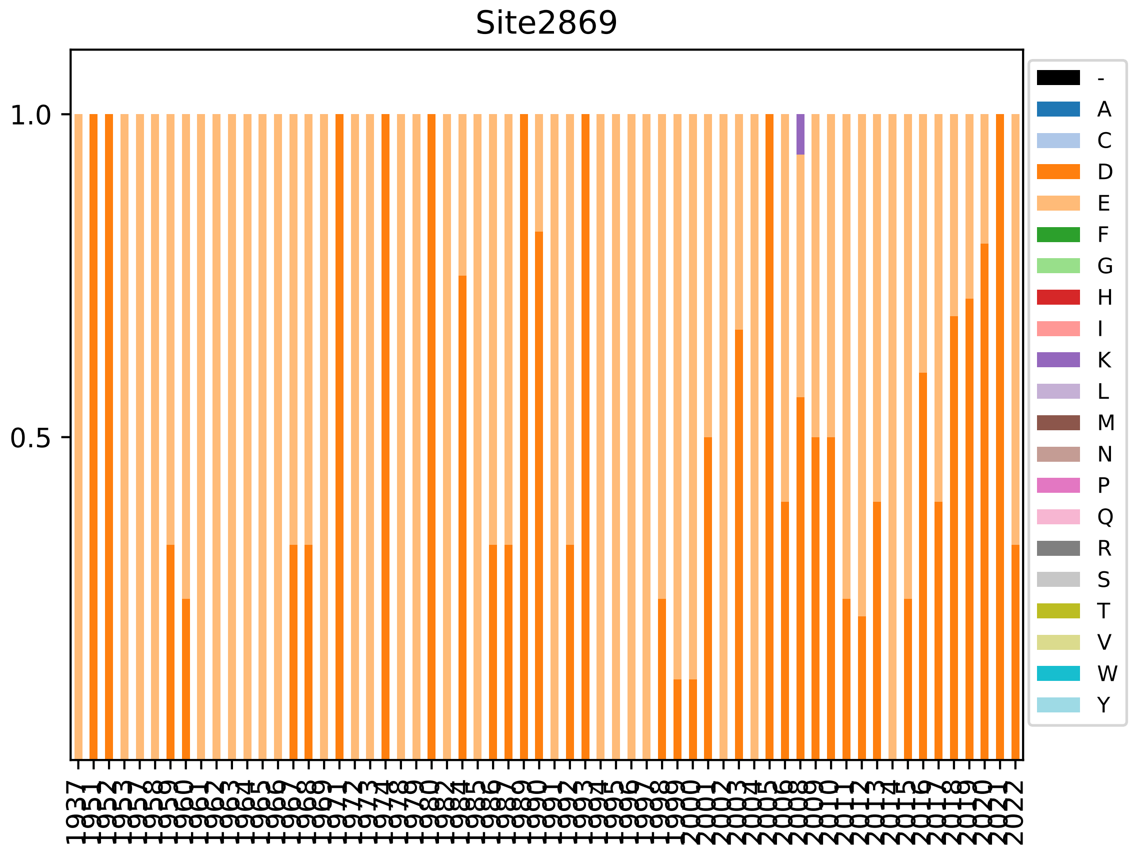

Site2869
1.0
0.5
-
A
C
D
E
F
G
H
I
K
L
M
N
P
Q
R
S
T
V
W
Y

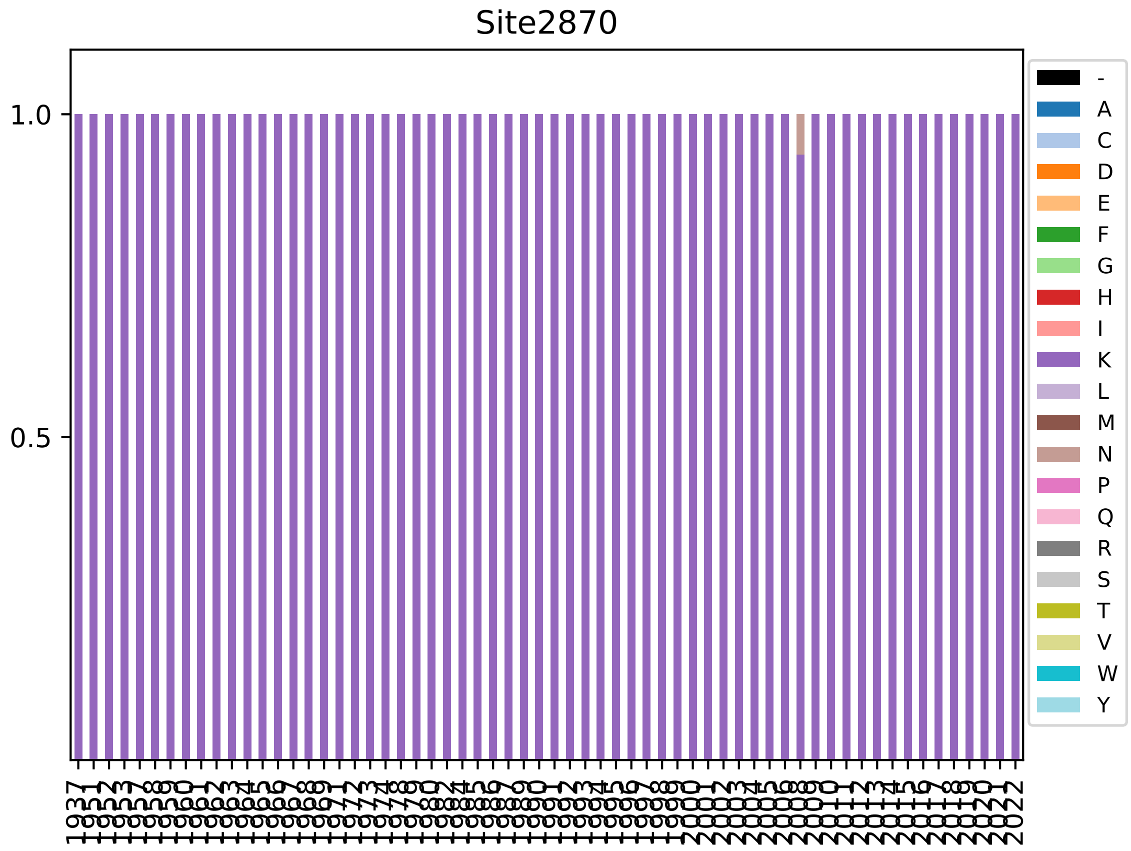

Site2870
1.0
0.5
-
A
C
D
E
F
G
H
I
K
L
M
N
P
Q
R
S
T
V
W
Y

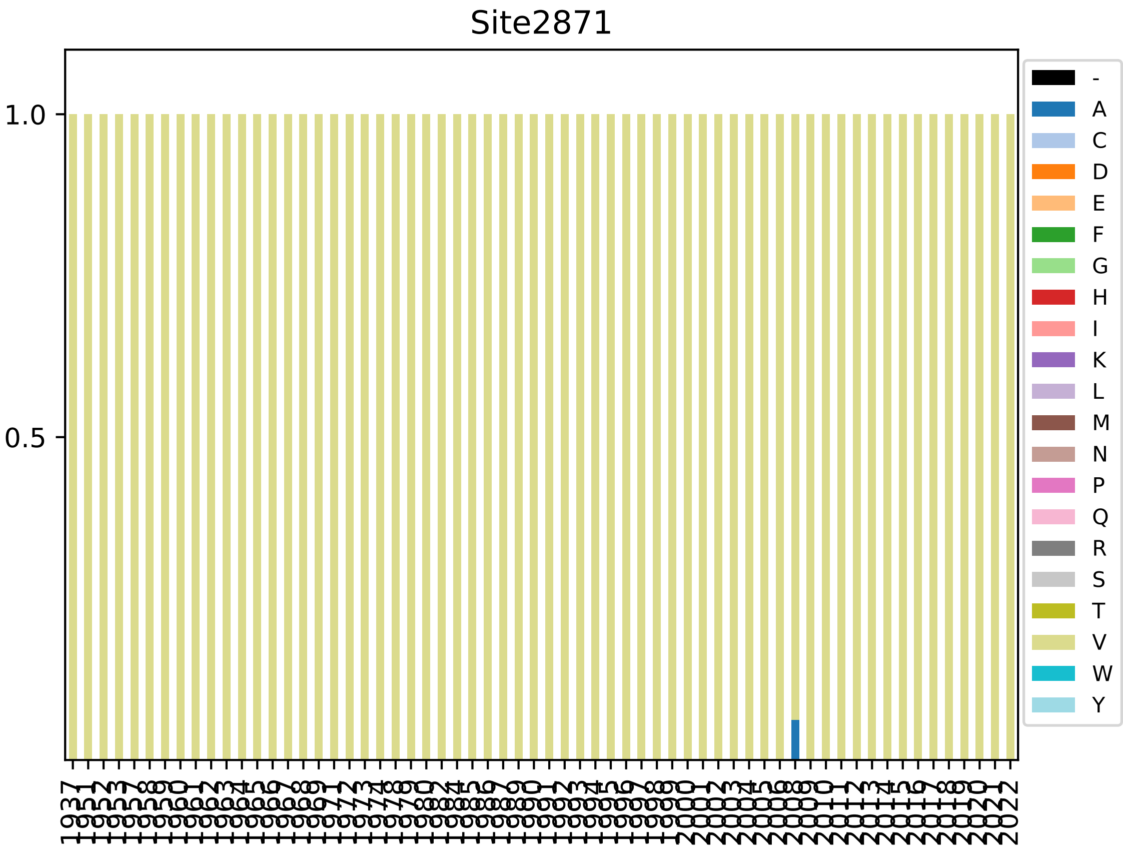
Site2871
1.0
0.5
-
A
C
D
E
F
G
H
I
K
L
M
N
P
Q
R
S
T
V
W
Y

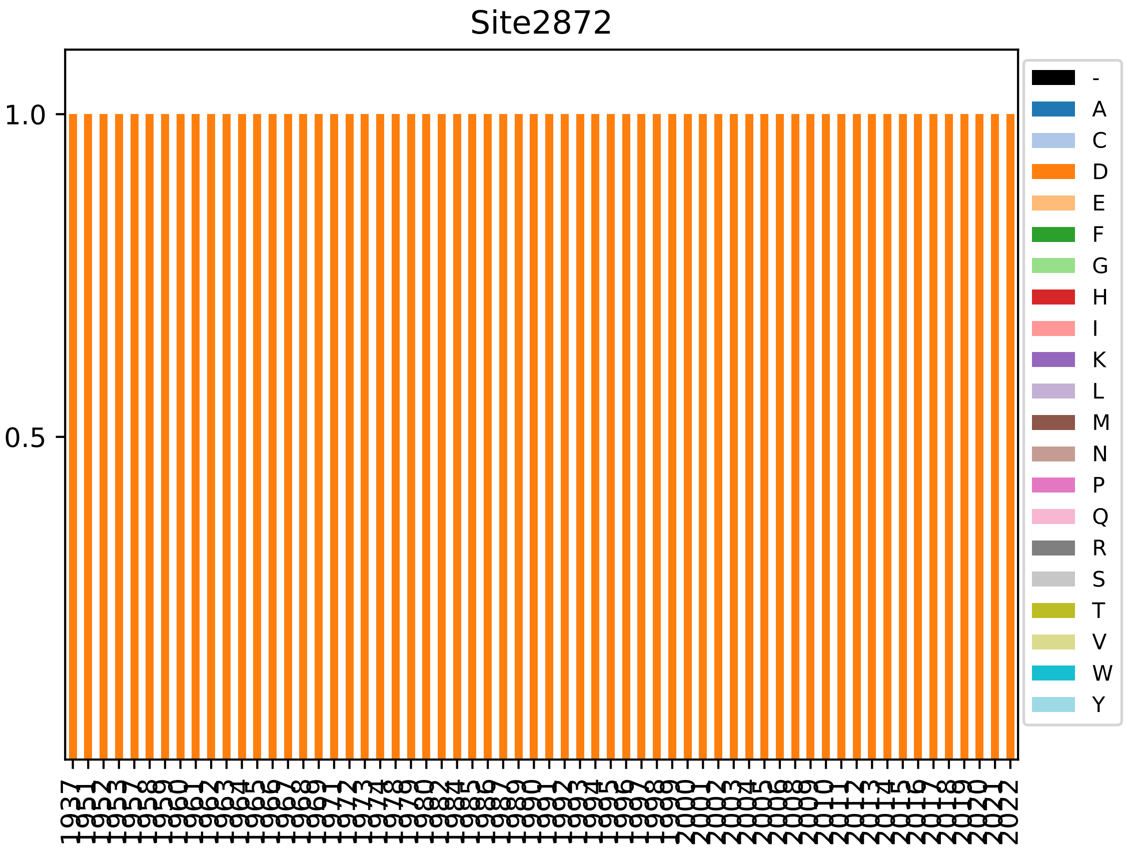
Site2872
1.0
0.5
-
A
C
D
E
F
G
H
I
K
L
M
N
P
Q
R
S
T
V
W
Y

Site2873

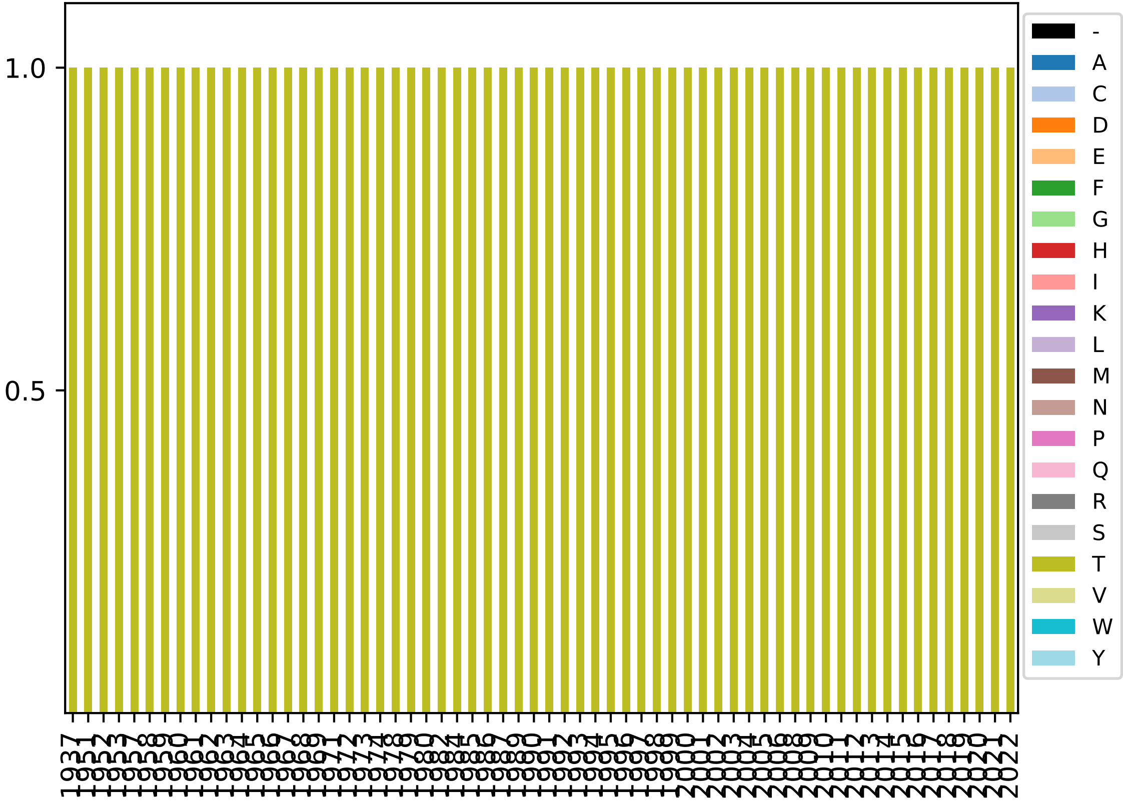

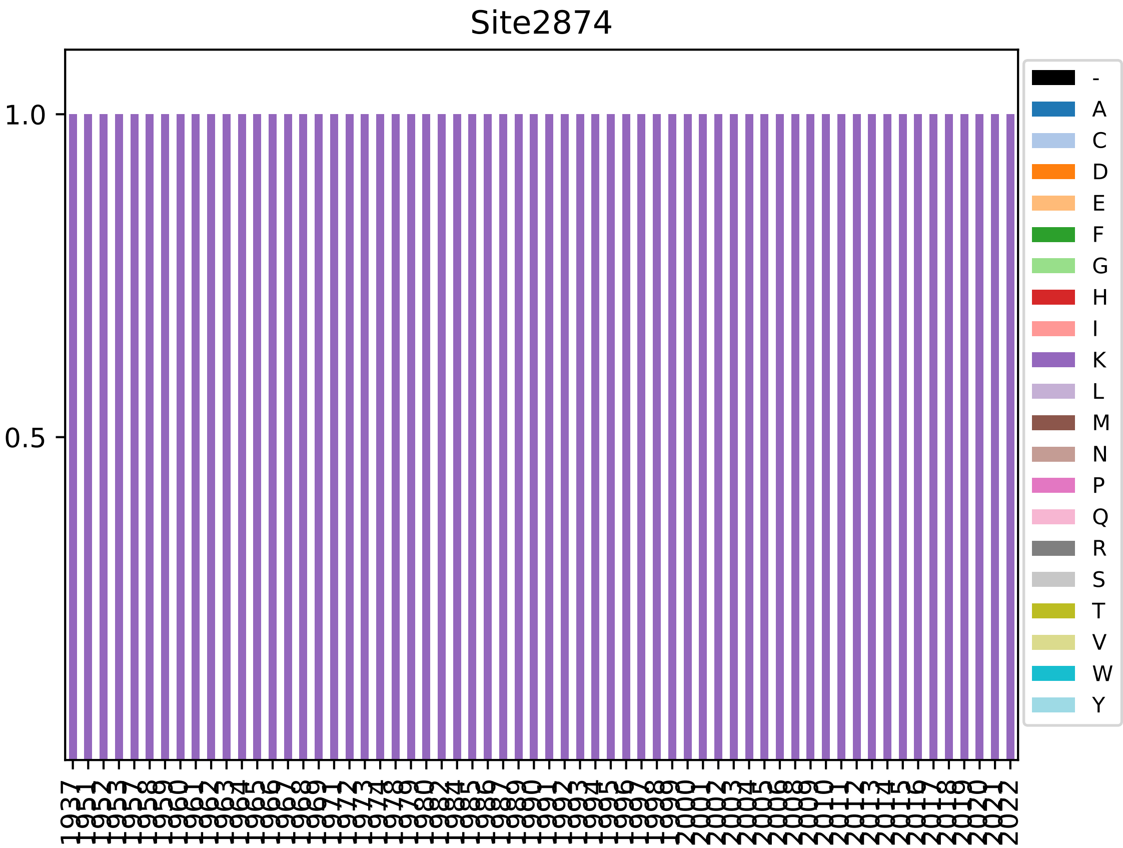
Site2874
1.0
0.5
-
A
C
D
E
F
G
H
I
K
L
M
N
P
Q
R
S
T
V
W
Y

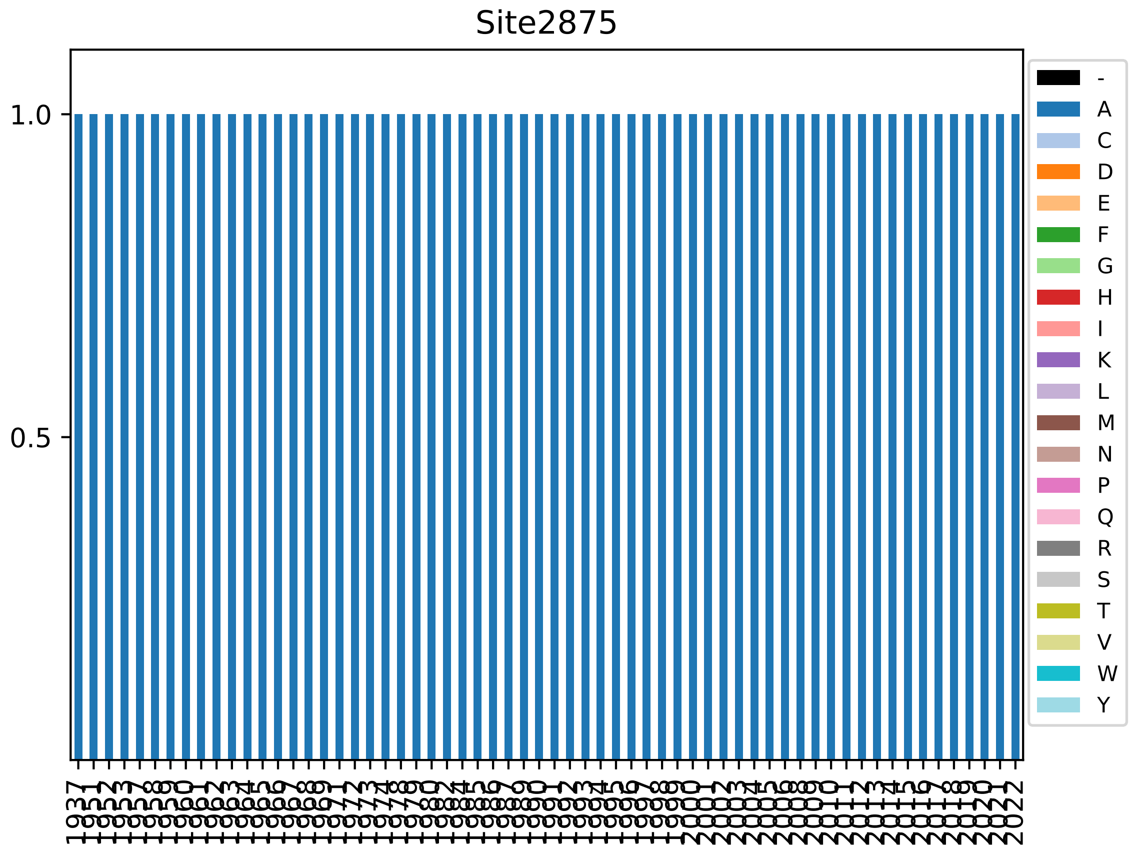

Site2875
1.0
0.5
-
A
C
D
E
F
G
H
I
K
L
M
N
P
Q
R
S
T
V
W
Y

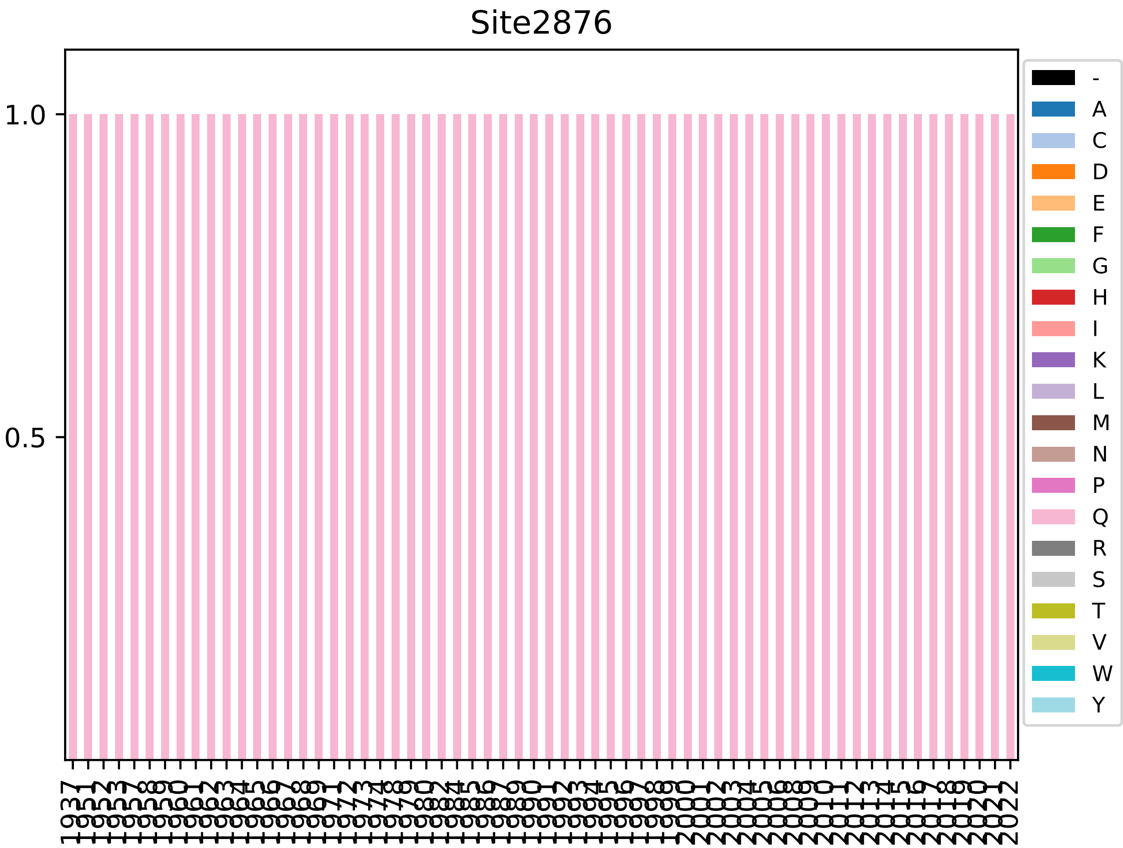
Site2876
1.0
0.5
-
A
C
D
E
F
G
H
I
K
L
M
N
P
Q
R
S
T
V
W
Y

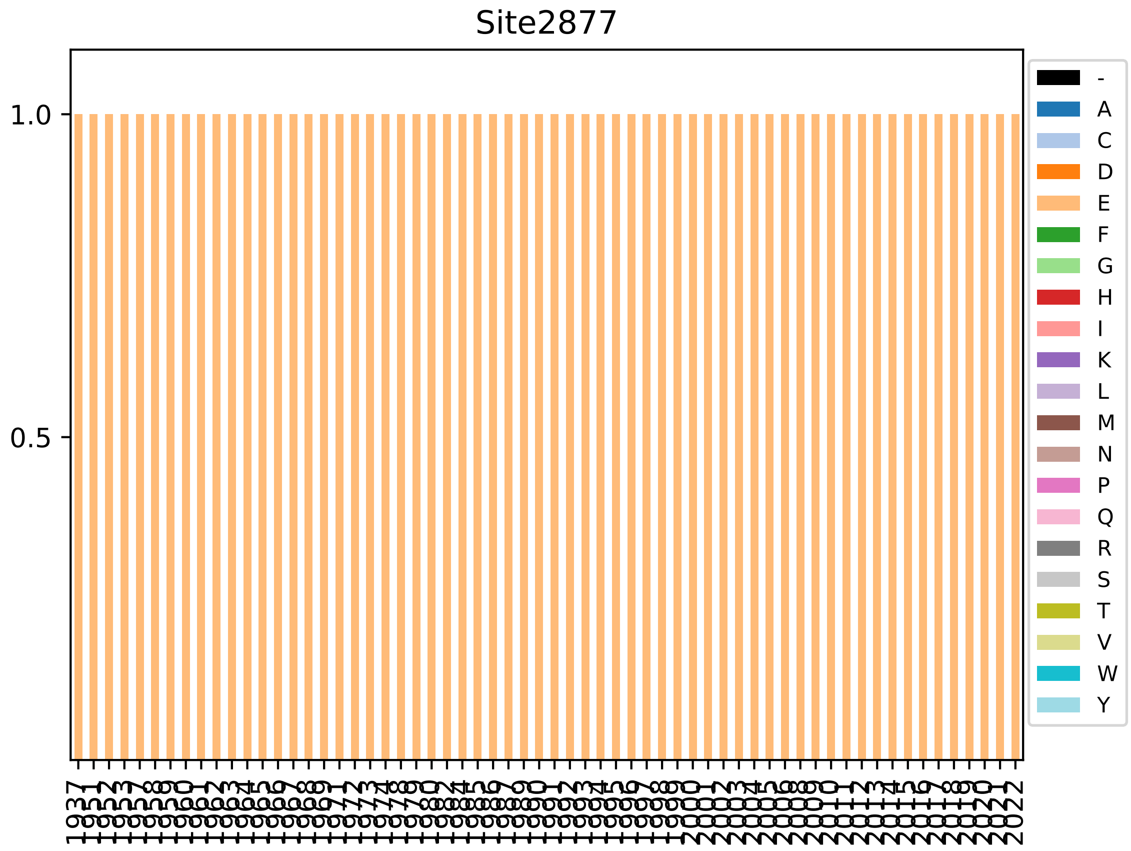
Site2877
1.0
0.5
-
A
C
D
E
F
G
H
I
K
L
M
N
P
Q
R
S
T
V
W
Y

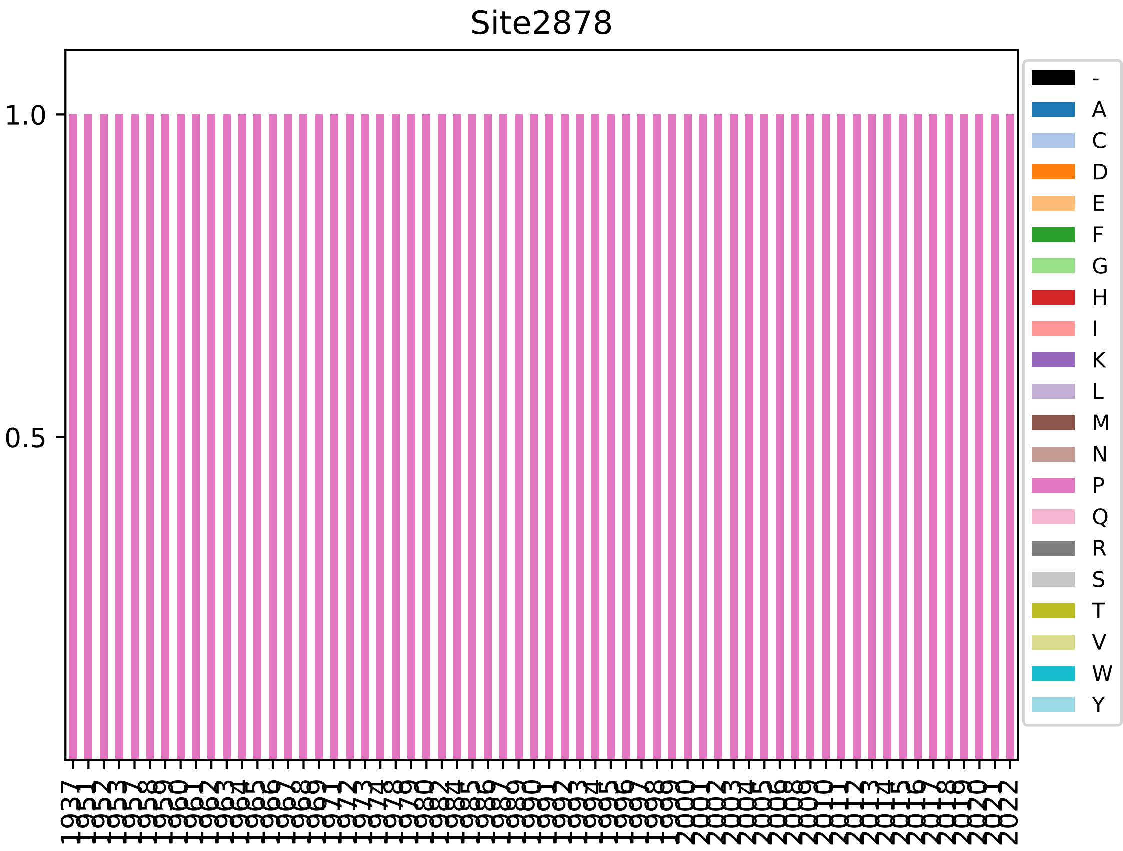
Site2878
1.0
0.5
-
A
C
D
E
F
G
H
I
K
L
M
N
P
Q
R
S
T
V
W
Y

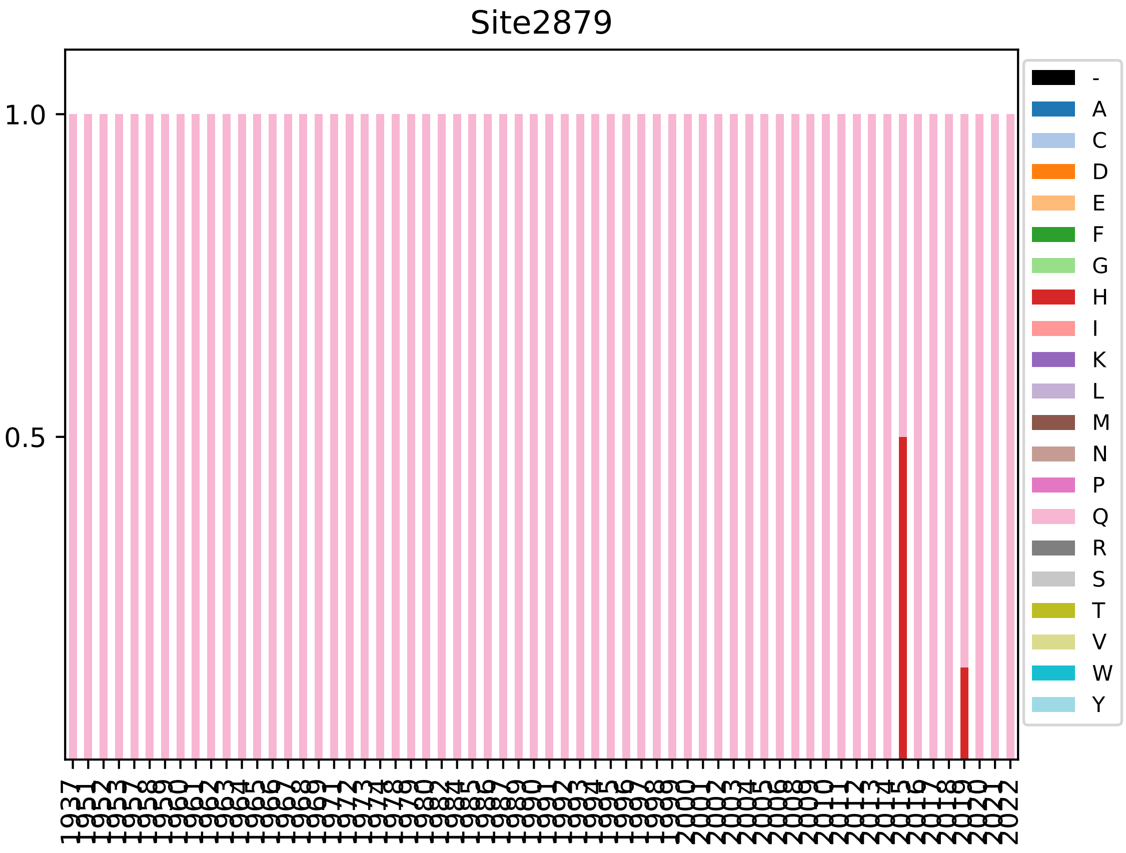
Site2879
1.0
0.5
-
A
C
D
E
F
G
H
I
K
L
M
N
P
Q
R
S
T
V
W
Y

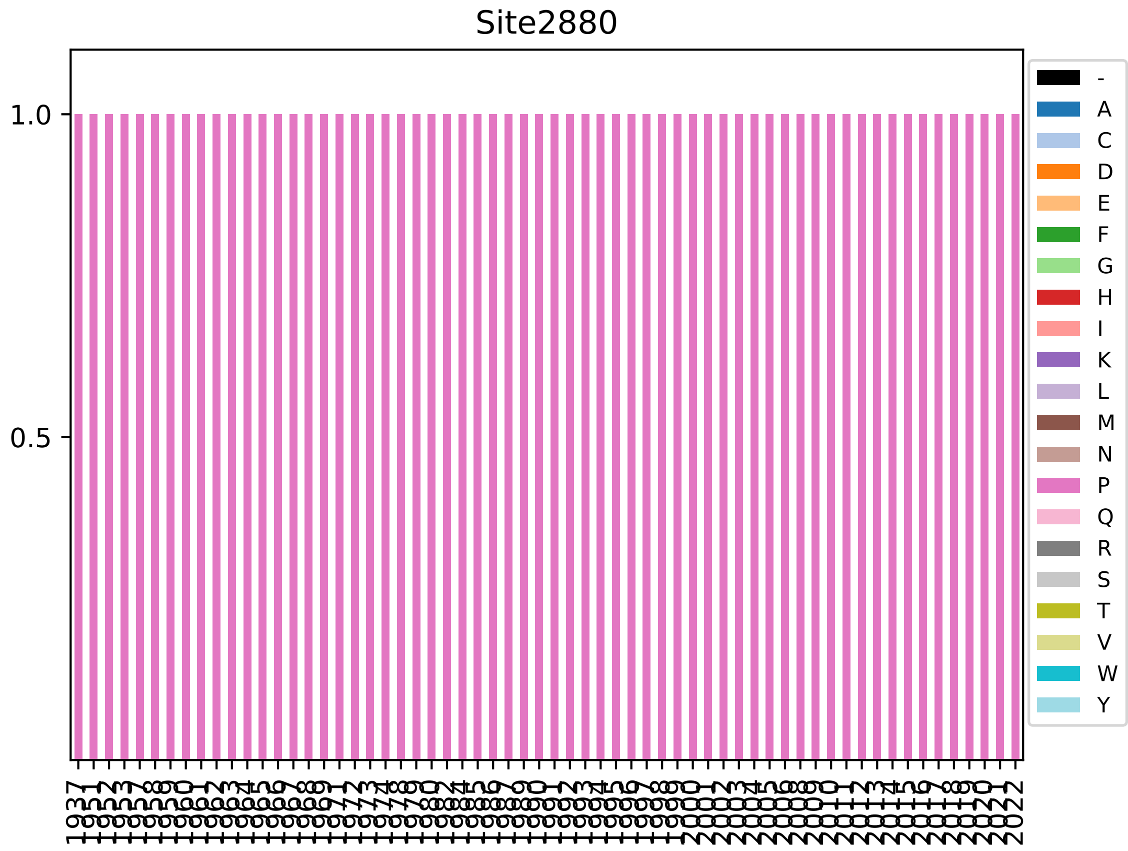
Site2880
1.0
0.5
-
A
C
D
E
F
G
H
I
K
L
M
N
P
Q
R
S
T
V
W
Y

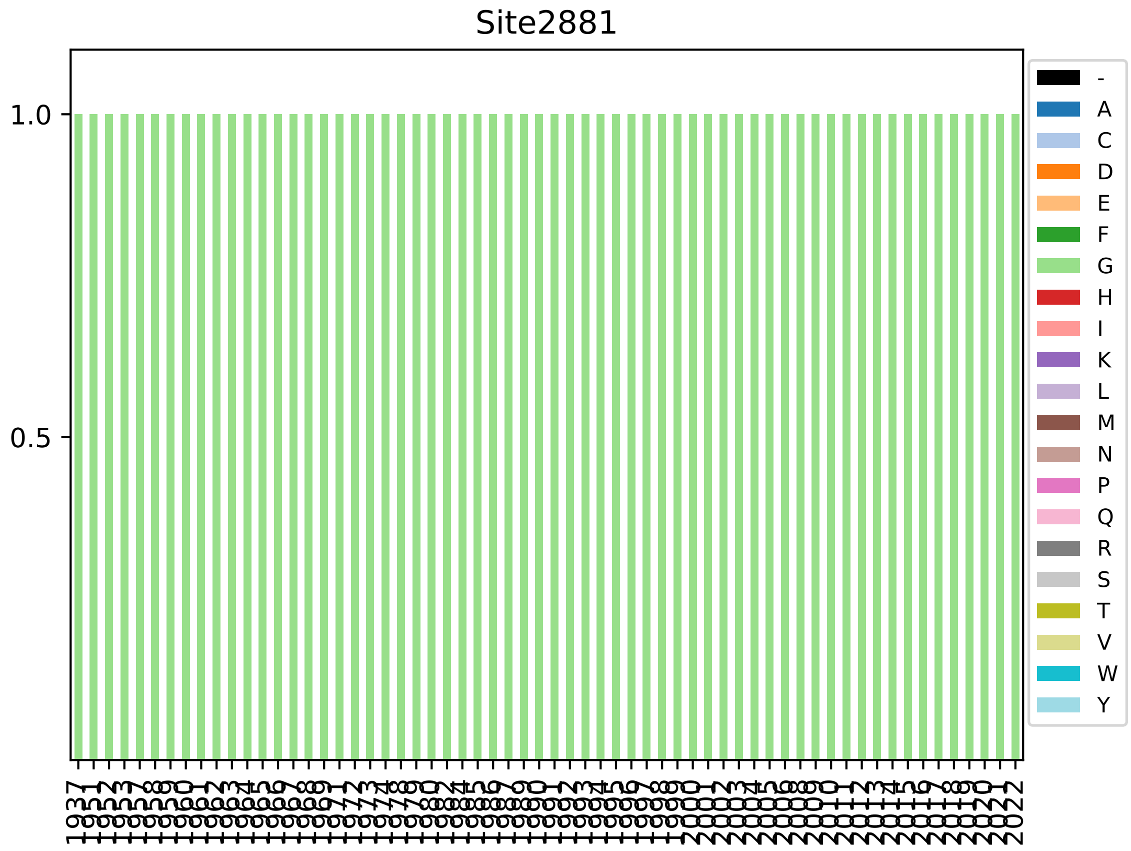
Site2881
1.0
0.5
-
A
C
D
E
F
G
H
I
K
L
M
N
P
Q
R
S
T
V
W
Y

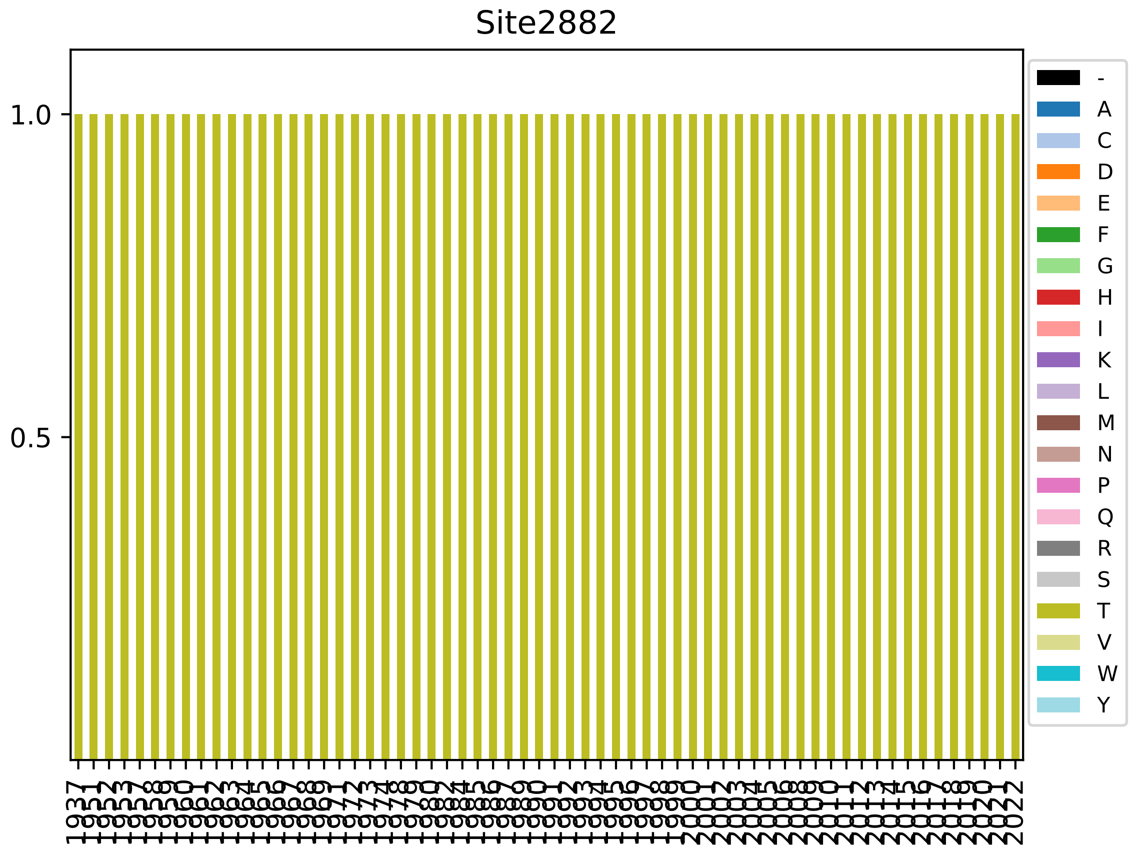
Site2882
1.0
0.5
-
A
C
D
E
F
G
H
I
K
L
M
N
P
Q
R
S
T
V
W
Y

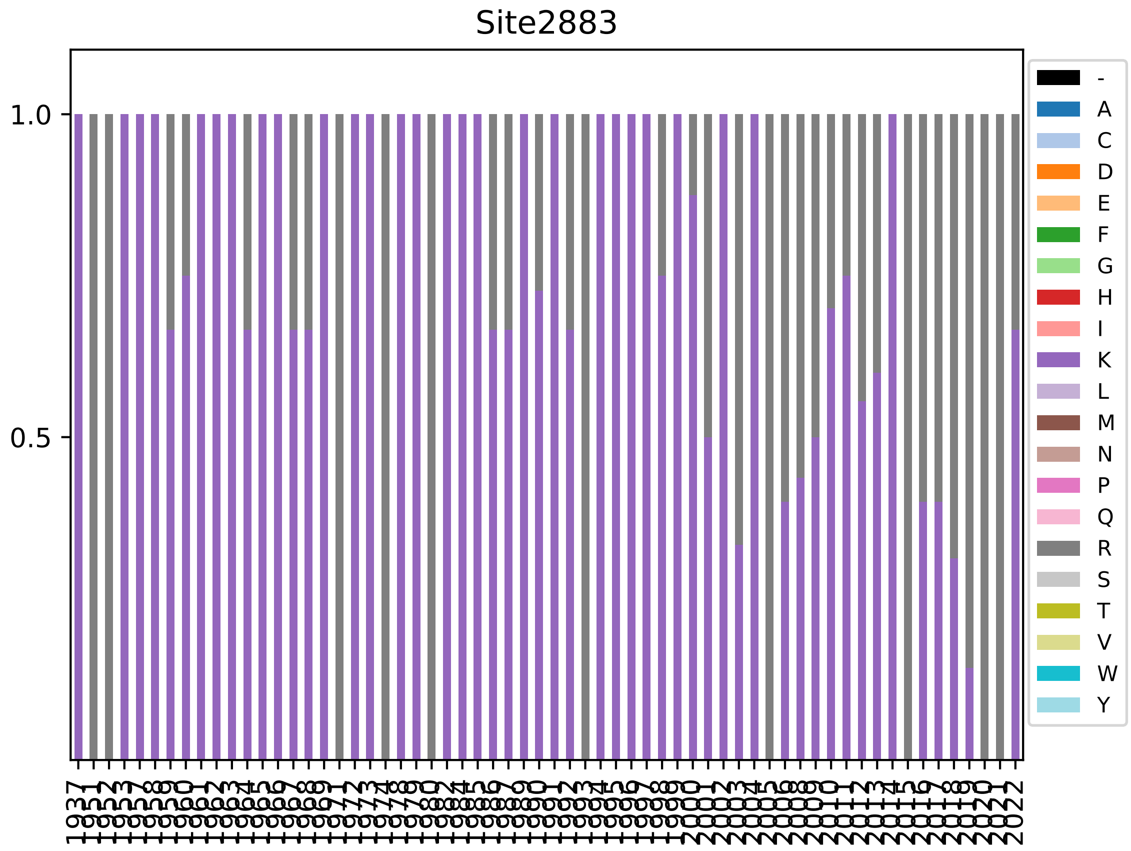
Site2883
1.0
0.5
-
A
C
D
E
F
G
H
I
K
L
M
N
P
Q
R
S
T
V
W
Y

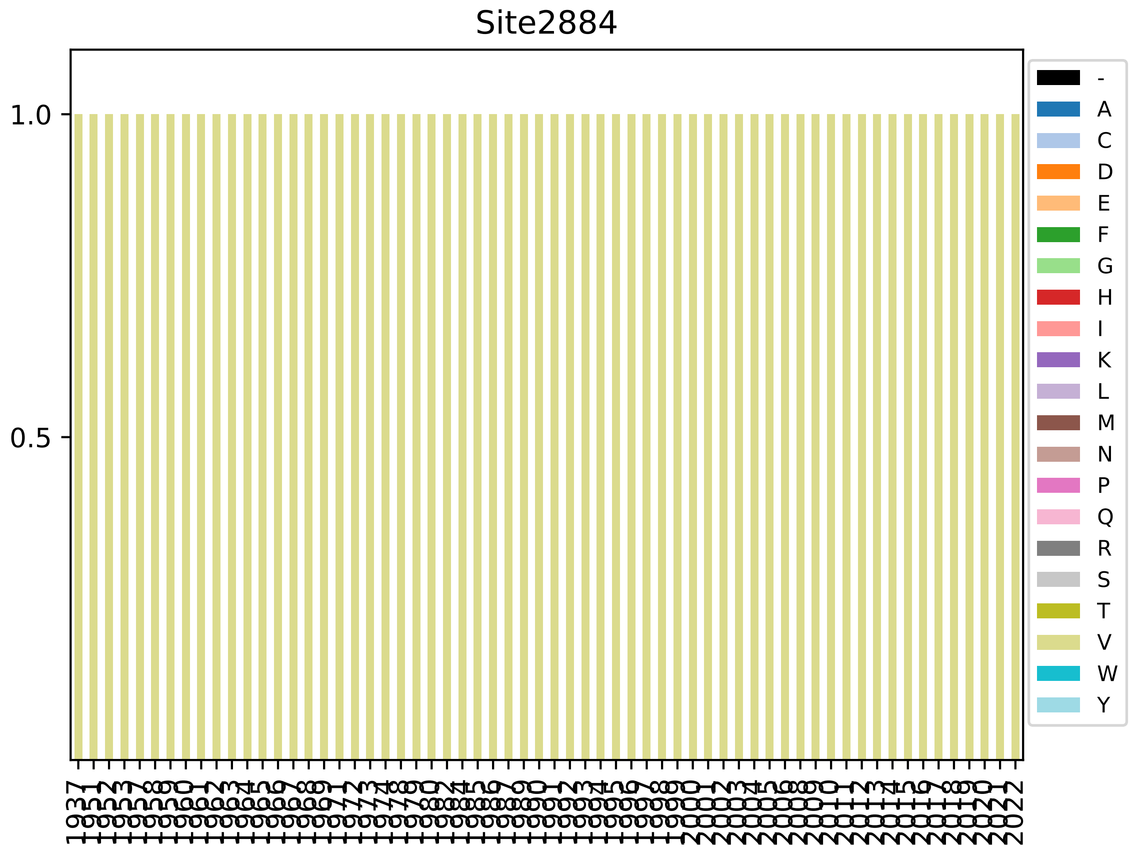
Site2884
1.0
0.5
-
A
C
D
E
F
G
H
I
K
L
M
N
P
Q
R
S
T
V
W
Y

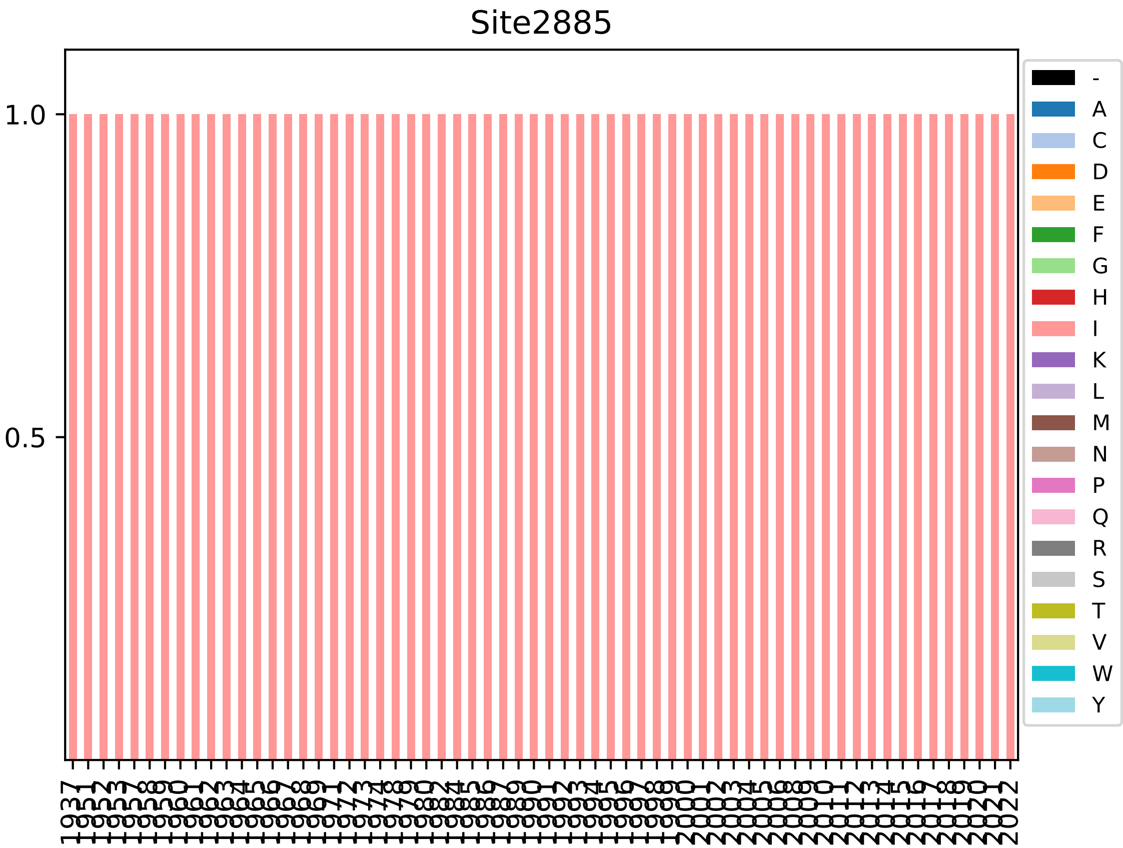
Site2885
1.0
0.5
-
A
C
D
E
F
G
H
I
K
L
M
N
P
Q
R
S
T
V
W
Y

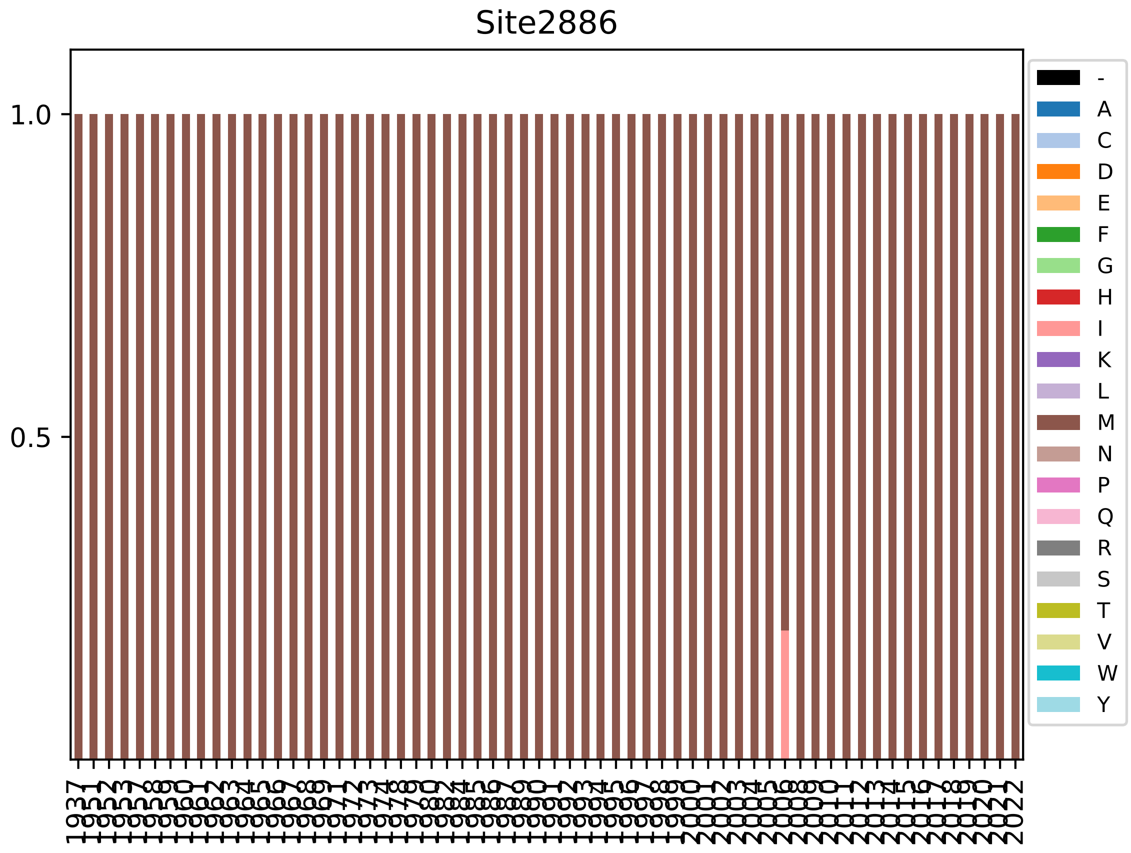

Site2886
1.0
0.5
-
A
C
D
E
F
G
H
I
K
L
M
N
P
Q
R
S
T
V
W
Y

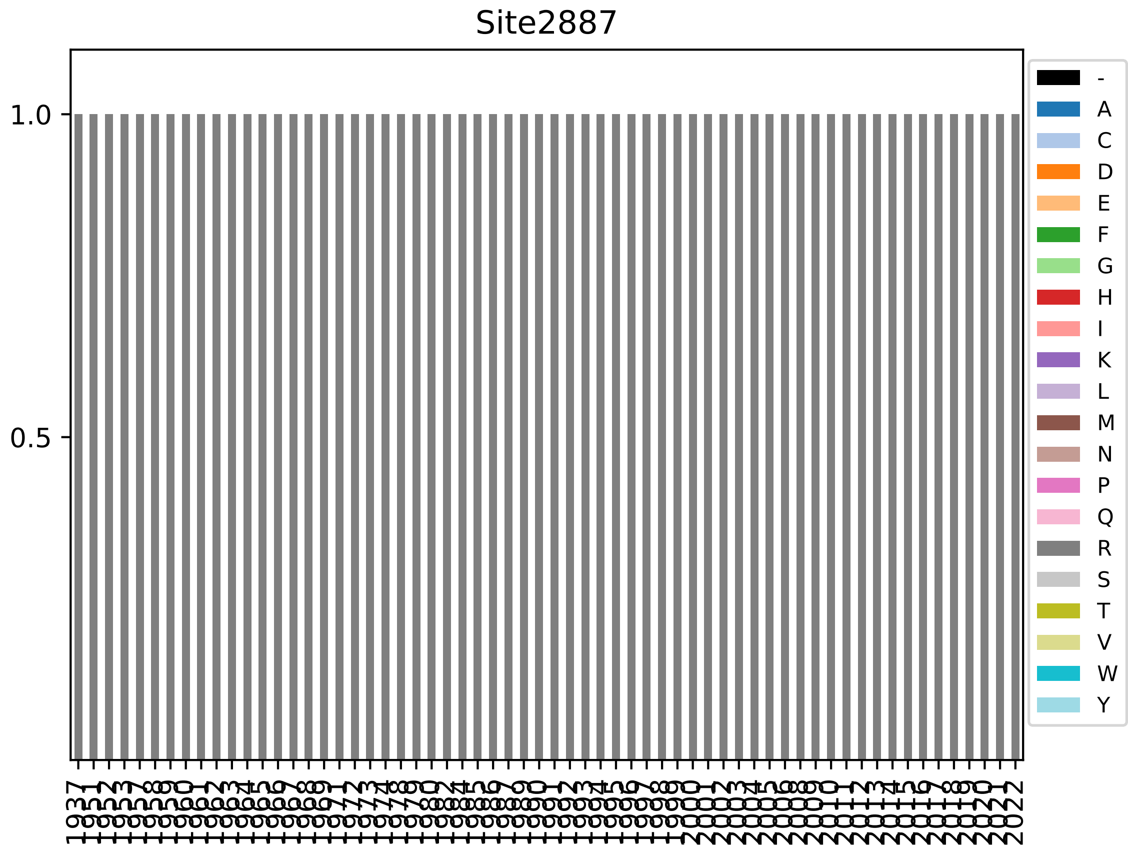

Site2887
1.0
0.5
-
A
C
D
E
F
G
H
I
K
L
M
N
P
Q
R
S
T
V
W
Y

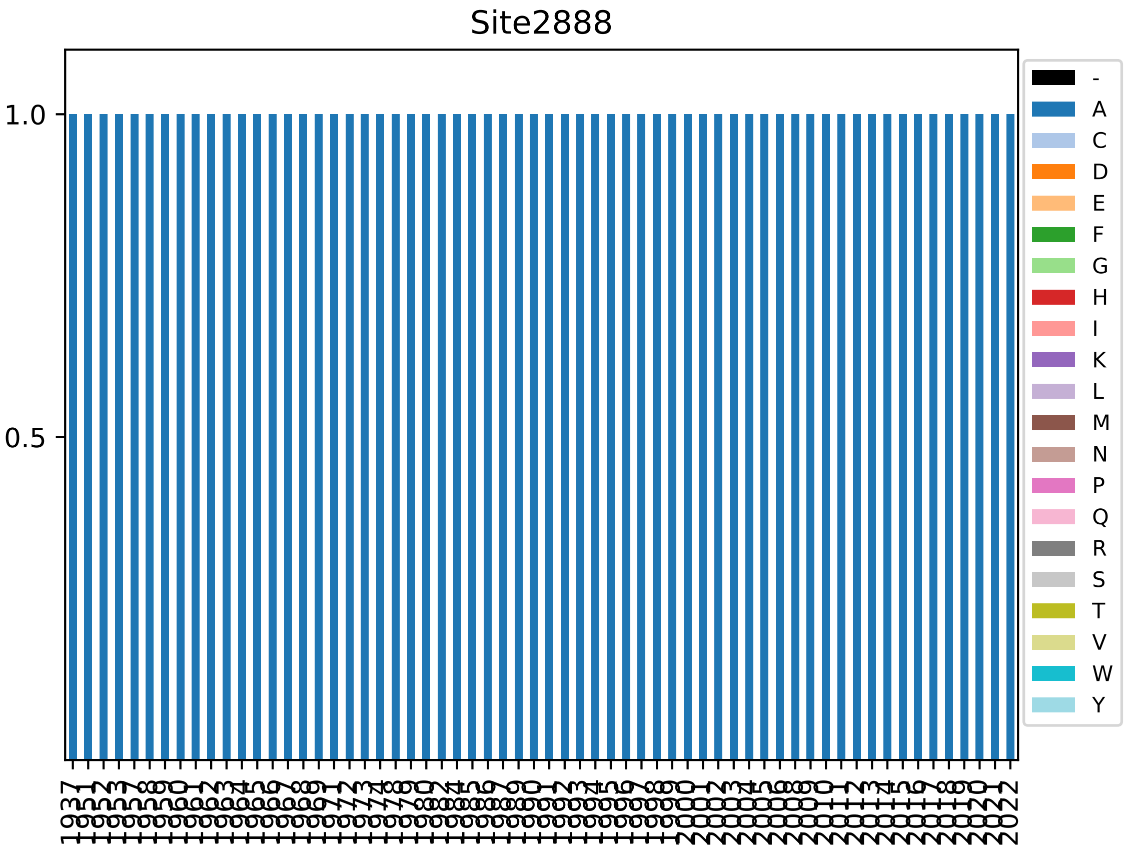
Site2888
1.0
0.5
-
A
C
D
E
F
G
H
I
K
L
M
N
P
Q
R
S
T
V
W
Y

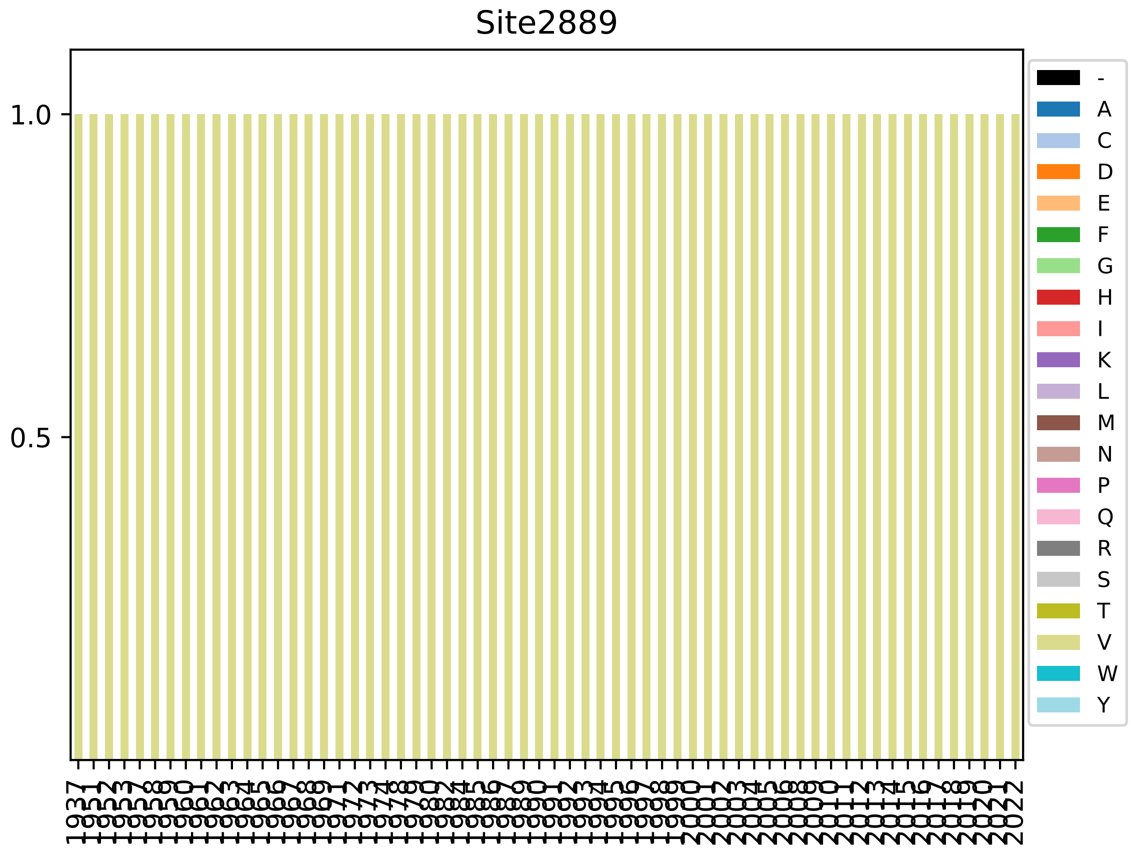
Site2889
1.0
0.5
-
A
C
D
E
F
G
H
I
K
L
M
N
P
Q
R
S
T
V
W
Y

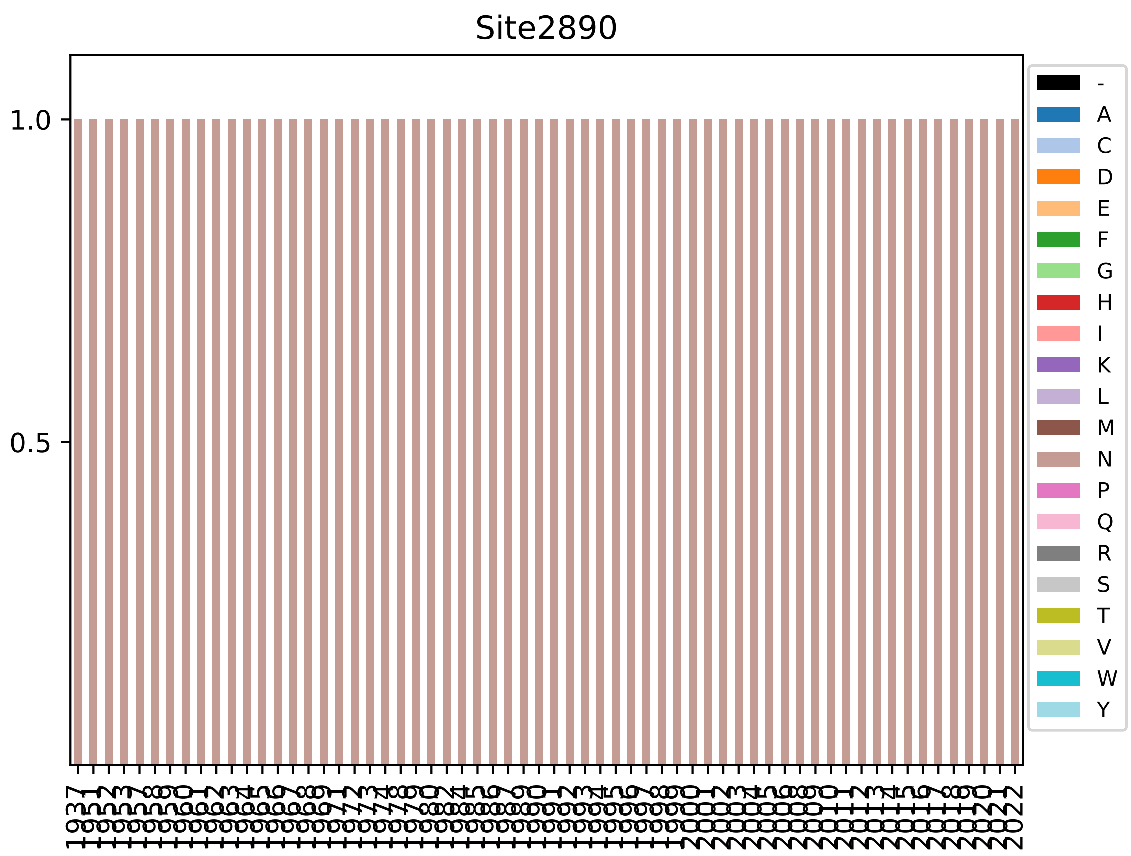
Site2890
1.0
0.5
-
A
C
D
E
F
G
H
I
K
L
M
N
P
Q
R
S
T
V
W
Y

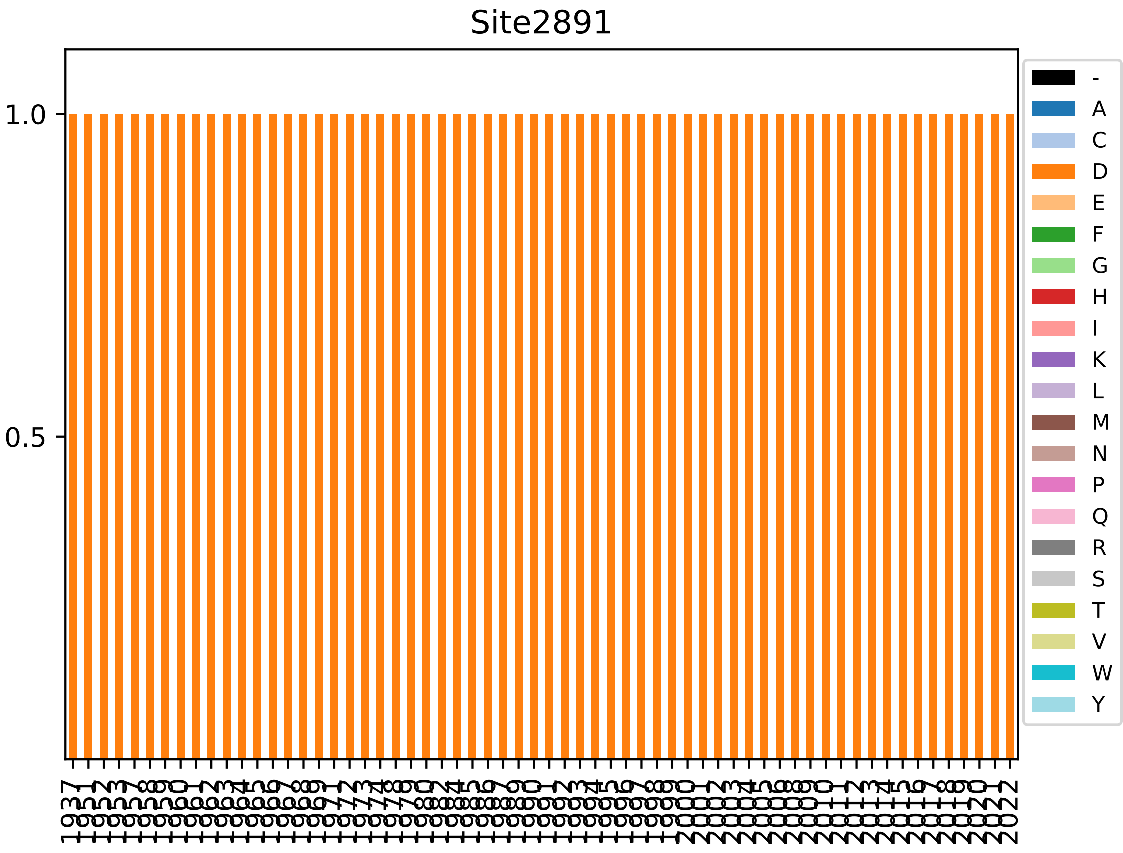
Site2891
1.0
0.5
-
A
C
D
E
F
G
H
I
K
L
M
N
P
Q
R
S
T
V
W
Y

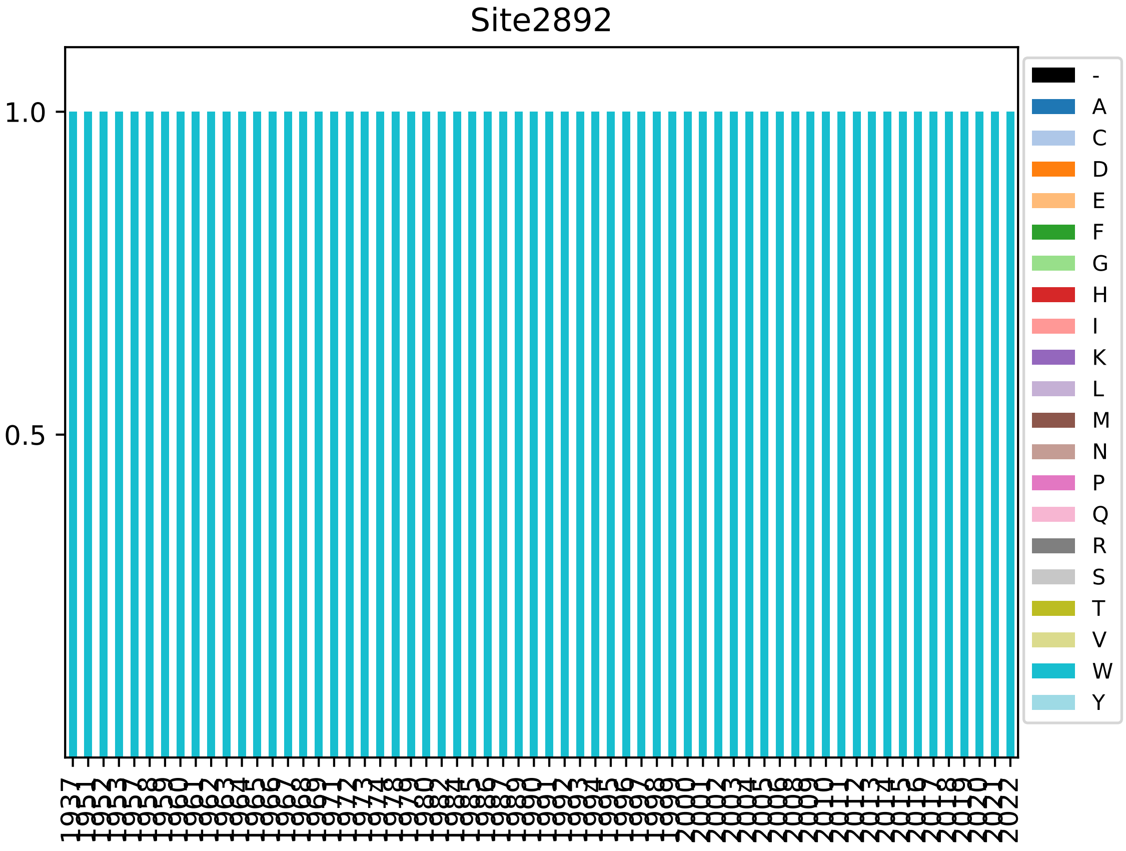
Site2892
1.0
0.5
-
A
C
D
E
F
G
H
I
K
L
M
N
P
Q
R
S
T
V
W
Y

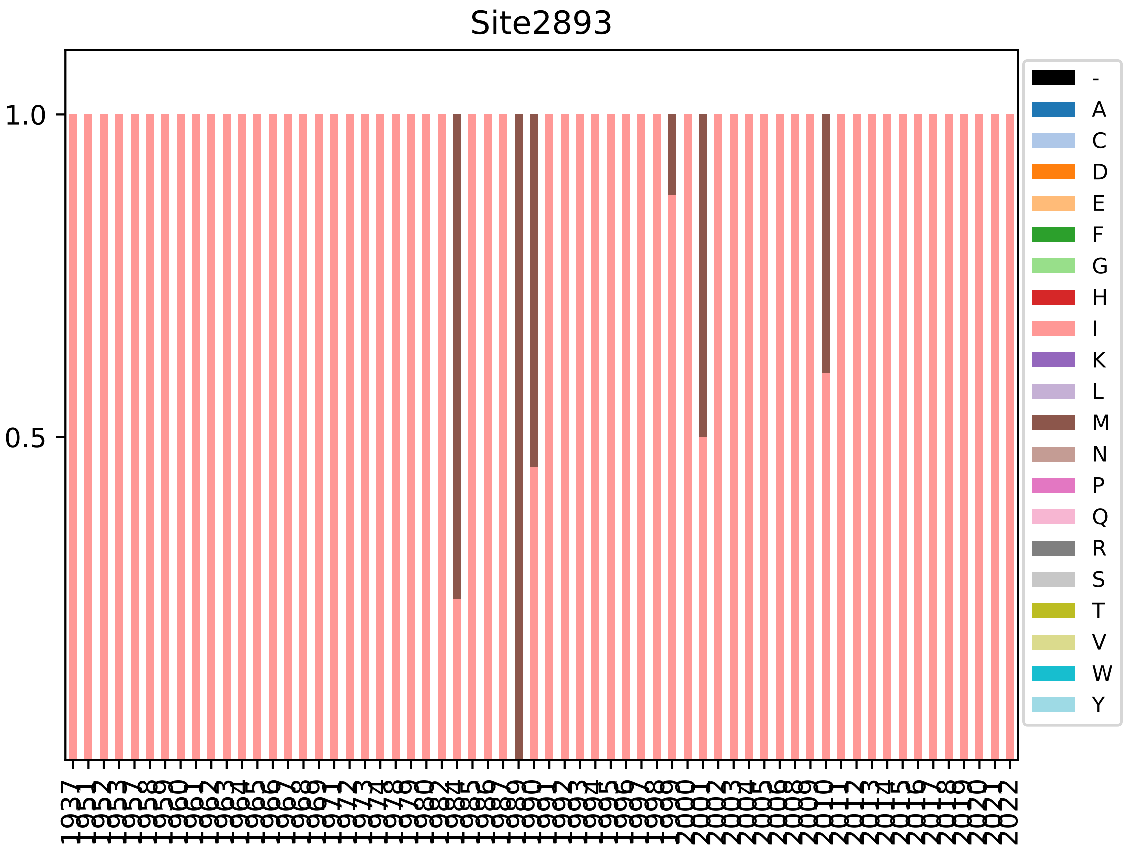
Site2893
1.0
0.5
-
A
C
D
E
F
G
H
I
K
L
M
N
P
Q
R
S
T
V
W
Y

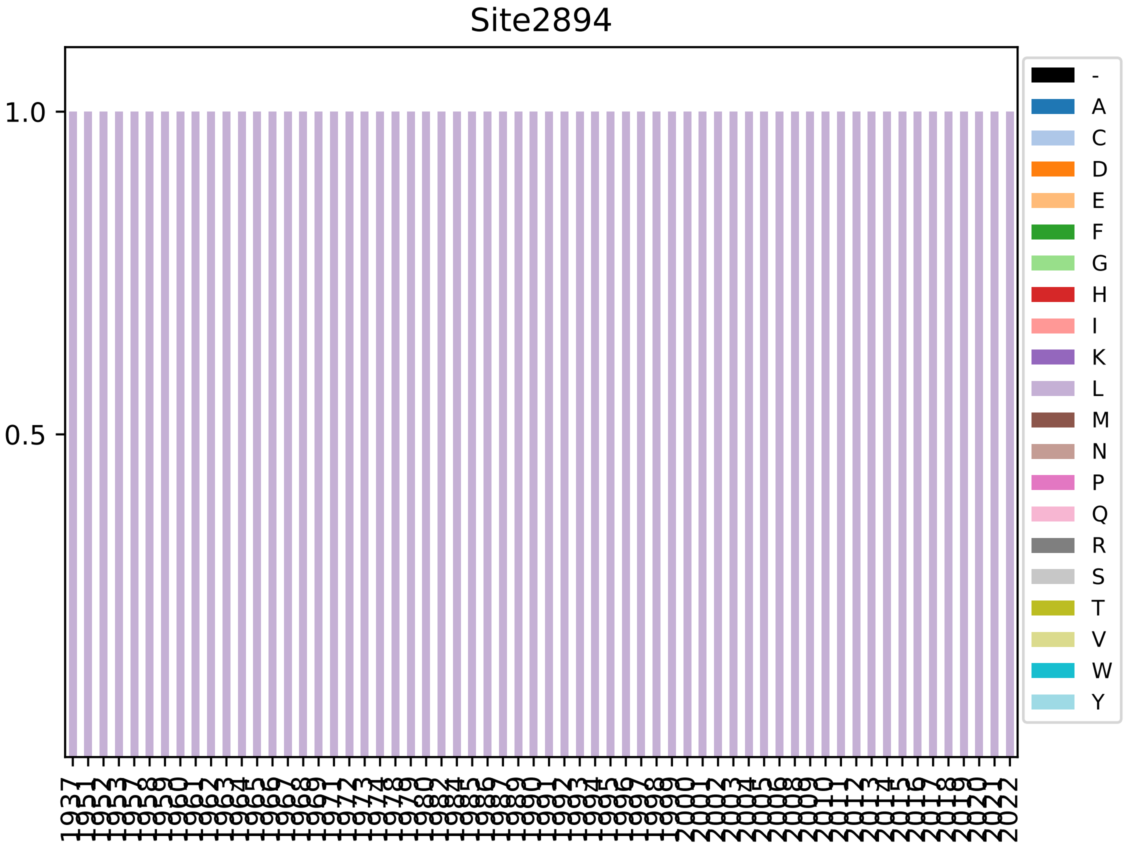
Site2894
1.0
0.5
-
A
C
D
E
F
G
H
I
K
L
M
N
P
Q
R
S
T
V
W
Y

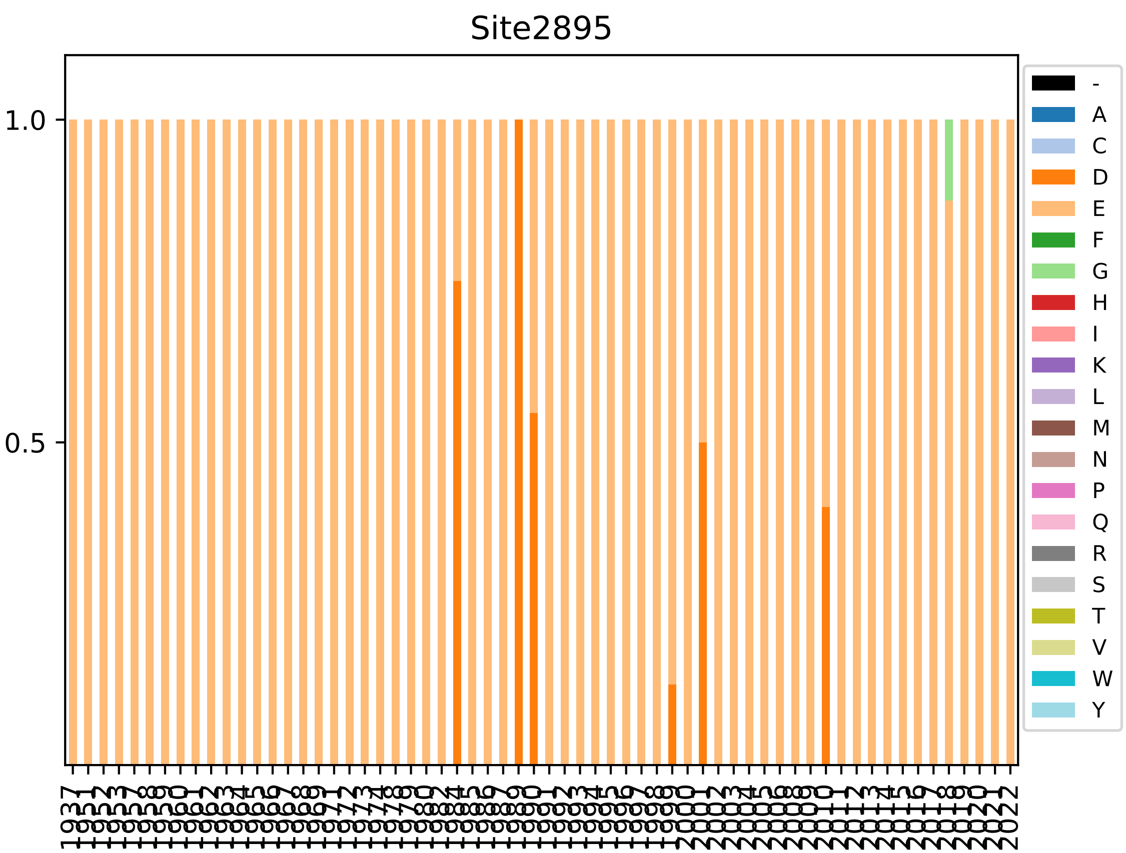
Site2895
1.0
0.5
-
A
C
D
E
F
G
H
I
K
L
M
N
P
Q
R
S
T
V
W
Y

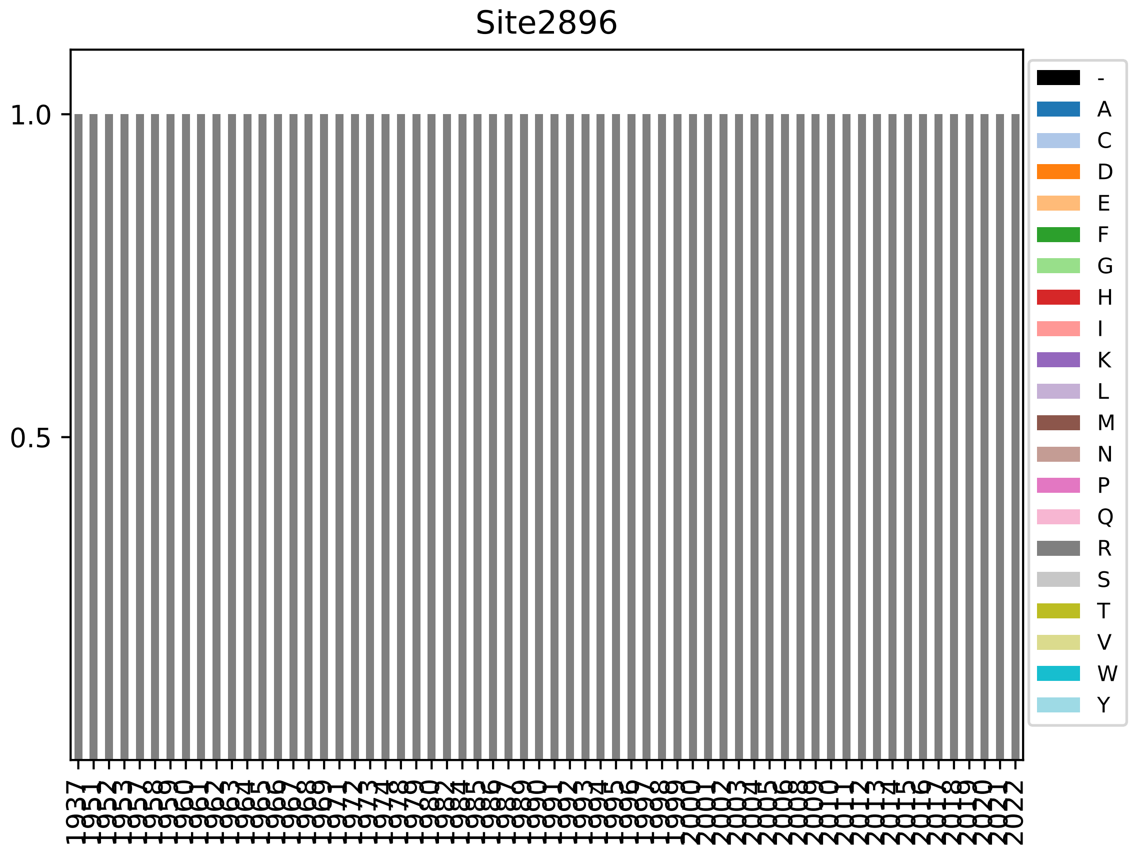
Site2896
1.0
0.5
-
A
C
D
E
F
G
H
I
K
L
M
N
P
Q
R
S
T
V
W
Y

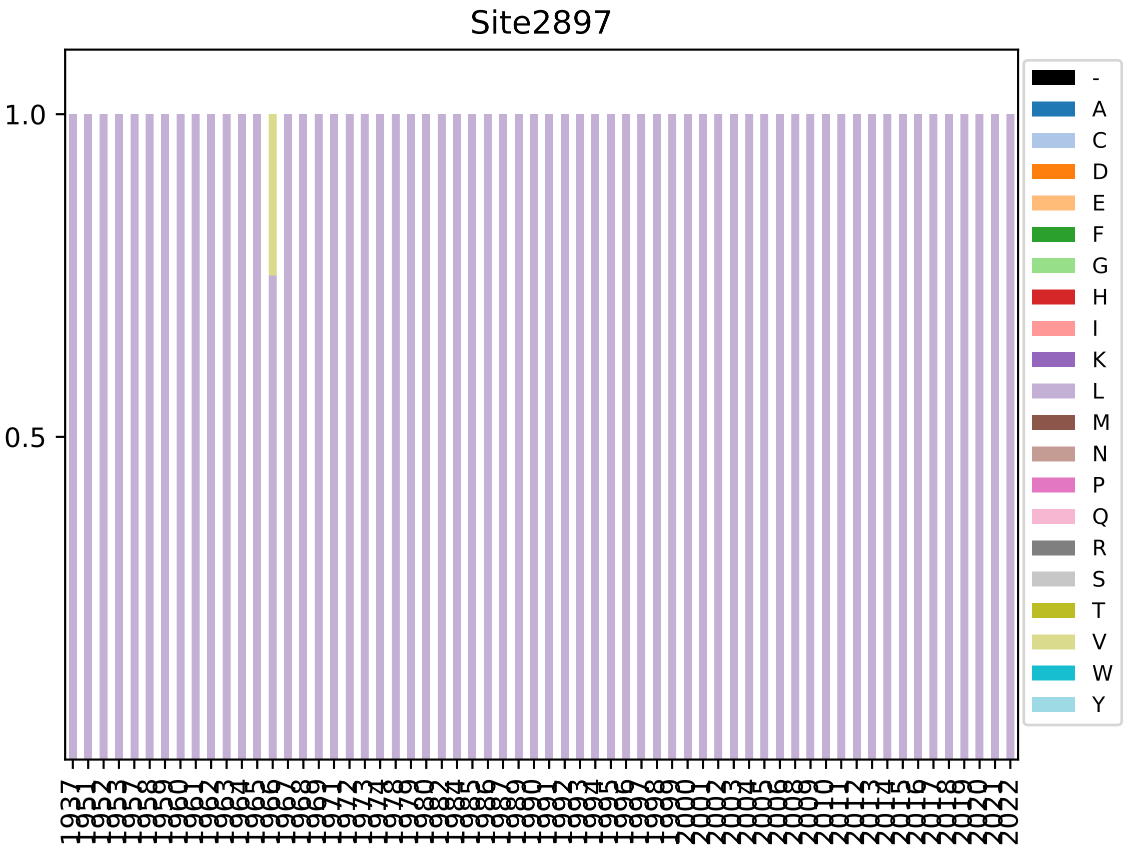
Site2897
1.0
0.5
-
A
C
D
E
F
G
H
I
K
L
M
N
P
Q
R
S
T
V
W
Y

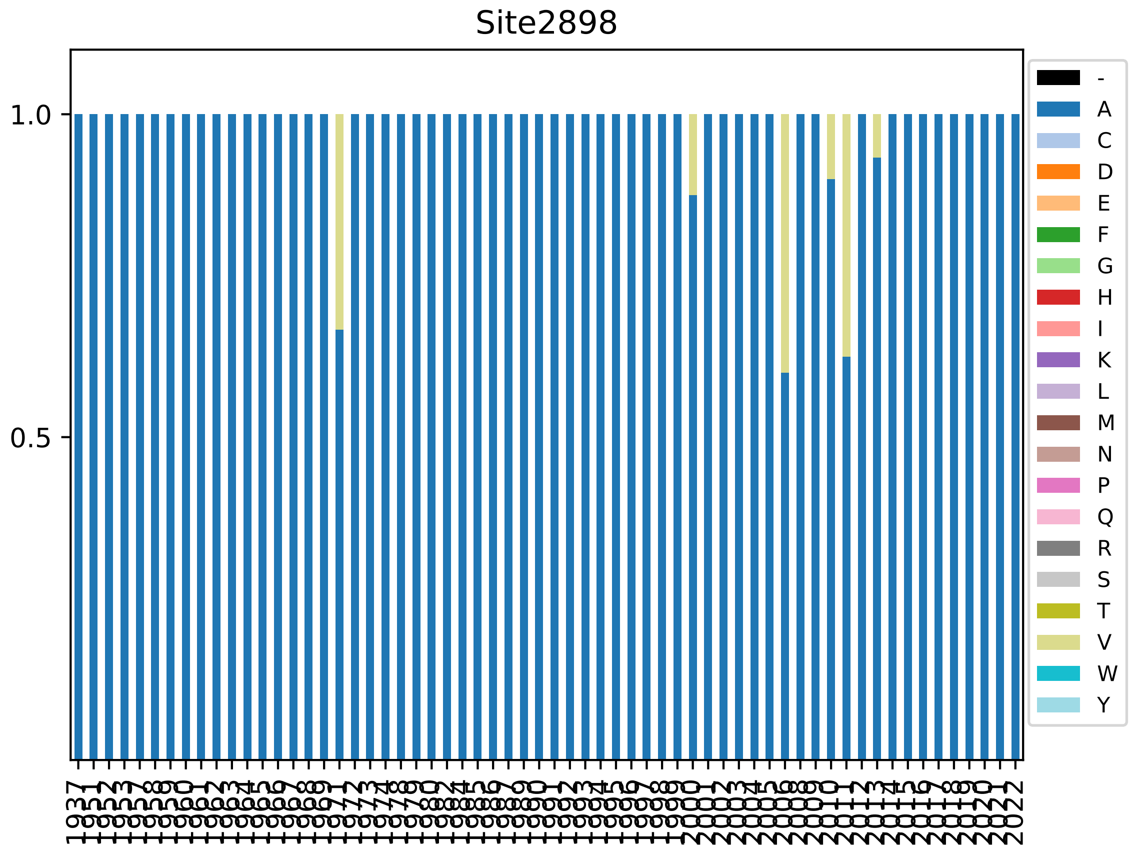
Site2898
1.0
0.5
-
A
C
D
E
F
G
H
I
K
L
M
N
P
Q
R
S
T
V
W
Y

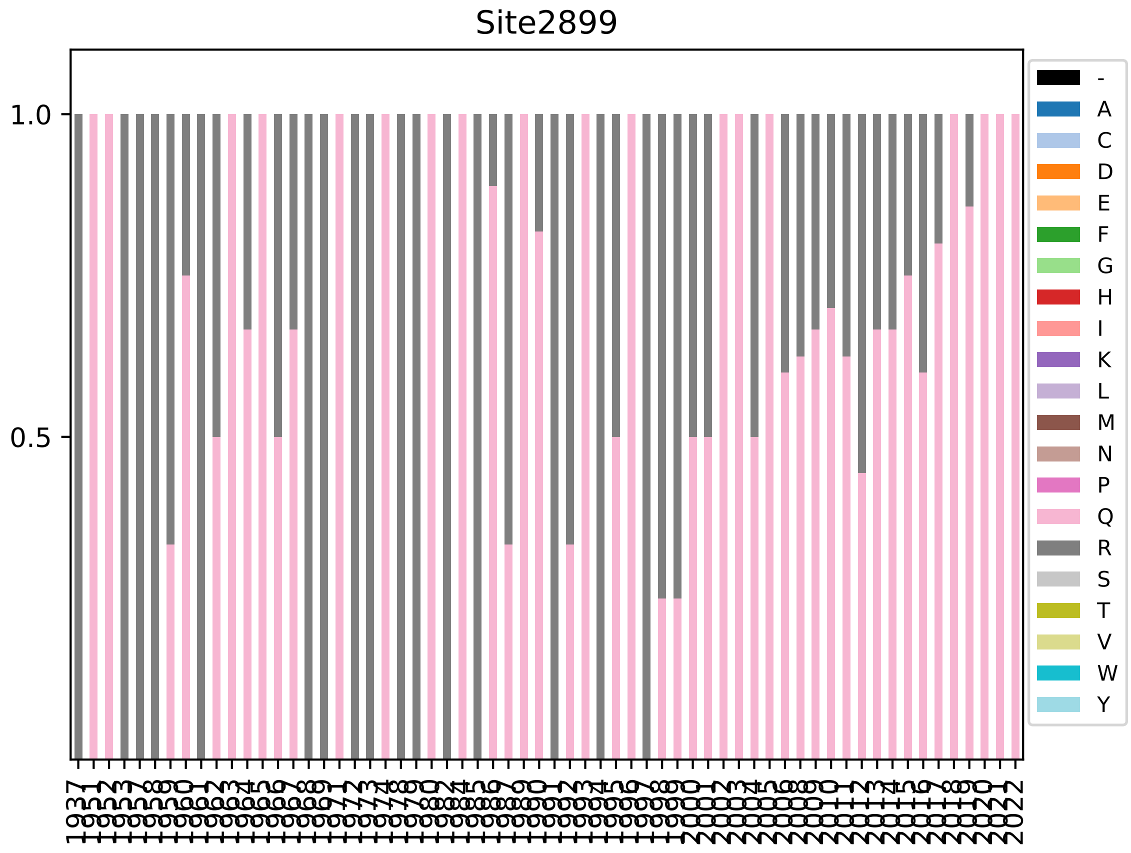

Site2899
1.0
0.5
-
A
C
D
E
F
G
H
I
K
L
M
N
P
Q
R
S
T
V
W
Y

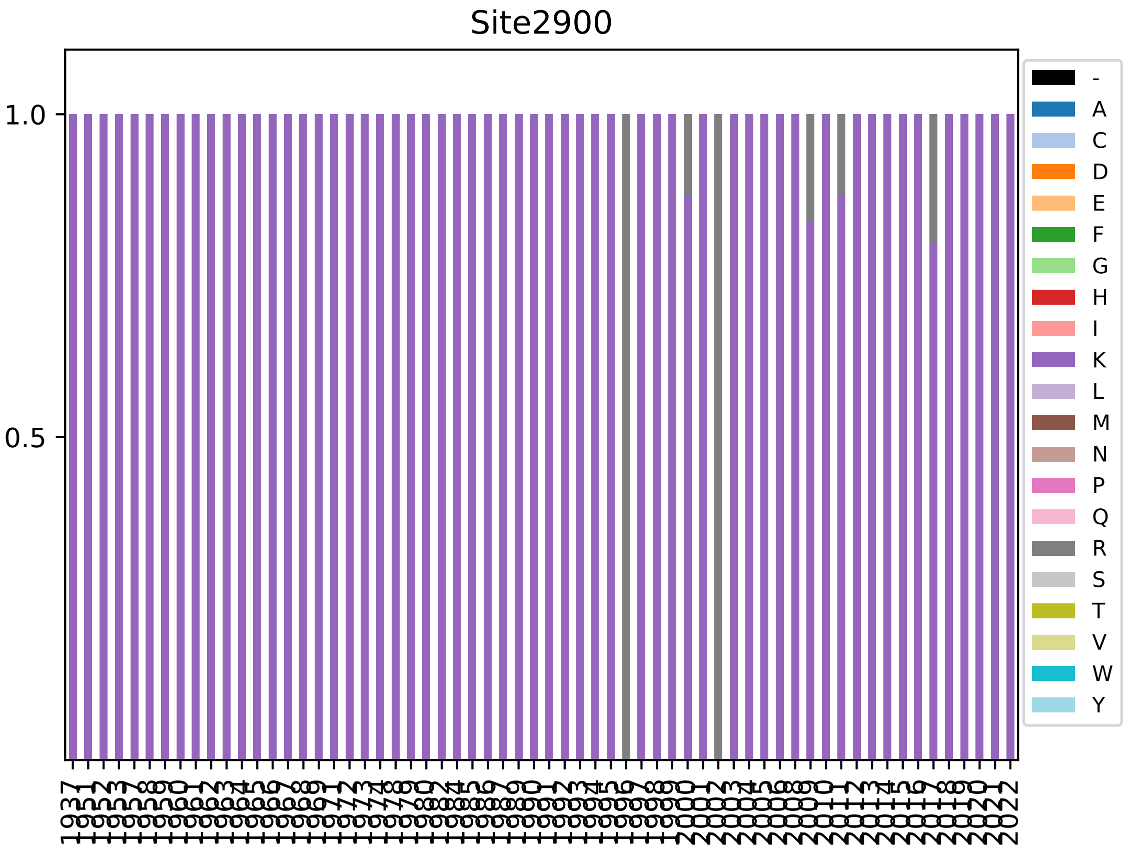
Site2900
1.0
0.5
-
A
C
D
E
F
G
H
I
K
L
M
N
P
Q
R
S
T
V
W
Y

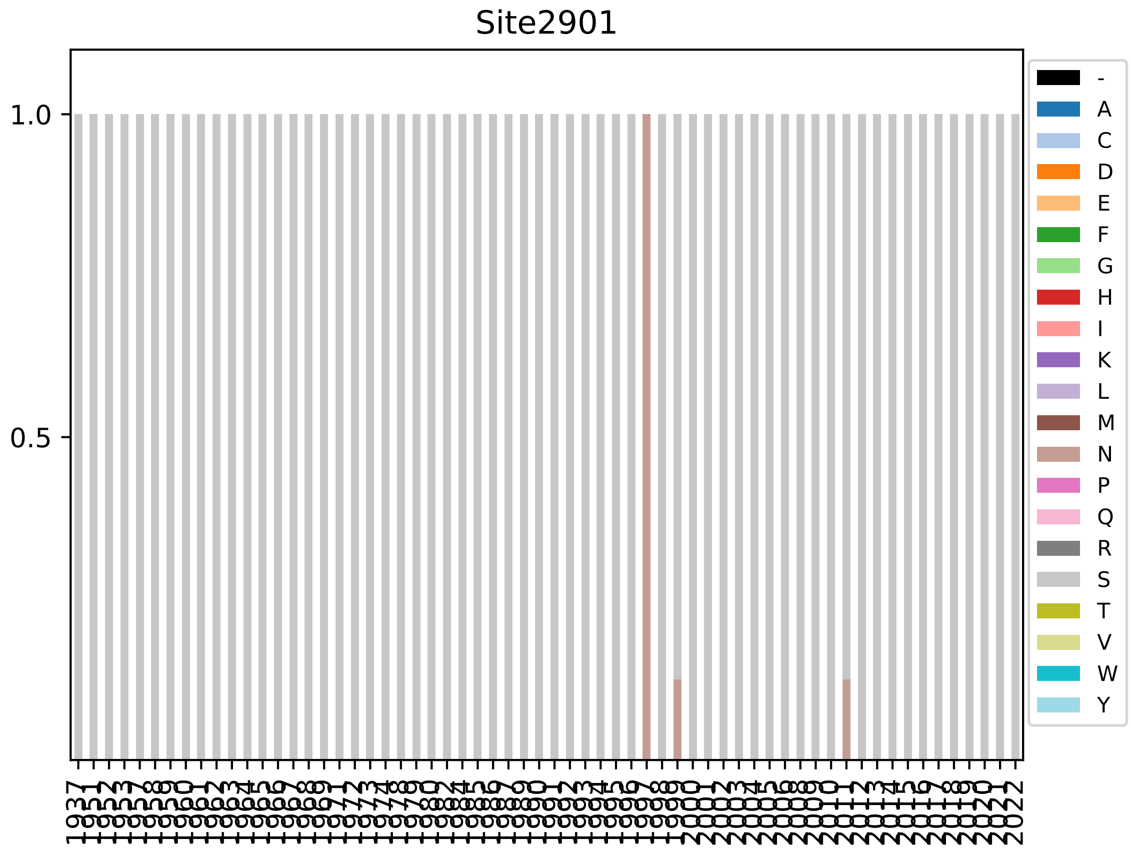
Site2901
1.0
0.5
-
A
C
D
E
F
G
H
I
K
L
M
N
P
Q
R
S
T
V
W
Y

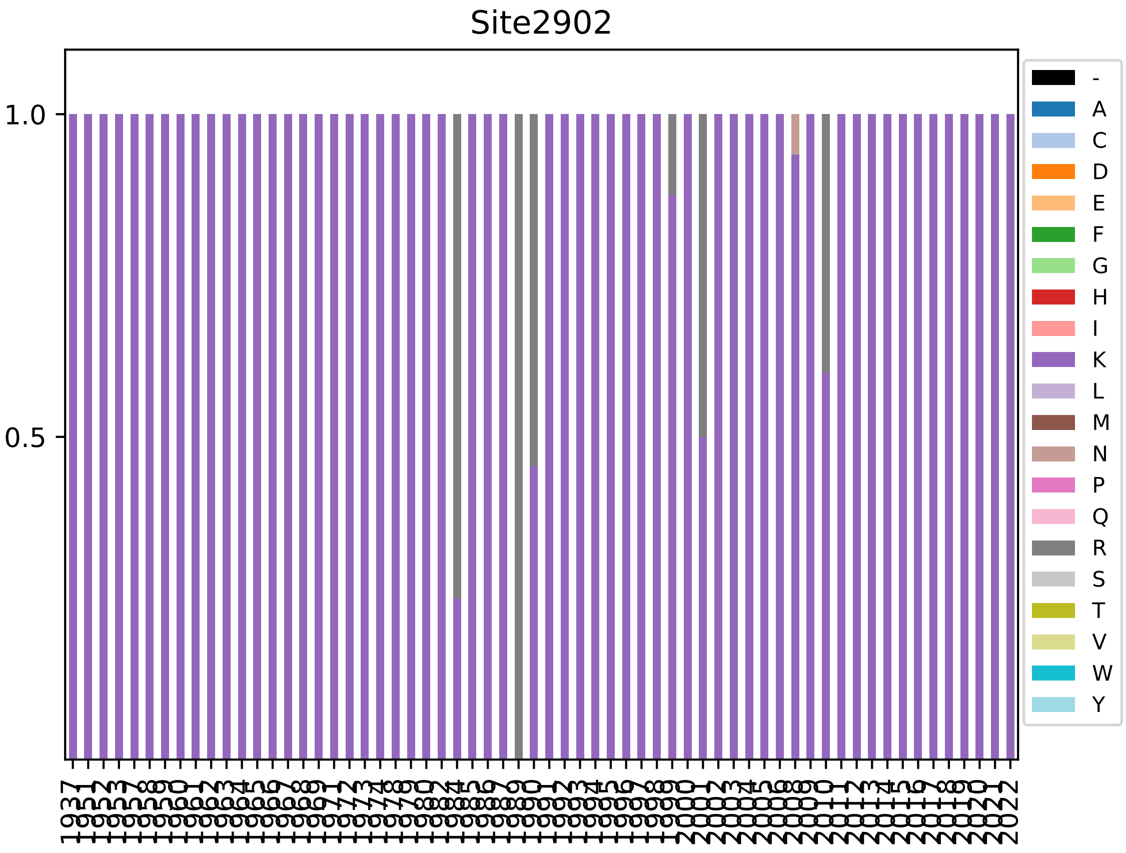

Site2902
1.0
0.5
-
A
C
D
E
F
G
H
I
K
L
M
N
P
Q
R
S
T
V
W
Y

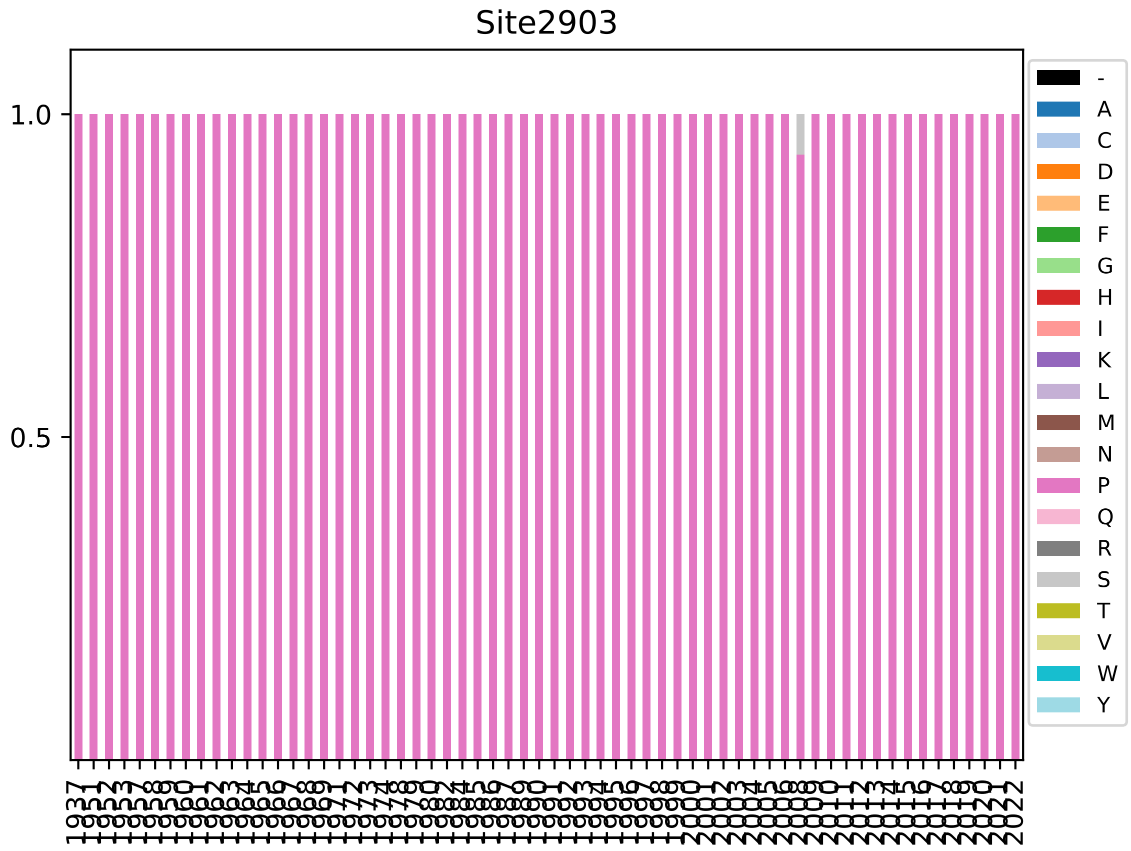
Site2903
1.0
0.5
-
A
C
D
E
F
G
H
I
K
L
M
N
P
Q
R
S
T
V
W
Y

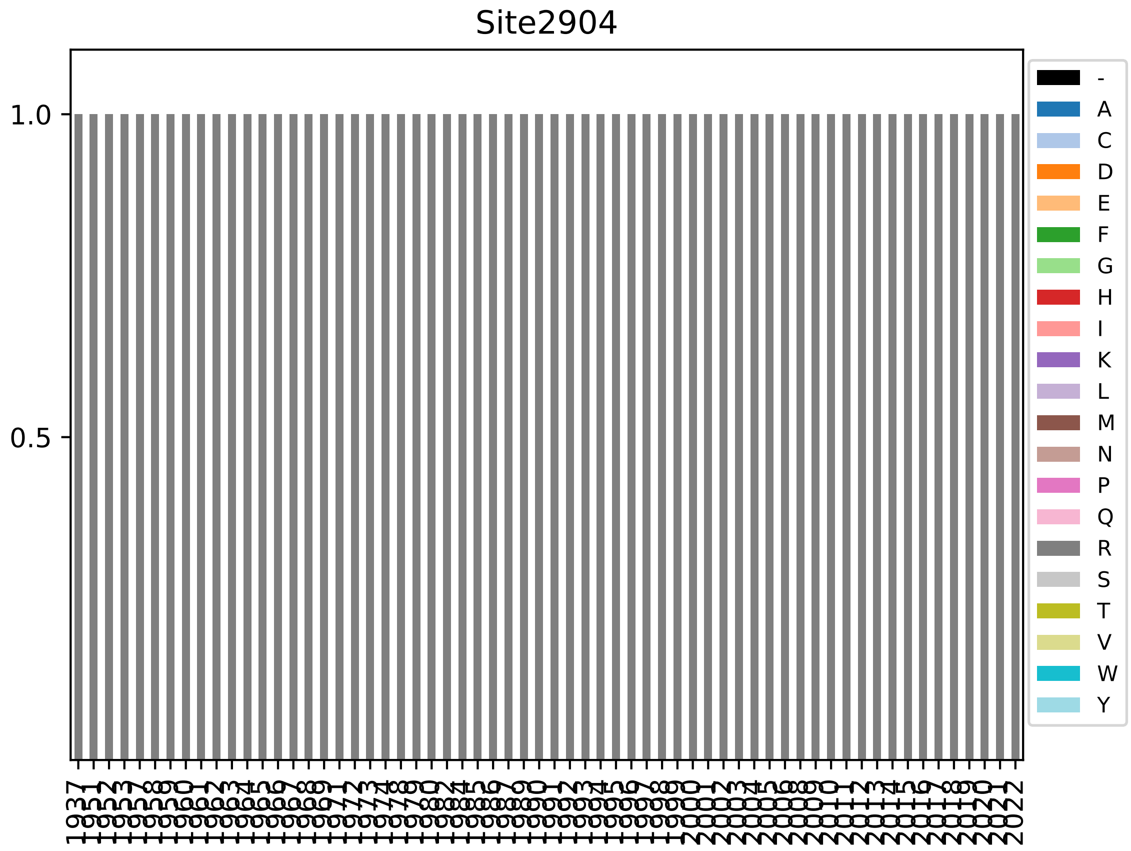
Site2904
1.0
0.5
-
A
C
D
E
F
G
H
I
K
L
M
N
P
Q
R
S
T
V
W
Y

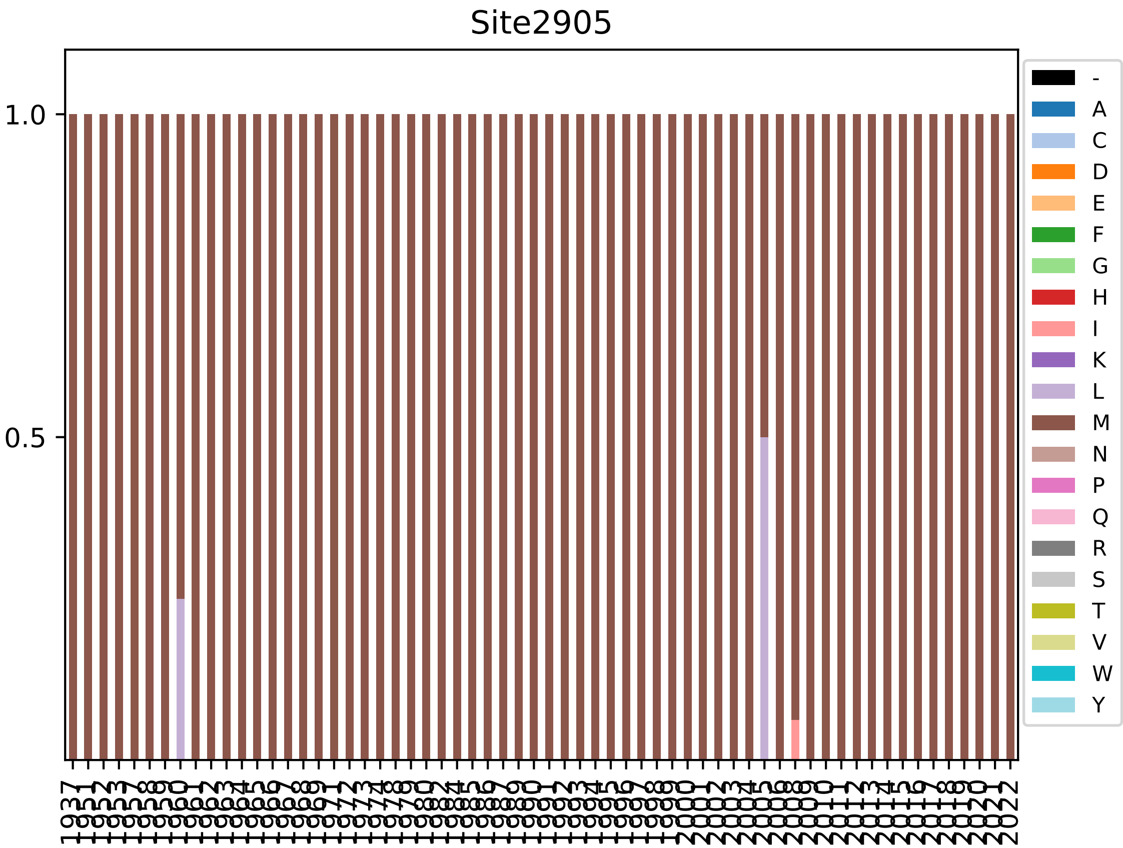
Site2905
1.0
0.5
-
A
C
D
E
F
G
H
I
K
L
M
N
P
Q
R
S
T
V
W
Y

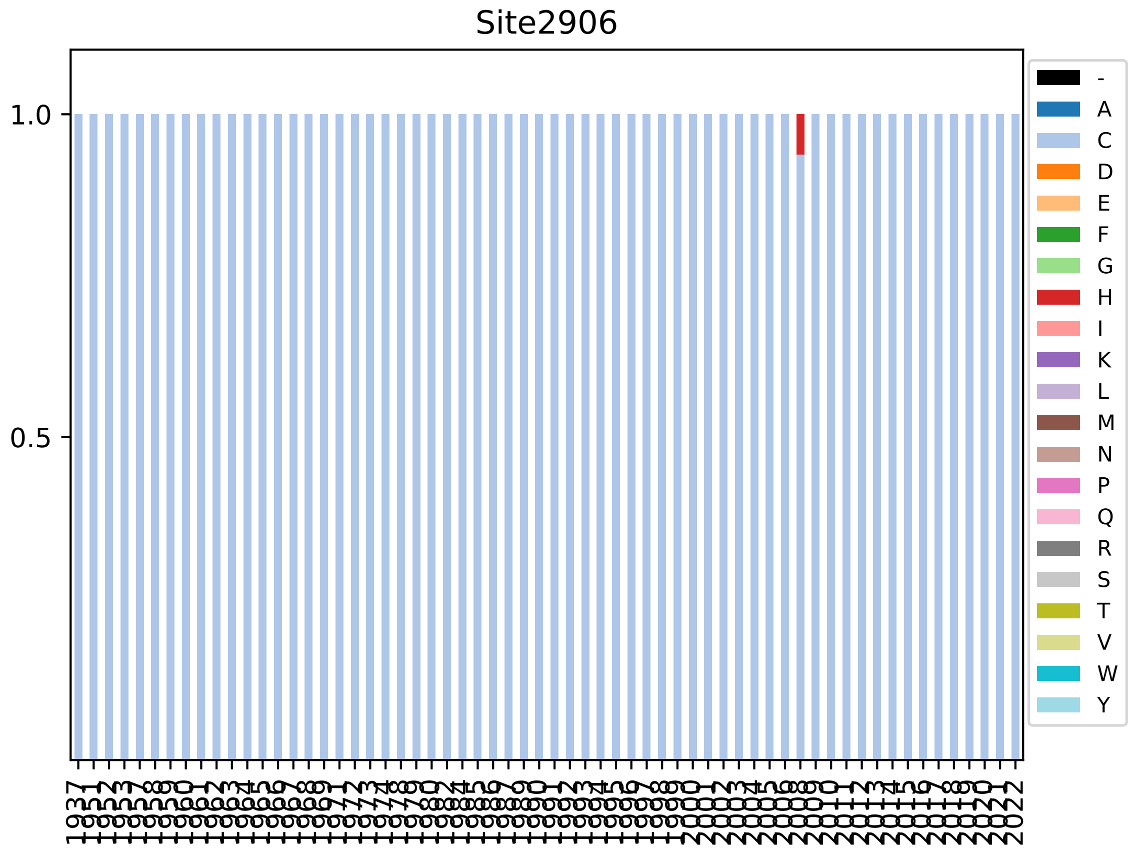
Site2906
1.0
0.5
-
A
C
D
E
F
G
H
I
K
L
M
N
P
Q
R
S
T
V
W
Y

Site2907

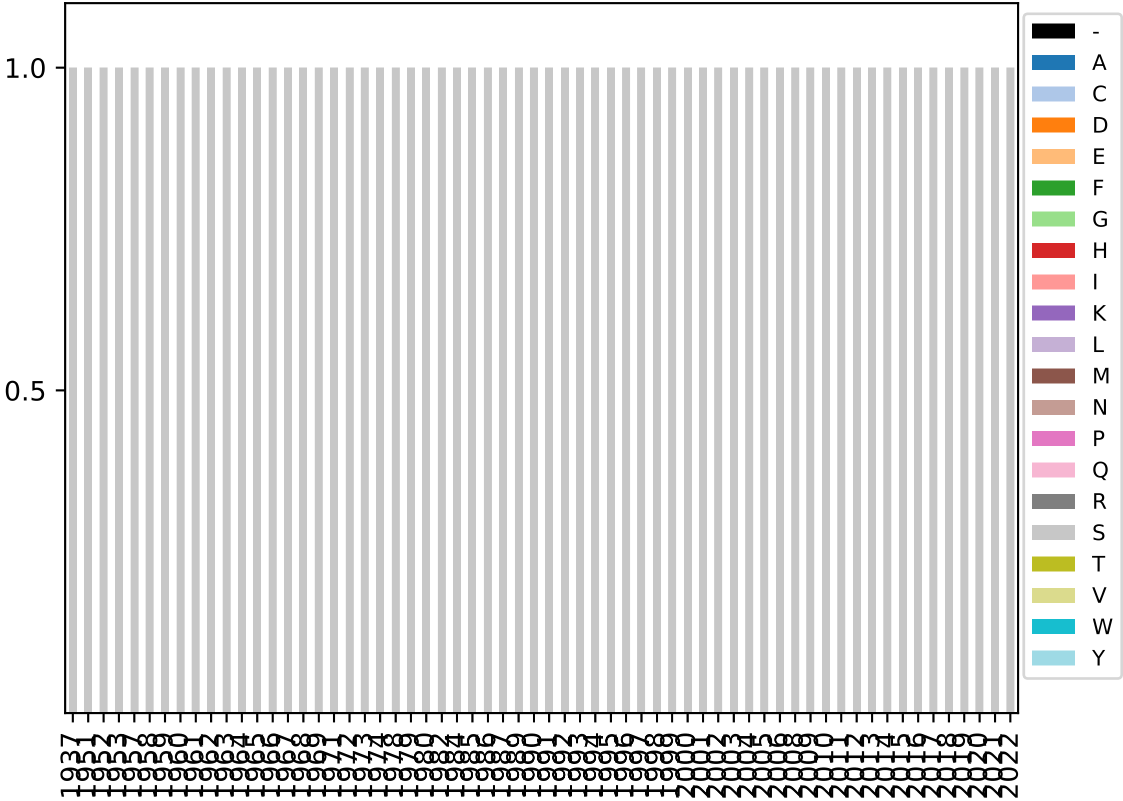

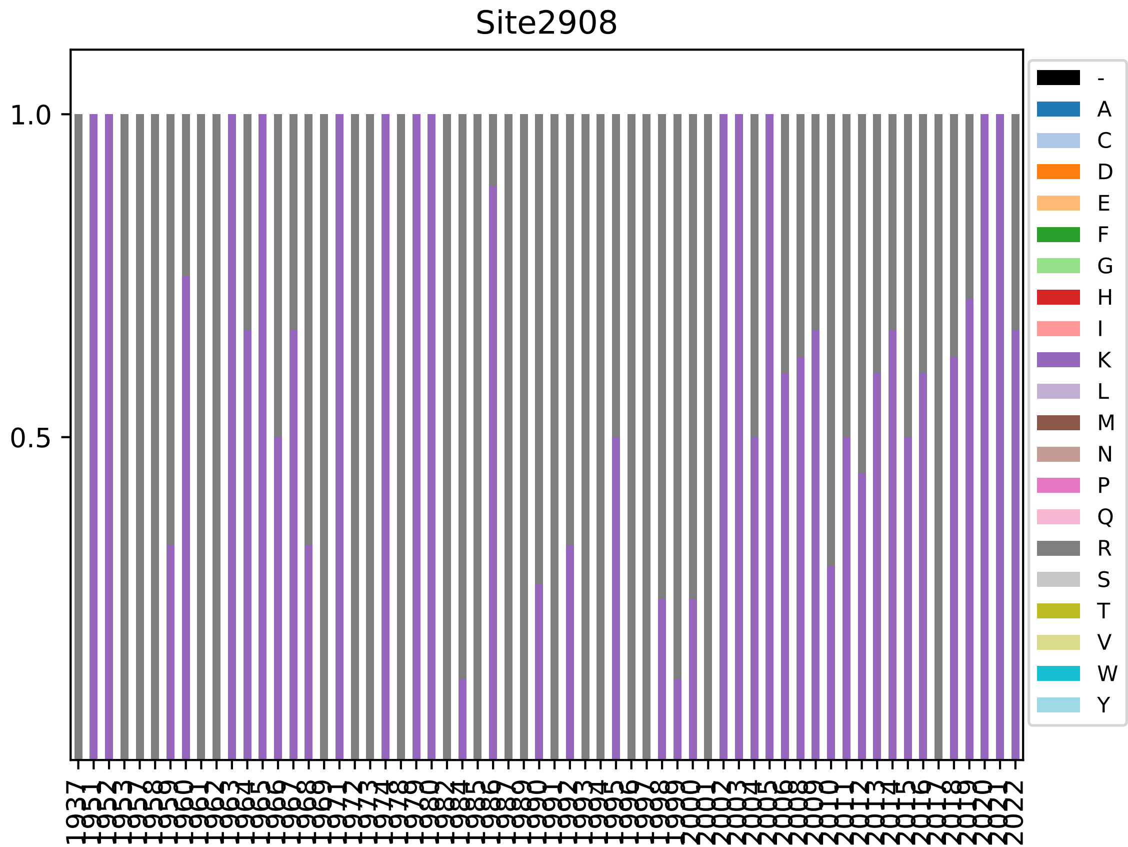

Site2908
1.0
0.5
-
A
C
D
E
F
G
H
I
K
L
M
N
P
Q
R
S
T
V
W
Y

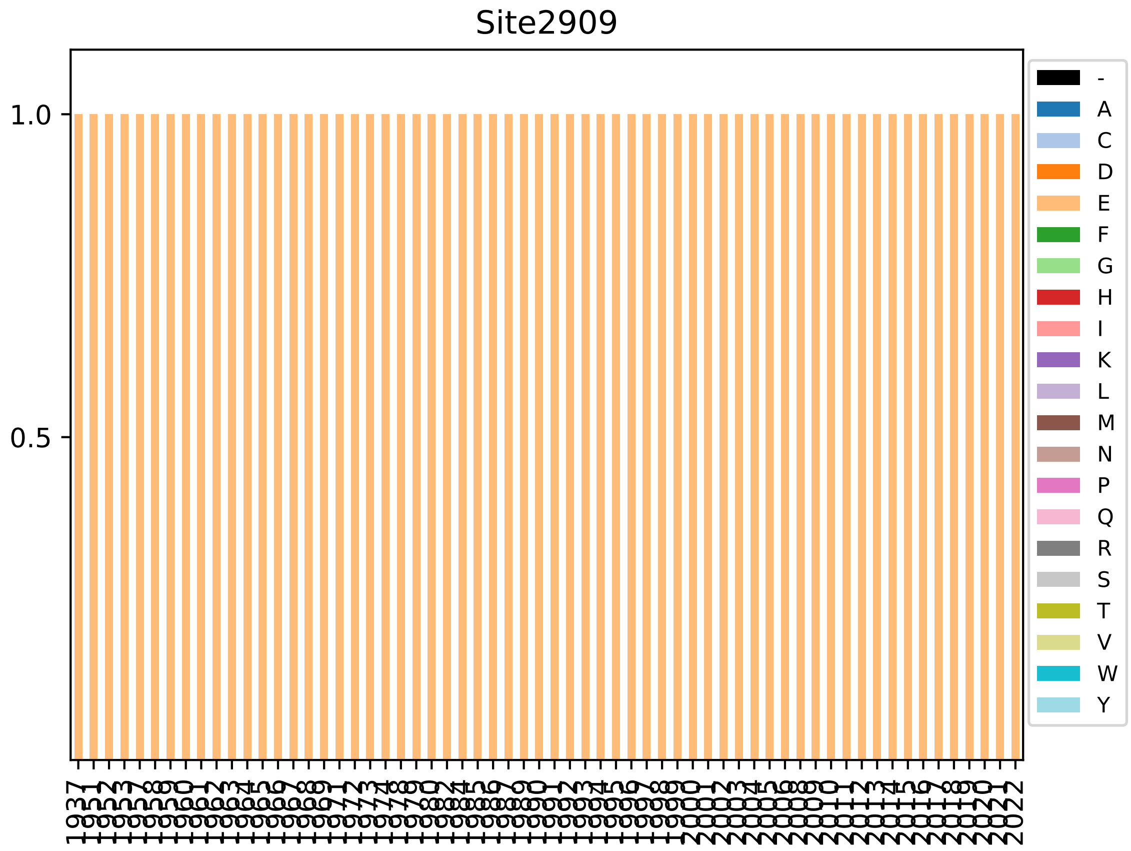
Site2909
1.0
0.5
-
A
C
D
E
F
G
H
I
K
L
M
N
P
Q
R
S
T
V
W
Y

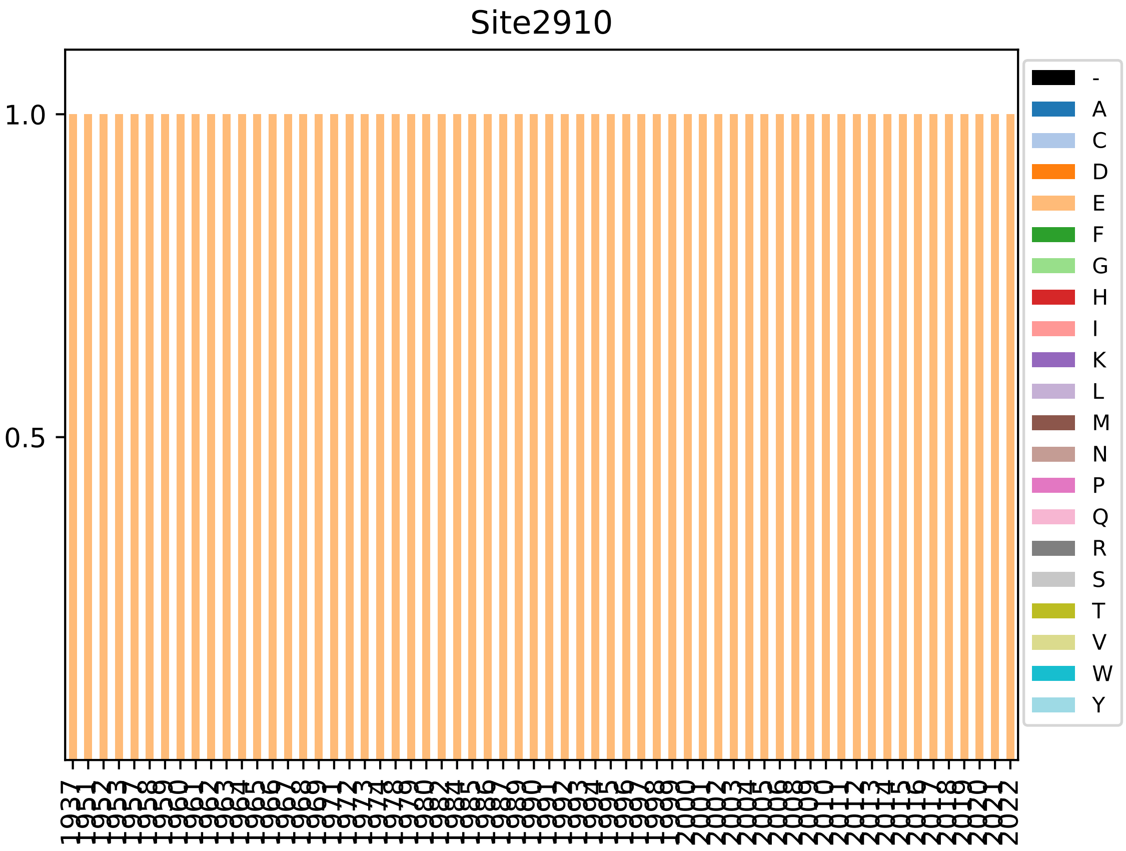
Site2910
1.0
0.5
-
A
C
D
E
F
G
H
I
K
L
M
N
P
Q
R
S
T
V
W
Y

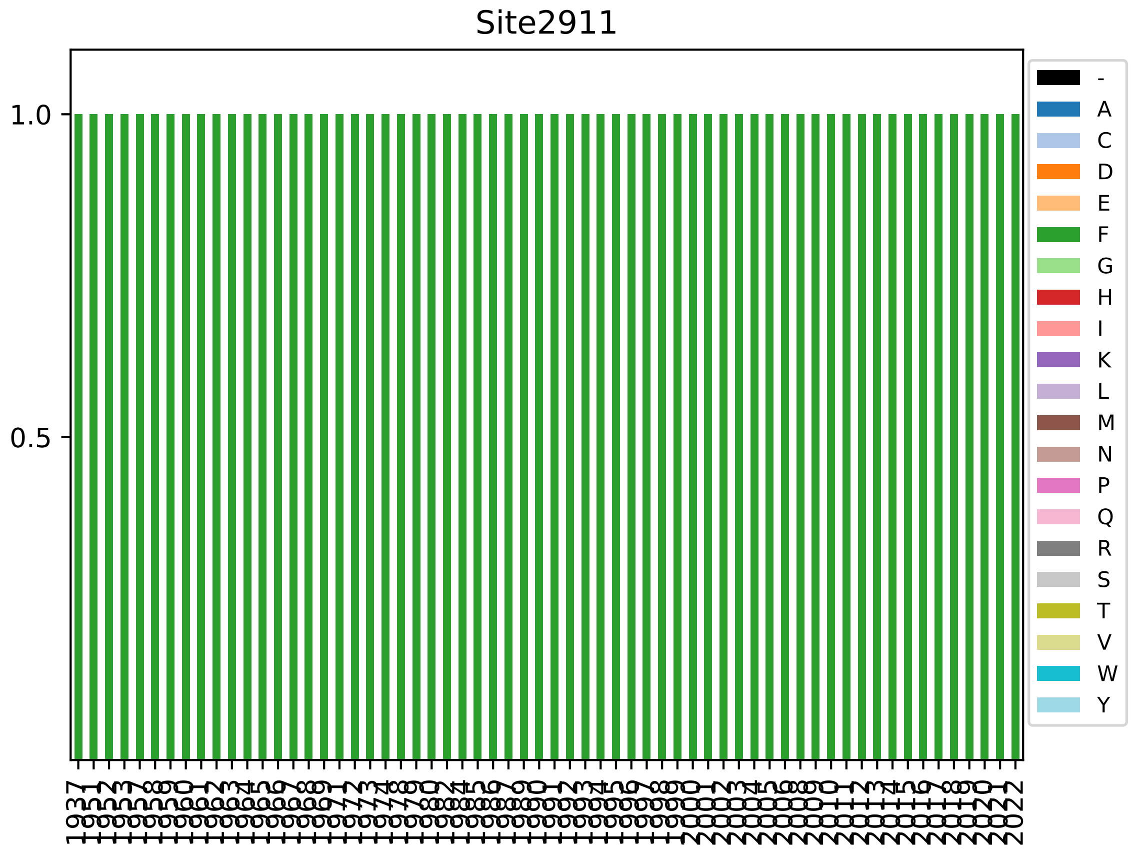

Site2911
1.0
0.5
-
A
C
D
E
F
G
H
I
K
L
M
N
P
Q
R
S
T
V
W
Y

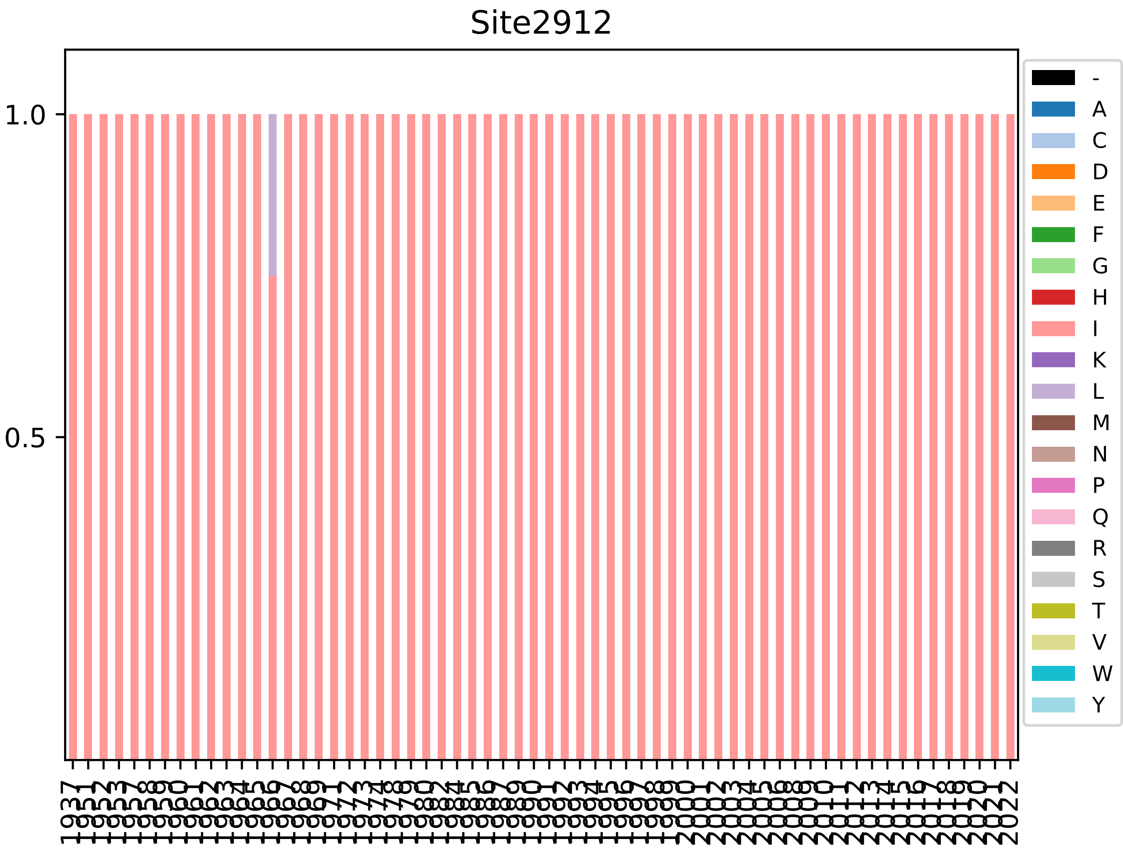
Site2912
1.0
0.5
-
A
C
D
E
F
G
H
I
K
L
M
N
P
Q
R
S
T
V
W
Y

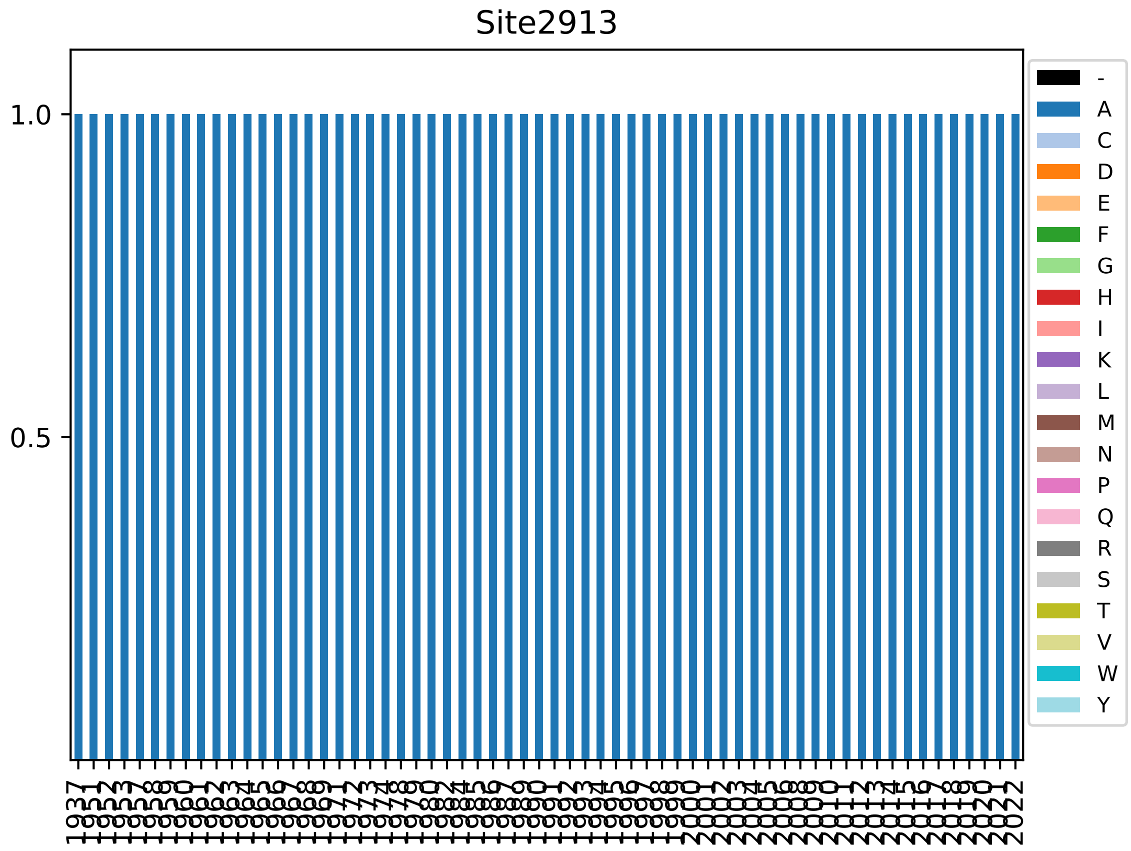
Site2913
1.0
0.5
-
A
C
D
E
F
G
H
I
K
L
M
N
P
Q
R
S
T
V
W
Y

Site2914

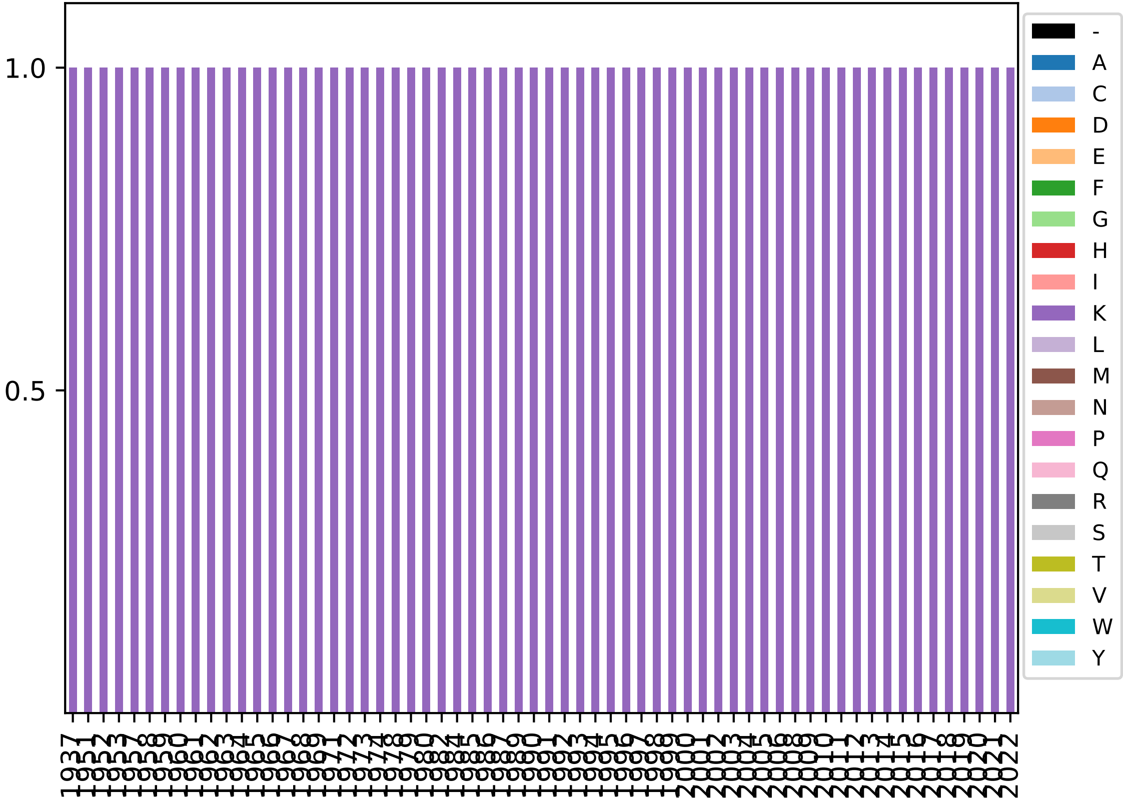

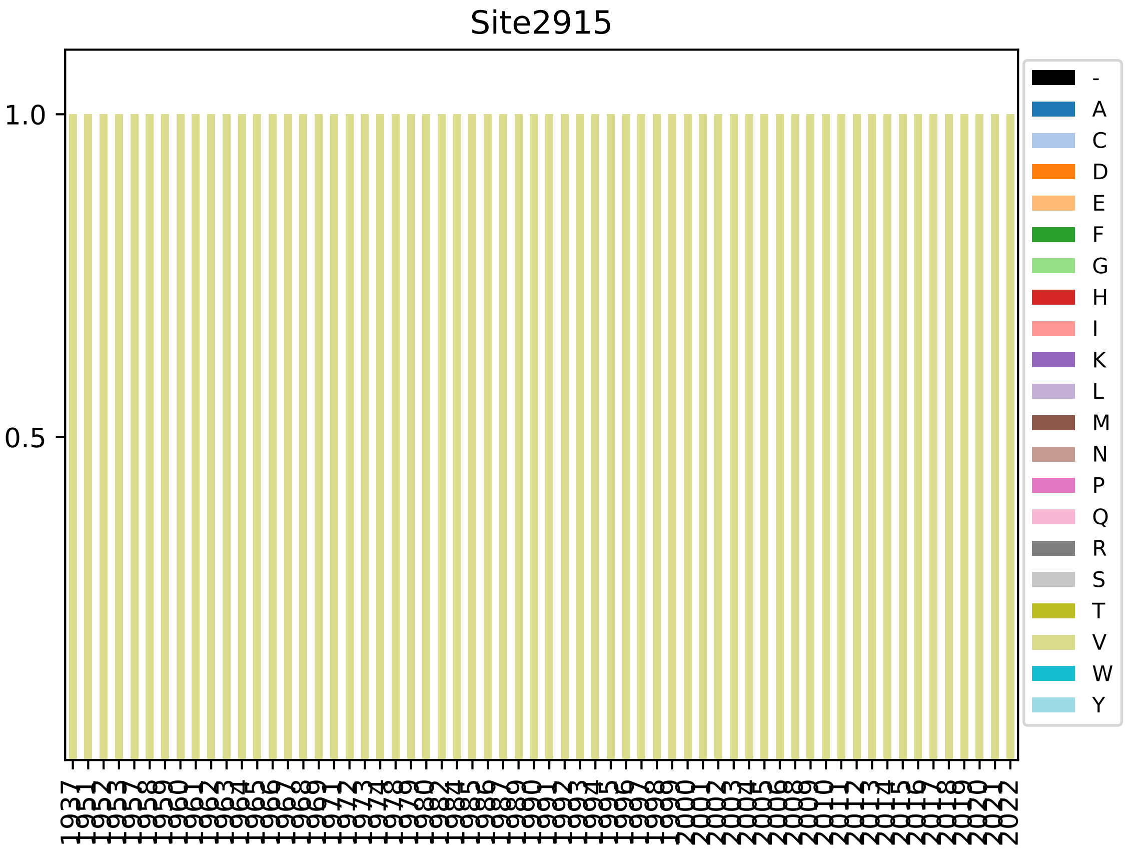
Site2915
1.0
0.5
-
A
C
D
E
F
G
H
I
K
L
M
N
P
Q
R
S
T
V
W
Y

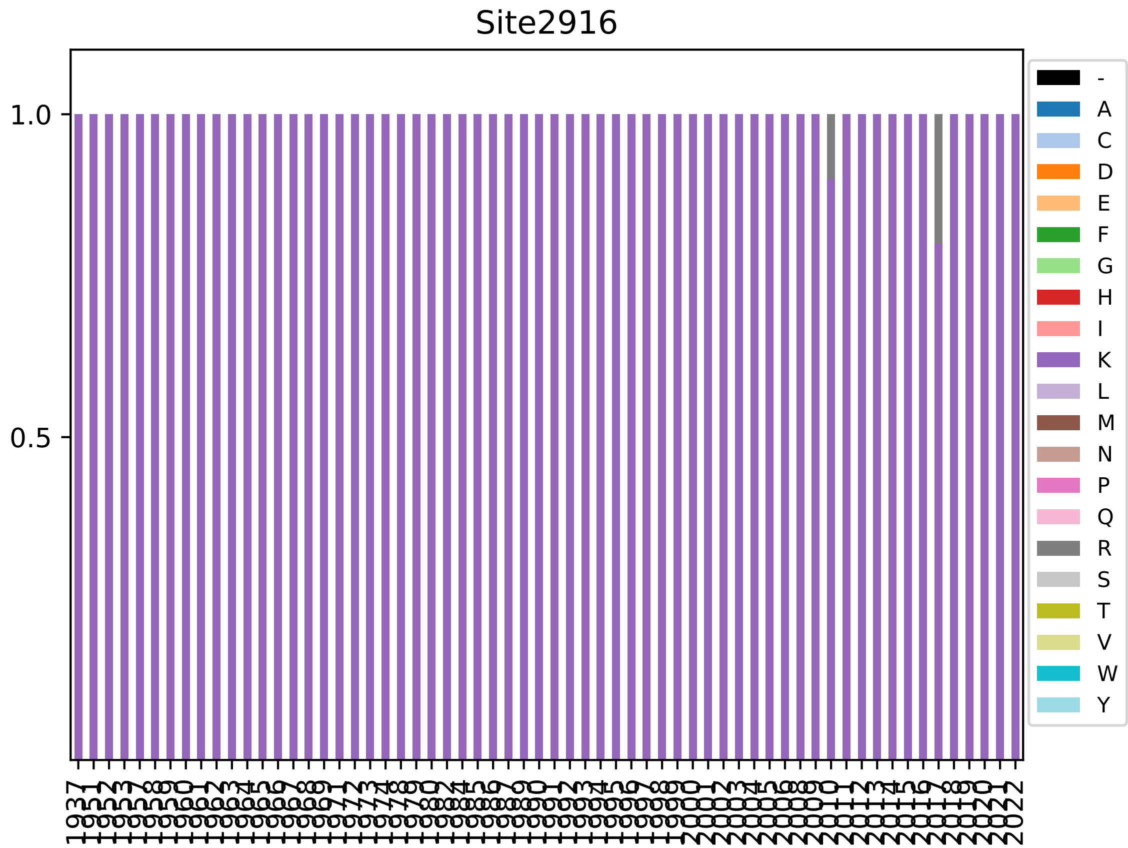
Site2916
1.0
0.5
-
A
C
D
E
F
G
H
I
K
L
M
N
P
Q
R
S
T
V
W
Y

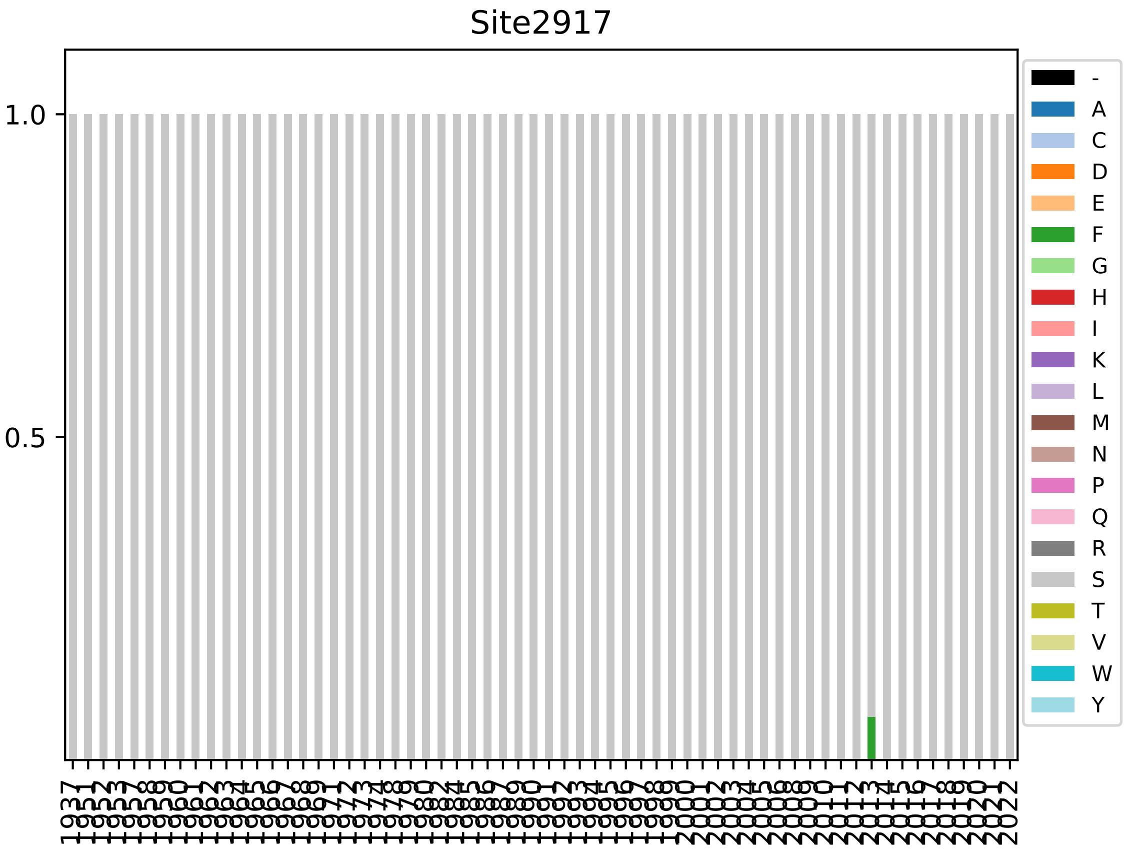
Site2917
1.0
0.5
-
A
C
D
E
F
G
H
I
K
L
M
N
P
Q
R
S
T
V
W
Y

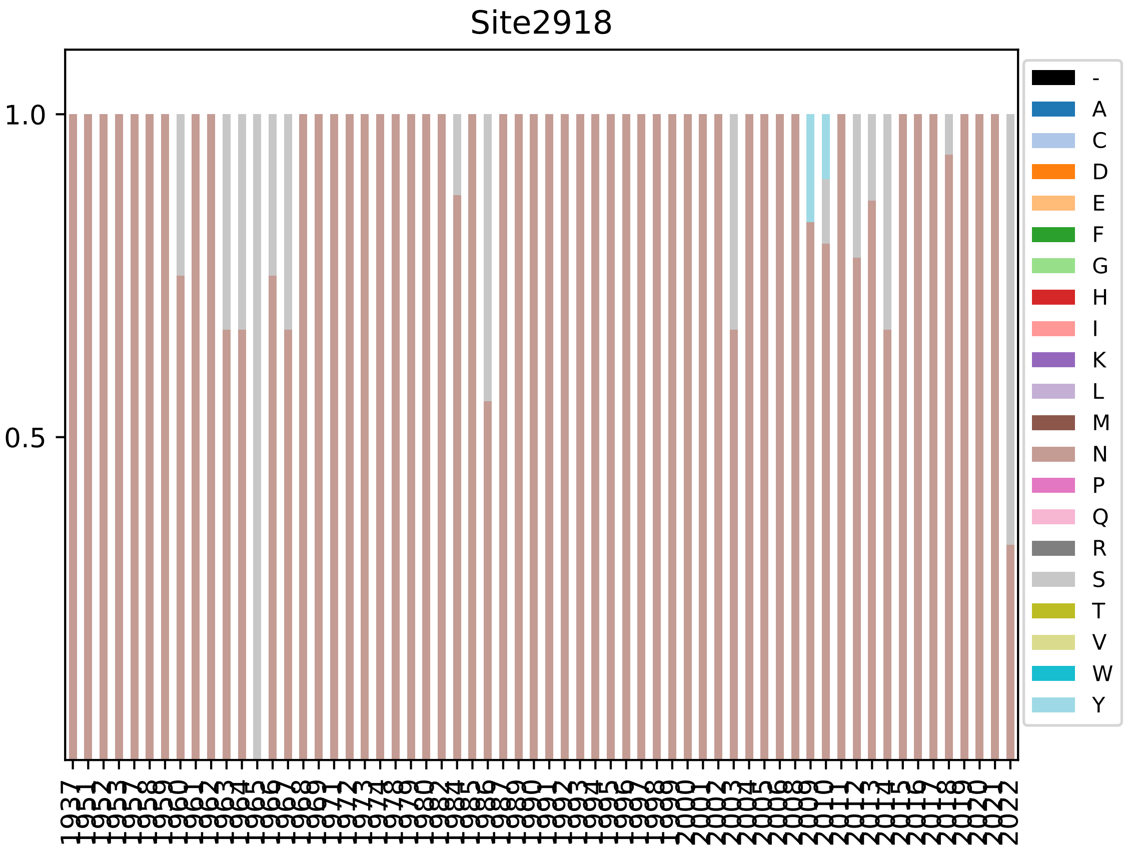
Site2918
1.0
0.5
-
A
C
D
E
F
G
H
I
K
L
M
N
P
Q
R
S
T
V
W
Y

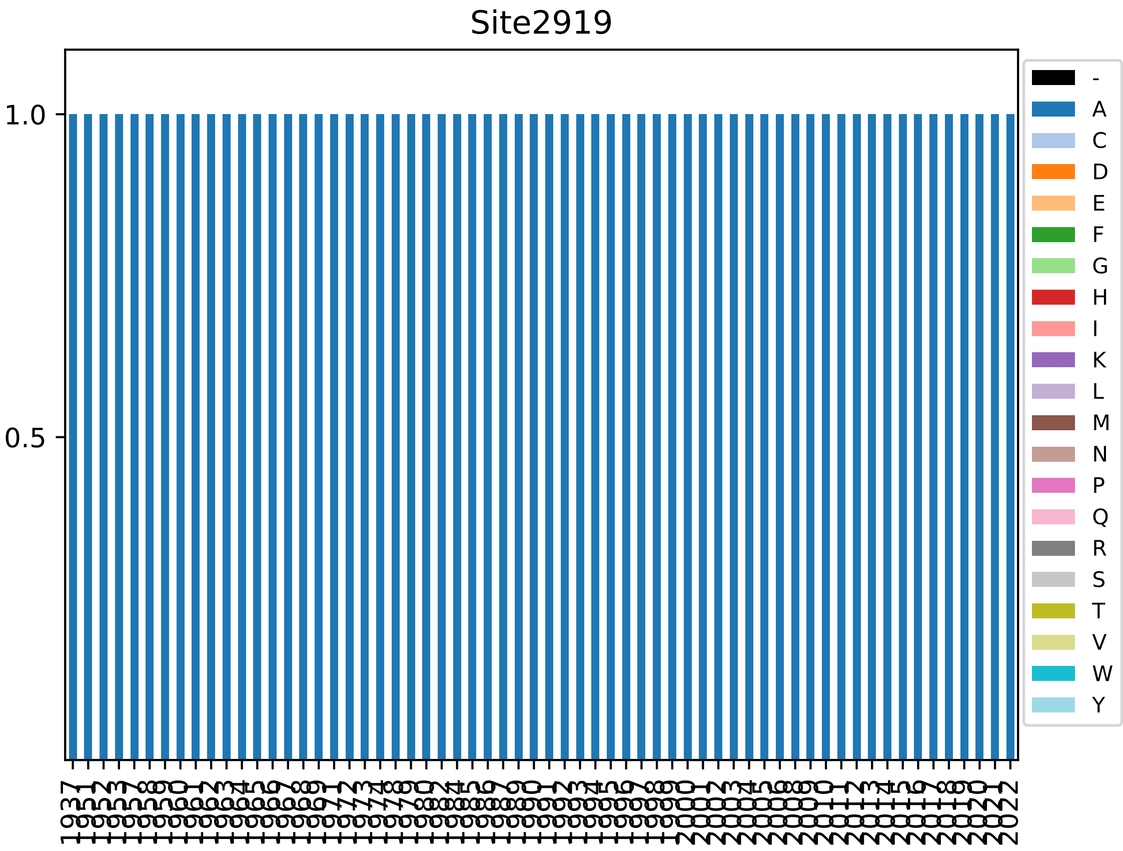
Site2919
1.0
0.5
-
A
C
D
E
F
G
H
I
K
L
M
N
P
Q
R
S
T
V
W
Y

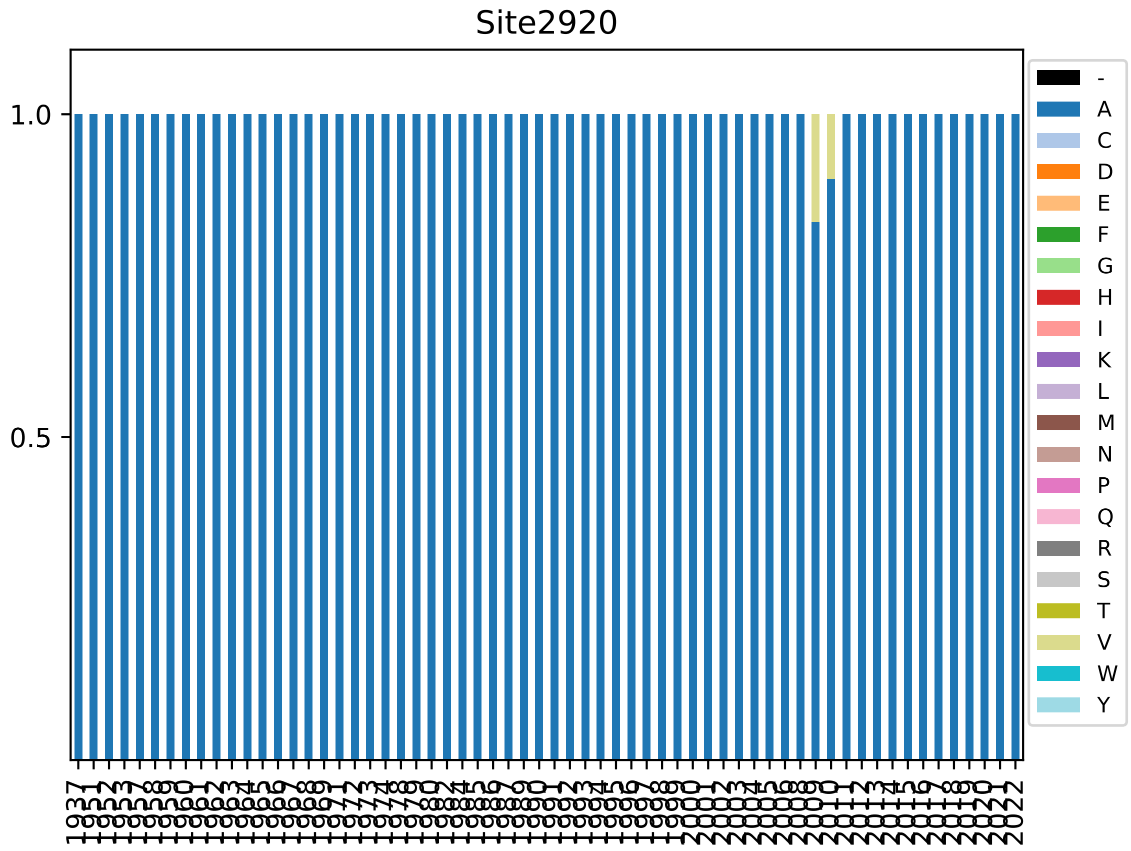
Site2920
1.0
0.5
-
A
C
D
E
F
G
H
I
K
L
M
N
P
Q
R
S
T
V
W
Y

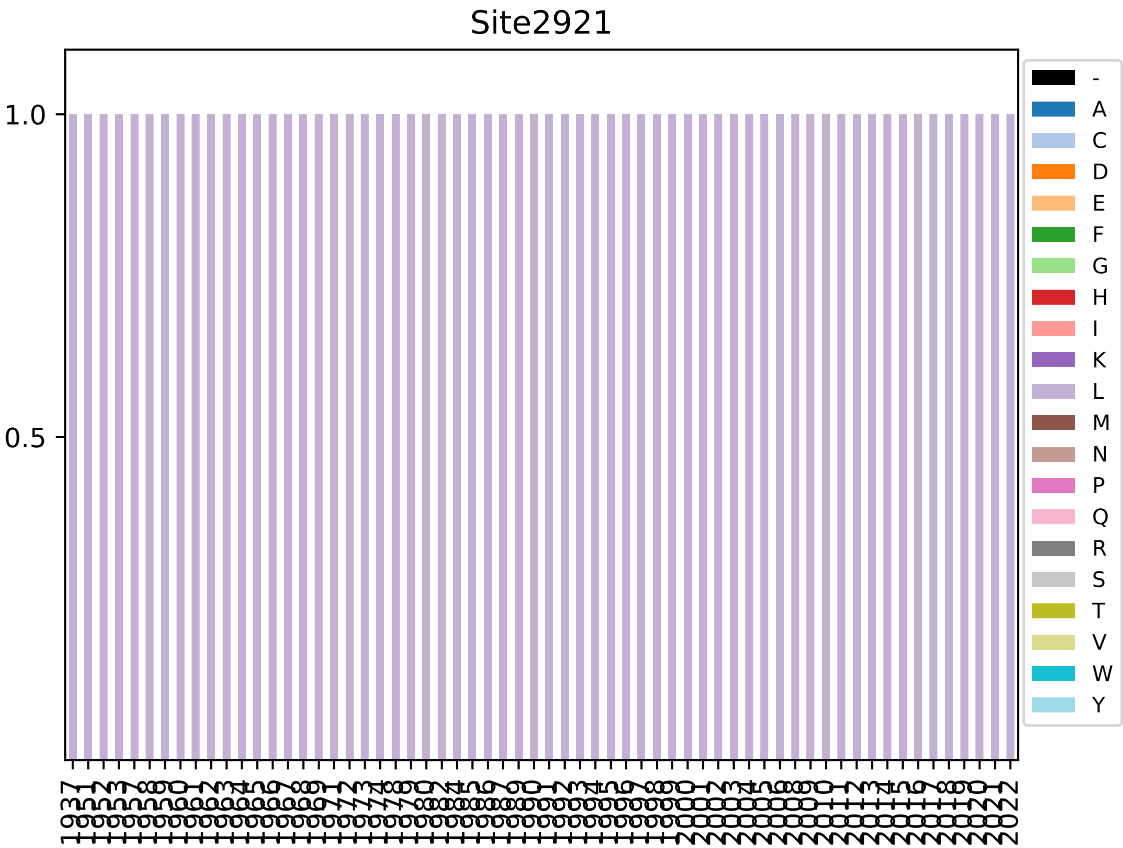
Site2921
1.0
0.5
-
A
C
D
E
F
G
H
I
K
L
M
N
P
Q
R
S
T
V
W
Y

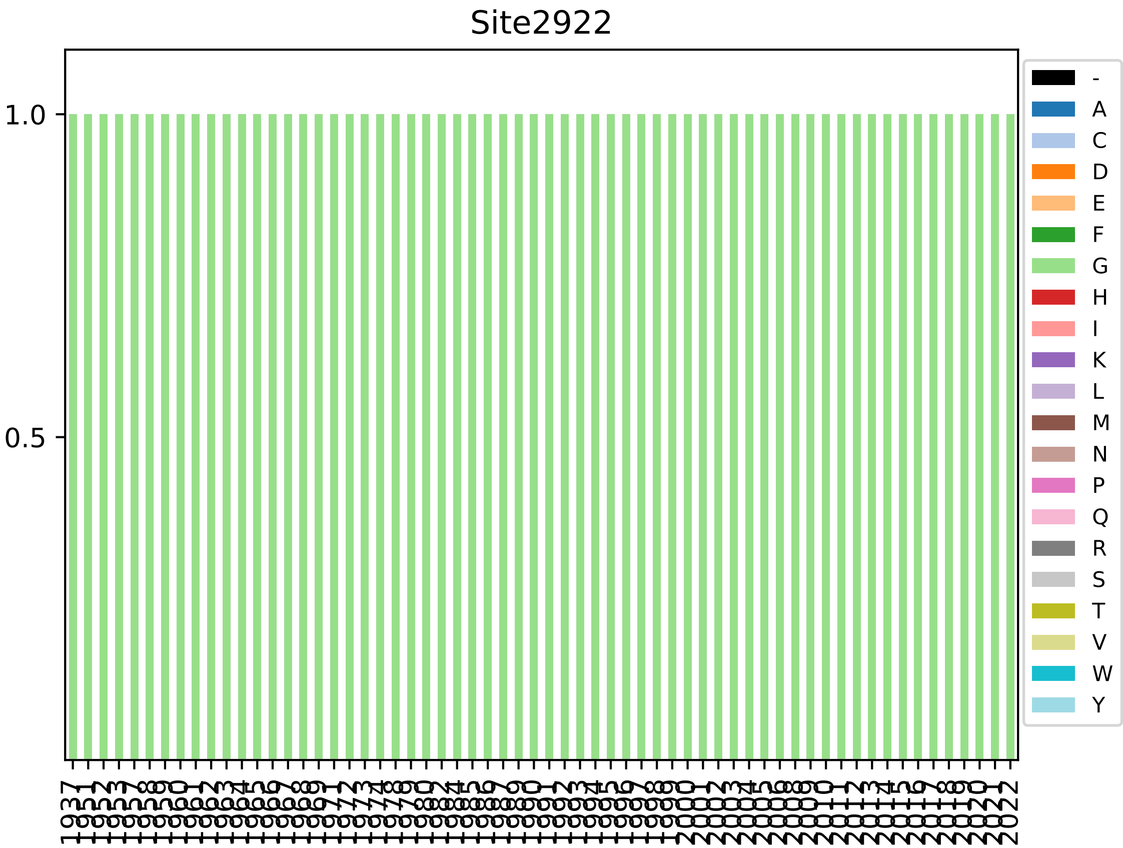
Site2922
1.0
0.5
-
A
C
D
E
F
G
H
I
K
L
M
N
P
Q
R
S
T
V
W
Y

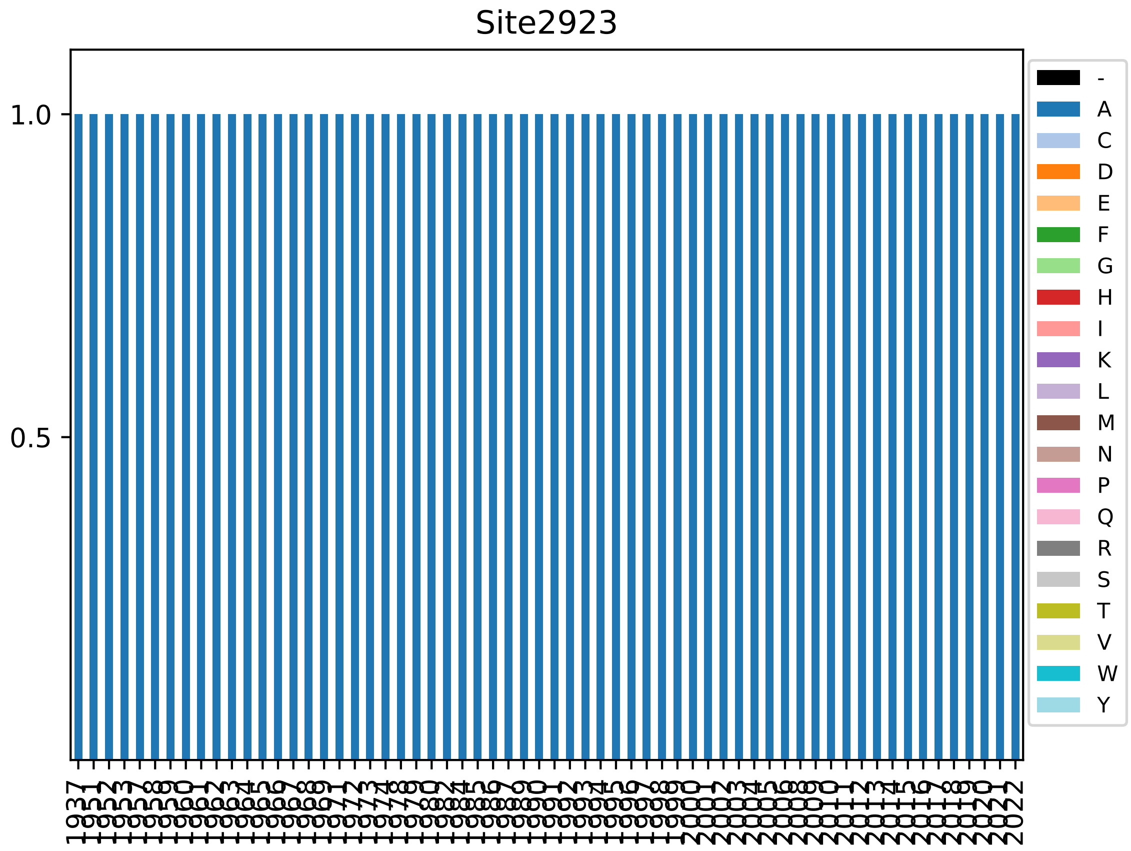
Site2923
1.0
0.5
-
A
C
D
E
F
G
H
I
K
L
M
N
P
Q
R
S
T
V
W
Y

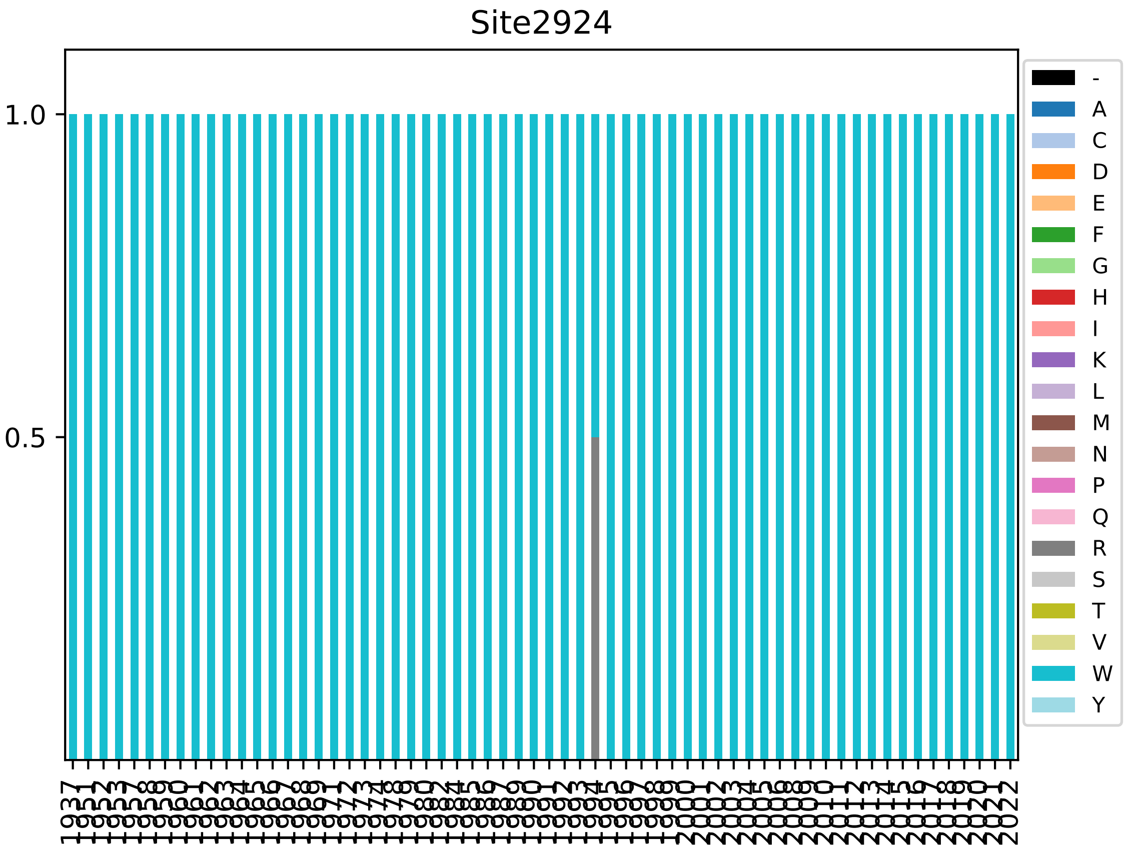
Site2924
1.0
0.5
-
A
C
D
E
F
G
H
I
K
L
M
N
P
Q
R
S
T
V
W
Y

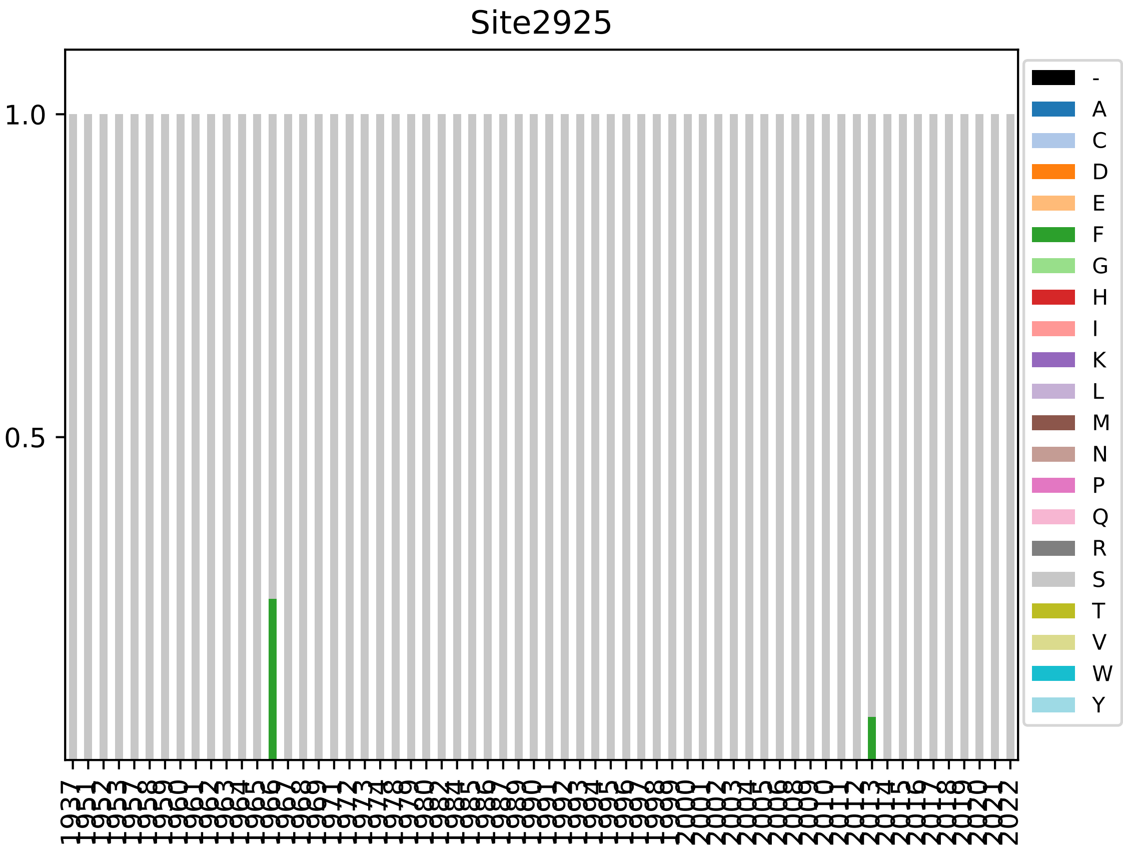

Site2925
1.0
0.5
-
A
C
D
E
F
G
H
I
K
L
M
N
P
Q
R
S
T
V
W
Y

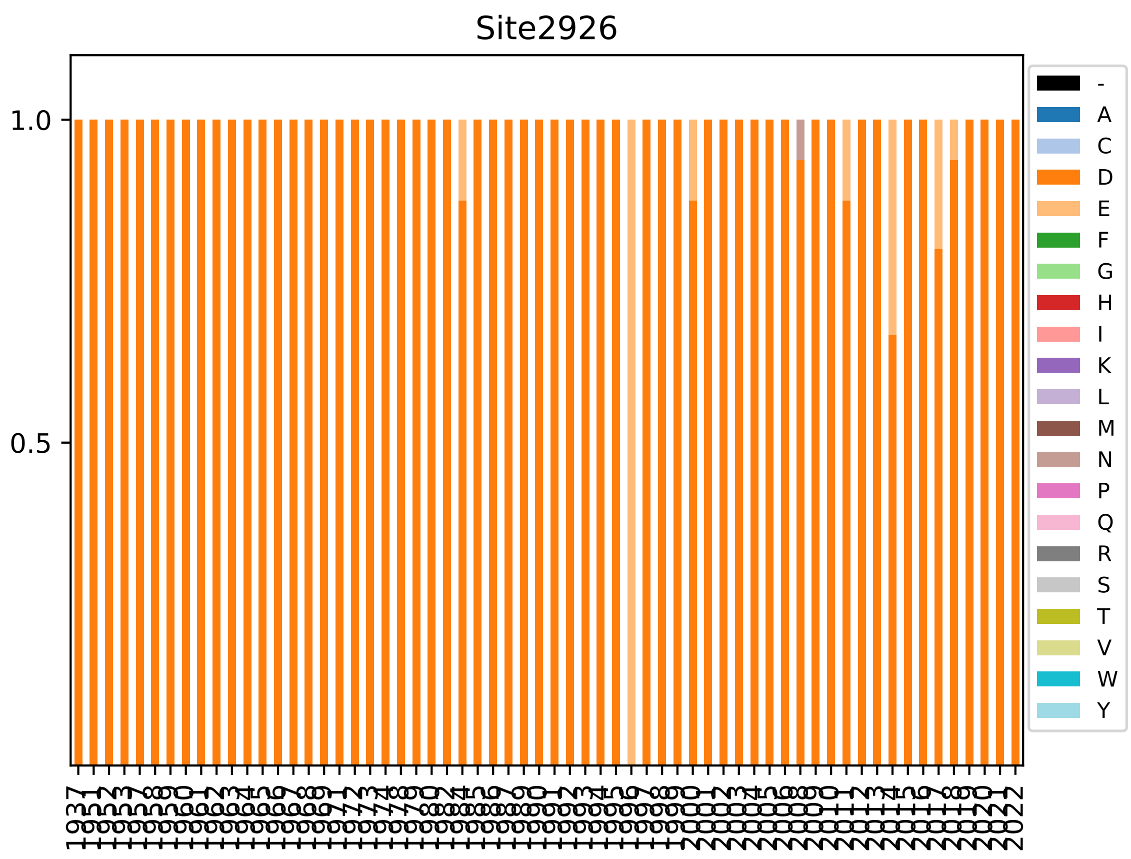
Site2926
1.0
0.5
-
A
C
D
E
F
G
H
I
K
L
M
N
P
Q
R
S
T
V
W
Y

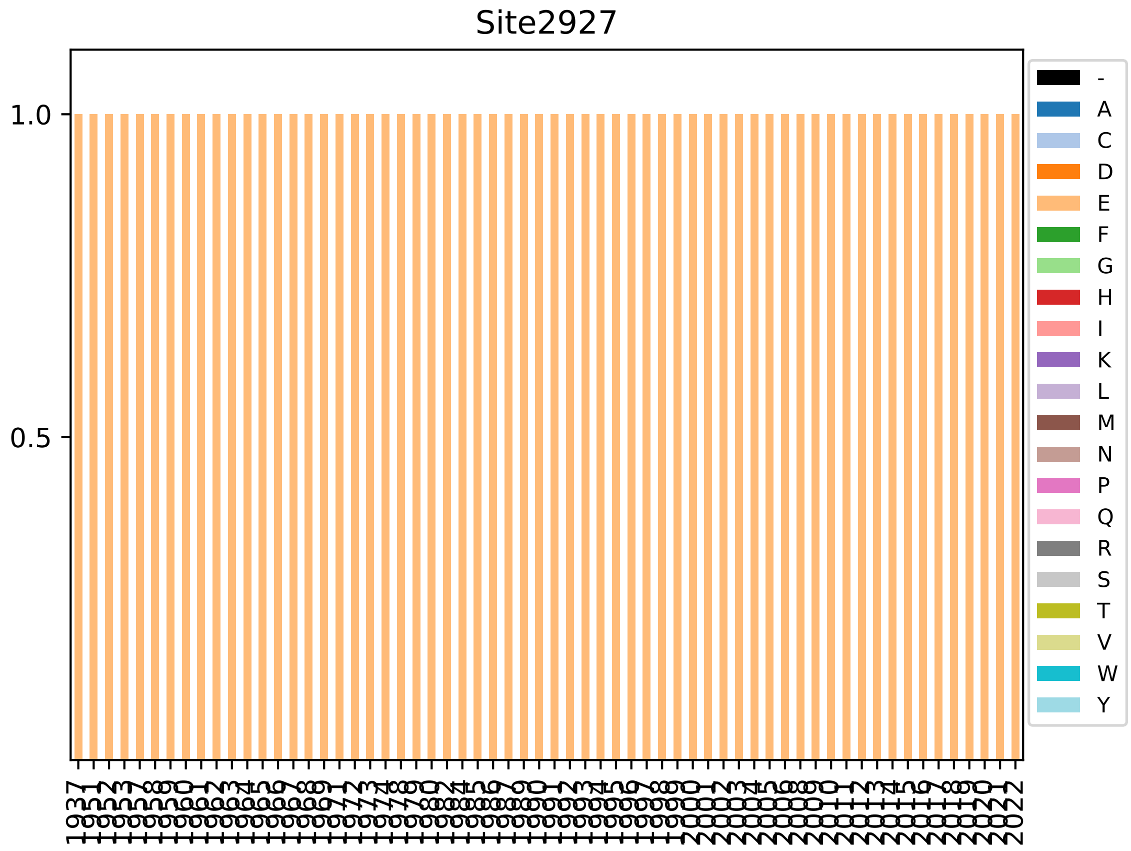
Site2927
1.0
0.5
-
A
C
D
E
F
G
H
I
K
L
M
N
P
Q
R
S
T
V
W
Y

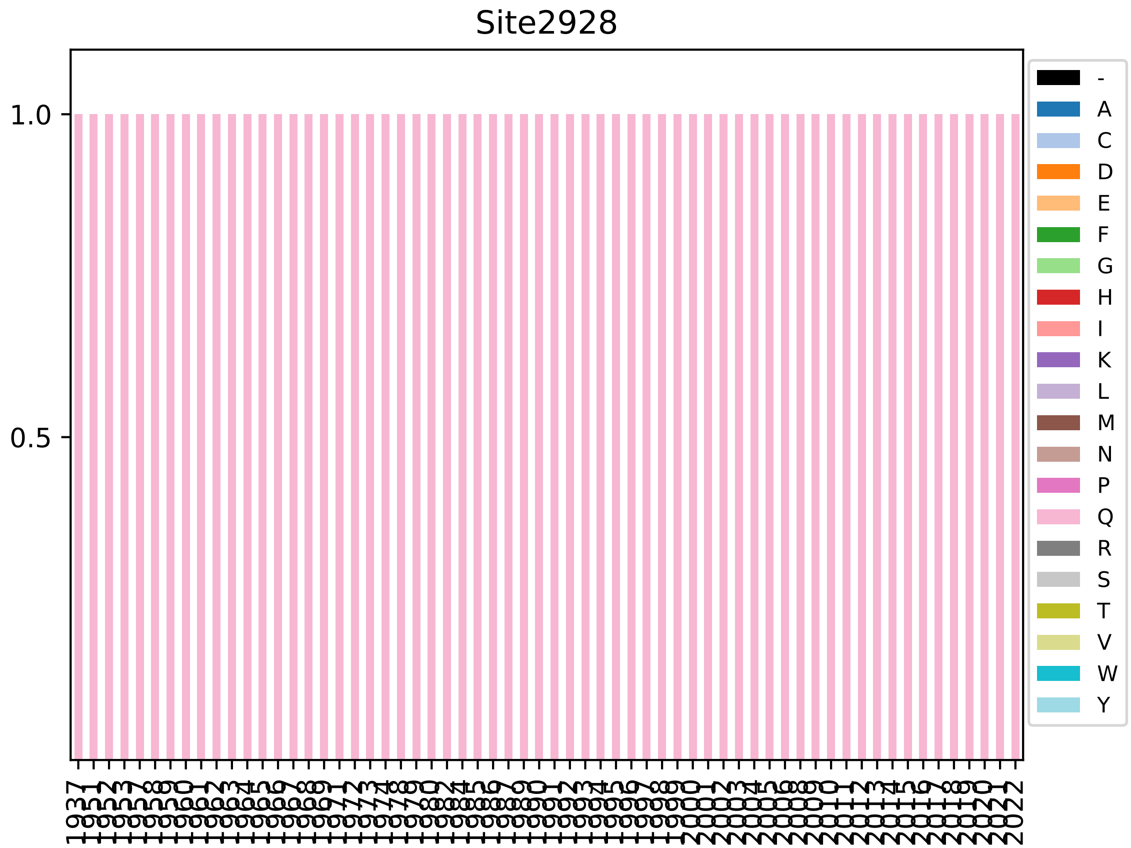
Site2928
1.0
0.5
-
A
C
D
E
F
G
H
I
K
L
M
N
P
Q
R
S
T
V
W
Y

Site2929

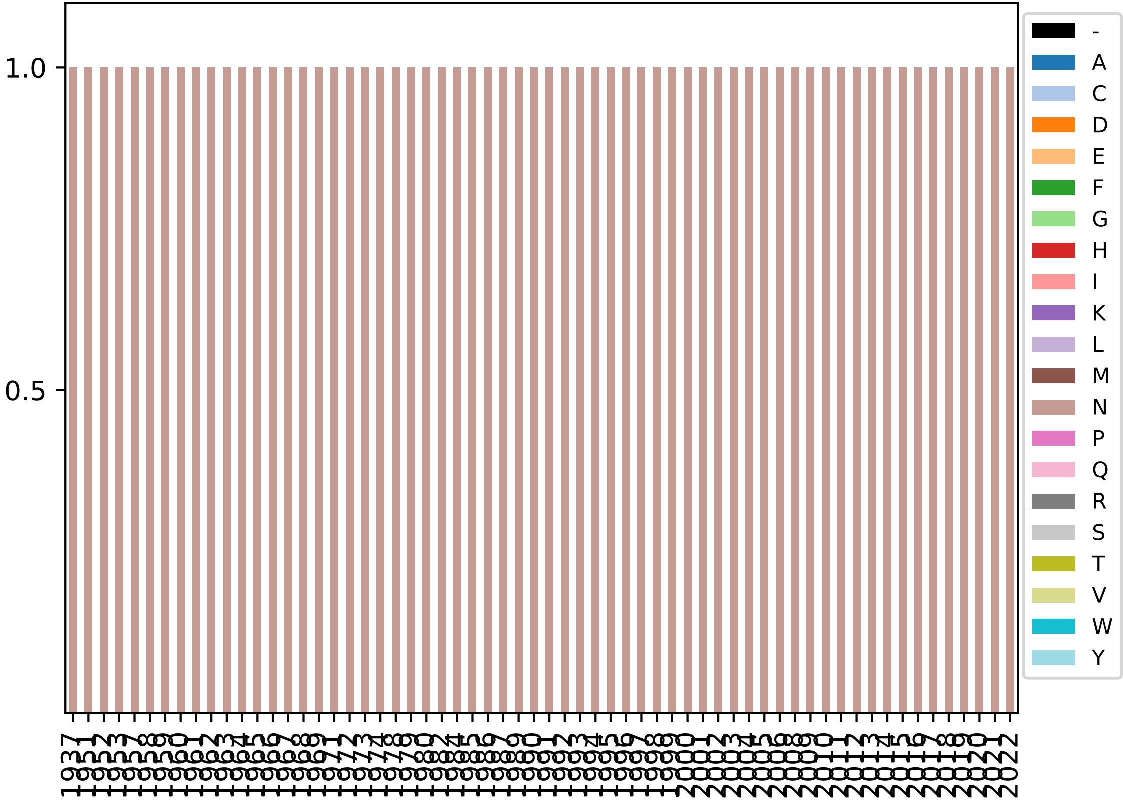

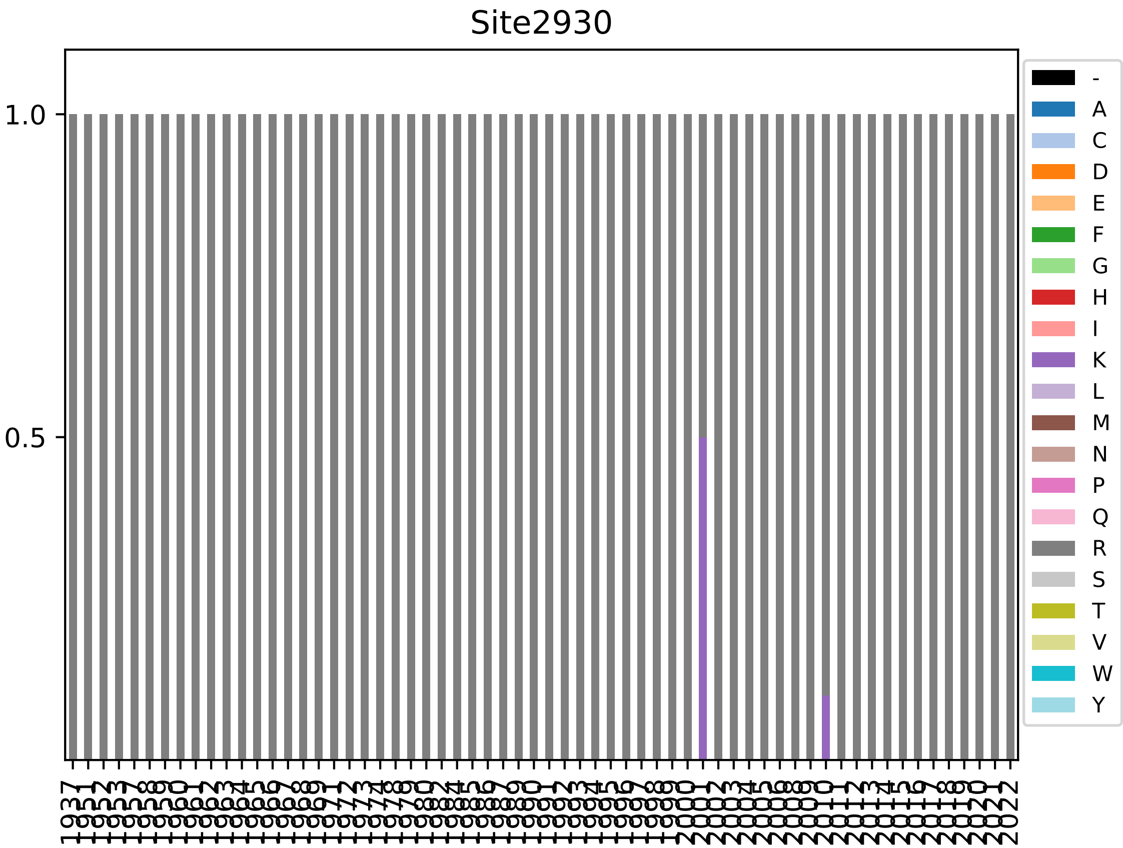
Site2930
1.0
0.5
-
A
C
D
E
F
G
H
I
K
L
M
N
P
Q
R
S
T
V
W
Y

Site2931

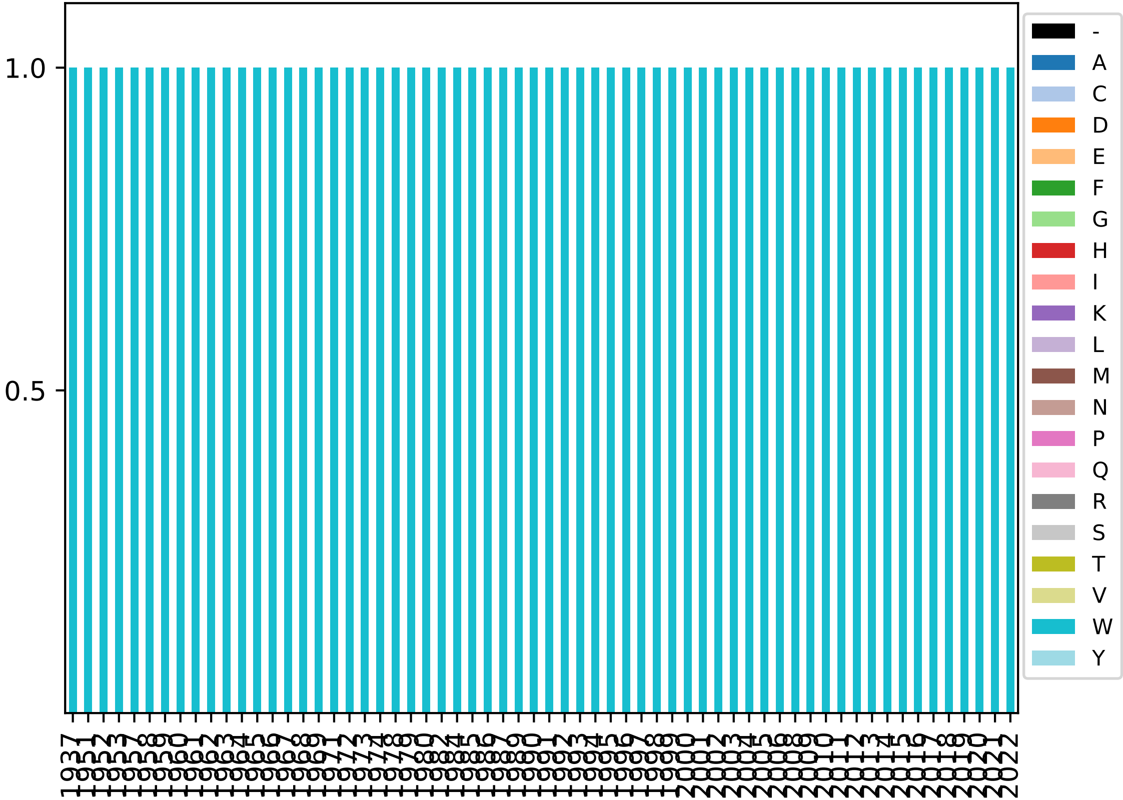

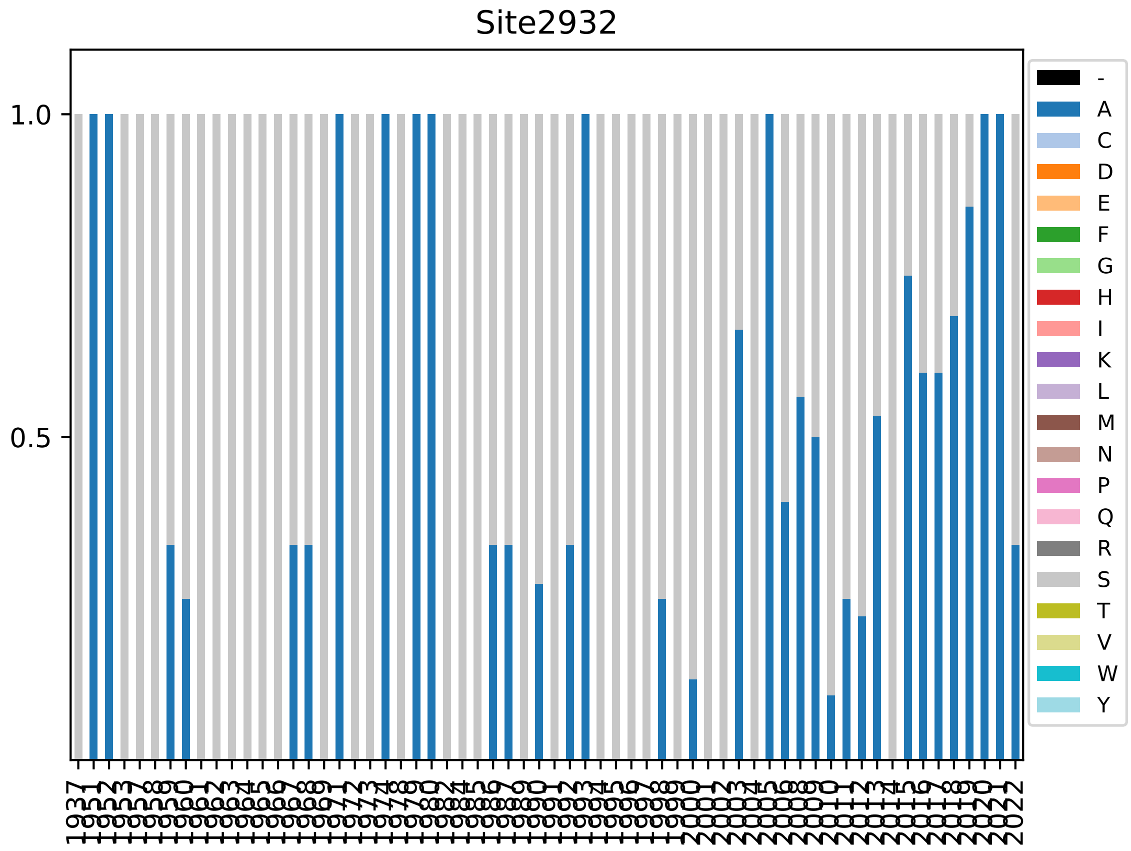
Site2932
1.0
0.5
-
A
C
D
E
F
G
H
I
K
L
M
N
P
Q
R
S
T
V
W
Y

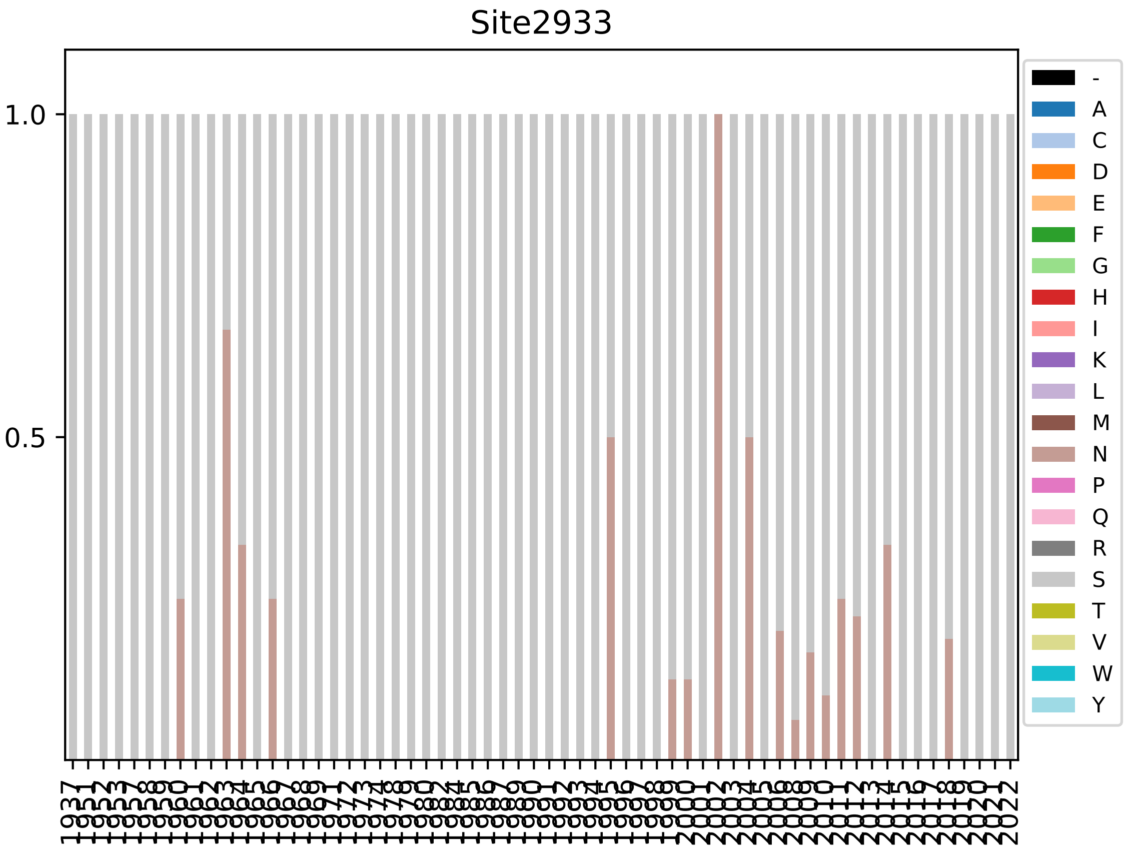

Site2933
1.0
0.5
-
A
C
D
E
F
G
H
I
K
L
M
N
P
Q
R
S
T
V
W
Y

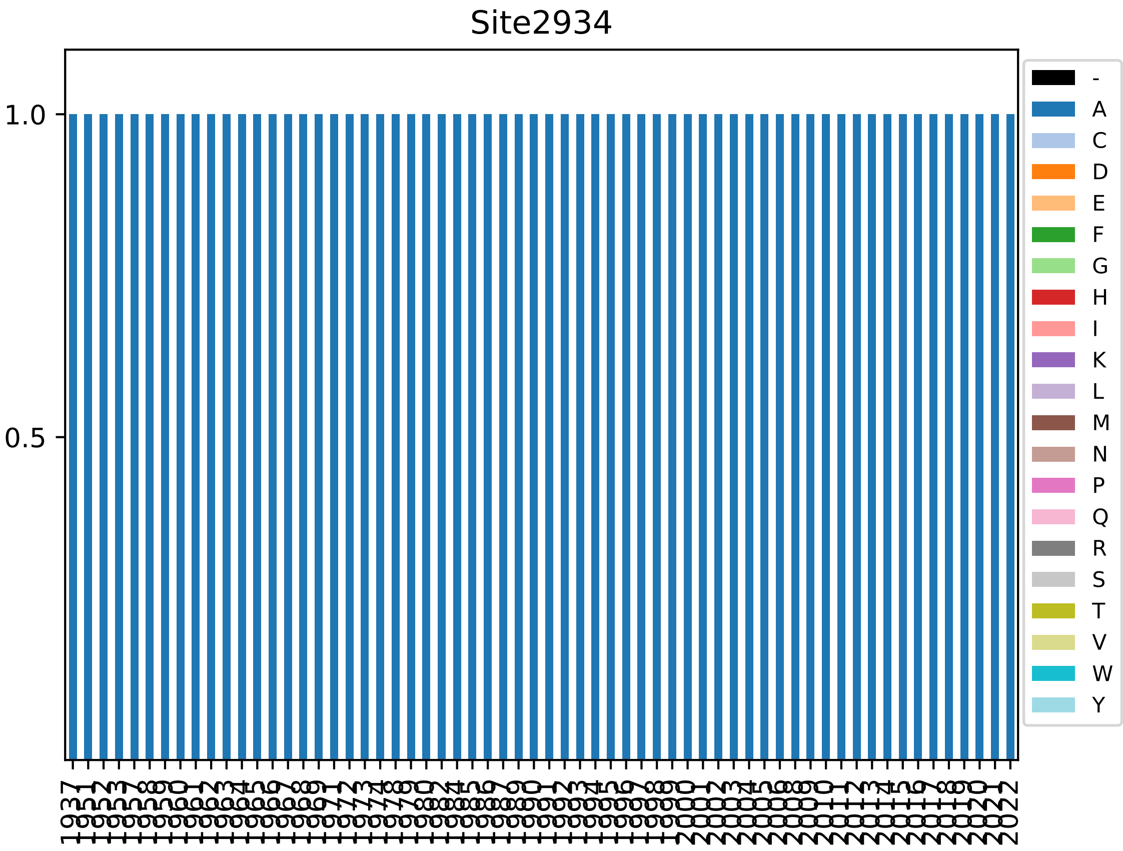
Site2934
1.0
0.5
-
A
C
D
E
F
G
H
I
K
L
M
N
P
Q
R
S
T
V
W
Y

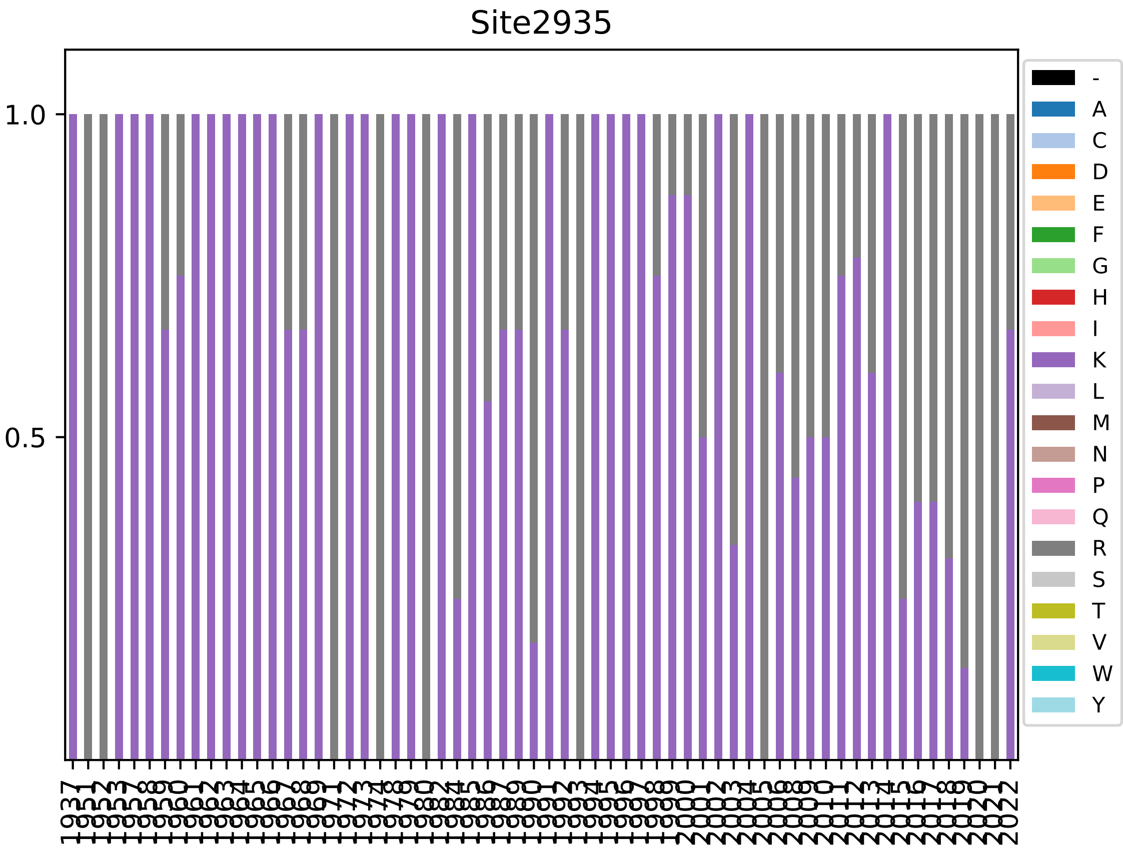
Site2935
1.0
0.5
-
A
C
D
E
F
G
H
I
K
L
M
N
P
Q
R
S
T
V
W
Y

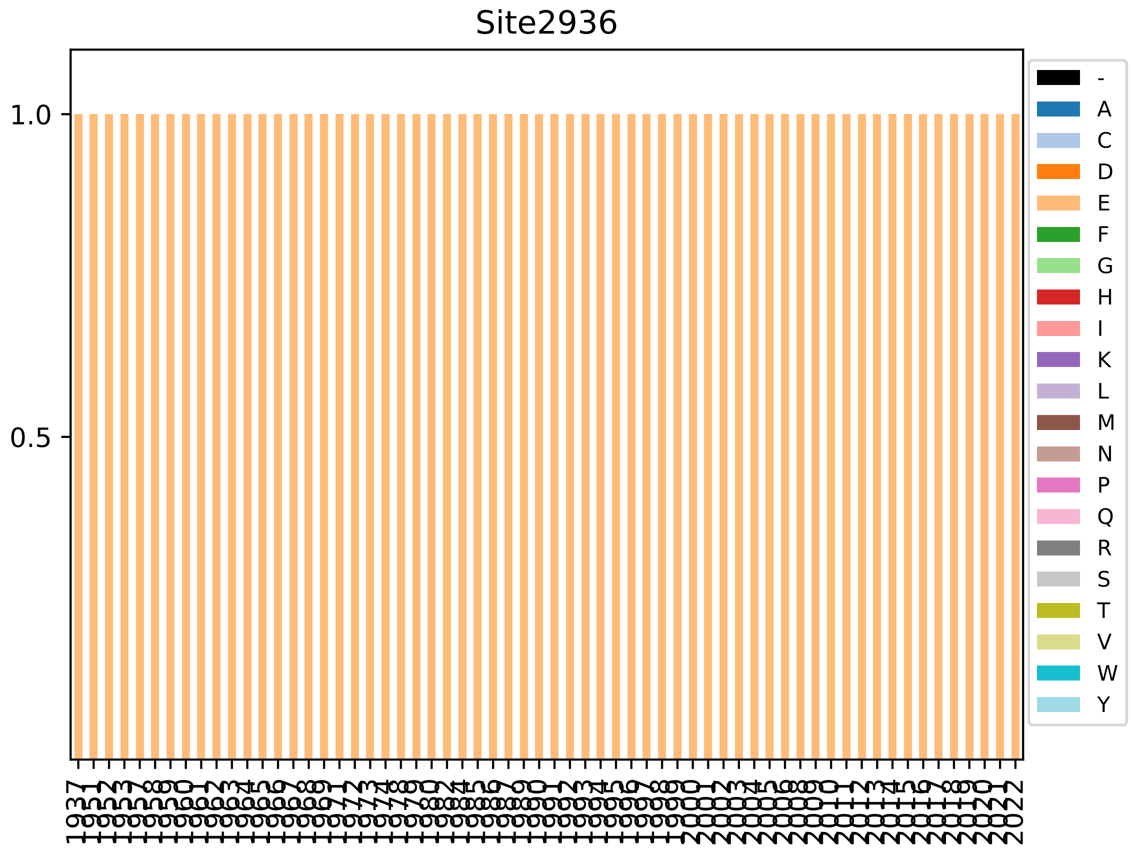
Site2936
1.0
0.5
-
A
C
D
E
F
G
H
I
K
L
M
N
P
Q
R
S
T
V
W
Y

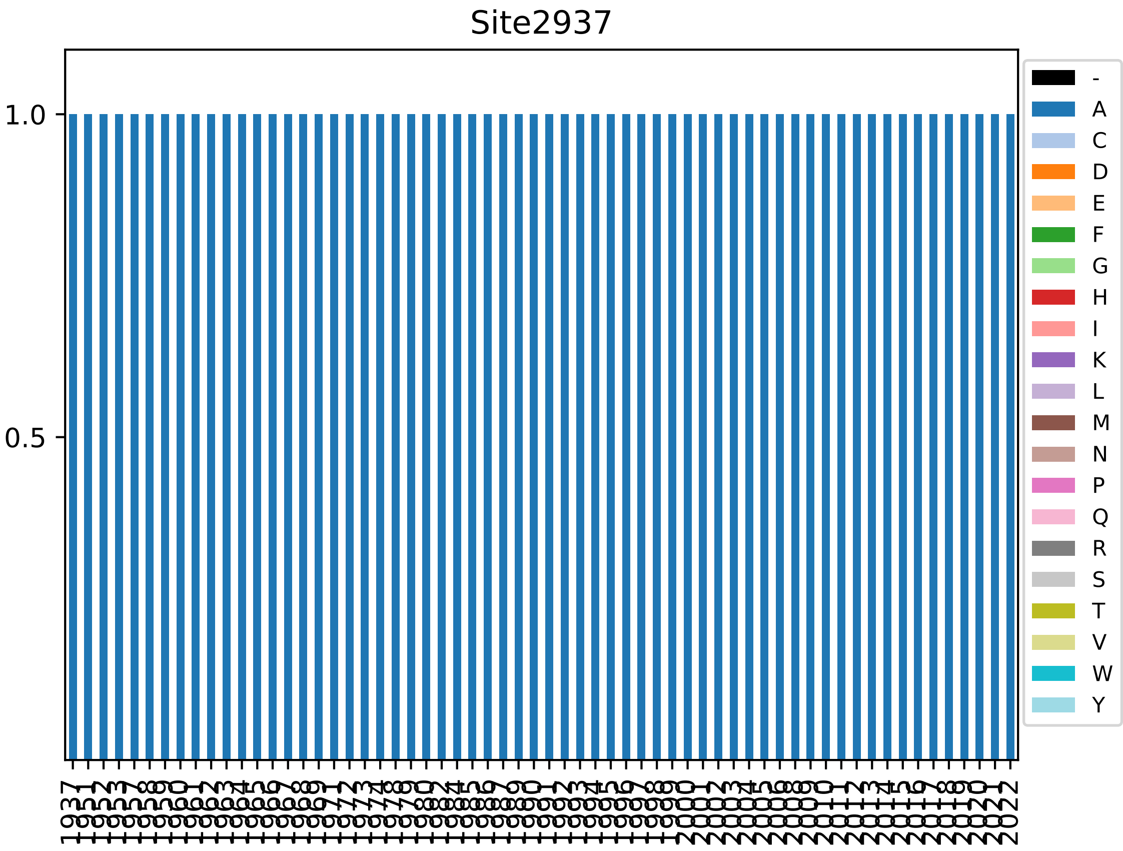
Site2937
1.0
0.5
-
A
C
D
E
F
G
H
I
K
L
M
N
P
Q
R
S
T
V
W
Y

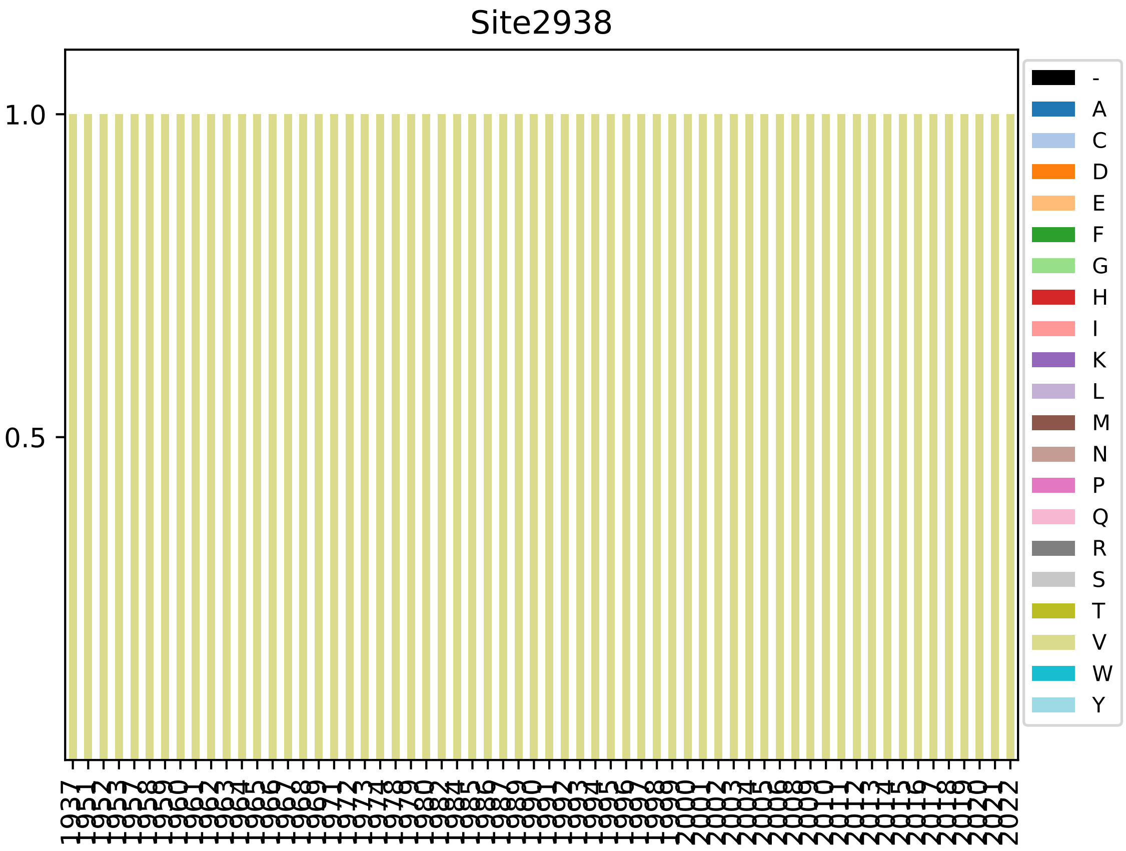
Site2938
1.0
0.5
-
A
C
D
E
F
G
H
I
K
L
M
N
P
Q
R
S
T
V
W
Y

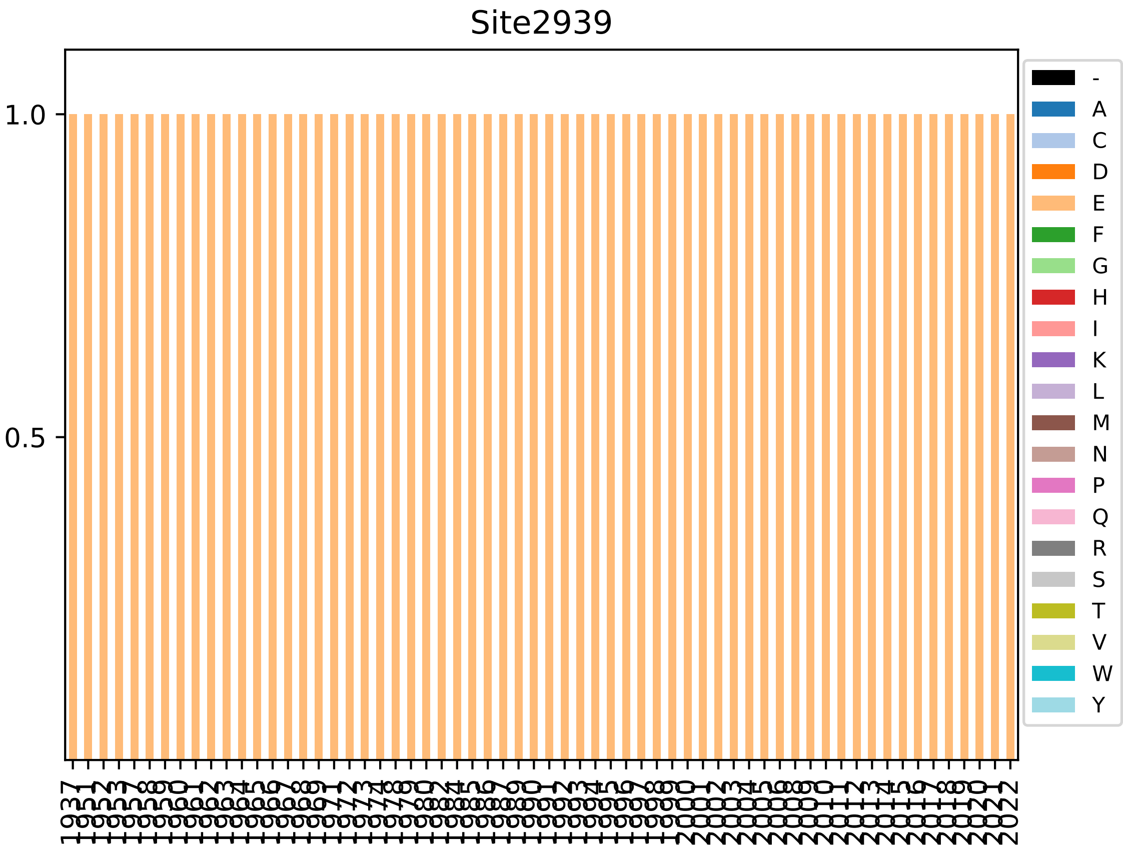
Site2939
1.0
0.5
-
A
C
D
E
F
G
H
I
K
L
M
N
P
Q
R
S
T
V
W
Y

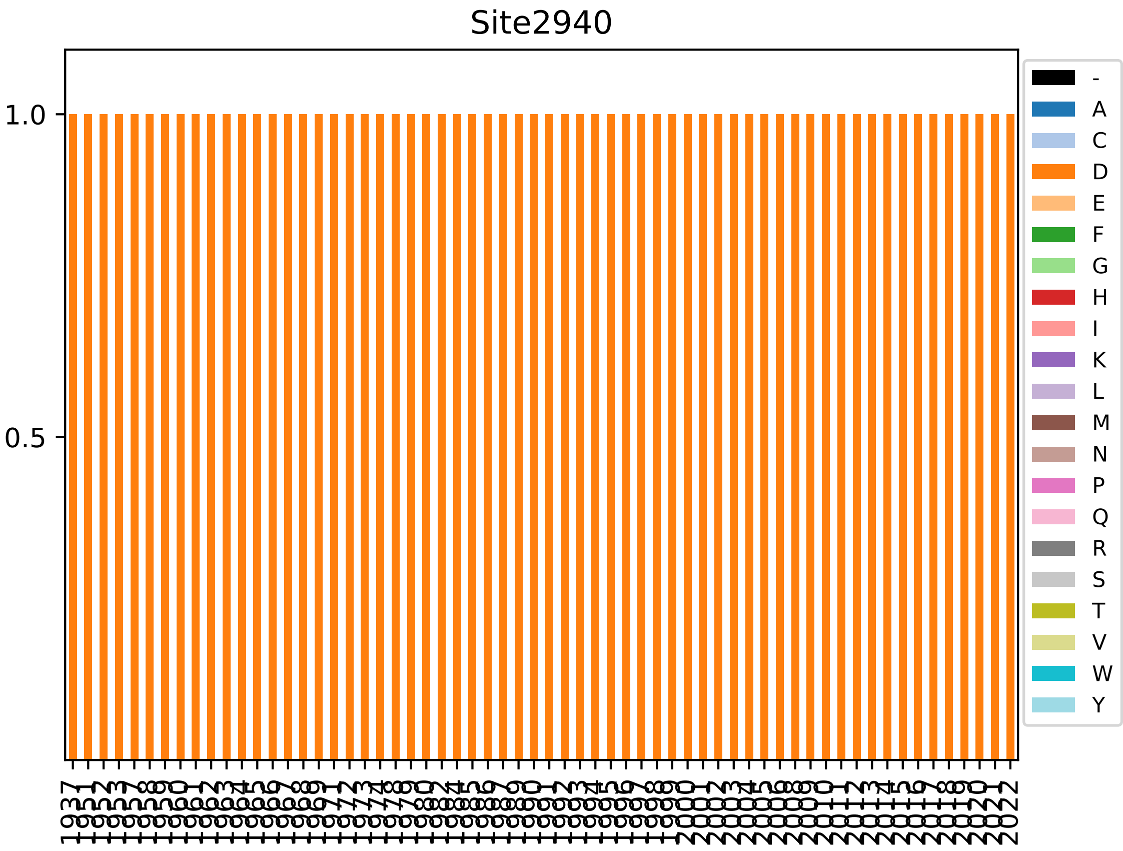
Site2940
1.0
0.5
-
A
C
D
E
F
G
H
I
K
L
M
N
P
Q
R
S
T
V
W
Y

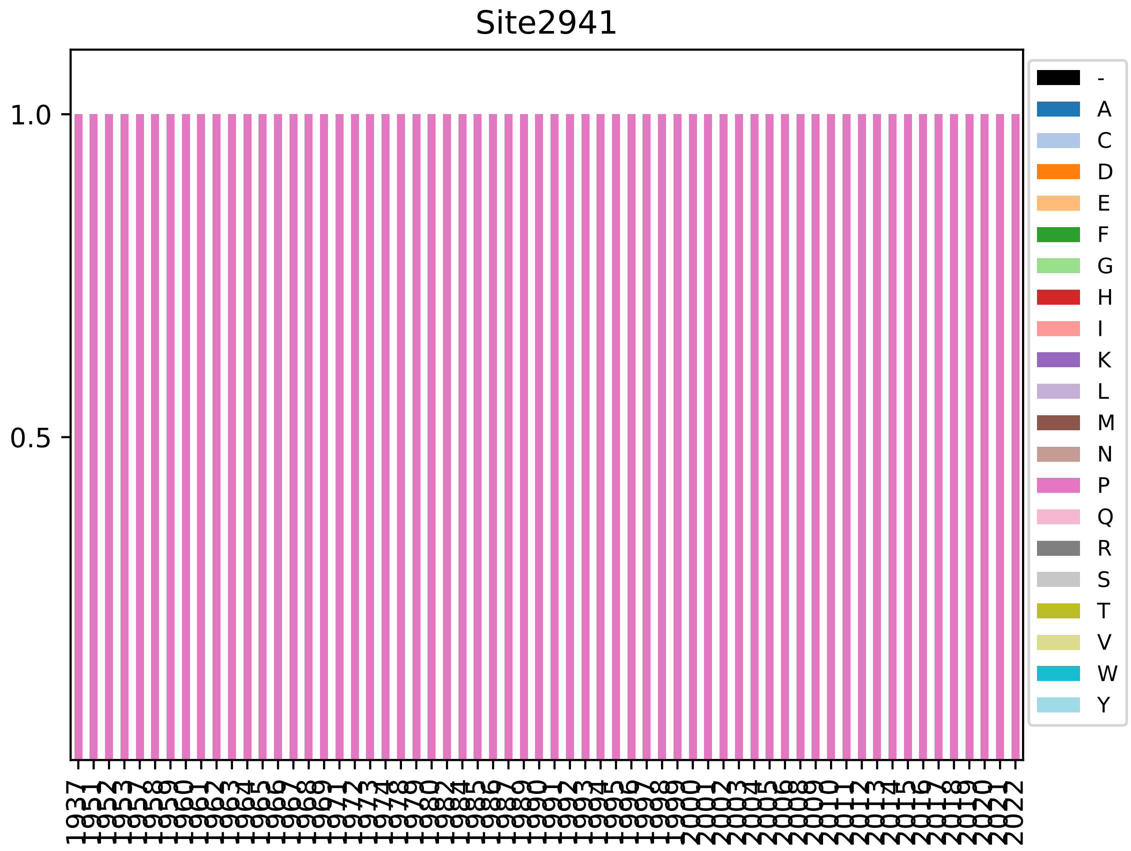
Site2941
1.0
0.5
-
A
C
D
E
F
G
H
I
K
L
M
N
P
Q
R
S
T
V
W
Y

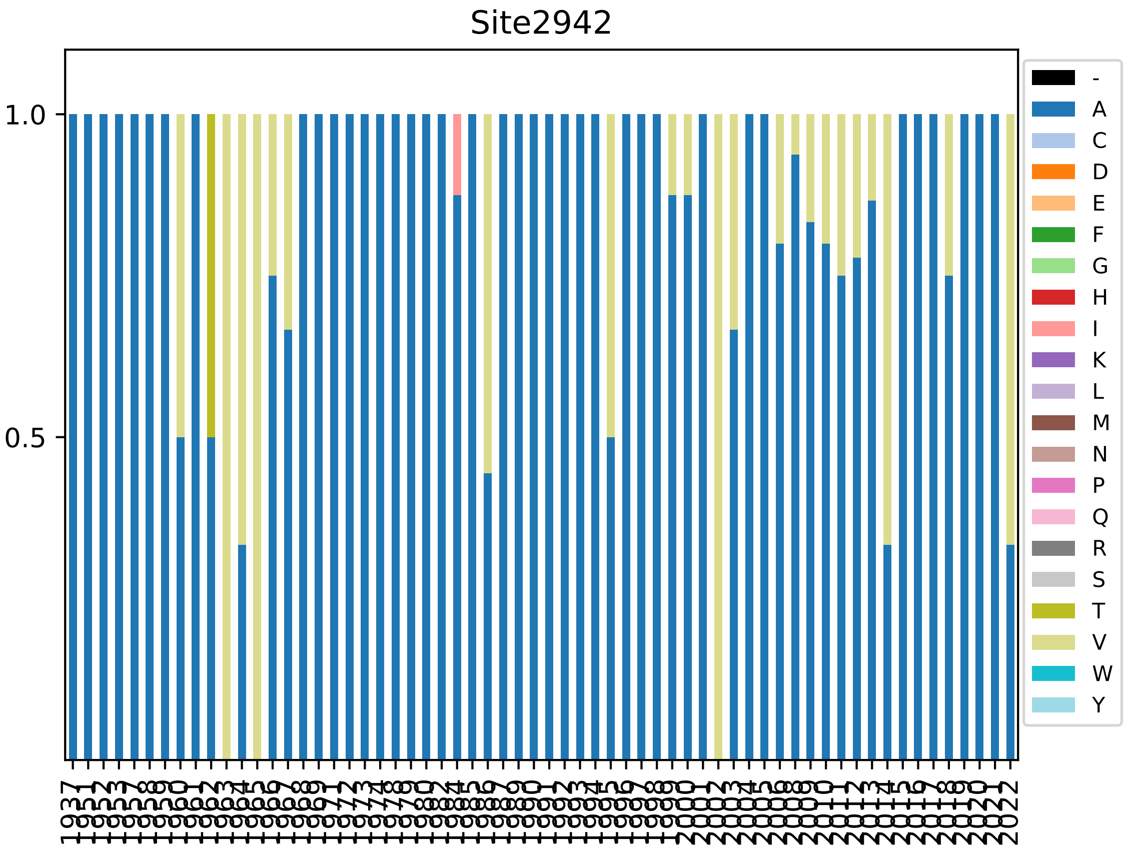
Site2942
1.0
0.5
-
A
C
D
E
F
G
H
I
K
L
M
N
P
Q
R
S
T
V
W
Y

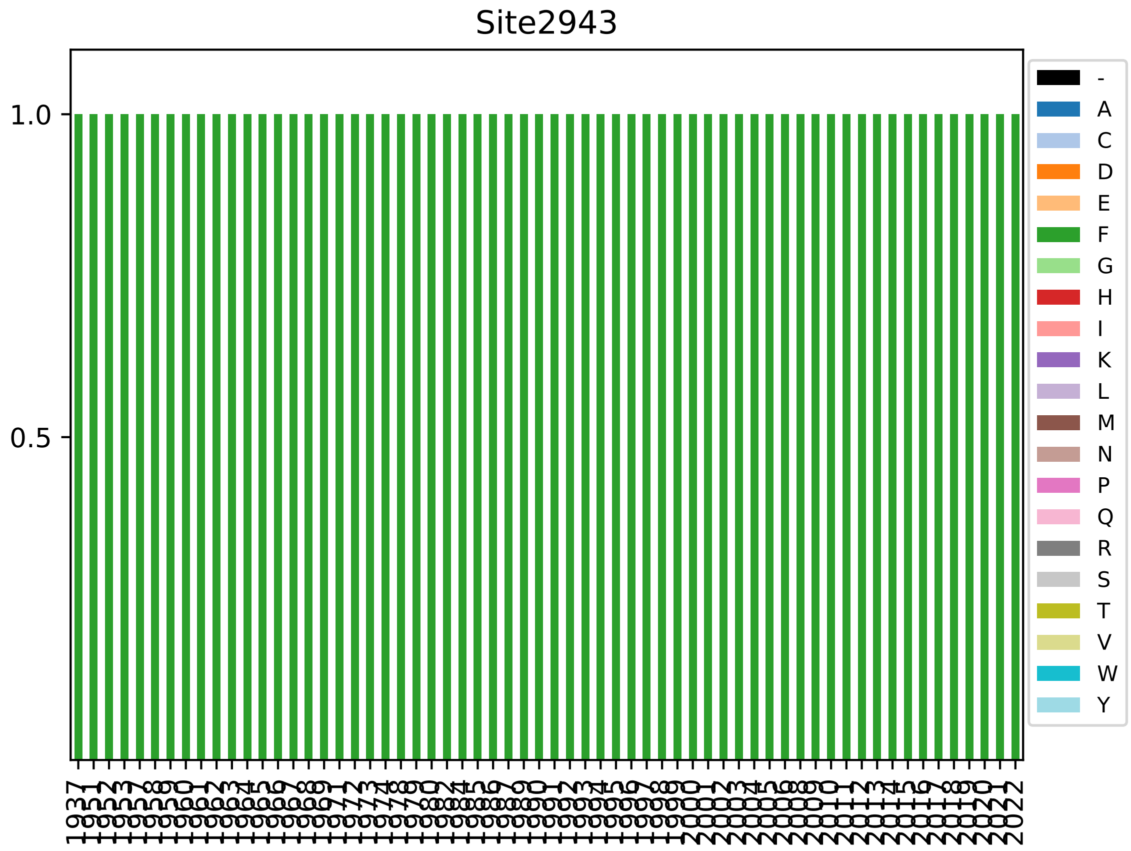
Site2943
1.0
0.5
-
A
C
D
E
F
G
H
I
K
L
M
N
P
Q
R
S
T
V
W
Y

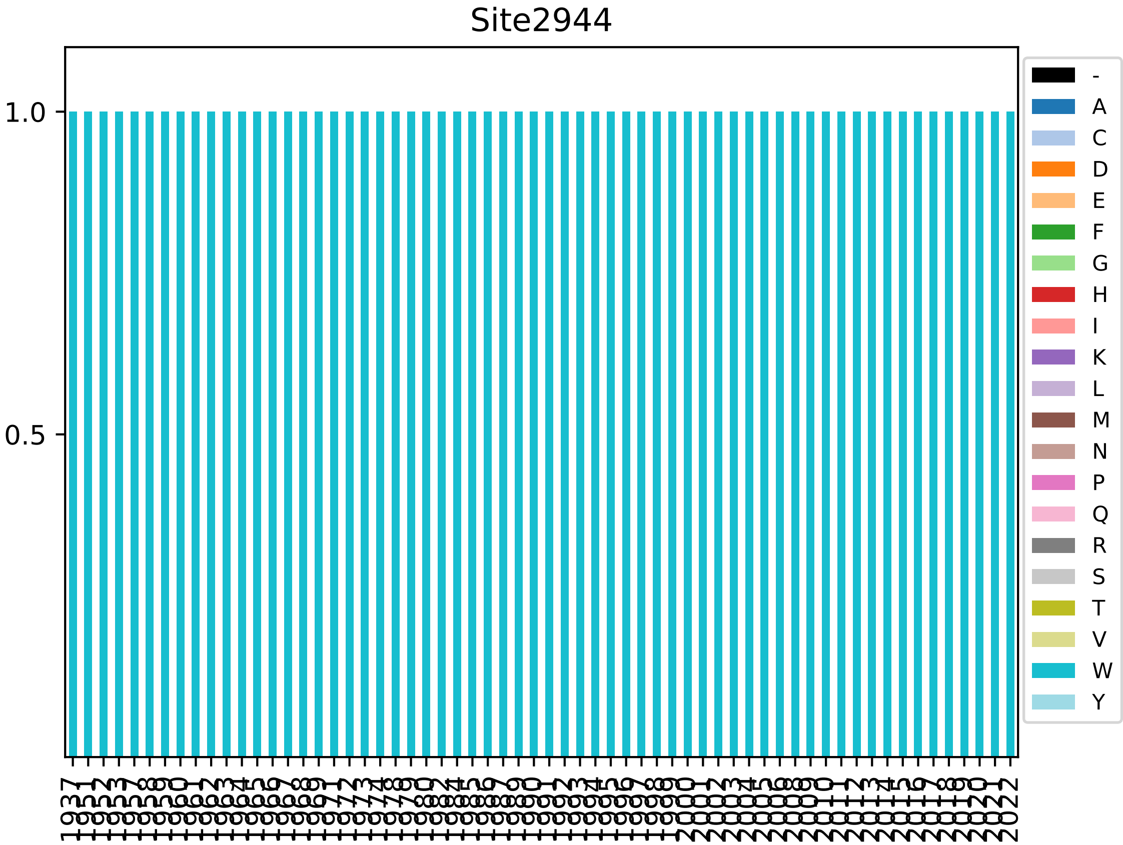
Site2944
1.0
0.5
-
A
C
D
E
F
G
H
I
K
L
M
N
P
Q
R
S
T
V
W
Y

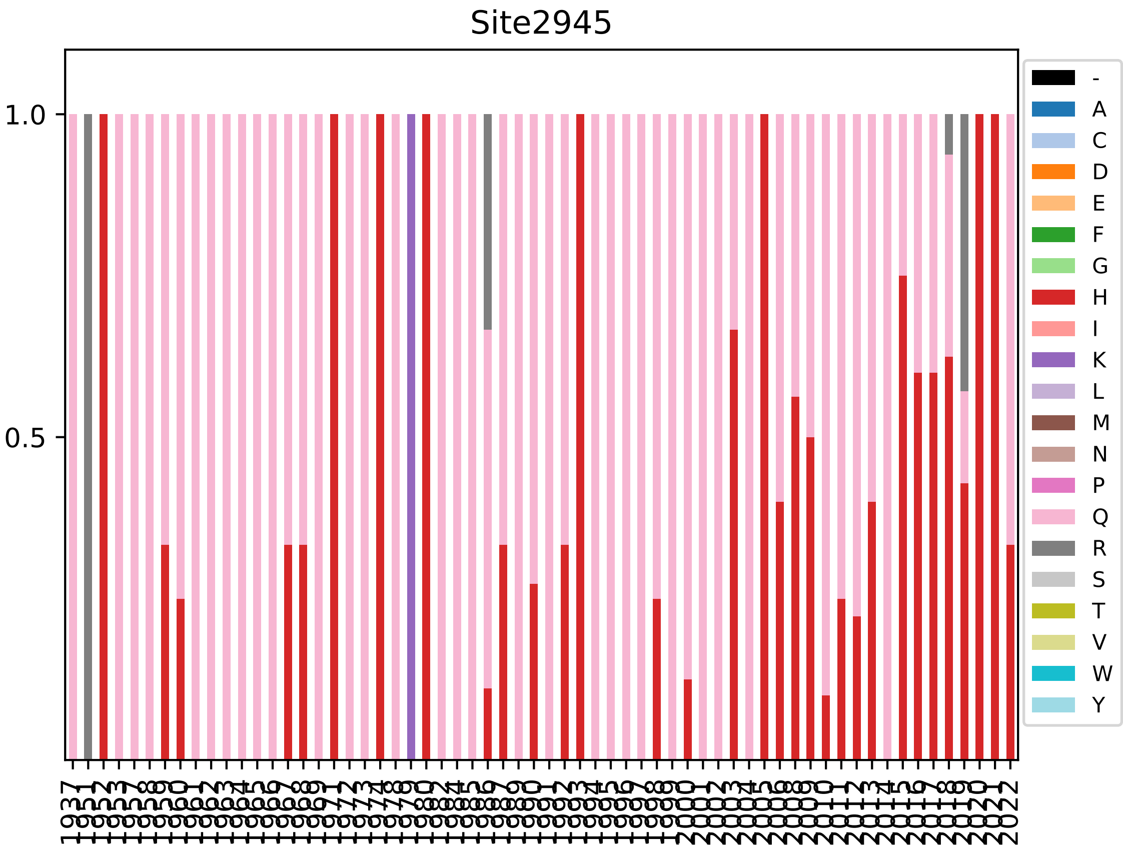
Site2945
1.0
0.5
-
A
C
D
E
F
G
H
I
K
L
M
N
P
Q
R
S
T
V
W
Y

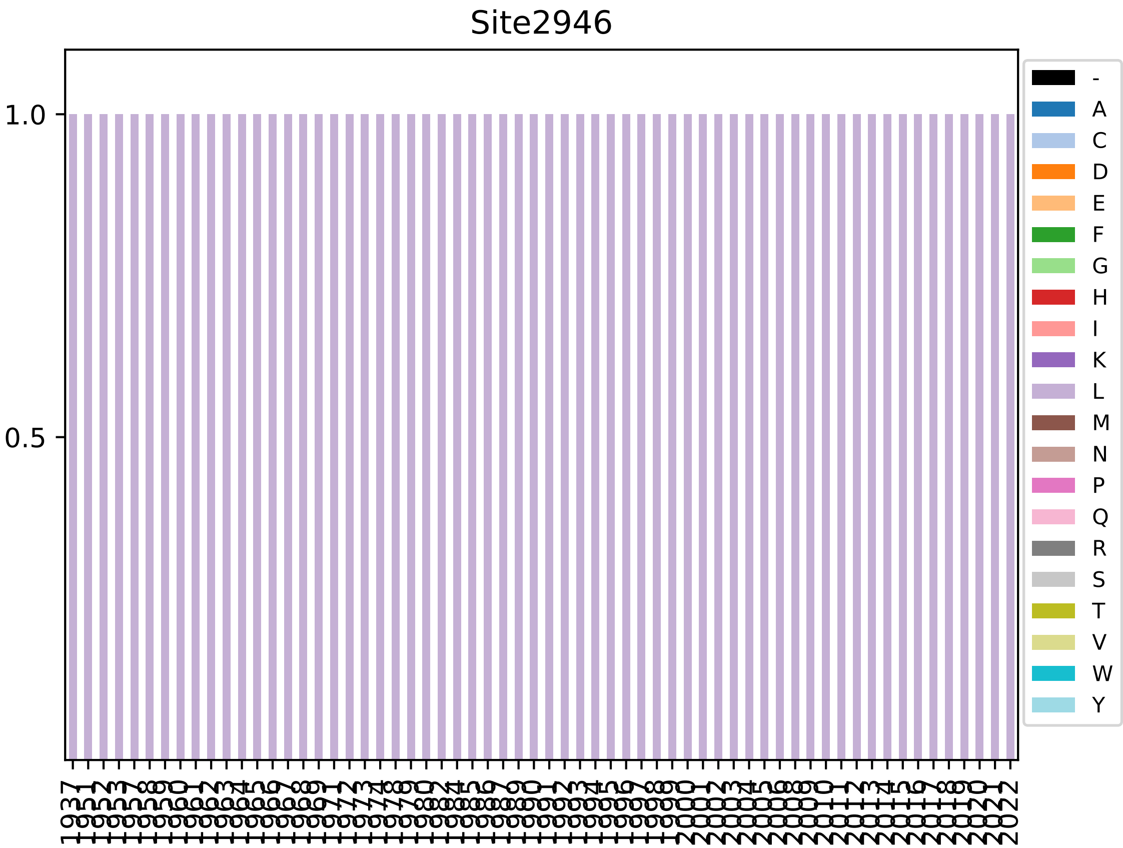
Site2946
1.0
0.5
-
A
C
D
E
F
G
H
I
K
L
M
N
P
Q
R
S
T
V
W
Y

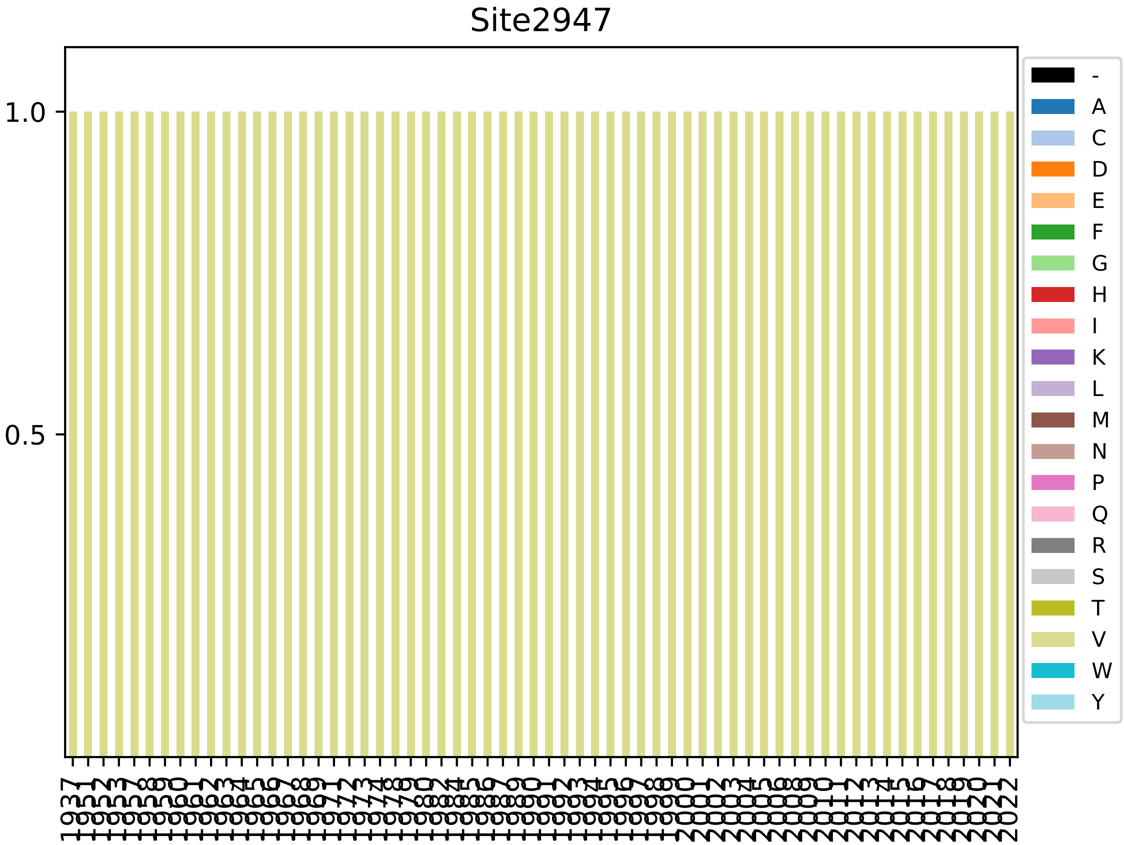
Site2947
1.0
0.5
-
A
C
D
E
F
G
H
I
K
L
M
N
P
Q
R
S
T
V
W
Y

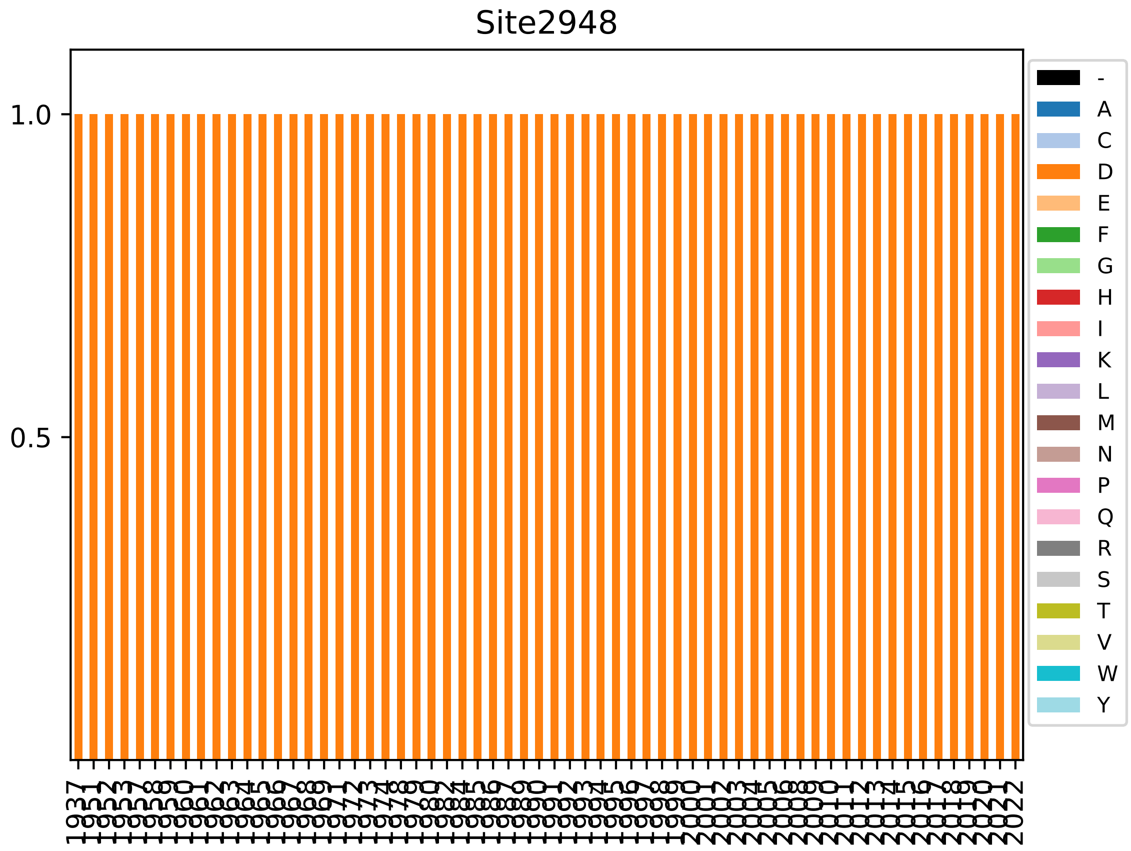
Site2948
1.0
0.5
-
A
C
D
E
F
G
H
I
K
L
M
N
P
Q
R
S
T
V
W
Y

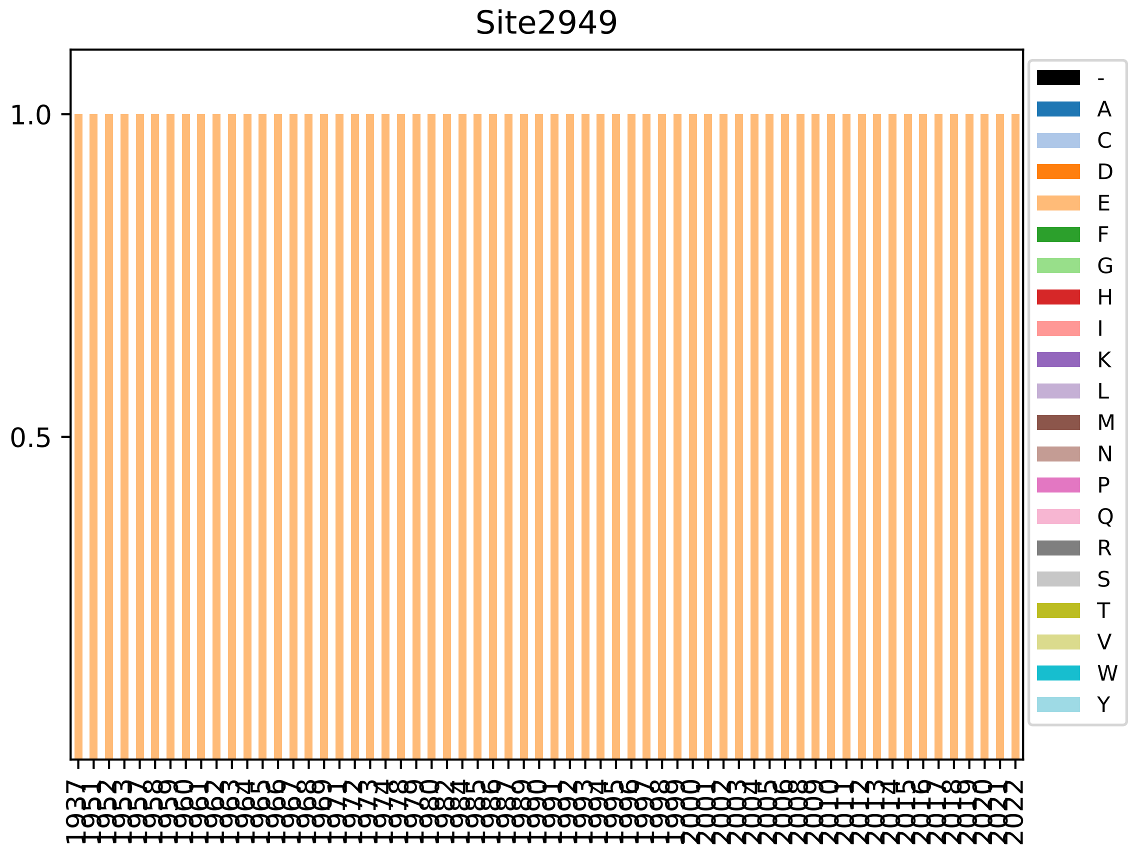
Site2949
1.0
0.5
-
A
C
D
E
F
G
H
I
K
L
M
N
P
Q
R
S
T
V
W
Y

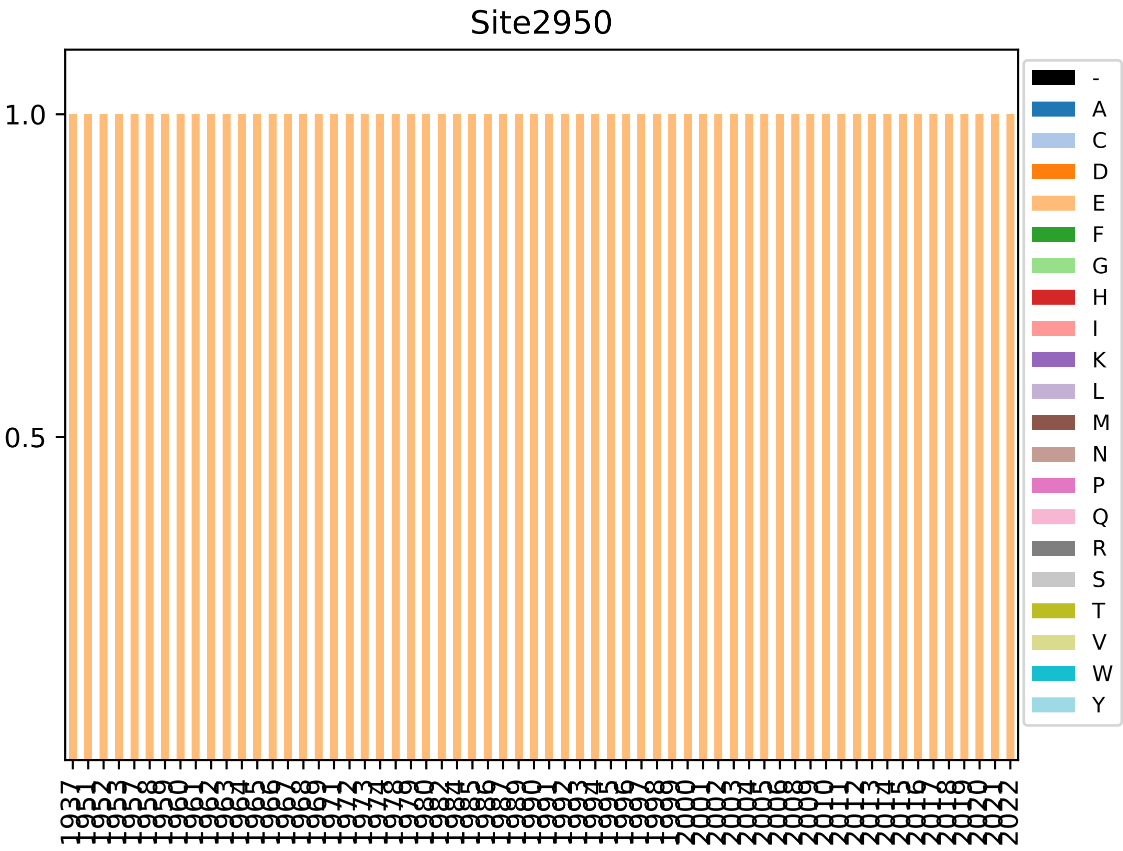
Site2950
1.0
0.5
-
A
C
D
E
F
G
H
I
K
L
M
N
P
Q
R
S
T
V
W
Y

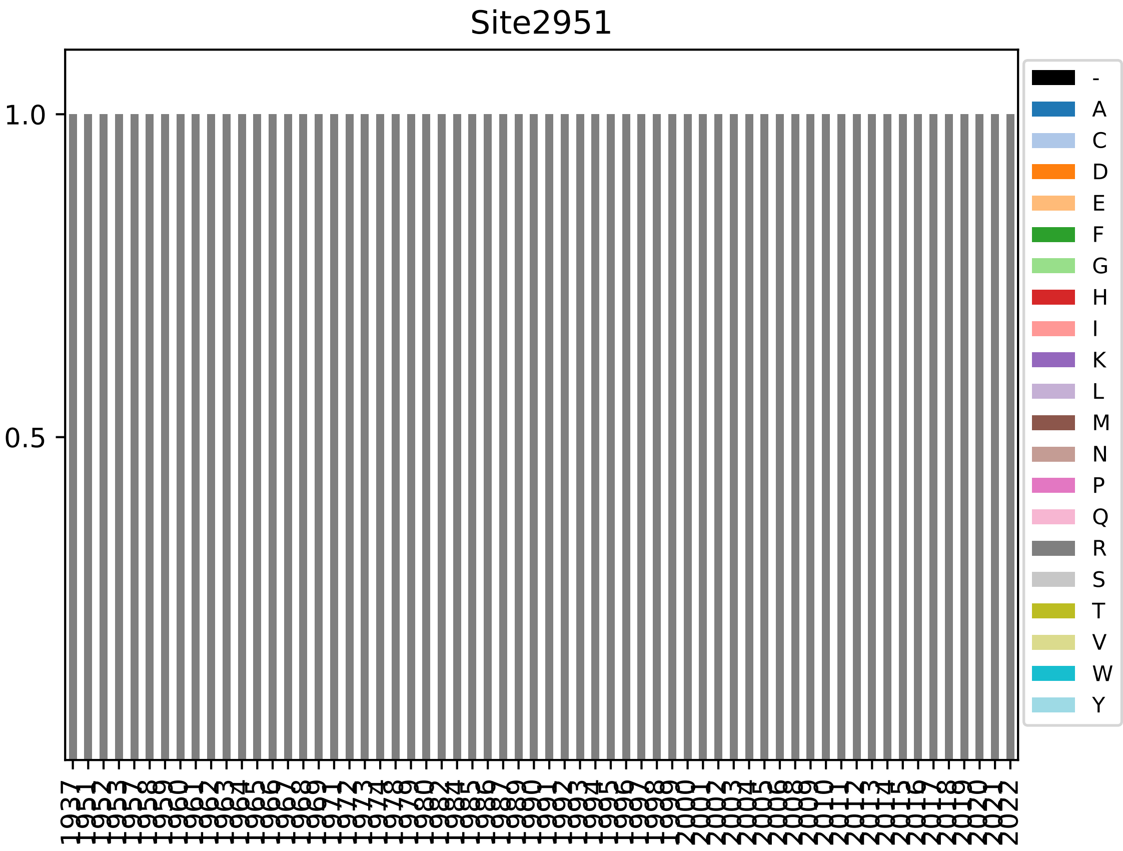
Site2951
1.0
0.5
-
A
C
D
E
F
G
H
I
K
L
M
N
P
Q
R
S
T
V
W
Y

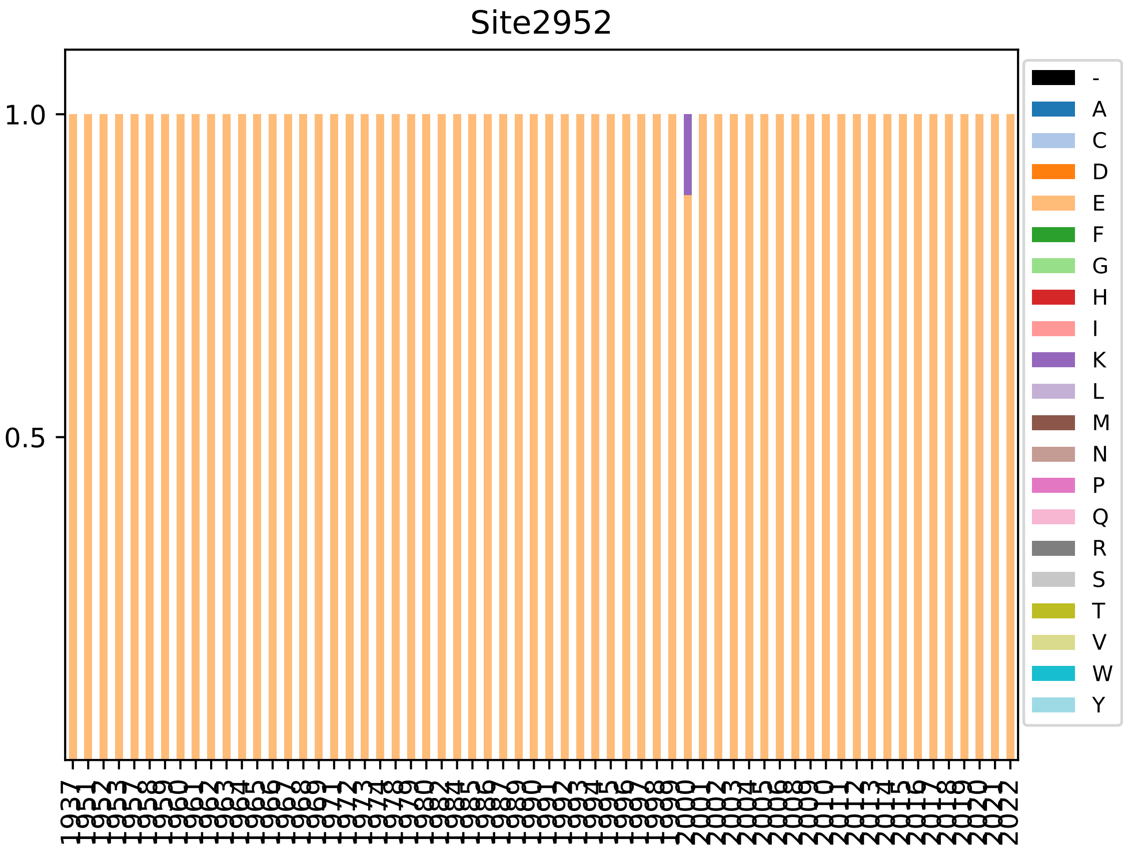
Site2952
1.0
0.5
-
A
C
D
E
F
G
H
I
K
L
M
N
P
Q
R
S
T
V
W
Y

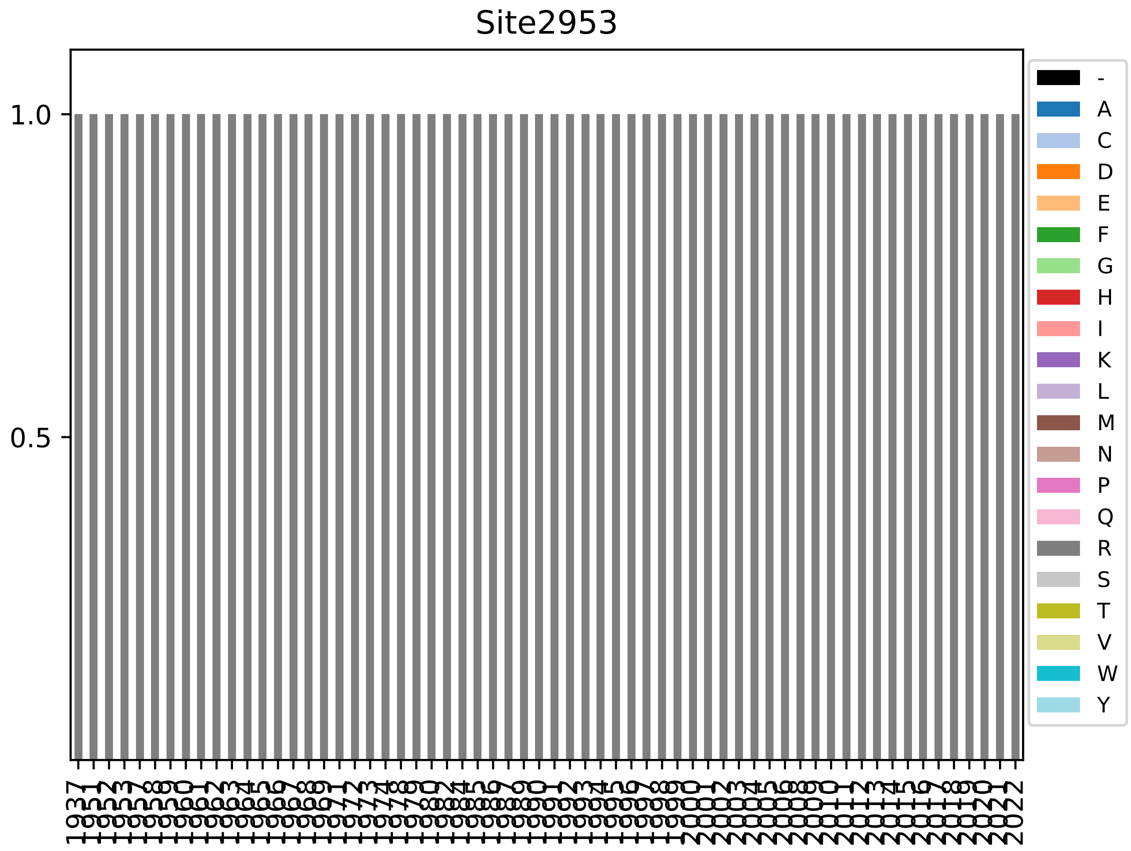
Site2953
1.0
0.5
-
A
C
D
E
F
G
H
I
K
L
M
N
P
Q
R
S
T
V
W
Y

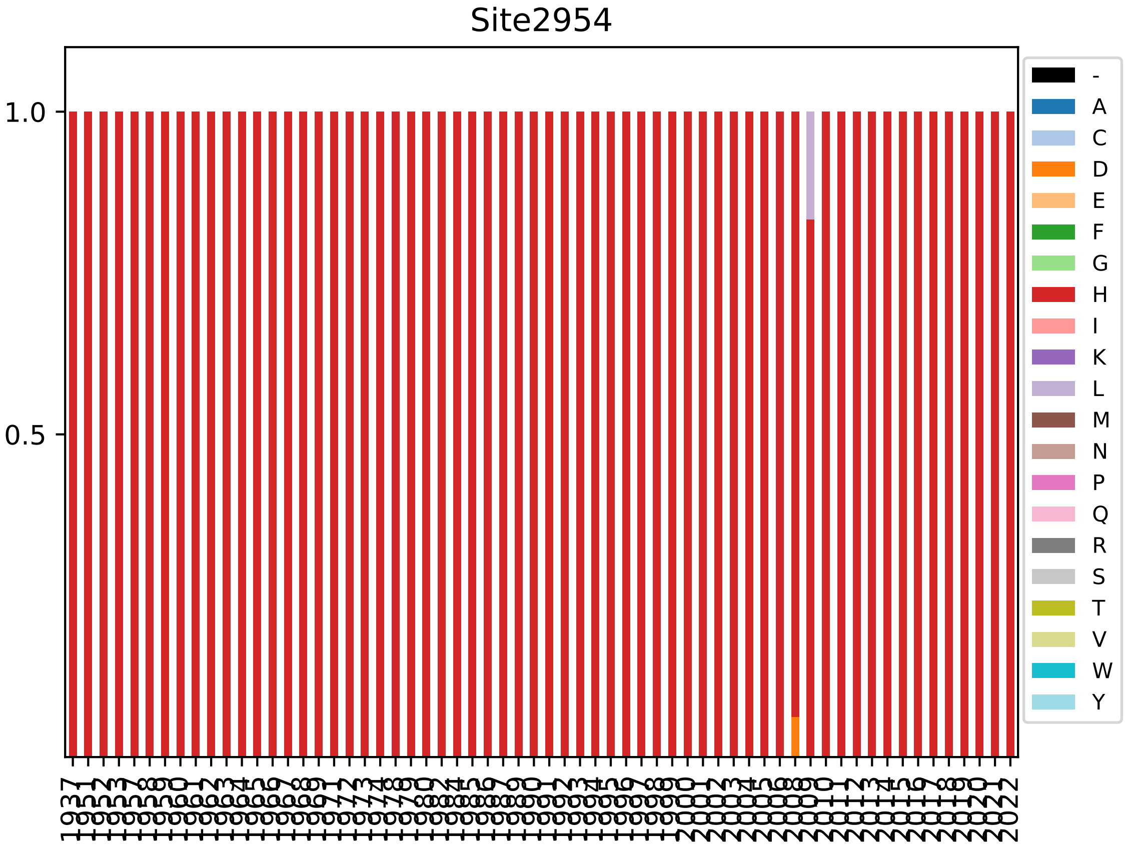
Site2954
1.0
0.5
-
A
C
D
E
F
G
H
I
K
L
M
N
P
Q
R
S
T
V
W
Y

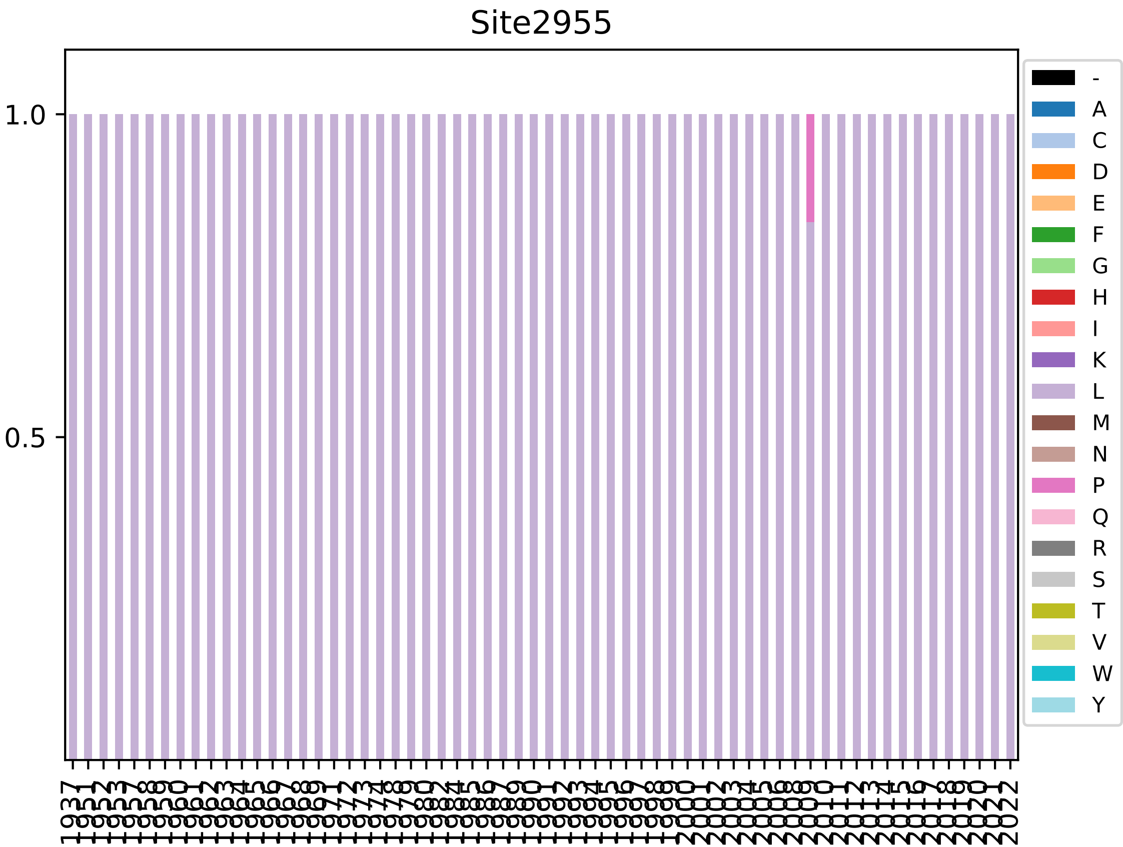
Site2955
1.0
0.5
-
A
C
D
E
F
G
H
I
K
L
M
N
P
Q
R
S
T
V
W
Y

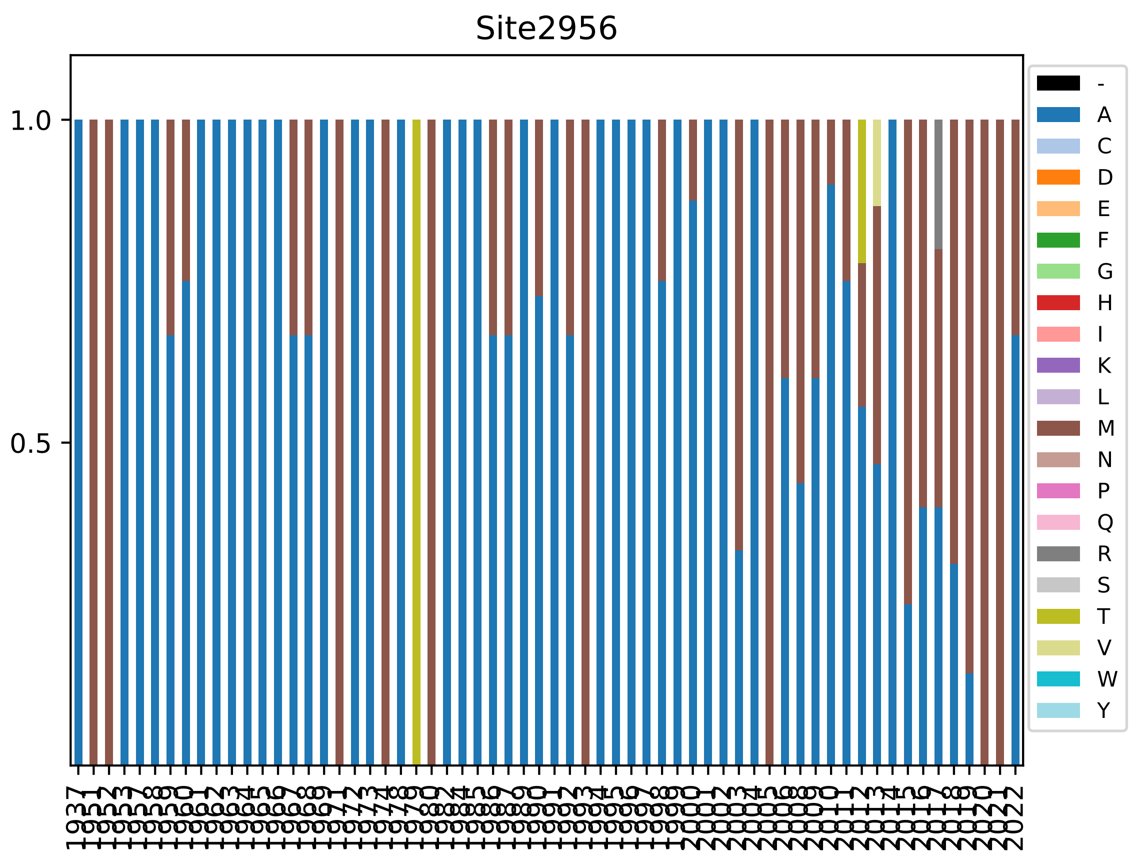
Site2956
1.0
0.5
-
A
C
D
E
F
G
H
I
K
L
M
N
P
Q
R
S
T
V
W
Y

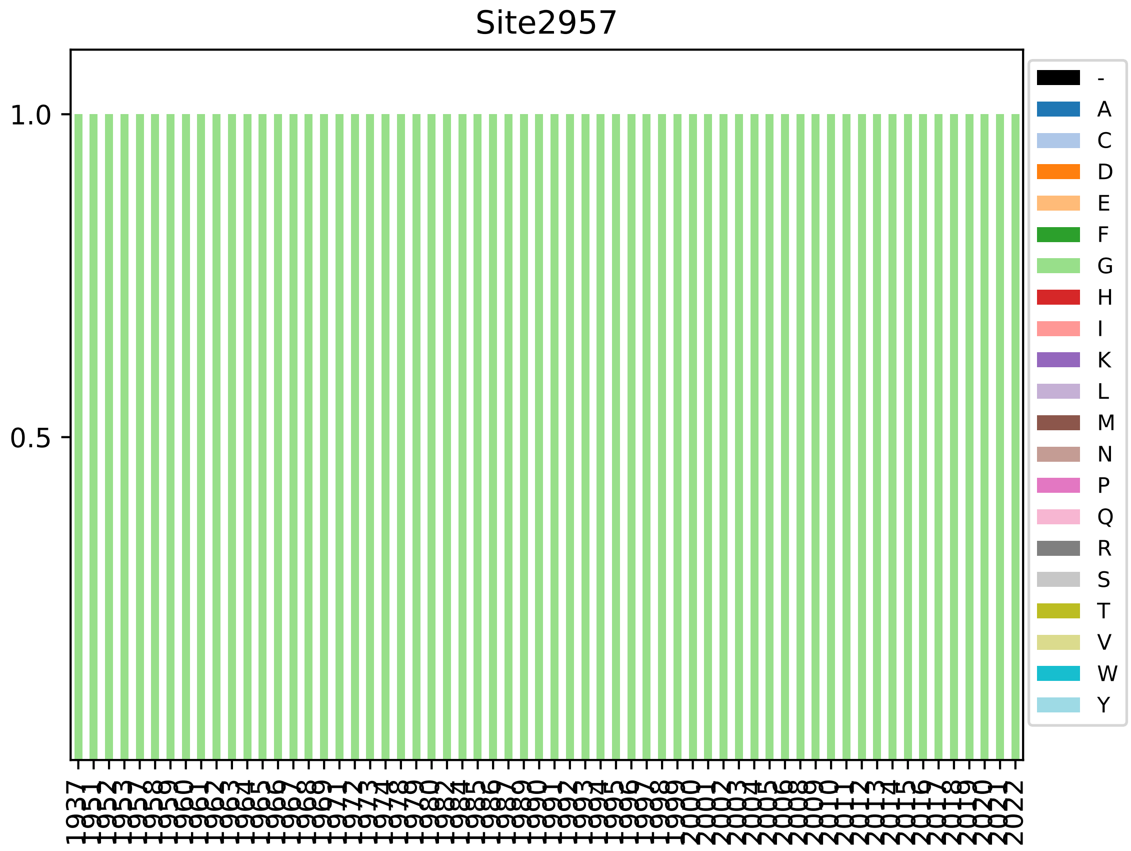
Site2957
1.0
0.5
-
A
C
D
E
F
G
H
I
K
L
M
N
P
Q
R
S
T
V
W
Y

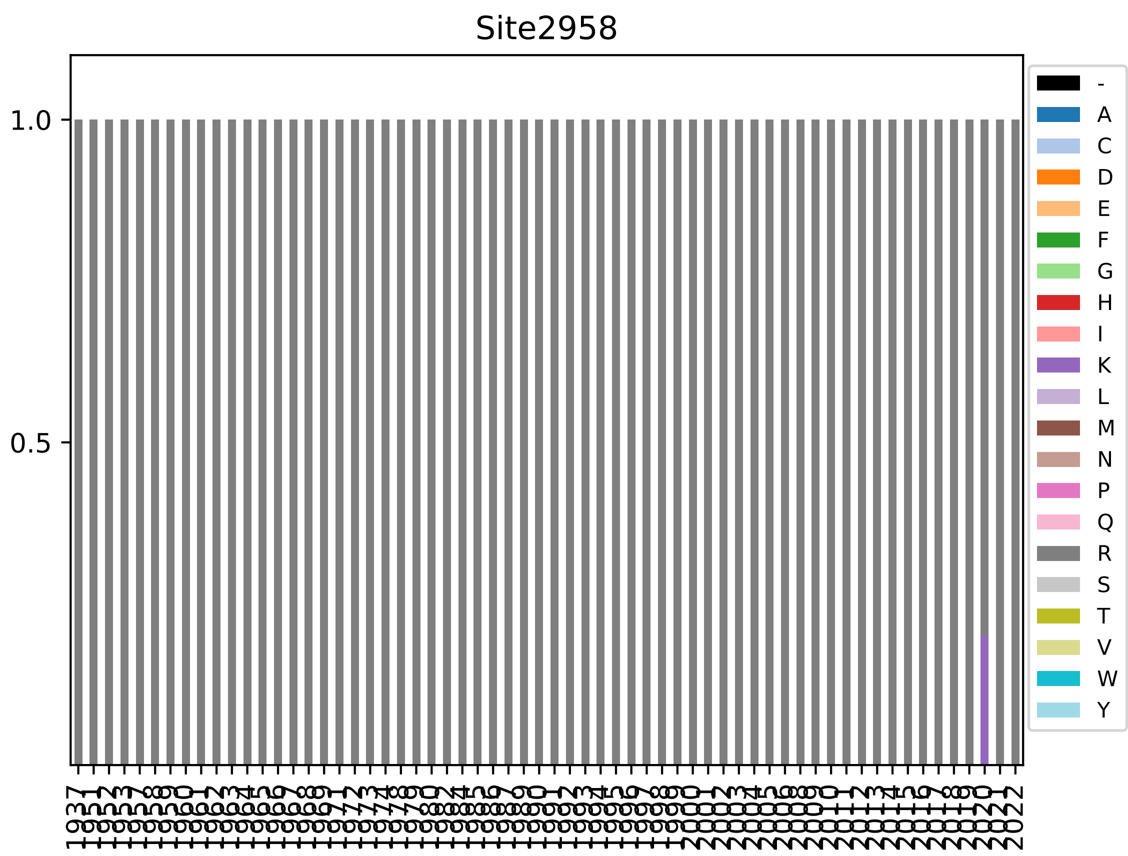
Site2958
1.0
0.5
-
A
C
D
E
F
G
H
I
K
L
M
N
P
Q
R
S
T
V
W
Y

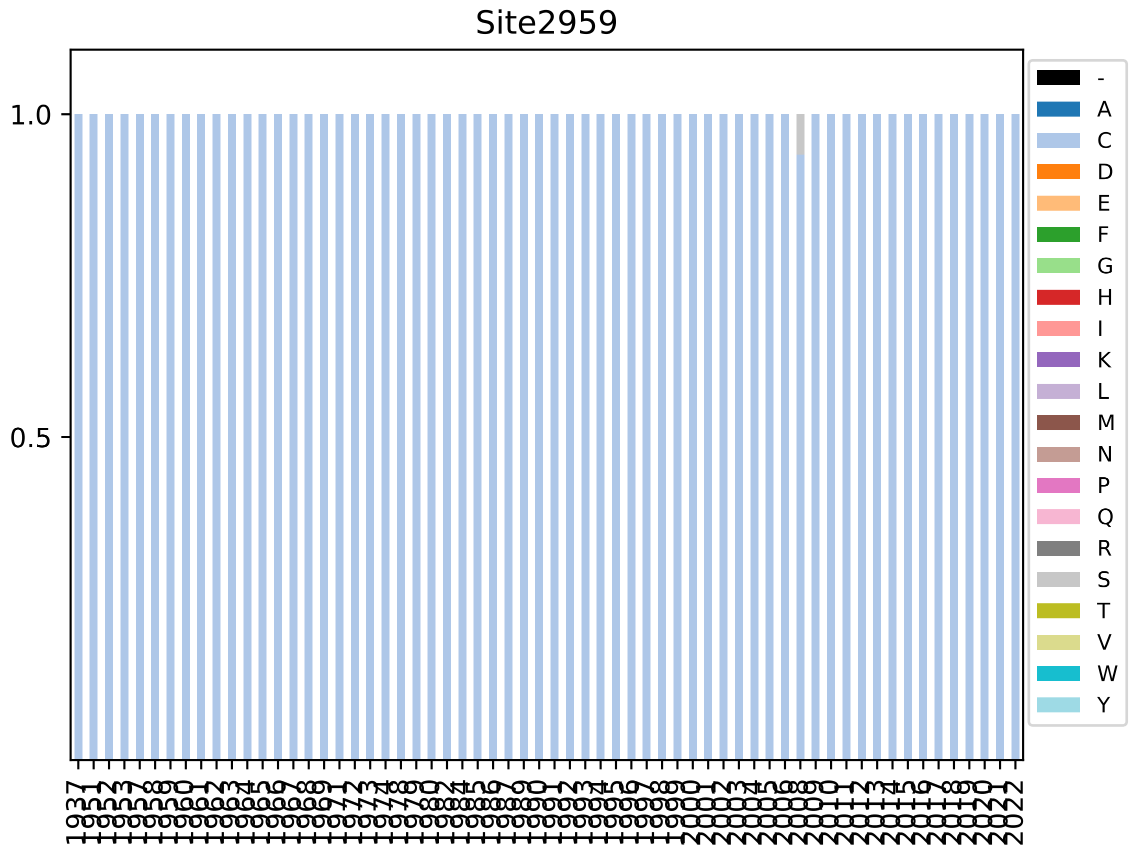
Site2959
1.0
0.5
-
A
C
D
E
F
G
H
I
K
L
M
N
P
Q
R
S
T
V
W
Y

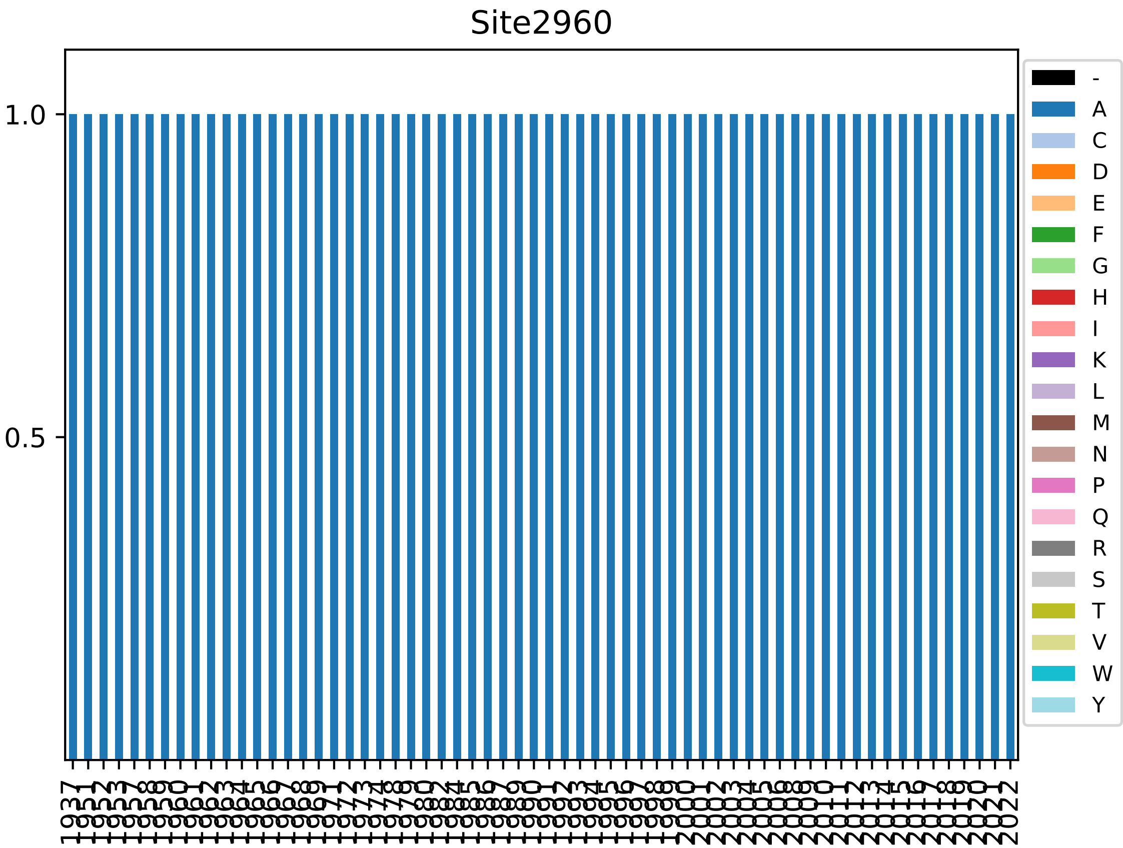
Site2960
1.0
0.5
-
A
C
D
E
F
G
H
I
K
L
M
N
P
Q
R
S
T
V
W
Y

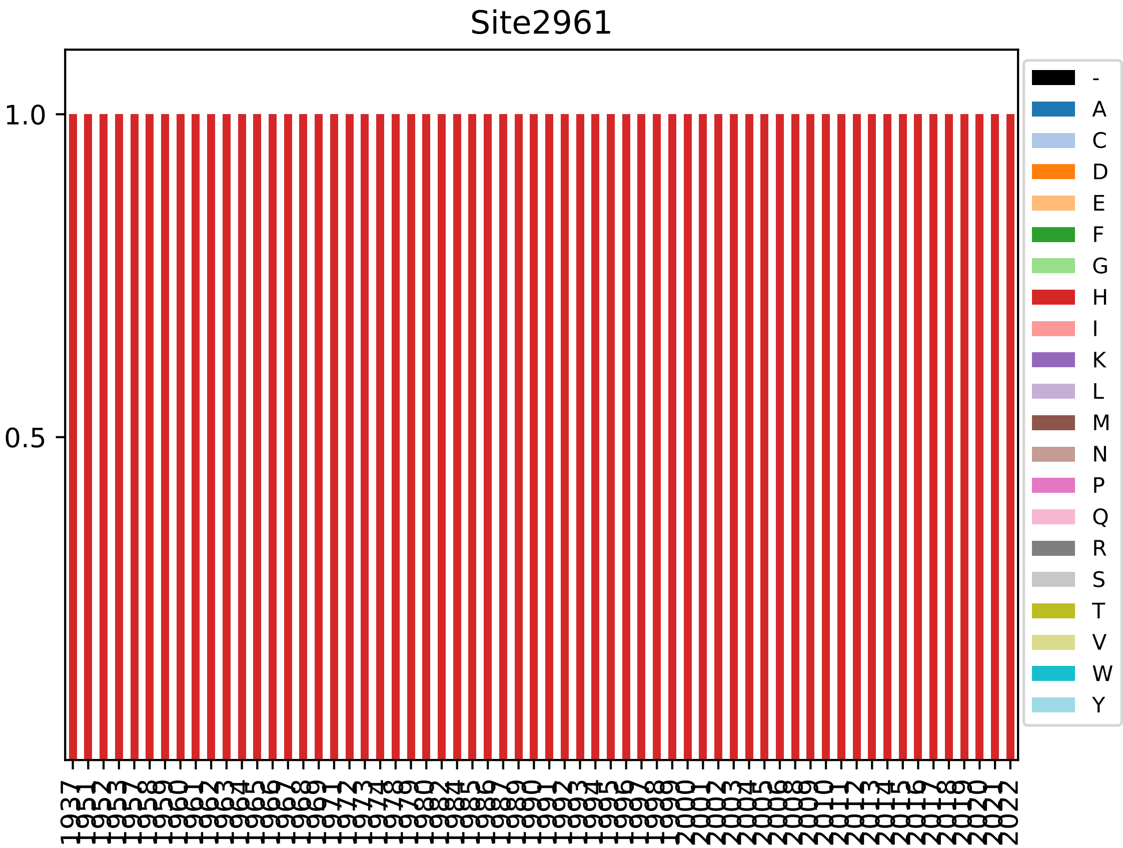
Site2961
1.0
0.5
-
A
C
D
E
F
G
H
I
K
L
M
N
P
Q
R
S
T
V
W
Y

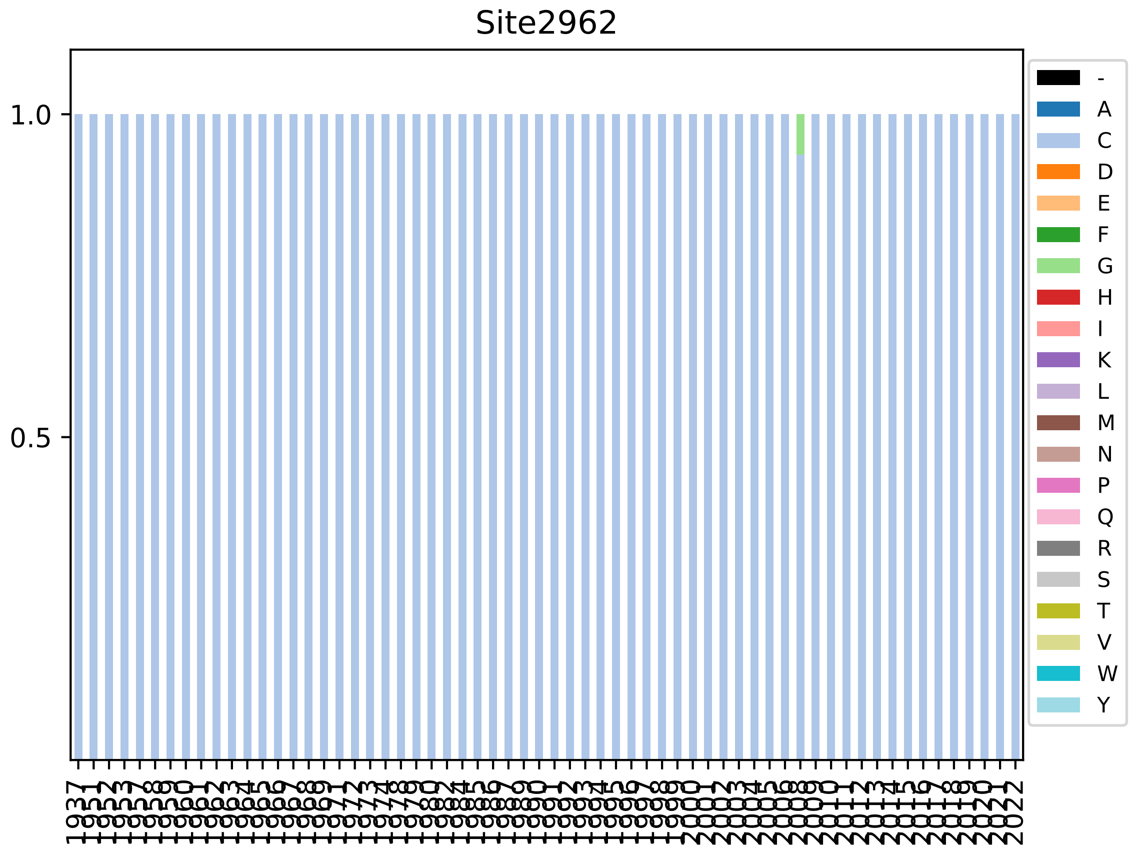
Site2962
1.0
0.5
-
A
C
D
E
F
G
H
I
K
L
M
N
P
Q
R
S
T
V
W
Y

Site2963

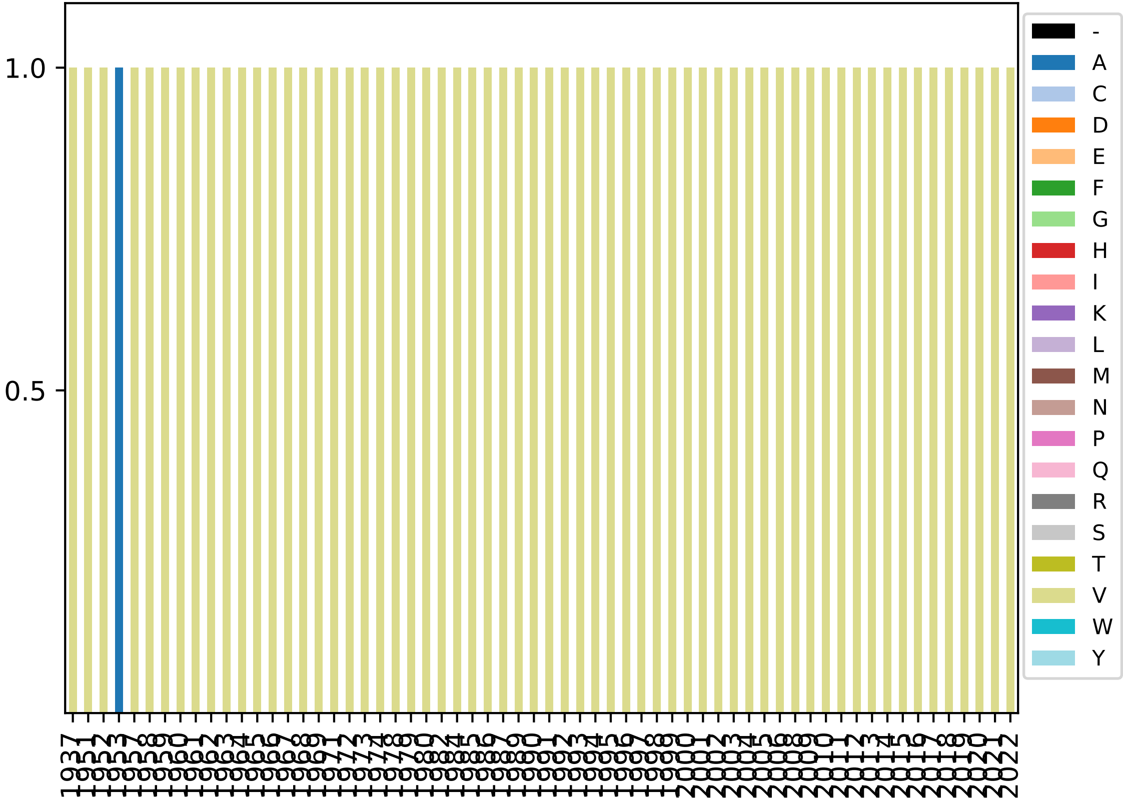

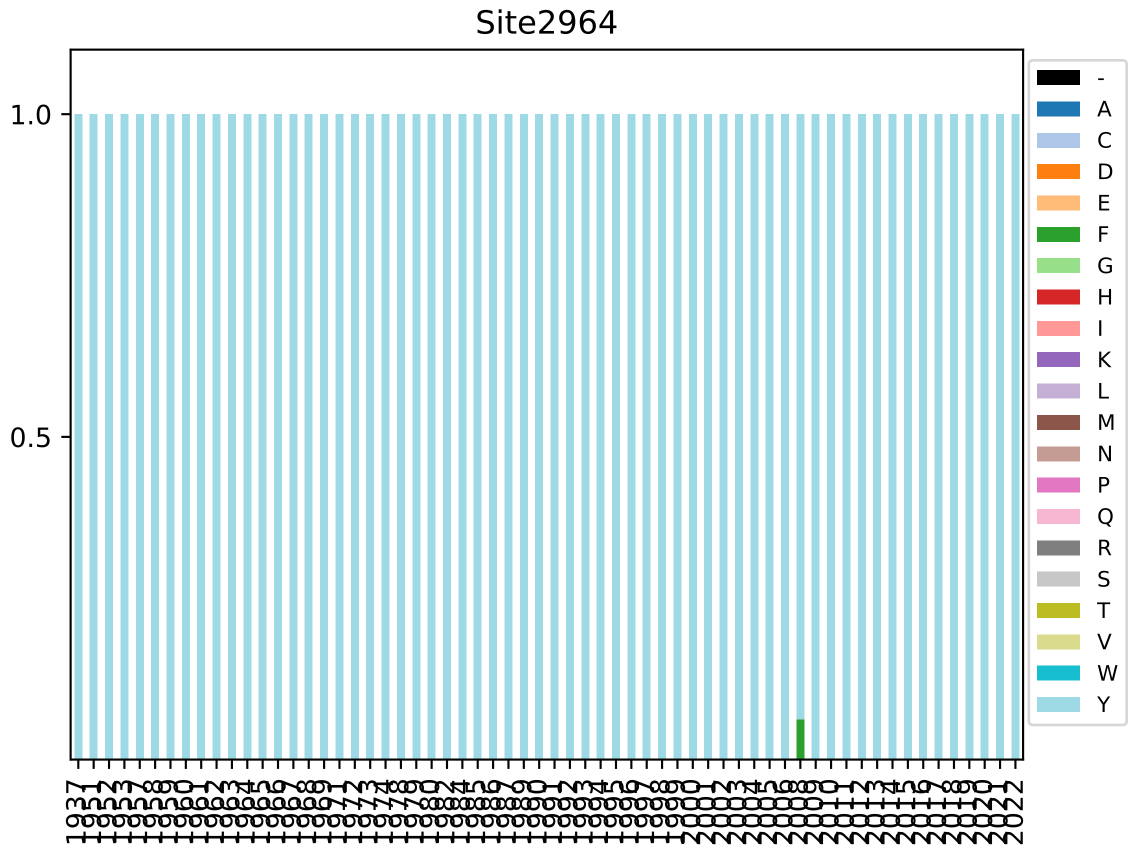
Site2964
1.0
0.5
-
A
C
D
E
F
G
H
I
K
L
M
N
P
Q
R
S
T
V
W
Y

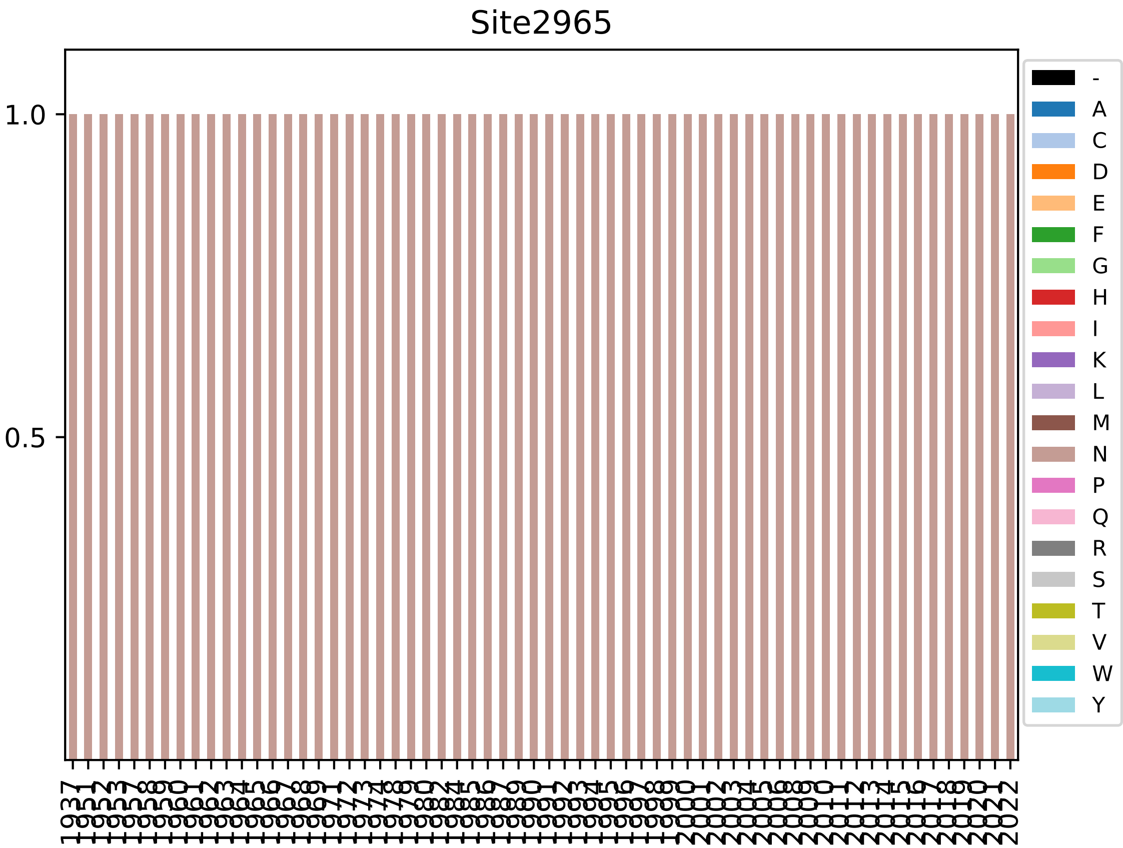
Site2965
1.0
0.5
-
A
C
D
E
F
G
H
I
K
L
M
N
P
Q
R
S
T
V
W
Y

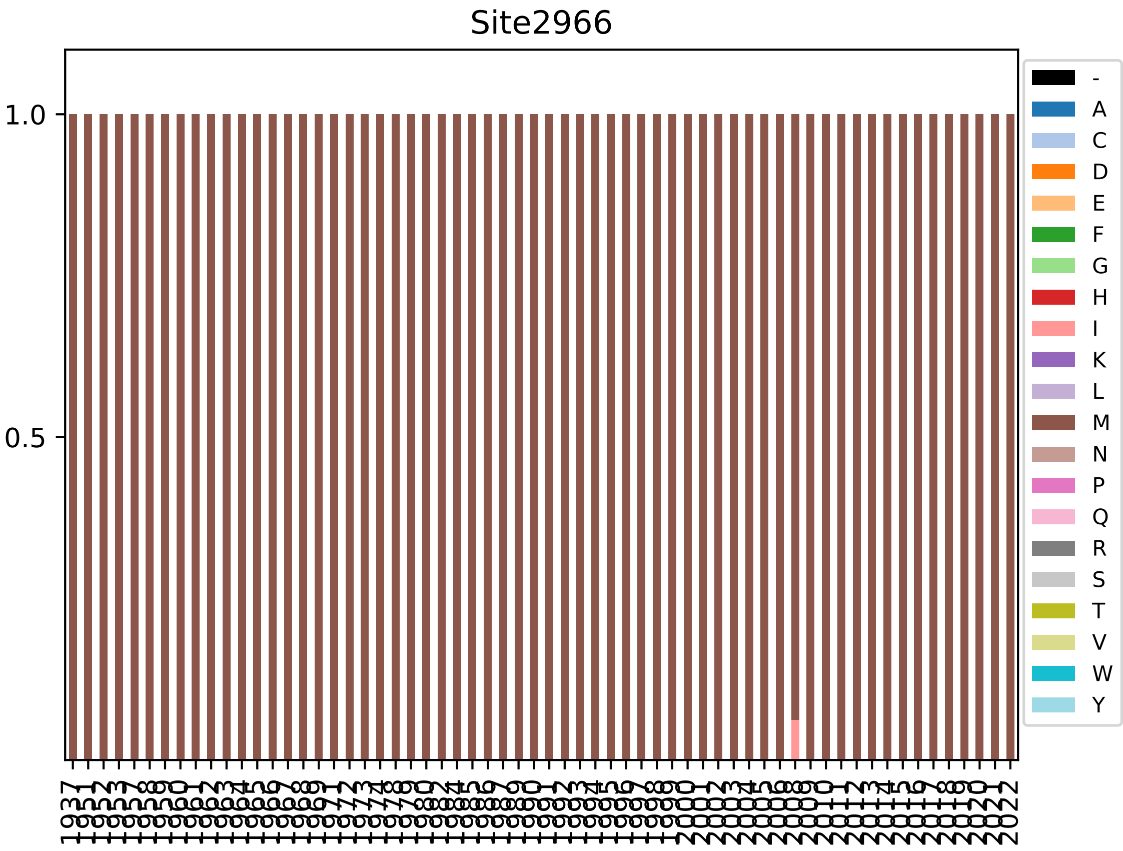
Site2966
1.0
0.5
-
A
C
D
E
F
G
H
I
K
L
M
N
P
Q
R
S
T
V
W
Y

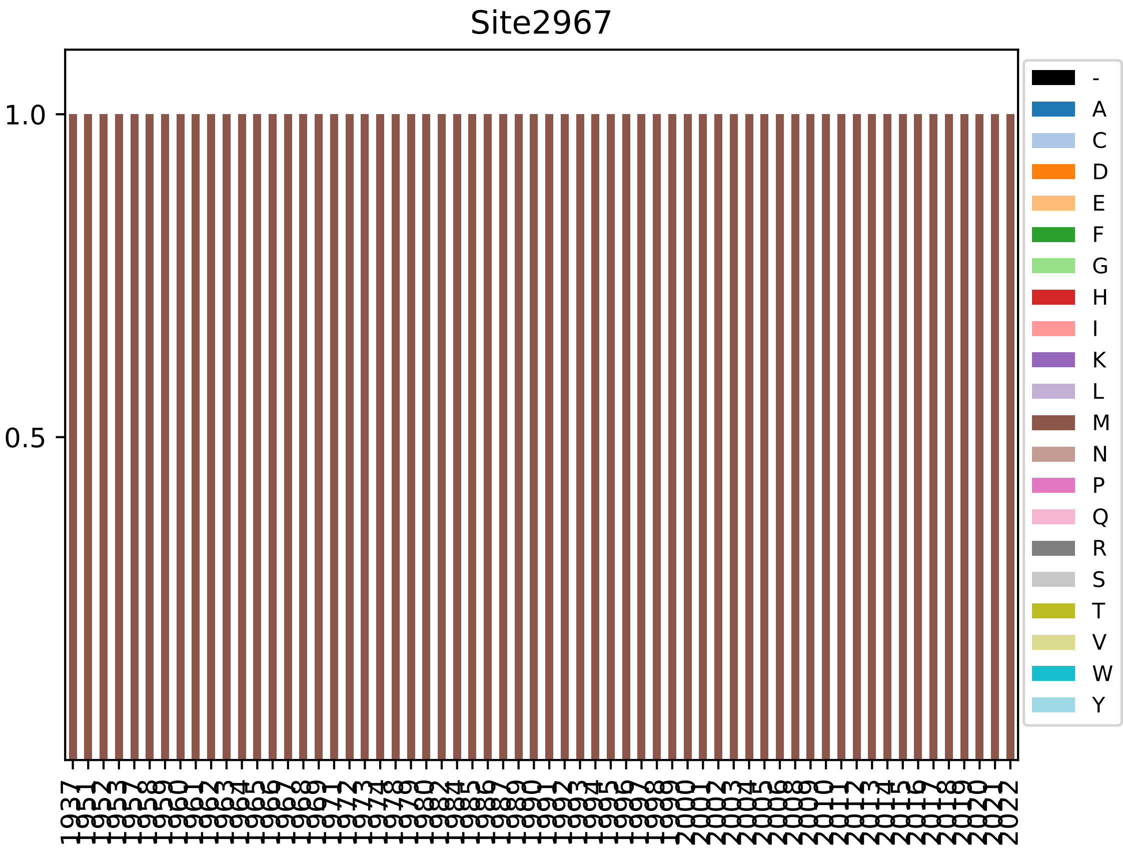
Site2967
1.0
0.5
-
A
C
D
E
F
G
H
I
K
L
M
N
P
Q
R
S
T
V
W
Y

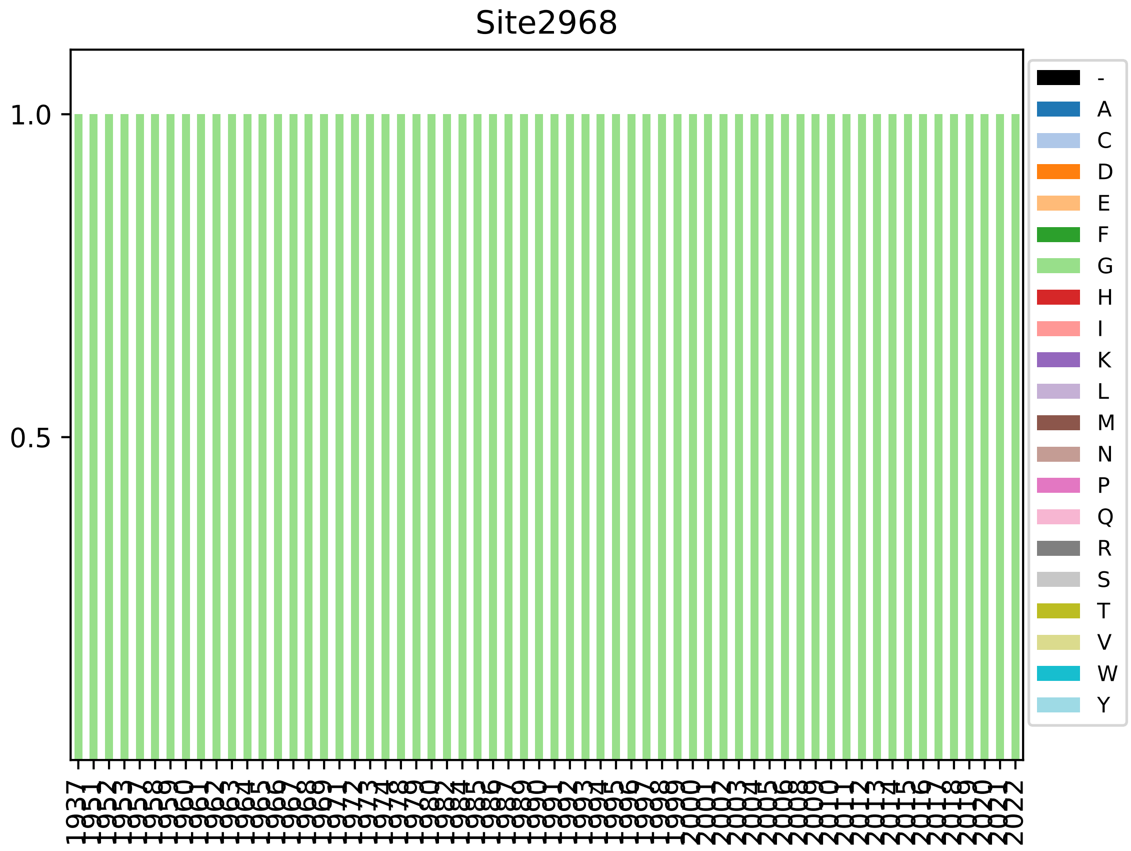
Site2968
1.0
0.5
-
A
C
D
E
F
G
H
I
K
L
M
N
P
Q
R
S
T
V
W
Y

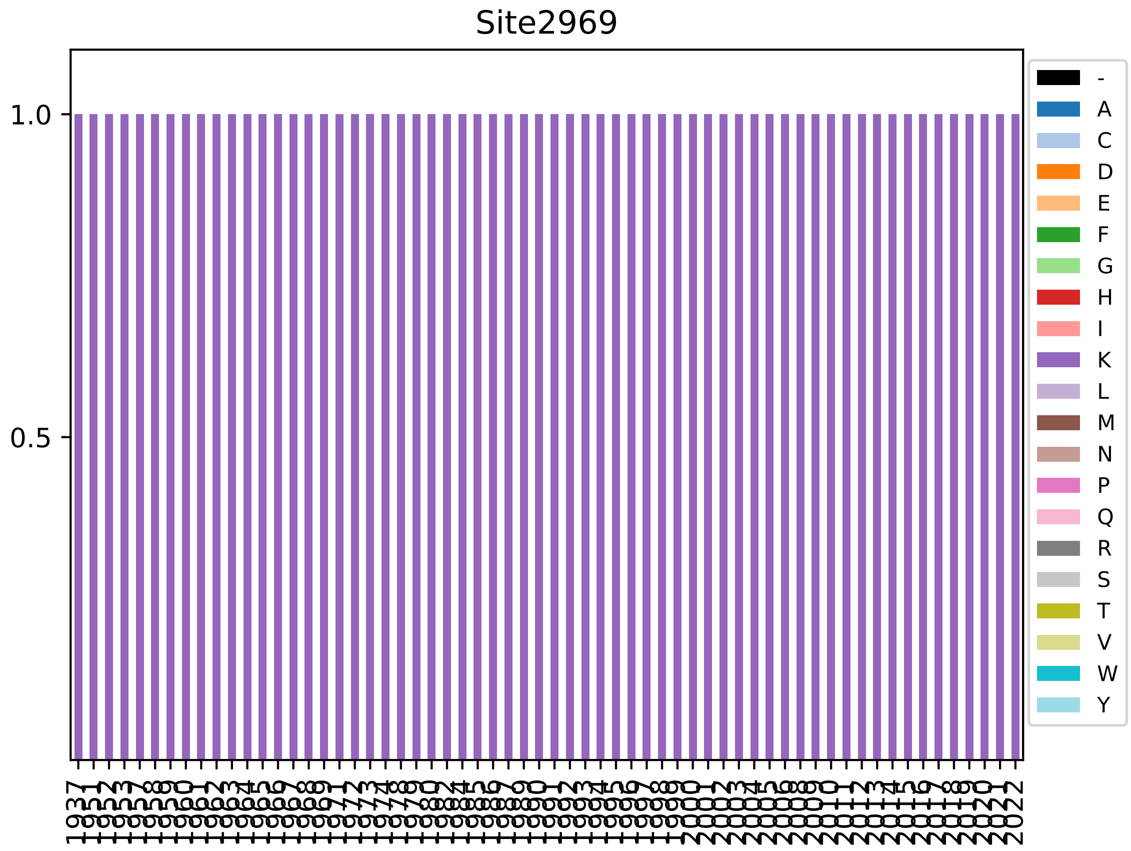
Site2969
1.0
0.5
-
A
C
D
E
F
G
H
I
K
L
M
N
P
Q
R
S
T
V
W
Y

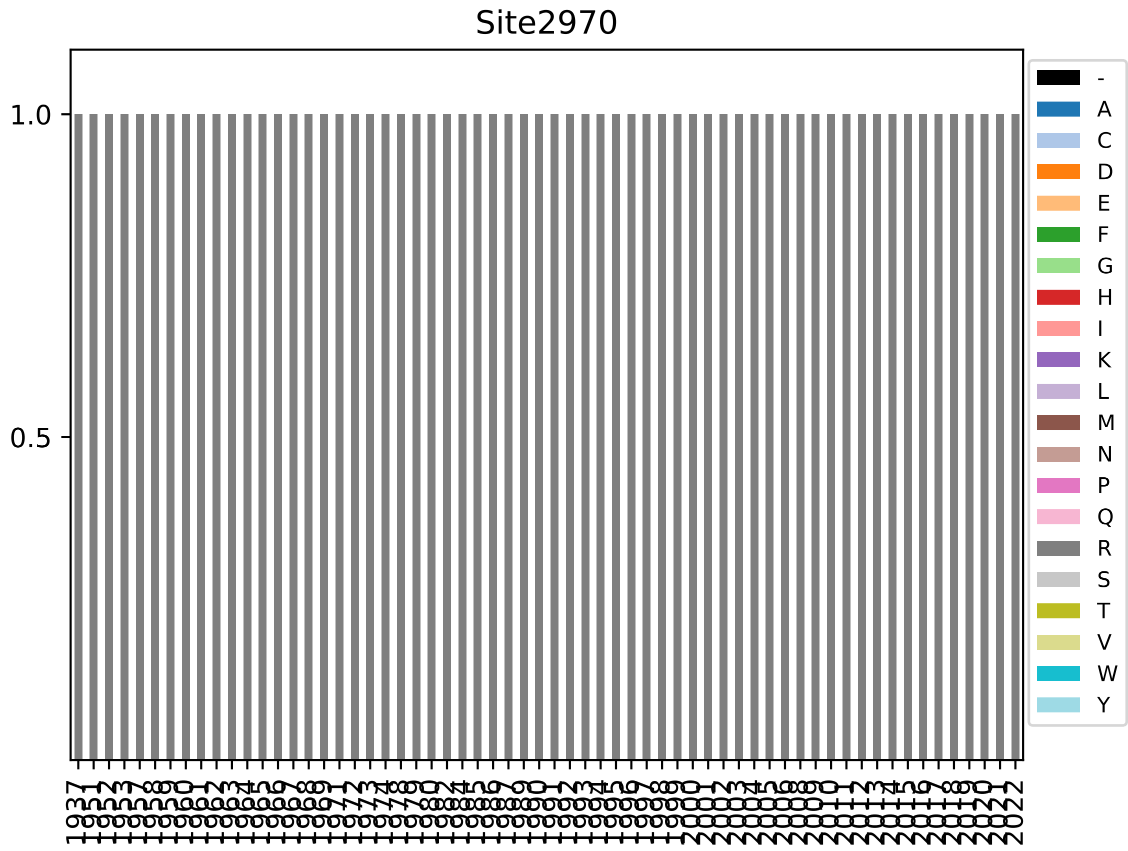
Site2970
1.0
0.5
-
A
C
D
E
F
G
H
I
K
L
M
N
P
Q
R
S
T
V
W
Y

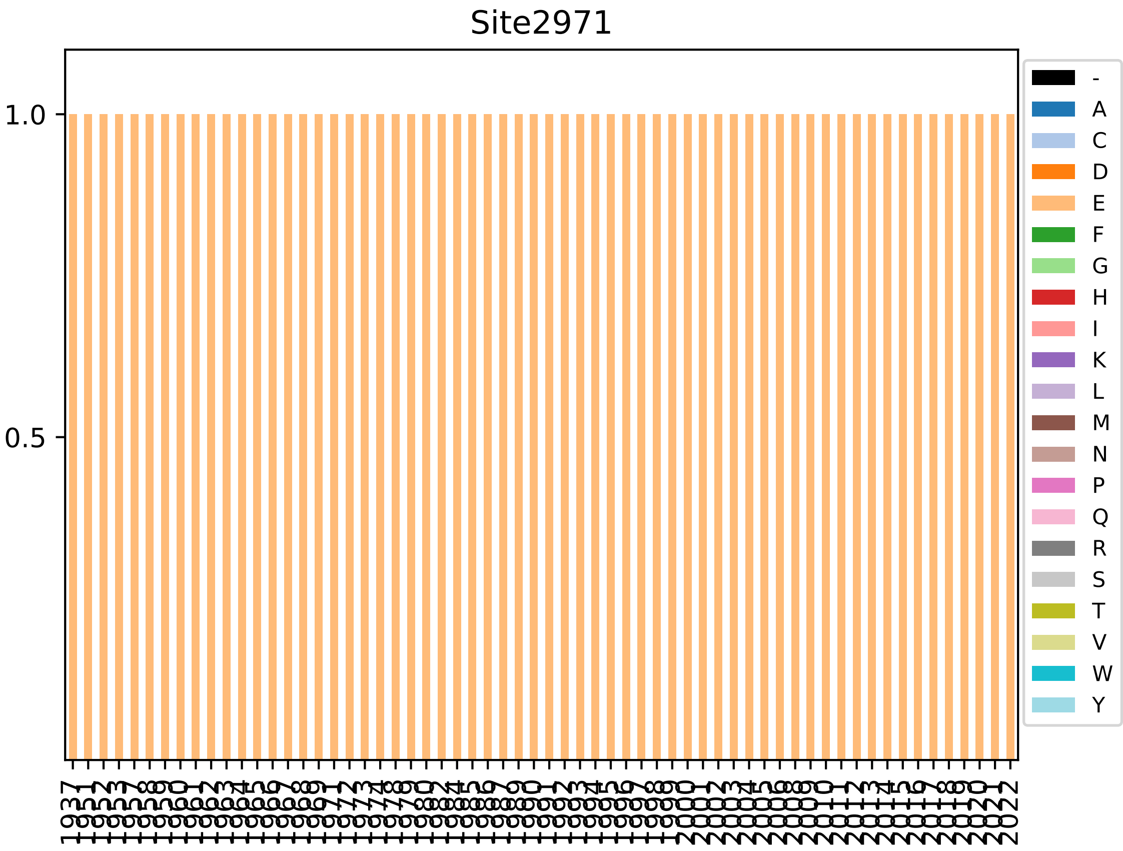
Site2971
1.0
0.5
-
A
C
D
E
F
G
H
I
K
L
M
N
P
Q
R
S
T
V
W
Y

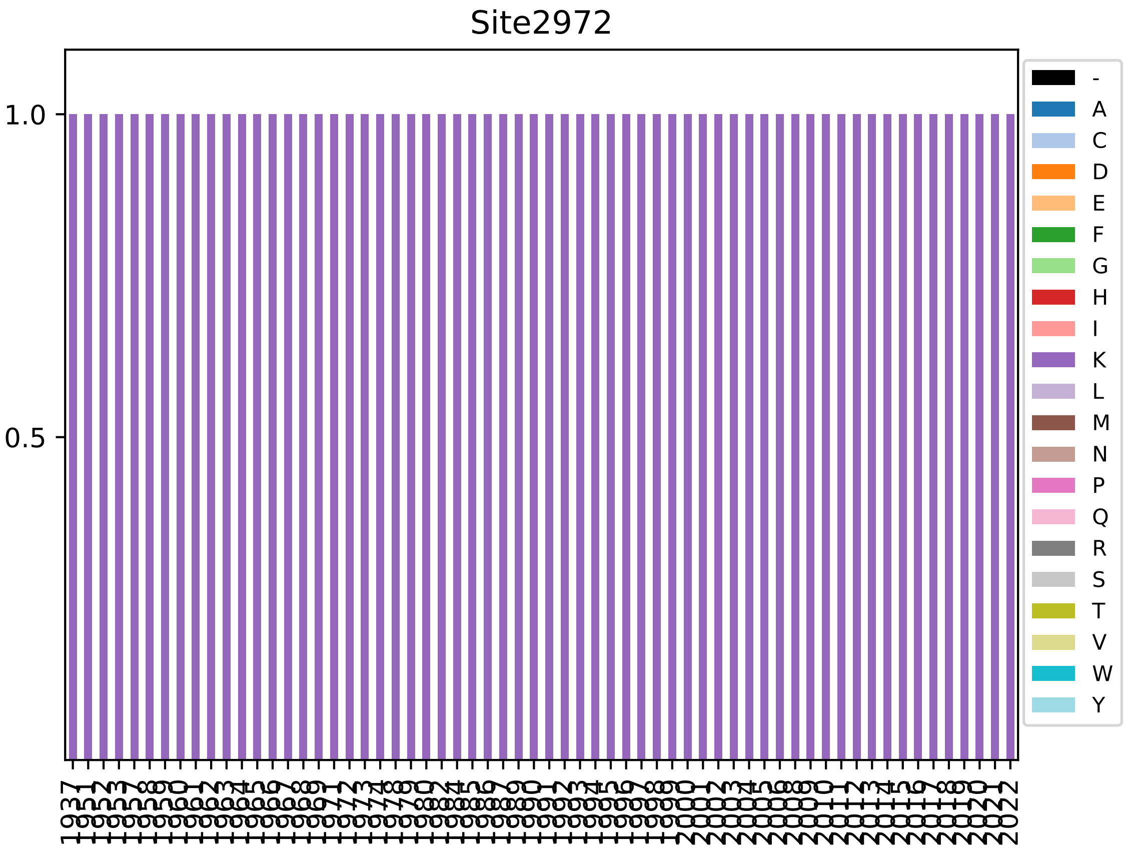
Site2972
1.0
0.5
-
A
C
D
E
F
G
H
I
K
L
M
N
P
Q
R
S
T
V
W
Y

Site2973

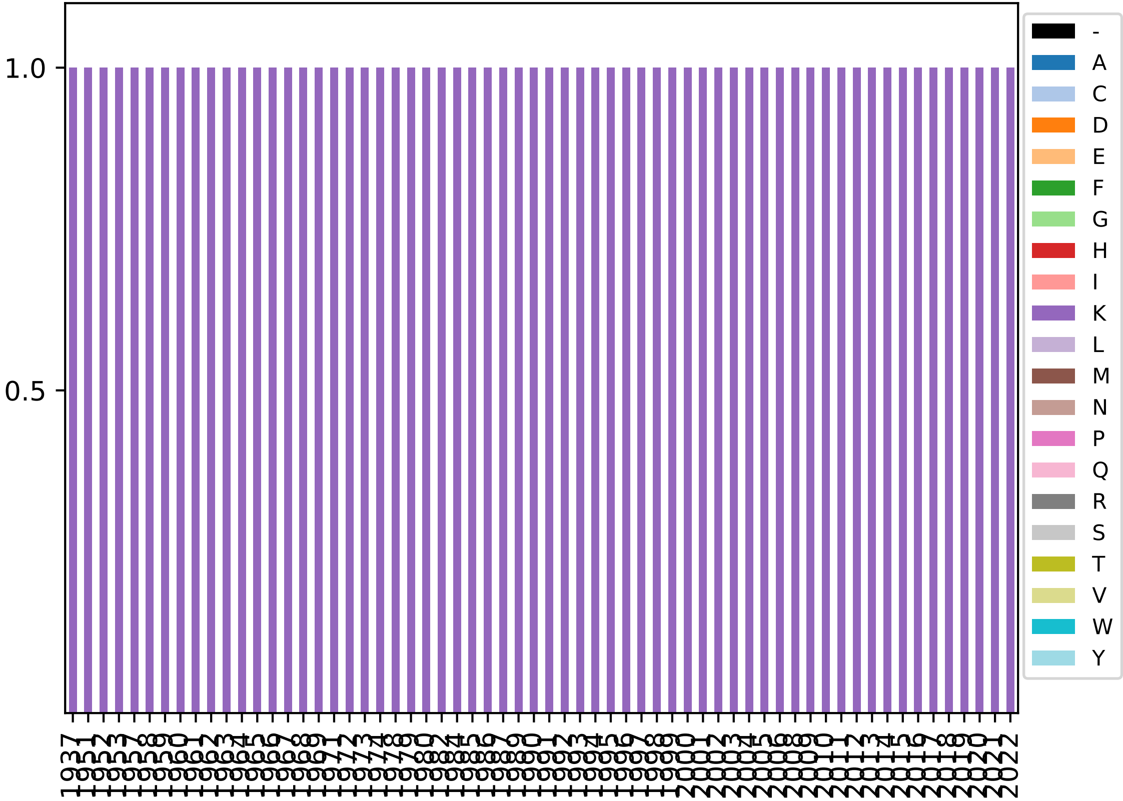

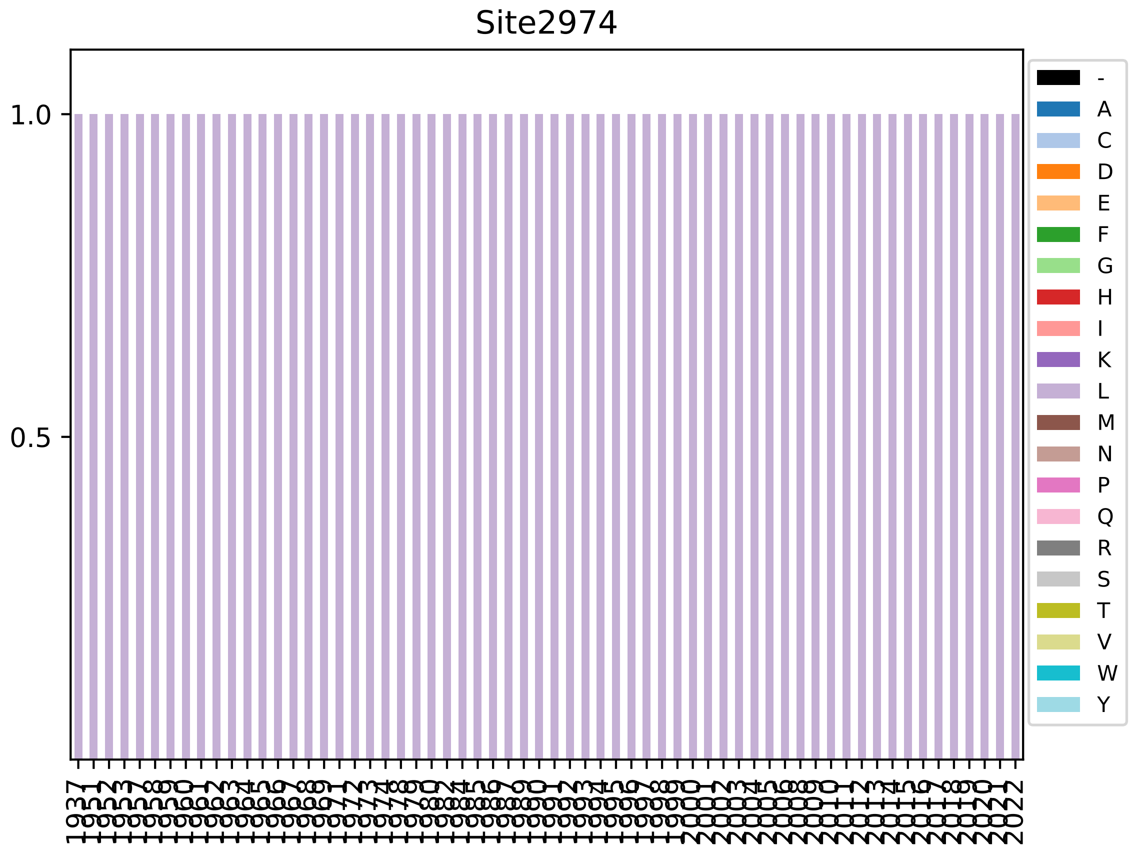
Site2974
1.0
0.5
-
A
C
D
E
F
G
H
I
K
L
M
N
P
Q
R
S
T
V
W
Y

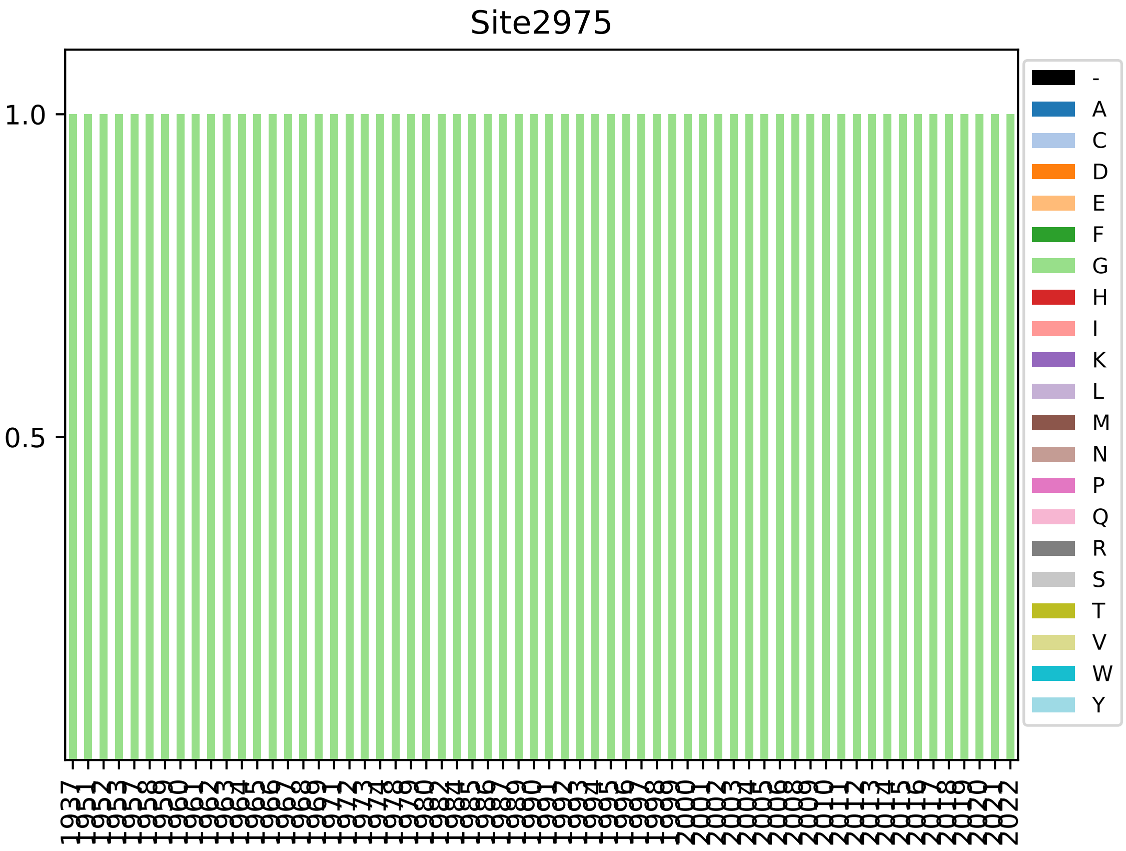
Site2975
1.0
0.5
-
A
C
D
E
F
G
H
I
K
L
M
N
P
Q
R
S
T
V
W
Y

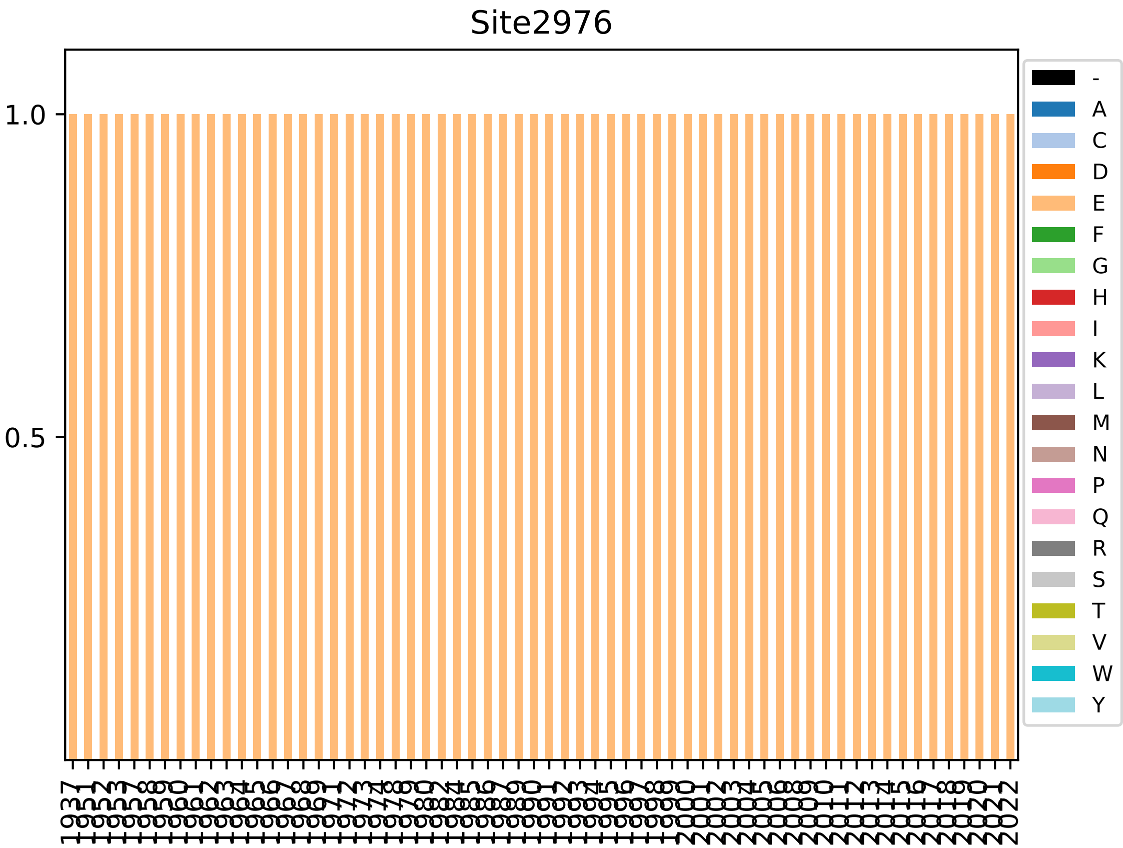
Site2976
1.0
0.5
-
A
C
D
E
F
G
H
I
K
L
M
N
P
Q
R
S
T
V
W
Y

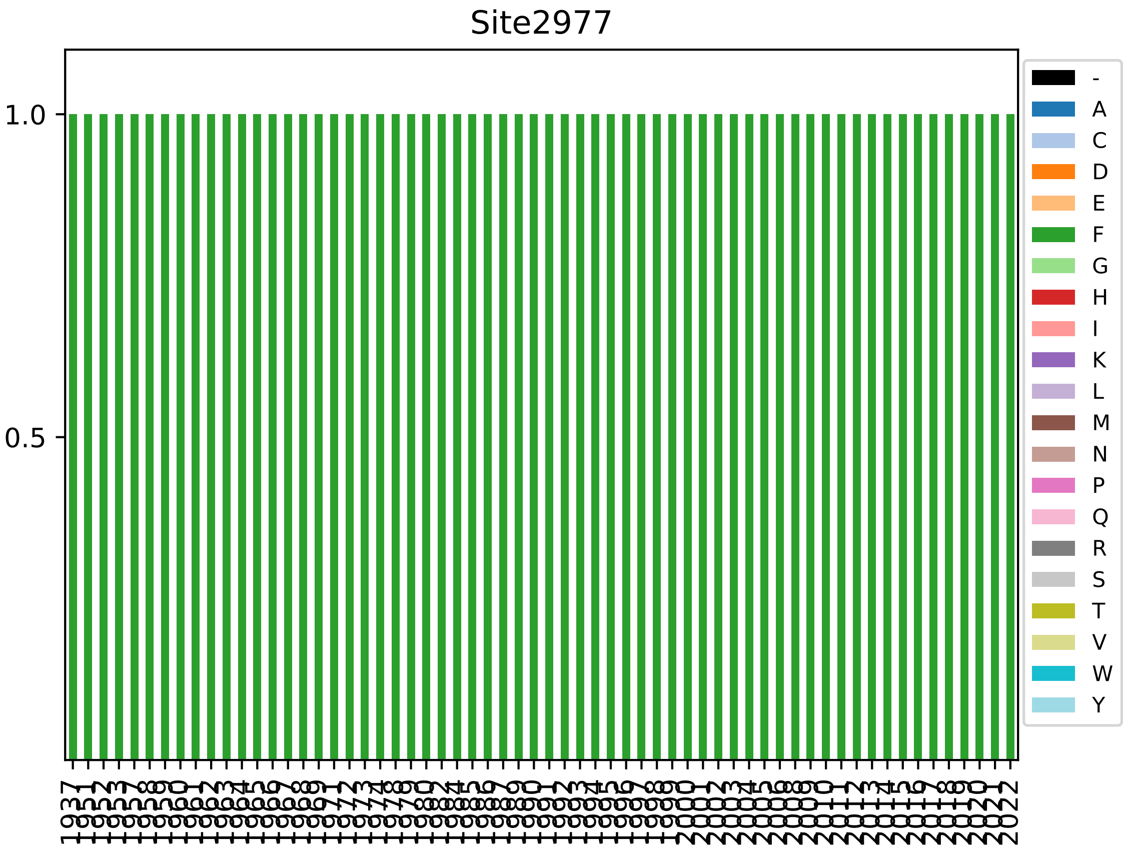
Site2977
1.0
0.5
-
A
C
D
E
F
G
H
I
K
L
M
N
P
Q
R
S
T
V
W
Y

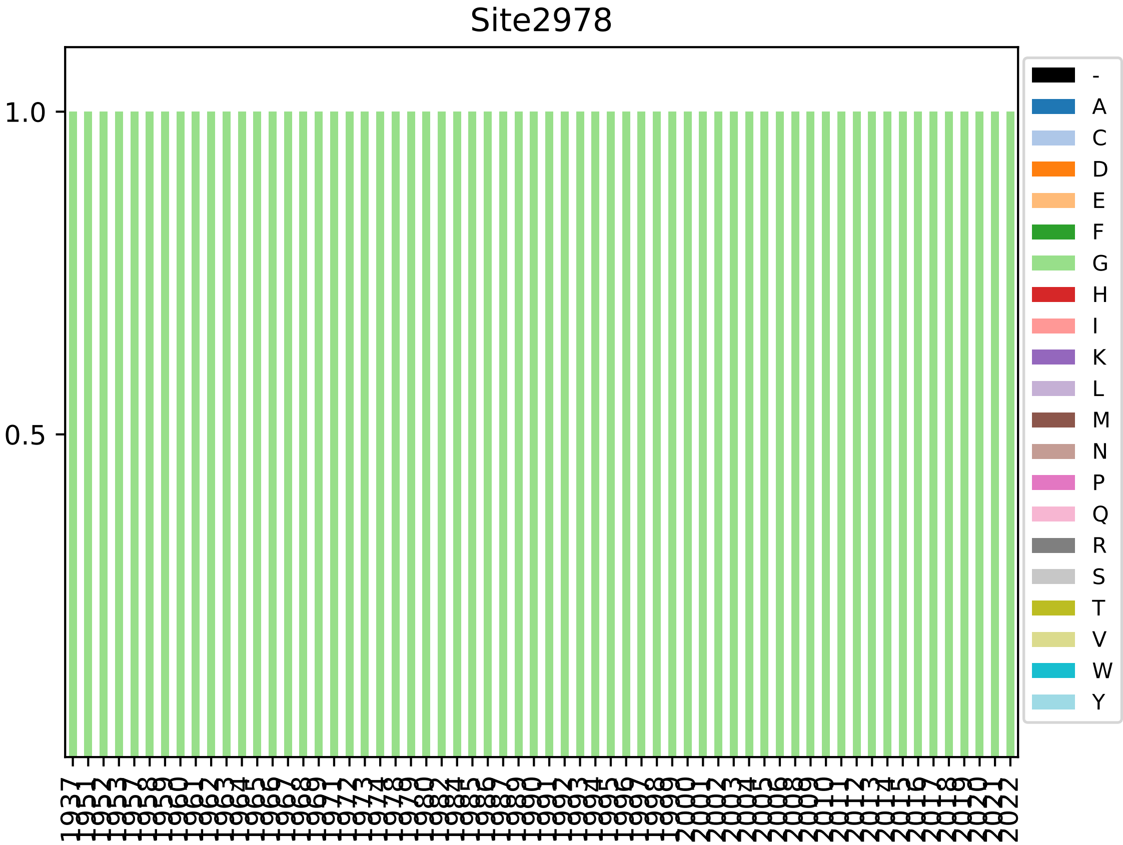
Site2978
1.0
0.5
-
A
C
D
E
F
G
H
I
K
L
M
N
P
Q
R
S
T
V
W
Y

Site2979

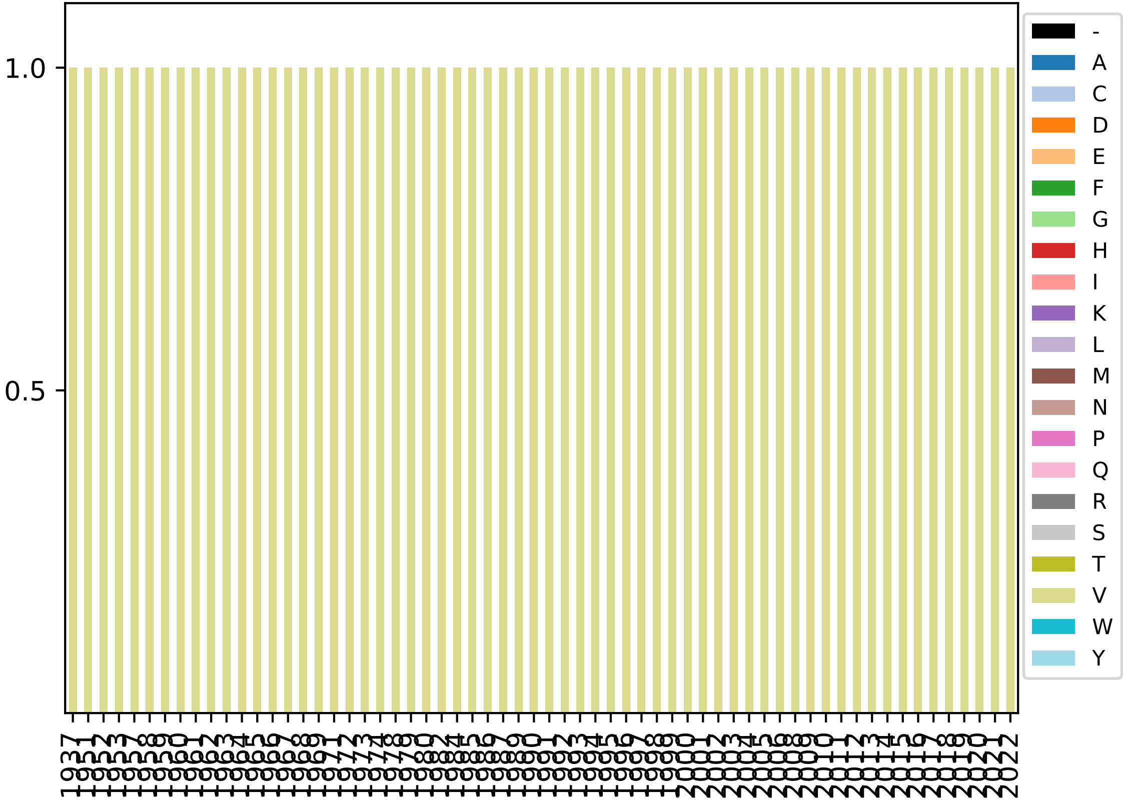

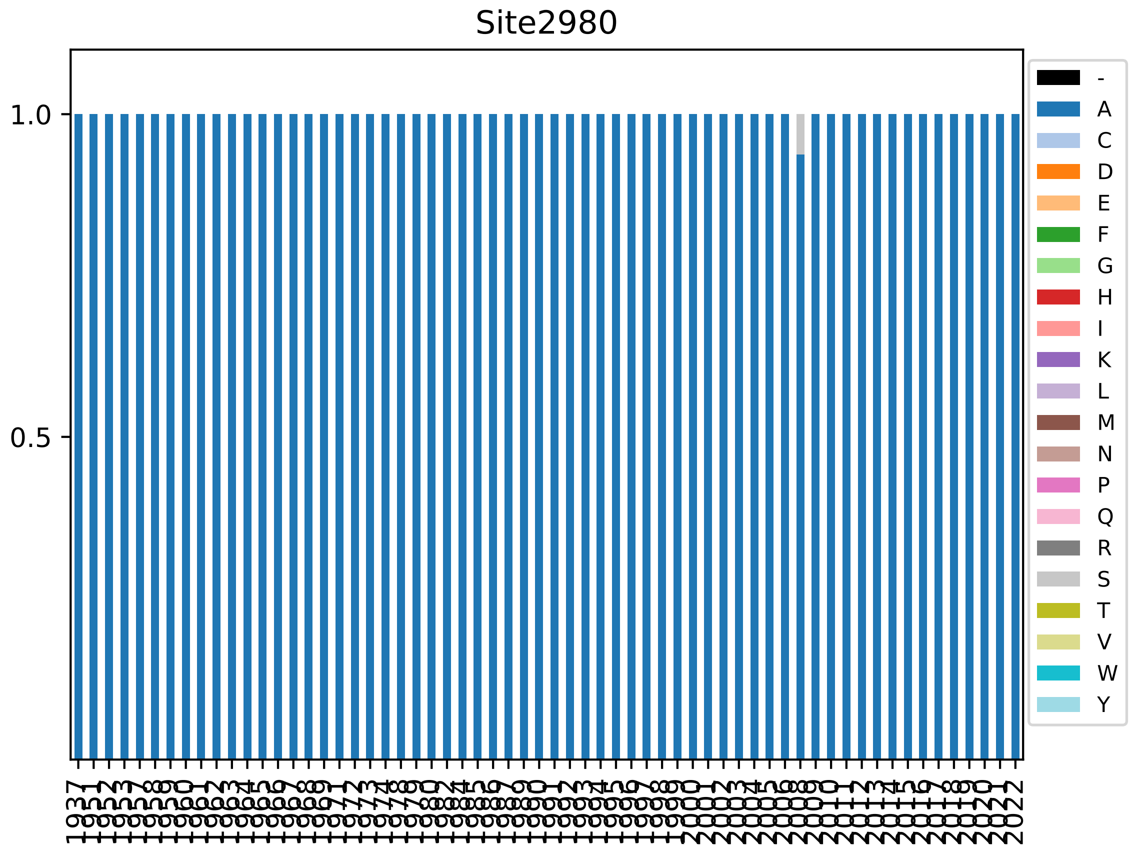
Site2980
1.0
0.5
-
A
C
D
E
F
G
H
I
K
L
M
N
P
Q
R
S
T
V
W
Y

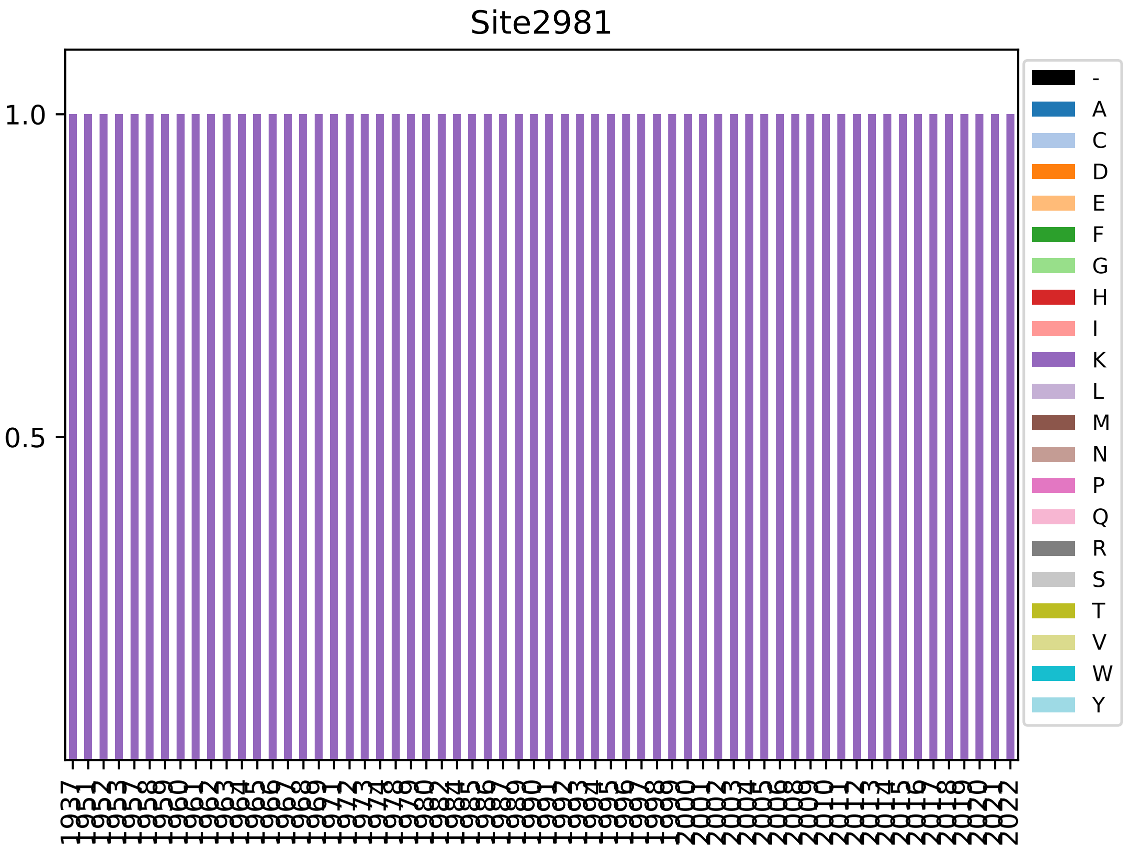

Site2981
1.0
0.5
-
A
C
D
E
F
G
H
I
K
L
M
N
P
Q
R
S
T
V
W
Y

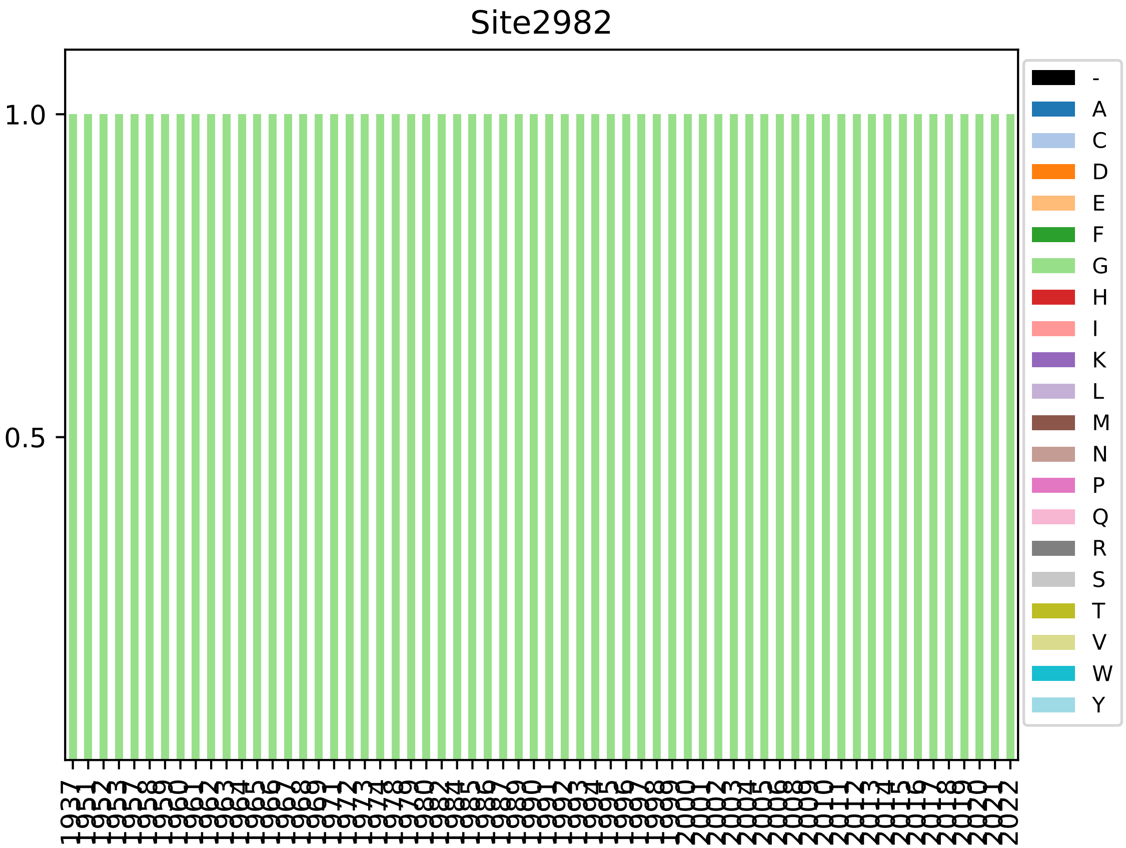
Site2982
1.0
0.5
-
A
C
D
E
F
G
H
I
K
L
M
N
P
Q
R
S
T
V
W
Y

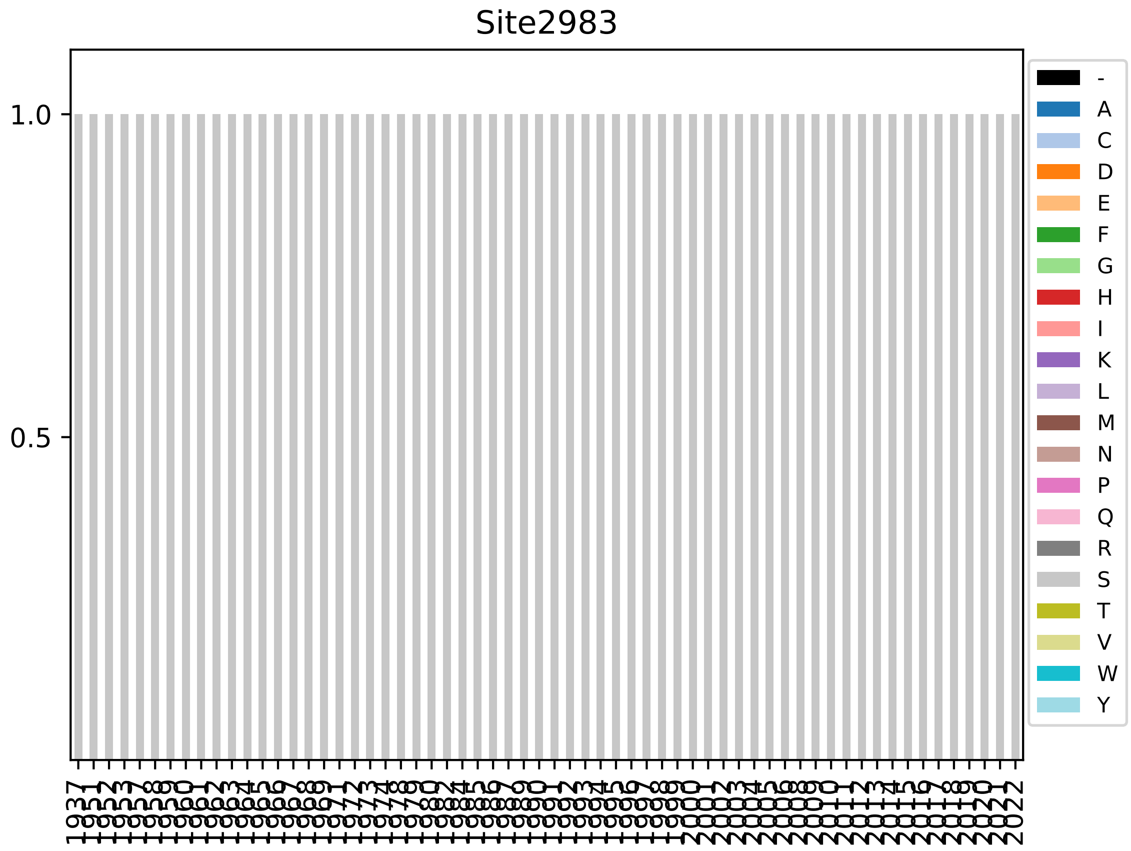
Site2983
1.0
0.5
-
A
C
D
E
F
G
H
I
K
L
M
N
P
Q
R
S
T
V
W
Y

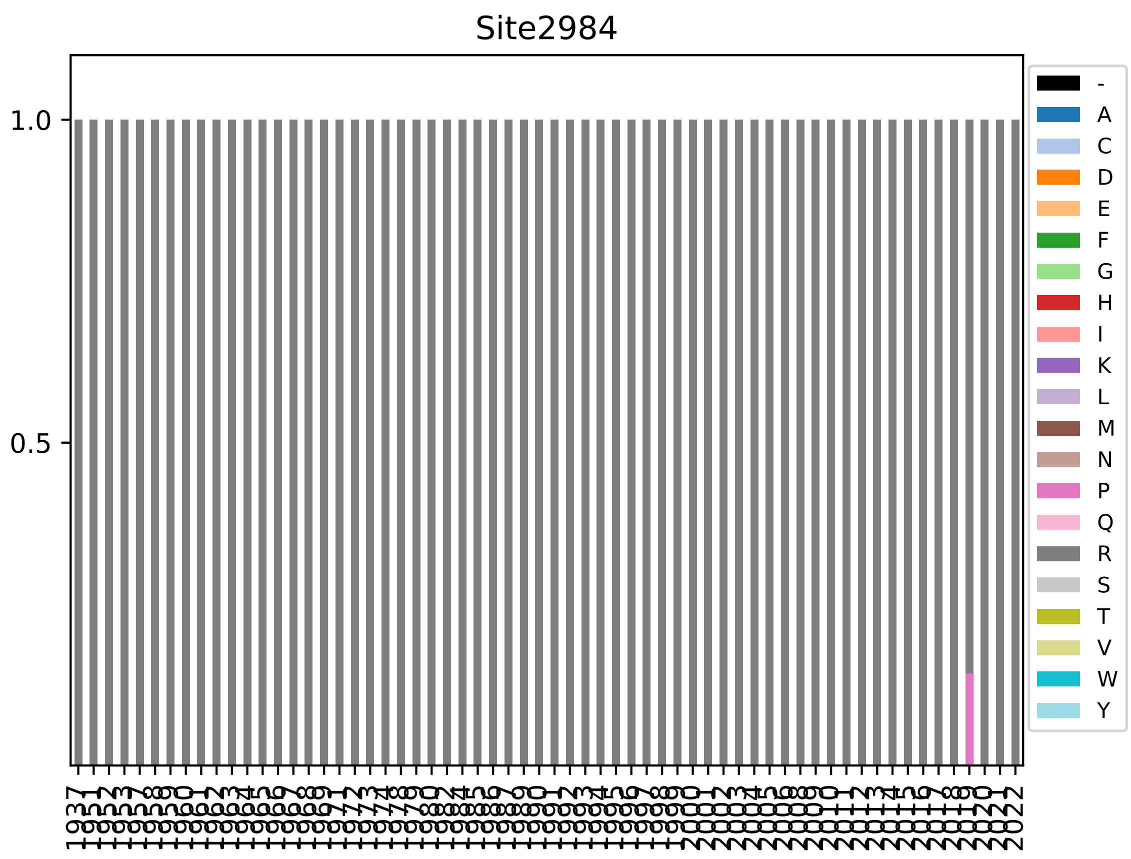

Site2984
1.0
0.5
-
A
C
D
E
F
G
H
I
K
L
M
N
P
Q
R
S
T
V
W
Y

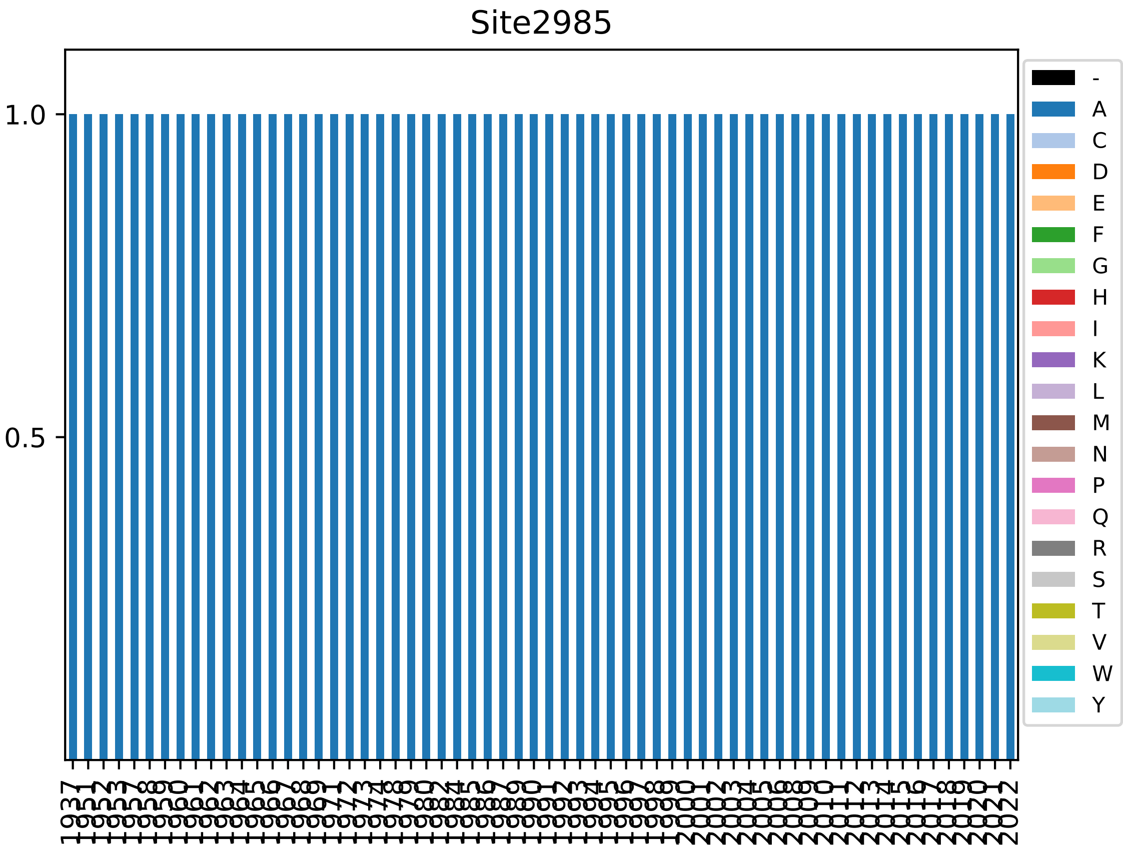
Site2985
1.0
0.5
-
A
C
D
E
F
G
H
I
K
L
M
N
P
Q
R
S
T
V
W
Y

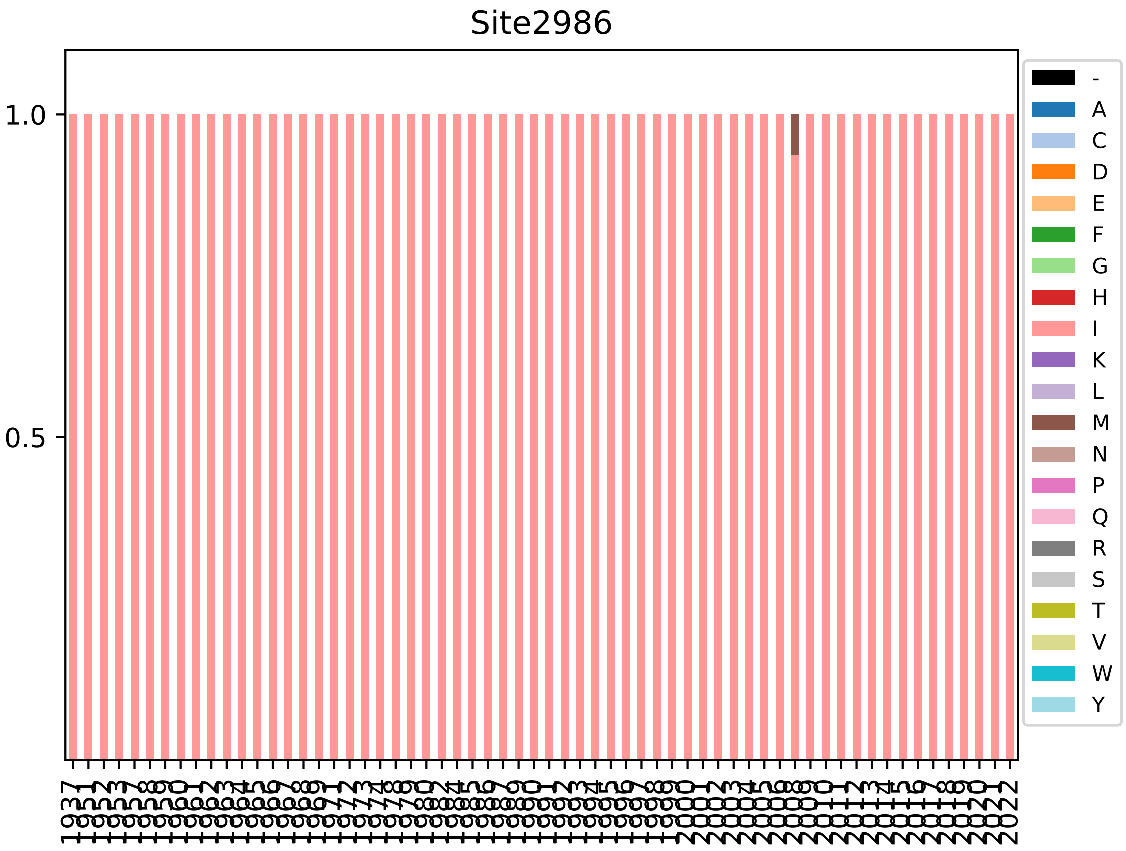
Site2986
1.0
0.5
-
A
C
D
E
F
G
H
I
K
L
M
N
P
Q
R
S
T
V
W
Y

Site2987

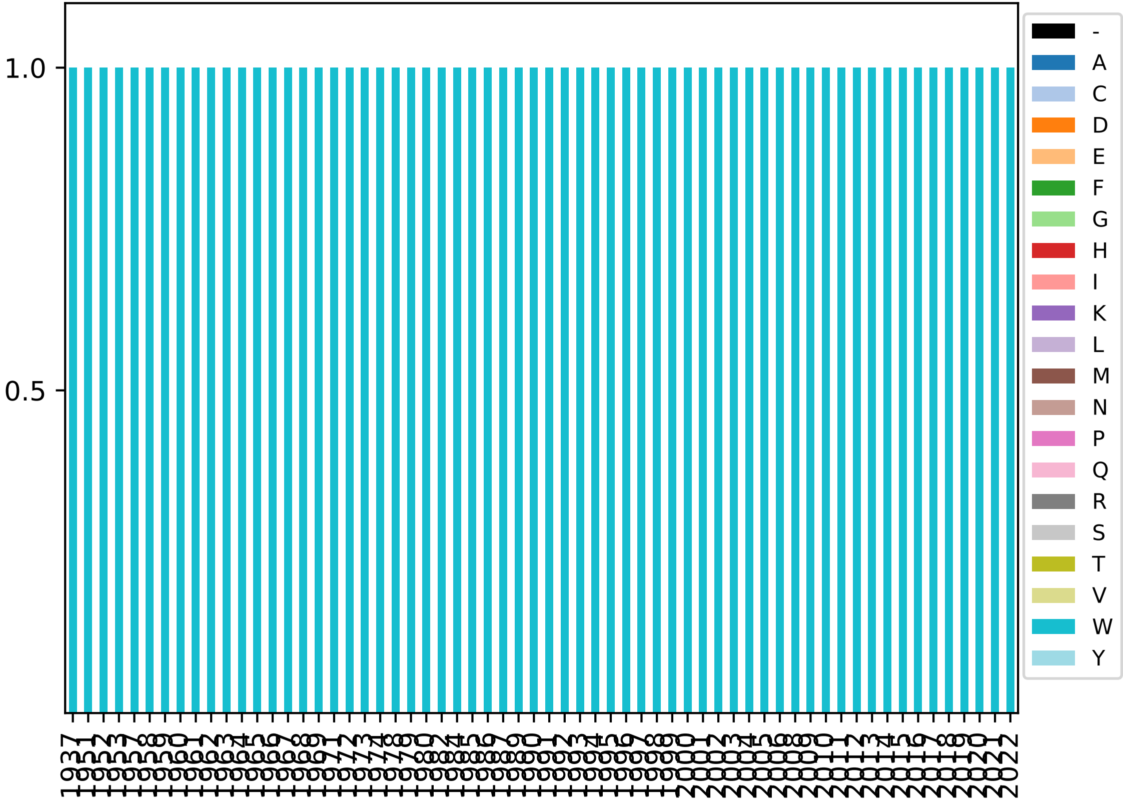

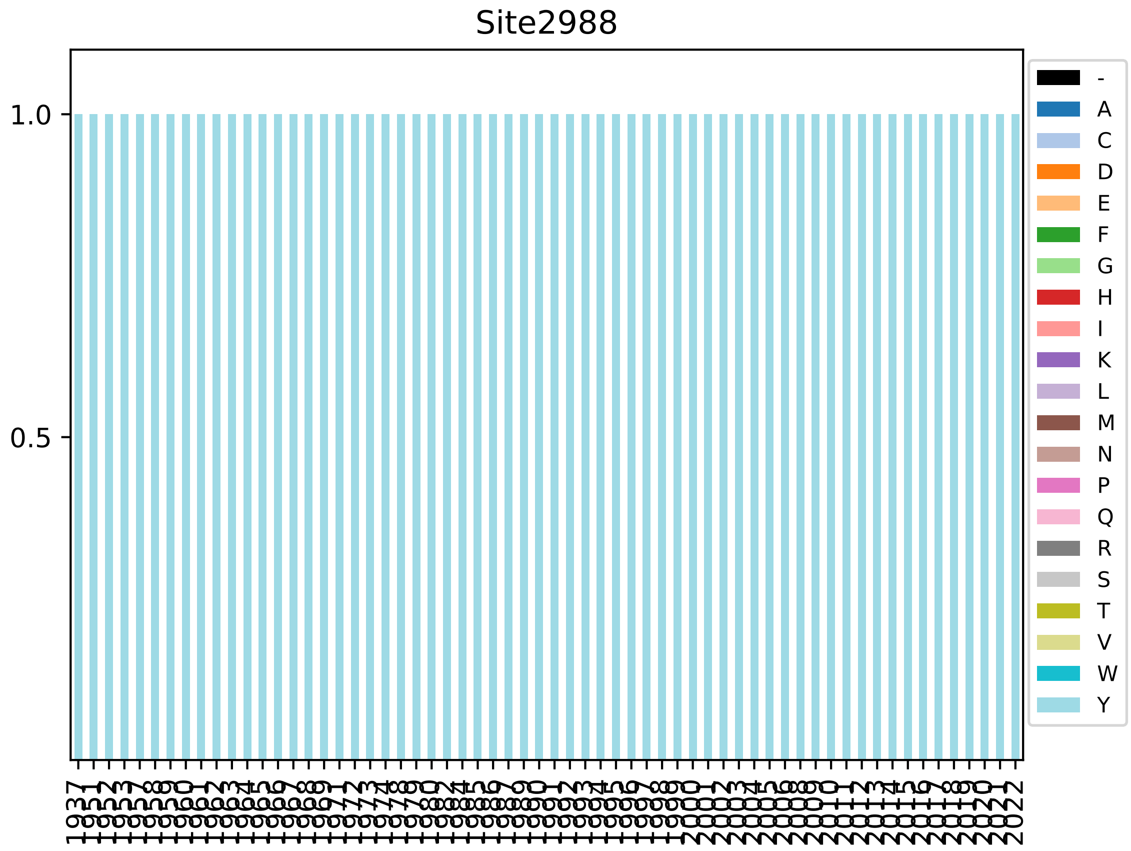
Site2988
1.0
0.5
-
A
C
D
E
F
G
H
I
K
L
M
N
P
Q
R
S
T
V
W
Y

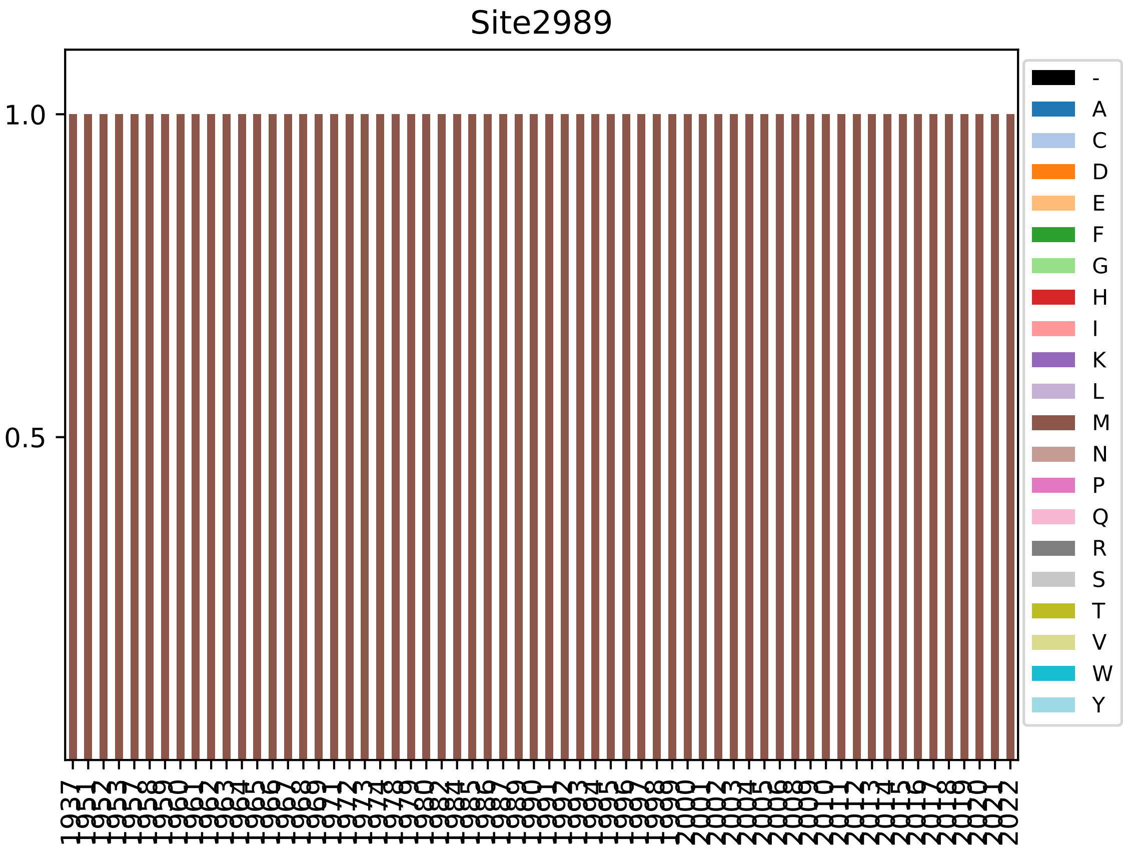
Site2989
1.0
0.5
-
A
C
D
E
F
G
H
I
K
L
M
N
P
Q
R
S
T
V
W
Y

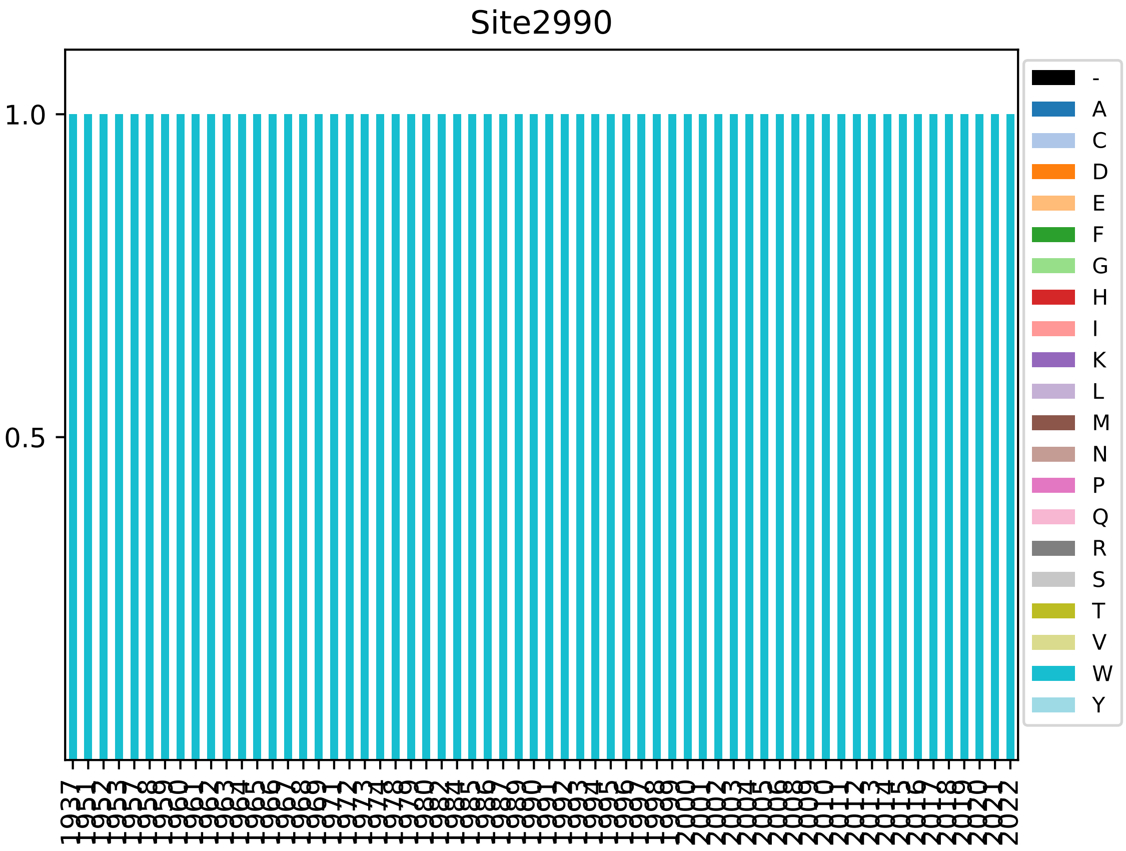
Site2990
1.0
0.5
-
A
C
D
E
F
G
H
I
K
L
M
N
P
Q
R
S
T
V
W
Y

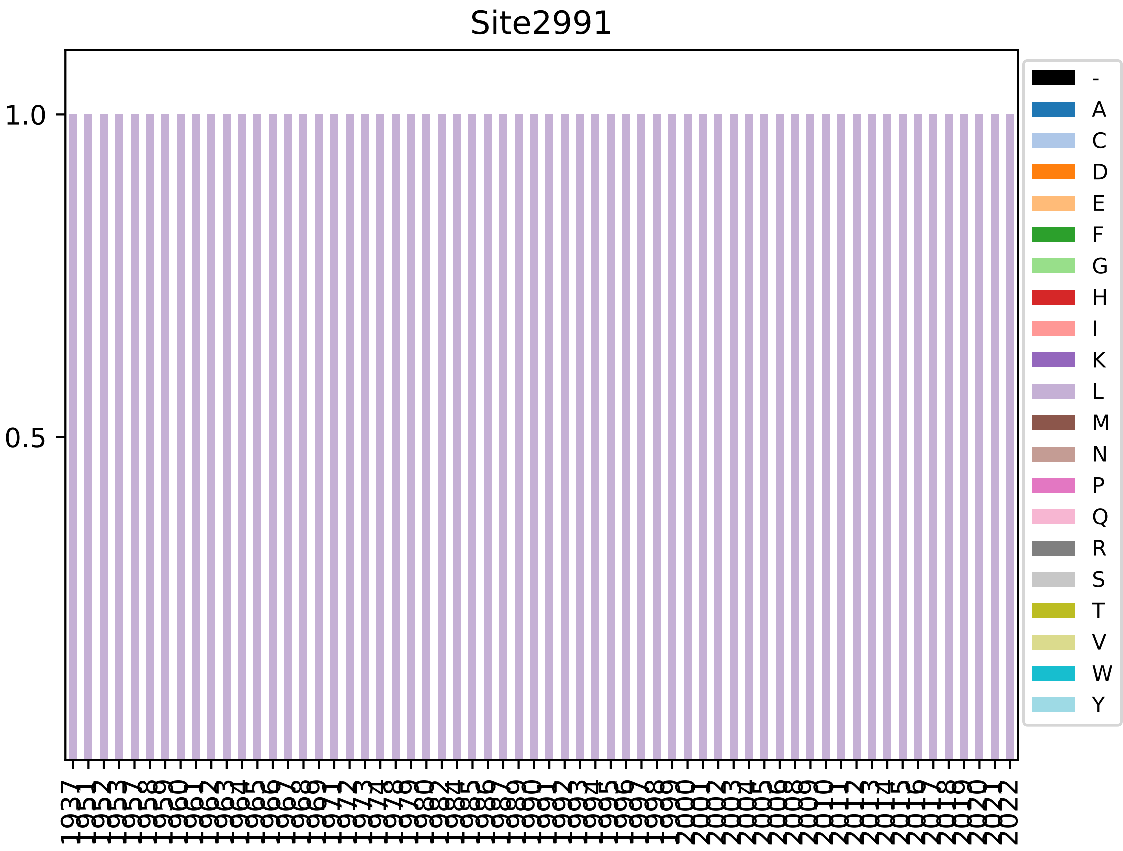
Site2991
1.0
0.5
-
A
C
D
E
F
G
H
I
K
L
M
N
P
Q
R
S
T
V
W
Y

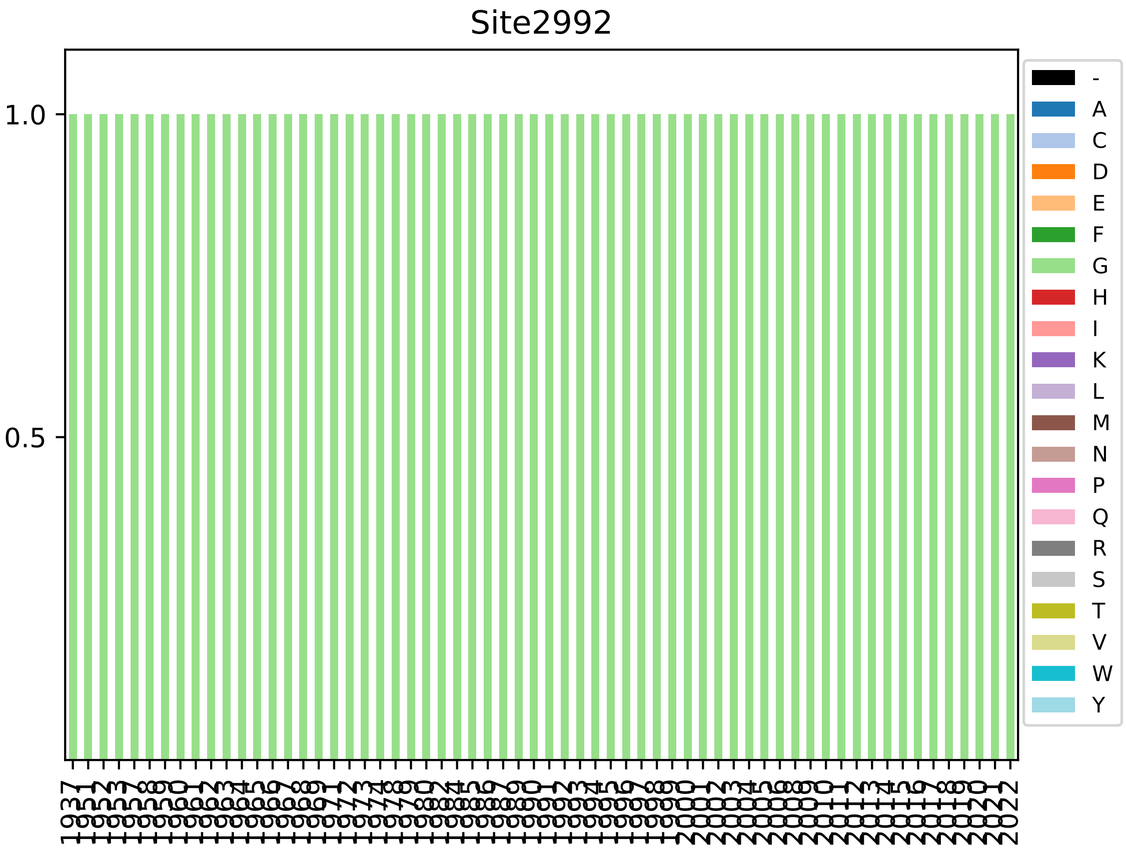

Site2992
1.0
0.5
-
A
C
D
E
F
G
H
I
K
L
M
N
P
Q
R
S
T
V
W
Y

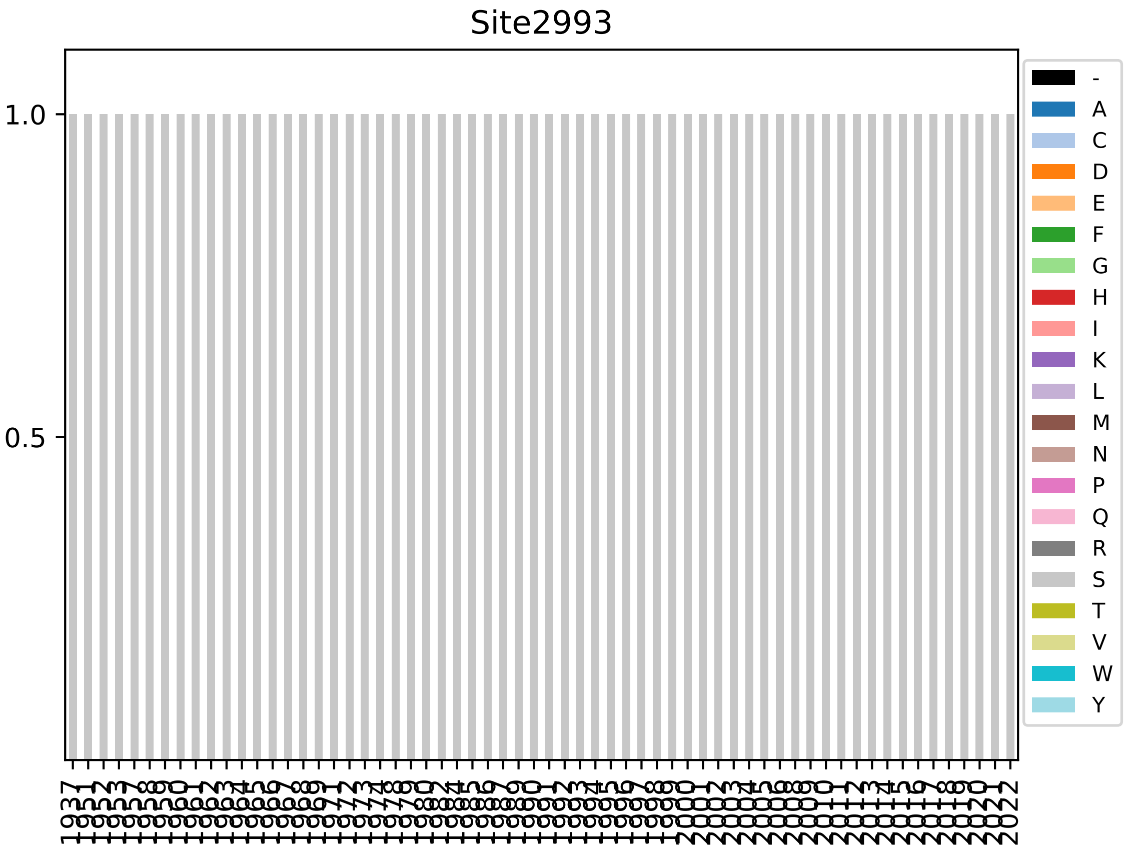
Site2993
1.0
0.5
-
A
C
D
E
F
G
H
I
K
L
M
N
P
Q
R
S
T
V
W
Y

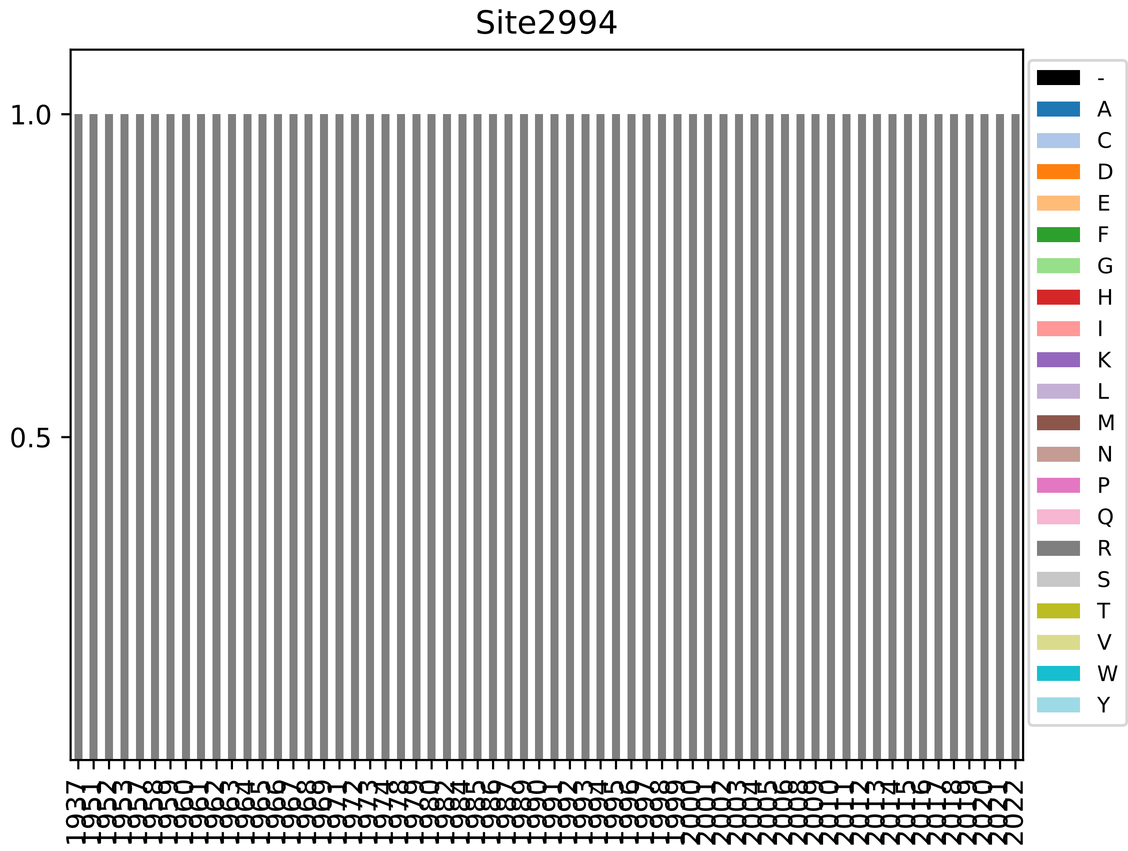
Site2994
1.0
0.5
-
A
C
D
E
F
G
H
I
K
L
M
N
P
Q
R
S
T
V
W
Y

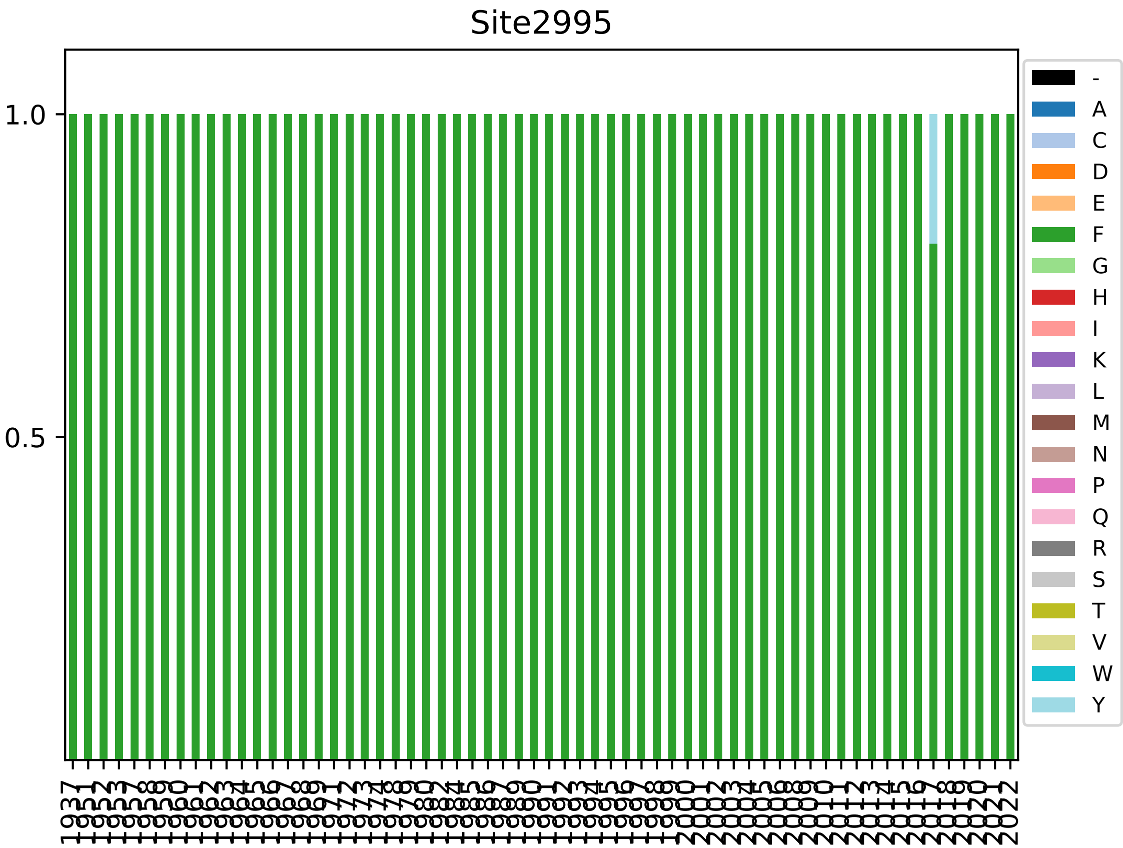

Site2995
1.0
0.5
-
A
C
D
E
F
G
H
I
K
L
M
N
P
Q
R
S
T
V
W
Y

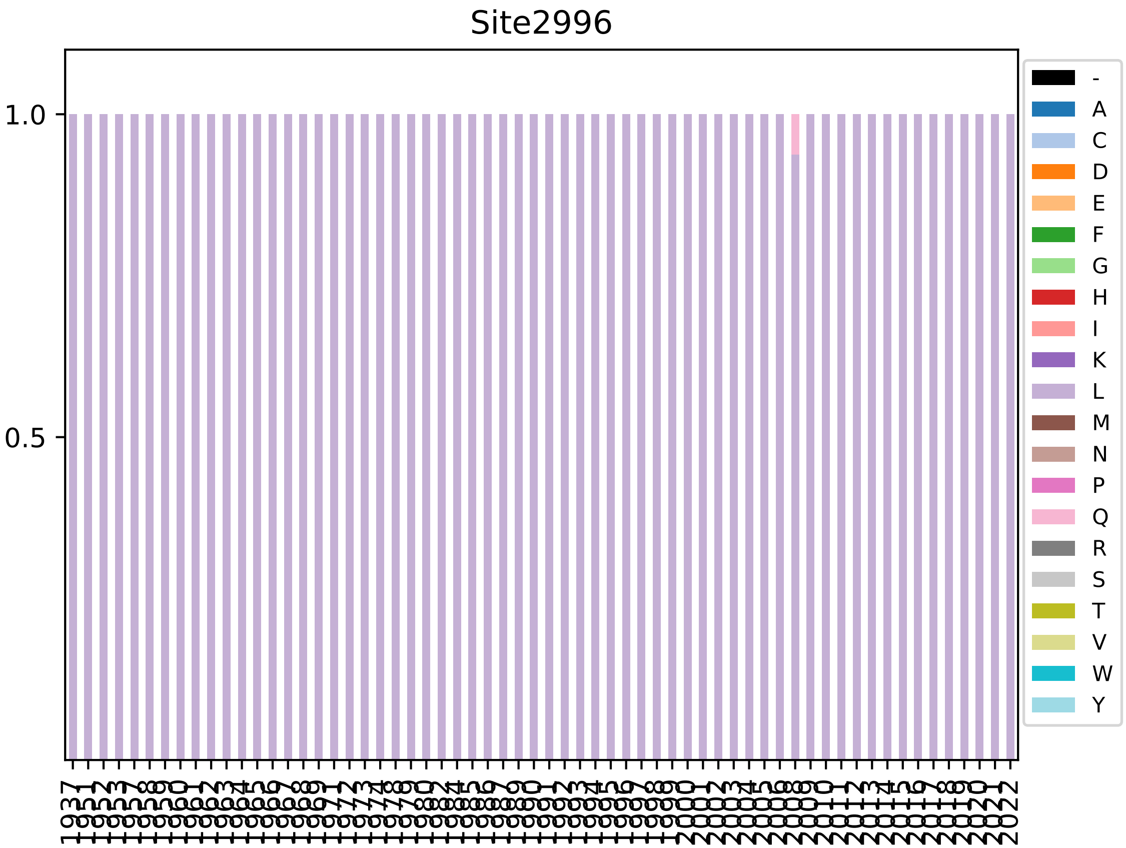
Site2996
1.0
0.5
-
A
C
D
E
F
G
H
I
K
L
M
N
P
Q
R
S
T
V
W
Y

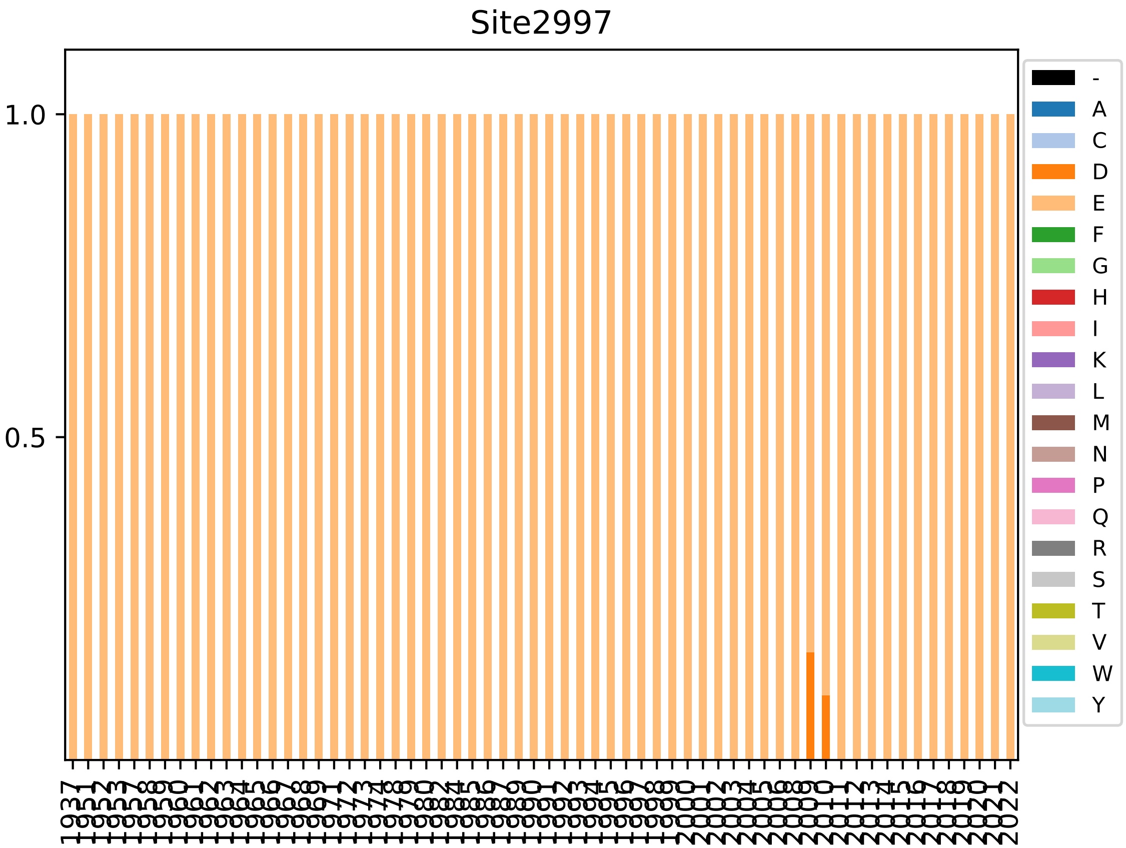
Site2997
1.0
0.5
-
A
C
D
E
F
G
H
I
K
L
M
N
P
Q
R
S
T
V
W
Y

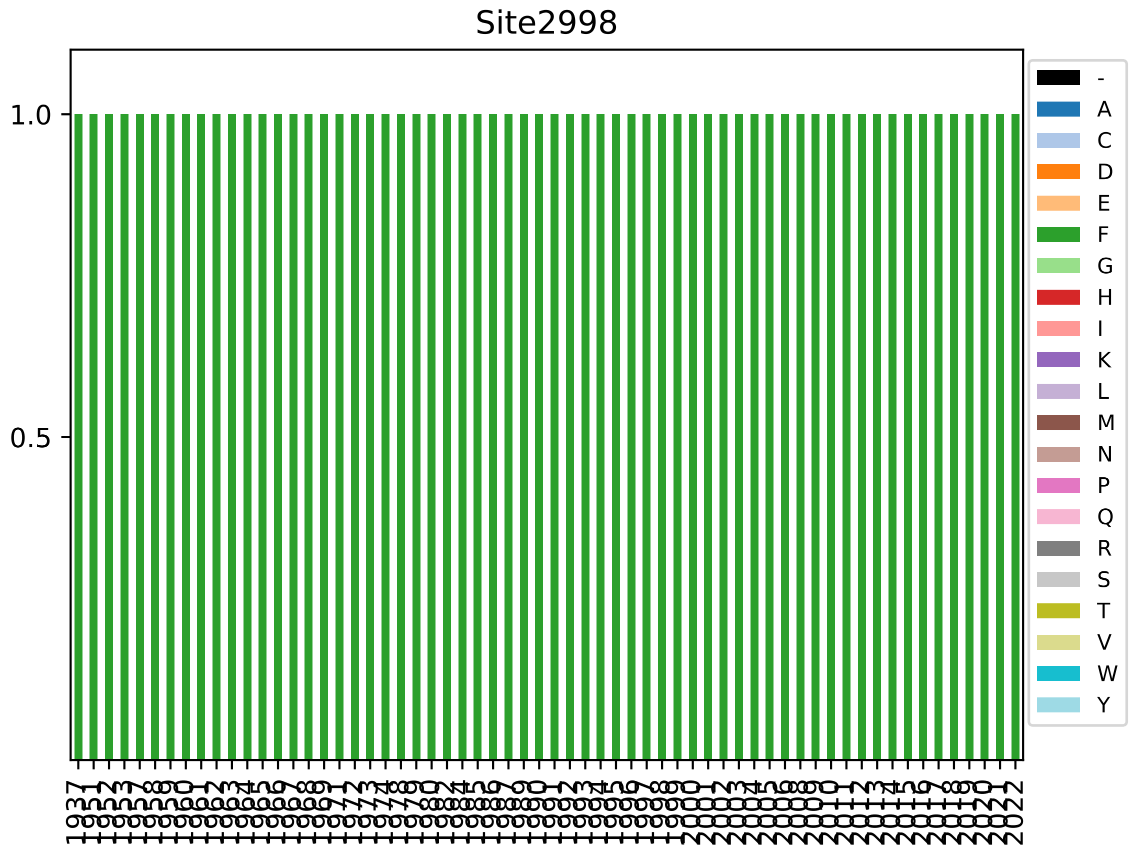
Site2998
1.0
0.5
-
A
C
D
E
F
G
H
I
K
L
M
N
P
Q
R
S
T
V
W
Y

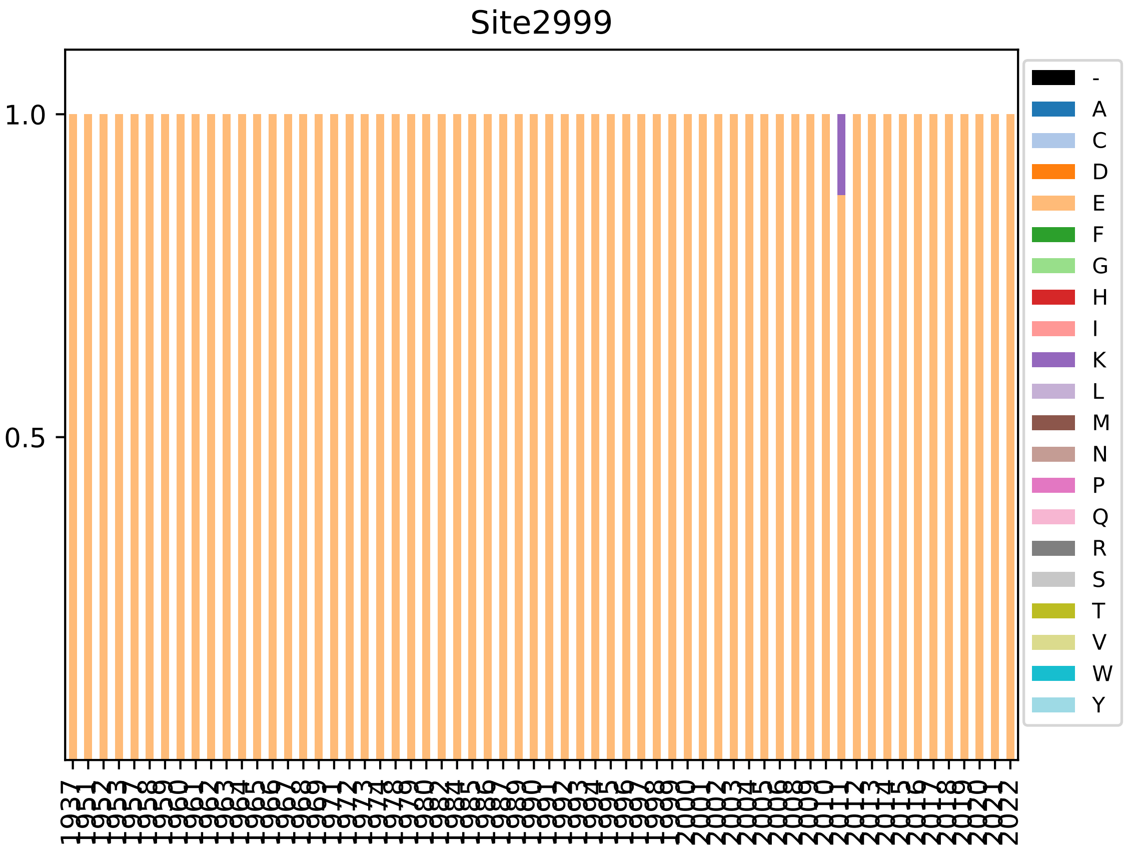
Site2999
1.0
0.5
-
A
C
D
E
F
G
H
I
K
L
M
N
P
Q
R
S
T
V
W
Y

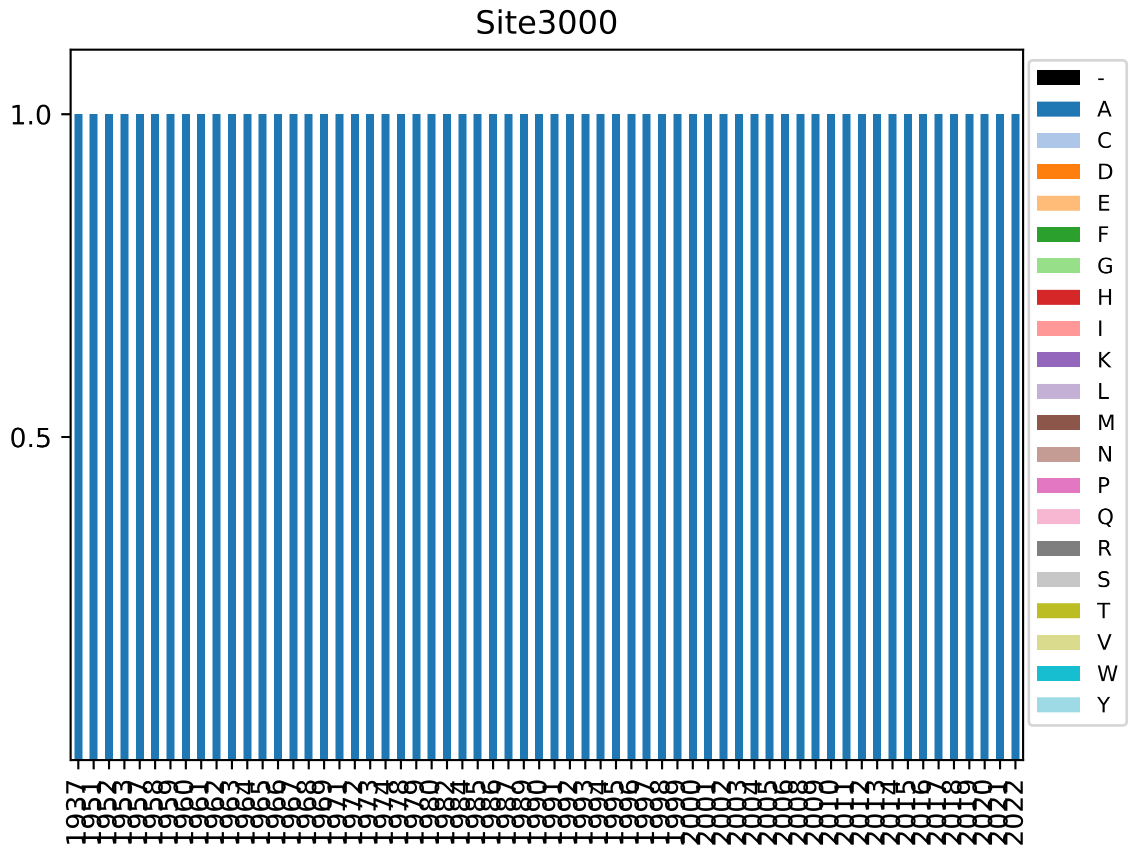
Site3000
1.0
0.5
-
A
C
D
E
F
G
H
I
K
L
M
N
P
Q
R
S
T
V
W
Y

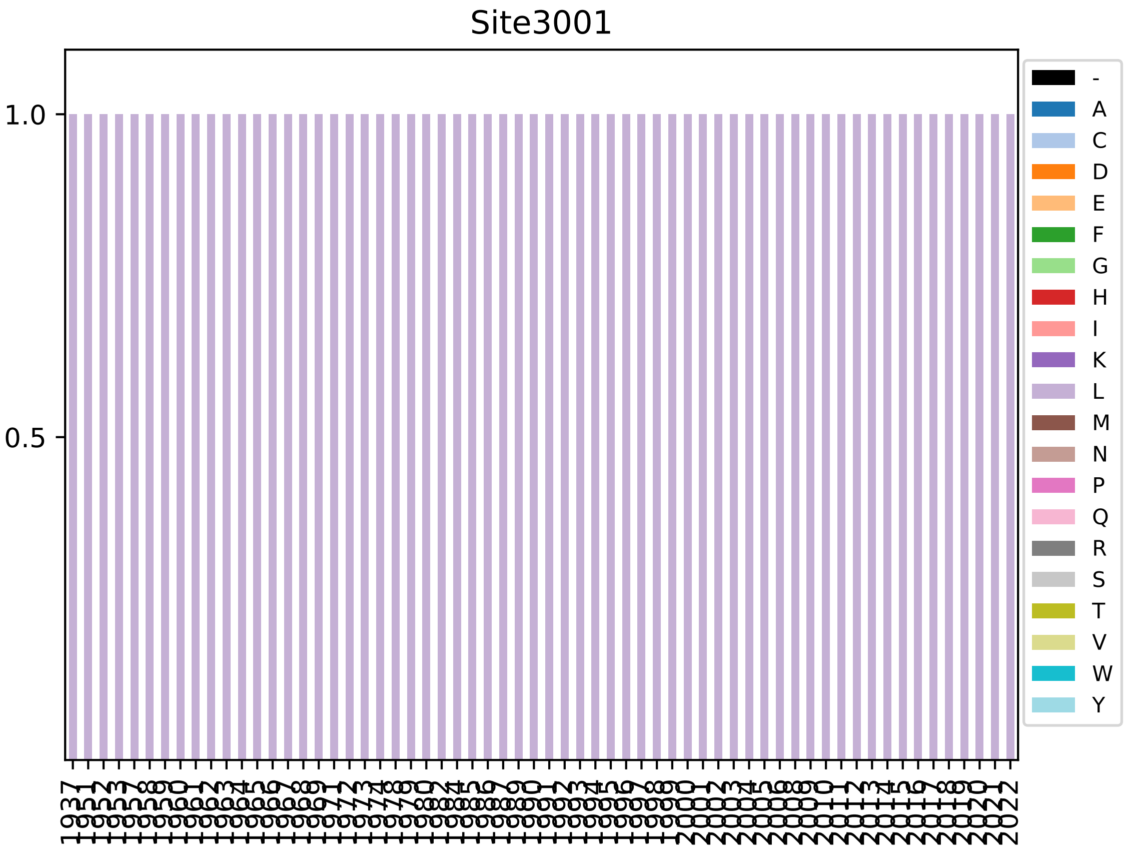
Site3001
1.0
0.5
-
A
C
D
E
F
G
H
I
K
L
M
N
P
Q
R
S
T
V
W
Y

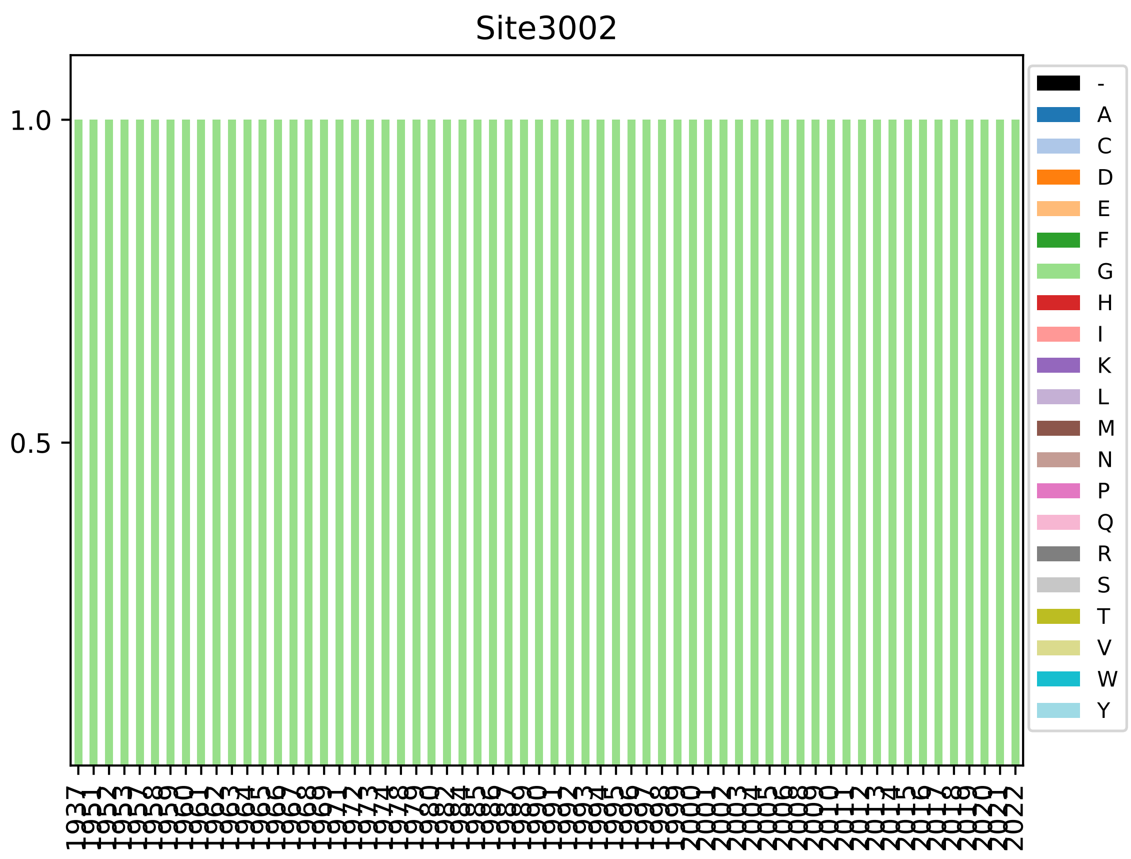
Site3002
1.0
0.5
-
A
C
D
E
F
G
H
I
K
L
M
N
P
Q
R
S
T
V
W
Y

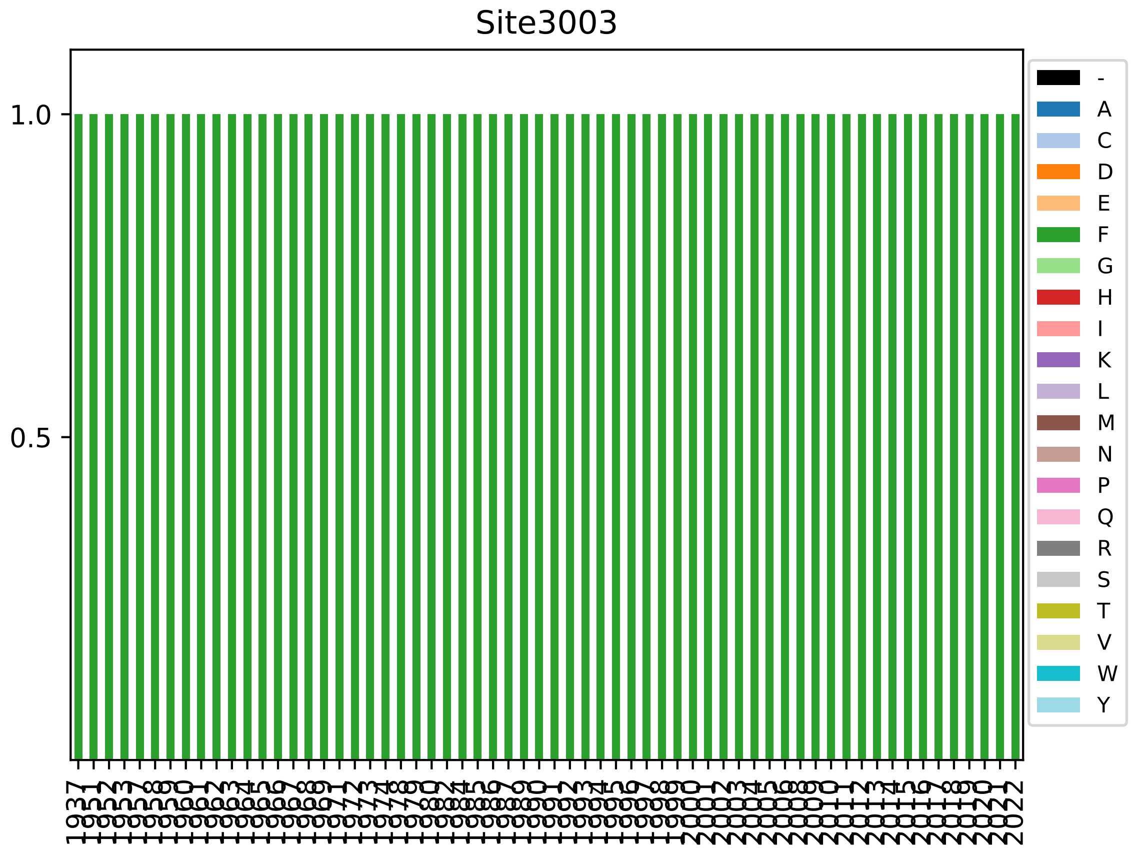
Site3003
1.0
0.5
-
A
C
D
E
F
G
H
I
K
L
M
N
P
Q
R
S
T
V
W
Y

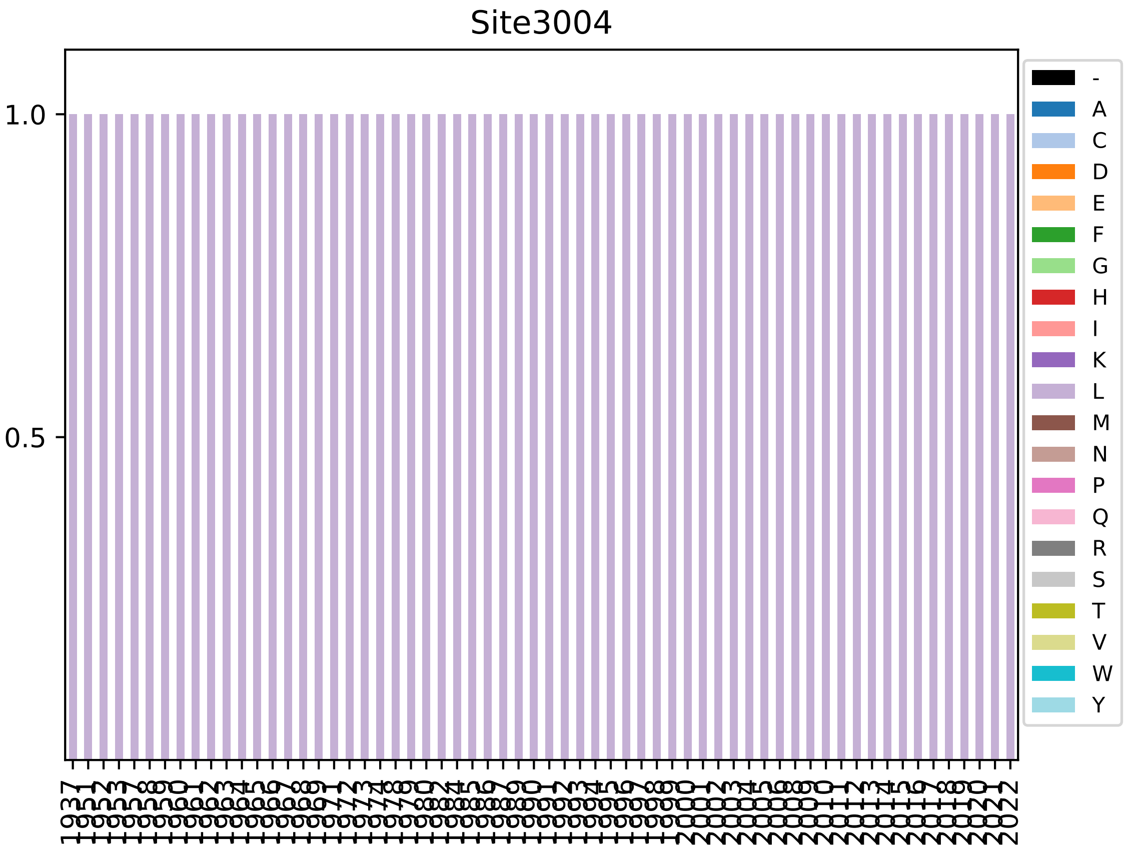

Site3004
1.0
0.5
-
A
C
D
E
F
G
H
I
K
L
M
N
P
Q
R
S
T
V
W
Y

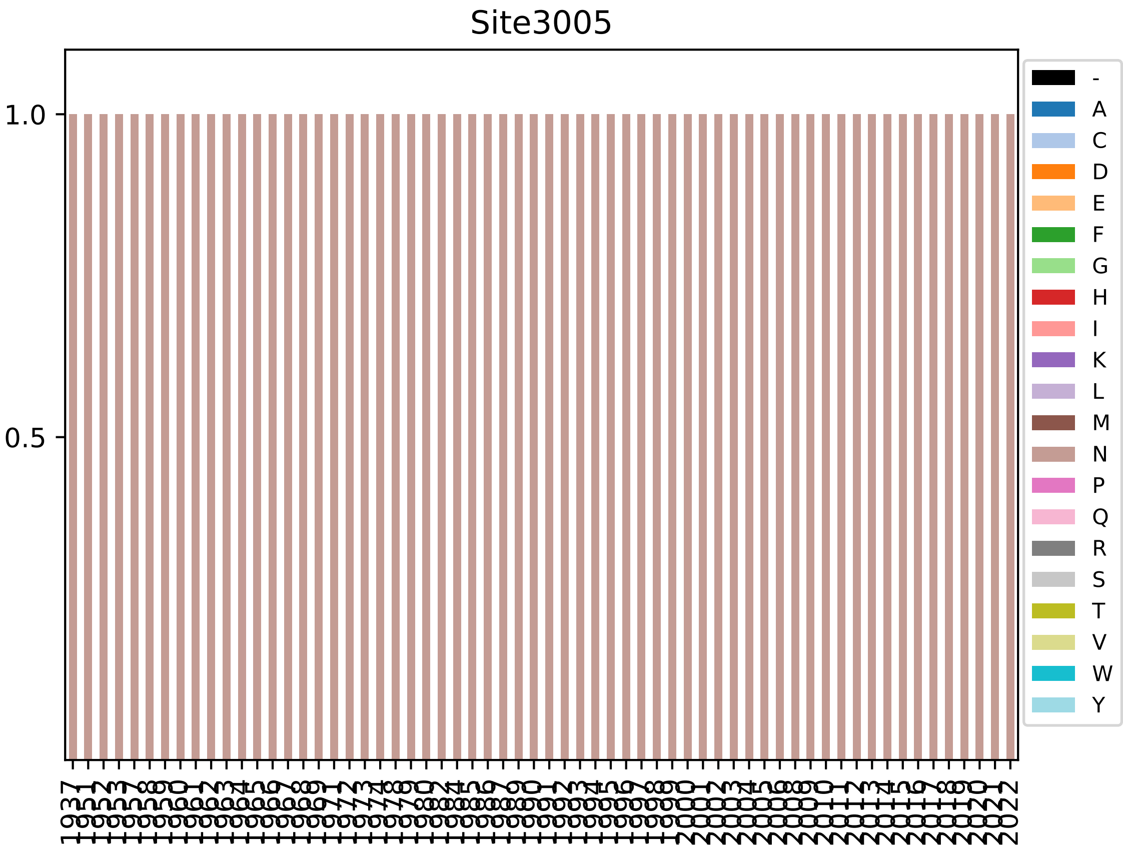
Site3005
1.0
0.5
-
A
C
D
E
F
G
H
I
K
L
M
N
P
Q
R
S
T
V
W
Y

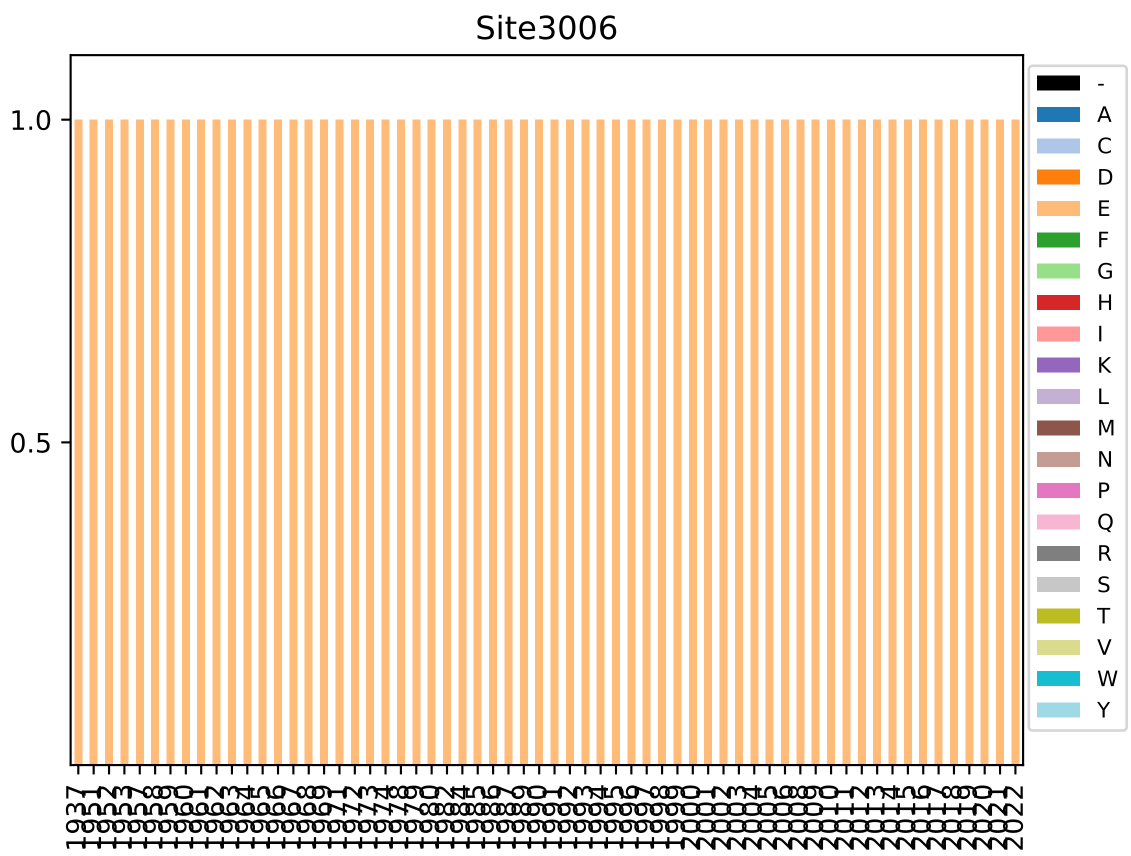
Site3006
1.0
0.5
-
A
C
D
E
F
G
H
I
K
L
M
N
P
Q
R
S
T
V
W
Y

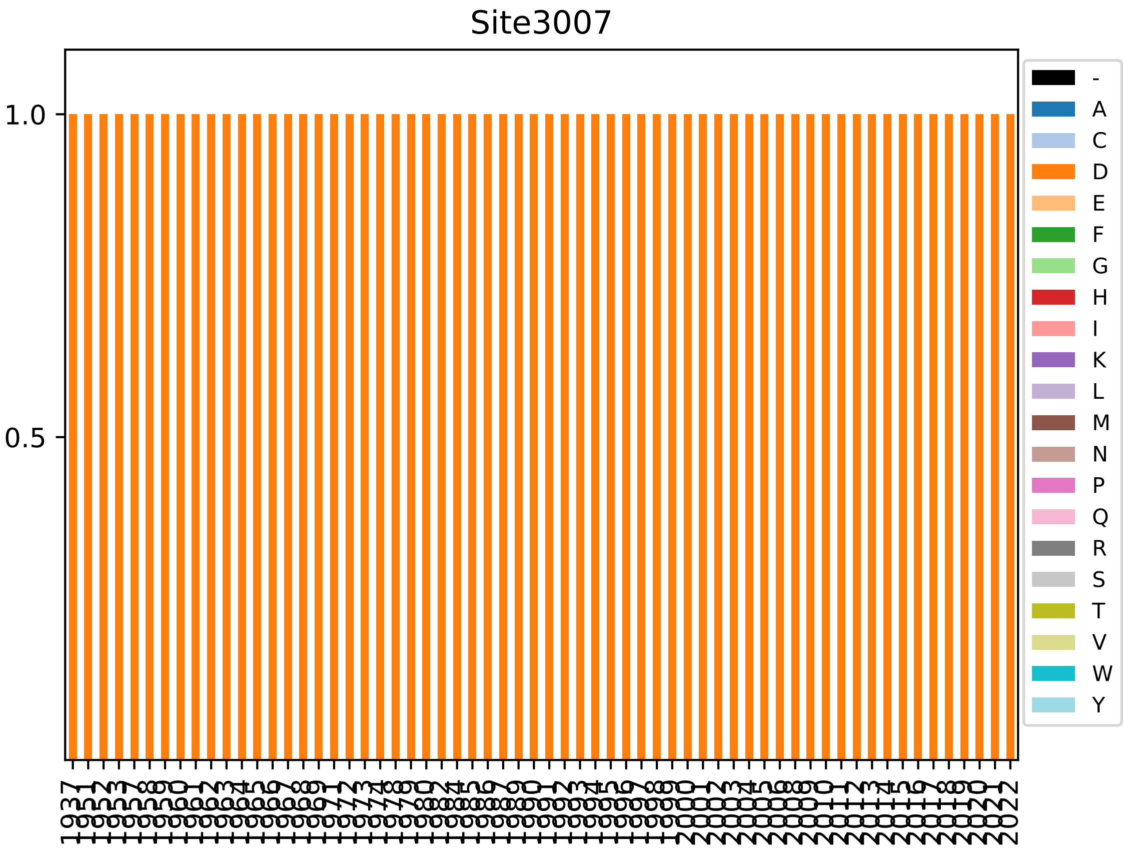

Site3007
1.0
0.5
-
A
C
D
E
F
G
H
I
K
L
M
N
P
Q
R
S
T
V
W
Y

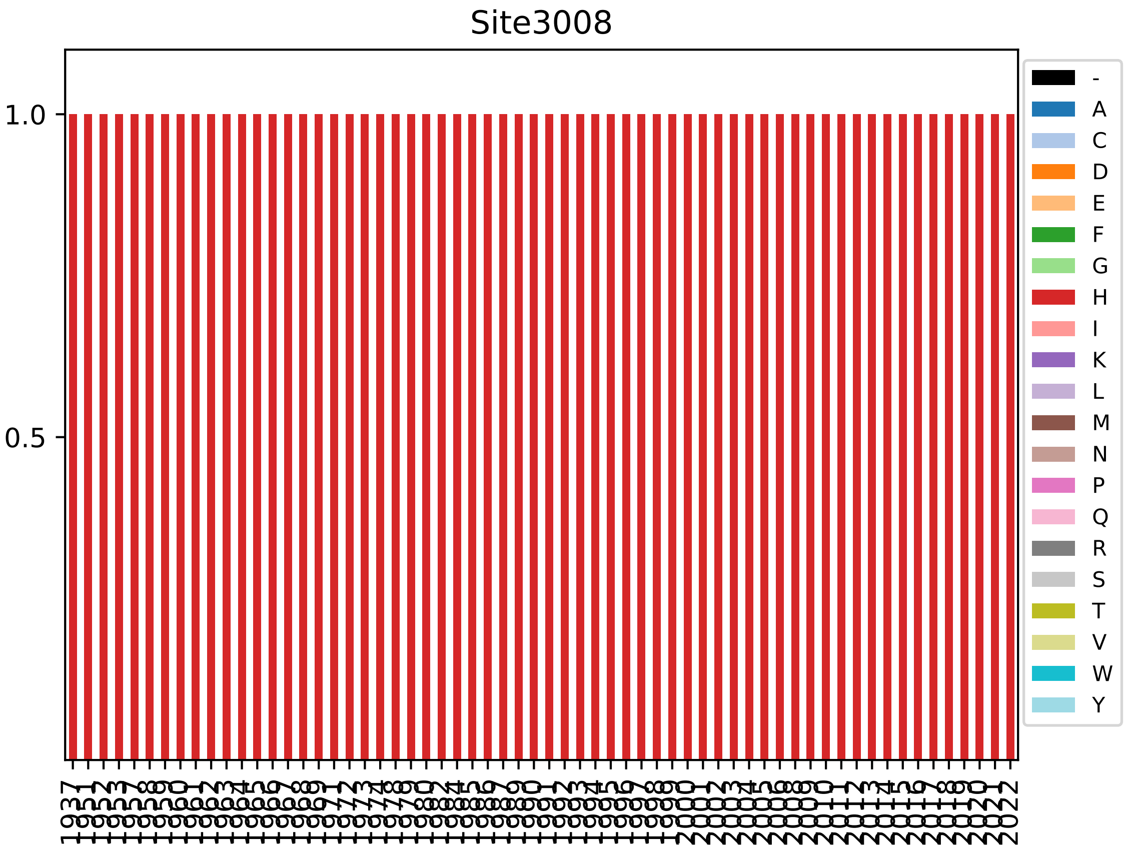
Site3008
1.0
0.5
-
A
C
D
E
F
G
H
I
K
L
M
N
P
Q
R
S
T
V
W
Y

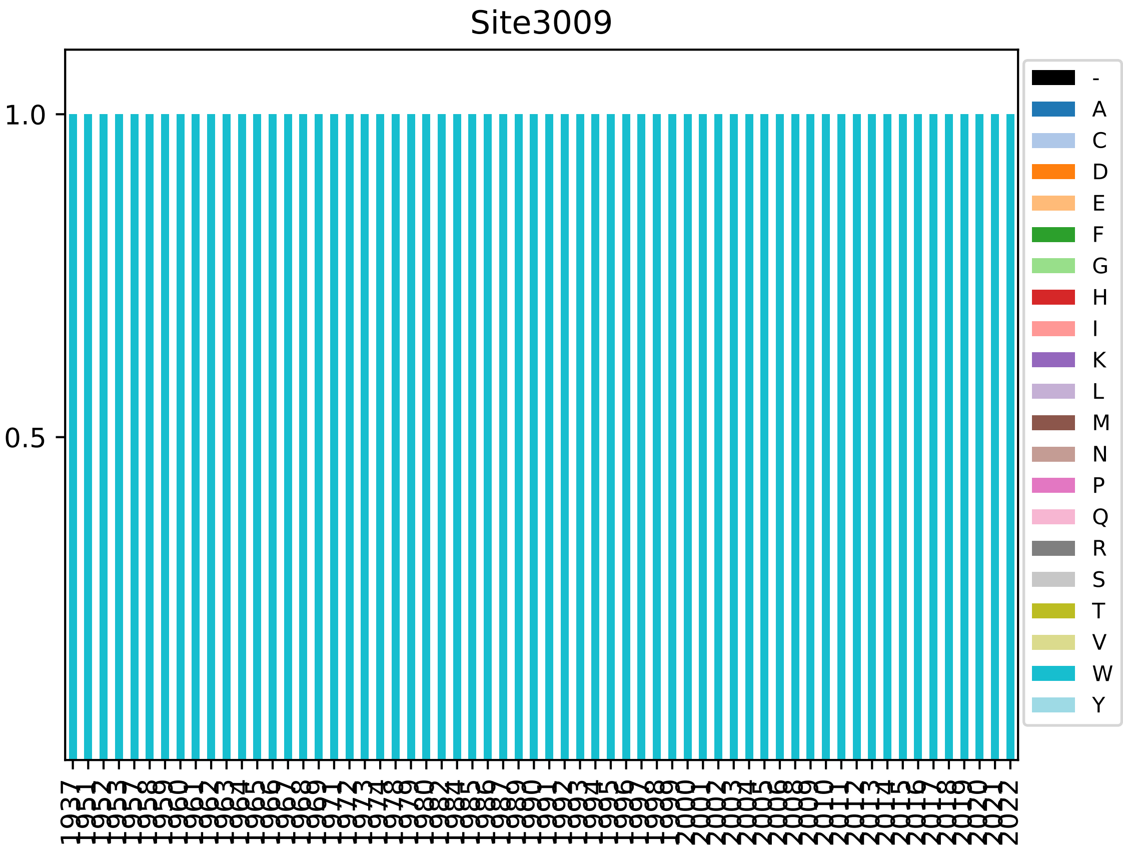
Site3009
1.0
0.5
-
A
C
D
E
F
G
H
I
K
L
M
N
P
Q
R
S
T
V
W
Y

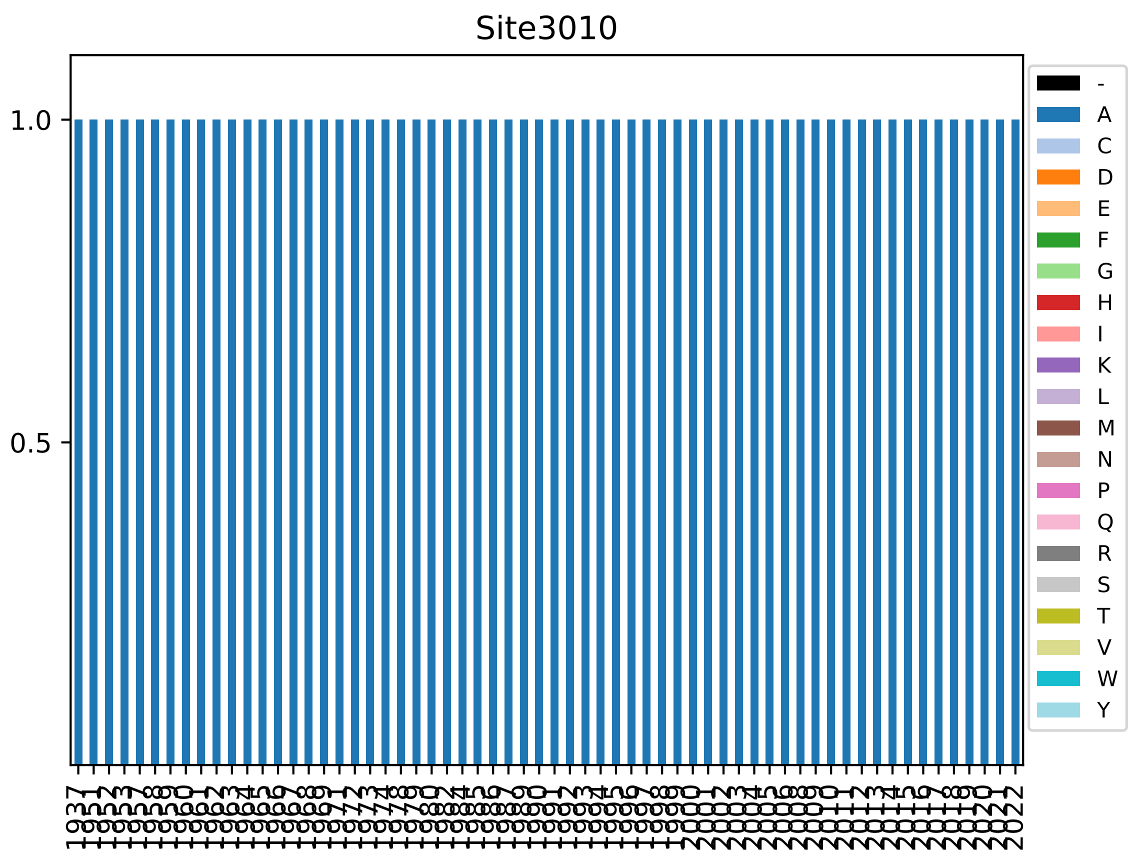
Site3010
1.0
0.5
-
A
C
D
E
F
G
H
I
K
L
M
N
P
Q
R
S
T
V
W
Y

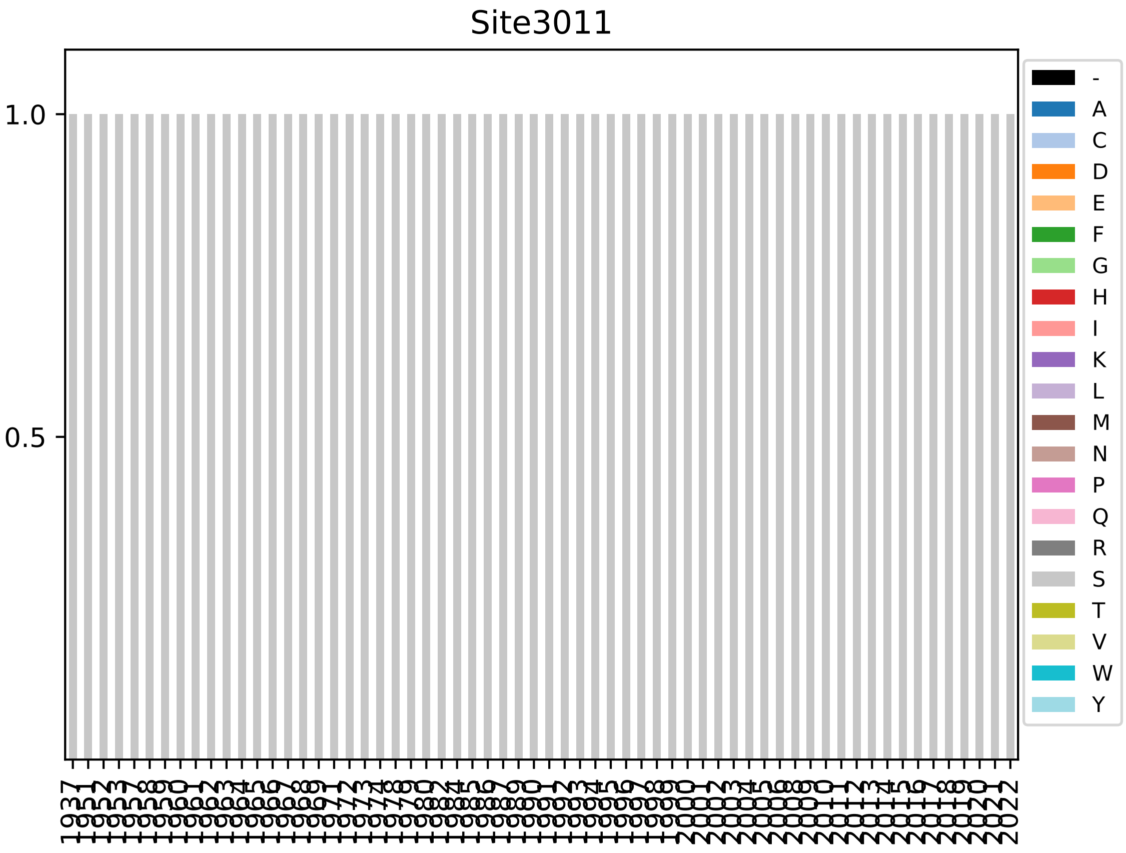
Site3011
1.0
0.5
-
A
C
D
E
F
G
H
I
K
L
M
N
P
Q
R
S
T
V
W
Y

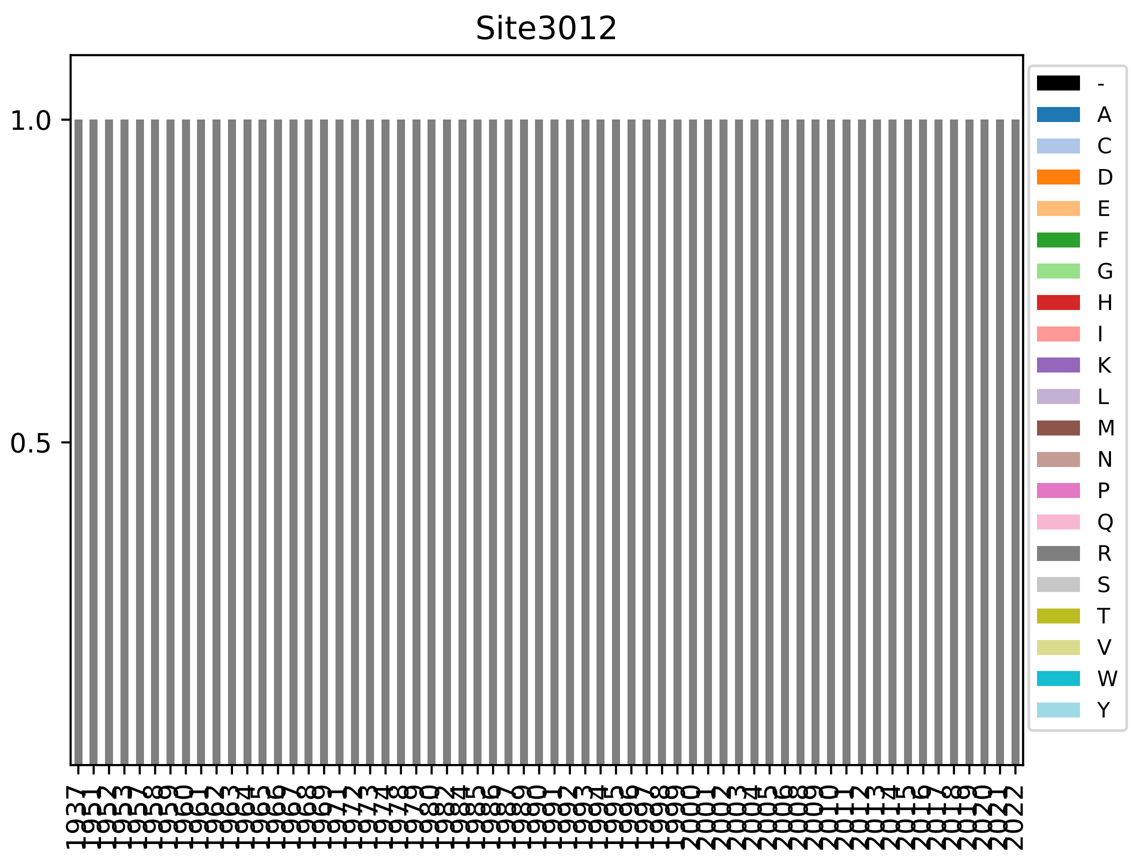
Site3012
1.0
0.5
-
A
C
D
E
F
G
H
I
K
L
M
N
P
Q
R
S
T
V
W
Y

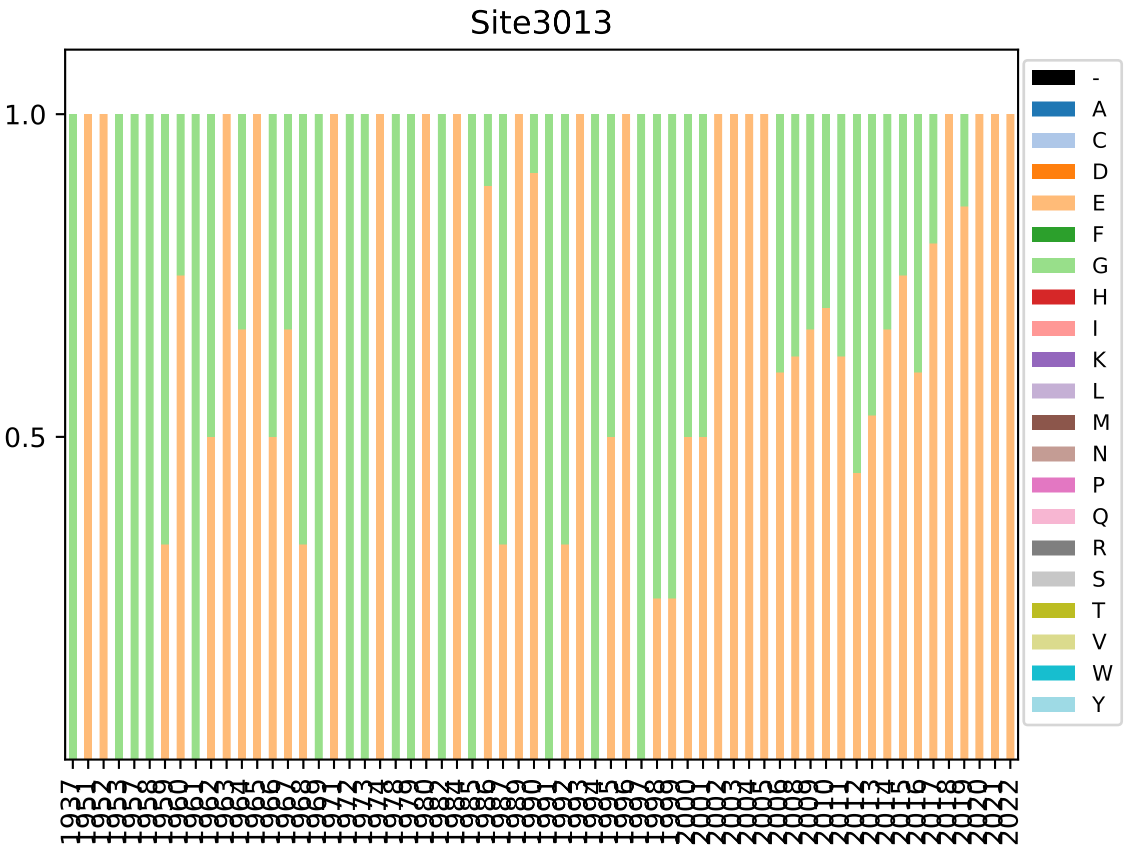
Site3013
1.0
0.5
-
A
C
D
E
F
G
H
I
K
L
M
N
P
Q
R
S
T
V
W
Y

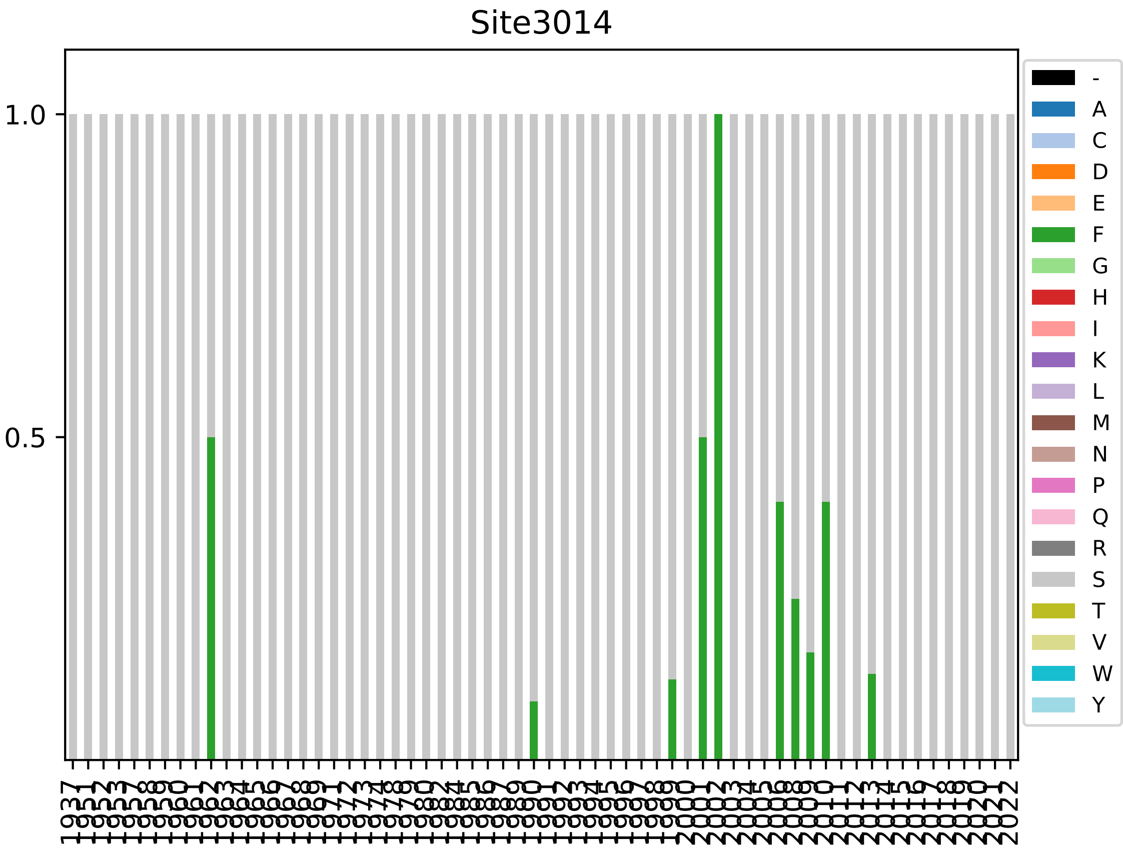
Site3014
1.0
0.5
-
A
C
D
E
F
G
H
I
K
L
M
N
P
Q
R
S
T
V
W
Y

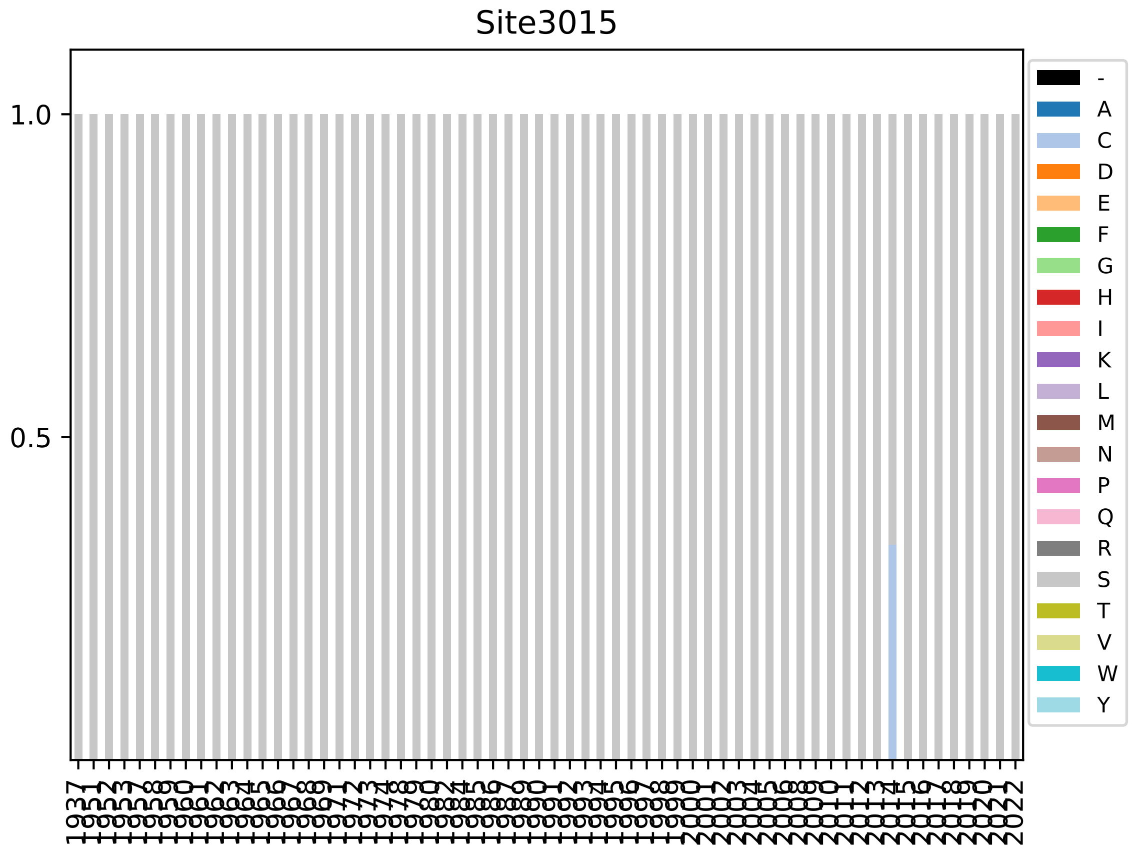
Site3015
1.0
0.5
-
A
C
D
E
F
G
H
I
K
L
M
N
P
Q
R
S
T
V
W
Y

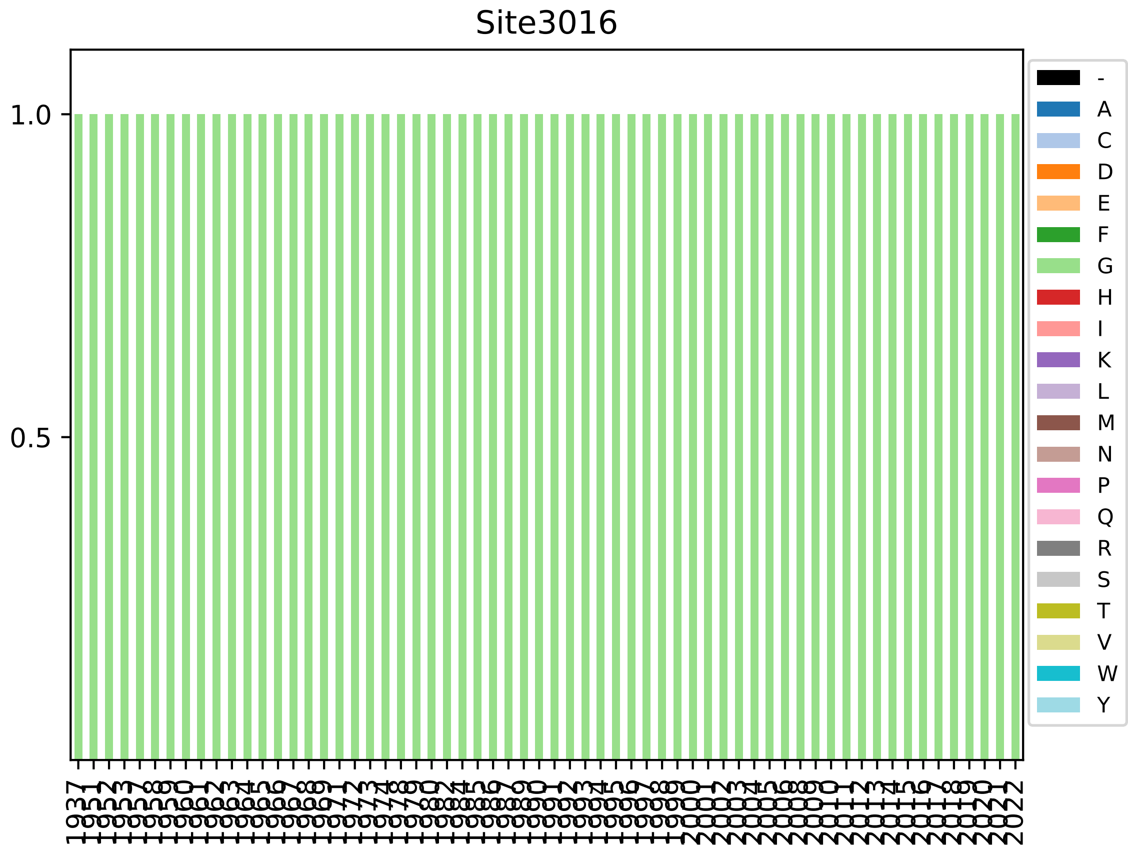
Site3016
1.0
0.5
-
A
C
D
E
F
G
H
I
K
L
M
N
P
Q
R
S
T
V
W
Y

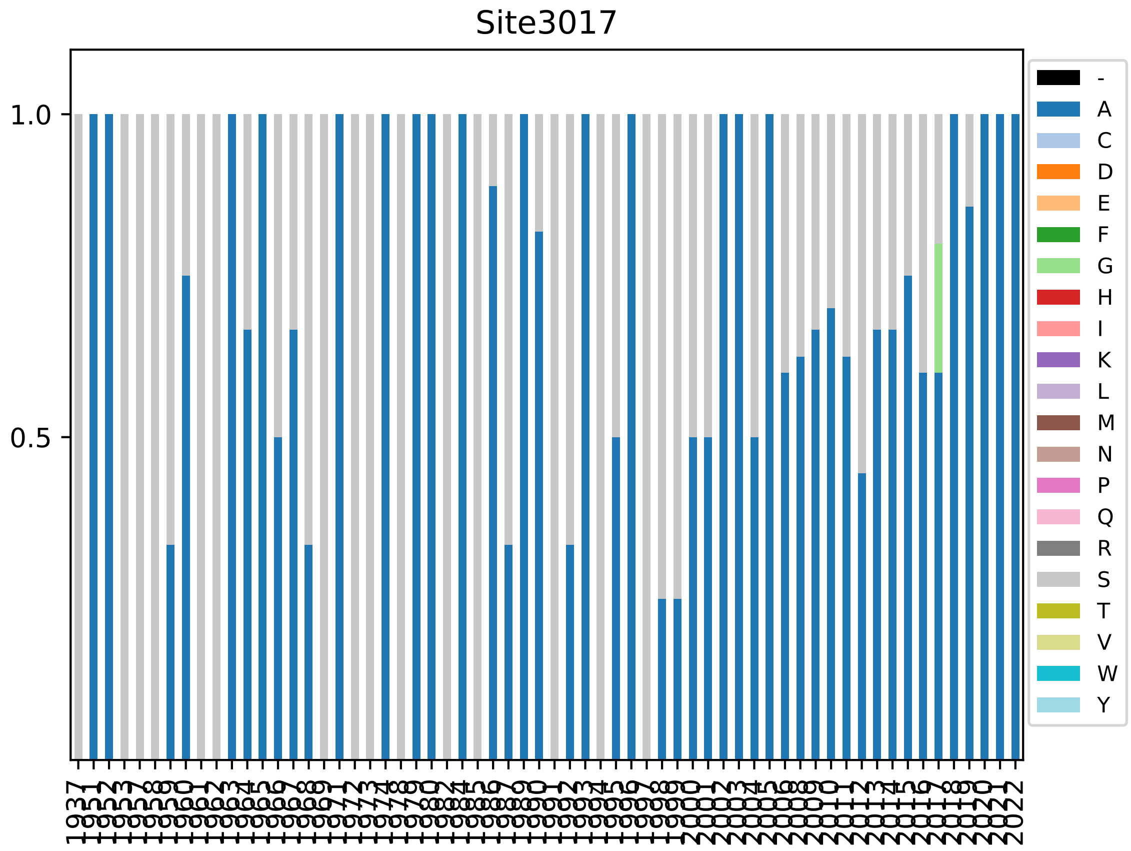
Site3017
1.0
0.5
-
A
C
D
E
F
G
H
I
K
L
M
N
P
Q
R
S
T
V
W
Y

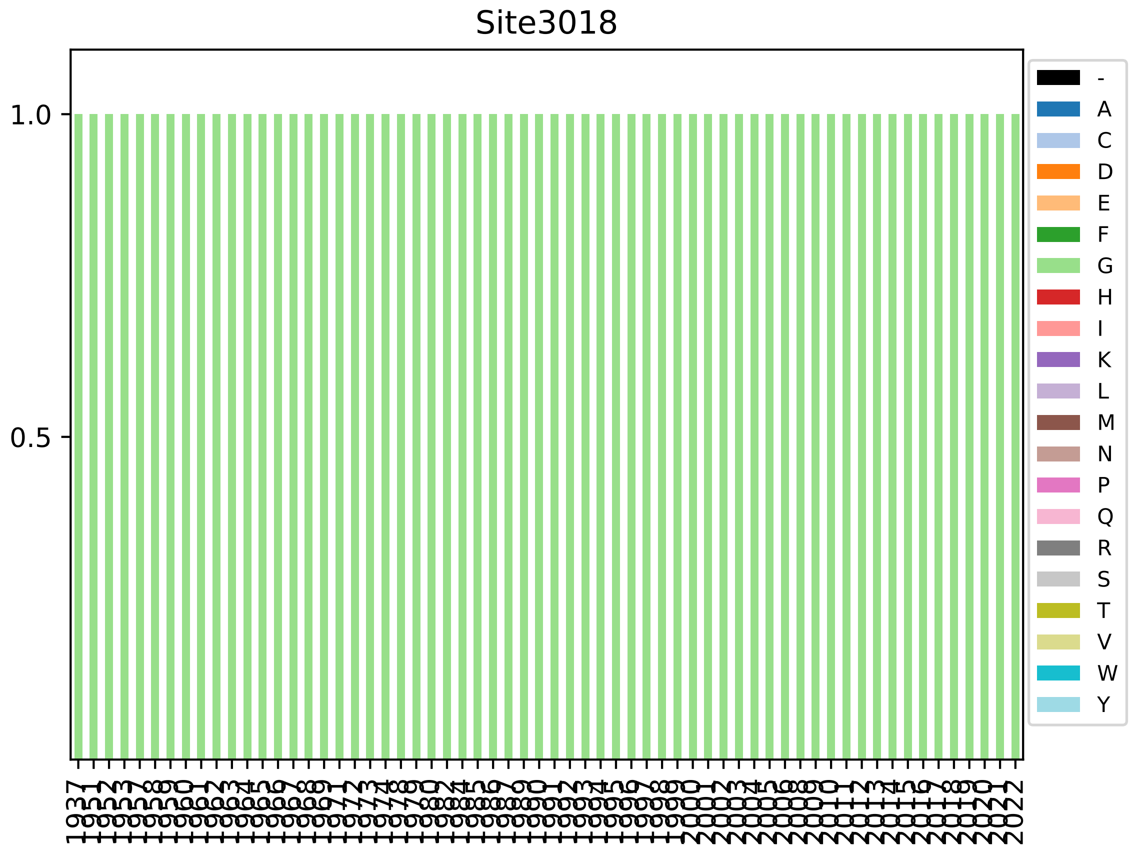
Site3018
1.0
0.5
-
A
C
D
E
F
G
H
I
K
L
M
N
P
Q
R
S
T
V
W
Y

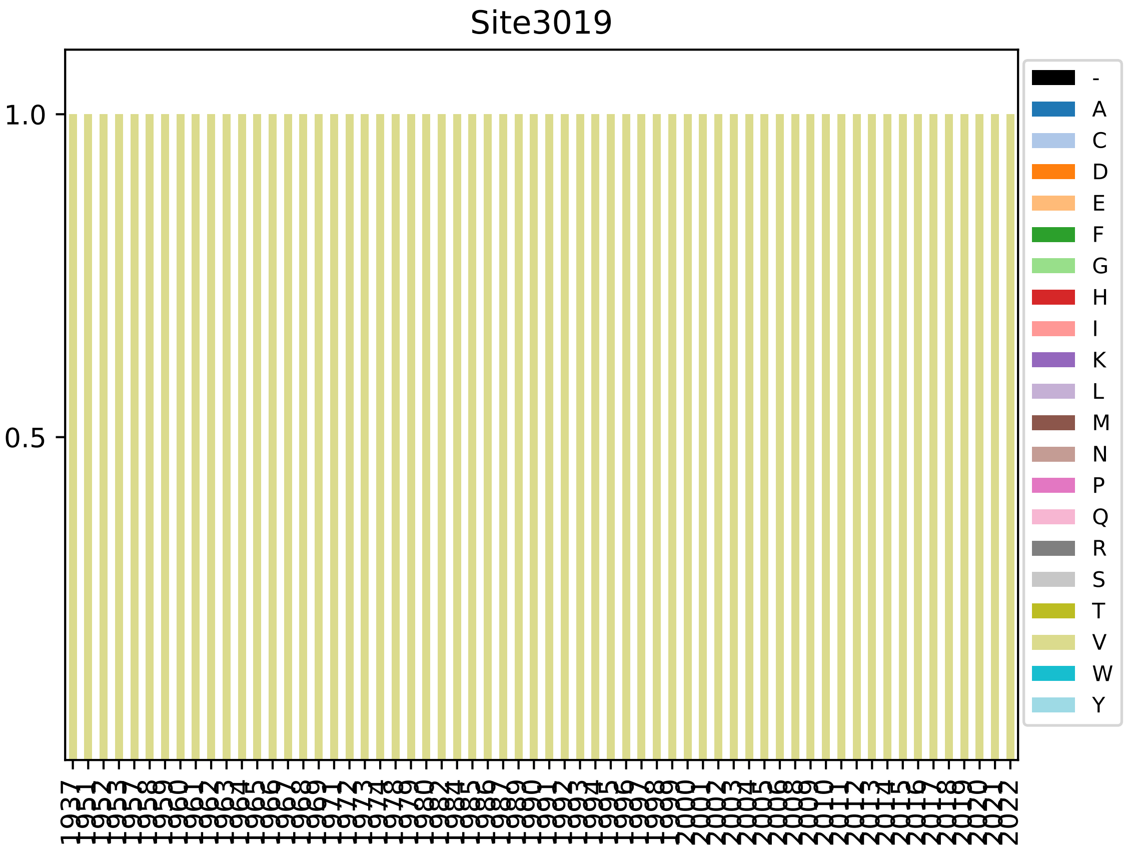
Site3019
1.0
0.5
-
A
C
D
E
F
G
H
I
K
L
M
N
P
Q
R
S
T
V
W
Y

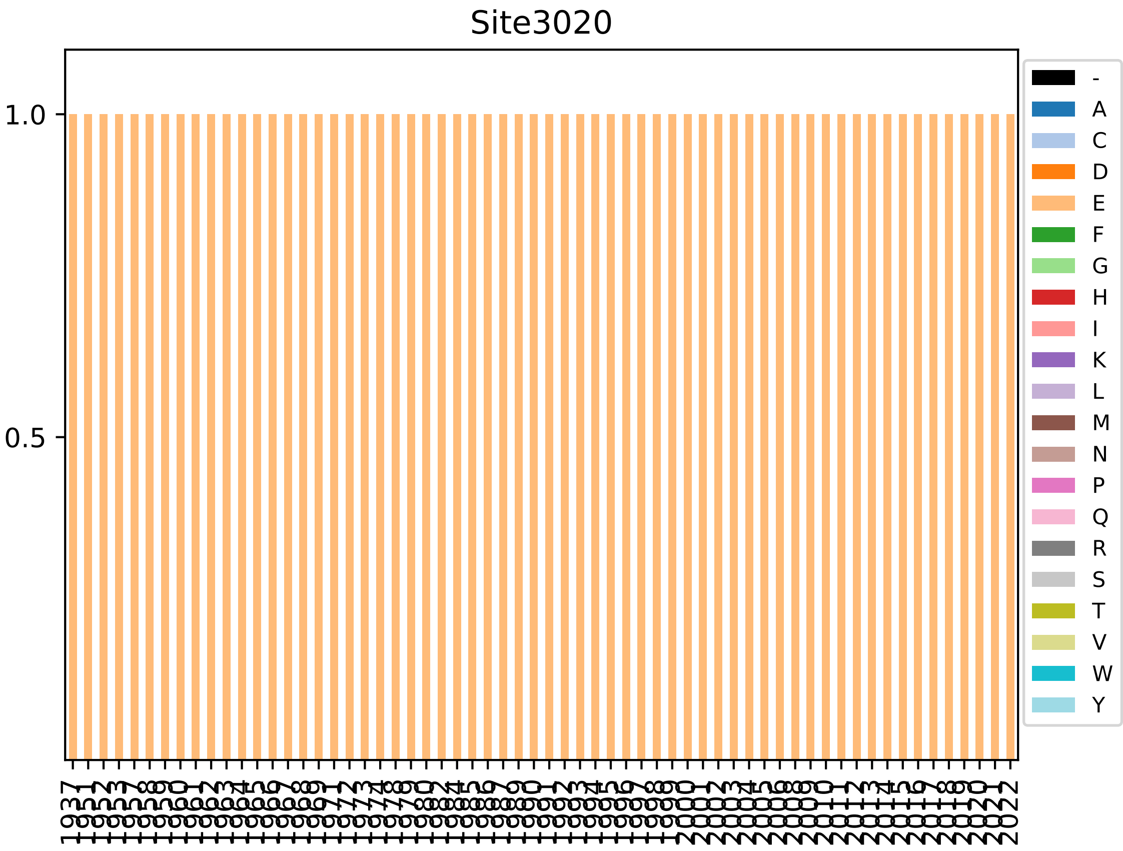
Site3020
1.0
0.5
-
A
C
D
E
F
G
H
I
K
L
M
N
P
Q
R
S
T
V
W
Y

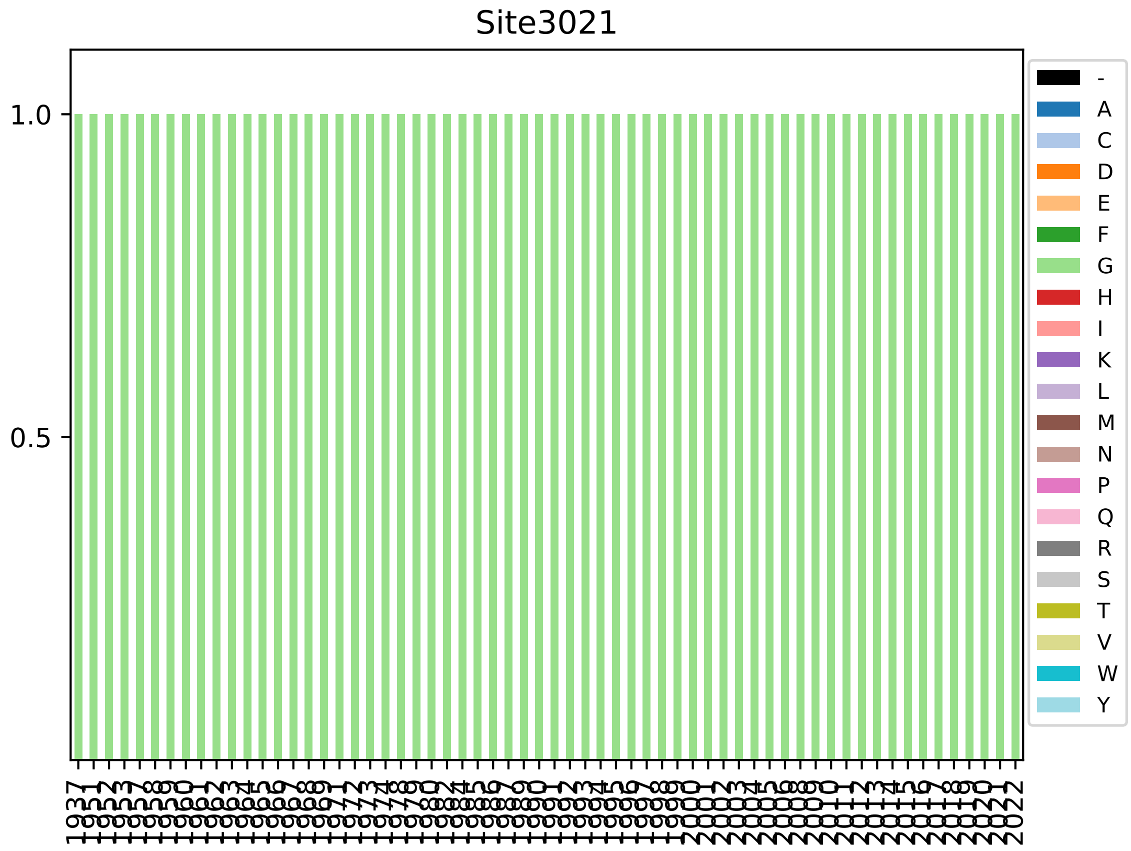
Site3021
1.0
0.5
-
A
C
D
E
F
G
H
I
K
L
M
N
P
Q
R
S
T
V
W
Y

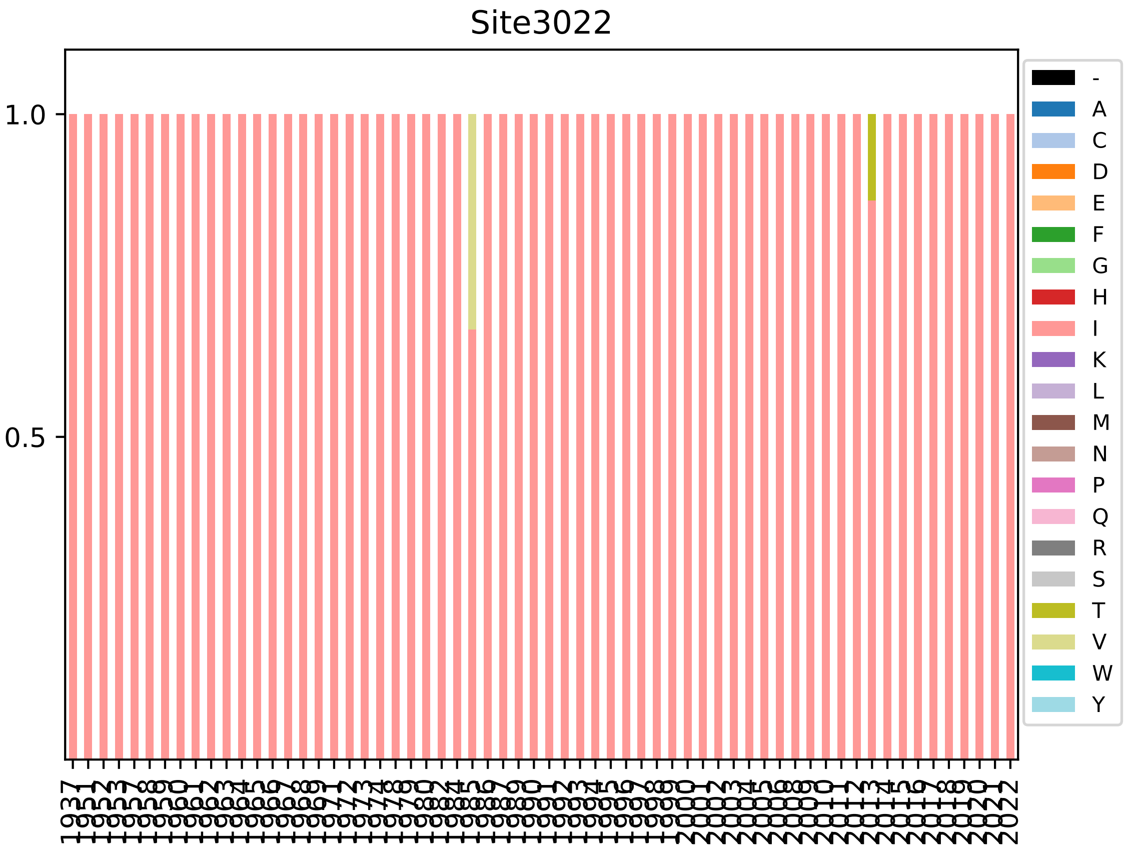
Site3022
1.0
0.5
-
A
C
D
E
F
G
H
I
K
L
M
N
P
Q
R
S
T
V
W
Y

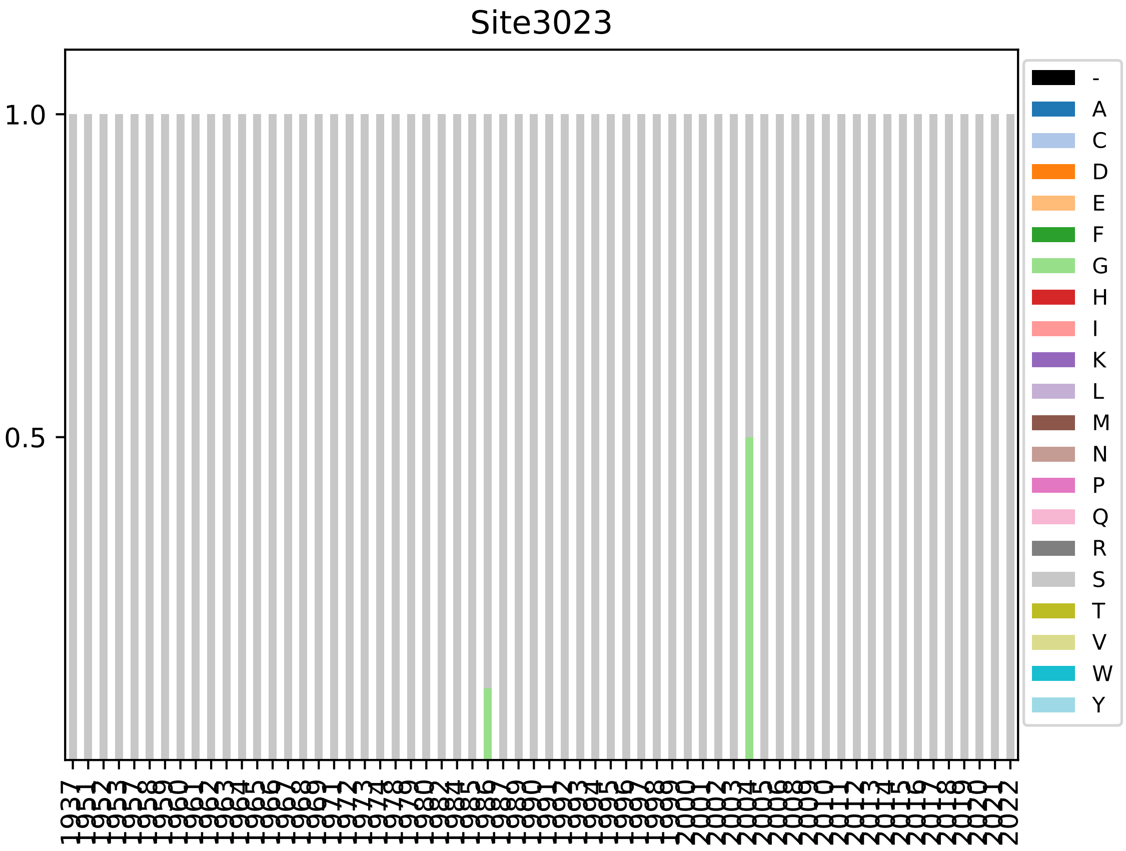
Site3023
1.0
0.5
-
A
C
D
E
F
G
H
I
K
L
M
N
P
Q
R
S
T
V
W
Y

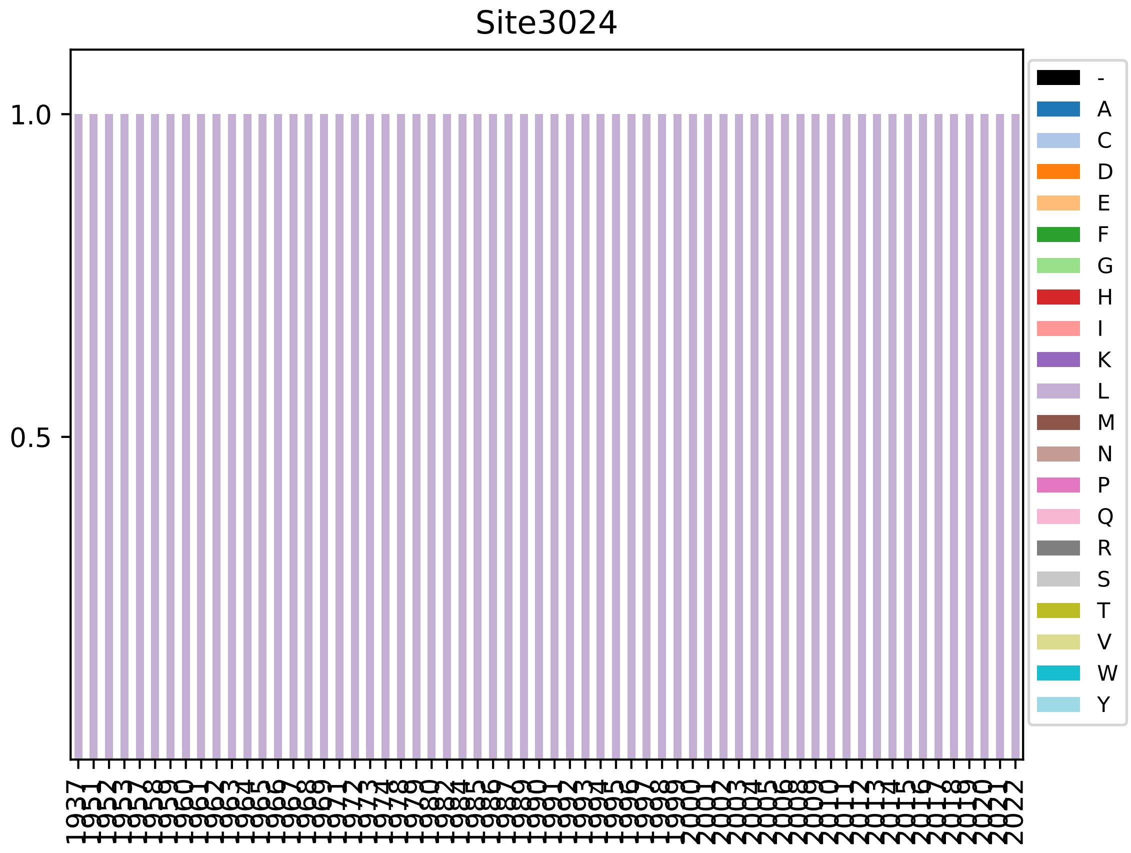
Site3024
1.0
0.5
-
A
C
D
E
F
G
H
I
K
L
M
N
P
Q
R
S
T
V
W
Y

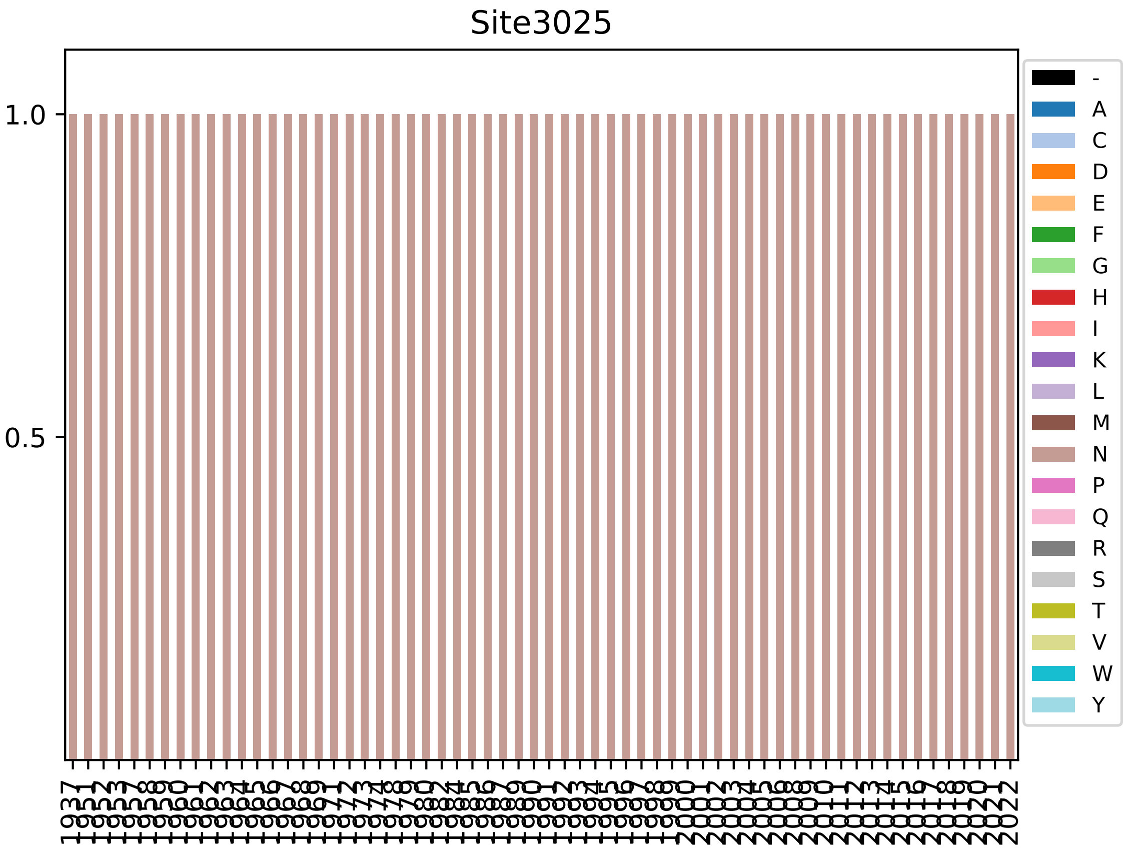
Site3025
1.0
0.5
-
A
C
D
E
F
G
H
I
K
L
M
N
P
Q
R
S
T
V
W
Y

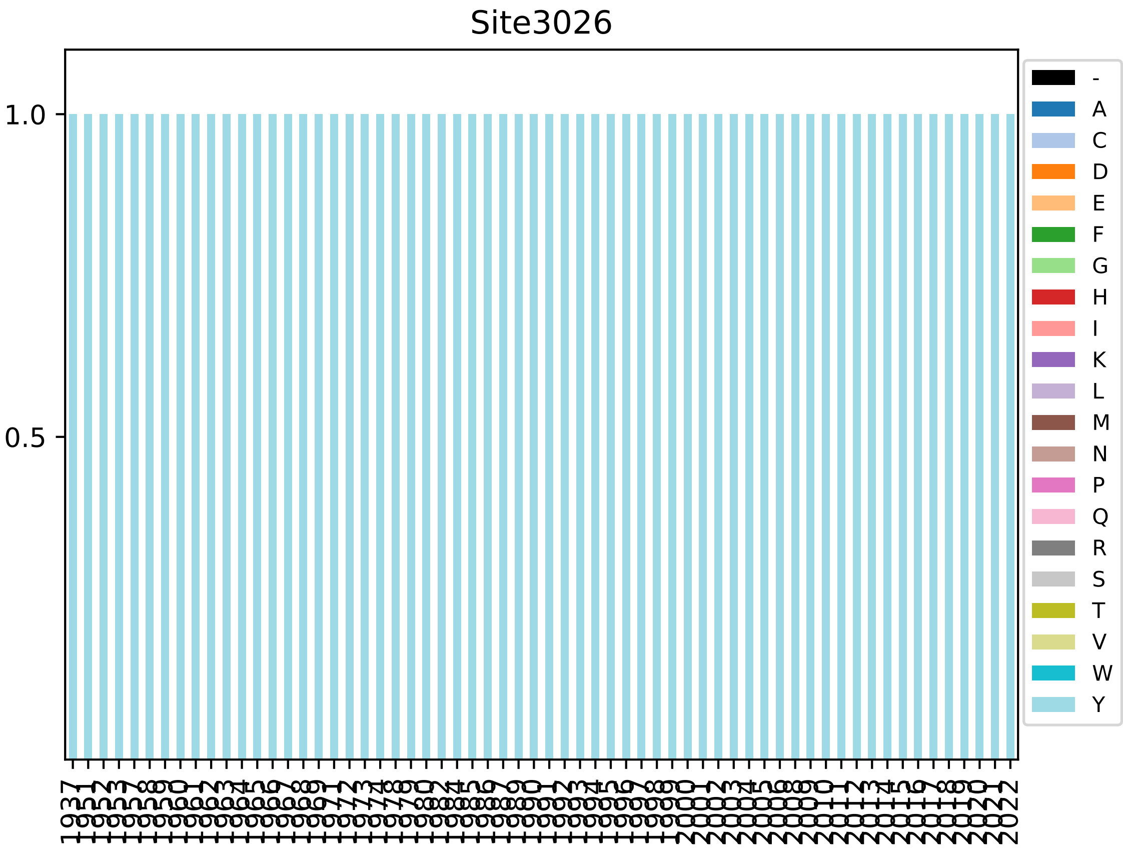
Site3026
1.0
0.5
-
A
C
D
E
F
G
H
I
K
L
M
N
P
Q
R
S
T
V
W
Y

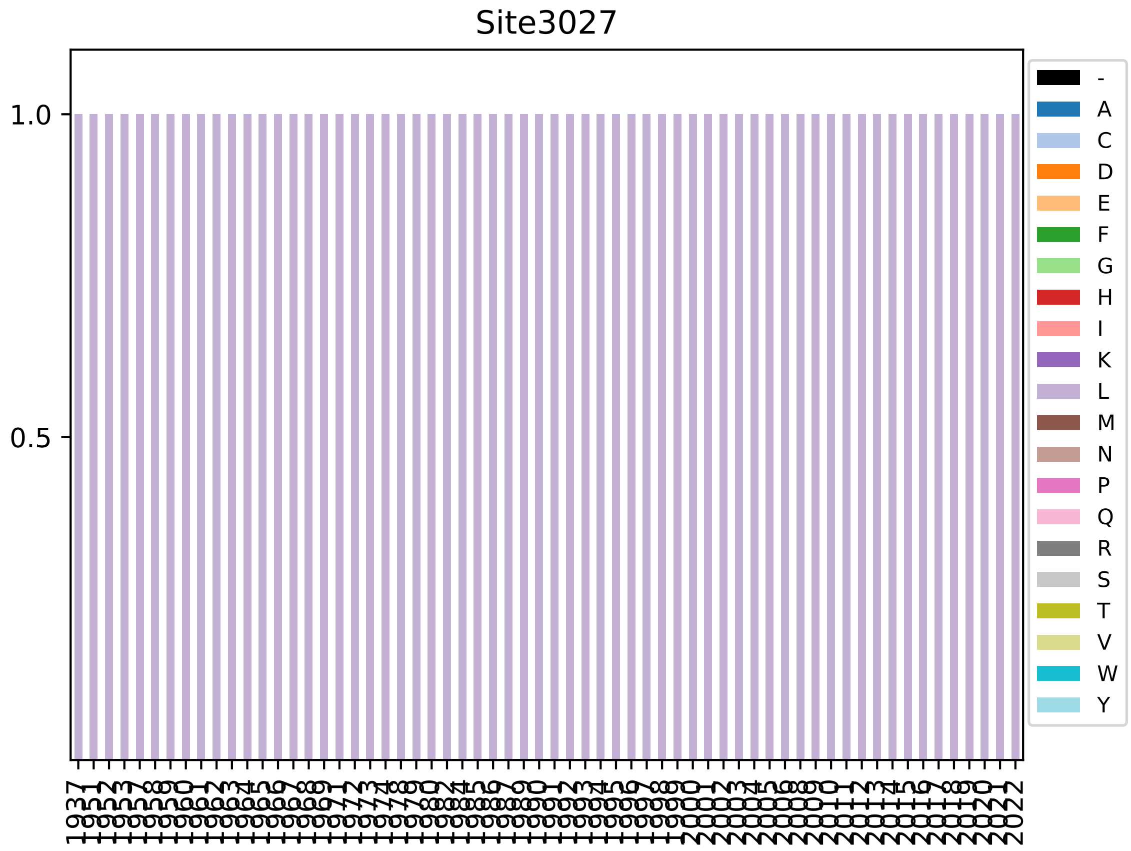
Site3027
1.0
0.5
-
A
C
D
E
F
G
H
I
K
L
M
N
P
Q
R
S
T
V
W
Y

Site3028

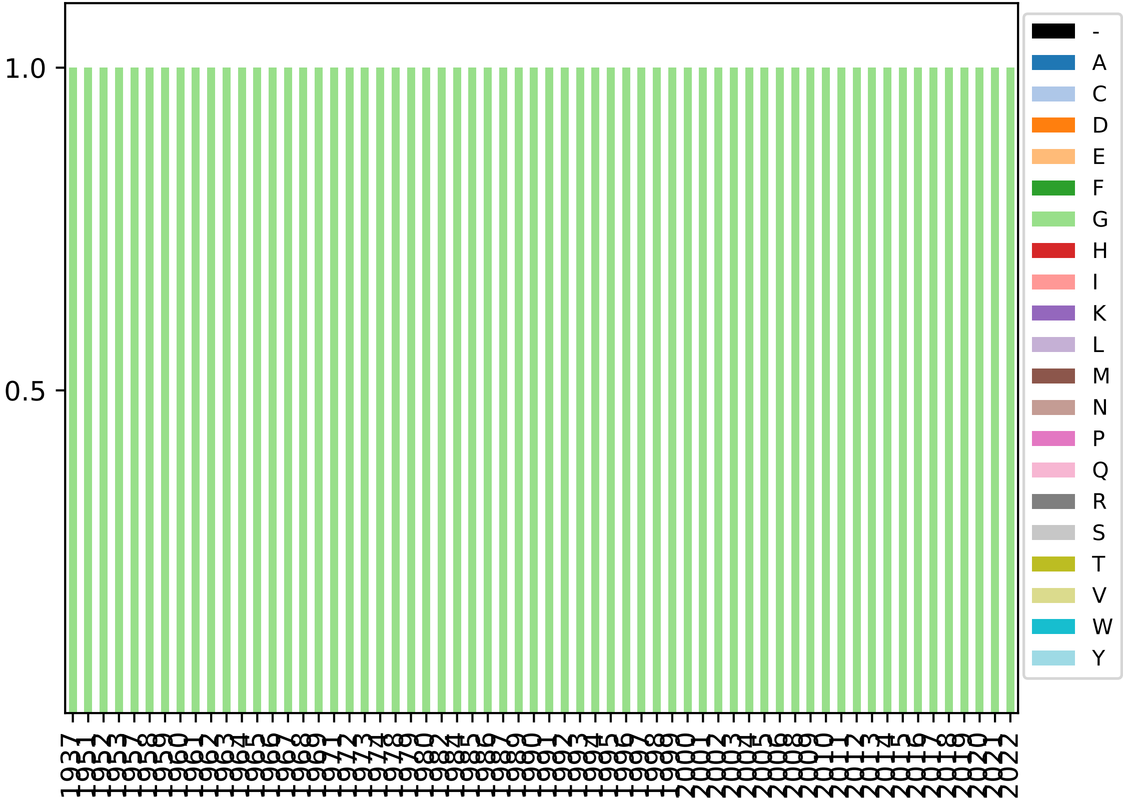

Site3029

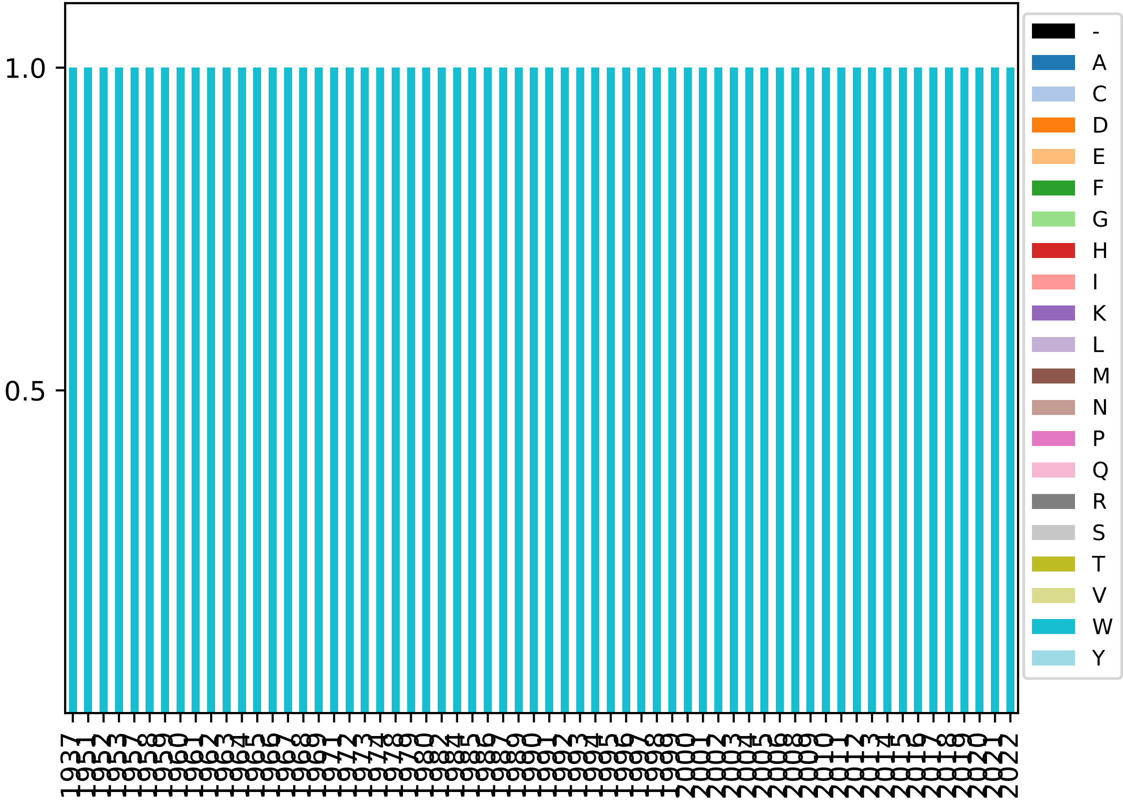

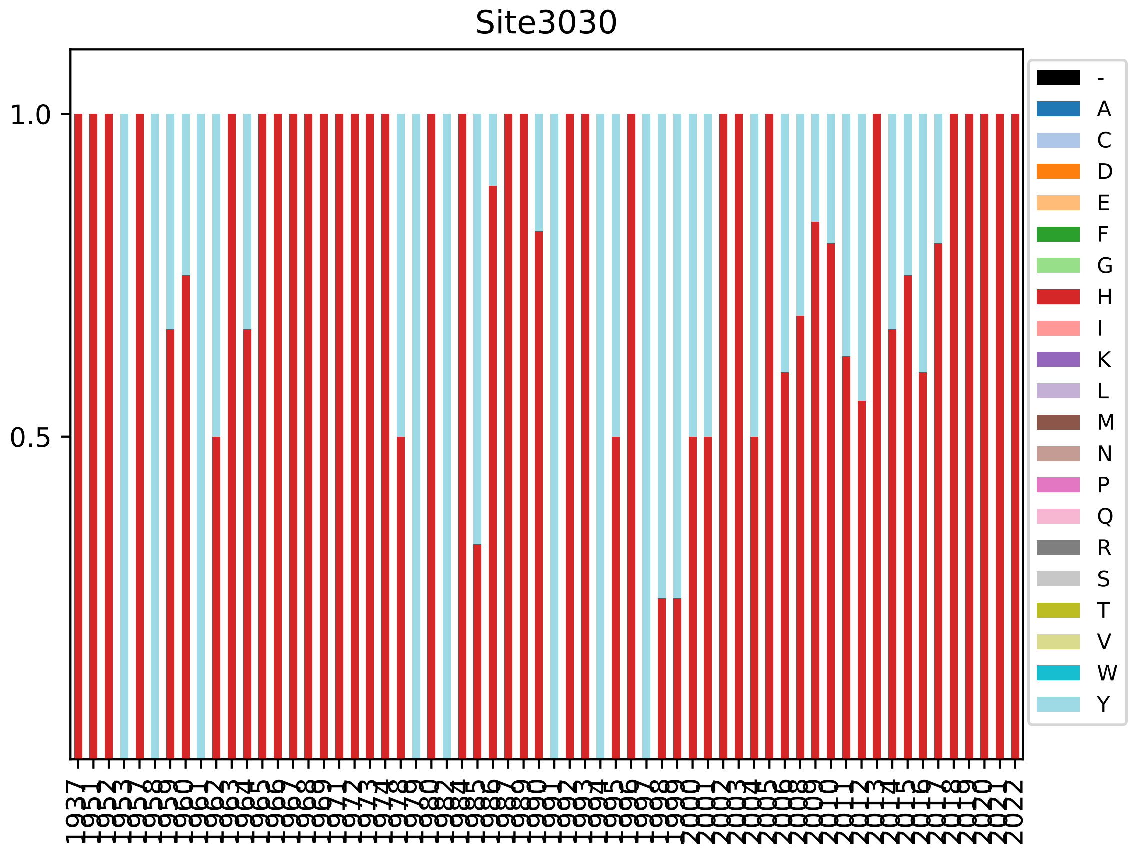

Site3030
1.0
0.5
-
A
C
D
E
F
G
H
I
K
L
M
N
P
Q
R
S
T
V
W
Y

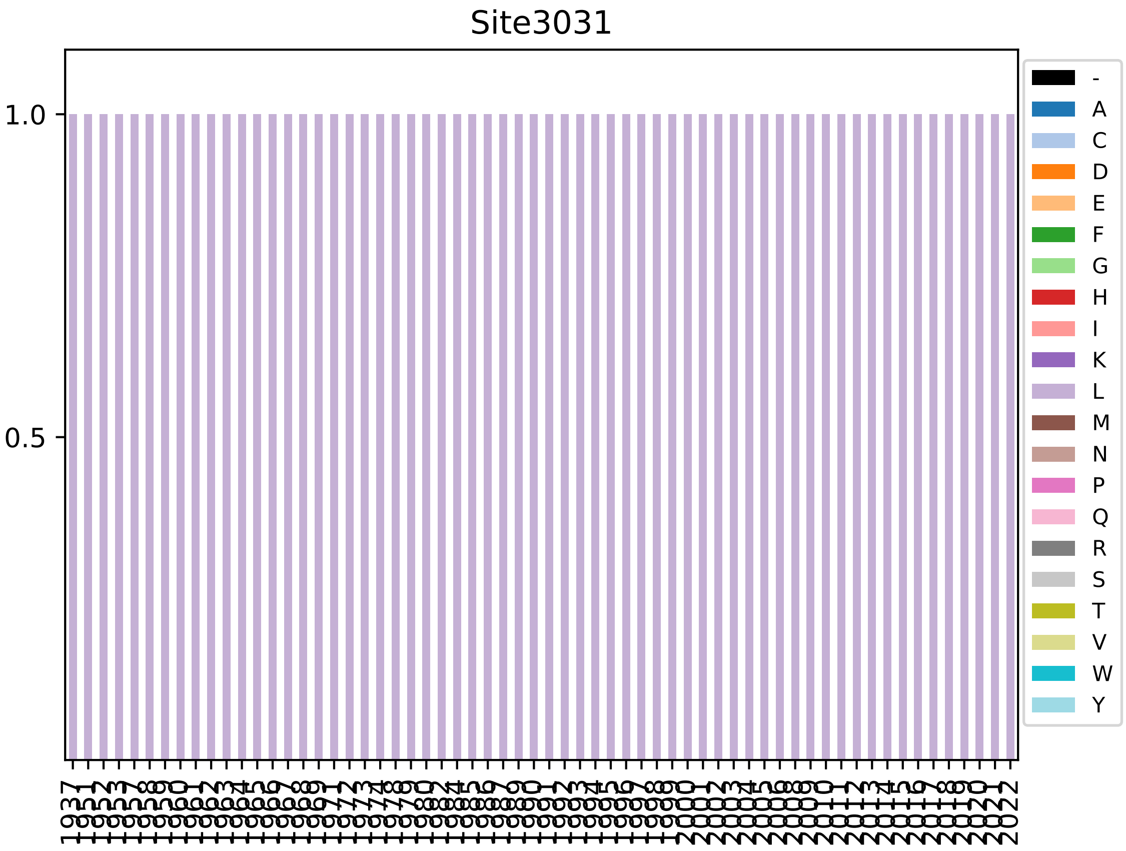
Site3031
1.0
0.5
-
A
C
D
E
F
G
H
I
K
L
M
N
P
Q
R
S
T
V
W
Y

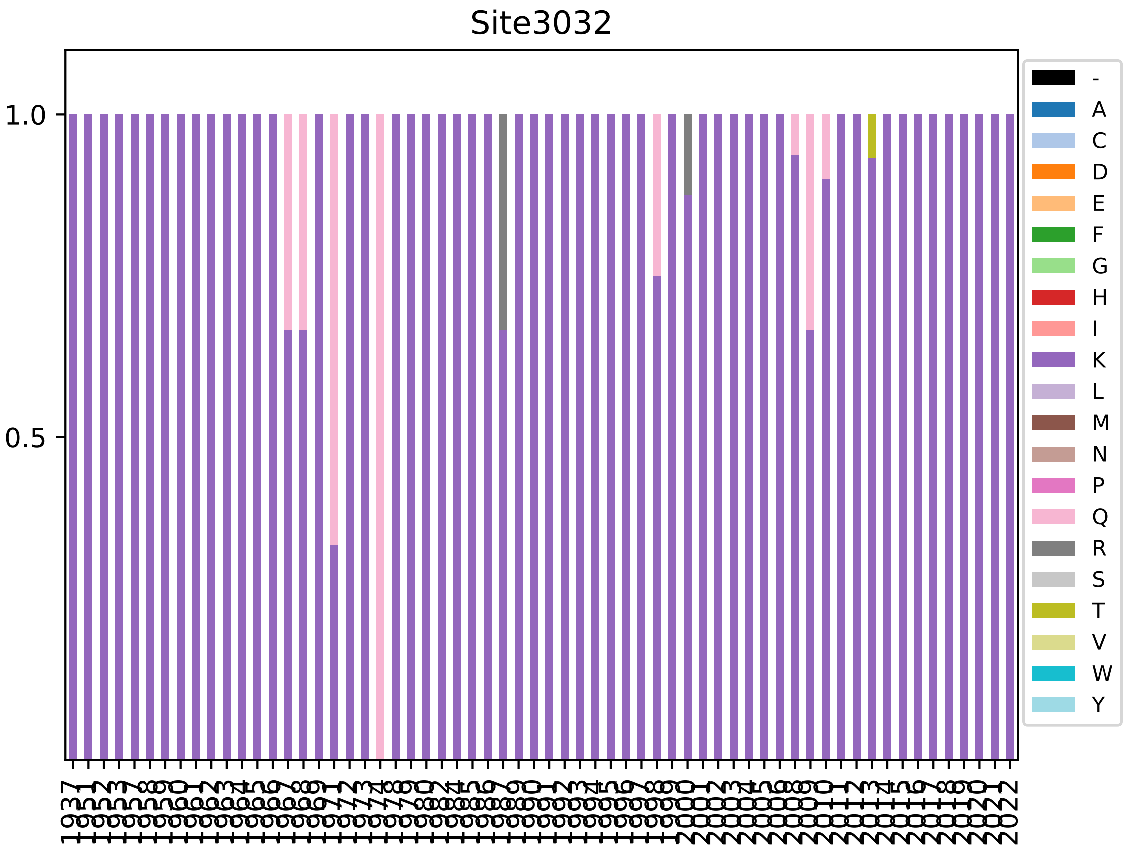
Site3032
1.0
0.5
-
A
C
D
E
F
G
H
I
K
L
M
N
P
Q
R
S
T
V
W
Y

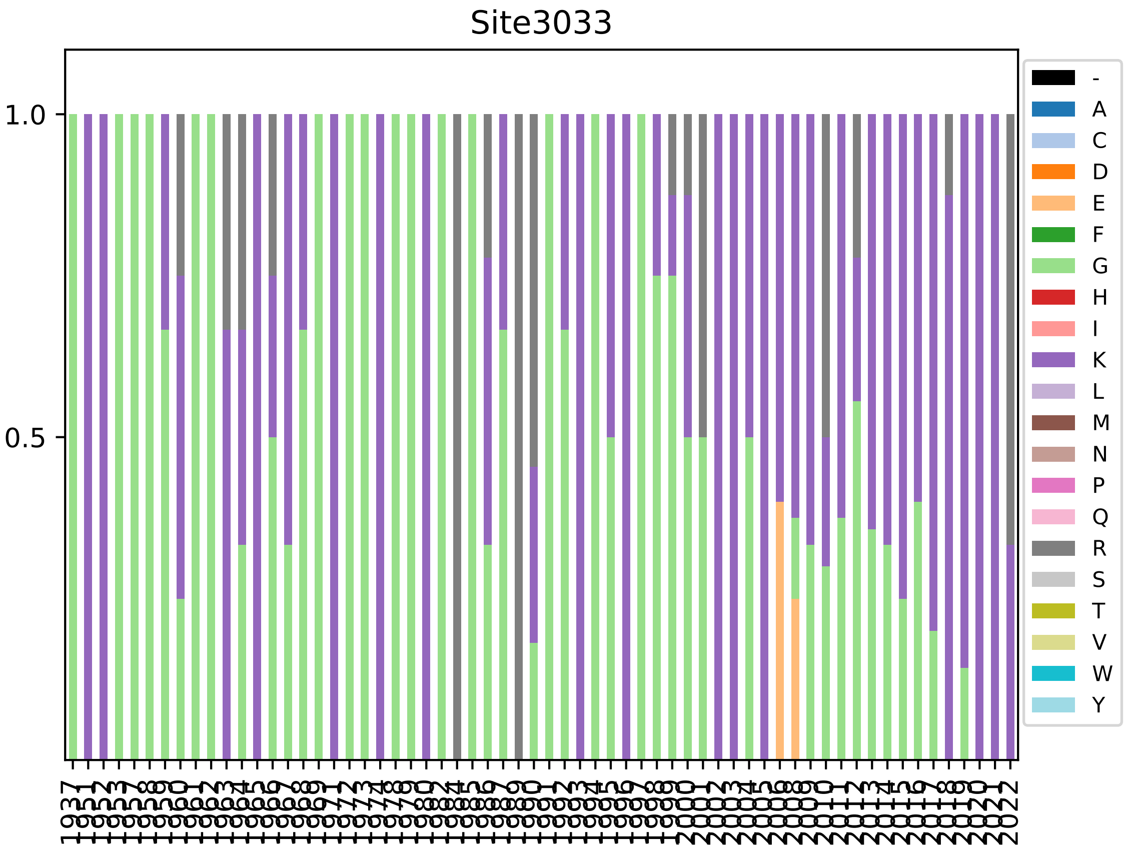

Site3033
1.0
0.5
-
A
C
D
E
F
G
H
I
K
L
M
N
P
Q
R
S
T
V
W
Y

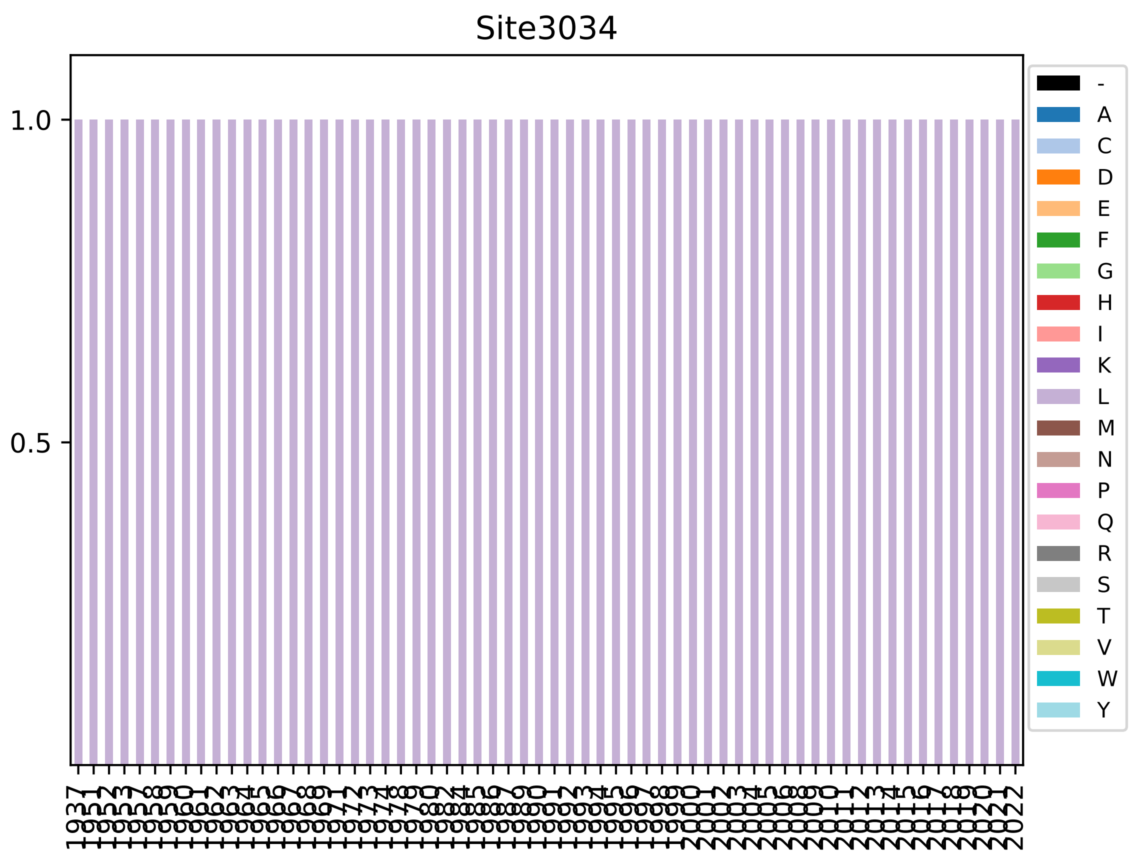
Site3034
1.0
0.5
-
A
C
D
E
F
G
H
I
K
L
M
N
P
Q
R
S
T
V
W
Y

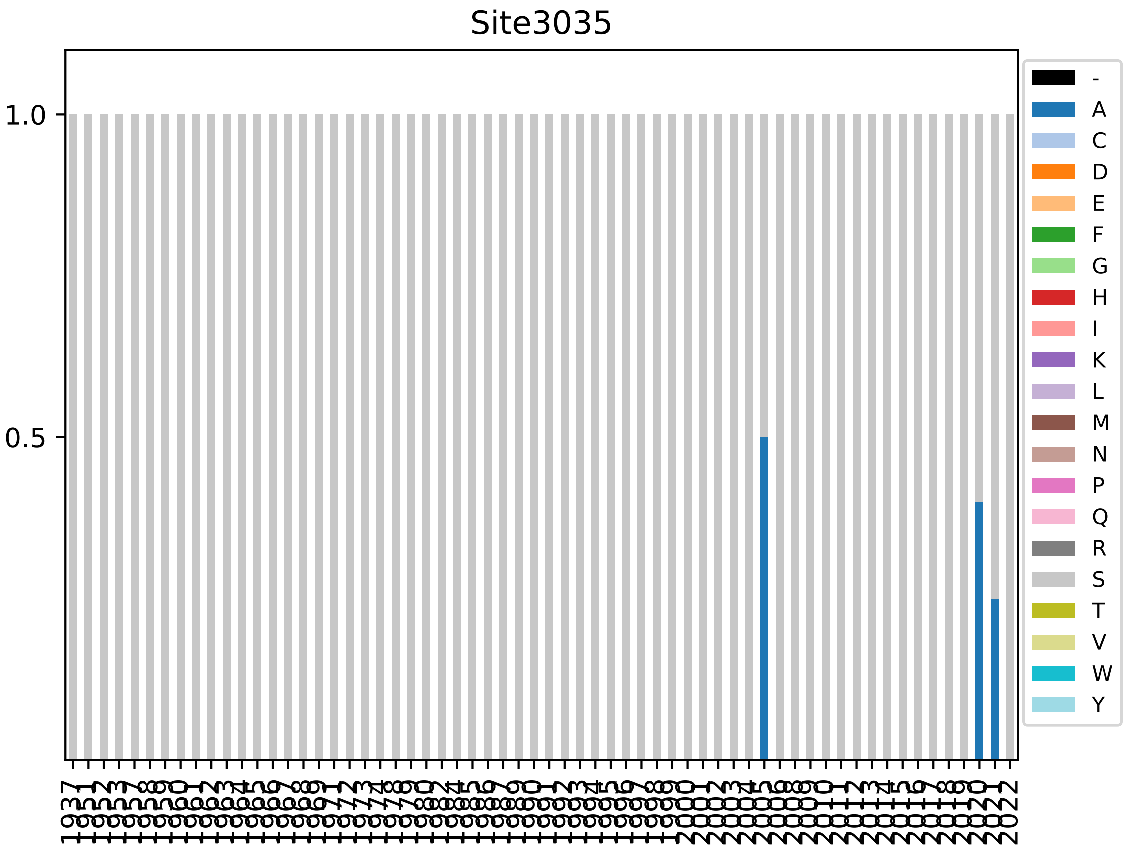

Site3035
1.0
0.5
-
A
C
D
E
F
G
H
I
K
L
M
N
P
Q
R
S
T
V
W
Y

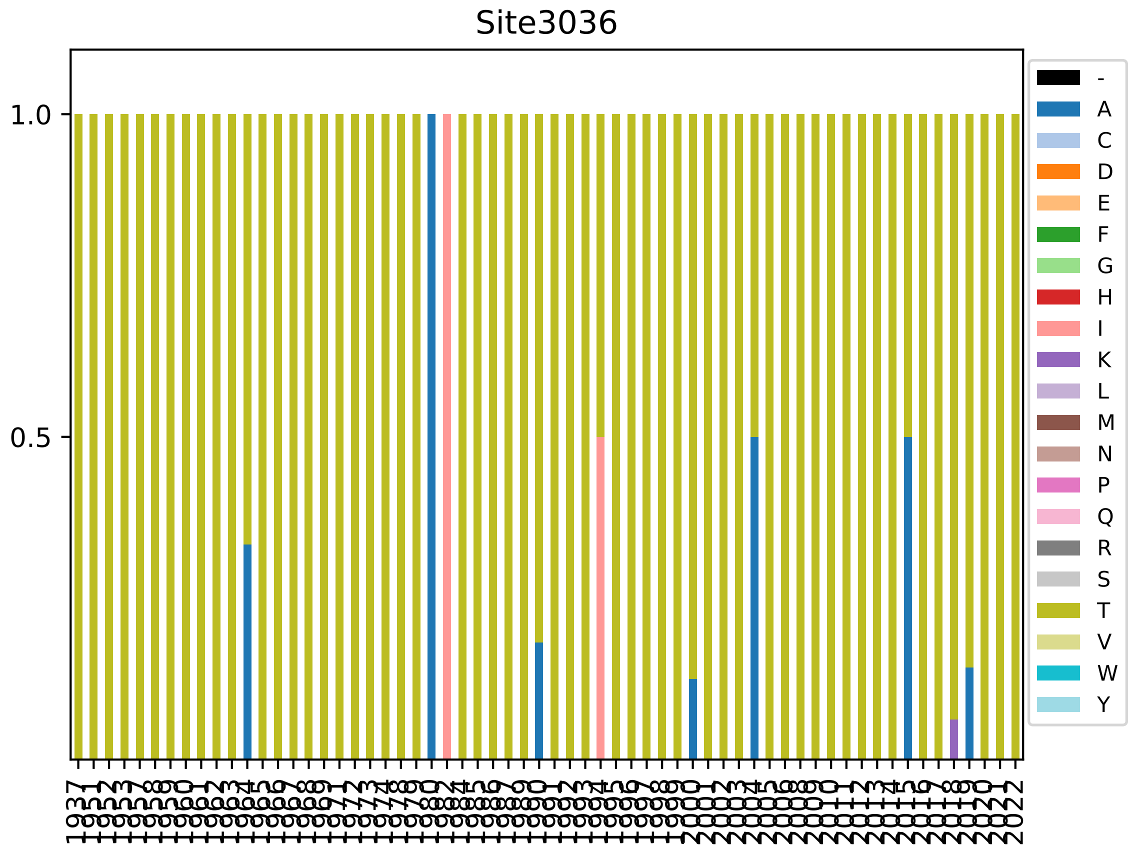
Site3036
1.0
0.5
-
A
C
D
E
F
G
H
I
K
L
M
N
P
Q
R
S
T
V
W
Y

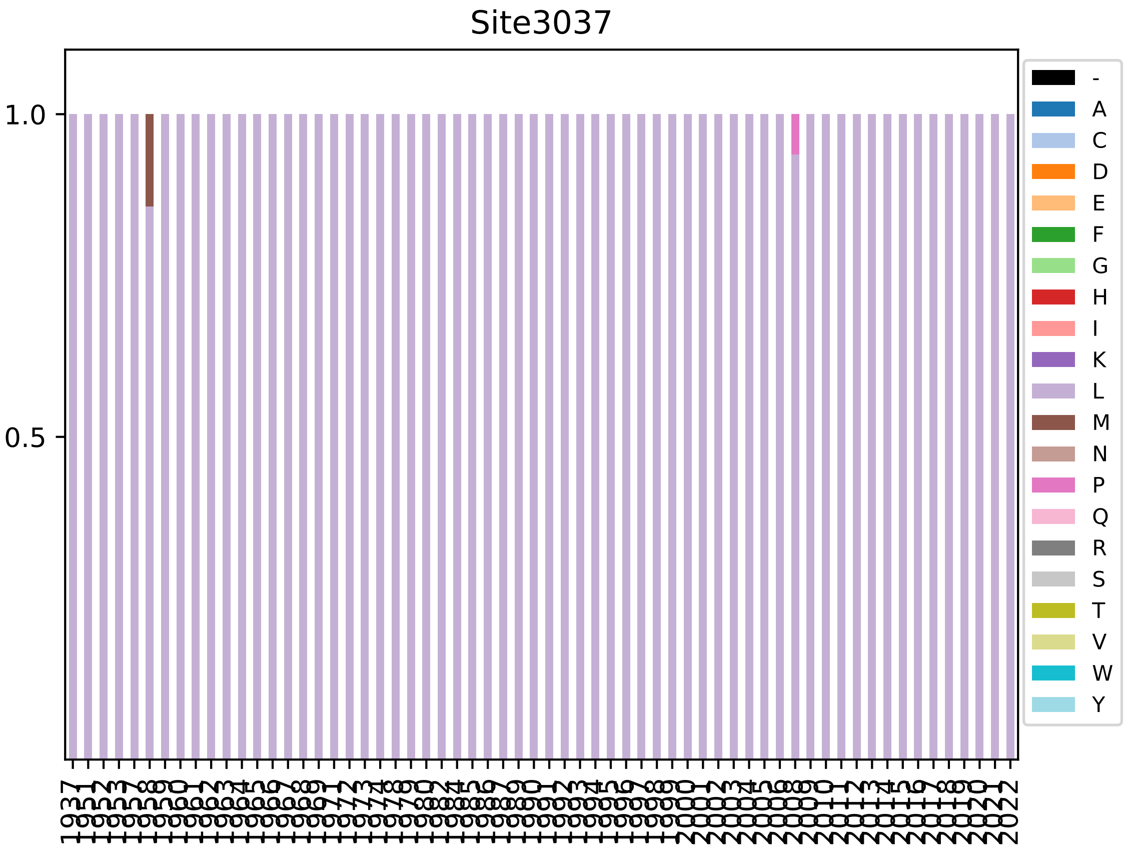
Site3037
1.0
0.5
-
A
C
D
E
F
G
H
I
K
L
M
N
P
Q
R
S
T
V
W
Y

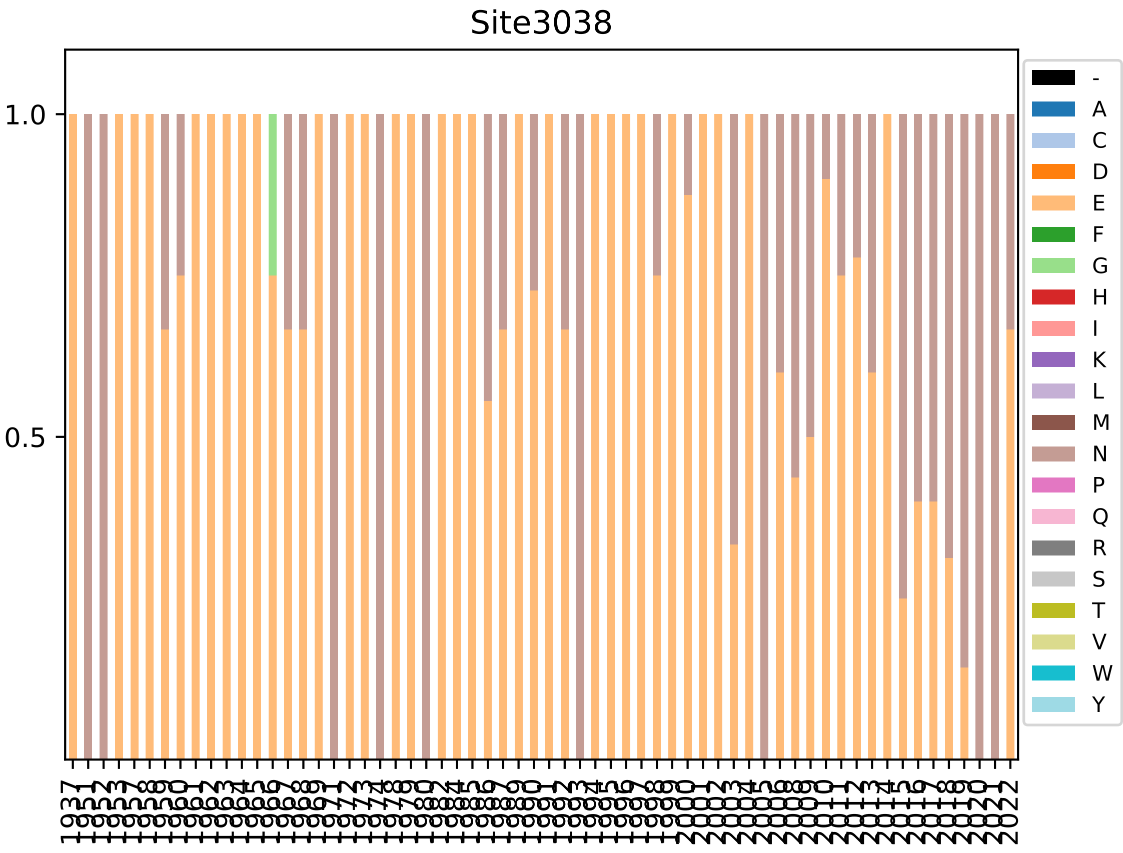
Site3038
1.0
0.5
-
A
C
D
E
F
G
H
I
K
L
M
N
P
Q
R
S
T
V
W
Y

Site3039

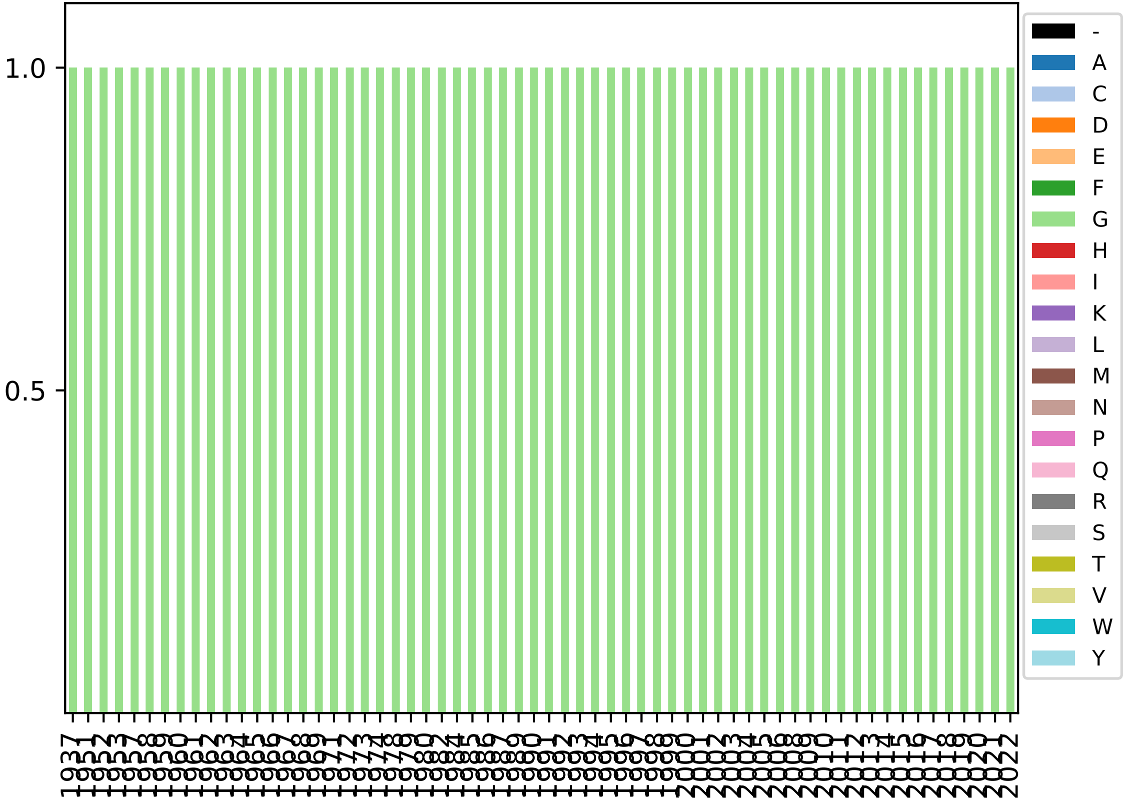

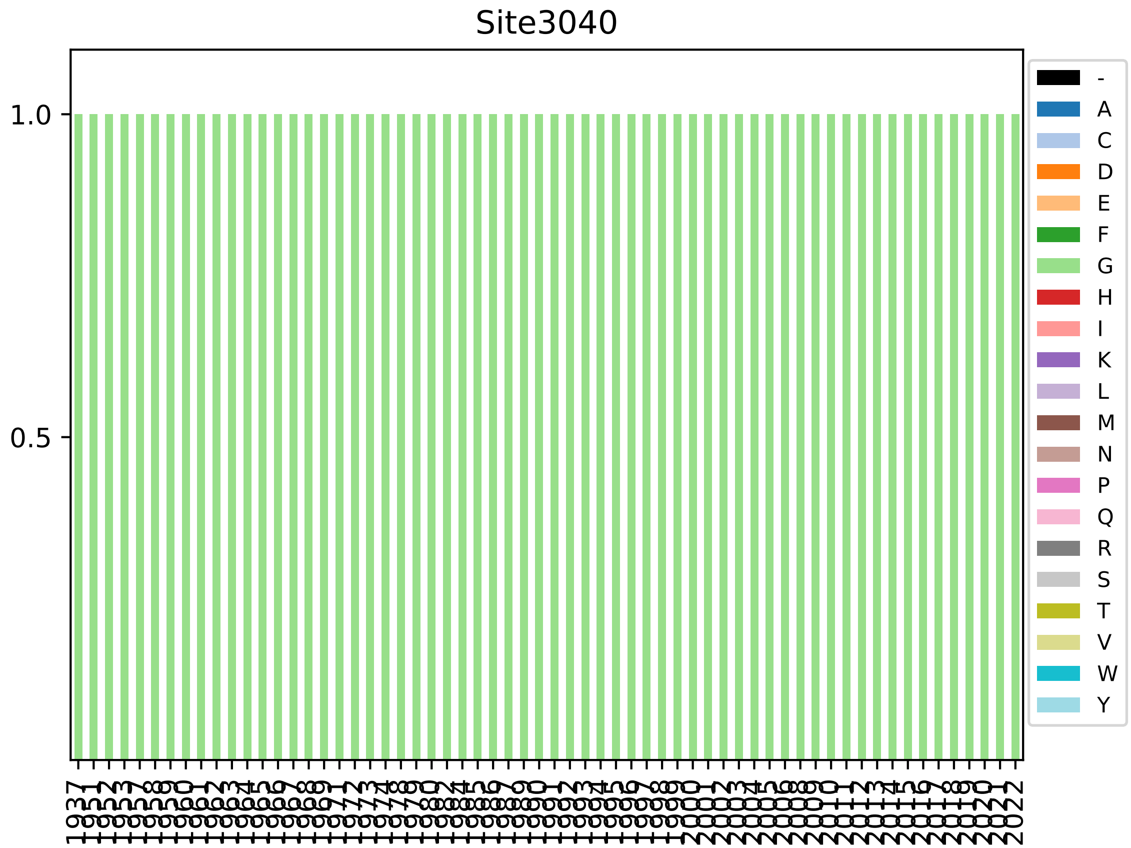
Site3040
1.0
0.5
-
A
C
D
E
F
G
H
I
K
L
M
N
P
Q
R
S
T
V
W
Y

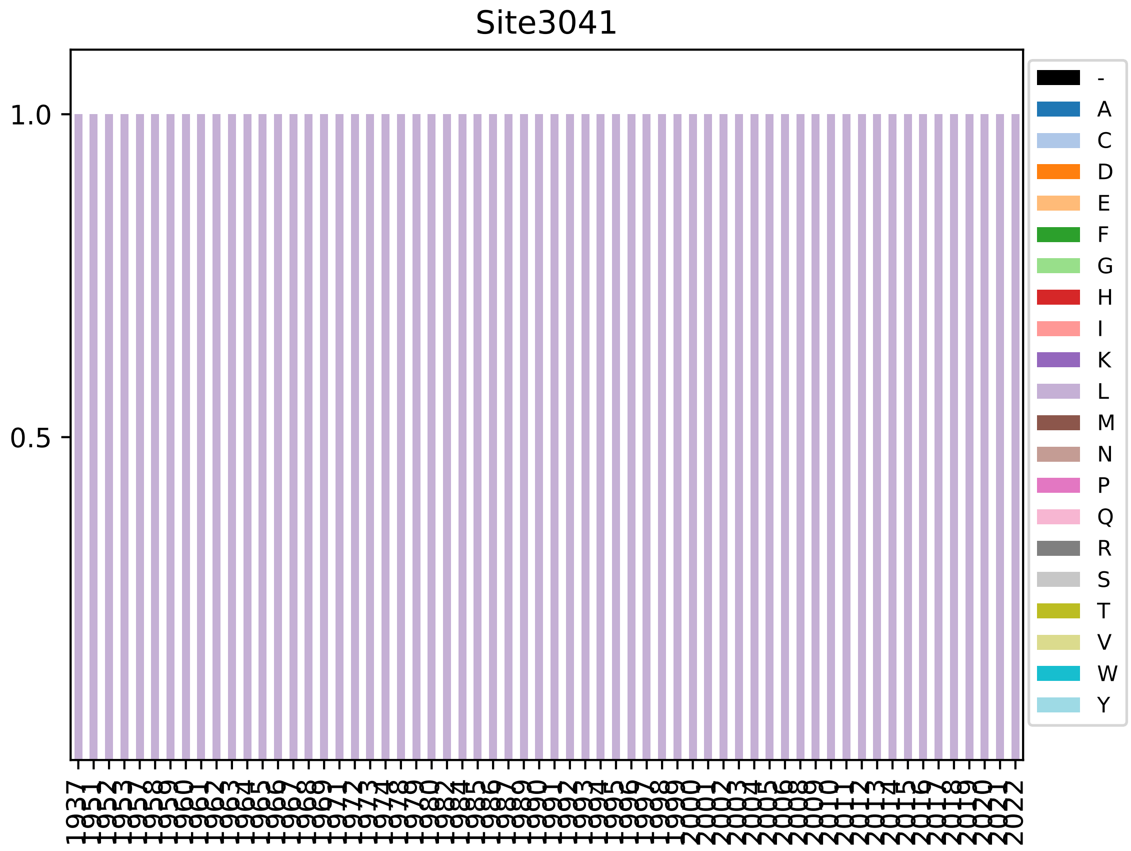
Site3041
1.0
0.5
-
A
C
D
E
F
G
H
I
K
L
M
N
P
Q
R
S
T
V
W
Y

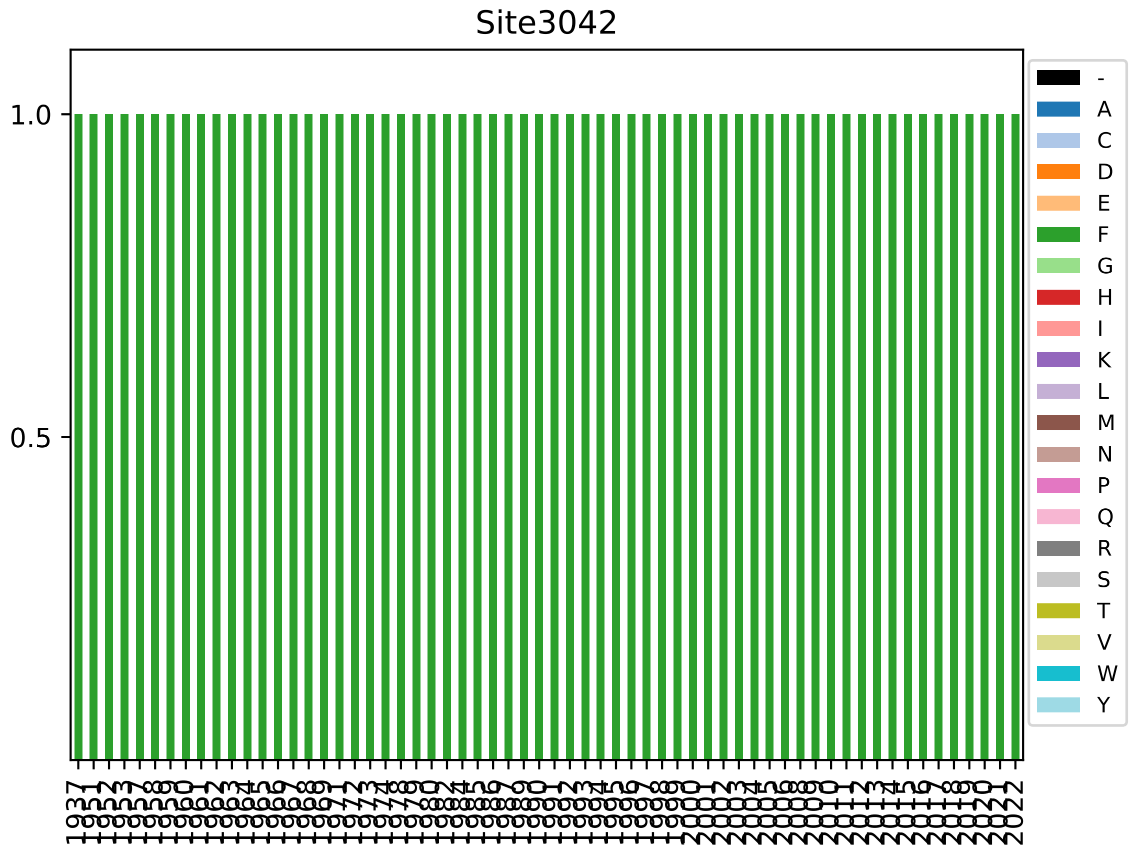
Site3042
1.0
0.5
-
A
C
D
E
F
G
H
I
K
L
M
N
P
Q
R
S
T
V
W
Y

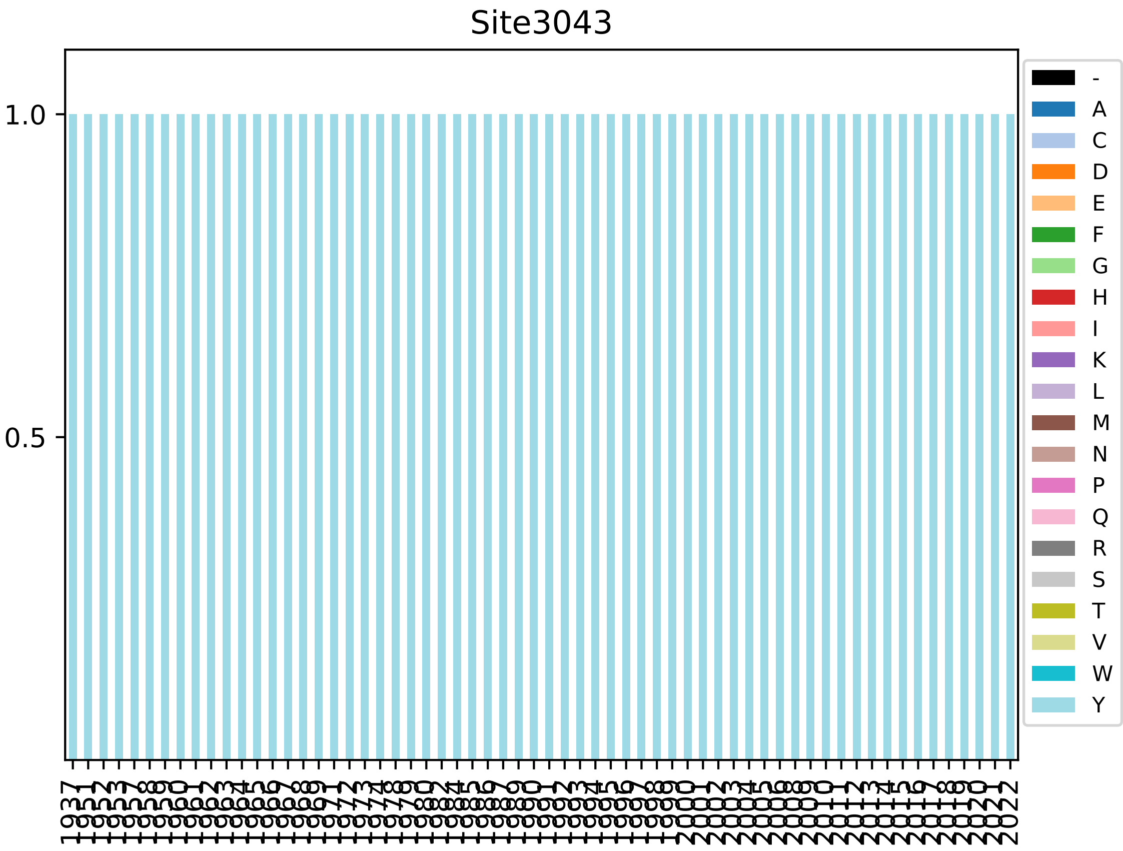
Site3043
1.0
0.5
-
A
C
D
E
F
G
H
I
K
L
M
N
P
Q
R
S
T
V
W
Y

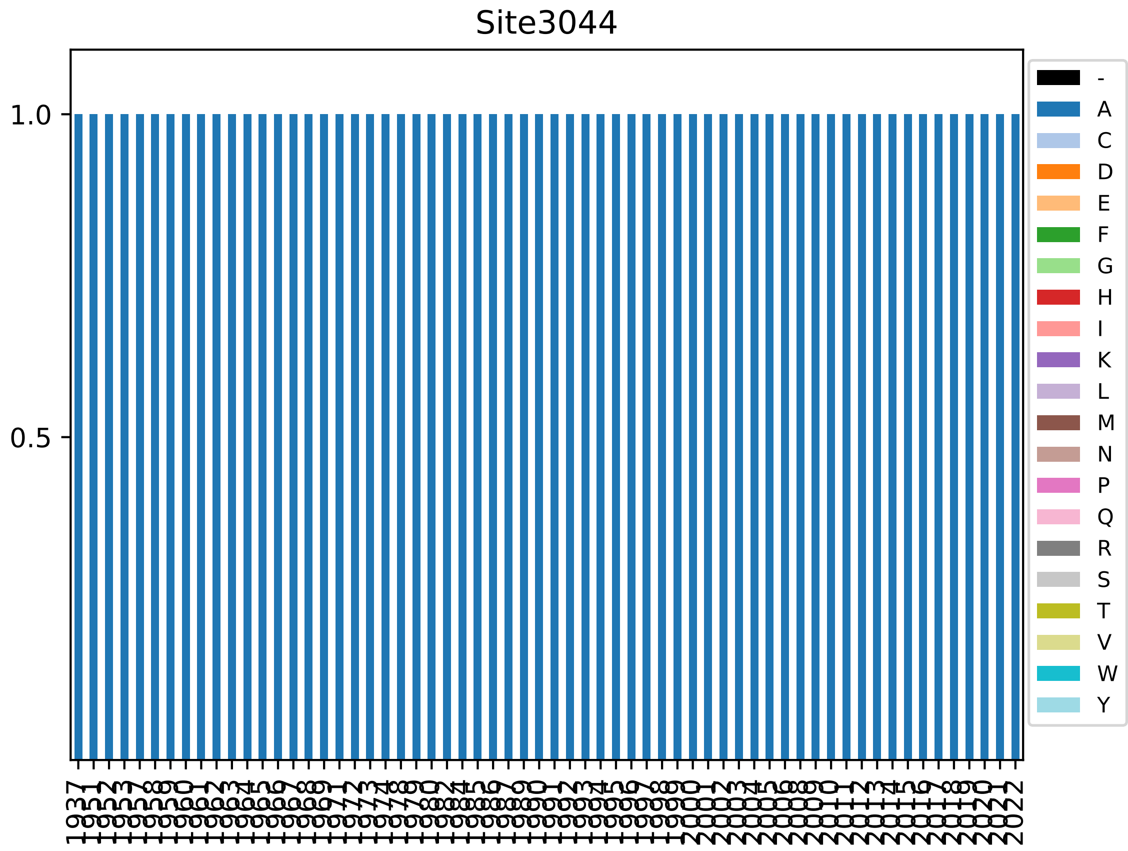
Site3044
1.0
0.5
-
A
C
D
E
F
G
H
I
K
L
M
N
P
Q
R
S
T
V
W
Y

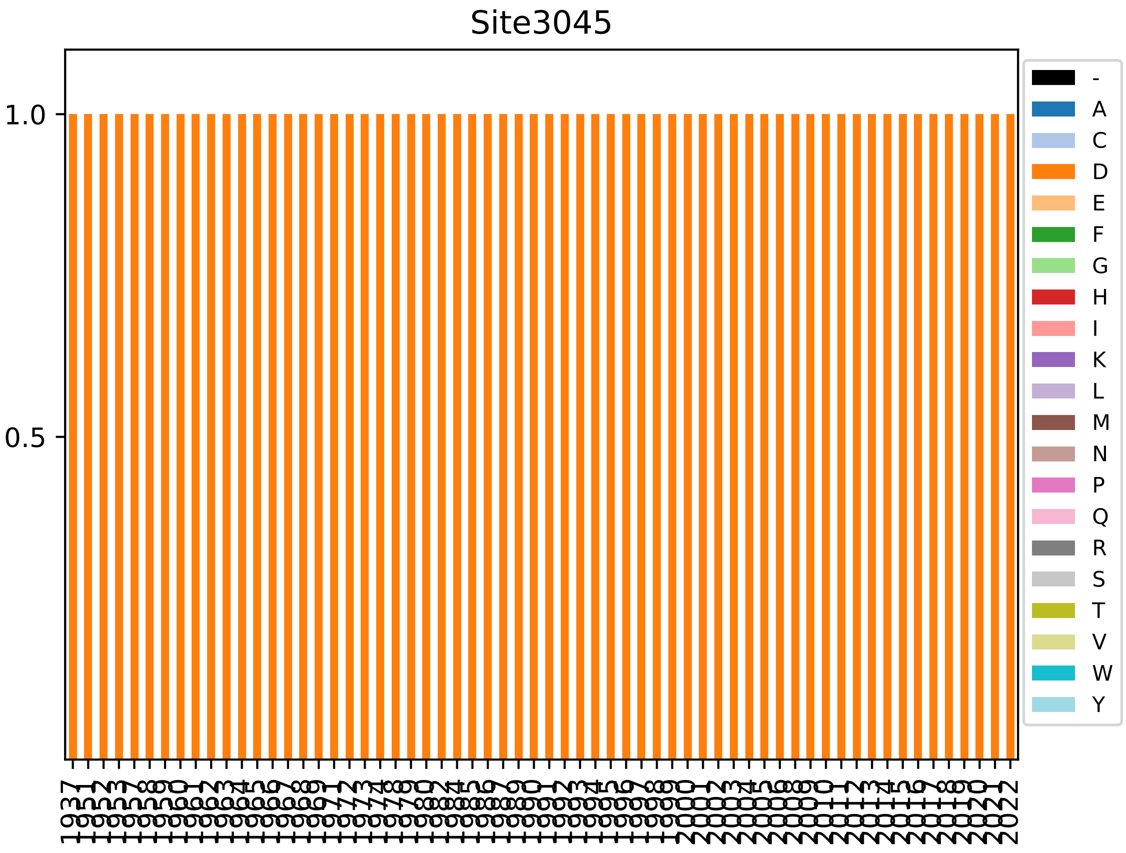
Site3045
1.0
0.5
-
A
C
D
E
F
G
H
I
K
L
M
N
P
Q
R
S
T
V
W
Y

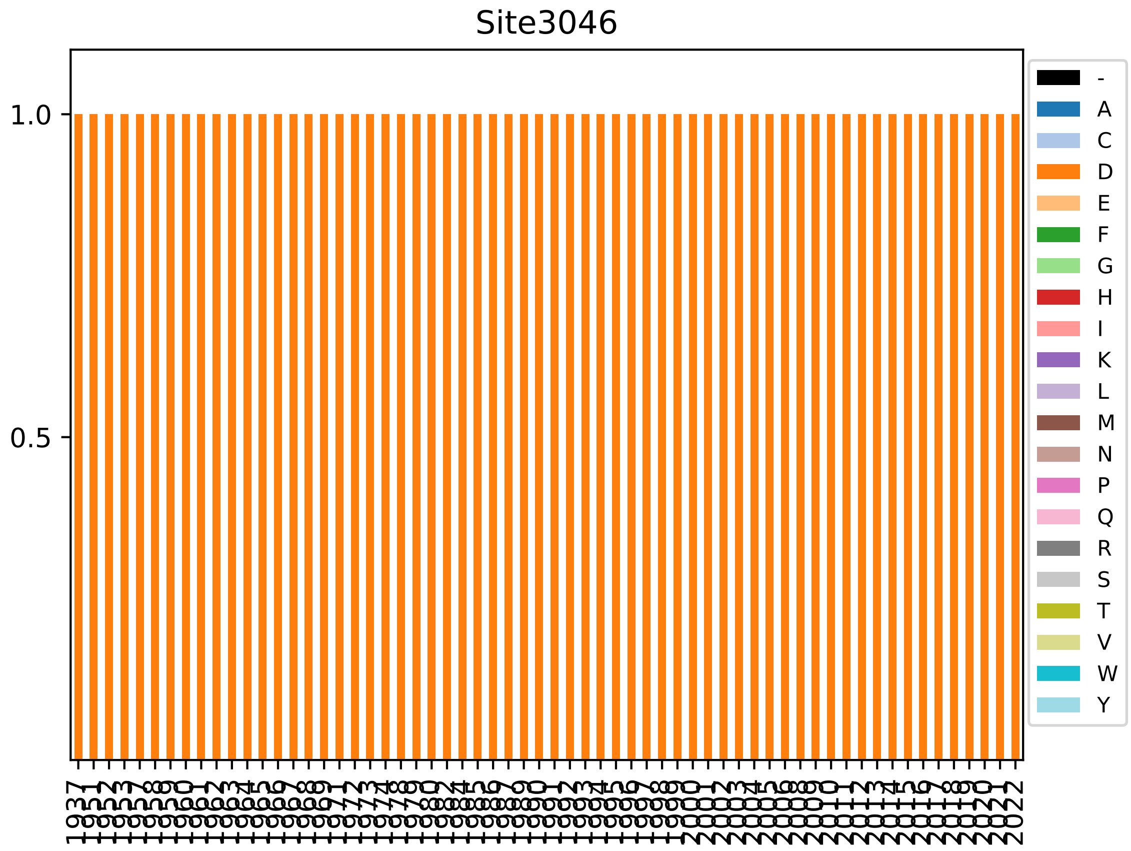
Site3046
1.0
0.5
-
A
C
D
E
F
G
H
I
K
L
M
N
P
Q
R
S
T
V
W
Y

Site3047

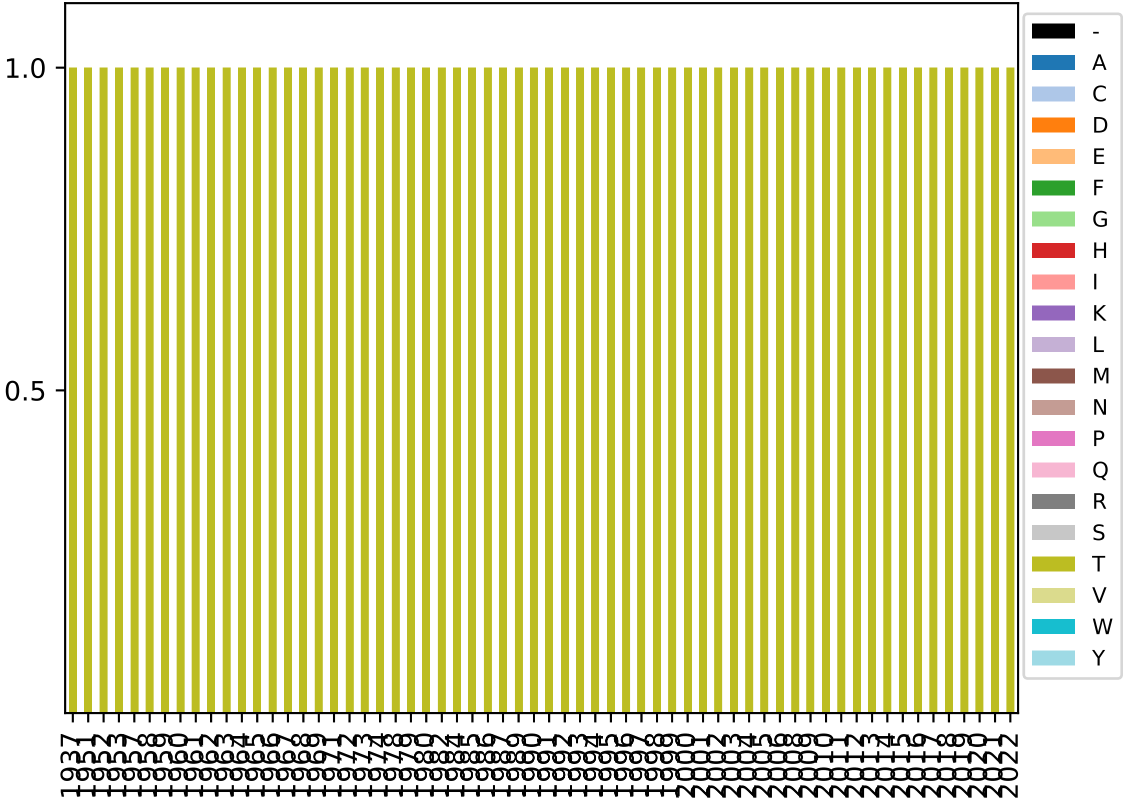

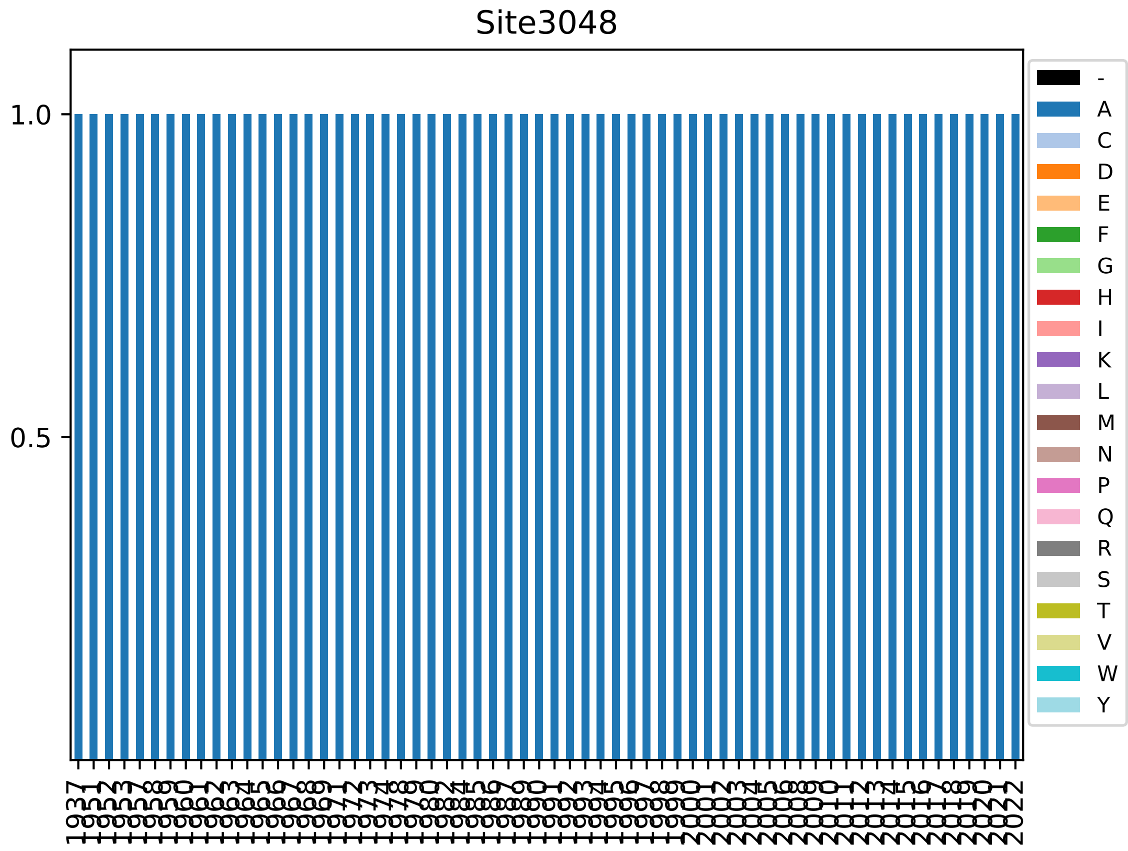
Site3048
1.0
0.5
-
A
C
D
E
F
G
H
I
K
L
M
N
P
Q
R
S
T
V
W
Y

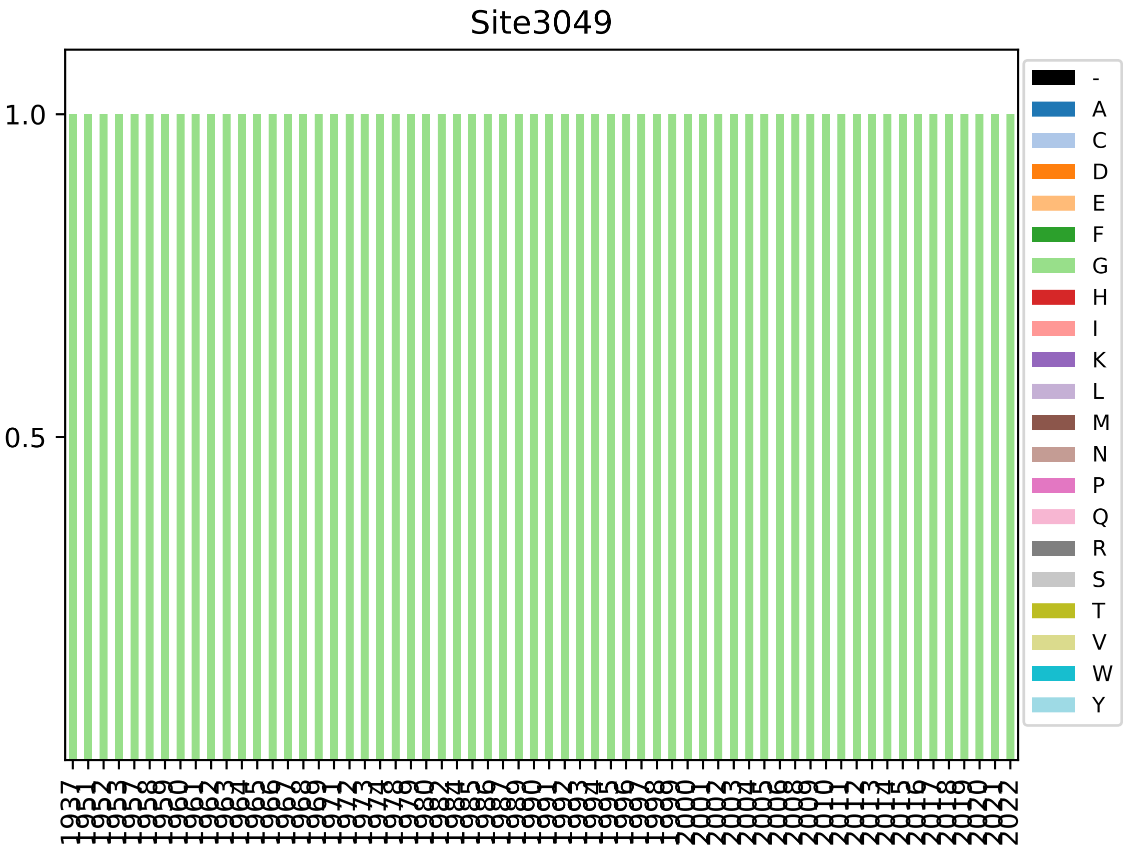
Site3049
1.0
0.5
-
A
C
D
E
F
G
H
I
K
L
M
N
P
Q
R
S
T
V
W
Y

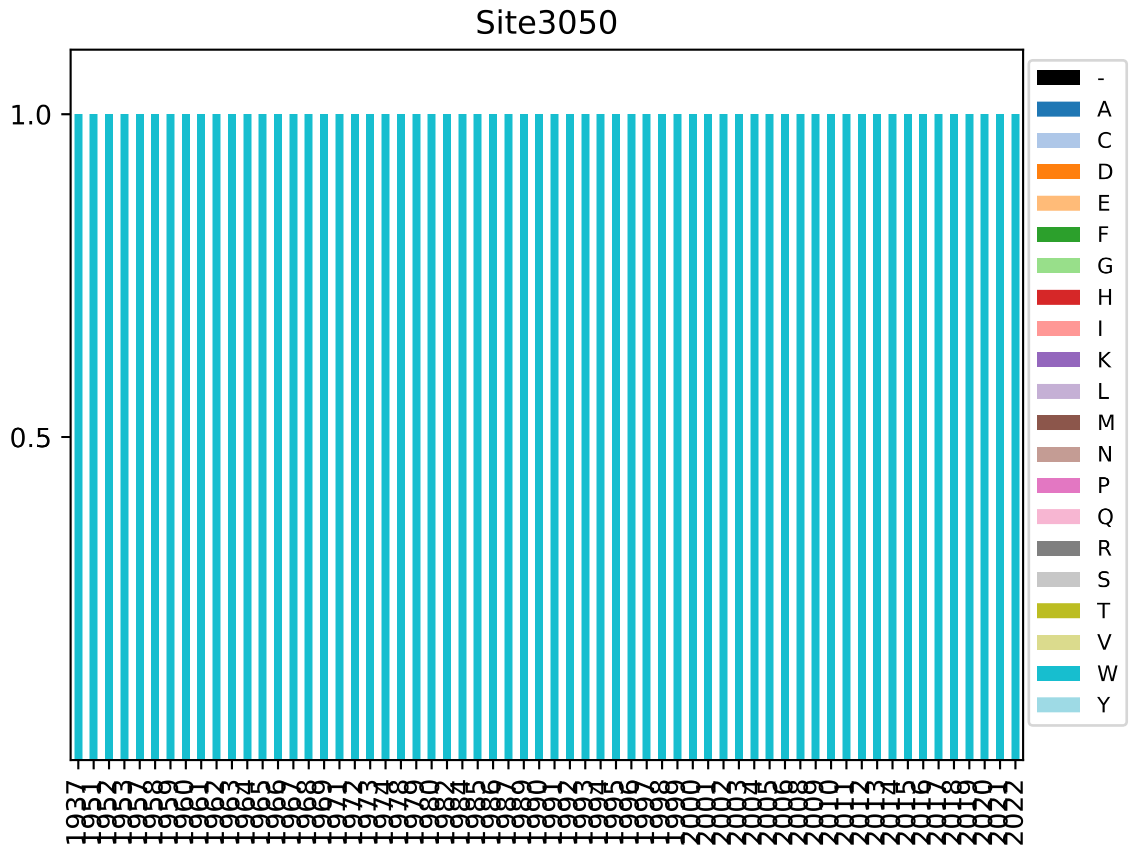
Site3050
1.0
0.5
-
A
C
D
E
F
G
H
I
K
L
M
N
P
Q
R
S
T
V
W
Y

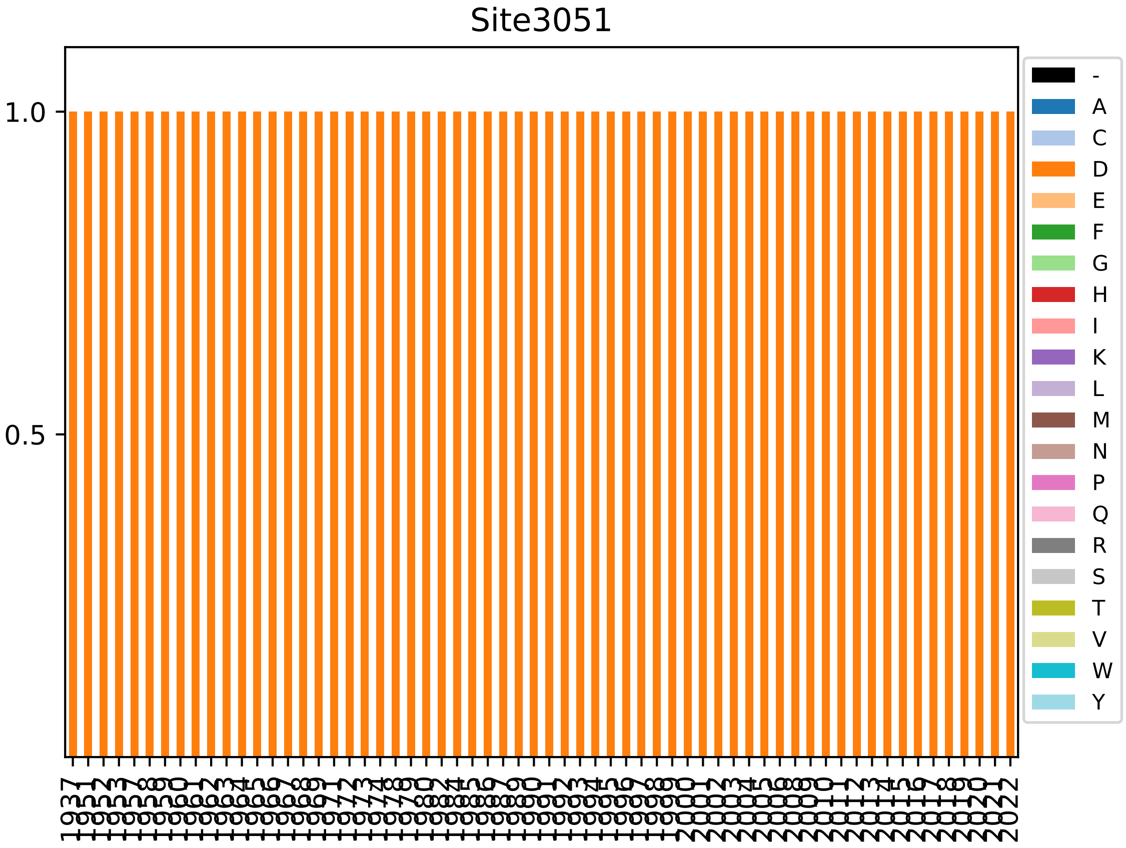
Site3051
1.0
0.5
-
A
C
D
E
F
G
H
I
K
L
M
N
P
Q
R
S
T
V
W
Y

Site3052

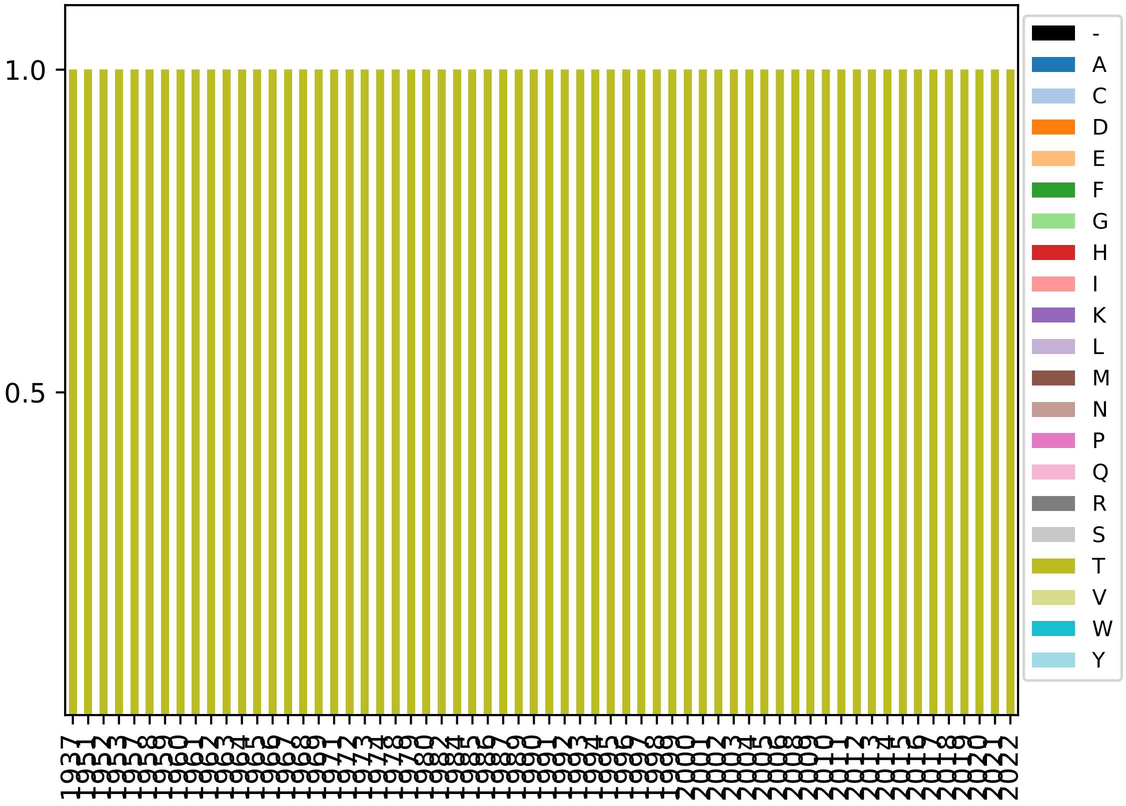

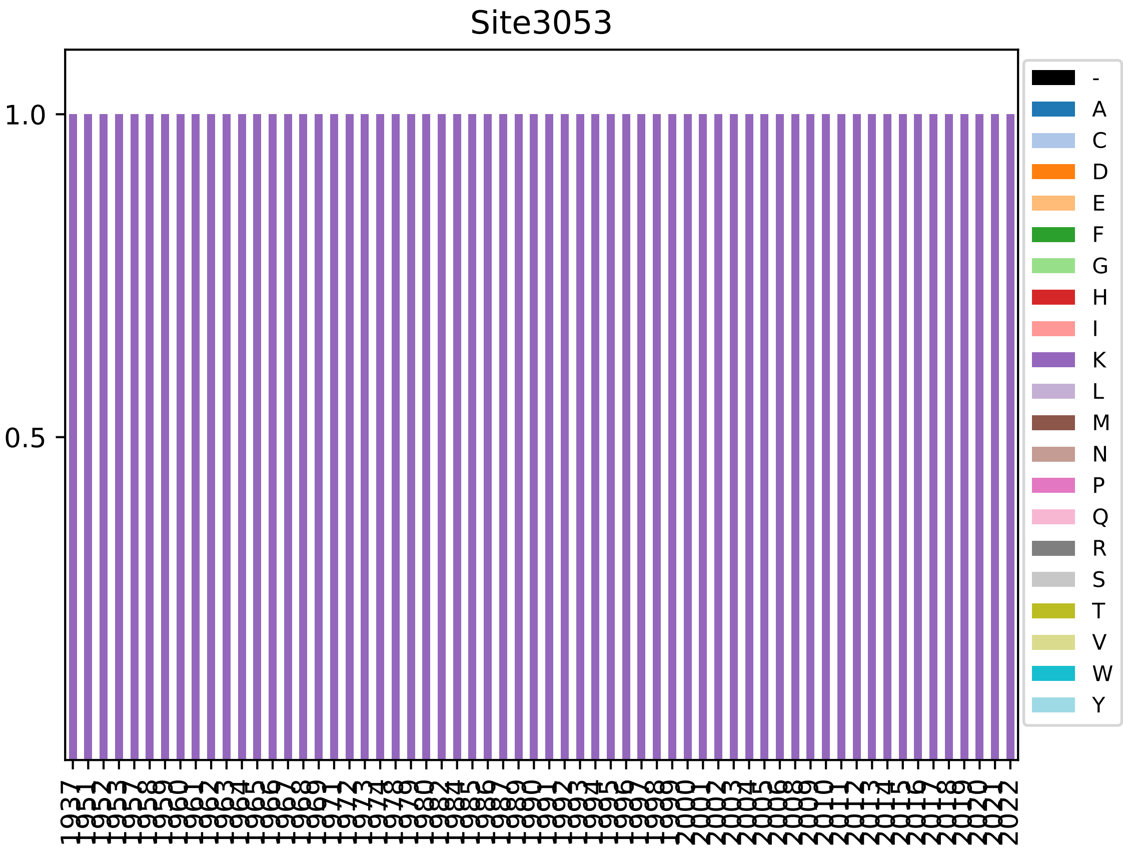

Site3053
1.0
0.5
-
A
C
D
E
F
G
H
I
K
L
M
N
P
Q
R
S
T
V
W
Y

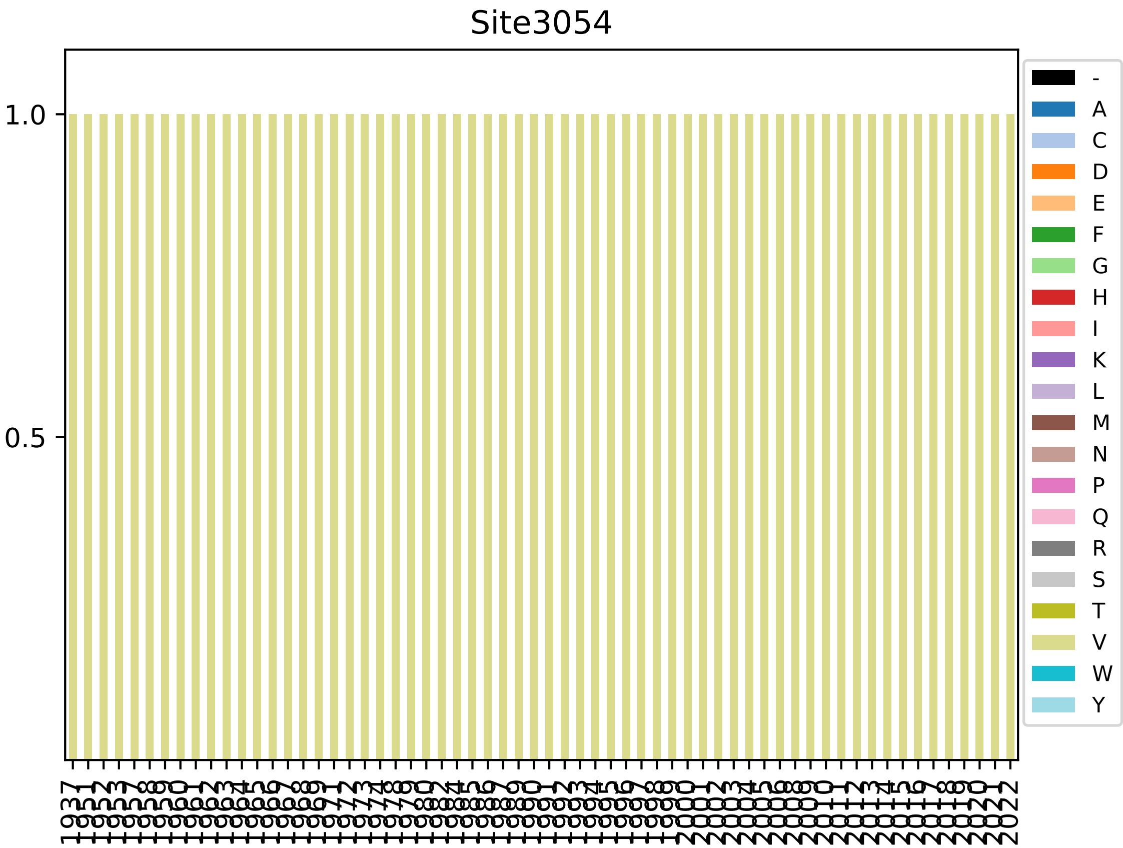
Site3054
1.0
0.5
-
A
C
D
E
F
G
H
I
K
L
M
N
P
Q
R
S
T
V
W
Y

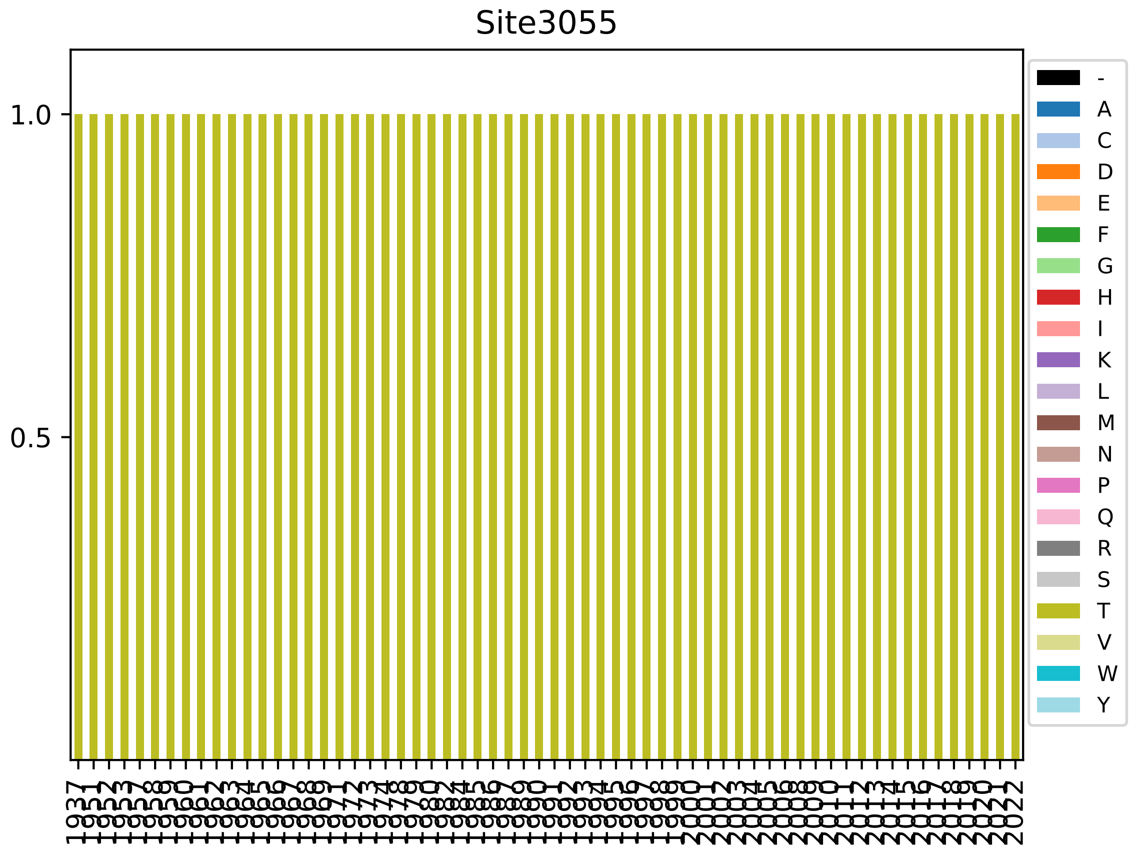
Site3055
1.0
0.5
-
A
C
D
E
F
G
H
I
K
L
M
N
P
Q
R
S
T
V
W
Y

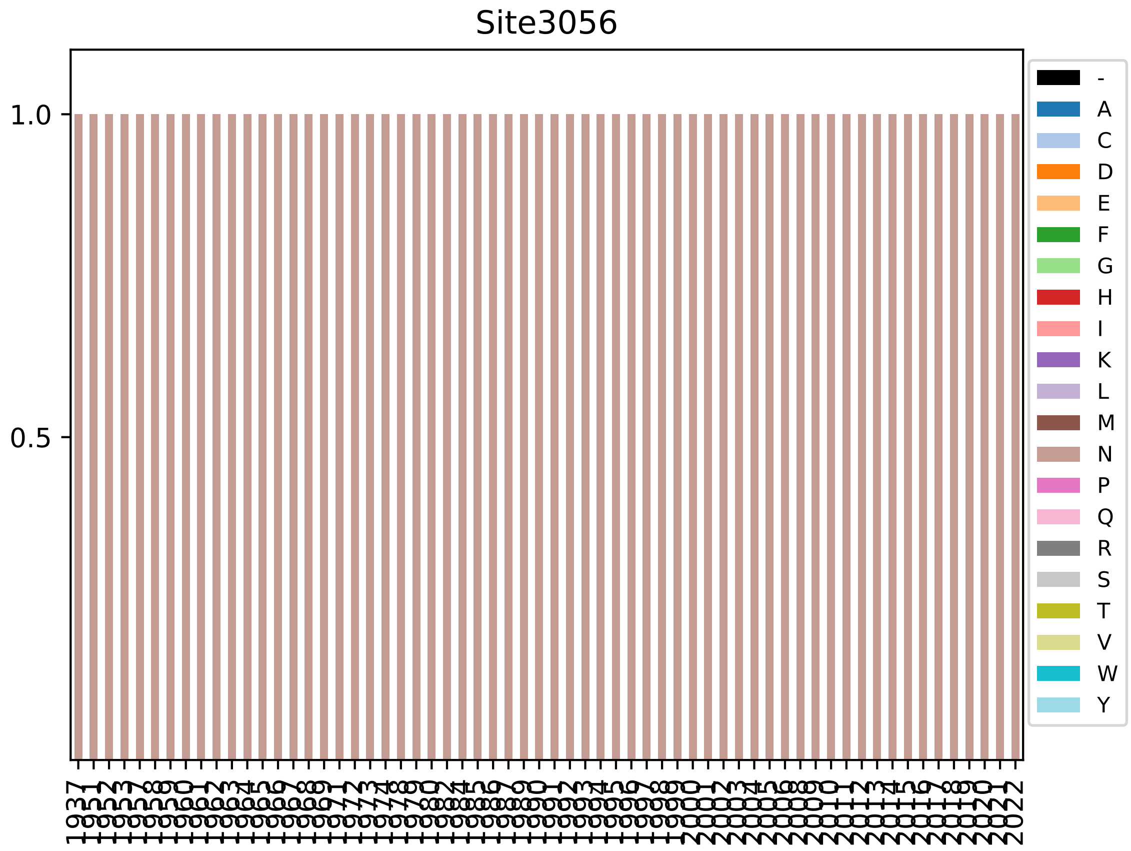
Site3056
1.0
0.5
-
A
C
D
E
F
G
H
I
K
L
M
N
P
Q
R
S
T
V
W
Y

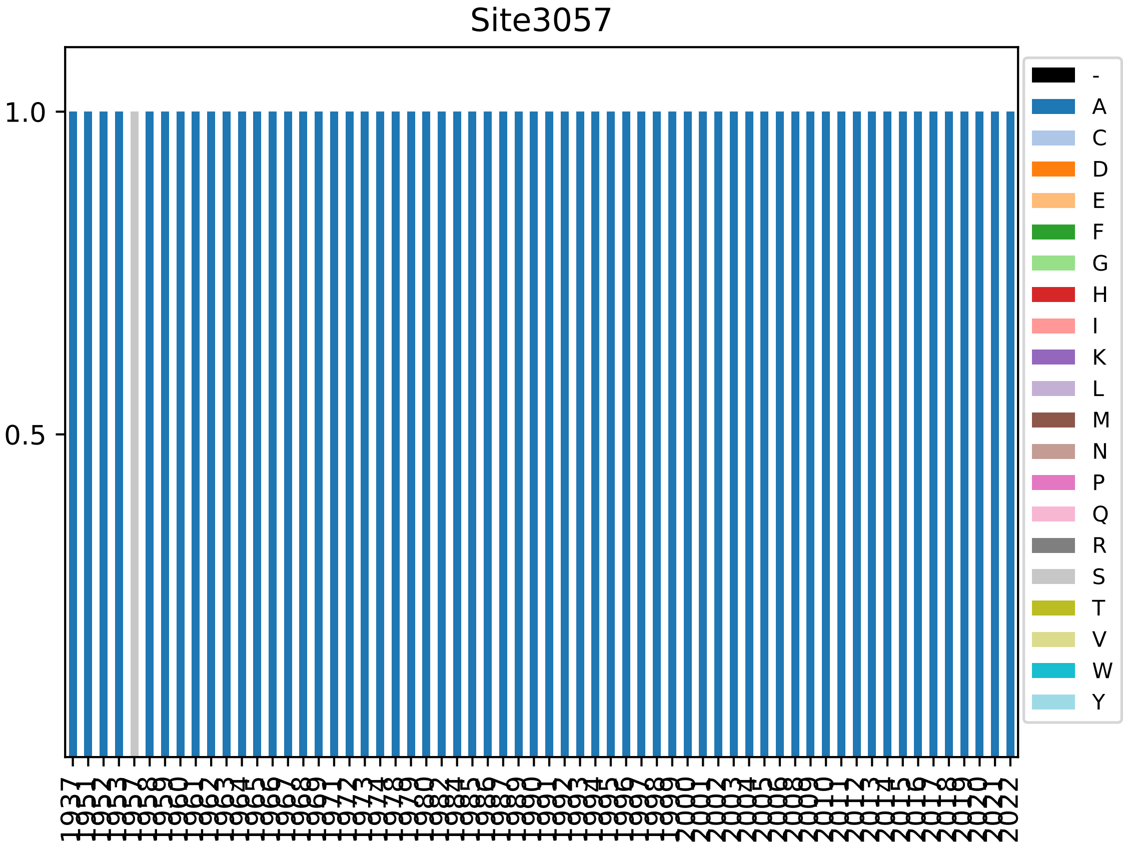
Site3057
1.0
0.5
-
A
C
D
E
F
G
H
I
K
L
M
N
P
Q
R
S
T
V
W
Y

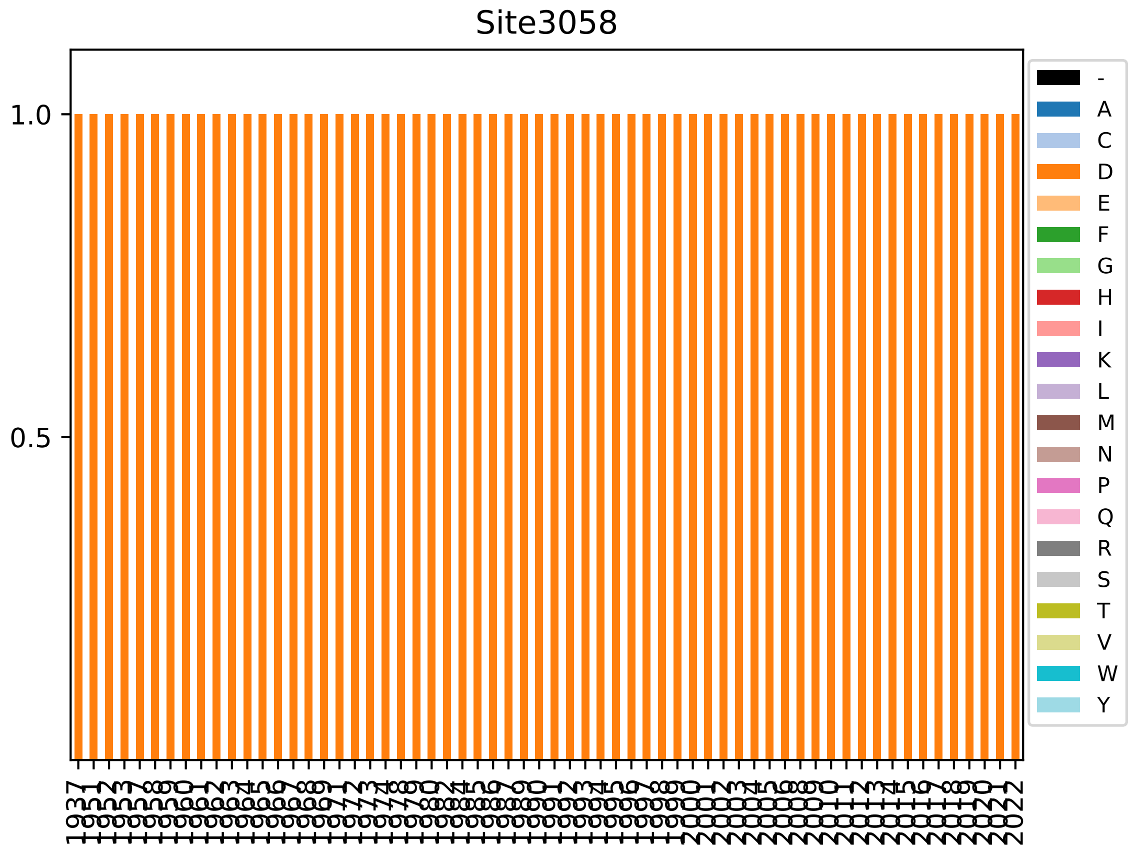
Site3058
1.0
0.5
-
A
C
D
E
F
G
H
I
K
L
M
N
P
Q
R
S
T
V
W
Y

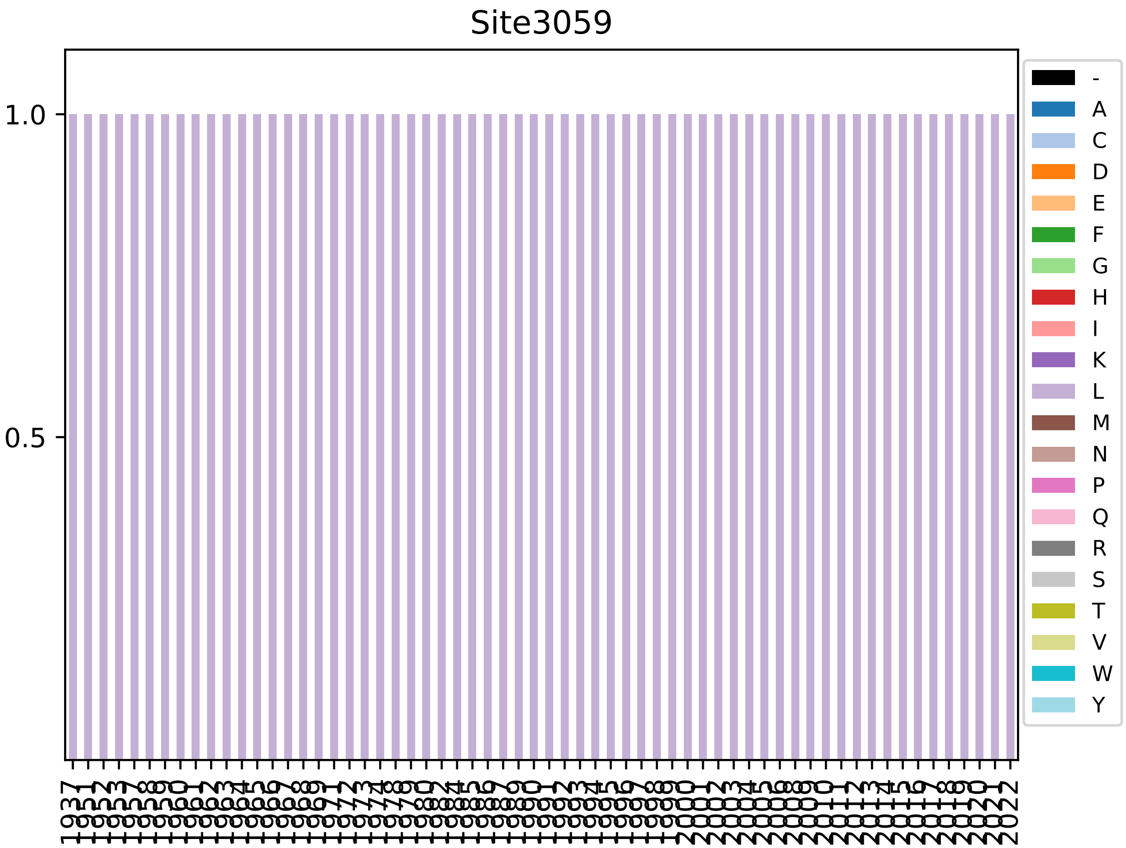
Site3059
1.0
0.5
-
A
C
D
E
F
G
H
I
K
L
M
N
P
Q
R
S
T
V
W
Y

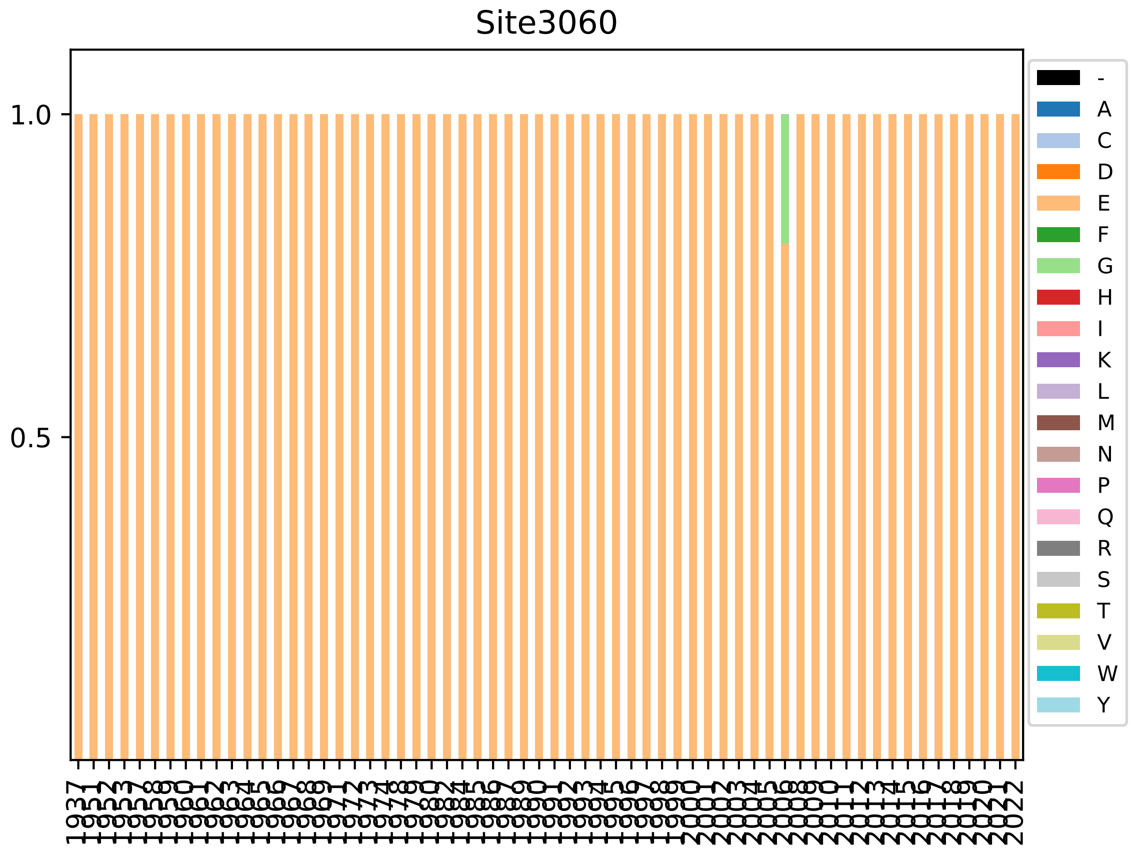
Site3060
1.0
0.5
-
A
C
D
E
F
G
H
I
K
L
M
N
P
Q
R
S
T
V
W
Y

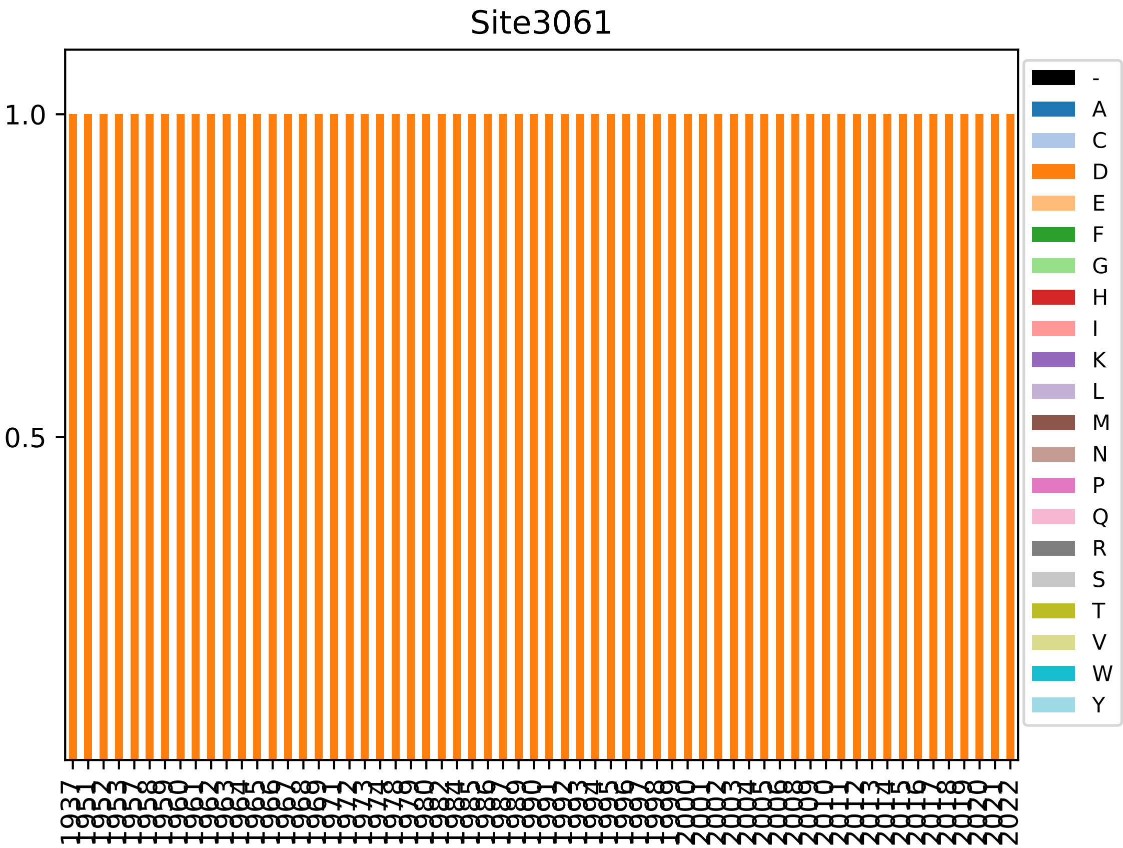
Site3061
1.0
0.5
-
A
C
D
E
F
G
H
I
K
L
M
N
P
Q
R
S
T
V
W
Y

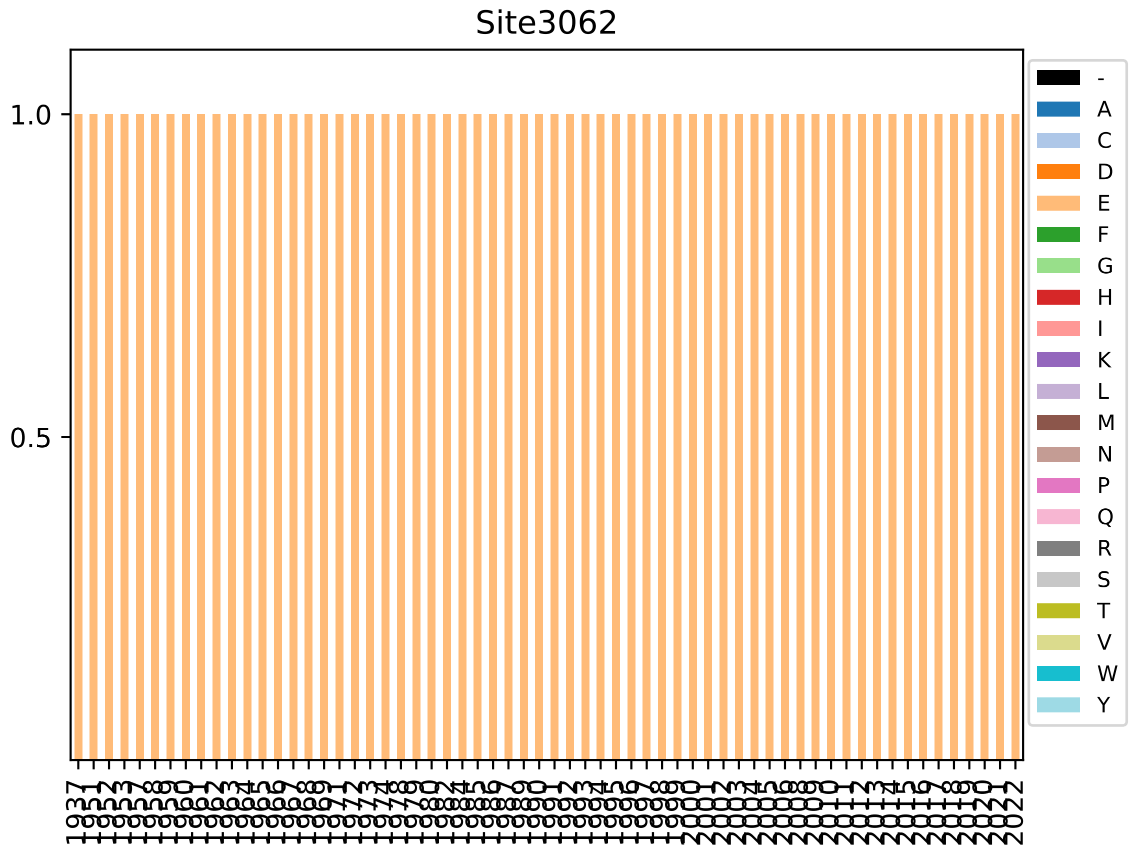
Site3062
1.0
0.5
-
A
C
D
E
F
G
H
I
K
L
M
N
P
Q
R
S
T
V
W
Y

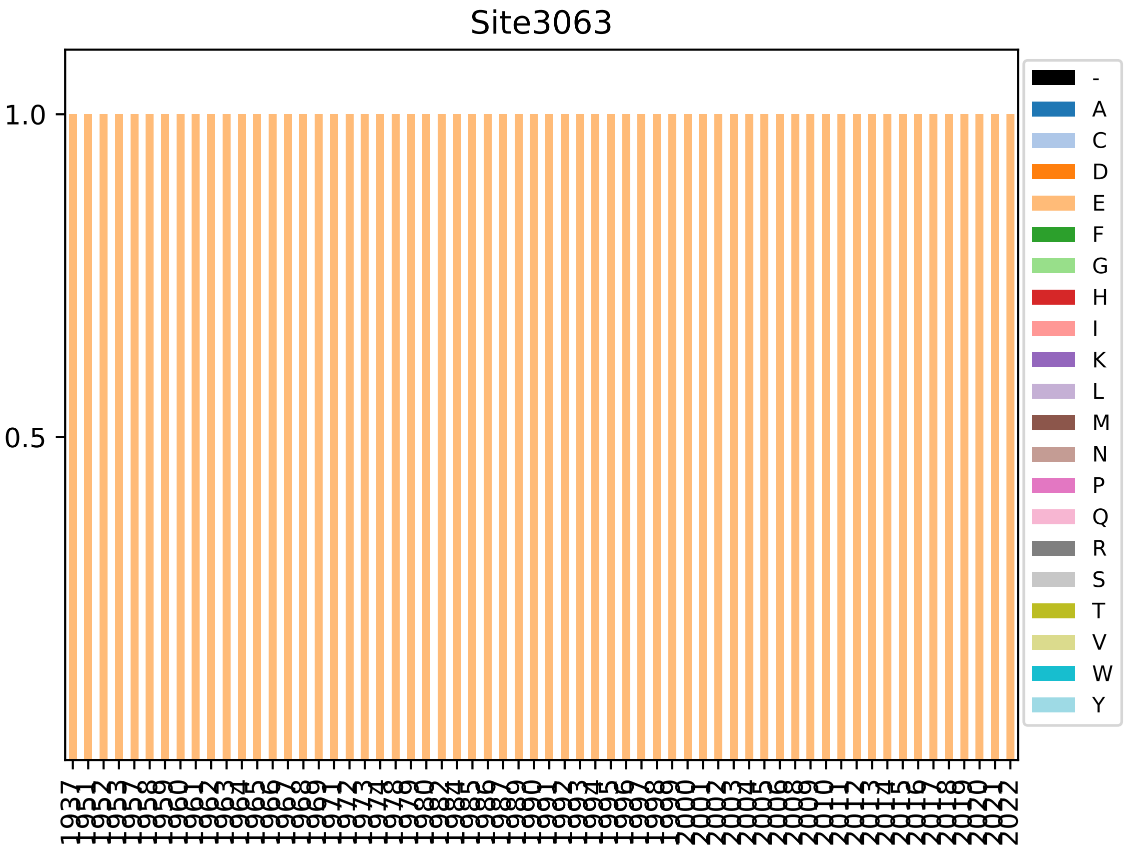
Site3063
1.0
0.5
-
A
C
D
E
F
G
H
I
K
L
M
N
P
Q
R
S
T
V
W
Y

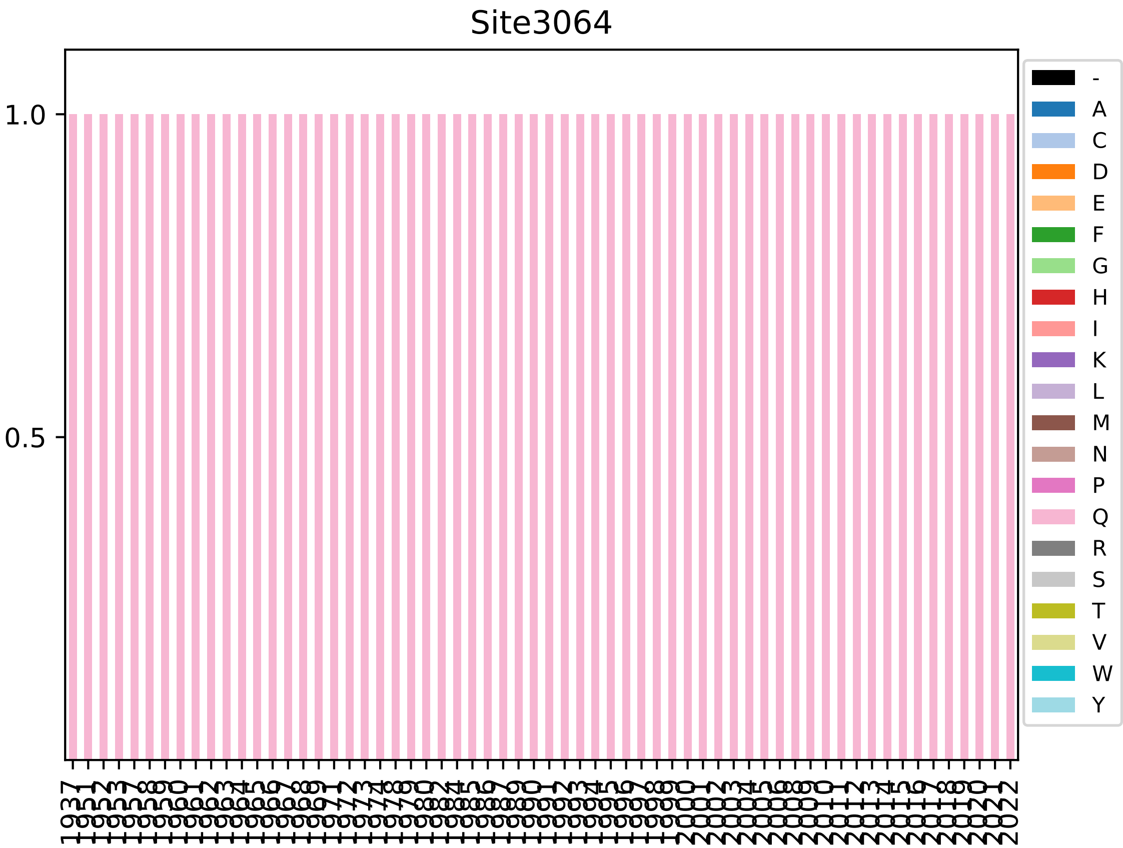
Site3064
1.0
0.5
-
A
C
D
E
F
G
H
I
K
L
M
N
P
Q
R
S
T
V
W
Y

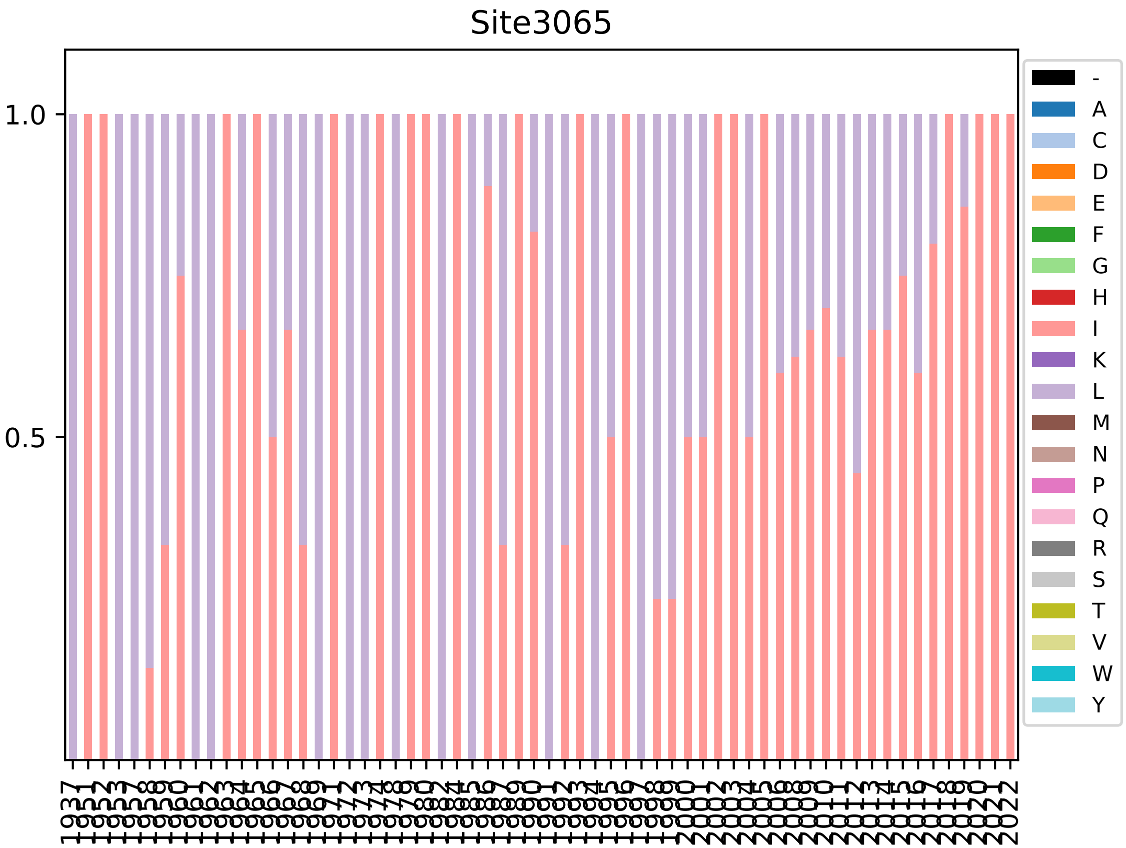
Site3065
1.0
0.5
-
A
C
D
E
F
G
H
I
K
L
M
N
P
Q
R
S
T
V
W
Y

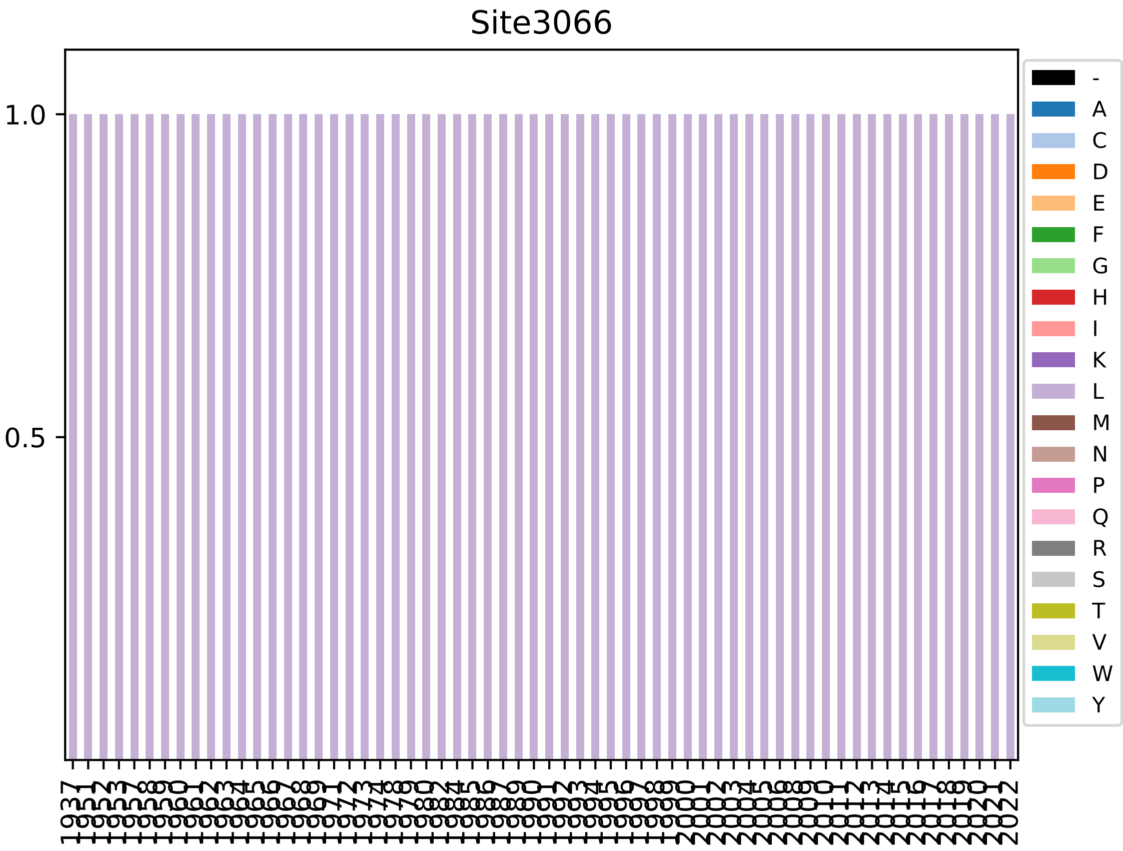
Site3066
1.0
0.5
-
A
C
D
E
F
G
H
I
K
L
M
N
P
Q
R
S
T
V
W
Y

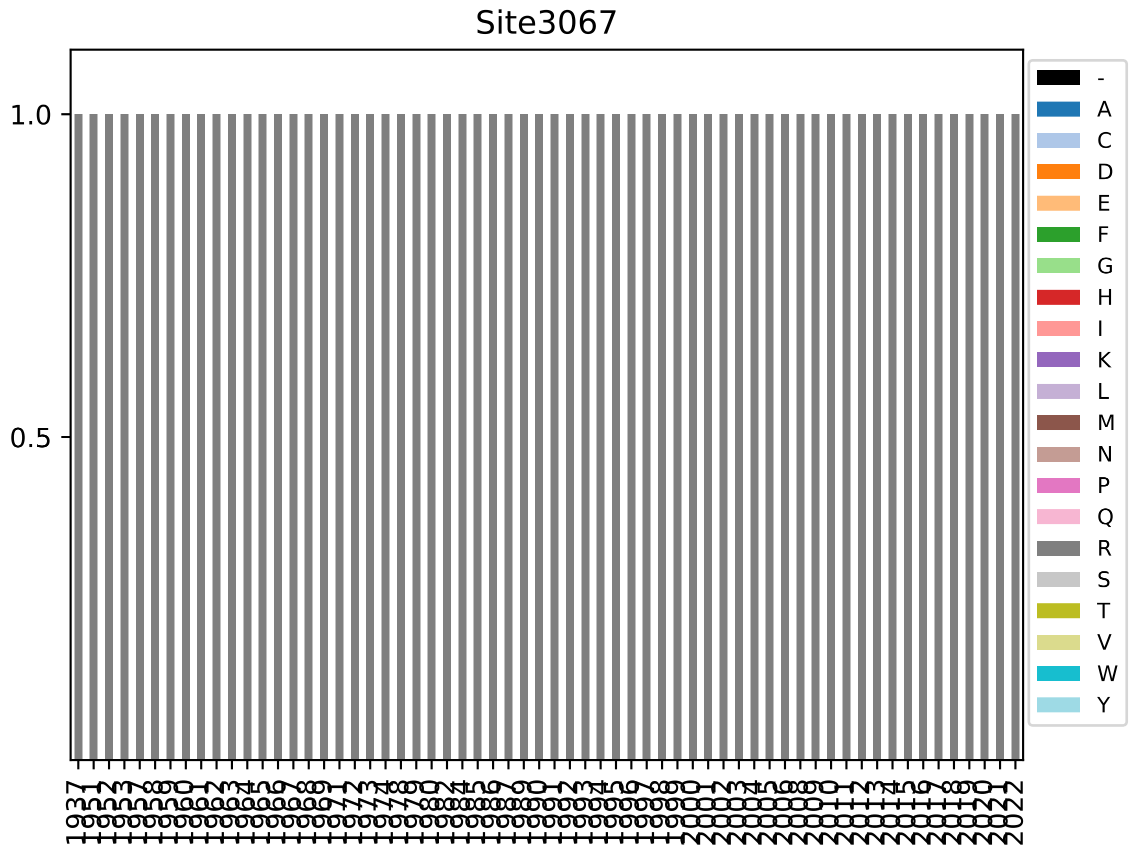
Site3067
1.0
0.5
-
A
C
D
E
F
G
H
I
K
L
M
N
P
Q
R
S
T
V
W
Y

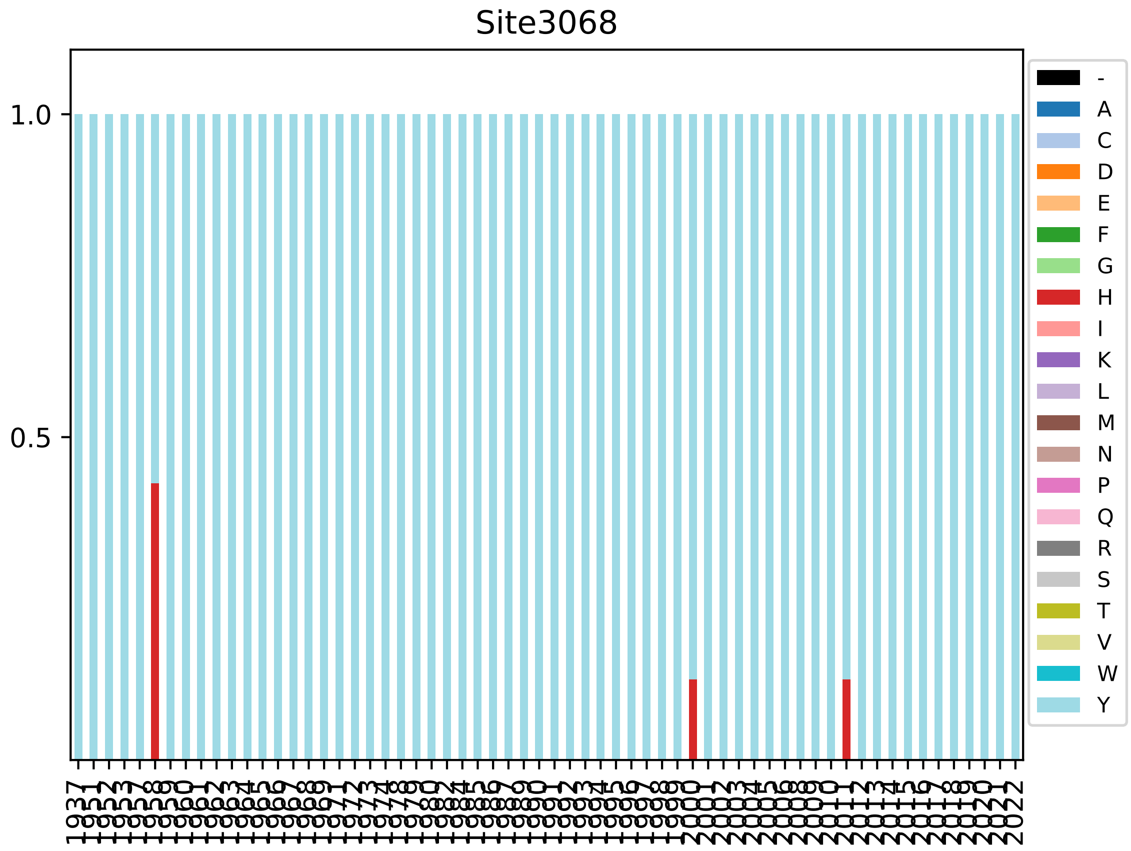
Site3068
1.0
0.5
-
A
C
D
E
F
G
H
I
K
L
M
N
P
Q
R
S
T
V
W
Y

Site3069

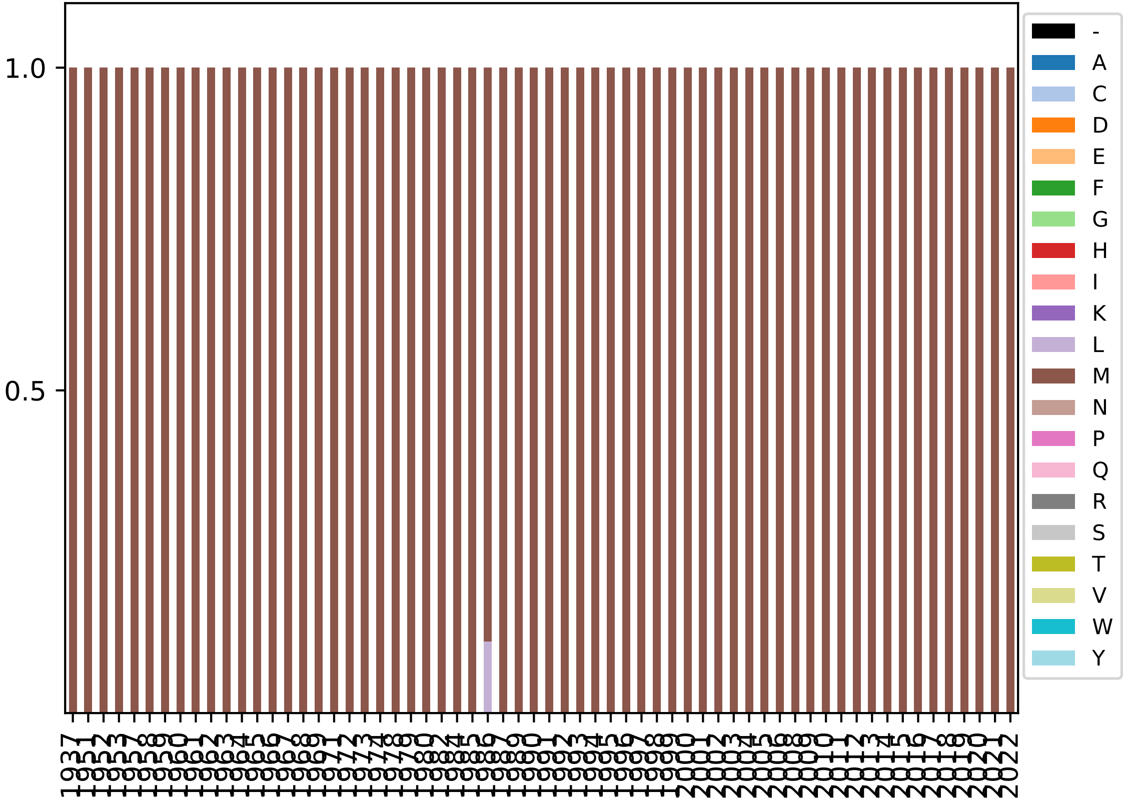

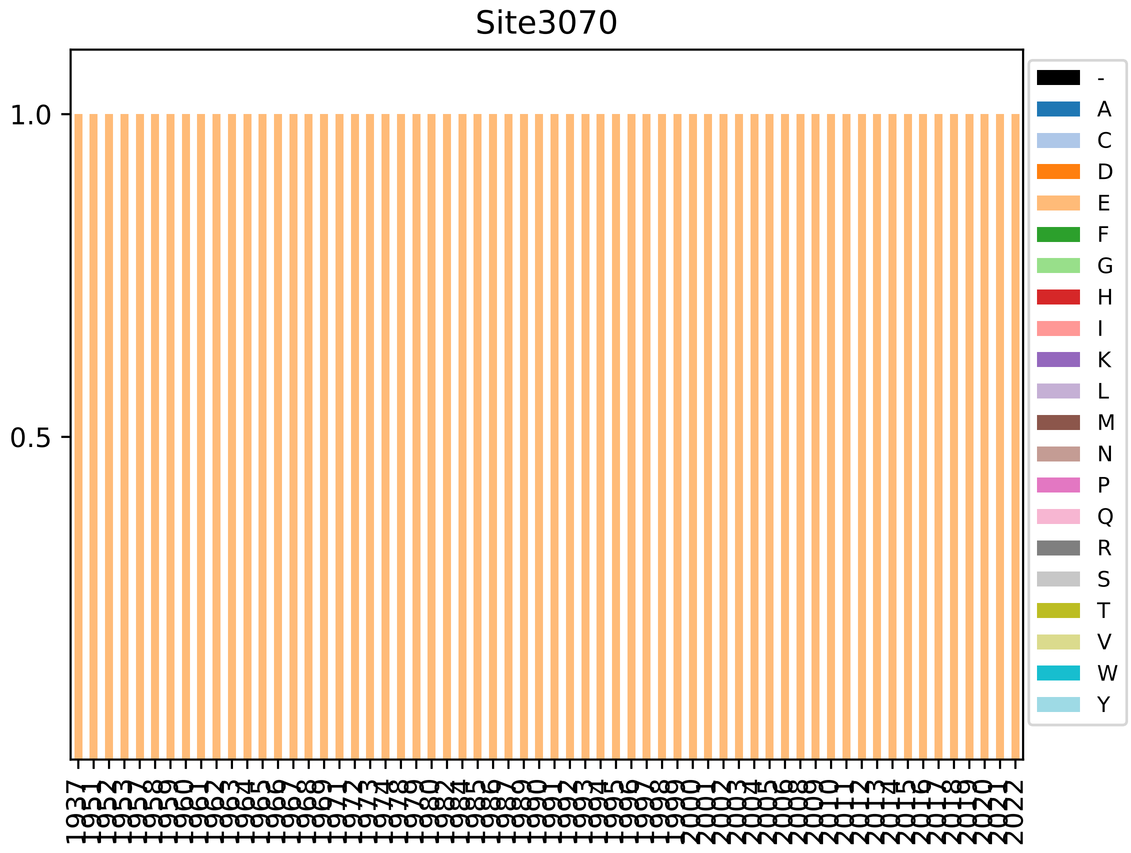
Site3070
1.0
0.5
-
A
C
D
E
F
G
H
I
K
L
M
N
P
Q
R
S
T
V
W
Y

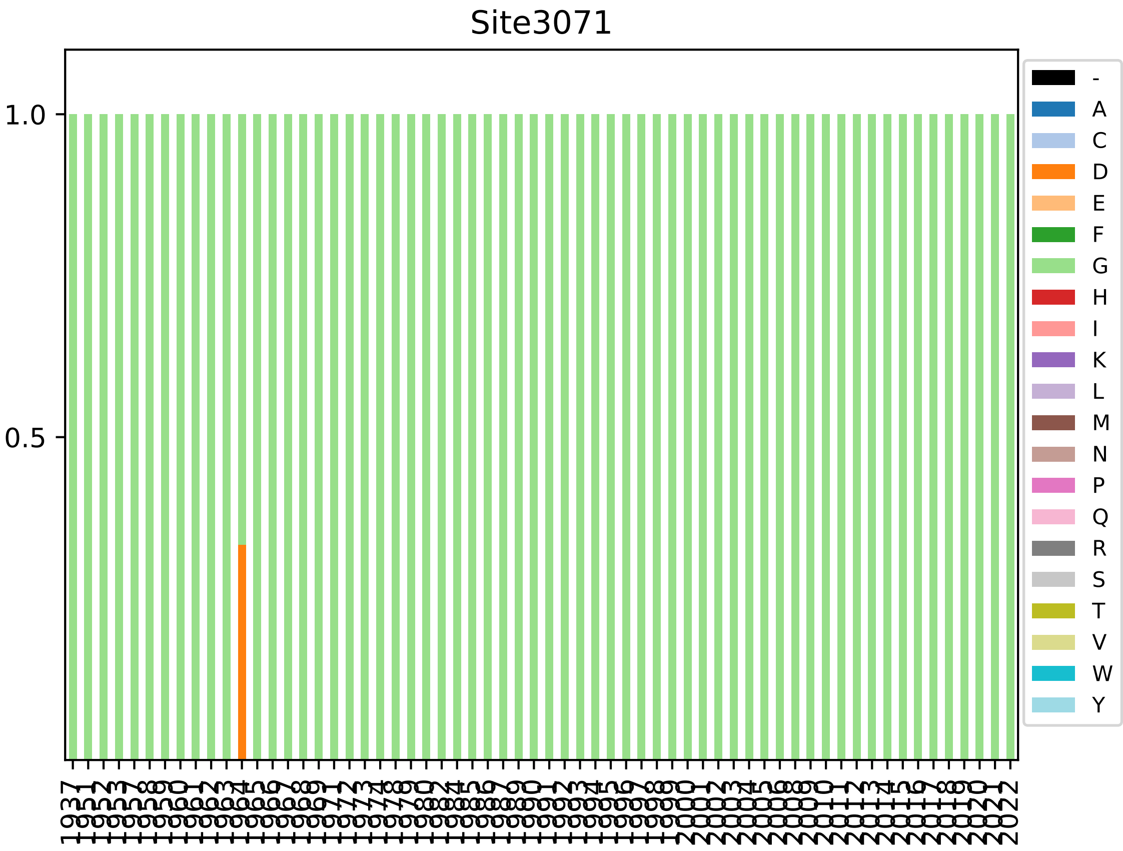
Site3071
1.0
0.5
-
A
C
D
E
F
G
H
I
K
L
M
N
P
Q
R
S
T
V
W
Y

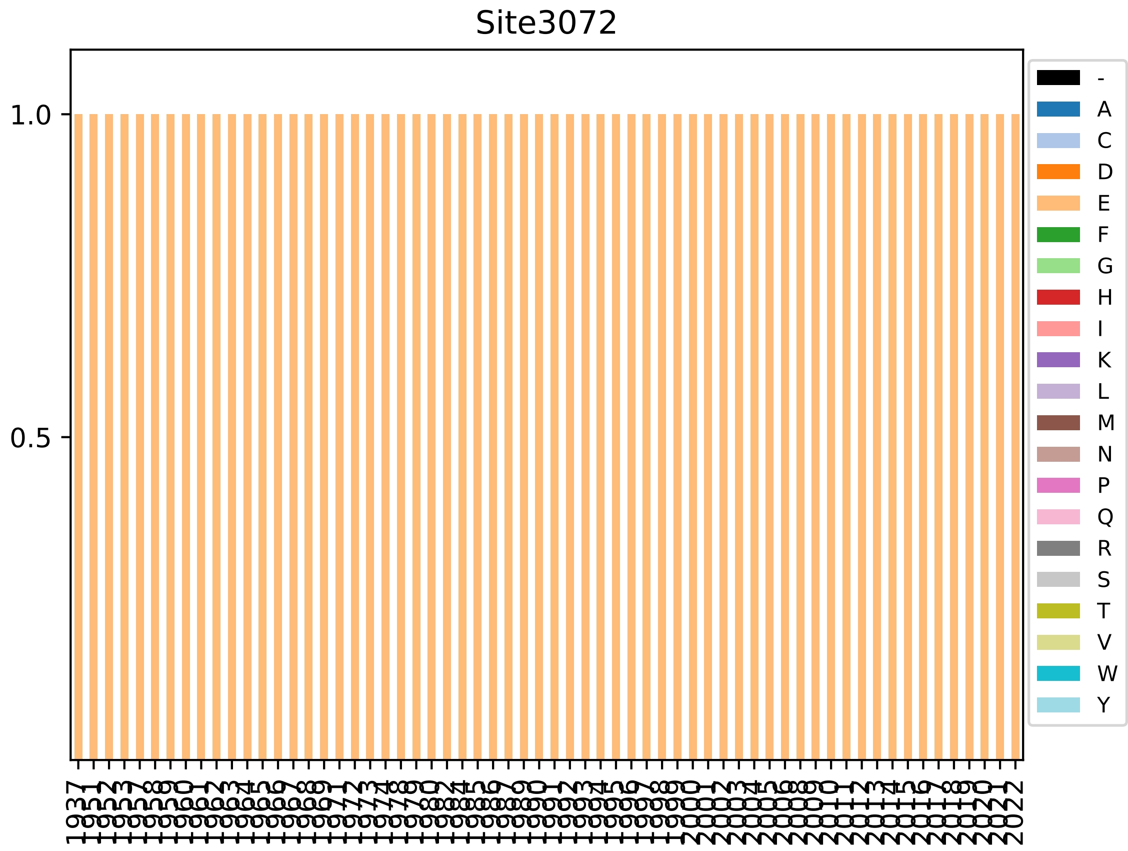

Site3072
1.0
0.5
-
A
C
D
E
F
G
H
I
K
L
M
N
P
Q
R
S
T
V
W
Y

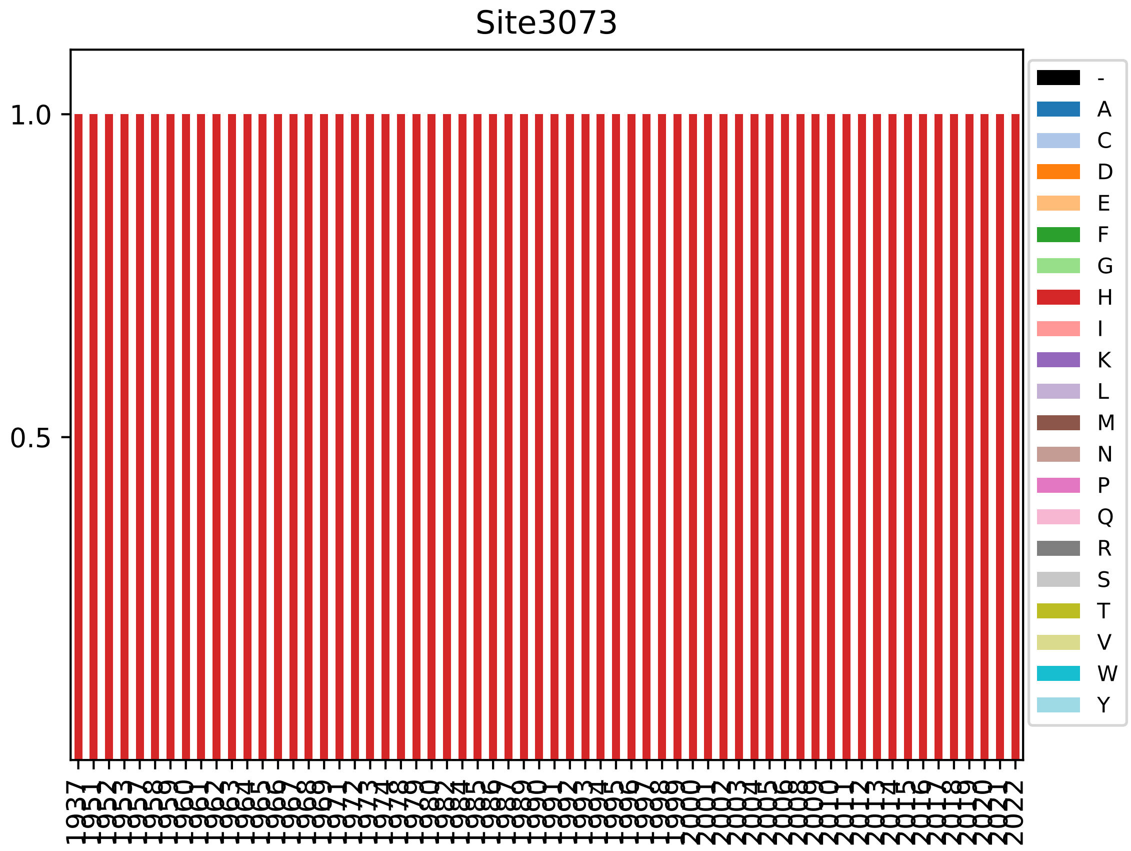
Site3073
1.0
0.5
-
A
C
D
E
F
G
H
I
K
L
M
N
P
Q
R
S
T
V
W
Y

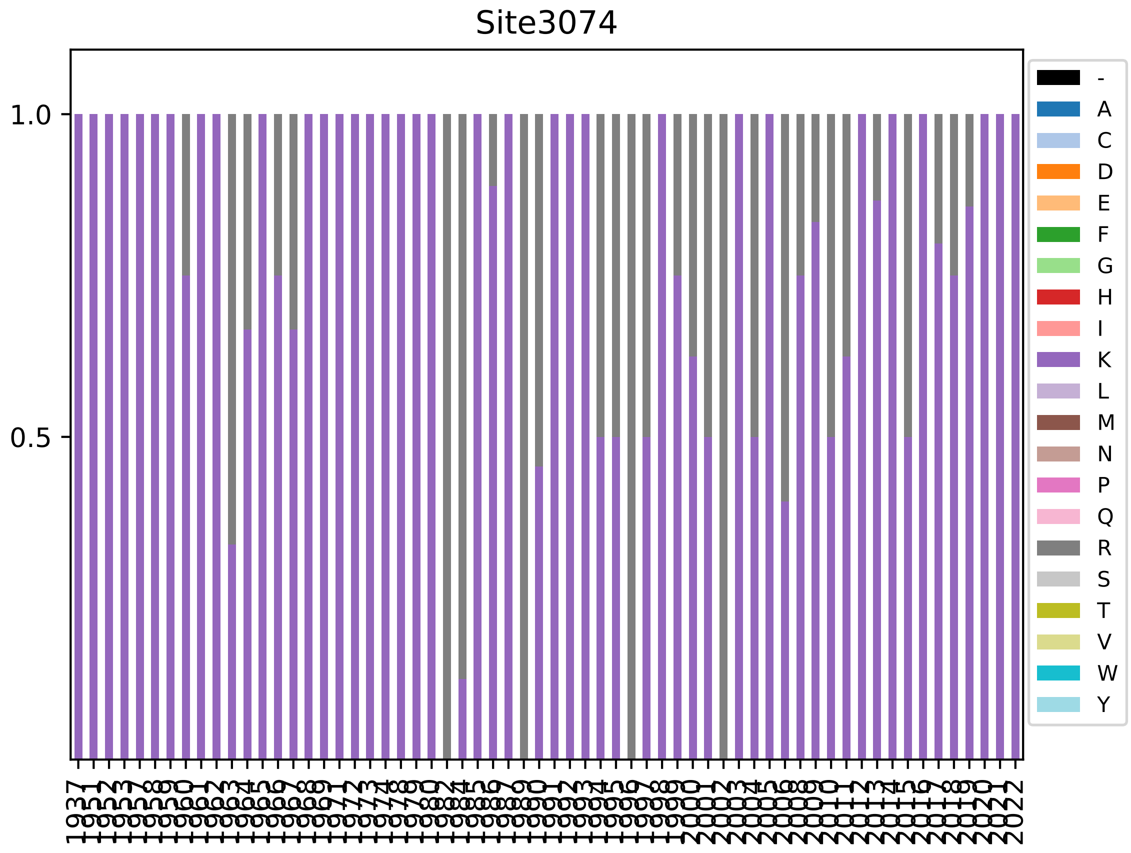
Site3074
1.0
0.5
-
A
C
D
E
F
G
H
I
K
L
M
N
P
Q
R
S
T
V
W
Y

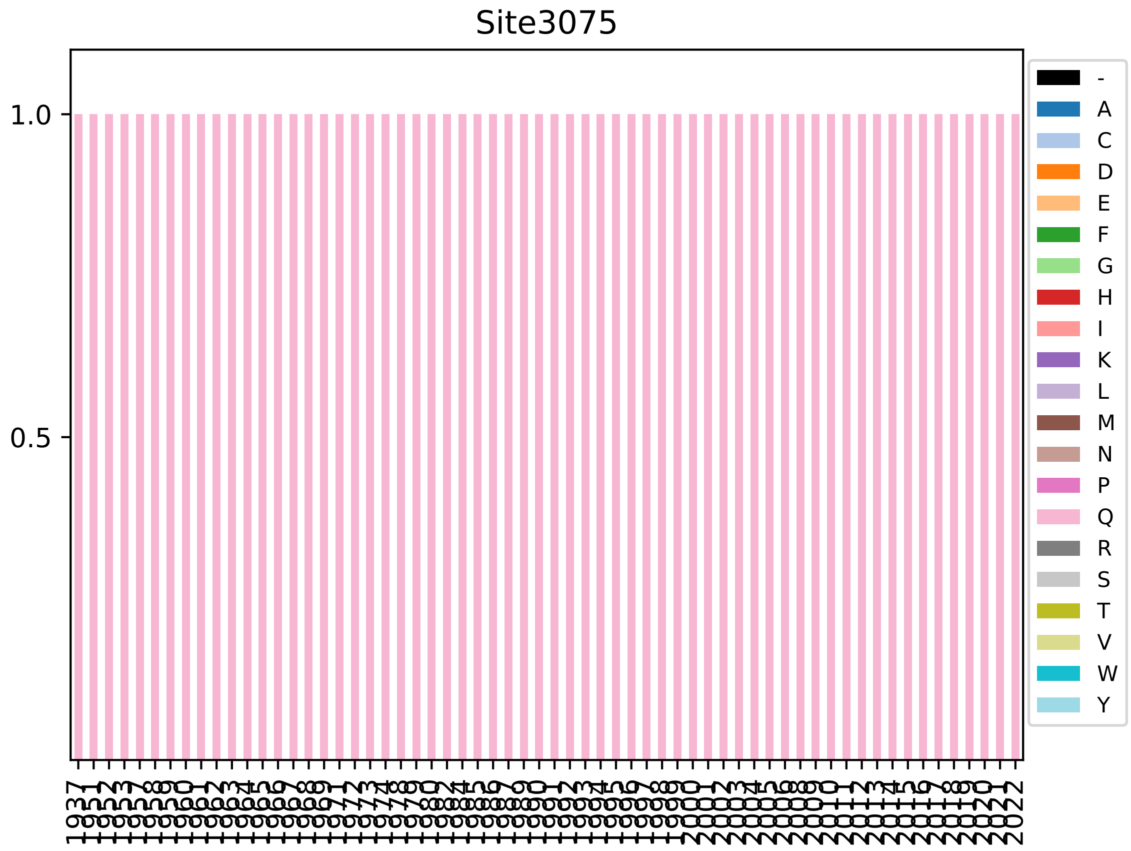
Site3075
1.0
0.5
-
A
C
D
E
F
G
H
I
K
L
M
N
P
Q
R
S
T
V
W
Y

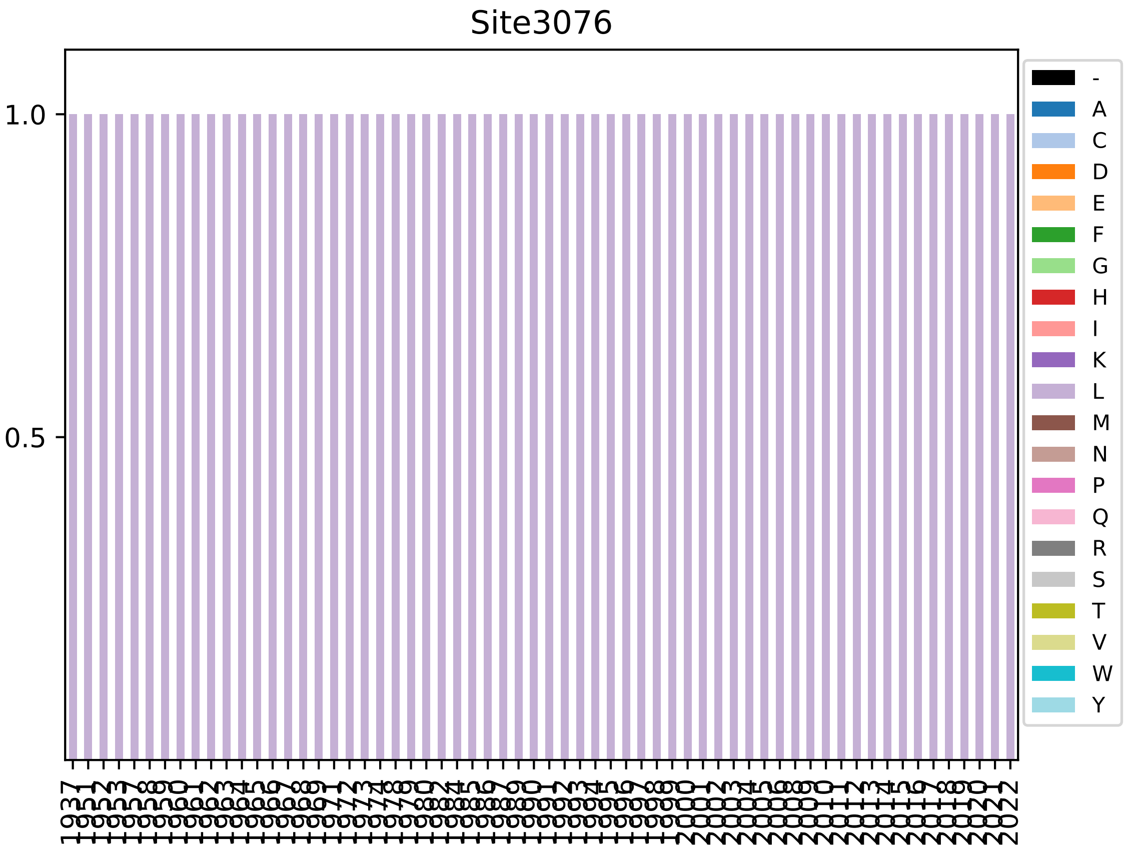
Site3076
1.0
0.5
-
A
C
D
E
F
G
H
I
K
L
M
N
P
Q
R
S
T
V
W
Y

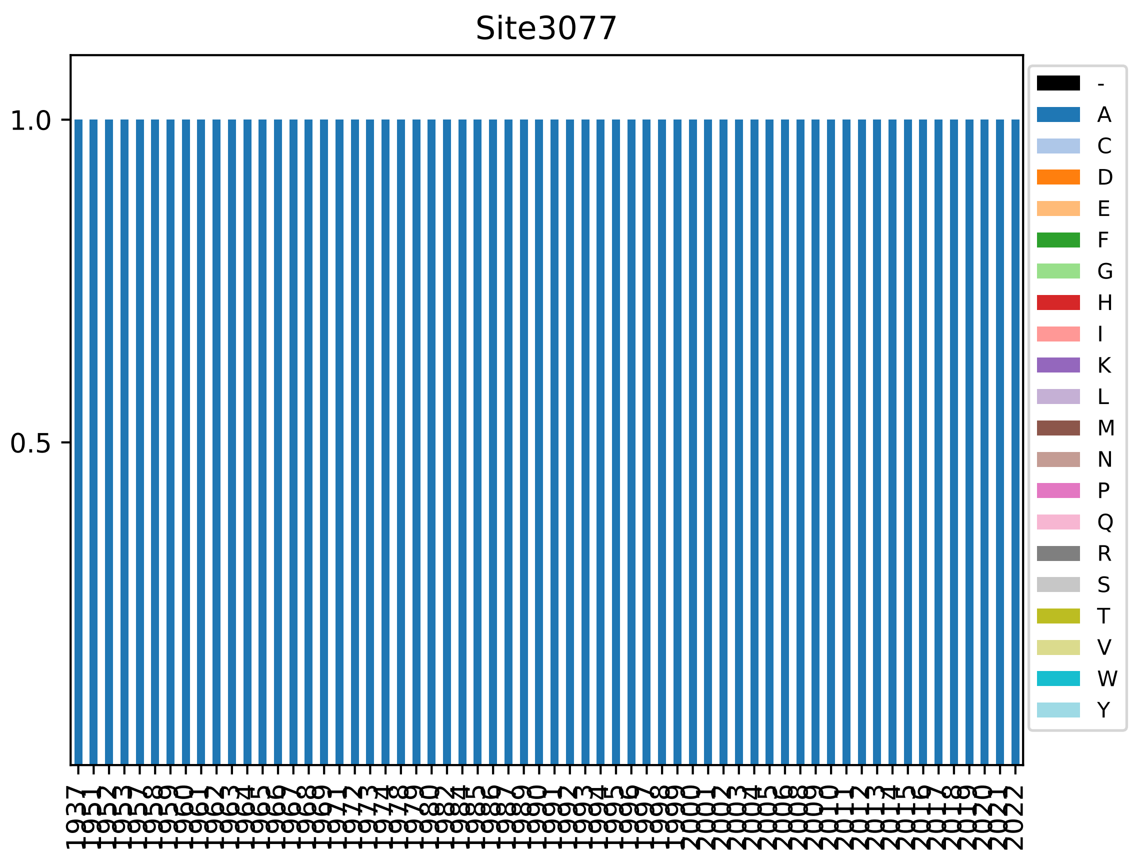
Site3077
1.0
0.5
-
A
C
D
E
F
G
H
I
K
L
M
N
P
Q
R
S
T
V
W
Y

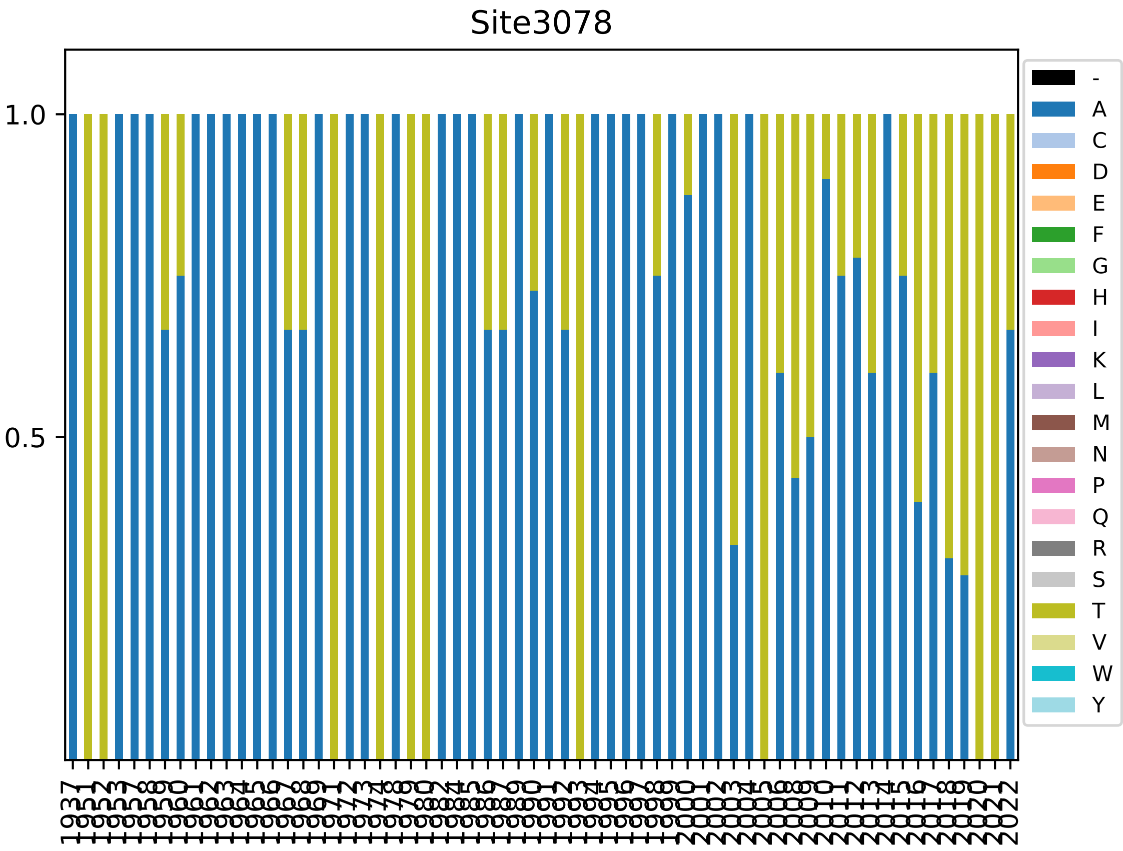
Site3078
1.0
0.5
-
A
C
D
E
F
G
H
I
K
L
M
N
P
Q
R
S
T
V
W
Y

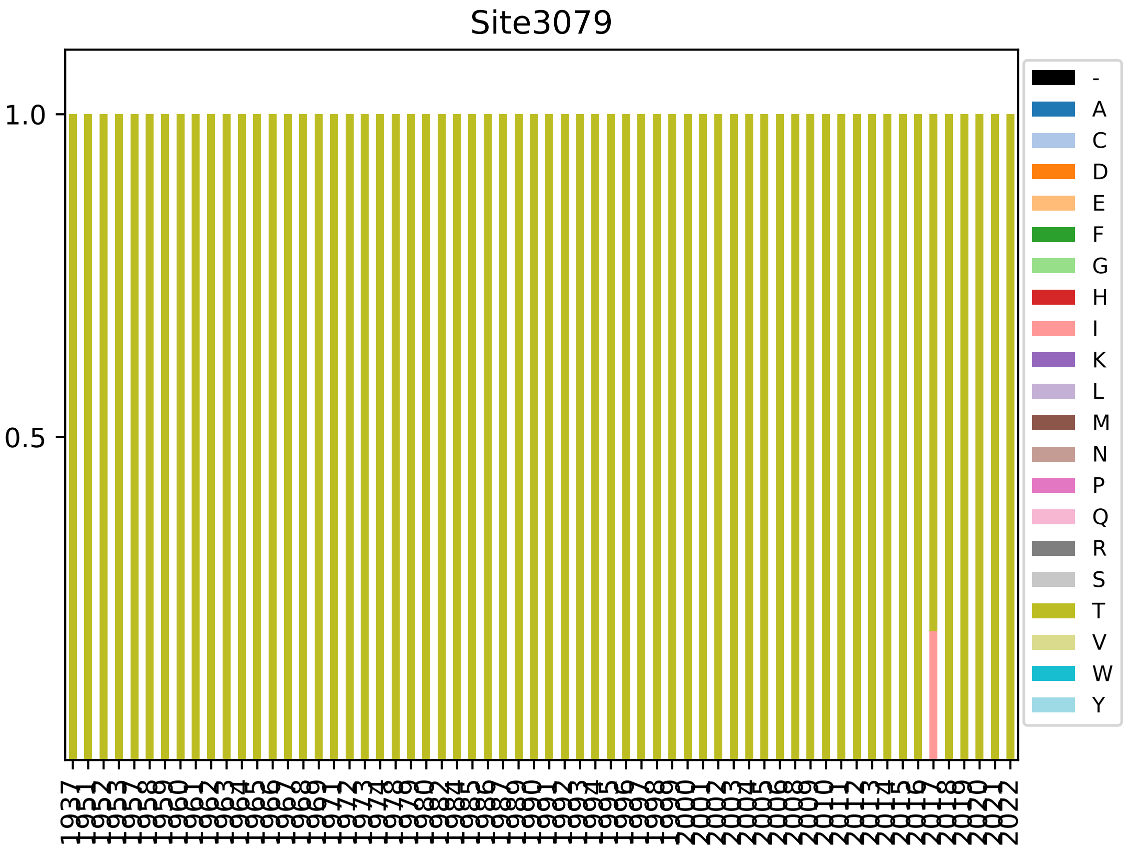
Site3079
1.0
0.5
-
A
C
D
E
F
G
H
I
K
L
M
N
P
Q
R
S
T
V
W
Y

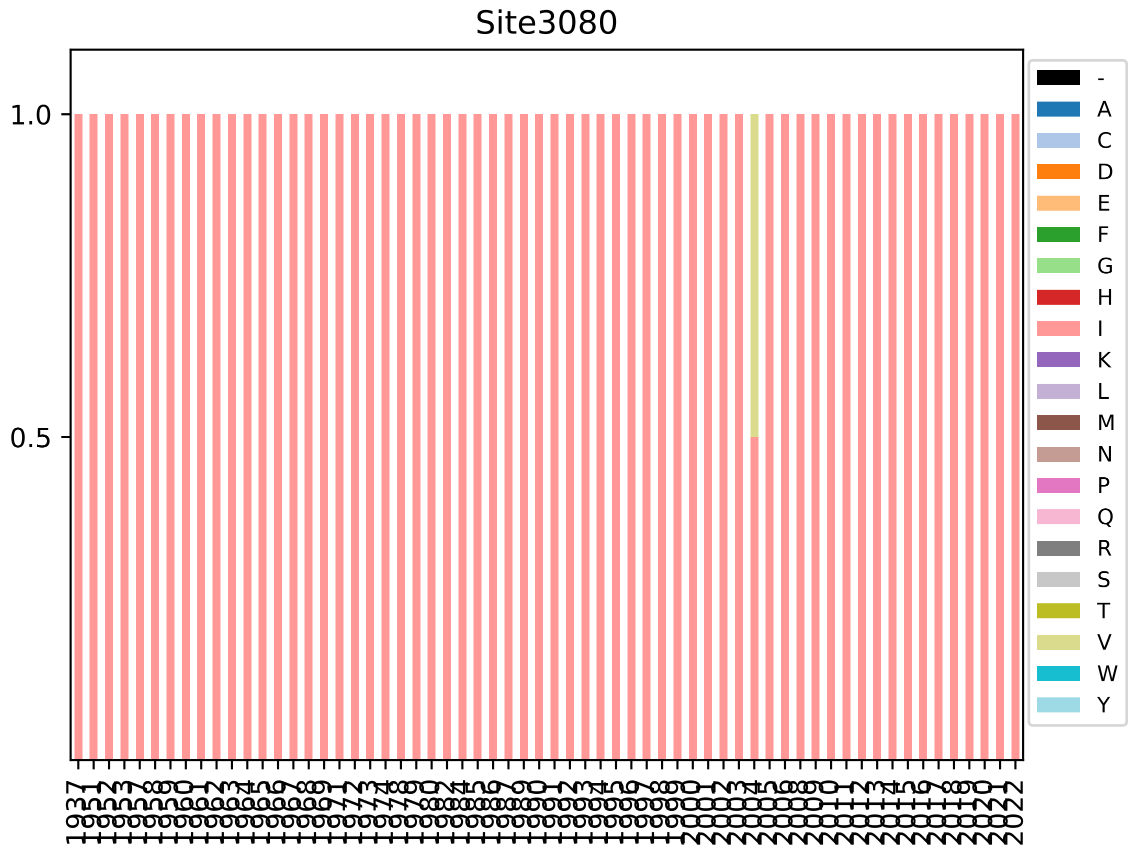
Site3080
1.0
0.5
-
A
C
D
E
F
G
H
I
K
L
M
N
P
Q
R
S
T
V
W
Y

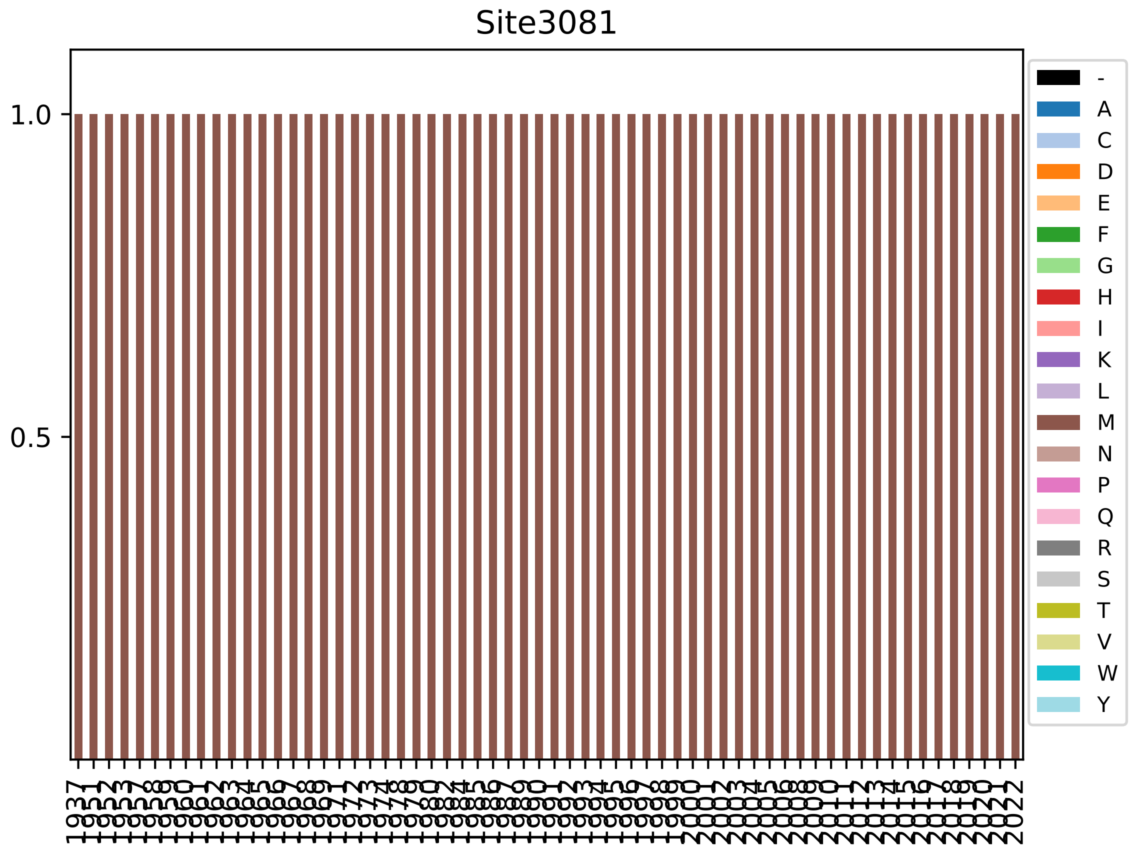
Site3081
1.0
0.5
-
A
C
D
E
F
G
H
I
K
L
M
N
P
Q
R
S
T
V
W
Y

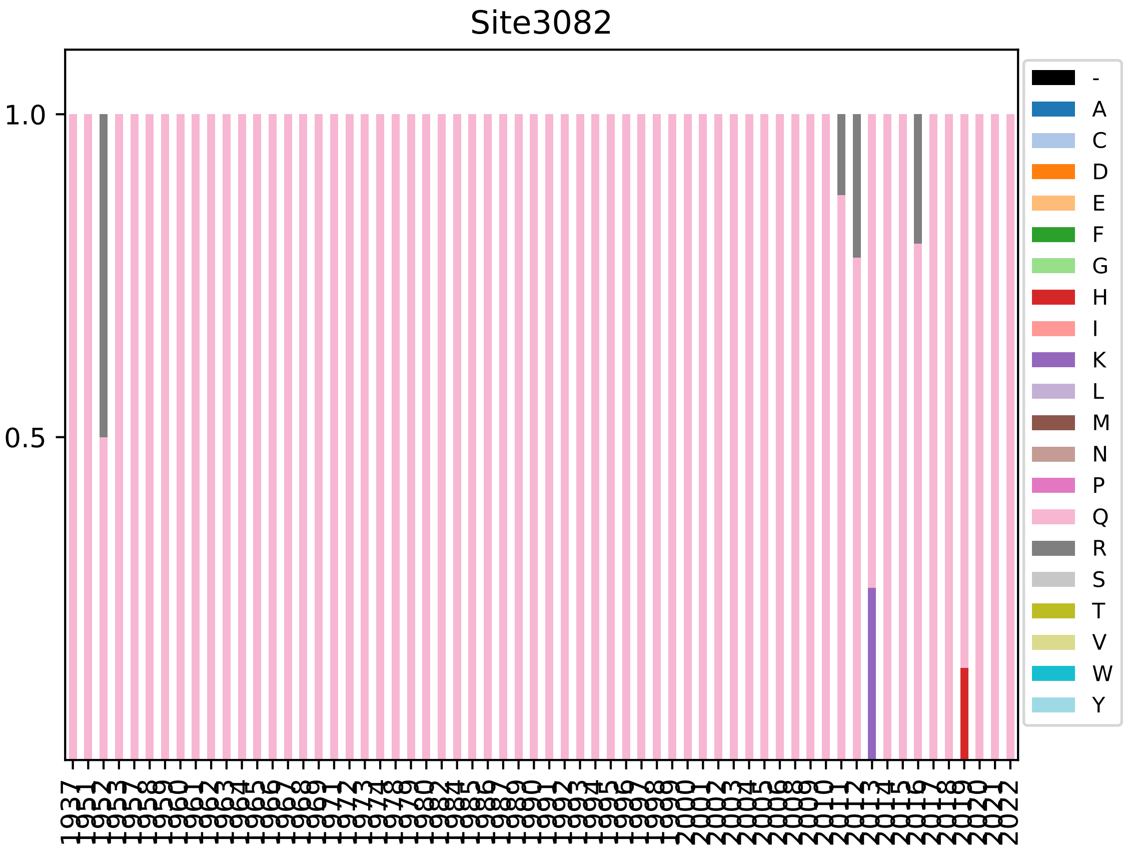
Site3082
1.0
0.5
-
A
C
D
E
F
G
H
I
K
L
M
N
P
Q
R
S
T
V
W
Y

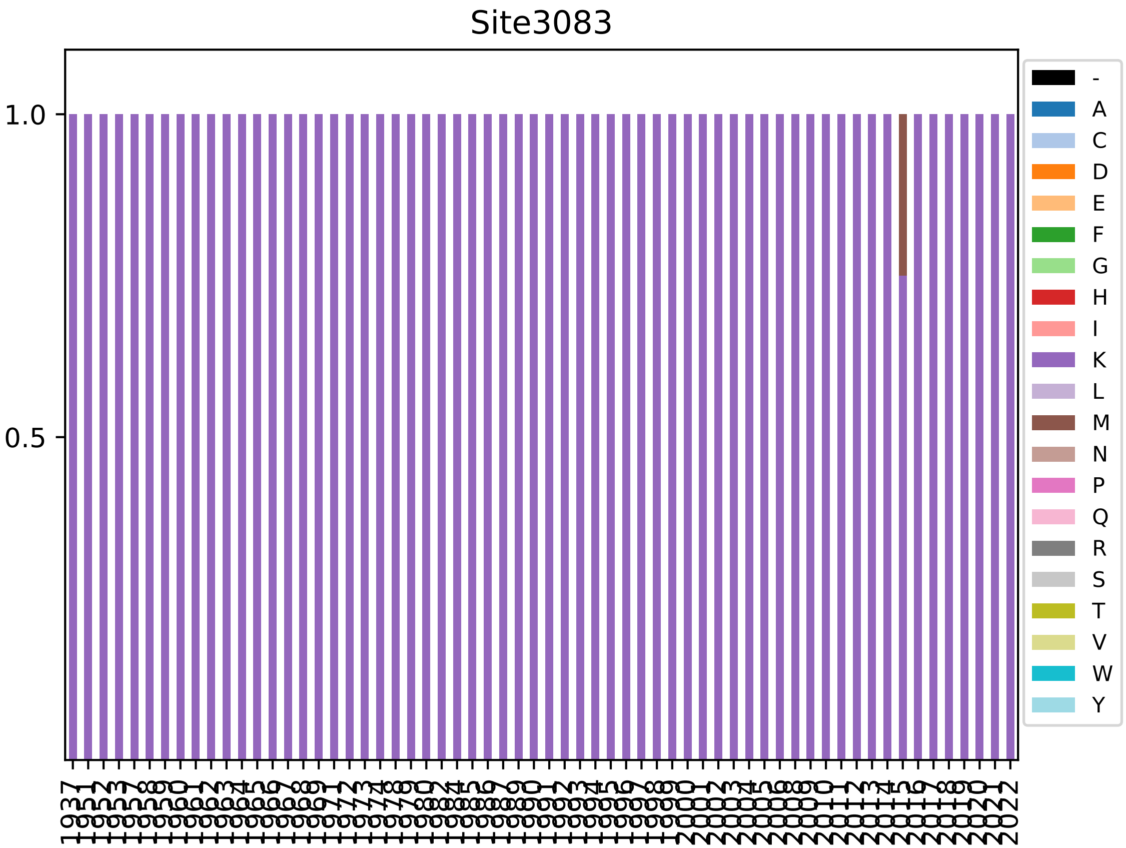

Site3083
1.0
0.5
-
A
C
D
E
F
G
H
I
K
L
M
N
P
Q
R
S
T
V
W
Y

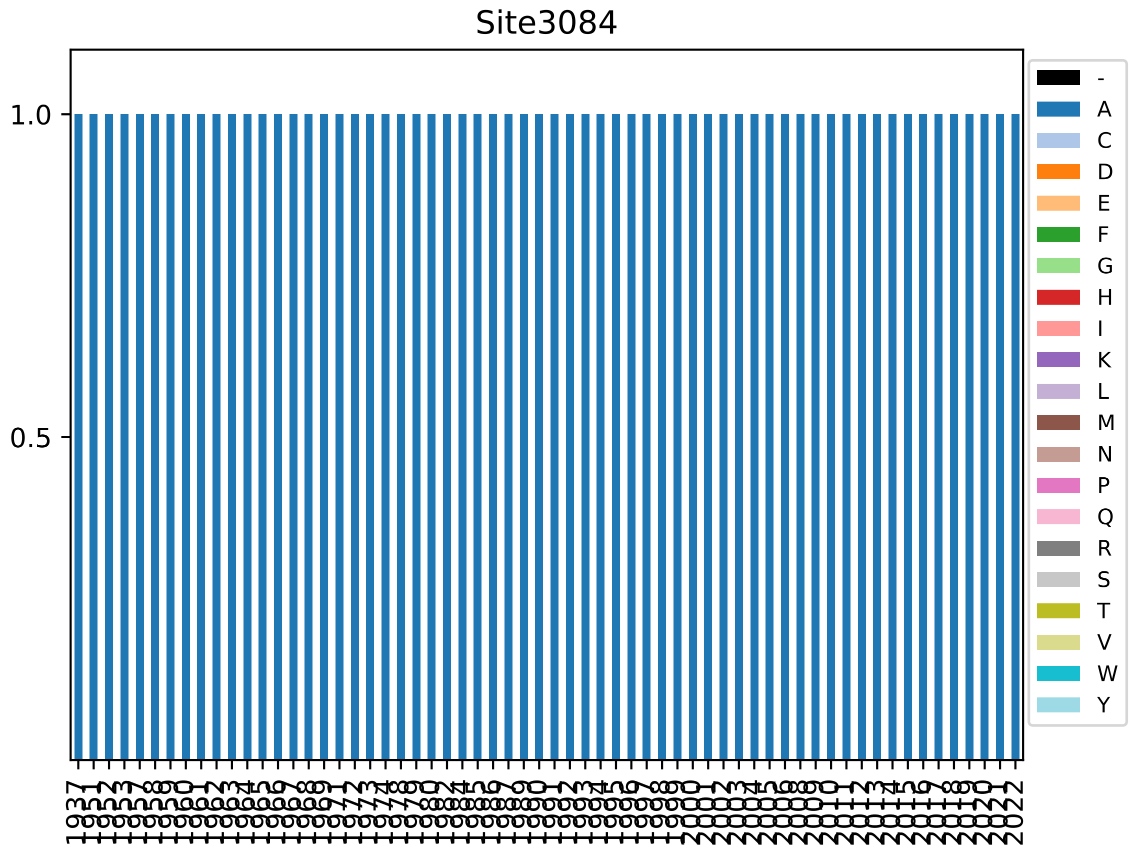
Site3084
1.0
0.5
-
A
C
D
E
F
G
H
I
K
L
M
N
P
Q
R
S
T
V
W
Y

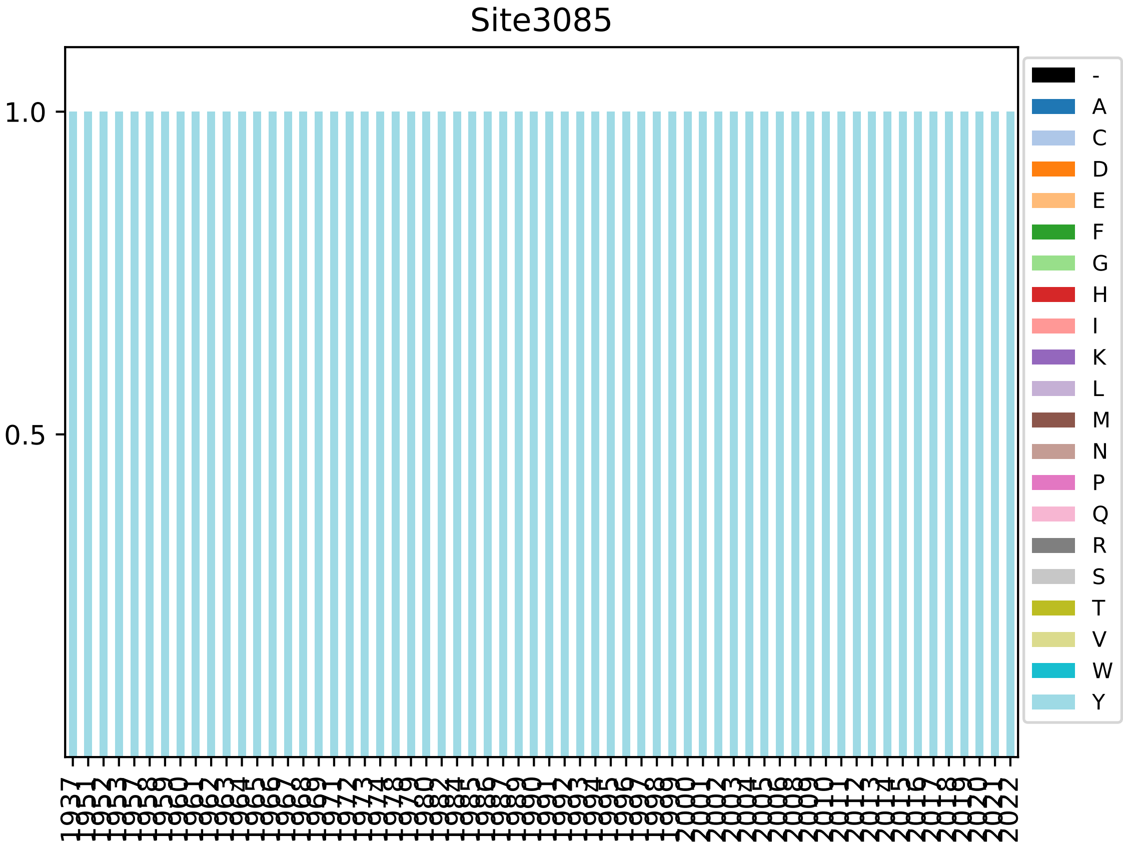
Site3085
1.0
0.5
-
A
C
D
E
F
G
H
I
K
L
M
N
P
Q
R
S
T
V
W
Y

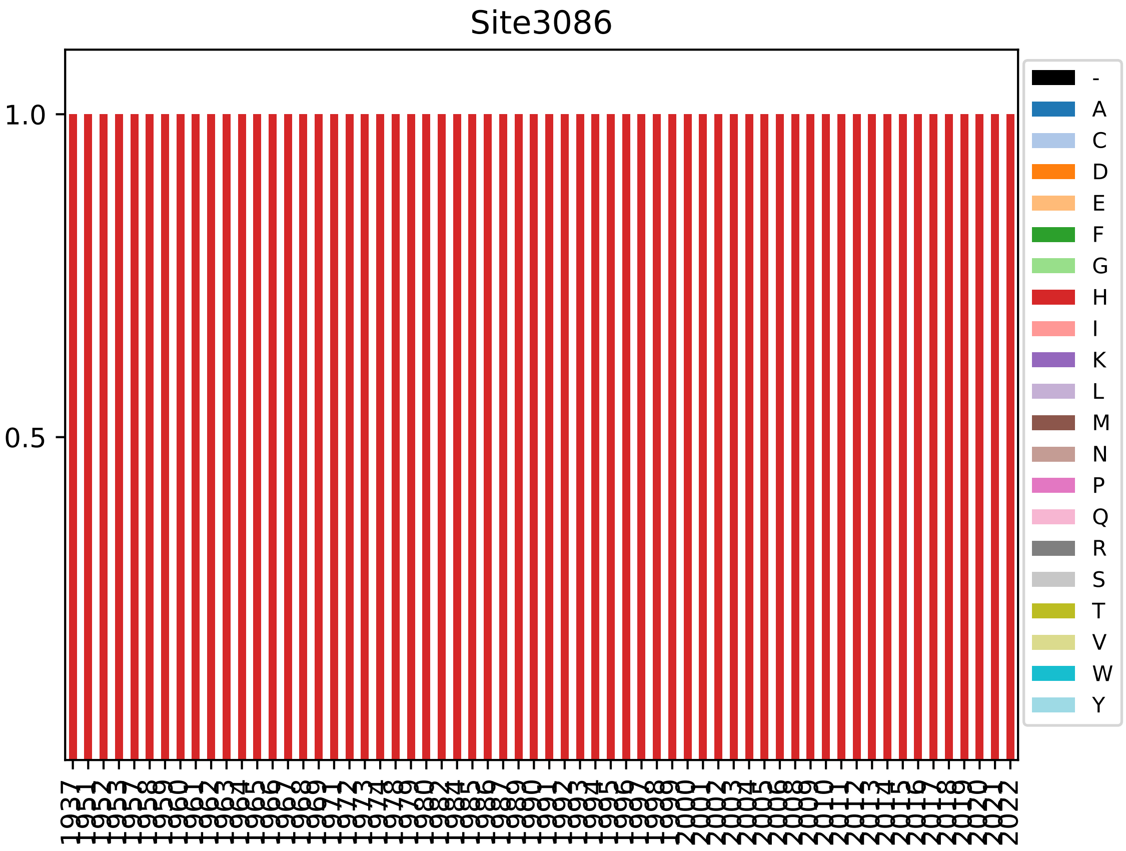

Site3086
1.0
0.5
-
A
C
D
E
F
G
H
I
K
L
M
N
P
Q
R
S
T
V
W
Y

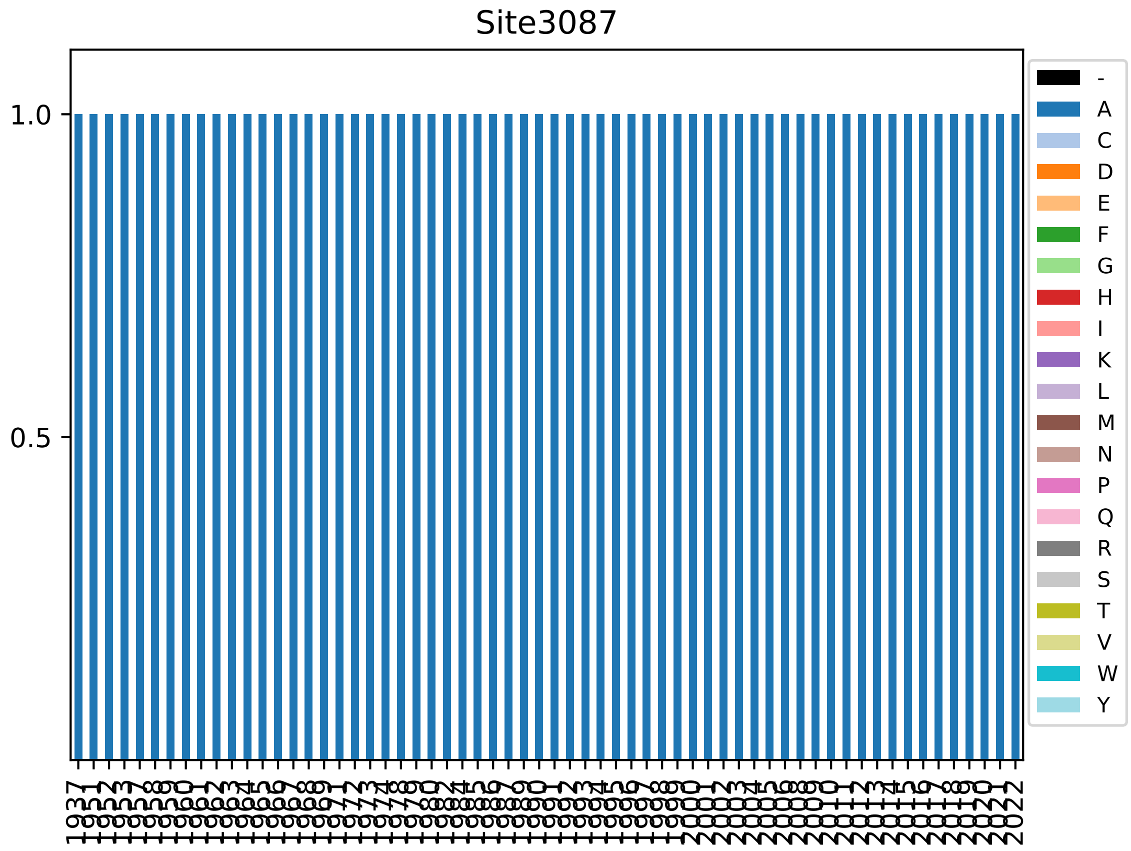
Site3087
1.0
0.5
-
A
C
D
E
F
G
H
I
K
L
M
N
P
Q
R
S
T
V
W
Y

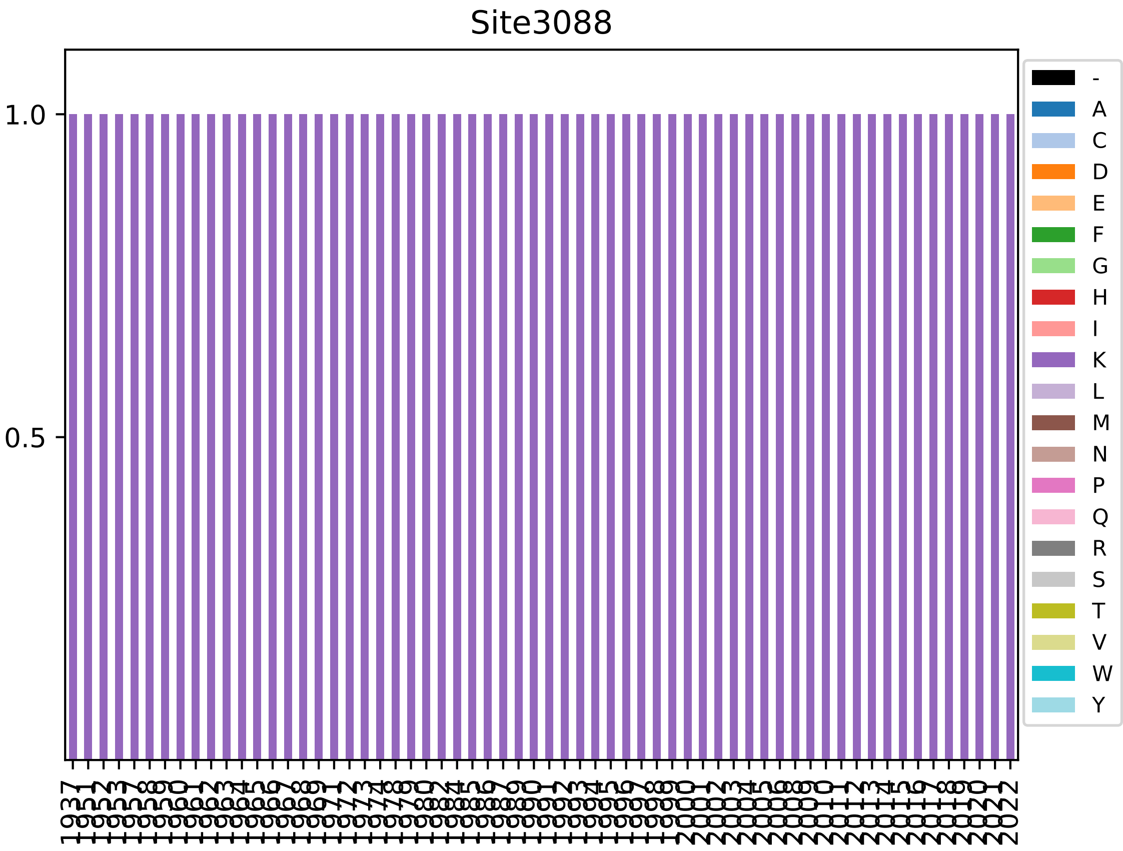
Site3088
1.0
0.5
-
A
C
D
E
F
G
H
I
K
L
M
N
P
Q
R
S
T
V
W
Y

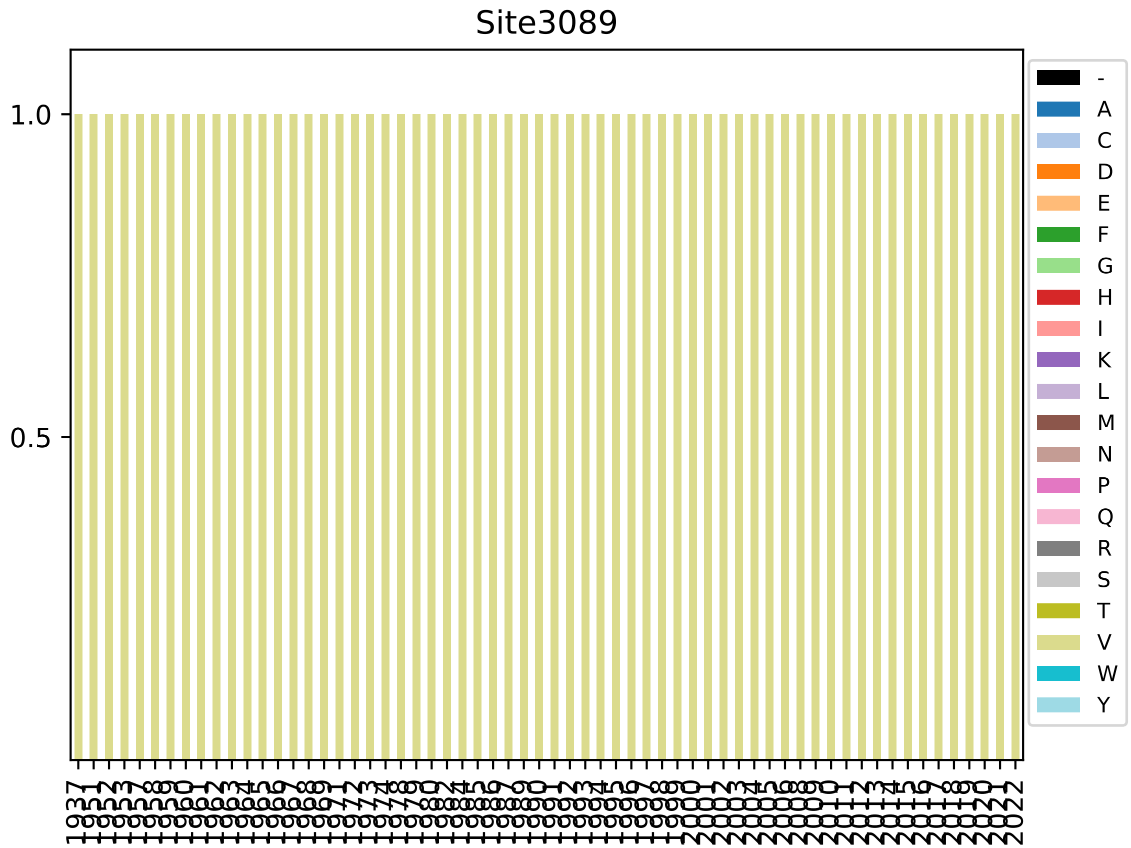
Site3089
1.0
0.5
-
A
C
D
E
F
G
H
I
K
L
M
N
P
Q
R
S
T
V
W
Y

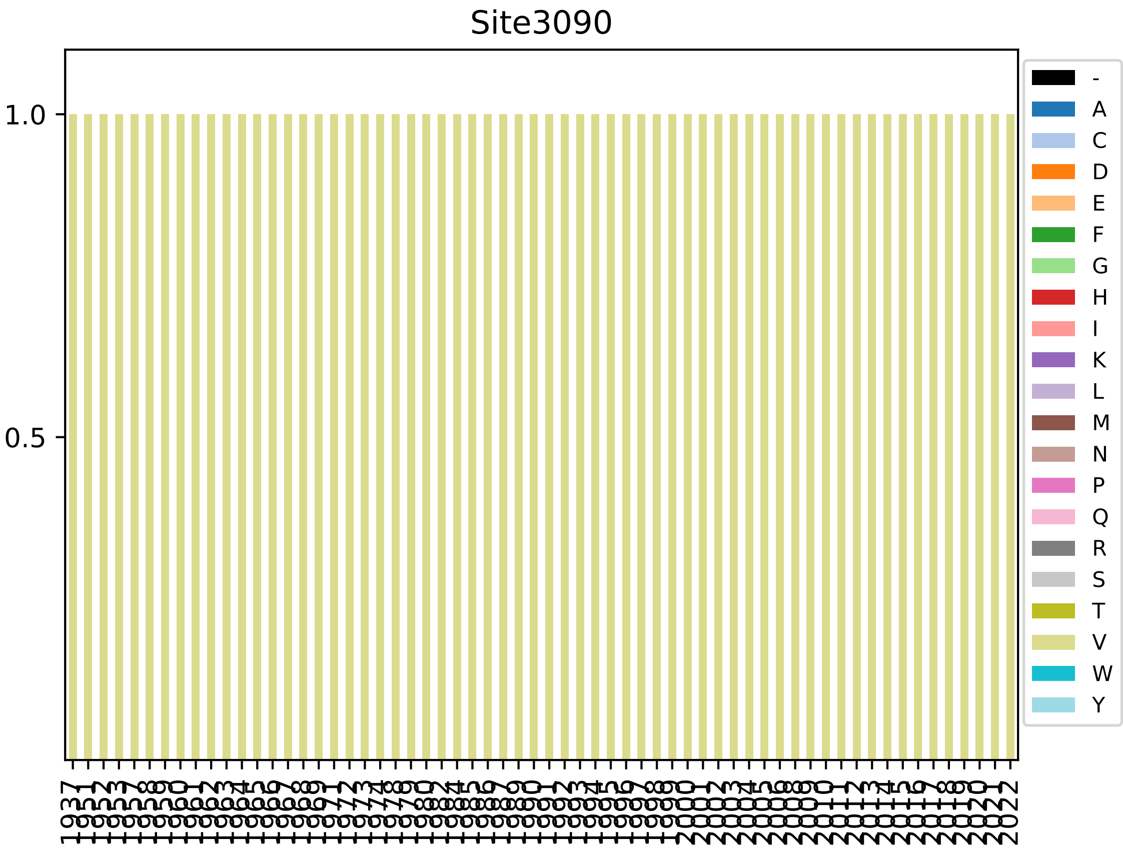
Site3090
1.0
0.5
-
A
C
D
E
F
G
H
I
K
L
M
N
P
Q
R
S
T
V
W
Y

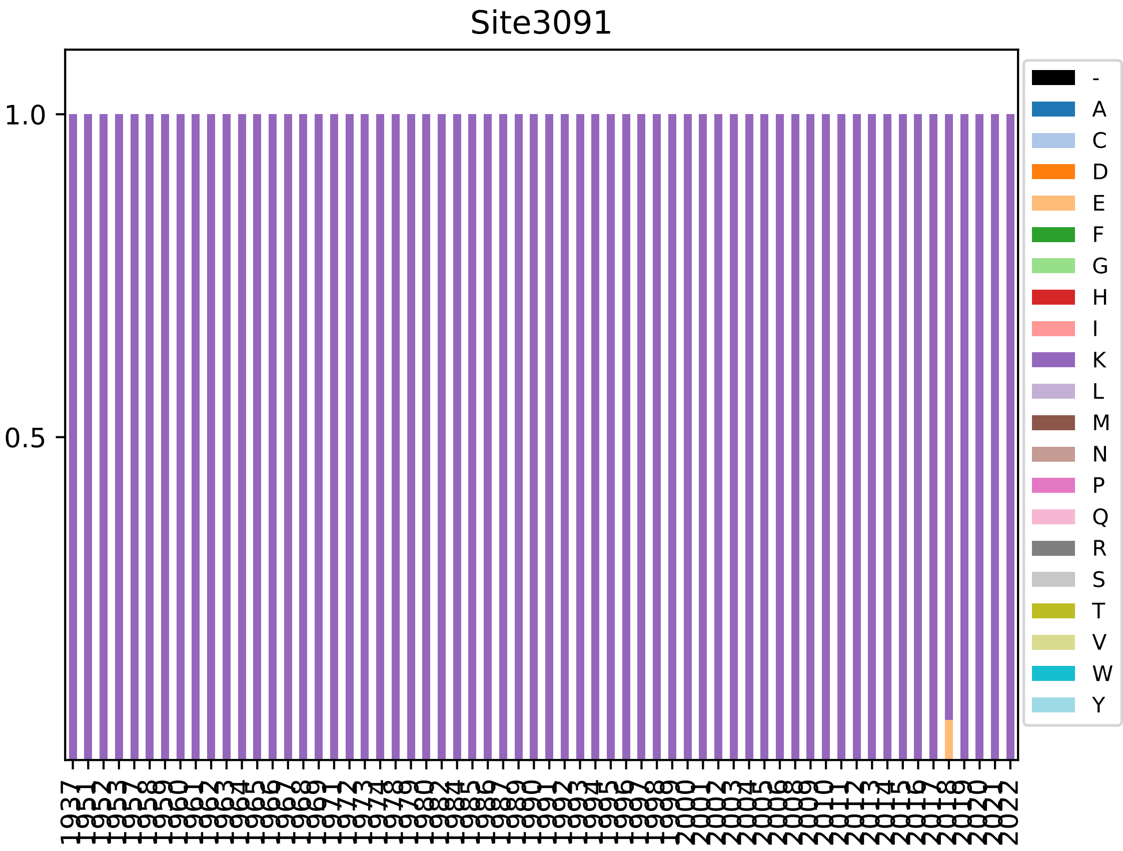
Site3091
1.0
0.5
-
A
C
D
E
F
G
H
I
K
L
M
N
P
Q
R
S
T
V
W
Y

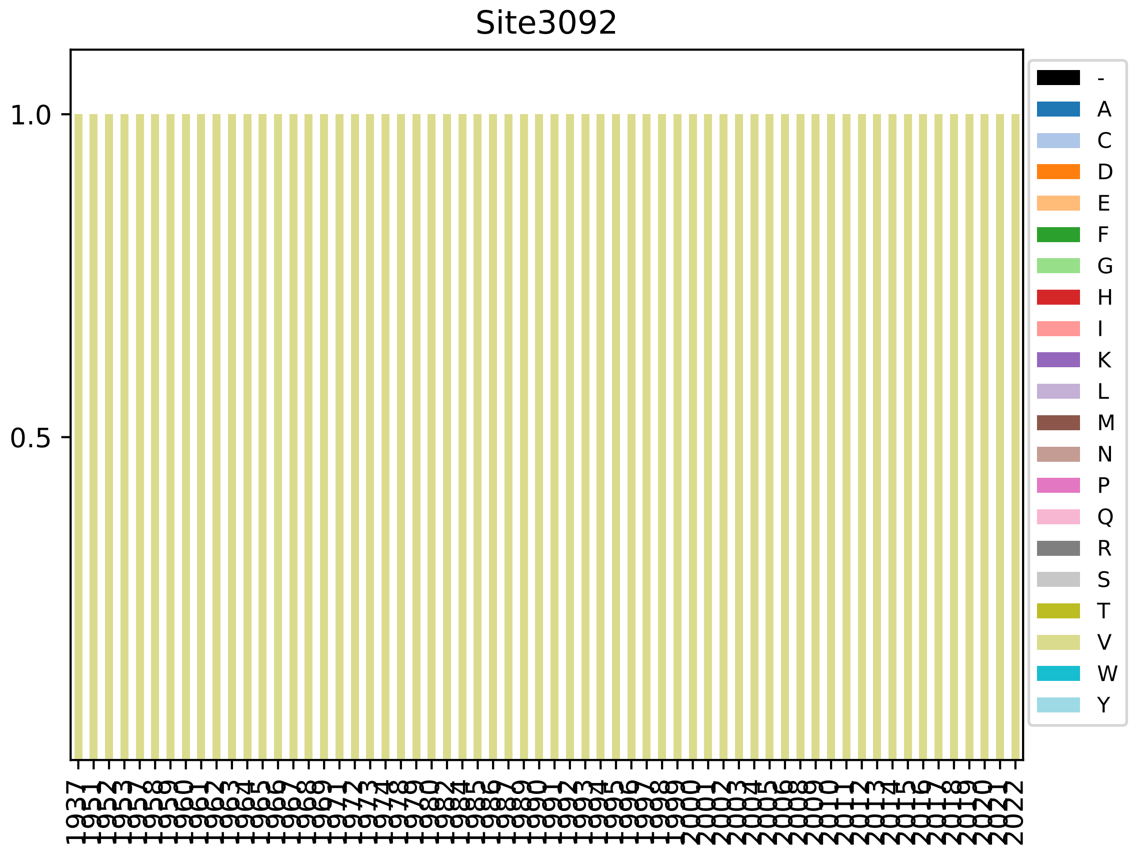

Site3092
1.0
0.5
-
A
C
D
E
F
G
H
I
K
L
M
N
P
Q
R
S
T
V
W
Y

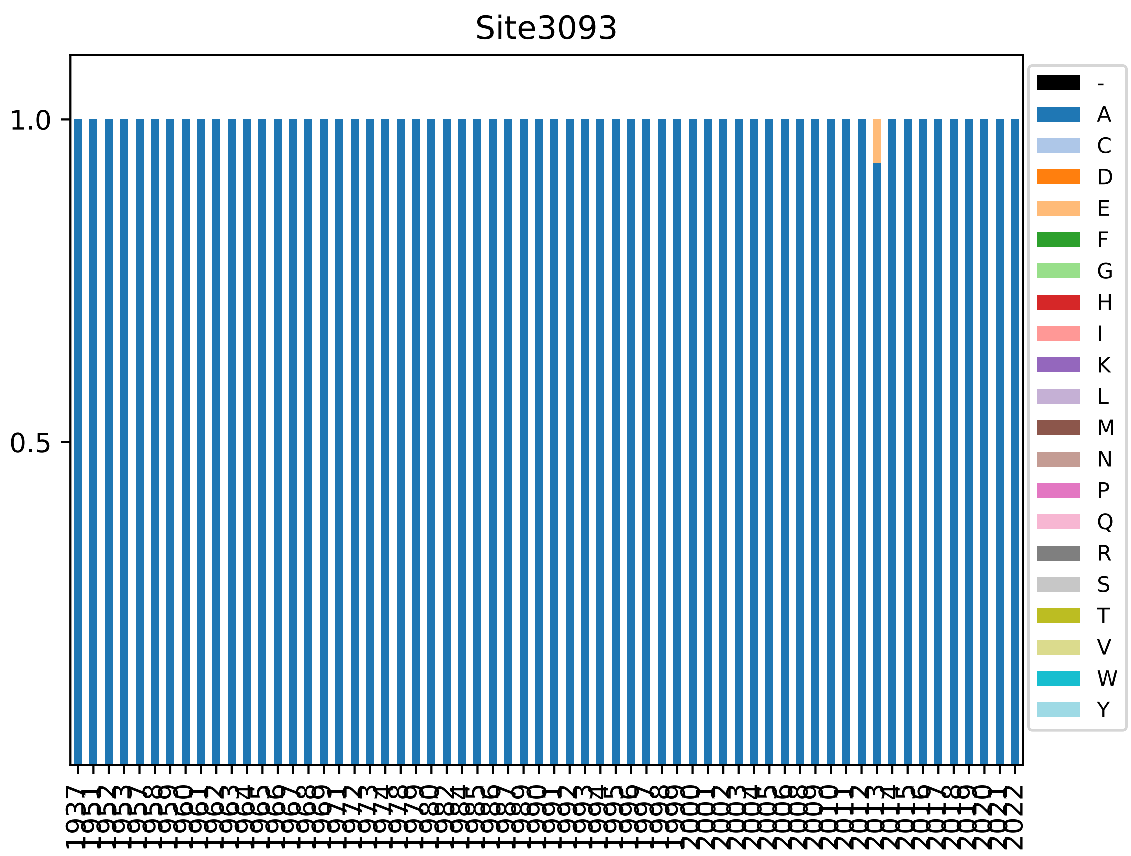
Site3093
1.0
0.5
-
A
C
D
E
F
G
H
I
K
L
M
N
P
Q
R
S
T
V
W
Y

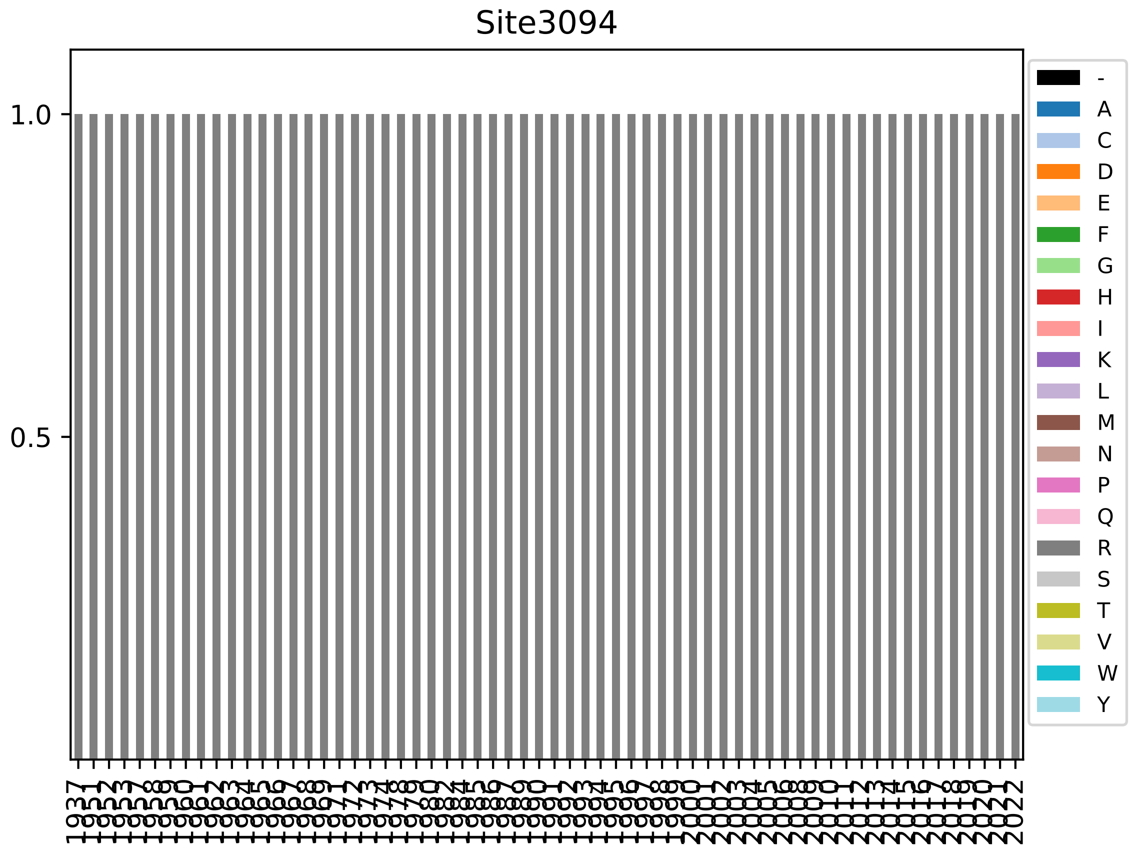

Site3094
1.0
0.5
-
A
C
D
E
F
G
H
I
K
L
M
N
P
Q
R
S
T
V
W
Y

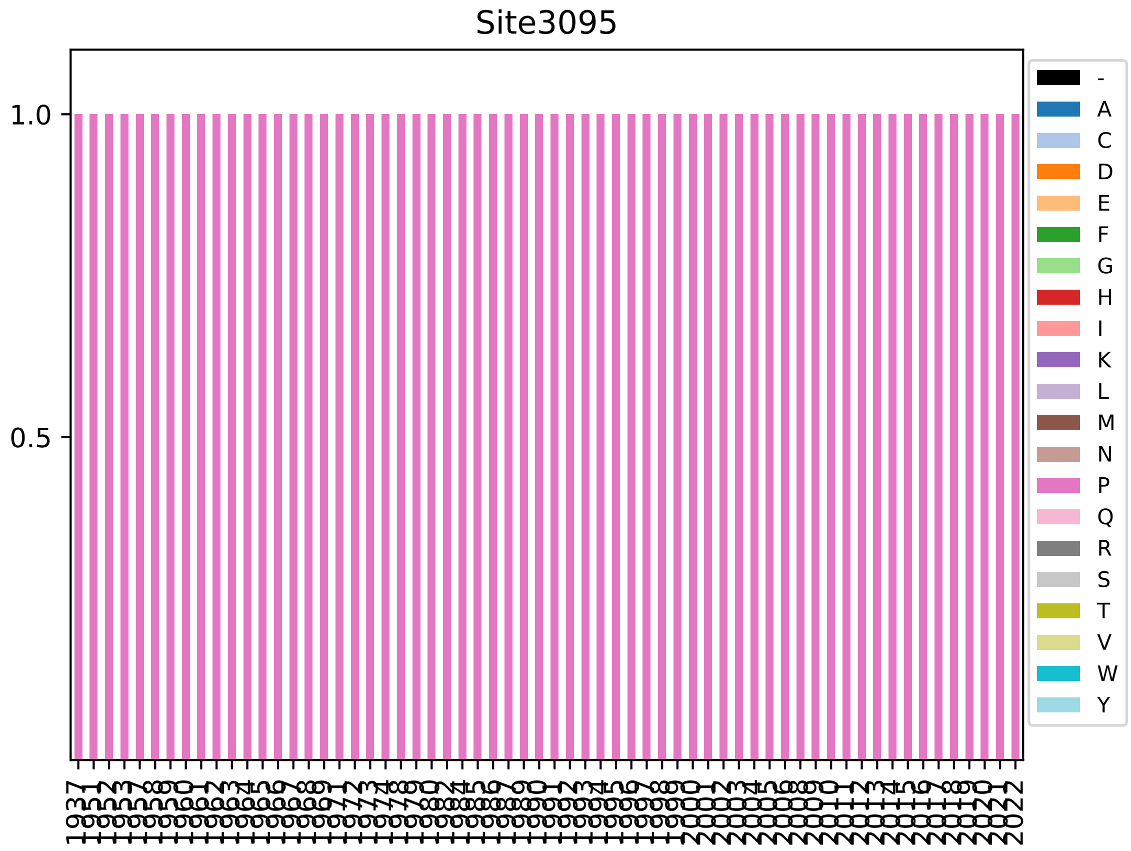
Site3095
1.0
0.5
-
A
C
D
E
F
G
H
I
K
L
M
N
P
Q
R
S
T
V
W
Y

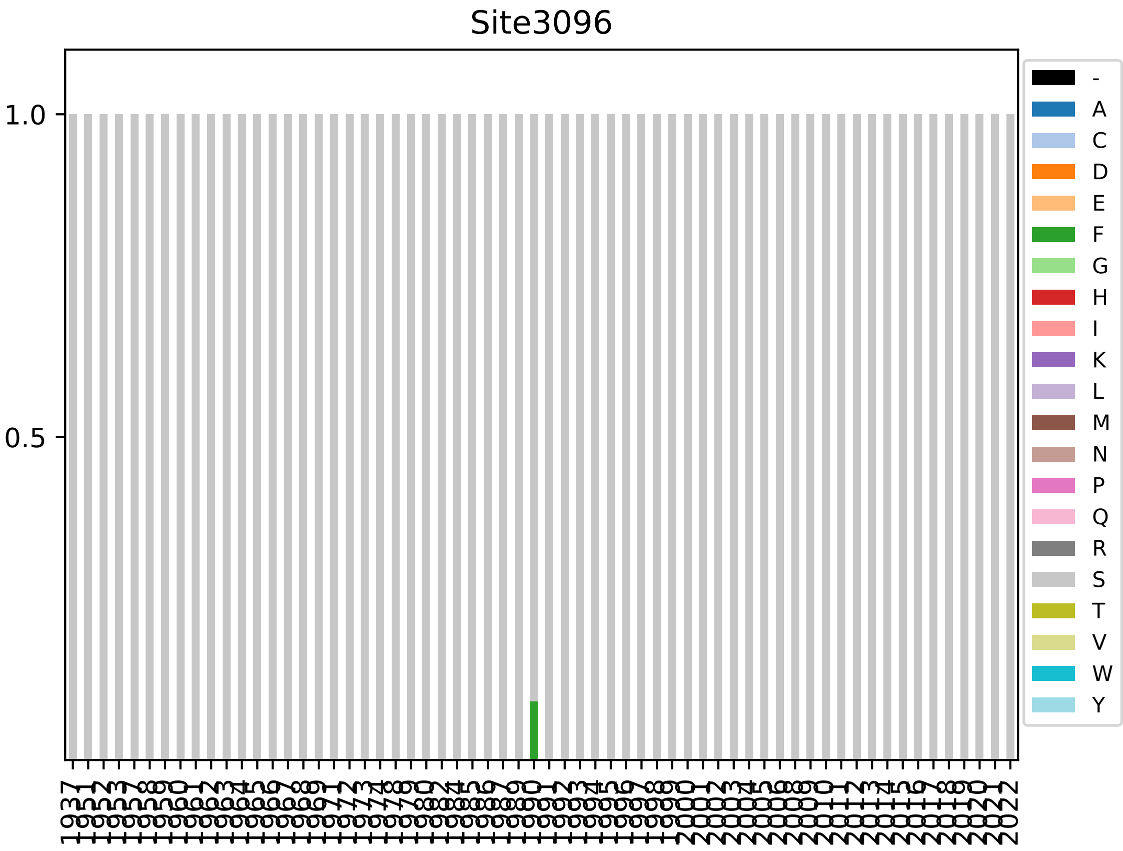
Site3096
1.0
0.5
-
A
C
D
E
F
G
H
I
K
L
M
N
P
Q
R
S
T
V
W
Y

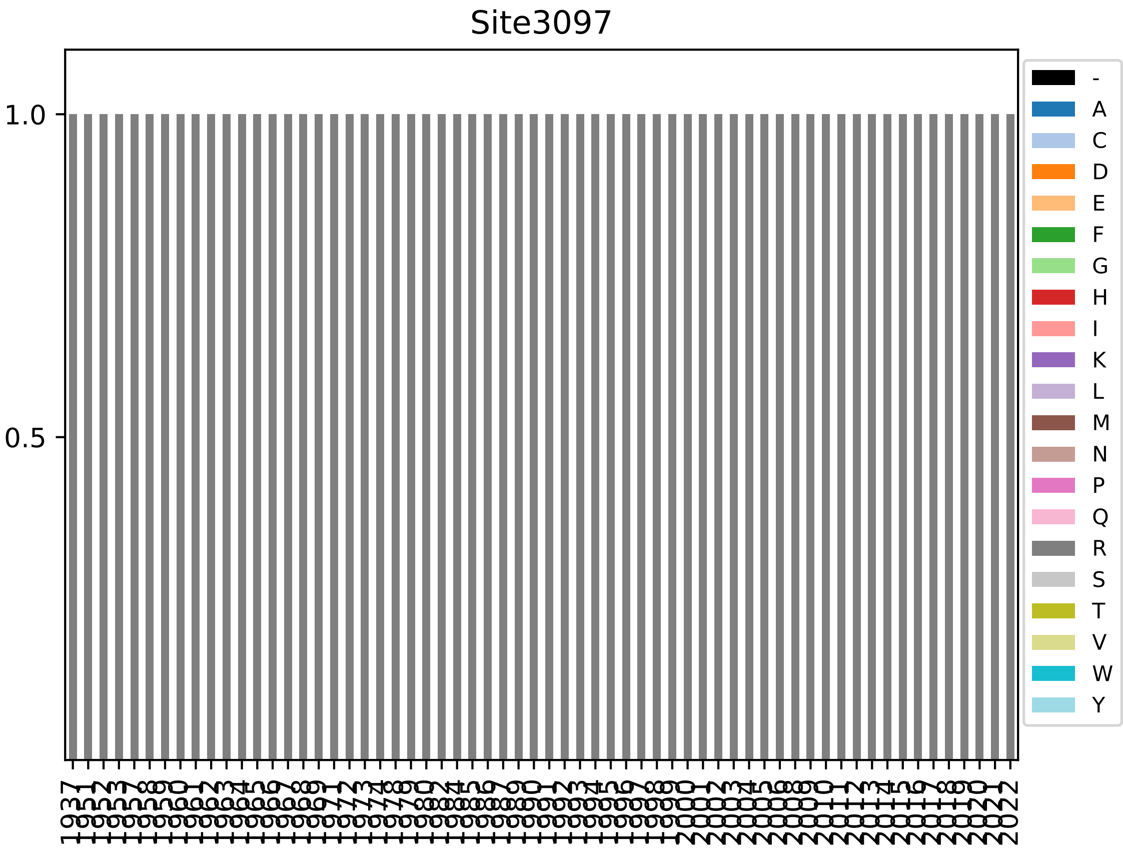
Site3097
1.0
0.5
-
A
C
D
E
F
G
H
I
K
L
M
N
P
Q
R
S
T
V
W
Y

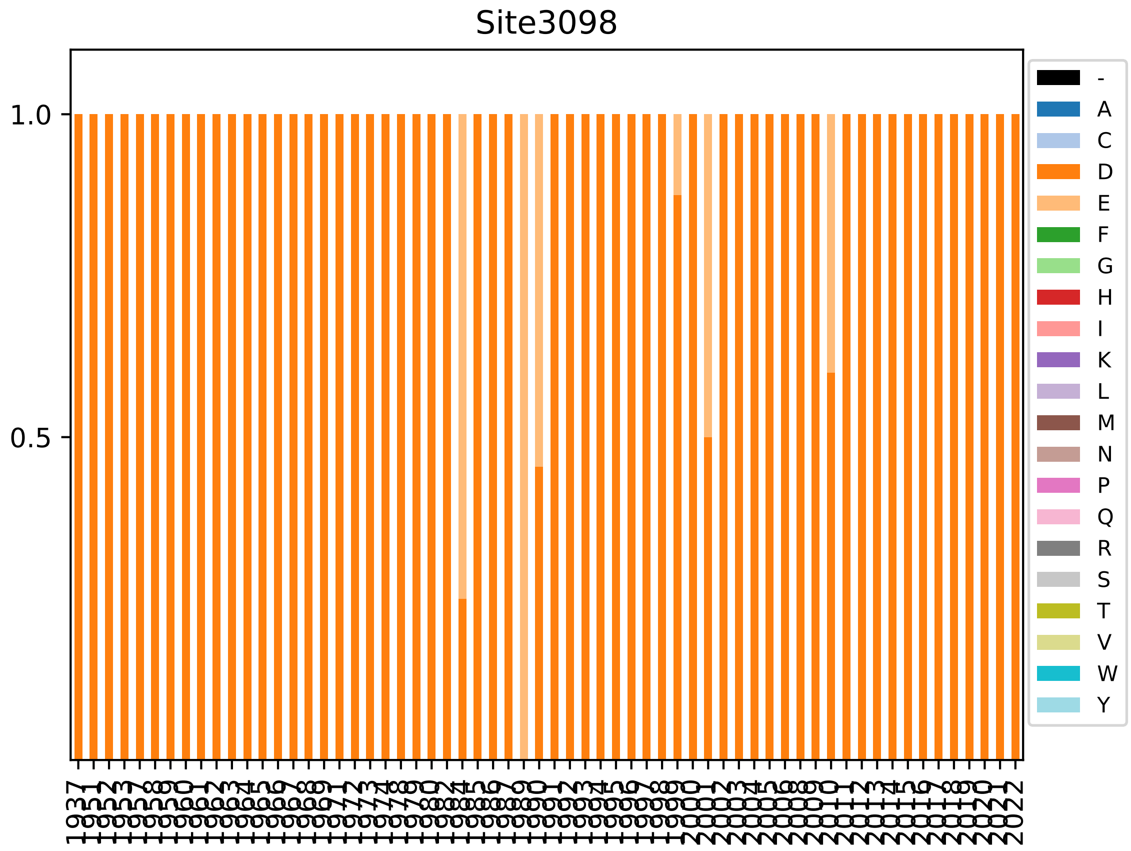
Site3098
1.0
0.5
-
A
C
D
E
F
G
H
I
K
L
M
N
P
Q
R
S
T
V
W
Y

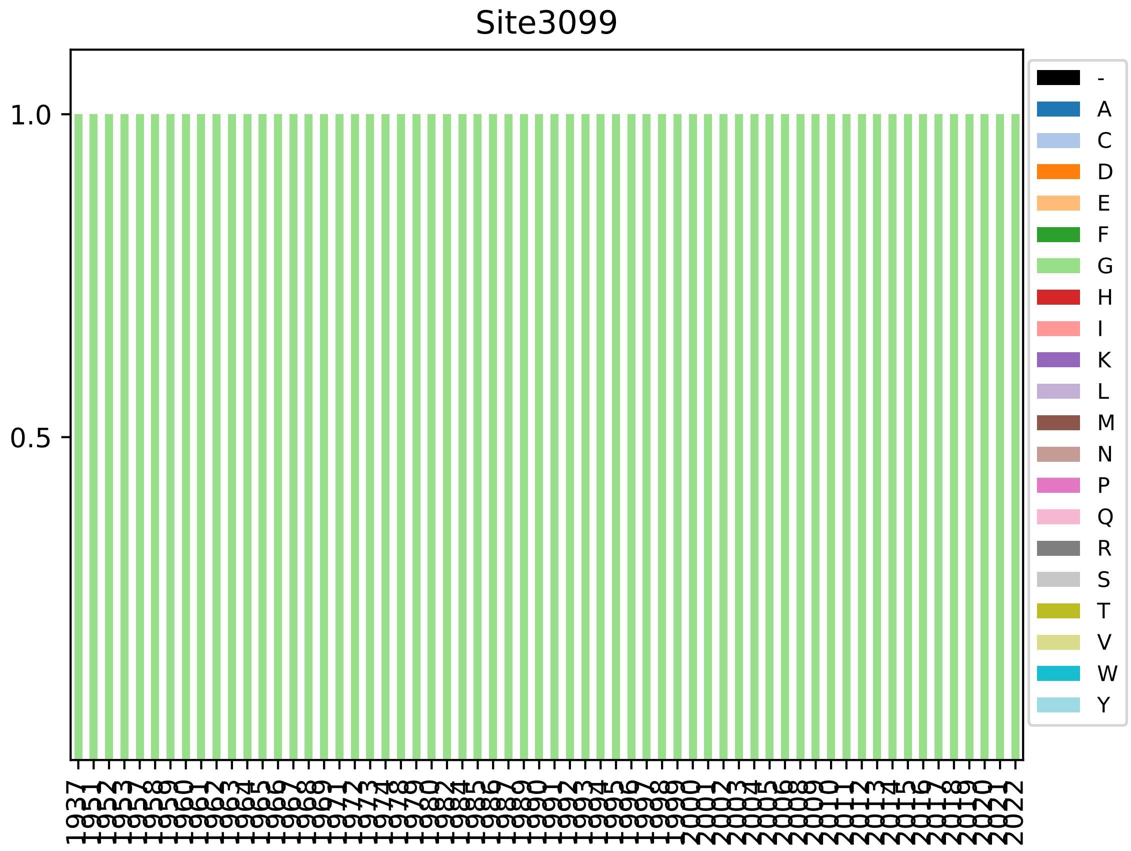
Site3099
1.0
0.5
-
A
C
D
E
F
G
H
I
K
L
M
N
P
Q
R
S
T
V
W
Y

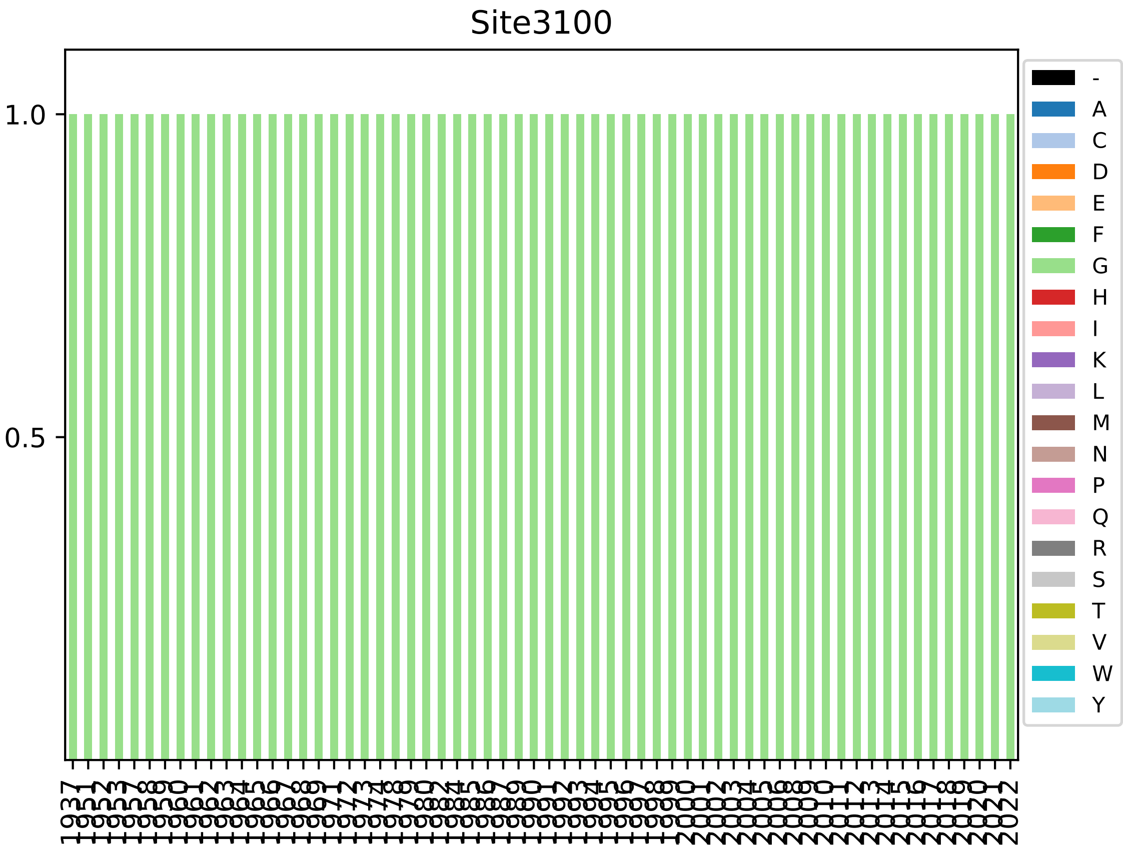
Site3100
1.0
0.5
-
A
C
D
E
F
G
H
I
K
L
M
N
P
Q
R
S
T
V
W
Y

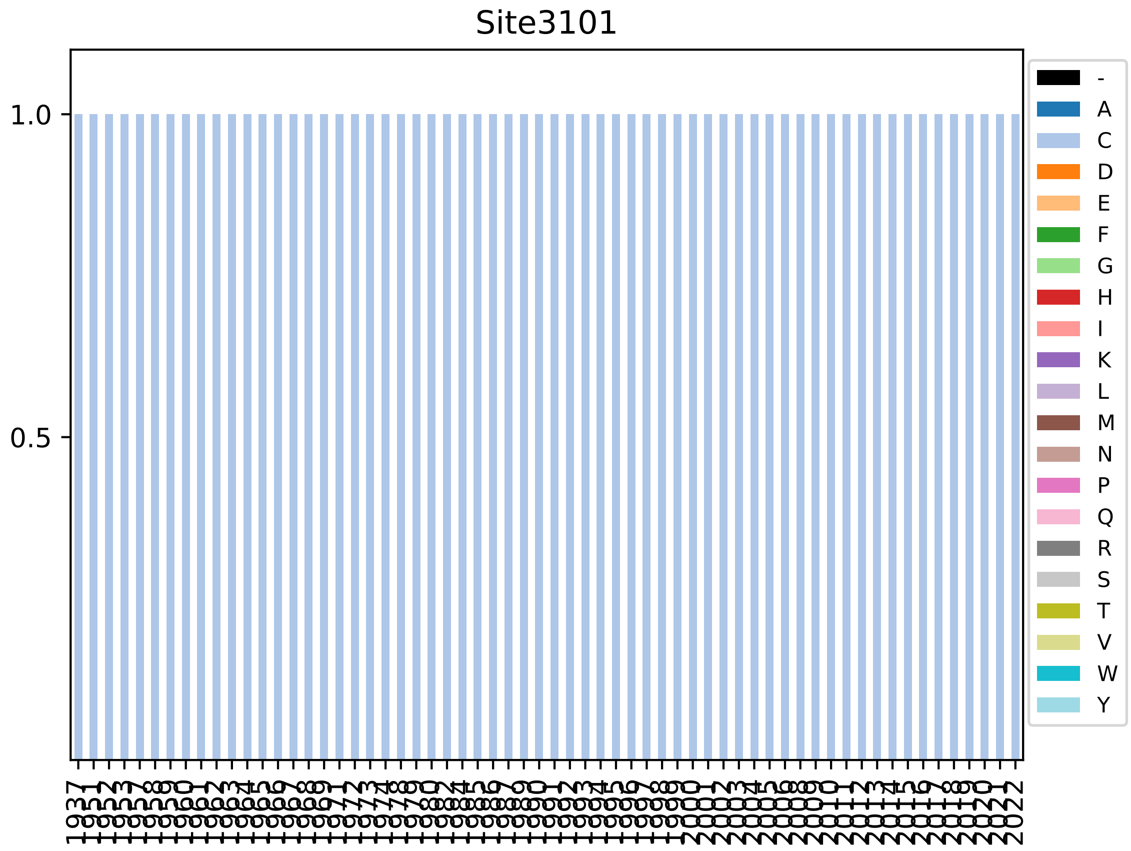
Site3101
1.0
0.5
-
A
C
D
E
F
G
H
I
K
L
M
N
P
Q
R
S
T
V
W
Y

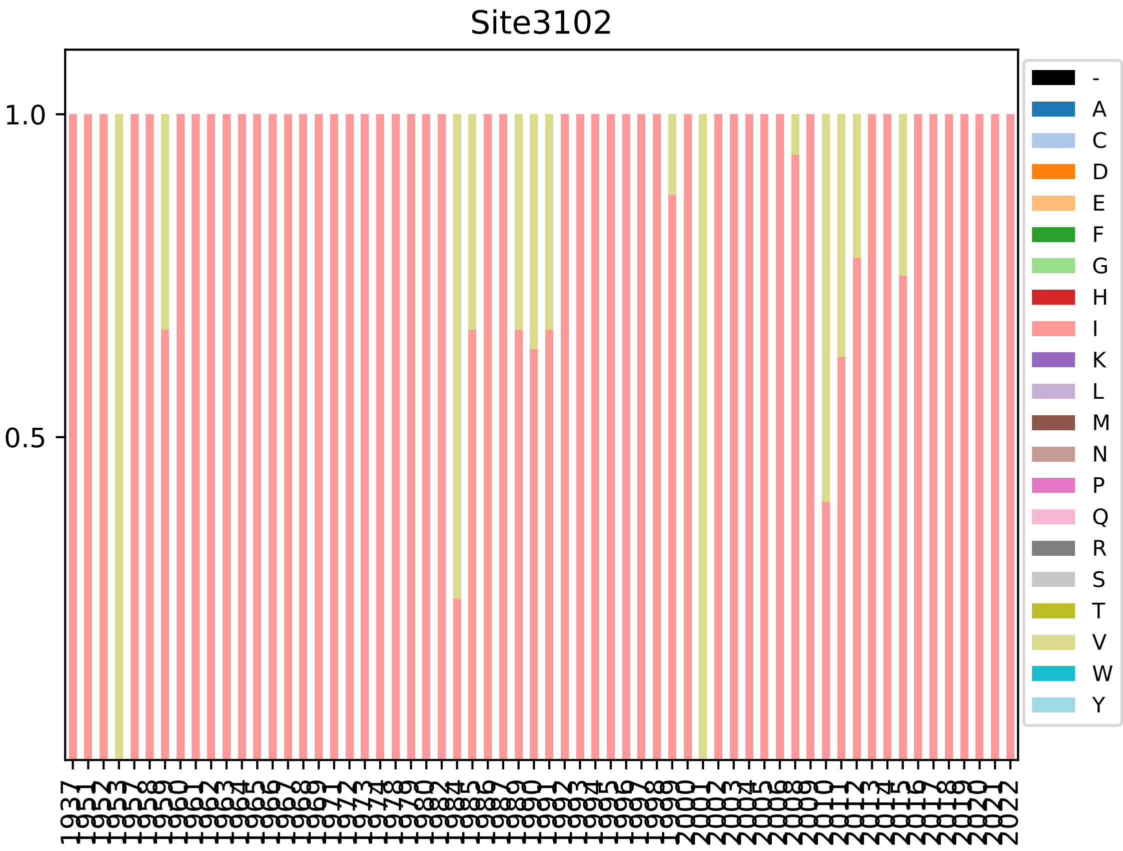
Site3102
1.0
0.5
-
A
C
D
E
F
G
H
I
K
L
M
N
P
Q
R
S
T
V
W
Y

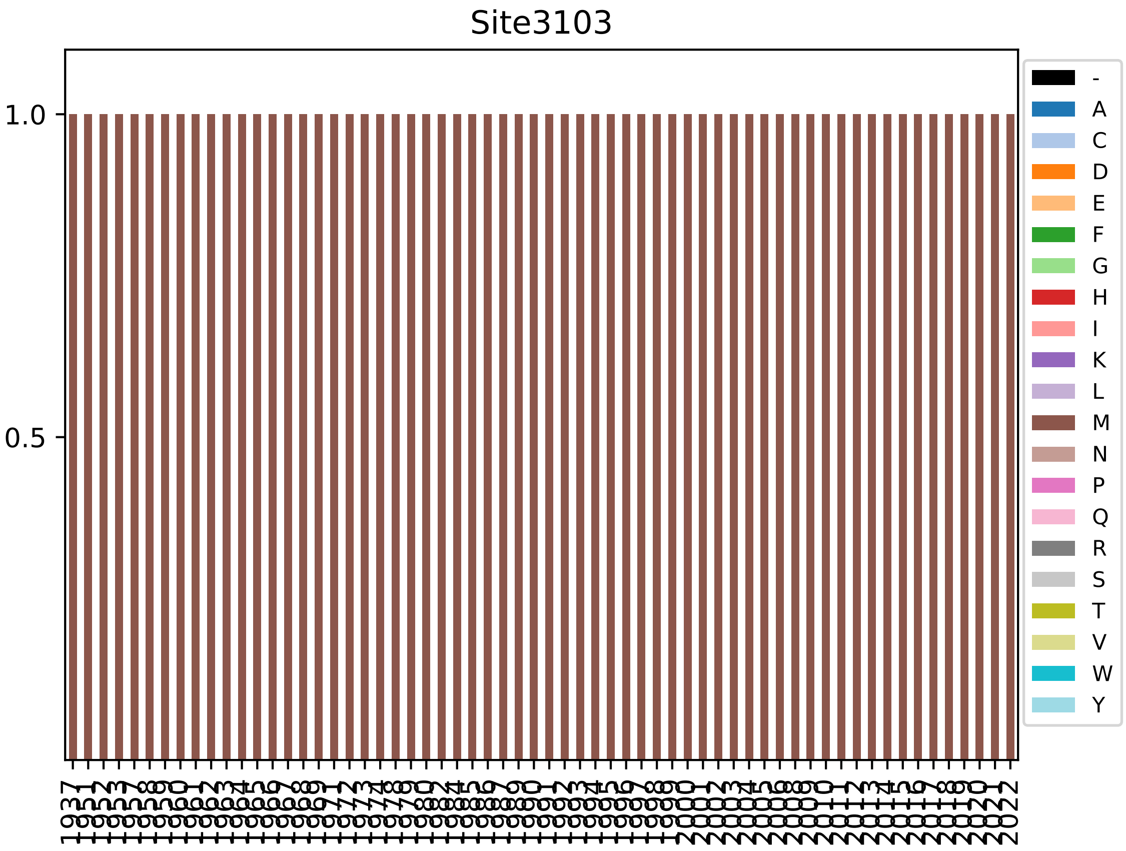
Site3103
1.0
0.5
-
A
C
D
E
F
G
H
I
K
L
M
N
P
Q
R
S
T
V
W
Y

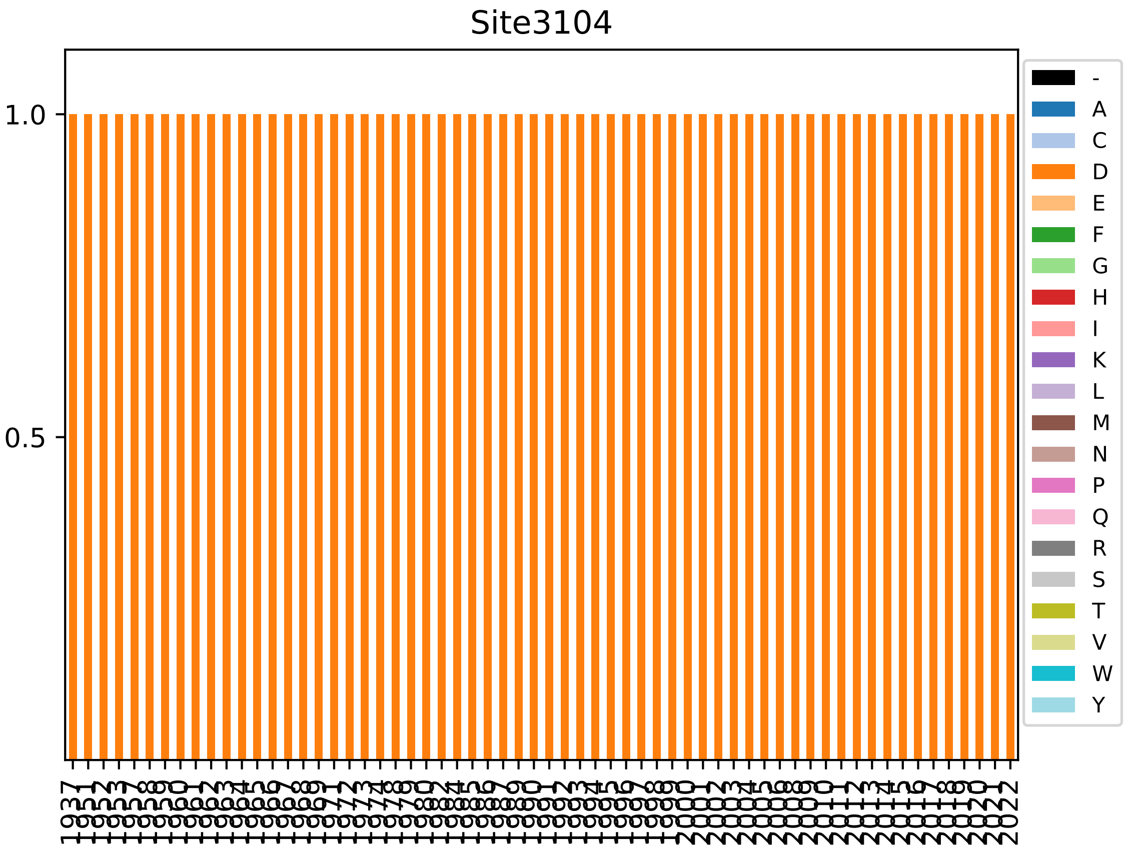
Site3104
1.0
0.5
-
A
C
D
E
F
G
H
I
K
L
M
N
P
Q
R
S
T
V
W
Y

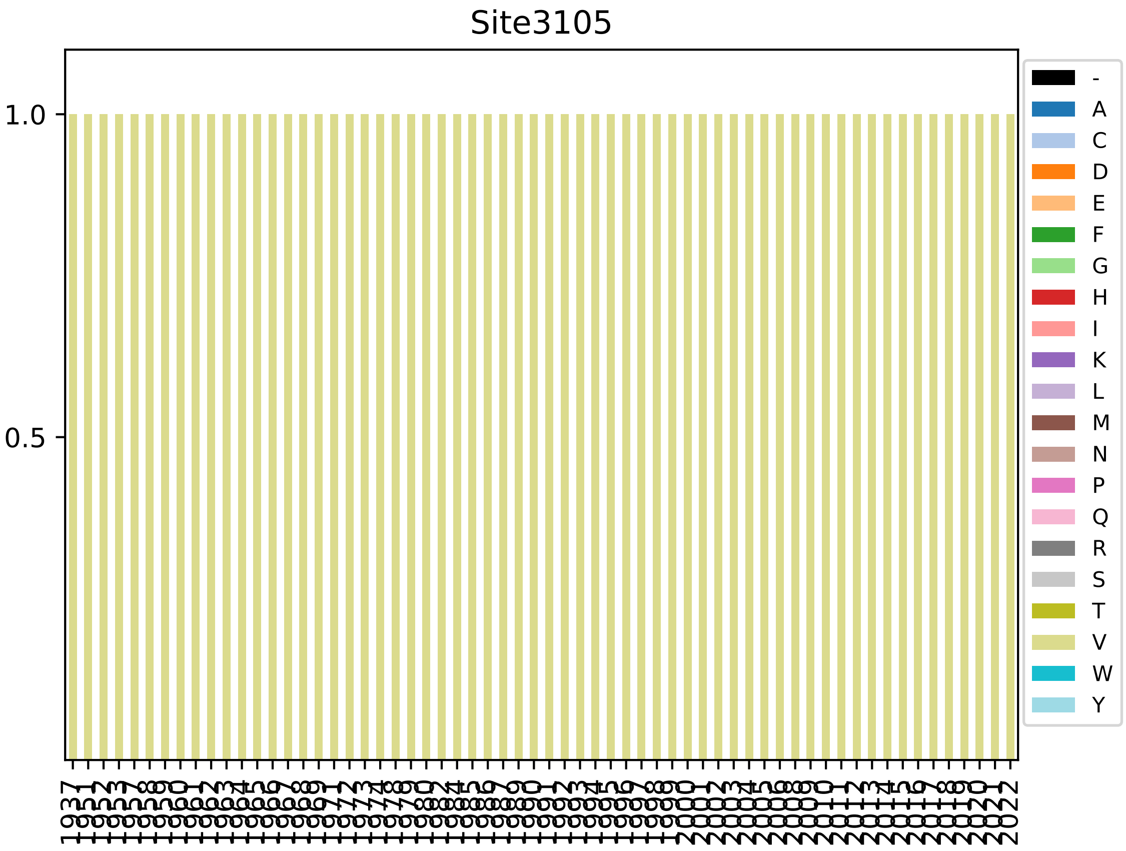
Site3105
1.0
0.5
-
A
C
D
E
F
G
H
I
K
L
M
N
P
Q
R
S
T
V
W
Y

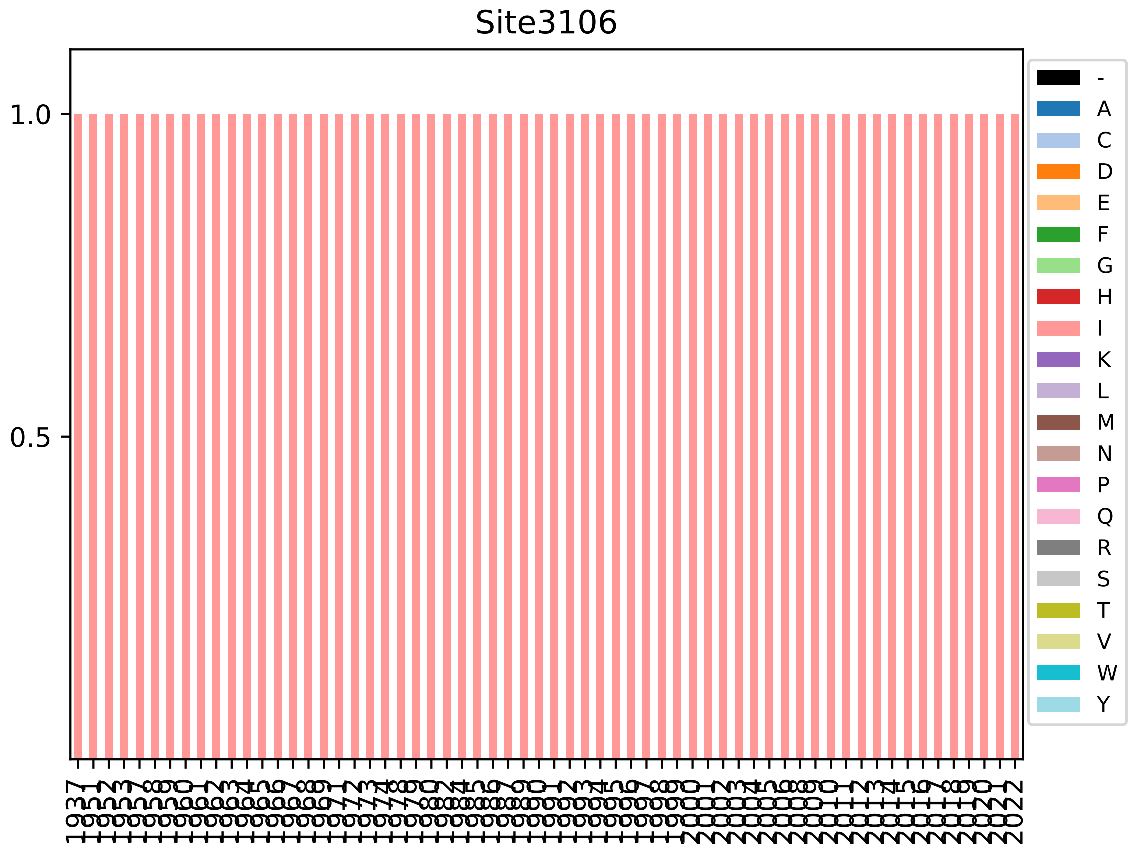
Site3106
1.0
0.5
-
A
C
D
E
F
G
H
I
K
L
M
N
P
Q
R
S
T
V
W
Y

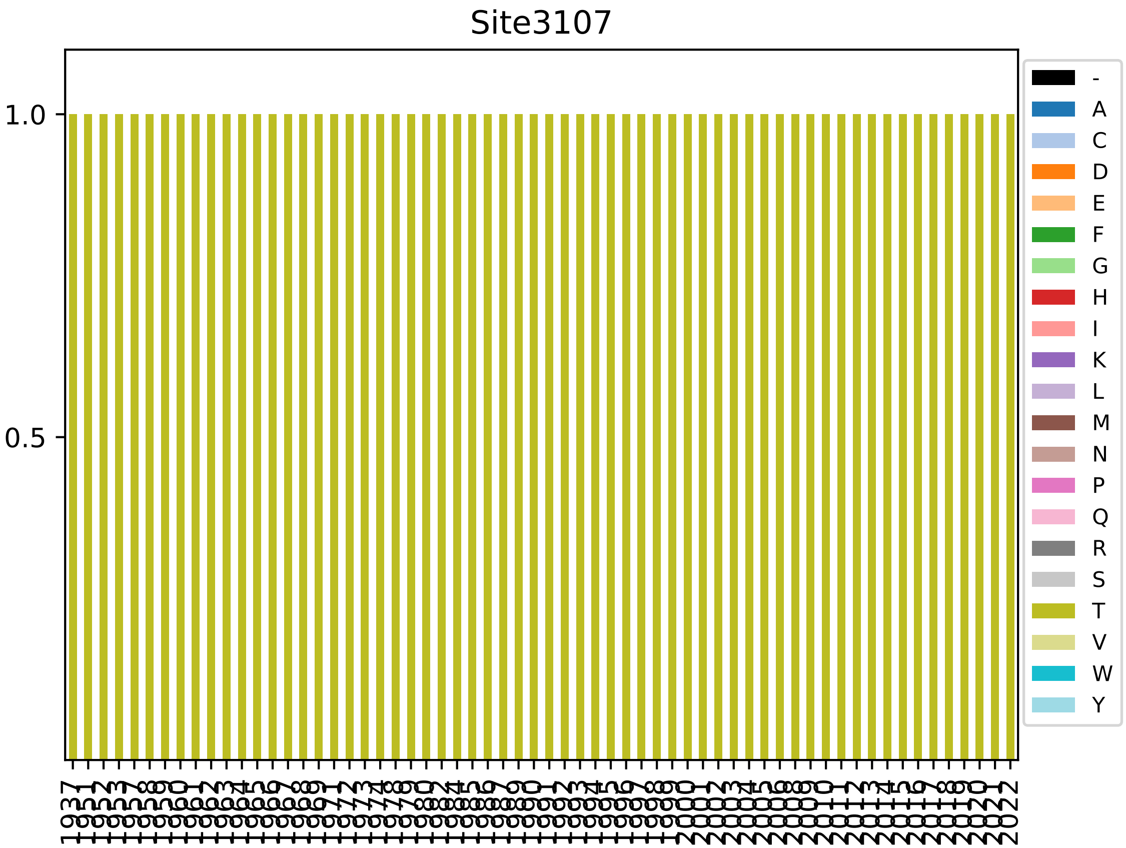
Site3107
1.0
0.5
-
A
C
D
E
F
G
H
I
K
L
M
N
P
Q
R
S
T
V
W
Y

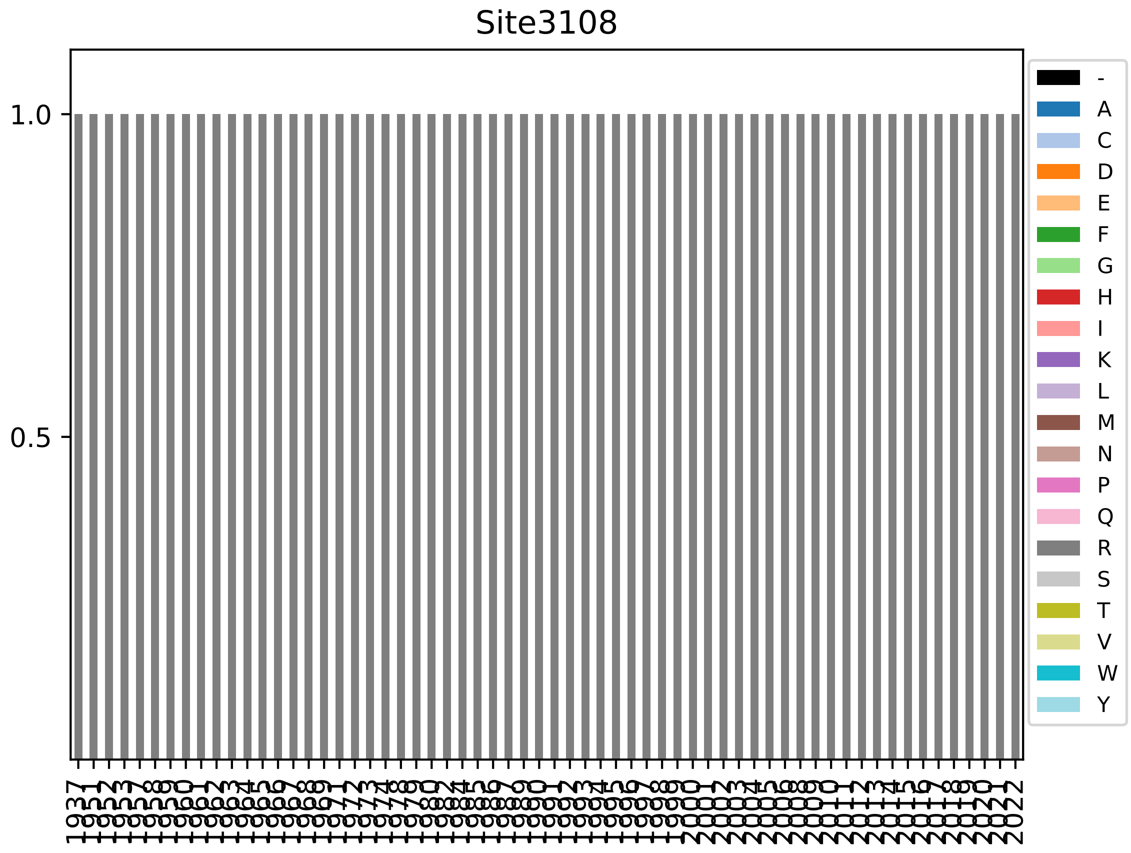
Site3108
1.0
0.5
-
A
C
D
E
F
G
H
I
K
L
M
N
P
Q
R
S
T
V
W
Y

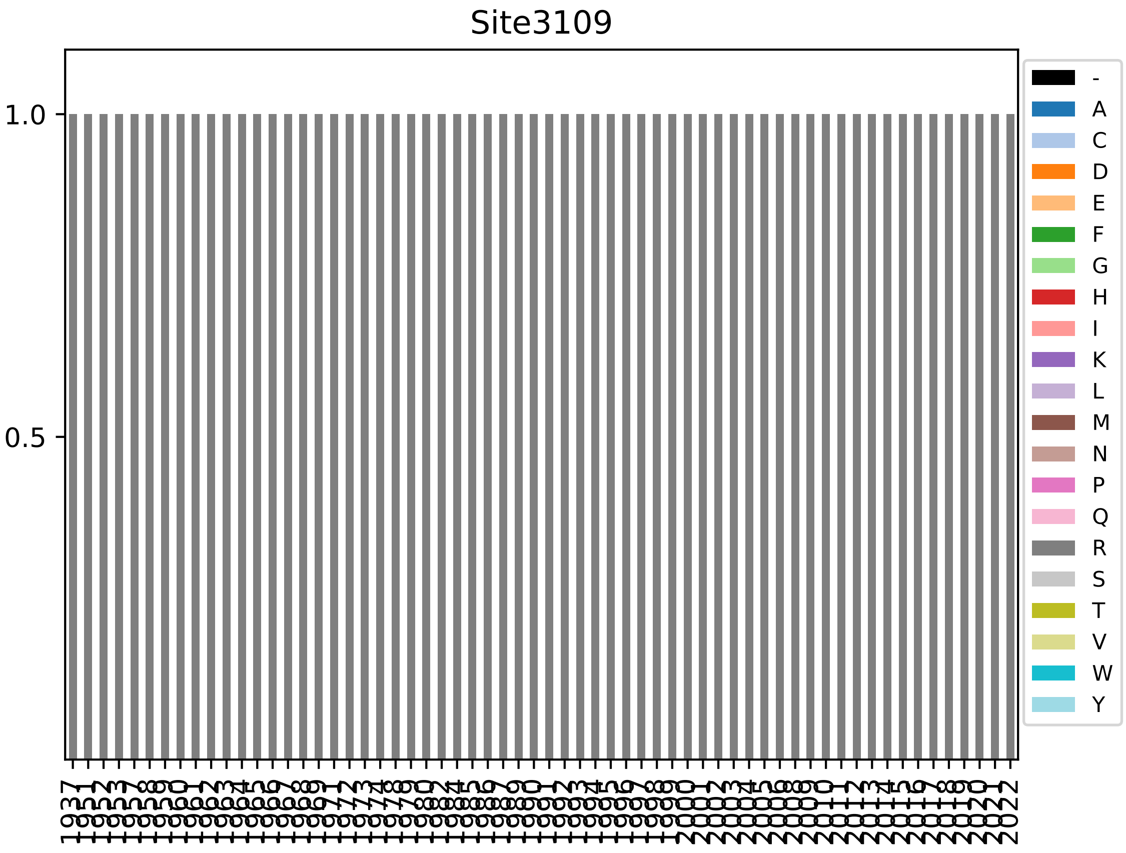
Site3109
1.0
0.5
-
A
C
D
E
F
G
H
I
K
L
M
N
P
Q
R
S
T
V
W
Y

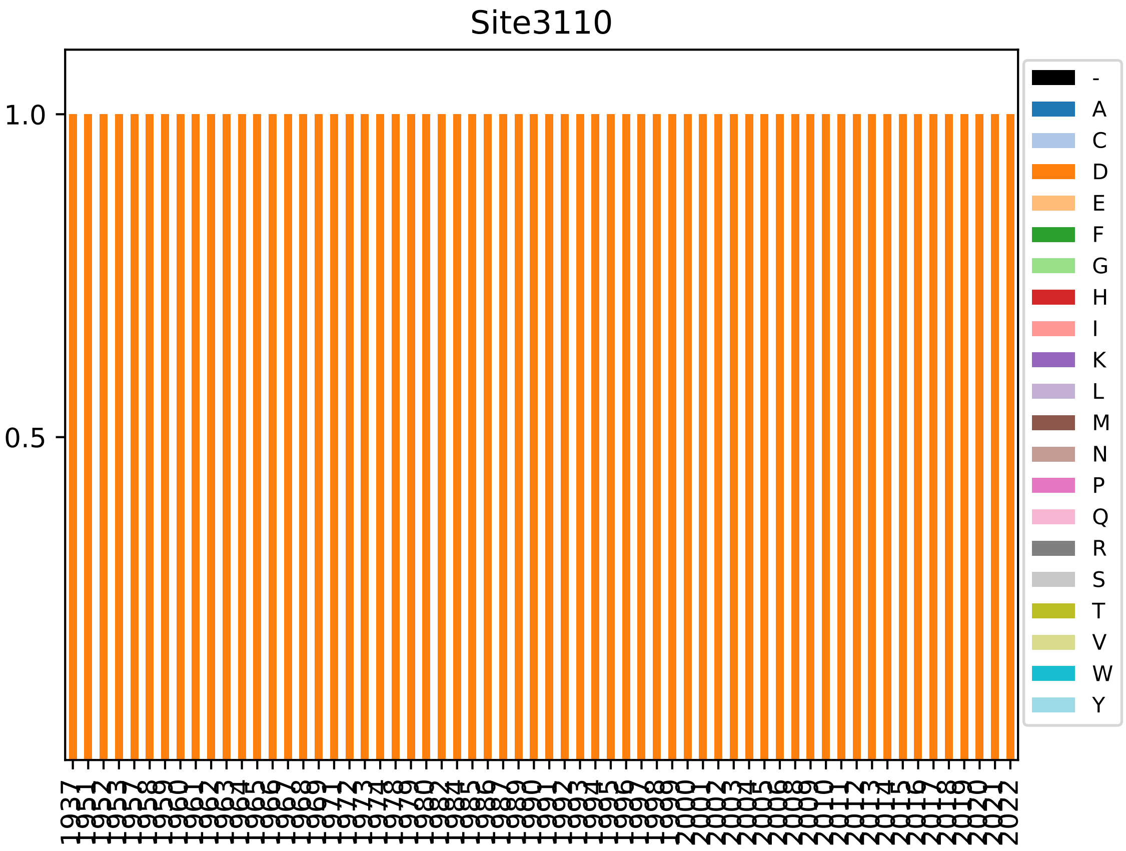
Site3110
1.0
0.5
-
A
C
D
E
F
G
H
I
K
L
M
N
P
Q
R
S
T
V
W
Y

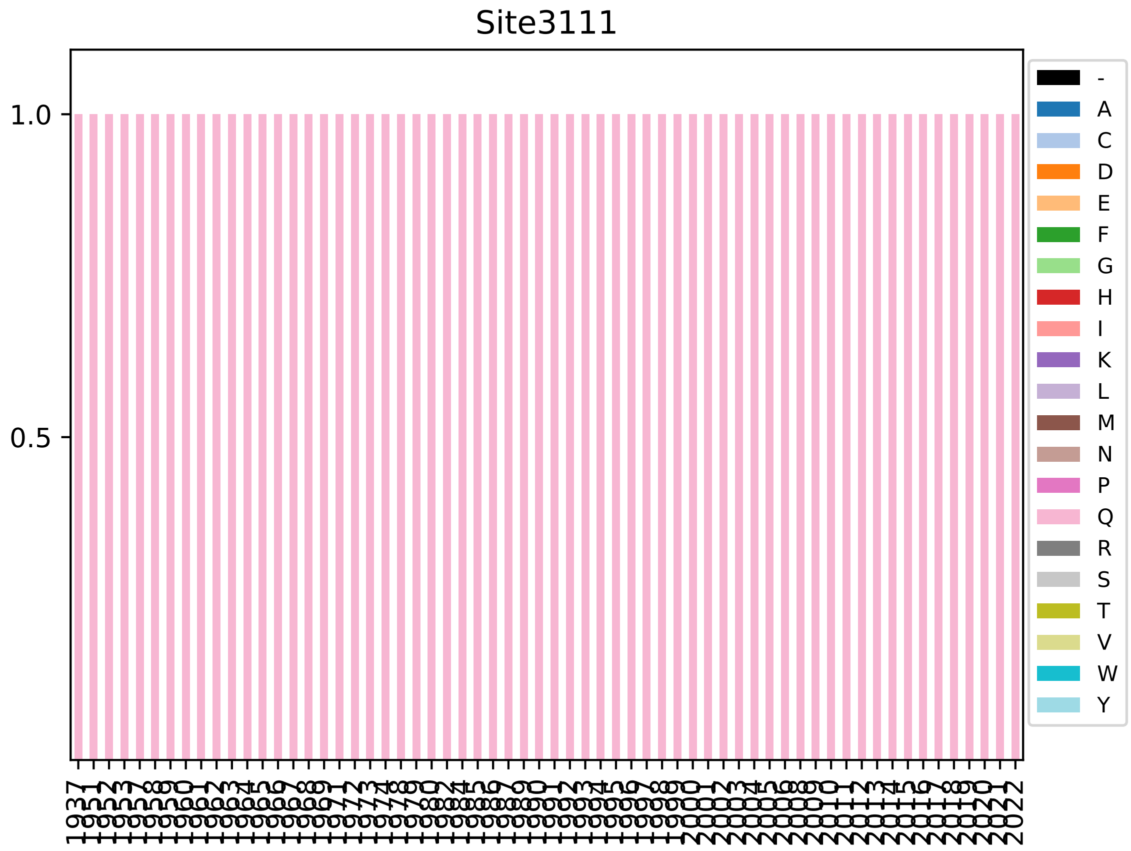
Site3111
1.0
0.5
-
A
C
D
E
F
G
H
I
K
L
M
N
P
Q
R
S
T
V
W
Y

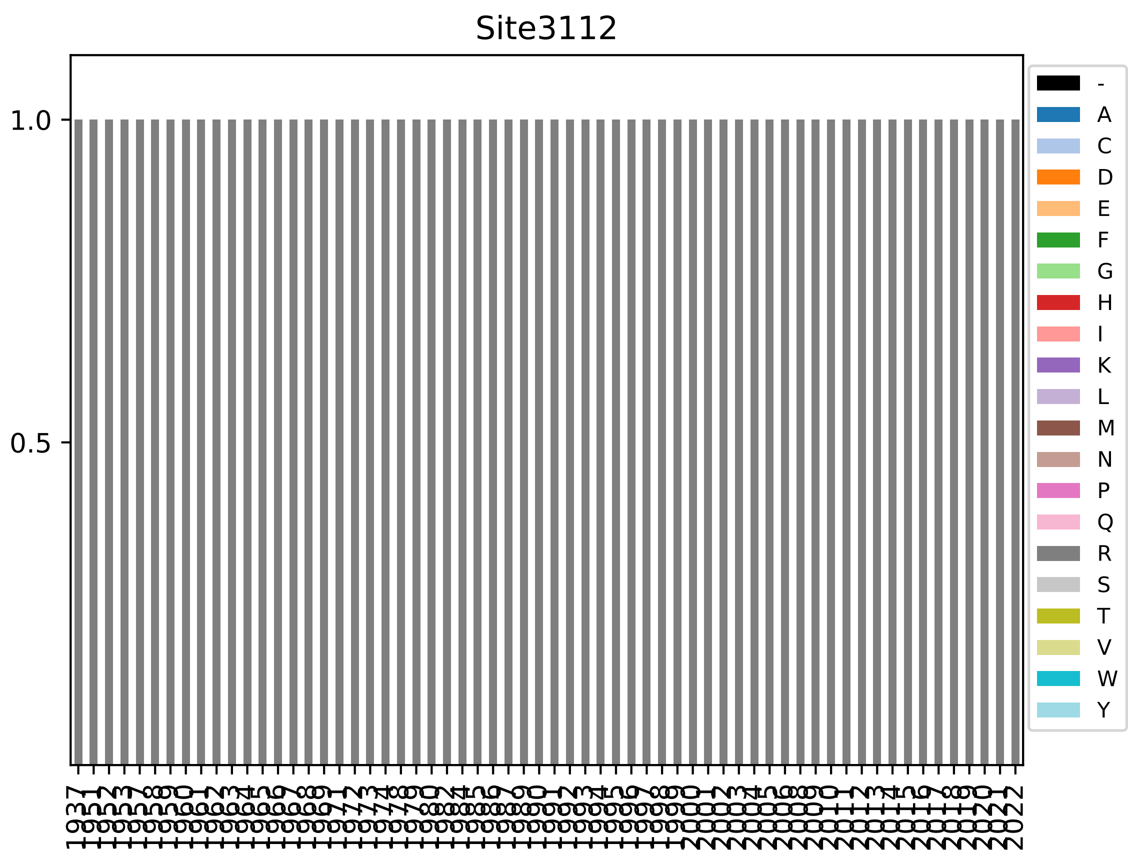

Site3112
1.0
0.5
-
A
C
D
E
F
G
H
I
K
L
M
N
P
Q
R
S
T
V
W
Y

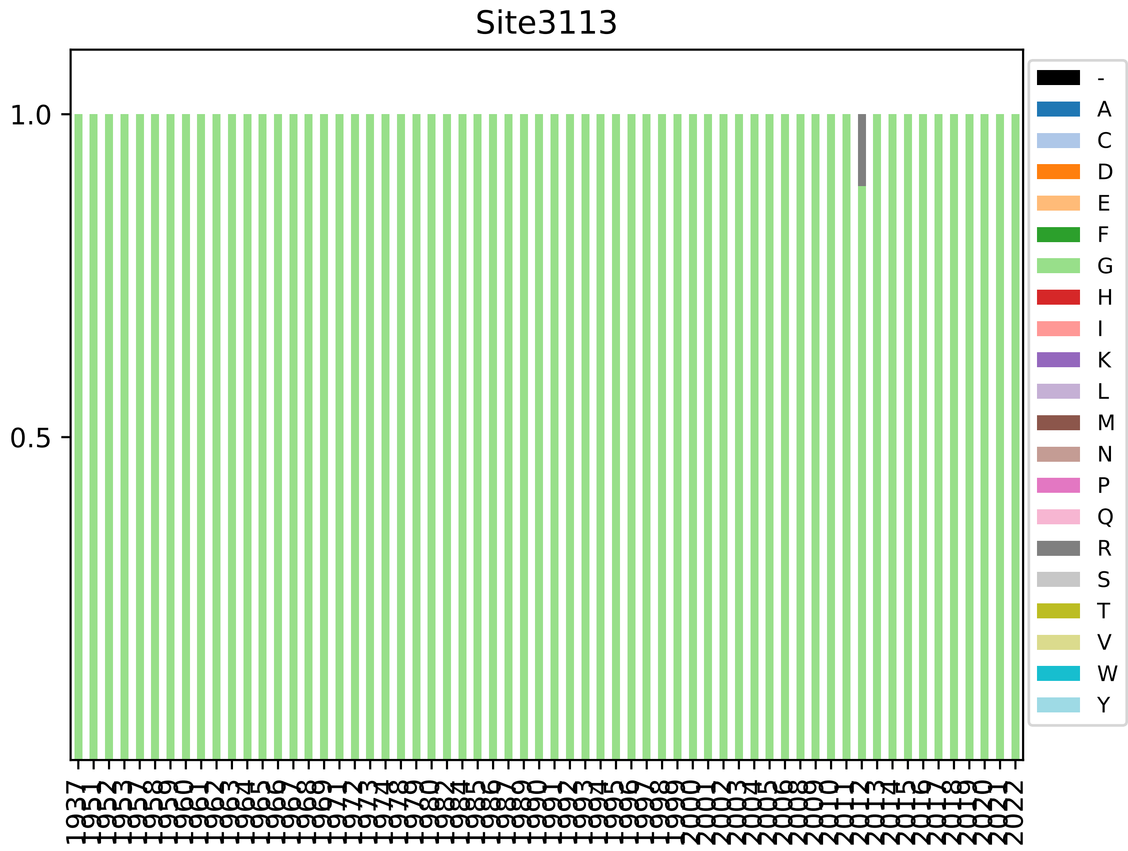
Site3113
1.0
0.5
-
A
C
D
E
F
G
H
I
K
L
M
N
P
Q
R
S
T
V
W
Y

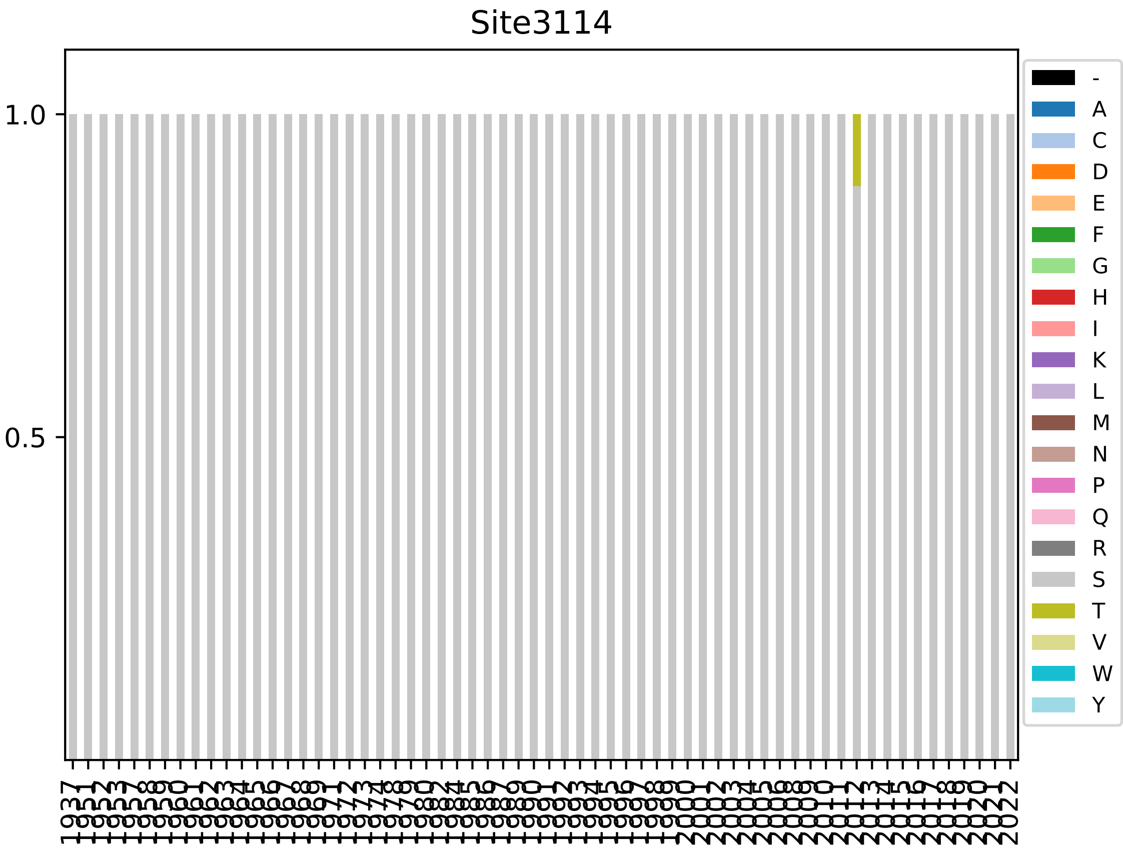
Site3114
1.0
0.5
-
A
C
D
E
F
G
H
I
K
L
M
N
P
Q
R
S
T
V
W
Y

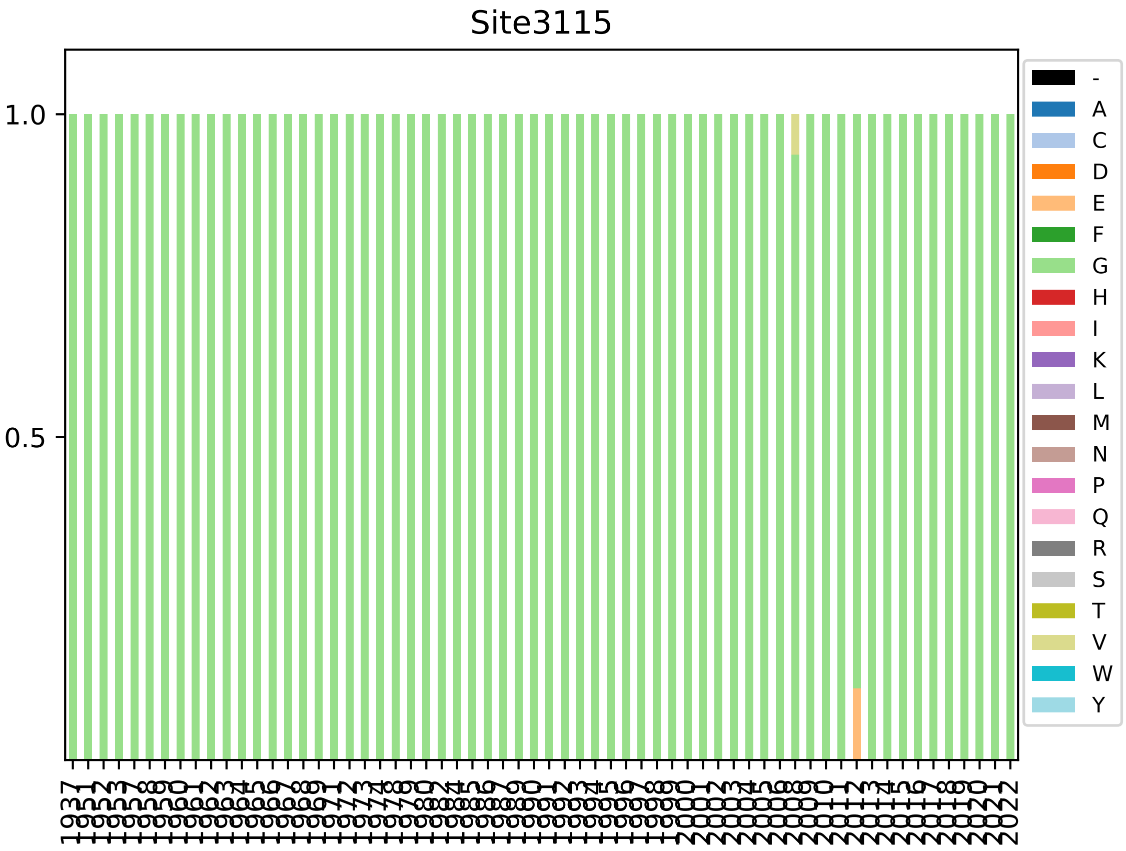
Site3115
1.0
0.5
-
A
C
D
E
F
G
H
I
K
L
M
N
P
Q
R
S
T
V
W
Y

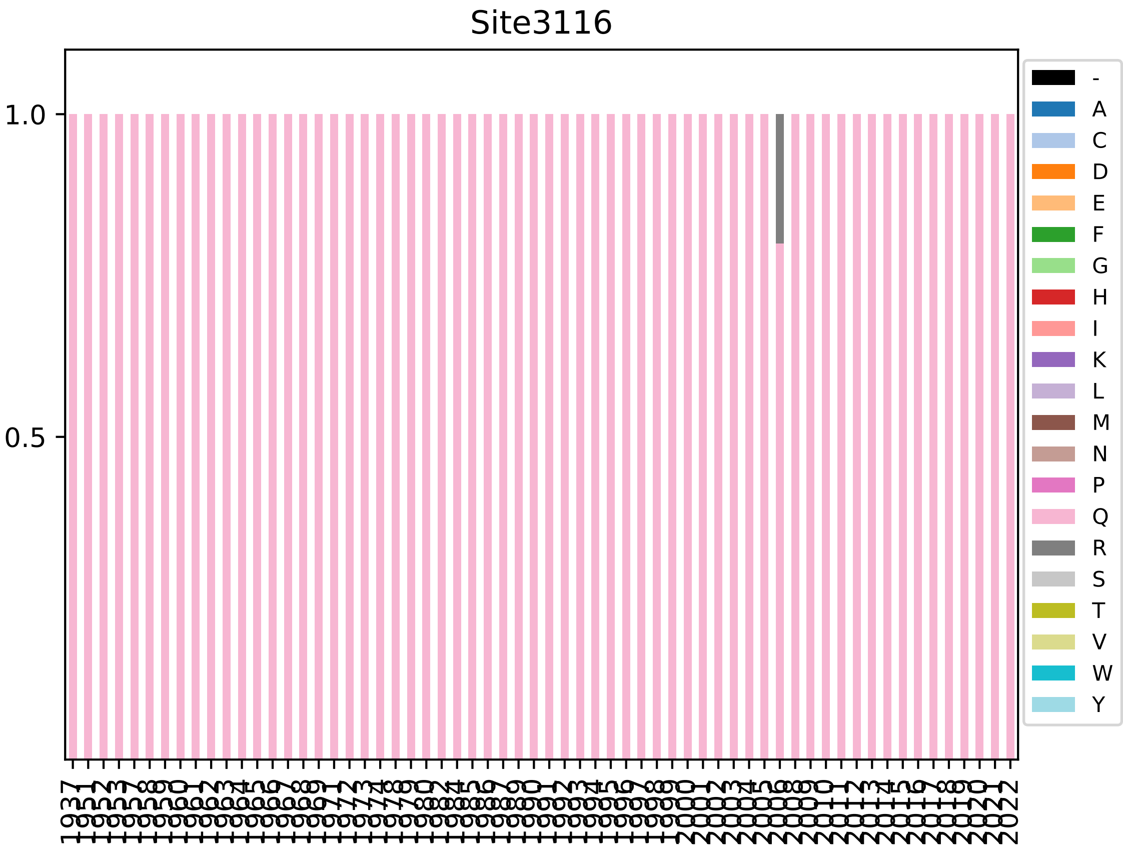
Site3116
1.0
0.5
-
A
C
D
E
F
G
H
I
K
L
M
N
P
Q
R
S
T
V
W
Y

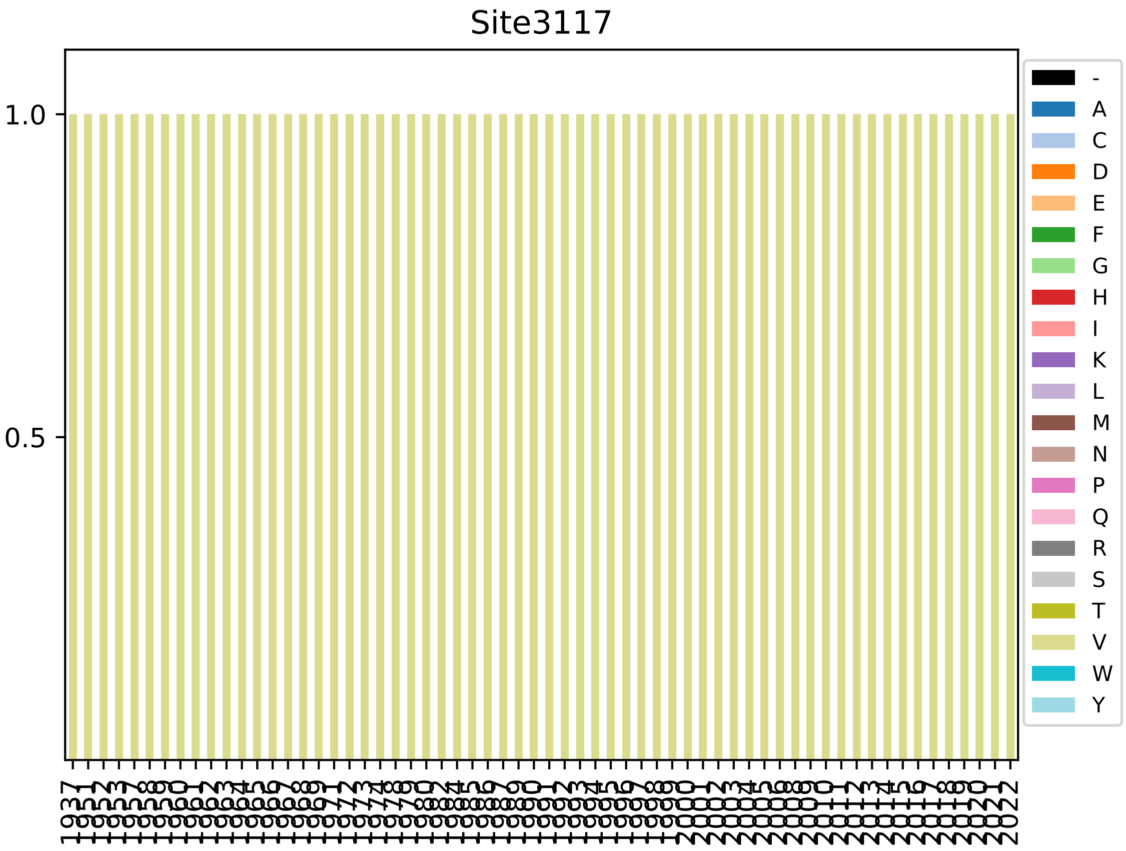

Site3117
1.0
0.5
-
A
C
D
E
F
G
H
I
K
L
M
N
P
Q
R
S
T
V
W
Y

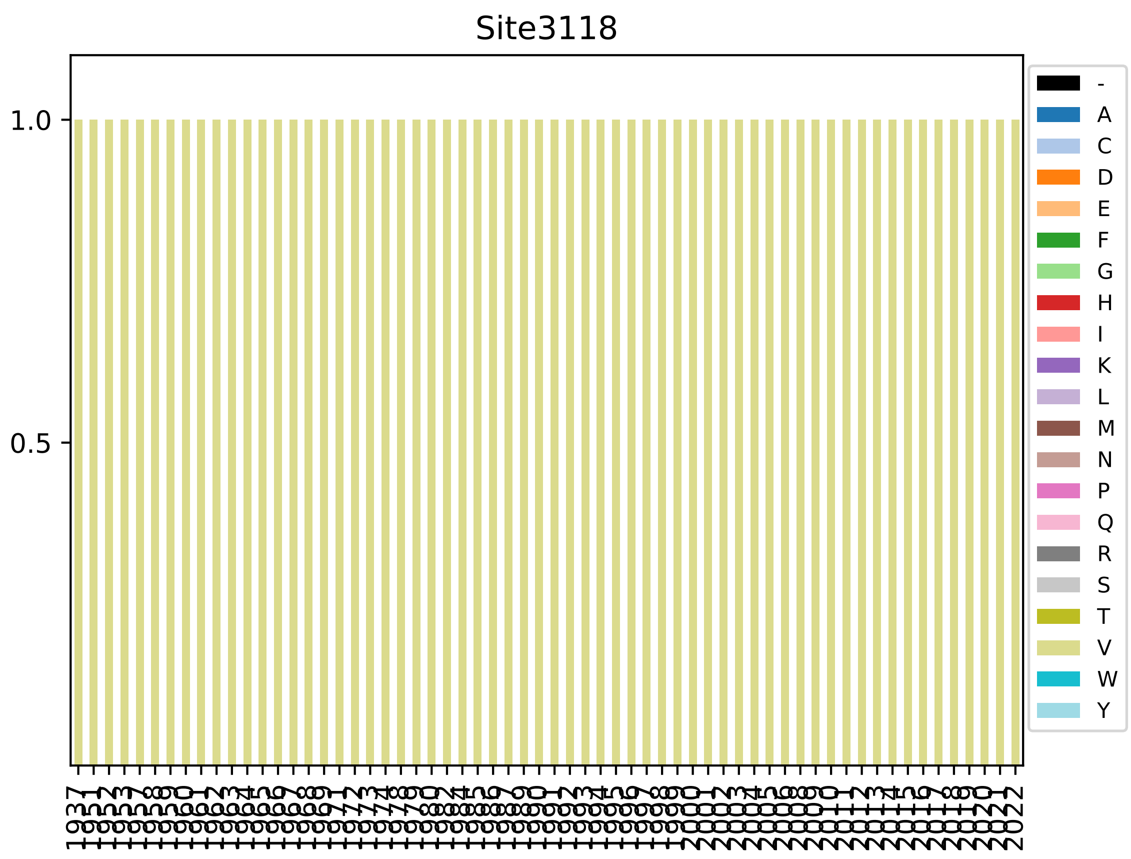
Site3118
1.0
0.5
-
A
C
D
E
F
G
H
I
K
L
M
N
P
Q
R
S
T
V
W
Y

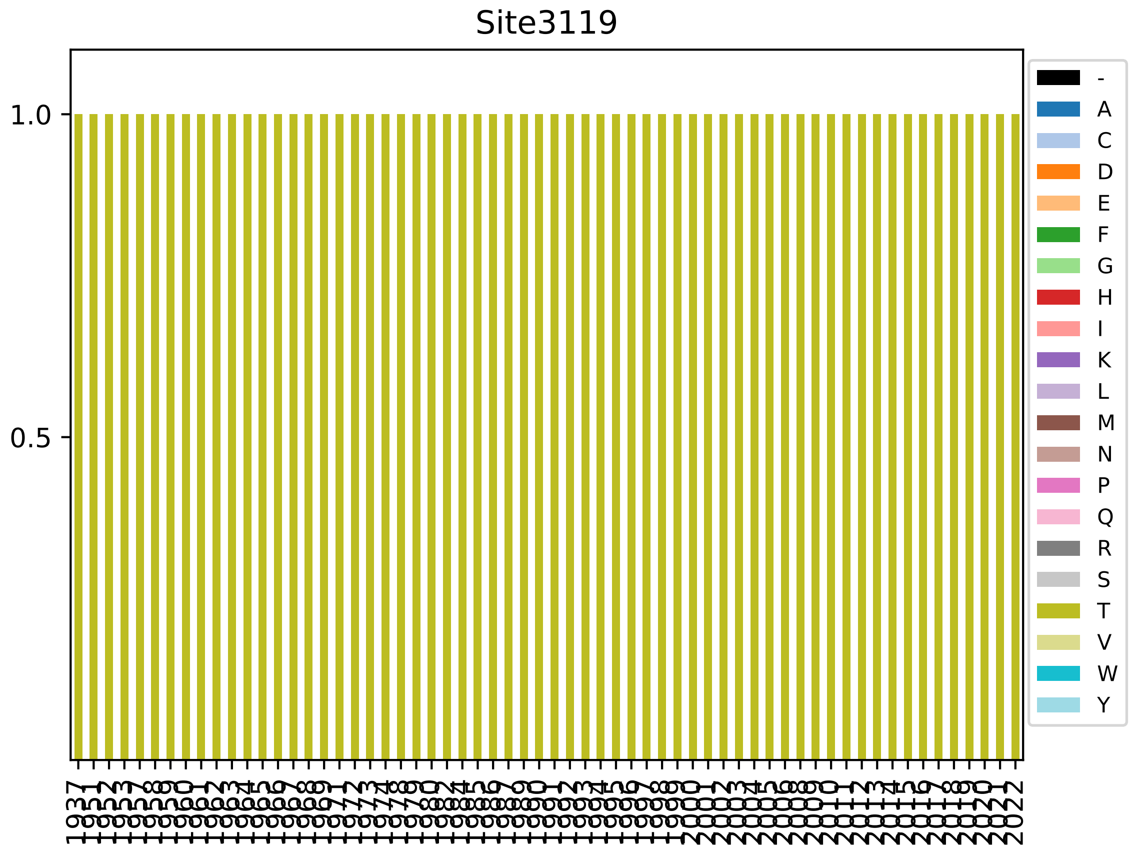
Site3119
1.0
0.5
-
A
C
D
E
F
G
H
I
K
L
M
N
P
Q
R
S
T
V
W
Y

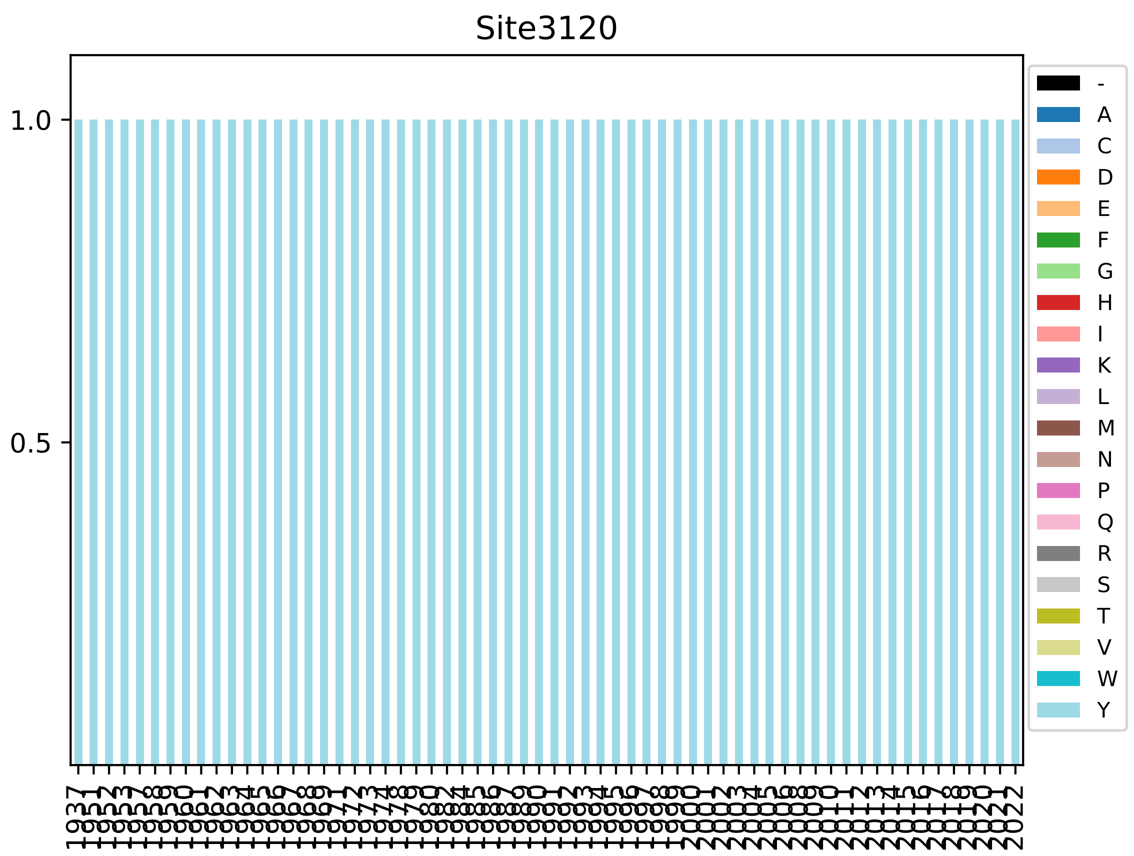
Site3120
1.0
0.5
-
A
C
D
E
F
G
H
I
K
L
M
N
P
Q
R
S
T
V
W
Y

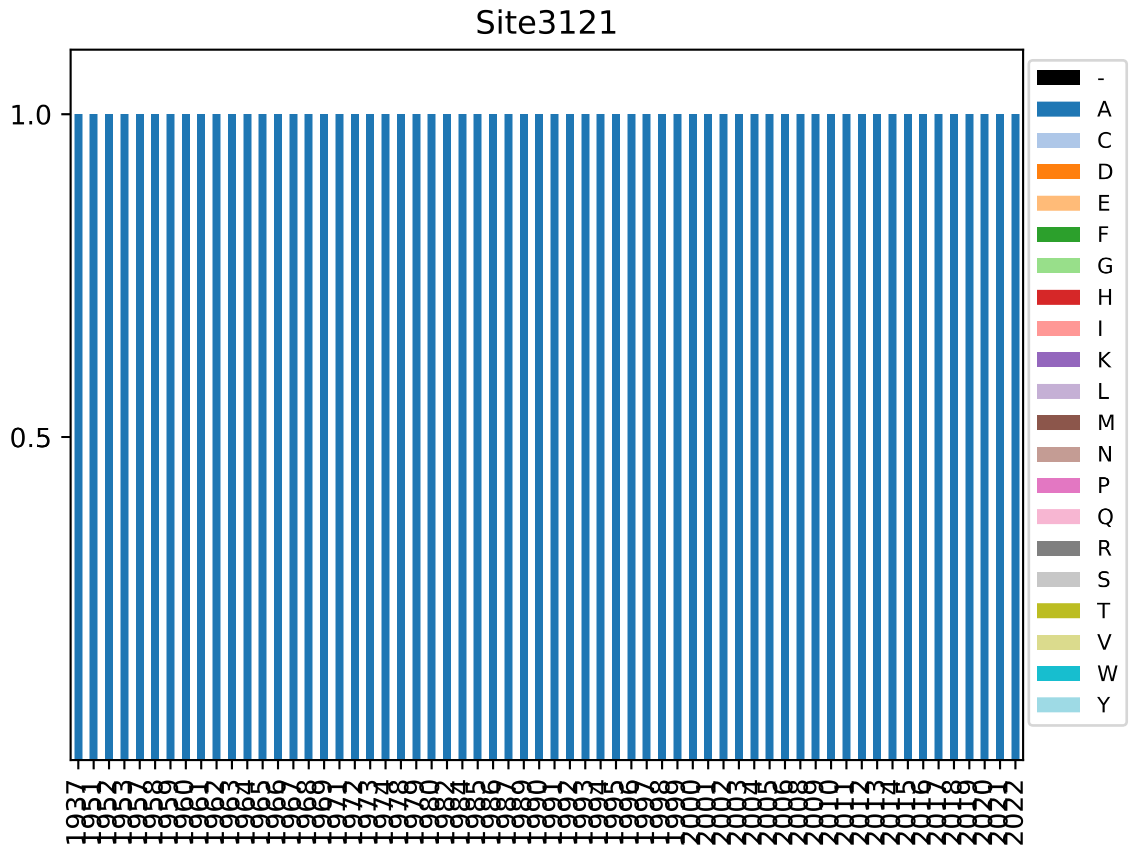

Site3121
1.0
0.5
-
A
C
D
E
F
G
H
I
K
L
M
N
P
Q
R
S
T
V
W
Y

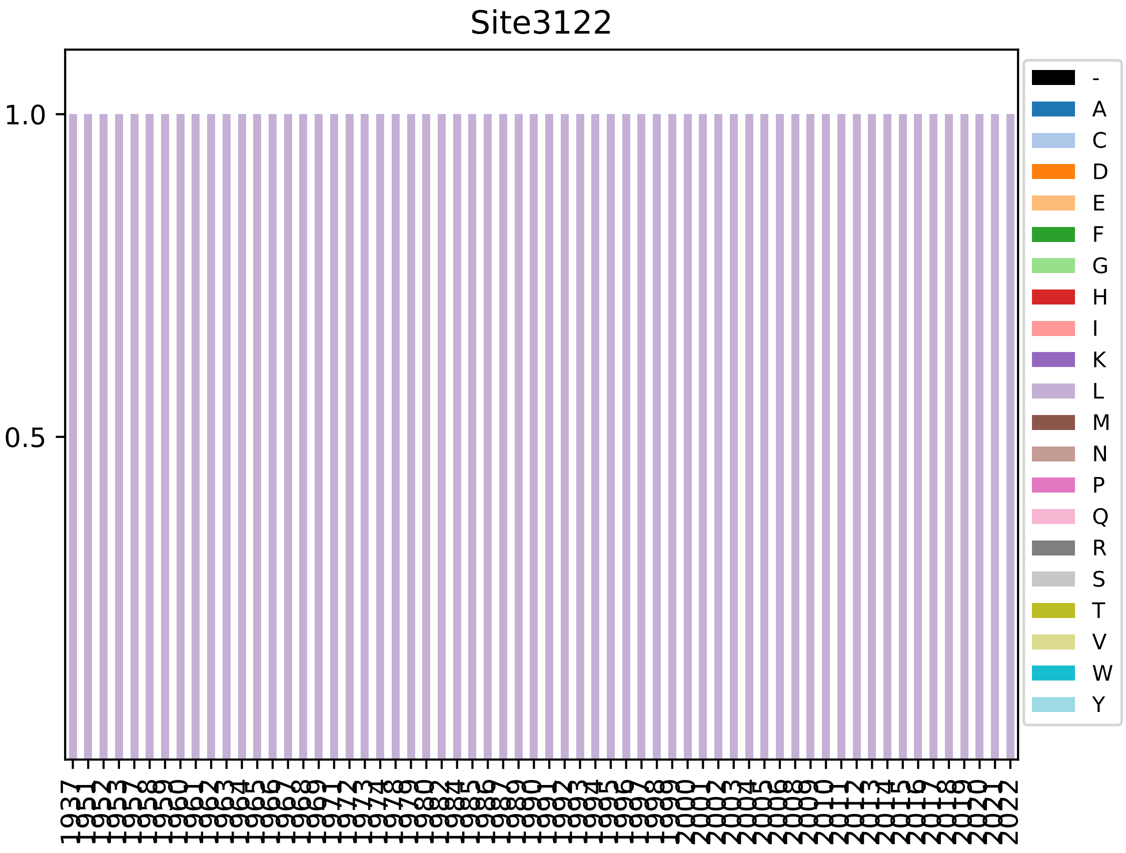
Site3122
1.0
0.5
-
A
C
D
E
F
G
H
I
K
L
M
N
P
Q
R
S
T
V
W
Y

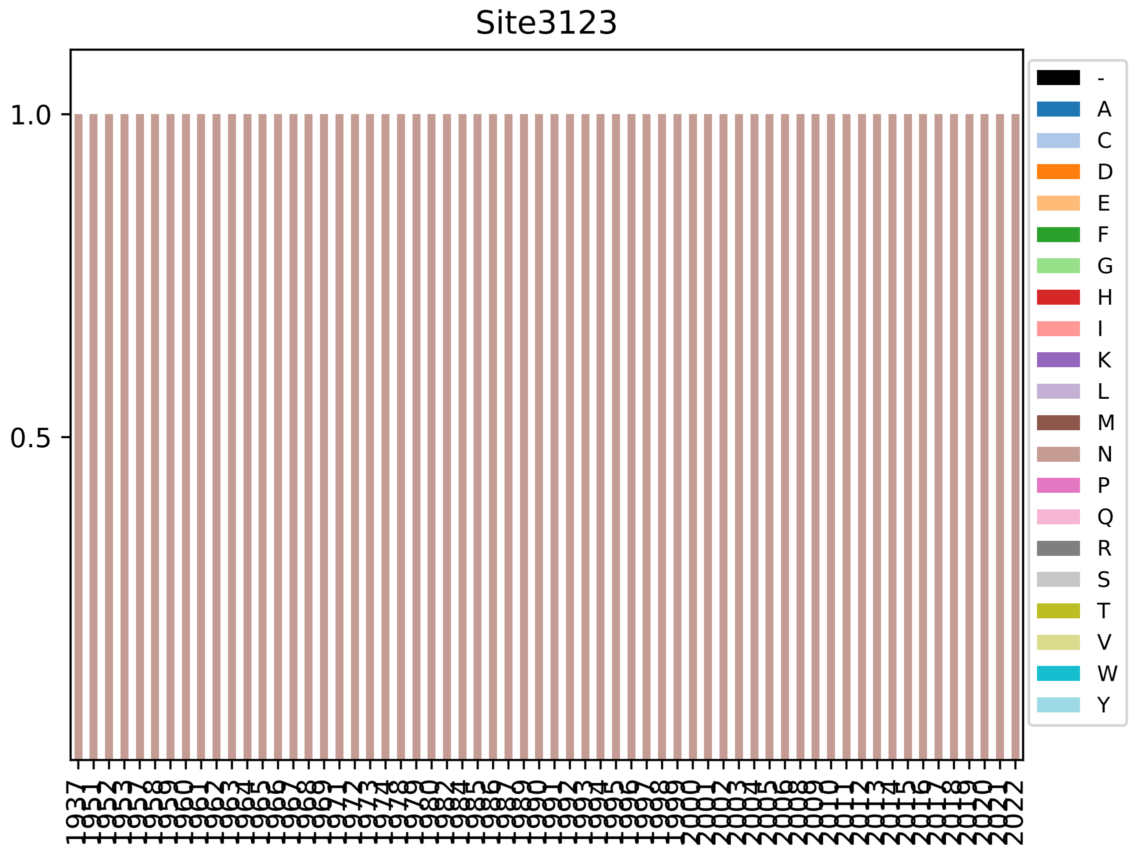

Site3123
1.0
0.5
-
A
C
D
E
F
G
H
I
K
L
M
N
P
Q
R
S
T
V
W
Y

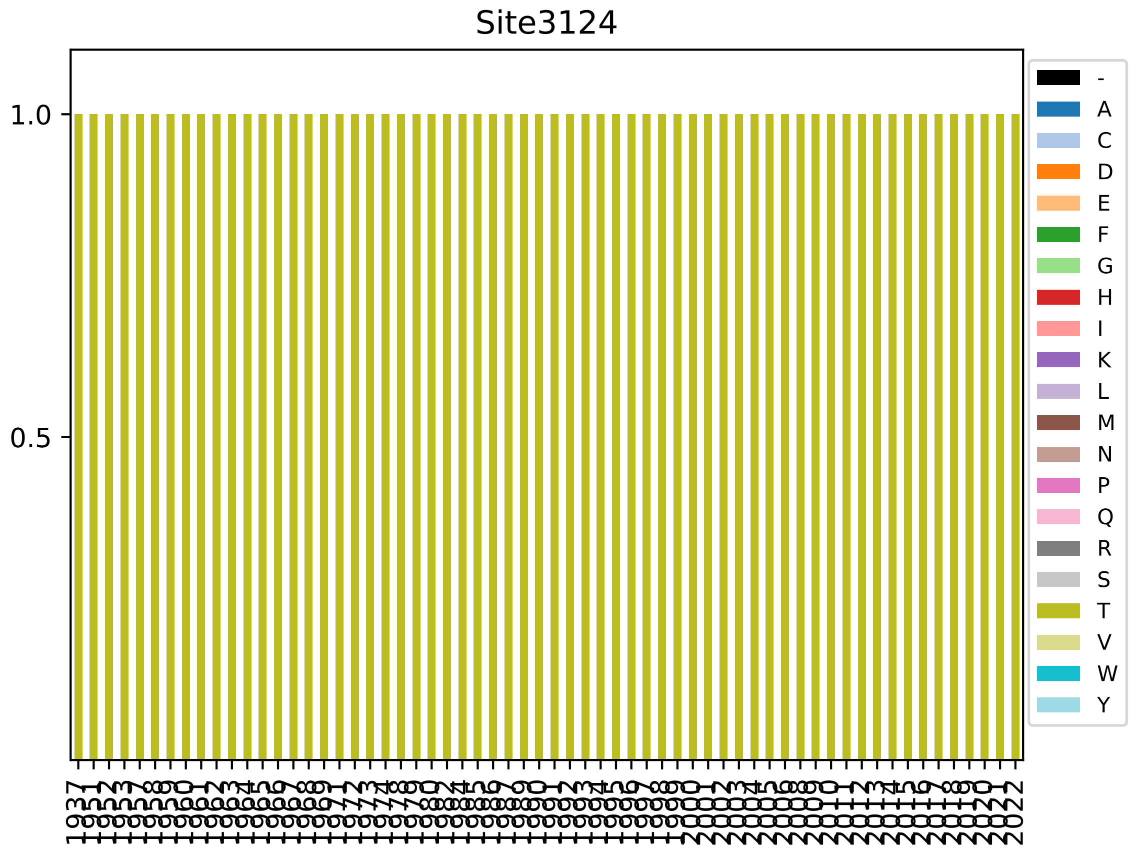
Site3124
1.0
0.5
-
A
C
D
E
F
G
H
I
K
L
M
N
P
Q
R
S
T
V
W
Y

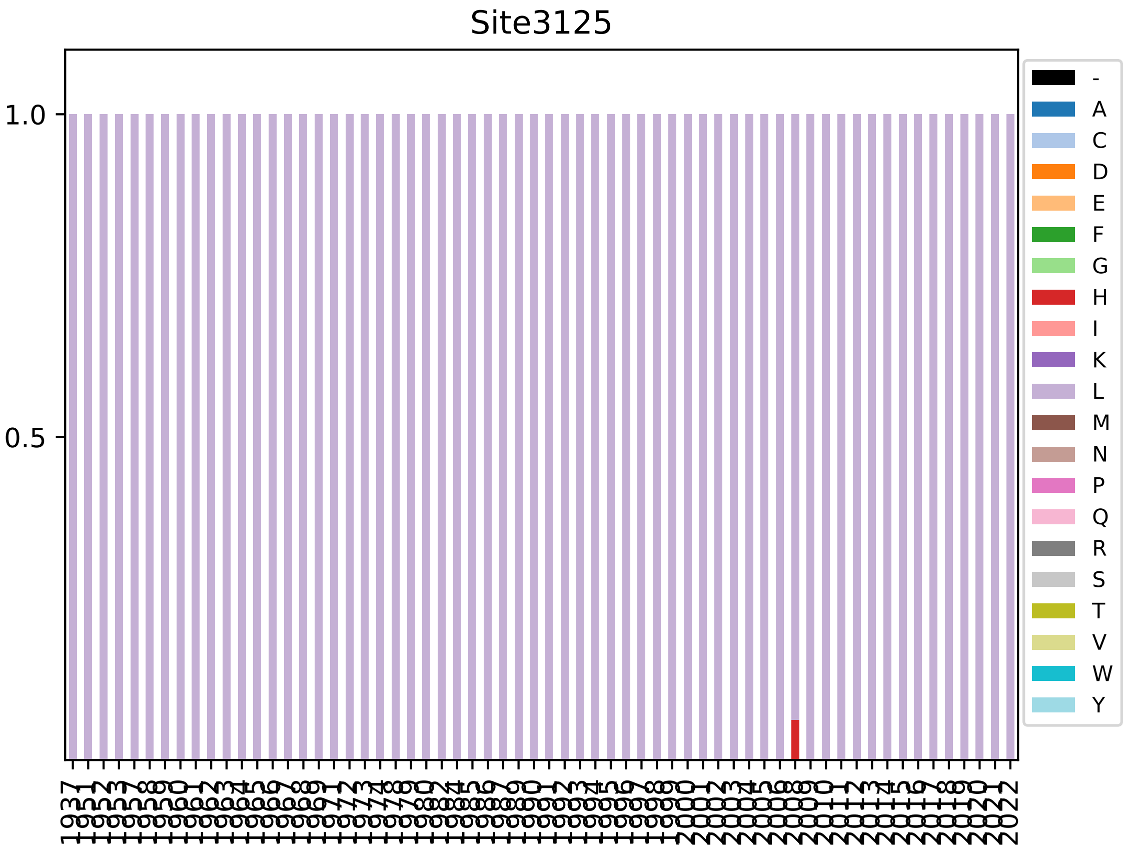
Site3125
1.0
0.5
-
A
C
D
E
F
G
H
I
K
L
M
N
P
Q
R
S
T
V
W
Y

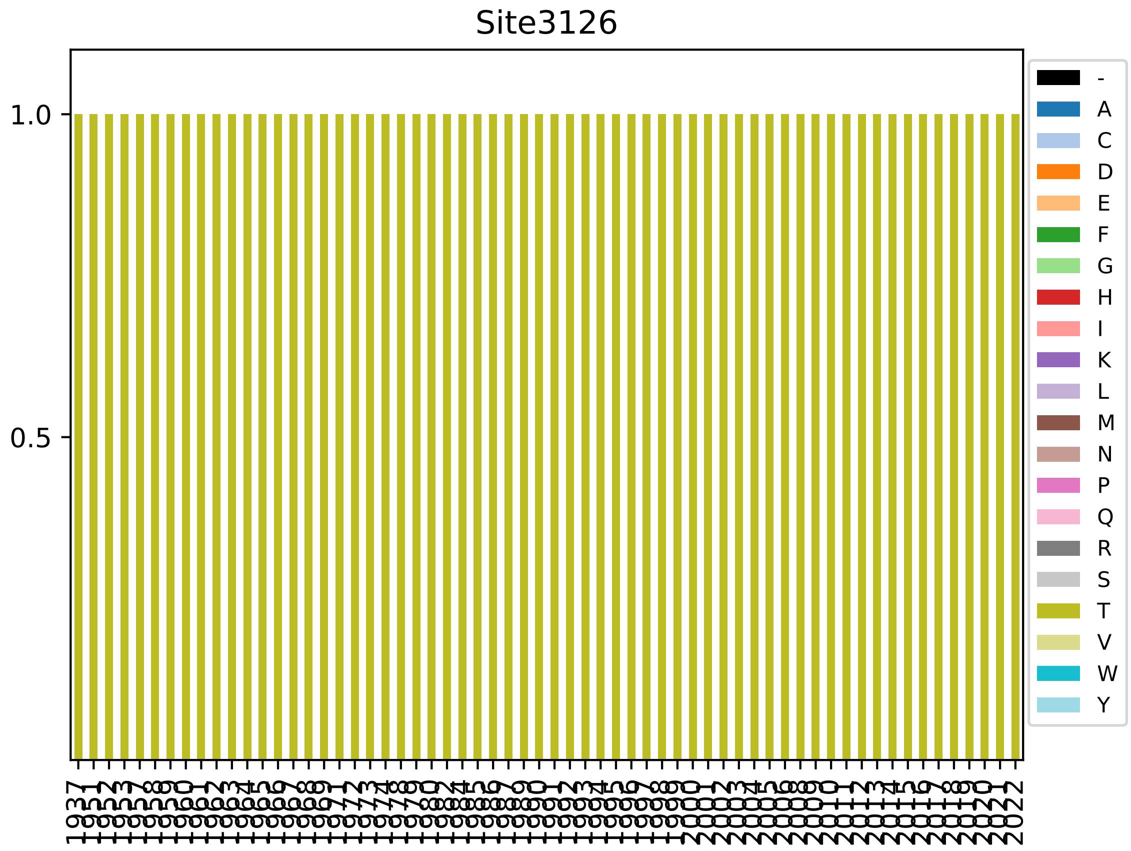
Site3126
1.0
0.5
-
A
C
D
E
F
G
H
I
K
L
M
N
P
Q
R
S
T
V
W
Y

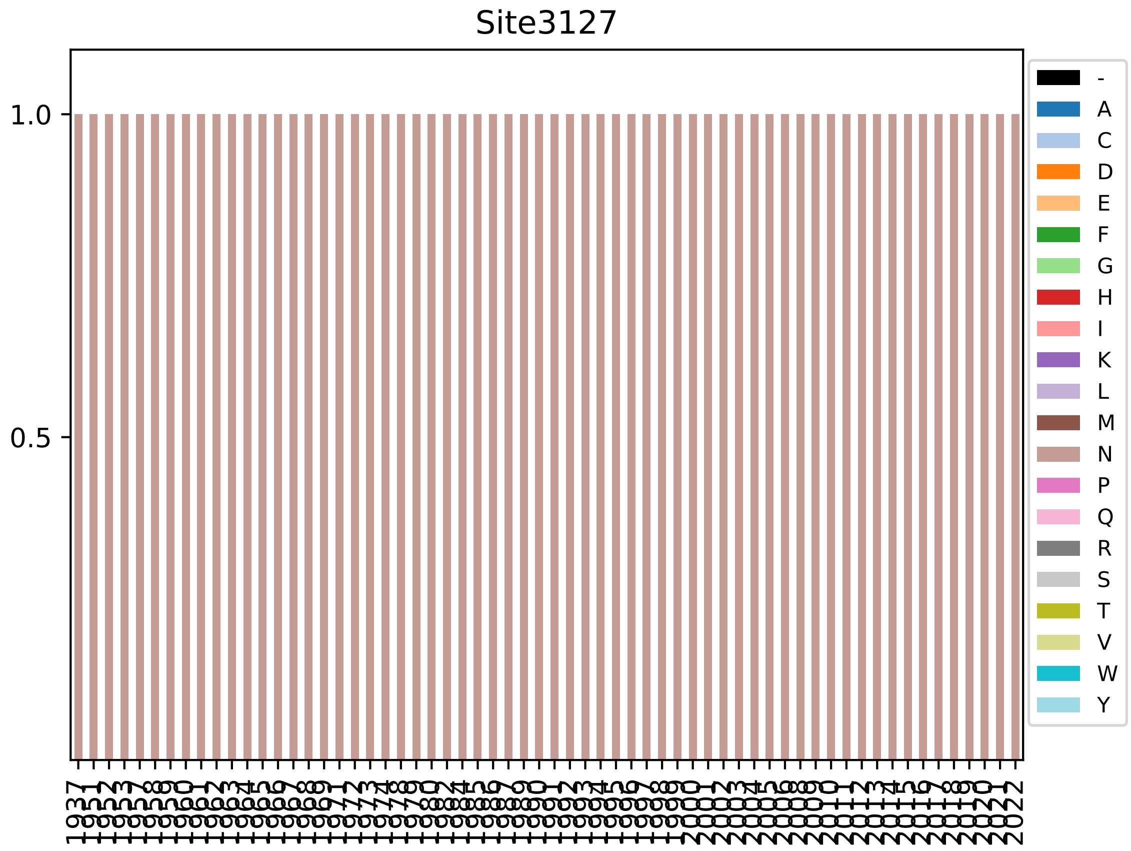
Site3127
1.0
0.5
-
A
C
D
E
F
G
H
I
K
L
M
N
P
Q
R
S
T
V
W
Y

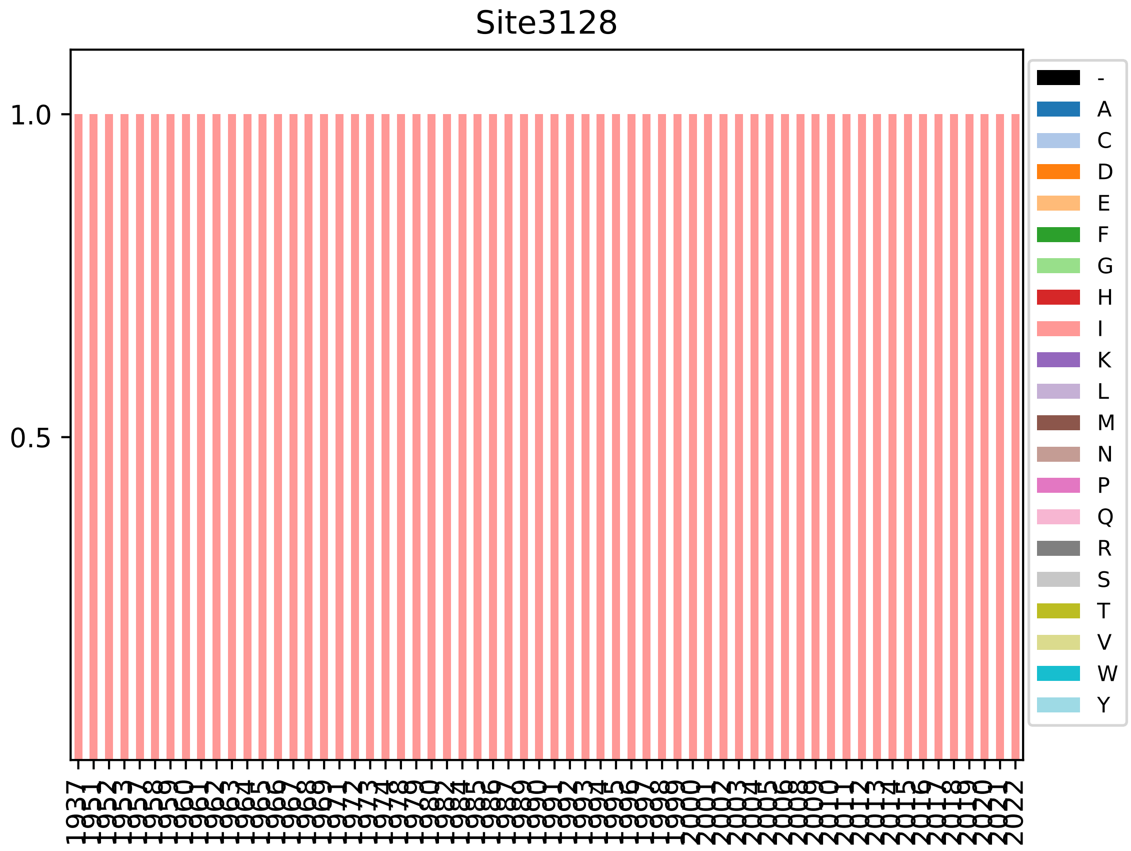
Site3128
1.0
0.5
-
A
C
D
E
F
G
H
I
K
L
M
N
P
Q
R
S
T
V
W
Y

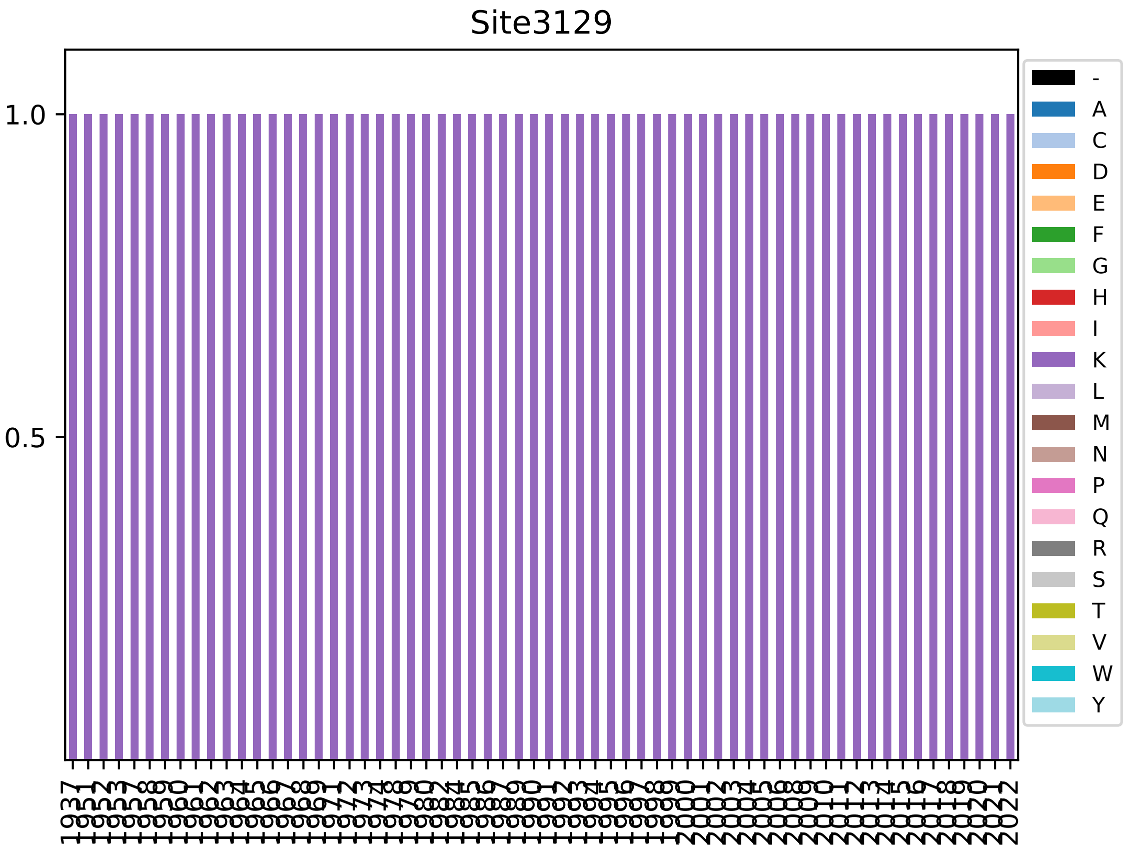
Site3129
1.0
0.5
-
A
C
D
E
F
G
H
I
K
L
M
N
P
Q
R
S
T
V
W
Y

Site3130

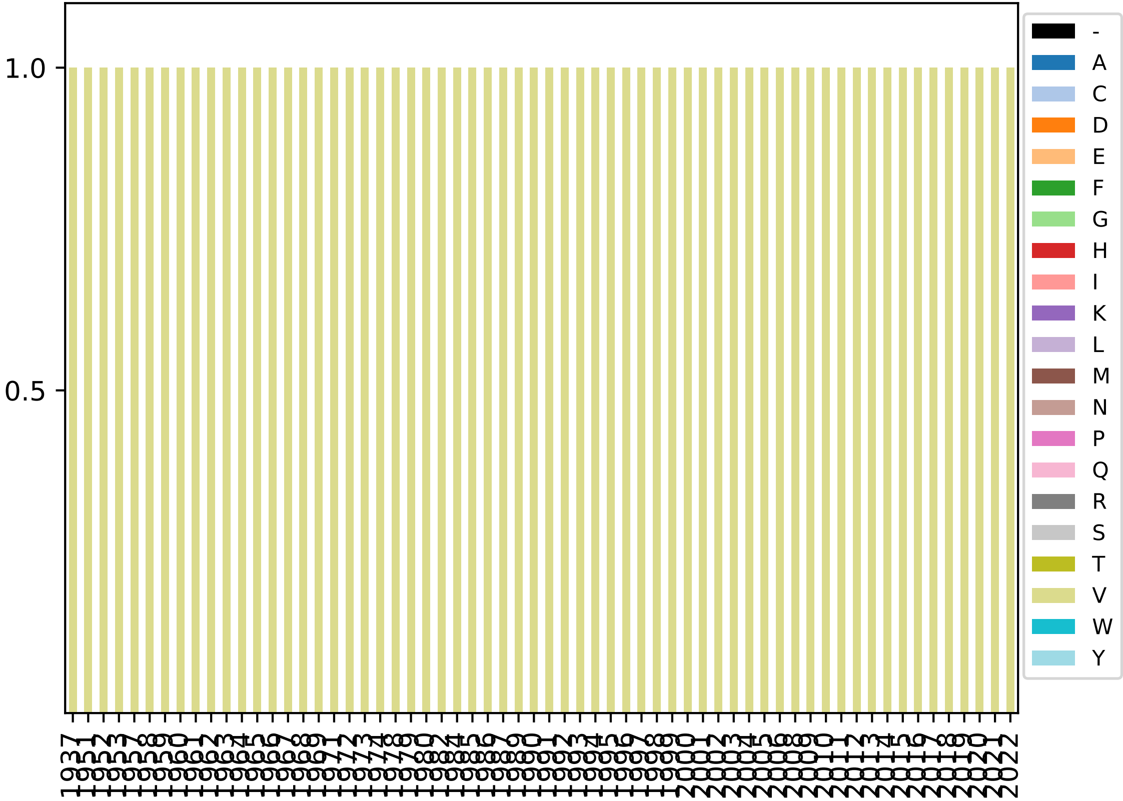

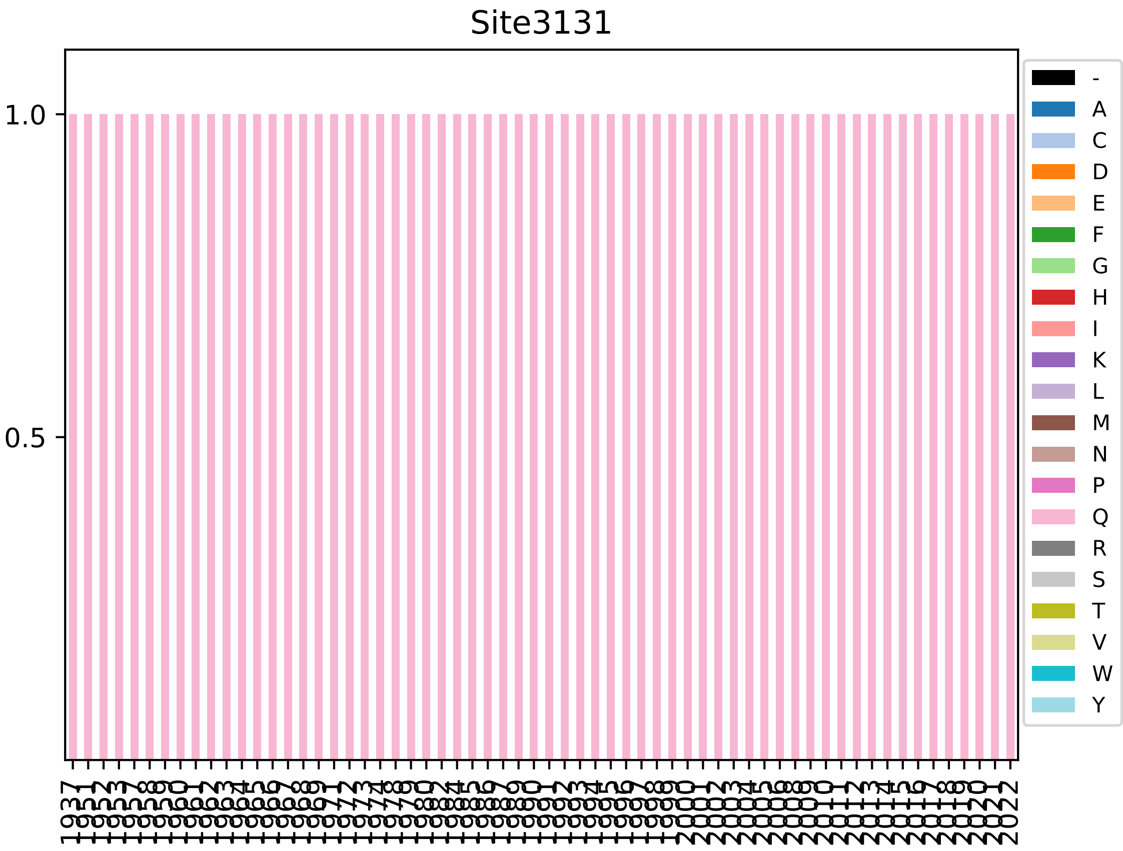
Site3131
1.0
0.5
-
A
C
D
E
F
G
H
I
K
L
M
N
P
Q
R
S
T
V
W
Y

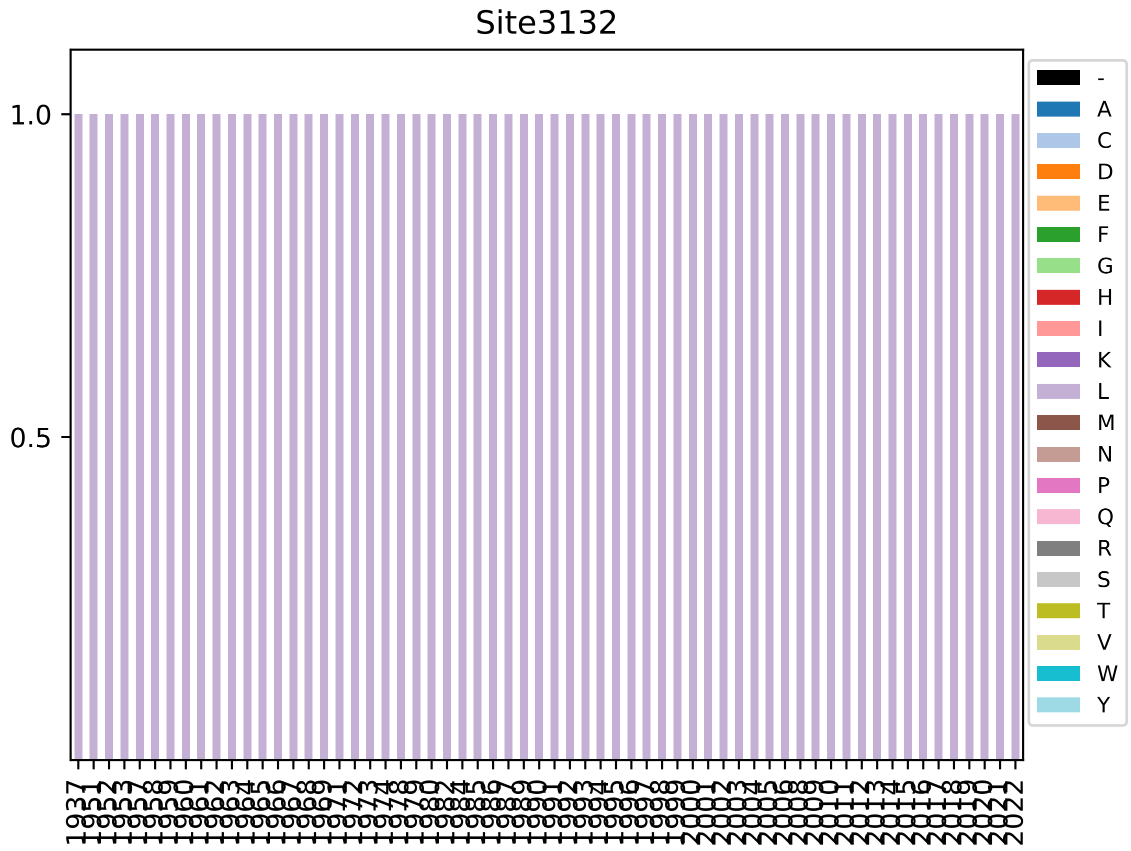
Site3132
1.0
0.5
-
A
C
D
E
F
G
H
I
K
L
M
N
P
Q
R
S
T
V
W
Y

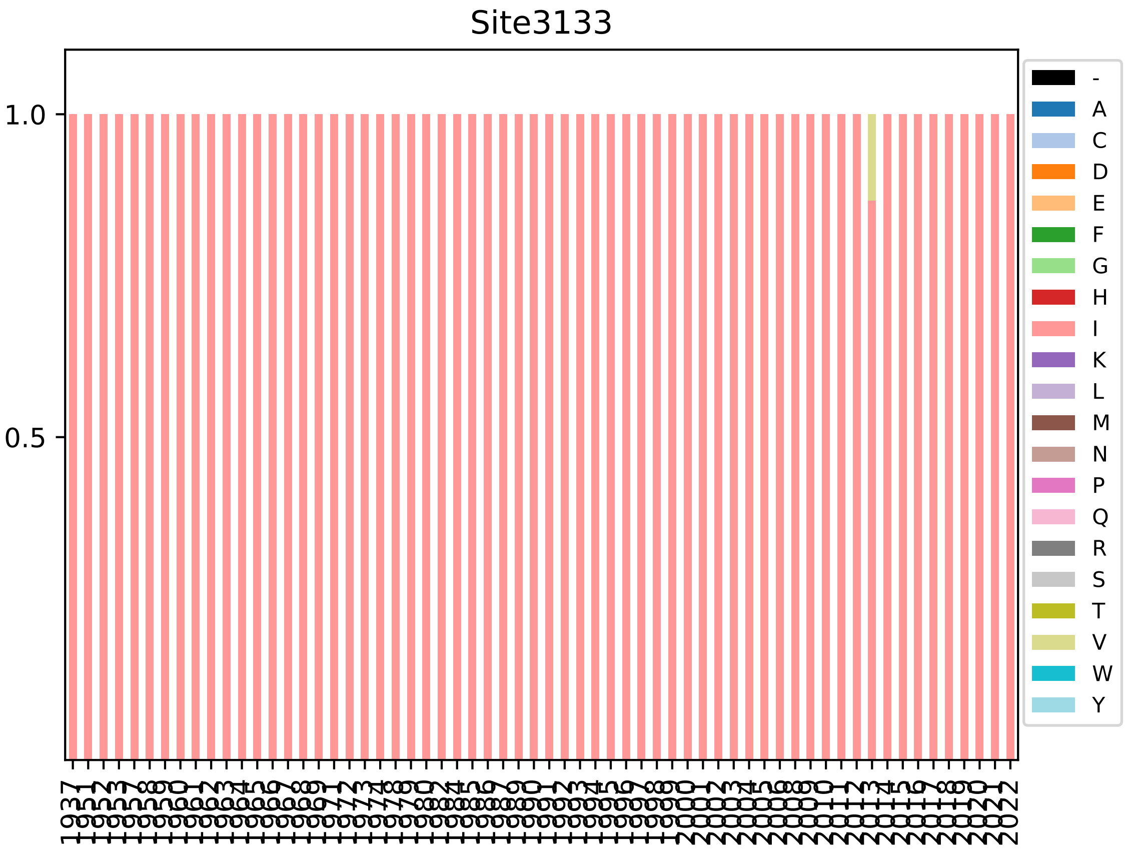
Site3133
1.0
0.5
-
A
C
D
E
F
G
H
I
K
L
M
N
P
Q
R
S
T
V
W
Y

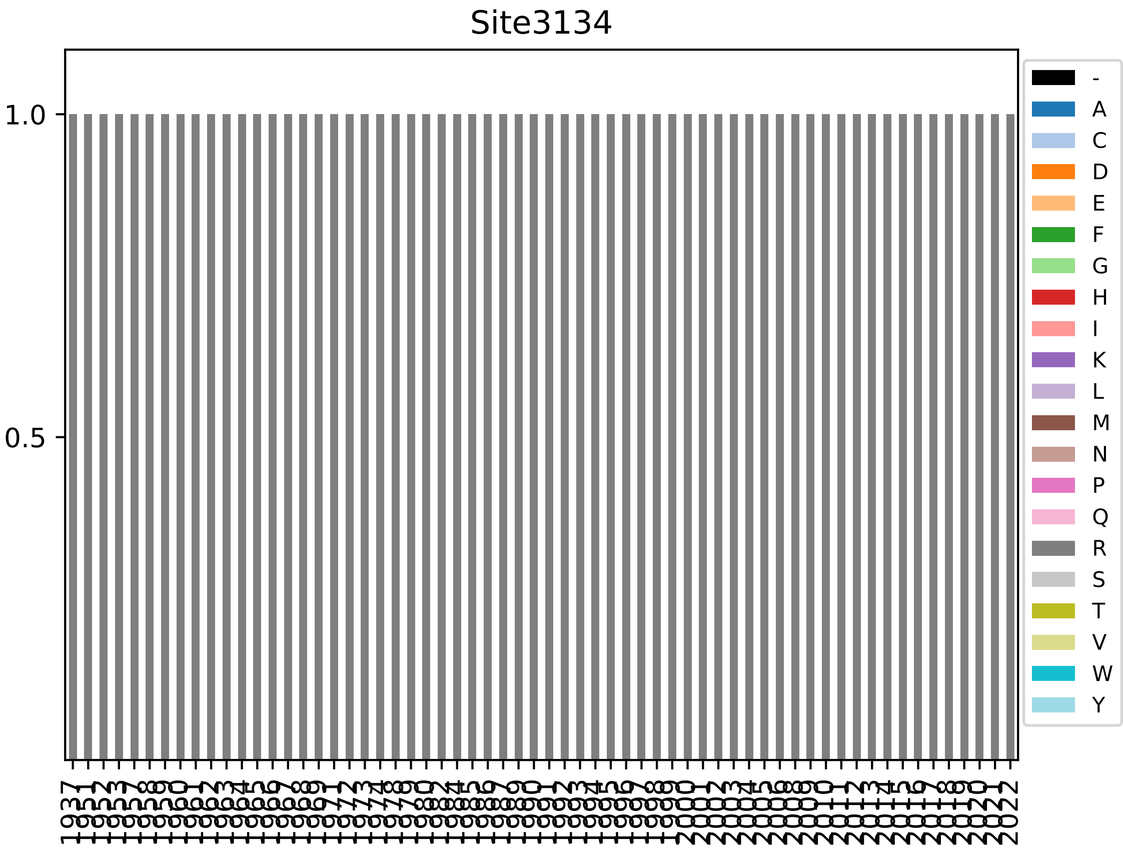
Site3134
1.0
0.5
-
A
C
D
E
F
G
H
I
K
L
M
N
P
Q
R
S
T
V
W
Y

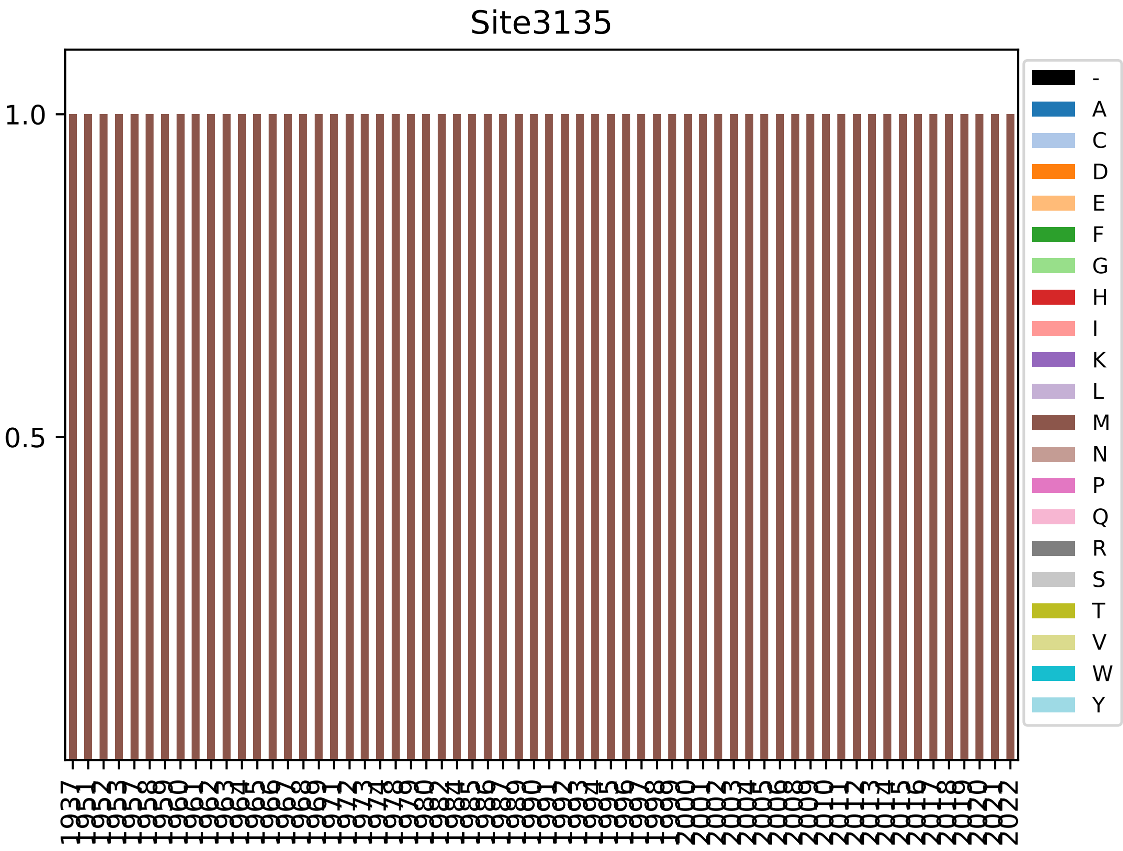
Site3135
1.0
0.5
-
A
C
D
E
F
G
H
I
K
L
M
N
P
Q
R
S
T
V
W
Y

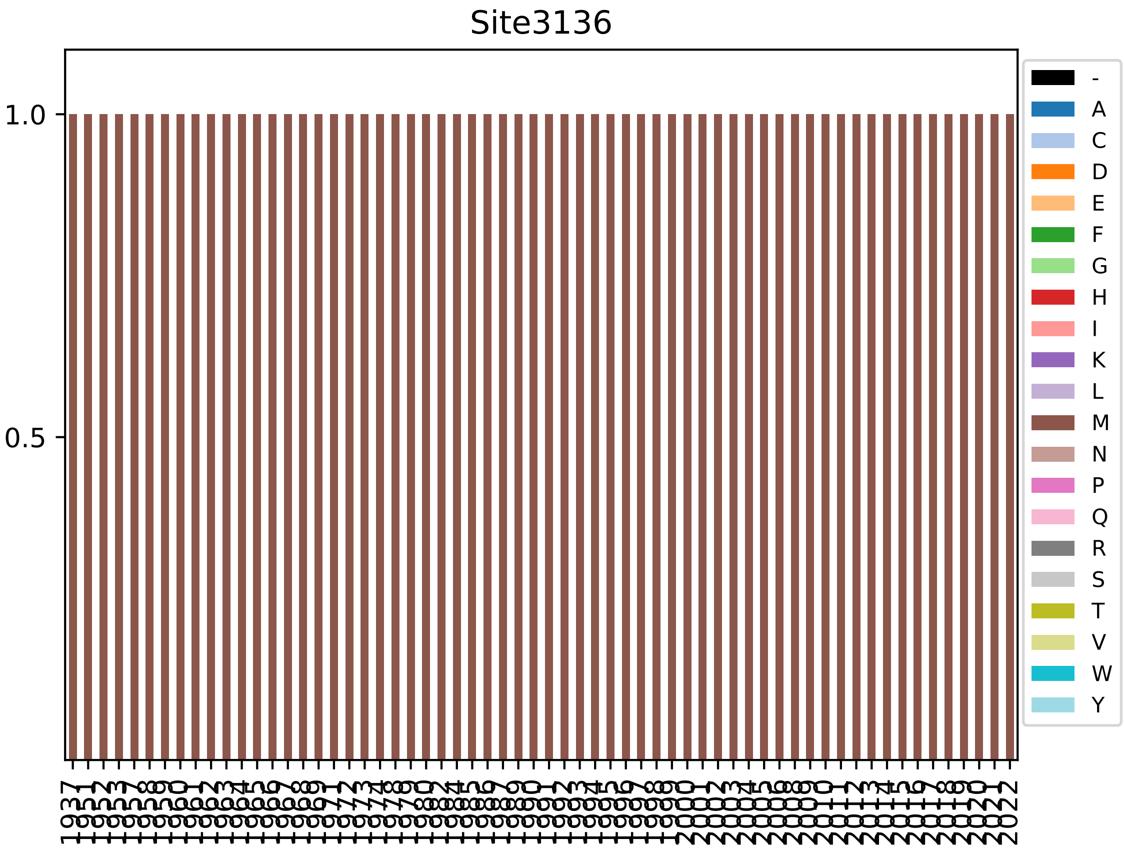
Site3136
1.0
0.5
-
A
C
D
E
F
G
H
I
K
L
M
N
P
Q
R
S
T
V
W
Y

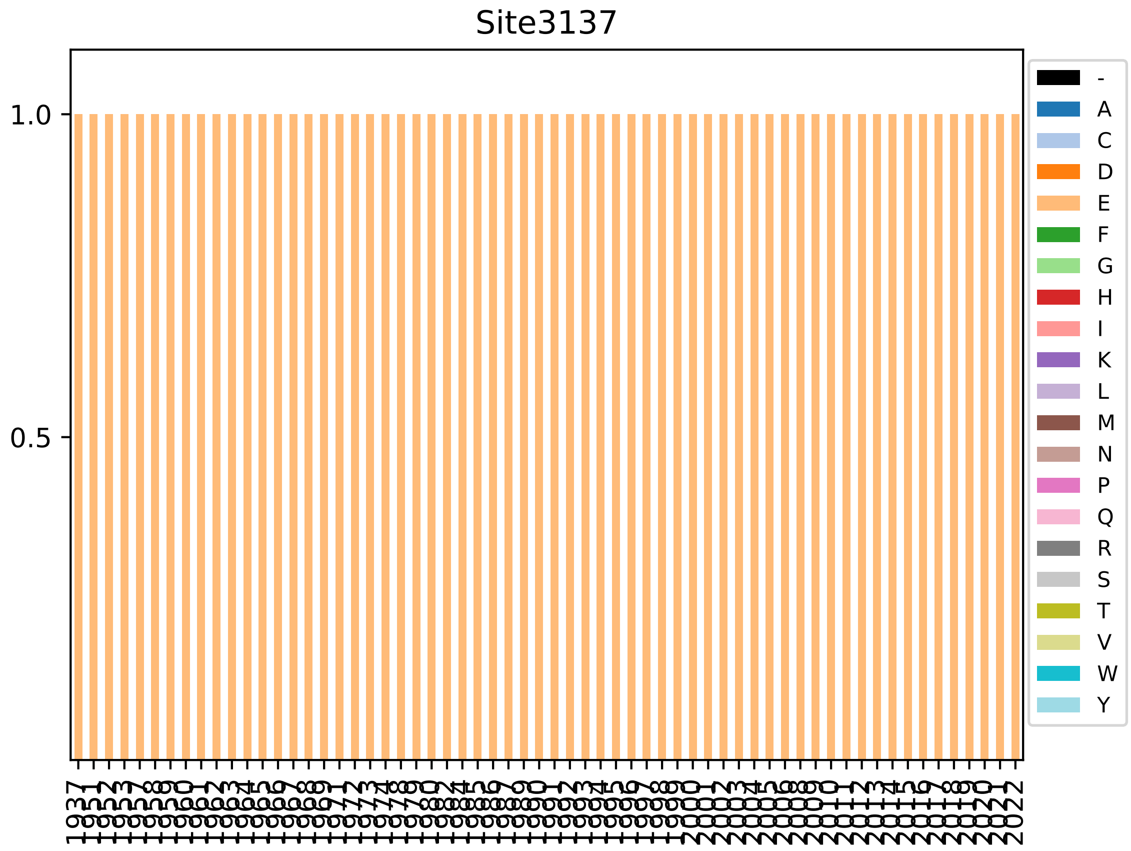
Site3137
1.0
0.5
-
A
C
D
E
F
G
H
I
K
L
M
N
P
Q
R
S
T
V
W
Y

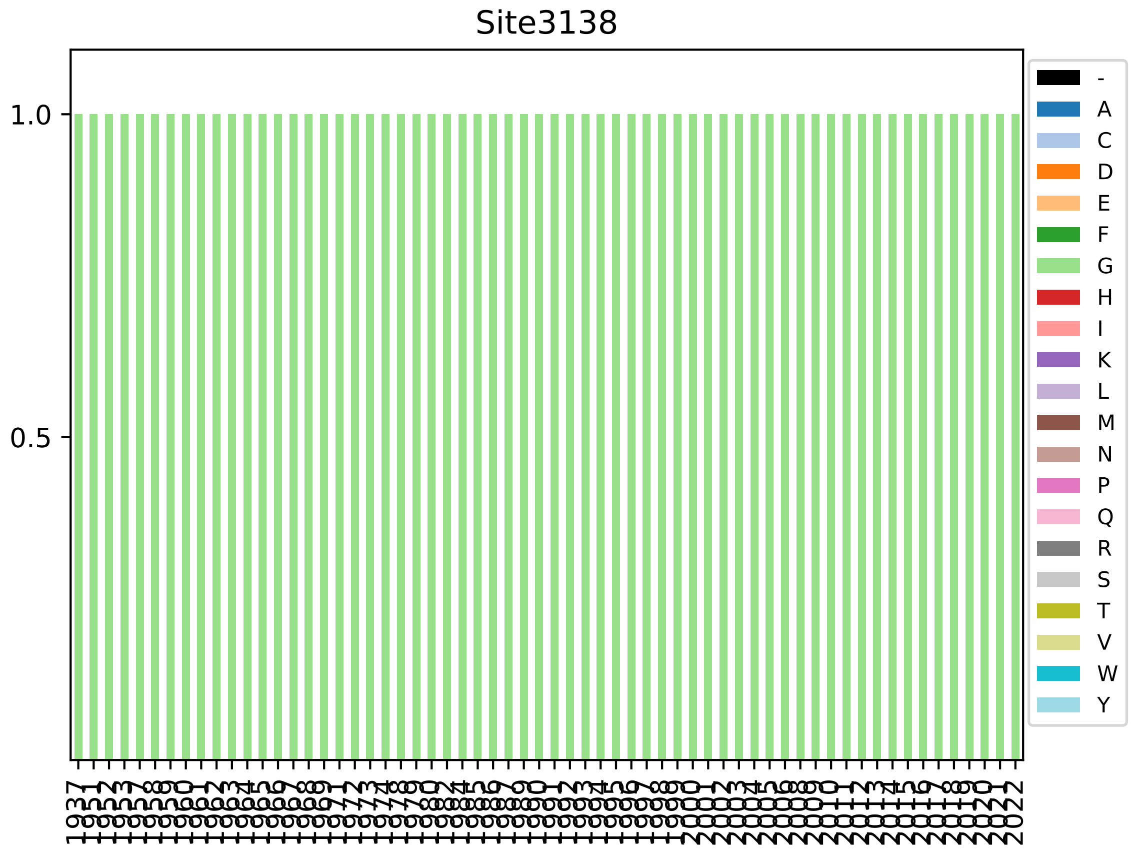
Site3138
1.0
0.5
-
A
C
D
E
F
G
H
I
K
L
M
N
P
Q
R
S
T
V
W
Y

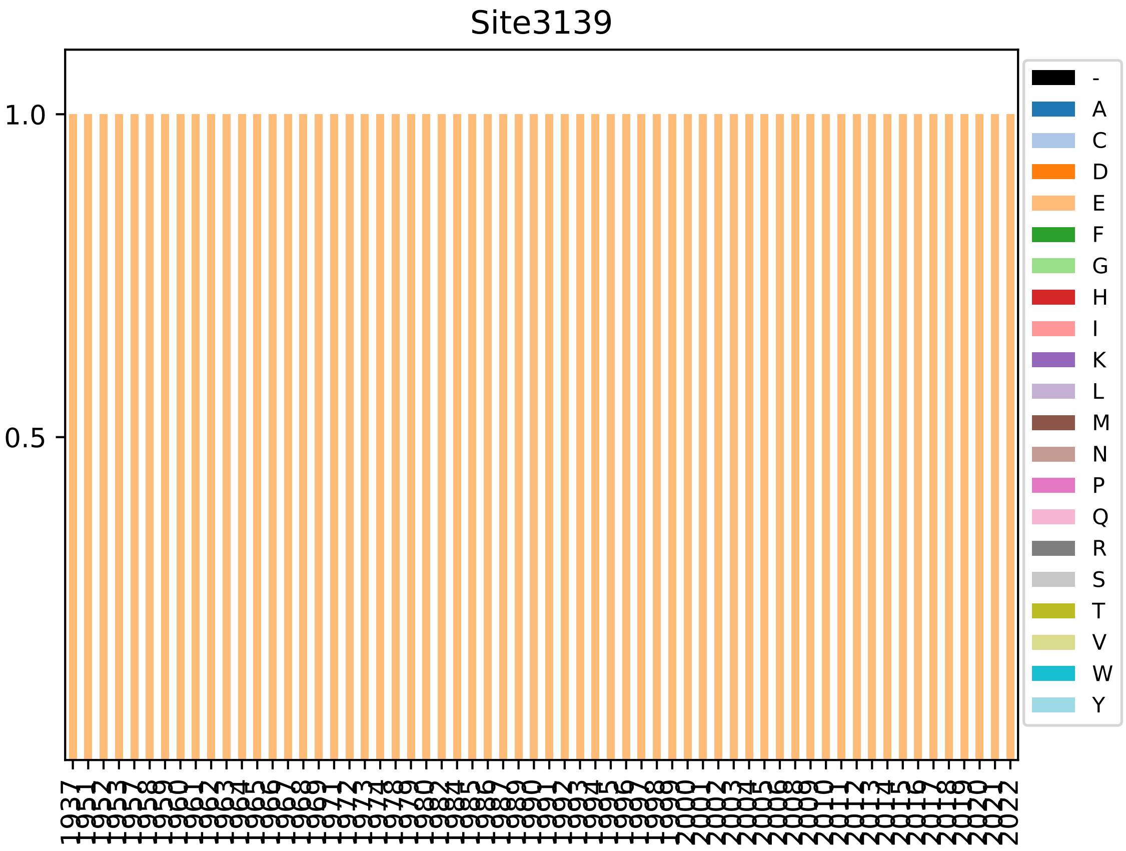

Site3139
1.0
0.5
-
A
C
D
E
F
G
H
I
K
L
M
N
P
Q
R
S
T
V
W
Y

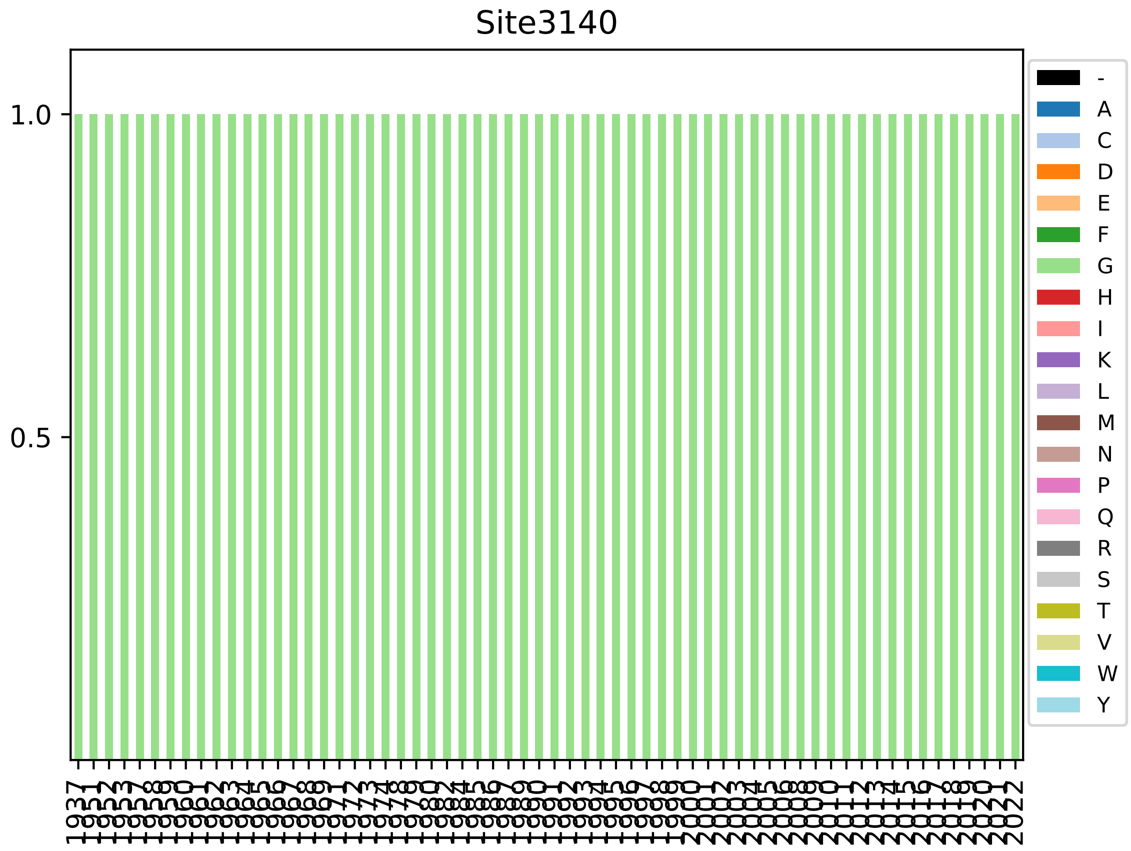
Site3140
1.0
0.5
-
A
C
D
E
F
G
H
I
K
L
M
N
P
Q
R
S
T
V
W
Y

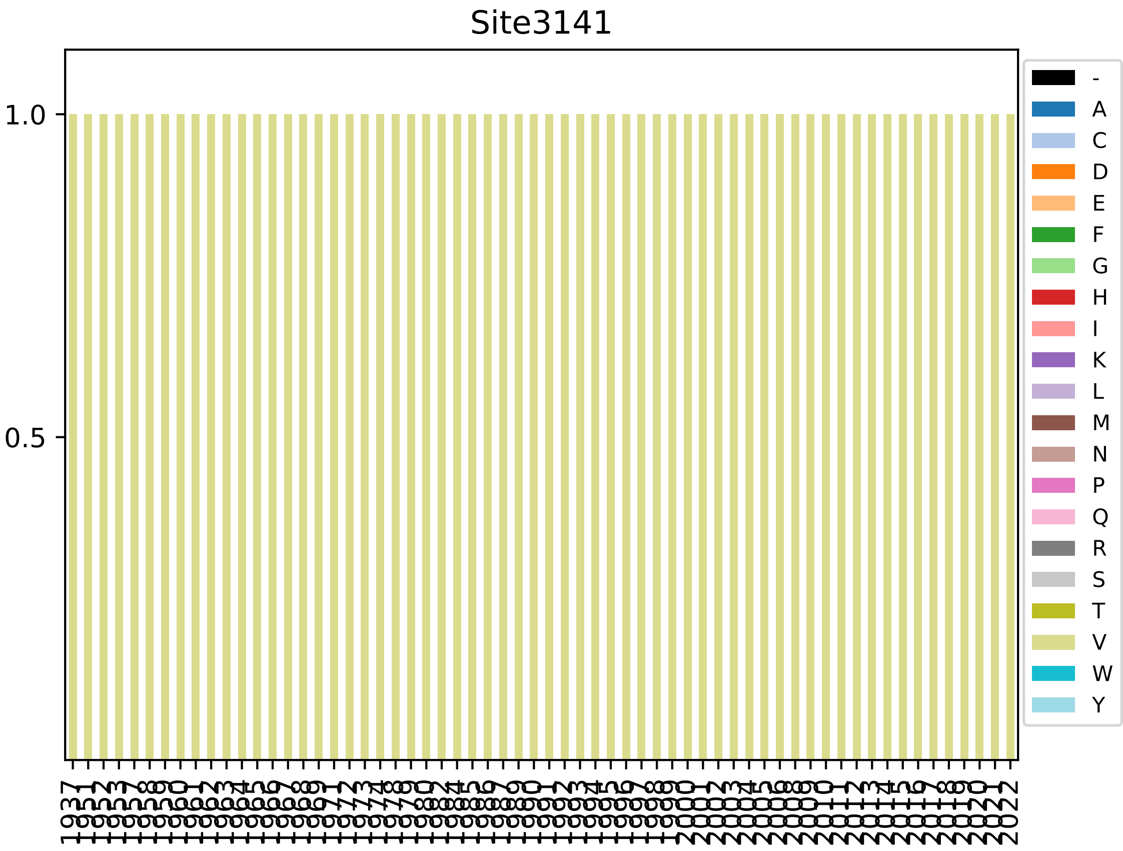
Site3141
1.0
0.5
-
A
C
D
E
F
G
H
I
K
L
M
N
P
Q
R
S
T
V
W
Y

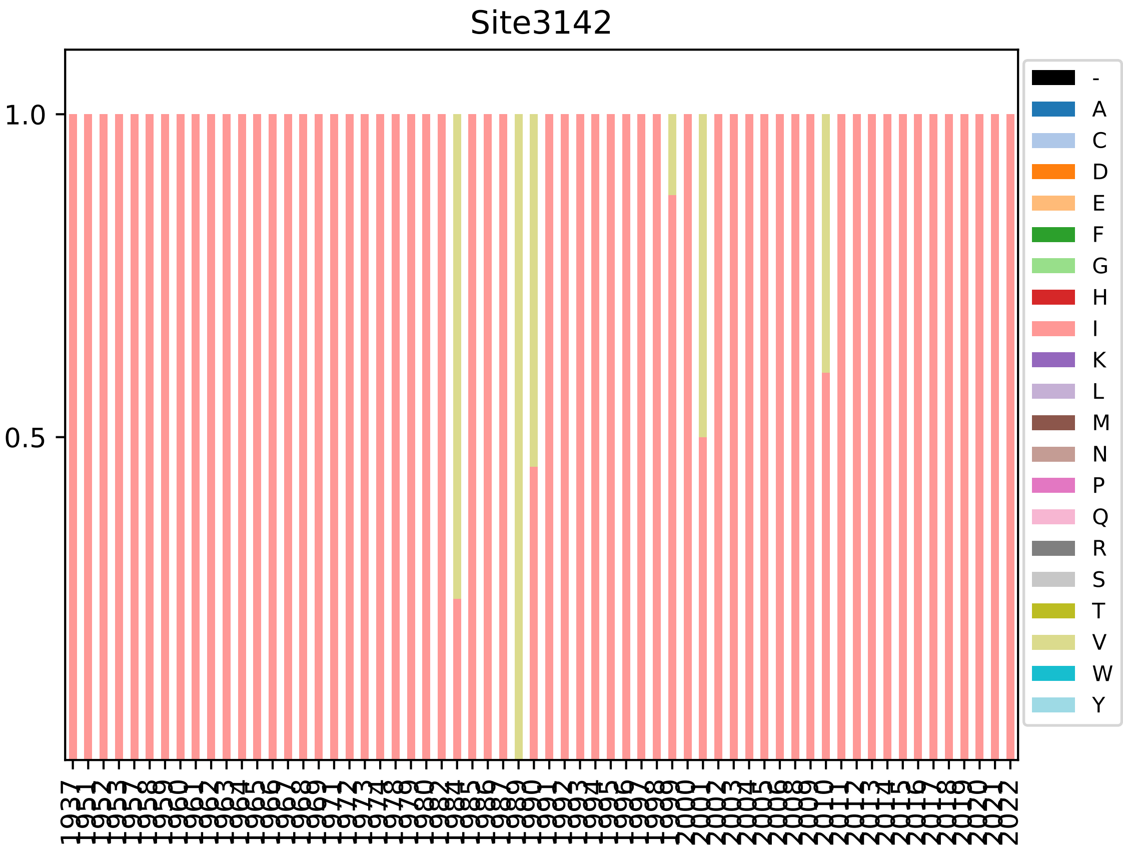
Site3142
1.0
0.5
-
A
C
D
E
F
G
H
I
K
L
M
N
P
Q
R
S
T
V
W
Y

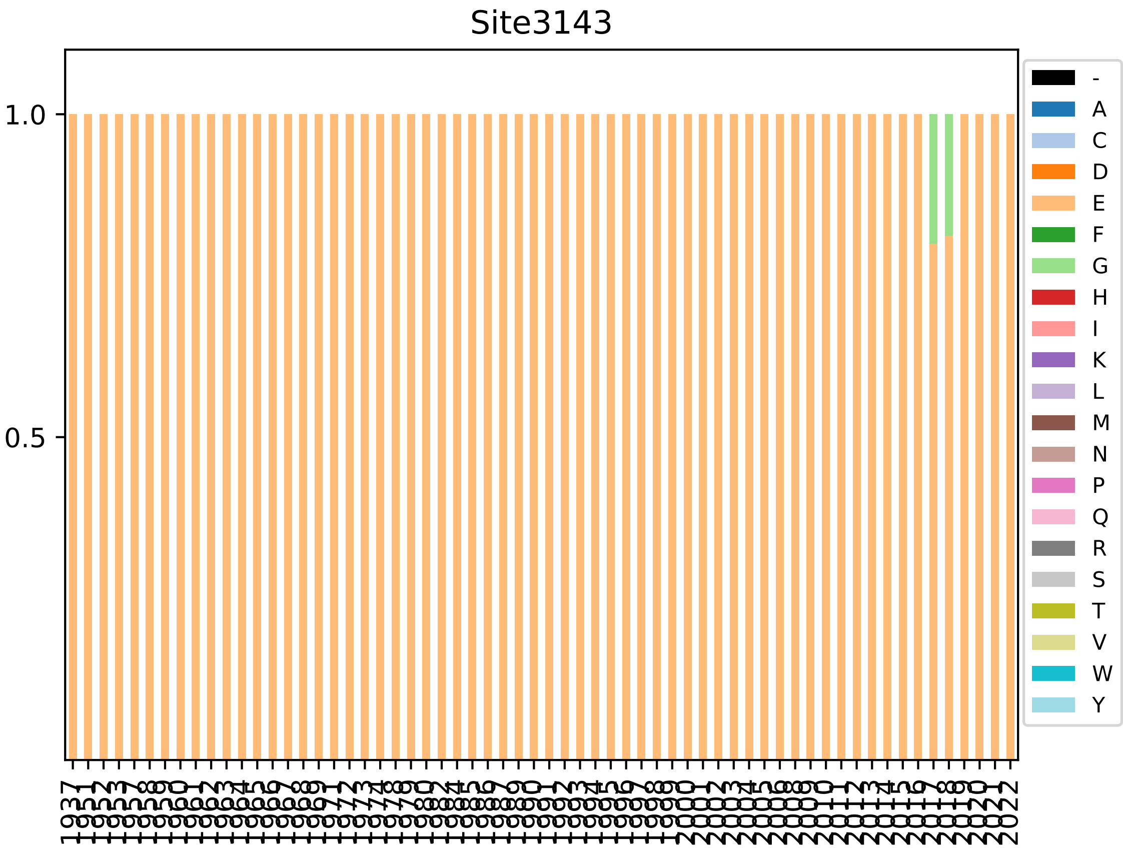
Site3143
1.0
0.5
-
A
C
D
E
F
G
H
I
K
L
M
N
P
Q
R
S
T
V
W
Y

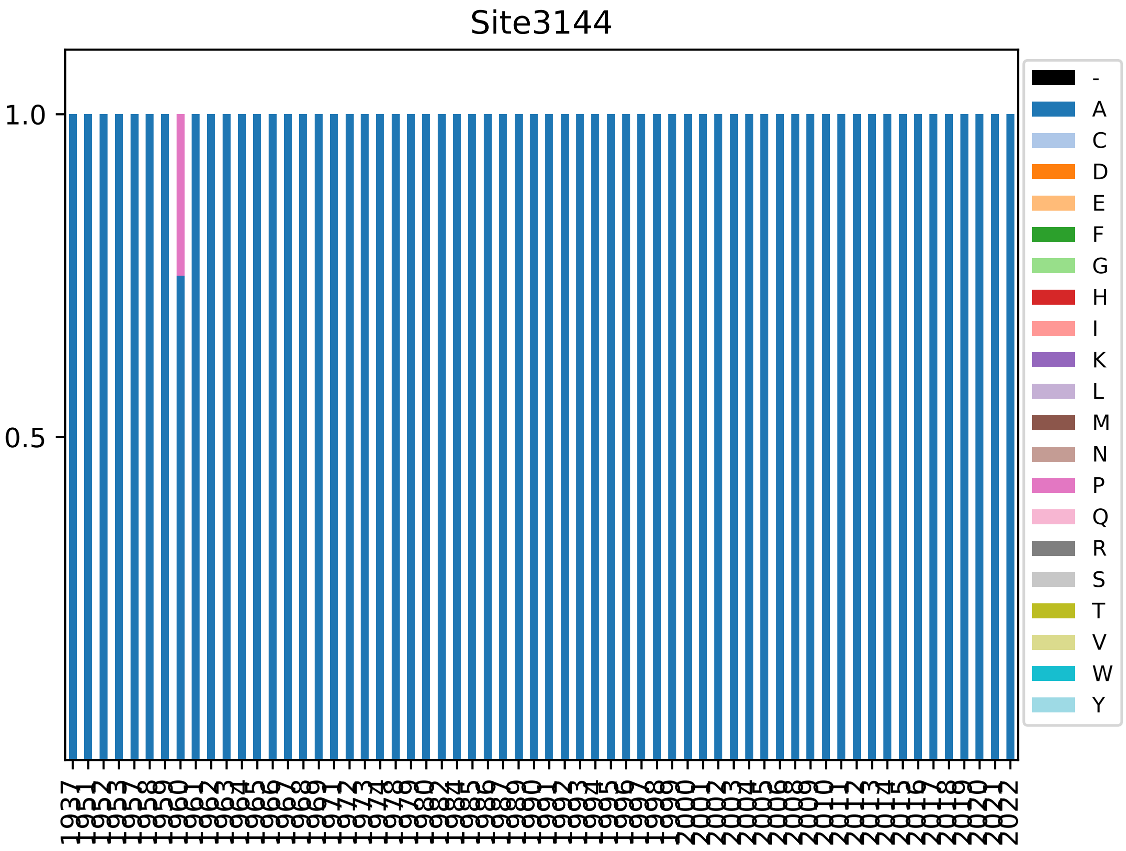
Site3144
1.0
0.5
-
A
C
D
E
F
G
H
I
K
L
M
N
P
Q
R
S
T
V
W
Y

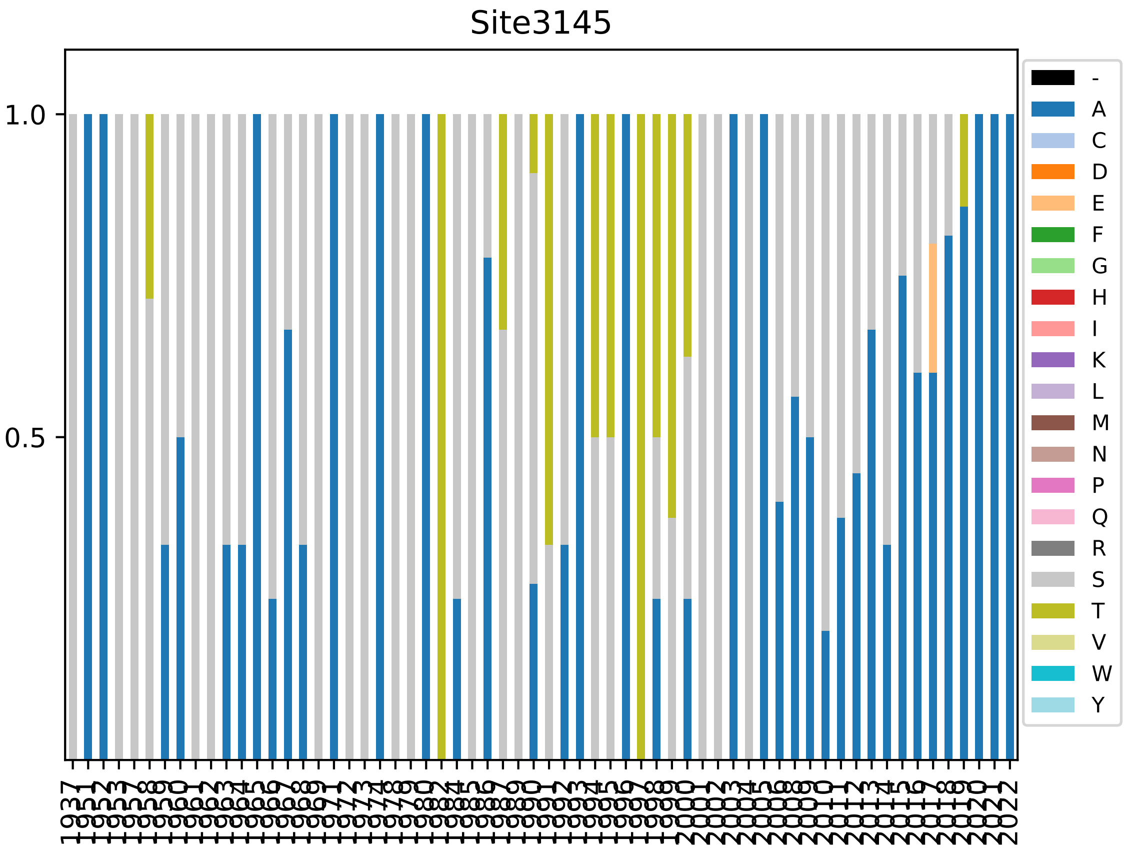
Site3145
1.0
0.5
-
A
C
D
E
F
G
H
I
K
L
M
N
P
Q
R
S
T
V
W
Y

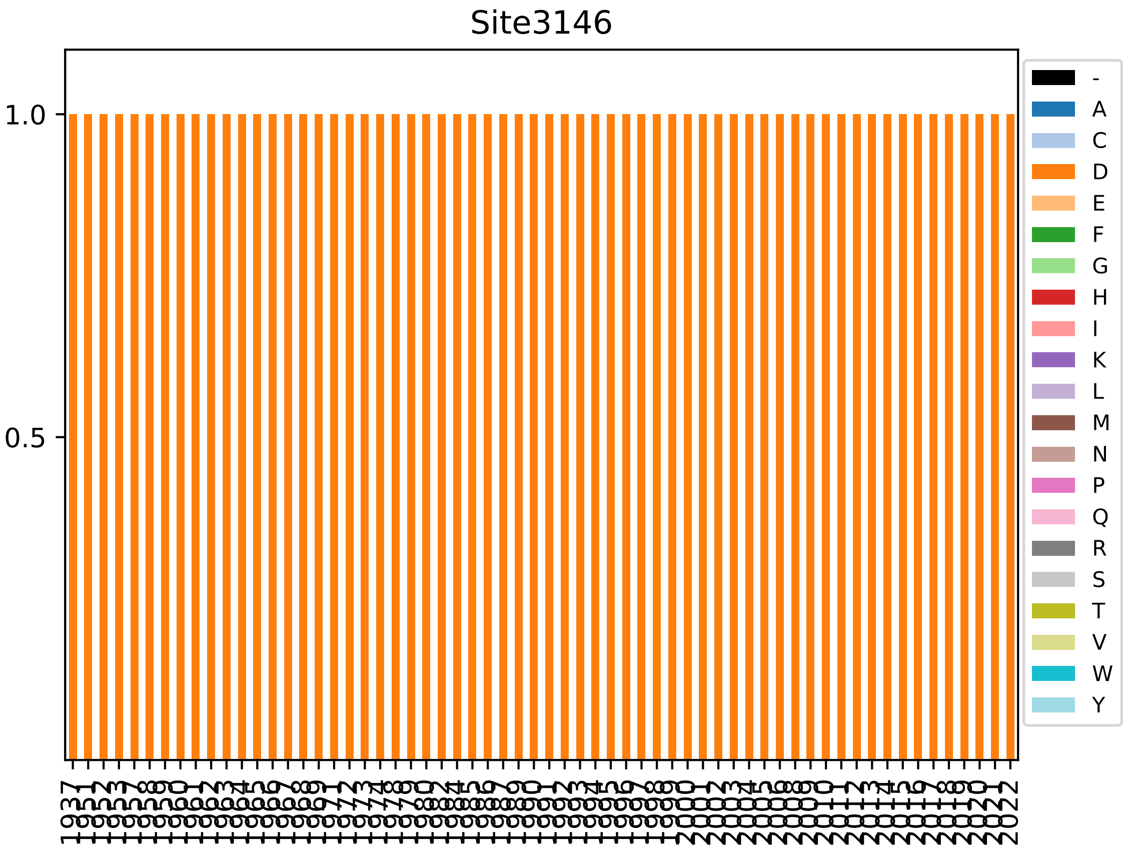
Site3146
1.0
0.5
-
A
C
D
E
F
G
H
I
K
L
M
N
P
Q
R
S
T
V
W
Y

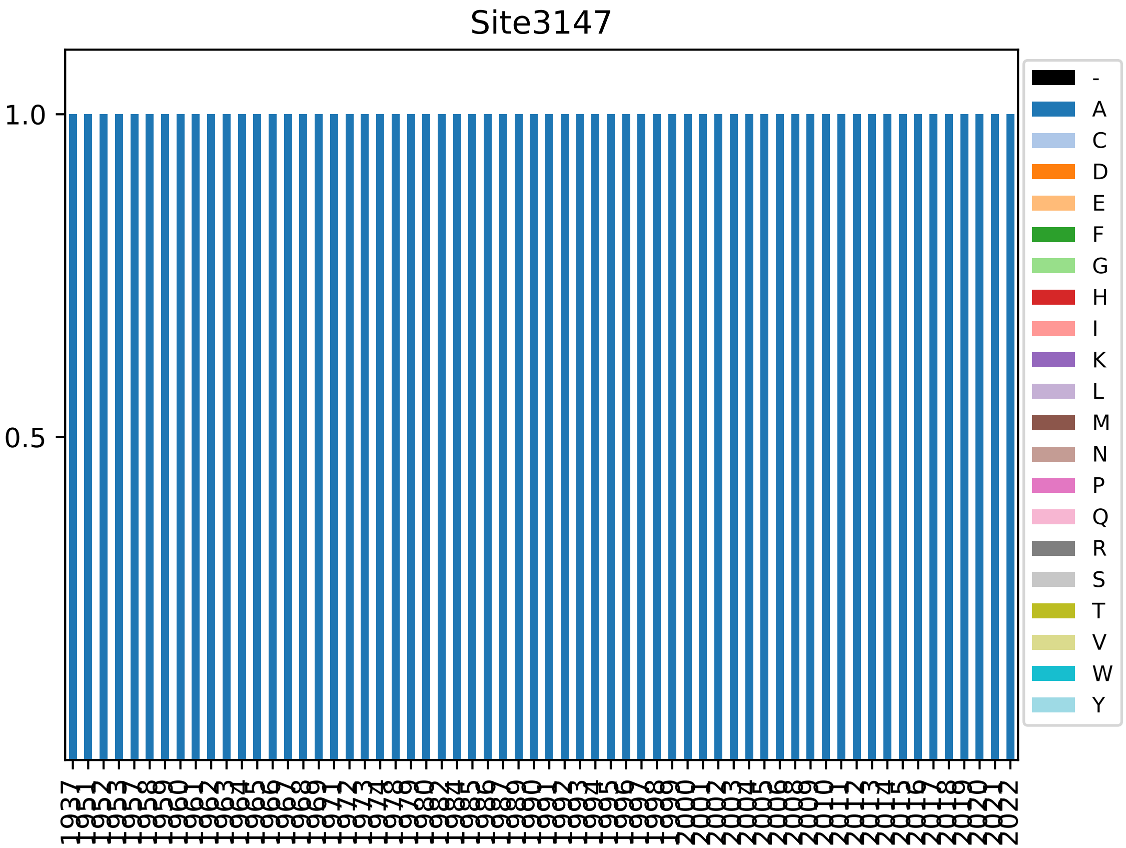
Site3147
1.0
0.5
-
A
C
D
E
F
G
H
I
K
L
M
N
P
Q
R
S
T
V
W
Y

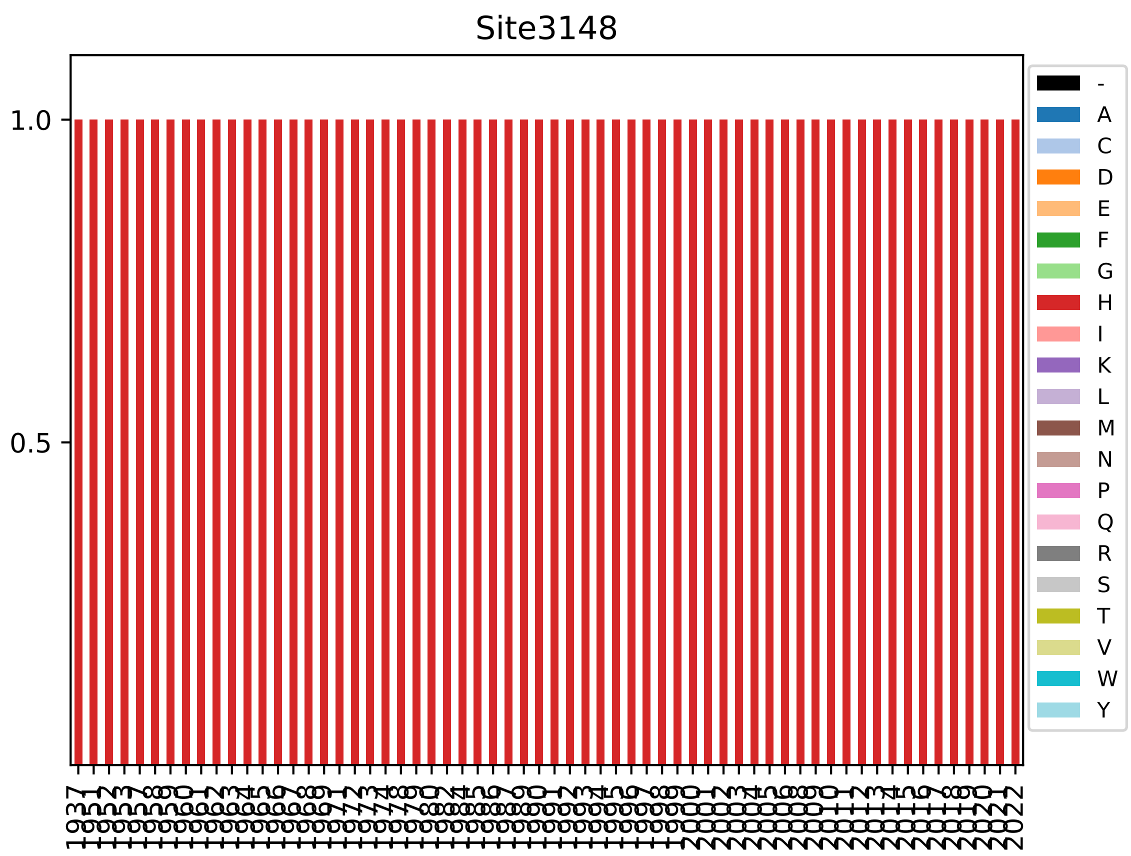

Site3148
1.0
0.5
-
A
C
D
E
F
G
H
I
K
L
M
N
P
Q
R
S
T
V
W
Y

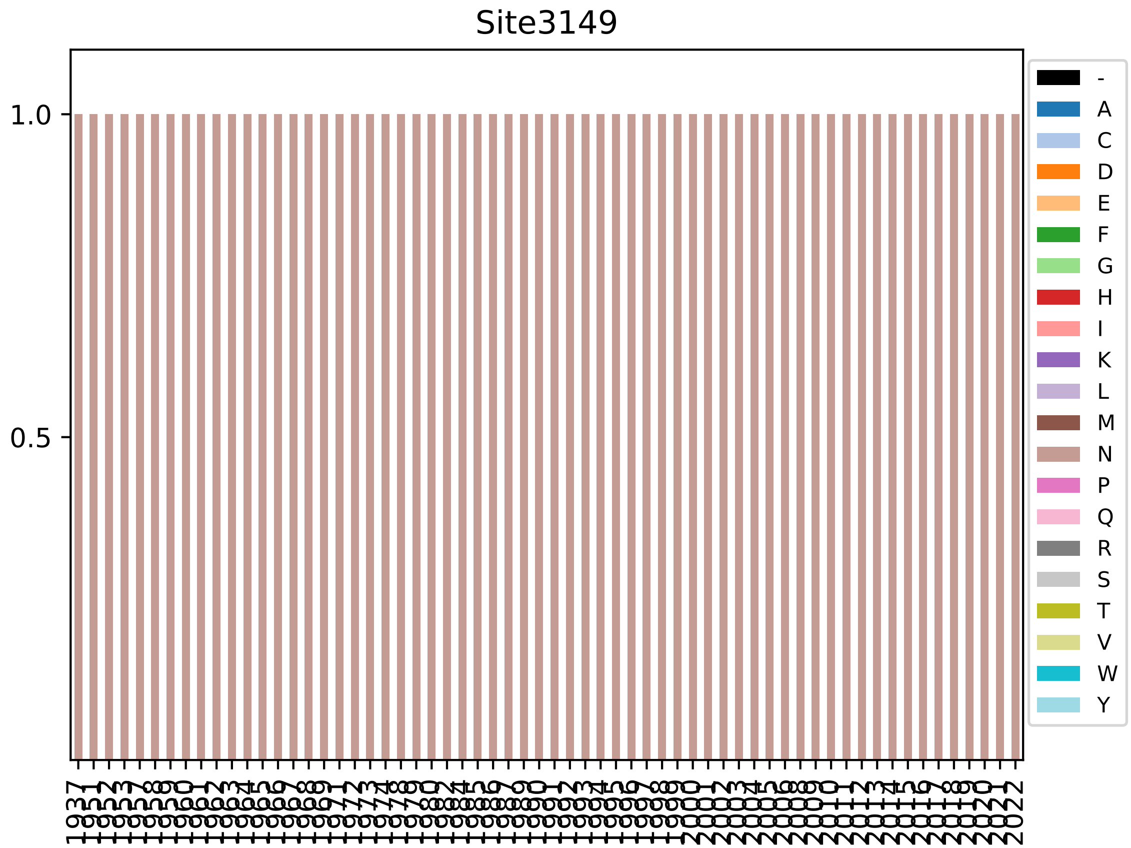
Site3149
1.0
0.5
-
A
C
D
E
F
G
H
I
K
L
M
N
P
Q
R
S
T
V
W
Y

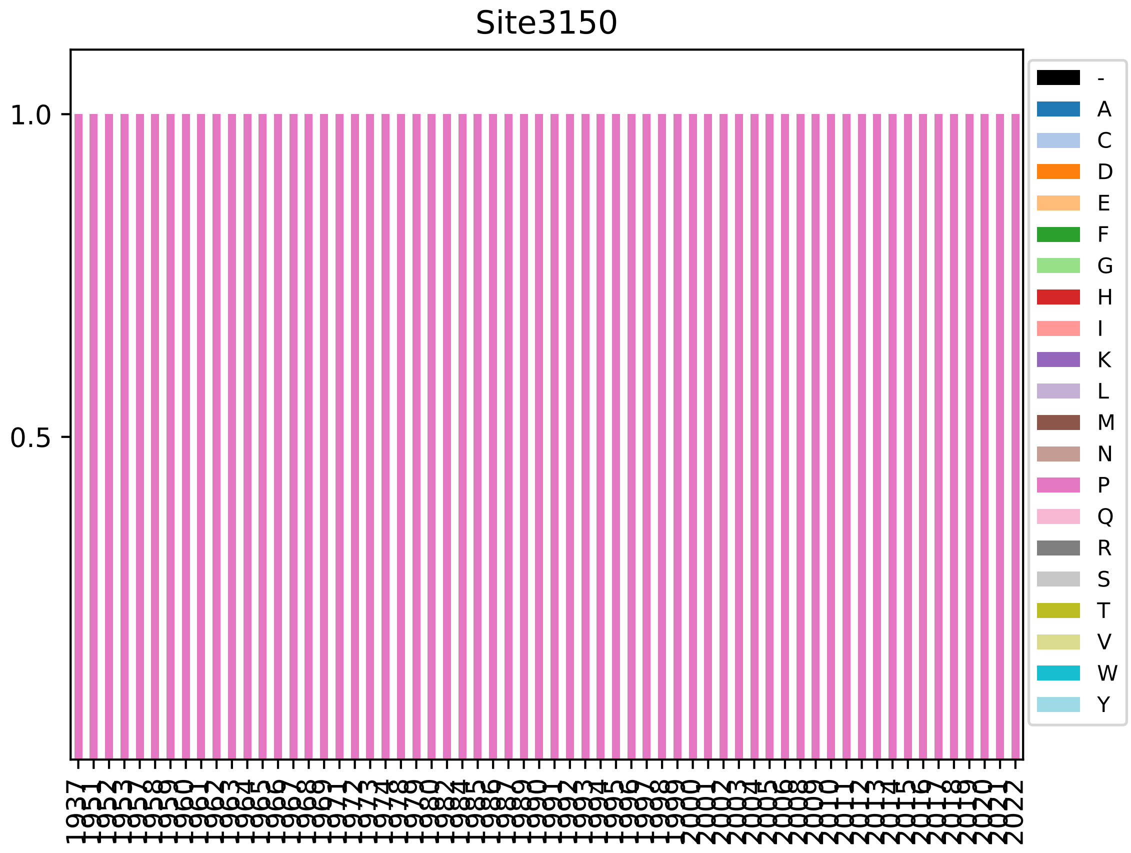
Site3150
1.0
0.5
-
A
C
D
E
F
G
H
I
K
L
M
N
P
Q
R
S
T
V
W
Y

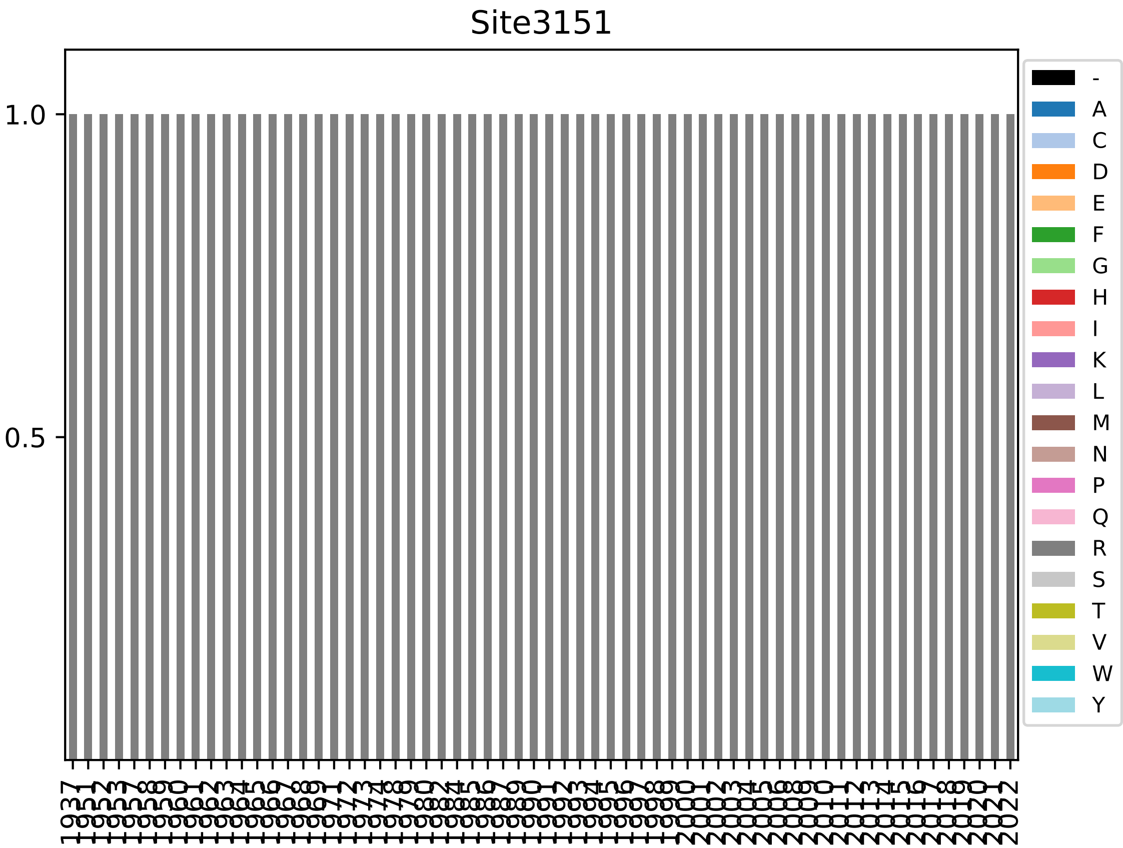
Site3151
1.0
0.5
-
A
C
D
E
F
G
H
I
K
L
M
N
P
Q
R
S
T
V
W
Y

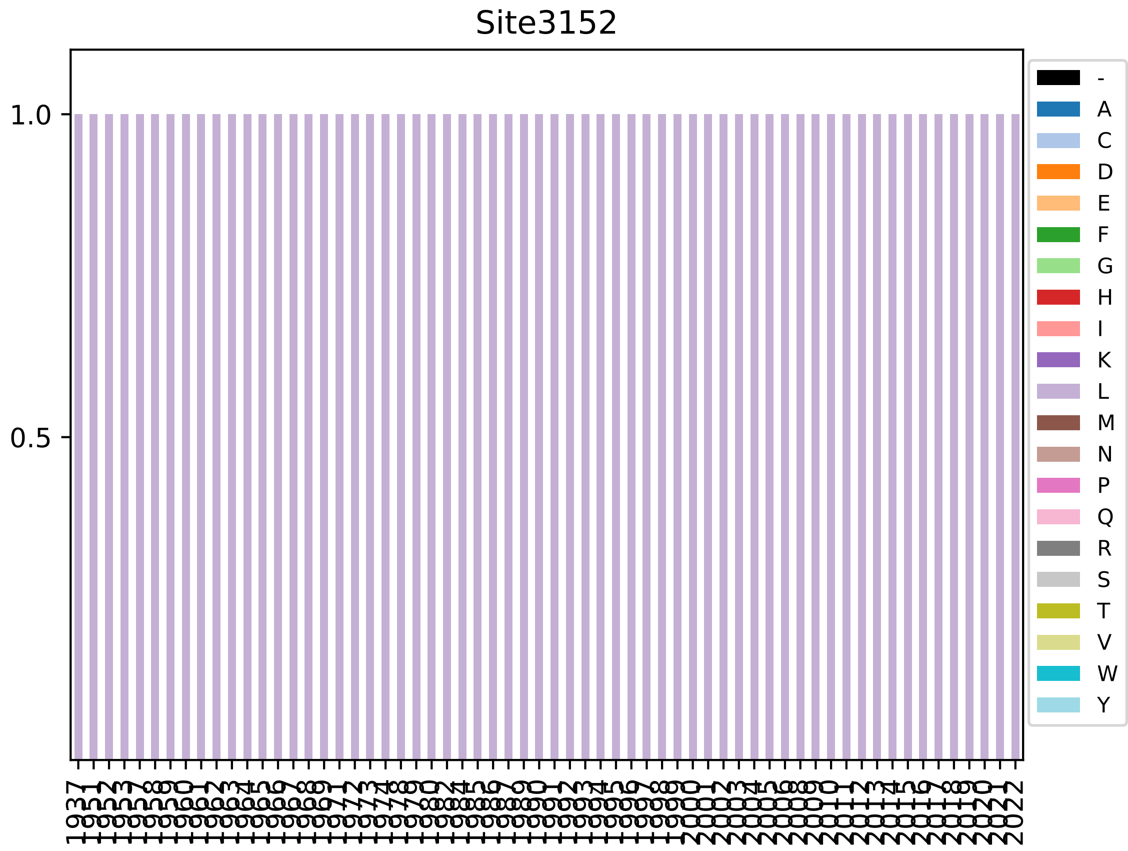
Site3152
1.0
0.5
-
A
C
D
E
F
G
H
I
K
L
M
N
P
Q
R
S
T
V
W
Y

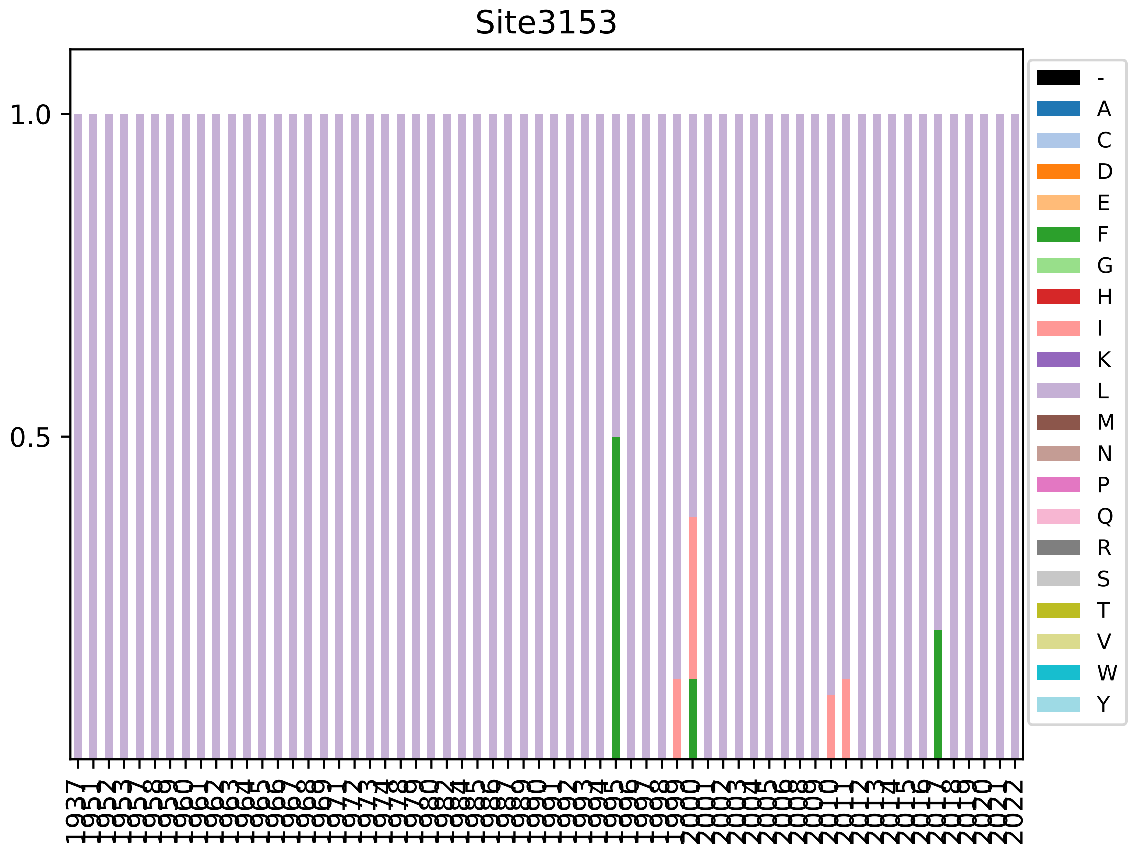
Site3153
1.0
0.5
-
A
C
D
E
F
G
H
I
K
L
M
N
P
Q
R
S
T
V
W
Y

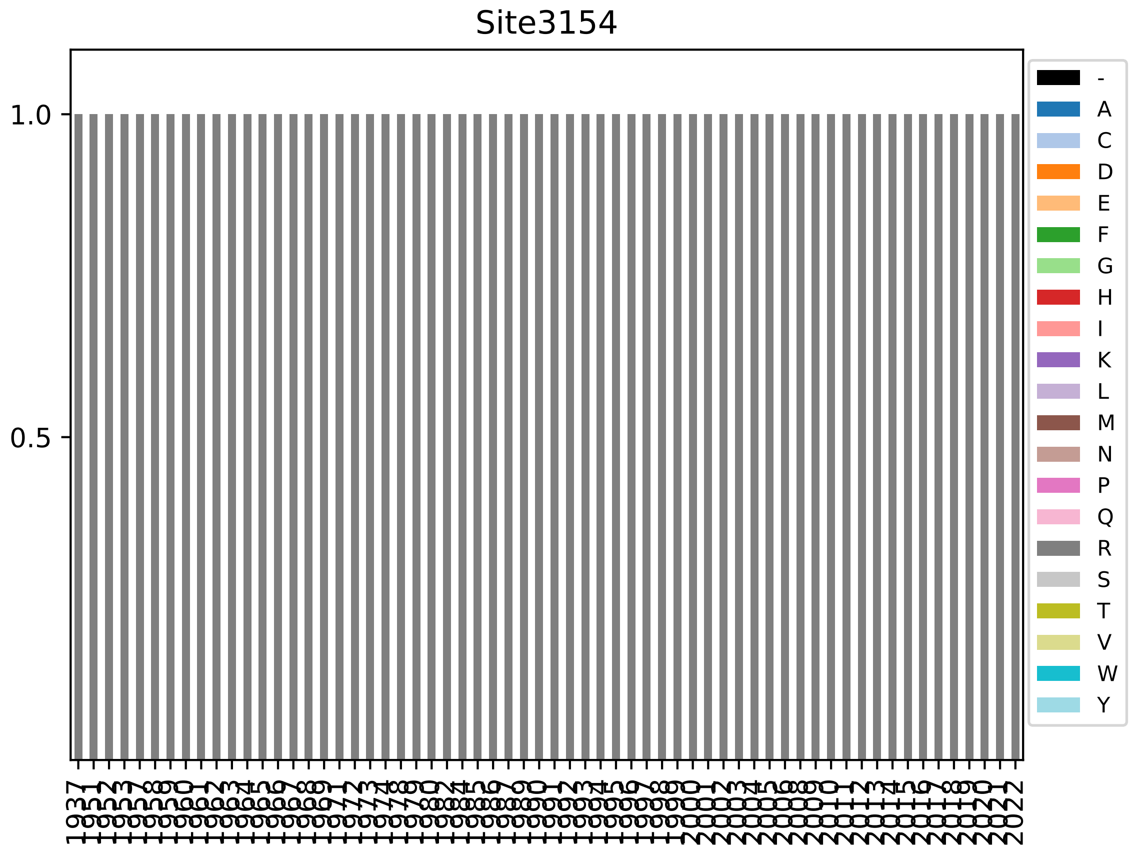
Site3154
1.0
0.5
-
A
C
D
E
F
G
H
I
K
L
M
N
P
Q
R
S
T
V
W
Y

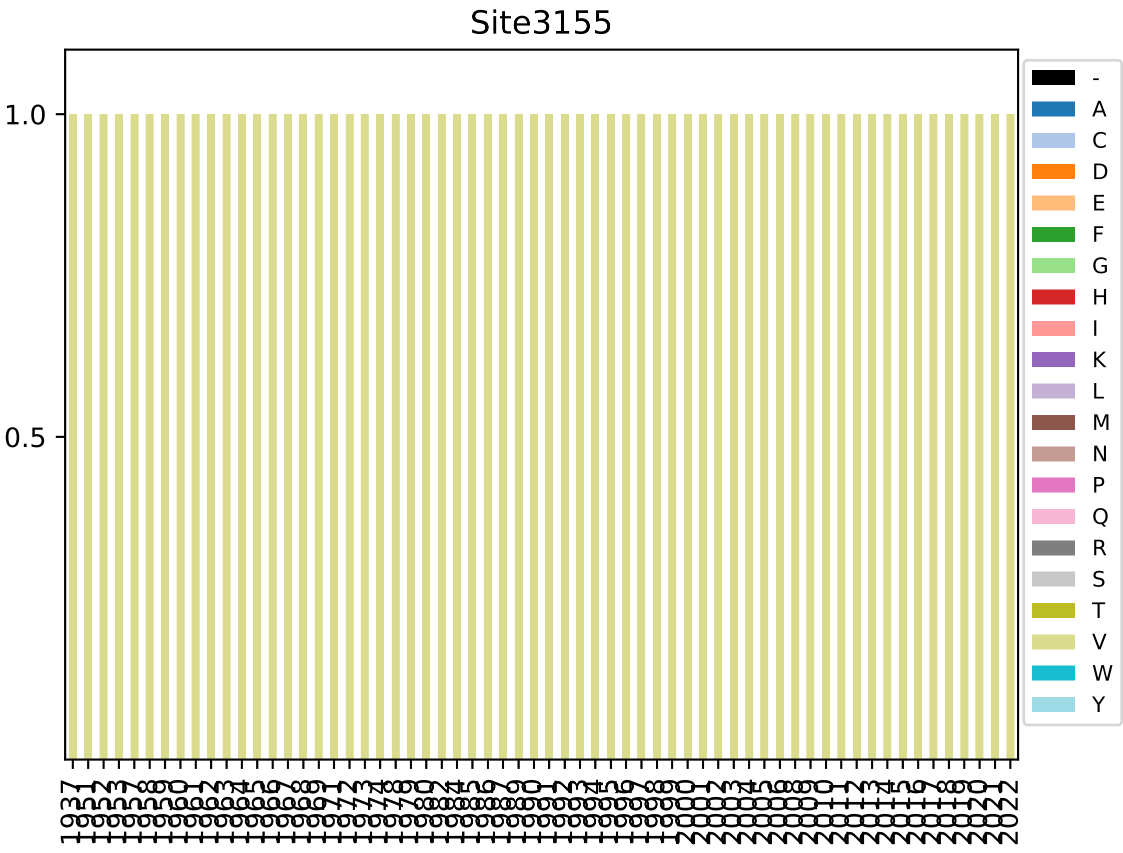
Site3155
1.0
0.5
-
A
C
D
E
F
G
H
I
K
L
M
N
P
Q
R
S
T
V
W
Y

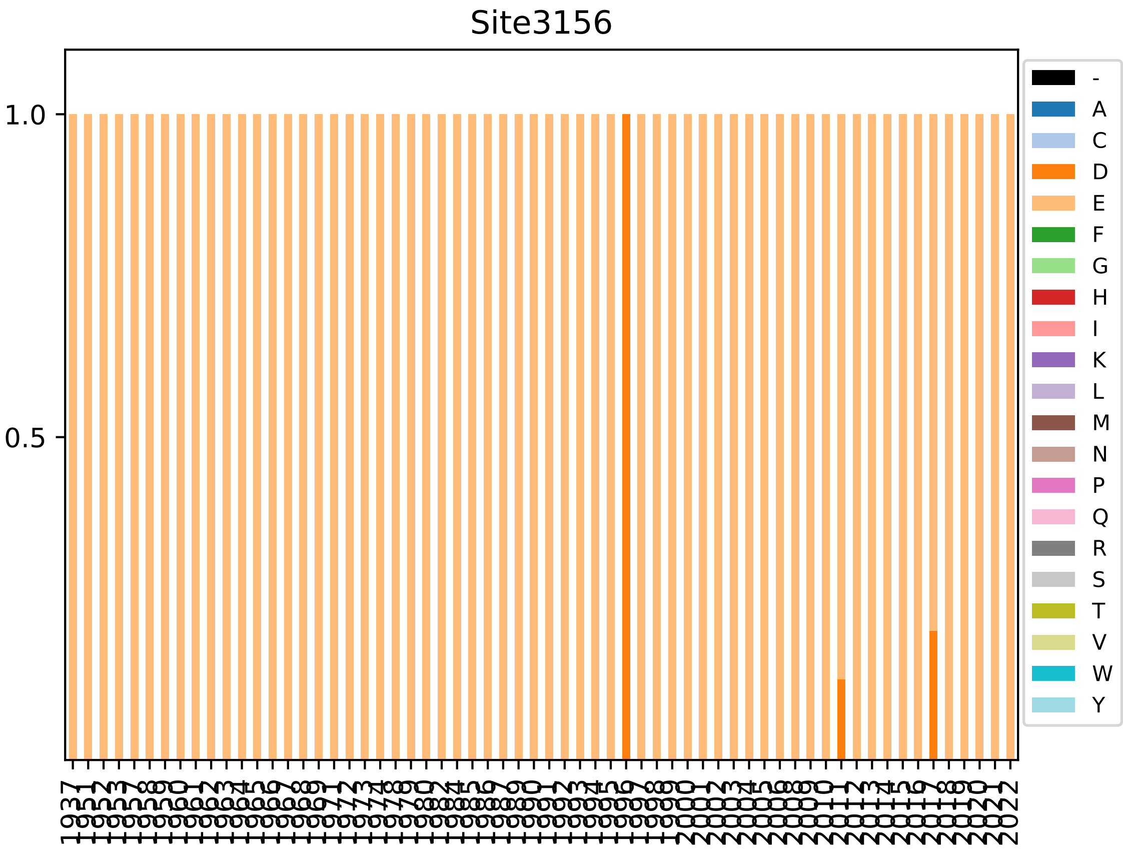
Site3156
1.0
0.5
-
A
C
D
E
F
G
H
I
K
L
M
N
P
Q
R
S
T
V
W
Y

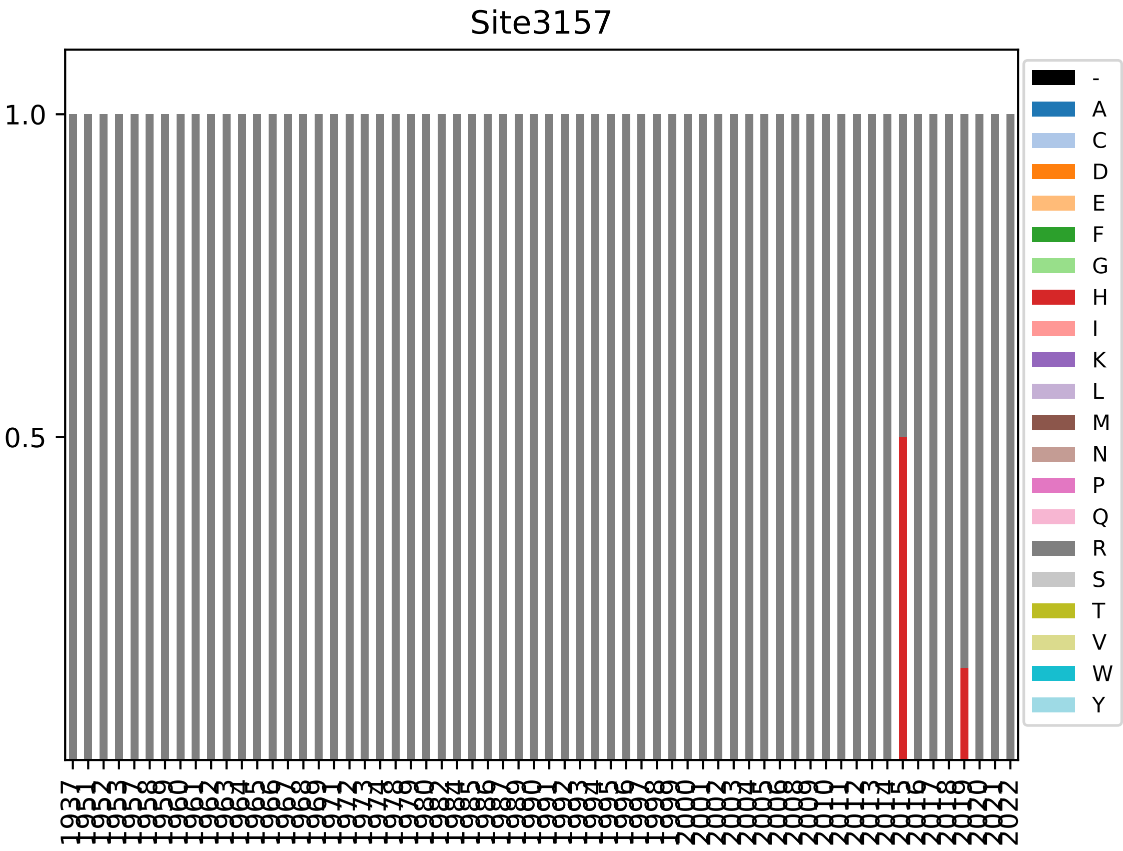
Site3157
1.0
0.5
-
A
C
D
E
F
G
H
I
K
L
M
N
P
Q
R
S
T
V
W
Y

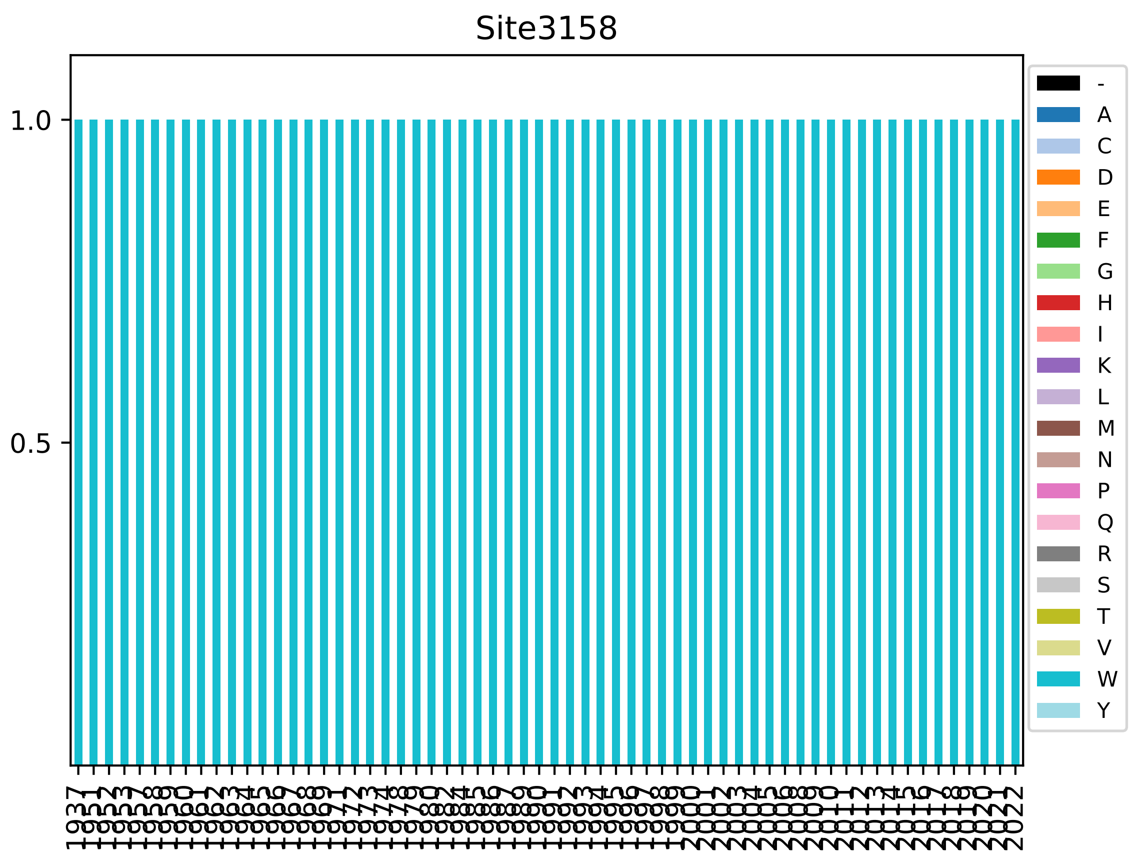
Site3158
1.0
0.5
-
A
C
D
E
F
G
H
I
K
L
M
N
P
Q
R
S
T
V
W
Y

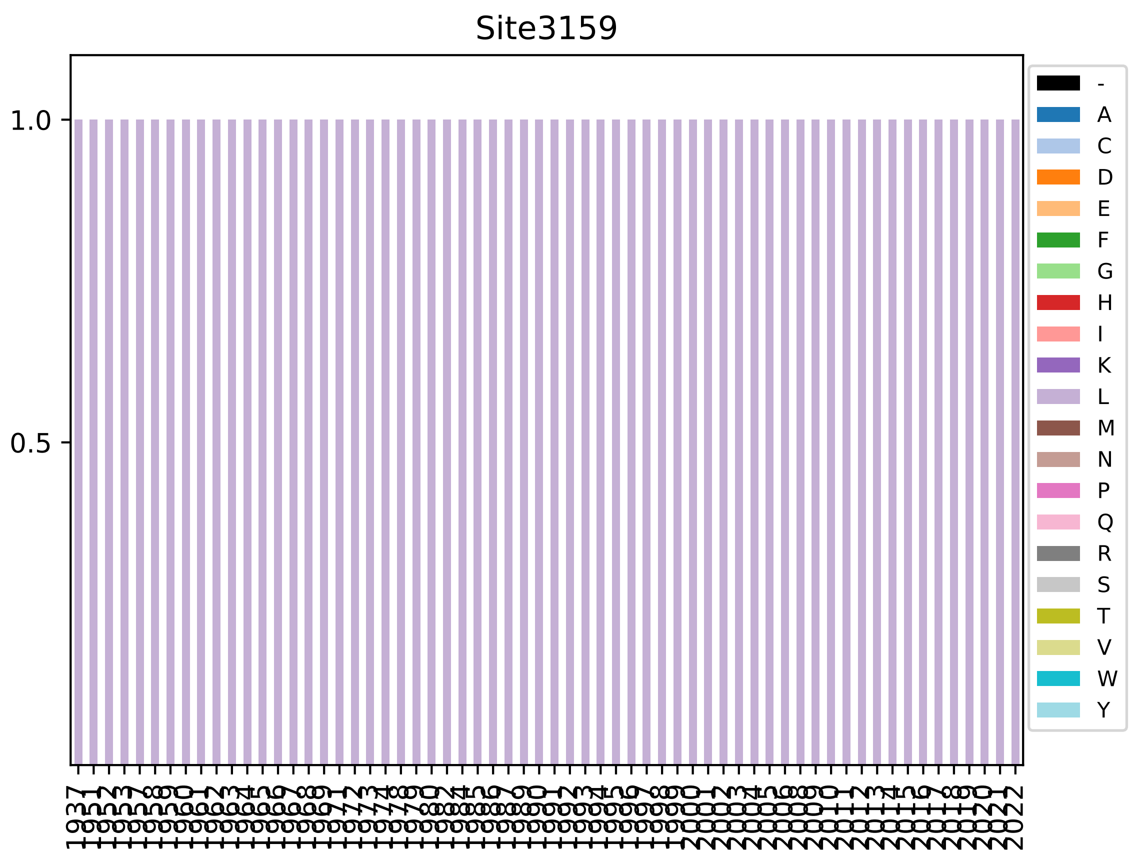
Site3159
1.0
0.5
-
A
C
D
E
F
G
H
I
K
L
M
N
P
Q
R
S
T
V
W
Y

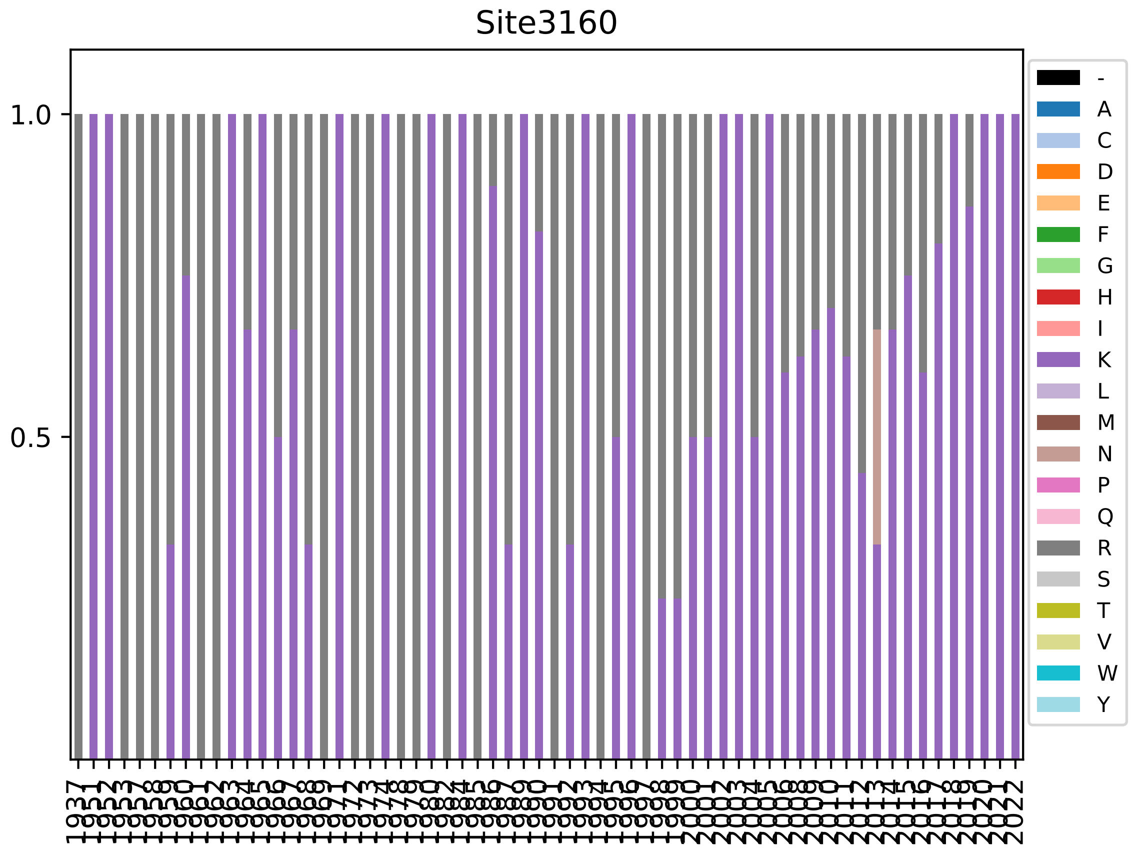
Site3160
1.0
0.5
-
A
C
D
E
F
G
H
I
K
L
M
N
P
Q
R
S
T
V
W
Y

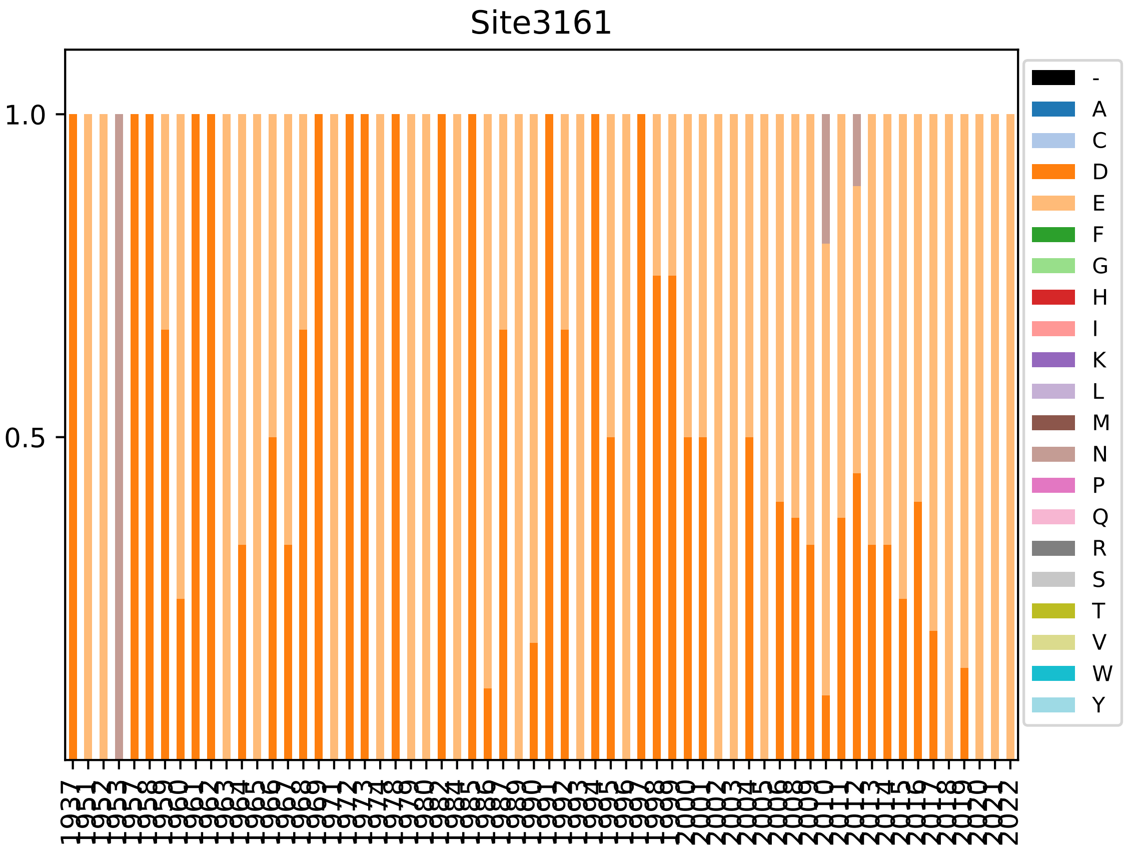
Site3161
1.0
0.5
-
A
C
D
E
F
G
H
I
K
L
M
N
P
Q
R
S
T
V
W
Y

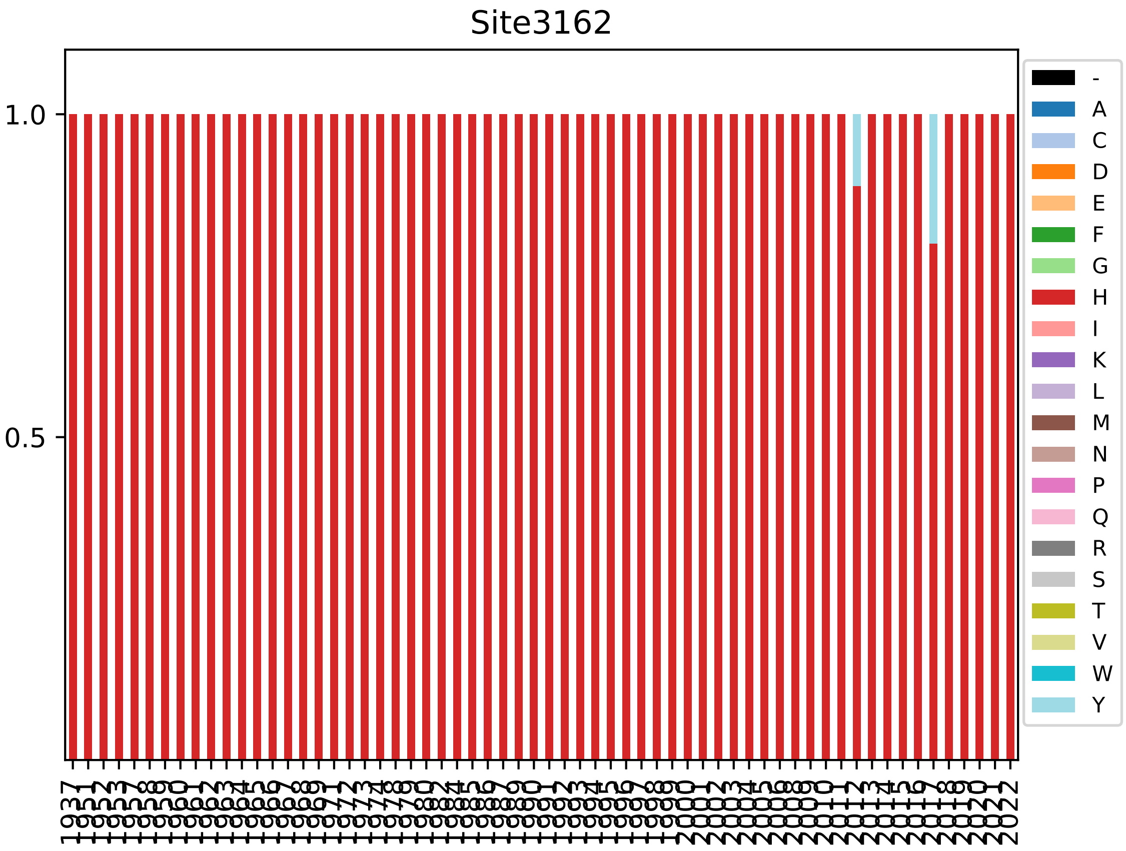
Site3162
1.0
0.5
-
A
C
D
E
F
G
H
I
K
L
M
N
P
Q
R
S
T
V
W
Y

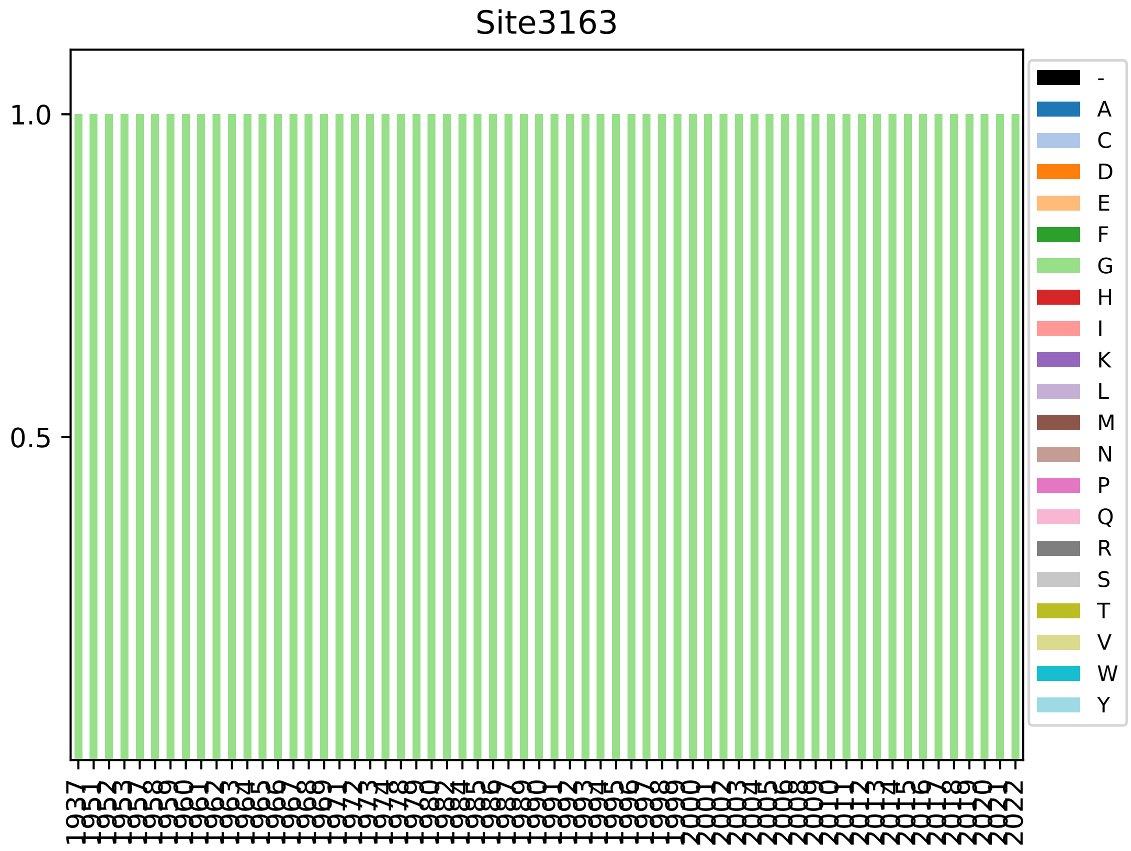

Site3163
1.0
0.5
-
A
C
D
E
F
G
H
I
K
L
M
N
P
Q
R
S
T
V
W
Y

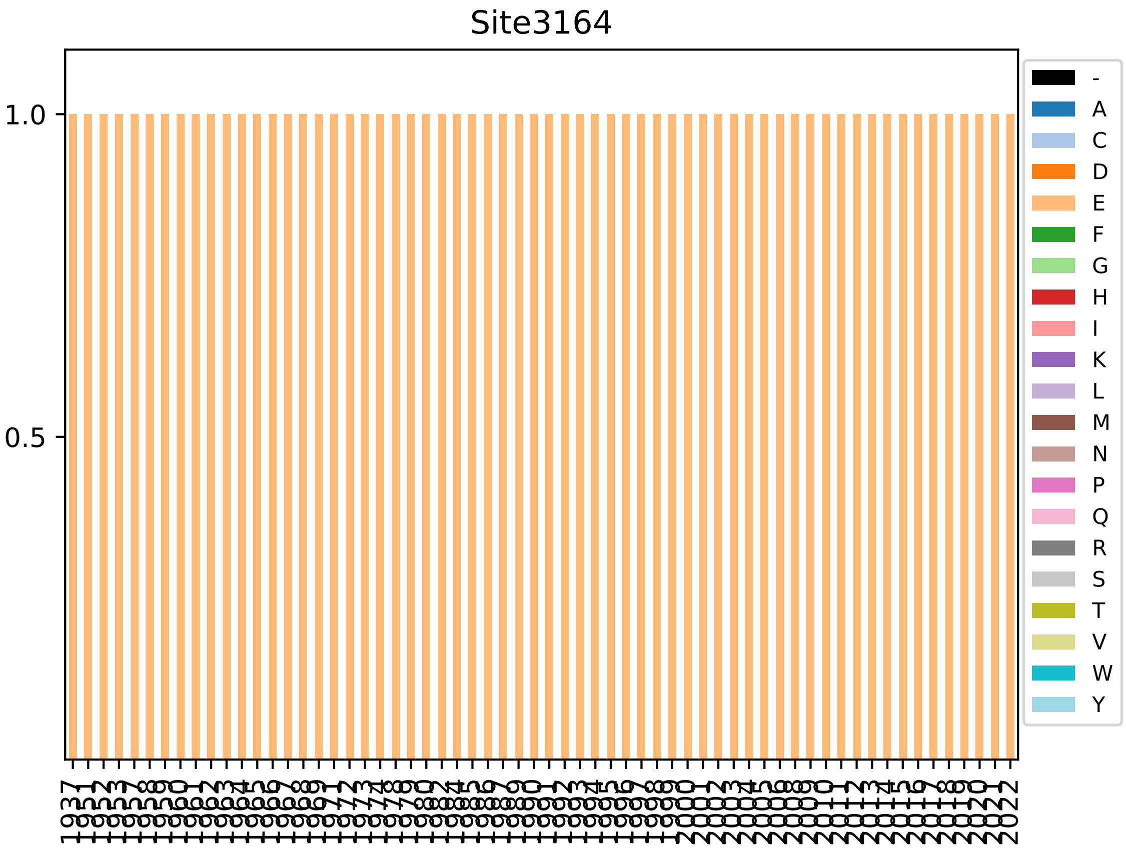
Site3164
1.0
0.5
-
A
C
D
E
F
G
H
I
K
L
M
N
P
Q
R
S
T
V
W
Y

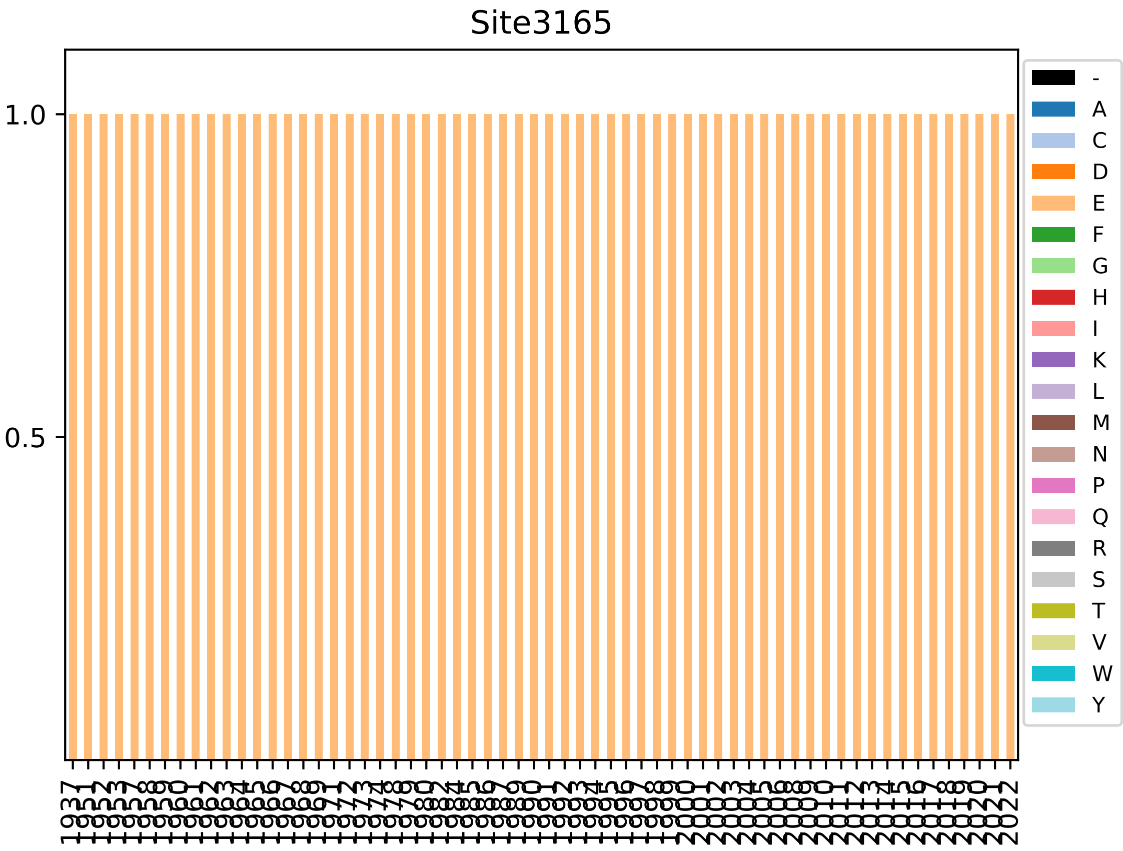
Site3165
1.0
0.5
-
A
C
D
E
F
G
H
I
K
L
M
N
P
Q
R
S
T
V
W
Y

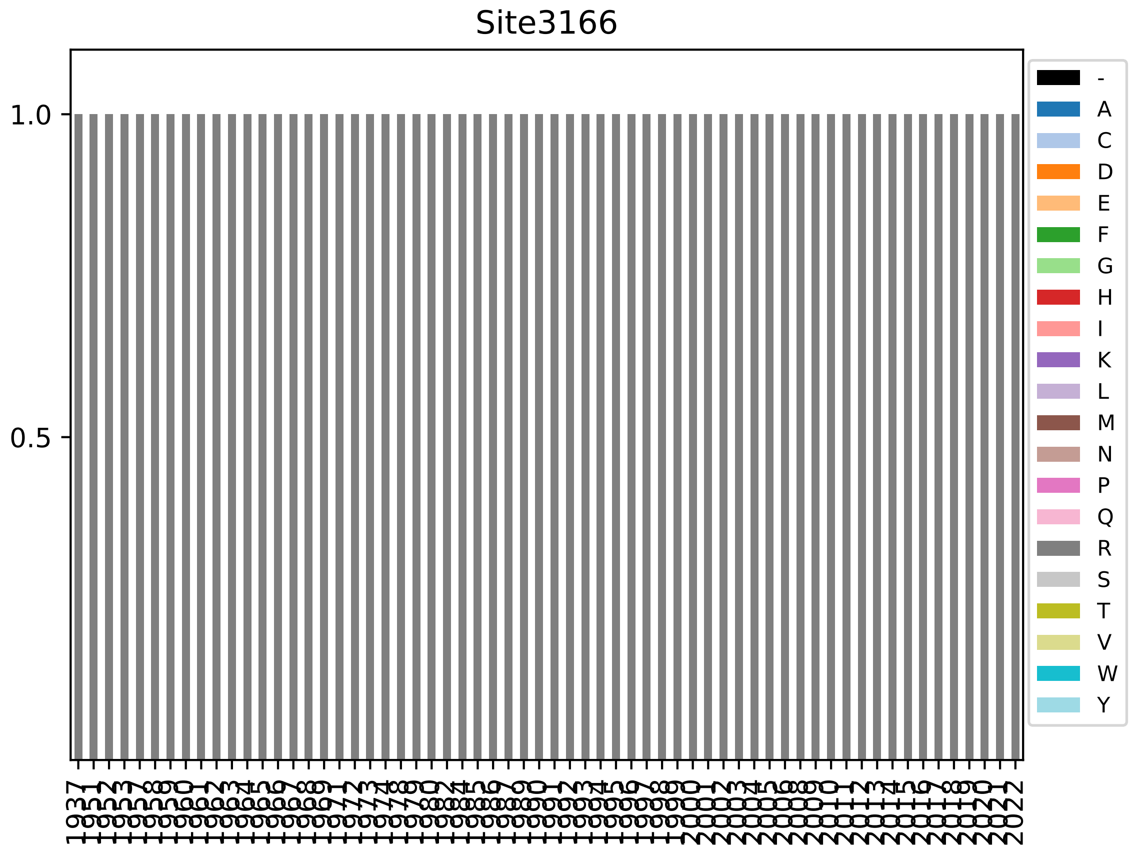
Site3166
1.0
0.5
-
A
C
D
E
F
G
H
I
K
L
M
N
P
Q
R
S
T
V
W
Y

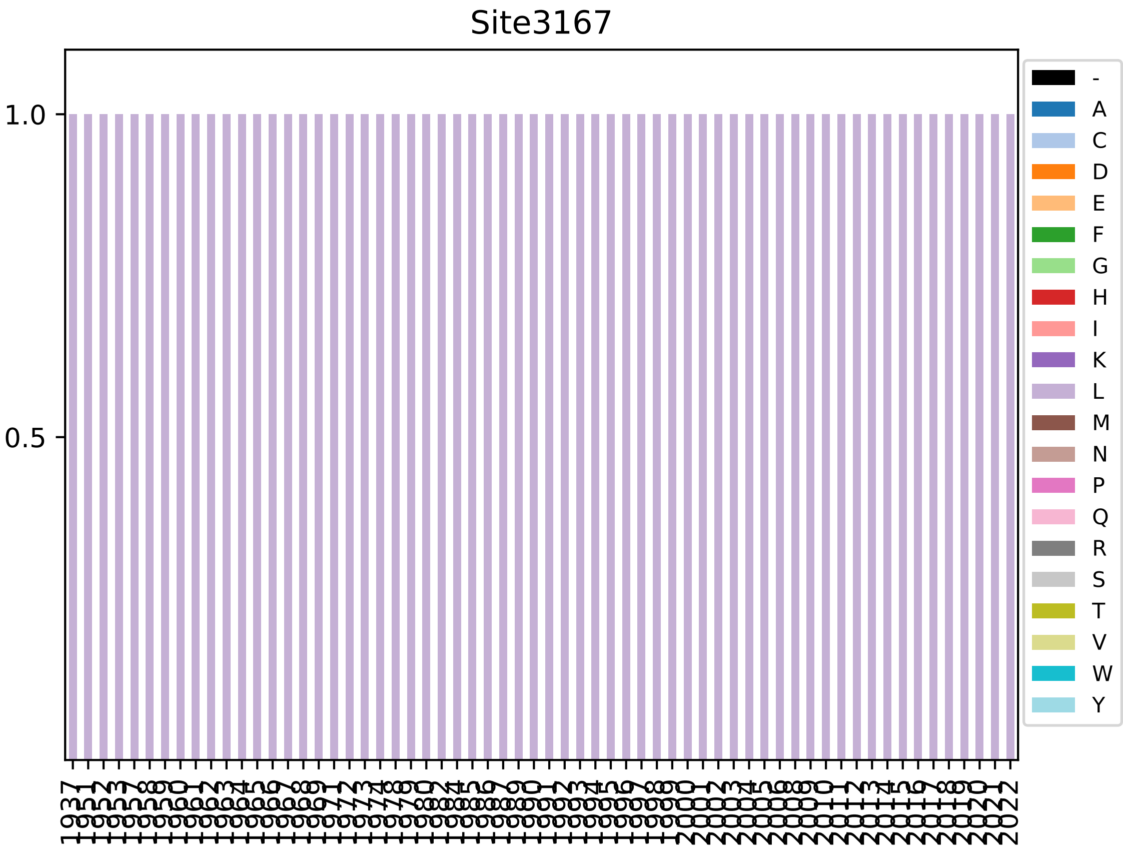

Site3167
1.0
0.5
-
A
C
D
E
F
G
H
I
K
L
M
N
P
Q
R
S
T
V
W
Y

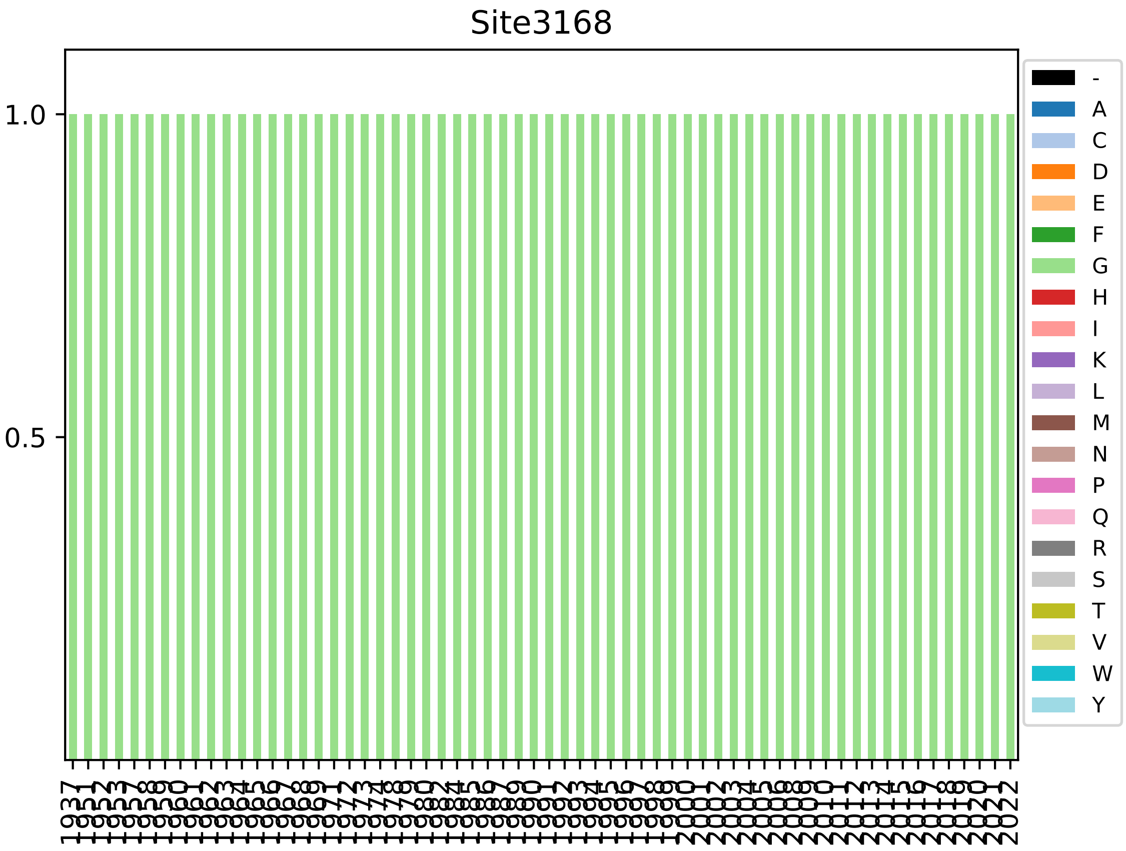
Site3168
1.0
0.5
-
A
C
D
E
F
G
H
I
K
L
M
N
P
Q
R
S
T
V
W
Y

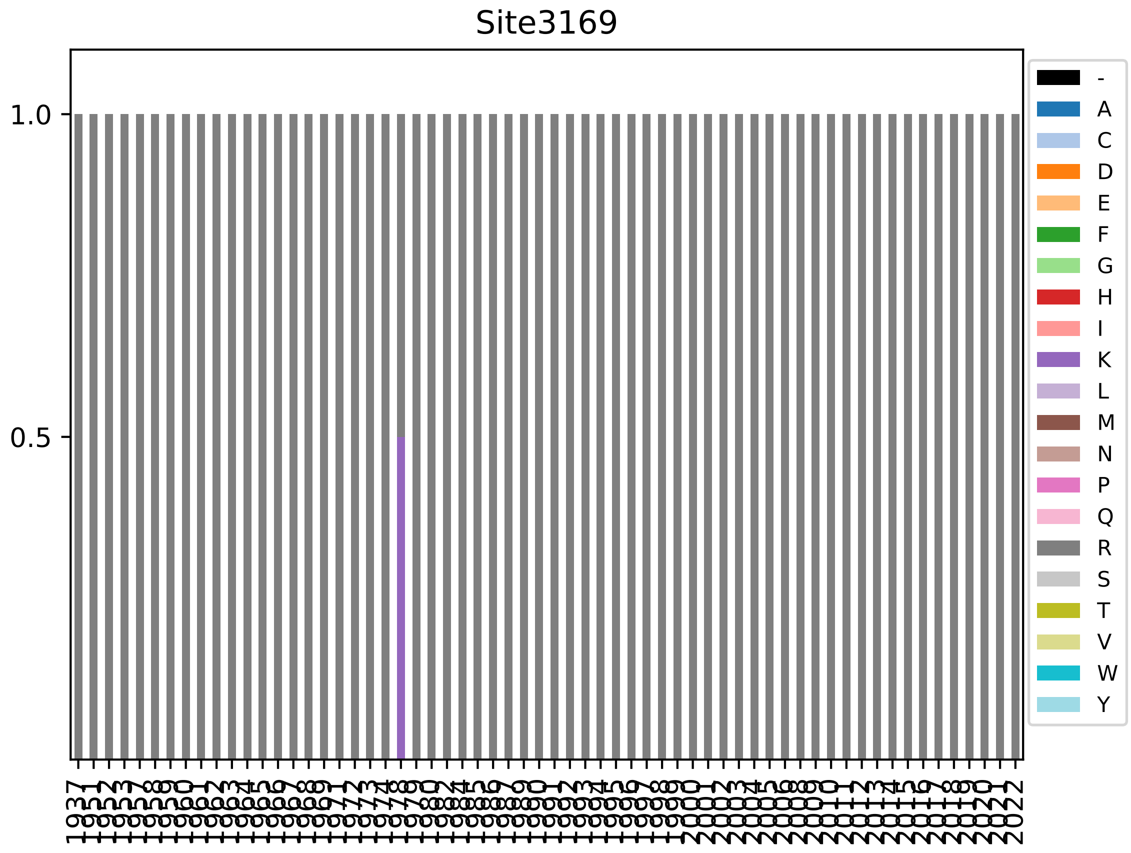
Site3169
1.0
0.5
-
A
C
D
E
F
G
H
I
K
L
M
N
P
Q
R
S
T
V
W
Y

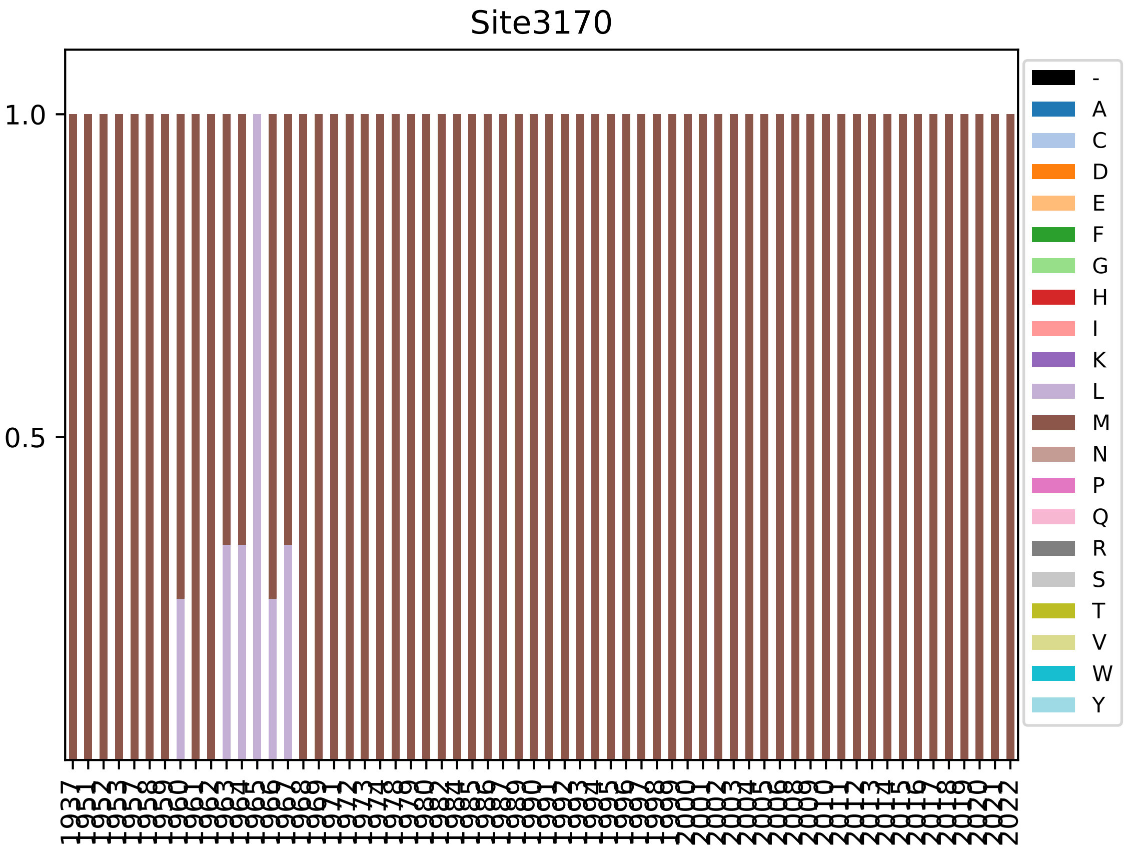
Site3170
1.0
0.5
-
A
C
D
E
F
G
H
I
K
L
M
N
P
Q
R
S
T
V
W
Y

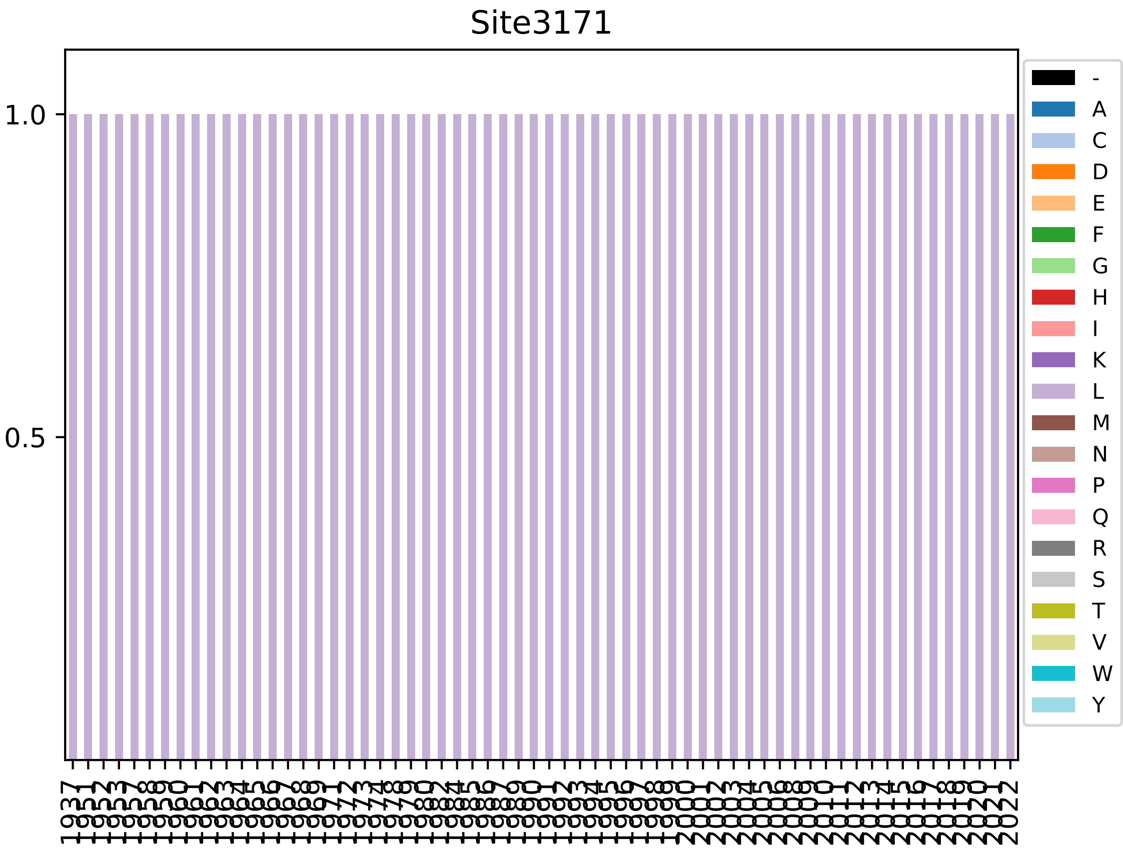
Site3171
1.0
0.5
-
A
C
D
E
F
G
H
I
K
L
M
N
P
Q
R
S
T
V
W
Y

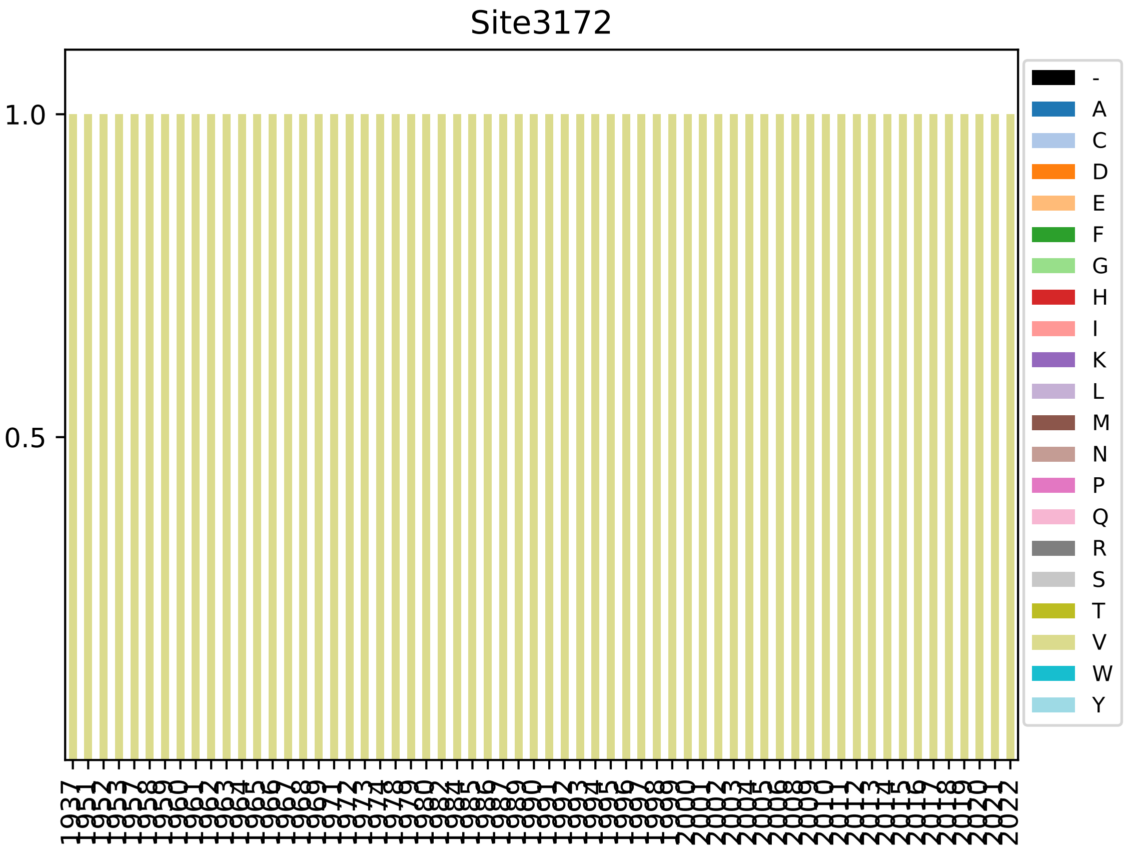
Site3172
1.0
0.5
-
A
C
D
E
F
G
H
I
K
L
M
N
P
Q
R
S
T
V
W
Y

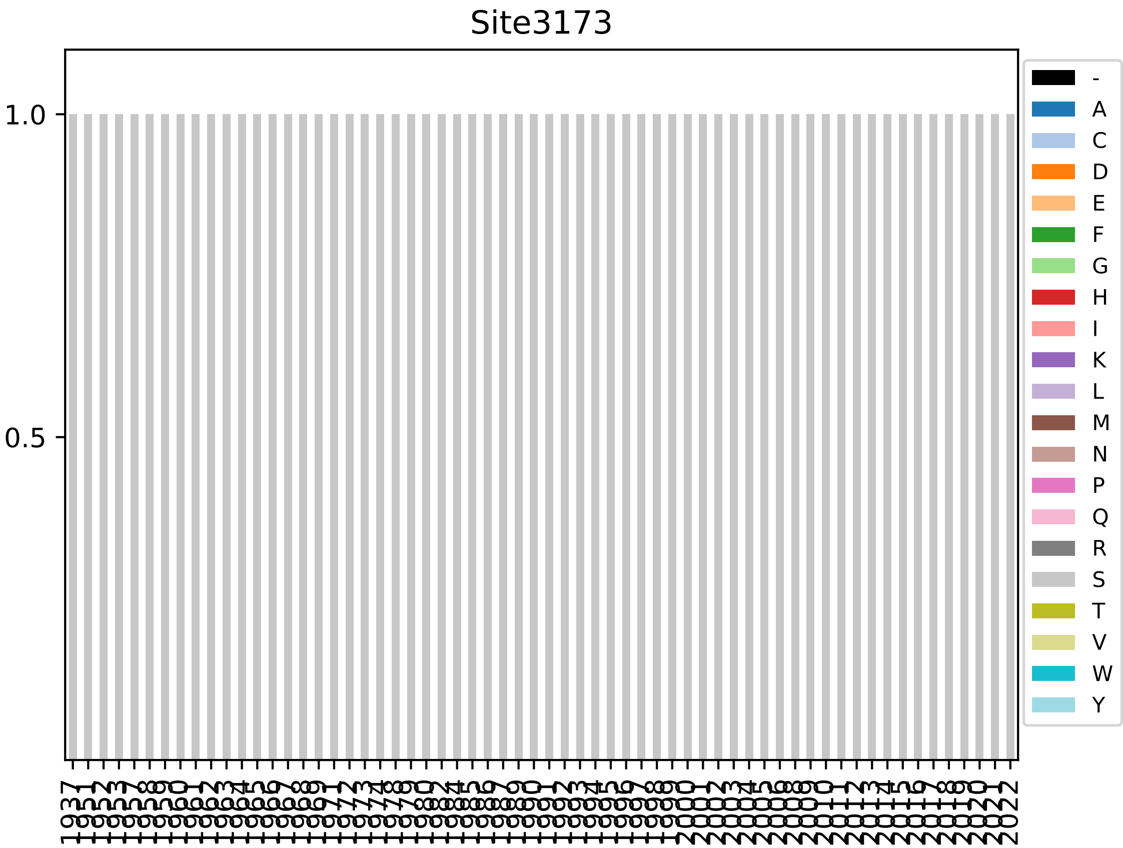
Site3173
1.0
0.5
-
A
C
D
E
F
G
H
I
K
L
M
N
P
Q
R
S
T
V
W
Y

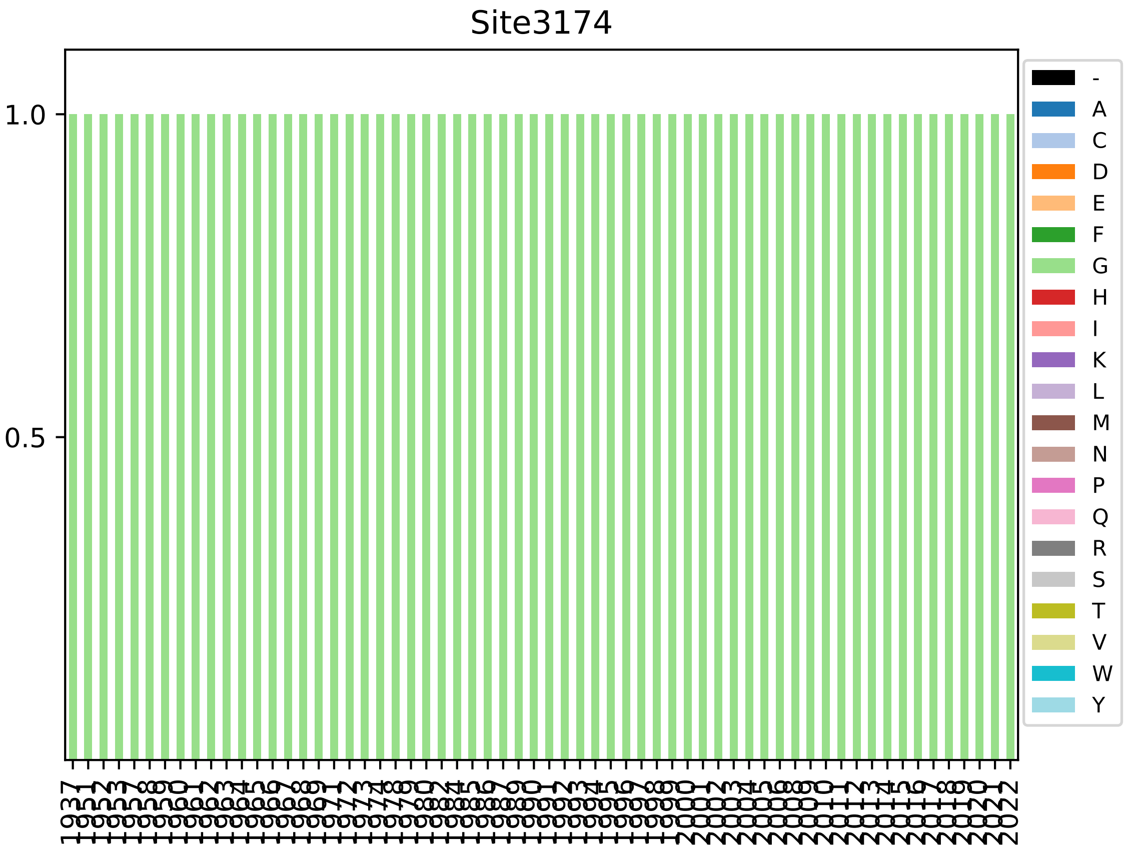

Site3174
1.0
0.5
-
A
C
D
E
F
G
H
I
K
L
M
N
P
Q
R
S
T
V
W
Y

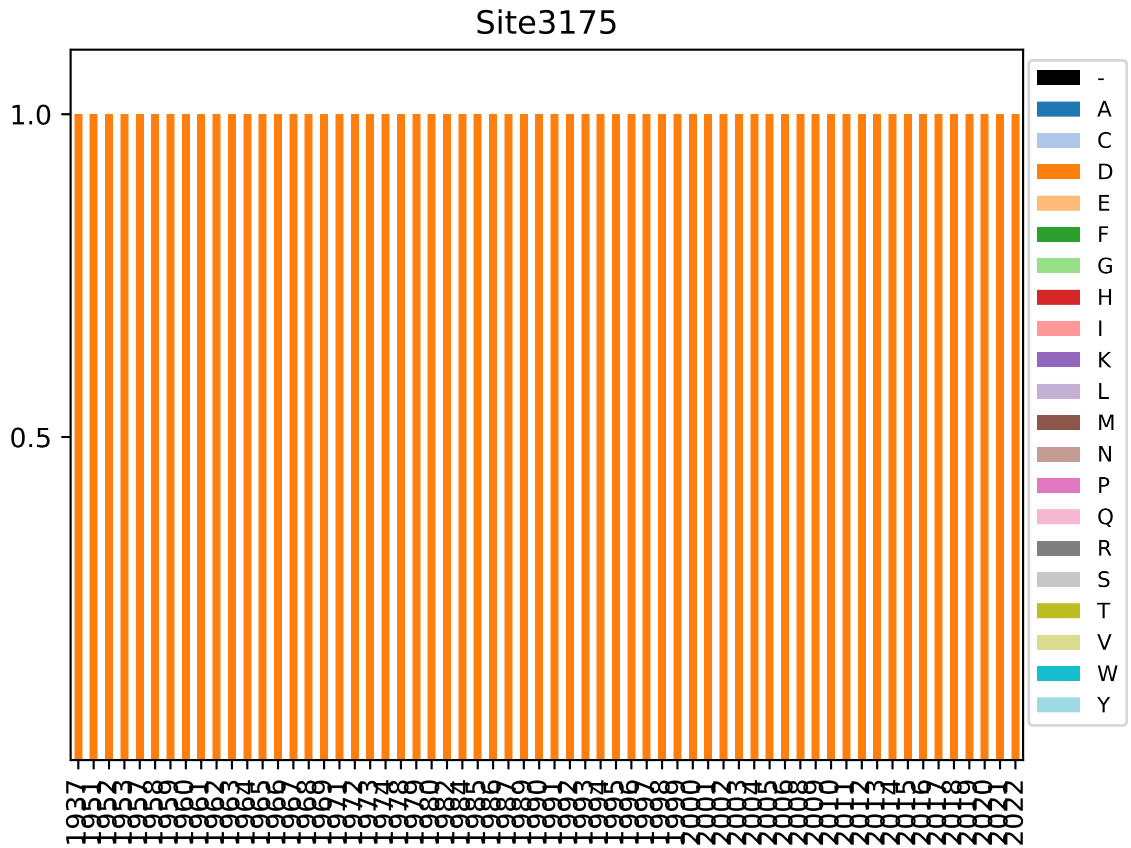
Site3175
1.0
0.5
-
A
C
D
E
F
G
H
I
K
L
M
N
P
Q
R
S
T
V
W
Y

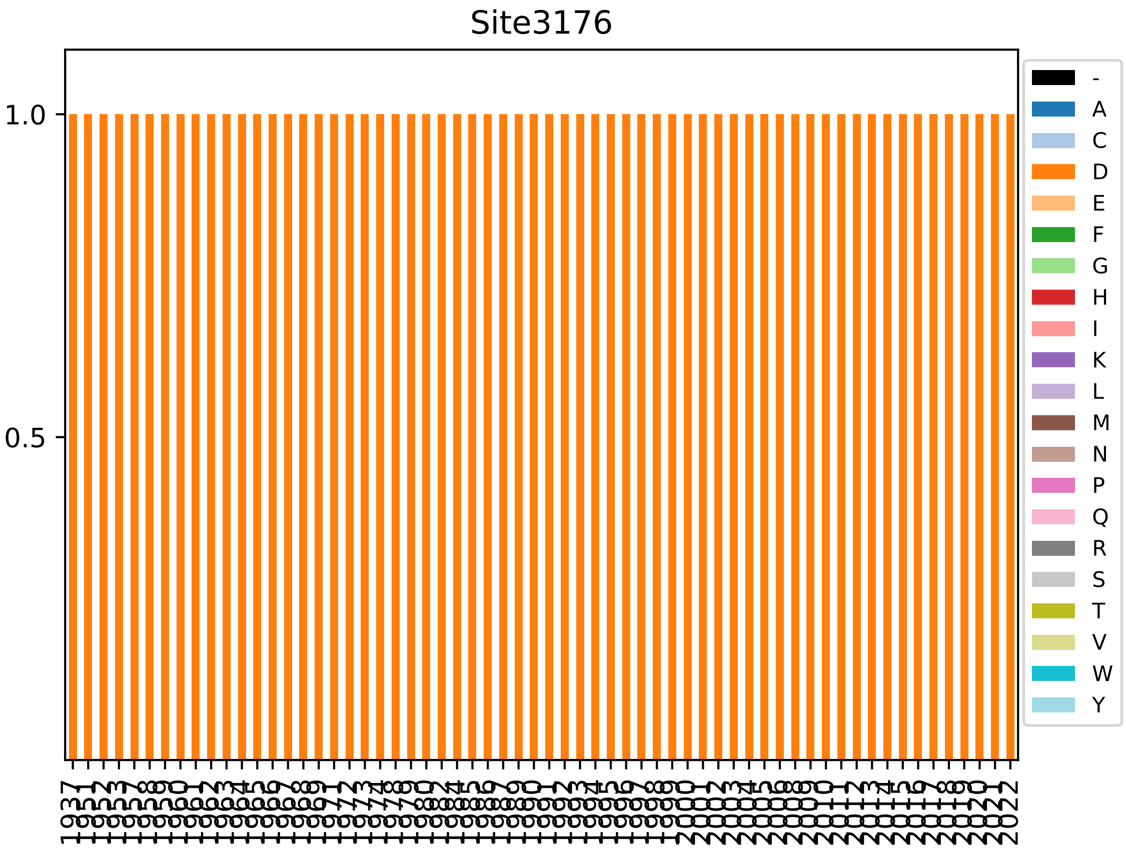
Site3176
1.0
0.5
-
A
C
D
E
F
G
H
I
K
L
M
N
P
Q
R
S
T
V
W
Y

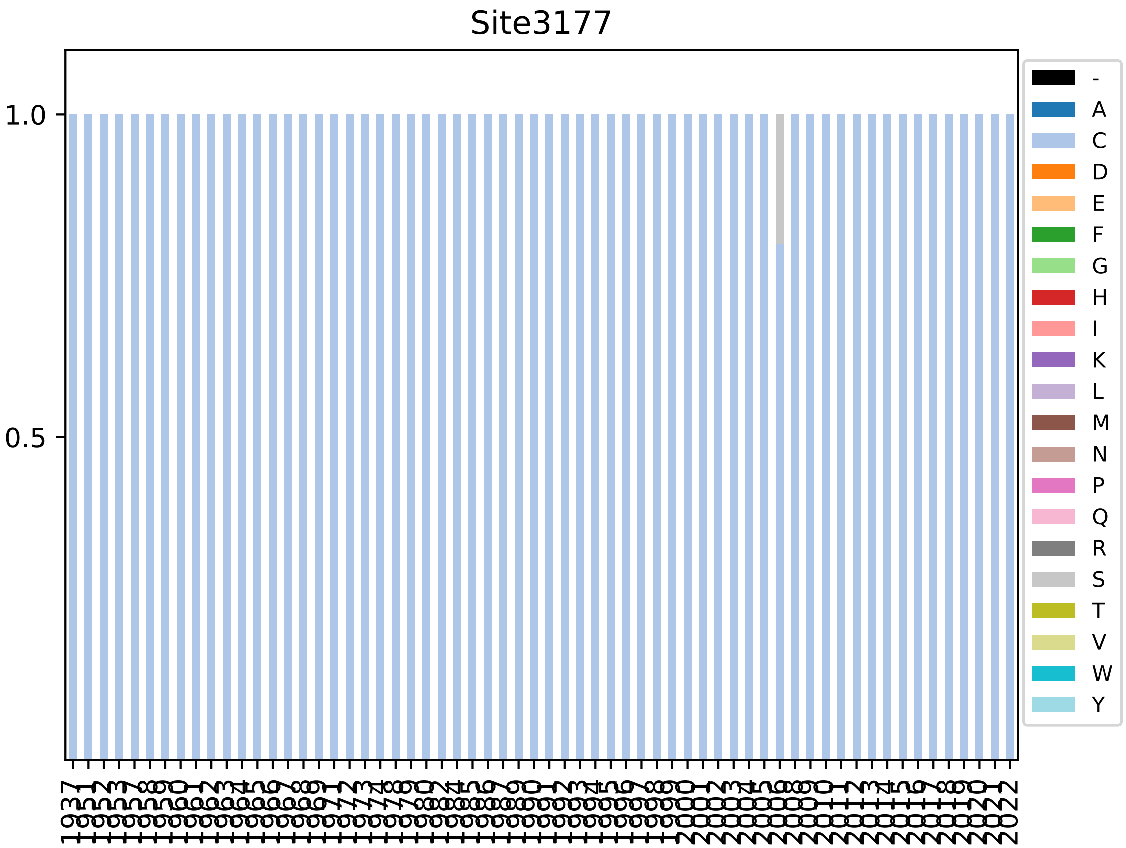
Site3177
1.0
0.5
-
A
C
D
E
F
G
H
I
K
L
M
N
P
Q
R
S
T
V
W
Y

Site3178

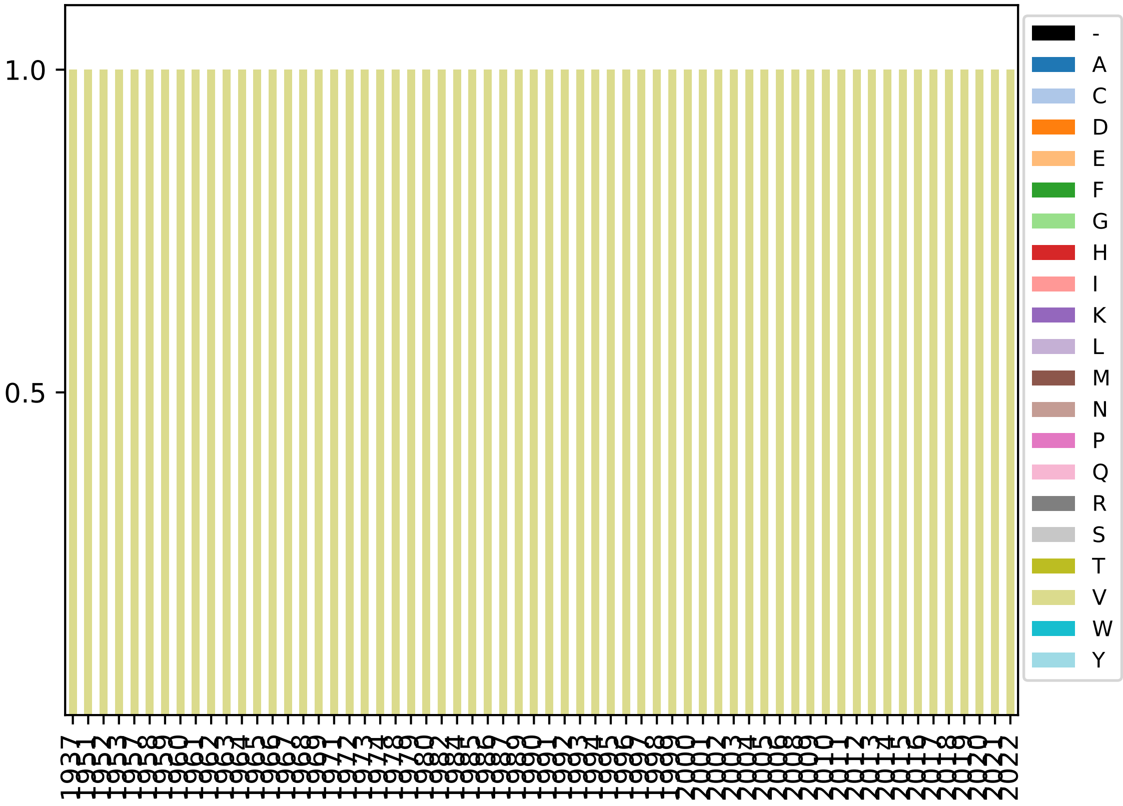

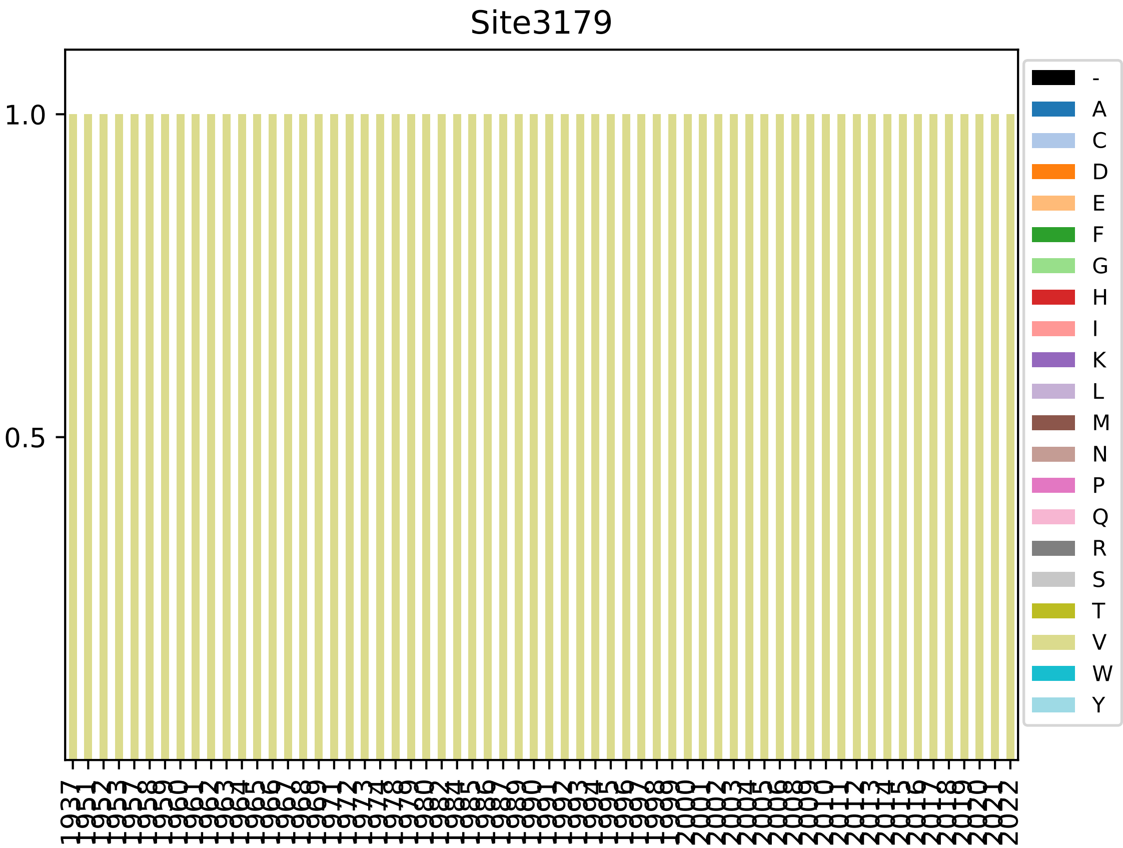
Site3179
1.0
0.5
-
A
C
D
E
F
G
H
I
K
L
M
N
P
Q
R
S
T
V
W
Y

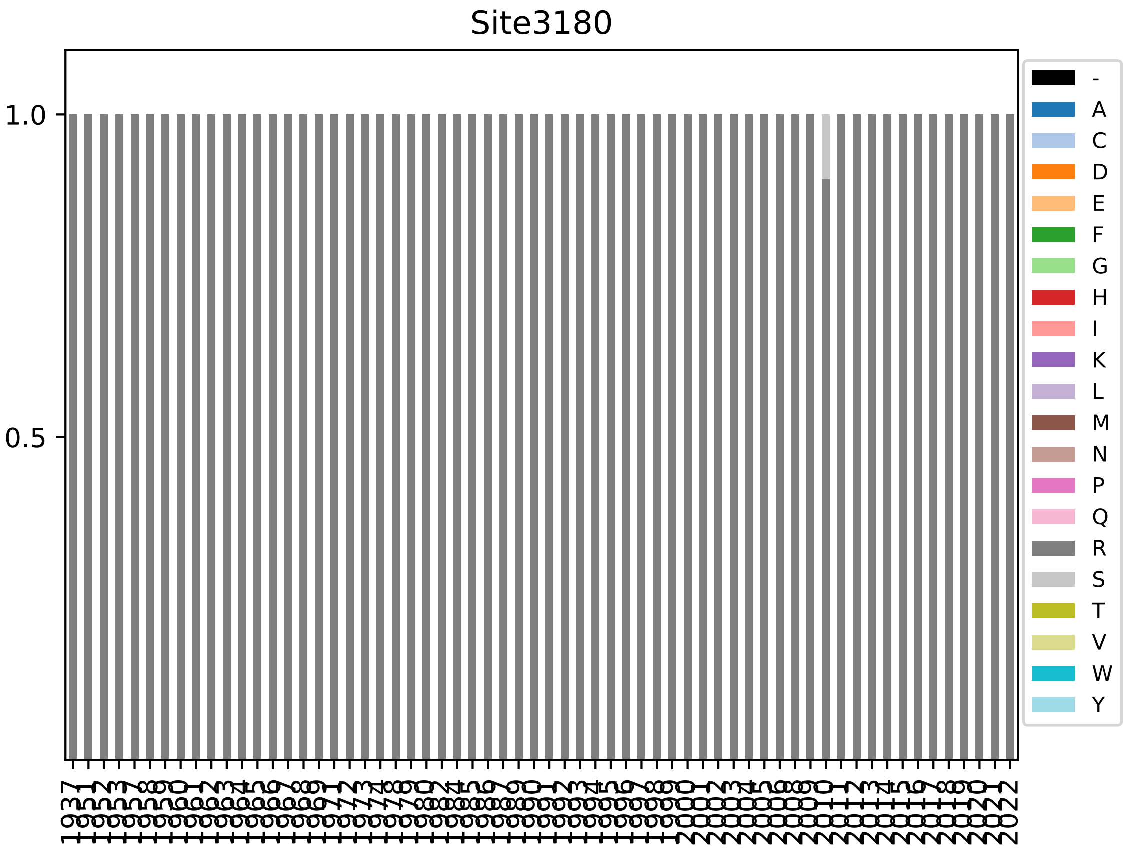
Site3180
1.0
0.5
-
A
C
D
E
F
G
H
I
K
L
M
N
P
Q
R
S
T
V
W
Y

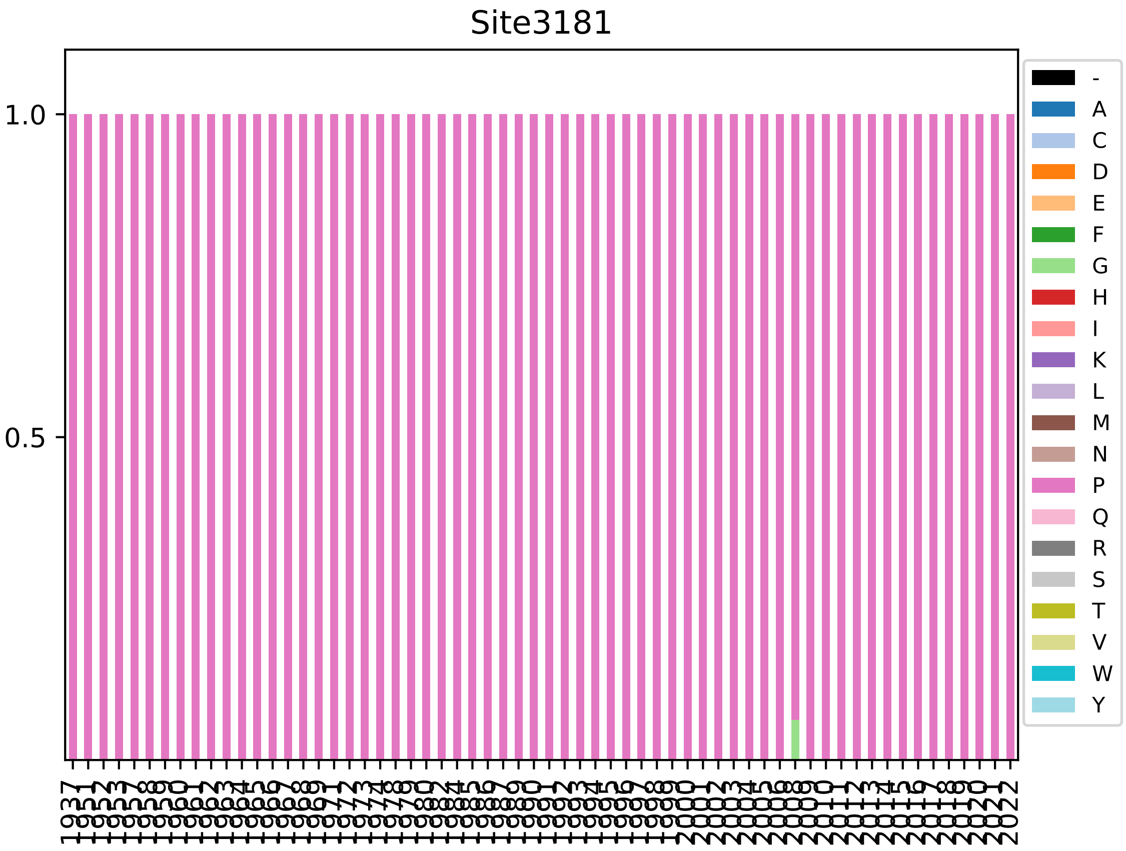
Site3181
1.0
0.5
-
A
C
D
E
F
G
H
I
K
L
M
N
P
Q
R
S
T
V
W
Y

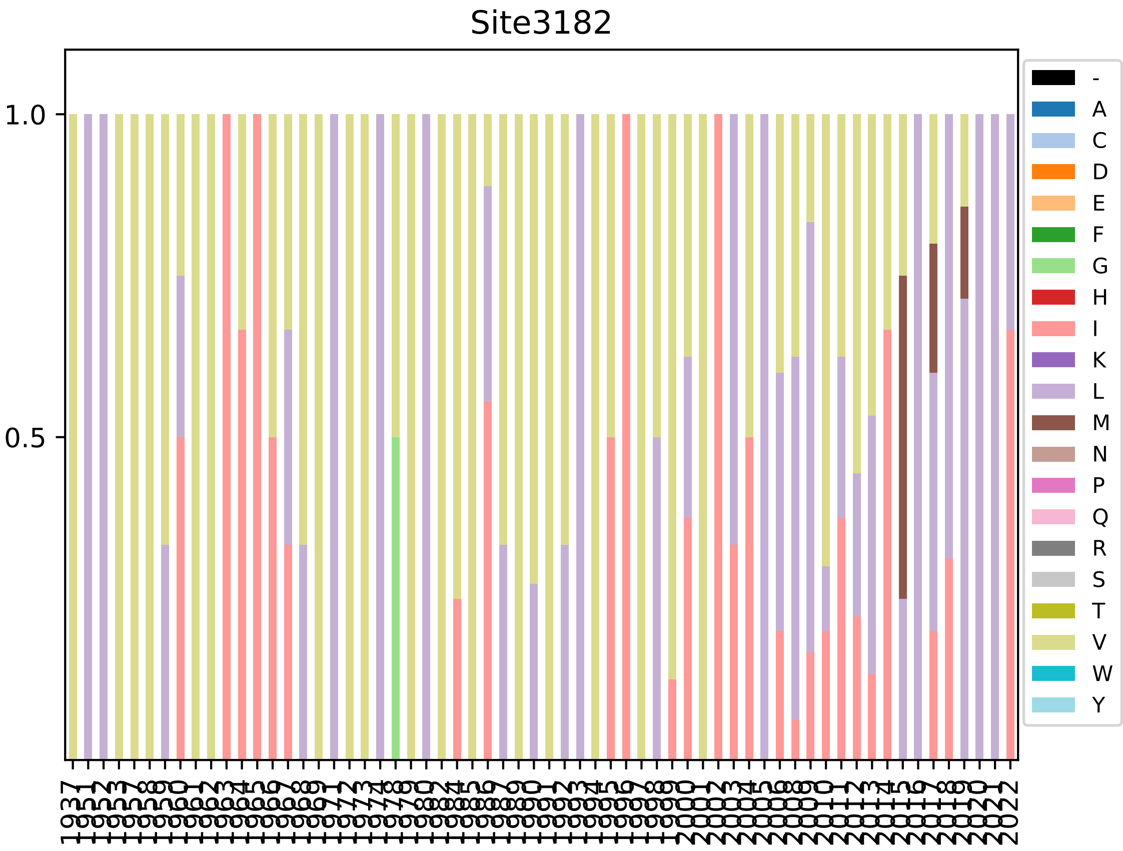
Site3182
1.0
0.5
-
A
C
D
E
F
G
H
I
K
L
M
N
P
Q
R
S
T
V
W
Y

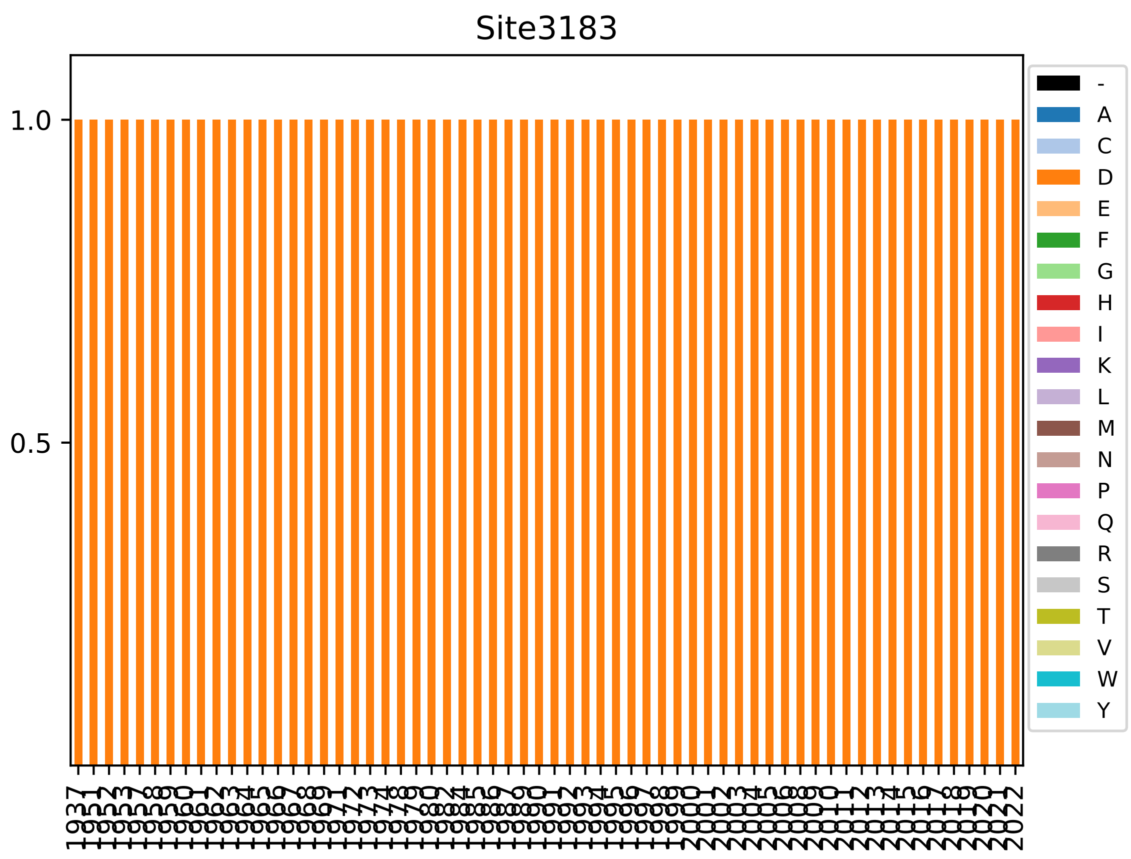

Site3183
1.0
0.5
-
A
C
D
E
F
G
H
I
K
L
M
N
P
Q
R
S
T
V
W
Y

Site3184

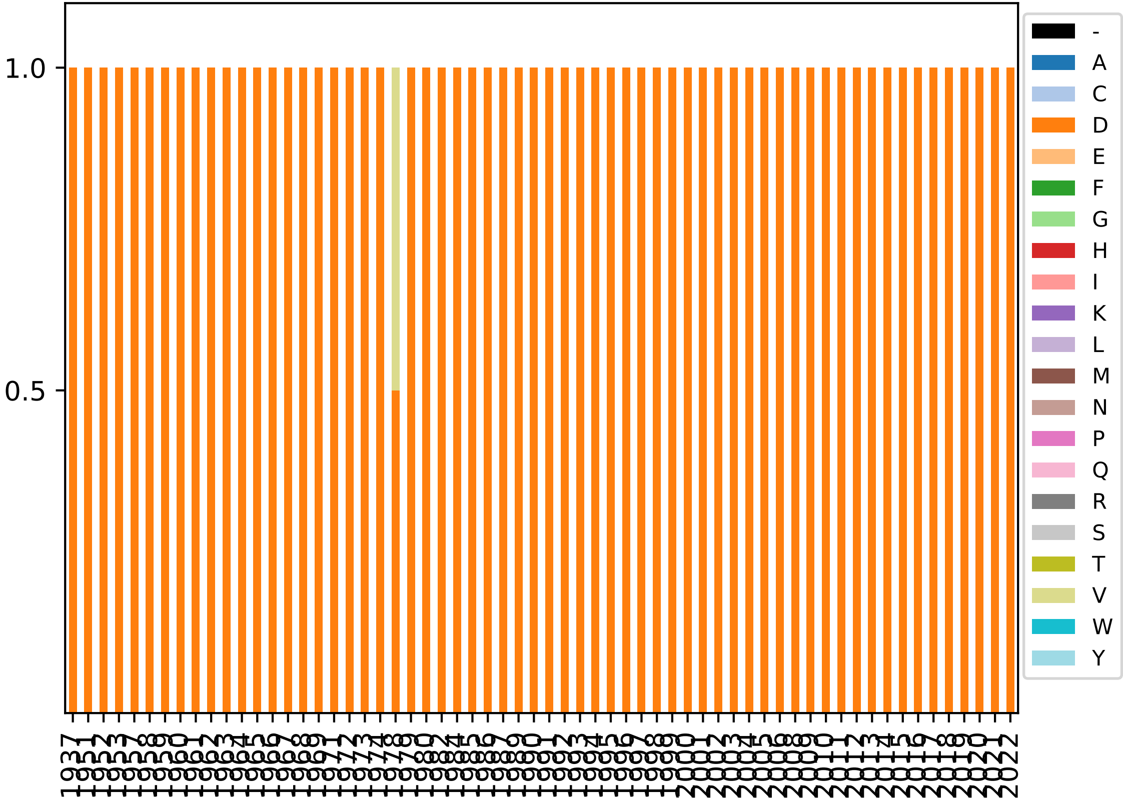

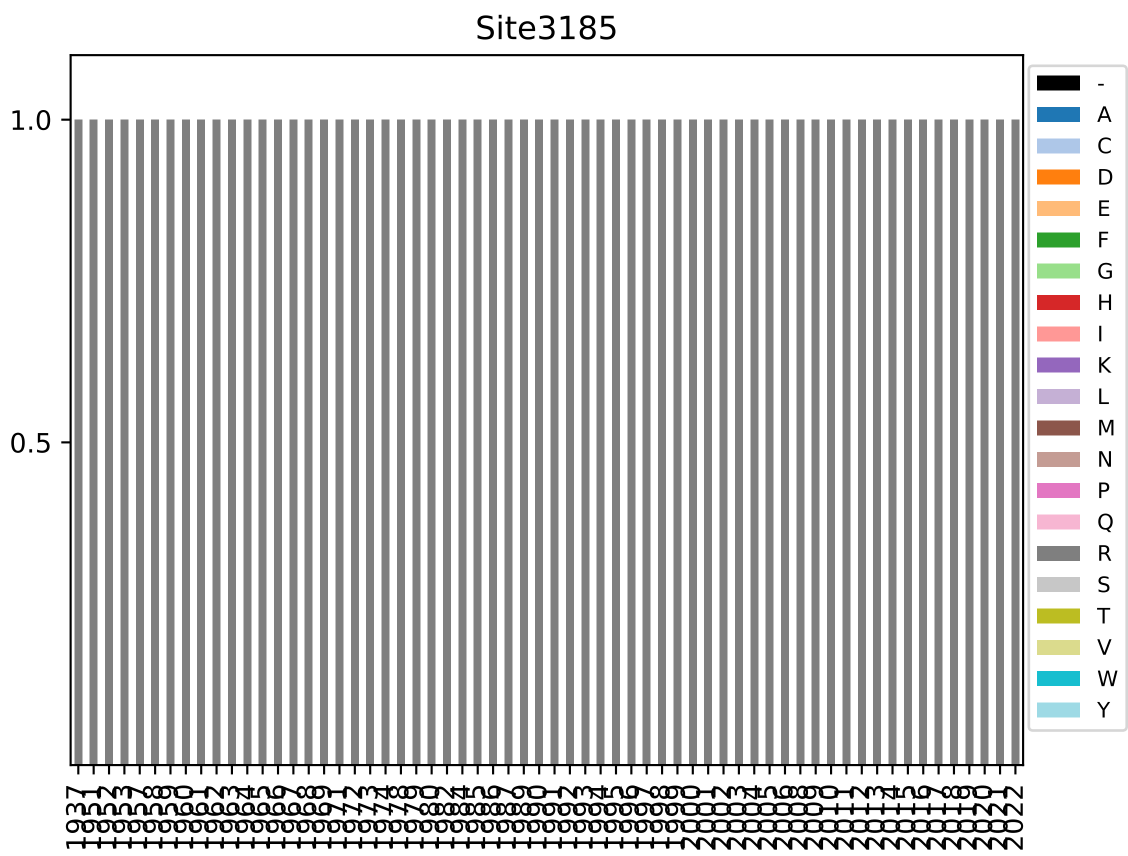
Site3185
1.0
0.5
-
A
C
D
E
F
G
H
I
K
L
M
N
P
Q
R
S
T
V
W
Y

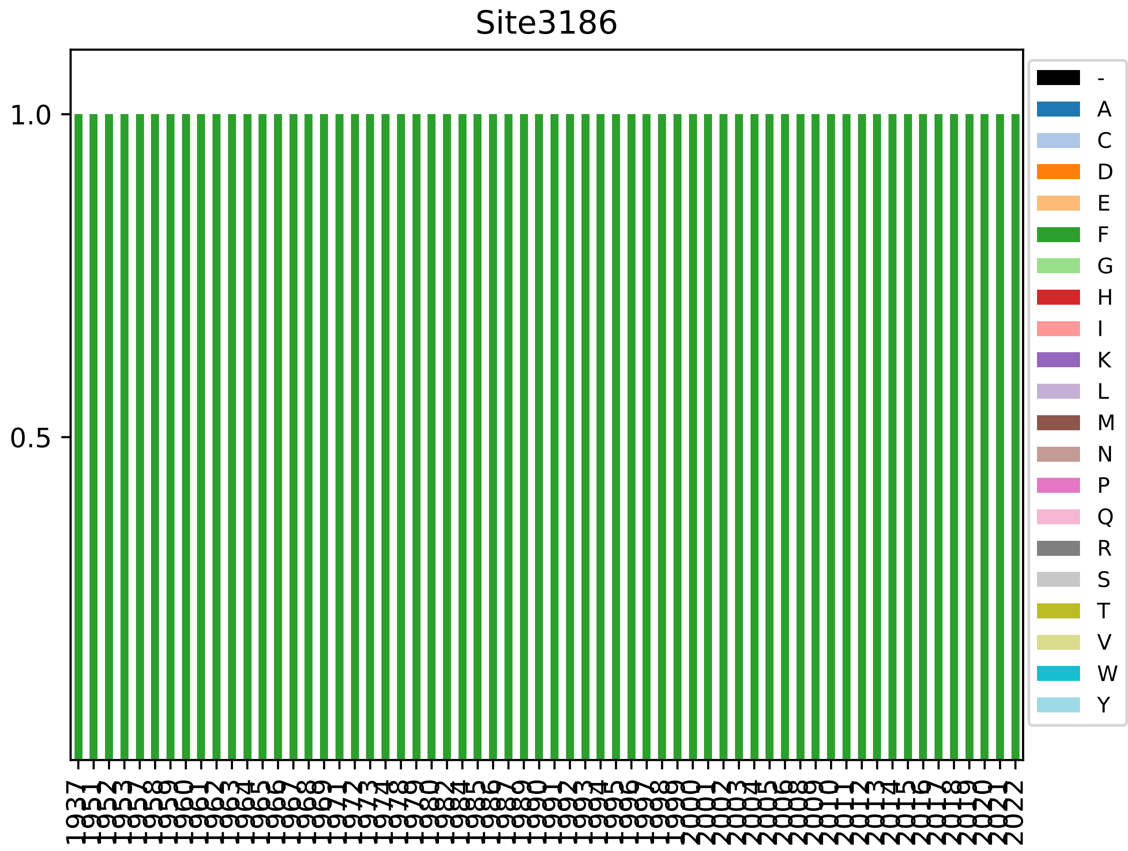
Site3186
1.0
0.5
-
A
C
D
E
F
G
H
I
K
L
M
N
P
Q
R
S
T
V
W
Y

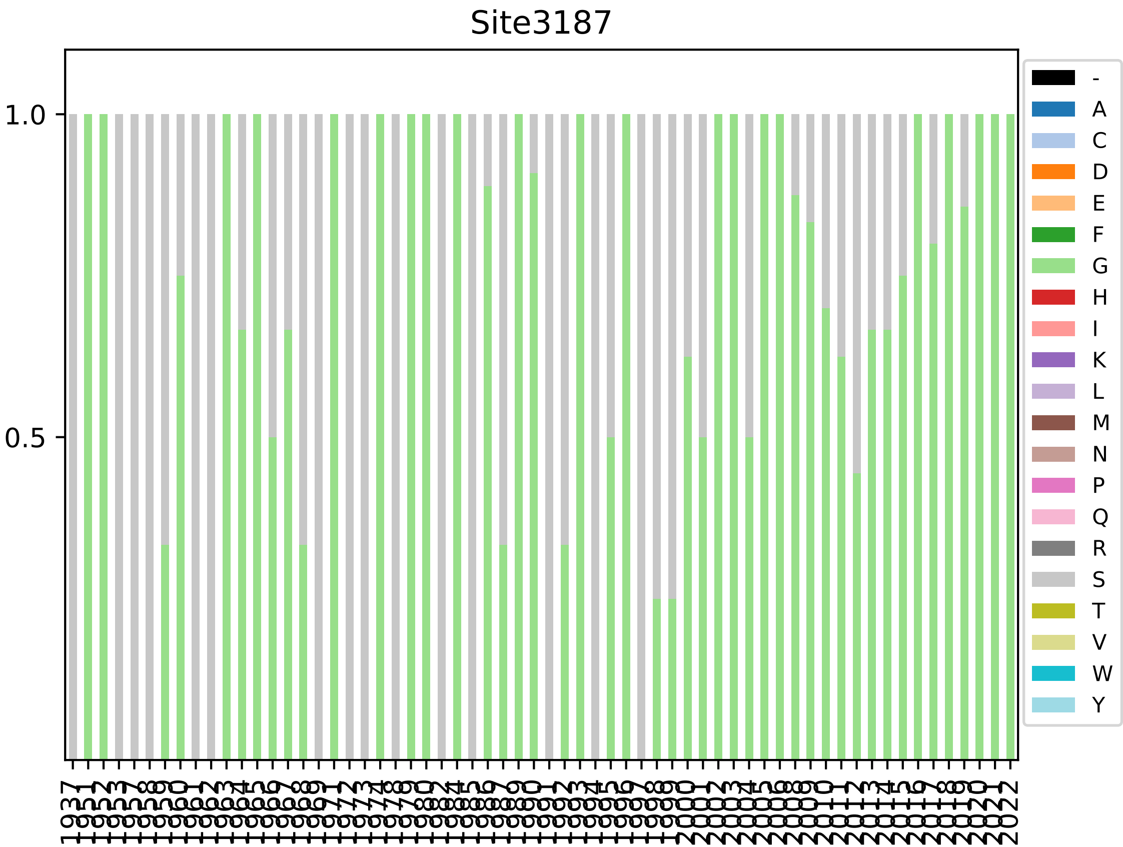
Site3187
1.0
0.5
-
A
C
D
E
F
G
H
I
K
L
M
N
P
Q
R
S
T
V
W
Y

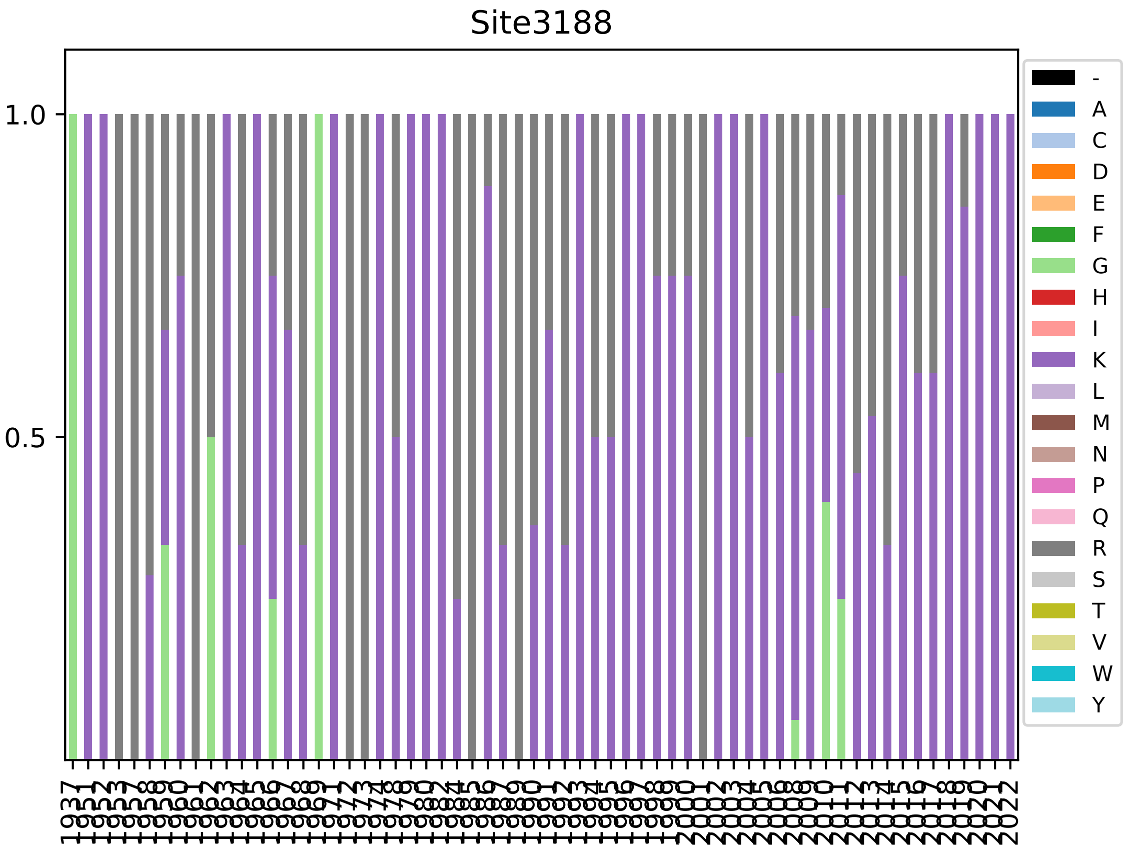

Site3188
1.0
0.5
-
A
C
D
E
F
G
H
I
K
L
M
N
P
Q
R
S
T
V
W
Y

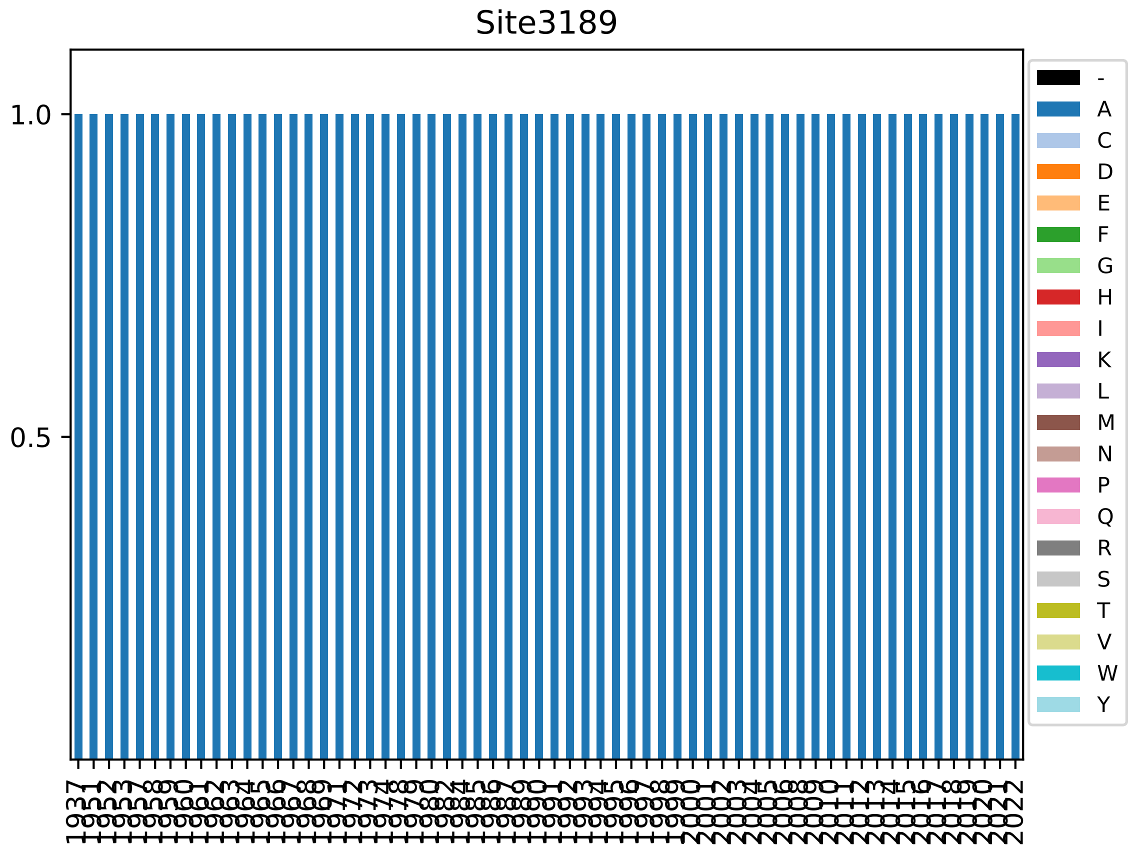
Site3189
1.0
0.5
-
A
C
D
E
F
G
H
I
K
L
M
N
P
Q
R
S
T
V
W
Y

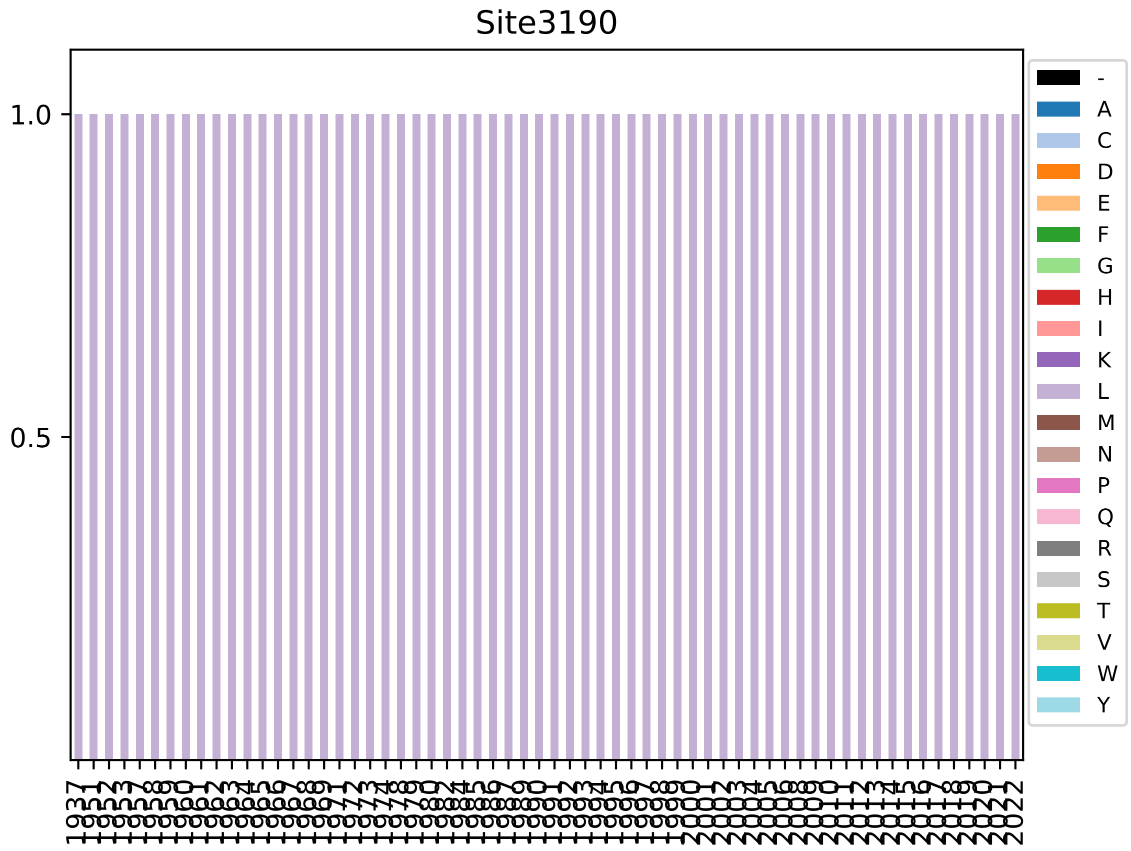
Site3190
1.0
0.5
-
A
C
D
E
F
G
H
I
K
L
M
N
P
Q
R
S
T
V
W
Y

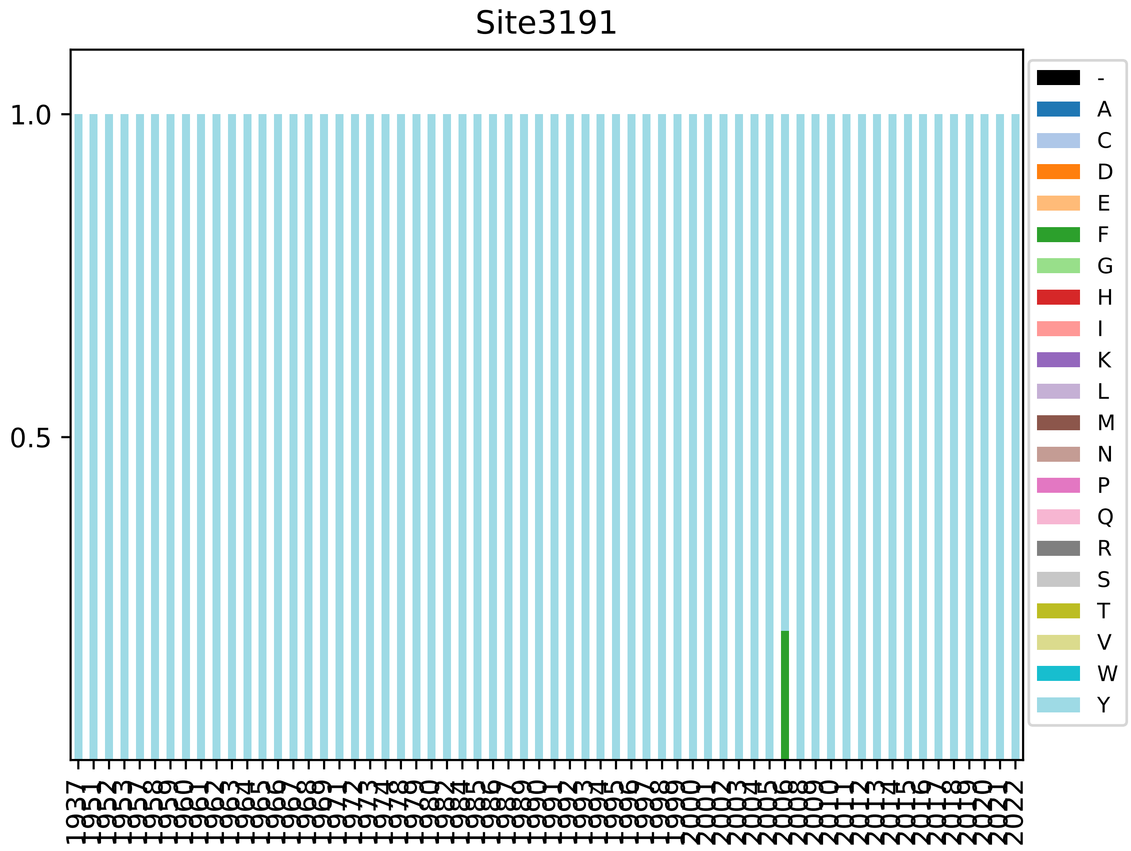
Site3191
1.0
0.5
-
A
C
D
E
F
G
H
I
K
L
M
N
P
Q
R
S
T
V
W
Y

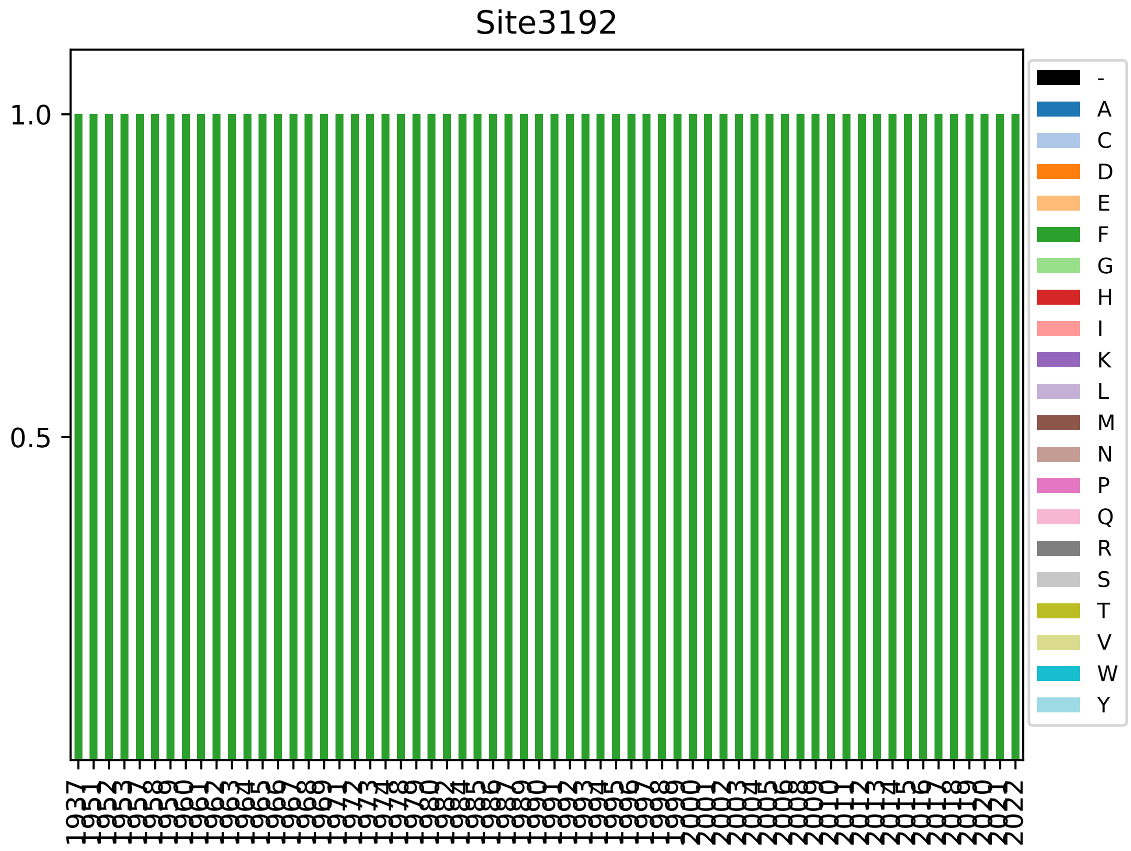
Site3192
1.0
0.5
-
A
C
D
E
F
G
H
I
K
L
M
N
P
Q
R
S
T
V
W
Y

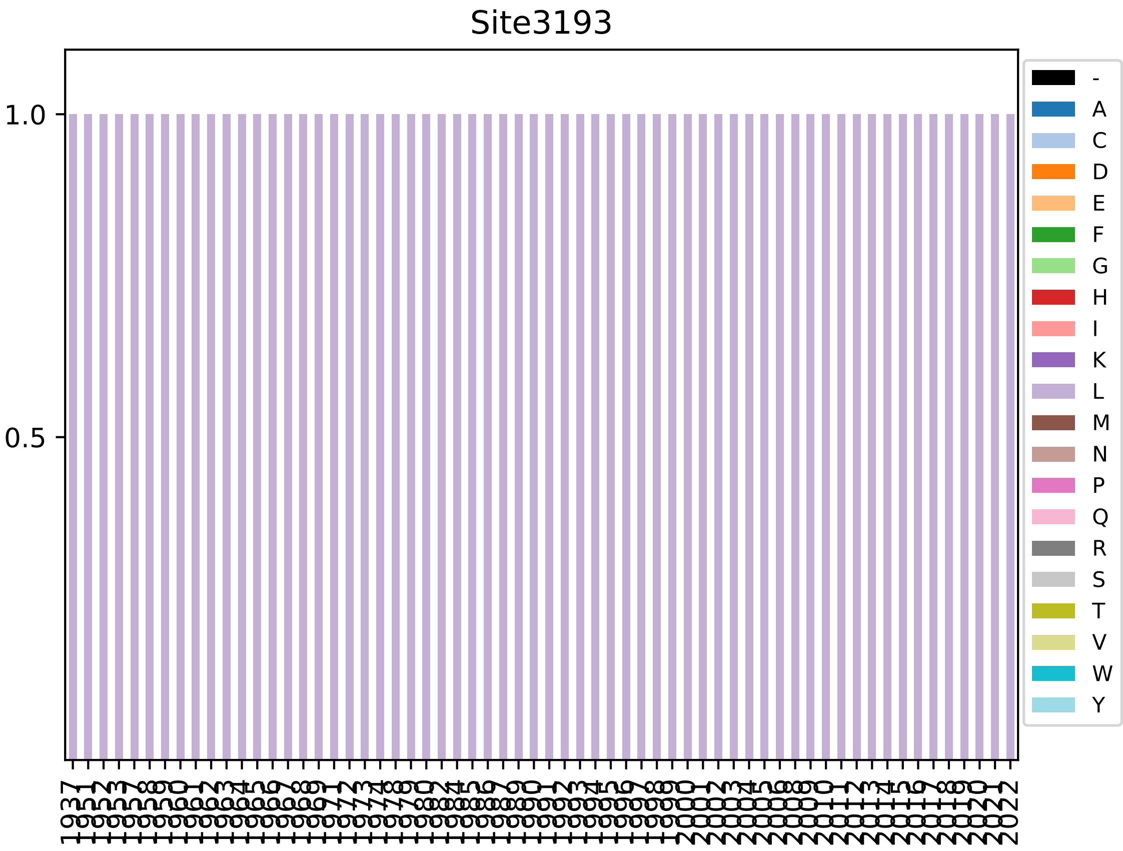
Site3193
1.0
0.5
-
A
C
D
E
F
G
H
I
K
L
M
N
P
Q
R
S
T
V
W
Y

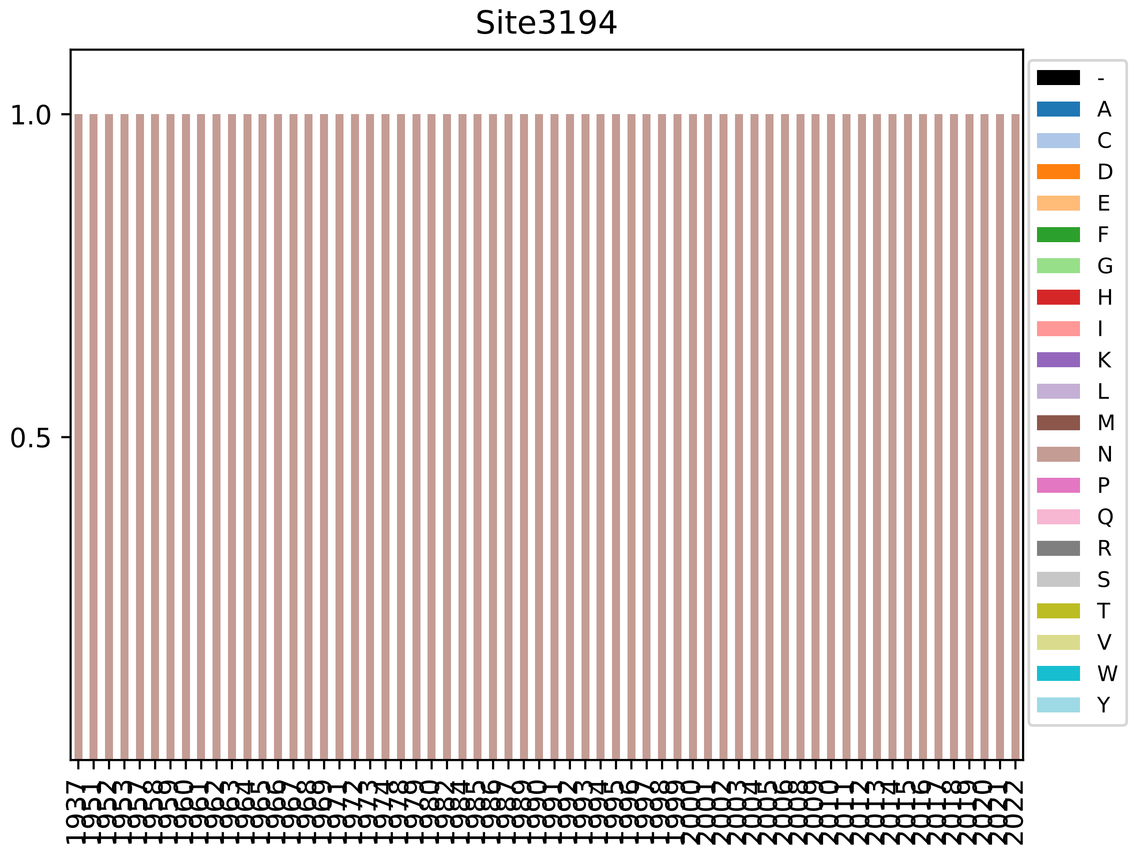
Site3194
1.0
0.5
-
A
C
D
E
F
G
H
I
K
L
M
N
P
Q
R
S
T
V
W
Y

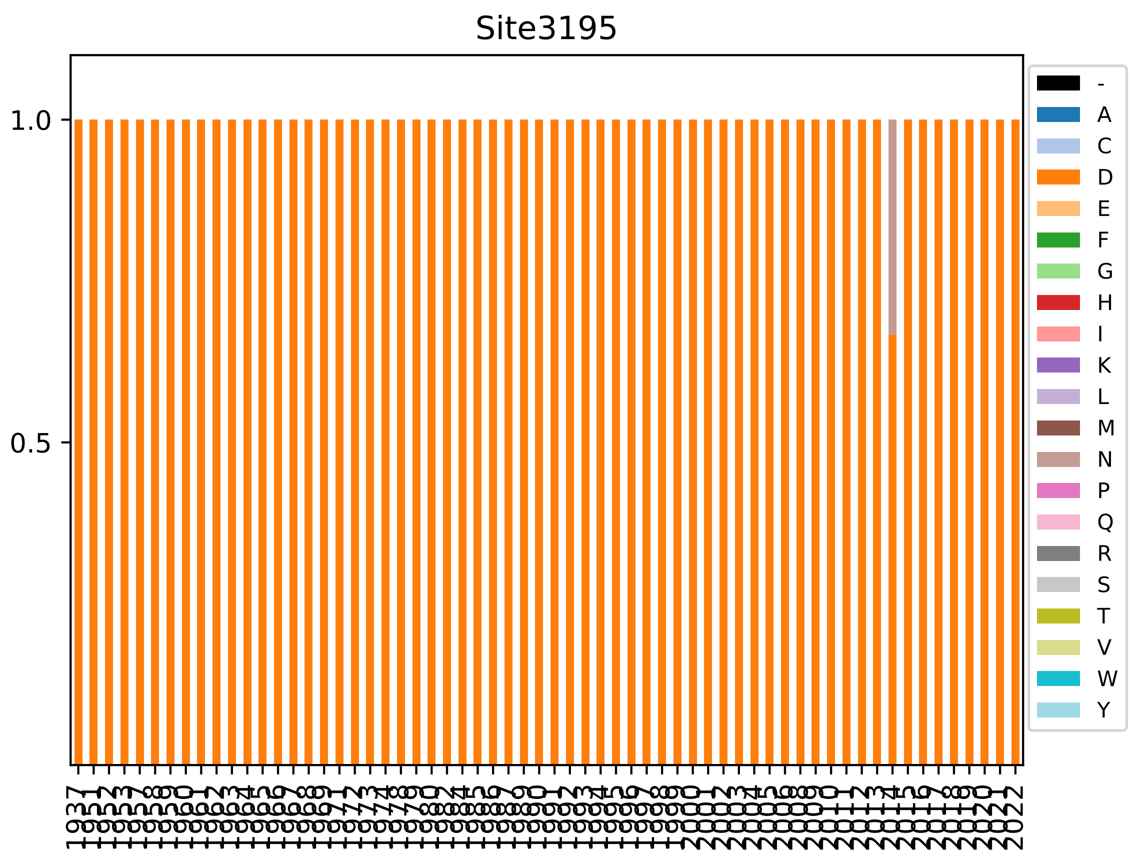
Site3195
1.0
0.5
-
A
C
D
E
F
G
H
I
K
L
M
N
P
Q
R
S
T
V
W
Y

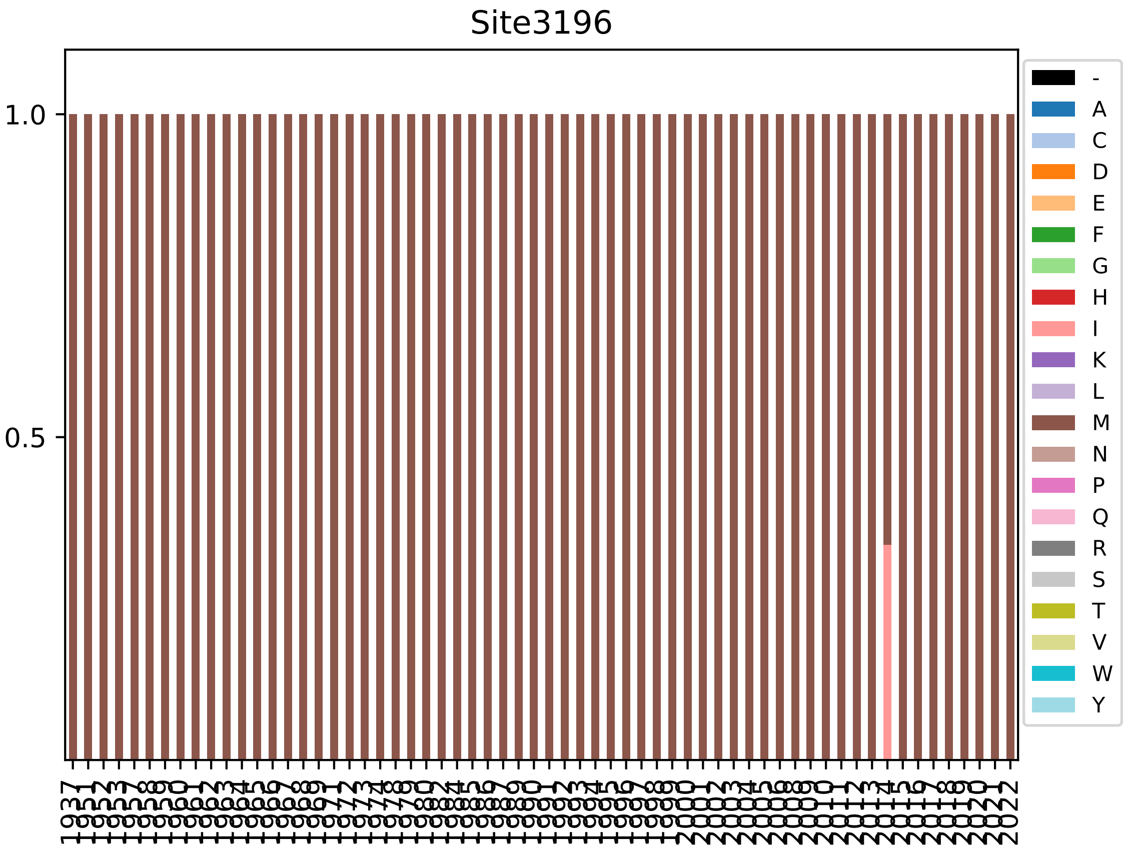
Site3196
1.0
0.5
-
A
C
D
E
F
G
H
I
K
L
M
N
P
Q
R
S
T
V
W
Y

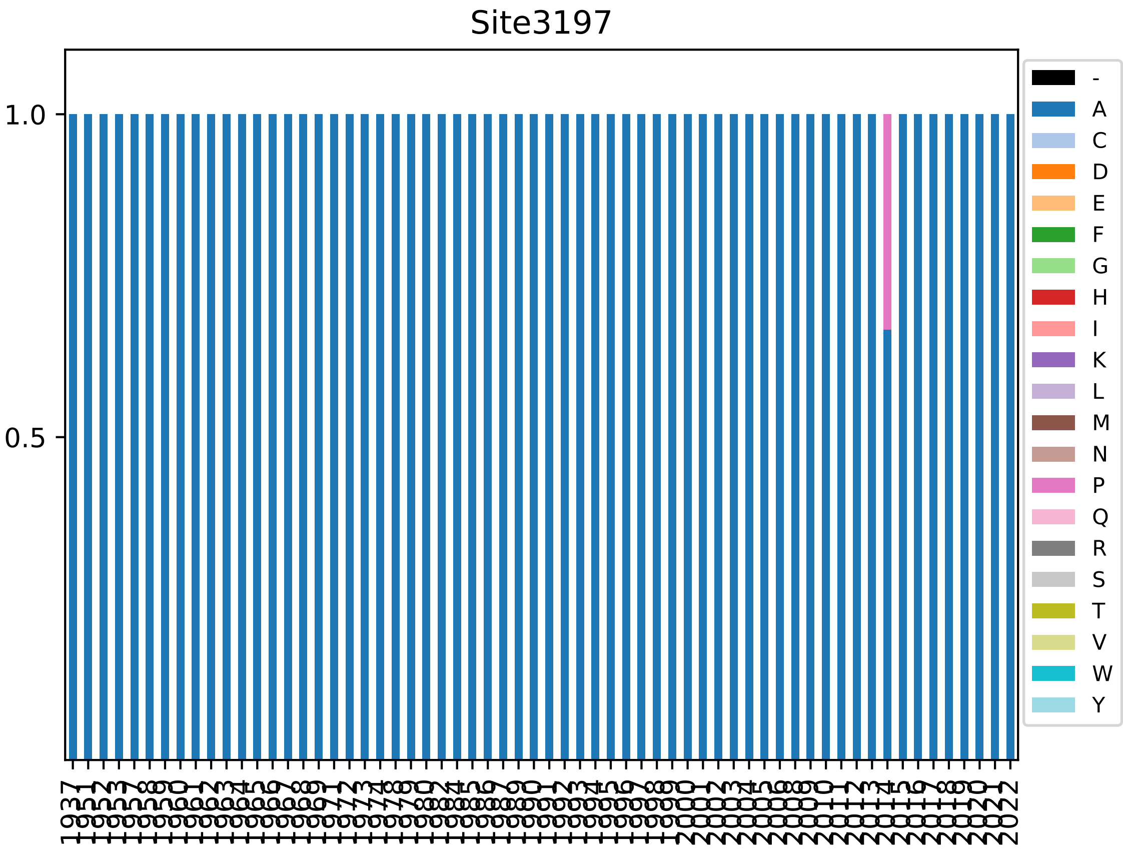

Site3197
1.0
0.5
-
A
C
D
E
F
G
H
I
K
L
M
N
P
Q
R
S
T
V
W
Y

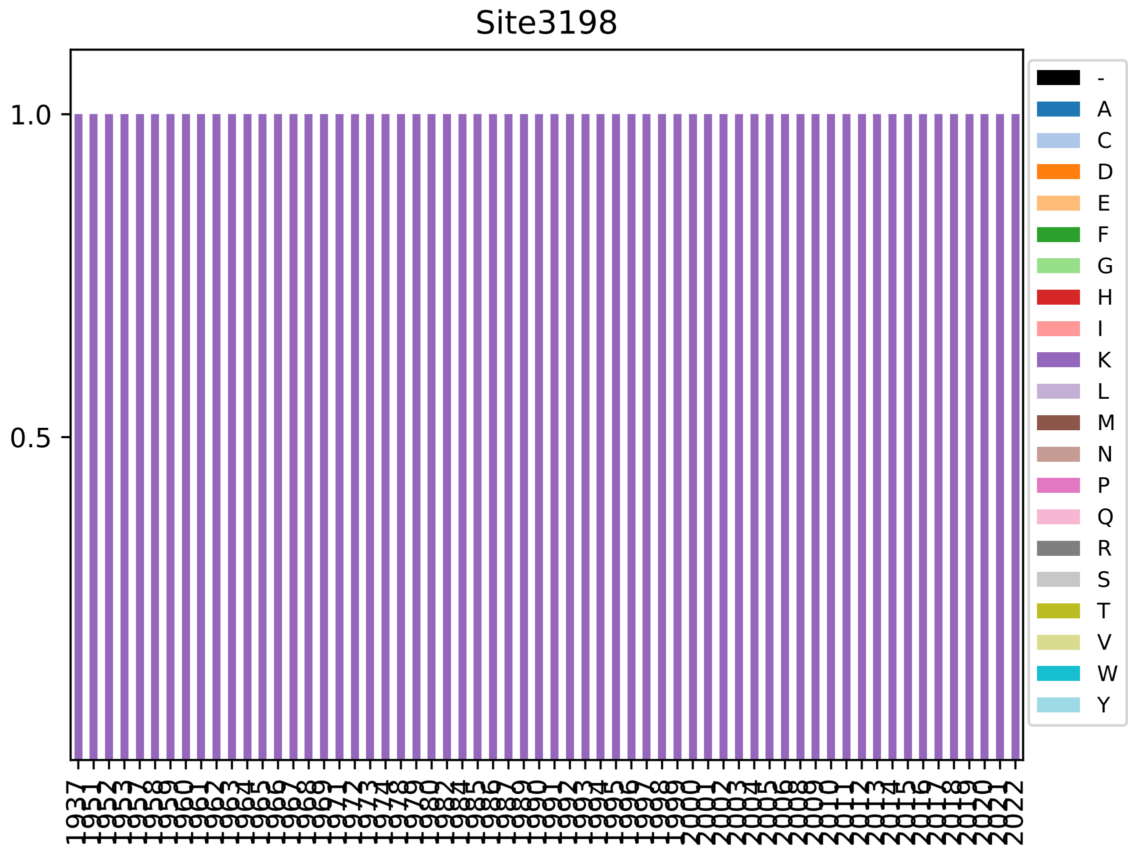
Site3198
1.0
0.5
-
A
C
D
E
F
G
H
I
K
L
M
N
P
Q
R
S
T
V
W
Y

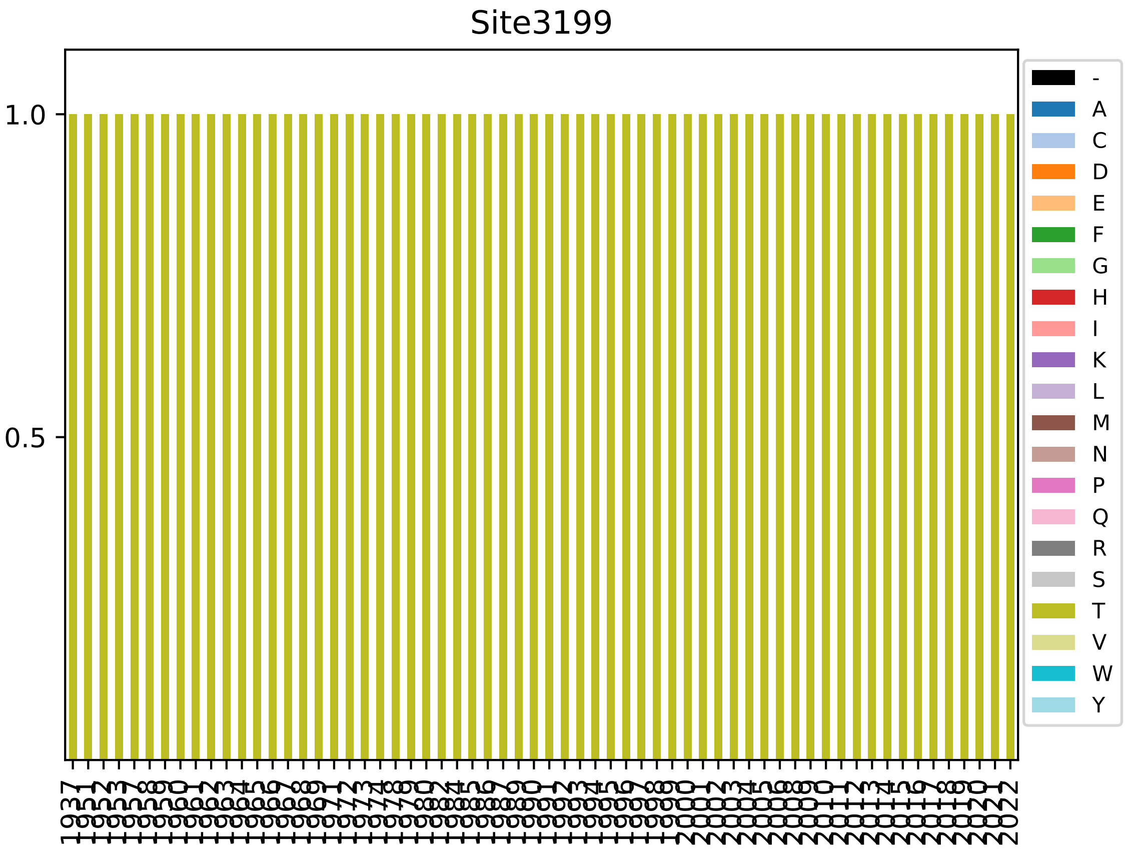
Site3199
1.0
0.5
-
A
C
D
E
F
G
H
I
K
L
M
N
P
Q
R
S
T
V
W
Y

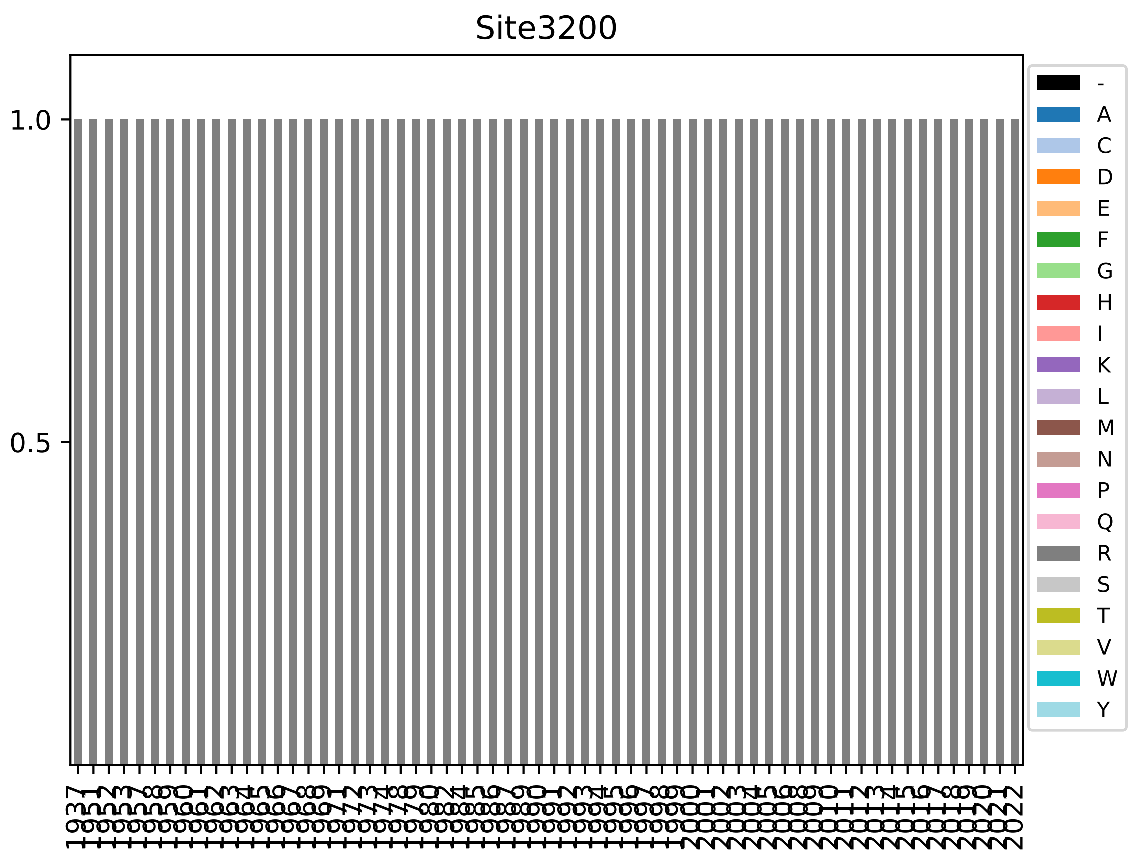
Site3200
1.0
0.5
-
A
C
D
E
F
G
H
I
K
L
M
N
P
Q
R
S
T
V
W
Y

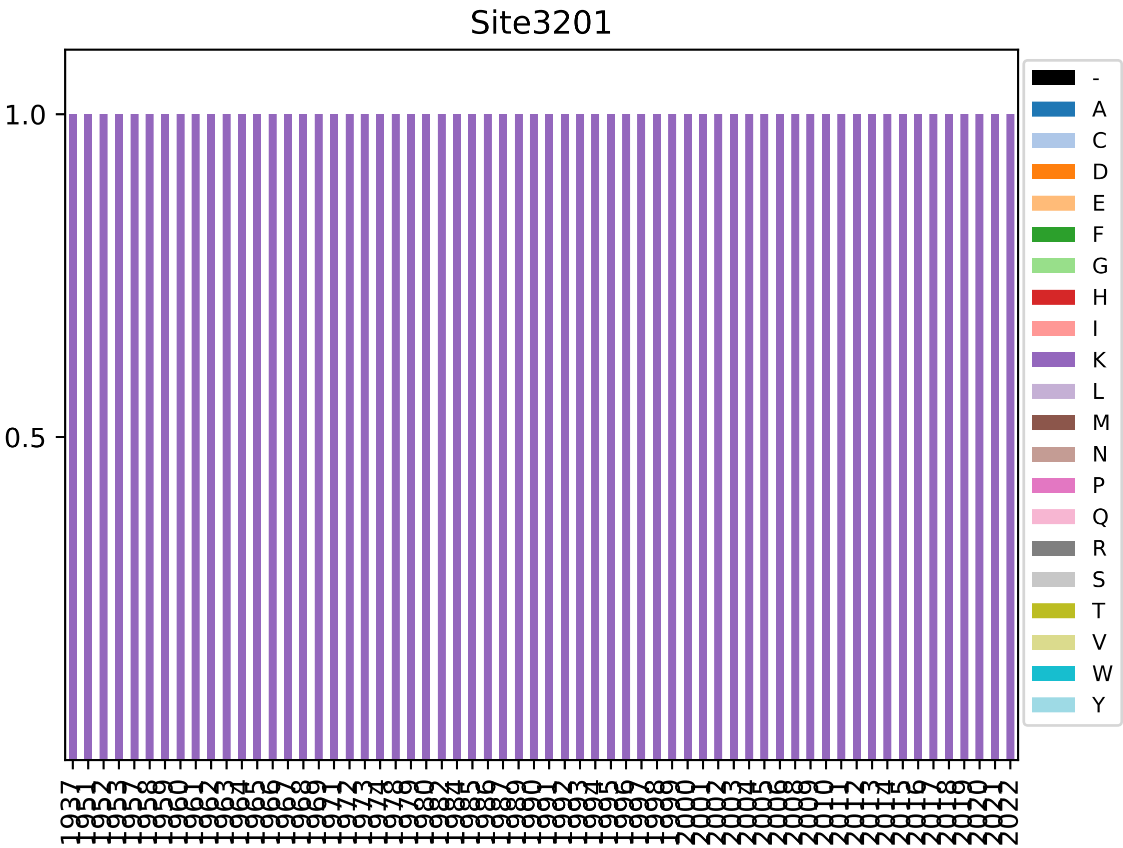
Site3201
1.0
0.5
-
A
C
D
E
F
G
H
I
K
L
M
N
P
Q
R
S
T
V
W
Y

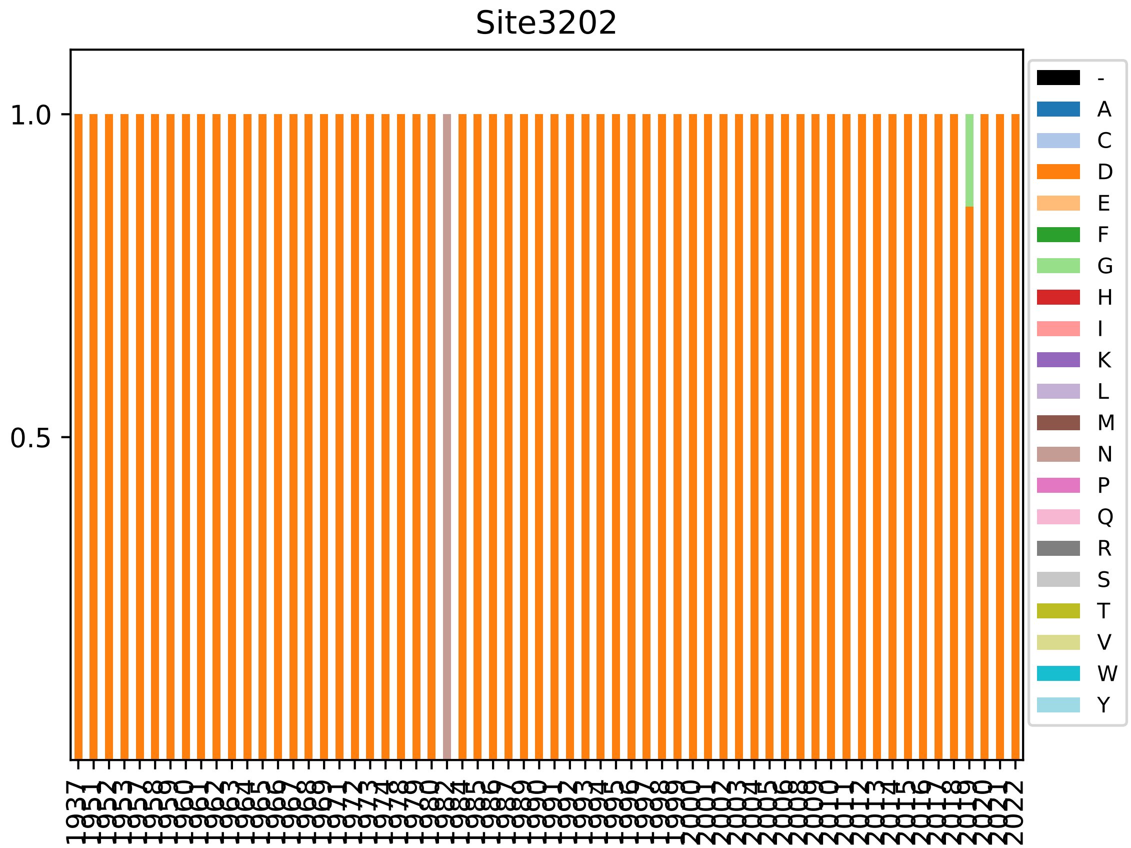
Site3202
1.0
0.5
-
A
C
D
E
F
G
H
I
K
L
M
N
P
Q
R
S
T
V
W
Y

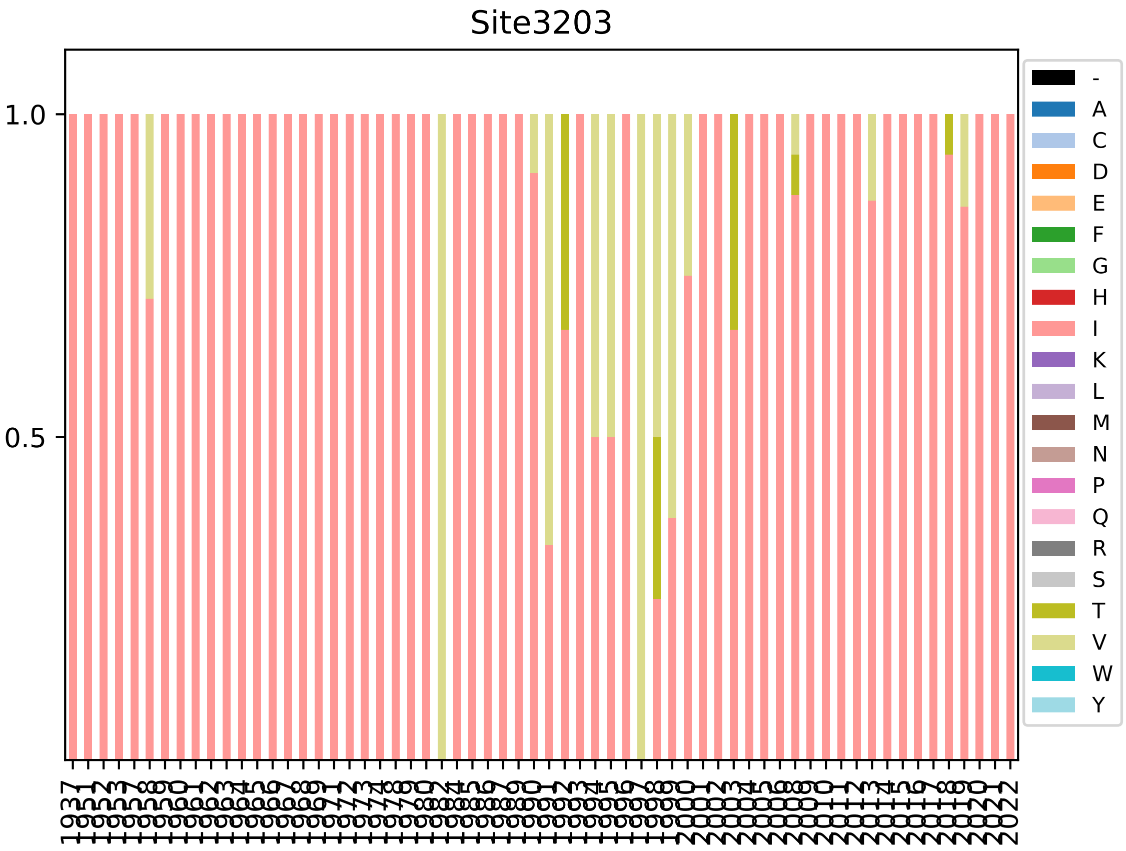
Site3203
1.0
0.5
-
A
C
D
E
F
G
H
I
K
L
M
N
P
Q
R
S
T
V
W
Y

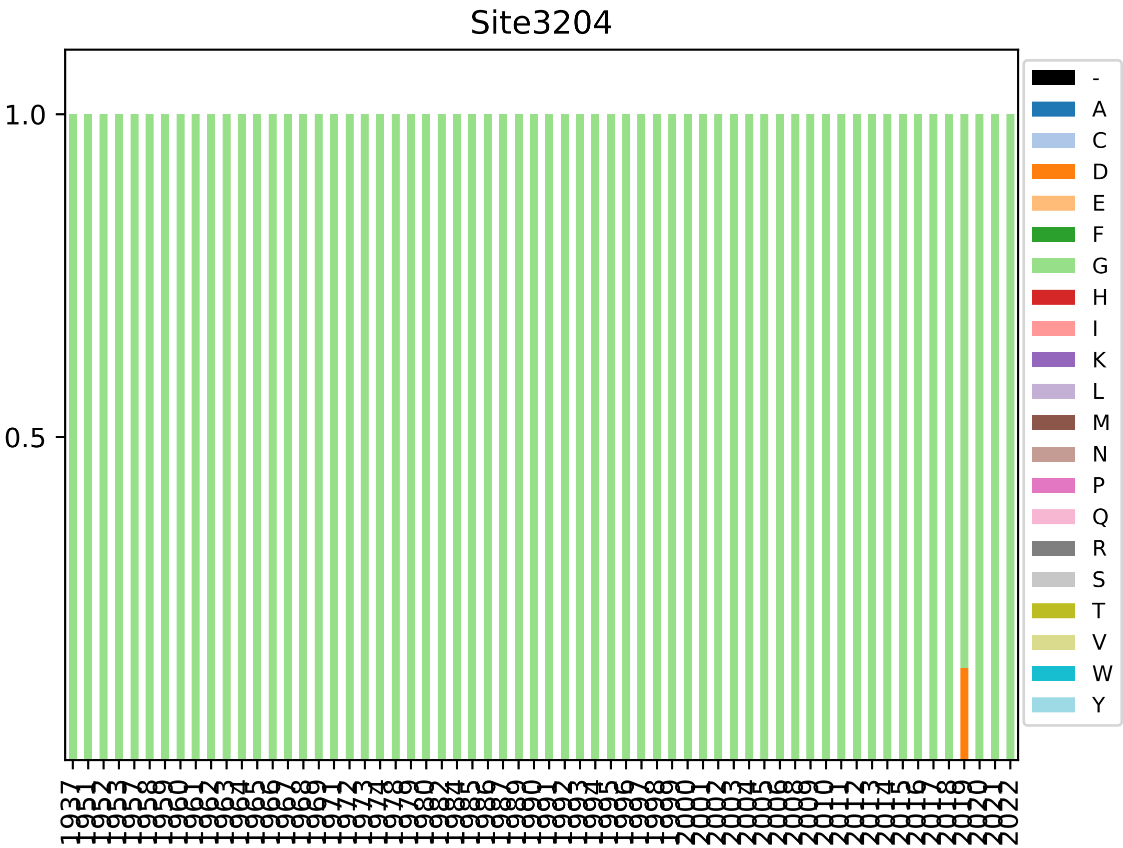
Site3204
1.0
0.5
-
A
C
D
E
F
G
H
I
K
L
M
N
P
Q
R
S
T
V
W
Y

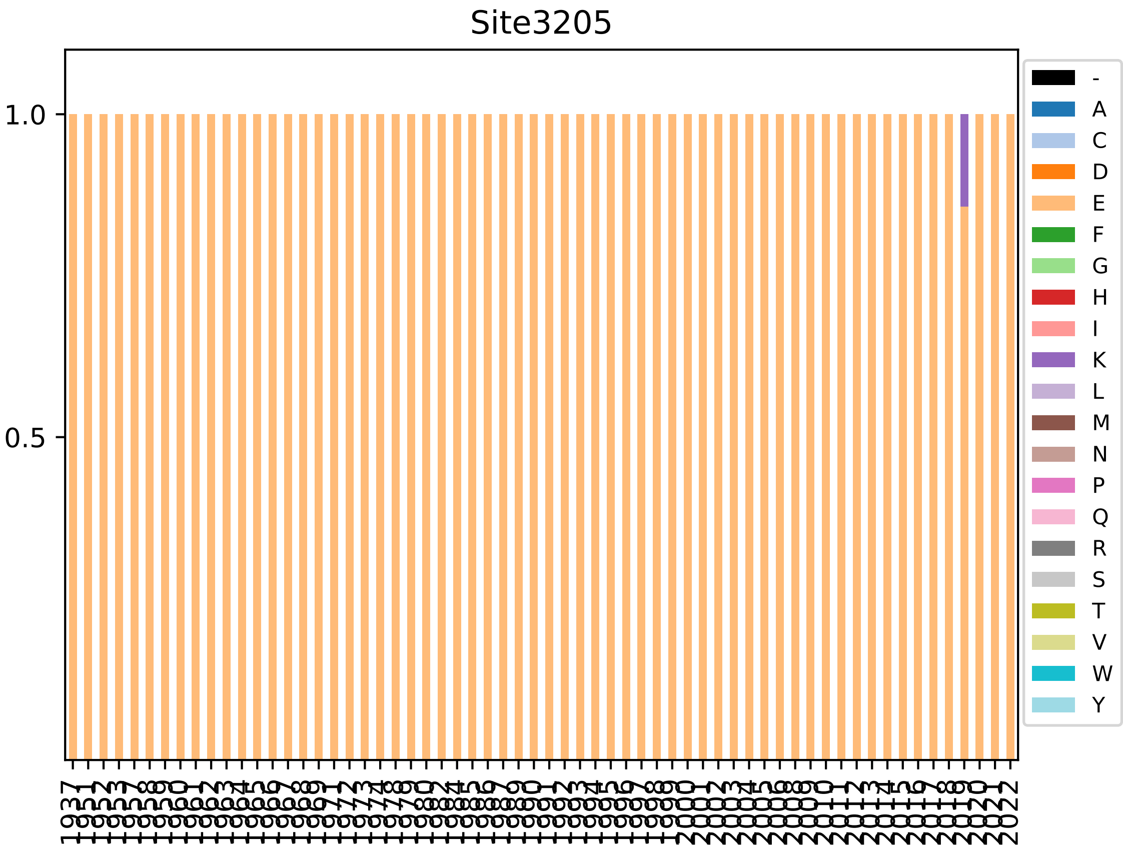
Site3205
1.0
0.5
-
A
C
D
E
F
G
H
I
K
L
M
N
P
Q
R
S
T
V
W
Y

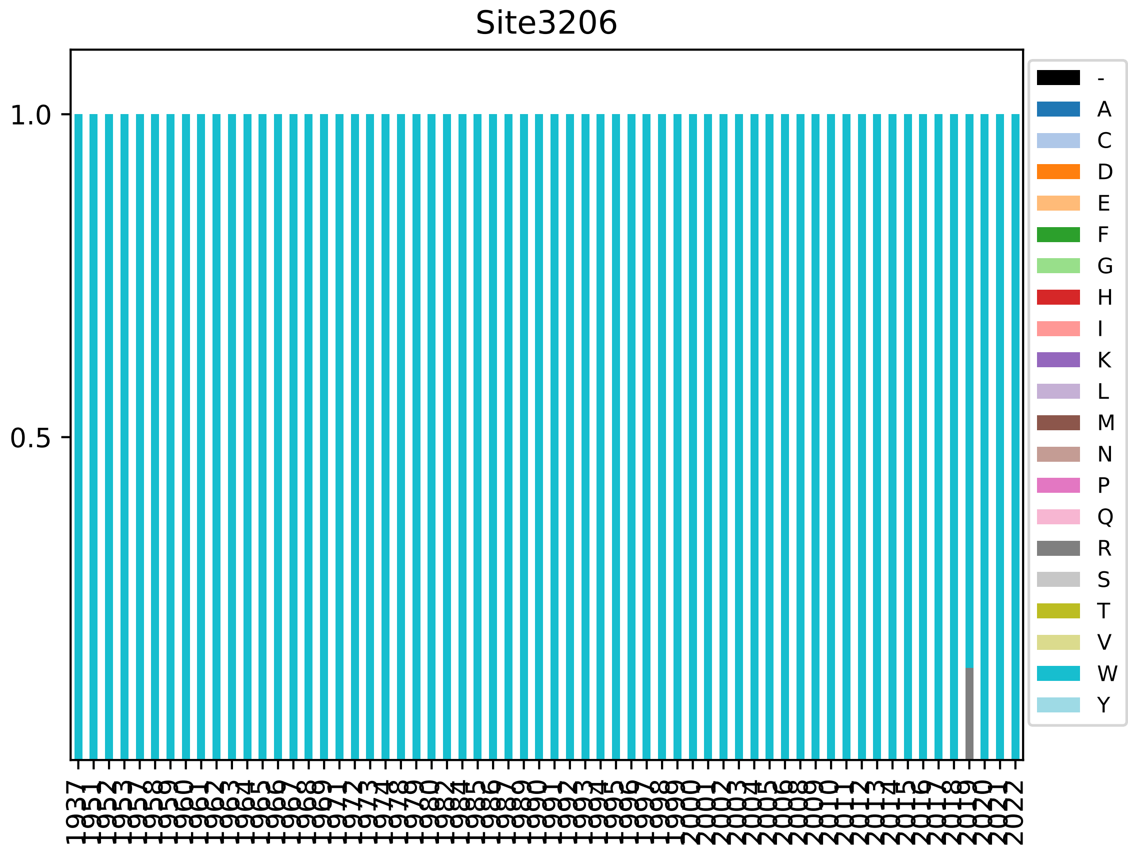
Site3206
1.0
0.5
-
A
C
D
E
F
G
H
I
K
L
M
N
P
Q
R
S
T
V
W
Y

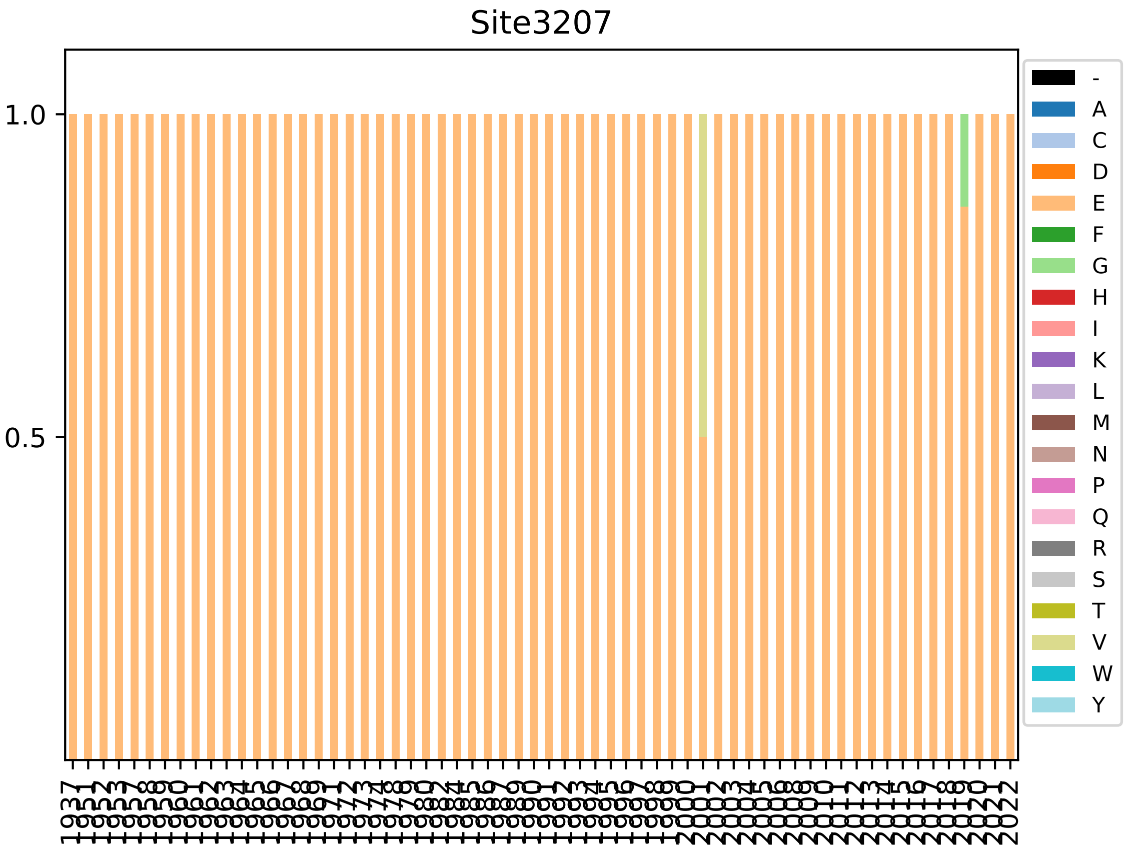

Site3207
1.0
0.5
-
A
C
D
E
F
G
H
I
K
L
M
N
P
Q
R
S
T
V
W
Y

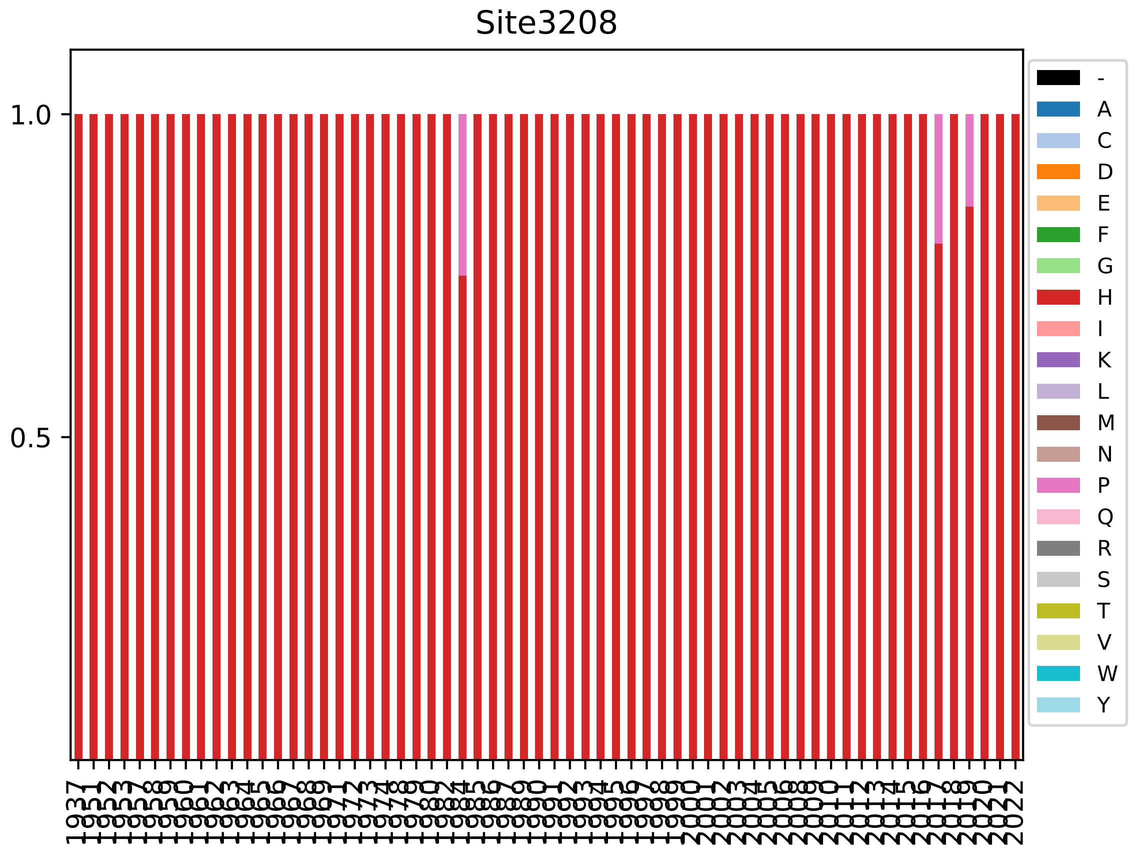
Site3208
1.0
0.5
-
A
C
D
E
F
G
H
I
K
L
M
N
P
Q
R
S
T
V
W
Y

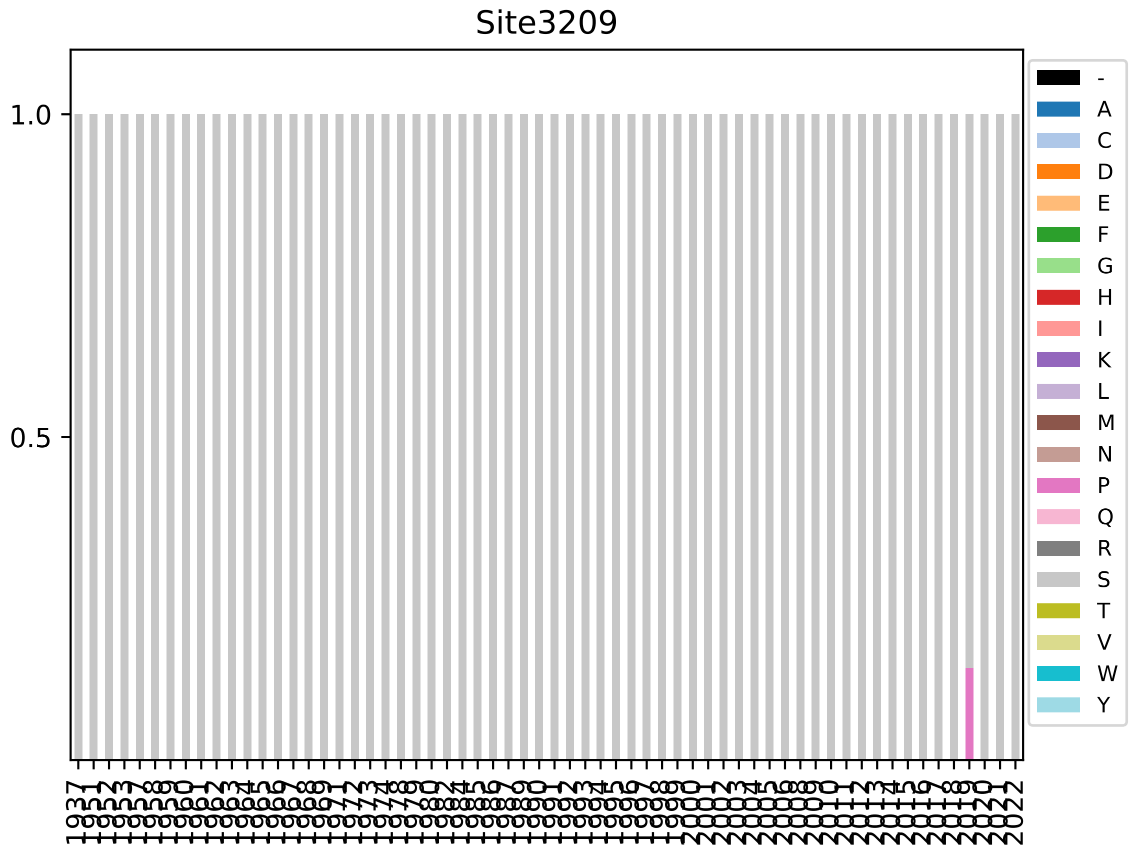
Site3209
1.0
0.5
-
A
C
D
E
F
G
H
I
K
L
M
N
P
Q
R
S
T
V
W
Y

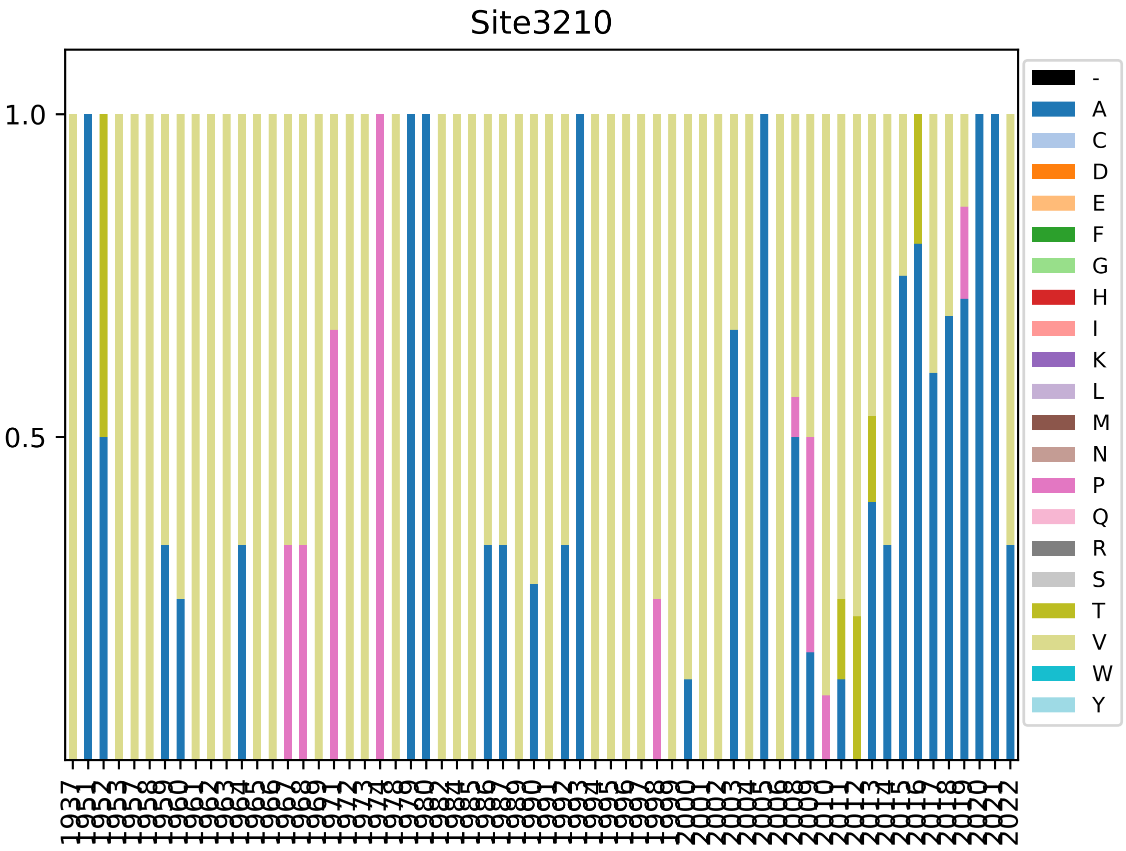

Site3210
1.0
0.5
-
A
C
D
E
F
G
H
I
K
L
M
N
P
Q
R
S
T
V
W
Y

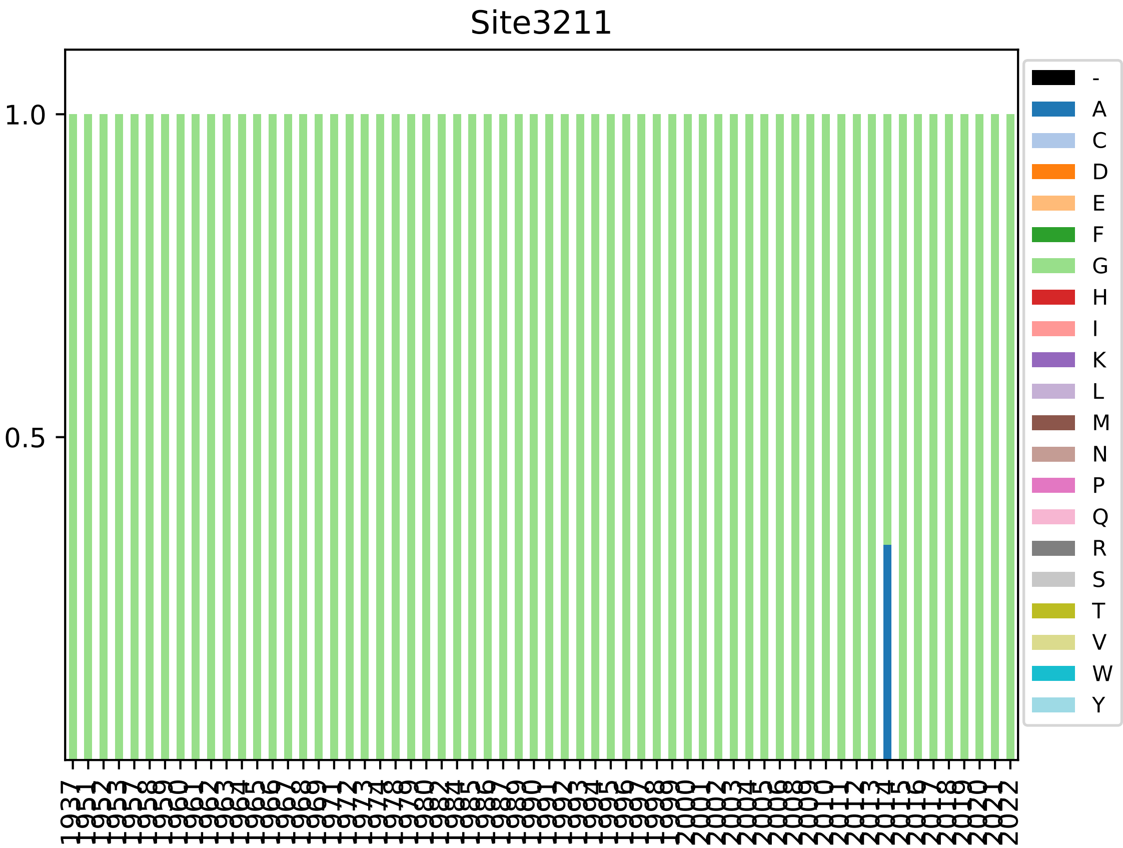
Site3211
1.0
0.5
-
A
C
D
E
F
G
H
I
K
L
M
N
P
Q
R
S
T
V
W
Y

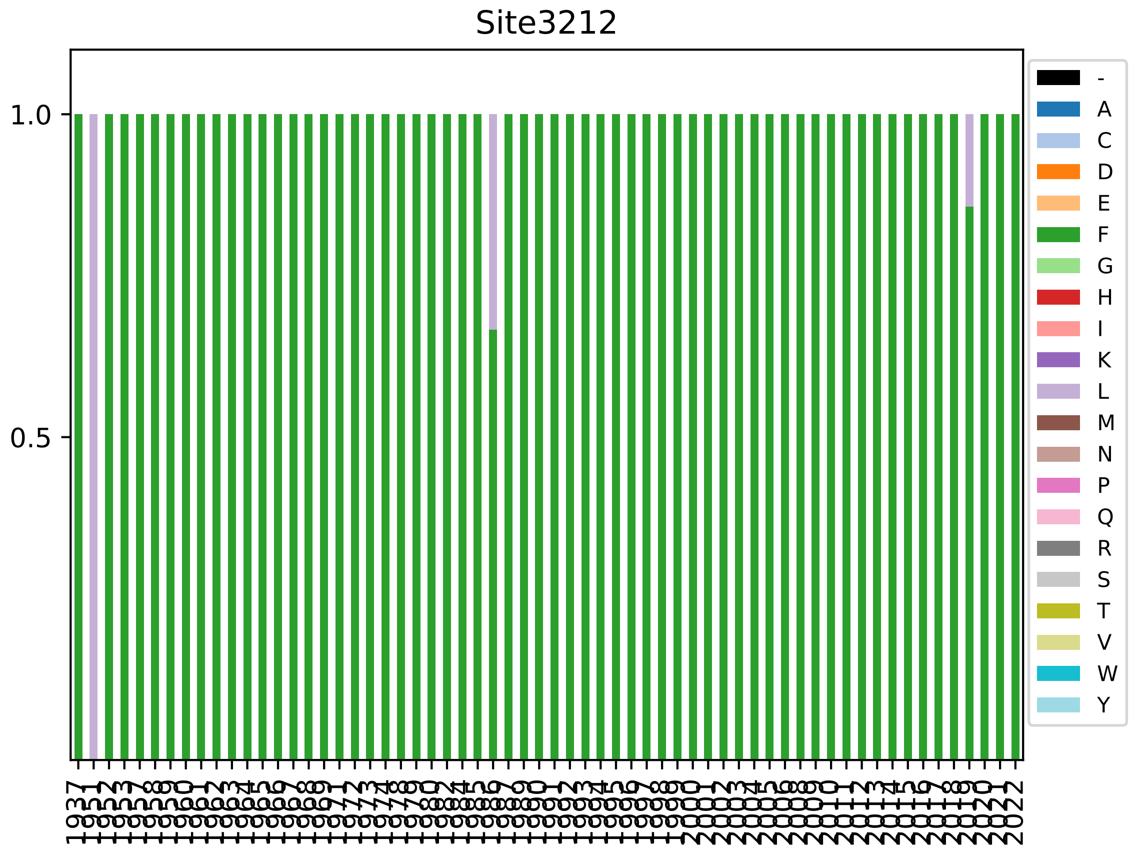
Site3212
1.0
0.5
-
A
C
D
E
F
G
H
I
K
L
M
N
P
Q
R
S
T
V
W
Y

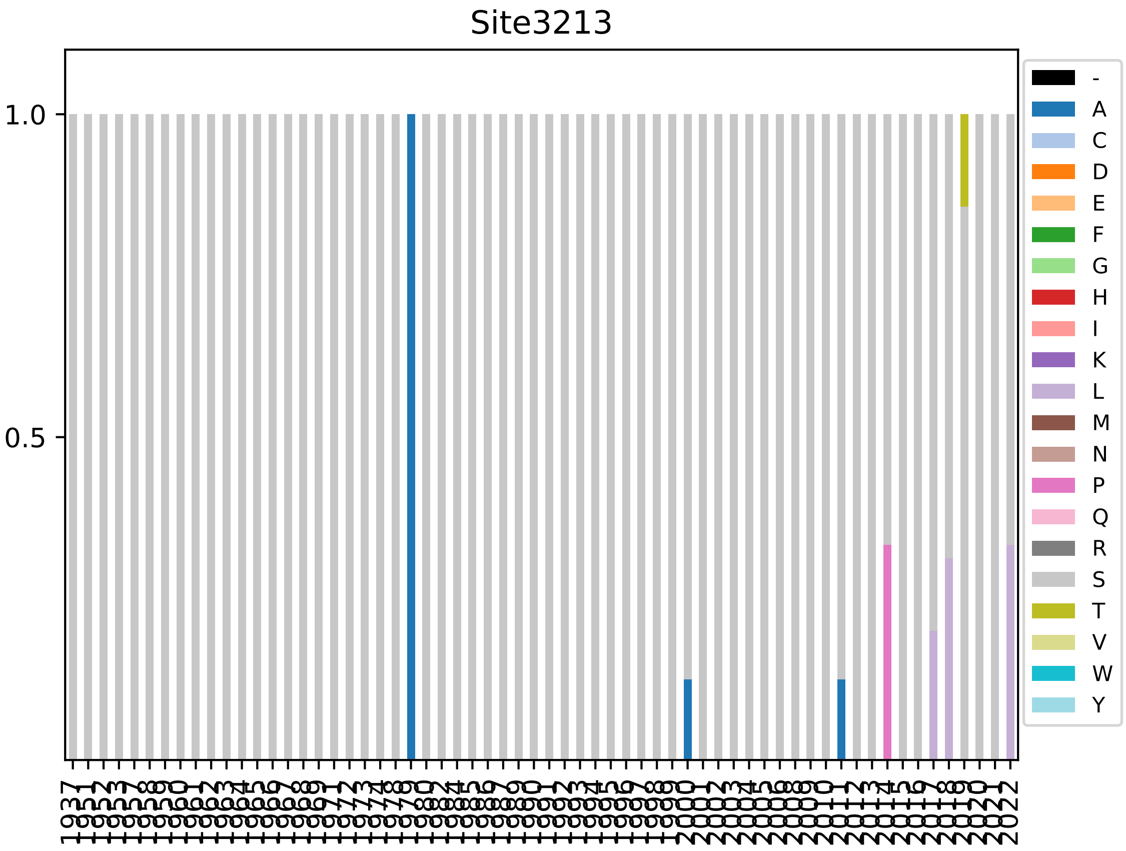
Site3213
1.0
0.5
-
A
C
D
E
F
G
H
I
K
L
M
N
P
Q
R
S
T
V
W
Y

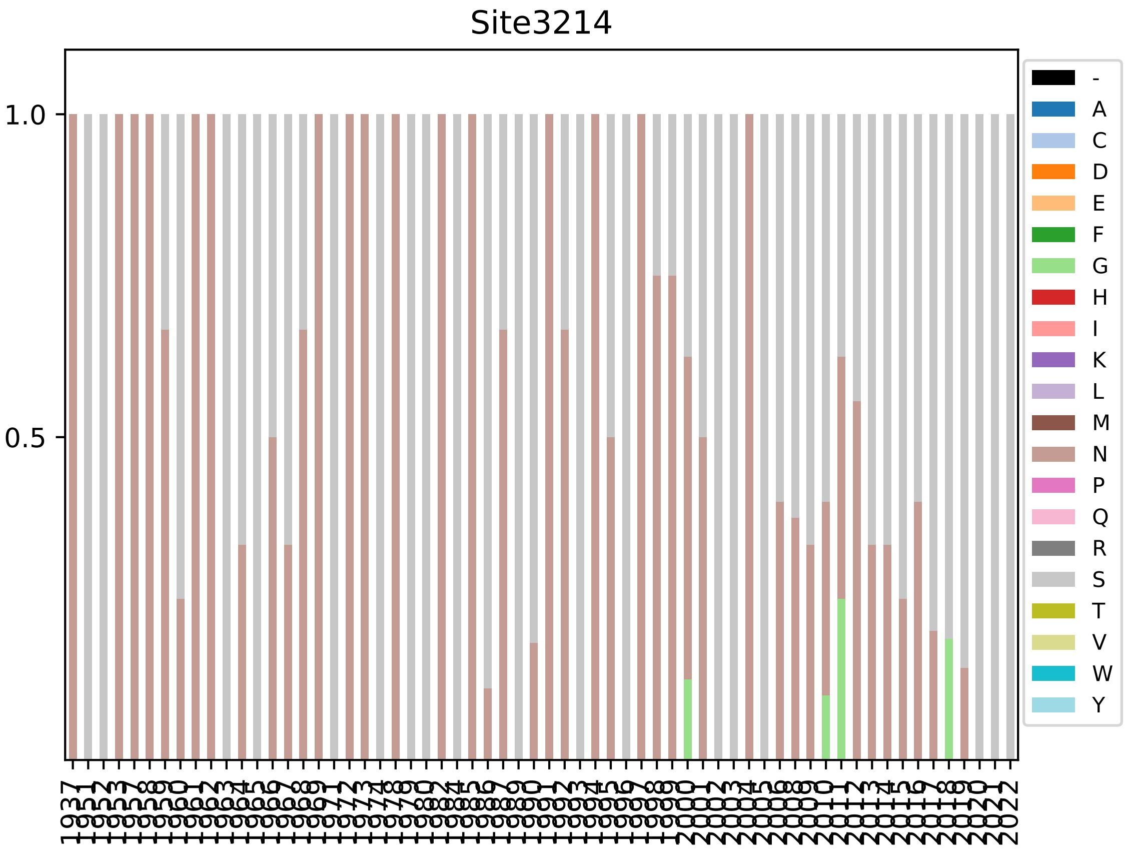
Site3214
1.0
0.5
-
A
C
D
E
F
G
H
I
K
L
M
N
P
Q
R
S
T
V
W
Y

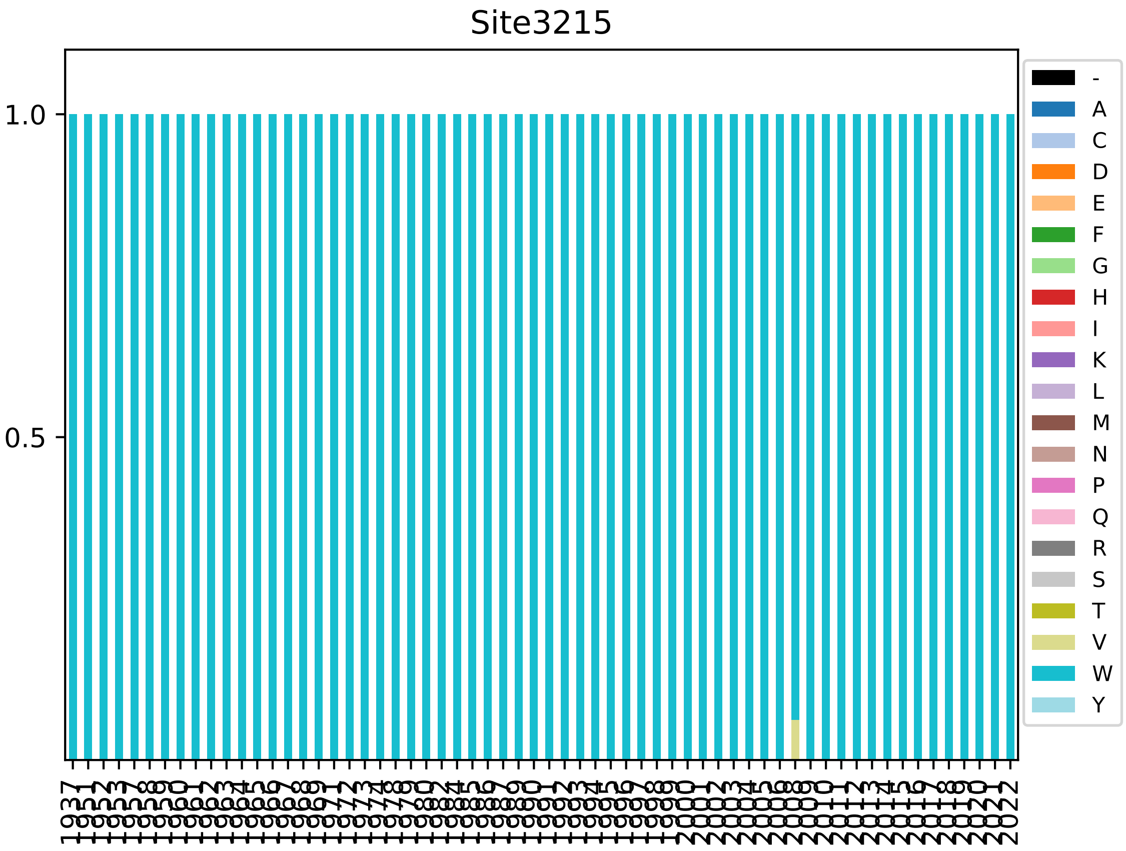
Site3215
1.0
0.5
-
A
C
D
E
F
G
H
I
K
L
M
N
P
Q
R
S
T
V
W
Y

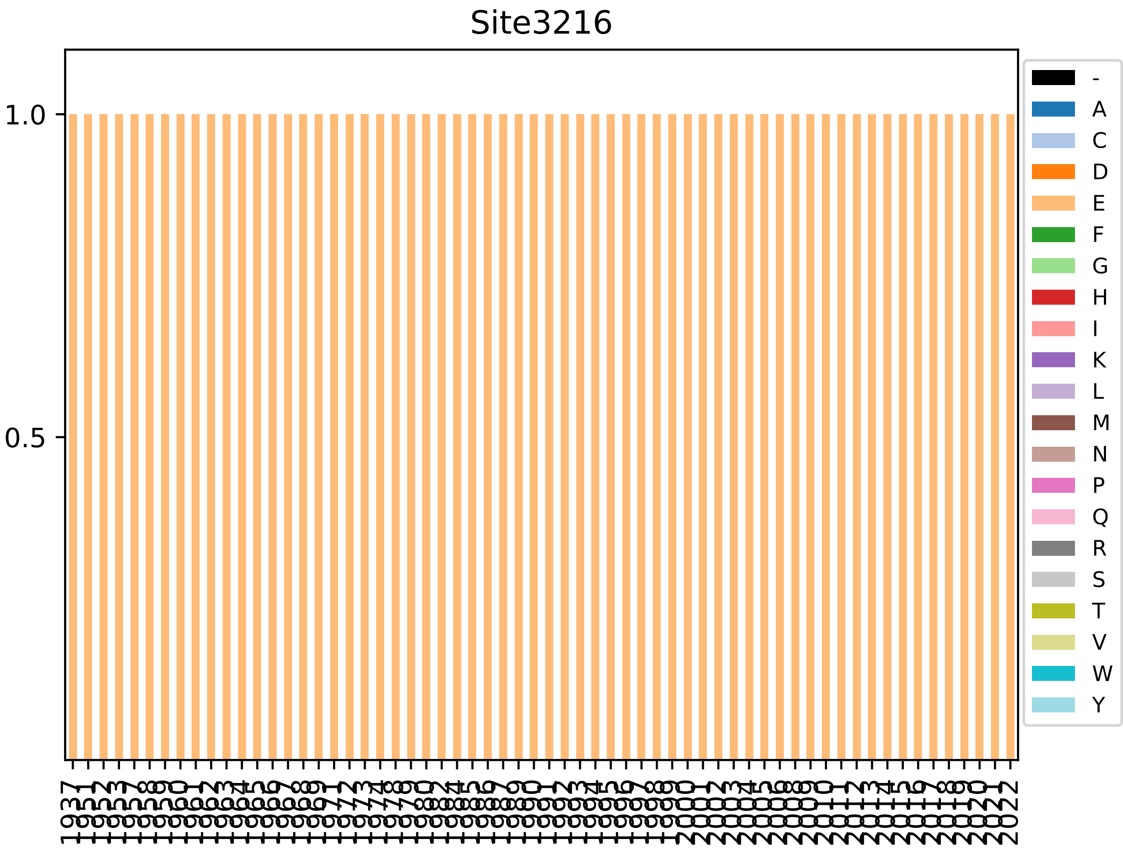
Site3216
1.0
0.5
-
A
C
D
E
F
G
H
I
K
L
M
N
P
Q
R
S
T
V
W
Y

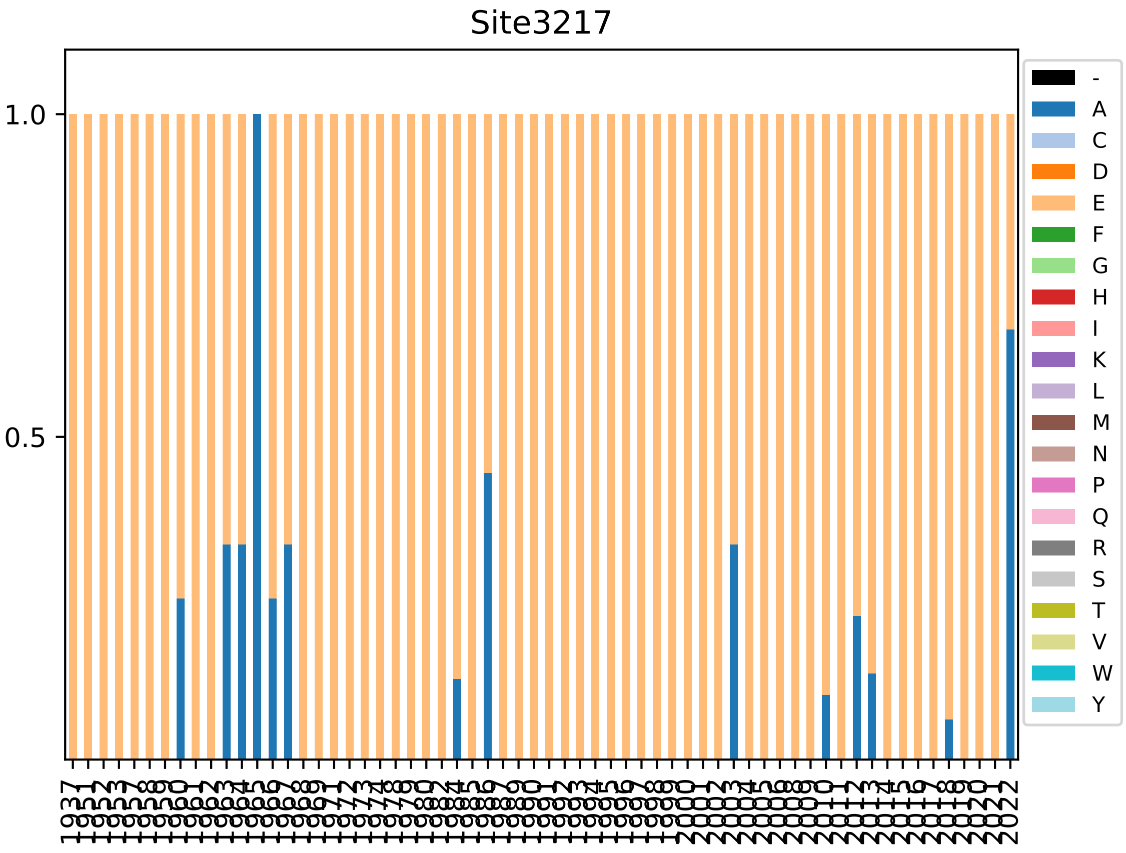
Site3217
1.0
0.5
-
A
C
D
E
F
G
H
I
K
L
M
N
P
Q
R
S
T
V
W
Y

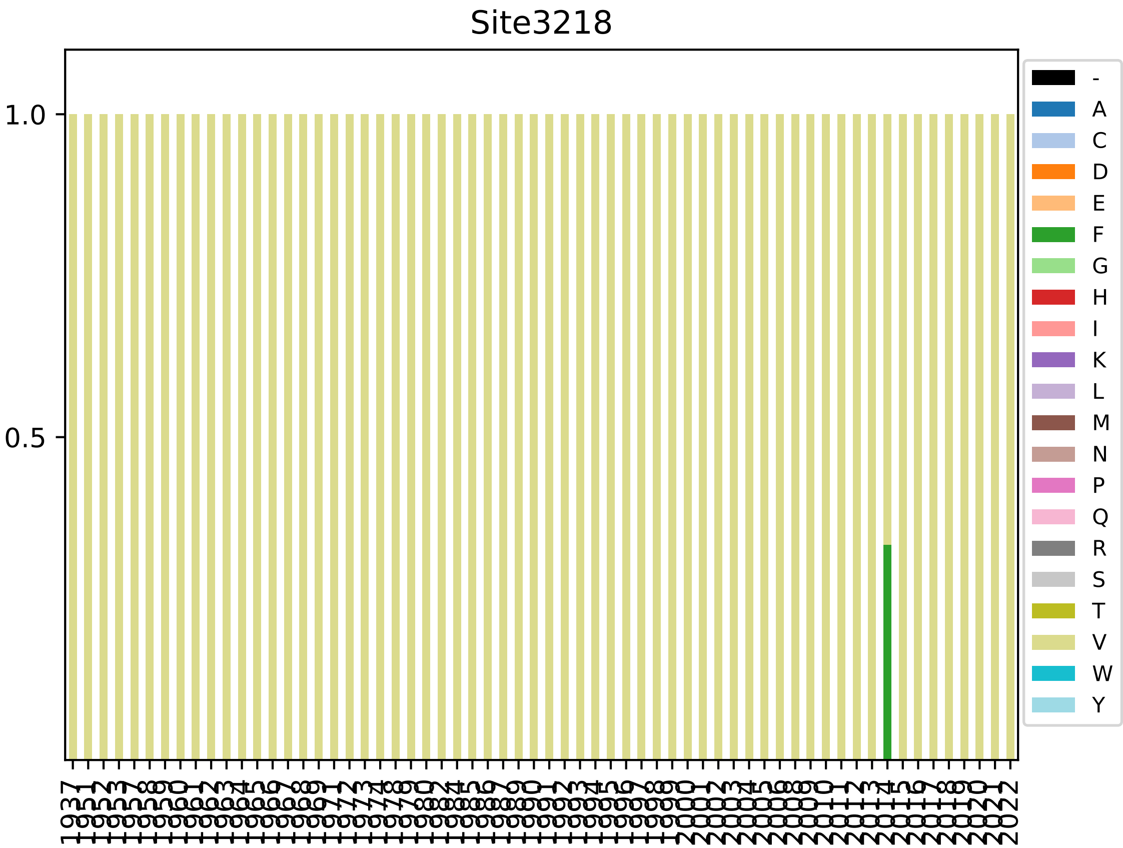
Site3218
1.0
0.5
-
A
C
D
E
F
G
H
I
K
L
M
N
P
Q
R
S
T
V
W
Y

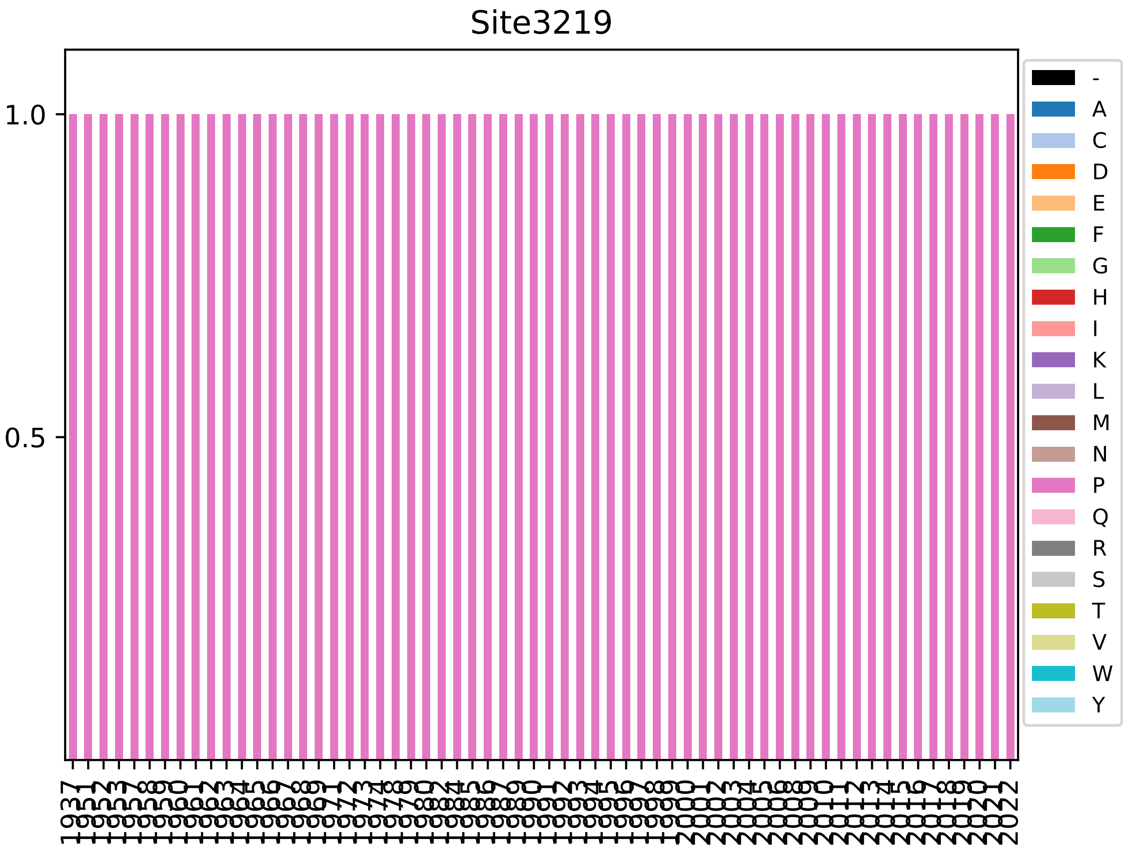

Site3219
1.0
0.5
-
A
C
D
E
F
G
H
I
K
L
M
N
P
Q
R
S
T
V
W
Y

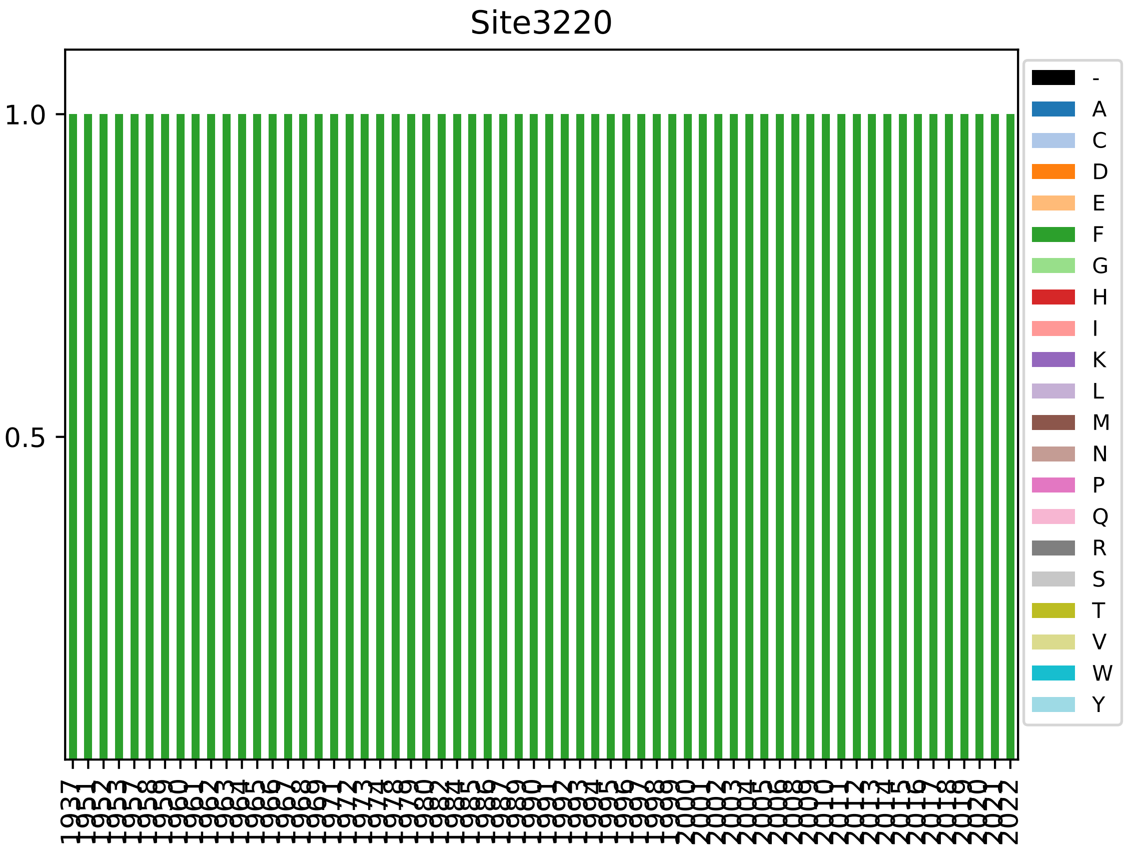
Site3220
1.0
0.5
-
A
C
D
E
F
G
H
I
K
L
M
N
P
Q
R
S
T
V
W
Y

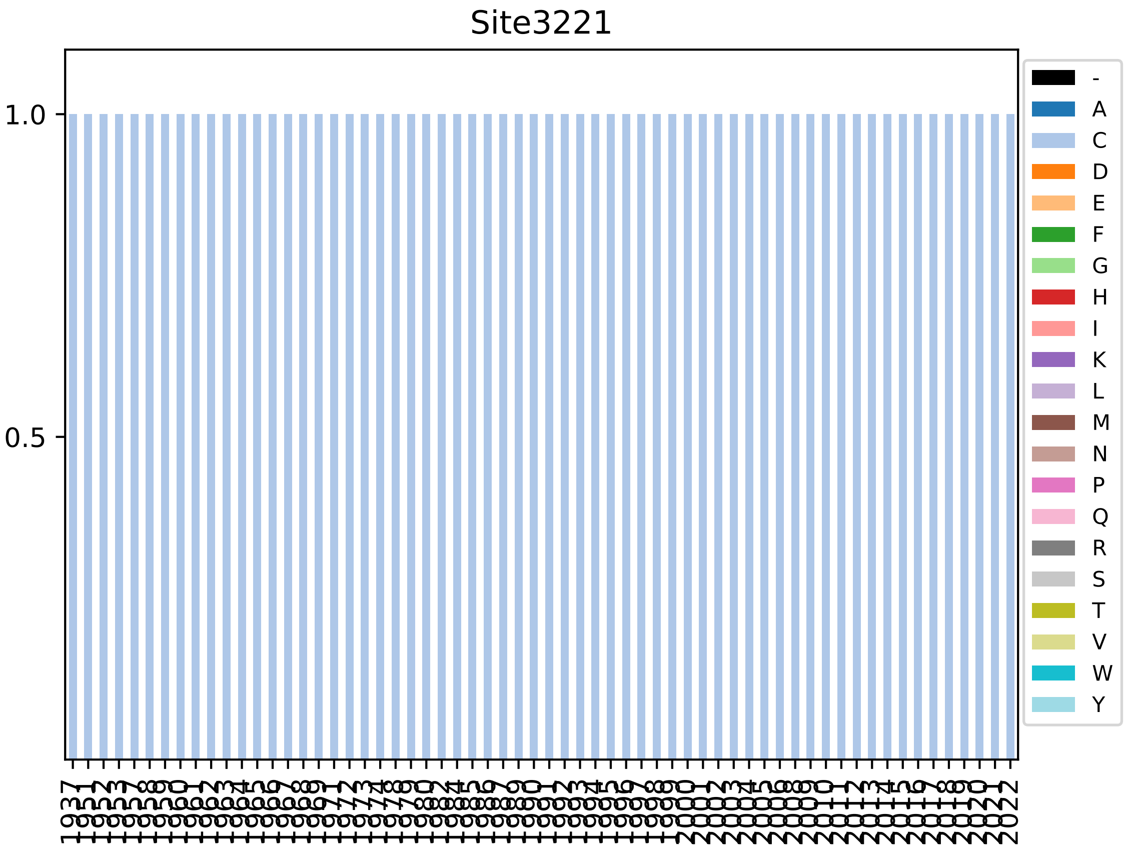
Site3221
1.0
0.5
-
A
C
D
E
F
G
H
I
K
L
M
N
P
Q
R
S
T
V
W
Y

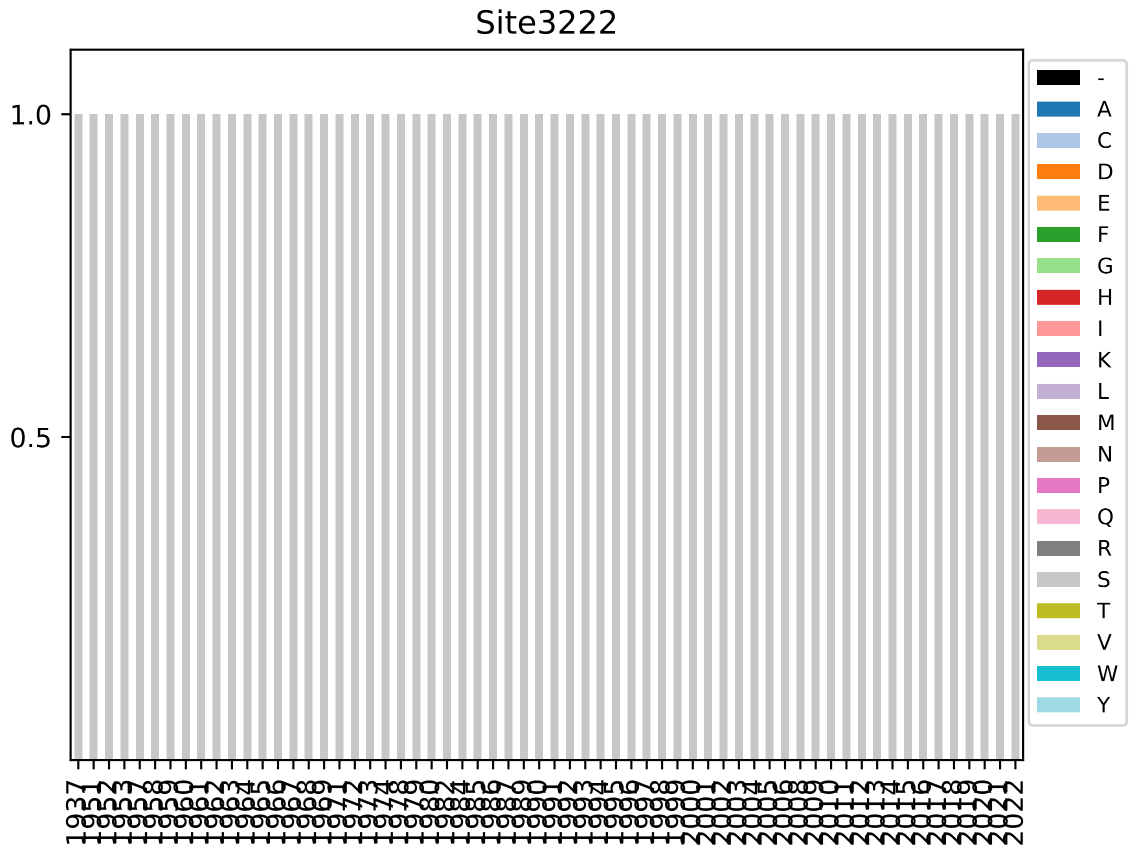
Site3222
1.0
0.5
-
A
C
D
E
F
G
H
I
K
L
M
N
P
Q
R
S
T
V
W
Y

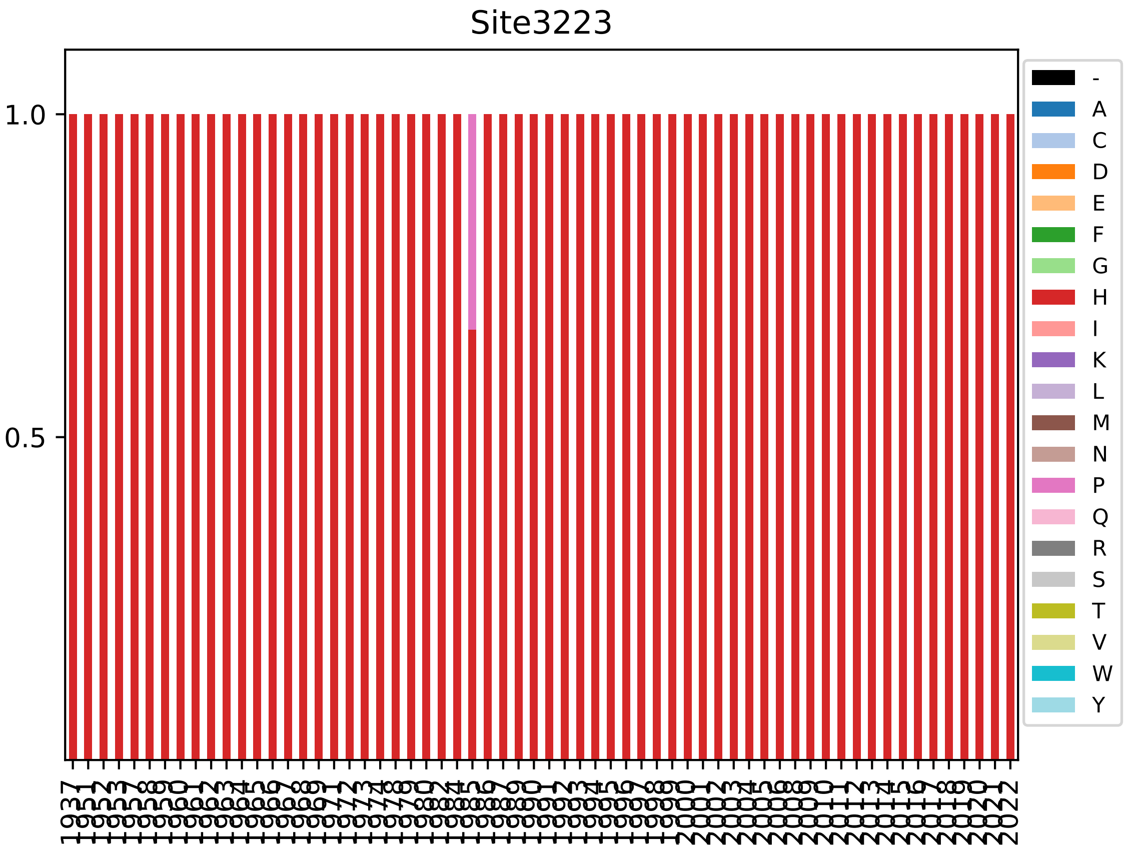
Site3223
1.0
0.5
-
A
C
D
E
F
G
H
I
K
L
M
N
P
Q
R
S
T
V
W
Y

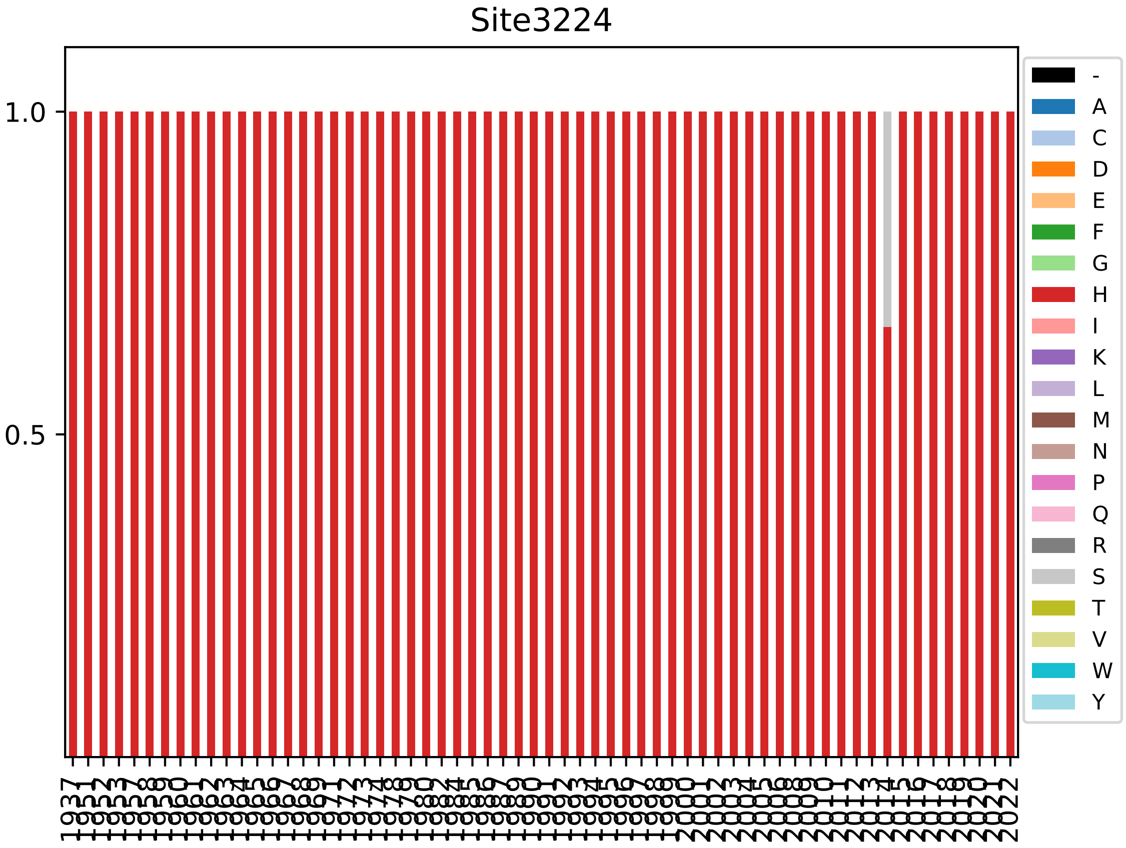
Site3224
1.0
0.5
-
A
C
D
E
F
G
H
I
K
L
M
N
P
Q
R
S
T
V
W
Y

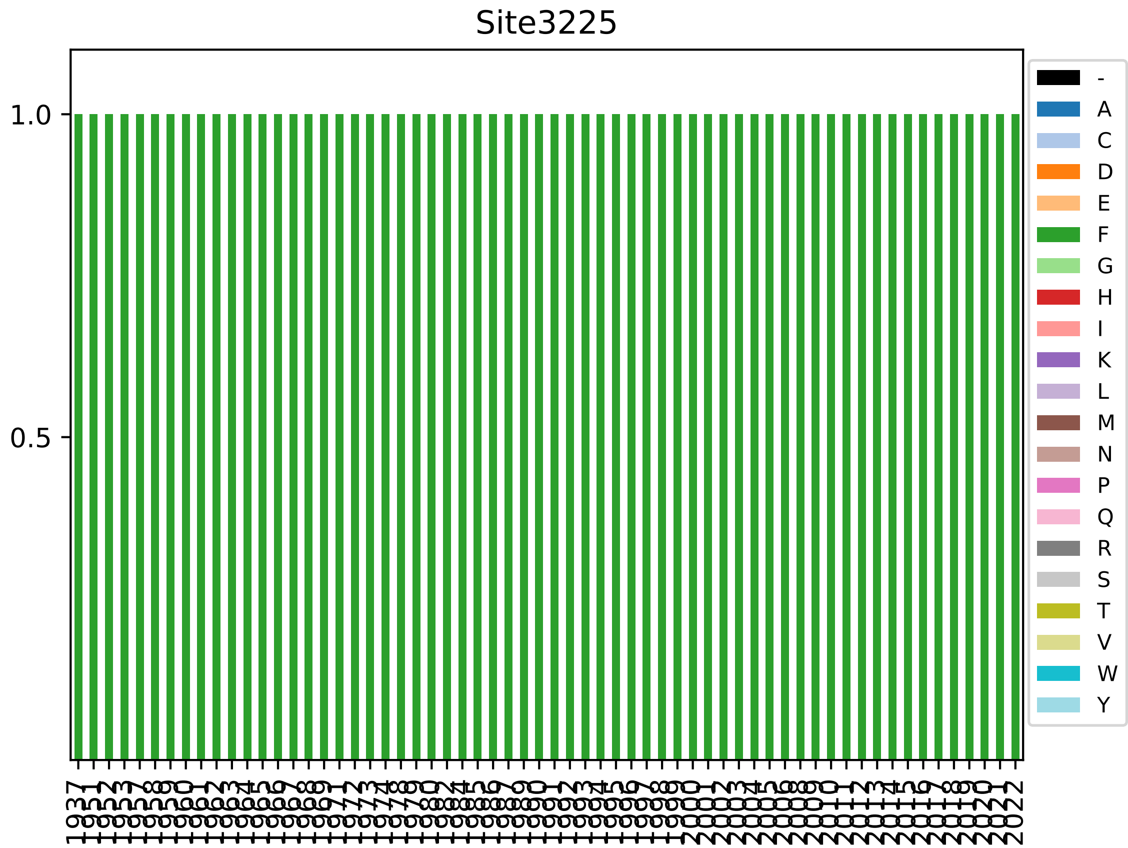
Site3225
1.0
0.5
-
A
C
D
E
F
G
H
I
K
L
M
N
P
Q
R
S
T
V
W
Y

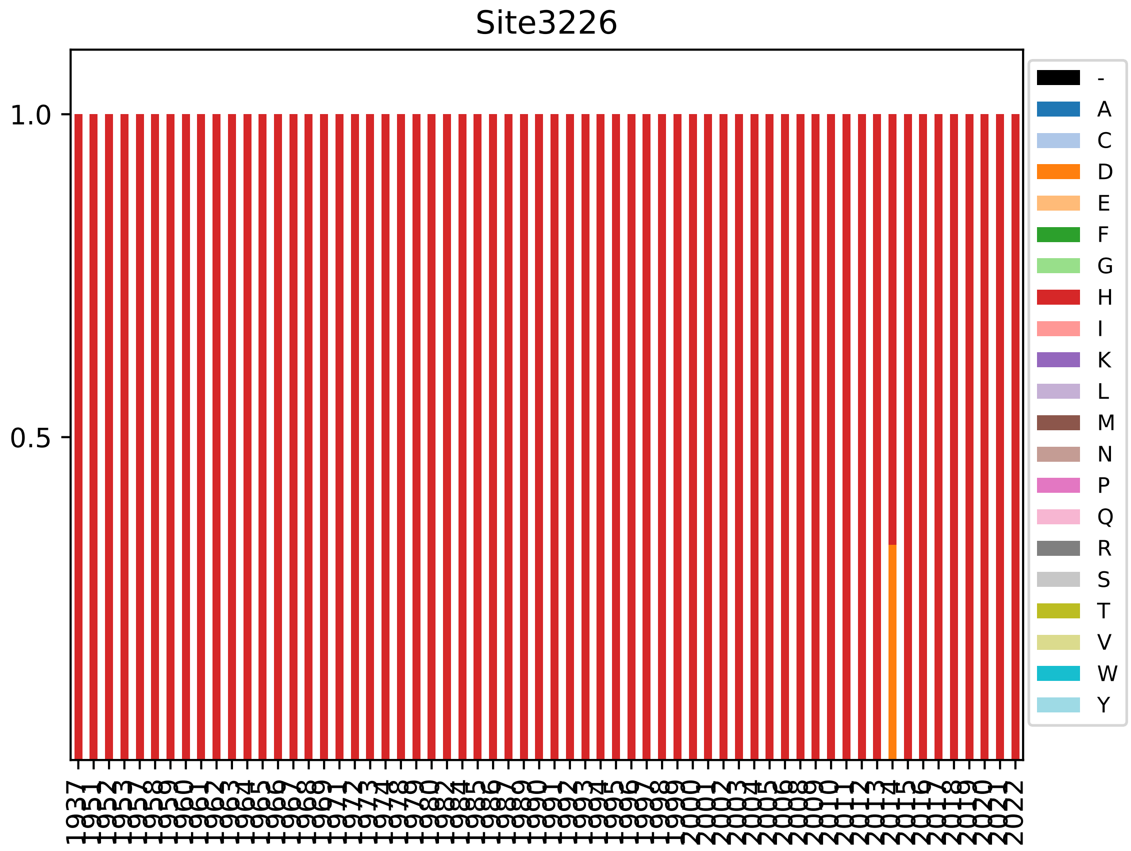
Site3226
1.0
0.5
-
A
C
D
E
F
G
H
I
K
L
M
N
P
Q
R
S
T
V
W
Y

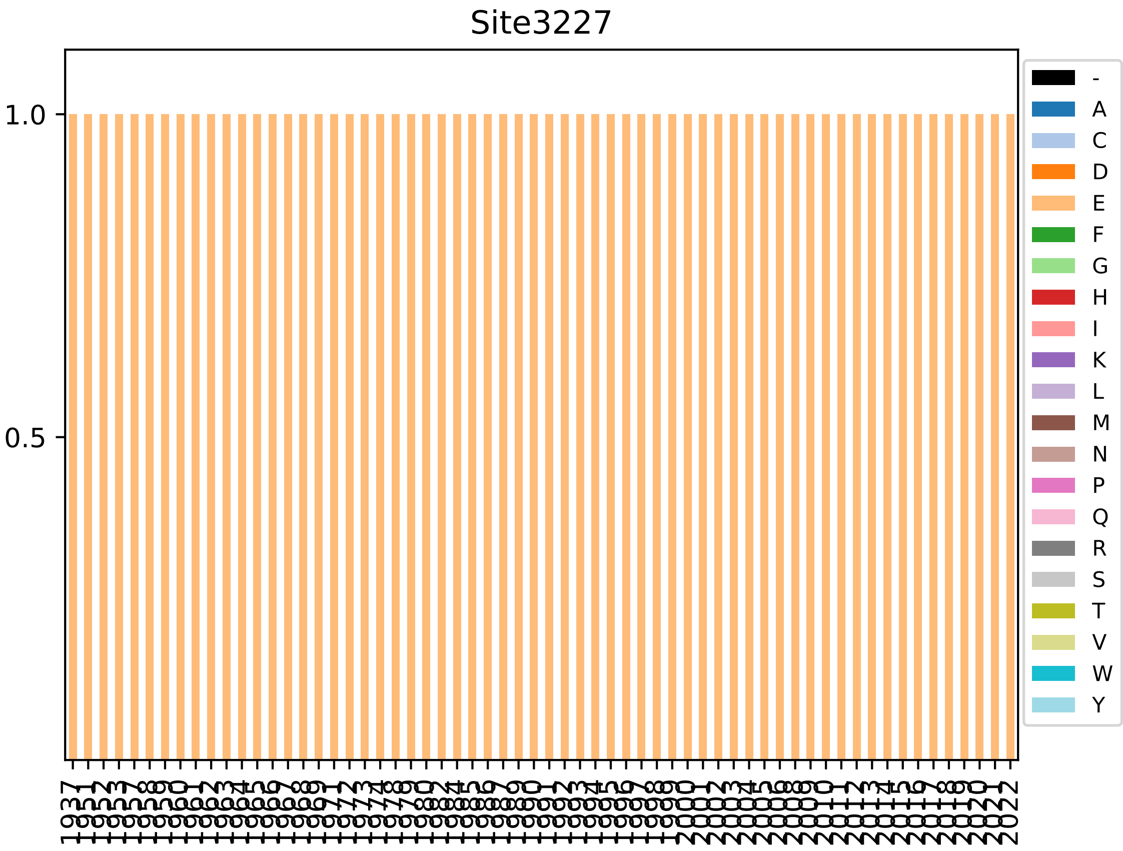
Site3227
1.0
0.5
-
A
C
D
E
F
G
H
I
K
L
M
N
P
Q
R
S
T
V
W
Y

Site3228

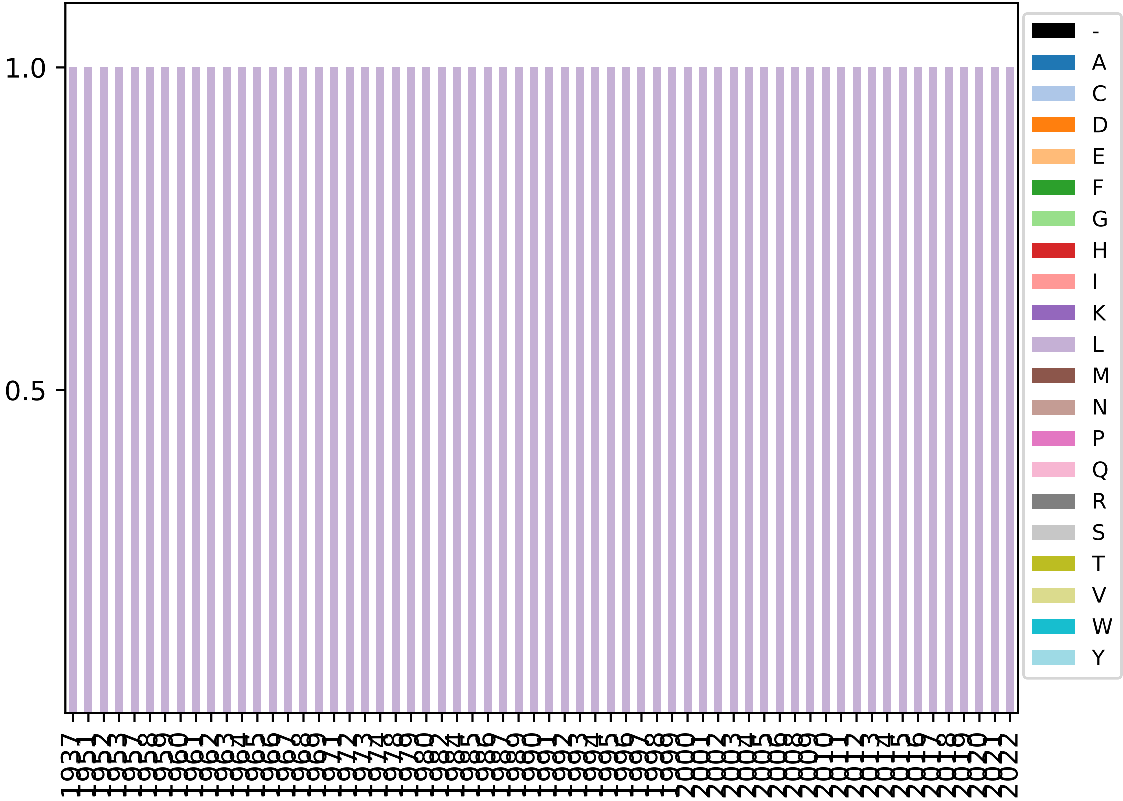

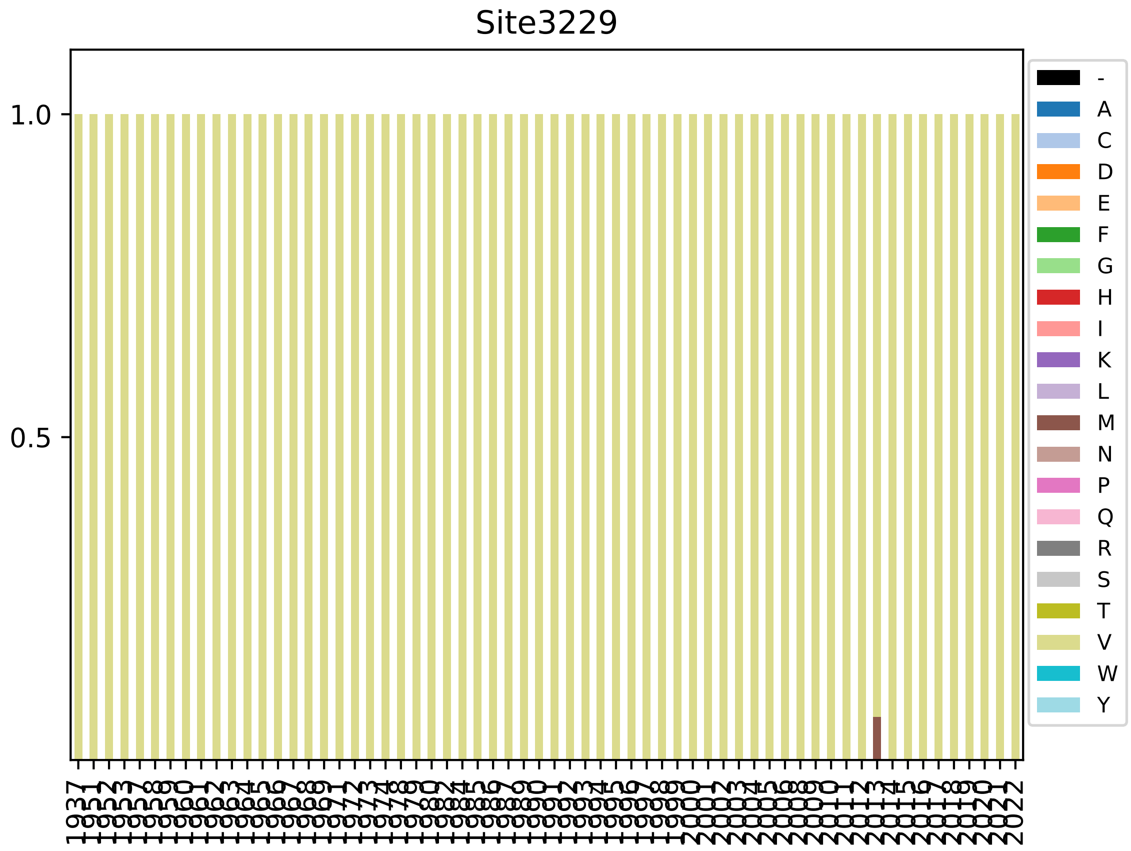

Site3229
1.0
0.5
-
A
C
D
E
F
G
H
I
K
L
M
N
P
Q
R
S
T
V
W
Y

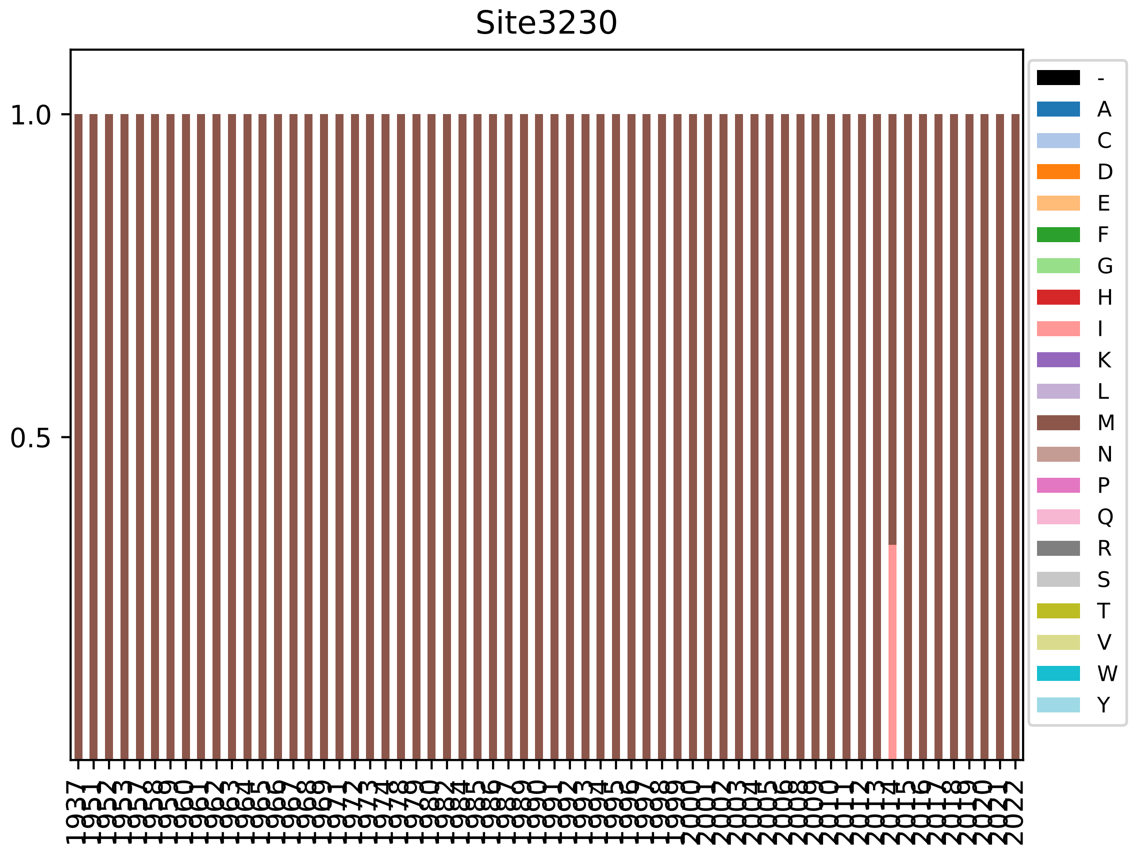
Site3230
1.0
0.5
-
A
C
D
E
F
G
H
I
K
L
M
N
P
Q
R
S
T
V
W
Y

Site3231

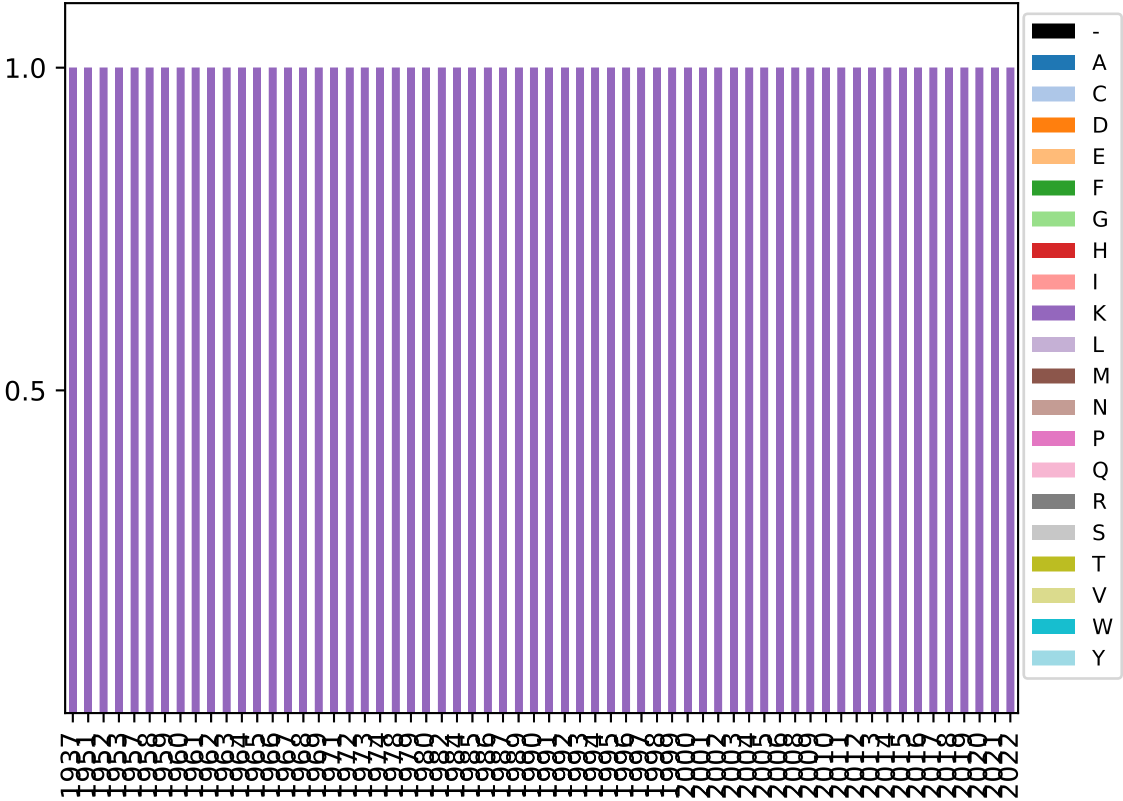

Site3232

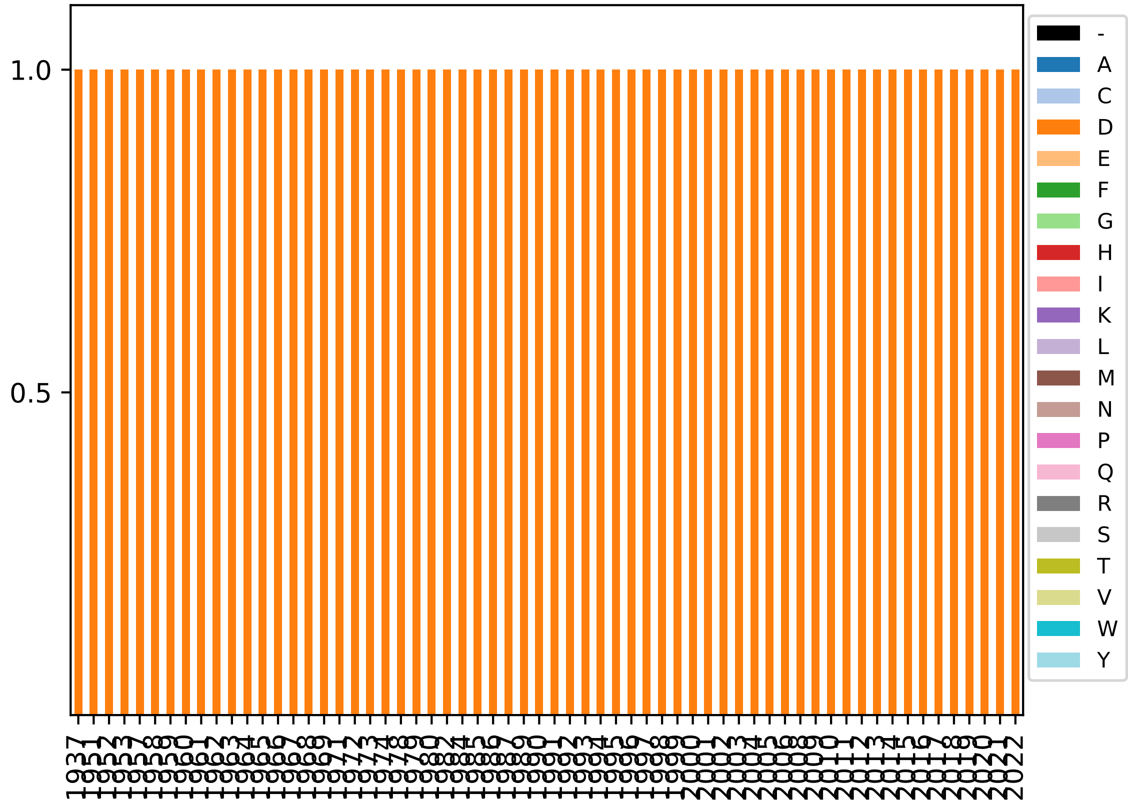

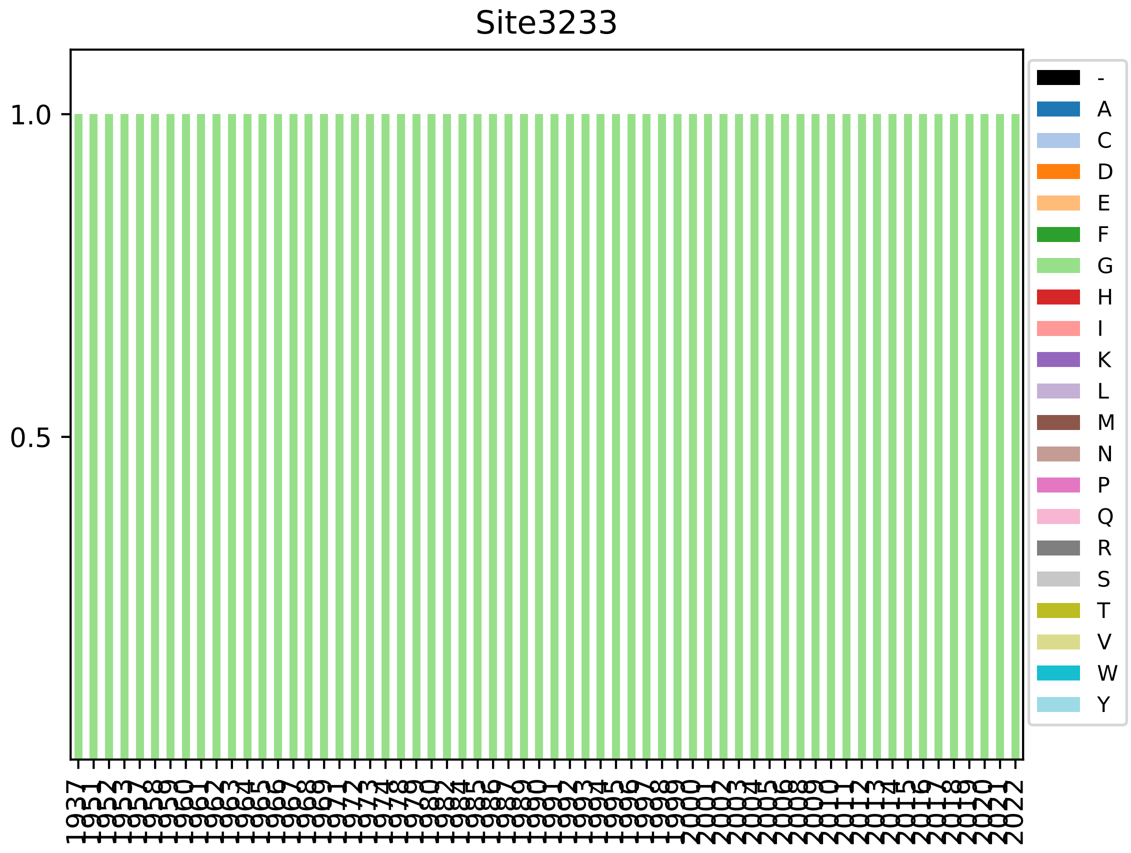
Site3233
1.0
0.5
-
A
C
D
E
F
G
H
I
K
L
M
N
P
Q
R
S
T
V
W
Y

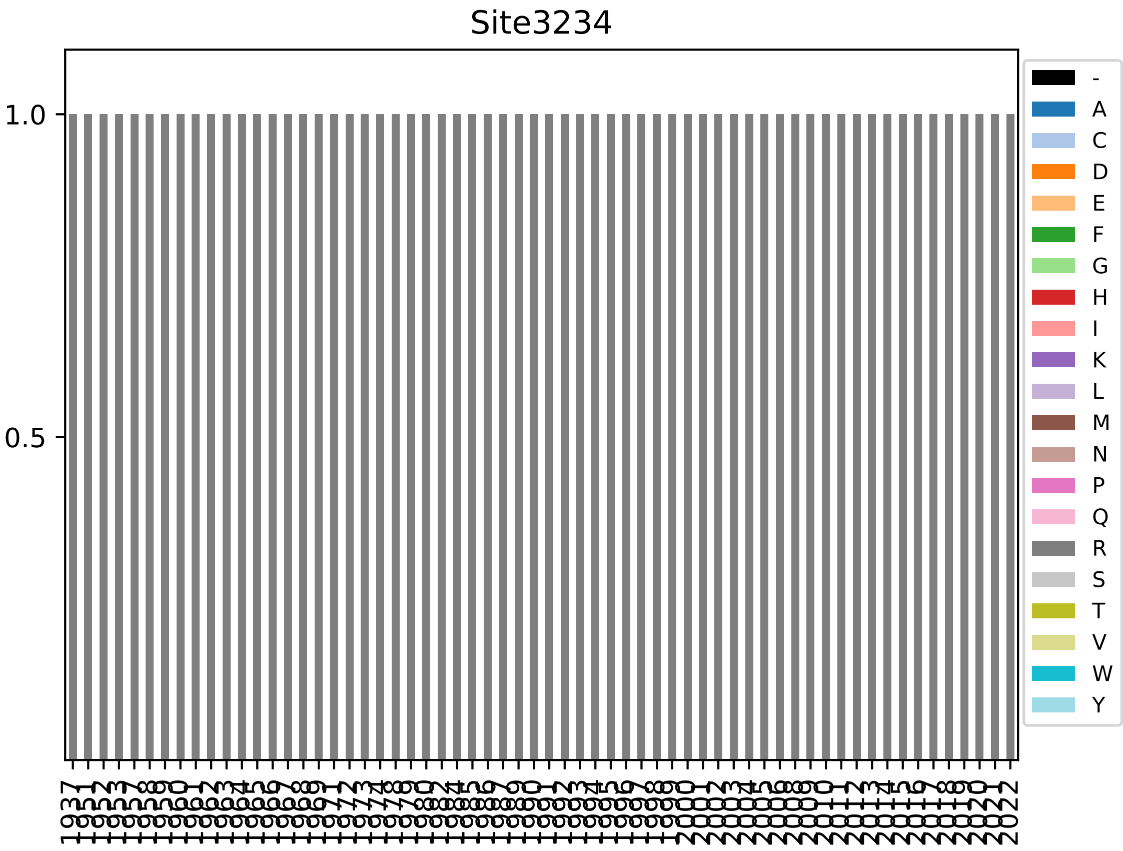
Site3234
1.0
0.5
-
A
C
D
E
F
G
H
I
K
L
M
N
P
Q
R
S
T
V
W
Y

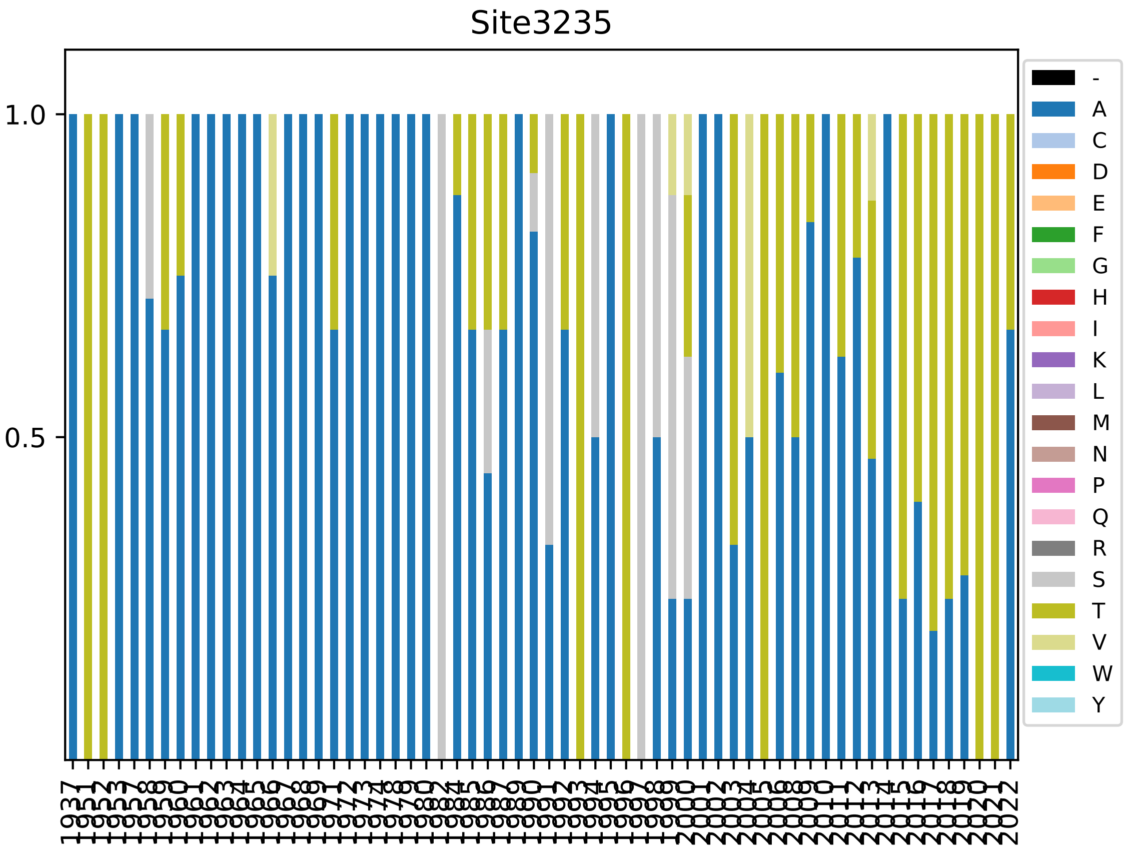
Site3235
1.0
0.5
-
A
C
D
E
F
G
H
I
K
L
M
N
P
Q
R
S
T
V
W
Y

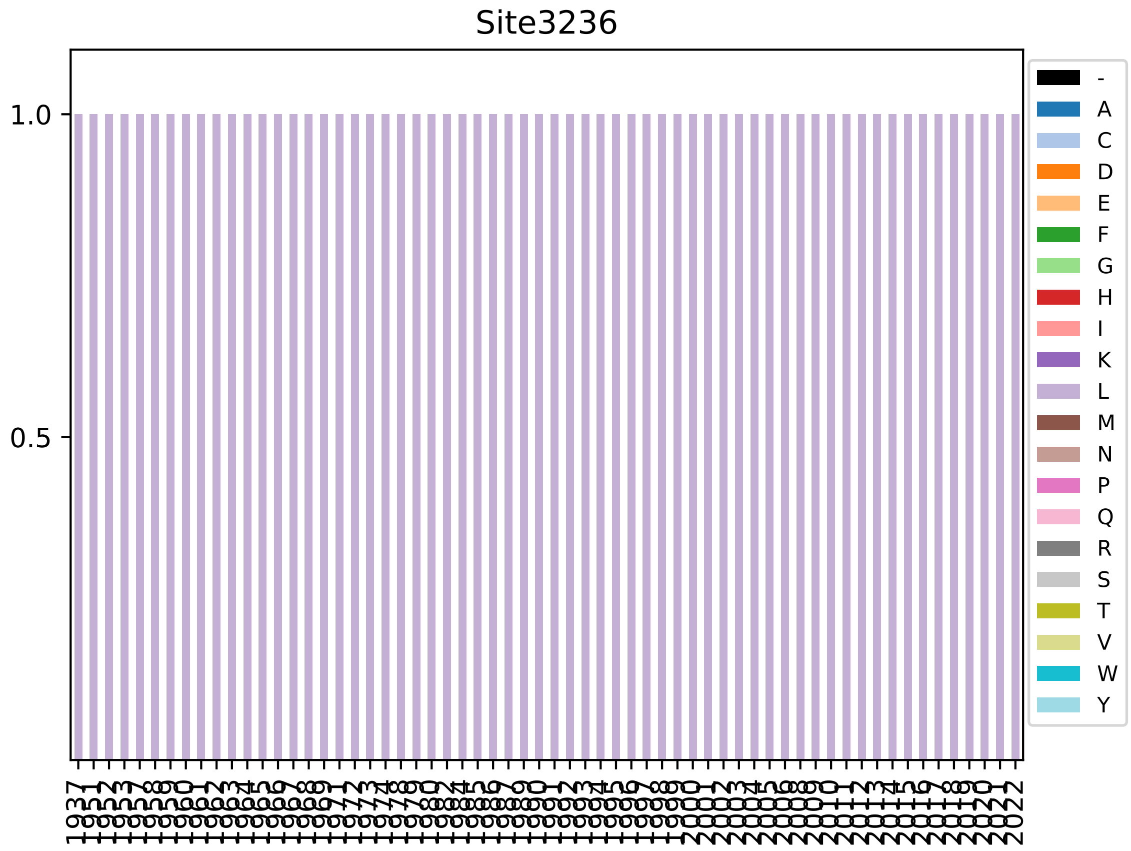
Site3236
1.0
0.5
-
A
C
D
E
F
G
H
I
K
L
M
N
P
Q
R
S
T
V
W
Y

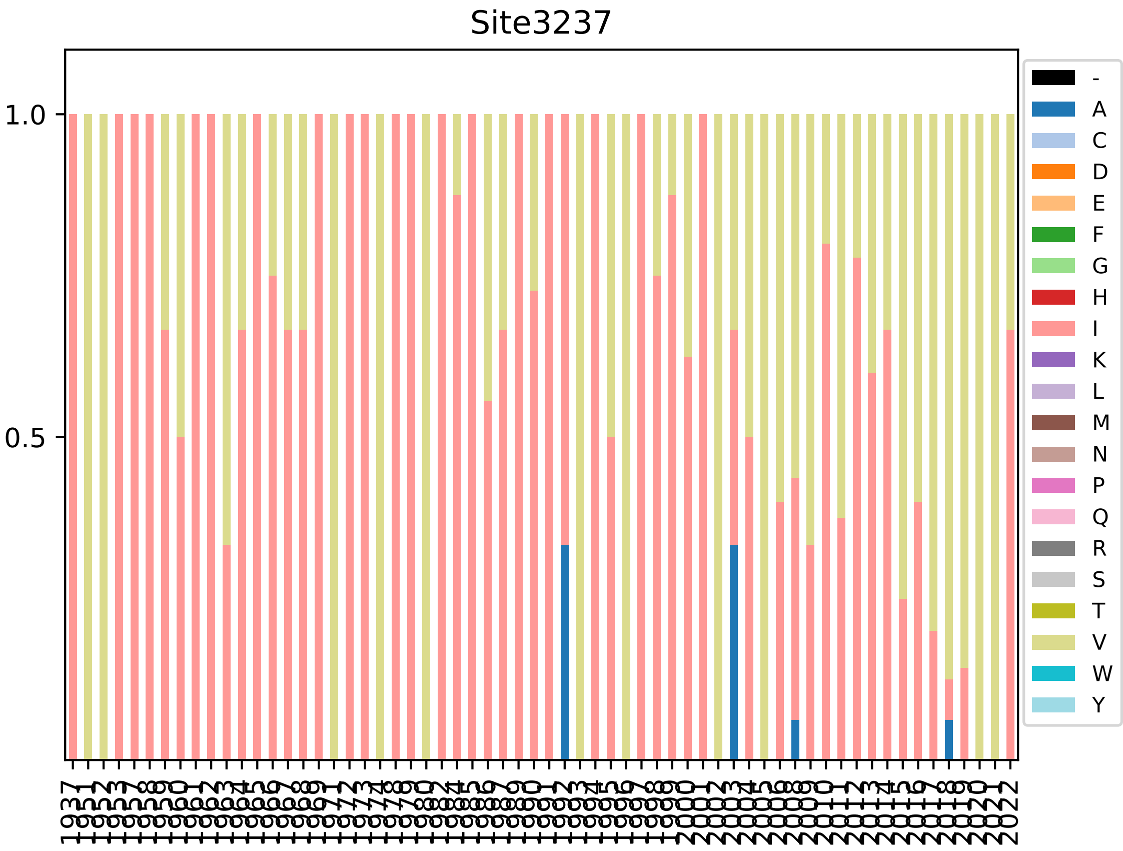
Site3237
1.0
0.5
-
A
C
D
E
F
G
H
I
K
L
M
N
P
Q
R
S
T
V
W
Y

Site3238

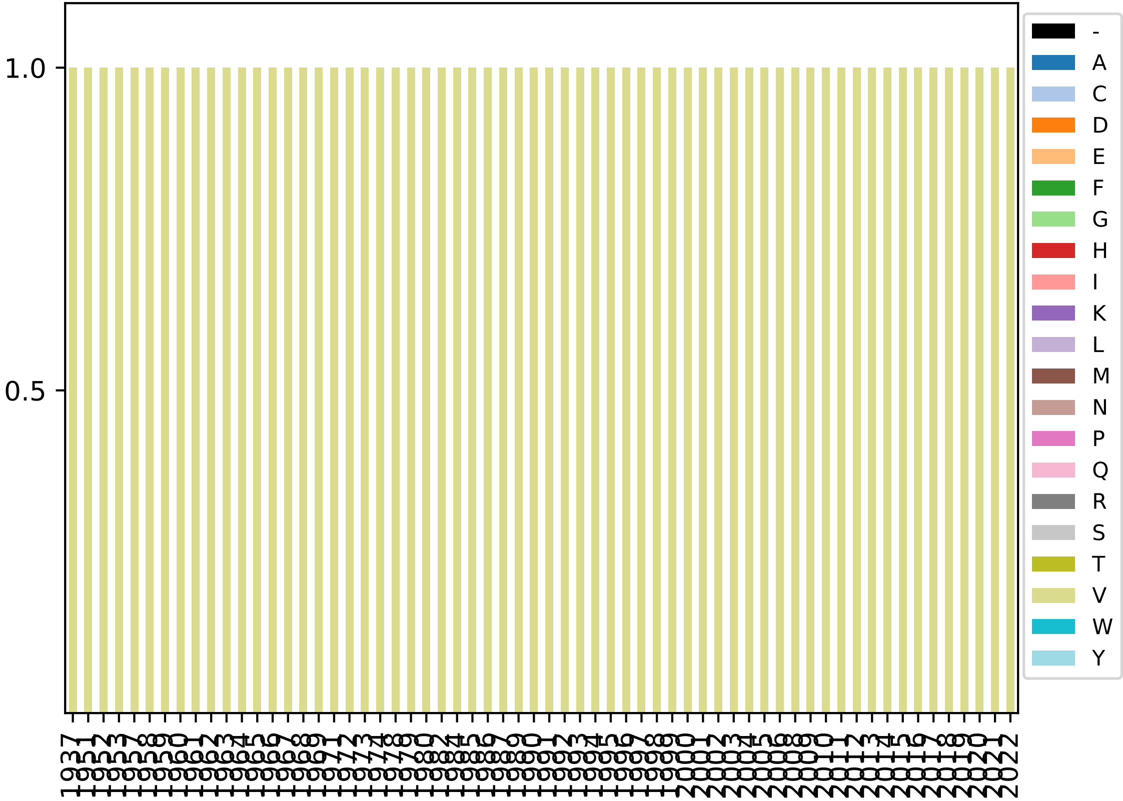

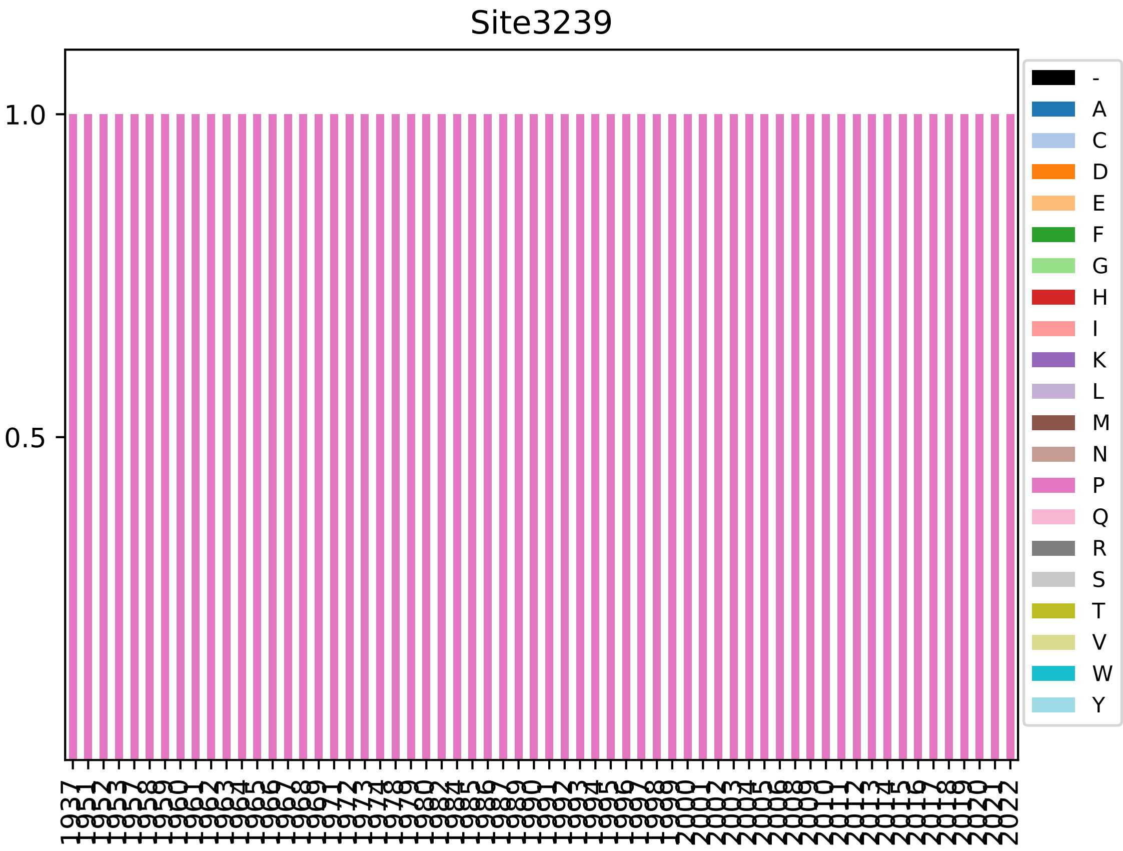
Site3239
1.0
0.5
-
A
C
D
E
F
G
H
I
K
L
M
N
P
Q
R
S
T
V
W
Y

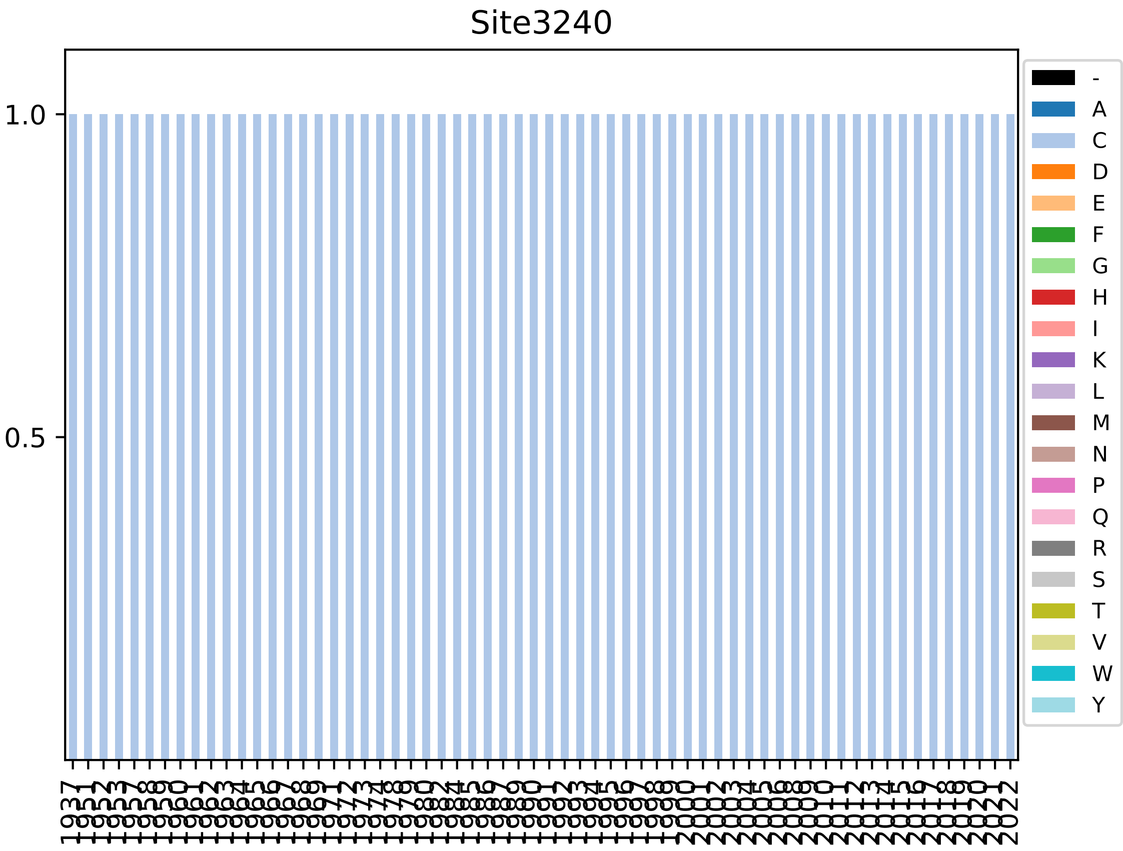
Site3240
1.0
0.5
-
A
C
D
E
F
G
H
I
K
L
M
N
P
Q
R
S
T
V
W
Y

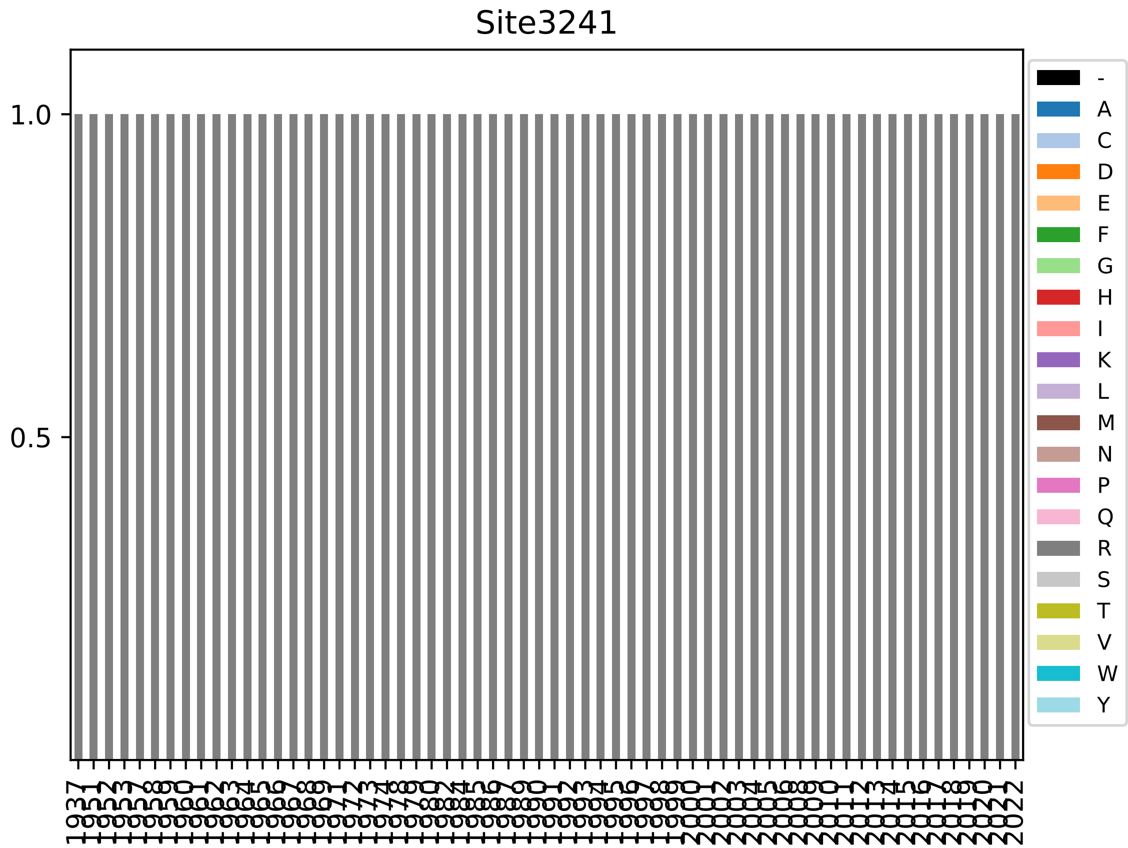
Site3241
1.0
0.5
-
A
C
D
E
F
G
H
I
K
L
M
N
P
Q
R
S
T
V
W
Y

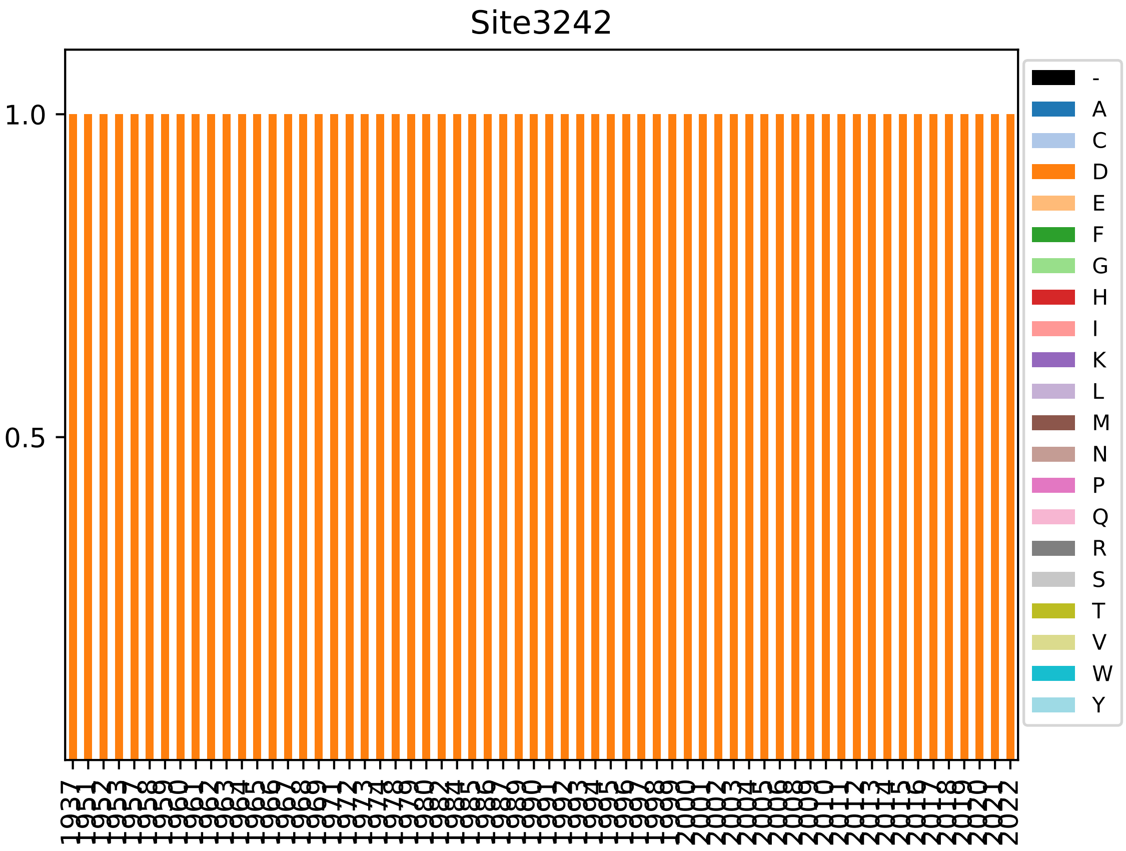

Site3242
1.0
0.5
-
A
C
D
E
F
G
H
I
K
L
M
N
P
Q
R
S
T
V
W
Y

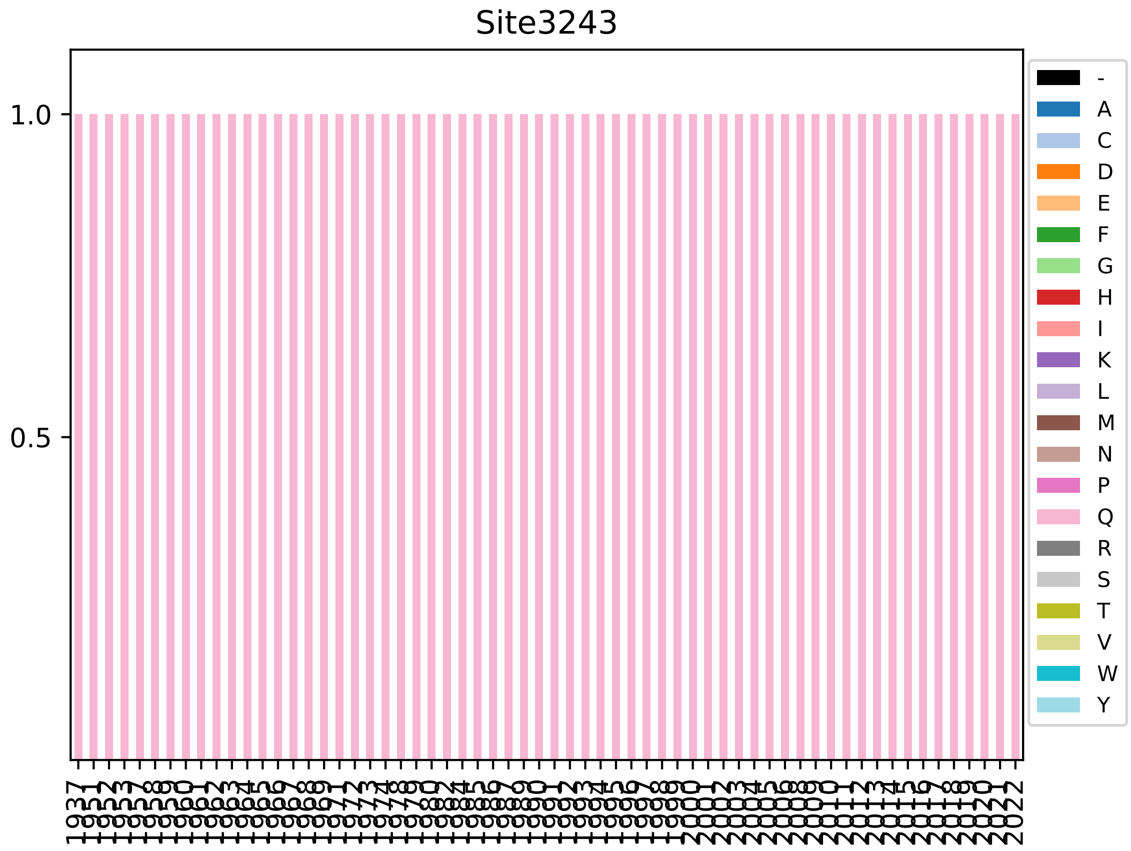
Site3243
1.0
0.5
-
A
C
D
E
F
G
H
I
K
L
M
N
P
Q
R
S
T
V
W
Y

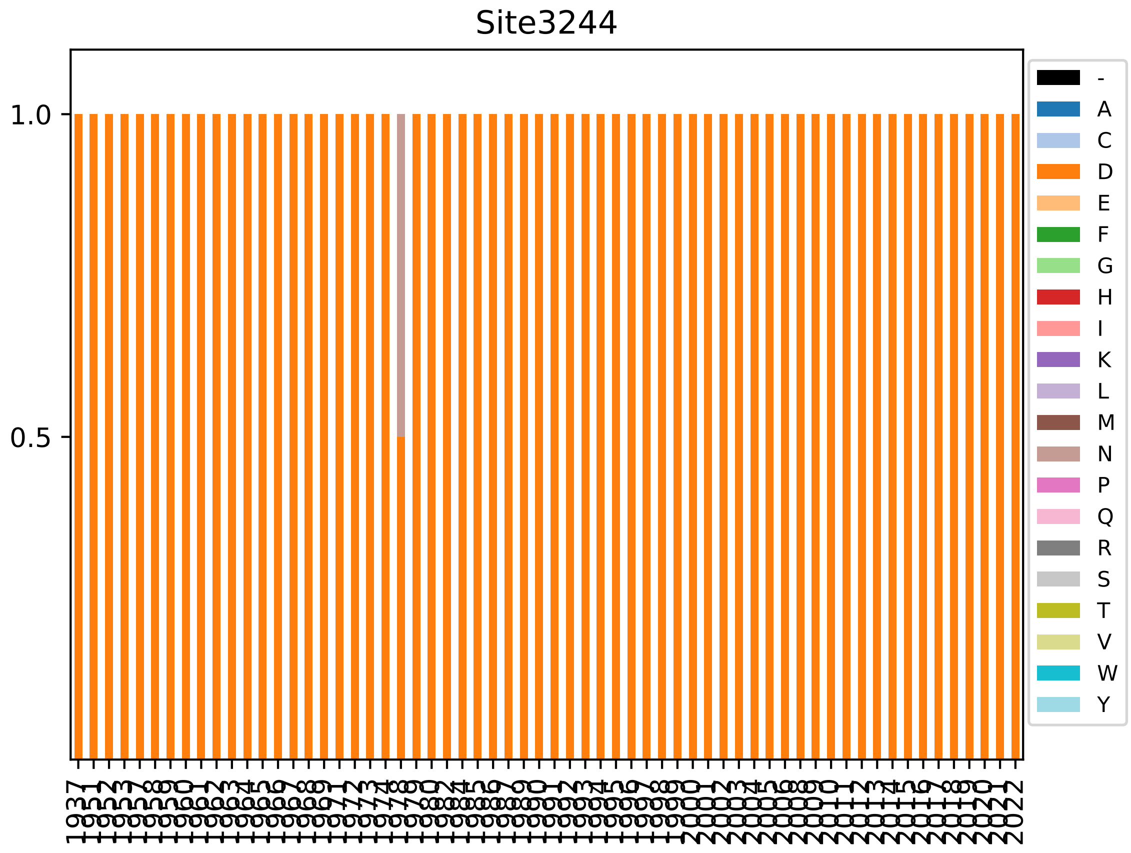
Site3244
1.0
0.5
-
A
C
D
E
F
G
H
I
K
L
M
N
P
Q
R
S
T
V
W
Y

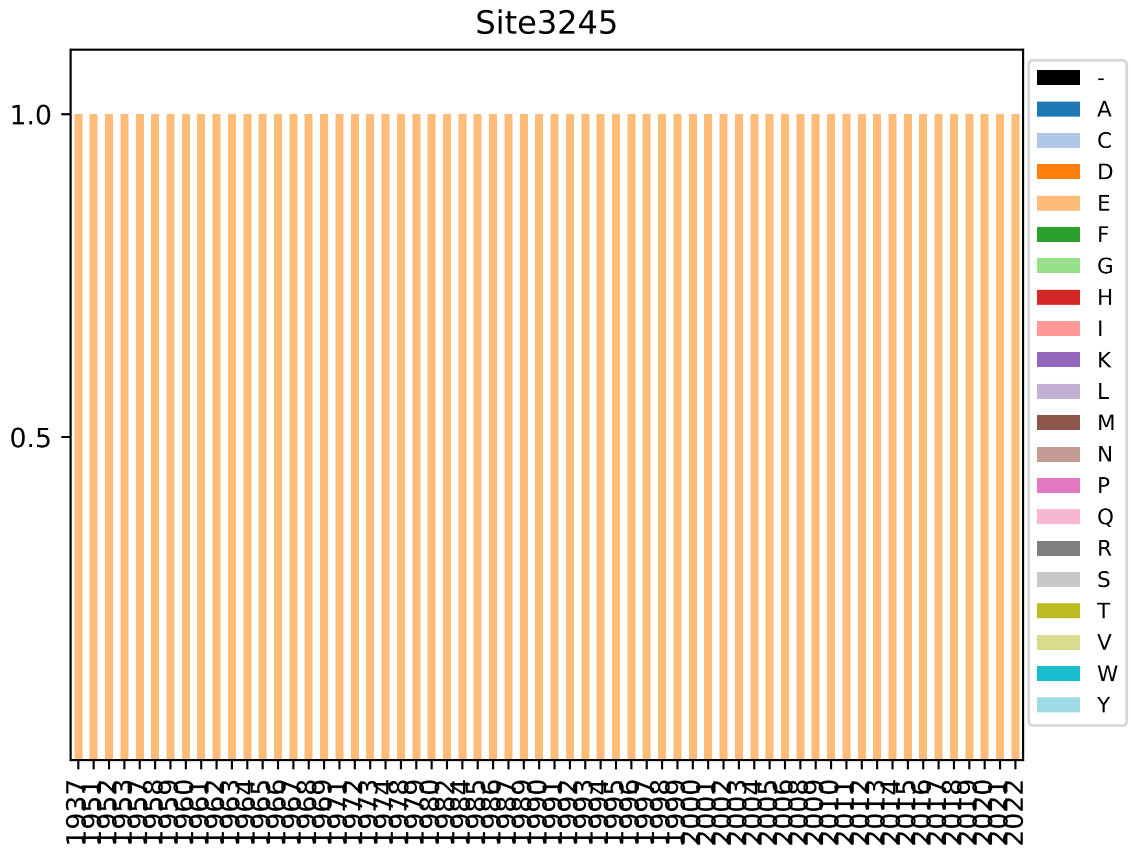
Site3245
1.0
0.5
-
A
C
D
E
F
G
H
I
K
L
M
N
P
Q
R
S
T
V
W
Y

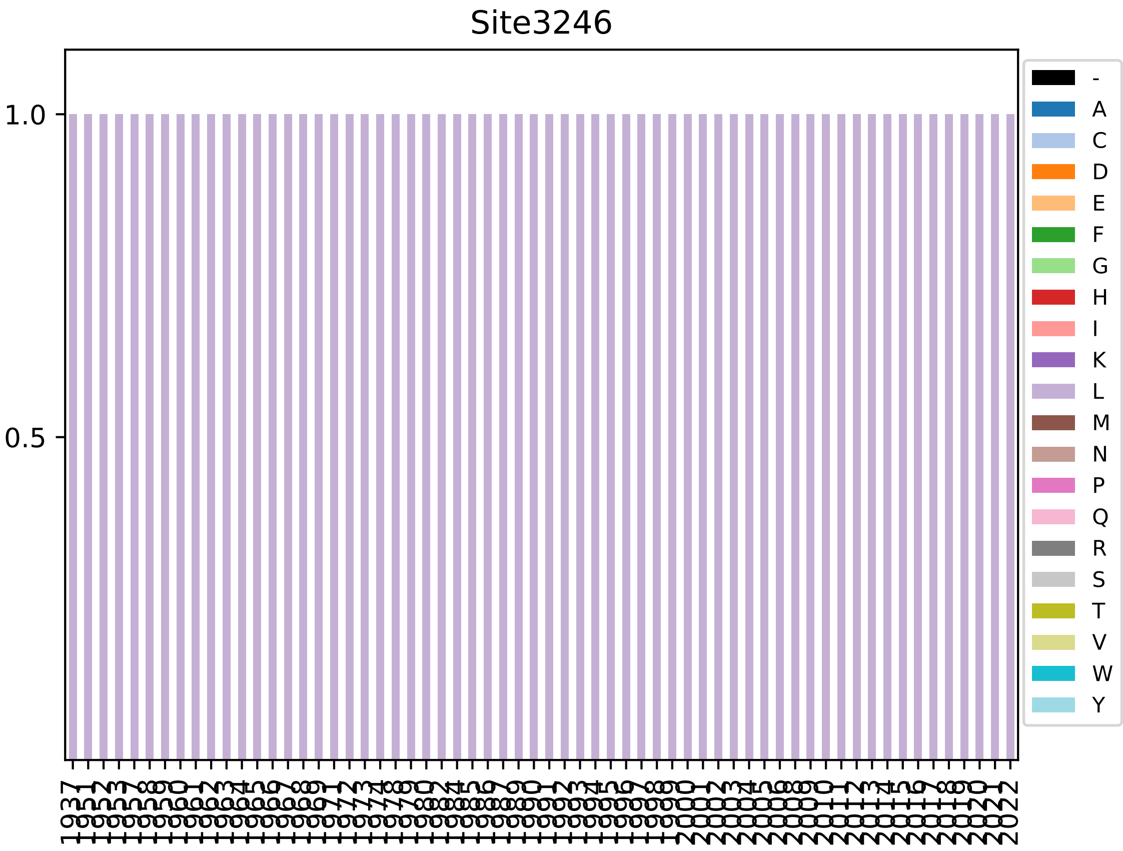
Site3246
1.0
0.5
-
A
C
D
E
F
G
H
I
K
L
M
N
P
Q
R
S
T
V
W
Y

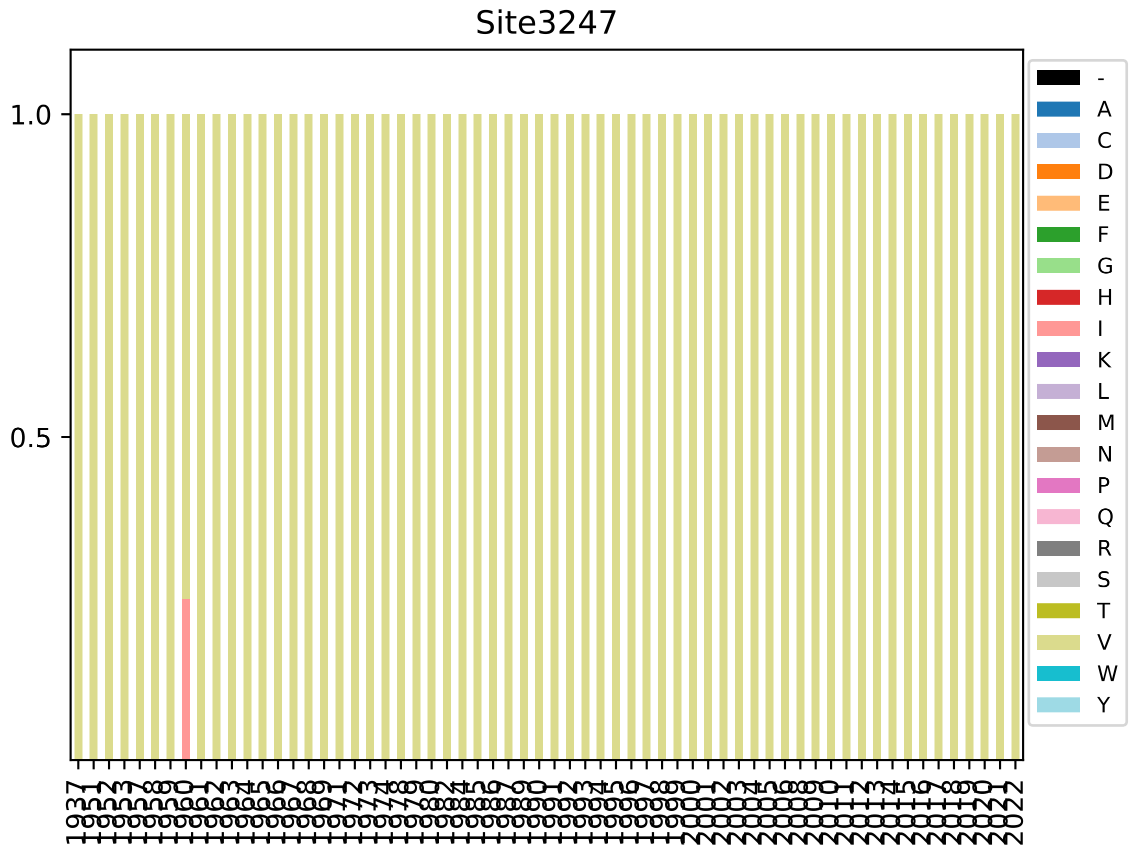
Site3247
1.0
0.5
-
A
C
D
E
F
G
H
I
K
L
M
N
P
Q
R
S
T
V
W
Y

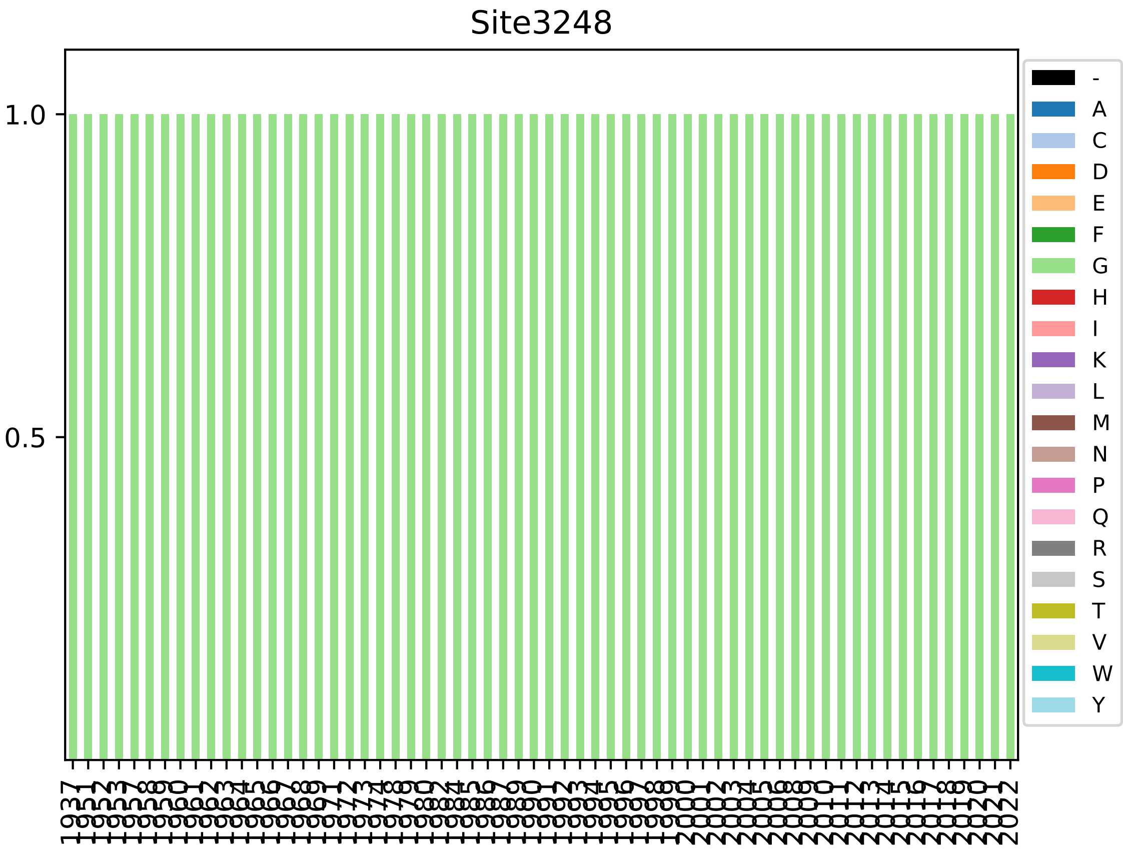
Site3248
1.0
0.5
-
A
C
D
E
F
G
H
I
K
L
M
N
P
Q
R
S
T
V
W
Y

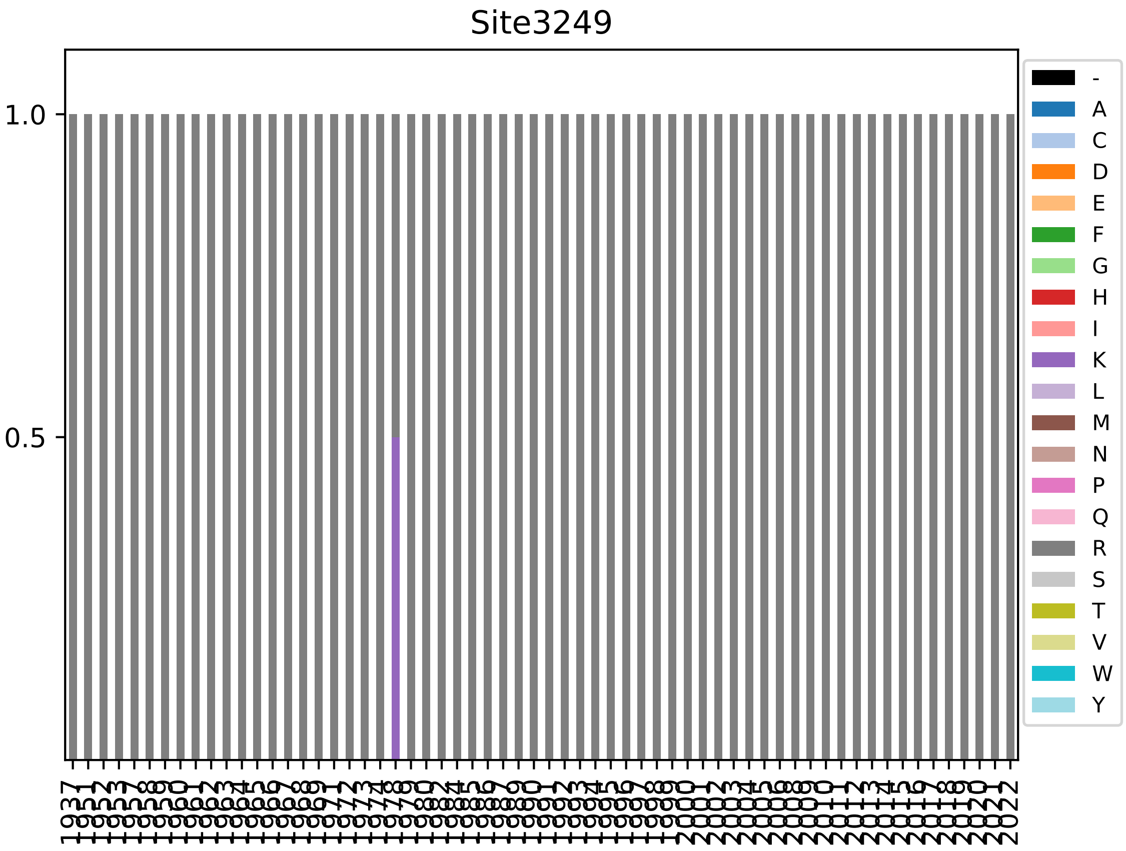

Site3249
1.0
0.5
-
A
C
D
E
F
G
H
I
K
L
M
N
P
Q
R
S
T
V
W
Y

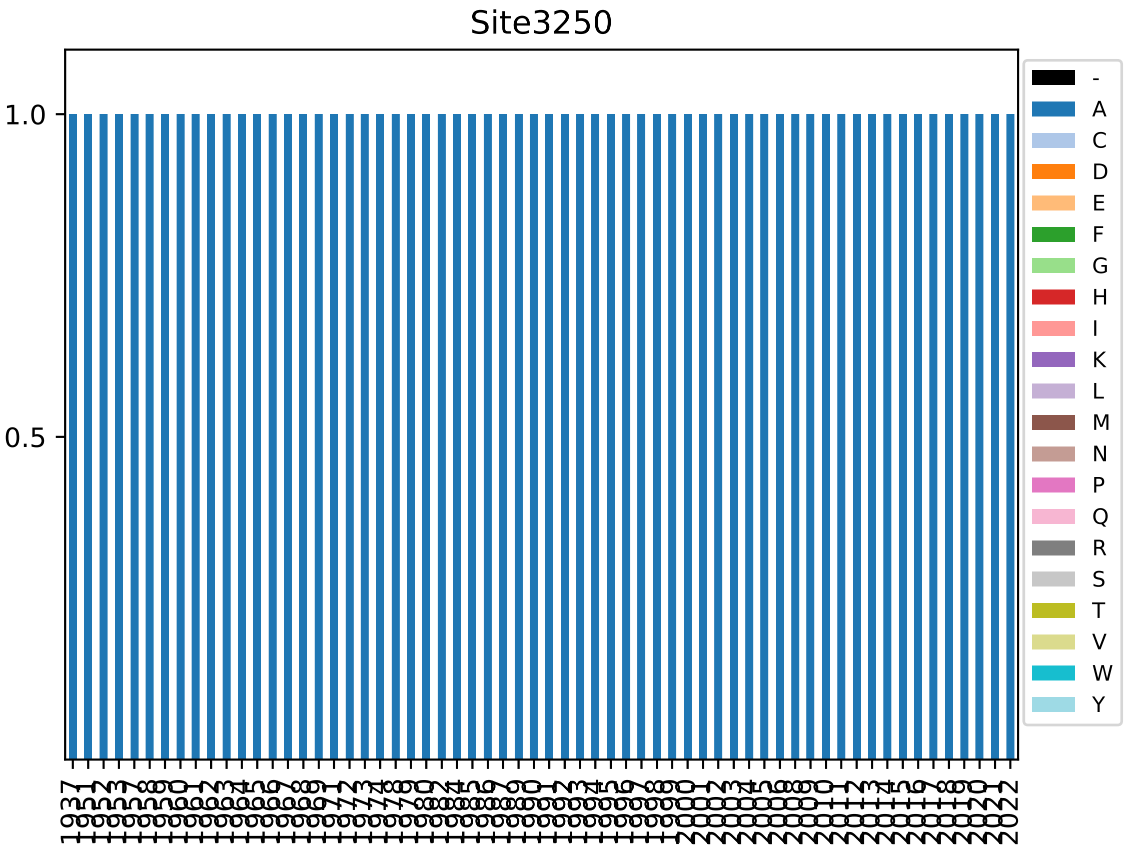
Site3250
1.0
0.5
-
A
C
D
E
F
G
H
I
K
L
M
N
P
Q
R
S
T
V
W
Y

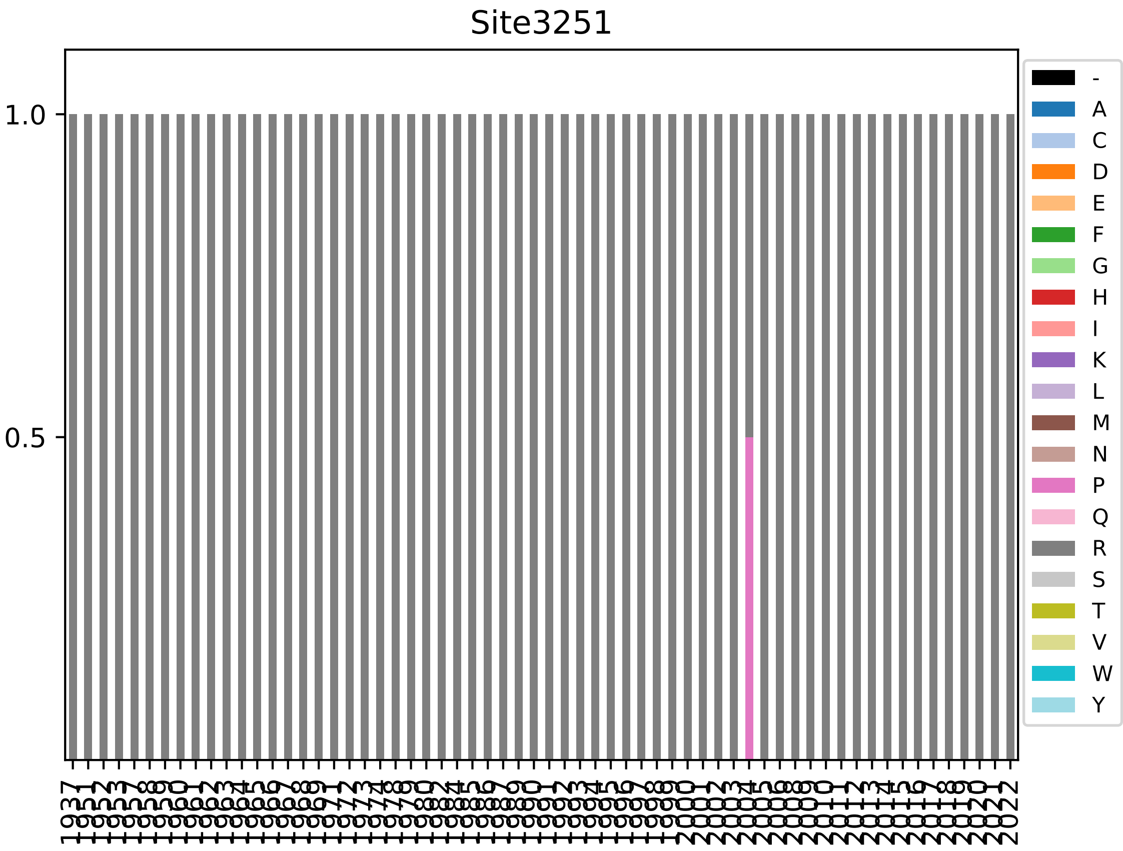
Site3251
1.0
0.5
-
A
C
D
E
F
G
H
I
K
L
M
N
P
Q
R
S
T
V
W
Y

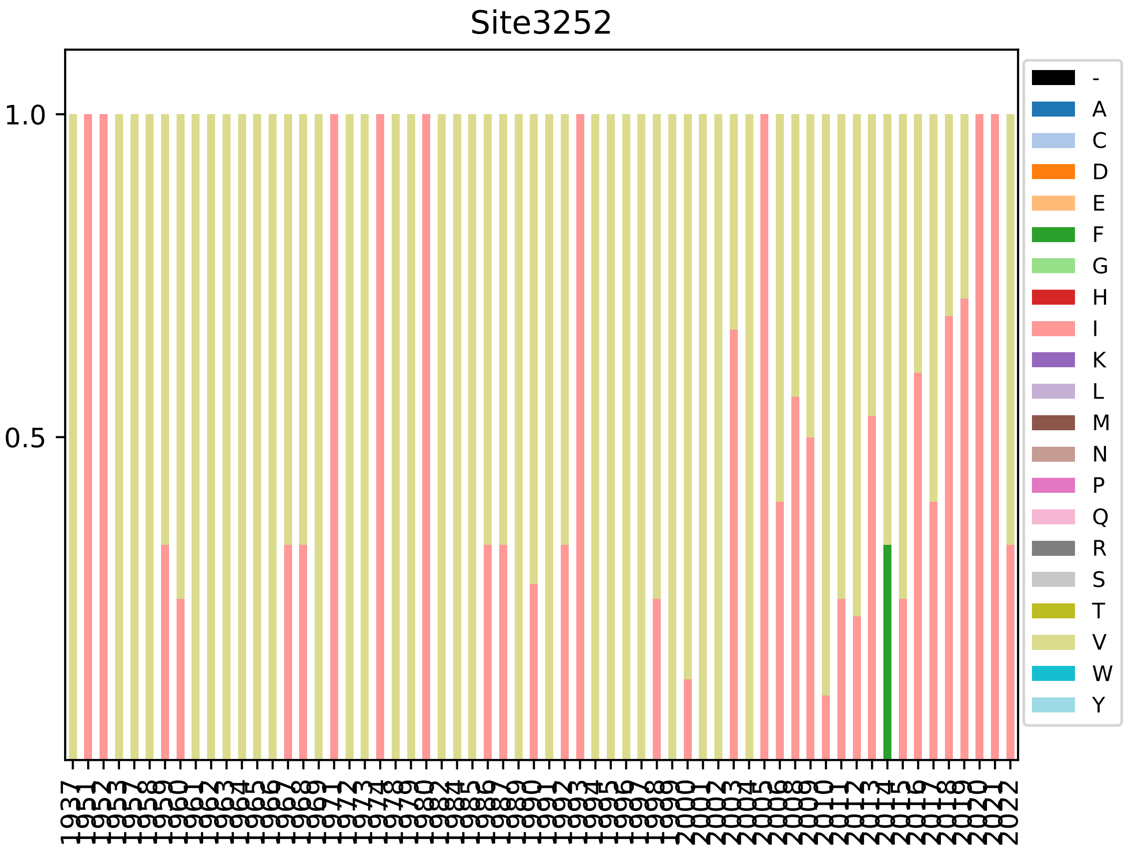

Site3252
1.0
0.5
-
A
C
D
E
F
G
H
I
K
L
M
N
P
Q
R
S
T
V
W
Y

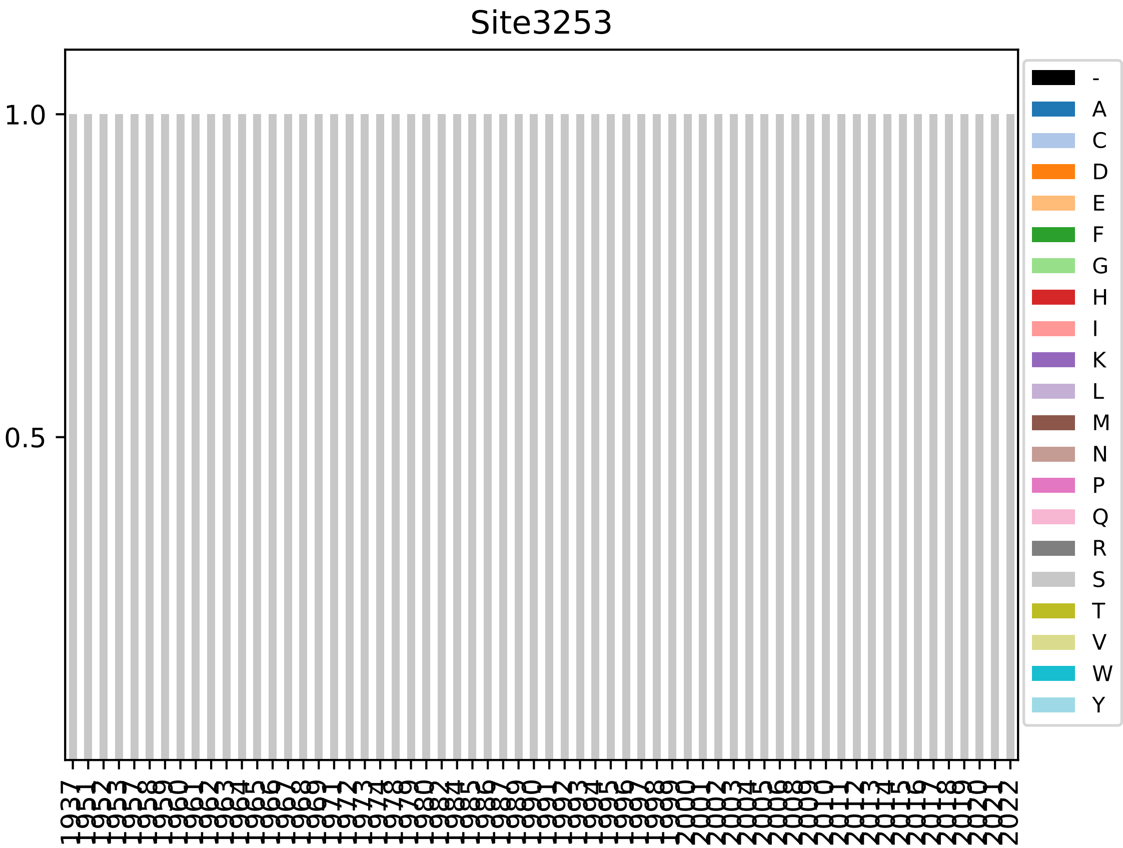
Site3253
1.0
0.5
-
A
C
D
E
F
G
H
I
K
L
M
N
P
Q
R
S
T
V
W
Y

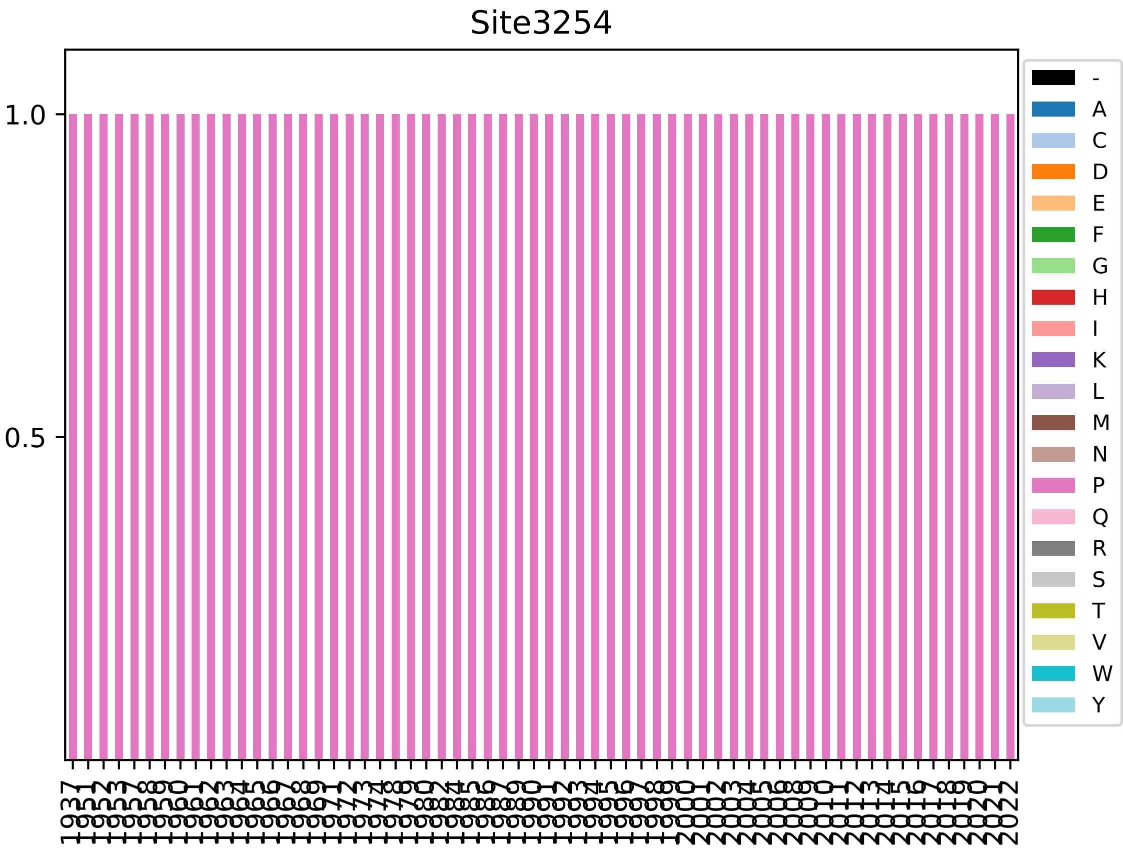
Site3254
1.0
0.5
-
A
C
D
E
F
G
H
I
K
L
M
N
P
Q
R
S
T
V
W
Y

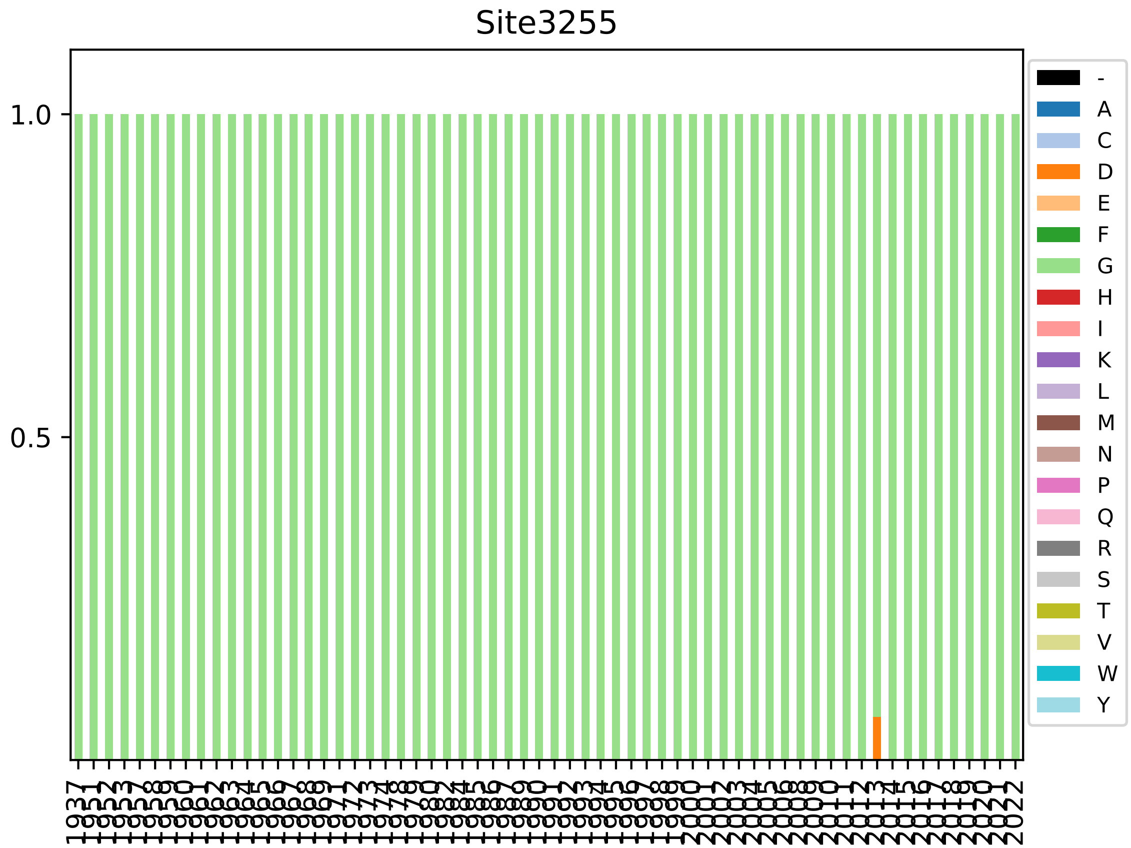
Site3255
1.0
0.5
-
A
C
D
E
F
G
H
I
K
L
M
N
P
Q
R
S
T
V
W
Y

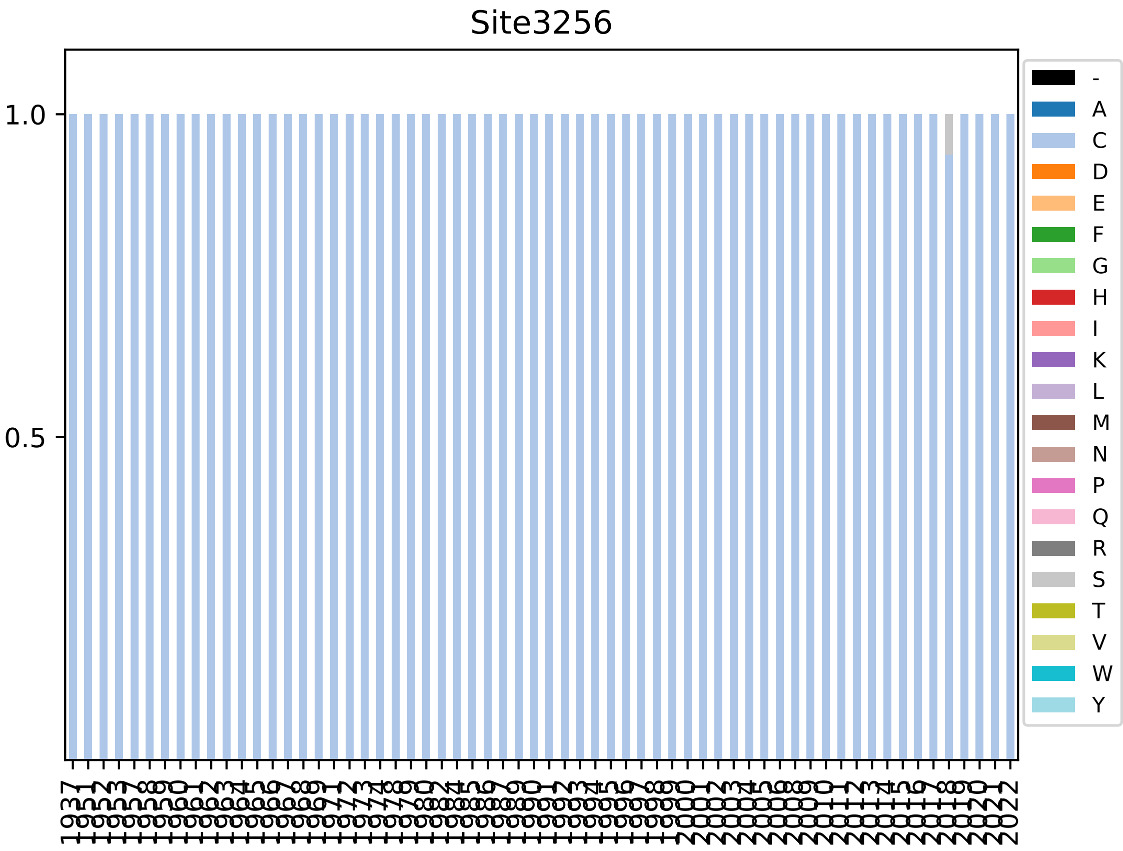
Site3256
1.0
0.5
-
A
C
D
E
F
G
H
I
K
L
M
N
P
Q
R
S
T
V
W
Y

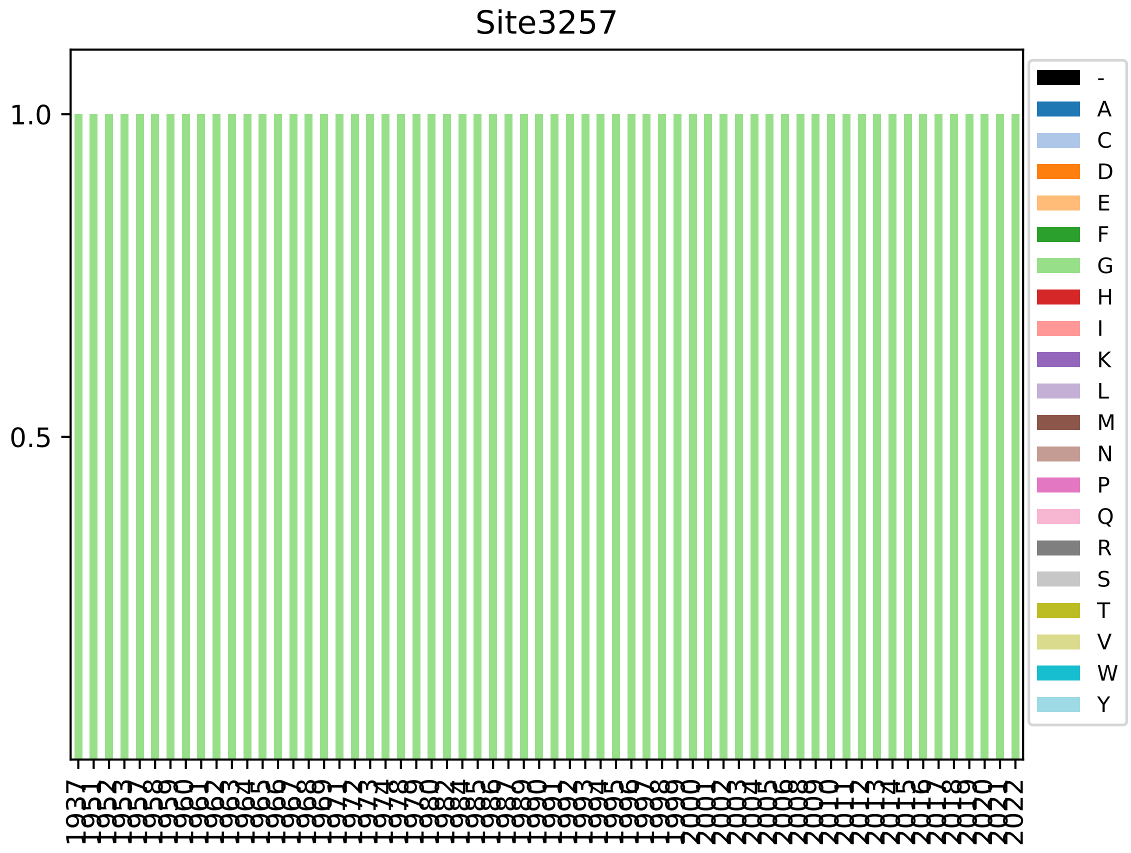
Site3257
1.0
0.5
-
A
C
D
E
F
G
H
I
K
L
M
N
P
Q
R
S
T
V
W
Y

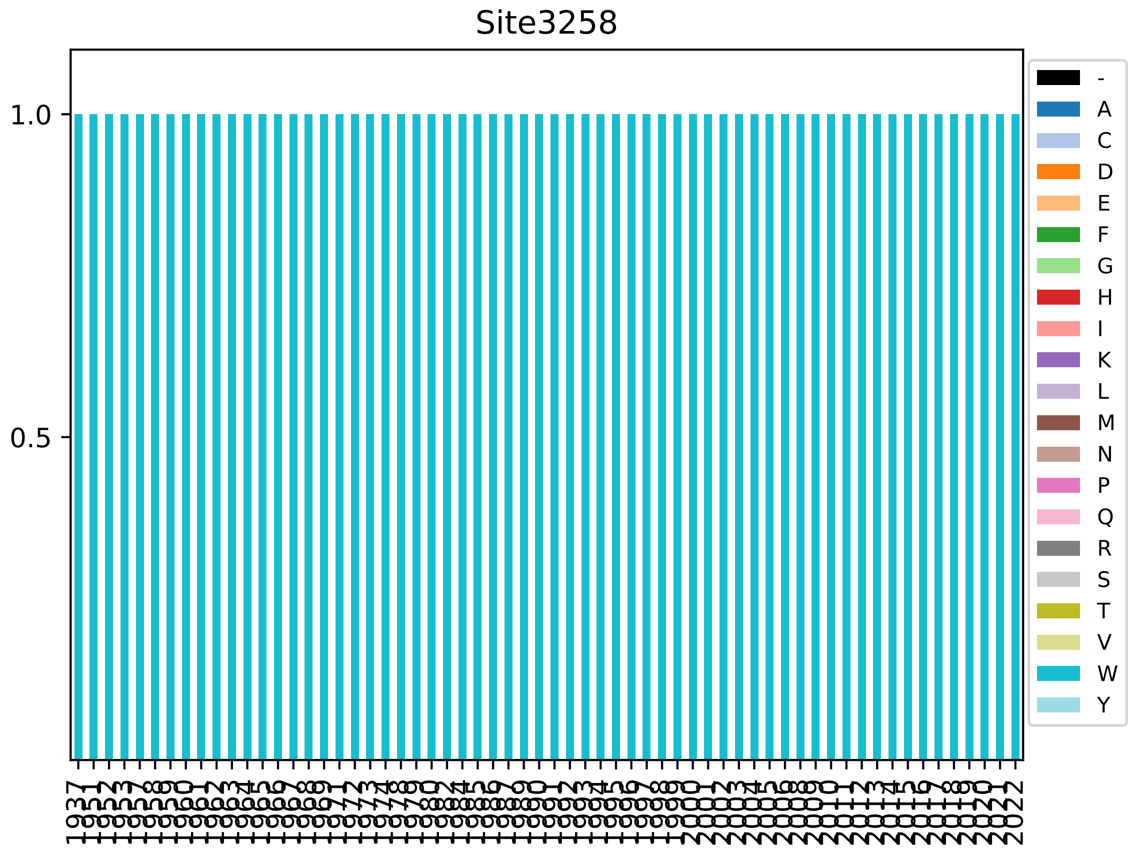
Site3258
1.0
0.5
-
A
C
D
E
F
G
H
I
K
L
M
N
P
Q
R
S
T
V
W
Y

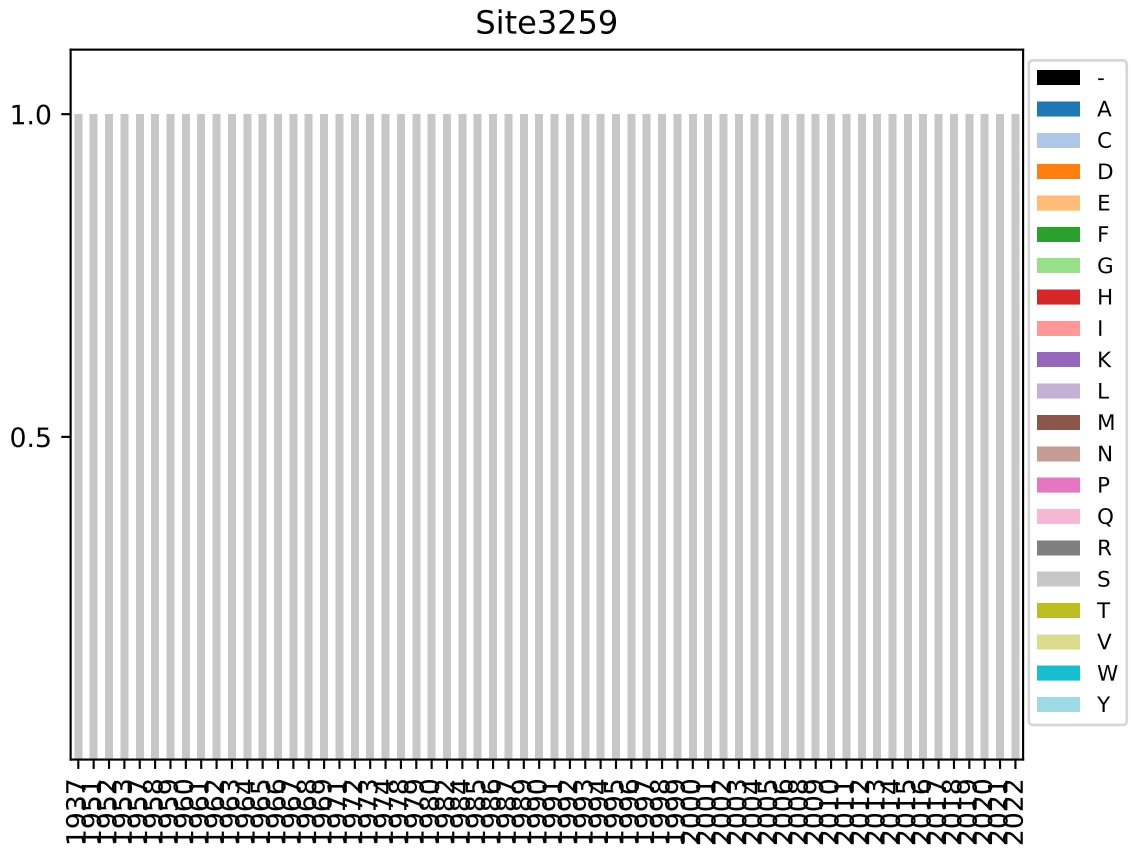
Site3259
1.0
0.5
-
A
C
D
E
F
G
H
I
K
L
M
N
P
Q
R
S
T
V
W
Y

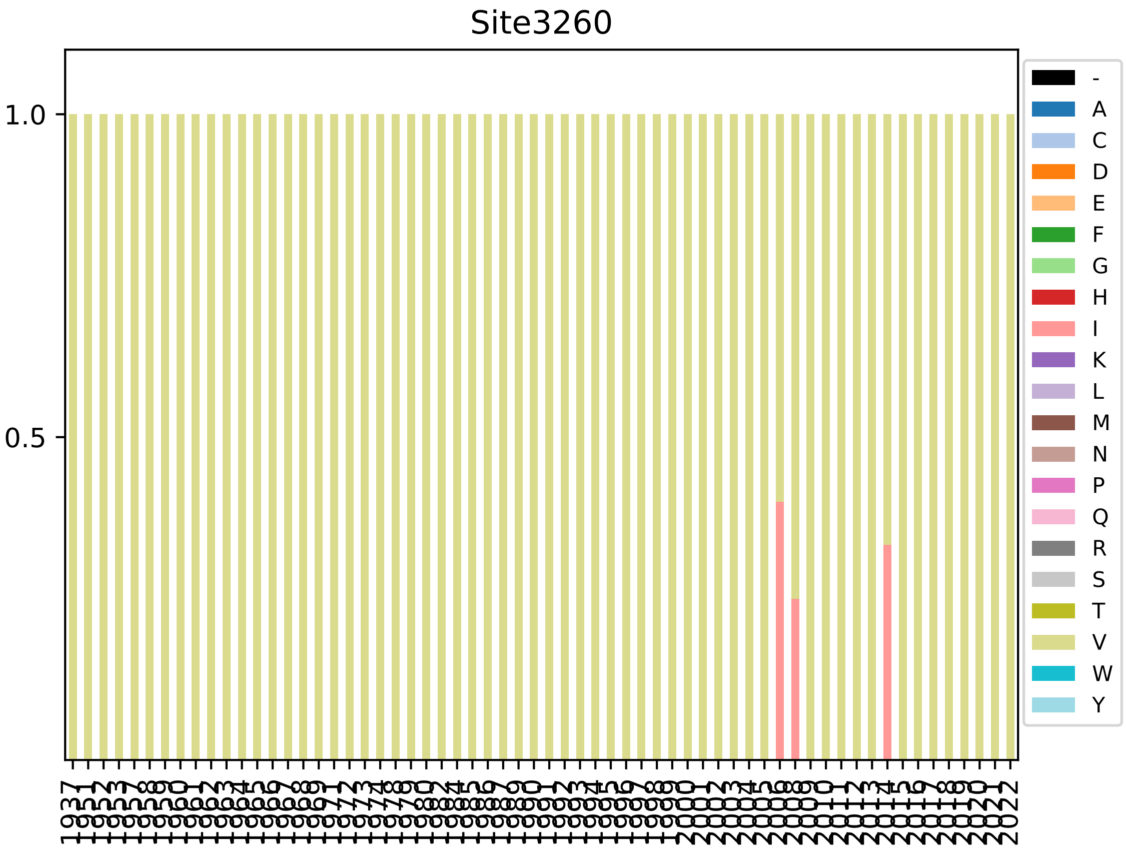
Site3260
1.0
0.5
-
A
C
D
E
F
G
H
I
K
L
M
N
P
Q
R
S
T
V
W
Y

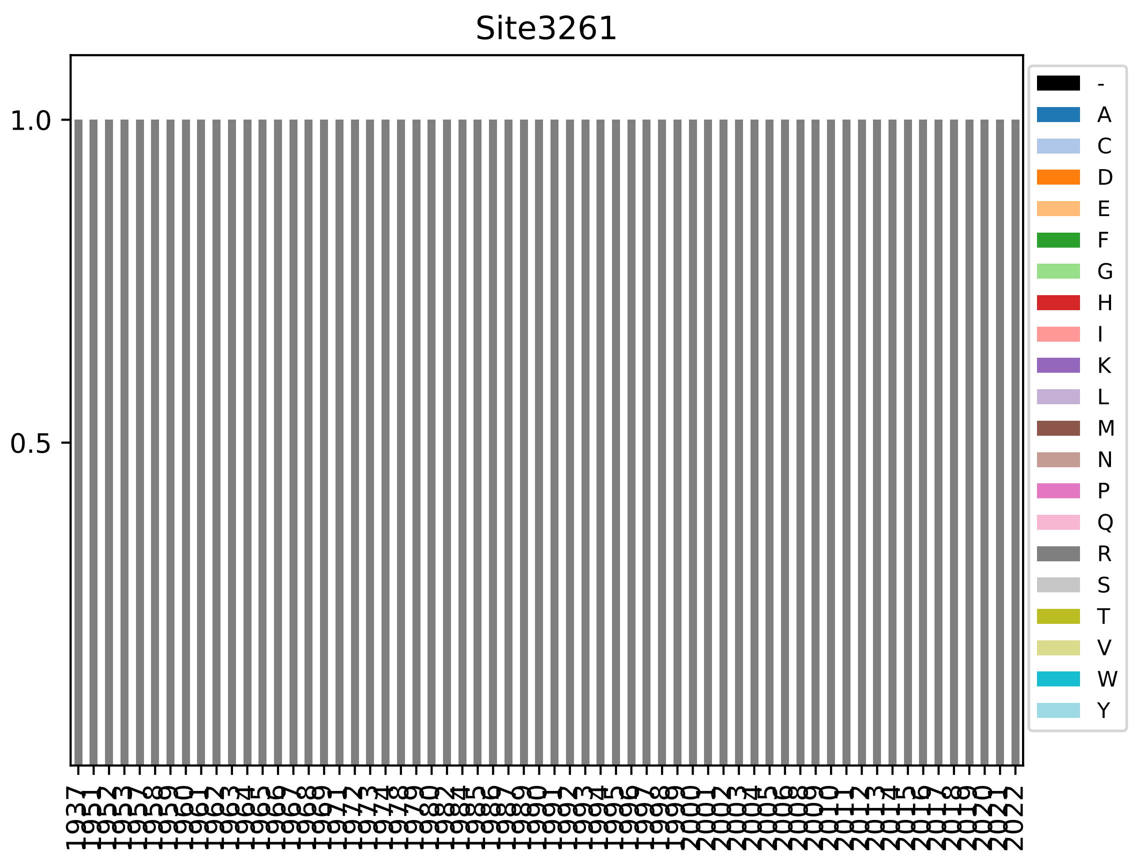
Site3261
1.0
0.5
-
A
C
D
E
F
G
H
I
K
L
M
N
P
Q
R
S
T
V
W
Y

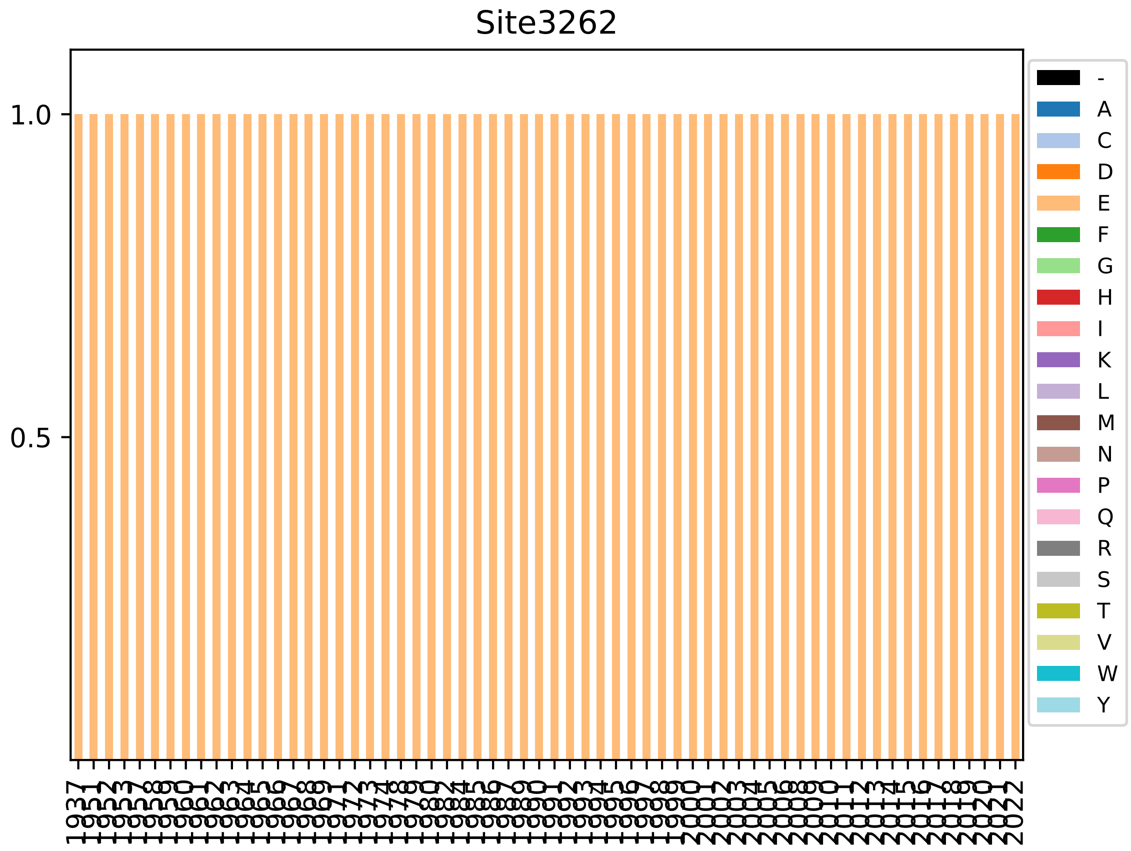
Site3262
1.0
0.5
-
A
C
D
E
F
G
H
I
K
L
M
N
P
Q
R
S
T
V
W
Y

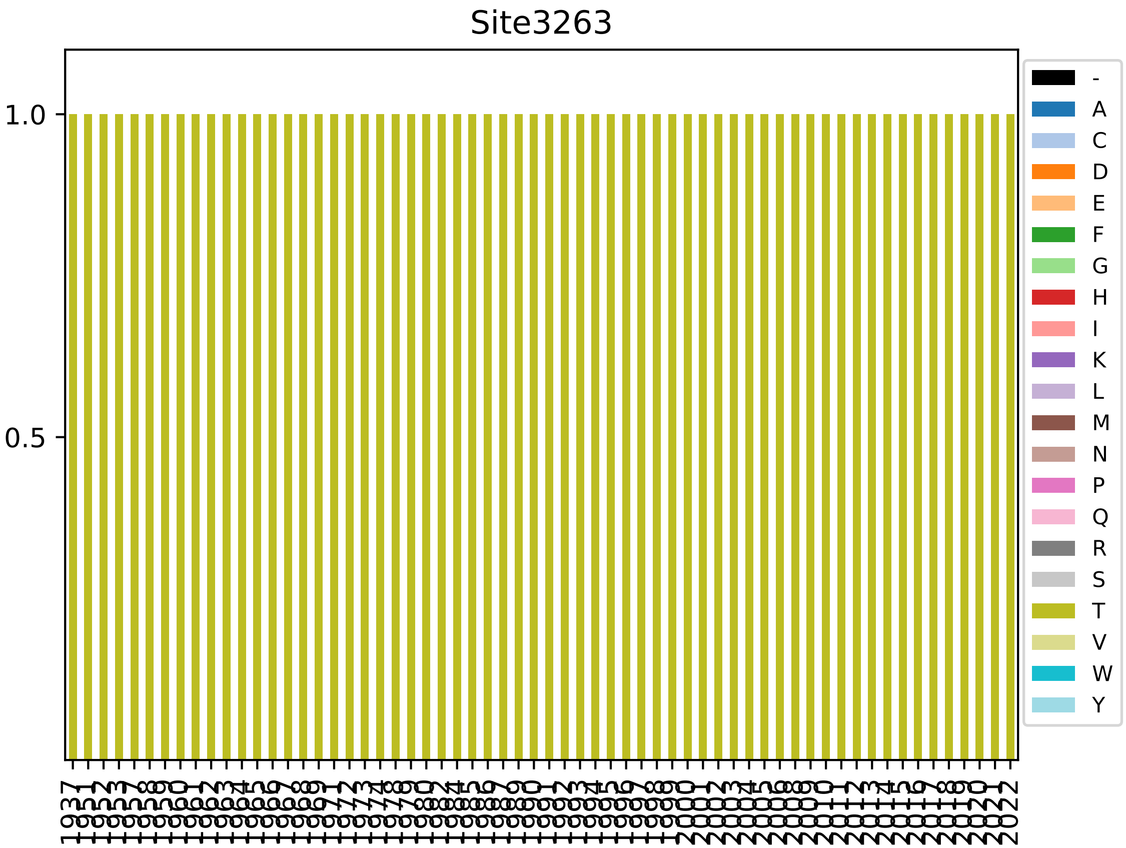
Site3263
1.0
0.5
-
A
C
D
E
F
G
H
I
K
L
M
N
P
Q
R
S
T
V
W
Y

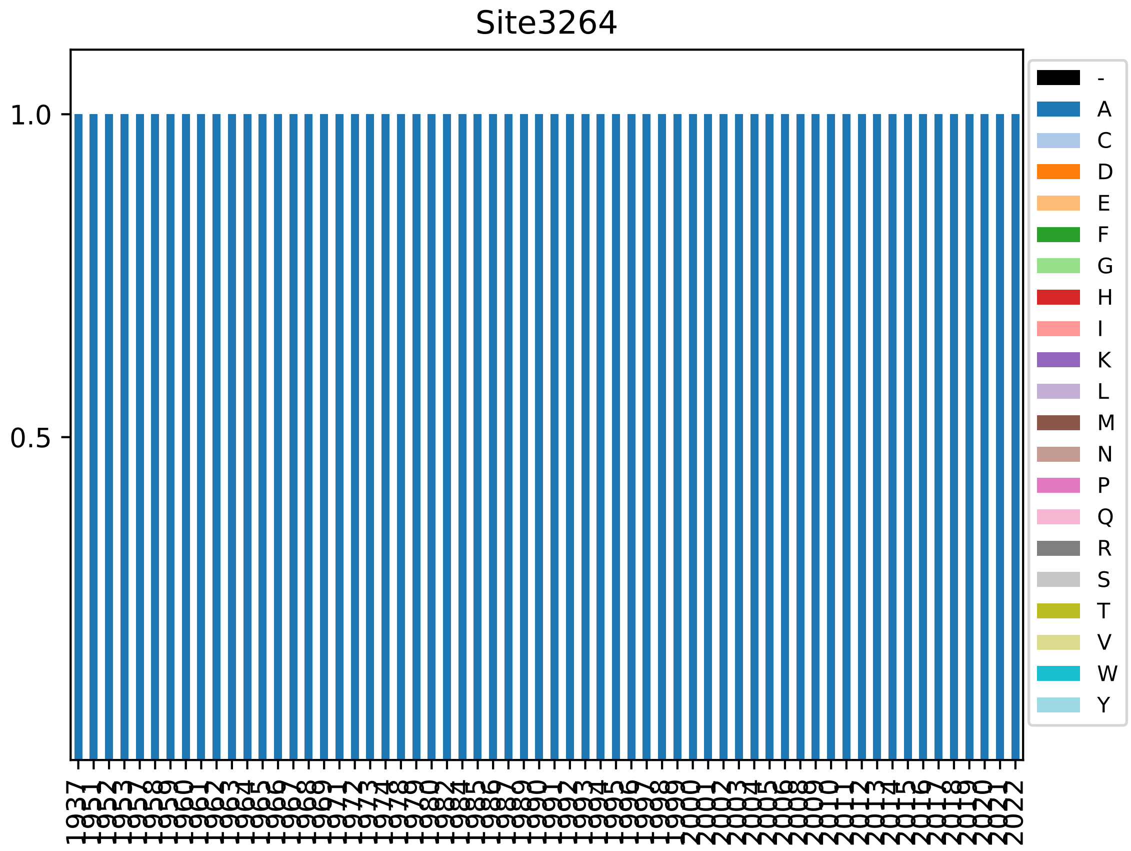
Site3264
1.0
0.5
-
A
C
D
E
F
G
H
I
K
L
M
N
P
Q
R
S
T
V
W
Y

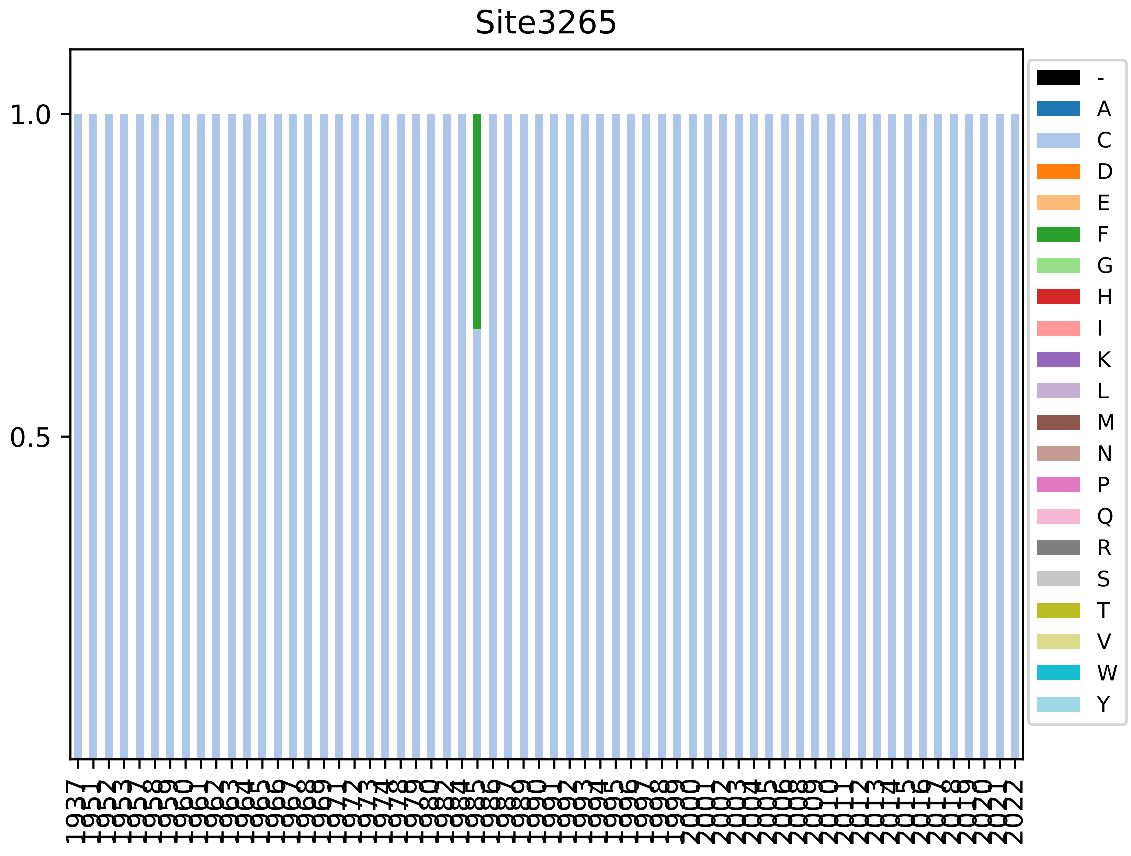
Site3265
1.0
0.5
-
A
C
D
E
F
G
H
I
K
L
M
N
P
Q
R
S
T
V
W
Y

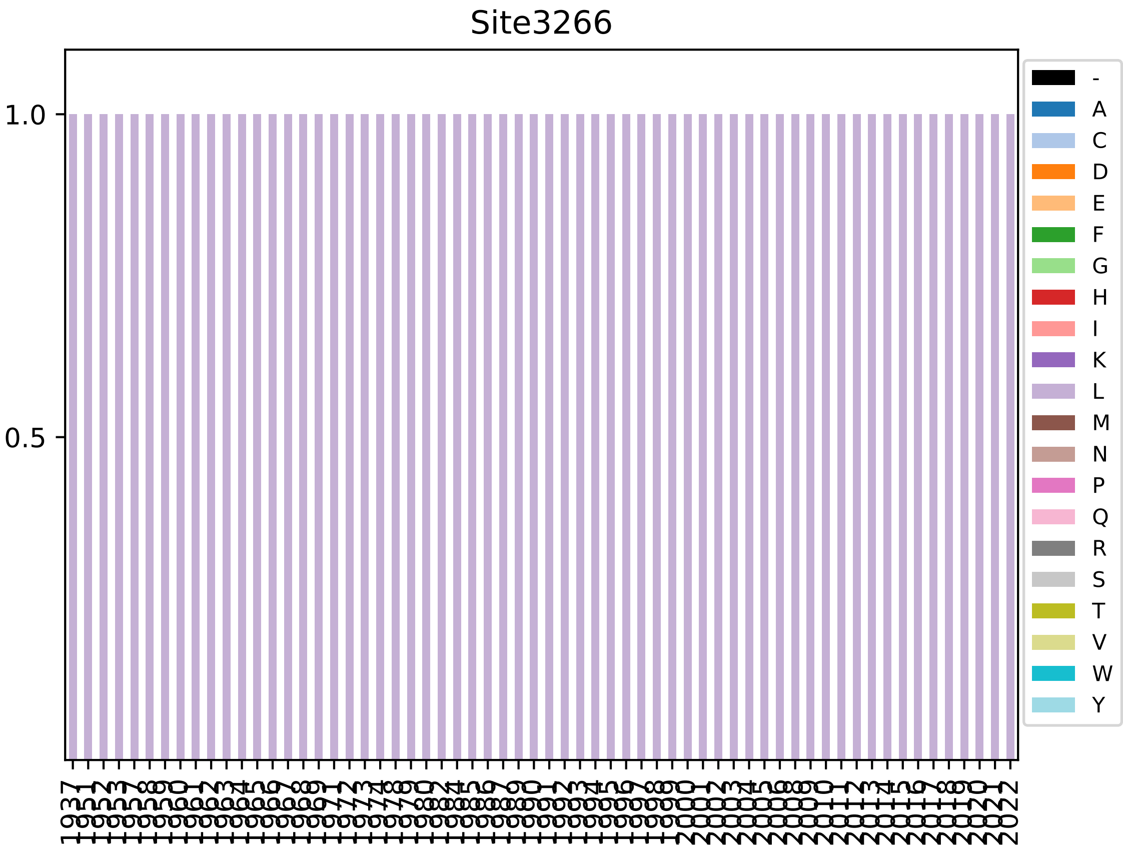
Site3266
1.0
0.5
-
A
C
D
E
F
G
H
I
K
L
M
N
P
Q
R
S
T
V
W
Y

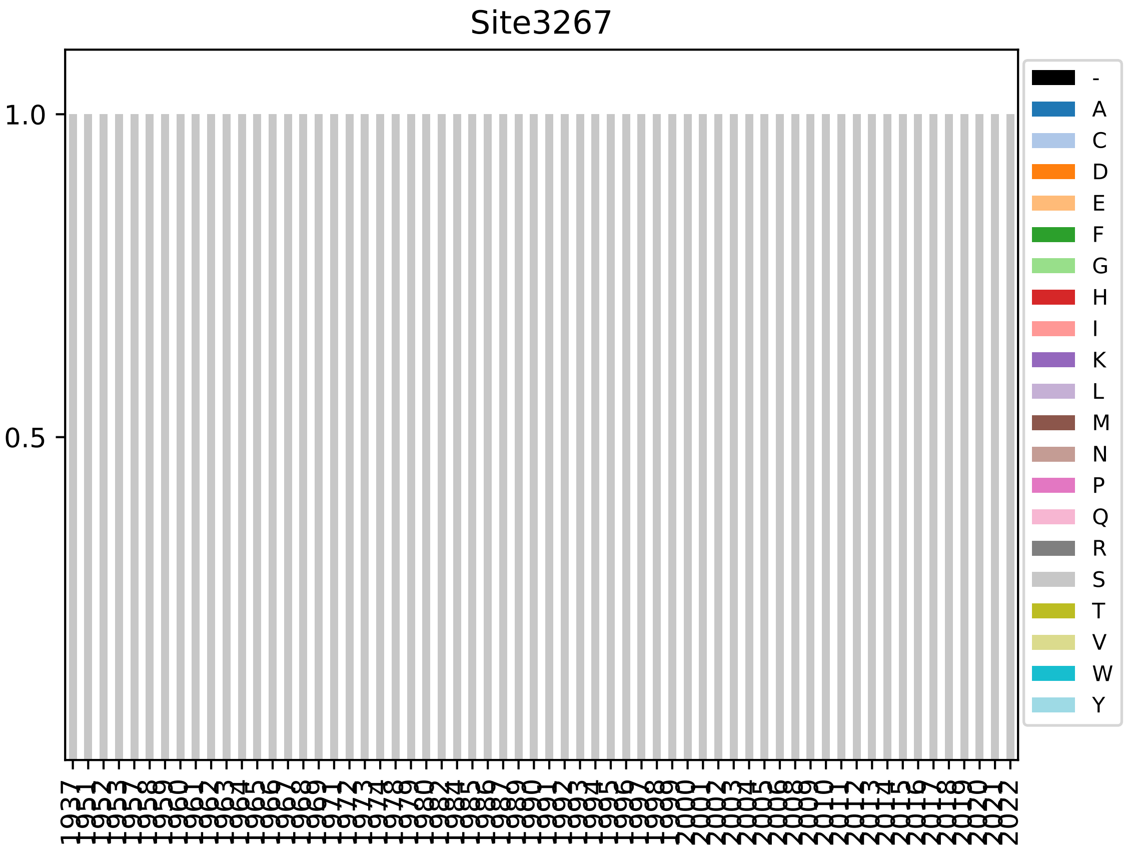
Site3267
1.0
0.5
-
A
C
D
E
F
G
H
I
K
L
M
N
P
Q
R
S
T
V
W
Y

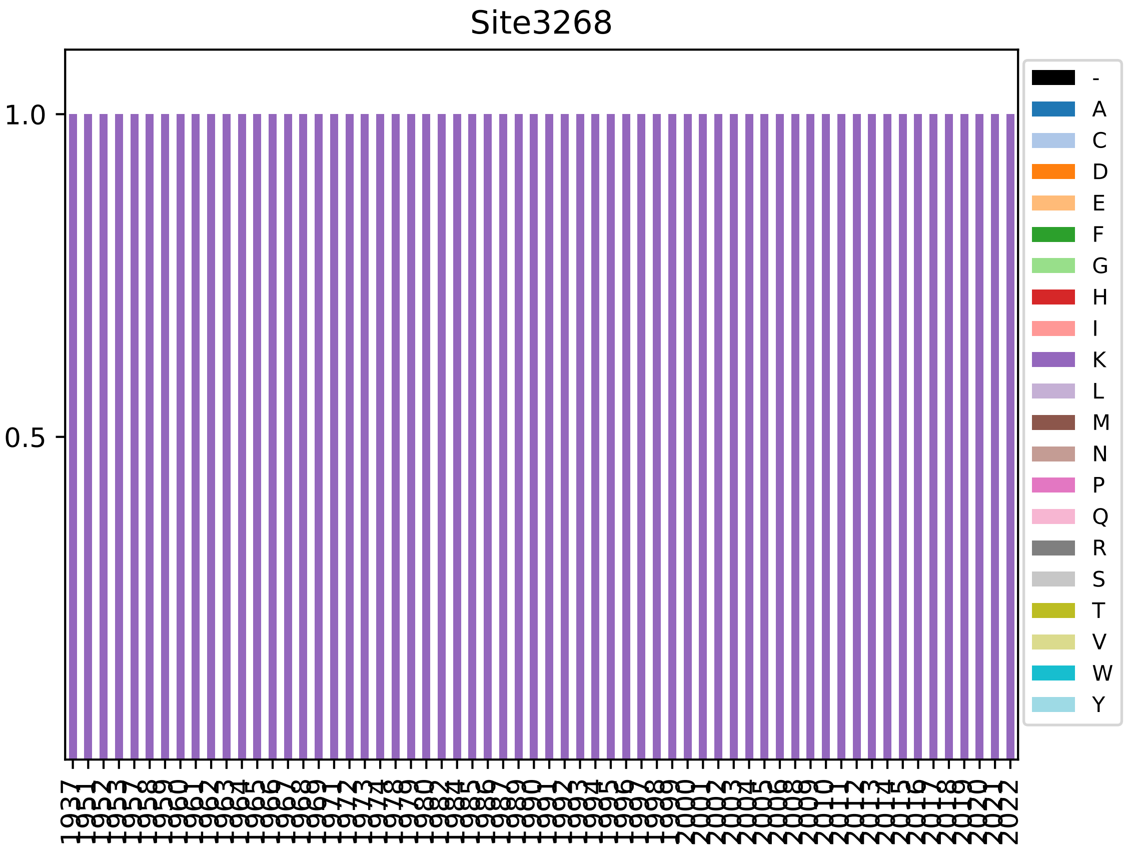
Site3268
1.0
0.5
-
A
C
D
E
F
G
H
I
K
L
M
N
P
Q
R
S
T
V
W
Y

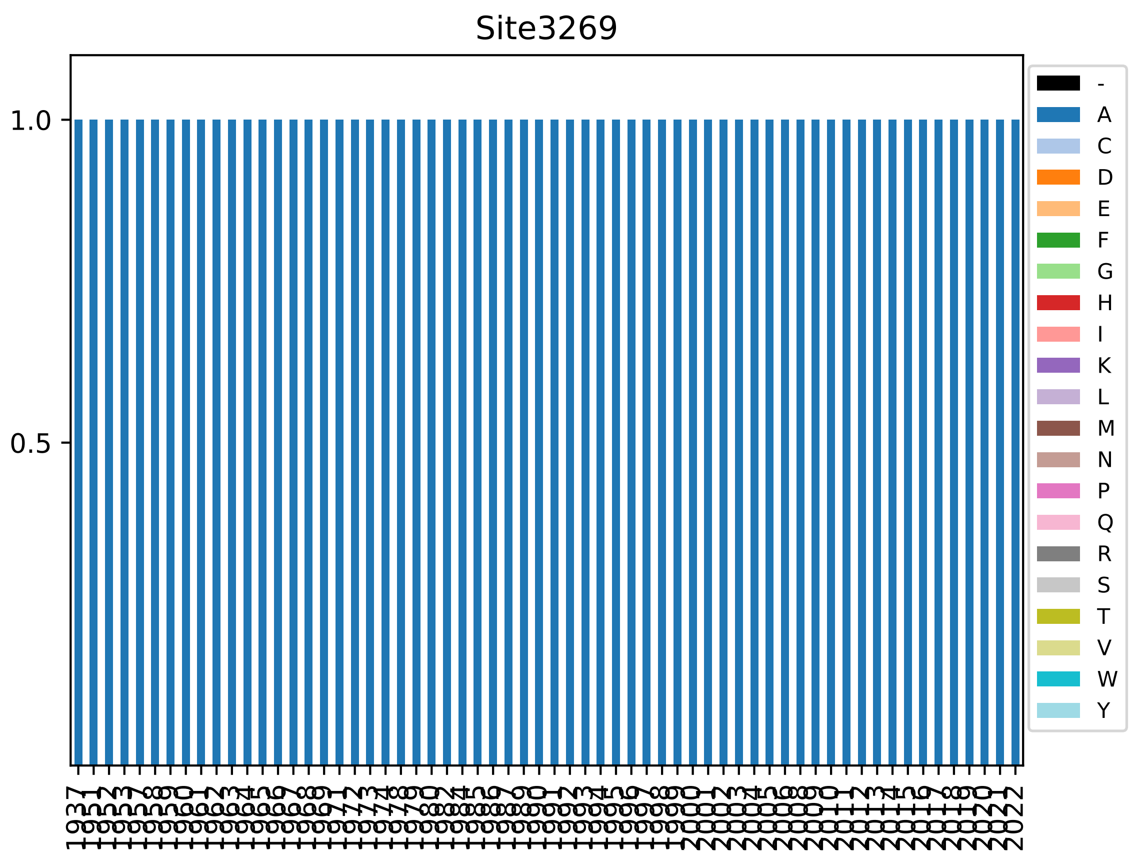
Site3269
1.0
0.5
-
A
C
D
E
F
G
H
I
K
L
M
N
P
Q
R
S
T
V
W
Y

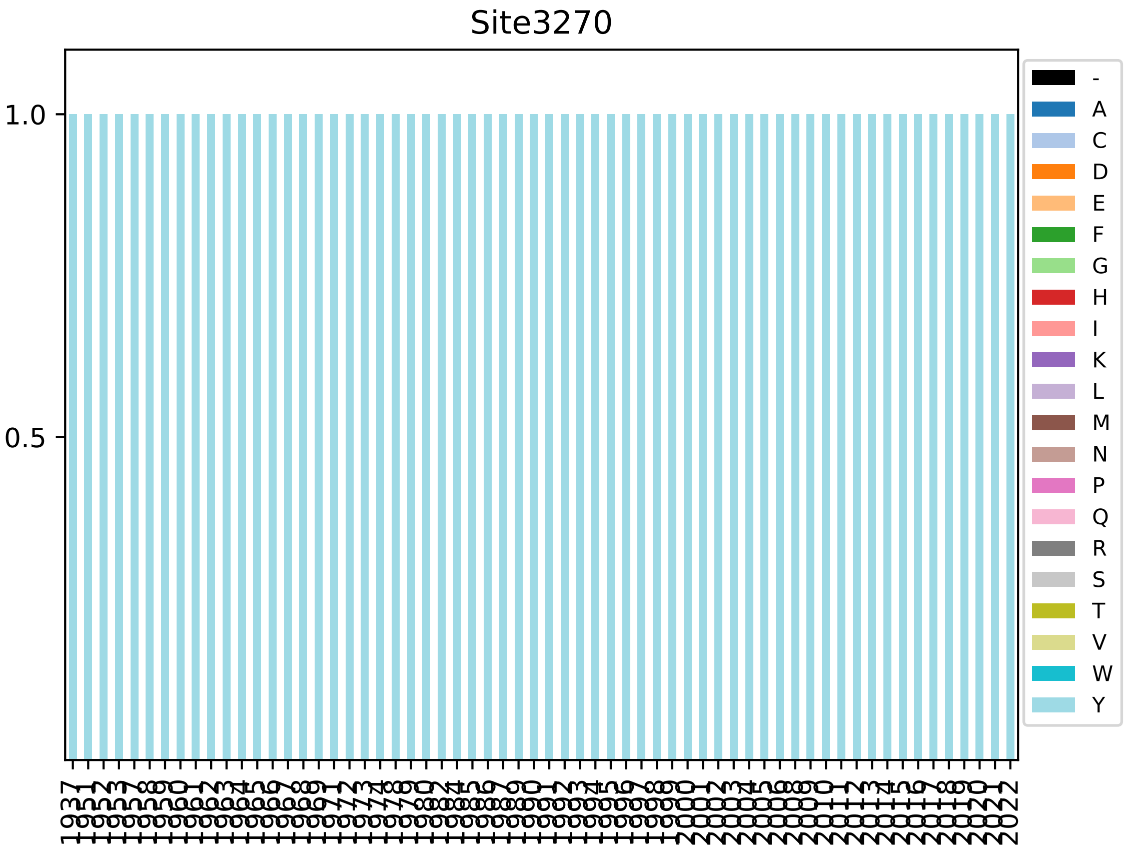

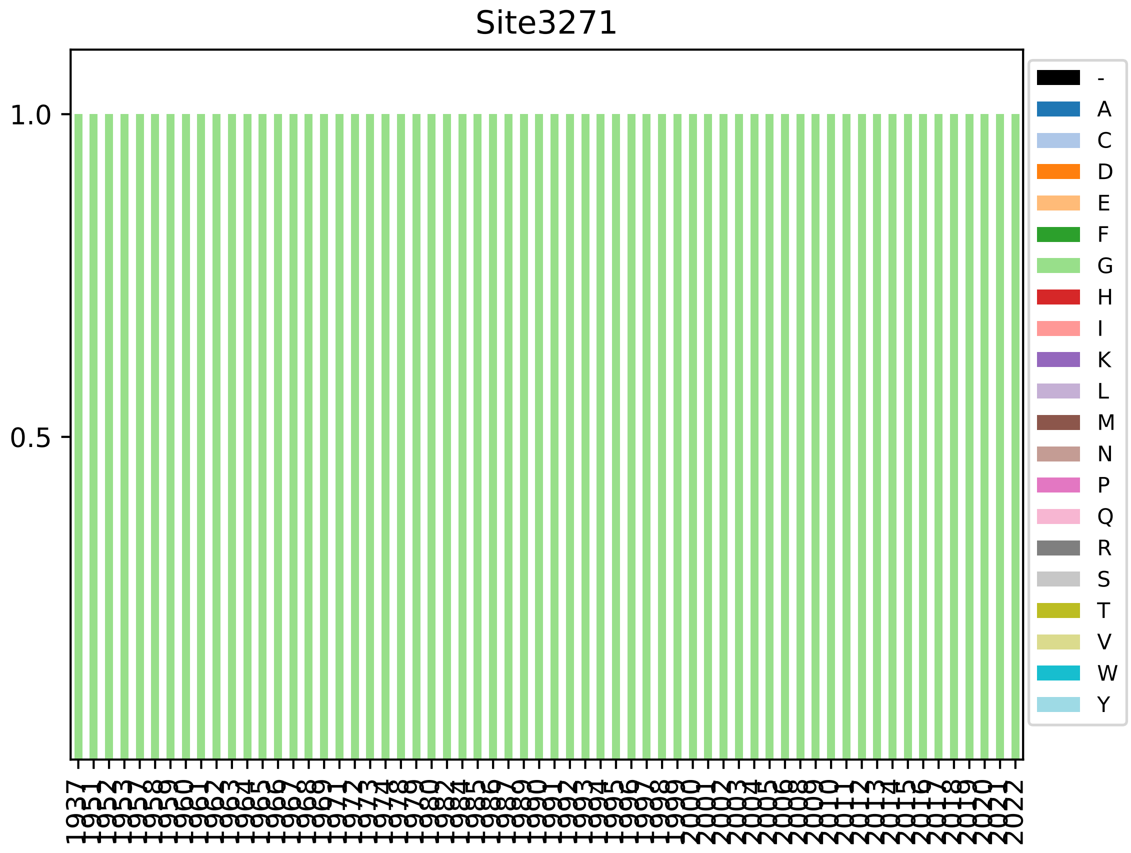
Site3271
1.0
0.5
-
A
C
D
E
F
G
H
I
K
L
M
N
P
Q
R
S
T
V
W
Y

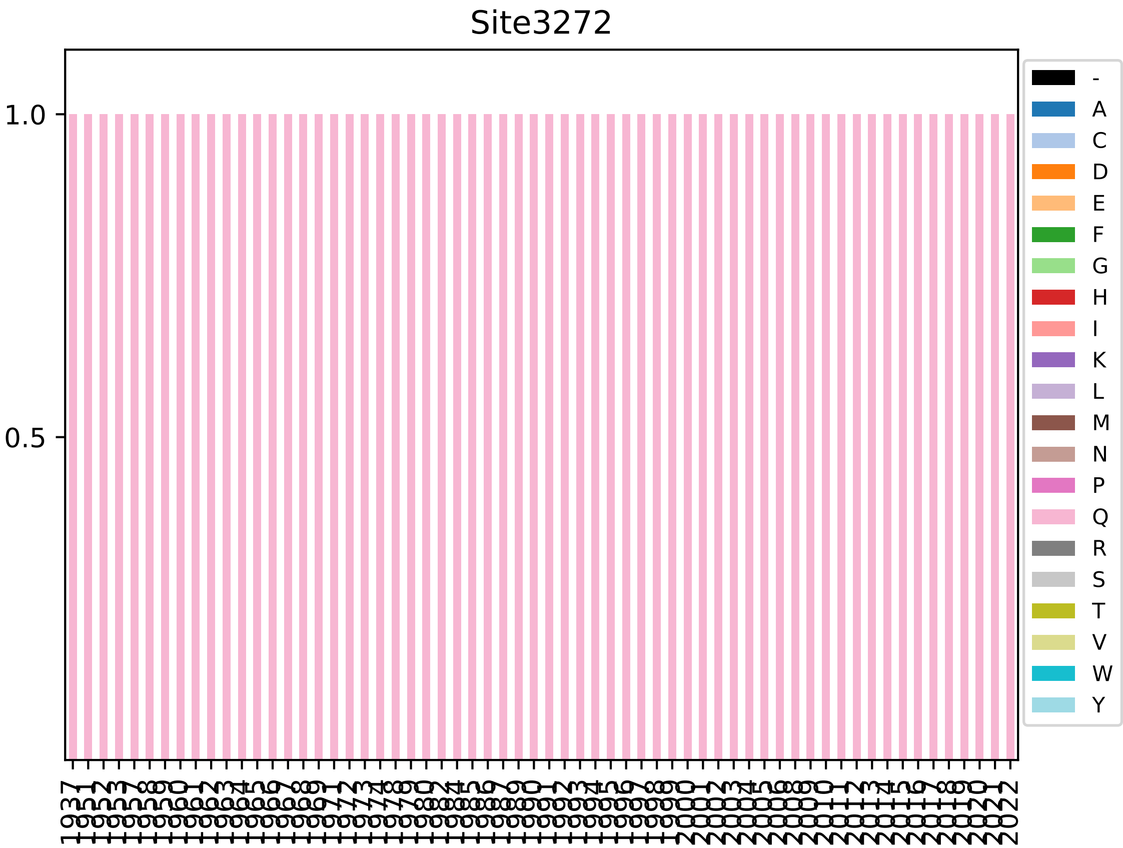
Site3272
1.0
0.5
-
A
C
D
E
F
G
H
I
K
L
M
N
P
Q
R
S
T
V
W
Y

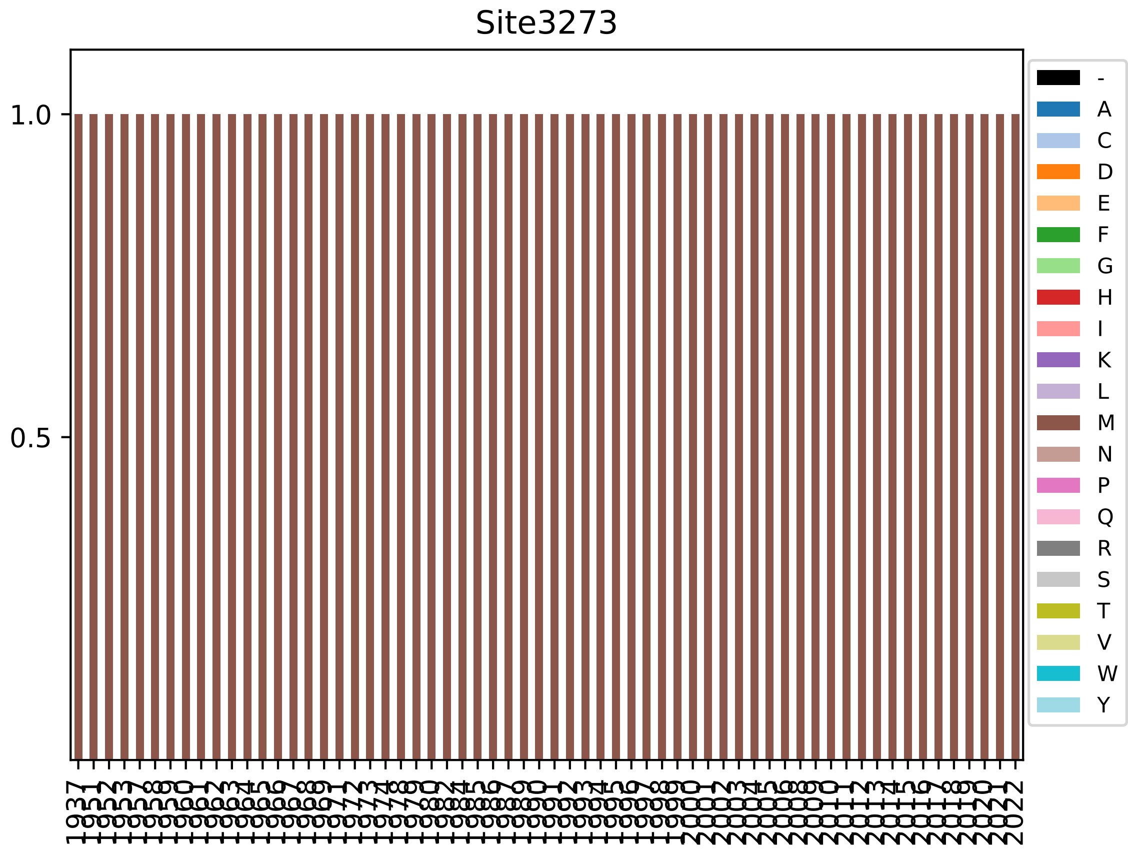
Site3273
1.0
0.5
-
A
C
D
E
F
G
H
I
K
L
M
N
P
Q
R
S
T
V
W
Y

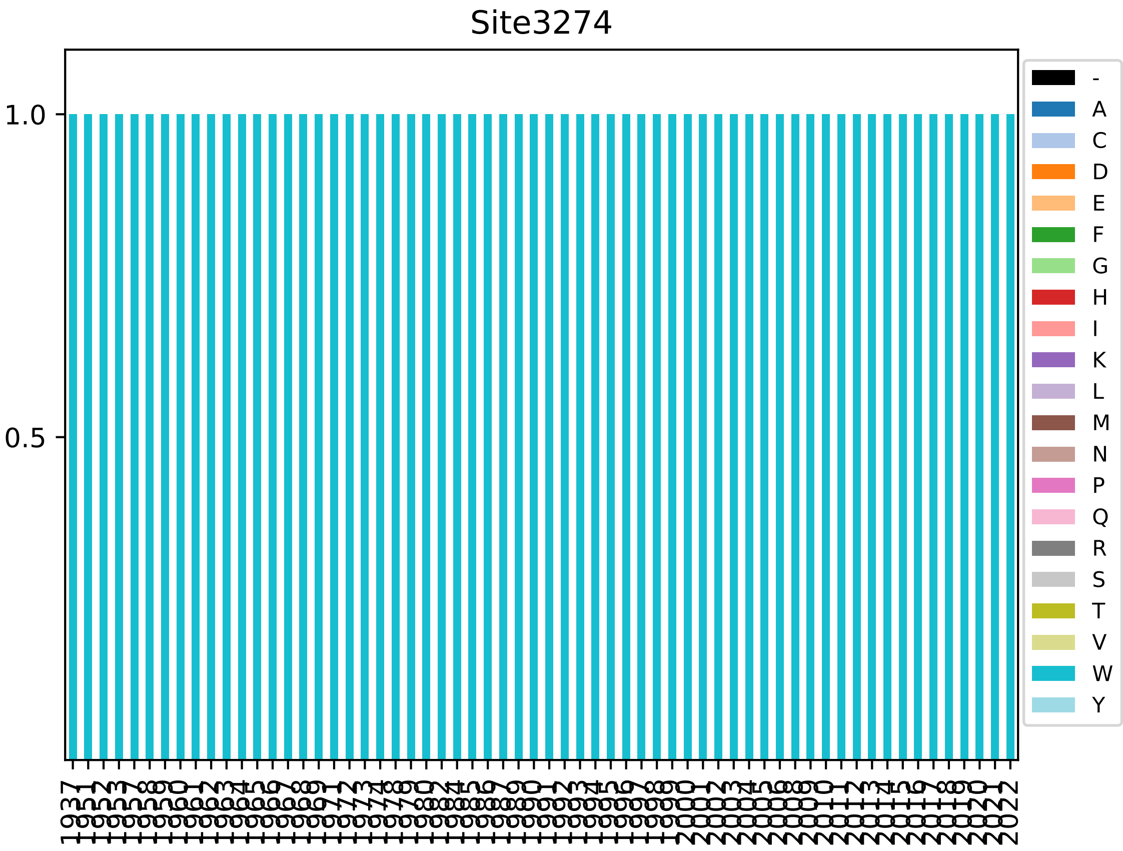
Site3274
1.0
0.5
-
A
C
D
E
F
G
H
I
K
L
M
N
P
Q
R
S
T
V
W
Y

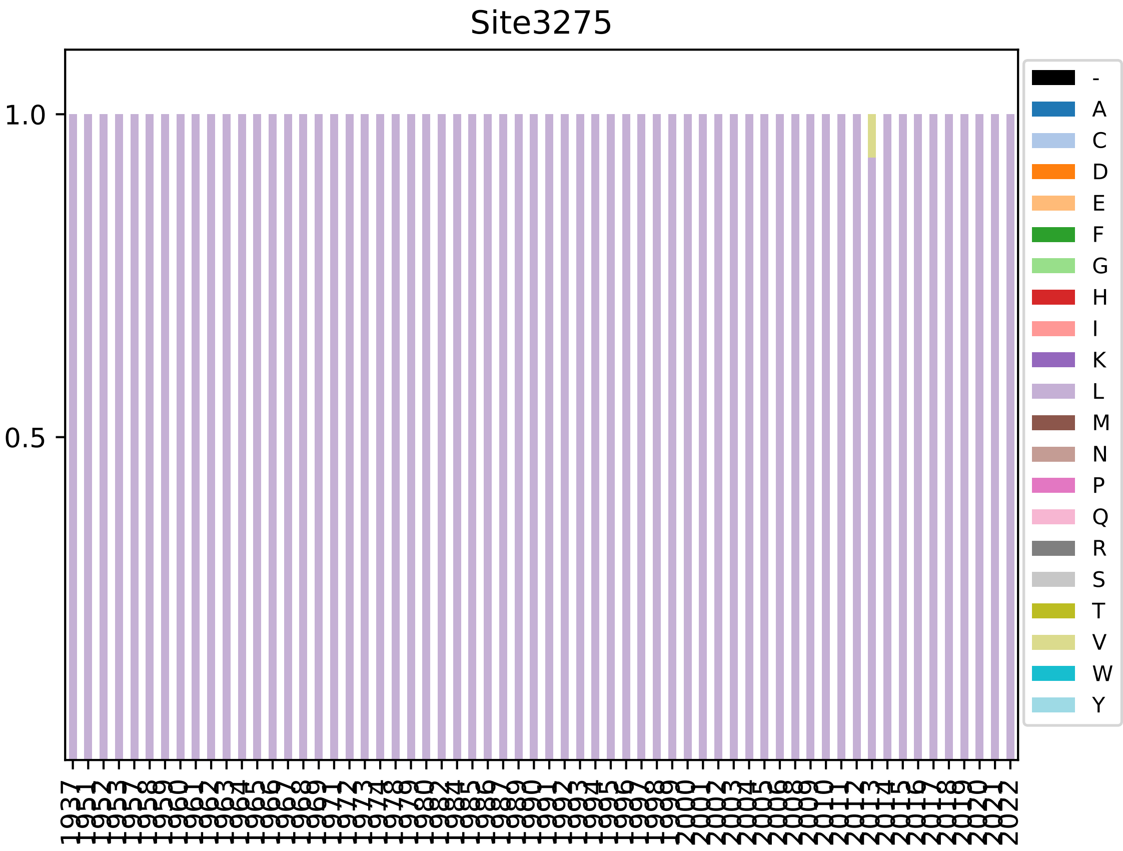
Site3275
1.0
0.5
-
A
C
D
E
F
G
H
I
K
L
M
N
P
Q
R
S
T
V
W
Y

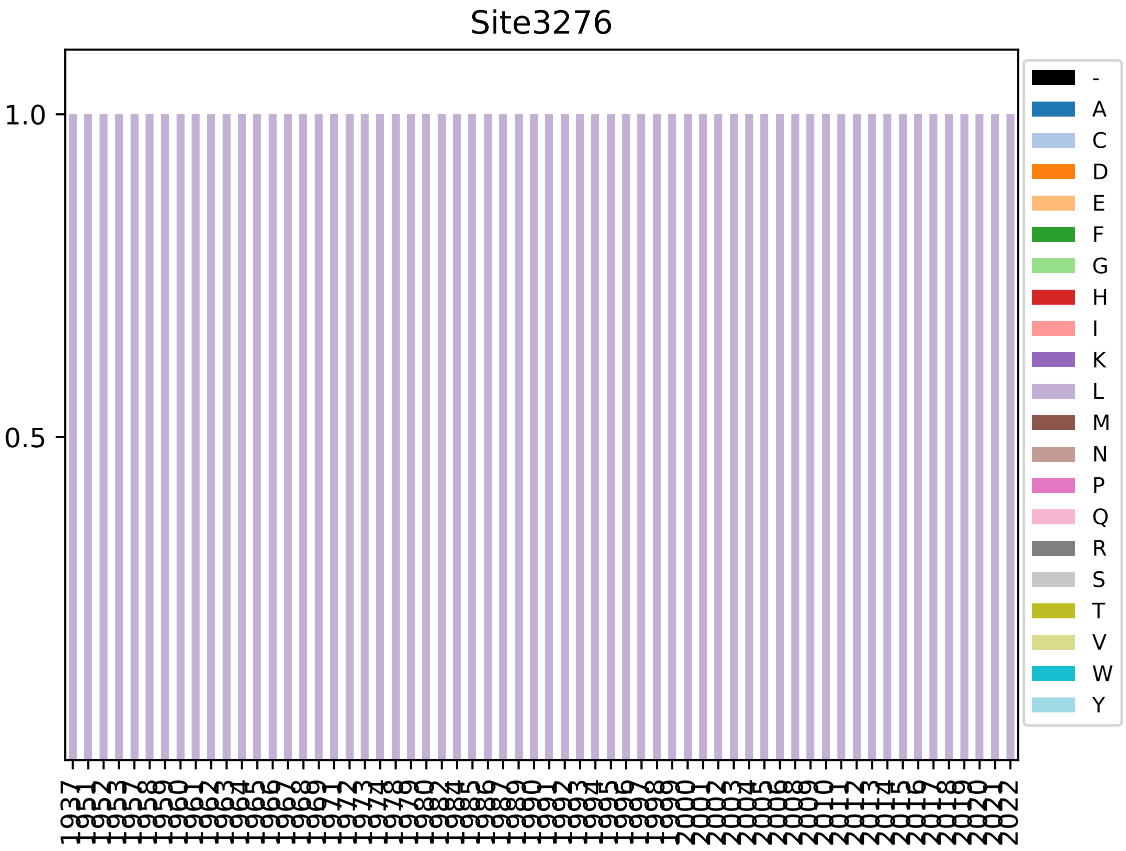
Site3276
1.0
0.5
-
A
C
D
E
F
G
H
I
K
L
M
N
P
Q
R
S
T
V
W
Y

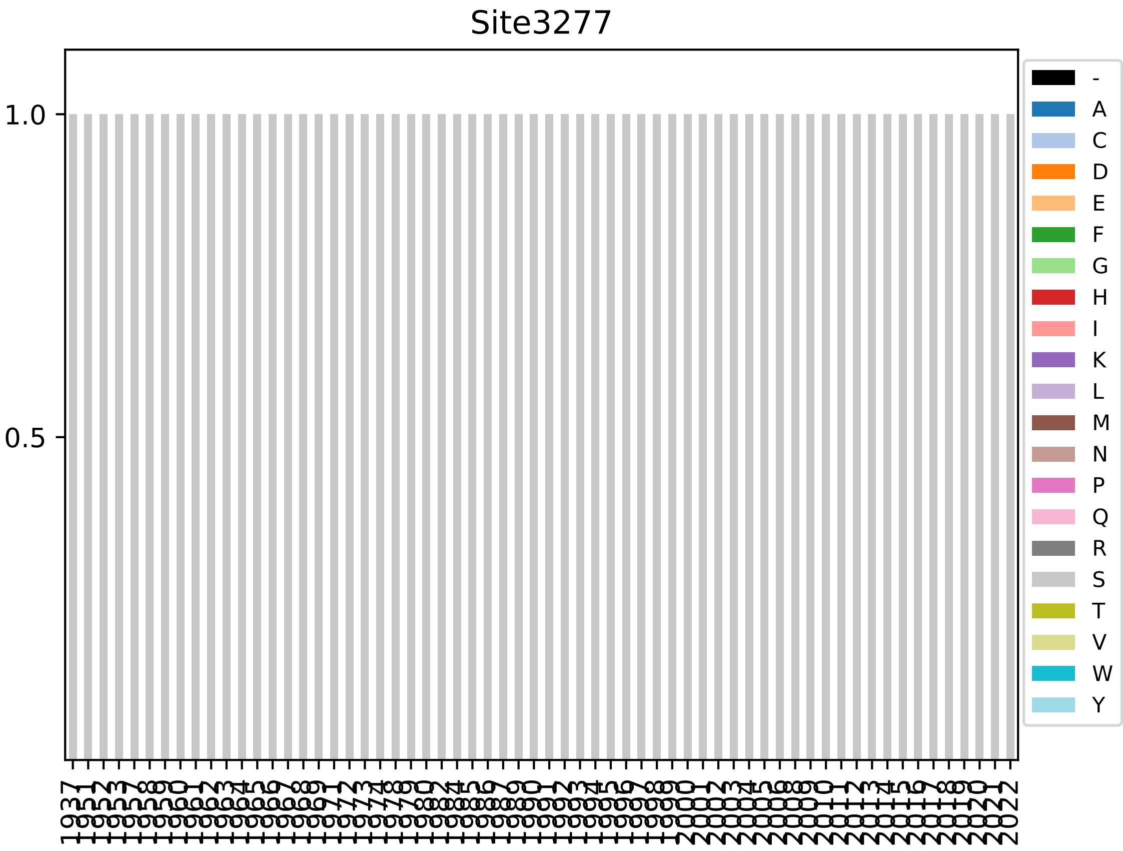
Site3277
1.0
0.5
-
A
C
D
E
F
G
H
I
K
L
M
N
P
Q
R
S
T
V
W
Y

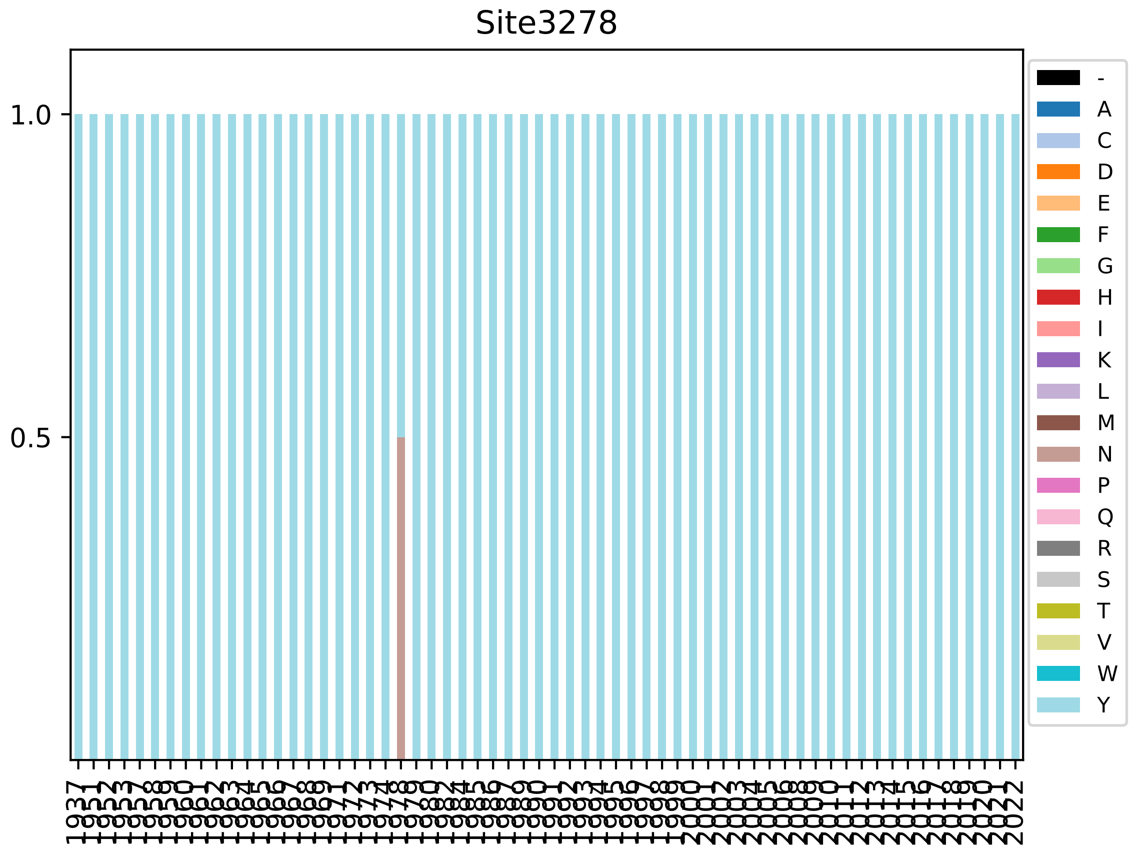
Site3278
1.0
0.5
-
A
C
D
E
F
G
H
I
K
L
M
N
P
Q
R
S
T
V
W
Y

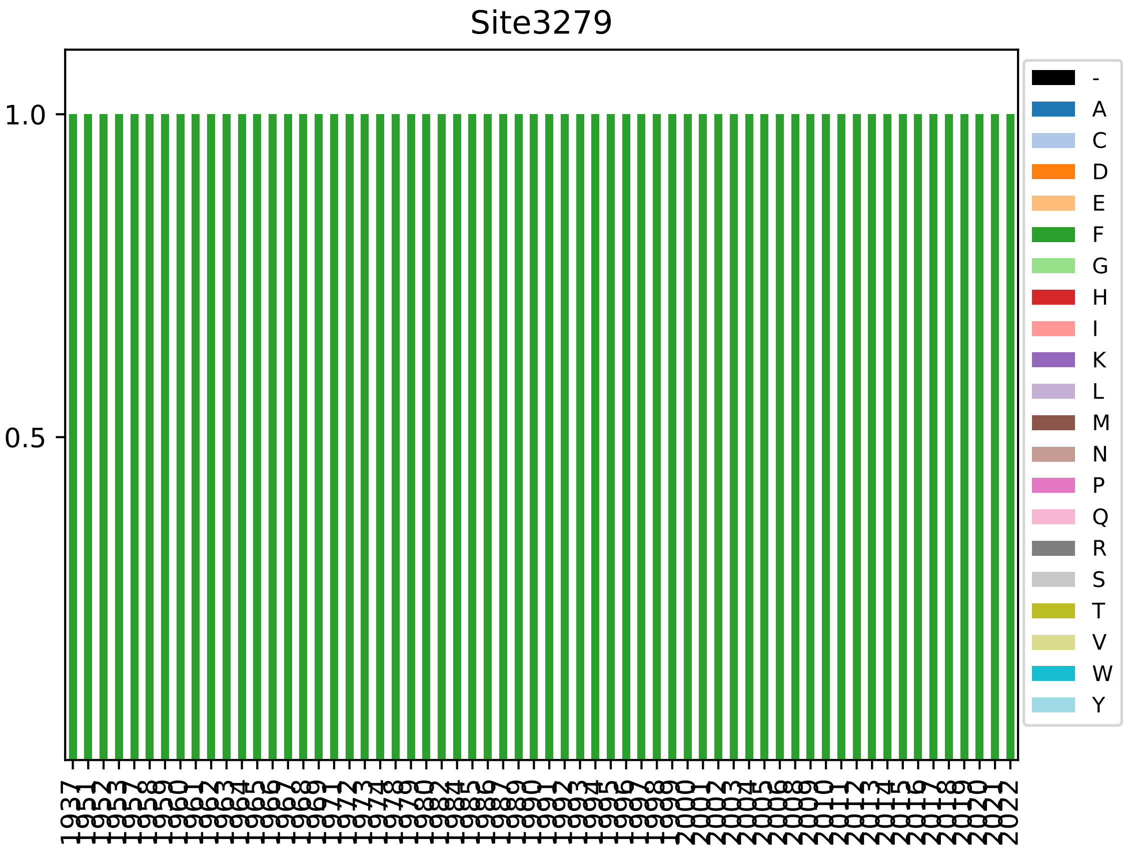
Site3279
1.0
0.5
-
A
C
D
E
F
G
H
I
K
L
M
N
P
Q
R
S
T
V
W
Y

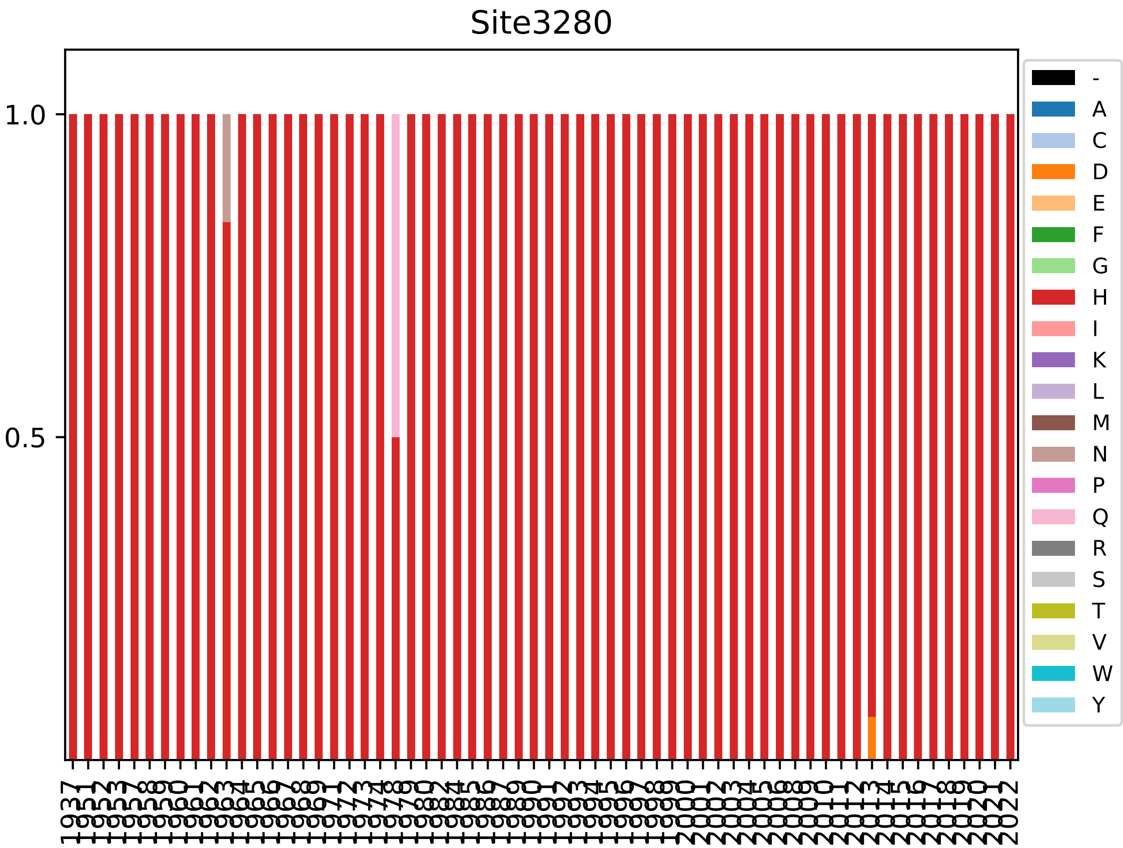
Site3280
1.0
0.5
-
A
C
D
E
F
G
H
I
K
L
M
N
P
Q
R
S
T
V
W
Y

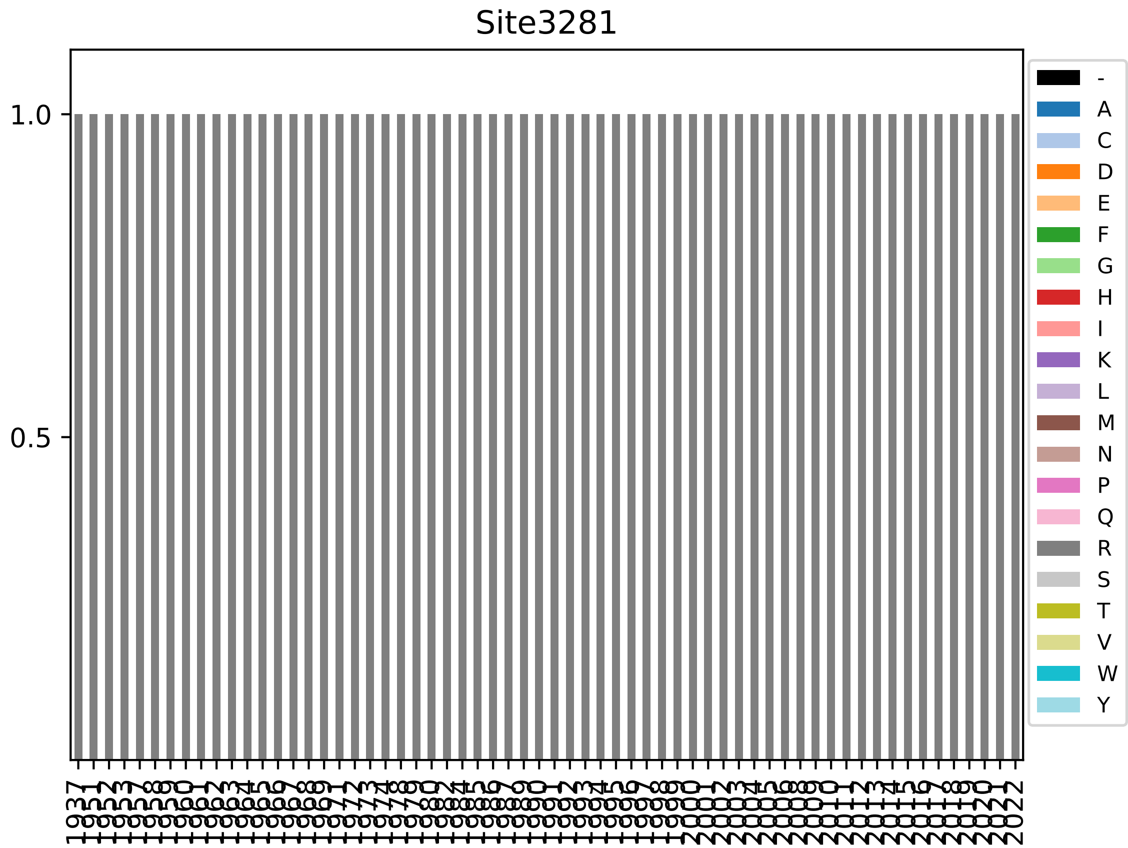
Site3281
1.0
0.5
-
A
C
D
E
F
G
H
I
K
L
M
N
P
Q
R
S
T
V
W
Y

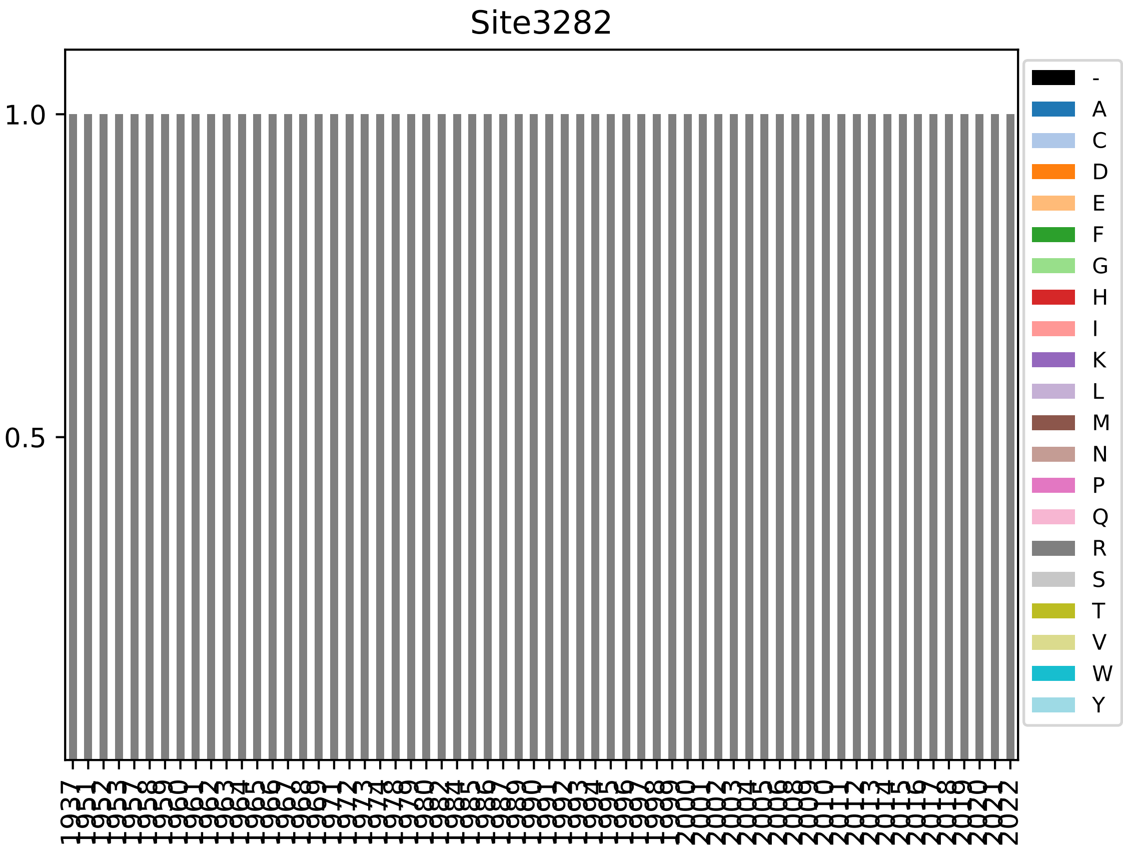
Site3282
1.0
0.5
-
A
C
D
E
F
G
H
I
K
L
M
N
P
Q
R
S
T
V
W
Y

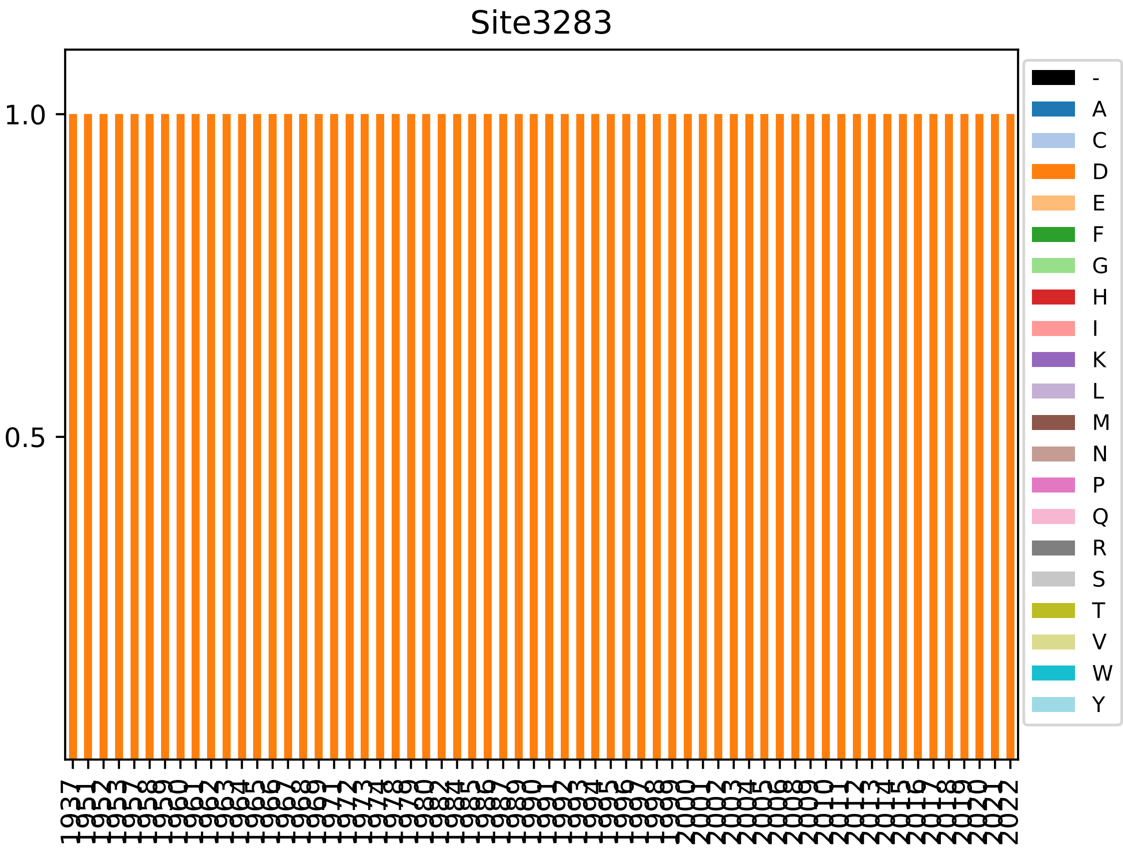
Site3283
1.0
0.5
-
A
C
D
E
F
G
H
I
K
L
M
N
P
Q
R
S
T
V
W
Y

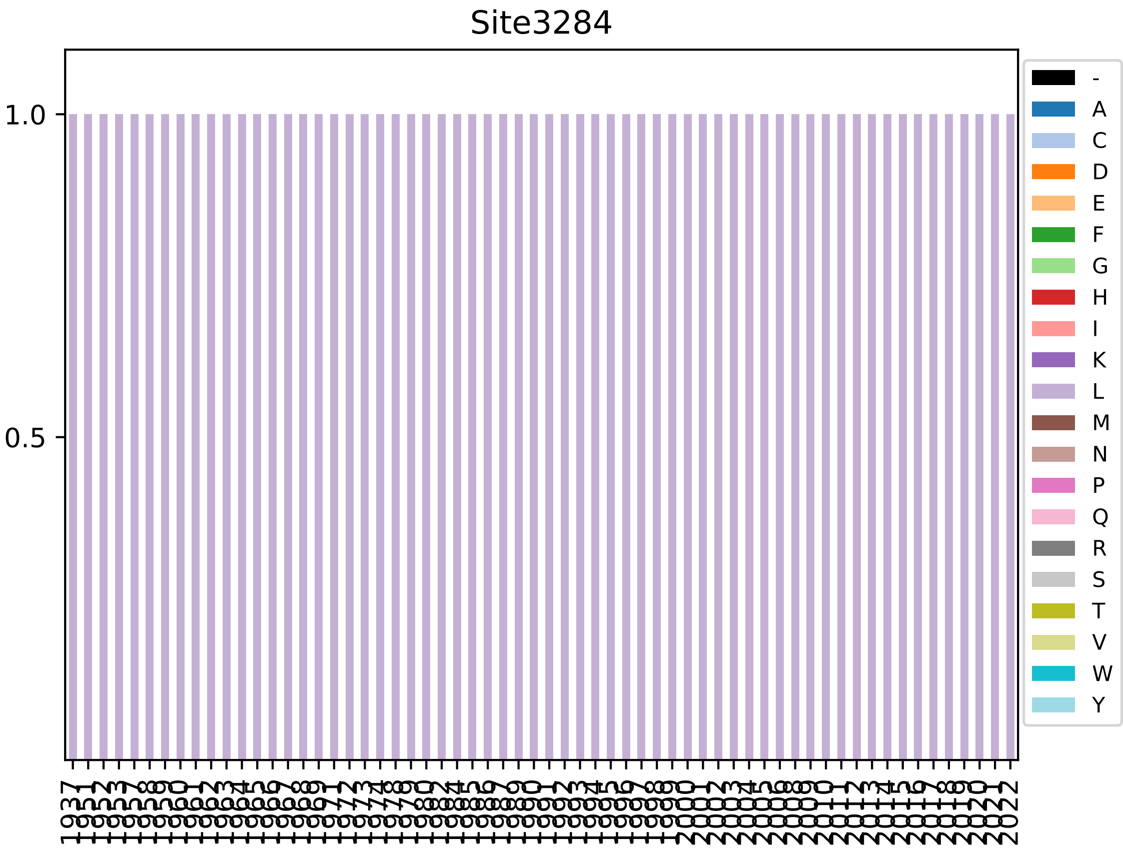
Site3284
1.0
0.5
-
A
C
D
E
F
G
H
I
K
L
M
N
P
Q
R
S
T
V
W
Y

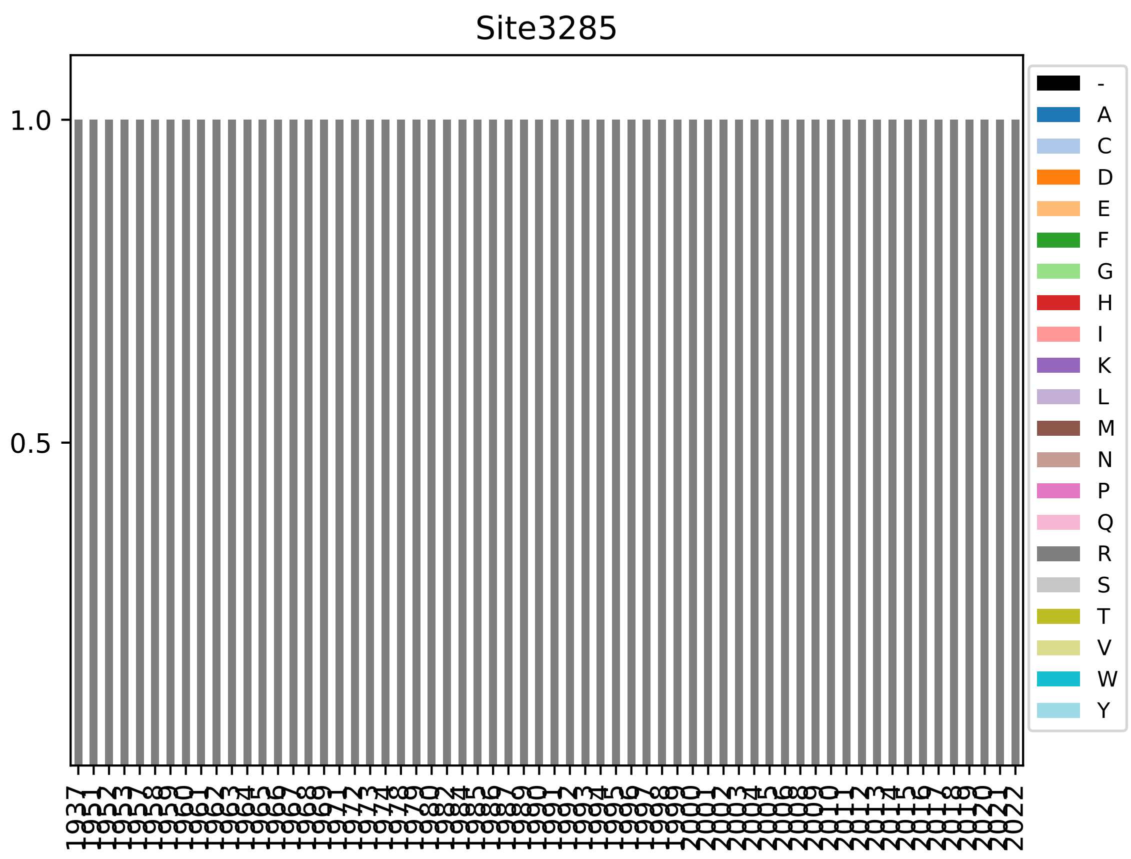
Site3285
1.0
0.5
-
A
C
D
E
F
G
H
I
K
L
M
N
P
Q
R
S
T
V
W
Y

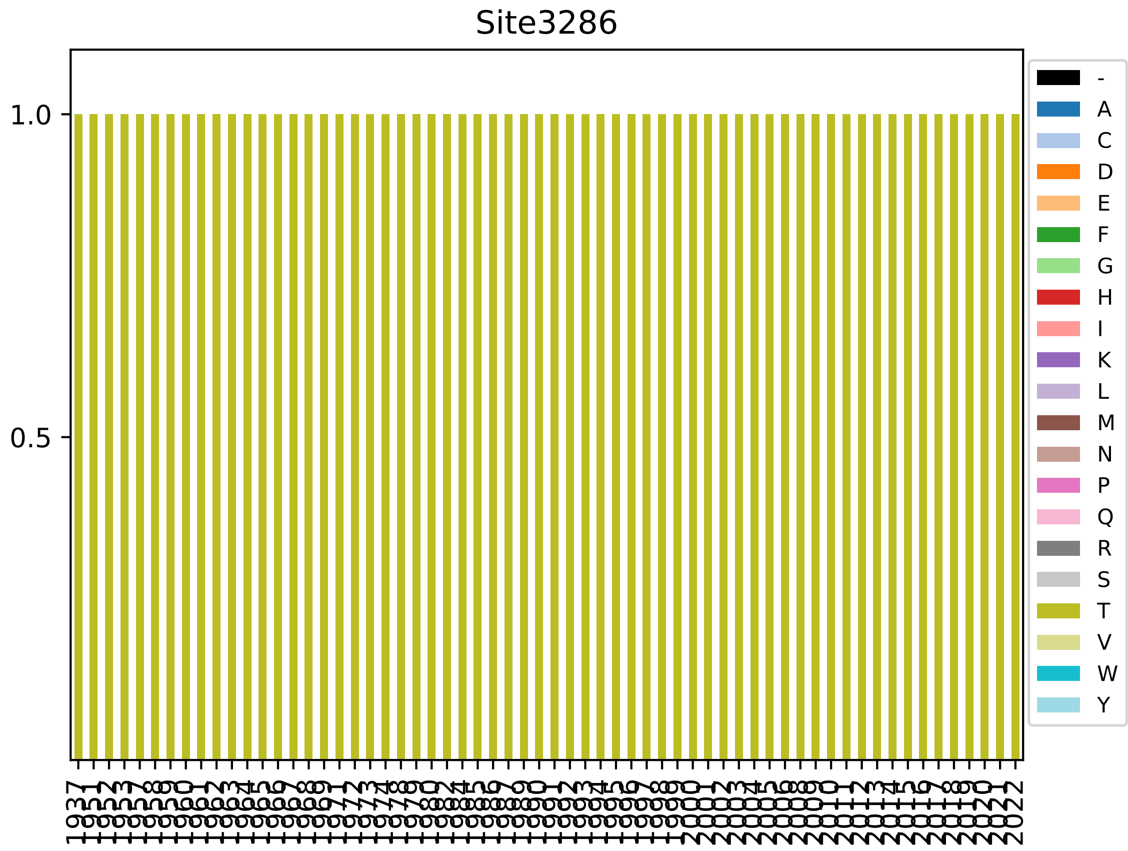
Site3286
1.0
0.5
-
A
C
D
E
F
G
H
I
K
L
M
N
P
Q
R
S
T
V
W
Y

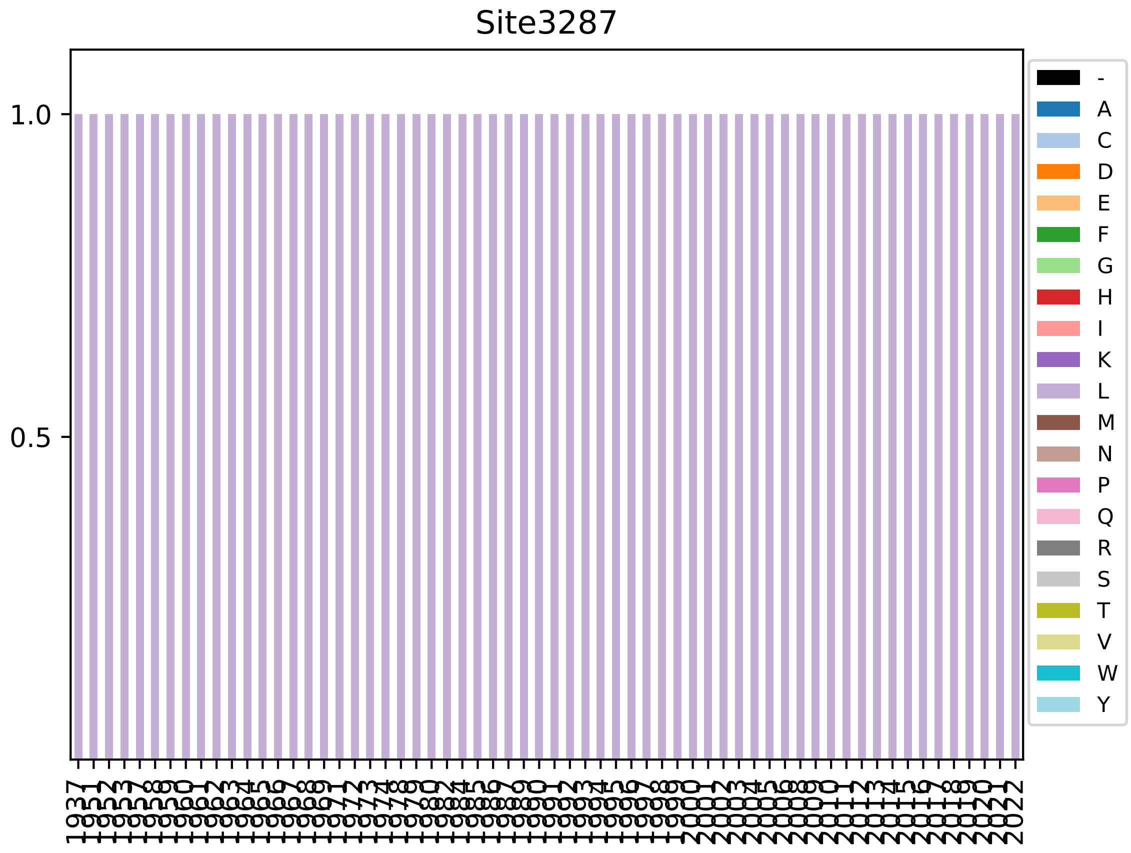
Site3287
1.0
0.5
-
A
C
D
E
F
G
H
I
K
L
M
N
P
Q
R
S
T
V
W
Y

Site3288

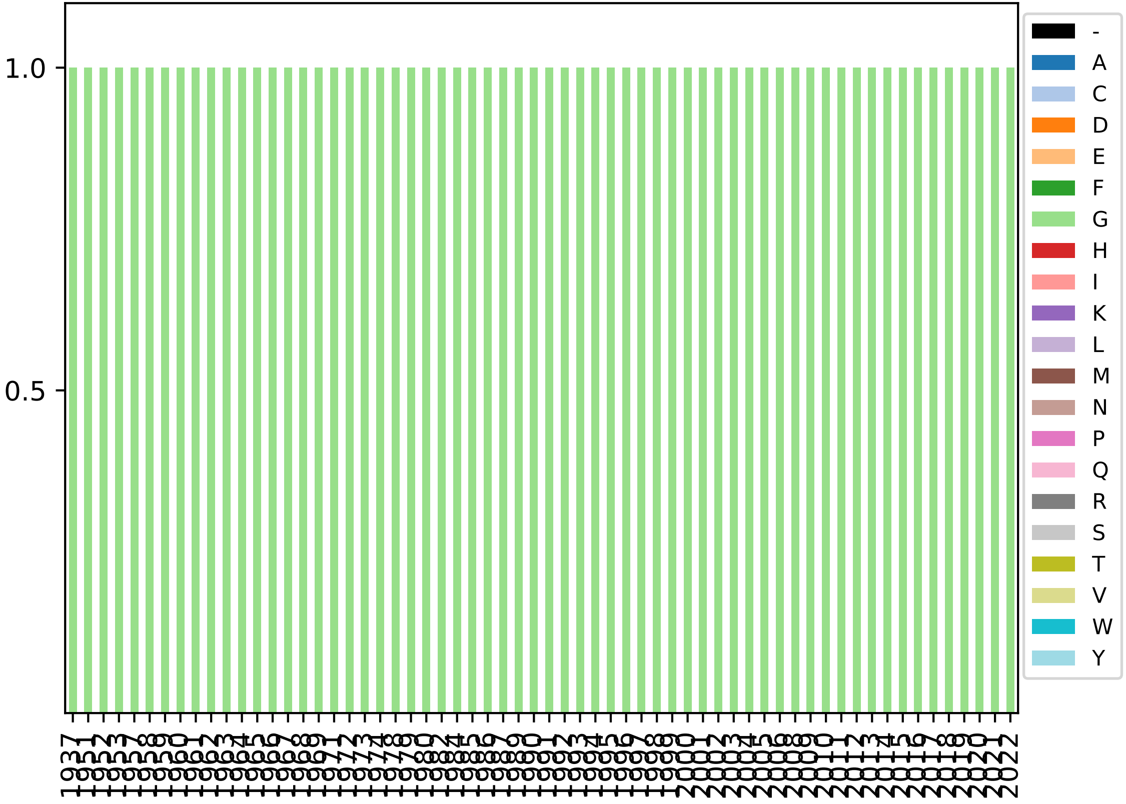

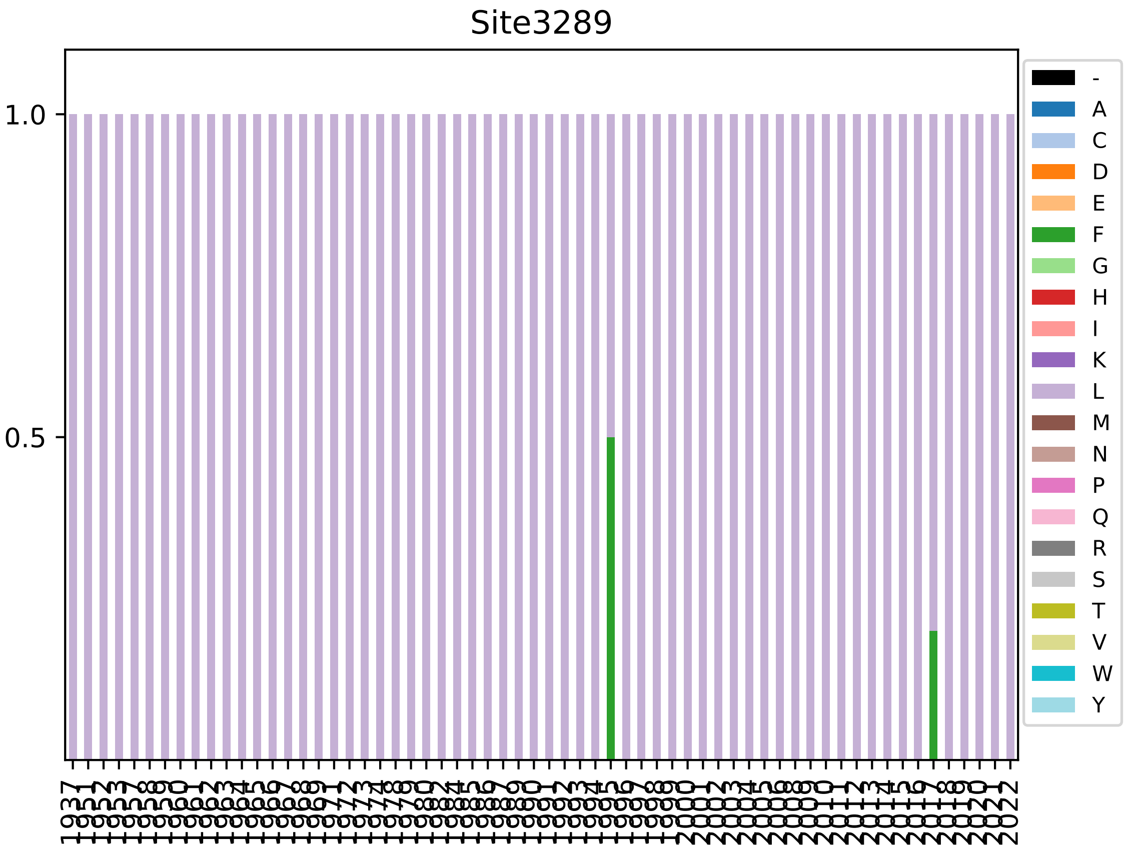
Site3289
1.0
0.5
-
A
C
D
E
F
G
H
I
K
L
M
N
P
Q
R
S
T
V
W
Y

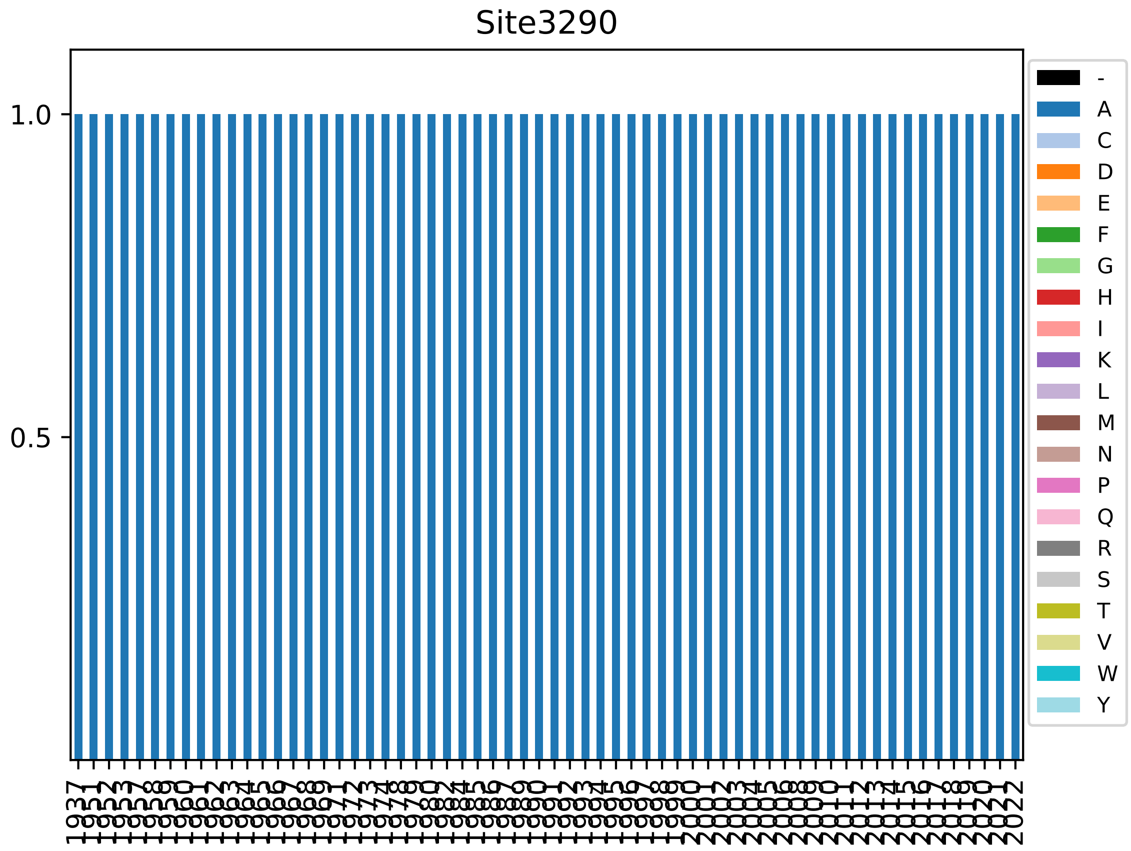
Site3290
1.0
0.5
-
A
C
D
E
F
G
H
I
K
L
M
N
P
Q
R
S
T
V
W
Y

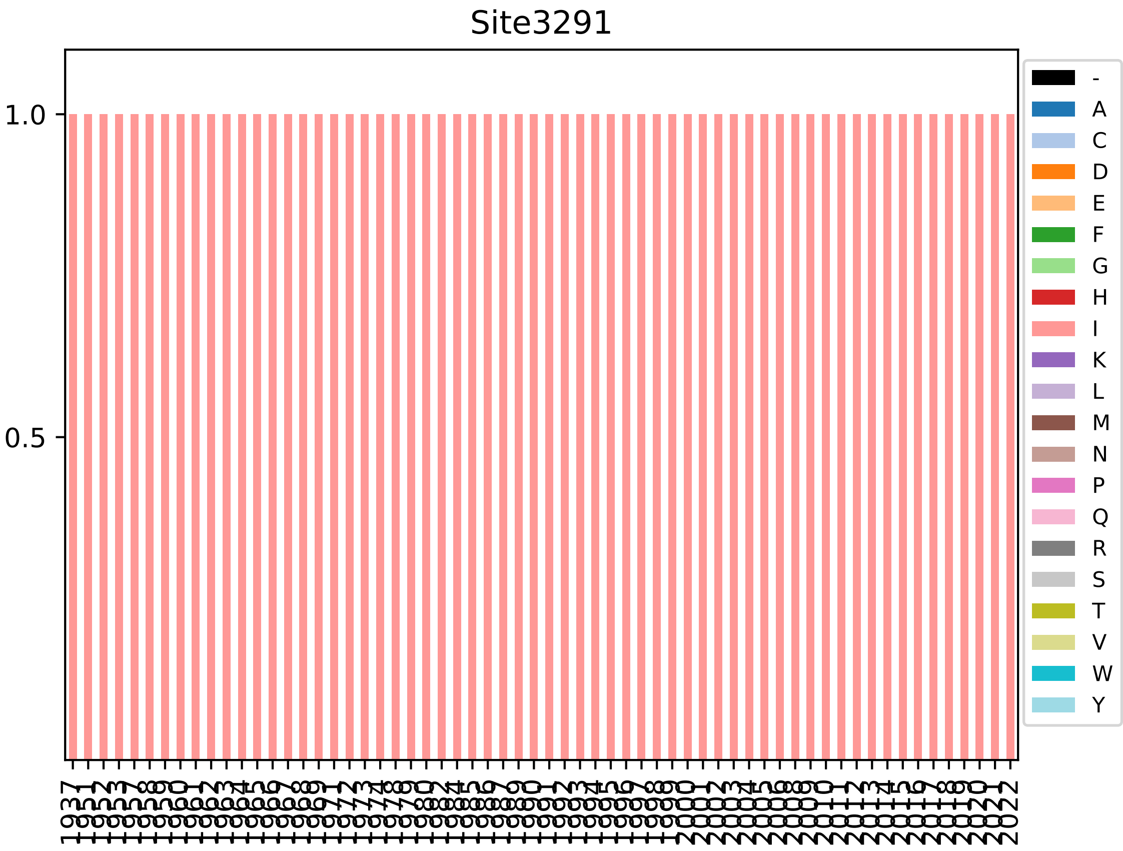
Site3291
1.0
0.5
-
A
C
D
E
F
G
H
I
K
L
M
N
P
Q
R
S
T
V
W
Y

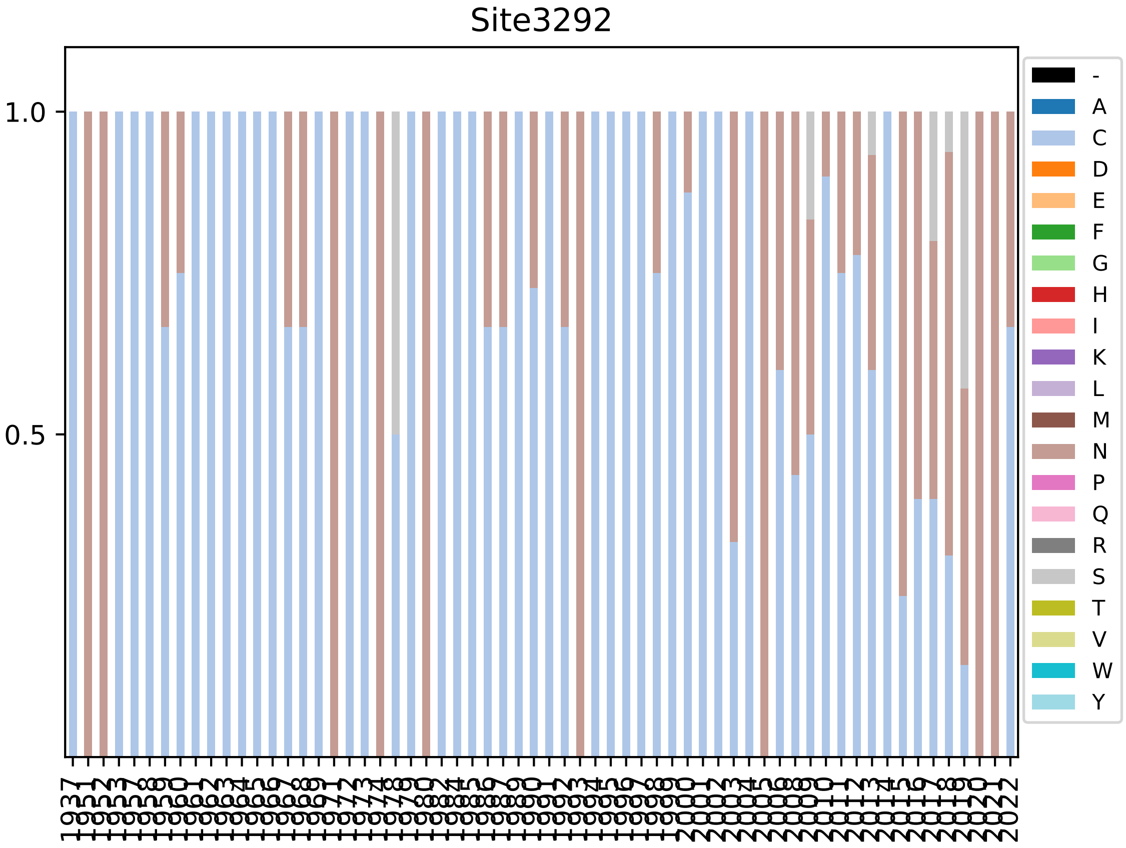
Site3292
1.0
0.5
-
A
C
D
E
F
G
H
I
K
L
M
N
P
Q
R
S
T
V
W
Y

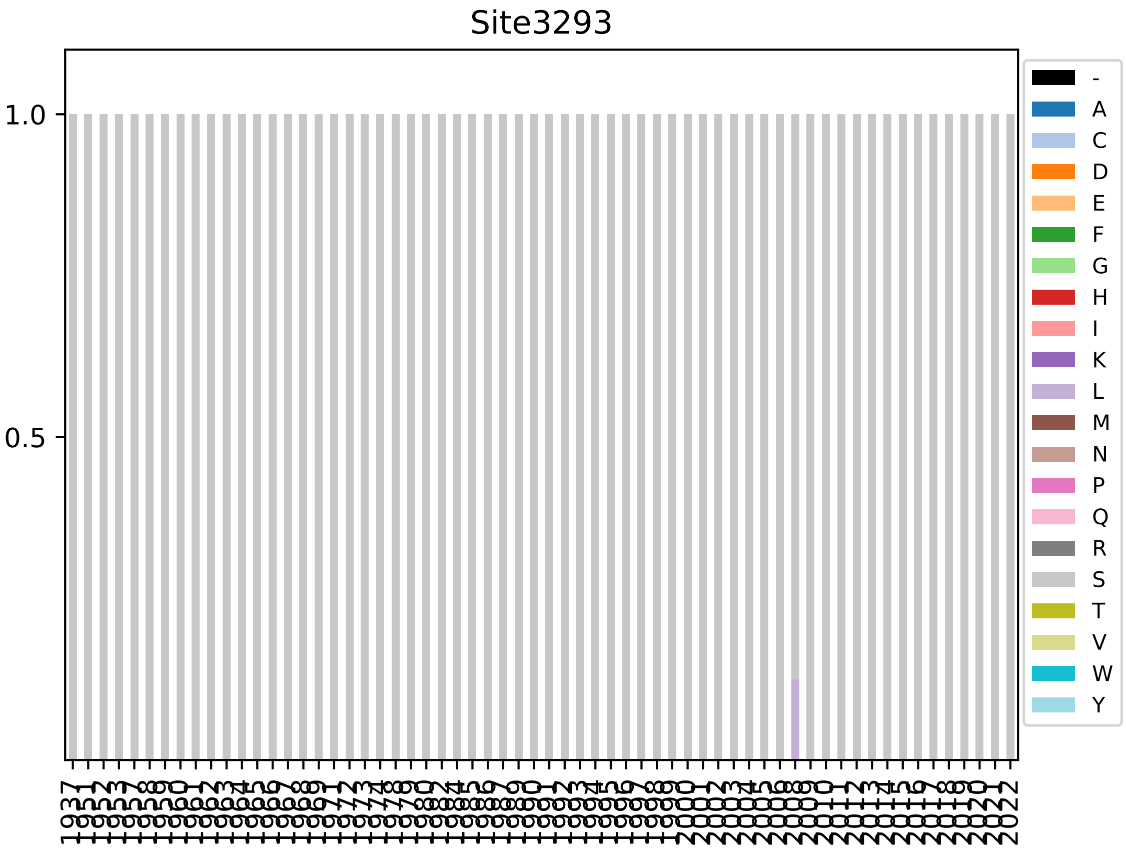
Site3293
1.0
0.5
-
A
C
D
E
F
G
H
I
K
L
M
N
P
Q
R
S
T
V
W
Y

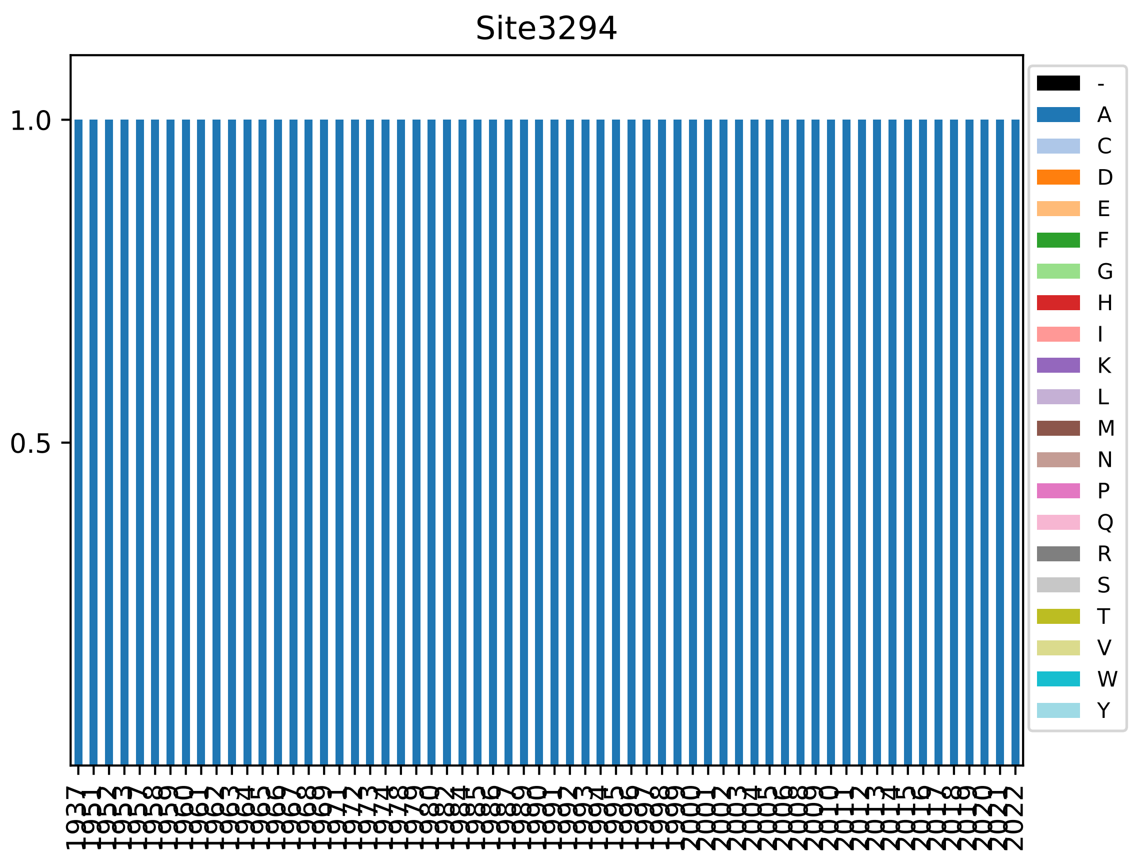
Site3294
1.0
0.5
-
A
C
D
E
F
G
H
I
K
L
M
N
P
Q
R
S
T
V
W
Y

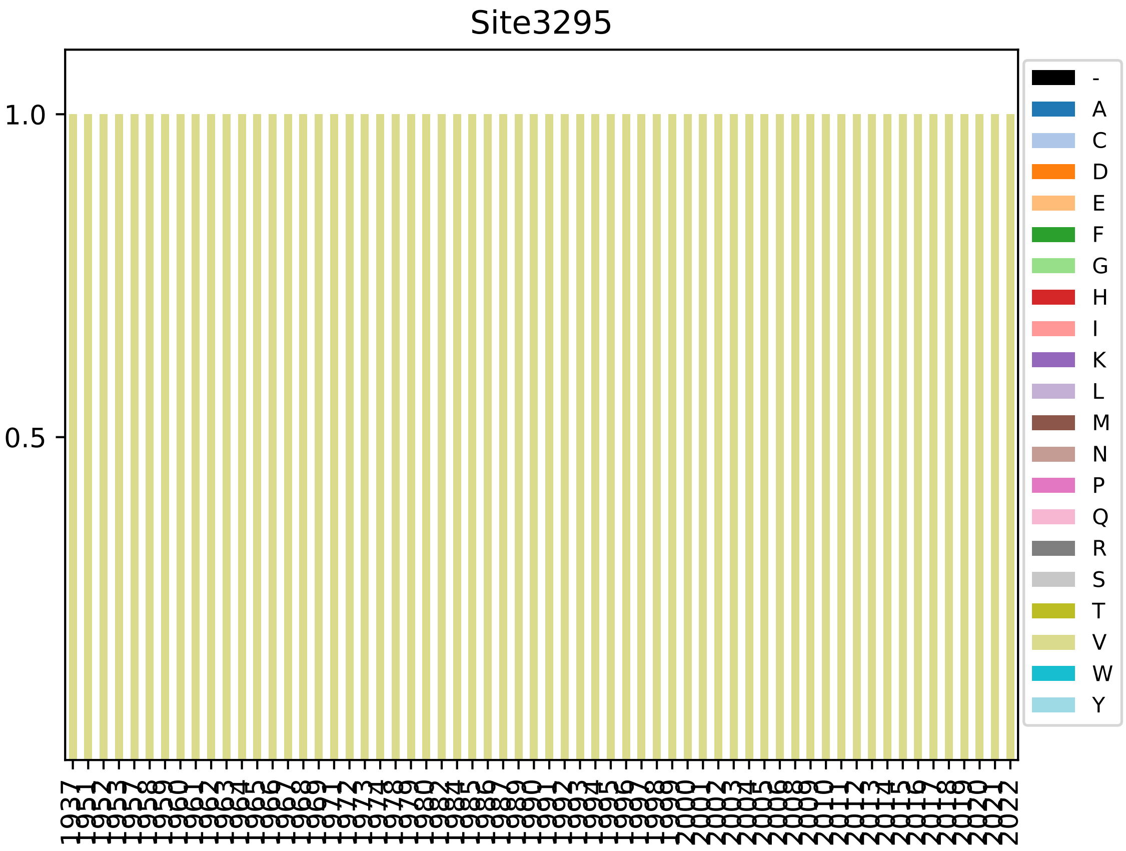
Site3295
1.0
0.5
-
A
C
D
E
F
G
H
I
K
L
M
N
P
Q
R
S
T
V
W
Y

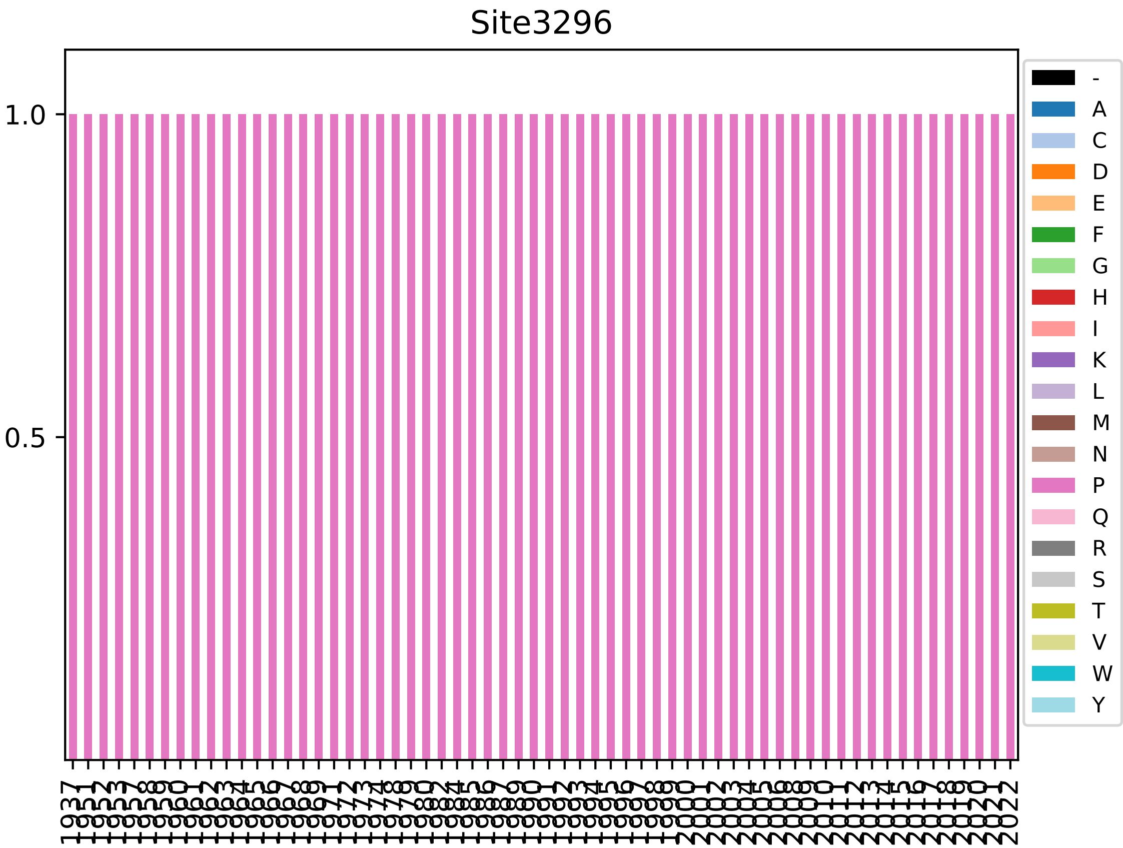
Site3296
1.0
0.5
-
A
C
D
E
F
G
H
I
K
L
M
N
P
Q
R
S
T
V
W
Y

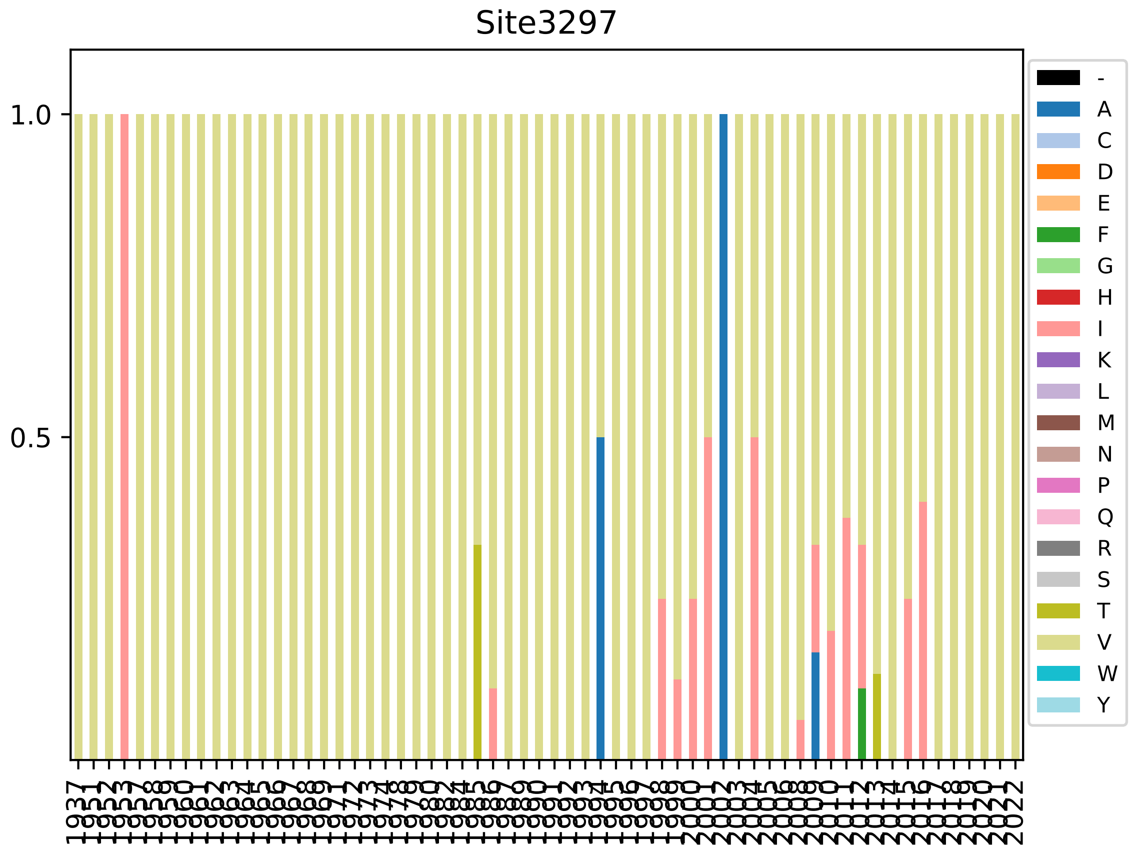
Site3297
1.0
0.5
-
A
C
D
E
F
G
H
I
K
L
M
N
P
Q
R
S
T
V
W
Y

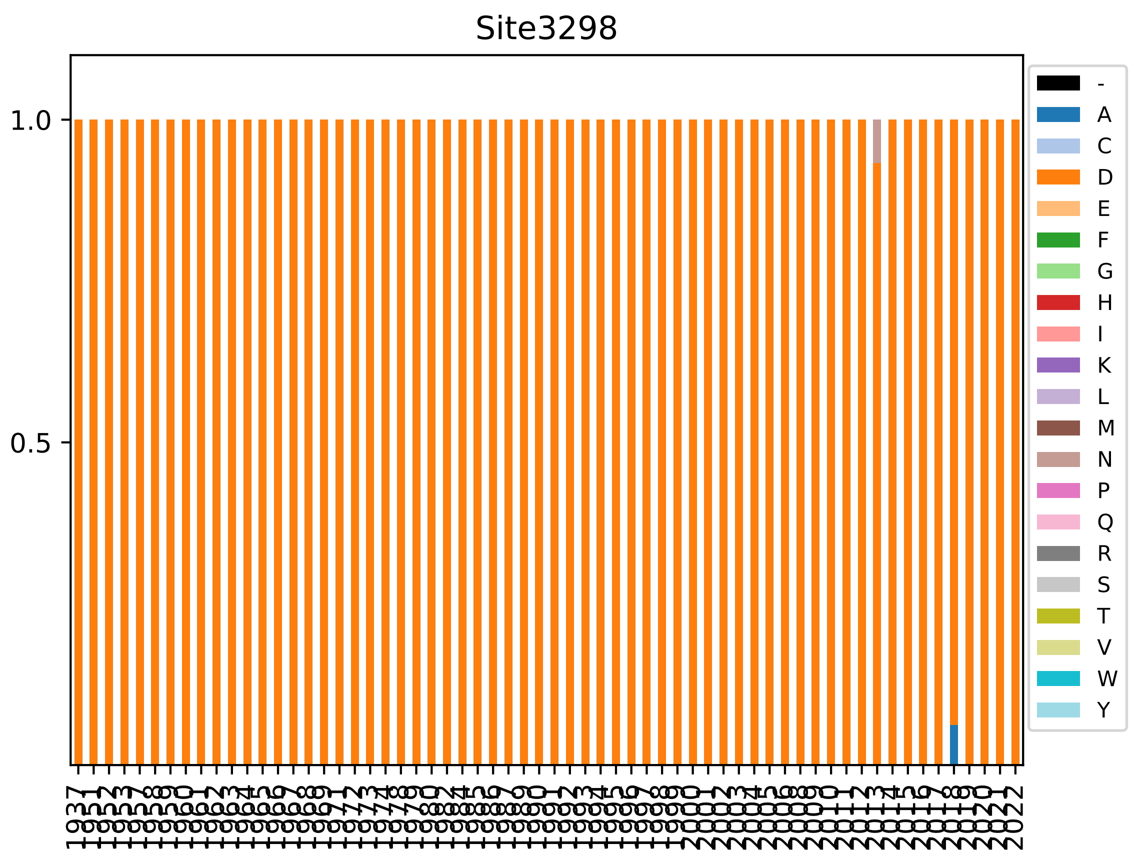
Site3298
1.0
0.5
-
A
C
D
E
F
G
H
I
K
L
M
N
P
Q
R
S
T
V
W
Y

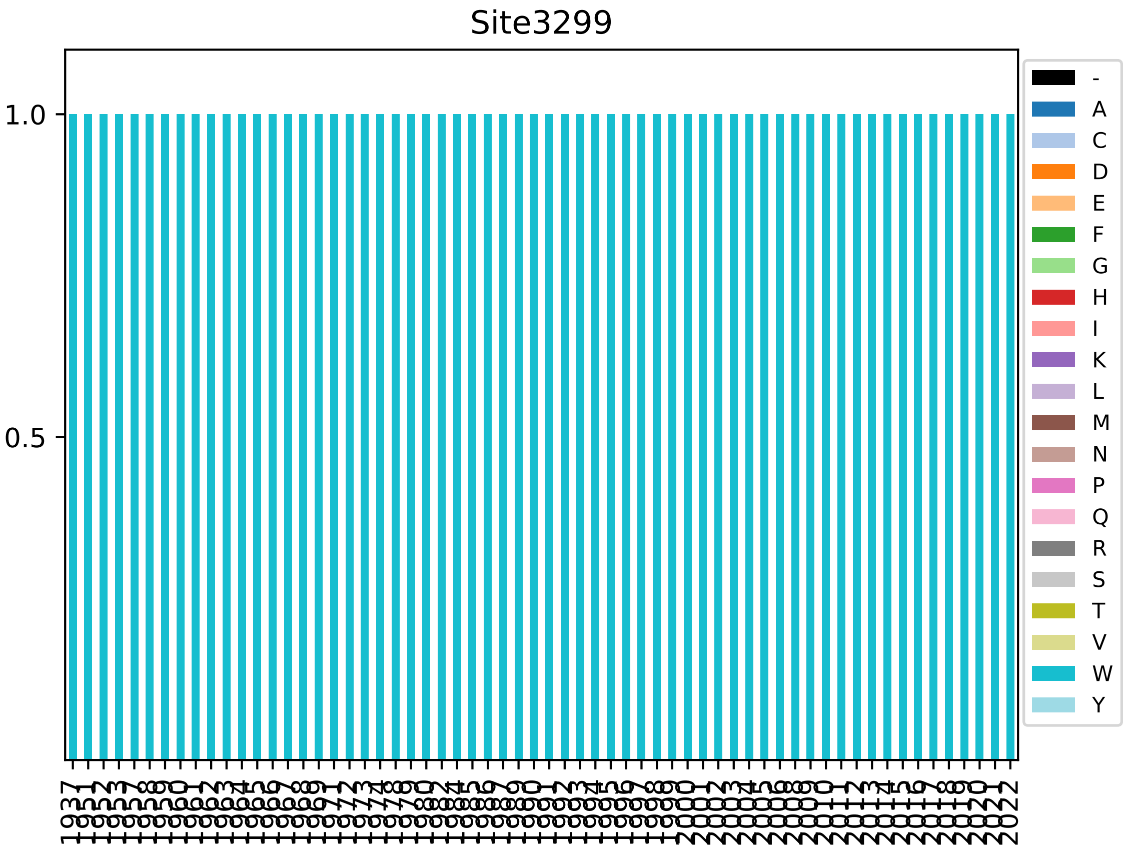
Site3299
1.0
0.5
-
A
C
D
E
F
G
H
I
K
L
M
N
P
Q
R
S
T
V
W
Y

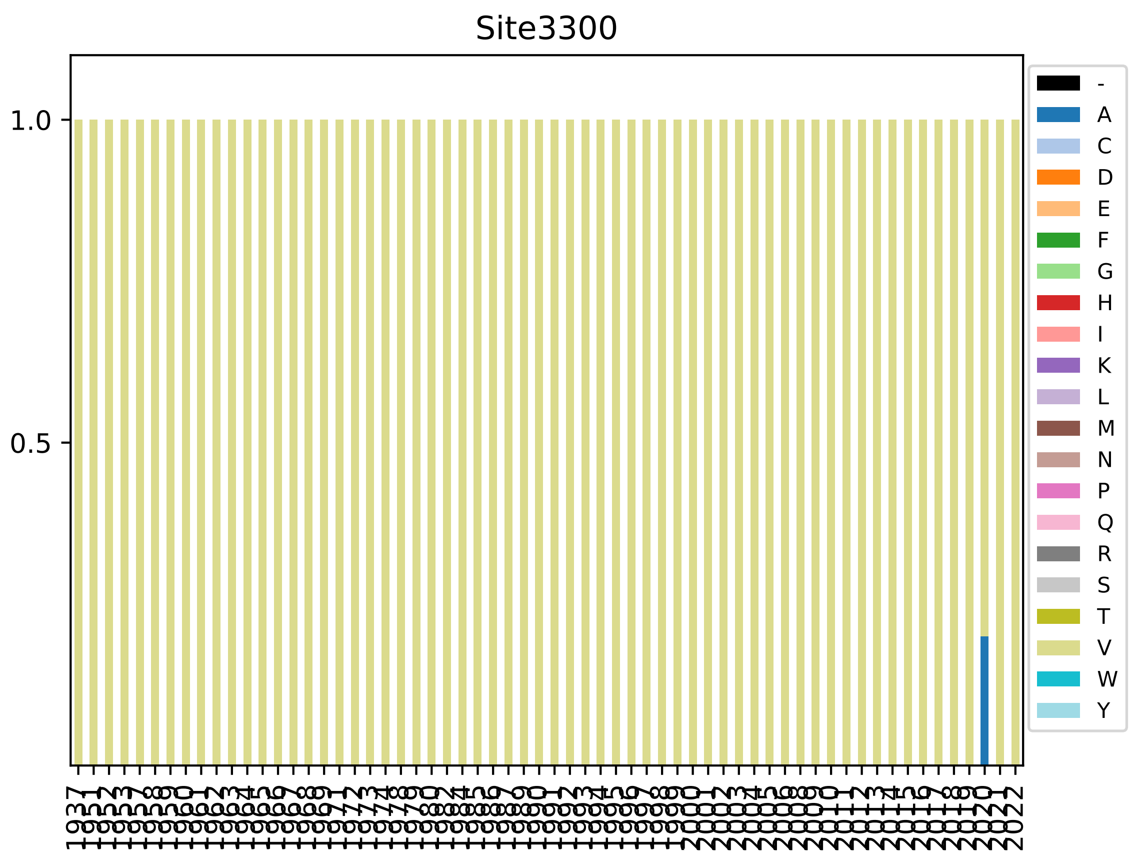
Site3300
1.0
0.5
-
A
C
D
E
F
G
H
I
K
L
M
N
P
Q
R
S
T
V
W
Y

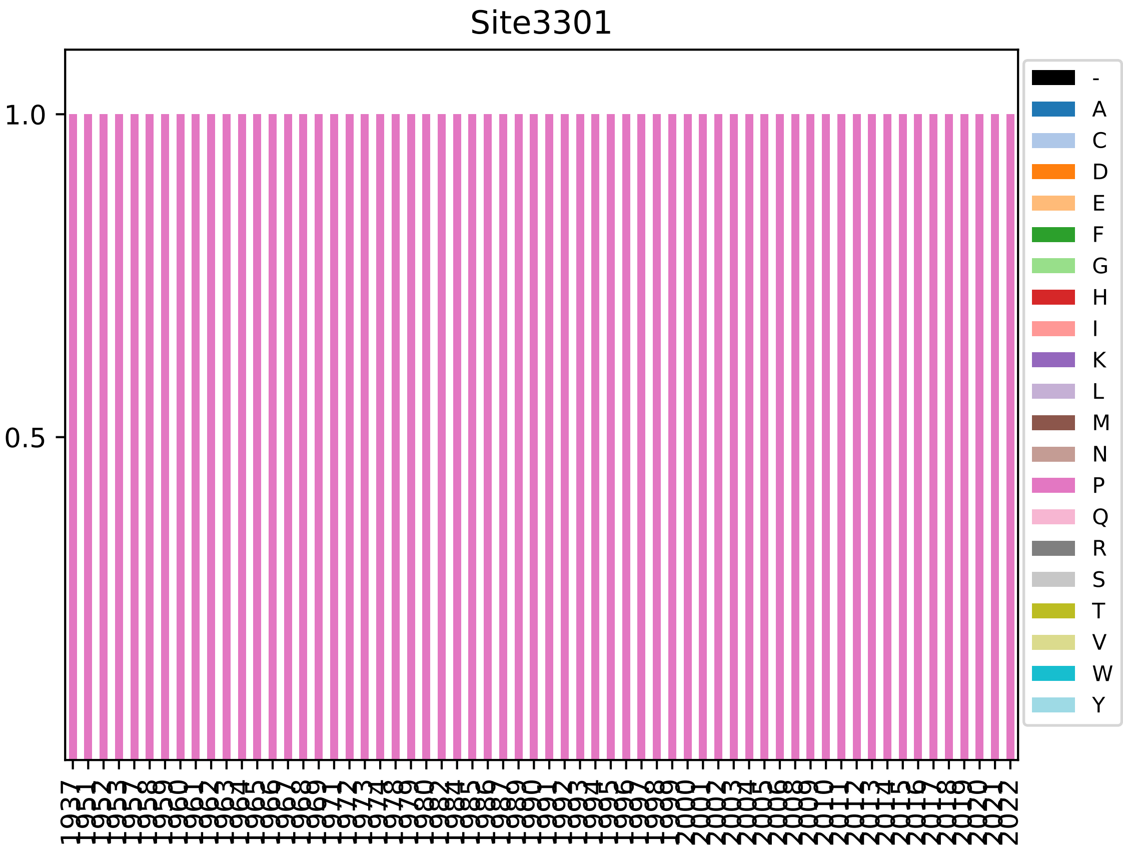
Site3301
1.0
0.5
-
A
C
D
E
F
G
H
I
K
L
M
N
P
Q
R
S
T
V
W
Y

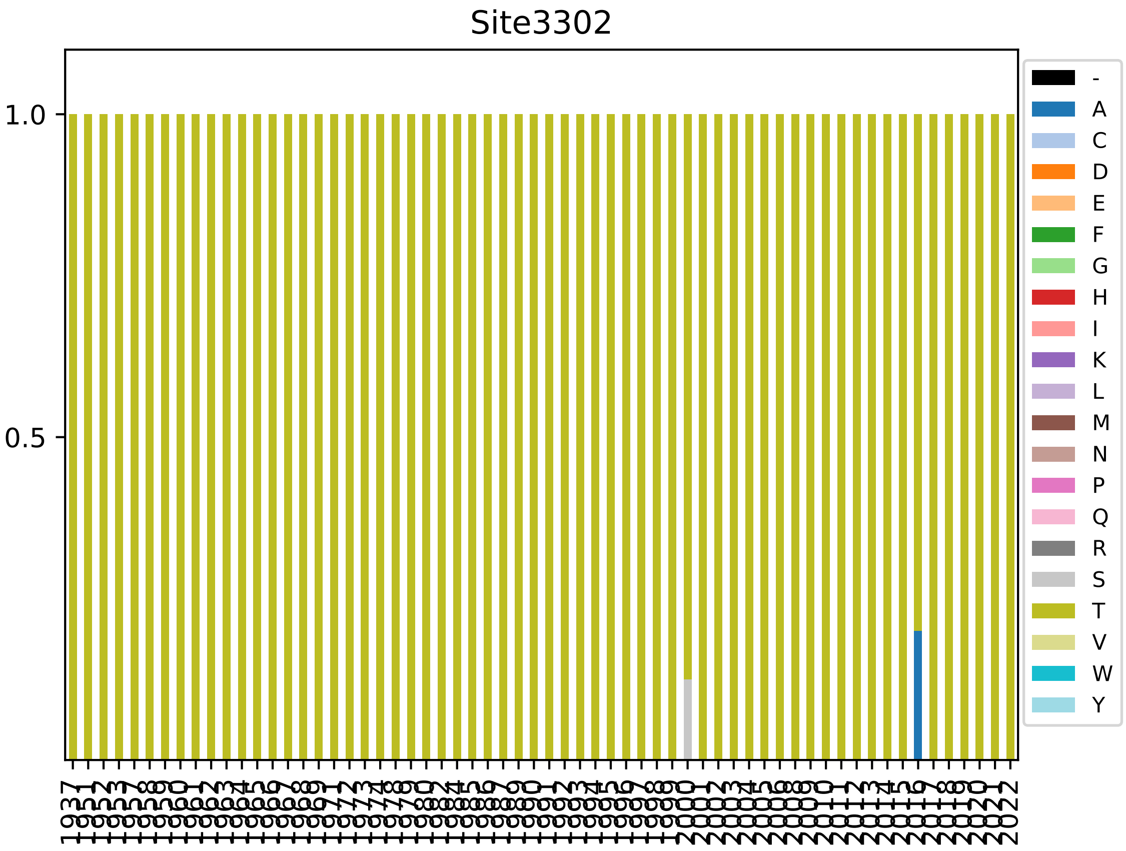
Site3302
1.0
0.5
-
A
C
D
E
F
G
H
I
K
L
M
N
P
Q
R
S
T
V
W
Y

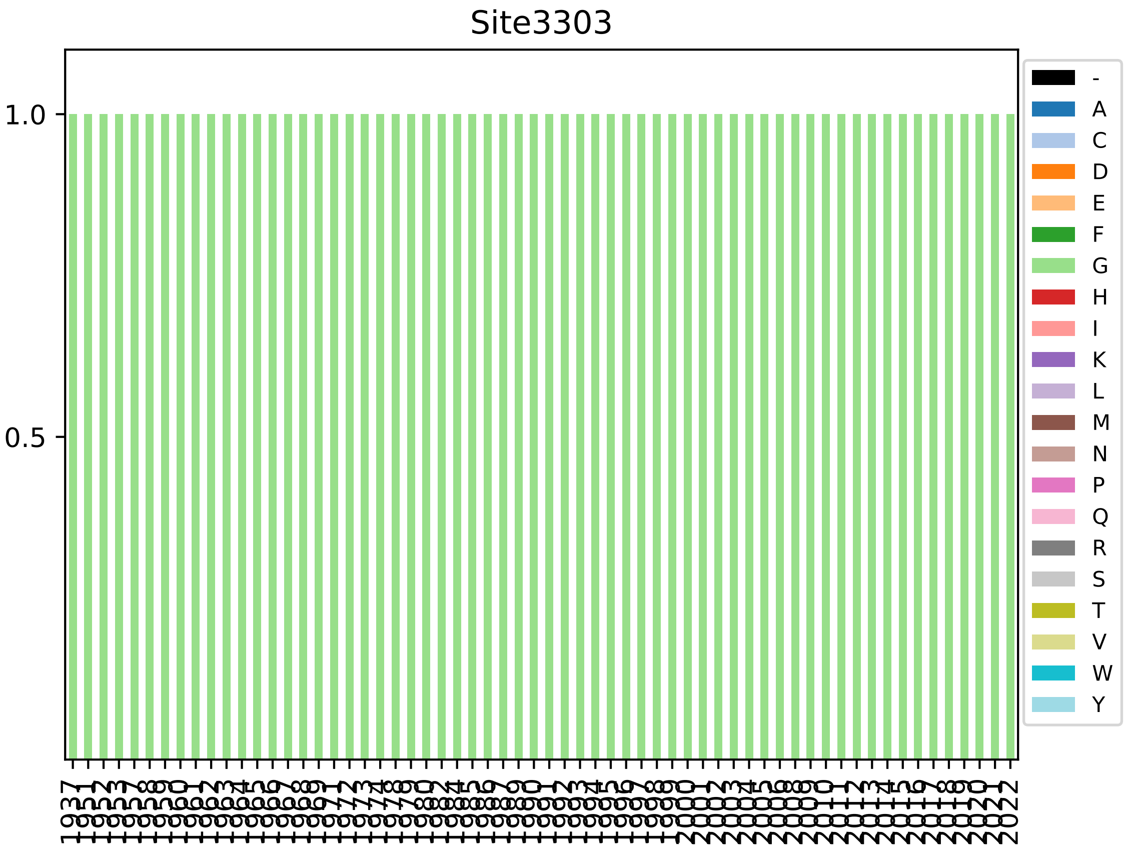
Site3303
1.0
0.5
-
A
C
D
E
F
G
H
I
K
L
M
N
P
Q
R
S
T
V
W
Y

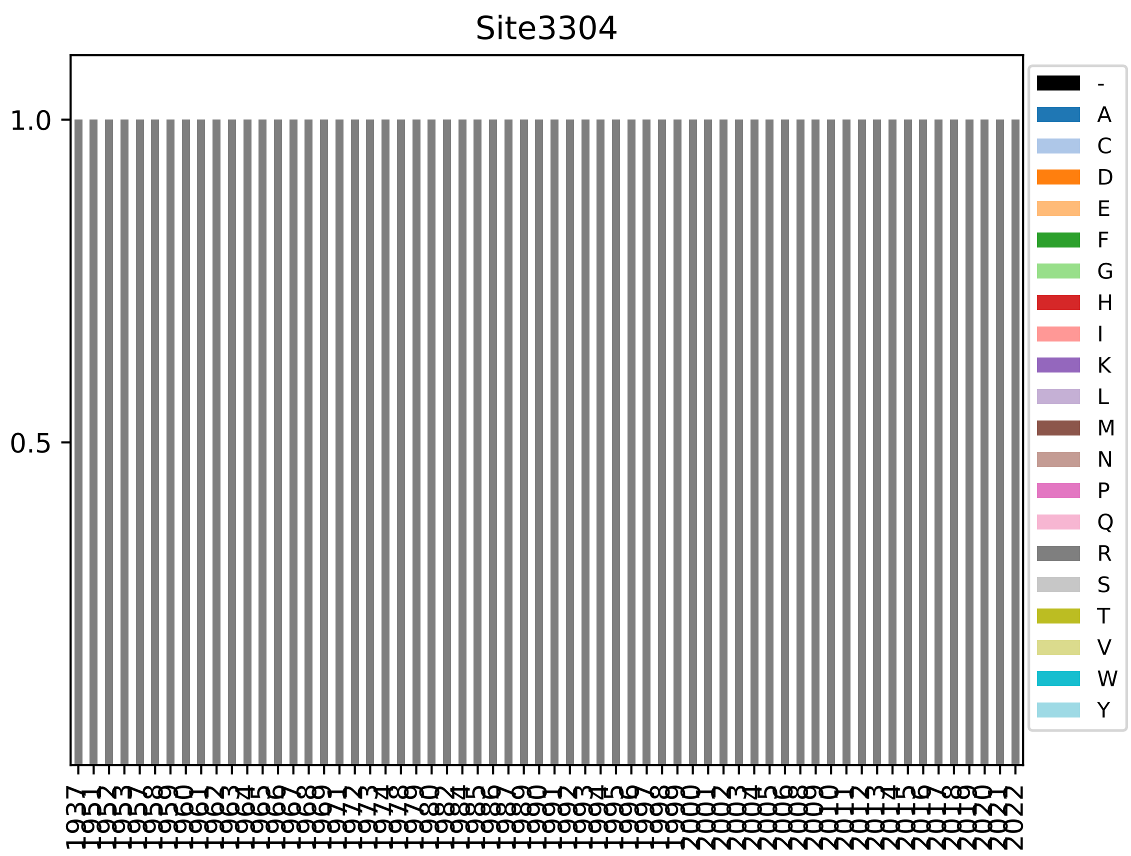
Site3304
1.0
0.5
-
A
C
D
E
F
G
H
I
K
L
M
N
P
Q
R
S
T
V
W
Y

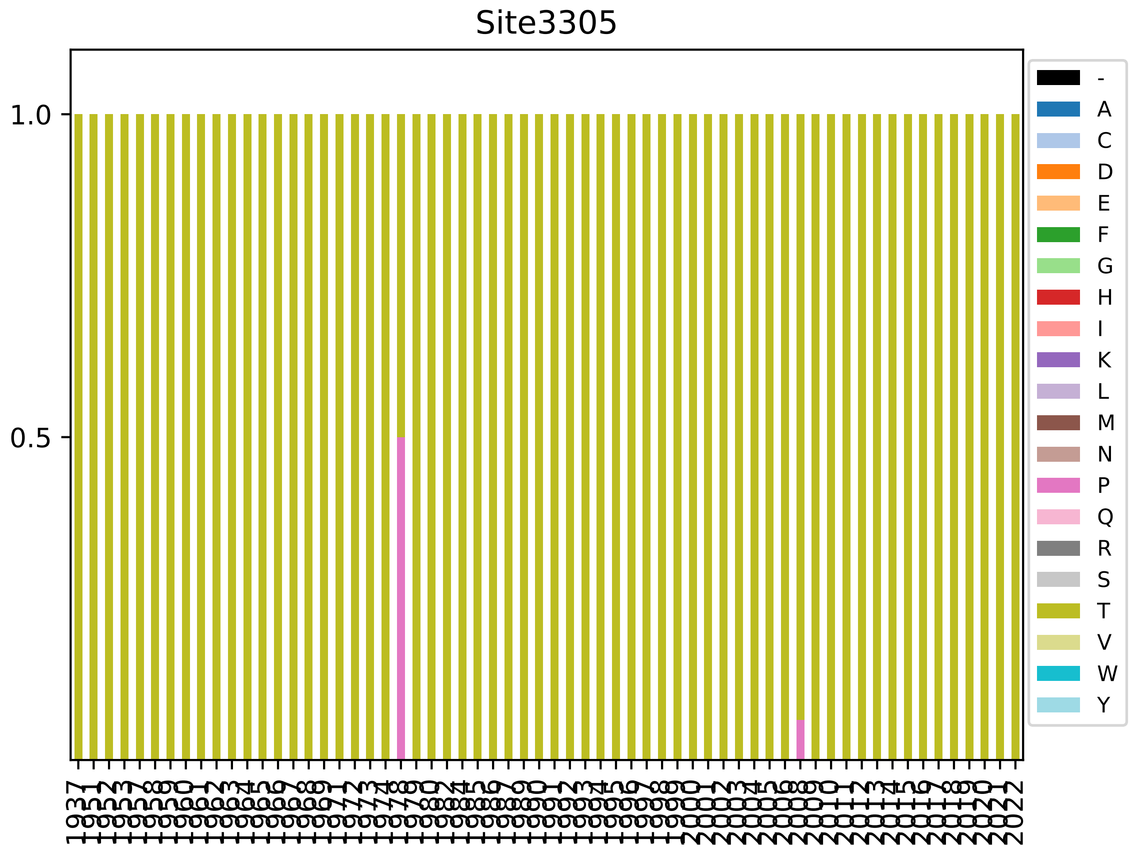
Site3305
1.0
0.5
-
A
C
D
E
F
G
H
I
K
L
M
N
P
Q
R
S
T
V
W
Y

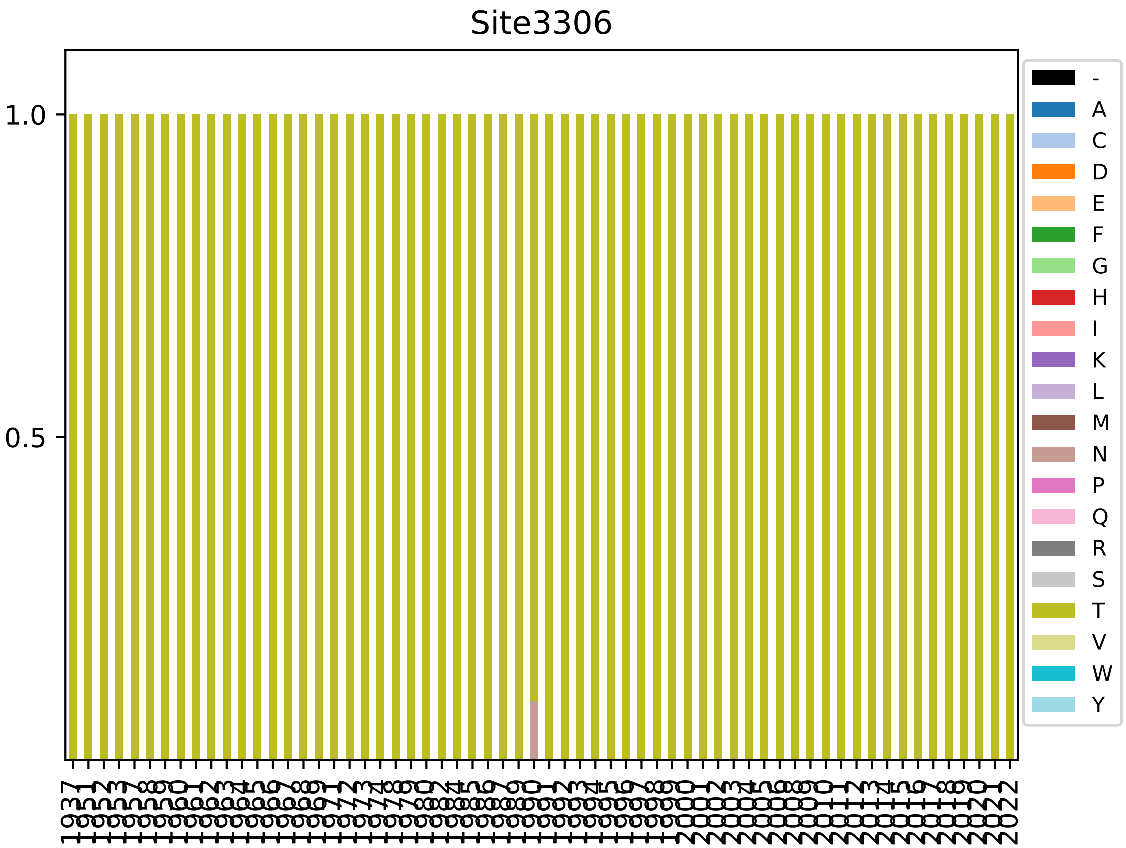
Site3306
1.0
0.5
-
A
C
D
E
F
G
H
I
K
L
M
N
P
Q
R
S
T
V
W
Y

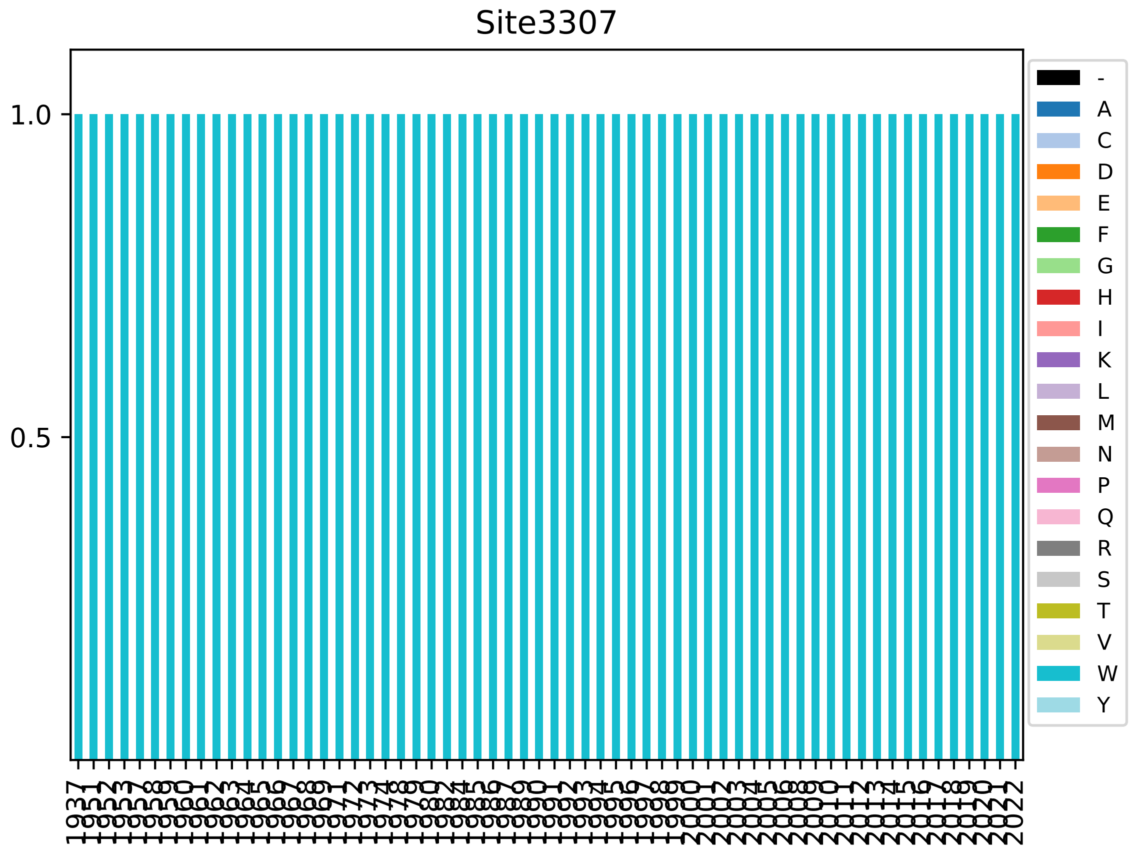
Site3307
1.0
0.5
-
A
C
D
E
F
G
H
I
K
L
M
N
P
Q
R
S
T
V
W
Y

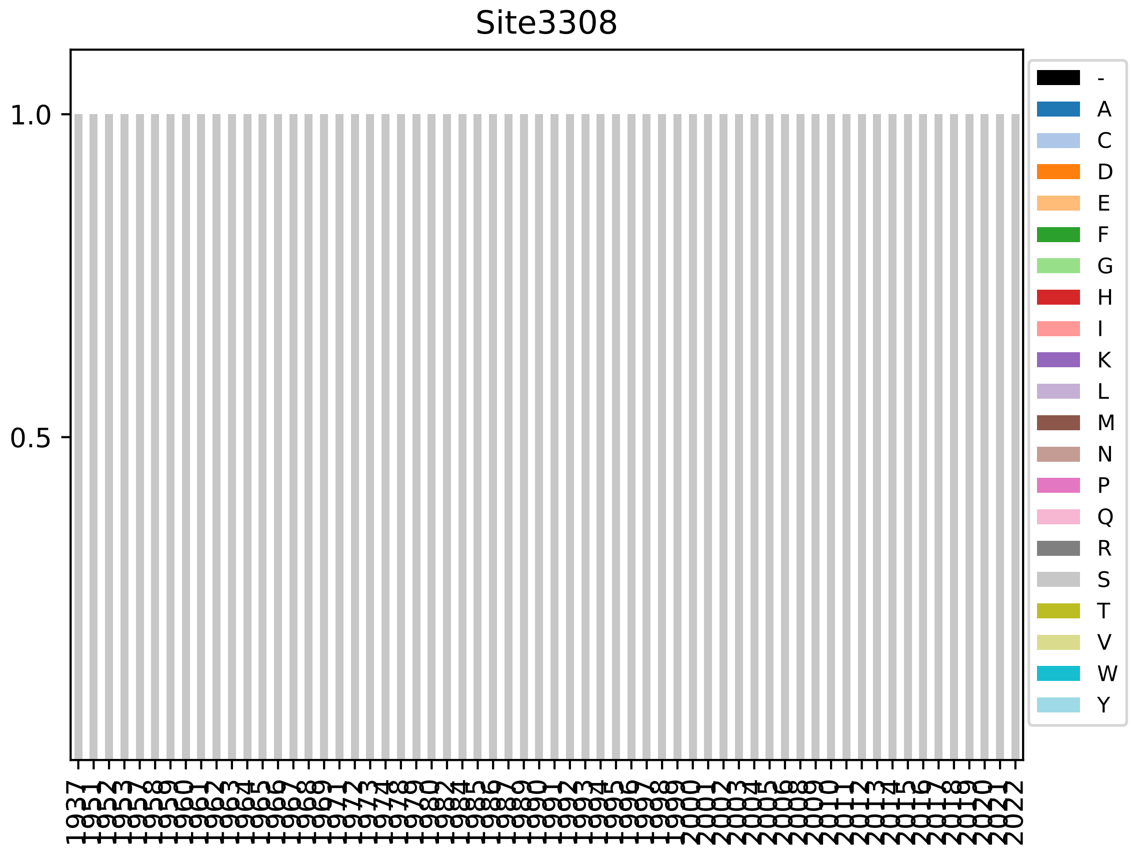
Site3308
1.0
0.5
-
A
C
D
E
F
G
H
I
K
L
M
N
P
Q
R
S
T
V
W
Y

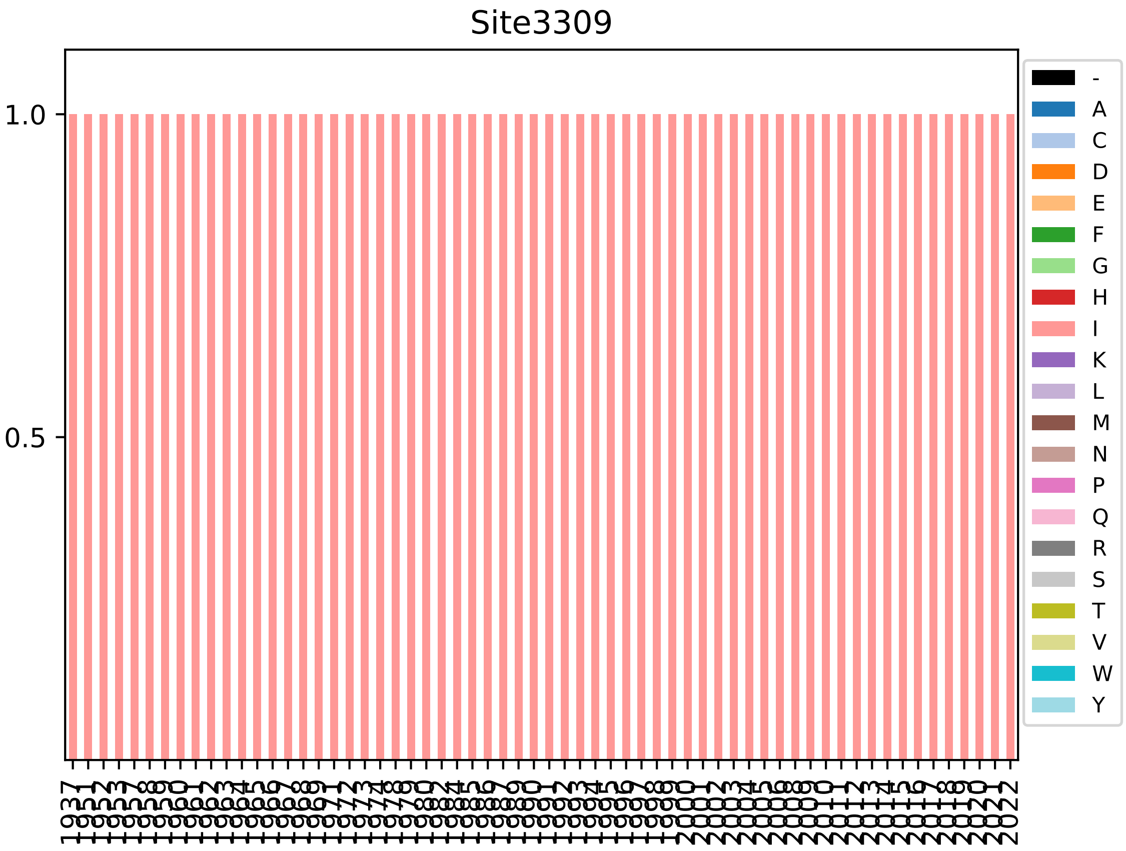

Site3309
1.0
0.5
-
A
C
D
E
F
G
H
I
K
L
M
N
P
Q
R
S
T
V
W
Y

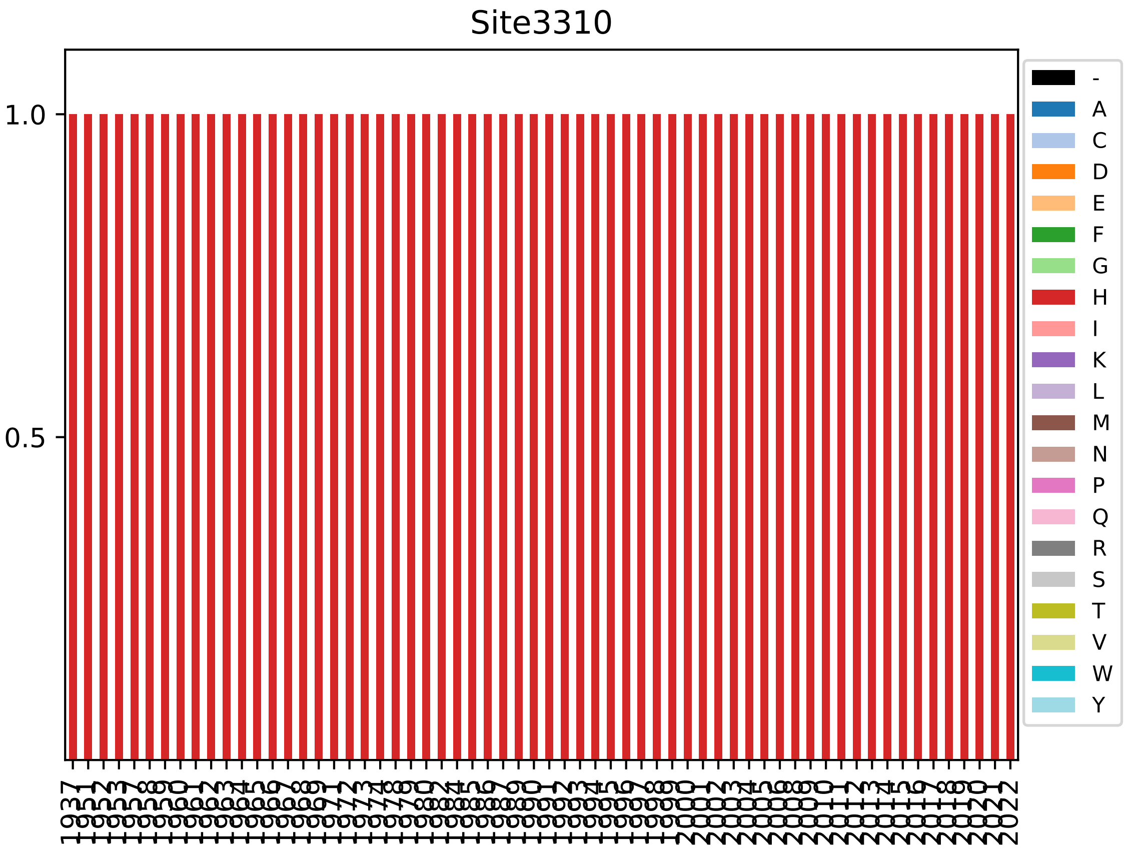
Site3310
1.0
0.5
-
A
C
D
E
F
G
H
I
K
L
M
N
P
Q
R
S
T
V
W
Y

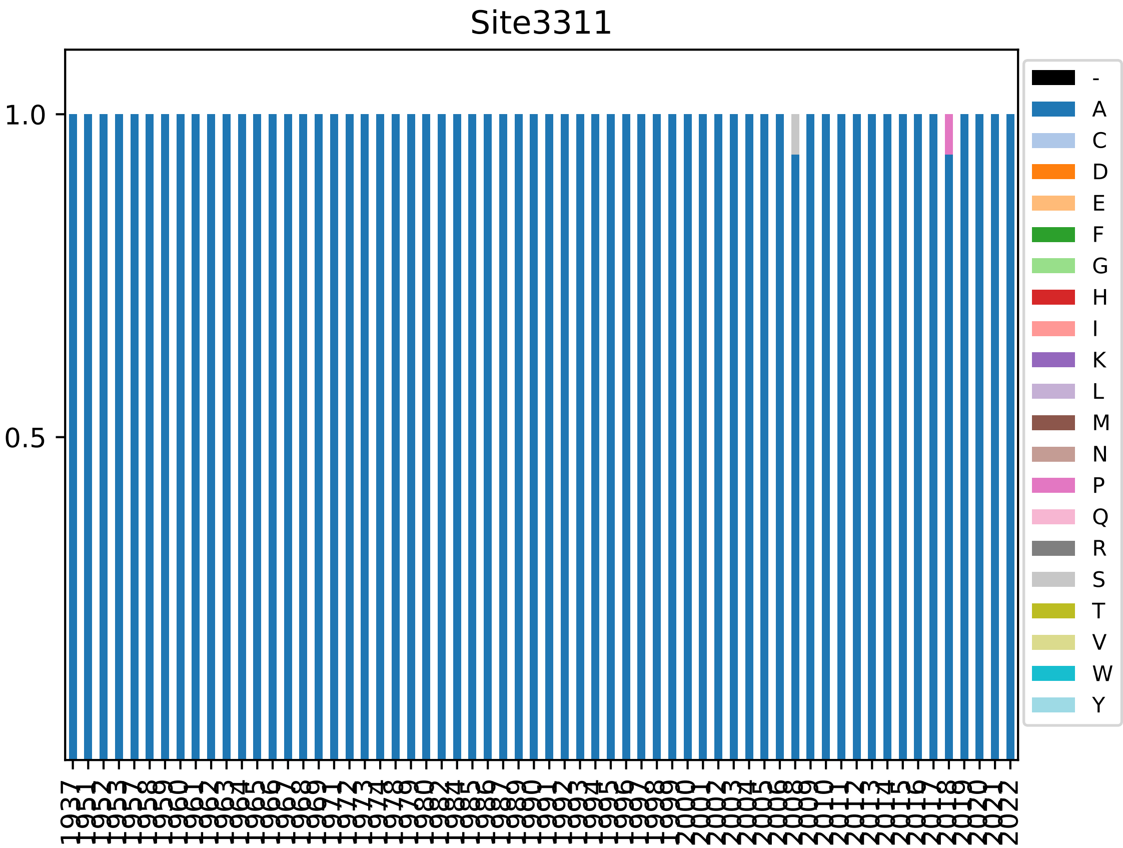
Site3311
1.0
0.5
-
A
C
D
E
F
G
H
I
K
L
M
N
P
Q
R
S
T
V
W
Y

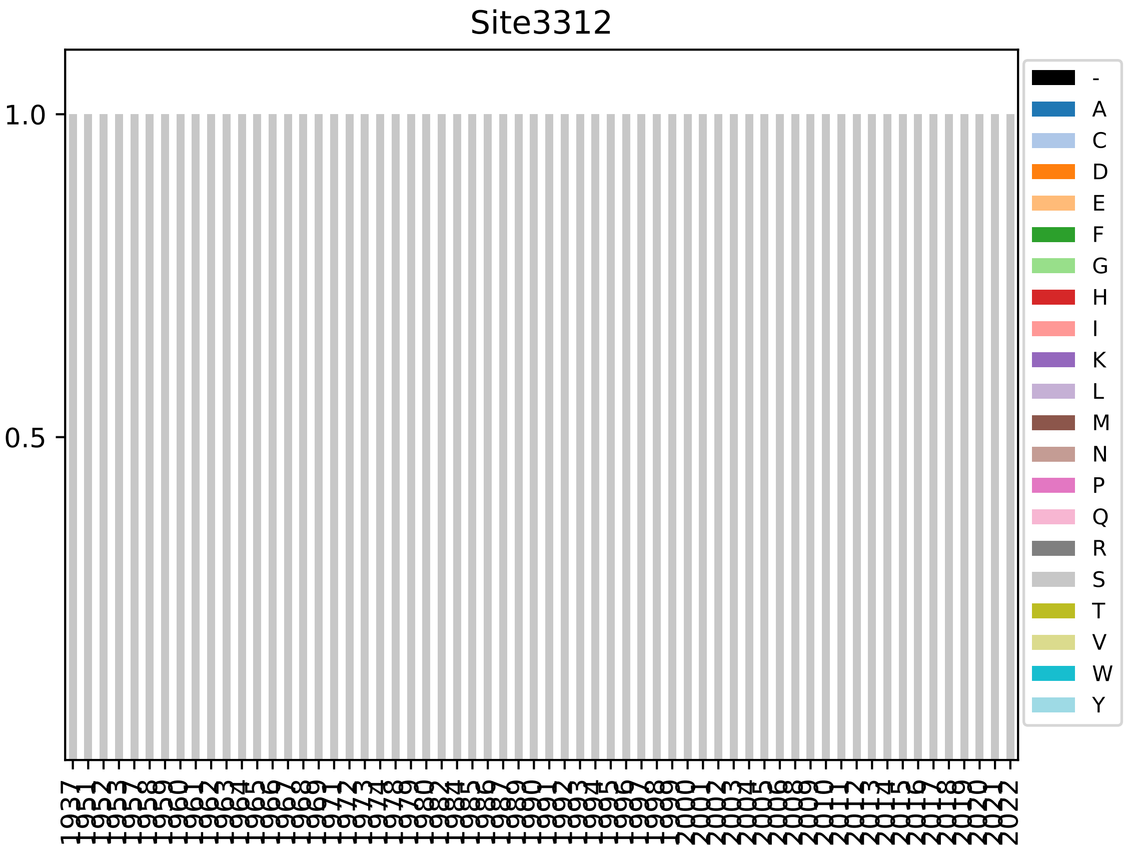
Site3312
1.0
0.5
-
A
C
D
E
F
G
H
I
K
L
M
N
P
Q
R
S
T
V
W
Y

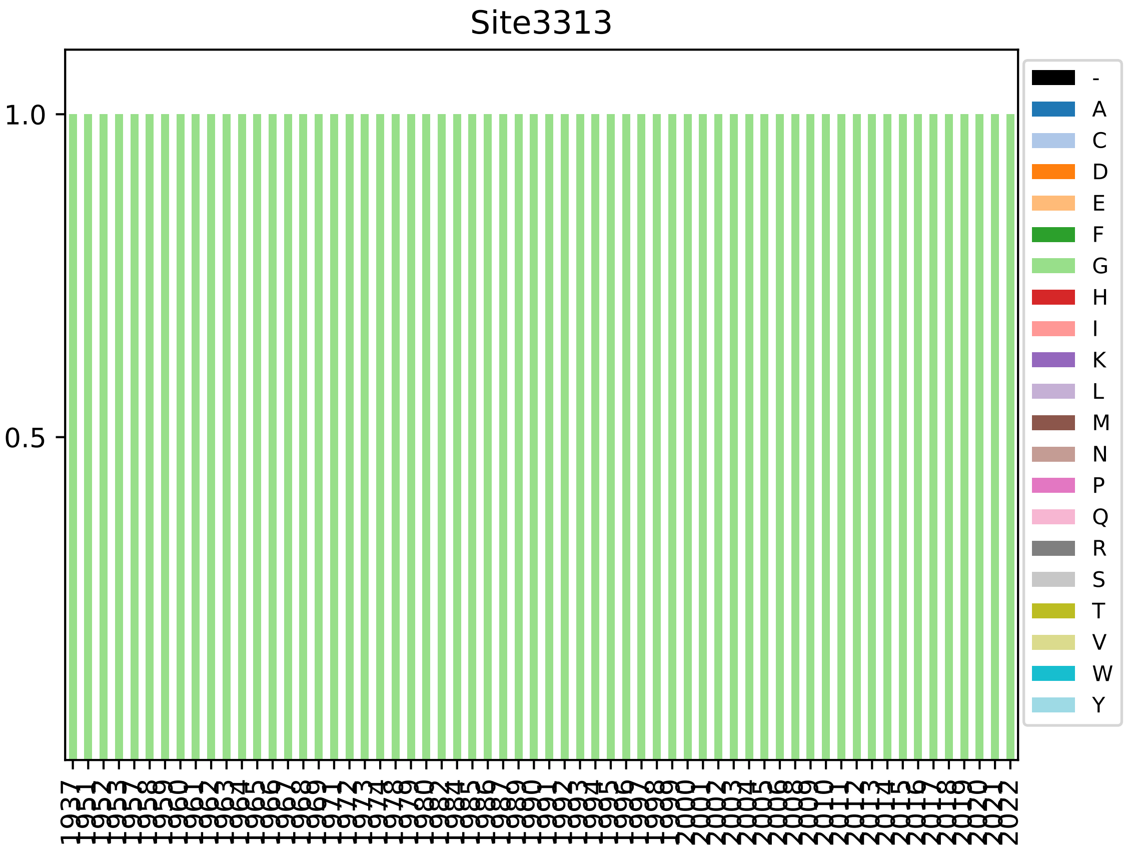
Site3313
1.0
0.5
-
A
C
D
E
F
G
H
I
K
L
M
N
P
Q
R
S
T
V
W
Y

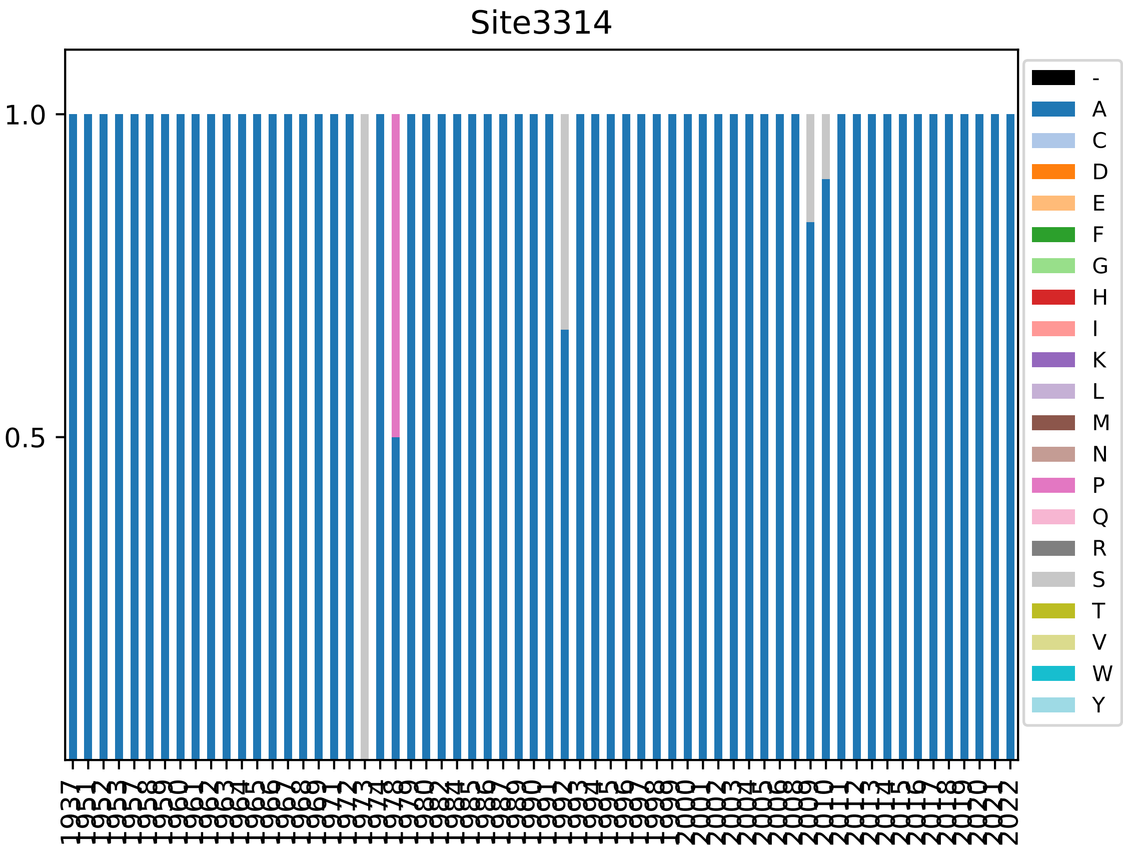
Site3314
1.0
0.5
-
A
C
D
E
F
G
H
I
K
L
M
N
P
Q
R
S
T
V
W
Y

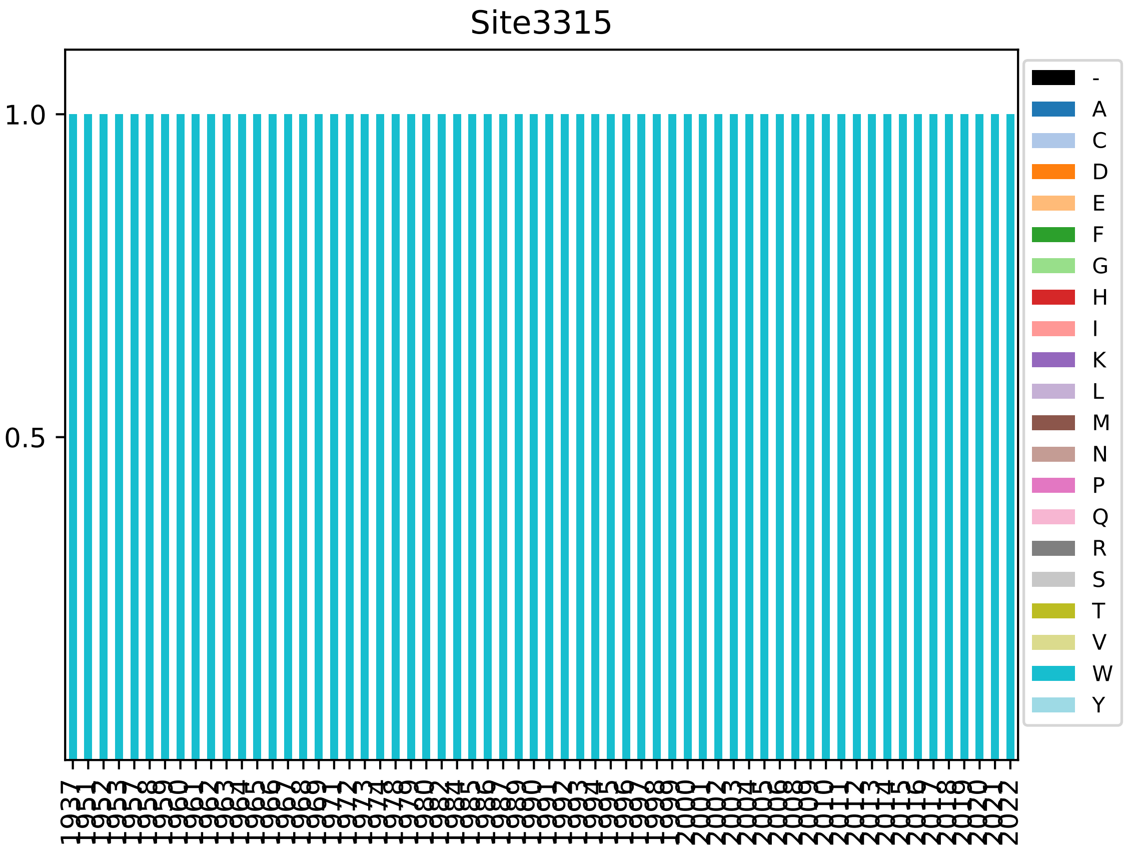
Site3315
1.0
0.5
-
A
C
D
E
F
G
H
I
K
L
M
N
P
Q
R
S
T
V
W
Y

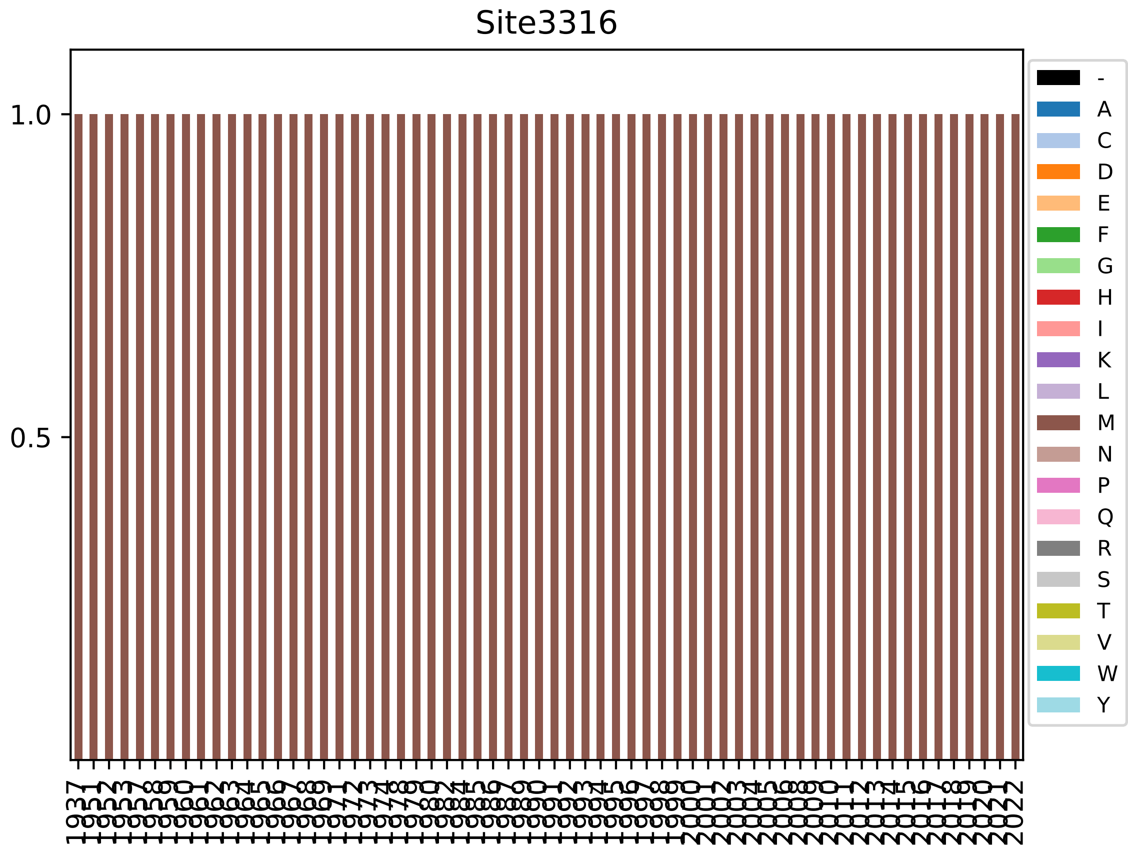

Site3316
1.0
0.5
-
A
C
D
E
F
G
H
I
K
L
M
N
P
Q
R
S
T
V
W
Y

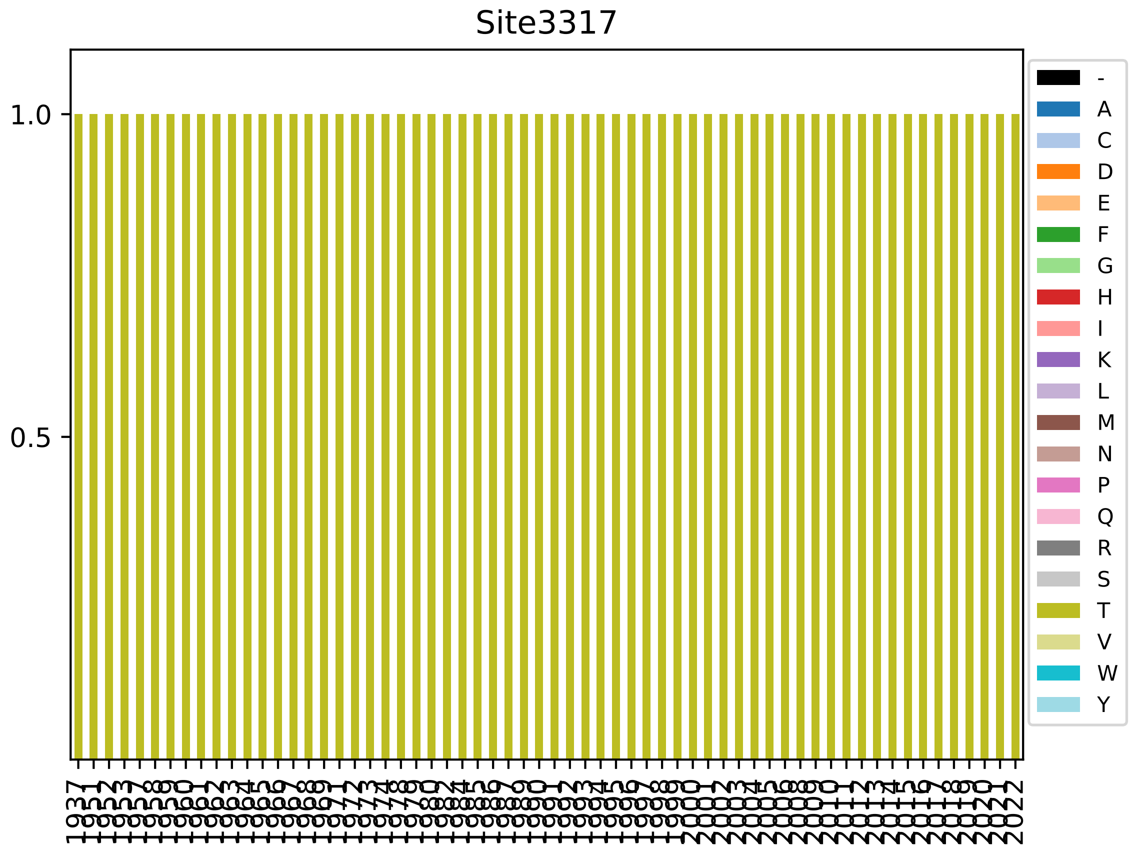
Site3317
1.0
0.5
-
A
C
D
E
F
G
H
I
K
L
M
N
P
Q
R
S
T
V
W
Y

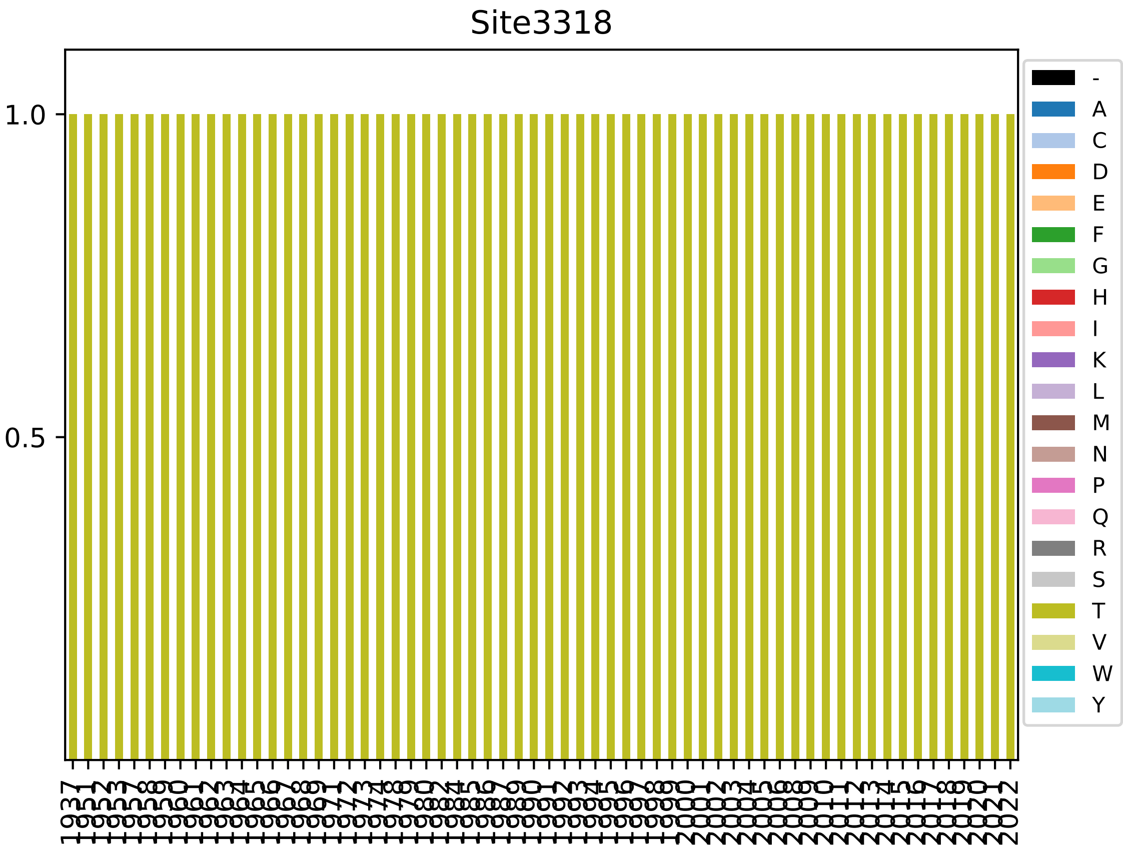
Site3318
1.0
0.5
-
A
C
D
E
F
G
H
I
K
L
M
N
P
Q
R
S
T
V
W
Y

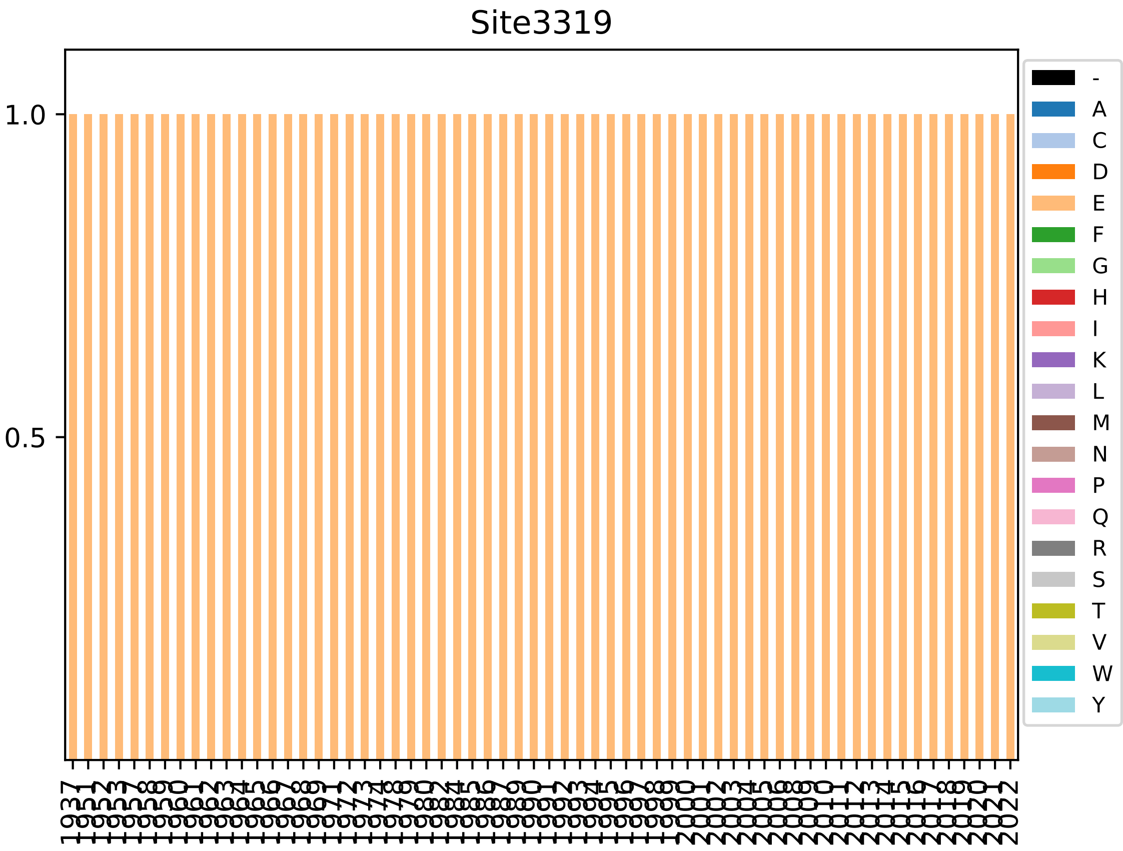
Site3319
1.0
0.5
-
A
C
D
E
F
G
H
I
K
L
M
N
P
Q
R
S
T
V
W
Y

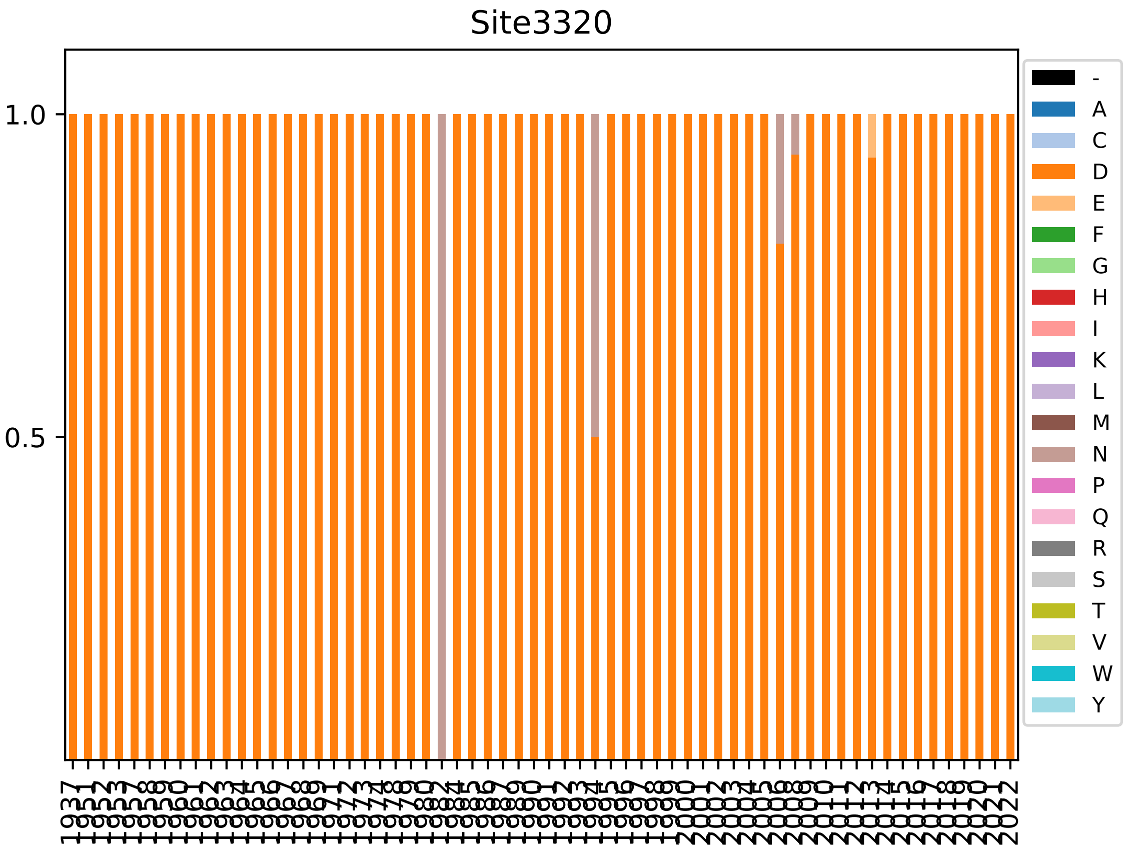
Site3320
1.0
0.5
-
A
C
D
E
F
G
H
I
K
L
M
N
P
Q
R
S
T
V
W
Y

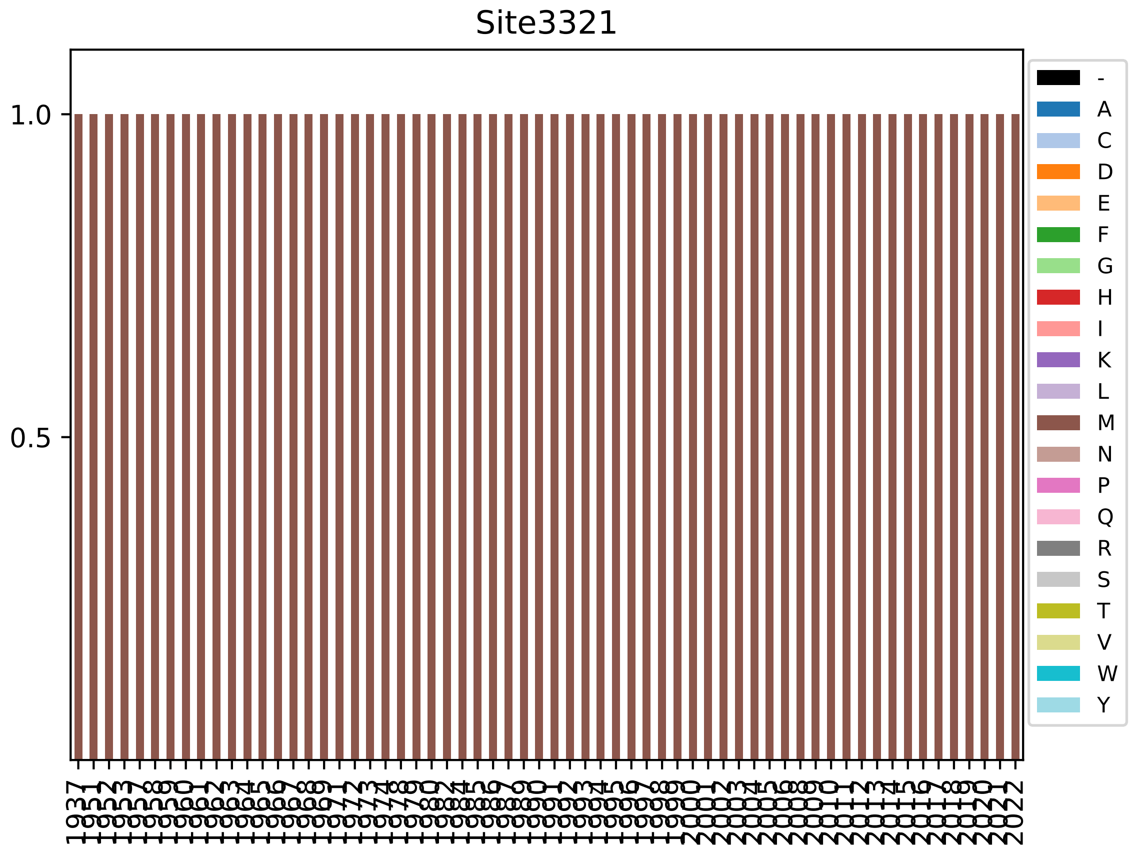
Site3321
1.0
0.5
-
A
C
D
E
F
G
H
I
K
L
M
N
P
Q
R
S
T
V
W
Y

Site3322

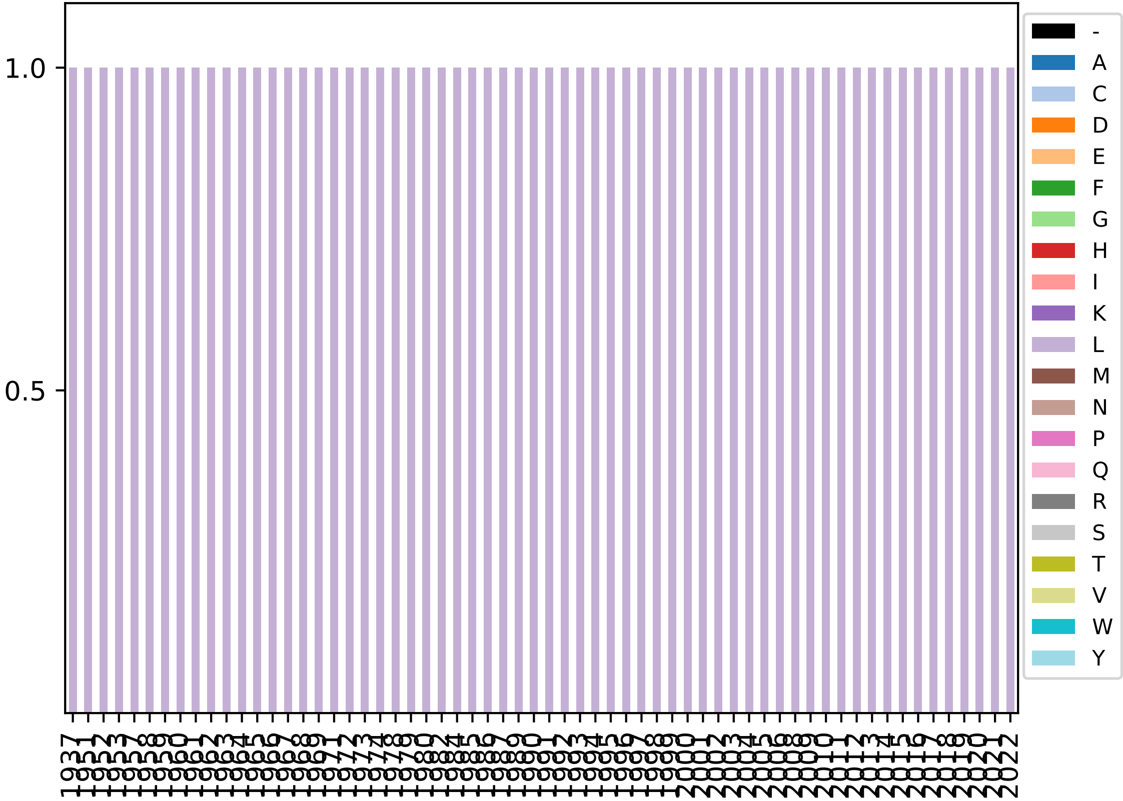

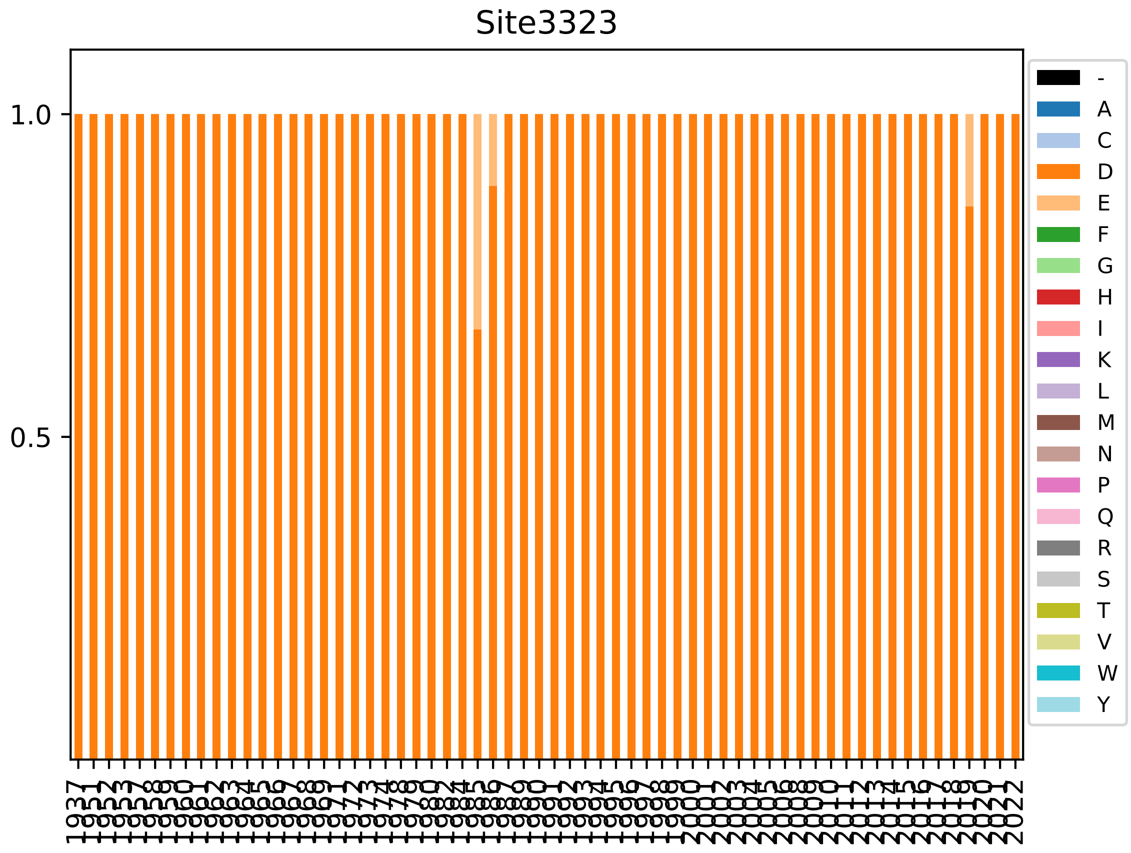

Site3323
1.0
0.5
-
A
C
D
E
F
G
H
I
K
L
M
N
P
Q
R
S
T
V
W
Y

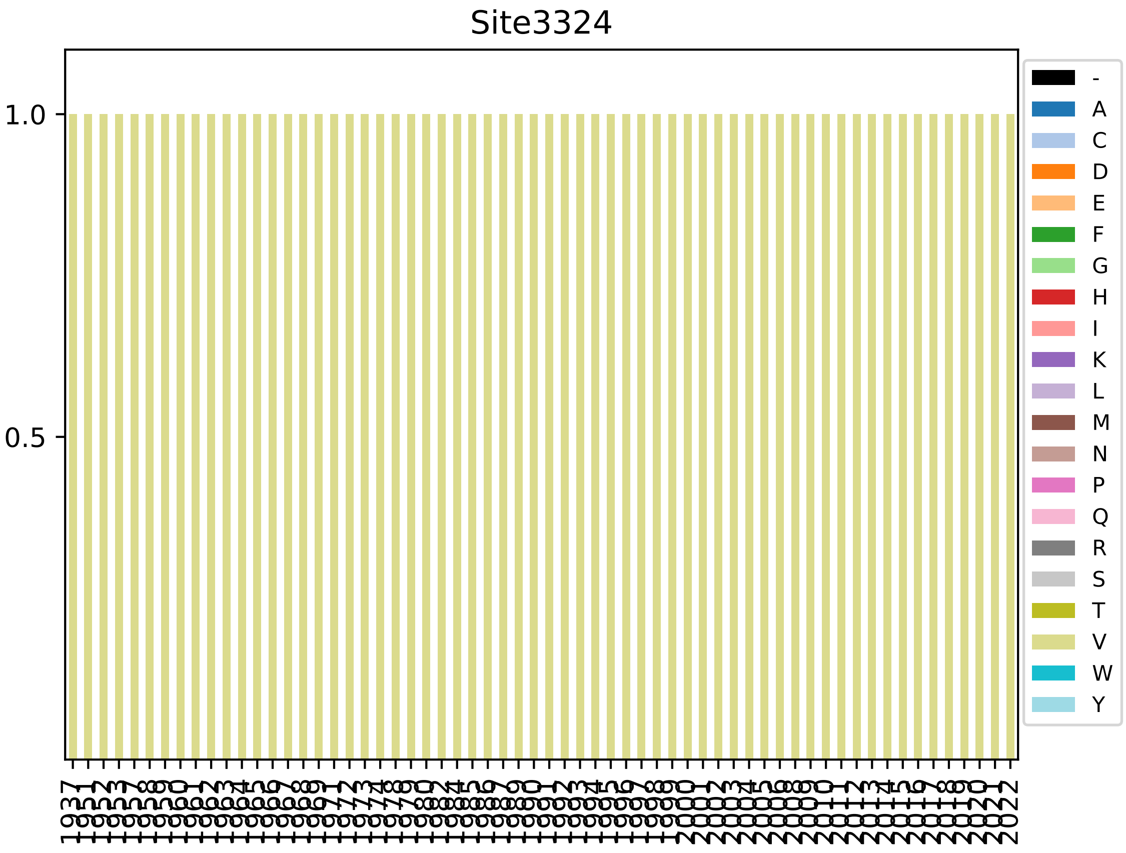
Site3324
1.0
0.5
-
A
C
D
E
F
G
H
I
K
L
M
N
P
Q
R
S
T
V
W
Y

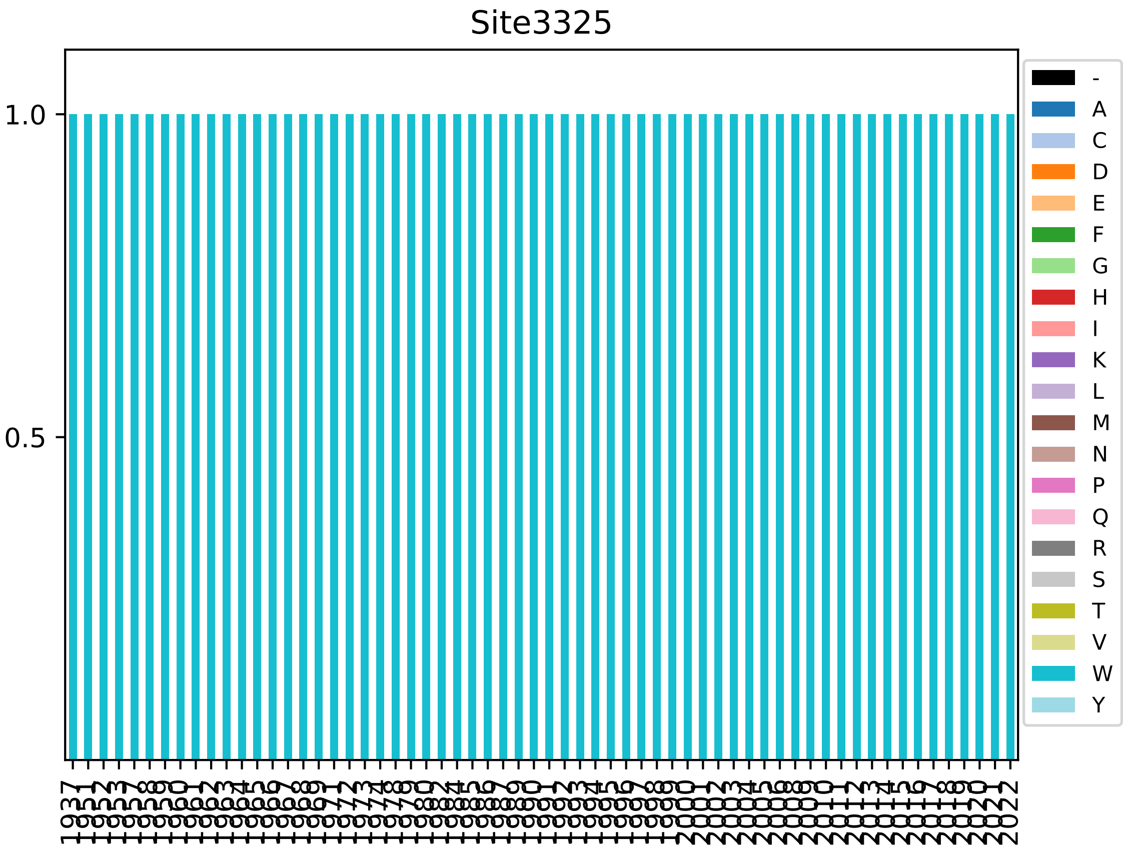
Site3325
1.0
0.5
-
A
C
D
E
F
G
H
I
K
L
M
N
P
Q
R
S
T
V
W
Y

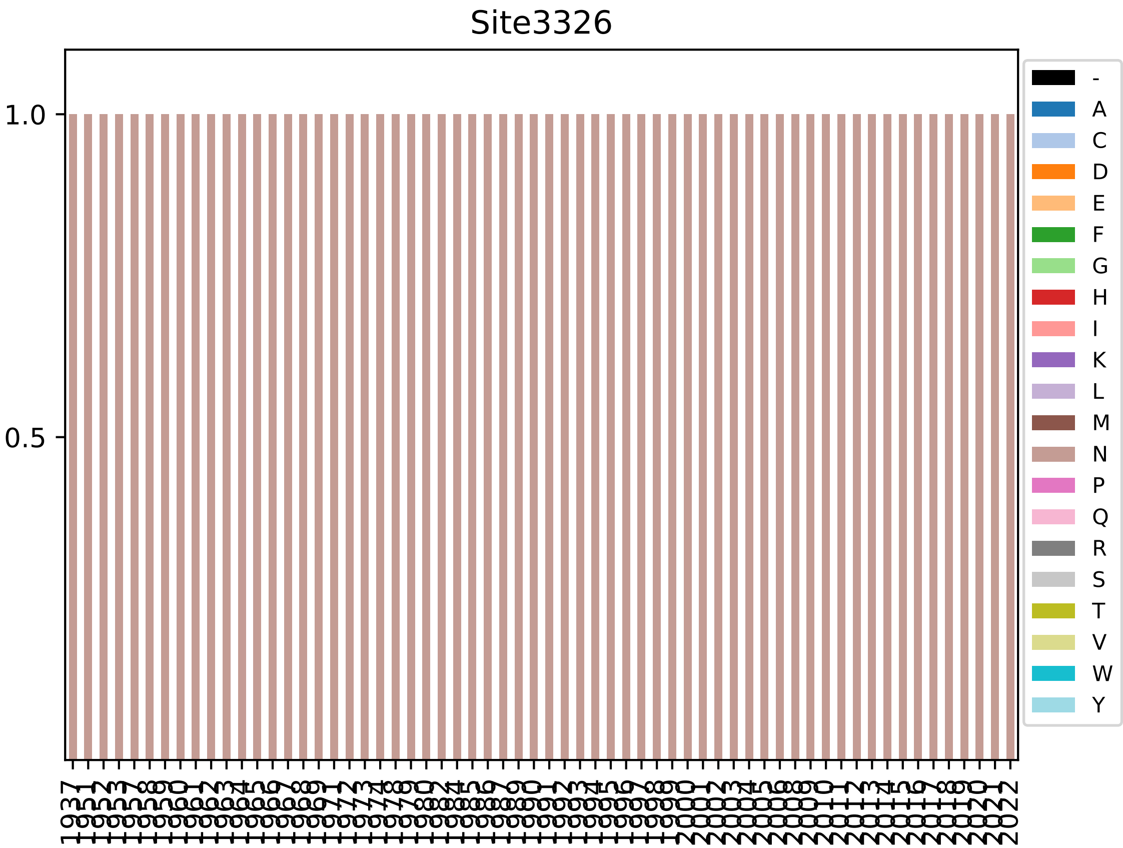
Site3326
1.0
0.5
-
A
C
D
E
F
G
H
I
K
L
M
N
P
Q
R
S
T
V
W
Y

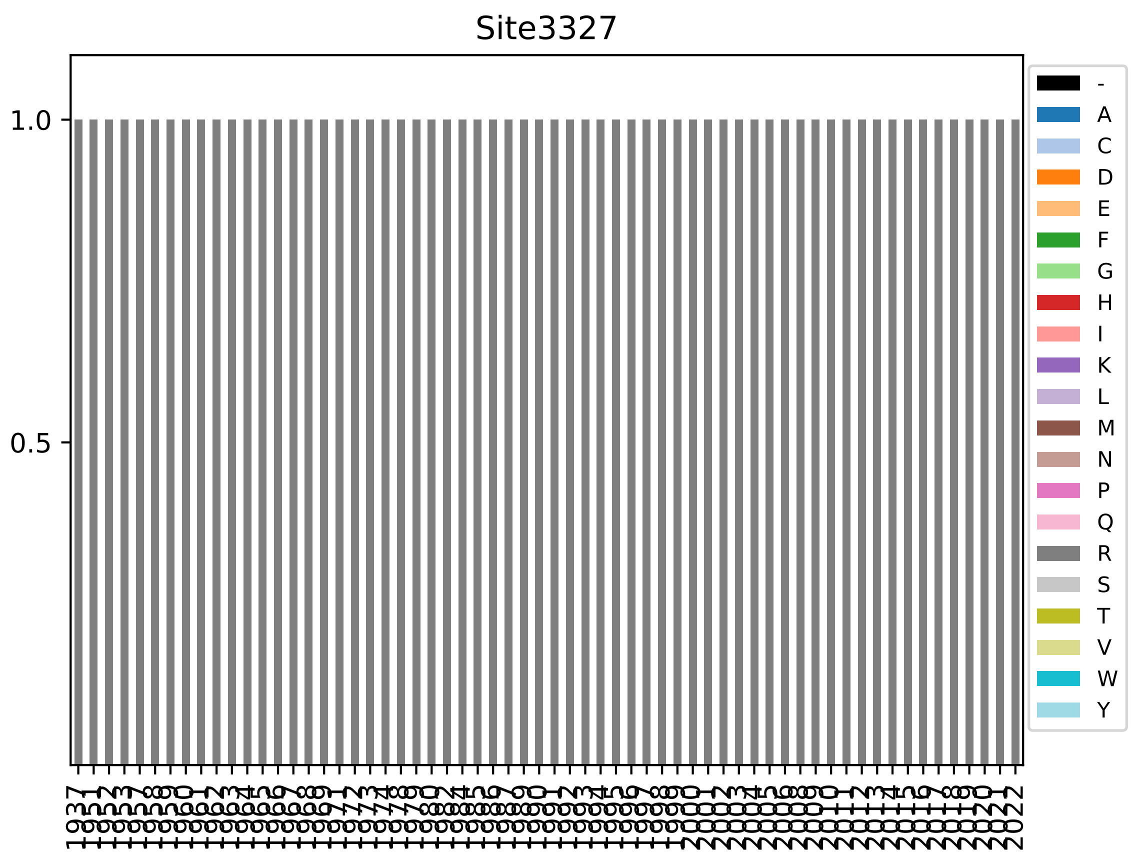
Site3327
1.0
0.5
-
A
C
D
E
F
G
H
I
K
L
M
N
P
Q
R
S
T
V
W
Y

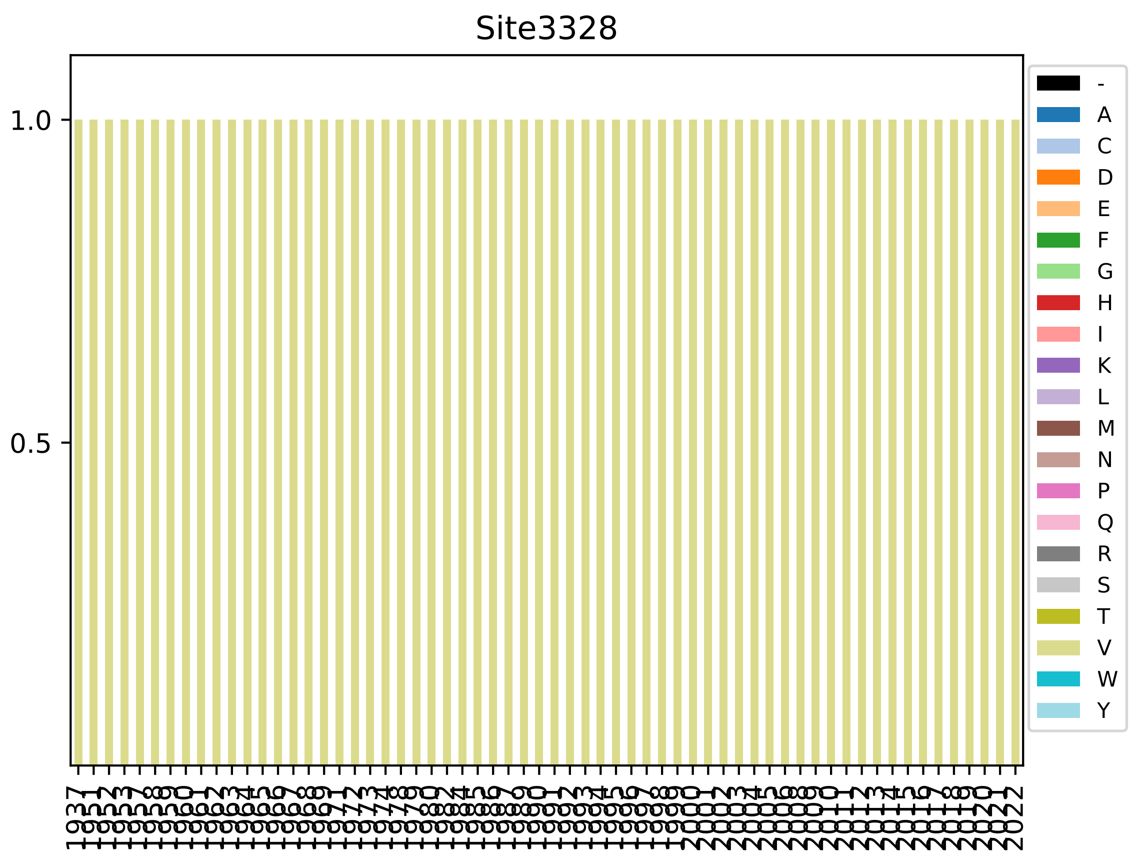
Site3328
1.0
0.5
-
A
C
D
E
F
G
H
I
K
L
M
N
P
Q
R
S
T
V
W
Y

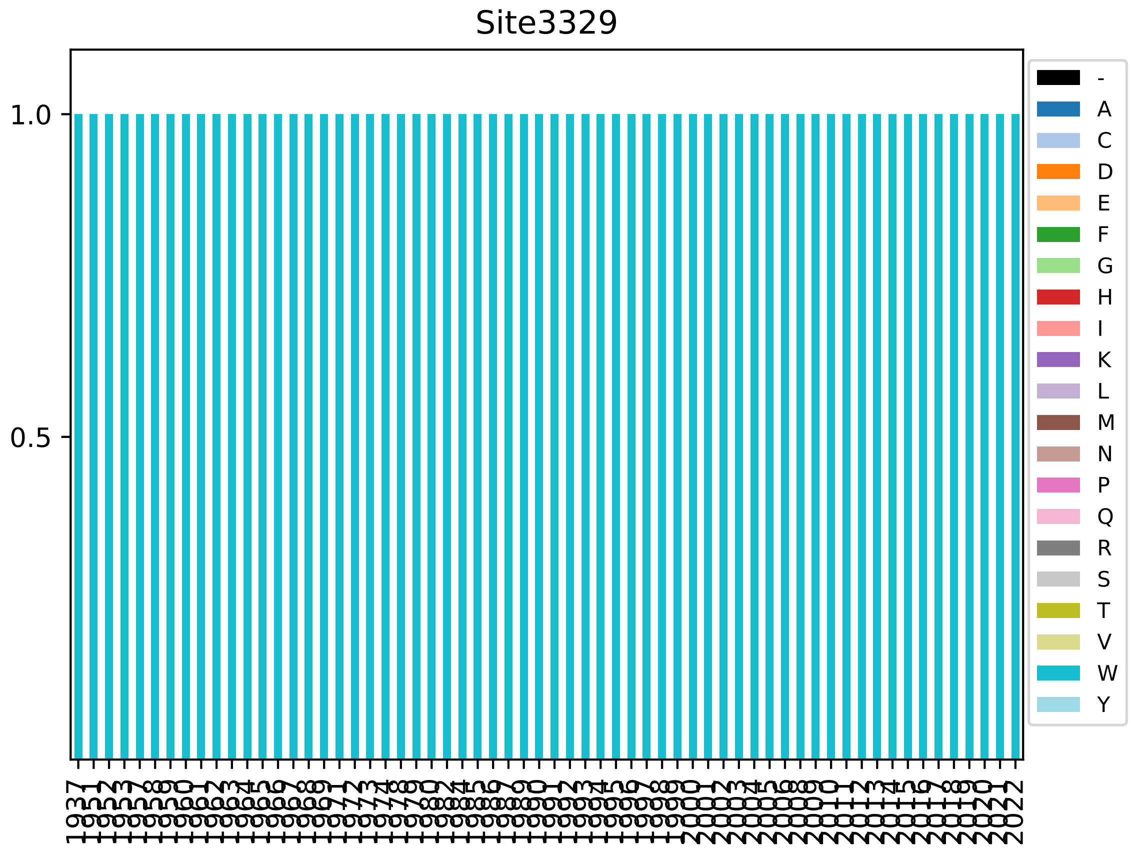
Site3329
1.0
0.5
-
A
C
D
E
F
G
H
I
K
L
M
N
P
Q
R
S
T
V
W
Y

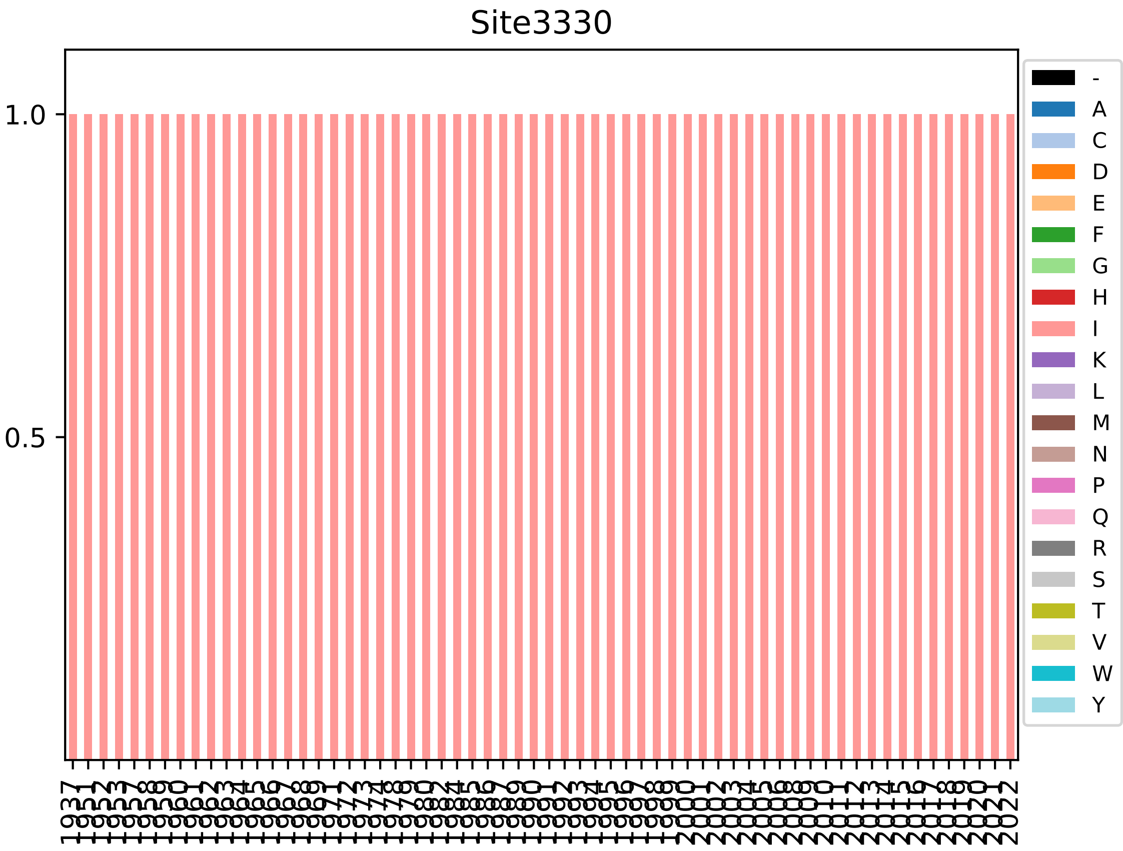
Site3330
1.0
0.5
-
A
C
D
E
F
G
H
I
K
L
M
N
P
Q
R
S
T
V
W
Y

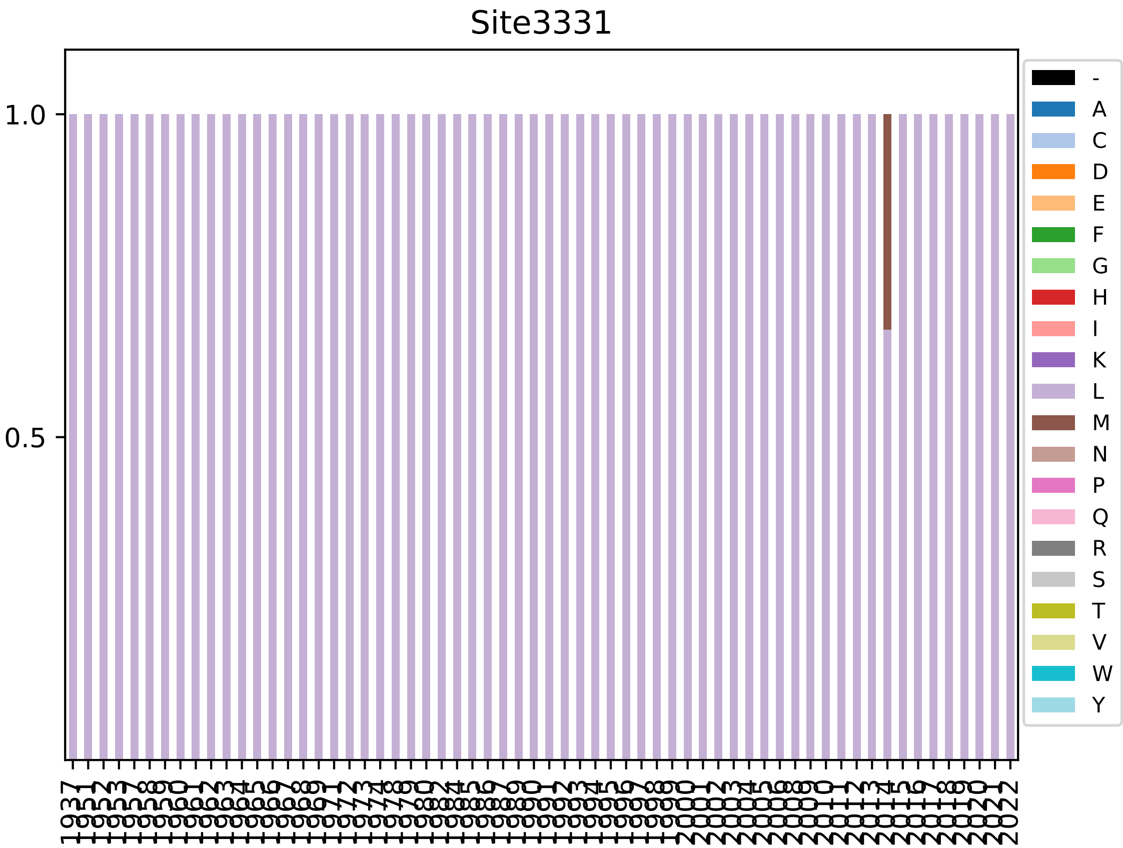
Site3331
1.0
0.5
-
A
C
D
E
F
G
H
I
K
L
M
N
P
Q
R
S
T
V
W
Y

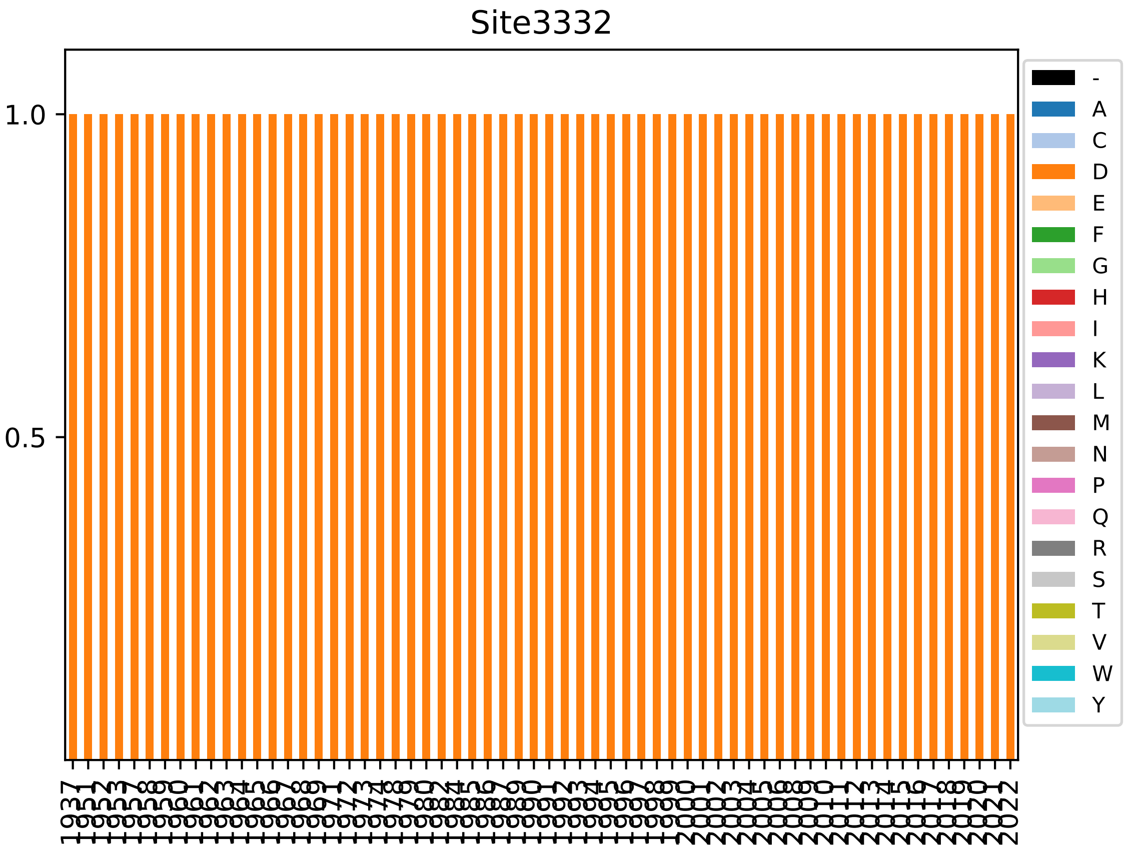
Site3332
1.0
0.5
-
A
C
D
E
F
G
H
I
K
L
M
N
P
Q
R
S
T
V
W
Y

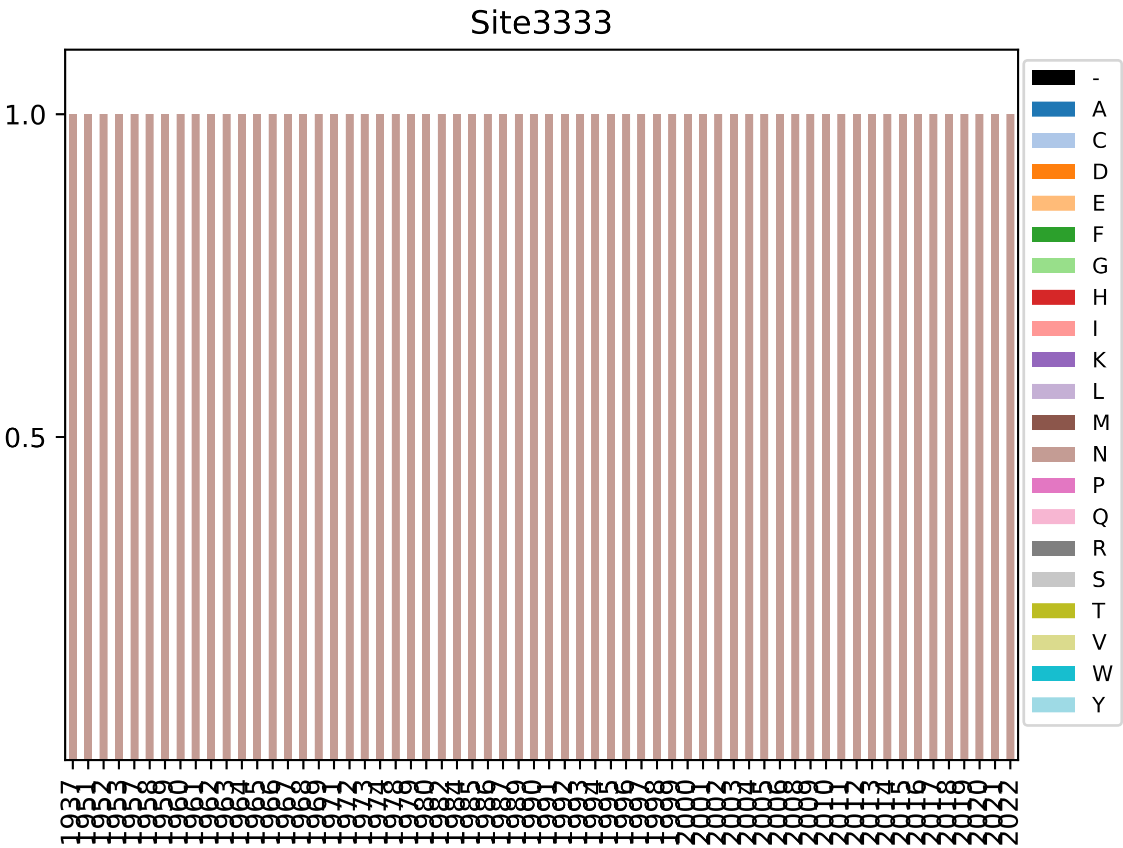
Site3333
1.0
0.5
-
A
C
D
E
F
G
H
I
K
L
M
N
P
Q
R
S
T
V
W
Y

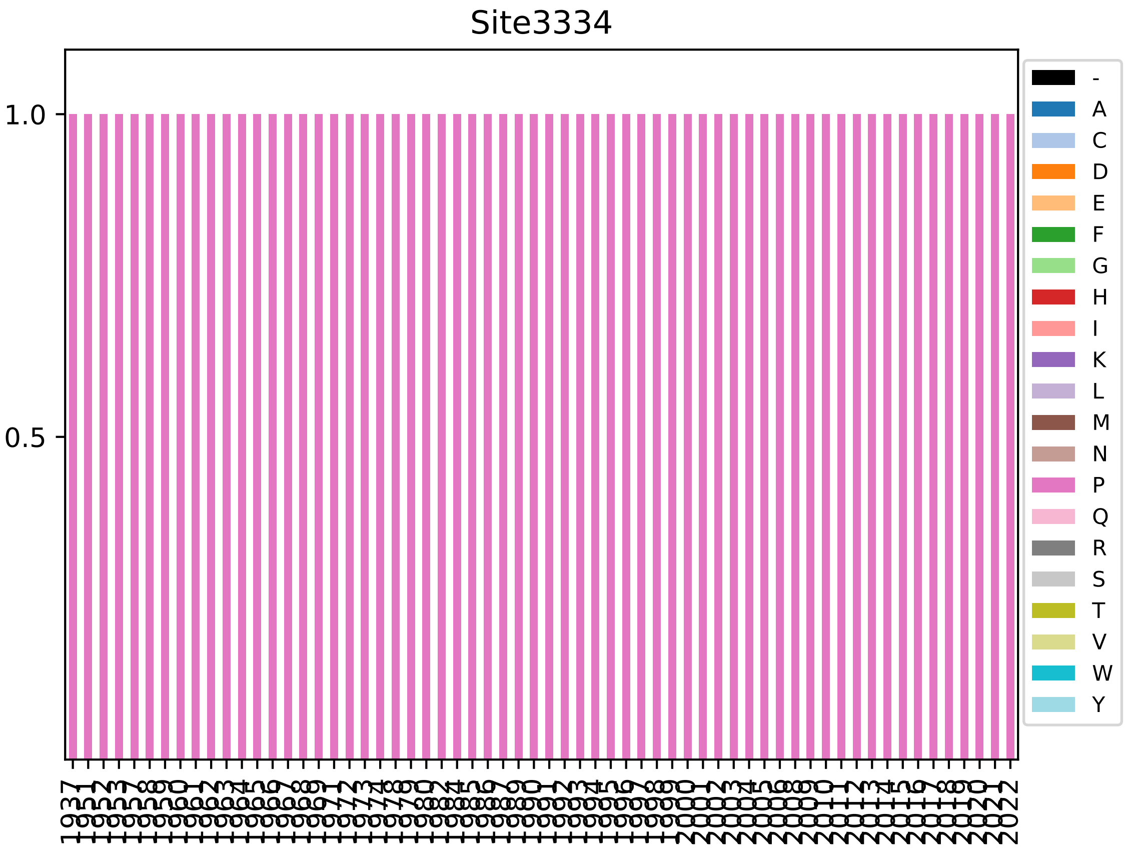
Site3334
1.0
0.5
-
A
C
D
E
F
G
H
I
K
L
M
N
P
Q
R
S
T
V
W
Y

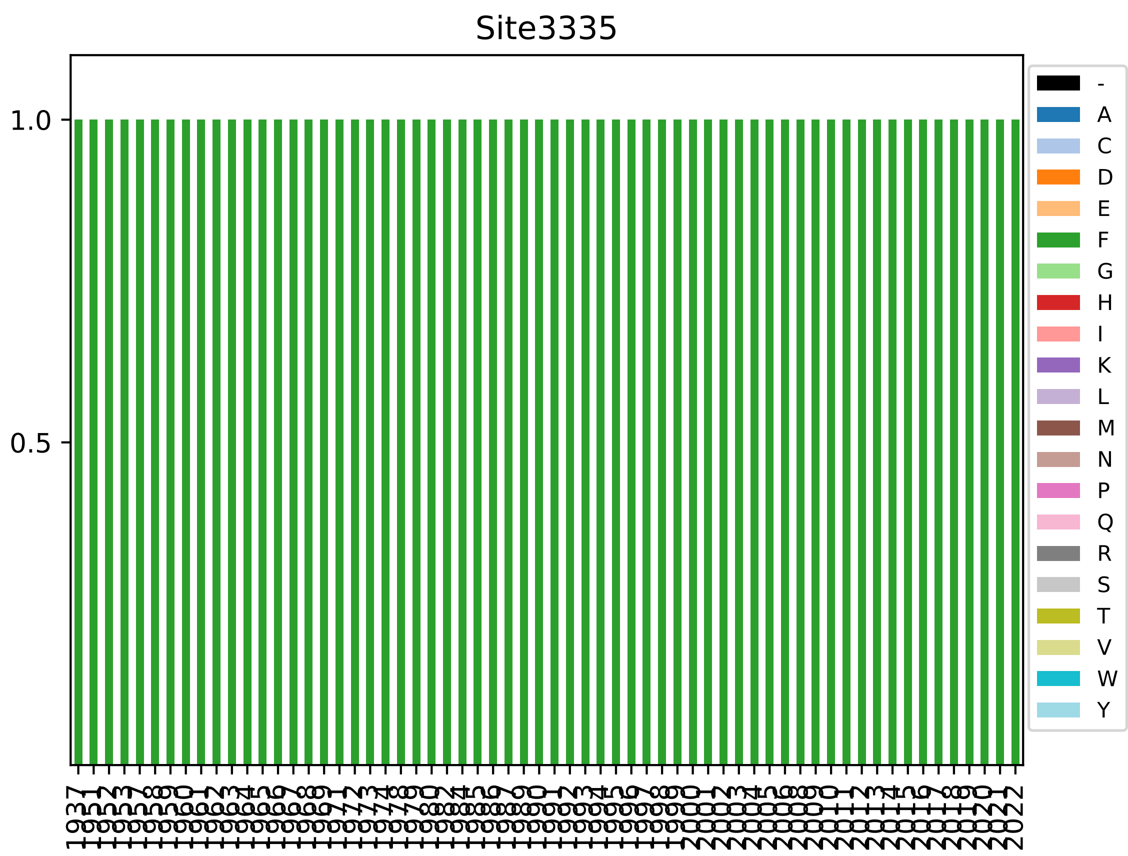
Site3335
1.0
0.5
-
A
C
D
E
F
G
H
I
K
L
M
N
P
Q
R
S
T
V
W
Y

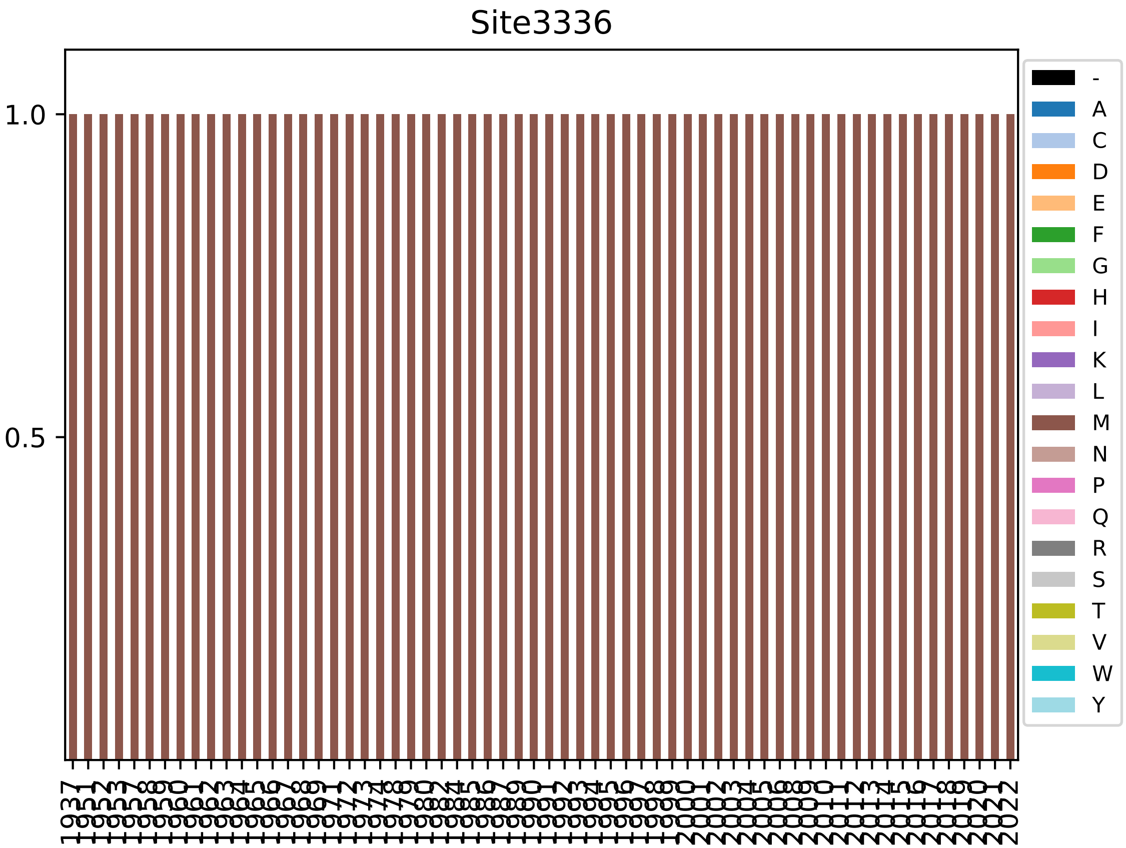
Site3336
1.0
0.5
-
A
C
D
E
F
G
H
I
K
L
M
N
P
Q
R
S
T
V
W
Y

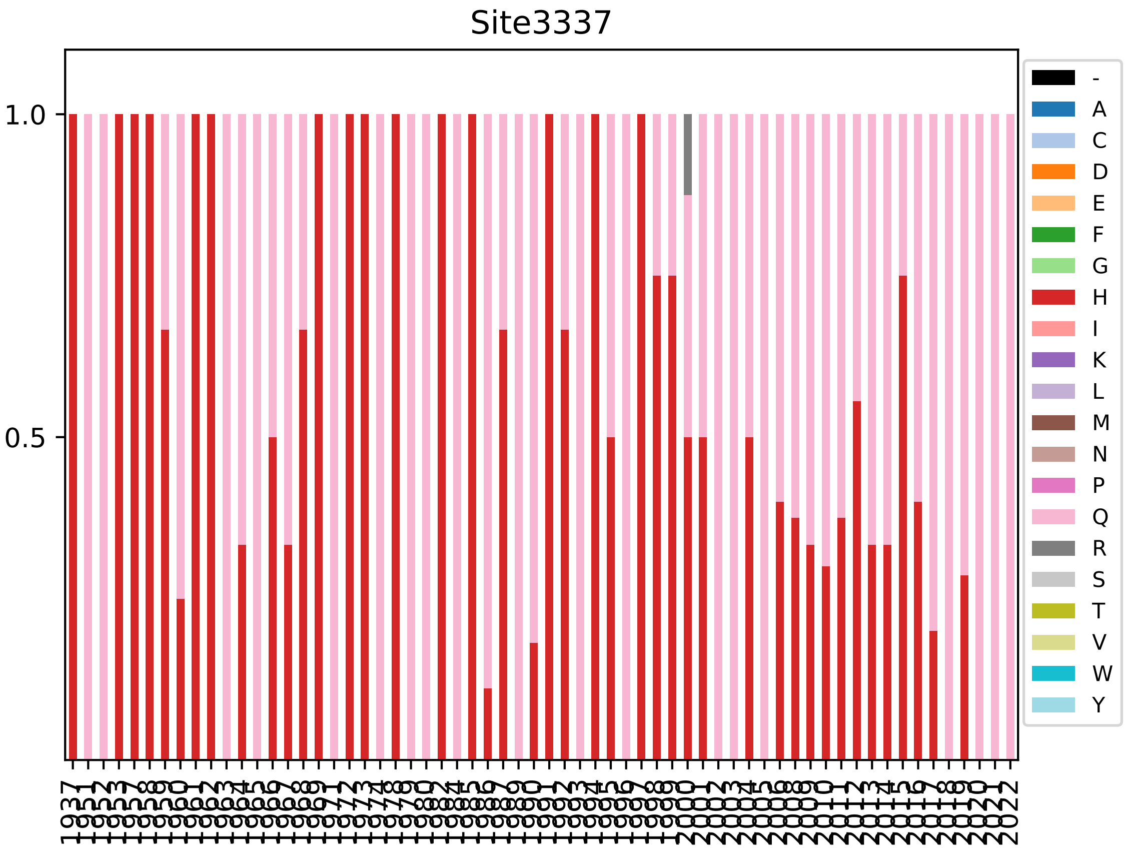
Site3337
1.0
0.5
-
A
C
D
E
F
G
H
I
K
L
M
N
P
Q
R
S
T
V
W
Y

Site3338

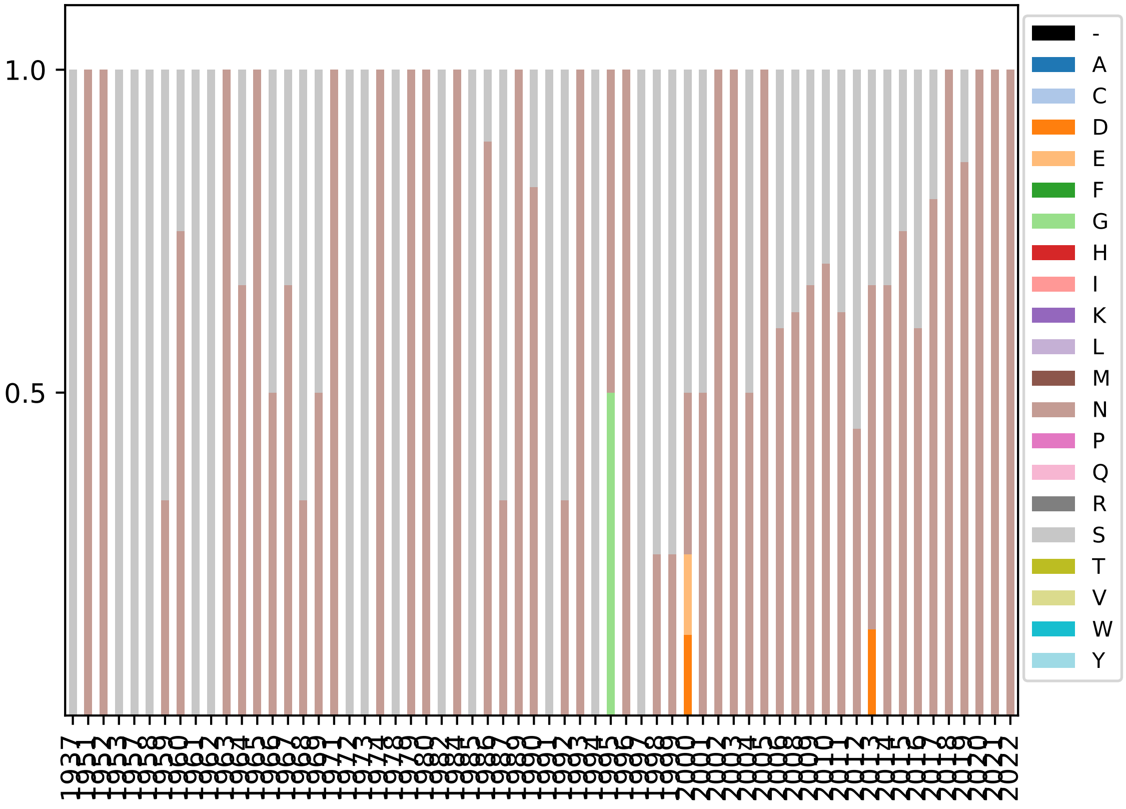

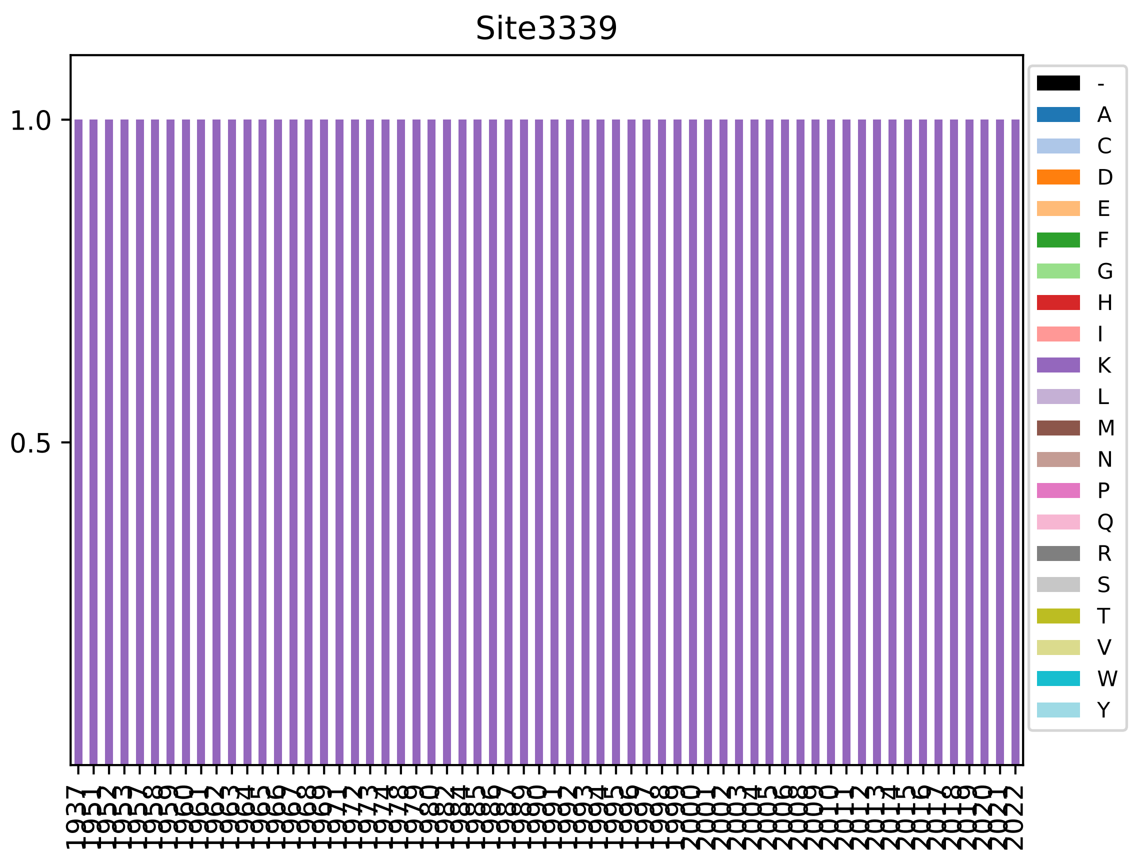
Site3339
1.0
0.5
-
A
C
D
E
F
G
H
I
K
L
M
N
P
Q
R
S
T
V
W
Y

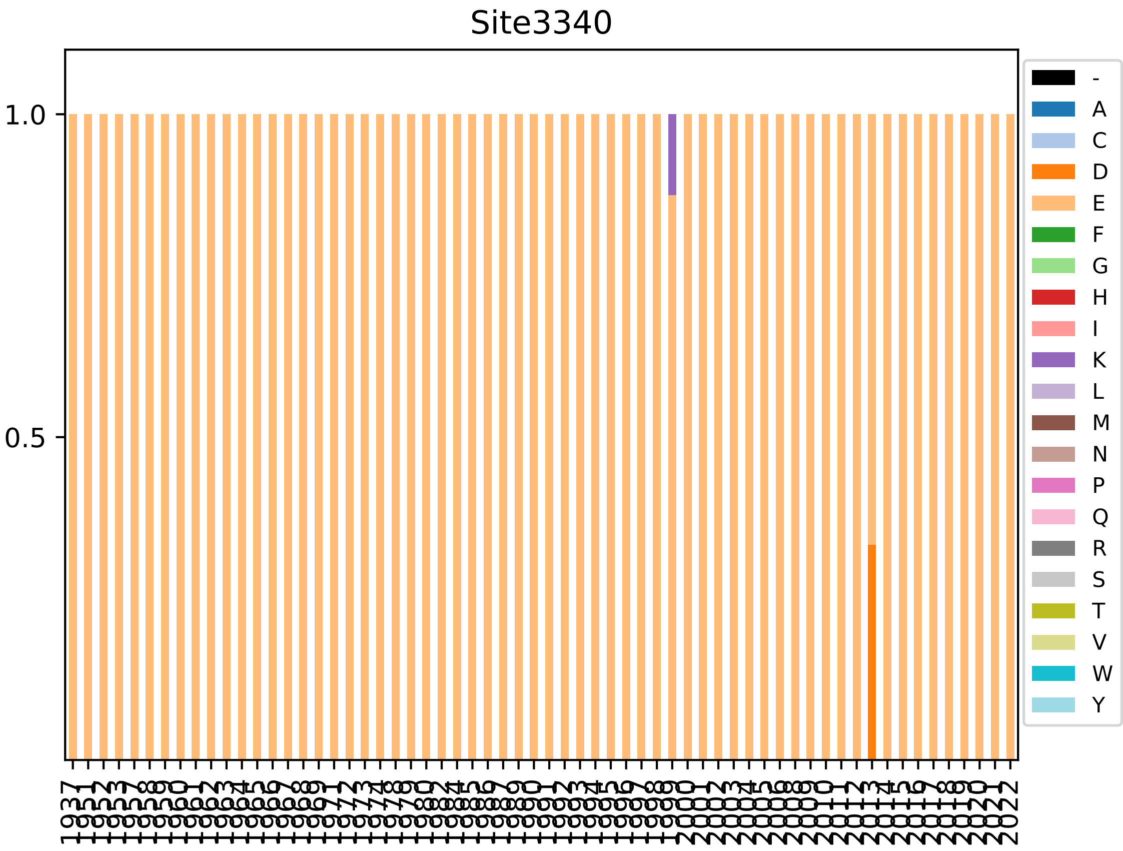
Site3340
1.0
0.5
-
A
C
D
E
F
G
H
I
K
L
M
N
P
Q
R
S
T
V
W
Y

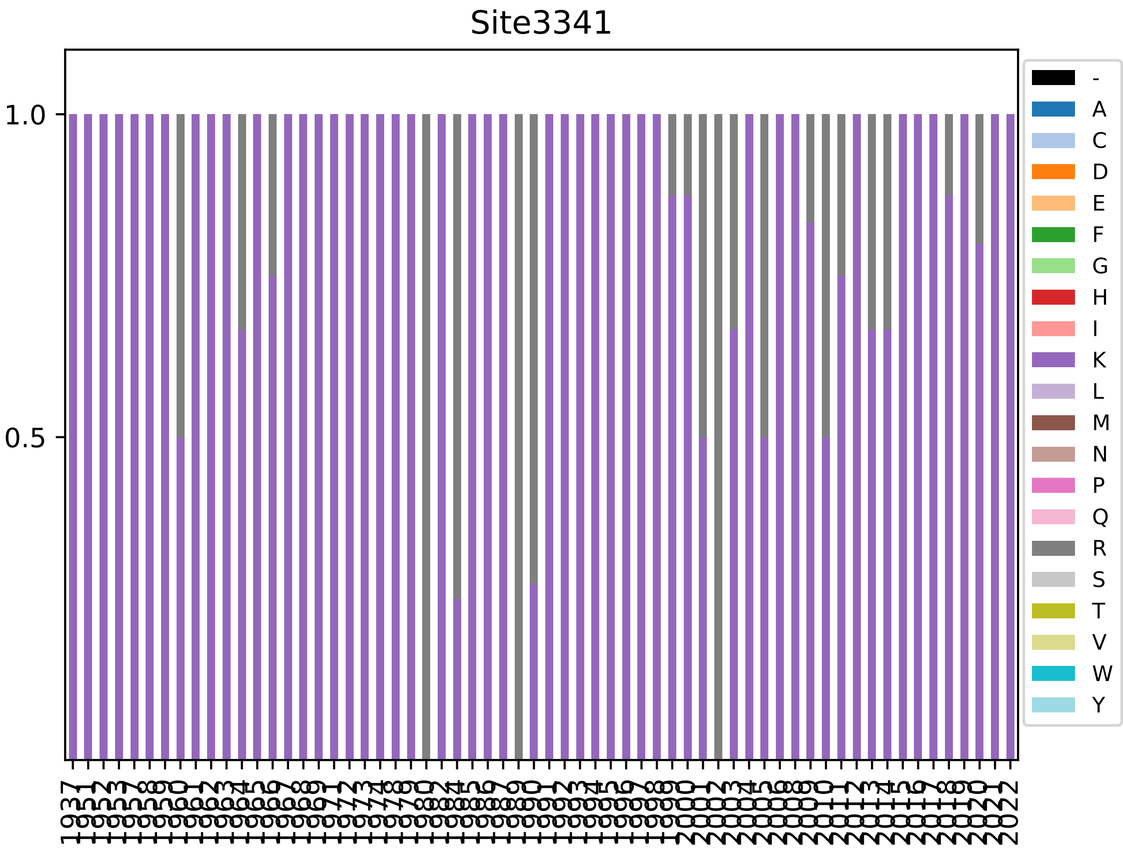
Site3341
1.0
0.5
-
A
C
D
E
F
G
H
I
K
L
M
N
P
Q
R
S
T
V
W
Y

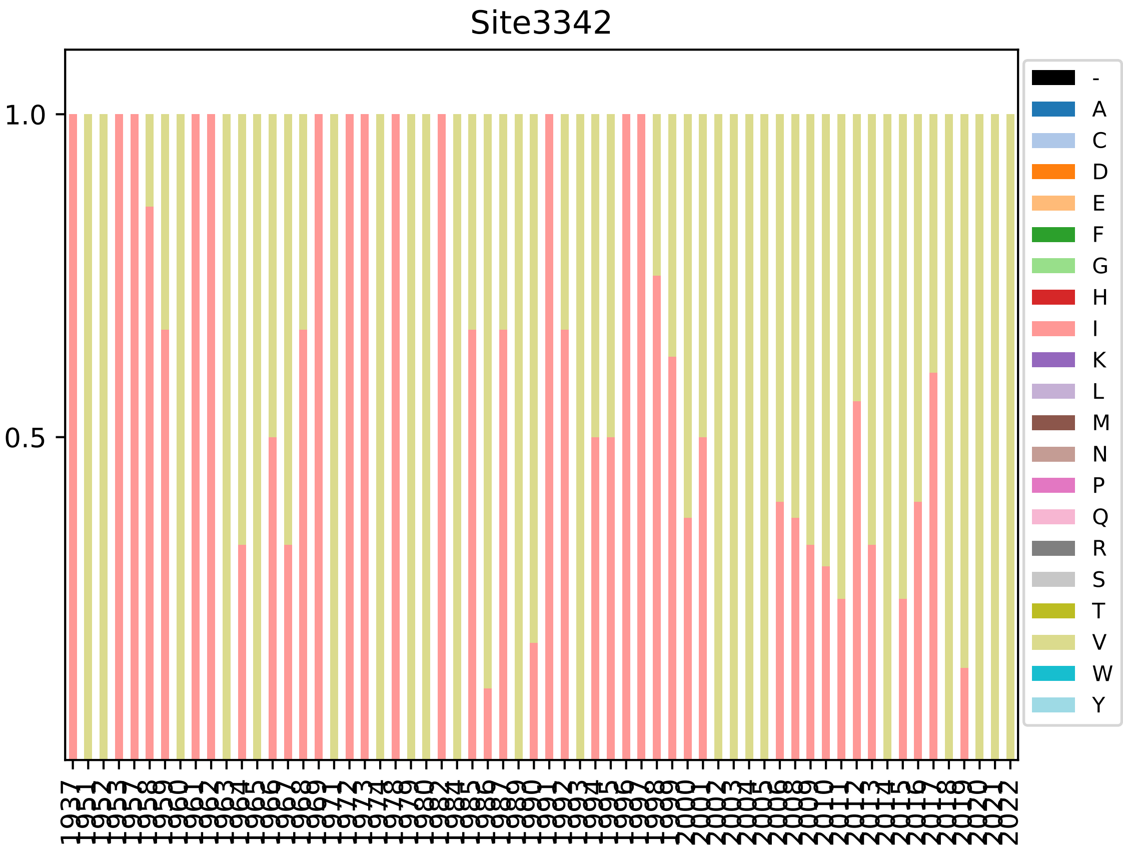
Site3342
1.0
0.5
-
A
C
D
E
F
G
H
I
K
L
M
N
P
Q
R
S
T
V
W
Y

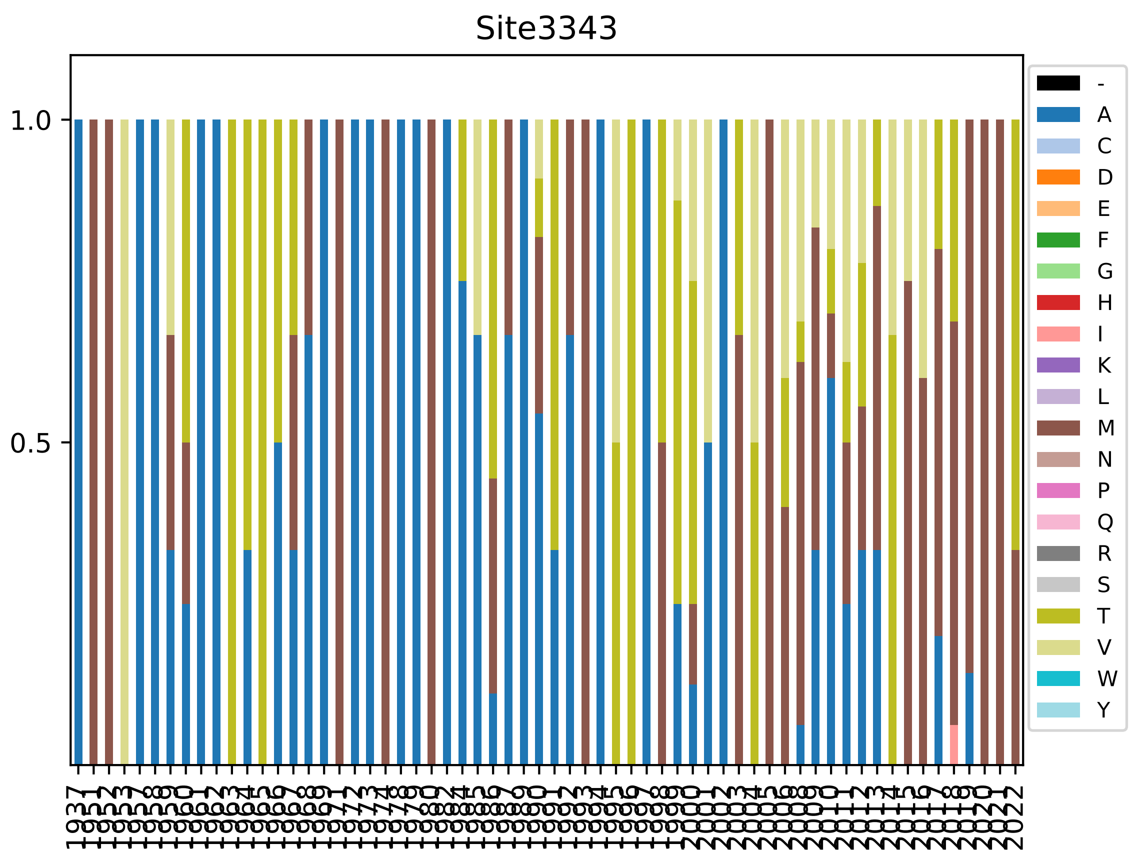
Site3343
1.0
0.5
-
A
C
D
E
F
G
H
I
K
L
M
N
P
Q
R
S
T
V
W
Y

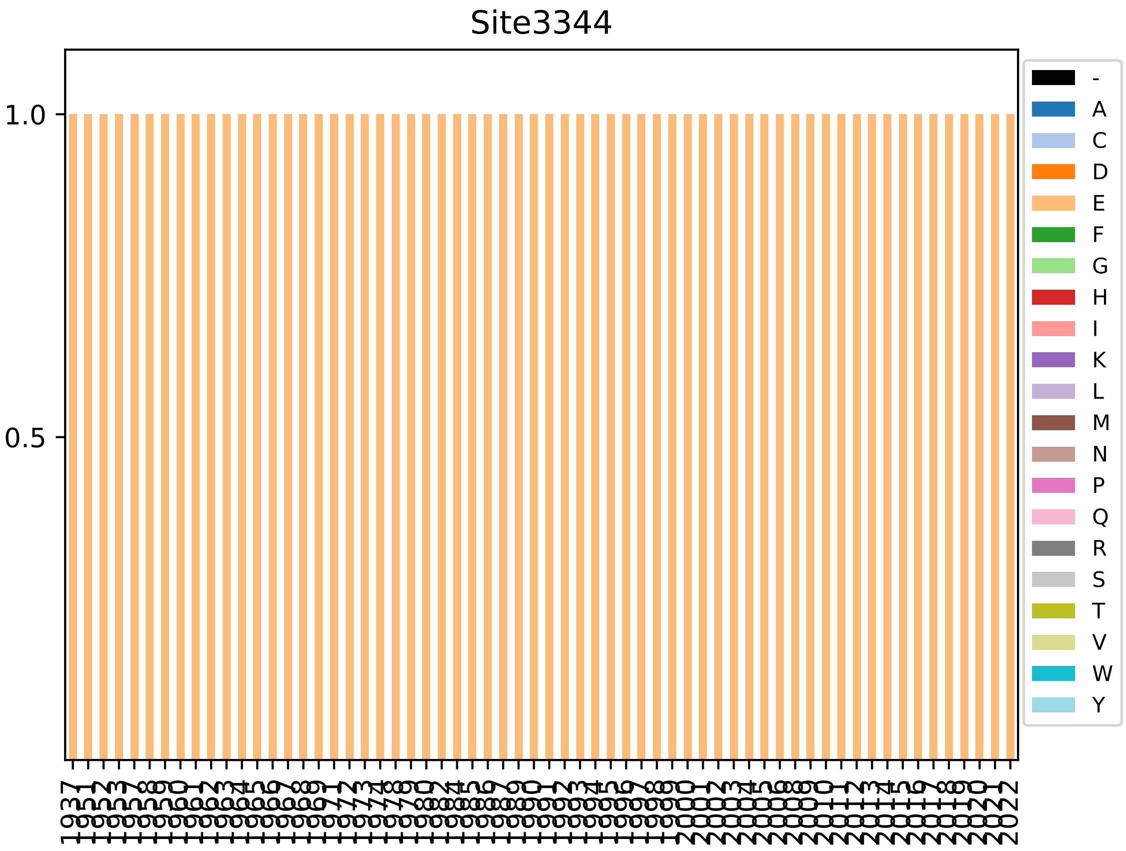
Site3344
1.0
0.5
-
A
C
D
E
F
G
H
I
K
L
M
N
P
Q
R
S
T
V
W
Y

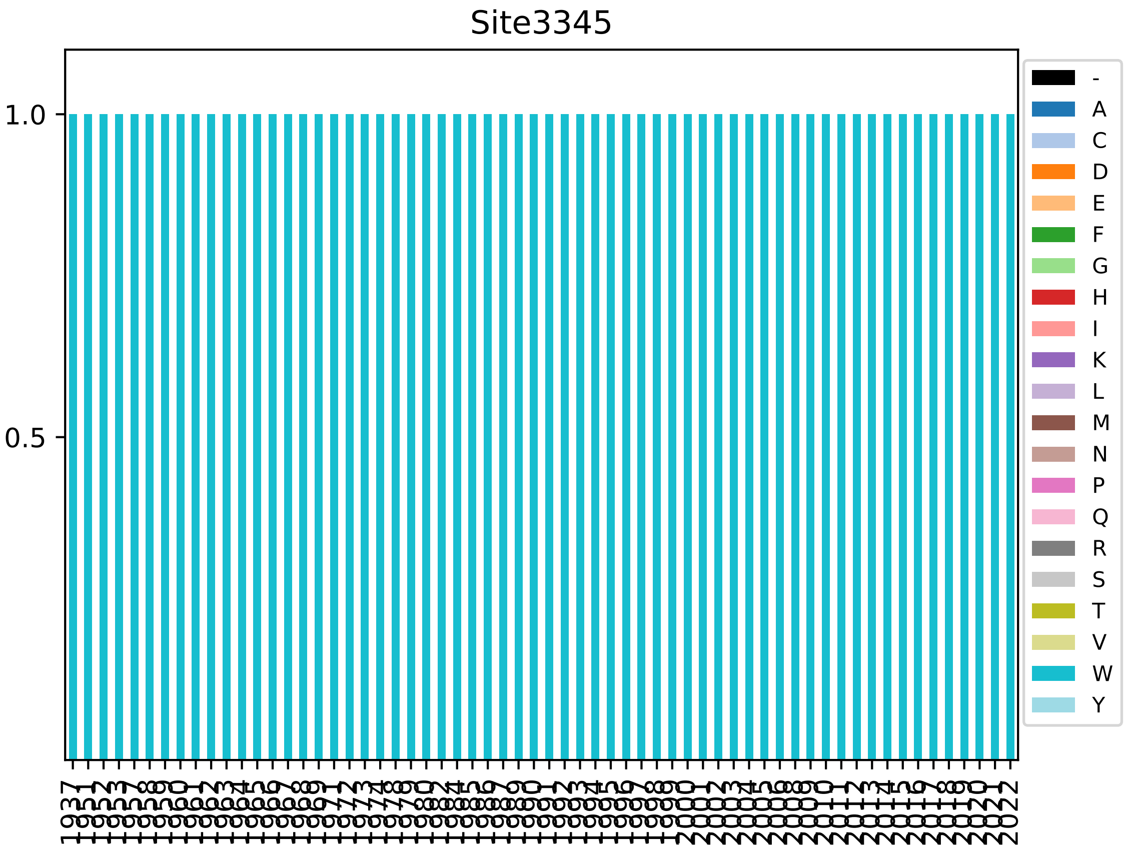
Site3345
1.0
0.5
-
A
C
D
E
F
G
H
I
K
L
M
N
P
Q
R
S
T
V
W
Y

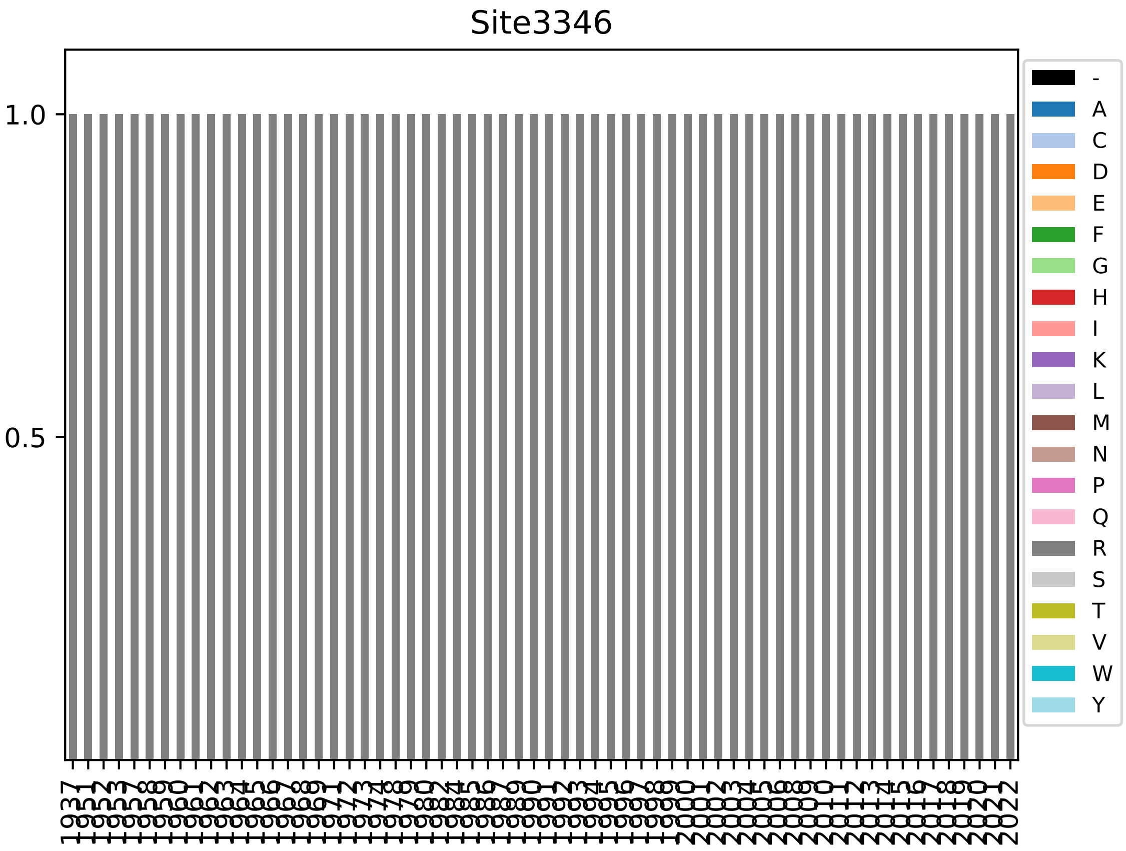
Site3346
1.0
0.5
-
A
C
D
E
F
G
H
I
K
L
M
N
P
Q
R
S
T
V
W
Y

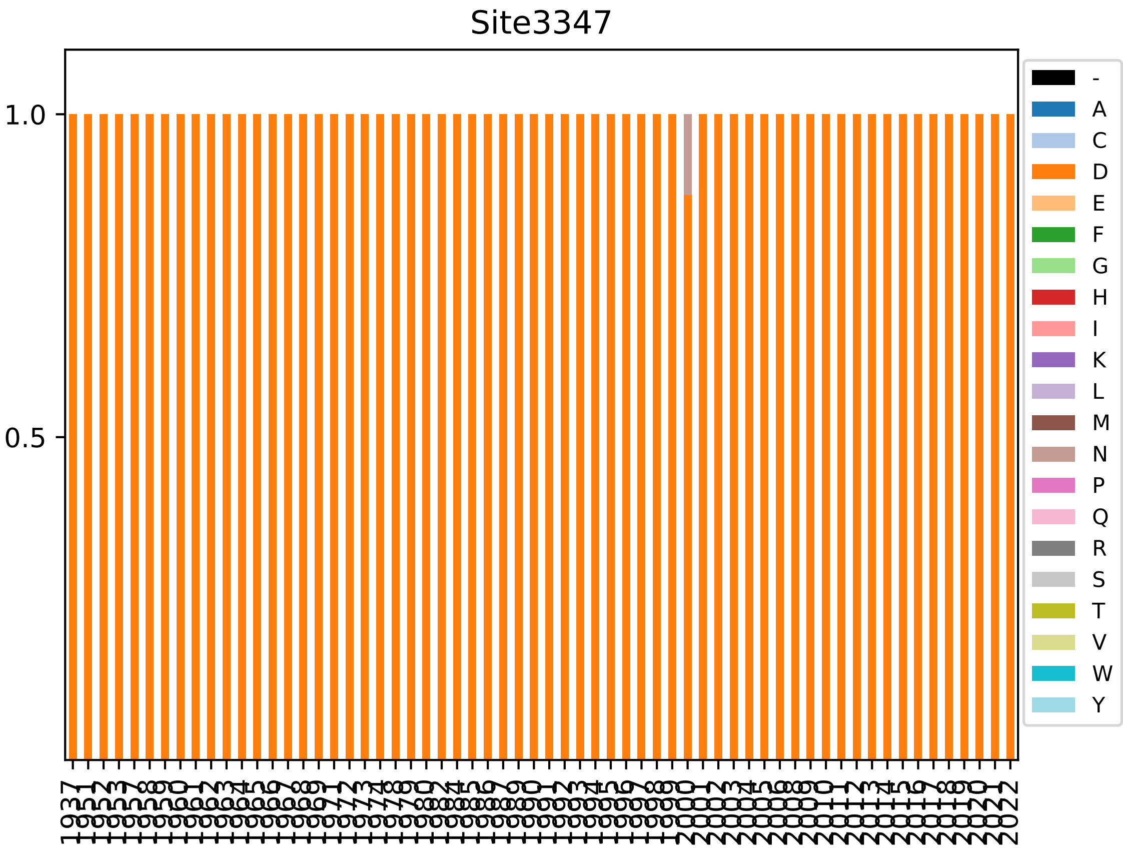
Site3347
1.0
0.5
-
A
C
D
E
F
G
H
I
K
L
M
N
P
Q
R
S
T
V
W
Y

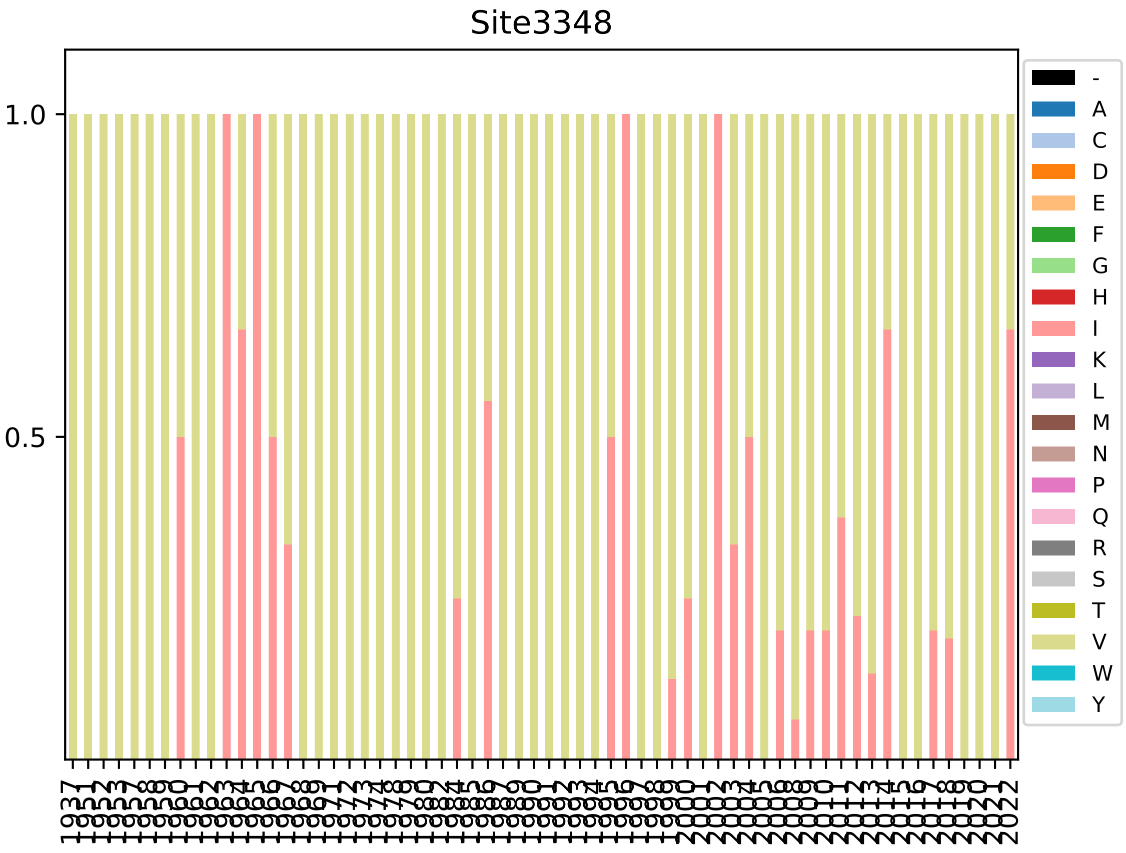
Site3348
1.0
0.5
-
A
C
D
E
F
G
H
I
K
L
M
N
P
Q
R
S
T
V
W
Y

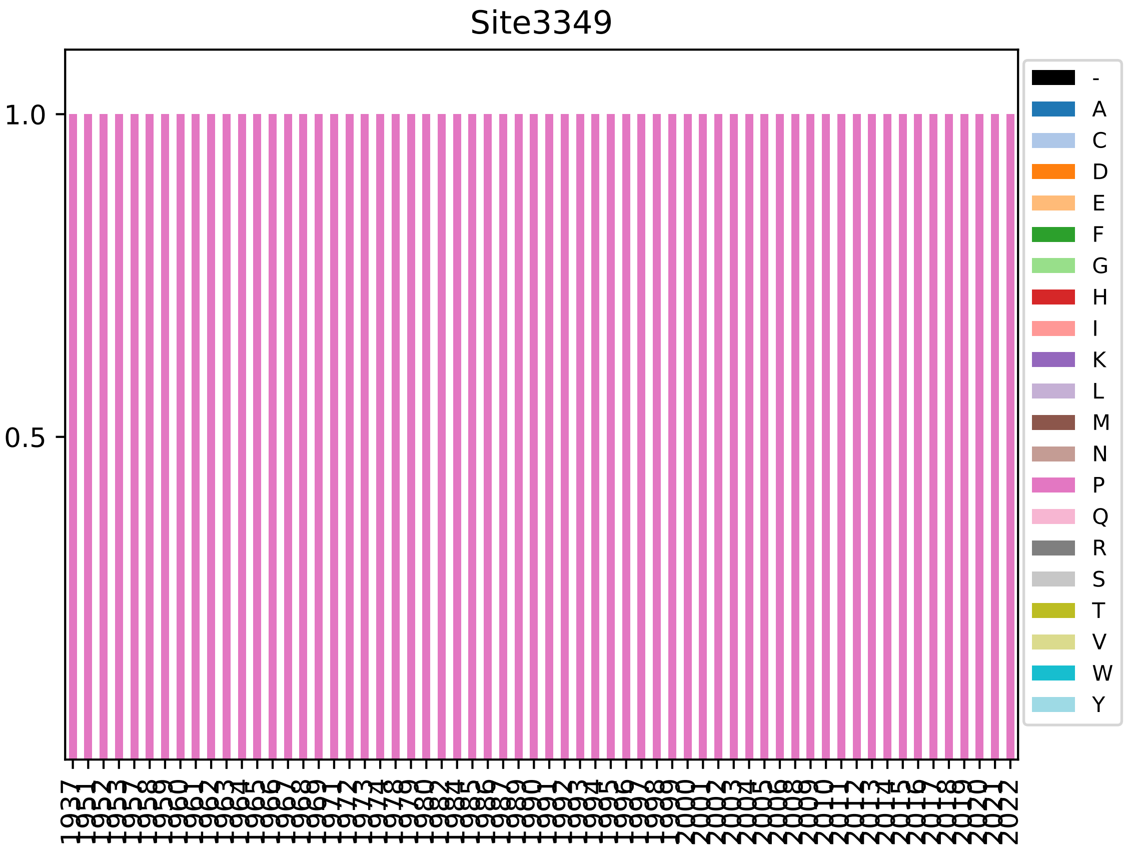
Site3349
1.0
0.5
-
A
C
D
E
F
G
H
I
K
L
M
N
P
Q
R
S
T
V
W
Y

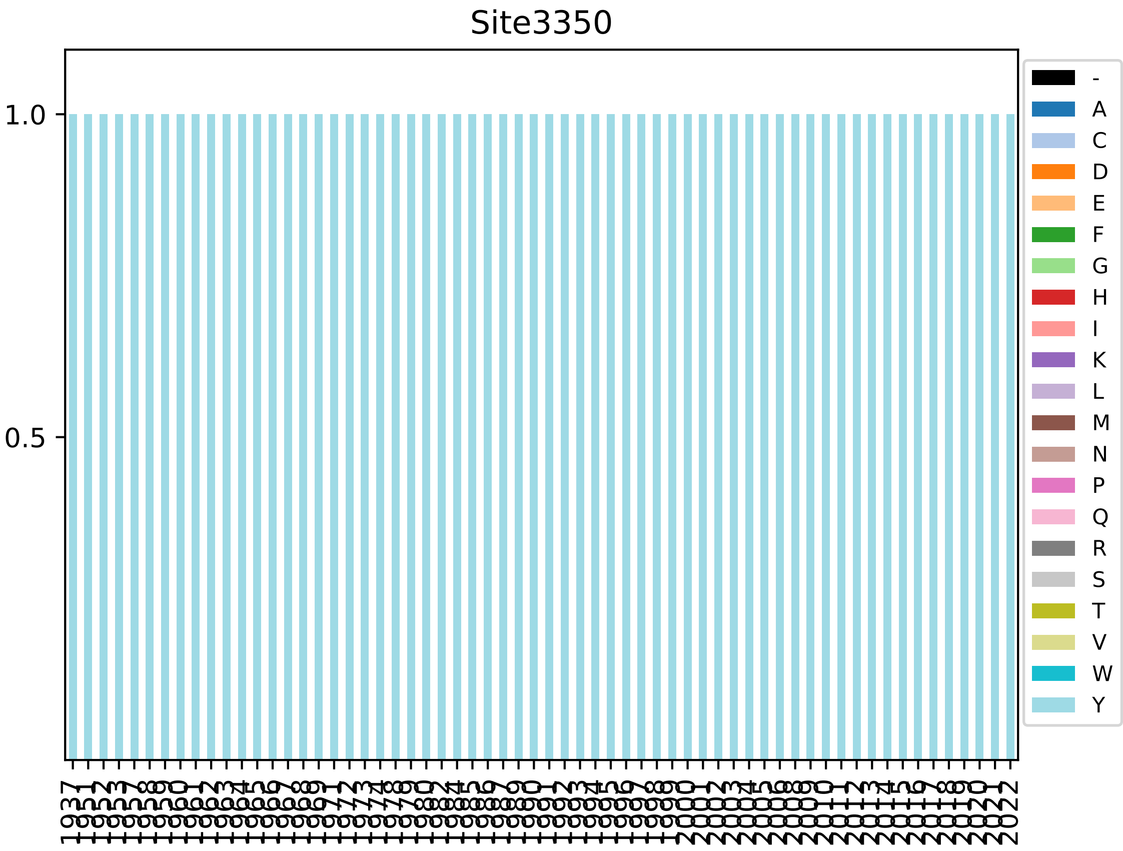
Site3350
1.0
0.5
-
A
C
D
E
F
G
H
I
K
L
M
N
P
Q
R
S
T
V
W
Y

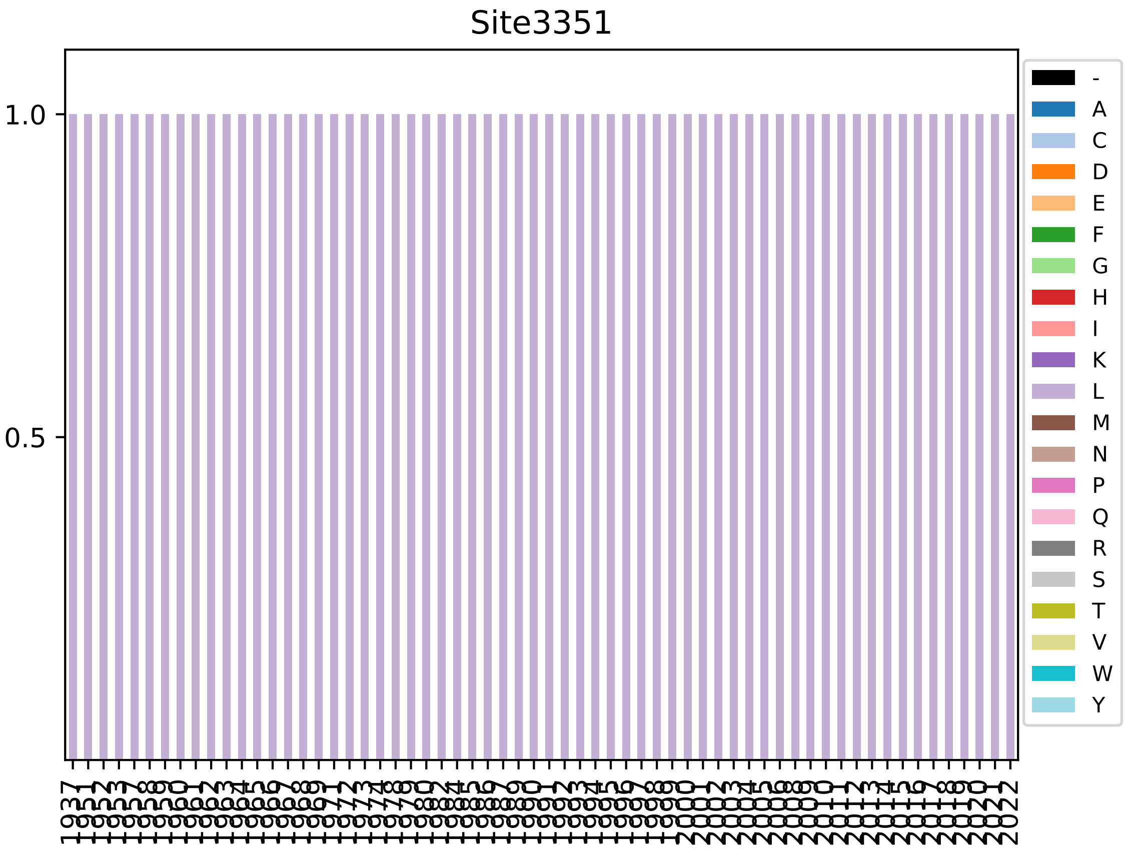
Site3351
1.0
0.5
-
A
C
D
E
F
G
H
I
K
L
M
N
P
Q
R
S
T
V
W
Y

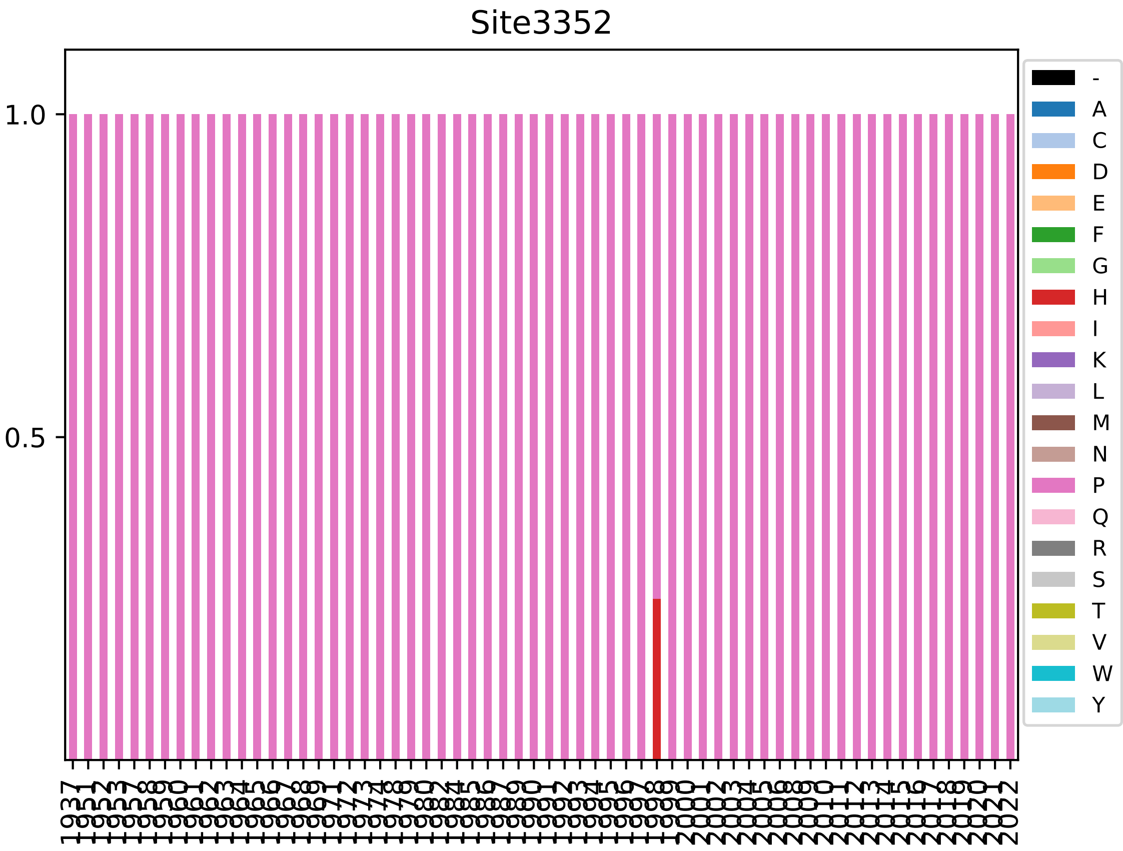
Site3352
1.0
0.5
-
A
C
D
E
F
G
H
I
K
L
M
N
P
Q
R
S
T
V
W
Y

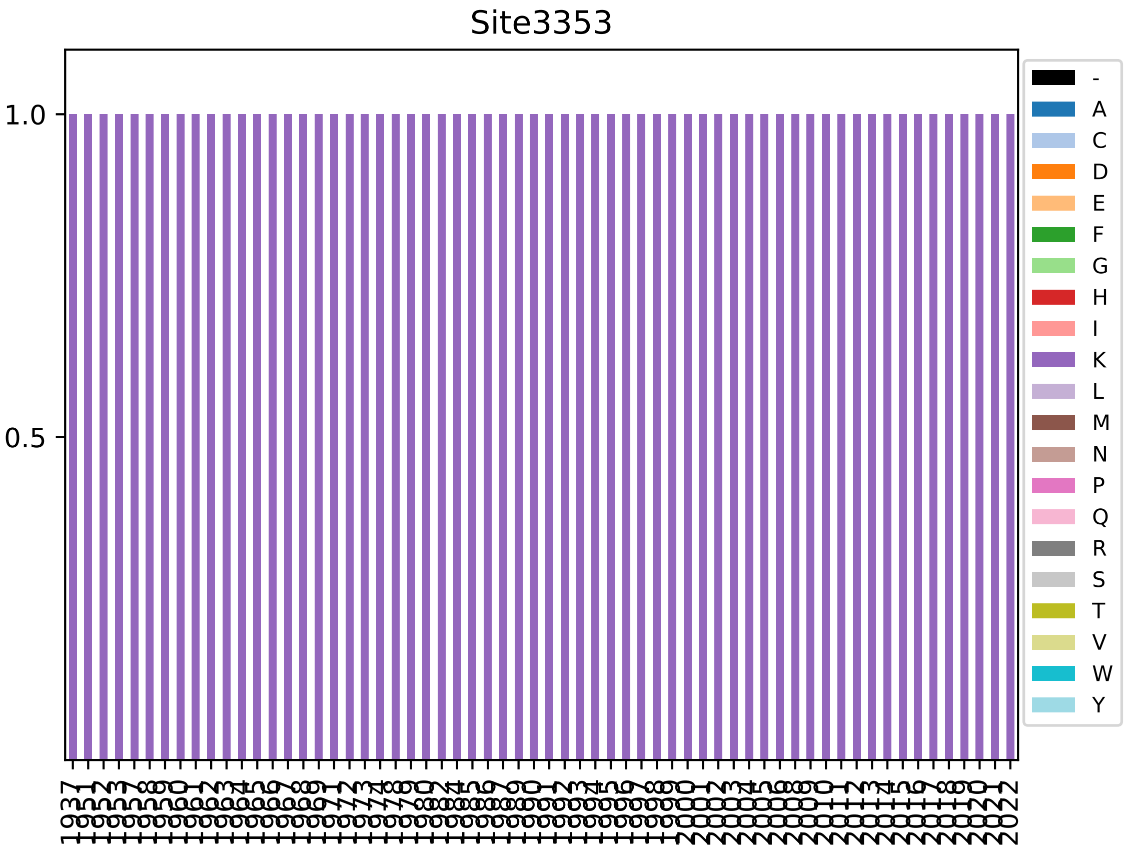
Site3353
1.0
0.5
-
A
C
D
E
F
G
H
I
K
L
M
N
P
Q
R
S
T
V
W
Y

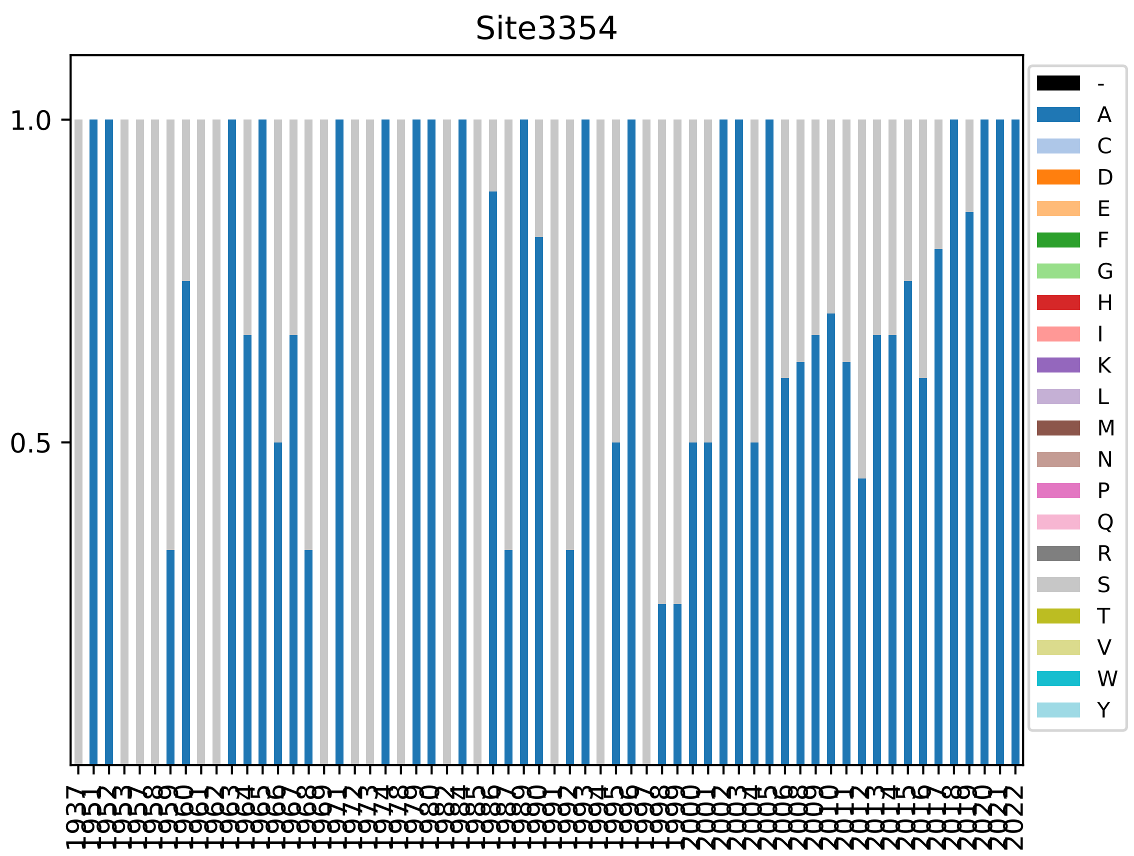
Site3354
1.0
0.5
-
A
C
D
E
F
G
H
I
K
L
M
N
P
Q
R
S
T
V
W
Y

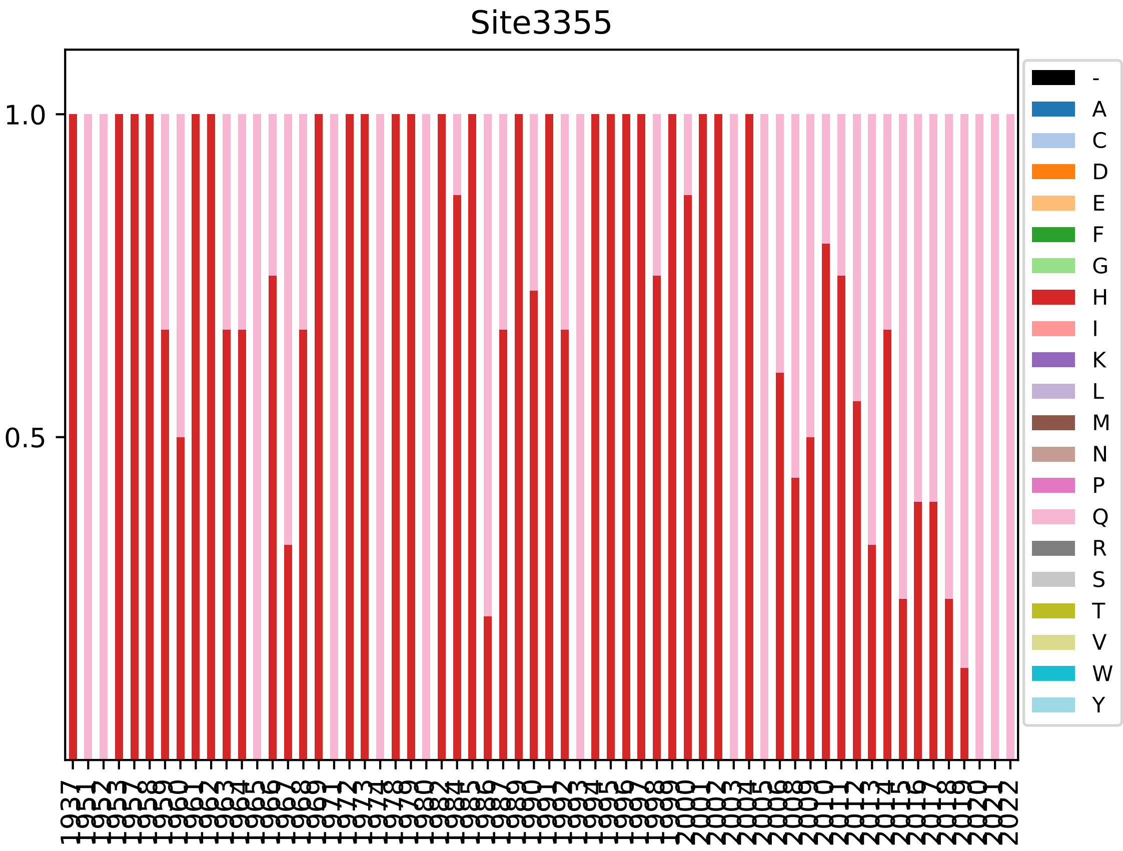
Site3355
1.0
0.5
-
A
C
D
E
F
G
H
I
K
L
M
N
P
Q
R
S
T
V
W
Y

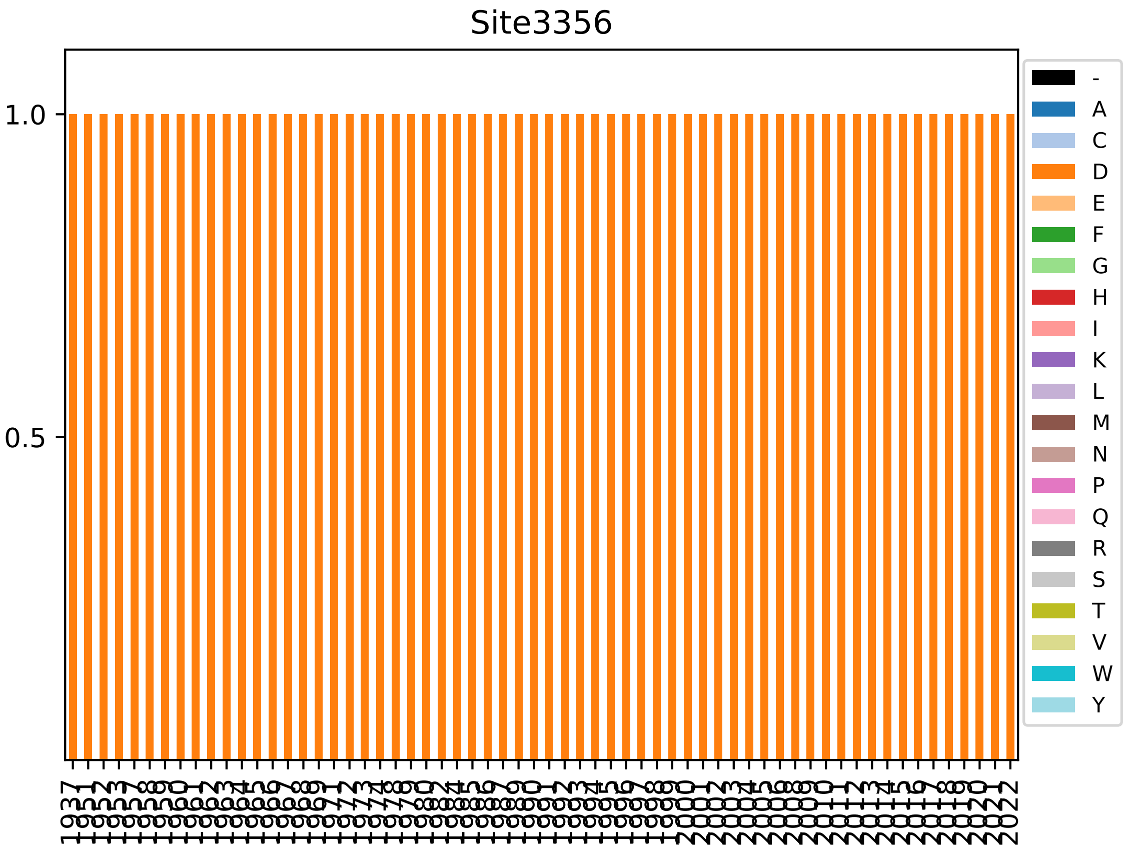
Site3356
1.0
0.5
-
A
C
D
E
F
G
H
I
K
L
M
N
P
Q
R
S
T
V
W
Y

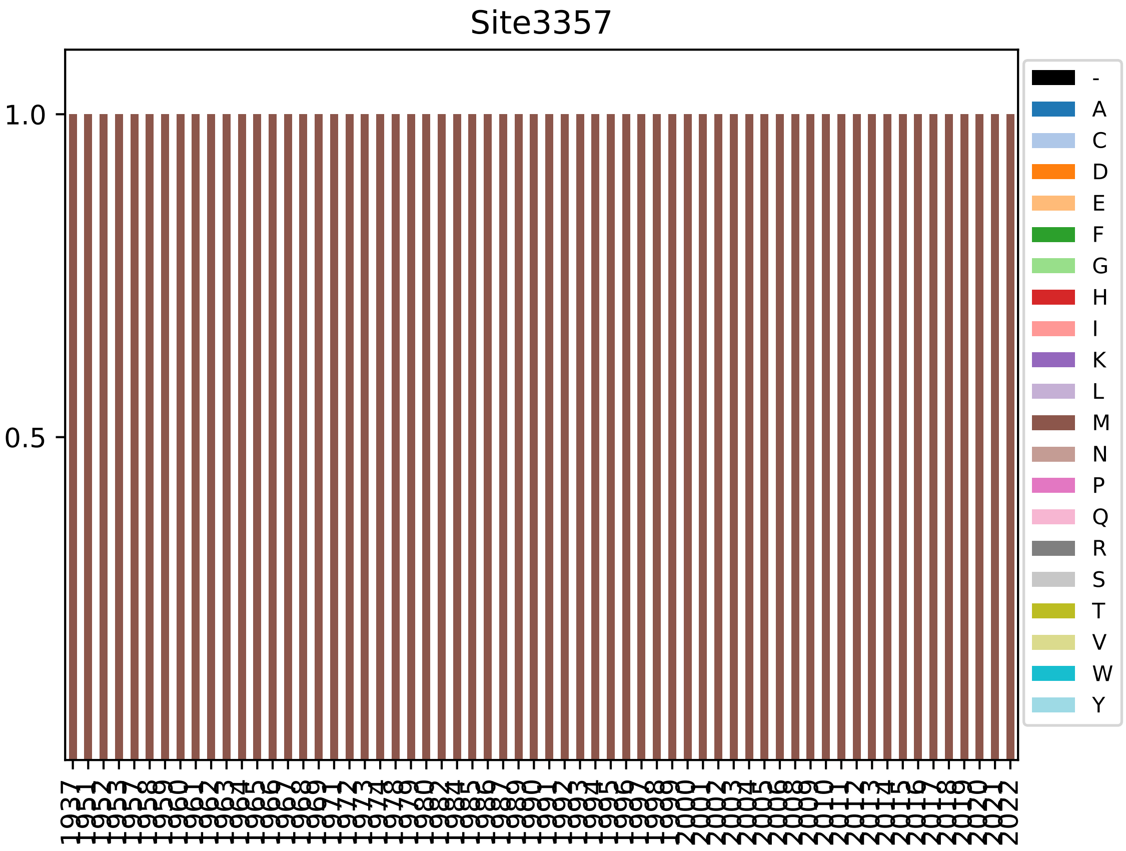
Site3357
1.0
0.5
-
A
C
D
E
F
G
H
I
K
L
M
N
P
Q
R
S
T
V
W
Y

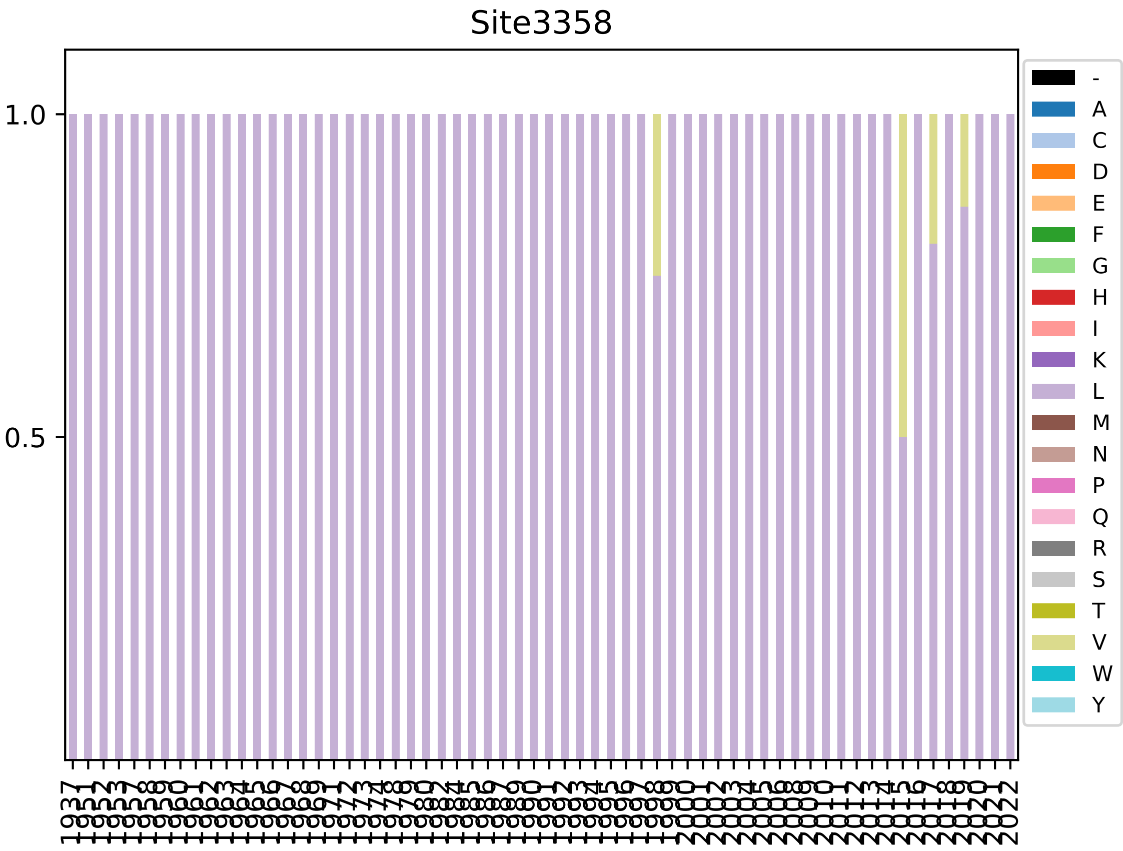
Site3358
1.0
0.5
-
A
C
D
E
F
G
H
I
K
L
M
N
P
Q
R
S
T
V
W
Y

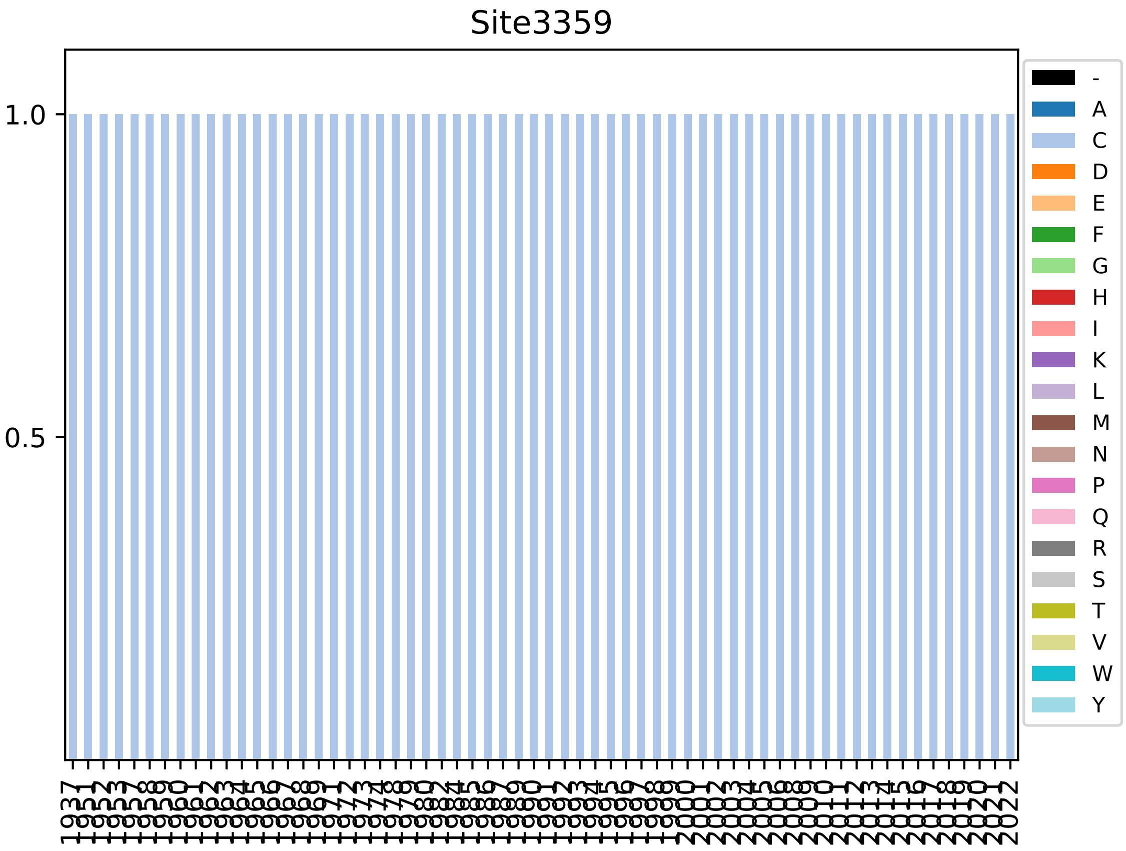
Site3359
1.0
0.5
-
A
C
D
E
F
G
H
I
K
L
M
N
P
Q
R
S
T
V
W
Y

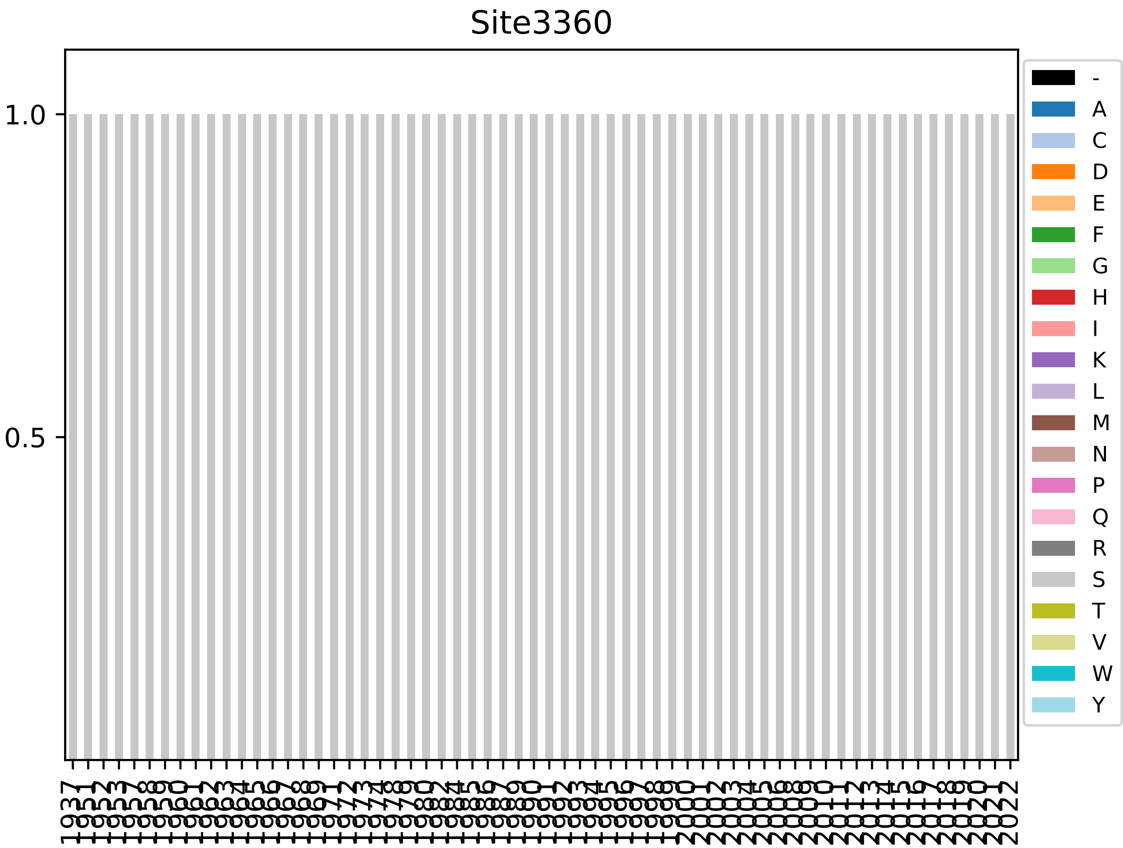
Site3360
1.0
0.5
-
A
C
D
E
F
G
H
I
K
L
M
N
P
Q
R
S
T
V
W
Y

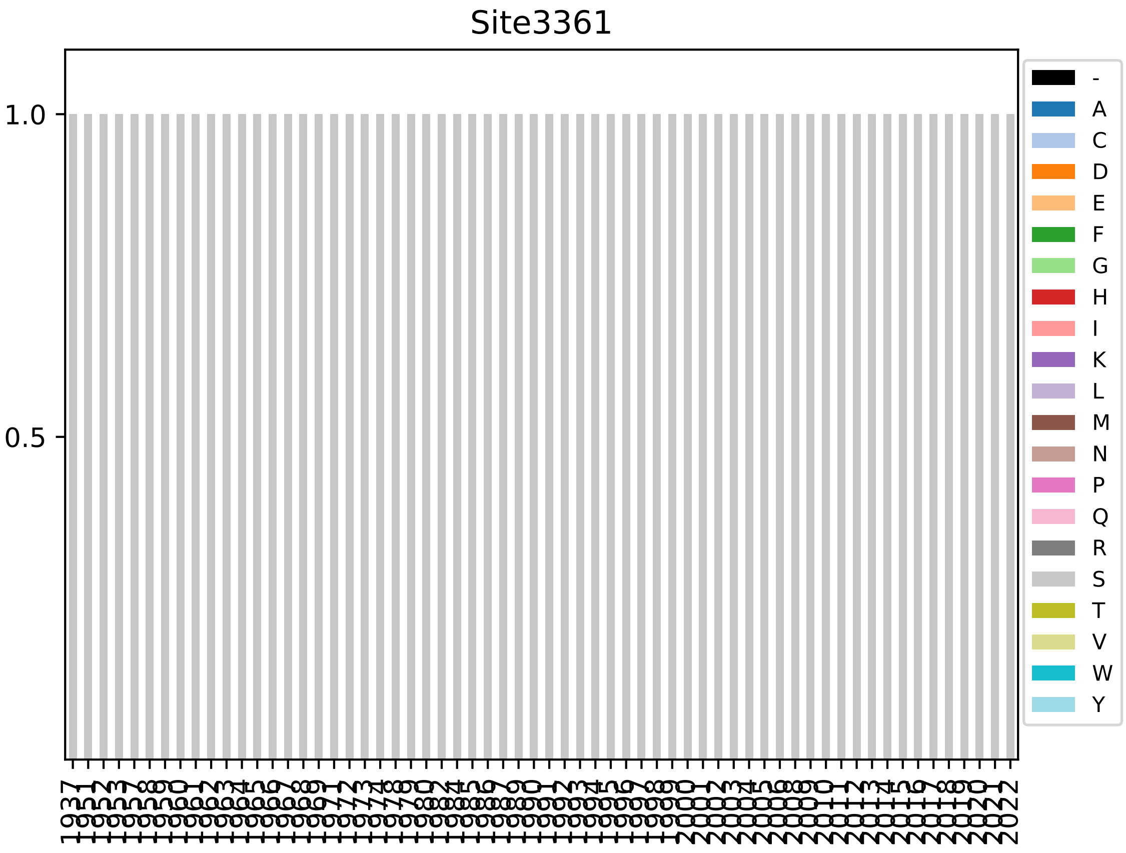
Site3361
1.0
0.5
-
A
C
D
E
F
G
H
I
K
L
M
N
P
Q
R
S
T
V
W
Y

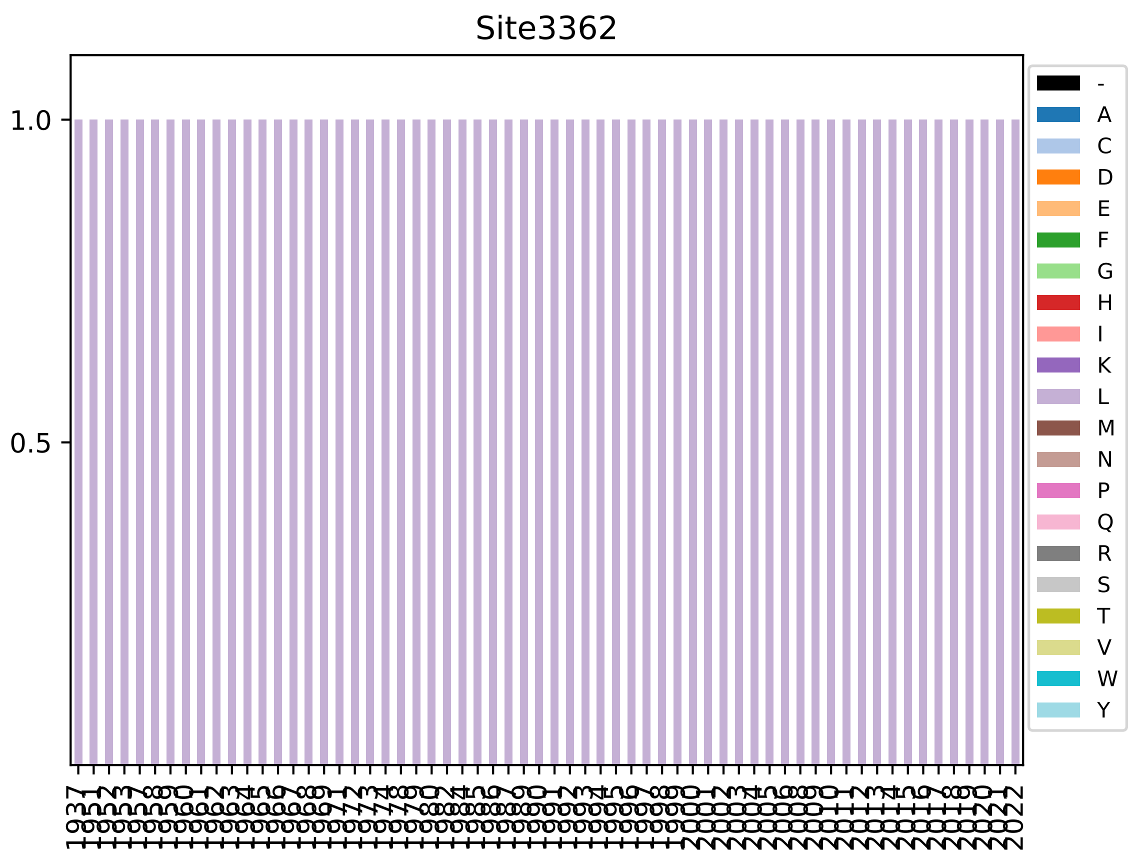
Site3362
1.0
0.5
-
A
C
D
E
F
G
H
I
K
L
M
N
P
Q
R
S
T
V
W
Y

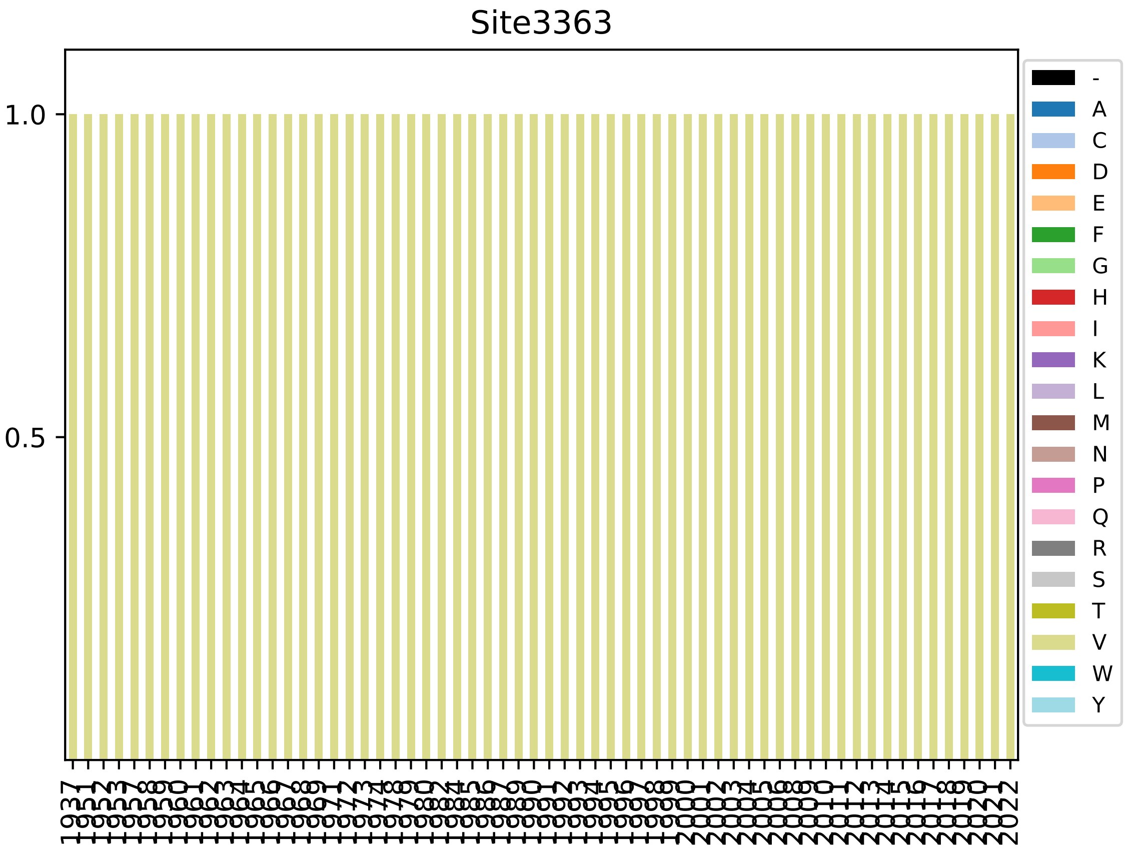
Site3363
1.0
0.5
-
A
C
D
E
F
G
H
I
K
L
M
N
P
Q
R
S
T
V
W
Y

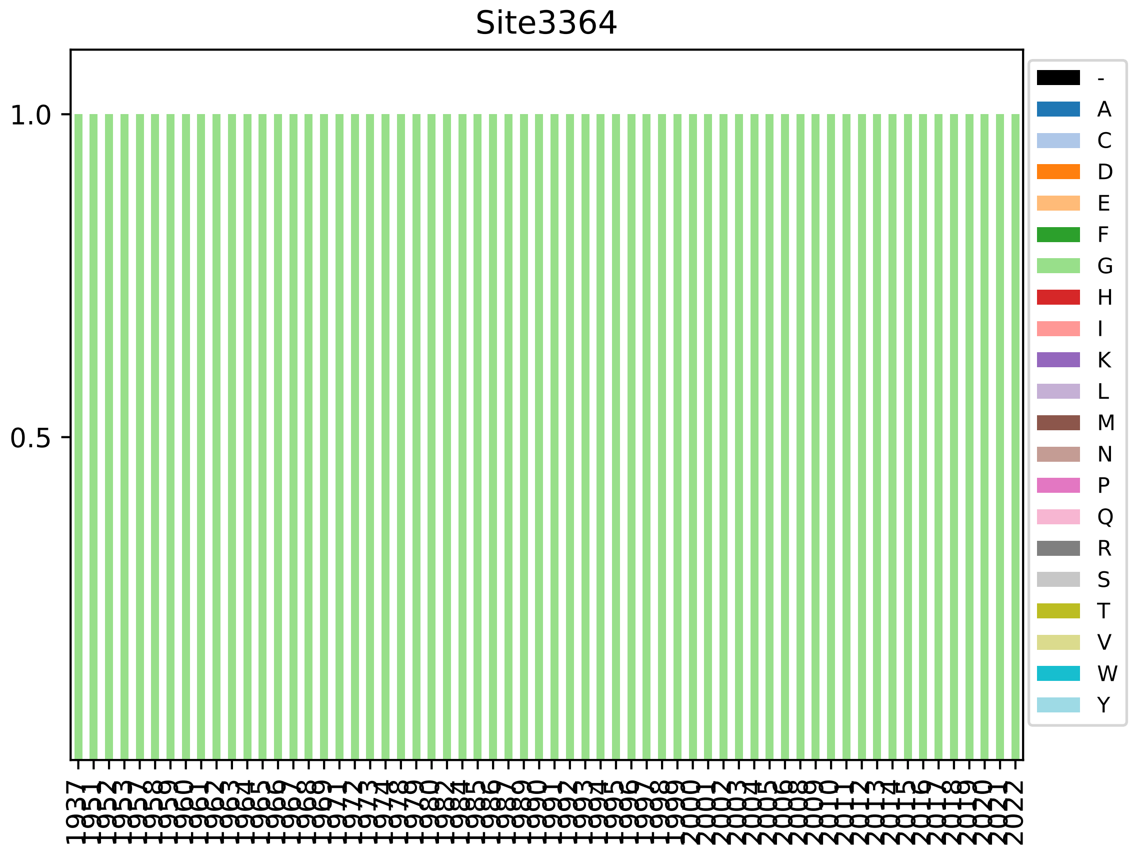
Site3364
1.0
0.5
-
A
C
D
E
F
G
H
I
K
L
M
N
P
Q
R
S
T
V
W
Y

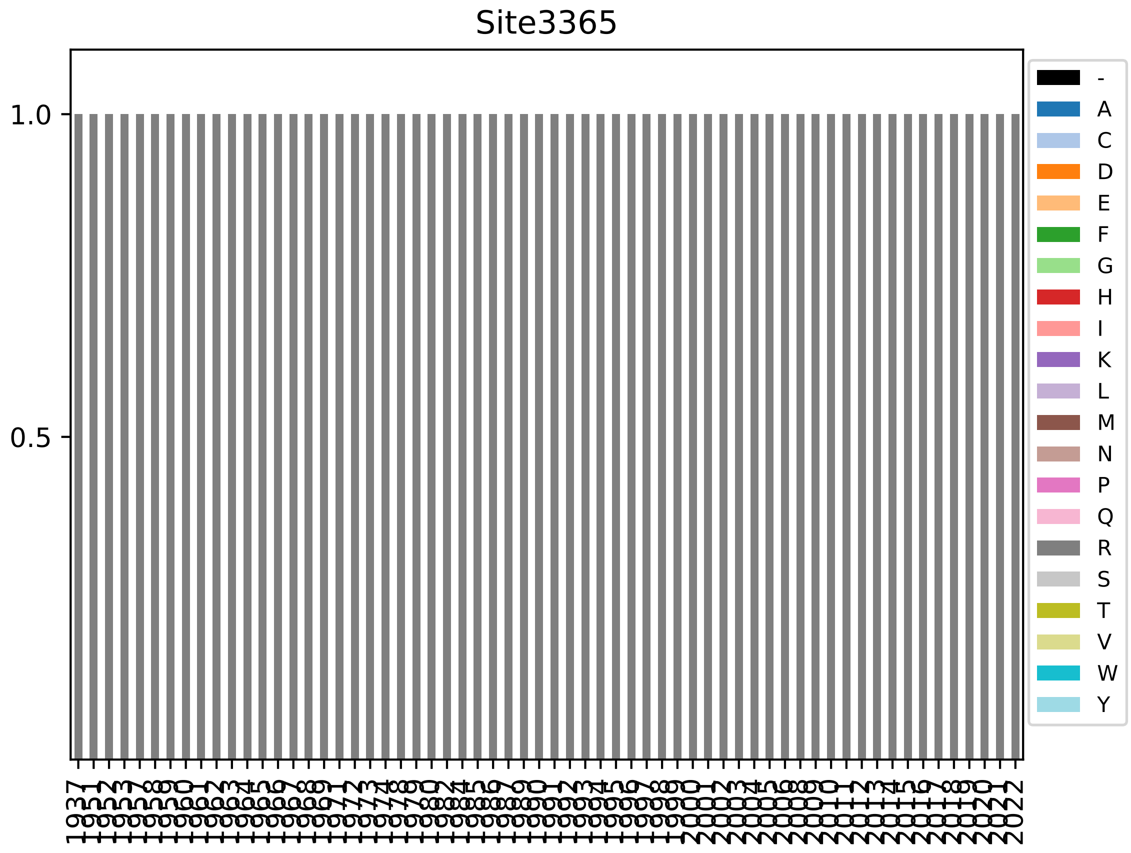
Site3365
1.0
0.5
-
A
C
D
E
F
G
H
I
K
L
M
N
P
Q
R
S
T
V
W
Y

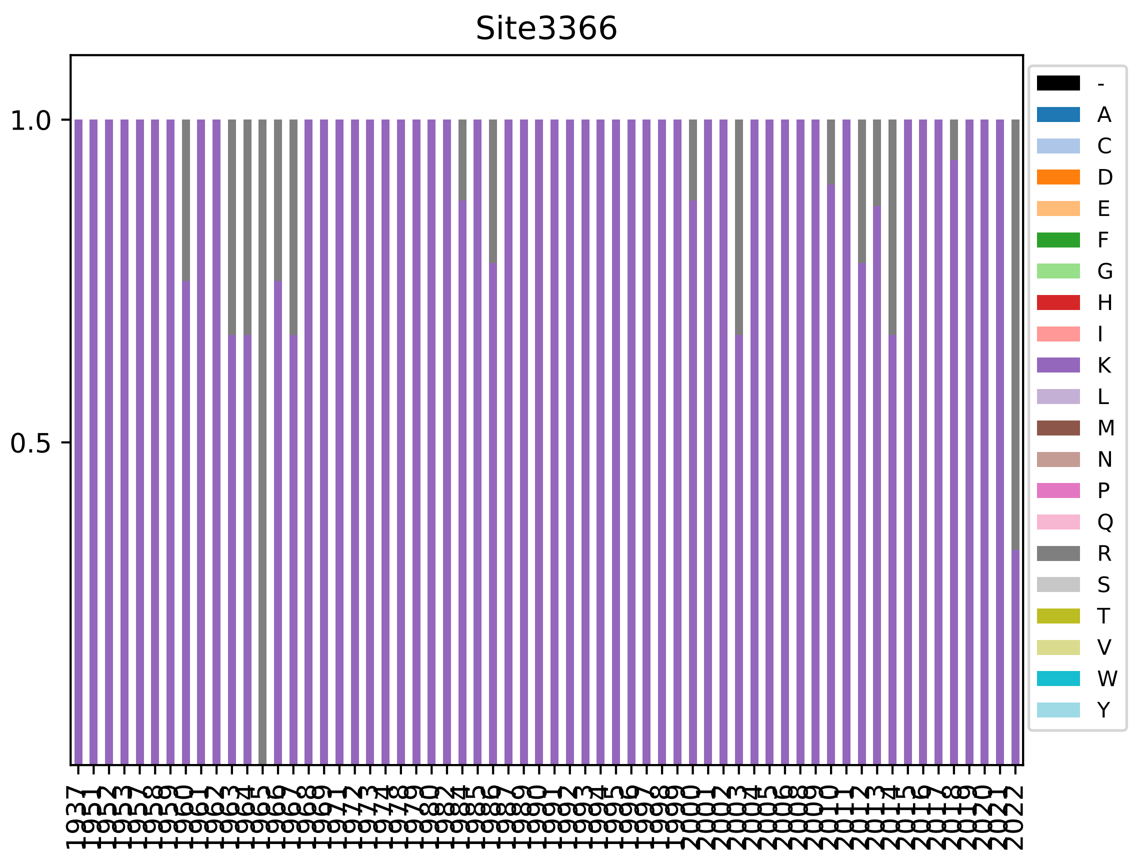
Site3366
1.0
0.5
-
A
C
D
E
F
G
H
I
K
L
M
N
P
Q
R
S
T
V
W
Y

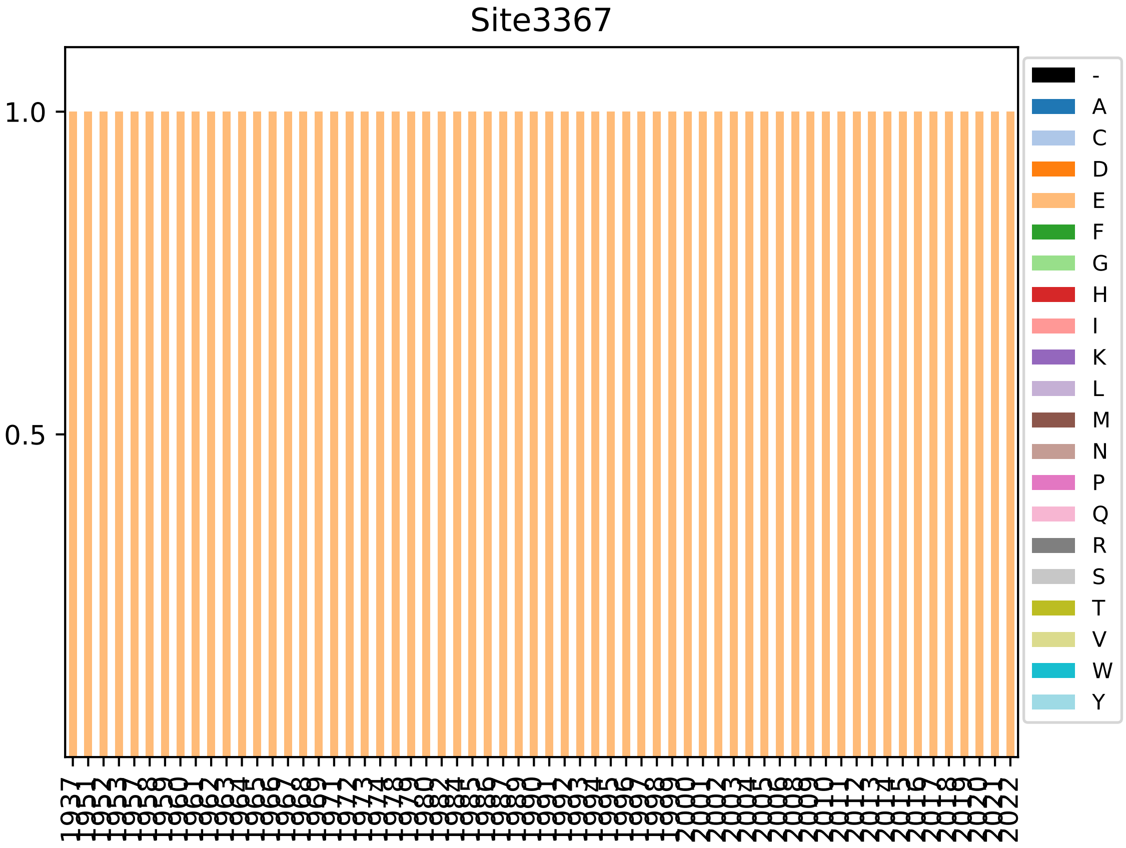

Site3367
1.0
0.5
-
A
C
D
E
F
G
H
I
K
L
M
N
P
Q
R
S
T
V
W
Y

Site3368

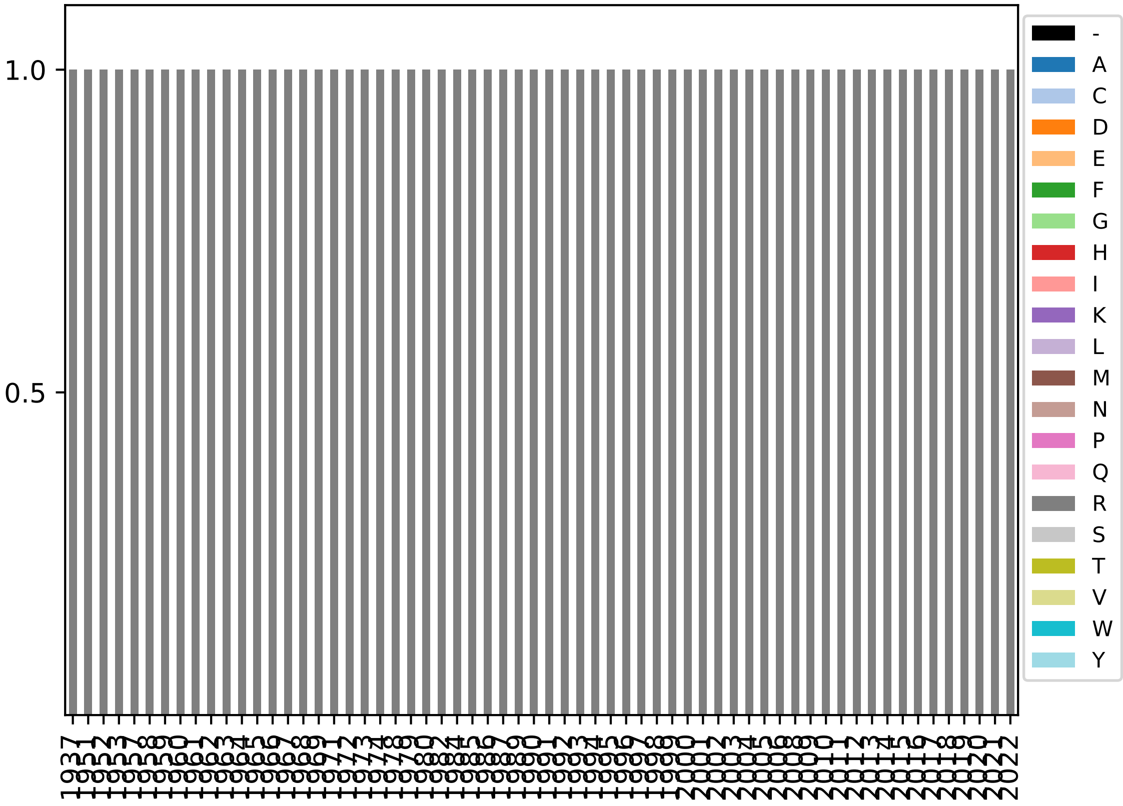

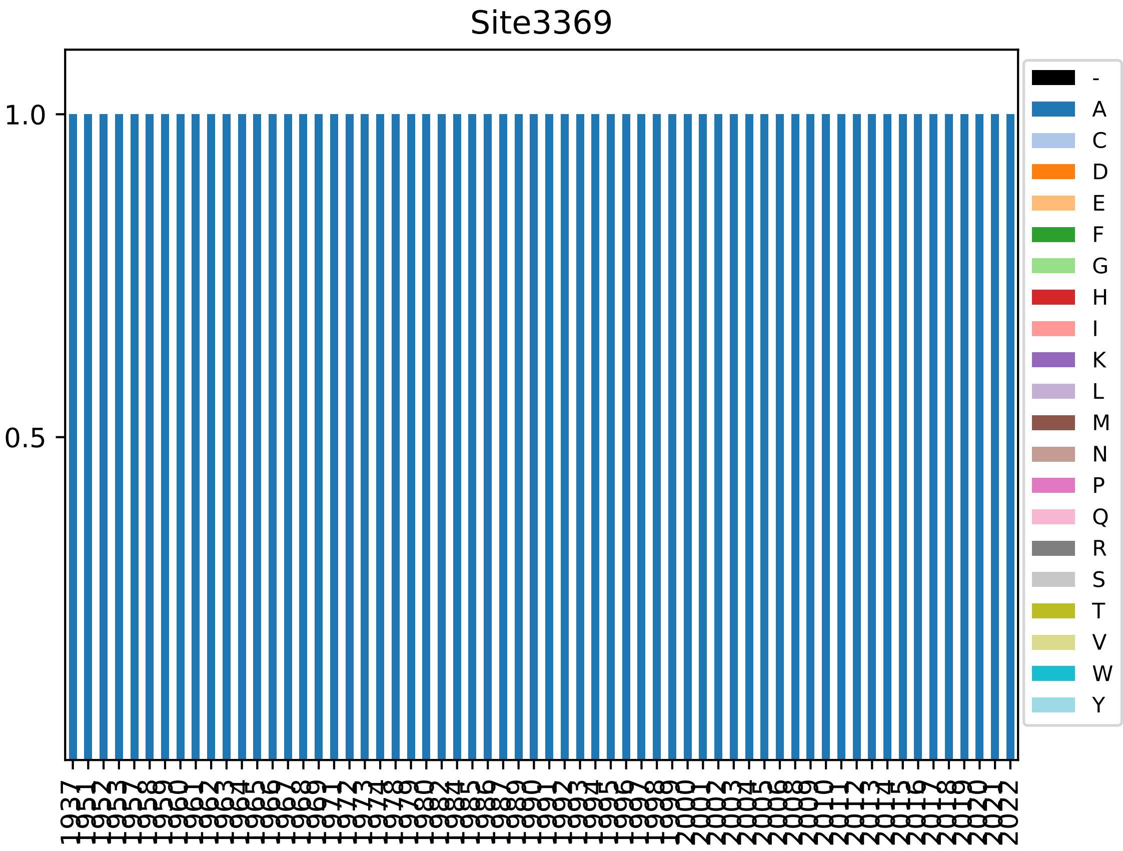
Site3369
1.0
0.5
-
A
C
D
E
F
G
H
I
K
L
M
N
P
Q
R
S
T
V
W
Y

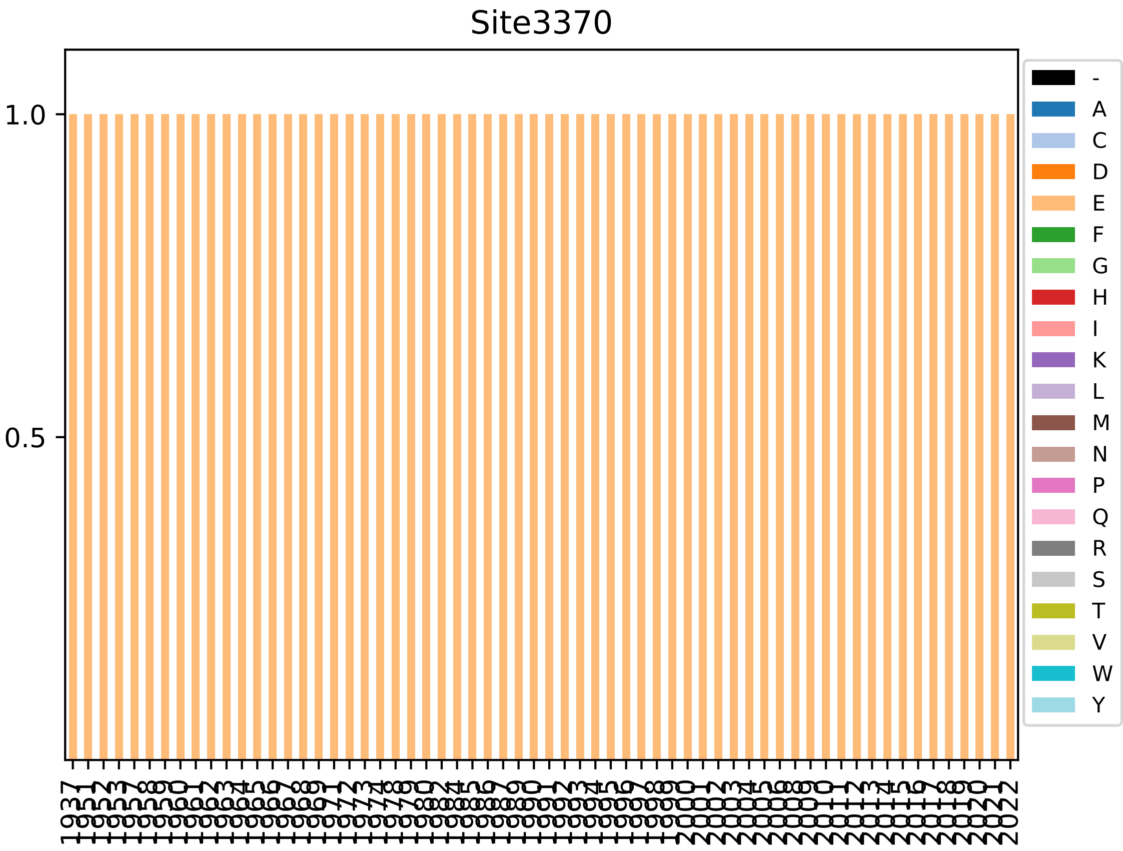
Site3370
1.0
0.5
-
A
C
D
E
F
G
H
I
K
L
M
N
P
Q
R
S
T
V
W
Y

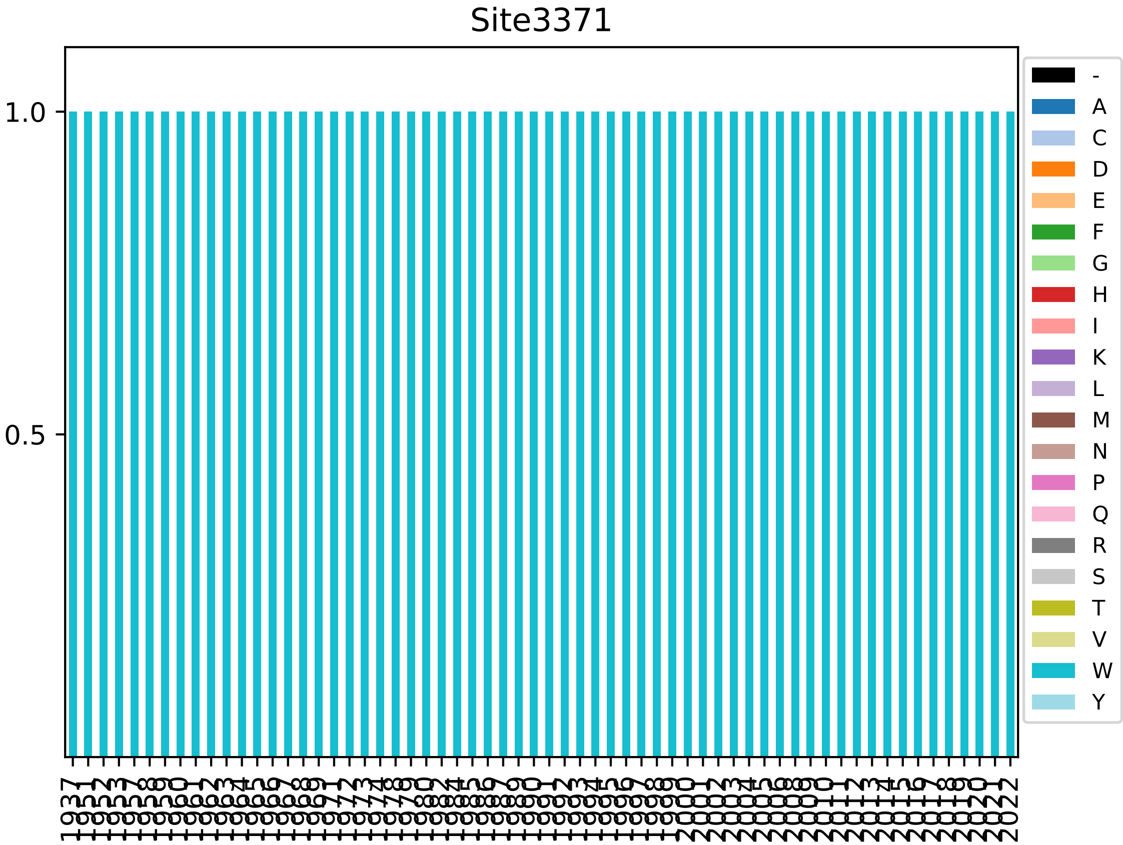
Site3371
1.0
0.5
-
A
C
D
E
F
G
H
I
K
L
M
N
P
Q
R
S
T
V
W
Y

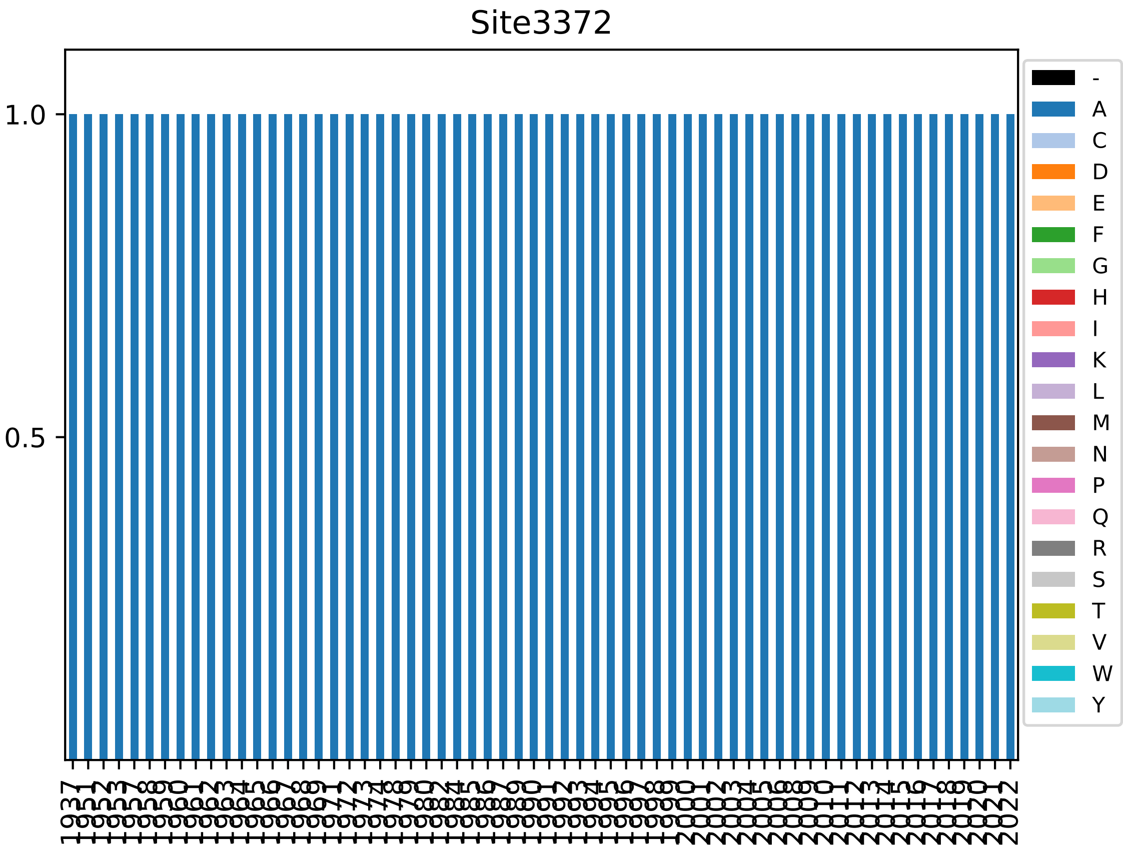
Site3372
1.0
0.5
-
A
C
D
E
F
G
H
I
K
L
M
N
P
Q
R
S
T
V
W
Y

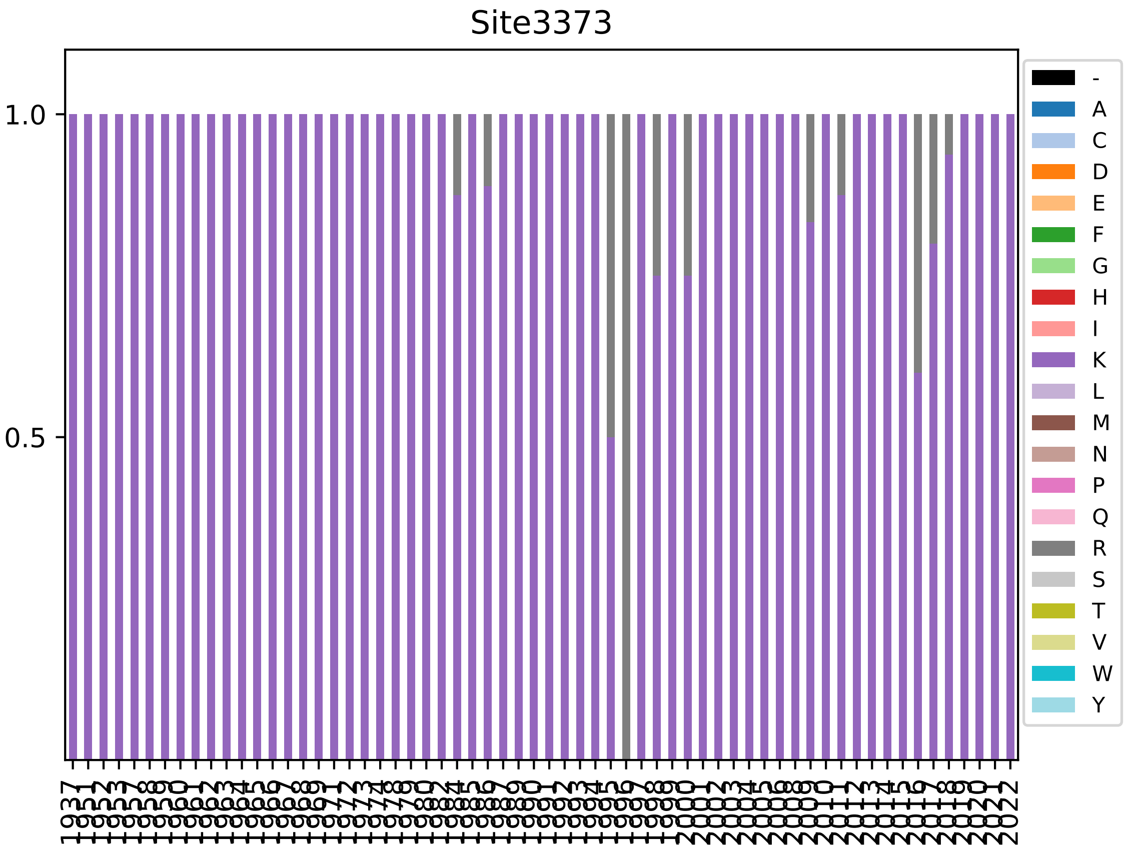
Site3373
1.0
0.5
-
A
C
D
E
F
G
H
I
K
L
M
N
P
Q
R
S
T
V
W
Y

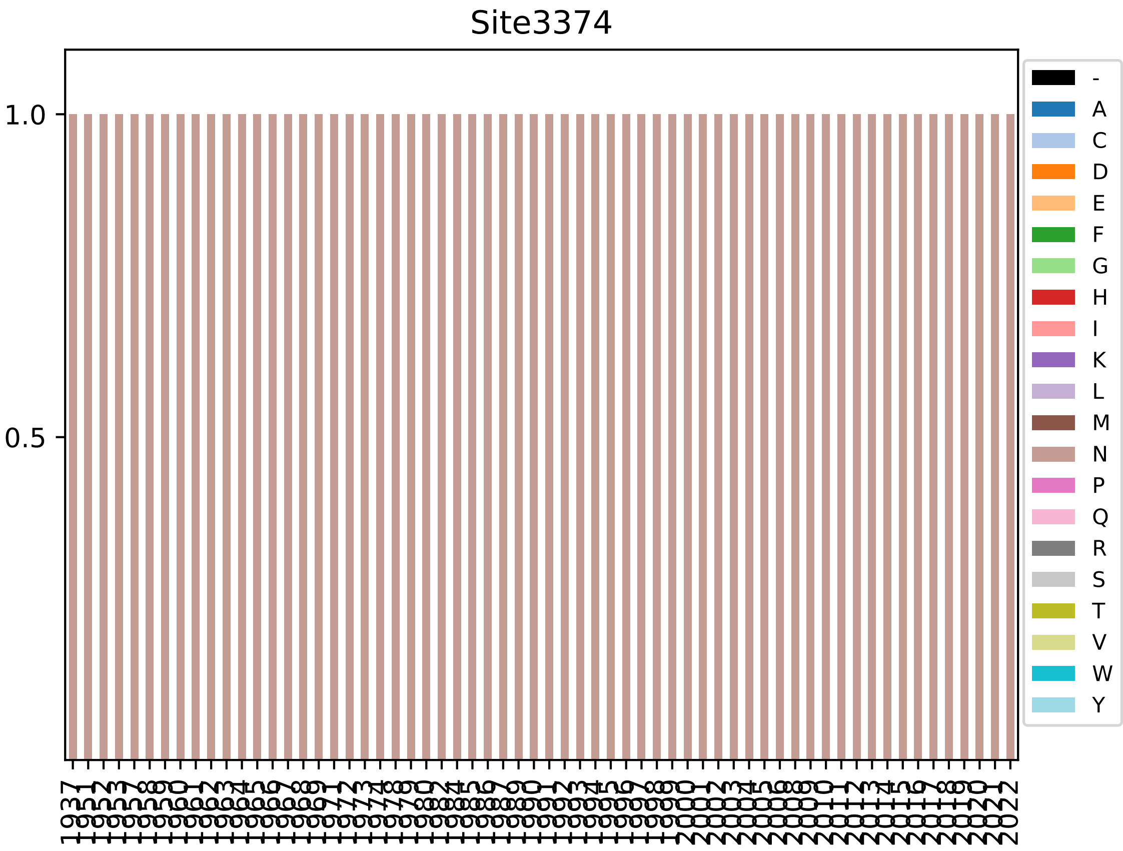

Site3374
1.0
0.5
-
A
C
D
E
F
G
H
I
K
L
M
N
P
Q
R
S
T
V
W
Y

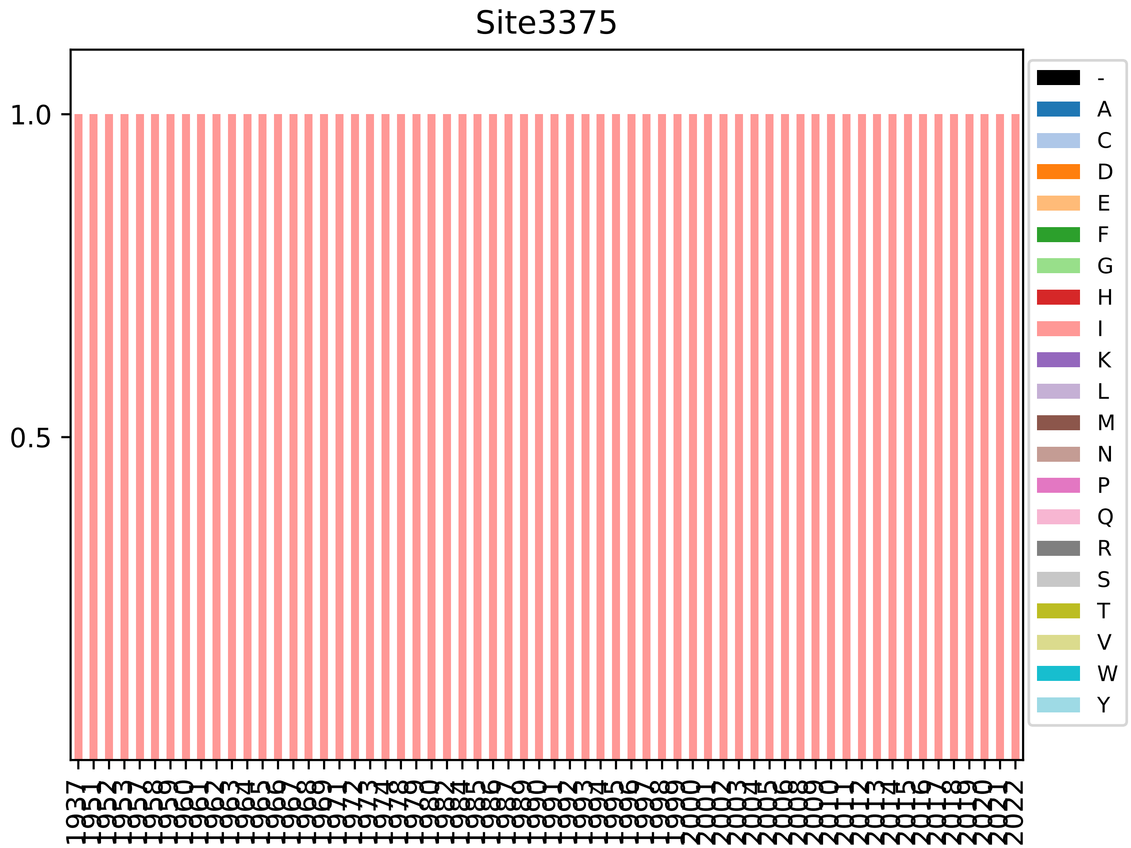

Site3375
1.0
0.5
-
A
C
D
E
F
G
H
I
K
L
M
N
P
Q
R
S
T
V
W
Y

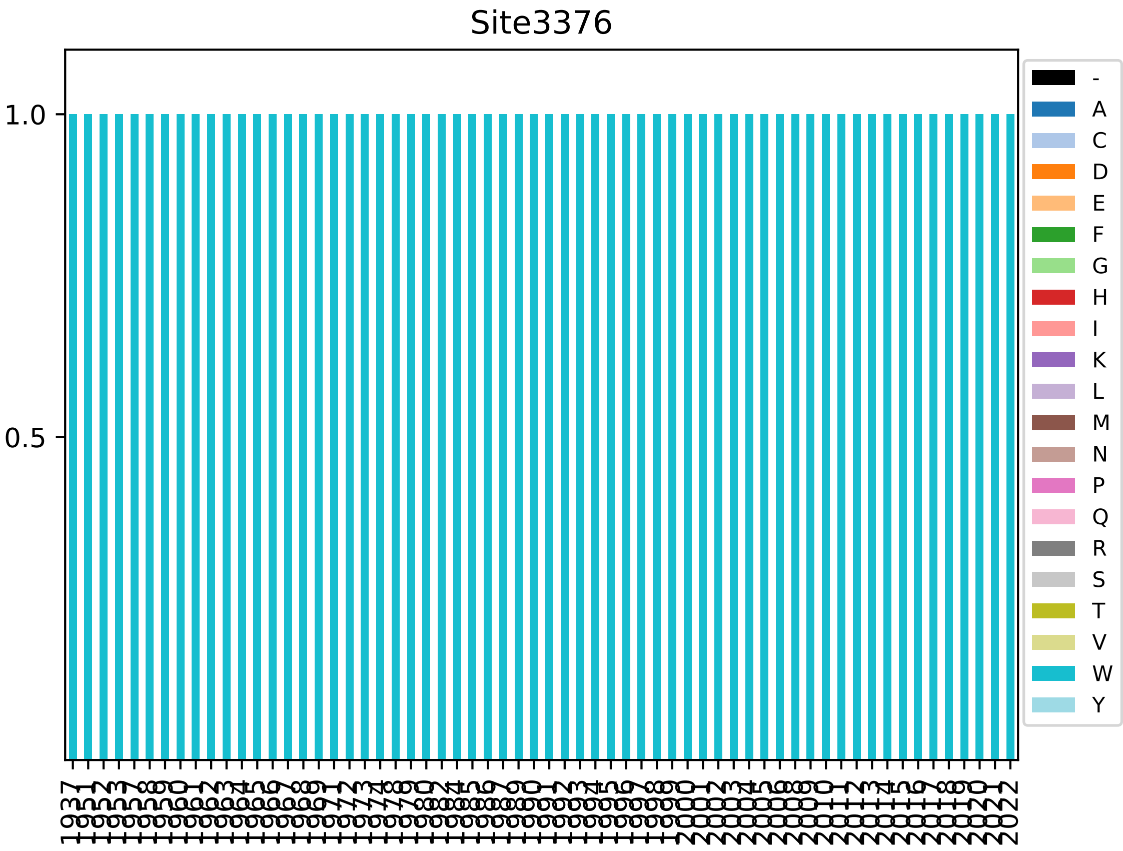
Site3376
1.0
0.5
-
A
C
D
E
F
G
H
I
K
L
M
N
P
Q
R
S
T
V
W
Y

Site3377

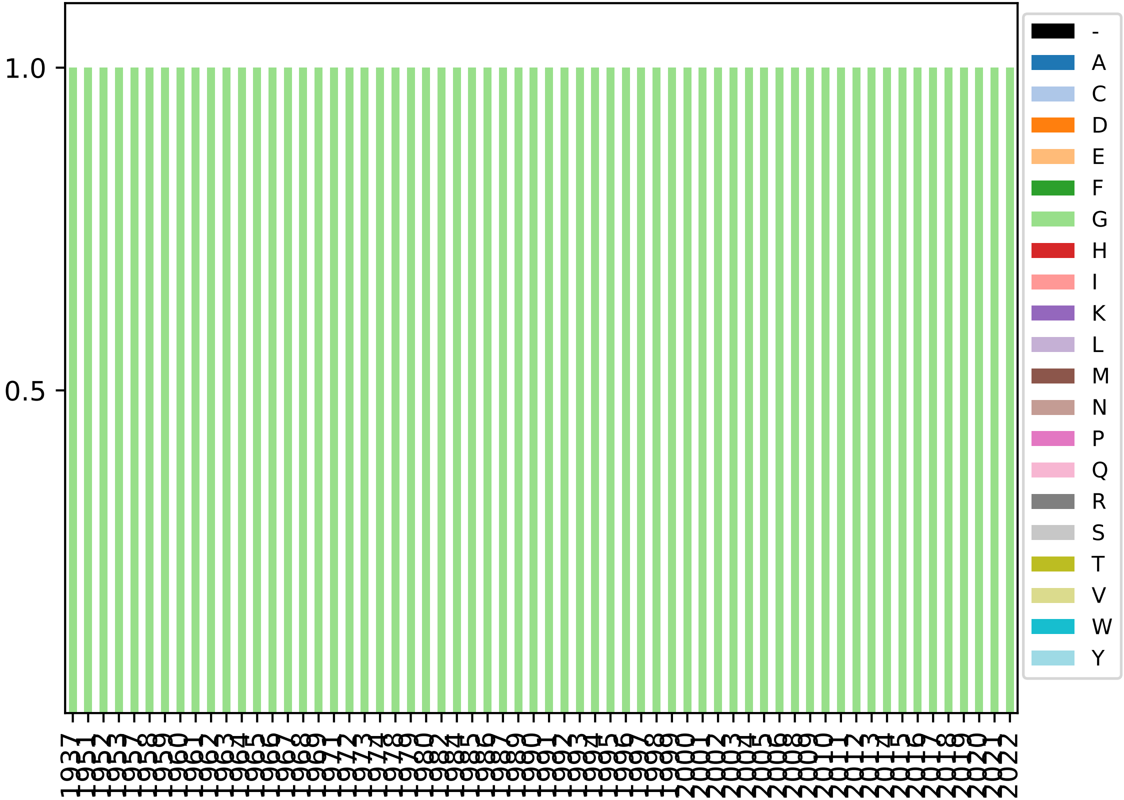

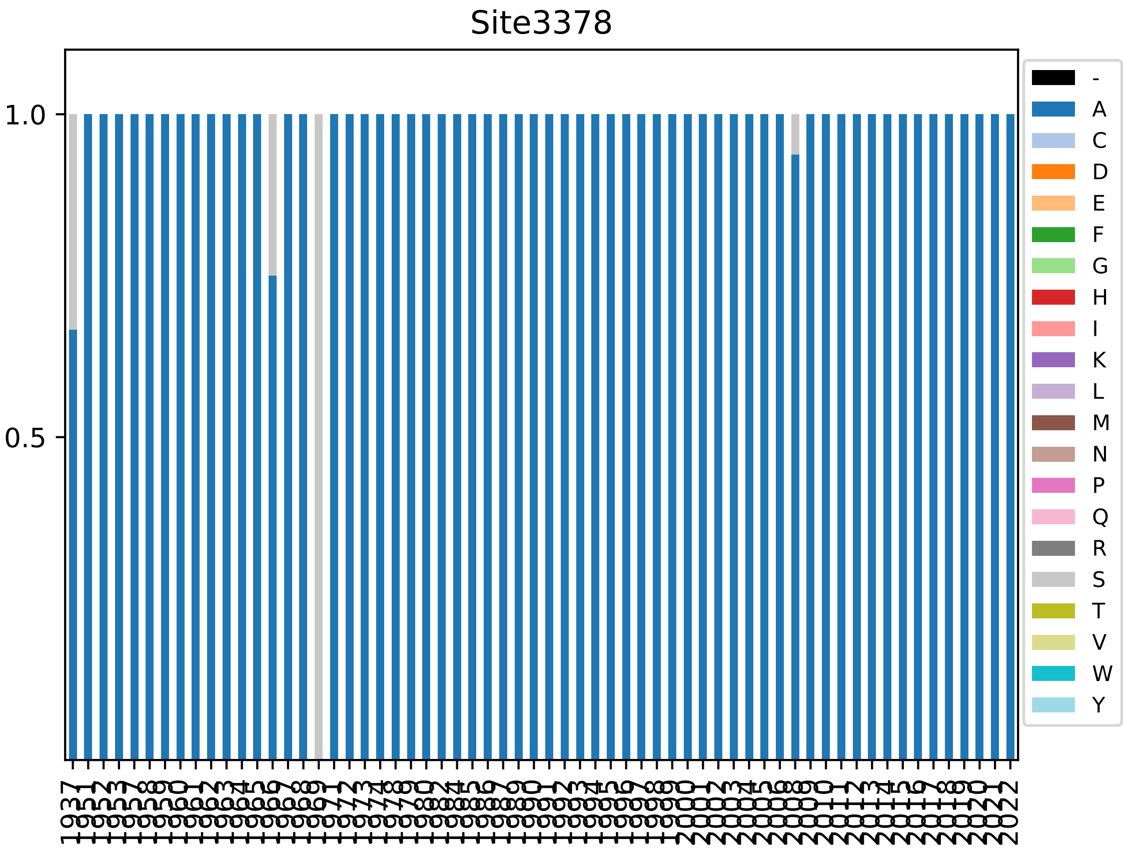
Site3378
1.0
0.5
-
A
C
D
E
F
G
H
I
K
L
M
N
P
Q
R
S
T
V
W
Y

Site3379

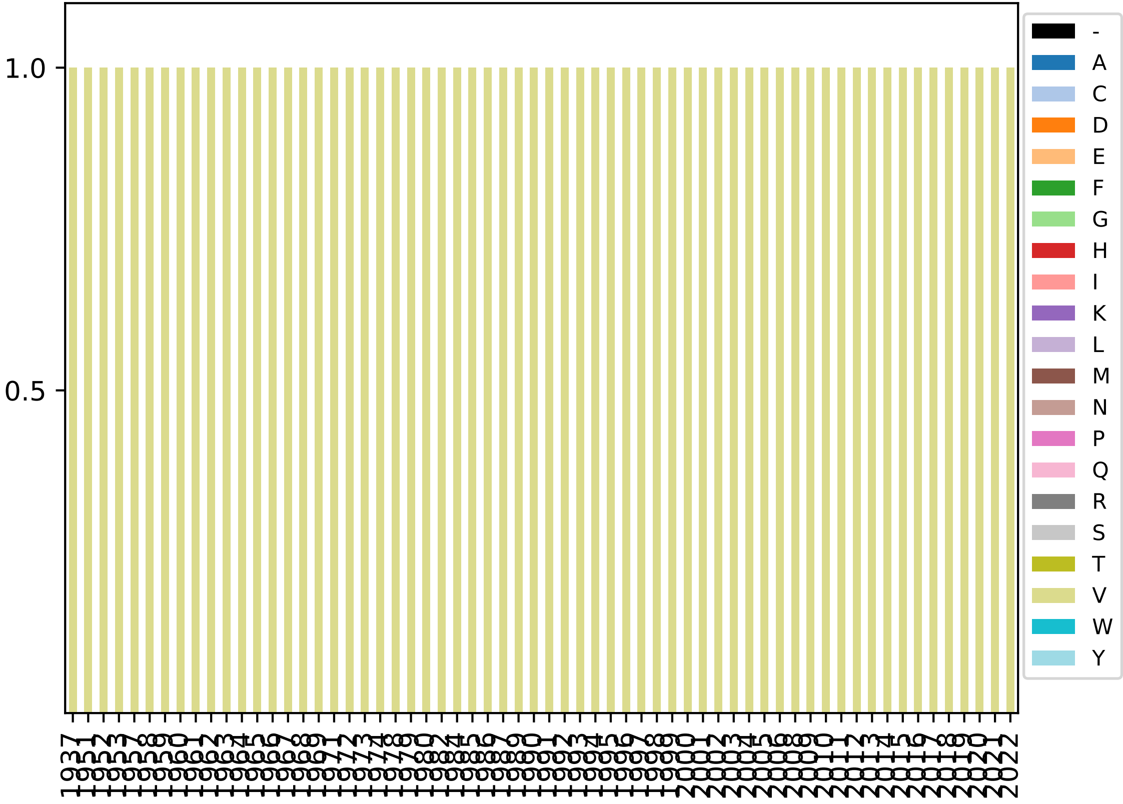

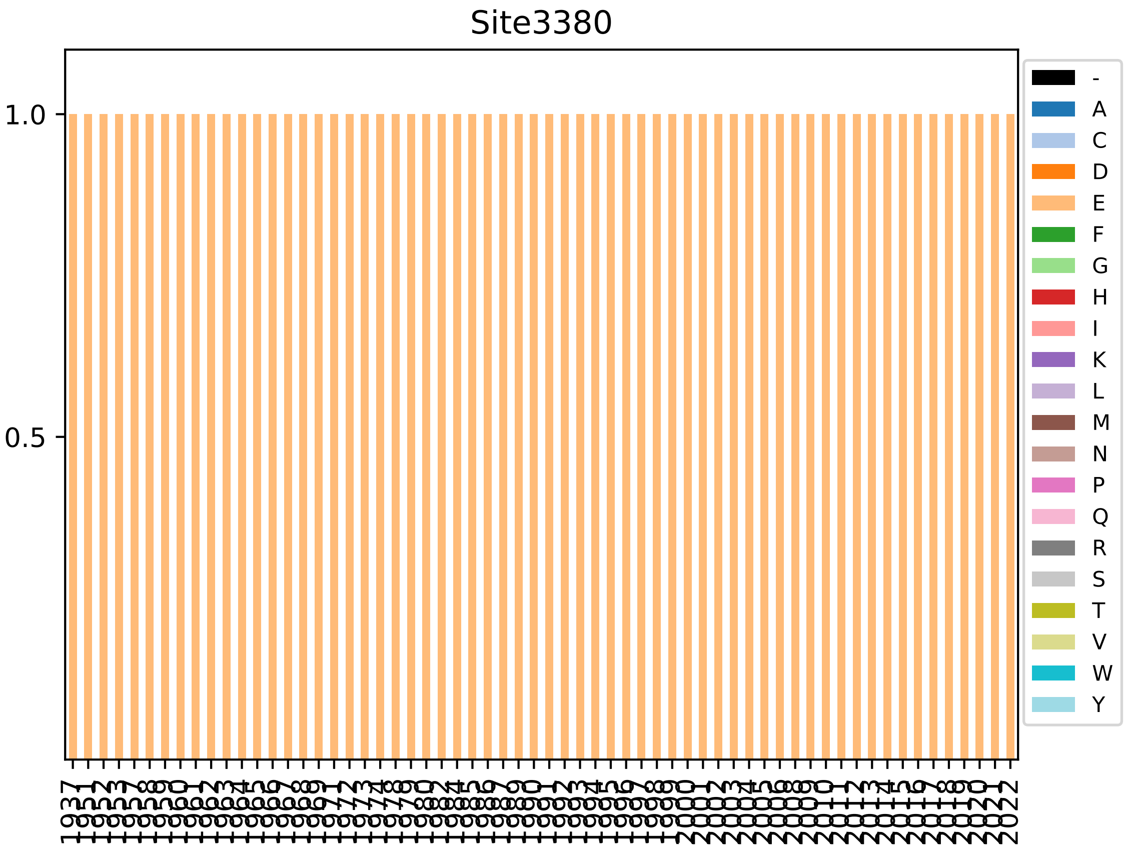
Site3380
1.0
0.5
-
A
C
D
E
F
G
H
I
K
L
M
N
P
Q
R
S
T
V
W
Y

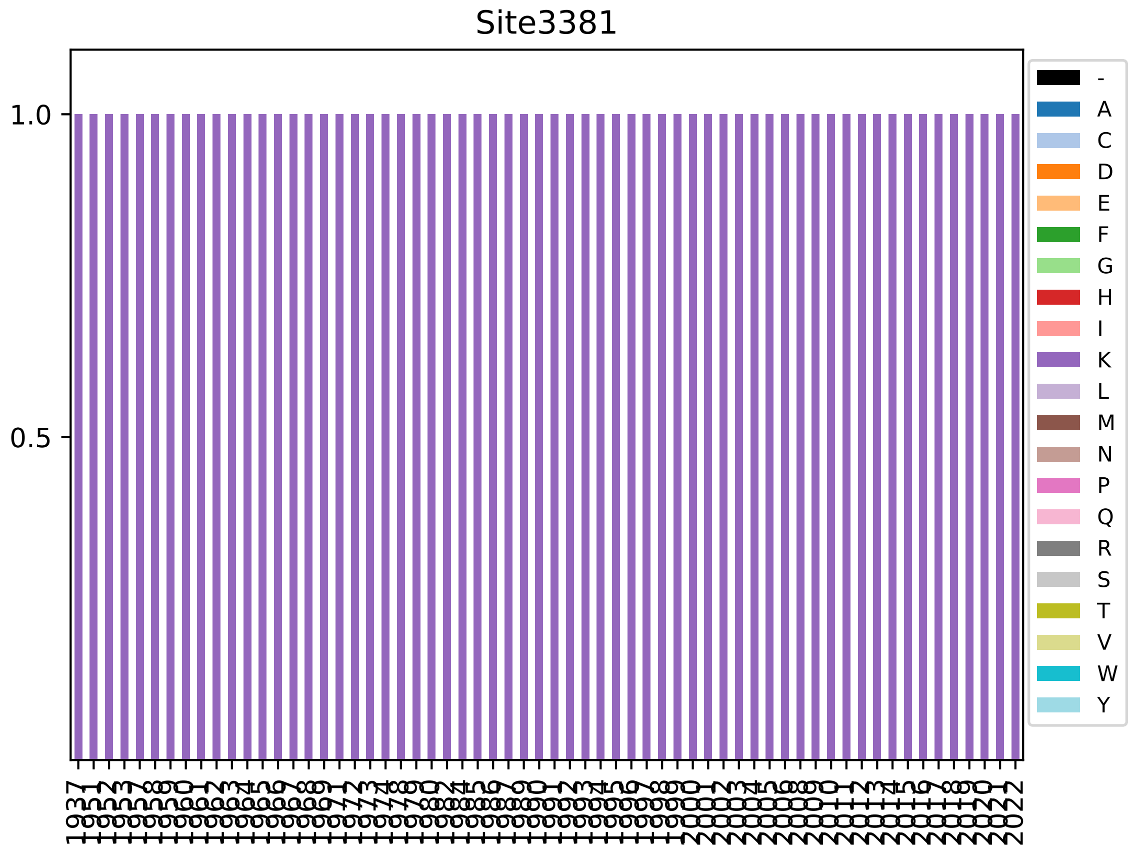
Site3381
1.0
0.5
-
A
C
D
E
F
G
H
I
K
L
M
N
P
Q
R
S
T
V
W
Y

Site3382

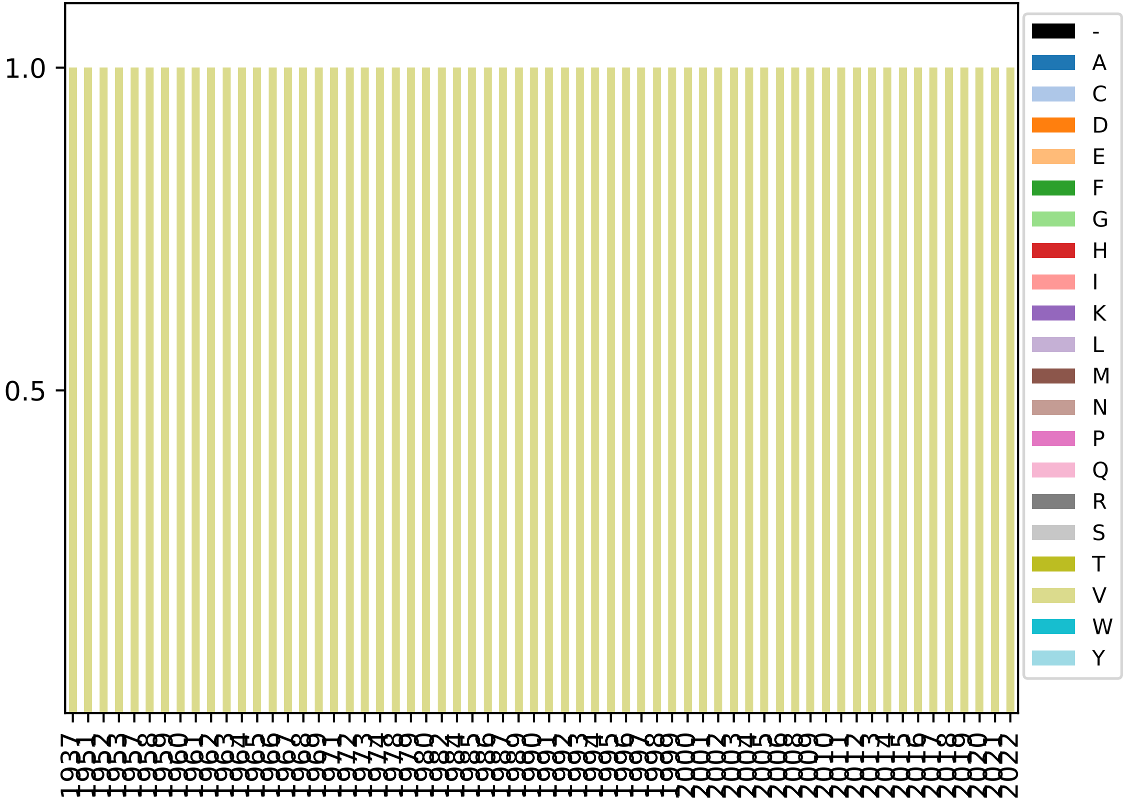

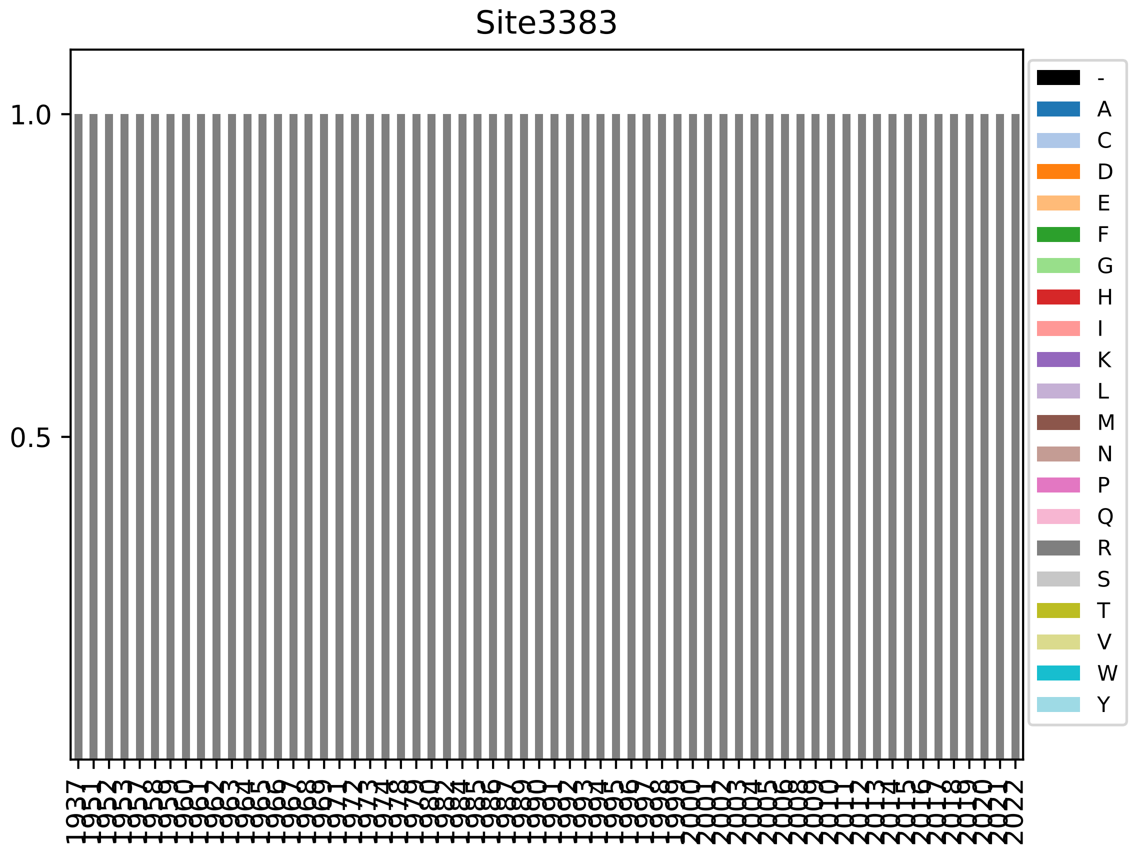
Site3383
1.0
0.5
-
A
C
D
E
F
G
H
I
K
L
M
N
P
Q
R
S
T
V
W
Y

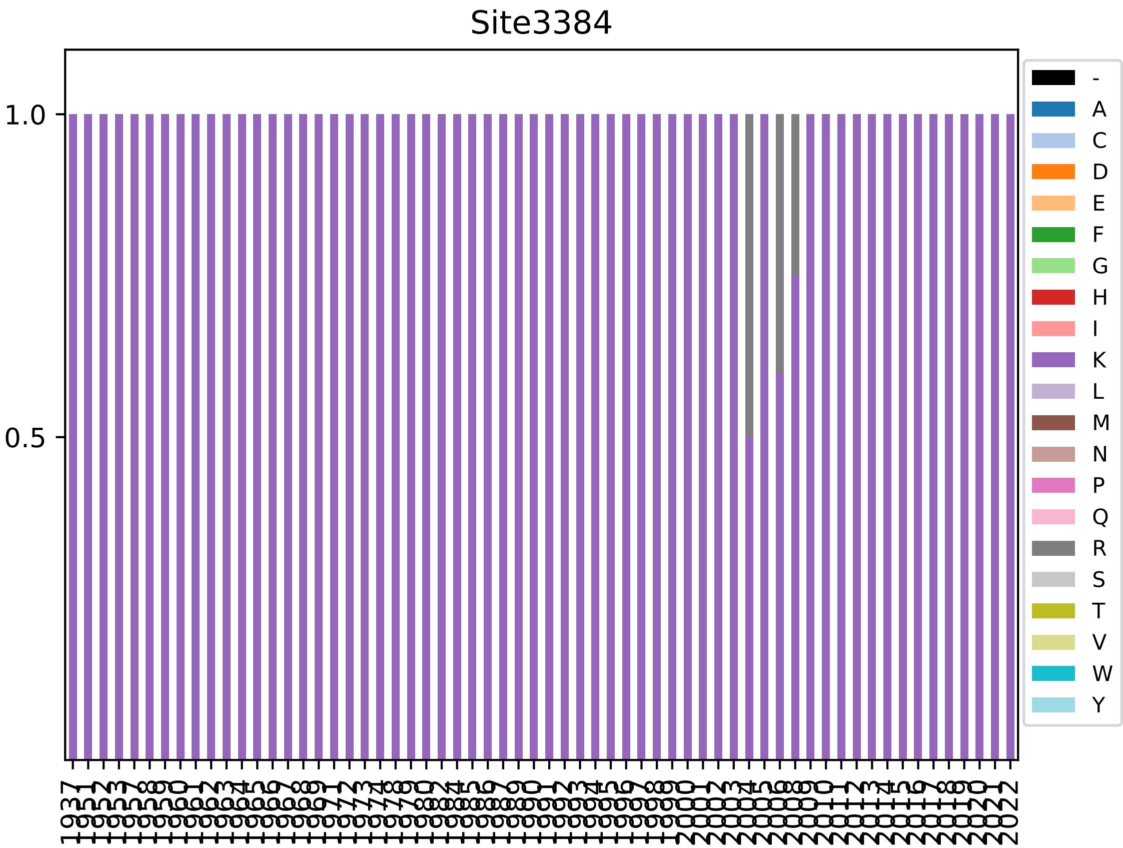
Site3384
1.0
0.5
-
A
C
D
E
F
G
H
I
K
L
M
N
P
Q
R
S
T
V
W
Y

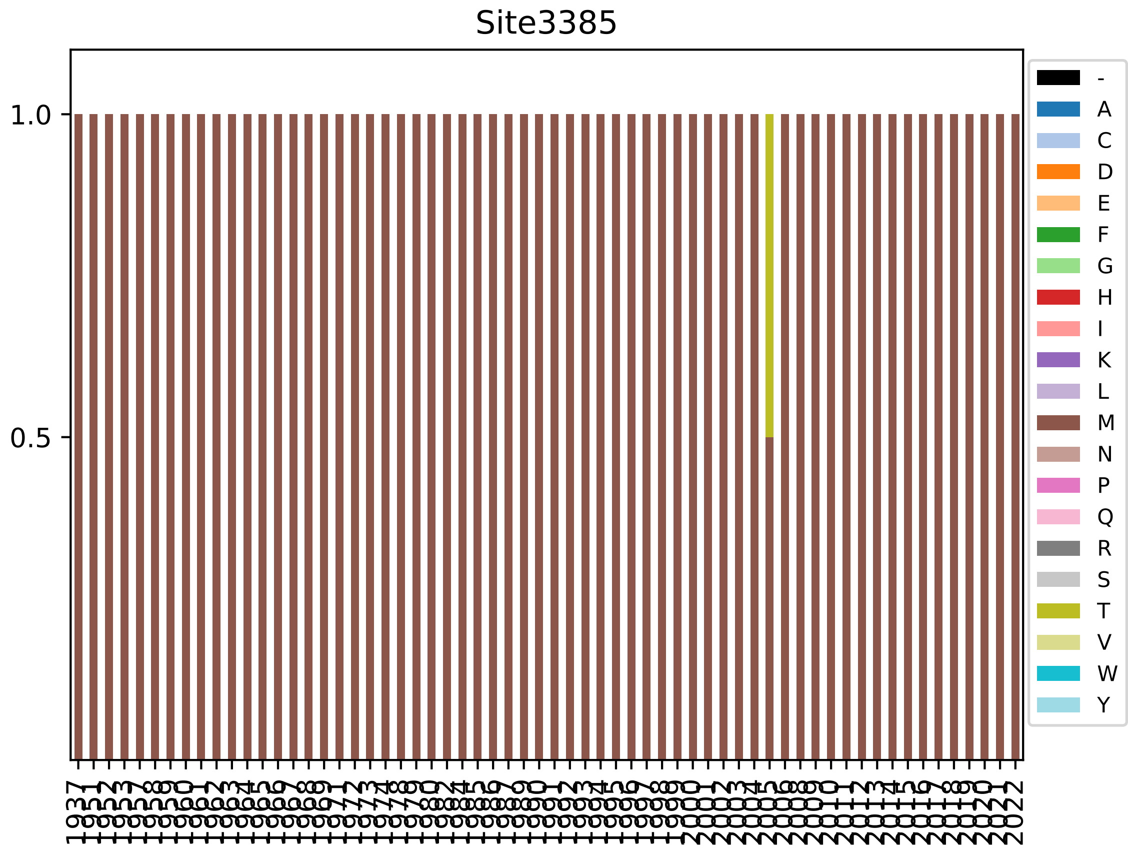
Site3385
1.0
0.5
-
A
C
D
E
F
G
H
I
K
L
M
N
P
Q
R
S
T
V
W
Y

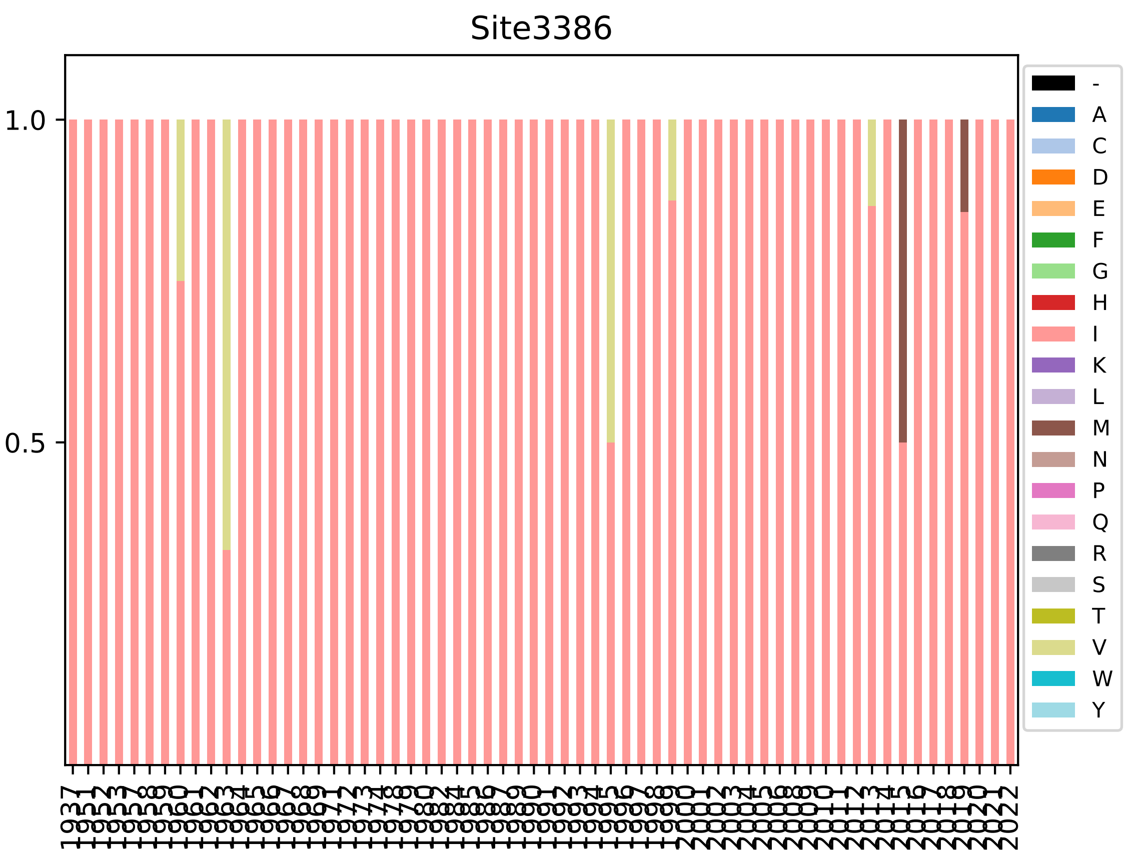
Site3386
1.0
0.5
-
A
C
D
E
F
G
H
I
K
L
M
N
P
Q
R
S
T
V
W
Y

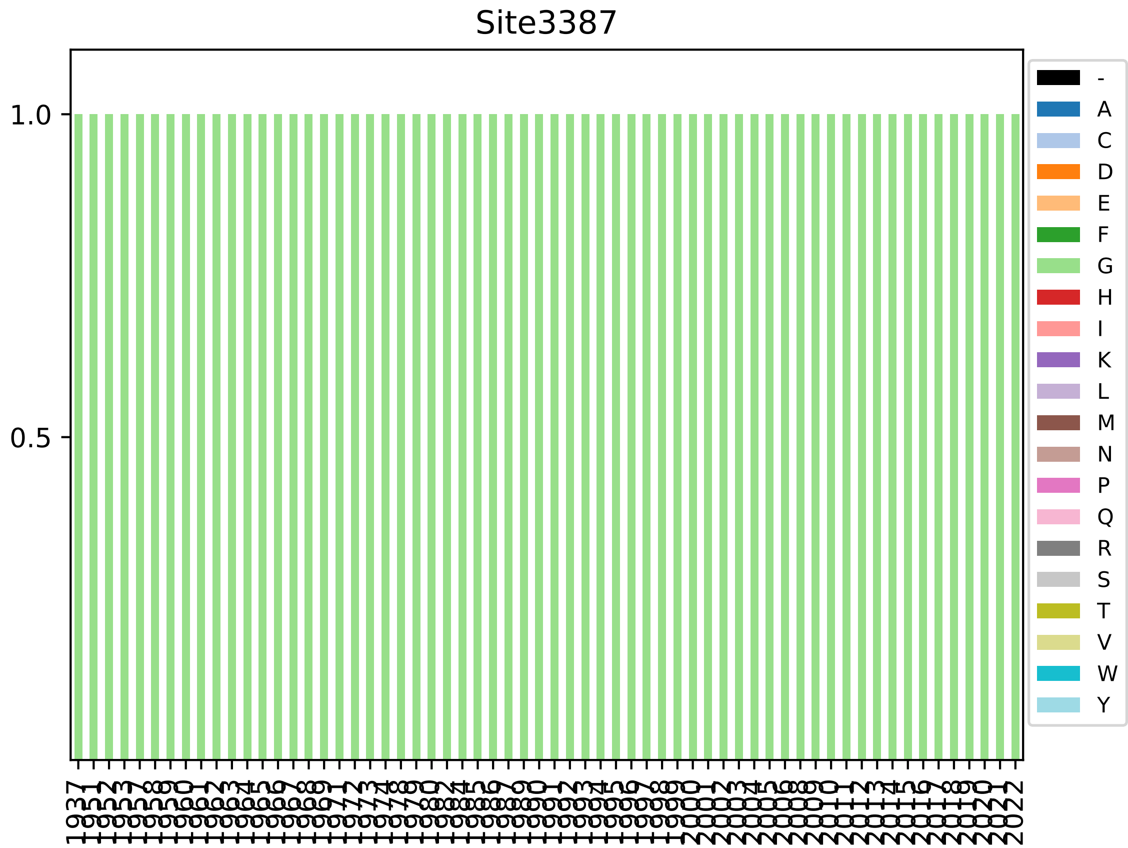
Site3387
1.0
0.5
-
A
C
D
E
F
G
H
I
K
L
M
N
P
Q
R
S
T
V
W
Y

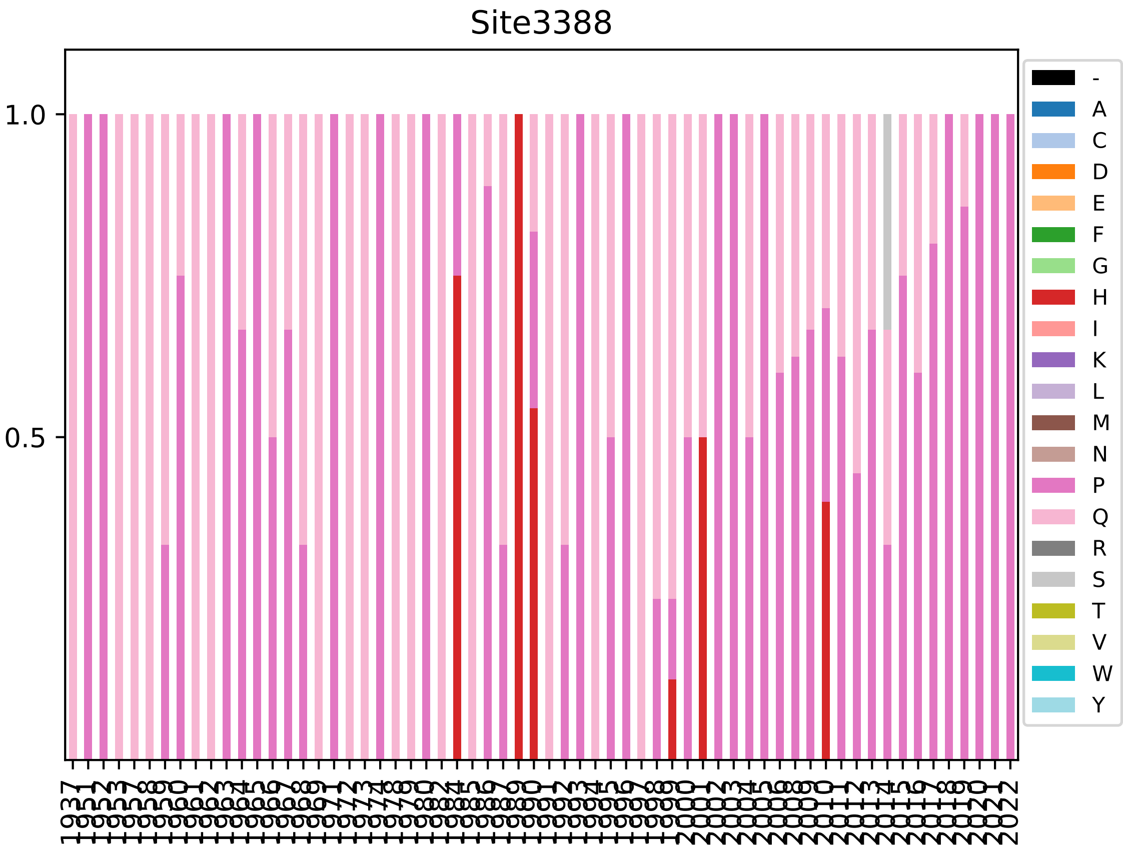
Site3388
1.0
0.5
-
A
C
D
E
F
G
H
I
K
L
M
N
P
Q
R
S
T
V
W
Y

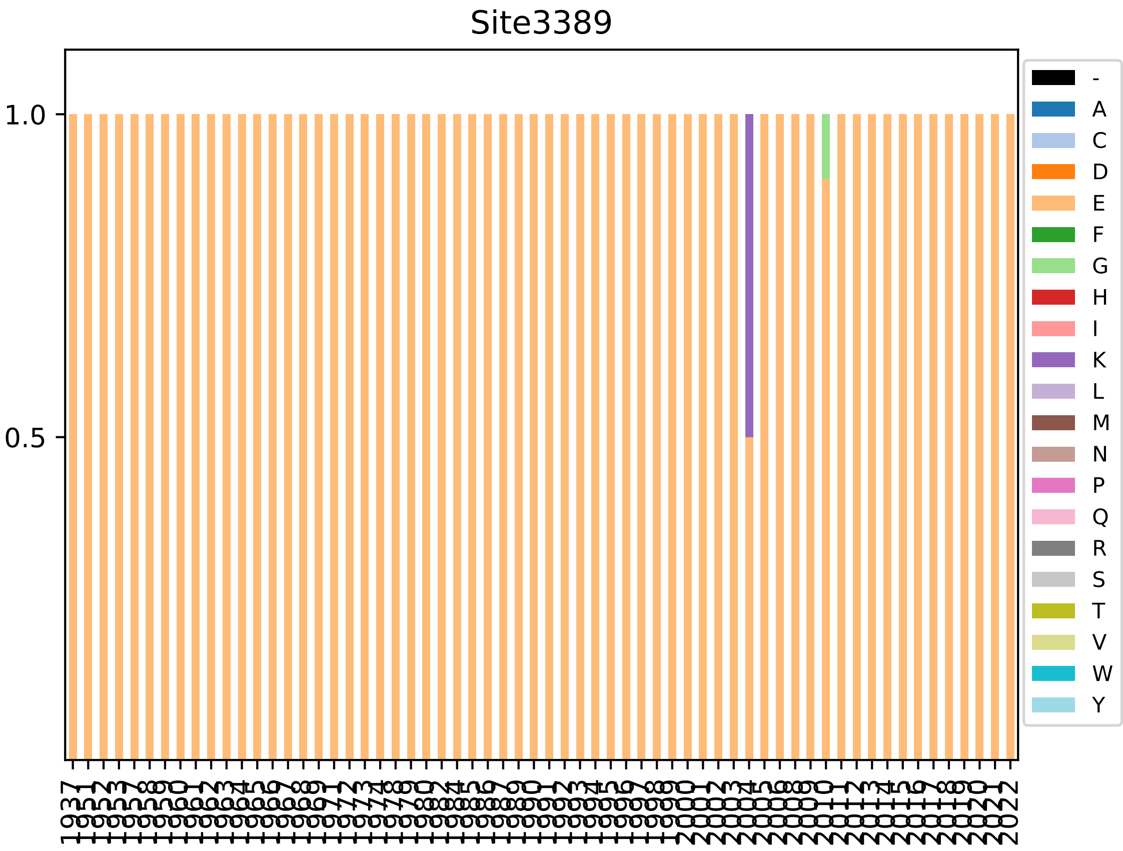
Site3389
1.0
0.5
-
A
C
D
E
F
G
H
I
K
L
M
N
P
Q
R
S
T
V
W
Y

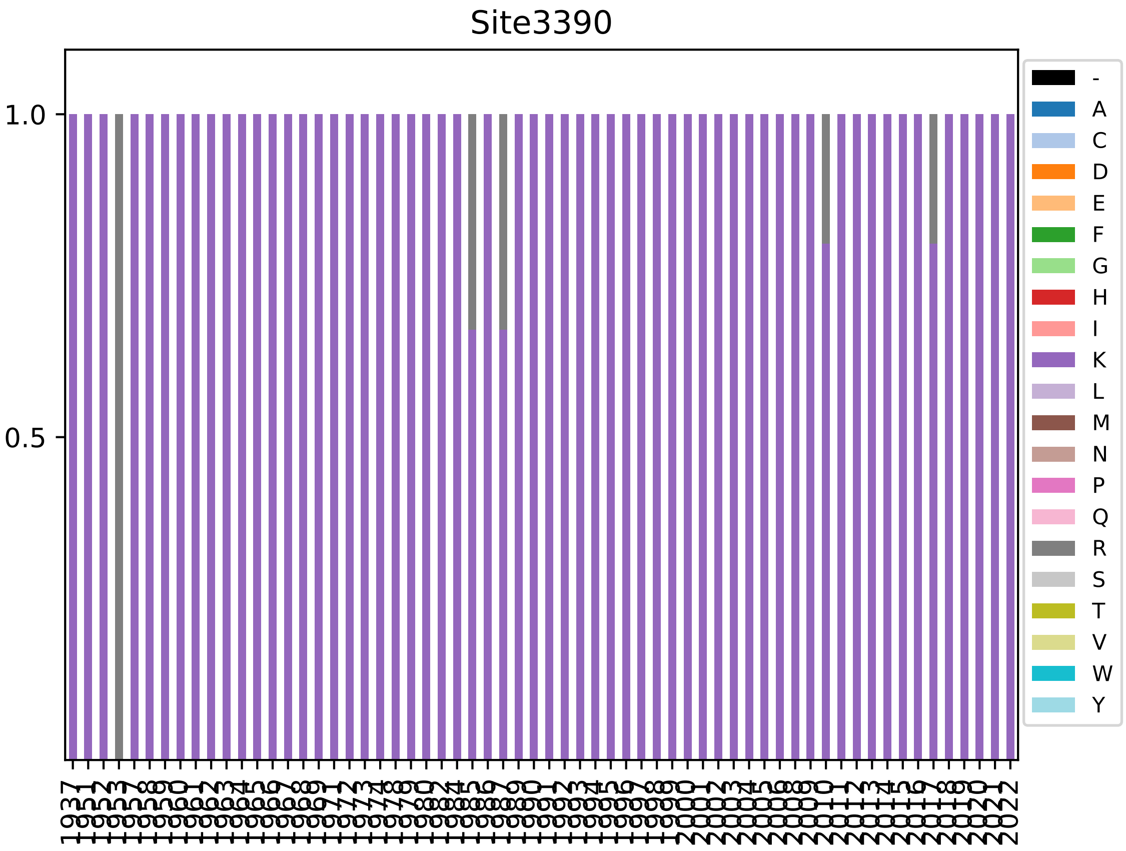
Site3390
1.0
0.5
-
A
C
D
E
F
G
H
I
K
L
M
N
P
Q
R
S
T
V
W
Y

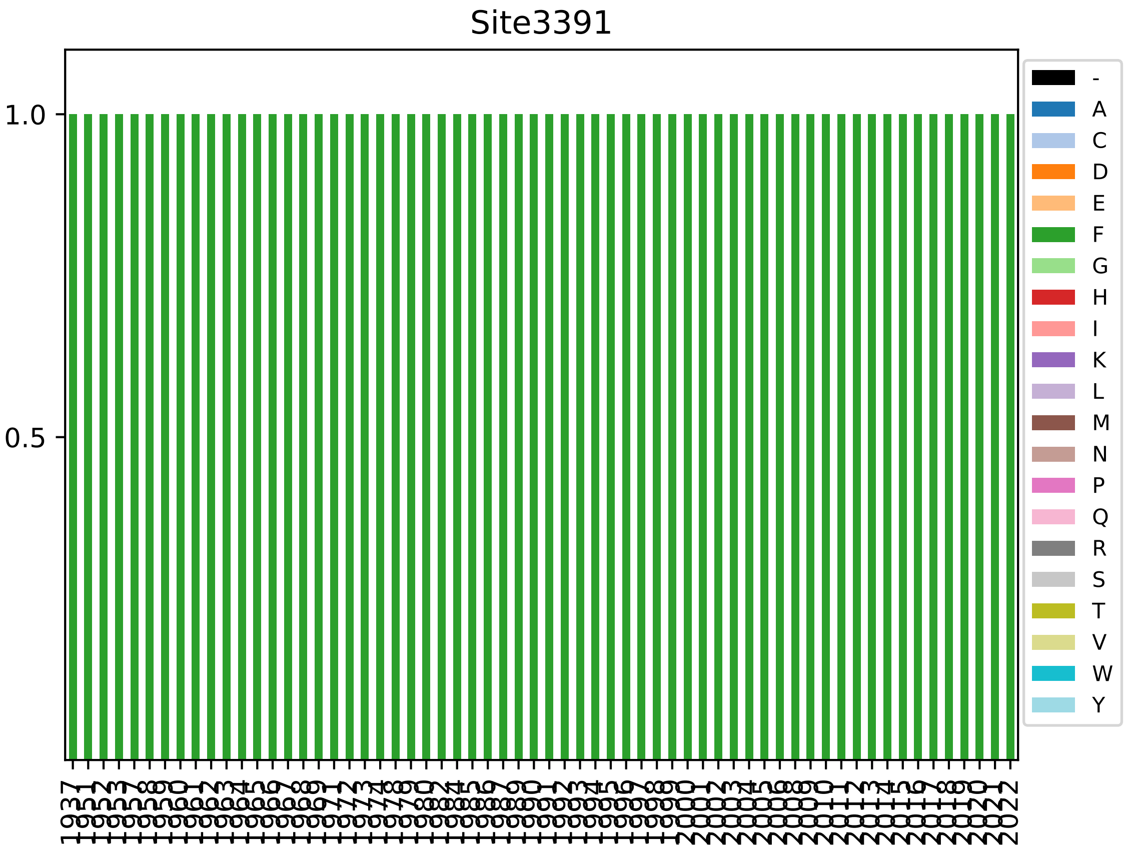
Site3391
1.0
0.5
-
A
C
D
E
F
G
H
I
K
L
M
N
P
Q
R
S
T
V
W
Y

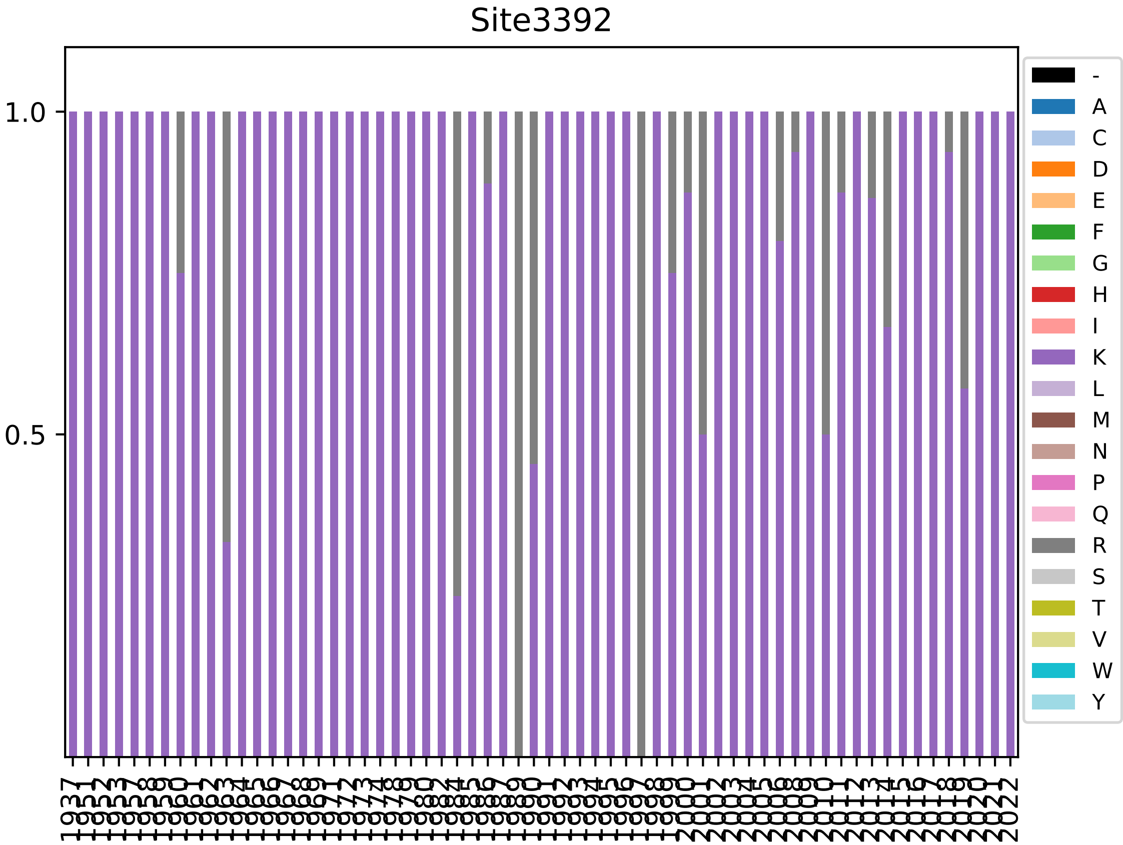
Site3392
1.0
0.5
-
A
C
D
E
F
G
H
I
K
L
M
N
P
Q
R
S
T
V
W
Y

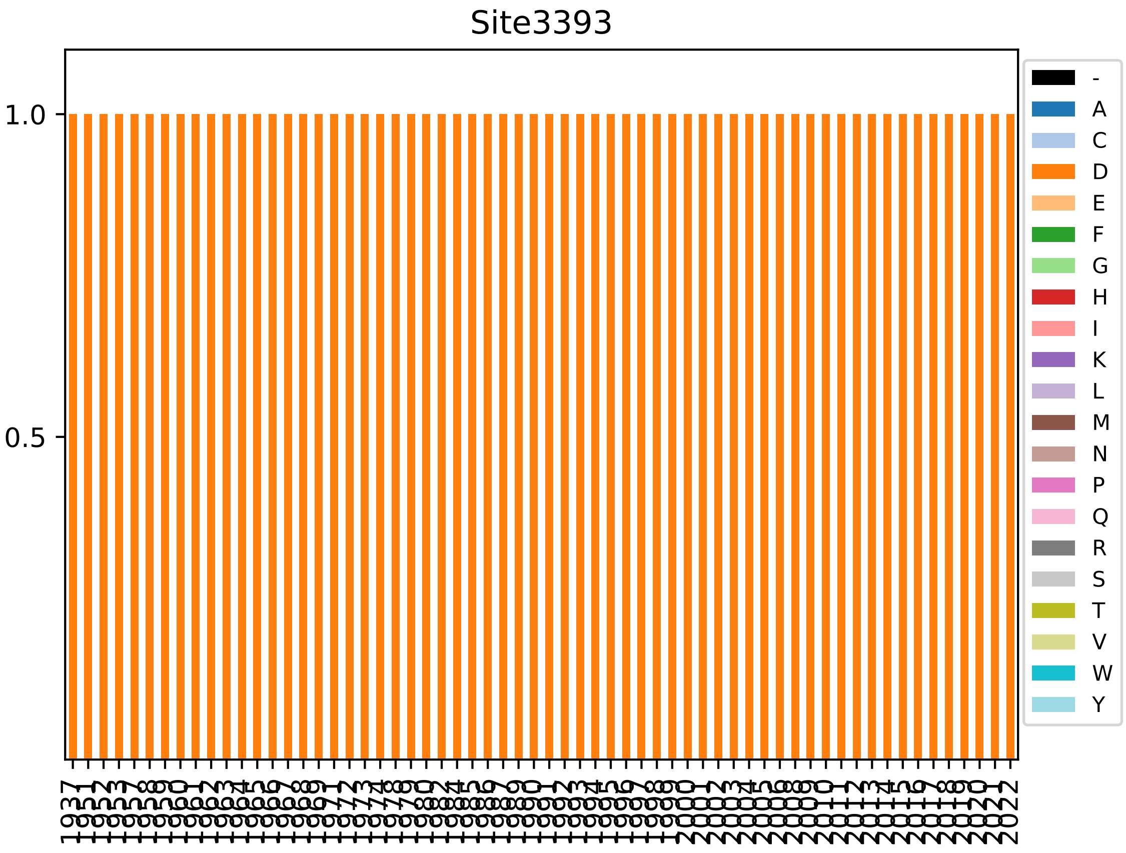
Site3393
1.0
0.5
-
A
C
D
E
F
G
H
I
K
L
M
N
P
Q
R
S
T
V
W
Y

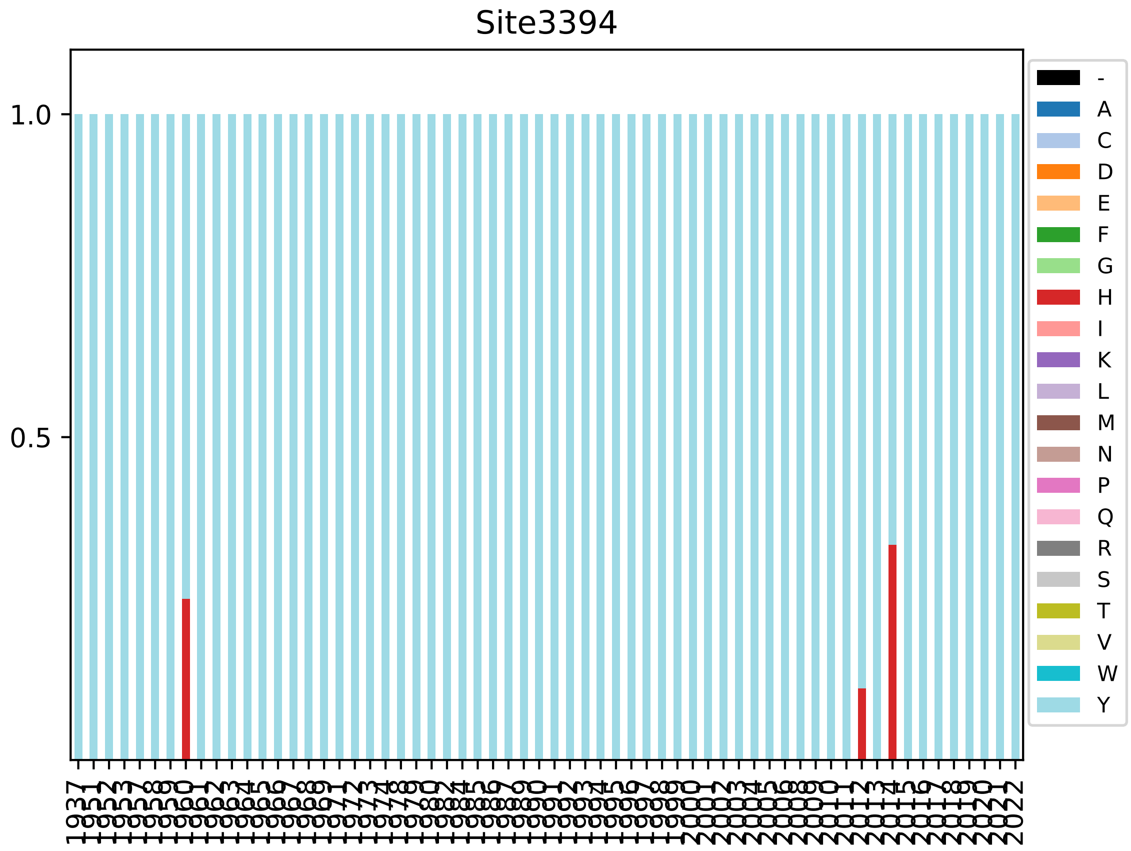
Site3394
1.0
0.5
-
A
C
D
E
F
G
H
I
K
L
M
N
P
Q
R
S
T
V
W
Y

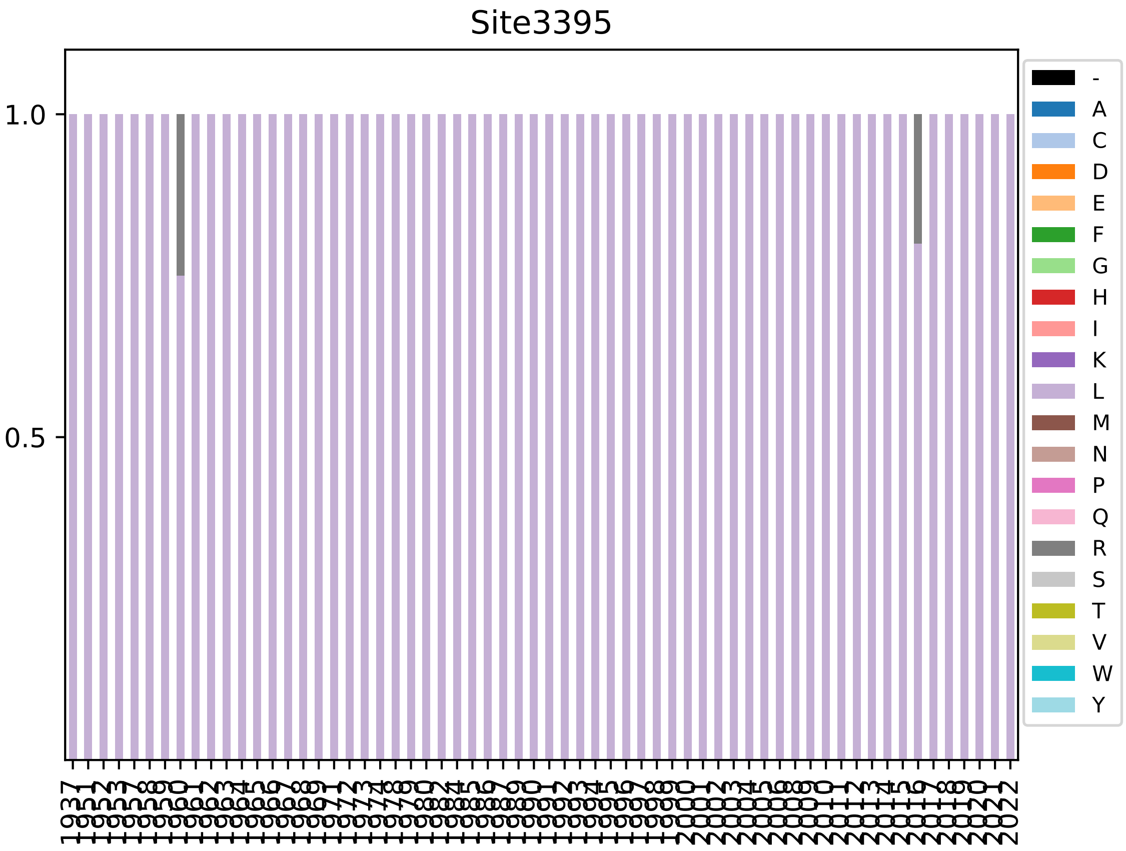
Site3395
1.0
0.5
-
A
C
D
E
F
G
H
I
K
L
M
N
P
Q
R
S
T
V
W
Y

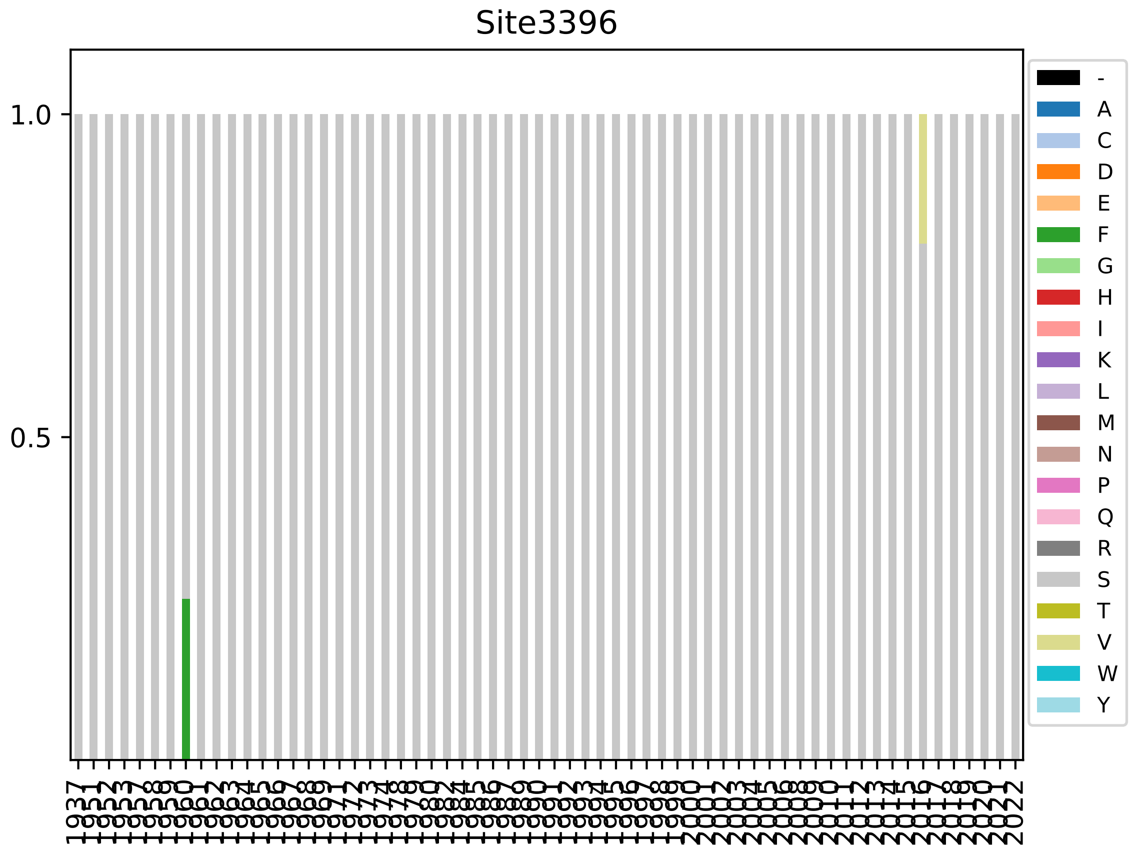
Site3396
1.0
0.5
-
A
C
D
E
F
G
H
I
K
L
M
N
P
Q
R
S
T
V
W
Y

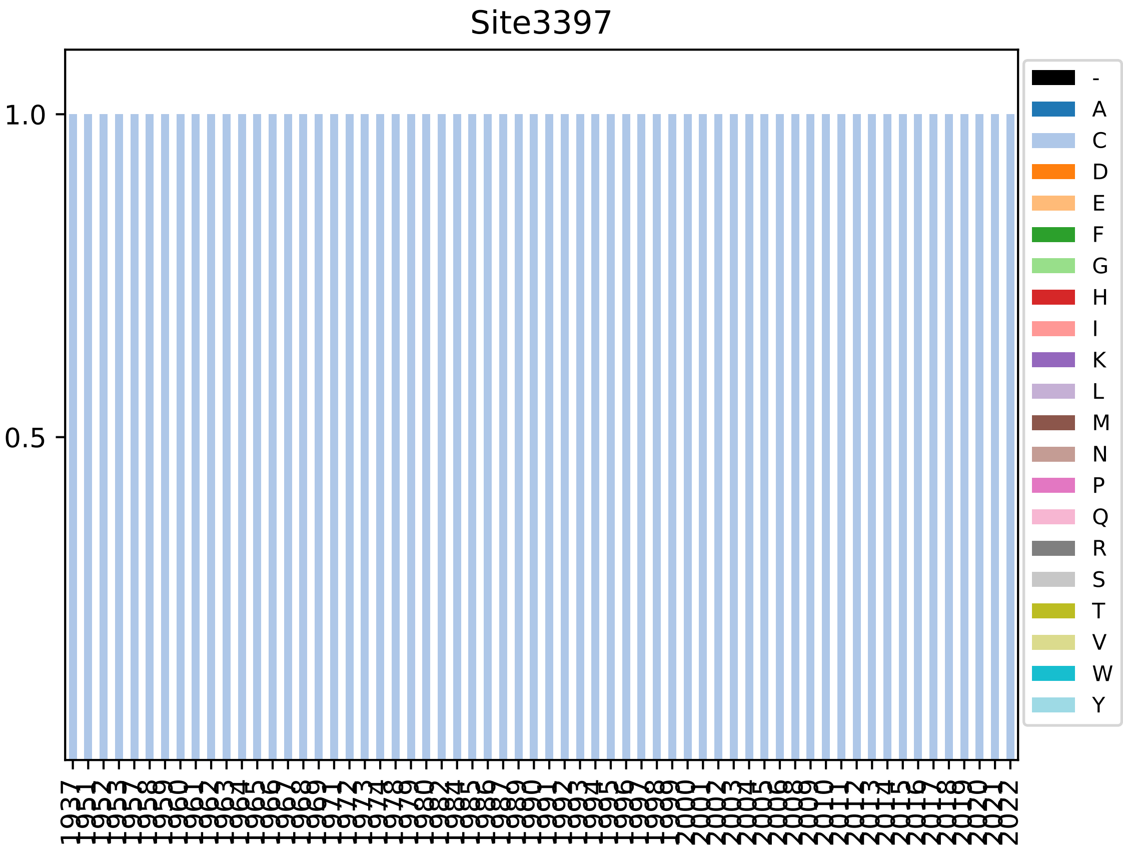
Site3397
1.0
0.5
-
A
C
D
E
F
G
H
I
K
L
M
N
P
Q
R
S
T
V
W
Y

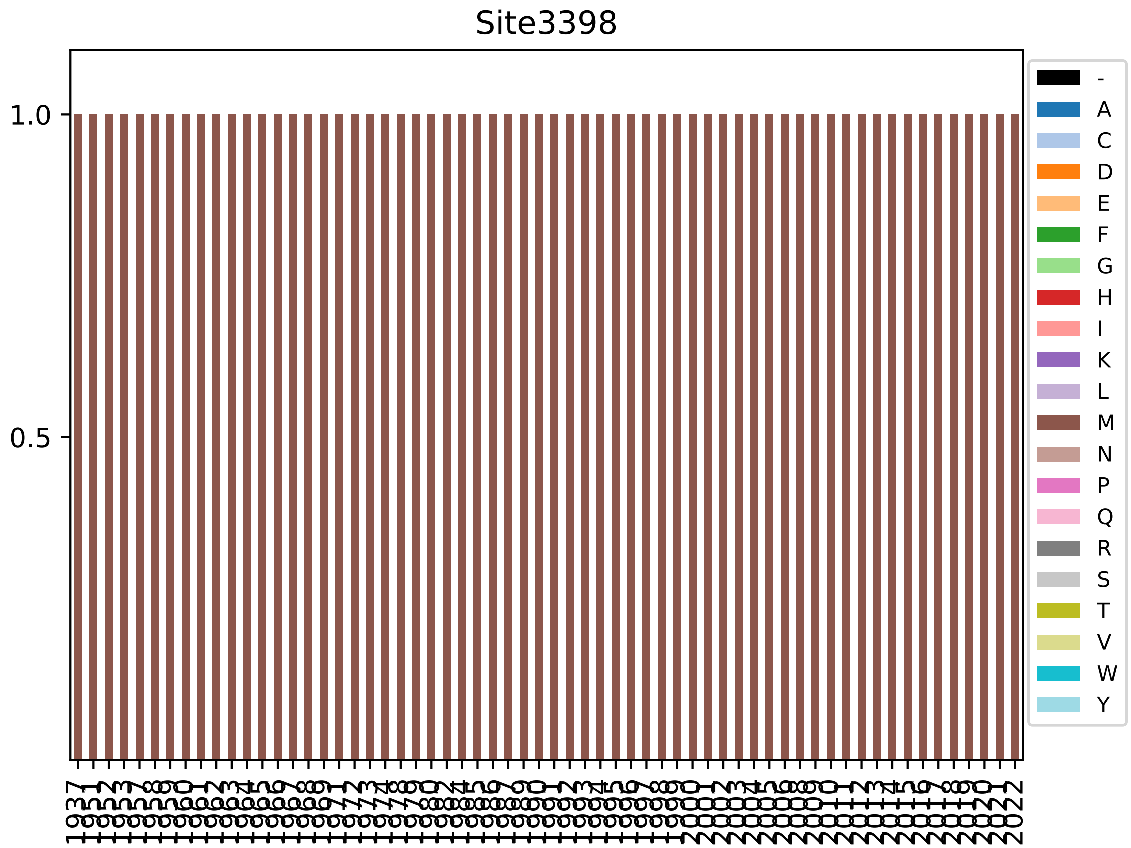
Site3398
1.0
0.5
-
A
C
D
E
F
G
H
I
K
L
M
N
P
Q
R
S
T
V
W
Y

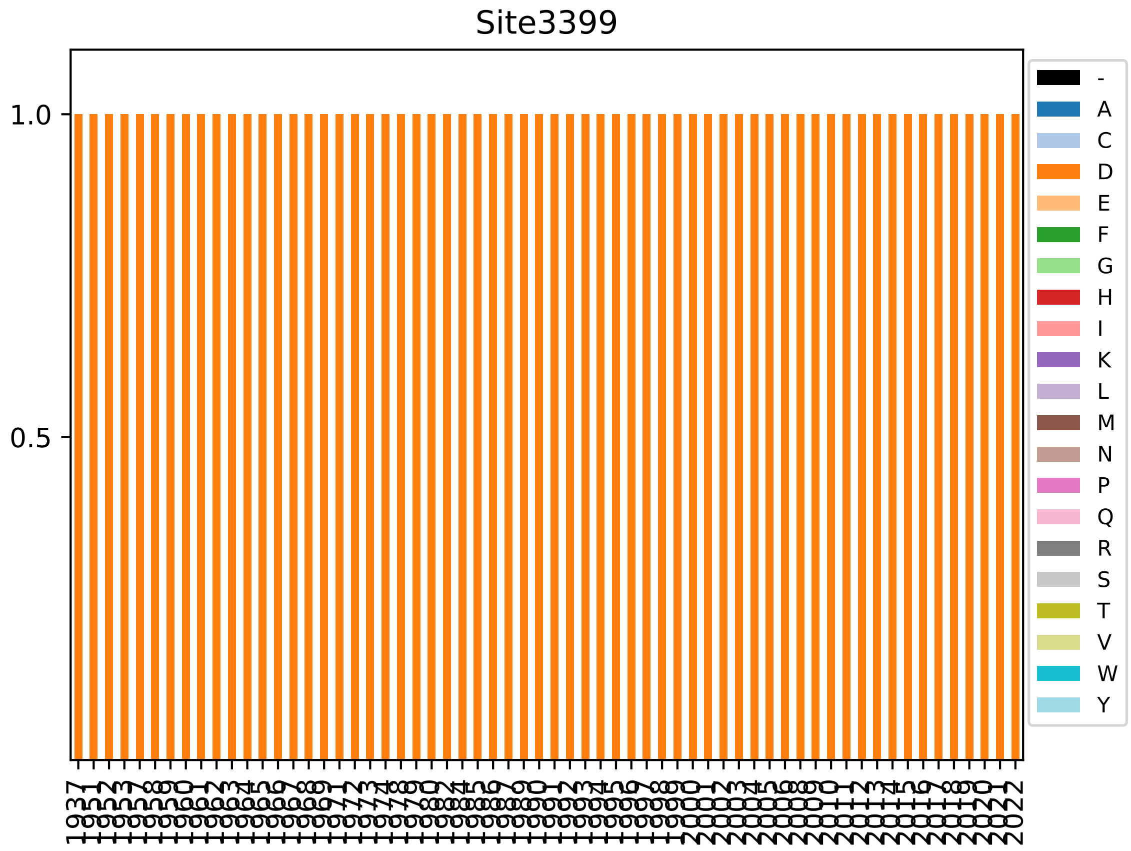
Site3399
1.0
0.5
-
A
C
D
E
F
G
H
I
K
L
M
N
P
Q
R
S
T
V
W
Y

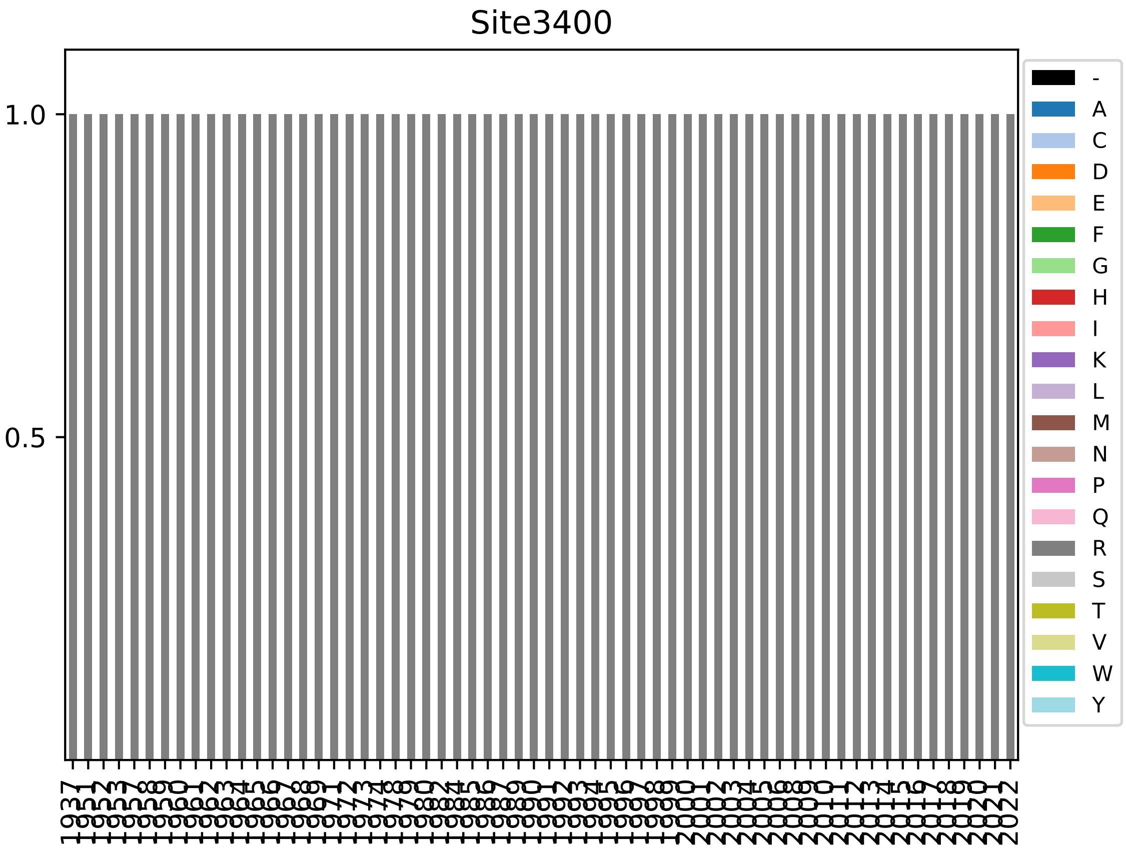
Site3400
1.0
0.5
-
A
C
D
E
F
G
H
I
K
L
M
N
P
Q
R
S
T
V
W
Y

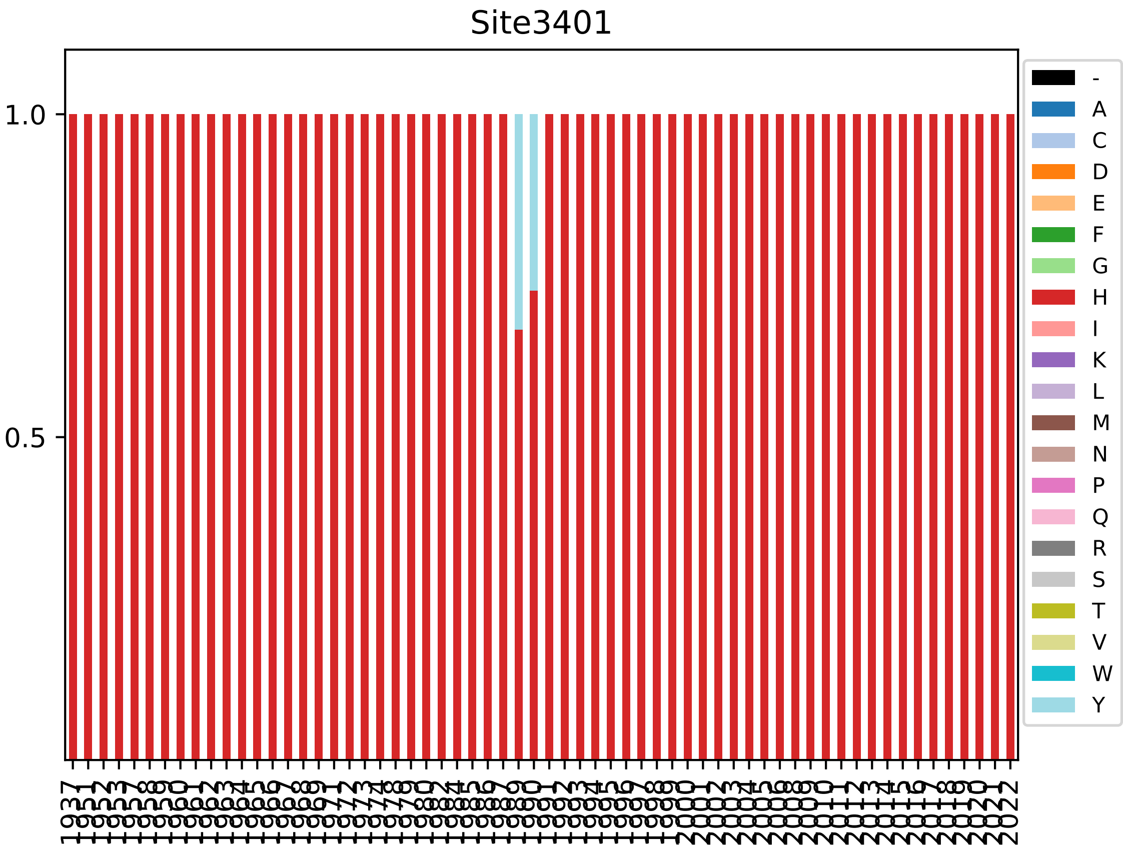
Site3401
1.0
0.5
-
A
C
D
E
F
G
H
I
K
L
M
N
P
Q
R
S
T
V
W
Y

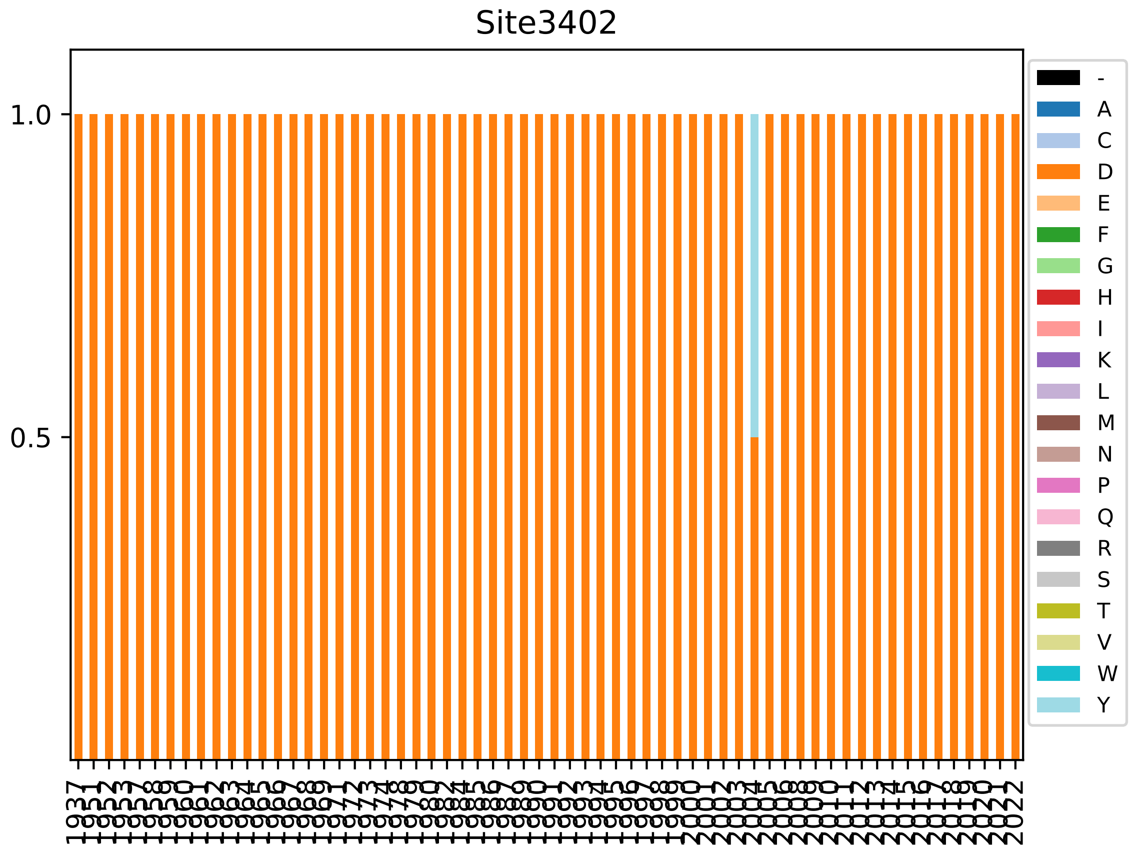

Site3402
1.0
0.5
-
A
C
D
E
F
G
H
I
K
L
M
N
P
Q
R
S
T
V
W
Y

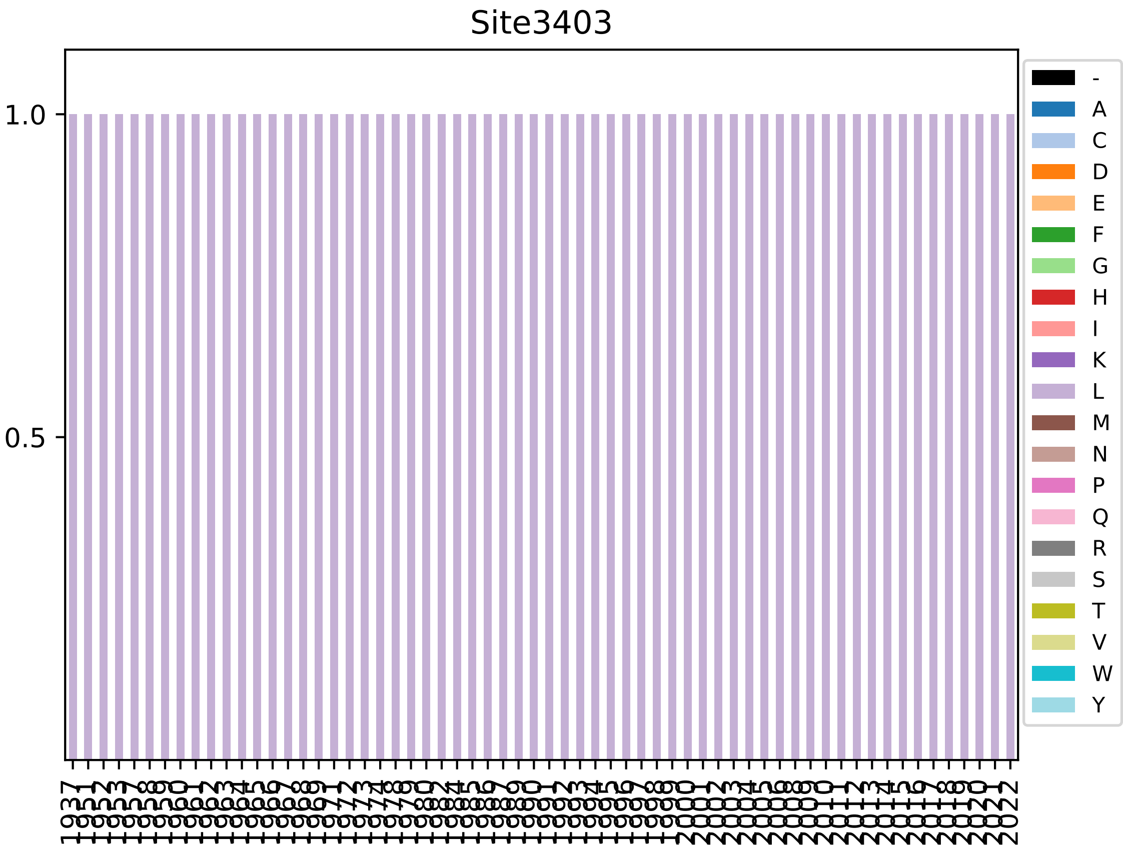
Site3403
1.0
0.5
-
A
C
D
E
F
G
H
I
K
L
M
N
P
Q
R
S
T
V
W
Y

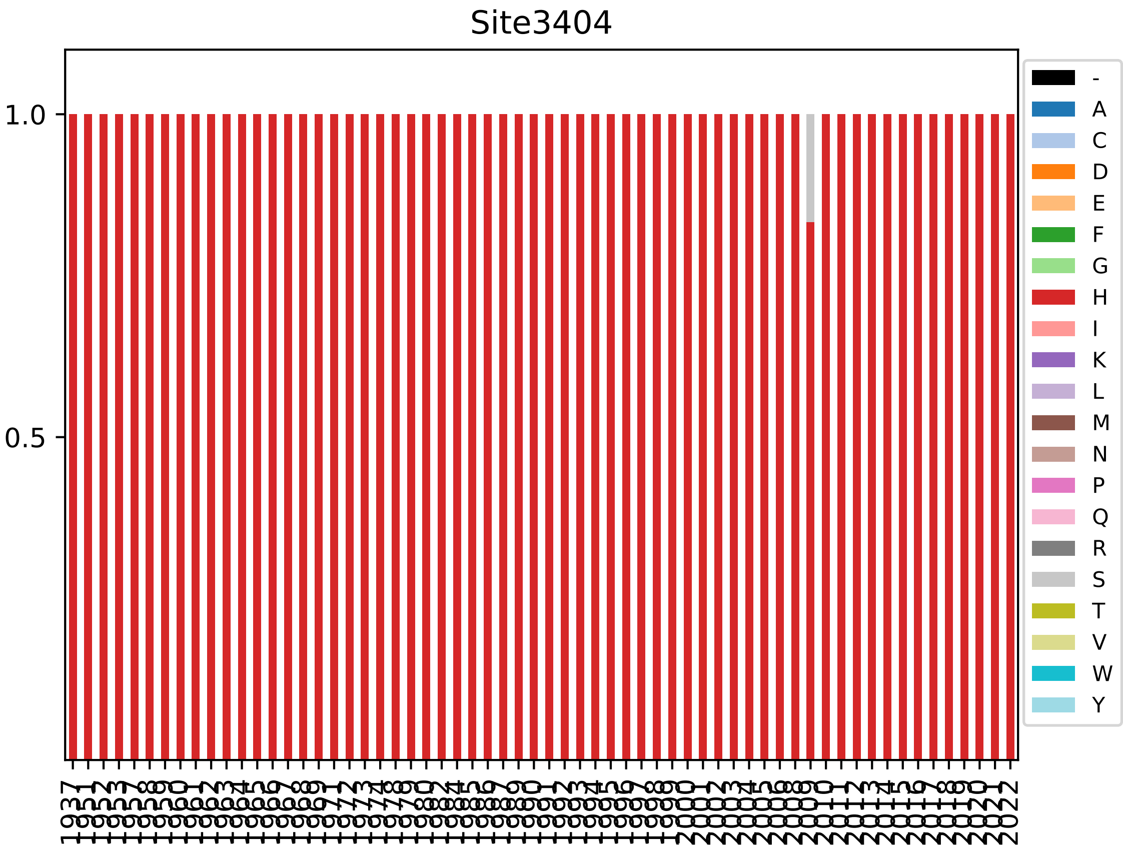
Site3404
1.0
0.5
-
A
C
D
E
F
G
H
I
K
L
M
N
P
Q
R
S
T
V
W
Y

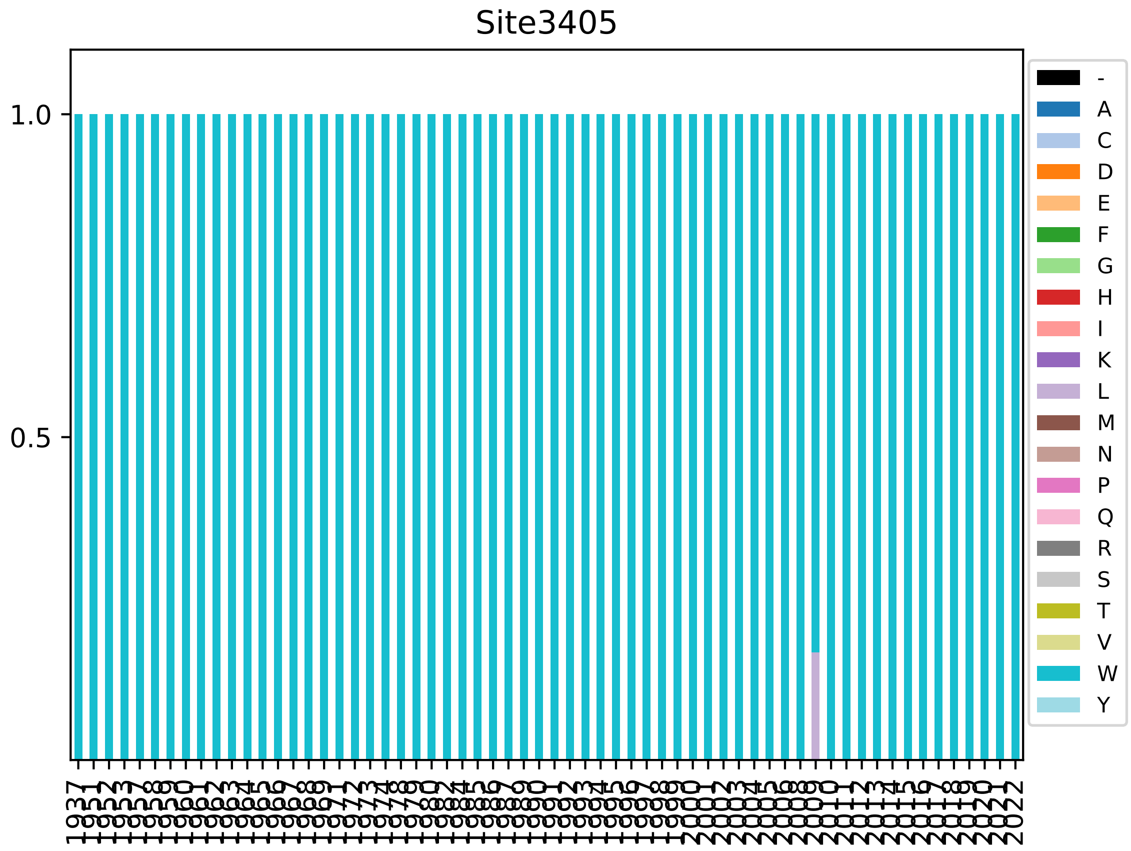
Site3405
1.0
0.5
-
A
C
D
E
F
G
H
I
K
L
M
N
P
Q
R
S
T
V
W
Y

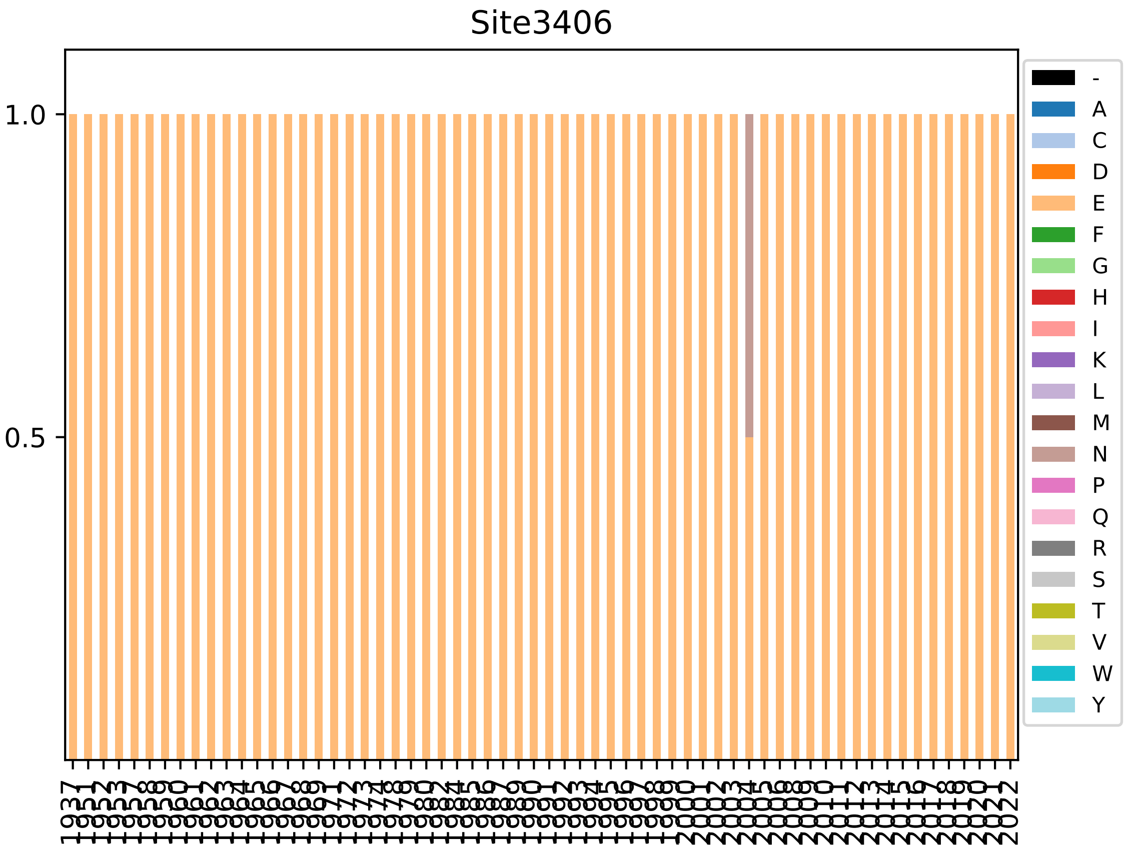
Site3406
1.0
0.5
-
A
C
D
E
F
G
H
I
K
L
M
N
P
Q
R
S
T
V
W
Y

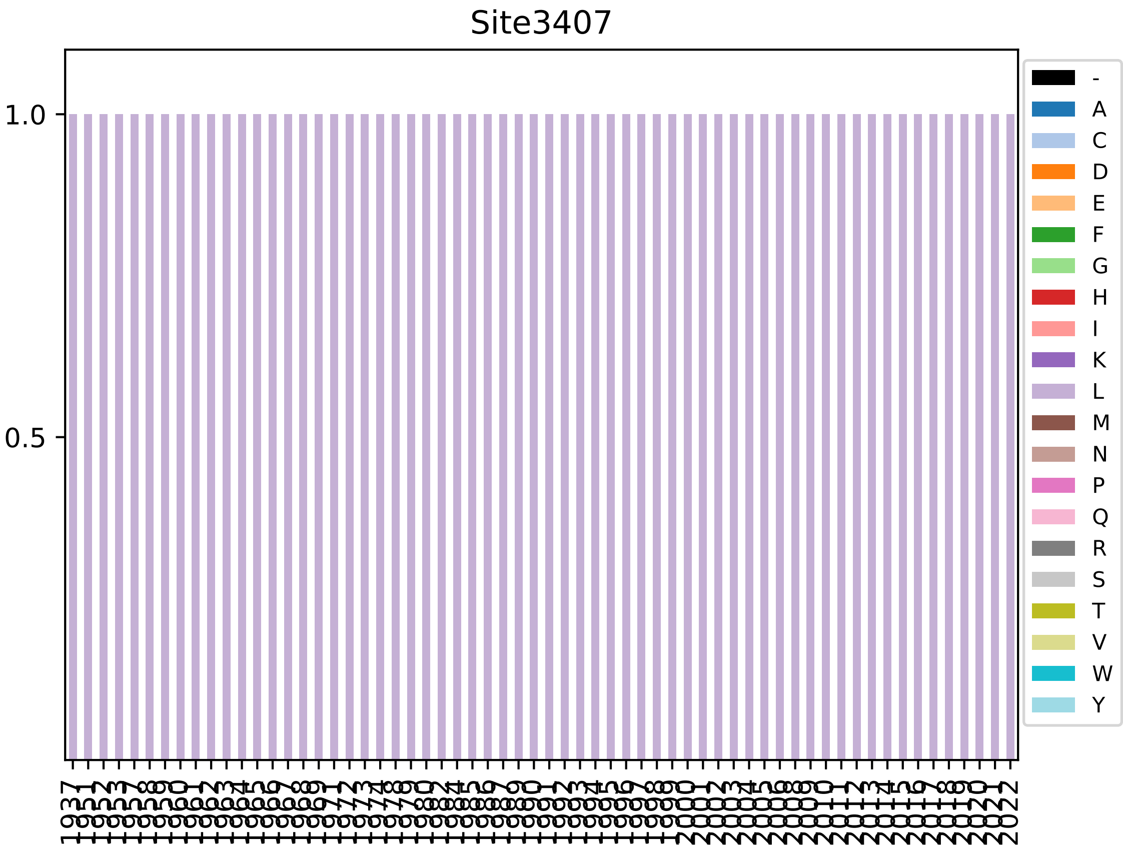

Site3407
1.0
0.5
-
A
C
D
E
F
G
H
I
K
L
M
N
P
Q
R
S
T
V
W
Y

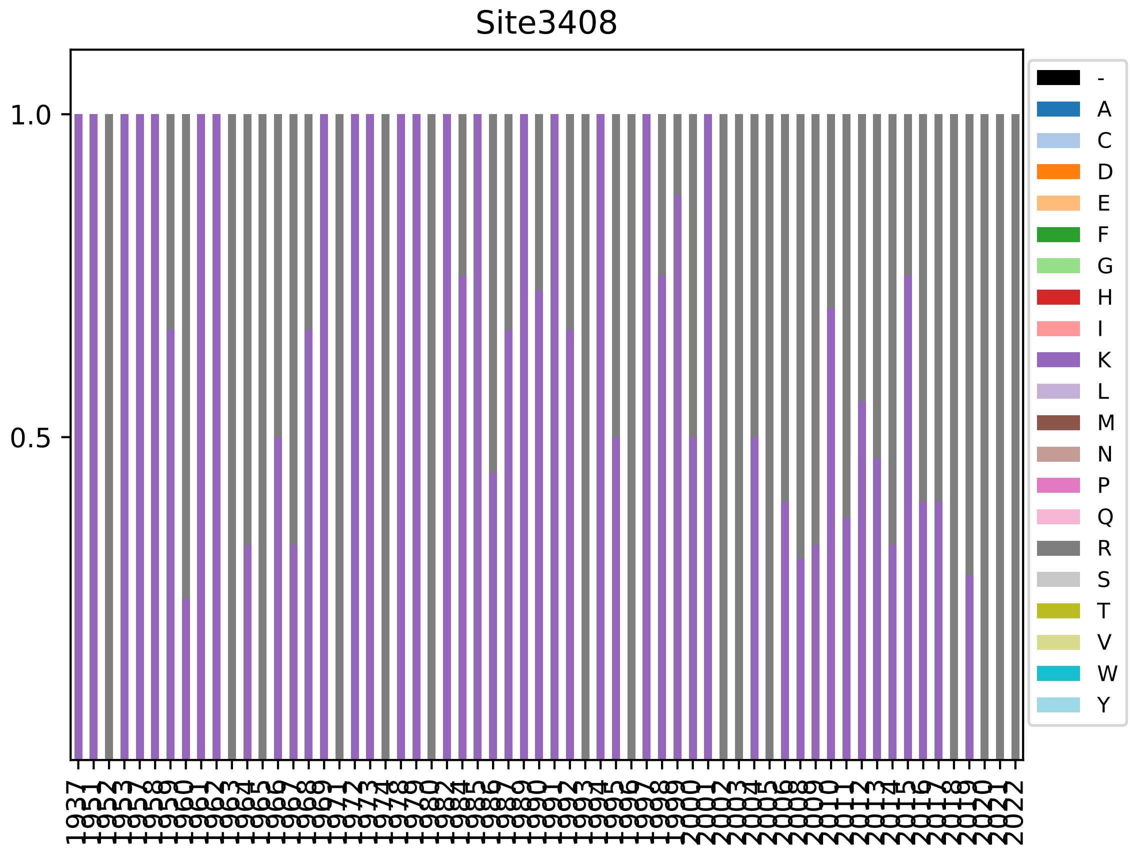
Site3408
1.0
0.5
-
A
C
D
E
F
G
H
I
K
L
M
N
P
Q
R
S
T
V
W
Y

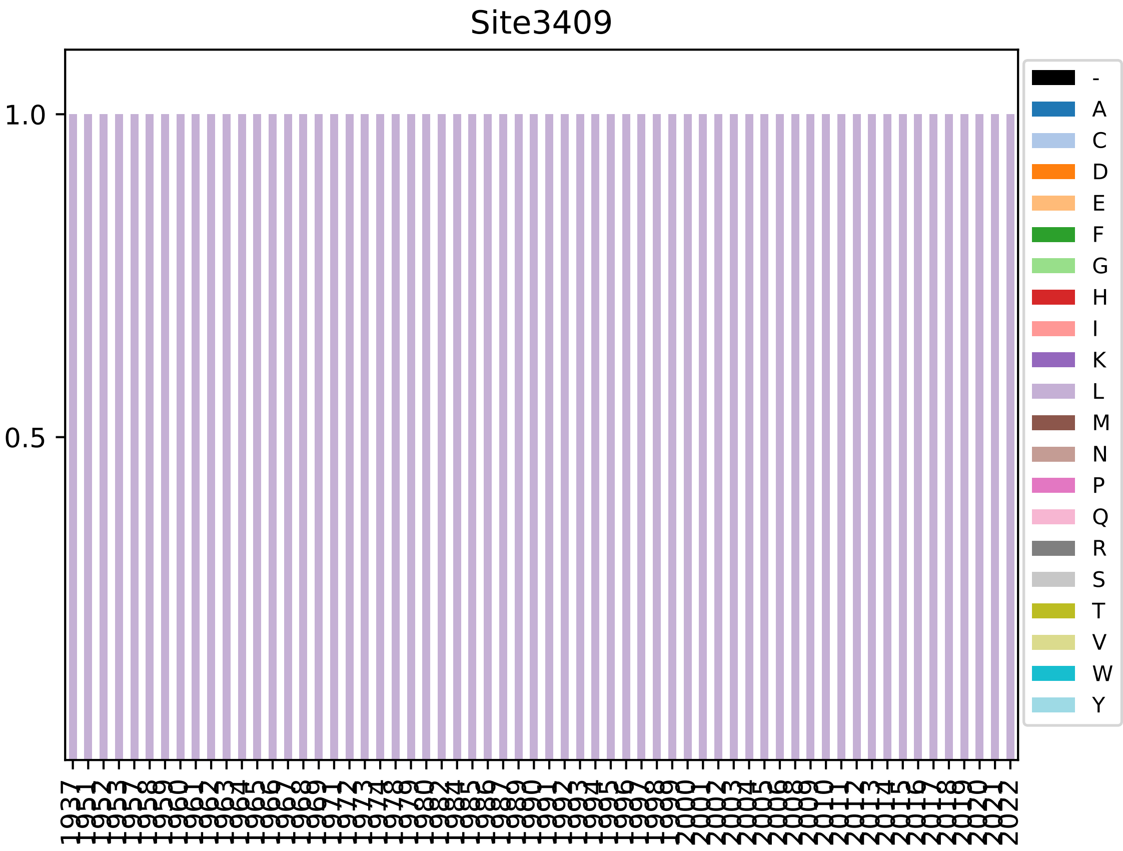
Site3409
1.0
0.5
-
A
C
D
E
F
G
H
I
K
L
M
N
P
Q
R
S
T
V
W
Y

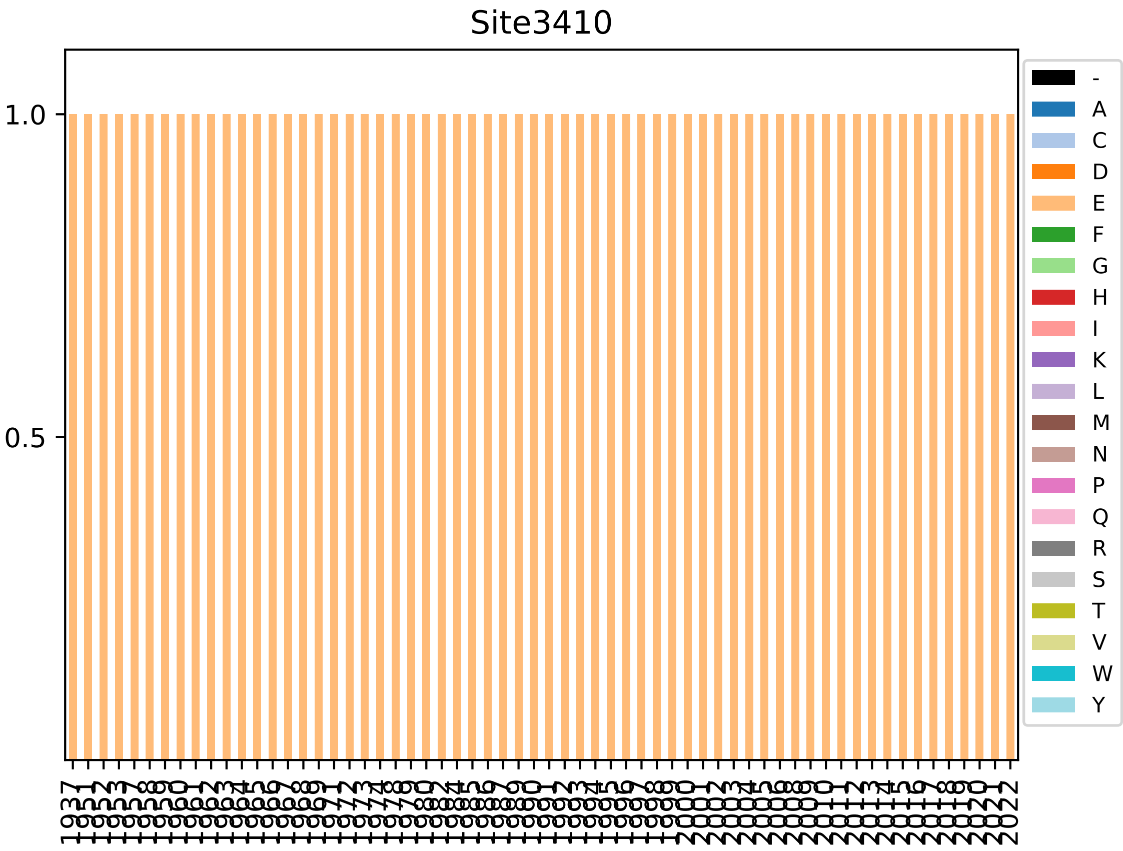
Site3410
1.0
0.5
-
A
C
D
E
F
G
H
I
K
L
M
N
P
Q
R
S
T
V
W
Y

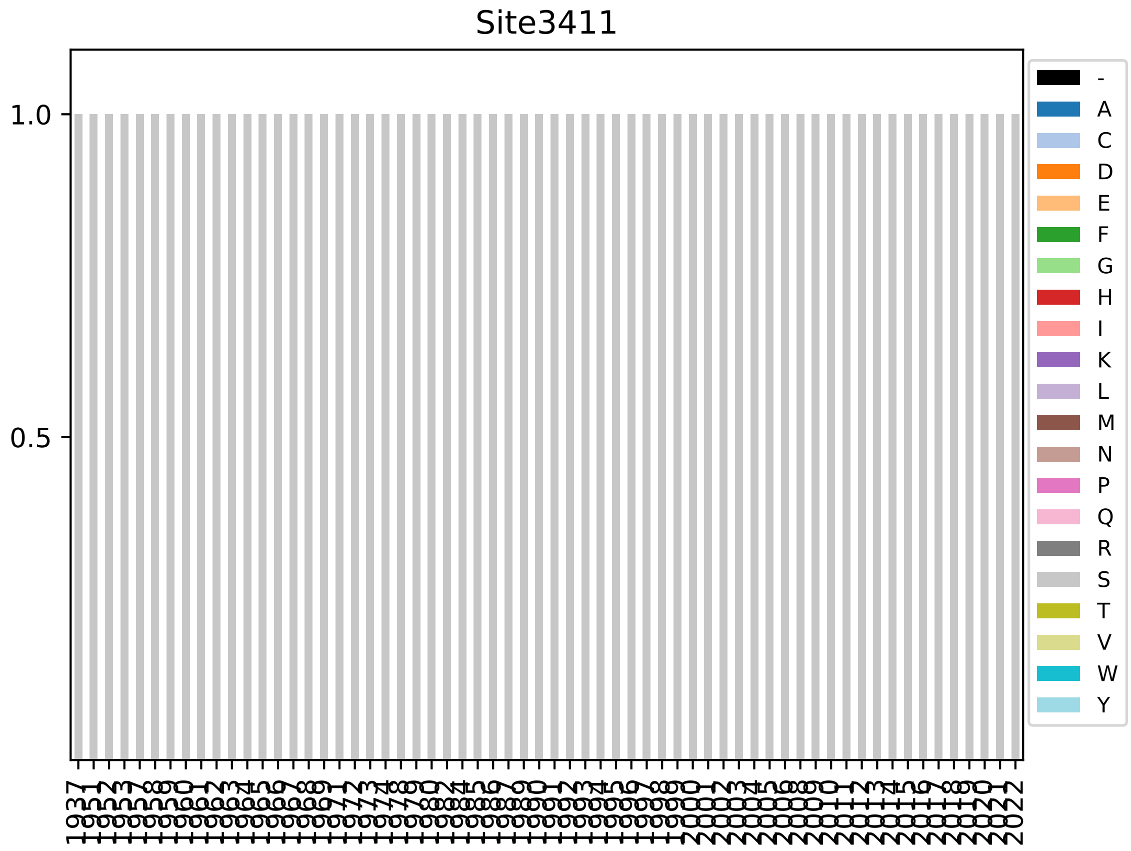
Site3411
1.0
0.5
-
A
C
D
E
F
G
H
I
K
L
M
N
P
Q
R
S
T
V
W
Y

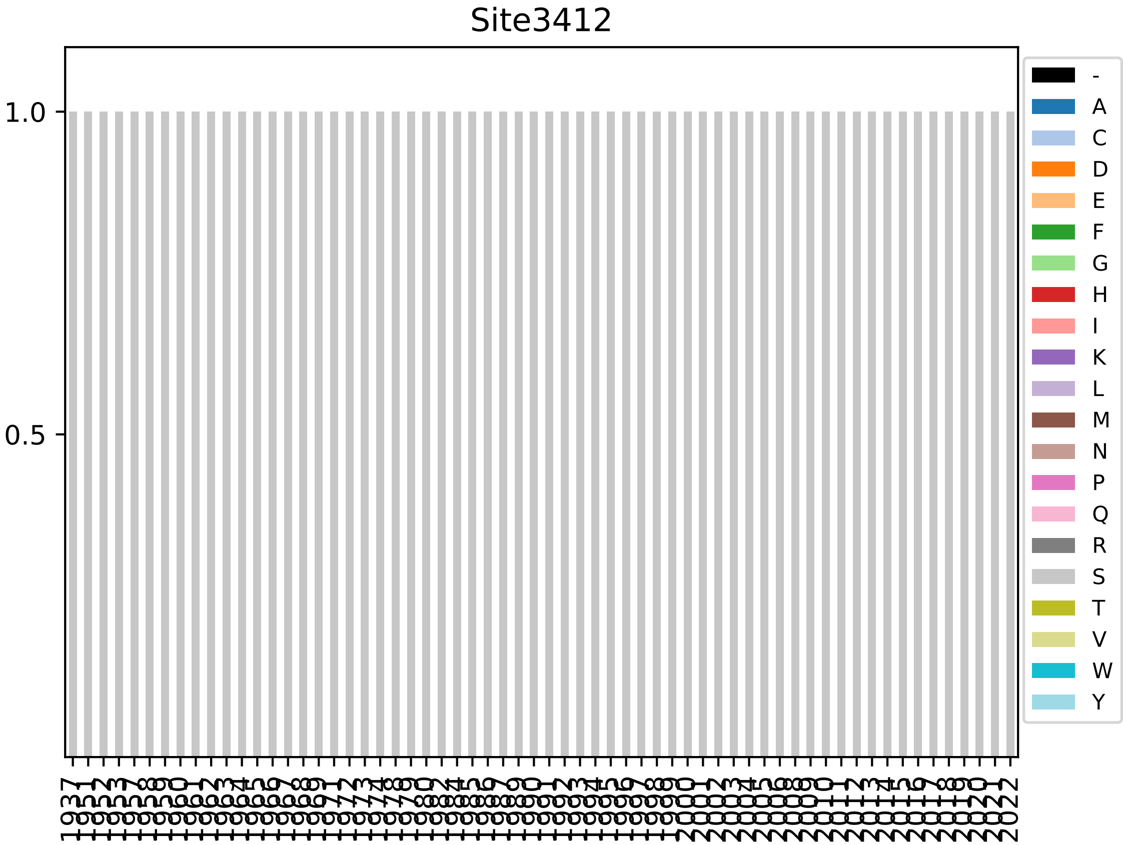
Site3412
1.0
0.5
-
A
C
D
E
F
G
H
I
K
L
M
N
P
Q
R
S
T
V
W
Y

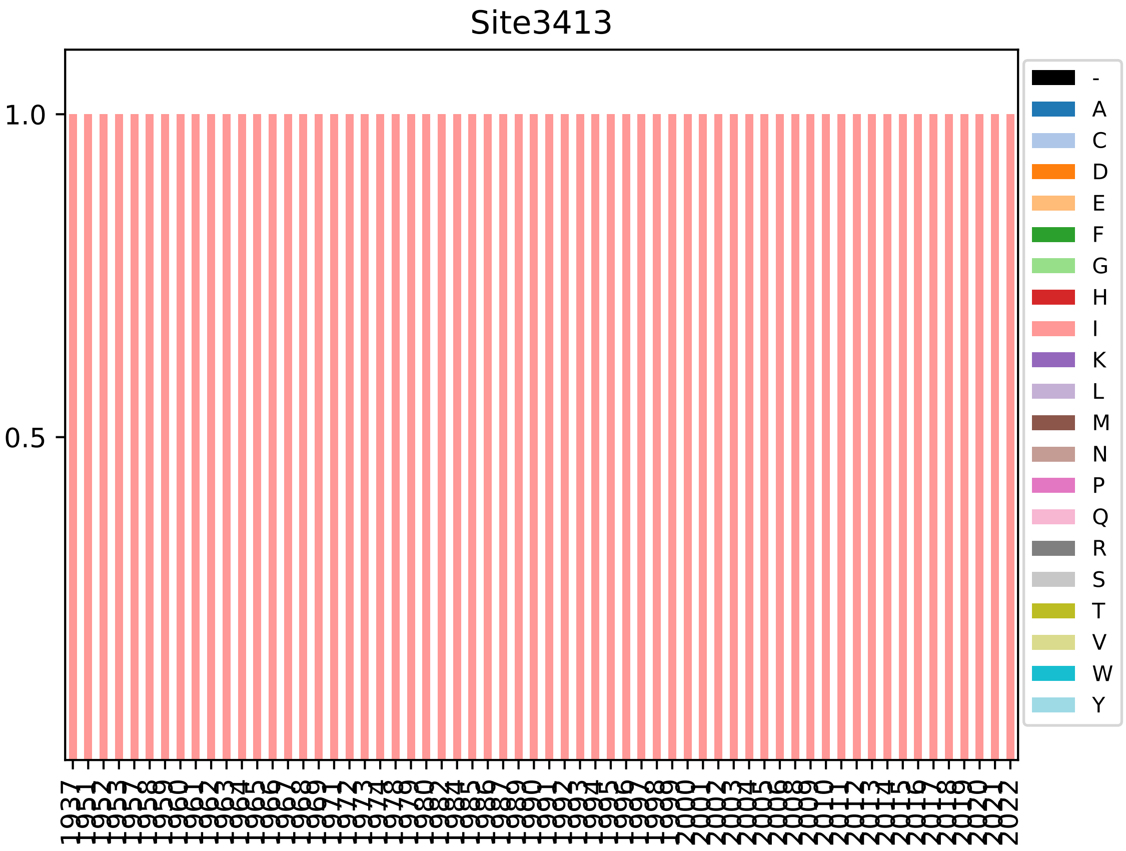
Site3413
1.0
0.5
-
A
C
D
E
F
G
H
I
K
L
M
N
P
Q
R
S
T
V
W
Y

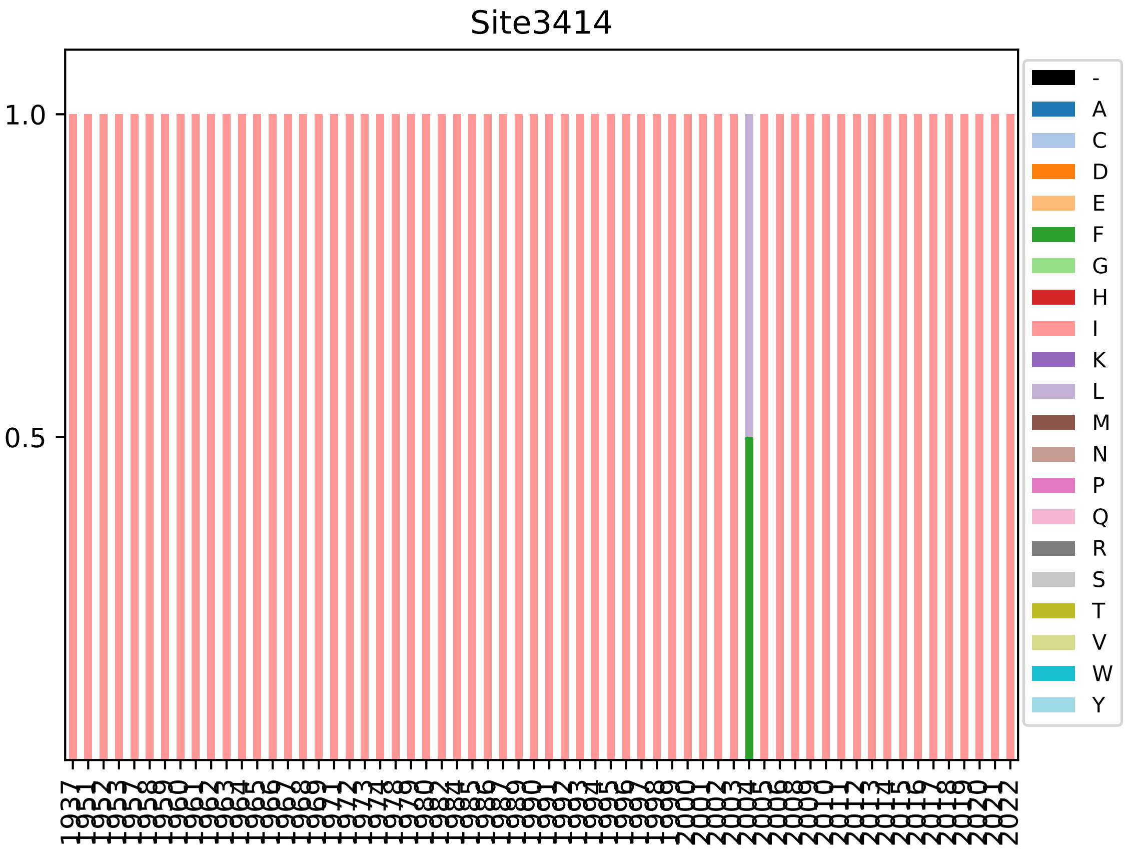
Site3414
1.0
0.5
-
A
C
D
E
F
G
H
I
K
L
M
N
P
Q
R
S
T
V
W
Y

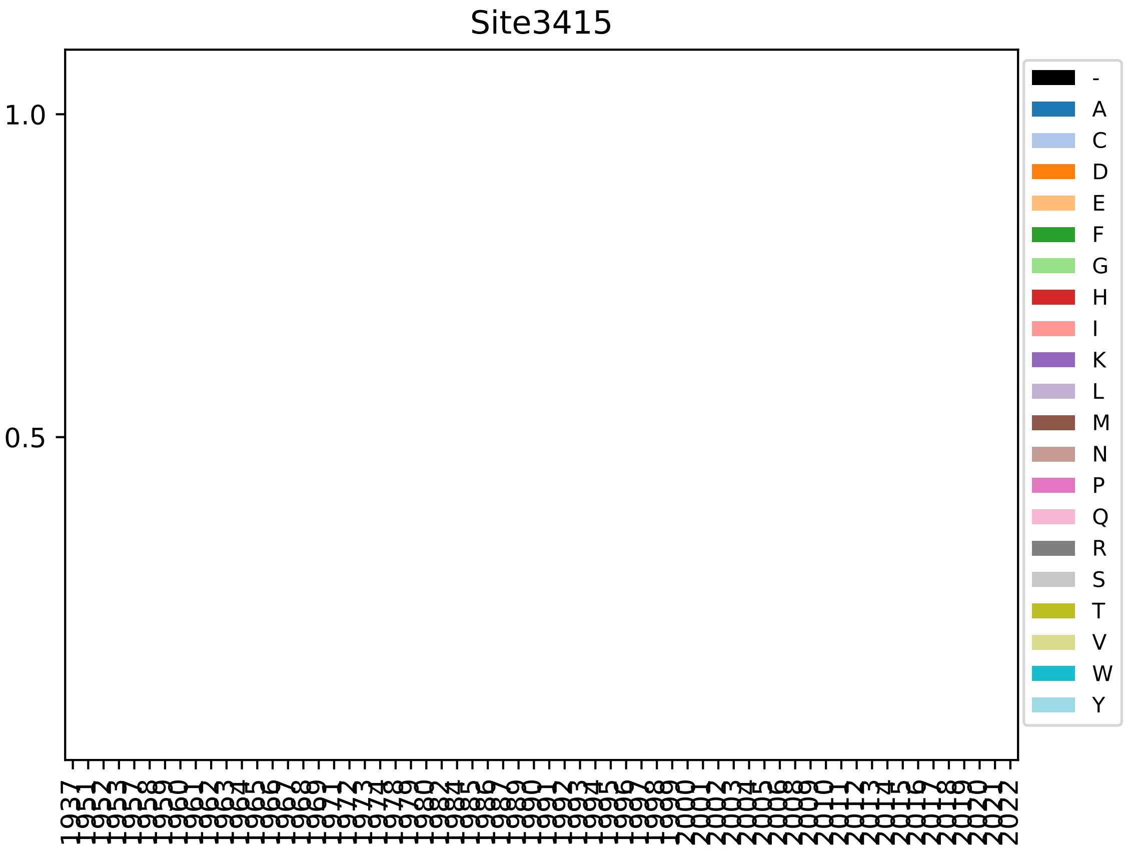
Site3415
1.0
0.5
-
A
C
D
E
F
G
H
I
K
L
M
N
P
Q
R
S
T
V
W
Y
